# Supplementary material for: Macroevolutionary dynamics of gene family gain and loss along multicellular eukaryotic lineages
Source: Nat Commun. 2024 Mar 26;15:2663. doi: 10.1038/s41467-024-47017-w (PMC10966110; doi:10.1038/s41467-024-47017-w)
Supplement: Supplementary file 16 — Supplementary Dataset 11 [file 41467_2024_47017_MOESM16_ESM.pdf]

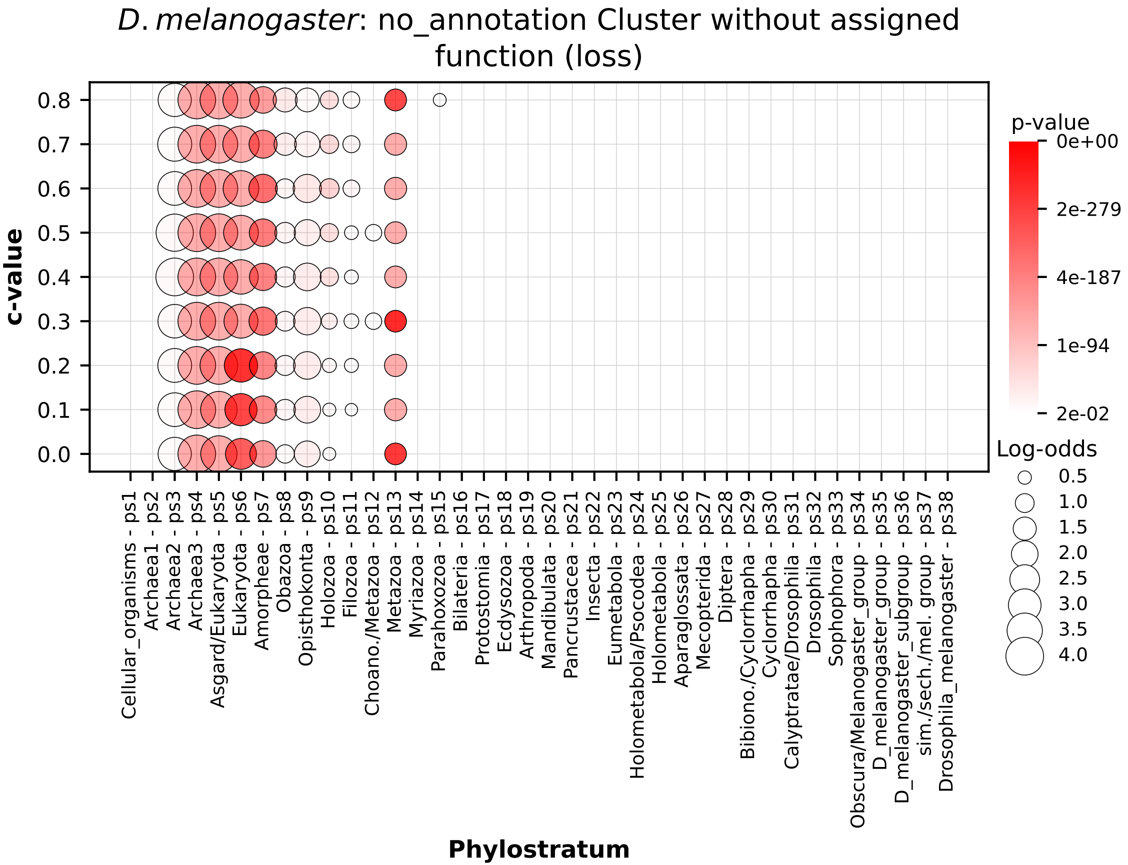

D. melanogaster: no_annotation Cluster without assigned function (loss)
c-value
0.8
0.7
0.6
0.5
0.4
0.3
0.2
0.1
0.0
Phylostratum
Cellular_organisms - ps1
Archaea1 - ps2
Archaea2 - ps3
Archaea3 - ps4
Asgard/Eukaryota - ps5
Eukaryota - ps6
Amorpheae - ps7
Obazoa - ps8
Opisthokonta - ps9
Holozoa - ps10
Filozoa - ps11
Choano./Metazoa - ps12
Metazoa - ps13
Myriazoa - ps14
Parahoxozoa - ps15
Bilateria - ps16
Protostomia - ps17
Ecdysozoa - ps18
Arthropoda - ps19
Mandibulata - ps20
Pancrustacea - ps21
Insecta - ps22
Eumetabola - ps23
Holometabola/Psocodea - ps24
Holometabola - ps25
Aparaglossata - ps26
Mecopterida - ps27
Diptera - ps28
Bibiono./Cyclorrhapha - ps29
Cyclorrhapha - ps30
Calyptratae/Drosophila - ps31
Drosophila - ps32
Sophophora - ps33
Obscura/Melanogaster_group - ps34
D_melanogaster_group - ps35
D_melanogaster_subgroup - ps36
sim./sech./mel. group - ps37
Drosophila_melanogaster - ps38
p-value
0e+00
2e-279
4e-187
1e-94
2e-02
Log-odds
0.5
1.0
1.5
2.0
2.5
3.0
3.5
4.0

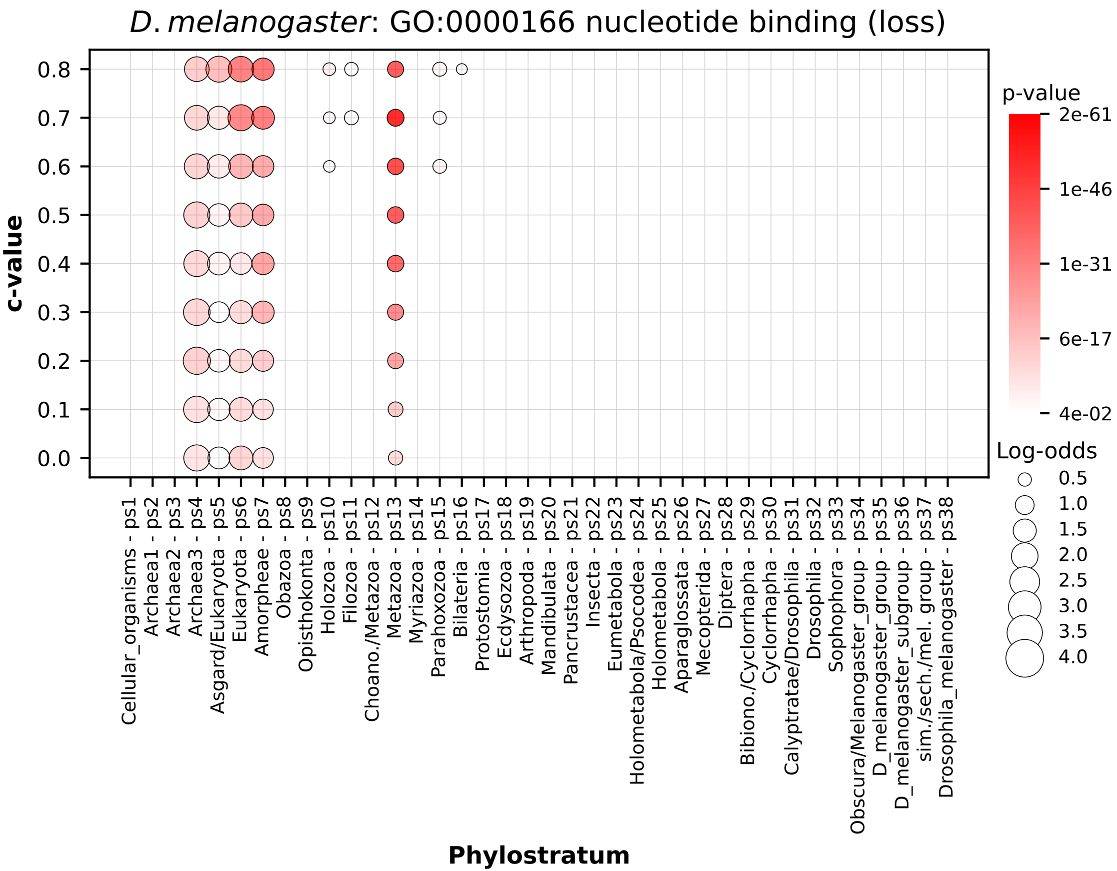

D. melanogaster: GO:0000166 nucleotide binding (loss)
c-value
0.8
0.7
0.6
0.5
0.4
0.3
0.2
0.1
0.0
p-value
2e-61
1e-46
1e-31
6e-17
4e-02
Log-odds
0.5
1.0
1.5
2.0
2.5
3.0
3.5
4.0
Cellular_organisms - ps1
Archaea1 - ps2
Archaea2 - ps3
Archaea3 - ps4
Asgard/Eukaryota - ps5
Eukaryota - ps6
Amorpheae - ps7
Obazoa - ps8
Opisthokonta - ps9
Holozoa - ps10
Filozoa - ps11
Choano./Metazoa - ps12
Metazoa - ps13
Myriazoa - ps14
Parahoxozoa - ps15
Bilateria - ps16
Protostomia - ps17
Ecdysozoa - ps18
Arthropoda - ps19
Mandibulata - ps20
Pancrustacea - ps21
Insecta - ps22
Eumetabola - ps23
Holometabola/Psocodea - ps24
Holometabola - ps25
Aparaglossata - ps26
Mecopterida - ps27
Diptera - ps28
Bibiono./Cyclorrhapha - ps29
Cyclorrhapha - ps30
Calyptratae/Drosophila - ps31
Drosophila - ps32
Sophophora - ps33
Obscura/Melanogaster_group - ps34
D_melanogaster_group - ps35
D_melanogaster_subgroup - ps36
sim./sech./mel. group - ps37
Drosophila_melanogaster - ps38
Phylostratum

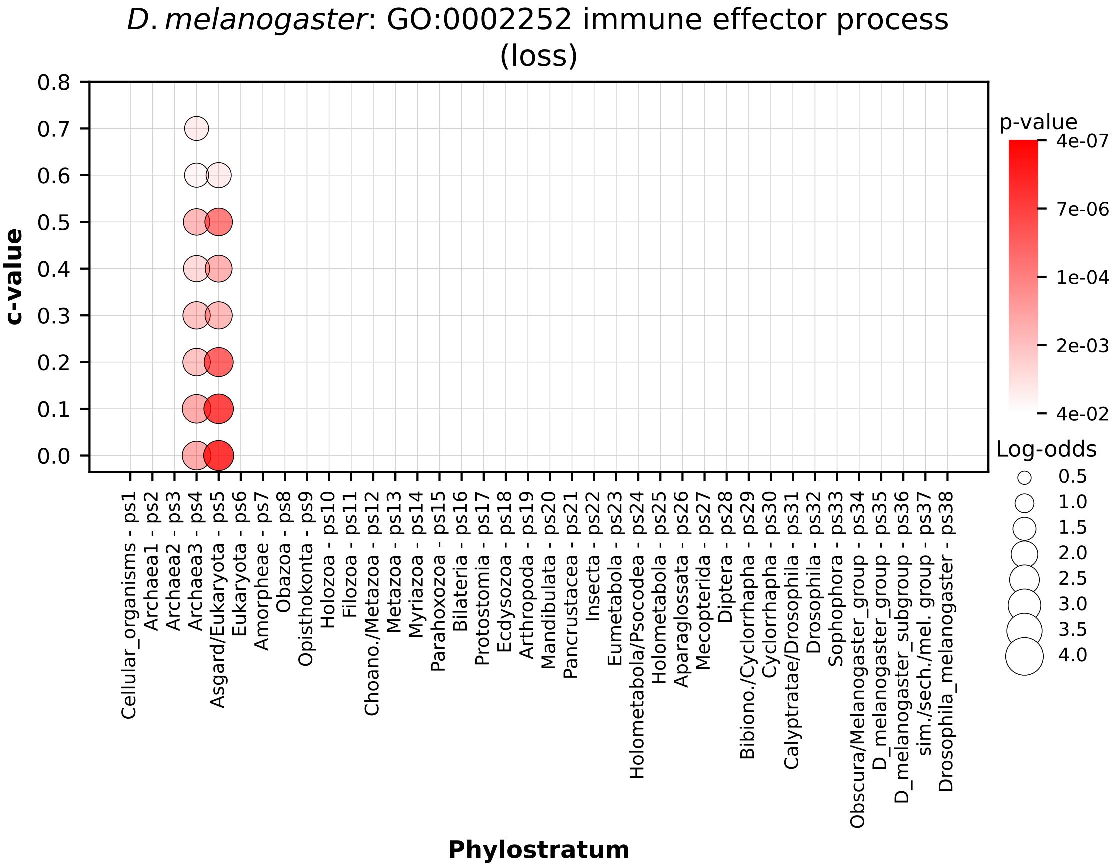

D. melanogaster: GO:0002252 immune effector process (loss)
c-value
0.8
0.7
0.6
0.5
0.4
0.3
0.2
0.1
0.0
Phylostratum
Cellular_organisms - ps1
Archaea1 - ps2
Archaea2 - ps3
Archaea3 - ps4
Asgard/Eukaryota - ps5
Eukaryota - ps6
Amorpheae - ps7
Obazoa - ps8
Opisthokonta - ps9
Holozoa - ps10
Filozoa - ps11
Choano./Metazoa - ps12
Metazoa - ps13
Myriazoa - ps14
Parahoxozoa - ps15
Bilateria - ps16
Protostomia - ps17
Ecdysozoa - ps18
Arthropoda - ps19
Mandibulata - ps20
Pancrustacea - ps21
Insecta - ps22
Eumetabola - ps23
Holometabola/Psocodea - ps24
Holometabola - ps25
Aparaglossata - ps26
Mecopterida - ps27
Diptera - ps28
Bibiono./Cyclorrhapha - ps29
Cyclorrhapha - ps30
Calyptratae/Drosophila - ps31
Drosophila - ps32
Sophophora - ps33
Obscura/Melanogaster_group - ps34
D_melanogaster_group - ps35
D_melanogaster_subgroup - ps36
sim./sech./mel. group - ps37
Drosophila_melanogaster - ps38
p-value
4e-07
7e-06
1e-04
2e-03
4e-02
Log-odds
0.5
1.0
1.5
2.0
2.5
3.0
3.5
4.0

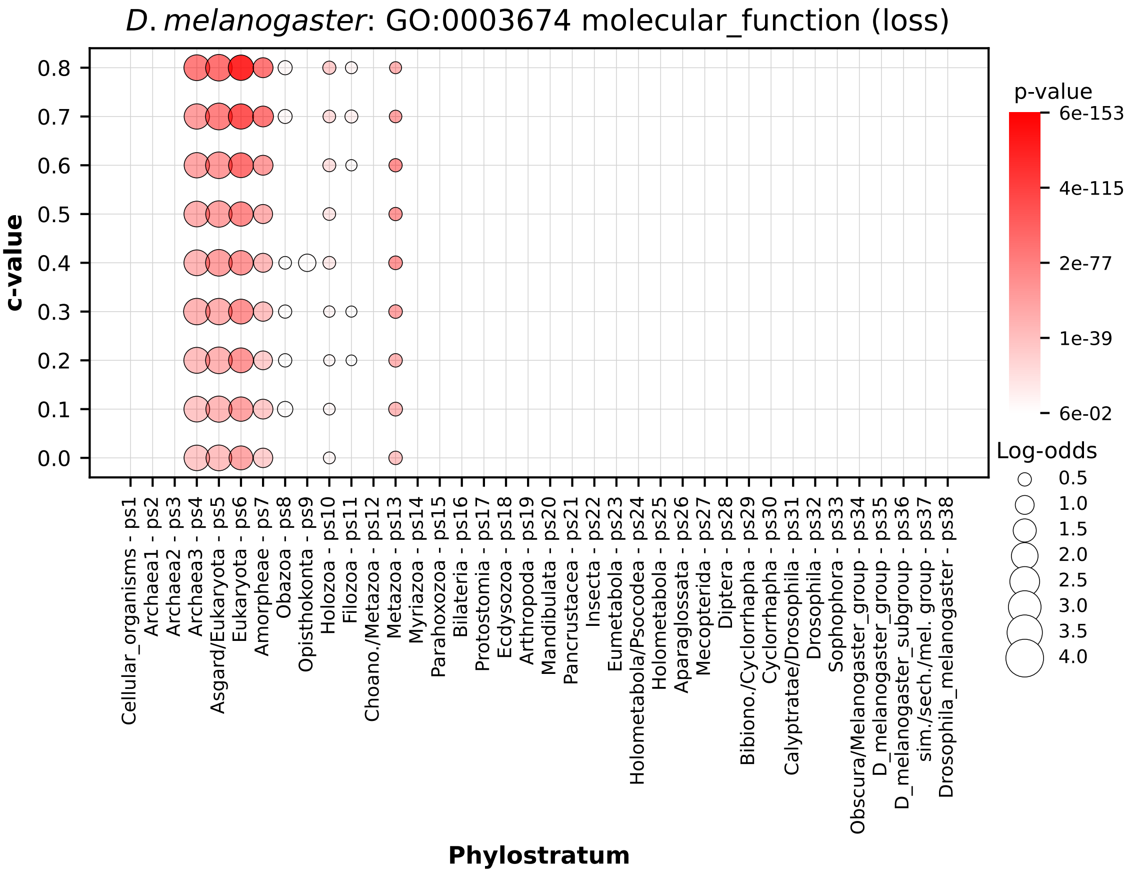
D. melanogaster: GO:0003674 molecular_function (loss)
c-value
0.8
0.7
0.6
0.5
0.4
0.3
0.2
0.1
0.0
Phylostratum
Cellular_organisms - ps1
Archaea1 - ps2
Archaea2 - ps3
Archaea3 - ps4
Asgard/Eukaryota - ps5
Eukaryota - ps6
Amorpheae - ps7
Obazoa - ps8
Opisthokonta - ps9
Holozoa - ps10
Filozoa - ps11
Choano./Metazoa - ps12
Metazoa - ps13
Myriazoa - ps14
Parahoxozoa - ps15
Bilateria - ps16
Protostomia - ps17
Ecdysozoa - ps18
Arthropoda - ps19
Mandibulata - ps20
Pancrustacea - ps21
Insecta - ps22
Eumetabola - ps23
Holometabola/Psocodea - ps24
Holometabola - ps25
Aparaglossata - ps26
Mecopterida - ps27
Diptera - ps28
Bibiono./Cyclorrhapha - ps29
Cyclorrhapha - ps30
Calyptratae/Drosophila - ps31
Drosophila - ps32
Sophophora - ps33
Obscura/Melanogaster_group - ps34
D_melanogaster_group - ps35
D_melanogaster_subgroup - ps36
sim./sech./mel. group - ps37
Drosophila_melanogaster - ps38
p-value
6e-153
4e-115
2e-77
1e-39
6e-02
Log-odds
0.5
1.0
1.5
2.0
2.5
3.0
3.5
4.0

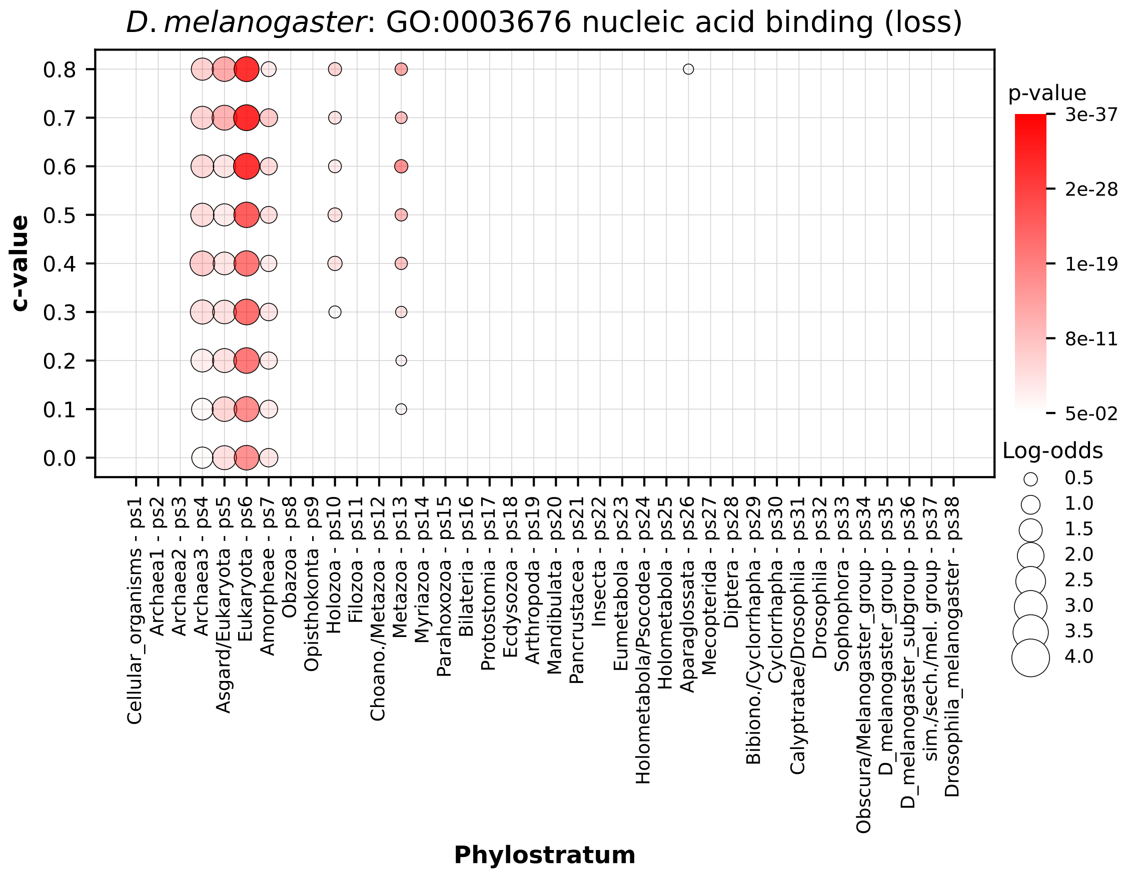

D. melanogaster: GO:0003676 nucleic acid binding (loss)
c-value
0.8
0.7
0.6
0.5
0.4
0.3
0.2
0.1
0.0
Phylostratum
Cellular_organisms - ps1
Archaea1 - ps2
Archaea2 - ps3
Archaea3 - ps4
Asgard/Eukaryota - ps5
Eukaryota - ps6
Amorpheae - ps7
Obazoa - ps8
Opisthokonta - ps9
Holozoa - ps10
Filozoa - ps11
Choano./Metazoa - ps12
Metazoa - ps13
Myriazoa - ps14
Parahoxozoa - ps15
Bilateria - ps16
Protostomia - ps17
Ecdysozoa - ps18
Arthropoda - ps19
Mandibulata - ps20
Pancrustacea - ps21
Insecta - ps22
Eumetabola - ps23
Holometabola/Psocodea - ps24
Holometabola - ps25
Aparaglossata - ps26
Mecopterida - ps27
Diptera - ps28
Bibiono./Cyclorrhapha - ps29
Cyclorrhapha - ps30
Calyptratae/Drosophila - ps31
Drosophila - ps32
Sophophora - ps33
Obscura/Melanogaster_group - ps34
D_melanogaster_group - ps35
D_melanogaster_subgroup - ps36
sim./sech./mel. group - ps37
Drosophila_melanogaster - ps38
p-value
3e-37
2e-28
1e-19
8e-11
5e-02
Log-odds
0.5
1.0
1.5
2.0
2.5
3.0
3.5
4.0

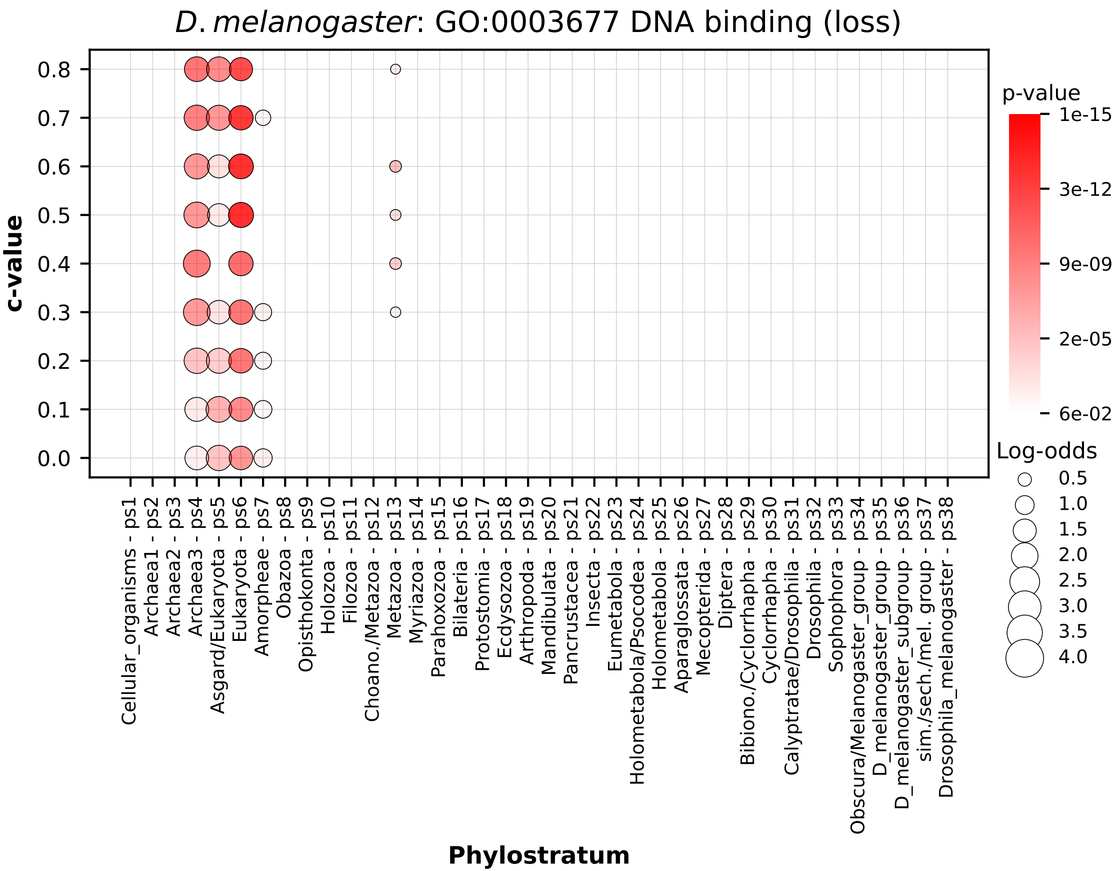

D. melanogaster: GO:0003677 DNA binding (loss)
c-value
0.8
0.7
0.6
0.5
0.4
0.3
0.2
0.1
0.0
Phylostratum
Cellular_organisms - ps1
Archaea1 - ps2
Archaea2 - ps3
Archaea3 - ps4
Asgard/Eukaryota - ps5
Eukaryota - ps6
Amorpheae - ps7
Obazoa - ps8
Opisthokonta - ps9
Holozoa - ps10
Filozoa - ps11
Choano./Metazoa - ps12
Metazoa - ps13
Myriazoa - ps14
Parahoxozoa - ps15
Bilateria - ps16
Protostomia - ps17
Ecdysozoa - ps18
Arthropoda - ps19
Mandibulata - ps20
Pancrustacea - ps21
Insecta - ps22
Eumetabola - ps23
Holometabola/Psocodea - ps24
Holometabola - ps25
Aparaglossata - ps26
Mecopterida - ps27
Diptera - ps28
Bibiono./Cyclorrhapha - ps29
Cyclorrhapha - ps30
Calyptratae/Drosophila - ps31
Drosophila - ps32
Sophophora - ps33
Obscura/Melanogaster_group - ps34
D_melanogaster_group - ps35
D_melanogaster_subgroup - ps36
sim./sech./mel. group - ps37
Drosophila_melanogaster - ps38
p-value
1e-15
3e-12
9e-09
2e-05
6e-02
Log-odds
0.5
1.0
1.5
2.0
2.5
3.0
3.5
4.0

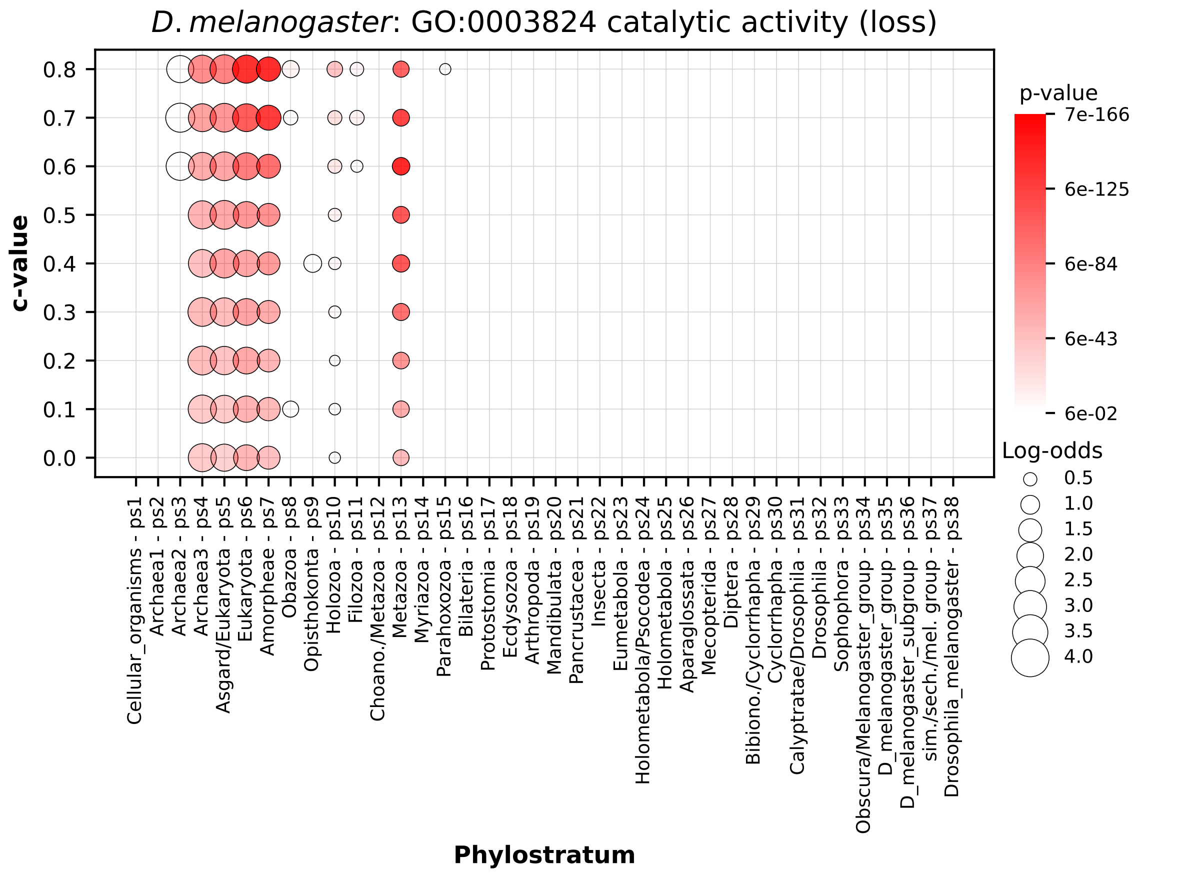

D. melanogaster: GO:0003824 catalytic activity (loss)
c-value
0.8
0.7
0.6
0.5
0.4
0.3
0.2
0.1
0.0
p-value
7e-166
6e-125
6e-84
6e-43
6e-02
Log-odds
0.5
1.0
1.5
2.0
2.5
3.0
3.5
4.0
Cellular_organisms - ps1
Archaea1 - ps2
Archaea2 - ps3
Archaea3 - ps4
Asgard/Eukaryota - ps5
Eukaryota - ps6
Amorpheae - ps7
Obazoa - ps8
Opisthokonta - ps9
Holozoa - ps10
Filozoa - ps11
Choano./Metazoa - ps12
Metazoa - ps13
Myriazoa - ps14
Parahoxozoa - ps15
Bilateria - ps16
Protostomia - ps17
Ecdysozoa - ps18
Arthropoda - ps19
Mandibulata - ps20
Pancrustacea - ps21
Insecta - ps22
Eumetabola - ps23
Holometabola/Psocodea - ps24
Holometabola - ps25
Aparaglossata - ps26
Mecopterida - ps27
Diptera - ps28
Bibiono./Cyclorrhapha - ps29
Cyclorrhapha - ps30
Calyptratae/Drosophila - ps31
Drosophila - ps32
Sophophora - ps33
Obscura/Melanogaster_group - ps34
D_melanogaster_group - ps35
D_melanogaster_subgroup - ps36
sim./sech./mel. group - ps37
Drosophila_melanogaster - ps38
Phylostratum

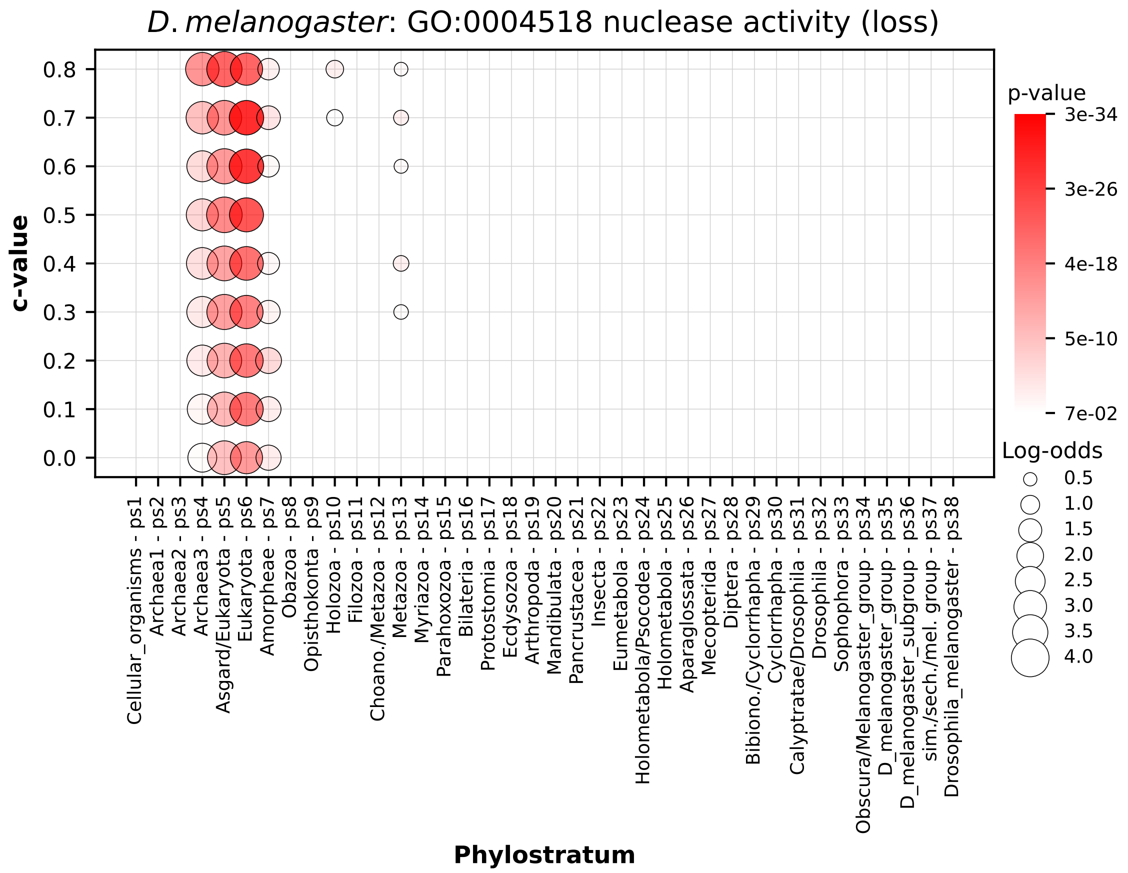

D. melanogaster: GO:0004518 nuclease activity (loss)
c-value
0.8
0.7
0.6
0.5
0.4
0.3
0.2
0.1
0.0
p-value
3e-34
3e-26
4e-18
5e-10
7e-02
Log-odds
0.5
1.0
1.5
2.0
2.5
3.0
3.5
4.0
Cellular_organisms - ps1
Archaea1 - ps2
Archaea2 - ps3
Archaea3 - ps4
Asgard/Eukaryota - ps5
Eukaryota - ps6
Amorpheae - ps7
Obazoa - ps8
Opisthokonta - ps9
Holozoa - ps10
Filozoa - ps11
Choano./Metazoa - ps12
Metazoa - ps13
Myriazoa - ps14
Parahoxozoa - ps15
Bilateria - ps16
Protostomia - ps17
Ecdysozoa - ps18
Arthropoda - ps19
Mandibulata - ps20
Pancrustacea - ps21
Insecta - ps22
Eumetabola - ps23
Holometabola/Psocodea - ps24
Holometabola - ps25
Aparaglossata - ps26
Mecopterida - ps27
Diptera - ps28
Bibiono./Cyclorrhapha - ps29
Cyclorrhapha - ps30
Calyptratae/Drosophila - ps31
Drosophila - ps32
Sophophora - ps33
Obscura/Melanogaster_group - ps34
D_melanogaster_group - ps35
D_melanogaster_subgroup - ps36
sim./sech./mel. group - ps37
Drosophila_melanogaster - ps38
Phylostratum

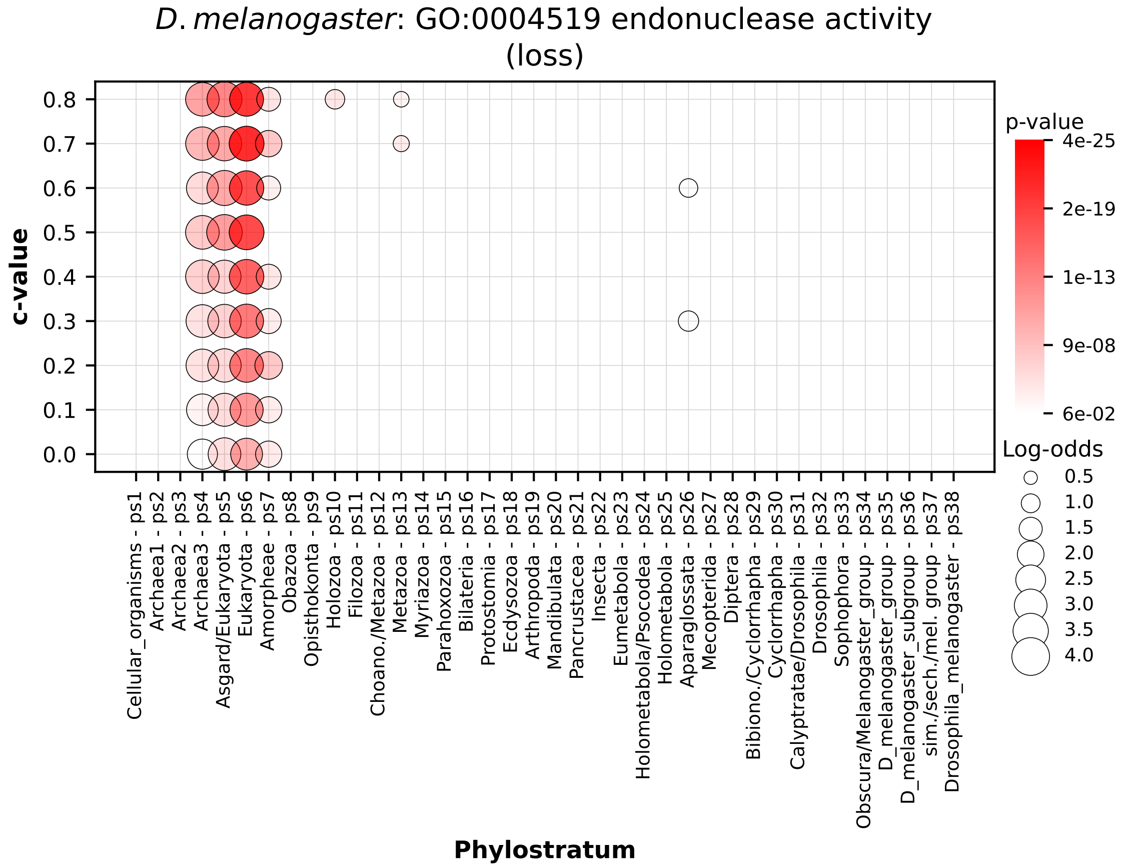

D. melanogaster: GO:0004519 endonuclease activity
(loss)
c-value
0.8
0.7
0.6
0.5
0.4
0.3
0.2
0.1
0.0
Phylostratum
Cellular_organisms - ps1
Archaea1 - ps2
Archaea2 - ps3
Archaea3 - ps4
Asgard/Eukaryota - ps5
Eukaryota - ps6
Amorpheae - ps7
Obazoa - ps8
Opisthokonta - ps9
Holozoa - ps10
Filozoa - ps11
Choano./Metazoa - ps12
Metazoa - ps13
Myriazoa - ps14
Parahoxozoa - ps15
Bilateria - ps16
Protostomia - ps17
Ecdysozoa - ps18
Arthropoda - ps19
Mandibulata - ps20
Pancrustacea - ps21
Insecta - ps22
Eumetabola - ps23
Holometabola/Psocodea - ps24
Holometabola - ps25
Aparaglossata - ps26
Mecopterida - ps27
Diptera - ps28
Bibiono./Cyclorrhapha - ps29
Cyclorrhapha - ps30
Calyptratae/Drosophila - ps31
Drosophila - ps32
Sophophora - ps33
Obscura/Melanogaster_group - ps34
D_melanogaster_group - ps35
D_melanogaster_subgroup - ps36
sim./sech./mel. group - ps37
Drosophila_melanogaster - ps38
p-value
4e-25
2e-19
1e-13
9e-08
6e-02
Log-odds
0.5
1.0
1.5
2.0
2.5
3.0
3.5
4.0

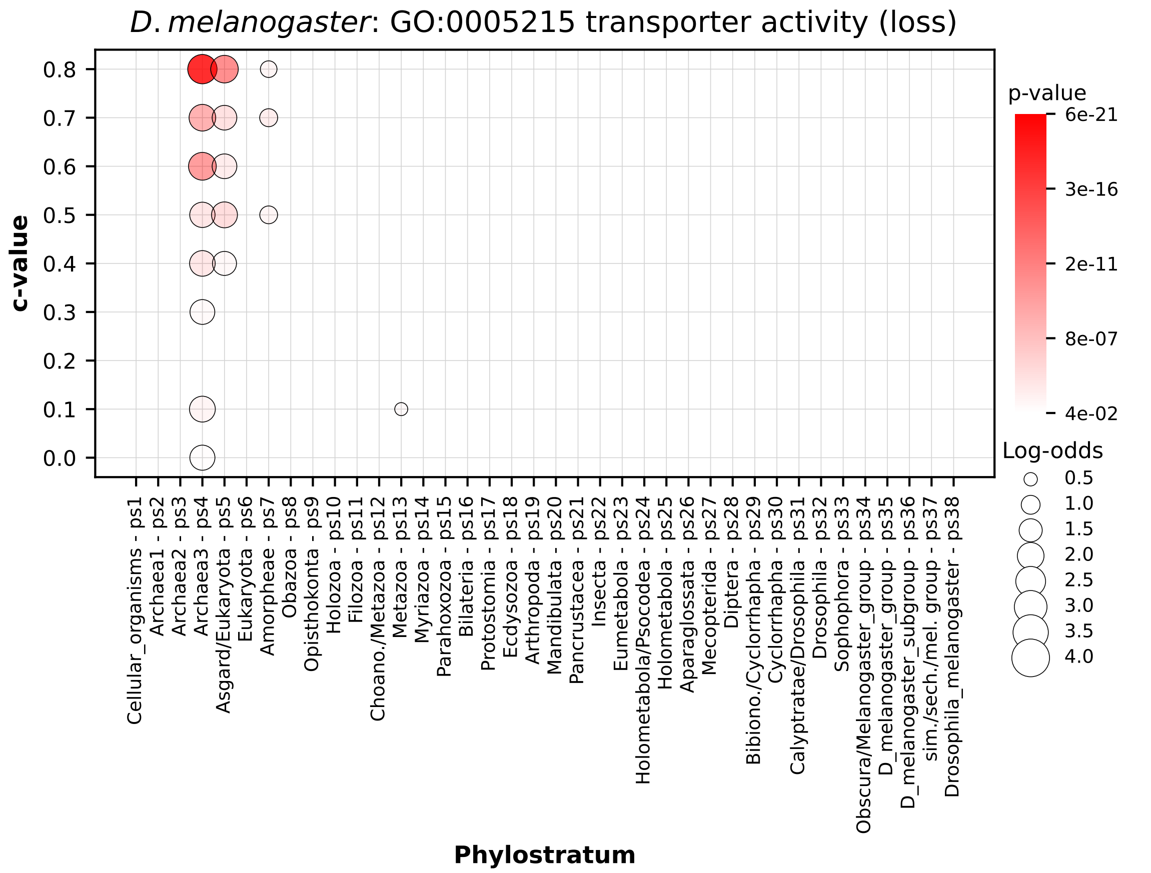

D. melanogaster: GO:0005215 transporter activity (loss)
c-value
0.8
0.7
0.6
0.5
0.4
0.3
0.2
0.1
0.0
p-value
6e-21
3e-16
2e-11
8e-07
4e-02
Log-odds
0.5
1.0
1.5
2.0
2.5
3.0
3.5
4.0
Cellular_organisms - ps1
Archaea1 - ps2
Archaea2 - ps3
Archaea3 - ps4
Asgard/Eukaryota - ps5
Eukaryota - ps6
Amorpheae - ps7
Obazoa - ps8
Opisthokonta - ps9
Holozoa - ps10
Filozoa - ps11
Choano./Metazoa - ps12
Metazoa - ps13
Myriazoa - ps14
Parahoxozoa - ps15
Bilateria - ps16
Protostomia - ps17
Ecdysozoa - ps18
Arthropoda - ps19
Mandibulata - ps20
Pancrustacea - ps21
Insecta - ps22
Eumetabola - ps23
Holometabola/Psocodea - ps24
Holometabola - ps25
Aparaglossata - ps26
Mecopterida - ps27
Diptera - ps28
Bibiono./Cyclorrhapha - ps29
Cyclorrhapha - ps30
Calyptratae/Drosophila - ps31
Drosophila - ps32
Sophophora - ps33
Obscura/Melanogaster_group - ps34
D_melanogaster_group - ps35
D_melanogaster_subgroup - ps36
sim./sech./mel. group - ps37
Drosophila_melanogaster - ps38
Phylostratum

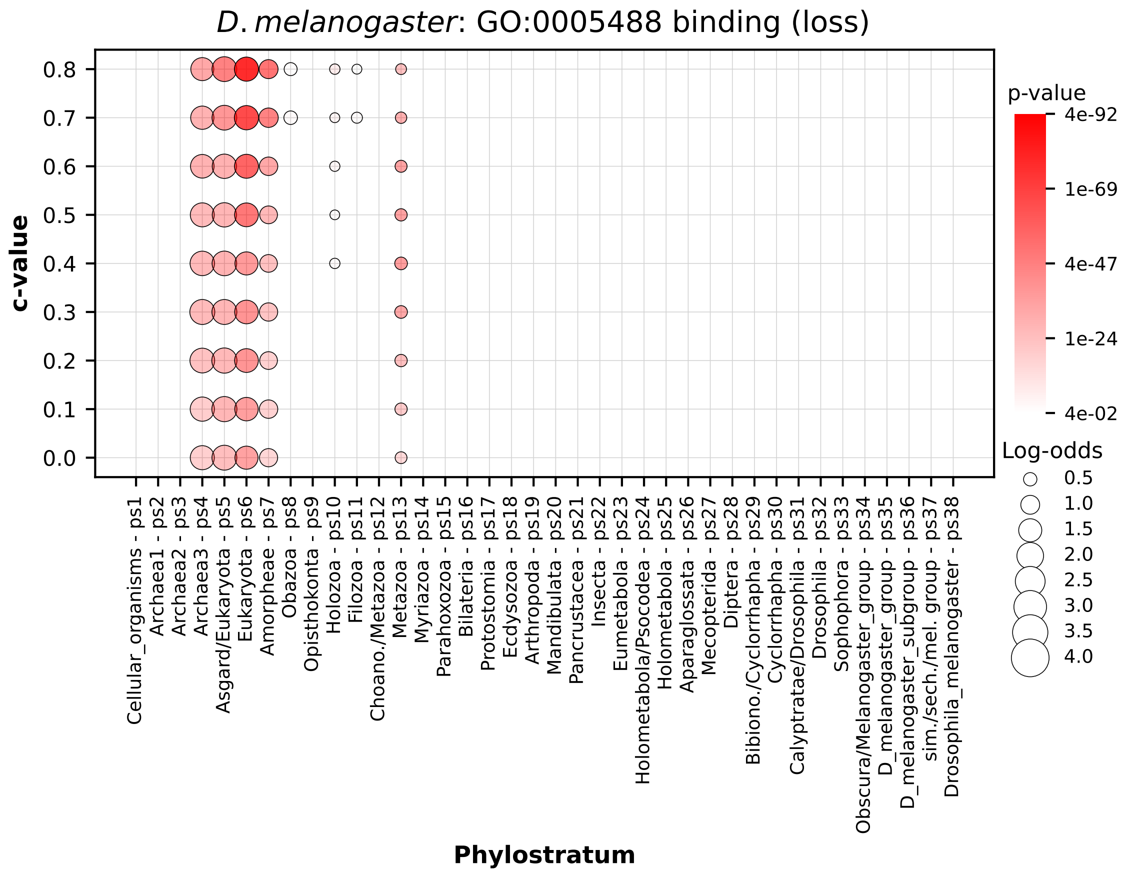

D. melanogaster: GO:0005488 binding (loss)
c-value
0.8
0.7
0.6
0.5
0.4
0.3
0.2
0.1
0.0
p-value
4e-92
1e-69
4e-47
1e-24
4e-02
Log-odds
0.5
1.0
1.5
2.0
2.5
3.0
3.5
4.0
Cellular_organisms - ps1
Archaea1 - ps2
Archaea2 - ps3
Archaea3 - ps4
Asgard/Eukaryota - ps5
Eukaryota - ps6
Amorpheae - ps7
Obazoa - ps8
Opisthokonta - ps9
Holozoa - ps10
Filozoa - ps11
Choano./Metazoa - ps12
Metazoa - ps13
Myriazoa - ps14
Parahoxozoa - ps15
Bilateria - ps16
Protostomia - ps17
Ecdysozoa - ps18
Arthropoda - ps19
Mandibulata - ps20
Pancrustacea - ps21
Insecta - ps22
Eumetabola - ps23
Holometabola/Psocodea - ps24
Holometabola - ps25
Aparaglossata - ps26
Mecopterida - ps27
Diptera - ps28
Bibiono./Cyclorrhapha - ps29
Cyclorrhapha - ps30
Calyptratae/Drosophila - ps31
Drosophila - ps32
Sophophora - ps33
Obscura/Melanogaster_group - ps34
D_melanogaster_group - ps35
D_melanogaster_subgroup - ps36
sim./sech./mel. group - ps37
Drosophila_melanogaster - ps38
Phylostratum

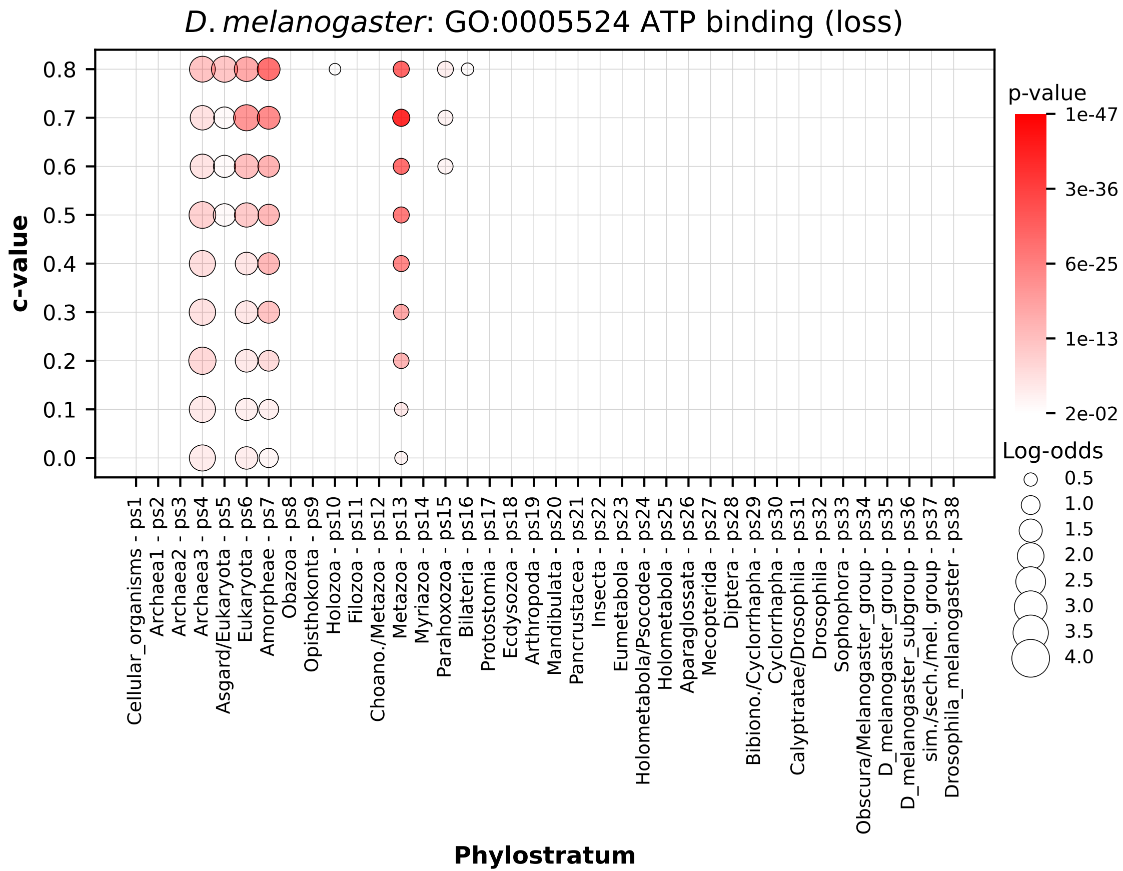

D. melanogaster: GO:0005524 ATP binding (loss)
c-value
0.8
0.7
0.6
0.5
0.4
0.3
0.2
0.1
0.0
p-value
1e-47
3e-36
6e-25
1e-13
2e-02
Log-odds
0.5
1.0
1.5
2.0
2.5
3.0
3.5
4.0
Cellular_organisms - ps1
Archaea1 - ps2
Archaea2 - ps3
Archaea3 - ps4
Asgard/Eukaryota - ps5
Eukaryota - ps6
Amorpheae - ps7
Obazoa - ps8
Opisthokonta - ps9
Holozoa - ps10
Filozoa - ps11
Choano./Metazoa - ps12
Metazoa - ps13
Myriazoa - ps14
Parahoxozoa - ps15
Bilateria - ps16
Protostomia - ps17
Ecdysozoa - ps18
Arthropoda - ps19
Mandibulata - ps20
Pancrustacea - ps21
Insecta - ps22
Eumetabola - ps23
Holometabola/Psocodea - ps24
Holometabola - ps25
Aparaglossata - ps26
Mecopterida - ps27
Diptera - ps28
Bibiono./Cyclorrhapha - ps29
Cyclorrhapha - ps30
Calyptratae/Drosophila - ps31
Drosophila - ps32
Sophophora - ps33
Obscura/Melanogaster_group - ps34
D_melanogaster_group - ps35
D_melanogaster_subgroup - ps36
sim./sech./mel. group - ps37
Drosophila_melanogaster - ps38
Phylostratum

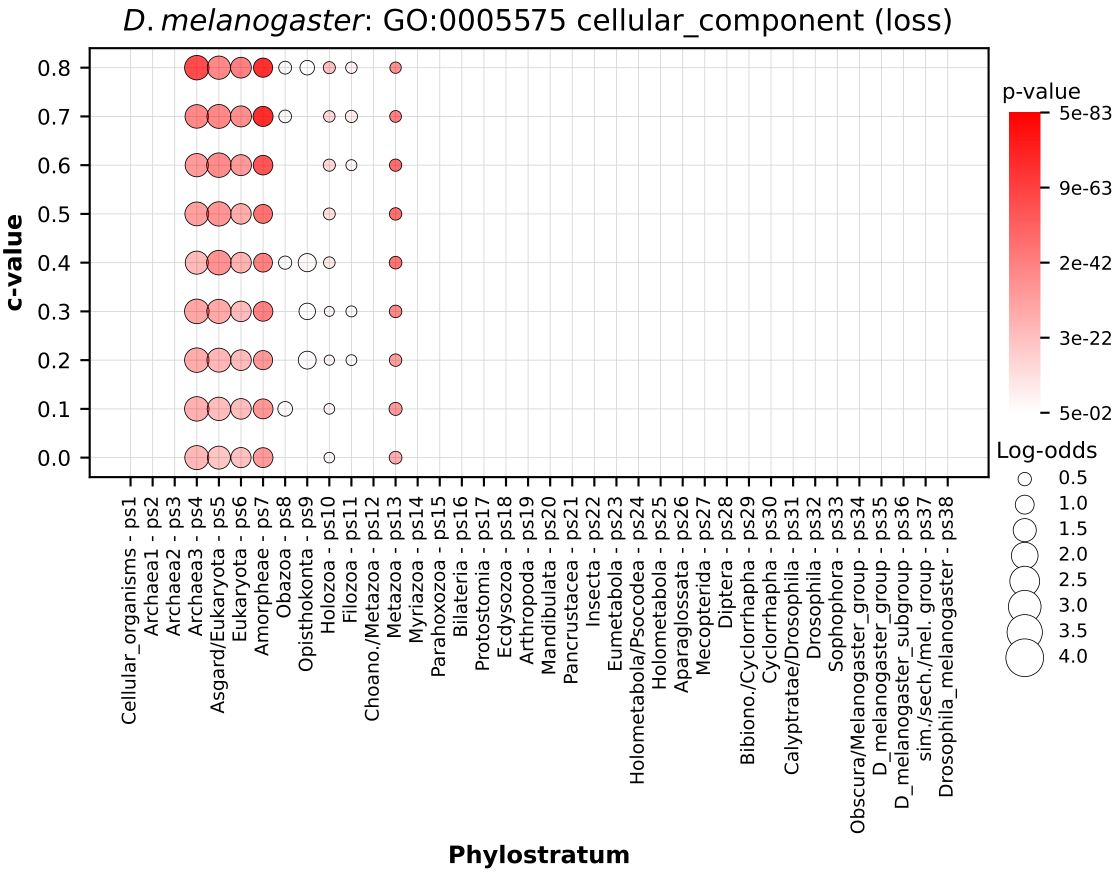

D. melanogaster: GO:0005575 cellular_component (loss)
c-value
0.8
0.7
0.6
0.5
0.4
0.3
0.2
0.1
0.0
Phylostratum
Cellular_organisms - ps1
Archaea1 - ps2
Archaea2 - ps3
Archaea3 - ps4
Asgard/Eukaryota - ps5
Eukaryota - ps6
Amorpheae - ps7
Obazoa - ps8
Opisthokonta - ps9
Holozoa - ps10
Filozoa - ps11
Choano./Metazoa - ps12
Metazoa - ps13
Myriazoa - ps14
Parahoxozoa - ps15
Bilateria - ps16
Protostomia - ps17
Ecdysozoa - ps18
Arthropoda - ps19
Mandibulata - ps20
Pancrustacea - ps21
Insecta - ps22
Eumetabola - ps23
Holometabola/Psocodea - ps24
Holometabola - ps25
Aparaglossata - ps26
Mecopterida - ps27
Diptera - ps28
Bibiono./Cyclorrhapha - ps29
Cyclorrhapha - ps30
Calyptratae/Drosophila - ps31
Drosophila - ps32
Sophophora - ps33
Obscura/Melanogaster_group - ps34
D_melanogaster_group - ps35
D_melanogaster_subgroup - ps36
sim./sech./mel. group - ps37
Drosophila_melanogaster - ps38
p-value
5e-83
9e-63
2e-42
3e-22
5e-02
Log-odds
0.5
1.0
1.5
2.0
2.5
3.0
3.5
4.0

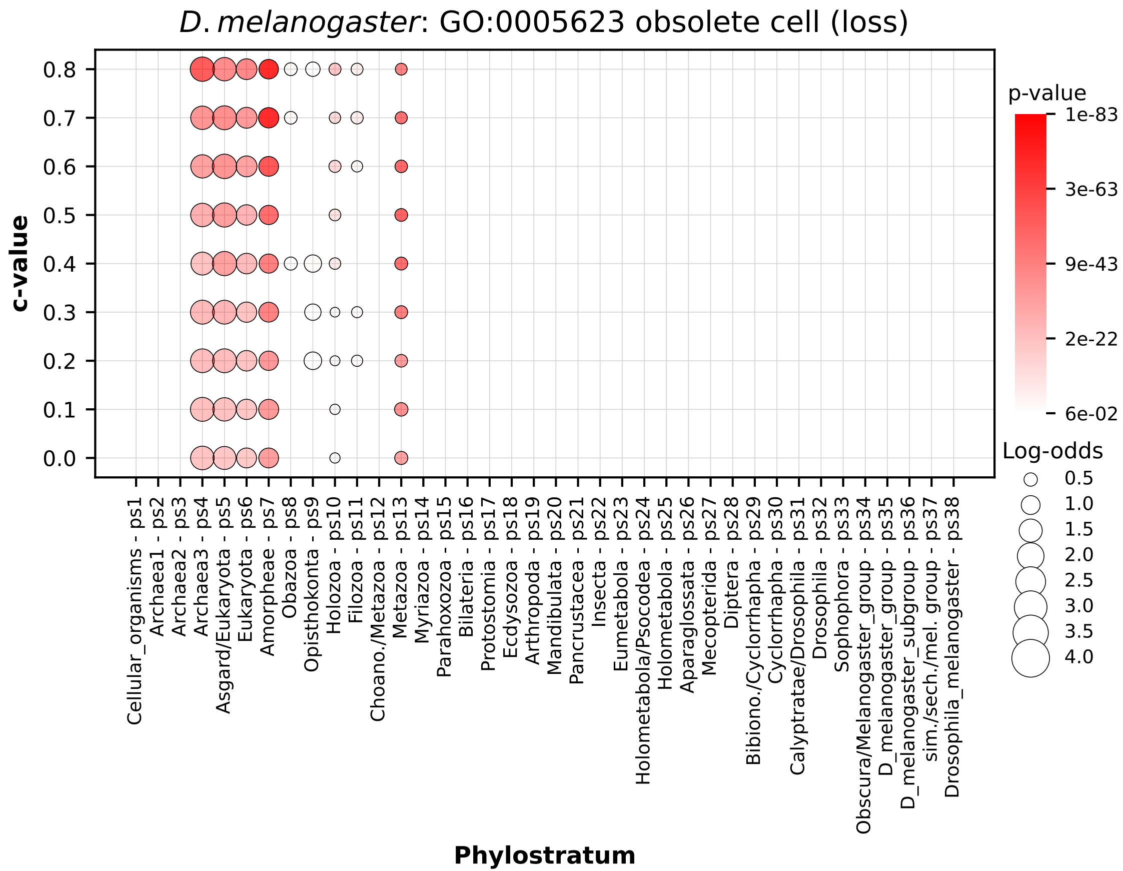

D. melanogaster: GO:0005623 obsolete cell (loss)
c-value
0.8
0.7
0.6
0.5
0.4
0.3
0.2
0.1
0.0
p-value
1e-83
3e-63
9e-43
2e-22
6e-02
Log-odds
0.5
1.0
1.5
2.0
2.5
3.0
3.5
4.0
Cellular_organisms - ps1
Archaea1 - ps2
Archaea2 - ps3
Archaea3 - ps4
Asgard/Eukaryota - ps5
Eukaryota - ps6
Amorpheae - ps7
Obazoa - ps8
Opisthokonta - ps9
Holozoa - ps10
Filozoa - ps11
Choano./Metazoa - ps12
Metazoa - ps13
Myriazoa - ps14
Parahoxozoa - ps15
Bilateria - ps16
Protostomia - ps17
Ecdysozoa - ps18
Arthropoda - ps19
Mandibulata - ps20
Pancrustacea - ps21
Insecta - ps22
Eumetabola - ps23
Holometabola/Psocodea - ps24
Holometabola - ps25
Aparaglossata - ps26
Mecopterida - ps27
Diptera - ps28
Bibiono./Cyclorrhapha - ps29
Cyclorrhapha - ps30
Calyptratae/Drosophila - ps31
Drosophila - ps32
Sophophora - ps33
Obscura/Melanogaster_group - ps34
D_melanogaster_group - ps35
D_melanogaster_subgroup - ps36
sim./sech./mel. group - ps37
Drosophila_melanogaster - ps38
Phylostratum

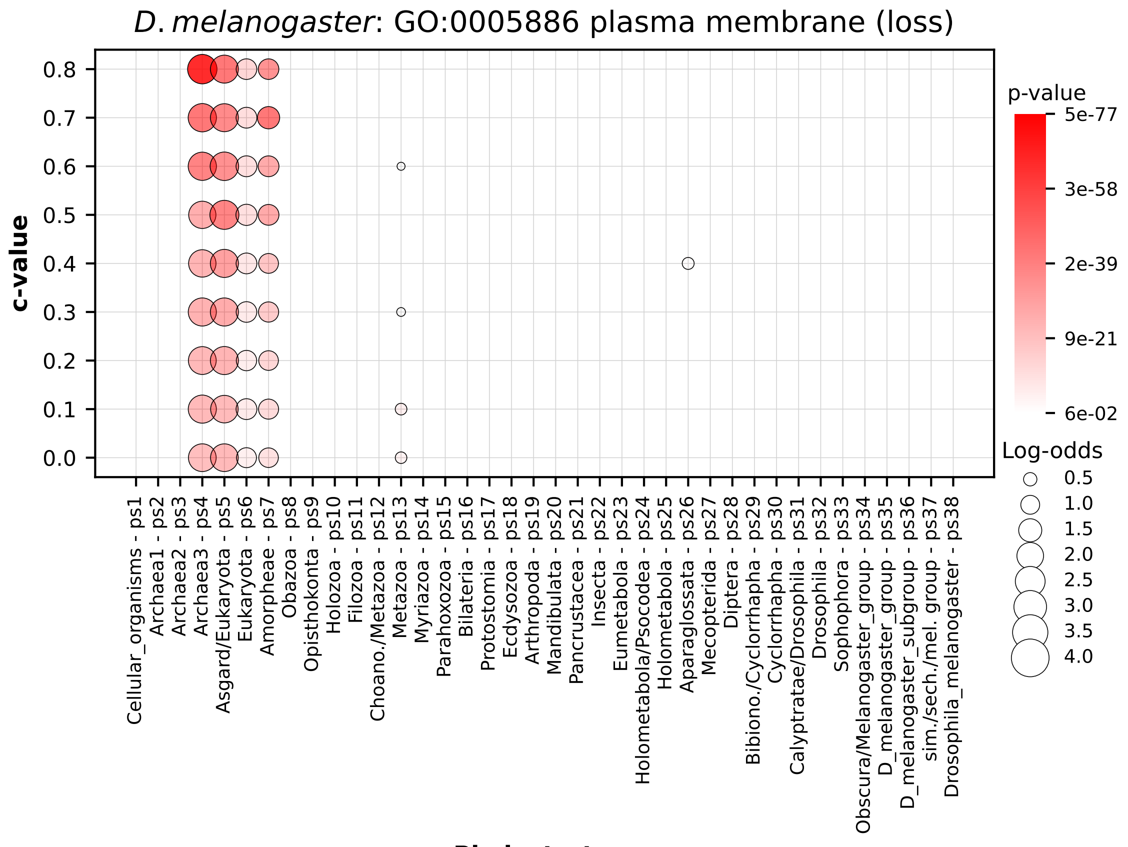

D. melanogaster: GO:0005886 plasma membrane (loss)
c-value
0.8
0.7
0.6
0.5
0.4
0.3
0.2
0.1
0.0
Phylostratum
Cellular_organisms - ps1
Archaea1 - ps2
Archaea2 - ps3
Archaea3 - ps4
Asgard/Eukaryota - ps5
Eukaryota - ps6
Amorpheae - ps7
Obazoa - ps8
Opisthokonta - ps9
Holozoa - ps10
Filozoa - ps11
Choano./Metazoa - ps12
Metazoa - ps13
Myriazoa - ps14
Parahoxozoa - ps15
Bilateria - ps16
Protostomia - ps17
Ecdysozoa - ps18
Arthropoda - ps19
Mandibulata - ps20
Pancrustacea - ps21
Insecta - ps22
Eumetabola - ps23
Holometabola/Psocodea - ps24
Holometabola - ps25
Aparaglossata - ps26
Mecopterida - ps27
Diptera - ps28
Bibiono./Cyclorrhapha - ps29
Cyclorrhapha - ps30
Calyptratae/Drosophila - ps31
Drosophila - ps32
Sophophora - ps33
Obscura/Melanogaster_group - ps34
D_melanogaster_group - ps35
D_melanogaster_subgroup - ps36
sim./sech./mel. group - ps37
Drosophila_melanogaster - ps38
p-value
5e-77
3e-58
2e-39
9e-21
6e-02
Log-odds
0.5
1.0
1.5
2.0
2.5
3.0
3.5
4.0

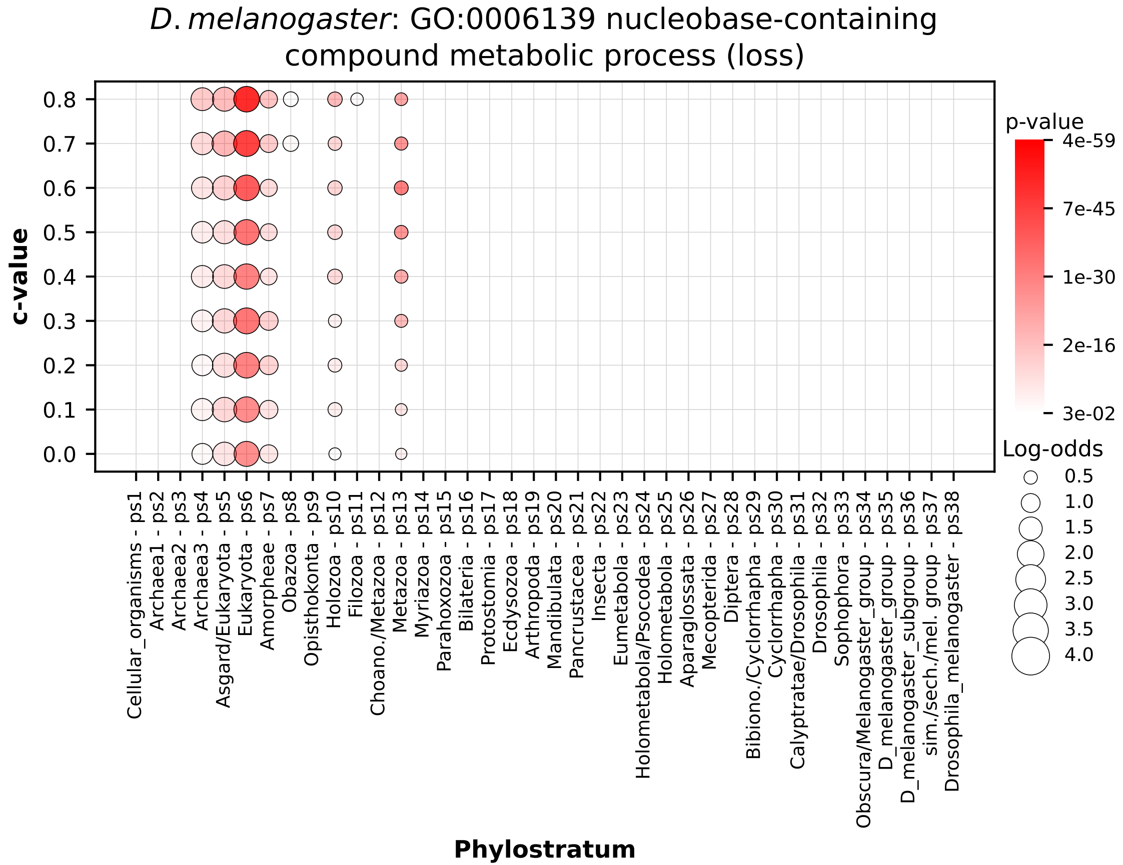
D. melanogaster: GO:0006139 nucleobase-containing compound metabolic process (loss)
c-value
0.8
0.7
0.6
0.5
0.4
0.3
0.2
0.1
0.0
p-value
4e-59
7e-45
1e-30
2e-16
3e-02
Log-odds
0.5
1.0
1.5
2.0
2.5
3.0
3.5
4.0
Cellular_organisms - ps1
Archaea1 - ps2
Archaea2 - ps3
Archaea3 - ps4
Asgard/Eukaryota - ps5
Eukaryota - ps6
Amorpheae - ps7
Obazoa - ps8
Opisthokonta - ps9
Holozoa - ps10
Filozoa - ps11
Choano./Metazoa - ps12
Metazoa - ps13
Myriazoa - ps14
Parahoxozoa - ps15
Bilateria - ps16
Protostomia - ps17
Ecdysozoa - ps18
Arthropoda - ps19
Mandibulata - ps20
Pancrustacea - ps21
Insecta - ps22
Eumetabola - ps23
Holometabola/Psocodea - ps24
Holometabola - ps25
Aparaglossata - ps26
Mecopterida - ps27
Diptera - ps28
Bibiono./Cyclorrhapha - ps29
Cyclorrhapha - ps30
Calyptratae/Drosophila - ps31
Drosophila - ps32
Sophophora - ps33
Obscura/Melanogaster_group - ps34
D_melanogaster_group - ps35
D_melanogaster_subgroup - ps36
sim./sech./mel. group - ps37
Drosophila_melanogaster - ps38
Phylostratum

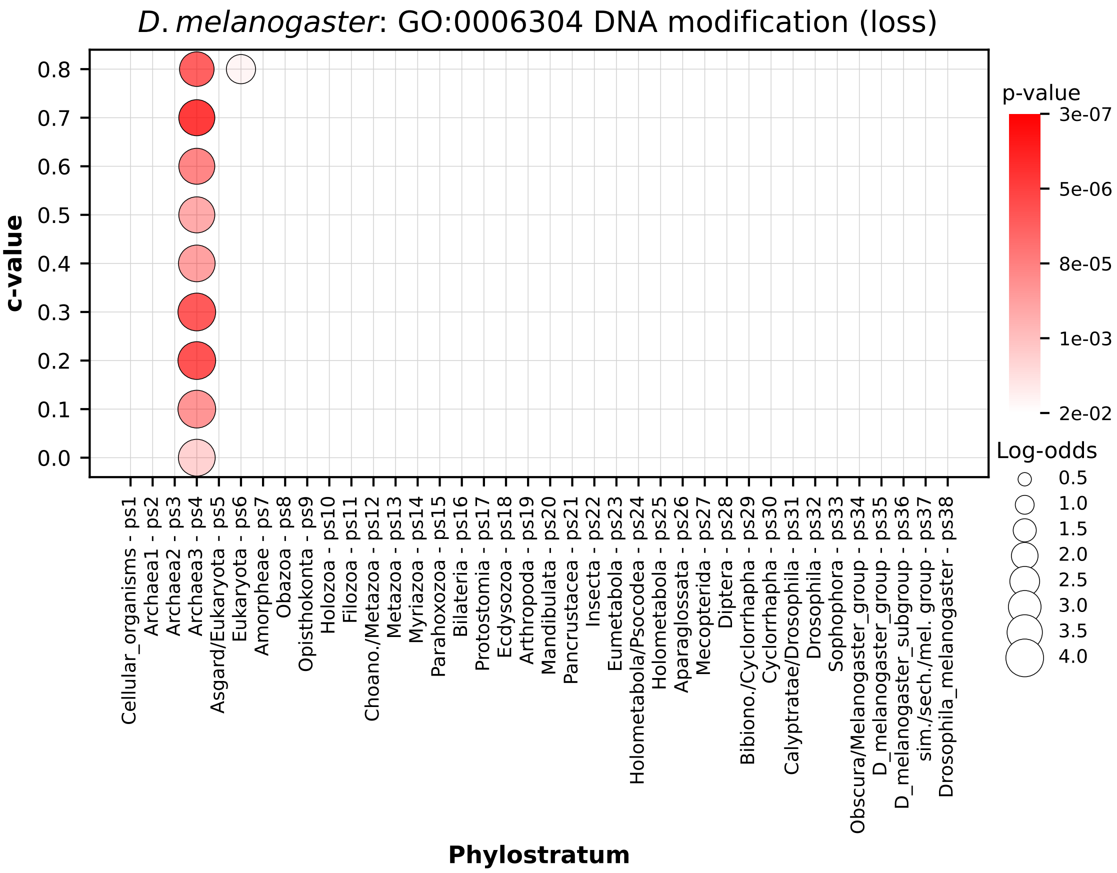

D. melanogaster: GO:0006304 DNA modification (loss)
c-value
0.8
0.7
0.6
0.5
0.4
0.3
0.2
0.1
0.0
p-value
3e-07
5e-06
8e-05
1e-03
2e-02
Log-odds
0.5
1.0
1.5
2.0
2.5
3.0
3.5
4.0
Cellular_organisms - ps1
Archaea1 - ps2
Archaea2 - ps3
Archaea3 - ps4
Asgard/Eukaryota - ps5
Eukaryota - ps6
Amorpheae - ps7
Obazoa - ps8
Opisthokonta - ps9
Holozoa - ps10
Filozoa - ps11
Choano./Metazoa - ps12
Metazoa - ps13
Myriazoa - ps14
Parahoxozoa - ps15
Bilateria - ps16
Protostomia - ps17
Ecdysozoa - ps18
Arthropoda - ps19
Mandibulata - ps20
Pancrustacea - ps21
Insecta - ps22
Eumetabola - ps23
Holometabola/Psocodea - ps24
Holometabola - ps25
Aparaglossata - ps26
Mecopterida - ps27
Diptera - ps28
Bibiono./Cyclorrhapha - ps29
Cyclorrhapha - ps30
Calyptratae/Drosophila - ps31
Drosophila - ps32
Sophophora - ps33
Obscura/Melanogaster_group - ps34
D_melanogaster_group - ps35
D_melanogaster_subgroup - ps36
sim./sech./mel. group - ps37
Drosophila_melanogaster - ps38
Phylostratum

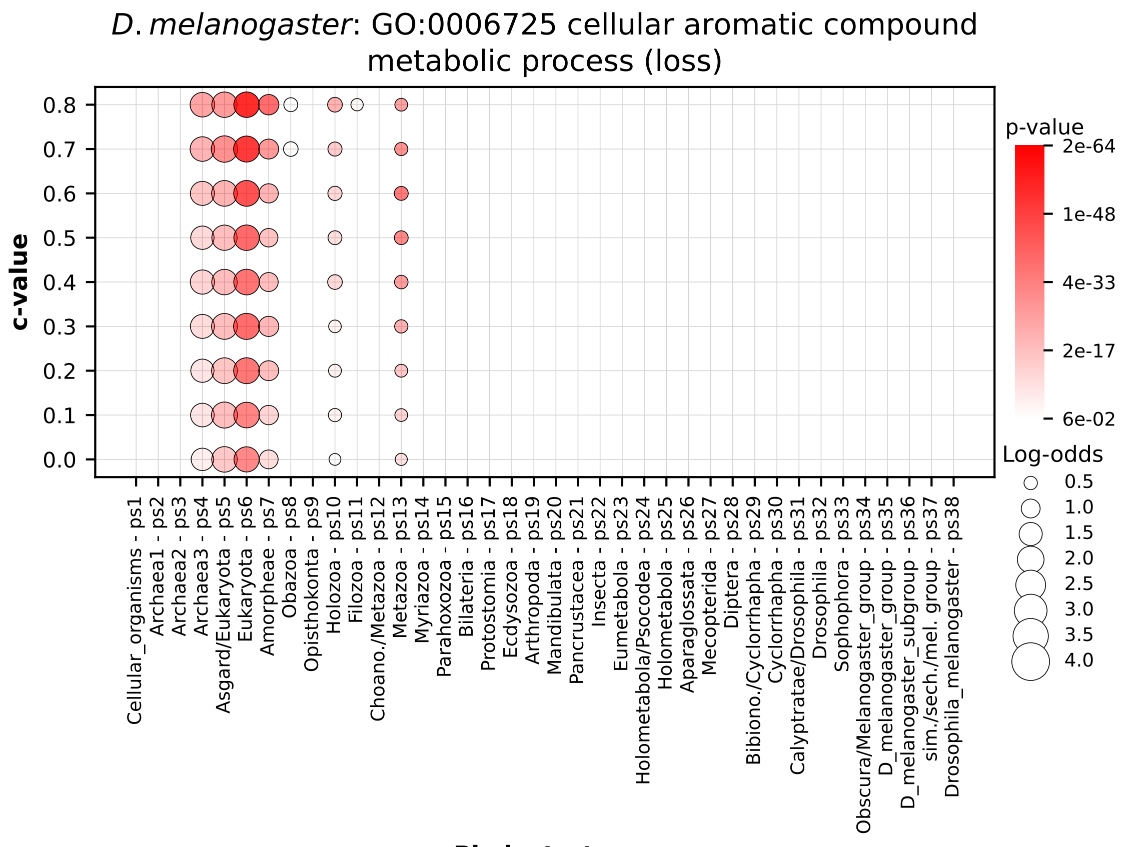

D. melanogaster: GO:0006725 cellular aromatic compound metabolic process (loss)
c-value
0.8
0.7
0.6
0.5
0.4
0.3
0.2
0.1
0.0
Phylostratum
Cellular_organisms - ps1
Archaea1 - ps2
Archaea2 - ps3
Archaea3 - ps4
Asgard/Eukaryota - ps5
Eukaryota - ps6
Amorpheae - ps7
Obazoa - ps8
Opisthokonta - ps9
Holozoa - ps10
Filozoa - ps11
Choano./Metazoa - ps12
Metazoa - ps13
Myriazoa - ps14
Parahoxozoa - ps15
Bilateria - ps16
Protostomia - ps17
Ecdysozoa - ps18
Arthropoda - ps19
Mandibulata - ps20
Pancrustacea - ps21
Insecta - ps22
Eumetabola - ps23
Holometabola/Psocodea - ps24
Holometabola - ps25
Aparaglossata - ps26
Mecopterida - ps27
Diptera - ps28
Bibiono./Cyclorrhapha - ps29
Cyclorrhapha - ps30
Calyptratae/Drosophila - ps31
Drosophila - ps32
Sophophora - ps33
Obscura/Melanogaster_group - ps34
D_melanogaster_group - ps35
D_melanogaster_subgroup - ps36
sim./sech./mel. group - ps37
Drosophila_melanogaster - ps38
p-value
2e-64
1e-48
4e-33
2e-17
6e-02
Log-odds
0.5
1.0
1.5
2.0
2.5
3.0
3.5
4.0

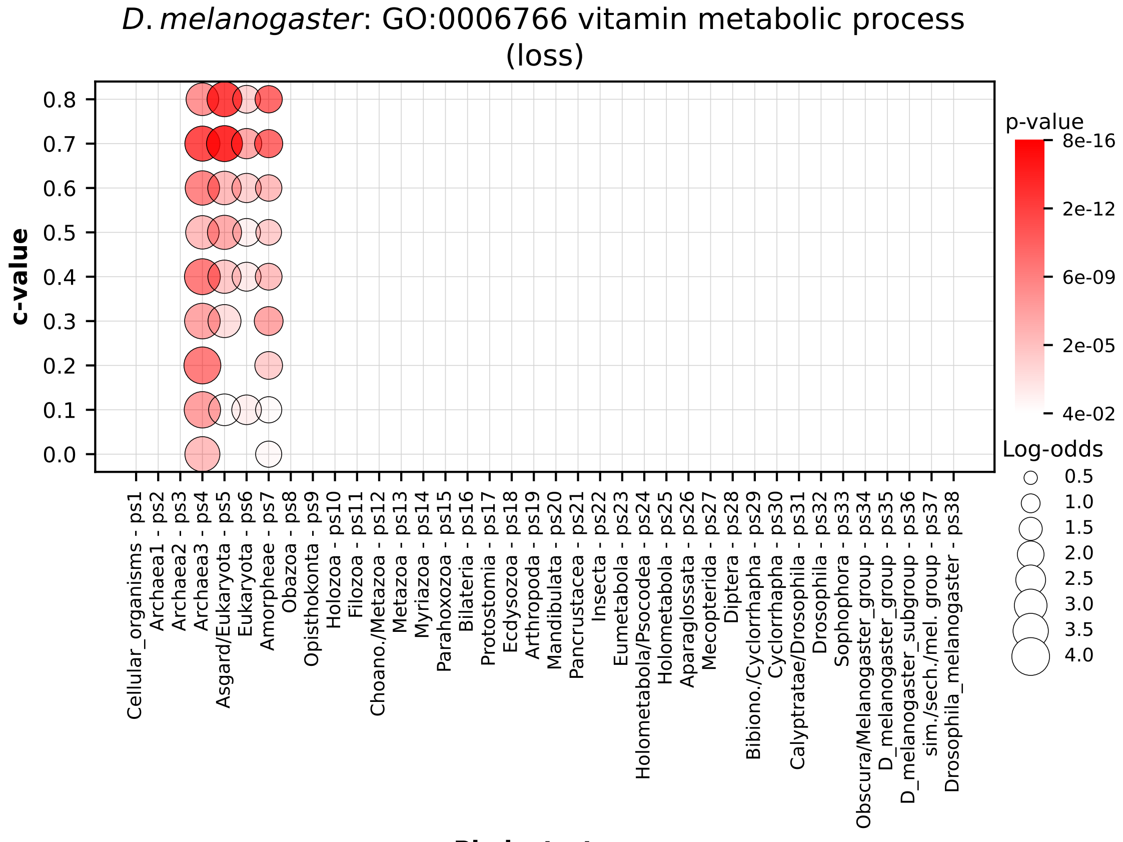

D. melanogaster: GO:0006766 vitamin metabolic process (loss)
c-value
0.8
0.7
0.6
0.5
0.4
0.3
0.2
0.1
0.0
Phylostratum
Cellular_organisms - ps1
Archaea1 - ps2
Archaea2 - ps3
Archaea3 - ps4
Asgard/Eukaryota - ps5
Eukaryota - ps6
Amorpheae - ps7
Obazoa - ps8
Opisthokonta - ps9
Holozoa - ps10
Filozoa - ps11
Choano./Metazoa - ps12
Metazoa - ps13
Myriazoa - ps14
Parahoxozoa - ps15
Bilateria - ps16
Protostomia - ps17
Ecdysozoa - ps18
Arthropoda - ps19
Mandibulata - ps20
Pancrustacea - ps21
Insecta - ps22
Eumetabola - ps23
Holometabola/Psocodea - ps24
Holometabola - ps25
Aparaglossata - ps26
Mecopterida - ps27
Diptera - ps28
Bibiono./Cyclorrhapha - ps29
Cyclorrhapha - ps30
Calyptratae/Drosophila - ps31
Drosophila - ps32
Sophophora - ps33
Obscura/Melanogaster_group - ps34
D_melanogaster_group - ps35
D_melanogaster_subgroup - ps36
sim./sech./mel. group - ps37
Drosophila_melanogaster - ps38
p-value
8e-16
2e-12
6e-09
2e-05
4e-02
Log-odds
0.5
1.0
1.5
2.0
2.5
3.0
3.5
4.0

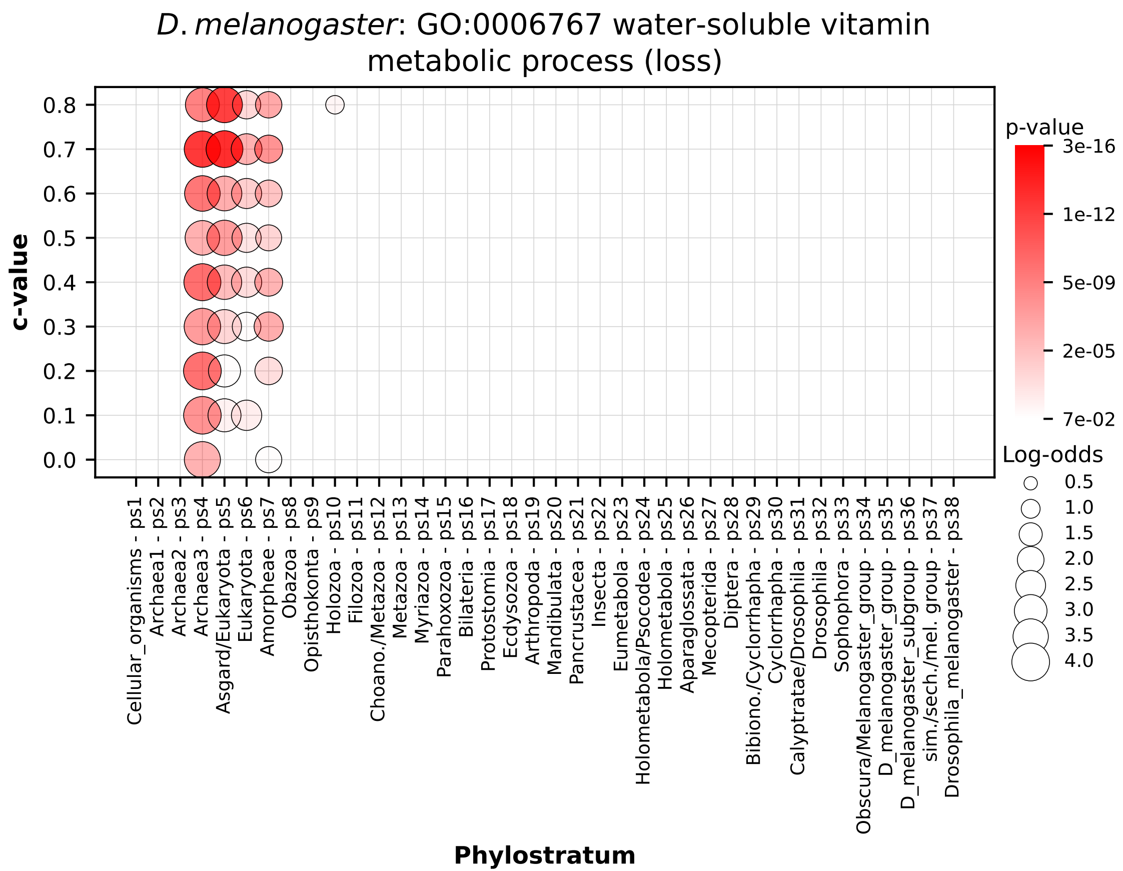
D. melanogaster: GO:0006767 water-soluble vitamin metabolic process (loss)
c-value
0.8
0.7
0.6
0.5
0.4
0.3
0.2
0.1
0.0
p-value
3e-16
1e-12
5e-09
2e-05
7e-02
Log-odds
0.5
1.0
1.5
2.0
2.5
3.0
3.5
4.0
Cellular_organisms - ps1
Archaea1 - ps2
Archaea2 - ps3
Archaea3 - ps4
Asgard/Eukaryota - ps5
Eukaryota - ps6
Amorpheae - ps7
Obazoa - ps8
Opisthokonta - ps9
Holozoa - ps10
Filozoa - ps11
Choano./Metazoa - ps12
Metazoa - ps13
Myriazoa - ps14
Parahoxozoa - ps15
Bilateria - ps16
Protostomia - ps17
Ecdysozoa - ps18
Arthropoda - ps19
Mandibulata - ps20
Pancrustacea - ps21
Insecta - ps22
Eumetabola - ps23
Holometabola/Psocodea - ps24
Holometabola - ps25
Aparaglossata - ps26
Mecopterida - ps27
Diptera - ps28
Bibiono./Cyclorrhapha - ps29
Cyclorrhapha - ps30
Calyptratae/Drosophila - ps31
Drosophila - ps32
Sophophora - ps33
Obscura/Melanogaster_group - ps34
D_melanogaster_group - ps35
D_melanogaster_subgroup - ps36
sim./sech./mel. group - ps37
Drosophila_melanogaster - ps38
Phylostratum

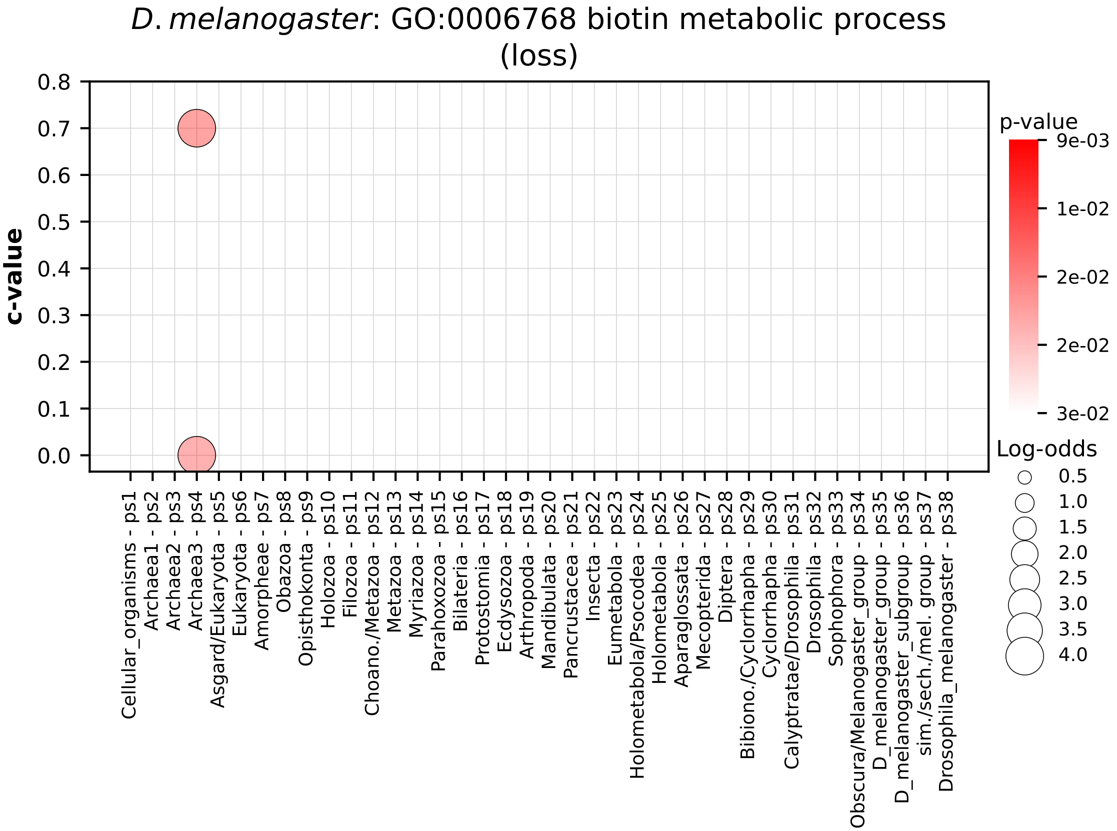

D. melanogaster: GO:0006768 biotin metabolic process
(loss)
c-value
0.8
0.7
0.6
0.5
0.4
0.3
0.2
0.1
0.0
Phylostratum
Cellular_organisms - ps1
Archaea1 - ps2
Archaea2 - ps3
Archaea3 - ps4
Asgard/Eukaryota - ps5
Eukaryota - ps6
Amorpheae - ps7
Obazoa - ps8
Opisthokonta - ps9
Holozoa - ps10
Filozoa - ps11
Choano./Metazoa - ps12
Metazoa - ps13
Myriazoa - ps14
Parahoxozoa - ps15
Bilateria - ps16
Protostomia - ps17
Ecdysozoa - ps18
Arthropoda - ps19
Mandibulata - ps20
Pancrustacea - ps21
Insecta - ps22
Eumetabola - ps23
Holometabola/Psocodea - ps24
Holometabola - ps25
Aparaglossata - ps26
Mecopterida - ps27
Diptera - ps28
Bibiono./Cyclorrhapha - ps29
Cyclorrhapha - ps30
Calyptratae/Drosophila - ps31
Drosophila - ps32
Sophophora - ps33
Obscura/Melanogaster_group - ps34
D_melanogaster_group - ps35
D_melanogaster_subgroup - ps36
sim./sech./mel. group - ps37
Drosophila_melanogaster - ps38
p-value
9e-03
1e-02
2e-02
2e-02
3e-02
Log-odds
0.5
1.0
1.5
2.0
2.5
3.0
3.5
4.0

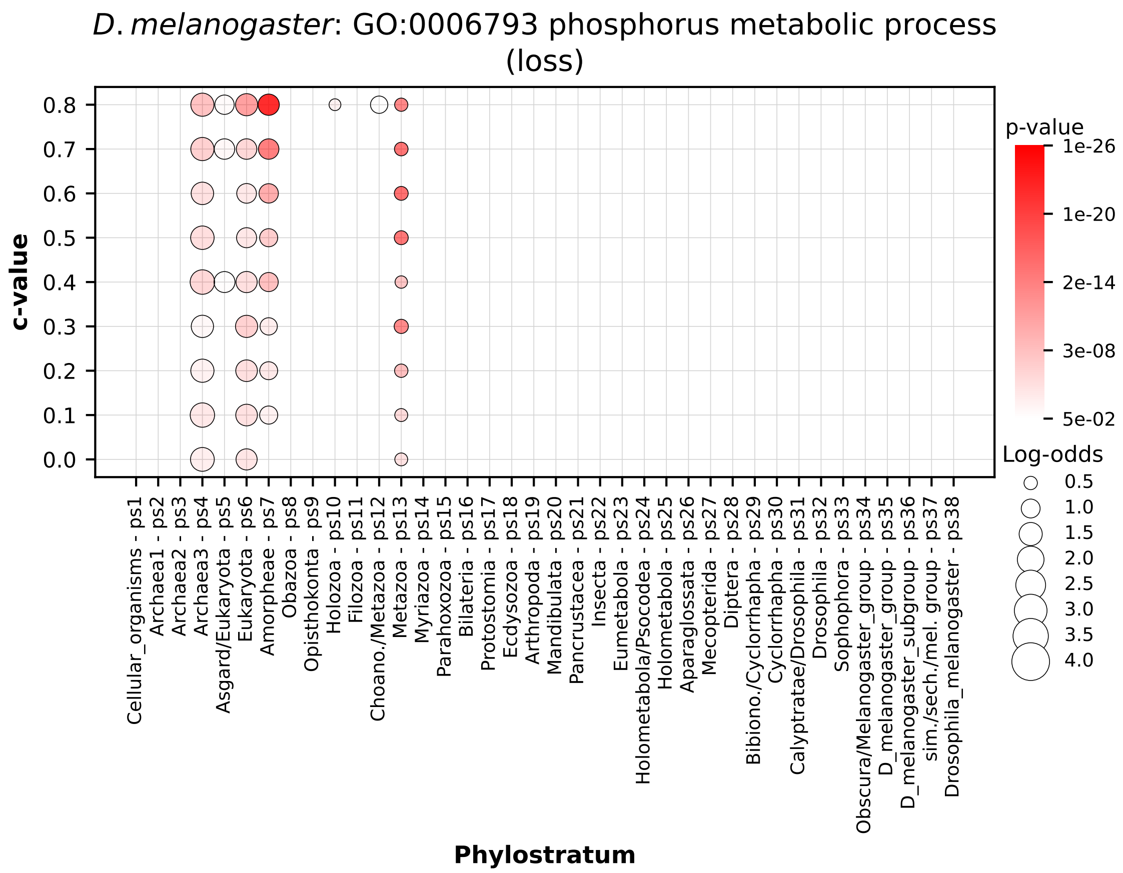

D. melanogaster: GO:0006793 phosphorus metabolic process (loss)
c-value
0.8
0.7
0.6
0.5
0.4
0.3
0.2
0.1
0.0
Phylostratum
Cellular_organisms - ps1
Archaea1 - ps2
Archaea2 - ps3
Archaea3 - ps4
Asgard/Eukaryota - ps5
Eukaryota - ps6
Amorpheae - ps7
Obazoa - ps8
Opisthokonta - ps9
Holozoa - ps10
Filozoa - ps11
Choano./Metazoa - ps12
Metazoa - ps13
Myriazoa - ps14
Parahoxozoa - ps15
Bilateria - ps16
Protostomia - ps17
Ecdysozoa - ps18
Arthropoda - ps19
Mandibulata - ps20
Pancrustacea - ps21
Insecta - ps22
Eumetabola - ps23
Holometabola/Psocodea - ps24
Holometabola - ps25
Aparaglossata - ps26
Mecopterida - ps27
Diptera - ps28
Bibiono./Cyclorrhapha - ps29
Cyclorrhapha - ps30
Calyptratae/Drosophila - ps31
Drosophila - ps32
Sophophora - ps33
Obscura/Melanogaster_group - ps34
D_melanogaster_group - ps35
D_melanogaster_subgroup - ps36
sim./sech./mel. group - ps37
Drosophila_melanogaster - ps38
p-value
1e-26
1e-20
2e-14
3e-08
5e-02
Log-odds
0.5
1.0
1.5
2.0
2.5
3.0
3.5
4.0

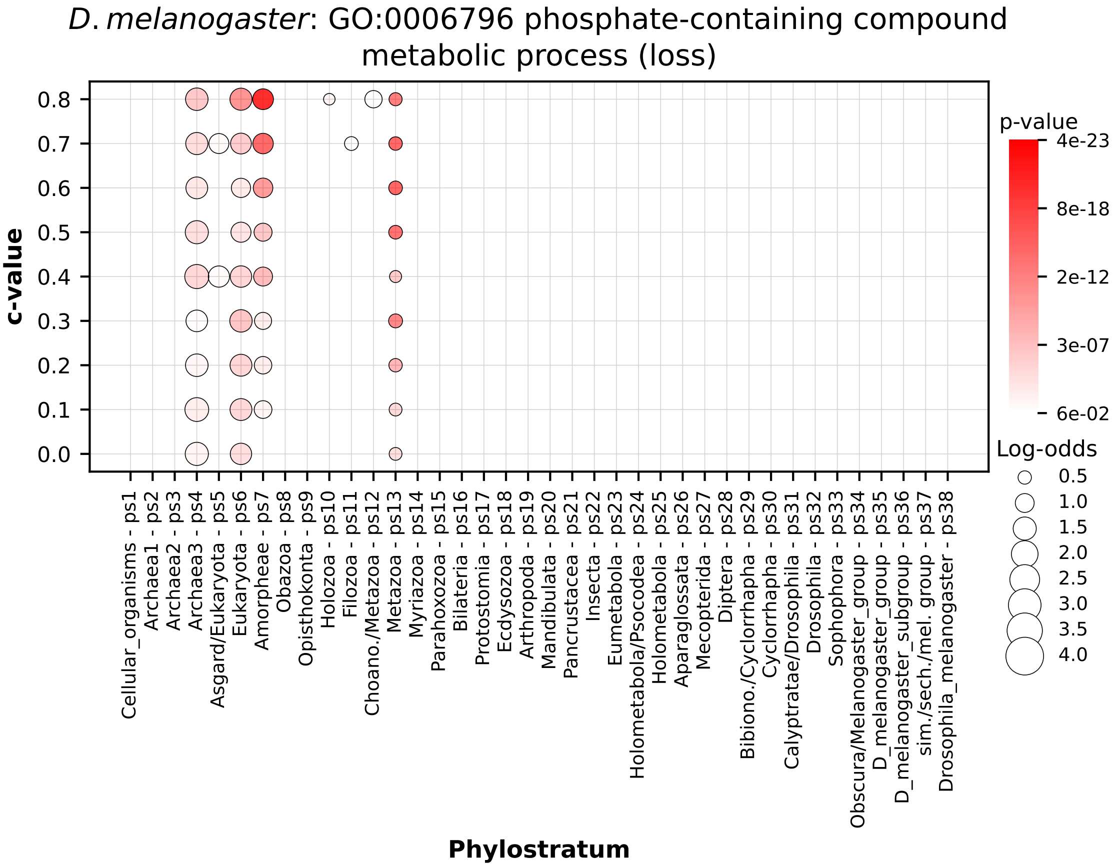
D. melanogaster: GO:0006796 phosphate-containing compound metabolic process (loss)
c-value
0.8
0.7
0.6
0.5
0.4
0.3
0.2
0.1
0.0
p-value
4e-23
8e-18
2e-12
3e-07
6e-02
Log-odds
0.5
1.0
1.5
2.0
2.5
3.0
3.5
4.0
Cellular_organisms - ps1
Archaea1 - ps2
Archaea2 - ps3
Archaea3 - ps4
Asgard/Eukaryota - ps5
Eukaryota - ps6
Amorpheae - ps7
Obazoa - ps8
Opisthokonta - ps9
Holozoa - ps10
Filozoa - ps11
Choano./Metazoa - ps12
Metazoa - ps13
Myriazoa - ps14
Parahoxozoa - ps15
Bilateria - ps16
Protostomia - ps17
Ecdysozoa - ps18
Arthropoda - ps19
Mandibulata - ps20
Pancrustacea - ps21
Insecta - ps22
Eumetabola - ps23
Holometabola/Psocodea - ps24
Holometabola - ps25
Aparaglossata - ps26
Mecopterida - ps27
Diptera - ps28
Bibiono./Cyclorrhapha - ps29
Cyclorrhapha - ps30
Calyptratae/Drosophila - ps31
Drosophila - ps32
Sophophora - ps33
Obscura/Melanogaster_group - ps34
D_melanogaster_group - ps35
D_melanogaster_subgroup - ps36
sim./sech./mel. group - ps37
Drosophila_melanogaster - ps38
Phylostratum

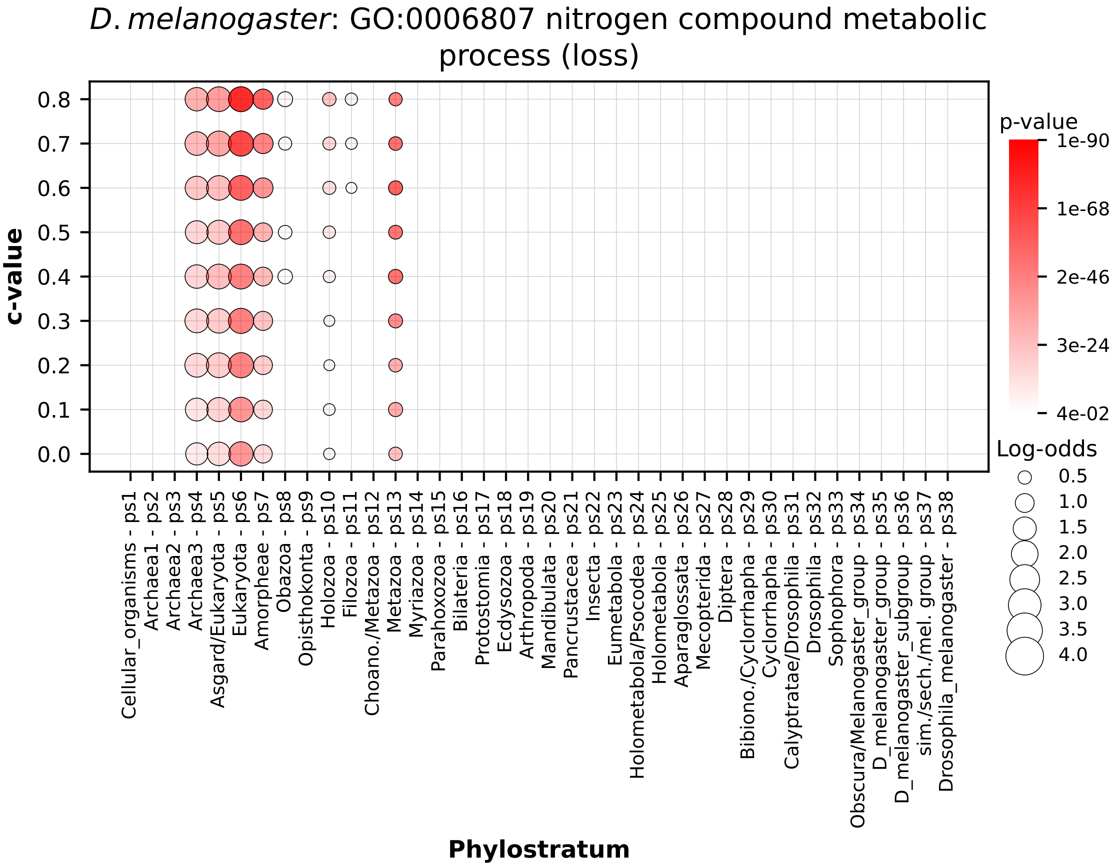

D. melanogaster: GO:0006807 nitrogen compound metabolic process (loss)
c-value
0.8
0.7
0.6
0.5
0.4
0.3
0.2
0.1
0.0
p-value
1e-90
1e-68
2e-46
3e-24
4e-02
Log-odds
0.5
1.0
1.5
2.0
2.5
3.0
3.5
4.0
Cellular_organisms - ps1
Archaea1 - ps2
Archaea2 - ps3
Archaea3 - ps4
Asgard/Eukaryota - ps5
Eukaryota - ps6
Amorpheae - ps7
Obazoa - ps8
Opisthokonta - ps9
Holozoa - ps10
Filozoa - ps11
Choano./Metazoa - ps12
Metazoa - ps13
Myriazoa - ps14
Parahoxozoa - ps15
Bilateria - ps16
Protostomia - ps17
Ecdysozoa - ps18
Arthropoda - ps19
Mandibulata - ps20
Pancrustacea - ps21
Insecta - ps22
Eumetabola - ps23
Holometabola/Psocodea - ps24
Holometabola - ps25
Aparaglossata - ps26
Mecopterida - ps27
Diptera - ps28
Bibiono./Cyclorrhapha - ps29
Cyclorrhapha - ps30
Calyptratae/Drosophila - ps31
Drosophila - ps32
Sophophora - ps33
Obscura/Melanogaster_group - ps34
D_melanogaster_group - ps35
D_melanogaster_subgroup - ps36
sim./sech./mel. group - ps37
Drosophila_melanogaster - ps38
Phylostratum

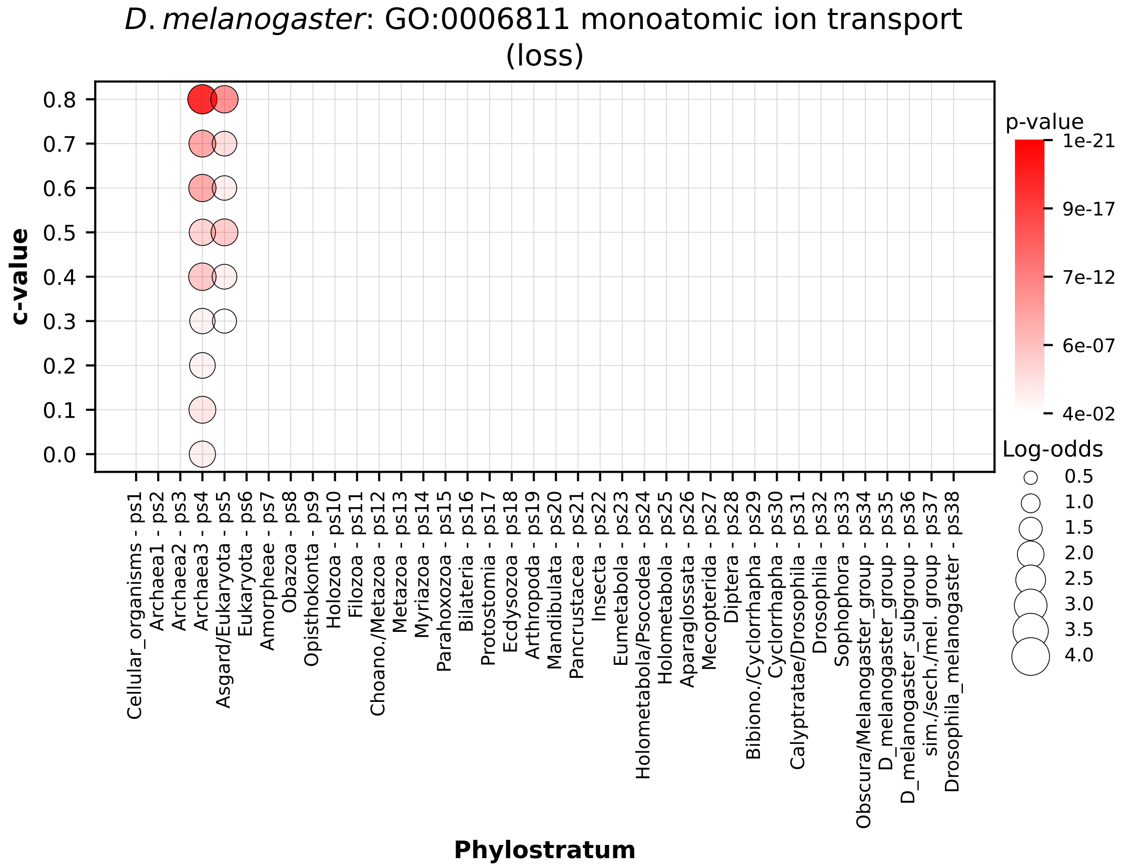

$D. melanogaster$: GO:0006811 monoatomic ion transport
(loss)
c-value
0.8
0.7
0.6
0.5
0.4
0.3
0.2
0.1
0.0
p-value
1e-21
9e-17
7e-12
6e-07
4e-02
Log-odds
0.5
1.0
1.5
2.0
2.5
3.0
3.5
4.0
Cellular_organisms - ps1
Archaea1 - ps2
Archaea2 - ps3
Archaea3 - ps4
Asgard/Eukaryota - ps5
Eukaryota - ps6
Amorpheae - ps7
Obazoa - ps8
Opisthokonta - ps9
Holozoa - ps10
Filozoa - ps11
Choano./Metazoa - ps12
Metazoa - ps13
Myriazoa - ps14
Parahoxozoa - ps15
Bilateria - ps16
Protostomia - ps17
Ecdysozoa - ps18
Arthropoda - ps19
Mandibulata - ps20
Pancrustacea - ps21
Insecta - ps22
Eumetabola - ps23
Holometabola/Psocodea - ps24
Holometabola - ps25
Aparaglossata - ps26
Mecopterida - ps27
Diptera - ps28
Bibiono./Cyclorrhapha - ps29
Cyclorrhapha - ps30
Calyptratae/Drosophila - ps31
Drosophila - ps32
Sophophora - ps33
Obscura/Melanogaster_group - ps34
D_melanogaster_group - ps35
D_melanogaster_subgroup - ps36
sim./sech./mel. group - ps37
Drosophila_melanogaster - ps38
Phylostratum

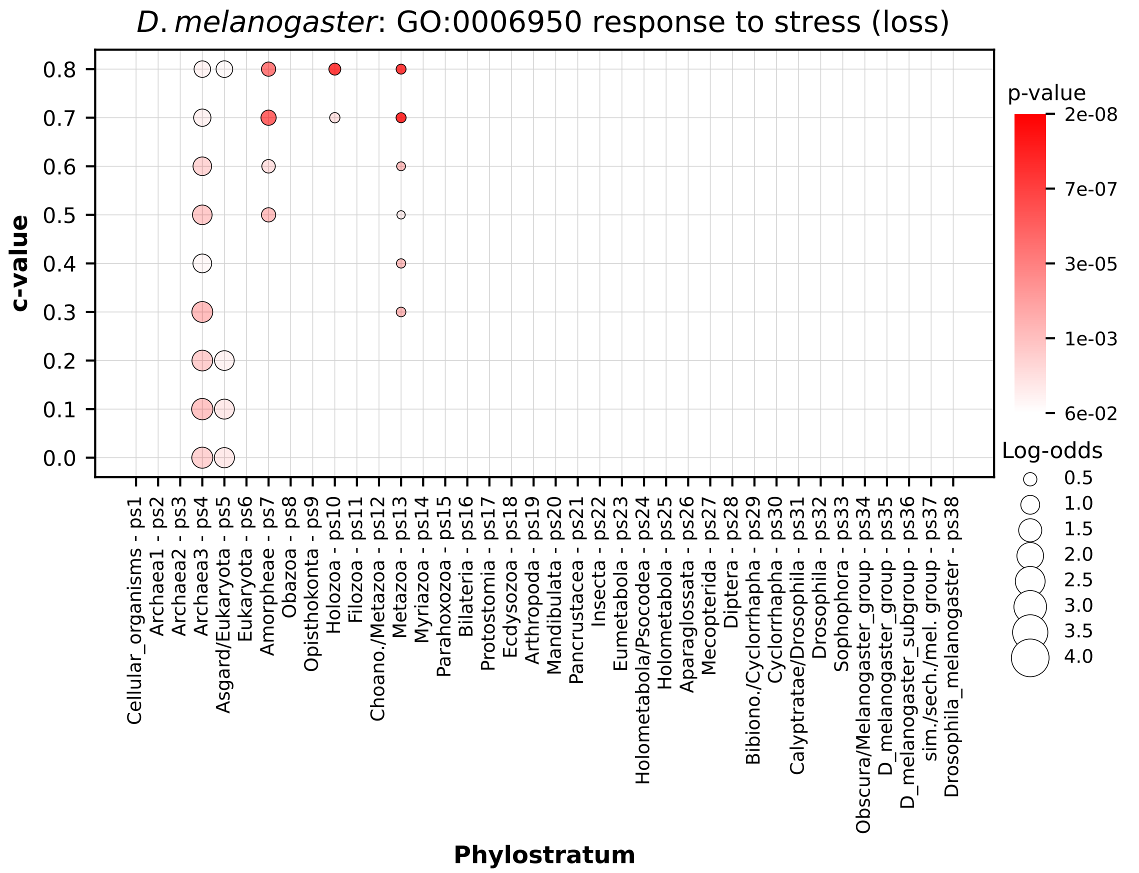

D. melanogaster: GO:0006950 response to stress (loss)
c-value
0.8
0.7
0.6
0.5
0.4
0.3
0.2
0.1
0.0
Phylostratum
Cellular_organisms - ps1
Archaea1 - ps2
Archaea2 - ps3
Archaea3 - ps4
Asgard/Eukaryota - ps5
Eukaryota - ps6
Amorpheae - ps7
Obazoa - ps8
Opisthokonta - ps9
Holozoa - ps10
Filozoa - ps11
Choano./Metazoa - ps12
Metazoa - ps13
Myriazoa - ps14
Parahoxozoa - ps15
Bilateria - ps16
Protostomia - ps17
Ecdysozoa - ps18
Arthropoda - ps19
Mandibulata - ps20
Pancrustacea - ps21
Insecta - ps22
Eumetabola - ps23
Holometabola/Psocodea - ps24
Holometabola - ps25
Aparaglossata - ps26
Mecopterida - ps27
Diptera - ps28
Bibiono./Cyclorrhapha - ps29
Cyclorrhapha - ps30
Calyptratae/Drosophila - ps31
Drosophila - ps32
Sophophora - ps33
Obscura/Melanogaster_group - ps34
D_melanogaster_group - ps35
D_melanogaster_subgroup - ps36
sim./sech./mel. group - ps37
Drosophila_melanogaster - ps38
p-value
2e-08
7e-07
3e-05
1e-03
6e-02
Log-odds
0.5
1.0
1.5
2.0
2.5
3.0
3.5
4.0

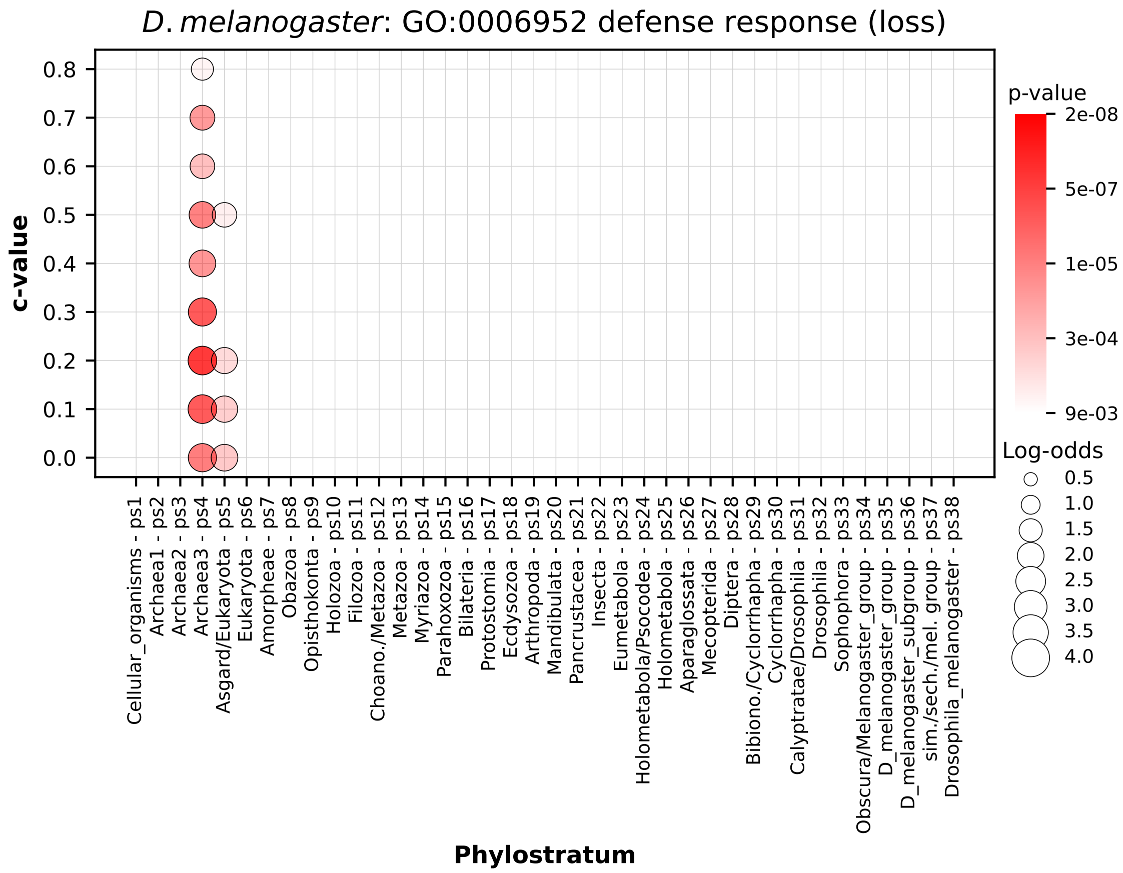

D. melanogaster: GO:0006952 defense response (loss)
c-value
0.8
0.7
0.6
0.5
0.4
0.3
0.2
0.1
0.0
p-value
2e-08
5e-07
1e-05
3e-04
9e-03
Log-odds
0.5
1.0
1.5
2.0
2.5
3.0
3.5
4.0
Cellular_organisms - ps1
Archaea1 - ps2
Archaea2 - ps3
Archaea3 - ps4
Asgard/Eukaryota - ps5
Eukaryota - ps6
Amorpheae - ps7
Obazoa - ps8
Opisthokonta - ps9
Holozoa - ps10
Filozoa - ps11
Choano./Metazoa - ps12
Metazoa - ps13
Myriazoa - ps14
Parahoxozoa - ps15
Bilateria - ps16
Protostomia - ps17
Ecdysozoa - ps18
Arthropoda - ps19
Mandibulata - ps20
Pancrustacea - ps21
Insecta - ps22
Eumetabola - ps23
Holometabola/Psocodea - ps24
Holometabola - ps25
Aparaglossata - ps26
Mecopterida - ps27
Diptera - ps28
Bibiono./Cyclorrhapha - ps29
Cyclorrhapha - ps30
Calyptratae/Drosophila - ps31
Drosophila - ps32
Sophophora - ps33
Obscura/Melanogaster_group - ps34
D_melanogaster_group - ps35
D_melanogaster_subgroup - ps36
sim./sech./mel. group - ps37
Drosophila_melanogaster - ps38
Phylostratum

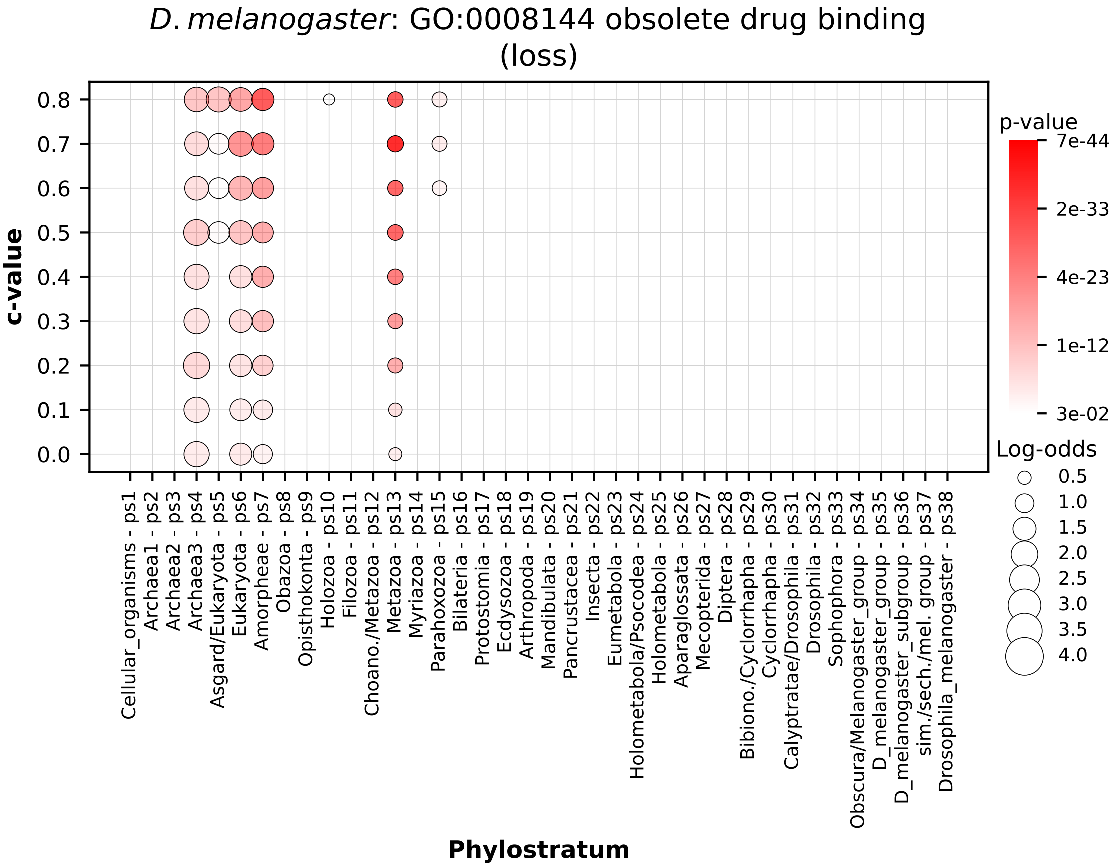
D. melanogaster: GO:0008144 obsolete drug binding
(loss)
c-value
0.8
0.7
0.6
0.5
0.4
0.3
0.2
0.1
0.0
p-value
7e-44
2e-33
4e-23
1e-12
3e-02
Log-odds
0.5
1.0
1.5
2.0
2.5
3.0
3.5
4.0
Cellular_organisms - ps1
Archaea1 - ps2
Archaea2 - ps3
Archaea3 - ps4
Asgard/Eukaryota - ps5
Eukaryota - ps6
Amorpheae - ps7
Obazoa - ps8
Opisthokonta - ps9
Holozoa - ps10
Filozoa - ps11
Choano./Metazoa - ps12
Metazoa - ps13
Myriazoa - ps14
Parahoxozoa - ps15
Bilateria - ps16
Protostomia - ps17
Ecdysozoa - ps18
Arthropoda - ps19
Mandibulata - ps20
Pancrustacea - ps21
Insecta - ps22
Eumetabola - ps23
Holometabola/Psocodea - ps24
Holometabola - ps25
Aparaglossata - ps26
Mecopterida - ps27
Diptera - ps28
Bibiono./Cyclorrhapha - ps29
Cyclorrhapha - ps30
Calyptratae/Drosophila - ps31
Drosophila - ps32
Sophophora - ps33
Obscura/Melanogaster_group - ps34
D_melanogaster_group - ps35
D_melanogaster_subgroup - ps36
sim./sech./mel. group - ps37
Drosophila_melanogaster - ps38
Phylostratum

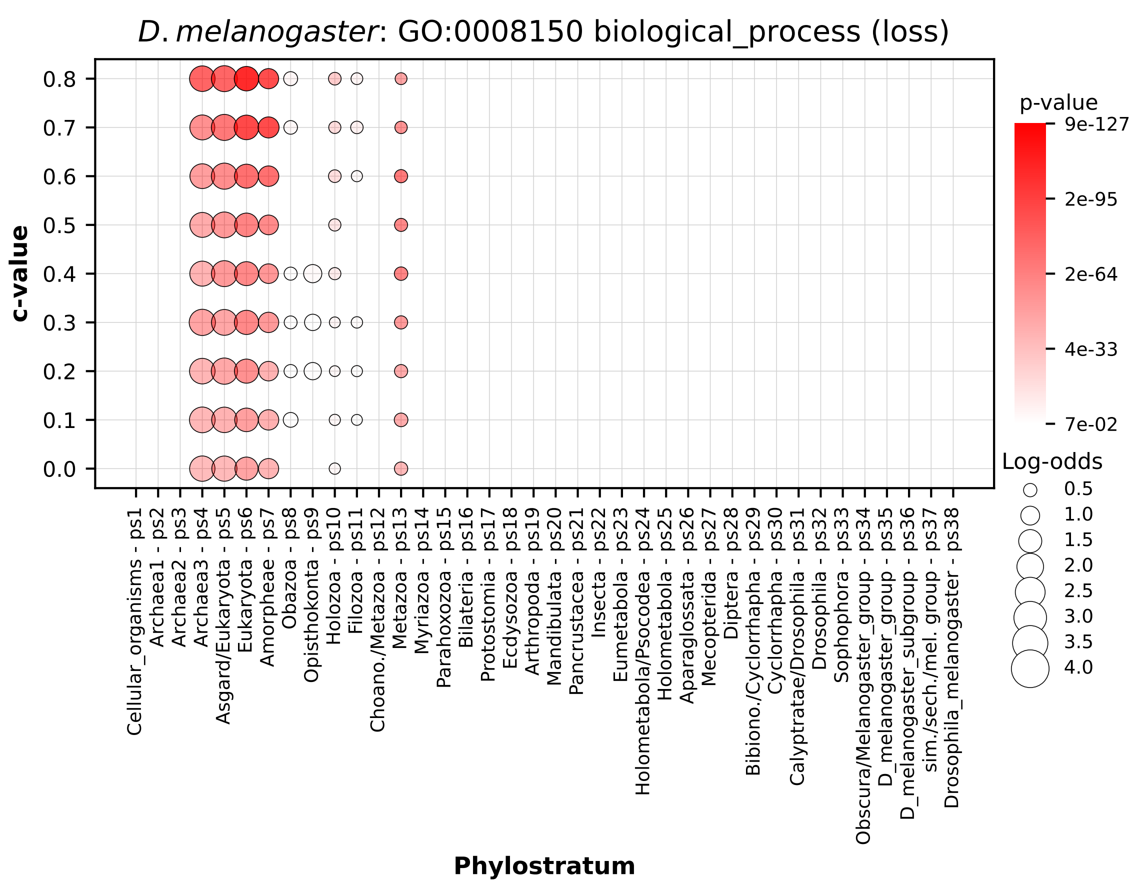

D. melanogaster: GO:0008150 biological_process (loss)
c-value
0.8
0.7
0.6
0.5
0.4
0.3
0.2
0.1
0.0
Phylostratum
Cellular_organisms - ps1
Archaea1 - ps2
Archaea2 - ps3
Archaea3 - ps4
Asgard/Eukaryota - ps5
Eukaryota - ps6
Amorpheae - ps7
Obazoa - ps8
Opisthokonta - ps9
Holozoa - ps10
Filozoa - ps11
Choano./Metazoa - ps12
Metazoa - ps13
Myriazoa - ps14
Parahoxozoa - ps15
Bilateria - ps16
Protostomia - ps17
Ecdysozoa - ps18
Arthropoda - ps19
Mandibulata - ps20
Pancrustacea - ps21
Insecta - ps22
Eumetabola - ps23
Holometabola/Psocodea - ps24
Holometabola - ps25
Aparaglossata - ps26
Mecopterida - ps27
Diptera - ps28
Bibiono./Cyclorrhapha - ps29
Cyclorrhapha - ps30
Calyptratae/Drosophila - ps31
Drosophila - ps32
Sophophora - ps33
Obscura/Melanogaster_group - ps34
D_melanogaster_group - ps35
D_melanogaster_subgroup - ps36
sim./sech./mel. group - ps37
Drosophila_melanogaster - ps38
p-value
9e-127
2e-95
2e-64
4e-33
7e-02
Log-odds
0.5
1.0
1.5
2.0
2.5
3.0
3.5
4.0

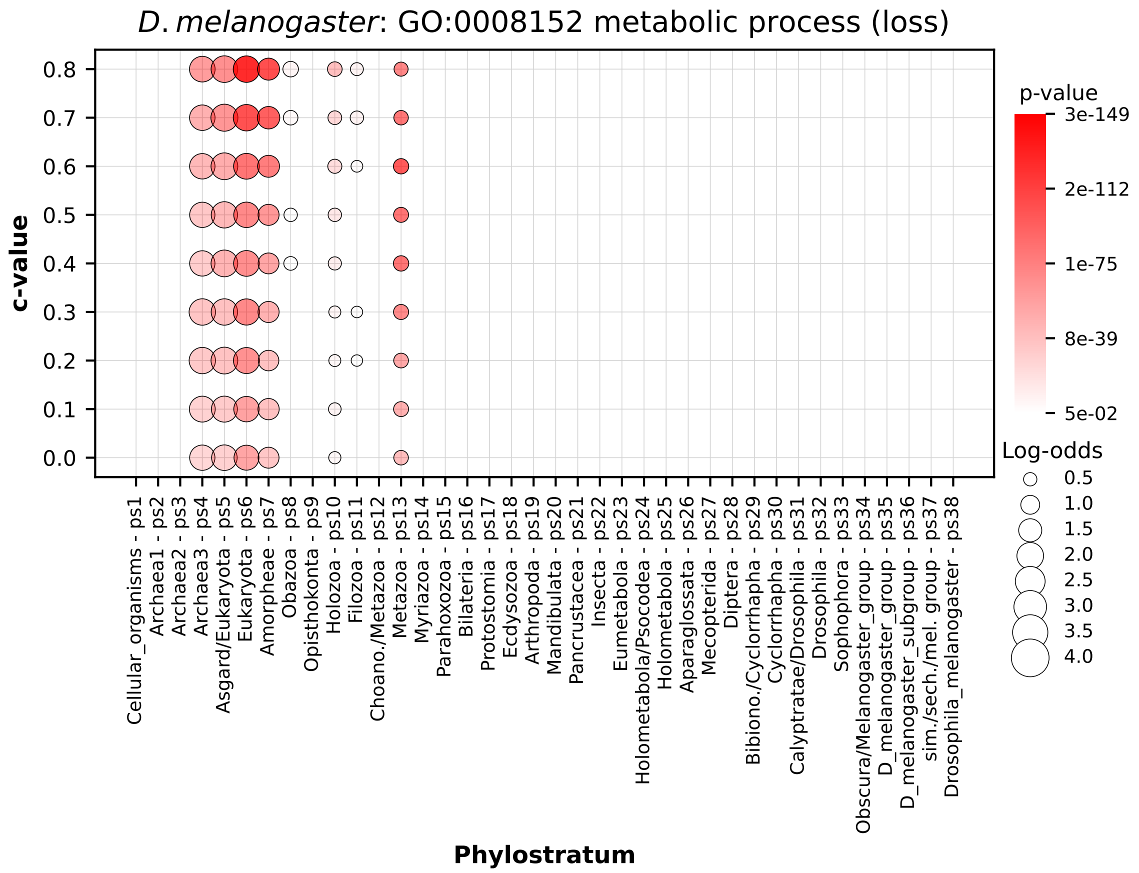

D. melanogaster: GO:0008152 metabolic process (loss)
c-value
0.8
0.7
0.6
0.5
0.4
0.3
0.2
0.1
0.0
Phylostratum
Cellular_organisms - ps1
Archaea1 - ps2
Archaea2 - ps3
Archaea3 - ps4
Asgard/Eukaryota - ps5
Eukaryota - ps6
Amorpheae - ps7
Obazoa - ps8
Opisthokonta - ps9
Holozoa - ps10
Filozoa - ps11
Choano./Metazoa - ps12
Metazoa - ps13
Myriazoa - ps14
Parahoxozoa - ps15
Bilateria - ps16
Protostomia - ps17
Ecdysozoa - ps18
Arthropoda - ps19
Mandibulata - ps20
Pancrustacea - ps21
Insecta - ps22
Eumetabola - ps23
Holometabola/Psocodea - ps24
Holometabola - ps25
Aparaglossata - ps26
Mecopterida - ps27
Diptera - ps28
Bibiono./Cyclorrhapha - ps29
Cyclorrhapha - ps30
Calyptratae/Drosophila - ps31
Drosophila - ps32
Sophophora - ps33
Obscura/Melanogaster_group - ps34
D_melanogaster_group - ps35
D_melanogaster_subgroup - ps36
sim./sech./mel. group - ps37
Drosophila_melanogaster - ps38
p-value
3e-149
2e-112
1e-75
8e-39
5e-02
Log-odds
0.5
1.0
1.5
2.0
2.5
3.0
3.5
4.0

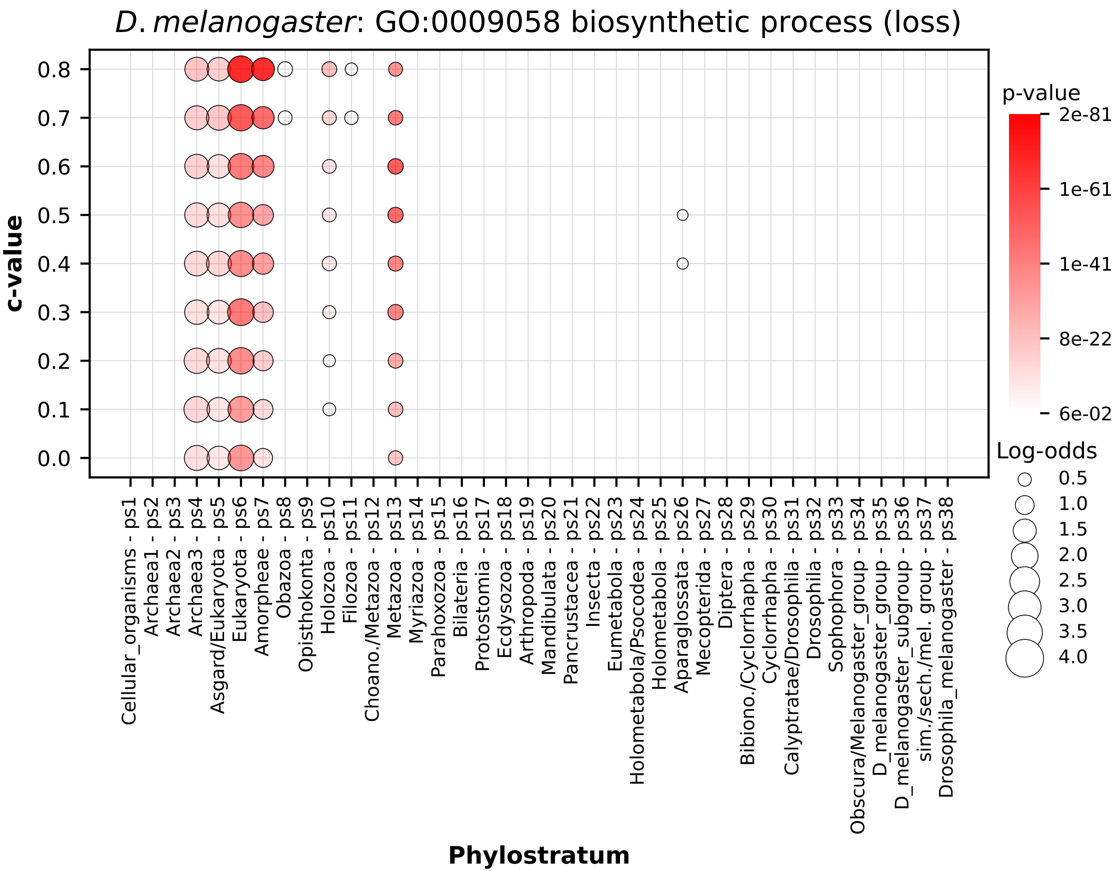
D. melanogaster: GO:0009058 biosynthetic process (loss)
c-value
0.8
0.7
0.6
0.5
0.4
0.3
0.2
0.1
0.0
p-value
2e-81
1e-61
1e-41
8e-22
6e-02
Log-odds
0.5
1.0
1.5
2.0
2.5
3.0
3.5
4.0
Cellular_organisms - ps1
Archaea1 - ps2
Archaea2 - ps3
Archaea3 - ps4
Asgard/Eukaryota - ps5
Eukaryota - ps6
Amorpheae - ps7
Obazoa - ps8
Opisthokonta - ps9
Holozoa - ps10
Filozoa - ps11
Choano./Metazoa - ps12
Metazoa - ps13
Myriazoa - ps14
Parahoxozoa - ps15
Bilateria - ps16
Protostomia - ps17
Ecdysozoa - ps18
Arthropoda - ps19
Mandibulata - ps20
Pancrustacea - ps21
Insecta - ps22
Eumetabola - ps23
Holometabola/Psocodea - ps24
Holometabola - ps25
Aparaglossata - ps26
Mecopterida - ps27
Diptera - ps28
Bibiono./Cyclorrhapha - ps29
Cyclorrhapha - ps30
Calyptratae/Drosophila - ps31
Drosophila - ps32
Sophophora - ps33
Obscura/Melanogaster_group - ps34
D_melanogaster_group - ps35
D_melanogaster_subgroup - ps36
sim./sech./mel. group - ps37
Drosophila_melanogaster - ps38
Phylostratum

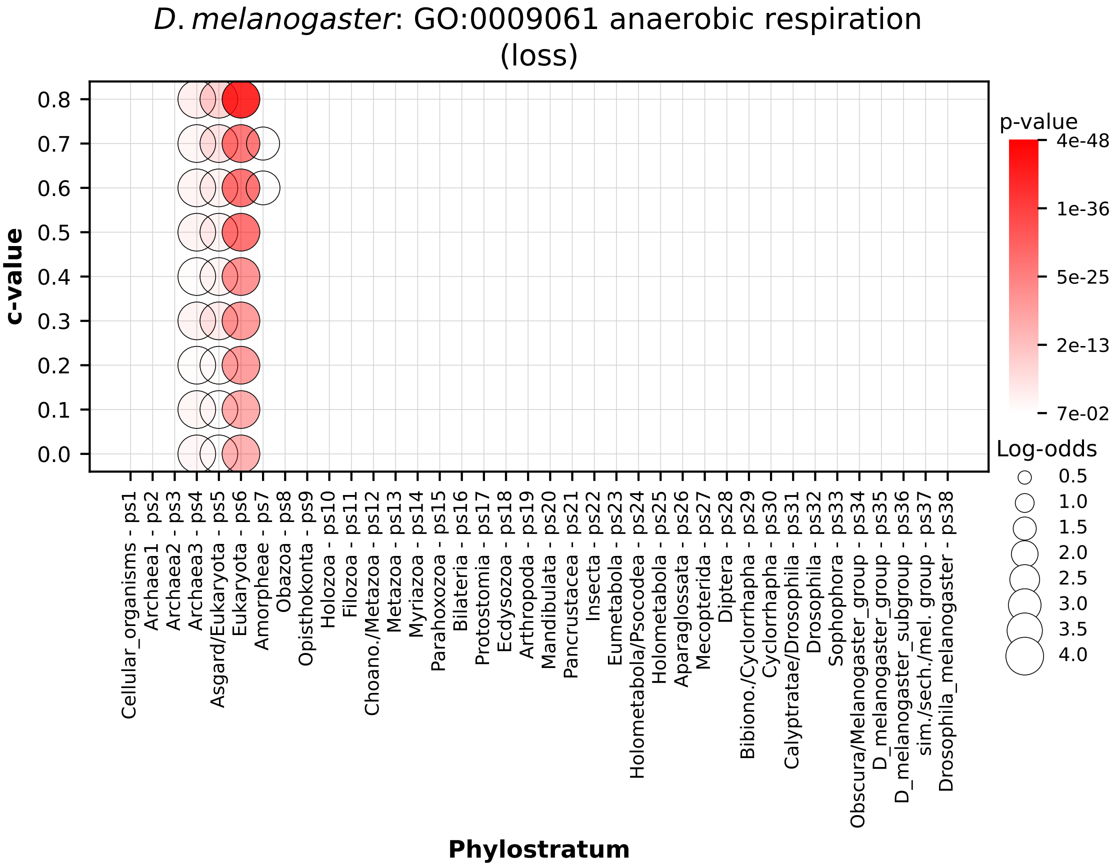

D. melanogaster: GO:0009061 anaerobic respiration (loss)
c-value
0.8
0.7
0.6
0.5
0.4
0.3
0.2
0.1
0.0
p-value
4e-48
1e-36
5e-25
2e-13
7e-02
Log-odds
0.5
1.0
1.5
2.0
2.5
3.0
3.5
4.0
Cellular_organisms - ps1
Archaea1 - ps2
Archaea2 - ps3
Archaea3 - ps4
Asgard/Eukaryota - ps5
Eukaryota - ps6
Amorpheae - ps7
Obazoa - ps8
Opisthokonta - ps9
Holozoa - ps10
Filozoa - ps11
Choano./Metazoa - ps12
Metazoa - ps13
Myriazoa - ps14
Parahoxozoa - ps15
Bilateria - ps16
Protostomia - ps17
Ecdysozoa - ps18
Arthropoda - ps19
Mandibulata - ps20
Pancrustacea - ps21
Insecta - ps22
Eumetabola - ps23
Holometabola/Psocodea - ps24
Holometabola - ps25
Aparaglossata - ps26
Mecopterida - ps27
Diptera - ps28
Bibiono./Cyclorrhapha - ps29
Cyclorrhapha - ps30
Calyptratae/Drosophila - ps31
Drosophila - ps32
Sophophora - ps33
Obscura/Melanogaster_group - ps34
D_melanogaster_group - ps35
D_melanogaster_subgroup - ps36
sim./sech./mel. group - ps37
Drosophila_melanogaster - ps38
Phylostratum

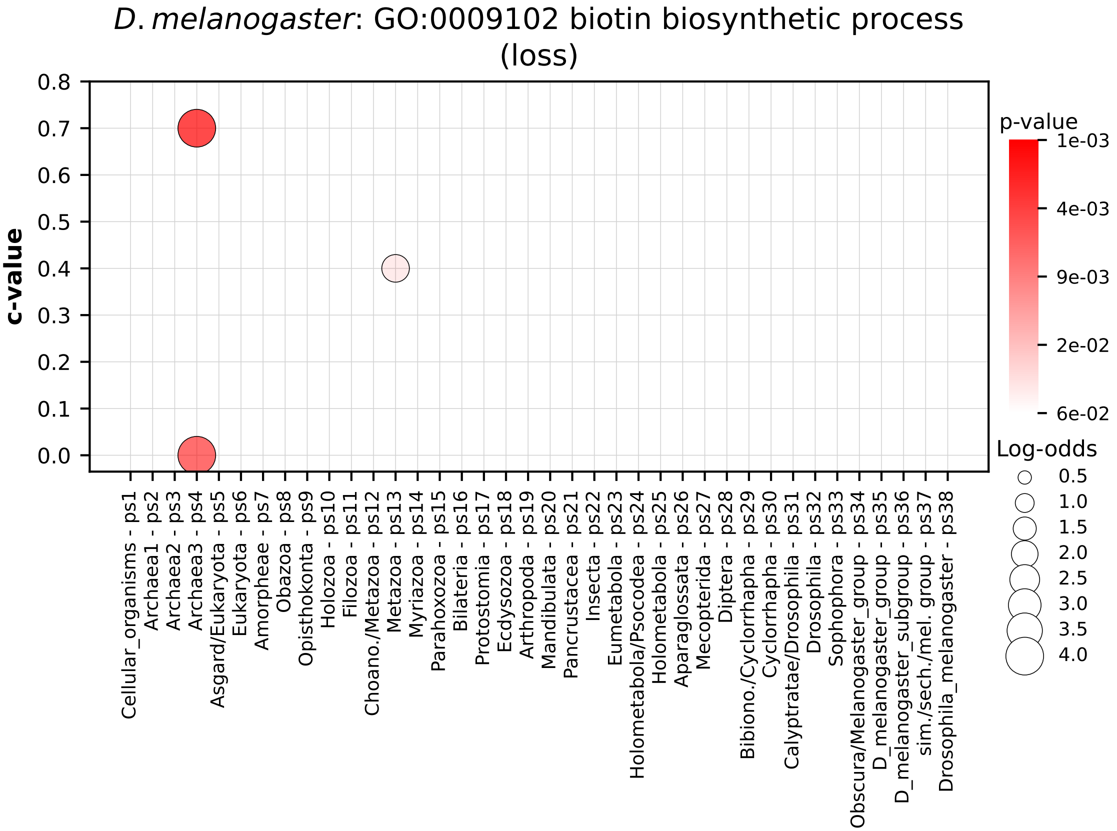

D. melanogaster: GO:0009102 biotin biosynthetic process
(loss)
c-value
0.8
0.7
0.6
0.5
0.4
0.3
0.2
0.1
0.0
Phylostratum
Cellular_organisms - ps1
Archaea1 - ps2
Archaea2 - ps3
Archaea3 - ps4
Asgard/Eukaryota - ps5
Eukaryota - ps6
Amorpheae - ps7
Obazoa - ps8
Opisthokonta - ps9
Holozoa - ps10
Filozoa - ps11
Choano./Metazoa - ps12
Metazoa - ps13
Myriazoa - ps14
Parahoxozoa - ps15
Bilateria - ps16
Protostomia - ps17
Ecdysozoa - ps18
Arthropoda - ps19
Mandibulata - ps20
Pancrustacea - ps21
Insecta - ps22
Eumetabola - ps23
Holometabola/Psocodea - ps24
Holometabola - ps25
Aparaglossata - ps26
Mecopterida - ps27
Diptera - ps28
Bibiono./Cyclorrhapha - ps29
Cyclorrhapha - ps30
Calyptratae/Drosophila - ps31
Drosophila - ps32
Sophophora - ps33
Obscura/Melanogaster_group - ps34
D_melanogaster_group - ps35
D_melanogaster_subgroup - ps36
sim./sech./mel. group - ps37
Drosophila_melanogaster - ps38
p-value
1e-03
4e-03
9e-03
2e-02
6e-02
Log-odds
0.5
1.0
1.5
2.0
2.5
3.0
3.5
4.0

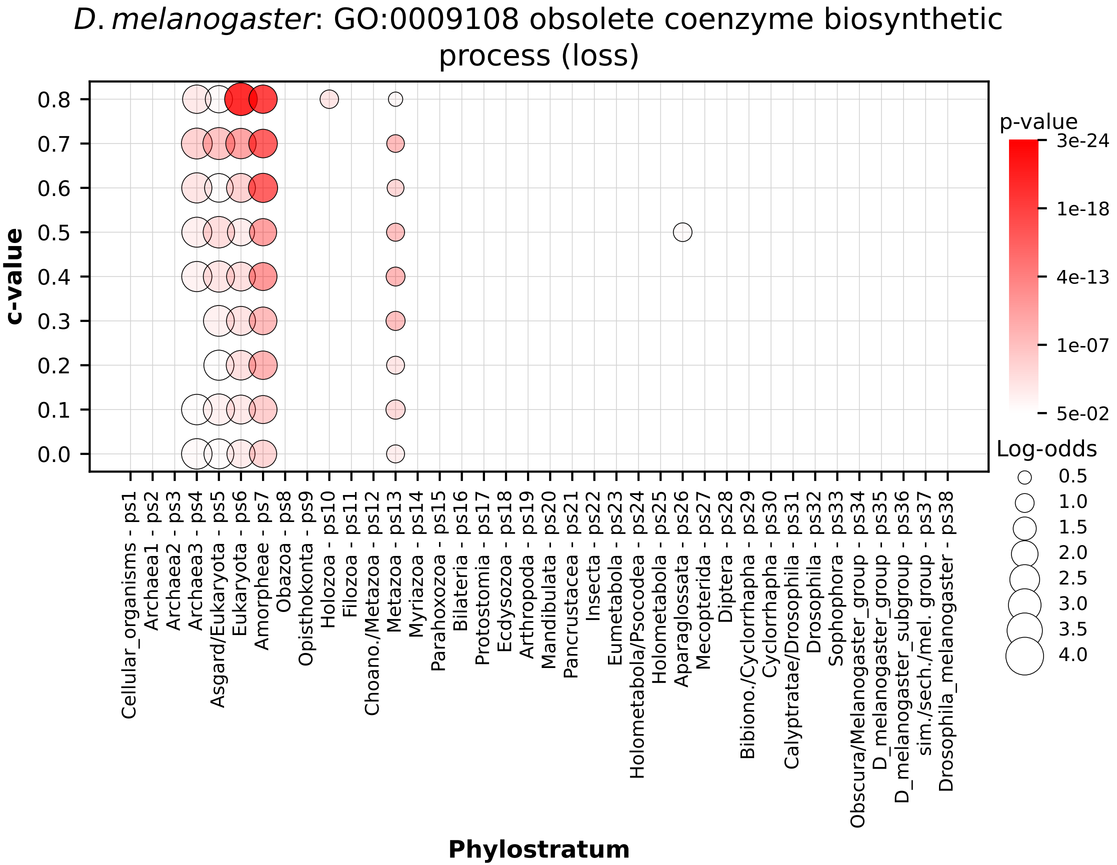

D. melanogaster: GO:0009108 obsolete coenzyme biosynthetic process (loss)
c-value
0.8
0.7
0.6
0.5
0.4
0.3
0.2
0.1
0.0
Phylostratum
Cellular_organisms - ps1
Archaea1 - ps2
Archaea2 - ps3
Archaea3 - ps4
Asgard/Eukaryota - ps5
Eukaryota - ps6
Amorpheae - ps7
Obazoa - ps8
Opisthokonta - ps9
Holozoa - ps10
Filozoa - ps11
Choano./Metazoa - ps12
Metazoa - ps13
Myriazoa - ps14
Parahoxozoa - ps15
Bilateria - ps16
Protostomia - ps17
Ecdysozoa - ps18
Arthropoda - ps19
Mandibulata - ps20
Pancrustacea - ps21
Insecta - ps22
Eumetabola - ps23
Holometabola/Psocodea - ps24
Holometabola - ps25
Aparaglossata - ps26
Mecopterida - ps27
Diptera - ps28
Bibiono./Cyclorrhapha - ps29
Cyclorrhapha - ps30
Calyptratae/Drosophila - ps31
Drosophila - ps32
Sophophora - ps33
Obscura/Melanogaster_group - ps34
D_melanogaster_group - ps35
D_melanogaster_subgroup - ps36
sim./sech./mel. group - ps37
Drosophila_melanogaster - ps38
p-value
3e-24
1e-18
4e-13
1e-07
5e-02
Log-odds
0.5
1.0
1.5
2.0
2.5
3.0
3.5
4.0

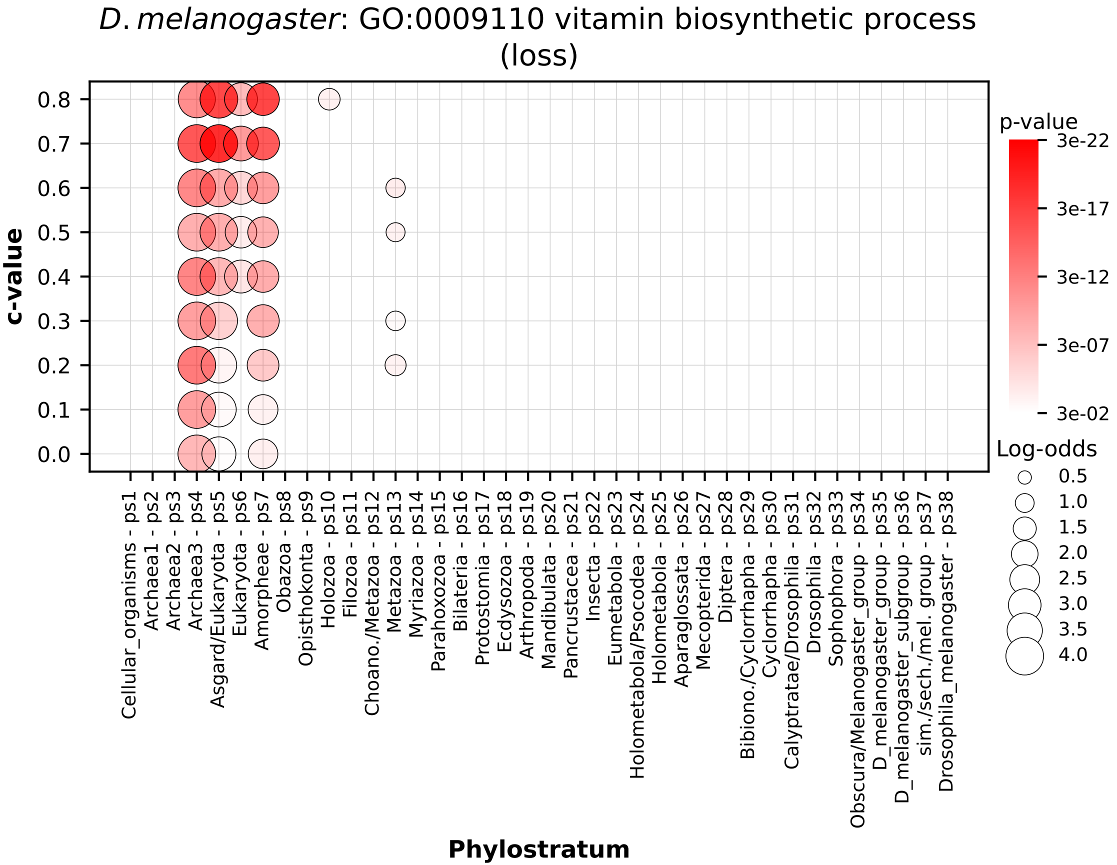

D. melanogaster: GO:0009110 vitamin biosynthetic process (loss)
c-value
0.8
0.7
0.6
0.5
0.4
0.3
0.2
0.1
0.0
p-value
3e-22
3e-17
3e-12
3e-07
3e-02
Log-odds
0.5
1.0
1.5
2.0
2.5
3.0
3.5
4.0
Cellular_organisms - ps1
Archaea1 - ps2
Archaea2 - ps3
Archaea3 - ps4
Asgard/Eukaryota - ps5
Eukaryota - ps6
Amorpheae - ps7
Obazoa - ps8
Opisthokonta - ps9
Holozoa - ps10
Filozoa - ps11
Choano./Metazoa - ps12
Metazoa - ps13
Myriazoa - ps14
Parahoxozoa - ps15
Bilateria - ps16
Protostomia - ps17
Ecdysozoa - ps18
Arthropoda - ps19
Mandibulata - ps20
Pancrustacea - ps21
Insecta - ps22
Eumetabola - ps23
Holometabola/Psocodea - ps24
Holometabola - ps25
Aparaglossata - ps26
Mecopterida - ps27
Diptera - ps28
Bibiono./Cyclorrhapha - ps29
Cyclorrhapha - ps30
Calyptratae/Drosophila - ps31
Drosophila - ps32
Sophophora - ps33
Obscura/Melanogaster_group - ps34
D_melanogaster_group - ps35
D_melanogaster_subgroup - ps36
sim./sech./mel. group - ps37
Drosophila_melanogaster - ps38
Phylostratum

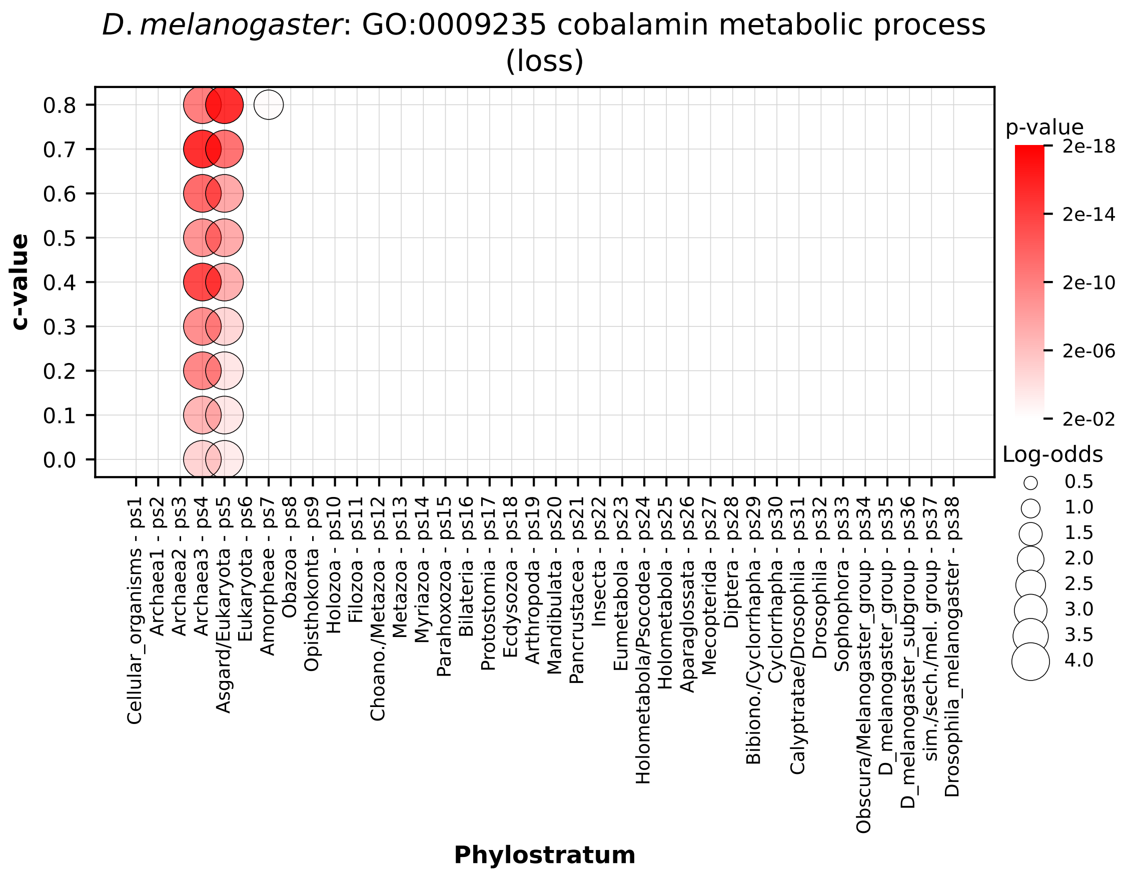

D. melanogaster: GO:0009235 cobalamin metabolic process
(loss)
c-value
0.8
0.7
0.6
0.5
0.4
0.3
0.2
0.1
0.0
Phylostratum
Cellular_organisms - ps1
Archaea1 - ps2
Archaea2 - ps3
Archaea3 - ps4
Asgard/Eukaryota - ps5
Eukaryota - ps6
Amorpheae - ps7
Obazoa - ps8
Opisthokonta - ps9
Holozoa - ps10
Filozoa - ps11
Choano./Metazoa - ps12
Metazoa - ps13
Myriazoa - ps14
Parahoxozoa - ps15
Bilateria - ps16
Protostomia - ps17
Ecdysozoa - ps18
Arthropoda - ps19
Mandibulata - ps20
Pancrustacea - ps21
Insecta - ps22
Eumetabola - ps23
Holometabola/Psocodea - ps24
Holometabola - ps25
Aparaglossata - ps26
Mecopterida - ps27
Diptera - ps28
Bibiono./Cyclorrhapha - ps29
Cyclorrhapha - ps30
Calyptratae/Drosophila - ps31
Drosophila - ps32
Sophophora - ps33
Obscura/Melanogaster_group - ps34
D_melanogaster_group - ps35
D_melanogaster_subgroup - ps36
sim./sech./mel. group - ps37
Drosophila_melanogaster - ps38
p-value
2e-18
2e-14
2e-10
2e-06
2e-02
Log-odds
0.5
1.0
1.5
2.0
2.5
3.0
3.5
4.0

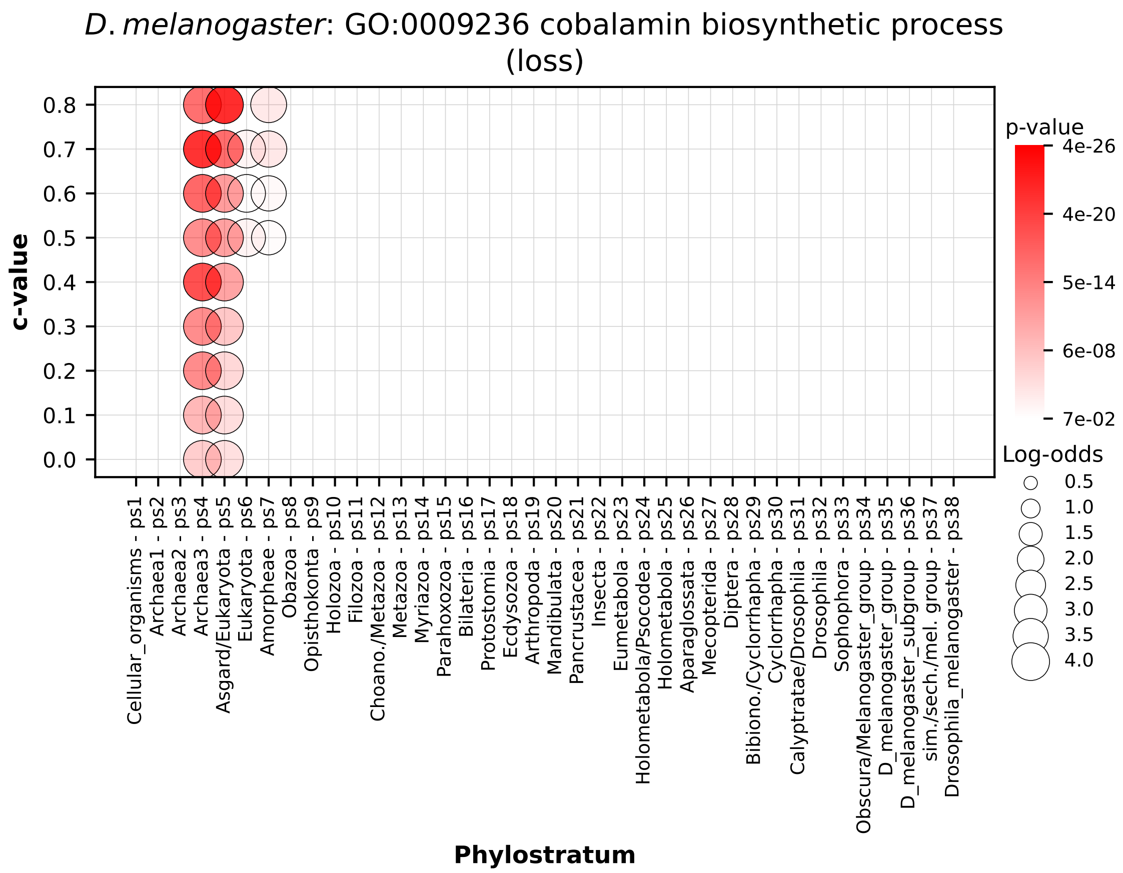

D. melanogaster: GO:0009236 cobalamin biosynthetic process (loss)
c-value
0.8
0.7
0.6
0.5
0.4
0.3
0.2
0.1
0.0
Phylostratum
Cellular_organisms - ps1
Archaea1 - ps2
Archaea2 - ps3
Archaea3 - ps4
Asgard/Eukaryota - ps5
Eukaryota - ps6
Amorpheae - ps7
Obazoa - ps8
Opisthokonta - ps9
Holozoa - ps10
Filozoa - ps11
Choano./Metazoa - ps12
Metazoa - ps13
Myriazoa - ps14
Parahoxozoa - ps15
Bilateria - ps16
Protostomia - ps17
Ecdysozoa - ps18
Arthropoda - ps19
Mandibulata - ps20
Pancrustacea - ps21
Insecta - ps22
Eumetabola - ps23
Holometabola/Psocodea - ps24
Holometabola - ps25
Aparaglossata - ps26
Mecopterida - ps27
Diptera - ps28
Bibiono./Cyclorrhapha - ps29
Cyclorrhapha - ps30
Calyptratae/Drosophila - ps31
Drosophila - ps32
Sophophora - ps33
Obscura/Melanogaster_group - ps34
D_melanogaster_group - ps35
D_melanogaster_subgroup - ps36
sim./sech./mel. group - ps37
Drosophila_melanogaster - ps38
p-value
4e-26
4e-20
5e-14
6e-08
7e-02
Log-odds
0.5
1.0
1.5
2.0
2.5
3.0
3.5
4.0

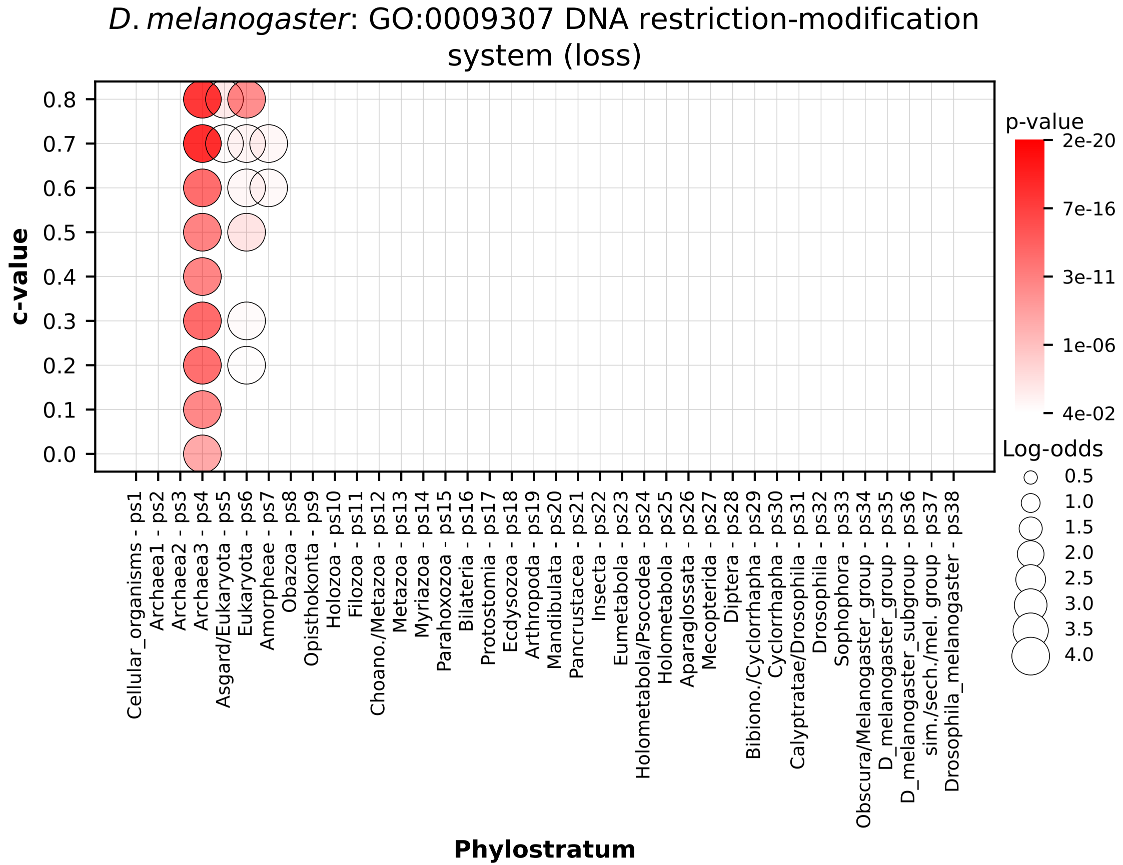

D. melanogaster: GO:0009307 DNA restriction-modification system (loss)
c-value
0.8
0.7
0.6
0.5
0.4
0.3
0.2
0.1
0.0
p-value
2e-20
7e-16
3e-11
1e-06
4e-02
Log-odds
0.5
1.0
1.5
2.0
2.5
3.0
3.5
4.0
Cellular_organisms - ps1
Archaea1 - ps2
Archaea2 - ps3
Archaea3 - ps4
Asgard/Eukaryota - ps5
Eukaryota - ps6
Amorpheae - ps7
Obazoa - ps8
Opisthokonta - ps9
Holozoa - ps10
Filozoa - ps11
Choano./Metazoa - ps12
Metazoa - ps13
Myriazoa - ps14
Parahoxozoa - ps15
Bilateria - ps16
Protostomia - ps17
Ecdysozoa - ps18
Arthropoda - ps19
Mandibulata - ps20
Pancrustacea - ps21
Insecta - ps22
Eumetabola - ps23
Holometabola/Psocodea - ps24
Holometabola - ps25
Aparaglossata - ps26
Mecopterida - ps27
Diptera - ps28
Bibiono./Cyclorrhapha - ps29
Cyclorrhapha - ps30
Calyptratae/Drosophila - ps31
Drosophila - ps32
Sophophora - ps33
Obscura/Melanogaster_group - ps34
D_melanogaster_group - ps35
D_melanogaster_subgroup - ps36
sim./sech./mel. group - ps37
Drosophila_melanogaster - ps38
Phylostratum

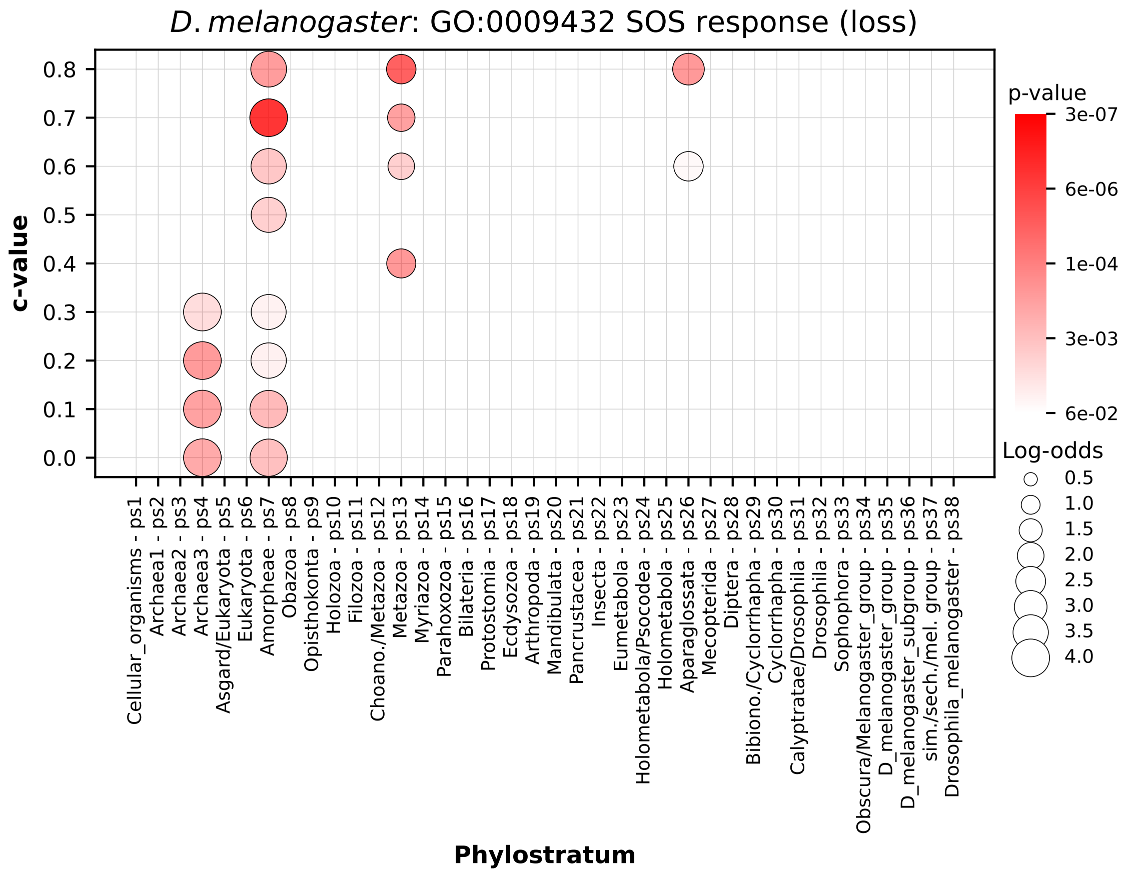

D. melanogaster: GO:0009432 SOS response (loss)
c-value
0.8
0.7
0.6
0.5
0.4
0.3
0.2
0.1
0.0
Phylostratum
Cellular_organisms - ps1
Archaea1 - ps2
Archaea2 - ps3
Archaea3 - ps4
Asgard/Eukaryota - ps5
Eukaryota - ps6
Amorpheae - ps7
Obazoa - ps8
Opisthokonta - ps9
Holozoa - ps10
Filozoa - ps11
Choano./Metazoa - ps12
Metazoa - ps13
Myriazoa - ps14
Parahoxozoa - ps15
Bilateria - ps16
Protostomia - ps17
Ecdysozoa - ps18
Arthropoda - ps19
Mandibulata - ps20
Pancrustacea - ps21
Insecta - ps22
Eumetabola - ps23
Holometabola/Psocodea - ps24
Holometabola - ps25
Aparaglossata - ps26
Mecopterida - ps27
Diptera - ps28
Bibiono./Cyclorrhapha - ps29
Cyclorrhapha - ps30
Calyptratae/Drosophila - ps31
Drosophila - ps32
Sophophora - ps33
Obscura/Melanogaster_group - ps34
D_melanogaster_group - ps35
D_melanogaster_subgroup - ps36
sim./sech./mel. group - ps37
Drosophila_melanogaster - ps38
p-value
3e-07
6e-06
1e-04
3e-03
6e-02
Log-odds
0.5
1.0
1.5
2.0
2.5
3.0
3.5
4.0

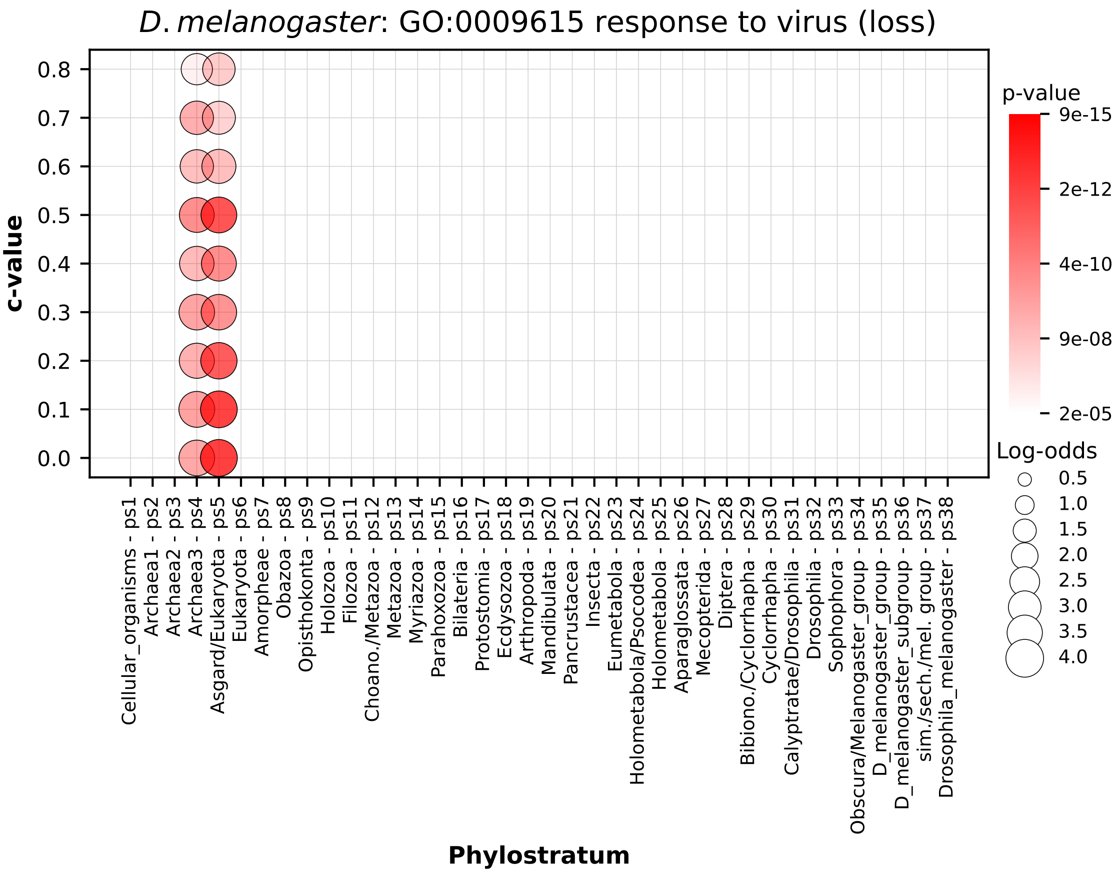

D. melanogaster: GO:0009615 response to virus (loss)
c-value
0.8
0.7
0.6
0.5
0.4
0.3
0.2
0.1
0.0
p-value
9e-15
2e-12
4e-10
9e-08
2e-05
Log-odds
0.5
1.0
1.5
2.0
2.5
3.0
3.5
4.0
Cellular_organisms - ps1
Archaea1 - ps2
Archaea2 - ps3
Archaea3 - ps4
Asgard/Eukaryota - ps5
Eukaryota - ps6
Amorpheae - ps7
Obazoa - ps8
Opisthokonta - ps9
Holozoa - ps10
Filozoa - ps11
Choano./Metazoa - ps12
Metazoa - ps13
Myriazoa - ps14
Parahoxozoa - ps15
Bilateria - ps16
Protostomia - ps17
Ecdysozoa - ps18
Arthropoda - ps19
Mandibulata - ps20
Pancrustacea - ps21
Insecta - ps22
Eumetabola - ps23
Holometabola/Psocodea - ps24
Holometabola - ps25
Aparaglossata - ps26
Mecopterida - ps27
Diptera - ps28
Bibiono./Cyclorrhapha - ps29
Cyclorrhapha - ps30
Calyptratae/Drosophila - ps31
Drosophila - ps32
Sophophora - ps33
Obscura/Melanogaster_group - ps34
D_melanogaster_group - ps35
D_melanogaster_subgroup - ps36
sim./sech./mel. group - ps37
Drosophila_melanogaster - ps38
Phylostratum

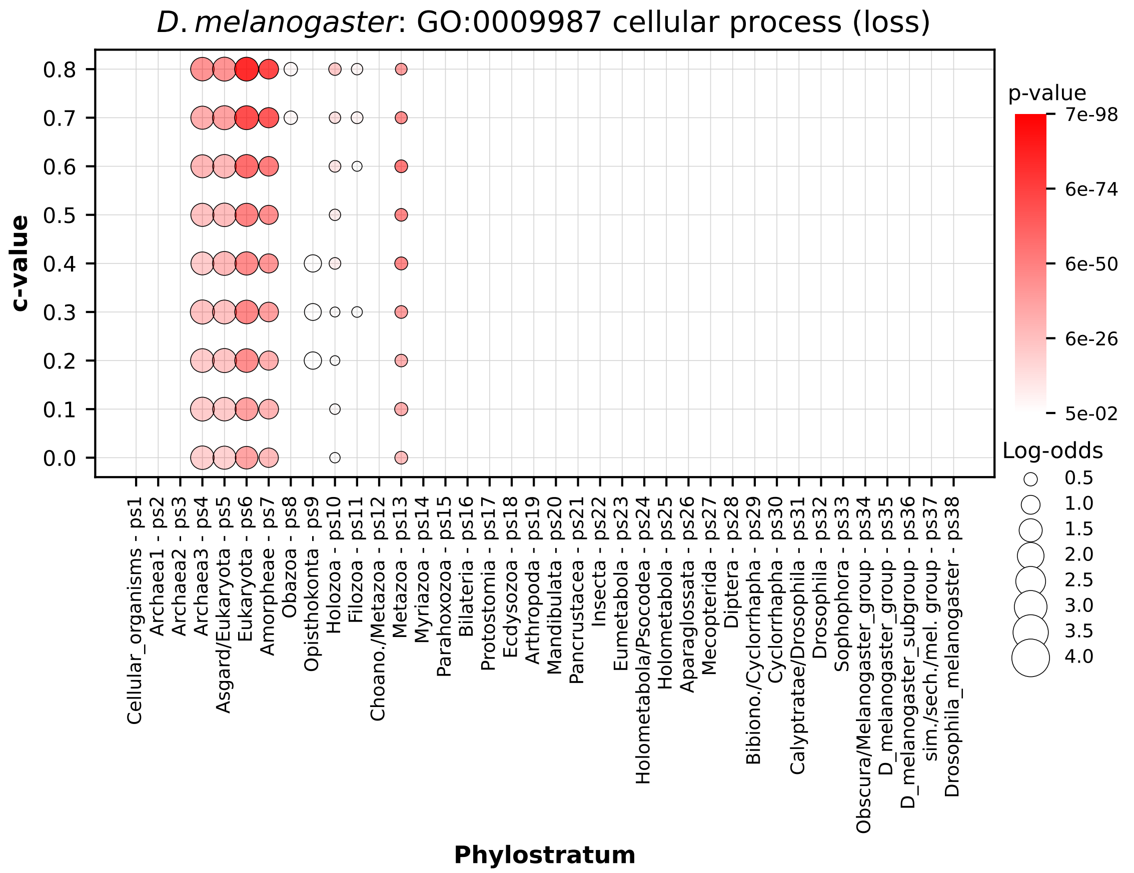

D. melanogaster: GO:0009987 cellular process (loss)
c-value
0.8
0.7
0.6
0.5
0.4
0.3
0.2
0.1
0.0
Phylostratum
Cellular_organisms - ps1
Archaea1 - ps2
Archaea2 - ps3
Archaea3 - ps4
Asgard/Eukaryota - ps5
Eukaryota - ps6
Amorpheae - ps7
Obazoa - ps8
Opisthokonta - ps9
Holozoa - ps10
Filozoa - ps11
Choano./Metazoa - ps12
Metazoa - ps13
Myriazoa - ps14
Parahoxozoa - ps15
Bilateria - ps16
Protostomia - ps17
Ecdysozoa - ps18
Arthropoda - ps19
Mandibulata - ps20
Pancrustacea - ps21
Insecta - ps22
Eumetabola - ps23
Holometabola/Psocodea - ps24
Holometabola - ps25
Aparaglossata - ps26
Mecopterida - ps27
Diptera - ps28
Bibiono./Cyclorrhapha - ps29
Cyclorrhapha - ps30
Calyptratae/Drosophila - ps31
Drosophila - ps32
Sophophora - ps33
Obscura/Melanogaster_group - ps34
D_melanogaster_group - ps35
D_melanogaster_subgroup - ps36
sim./sech./mel. group - ps37
Drosophila_melanogaster - ps38
p-value
7e-98
6e-74
6e-50
6e-26
5e-02
Log-odds
0.5
1.0
1.5
2.0
2.5
3.0
3.5
4.0

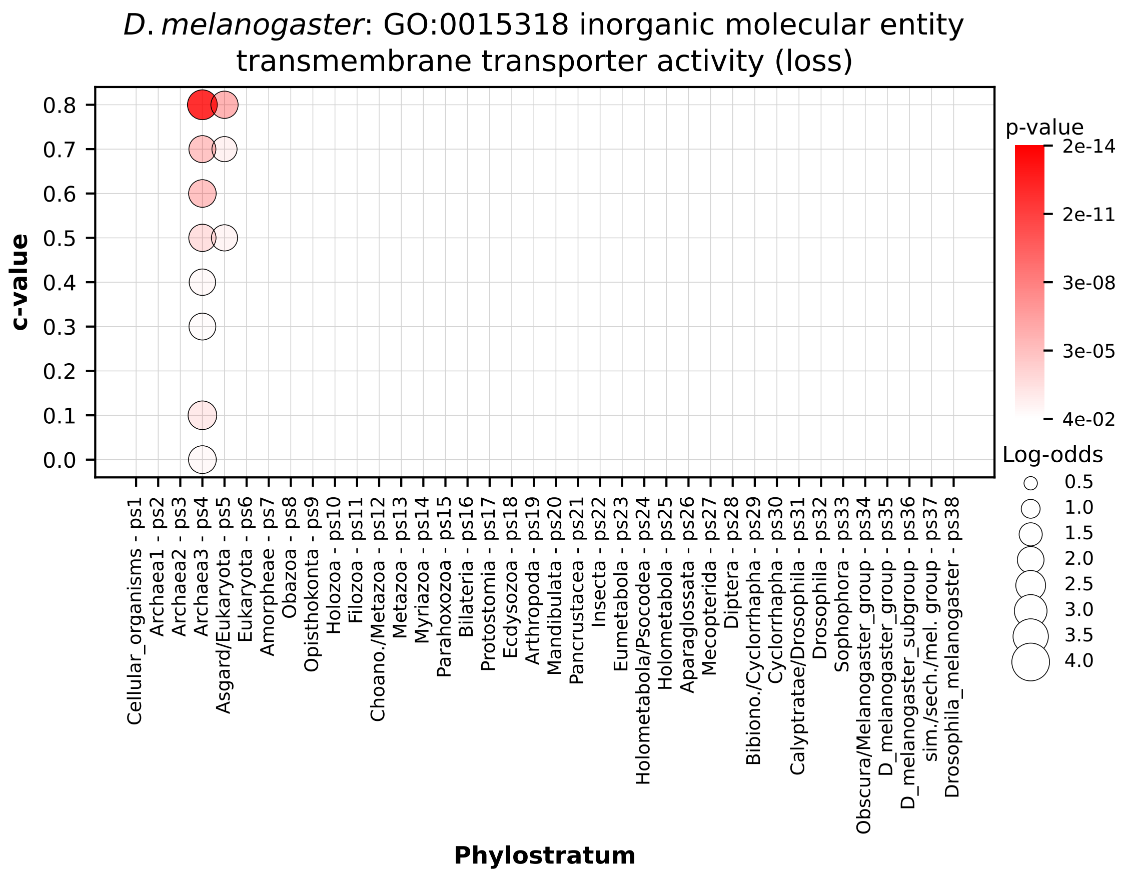
D. melanogaster: GO:0015318 inorganic molecular entity transmembrane transporter activity (loss)
c-value
Phylostratum
p-value
2e-14
2e-11
3e-08
3e-05
4e-02
Log-odds
0.5
1.0
1.5
2.0
2.5
3.0
3.5
4.0
Cellular_organisms - ps1
Archaea1 - ps2
Archaea2 - ps3
Archaea3 - ps4
Asgard/Eukaryota - ps5
Eukaryota - ps6
Amorpheae - ps7
Obazoa - ps8
Opisthokonta - ps9
Holozoa - ps10
Filozoa - ps11
Choano./Metazoa - ps12
Metazoa - ps13
Myriazoa - ps14
Parahoxozoa - ps15
Bilateria - ps16
Protostomia - ps17
Ecdysozoa - ps18
Arthropoda - ps19
Mandibulata - ps20
Pancrustacea - ps21
Insecta - ps22
Eumetabola - ps23
Holometabola/Psocodea - ps24
Holometabola - ps25
Aparaglossata - ps26
Mecopterida - ps27
Diptera - ps28
Bibiono./Cyclorrhapha - ps29
Cyclorrhapha - ps30
Calyptratae/Drosophila - ps31
Drosophila - ps32
Sophophora - ps33
Obscura/Melanogaster_group - ps34
D_melanogaster_group - ps35
D_melanogaster_subgroup - ps36
sim./sech./mel. group - ps37
Drosophila_melanogaster - ps38

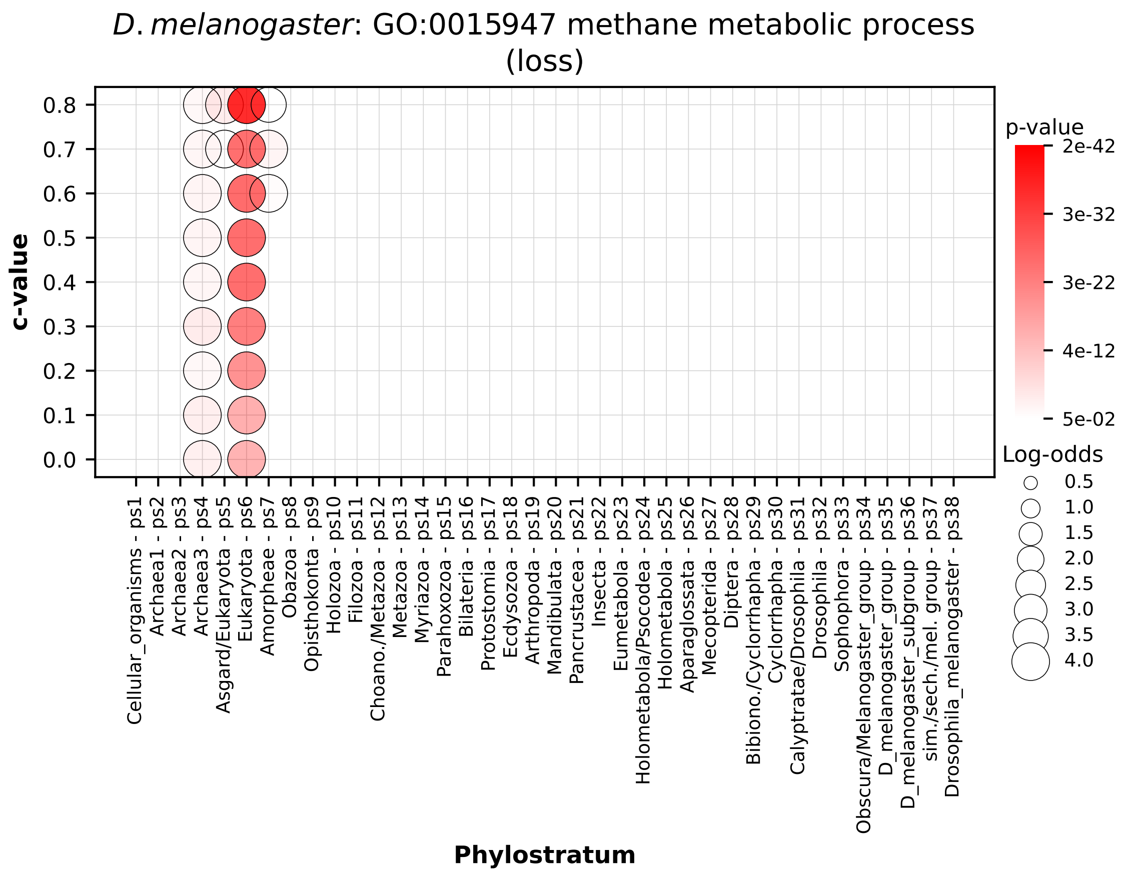

D. melanogaster: GO:0015947 methane metabolic process (loss)
c-value
0.8
0.7
0.6
0.5
0.4
0.3
0.2
0.1
0.0
p-value
2e-42
3e-32
3e-22
4e-12
5e-02
Log-odds
0.5
1.0
1.5
2.0
2.5
3.0
3.5
4.0
Cellular_organisms - ps1
Archaea1 - ps2
Archaea2 - ps3
Archaea3 - ps4
Asgard/Eukaryota - ps5
Eukaryota - ps6
Amorpheae - ps7
Obazoa - ps8
Opisthokonta - ps9
Holozoa - ps10
Filozoa - ps11
Choano./Metazoa - ps12
Metazoa - ps13
Myriazoa - ps14
Parahoxozoa - ps15
Bilateria - ps16
Protostomia - ps17
Ecdysozoa - ps18
Arthropoda - ps19
Mandibulata - ps20
Pancrustacea - ps21
Insecta - ps22
Eumetabola - ps23
Holometabola/Psocodea - ps24
Holometabola - ps25
Aparaglossata - ps26
Mecopterida - ps27
Diptera - ps28
Bibiono./Cyclorrhapha - ps29
Cyclorrhapha - ps30
Calyptratae/Drosophila - ps31
Drosophila - ps32
Sophophora - ps33
Obscura/Melanogaster_group - ps34
D_melanogaster_group - ps35
D_melanogaster_subgroup - ps36
sim./sech./mel. group - ps37
Drosophila_melanogaster - ps38
Phylostratum

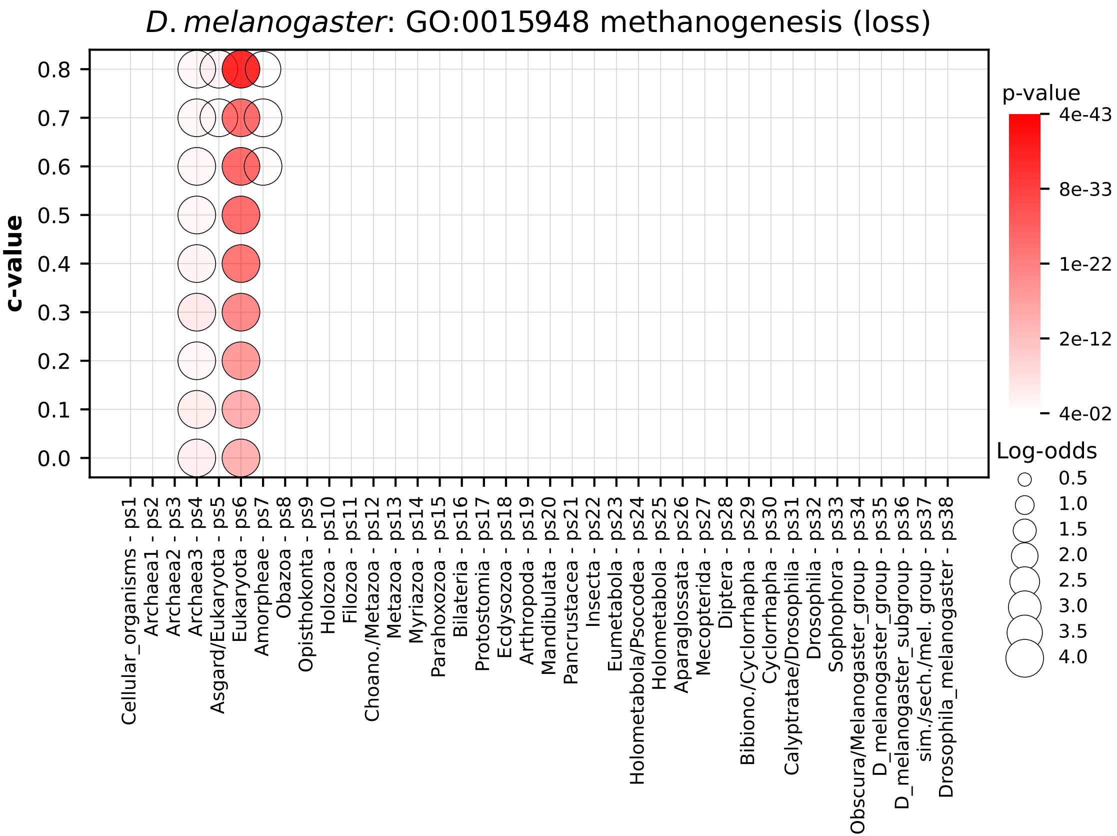

D. melanogaster: GO:0015948 methanogenesis (loss)
c-value
0.0
0.1
0.2
0.3
0.4
0.5
0.6
0.7
0.8
Phylostratum
Cellular_organisms - ps1
Archaea1 - ps2
Archaea2 - ps3
Archaea3 - ps4
Asgard/Eukaryota - ps5
Eukaryota - ps6
Amorpheae - ps7
Obazoa - ps8
Opisthokonta - ps9
Holozoa - ps10
Filozoa - ps11
Choano./Metazoa - ps12
Metazoa - ps13
Myriazoa - ps14
Parahoxozoa - ps15
Bilateria - ps16
Protostomia - ps17
Ecdysozoa - ps18
Arthropoda - ps19
Mandibulata - ps20
Pancrustacea - ps21
Insecta - ps22
Eumetabola - ps23
Holometabola/Psocodea - ps24
Holometabola - ps25
Aparaglossata - ps26
Mecopterida - ps27
Diptera - ps28
Bibiono./Cyclorrhapha - ps29
Cyclorrhapha - ps30
Calyptratae/Drosophila - ps31
Drosophila - ps32
Sophophora - ps33
Obscura/Melanogaster_group - ps34
D_melanogaster_group - ps35
D_melanogaster_subgroup - ps36
sim./sech./mel. group - ps37
Drosophila_melanogaster - ps38
p-value
4e-43
8e-33
1e-22
2e-12
4e-02
Log-odds
0.5
1.0
1.5
2.0
2.5
3.0
3.5
4.0

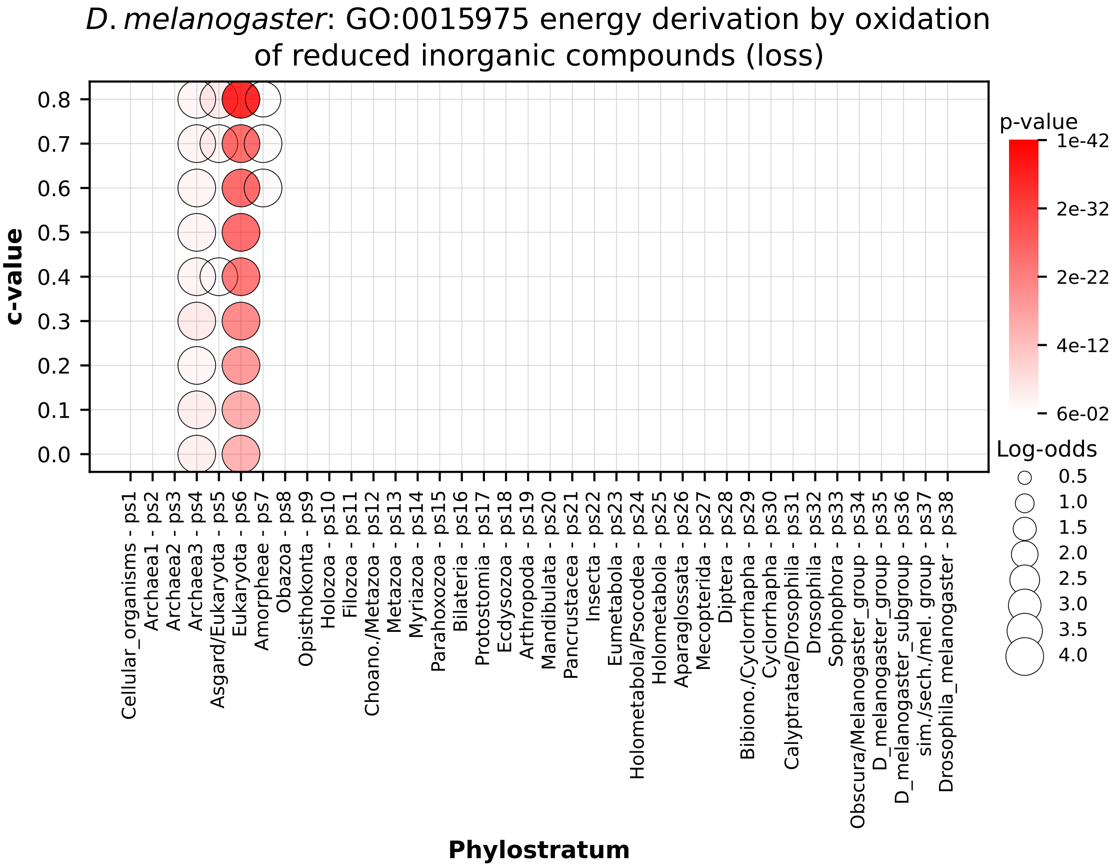
D. melanogaster: GO:0015975 energy derivation by oxidation of reduced inorganic compounds (loss)
c-value
0.8
0.7
0.6
0.5
0.4
0.3
0.2
0.1
0.0
p-value
1e-42
2e-32
2e-22
4e-12
6e-02
Log-odds
0.5
1.0
1.5
2.0
2.5
3.0
3.5
4.0
Cellular_organisms - ps1
Archaea1 - ps2
Archaea2 - ps3
Archaea3 - ps4
Asgard/Eukaryota - ps5
Eukaryota - ps6
Amorpheae - ps7
Obazoa - ps8
Opisthokonta - ps9
Holozoa - ps10
Filozoa - ps11
Choano./Metazoa - ps12
Metazoa - ps13
Myriazoa - ps14
Parahoxozoa - ps15
Bilateria - ps16
Protostomia - ps17
Ecdysozoa - ps18
Arthropoda - ps19
Mandibulata - ps20
Pancrustacea - ps21
Insecta - ps22
Eumetabola - ps23
Holometabola/Psocodea - ps24
Holometabola - ps25
Aparaglossata - ps26
Mecopterida - ps27
Diptera - ps28
Bibiono./Cyclorrhapha - ps29
Cyclorrhapha - ps30
Calyptratae/Drosophila - ps31
Drosophila - ps32
Sophophora - ps33
Obscura/Melanogaster_group - ps34
D_melanogaster_group - ps35
D_melanogaster_subgroup - ps36
sim./sech./mel. group - ps37
Drosophila_melanogaster - ps38
Phylostratum

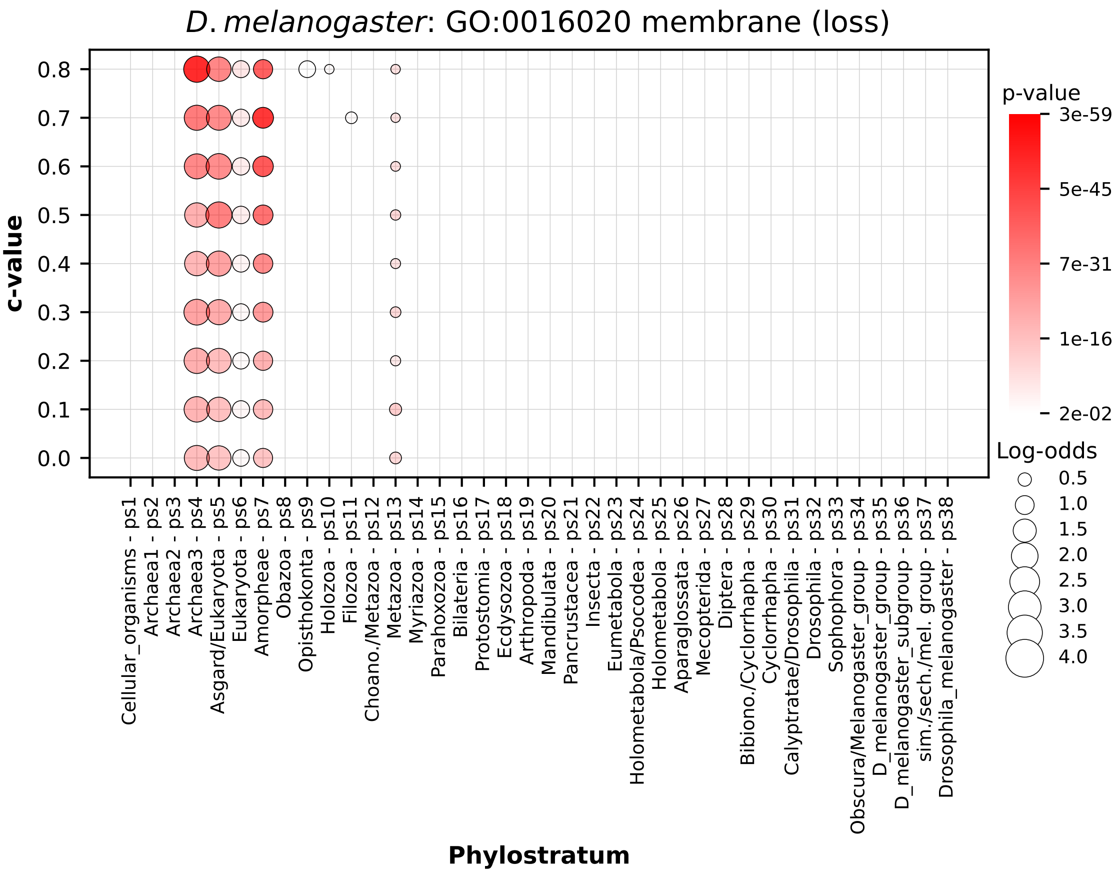
D. melanogaster: GO:0016020 membrane (loss)
c-value
0.8
0.7
0.6
0.5
0.4
0.3
0.2
0.1
0.0
p-value
3e-59
5e-45
7e-31
1e-16
2e-02
Log-odds
0.5
1.0
1.5
2.0
2.5
3.0
3.5
4.0
Cellular_organisms - ps1
Archaea1 - ps2
Archaea2 - ps3
Archaea3 - ps4
Asgard/Eukaryota - ps5
Eukaryota - ps6
Amorpheae - ps7
Obazoa - ps8
Opisthokonta - ps9
Holozoa - ps10
Filozoa - ps11
Choano./Metazoa - ps12
Metazoa - ps13
Myriazoa - ps14
Parahoxozoa - ps15
Bilateria - ps16
Protostomia - ps17
Ecdysozoa - ps18
Arthropoda - ps19
Mandibulata - ps20
Pancrustacea - ps21
Insecta - ps22
Eumetabola - ps23
Holometabola/Psocodea - ps24
Holometabola - ps25
Aparaglossata - ps26
Mecopterida - ps27
Diptera - ps28
Bibiono./Cyclorrhapha - ps29
Cyclorrhapha - ps30
Calyptratae/Drosophila - ps31
Drosophila - ps32
Sophophora - ps33
Obscura/Melanogaster_group - ps34
D_melanogaster_group - ps35
D_melanogaster_subgroup - ps36
sim./sech./mel. group - ps37
Drosophila_melanogaster - ps38
Phylostratum

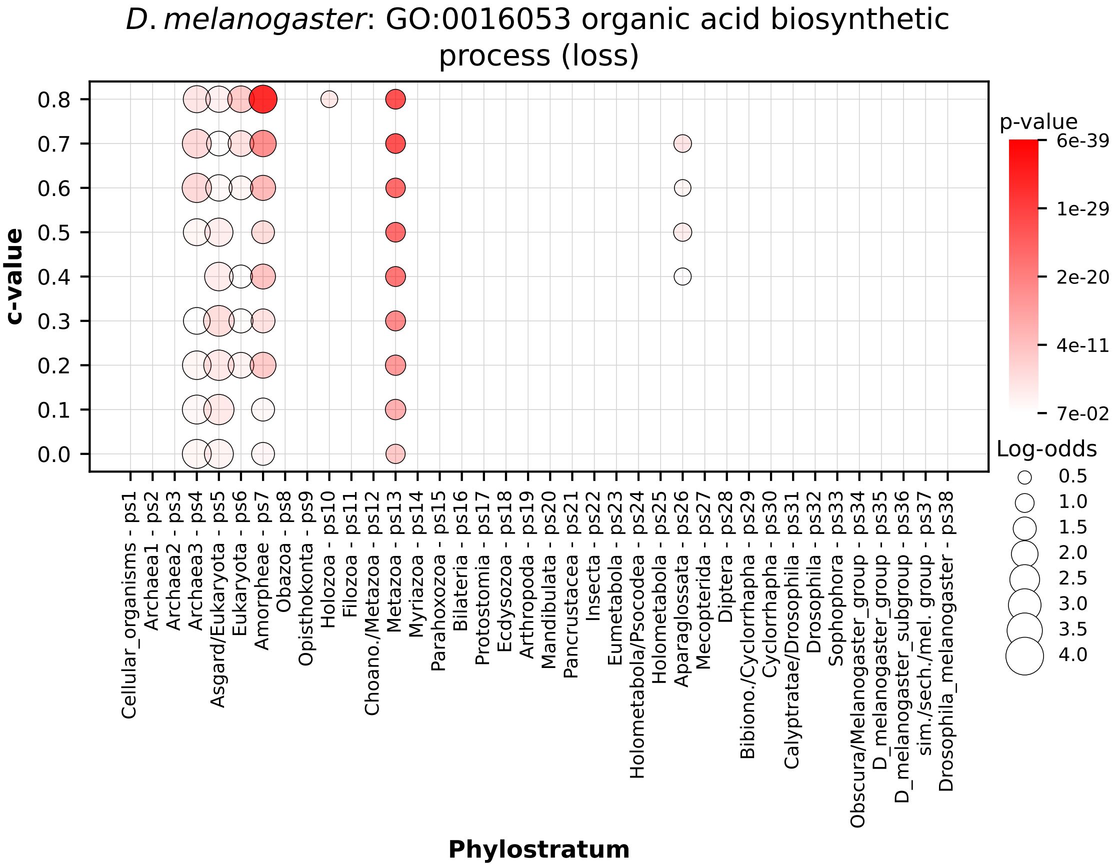

D. melanogaster: GO:0016053 organic acid biosynthetic process (loss)
c-value
0.8
0.7
0.6
0.5
0.4
0.3
0.2
0.1
0.0
Phylostratum
Cellular_organisms - ps1
Archaea1 - ps2
Archaea2 - ps3
Archaea3 - ps4
Asgard/Eukaryota - ps5
Eukaryota - ps6
Amorpheae - ps7
Obazoa - ps8
Opisthokonta - ps9
Holozoa - ps10
Filozoa - ps11
Choano./Metazoa - ps12
Metazoa - ps13
Myriazoa - ps14
Parahoxozoa - ps15
Bilateria - ps16
Protostomia - ps17
Ecdysozoa - ps18
Arthropoda - ps19
Mandibulata - ps20
Pancrustacea - ps21
Insecta - ps22
Eumetabola - ps23
Holometabola/Psocodea - ps24
Holometabola - ps25
Aparaglossata - ps26
Mecopterida - ps27
Diptera - ps28
Bibiono./Cyclorrhapha - ps29
Cyclorrhapha - ps30
Calyptratae/Drosophila - ps31
Drosophila - ps32
Sophophora - ps33
Obscura/Melanogaster_group - ps34
D_melanogaster_group - ps35
D_melanogaster_subgroup - ps36
sim./sech./mel. group - ps37
Drosophila_melanogaster - ps38
p-value
6e-39
1e-29
2e-20
4e-11
7e-02
Log-odds
0.5
1.0
1.5
2.0
2.5
3.0
3.5
4.0

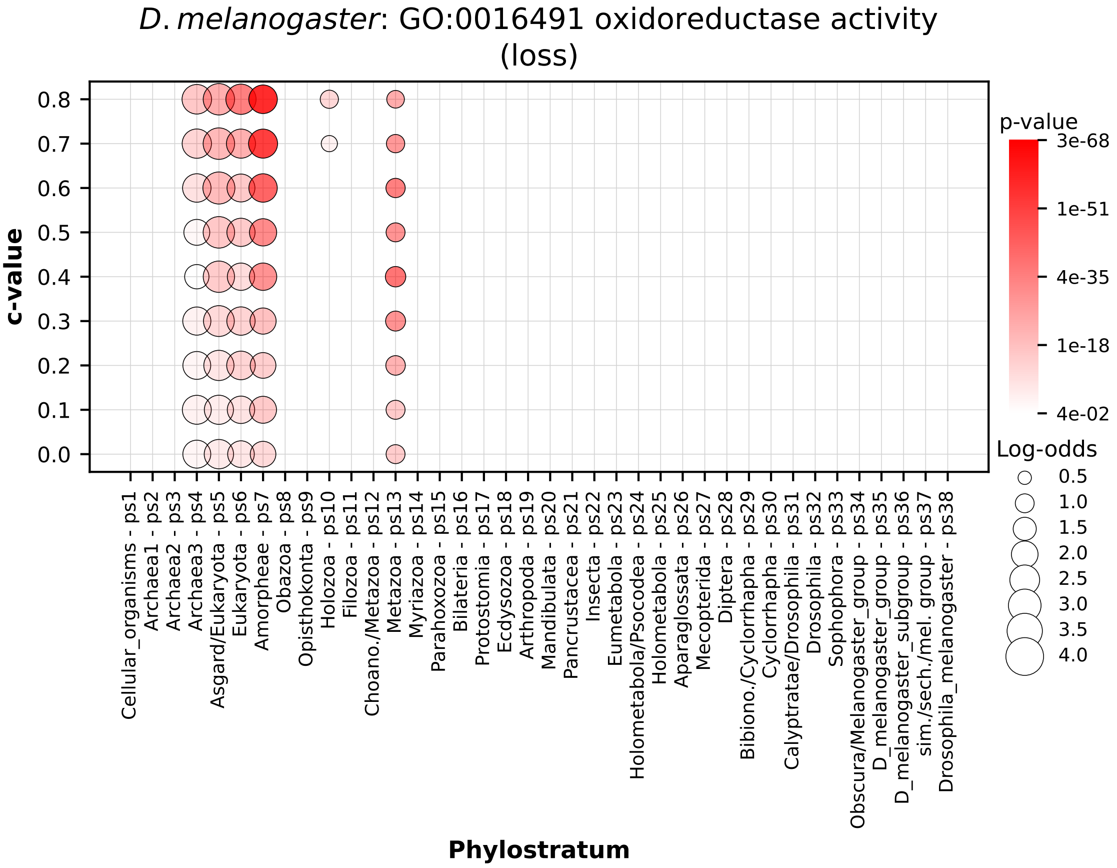

D. melanogaster: GO:0016491 oxidoreductase activity
(loss)
c-value
0.8
0.7
0.6
0.5
0.4
0.3
0.2
0.1
0.0
Phylostratum
Cellular_organisms - ps1
Archaea1 - ps2
Archaea2 - ps3
Archaea3 - ps4
Asgard/Eukaryota - ps5
Eukaryota - ps6
Amorpheae - ps7
Obazoa - ps8
Opisthokonta - ps9
Holozoa - ps10
Filozoa - ps11
Choano./Metazoa - ps12
Metazoa - ps13
Myriazoa - ps14
Parahoxozoa - ps15
Bilateria - ps16
Protostomia - ps17
Ecdysozoa - ps18
Arthropoda - ps19
Mandibulata - ps20
Pancrustacea - ps21
Insecta - ps22
Eumetabola - ps23
Holometabola/Psocodea - ps24
Holometabola - ps25
Aparaglossata - ps26
Mecopterida - ps27
Diptera - ps28
Bibiono./Cyclorrhapha - ps29
Cyclorrhapha - ps30
Calyptratae/Drosophila - ps31
Drosophila - ps32
Sophophora - ps33
Obscura/Melanogaster_group - ps34
D_melanogaster_group - ps35
D_melanogaster_subgroup - ps36
sim./sech./mel. group - ps37
Drosophila_melanogaster - ps38
p-value
3e-68
1e-51
4e-35
1e-18
4e-02
Log-odds
0.5
1.0
1.5
2.0
2.5
3.0
3.5
4.0

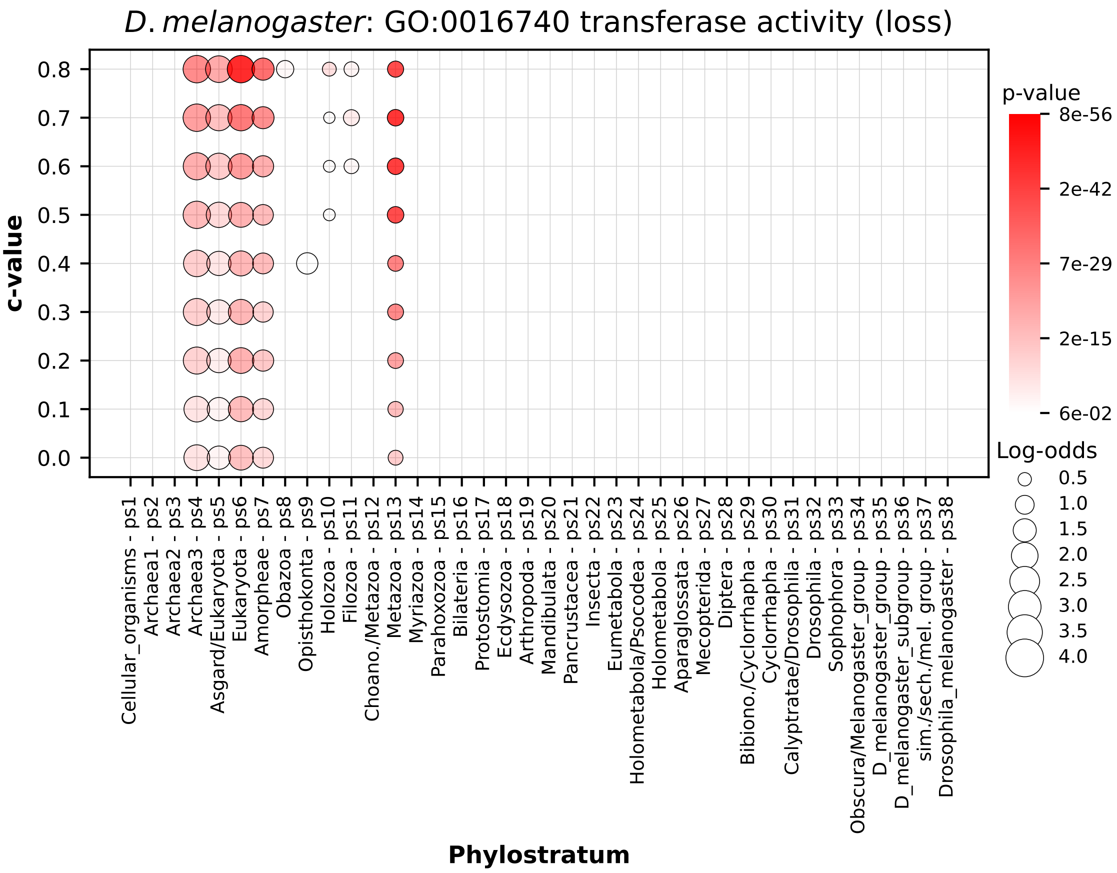

D. melanogaster: GO:0016740 transferase activity (loss)
c-value
0.8
0.7
0.6
0.5
0.4
0.3
0.2
0.1
0.0
p-value
8e-56
2e-42
7e-29
2e-15
6e-02
Log-odds
0.5
1.0
1.5
2.0
2.5
3.0
3.5
4.0
Cellular_organisms - ps1
Archaea1 - ps2
Archaea2 - ps3
Archaea3 - ps4
Asgard/Eukaryota - ps5
Eukaryota - ps6
Amorpheae - ps7
Obazoa - ps8
Opisthokonta - ps9
Holozoa - ps10
Filozoa - ps11
Choano./Metazoa - ps12
Metazoa - ps13
Myriazoa - ps14
Parahoxozoa - ps15
Bilateria - ps16
Protostomia - ps17
Ecdysozoa - ps18
Arthropoda - ps19
Mandibulata - ps20
Pancrustacea - ps21
Insecta - ps22
Eumetabola - ps23
Holometabola/Psocodea - ps24
Holometabola - ps25
Aparaglossata - ps26
Mecopterida - ps27
Diptera - ps28
Bibiono./Cyclorrhapha - ps29
Cyclorrhapha - ps30
Calyptratae/Drosophila - ps31
Drosophila - ps32
Sophophora - ps33
Obscura/Melanogaster_group - ps34
D_melanogaster_group - ps35
D_melanogaster_subgroup - ps36
sim./sech./mel. group - ps37
Drosophila_melanogaster - ps38
Phylostratum

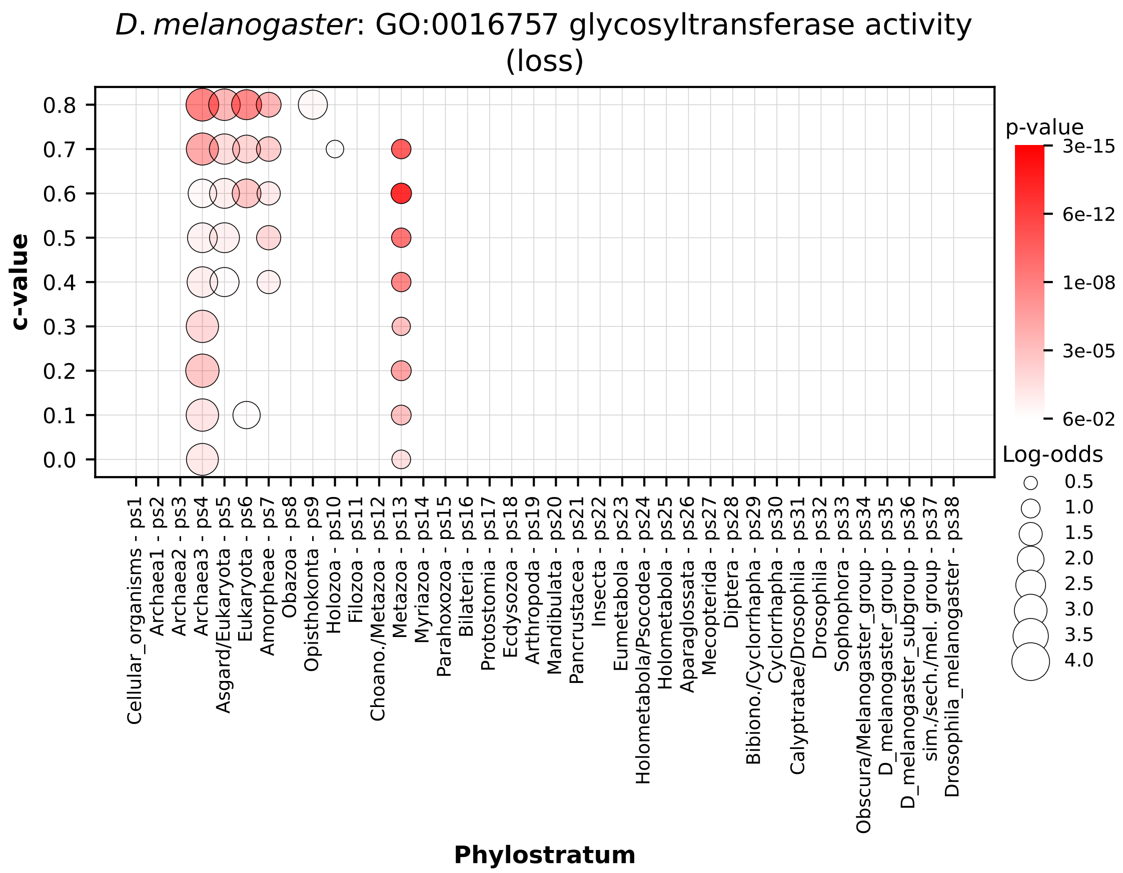

D. melanogaster: GO:0016757 glycosyltransferase activity (loss)
c-value
0.8
0.7
0.6
0.5
0.4
0.3
0.2
0.1
0.0
Phylostratum
Cellular_organisms - ps1
Archaea1 - ps2
Archaea2 - ps3
Archaea3 - ps4
Asgard/Eukaryota - ps5
Eukaryota - ps6
Amorpheae - ps7
Obazoa - ps8
Opisthokonta - ps9
Holozoa - ps10
Filozoa - ps11
Choano./Metazoa - ps12
Metazoa - ps13
Myriazoa - ps14
Parahoxozoa - ps15
Bilateria - ps16
Protostomia - ps17
Ecdysozoa - ps18
Arthropoda - ps19
Mandibulata - ps20
Pancrustacea - ps21
Insecta - ps22
Eumetabola - ps23
Holometabola/Psocodea - ps24
Holometabola - ps25
Aparaglossata - ps26
Mecopterida - ps27
Diptera - ps28
Bibiono./Cyclorrhapha - ps29
Cyclorrhapha - ps30
Calyptratae/Drosophila - ps31
Drosophila - ps32
Sophophora - ps33
Obscura/Melanogaster_group - ps34
D_melanogaster_group - ps35
D_melanogaster_subgroup - ps36
sim./sech./mel. group - ps37
Drosophila_melanogaster - ps38
p-value
3e-15
6e-12
1e-08
3e-05
6e-02
Log-odds
0.5
1.0
1.5
2.0
2.5
3.0
3.5
4.0

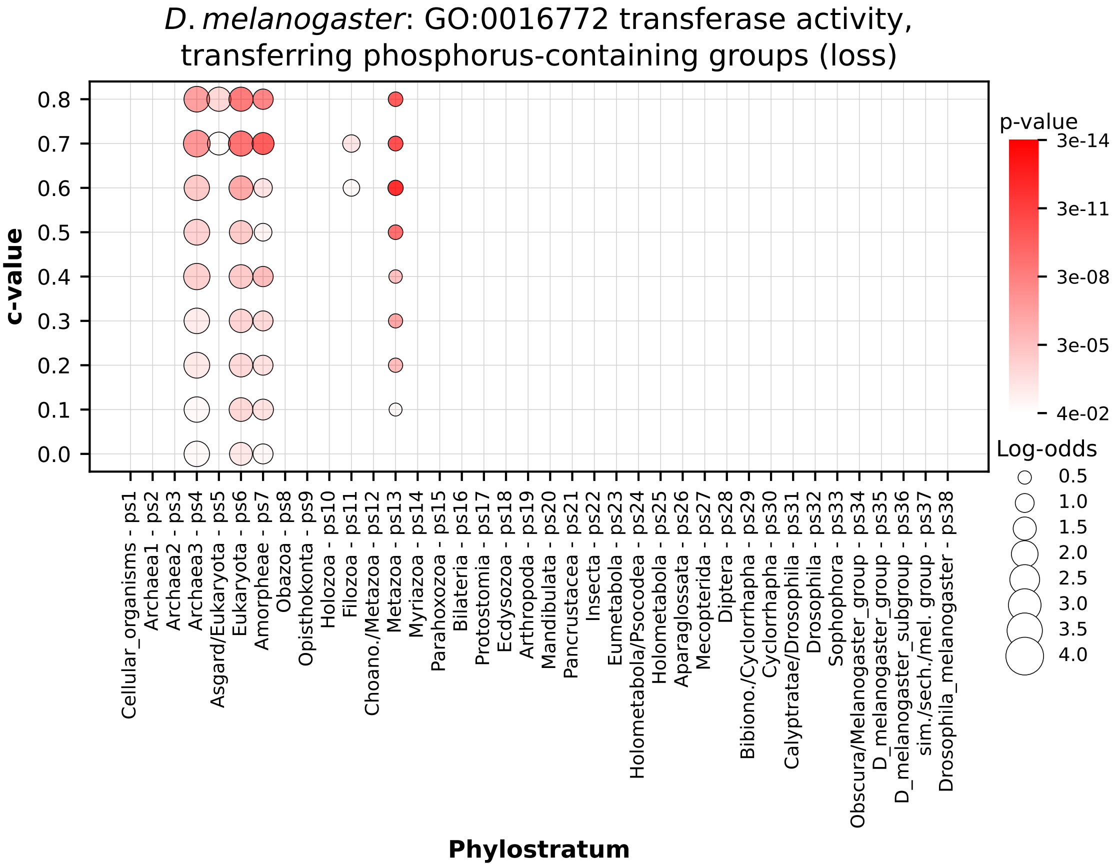
D. melanogaster: GO:0016772 transferase activity, transferring phosphorus-containing groups (loss)
c-value
0.0
0.1
0.2
0.3
0.4
0.5
0.6
0.7
0.8
Phylostratum
Cellular_organisms - ps1
Archaea1 - ps2
Archaea2 - ps3
Archaea3 - ps4
Asgard/Eukaryota - ps5
Eukaryota - ps6
Amorpheae - ps7
Obazoa - ps8
Opisthokonta - ps9
Holozoa - ps10
Filozoa - ps11
Choano./Metazoa - ps12
Metazoa - ps13
Myriazoa - ps14
Parahoxozoa - ps15
Bilateria - ps16
Protostomia - ps17
Ecdysozoa - ps18
Arthropoda - ps19
Mandibulata - ps20
Pancrustacea - ps21
Insecta - ps22
Eumetabola - ps23
Holometabola/Psocodea - ps24
Holometabola - ps25
Aparaglossata - ps26
Mecopterida - ps27
Diptera - ps28
Bibiono./Cyclorrhapha - ps29
Cyclorrhapha - ps30
Calyptratae/Drosophila - ps31
Drosophila - ps32
Sophophora - ps33
Obscura/Melanogaster_group - ps34
D_melanogaster_group - ps35
D_melanogaster_subgroup - ps36
sim./sech./mel. group - ps37
Drosophila_melanogaster - ps38
p-value
3e-14
3e-11
3e-08
3e-05
4e-02
Log-odds
0.5
1.0
1.5
2.0
2.5
3.0
3.5
4.0

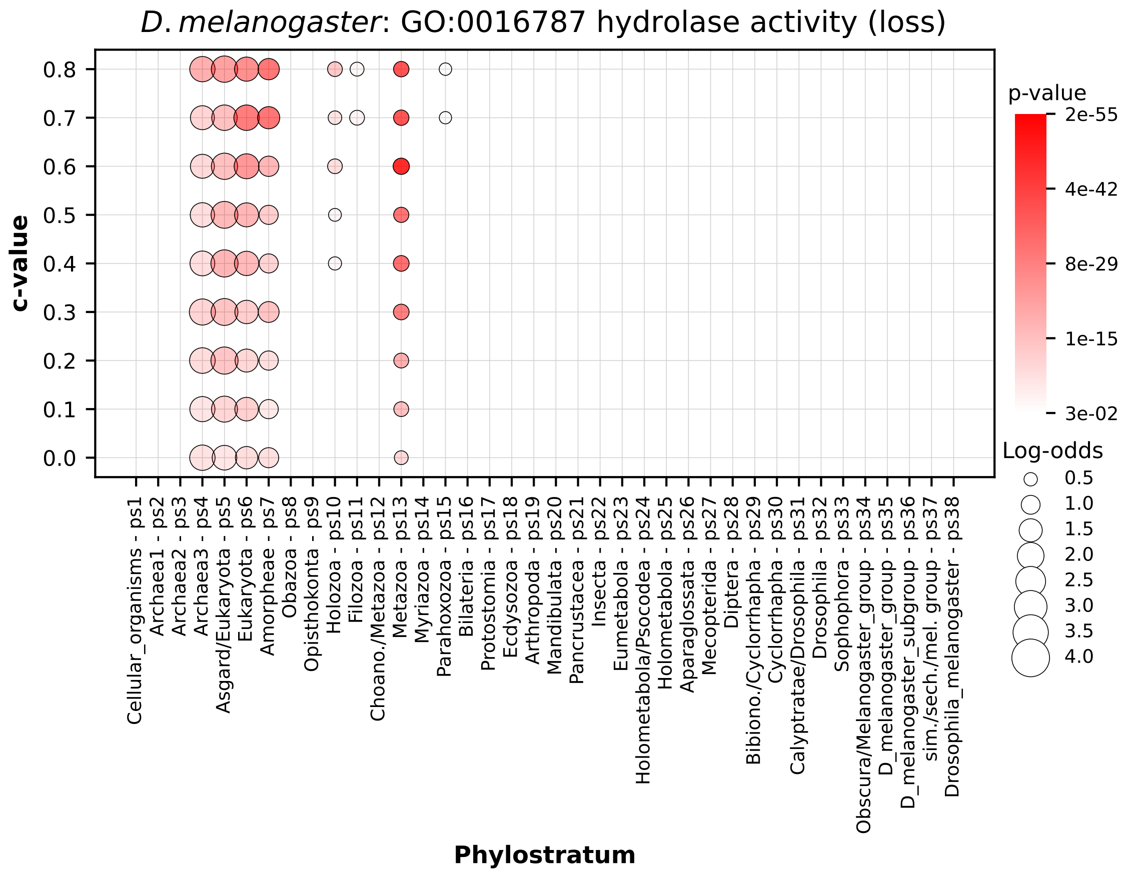

D. melanogaster: GO:0016787 hydrolase activity (loss)
c-value
0.8
0.7
0.6
0.5
0.4
0.3
0.2
0.1
0.0
p-value
2e-55
4e-42
8e-29
1e-15
3e-02
Log-odds
0.5
1.0
1.5
2.0
2.5
3.0
3.5
4.0
Cellular_organisms - ps1
Archaea1 - ps2
Archaea2 - ps3
Archaea3 - ps4
Asgard/Eukaryota - ps5
Eukaryota - ps6
Amorpheae - ps7
Obazoa - ps8
Opisthokonta - ps9
Holozoa - ps10
Filozoa - ps11
Choano./Metazoa - ps12
Metazoa - ps13
Myriazoa - ps14
Parahoxozoa - ps15
Bilateria - ps16
Protostomia - ps17
Ecdysozoa - ps18
Arthropoda - ps19
Mandibulata - ps20
Pancrustacea - ps21
Insecta - ps22
Eumetabola - ps23
Holometabola/Psocodea - ps24
Holometabola - ps25
Aparaglossata - ps26
Mecopterida - ps27
Diptera - ps28
Bibiono./Cyclorrhapha - ps29
Cyclorrhapha - ps30
Calyptratae/Drosophila - ps31
Drosophila - ps32
Sophophora - ps33
Obscura/Melanogaster_group - ps34
D_melanogaster_group - ps35
D_melanogaster_subgroup - ps36
sim./sech./mel. group - ps37
Drosophila_melanogaster - ps38
Phylostratum

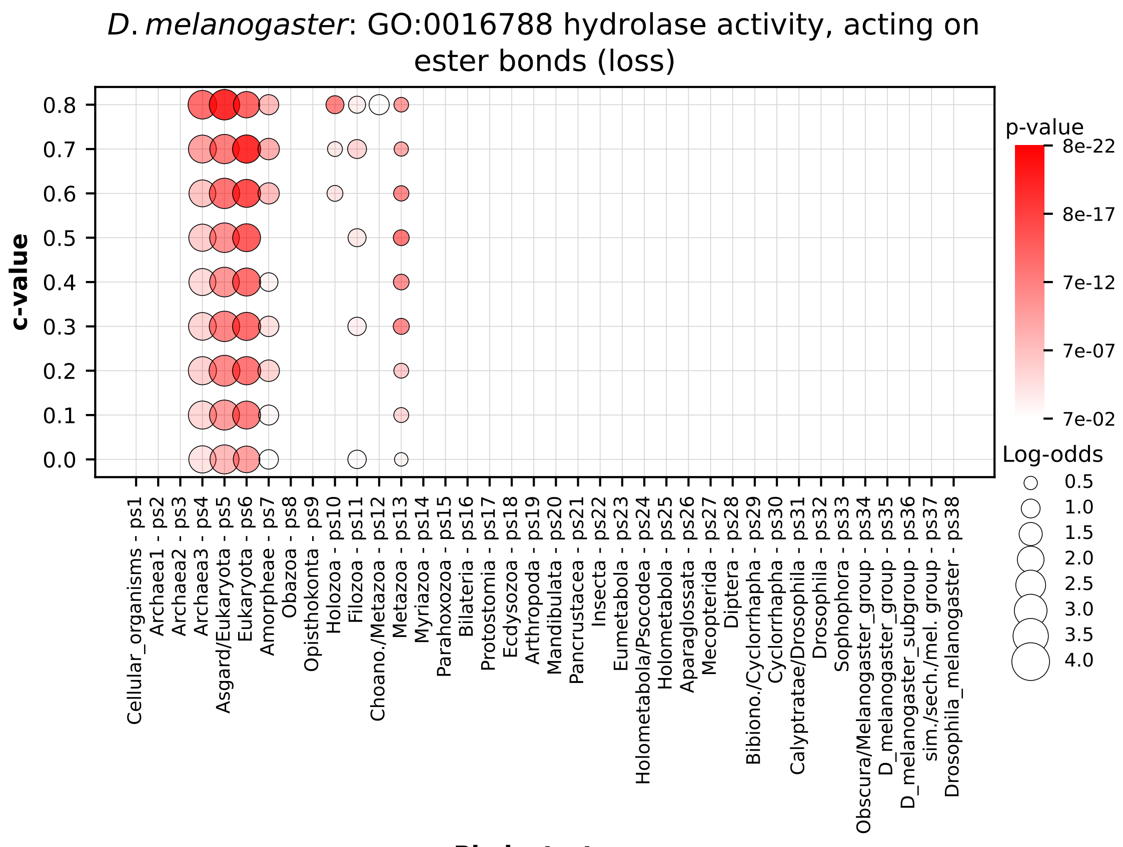

D. melanogaster: GO:0016788 hydrolase activity, acting on ester bonds (loss)
c-value
0.8
0.7
0.6
0.5
0.4
0.3
0.2
0.1
0.0
p-value
8e-22
8e-17
7e-12
7e-07
7e-02
Log-odds
0.5
1.0
1.5
2.0
2.5
3.0
3.5
4.0
Cellular_organisms - ps1
Archaea1 - ps2
Archaea2 - ps3
Archaea3 - ps4
Asgard/Eukaryota - ps5
Eukaryota - ps6
Amorpheae - ps7
Obazoa - ps8
Opisthokonta - ps9
Holozoa - ps10
Filozoa - ps11
Choano./Metazoa - ps12
Metazoa - ps13
Myriazoa - ps14
Parahoxozoa - ps15
Bilateria - ps16
Protostomia - ps17
Ecdysozoa - ps18
Arthropoda - ps19
Mandibulata - ps20
Pancrustacea - ps21
Insecta - ps22
Eumetabola - ps23
Holometabola/Psocodea - ps24
Holometabola - ps25
Aparaglossata - ps26
Mecopterida - ps27
Diptera - ps28
Bibiono./Cyclorrhapha - ps29
Cyclorrhapha - ps30
Calyptratae/Drosophila - ps31
Drosophila - ps32
Sophophora - ps33
Obscura/Melanogaster_group - ps34
D_melanogaster_group - ps35
D_melanogaster_subgroup - ps36
sim./sech./mel. group - ps37
Drosophila_melanogaster - ps38
Phylostratum

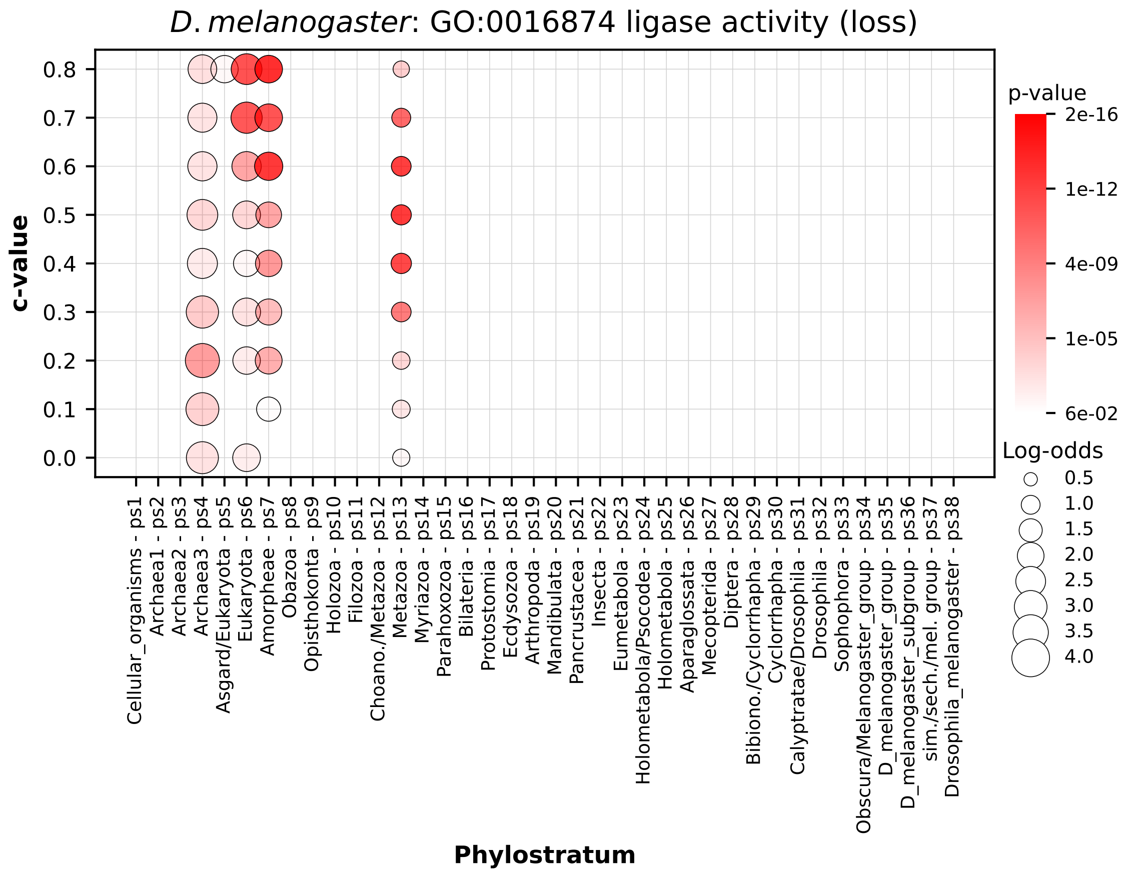

D. melanogaster: GO:0016874 ligase activity (loss)
c-value
0.0
0.1
0.2
0.3
0.4
0.5
0.6
0.7
0.8
Phylostratum
Cellular_organisms - ps1
Archaea1 - ps2
Archaea2 - ps3
Archaea3 - ps4
Asgard/Eukaryota - ps5
Eukaryota - ps6
Amorpheae - ps7
Obazoa - ps8
Opisthokonta - ps9
Holozoa - ps10
Filozoa - ps11
Choano./Metazoa - ps12
Metazoa - ps13
Myriazoa - ps14
Parahoxozoa - ps15
Bilateria - ps16
Protostomia - ps17
Ecdysozoa - ps18
Arthropoda - ps19
Mandibulata - ps20
Pancrustacea - ps21
Insecta - ps22
Eumetabola - ps23
Holometabola/Psocodea - ps24
Holometabola - ps25
Aparaglossata - ps26
Mecopterida - ps27
Diptera - ps28
Bibiono./Cyclorrhapha - ps29
Cyclorrhapha - ps30
Calyptratae/Drosophila - ps31
Drosophila - ps32
Sophophora - ps33
Obscura/Melanogaster_group - ps34
D_melanogaster_group - ps35
D_melanogaster_subgroup - ps36
sim./sech./mel. group - ps37
Drosophila_melanogaster - ps38
p-value
2e-16
1e-12
4e-09
1e-05
6e-02
Log-odds
0.5
1.0
1.5
2.0
2.5
3.0
3.5
4.0

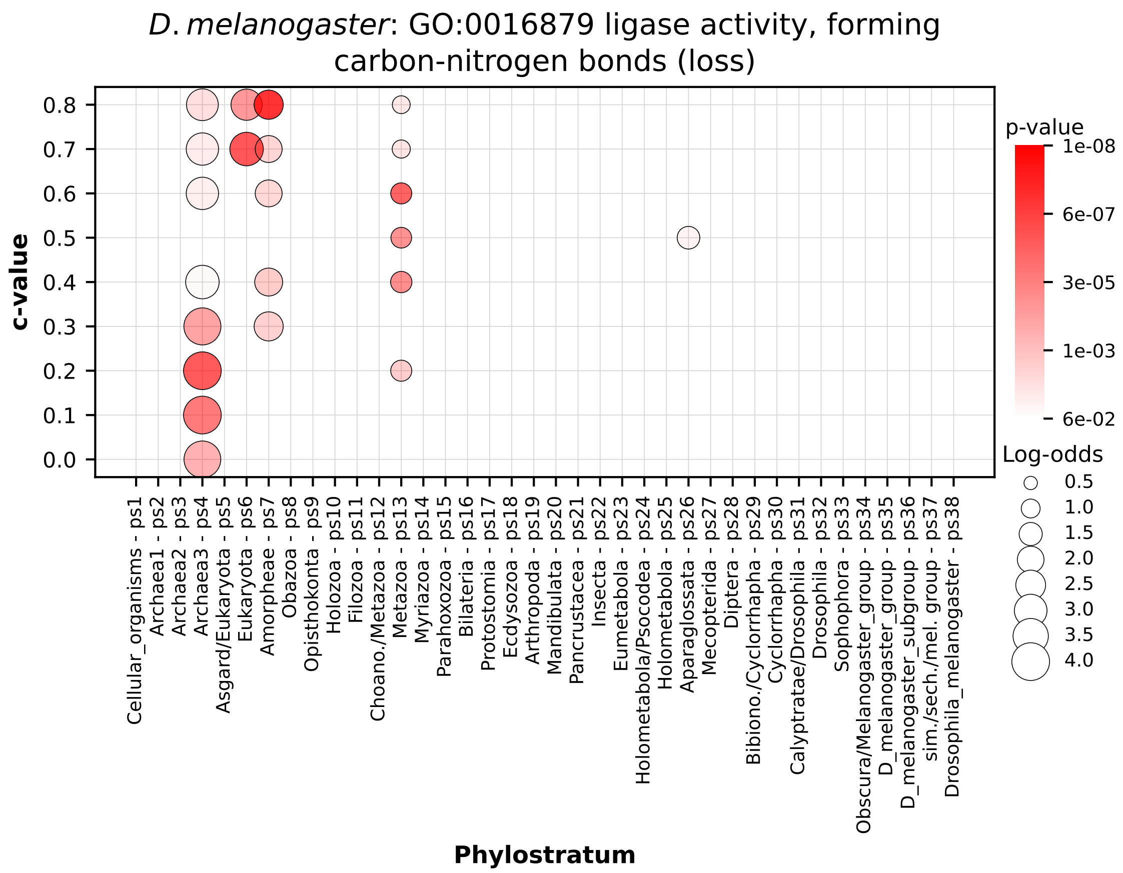

D. melanogaster: GO:0016879 ligase activity, forming carbon-nitrogen bonds (loss)
c-value
0.8
0.7
0.6
0.5
0.4
0.3
0.2
0.1
0.0
p-value
1e-08
6e-07
3e-05
1e-03
6e-02
Log-odds
0.5
1.0
1.5
2.0
2.5
3.0
3.5
4.0
Cellular_organisms - ps1
Archaea1 - ps2
Archaea2 - ps3
Archaea3 - ps4
Asgard/Eukaryota - ps5
Eukaryota - ps6
Amorpheae - ps7
Obazoa - ps8
Opisthokonta - ps9
Holozoa - ps10
Filozoa - ps11
Choano./Metazoa - ps12
Metazoa - ps13
Myriazoa - ps14
Parahoxozoa - ps15
Bilateria - ps16
Protostomia - ps17
Ecdysozoa - ps18
Arthropoda - ps19
Mandibulata - ps20
Pancrustacea - ps21
Insecta - ps22
Eumetabola - ps23
Holometabola/Psocodea - ps24
Holometabola - ps25
Aparaglossata - ps26
Mecopterida - ps27
Diptera - ps28
Bibiono./Cyclorrhapha - ps29
Cyclorrhapha - ps30
Calyptratae/Drosophila - ps31
Drosophila - ps32
Sophophora - ps33
Obscura/Melanogaster_group - ps34
D_melanogaster_group - ps35
D_melanogaster_subgroup - ps36
sim./sech./mel. group - ps37
Drosophila_melanogaster - ps38
Phylostratum

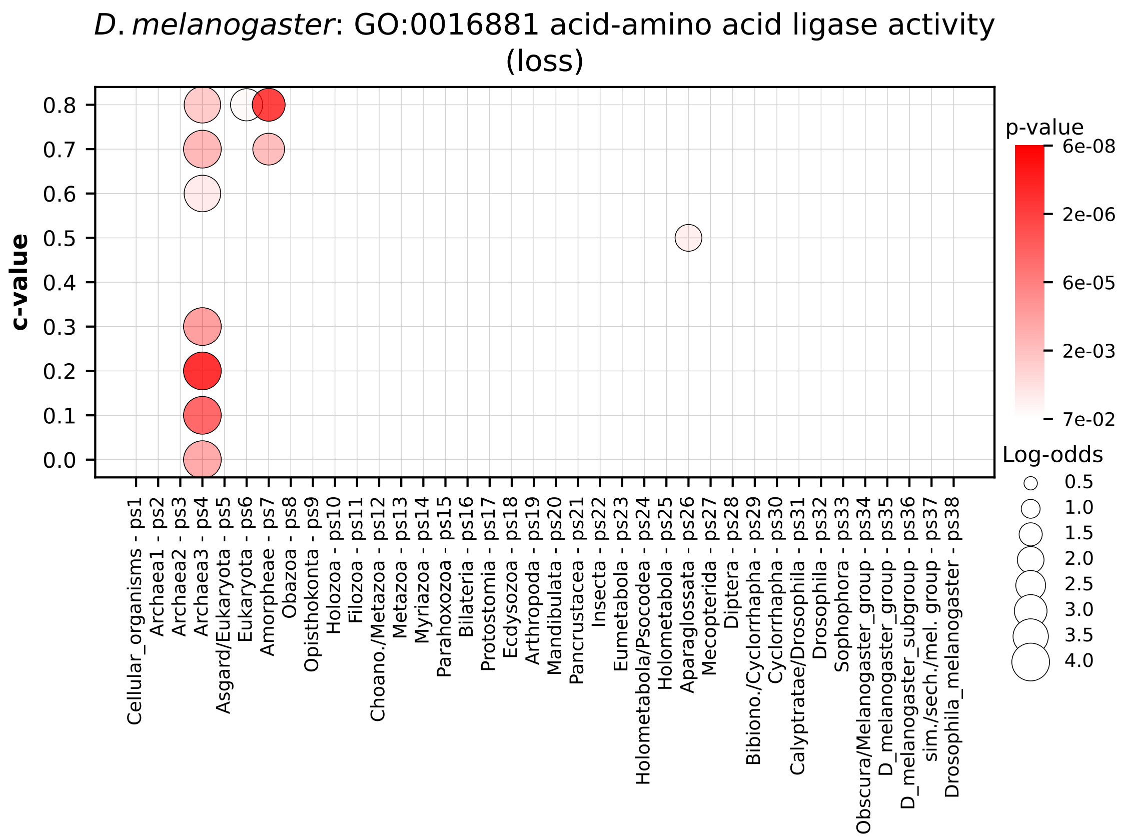
D. melanogaster: GO:0016881 acid-amino acid ligase activity (loss)
c-value
Phylostratum
p-value
6e-08
2e-06
6e-05
2e-03
7e-02
Log-odds
0.5
1.0
1.5
2.0
2.5
3.0
3.5
4.0
Cellular_organisms - ps1
Archaea1 - ps2
Archaea2 - ps3
Archaea3 - ps4
Asgard/Eukaryota - ps5
Eukaryota - ps6
Amorpheae - ps7
Obazoa - ps8
Opisthokonta - ps9
Holozoa - ps10
Filozoa - ps11
Choano./Metazoa - ps12
Metazoa - ps13
Myriazoa - ps14
Parahoxozoa - ps15
Bilateria - ps16
Protostomia - ps17
Ecdysozoa - ps18
Arthropoda - ps19
Mandibulata - ps20
Pancrustacea - ps21
Insecta - ps22
Eumetabola - ps23
Holometabola/Psocodea - ps24
Holometabola - ps25
Aparaglossata - ps26
Mecopterida - ps27
Diptera - ps28
Bibiono./Cyclorrhapha - ps29
Cyclorrhapha - ps30
Calyptratae/Drosophila - ps31
Drosophila - ps32
Sophophora - ps33
Obscura/Melanogaster_group - ps34
D_melanogaster_group - ps35
D_melanogaster_subgroup - ps36
sim./sech./mel. group - ps37
Drosophila_melanogaster - ps38

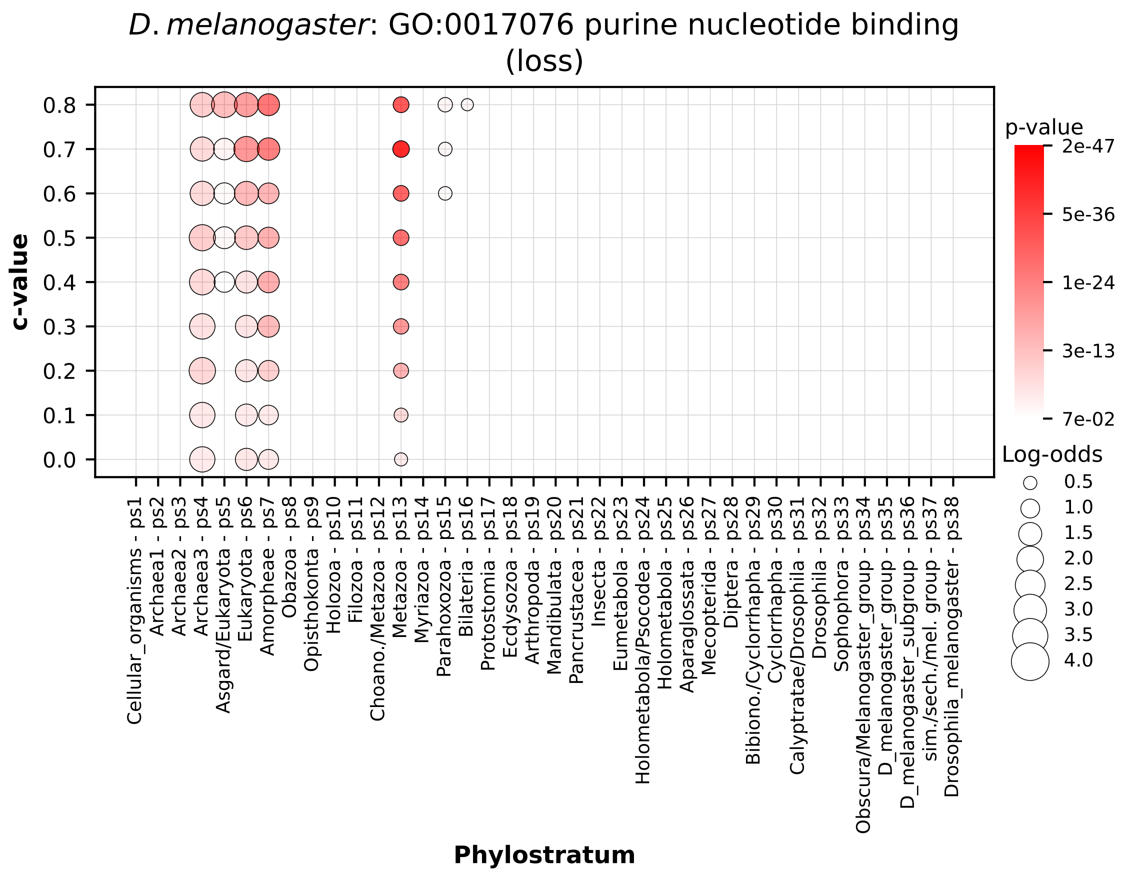

D. melanogaster: GO:0017076 purine nucleotide binding (loss)
c-value
0.8
0.7
0.6
0.5
0.4
0.3
0.2
0.1
0.0
p-value
2e-47
5e-36
1e-24
3e-13
7e-02
Log-odds
0.5
1.0
1.5
2.0
2.5
3.0
3.5
4.0
Cellular_organisms - ps1
Archaea1 - ps2
Archaea2 - ps3
Archaea3 - ps4
Asgard/Eukaryota - ps5
Eukaryota - ps6
Amorpheae - ps7
Obazoa - ps8
Opisthokonta - ps9
Holozoa - ps10
Filozoa - ps11
Choano./Metazoa - ps12
Metazoa - ps13
Myriazoa - ps14
Parahoxozoa - ps15
Bilateria - ps16
Protostomia - ps17
Ecdysozoa - ps18
Arthropoda - ps19
Mandibulata - ps20
Pancrustacea - ps21
Insecta - ps22
Eumetabola - ps23
Holometabola/Psocodea - ps24
Holometabola - ps25
Aparaglossata - ps26
Mecopterida - ps27
Diptera - ps28
Bibiono./Cyclorrhapha - ps29
Cyclorrhapha - ps30
Calyptratae/Drosophila - ps31
Drosophila - ps32
Sophophora - ps33
Obscura/Melanogaster_group - ps34
D_melanogaster_group - ps35
D_melanogaster_subgroup - ps36
sim./sech./mel. group - ps37
Drosophila_melanogaster - ps38
Phylostratum

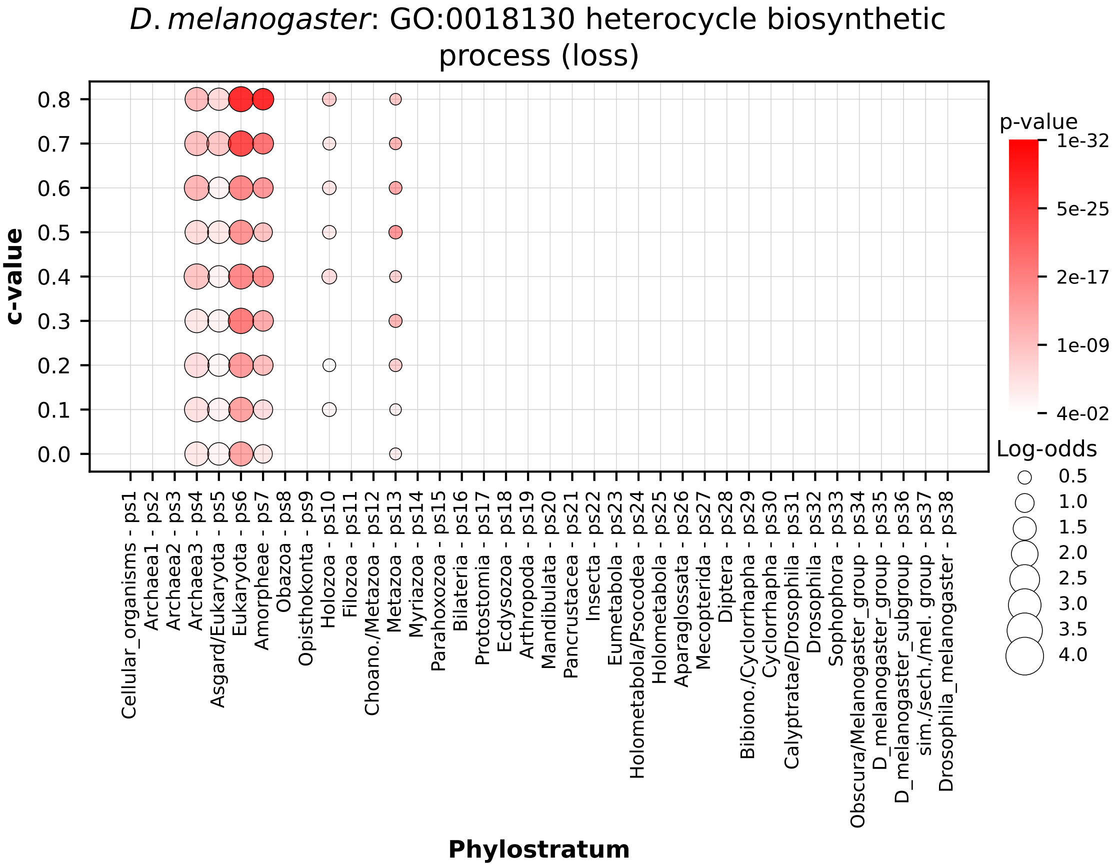

D. melanogaster: GO:0018130 heterocycle biosynthetic process (loss)
c-value
0.8
0.7
0.6
0.5
0.4
0.3
0.2
0.1
0.0
Phylostratum
Cellular_organisms - ps1
Archaea1 - ps2
Archaea2 - ps3
Archaea3 - ps4
Asgard/Eukaryota - ps5
Eukaryota - ps6
Amorpheae - ps7
Obazoa - ps8
Opisthokonta - ps9
Holozoa - ps10
Filozoa - ps11
Choano./Metazoa - ps12
Metazoa - ps13
Myriazoa - ps14
Parahoxozoa - ps15
Bilateria - ps16
Protostomia - ps17
Ecdysozoa - ps18
Arthropoda - ps19
Mandibulata - ps20
Pancrustacea - ps21
Insecta - ps22
Eumetabola - ps23
Holometabola/Psocodea - ps24
Holometabola - ps25
Aparaglossata - ps26
Mecopterida - ps27
Diptera - ps28
Bibiono./Cyclorrhapha - ps29
Cyclorrhapha - ps30
Calyptratae/Drosophila - ps31
Drosophila - ps32
Sophophora - ps33
Obscura/Melanogaster_group - ps34
D_melanogaster_group - ps35
D_melanogaster_subgroup - ps36
sim./sech./mel. group - ps37
Drosophila_melanogaster - ps38
p-value
1e-32
5e-25
2e-17
1e-09
4e-02
Log-odds
0.5
1.0
1.5
2.0
2.5
3.0
3.5
4.0

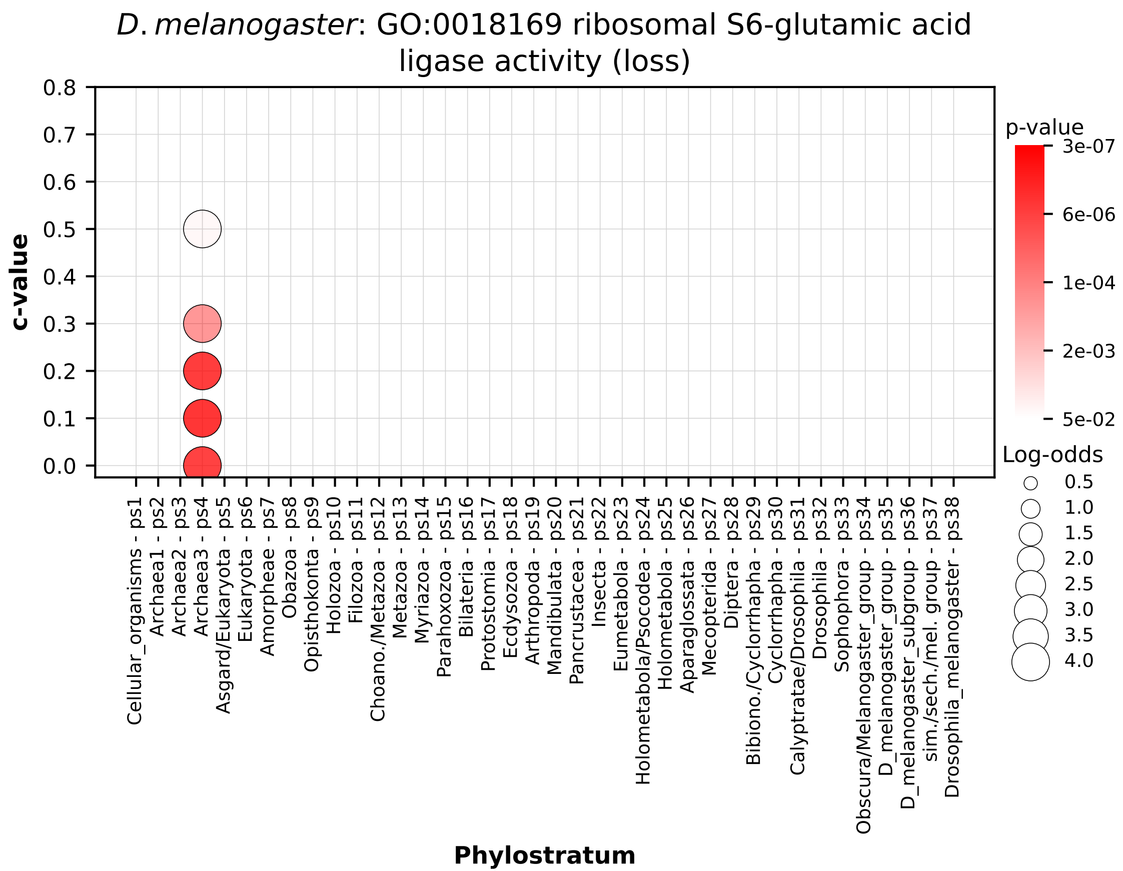

D. melanogaster: GO:0018169 ribosomal S6-glutamic acid ligase activity (loss)
c-value
Phylostratum
p-value
3e-07
6e-06
1e-04
2e-03
5e-02
Log-odds
0.5
1.0
1.5
2.0
2.5
3.0
3.5
4.0
Cellular_organisms - ps1
Archaea1 - ps2
Archaea2 - ps3
Archaea3 - ps4
Asgard/Eukaryota - ps5
Eukaryota - ps6
Amorpheae - ps7
Obazoa - ps8
Opisthokonta - ps9
Holozoa - ps10
Filozoa - ps11
Choano./Metazoa - ps12
Metazoa - ps13
Myriazoa - ps14
Parahoxozoa - ps15
Bilateria - ps16
Protostomia - ps17
Ecdysozoa - ps18
Arthropoda - ps19
Mandibulata - ps20
Pancrustacea - ps21
Insecta - ps22
Eumetabola - ps23
Holometabola/Psocodea - ps24
Holometabola - ps25
Aparaglossata - ps26
Mecopterida - ps27
Diptera - ps28
Bibiono./Cyclorrhapha - ps29
Cyclorrhapha - ps30
Calyptratae/Drosophila - ps31
Drosophila - ps32
Sophophora - ps33
Obscura/Melanogaster_group - ps34
D_melanogaster_group - ps35
D_melanogaster_subgroup - ps36
sim./sech./mel. group - ps37
Drosophila_melanogaster - ps38

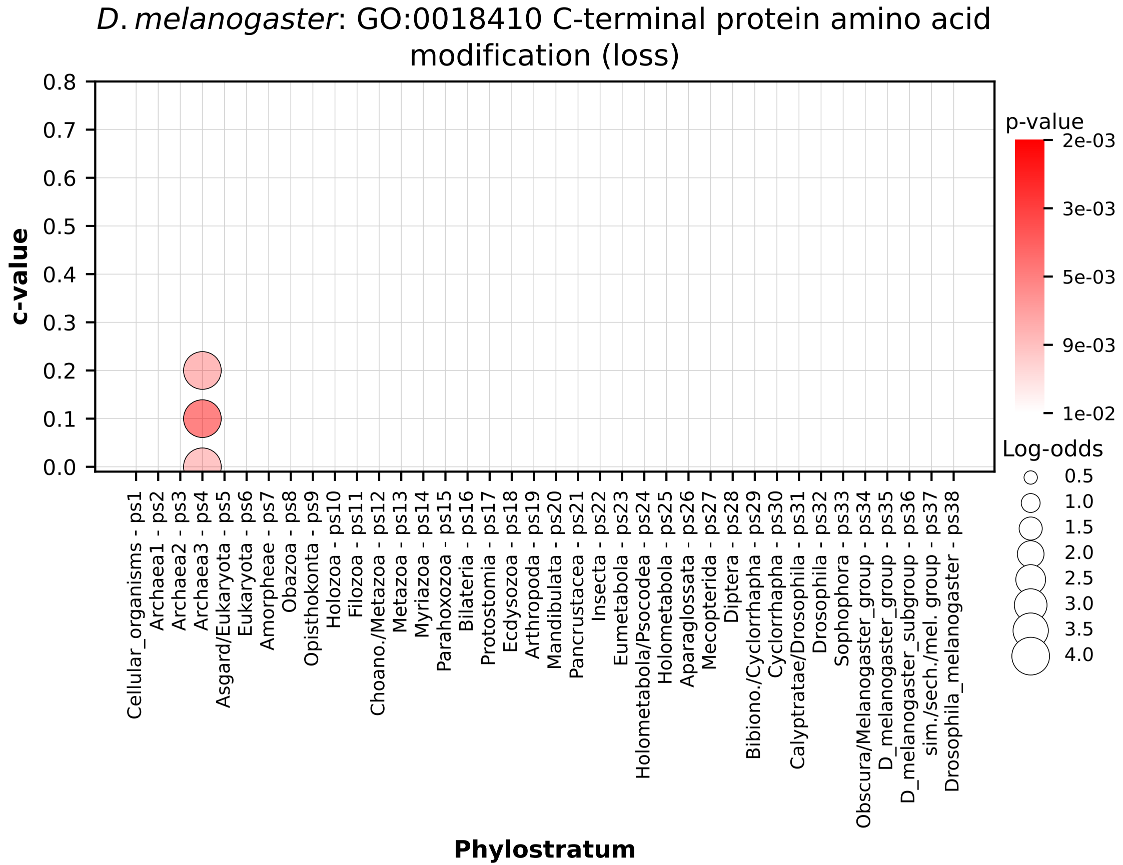

D. melanogaster: GO:0018410 C-terminal protein amino acid modification (loss)
c-value
0.8
0.7
0.6
0.5
0.4
0.3
0.2
0.1
0.0
p-value
2e-03
3e-03
5e-03
9e-03
1e-02
Log-odds
0.5
1.0
1.5
2.0
2.5
3.0
3.5
4.0
Cellular_organisms - ps1
Archaea1 - ps2
Archaea2 - ps3
Archaea3 - ps4
Asgard/Eukaryota - ps5
Eukaryota - ps6
Amorpheae - ps7
Obazoa - ps8
Opisthokonta - ps9
Holozoa - ps10
Filozoa - ps11
Choano./Metazoa - ps12
Metazoa - ps13
Myriazoa - ps14
Parahoxozoa - ps15
Bilateria - ps16
Protostomia - ps17
Ecdysozoa - ps18
Arthropoda - ps19
Mandibulata - ps20
Pancrustacea - ps21
Insecta - ps22
Eumetabola - ps23
Holometabola/Psocodea - ps24
Holometabola - ps25
Aparaglossata - ps26
Mecopterida - ps27
Diptera - ps28
Bibiono./Cyclorrhapha - ps29
Cyclorrhapha - ps30
Calyptratae/Drosophila - ps31
Drosophila - ps32
Sophophora - ps33
Obscura/Melanogaster_group - ps34
D_melanogaster_group - ps35
D_melanogaster_subgroup - ps36
sim./sech./mel. group - ps37
Drosophila_melanogaster - ps38
Phylostratum

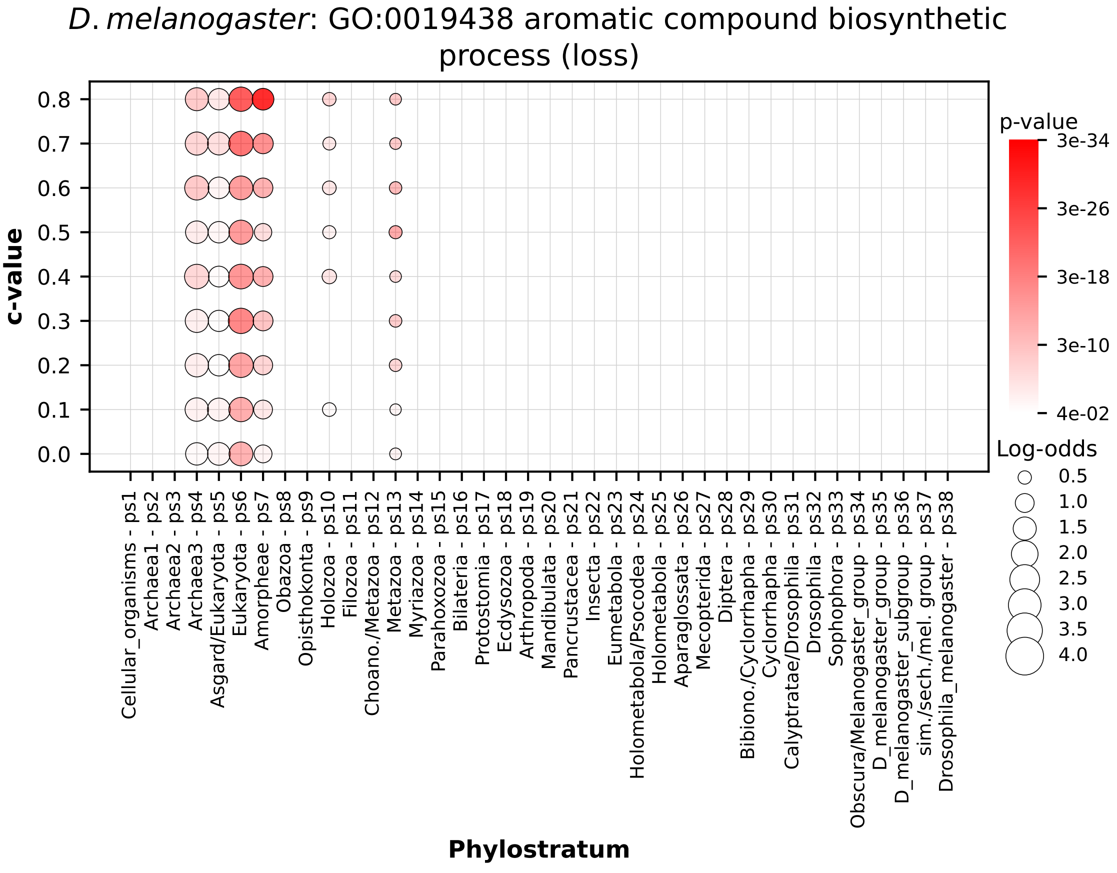

D. melanogaster: GO:0019438 aromatic compound biosynthetic process (loss)
c-value
Phylostratum
p-value
3e-34
3e-26
3e-18
3e-10
4e-02
Log-odds
0.5
1.0
1.5
2.0
2.5
3.0
3.5
4.0
Cellular_organisms - ps1
Archaea1 - ps2
Archaea2 - ps3
Archaea3 - ps4
Asgard/Eukaryota - ps5
Eukaryota - ps6
Amorpheae - ps7
Obazoa - ps8
Opisthokonta - ps9
Holozoa - ps10
Filozoa - ps11
Choano./Metazoa - ps12
Metazoa - ps13
Myriazoa - ps14
Parahoxozoa - ps15
Bilateria - ps16
Protostomia - ps17
Ecdysozoa - ps18
Arthropoda - ps19
Mandibulata - ps20
Pancrustacea - ps21
Insecta - ps22
Eumetabola - ps23
Holometabola/Psocodea - ps24
Holometabola - ps25
Aparaglossata - ps26
Mecopterida - ps27
Diptera - ps28
Bibiono./Cyclorrhapha - ps29
Cyclorrhapha - ps30
Calyptratae/Drosophila - ps31
Drosophila - ps32
Sophophora - ps33
Obscura/Melanogaster_group - ps34
D_melanogaster_group - ps35
D_melanogaster_subgroup - ps36
sim./sech./mel. group - ps37
Drosophila_melanogaster - ps38

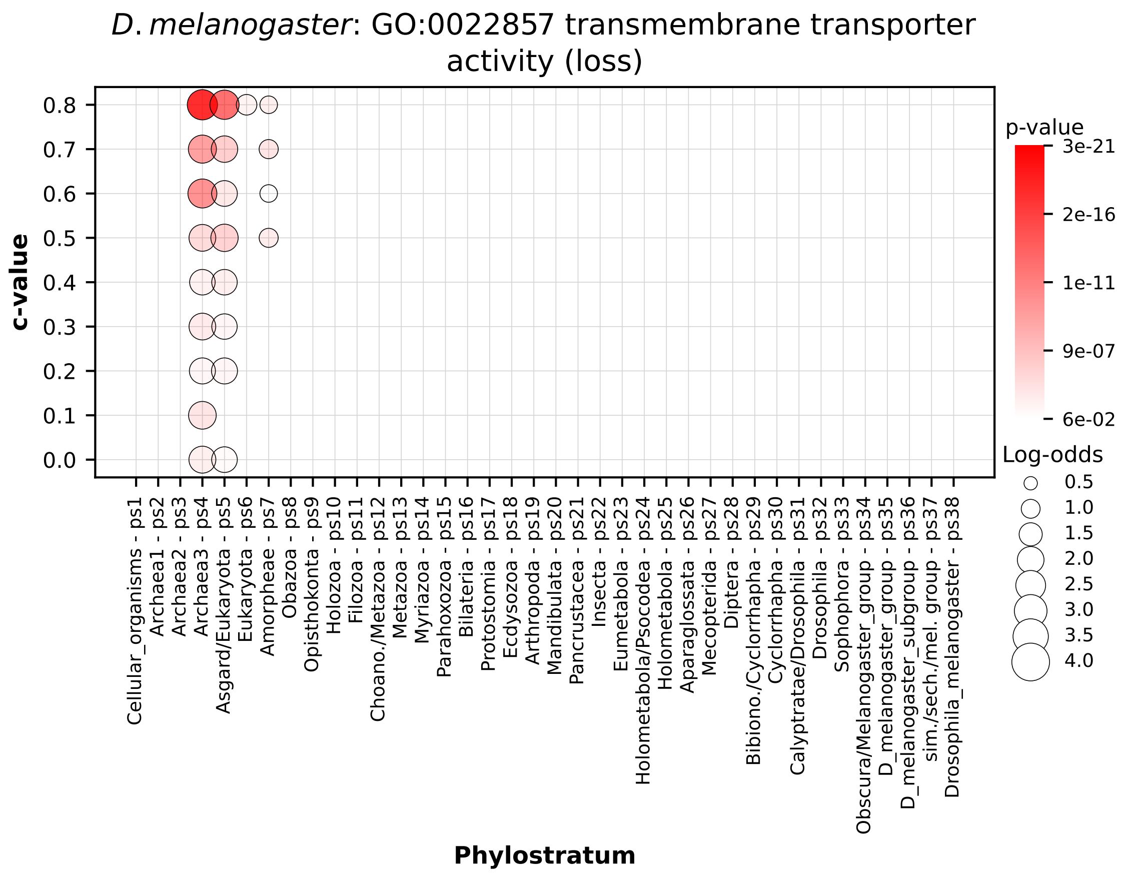

D. melanogaster: GO:0022857 transmembrane transporter activity (loss)
c-value
0.8
0.7
0.6
0.5
0.4
0.3
0.2
0.1
0.0
Phylostratum
Cellular_organisms - ps1
Archaea1 - ps2
Archaea2 - ps3
Archaea3 - ps4
Asgard/Eukaryota - ps5
Eukaryota - ps6
Amorpheae - ps7
Obazoa - ps8
Opisthokonta - ps9
Holozoa - ps10
Filozoa - ps11
Choano./Metazoa - ps12
Metazoa - ps13
Myriazoa - ps14
Parahoxozoa - ps15
Bilateria - ps16
Protostomia - ps17
Ecdysozoa - ps18
Arthropoda - ps19
Mandibulata - ps20
Pancrustacea - ps21
Insecta - ps22
Eumetabola - ps23
Holometabola/Psocodea - ps24
Holometabola - ps25
Aparaglossata - ps26
Mecopterida - ps27
Diptera - ps28
Bibiono./Cyclorrhapha - ps29
Cyclorrhapha - ps30
Calyptratae/Drosophila - ps31
Drosophila - ps32
Sophophora - ps33
Obscura/Melanogaster_group - ps34
D_melanogaster_group - ps35
D_melanogaster_subgroup - ps36
sim./sech./mel. group - ps37
Drosophila_melanogaster - ps38
p-value
3e-21
2e-16
1e-11
9e-07
6e-02
Log-odds
0.5
1.0
1.5
2.0
2.5
3.0
3.5
4.0

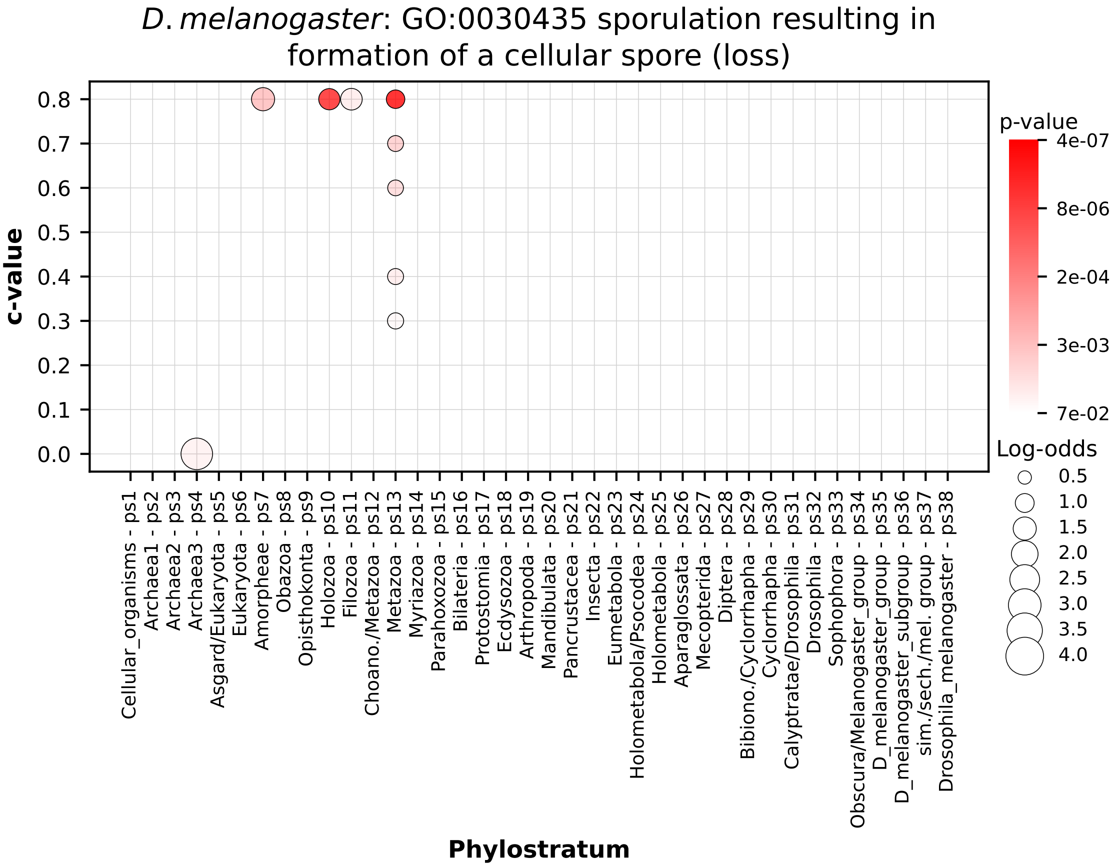

D. melanogaster: GO:0030435 sporulation resulting in formation of a cellular spore (loss)
c-value
0.8
0.7
0.6
0.5
0.4
0.3
0.2
0.1
0.0
p-value
4e-07
8e-06
2e-04
3e-03
7e-02
Log-odds
0.5
1.0
1.5
2.0
2.5
3.0
3.5
4.0
Cellular_organisms - ps1
Archaea1 - ps2
Archaea2 - ps3
Archaea3 - ps4
Asgard/Eukaryota - ps5
Eukaryota - ps6
Amorpheae - ps7
Obazoa - ps8
Opisthokonta - ps9
Holozoa - ps10
Filozoa - ps11
Choano./Metazoa - ps12
Metazoa - ps13
Myriazoa - ps14
Parahoxozoa - ps15
Bilateria - ps16
Protostomia - ps17
Ecdysozoa - ps18
Arthropoda - ps19
Mandibulata - ps20
Pancrustacea - ps21
Insecta - ps22
Eumetabola - ps23
Holometabola/Psocodea - ps24
Holometabola - ps25
Aparaglossata - ps26
Mecopterida - ps27
Diptera - ps28
Bibiono./Cyclorrhapha - ps29
Cyclorrhapha - ps30
Calyptratae/Drosophila - ps31
Drosophila - ps32
Sophophora - ps33
Obscura/Melanogaster_group - ps34
D_melanogaster_group - ps35
D_melanogaster_subgroup - ps36
sim./sech./mel. group - ps37
Drosophila_melanogaster - ps38
Phylostratum

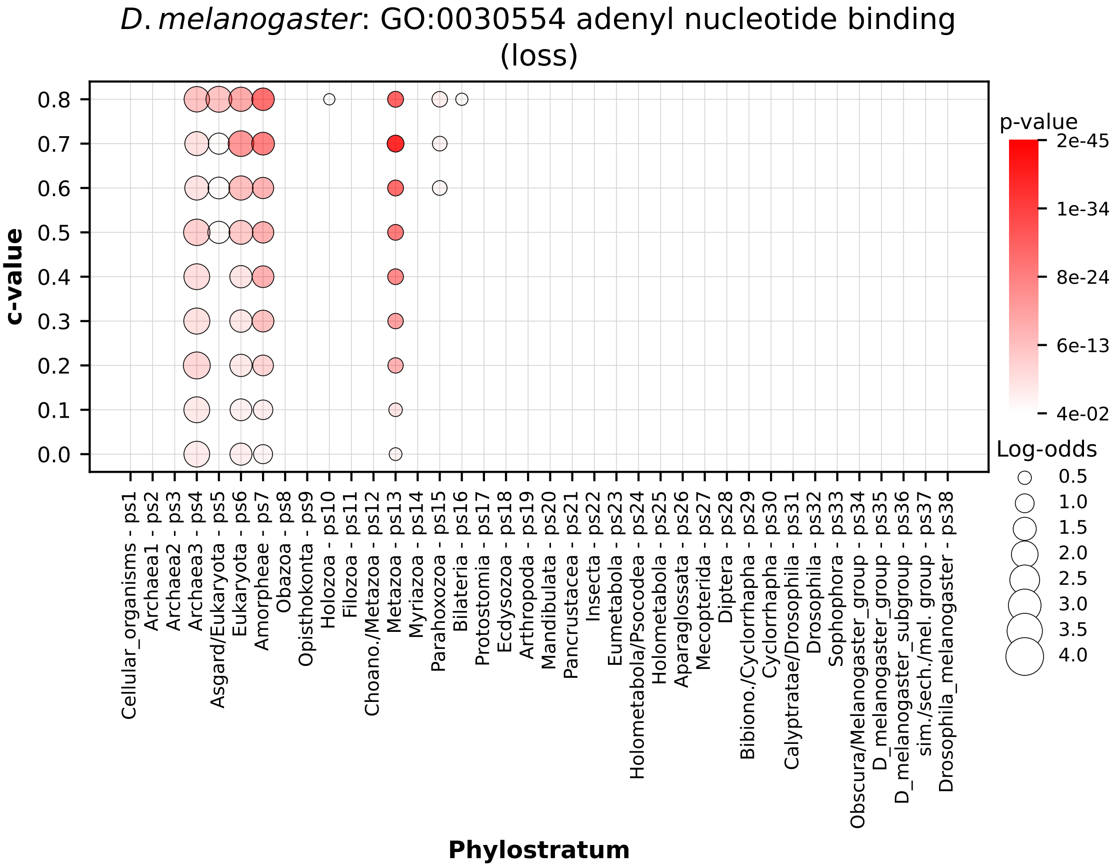

D. melanogaster: GO:0030554 adenyl nucleotide binding
(loss)
c-value
0.8
0.7
0.6
0.5
0.4
0.3
0.2
0.1
0.0
Phylostratum
Cellular_organisms - ps1
Archaea1 - ps2
Archaea2 - ps3
Archaea3 - ps4
Asgard/Eukaryota - ps5
Eukaryota - ps6
Amorpheae - ps7
Obazoa - ps8
Opisthokonta - ps9
Holozoa - ps10
Filozoa - ps11
Choano./Metazoa - ps12
Metazoa - ps13
Myriazoa - ps14
Parahoxozoa - ps15
Bilateria - ps16
Protostomia - ps17
Ecdysozoa - ps18
Arthropoda - ps19
Mandibulata - ps20
Pancrustacea - ps21
Insecta - ps22
Eumetabola - ps23
Holometabola/Psocodea - ps24
Holometabola - ps25
Aparaglossata - ps26
Mecopterida - ps27
Diptera - ps28
Bibiono./Cyclorrhapha - ps29
Cyclorrhapha - ps30
Calyptratae/Drosophila - ps31
Drosophila - ps32
Sophophora - ps33
Obscura/Melanogaster_group - ps34
D_melanogaster_group - ps35
D_melanogaster_subgroup - ps36
sim./sech./mel. group - ps37
Drosophila_melanogaster - ps38
p-value
2e-45
1e-34
8e-24
6e-13
4e-02
Log-odds
0.5
1.0
1.5
2.0
2.5
3.0
3.5
4.0

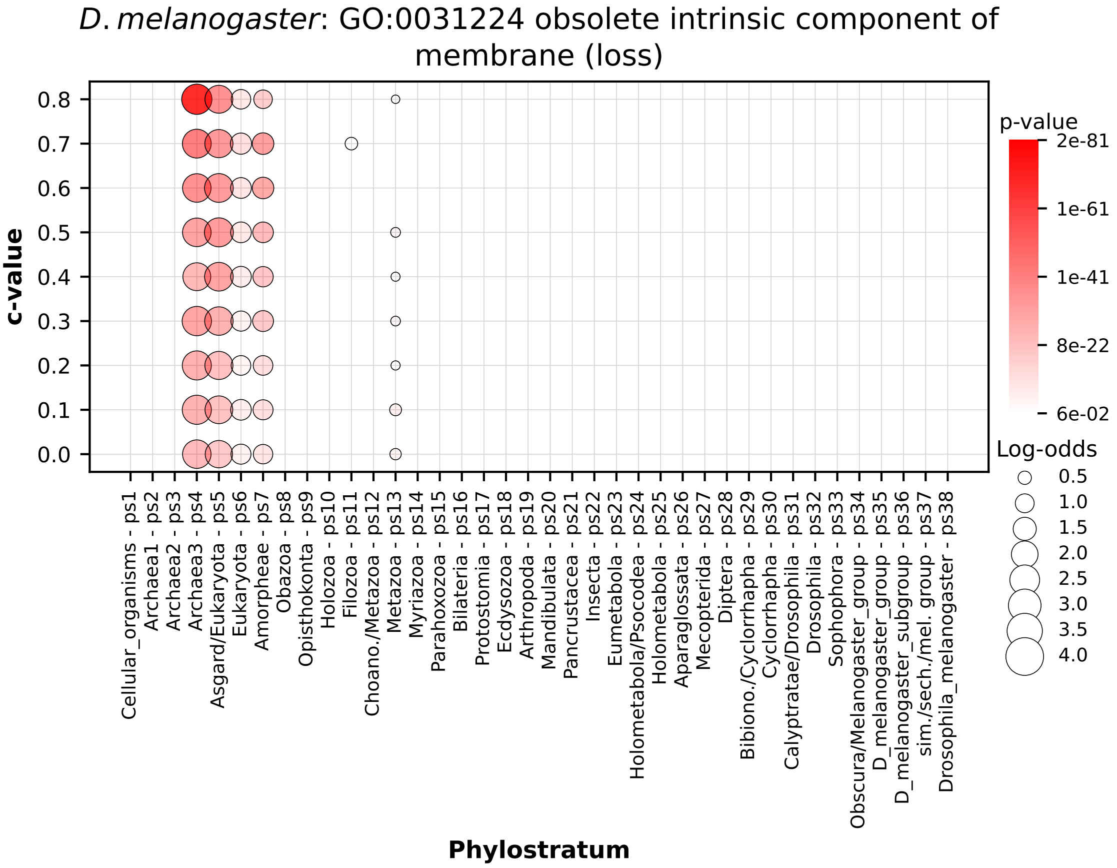

D. melanogaster: GO:0031224 obsolete intrinsic component of membrane (loss)
c-value
0.8
0.7
0.6
0.5
0.4
0.3
0.2
0.1
0.0
Phylostratum
Cellular_organisms - ps1
Archaea1 - ps2
Archaea2 - ps3
Archaea3 - ps4
Asgard/Eukaryota - ps5
Eukaryota - ps6
Amorpheae - ps7
Obazoa - ps8
Opisthokonta - ps9
Holozoa - ps10
Filozoa - ps11
Choano./Metazoa - ps12
Metazoa - ps13
Myriazoa - ps14
Parahoxozoa - ps15
Bilateria - ps16
Protostomia - ps17
Ecdysozoa - ps18
Arthropoda - ps19
Mandibulata - ps20
Pancrustacea - ps21
Insecta - ps22
Eumetabola - ps23
Holometabola/Psocodea - ps24
Holometabola - ps25
Aparaglossata - ps26
Mecopterida - ps27
Diptera - ps28
Bibiono./Cyclorrhapha - ps29
Cyclorrhapha - ps30
Calyptratae/Drosophila - ps31
Drosophila - ps32
Sophophora - ps33
Obscura/Melanogaster_group - ps34
D_melanogaster_group - ps35
D_melanogaster_subgroup - ps36
sim./sech./mel. group - ps37
Drosophila_melanogaster - ps38
p-value
2e-81
1e-61
1e-41
8e-22
6e-02
Log-odds
0.5
1.0
1.5
2.0
2.5
3.0
3.5
4.0

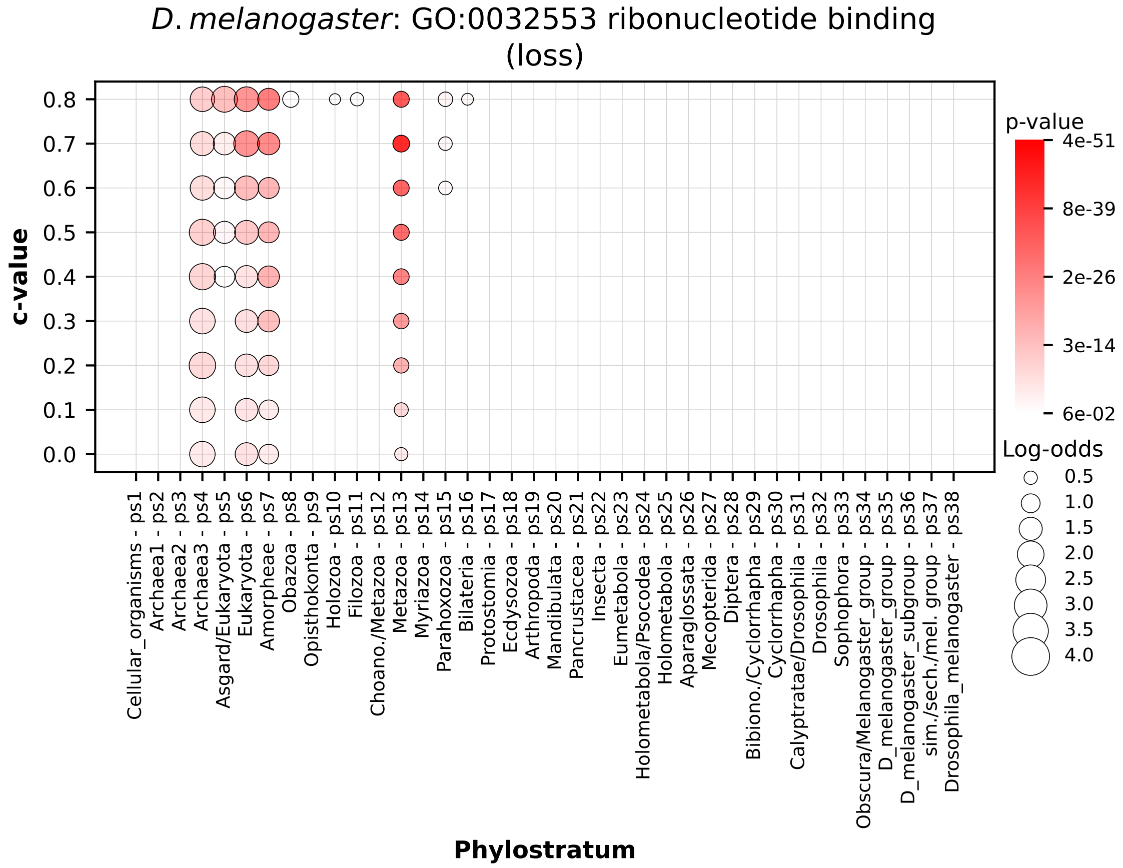

D. melanogaster: GO:0032553 ribonucleotide binding (loss)
c-value
0.8
0.7
0.6
0.5
0.4
0.3
0.2
0.1
0.0
p-value
4e-51
8e-39
2e-26
3e-14
6e-02
Log-odds
0.5
1.0
1.5
2.0
2.5
3.0
3.5
4.0
Cellular_organisms - ps1
Archaea1 - ps2
Archaea2 - ps3
Archaea3 - ps4
Asgard/Eukaryota - ps5
Eukaryota - ps6
Amorpheae - ps7
Obazoa - ps8
Opisthokonta - ps9
Holozoa - ps10
Filozoa - ps11
Choano./Metazoa - ps12
Metazoa - ps13
Myriazoa - ps14
Parahoxozoa - ps15
Bilateria - ps16
Protostomia - ps17
Ecdysozoa - ps18
Arthropoda - ps19
Mandibulata - ps20
Pancrustacea - ps21
Insecta - ps22
Eumetabola - ps23
Holometabola/Psocodea - ps24
Holometabola - ps25
Aparaglossata - ps26
Mecopterida - ps27
Diptera - ps28
Bibiono./Cyclorrhapha - ps29
Cyclorrhapha - ps30
Calyptratae/Drosophila - ps31
Drosophila - ps32
Sophophora - ps33
Obscura/Melanogaster_group - ps34
D_melanogaster_group - ps35
D_melanogaster_subgroup - ps36
sim./sech./mel. group - ps37
Drosophila_melanogaster - ps38
Phylostratum

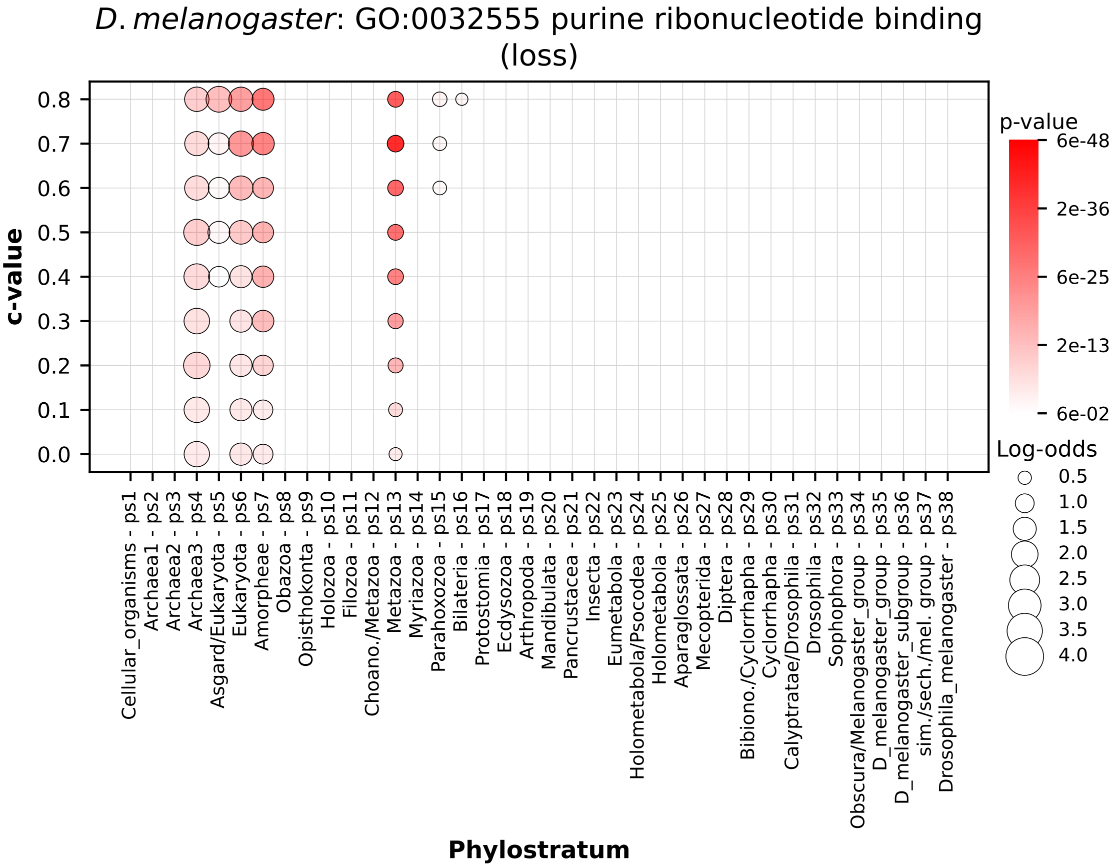

D. melanogaster: GO:0032555 purine ribonucleotide binding (loss)
c-value
0.8
0.7
0.6
0.5
0.4
0.3
0.2
0.1
0.0
Phylostratum
Cellular_organisms - ps1
Archaea1 - ps2
Archaea2 - ps3
Archaea3 - ps4
Asgard/Eukaryota - ps5
Eukaryota - ps6
Amorpheae - ps7
Obazoa - ps8
Opisthokonta - ps9
Holozoa - ps10
Filozoa - ps11
Choano./Metazoa - ps12
Metazoa - ps13
Myriazoa - ps14
Parahoxozoa - ps15
Bilateria - ps16
Protostomia - ps17
Ecdysozoa - ps18
Arthropoda - ps19
Mandibulata - ps20
Pancrustacea - ps21
Insecta - ps22
Eumetabola - ps23
Holometabola/Psocodea - ps24
Holometabola - ps25
Aparaglossata - ps26
Mecopterida - ps27
Diptera - ps28
Bibiono./Cyclorrhapha - ps29
Cyclorrhapha - ps30
Calyptratae/Drosophila - ps31
Drosophila - ps32
Sophophora - ps33
Obscura/Melanogaster_group - ps34
D_melanogaster_group - ps35
D_melanogaster_subgroup - ps36
sim./sech./mel. group - ps37
Drosophila_melanogaster - ps38
p-value
6e-48
2e-36
6e-25
2e-13
6e-02
Log-odds
0.5
1.0
1.5
2.0
2.5
3.0
3.5
4.0

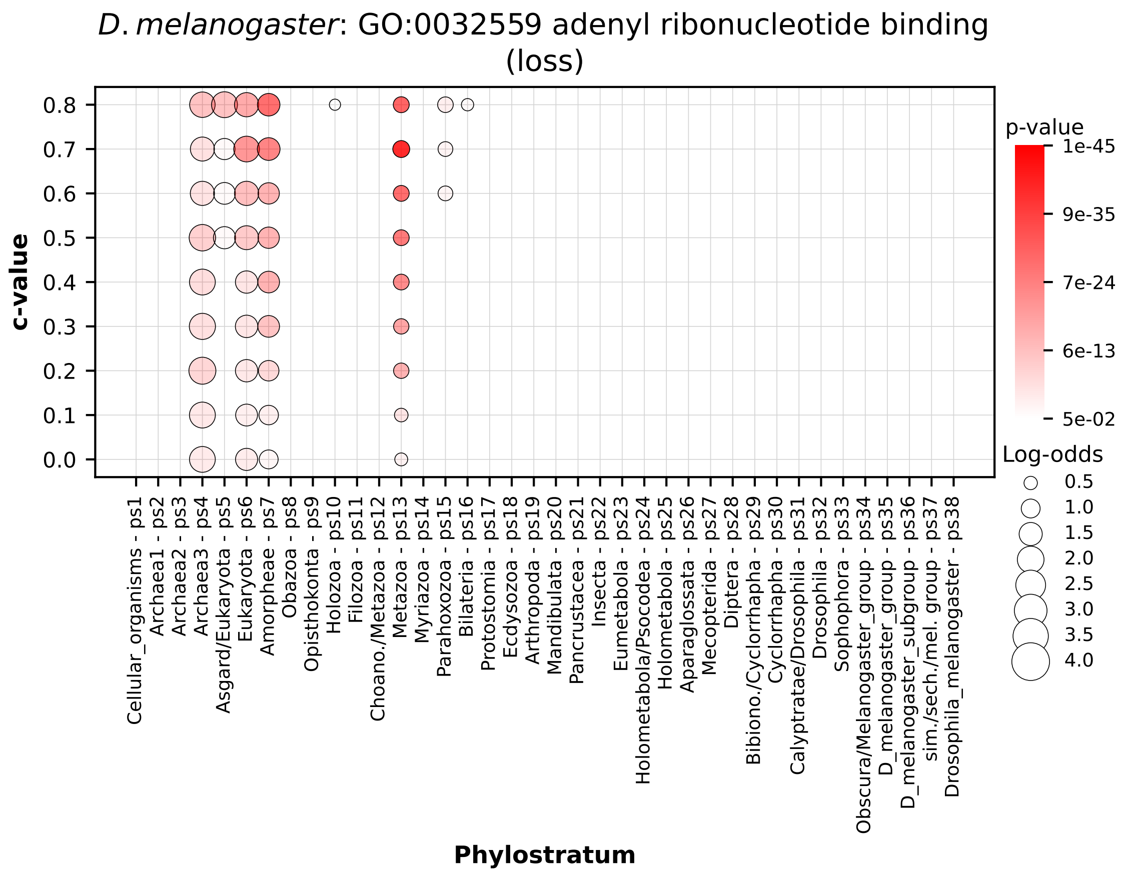

D. melanogaster: GO:0032559 adenyl ribonucleotide binding (loss)
c-value
0.8
0.7
0.6
0.5
0.4
0.3
0.2
0.1
0.0
p-value
1e-45
9e-35
7e-24
6e-13
5e-02
Log-odds
0.5
1.0
1.5
2.0
2.5
3.0
3.5
4.0
Cellular_organisms - ps1
Archaea1 - ps2
Archaea2 - ps3
Archaea3 - ps4
Asgard/Eukaryota - ps5
Eukaryota - ps6
Amorpheae - ps7
Obazoa - ps8
Opisthokonta - ps9
Holozoa - ps10
Filozoa - ps11
Choano./Metazoa - ps12
Metazoa - ps13
Myriazoa - ps14
Parahoxozoa - ps15
Bilateria - ps16
Protostomia - ps17
Ecdysozoa - ps18
Arthropoda - ps19
Mandibulata - ps20
Pancrustacea - ps21
Insecta - ps22
Eumetabola - ps23
Holometabola/Psocodea - ps24
Holometabola - ps25
Aparaglossata - ps26
Mecopterida - ps27
Diptera - ps28
Bibiono./Cyclorrhapha - ps29
Cyclorrhapha - ps30
Calyptratae/Drosophila - ps31
Drosophila - ps32
Sophophora - ps33
Obscura/Melanogaster_group - ps34
D_melanogaster_group - ps35
D_melanogaster_subgroup - ps36
sim./sech./mel. group - ps37
Drosophila_melanogaster - ps38
Phylostratum

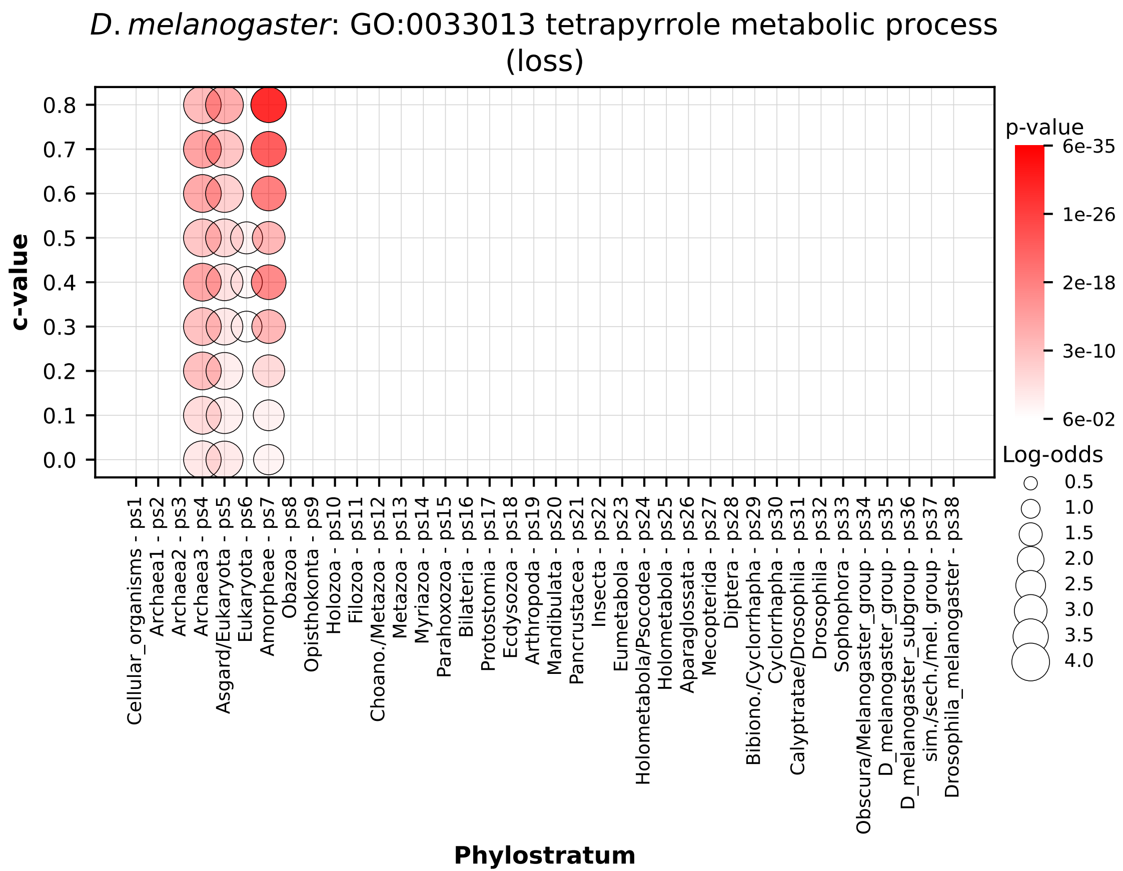

D. melanogaster: GO:0033013 tetrapyrrole metabolic process (loss)
c-value
0.8
0.7
0.6
0.5
0.4
0.3
0.2
0.1
0.0
p-value
6e-35
1e-26
2e-18
3e-10
6e-02
Log-odds
0.5
1.0
1.5
2.0
2.5
3.0
3.5
4.0
Cellular_organisms - ps1
Archaea1 - ps2
Archaea2 - ps3
Archaea3 - ps4
Asgard/Eukaryota - ps5
Eukaryota - ps6
Amorpheae - ps7
Obazoa - ps8
Opisthokonta - ps9
Holozoa - ps10
Filozoa - ps11
Choano./Metazoa - ps12
Metazoa - ps13
Myriazoa - ps14
Parahoxozoa - ps15
Bilateria - ps16
Protostomia - ps17
Ecdysozoa - ps18
Arthropoda - ps19
Mandibulata - ps20
Pancrustacea - ps21
Insecta - ps22
Eumetabola - ps23
Holometabola/Psocodea - ps24
Holometabola - ps25
Aparaglossata - ps26
Mecopterida - ps27
Diptera - ps28
Bibiono./Cyclorrhapha - ps29
Cyclorrhapha - ps30
Calyptratae/Drosophila - ps31
Drosophila - ps32
Sophophora - ps33
Obscura/Melanogaster_group - ps34
D_melanogaster_group - ps35
D_melanogaster_subgroup - ps36
sim./sech./mel. group - ps37
Drosophila_melanogaster - ps38
Phylostratum

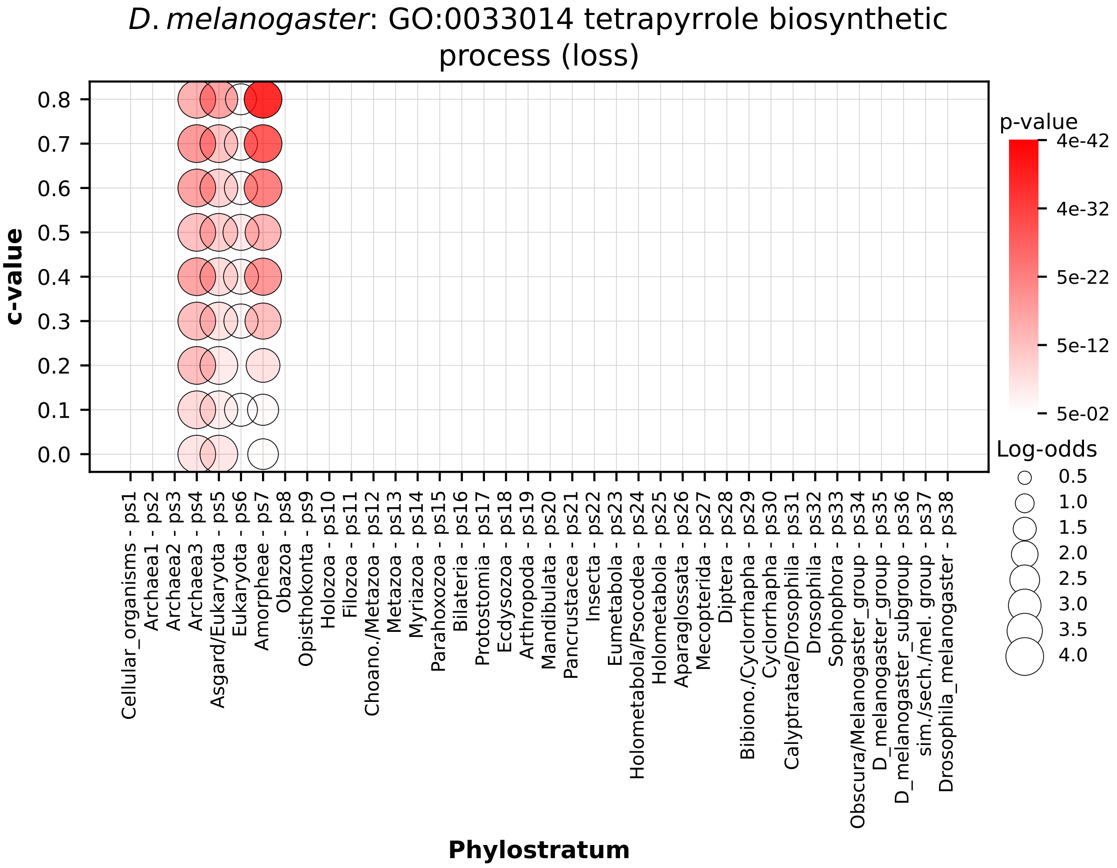

D. melanogaster: GO:0033014 tetrapyrrole biosynthetic process (loss)
c-value
0.8
0.7
0.6
0.5
0.4
0.3
0.2
0.1
0.0
p-value
4e-42
4e-32
5e-22
5e-12
5e-02
Log-odds
0.5
1.0
1.5
2.0
2.5
3.0
3.5
4.0
Cellular_organisms - ps1
Archaea1 - ps2
Archaea2 - ps3
Archaea3 - ps4
Asgard/Eukaryota - ps5
Eukaryota - ps6
Amorpheae - ps7
Obazoa - ps8
Opisthokonta - ps9
Holozoa - ps10
Filozoa - ps11
Choano./Metazoa - ps12
Metazoa - ps13
Myriazoa - ps14
Parahoxozoa - ps15
Bilateria - ps16
Protostomia - ps17
Ecdysozoa - ps18
Arthropoda - ps19
Mandibulata - ps20
Pancrustacea - ps21
Insecta - ps22
Eumetabola - ps23
Holometabola/Psocodea - ps24
Holometabola - ps25
Aparaglossata - ps26
Mecopterida - ps27
Diptera - ps28
Bibiono./Cyclorrhapha - ps29
Cyclorrhapha - ps30
Calyptratae/Drosophila - ps31
Drosophila - ps32
Sophophora - ps33
Obscura/Melanogaster_group - ps34
D_melanogaster_group - ps35
D_melanogaster_subgroup - ps36
sim./sech./mel. group - ps37
Drosophila_melanogaster - ps38
Phylostratum

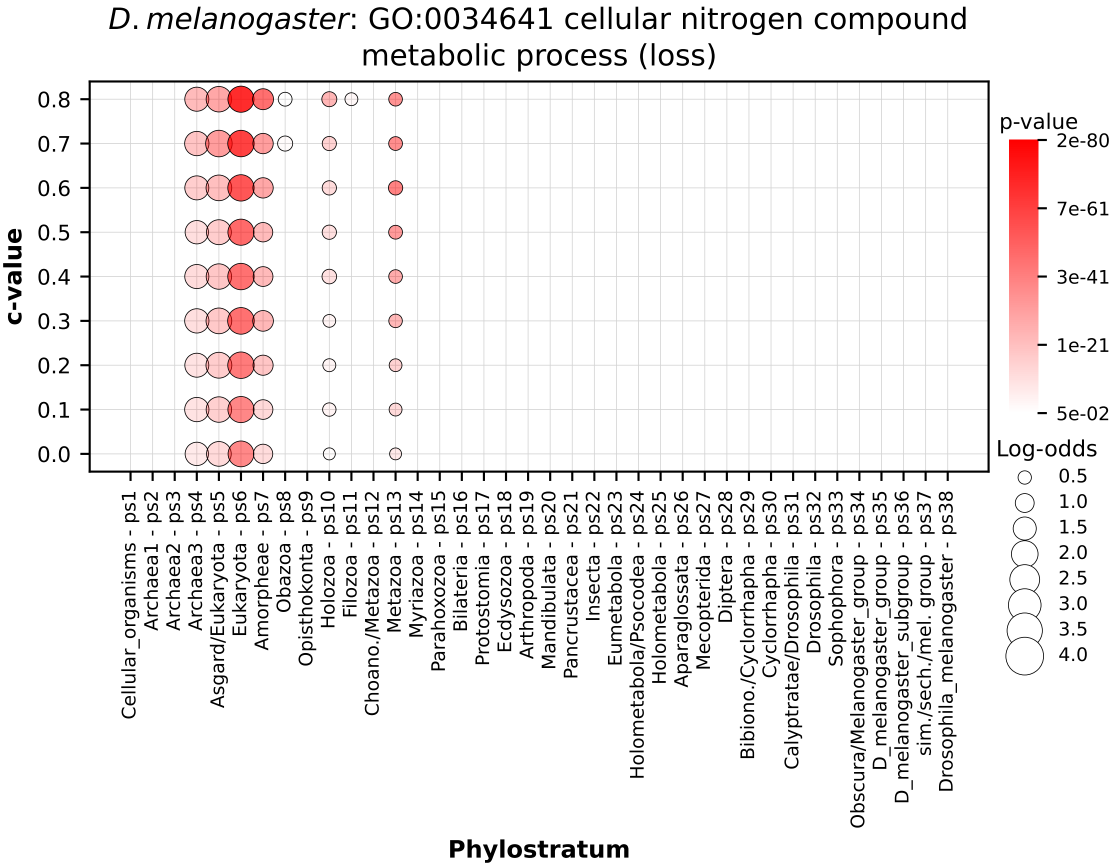

D. melanogaster: GO:0034641 cellular nitrogen compound metabolic process (loss)
c-value
0.8
0.7
0.6
0.5
0.4
0.3
0.2
0.1
0.0
p-value
2e-80
7e-61
3e-41
1e-21
5e-02
Log-odds
0.5
1.0
1.5
2.0
2.5
3.0
3.5
4.0
Cellular_organisms - ps1
Archaea1 - ps2
Archaea2 - ps3
Archaea3 - ps4
Asgard/Eukaryota - ps5
Eukaryota - ps6
Amorpheae - ps7
Obazoa - ps8
Opisthokonta - ps9
Holozoa - ps10
Filozoa - ps11
Choano./Metazoa - ps12
Metazoa - ps13
Myriazoa - ps14
Parahoxozoa - ps15
Bilateria - ps16
Protostomia - ps17
Ecdysozoa - ps18
Arthropoda - ps19
Mandibulata - ps20
Pancrustacea - ps21
Insecta - ps22
Eumetabola - ps23
Holometabola/Psocodea - ps24
Holometabola - ps25
Aparaglossata - ps26
Mecopterida - ps27
Diptera - ps28
Bibiono./Cyclorrhapha - ps29
Cyclorrhapha - ps30
Calyptratae/Drosophila - ps31
Drosophila - ps32
Sophophora - ps33
Obscura/Melanogaster_group - ps34
D_melanogaster_group - ps35
D_melanogaster_subgroup - ps36
sim./sech./mel. group - ps37
Drosophila_melanogaster - ps38
Phylostratum

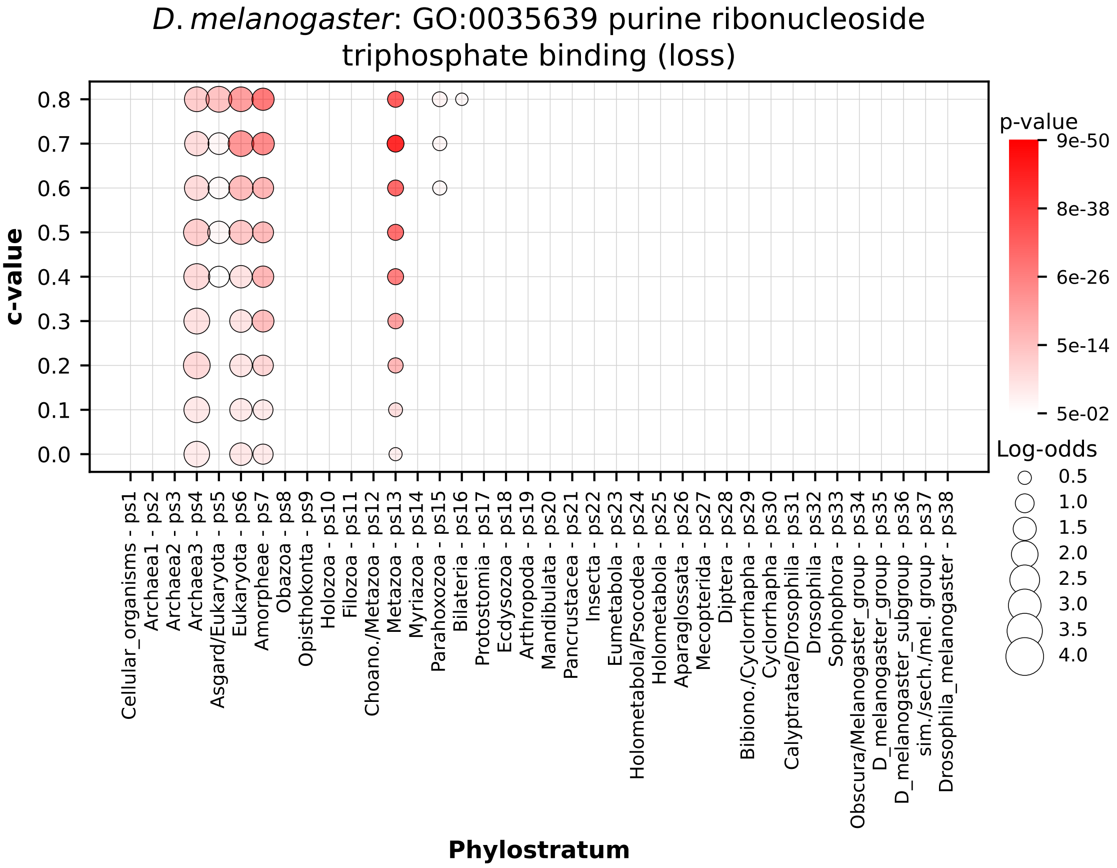
D. melanogaster: GO:0035639 purine ribonucleoside triphosphate binding (loss)
c-value
0.8
0.7
0.6
0.5
0.4
0.3
0.2
0.1
0.0
p-value
9e-50
8e-38
6e-26
5e-14
5e-02
Log-odds
0.5
1.0
1.5
2.0
2.5
3.0
3.5
4.0
Cellular_organisms - ps1
Archaea1 - ps2
Archaea2 - ps3
Archaea3 - ps4
Asgard/Eukaryota - ps5
Eukaryota - ps6
Amorpheae - ps7
Obazoa - ps8
Opisthokonta - ps9
Holozoa - ps10
Filozoa - ps11
Choano./Metazoa - ps12
Metazoa - ps13
Myriazoa - ps14
Parahoxozoa - ps15
Bilateria - ps16
Protostomia - ps17
Ecdysozoa - ps18
Arthropoda - ps19
Mandibulata - ps20
Pancrustacea - ps21
Insecta - ps22
Eumetabola - ps23
Holometabola/Psocodea - ps24
Holometabola - ps25
Aparaglossata - ps26
Mecopterida - ps27
Diptera - ps28
Bibiono./Cyclorrhapha - ps29
Cyclorrhapha - ps30
Calyptratae/Drosophila - ps31
Drosophila - ps32
Sophophora - ps33
Obscura/Melanogaster_group - ps34
D_melanogaster_group - ps35
D_melanogaster_subgroup - ps36
sim./sech./mel. group - ps37
Drosophila_melanogaster - ps38
Phylostratum

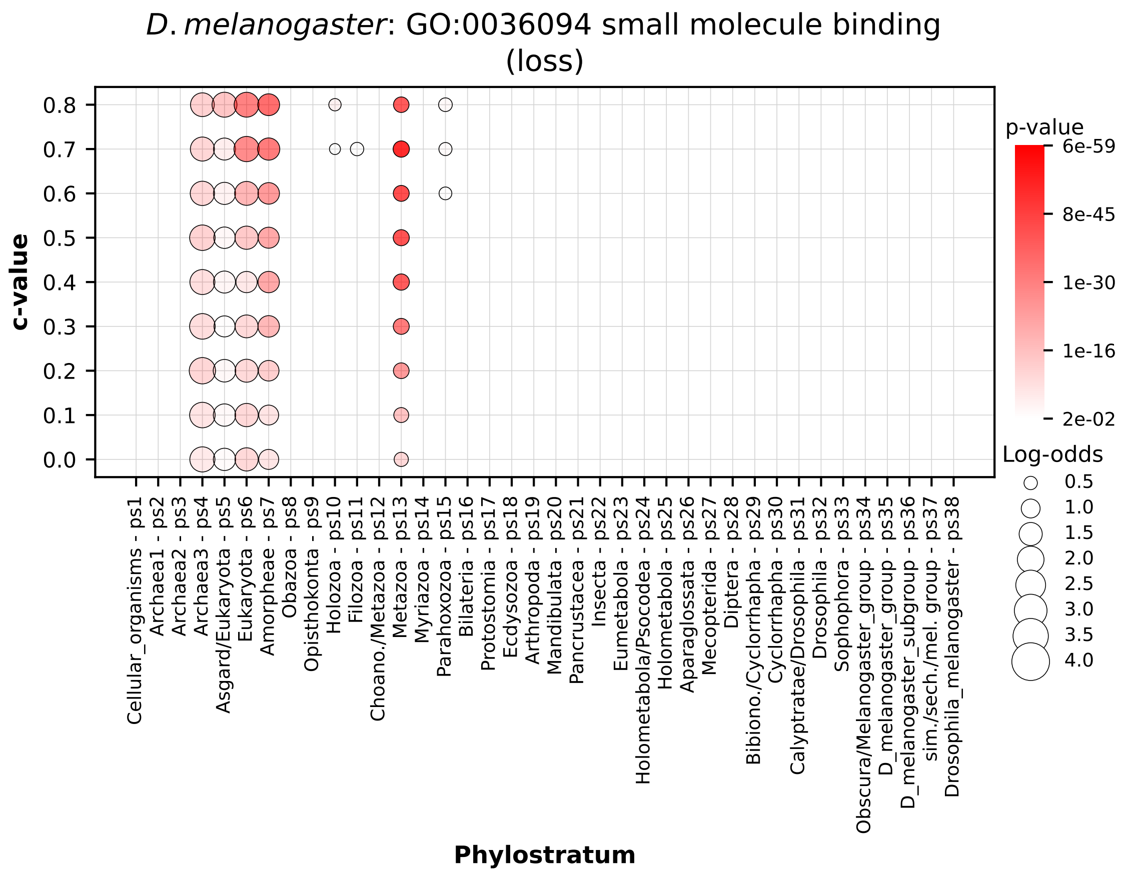

D. melanogaster: GO:0036094 small molecule binding (loss)
c-value
0.8
0.7
0.6
0.5
0.4
0.3
0.2
0.1
0.0
p-value
6e-59
8e-45
1e-30
1e-16
2e-02
Log-odds
0.5
1.0
1.5
2.0
2.5
3.0
3.5
4.0
Cellular_organisms - ps1
Archaea1 - ps2
Archaea2 - ps3
Archaea3 - ps4
Asgard/Eukaryota - ps5
Eukaryota - ps6
Amorpheae - ps7
Obazoa - ps8
Opisthokonta - ps9
Holozoa - ps10
Filozoa - ps11
Choano./Metazoa - ps12
Metazoa - ps13
Myriazoa - ps14
Parahoxozoa - ps15
Bilateria - ps16
Protostomia - ps17
Ecdysozoa - ps18
Arthropoda - ps19
Mandibulata - ps20
Pancrustacea - ps21
Insecta - ps22
Eumetabola - ps23
Holometabola/Psocodea - ps24
Holometabola - ps25
Aparaglossata - ps26
Mecopterida - ps27
Diptera - ps28
Bibiono./Cyclorrhapha - ps29
Cyclorrhapha - ps30
Calyptratae/Drosophila - ps31
Drosophila - ps32
Sophophora - ps33
Obscura/Melanogaster_group - ps34
D_melanogaster_group - ps35
D_melanogaster_subgroup - ps36
sim./sech./mel. group - ps37
Drosophila_melanogaster - ps38
Phylostratum

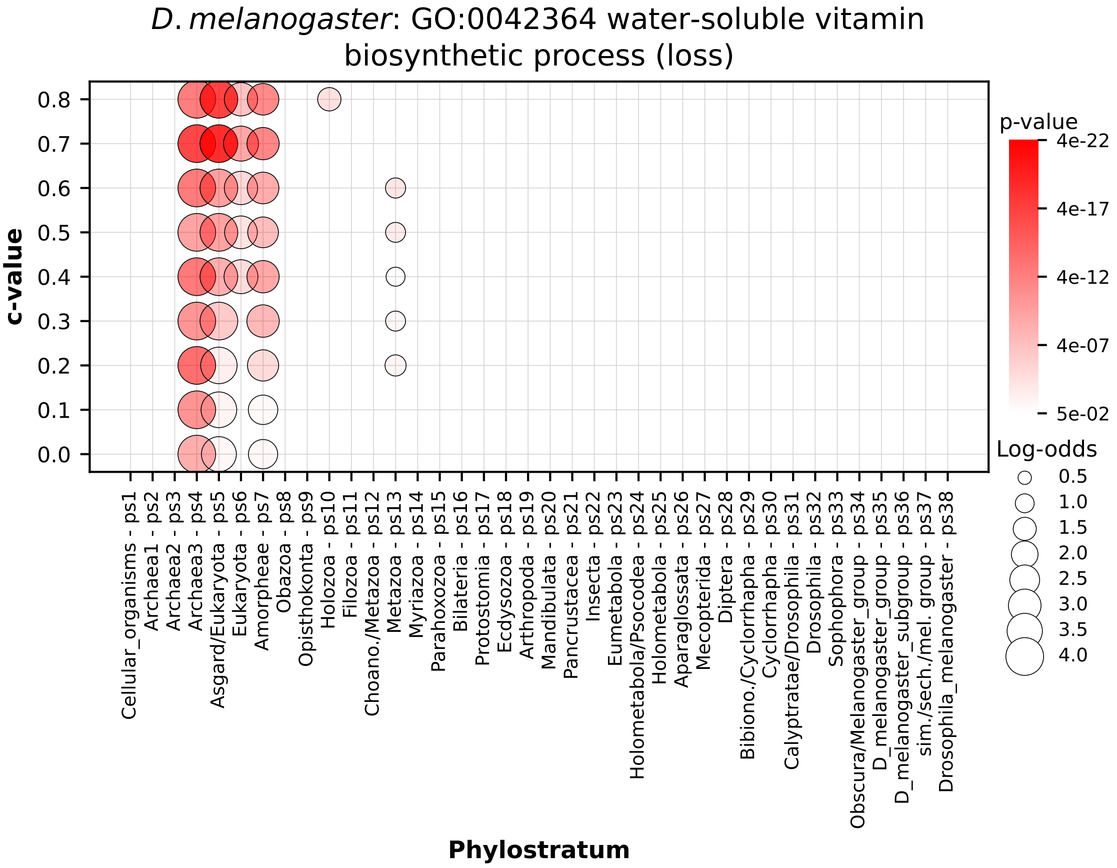
D. melanogaster: GO:0042364 water-soluble vitamin biosynthetic process (loss)
c-value
0.8
0.7
0.6
0.5
0.4
0.3
0.2
0.1
0.0
p-value
4e-22
4e-17
4e-12
4e-07
5e-02
Log-odds
0.5
1.0
1.5
2.0
2.5
3.0
3.5
4.0
Cellular_organisms - ps1
Archaea1 - ps2
Archaea2 - ps3
Archaea3 - ps4
Asgard/Eukaryota - ps5
Eukaryota - ps6
Amorpheae - ps7
Obazoa - ps8
Opisthokonta - ps9
Holozoa - ps10
Filozoa - ps11
Choano./Metazoa - ps12
Metazoa - ps13
Myriazoa - ps14
Parahoxozoa - ps15
Bilateria - ps16
Protostomia - ps17
Ecdysozoa - ps18
Arthropoda - ps19
Mandibulata - ps20
Pancrustacea - ps21
Insecta - ps22
Eumetabola - ps23
Holometabola/Psocodea - ps24
Holometabola - ps25
Aparaglossata - ps26
Mecopterida - ps27
Diptera - ps28
Bibiono./Cyclorrhapha - ps29
Cyclorrhapha - ps30
Calyptratae/Drosophila - ps31
Drosophila - ps32
Sophophora - ps33
Obscura/Melanogaster_group - ps34
D_melanogaster_group - ps35
D_melanogaster_subgroup - ps36
sim./sech./mel. group - ps37
Drosophila_melanogaster - ps38
Phylostratum

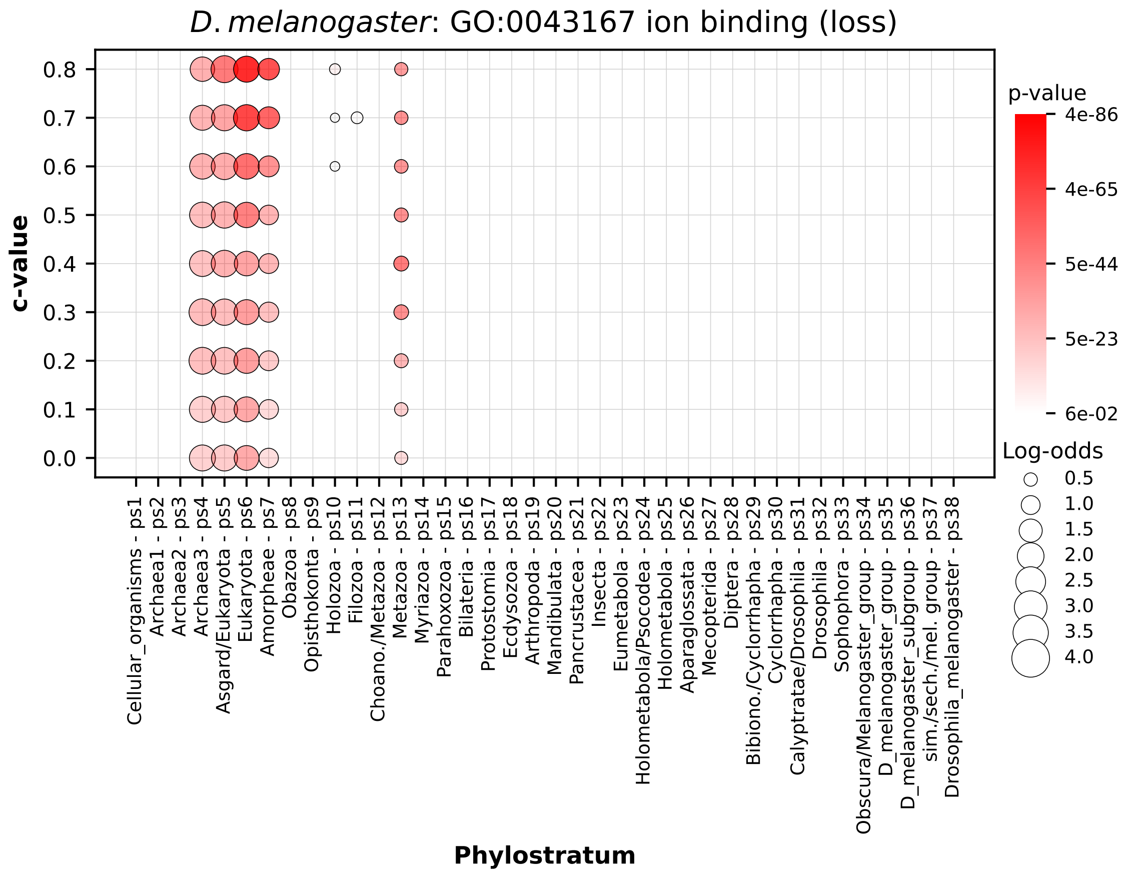
D. melanogaster: GO:0043167 ion binding (loss)
c-value
0.8
0.7
0.6
0.5
0.4
0.3
0.2
0.1
0.0
Phylostratum
Cellular_organisms - ps1
Archaea1 - ps2
Archaea2 - ps3
Archaea3 - ps4
Asgard/Eukaryota - ps5
Eukaryota - ps6
Amorpheae - ps7
Obazoa - ps8
Opisthokonta - ps9
Holozoa - ps10
Filozoa - ps11
Choano./Metazoa - ps12
Metazoa - ps13
Myriazoa - ps14
Parahoxozoa - ps15
Bilateria - ps16
Protostomia - ps17
Ecdysozoa - ps18
Arthropoda - ps19
Mandibulata - ps20
Pancrustacea - ps21
Insecta - ps22
Eumetabola - ps23
Holometabola/Psocodea - ps24
Holometabola - ps25
Aparaglossata - ps26
Mecopterida - ps27
Diptera - ps28
Bibiono./Cyclorrhapha - ps29
Cyclorrhapha - ps30
Calyptratae/Drosophila - ps31
Drosophila - ps32
Sophophora - ps33
Obscura/Melanogaster_group - ps34
D_melanogaster_group - ps35
D_melanogaster_subgroup - ps36
sim./sech./mel. group - ps37
Drosophila_melanogaster - ps38
p-value
4e-86
4e-65
5e-44
5e-23
6e-02
Log-odds
0.5
1.0
1.5
2.0
2.5
3.0
3.5
4.0

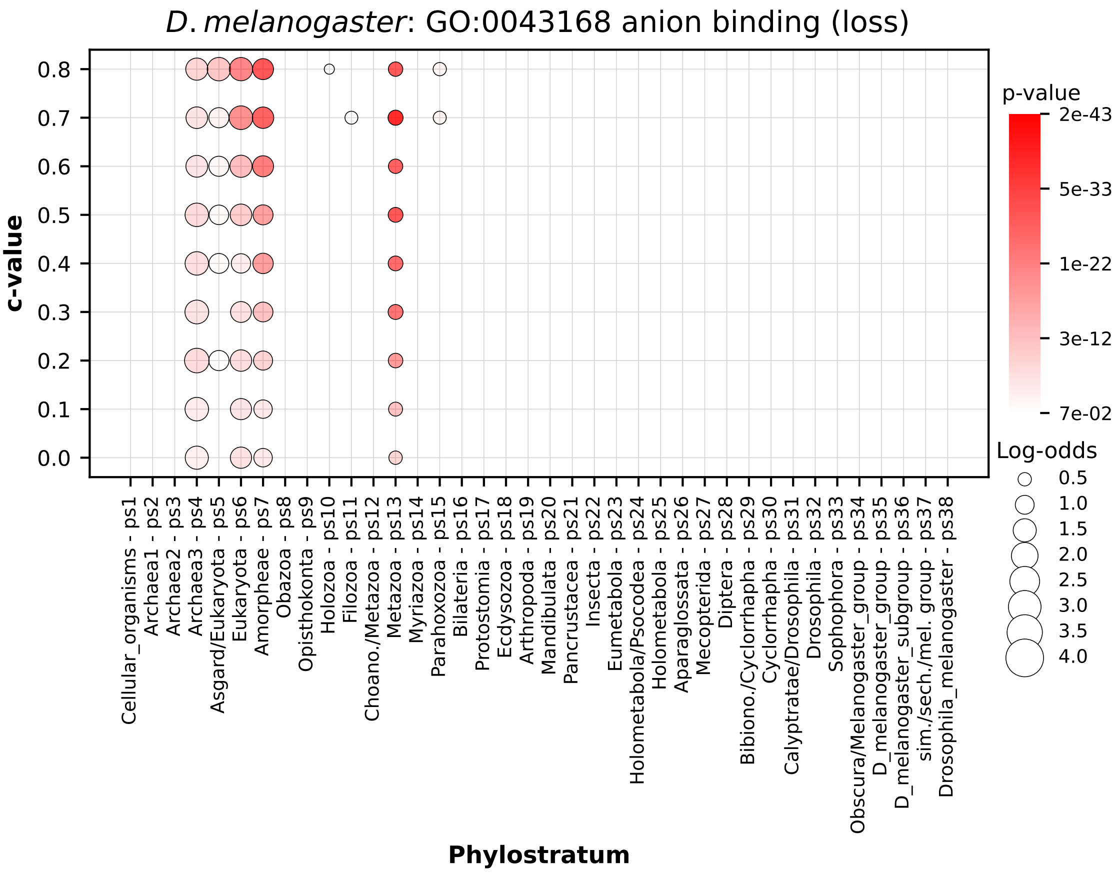
D. melanogaster: GO:0043168 anion binding (loss)
c-value
0.8
0.7
0.6
0.5
0.4
0.3
0.2
0.1
0.0
Phylostratum
Cellular_organisms - ps1
Archaea1 - ps2
Archaea2 - ps3
Archaea3 - ps4
Asgard/Eukaryota - ps5
Eukaryota - ps6
Amorpheae - ps7
Obazoa - ps8
Opisthokonta - ps9
Holozoa - ps10
Filozoa - ps11
Choano./Metazoa - ps12
Metazoa - ps13
Myriazoa - ps14
Parahoxozoa - ps15
Bilateria - ps16
Protostomia - ps17
Ecdysozoa - ps18
Arthropoda - ps19
Mandibulata - ps20
Pancrustacea - ps21
Insecta - ps22
Eumetabola - ps23
Holometabola/Psocodea - ps24
Holometabola - ps25
Aparaglossata - ps26
Mecopterida - ps27
Diptera - ps28
Bibiono./Cyclorrhapha - ps29
Cyclorrhapha - ps30
Calyptratae/Drosophila - ps31
Drosophila - ps32
Sophophora - ps33
Obscura/Melanogaster_group - ps34
D_melanogaster_group - ps35
D_melanogaster_subgroup - ps36
sim./sech./mel. group - ps37
Drosophila_melanogaster - ps38
p-value
2e-43
5e-33
1e-22
3e-12
7e-02
Log-odds
0.5
1.0
1.5
2.0
2.5
3.0
3.5
4.0

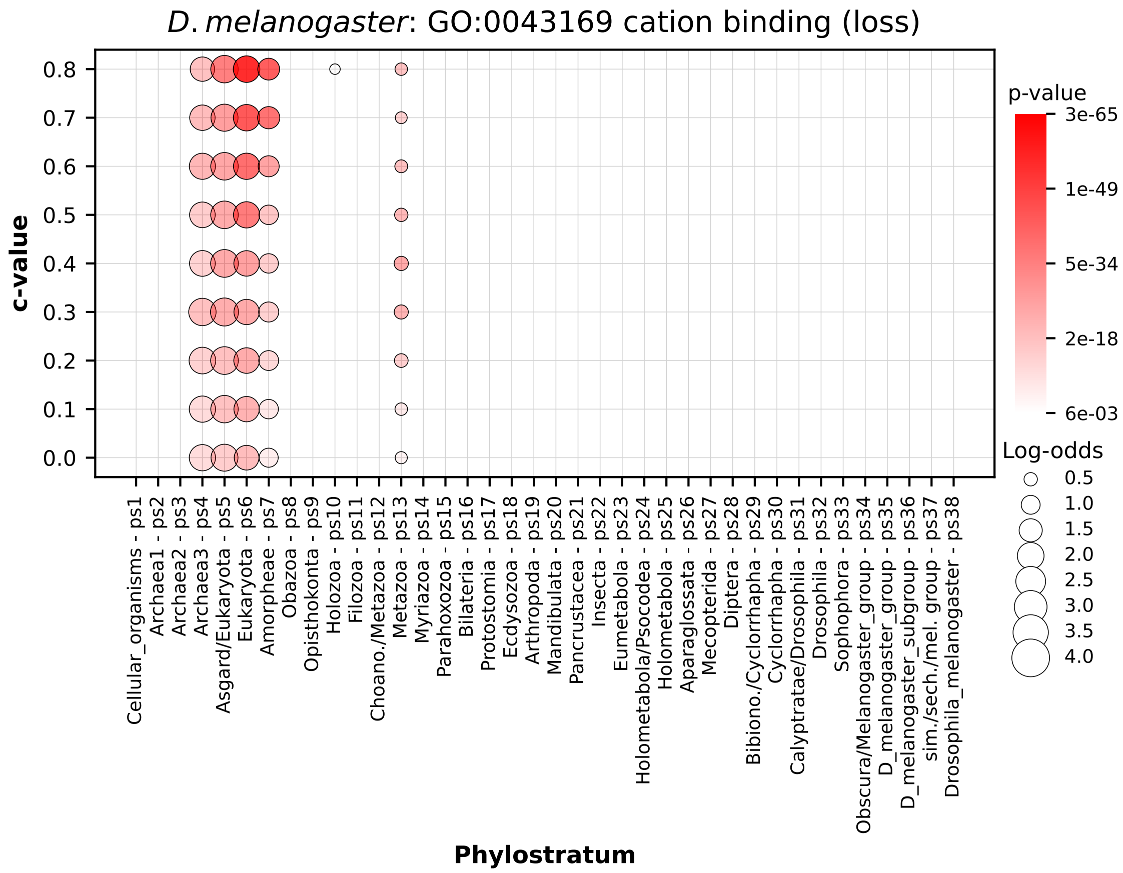
D. melanogaster: GO:0043169 cation binding (loss)
c-value
0.0
0.1
0.2
0.3
0.4
0.5
0.6
0.7
0.8
p-value
3e-65
1e-49
5e-34
2e-18
6e-03
Log-odds
0.5
1.0
1.5
2.0
2.5
3.0
3.5
4.0
Cellular_organisms - ps1
Archaea1 - ps2
Archaea2 - ps3
Archaea3 - ps4
Asgard/Eukaryota - ps5
Eukaryota - ps6
Amorpheae - ps7
Obazoa - ps8
Opisthokonta - ps9
Holozoa - ps10
Filozoa - ps11
Choano./Metazoa - ps12
Metazoa - ps13
Myriazoa - ps14
Parahoxozoa - ps15
Bilateria - ps16
Protostomia - ps17
Ecdysozoa - ps18
Arthropoda - ps19
Mandibulata - ps20
Pancrustacea - ps21
Insecta - ps22
Eumetabola - ps23
Holometabola/Psocodea - ps24
Holometabola - ps25
Aparaglossata - ps26
Mecopterida - ps27
Diptera - ps28
Bibiono./Cyclorrhapha - ps29
Cyclorrhapha - ps30
Calyptratae/Drosophila - ps31
Drosophila - ps32
Sophophora - ps33
Obscura/Melanogaster_group - ps34
D_melanogaster_group - ps35
D_melanogaster_subgroup - ps36
sim./sech./mel. group - ps37
Drosophila_melanogaster - ps38
Phylostratum

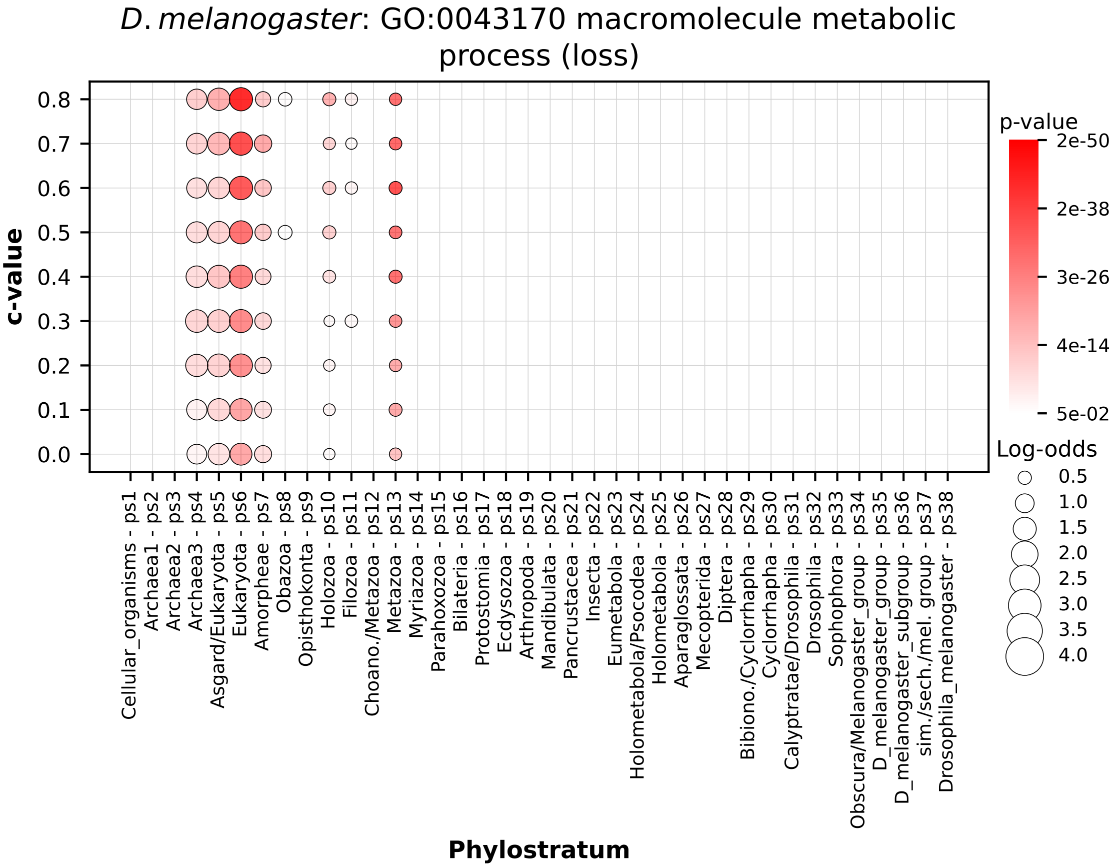

D. melanogaster: GO:0043170 macromolecule metabolic process (loss)
c-value
0.8
0.7
0.6
0.5
0.4
0.3
0.2
0.1
0.0
Phylostratum
Cellular_organisms - ps1
Archaea1 - ps2
Archaea2 - ps3
Archaea3 - ps4
Asgard/Eukaryota - ps5
Eukaryota - ps6
Amorpheae - ps7
Obazoa - ps8
Opisthokonta - ps9
Holozoa - ps10
Filozoa - ps11
Choano./Metazoa - ps12
Metazoa - ps13
Myriazoa - ps14
Parahoxozoa - ps15
Bilateria - ps16
Protostomia - ps17
Ecdysozoa - ps18
Arthropoda - ps19
Mandibulata - ps20
Pancrustacea - ps21
Insecta - ps22
Eumetabola - ps23
Holometabola/Psocodea - ps24
Holometabola - ps25
Aparaglossata - ps26
Mecopterida - ps27
Diptera - ps28
Bibiono./Cyclorrhapha - ps29
Cyclorrhapha - ps30
Calyptratae/Drosophila - ps31
Drosophila - ps32
Sophophora - ps33
Obscura/Melanogaster_group - ps34
D_melanogaster_group - ps35
D_melanogaster_subgroup - ps36
sim./sech./mel. group - ps37
Drosophila_melanogaster - ps38
p-value
2e-50
2e-38
3e-26
4e-14
5e-02
Log-odds
0.5
1.0
1.5
2.0
2.5
3.0
3.5
4.0

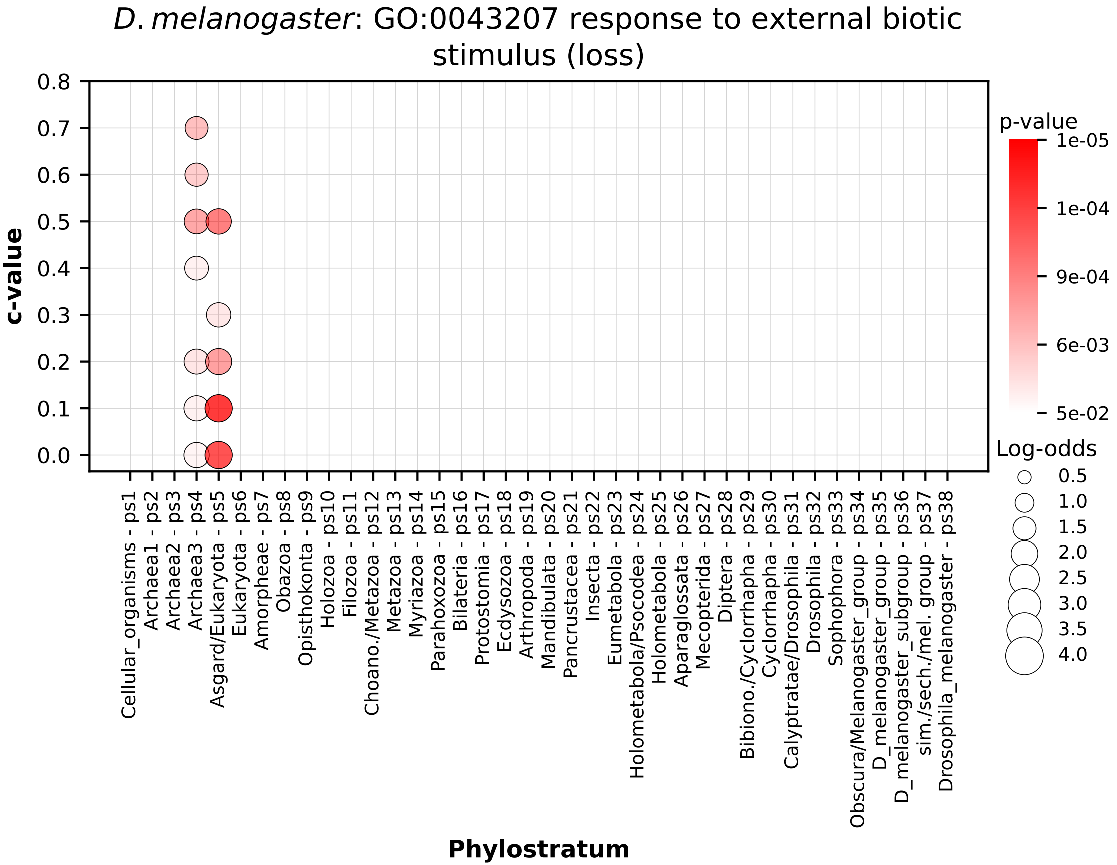

D. melanogaster: GO:0043207 response to external biotic stimulus (loss)
c-value
0.8
0.7
0.6
0.5
0.4
0.3
0.2
0.1
0.0
p-value
1e-05
1e-04
9e-04
6e-03
5e-02
Log-odds
0.5
1.0
1.5
2.0
2.5
3.0
3.5
4.0
Cellular_organisms - ps1
Archaea1 - ps2
Archaea2 - ps3
Archaea3 - ps4
Asgard/Eukaryota - ps5
Eukaryota - ps6
Amorpheae - ps7
Obazoa - ps8
Opisthokonta - ps9
Holozoa - ps10
Filozoa - ps11
Choano./Metazoa - ps12
Metazoa - ps13
Myriazoa - ps14
Parahoxozoa - ps15
Bilateria - ps16
Protostomia - ps17
Ecdysozoa - ps18
Arthropoda - ps19
Mandibulata - ps20
Pancrustacea - ps21
Insecta - ps22
Eumetabola - ps23
Holometabola/Psocodea - ps24
Holometabola - ps25
Aparaglossata - ps26
Mecopterida - ps27
Diptera - ps28
Bibiono./Cyclorrhapha - ps29
Cyclorrhapha - ps30
Calyptratae/Drosophila - ps31
Drosophila - ps32
Sophophora - ps33
Obscura/Melanogaster_group - ps34
D_melanogaster_group - ps35
D_melanogaster_subgroup - ps36
sim./sech./mel. group - ps37
Drosophila_melanogaster - ps38
Phylostratum

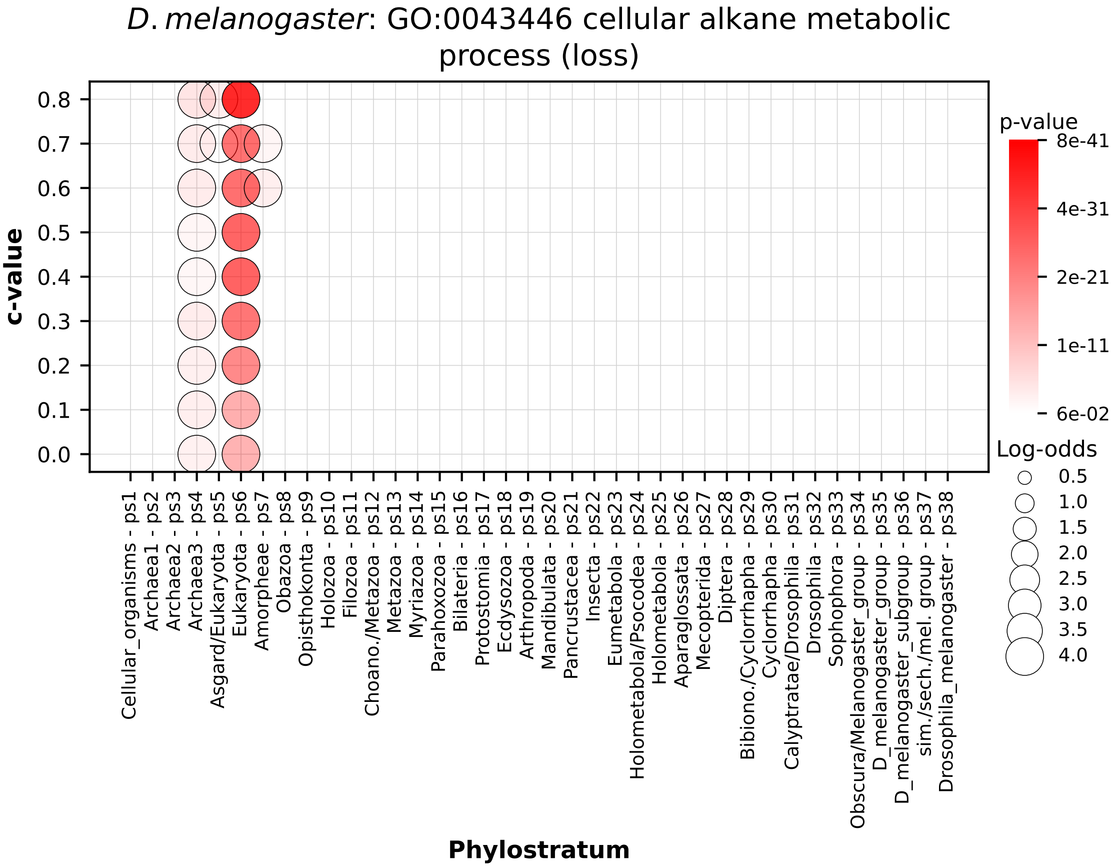

D. melanogaster: GO:0043446 cellular alkane metabolic process (loss)
c-value
0.8
0.7
0.6
0.5
0.4
0.3
0.2
0.1
0.0
p-value
8e-41
4e-31
2e-21
1e-11
6e-02
Log-odds
0.5
1.0
1.5
2.0
2.5
3.0
3.5
4.0
Cellular_organisms - ps1
Archaea1 - ps2
Archaea2 - ps3
Archaea3 - ps4
Asgard/Eukaryota - ps5
Eukaryota - ps6
Amorpheae - ps7
Obazoa - ps8
Opisthokonta - ps9
Holozoa - ps10
Filozoa - ps11
Choano./Metazoa - ps12
Metazoa - ps13
Myriazoa - ps14
Parahoxozoa - ps15
Bilateria - ps16
Protostomia - ps17
Ecdysozoa - ps18
Arthropoda - ps19
Mandibulata - ps20
Pancrustacea - ps21
Insecta - ps22
Eumetabola - ps23
Holometabola/Psocodea - ps24
Holometabola - ps25
Aparaglossata - ps26
Mecopterida - ps27
Diptera - ps28
Bibiono./Cyclorrhapha - ps29
Cyclorrhapha - ps30
Calyptratae/Drosophila - ps31
Drosophila - ps32
Sophophora - ps33
Obscura/Melanogaster_group - ps34
D_melanogaster_group - ps35
D_melanogaster_subgroup - ps36
sim./sech./mel. group - ps37
Drosophila_melanogaster - ps38
Phylostratum

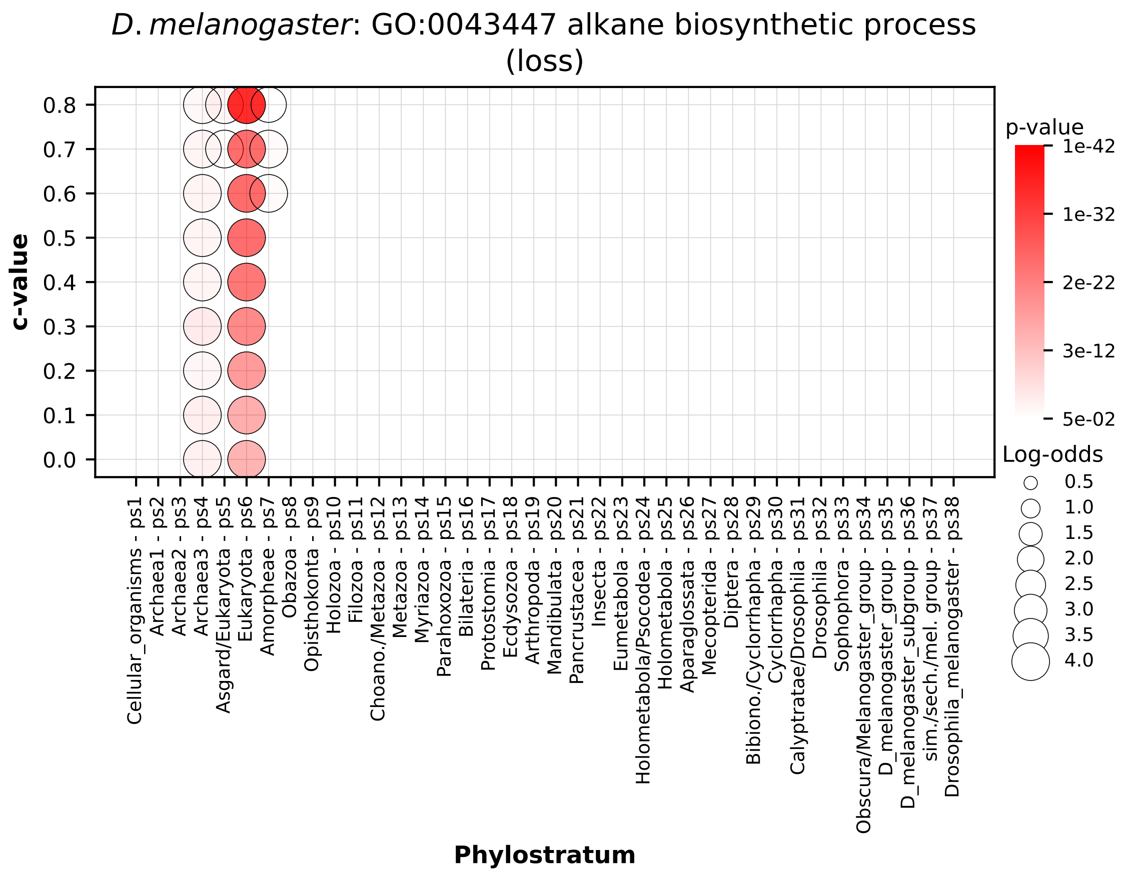

$D. melanogaster$: GO:0043447 alkane biosynthetic process (loss)
c-value
0.8
0.7
0.6
0.5
0.4
0.3
0.2
0.1
0.0
Phylostratum
Cellular_organisms - ps1
Archaea1 - ps2
Archaea2 - ps3
Archaea3 - ps4
Asgard/Eukaryota - ps5
Eukaryota - ps6
Amorpheae - ps7
Obazoa - ps8
Opisthokonta - ps9
Holozoa - ps10
Filozoa - ps11
Choano./Metazoa - ps12
Metazoa - ps13
Myriazoa - ps14
Parahoxozoa - ps15
Bilateria - ps16
Protostomia - ps17
Ecdysozoa - ps18
Arthropoda - ps19
Mandibulata - ps20
Pancrustacea - ps21
Insecta - ps22
Eumetabola - ps23
Holometabola/Psocodea - ps24
Holometabola - ps25
Aparaglossata - ps26
Mecopterida - ps27
Diptera - ps28
Bibiono./Cyclorrhapha - ps29
Cyclorrhapha - ps30
Calyptratae/Drosophila - ps31
Drosophila - ps32
Sophophora - ps33
Obscura/Melanogaster_group - ps34
D_melanogaster_group - ps35
D_melanogaster_subgroup - ps36
sim./sech./mel. group - ps37
Drosophila_melanogaster - ps38
p-value
1e-42
1e-32
2e-22
3e-12
5e-02
Log-odds
0.5
1.0
1.5
2.0
2.5
3.0
3.5
4.0

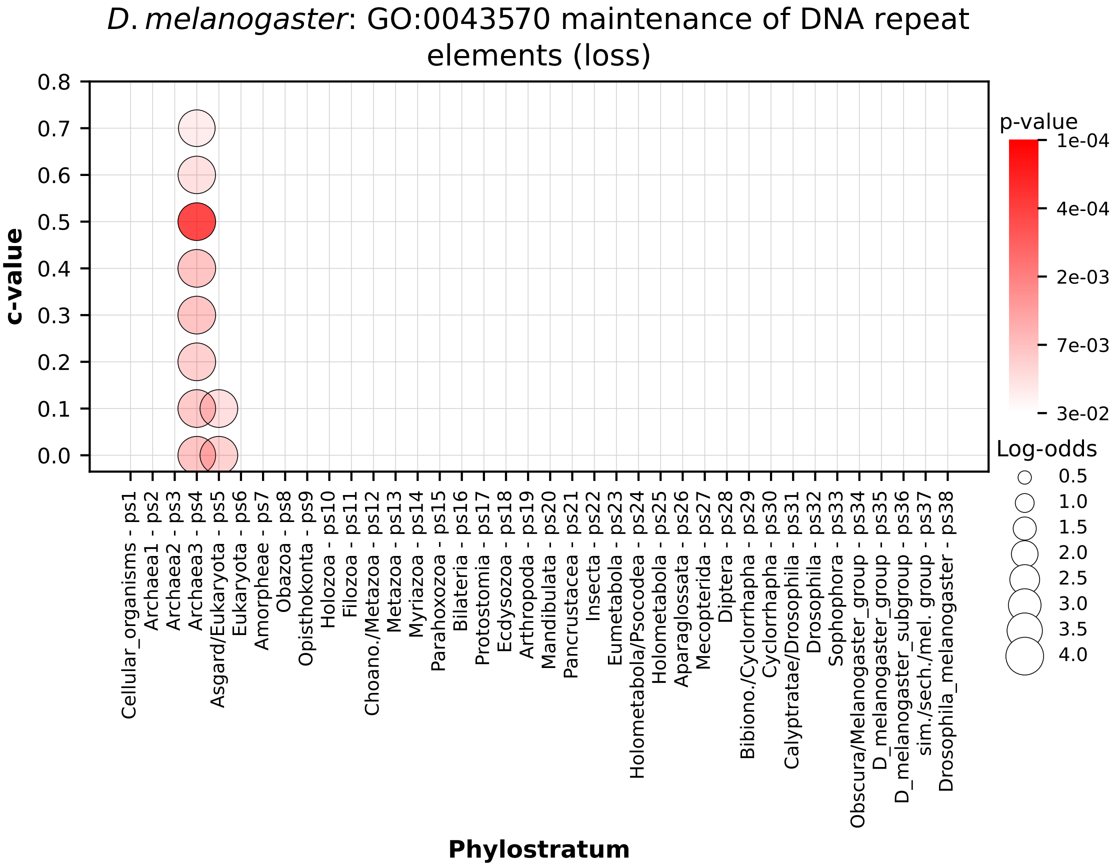

D. melanogaster: GO:0043570 maintenance of DNA repeat elements (loss)
c-value
0.8
0.7
0.6
0.5
0.4
0.3
0.2
0.1
0.0
p-value
1e-04
4e-04
2e-03
7e-03
3e-02
Log-odds
0.5
1.0
1.5
2.0
2.5
3.0
3.5
4.0
Cellular_organisms - ps1
Archaea1 - ps2
Archaea2 - ps3
Archaea3 - ps4
Asgard/Eukaryota - ps5
Eukaryota - ps6
Amorpheae - ps7
Obazoa - ps8
Opisthokonta - ps9
Holozoa - ps10
Filozoa - ps11
Choano./Metazoa - ps12
Metazoa - ps13
Myriazoa - ps14
Parahoxozoa - ps15
Bilateria - ps16
Protostomia - ps17
Ecdysozoa - ps18
Arthropoda - ps19
Mandibulata - ps20
Pancrustacea - ps21
Insecta - ps22
Eumetabola - ps23
Holometabola/Psocodea - ps24
Holometabola - ps25
Aparaglossata - ps26
Mecopterida - ps27
Diptera - ps28
Bibiono./Cyclorrhapha - ps29
Cyclorrhapha - ps30
Calyptratae/Drosophila - ps31
Drosophila - ps32
Sophophora - ps33
Obscura/Melanogaster_group - ps34
D_melanogaster_group - ps35
D_melanogaster_subgroup - ps36
sim./sech./mel. group - ps37
Drosophila_melanogaster - ps38
Phylostratum

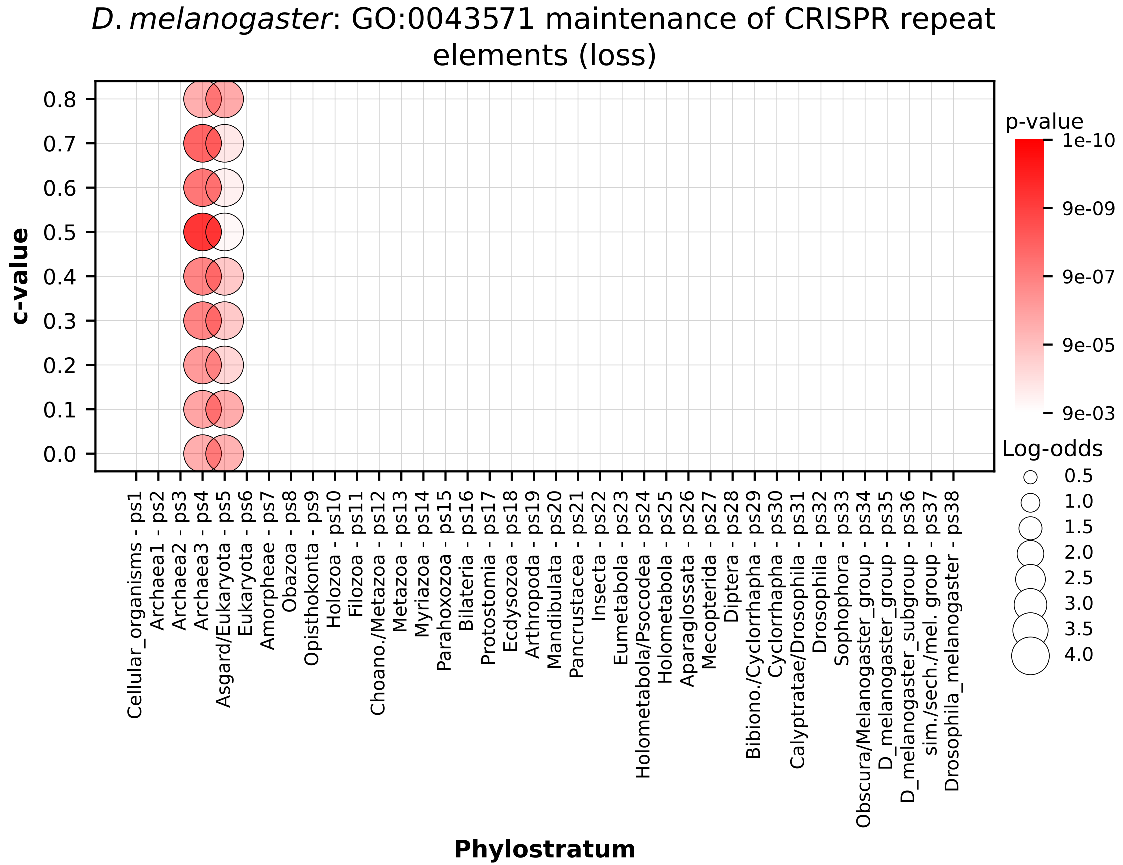
D. melanogaster: GO:0043571 maintenance of CRISPR repeat elements (loss)
c-value
0.8
0.7
0.6
0.5
0.4
0.3
0.2
0.1
0.0
p-value
1e-10
9e-09
9e-07
9e-05
9e-03
Log-odds
0.5
1.0
1.5
2.0
2.5
3.0
3.5
4.0
Cellular_organisms - ps1
Archaea1 - ps2
Archaea2 - ps3
Archaea3 - ps4
Asgard/Eukaryota - ps5
Eukaryota - ps6
Amorpheae - ps7
Obazoa - ps8
Opisthokonta - ps9
Holozoa - ps10
Filozoa - ps11
Choano./Metazoa - ps12
Metazoa - ps13
Myriazoa - ps14
Parahoxozoa - ps15
Bilateria - ps16
Protostomia - ps17
Ecdysozoa - ps18
Arthropoda - ps19
Mandibulata - ps20
Pancrustacea - ps21
Insecta - ps22
Eumetabola - ps23
Holometabola/Psocodea - ps24
Holometabola - ps25
Aparaglossata - ps26
Mecopterida - ps27
Diptera - ps28
Bibiono./Cyclorrhapha - ps29
Cyclorrhapha - ps30
Calyptratae/Drosophila - ps31
Drosophila - ps32
Sophophora - ps33
Obscura/Melanogaster_group - ps34
D_melanogaster_group - ps35
D_melanogaster_subgroup - ps36
sim./sech./mel. group - ps37
Drosophila_melanogaster - ps38
Phylostratum

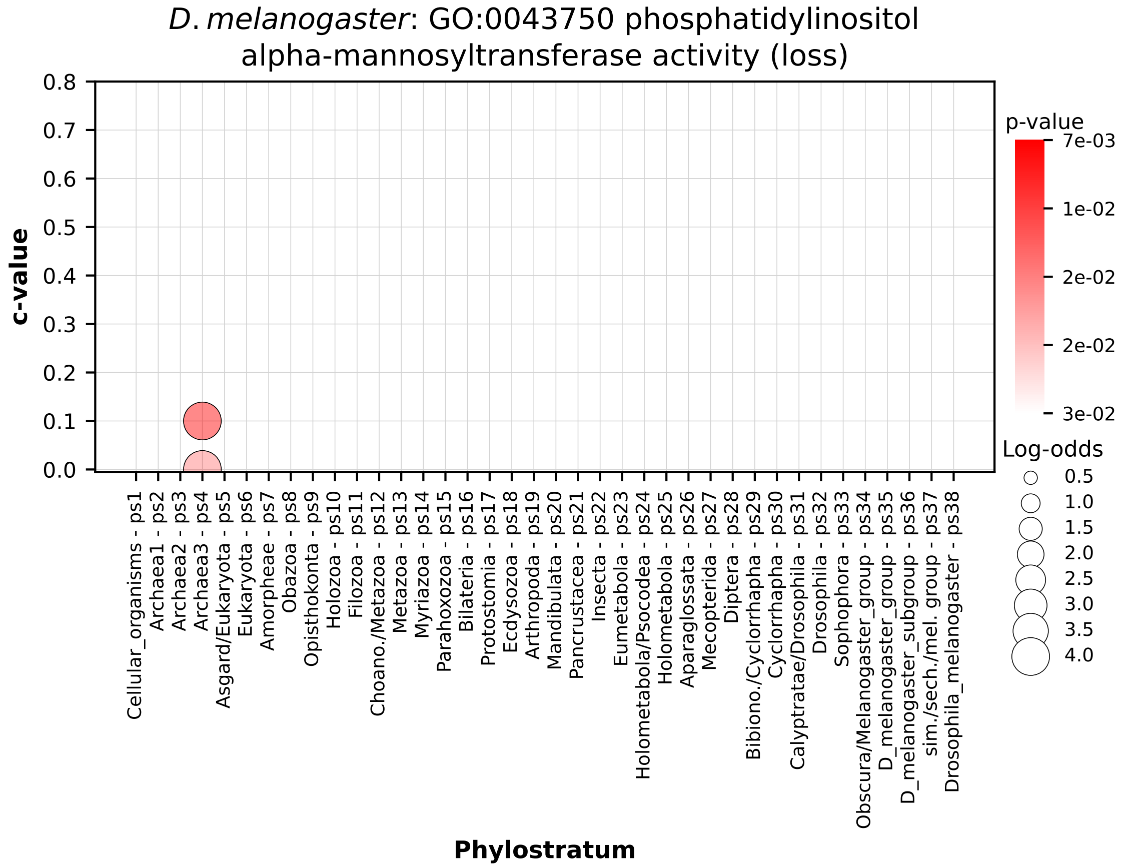

D. melanogaster: GO:0043750 phosphatidylinositol alpha-mannosyltransferase activity (loss)
c-value
0.8
0.7
0.6
0.5
0.4
0.3
0.2
0.1
0.0
Phylostratum
Cellular_organisms - ps1
Archaea1 - ps2
Archaea2 - ps3
Archaea3 - ps4
Asgard/Eukaryota - ps5
Eukaryota - ps6
Amorpheae - ps7
Obazoa - ps8
Opisthokonta - ps9
Holozoa - ps10
Filozoa - ps11
Choano./Metazoa - ps12
Metazoa - ps13
Myriazoa - ps14
Parahoxozoa - ps15
Bilateria - ps16
Protostomia - ps17
Ecdysozoa - ps18
Arthropoda - ps19
Mandibulata - ps20
Pancrustacea - ps21
Insecta - ps22
Eumetabola - ps23
Holometabola/Psocodea - ps24
Holometabola - ps25
Aparaglossata - ps26
Mecopterida - ps27
Diptera - ps28
Bibiono./Cyclorrhapha - ps29
Cyclorrhapha - ps30
Calyptratae/Drosophila - ps31
Drosophila - ps32
Sophophora - ps33
Obscura/Melanogaster_group - ps34
D_melanogaster_group - ps35
D_melanogaster_subgroup - ps36
sim./sech./mel. group - ps37
Drosophila_melanogaster - ps38
p-value
7e-03
1e-02
2e-02
2e-02
3e-02
Log-odds
0.5
1.0
1.5
2.0
2.5
3.0
3.5
4.0

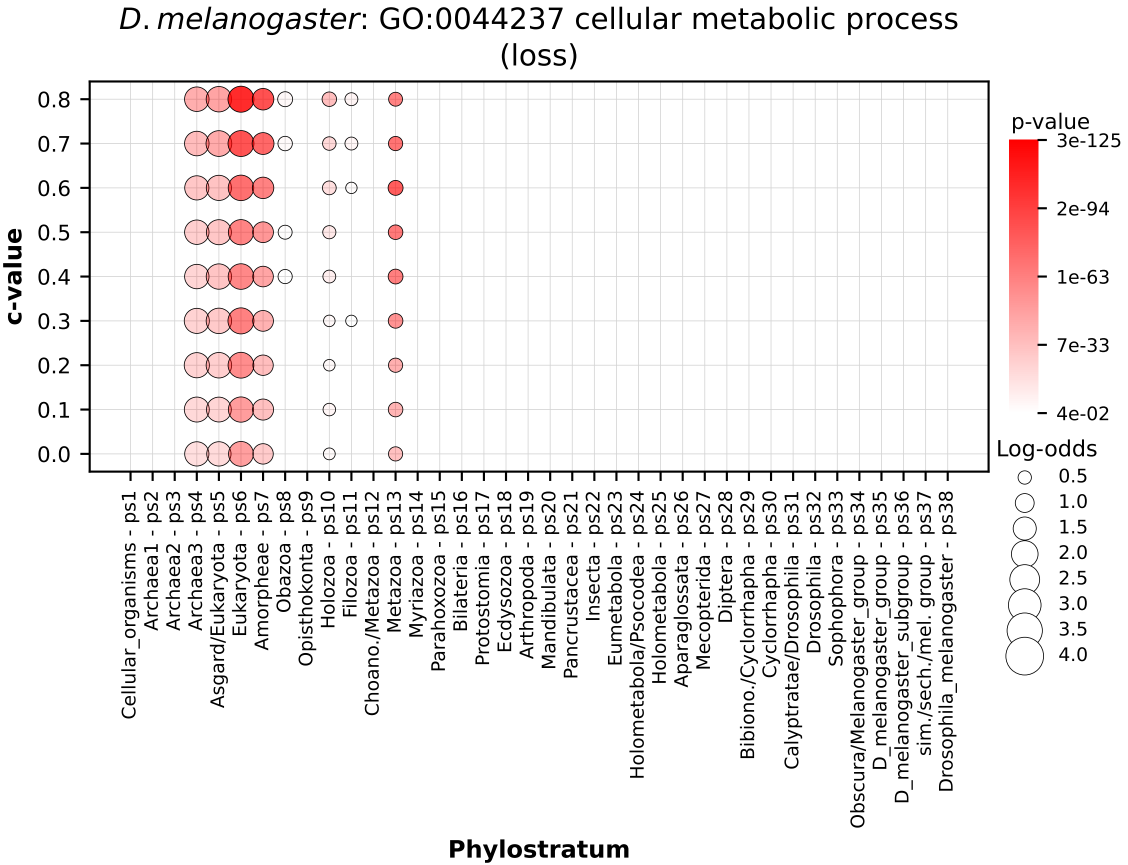

D. melanogaster: GO:0044237 cellular metabolic process (loss)
c-value
0.8
0.7
0.6
0.5
0.4
0.3
0.2
0.1
0.0
p-value
3e-125
2e-94
1e-63
7e-33
4e-02
Log-odds
0.5
1.0
1.5
2.0
2.5
3.0
3.5
4.0
Cellular_organisms - ps1
Archaea1 - ps2
Archaea2 - ps3
Archaea3 - ps4
Asgard/Eukaryota - ps5
Eukaryota - ps6
Amorpheae - ps7
Obazoa - ps8
Opisthokonta - ps9
Holozoa - ps10
Filozoa - ps11
Choano./Metazoa - ps12
Metazoa - ps13
Myriazoa - ps14
Parahoxozoa - ps15
Bilateria - ps16
Protostomia - ps17
Ecdysozoa - ps18
Arthropoda - ps19
Mandibulata - ps20
Pancrustacea - ps21
Insecta - ps22
Eumetabola - ps23
Holometabola/Psocodea - ps24
Holometabola - ps25
Aparaglossata - ps26
Mecopterida - ps27
Diptera - ps28
Bibiono./Cyclorrhapha - ps29
Cyclorrhapha - ps30
Calyptratae/Drosophila - ps31
Drosophila - ps32
Sophophora - ps33
Obscura/Melanogaster_group - ps34
D_melanogaster_group - ps35
D_melanogaster_subgroup - ps36
sim./sech./mel. group - ps37
Drosophila_melanogaster - ps38
Phylostratum

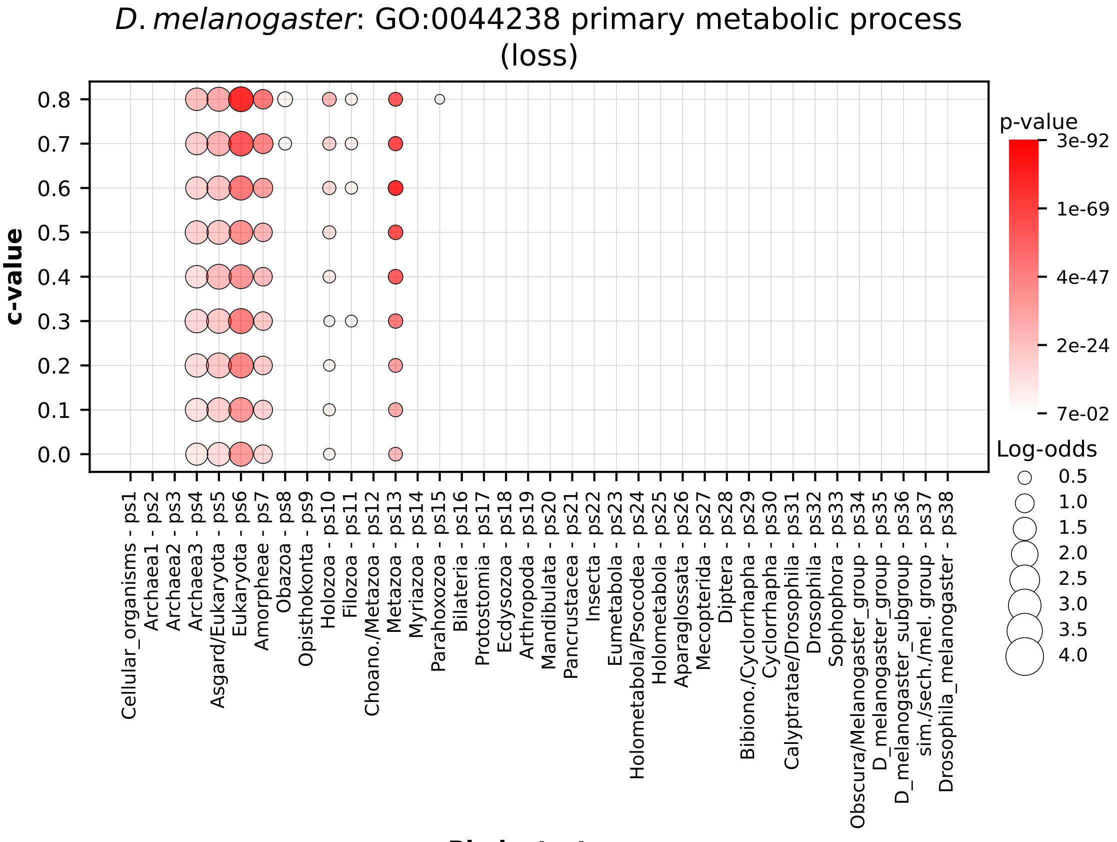
D. melanogaster: GO:0044238 primary metabolic process (loss)
c-value
0.8
0.7
0.6
0.5
0.4
0.3
0.2
0.1
0.0
Phylostratum
Cellular_organisms - ps1
Archaea1 - ps2
Archaea2 - ps3
Archaea3 - ps4
Asgard/Eukaryota - ps5
Eukaryota - ps6
Amorpheae - ps7
Obazoa - ps8
Opisthokonta - ps9
Holozoa - ps10
Filozoa - ps11
Choano./Metazoa - ps12
Metazoa - ps13
Myriazoa - ps14
Parahoxozoa - ps15
Bilateria - ps16
Protostomia - ps17
Ecdysozoa - ps18
Arthropoda - ps19
Mandibulata - ps20
Pancrustacea - ps21
Insecta - ps22
Eumetabola - ps23
Holometabola/Psocodea - ps24
Holometabola - ps25
Aparaglossata - ps26
Mecopterida - ps27
Diptera - ps28
Bibiono./Cyclorrhapha - ps29
Cyclorrhapha - ps30
Calyptratae/Drosophila - ps31
Drosophila - ps32
Sophophora - ps33
Obscura/Melanogaster_group - ps34
D_melanogaster_group - ps35
D_melanogaster_subgroup - ps36
sim./sech./mel. group - ps37
Drosophila_melanogaster - ps38
p-value
3e-92
1e-69
4e-47
2e-24
7e-02
Log-odds
0.5
1.0
1.5
2.0
2.5
3.0
3.5
4.0

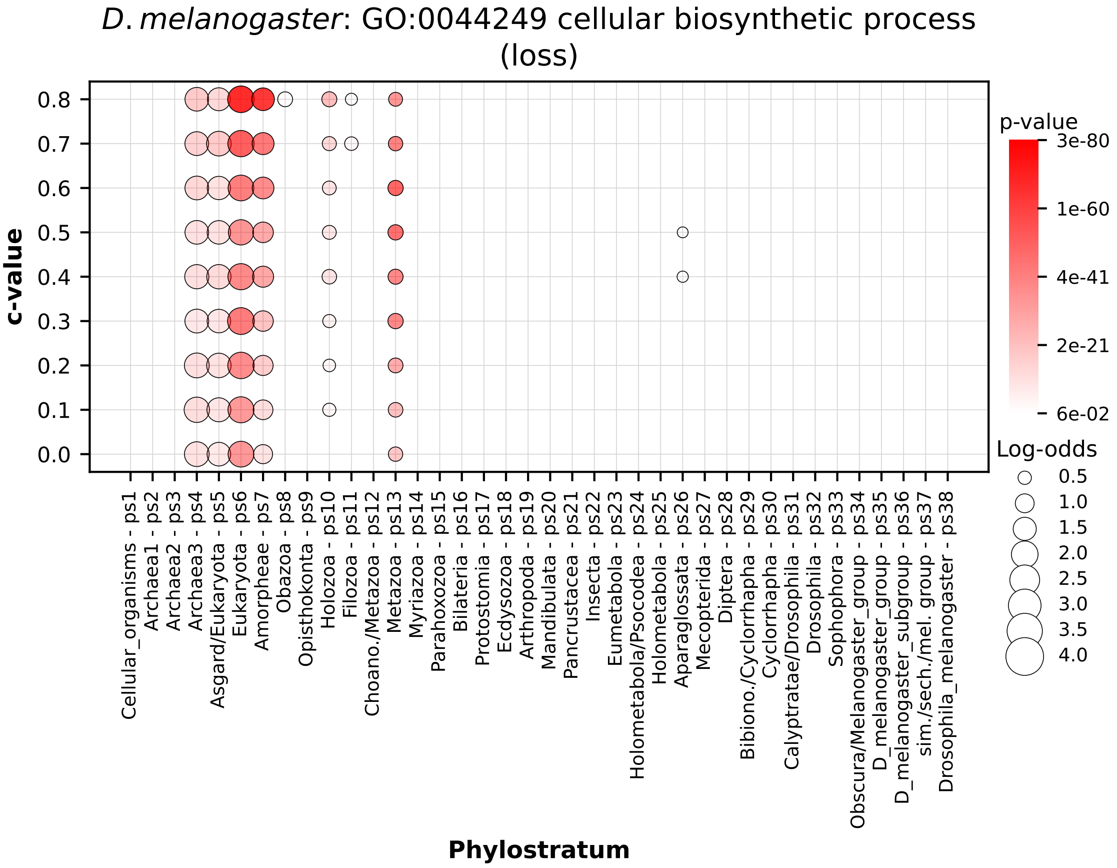

D. melanogaster: GO:0044249 cellular biosynthetic process (loss)
c-value
0.8
0.7
0.6
0.5
0.4
0.3
0.2
0.1
0.0
Phylostratum
Cellular_organisms - ps1
Archaea1 - ps2
Archaea2 - ps3
Archaea3 - ps4
Asgard/Eukaryota - ps5
Eukaryota - ps6
Amorpheae - ps7
Obazoa - ps8
Opisthokonta - ps9
Holozoa - ps10
Filozoa - ps11
Choano./Metazoa - ps12
Metazoa - ps13
Myriazoa - ps14
Parahoxozoa - ps15
Bilateria - ps16
Protostomia - ps17
Ecdysozoa - ps18
Arthropoda - ps19
Mandibulata - ps20
Pancrustacea - ps21
Insecta - ps22
Eumetabola - ps23
Holometabola/Psocodea - ps24
Holometabola - ps25
Aparaglossata - ps26
Mecopterida - ps27
Diptera - ps28
Bibiono./Cyclorrhapha - ps29
Cyclorrhapha - ps30
Calyptratae/Drosophila - ps31
Drosophila - ps32
Sophophora - ps33
Obscura/Melanogaster_group - ps34
D_melanogaster_group - ps35
D_melanogaster_subgroup - ps36
sim./sech./mel. group - ps37
Drosophila_melanogaster - ps38
p-value
3e-80
1e-60
4e-41
2e-21
6e-02
Log-odds
0.5
1.0
1.5
2.0
2.5
3.0
3.5
4.0

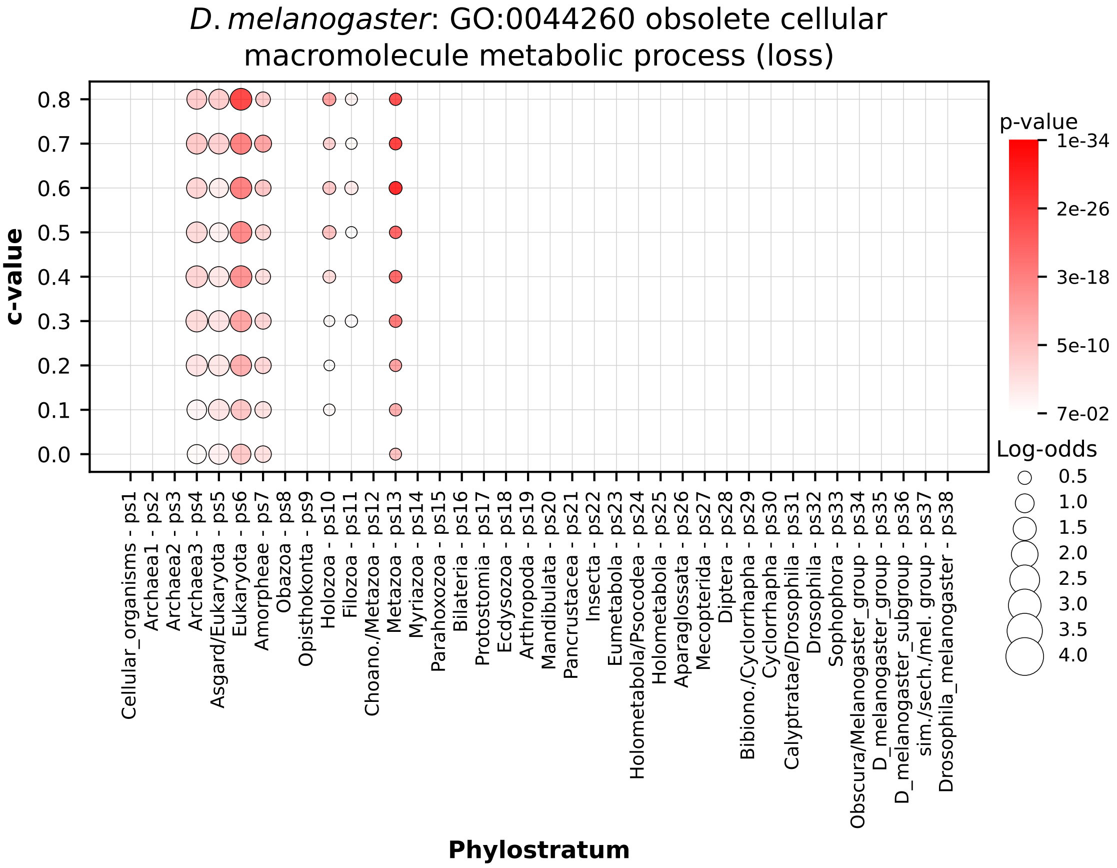

D. melanogaster: GO:0044260 obsolete cellular macromolecule metabolic process (loss)
c-value
0.8
0.7
0.6
0.5
0.4
0.3
0.2
0.1
0.0
Phylostratum
Cellular_organisms - ps1
Archaea1 - ps2
Archaea2 - ps3
Archaea3 - ps4
Asgard/Eukaryota - ps5
Eukaryota - ps6
Amorpheae - ps7
Obazoa - ps8
Opisthokonta - ps9
Holozoa - ps10
Filozoa - ps11
Choano./Metazoa - ps12
Metazoa - ps13
Myriazoa - ps14
Parahoxozoa - ps15
Bilateria - ps16
Protostomia - ps17
Ecdysozoa - ps18
Arthropoda - ps19
Mandibulata - ps20
Pancrustacea - ps21
Insecta - ps22
Eumetabola - ps23
Holometabola/Psocodea - ps24
Holometabola - ps25
Aparaglossata - ps26
Mecopterida - ps27
Diptera - ps28
Bibiono./Cyclorrhapha - ps29
Cyclorrhapha - ps30
Calyptratae/Drosophila - ps31
Drosophila - ps32
Sophophora - ps33
Obscura/Melanogaster_group - ps34
D_melanogaster_group - ps35
D_melanogaster_subgroup - ps36
sim./sech./mel. group - ps37
Drosophila_melanogaster - ps38
p-value
1e-34
2e-26
3e-18
5e-10
7e-02
Log-odds
0.5
1.0
1.5
2.0
2.5
3.0
3.5
4.0

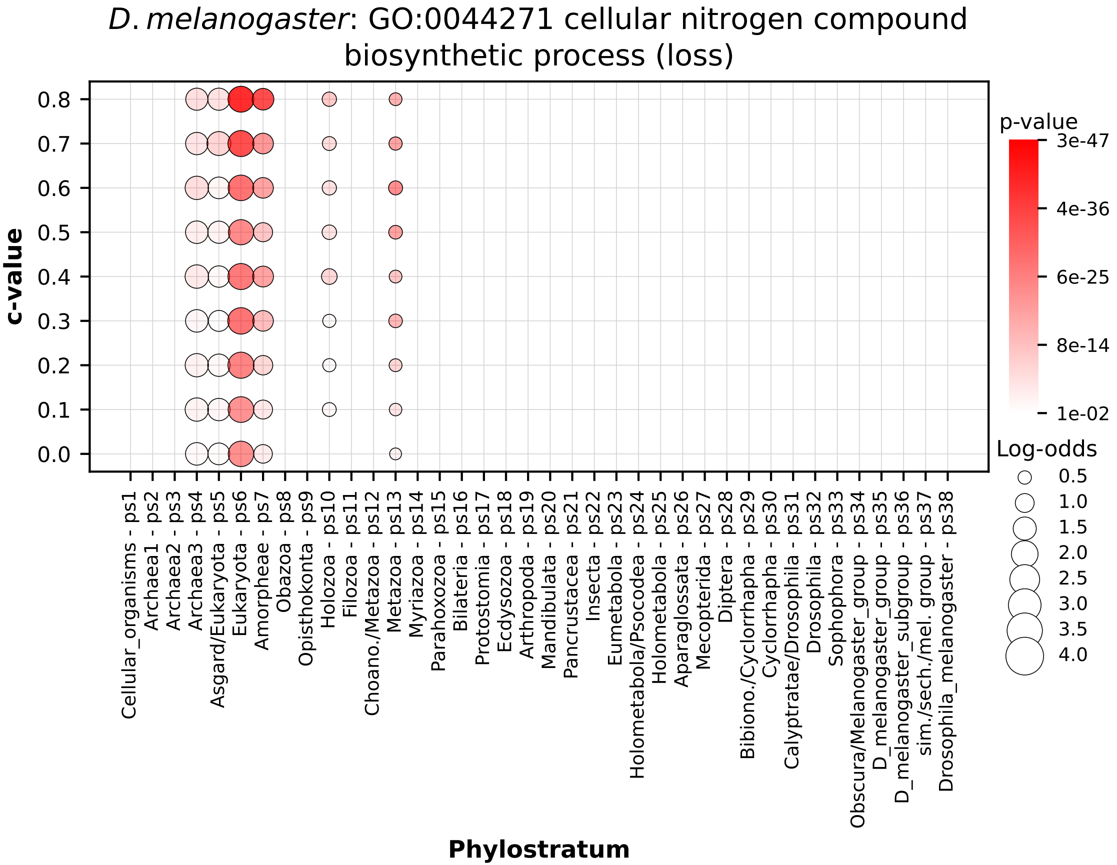

D. melanogaster: GO:0044271 cellular nitrogen compound biosynthetic process (loss)
c-value
0.8
0.7
0.6
0.5
0.4
0.3
0.2
0.1
0.0
p-value
3e-47
4e-36
6e-25
8e-14
1e-02
Log-odds
0.5
1.0
1.5
2.0
2.5
3.0
3.5
4.0
Cellular_organisms - ps1
Archaea1 - ps2
Archaea2 - ps3
Archaea3 - ps4
Asgard/Eukaryota - ps5
Eukaryota - ps6
Amorpheae - ps7
Obazoa - ps8
Opisthokonta - ps9
Holozoa - ps10
Filozoa - ps11
Choano./Metazoa - ps12
Metazoa - ps13
Myriazoa - ps14
Parahoxozoa - ps15
Bilateria - ps16
Protostomia - ps17
Ecdysozoa - ps18
Arthropoda - ps19
Mandibulata - ps20
Pancrustacea - ps21
Insecta - ps22
Eumetabola - ps23
Holometabola/Psocodea - ps24
Holometabola - ps25
Aparaglossata - ps26
Mecopterida - ps27
Diptera - ps28
Bibiono./Cyclorrhapha - ps29
Cyclorrhapha - ps30
Calyptratae/Drosophila - ps31
Drosophila - ps32
Sophophora - ps33
Obscura/Melanogaster_group - ps34
D_melanogaster_group - ps35
D_melanogaster_subgroup - ps36
sim./sech./mel. group - ps37
Drosophila_melanogaster - ps38
Phylostratum

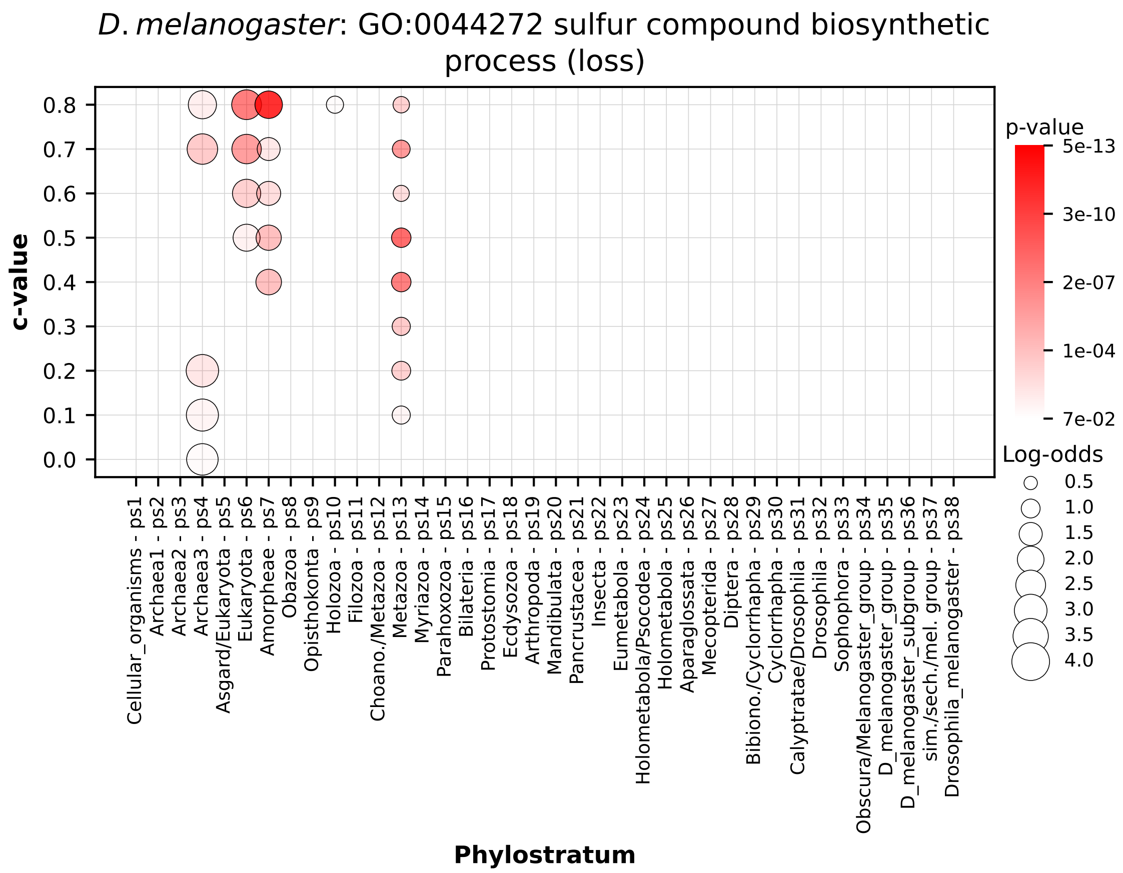

D. melanogaster: GO:0044272 sulfur compound biosynthetic process (loss)
c-value
0.8
0.7
0.6
0.5
0.4
0.3
0.2
0.1
0.0
p-value
5e-13
3e-10
2e-07
1e-04
7e-02
Log-odds
0.5
1.0
1.5
2.0
2.5
3.0
3.5
4.0
Cellular_organisms - ps1
Archaea1 - ps2
Archaea2 - ps3
Archaea3 - ps4
Asgard/Eukaryota - ps5
Eukaryota - ps6
Amorpheae - ps7
Obazoa - ps8
Opisthokonta - ps9
Holozoa - ps10
Filozoa - ps11
Choano./Metazoa - ps12
Metazoa - ps13
Myriazoa - ps14
Parahoxozoa - ps15
Bilateria - ps16
Protostomia - ps17
Ecdysozoa - ps18
Arthropoda - ps19
Mandibulata - ps20
Pancrustacea - ps21
Insecta - ps22
Eumetabola - ps23
Holometabola/Psocodea - ps24
Holometabola - ps25
Aparaglossata - ps26
Mecopterida - ps27
Diptera - ps28
Bibiono./Cyclorrhapha - ps29
Cyclorrhapha - ps30
Calyptratae/Drosophila - ps31
Drosophila - ps32
Sophophora - ps33
Obscura/Melanogaster_group - ps34
D_melanogaster_group - ps35
D_melanogaster_subgroup - ps36
sim./sech./mel. group - ps37
Drosophila_melanogaster - ps38
Phylostratum

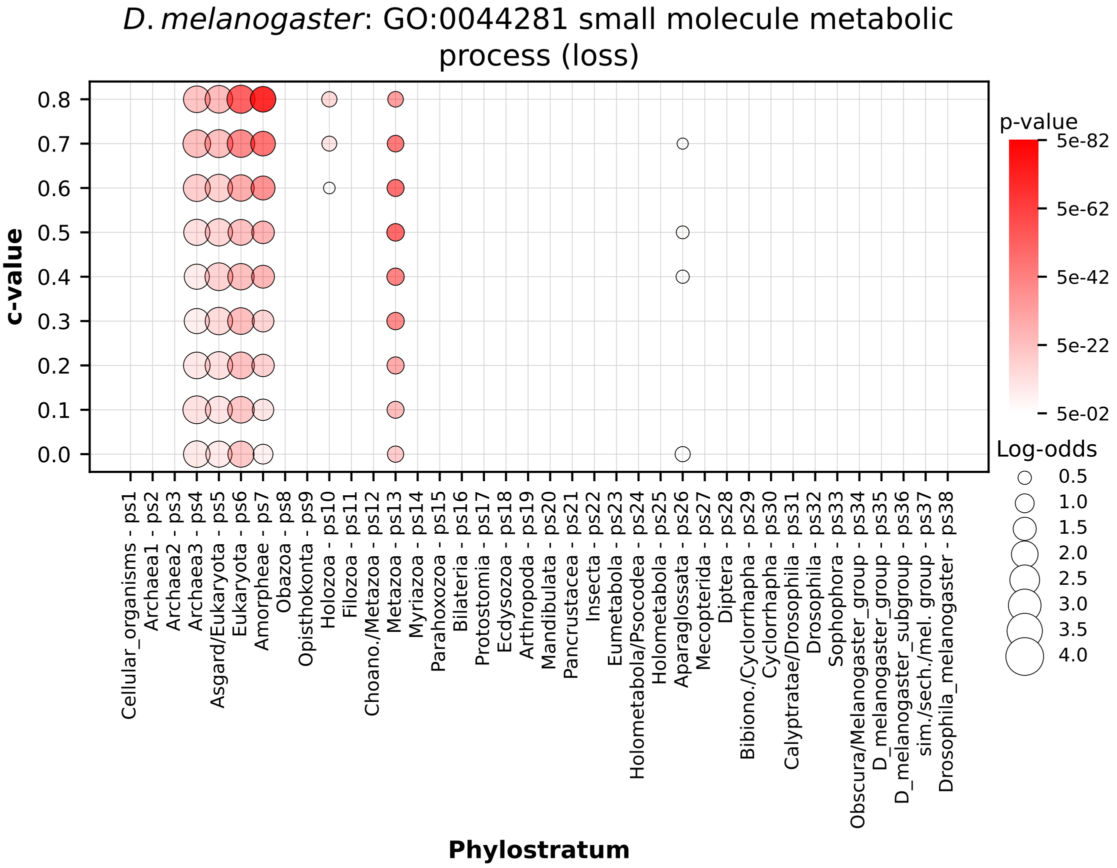

D. melanogaster: GO:0044281 small molecule metabolic process (loss)
c-value
0.8
0.7
0.6
0.5
0.4
0.3
0.2
0.1
0.0
Phylostratum
Cellular_organisms - ps1
Archaea1 - ps2
Archaea2 - ps3
Archaea3 - ps4
Asgard/Eukaryota - ps5
Eukaryota - ps6
Amorpheae - ps7
Obazoa - ps8
Opisthokonta - ps9
Holozoa - ps10
Filozoa - ps11
Choano./Metazoa - ps12
Metazoa - ps13
Myriazoa - ps14
Parahoxozoa - ps15
Bilateria - ps16
Protostomia - ps17
Ecdysozoa - ps18
Arthropoda - ps19
Mandibulata - ps20
Pancrustacea - ps21
Insecta - ps22
Eumetabola - ps23
Holometabola/Psocodea - ps24
Holometabola - ps25
Aparaglossata - ps26
Mecopterida - ps27
Diptera - ps28
Bibiono./Cyclorrhapha - ps29
Cyclorrhapha - ps30
Calyptratae/Drosophila - ps31
Drosophila - ps32
Sophophora - ps33
Obscura/Melanogaster_group - ps34
D_melanogaster_group - ps35
D_melanogaster_subgroup - ps36
sim./sech./mel. group - ps37
Drosophila_melanogaster - ps38
p-value
5e-82
5e-62
5e-42
5e-22
5e-02
Log-odds
0.5
1.0
1.5
2.0
2.5
3.0
3.5
4.0

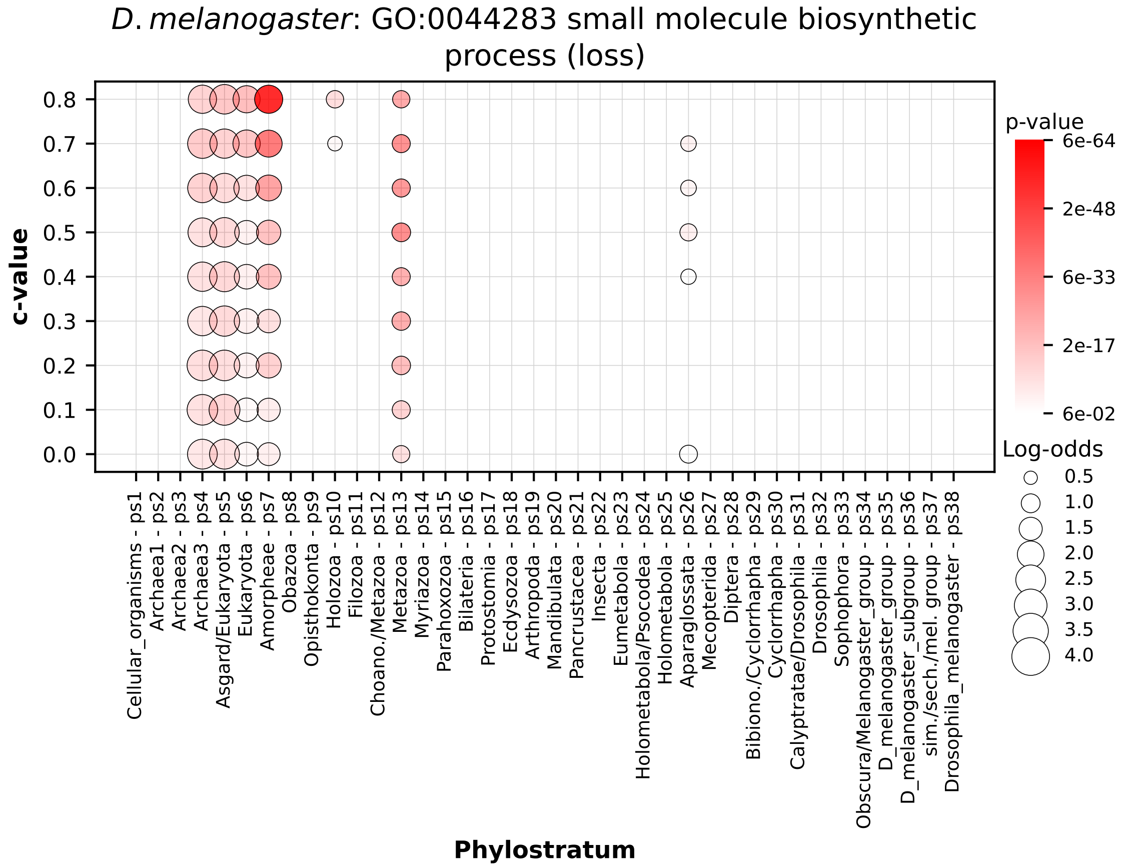
D. melanogaster: GO:0044283 small molecule biosynthetic process (loss)
c-value
0.8
0.7
0.6
0.5
0.4
0.3
0.2
0.1
0.0
Phylostratum
Cellular_organisms - ps1
Archaea1 - ps2
Archaea2 - ps3
Archaea3 - ps4
Asgard/Eukaryota - ps5
Eukaryota - ps6
Amorpheae - ps7
Obazoa - ps8
Opisthokonta - ps9
Holozoa - ps10
Filozoa - ps11
Choano./Metazoa - ps12
Metazoa - ps13
Myriazoa - ps14
Parahoxozoa - ps15
Bilateria - ps16
Protostomia - ps17
Ecdysozoa - ps18
Arthropoda - ps19
Mandibulata - ps20
Pancrustacea - ps21
Insecta - ps22
Eumetabola - ps23
Holometabola/Psocodea - ps24
Holometabola - ps25
Aparaglossata - ps26
Mecopterida - ps27
Diptera - ps28
Bibiono./Cyclorrhapha - ps29
Cyclorrhapha - ps30
Calyptratae/Drosophila - ps31
Drosophila - ps32
Sophophora - ps33
Obscura/Melanogaster_group - ps34
D_melanogaster_group - ps35
D_melanogaster_subgroup - ps36
sim./sech./mel. group - ps37
Drosophila_melanogaster - ps38
p-value
6e-64
2e-48
6e-33
2e-17
6e-02
Log-odds
0.5
1.0
1.5
2.0
2.5
3.0
3.5
4.0

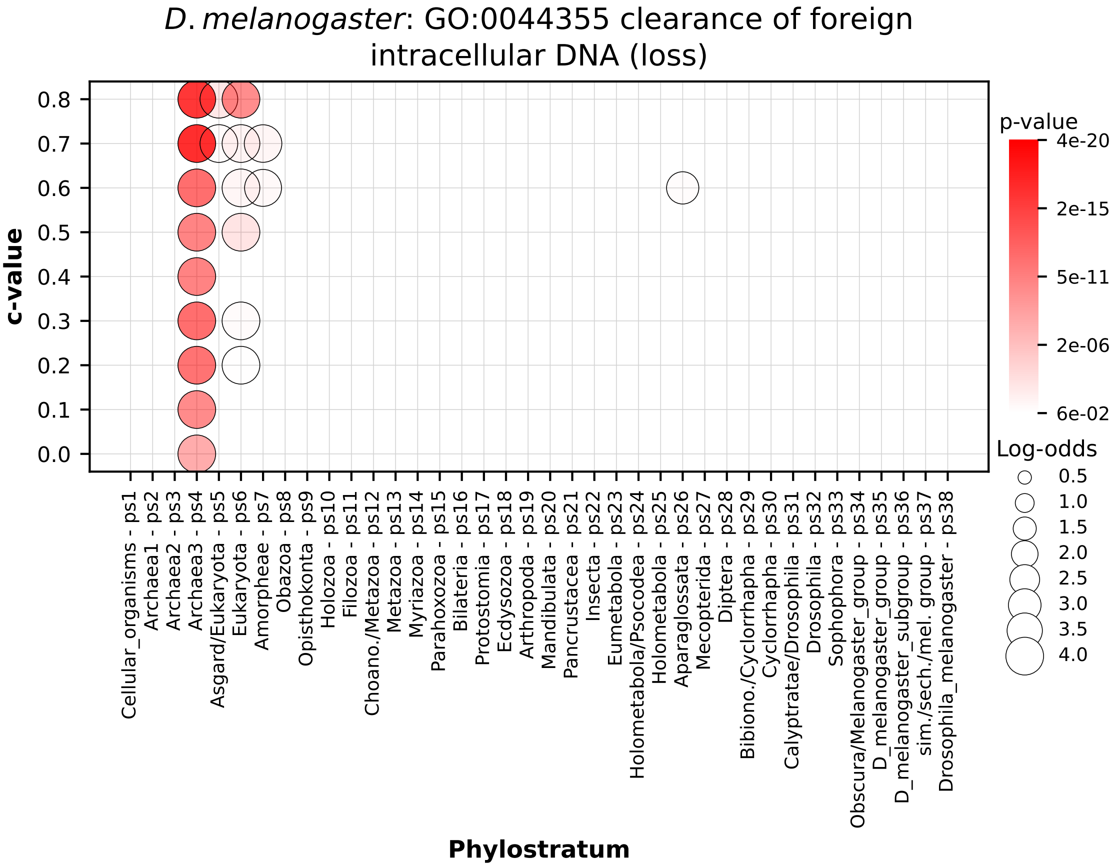

D. melanogaster: GO:0044355 clearance of foreign intracellular DNA (loss)
c-value
0.8
0.7
0.6
0.5
0.4
0.3
0.2
0.1
0.0
Phylostratum
Cellular_organisms - ps1
Archaea1 - ps2
Archaea2 - ps3
Archaea3 - ps4
Asgard/Eukaryota - ps5
Eukaryota - ps6
Amorpheae - ps7
Obazoa - ps8
Opisthokonta - ps9
Holozoa - ps10
Filozoa - ps11
Choano./Metazoa - ps12
Metazoa - ps13
Myriazoa - ps14
Parahoxozoa - ps15
Bilateria - ps16
Protostomia - ps17
Ecdysozoa - ps18
Arthropoda - ps19
Mandibulata - ps20
Pancrustacea - ps21
Insecta - ps22
Eumetabola - ps23
Holometabola/Psocodea - ps24
Holometabola - ps25
Aparaglossata - ps26
Mecopterida - ps27
Diptera - ps28
Bibiono./Cyclorrhapha - ps29
Cyclorrhapha - ps30
Calyptratae/Drosophila - ps31
Drosophila - ps32
Sophophora - ps33
Obscura/Melanogaster_group - ps34
D_melanogaster_group - ps35
D_melanogaster_subgroup - ps36
sim./sech./mel. group - ps37
Drosophila_melanogaster - ps38
p-value
4e-20
2e-15
5e-11
2e-06
6e-02
Log-odds
0.5
1.0
1.5
2.0
2.5
3.0
3.5
4.0

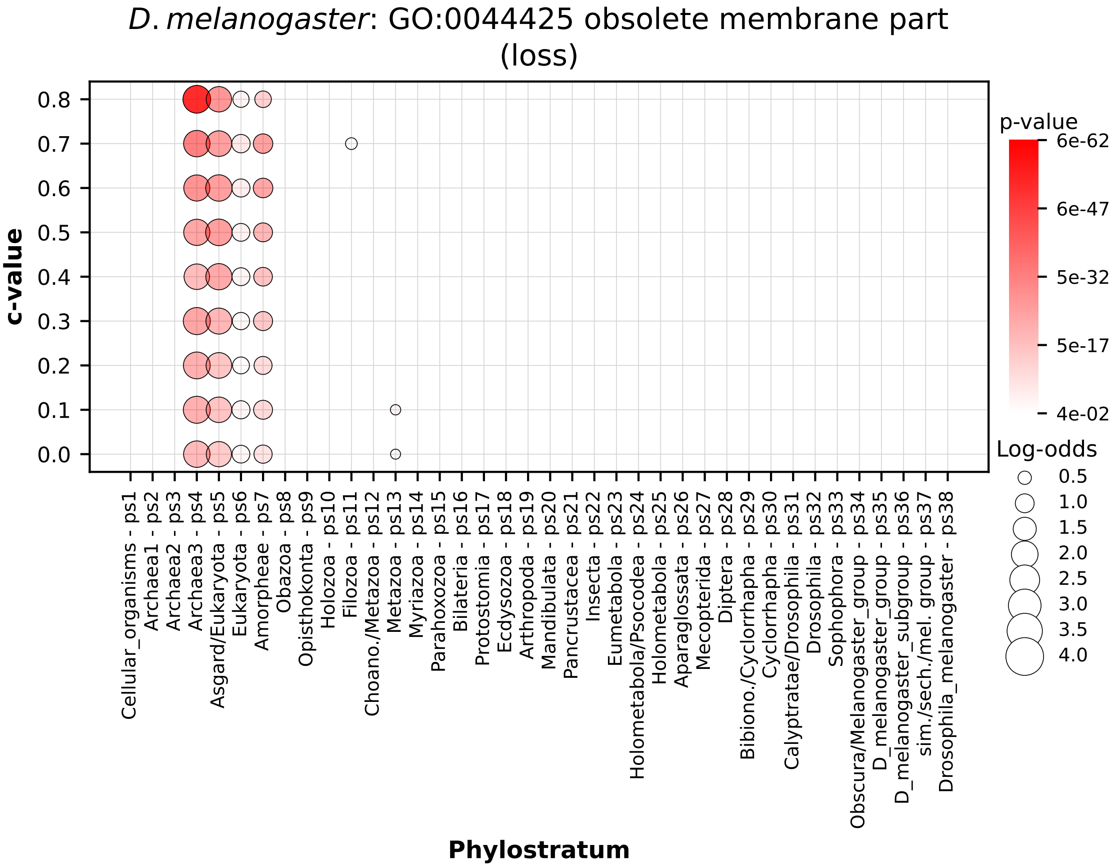

D. melanogaster: GO:0044425 obsolete membrane part (loss)
c-value
0.8
0.7
0.6
0.5
0.4
0.3
0.2
0.1
0.0
p-value
6e-62
6e-47
5e-32
5e-17
4e-02
Log-odds
0.5
1.0
1.5
2.0
2.5
3.0
3.5
4.0
Cellular_organisms - ps1
Archaea1 - ps2
Archaea2 - ps3
Archaea3 - ps4
Asgard/Eukaryota - ps5
Eukaryota - ps6
Amorpheae - ps7
Obazoa - ps8
Opisthokonta - ps9
Holozoa - ps10
Filozoa - ps11
Choano./Metazoa - ps12
Metazoa - ps13
Myriazoa - ps14
Parahoxozoa - ps15
Bilateria - ps16
Protostomia - ps17
Ecdysozoa - ps18
Arthropoda - ps19
Mandibulata - ps20
Pancrustacea - ps21
Insecta - ps22
Eumetabola - ps23
Holometabola/Psocodea - ps24
Holometabola - ps25
Aparaglossata - ps26
Mecopterida - ps27
Diptera - ps28
Bibiono./Cyclorrhapha - ps29
Cyclorrhapha - ps30
Calyptratae/Drosophila - ps31
Drosophila - ps32
Sophophora - ps33
Obscura/Melanogaster_group - ps34
D_melanogaster_group - ps35
D_melanogaster_subgroup - ps36
sim./sech./mel. group - ps37
Drosophila_melanogaster - ps38
Phylostratum

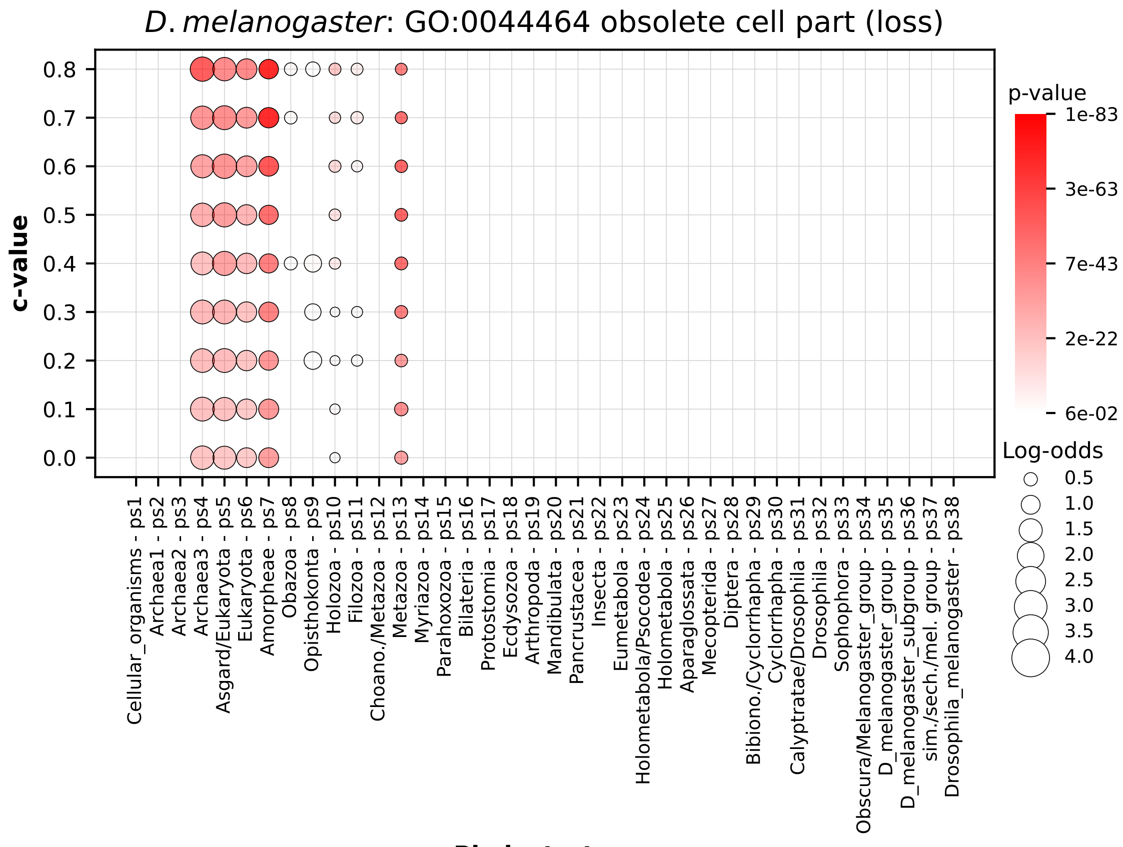

D. melanogaster: GO:0044464 obsolete cell part (loss)
c-value
0.8
0.7
0.6
0.5
0.4
0.3
0.2
0.1
0.0
Phylostratum
Cellular_organisms - ps1
Archaea1 - ps2
Archaea2 - ps3
Archaea3 - ps4
Asgard/Eukaryota - ps5
Eukaryota - ps6
Amorpheae - ps7
Obazoa - ps8
Opisthokonta - ps9
Holozoa - ps10
Filozoa - ps11
Choano./Metazoa - ps12
Metazoa - ps13
Myriazoa - ps14
Parahoxozoa - ps15
Bilateria - ps16
Protostomia - ps17
Ecdysozoa - ps18
Arthropoda - ps19
Mandibulata - ps20
Pancrustacea - ps21
Insecta - ps22
Eumetabola - ps23
Holometabola/Psocodea - ps24
Holometabola - ps25
Aparaglossata - ps26
Mecopterida - ps27
Diptera - ps28
Bibiono./Cyclorrhapha - ps29
Cyclorrhapha - ps30
Calyptratae/Drosophila - ps31
Drosophila - ps32
Sophophora - ps33
Obscura/Melanogaster_group - ps34
D_melanogaster_group - ps35
D_melanogaster_subgroup - ps36
sim./sech./mel. group - ps37
Drosophila_melanogaster - ps38
p-value
1e-83
3e-63
7e-43
2e-22
6e-02
Log-odds
0.5
1.0
1.5
2.0
2.5
3.0
3.5
4.0

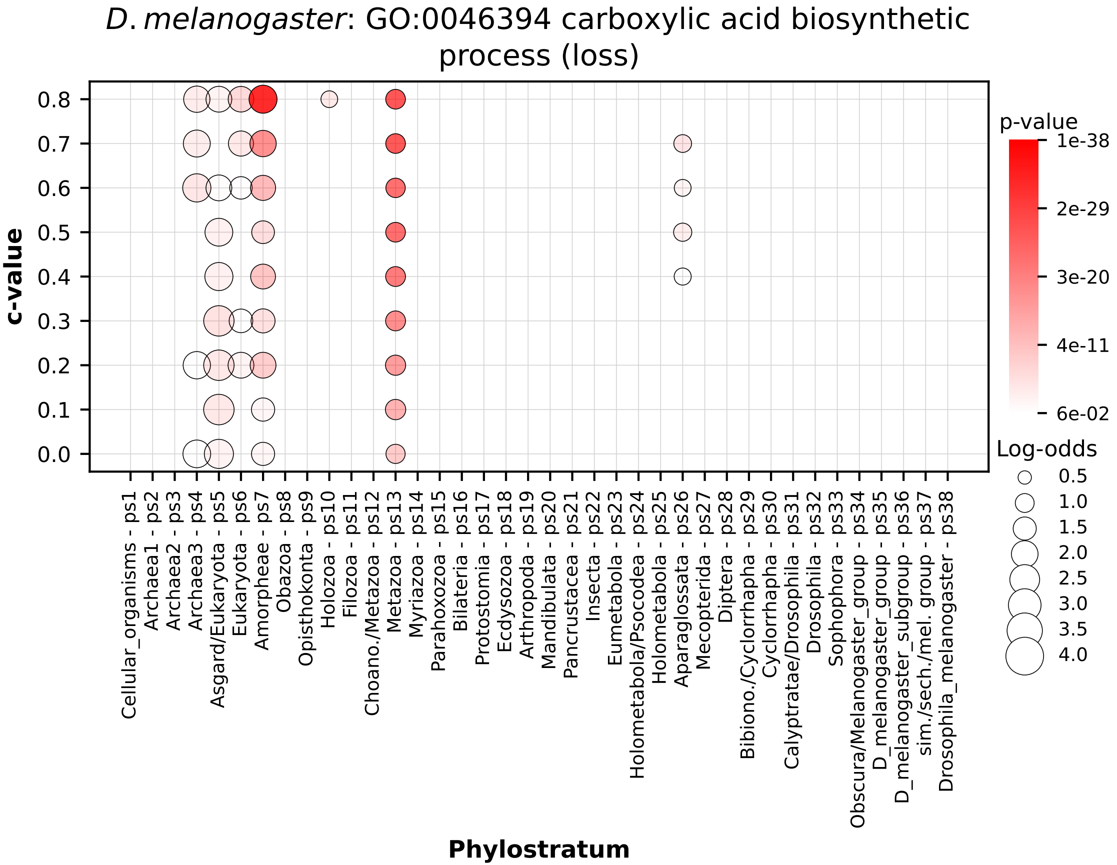
D. melanogaster: GO:0046394 carboxylic acid biosynthetic process (loss)
c-value
0.8
0.7
0.6
0.5
0.4
0.3
0.2
0.1
0.0
Phylostratum
Cellular_organisms - ps1
Archaea1 - ps2
Archaea2 - ps3
Archaea3 - ps4
Asgard/Eukaryota - ps5
Eukaryota - ps6
Amorpheae - ps7
Obazoa - ps8
Opisthokonta - ps9
Holozoa - ps10
Filozoa - ps11
Choano./Metazoa - ps12
Metazoa - ps13
Myriazoa - ps14
Parahoxozoa - ps15
Bilateria - ps16
Protostomia - ps17
Ecdysozoa - ps18
Arthropoda - ps19
Mandibulata - ps20
Pancrustacea - ps21
Insecta - ps22
Eumetabola - ps23
Holometabola/Psocodea - ps24
Holometabola - ps25
Aparaglossata - ps26
Mecopterida - ps27
Diptera - ps28
Bibiono./Cyclorrhapha - ps29
Cyclorrhapha - ps30
Calyptratae/Drosophila - ps31
Drosophila - ps32
Sophophora - ps33
Obscura/Melanogaster_group - ps34
D_melanogaster_group - ps35
D_melanogaster_subgroup - ps36
sim./sech./mel. group - ps37
Drosophila_melanogaster - ps38
p-value
1e-38
2e-29
3e-20
4e-11
6e-02
Log-odds
0.5
1.0
1.5
2.0
2.5
3.0
3.5
4.0

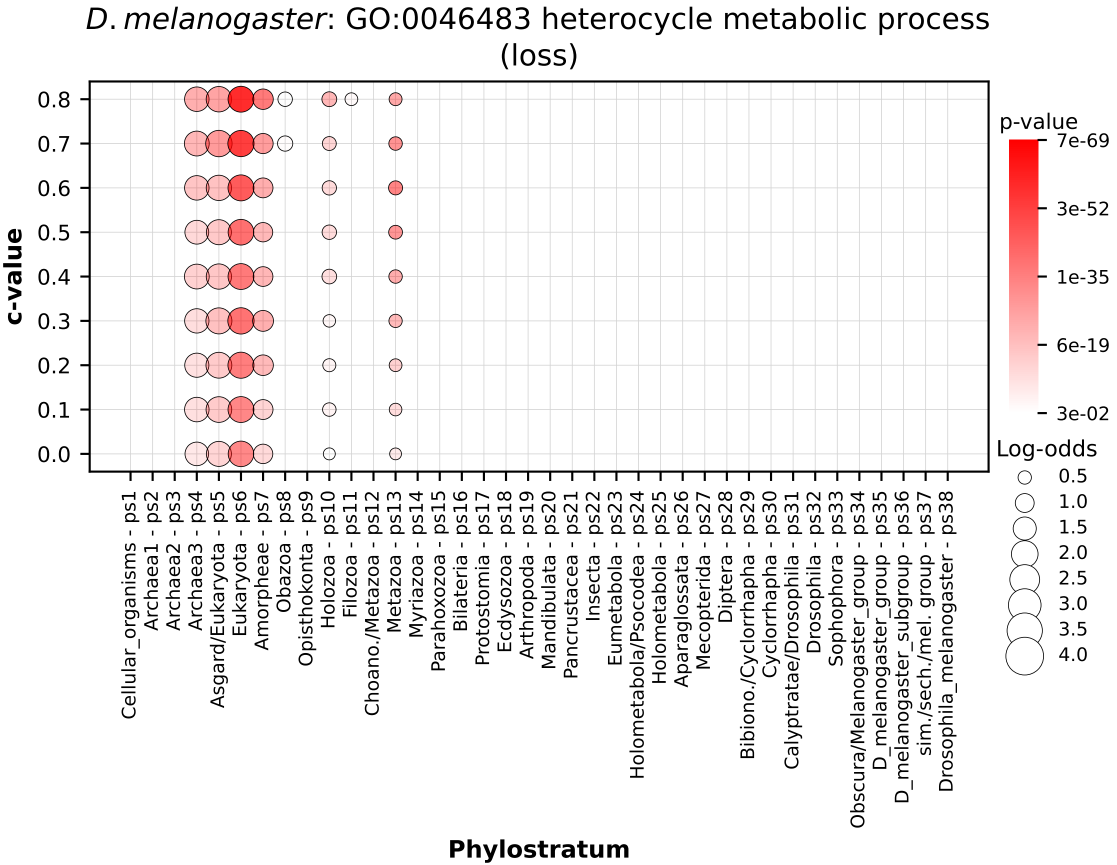

D. melanogaster: GO:0046483 heterocycle metabolic process (loss)
c-value
0.8
0.7
0.6
0.5
0.4
0.3
0.2
0.1
0.0
Phylostratum
Cellular_organisms - ps1
Archaea1 - ps2
Archaea2 - ps3
Archaea3 - ps4
Asgard/Eukaryota - ps5
Eukaryota - ps6
Amorpheae - ps7
Obazoa - ps8
Opisthokonta - ps9
Holozoa - ps10
Filozoa - ps11
Choano./Metazoa - ps12
Metazoa - ps13
Myriazoa - ps14
Parahoxozoa - ps15
Bilateria - ps16
Protostomia - ps17
Ecdysozoa - ps18
Arthropoda - ps19
Mandibulata - ps20
Pancrustacea - ps21
Insecta - ps22
Eumetabola - ps23
Holometabola/Psocodea - ps24
Holometabola - ps25
Aparaglossata - ps26
Mecopterida - ps27
Diptera - ps28
Bibiono./Cyclorrhapha - ps29
Cyclorrhapha - ps30
Calyptratae/Drosophila - ps31
Drosophila - ps32
Sophophora - ps33
Obscura/Melanogaster_group - ps34
D_melanogaster_group - ps35
D_melanogaster_subgroup - ps36
sim./sech./mel. group - ps37
Drosophila_melanogaster - ps38
p-value
7e-69
3e-52
1e-35
6e-19
3e-02
Log-odds
0.5
1.0
1.5
2.0
2.5
3.0
3.5
4.0

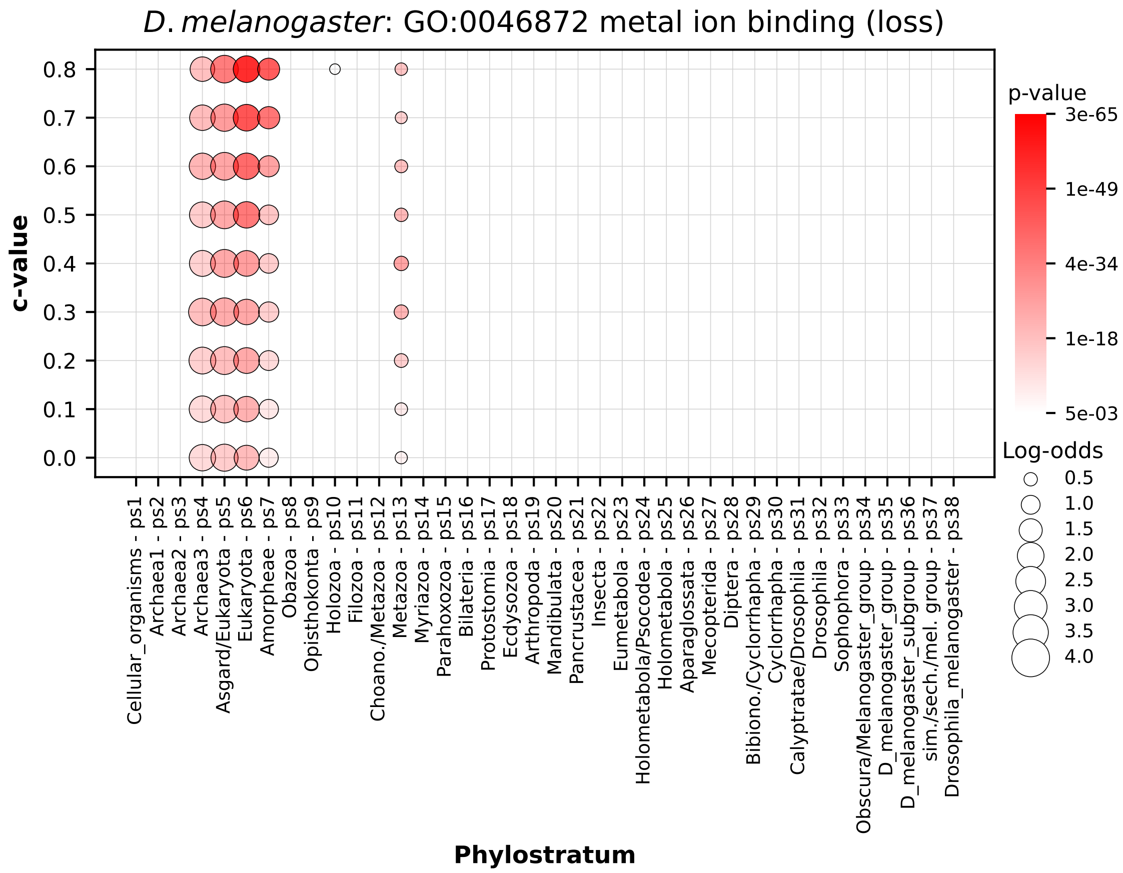

D. melanogaster: GO:0046872 metal ion binding (loss)
c-value
0.8
0.7
0.6
0.5
0.4
0.3
0.2
0.1
0.0
Phylostratum
Cellular_organisms - ps1
Archaea1 - ps2
Archaea2 - ps3
Archaea3 - ps4
Asgard/Eukaryota - ps5
Eukaryota - ps6
Amorpheae - ps7
Obazoa - ps8
Opisthokonta - ps9
Holozoa - ps10
Filozoa - ps11
Choano./Metazoa - ps12
Metazoa - ps13
Myriazoa - ps14
Parahoxozoa - ps15
Bilateria - ps16
Protostomia - ps17
Ecdysozoa - ps18
Arthropoda - ps19
Mandibulata - ps20
Pancrustacea - ps21
Insecta - ps22
Eumetabola - ps23
Holometabola/Psocodea - ps24
Holometabola - ps25
Aparaglossata - ps26
Mecopterida - ps27
Diptera - ps28
Bibiono./Cyclorrhapha - ps29
Cyclorrhapha - ps30
Calyptratae/Drosophila - ps31
Drosophila - ps32
Sophophora - ps33
Obscura/Melanogaster_group - ps34
D_melanogaster_group - ps35
D_melanogaster_subgroup - ps36
sim./sech./mel. group - ps37
Drosophila_melanogaster - ps38
p-value
3e-65
1e-49
4e-34
1e-18
5e-03
Log-odds
0.5
1.0
1.5
2.0
2.5
3.0
3.5
4.0

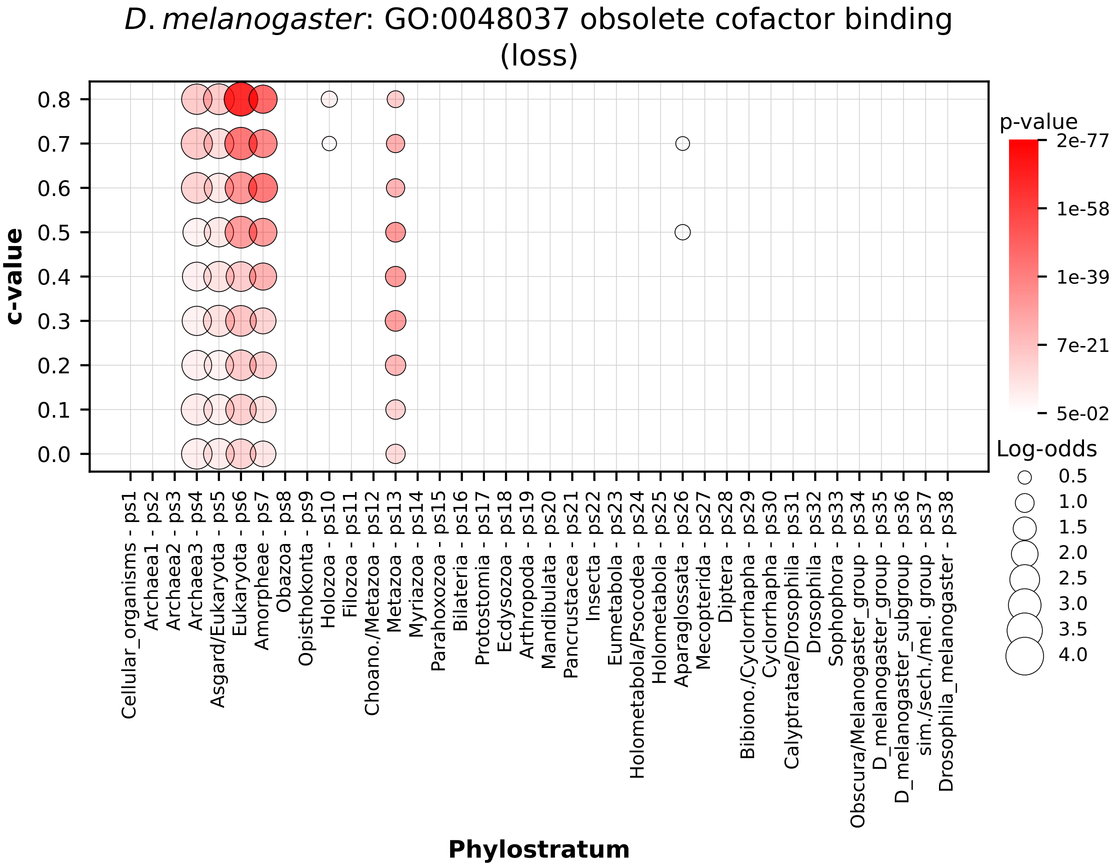

D. melanogaster: GO:0048037 obsolete cofactor binding (loss)
c-value
0.8
0.7
0.6
0.5
0.4
0.3
0.2
0.1
0.0
p-value
2e-77
1e-58
1e-39
7e-21
5e-02
Log-odds
0.5
1.0
1.5
2.0
2.5
3.0
3.5
4.0
Cellular_organisms - ps1
Archaea1 - ps2
Archaea2 - ps3
Archaea3 - ps4
Asgard/Eukaryota - ps5
Eukaryota - ps6
Amorpheae - ps7
Obazoa - ps8
Opisthokonta - ps9
Holozoa - ps10
Filozoa - ps11
Choano./Metazoa - ps12
Metazoa - ps13
Myriazoa - ps14
Parahoxozoa - ps15
Bilateria - ps16
Protostomia - ps17
Ecdysozoa - ps18
Arthropoda - ps19
Mandibulata - ps20
Pancrustacea - ps21
Insecta - ps22
Eumetabola - ps23
Holometabola/Psocodea - ps24
Holometabola - ps25
Aparaglossata - ps26
Mecopterida - ps27
Diptera - ps28
Bibiono./Cyclorrhapha - ps29
Cyclorrhapha - ps30
Calyptratae/Drosophila - ps31
Drosophila - ps32
Sophophora - ps33
Obscura/Melanogaster_group - ps34
D_melanogaster_group - ps35
D_melanogaster_subgroup - ps36
sim./sech./mel. group - ps37
Drosophila_melanogaster - ps38
Phylostratum

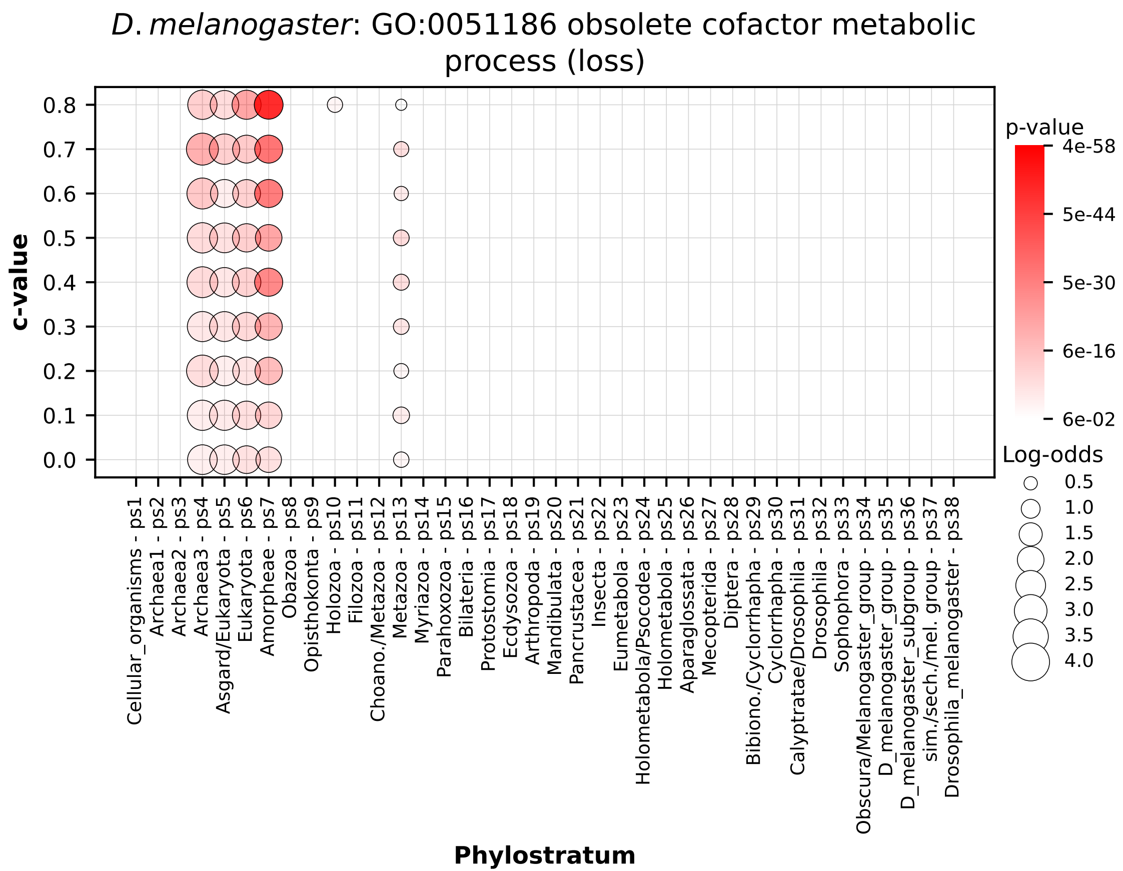
D. melanogaster: GO:0051186 obsolete cofactor metabolic process (loss)
c-value
0.8
0.7
0.6
0.5
0.4
0.3
0.2
0.1
0.0
p-value
4e-58
5e-44
5e-30
6e-16
6e-02
Log-odds
0.5
1.0
1.5
2.0
2.5
3.0
3.5
4.0
Cellular_organisms - ps1
Archaea1 - ps2
Archaea2 - ps3
Archaea3 - ps4
Asgard/Eukaryota - ps5
Eukaryota - ps6
Amorpheae - ps7
Obazoa - ps8
Opisthokonta - ps9
Holozoa - ps10
Filozoa - ps11
Choano./Metazoa - ps12
Metazoa - ps13
Myriazoa - ps14
Parahoxozoa - ps15
Bilateria - ps16
Protostomia - ps17
Ecdysozoa - ps18
Arthropoda - ps19
Mandibulata - ps20
Pancrustacea - ps21
Insecta - ps22
Eumetabola - ps23
Holometabola/Psocodea - ps24
Holometabola - ps25
Aparaglossata - ps26
Mecopterida - ps27
Diptera - ps28
Bibiono./Cyclorrhapha - ps29
Cyclorrhapha - ps30
Calyptratae/Drosophila - ps31
Drosophila - ps32
Sophophora - ps33
Obscura/Melanogaster_group - ps34
D_melanogaster_group - ps35
D_melanogaster_subgroup - ps36
sim./sech./mel. group - ps37
Drosophila_melanogaster - ps38
Phylostratum

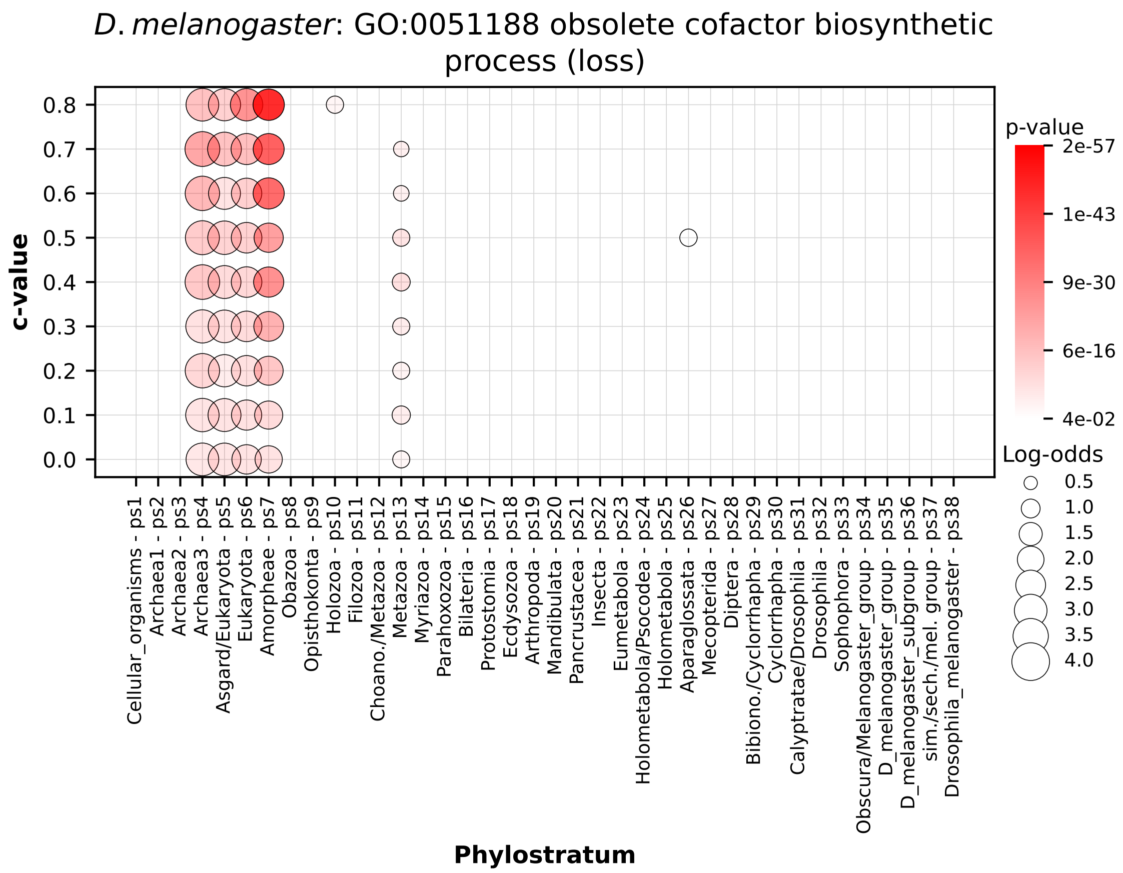

D. melanogaster: GO:0051188 obsolete cofactor biosynthetic process (loss)
c-value
0.8
0.7
0.6
0.5
0.4
0.3
0.2
0.1
0.0
Phylostratum
Cellular_organisms - ps1
Archaea1 - ps2
Archaea2 - ps3
Archaea3 - ps4
Asgard/Eukaryota - ps5
Eukaryota - ps6
Amorpheae - ps7
Obazoa - ps8
Opisthokonta - ps9
Holozoa - ps10
Filozoa - ps11
Choano./Metazoa - ps12
Metazoa - ps13
Myriazoa - ps14
Parahoxozoa - ps15
Bilateria - ps16
Protostomia - ps17
Ecdysozoa - ps18
Arthropoda - ps19
Mandibulata - ps20
Pancrustacea - ps21
Insecta - ps22
Eumetabola - ps23
Holometabola/Psocodea - ps24
Holometabola - ps25
Aparaglossata - ps26
Mecopterida - ps27
Diptera - ps28
Bibiono./Cyclorrhapha - ps29
Cyclorrhapha - ps30
Calyptratae/Drosophila - ps31
Drosophila - ps32
Sophophora - ps33
Obscura/Melanogaster_group - ps34
D_melanogaster_group - ps35
D_melanogaster_subgroup - ps36
sim./sech./mel. group - ps37
Drosophila_melanogaster - ps38
p-value
2e-57
1e-43
9e-30
6e-16
4e-02
Log-odds
0.5
1.0
1.5
2.0
2.5
3.0
3.5
4.0

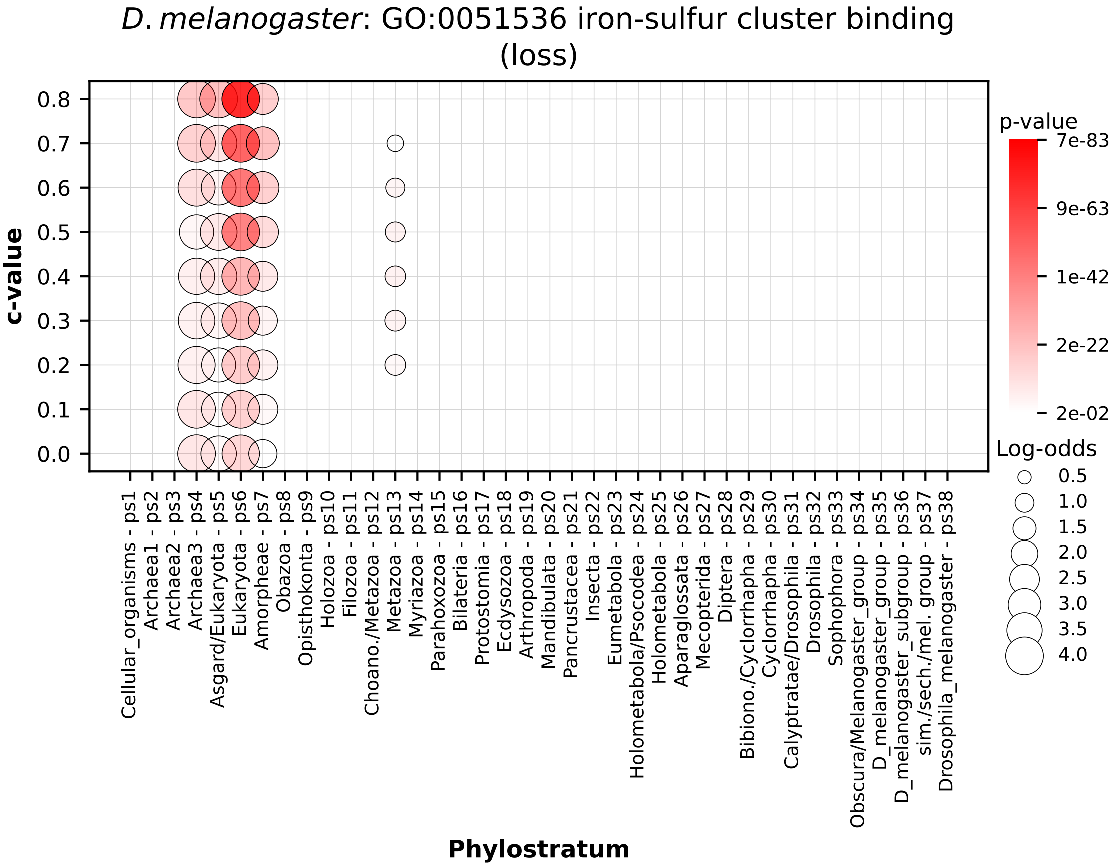

D. melanogaster: GO:0051536 iron-sulfur cluster binding
(loss)
c-value
0.8
0.7
0.6
0.5
0.4
0.3
0.2
0.1
0.0
p-value
7e-83
9e-63
1e-42
2e-22
2e-02
Log-odds
0.5
1.0
1.5
2.0
2.5
3.0
3.5
4.0
Cellular_organisms - ps1
Archaea1 - ps2
Archaea2 - ps3
Archaea3 - ps4
Asgard/Eukaryota - ps5
Eukaryota - ps6
Amorpheae - ps7
Obazoa - ps8
Opisthokonta - ps9
Holozoa - ps10
Filozoa - ps11
Choano./Metazoa - ps12
Metazoa - ps13
Myriazoa - ps14
Parahoxozoa - ps15
Bilateria - ps16
Protostomia - ps17
Ecdysozoa - ps18
Arthropoda - ps19
Mandibulata - ps20
Pancrustacea - ps21
Insecta - ps22
Eumetabola - ps23
Holometabola/Psocodea - ps24
Holometabola - ps25
Aparaglossata - ps26
Mecopterida - ps27
Diptera - ps28
Bibiono./Cyclorrhapha - ps29
Cyclorrhapha - ps30
Calyptratae/Drosophila - ps31
Drosophila - ps32
Sophophora - ps33
Obscura/Melanogaster_group - ps34
D_melanogaster_group - ps35
D_melanogaster_subgroup - ps36
sim./sech./mel. group - ps37
Drosophila_melanogaster - ps38
Phylostratum

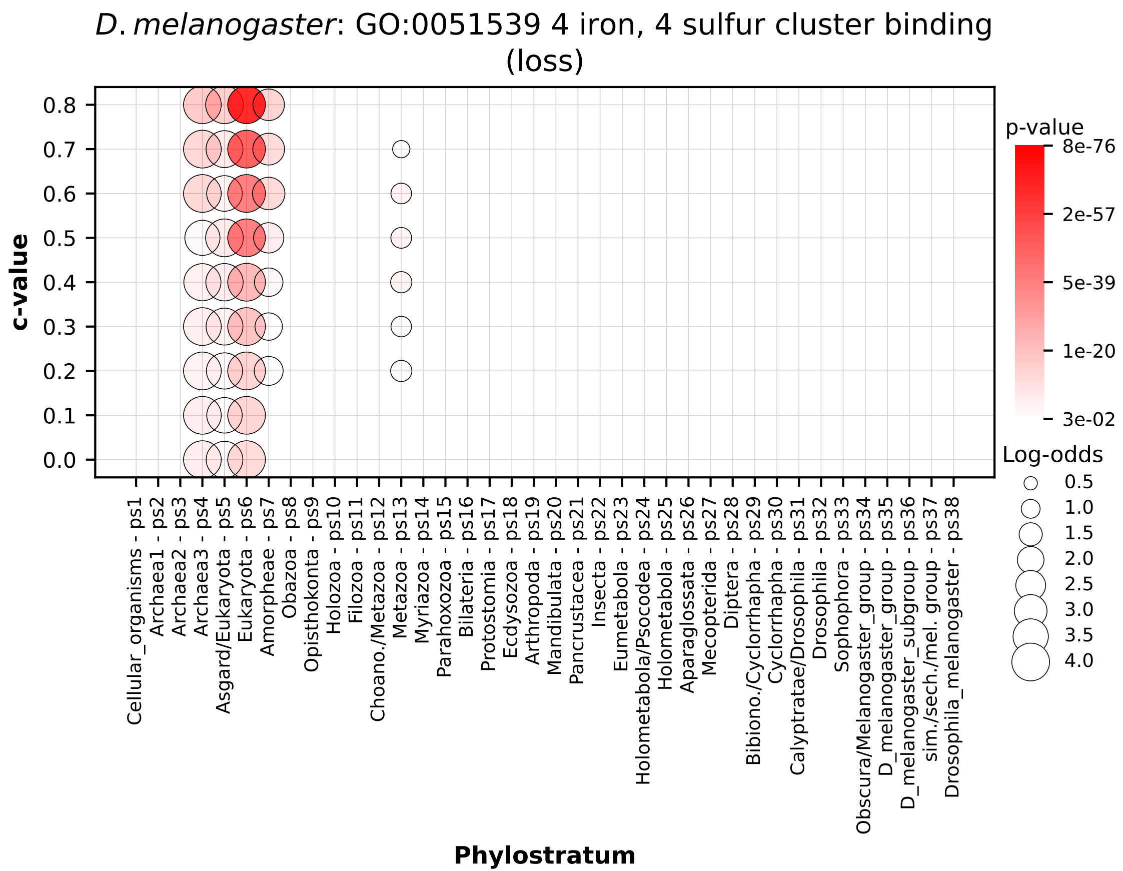

D. melanogaster: GO:0051539 4 iron, 4 sulfur cluster binding (loss)
c-value
0.8
0.7
0.6
0.5
0.4
0.3
0.2
0.1
0.0
p-value
8e-76
2e-57
5e-39
1e-20
3e-02
Log-odds
0.5
1.0
1.5
2.0
2.5
3.0
3.5
4.0
Cellular_organisms - ps1
Archaea1 - ps2
Archaea2 - ps3
Archaea3 - ps4
Asgard/Eukaryota - ps5
Eukaryota - ps6
Amorpheae - ps7
Obazoa - ps8
Opisthokonta - ps9
Holozoa - ps10
Filozoa - ps11
Choano./Metazoa - ps12
Metazoa - ps13
Myriazoa - ps14
Parahoxozoa - ps15
Bilateria - ps16
Protostomia - ps17
Ecdysozoa - ps18
Arthropoda - ps19
Mandibulata - ps20
Pancrustacea - ps21
Insecta - ps22
Eumetabola - ps23
Holometabola/Psocodea - ps24
Holometabola - ps25
Aparaglossata - ps26
Mecopterida - ps27
Diptera - ps28
Bibiono./Cyclorrhapha - ps29
Cyclorrhapha - ps30
Calyptratae/Drosophila - ps31
Drosophila - ps32
Sophophora - ps33
Obscura/Melanogaster_group - ps34
D_melanogaster_group - ps35
D_melanogaster_subgroup - ps36
sim./sech./mel. group - ps37
Drosophila_melanogaster - ps38
Phylostratum

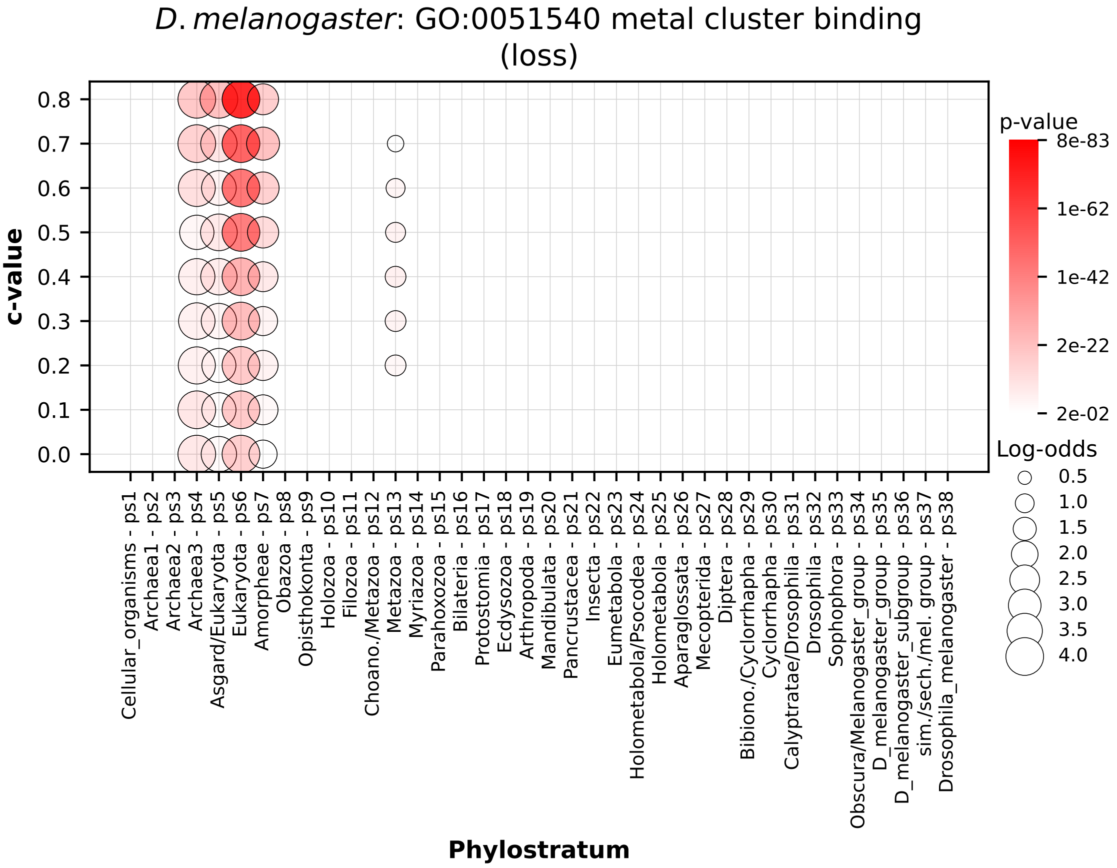

D. melanogaster: GO:0051540 metal cluster binding
(loss)
c-value
0.8
0.7
0.6
0.5
0.4
0.3
0.2
0.1
0.0
Phylostratum
Cellular_organisms - ps1
Archaea1 - ps2
Archaea2 - ps3
Archaea3 - ps4
Asgard/Eukaryota - ps5
Eukaryota - ps6
Amorpheae - ps7
Obazoa - ps8
Opisthokonta - ps9
Holozoa - ps10
Filozoa - ps11
Choano./Metazoa - ps12
Metazoa - ps13
Myriazoa - ps14
Parahoxozoa - ps15
Bilateria - ps16
Protostomia - ps17
Ecdysozoa - ps18
Arthropoda - ps19
Mandibulata - ps20
Pancrustacea - ps21
Insecta - ps22
Eumetabola - ps23
Holometabola/Psocodea - ps24
Holometabola - ps25
Aparaglossata - ps26
Mecopterida - ps27
Diptera - ps28
Bibiono./Cyclorrhapha - ps29
Cyclorrhapha - ps30
Calyptratae/Drosophila - ps31
Drosophila - ps32
Sophophora - ps33
Obscura/Melanogaster_group - ps34
D_melanogaster_group - ps35
D_melanogaster_subgroup - ps36
sim./sech./mel. group - ps37
Drosophila_melanogaster - ps38
p-value
8e-83
1e-62
1e-42
2e-22
2e-02
Log-odds
0.5
1.0
1.5
2.0
2.5
3.0
3.5
4.0

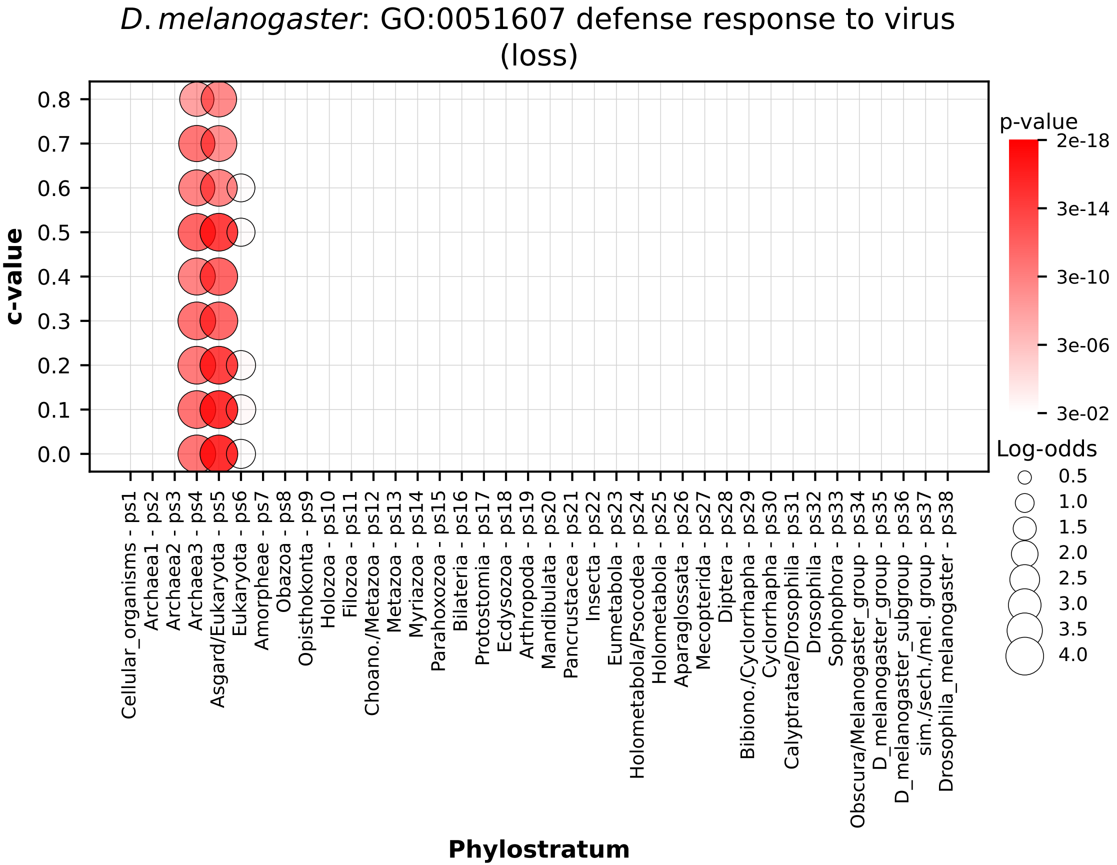

D. melanogaster: GO:0051607 defense response to virus
(loss)
c-value
0.8
0.7
0.6
0.5
0.4
0.3
0.2
0.1
0.0
p-value
2e-18
3e-14
3e-10
3e-06
3e-02
Log-odds
0.5
1.0
1.5
2.0
2.5
3.0
3.5
4.0
Cellular_organisms - ps1
Archaea1 - ps2
Archaea2 - ps3
Archaea3 - ps4
Asgard/Eukaryota - ps5
Eukaryota - ps6
Amorpheae - ps7
Obazoa - ps8
Opisthokonta - ps9
Holozoa - ps10
Filozoa - ps11
Choano./Metazoa - ps12
Metazoa - ps13
Myriazoa - ps14
Parahoxozoa - ps15
Bilateria - ps16
Protostomia - ps17
Ecdysozoa - ps18
Arthropoda - ps19
Mandibulata - ps20
Pancrustacea - ps21
Insecta - ps22
Eumetabola - ps23
Holometabola/Psocodea - ps24
Holometabola - ps25
Aparaglossata - ps26
Mecopterida - ps27
Diptera - ps28
Bibiono./Cyclorrhapha - ps29
Cyclorrhapha - ps30
Calyptratae/Drosophila - ps31
Drosophila - ps32
Sophophora - ps33
Obscura/Melanogaster_group - ps34
D_melanogaster_group - ps35
D_melanogaster_subgroup - ps36
sim./sech./mel. group - ps37
Drosophila_melanogaster - ps38
Phylostratum

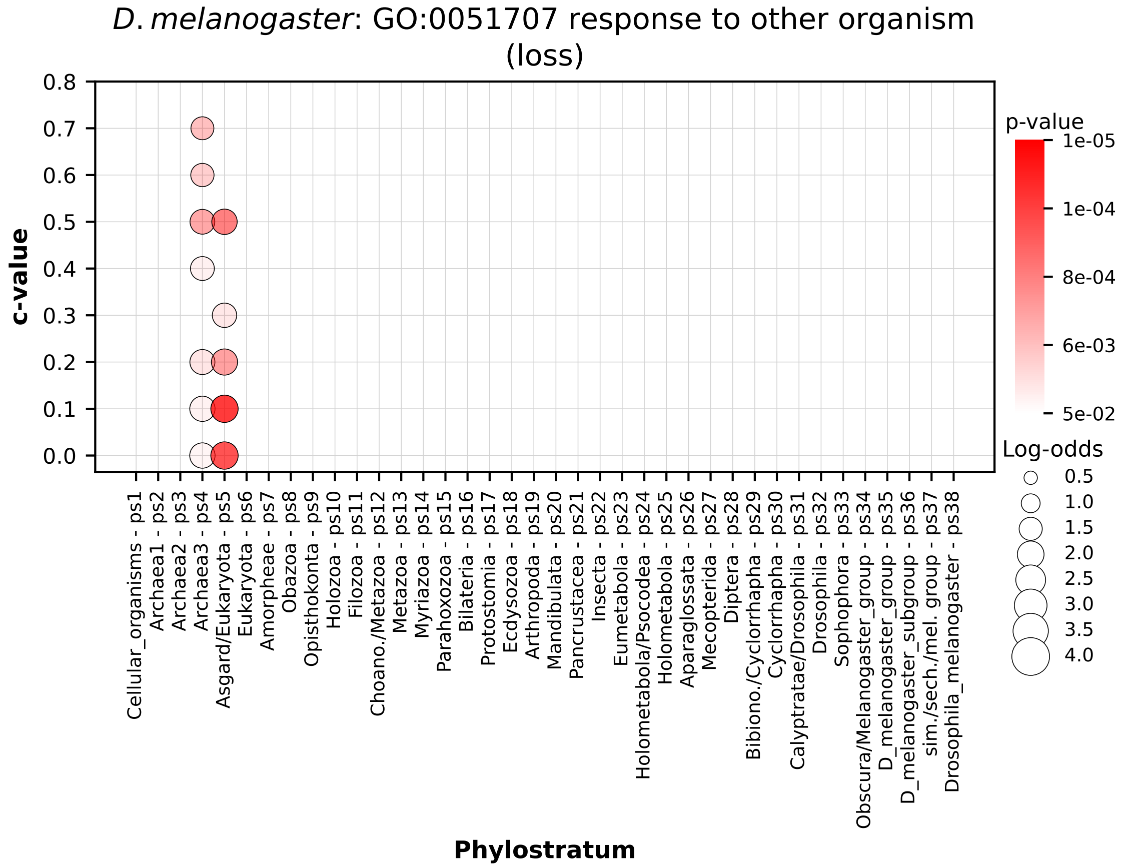

D. melanogaster: GO:0051707 response to other organism (loss)
c-value
0.8
0.7
0.6
0.5
0.4
0.3
0.2
0.1
0.0
p-value
1e-05
1e-04
8e-04
6e-03
5e-02
Log-odds
0.5
1.0
1.5
2.0
2.5
3.0
3.5
4.0
Cellular_organisms - ps1
Archaea1 - ps2
Archaea2 - ps3
Archaea3 - ps4
Asgard/Eukaryota - ps5
Eukaryota - ps6
Amorpheae - ps7
Obazoa - ps8
Opisthokonta - ps9
Holozoa - ps10
Filozoa - ps11
Choano./Metazoa - ps12
Metazoa - ps13
Myriazoa - ps14
Parahoxozoa - ps15
Bilateria - ps16
Protostomia - ps17
Ecdysozoa - ps18
Arthropoda - ps19
Mandibulata - ps20
Pancrustacea - ps21
Insecta - ps22
Eumetabola - ps23
Holometabola/Psocodea - ps24
Holometabola - ps25
Aparaglossata - ps26
Mecopterida - ps27
Diptera - ps28
Bibiono./Cyclorrhapha - ps29
Cyclorrhapha - ps30
Calyptratae/Drosophila - ps31
Drosophila - ps32
Sophophora - ps33
Obscura/Melanogaster_group - ps34
D_melanogaster_group - ps35
D_melanogaster_subgroup - ps36
sim./sech./mel. group - ps37
Drosophila_melanogaster - ps38
Phylostratum

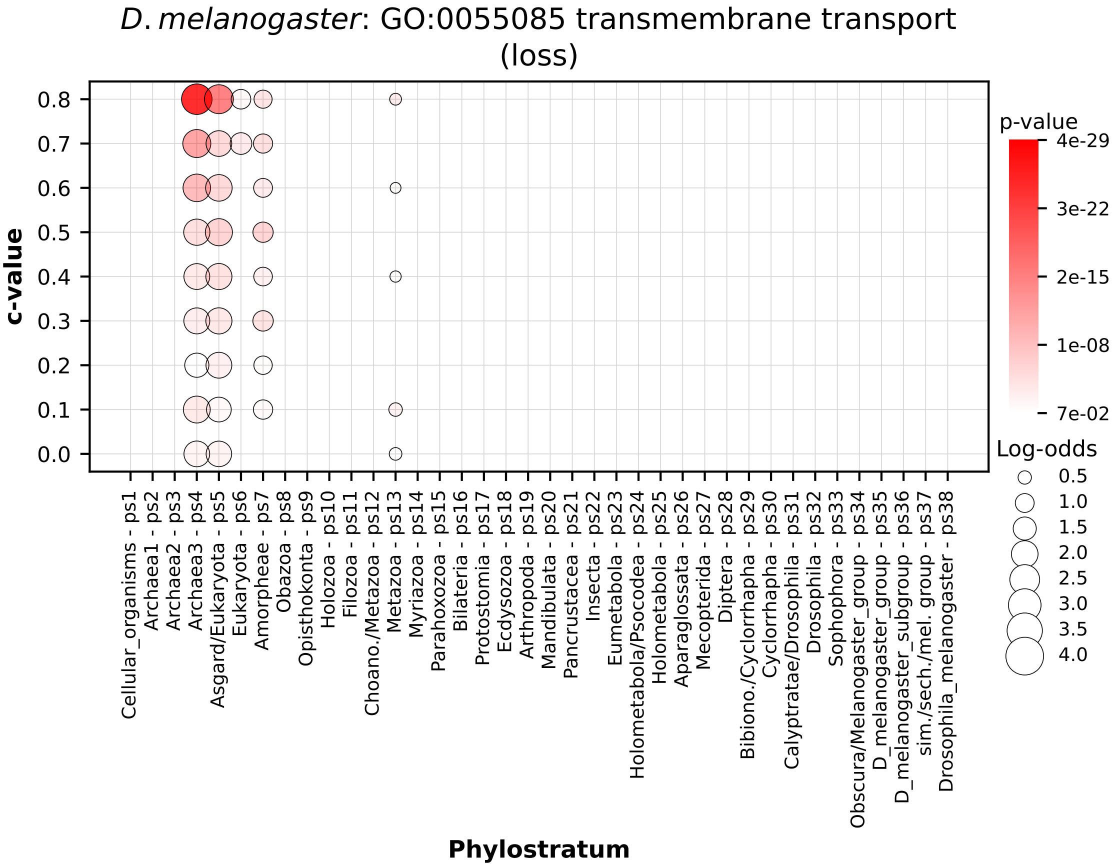

D. melanogaster: GO:0055085 transmembrane transport
(loss)
c-value
0.8
0.7
0.6
0.5
0.4
0.3
0.2
0.1
0.0
p-value
4e-29
3e-22
2e-15
1e-08
7e-02
Log-odds
0.5
1.0
1.5
2.0
2.5
3.0
3.5
4.0
Cellular_organisms - ps1
Archaea1 - ps2
Archaea2 - ps3
Archaea3 - ps4
Asgard/Eukaryota - ps5
Eukaryota - ps6
Amorpheae - ps7
Obazoa - ps8
Opisthokonta - ps9
Holozoa - ps10
Filozoa - ps11
Choano./Metazoa - ps12
Metazoa - ps13
Myriazoa - ps14
Parahoxozoa - ps15
Bilateria - ps16
Protostomia - ps17
Ecdysozoa - ps18
Arthropoda - ps19
Mandibulata - ps20
Pancrustacea - ps21
Insecta - ps22
Eumetabola - ps23
Holometabola/Psocodea - ps24
Holometabola - ps25
Aparaglossata - ps26
Mecopterida - ps27
Diptera - ps28
Bibiono./Cyclorrhapha - ps29
Cyclorrhapha - ps30
Calyptratae/Drosophila - ps31
Drosophila - ps32
Sophophora - ps33
Obscura/Melanogaster_group - ps34
D_melanogaster_group - ps35
D_melanogaster_subgroup - ps36
sim./sech./mel. group - ps37
Drosophila_melanogaster - ps38
Phylostratum

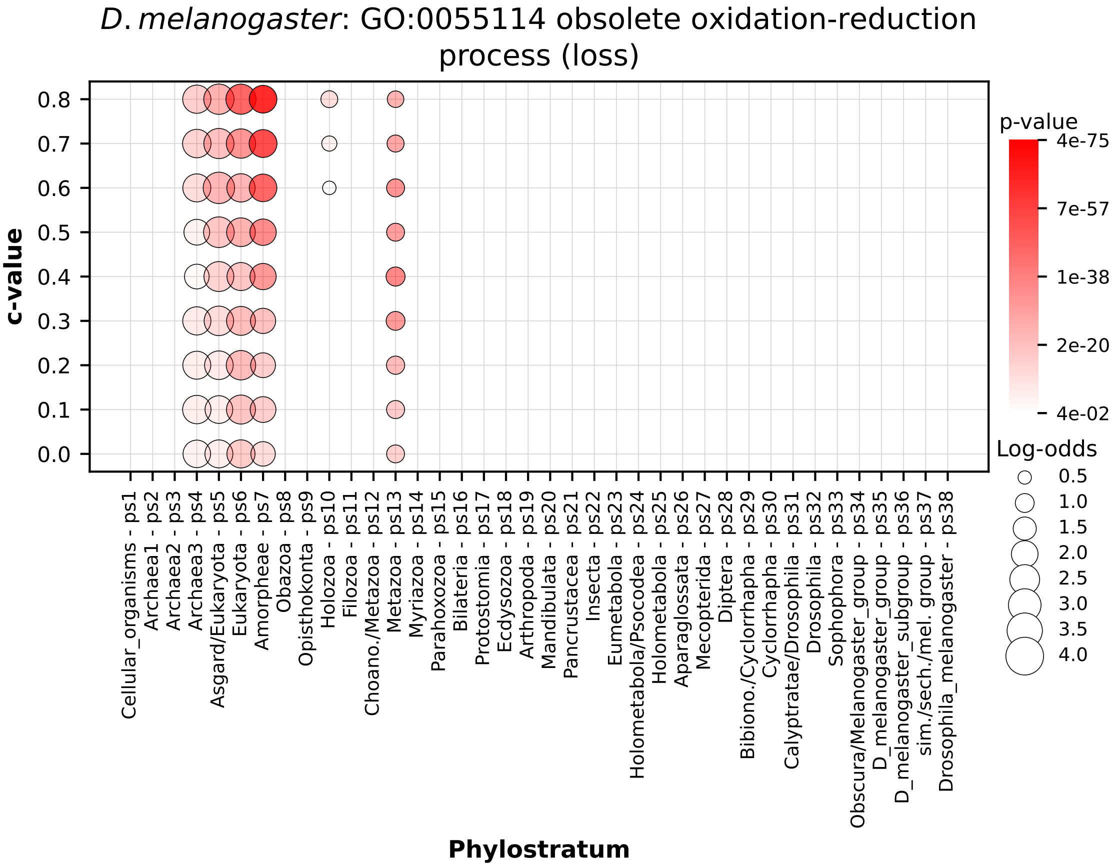

D. melanogaster: GO:0055114 obsolete oxidation-reduction process (loss)
c-value
0.8
0.7
0.6
0.5
0.4
0.3
0.2
0.1
0.0
Phylostratum
Cellular_organisms - ps1
Archaea1 - ps2
Archaea2 - ps3
Archaea3 - ps4
Asgard/Eukaryota - ps5
Eukaryota - ps6
Amorpheae - ps7
Obazoa - ps8
Opisthokonta - ps9
Holozoa - ps10
Filozoa - ps11
Choano./Metazoa - ps12
Metazoa - ps13
Myriazoa - ps14
Parahoxozoa - ps15
Bilateria - ps16
Protostomia - ps17
Ecdysozoa - ps18
Arthropoda - ps19
Mandibulata - ps20
Pancrustacea - ps21
Insecta - ps22
Eumetabola - ps23
Holometabola/Psocodea - ps24
Holometabola - ps25
Aparaglossata - ps26
Mecopterida - ps27
Diptera - ps28
Bibiono./Cyclorrhapha - ps29
Cyclorrhapha - ps30
Calyptratae/Drosophila - ps31
Drosophila - ps32
Sophophora - ps33
Obscura/Melanogaster_group - ps34
D_melanogaster_group - ps35
D_melanogaster_subgroup - ps36
sim./sech./mel. group - ps37
Drosophila_melanogaster - ps38
p-value
4e-75
7e-57
1e-38
2e-20
4e-02
Log-odds
0.5
1.0
1.5
2.0
2.5
3.0
3.5
4.0

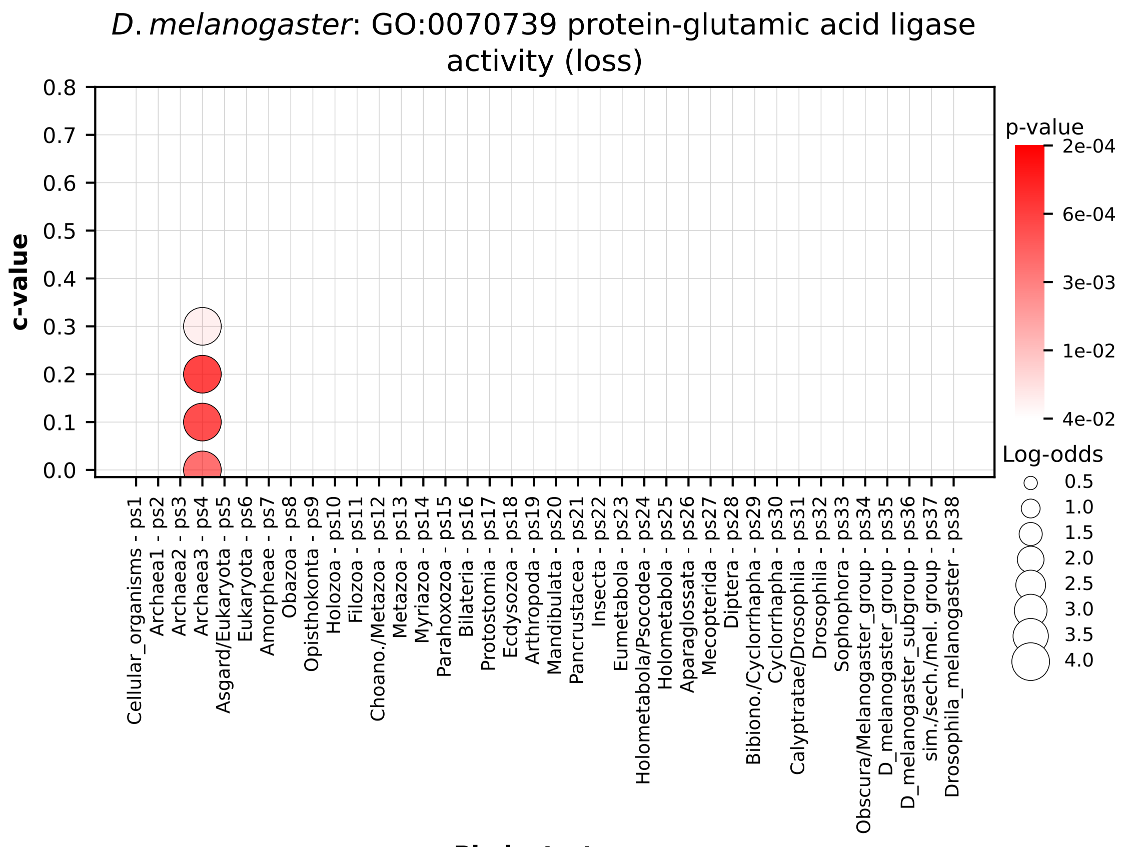

D. melanogaster: GO:0070739 protein-glutamic acid ligase activity (loss)
c-value
0.8
0.7
0.6
0.5
0.4
0.3
0.2
0.1
0.0
p-value
2e-04
6e-04
3e-03
1e-02
4e-02
Log-odds
0.5
1.0
1.5
2.0
2.5
3.0
3.5
4.0
Cellular_organisms - ps1
Archaea1 - ps2
Archaea2 - ps3
Archaea3 - ps4
Asgard/Eukaryota - ps5
Eukaryota - ps6
Amorpheae - ps7
Obazoa - ps8
Opisthokonta - ps9
Holozoa - ps10
Filozoa - ps11
Choano./Metazoa - ps12
Metazoa - ps13
Myriazoa - ps14
Parahoxozoa - ps15
Bilateria - ps16
Protostomia - ps17
Ecdysozoa - ps18
Arthropoda - ps19
Mandibulata - ps20
Pancrustacea - ps21
Insecta - ps22
Eumetabola - ps23
Holometabola/Psocodea - ps24
Holometabola - ps25
Aparaglossata - ps26
Mecopterida - ps27
Diptera - ps28
Bibiono./Cyclorrhapha - ps29
Cyclorrhapha - ps30
Calyptratae/Drosophila - ps31
Drosophila - ps32
Sophophora - ps33
Obscura/Melanogaster_group - ps34
D_melanogaster_group - ps35
D_melanogaster_subgroup - ps36
sim./sech./mel. group - ps37
Drosophila_melanogaster - ps38
Phylostratum

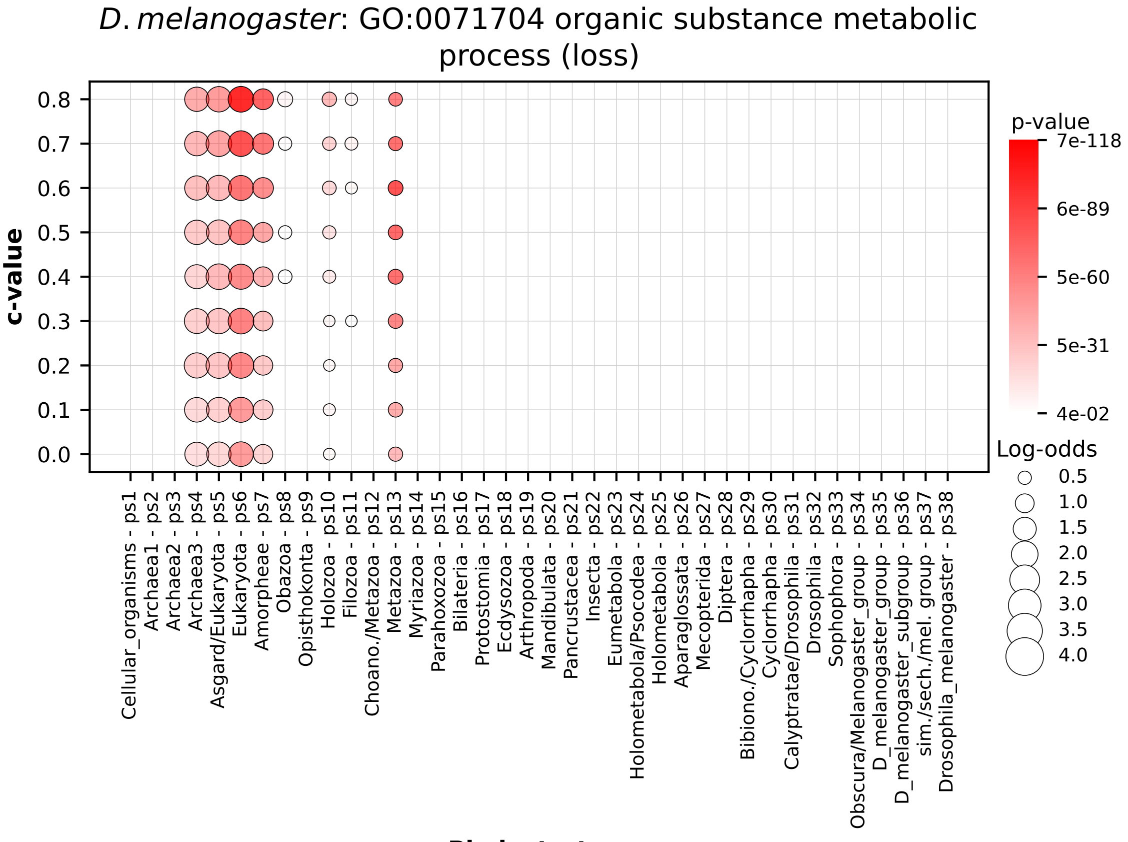

D. melanogaster: GO:0071704 organic substance metabolic process (loss)
c-value
0.8
0.7
0.6
0.5
0.4
0.3
0.2
0.1
0.0
Phylostratum
Cellular_organisms - ps1
Archaea1 - ps2
Archaea2 - ps3
Archaea3 - ps4
Asgard/Eukaryota - ps5
Eukaryota - ps6
Amorpheae - ps7
Obazoa - ps8
Opisthokonta - ps9
Holozoa - ps10
Filozoa - ps11
Choano./Metazoa - ps12
Metazoa - ps13
Myriazoa - ps14
Parahoxozoa - ps15
Bilateria - ps16
Protostomia - ps17
Ecdysozoa - ps18
Arthropoda - ps19
Mandibulata - ps20
Pancrustacea - ps21
Insecta - ps22
Eumetabola - ps23
Holometabola/Psocodea - ps24
Holometabola - ps25
Aparaglossata - ps26
Mecopterida - ps27
Diptera - ps28
Bibiono./Cyclorrhapha - ps29
Cyclorrhapha - ps30
Calyptratae/Drosophila - ps31
Drosophila - ps32
Sophophora - ps33
Obscura/Melanogaster_group - ps34
D_melanogaster_group - ps35
D_melanogaster_subgroup - ps36
sim./sech./mel. group - ps37
Drosophila_melanogaster - ps38
p-value
7e-118
6e-89
5e-60
5e-31
4e-02
Log-odds
0.5
1.0
1.5
2.0
2.5
3.0
3.5
4.0

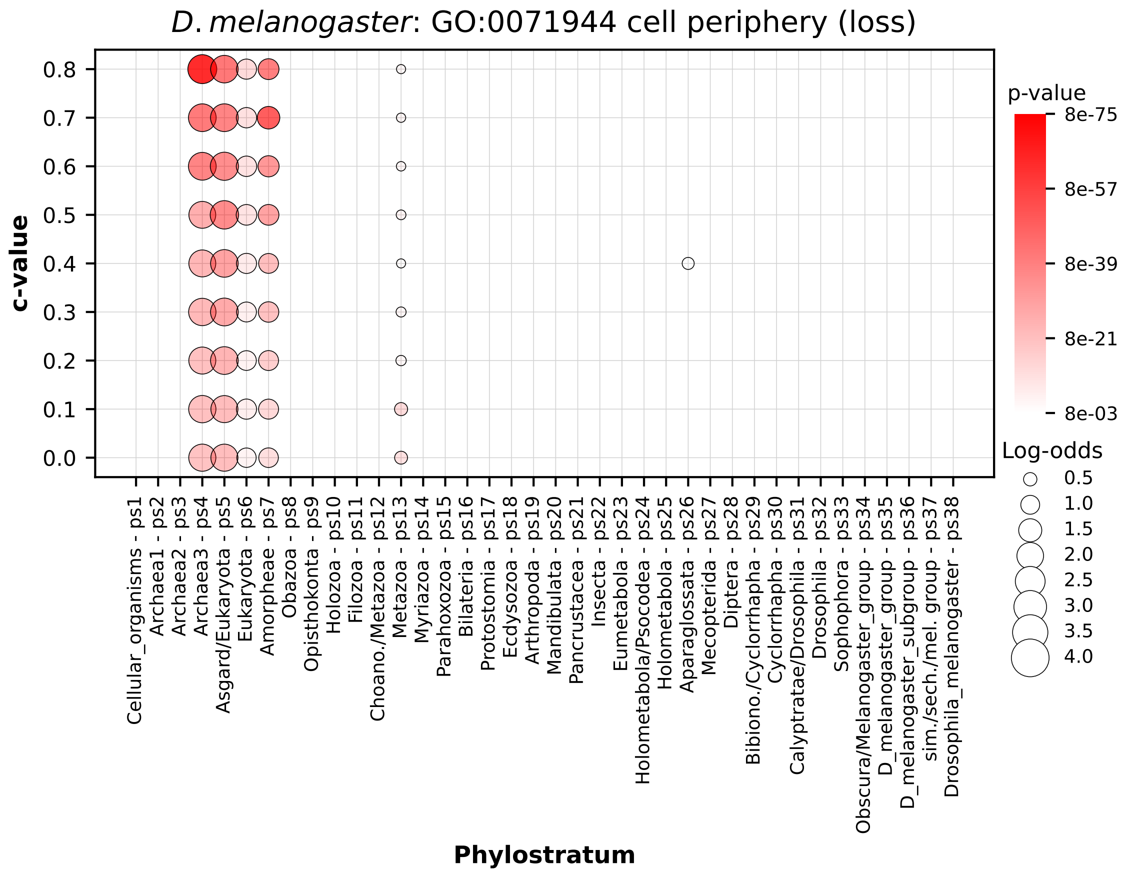

D. melanogaster: GO:0071944 cell periphery (loss)
c-value
0.8
0.7
0.6
0.5
0.4
0.3
0.2
0.1
0.0
p-value
8e-75
8e-57
8e-39
8e-21
8e-03
Log-odds
0.5
1.0
1.5
2.0
2.5
3.0
3.5
4.0
Cellular_organisms - ps1
Archaea1 - ps2
Archaea2 - ps3
Archaea3 - ps4
Asgard/Eukaryota - ps5
Eukaryota - ps6
Amorpheae - ps7
Obazoa - ps8
Opisthokonta - ps9
Holozoa - ps10
Filozoa - ps11
Choano./Metazoa - ps12
Metazoa - ps13
Myriazoa - ps14
Parahoxozoa - ps15
Bilateria - ps16
Protostomia - ps17
Ecdysozoa - ps18
Arthropoda - ps19
Mandibulata - ps20
Pancrustacea - ps21
Insecta - ps22
Eumetabola - ps23
Holometabola/Psocodea - ps24
Holometabola - ps25
Aparaglossata - ps26
Mecopterida - ps27
Diptera - ps28
Bibiono./Cyclorrhapha - ps29
Cyclorrhapha - ps30
Calyptratae/Drosophila - ps31
Drosophila - ps32
Sophophora - ps33
Obscura/Melanogaster_group - ps34
D_melanogaster_group - ps35
D_melanogaster_subgroup - ps36
sim./sech./mel. group - ps37
Drosophila_melanogaster - ps38
Phylostratum

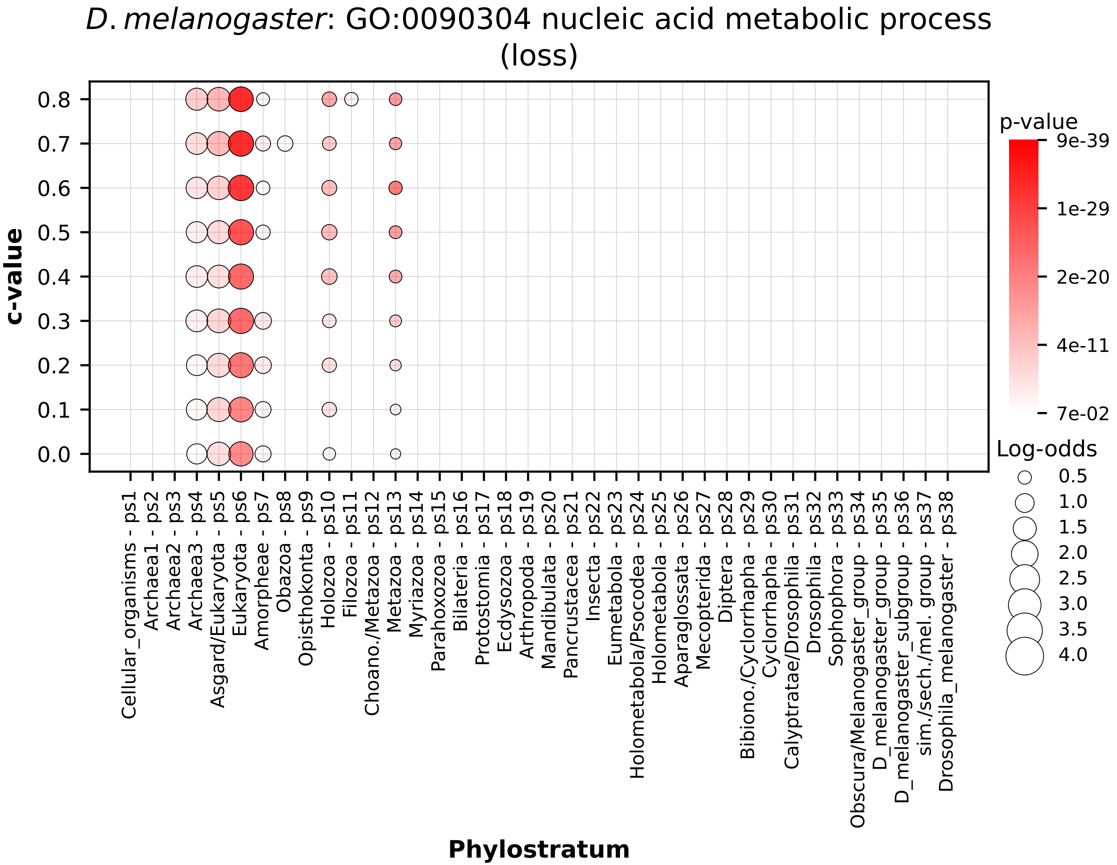
D. melanogaster: GO:0090304 nucleic acid metabolic process (loss)
c-value
0.8
0.7
0.6
0.5
0.4
0.3
0.2
0.1
0.0
p-value
9e-39
1e-29
2e-20
4e-11
7e-02
Log-odds
0.5
1.0
1.5
2.0
2.5
3.0
3.5
4.0
Cellular_organisms - ps1
Archaea1 - ps2
Archaea2 - ps3
Archaea3 - ps4
Asgard/Eukaryota - ps5
Eukaryota - ps6
Amorpheae - ps7
Obazoa - ps8
Opisthokonta - ps9
Holozoa - ps10
Filozoa - ps11
Choano./Metazoa - ps12
Metazoa - ps13
Myriazoa - ps14
Parahoxozoa - ps15
Bilateria - ps16
Protostomia - ps17
Ecdysozoa - ps18
Arthropoda - ps19
Mandibulata - ps20
Pancrustacea - ps21
Insecta - ps22
Eumetabola - ps23
Holometabola/Psocodea - ps24
Holometabola - ps25
Aparaglossata - ps26
Mecopterida - ps27
Diptera - ps28
Bibiono./Cyclorrhapha - ps29
Cyclorrhapha - ps30
Calyptratae/Drosophila - ps31
Drosophila - ps32
Sophophora - ps33
Obscura/Melanogaster_group - ps34
D_melanogaster_group - ps35
D_melanogaster_subgroup - ps36
sim./sech./mel. group - ps37
Drosophila_melanogaster - ps38
Phylostratum

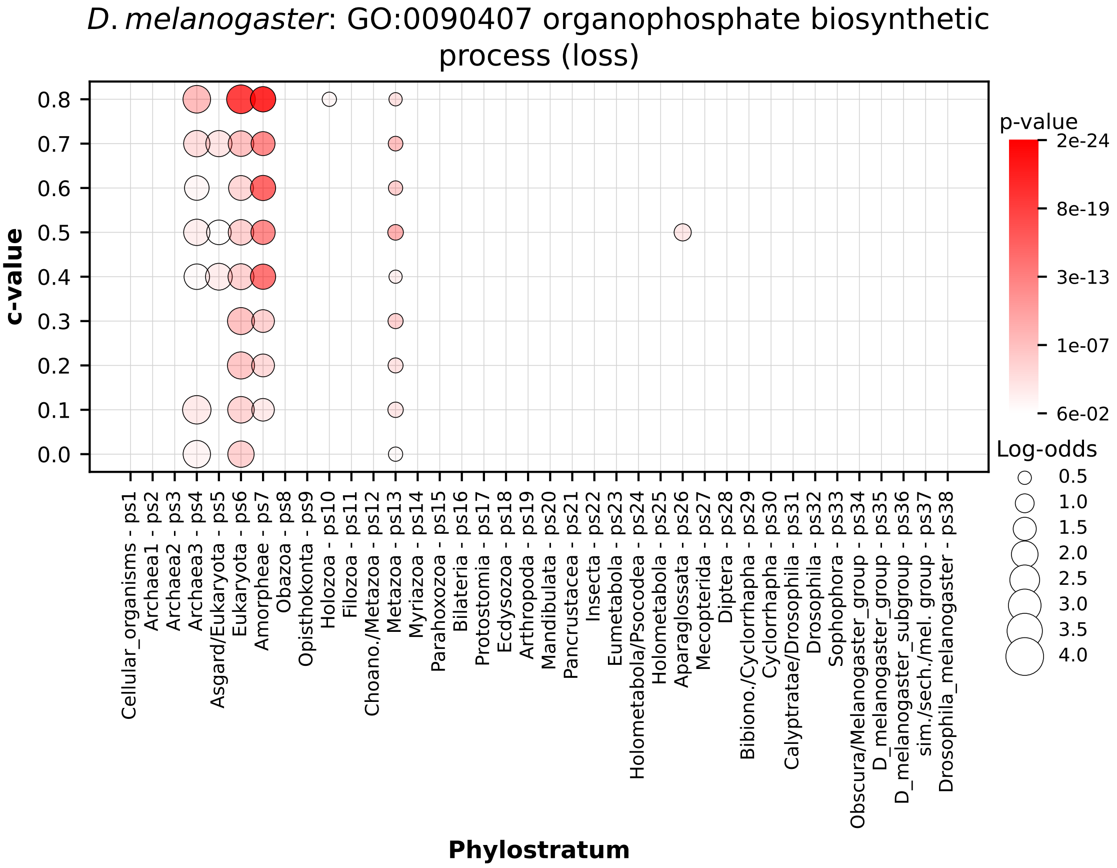

D. melanogaster: GO:0090407 organophosphate biosynthetic process (loss)
c-value
0.8
0.7
0.6
0.5
0.4
0.3
0.2
0.1
0.0
p-value
2e-24
8e-19
3e-13
1e-07
6e-02
Log-odds
0.5
1.0
1.5
2.0
2.5
3.0
3.5
4.0
Cellular_organisms - ps1
Archaea1 - ps2
Archaea2 - ps3
Archaea3 - ps4
Asgard/Eukaryota - ps5
Eukaryota - ps6
Amorpheae - ps7
Obazoa - ps8
Opisthokonta - ps9
Holozoa - ps10
Filozoa - ps11
Choano./Metazoa - ps12
Metazoa - ps13
Myriazoa - ps14
Parahoxozoa - ps15
Bilateria - ps16
Protostomia - ps17
Ecdysozoa - ps18
Arthropoda - ps19
Mandibulata - ps20
Pancrustacea - ps21
Insecta - ps22
Eumetabola - ps23
Holometabola/Psocodea - ps24
Holometabola - ps25
Aparaglossata - ps26
Mecopterida - ps27
Diptera - ps28
Bibiono./Cyclorrhapha - ps29
Cyclorrhapha - ps30
Calyptratae/Drosophila - ps31
Drosophila - ps32
Sophophora - ps33
Obscura/Melanogaster_group - ps34
D_melanogaster_group - ps35
D_melanogaster_subgroup - ps36
sim./sech./mel. group - ps37
Drosophila_melanogaster - ps38
Phylostratum

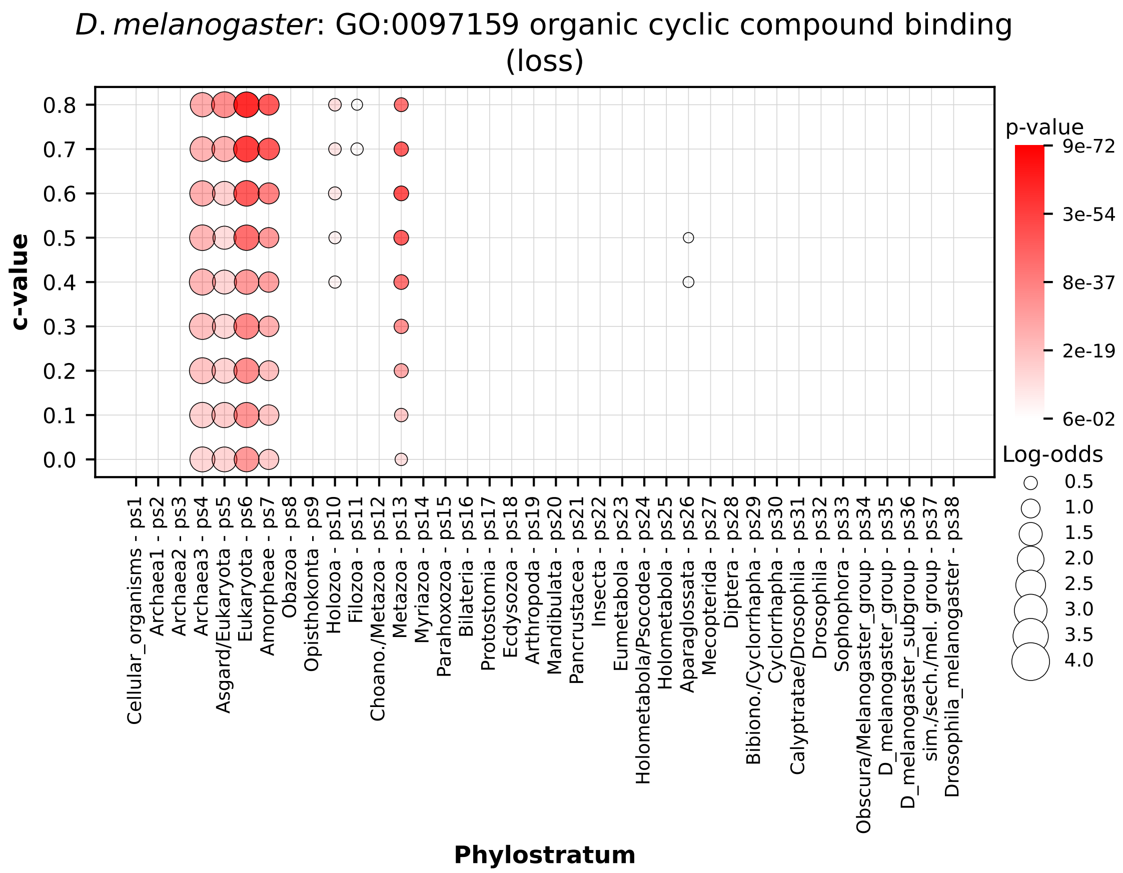

D. melanogaster: GO:0097159 organic cyclic compound binding (loss)
c-value
0.8
0.7
0.6
0.5
0.4
0.3
0.2
0.1
0.0
p-value
9e-72
3e-54
8e-37
2e-19
6e-02
Log-odds
0.5
1.0
1.5
2.0
2.5
3.0
3.5
4.0
Cellular_organisms - ps1
Archaea1 - ps2
Archaea2 - ps3
Archaea3 - ps4
Asgard/Eukaryota - ps5
Eukaryota - ps6
Amorpheae - ps7
Obazoa - ps8
Opisthokonta - ps9
Holozoa - ps10
Filozoa - ps11
Choano./Metazoa - ps12
Metazoa - ps13
Myriazoa - ps14
Parahoxozoa - ps15
Bilateria - ps16
Protostomia - ps17
Ecdysozoa - ps18
Arthropoda - ps19
Mandibulata - ps20
Pancrustacea - ps21
Insecta - ps22
Eumetabola - ps23
Holometabola/Psocodea - ps24
Holometabola - ps25
Aparaglossata - ps26
Mecopterida - ps27
Diptera - ps28
Bibiono./Cyclorrhapha - ps29
Cyclorrhapha - ps30
Calyptratae/Drosophila - ps31
Drosophila - ps32
Sophophora - ps33
Obscura/Melanogaster_group - ps34
D_melanogaster_group - ps35
D_melanogaster_subgroup - ps36
sim./sech./mel. group - ps37
Drosophila_melanogaster - ps38
Phylostratum

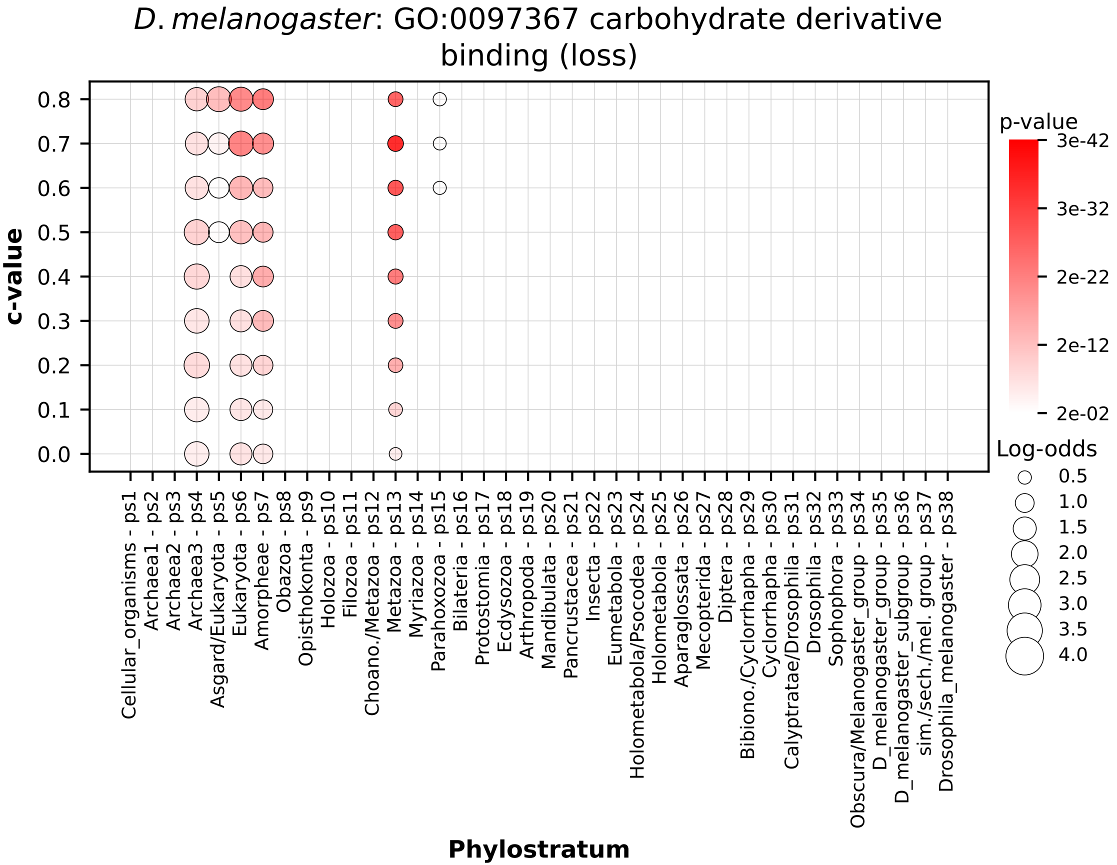

D. melanogaster: GO:0097367 carbohydrate derivative binding (loss)
c-value
0.8
0.7
0.6
0.5
0.4
0.3
0.2
0.1
0.0
Phylostratum
Cellular_organisms - ps1
Archaea1 - ps2
Archaea2 - ps3
Archaea3 - ps4
Asgard/Eukaryota - ps5
Eukaryota - ps6
Amorpheae - ps7
Obazoa - ps8
Opisthokonta - ps9
Holozoa - ps10
Filozoa - ps11
Choano./Metazoa - ps12
Metazoa - ps13
Myriazoa - ps14
Parahoxozoa - ps15
Bilateria - ps16
Protostomia - ps17
Ecdysozoa - ps18
Arthropoda - ps19
Mandibulata - ps20
Pancrustacea - ps21
Insecta - ps22
Eumetabola - ps23
Holometabola/Psocodea - ps24
Holometabola - ps25
Aparaglossata - ps26
Mecopterida - ps27
Diptera - ps28
Bibiono./Cyclorrhapha - ps29
Cyclorrhapha - ps30
Calyptratae/Drosophila - ps31
Drosophila - ps32
Sophophora - ps33
Obscura/Melanogaster_group - ps34
D_melanogaster_group - ps35
D_melanogaster_subgroup - ps36
sim./sech./mel. group - ps37
Drosophila_melanogaster - ps38
p-value
3e-42
3e-32
2e-22
2e-12
2e-02
Log-odds
0.5
1.0
1.5
2.0
2.5
3.0
3.5
4.0

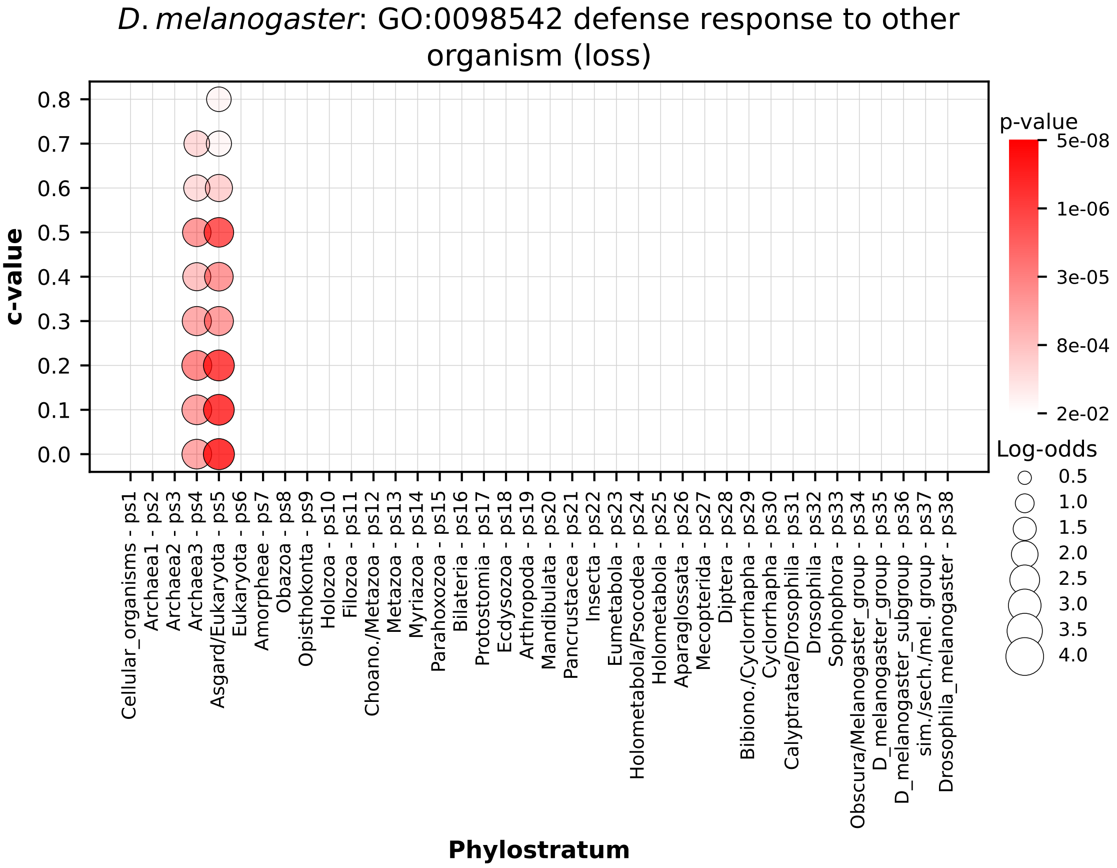
D. melanogaster: GO:0098542 defense response to other organism (loss)
c-value
0.8
0.7
0.6
0.5
0.4
0.3
0.2
0.1
0.0
p-value
5e-08
1e-06
3e-05
8e-04
2e-02
Log-odds
0.5
1.0
1.5
2.0
2.5
3.0
3.5
4.0
Cellular_organisms - ps1
Archaea1 - ps2
Archaea2 - ps3
Archaea3 - ps4
Asgard/Eukaryota - ps5
Eukaryota - ps6
Amorpheae - ps7
Obazoa - ps8
Opisthokonta - ps9
Holozoa - ps10
Filozoa - ps11
Choano./Metazoa - ps12
Metazoa - ps13
Myriazoa - ps14
Parahoxozoa - ps15
Bilateria - ps16
Protostomia - ps17
Ecdysozoa - ps18
Arthropoda - ps19
Mandibulata - ps20
Pancrustacea - ps21
Insecta - ps22
Eumetabola - ps23
Holometabola/Psocodea - ps24
Holometabola - ps25
Aparaglossata - ps26
Mecopterida - ps27
Diptera - ps28
Bibiono./Cyclorrhapha - ps29
Cyclorrhapha - ps30
Calyptratae/Drosophila - ps31
Drosophila - ps32
Sophophora - ps33
Obscura/Melanogaster_group - ps34
D_melanogaster_group - ps35
D_melanogaster_subgroup - ps36
sim./sech./mel. group - ps37
Drosophila_melanogaster - ps38
Phylostratum

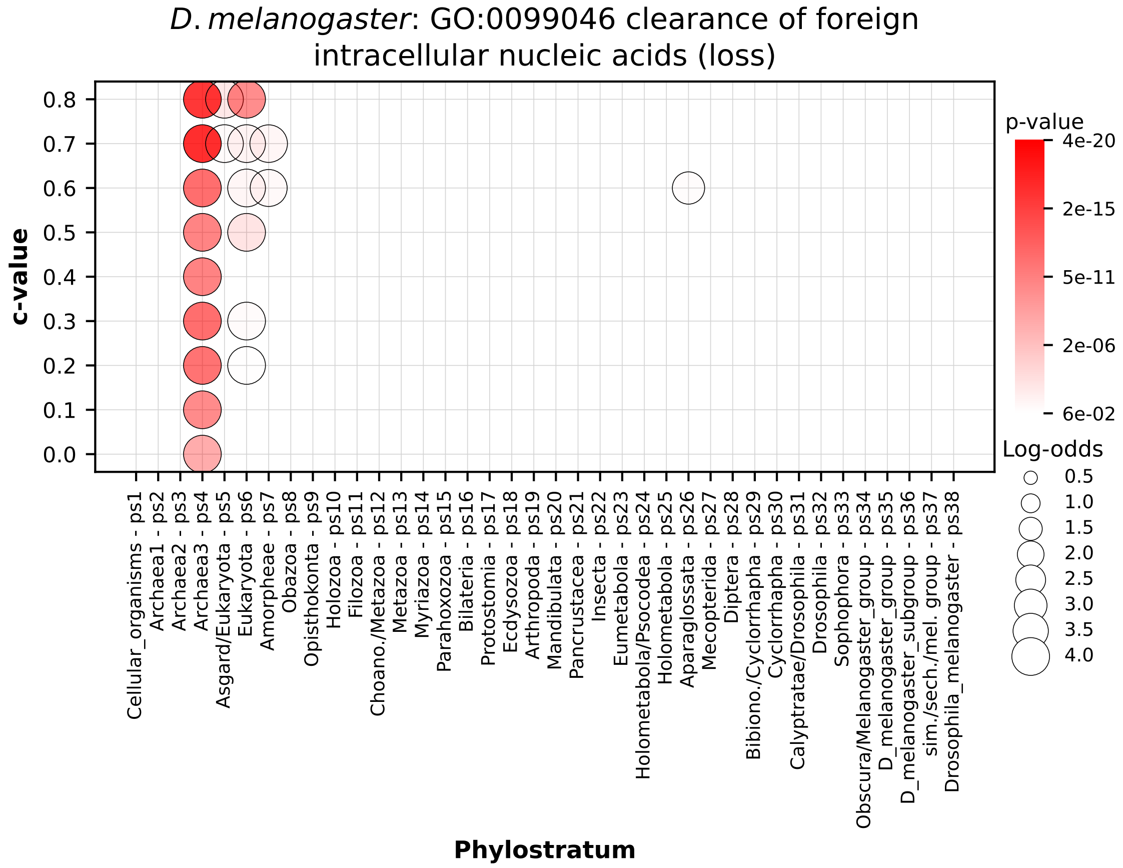

D. melanogaster: GO:0099046 clearance of foreign intracellular nucleic acids (loss)
c-value
0.8
0.7
0.6
0.5
0.4
0.3
0.2
0.1
0.0
p-value
4e-20
2e-15
5e-11
2e-06
6e-02
Log-odds
0.5
1.0
1.5
2.0
2.5
3.0
3.5
4.0
Cellular_organisms - ps1
Archaea1 - ps2
Archaea2 - ps3
Archaea3 - ps4
Asgard/Eukaryota - ps5
Eukaryota - ps6
Amorpheae - ps7
Obazoa - ps8
Opisthokonta - ps9
Holozoa - ps10
Filozoa - ps11
Choano./Metazoa - ps12
Metazoa - ps13
Myriazoa - ps14
Parahoxozoa - ps15
Bilateria - ps16
Protostomia - ps17
Ecdysozoa - ps18
Arthropoda - ps19
Mandibulata - ps20
Pancrustacea - ps21
Insecta - ps22
Eumetabola - ps23
Holometabola/Psocodea - ps24
Holometabola - ps25
Aparaglossata - ps26
Mecopterida - ps27
Diptera - ps28
Bibiono./Cyclorrhapha - ps29
Cyclorrhapha - ps30
Calyptratae/Drosophila - ps31
Drosophila - ps32
Sophophora - ps33
Obscura/Melanogaster_group - ps34
D_melanogaster_group - ps35
D_melanogaster_subgroup - ps36
sim./sech./mel. group - ps37
Drosophila_melanogaster - ps38
Phylostratum

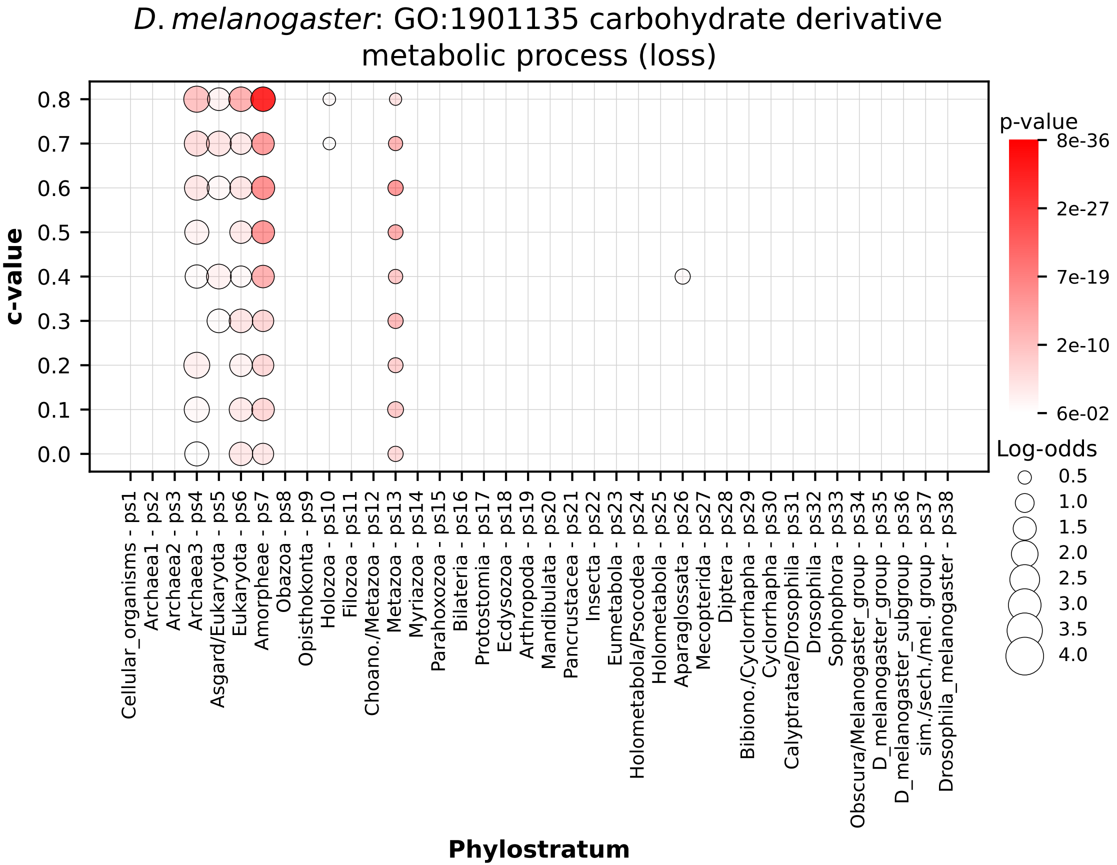

D. melanogaster: GO:1901135 carbohydrate derivative metabolic process (loss)
c-value
0.8
0.7
0.6
0.5
0.4
0.3
0.2
0.1
0.0
p-value
8e-36
2e-27
7e-19
2e-10
6e-02
Log-odds
0.5
1.0
1.5
2.0
2.5
3.0
3.5
4.0
Cellular_organisms - ps1
Archaea1 - ps2
Archaea2 - ps3
Archaea3 - ps4
Asgard/Eukaryota - ps5
Eukaryota - ps6
Amorpheae - ps7
Obazoa - ps8
Opisthokonta - ps9
Holozoa - ps10
Filozoa - ps11
Choano./Metazoa - ps12
Metazoa - ps13
Myriazoa - ps14
Parahoxozoa - ps15
Bilateria - ps16
Protostomia - ps17
Ecdysozoa - ps18
Arthropoda - ps19
Mandibulata - ps20
Pancrustacea - ps21
Insecta - ps22
Eumetabola - ps23
Holometabola/Psocodea - ps24
Holometabola - ps25
Aparaglossata - ps26
Mecopterida - ps27
Diptera - ps28
Bibiono./Cyclorrhapha - ps29
Cyclorrhapha - ps30
Calyptratae/Drosophila - ps31
Drosophila - ps32
Sophophora - ps33
Obscura/Melanogaster_group - ps34
D_melanogaster_group - ps35
D_melanogaster_subgroup - ps36
sim./sech./mel. group - ps37
Drosophila_melanogaster - ps38
Phylostratum

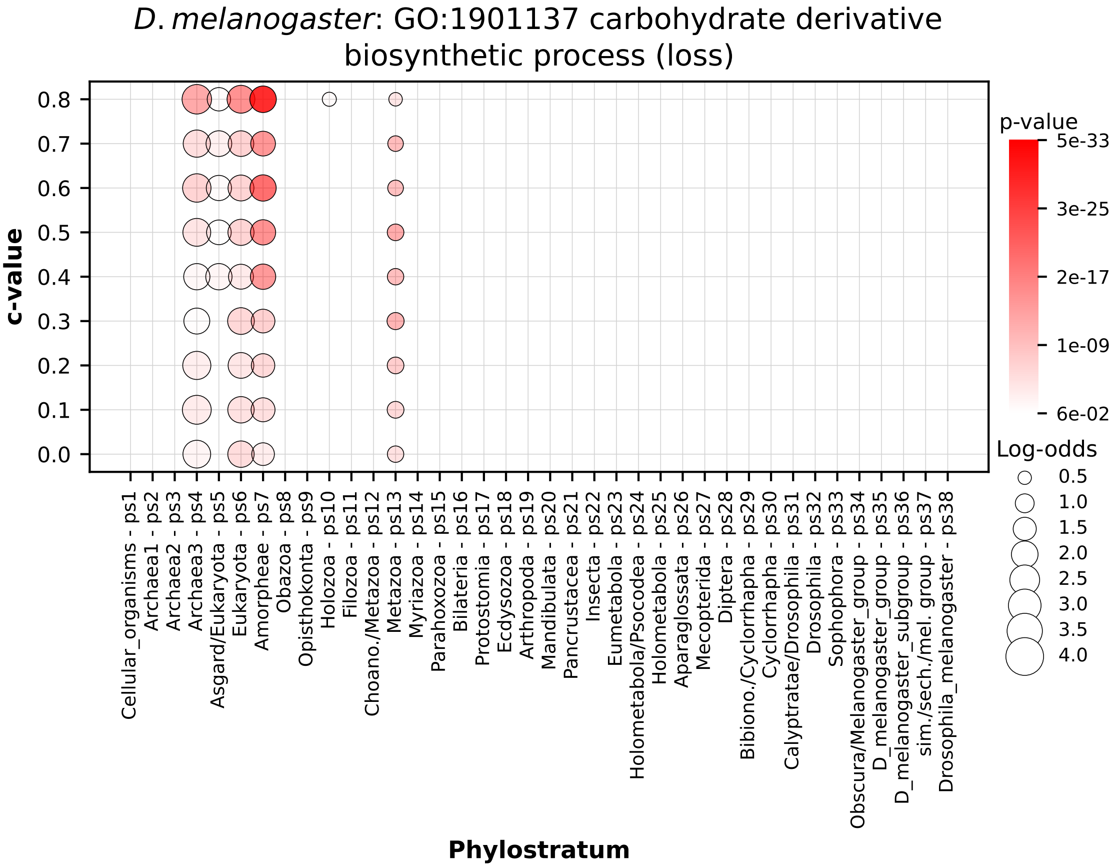

D. melanogaster: GO:1901137 carbohydrate derivative biosynthetic process (loss)
c-value
0.8
0.7
0.6
0.5
0.4
0.3
0.2
0.1
0.0
p-value
5e-33
3e-25
2e-17
1e-09
6e-02
Log-odds
0.5
1.0
1.5
2.0
2.5
3.0
3.5
4.0
Cellular_organisms - ps1
Archaea1 - ps2
Archaea2 - ps3
Archaea3 - ps4
Asgard/Eukaryota - ps5
Eukaryota - ps6
Amorpheae - ps7
Obazoa - ps8
Opisthokonta - ps9
Holozoa - ps10
Filozoa - ps11
Choano./Metazoa - ps12
Metazoa - ps13
Myriazoa - ps14
Parahoxozoa - ps15
Bilateria - ps16
Protostomia - ps17
Ecdysozoa - ps18
Arthropoda - ps19
Mandibulata - ps20
Pancrustacea - ps21
Insecta - ps22
Eumetabola - ps23
Holometabola/Psocodea - ps24
Holometabola - ps25
Aparaglossata - ps26
Mecopterida - ps27
Diptera - ps28
Bibiono./Cyclorrhapha - ps29
Cyclorrhapha - ps30
Calyptratae/Drosophila - ps31
Drosophila - ps32
Sophophora - ps33
Obscura/Melanogaster_group - ps34
D_melanogaster_group - ps35
D_melanogaster_subgroup - ps36
sim./sech./mel. group - ps37
Drosophila_melanogaster - ps38
Phylostratum

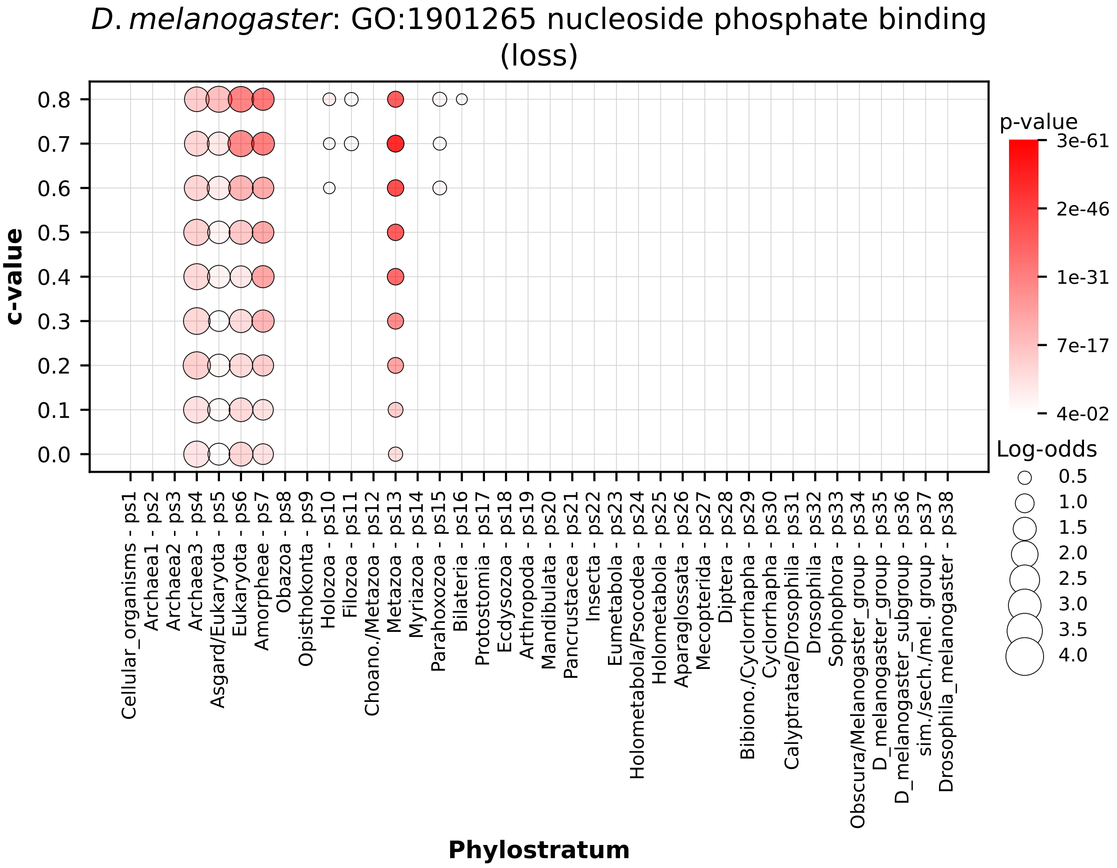

D. melanogaster: GO:1901265 nucleoside phosphate binding (loss)
c-value
0.8
0.7
0.6
0.5
0.4
0.3
0.2
0.1
0.0
p-value
3e-61
2e-46
1e-31
7e-17
4e-02
Log-odds
0.5
1.0
1.5
2.0
2.5
3.0
3.5
4.0
Cellular_organisms - ps1
Archaea1 - ps2
Archaea2 - ps3
Archaea3 - ps4
Asgard/Eukaryota - ps5
Eukaryota - ps6
Amorpheae - ps7
Obazoa - ps8
Opisthokonta - ps9
Holozoa - ps10
Filozoa - ps11
Choano./Metazoa - ps12
Metazoa - ps13
Myriazoa - ps14
Parahoxozoa - ps15
Bilateria - ps16
Protostomia - ps17
Ecdysozoa - ps18
Arthropoda - ps19
Mandibulata - ps20
Pancrustacea - ps21
Insecta - ps22
Eumetabola - ps23
Holometabola/Psocodea - ps24
Holometabola - ps25
Aparaglossata - ps26
Mecopterida - ps27
Diptera - ps28
Bibiono./Cyclorrhapha - ps29
Cyclorrhapha - ps30
Calyptratae/Drosophila - ps31
Drosophila - ps32
Sophophora - ps33
Obscura/Melanogaster_group - ps34
D_melanogaster_group - ps35
D_melanogaster_subgroup - ps36
sim./sech./mel. group - ps37
Drosophila_melanogaster - ps38
Phylostratum

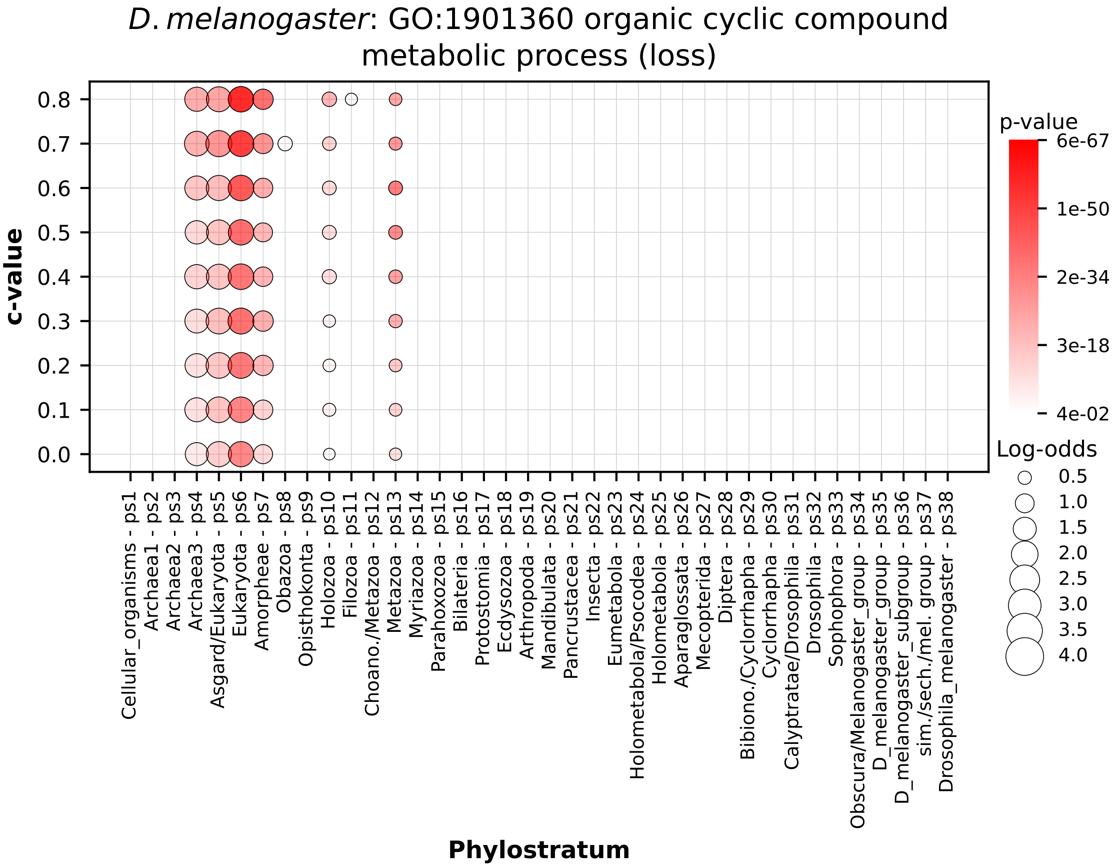

D. melanogaster: GO:1901360 organic cyclic compound metabolic process (loss)
c-value
0.8
0.7
0.6
0.5
0.4
0.3
0.2
0.1
0.0
p-value
6e-67
1e-50
2e-34
3e-18
4e-02
Log-odds
0.5
1.0
1.5
2.0
2.5
3.0
3.5
4.0
Cellular_organisms - ps1
Archaea1 - ps2
Archaea2 - ps3
Archaea3 - ps4
Asgard/Eukaryota - ps5
Eukaryota - ps6
Amorpheae - ps7
Obazoa - ps8
Opisthokonta - ps9
Holozoa - ps10
Filozoa - ps11
Choano./Metazoa - ps12
Metazoa - ps13
Myriazoa - ps14
Parahoxozoa - ps15
Bilateria - ps16
Protostomia - ps17
Ecdysozoa - ps18
Arthropoda - ps19
Mandibulata - ps20
Pancrustacea - ps21
Insecta - ps22
Eumetabola - ps23
Holometabola/Psocodea - ps24
Holometabola - ps25
Aparaglossata - ps26
Mecopterida - ps27
Diptera - ps28
Bibiono./Cyclorrhapha - ps29
Cyclorrhapha - ps30
Calyptratae/Drosophila - ps31
Drosophila - ps32
Sophophora - ps33
Obscura/Melanogaster_group - ps34
D_melanogaster_group - ps35
D_melanogaster_subgroup - ps36
sim./sech./mel. group - ps37
Drosophila_melanogaster - ps38
Phylostratum

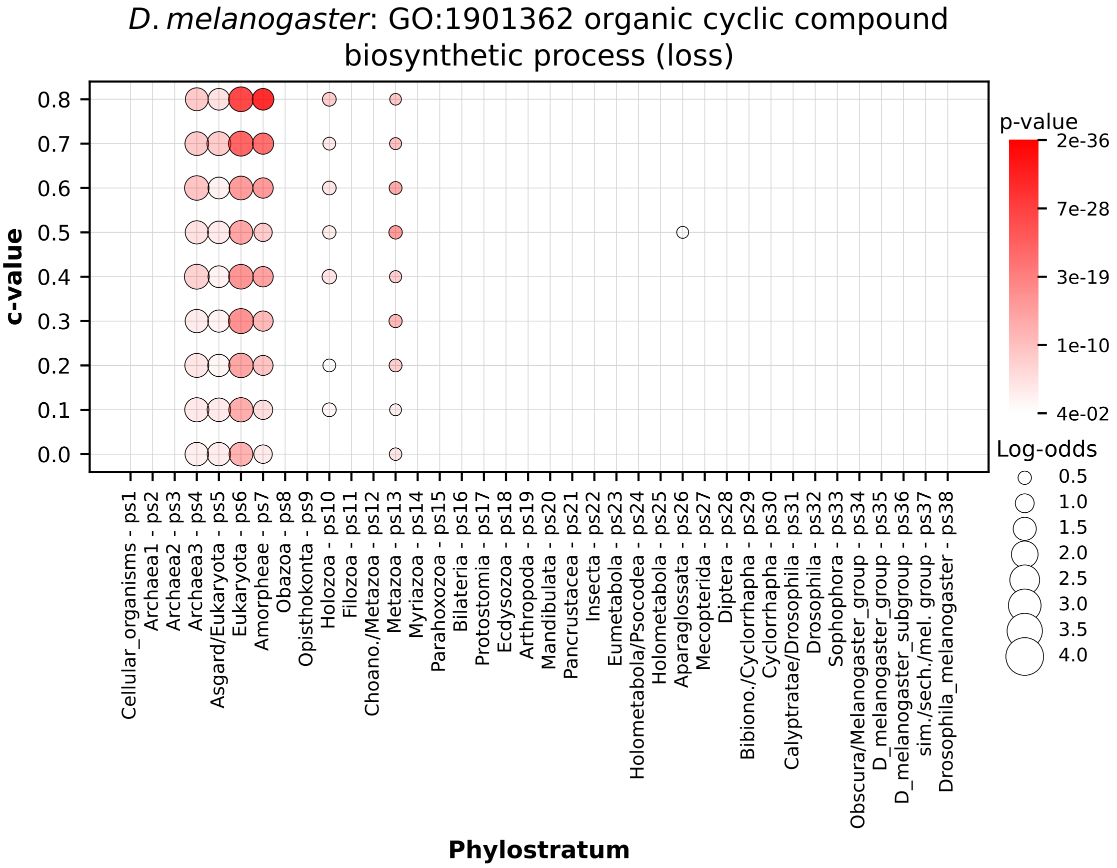

D. melanogaster: GO:1901362 organic cyclic compound biosynthetic process (loss)
c-value
0.8
0.7
0.6
0.5
0.4
0.3
0.2
0.1
0.0
p-value
2e-36
7e-28
3e-19
1e-10
4e-02
Log-odds
0.5
1.0
1.5
2.0
2.5
3.0
3.5
4.0
Cellular_organisms - ps1
Archaea1 - ps2
Archaea2 - ps3
Archaea3 - ps4
Asgard/Eukaryota - ps5
Eukaryota - ps6
Amorpheae - ps7
Obazoa - ps8
Opisthokonta - ps9
Holozoa - ps10
Filozoa - ps11
Choano./Metazoa - ps12
Metazoa - ps13
Myriazoa - ps14
Parahoxozoa - ps15
Bilateria - ps16
Protostomia - ps17
Ecdysozoa - ps18
Arthropoda - ps19
Mandibulata - ps20
Pancrustacea - ps21
Insecta - ps22
Eumetabola - ps23
Holometabola/Psocodea - ps24
Holometabola - ps25
Aparaglossata - ps26
Mecopterida - ps27
Diptera - ps28
Bibiono./Cyclorrhapha - ps29
Cyclorrhapha - ps30
Calyptratae/Drosophila - ps31
Drosophila - ps32
Sophophora - ps33
Obscura/Melanogaster_group - ps34
D_melanogaster_group - ps35
D_melanogaster_subgroup - ps36
sim./sech./mel. group - ps37
Drosophila_melanogaster - ps38
Phylostratum

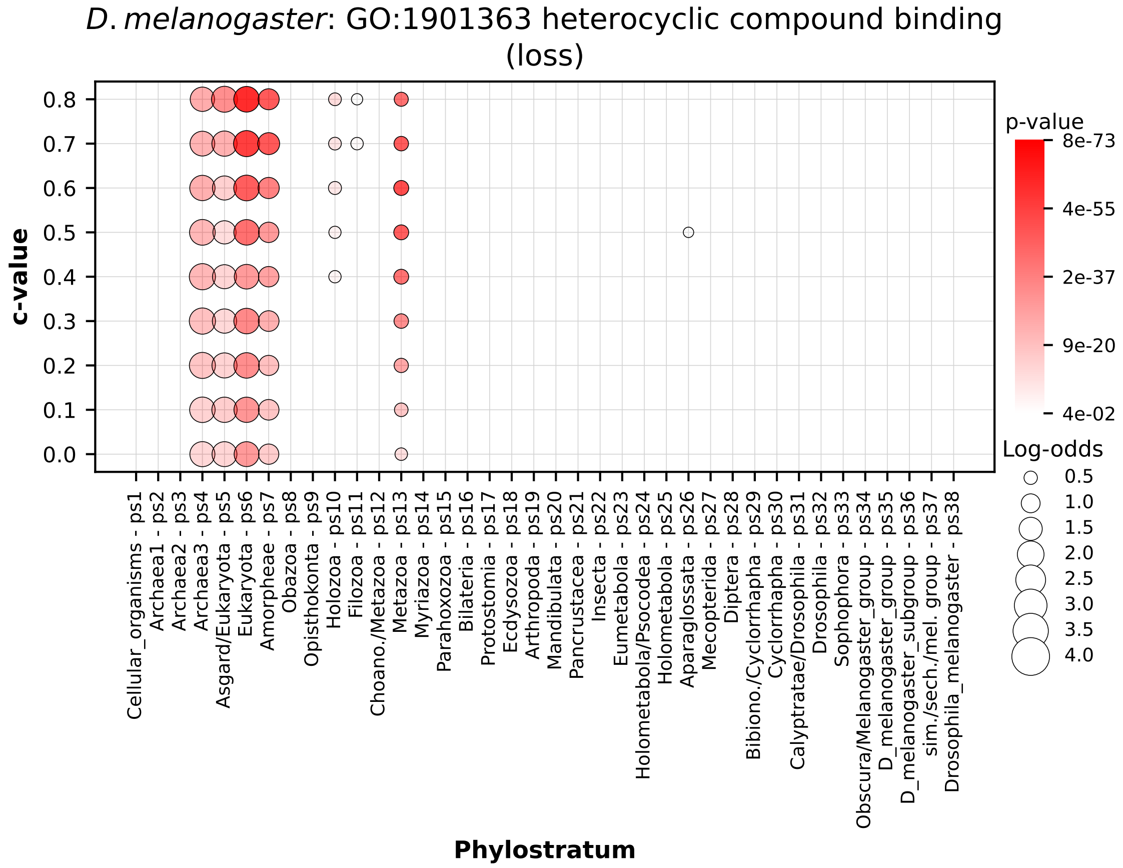

D. melanogaster: GO:1901363 heterocyclic compound binding
(loss)
c-value
0.8
0.7
0.6
0.5
0.4
0.3
0.2
0.1
0.0
p-value
8e-73
4e-55
2e-37
9e-20
4e-02
Log-odds
0.5
1.0
1.5
2.0
2.5
3.0
3.5
4.0
Cellular_organisms - ps1
Archaea1 - ps2
Archaea2 - ps3
Archaea3 - ps4
Asgard/Eukaryota - ps5
Eukaryota - ps6
Amorpheae - ps7
Obazoa - ps8
Opisthokonta - ps9
Holozoa - ps10
Filozoa - ps11
Choano./Metazoa - ps12
Metazoa - ps13
Myriazoa - ps14
Parahoxozoa - ps15
Bilateria - ps16
Protostomia - ps17
Ecdysozoa - ps18
Arthropoda - ps19
Mandibulata - ps20
Pancrustacea - ps21
Insecta - ps22
Eumetabola - ps23
Holometabola/Psocodea - ps24
Holometabola - ps25
Aparaglossata - ps26
Mecopterida - ps27
Diptera - ps28
Bibiono./Cyclorrhapha - ps29
Cyclorrhapha - ps30
Calyptratae/Drosophila - ps31
Drosophila - ps32
Sophophora - ps33
Obscura/Melanogaster_group - ps34
D_melanogaster_group - ps35
D_melanogaster_subgroup - ps36
sim./sech./mel. group - ps37
Drosophila_melanogaster - ps38
Phylostratum

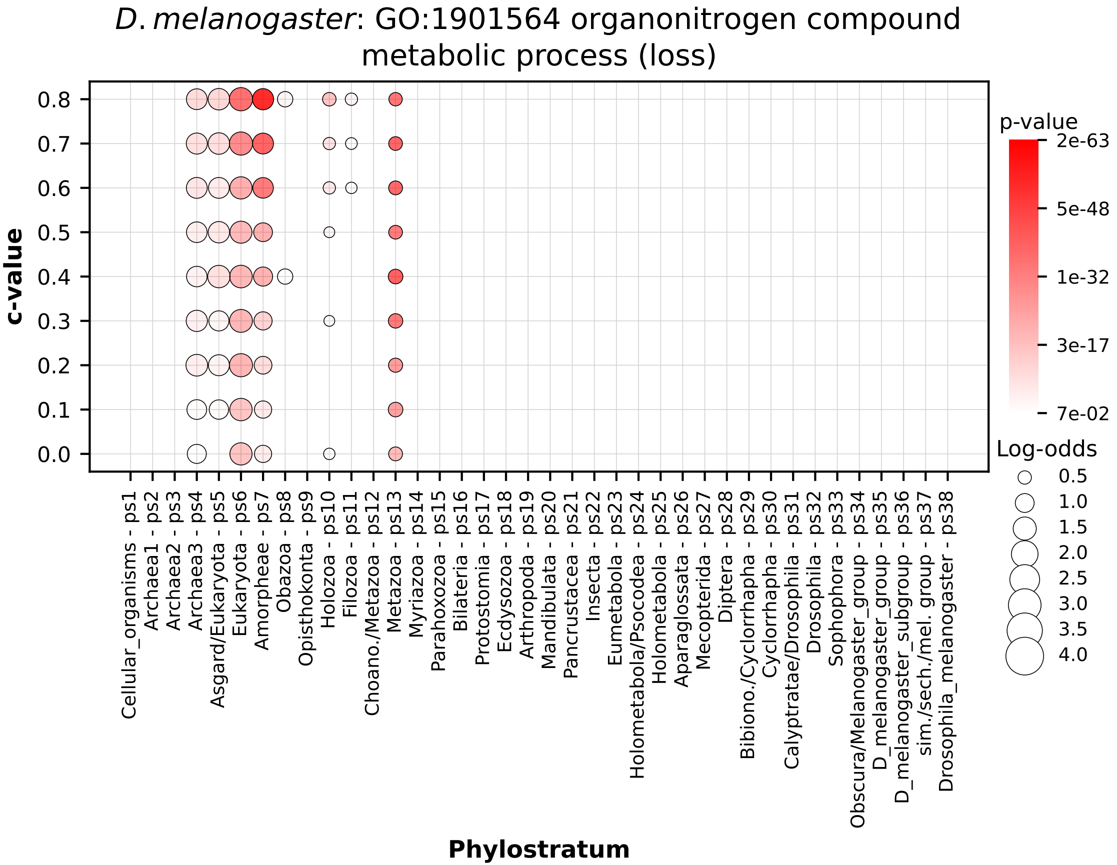

D. melanogaster: GO:1901564 organonitrogen compound metabolic process (loss)
c-value
0.8
0.7
0.6
0.5
0.4
0.3
0.2
0.1
0.0
p-value
2e-63
5e-48
1e-32
3e-17
7e-02
Log-odds
0.5
1.0
1.5
2.0
2.5
3.0
3.5
4.0
Cellular_organisms - ps1
Archaea1 - ps2
Archaea2 - ps3
Archaea3 - ps4
Asgard/Eukaryota - ps5
Eukaryota - ps6
Amorpheae - ps7
Obazoa - ps8
Opisthokonta - ps9
Holozoa - ps10
Filozoa - ps11
Choano./Metazoa - ps12
Metazoa - ps13
Myriazoa - ps14
Parahoxozoa - ps15
Bilateria - ps16
Protostomia - ps17
Ecdysozoa - ps18
Arthropoda - ps19
Mandibulata - ps20
Pancrustacea - ps21
Insecta - ps22
Eumetabola - ps23
Holometabola/Psocodea - ps24
Holometabola - ps25
Aparaglossata - ps26
Mecopterida - ps27
Diptera - ps28
Bibiono./Cyclorrhapha - ps29
Cyclorrhapha - ps30
Calyptratae/Drosophila - ps31
Drosophila - ps32
Sophophora - ps33
Obscura/Melanogaster_group - ps34
D_melanogaster_group - ps35
D_melanogaster_subgroup - ps36
sim./sech./mel. group - ps37
Drosophila_melanogaster - ps38
Phylostratum

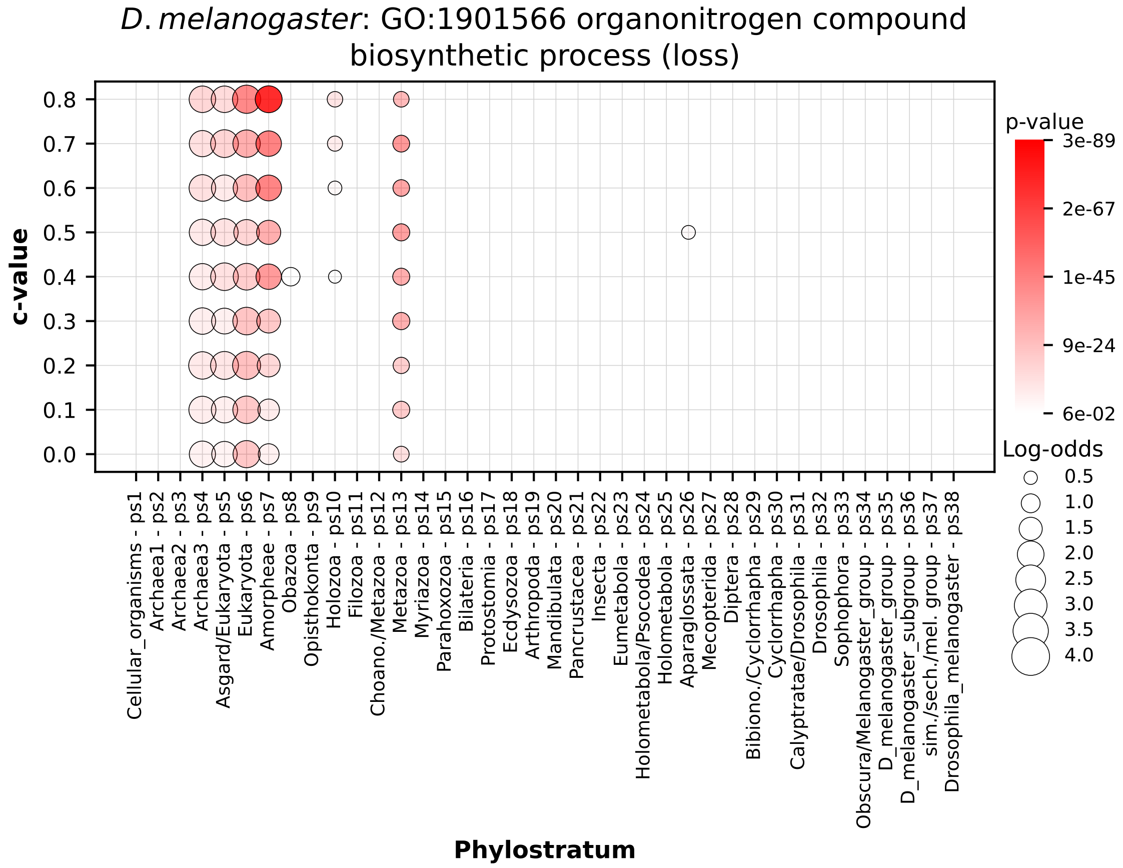

D. melanogaster: GO:1901566 organonitrogen compound biosynthetic process (loss)
c-value
0.8
0.7
0.6
0.5
0.4
0.3
0.2
0.1
0.0
p-value
3e-89
2e-67
1e-45
9e-24
6e-02
Log-odds
0.5
1.0
1.5
2.0
2.5
3.0
3.5
4.0
Cellular_organisms - ps1
Archaea1 - ps2
Archaea2 - ps3
Archaea3 - ps4
Asgard/Eukaryota - ps5
Eukaryota - ps6
Amorpheae - ps7
Obazoa - ps8
Opisthokonta - ps9
Holozoa - ps10
Filozoa - ps11
Choano./Metazoa - ps12
Metazoa - ps13
Myriazoa - ps14
Parahoxozoa - ps15
Bilateria - ps16
Protostomia - ps17
Ecdysozoa - ps18
Arthropoda - ps19
Mandibulata - ps20
Pancrustacea - ps21
Insecta - ps22
Eumetabola - ps23
Holometabola/Psocodea - ps24
Holometabola - ps25
Aparaglossata - ps26
Mecopterida - ps27
Diptera - ps28
Bibiono./Cyclorrhapha - ps29
Cyclorrhapha - ps30
Calyptratae/Drosophila - ps31
Drosophila - ps32
Sophophora - ps33
Obscura/Melanogaster_group - ps34
D_melanogaster_group - ps35
D_melanogaster_subgroup - ps36
sim./sech./mel. group - ps37
Drosophila_melanogaster - ps38
Phylostratum

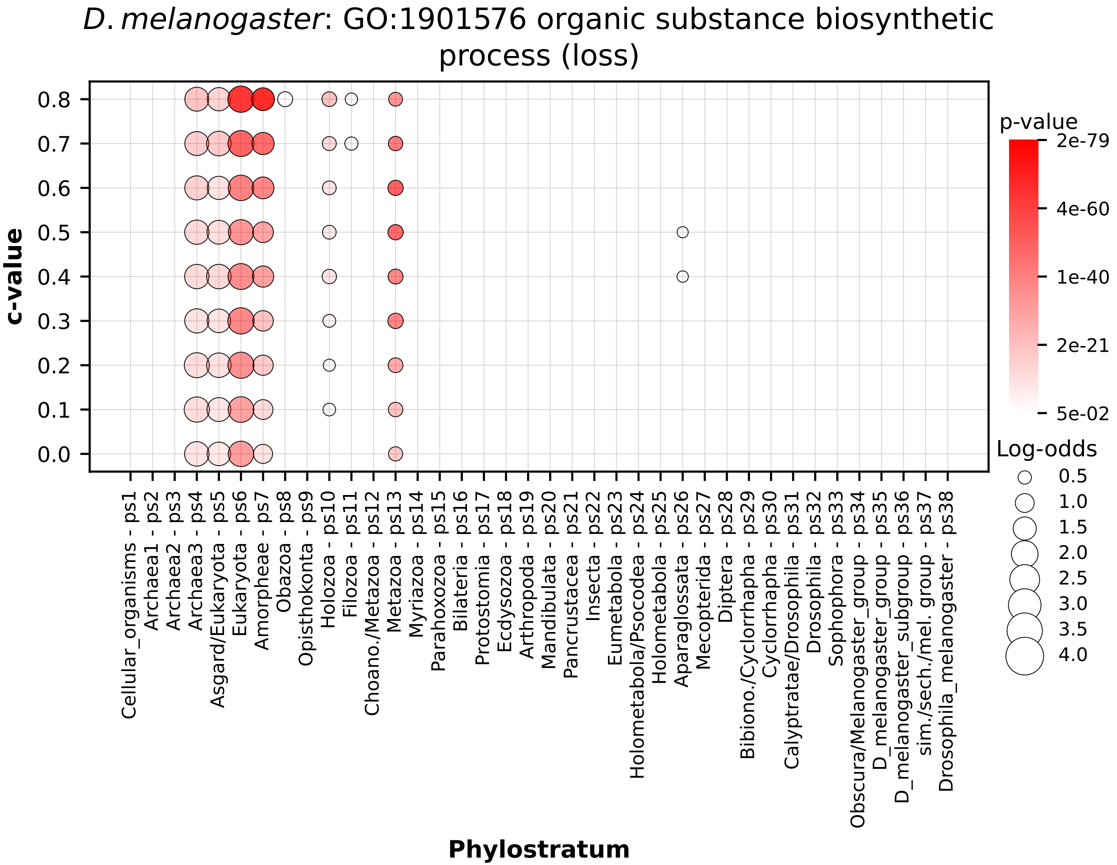

D. melanogaster: GO:1901576 organic substance biosynthetic process (loss)
c-value
0.8
0.7
0.6
0.5
0.4
0.3
0.2
0.1
0.0
p-value
2e-79
4e-60
1e-40
2e-21
5e-02
Log-odds
0.5
1.0
1.5
2.0
2.5
3.0
3.5
4.0
Cellular_organisms - ps1
Archaea1 - ps2
Archaea2 - ps3
Archaea3 - ps4
Asgard/Eukaryota - ps5
Eukaryota - ps6
Amorpheae - ps7
Obazoa - ps8
Opisthokonta - ps9
Holozoa - ps10
Filozoa - ps11
Choano./Metazoa - ps12
Metazoa - ps13
Myriazoa - ps14
Parahoxozoa - ps15
Bilateria - ps16
Protostomia - ps17
Ecdysozoa - ps18
Arthropoda - ps19
Mandibulata - ps20
Pancrustacea - ps21
Insecta - ps22
Eumetabola - ps23
Holometabola/Psocodea - ps24
Holometabola - ps25
Aparaglossata - ps26
Mecopterida - ps27
Diptera - ps28
Bibiono./Cyclorrhapha - ps29
Cyclorrhapha - ps30
Calyptratae/Drosophila - ps31
Drosophila - ps32
Sophophora - ps33
Obscura/Melanogaster_group - ps34
D_melanogaster_group - ps35
D_melanogaster_subgroup - ps36
sim./sech./mel. group - ps37
Drosophila_melanogaster - ps38
Phylostratum

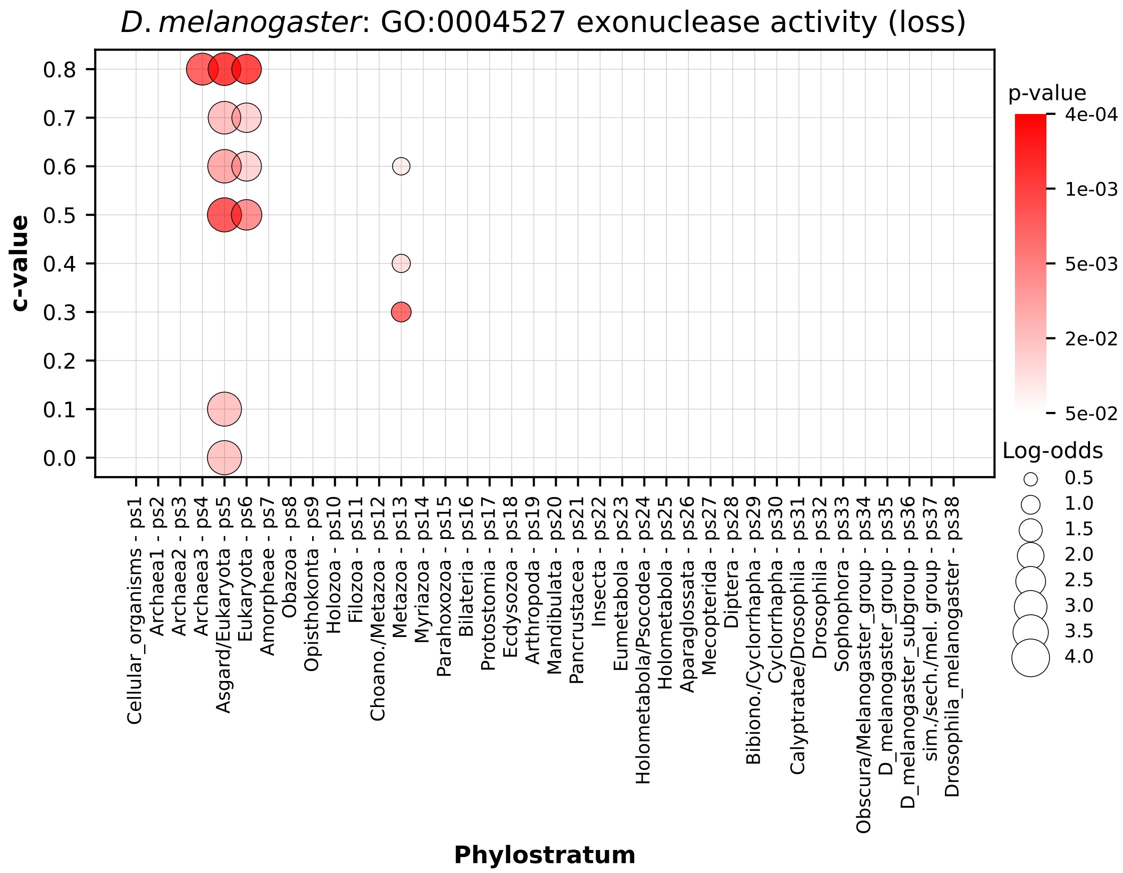

D. melanogaster: GO:0004527 exonuclease activity (loss)
c-value
0.8
0.7
0.6
0.5
0.4
0.3
0.2
0.1
0.0
Phylostratum
Cellular_organisms - ps1
Archaea1 - ps2
Archaea2 - ps3
Archaea3 - ps4
Asgard/Eukaryota - ps5
Eukaryota - ps6
Amorpheae - ps7
Obazoa - ps8
Opisthokonta - ps9
Holozoa - ps10
Filozoa - ps11
Choano./Metazoa - ps12
Metazoa - ps13
Myriazoa - ps14
Parahoxozoa - ps15
Bilateria - ps16
Protostomia - ps17
Ecdysozoa - ps18
Arthropoda - ps19
Mandibulata - ps20
Pancrustacea - ps21
Insecta - ps22
Eumetabola - ps23
Holometabola/Psocodea - ps24
Holometabola - ps25
Aparaglossata - ps26
Mecopterida - ps27
Diptera - ps28
Bibiono./Cyclorrhapha - ps29
Cyclorrhapha - ps30
Calyptratae/Drosophila - ps31
Drosophila - ps32
Sophophora - ps33
Obscura/Melanogaster_group - ps34
D_melanogaster_group - ps35
D_melanogaster_subgroup - ps36
sim./sech./mel. group - ps37
Drosophila_melanogaster - ps38
p-value
4e-04
1e-03
5e-03
2e-02
5e-02
Log-odds
0.5
1.0
1.5
2.0
2.5
3.0
3.5
4.0

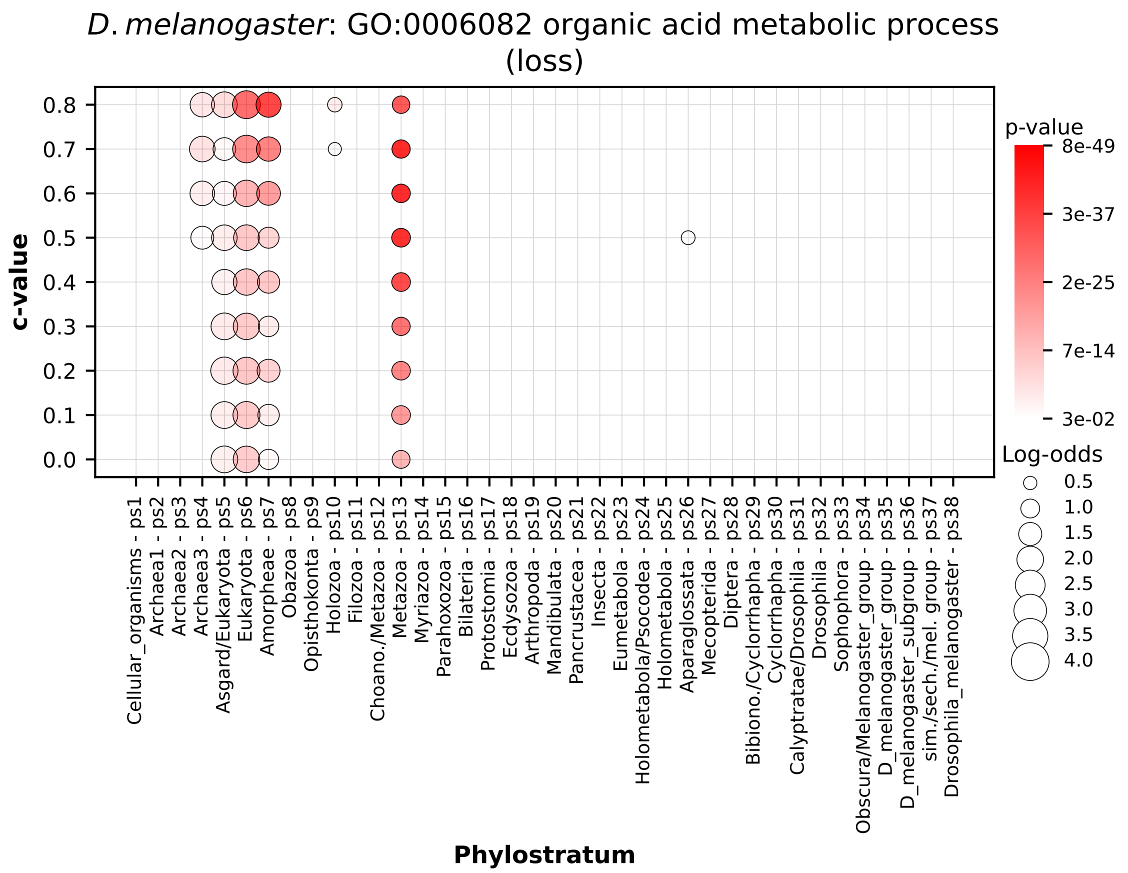
D. melanogaster: GO:0006082 organic acid metabolic process
(loss)
c-value
0.8
0.7
0.6
0.5
0.4
0.3
0.2
0.1
0.0
p-value
8e-49
3e-37
2e-25
7e-14
3e-02
Log-odds
0.5
1.0
1.5
2.0
2.5
3.0
3.5
4.0
Cellular_organisms - ps1
Archaea1 - ps2
Archaea2 - ps3
Archaea3 - ps4
Asgard/Eukaryota - ps5
Eukaryota - ps6
Amorpheae - ps7
Obazoa - ps8
Opisthokonta - ps9
Holozoa - ps10
Filozoa - ps11
Choano./Metazoa - ps12
Metazoa - ps13
Myriazoa - ps14
Parahoxozoa - ps15
Bilateria - ps16
Protostomia - ps17
Ecdysozoa - ps18
Arthropoda - ps19
Mandibulata - ps20
Pancrustacea - ps21
Insecta - ps22
Eumetabola - ps23
Holometabola/Psocodea - ps24
Holometabola - ps25
Aparaglossata - ps26
Mecopterida - ps27
Diptera - ps28
Bibiono./Cyclorrhapha - ps29
Cyclorrhapha - ps30
Calyptratae/Drosophila - ps31
Drosophila - ps32
Sophophora - ps33
Obscura/Melanogaster_group - ps34
D_melanogaster_group - ps35
D_melanogaster_subgroup - ps36
sim./sech./mel. group - ps37
Drosophila_melanogaster - ps38
Phylostratum

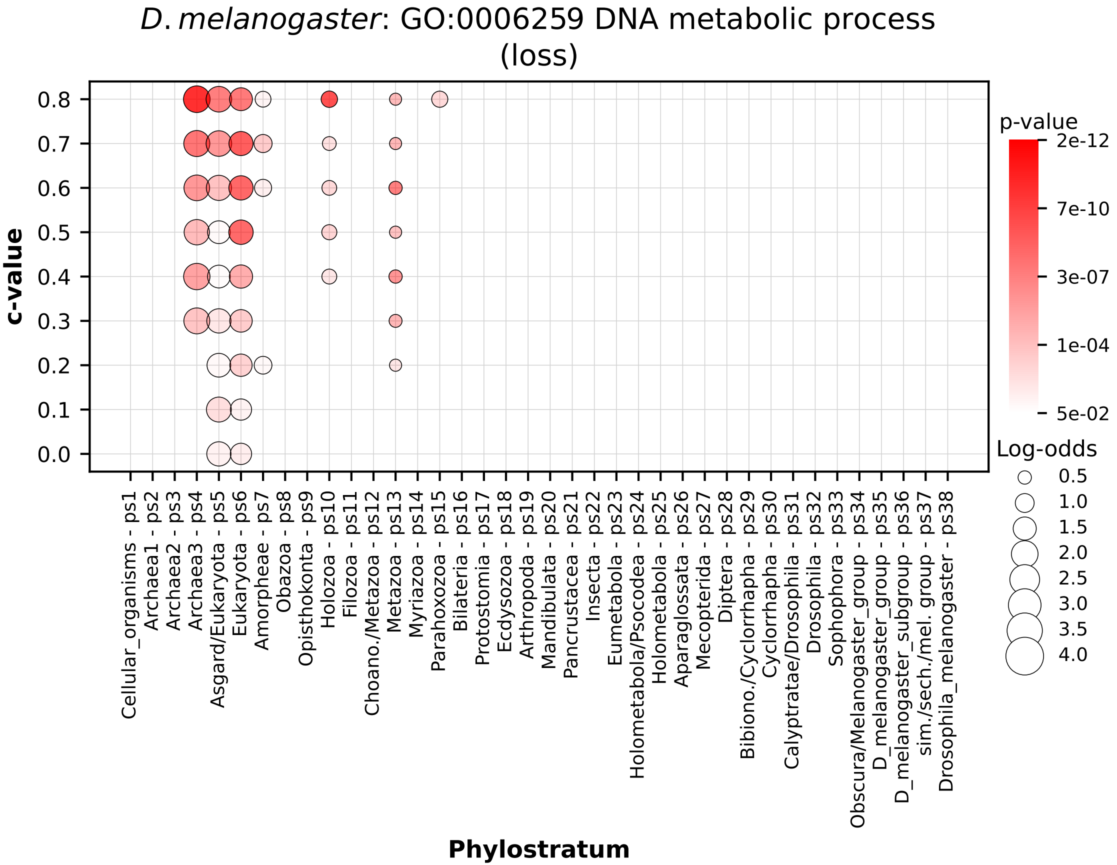

D. melanogaster: GO:0006259 DNA metabolic process
(loss)
c-value
0.8
0.7
0.6
0.5
0.4
0.3
0.2
0.1
0.0
p-value
2e-12
7e-10
3e-07
1e-04
5e-02
Log-odds
0.5
1.0
1.5
2.0
2.5
3.0
3.5
4.0
Cellular_organisms - ps1
Archaea1 - ps2
Archaea2 - ps3
Archaea3 - ps4
Asgard/Eukaryota - ps5
Eukaryota - ps6
Amorpheae - ps7
Obazoa - ps8
Opisthokonta - ps9
Holozoa - ps10
Filozoa - ps11
Choano./Metazoa - ps12
Metazoa - ps13
Myriazoa - ps14
Parahoxozoa - ps15
Bilateria - ps16
Protostomia - ps17
Ecdysozoa - ps18
Arthropoda - ps19
Mandibulata - ps20
Pancrustacea - ps21
Insecta - ps22
Eumetabola - ps23
Holometabola/Psocodea - ps24
Holometabola - ps25
Aparaglossata - ps26
Mecopterida - ps27
Diptera - ps28
Bibiono./Cyclorrhapha - ps29
Cyclorrhapha - ps30
Calyptratae/Drosophila - ps31
Drosophila - ps32
Sophophora - ps33
Obscura/Melanogaster_group - ps34
D_melanogaster_group - ps35
D_melanogaster_subgroup - ps36
sim./sech./mel. group - ps37
Drosophila_melanogaster - ps38
Phylostratum

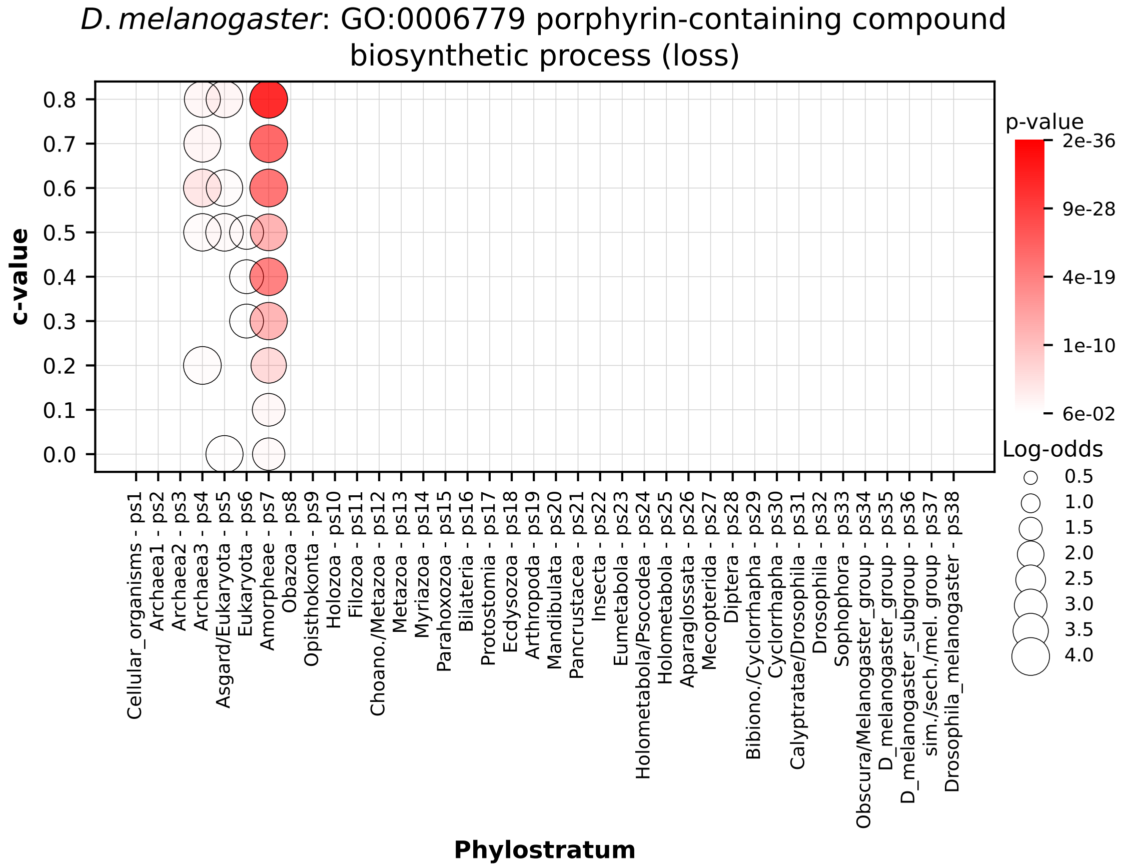

D. melanogaster: GO:0006779 porphyrin-containing compound biosynthetic process (loss)
c-value
0.8
0.7
0.6
0.5
0.4
0.3
0.2
0.1
0.0
p-value
2e-36
9e-28
4e-19
1e-10
6e-02
Log-odds
0.5
1.0
1.5
2.0
2.5
3.0
3.5
4.0
Cellular_organisms - ps1
Archaea1 - ps2
Archaea2 - ps3
Archaea3 - ps4
Asgard/Eukaryota - ps5
Eukaryota - ps6
Amorpheae - ps7
Obazoa - ps8
Opisthokonta - ps9
Holozoa - ps10
Filozoa - ps11
Choano./Metazoa - ps12
Metazoa - ps13
Myriazoa - ps14
Parahoxozoa - ps15
Bilateria - ps16
Protostomia - ps17
Ecdysozoa - ps18
Arthropoda - ps19
Mandibulata - ps20
Pancrustacea - ps21
Insecta - ps22
Eumetabola - ps23
Holometabola/Psocodea - ps24
Holometabola - ps25
Aparaglossata - ps26
Mecopterida - ps27
Diptera - ps28
Bibiono./Cyclorrhapha - ps29
Cyclorrhapha - ps30
Calyptratae/Drosophila - ps31
Drosophila - ps32
Sophophora - ps33
Obscura/Melanogaster_group - ps34
D_melanogaster_group - ps35
D_melanogaster_subgroup - ps36
sim./sech./mel. group - ps37
Drosophila_melanogaster - ps38
Phylostratum

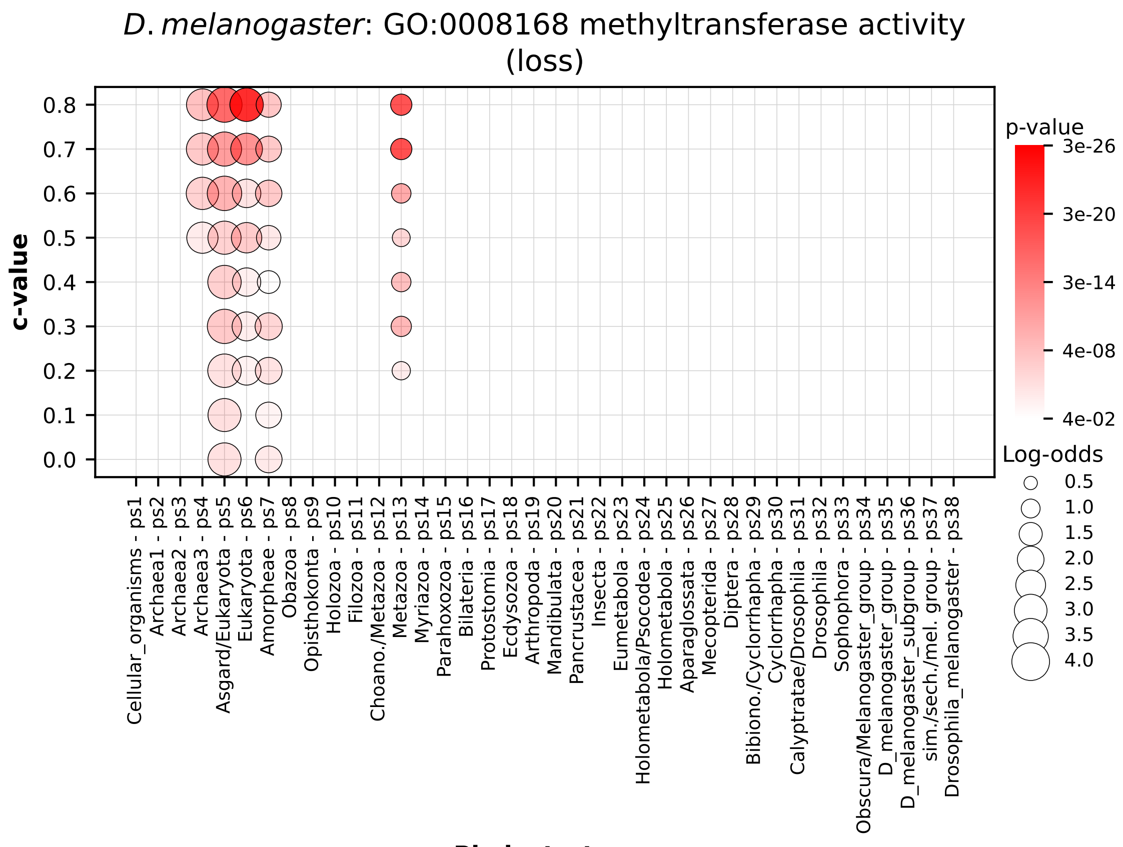

D. melanogaster: GO:0008168 methyltransferase activity (loss)
c-value
0.8
0.7
0.6
0.5
0.4
0.3
0.2
0.1
0.0
Phylostratum
Cellular_organisms - ps1
Archaea1 - ps2
Archaea2 - ps3
Archaea3 - ps4
Asgard/Eukaryota - ps5
Eukaryota - ps6
Amorpheae - ps7
Obazoa - ps8
Opisthokonta - ps9
Holozoa - ps10
Filozoa - ps11
Choano./Metazoa - ps12
Metazoa - ps13
Myriazoa - ps14
Parahoxozoa - ps15
Bilateria - ps16
Protostomia - ps17
Ecdysozoa - ps18
Arthropoda - ps19
Mandibulata - ps20
Pancrustacea - ps21
Insecta - ps22
Eumetabola - ps23
Holometabola/Psocodea - ps24
Holometabola - ps25
Aparaglossata - ps26
Mecopterida - ps27
Diptera - ps28
Bibiono./Cyclorrhapha - ps29
Cyclorrhapha - ps30
Calyptratae/Drosophila - ps31
Drosophila - ps32
Sophophora - ps33
Obscura/Melanogaster_group - ps34
D_melanogaster_group - ps35
D_melanogaster_subgroup - ps36
sim./sech./mel. group - ps37
Drosophila_melanogaster - ps38
p-value
3e-26
3e-20
3e-14
4e-08
4e-02
Log-odds
0.5
1.0
1.5
2.0
2.5
3.0
3.5
4.0

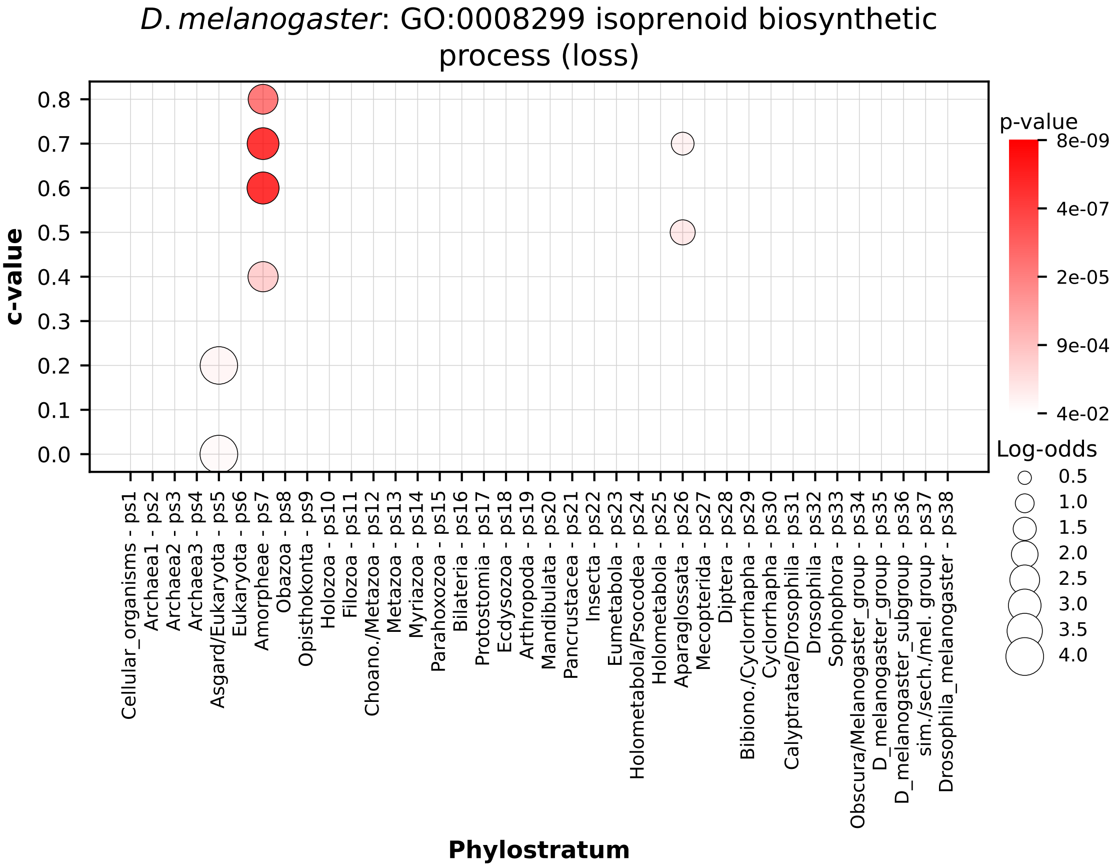

D. melanogaster: GO:0008299 isoprenoid biosynthetic process (loss)
c-value
0.0
0.1
0.2
0.3
0.4
0.5
0.6
0.7
0.8
Phylostratum
Cellular_organisms - ps1
Archaea1 - ps2
Archaea2 - ps3
Archaea3 - ps4
Asgard/Eukaryota - ps5
Eukaryota - ps6
Amorpheae - ps7
Obazoa - ps8
Opisthokonta - ps9
Holozoa - ps10
Filozoa - ps11
Choano./Metazoa - ps12
Metazoa - ps13
Myriazoa - ps14
Parahoxozoa - ps15
Bilateria - ps16
Protostomia - ps17
Ecdysozoa - ps18
Arthropoda - ps19
Mandibulata - ps20
Pancrustacea - ps21
Insecta - ps22
Eumetabola - ps23
Holometabola/Psocodea - ps24
Holometabola - ps25
Aparaglossata - ps26
Mecopterida - ps27
Diptera - ps28
Bibiono./Cyclorrhapha - ps29
Cyclorrhapha - ps30
Calyptratae/Drosophila - ps31
Drosophila - ps32
Sophophora - ps33
Obscura/Melanogaster_group - ps34
D_melanogaster_group - ps35
D_melanogaster_subgroup - ps36
sim./sech./mel. group - ps37
Drosophila_melanogaster - ps38
p-value
8e-09
4e-07
2e-05
9e-04
4e-02
Log-odds
0.5
1.0
1.5
2.0
2.5
3.0
3.5
4.0

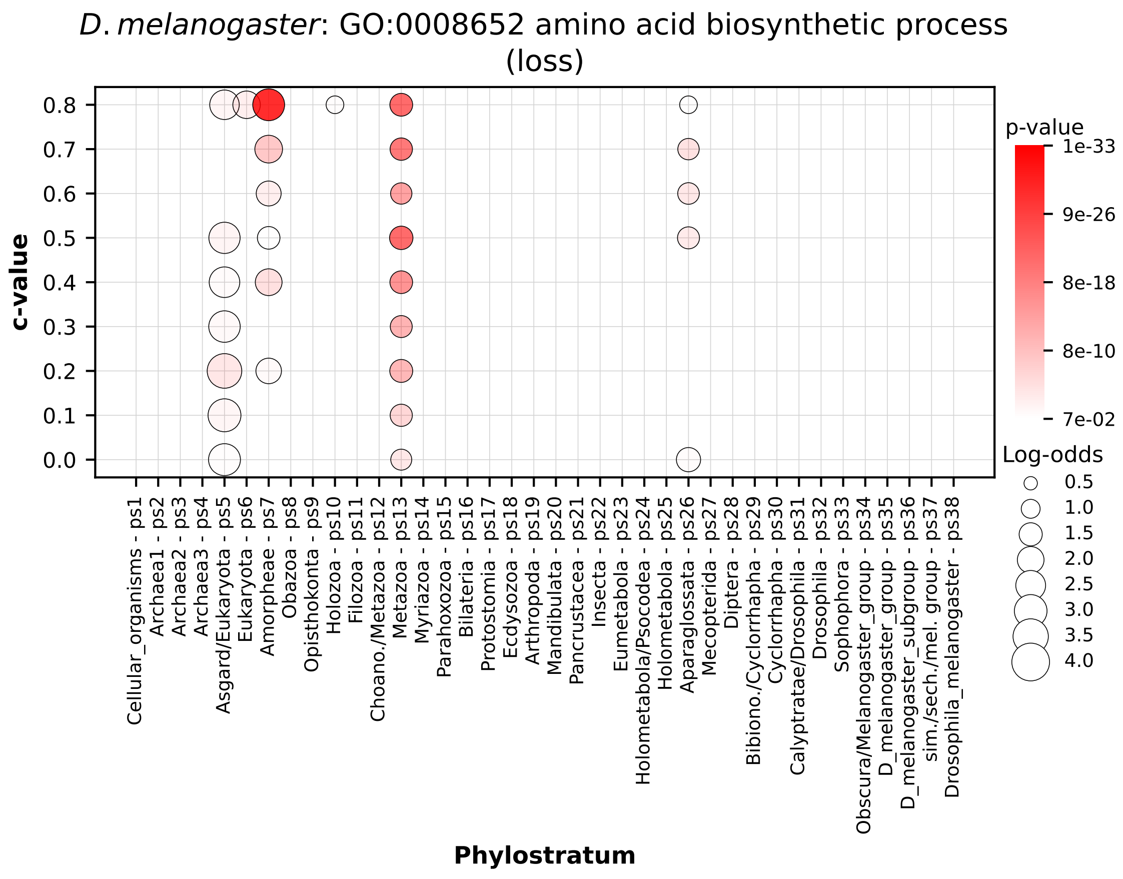

D. melanogaster: GO:0008652 amino acid biosynthetic process
(loss)
c-value
Phylostratum
p-value
1e-33
9e-26
8e-18
8e-10
7e-02
Log-odds
0.5
1.0
1.5
2.0
2.5
3.0
3.5
4.0
Cellular_organisms - ps1
Archaea1 - ps2
Archaea2 - ps3
Archaea3 - ps4
Asgard/Eukaryota - ps5
Eukaryota - ps6
Amorpheae - ps7
Obazoa - ps8
Opisthokonta - ps9
Holozoa - ps10
Filozoa - ps11
Choano./Metazoa - ps12
Metazoa - ps13
Myriazoa - ps14
Parahoxozoa - ps15
Bilateria - ps16
Protostomia - ps17
Ecdysozoa - ps18
Arthropoda - ps19
Mandibulata - ps20
Pancrustacea - ps21
Insecta - ps22
Eumetabola - ps23
Holometabola/Psocodea - ps24
Holometabola - ps25
Aparaglossata - ps26
Mecopterida - ps27
Diptera - ps28
Bibiono./Cyclorrhapha - ps29
Cyclorrhapha - ps30
Calyptratae/Drosophila - ps31
Drosophila - ps32
Sophophora - ps33
Obscura/Melanogaster_group - ps34
D_melanogaster_group - ps35
D_melanogaster_subgroup - ps36
sim./sech./mel. group - ps37
Drosophila_melanogaster - ps38

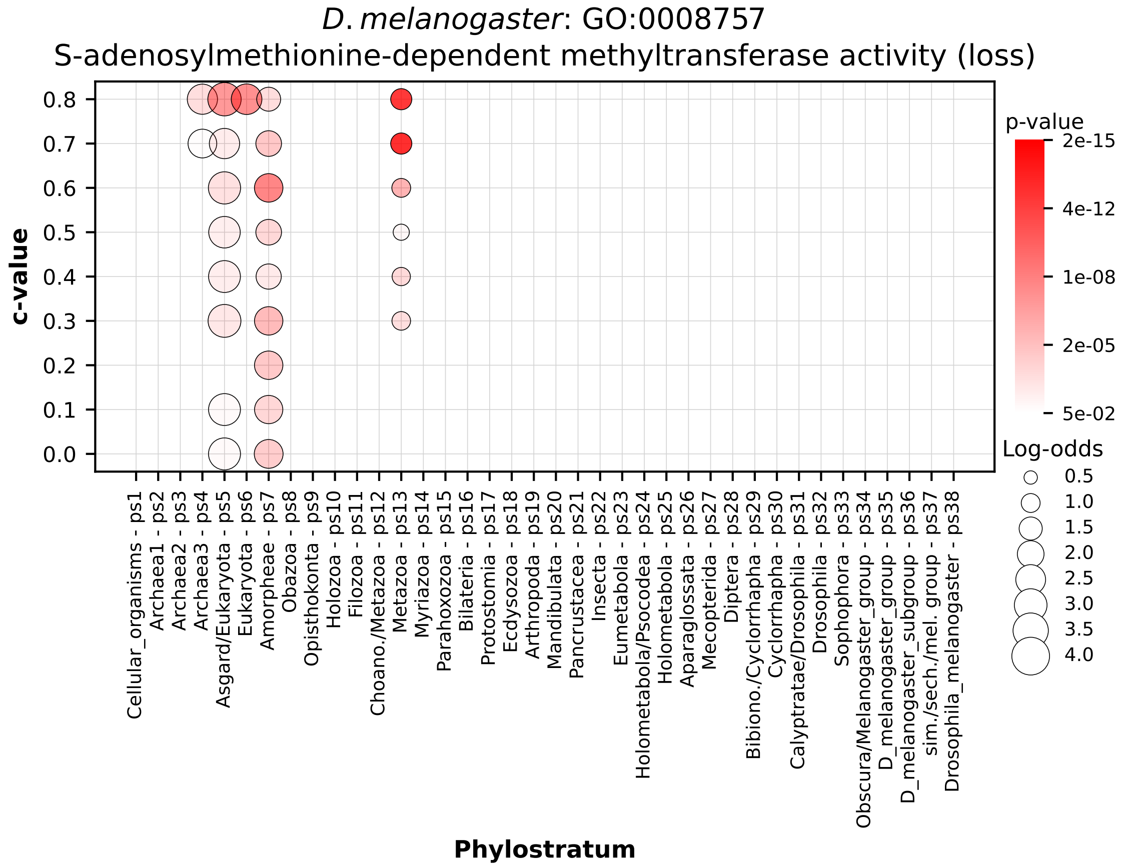

D. melanogaster: GO:0008757
S-adenosylmethionine-dependent methyltransferase activity (loss)
c-value
0.8
0.7
0.6
0.5
0.4
0.3
0.2
0.1
0.0
p-value
2e-15
4e-12
1e-08
2e-05
5e-02
Log-odds
0.5
1.0
1.5
2.0
2.5
3.0
3.5
4.0
Cellular_organisms - ps1
Archaea1 - ps2
Archaea2 - ps3
Archaea3 - ps4
Asgard/Eukaryota - ps5
Eukaryota - ps6
Amorpheae - ps7
Obazoa - ps8
Opisthokonta - ps9
Holozoa - ps10
Filozoa - ps11
Choano./Metazoa - ps12
Metazoa - ps13
Myriazoa - ps14
Parahoxozoa - ps15
Bilateria - ps16
Protostomia - ps17
Ecdysozoa - ps18
Arthropoda - ps19
Mandibulata - ps20
Pancrustacea - ps21
Insecta - ps22
Eumetabola - ps23
Holometabola/Psocodea - ps24
Holometabola - ps25
Aparaglossata - ps26
Mecopterida - ps27
Diptera - ps28
Bibiono./Cyclorrhapha - ps29
Cyclorrhapha - ps30
Calyptratae/Drosophila - ps31
Drosophila - ps32
Sophophora - ps33
Obscura/Melanogaster_group - ps34
D_melanogaster_group - ps35
D_melanogaster_subgroup - ps36
sim./sech./mel. group - ps37
Drosophila_melanogaster - ps38
Phylostratum

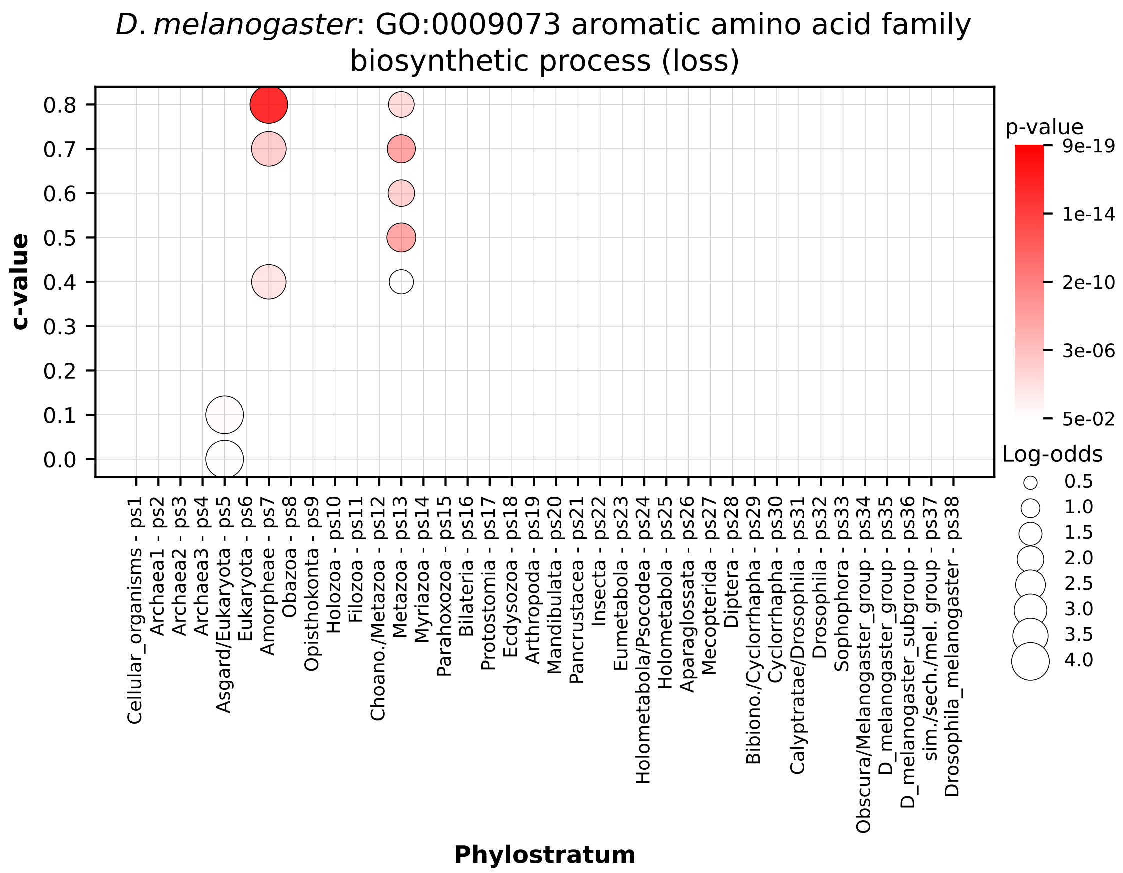

D. melanogaster: GO:0009073 aromatic amino acid family biosynthetic process (loss)
c-value
0.8
0.7
0.6
0.5
0.4
0.3
0.2
0.1
0.0
p-value
9e-19
1e-14
2e-10
3e-06
5e-02
Log-odds
0.5
1.0
1.5
2.0
2.5
3.0
3.5
4.0
Cellular_organisms - ps1
Archaea1 - ps2
Archaea2 - ps3
Archaea3 - ps4
Asgard/Eukaryota - ps5
Eukaryota - ps6
Amorpheae - ps7
Obazoa - ps8
Opisthokonta - ps9
Holozoa - ps10
Filozoa - ps11
Choano./Metazoa - ps12
Metazoa - ps13
Myriazoa - ps14
Parahoxozoa - ps15
Bilateria - ps16
Protostomia - ps17
Ecdysozoa - ps18
Arthropoda - ps19
Mandibulata - ps20
Pancrustacea - ps21
Insecta - ps22
Eumetabola - ps23
Holometabola/Psocodea - ps24
Holometabola - ps25
Aparaglossata - ps26
Mecopterida - ps27
Diptera - ps28
Bibiono./Cyclorrhapha - ps29
Cyclorrhapha - ps30
Calyptratae/Drosophila - ps31
Drosophila - ps32
Sophophora - ps33
Obscura/Melanogaster_group - ps34
D_melanogaster_group - ps35
D_melanogaster_subgroup - ps36
sim./sech./mel. group - ps37
Drosophila_melanogaster - ps38
Phylostratum

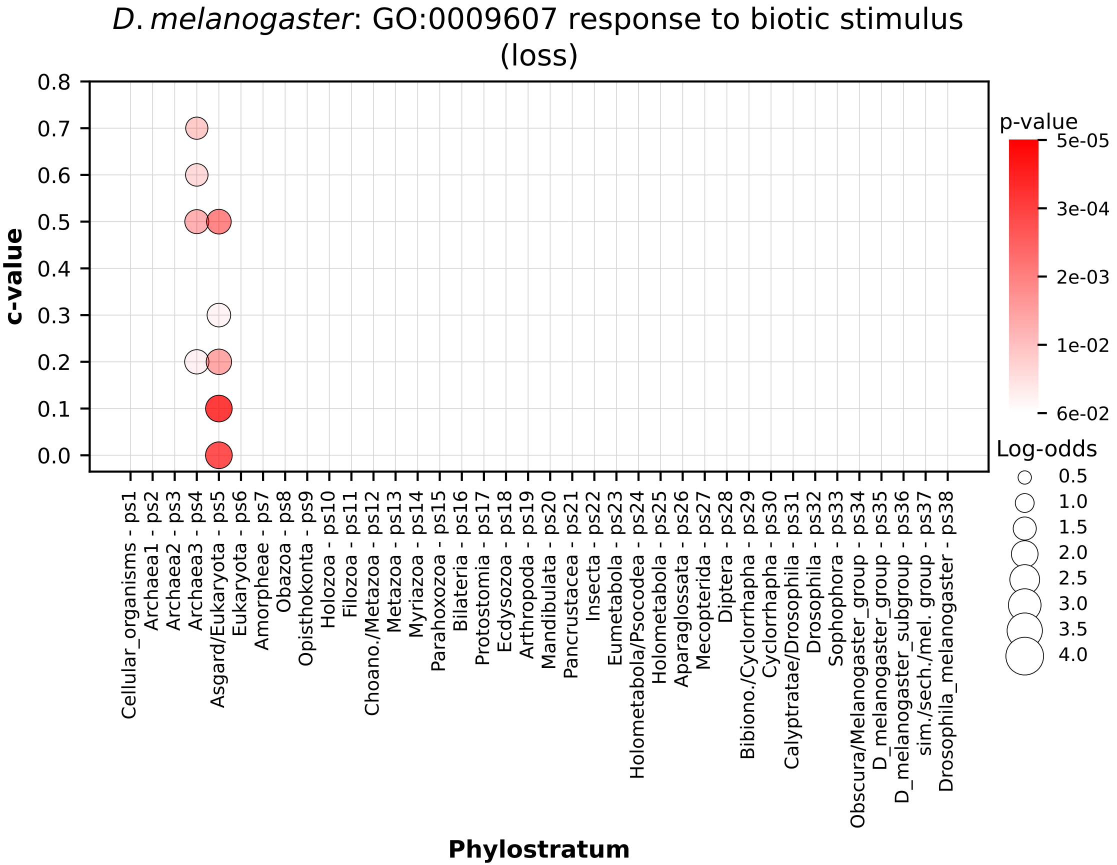

D. melanogaster: GO:0009607 response to biotic stimulus
(loss)
c-value
0.8
0.7
0.6
0.5
0.4
0.3
0.2
0.1
0.0
Phylostratum
Cellular_organisms - ps1
Archaea1 - ps2
Archaea2 - ps3
Archaea3 - ps4
Asgard/Eukaryota - ps5
Eukaryota - ps6
Amorpheae - ps7
Obazoa - ps8
Opisthokonta - ps9
Holozoa - ps10
Filozoa - ps11
Choano./Metazoa - ps12
Metazoa - ps13
Myriazoa - ps14
Parahoxozoa - ps15
Bilateria - ps16
Protostomia - ps17
Ecdysozoa - ps18
Arthropoda - ps19
Mandibulata - ps20
Pancrustacea - ps21
Insecta - ps22
Eumetabola - ps23
Holometabola/Psocodea - ps24
Holometabola - ps25
Aparaglossata - ps26
Mecopterida - ps27
Diptera - ps28
Bibiono./Cyclorrhapha - ps29
Cyclorrhapha - ps30
Calyptratae/Drosophila - ps31
Drosophila - ps32
Sophophora - ps33
Obscura/Melanogaster_group - ps34
D_melanogaster_group - ps35
D_melanogaster_subgroup - ps36
sim./sech./mel. group - ps37
Drosophila_melanogaster - ps38
p-value
5e-05
3e-04
2e-03
1e-02
6e-02
Log-odds
0.5
1.0
1.5
2.0
2.5
3.0
3.5
4.0

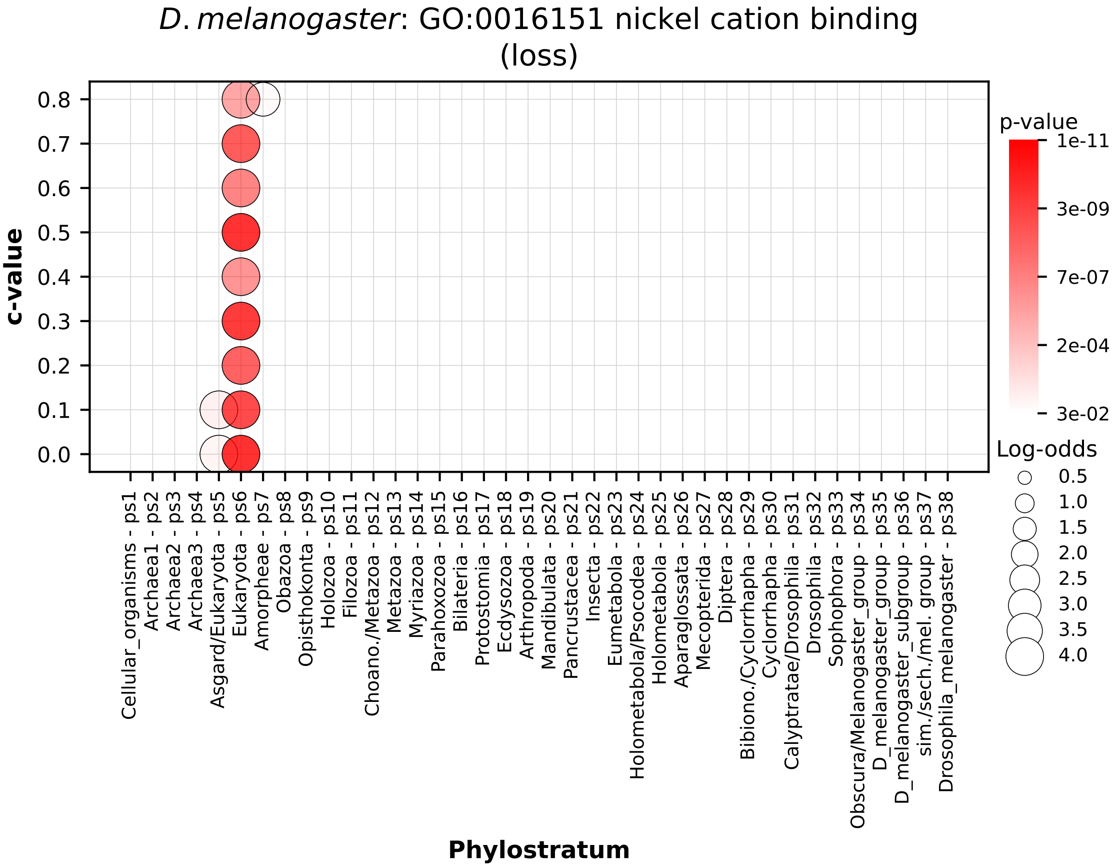

D. melanogaster: GO:0016151 nickel cation binding
(loss)
c-value
0.8
0.7
0.6
0.5
0.4
0.3
0.2
0.1
0.0
p-value
1e-11
3e-09
7e-07
2e-04
3e-02
Log-odds
0.5
1.0
1.5
2.0
2.5
3.0
3.5
4.0
Cellular_organisms - ps1
Archaea1 - ps2
Archaea2 - ps3
Archaea3 - ps4
Asgard/Eukaryota - ps5
Eukaryota - ps6
Amorpheae - ps7
Obazoa - ps8
Opisthokonta - ps9
Holozoa - ps10
Filozoa - ps11
Choano./Metazoa - ps12
Metazoa - ps13
Myriazoa - ps14
Parahoxozoa - ps15
Bilateria - ps16
Protostomia - ps17
Ecdysozoa - ps18
Arthropoda - ps19
Mandibulata - ps20
Pancrustacea - ps21
Insecta - ps22
Eumetabola - ps23
Holometabola/Psocodea - ps24
Holometabola - ps25
Aparaglossata - ps26
Mecopterida - ps27
Diptera - ps28
Bibiono./Cyclorrhapha - ps29
Cyclorrhapha - ps30
Calyptratae/Drosophila - ps31
Drosophila - ps32
Sophophora - ps33
Obscura/Melanogaster_group - ps34
D_melanogaster_group - ps35
D_melanogaster_subgroup - ps36
sim./sech./mel. group - ps37
Drosophila_melanogaster - ps38
Phylostratum

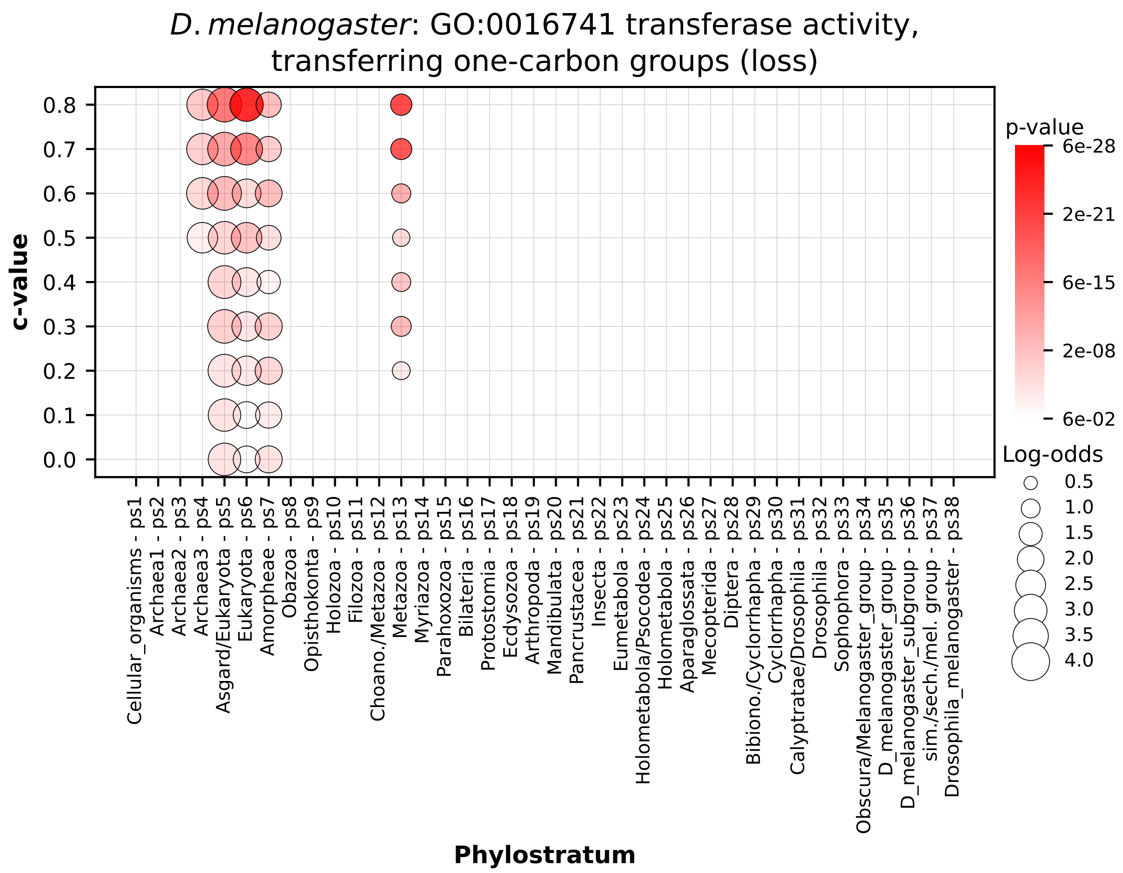
D. melanogaster: GO:0016741 transferase activity, transferring one-carbon groups (loss)
c-value
0.8
0.7
0.6
0.5
0.4
0.3
0.2
0.1
0.0
p-value
6e-28
2e-21
6e-15
2e-08
6e-02
Log-odds
0.5
1.0
1.5
2.0
2.5
3.0
3.5
4.0
Cellular_organisms - ps1
Archaea1 - ps2
Archaea2 - ps3
Archaea3 - ps4
Asgard/Eukaryota - ps5
Eukaryota - ps6
Amorpheae - ps7
Obazoa - ps8
Opisthokonta - ps9
Holozoa - ps10
Filozoa - ps11
Choano./Metazoa - ps12
Metazoa - ps13
Myriazoa - ps14
Parahoxozoa - ps15
Bilateria - ps16
Protostomia - ps17
Ecdysozoa - ps18
Arthropoda - ps19
Mandibulata - ps20
Pancrustacea - ps21
Insecta - ps22
Eumetabola - ps23
Holometabola/Psocodea - ps24
Holometabola - ps25
Aparaglossata - ps26
Mecopterida - ps27
Diptera - ps28
Bibiono./Cyclorrhapha - ps29
Cyclorrhapha - ps30
Calyptratae/Drosophila - ps31
Drosophila - ps32
Sophophora - ps33
Obscura/Melanogaster_group - ps34
D_melanogaster_group - ps35
D_melanogaster_subgroup - ps36
sim./sech./mel. group - ps37
Drosophila_melanogaster - ps38
Phylostratum

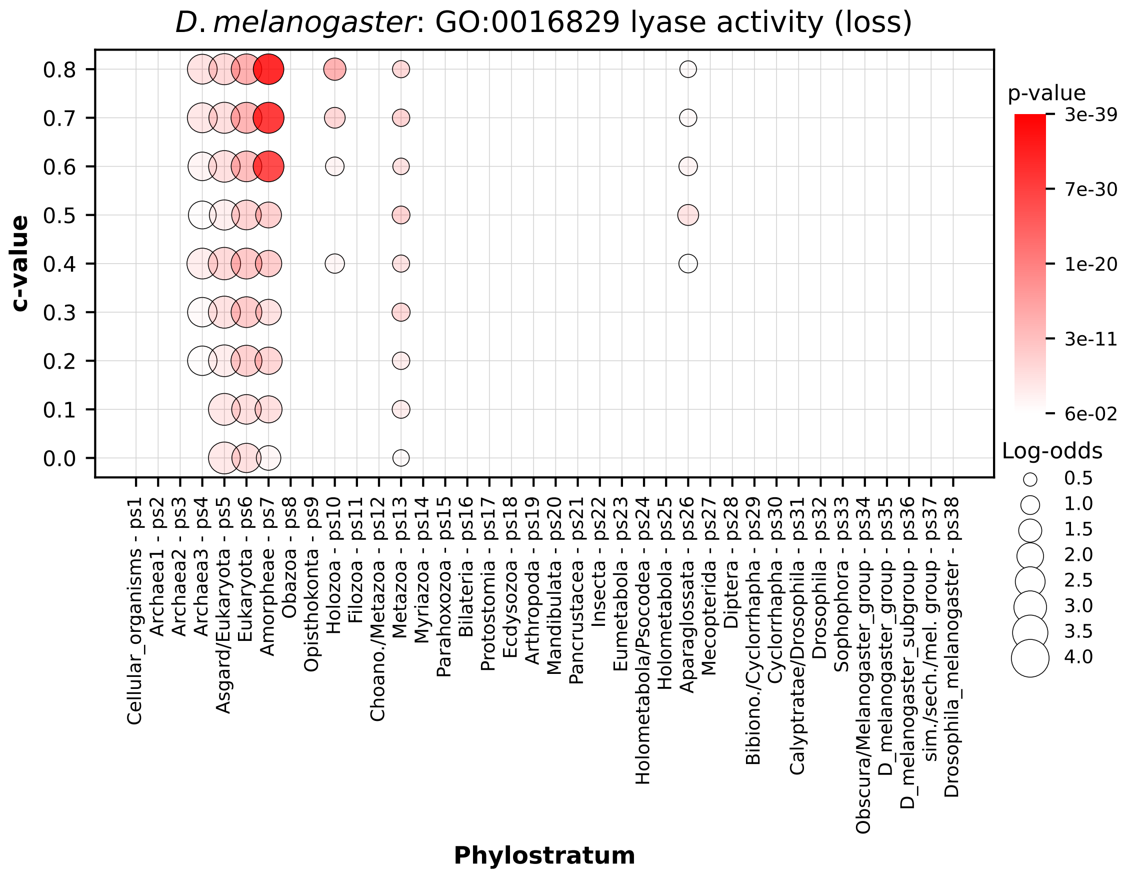
D. melanogaster: GO:0016829 lyase activity (loss)
c-value
0.0
0.1
0.2
0.3
0.4
0.5
0.6
0.7
0.8
Phylostratum
Cellular_organisms - ps1
Archaea1 - ps2
Archaea2 - ps3
Archaea3 - ps4
Asgard/Eukaryota - ps5
Eukaryota - ps6
Amorpheae - ps7
Obazoa - ps8
Opisthokonta - ps9
Holozoa - ps10
Filozoa - ps11
Choano./Metazoa - ps12
Metazoa - ps13
Myriazoa - ps14
Parahoxozoa - ps15
Bilateria - ps16
Protostomia - ps17
Ecdysozoa - ps18
Arthropoda - ps19
Mandibulata - ps20
Pancrustacea - ps21
Insecta - ps22
Eumetabola - ps23
Holometabola/Psocodea - ps24
Holometabola - ps25
Aparaglossata - ps26
Mecopterida - ps27
Diptera - ps28
Bibiono./Cyclorrhapha - ps29
Cyclorrhapha - ps30
Calyptratae/Drosophila - ps31
Drosophila - ps32
Sophophora - ps33
Obscura/Melanogaster_group - ps34
D_melanogaster_group - ps35
D_melanogaster_subgroup - ps36
sim./sech./mel. group - ps37
Drosophila_melanogaster - ps38
p-value
3e-39
7e-30
1e-20
3e-11
6e-02
Log-odds
0.5
1.0
1.5
2.0
2.5
3.0
3.5
4.0

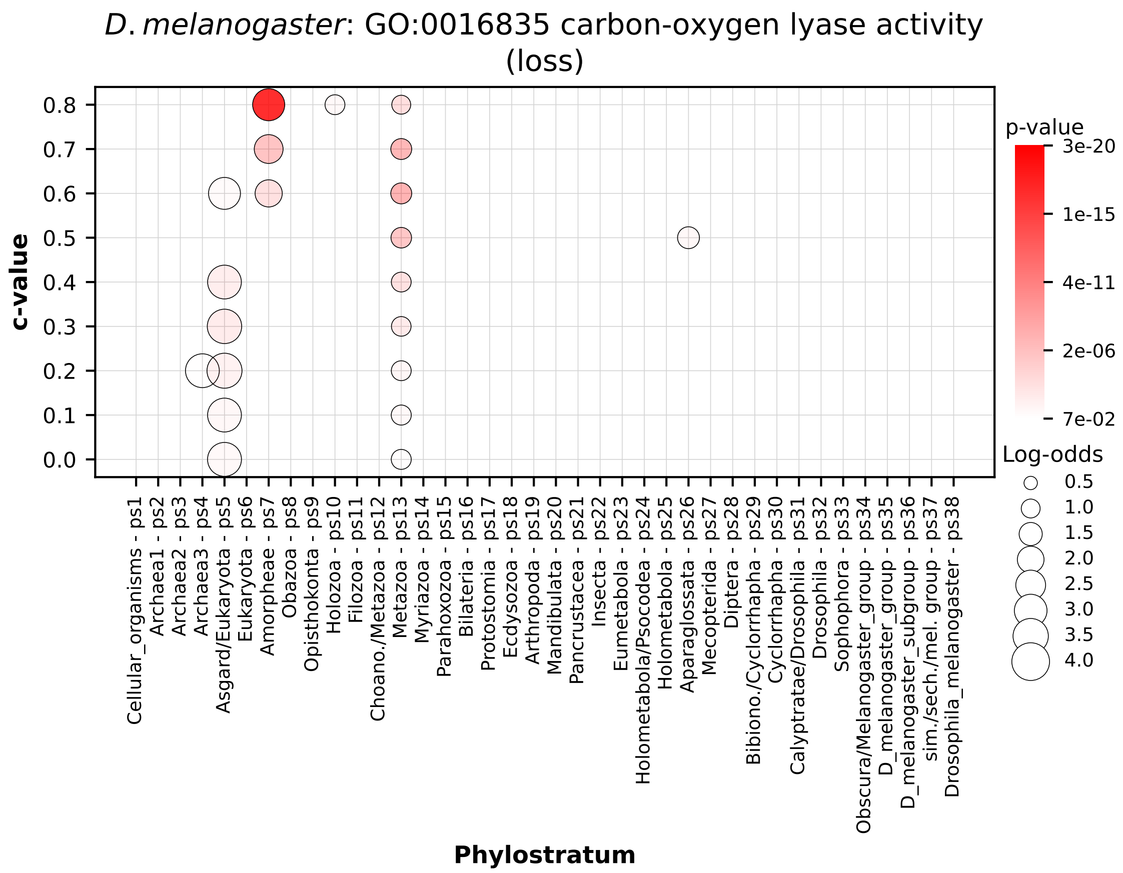

D. melanogaster: GO:0016835 carbon-oxygen lyase activity (loss)
c-value
0.8
0.7
0.6
0.5
0.4
0.3
0.2
0.1
0.0
Phylostratum
Cellular_organisms - ps1
Archaea1 - ps2
Archaea2 - ps3
Archaea3 - ps4
Asgard/Eukaryota - ps5
Eukaryota - ps6
Amorpheae - ps7
Obazoa - ps8
Opisthokonta - ps9
Holozoa - ps10
Filozoa - ps11
Choano./Metazoa - ps12
Metazoa - ps13
Myriazoa - ps14
Parahoxozoa - ps15
Bilateria - ps16
Protostomia - ps17
Ecdysozoa - ps18
Arthropoda - ps19
Mandibulata - ps20
Pancrustacea - ps21
Insecta - ps22
Eumetabola - ps23
Holometabola/Psocodea - ps24
Holometabola - ps25
Aparaglossata - ps26
Mecopterida - ps27
Diptera - ps28
Bibiono./Cyclorrhapha - ps29
Cyclorrhapha - ps30
Calyptratae/Drosophila - ps31
Drosophila - ps32
Sophophora - ps33
Obscura/Melanogaster_group - ps34
D_melanogaster_group - ps35
D_melanogaster_subgroup - ps36
sim./sech./mel. group - ps37
Drosophila_melanogaster - ps38
p-value
3e-20
1e-15
4e-11
2e-06
7e-02
Log-odds
0.5
1.0
1.5
2.0
2.5
3.0
3.5
4.0

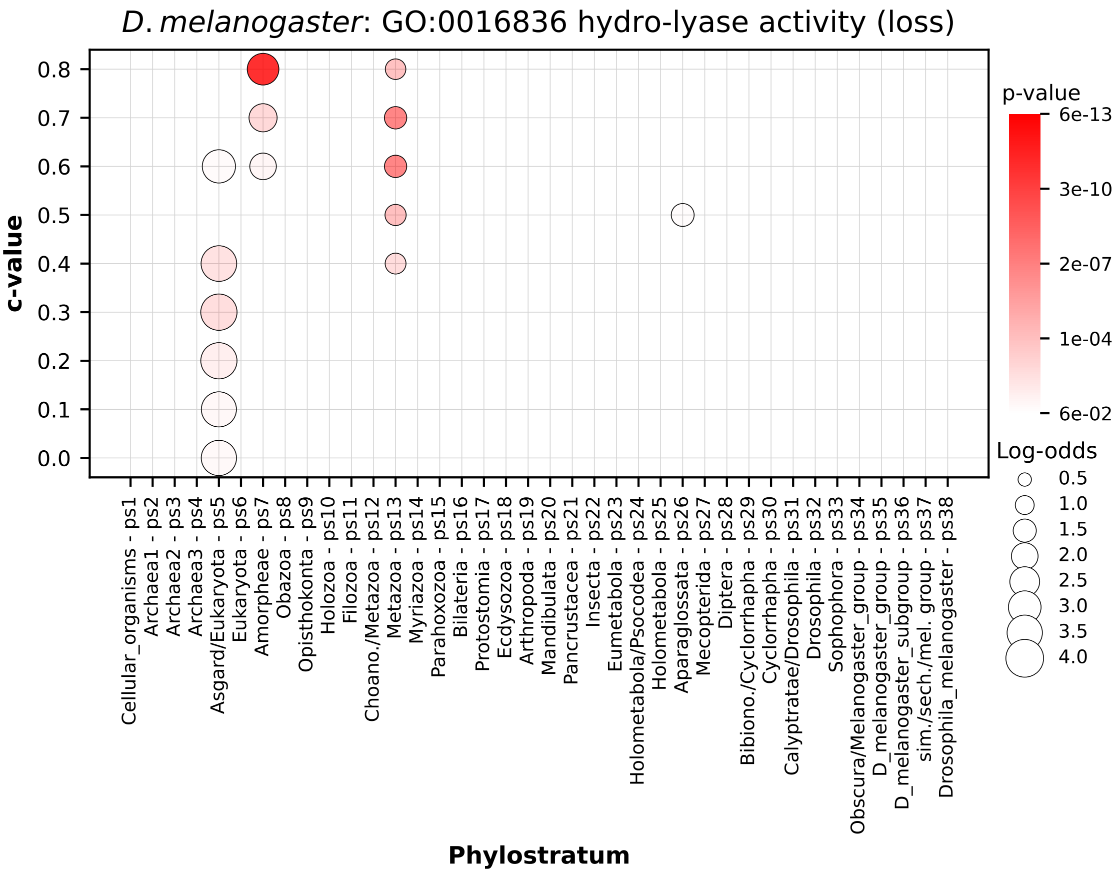

D. melanogaster: GO:0016836 hydro-lyase activity (loss)
c-value
0.0
0.1
0.2
0.3
0.4
0.5
0.6
0.7
0.8
Phylostratum
Cellular_organisms - ps1
Archaea1 - ps2
Archaea2 - ps3
Archaea3 - ps4
Asgard/Eukaryota - ps5
Eukaryota - ps6
Amorpheae - ps7
Obazoa - ps8
Opisthokonta - ps9
Holozoa - ps10
Filozoa - ps11
Choano./Metazoa - ps12
Metazoa - ps13
Myriazoa - ps14
Parahoxozoa - ps15
Bilateria - ps16
Protostomia - ps17
Ecdysozoa - ps18
Arthropoda - ps19
Mandibulata - ps20
Pancrustacea - ps21
Insecta - ps22
Eumetabola - ps23
Holometabola/Psocodea - ps24
Holometabola - ps25
Aparaglossata - ps26
Mecopterida - ps27
Diptera - ps28
Bibiono./Cyclorrhapha - ps29
Cyclorrhapha - ps30
Calyptratae/Drosophila - ps31
Drosophila - ps32
Sophophora - ps33
Obscura/Melanogaster_group - ps34
D_melanogaster_group - ps35
D_melanogaster_subgroup - ps36
sim./sech./mel. group - ps37
Drosophila_melanogaster - ps38
p-value
6e-13
3e-10
2e-07
1e-04
6e-02
Log-odds
0.5
1.0
1.5
2.0
2.5
3.0
3.5
4.0

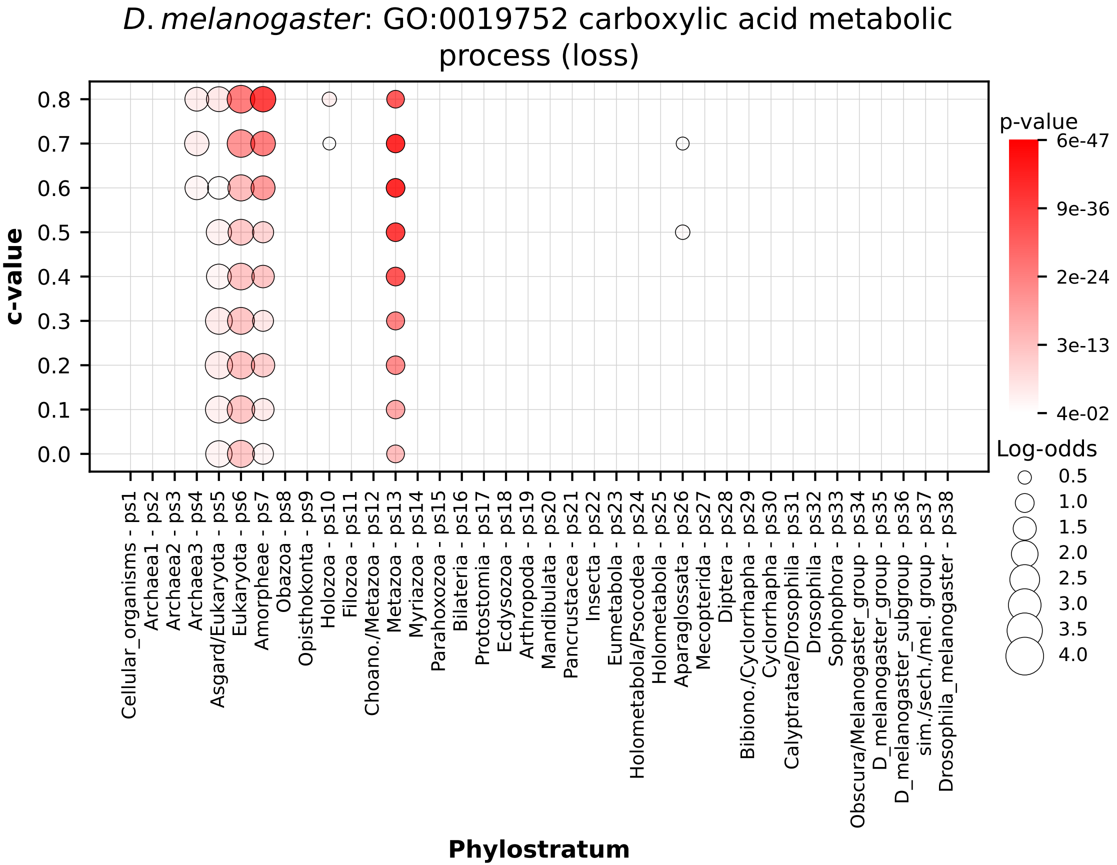
D. melanogaster: GO:0019752 carboxylic acid metabolic process (loss)
c-value
0.8
0.7
0.6
0.5
0.4
0.3
0.2
0.1
0.0
p-value
6e-47
9e-36
2e-24
3e-13
4e-02
Log-odds
0.5
1.0
1.5
2.0
2.5
3.0
3.5
4.0
Cellular_organisms - ps1
Archaea1 - ps2
Archaea2 - ps3
Archaea3 - ps4
Asgard/Eukaryota - ps5
Eukaryota - ps6
Amorpheae - ps7
Obazoa - ps8
Opisthokonta - ps9
Holozoa - ps10
Filozoa - ps11
Choano./Metazoa - ps12
Metazoa - ps13
Myriazoa - ps14
Parahoxozoa - ps15
Bilateria - ps16
Protostomia - ps17
Ecdysozoa - ps18
Arthropoda - ps19
Mandibulata - ps20
Pancrustacea - ps21
Insecta - ps22
Eumetabola - ps23
Holometabola/Psocodea - ps24
Holometabola - ps25
Aparaglossata - ps26
Mecopterida - ps27
Diptera - ps28
Bibiono./Cyclorrhapha - ps29
Cyclorrhapha - ps30
Calyptratae/Drosophila - ps31
Drosophila - ps32
Sophophora - ps33
Obscura/Melanogaster_group - ps34
D_melanogaster_group - ps35
D_melanogaster_subgroup - ps36
sim./sech./mel. group - ps37
Drosophila_melanogaster - ps38
Phylostratum

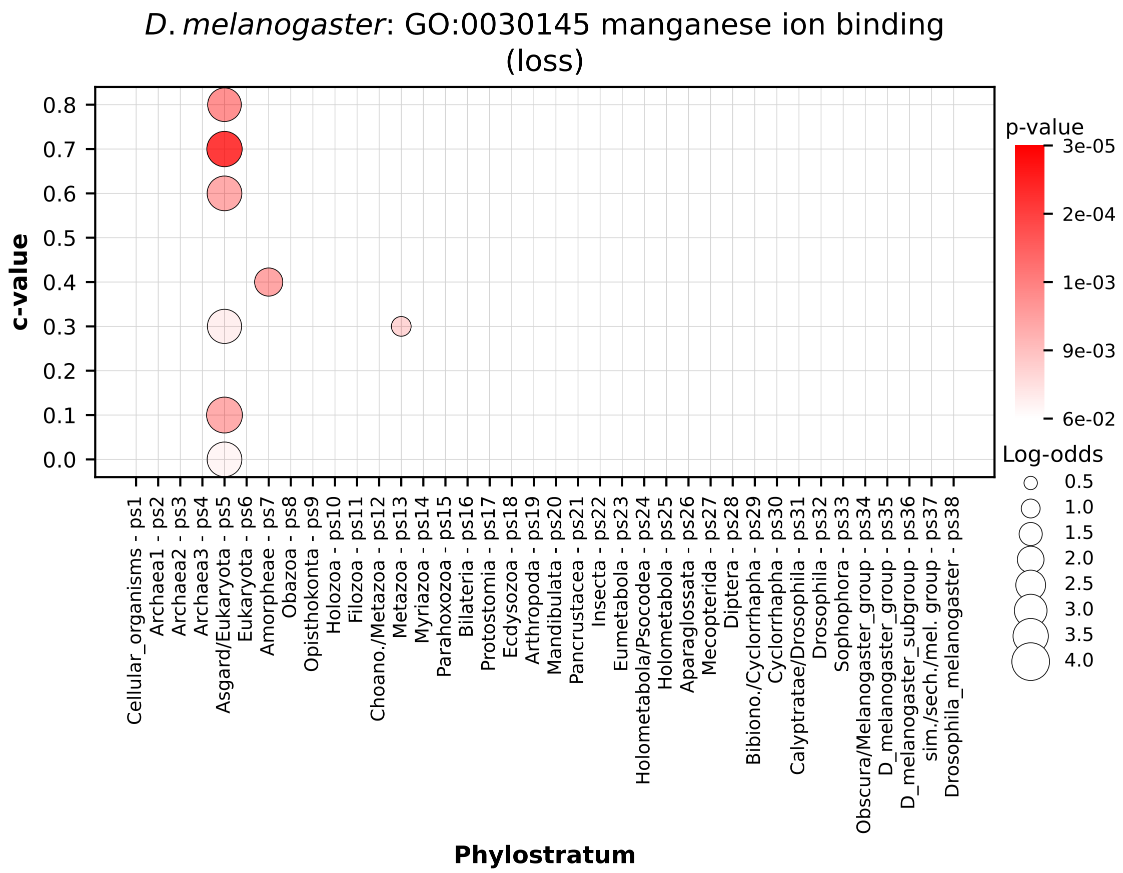
D. melanogaster: GO:0030145 manganese ion binding
(loss)
c-value
0.8
0.7
0.6
0.5
0.4
0.3
0.2
0.1
0.0
Phylostratum
Cellular_organisms - ps1
Archaea1 - ps2
Archaea2 - ps3
Archaea3 - ps4
Asgard/Eukaryota - ps5
Eukaryota - ps6
Amorpheae - ps7
Obazoa - ps8
Opisthokonta - ps9
Holozoa - ps10
Filozoa - ps11
Choano./Metazoa - ps12
Metazoa - ps13
Myriazoa - ps14
Parahoxozoa - ps15
Bilateria - ps16
Protostomia - ps17
Ecdysozoa - ps18
Arthropoda - ps19
Mandibulata - ps20
Pancrustacea - ps21
Insecta - ps22
Eumetabola - ps23
Holometabola/Psocodea - ps24
Holometabola - ps25
Aparaglossata - ps26
Mecopterida - ps27
Diptera - ps28
Bibiono./Cyclorrhapha - ps29
Cyclorrhapha - ps30
Calyptratae/Drosophila - ps31
Drosophila - ps32
Sophophora - ps33
Obscura/Melanogaster_group - ps34
D_melanogaster_group - ps35
D_melanogaster_subgroup - ps36
sim./sech./mel. group - ps37
Drosophila_melanogaster - ps38
p-value
3e-05
2e-04
1e-03
9e-03
6e-02
Log-odds
0.5
1.0
1.5
2.0
2.5
3.0
3.5
4.0

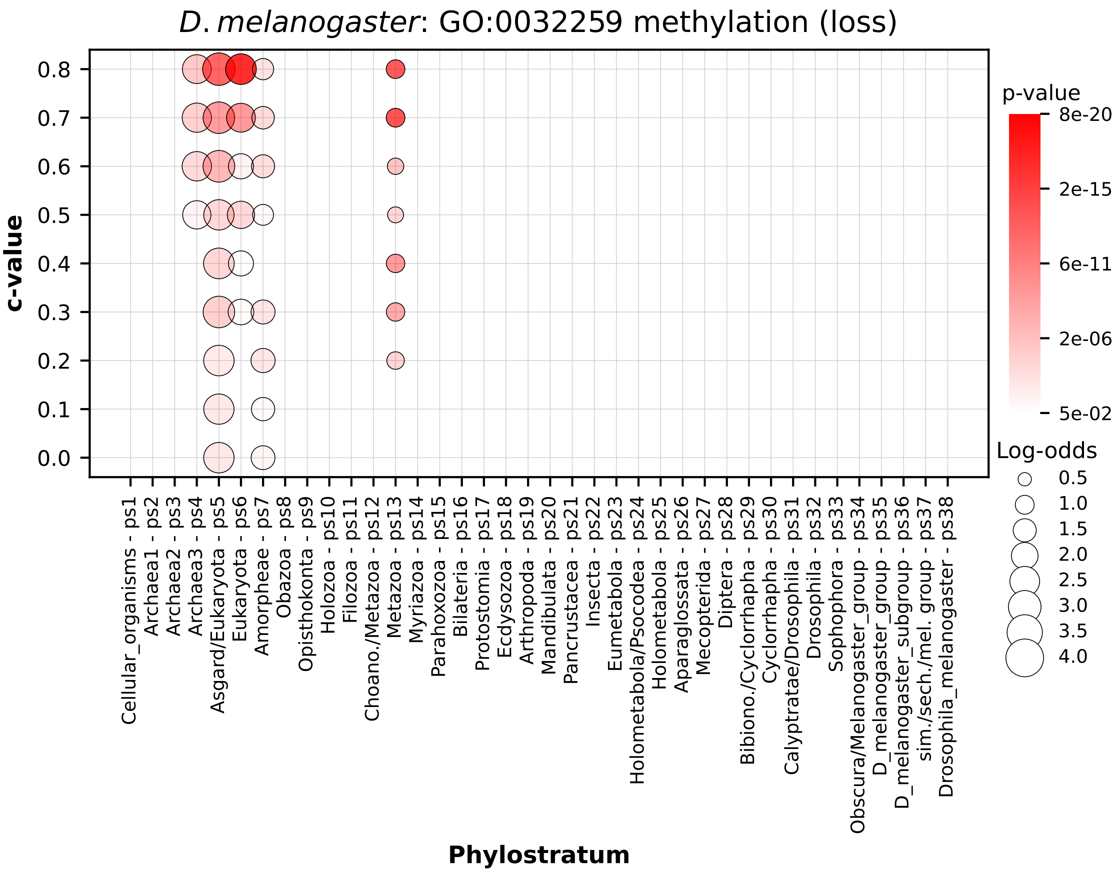
D. melanogaster: GO:0032259 methylation (loss)
c-value
0.8
0.7
0.6
0.5
0.4
0.3
0.2
0.1
0.0
Phylostratum
Cellular_organisms - ps1
Archaea1 - ps2
Archaea2 - ps3
Archaea3 - ps4
Asgard/Eukaryota - ps5
Eukaryota - ps6
Amorpheae - ps7
Obazoa - ps8
Opisthokonta - ps9
Holozoa - ps10
Filozoa - ps11
Choano./Metazoa - ps12
Metazoa - ps13
Myriazoa - ps14
Parahoxozoa - ps15
Bilateria - ps16
Protostomia - ps17
Ecdysozoa - ps18
Arthropoda - ps19
Mandibulata - ps20
Pancrustacea - ps21
Insecta - ps22
Eumetabola - ps23
Holometabola/Psocodea - ps24
Holometabola - ps25
Aparaglossata - ps26
Mecopterida - ps27
Diptera - ps28
Bibiono./Cyclorrhapha - ps29
Cyclorrhapha - ps30
Calyptratae/Drosophila - ps31
Drosophila - ps32
Sophophora - ps33
Obscura/Melanogaster_group - ps34
D_melanogaster_group - ps35
D_melanogaster_subgroup - ps36
sim./sech./mel. group - ps37
Drosophila_melanogaster - ps38
p-value
8e-20
2e-15
6e-11
2e-06
5e-02
Log-odds
0.5
1.0
1.5
2.0
2.5
3.0
3.5
4.0

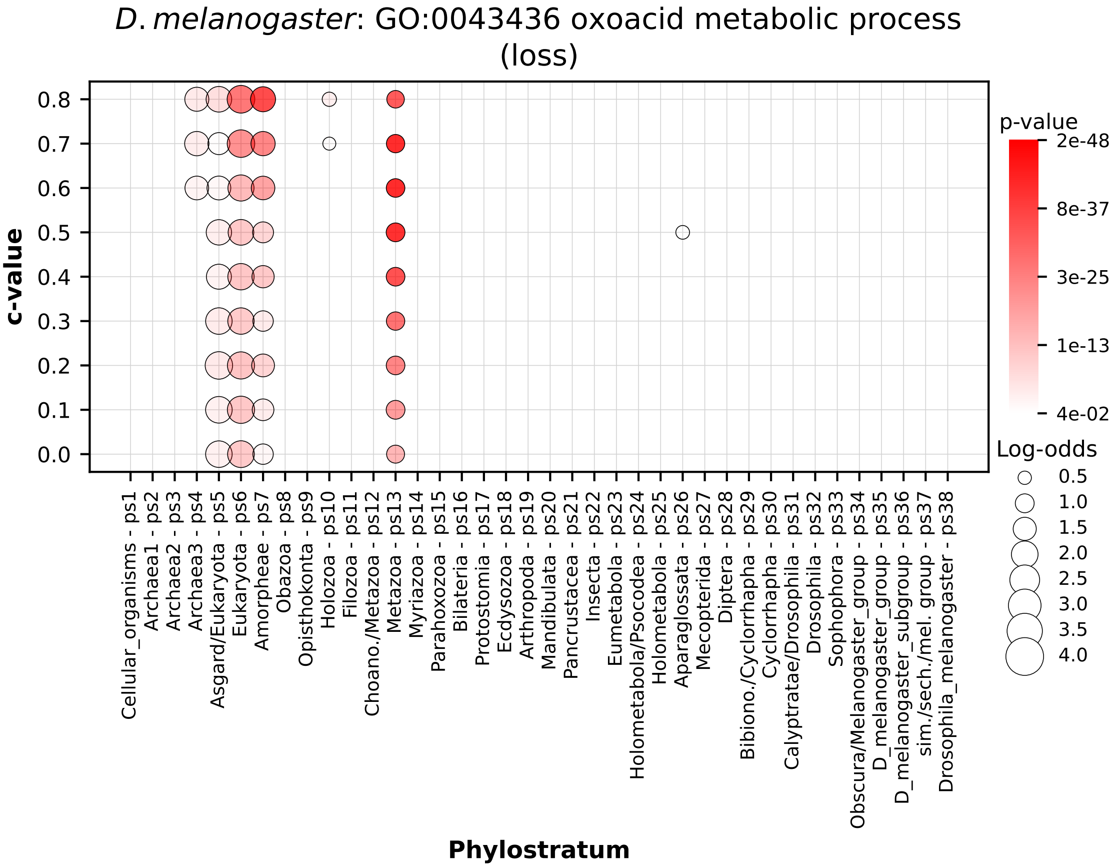

D. melanogaster: GO:0043436 oxoacid metabolic process
(loss)
c-value
0.8
0.7
0.6
0.5
0.4
0.3
0.2
0.1
0.0
Phylostratum
Cellular_organisms - ps1
Archaea1 - ps2
Archaea2 - ps3
Archaea3 - ps4
Asgard/Eukaryota - ps5
Eukaryota - ps6
Amorpheae - ps7
Obazoa - ps8
Opisthokonta - ps9
Holozoa - ps10
Filozoa - ps11
Choano./Metazoa - ps12
Metazoa - ps13
Myriazoa - ps14
Parahoxozoa - ps15
Bilateria - ps16
Protostomia - ps17
Ecdysozoa - ps18
Arthropoda - ps19
Mandibulata - ps20
Pancrustacea - ps21
Insecta - ps22
Eumetabola - ps23
Holometabola/Psocodea - ps24
Holometabola - ps25
Aparaglossata - ps26
Mecopterida - ps27
Diptera - ps28
Bibiono./Cyclorrhapha - ps29
Cyclorrhapha - ps30
Calyptratae/Drosophila - ps31
Drosophila - ps32
Sophophora - ps33
Obscura/Melanogaster_group - ps34
D_melanogaster_group - ps35
D_melanogaster_subgroup - ps36
sim./sech./mel. group - ps37
Drosophila_melanogaster - ps38
p-value
2e-48
8e-37
3e-25
1e-13
4e-02
Log-odds
0.5
1.0
1.5
2.0
2.5
3.0
3.5
4.0

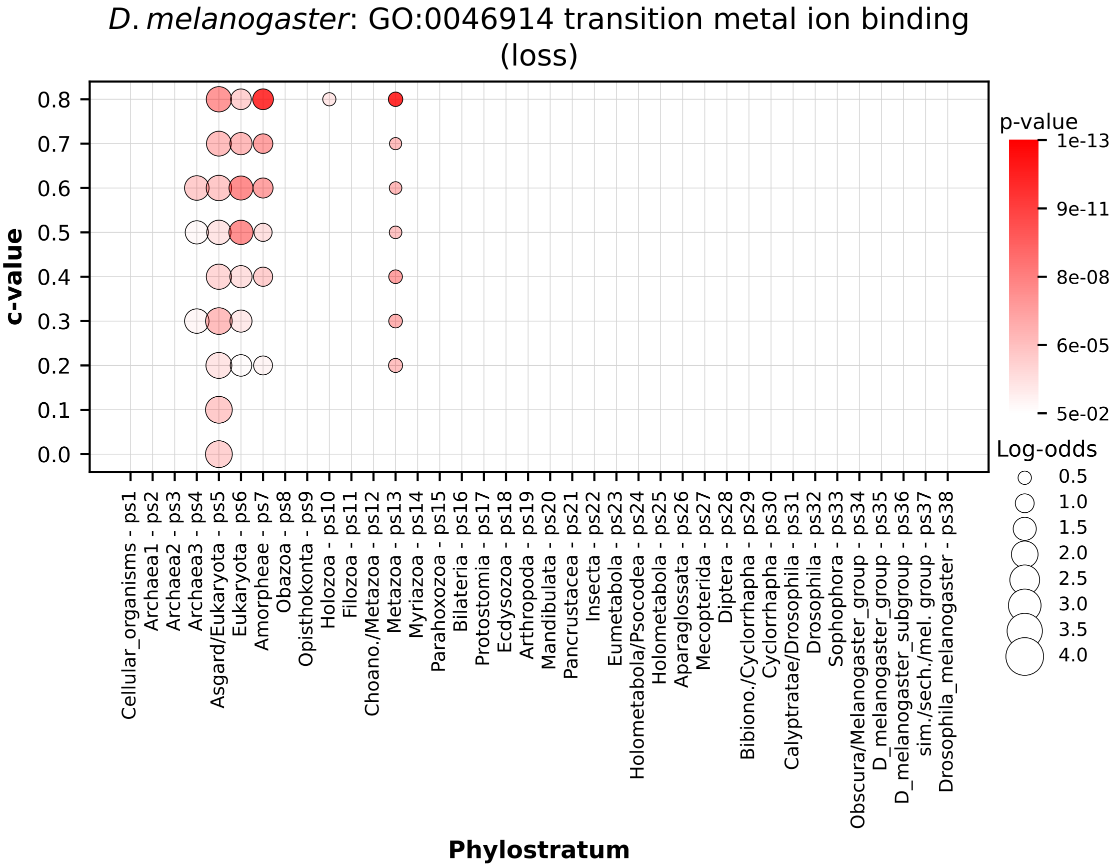

D. melanogaster: GO:0046914 transition metal ion binding (loss)
c-value
0.8
0.7
0.6
0.5
0.4
0.3
0.2
0.1
0.0
Phylostratum
Cellular_organisms - ps1
Archaea1 - ps2
Archaea2 - ps3
Archaea3 - ps4
Asgard/Eukaryota - ps5
Eukaryota - ps6
Amorpheae - ps7
Obazoa - ps8
Opisthokonta - ps9
Holozoa - ps10
Filozoa - ps11
Choano./Metazoa - ps12
Metazoa - ps13
Myriazoa - ps14
Parahoxozoa - ps15
Bilateria - ps16
Protostomia - ps17
Ecdysozoa - ps18
Arthropoda - ps19
Mandibulata - ps20
Pancrustacea - ps21
Insecta - ps22
Eumetabola - ps23
Holometabola/Psocodea - ps24
Holometabola - ps25
Aparaglossata - ps26
Mecopterida - ps27
Diptera - ps28
Bibiono./Cyclorrhapha - ps29
Cyclorrhapha - ps30
Calyptratae/Drosophila - ps31
Drosophila - ps32
Sophophora - ps33
Obscura/Melanogaster_group - ps34
D_melanogaster_group - ps35
D_melanogaster_subgroup - ps36
sim./sech./mel. group - ps37
Drosophila_melanogaster - ps38
p-value
1e-13
9e-11
8e-08
6e-05
5e-02
Log-odds
0.5
1.0
1.5
2.0
2.5
3.0
3.5
4.0

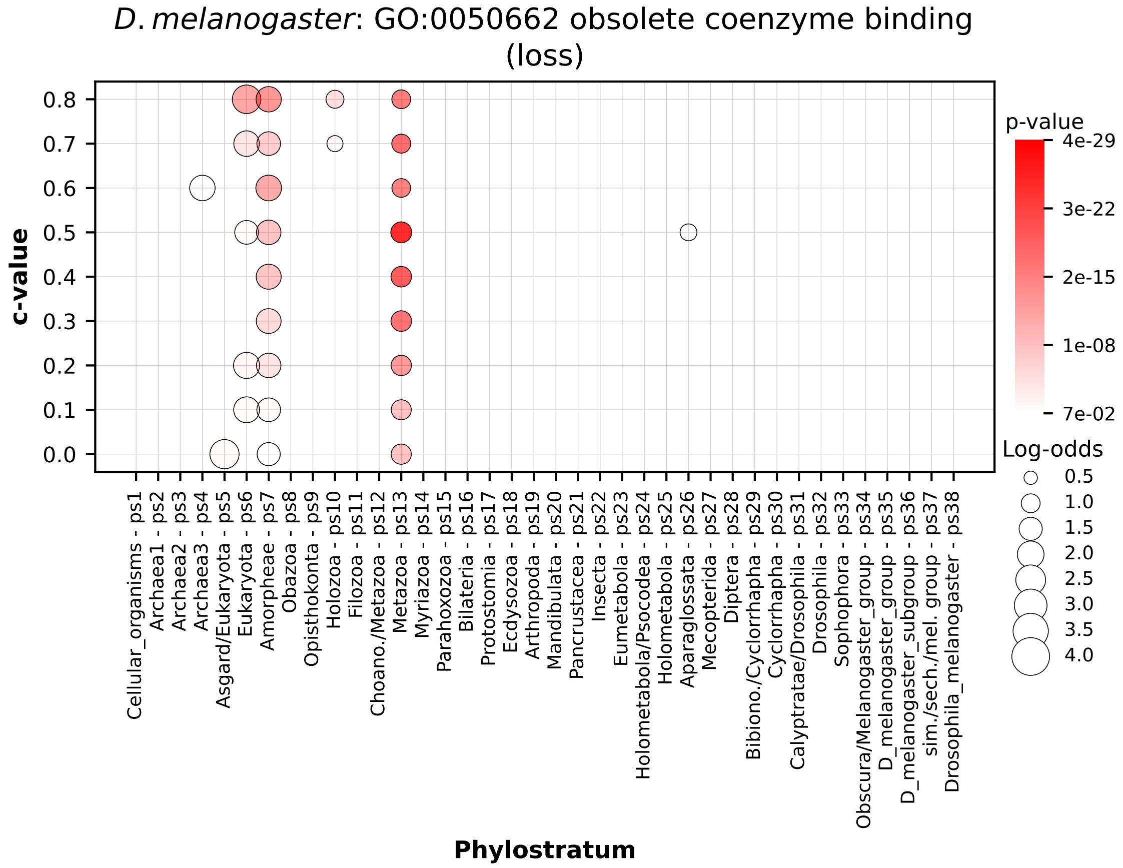

D. melanogaster: GO:0050662 obsolete coenzyme binding (loss)
c-value
0.8
0.7
0.6
0.5
0.4
0.3
0.2
0.1
0.0
Phylostratum
Cellular_organisms - ps1
Archaea1 - ps2
Archaea2 - ps3
Archaea3 - ps4
Asgard/Eukaryota - ps5
Eukaryota - ps6
Amorpheae - ps7
Obazoa - ps8
Opisthokonta - ps9
Holozoa - ps10
Filozoa - ps11
Choano./Metazoa - ps12
Metazoa - ps13
Myriazoa - ps14
Parahoxozoa - ps15
Bilateria - ps16
Protostomia - ps17
Ecdysozoa - ps18
Arthropoda - ps19
Mandibulata - ps20
Pancrustacea - ps21
Insecta - ps22
Eumetabola - ps23
Holometabola/Psocodea - ps24
Holometabola - ps25
Aparaglossata - ps26
Mecopterida - ps27
Diptera - ps28
Bibiono./Cyclorrhapha - ps29
Cyclorrhapha - ps30
Calyptratae/Drosophila - ps31
Drosophila - ps32
Sophophora - ps33
Obscura/Melanogaster_group - ps34
D_melanogaster_group - ps35
D_melanogaster_subgroup - ps36
sim./sech./mel. group - ps37
Drosophila_melanogaster - ps38
p-value
4e-29
3e-22
2e-15
1e-08
7e-02
Log-odds
0.5
1.0
1.5
2.0
2.5
3.0
3.5
4.0

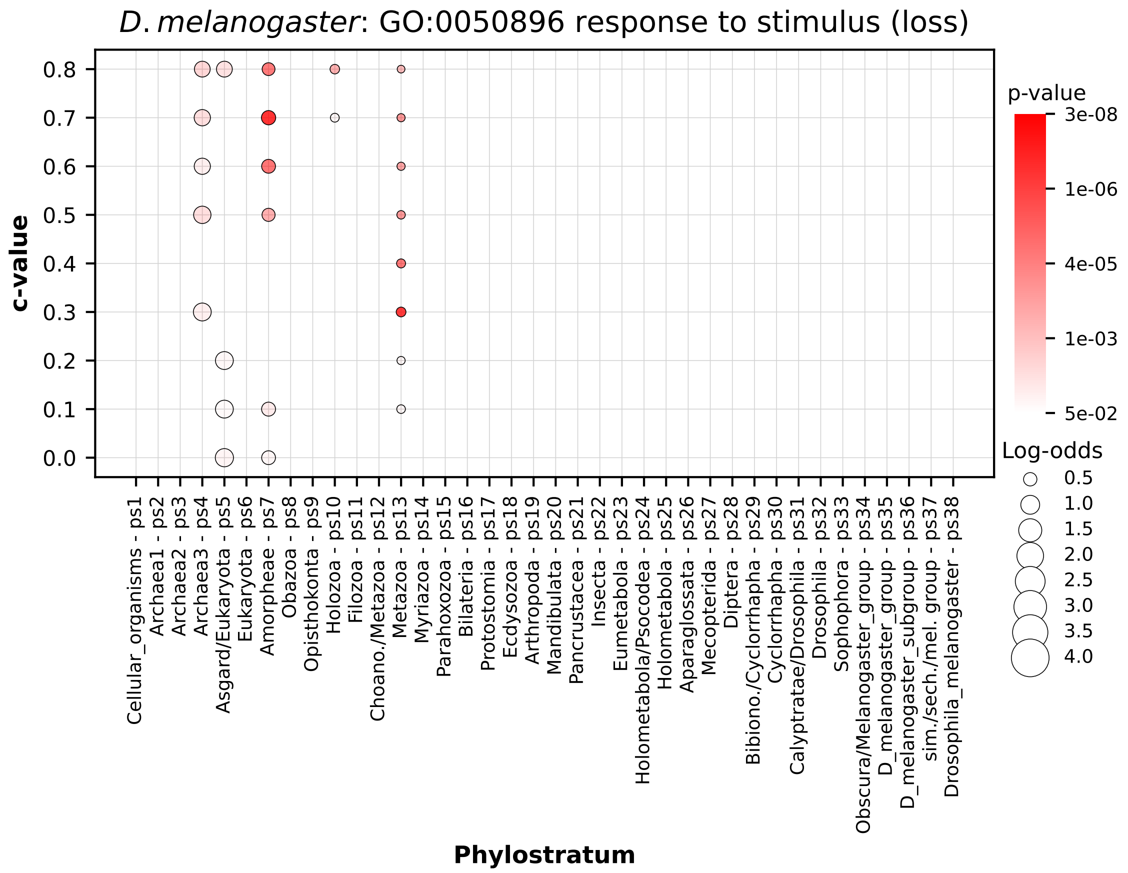
D. melanogaster: GO:0050896 response to stimulus (loss)
c-value
0.8
0.7
0.6
0.5
0.4
0.3
0.2
0.1
0.0
Phylostratum
Cellular_organisms - ps1
Archaea1 - ps2
Archaea2 - ps3
Archaea3 - ps4
Asgard/Eukaryota - ps5
Eukaryota - ps6
Amorpheae - ps7
Obazoa - ps8
Opisthokonta - ps9
Holozoa - ps10
Filozoa - ps11
Choano./Metazoa - ps12
Metazoa - ps13
Myriazoa - ps14
Parahoxozoa - ps15
Bilateria - ps16
Protostomia - ps17
Ecdysozoa - ps18
Arthropoda - ps19
Mandibulata - ps20
Pancrustacea - ps21
Insecta - ps22
Eumetabola - ps23
Holometabola/Psocodea - ps24
Holometabola - ps25
Aparaglossata - ps26
Mecopterida - ps27
Diptera - ps28
Bibiono./Cyclorrhapha - ps29
Cyclorrhapha - ps30
Calyptratae/Drosophila - ps31
Drosophila - ps32
Sophophora - ps33
Obscura/Melanogaster_group - ps34
D_melanogaster_group - ps35
D_melanogaster_subgroup - ps36
sim./sech./mel. group - ps37
Drosophila_melanogaster - ps38
p-value
3e-08
1e-06
4e-05
1e-03
5e-02
Log-odds
0.5
1.0
1.5
2.0
2.5
3.0
3.5
4.0

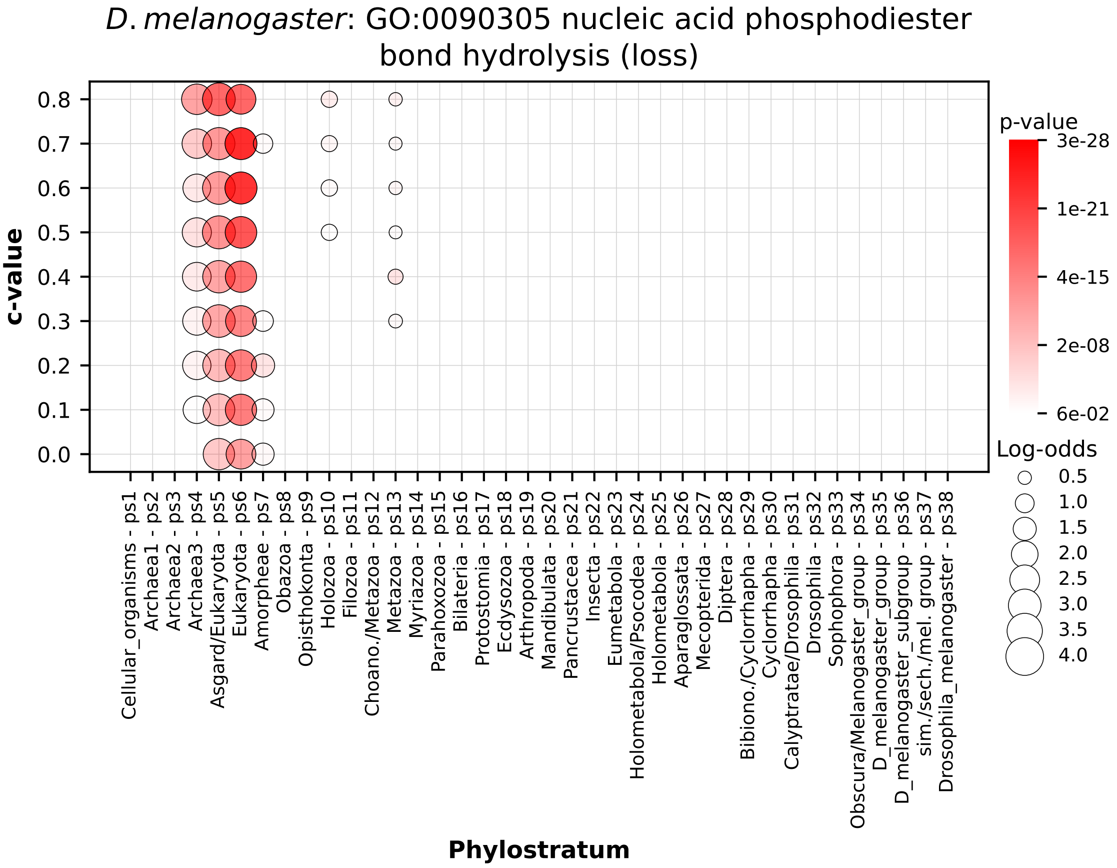
D. melanogaster: GO:0090305 nucleic acid phosphodiester bond hydrolysis (loss)
c-value
0.8
0.7
0.6
0.5
0.4
0.3
0.2
0.1
0.0
Phylostratum
Cellular_organisms - ps1
Archaea1 - ps2
Archaea2 - ps3
Archaea3 - ps4
Asgard/Eukaryota - ps5
Eukaryota - ps6
Amorpheae - ps7
Obazoa - ps8
Opisthokonta - ps9
Holozoa - ps10
Filozoa - ps11
Choano./Metazoa - ps12
Metazoa - ps13
Myriazoa - ps14
Parahoxozoa - ps15
Bilateria - ps16
Protostomia - ps17
Ecdysozoa - ps18
Arthropoda - ps19
Mandibulata - ps20
Pancrustacea - ps21
Insecta - ps22
Eumetabola - ps23
Holometabola/Psocodea - ps24
Holometabola - ps25
Aparaglossata - ps26
Mecopterida - ps27
Diptera - ps28
Bibiono./Cyclorrhapha - ps29
Cyclorrhapha - ps30
Calyptratae/Drosophila - ps31
Drosophila - ps32
Sophophora - ps33
Obscura/Melanogaster_group - ps34
D_melanogaster_group - ps35
D_melanogaster_subgroup - ps36
sim./sech./mel. group - ps37
Drosophila_melanogaster - ps38
p-value
3e-28
1e-21
4e-15
2e-08
6e-02
Log-odds
0.5
1.0
1.5
2.0
2.5
3.0
3.5
4.0

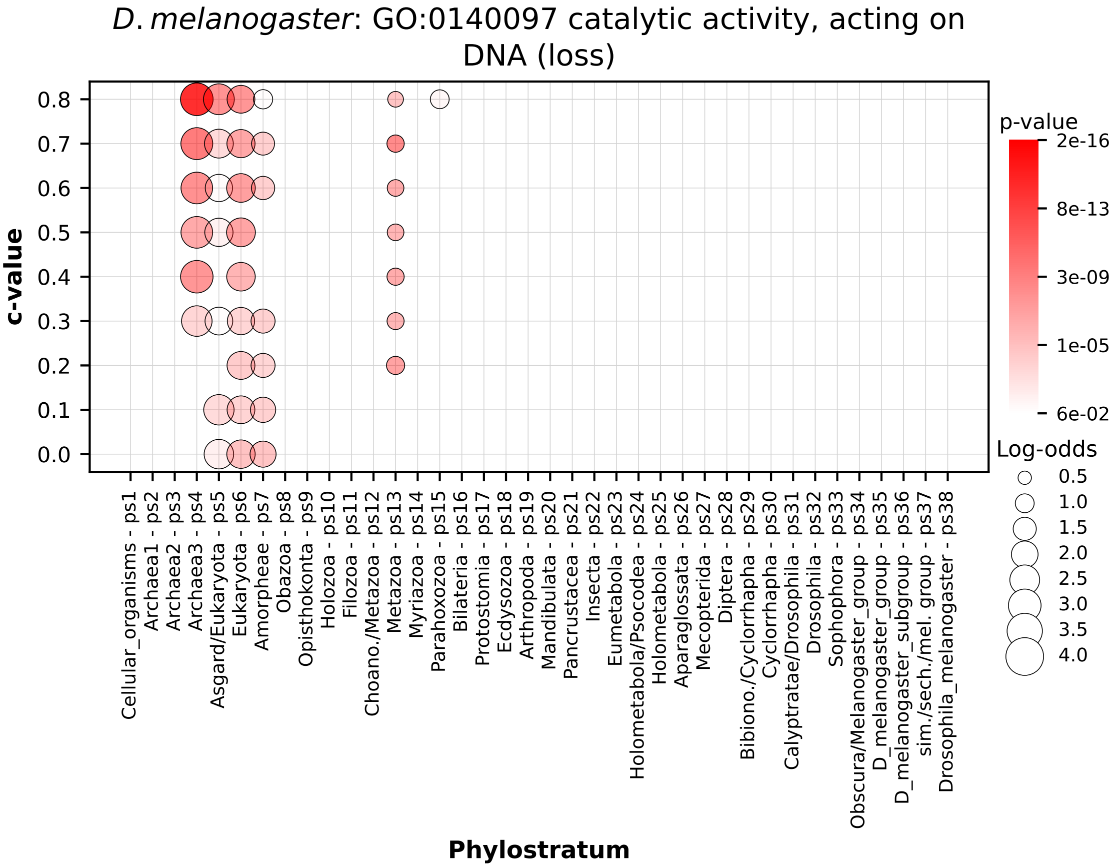

D. melanogaster: GO:0140097 catalytic activity, acting on DNA (loss)
c-value
0.8
0.7
0.6
0.5
0.4
0.3
0.2
0.1
0.0
p-value
2e-16
8e-13
3e-09
1e-05
6e-02
Log-odds
0.5
1.0
1.5
2.0
2.5
3.0
3.5
4.0
Cellular_organisms - ps1
Archaea1 - ps2
Archaea2 - ps3
Archaea3 - ps4
Asgard/Eukaryota - ps5
Eukaryota - ps6
Amorpheae - ps7
Obazoa - ps8
Opisthokonta - ps9
Holozoa - ps10
Filozoa - ps11
Choano./Metazoa - ps12
Metazoa - ps13
Myriazoa - ps14
Parahoxozoa - ps15
Bilateria - ps16
Protostomia - ps17
Ecdysozoa - ps18
Arthropoda - ps19
Mandibulata - ps20
Pancrustacea - ps21
Insecta - ps22
Eumetabola - ps23
Holometabola/Psocodea - ps24
Holometabola - ps25
Aparaglossata - ps26
Mecopterida - ps27
Diptera - ps28
Bibiono./Cyclorrhapha - ps29
Cyclorrhapha - ps30
Calyptratae/Drosophila - ps31
Drosophila - ps32
Sophophora - ps33
Obscura/Melanogaster_group - ps34
D_melanogaster_group - ps35
D_melanogaster_subgroup - ps36
sim./sech./mel. group - ps37
Drosophila_melanogaster - ps38
Phylostratum

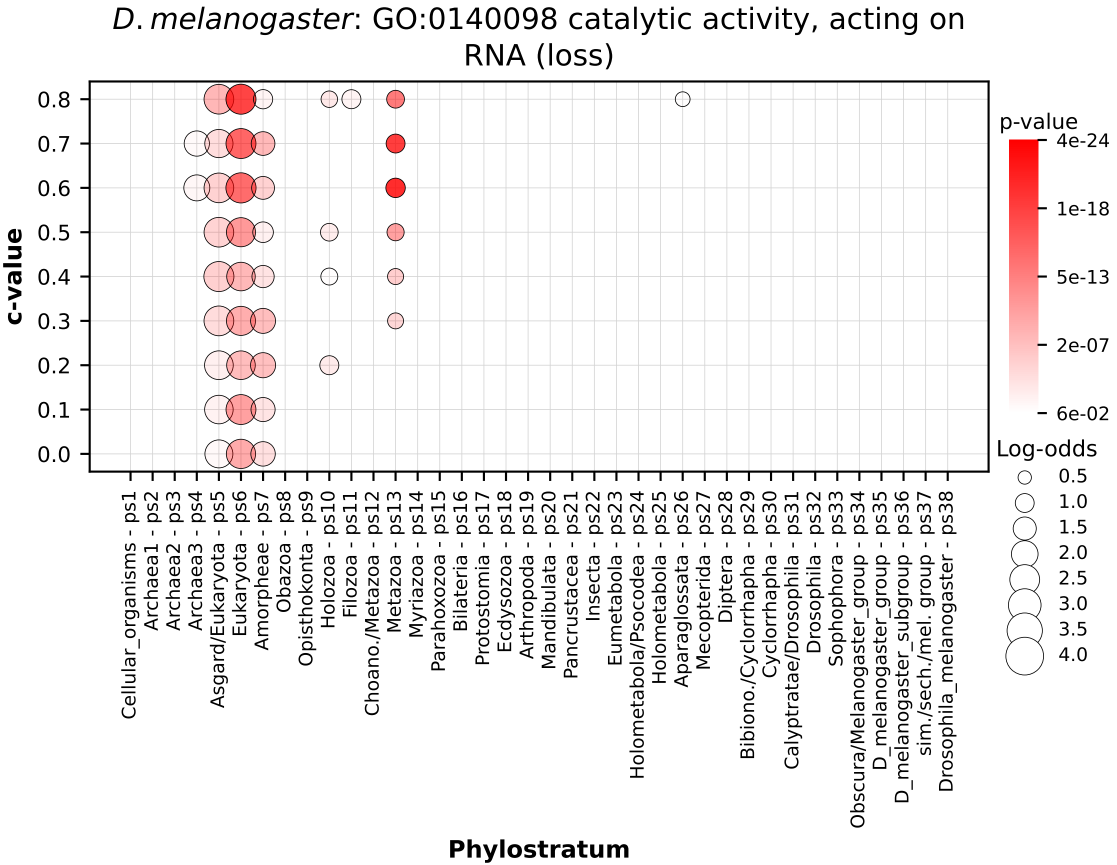

D. melanogaster: GO:0140098 catalytic activity, acting on RNA (loss)
c-value
0.8
0.7
0.6
0.5
0.4
0.3
0.2
0.1
0.0
Phylostratum
Cellular_organisms - ps1
Archaea1 - ps2
Archaea2 - ps3
Archaea3 - ps4
Asgard/Eukaryota - ps5
Eukaryota - ps6
Amorpheae - ps7
Obazoa - ps8
Opisthokonta - ps9
Holozoa - ps10
Filozoa - ps11
Choano./Metazoa - ps12
Metazoa - ps13
Myriazoa - ps14
Parahoxozoa - ps15
Bilateria - ps16
Protostomia - ps17
Ecdysozoa - ps18
Arthropoda - ps19
Mandibulata - ps20
Pancrustacea - ps21
Insecta - ps22
Eumetabola - ps23
Holometabola/Psocodea - ps24
Holometabola - ps25
Aparaglossata - ps26
Mecopterida - ps27
Diptera - ps28
Bibiono./Cyclorrhapha - ps29
Cyclorrhapha - ps30
Calyptratae/Drosophila - ps31
Drosophila - ps32
Sophophora - ps33
Obscura/Melanogaster_group - ps34
D_melanogaster_group - ps35
D_melanogaster_subgroup - ps36
sim./sech./mel. group - ps37
Drosophila_melanogaster - ps38
p-value
4e-24
1e-18
5e-13
2e-07
6e-02
Log-odds
0.5
1.0
1.5
2.0
2.5
3.0
3.5
4.0

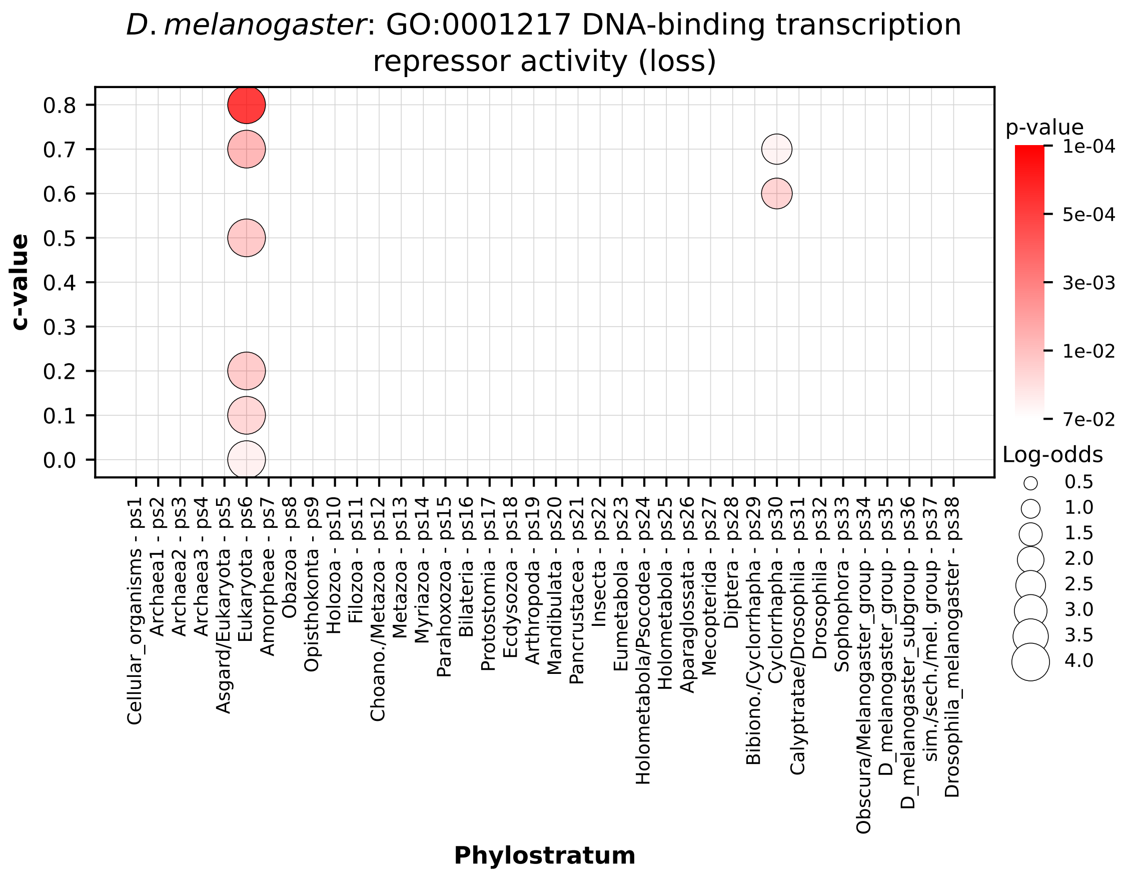

D. melanogaster: GO:0001217 DNA-binding transcription repressor activity (loss)
c-value
0.0
0.1
0.2
0.3
0.4
0.5
0.6
0.7
0.8
Phylostratum
Cellular_organisms - ps1
Archaea1 - ps2
Archaea2 - ps3
Archaea3 - ps4
Asgard/Eukaryota - ps5
Eukaryota - ps6
Amorpheae - ps7
Obazoa - ps8
Opisthokonta - ps9
Holozoa - ps10
Filozoa - ps11
Choano./Metazoa - ps12
Metazoa - ps13
Myriazoa - ps14
Parahoxozoa - ps15
Bilateria - ps16
Protostomia - ps17
Ecdysozoa - ps18
Arthropoda - ps19
Mandibulata - ps20
Pancrustacea - ps21
Insecta - ps22
Eumetabola - ps23
Holometabola/Psocodea - ps24
Holometabola - ps25
Aparaglossata - ps26
Mecopterida - ps27
Diptera - ps28
Bibiono./Cyclorrhapha - ps29
Cyclorrhapha - ps30
Calyptratae/Drosophila - ps31
Drosophila - ps32
Sophophora - ps33
Obscura/Melanogaster_group - ps34
D_melanogaster_group - ps35
D_melanogaster_subgroup - ps36
sim./sech./mel. group - ps37
Drosophila_melanogaster - ps38
p-value
1e-04
5e-04
3e-03
1e-02
7e-02
Log-odds
0.5
1.0
1.5
2.0
2.5
3.0
3.5
4.0

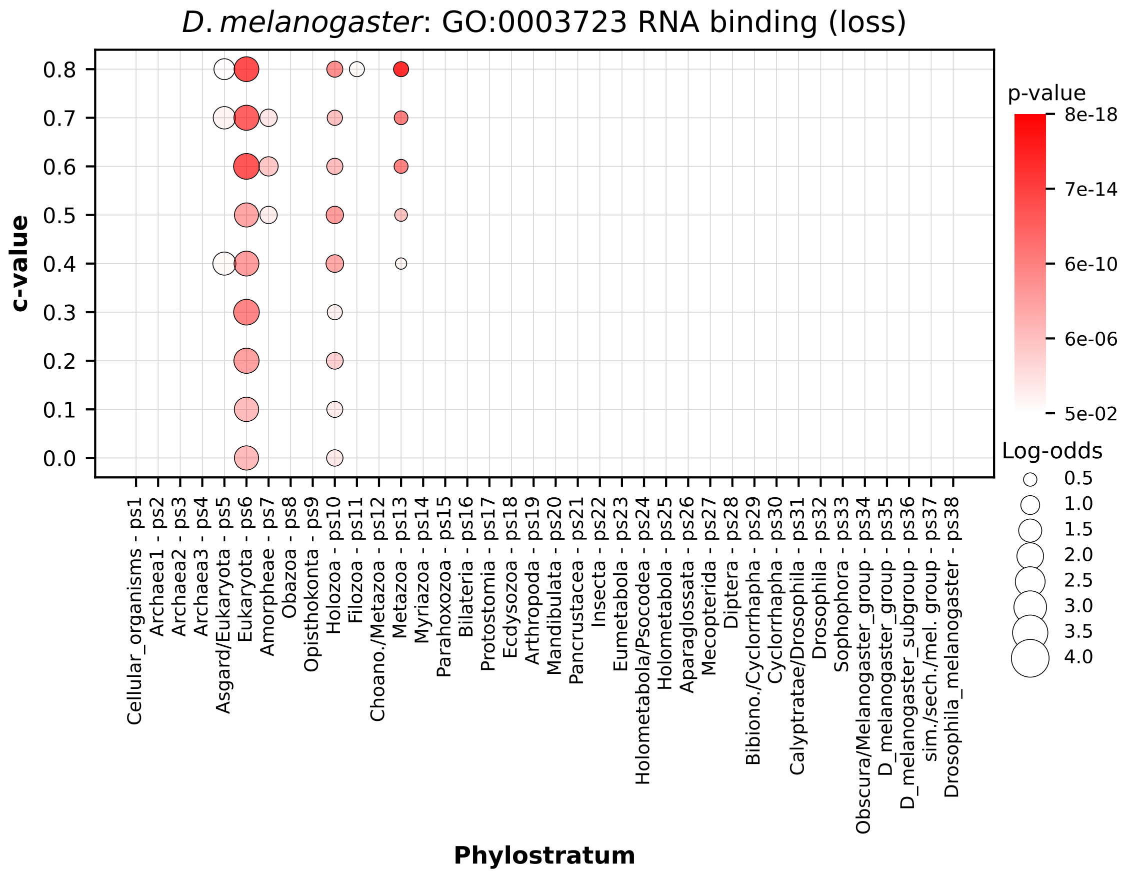

D. melanogaster: GO:0003723 RNA binding (loss)
c-value
0.8
0.7
0.6
0.5
0.4
0.3
0.2
0.1
0.0
Phylostratum
Cellular_organisms - ps1
Archaea1 - ps2
Archaea2 - ps3
Archaea3 - ps4
Asgard/Eukaryota - ps5
Eukaryota - ps6
Amorpheae - ps7
Obazoa - ps8
Opisthokonta - ps9
Holozoa - ps10
Filozoa - ps11
Choano./Metazoa - ps12
Metazoa - ps13
Myriazoa - ps14
Parahoxozoa - ps15
Bilateria - ps16
Protostomia - ps17
Ecdysozoa - ps18
Arthropoda - ps19
Mandibulata - ps20
Pancrustacea - ps21
Insecta - ps22
Eumetabola - ps23
Holometabola/Psocodea - ps24
Holometabola - ps25
Aparaglossata - ps26
Mecopterida - ps27
Diptera - ps28
Bibiono./Cyclorrhapha - ps29
Cyclorrhapha - ps30
Calyptratae/Drosophila - ps31
Drosophila - ps32
Sophophora - ps33
Obscura/Melanogaster_group - ps34
D_melanogaster_group - ps35
D_melanogaster_subgroup - ps36
sim./sech./mel. group - ps37
Drosophila_melanogaster - ps38
p-value
8e-18
7e-14
6e-10
6e-06
5e-02
Log-odds
0.5
1.0
1.5
2.0
2.5
3.0
3.5
4.0

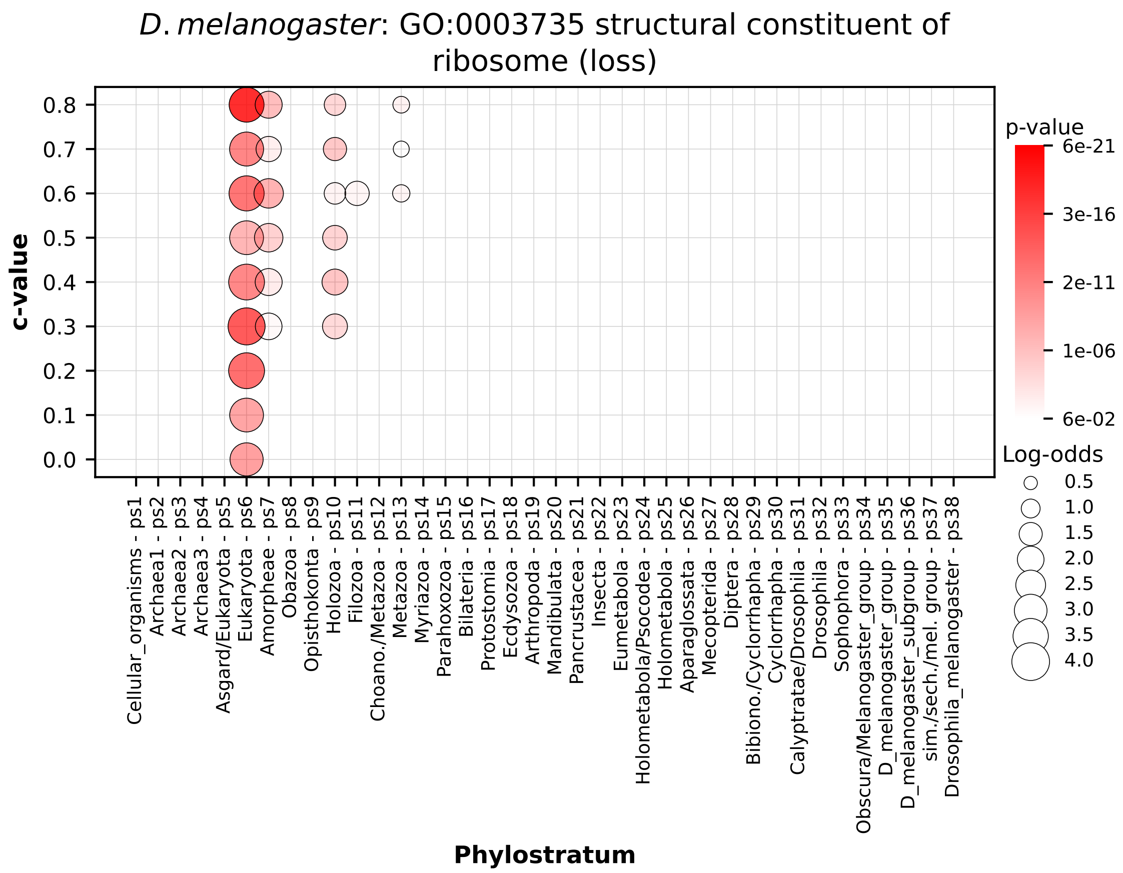

D. melanogaster: GO:0003735 structural constituent of ribosome (loss)
c-value
0.8
0.7
0.6
0.5
0.4
0.3
0.2
0.1
0.0
Phylostratum
Cellular_organisms - ps1
Archaea1 - ps2
Archaea2 - ps3
Archaea3 - ps4
Asgard/Eukaryota - ps5
Eukaryota - ps6
Amorpheae - ps7
Obazoa - ps8
Opisthokonta - ps9
Holozoa - ps10
Filozoa - ps11
Choano./Metazoa - ps12
Metazoa - ps13
Myriazoa - ps14
Parahoxozoa - ps15
Bilateria - ps16
Protostomia - ps17
Ecdysozoa - ps18
Arthropoda - ps19
Mandibulata - ps20
Pancrustacea - ps21
Insecta - ps22
Eumetabola - ps23
Holometabola/Psocodea - ps24
Holometabola - ps25
Aparaglossata - ps26
Mecopterida - ps27
Diptera - ps28
Bibiono./Cyclorrhapha - ps29
Cyclorrhapha - ps30
Calyptratae/Drosophila - ps31
Drosophila - ps32
Sophophora - ps33
Obscura/Melanogaster_group - ps34
D_melanogaster_group - ps35
D_melanogaster_subgroup - ps36
sim./sech./mel. group - ps37
Drosophila_melanogaster - ps38
p-value
6e-21
3e-16
2e-11
1e-06
6e-02
Log-odds
0.5
1.0
1.5
2.0
2.5
3.0
3.5
4.0

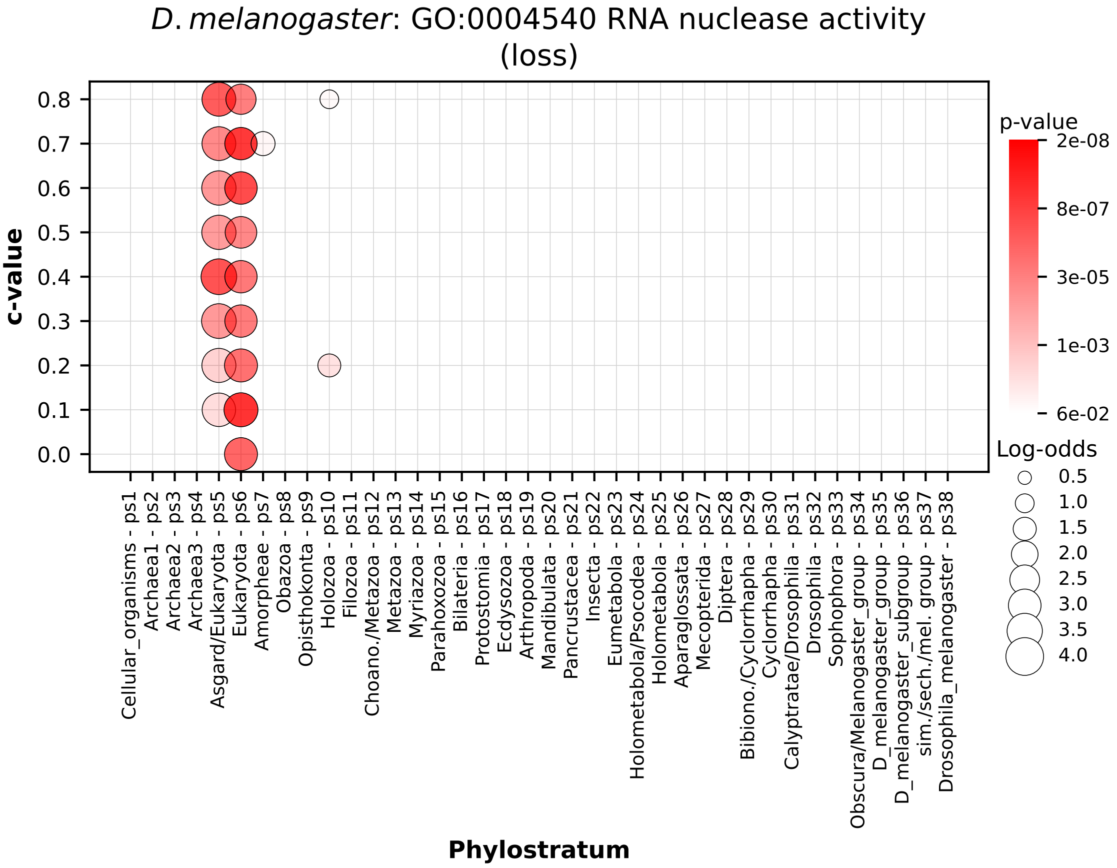
D. melanogaster: GO:0004540 RNA nuclease activity
(loss)
c-value
0.8
0.7
0.6
0.5
0.4
0.3
0.2
0.1
0.0
Phylostratum
Cellular_organisms - ps1
Archaea1 - ps2
Archaea2 - ps3
Archaea3 - ps4
Asgard/Eukaryota - ps5
Eukaryota - ps6
Amorpheae - ps7
Obazoa - ps8
Opisthokonta - ps9
Holozoa - ps10
Filozoa - ps11
Choano./Metazoa - ps12
Metazoa - ps13
Myriazoa - ps14
Parahoxozoa - ps15
Bilateria - ps16
Protostomia - ps17
Ecdysozoa - ps18
Arthropoda - ps19
Mandibulata - ps20
Pancrustacea - ps21
Insecta - ps22
Eumetabola - ps23
Holometabola/Psocodea - ps24
Holometabola - ps25
Aparaglossata - ps26
Mecopterida - ps27
Diptera - ps28
Bibiono./Cyclorrhapha - ps29
Cyclorrhapha - ps30
Calyptratae/Drosophila - ps31
Drosophila - ps32
Sophophora - ps33
Obscura/Melanogaster_group - ps34
D_melanogaster_group - ps35
D_melanogaster_subgroup - ps36
sim./sech./mel. group - ps37
Drosophila_melanogaster - ps38
p-value
2e-08
8e-07
3e-05
1e-03
6e-02
Log-odds
0.5
1.0
1.5
2.0
2.5
3.0
3.5
4.0

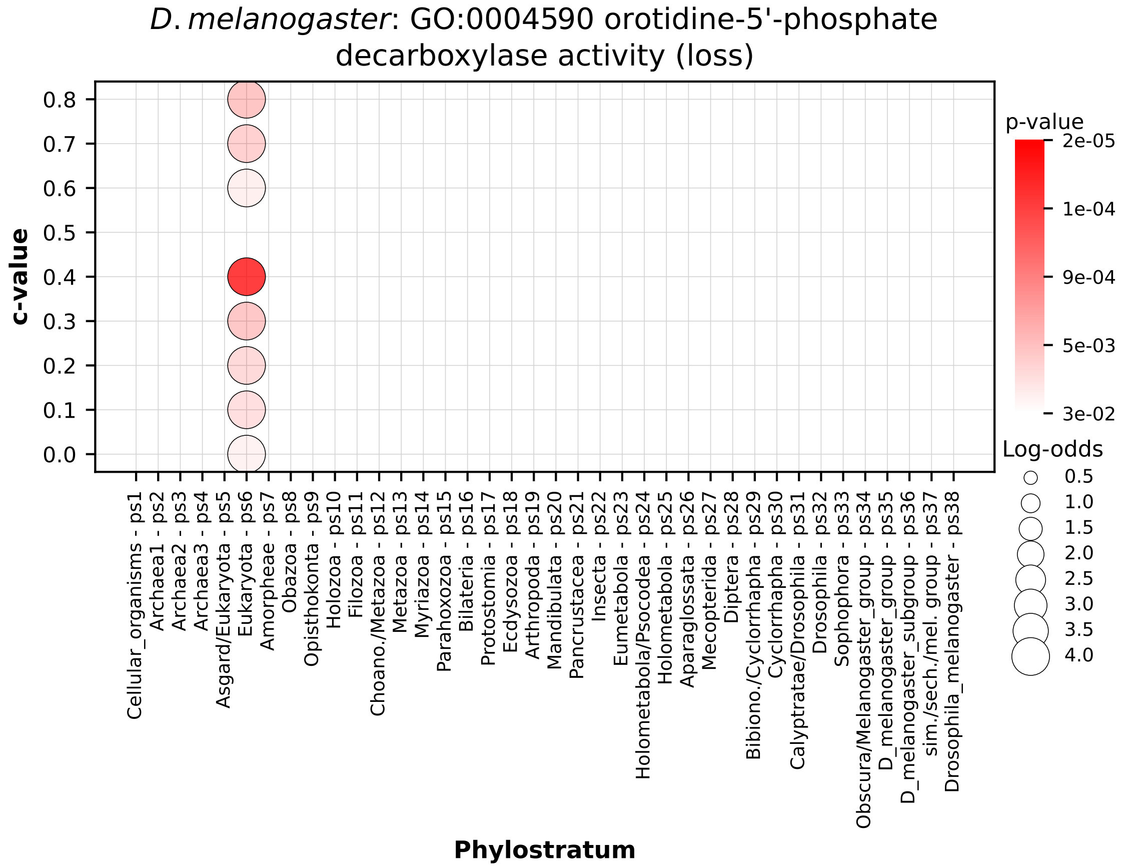

D. melanogaster: GO:0004590 orotidine-5'-phosphate decarboxylase activity (loss)
c-value
0.0
0.1
0.2
0.3
0.4
0.5
0.6
0.7
0.8
Phylostratum
Cellular_organisms - ps1
Archaea1 - ps2
Archaea2 - ps3
Archaea3 - ps4
Asgard/Eukaryota - ps5
Eukaryota - ps6
Amorpheae - ps7
Obazoa - ps8
Opisthokonta - ps9
Holozoa - ps10
Filozoa - ps11
Choano./Metazoa - ps12
Metazoa - ps13
Myriazoa - ps14
Parahoxozoa - ps15
Bilateria - ps16
Protostomia - ps17
Ecdysozoa - ps18
Arthropoda - ps19
Mandibulata - ps20
Pancrustacea - ps21
Insecta - ps22
Eumetabola - ps23
Holometabola/Psocodea - ps24
Holometabola - ps25
Aparaglossata - ps26
Mecopterida - ps27
Diptera - ps28
Bibiono./Cyclorrhapha - ps29
Cyclorrhapha - ps30
Calyptratae/Drosophila - ps31
Drosophila - ps32
Sophophora - ps33
Obscura/Melanogaster_group - ps34
D_melanogaster_group - ps35
D_melanogaster_subgroup - ps36
sim./sech./mel. group - ps37
Drosophila_melanogaster - ps38
p-value
2e-05
1e-04
9e-04
5e-03
3e-02
Log-odds
0.5
1.0
1.5
2.0
2.5
3.0
3.5
4.0

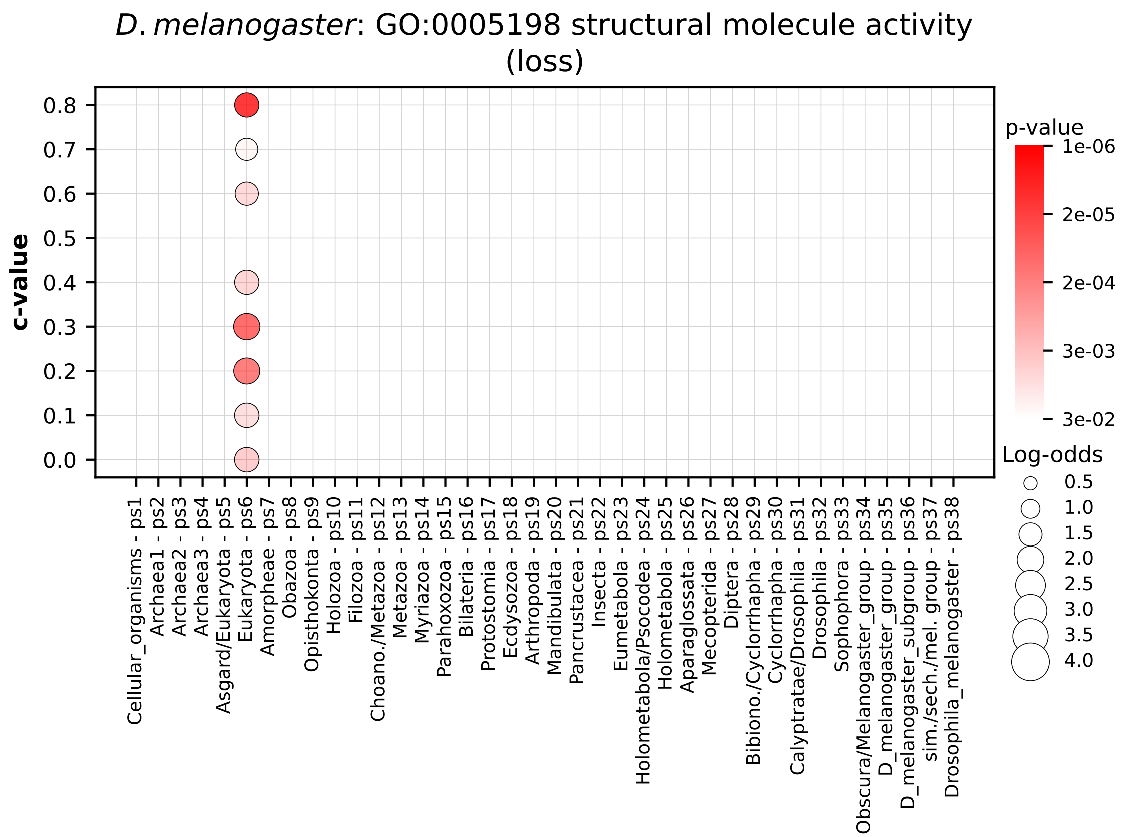
D. melanogaster: GO:0005198 structural molecule activity (loss)
c-value
Phylostratum
p-value
1e-06
2e-05
2e-04
3e-03
3e-02
Log-odds
0.5
1.0
1.5
2.0
2.5
3.0
3.5
4.0
Cellular_organisms - ps1
Archaea1 - ps2
Archaea2 - ps3
Archaea3 - ps4
Asgard/Eukaryota - ps5
Eukaryota - ps6
Amorpheae - ps7
Obazoa - ps8
Opisthokonta - ps9
Holozoa - ps10
Filozoa - ps11
Choano./Metazoa - ps12
Metazoa - ps13
Myriazoa - ps14
Parahoxozoa - ps15
Bilateria - ps16
Protostomia - ps17
Ecdysozoa - ps18
Arthropoda - ps19
Mandibulata - ps20
Pancrustacea - ps21
Insecta - ps22
Eumetabola - ps23
Holometabola/Psocodea - ps24
Holometabola - ps25
Aparaglossata - ps26
Mecopterida - ps27
Diptera - ps28
Bibiono./Cyclorrhapha - ps29
Cyclorrhapha - ps30
Calyptratae/Drosophila - ps31
Drosophila - ps32
Sophophora - ps33
Obscura/Melanogaster_group - ps34
D_melanogaster_group - ps35
D_melanogaster_subgroup - ps36
sim./sech./mel. group - ps37
Drosophila_melanogaster - ps38

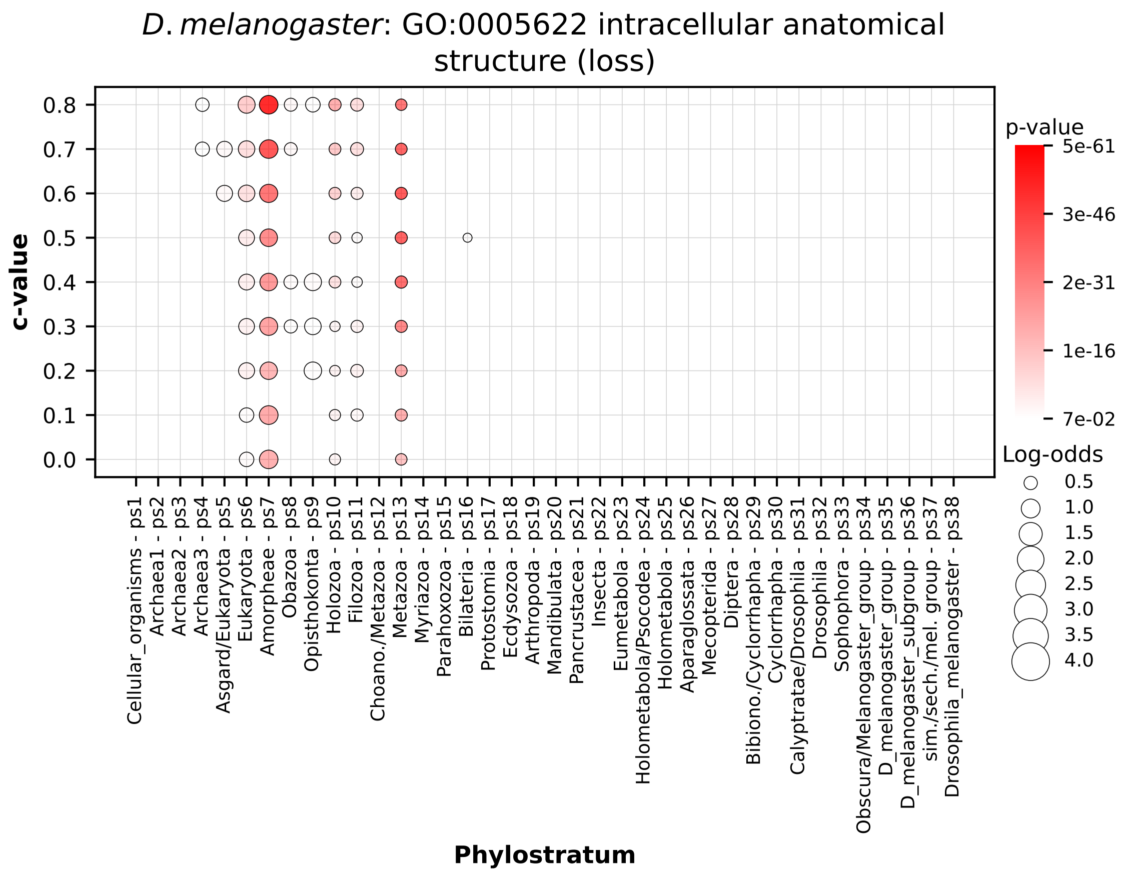

D. melanogaster: GO:0005622 intracellular anatomical structure (loss)
c-value
0.8
0.7
0.6
0.5
0.4
0.3
0.2
0.1
0.0
Phylostratum
Cellular_organisms - ps1
Archaea1 - ps2
Archaea2 - ps3
Archaea3 - ps4
Asgard/Eukaryota - ps5
Eukaryota - ps6
Amorpheae - ps7
Obazoa - ps8
Opisthokonta - ps9
Holozoa - ps10
Filozoa - ps11
Choano./Metazoa - ps12
Metazoa - ps13
Myriazoa - ps14
Parahoxozoa - ps15
Bilateria - ps16
Protostomia - ps17
Ecdysozoa - ps18
Arthropoda - ps19
Mandibulata - ps20
Pancrustacea - ps21
Insecta - ps22
Eumetabola - ps23
Holometabola/Psocodea - ps24
Holometabola - ps25
Aparaglossata - ps26
Mecopterida - ps27
Diptera - ps28
Bibiono./Cyclorrhapha - ps29
Cyclorrhapha - ps30
Calyptratae/Drosophila - ps31
Drosophila - ps32
Sophophora - ps33
Obscura/Melanogaster_group - ps34
D_melanogaster_group - ps35
D_melanogaster_subgroup - ps36
sim./sech./mel. group - ps37
Drosophila_melanogaster - ps38
p-value
5e-61
3e-46
2e-31
1e-16
7e-02
Log-odds
0.5
1.0
1.5
2.0
2.5
3.0
3.5
4.0

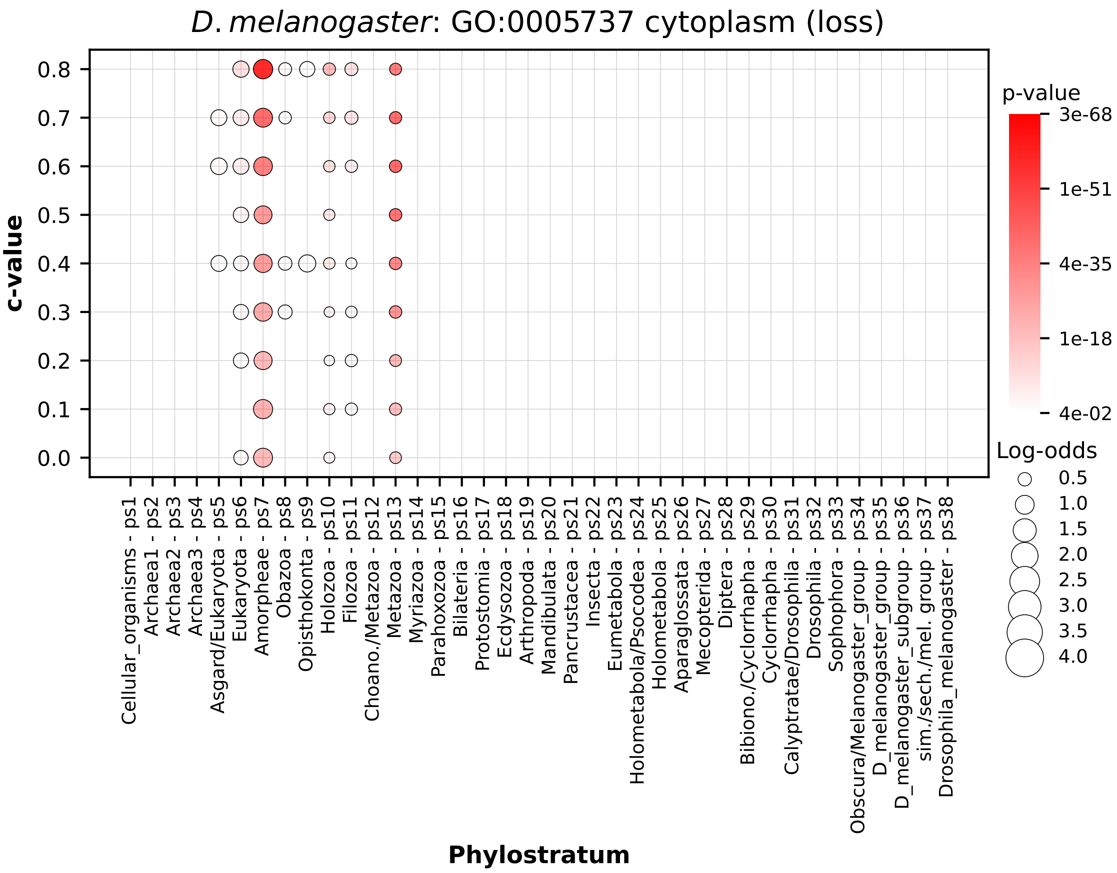
D. melanogaster: GO:0005737 cytoplasm (loss)
c-value
0.8
0.7
0.6
0.5
0.4
0.3
0.2
0.1
0.0
Phylostratum
Cellular_organisms - ps1
Archaea1 - ps2
Archaea2 - ps3
Archaea3 - ps4
Asgard/Eukaryota - ps5
Eukaryota - ps6
Amorpheae - ps7
Obazoa - ps8
Opisthokonta - ps9
Holozoa - ps10
Filozoa - ps11
Choano./Metazoa - ps12
Metazoa - ps13
Myriazoa - ps14
Parahoxozoa - ps15
Bilateria - ps16
Protostomia - ps17
Ecdysozoa - ps18
Arthropoda - ps19
Mandibulata - ps20
Pancrustacea - ps21
Insecta - ps22
Eumetabola - ps23
Holometabola/Psocodea - ps24
Holometabola - ps25
Aparaglossata - ps26
Mecopterida - ps27
Diptera - ps28
Bibiono./Cyclorrhapha - ps29
Cyclorrhapha - ps30
Calyptratae/Drosophila - ps31
Drosophila - ps32
Sophophora - ps33
Obscura/Melanogaster_group - ps34
D_melanogaster_group - ps35
D_melanogaster_subgroup - ps36
sim./sech./mel. group - ps37
Drosophila_melanogaster - ps38
p-value
3e-68
1e-51
4e-35
1e-18
4e-02
Log-odds
0.5
1.0
1.5
2.0
2.5
3.0
3.5
4.0

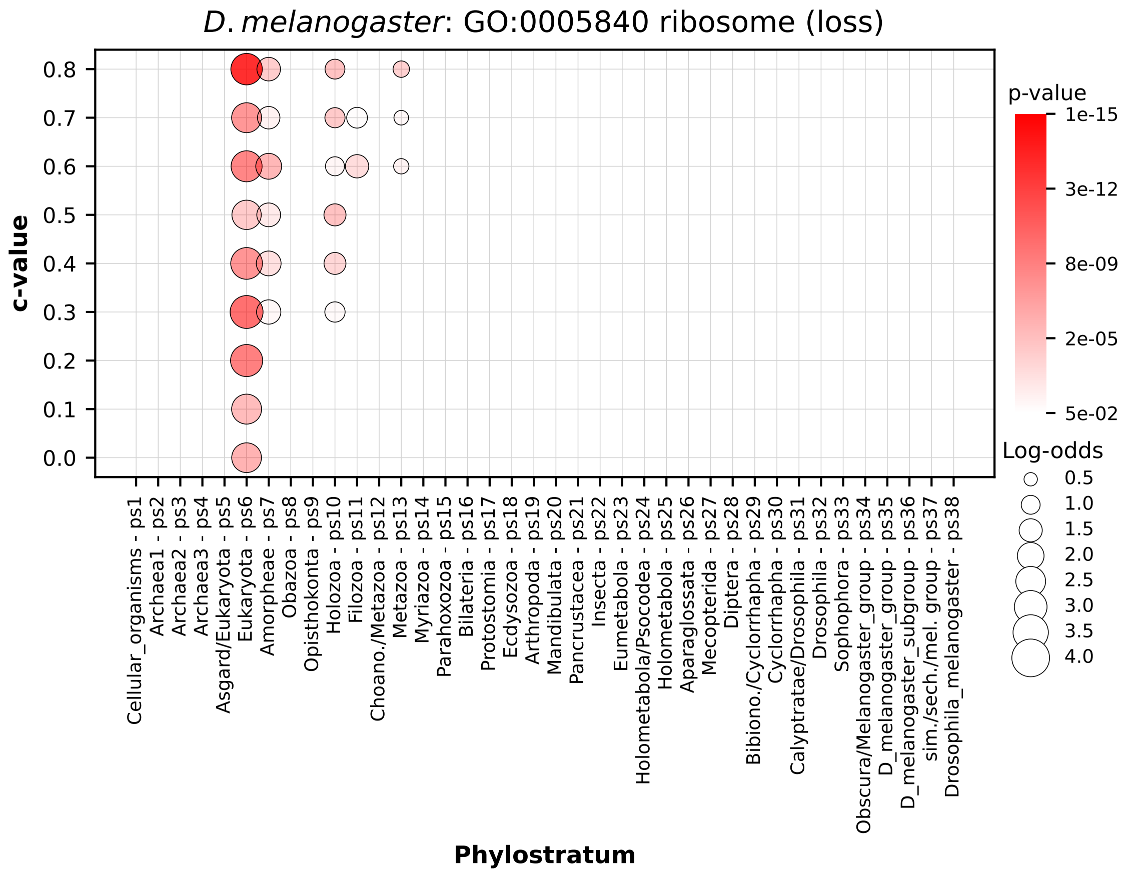

D. melanogaster: GO:0005840 ribosome (loss)
c-value
0.8
0.7
0.6
0.5
0.4
0.3
0.2
0.1
0.0
p-value
1e-15
3e-12
8e-09
2e-05
5e-02
Log-odds
0.5
1.0
1.5
2.0
2.5
3.0
3.5
4.0
Cellular_organisms - ps1
Archaea1 - ps2
Archaea2 - ps3
Archaea3 - ps4
Asgard/Eukaryota - ps5
Eukaryota - ps6
Amorpheae - ps7
Obazoa - ps8
Opisthokonta - ps9
Holozoa - ps10
Filozoa - ps11
Choano./Metazoa - ps12
Metazoa - ps13
Myriazoa - ps14
Parahoxozoa - ps15
Bilateria - ps16
Protostomia - ps17
Ecdysozoa - ps18
Arthropoda - ps19
Mandibulata - ps20
Pancrustacea - ps21
Insecta - ps22
Eumetabola - ps23
Holometabola/Psocodea - ps24
Holometabola - ps25
Aparaglossata - ps26
Mecopterida - ps27
Diptera - ps28
Bibiono./Cyclorrhapha - ps29
Cyclorrhapha - ps30
Calyptratae/Drosophila - ps31
Drosophila - ps32
Sophophora - ps33
Obscura/Melanogaster_group - ps34
D_melanogaster_group - ps35
D_melanogaster_subgroup - ps36
sim./sech./mel. group - ps37
Drosophila_melanogaster - ps38
Phylostratum

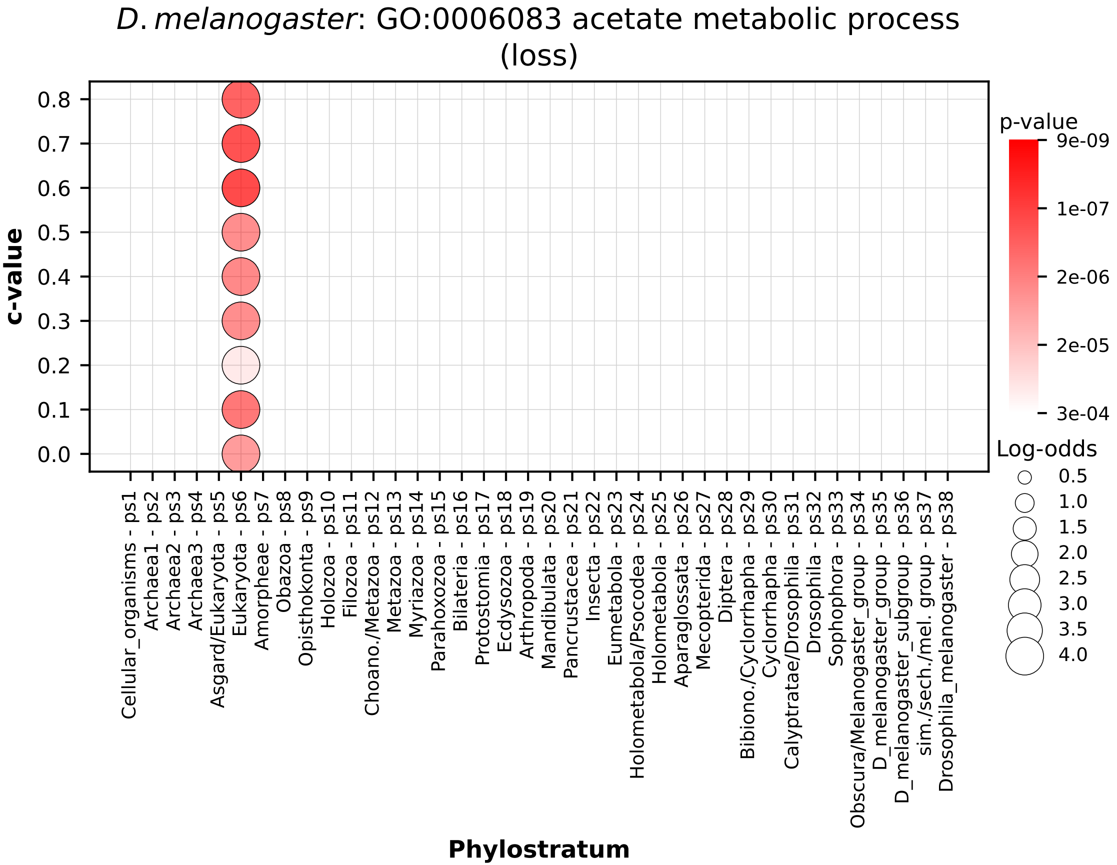

D. melanogaster: GO:0006083 acetate metabolic process (loss)
c-value
0.8
0.7
0.6
0.5
0.4
0.3
0.2
0.1
0.0
p-value
9e-09
1e-07
2e-06
2e-05
3e-04
Log-odds
0.5
1.0
1.5
2.0
2.5
3.0
3.5
4.0
Cellular_organisms - ps1
Archaea1 - ps2
Archaea2 - ps3
Archaea3 - ps4
Asgard/Eukaryota - ps5
Eukaryota - ps6
Amorpheae - ps7
Obazoa - ps8
Opisthokonta - ps9
Holozoa - ps10
Filozoa - ps11
Choano./Metazoa - ps12
Metazoa - ps13
Myriazoa - ps14
Parahoxozoa - ps15
Bilateria - ps16
Protostomia - ps17
Ecdysozoa - ps18
Arthropoda - ps19
Mandibulata - ps20
Pancrustacea - ps21
Insecta - ps22
Eumetabola - ps23
Holometabola/Psocodea - ps24
Holometabola - ps25
Aparaglossata - ps26
Mecopterida - ps27
Diptera - ps28
Bibiono./Cyclorrhapha - ps29
Cyclorrhapha - ps30
Calyptratae/Drosophila - ps31
Drosophila - ps32
Sophophora - ps33
Obscura/Melanogaster_group - ps34
D_melanogaster_group - ps35
D_melanogaster_subgroup - ps36
sim./sech./mel. group - ps37
Drosophila_melanogaster - ps38
Phylostratum

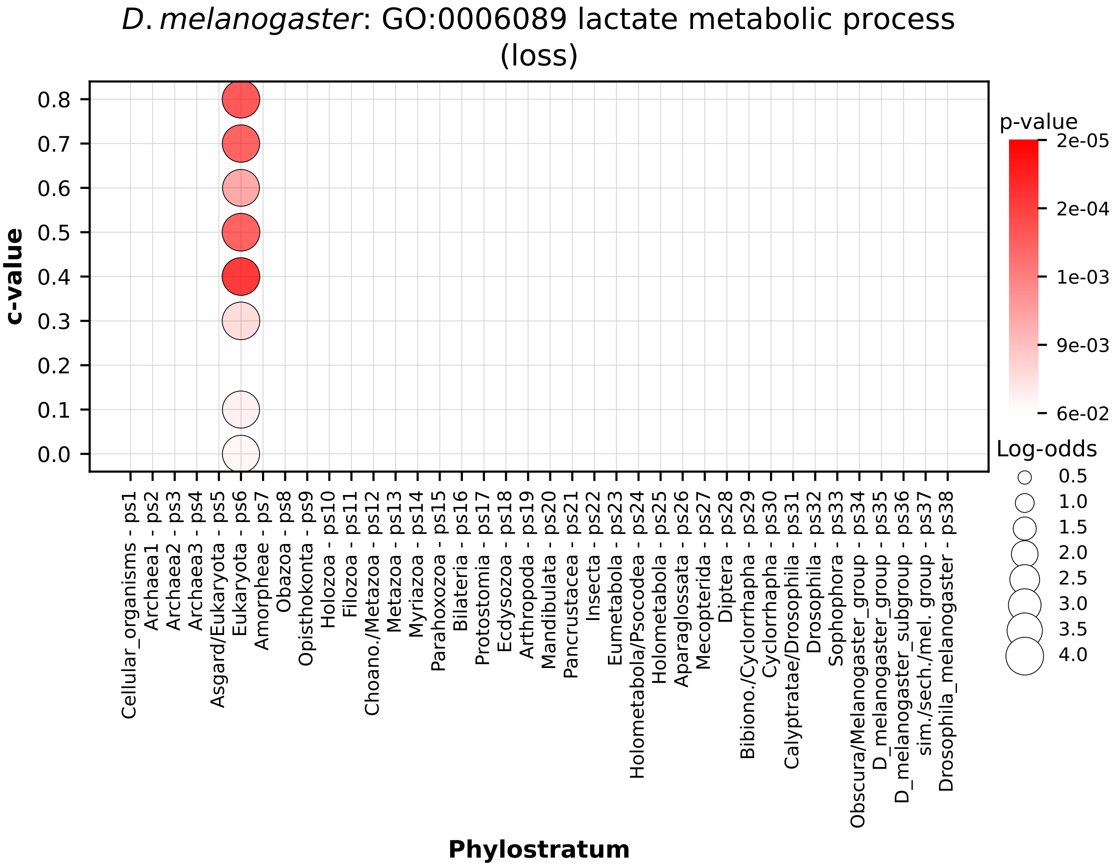

D. melanogaster: GO:0006089 lactate metabolic process (loss)
c-value
0.8
0.7
0.6
0.5
0.4
0.3
0.2
0.1
0.0
Phylostratum
Cellular_organisms - ps1
Archaea1 - ps2
Archaea2 - ps3
Archaea3 - ps4
Asgard/Eukaryota - ps5
Eukaryota - ps6
Amorpheae - ps7
Obazoa - ps8
Opisthokonta - ps9
Holozoa - ps10
Filozoa - ps11
Choano./Metazoa - ps12
Metazoa - ps13
Myriazoa - ps14
Parahoxozoa - ps15
Bilateria - ps16
Protostomia - ps17
Ecdysozoa - ps18
Arthropoda - ps19
Mandibulata - ps20
Pancrustacea - ps21
Insecta - ps22
Eumetabola - ps23
Holometabola/Psocodea - ps24
Holometabola - ps25
Aparaglossata - ps26
Mecopterida - ps27
Diptera - ps28
Bibiono./Cyclorrhapha - ps29
Cyclorrhapha - ps30
Calyptratae/Drosophila - ps31
Drosophila - ps32
Sophophora - ps33
Obscura/Melanogaster_group - ps34
D_melanogaster_group - ps35
D_melanogaster_subgroup - ps36
sim./sech./mel. group - ps37
Drosophila_melanogaster - ps38
p-value
2e-05
2e-04
1e-03
9e-03
6e-02
Log-odds
0.5
1.0
1.5
2.0
2.5
3.0
3.5
4.0

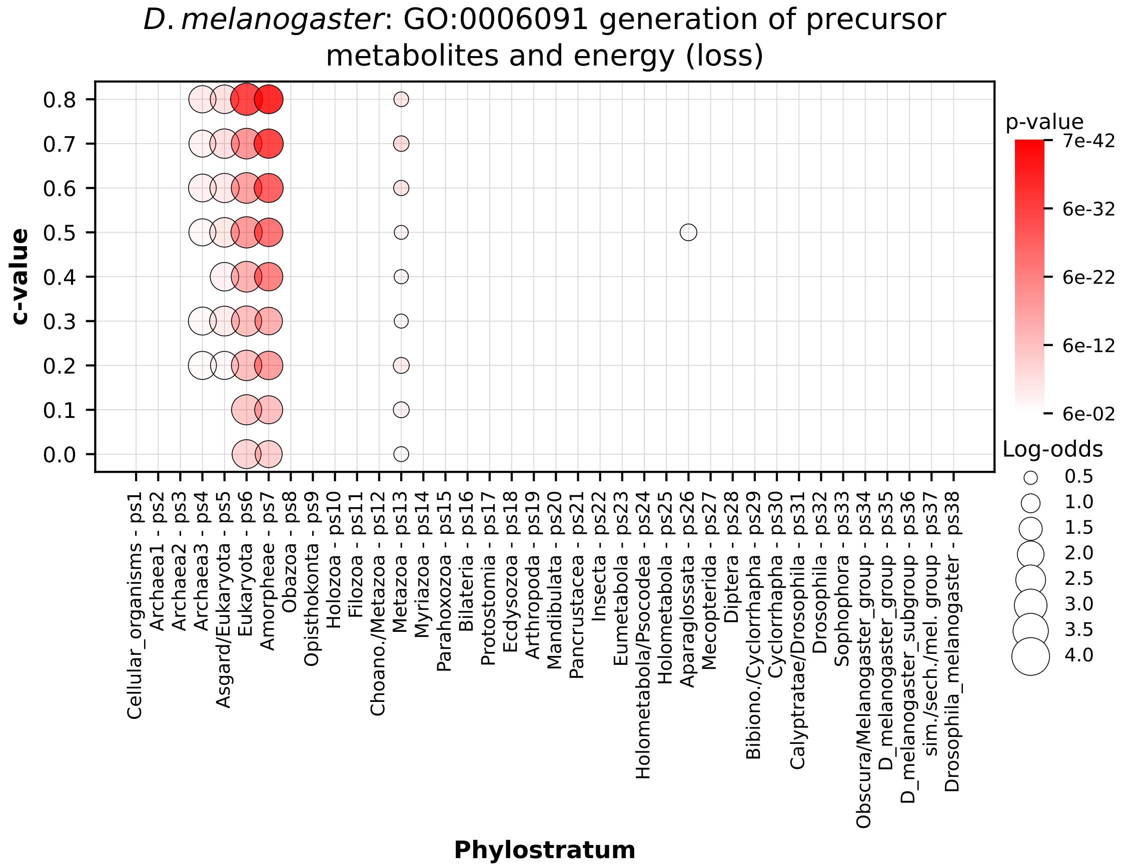
D. melanogaster: GO:0006091 generation of precursor metabolites and energy (loss)
c-value
0.8
0.7
0.6
0.5
0.4
0.3
0.2
0.1
0.0
Phylostratum
Cellular_organisms - ps1
Archaea1 - ps2
Archaea2 - ps3
Archaea3 - ps4
Asgard/Eukaryota - ps5
Eukaryota - ps6
Amorpheae - ps7
Obazoa - ps8
Opisthokonta - ps9
Holozoa - ps10
Filozoa - ps11
Choano./Metazoa - ps12
Metazoa - ps13
Myriazoa - ps14
Parahoxozoa - ps15
Bilateria - ps16
Protostomia - ps17
Ecdysozoa - ps18
Arthropoda - ps19
Mandibulata - ps20
Pancrustacea - ps21
Insecta - ps22
Eumetabola - ps23
Holometabola/Psocodea - ps24
Holometabola - ps25
Aparaglossata - ps26
Mecopterida - ps27
Diptera - ps28
Bibiono./Cyclorrhapha - ps29
Cyclorrhapha - ps30
Calyptratae/Drosophila - ps31
Drosophila - ps32
Sophophora - ps33
Obscura/Melanogaster_group - ps34
D_melanogaster_group - ps35
D_melanogaster_subgroup - ps36
sim./sech./mel. group - ps37
Drosophila_melanogaster - ps38
p-value
7e-42
6e-32
6e-22
6e-12
6e-02
Log-odds
0.5
1.0
1.5
2.0
2.5
3.0
3.5
4.0

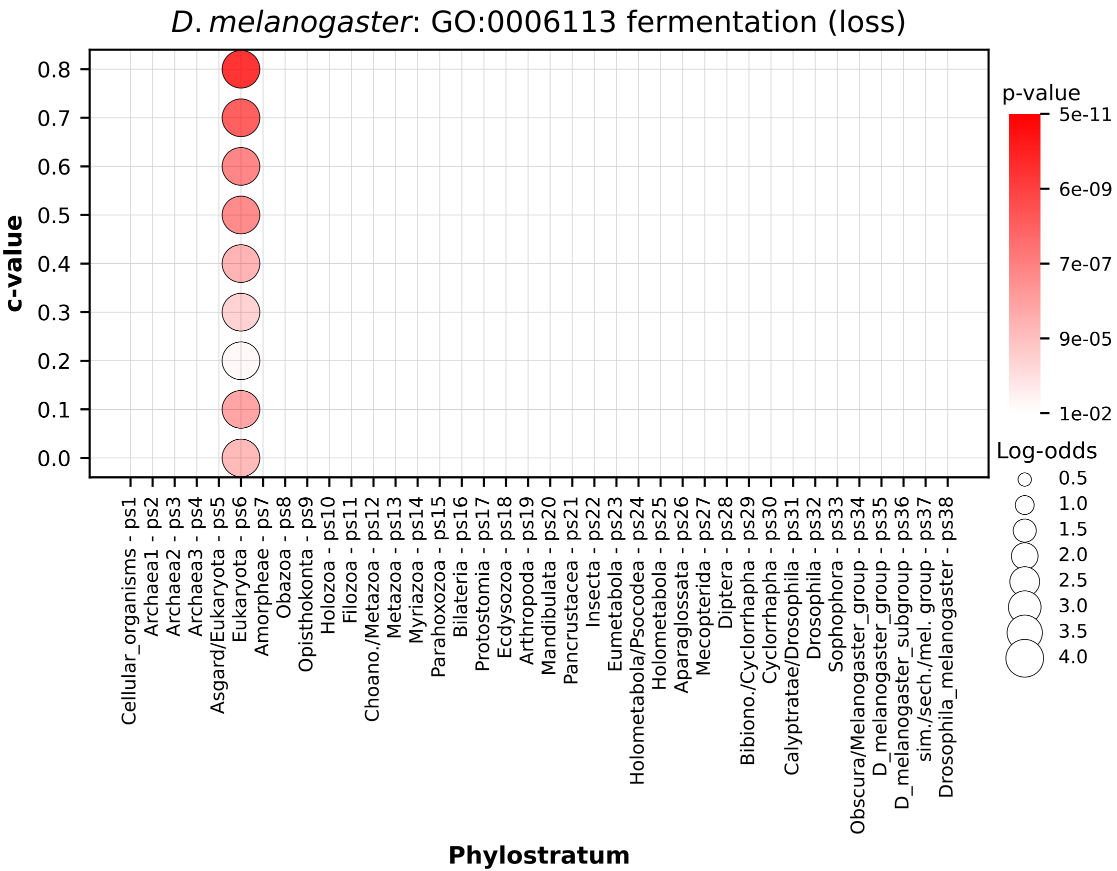

D. melanogaster: GO:0006113 fermentation (loss)
c-value
0.8
0.7
0.6
0.5
0.4
0.3
0.2
0.1
0.0
p-value
5e-11
6e-09
7e-07
9e-05
1e-02
Log-odds
0.5
1.0
1.5
2.0
2.5
3.0
3.5
4.0
Cellular_organisms - ps1
Archaea1 - ps2
Archaea2 - ps3
Archaea3 - ps4
Asgard/Eukaryota - ps5
Eukaryota - ps6
Amorpheae - ps7
Obazoa - ps8
Opisthokonta - ps9
Holozoa - ps10
Filozoa - ps11
Choano./Metazoa - ps12
Metazoa - ps13
Myriazoa - ps14
Parahoxozoa - ps15
Bilateria - ps16
Protostomia - ps17
Ecdysozoa - ps18
Arthropoda - ps19
Mandibulata - ps20
Pancrustacea - ps21
Insecta - ps22
Eumetabola - ps23
Holometabola/Psocodea - ps24
Holometabola - ps25
Aparaglossata - ps26
Mecopterida - ps27
Diptera - ps28
Bibiono./Cyclorrhapha - ps29
Cyclorrhapha - ps30
Calyptratae/Drosophila - ps31
Drosophila - ps32
Sophophora - ps33
Obscura/Melanogaster_group - ps34
D_melanogaster_group - ps35
D_melanogaster_subgroup - ps36
sim./sech./mel. group - ps37
Drosophila_melanogaster - ps38
Phylostratum

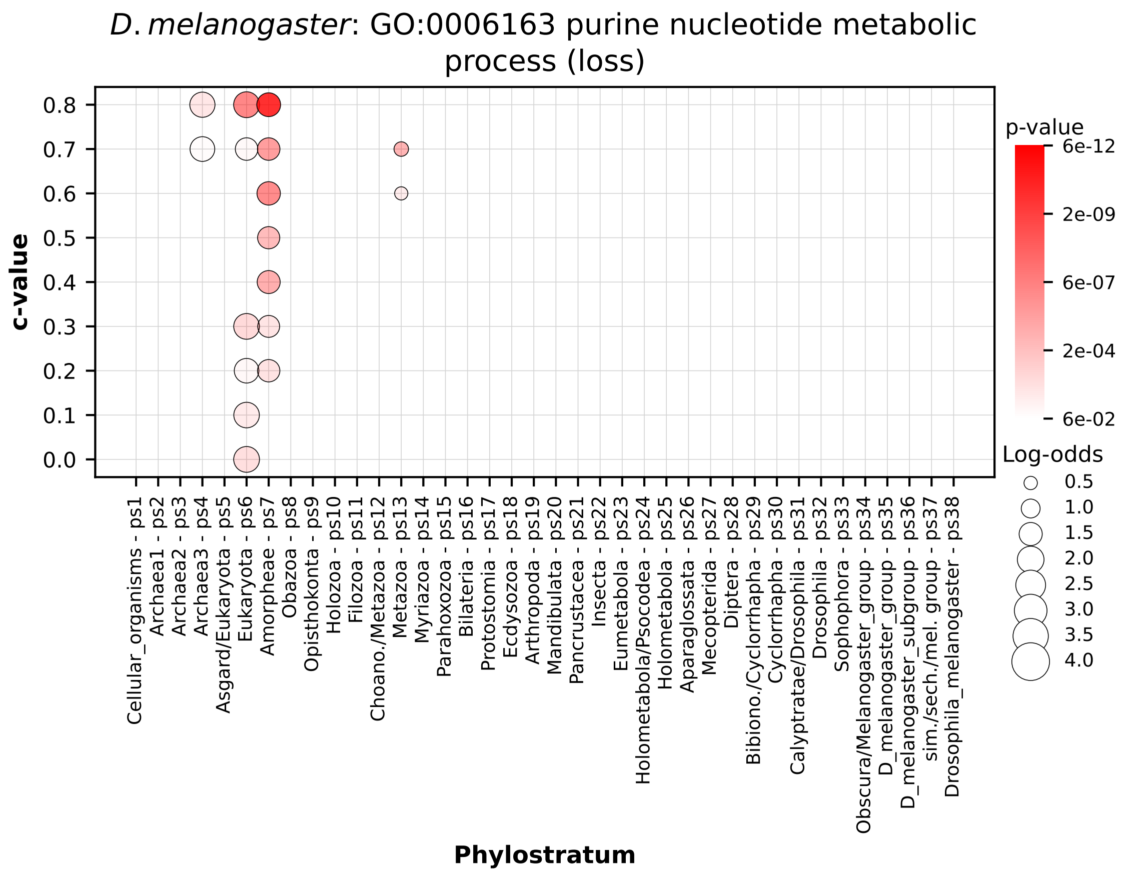

D. melanogaster: GO:0006163 purine nucleotide metabolic process (loss)
c-value
0.8
0.7
0.6
0.5
0.4
0.3
0.2
0.1
0.0
Phylostratum
Cellular_organisms - ps1
Archaea1 - ps2
Archaea2 - ps3
Archaea3 - ps4
Asgard/Eukaryota - ps5
Eukaryota - ps6
Amorpheae - ps7
Obazoa - ps8
Opisthokonta - ps9
Holozoa - ps10
Filozoa - ps11
Choano./Metazoa - ps12
Metazoa - ps13
Myriazoa - ps14
Parahoxozoa - ps15
Bilateria - ps16
Protostomia - ps17
Ecdysozoa - ps18
Arthropoda - ps19
Mandibulata - ps20
Pancrustacea - ps21
Insecta - ps22
Eumetabola - ps23
Holometabola/Psocodea - ps24
Holometabola - ps25
Aparaglossata - ps26
Mecopterida - ps27
Diptera - ps28
Bibiono./Cyclorrhapha - ps29
Cyclorrhapha - ps30
Calyptratae/Drosophila - ps31
Drosophila - ps32
Sophophora - ps33
Obscura/Melanogaster_group - ps34
D_melanogaster_group - ps35
D_melanogaster_subgroup - ps36
sim./sech./mel. group - ps37
Drosophila_melanogaster - ps38
p-value
6e-12
2e-09
6e-07
2e-04
6e-02
Log-odds
0.5
1.0
1.5
2.0
2.5
3.0
3.5
4.0

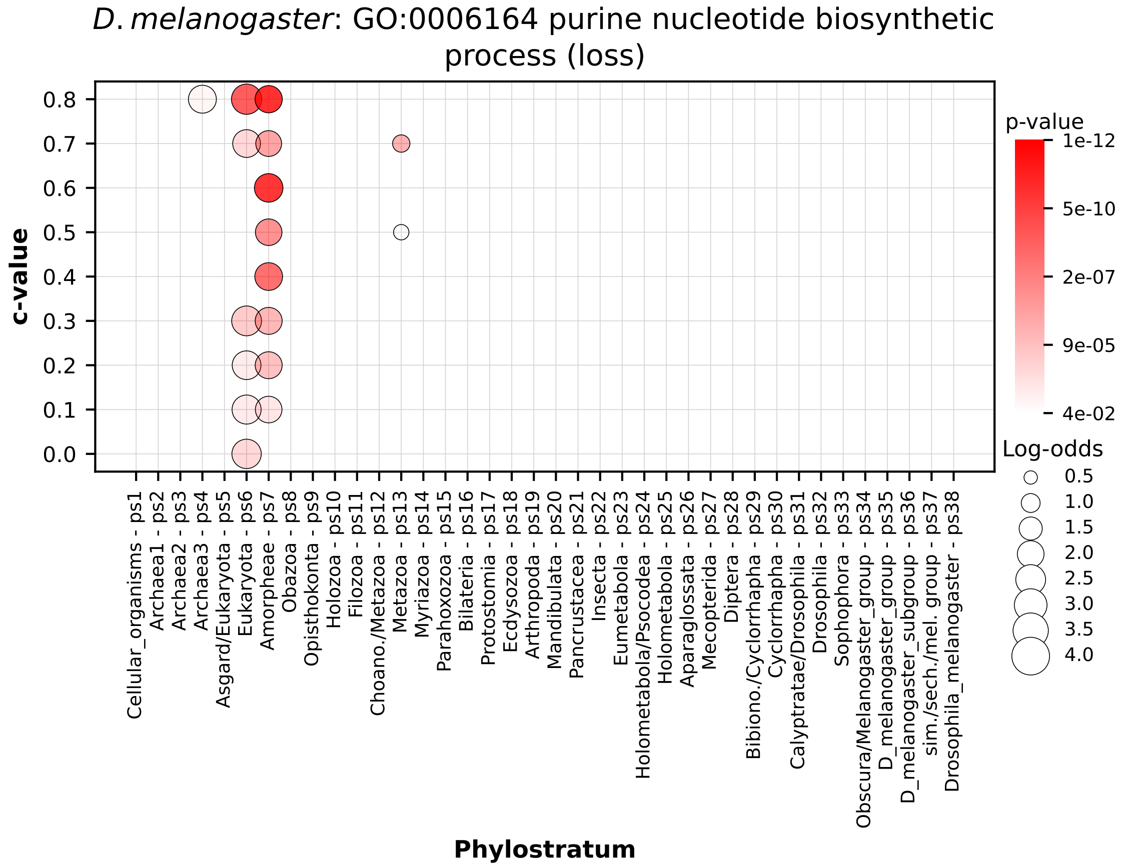
D. melanogaster: GO:0006164 purine nucleotide biosynthetic process (loss)
c-value
0.8
0.7
0.6
0.5
0.4
0.3
0.2
0.1
0.0
Phylostratum
Cellular_organisms - ps1
Archaea1 - ps2
Archaea2 - ps3
Archaea3 - ps4
Asgard/Eukaryota - ps5
Eukaryota - ps6
Amorpheae - ps7
Obazoa - ps8
Opisthokonta - ps9
Holozoa - ps10
Filozoa - ps11
Choano./Metazoa - ps12
Metazoa - ps13
Myriazoa - ps14
Parahoxozoa - ps15
Bilateria - ps16
Protostomia - ps17
Ecdysozoa - ps18
Arthropoda - ps19
Mandibulata - ps20
Pancrustacea - ps21
Insecta - ps22
Eumetabola - ps23
Holometabola/Psocodea - ps24
Holometabola - ps25
Aparaglossata - ps26
Mecopterida - ps27
Diptera - ps28
Bibiono./Cyclorrhapha - ps29
Cyclorrhapha - ps30
Calyptratae/Drosophila - ps31
Drosophila - ps32
Sophophora - ps33
Obscura/Melanogaster_group - ps34
D_melanogaster_group - ps35
D_melanogaster_subgroup - ps36
sim./sech./mel. group - ps37
Drosophila_melanogaster - ps38
p-value
1e-12
5e-10
2e-07
9e-05
4e-02
Log-odds
0.5
1.0
1.5
2.0
2.5
3.0
3.5
4.0

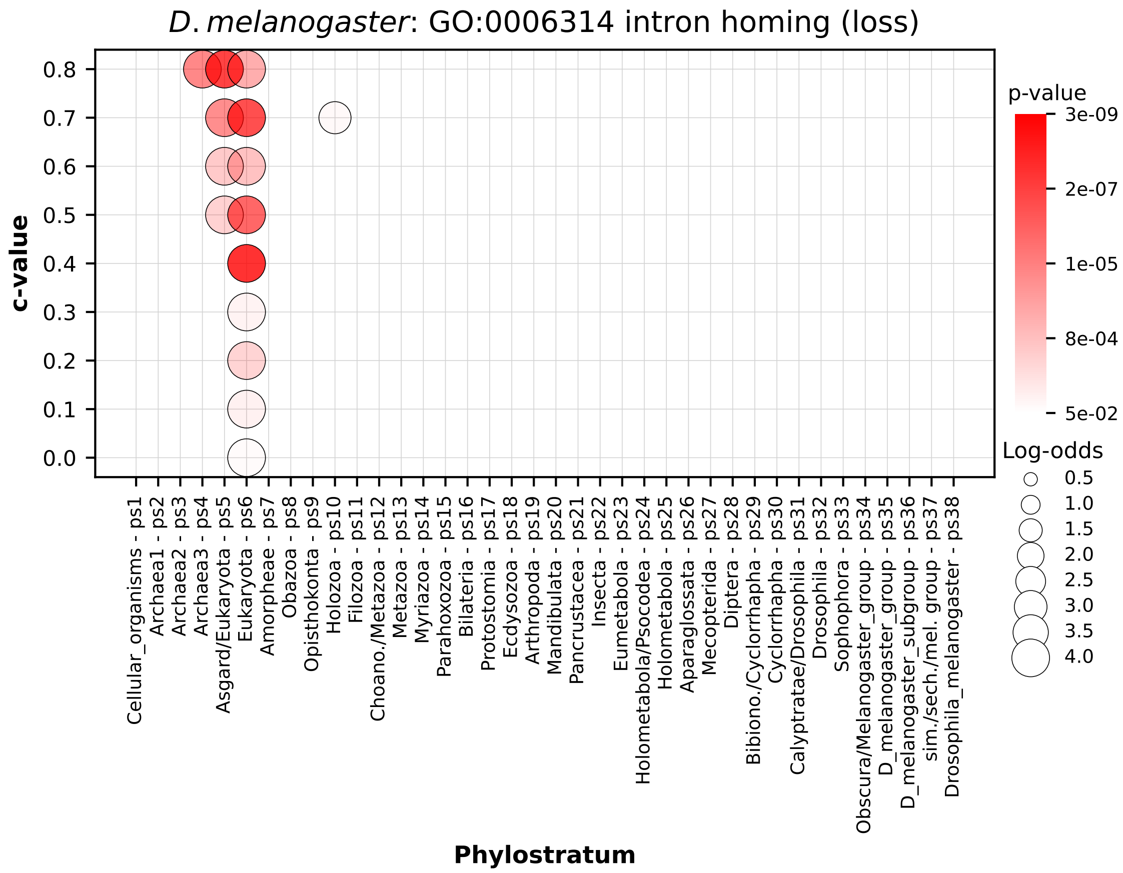
D. melanogaster: GO:0006314 intron homing (loss)
c-value
0.8
0.7
0.6
0.5
0.4
0.3
0.2
0.1
0.0
Phylostratum
Cellular_organisms - ps1
Archaea1 - ps2
Archaea2 - ps3
Archaea3 - ps4
Asgard/Eukaryota - ps5
Eukaryota - ps6
Amorpheae - ps7
Obazoa - ps8
Opisthokonta - ps9
Holozoa - ps10
Filozoa - ps11
Choano./Metazoa - ps12
Metazoa - ps13
Myriazoa - ps14
Parahoxozoa - ps15
Bilateria - ps16
Protostomia - ps17
Ecdysozoa - ps18
Arthropoda - ps19
Mandibulata - ps20
Pancrustacea - ps21
Insecta - ps22
Eumetabola - ps23
Holometabola/Psocodea - ps24
Holometabola - ps25
Aparaglossata - ps26
Mecopterida - ps27
Diptera - ps28
Bibiono./Cyclorrhapha - ps29
Cyclorrhapha - ps30
Calyptratae/Drosophila - ps31
Drosophila - ps32
Sophophora - ps33
Obscura/Melanogaster_group - ps34
D_melanogaster_group - ps35
D_melanogaster_subgroup - ps36
sim./sech./mel. group - ps37
Drosophila_melanogaster - ps38
p-value
3e-09
2e-07
1e-05
8e-04
5e-02
Log-odds
0.5
1.0
1.5
2.0
2.5
3.0
3.5
4.0

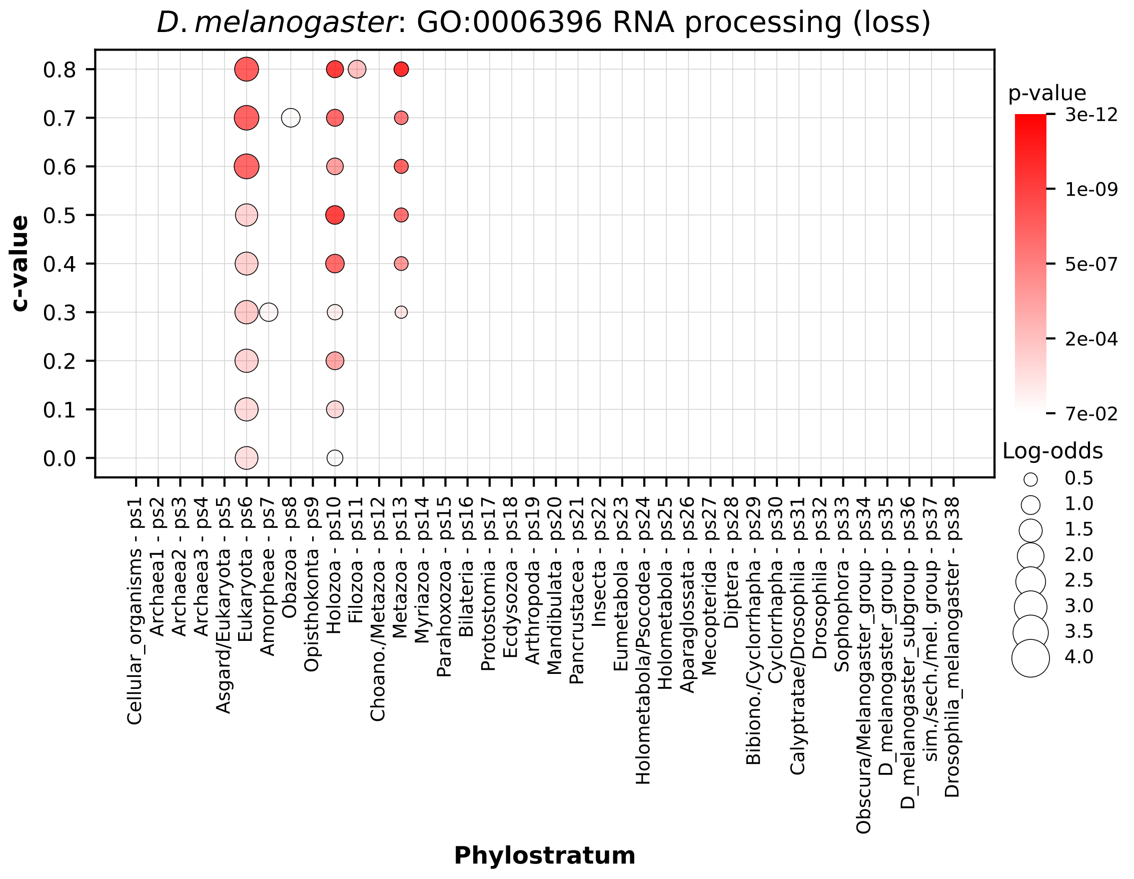

D. melanogaster: GO:0006396 RNA processing (loss)
c-value
0.8
0.7
0.6
0.5
0.4
0.3
0.2
0.1
0.0
p-value
3e-12
1e-09
5e-07
2e-04
7e-02
Log-odds
0.5
1.0
1.5
2.0
2.5
3.0
3.5
4.0
Cellular_organisms - ps1
Archaea1 - ps2
Archaea2 - ps3
Archaea3 - ps4
Asgard/Eukaryota - ps5
Eukaryota - ps6
Amorpheae - ps7
Obazoa - ps8
Opisthokonta - ps9
Holozoa - ps10
Filozoa - ps11
Choano./Metazoa - ps12
Metazoa - ps13
Myriazoa - ps14
Parahoxozoa - ps15
Bilateria - ps16
Protostomia - ps17
Ecdysozoa - ps18
Arthropoda - ps19
Mandibulata - ps20
Pancrustacea - ps21
Insecta - ps22
Eumetabola - ps23
Holometabola/Psocodea - ps24
Holometabola - ps25
Aparaglossata - ps26
Mecopterida - ps27
Diptera - ps28
Bibiono./Cyclorrhapha - ps29
Cyclorrhapha - ps30
Calyptratae/Drosophila - ps31
Drosophila - ps32
Sophophora - ps33
Obscura/Melanogaster_group - ps34
D_melanogaster_group - ps35
D_melanogaster_subgroup - ps36
sim./sech./mel. group - ps37
Drosophila_melanogaster - ps38
Phylostratum

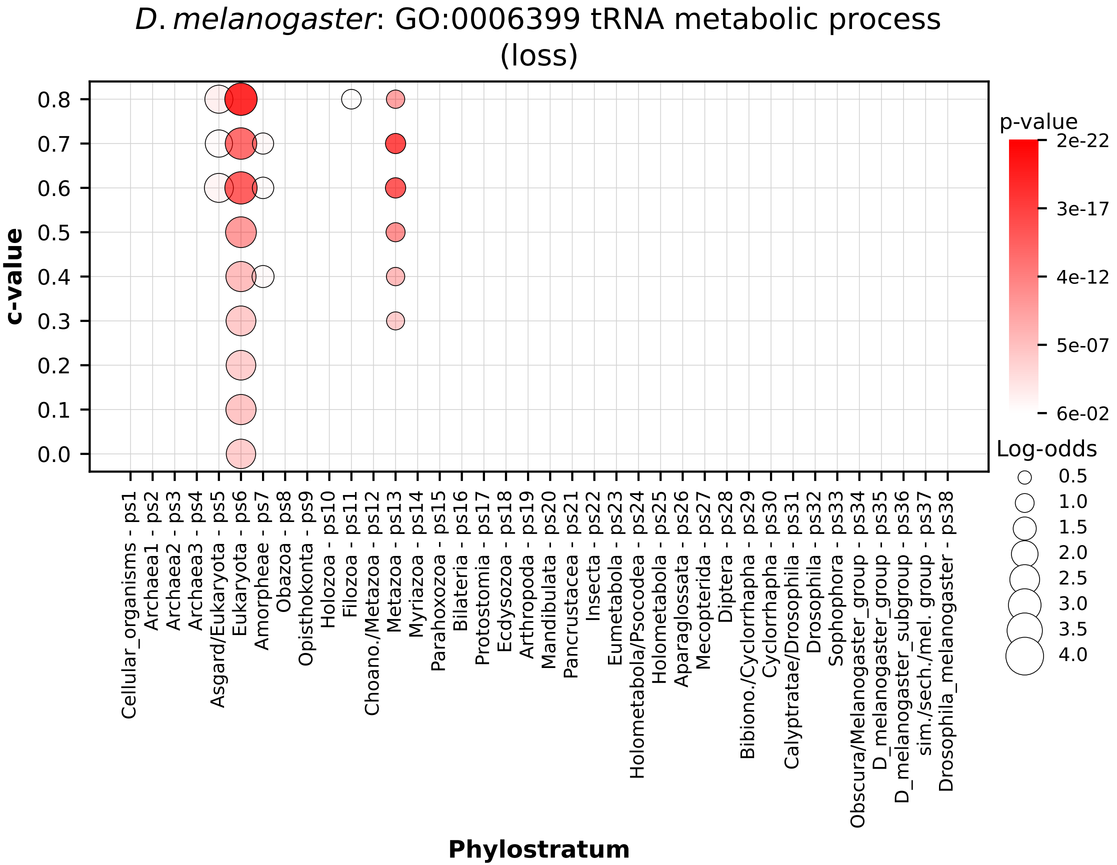

D. melanogaster: GO:0006399 tRNA metabolic process
(loss)
c-value
0.8
0.7
0.6
0.5
0.4
0.3
0.2
0.1
0.0
p-value
2e-22
3e-17
4e-12
5e-07
6e-02
Log-odds
0.5
1.0
1.5
2.0
2.5
3.0
3.5
4.0
Cellular_organisms - ps1
Archaea1 - ps2
Archaea2 - ps3
Archaea3 - ps4
Asgard/Eukaryota - ps5
Eukaryota - ps6
Amorpheae - ps7
Obazoa - ps8
Opisthokonta - ps9
Holozoa - ps10
Filozoa - ps11
Choano./Metazoa - ps12
Metazoa - ps13
Myriazoa - ps14
Parahoxozoa - ps15
Bilateria - ps16
Protostomia - ps17
Ecdysozoa - ps18
Arthropoda - ps19
Mandibulata - ps20
Pancrustacea - ps21
Insecta - ps22
Eumetabola - ps23
Holometabola/Psocodea - ps24
Holometabola - ps25
Aparaglossata - ps26
Mecopterida - ps27
Diptera - ps28
Bibiono./Cyclorrhapha - ps29
Cyclorrhapha - ps30
Calyptratae/Drosophila - ps31
Drosophila - ps32
Sophophora - ps33
Obscura/Melanogaster_group - ps34
D_melanogaster_group - ps35
D_melanogaster_subgroup - ps36
sim./sech./mel. group - ps37
Drosophila_melanogaster - ps38
Phylostratum

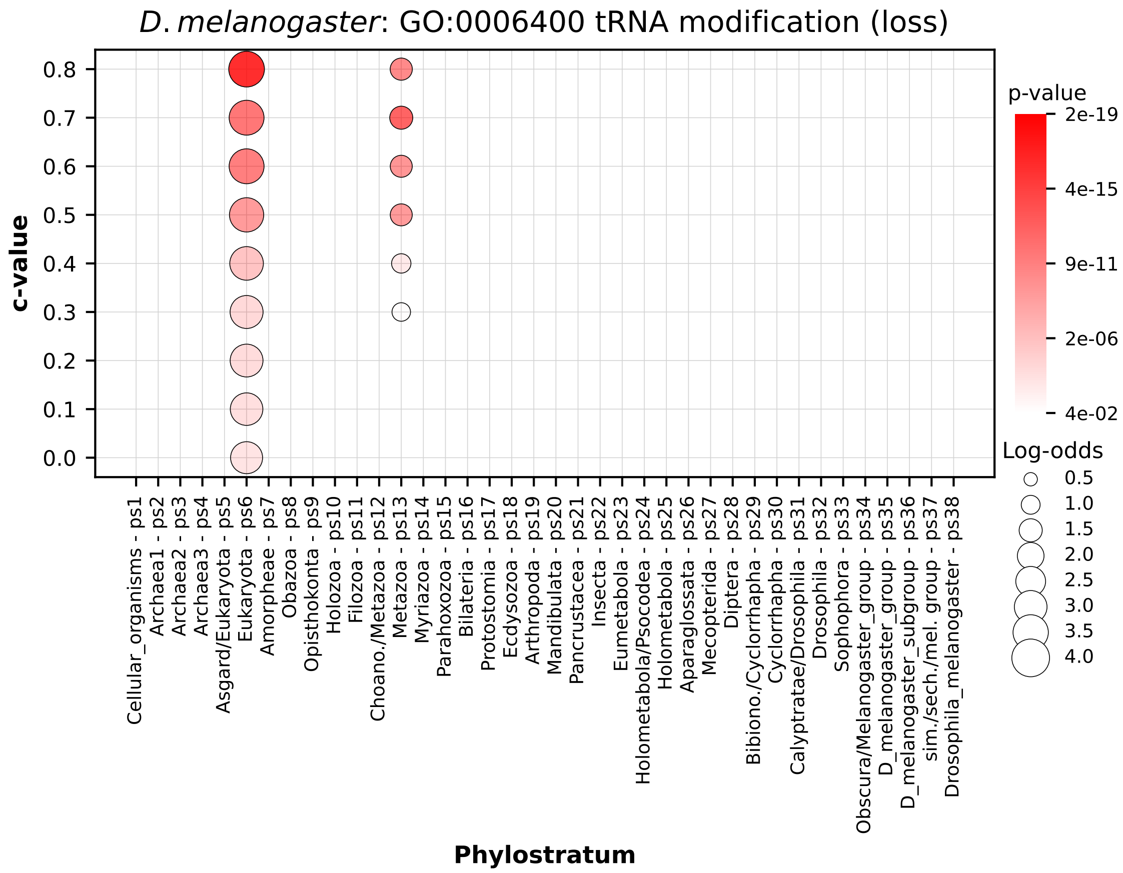

D. melanogaster: GO:0006400 tRNA modification (loss)
c-value
0.8
0.7
0.6
0.5
0.4
0.3
0.2
0.1
0.0
p-value
2e-19
4e-15
9e-11
2e-06
4e-02
Log-odds
0.5
1.0
1.5
2.0
2.5
3.0
3.5
4.0
Cellular_organisms - ps1
Archaea1 - ps2
Archaea2 - ps3
Archaea3 - ps4
Asgard/Eukaryota - ps5
Eukaryota - ps6
Amorpheae - ps7
Obazoa - ps8
Opisthokonta - ps9
Holozoa - ps10
Filozoa - ps11
Choano./Metazoa - ps12
Metazoa - ps13
Myriazoa - ps14
Parahoxozoa - ps15
Bilateria - ps16
Protostomia - ps17
Ecdysozoa - ps18
Arthropoda - ps19
Mandibulata - ps20
Pancrustacea - ps21
Insecta - ps22
Eumetabola - ps23
Holometabola/Psocodea - ps24
Holometabola - ps25
Aparaglossata - ps26
Mecopterida - ps27
Diptera - ps28
Bibiono./Cyclorrhapha - ps29
Cyclorrhapha - ps30
Calyptratae/Drosophila - ps31
Drosophila - ps32
Sophophora - ps33
Obscura/Melanogaster_group - ps34
D_melanogaster_group - ps35
D_melanogaster_subgroup - ps36
sim./sech./mel. group - ps37
Drosophila_melanogaster - ps38
Phylostratum

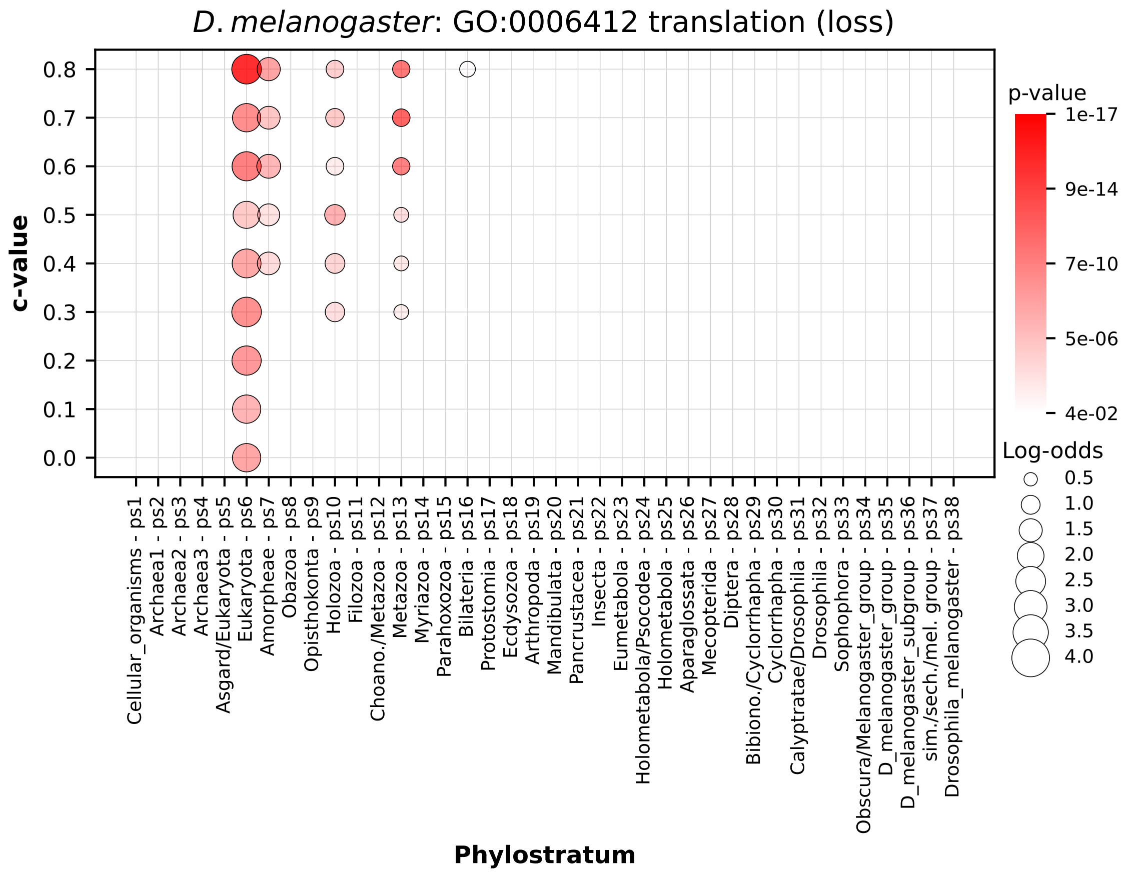

D. melanogaster: GO:0006412 translation (loss)
c-value
0.8
0.7
0.6
0.5
0.4
0.3
0.2
0.1
0.0
Phylostratum
Cellular_organisms - ps1
Archaea1 - ps2
Archaea2 - ps3
Archaea3 - ps4
Asgard/Eukaryota - ps5
Eukaryota - ps6
Amorpheae - ps7
Obazoa - ps8
Opisthokonta - ps9
Holozoa - ps10
Filozoa - ps11
Choano./Metazoa - ps12
Metazoa - ps13
Myriazoa - ps14
Parahoxozoa - ps15
Bilateria - ps16
Protostomia - ps17
Ecdysozoa - ps18
Arthropoda - ps19
Mandibulata - ps20
Pancrustacea - ps21
Insecta - ps22
Eumetabola - ps23
Holometabola/Psocodea - ps24
Holometabola - ps25
Aparaglossata - ps26
Mecopterida - ps27
Diptera - ps28
Bibiono./Cyclorrhapha - ps29
Cyclorrhapha - ps30
Calyptratae/Drosophila - ps31
Drosophila - ps32
Sophophora - ps33
Obscura/Melanogaster_group - ps34
D_melanogaster_group - ps35
D_melanogaster_subgroup - ps36
sim./sech./mel. group - ps37
Drosophila_melanogaster - ps38
p-value
1e-17
9e-14
7e-10
5e-06
4e-02
Log-odds
0.5
1.0
1.5
2.0
2.5
3.0
3.5
4.0

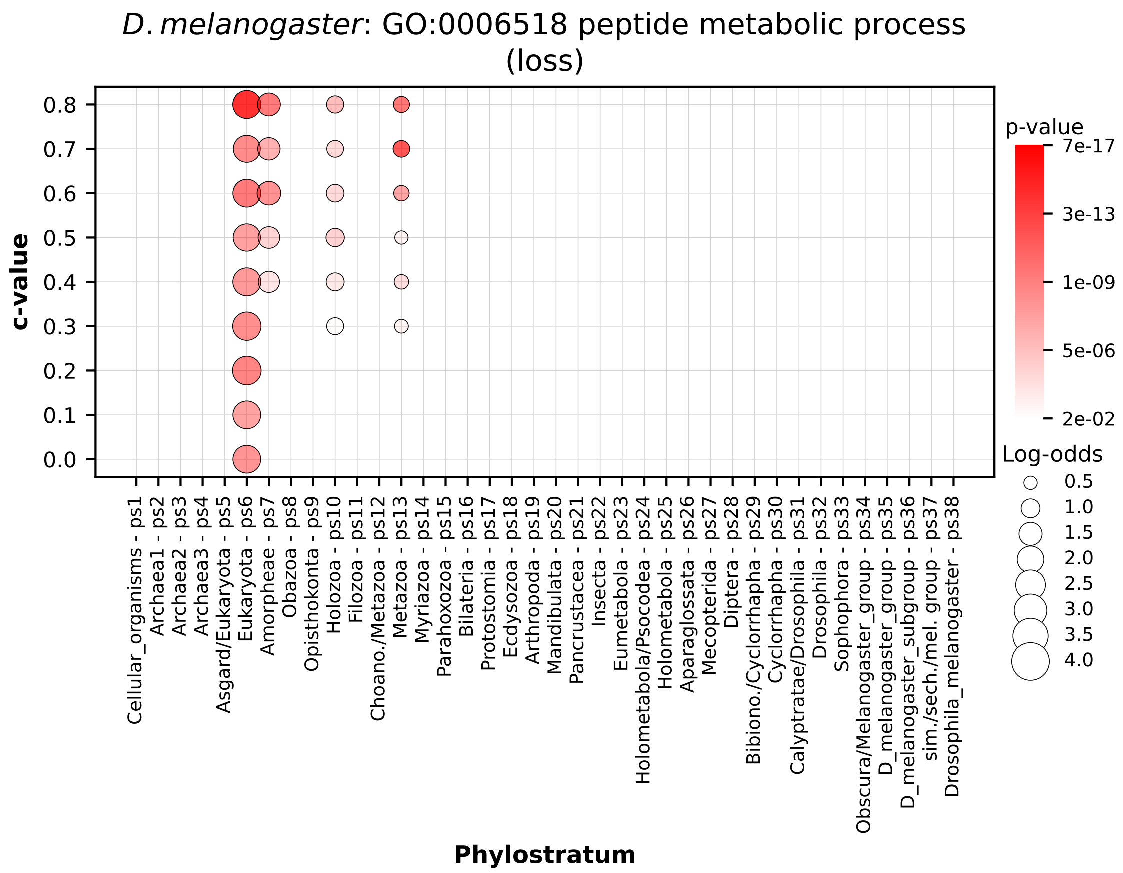

D. melanogaster: GO:0006518 peptide metabolic process
(loss)
c-value
0.8
0.7
0.6
0.5
0.4
0.3
0.2
0.1
0.0
p-value
7e-17
3e-13
1e-09
5e-06
2e-02
Log-odds
0.5
1.0
1.5
2.0
2.5
3.0
3.5
4.0
Cellular_organisms - ps1
Archaea1 - ps2
Archaea2 - ps3
Archaea3 - ps4
Asgard/Eukaryota - ps5
Eukaryota - ps6
Amorpheae - ps7
Obazoa - ps8
Opisthokonta - ps9
Holozoa - ps10
Filozoa - ps11
Choano./Metazoa - ps12
Metazoa - ps13
Myriazoa - ps14
Parahoxozoa - ps15
Bilateria - ps16
Protostomia - ps17
Ecdysozoa - ps18
Arthropoda - ps19
Mandibulata - ps20
Pancrustacea - ps21
Insecta - ps22
Eumetabola - ps23
Holometabola/Psocodea - ps24
Holometabola - ps25
Aparaglossata - ps26
Mecopterida - ps27
Diptera - ps28
Bibiono./Cyclorrhapha - ps29
Cyclorrhapha - ps30
Calyptratae/Drosophila - ps31
Drosophila - ps32
Sophophora - ps33
Obscura/Melanogaster_group - ps34
D_melanogaster_group - ps35
D_melanogaster_subgroup - ps36
sim./sech./mel. group - ps37
Drosophila_melanogaster - ps38
Phylostratum

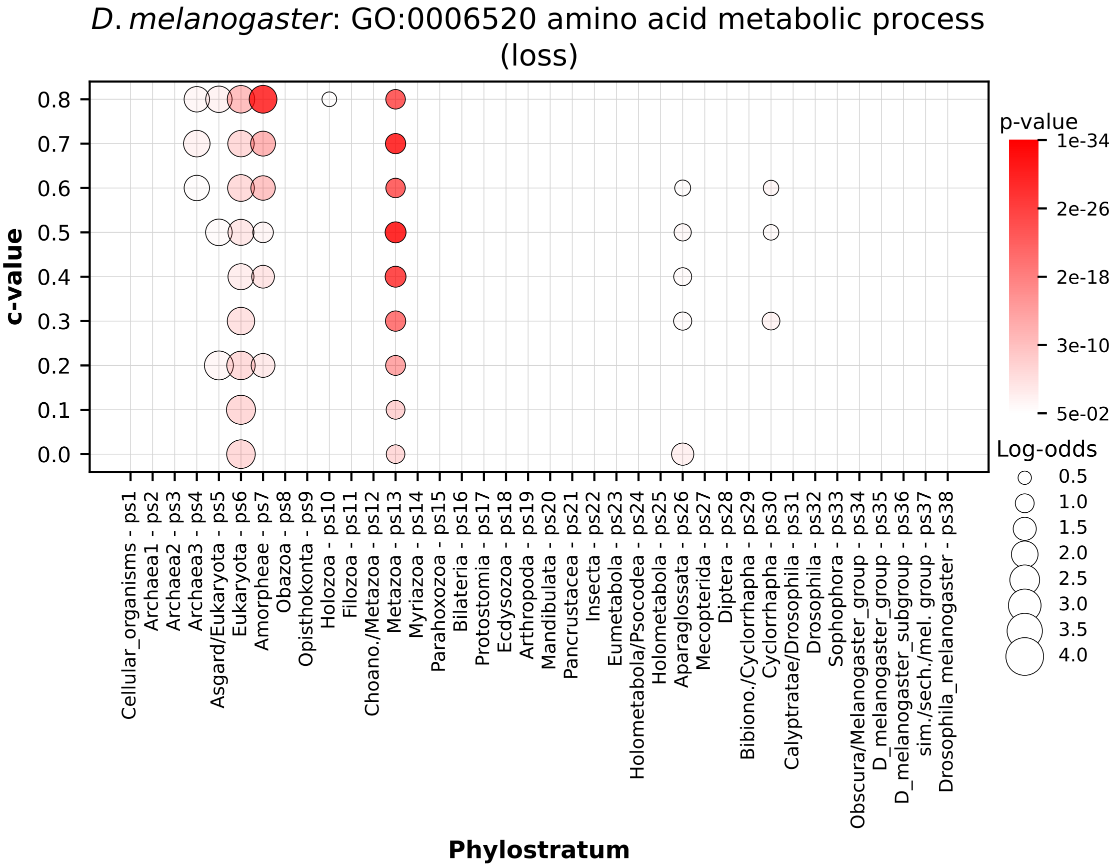
D. melanogaster: GO:0006520 amino acid metabolic process
(loss)
c-value
0.8
0.7
0.6
0.5
0.4
0.3
0.2
0.1
0.0
p-value
1e-34
2e-26
2e-18
3e-10
5e-02
Log-odds
0.5
1.0
1.5
2.0
2.5
3.0
3.5
4.0
Cellular_organisms - ps1
Archaea1 - ps2
Archaea2 - ps3
Archaea3 - ps4
Asgard/Eukaryota - ps5
Eukaryota - ps6
Amorpheae - ps7
Obazoa - ps8
Opisthokonta - ps9
Holozoa - ps10
Filozoa - ps11
Choano./Metazoa - ps12
Metazoa - ps13
Myriazoa - ps14
Parahoxozoa - ps15
Bilateria - ps16
Protostomia - ps17
Ecdysozoa - ps18
Arthropoda - ps19
Mandibulata - ps20
Pancrustacea - ps21
Insecta - ps22
Eumetabola - ps23
Holometabola/Psocodea - ps24
Holometabola - ps25
Aparaglossata - ps26
Mecopterida - ps27
Diptera - ps28
Bibiono./Cyclorrhapha - ps29
Cyclorrhapha - ps30
Calyptratae/Drosophila - ps31
Drosophila - ps32
Sophophora - ps33
Obscura/Melanogaster_group - ps34
D_melanogaster_group - ps35
D_melanogaster_subgroup - ps36
sim./sech./mel. group - ps37
Drosophila_melanogaster - ps38
Phylostratum

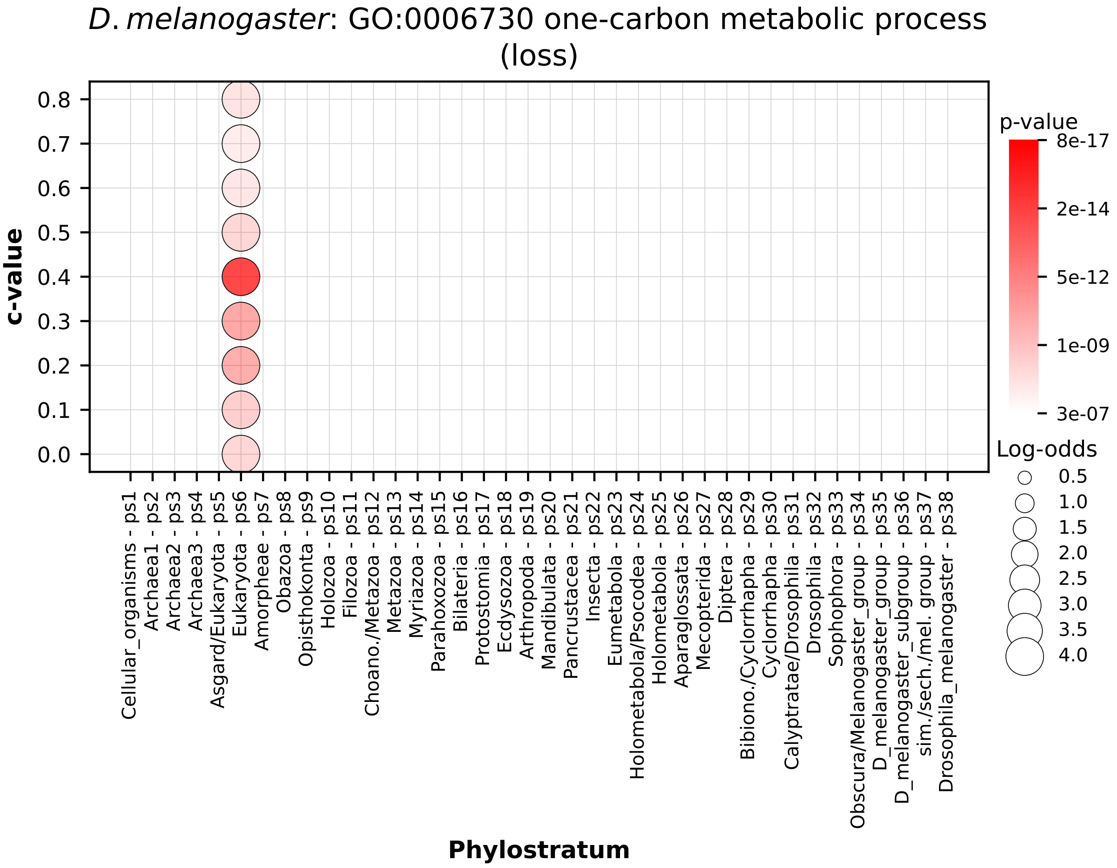

D. melanogaster: GO:0006730 one-carbon metabolic process (loss)
c-value
0.0
0.1
0.2
0.3
0.4
0.5
0.6
0.7
0.8
Phylostratum
Cellular_organisms - ps1
Archaea1 - ps2
Archaea2 - ps3
Archaea3 - ps4
Asgard/Eukaryota - ps5
Eukaryota - ps6
Amorpheae - ps7
Obazoa - ps8
Opisthokonta - ps9
Holozoa - ps10
Filozoa - ps11
Choano./Metazoa - ps12
Metazoa - ps13
Myriazoa - ps14
Parahoxozoa - ps15
Bilateria - ps16
Protostomia - ps17
Ecdysozoa - ps18
Arthropoda - ps19
Mandibulata - ps20
Pancrustacea - ps21
Insecta - ps22
Eumetabola - ps23
Holometabola/Psocodea - ps24
Holometabola - ps25
Aparaglossata - ps26
Mecopterida - ps27
Diptera - ps28
Bibiono./Cyclorrhapha - ps29
Cyclorrhapha - ps30
Calyptratae/Drosophila - ps31
Drosophila - ps32
Sophophora - ps33
Obscura/Melanogaster_group - ps34
D_melanogaster_group - ps35
D_melanogaster_subgroup - ps36
sim./sech./mel. group - ps37
Drosophila_melanogaster - ps38
p-value
8e-17
2e-14
5e-12
1e-09
3e-07
Log-odds
0.5
1.0
1.5
2.0
2.5
3.0
3.5
4.0

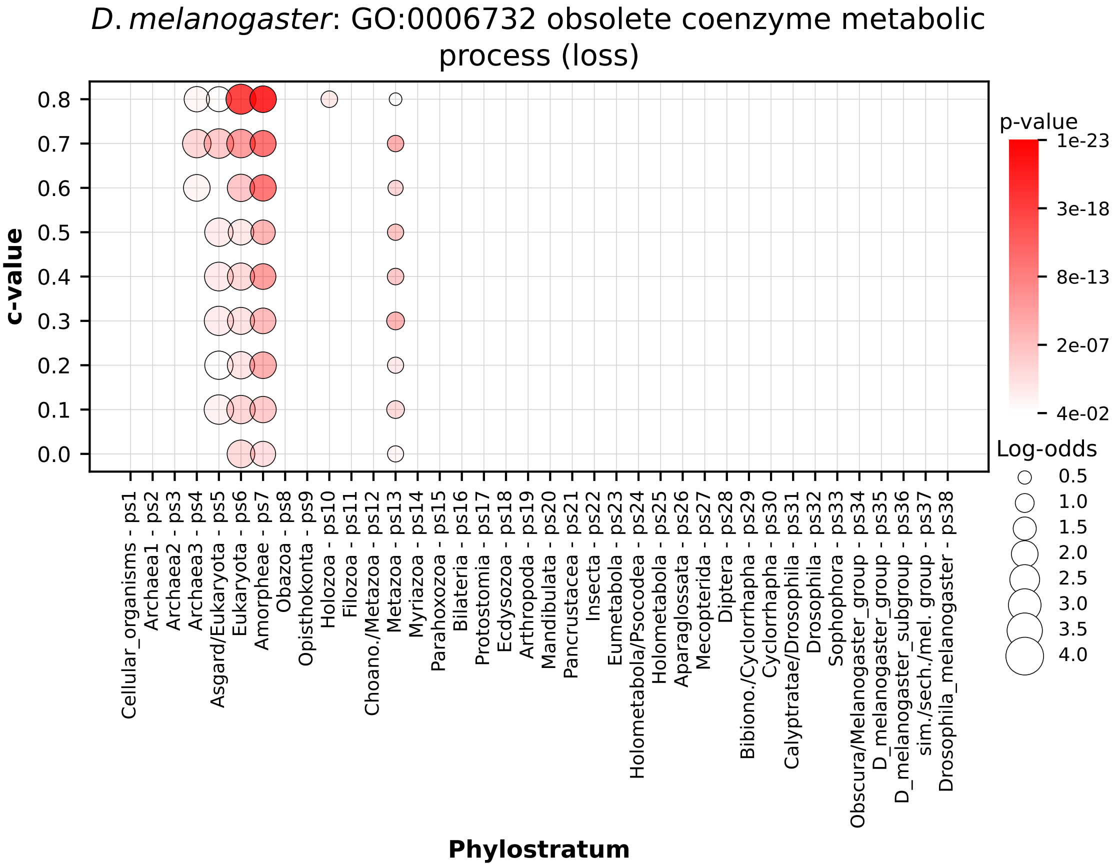

D. melanogaster: GO:0006732 obsolete coenzyme metabolic process (loss)
c-value
0.8
0.7
0.6
0.5
0.4
0.3
0.2
0.1
0.0
p-value
1e-23
3e-18
8e-13
2e-07
4e-02
Log-odds
0.5
1.0
1.5
2.0
2.5
3.0
3.5
4.0
Cellular_organisms - ps1
Archaea1 - ps2
Archaea2 - ps3
Archaea3 - ps4
Asgard/Eukaryota - ps5
Eukaryota - ps6
Amorpheae - ps7
Obazoa - ps8
Opisthokonta - ps9
Holozoa - ps10
Filozoa - ps11
Choano./Metazoa - ps12
Metazoa - ps13
Myriazoa - ps14
Parahoxozoa - ps15
Bilateria - ps16
Protostomia - ps17
Ecdysozoa - ps18
Arthropoda - ps19
Mandibulata - ps20
Pancrustacea - ps21
Insecta - ps22
Eumetabola - ps23
Holometabola/Psocodea - ps24
Holometabola - ps25
Aparaglossata - ps26
Mecopterida - ps27
Diptera - ps28
Bibiono./Cyclorrhapha - ps29
Cyclorrhapha - ps30
Calyptratae/Drosophila - ps31
Drosophila - ps32
Sophophora - ps33
Obscura/Melanogaster_group - ps34
D_melanogaster_group - ps35
D_melanogaster_subgroup - ps36
sim./sech./mel. group - ps37
Drosophila_melanogaster - ps38
Phylostratum

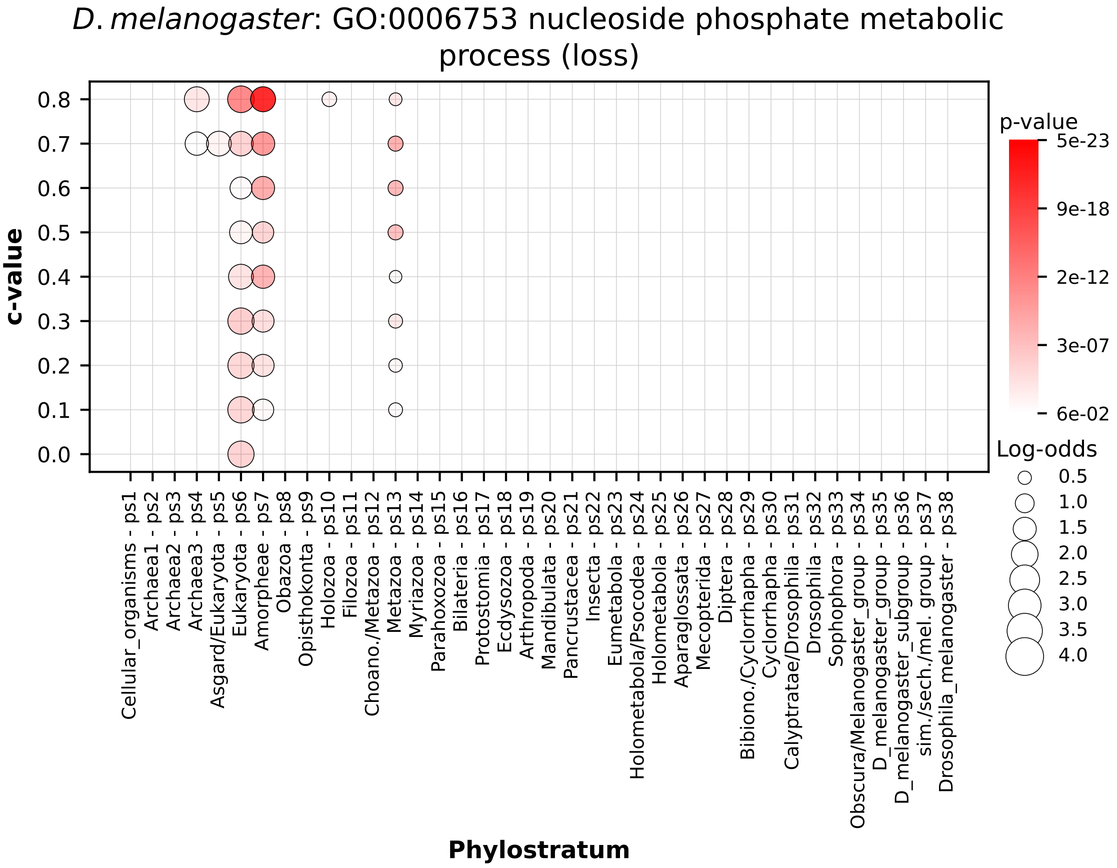

D. melanogaster: GO:0006753 nucleoside phosphate metabolic process (loss)
c-value
0.8
0.7
0.6
0.5
0.4
0.3
0.2
0.1
0.0
Phylostratum
Cellular_organisms - ps1
Archaea1 - ps2
Archaea2 - ps3
Archaea3 - ps4
Asgard/Eukaryota - ps5
Eukaryota - ps6
Amorpheae - ps7
Obazoa - ps8
Opisthokonta - ps9
Holozoa - ps10
Filozoa - ps11
Choano./Metazoa - ps12
Metazoa - ps13
Myriazoa - ps14
Parahoxozoa - ps15
Bilateria - ps16
Protostomia - ps17
Ecdysozoa - ps18
Arthropoda - ps19
Mandibulata - ps20
Pancrustacea - ps21
Insecta - ps22
Eumetabola - ps23
Holometabola/Psocodea - ps24
Holometabola - ps25
Aparaglossata - ps26
Mecopterida - ps27
Diptera - ps28
Bibiono./Cyclorrhapha - ps29
Cyclorrhapha - ps30
Calyptratae/Drosophila - ps31
Drosophila - ps32
Sophophora - ps33
Obscura/Melanogaster_group - ps34
D_melanogaster_group - ps35
D_melanogaster_subgroup - ps36
sim./sech./mel. group - ps37
Drosophila_melanogaster - ps38
p-value
5e-23
9e-18
2e-12
3e-07
6e-02
Log-odds
0.5
1.0
1.5
2.0
2.5
3.0
3.5
4.0

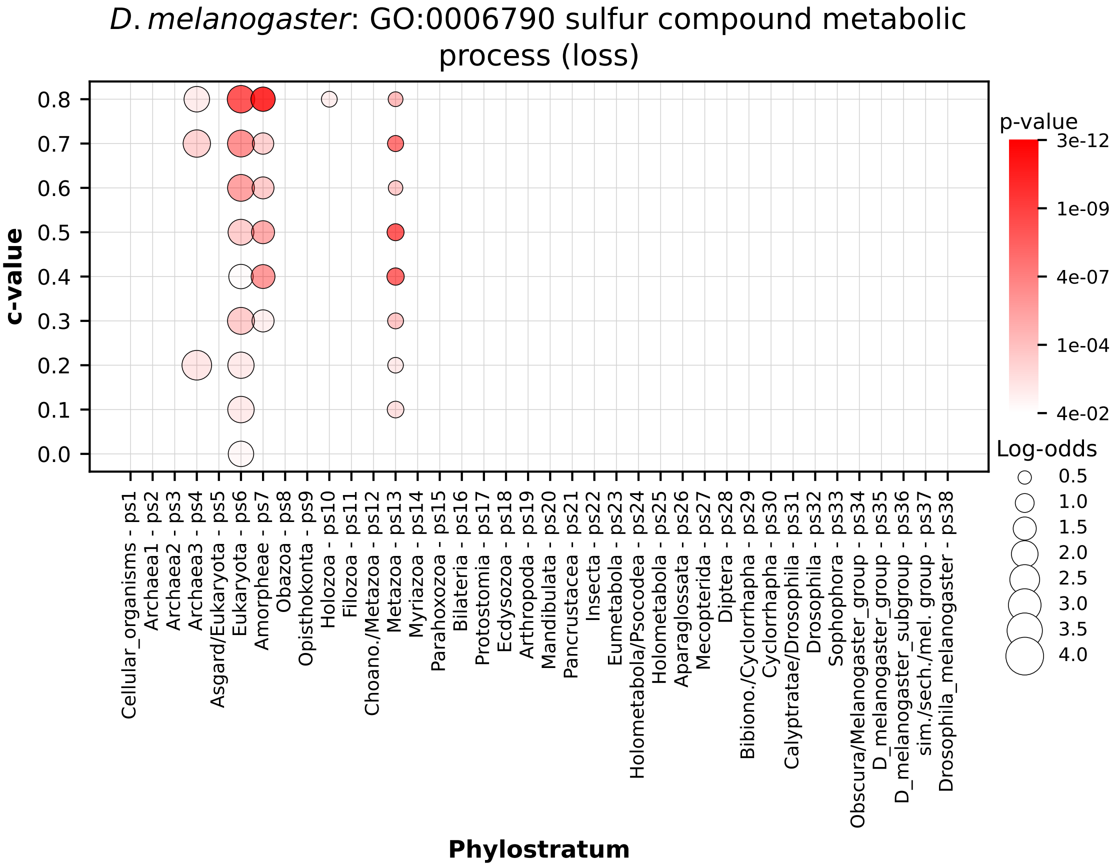

D. melanogaster: GO:0006790 sulfur compound metabolic process (loss)
c-value
0.8
0.7
0.6
0.5
0.4
0.3
0.2
0.1
0.0
p-value
3e-12
1e-09
4e-07
1e-04
4e-02
Log-odds
0.5
1.0
1.5
2.0
2.5
3.0
3.5
4.0
Cellular_organisms - ps1
Archaea1 - ps2
Archaea2 - ps3
Archaea3 - ps4
Asgard/Eukaryota - ps5
Eukaryota - ps6
Amorpheae - ps7
Obazoa - ps8
Opisthokonta - ps9
Holozoa - ps10
Filozoa - ps11
Choano./Metazoa - ps12
Metazoa - ps13
Myriazoa - ps14
Parahoxozoa - ps15
Bilateria - ps16
Protostomia - ps17
Ecdysozoa - ps18
Arthropoda - ps19
Mandibulata - ps20
Pancrustacea - ps21
Insecta - ps22
Eumetabola - ps23
Holometabola/Psocodea - ps24
Holometabola - ps25
Aparaglossata - ps26
Mecopterida - ps27
Diptera - ps28
Bibiono./Cyclorrhapha - ps29
Cyclorrhapha - ps30
Calyptratae/Drosophila - ps31
Drosophila - ps32
Sophophora - ps33
Obscura/Melanogaster_group - ps34
D_melanogaster_group - ps35
D_melanogaster_subgroup - ps36
sim./sech./mel. group - ps37
Drosophila_melanogaster - ps38
Phylostratum

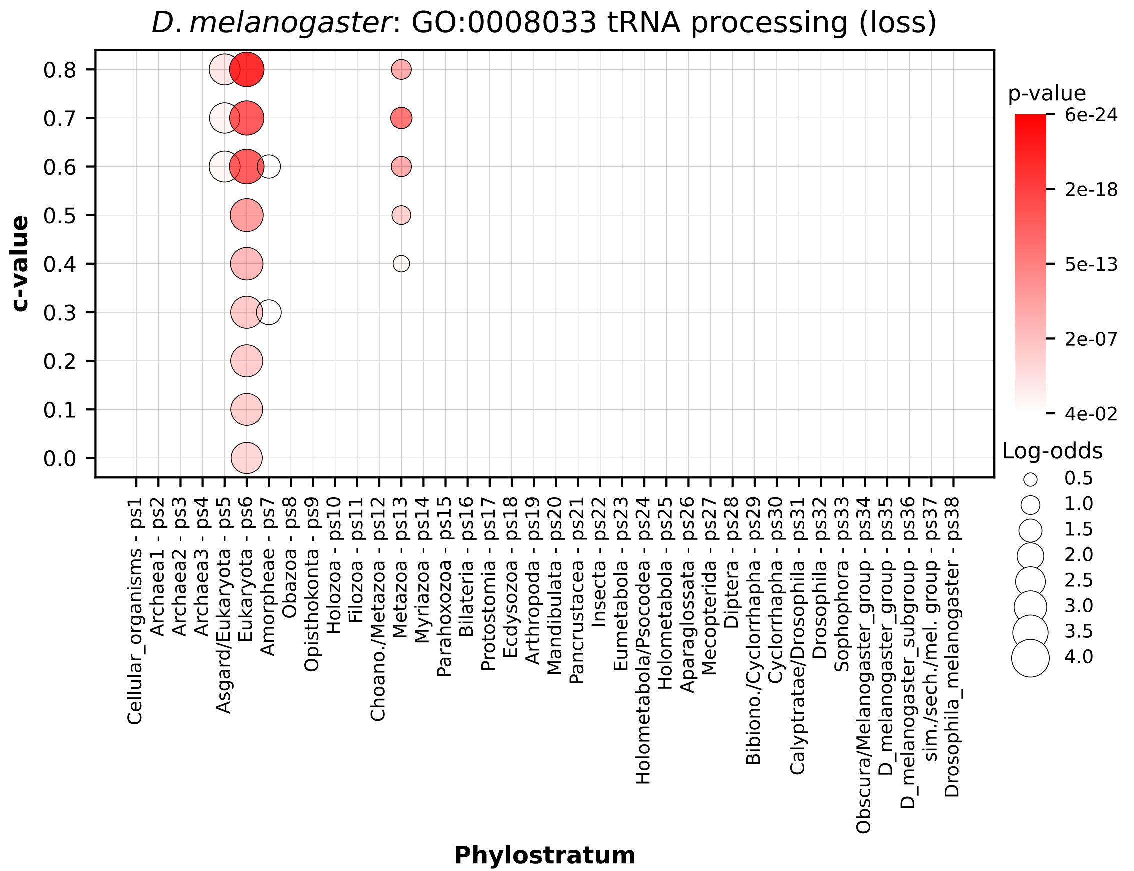
D. melanogaster: GO:0008033 tRNA processing (loss)
c-value
0.8
0.7
0.6
0.5
0.4
0.3
0.2
0.1
0.0
p-value
6e-24
2e-18
5e-13
2e-07
4e-02
Log-odds
0.5
1.0
1.5
2.0
2.5
3.0
3.5
4.0
Cellular_organisms - ps1
Archaea1 - ps2
Archaea2 - ps3
Archaea3 - ps4
Asgard/Eukaryota - ps5
Eukaryota - ps6
Amorpheae - ps7
Obazoa - ps8
Opisthokonta - ps9
Holozoa - ps10
Filozoa - ps11
Choano./Metazoa - ps12
Metazoa - ps13
Myriazoa - ps14
Parahoxozoa - ps15
Bilateria - ps16
Protostomia - ps17
Ecdysozoa - ps18
Arthropoda - ps19
Mandibulata - ps20
Pancrustacea - ps21
Insecta - ps22
Eumetabola - ps23
Holometabola/Psocodea - ps24
Holometabola - ps25
Aparaglossata - ps26
Mecopterida - ps27
Diptera - ps28
Bibiono./Cyclorrhapha - ps29
Cyclorrhapha - ps30
Calyptratae/Drosophila - ps31
Drosophila - ps32
Sophophora - ps33
Obscura/Melanogaster_group - ps34
D_melanogaster_group - ps35
D_melanogaster_subgroup - ps36
sim./sech./mel. group - ps37
Drosophila_melanogaster - ps38
Phylostratum

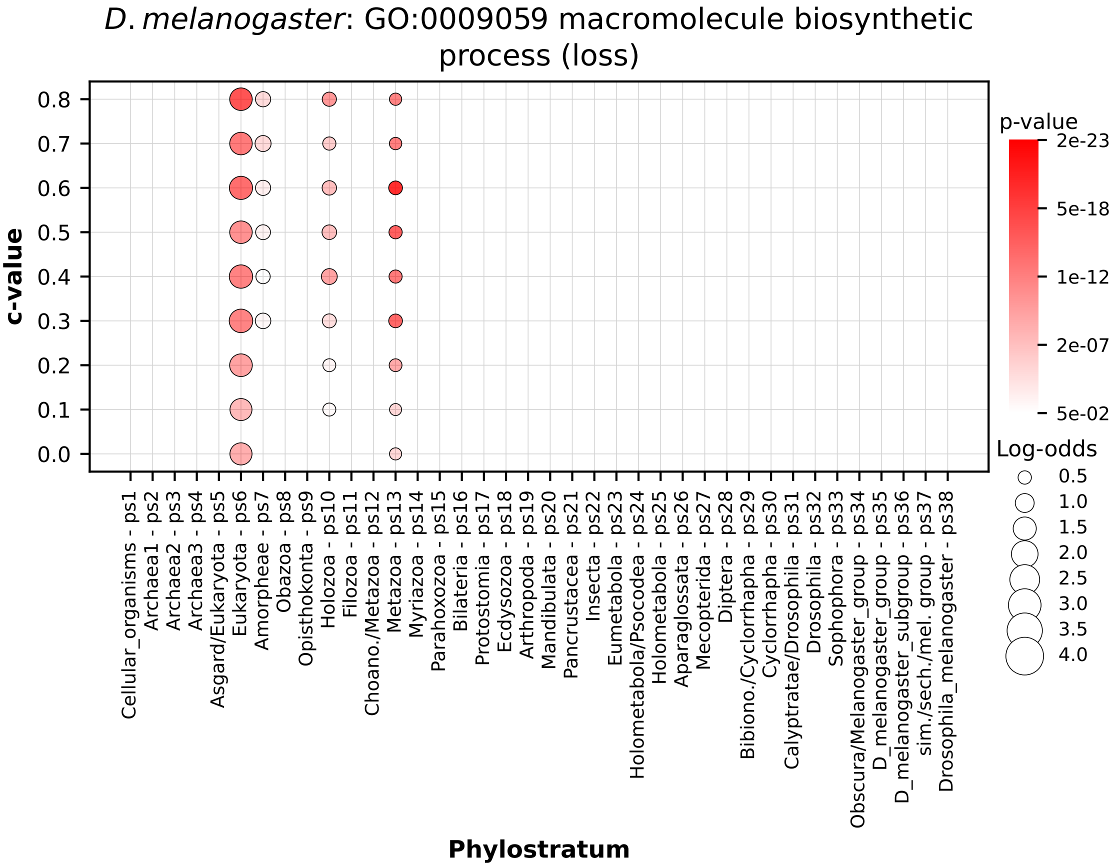

D. melanogaster: GO:0009059 macromolecule biosynthetic process (loss)
c-value
0.8
0.7
0.6
0.5
0.4
0.3
0.2
0.1
0.0
Phylostratum
Cellular_organisms - ps1
Archaea1 - ps2
Archaea2 - ps3
Archaea3 - ps4
Asgard/Eukaryota - ps5
Eukaryota - ps6
Amorpheae - ps7
Obazoa - ps8
Opisthokonta - ps9
Holozoa - ps10
Filozoa - ps11
Choano./Metazoa - ps12
Metazoa - ps13
Myriazoa - ps14
Parahoxozoa - ps15
Bilateria - ps16
Protostomia - ps17
Ecdysozoa - ps18
Arthropoda - ps19
Mandibulata - ps20
Pancrustacea - ps21
Insecta - ps22
Eumetabola - ps23
Holometabola/Psocodea - ps24
Holometabola - ps25
Aparaglossata - ps26
Mecopterida - ps27
Diptera - ps28
Bibiono./Cyclorrhapha - ps29
Cyclorrhapha - ps30
Calyptratae/Drosophila - ps31
Drosophila - ps32
Sophophora - ps33
Obscura/Melanogaster_group - ps34
D_melanogaster_group - ps35
D_melanogaster_subgroup - ps36
sim./sech./mel. group - ps37
Drosophila_melanogaster - ps38
p-value
2e-23
5e-18
1e-12
2e-07
5e-02
Log-odds
0.5
1.0
1.5
2.0
2.5
3.0
3.5
4.0

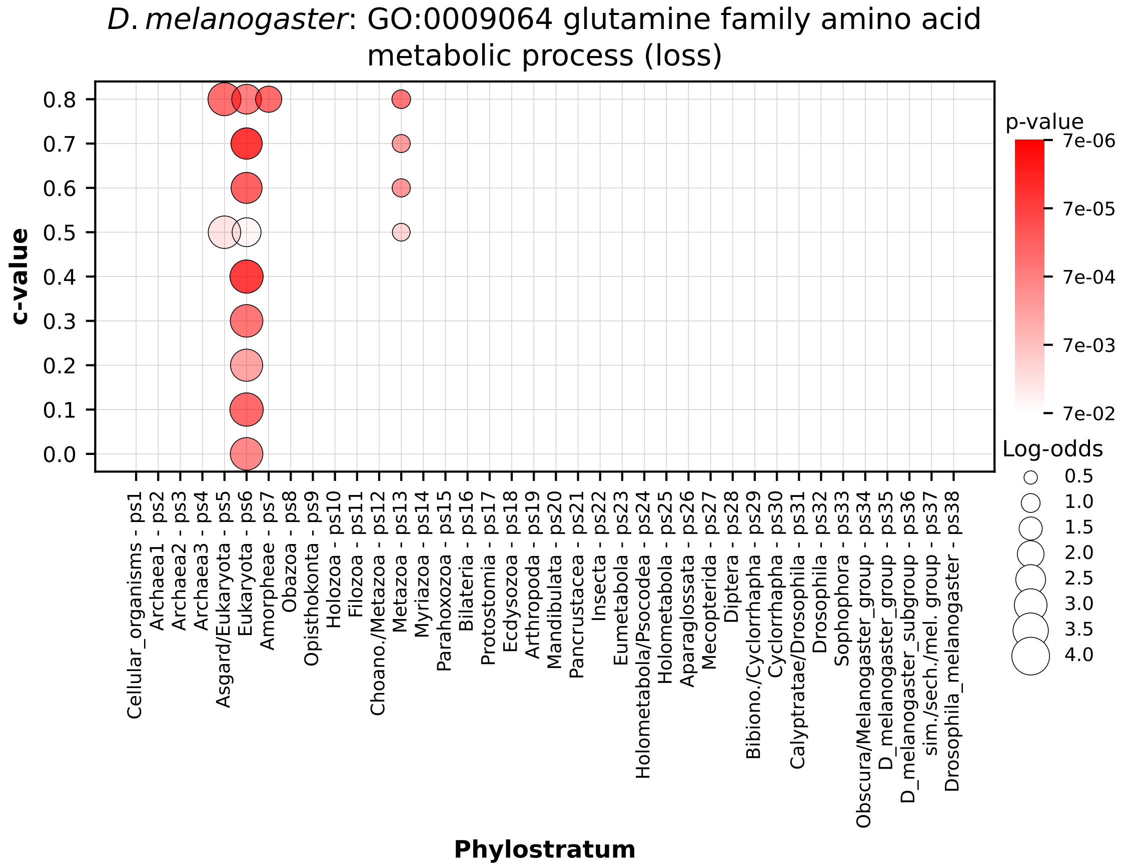

D. melanogaster: GO:0009064 glutamine family amino acid metabolic process (loss)
c-value
0.0
0.1
0.2
0.3
0.4
0.5
0.6
0.7
0.8
p-value
7e-06
7e-05
7e-04
7e-03
7e-02
Log-odds
0.5
1.0
1.5
2.0
2.5
3.0
3.5
4.0
Cellular_organisms - ps1
Archaea1 - ps2
Archaea2 - ps3
Archaea3 - ps4
Asgard/Eukaryota - ps5
Eukaryota - ps6
Amorpheae - ps7
Obazoa - ps8
Opisthokonta - ps9
Holozoa - ps10
Filozoa - ps11
Choano./Metazoa - ps12
Metazoa - ps13
Myriazoa - ps14
Parahoxozoa - ps15
Bilateria - ps16
Protostomia - ps17
Ecdysozoa - ps18
Arthropoda - ps19
Mandibulata - ps20
Pancrustacea - ps21
Insecta - ps22
Eumetabola - ps23
Holometabola/Psocodea - ps24
Holometabola - ps25
Aparaglossata - ps26
Mecopterida - ps27
Diptera - ps28
Bibiono./Cyclorrhapha - ps29
Cyclorrhapha - ps30
Calyptratae/Drosophila - ps31
Drosophila - ps32
Sophophora - ps33
Obscura/Melanogaster_group - ps34
D_melanogaster_group - ps35
D_melanogaster_subgroup - ps36
sim./sech./mel. group - ps37
Drosophila_melanogaster - ps38
Phylostratum

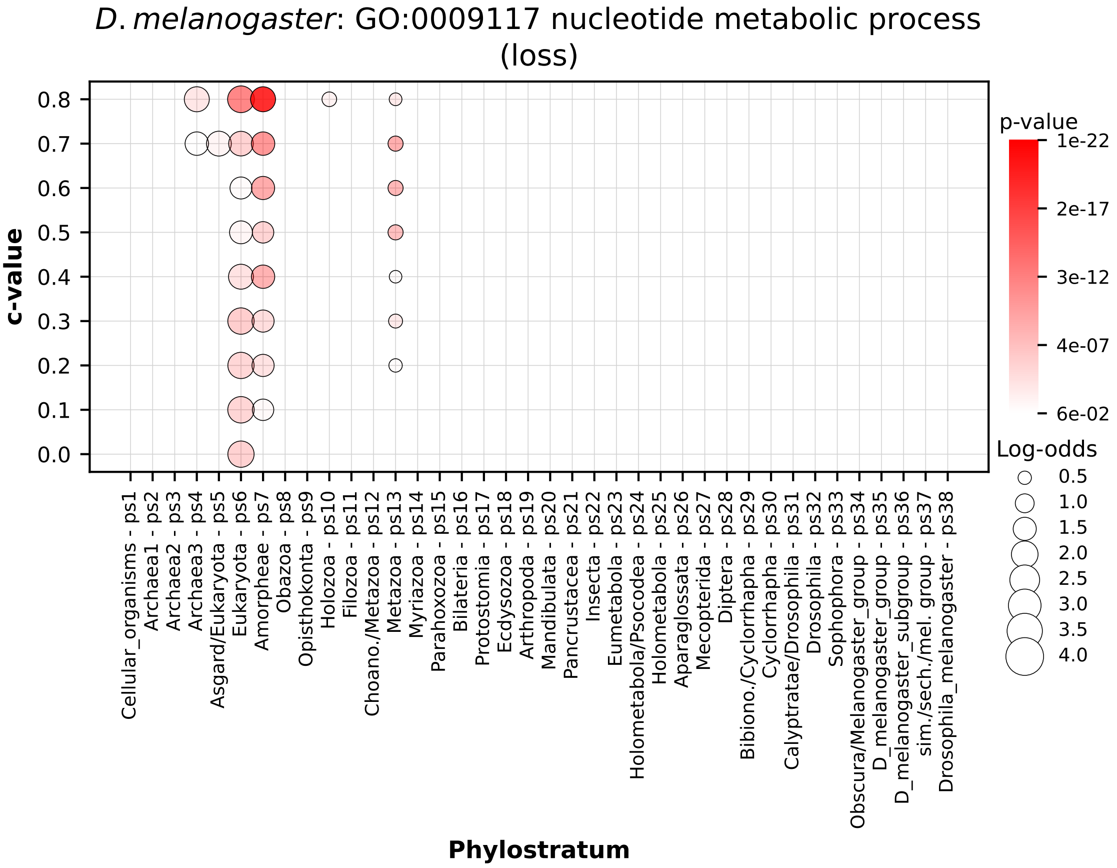

D. melanogaster: GO:0009117 nucleotide metabolic process
(loss)
c-value
0.8
0.7
0.6
0.5
0.4
0.3
0.2
0.1
0.0
p-value
1e-22
2e-17
3e-12
4e-07
6e-02
Log-odds
0.5
1.0
1.5
2.0
2.5
3.0
3.5
4.0
Cellular_organisms - ps1
Archaea1 - ps2
Archaea2 - ps3
Archaea3 - ps4
Asgard/Eukaryota - ps5
Eukaryota - ps6
Amorpheae - ps7
Obazoa - ps8
Opisthokonta - ps9
Holozoa - ps10
Filozoa - ps11
Choano./Metazoa - ps12
Metazoa - ps13
Myriazoa - ps14
Parahoxozoa - ps15
Bilateria - ps16
Protostomia - ps17
Ecdysozoa - ps18
Arthropoda - ps19
Mandibulata - ps20
Pancrustacea - ps21
Insecta - ps22
Eumetabola - ps23
Holometabola/Psocodea - ps24
Holometabola - ps25
Aparaglossata - ps26
Mecopterida - ps27
Diptera - ps28
Bibiono./Cyclorrhapha - ps29
Cyclorrhapha - ps30
Calyptratae/Drosophila - ps31
Drosophila - ps32
Sophophora - ps33
Obscura/Melanogaster_group - ps34
D_melanogaster_group - ps35
D_melanogaster_subgroup - ps36
sim./sech./mel. group - ps37
Drosophila_melanogaster - ps38
Phylostratum

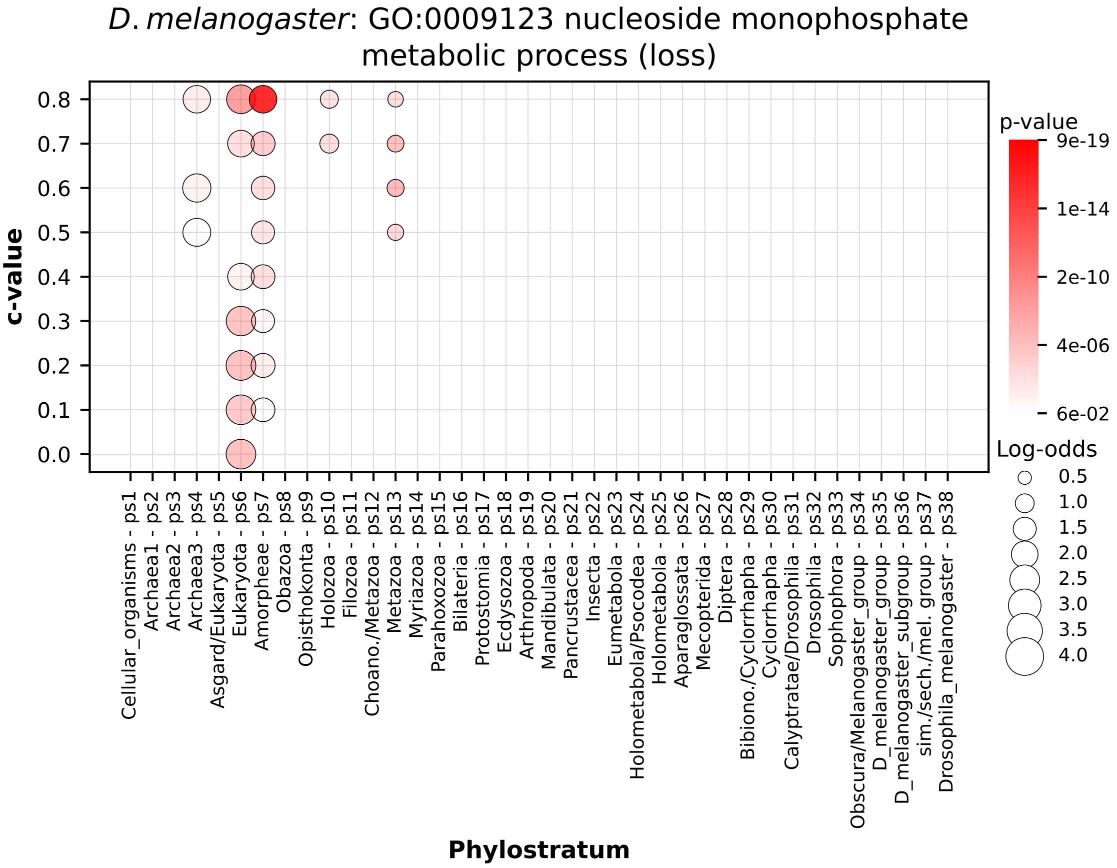

D. melanogaster: GO:0009123 nucleoside monophosphate metabolic process (loss)
c-value
0.8
0.7
0.6
0.5
0.4
0.3
0.2
0.1
0.0
p-value
9e-19
1e-14
2e-10
4e-06
6e-02
Log-odds
0.5
1.0
1.5
2.0
2.5
3.0
3.5
4.0
Cellular_organisms - ps1
Archaea1 - ps2
Archaea2 - ps3
Archaea3 - ps4
Asgard/Eukaryota - ps5
Eukaryota - ps6
Amorpheae - ps7
Obazoa - ps8
Opisthokonta - ps9
Holozoa - ps10
Filozoa - ps11
Choano./Metazoa - ps12
Metazoa - ps13
Myriazoa - ps14
Parahoxozoa - ps15
Bilateria - ps16
Protostomia - ps17
Ecdysozoa - ps18
Arthropoda - ps19
Mandibulata - ps20
Pancrustacea - ps21
Insecta - ps22
Eumetabola - ps23
Holometabola/Psocodea - ps24
Holometabola - ps25
Aparaglossata - ps26
Mecopterida - ps27
Diptera - ps28
Bibiono./Cyclorrhapha - ps29
Cyclorrhapha - ps30
Calyptratae/Drosophila - ps31
Drosophila - ps32
Sophophora - ps33
Obscura/Melanogaster_group - ps34
D_melanogaster_group - ps35
D_melanogaster_subgroup - ps36
sim./sech./mel. group - ps37
Drosophila_melanogaster - ps38
Phylostratum

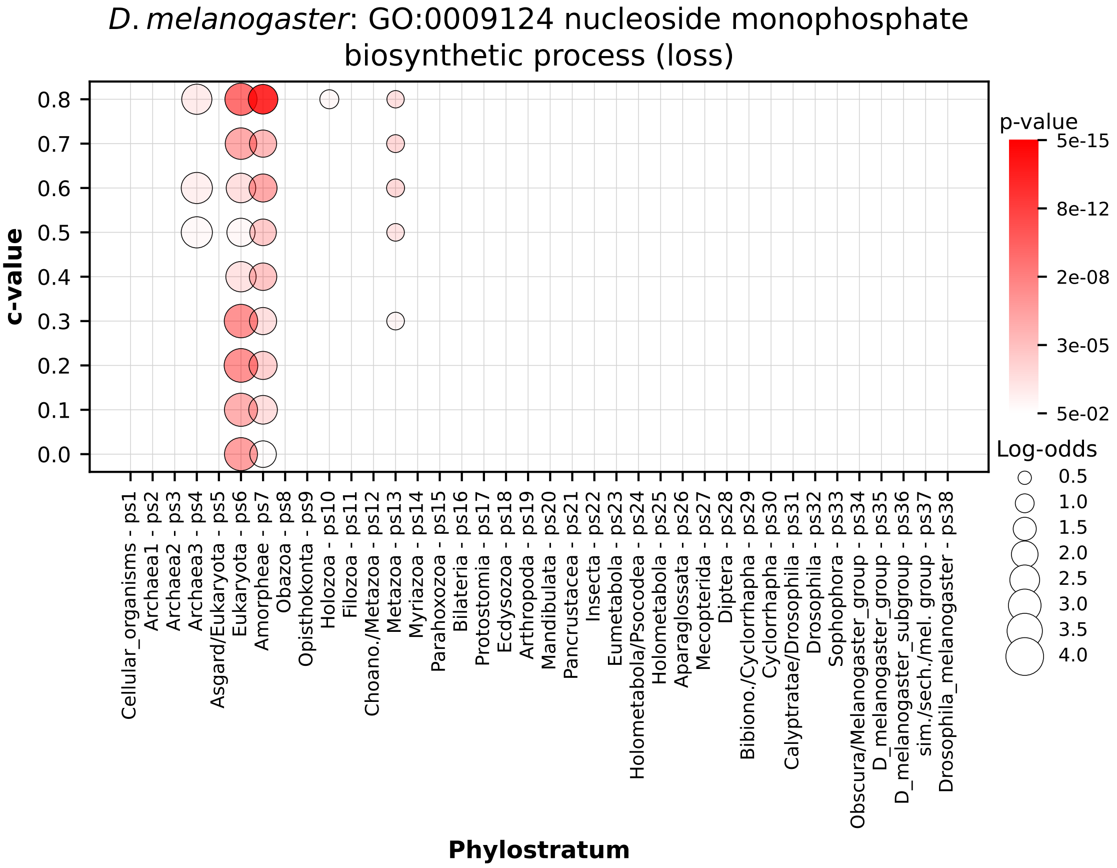
D. melanogaster: GO:0009124 nucleoside monophosphate biosynthetic process (loss)
c-value
0.8
0.7
0.6
0.5
0.4
0.3
0.2
0.1
0.0
Phylostratum
Cellular_organisms - ps1
Archaea1 - ps2
Archaea2 - ps3
Archaea3 - ps4
Asgard/Eukaryota - ps5
Eukaryota - ps6
Amorpheae - ps7
Obazoa - ps8
Opisthokonta - ps9
Holozoa - ps10
Filozoa - ps11
Choano./Metazoa - ps12
Metazoa - ps13
Myriazoa - ps14
Parahoxozoa - ps15
Bilateria - ps16
Protostomia - ps17
Ecdysozoa - ps18
Arthropoda - ps19
Mandibulata - ps20
Pancrustacea - ps21
Insecta - ps22
Eumetabola - ps23
Holometabola/Psocodea - ps24
Holometabola - ps25
Aparaglossata - ps26
Mecopterida - ps27
Diptera - ps28
Bibiono./Cyclorrhapha - ps29
Cyclorrhapha - ps30
Calyptratae/Drosophila - ps31
Drosophila - ps32
Sophophora - ps33
Obscura/Melanogaster_group - ps34
D_melanogaster_group - ps35
D_melanogaster_subgroup - ps36
sim./sech./mel. group - ps37
Drosophila_melanogaster - ps38
p-value
5e-15
8e-12
2e-08
3e-05
5e-02
Log-odds
0.5
1.0
1.5
2.0
2.5
3.0
3.5
4.0

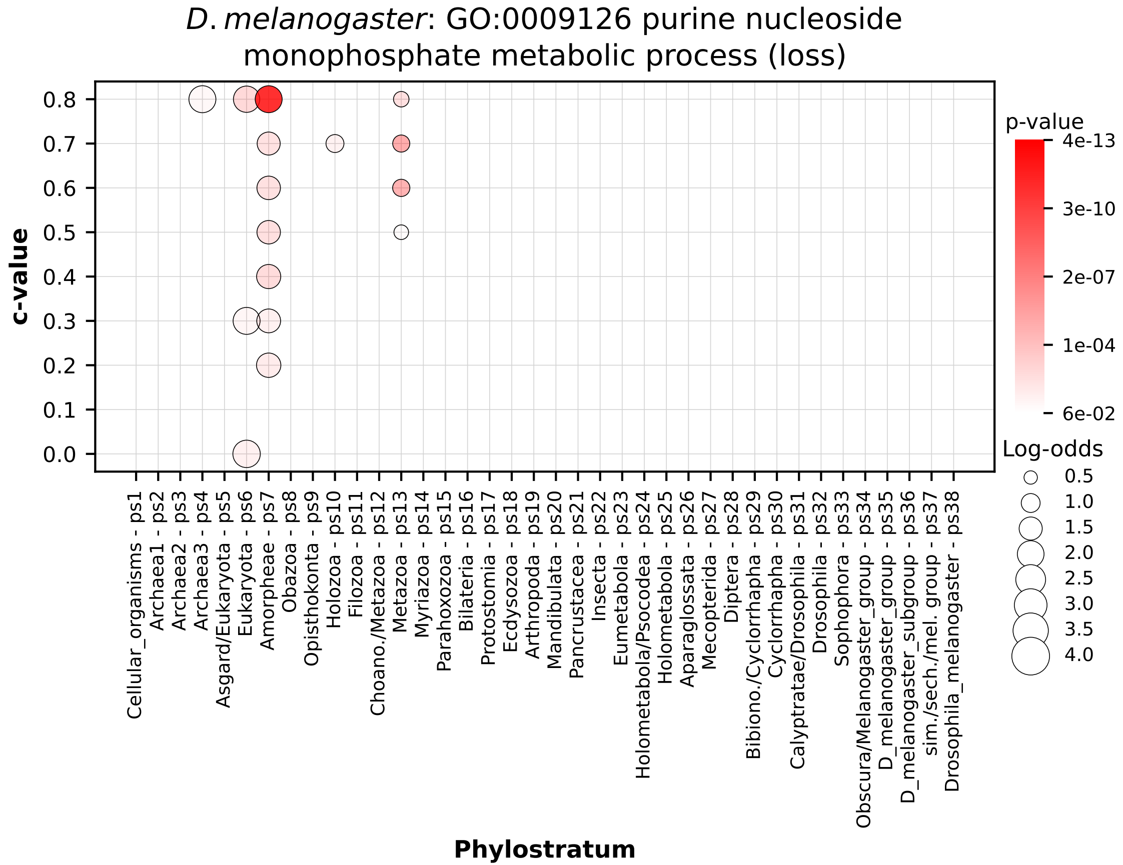

D. melanogaster: GO:0009126 purine nucleoside monophosphate metabolic process (loss)
c-value
0.8
0.7
0.6
0.5
0.4
0.3
0.2
0.1
0.0
p-value
4e-13
3e-10
2e-07
1e-04
6e-02
Log-odds
0.5
1.0
1.5
2.0
2.5
3.0
3.5
4.0
Cellular_organisms - ps1
Archaea1 - ps2
Archaea2 - ps3
Archaea3 - ps4
Asgard/Eukaryota - ps5
Eukaryota - ps6
Amorpheae - ps7
Obazoa - ps8
Opisthokonta - ps9
Holozoa - ps10
Filozoa - ps11
Choano./Metazoa - ps12
Metazoa - ps13
Myriazoa - ps14
Parahoxozoa - ps15
Bilateria - ps16
Protostomia - ps17
Ecdysozoa - ps18
Arthropoda - ps19
Mandibulata - ps20
Pancrustacea - ps21
Insecta - ps22
Eumetabola - ps23
Holometabola/Psocodea - ps24
Holometabola - ps25
Aparaglossata - ps26
Mecopterida - ps27
Diptera - ps28
Bibiono./Cyclorrhapha - ps29
Cyclorrhapha - ps30
Calyptratae/Drosophila - ps31
Drosophila - ps32
Sophophora - ps33
Obscura/Melanogaster_group - ps34
D_melanogaster_group - ps35
D_melanogaster_subgroup - ps36
sim./sech./mel. group - ps37
Drosophila_melanogaster - ps38
Phylostratum

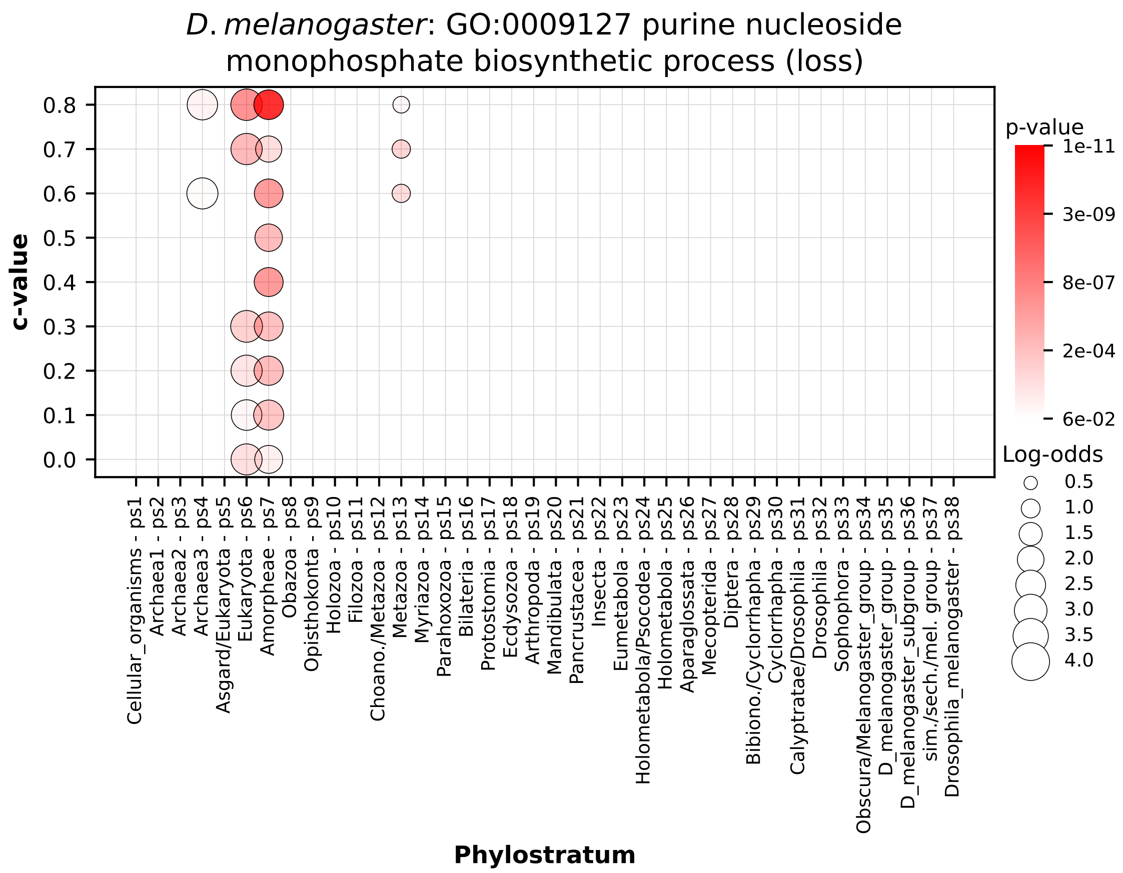
D. melanogaster: GO:0009127 purine nucleoside monophosphate biosynthetic process (loss)
c-value
0.8
0.7
0.6
0.5
0.4
0.3
0.2
0.1
0.0
p-value
1e-11
3e-09
8e-07
2e-04
6e-02
Log-odds
0.5
1.0
1.5
2.0
2.5
3.0
3.5
4.0
Cellular_organisms - ps1
Archaea1 - ps2
Archaea2 - ps3
Archaea3 - ps4
Asgard/Eukaryota - ps5
Eukaryota - ps6
Amorpheae - ps7
Obazoa - ps8
Opisthokonta - ps9
Holozoa - ps10
Filozoa - ps11
Choano./Metazoa - ps12
Metazoa - ps13
Myriazoa - ps14
Parahoxozoa - ps15
Bilateria - ps16
Protostomia - ps17
Ecdysozoa - ps18
Arthropoda - ps19
Mandibulata - ps20
Pancrustacea - ps21
Insecta - ps22
Eumetabola - ps23
Holometabola/Psocodea - ps24
Holometabola - ps25
Aparaglossata - ps26
Mecopterida - ps27
Diptera - ps28
Bibiono./Cyclorrhapha - ps29
Cyclorrhapha - ps30
Calyptratae/Drosophila - ps31
Drosophila - ps32
Sophophora - ps33
Obscura/Melanogaster_group - ps34
D_melanogaster_group - ps35
D_melanogaster_subgroup - ps36
sim./sech./mel. group - ps37
Drosophila_melanogaster - ps38
Phylostratum

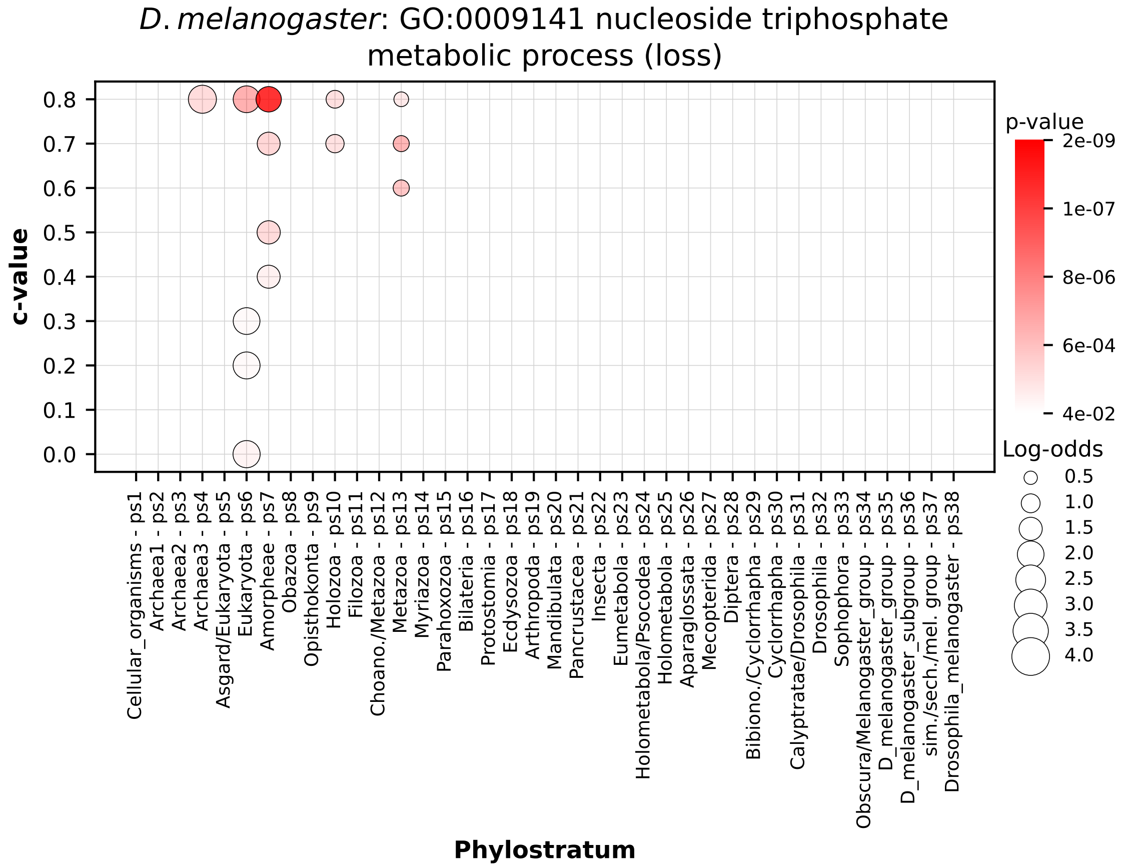

D. melanogaster: GO:0009141 nucleoside triphosphate metabolic process (loss)
c-value
0.8
0.7
0.6
0.5
0.4
0.3
0.2
0.1
0.0
p-value
2e-09
1e-07
8e-06
6e-04
4e-02
Log-odds
0.5
1.0
1.5
2.0
2.5
3.0
3.5
4.0
Cellular_organisms - ps1
Archaea1 - ps2
Archaea2 - ps3
Archaea3 - ps4
Asgard/Eukaryota - ps5
Eukaryota - ps6
Amorpheae - ps7
Obazoa - ps8
Opisthokonta - ps9
Holozoa - ps10
Filozoa - ps11
Choano./Metazoa - ps12
Metazoa - ps13
Myriazoa - ps14
Parahoxozoa - ps15
Bilateria - ps16
Protostomia - ps17
Ecdysozoa - ps18
Arthropoda - ps19
Mandibulata - ps20
Pancrustacea - ps21
Insecta - ps22
Eumetabola - ps23
Holometabola/Psocodea - ps24
Holometabola - ps25
Aparaglossata - ps26
Mecopterida - ps27
Diptera - ps28
Bibiono./Cyclorrhapha - ps29
Cyclorrhapha - ps30
Calyptratae/Drosophila - ps31
Drosophila - ps32
Sophophora - ps33
Obscura/Melanogaster_group - ps34
D_melanogaster_group - ps35
D_melanogaster_subgroup - ps36
sim./sech./mel. group - ps37
Drosophila_melanogaster - ps38
Phylostratum

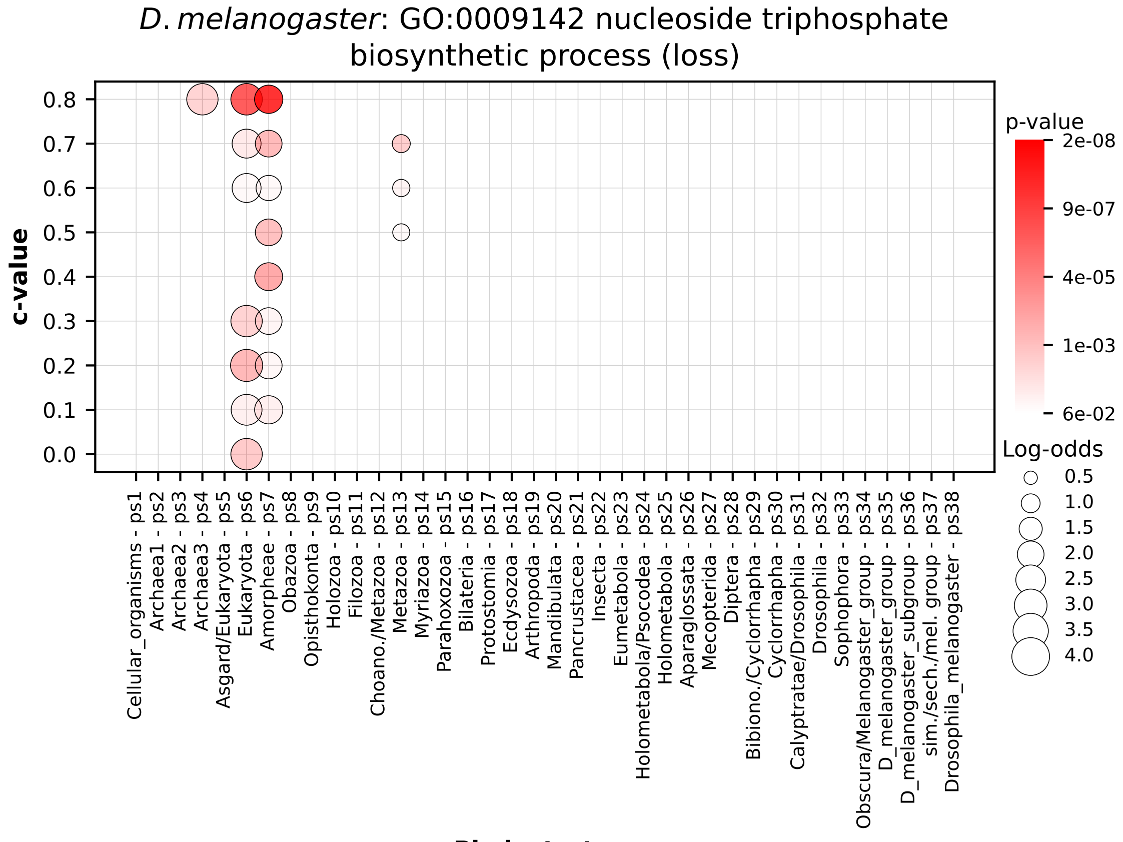
D. melanogaster: GO:0009142 nucleoside triphosphate biosynthetic process (loss)
c-value
0.8
0.7
0.6
0.5
0.4
0.3
0.2
0.1
0.0
p-value
2e-08
9e-07
4e-05
1e-03
6e-02
Log-odds
0.5
1.0
1.5
2.0
2.5
3.0
3.5
4.0
Cellular_organisms - ps1
Archaea1 - ps2
Archaea2 - ps3
Archaea3 - ps4
Asgard/Eukaryota - ps5
Eukaryota - ps6
Amorpheae - ps7
Obazoa - ps8
Opisthokonta - ps9
Holozoa - ps10
Filozoa - ps11
Choano./Metazoa - ps12
Metazoa - ps13
Myriazoa - ps14
Parahoxozoa - ps15
Bilateria - ps16
Protostomia - ps17
Ecdysozoa - ps18
Arthropoda - ps19
Mandibulata - ps20
Pancrustacea - ps21
Insecta - ps22
Eumetabola - ps23
Holometabola/Psocodea - ps24
Holometabola - ps25
Aparaglossata - ps26
Mecopterida - ps27
Diptera - ps28
Bibiono./Cyclorrhapha - ps29
Cyclorrhapha - ps30
Calyptratae/Drosophila - ps31
Drosophila - ps32
Sophophora - ps33
Obscura/Melanogaster_group - ps34
D_melanogaster_group - ps35
D_melanogaster_subgroup - ps36
sim./sech./mel. group - ps37
Drosophila_melanogaster - ps38
Phylostratum

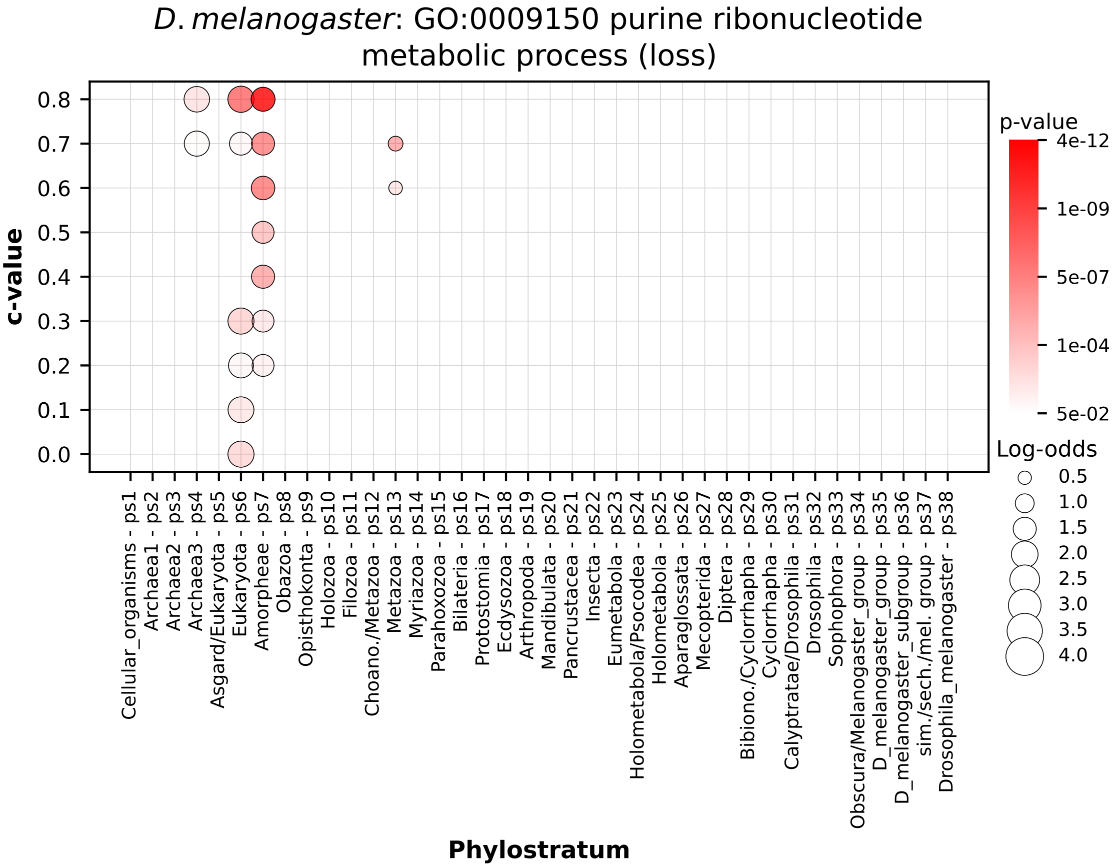
D. melanogaster: GO:0009150 purine ribonucleotide metabolic process (loss)
c-value
0.8
0.7
0.6
0.5
0.4
0.3
0.2
0.1
0.0
Phylostratum
Cellular_organisms - ps1
Archaea1 - ps2
Archaea2 - ps3
Archaea3 - ps4
Asgard/Eukaryota - ps5
Eukaryota - ps6
Amorpheae - ps7
Obazoa - ps8
Opisthokonta - ps9
Holozoa - ps10
Filozoa - ps11
Choano./Metazoa - ps12
Metazoa - ps13
Myriazoa - ps14
Parahoxozoa - ps15
Bilateria - ps16
Protostomia - ps17
Ecdysozoa - ps18
Arthropoda - ps19
Mandibulata - ps20
Pancrustacea - ps21
Insecta - ps22
Eumetabola - ps23
Holometabola/Psocodea - ps24
Holometabola - ps25
Aparaglossata - ps26
Mecopterida - ps27
Diptera - ps28
Bibiono./Cyclorrhapha - ps29
Cyclorrhapha - ps30
Calyptratae/Drosophila - ps31
Drosophila - ps32
Sophophora - ps33
Obscura/Melanogaster_group - ps34
D_melanogaster_group - ps35
D_melanogaster_subgroup - ps36
sim./sech./mel. group - ps37
Drosophila_melanogaster - ps38
p-value
4e-12
1e-09
5e-07
1e-04
5e-02
Log-odds
0.5
1.0
1.5
2.0
2.5
3.0
3.5
4.0

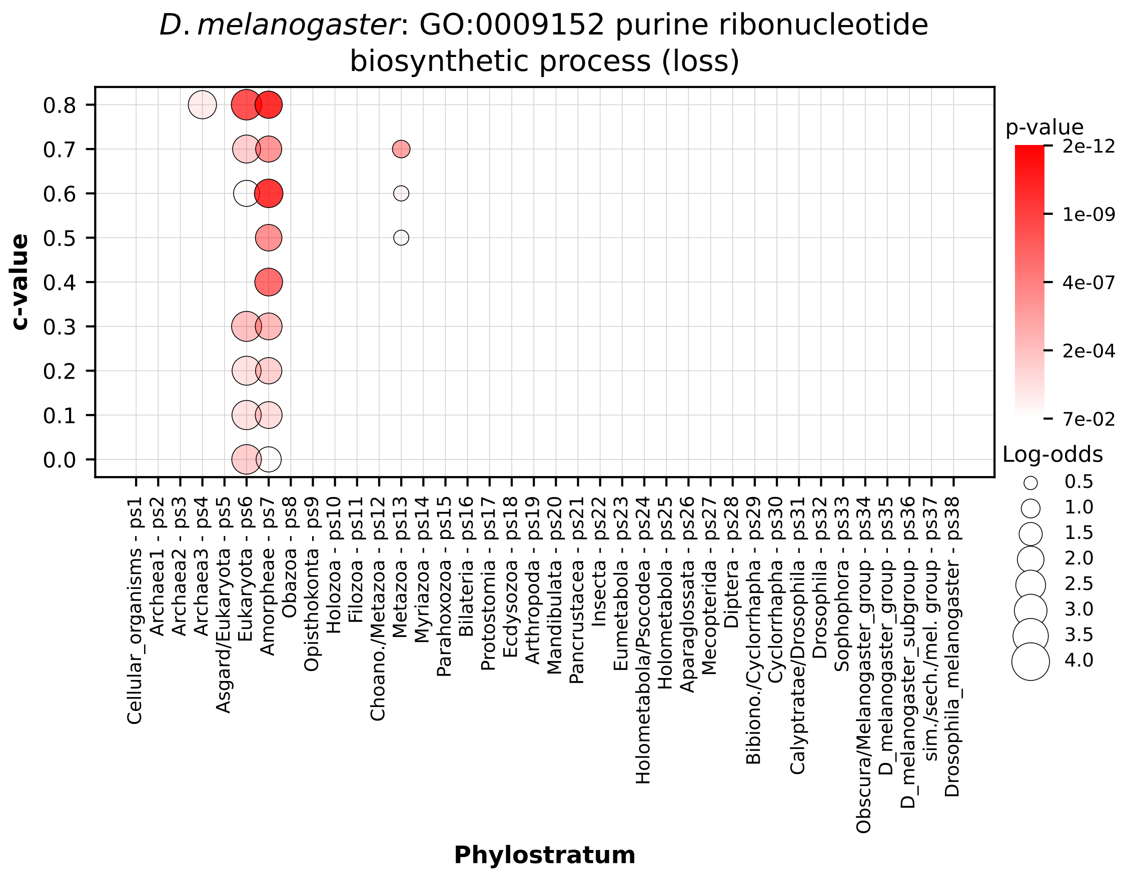
D. melanogaster: GO:0009152 purine ribonucleotide biosynthetic process (loss)
c-value
0.8
0.7
0.6
0.5
0.4
0.3
0.2
0.1
0.0
p-value
2e-12
1e-09
4e-07
2e-04
7e-02
Log-odds
0.5
1.0
1.5
2.0
2.5
3.0
3.5
4.0
Cellular_organisms - ps1
Archaea1 - ps2
Archaea2 - ps3
Archaea3 - ps4
Asgard/Eukaryota - ps5
Eukaryota - ps6
Amorpheae - ps7
Obazoa - ps8
Opisthokonta - ps9
Holozoa - ps10
Filozoa - ps11
Choano./Metazoa - ps12
Metazoa - ps13
Myriazoa - ps14
Parahoxozoa - ps15
Bilateria - ps16
Protostomia - ps17
Ecdysozoa - ps18
Arthropoda - ps19
Mandibulata - ps20
Pancrustacea - ps21
Insecta - ps22
Eumetabola - ps23
Holometabola/Psocodea - ps24
Holometabola - ps25
Aparaglossata - ps26
Mecopterida - ps27
Diptera - ps28
Bibiono./Cyclorrhapha - ps29
Cyclorrhapha - ps30
Calyptratae/Drosophila - ps31
Drosophila - ps32
Sophophora - ps33
Obscura/Melanogaster_group - ps34
D_melanogaster_group - ps35
D_melanogaster_subgroup - ps36
sim./sech./mel. group - ps37
Drosophila_melanogaster - ps38
Phylostratum

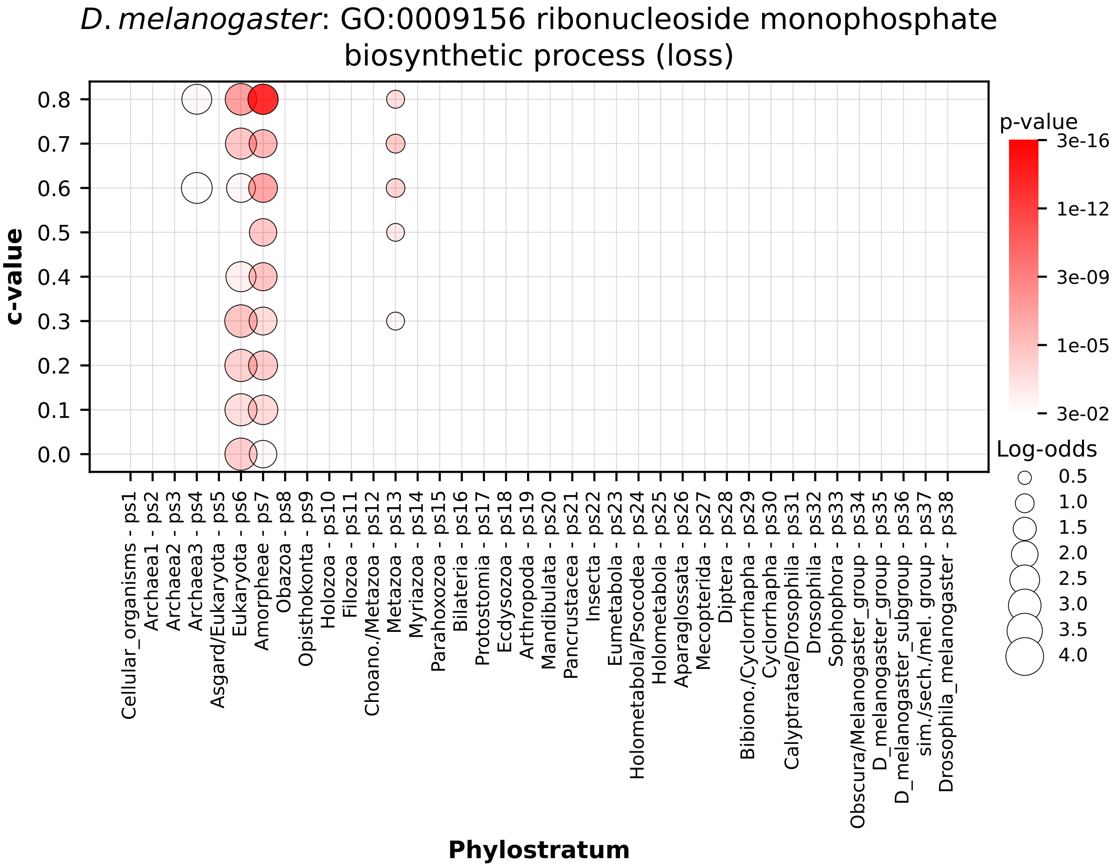

D. melanogaster: GO:0009156 ribonucleoside monophosphate biosynthetic process (loss)
c-value
0.8
0.7
0.6
0.5
0.4
0.3
0.2
0.1
0.0
p-value
3e-16
1e-12
3e-09
1e-05
3e-02
Log-odds
0.5
1.0
1.5
2.0
2.5
3.0
3.5
4.0
Cellular_organisms - ps1
Archaea1 - ps2
Archaea2 - ps3
Archaea3 - ps4
Asgard/Eukaryota - ps5
Eukaryota - ps6
Amorpheae - ps7
Obazoa - ps8
Opisthokonta - ps9
Holozoa - ps10
Filozoa - ps11
Choano./Metazoa - ps12
Metazoa - ps13
Myriazoa - ps14
Parahoxozoa - ps15
Bilateria - ps16
Protostomia - ps17
Ecdysozoa - ps18
Arthropoda - ps19
Mandibulata - ps20
Pancrustacea - ps21
Insecta - ps22
Eumetabola - ps23
Holometabola/Psocodea - ps24
Holometabola - ps25
Aparaglossata - ps26
Mecopterida - ps27
Diptera - ps28
Bibiono./Cyclorrhapha - ps29
Cyclorrhapha - ps30
Calyptratae/Drosophila - ps31
Drosophila - ps32
Sophophora - ps33
Obscura/Melanogaster_group - ps34
D_melanogaster_group - ps35
D_melanogaster_subgroup - ps36
sim./sech./mel. group - ps37
Drosophila_melanogaster - ps38
Phylostratum

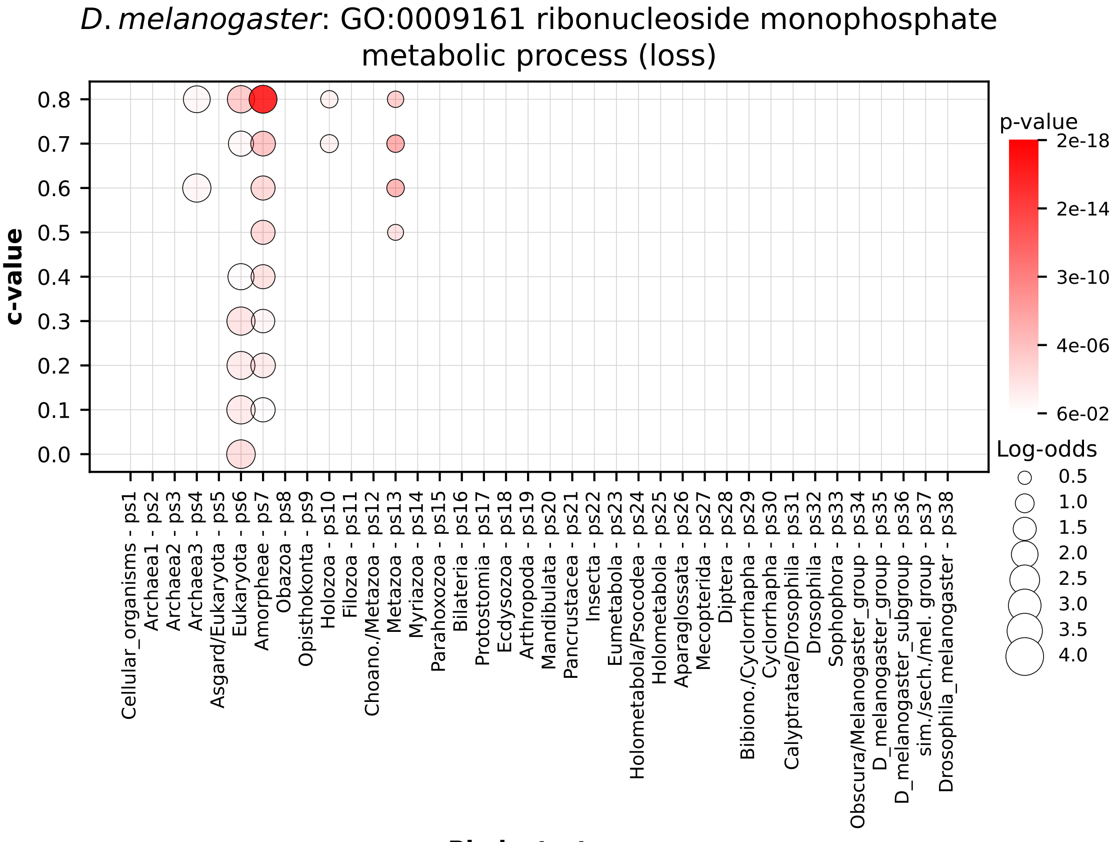

D. melanogaster: GO:0009161 ribonucleoside monophosphate metabolic process (loss)
c-value
0.8
0.7
0.6
0.5
0.4
0.3
0.2
0.1
0.0
Phylostratum
Cellular_organisms - ps1
Archaea1 - ps2
Archaea2 - ps3
Archaea3 - ps4
Asgard/Eukaryota - ps5
Eukaryota - ps6
Amorpheae - ps7
Obazoa - ps8
Opisthokonta - ps9
Holozoa - ps10
Filozoa - ps11
Choano./Metazoa - ps12
Metazoa - ps13
Myriazoa - ps14
Parahoxozoa - ps15
Bilateria - ps16
Protostomia - ps17
Ecdysozoa - ps18
Arthropoda - ps19
Mandibulata - ps20
Pancrustacea - ps21
Insecta - ps22
Eumetabola - ps23
Holometabola/Psocodea - ps24
Holometabola - ps25
Aparaglossata - ps26
Mecopterida - ps27
Diptera - ps28
Bibiono./Cyclorrhapha - ps29
Cyclorrhapha - ps30
Calyptratae/Drosophila - ps31
Drosophila - ps32
Sophophora - ps33
Obscura/Melanogaster_group - ps34
D_melanogaster_group - ps35
D_melanogaster_subgroup - ps36
sim./sech./mel. group - ps37
Drosophila_melanogaster - ps38
p-value
2e-18
2e-14
3e-10
4e-06
6e-02
Log-odds
0.5
1.0
1.5
2.0
2.5
3.0
3.5
4.0

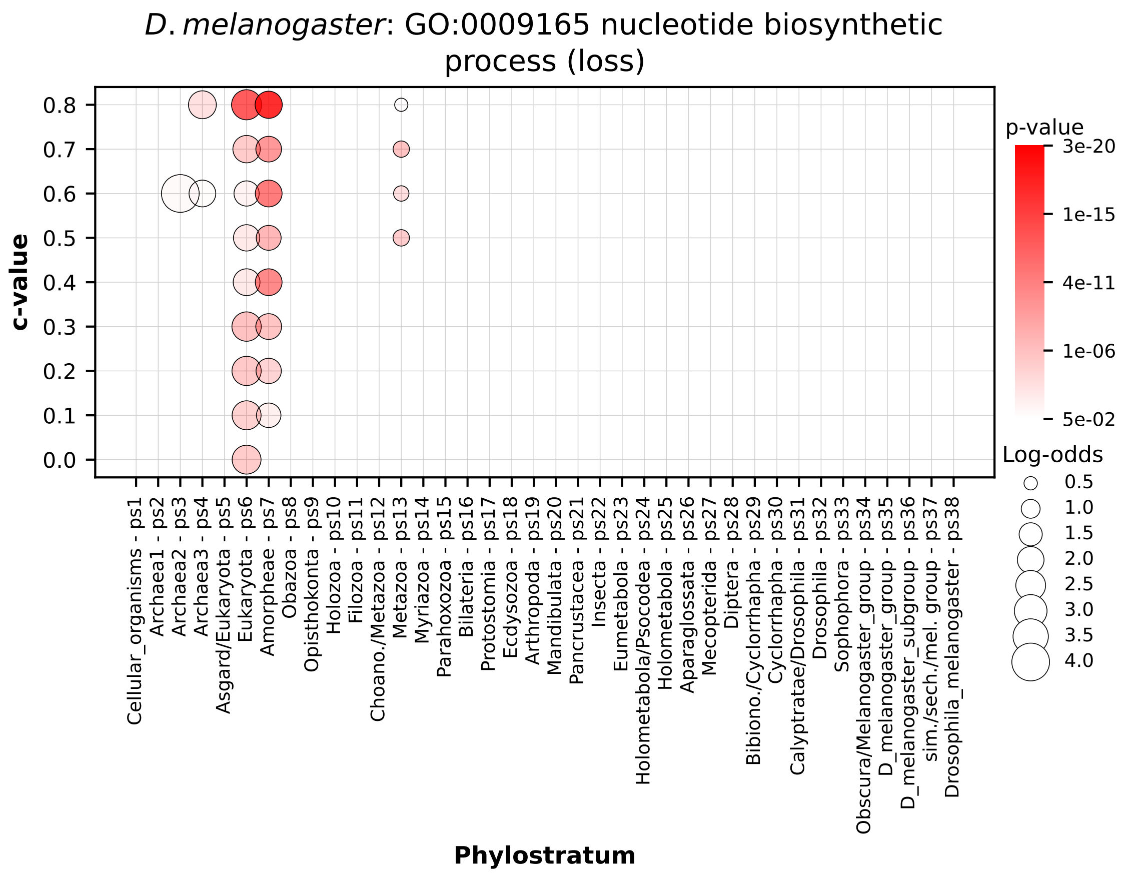

D. melanogaster: GO:0009165 nucleotide biosynthetic process (loss)
c-value
0.8
0.7
0.6
0.5
0.4
0.3
0.2
0.1
0.0
p-value
3e-20
1e-15
4e-11
1e-06
5e-02
Log-odds
0.5
1.0
1.5
2.0
2.5
3.0
3.5
4.0
Cellular_organisms - ps1
Archaea1 - ps2
Archaea2 - ps3
Archaea3 - ps4
Asgard/Eukaryota - ps5
Eukaryota - ps6
Amorpheae - ps7
Obazoa - ps8
Opisthokonta - ps9
Holozoa - ps10
Filozoa - ps11
Choano./Metazoa - ps12
Metazoa - ps13
Myriazoa - ps14
Parahoxozoa - ps15
Bilateria - ps16
Protostomia - ps17
Ecdysozoa - ps18
Arthropoda - ps19
Mandibulata - ps20
Pancrustacea - ps21
Insecta - ps22
Eumetabola - ps23
Holometabola/Psocodea - ps24
Holometabola - ps25
Aparaglossata - ps26
Mecopterida - ps27
Diptera - ps28
Bibiono./Cyclorrhapha - ps29
Cyclorrhapha - ps30
Calyptratae/Drosophila - ps31
Drosophila - ps32
Sophophora - ps33
Obscura/Melanogaster_group - ps34
D_melanogaster_group - ps35
D_melanogaster_subgroup - ps36
sim./sech./mel. group - ps37
Drosophila_melanogaster - ps38
Phylostratum

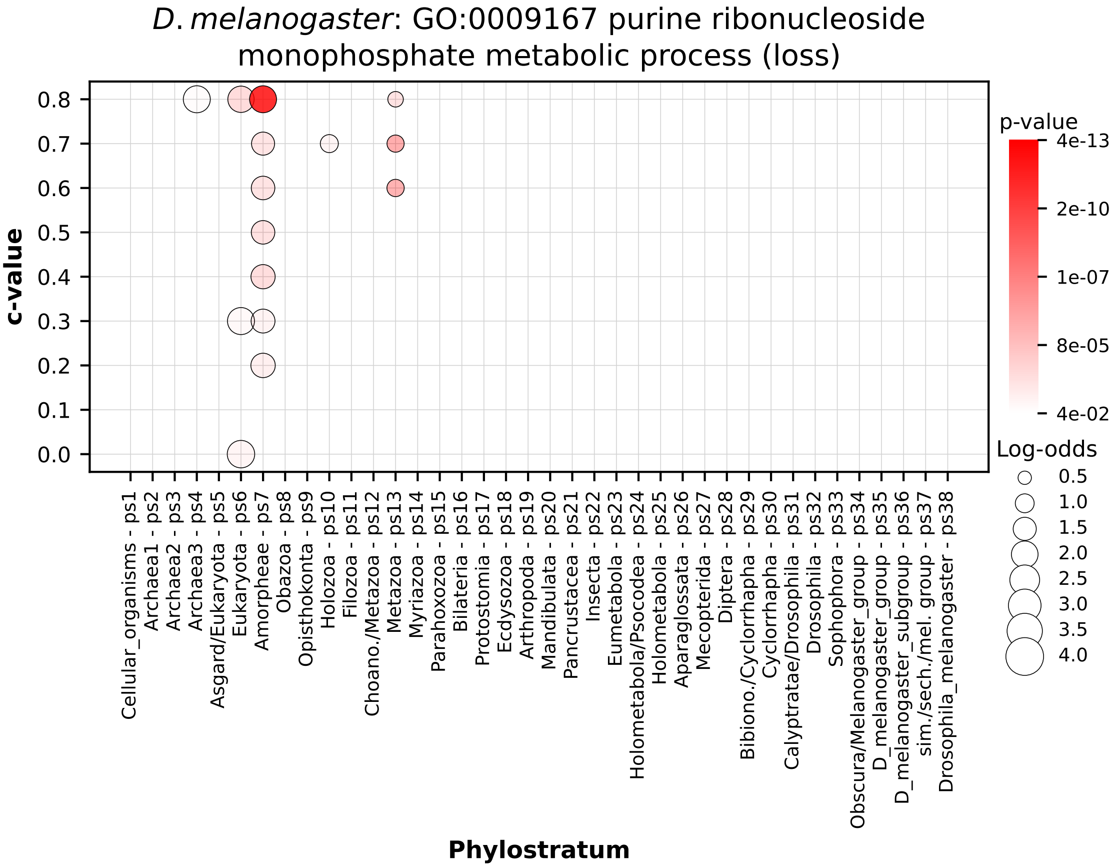

D. melanogaster: GO:0009167 purine ribonucleoside monophosphate metabolic process (loss)
c-value
0.8
0.7
0.6
0.5
0.4
0.3
0.2
0.1
0.0
p-value
4e-13
2e-10
1e-07
8e-05
4e-02
Log-odds
0.5
1.0
1.5
2.0
2.5
3.0
3.5
4.0
Cellular_organisms - ps1
Archaea1 - ps2
Archaea2 - ps3
Archaea3 - ps4
Asgard/Eukaryota - ps5
Eukaryota - ps6
Amorpheae - ps7
Obazoa - ps8
Opisthokonta - ps9
Holozoa - ps10
Filozoa - ps11
Choano./Metazoa - ps12
Metazoa - ps13
Myriazoa - ps14
Parahoxozoa - ps15
Bilateria - ps16
Protostomia - ps17
Ecdysozoa - ps18
Arthropoda - ps19
Mandibulata - ps20
Pancrustacea - ps21
Insecta - ps22
Eumetabola - ps23
Holometabola/Psocodea - ps24
Holometabola - ps25
Aparaglossata - ps26
Mecopterida - ps27
Diptera - ps28
Bibiono./Cyclorrhapha - ps29
Cyclorrhapha - ps30
Calyptratae/Drosophila - ps31
Drosophila - ps32
Sophophora - ps33
Obscura/Melanogaster_group - ps34
D_melanogaster_group - ps35
D_melanogaster_subgroup - ps36
sim./sech./mel. group - ps37
Drosophila_melanogaster - ps38
Phylostratum

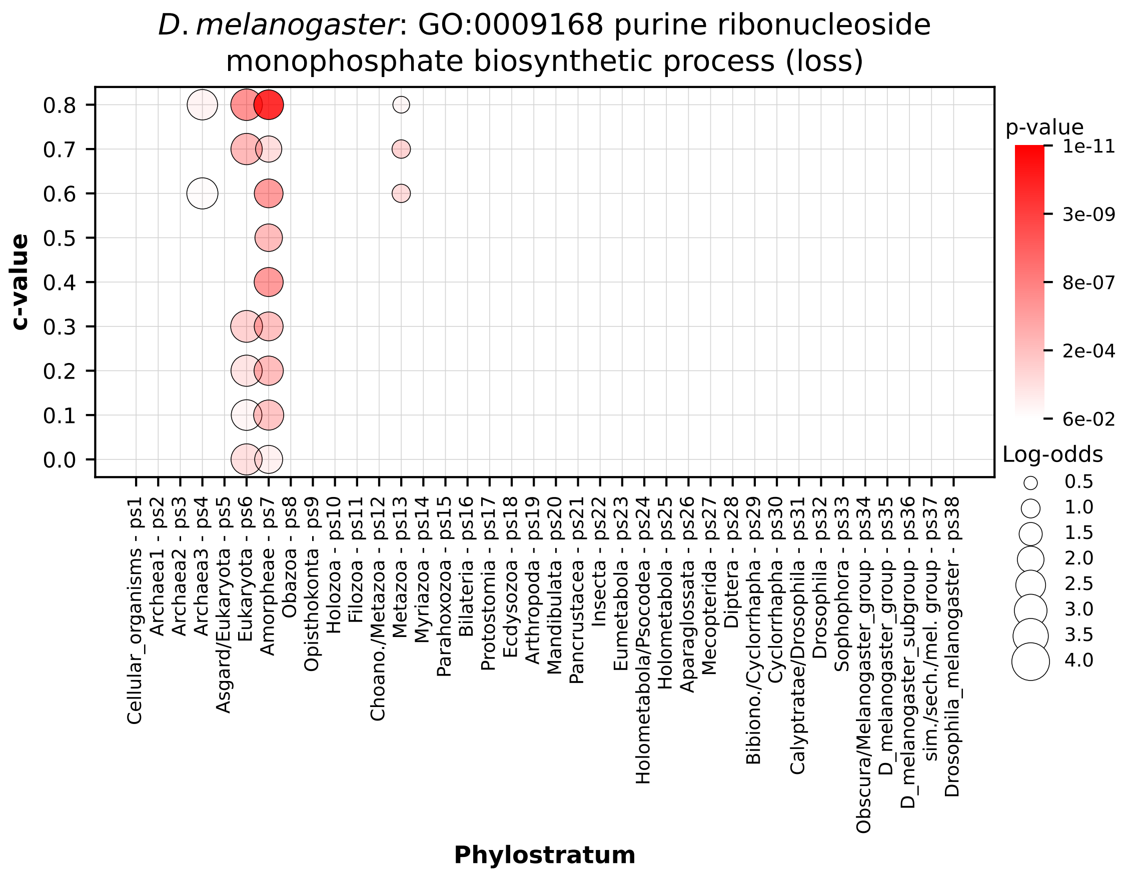

D. melanogaster: GO:0009168 purine ribonucleoside monophosphate biosynthetic process (loss)
c-value
0.8
0.7
0.6
0.5
0.4
0.3
0.2
0.1
0.0
p-value
1e-11
3e-09
8e-07
2e-04
6e-02
Log-odds
0.5
1.0
1.5
2.0
2.5
3.0
3.5
4.0
Cellular_organisms - ps1
Archaea1 - ps2
Archaea2 - ps3
Archaea3 - ps4
Asgard/Eukaryota - ps5
Eukaryota - ps6
Amorpheae - ps7
Obazoa - ps8
Opisthokonta - ps9
Holozoa - ps10
Filozoa - ps11
Choano./Metazoa - ps12
Metazoa - ps13
Myriazoa - ps14
Parahoxozoa - ps15
Bilateria - ps16
Protostomia - ps17
Ecdysozoa - ps18
Arthropoda - ps19
Mandibulata - ps20
Pancrustacea - ps21
Insecta - ps22
Eumetabola - ps23
Holometabola/Psocodea - ps24
Holometabola - ps25
Aparaglossata - ps26
Mecopterida - ps27
Diptera - ps28
Bibiono./Cyclorrhapha - ps29
Cyclorrhapha - ps30
Calyptratae/Drosophila - ps31
Drosophila - ps32
Sophophora - ps33
Obscura/Melanogaster_group - ps34
D_melanogaster_group - ps35
D_melanogaster_subgroup - ps36
sim./sech./mel. group - ps37
Drosophila_melanogaster - ps38
Phylostratum

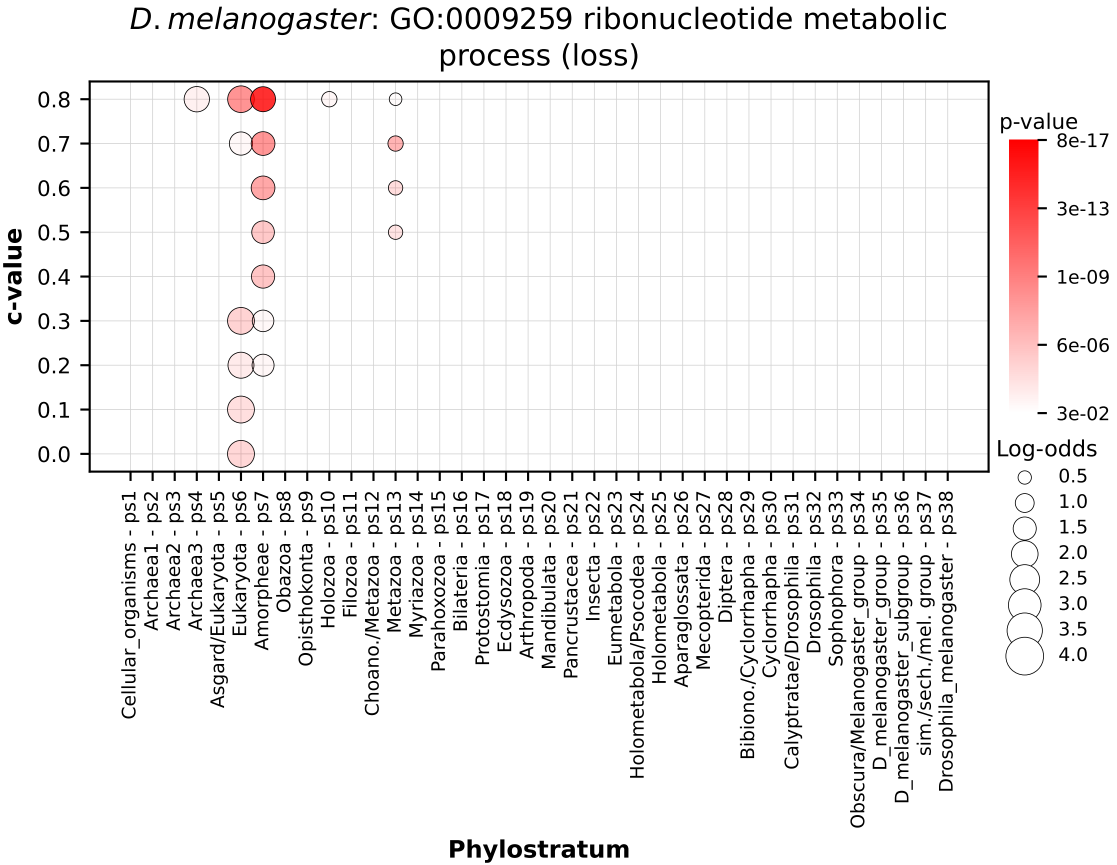

D. melanogaster: GO:0009259 ribonucleotide metabolic process (loss)
c-value
0.8
0.7
0.6
0.5
0.4
0.3
0.2
0.1
0.0
Phylostratum
Cellular_organisms - ps1
Archaea1 - ps2
Archaea2 - ps3
Archaea3 - ps4
Asgard/Eukaryota - ps5
Eukaryota - ps6
Amorpheae - ps7
Obazoa - ps8
Opisthokonta - ps9
Holozoa - ps10
Filozoa - ps11
Choano./Metazoa - ps12
Metazoa - ps13
Myriazoa - ps14
Parahoxozoa - ps15
Bilateria - ps16
Protostomia - ps17
Ecdysozoa - ps18
Arthropoda - ps19
Mandibulata - ps20
Pancrustacea - ps21
Insecta - ps22
Eumetabola - ps23
Holometabola/Psocodea - ps24
Holometabola - ps25
Aparaglossata - ps26
Mecopterida - ps27
Diptera - ps28
Bibiono./Cyclorrhapha - ps29
Cyclorrhapha - ps30
Calyptratae/Drosophila - ps31
Drosophila - ps32
Sophophora - ps33
Obscura/Melanogaster_group - ps34
D_melanogaster_group - ps35
D_melanogaster_subgroup - ps36
sim./sech./mel. group - ps37
Drosophila_melanogaster - ps38
p-value
8e-17
3e-13
1e-09
6e-06
3e-02
Log-odds
0.5
1.0
1.5
2.0
2.5
3.0
3.5
4.0

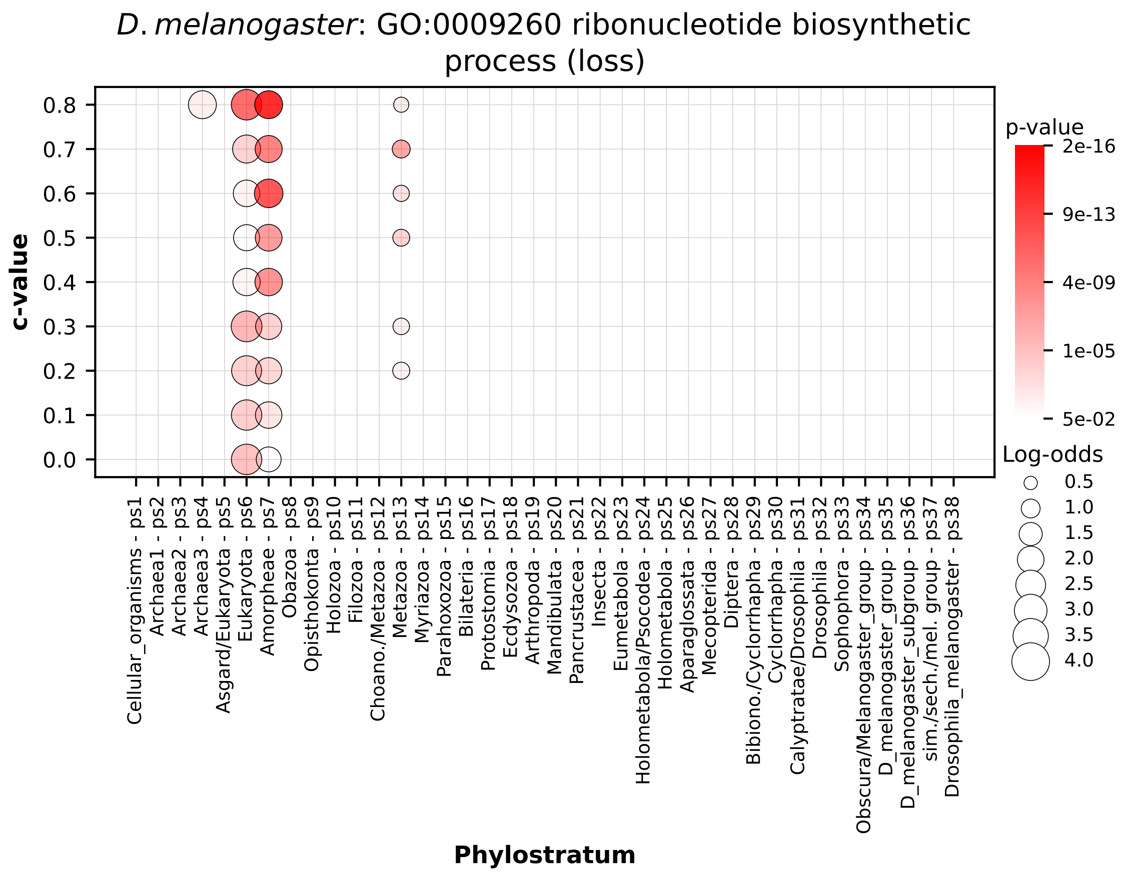

D. melanogaster: GO:0009260 ribonucleotide biosynthetic process (loss)
c-value
0.8
0.7
0.6
0.5
0.4
0.3
0.2
0.1
0.0
p-value
2e-16
9e-13
4e-09
1e-05
5e-02
Log-odds
0.5
1.0
1.5
2.0
2.5
3.0
3.5
4.0
Cellular_organisms - ps1
Archaea1 - ps2
Archaea2 - ps3
Archaea3 - ps4
Asgard/Eukaryota - ps5
Eukaryota - ps6
Amorpheae - ps7
Obazoa - ps8
Opisthokonta - ps9
Holozoa - ps10
Filozoa - ps11
Choano./Metazoa - ps12
Metazoa - ps13
Myriazoa - ps14
Parahoxozoa - ps15
Bilateria - ps16
Protostomia - ps17
Ecdysozoa - ps18
Arthropoda - ps19
Mandibulata - ps20
Pancrustacea - ps21
Insecta - ps22
Eumetabola - ps23
Holometabola/Psocodea - ps24
Holometabola - ps25
Aparaglossata - ps26
Mecopterida - ps27
Diptera - ps28
Bibiono./Cyclorrhapha - ps29
Cyclorrhapha - ps30
Calyptratae/Drosophila - ps31
Drosophila - ps32
Sophophora - ps33
Obscura/Melanogaster_group - ps34
D_melanogaster_group - ps35
D_melanogaster_subgroup - ps36
sim./sech./mel. group - ps37
Drosophila_melanogaster - ps38
Phylostratum

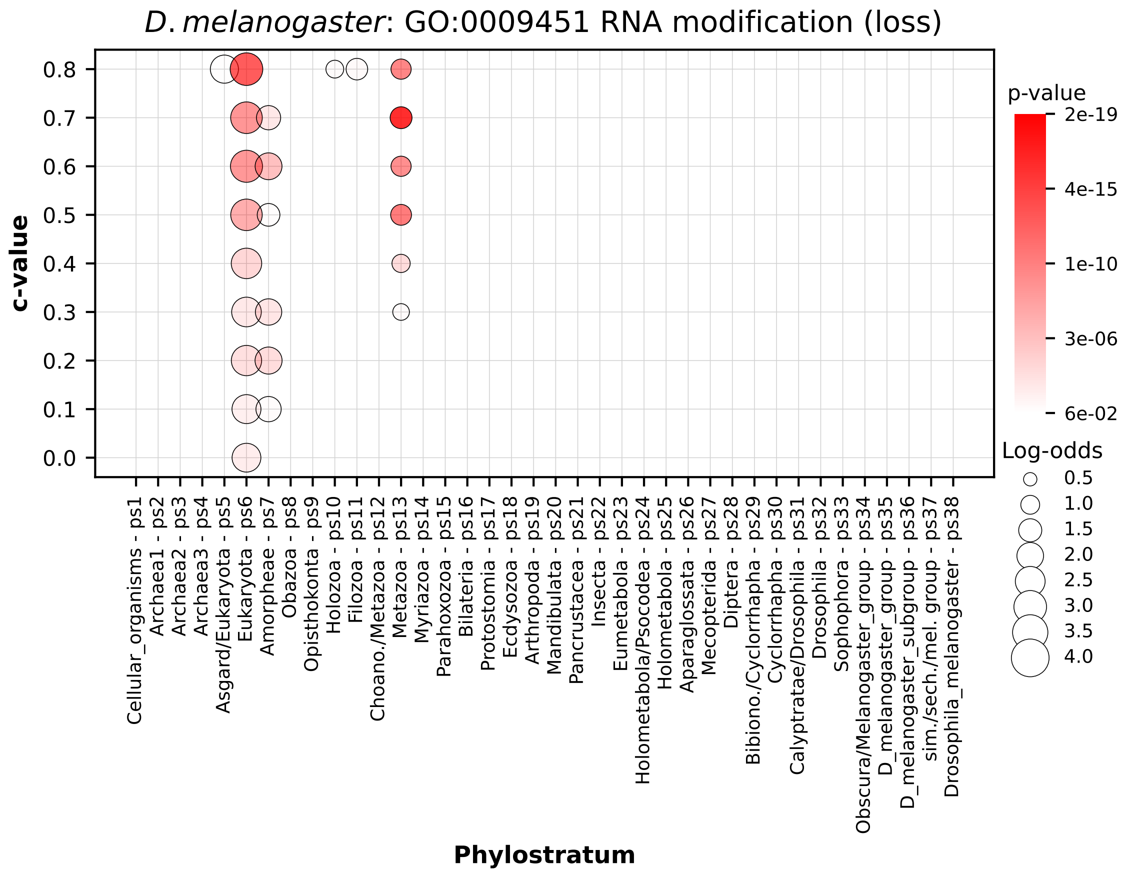

D. melanogaster: GO:0009451 RNA modification (loss)
c-value
0.0
0.1
0.2
0.3
0.4
0.5
0.6
0.7
0.8
Phylostratum
Cellular_organisms - ps1
Archaea1 - ps2
Archaea2 - ps3
Archaea3 - ps4
Asgard/Eukaryota - ps5
Eukaryota - ps6
Amorpheae - ps7
Obazoa - ps8
Opisthokonta - ps9
Holozoa - ps10
Filozoa - ps11
Choano./Metazoa - ps12
Metazoa - ps13
Myriazoa - ps14
Parahoxozoa - ps15
Bilateria - ps16
Protostomia - ps17
Ecdysozoa - ps18
Arthropoda - ps19
Mandibulata - ps20
Pancrustacea - ps21
Insecta - ps22
Eumetabola - ps23
Holometabola/Psocodea - ps24
Holometabola - ps25
Aparaglossata - ps26
Mecopterida - ps27
Diptera - ps28
Bibiono./Cyclorrhapha - ps29
Cyclorrhapha - ps30
Calyptratae/Drosophila - ps31
Drosophila - ps32
Sophophora - ps33
Obscura/Melanogaster_group - ps34
D_melanogaster_group - ps35
D_melanogaster_subgroup - ps36
sim./sech./mel. group - ps37
Drosophila_melanogaster - ps38
p-value
2e-19
4e-15
1e-10
3e-06
6e-02
Log-odds
0.5
1.0
1.5
2.0
2.5
3.0
3.5
4.0

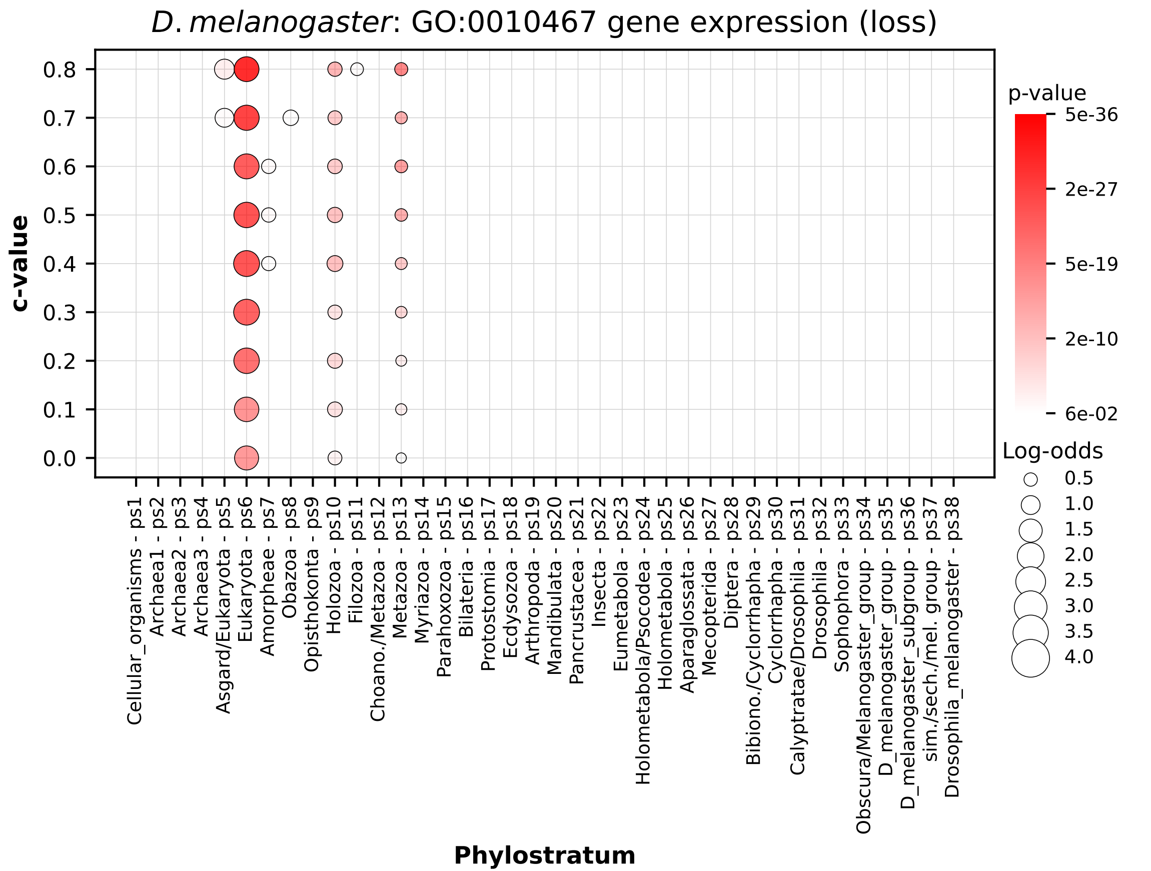

D. melanogaster: GO:0010467 gene expression (loss)
c-value
0.0
0.1
0.2
0.3
0.4
0.5
0.6
0.7
0.8
Phylostratum
Cellular_organisms - ps1
Archaea1 - ps2
Archaea2 - ps3
Archaea3 - ps4
Asgard/Eukaryota - ps5
Eukaryota - ps6
Amorpheae - ps7
Obazoa - ps8
Opisthokonta - ps9
Holozoa - ps10
Filozoa - ps11
Choano./Metazoa - ps12
Metazoa - ps13
Myriazoa - ps14
Parahoxozoa - ps15
Bilateria - ps16
Protostomia - ps17
Ecdysozoa - ps18
Arthropoda - ps19
Mandibulata - ps20
Pancrustacea - ps21
Insecta - ps22
Eumetabola - ps23
Holometabola/Psocodea - ps24
Holometabola - ps25
Aparaglossata - ps26
Mecopterida - ps27
Diptera - ps28
Bibiono./Cyclorrhapha - ps29
Cyclorrhapha - ps30
Calyptratae/Drosophila - ps31
Drosophila - ps32
Sophophora - ps33
Obscura/Melanogaster_group - ps34
D_melanogaster_group - ps35
D_melanogaster_subgroup - ps36
sim./sech./mel. group - ps37
Drosophila_melanogaster - ps38
p-value
5e-36
2e-27
5e-19
2e-10
6e-02
Log-odds
0.5
1.0
1.5
2.0
2.5
3.0
3.5
4.0

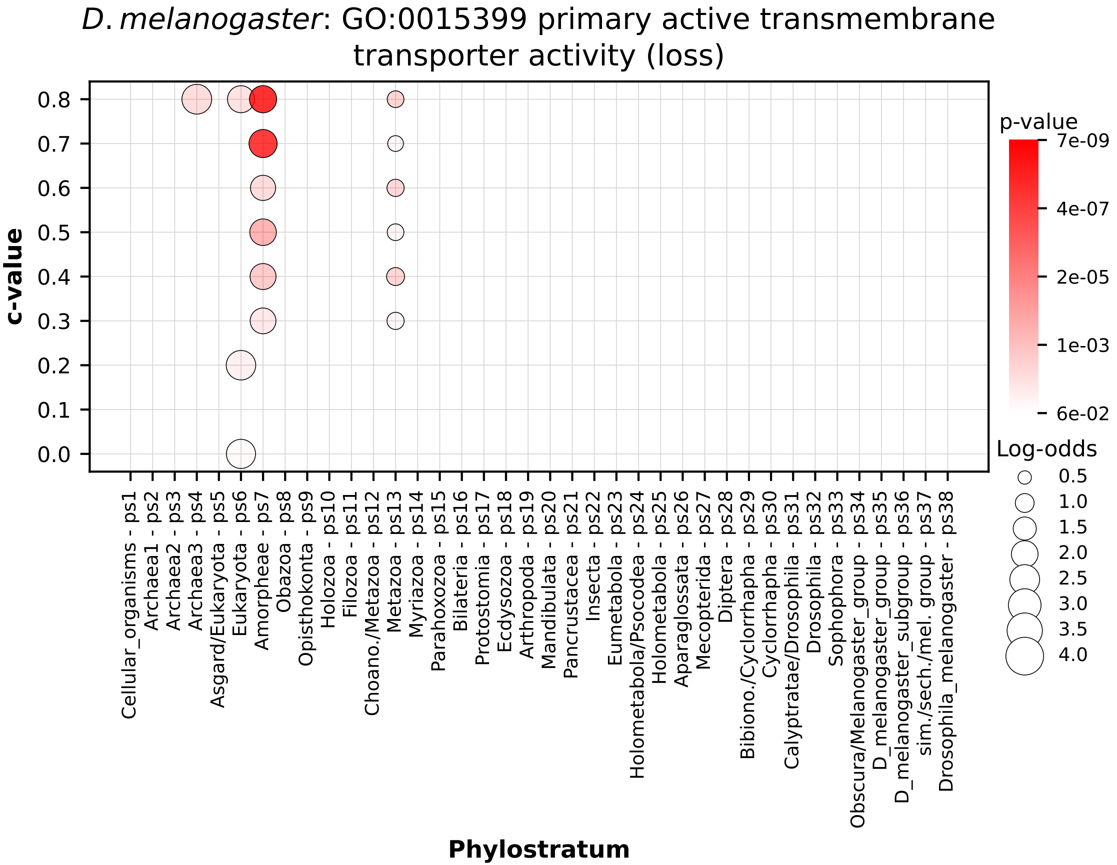

D. melanogaster: GO:0015399 primary active transmembrane transporter activity (loss)
c-value
0.8
0.7
0.6
0.5
0.4
0.3
0.2
0.1
0.0
p-value
7e-09
4e-07
2e-05
1e-03
6e-02
Log-odds
0.5
1.0
1.5
2.0
2.5
3.0
3.5
4.0
Cellular_organisms - ps1
Archaea1 - ps2
Archaea2 - ps3
Archaea3 - ps4
Asgard/Eukaryota - ps5
Eukaryota - ps6
Amorpheae - ps7
Obazoa - ps8
Opisthokonta - ps9
Holozoa - ps10
Filozoa - ps11
Choano./Metazoa - ps12
Metazoa - ps13
Myriazoa - ps14
Parahoxozoa - ps15
Bilateria - ps16
Protostomia - ps17
Ecdysozoa - ps18
Arthropoda - ps19
Mandibulata - ps20
Pancrustacea - ps21
Insecta - ps22
Eumetabola - ps23
Holometabola/Psocodea - ps24
Holometabola - ps25
Aparaglossata - ps26
Mecopterida - ps27
Diptera - ps28
Bibiono./Cyclorrhapha - ps29
Cyclorrhapha - ps30
Calyptratae/Drosophila - ps31
Drosophila - ps32
Sophophora - ps33
Obscura/Melanogaster_group - ps34
D_melanogaster_group - ps35
D_melanogaster_subgroup - ps36
sim./sech./mel. group - ps37
Drosophila_melanogaster - ps38
Phylostratum

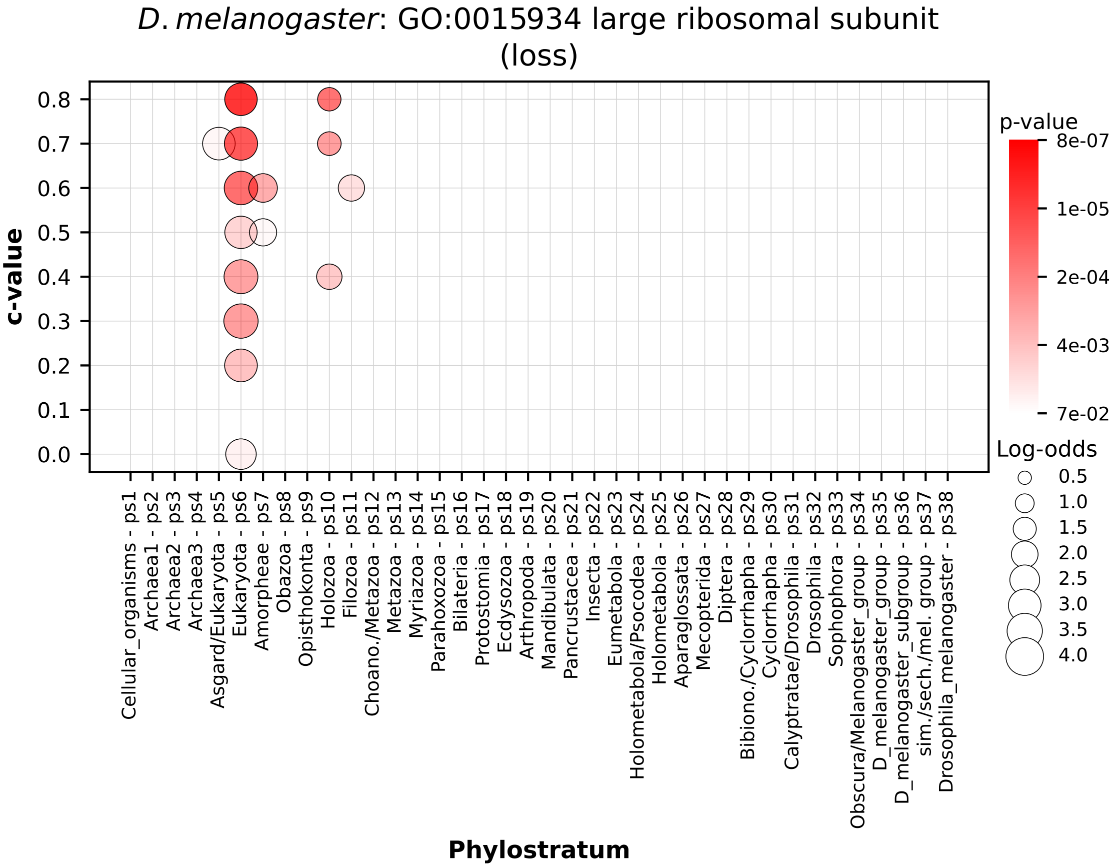

D. melanogaster: GO:0015934 large ribosomal subunit
(loss)
c-value
0.8
0.7
0.6
0.5
0.4
0.3
0.2
0.1
0.0
p-value
8e-07
1e-05
2e-04
4e-03
7e-02
Log-odds
0.5
1.0
1.5
2.0
2.5
3.0
3.5
4.0
Cellular_organisms - ps1
Archaea1 - ps2
Archaea2 - ps3
Archaea3 - ps4
Asgard/Eukaryota - ps5
Eukaryota - ps6
Amorpheae - ps7
Obazoa - ps8
Opisthokonta - ps9
Holozoa - ps10
Filozoa - ps11
Choano./Metazoa - ps12
Metazoa - ps13
Myriazoa - ps14
Parahoxozoa - ps15
Bilateria - ps16
Protostomia - ps17
Ecdysozoa - ps18
Arthropoda - ps19
Mandibulata - ps20
Pancrustacea - ps21
Insecta - ps22
Eumetabola - ps23
Holometabola/Psocodea - ps24
Holometabola - ps25
Aparaglossata - ps26
Mecopterida - ps27
Diptera - ps28
Bibiono./Cyclorrhapha - ps29
Cyclorrhapha - ps30
Calyptratae/Drosophila - ps31
Drosophila - ps32
Sophophora - ps33
Obscura/Melanogaster_group - ps34
D_melanogaster_group - ps35
D_melanogaster_subgroup - ps36
sim./sech./mel. group - ps37
Drosophila_melanogaster - ps38
Phylostratum

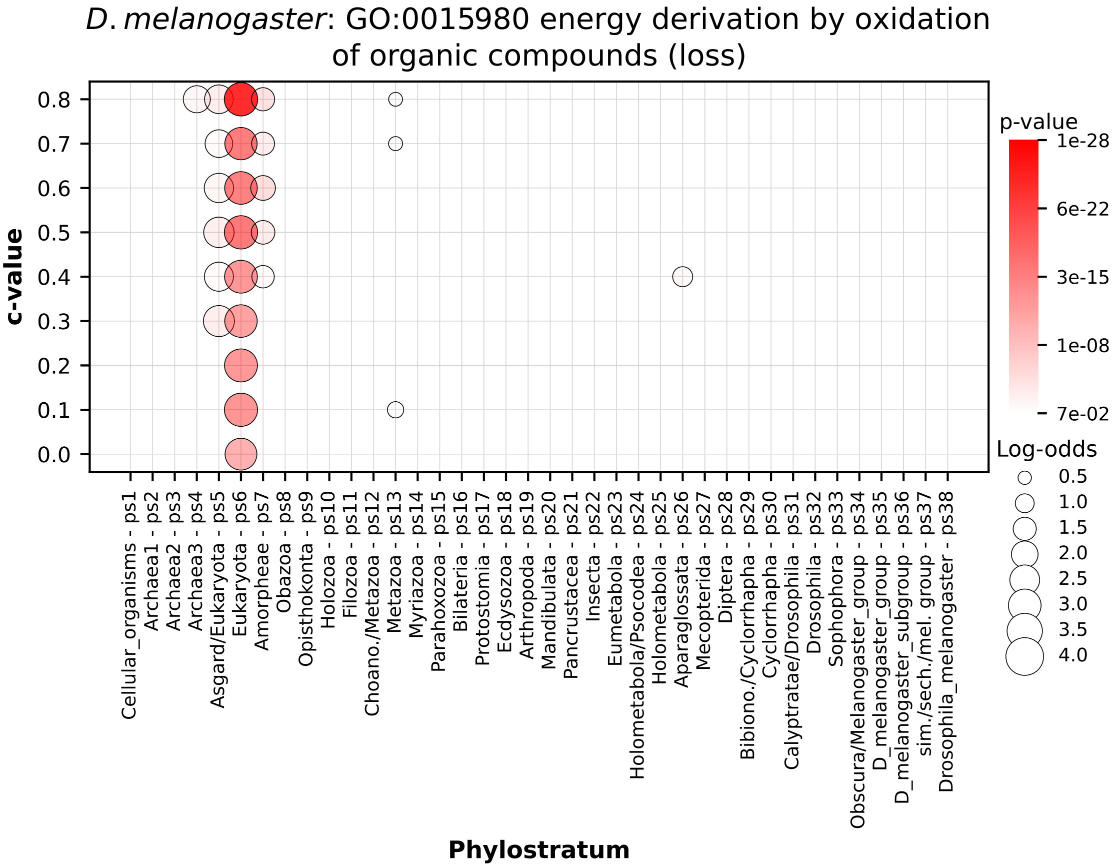

D. melanogaster: GO:0015980 energy derivation by oxidation of organic compounds (loss)
c-value
0.8
0.7
0.6
0.5
0.4
0.3
0.2
0.1
0.0
p-value
1e-28
6e-22
3e-15
1e-08
7e-02
Log-odds
0.5
1.0
1.5
2.0
2.5
3.0
3.5
4.0
Cellular_organisms - ps1
Archaea1 - ps2
Archaea2 - ps3
Archaea3 - ps4
Asgard/Eukaryota - ps5
Eukaryota - ps6
Amorpheae - ps7
Obazoa - ps8
Opisthokonta - ps9
Holozoa - ps10
Filozoa - ps11
Choano./Metazoa - ps12
Metazoa - ps13
Myriazoa - ps14
Parahoxozoa - ps15
Bilateria - ps16
Protostomia - ps17
Ecdysozoa - ps18
Arthropoda - ps19
Mandibulata - ps20
Pancrustacea - ps21
Insecta - ps22
Eumetabola - ps23
Holometabola/Psocodea - ps24
Holometabola - ps25
Aparaglossata - ps26
Mecopterida - ps27
Diptera - ps28
Bibiono./Cyclorrhapha - ps29
Cyclorrhapha - ps30
Calyptratae/Drosophila - ps31
Drosophila - ps32
Sophophora - ps33
Obscura/Melanogaster_group - ps34
D_melanogaster_group - ps35
D_melanogaster_subgroup - ps36
sim./sech./mel. group - ps37
Drosophila_melanogaster - ps38
Phylostratum

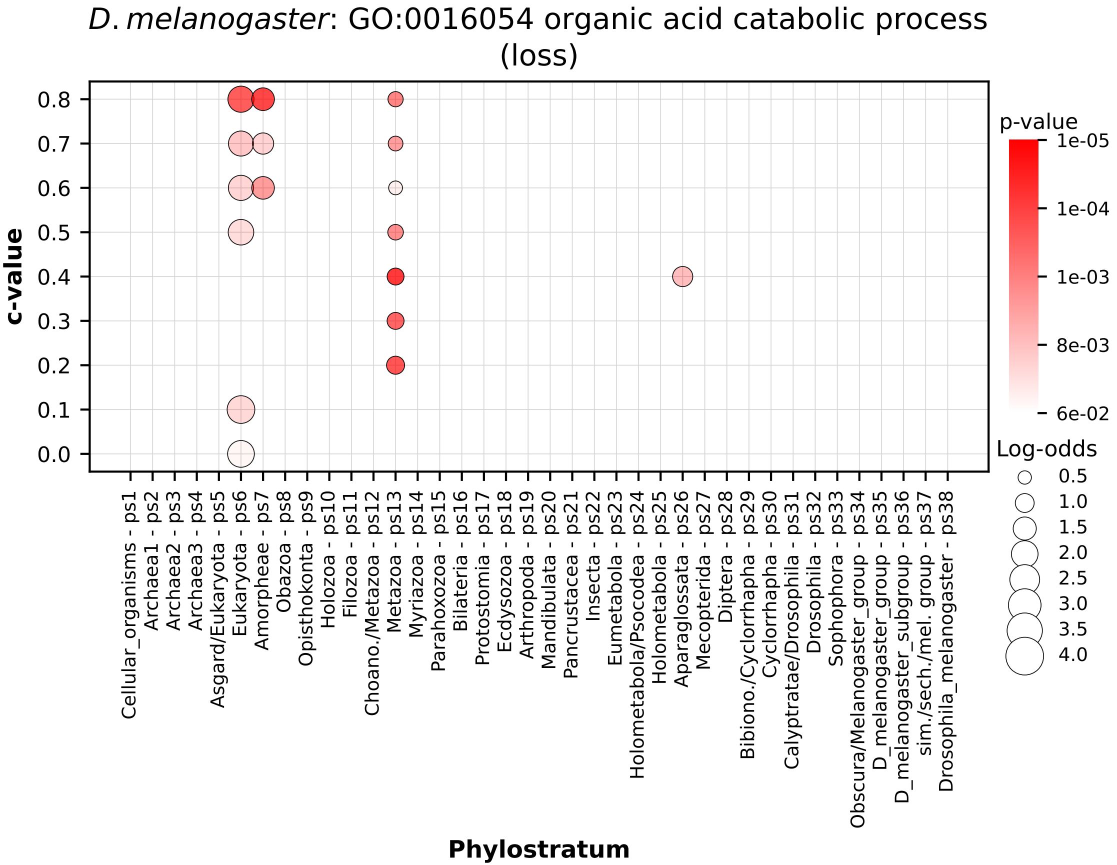

D. melanogaster: GO:0016054 organic acid catabolic process (loss)
c-value
0.8
0.7
0.6
0.5
0.4
0.3
0.2
0.1
0.0
Phylostratum
Cellular_organisms - ps1
Archaea1 - ps2
Archaea2 - ps3
Archaea3 - ps4
Asgard/Eukaryota - ps5
Eukaryota - ps6
Amorpheae - ps7
Obazoa - ps8
Opisthokonta - ps9
Holozoa - ps10
Filozoa - ps11
Choano./Metazoa - ps12
Metazoa - ps13
Myriazoa - ps14
Parahoxozoa - ps15
Bilateria - ps16
Protostomia - ps17
Ecdysozoa - ps18
Arthropoda - ps19
Mandibulata - ps20
Pancrustacea - ps21
Insecta - ps22
Eumetabola - ps23
Holometabola/Psocodea - ps24
Holometabola - ps25
Aparaglossata - ps26
Mecopterida - ps27
Diptera - ps28
Bibiono./Cyclorrhapha - ps29
Cyclorrhapha - ps30
Calyptratae/Drosophila - ps31
Drosophila - ps32
Sophophora - ps33
Obscura/Melanogaster_group - ps34
D_melanogaster_group - ps35
D_melanogaster_subgroup - ps36
sim./sech./mel. group - ps37
Drosophila_melanogaster - ps38
p-value
1e-05
1e-04
1e-03
8e-03
6e-02
Log-odds
0.5
1.0
1.5
2.0
2.5
3.0
3.5
4.0

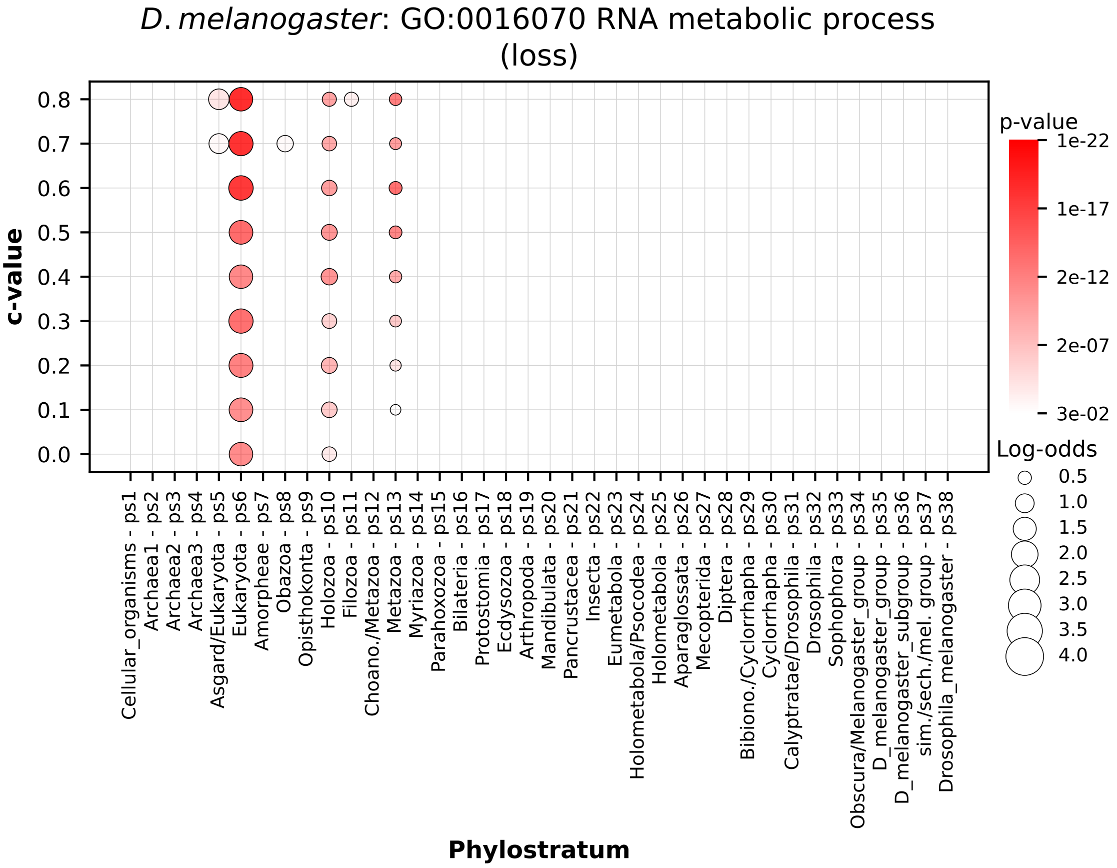

D. melanogaster: GO:0016070 RNA metabolic process (loss)
c-value
0.8
0.7
0.6
0.5
0.4
0.3
0.2
0.1
0.0
p-value
1e-22
1e-17
2e-12
2e-07
3e-02
Log-odds
0.5
1.0
1.5
2.0
2.5
3.0
3.5
4.0
Cellular_organisms - ps1
Archaea1 - ps2
Archaea2 - ps3
Archaea3 - ps4
Asgard/Eukaryota - ps5
Eukaryota - ps6
Amorpheae - ps7
Obazoa - ps8
Opisthokonta - ps9
Holozoa - ps10
Filozoa - ps11
Choano./Metazoa - ps12
Metazoa - ps13
Myriazoa - ps14
Parahoxozoa - ps15
Bilateria - ps16
Protostomia - ps17
Ecdysozoa - ps18
Arthropoda - ps19
Mandibulata - ps20
Pancrustacea - ps21
Insecta - ps22
Eumetabola - ps23
Holometabola/Psocodea - ps24
Holometabola - ps25
Aparaglossata - ps26
Mecopterida - ps27
Diptera - ps28
Bibiono./Cyclorrhapha - ps29
Cyclorrhapha - ps30
Calyptratae/Drosophila - ps31
Drosophila - ps32
Sophophora - ps33
Obscura/Melanogaster_group - ps34
D_melanogaster_group - ps35
D_melanogaster_subgroup - ps36
sim./sech./mel. group - ps37
Drosophila_melanogaster - ps38
Phylostratum

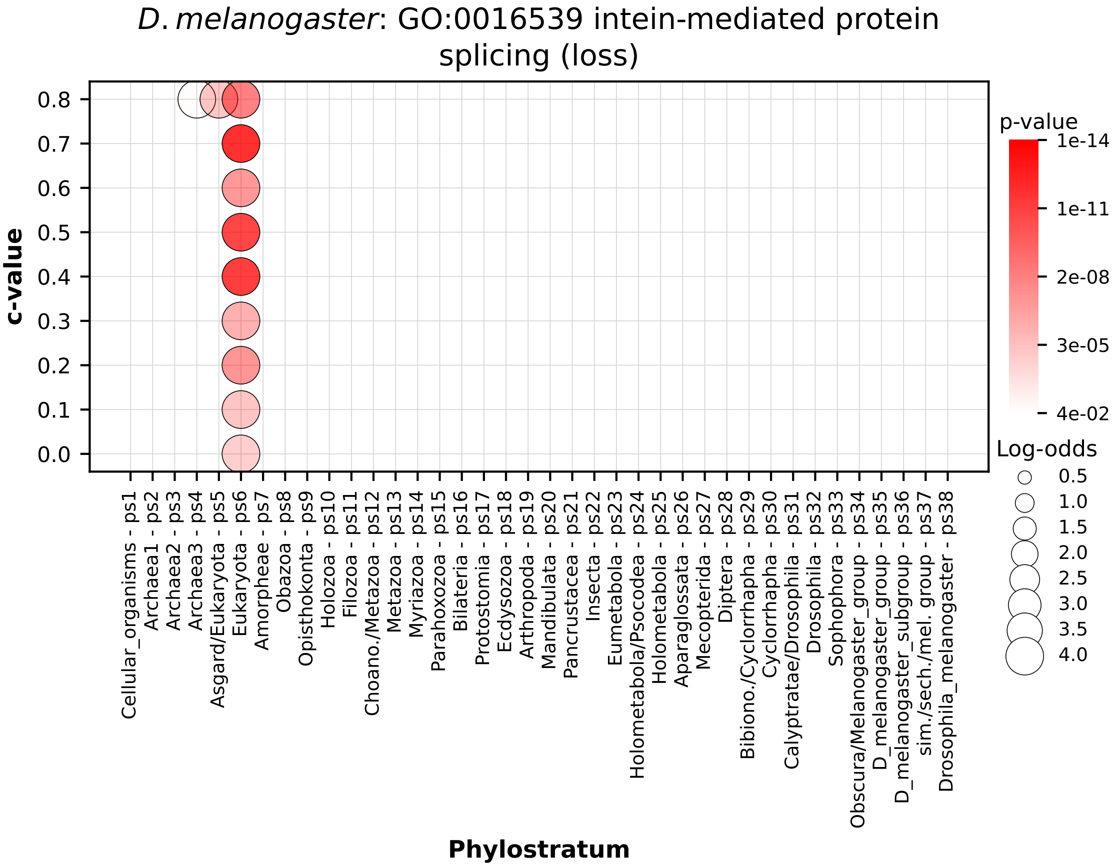

D. melanogaster: GO:0016539 intein-mediated protein splicing (loss)
c-value
0.8
0.7
0.6
0.5
0.4
0.3
0.2
0.1
0.0
Phylostratum
Cellular_organisms - ps1
Archaea1 - ps2
Archaea2 - ps3
Archaea3 - ps4
Asgard/Eukaryota - ps5
Eukaryota - ps6
Amorpheae - ps7
Obazoa - ps8
Opisthokonta - ps9
Holozoa - ps10
Filozoa - ps11
Choano./Metazoa - ps12
Metazoa - ps13
Myriazoa - ps14
Parahoxozoa - ps15
Bilateria - ps16
Protostomia - ps17
Ecdysozoa - ps18
Arthropoda - ps19
Mandibulata - ps20
Pancrustacea - ps21
Insecta - ps22
Eumetabola - ps23
Holometabola/Psocodea - ps24
Holometabola - ps25
Aparaglossata - ps26
Mecopterida - ps27
Diptera - ps28
Bibiono./Cyclorrhapha - ps29
Cyclorrhapha - ps30
Calyptratae/Drosophila - ps31
Drosophila - ps32
Sophophora - ps33
Obscura/Melanogaster_group - ps34
D_melanogaster_group - ps35
D_melanogaster_subgroup - ps36
sim./sech./mel. group - ps37
Drosophila_melanogaster - ps38
p-value
1e-14
1e-11
2e-08
3e-05
4e-02
Log-odds
0.5
1.0
1.5
2.0
2.5
3.0
3.5
4.0

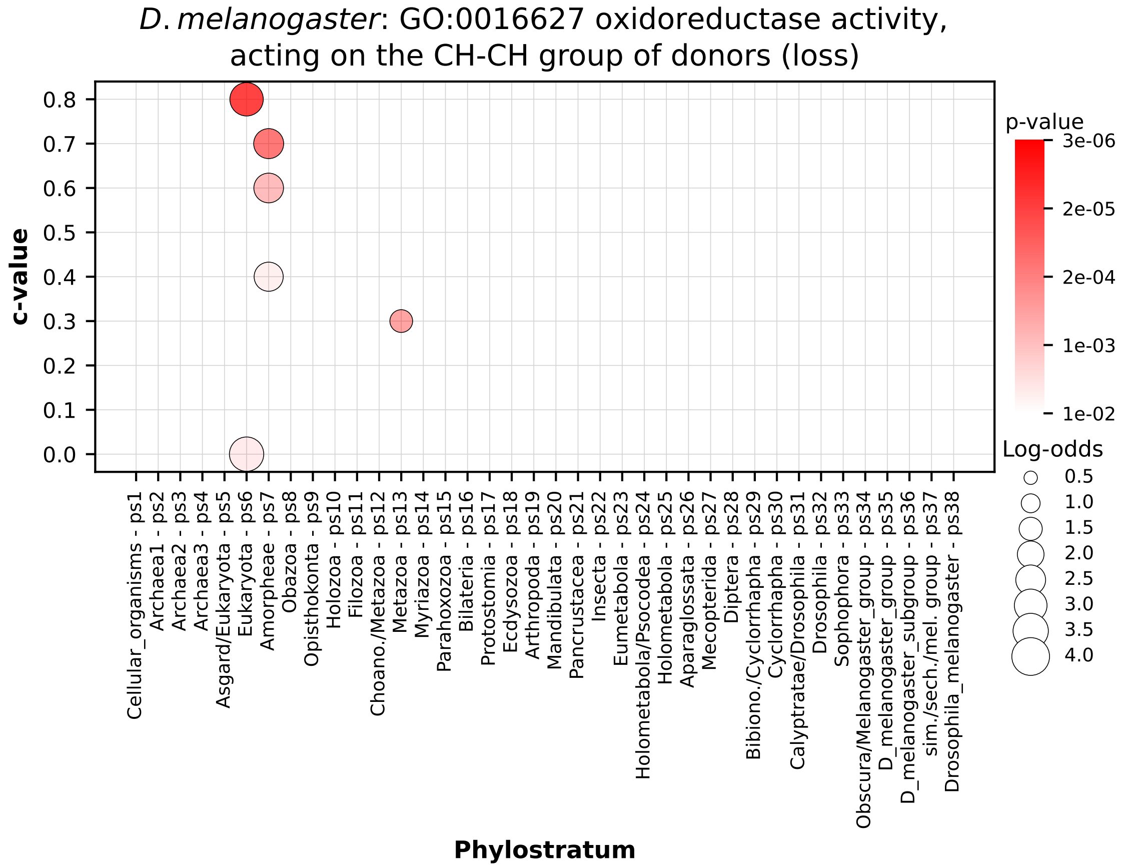
D. melanogaster: GO:0016627 oxidoreductase activity, acting on the CH-CH group of donors (loss)
c-value
0.8
0.7
0.6
0.5
0.4
0.3
0.2
0.1
0.0
p-value
3e-06
2e-05
2e-04
1e-03
1e-02
Log-odds
0.5
1.0
1.5
2.0
2.5
3.0
3.5
4.0
Cellular_organisms - ps1
Archaea1 - ps2
Archaea2 - ps3
Archaea3 - ps4
Asgard/Eukaryota - ps5
Eukaryota - ps6
Amorpheae - ps7
Obazoa - ps8
Opisthokonta - ps9
Holozoa - ps10
Filozoa - ps11
Choano./Metazoa - ps12
Metazoa - ps13
Myriazoa - ps14
Parahoxozoa - ps15
Bilateria - ps16
Protostomia - ps17
Ecdysozoa - ps18
Arthropoda - ps19
Mandibulata - ps20
Pancrustacea - ps21
Insecta - ps22
Eumetabola - ps23
Holometabola/Psocodea - ps24
Holometabola - ps25
Aparaglossata - ps26
Mecopterida - ps27
Diptera - ps28
Bibiono./Cyclorrhapha - ps29
Cyclorrhapha - ps30
Calyptratae/Drosophila - ps31
Drosophila - ps32
Sophophora - ps33
Obscura/Melanogaster_group - ps34
D_melanogaster_group - ps35
D_melanogaster_subgroup - ps36
sim./sech./mel. group - ps37
Drosophila_melanogaster - ps38
Phylostratum

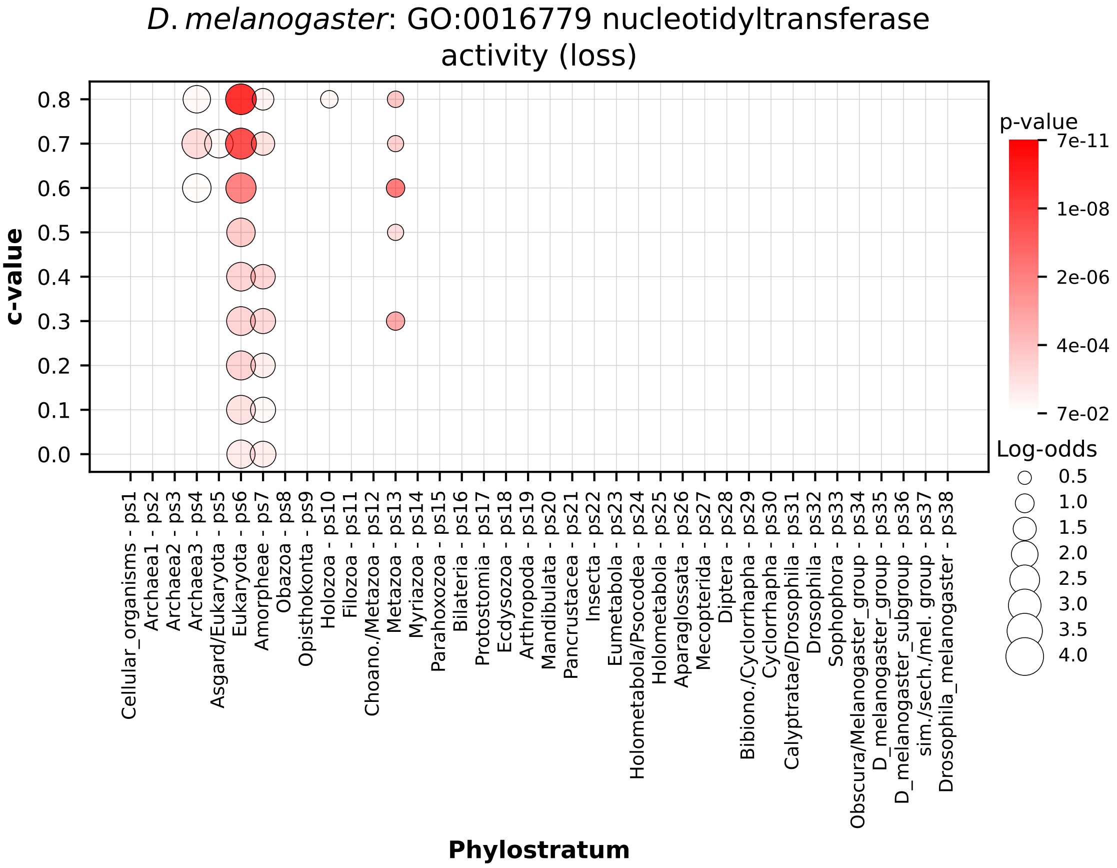

D. melanogaster: GO:0016779 nucleotidyltransferase activity (loss)
c-value
0.8
0.7
0.6
0.5
0.4
0.3
0.2
0.1
0.0
p-value
7e-11
1e-08
2e-06
4e-04
7e-02
Log-odds
0.5
1.0
1.5
2.0
2.5
3.0
3.5
4.0
Cellular_organisms - ps1
Archaea1 - ps2
Archaea2 - ps3
Archaea3 - ps4
Asgard/Eukaryota - ps5
Eukaryota - ps6
Amorpheae - ps7
Obazoa - ps8
Opisthokonta - ps9
Holozoa - ps10
Filozoa - ps11
Choano./Metazoa - ps12
Metazoa - ps13
Myriazoa - ps14
Parahoxozoa - ps15
Bilateria - ps16
Protostomia - ps17
Ecdysozoa - ps18
Arthropoda - ps19
Mandibulata - ps20
Pancrustacea - ps21
Insecta - ps22
Eumetabola - ps23
Holometabola/Psocodea - ps24
Holometabola - ps25
Aparaglossata - ps26
Mecopterida - ps27
Diptera - ps28
Bibiono./Cyclorrhapha - ps29
Cyclorrhapha - ps30
Calyptratae/Drosophila - ps31
Drosophila - ps32
Sophophora - ps33
Obscura/Melanogaster_group - ps34
D_melanogaster_group - ps35
D_melanogaster_subgroup - ps36
sim./sech./mel. group - ps37
Drosophila_melanogaster - ps38
Phylostratum

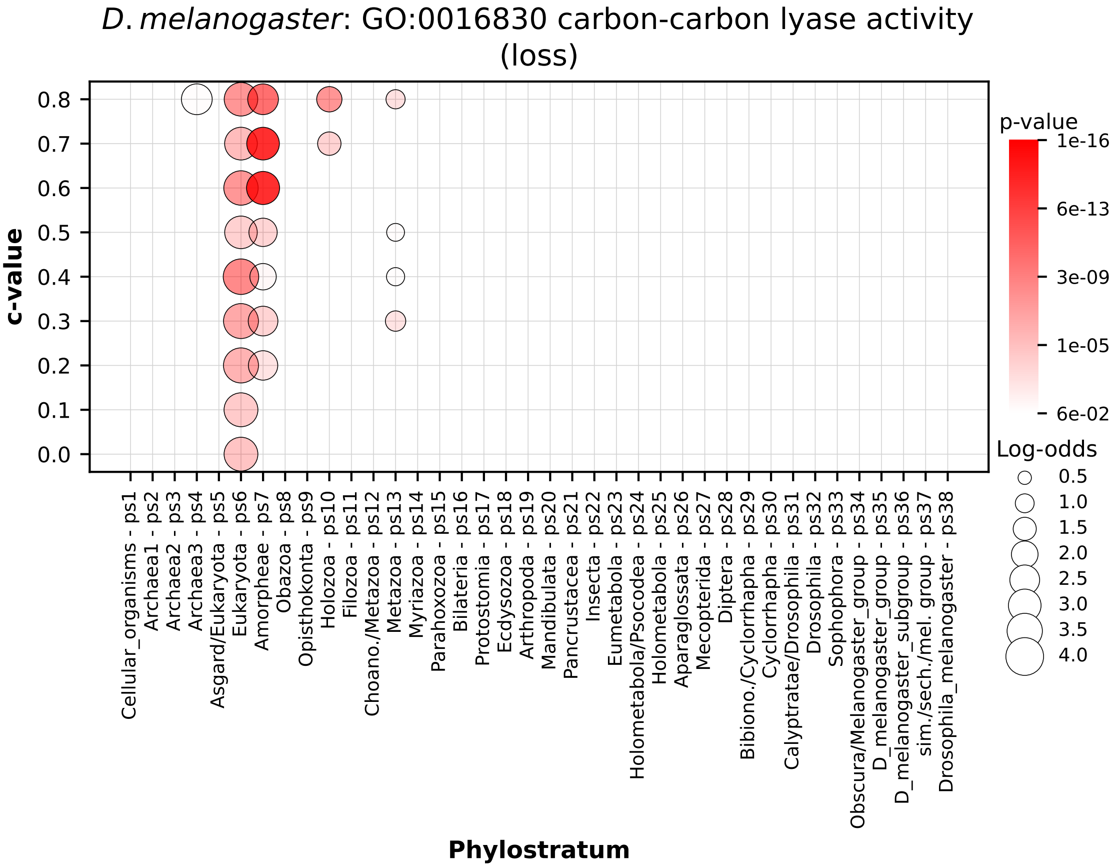

D. melanogaster: GO:0016830 carbon-carbon lyase activity (loss)
c-value
0.8
0.7
0.6
0.5
0.4
0.3
0.2
0.1
0.0
Phylostratum
Cellular_organisms - ps1
Archaea1 - ps2
Archaea2 - ps3
Archaea3 - ps4
Asgard/Eukaryota - ps5
Eukaryota - ps6
Amorpheae - ps7
Obazoa - ps8
Opisthokonta - ps9
Holozoa - ps10
Filozoa - ps11
Choano./Metazoa - ps12
Metazoa - ps13
Myriazoa - ps14
Parahoxozoa - ps15
Bilateria - ps16
Protostomia - ps17
Ecdysozoa - ps18
Arthropoda - ps19
Mandibulata - ps20
Pancrustacea - ps21
Insecta - ps22
Eumetabola - ps23
Holometabola/Psocodea - ps24
Holometabola - ps25
Aparaglossata - ps26
Mecopterida - ps27
Diptera - ps28
Bibiono./Cyclorrhapha - ps29
Cyclorrhapha - ps30
Calyptratae/Drosophila - ps31
Drosophila - ps32
Sophophora - ps33
Obscura/Melanogaster_group - ps34
D_melanogaster_group - ps35
D_melanogaster_subgroup - ps36
sim./sech./mel. group - ps37
Drosophila_melanogaster - ps38
p-value
1e-16
6e-13
3e-09
1e-05
6e-02
Log-odds
0.5
1.0
1.5
2.0
2.5
3.0
3.5
4.0

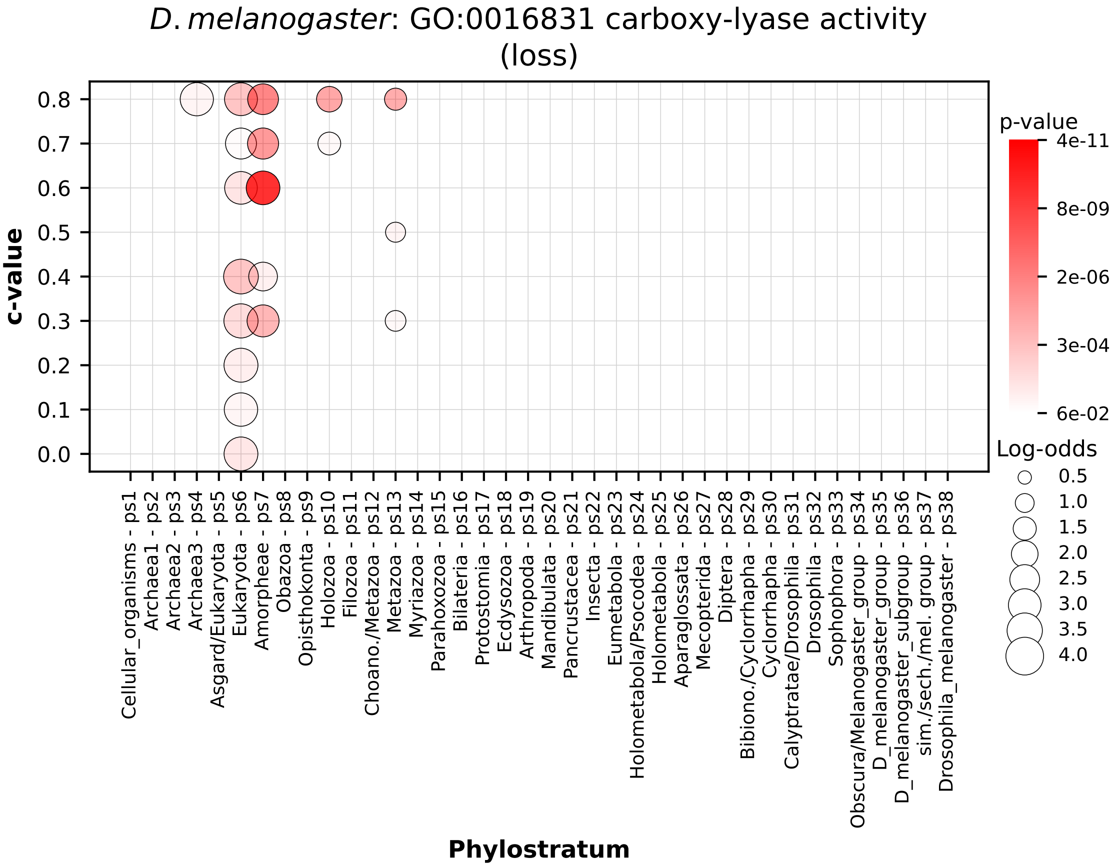

D. melanogaster: GO:0016831 carboxy-lyase activity (loss)
c-value
0.8
0.7
0.6
0.5
0.4
0.3
0.2
0.1
0.0
Phylostratum
Cellular_organisms - ps1
Archaea1 - ps2
Archaea2 - ps3
Archaea3 - ps4
Asgard/Eukaryota - ps5
Eukaryota - ps6
Amorpheae - ps7
Obazoa - ps8
Opisthokonta - ps9
Holozoa - ps10
Filozoa - ps11
Choano./Metazoa - ps12
Metazoa - ps13
Myriazoa - ps14
Parahoxozoa - ps15
Bilateria - ps16
Protostomia - ps17
Ecdysozoa - ps18
Arthropoda - ps19
Mandibulata - ps20
Pancrustacea - ps21
Insecta - ps22
Eumetabola - ps23
Holometabola/Psocodea - ps24
Holometabola - ps25
Aparaglossata - ps26
Mecopterida - ps27
Diptera - ps28
Bibiono./Cyclorrhapha - ps29
Cyclorrhapha - ps30
Calyptratae/Drosophila - ps31
Drosophila - ps32
Sophophora - ps33
Obscura/Melanogaster_group - ps34
D_melanogaster_group - ps35
D_melanogaster_subgroup - ps36
sim./sech./mel. group - ps37
Drosophila_melanogaster - ps38
p-value
4e-11
8e-09
2e-06
3e-04
6e-02
Log-odds
0.5
1.0
1.5
2.0
2.5
3.0
3.5
4.0

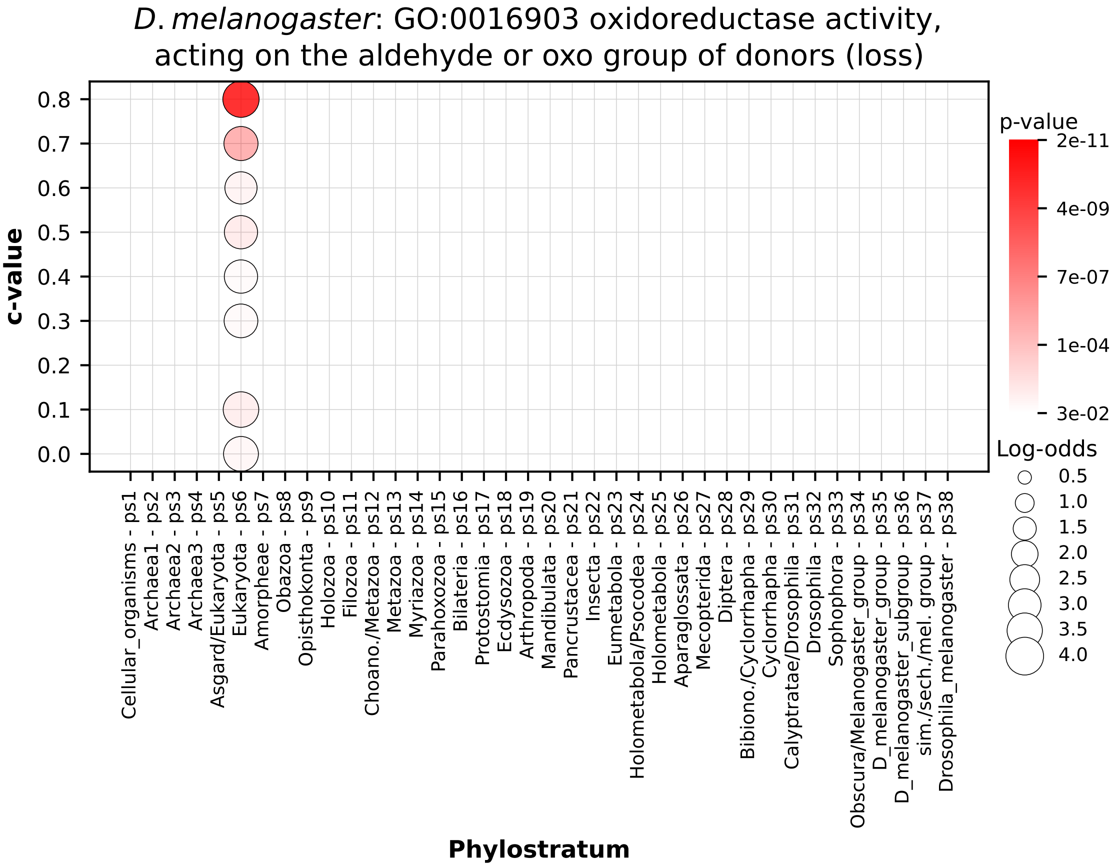

D. melanogaster: GO:0016903 oxidoreductase activity, acting on the aldehyde or oxo group of donors (loss)
c-value
0.8
0.7
0.6
0.5
0.4
0.3
0.2
0.1
0.0
p-value
2e-11
4e-09
7e-07
1e-04
3e-02
Log-odds
0.5
1.0
1.5
2.0
2.5
3.0
3.5
4.0
Cellular_organisms - ps1
Archaea1 - ps2
Archaea2 - ps3
Archaea3 - ps4
Asgard/Eukaryota - ps5
Eukaryota - ps6
Amorpheae - ps7
Obazoa - ps8
Opisthokonta - ps9
Holozoa - ps10
Filozoa - ps11
Choano./Metazoa - ps12
Metazoa - ps13
Myriazoa - ps14
Parahoxozoa - ps15
Bilateria - ps16
Protostomia - ps17
Ecdysozoa - ps18
Arthropoda - ps19
Mandibulata - ps20
Pancrustacea - ps21
Insecta - ps22
Eumetabola - ps23
Holometabola/Psocodea - ps24
Holometabola - ps25
Aparaglossata - ps26
Mecopterida - ps27
Diptera - ps28
Bibiono./Cyclorrhapha - ps29
Cyclorrhapha - ps30
Calyptratae/Drosophila - ps31
Drosophila - ps32
Sophophora - ps33
Obscura/Melanogaster_group - ps34
D_melanogaster_group - ps35
D_melanogaster_subgroup - ps36
sim./sech./mel. group - ps37
Drosophila_melanogaster - ps38
Phylostratum

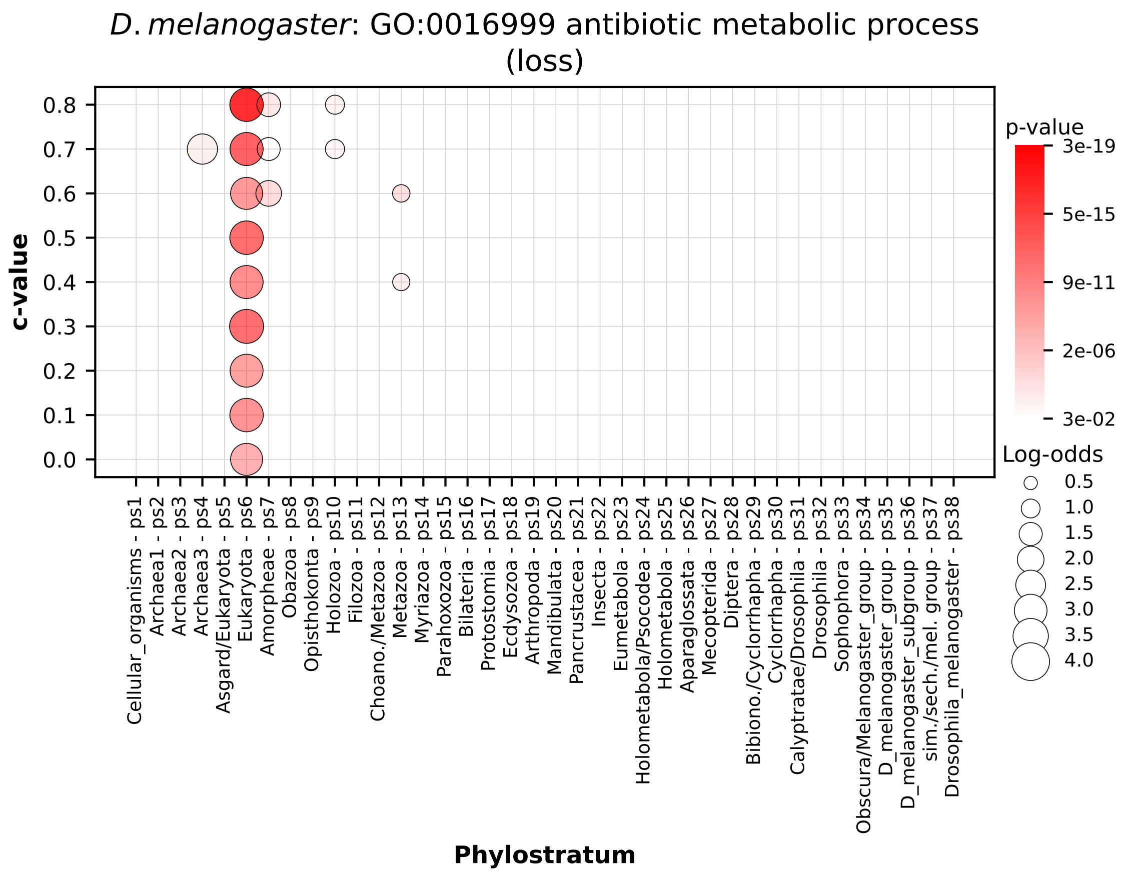

D. melanogaster: GO:0016999 antibiotic metabolic process
(loss)
c-value
0.8
0.7
0.6
0.5
0.4
0.3
0.2
0.1
0.0
p-value
3e-19
5e-15
9e-11
2e-06
3e-02
Log-odds
0.5
1.0
1.5
2.0
2.5
3.0
3.5
4.0
Cellular_organisms - ps1
Archaea1 - ps2
Archaea2 - ps3
Archaea3 - ps4
Asgard/Eukaryota - ps5
Eukaryota - ps6
Amorpheae - ps7
Obazoa - ps8
Opisthokonta - ps9
Holozoa - ps10
Filozoa - ps11
Choano./Metazoa - ps12
Metazoa - ps13
Myriazoa - ps14
Parahoxozoa - ps15
Bilateria - ps16
Protostomia - ps17
Ecdysozoa - ps18
Arthropoda - ps19
Mandibulata - ps20
Pancrustacea - ps21
Insecta - ps22
Eumetabola - ps23
Holometabola/Psocodea - ps24
Holometabola - ps25
Aparaglossata - ps26
Mecopterida - ps27
Diptera - ps28
Bibiono./Cyclorrhapha - ps29
Cyclorrhapha - ps30
Calyptratae/Drosophila - ps31
Drosophila - ps32
Sophophora - ps33
Obscura/Melanogaster_group - ps34
D_melanogaster_group - ps35
D_melanogaster_subgroup - ps36
sim./sech./mel. group - ps37
Drosophila_melanogaster - ps38
Phylostratum

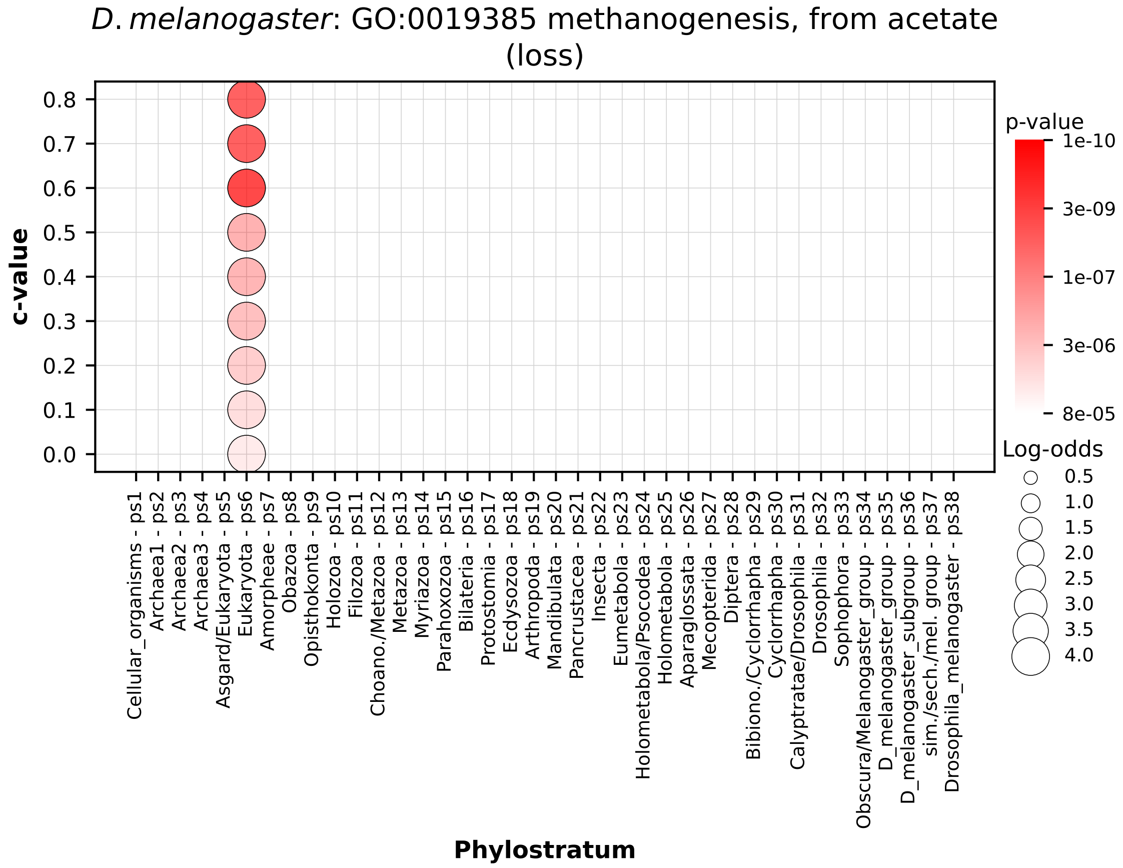

D. melanogaster: GO:0019385 methanogenesis, from acetate (loss)
c-value
0.8
0.7
0.6
0.5
0.4
0.3
0.2
0.1
0.0
p-value
1e-10
3e-09
1e-07
3e-06
8e-05
Log-odds
0.5
1.0
1.5
2.0
2.5
3.0
3.5
4.0
Cellular_organisms - ps1
Archaea1 - ps2
Archaea2 - ps3
Archaea3 - ps4
Asgard/Eukaryota - ps5
Eukaryota - ps6
Amorpheae - ps7
Obazoa - ps8
Opisthokonta - ps9
Holozoa - ps10
Filozoa - ps11
Choano./Metazoa - ps12
Metazoa - ps13
Myriazoa - ps14
Parahoxozoa - ps15
Bilateria - ps16
Protostomia - ps17
Ecdysozoa - ps18
Arthropoda - ps19
Mandibulata - ps20
Pancrustacea - ps21
Insecta - ps22
Eumetabola - ps23
Holometabola/Psocodea - ps24
Holometabola - ps25
Aparaglossata - ps26
Mecopterida - ps27
Diptera - ps28
Bibiono./Cyclorrhapha - ps29
Cyclorrhapha - ps30
Calyptratae/Drosophila - ps31
Drosophila - ps32
Sophophora - ps33
Obscura/Melanogaster_group - ps34
D_melanogaster_group - ps35
D_melanogaster_subgroup - ps36
sim./sech./mel. group - ps37
Drosophila_melanogaster - ps38
Phylostratum

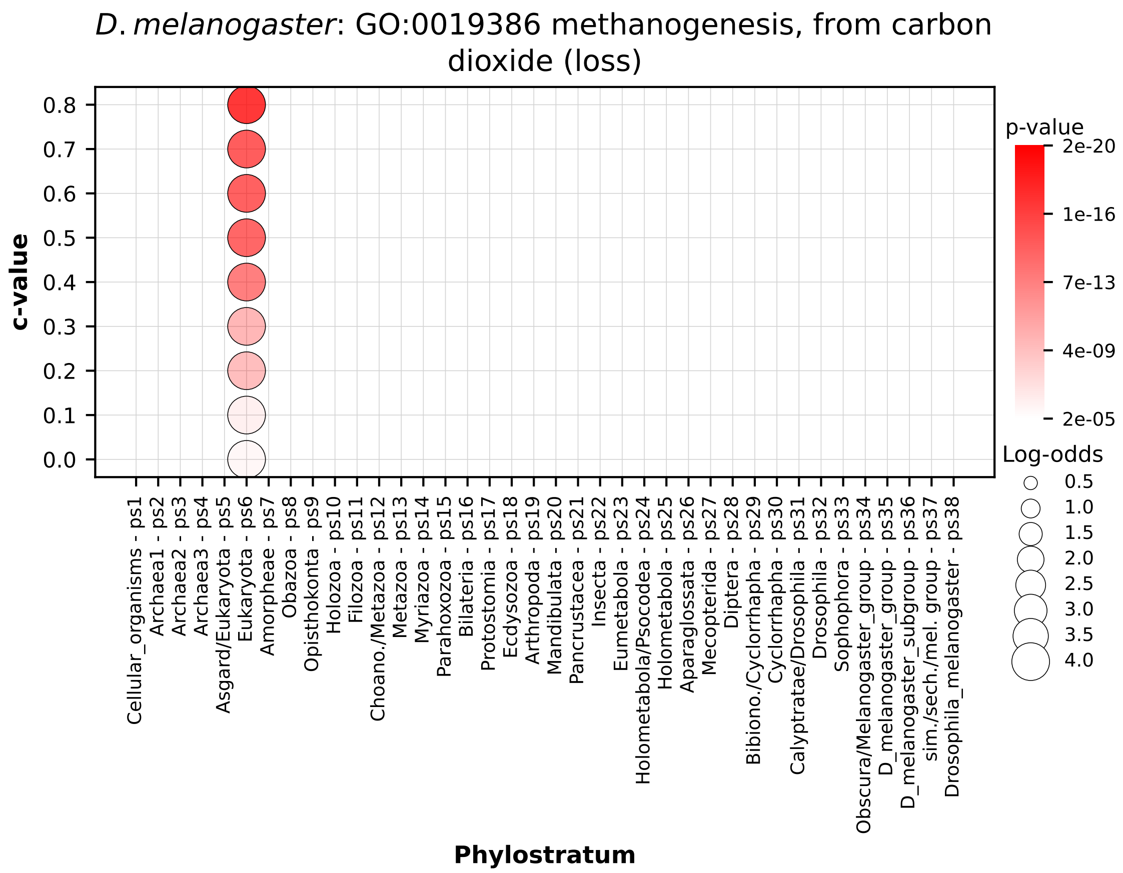

D. melanogaster: GO:0019386 methanogenesis, from carbon dioxide (loss)
c-value
0.8
0.7
0.6
0.5
0.4
0.3
0.2
0.1
0.0
p-value
2e-20
1e-16
7e-13
4e-09
2e-05
Log-odds
0.5
1.0
1.5
2.0
2.5
3.0
3.5
4.0
Cellular_organisms - ps1
Archaea1 - ps2
Archaea2 - ps3
Archaea3 - ps4
Asgard/Eukaryota - ps5
Eukaryota - ps6
Amorpheae - ps7
Obazoa - ps8
Opisthokonta - ps9
Holozoa - ps10
Filozoa - ps11
Choano./Metazoa - ps12
Metazoa - ps13
Myriazoa - ps14
Parahoxozoa - ps15
Bilateria - ps16
Protostomia - ps17
Ecdysozoa - ps18
Arthropoda - ps19
Mandibulata - ps20
Pancrustacea - ps21
Insecta - ps22
Eumetabola - ps23
Holometabola/Psocodea - ps24
Holometabola - ps25
Aparaglossata - ps26
Mecopterida - ps27
Diptera - ps28
Bibiono./Cyclorrhapha - ps29
Cyclorrhapha - ps30
Calyptratae/Drosophila - ps31
Drosophila - ps32
Sophophora - ps33
Obscura/Melanogaster_group - ps34
D_melanogaster_group - ps35
D_melanogaster_subgroup - ps36
sim./sech./mel. group - ps37
Drosophila_melanogaster - ps38
Phylostratum

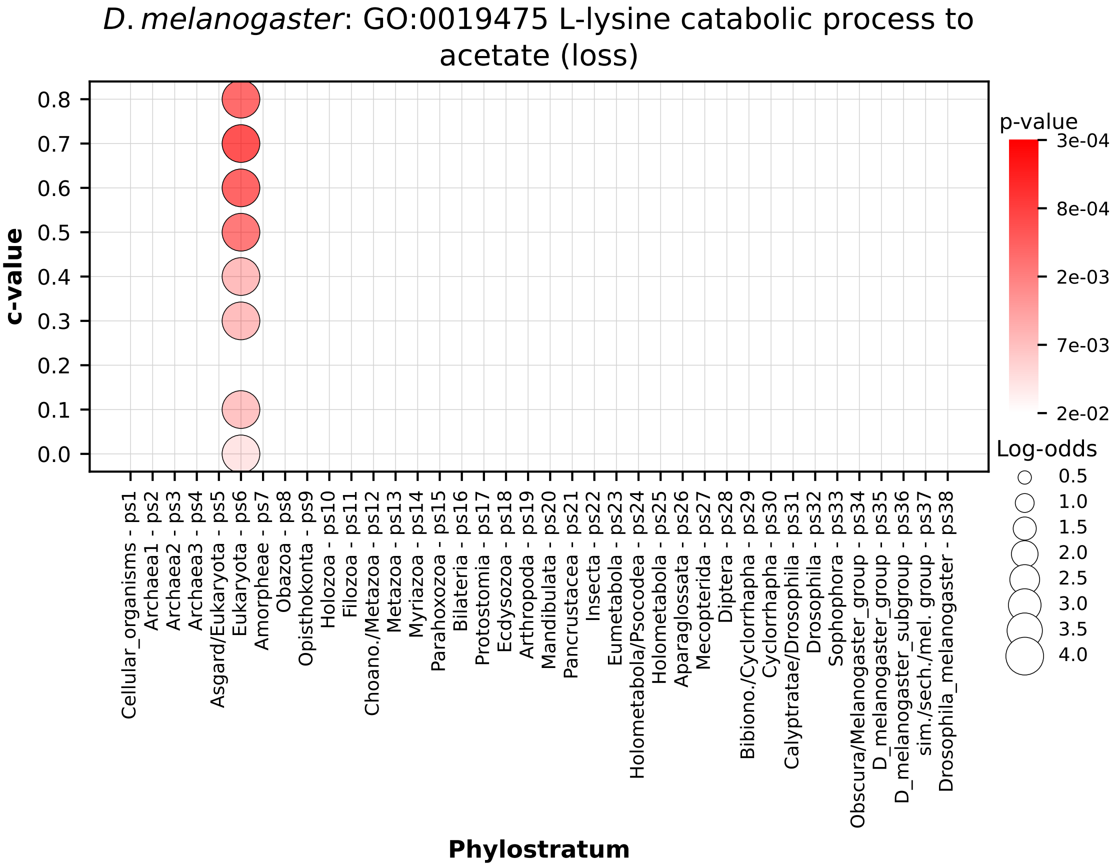

D. melanogaster: GO:0019475 L-lysine catabolic process to acetate (loss)
c-value
0.8
0.7
0.6
0.5
0.4
0.3
0.2
0.1
0.0
p-value
3e-04
8e-04
2e-03
7e-03
2e-02
Log-odds
0.5
1.0
1.5
2.0
2.5
3.0
3.5
4.0
Cellular_organisms - ps1
Archaea1 - ps2
Archaea2 - ps3
Archaea3 - ps4
Asgard/Eukaryota - ps5
Eukaryota - ps6
Amorpheae - ps7
Obazoa - ps8
Opisthokonta - ps9
Holozoa - ps10
Filozoa - ps11
Choano./Metazoa - ps12
Metazoa - ps13
Myriazoa - ps14
Parahoxozoa - ps15
Bilateria - ps16
Protostomia - ps17
Ecdysozoa - ps18
Arthropoda - ps19
Mandibulata - ps20
Pancrustacea - ps21
Insecta - ps22
Eumetabola - ps23
Holometabola/Psocodea - ps24
Holometabola - ps25
Aparaglossata - ps26
Mecopterida - ps27
Diptera - ps28
Bibiono./Cyclorrhapha - ps29
Cyclorrhapha - ps30
Calyptratae/Drosophila - ps31
Drosophila - ps32
Sophophora - ps33
Obscura/Melanogaster_group - ps34
D_melanogaster_group - ps35
D_melanogaster_subgroup - ps36
sim./sech./mel. group - ps37
Drosophila_melanogaster - ps38
Phylostratum

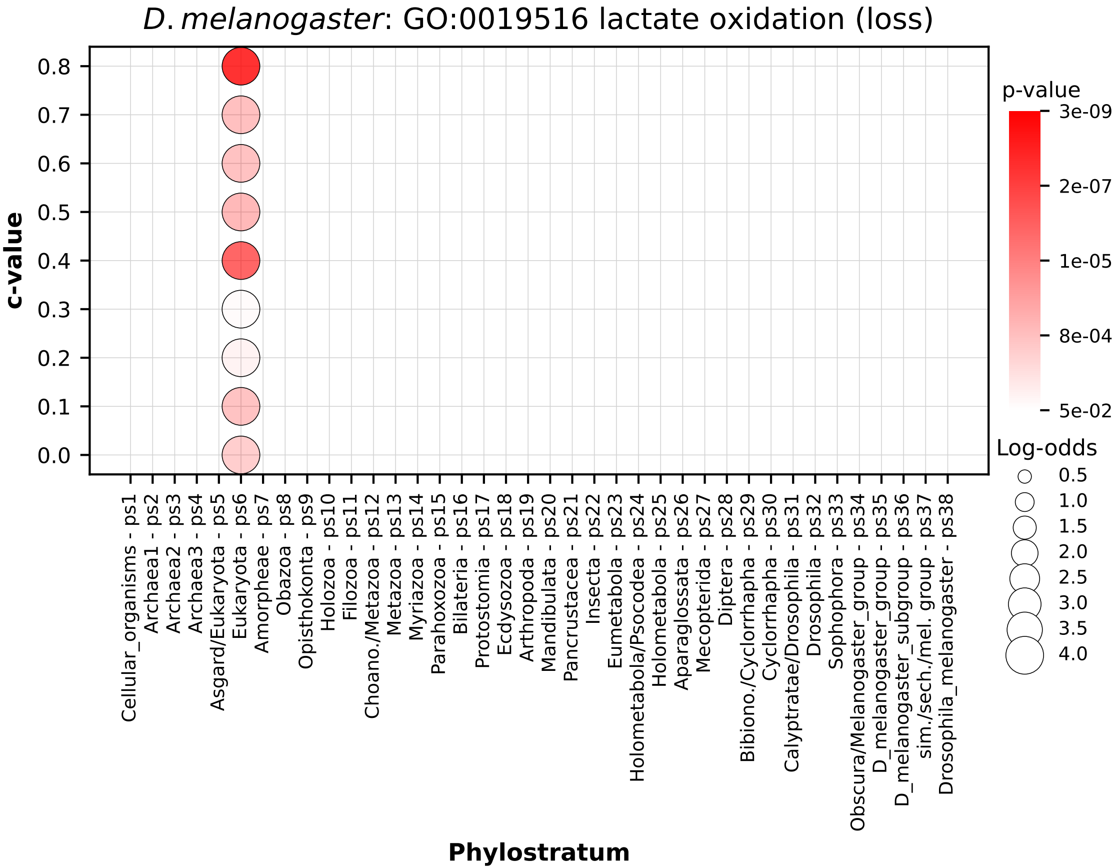
D. melanogaster: GO:0019516 lactate oxidation (loss)
c-value
0.8
0.7
0.6
0.5
0.4
0.3
0.2
0.1
0.0
p-value
3e-09
2e-07
1e-05
8e-04
5e-02
Log-odds
0.5
1.0
1.5
2.0
2.5
3.0
3.5
4.0
Cellular_organisms - ps1
Archaea1 - ps2
Archaea2 - ps3
Archaea3 - ps4
Asgard/Eukaryota - ps5
Eukaryota - ps6
Amorpheae - ps7
Obazoa - ps8
Opisthokonta - ps9
Holozoa - ps10
Filozoa - ps11
Choano./Metazoa - ps12
Metazoa - ps13
Myriazoa - ps14
Parahoxozoa - ps15
Bilateria - ps16
Protostomia - ps17
Ecdysozoa - ps18
Arthropoda - ps19
Mandibulata - ps20
Pancrustacea - ps21
Insecta - ps22
Eumetabola - ps23
Holometabola/Psocodea - ps24
Holometabola - ps25
Aparaglossata - ps26
Mecopterida - ps27
Diptera - ps28
Bibiono./Cyclorrhapha - ps29
Cyclorrhapha - ps30
Calyptratae/Drosophila - ps31
Drosophila - ps32
Sophophora - ps33
Obscura/Melanogaster_group - ps34
D_melanogaster_group - ps35
D_melanogaster_subgroup - ps36
sim./sech./mel. group - ps37
Drosophila_melanogaster - ps38
Phylostratum

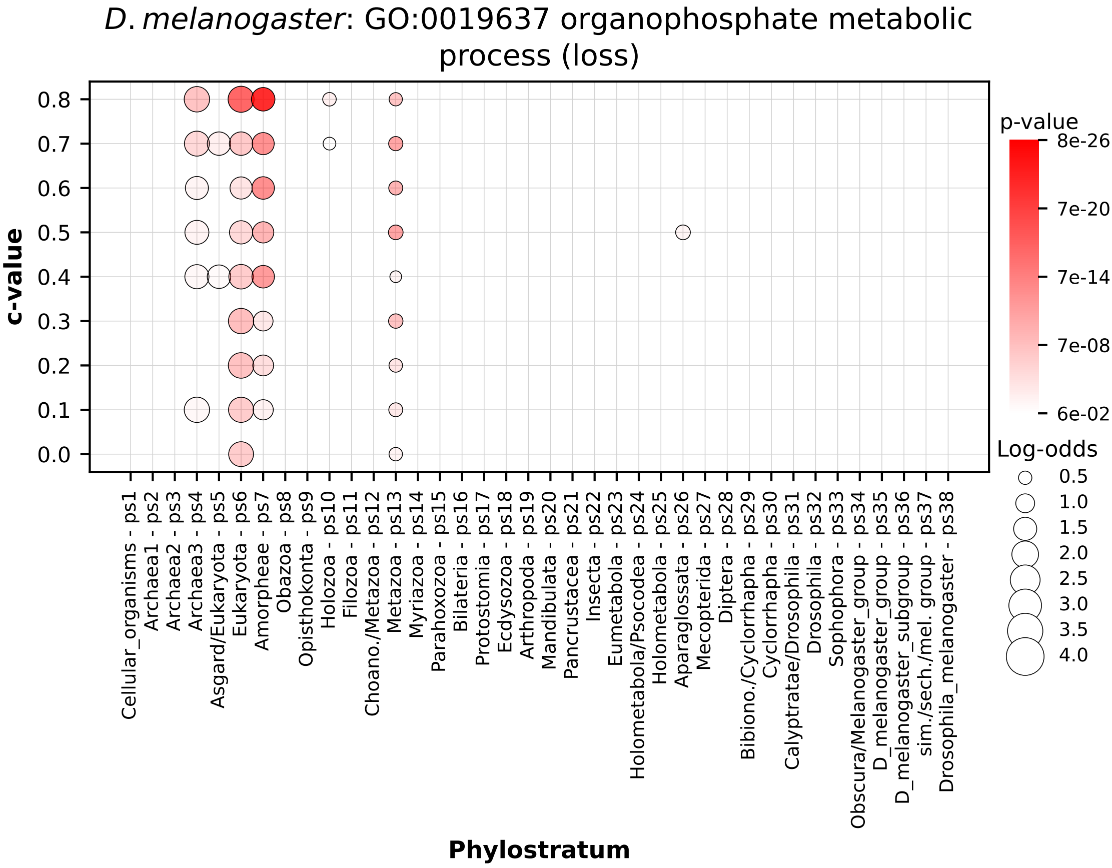
D. melanogaster: GO:0019637 organophosphate metabolic process (loss)
c-value
0.8
0.7
0.6
0.5
0.4
0.3
0.2
0.1
0.0
Phylostratum
Cellular_organisms - ps1
Archaea1 - ps2
Archaea2 - ps3
Archaea3 - ps4
Asgard/Eukaryota - ps5
Eukaryota - ps6
Amorpheae - ps7
Obazoa - ps8
Opisthokonta - ps9
Holozoa - ps10
Filozoa - ps11
Choano./Metazoa - ps12
Metazoa - ps13
Myriazoa - ps14
Parahoxozoa - ps15
Bilateria - ps16
Protostomia - ps17
Ecdysozoa - ps18
Arthropoda - ps19
Mandibulata - ps20
Pancrustacea - ps21
Insecta - ps22
Eumetabola - ps23
Holometabola/Psocodea - ps24
Holometabola - ps25
Aparaglossata - ps26
Mecopterida - ps27
Diptera - ps28
Bibiono./Cyclorrhapha - ps29
Cyclorrhapha - ps30
Calyptratae/Drosophila - ps31
Drosophila - ps32
Sophophora - ps33
Obscura/Melanogaster_group - ps34
D_melanogaster_group - ps35
D_melanogaster_subgroup - ps36
sim./sech./mel. group - ps37
Drosophila_melanogaster - ps38
p-value
8e-26
7e-20
7e-14
7e-08
6e-02
Log-odds
0.5
1.0
1.5
2.0
2.5
3.0
3.5
4.0

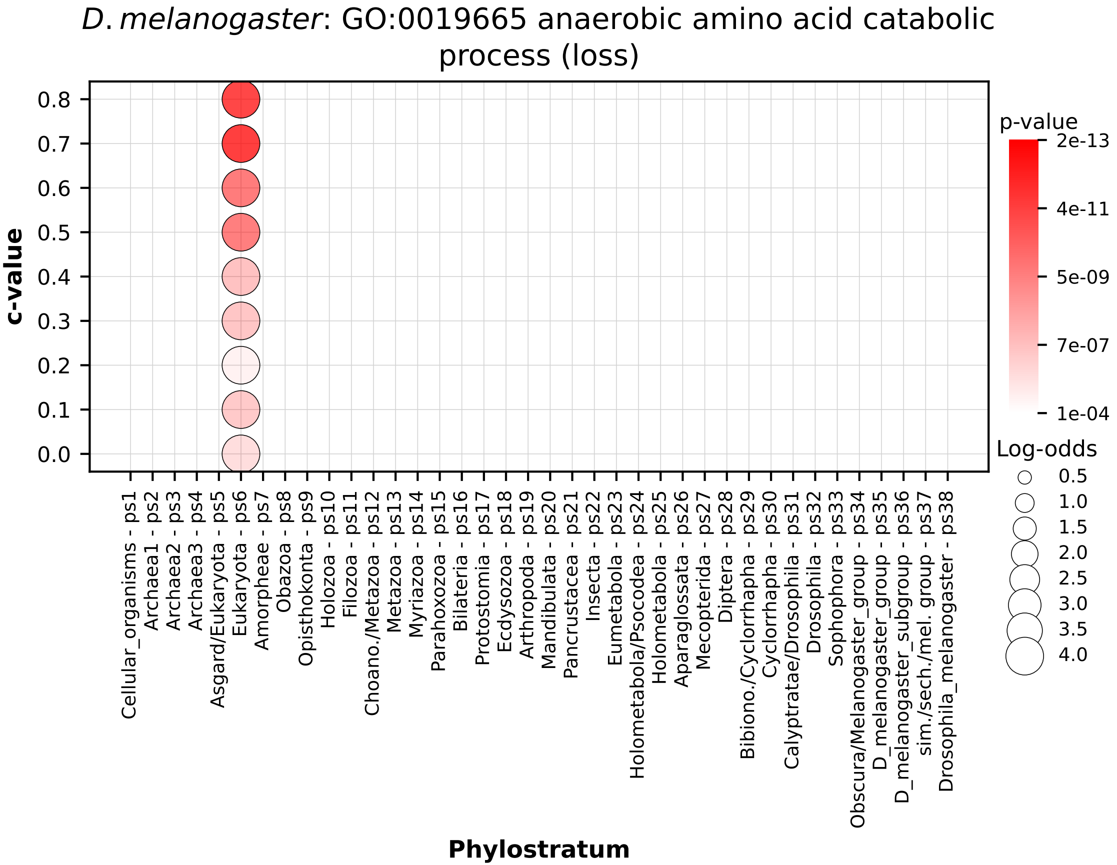

D. melanogaster: GO:0019665 anaerobic amino acid catabolic process (loss)
c-value
0.8
0.7
0.6
0.5
0.4
0.3
0.2
0.1
0.0
p-value
2e-13
4e-11
5e-09
7e-07
1e-04
Log-odds
0.5
1.0
1.5
2.0
2.5
3.0
3.5
4.0
Cellular_organisms - ps1
Archaea1 - ps2
Archaea2 - ps3
Archaea3 - ps4
Asgard/Eukaryota - ps5
Eukaryota - ps6
Amorpheae - ps7
Obazoa - ps8
Opisthokonta - ps9
Holozoa - ps10
Filozoa - ps11
Choano./Metazoa - ps12
Metazoa - ps13
Myriazoa - ps14
Parahoxozoa - ps15
Bilateria - ps16
Protostomia - ps17
Ecdysozoa - ps18
Arthropoda - ps19
Mandibulata - ps20
Pancrustacea - ps21
Insecta - ps22
Eumetabola - ps23
Holometabola/Psocodea - ps24
Holometabola - ps25
Aparaglossata - ps26
Mecopterida - ps27
Diptera - ps28
Bibiono./Cyclorrhapha - ps29
Cyclorrhapha - ps30
Calyptratae/Drosophila - ps31
Drosophila - ps32
Sophophora - ps33
Obscura/Melanogaster_group - ps34
D_melanogaster_group - ps35
D_melanogaster_subgroup - ps36
sim./sech./mel. group - ps37
Drosophila_melanogaster - ps38
Phylostratum

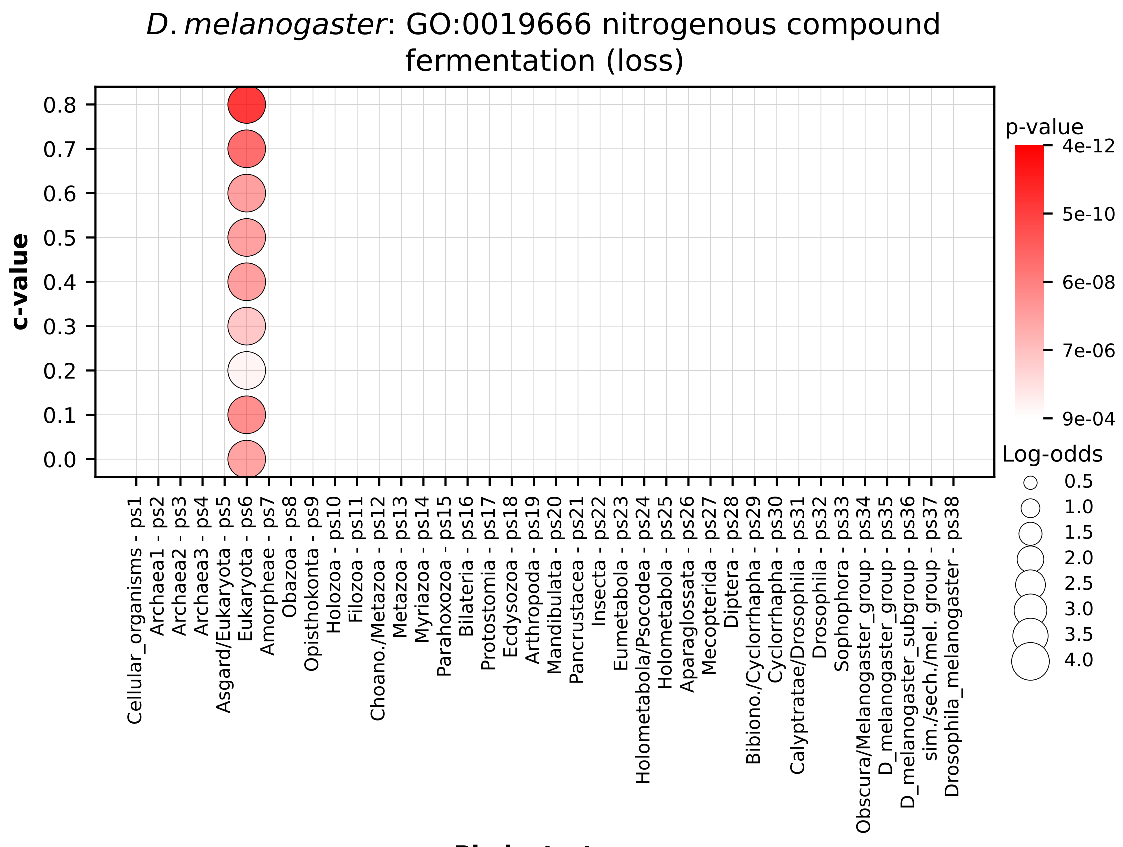

D. melanogaster: GO:0019666 nitrogenous compound fermentation (loss)
c-value
0.8
0.7
0.6
0.5
0.4
0.3
0.2
0.1
0.0
Phylostratum
Cellular_organisms - ps1
Archaea1 - ps2
Archaea2 - ps3
Archaea3 - ps4
Asgard/Eukaryota - ps5
Eukaryota - ps6
Amorpheae - ps7
Obazoa - ps8
Opisthokonta - ps9
Holozoa - ps10
Filozoa - ps11
Choano./Metazoa - ps12
Metazoa - ps13
Myriazoa - ps14
Parahoxozoa - ps15
Bilateria - ps16
Protostomia - ps17
Ecdysozoa - ps18
Arthropoda - ps19
Mandibulata - ps20
Pancrustacea - ps21
Insecta - ps22
Eumetabola - ps23
Holometabola/Psocodea - ps24
Holometabola - ps25
Aparaglossata - ps26
Mecopterida - ps27
Diptera - ps28
Bibiono./Cyclorrhapha - ps29
Cyclorrhapha - ps30
Calyptratae/Drosophila - ps31
Drosophila - ps32
Sophophora - ps33
Obscura/Melanogaster_group - ps34
D_melanogaster_group - ps35
D_melanogaster_subgroup - ps36
sim./sech./mel. group - ps37
Drosophila_melanogaster - ps38
p-value
4e-12
5e-10
6e-08
7e-06
9e-04
Log-odds
0.5
1.0
1.5
2.0
2.5
3.0
3.5
4.0

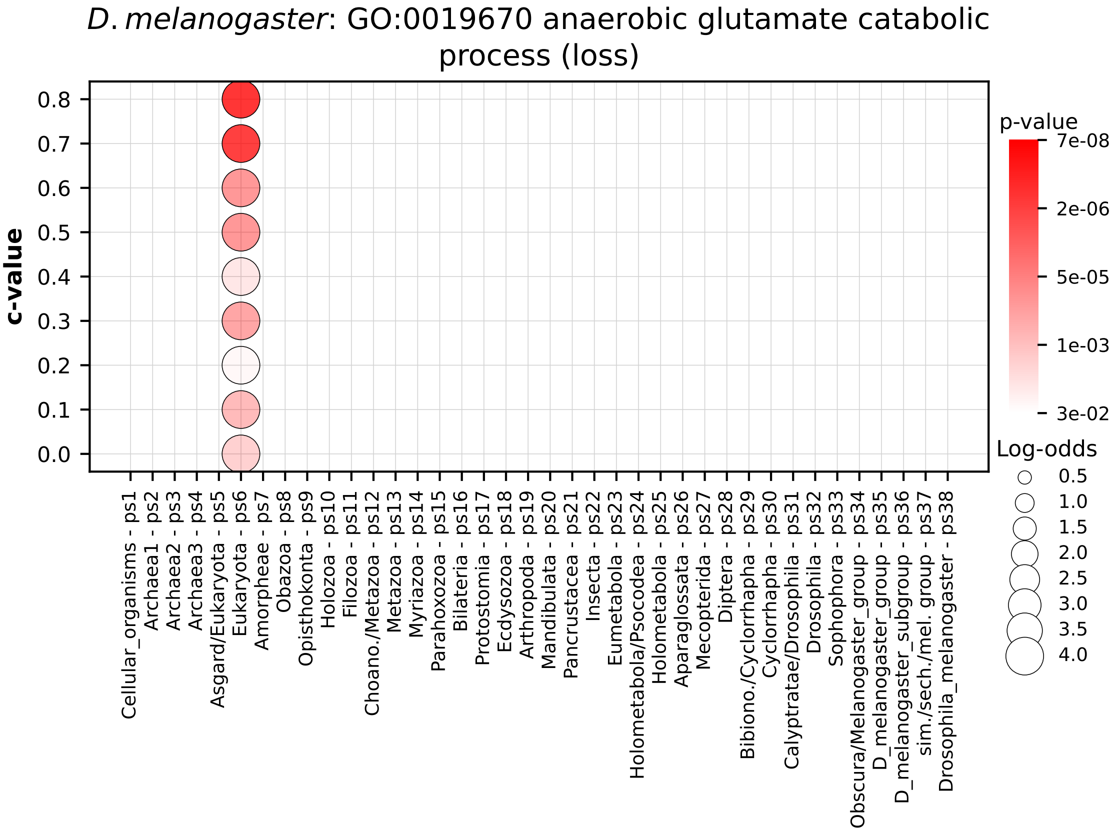
D. melanogaster: GO:0019670 anaerobic glutamate catabolic process (loss)
c-value
0.8
0.7
0.6
0.5
0.4
0.3
0.2
0.1
0.0
p-value
7e-08
2e-06
5e-05
1e-03
3e-02
Log-odds
0.5
1.0
1.5
2.0
2.5
3.0
3.5
4.0
Cellular_organisms - ps1
Archaea1 - ps2
Archaea2 - ps3
Archaea3 - ps4
Asgard/Eukaryota - ps5
Eukaryota - ps6
Amorpheae - ps7
Obazoa - ps8
Opisthokonta - ps9
Holozoa - ps10
Filozoa - ps11
Choano./Metazoa - ps12
Metazoa - ps13
Myriazoa - ps14
Parahoxozoa - ps15
Bilateria - ps16
Protostomia - ps17
Ecdysozoa - ps18
Arthropoda - ps19
Mandibulata - ps20
Pancrustacea - ps21
Insecta - ps22
Eumetabola - ps23
Holometabola/Psocodea - ps24
Holometabola - ps25
Aparaglossata - ps26
Mecopterida - ps27
Diptera - ps28
Bibiono./Cyclorrhapha - ps29
Cyclorrhapha - ps30
Calyptratae/Drosophila - ps31
Drosophila - ps32
Sophophora - ps33
Obscura/Melanogaster_group - ps34
D_melanogaster_group - ps35
D_melanogaster_subgroup - ps36
sim./sech./mel. group - ps37
Drosophila_melanogaster - ps38
Phylostratum

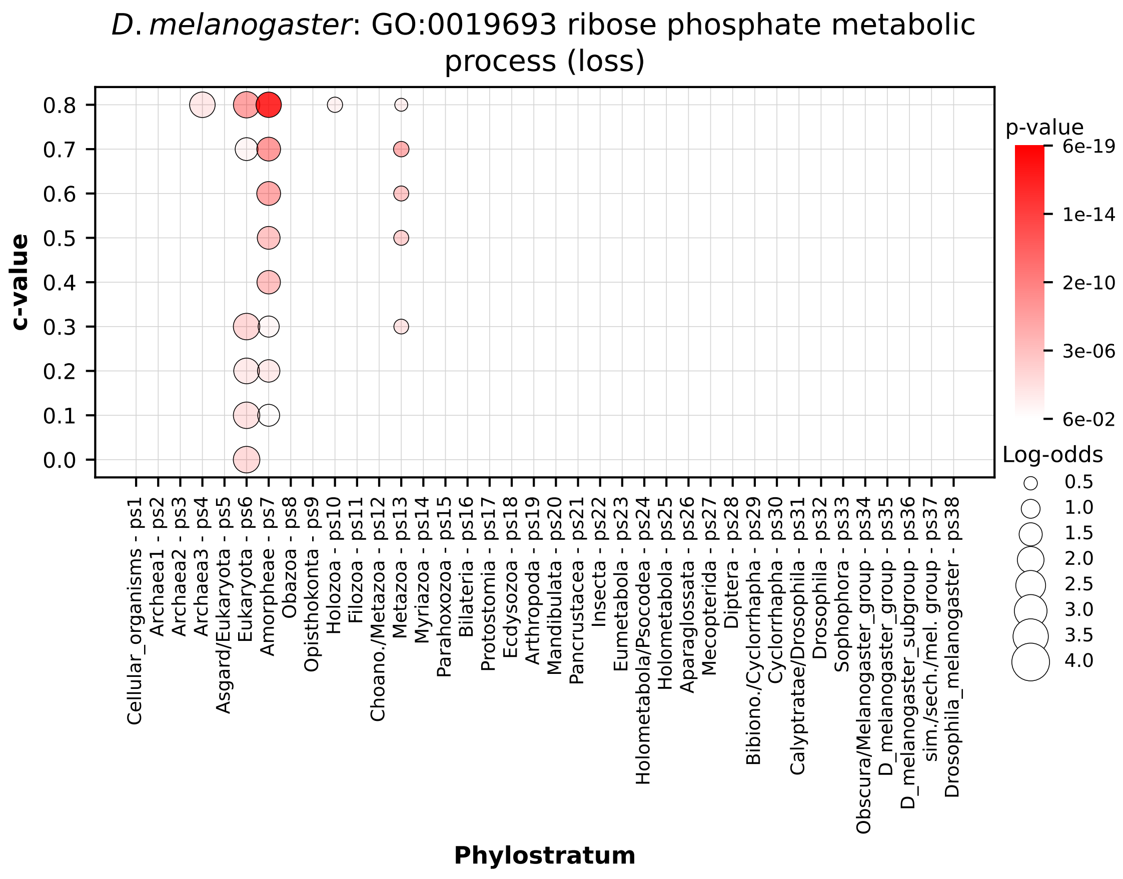

D. melanogaster: GO:0019693 ribose phosphate metabolic process (loss)
c-value
0.8
0.7
0.6
0.5
0.4
0.3
0.2
0.1
0.0
p-value
6e-19
1e-14
2e-10
3e-06
6e-02
Log-odds
0.5
1.0
1.5
2.0
2.5
3.0
3.5
4.0
Cellular_organisms - ps1
Archaea1 - ps2
Archaea2 - ps3
Archaea3 - ps4
Asgard/Eukaryota - ps5
Eukaryota - ps6
Amorpheae - ps7
Obazoa - ps8
Opisthokonta - ps9
Holozoa - ps10
Filozoa - ps11
Choano./Metazoa - ps12
Metazoa - ps13
Myriazoa - ps14
Parahoxozoa - ps15
Bilateria - ps16
Protostomia - ps17
Ecdysozoa - ps18
Arthropoda - ps19
Mandibulata - ps20
Pancrustacea - ps21
Insecta - ps22
Eumetabola - ps23
Holometabola/Psocodea - ps24
Holometabola - ps25
Aparaglossata - ps26
Mecopterida - ps27
Diptera - ps28
Bibiono./Cyclorrhapha - ps29
Cyclorrhapha - ps30
Calyptratae/Drosophila - ps31
Drosophila - ps32
Sophophora - ps33
Obscura/Melanogaster_group - ps34
D_melanogaster_group - ps35
D_melanogaster_subgroup - ps36
sim./sech./mel. group - ps37
Drosophila_melanogaster - ps38
Phylostratum

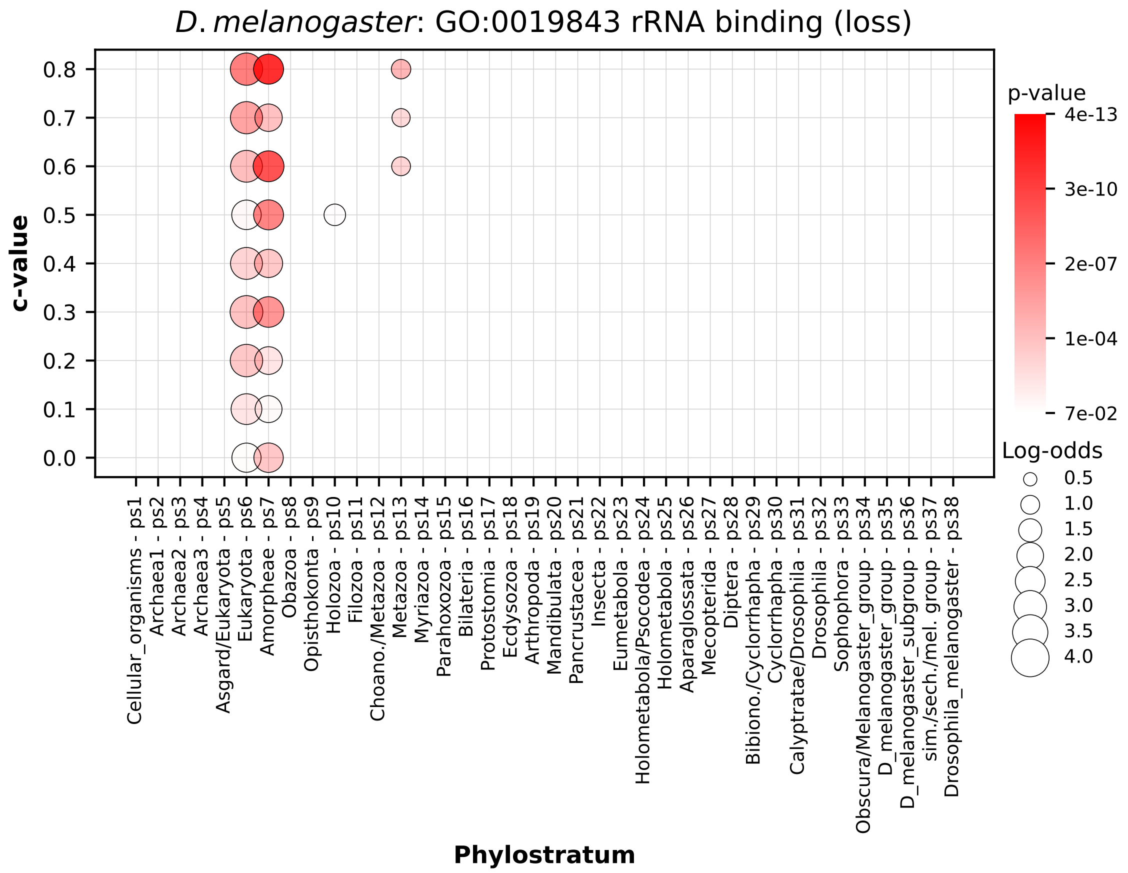

D. melanogaster: GO:0019843 rRNA binding (loss)
c-value
0.8
0.7
0.6
0.5
0.4
0.3
0.2
0.1
0.0
Phylostratum
Cellular_organisms - ps1
Archaea1 - ps2
Archaea2 - ps3
Archaea3 - ps4
Asgard/Eukaryota - ps5
Eukaryota - ps6
Amorpheae - ps7
Obazoa - ps8
Opisthokonta - ps9
Holozoa - ps10
Filozoa - ps11
Choano./Metazoa - ps12
Metazoa - ps13
Myriazoa - ps14
Parahoxozoa - ps15
Bilateria - ps16
Protostomia - ps17
Ecdysozoa - ps18
Arthropoda - ps19
Mandibulata - ps20
Pancrustacea - ps21
Insecta - ps22
Eumetabola - ps23
Holometabola/Psocodea - ps24
Holometabola - ps25
Aparaglossata - ps26
Mecopterida - ps27
Diptera - ps28
Bibiono./Cyclorrhapha - ps29
Cyclorrhapha - ps30
Calyptratae/Drosophila - ps31
Drosophila - ps32
Sophophora - ps33
Obscura/Melanogaster_group - ps34
D_melanogaster_group - ps35
D_melanogaster_subgroup - ps36
sim./sech./mel. group - ps37
Drosophila_melanogaster - ps38
p-value
4e-13
3e-10
2e-07
1e-04
7e-02
Log-odds
0.5
1.0
1.5
2.0
2.5
3.0
3.5
4.0

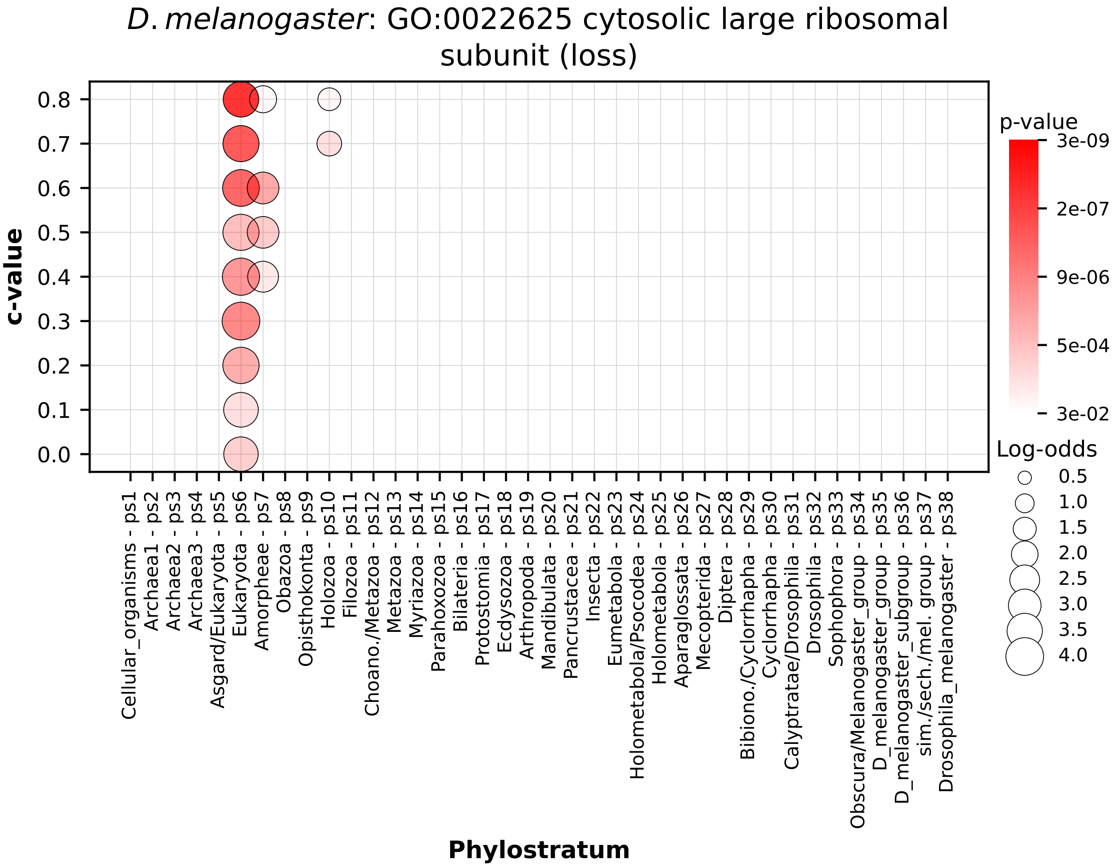

D. melanogaster: GO:0022625 cytosolic large ribosomal subunit (loss)
c-value
0.8
0.7
0.6
0.5
0.4
0.3
0.2
0.1
0.0
Phylostratum
Cellular_organisms - ps1
Archaea1 - ps2
Archaea2 - ps3
Archaea3 - ps4
Asgard/Eukaryota - ps5
Eukaryota - ps6
Amorpheae - ps7
Obazoa - ps8
Opisthokonta - ps9
Holozoa - ps10
Filozoa - ps11
Choano./Metazoa - ps12
Metazoa - ps13
Myriazoa - ps14
Parahoxozoa - ps15
Bilateria - ps16
Protostomia - ps17
Ecdysozoa - ps18
Arthropoda - ps19
Mandibulata - ps20
Pancrustacea - ps21
Insecta - ps22
Eumetabola - ps23
Holometabola/Psocodea - ps24
Holometabola - ps25
Aparaglossata - ps26
Mecopterida - ps27
Diptera - ps28
Bibiono./Cyclorrhapha - ps29
Cyclorrhapha - ps30
Calyptratae/Drosophila - ps31
Drosophila - ps32
Sophophora - ps33
Obscura/Melanogaster_group - ps34
D_melanogaster_group - ps35
D_melanogaster_subgroup - ps36
sim./sech./mel. group - ps37
Drosophila_melanogaster - ps38
p-value
3e-09
2e-07
9e-06
5e-04
3e-02
Log-odds
0.5
1.0
1.5
2.0
2.5
3.0
3.5
4.0

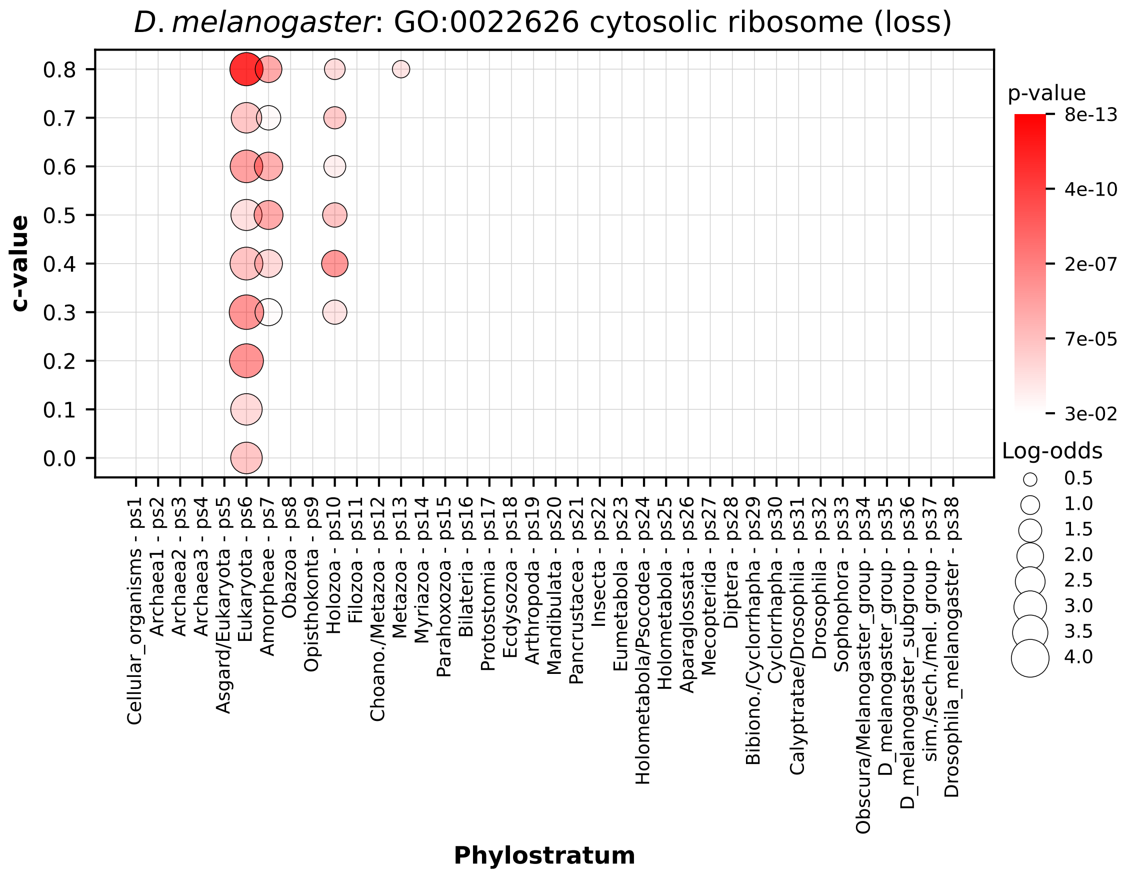
D. melanogaster: GO:0022626 cytosolic ribosome (loss)
c-value
0.0
0.1
0.2
0.3
0.4
0.5
0.6
0.7
0.8
Phylostratum
Cellular_organisms - ps1
Archaea1 - ps2
Archaea2 - ps3
Archaea3 - ps4
Asgard/Eukaryota - ps5
Eukaryota - ps6
Amorpheae - ps7
Obazoa - ps8
Opisthokonta - ps9
Holozoa - ps10
Filozoa - ps11
Choano./Metazoa - ps12
Metazoa - ps13
Myriazoa - ps14
Parahoxozoa - ps15
Bilateria - ps16
Protostomia - ps17
Ecdysozoa - ps18
Arthropoda - ps19
Mandibulata - ps20
Pancrustacea - ps21
Insecta - ps22
Eumetabola - ps23
Holometabola/Psocodea - ps24
Holometabola - ps25
Aparaglossata - ps26
Mecopterida - ps27
Diptera - ps28
Bibiono./Cyclorrhapha - ps29
Cyclorrhapha - ps30
Calyptratae/Drosophila - ps31
Drosophila - ps32
Sophophora - ps33
Obscura/Melanogaster_group - ps34
D_melanogaster_group - ps35
D_melanogaster_subgroup - ps36
sim./sech./mel. group - ps37
Drosophila_melanogaster - ps38
p-value
8e-13
4e-10
2e-07
7e-05
3e-02
Log-odds
0.5
1.0
1.5
2.0
2.5
3.0
3.5
4.0

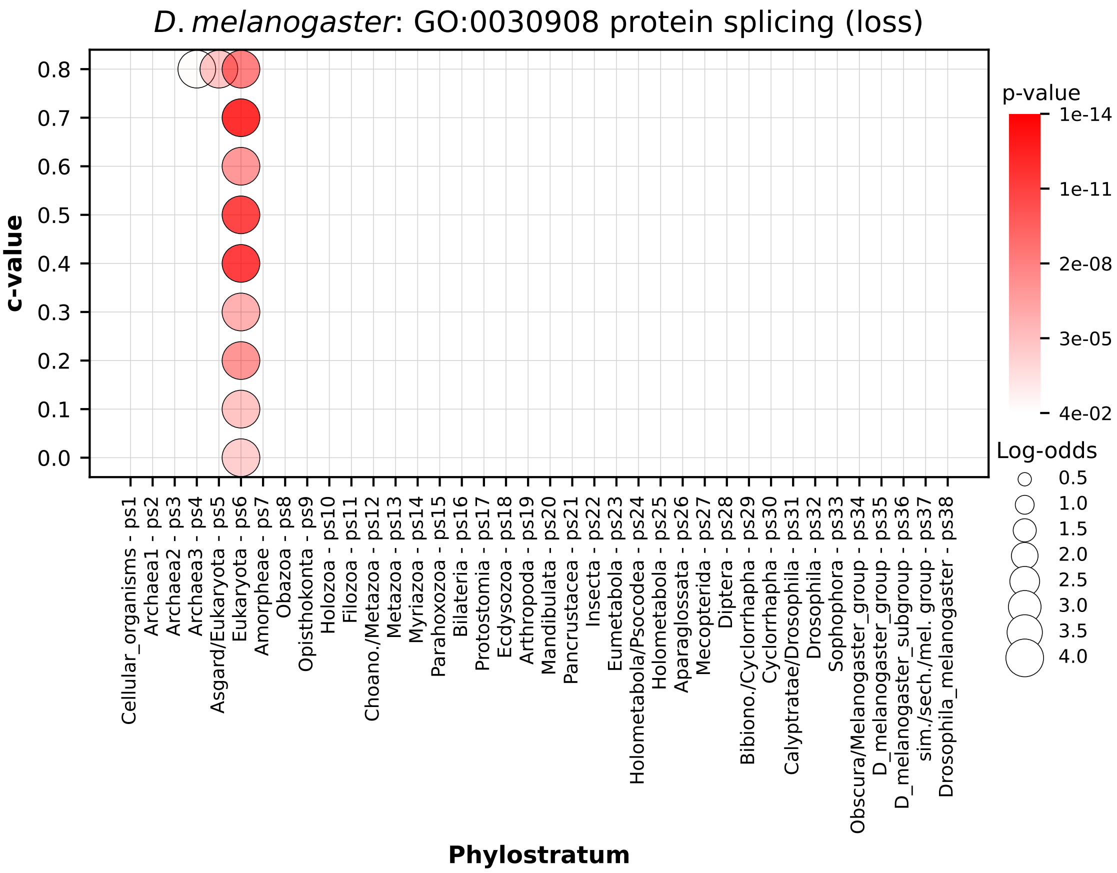
D. melanogaster: GO:0030908 protein splicing (loss)
c-value
0.8
0.7
0.6
0.5
0.4
0.3
0.2
0.1
0.0
p-value
1e-14
1e-11
2e-08
3e-05
4e-02
Log-odds
0.5
1.0
1.5
2.0
2.5
3.0
3.5
4.0
Cellular_organisms - ps1
Archaea1 - ps2
Archaea2 - ps3
Archaea3 - ps4
Asgard/Eukaryota - ps5
Eukaryota - ps6
Amorpheae - ps7
Obazoa - ps8
Opisthokonta - ps9
Holozoa - ps10
Filozoa - ps11
Choano./Metazoa - ps12
Metazoa - ps13
Myriazoa - ps14
Parahoxozoa - ps15
Bilateria - ps16
Protostomia - ps17
Ecdysozoa - ps18
Arthropoda - ps19
Mandibulata - ps20
Pancrustacea - ps21
Insecta - ps22
Eumetabola - ps23
Holometabola/Psocodea - ps24
Holometabola - ps25
Aparaglossata - ps26
Mecopterida - ps27
Diptera - ps28
Bibiono./Cyclorrhapha - ps29
Cyclorrhapha - ps30
Calyptratae/Drosophila - ps31
Drosophila - ps32
Sophophora - ps33
Obscura/Melanogaster_group - ps34
D_melanogaster_group - ps35
D_melanogaster_subgroup - ps36
sim./sech./mel. group - ps37
Drosophila_melanogaster - ps38
Phylostratum

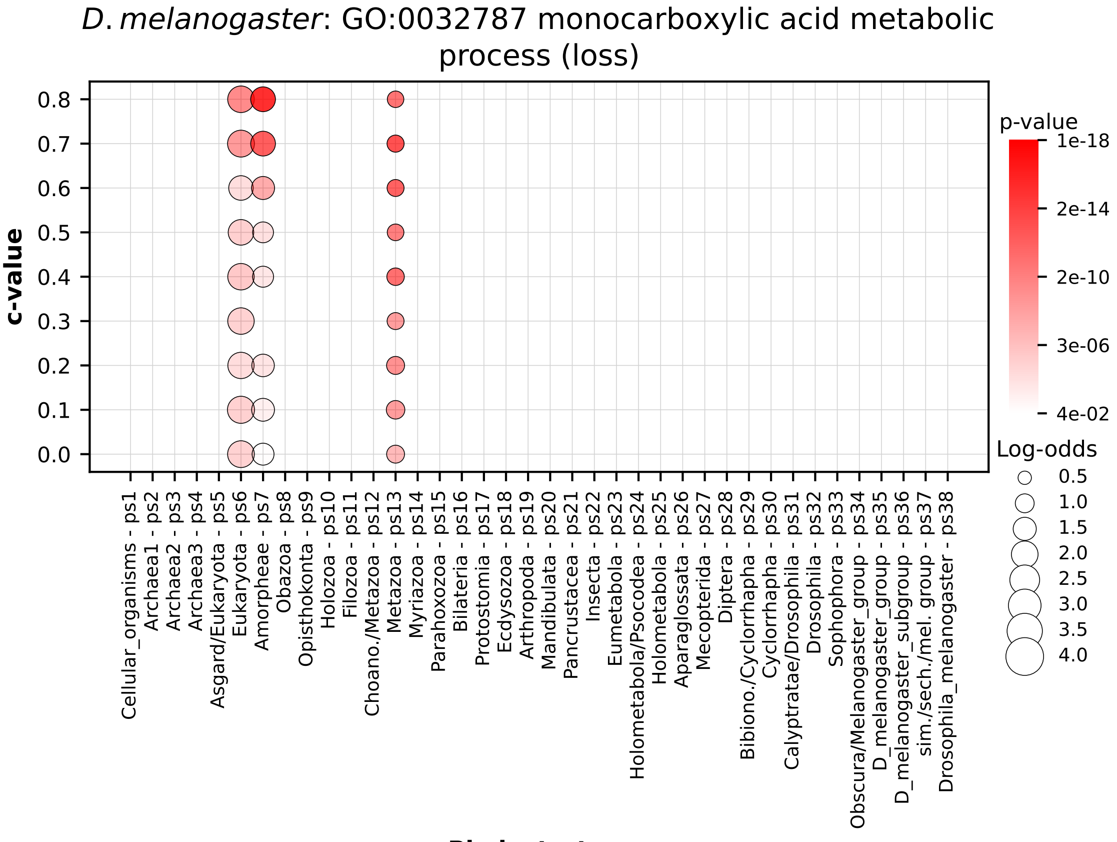

D. melanogaster: GO:0032787 monocarboxylic acid metabolic process (loss)
c-value
0.8
0.7
0.6
0.5
0.4
0.3
0.2
0.1
0.0
Phylostratum
Cellular_organisms - ps1
Archaea1 - ps2
Archaea2 - ps3
Archaea3 - ps4
Asgard/Eukaryota - ps5
Eukaryota - ps6
Amorpheae - ps7
Obazoa - ps8
Opisthokonta - ps9
Holozoa - ps10
Filozoa - ps11
Choano./Metazoa - ps12
Metazoa - ps13
Myriazoa - ps14
Parahoxozoa - ps15
Bilateria - ps16
Protostomia - ps17
Ecdysozoa - ps18
Arthropoda - ps19
Mandibulata - ps20
Pancrustacea - ps21
Insecta - ps22
Eumetabola - ps23
Holometabola/Psocodea - ps24
Holometabola - ps25
Aparaglossata - ps26
Mecopterida - ps27
Diptera - ps28
Bibiono./Cyclorrhapha - ps29
Cyclorrhapha - ps30
Calyptratae/Drosophila - ps31
Drosophila - ps32
Sophophora - ps33
Obscura/Melanogaster_group - ps34
D_melanogaster_group - ps35
D_melanogaster_subgroup - ps36
sim./sech./mel. group - ps37
Drosophila_melanogaster - ps38
p-value
1e-18
2e-14
2e-10
3e-06
4e-02
Log-odds
0.5
1.0
1.5
2.0
2.5
3.0
3.5
4.0

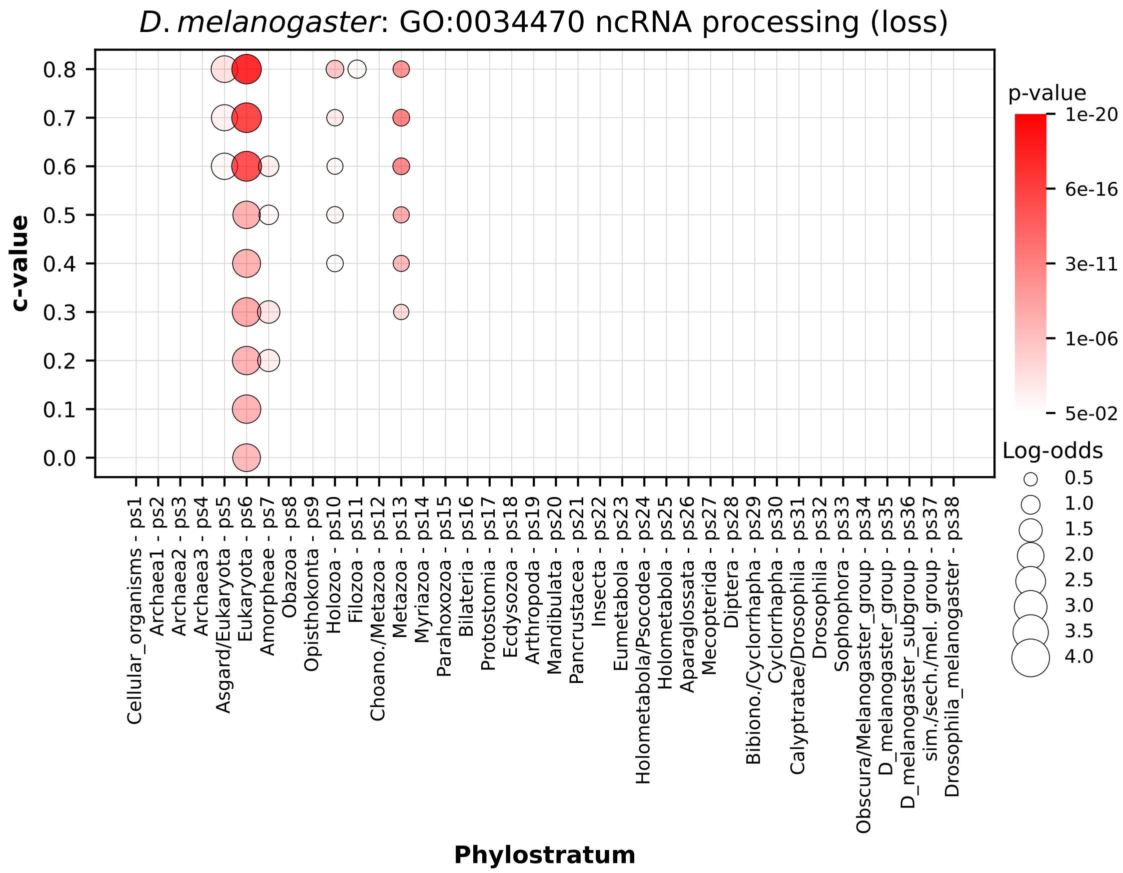

D. melanogaster: GO:0034470 ncRNA processing (loss)
c-value
0.8
0.7
0.6
0.5
0.4
0.3
0.2
0.1
0.0
Phylostratum
Cellular_organisms - ps1
Archaea1 - ps2
Archaea2 - ps3
Archaea3 - ps4
Asgard/Eukaryota - ps5
Eukaryota - ps6
Amorpheae - ps7
Obazoa - ps8
Opisthokonta - ps9
Holozoa - ps10
Filozoa - ps11
Choano./Metazoa - ps12
Metazoa - ps13
Myriazoa - ps14
Parahoxozoa - ps15
Bilateria - ps16
Protostomia - ps17
Ecdysozoa - ps18
Arthropoda - ps19
Mandibulata - ps20
Pancrustacea - ps21
Insecta - ps22
Eumetabola - ps23
Holometabola/Psocodea - ps24
Holometabola - ps25
Aparaglossata - ps26
Mecopterida - ps27
Diptera - ps28
Bibiono./Cyclorrhapha - ps29
Cyclorrhapha - ps30
Calyptratae/Drosophila - ps31
Drosophila - ps32
Sophophora - ps33
Obscura/Melanogaster_group - ps34
D_melanogaster_group - ps35
D_melanogaster_subgroup - ps36
sim./sech./mel. group - ps37
Drosophila_melanogaster - ps38
p-value
1e-20
6e-16
3e-11
1e-06
5e-02
Log-odds
0.5
1.0
1.5
2.0
2.5
3.0
3.5
4.0

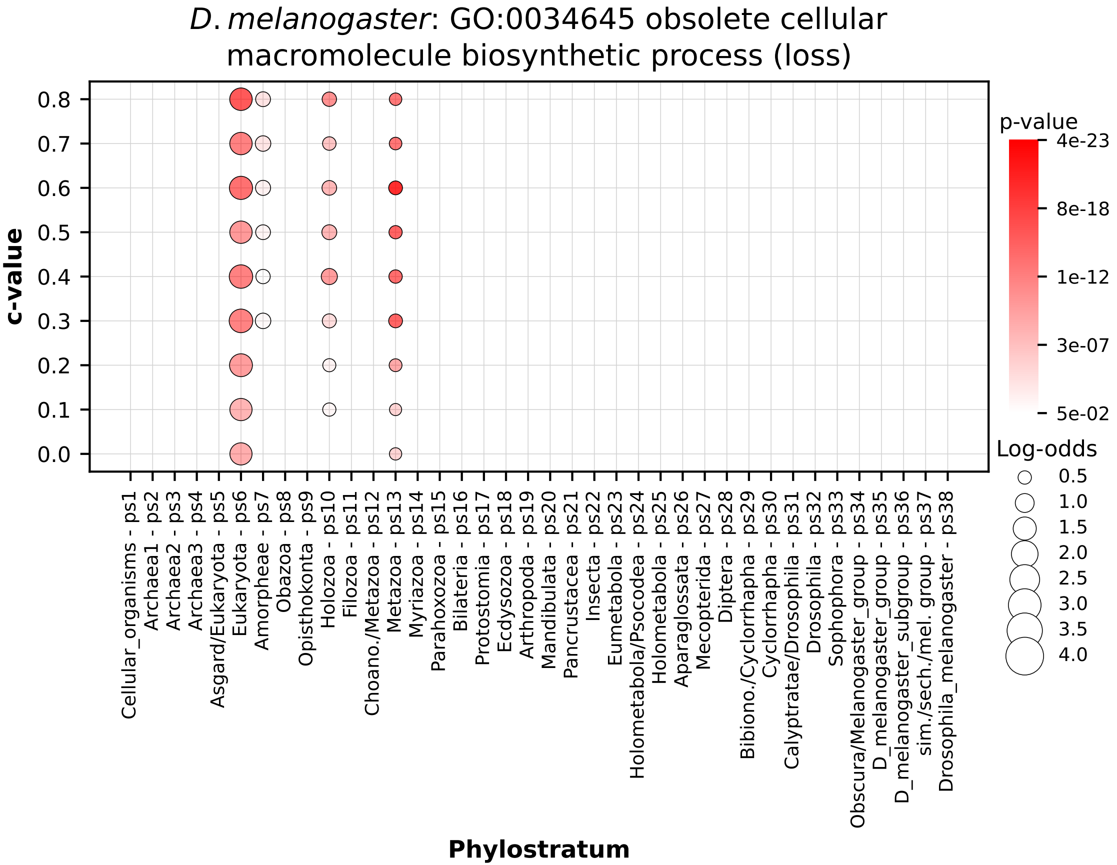
D. melanogaster: GO:0034645 obsolete cellular macromolecule biosynthetic process (loss)
c-value
0.8
0.7
0.6
0.5
0.4
0.3
0.2
0.1
0.0
p-value
4e-23
8e-18
1e-12
3e-07
5e-02
Log-odds
0.5
1.0
1.5
2.0
2.5
3.0
3.5
4.0
Cellular_organisms - ps1
Archaea1 - ps2
Archaea2 - ps3
Archaea3 - ps4
Asgard/Eukaryota - ps5
Eukaryota - ps6
Amorpheae - ps7
Obazoa - ps8
Opisthokonta - ps9
Holozoa - ps10
Filozoa - ps11
Choano./Metazoa - ps12
Metazoa - ps13
Myriazoa - ps14
Parahoxozoa - ps15
Bilateria - ps16
Protostomia - ps17
Ecdysozoa - ps18
Arthropoda - ps19
Mandibulata - ps20
Pancrustacea - ps21
Insecta - ps22
Eumetabola - ps23
Holometabola/Psocodea - ps24
Holometabola - ps25
Aparaglossata - ps26
Mecopterida - ps27
Diptera - ps28
Bibiono./Cyclorrhapha - ps29
Cyclorrhapha - ps30
Calyptratae/Drosophila - ps31
Drosophila - ps32
Sophophora - ps33
Obscura/Melanogaster_group - ps34
D_melanogaster_group - ps35
D_melanogaster_subgroup - ps36
sim./sech./mel. group - ps37
Drosophila_melanogaster - ps38
Phylostratum

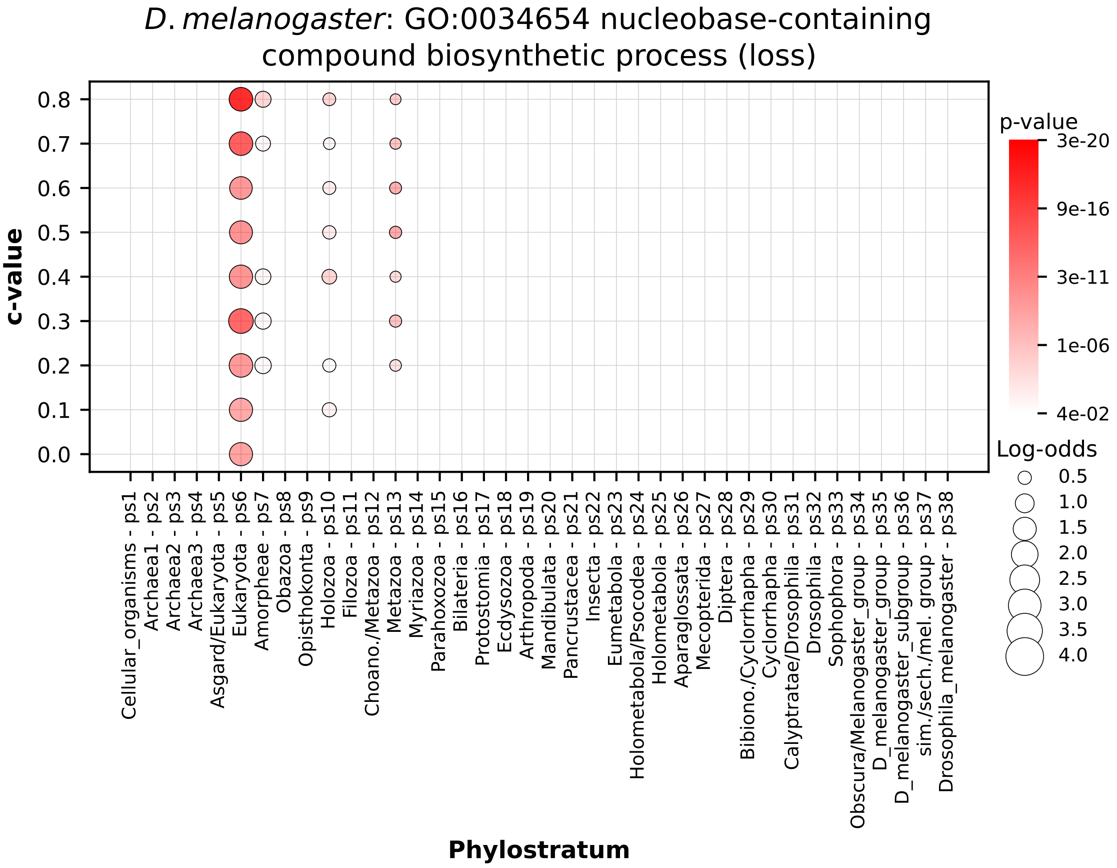

D. melanogaster: GO:0034654 nucleobase-containing compound biosynthetic process (loss)
c-value
0.8
0.7
0.6
0.5
0.4
0.3
0.2
0.1
0.0
Phylostratum
Cellular_organisms - ps1
Archaea1 - ps2
Archaea2 - ps3
Archaea3 - ps4
Asgard/Eukaryota - ps5
Eukaryota - ps6
Amorpheae - ps7
Obazoa - ps8
Opisthokonta - ps9
Holozoa - ps10
Filozoa - ps11
Choano./Metazoa - ps12
Metazoa - ps13
Myriazoa - ps14
Parahoxozoa - ps15
Bilateria - ps16
Protostomia - ps17
Ecdysozoa - ps18
Arthropoda - ps19
Mandibulata - ps20
Pancrustacea - ps21
Insecta - ps22
Eumetabola - ps23
Holometabola/Psocodea - ps24
Holometabola - ps25
Aparaglossata - ps26
Mecopterida - ps27
Diptera - ps28
Bibiono./Cyclorrhapha - ps29
Cyclorrhapha - ps30
Calyptratae/Drosophila - ps31
Drosophila - ps32
Sophophora - ps33
Obscura/Melanogaster_group - ps34
D_melanogaster_group - ps35
D_melanogaster_subgroup - ps36
sim./sech./mel. group - ps37
Drosophila_melanogaster - ps38
p-value
3e-20
9e-16
3e-11
1e-06
4e-02
Log-odds
0.5
1.0
1.5
2.0
2.5
3.0
3.5
4.0

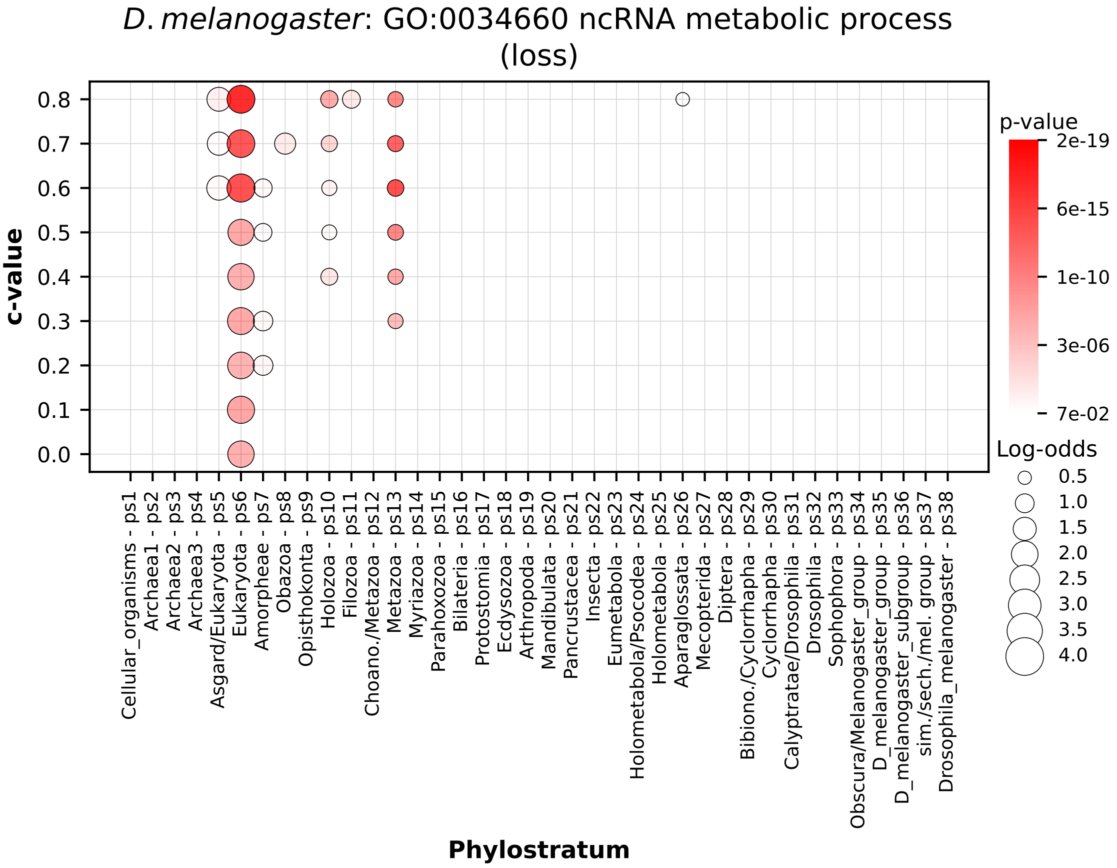

D. melanogaster: GO:0034660 ncRNA metabolic process (loss)
c-value
0.8
0.7
0.6
0.5
0.4
0.3
0.2
0.1
0.0
Phylostratum
Cellular_organisms - ps1
Archaea1 - ps2
Archaea2 - ps3
Archaea3 - ps4
Asgard/Eukaryota - ps5
Eukaryota - ps6
Amorpheae - ps7
Obazoa - ps8
Opisthokonta - ps9
Holozoa - ps10
Filozoa - ps11
Choano./Metazoa - ps12
Metazoa - ps13
Myriazoa - ps14
Parahoxozoa - ps15
Bilateria - ps16
Protostomia - ps17
Ecdysozoa - ps18
Arthropoda - ps19
Mandibulata - ps20
Pancrustacea - ps21
Insecta - ps22
Eumetabola - ps23
Holometabola/Psocodea - ps24
Holometabola - ps25
Aparaglossata - ps26
Mecopterida - ps27
Diptera - ps28
Bibiono./Cyclorrhapha - ps29
Cyclorrhapha - ps30
Calyptratae/Drosophila - ps31
Drosophila - ps32
Sophophora - ps33
Obscura/Melanogaster_group - ps34
D_melanogaster_group - ps35
D_melanogaster_subgroup - ps36
sim./sech./mel. group - ps37
Drosophila_melanogaster - ps38
p-value
2e-19
6e-15
1e-10
3e-06
7e-02
Log-odds
0.5
1.0
1.5
2.0
2.5
3.0
3.5
4.0

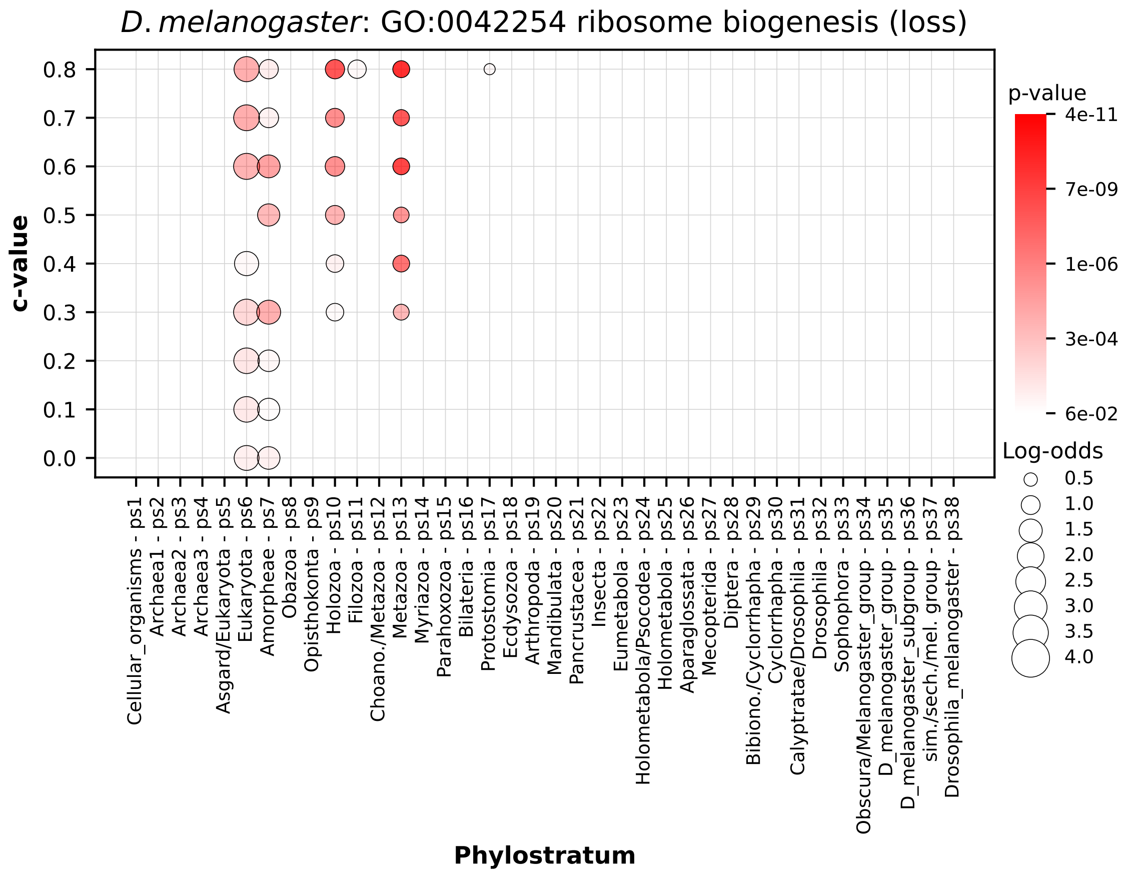

D. melanogaster: GO:0042254 ribosome biogenesis (loss)
c-value
0.8
0.7
0.6
0.5
0.4
0.3
0.2
0.1
0.0
Phylostratum
Cellular_organisms - ps1
Archaea1 - ps2
Archaea2 - ps3
Archaea3 - ps4
Asgard/Eukaryota - ps5
Eukaryota - ps6
Amorpheae - ps7
Obazoa - ps8
Opisthokonta - ps9
Holozoa - ps10
Filozoa - ps11
Choano./Metazoa - ps12
Metazoa - ps13
Myriazoa - ps14
Parahoxozoa - ps15
Bilateria - ps16
Protostomia - ps17
Ecdysozoa - ps18
Arthropoda - ps19
Mandibulata - ps20
Pancrustacea - ps21
Insecta - ps22
Eumetabola - ps23
Holometabola/Psocodea - ps24
Holometabola - ps25
Aparaglossata - ps26
Mecopterida - ps27
Diptera - ps28
Bibiono./Cyclorrhapha - ps29
Cyclorrhapha - ps30
Calyptratae/Drosophila - ps31
Drosophila - ps32
Sophophora - ps33
Obscura/Melanogaster_group - ps34
D_melanogaster_group - ps35
D_melanogaster_subgroup - ps36
sim./sech./mel. group - ps37
Drosophila_melanogaster - ps38
p-value
4e-11
7e-09
1e-06
3e-04
6e-02
Log-odds
0.5
1.0
1.5
2.0
2.5
3.0
3.5
4.0

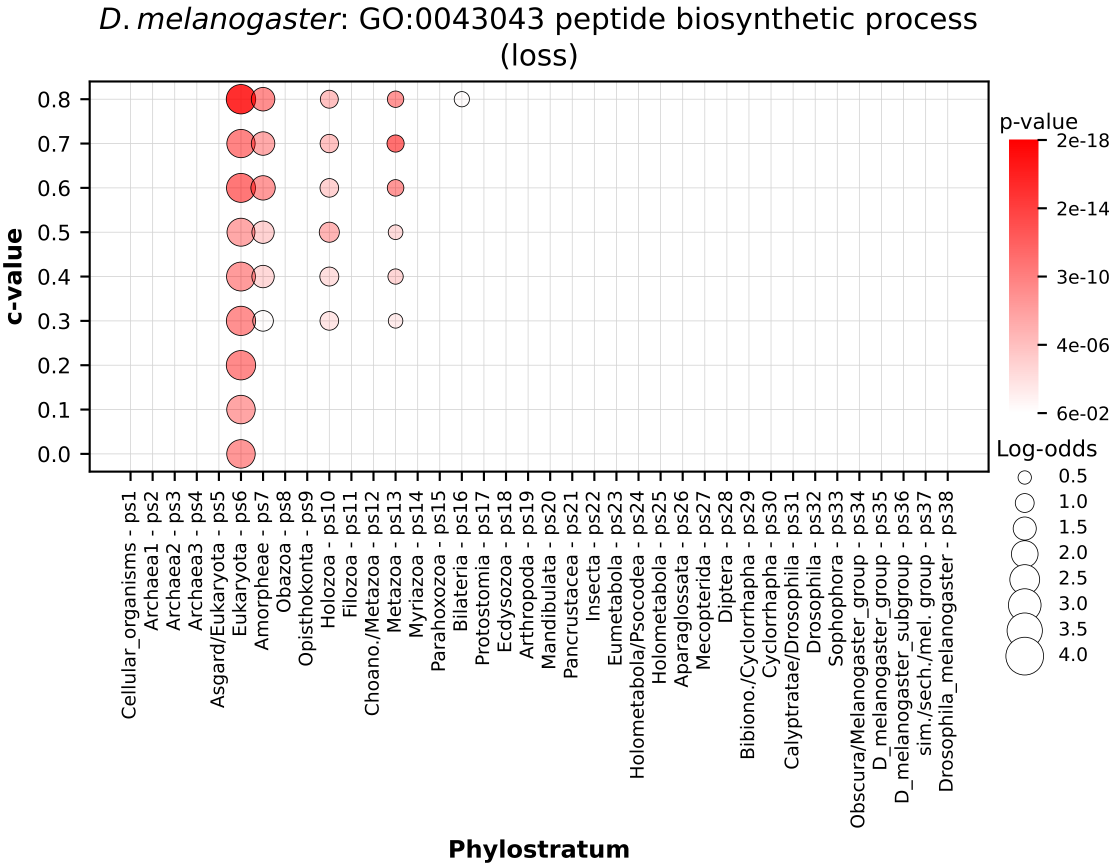

D. melanogaster: GO:0043043 peptide biosynthetic process (loss)
c-value
0.8
0.7
0.6
0.5
0.4
0.3
0.2
0.1
0.0
Phylostratum
Cellular_organisms - ps1
Archaea1 - ps2
Archaea2 - ps3
Archaea3 - ps4
Asgard/Eukaryota - ps5
Eukaryota - ps6
Amorpheae - ps7
Obazoa - ps8
Opisthokonta - ps9
Holozoa - ps10
Filozoa - ps11
Choano./Metazoa - ps12
Metazoa - ps13
Myriazoa - ps14
Parahoxozoa - ps15
Bilateria - ps16
Protostomia - ps17
Ecdysozoa - ps18
Arthropoda - ps19
Mandibulata - ps20
Pancrustacea - ps21
Insecta - ps22
Eumetabola - ps23
Holometabola/Psocodea - ps24
Holometabola - ps25
Aparaglossata - ps26
Mecopterida - ps27
Diptera - ps28
Bibiono./Cyclorrhapha - ps29
Cyclorrhapha - ps30
Calyptratae/Drosophila - ps31
Drosophila - ps32
Sophophora - ps33
Obscura/Melanogaster_group - ps34
D_melanogaster_group - ps35
D_melanogaster_subgroup - ps36
sim./sech./mel. group - ps37
Drosophila_melanogaster - ps38
p-value
2e-18
2e-14
3e-10
4e-06
6e-02
Log-odds
0.5
1.0
1.5
2.0
2.5
3.0
3.5
4.0

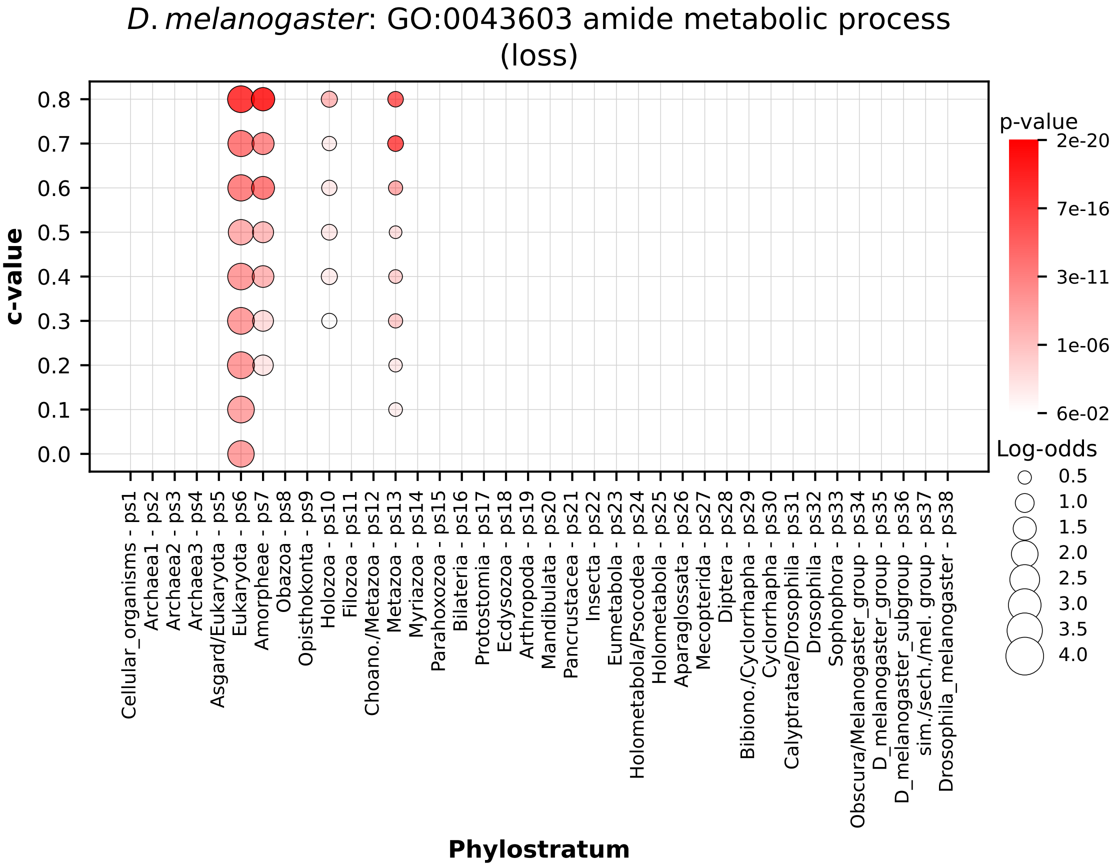

D. melanogaster: GO:0043603 amide metabolic process (loss)
c-value
0.8
0.7
0.6
0.5
0.4
0.3
0.2
0.1
0.0
p-value
2e-20
7e-16
3e-11
1e-06
6e-02
Log-odds
0.5
1.0
1.5
2.0
2.5
3.0
3.5
4.0
Cellular_organisms - ps1
Archaea1 - ps2
Archaea2 - ps3
Archaea3 - ps4
Asgard/Eukaryota - ps5
Eukaryota - ps6
Amorpheae - ps7
Obazoa - ps8
Opisthokonta - ps9
Holozoa - ps10
Filozoa - ps11
Choano./Metazoa - ps12
Metazoa - ps13
Myriazoa - ps14
Parahoxozoa - ps15
Bilateria - ps16
Protostomia - ps17
Ecdysozoa - ps18
Arthropoda - ps19
Mandibulata - ps20
Pancrustacea - ps21
Insecta - ps22
Eumetabola - ps23
Holometabola/Psocodea - ps24
Holometabola - ps25
Aparaglossata - ps26
Mecopterida - ps27
Diptera - ps28
Bibiono./Cyclorrhapha - ps29
Cyclorrhapha - ps30
Calyptratae/Drosophila - ps31
Drosophila - ps32
Sophophora - ps33
Obscura/Melanogaster_group - ps34
D_melanogaster_group - ps35
D_melanogaster_subgroup - ps36
sim./sech./mel. group - ps37
Drosophila_melanogaster - ps38
Phylostratum

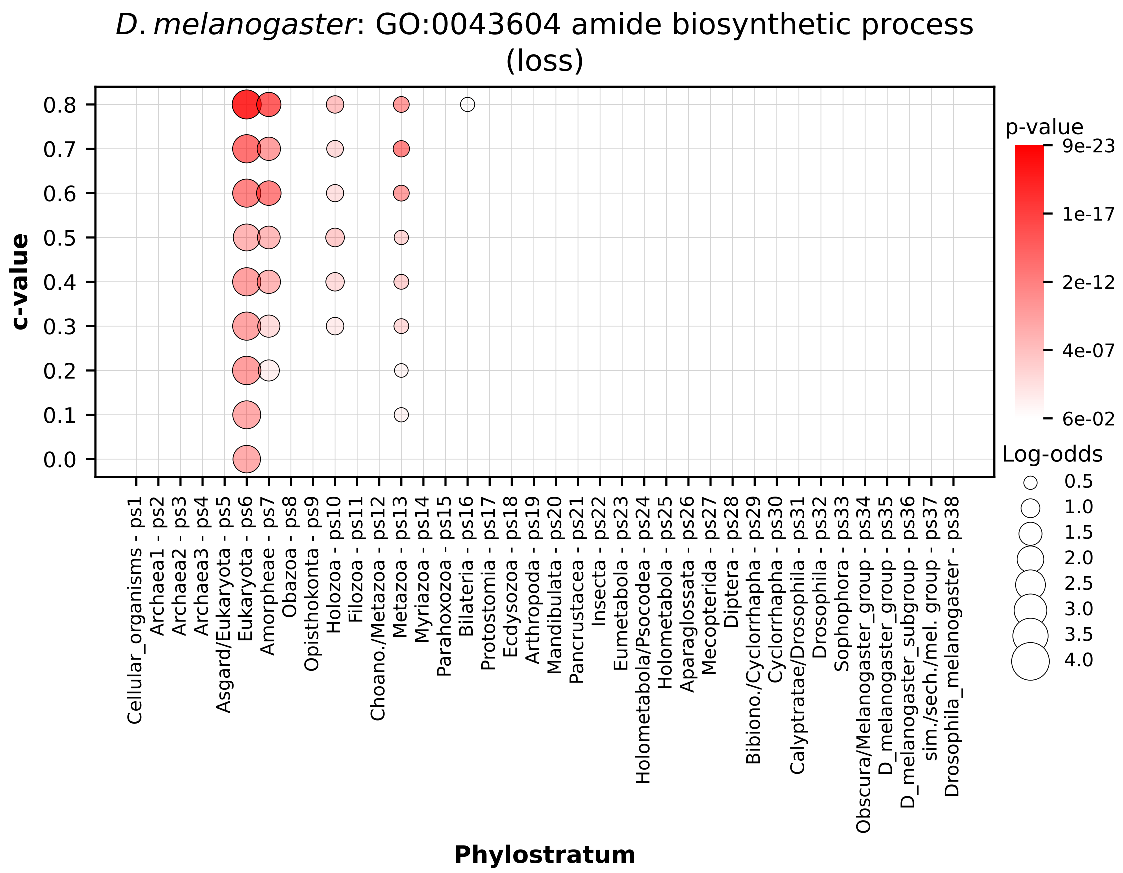
D. melanogaster: GO:0043604 amide biosynthetic process
(loss)
c-value
0.8
0.7
0.6
0.5
0.4
0.3
0.2
0.1
0.0
p-value
9e-23
1e-17
2e-12
4e-07
6e-02
Log-odds
0.5
1.0
1.5
2.0
2.5
3.0
3.5
4.0
Cellular_organisms - ps1
Archaea1 - ps2
Archaea2 - ps3
Archaea3 - ps4
Asgard/Eukaryota - ps5
Eukaryota - ps6
Amorpheae - ps7
Obazoa - ps8
Opisthokonta - ps9
Holozoa - ps10
Filozoa - ps11
Choano./Metazoa - ps12
Metazoa - ps13
Myriazoa - ps14
Parahoxozoa - ps15
Bilateria - ps16
Protostomia - ps17
Ecdysozoa - ps18
Arthropoda - ps19
Mandibulata - ps20
Pancrustacea - ps21
Insecta - ps22
Eumetabola - ps23
Holometabola/Psocodea - ps24
Holometabola - ps25
Aparaglossata - ps26
Mecopterida - ps27
Diptera - ps28
Bibiono./Cyclorrhapha - ps29
Cyclorrhapha - ps30
Calyptratae/Drosophila - ps31
Drosophila - ps32
Sophophora - ps33
Obscura/Melanogaster_group - ps34
D_melanogaster_group - ps35
D_melanogaster_subgroup - ps36
sim./sech./mel. group - ps37
Drosophila_melanogaster - ps38
Phylostratum

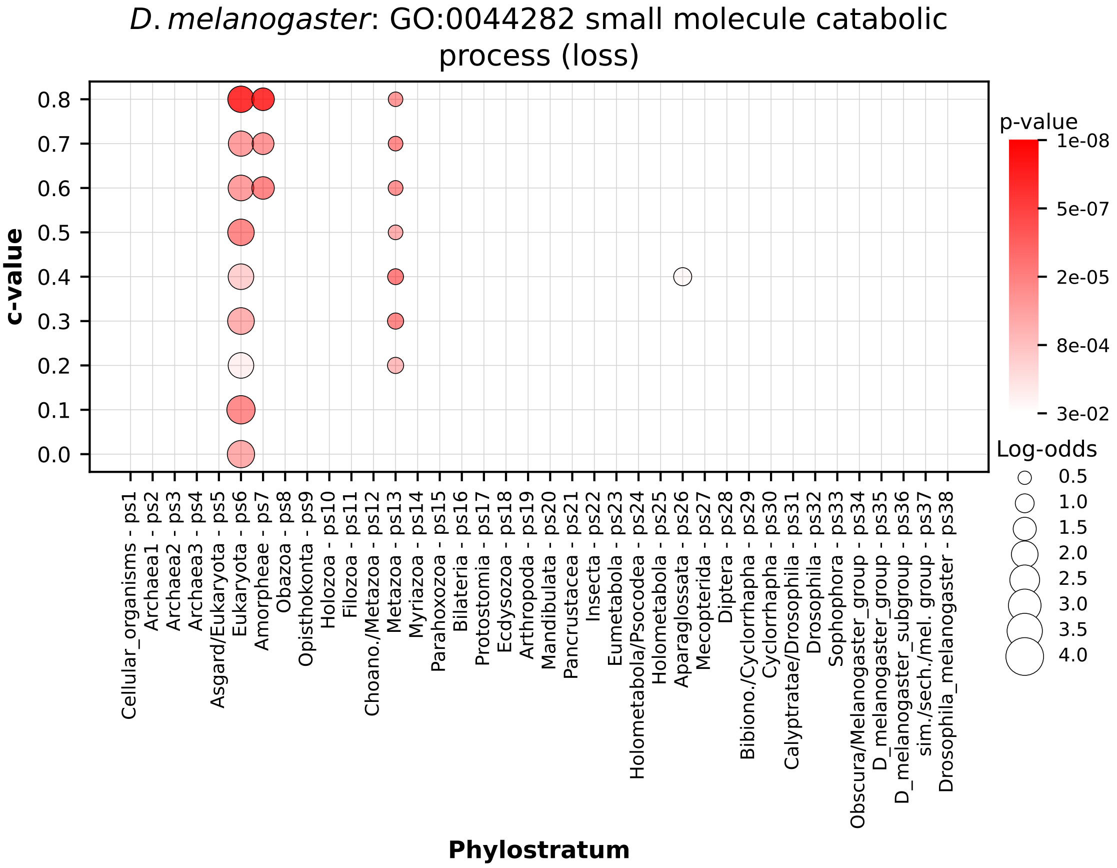

D. melanogaster: GO:0044282 small molecule catabolic process (loss)
c-value
Phylostratum
p-value
1e-08
5e-07
2e-05
8e-04
3e-02
Log-odds
0.5
1.0
1.5
2.0
2.5
3.0
3.5
4.0
Cellular_organisms - ps1
Archaea1 - ps2
Archaea2 - ps3
Archaea3 - ps4
Asgard/Eukaryota - ps5
Eukaryota - ps6
Amorpheae - ps7
Obazoa - ps8
Opisthokonta - ps9
Holozoa - ps10
Filozoa - ps11
Choano./Metazoa - ps12
Metazoa - ps13
Myriazoa - ps14
Parahoxozoa - ps15
Bilateria - ps16
Protostomia - ps17
Ecdysozoa - ps18
Arthropoda - ps19
Mandibulata - ps20
Pancrustacea - ps21
Insecta - ps22
Eumetabola - ps23
Holometabola/Psocodea - ps24
Holometabola - ps25
Aparaglossata - ps26
Mecopterida - ps27
Diptera - ps28
Bibiono./Cyclorrhapha - ps29
Cyclorrhapha - ps30
Calyptratae/Drosophila - ps31
Drosophila - ps32
Sophophora - ps33
Obscura/Melanogaster_group - ps34
D_melanogaster_group - ps35
D_melanogaster_subgroup - ps36
sim./sech./mel. group - ps37
Drosophila_melanogaster - ps38

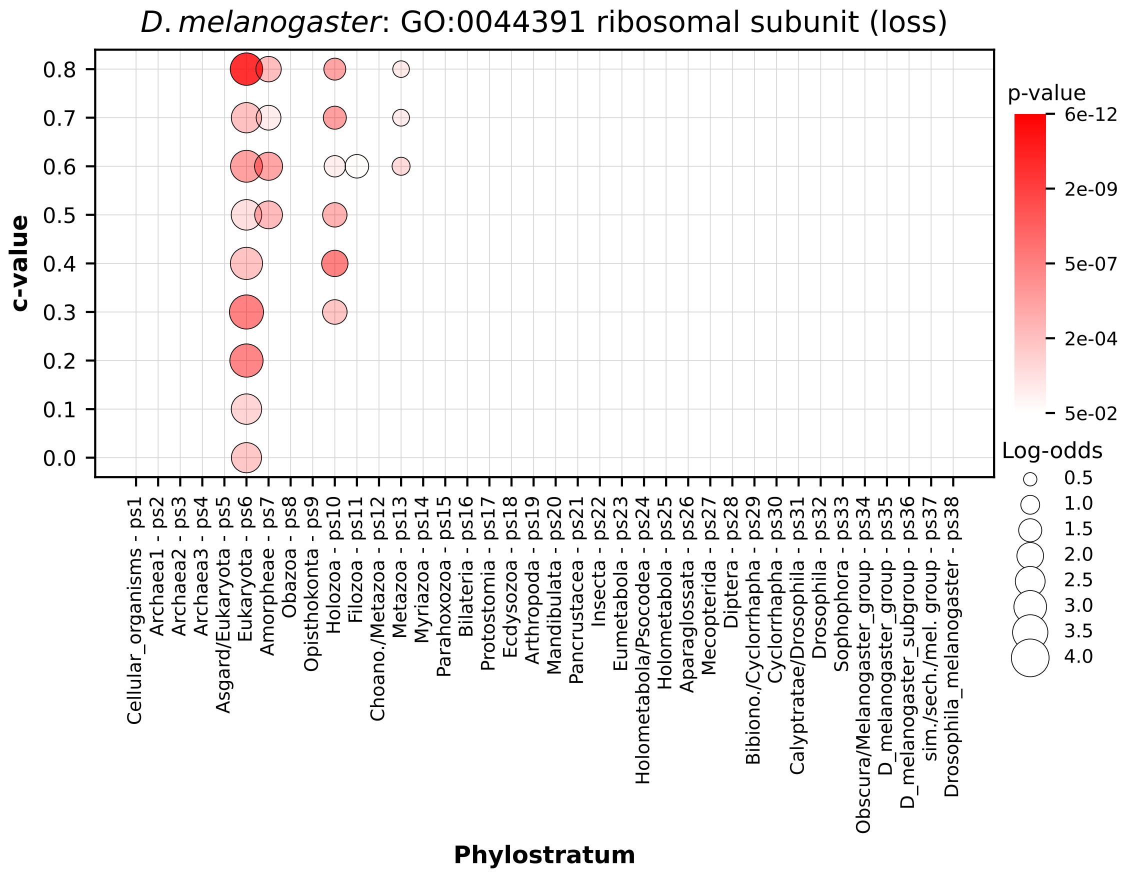

D. melanogaster: GO:0044391 ribosomal subunit (loss)
c-value
0.8
0.7
0.6
0.5
0.4
0.3
0.2
0.1
0.0
p-value
6e-12
2e-09
5e-07
2e-04
5e-02
Log-odds
0.5
1.0
1.5
2.0
2.5
3.0
3.5
4.0
Cellular_organisms - ps1
Archaea1 - ps2
Archaea2 - ps3
Archaea3 - ps4
Asgard/Eukaryota - ps5
Eukaryota - ps6
Amorpheae - ps7
Obazoa - ps8
Opisthokonta - ps9
Holozoa - ps10
Filozoa - ps11
Choano./Metazoa - ps12
Metazoa - ps13
Myriazoa - ps14
Parahoxozoa - ps15
Bilateria - ps16
Protostomia - ps17
Ecdysozoa - ps18
Arthropoda - ps19
Mandibulata - ps20
Pancrustacea - ps21
Insecta - ps22
Eumetabola - ps23
Holometabola/Psocodea - ps24
Holometabola - ps25
Aparaglossata - ps26
Mecopterida - ps27
Diptera - ps28
Bibiono./Cyclorrhapha - ps29
Cyclorrhapha - ps30
Calyptratae/Drosophila - ps31
Drosophila - ps32
Sophophora - ps33
Obscura/Melanogaster_group - ps34
D_melanogaster_group - ps35
D_melanogaster_subgroup - ps36
sim./sech./mel. group - ps37
Drosophila_melanogaster - ps38
Phylostratum

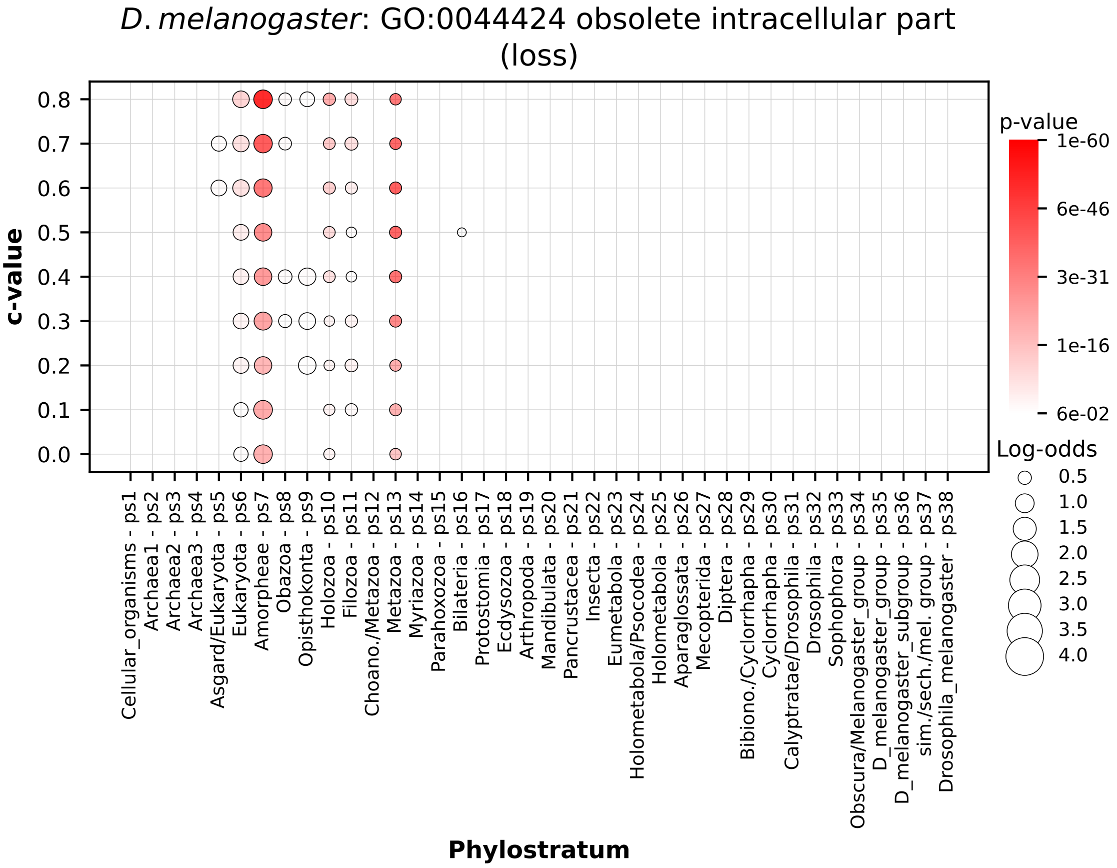

D. melanogaster: GO:0044424 obsolete intracellular part
(loss)
c-value
0.8
0.7
0.6
0.5
0.4
0.3
0.2
0.1
0.0
Phylostratum
Cellular_organisms - ps1
Archaea1 - ps2
Archaea2 - ps3
Archaea3 - ps4
Asgard/Eukaryota - ps5
Eukaryota - ps6
Amorpheae - ps7
Obazoa - ps8
Opisthokonta - ps9
Holozoa - ps10
Filozoa - ps11
Choano./Metazoa - ps12
Metazoa - ps13
Myriazoa - ps14
Parahoxozoa - ps15
Bilateria - ps16
Protostomia - ps17
Ecdysozoa - ps18
Arthropoda - ps19
Mandibulata - ps20
Pancrustacea - ps21
Insecta - ps22
Eumetabola - ps23
Holometabola/Psocodea - ps24
Holometabola - ps25
Aparaglossata - ps26
Mecopterida - ps27
Diptera - ps28
Bibiono./Cyclorrhapha - ps29
Cyclorrhapha - ps30
Calyptratae/Drosophila - ps31
Drosophila - ps32
Sophophora - ps33
Obscura/Melanogaster_group - ps34
D_melanogaster_group - ps35
D_melanogaster_subgroup - ps36
sim./sech./mel. group - ps37
Drosophila_melanogaster - ps38
p-value
1e-60
6e-46
3e-31
1e-16
6e-02
Log-odds
0.5
1.0
1.5
2.0
2.5
3.0
3.5
4.0

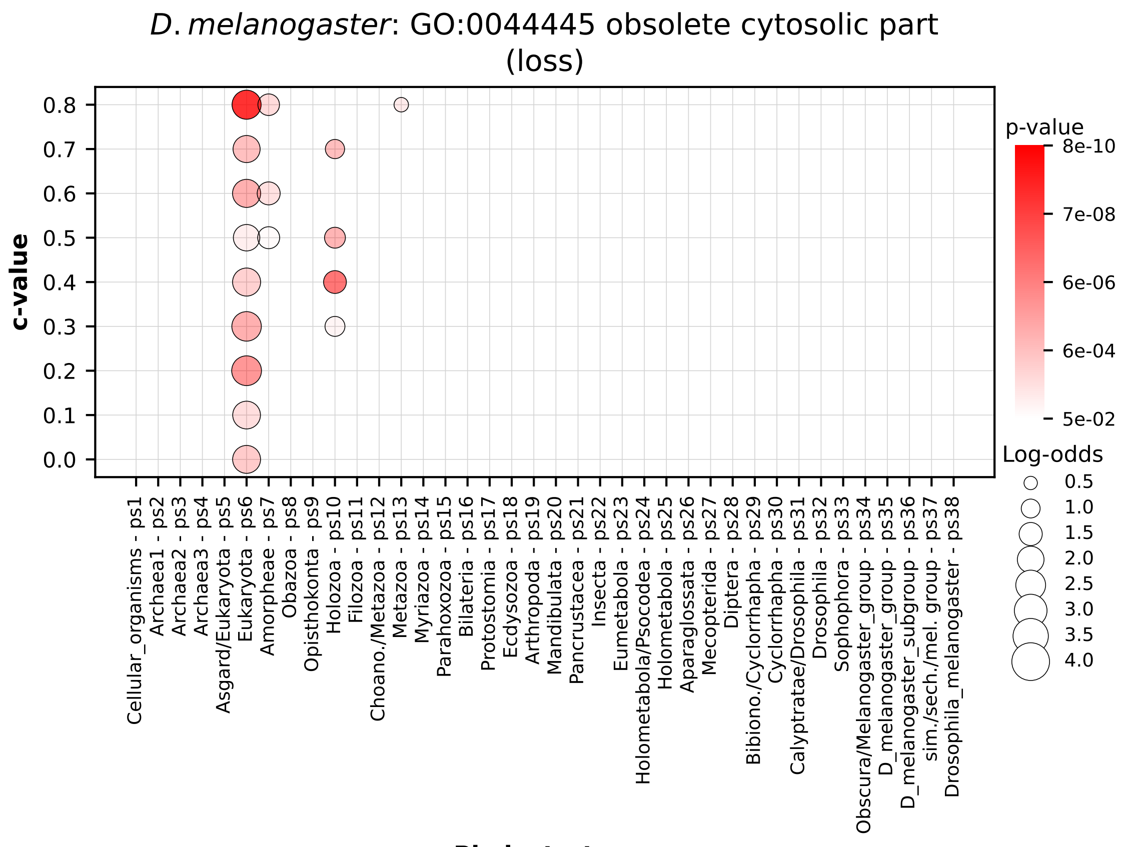

D. melanogaster: GO:0044445 obsolete cytosolic part
(loss)
c-value
0.8
0.7
0.6
0.5
0.4
0.3
0.2
0.1
0.0
p-value
8e-10
7e-08
6e-06
6e-04
5e-02
Log-odds
0.5
1.0
1.5
2.0
2.5
3.0
3.5
4.0
Cellular_organisms - ps1
Archaea1 - ps2
Archaea2 - ps3
Archaea3 - ps4
Asgard/Eukaryota - ps5
Eukaryota - ps6
Amorpheae - ps7
Obazoa - ps8
Opisthokonta - ps9
Holozoa - ps10
Filozoa - ps11
Choano./Metazoa - ps12
Metazoa - ps13
Myriazoa - ps14
Parahoxozoa - ps15
Bilateria - ps16
Protostomia - ps17
Ecdysozoa - ps18
Arthropoda - ps19
Mandibulata - ps20
Pancrustacea - ps21
Insecta - ps22
Eumetabola - ps23
Holometabola/Psocodea - ps24
Holometabola - ps25
Aparaglossata - ps26
Mecopterida - ps27
Diptera - ps28
Bibiono./Cyclorrhapha - ps29
Cyclorrhapha - ps30
Calyptratae/Drosophila - ps31
Drosophila - ps32
Sophophora - ps33
Obscura/Melanogaster_group - ps34
D_melanogaster_group - ps35
D_melanogaster_subgroup - ps36
sim./sech./mel. group - ps37
Drosophila_melanogaster - ps38
Phylostratum

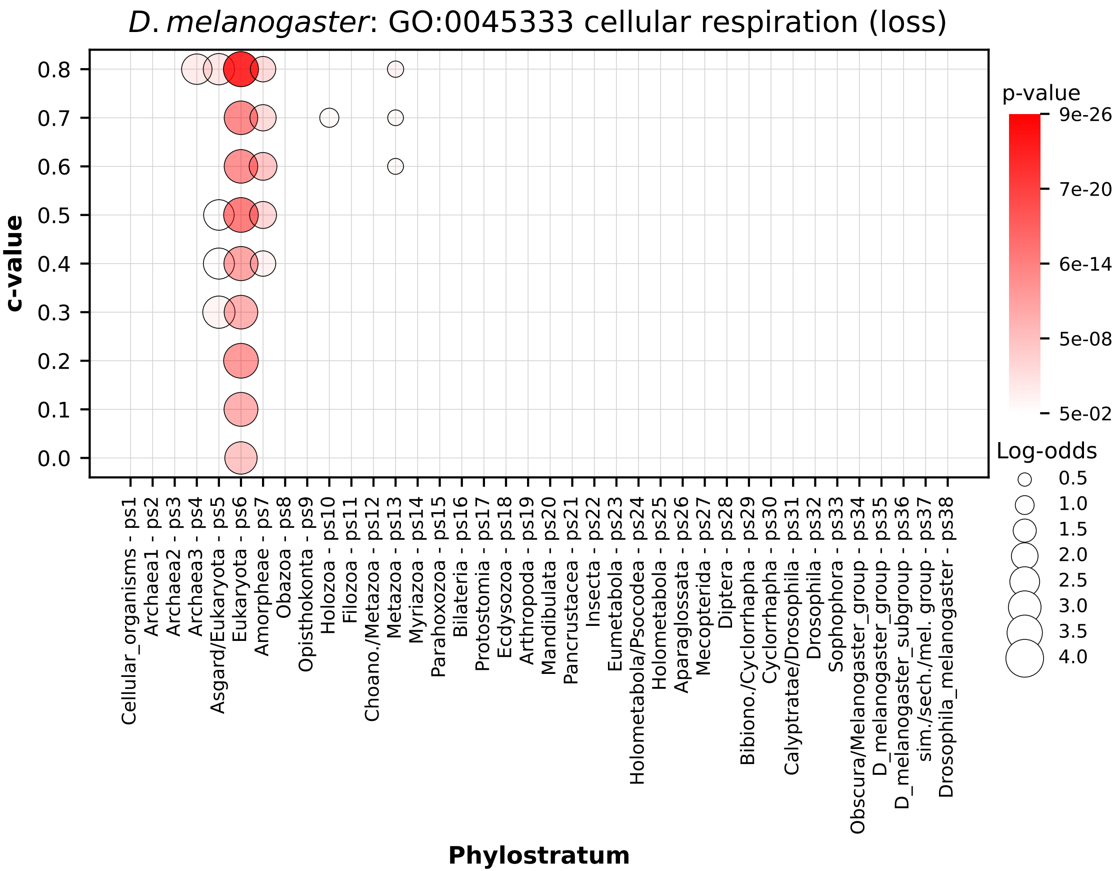

D. melanogaster: GO:0045333 cellular respiration (loss)
c-value
0.8
0.7
0.6
0.5
0.4
0.3
0.2
0.1
0.0
Phylostratum
Cellular_organisms - ps1
Archaea1 - ps2
Archaea2 - ps3
Archaea3 - ps4
Asgard/Eukaryota - ps5
Eukaryota - ps6
Amorpheae - ps7
Obazoa - ps8
Opisthokonta - ps9
Holozoa - ps10
Filozoa - ps11
Choano./Metazoa - ps12
Metazoa - ps13
Myriazoa - ps14
Parahoxozoa - ps15
Bilateria - ps16
Protostomia - ps17
Ecdysozoa - ps18
Arthropoda - ps19
Mandibulata - ps20
Pancrustacea - ps21
Insecta - ps22
Eumetabola - ps23
Holometabola/Psocodea - ps24
Holometabola - ps25
Aparaglossata - ps26
Mecopterida - ps27
Diptera - ps28
Bibiono./Cyclorrhapha - ps29
Cyclorrhapha - ps30
Calyptratae/Drosophila - ps31
Drosophila - ps32
Sophophora - ps33
Obscura/Melanogaster_group - ps34
D_melanogaster_group - ps35
D_melanogaster_subgroup - ps36
sim./sech./mel. group - ps37
Drosophila_melanogaster - ps38
p-value
9e-26
7e-20
6e-14
5e-08
5e-02
Log-odds
0.5
1.0
1.5
2.0
2.5
3.0
3.5
4.0

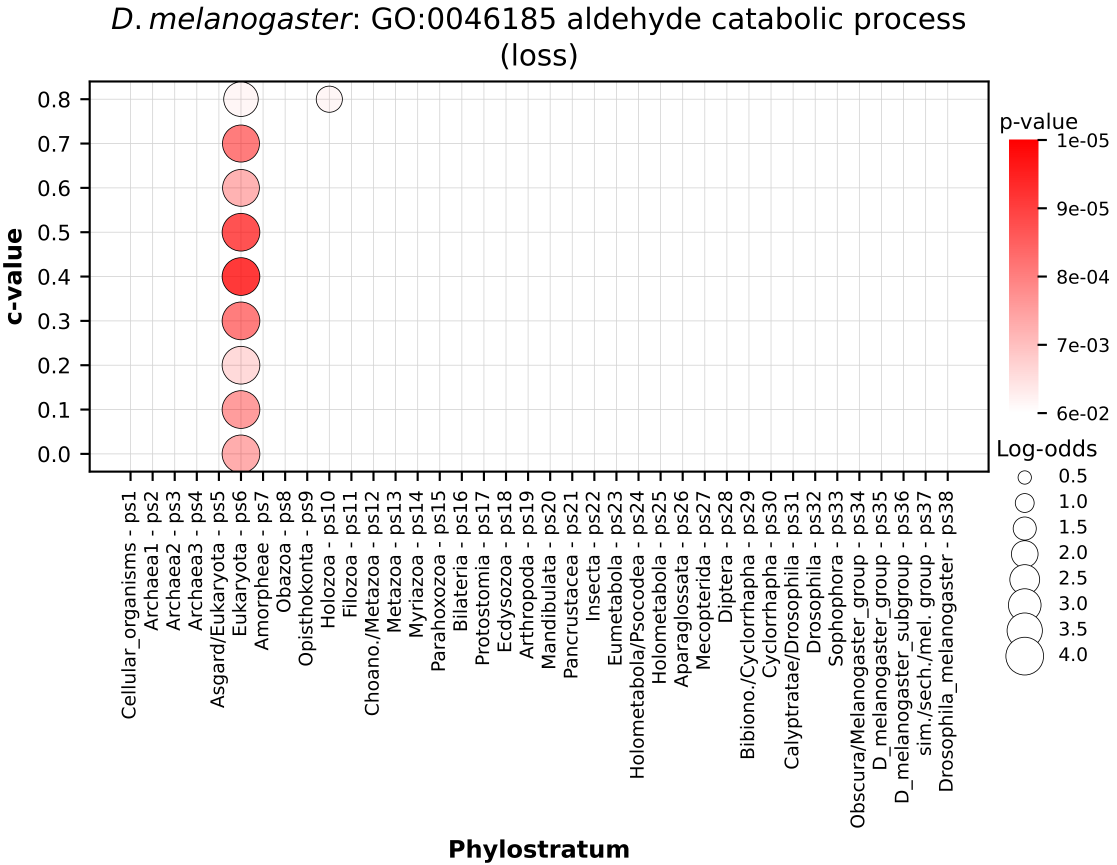
D. melanogaster: GO:0046185 aldehyde catabolic process
(loss)
c-value
0.8
0.7
0.6
0.5
0.4
0.3
0.2
0.1
0.0
Phylostratum
Cellular_organisms - ps1
Archaea1 - ps2
Archaea2 - ps3
Archaea3 - ps4
Asgard/Eukaryota - ps5
Eukaryota - ps6
Amorpheae - ps7
Obazoa - ps8
Opisthokonta - ps9
Holozoa - ps10
Filozoa - ps11
Choano./Metazoa - ps12
Metazoa - ps13
Myriazoa - ps14
Parahoxozoa - ps15
Bilateria - ps16
Protostomia - ps17
Ecdysozoa - ps18
Arthropoda - ps19
Mandibulata - ps20
Pancrustacea - ps21
Insecta - ps22
Eumetabola - ps23
Holometabola/Psocodea - ps24
Holometabola - ps25
Aparaglossata - ps26
Mecopterida - ps27
Diptera - ps28
Bibiono./Cyclorrhapha - ps29
Cyclorrhapha - ps30
Calyptratae/Drosophila - ps31
Drosophila - ps32
Sophophora - ps33
Obscura/Melanogaster_group - ps34
D_melanogaster_group - ps35
D_melanogaster_subgroup - ps36
sim./sech./mel. group - ps37
Drosophila_melanogaster - ps38
p-value
1e-05
9e-05
8e-04
7e-03
6e-02
Log-odds
0.5
1.0
1.5
2.0
2.5
3.0
3.5
4.0

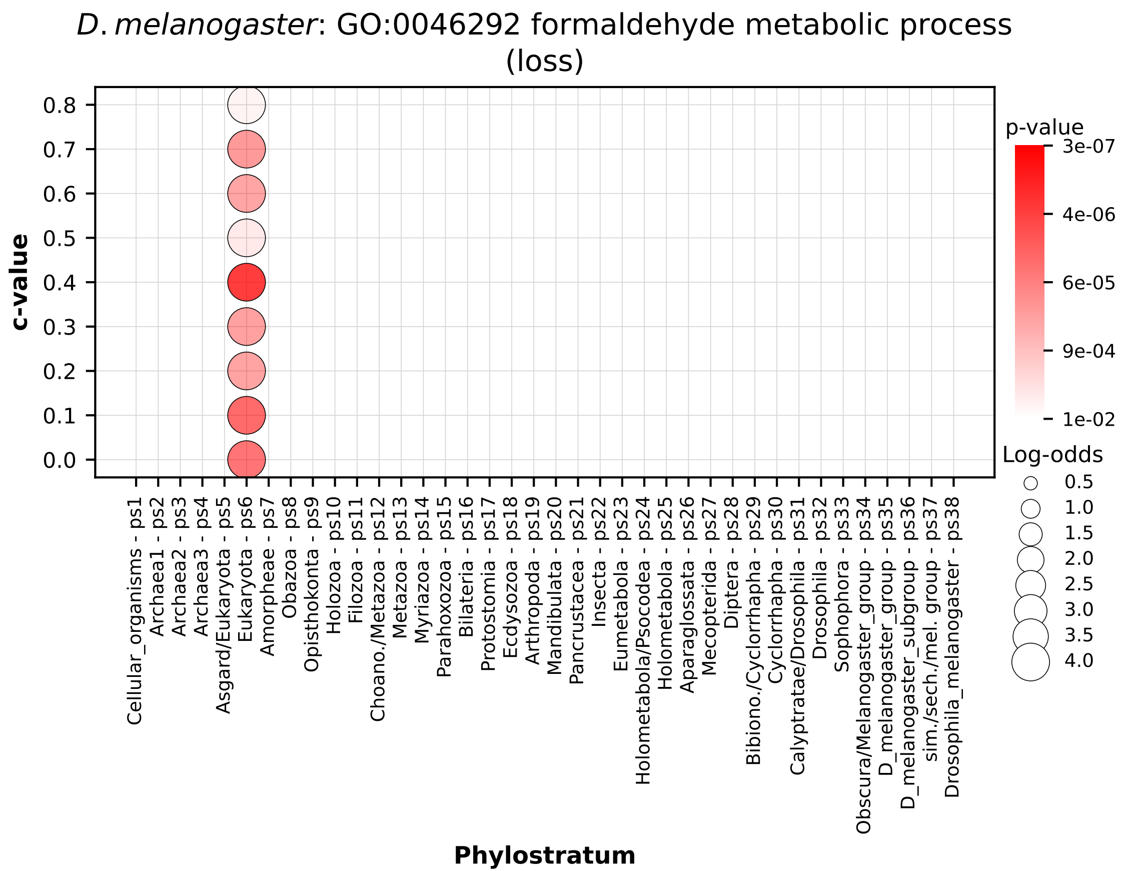

D. melanogaster: GO:0046292 formaldehyde metabolic process (loss)
c-value
0.8
0.7
0.6
0.5
0.4
0.3
0.2
0.1
0.0
Phylostratum
Cellular_organisms - ps1
Archaea1 - ps2
Archaea2 - ps3
Archaea3 - ps4
Asgard/Eukaryota - ps5
Eukaryota - ps6
Amorpheae - ps7
Obazoa - ps8
Opisthokonta - ps9
Holozoa - ps10
Filozoa - ps11
Choano./Metazoa - ps12
Metazoa - ps13
Myriazoa - ps14
Parahoxozoa - ps15
Bilateria - ps16
Protostomia - ps17
Ecdysozoa - ps18
Arthropoda - ps19
Mandibulata - ps20
Pancrustacea - ps21
Insecta - ps22
Eumetabola - ps23
Holometabola/Psocodea - ps24
Holometabola - ps25
Aparaglossata - ps26
Mecopterida - ps27
Diptera - ps28
Bibiono./Cyclorrhapha - ps29
Cyclorrhapha - ps30
Calyptratae/Drosophila - ps31
Drosophila - ps32
Sophophora - ps33
Obscura/Melanogaster_group - ps34
D_melanogaster_group - ps35
D_melanogaster_subgroup - ps36
sim./sech./mel. group - ps37
Drosophila_melanogaster - ps38
p-value
3e-07
4e-06
6e-05
9e-04
1e-02
Log-odds
0.5
1.0
1.5
2.0
2.5
3.0
3.5
4.0

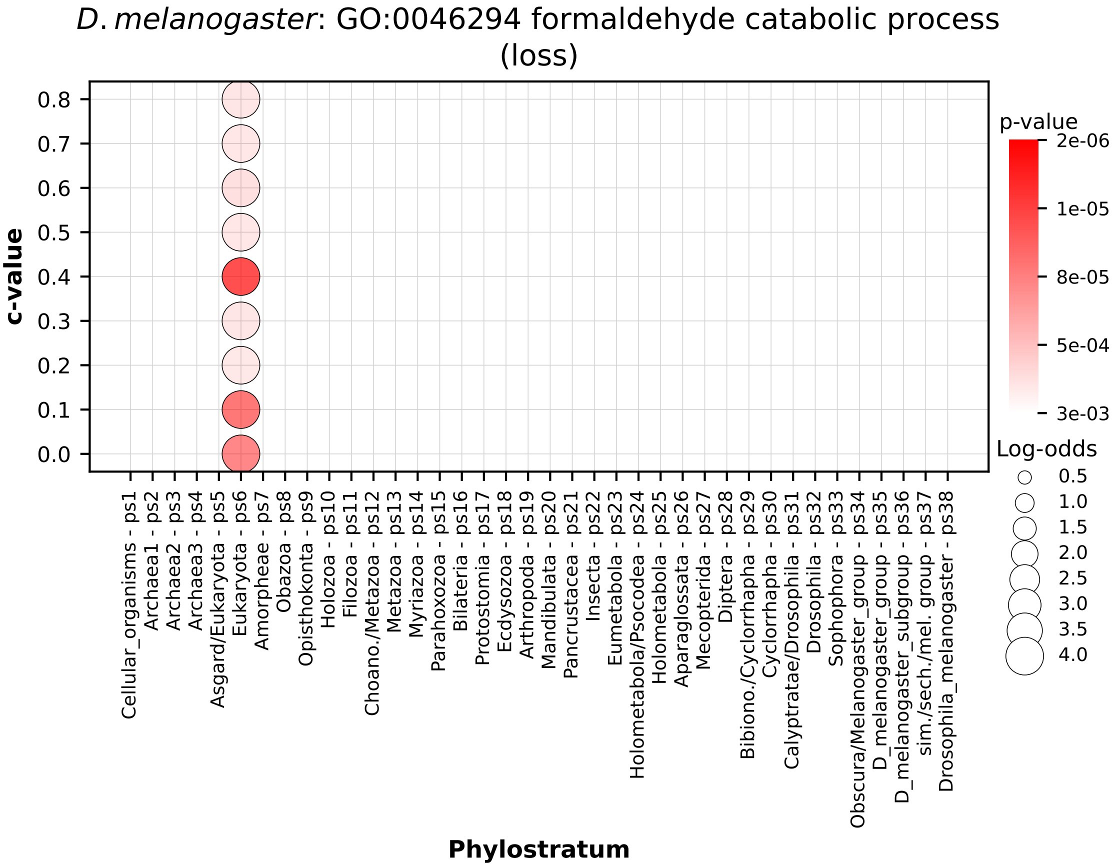

D. melanogaster: GO:0046294 formaldehyde catabolic process (loss)
c-value
0.8
0.7
0.6
0.5
0.4
0.3
0.2
0.1
0.0
p-value
2e-06
1e-05
8e-05
5e-04
3e-03
Log-odds
0.5
1.0
1.5
2.0
2.5
3.0
3.5
4.0
Cellular_organisms - ps1
Archaea1 - ps2
Archaea2 - ps3
Archaea3 - ps4
Asgard/Eukaryota - ps5
Eukaryota - ps6
Amorpheae - ps7
Obazoa - ps8
Opisthokonta - ps9
Holozoa - ps10
Filozoa - ps11
Choano./Metazoa - ps12
Metazoa - ps13
Myriazoa - ps14
Parahoxozoa - ps15
Bilateria - ps16
Protostomia - ps17
Ecdysozoa - ps18
Arthropoda - ps19
Mandibulata - ps20
Pancrustacea - ps21
Insecta - ps22
Eumetabola - ps23
Holometabola/Psocodea - ps24
Holometabola - ps25
Aparaglossata - ps26
Mecopterida - ps27
Diptera - ps28
Bibiono./Cyclorrhapha - ps29
Cyclorrhapha - ps30
Calyptratae/Drosophila - ps31
Drosophila - ps32
Sophophora - ps33
Obscura/Melanogaster_group - ps34
D_melanogaster_group - ps35
D_melanogaster_subgroup - ps36
sim./sech./mel. group - ps37
Drosophila_melanogaster - ps38
Phylostratum

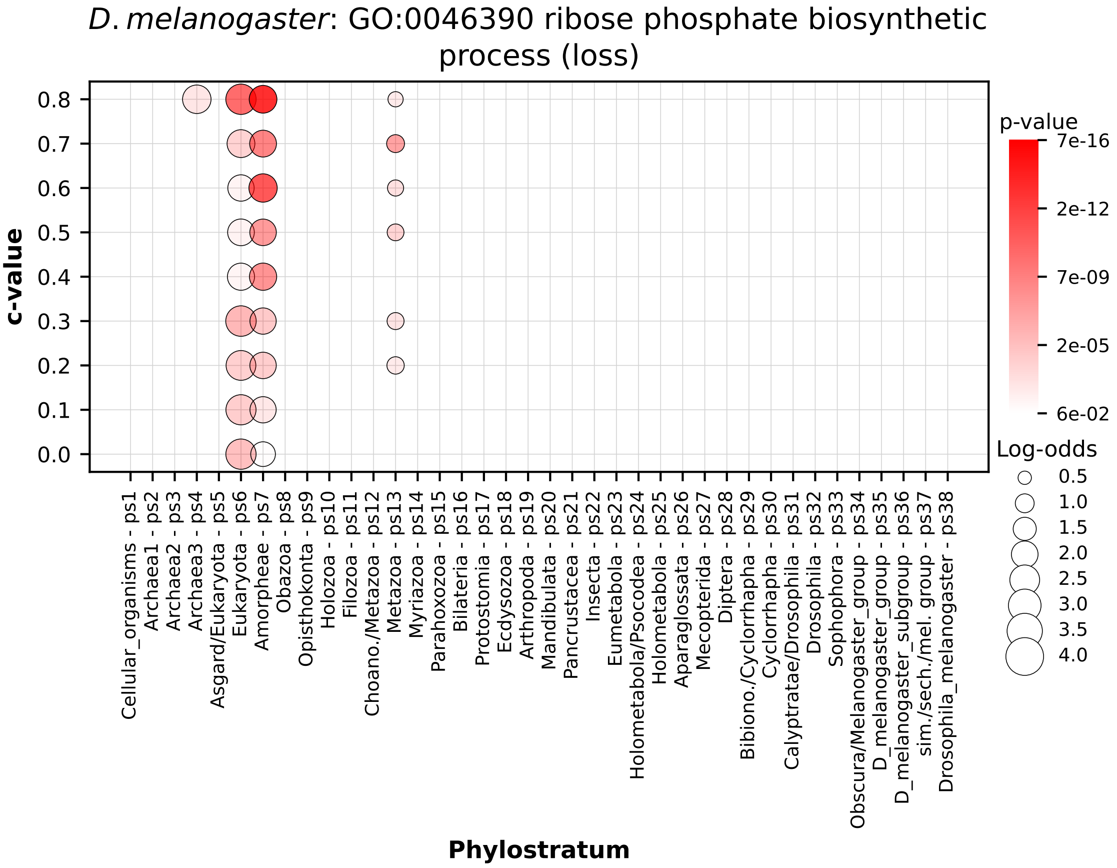

D. melanogaster: GO:0046390 ribose phosphate biosynthetic process (loss)
c-value
0.8
0.7
0.6
0.5
0.4
0.3
0.2
0.1
0.0
Phylostratum
Cellular_organisms - ps1
Archaea1 - ps2
Archaea2 - ps3
Archaea3 - ps4
Asgard/Eukaryota - ps5
Eukaryota - ps6
Amorpheae - ps7
Obazoa - ps8
Opisthokonta - ps9
Holozoa - ps10
Filozoa - ps11
Choano./Metazoa - ps12
Metazoa - ps13
Myriazoa - ps14
Parahoxozoa - ps15
Bilateria - ps16
Protostomia - ps17
Ecdysozoa - ps18
Arthropoda - ps19
Mandibulata - ps20
Pancrustacea - ps21
Insecta - ps22
Eumetabola - ps23
Holometabola/Psocodea - ps24
Holometabola - ps25
Aparaglossata - ps26
Mecopterida - ps27
Diptera - ps28
Bibiono./Cyclorrhapha - ps29
Cyclorrhapha - ps30
Calyptratae/Drosophila - ps31
Drosophila - ps32
Sophophora - ps33
Obscura/Melanogaster_group - ps34
D_melanogaster_group - ps35
D_melanogaster_subgroup - ps36
sim./sech./mel. group - ps37
Drosophila_melanogaster - ps38
p-value
7e-16
2e-12
7e-09
2e-05
6e-02
Log-odds
0.5
1.0
1.5
2.0
2.5
3.0
3.5
4.0

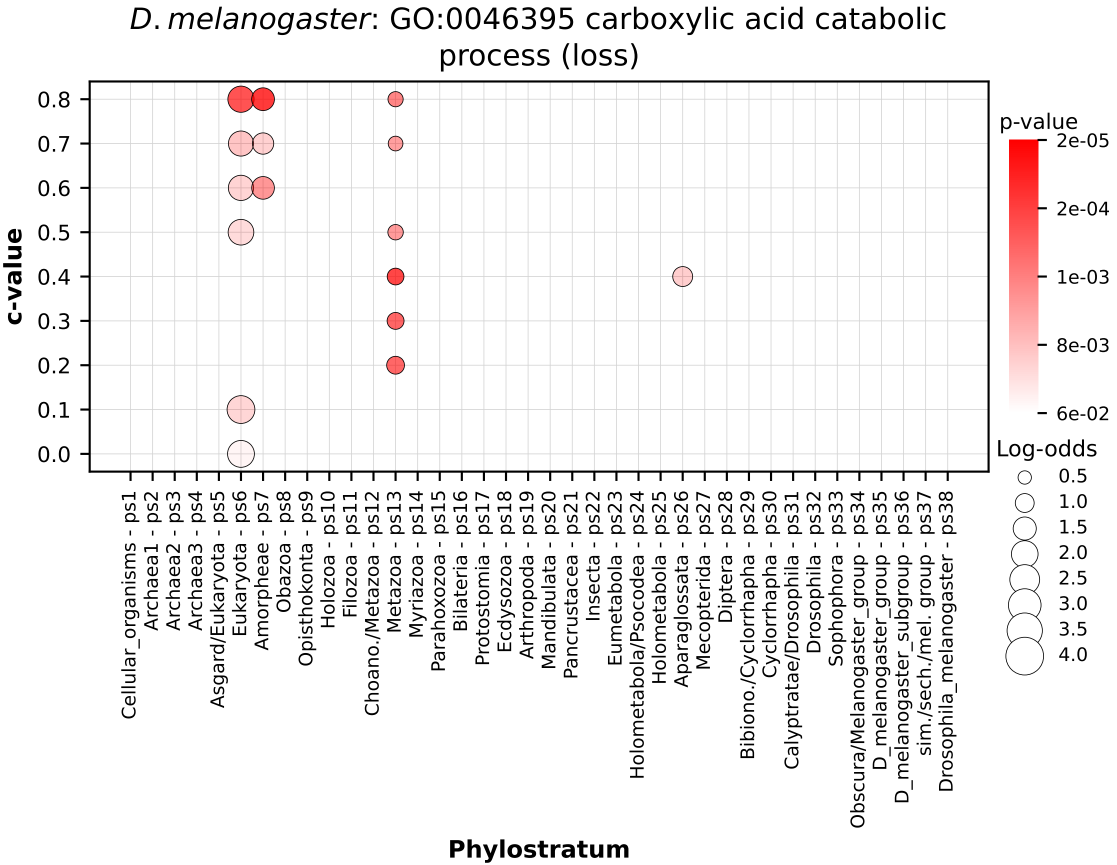

D. melanogaster: GO:0046395 carboxylic acid catabolic process (loss)
c-value
0.8
0.7
0.6
0.5
0.4
0.3
0.2
0.1
0.0
Phylostratum
Cellular_organisms - ps1
Archaea1 - ps2
Archaea2 - ps3
Archaea3 - ps4
Asgard/Eukaryota - ps5
Eukaryota - ps6
Amorpheae - ps7
Obazoa - ps8
Opisthokonta - ps9
Holozoa - ps10
Filozoa - ps11
Choano./Metazoa - ps12
Metazoa - ps13
Myriazoa - ps14
Parahoxozoa - ps15
Bilateria - ps16
Protostomia - ps17
Ecdysozoa - ps18
Arthropoda - ps19
Mandibulata - ps20
Pancrustacea - ps21
Insecta - ps22
Eumetabola - ps23
Holometabola/Psocodea - ps24
Holometabola - ps25
Aparaglossata - ps26
Mecopterida - ps27
Diptera - ps28
Bibiono./Cyclorrhapha - ps29
Cyclorrhapha - ps30
Calyptratae/Drosophila - ps31
Drosophila - ps32
Sophophora - ps33
Obscura/Melanogaster_group - ps34
D_melanogaster_group - ps35
D_melanogaster_subgroup - ps36
sim./sech./mel. group - ps37
Drosophila_melanogaster - ps38
p-value
2e-05
2e-04
1e-03
8e-03
6e-02
Log-odds
0.5
1.0
1.5
2.0
2.5
3.0
3.5
4.0

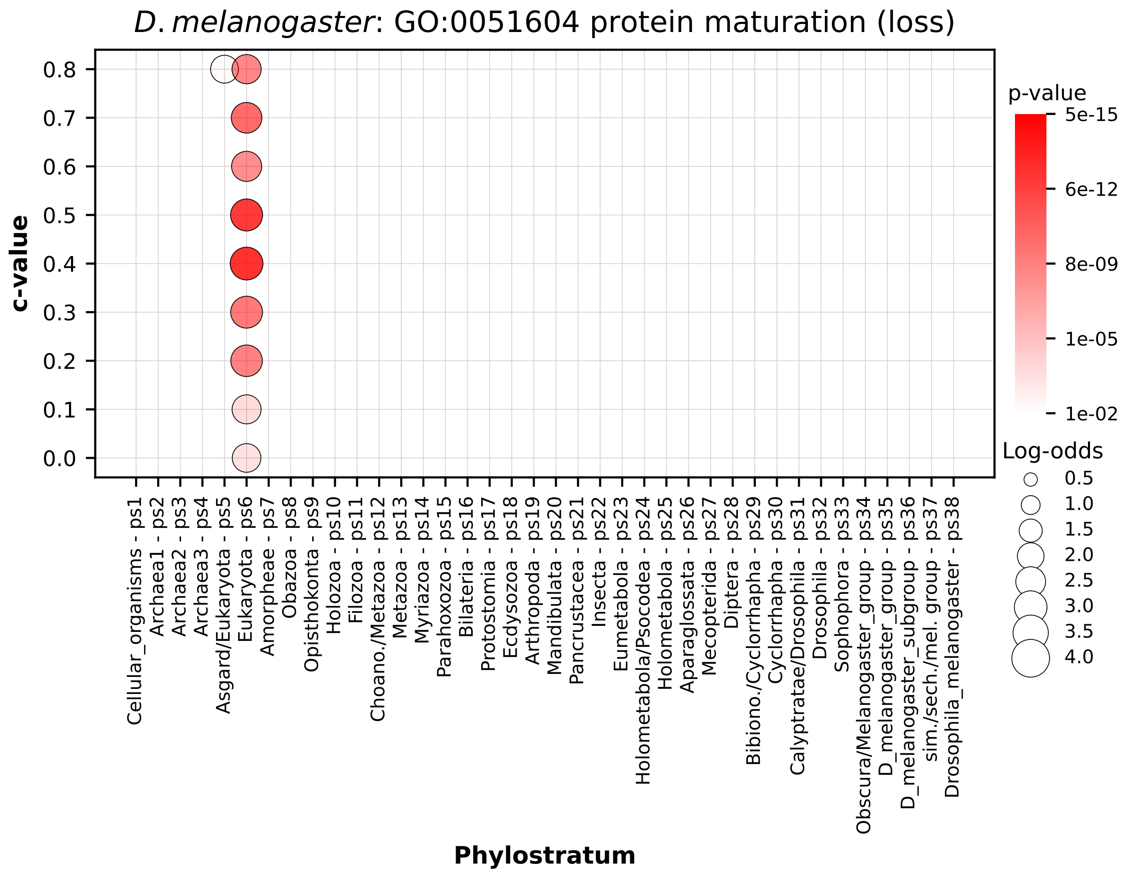
D. melanogaster: GO:0051604 protein maturation (loss)
c-value
0.0
0.1
0.2
0.3
0.4
0.5
0.6
0.7
0.8
Phylostratum
Cellular_organisms - ps1
Archaea1 - ps2
Archaea2 - ps3
Archaea3 - ps4
Asgard/Eukaryota - ps5
Eukaryota - ps6
Amorpheae - ps7
Obazoa - ps8
Opisthokonta - ps9
Holozoa - ps10
Filozoa - ps11
Choano./Metazoa - ps12
Metazoa - ps13
Myriazoa - ps14
Parahoxozoa - ps15
Bilateria - ps16
Protostomia - ps17
Ecdysozoa - ps18
Arthropoda - ps19
Mandibulata - ps20
Pancrustacea - ps21
Insecta - ps22
Eumetabola - ps23
Holometabola/Psocodea - ps24
Holometabola - ps25
Aparaglossata - ps26
Mecopterida - ps27
Diptera - ps28
Bibiono./Cyclorrhapha - ps29
Cyclorrhapha - ps30
Calyptratae/Drosophila - ps31
Drosophila - ps32
Sophophora - ps33
Obscura/Melanogaster_group - ps34
D_melanogaster_group - ps35
D_melanogaster_subgroup - ps36
sim./sech./mel. group - ps37
Drosophila_melanogaster - ps38
p-value
5e-15
6e-12
8e-09
1e-05
1e-02
Log-odds
0.5
1.0
1.5
2.0
2.5
3.0
3.5
4.0

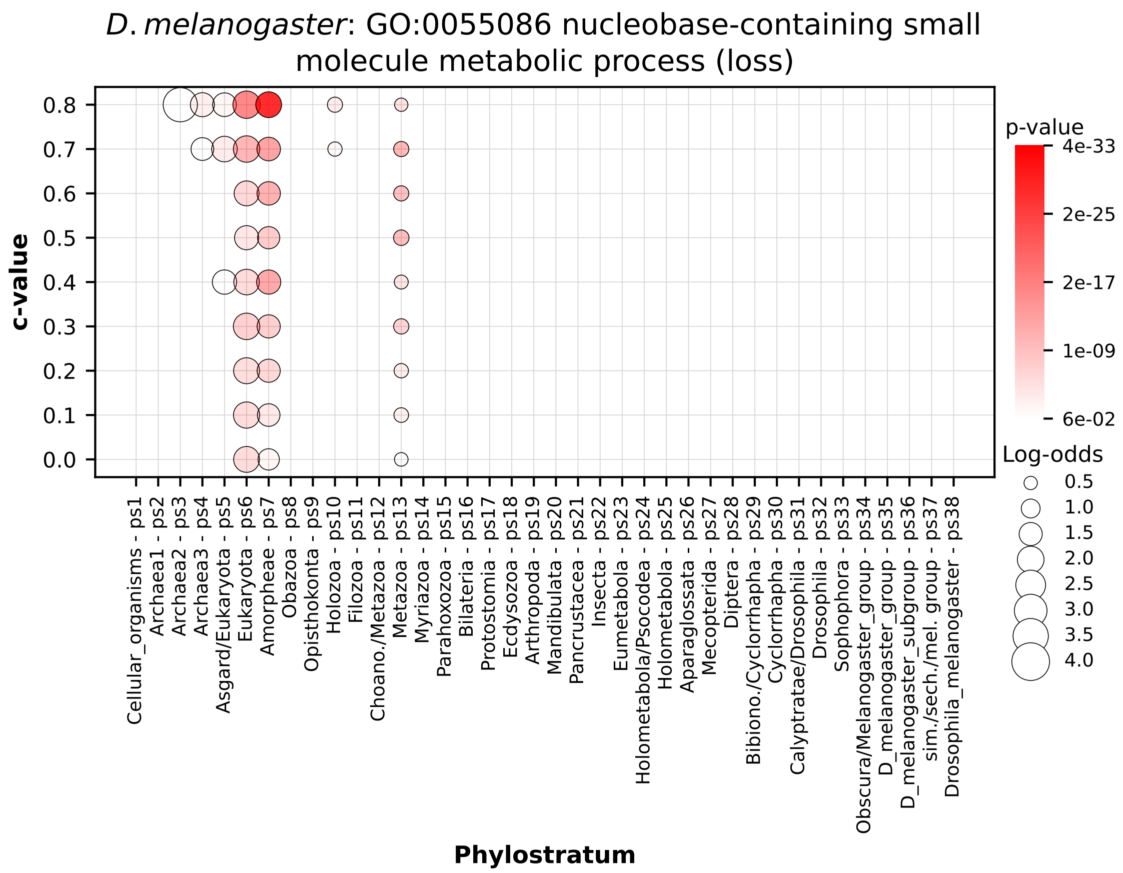

D. melanogaster: GO:0055086 nucleobase-containing small molecule metabolic process (loss)
c-value
0.8
0.7
0.6
0.5
0.4
0.3
0.2
0.1
0.0
p-value
4e-33
2e-25
2e-17
1e-09
6e-02
Log-odds
0.5
1.0
1.5
2.0
2.5
3.0
3.5
4.0
Cellular_organisms - ps1
Archaea1 - ps2
Archaea2 - ps3
Archaea3 - ps4
Asgard/Eukaryota - ps5
Eukaryota - ps6
Amorpheae - ps7
Obazoa - ps8
Opisthokonta - ps9
Holozoa - ps10
Filozoa - ps11
Choano./Metazoa - ps12
Metazoa - ps13
Myriazoa - ps14
Parahoxozoa - ps15
Bilateria - ps16
Protostomia - ps17
Ecdysozoa - ps18
Arthropoda - ps19
Mandibulata - ps20
Pancrustacea - ps21
Insecta - ps22
Eumetabola - ps23
Holometabola/Psocodea - ps24
Holometabola - ps25
Aparaglossata - ps26
Mecopterida - ps27
Diptera - ps28
Bibiono./Cyclorrhapha - ps29
Cyclorrhapha - ps30
Calyptratae/Drosophila - ps31
Drosophila - ps32
Sophophora - ps33
Obscura/Melanogaster_group - ps34
D_melanogaster_group - ps35
D_melanogaster_subgroup - ps36
sim./sech./mel. group - ps37
Drosophila_melanogaster - ps38
Phylostratum

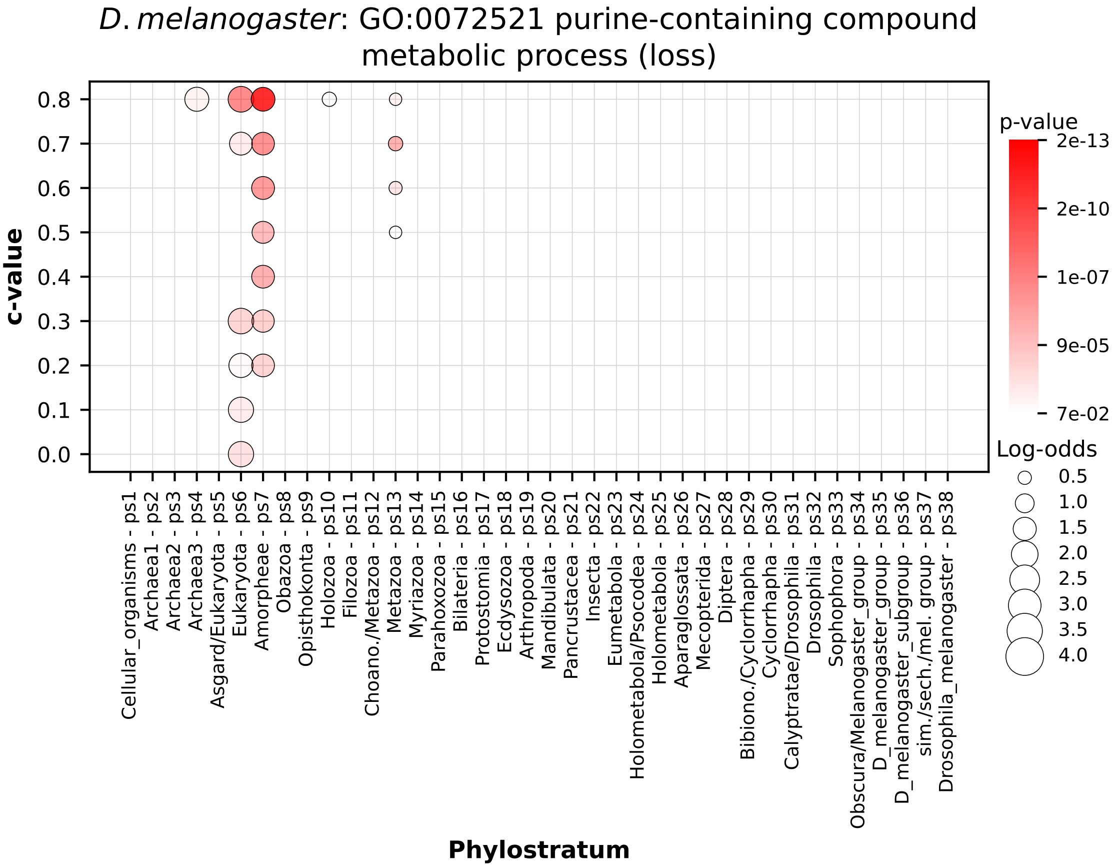

D. melanogaster: GO:0072521 purine-containing compound metabolic process (loss)
c-value
0.8
0.7
0.6
0.5
0.4
0.3
0.2
0.1
0.0
Phylostratum
Cellular_organisms - ps1
Archaea1 - ps2
Archaea2 - ps3
Archaea3 - ps4
Asgard/Eukaryota - ps5
Eukaryota - ps6
Amorpheae - ps7
Obazoa - ps8
Opisthokonta - ps9
Holozoa - ps10
Filozoa - ps11
Choano./Metazoa - ps12
Metazoa - ps13
Myriazoa - ps14
Parahoxozoa - ps15
Bilateria - ps16
Protostomia - ps17
Ecdysozoa - ps18
Arthropoda - ps19
Mandibulata - ps20
Pancrustacea - ps21
Insecta - ps22
Eumetabola - ps23
Holometabola/Psocodea - ps24
Holometabola - ps25
Aparaglossata - ps26
Mecopterida - ps27
Diptera - ps28
Bibiono./Cyclorrhapha - ps29
Cyclorrhapha - ps30
Calyptratae/Drosophila - ps31
Drosophila - ps32
Sophophora - ps33
Obscura/Melanogaster_group - ps34
D_melanogaster_group - ps35
D_melanogaster_subgroup - ps36
sim./sech./mel. group - ps37
Drosophila_melanogaster - ps38
p-value
2e-13
2e-10
1e-07
9e-05
7e-02
Log-odds
0.5
1.0
1.5
2.0
2.5
3.0
3.5
4.0

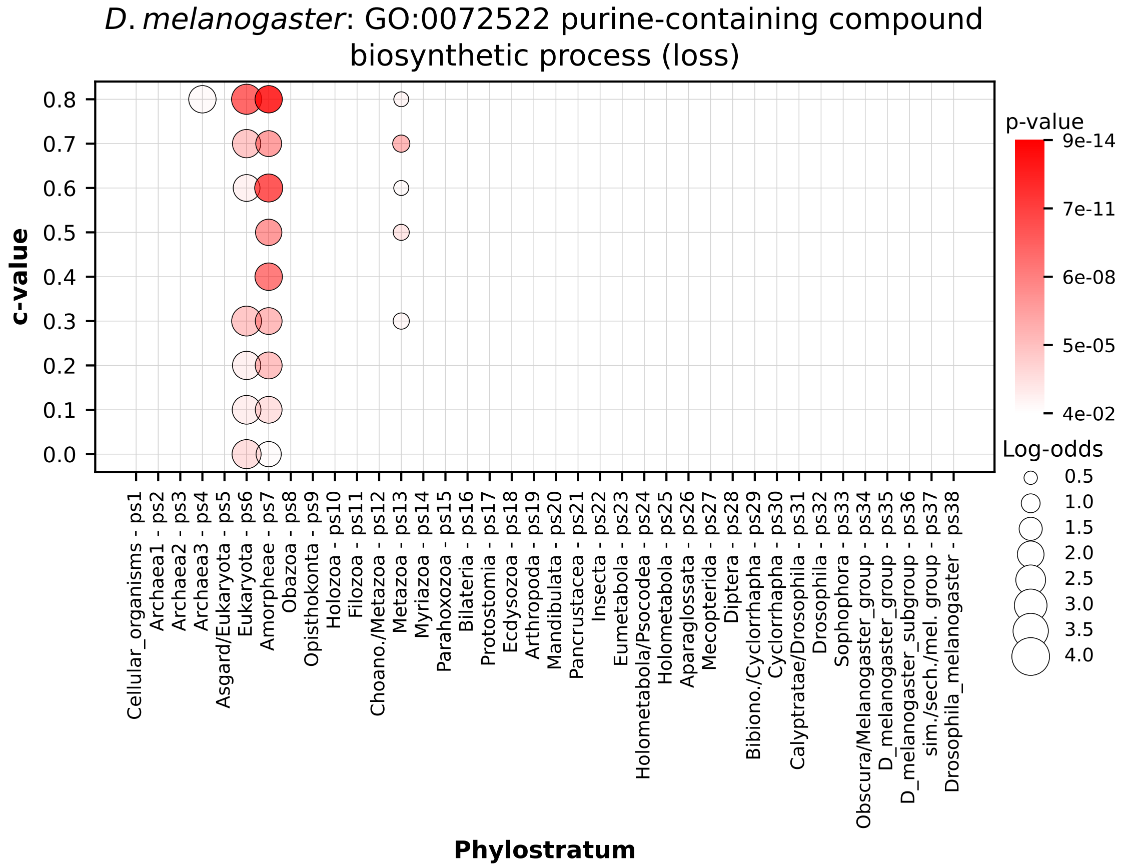

D. melanogaster: GO:0072522 purine-containing compound biosynthetic process (loss)
c-value
0.8
0.7
0.6
0.5
0.4
0.3
0.2
0.1
0.0
Phylostratum
Cellular_organisms - ps1
Archaea1 - ps2
Archaea2 - ps3
Archaea3 - ps4
Asgard/Eukaryota - ps5
Eukaryota - ps6
Amorpheae - ps7
Obazoa - ps8
Opisthokonta - ps9
Holozoa - ps10
Filozoa - ps11
Choano./Metazoa - ps12
Metazoa - ps13
Myriazoa - ps14
Parahoxozoa - ps15
Bilateria - ps16
Protostomia - ps17
Ecdysozoa - ps18
Arthropoda - ps19
Mandibulata - ps20
Pancrustacea - ps21
Insecta - ps22
Eumetabola - ps23
Holometabola/Psocodea - ps24
Holometabola - ps25
Aparaglossata - ps26
Mecopterida - ps27
Diptera - ps28
Bibiono./Cyclorrhapha - ps29
Cyclorrhapha - ps30
Calyptratae/Drosophila - ps31
Drosophila - ps32
Sophophora - ps33
Obscura/Melanogaster_group - ps34
D_melanogaster_group - ps35
D_melanogaster_subgroup - ps36
sim./sech./mel. group - ps37
Drosophila_melanogaster - ps38
p-value
9e-14
7e-11
6e-08
5e-05
4e-02
Log-odds
0.5
1.0
1.5
2.0
2.5
3.0
3.5
4.0

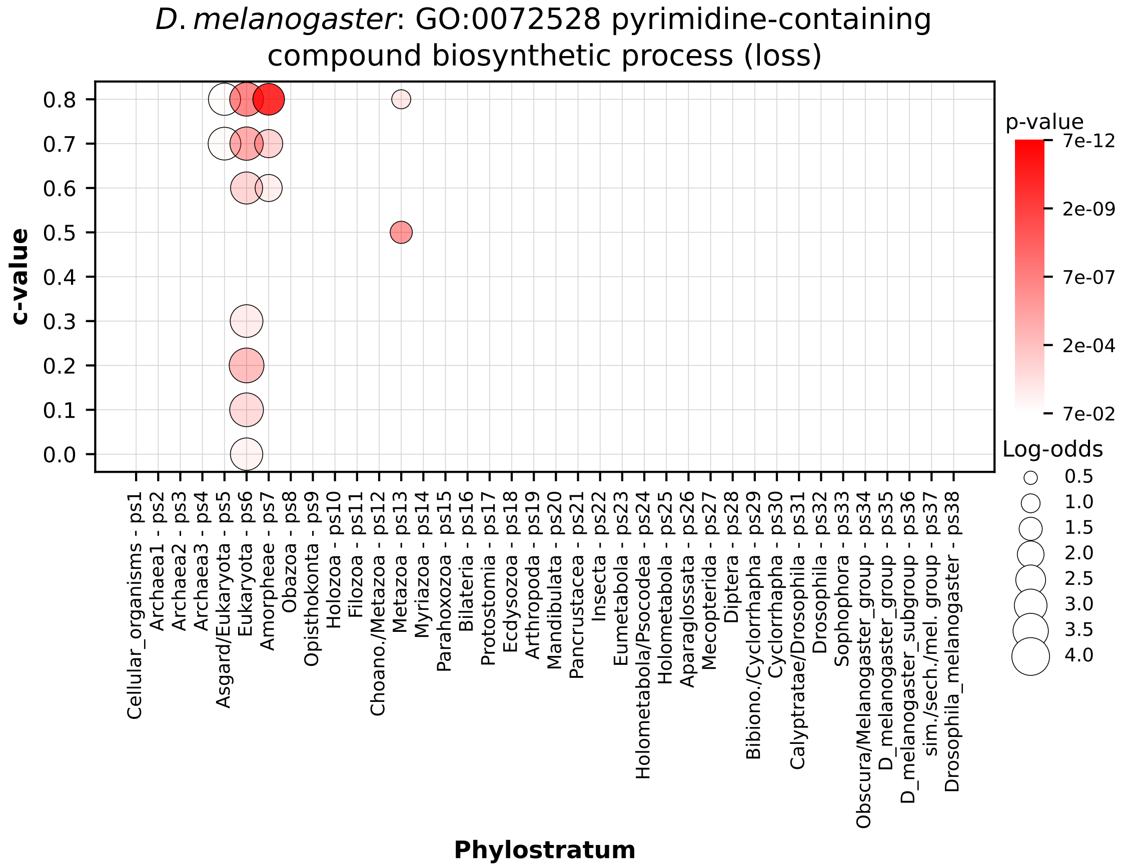

D. melanogaster: GO:0072528 pyrimidine-containing compound biosynthetic process (loss)
c-value
0.8
0.7
0.6
0.5
0.4
0.3
0.2
0.1
0.0
p-value
7e-12
2e-09
7e-07
2e-04
7e-02
Log-odds
0.5
1.0
1.5
2.0
2.5
3.0
3.5
4.0
Cellular_organisms - ps1
Archaea1 - ps2
Archaea2 - ps3
Archaea3 - ps4
Asgard/Eukaryota - ps5
Eukaryota - ps6
Amorpheae - ps7
Obazoa - ps8
Opisthokonta - ps9
Holozoa - ps10
Filozoa - ps11
Choano./Metazoa - ps12
Metazoa - ps13
Myriazoa - ps14
Parahoxozoa - ps15
Bilateria - ps16
Protostomia - ps17
Ecdysozoa - ps18
Arthropoda - ps19
Mandibulata - ps20
Pancrustacea - ps21
Insecta - ps22
Eumetabola - ps23
Holometabola/Psocodea - ps24
Holometabola - ps25
Aparaglossata - ps26
Mecopterida - ps27
Diptera - ps28
Bibiono./Cyclorrhapha - ps29
Cyclorrhapha - ps30
Calyptratae/Drosophila - ps31
Drosophila - ps32
Sophophora - ps33
Obscura/Melanogaster_group - ps34
D_melanogaster_group - ps35
D_melanogaster_subgroup - ps36
sim./sech./mel. group - ps37
Drosophila_melanogaster - ps38
Phylostratum

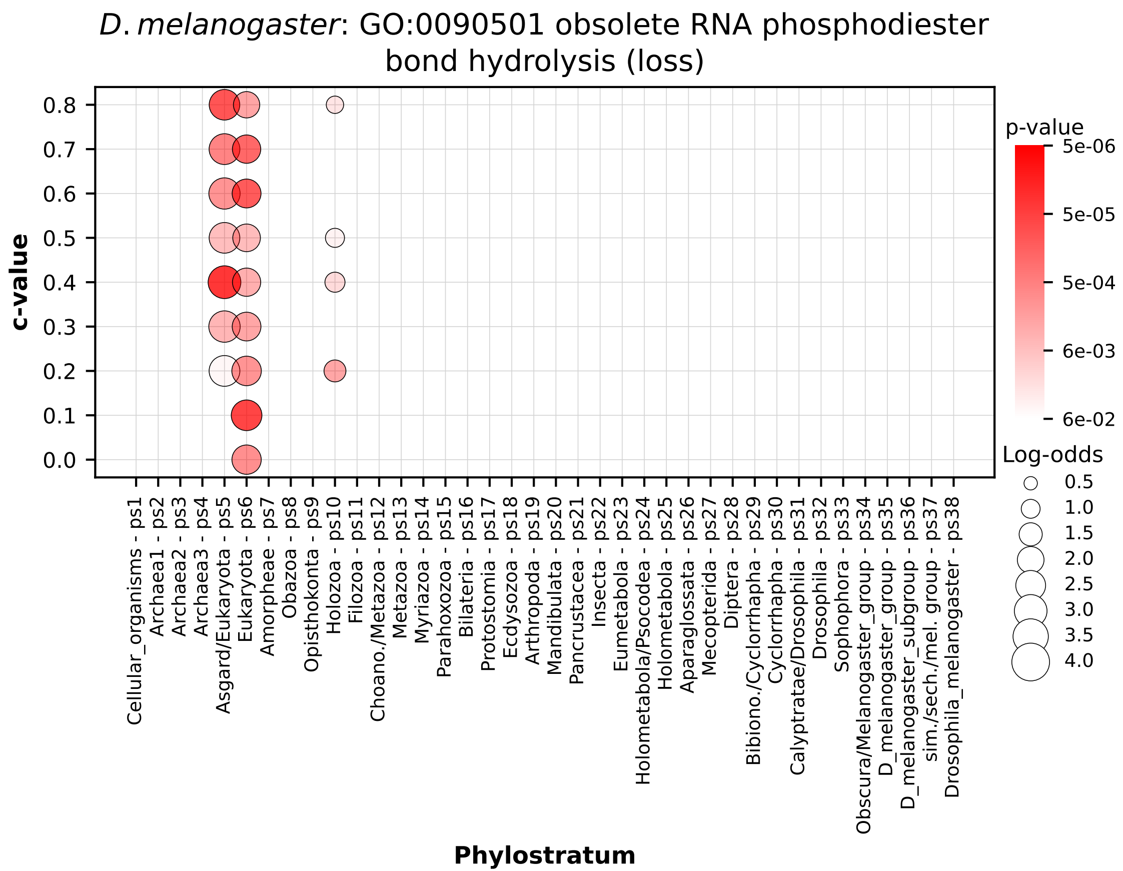

D. melanogaster: GO:0090501 obsolete RNA phosphodiester bond hydrolysis (loss)
c-value
0.8
0.7
0.6
0.5
0.4
0.3
0.2
0.1
0.0
p-value
5e-06
5e-05
5e-04
6e-03
6e-02
Log-odds
0.5
1.0
1.5
2.0
2.5
3.0
3.5
4.0
Cellular_organisms - ps1
Archaea1 - ps2
Archaea2 - ps3
Archaea3 - ps4
Asgard/Eukaryota - ps5
Eukaryota - ps6
Amorpheae - ps7
Obazoa - ps8
Opisthokonta - ps9
Holozoa - ps10
Filozoa - ps11
Choano./Metazoa - ps12
Metazoa - ps13
Myriazoa - ps14
Parahoxozoa - ps15
Bilateria - ps16
Protostomia - ps17
Ecdysozoa - ps18
Arthropoda - ps19
Mandibulata - ps20
Pancrustacea - ps21
Insecta - ps22
Eumetabola - ps23
Holometabola/Psocodea - ps24
Holometabola - ps25
Aparaglossata - ps26
Mecopterida - ps27
Diptera - ps28
Bibiono./Cyclorrhapha - ps29
Cyclorrhapha - ps30
Calyptratae/Drosophila - ps31
Drosophila - ps32
Sophophora - ps33
Obscura/Melanogaster_group - ps34
D_melanogaster_group - ps35
D_melanogaster_subgroup - ps36
sim./sech./mel. group - ps37
Drosophila_melanogaster - ps38
Phylostratum

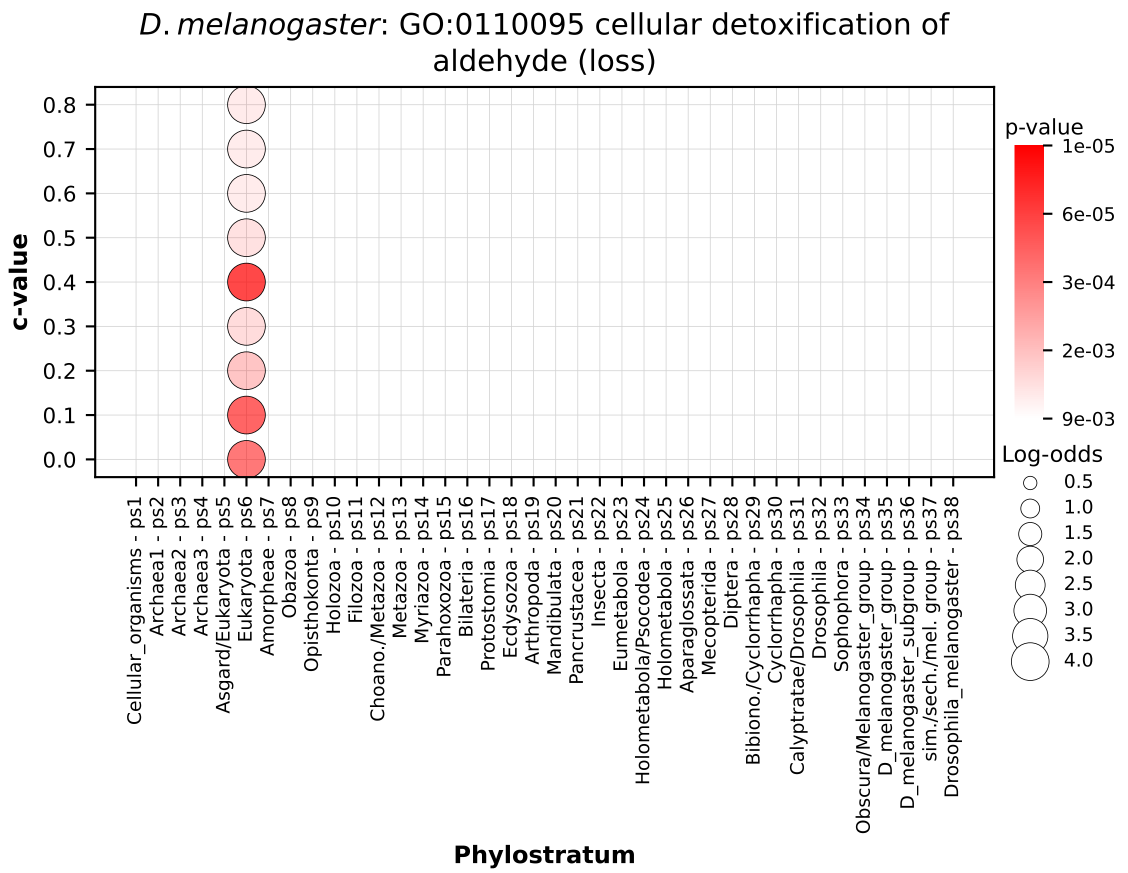

D. melanogaster: GO:0110095 cellular detoxification of aldehyde (loss)
c-value
0.0
0.1
0.2
0.3
0.4
0.5
0.6
0.7
0.8
Phylostratum
Cellular_organisms - ps1
Archaea1 - ps2
Archaea2 - ps3
Archaea3 - ps4
Asgard/Eukaryota - ps5
Eukaryota - ps6
Amorpheae - ps7
Obazoa - ps8
Opisthokonta - ps9
Holozoa - ps10
Filozoa - ps11
Choano./Metazoa - ps12
Metazoa - ps13
Myriazoa - ps14
Parahoxozoa - ps15
Bilateria - ps16
Protostomia - ps17
Ecdysozoa - ps18
Arthropoda - ps19
Mandibulata - ps20
Pancrustacea - ps21
Insecta - ps22
Eumetabola - ps23
Holometabola/Psocodea - ps24
Holometabola - ps25
Aparaglossata - ps26
Mecopterida - ps27
Diptera - ps28
Bibiono./Cyclorrhapha - ps29
Cyclorrhapha - ps30
Calyptratae/Drosophila - ps31
Drosophila - ps32
Sophophora - ps33
Obscura/Melanogaster_group - ps34
D_melanogaster_group - ps35
D_melanogaster_subgroup - ps36
sim./sech./mel. group - ps37
Drosophila_melanogaster - ps38
p-value
1e-05
6e-05
3e-04
2e-03
9e-03
Log-odds
0.5
1.0
1.5
2.0
2.5
3.0
3.5
4.0

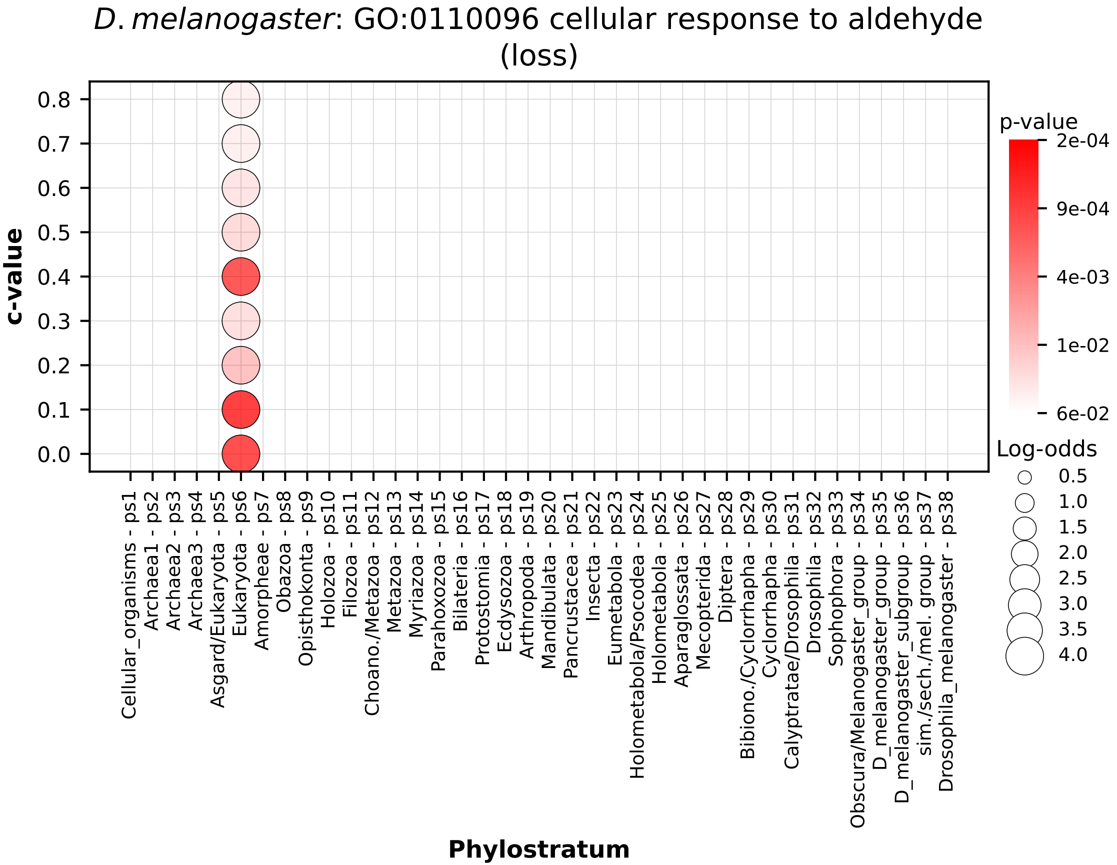

D. melanogaster: GO:0110096 cellular response to aldehyde
(loss)
c-value
0.0
0.1
0.2
0.3
0.4
0.5
0.6
0.7
0.8
Phylostratum
Cellular_organisms - ps1
Archaea1 - ps2
Archaea2 - ps3
Archaea3 - ps4
Asgard/Eukaryota - ps5
Eukaryota - ps6
Amorpheae - ps7
Obazoa - ps8
Opisthokonta - ps9
Holozoa - ps10
Filozoa - ps11
Choano./Metazoa - ps12
Metazoa - ps13
Myriazoa - ps14
Parahoxozoa - ps15
Bilateria - ps16
Protostomia - ps17
Ecdysozoa - ps18
Arthropoda - ps19
Mandibulata - ps20
Pancrustacea - ps21
Insecta - ps22
Eumetabola - ps23
Holometabola/Psocodea - ps24
Holometabola - ps25
Aparaglossata - ps26
Mecopterida - ps27
Diptera - ps28
Bibiono./Cyclorrhapha - ps29
Cyclorrhapha - ps30
Calyptratae/Drosophila - ps31
Drosophila - ps32
Sophophora - ps33
Obscura/Melanogaster_group - ps34
D_melanogaster_group - ps35
D_melanogaster_subgroup - ps36
sim./sech./mel. group - ps37
Drosophila_melanogaster - ps38
p-value
2e-04
9e-04
4e-03
1e-02
6e-02
Log-odds
0.5
1.0
1.5
2.0
2.5
3.0
3.5
4.0

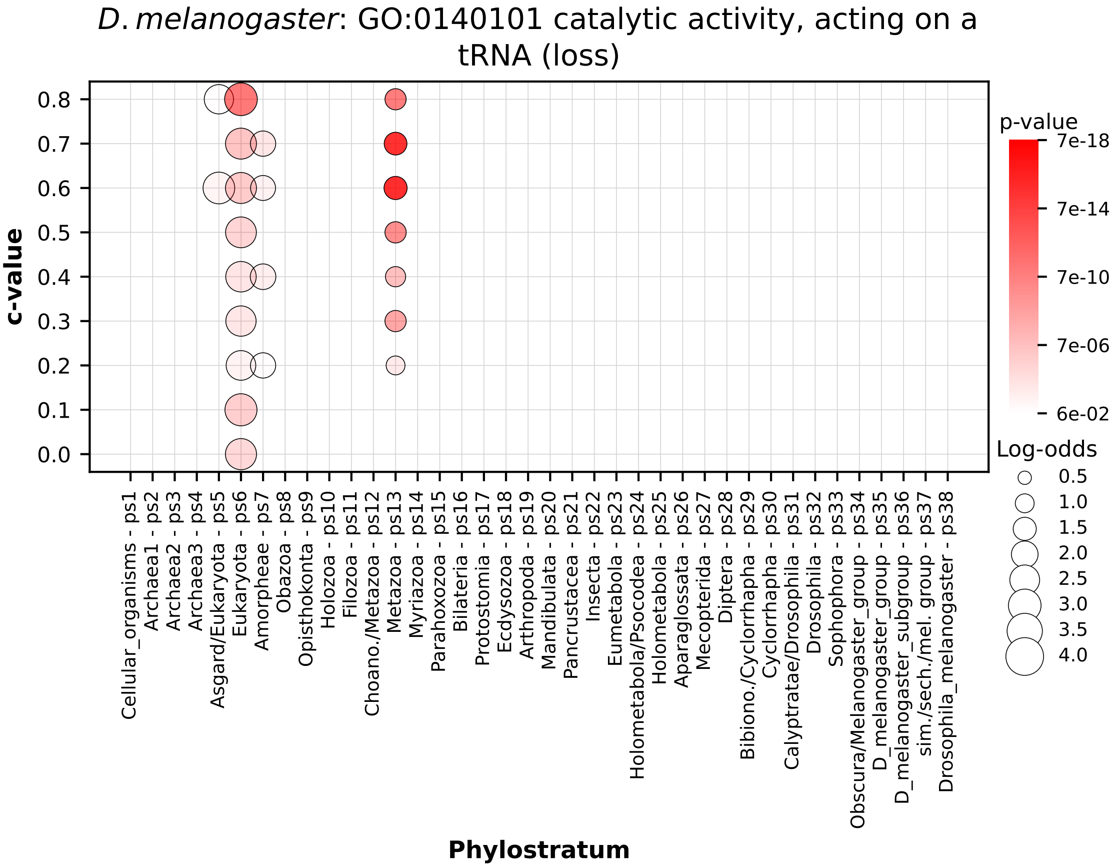

D. melanogaster: GO:0140101 catalytic activity, acting on a tRNA (loss)
c-value
0.8
0.7
0.6
0.5
0.4
0.3
0.2
0.1
0.0
p-value
7e-18
7e-14
7e-10
7e-06
6e-02
Log-odds
0.5
1.0
1.5
2.0
2.5
3.0
3.5
4.0
Cellular_organisms - ps1
Archaea1 - ps2
Archaea2 - ps3
Archaea3 - ps4
Asgard/Eukaryota - ps5
Eukaryota - ps6
Amorpheae - ps7
Obazoa - ps8
Opisthokonta - ps9
Holozoa - ps10
Filozoa - ps11
Choano./Metazoa - ps12
Metazoa - ps13
Myriazoa - ps14
Parahoxozoa - ps15
Bilateria - ps16
Protostomia - ps17
Ecdysozoa - ps18
Arthropoda - ps19
Mandibulata - ps20
Pancrustacea - ps21
Insecta - ps22
Eumetabola - ps23
Holometabola/Psocodea - ps24
Holometabola - ps25
Aparaglossata - ps26
Mecopterida - ps27
Diptera - ps28
Bibiono./Cyclorrhapha - ps29
Cyclorrhapha - ps30
Calyptratae/Drosophila - ps31
Drosophila - ps32
Sophophora - ps33
Obscura/Melanogaster_group - ps34
D_melanogaster_group - ps35
D_melanogaster_subgroup - ps36
sim./sech./mel. group - ps37
Drosophila_melanogaster - ps38
Phylostratum

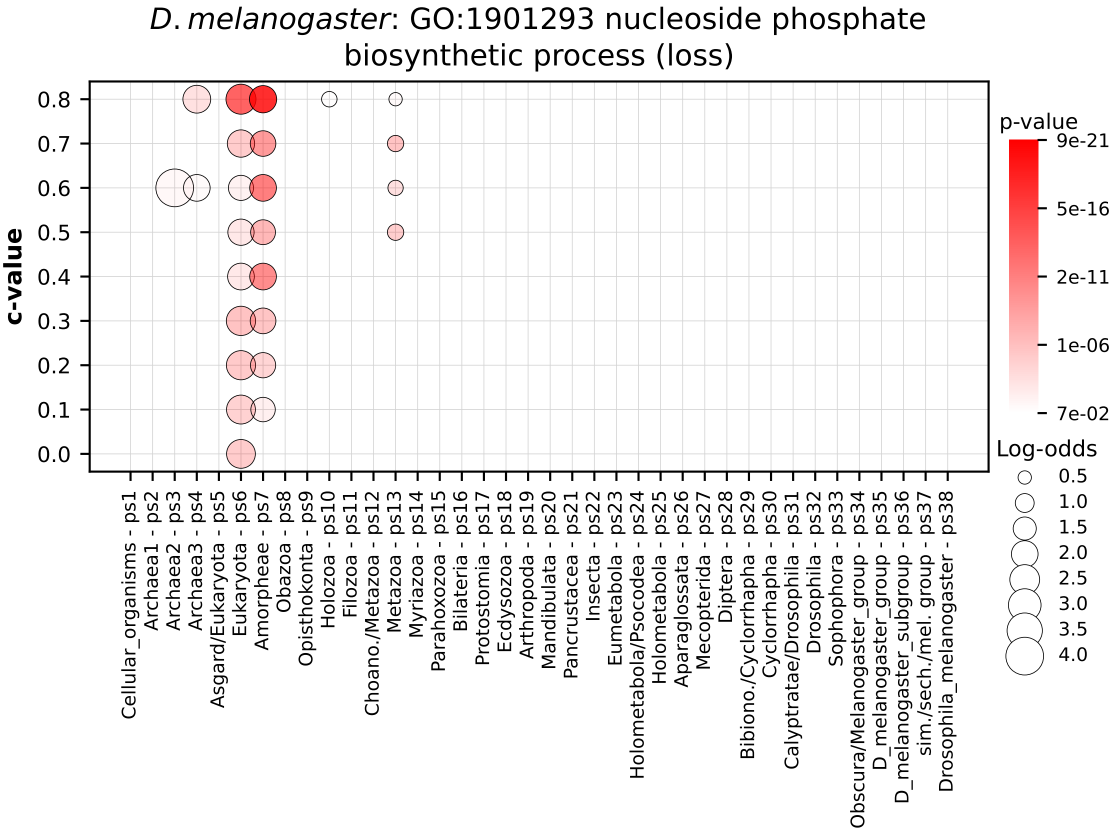

D. melanogaster: GO:1901293 nucleoside phosphate biosynthetic process (loss)
c-value
0.8
0.7
0.6
0.5
0.4
0.3
0.2
0.1
0.0
p-value
9e-21
5e-16
2e-11
1e-06
7e-02
Log-odds
0.5
1.0
1.5
2.0
2.5
3.0
3.5
4.0
Cellular_organisms - ps1
Archaea1 - ps2
Archaea2 - ps3
Archaea3 - ps4
Asgard/Eukaryota - ps5
Eukaryota - ps6
Amorpheae - ps7
Obazoa - ps8
Opisthokonta - ps9
Holozoa - ps10
Filozoa - ps11
Choano./Metazoa - ps12
Metazoa - ps13
Myriazoa - ps14
Parahoxozoa - ps15
Bilateria - ps16
Protostomia - ps17
Ecdysozoa - ps18
Arthropoda - ps19
Mandibulata - ps20
Pancrustacea - ps21
Insecta - ps22
Eumetabola - ps23
Holometabola/Psocodea - ps24
Holometabola - ps25
Aparaglossata - ps26
Mecopterida - ps27
Diptera - ps28
Bibiono./Cyclorrhapha - ps29
Cyclorrhapha - ps30
Calyptratae/Drosophila - ps31
Drosophila - ps32
Sophophora - ps33
Obscura/Melanogaster_group - ps34
D_melanogaster_group - ps35
D_melanogaster_subgroup - ps36
sim./sech./mel. group - ps37
Drosophila_melanogaster - ps38
Phylostratum

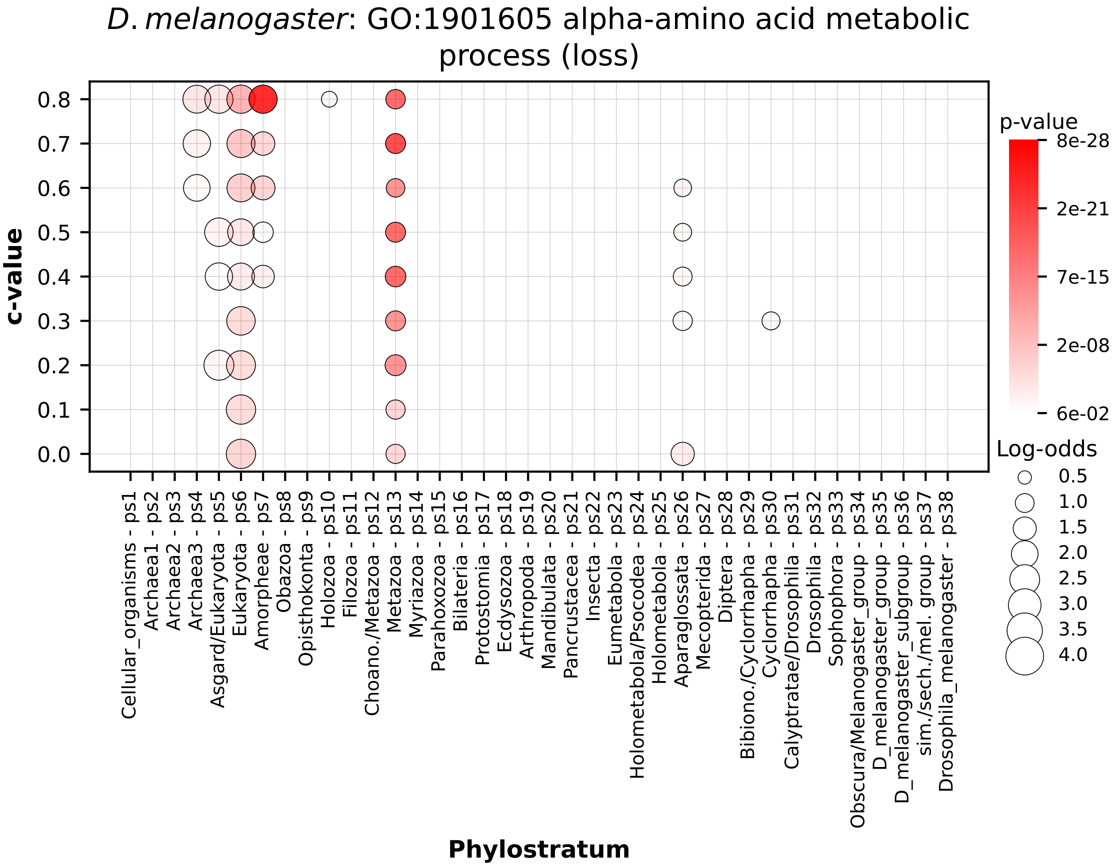

D. melanogaster: GO:1901605 alpha-amino acid metabolic process (loss)
c-value
0.8
0.7
0.6
0.5
0.4
0.3
0.2
0.1
0.0
Phylostratum
Cellular_organisms - ps1
Archaea1 - ps2
Archaea2 - ps3
Archaea3 - ps4
Asgard/Eukaryota - ps5
Eukaryota - ps6
Amorpheae - ps7
Obazoa - ps8
Opisthokonta - ps9
Holozoa - ps10
Filozoa - ps11
Choano./Metazoa - ps12
Metazoa - ps13
Myriazoa - ps14
Parahoxozoa - ps15
Bilateria - ps16
Protostomia - ps17
Ecdysozoa - ps18
Arthropoda - ps19
Mandibulata - ps20
Pancrustacea - ps21
Insecta - ps22
Eumetabola - ps23
Holometabola/Psocodea - ps24
Holometabola - ps25
Aparaglossata - ps26
Mecopterida - ps27
Diptera - ps28
Bibiono./Cyclorrhapha - ps29
Cyclorrhapha - ps30
Calyptratae/Drosophila - ps31
Drosophila - ps32
Sophophora - ps33
Obscura/Melanogaster_group - ps34
D_melanogaster_group - ps35
D_melanogaster_subgroup - ps36
sim./sech./mel. group - ps37
Drosophila_melanogaster - ps38
p-value
8e-28
2e-21
7e-15
2e-08
6e-02
Log-odds
0.5
1.0
1.5
2.0
2.5
3.0
3.5
4.0

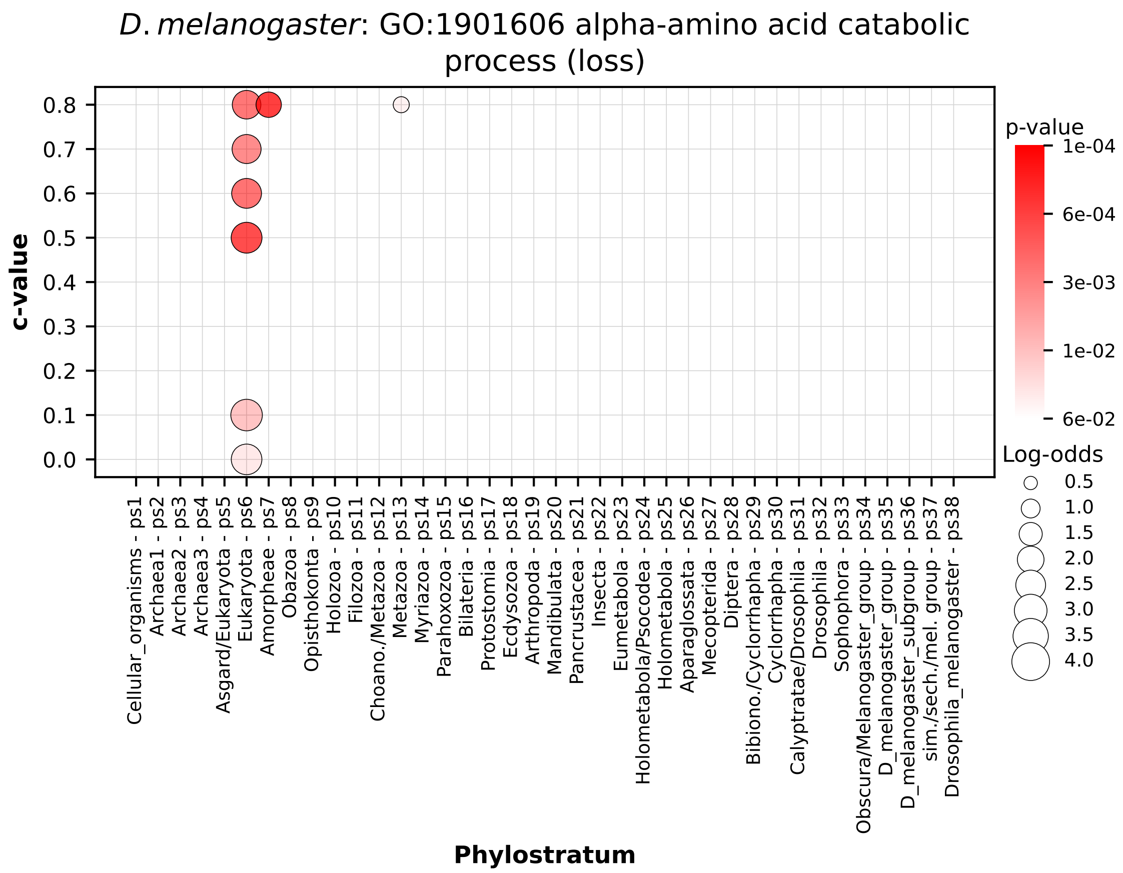

D. melanogaster: GO:1901606 alpha-amino acid catabolic process (loss)
c-value
0.8
0.7
0.6
0.5
0.4
0.3
0.2
0.1
0.0
p-value
1e-04
6e-04
3e-03
1e-02
6e-02
Log-odds
0.5
1.0
1.5
2.0
2.5
3.0
3.5
4.0
Cellular_organisms - ps1
Archaea1 - ps2
Archaea2 - ps3
Archaea3 - ps4
Asgard/Eukaryota - ps5
Eukaryota - ps6
Amorpheae - ps7
Obazoa - ps8
Opisthokonta - ps9
Holozoa - ps10
Filozoa - ps11
Choano./Metazoa - ps12
Metazoa - ps13
Myriazoa - ps14
Parahoxozoa - ps15
Bilateria - ps16
Protostomia - ps17
Ecdysozoa - ps18
Arthropoda - ps19
Mandibulata - ps20
Pancrustacea - ps21
Insecta - ps22
Eumetabola - ps23
Holometabola/Psocodea - ps24
Holometabola - ps25
Aparaglossata - ps26
Mecopterida - ps27
Diptera - ps28
Bibiono./Cyclorrhapha - ps29
Cyclorrhapha - ps30
Calyptratae/Drosophila - ps31
Drosophila - ps32
Sophophora - ps33
Obscura/Melanogaster_group - ps34
D_melanogaster_group - ps35
D_melanogaster_subgroup - ps36
sim./sech./mel. group - ps37
Drosophila_melanogaster - ps38
Phylostratum

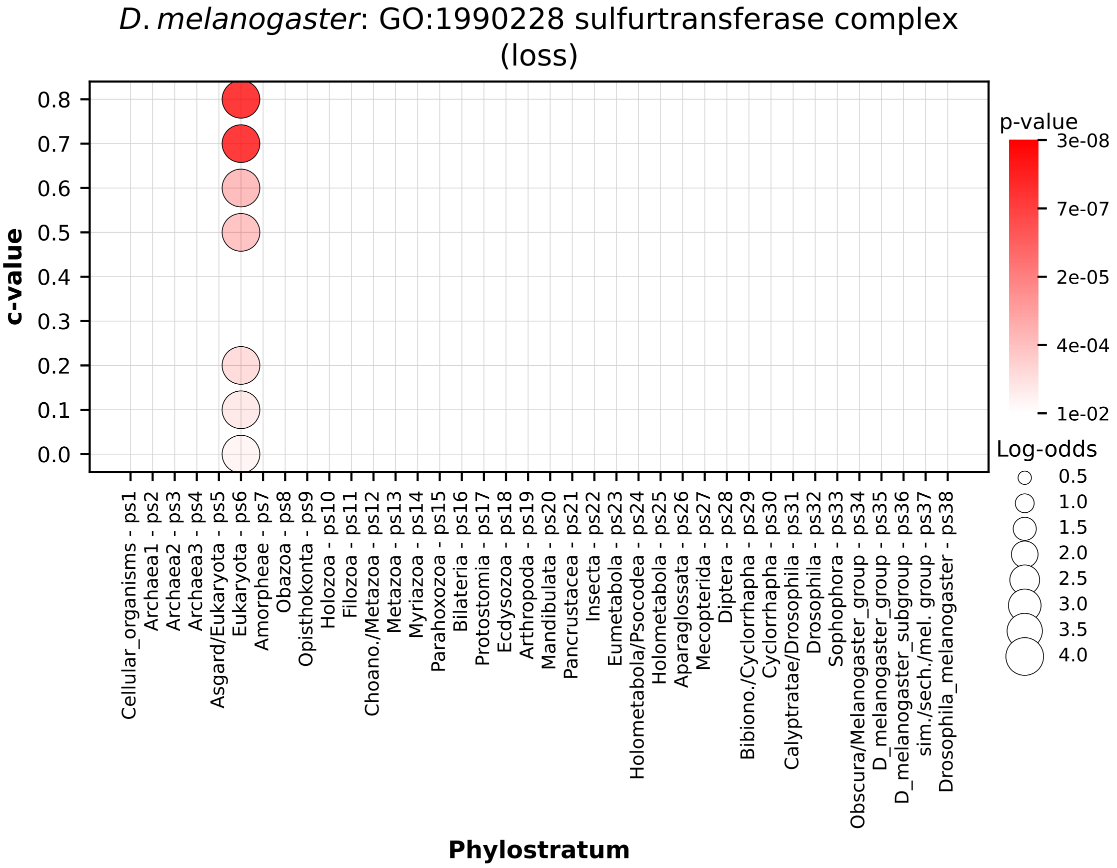
D. melanogaster: GO:1990228 sulfurtransferase complex
(loss)
c-value
0.8
0.7
0.6
0.5
0.4
0.3
0.2
0.1
0.0
Phylostratum
Cellular_organisms - ps1
Archaea1 - ps2
Archaea2 - ps3
Archaea3 - ps4
Asgard/Eukaryota - ps5
Eukaryota - ps6
Amorpheae - ps7
Obazoa - ps8
Opisthokonta - ps9
Holozoa - ps10
Filozoa - ps11
Choano./Metazoa - ps12
Metazoa - ps13
Myriazoa - ps14
Parahoxozoa - ps15
Bilateria - ps16
Protostomia - ps17
Ecdysozoa - ps18
Arthropoda - ps19
Mandibulata - ps20
Pancrustacea - ps21
Insecta - ps22
Eumetabola - ps23
Holometabola/Psocodea - ps24
Holometabola - ps25
Aparaglossata - ps26
Mecopterida - ps27
Diptera - ps28
Bibiono./Cyclorrhapha - ps29
Cyclorrhapha - ps30
Calyptratae/Drosophila - ps31
Drosophila - ps32
Sophophora - ps33
Obscura/Melanogaster_group - ps34
D_melanogaster_group - ps35
D_melanogaster_subgroup - ps36
sim./sech./mel. group - ps37
Drosophila_melanogaster - ps38
p-value
3e-08
7e-07
2e-05
4e-04
1e-02
Log-odds
0.5
1.0
1.5
2.0
2.5
3.0
3.5
4.0

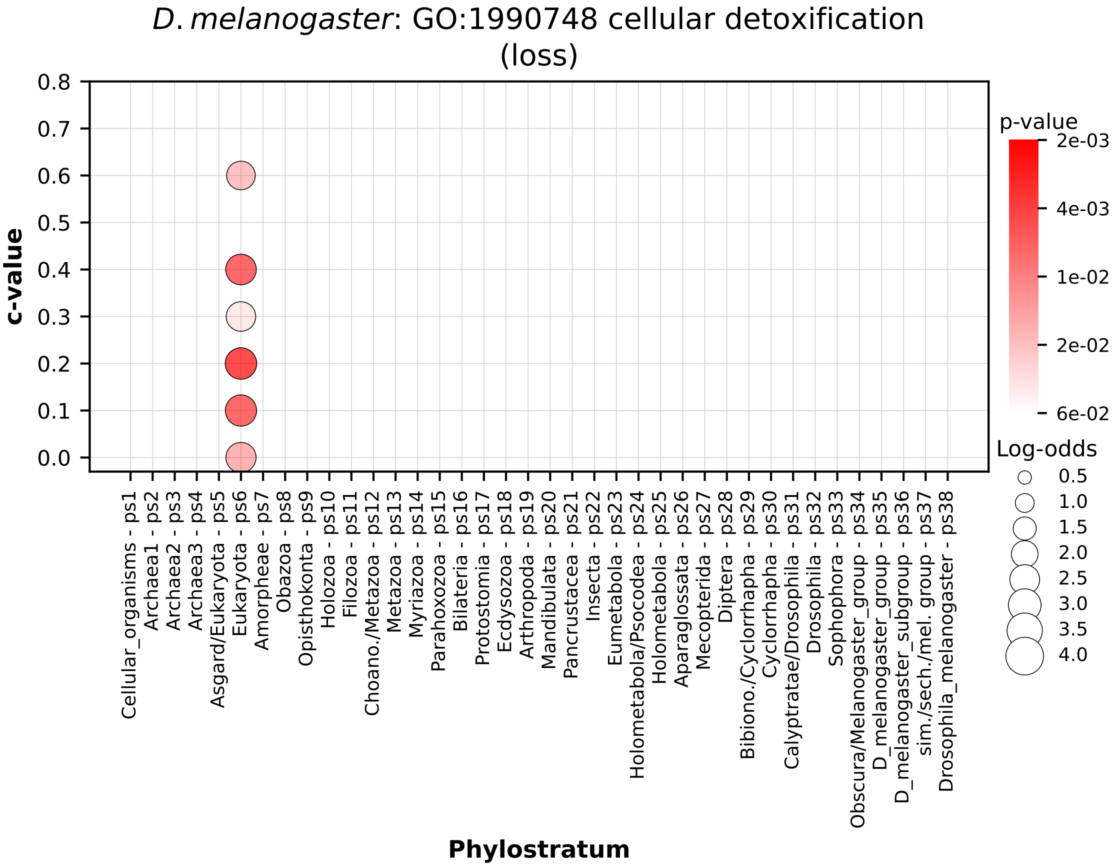

D. melanogaster: GO:1990748 cellular detoxification (loss)
c-value
0.8
0.7
0.6
0.5
0.4
0.3
0.2
0.1
0.0
Phylostratum
Cellular_organisms - ps1
Archaea1 - ps2
Archaea2 - ps3
Archaea3 - ps4
Asgard/Eukaryota - ps5
Eukaryota - ps6
Amorpheae - ps7
Obazoa - ps8
Opisthokonta - ps9
Holozoa - ps10
Filozoa - ps11
Choano./Metazoa - ps12
Metazoa - ps13
Myriazoa - ps14
Parahoxozoa - ps15
Bilateria - ps16
Protostomia - ps17
Ecdysozoa - ps18
Arthropoda - ps19
Mandibulata - ps20
Pancrustacea - ps21
Insecta - ps22
Eumetabola - ps23
Holometabola/Psocodea - ps24
Holometabola - ps25
Aparaglossata - ps26
Mecopterida - ps27
Diptera - ps28
Bibiono./Cyclorrhapha - ps29
Cyclorrhapha - ps30
Calyptratae/Drosophila - ps31
Drosophila - ps32
Sophophora - ps33
Obscura/Melanogaster_group - ps34
D_melanogaster_group - ps35
D_melanogaster_subgroup - ps36
sim./sech./mel. group - ps37
Drosophila_melanogaster - ps38
p-value
2e-03
4e-03
1e-02
2e-02
6e-02
Log-odds
0.5
1.0
1.5
2.0
2.5
3.0
3.5
4.0

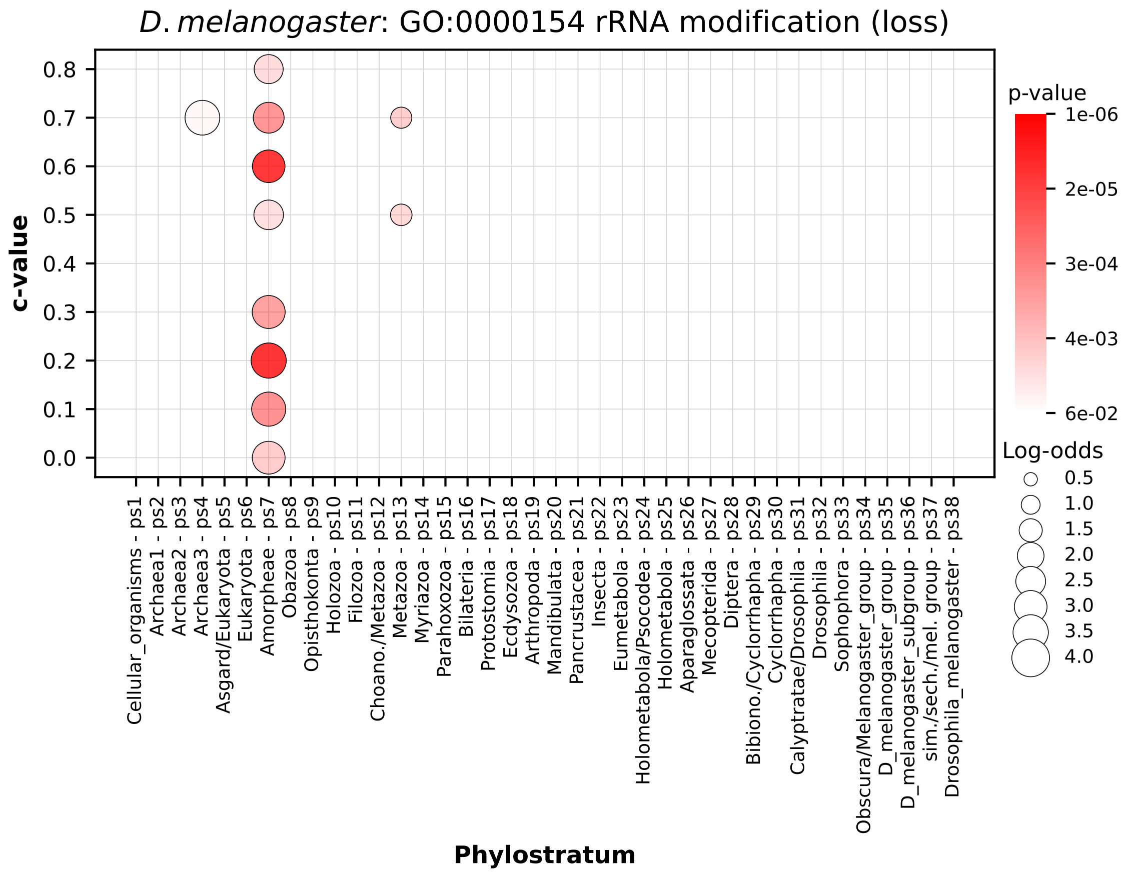
D. melanogaster: GO:0000154 rRNA modification (loss)
c-value
0.8
0.7
0.6
0.5
0.4
0.3
0.2
0.1
0.0
p-value
1e-06
2e-05
3e-04
4e-03
6e-02
Log-odds
0.5
1.0
1.5
2.0
2.5
3.0
3.5
4.0
Cellular_organisms - ps1
Archaea1 - ps2
Archaea2 - ps3
Archaea3 - ps4
Asgard/Eukaryota - ps5
Eukaryota - ps6
Amorpheae - ps7
Obazoa - ps8
Opisthokonta - ps9
Holozoa - ps10
Filozoa - ps11
Choano./Metazoa - ps12
Metazoa - ps13
Myriazoa - ps14
Parahoxozoa - ps15
Bilateria - ps16
Protostomia - ps17
Ecdysozoa - ps18
Arthropoda - ps19
Mandibulata - ps20
Pancrustacea - ps21
Insecta - ps22
Eumetabola - ps23
Holometabola/Psocodea - ps24
Holometabola - ps25
Aparaglossata - ps26
Mecopterida - ps27
Diptera - ps28
Bibiono./Cyclorrhapha - ps29
Cyclorrhapha - ps30
Calyptratae/Drosophila - ps31
Drosophila - ps32
Sophophora - ps33
Obscura/Melanogaster_group - ps34
D_melanogaster_group - ps35
D_melanogaster_subgroup - ps36
sim./sech./mel. group - ps37
Drosophila_melanogaster - ps38
Phylostratum

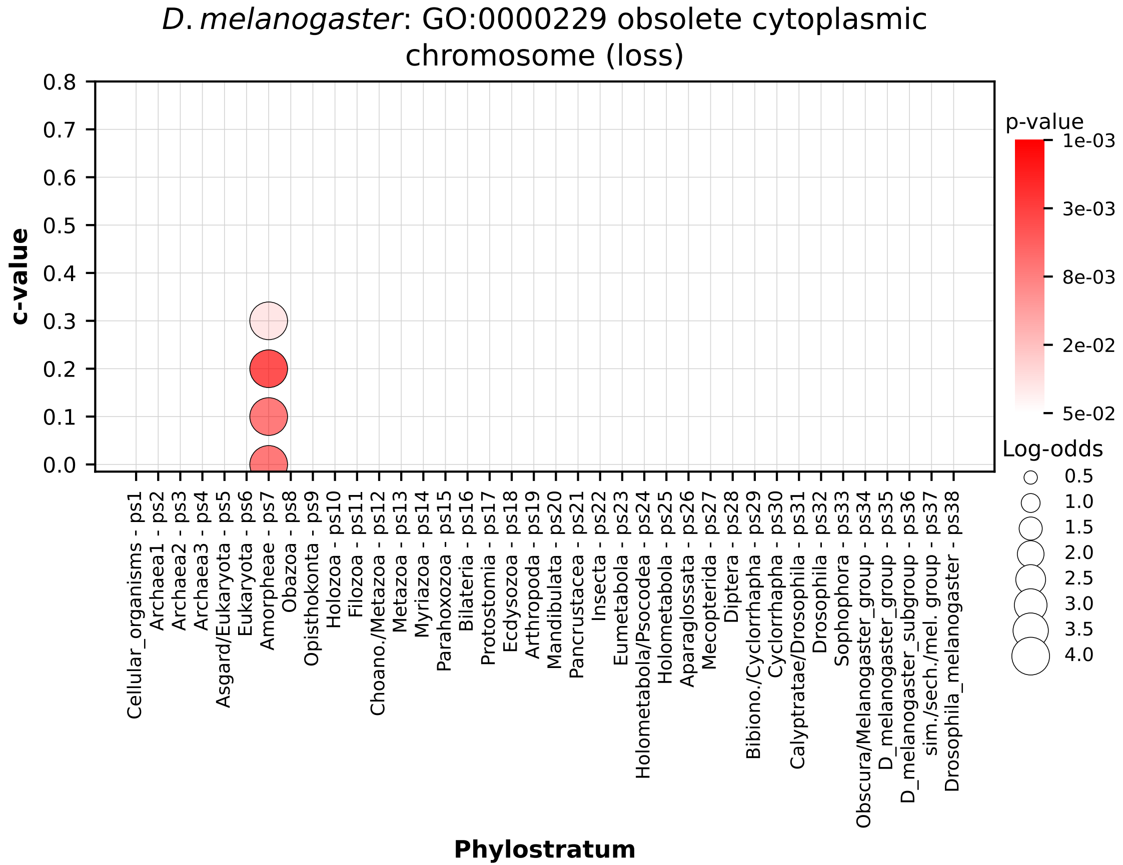

D. melanogaster: GO:0000229 obsolete cytoplasmic chromosome (loss)
c-value
0.8
0.7
0.6
0.5
0.4
0.3
0.2
0.1
0.0
Phylostratum
Cellular_organisms - ps1
Archaea1 - ps2
Archaea2 - ps3
Archaea3 - ps4
Asgard/Eukaryota - ps5
Eukaryota - ps6
Amorpheae - ps7
Obazoa - ps8
Opisthokonta - ps9
Holozoa - ps10
Filozoa - ps11
Choano./Metazoa - ps12
Metazoa - ps13
Myriazoa - ps14
Parahoxozoa - ps15
Bilateria - ps16
Protostomia - ps17
Ecdysozoa - ps18
Arthropoda - ps19
Mandibulata - ps20
Pancrustacea - ps21
Insecta - ps22
Eumetabola - ps23
Holometabola/Psocodea - ps24
Holometabola - ps25
Aparaglossata - ps26
Mecopterida - ps27
Diptera - ps28
Bibiono./Cyclorrhapha - ps29
Cyclorrhapha - ps30
Calyptratae/Drosophila - ps31
Drosophila - ps32
Sophophora - ps33
Obscura/Melanogaster_group - ps34
D_melanogaster_group - ps35
D_melanogaster_subgroup - ps36
sim./sech./mel. group - ps37
Drosophila_melanogaster - ps38
p-value
1e-03
3e-03
8e-03
2e-02
5e-02
Log-odds
0.5
1.0
1.5
2.0
2.5
3.0
3.5
4.0

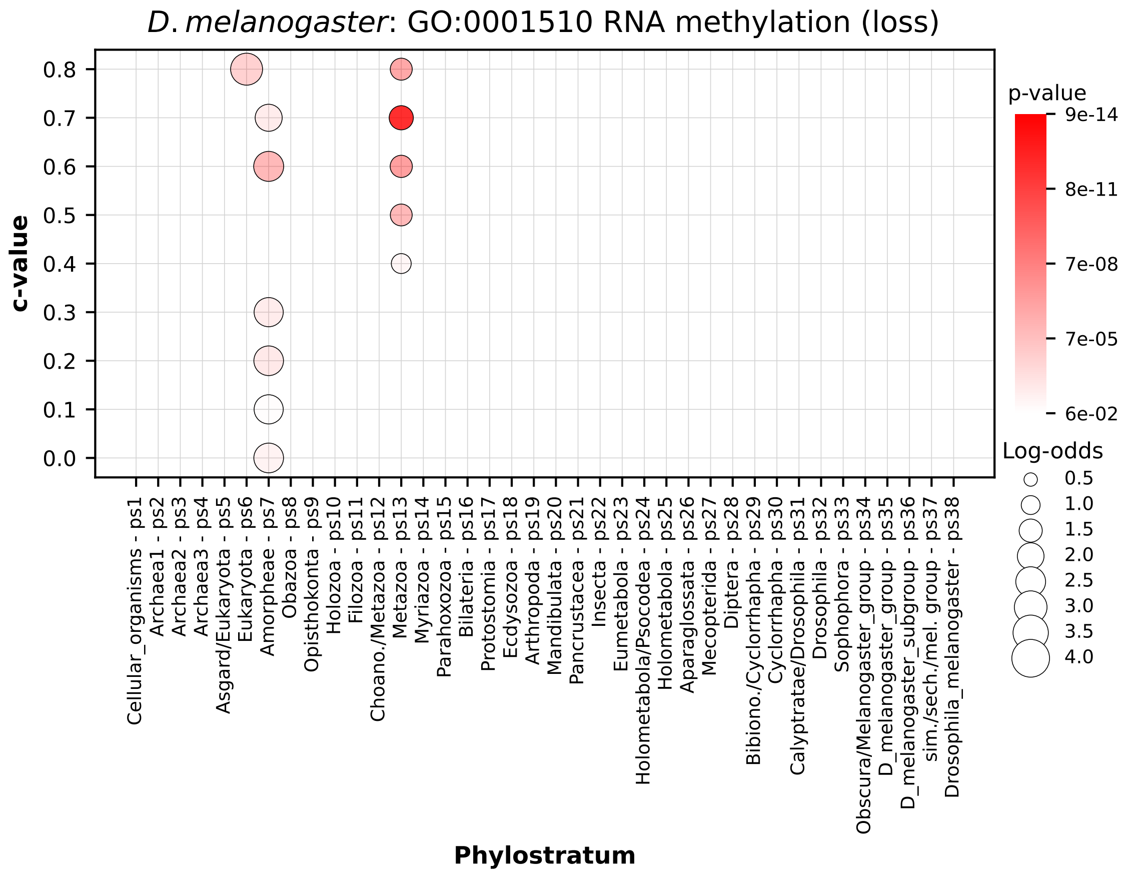
D. melanogaster: GO:0001510 RNA methylation (loss)
c-value
0.0
0.1
0.2
0.3
0.4
0.5
0.6
0.7
0.8
p-value
9e-14
8e-11
7e-08
7e-05
6e-02
Log-odds
0.5
1.0
1.5
2.0
2.5
3.0
3.5
4.0
Cellular_organisms - ps1
Archaea1 - ps2
Archaea2 - ps3
Archaea3 - ps4
Asgard/Eukaryota - ps5
Eukaryota - ps6
Amorpheae - ps7
Obazoa - ps8
Opisthokonta - ps9
Holozoa - ps10
Filozoa - ps11
Choano./Metazoa - ps12
Metazoa - ps13
Myriazoa - ps14
Parahoxozoa - ps15
Bilateria - ps16
Protostomia - ps17
Ecdysozoa - ps18
Arthropoda - ps19
Mandibulata - ps20
Pancrustacea - ps21
Insecta - ps22
Eumetabola - ps23
Holometabola/Psocodea - ps24
Holometabola - ps25
Aparaglossata - ps26
Mecopterida - ps27
Diptera - ps28
Bibiono./Cyclorrhapha - ps29
Cyclorrhapha - ps30
Calyptratae/Drosophila - ps31
Drosophila - ps32
Sophophora - ps33
Obscura/Melanogaster_group - ps34
D_melanogaster_group - ps35
D_melanogaster_subgroup - ps36
sim./sech./mel. group - ps37
Drosophila_melanogaster - ps38
Phylostratum

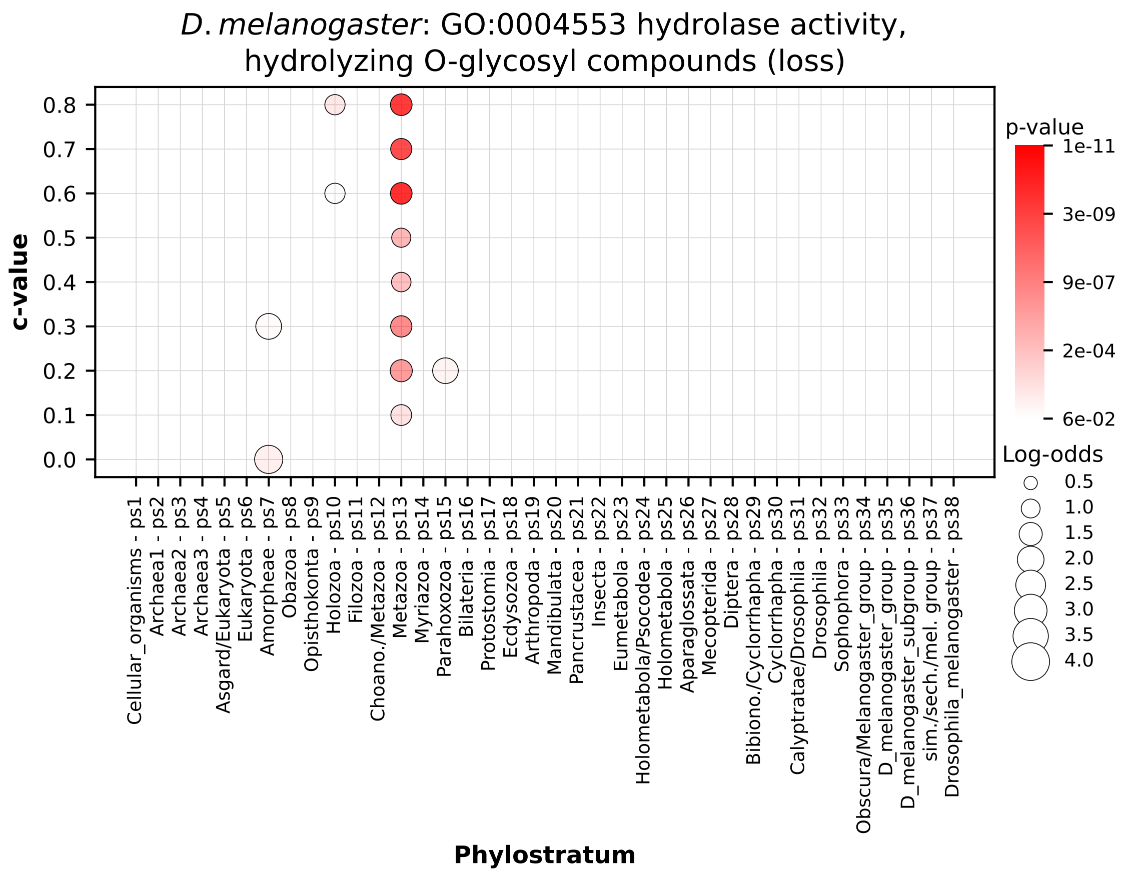

D. melanogaster: GO:0004553 hydrolase activity, hydrolyzing O-glycosyl compounds (loss)
c-value
0.8
0.7
0.6
0.5
0.4
0.3
0.2
0.1
0.0
Phylostratum
Cellular_organisms - ps1
Archaea1 - ps2
Archaea2 - ps3
Archaea3 - ps4
Asgard/Eukaryota - ps5
Eukaryota - ps6
Amorpheae - ps7
Obazoa - ps8
Opisthokonta - ps9
Holozoa - ps10
Filozoa - ps11
Choano./Metazoa - ps12
Metazoa - ps13
Myriazoa - ps14
Parahoxozoa - ps15
Bilateria - ps16
Protostomia - ps17
Ecdysozoa - ps18
Arthropoda - ps19
Mandibulata - ps20
Pancrustacea - ps21
Insecta - ps22
Eumetabola - ps23
Holometabola/Psocodea - ps24
Holometabola - ps25
Aparaglossata - ps26
Mecopterida - ps27
Diptera - ps28
Bibiono./Cyclorrhapha - ps29
Cyclorrhapha - ps30
Calyptratae/Drosophila - ps31
Drosophila - ps32
Sophophora - ps33
Obscura/Melanogaster_group - ps34
D_melanogaster_group - ps35
D_melanogaster_subgroup - ps36
sim./sech./mel. group - ps37
Drosophila_melanogaster - ps38
p-value
1e-11
3e-09
9e-07
2e-04
6e-02
Log-odds
0.5
1.0
1.5
2.0
2.5
3.0
3.5
4.0

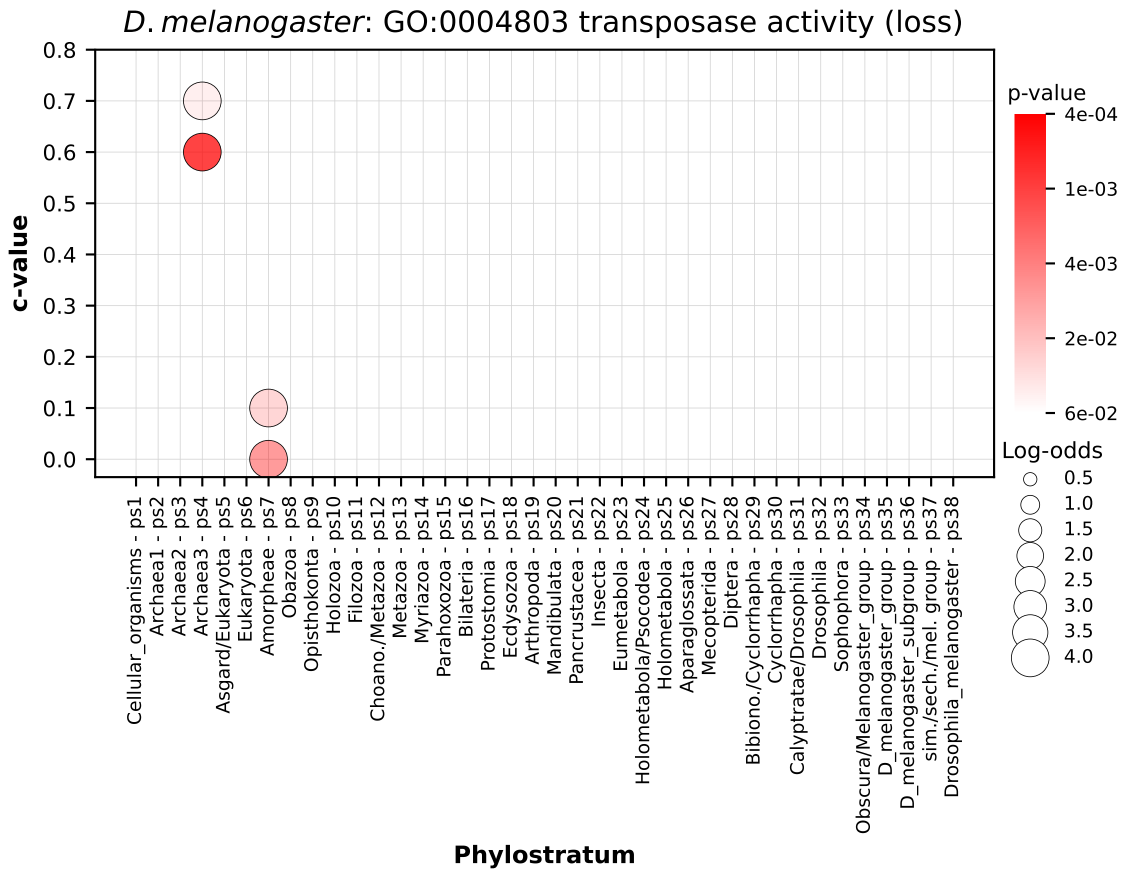

D. melanogaster: GO:0004803 transposase activity (loss)
c-value
0.8
0.7
0.6
0.5
0.4
0.3
0.2
0.1
0.0
Phylostratum
Cellular_organisms - ps1
Archaea1 - ps2
Archaea2 - ps3
Archaea3 - ps4
Asgard/Eukaryota - ps5
Eukaryota - ps6
Amorpheae - ps7
Obazoa - ps8
Opisthokonta - ps9
Holozoa - ps10
Filozoa - ps11
Choano./Metazoa - ps12
Metazoa - ps13
Myriazoa - ps14
Parahoxozoa - ps15
Bilateria - ps16
Protostomia - ps17
Ecdysozoa - ps18
Arthropoda - ps19
Mandibulata - ps20
Pancrustacea - ps21
Insecta - ps22
Eumetabola - ps23
Holometabola/Psocodea - ps24
Holometabola - ps25
Aparaglossata - ps26
Mecopterida - ps27
Diptera - ps28
Bibiono./Cyclorrhapha - ps29
Cyclorrhapha - ps30
Calyptratae/Drosophila - ps31
Drosophila - ps32
Sophophora - ps33
Obscura/Melanogaster_group - ps34
D_melanogaster_group - ps35
D_melanogaster_subgroup - ps36
sim./sech./mel. group - ps37
Drosophila_melanogaster - ps38
p-value
4e-04
1e-03
4e-03
2e-02
6e-02
Log-odds
0.5
1.0
1.5
2.0
2.5
3.0
3.5
4.0

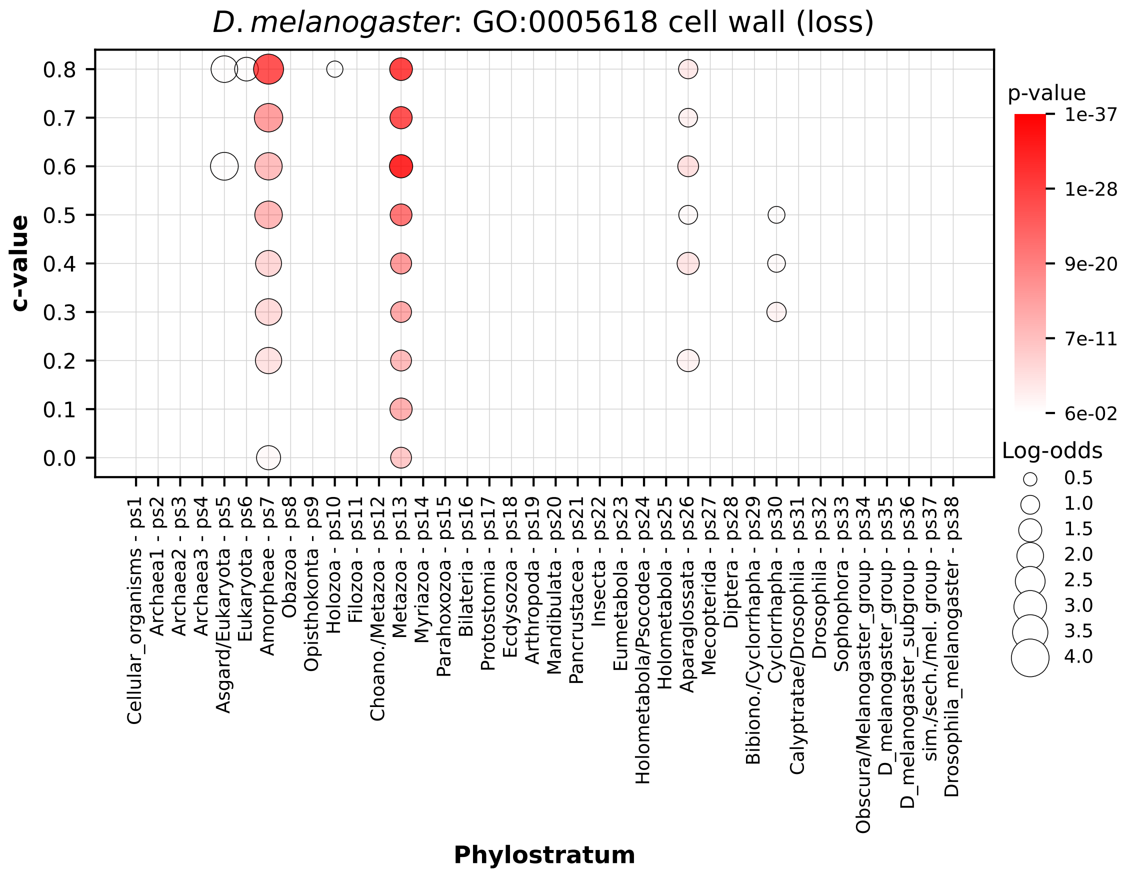

D. melanogaster: GO:0005618 cell wall (loss)
c-value
0.8
0.7
0.6
0.5
0.4
0.3
0.2
0.1
0.0
Phylostratum
Cellular_organisms - ps1
Archaea1 - ps2
Archaea2 - ps3
Archaea3 - ps4
Asgard/Eukaryota - ps5
Eukaryota - ps6
Amorpheae - ps7
Obazoa - ps8
Opisthokonta - ps9
Holozoa - ps10
Filozoa - ps11
Choano./Metazoa - ps12
Metazoa - ps13
Myriazoa - ps14
Parahoxozoa - ps15
Bilateria - ps16
Protostomia - ps17
Ecdysozoa - ps18
Arthropoda - ps19
Mandibulata - ps20
Pancrustacea - ps21
Insecta - ps22
Eumetabola - ps23
Holometabola/Psocodea - ps24
Holometabola - ps25
Aparaglossata - ps26
Mecopterida - ps27
Diptera - ps28
Bibiono./Cyclorrhapha - ps29
Cyclorrhapha - ps30
Calyptratae/Drosophila - ps31
Drosophila - ps32
Sophophora - ps33
Obscura/Melanogaster_group - ps34
D_melanogaster_group - ps35
D_melanogaster_subgroup - ps36
sim./sech./mel. group - ps37
Drosophila_melanogaster - ps38
p-value
1e-37
1e-28
9e-20
7e-11
6e-02
Log-odds
0.5
1.0
1.5
2.0
2.5
3.0
3.5
4.0

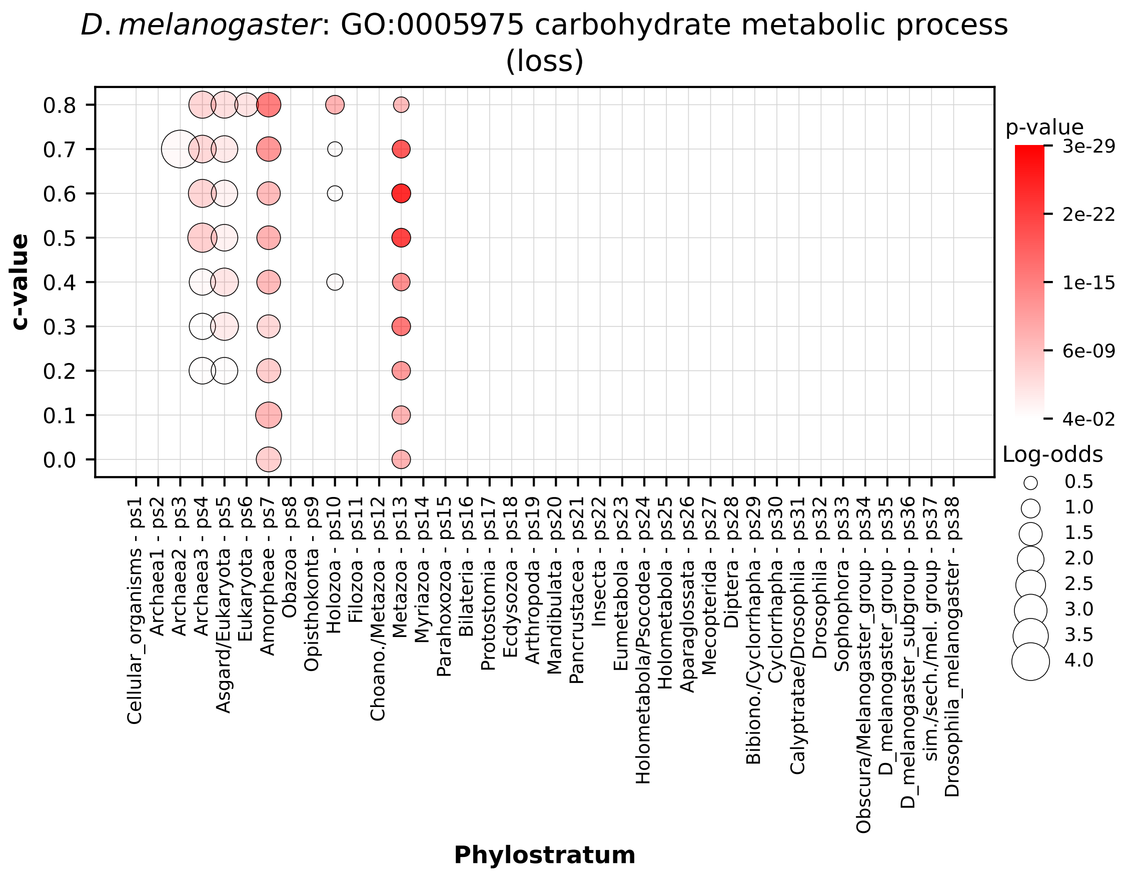

D. melanogaster: GO:0005975 carbohydrate metabolic process (loss)
c-value
0.8
0.7
0.6
0.5
0.4
0.3
0.2
0.1
0.0
p-value
3e-29
2e-22
1e-15
6e-09
4e-02
Log-odds
0.5
1.0
1.5
2.0
2.5
3.0
3.5
4.0
Cellular_organisms - ps1
Archaea1 - ps2
Archaea2 - ps3
Archaea3 - ps4
Asgard/Eukaryota - ps5
Eukaryota - ps6
Amorpheae - ps7
Obazoa - ps8
Opisthokonta - ps9
Holozoa - ps10
Filozoa - ps11
Choano./Metazoa - ps12
Metazoa - ps13
Myriazoa - ps14
Parahoxozoa - ps15
Bilateria - ps16
Protostomia - ps17
Ecdysozoa - ps18
Arthropoda - ps19
Mandibulata - ps20
Pancrustacea - ps21
Insecta - ps22
Eumetabola - ps23
Holometabola/Psocodea - ps24
Holometabola - ps25
Aparaglossata - ps26
Mecopterida - ps27
Diptera - ps28
Bibiono./Cyclorrhapha - ps29
Cyclorrhapha - ps30
Calyptratae/Drosophila - ps31
Drosophila - ps32
Sophophora - ps33
Obscura/Melanogaster_group - ps34
D_melanogaster_group - ps35
D_melanogaster_subgroup - ps36
sim./sech./mel. group - ps37
Drosophila_melanogaster - ps38
Phylostratum

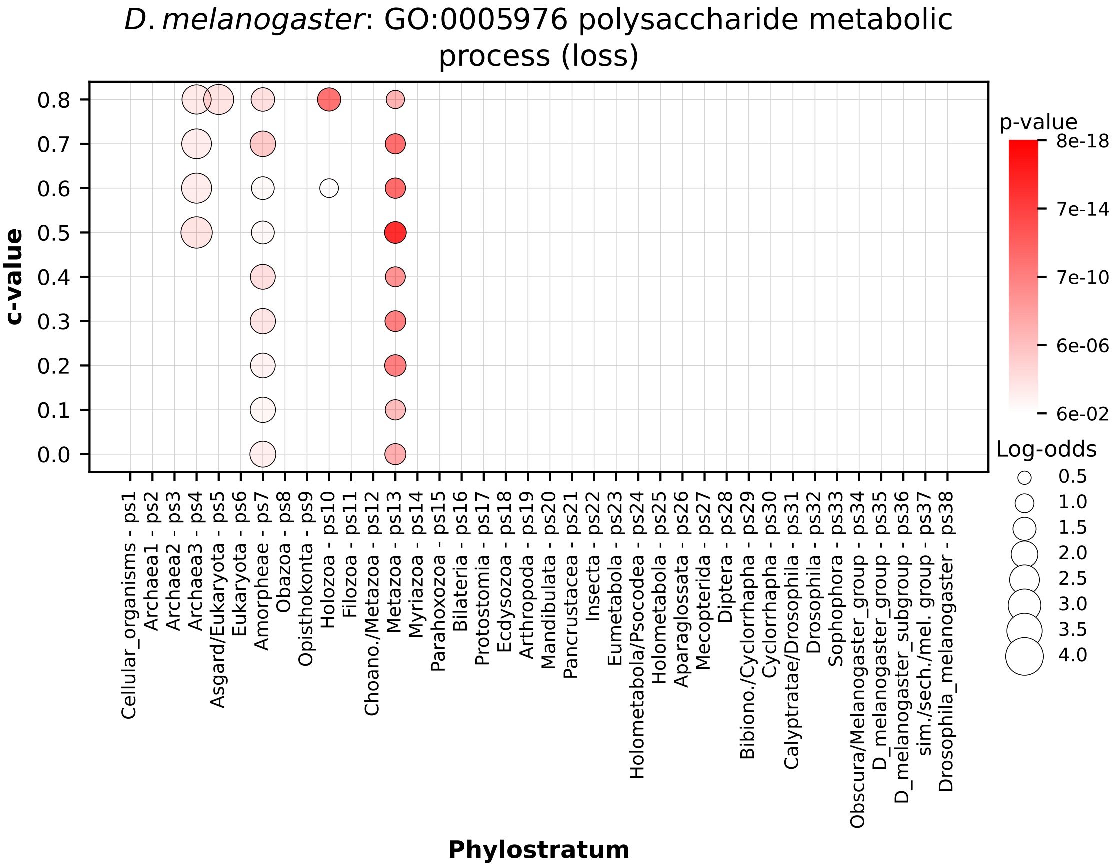
D. melanogaster: GO:0005976 polysaccharide metabolic process (loss)
c-value
0.8
0.7
0.6
0.5
0.4
0.3
0.2
0.1
0.0
Phylostratum
Cellular_organisms - ps1
Archaea1 - ps2
Archaea2 - ps3
Archaea3 - ps4
Asgard/Eukaryota - ps5
Eukaryota - ps6
Amorpheae - ps7
Obazoa - ps8
Opisthokonta - ps9
Holozoa - ps10
Filozoa - ps11
Choano./Metazoa - ps12
Metazoa - ps13
Myriazoa - ps14
Parahoxozoa - ps15
Bilateria - ps16
Protostomia - ps17
Ecdysozoa - ps18
Arthropoda - ps19
Mandibulata - ps20
Pancrustacea - ps21
Insecta - ps22
Eumetabola - ps23
Holometabola/Psocodea - ps24
Holometabola - ps25
Aparaglossata - ps26
Mecopterida - ps27
Diptera - ps28
Bibiono./Cyclorrhapha - ps29
Cyclorrhapha - ps30
Calyptratae/Drosophila - ps31
Drosophila - ps32
Sophophora - ps33
Obscura/Melanogaster_group - ps34
D_melanogaster_group - ps35
D_melanogaster_subgroup - ps36
sim./sech./mel. group - ps37
Drosophila_melanogaster - ps38
p-value
8e-18
7e-14
7e-10
6e-06
6e-02
Log-odds
0.5
1.0
1.5
2.0
2.5
3.0
3.5
4.0

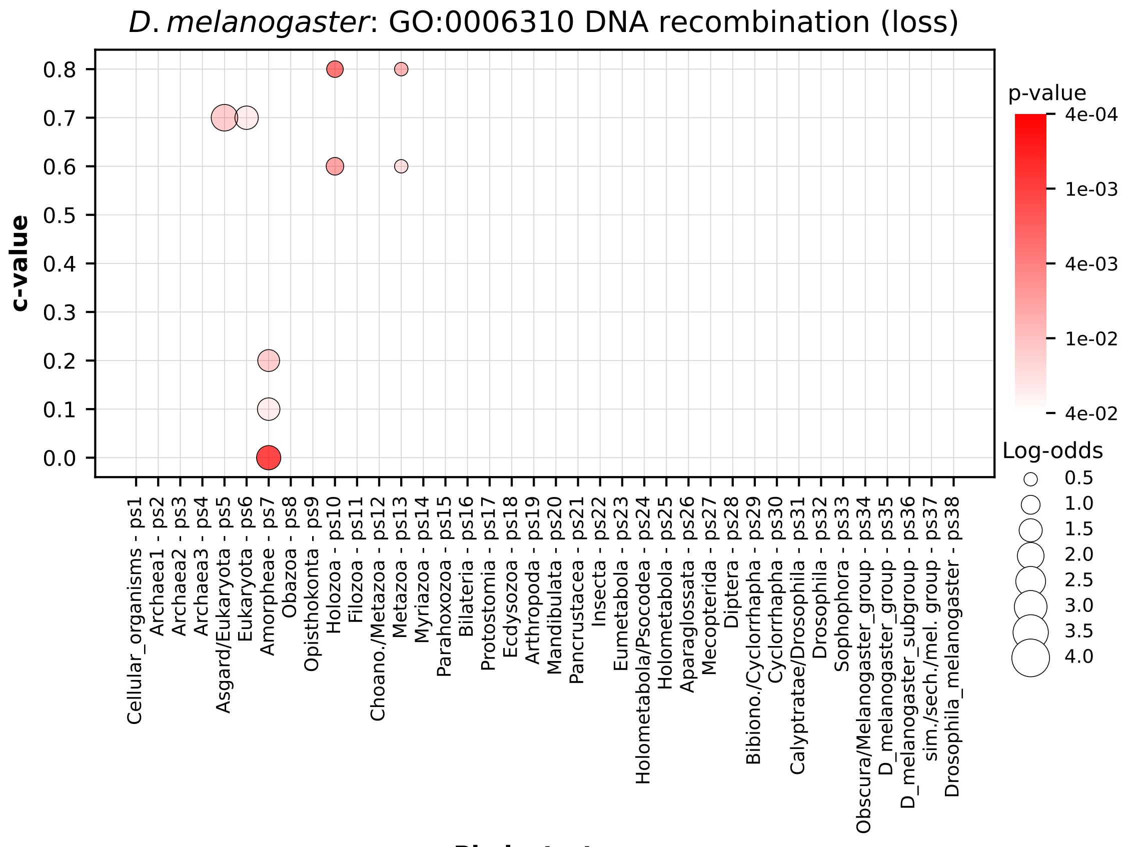
D. melanogaster: GO:0006310 DNA recombination (loss)
c-value
0.0
0.1
0.2
0.3
0.4
0.5
0.6
0.7
0.8
Phylostratum
Cellular_organisms - ps1
Archaea1 - ps2
Archaea2 - ps3
Archaea3 - ps4
Asgard/Eukaryota - ps5
Eukaryota - ps6
Amorpheae - ps7
Obazoa - ps8
Opisthokonta - ps9
Holozoa - ps10
Filozoa - ps11
Choano./Metazoa - ps12
Metazoa - ps13
Myriazoa - ps14
Parahoxozoa - ps15
Bilateria - ps16
Protostomia - ps17
Ecdysozoa - ps18
Arthropoda - ps19
Mandibulata - ps20
Pancrustacea - ps21
Insecta - ps22
Eumetabola - ps23
Holometabola/Psocodea - ps24
Holometabola - ps25
Aparaglossata - ps26
Mecopterida - ps27
Diptera - ps28
Bibiono./Cyclorrhapha - ps29
Cyclorrhapha - ps30
Calyptratae/Drosophila - ps31
Drosophila - ps32
Sophophora - ps33
Obscura/Melanogaster_group - ps34
D_melanogaster_group - ps35
D_melanogaster_subgroup - ps36
sim./sech./mel. group - ps37
Drosophila_melanogaster - ps38
p-value
4e-04
1e-03
4e-03
1e-02
4e-02
Log-odds
0.5
1.0
1.5
2.0
2.5
3.0
3.5
4.0

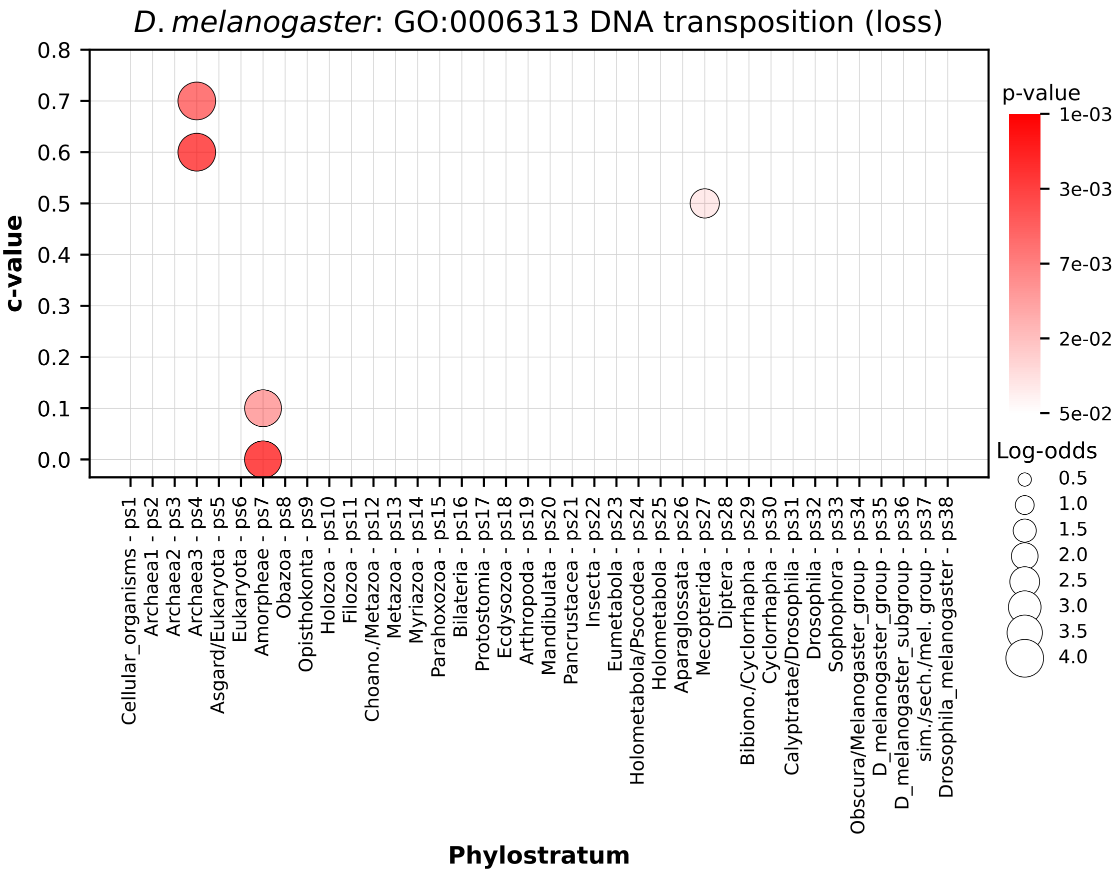

D. melanogaster: GO:0006313 DNA transposition (loss)
c-value
0.0
0.1
0.2
0.3
0.4
0.5
0.6
0.7
0.8
Phylostratum
Cellular_organisms - ps1
Archaea1 - ps2
Archaea2 - ps3
Archaea3 - ps4
Asgard/Eukaryota - ps5
Eukaryota - ps6
Amorpheae - ps7
Obazoa - ps8
Opisthokonta - ps9
Holozoa - ps10
Filozoa - ps11
Choano./Metazoa - ps12
Metazoa - ps13
Myriazoa - ps14
Parahoxozoa - ps15
Bilateria - ps16
Protostomia - ps17
Ecdysozoa - ps18
Arthropoda - ps19
Mandibulata - ps20
Pancrustacea - ps21
Insecta - ps22
Eumetabola - ps23
Holometabola/Psocodea - ps24
Holometabola - ps25
Aparaglossata - ps26
Mecopterida - ps27
Diptera - ps28
Bibiono./Cyclorrhapha - ps29
Cyclorrhapha - ps30
Calyptratae/Drosophila - ps31
Drosophila - ps32
Sophophora - ps33
Obscura/Melanogaster_group - ps34
D_melanogaster_group - ps35
D_melanogaster_subgroup - ps36
sim./sech./mel. group - ps37
Drosophila_melanogaster - ps38
p-value
1e-03
3e-03
7e-03
2e-02
5e-02
Log-odds
0.5
1.0
1.5
2.0
2.5
3.0
3.5
4.0

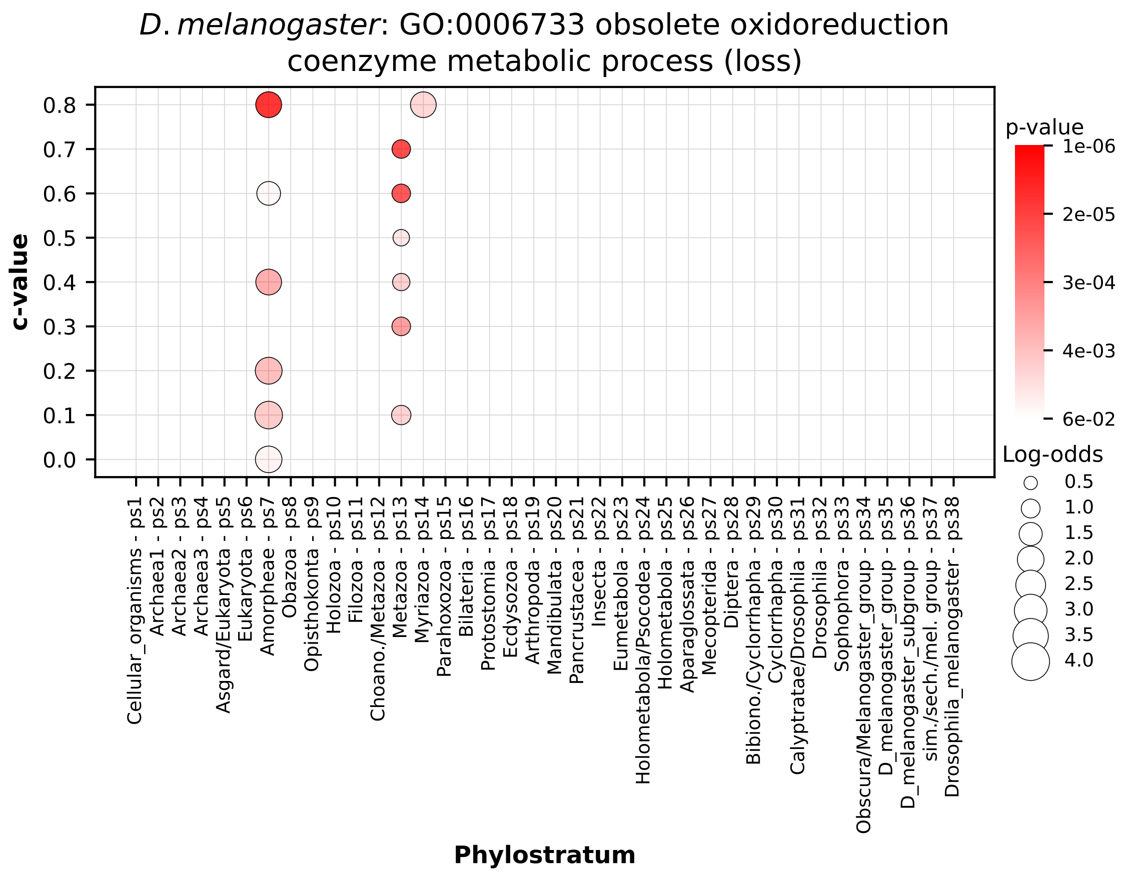
D. melanogaster: GO:0006733 obsolete oxidoreduction coenzyme metabolic process (loss)
c-value
0.8
0.7
0.6
0.5
0.4
0.3
0.2
0.1
0.0
Phylostratum
Cellular_organisms - ps1
Archaea1 - ps2
Archaea2 - ps3
Archaea3 - ps4
Asgard/Eukaryota - ps5
Eukaryota - ps6
Amorpheae - ps7
Obazoa - ps8
Opisthokonta - ps9
Holozoa - ps10
Filozoa - ps11
Choano./Metazoa - ps12
Metazoa - ps13
Myriazoa - ps14
Parahoxozoa - ps15
Bilateria - ps16
Protostomia - ps17
Ecdysozoa - ps18
Arthropoda - ps19
Mandibulata - ps20
Pancrustacea - ps21
Insecta - ps22
Eumetabola - ps23
Holometabola/Psocodea - ps24
Holometabola - ps25
Aparaglossata - ps26
Mecopterida - ps27
Diptera - ps28
Bibiono./Cyclorrhapha - ps29
Cyclorrhapha - ps30
Calyptratae/Drosophila - ps31
Drosophila - ps32
Sophophora - ps33
Obscura/Melanogaster_group - ps34
D_melanogaster_group - ps35
D_melanogaster_subgroup - ps36
sim./sech./mel. group - ps37
Drosophila_melanogaster - ps38
p-value
1e-06
2e-05
3e-04
4e-03
6e-02
Log-odds
0.5
1.0
1.5
2.0
2.5
3.0
3.5
4.0

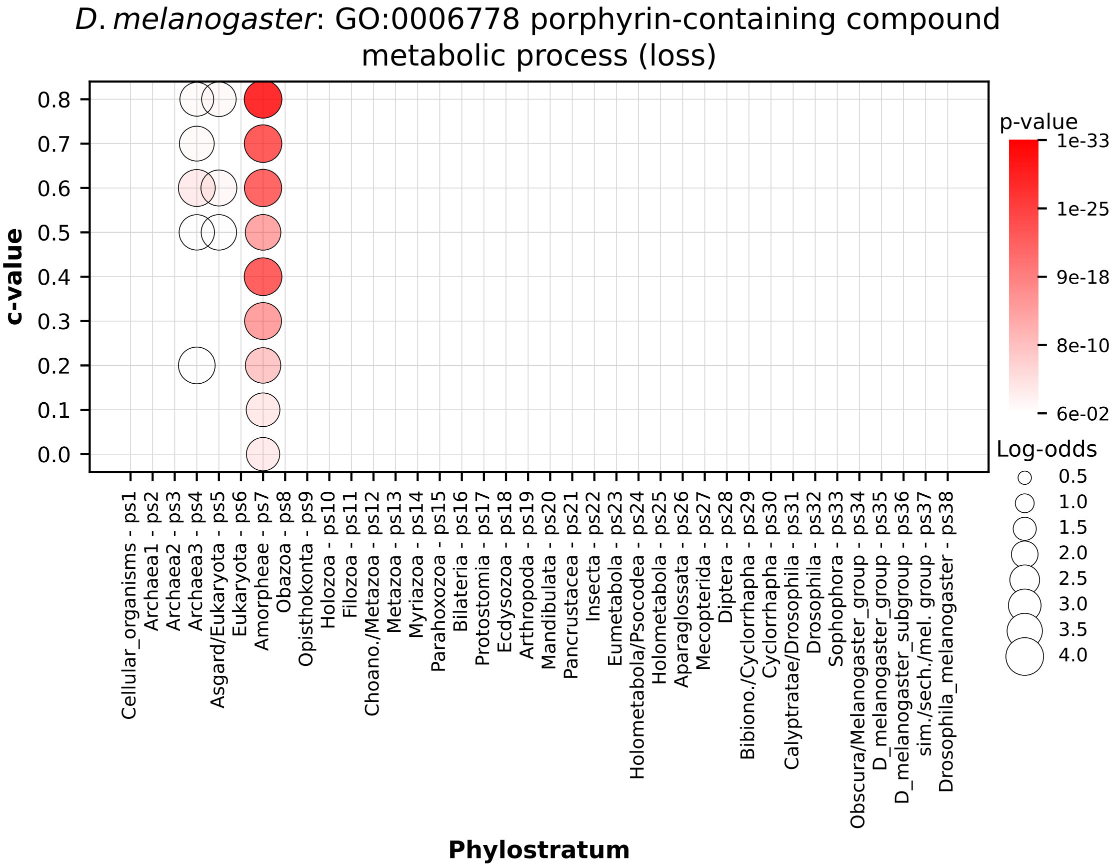

D. melanogaster: GO:0006778 porphyrin-containing compound metabolic process (loss)
c-value
Phylostratum
p-value
1e-33
1e-25
9e-18
8e-10
6e-02
Log-odds
0.5
1.0
1.5
2.0
2.5
3.0
3.5
4.0
Cellular_organisms - ps1
Archaea1 - ps2
Archaea2 - ps3
Archaea3 - ps4
Asgard/Eukaryota - ps5
Eukaryota - ps6
Amorpheae - ps7
Obazoa - ps8
Opisthokonta - ps9
Holozoa - ps10
Filozoa - ps11
Choano./Metazoa - ps12
Metazoa - ps13
Myriazoa - ps14
Parahoxozoa - ps15
Bilateria - ps16
Protostomia - ps17
Ecdysozoa - ps18
Arthropoda - ps19
Mandibulata - ps20
Pancrustacea - ps21
Insecta - ps22
Eumetabola - ps23
Holometabola/Psocodea - ps24
Holometabola - ps25
Aparaglossata - ps26
Mecopterida - ps27
Diptera - ps28
Bibiono./Cyclorrhapha - ps29
Cyclorrhapha - ps30
Calyptratae/Drosophila - ps31
Drosophila - ps32
Sophophora - ps33
Obscura/Melanogaster_group - ps34
D_melanogaster_group - ps35
D_melanogaster_subgroup - ps36
sim./sech./mel. group - ps37
Drosophila_melanogaster - ps38

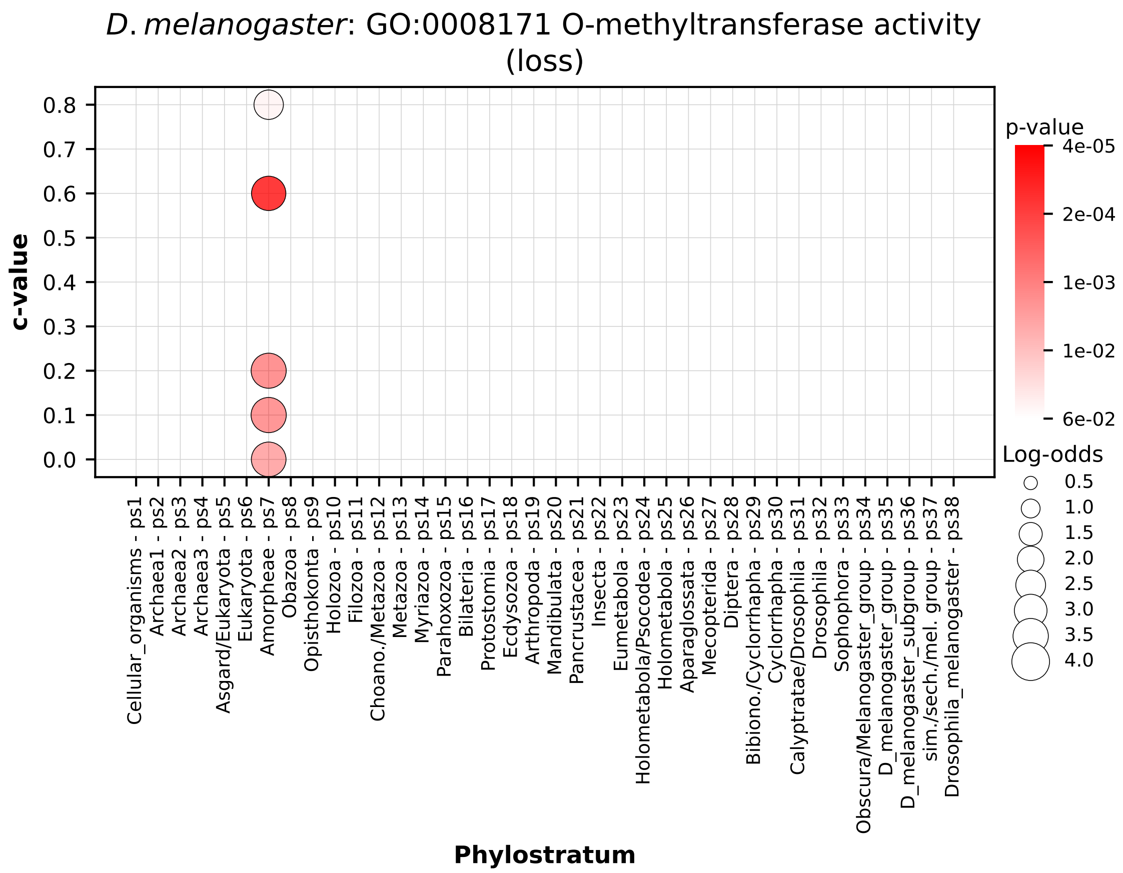

D. melanogaster: GO:0008171 O-methyltransferase activity (loss)
c-value
0.8
0.7
0.6
0.5
0.4
0.3
0.2
0.1
0.0
p-value
4e-05
2e-04
1e-03
1e-02
6e-02
Log-odds
0.5
1.0
1.5
2.0
2.5
3.0
3.5
4.0
Cellular_organisms - ps1
Archaea1 - ps2
Archaea2 - ps3
Archaea3 - ps4
Asgard/Eukaryota - ps5
Eukaryota - ps6
Amorpheae - ps7
Obazoa - ps8
Opisthokonta - ps9
Holozoa - ps10
Filozoa - ps11
Choano./Metazoa - ps12
Metazoa - ps13
Myriazoa - ps14
Parahoxozoa - ps15
Bilateria - ps16
Protostomia - ps17
Ecdysozoa - ps18
Arthropoda - ps19
Mandibulata - ps20
Pancrustacea - ps21
Insecta - ps22
Eumetabola - ps23
Holometabola/Psocodea - ps24
Holometabola - ps25
Aparaglossata - ps26
Mecopterida - ps27
Diptera - ps28
Bibiono./Cyclorrhapha - ps29
Cyclorrhapha - ps30
Calyptratae/Drosophila - ps31
Drosophila - ps32
Sophophora - ps33
Obscura/Melanogaster_group - ps34
D_melanogaster_group - ps35
D_melanogaster_subgroup - ps36
sim./sech./mel. group - ps37
Drosophila_melanogaster - ps38
Phylostratum

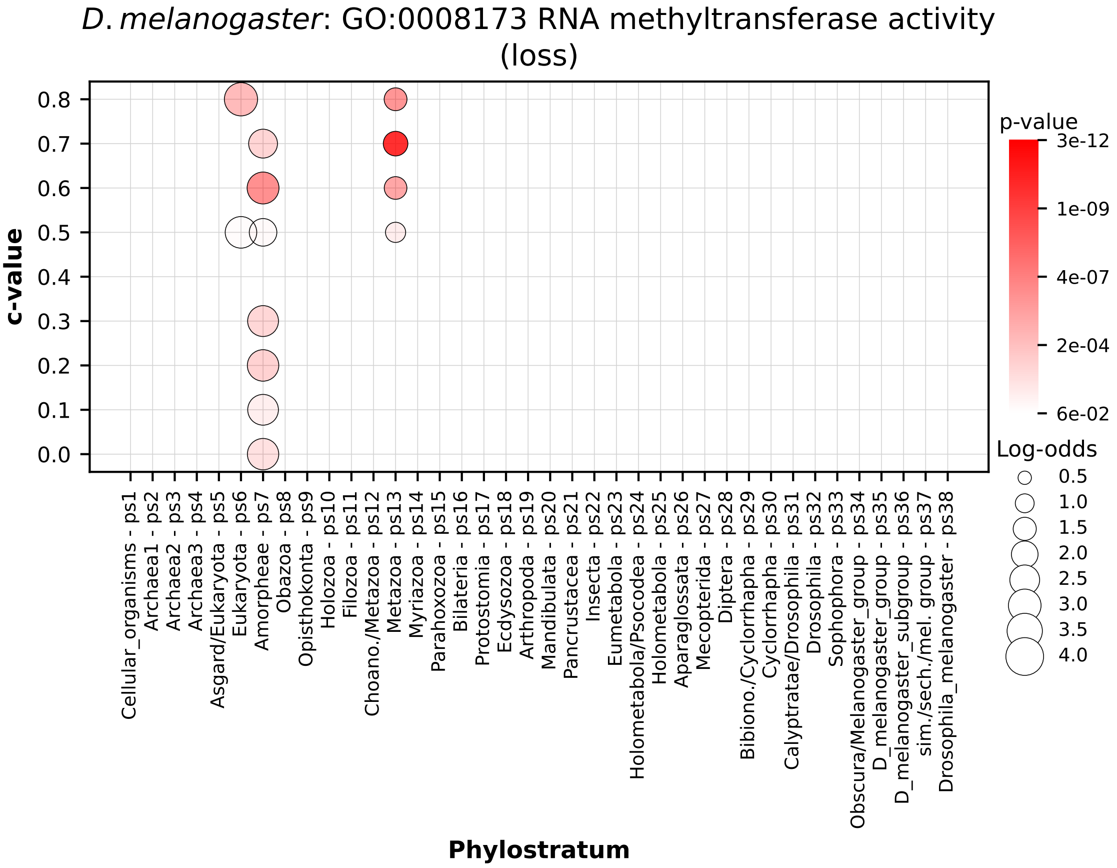

D. melanogaster: GO:0008173 RNA methyltransferase activity (loss)
c-value
0.8
0.7
0.6
0.5
0.4
0.3
0.2
0.1
0.0
p-value
3e-12
1e-09
4e-07
2e-04
6e-02
Log-odds
0.5
1.0
1.5
2.0
2.5
3.0
3.5
4.0
Cellular_organisms - ps1
Archaea1 - ps2
Archaea2 - ps3
Archaea3 - ps4
Asgard/Eukaryota - ps5
Eukaryota - ps6
Amorpheae - ps7
Obazoa - ps8
Opisthokonta - ps9
Holozoa - ps10
Filozoa - ps11
Choano./Metazoa - ps12
Metazoa - ps13
Myriazoa - ps14
Parahoxozoa - ps15
Bilateria - ps16
Protostomia - ps17
Ecdysozoa - ps18
Arthropoda - ps19
Mandibulata - ps20
Pancrustacea - ps21
Insecta - ps22
Eumetabola - ps23
Holometabola/Psocodea - ps24
Holometabola - ps25
Aparaglossata - ps26
Mecopterida - ps27
Diptera - ps28
Bibiono./Cyclorrhapha - ps29
Cyclorrhapha - ps30
Calyptratae/Drosophila - ps31
Drosophila - ps32
Sophophora - ps33
Obscura/Melanogaster_group - ps34
D_melanogaster_group - ps35
D_melanogaster_subgroup - ps36
sim./sech./mel. group - ps37
Drosophila_melanogaster - ps38
Phylostratum

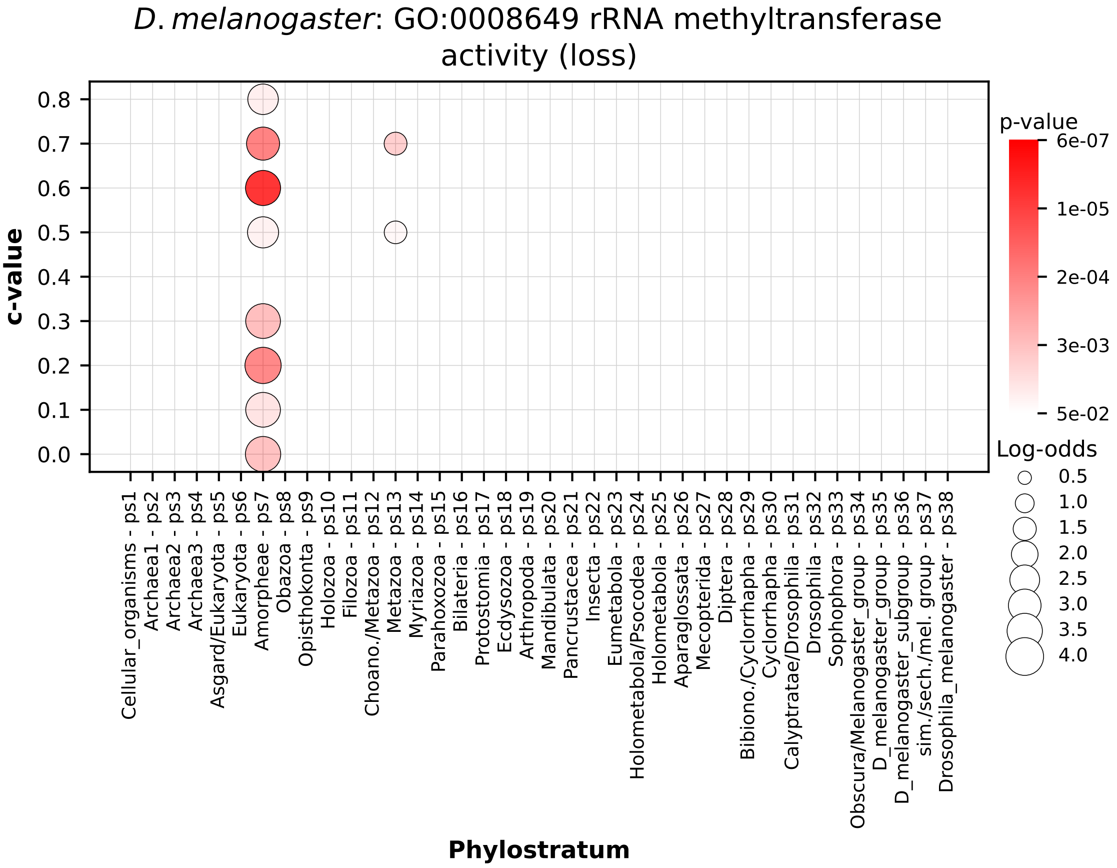
D. melanogaster: GO:0008649 rRNA methyltransferase activity (loss)
c-value
0.8
0.7
0.6
0.5
0.4
0.3
0.2
0.1
0.0
p-value
6e-07
1e-05
2e-04
3e-03
5e-02
Log-odds
0.5
1.0
1.5
2.0
2.5
3.0
3.5
4.0
Cellular_organisms - ps1
Archaea1 - ps2
Archaea2 - ps3
Archaea3 - ps4
Asgard/Eukaryota - ps5
Eukaryota - ps6
Amorpheae - ps7
Obazoa - ps8
Opisthokonta - ps9
Holozoa - ps10
Filozoa - ps11
Choano./Metazoa - ps12
Metazoa - ps13
Myriazoa - ps14
Parahoxozoa - ps15
Bilateria - ps16
Protostomia - ps17
Ecdysozoa - ps18
Arthropoda - ps19
Mandibulata - ps20
Pancrustacea - ps21
Insecta - ps22
Eumetabola - ps23
Holometabola/Psocodea - ps24
Holometabola - ps25
Aparaglossata - ps26
Mecopterida - ps27
Diptera - ps28
Bibiono./Cyclorrhapha - ps29
Cyclorrhapha - ps30
Calyptratae/Drosophila - ps31
Drosophila - ps32
Sophophora - ps33
Obscura/Melanogaster_group - ps34
D_melanogaster_group - ps35
D_melanogaster_subgroup - ps36
sim./sech./mel. group - ps37
Drosophila_melanogaster - ps38
Phylostratum

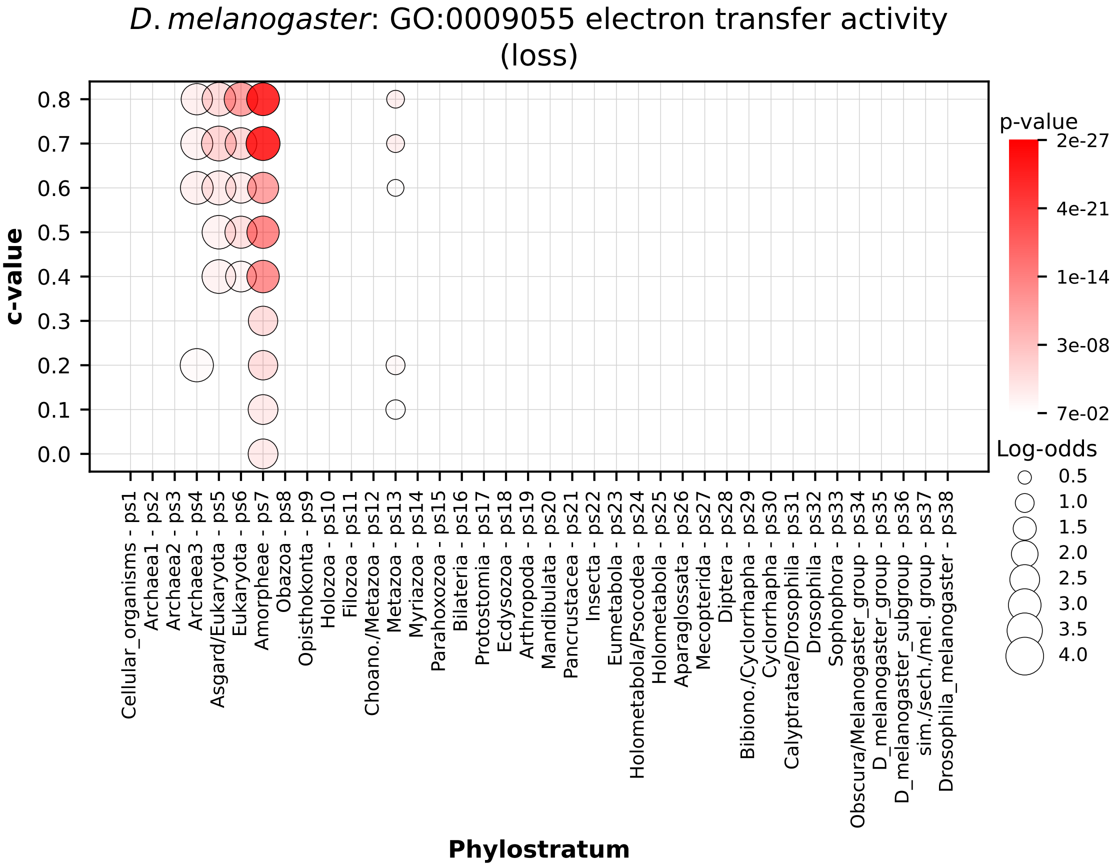

D. melanogaster: GO:0009055 electron transfer activity (loss)
c-value
Phylostratum
p-value
2e-27
4e-21
1e-14
3e-08
7e-02
Log-odds
0.5
1.0
1.5
2.0
2.5
3.0
3.5
4.0
0.8
0.7
0.6
0.5
0.4
0.3
0.2
0.1
0.0
Cellular_organisms - ps1
Archaea1 - ps2
Archaea2 - ps3
Archaea3 - ps4
Asgard/Eukaryota - ps5
Eukaryota - ps6
Amorpheae - ps7
Obazoa - ps8
Opisthokonta - ps9
Holozoa - ps10
Filozoa - ps11
Choano./Metazoa - ps12
Metazoa - ps13
Myriazoa - ps14
Parahoxozoa - ps15
Bilateria - ps16
Protostomia - ps17
Ecdysozoa - ps18
Arthropoda - ps19
Mandibulata - ps20
Pancrustacea - ps21
Insecta - ps22
Eumetabola - ps23
Holometabola/Psocodea - ps24
Holometabola - ps25
Aparaglossata - ps26
Mecopterida - ps27
Diptera - ps28
Bibiono./Cyclorrhapha - ps29
Cyclorrhapha - ps30
Calyptratae/Drosophila - ps31
Drosophila - ps32
Sophophora - ps33
Obscura/Melanogaster_group - ps34
D_melanogaster_group - ps35
D_melanogaster_subgroup - ps36
sim./sech./mel. group - ps37
Drosophila_melanogaster - ps38

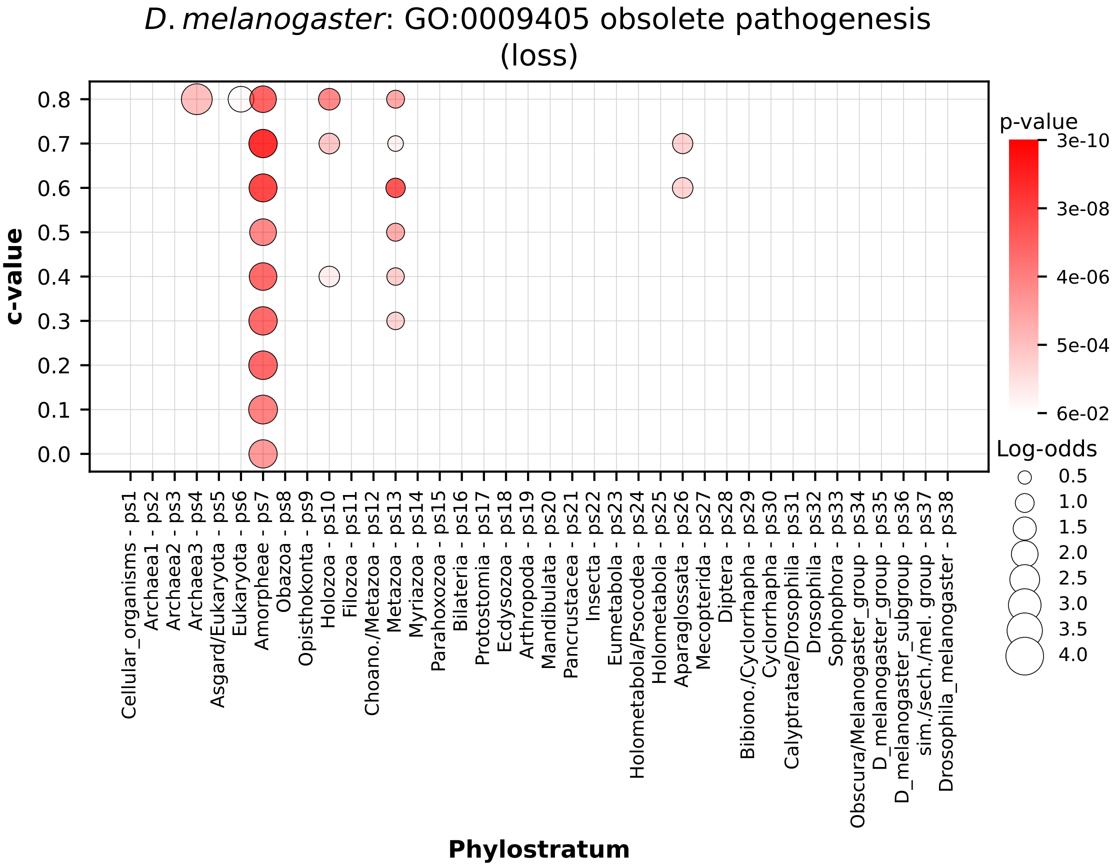

D. melanogaster: GO:0009405 obsolete pathogenesis (loss)
c-value
0.8
0.7
0.6
0.5
0.4
0.3
0.2
0.1
0.0
p-value
3e-10
3e-08
4e-06
5e-04
6e-02
Log-odds
0.5
1.0
1.5
2.0
2.5
3.0
3.5
4.0
Cellular_organisms - ps1
Archaea1 - ps2
Archaea2 - ps3
Archaea3 - ps4
Asgard/Eukaryota - ps5
Eukaryota - ps6
Amorpheae - ps7
Obazoa - ps8
Opisthokonta - ps9
Holozoa - ps10
Filozoa - ps11
Choano./Metazoa - ps12
Metazoa - ps13
Myriazoa - ps14
Parahoxozoa - ps15
Bilateria - ps16
Protostomia - ps17
Ecdysozoa - ps18
Arthropoda - ps19
Mandibulata - ps20
Pancrustacea - ps21
Insecta - ps22
Eumetabola - ps23
Holometabola/Psocodea - ps24
Holometabola - ps25
Aparaglossata - ps26
Mecopterida - ps27
Diptera - ps28
Bibiono./Cyclorrhapha - ps29
Cyclorrhapha - ps30
Calyptratae/Drosophila - ps31
Drosophila - ps32
Sophophora - ps33
Obscura/Melanogaster_group - ps34
D_melanogaster_group - ps35
D_melanogaster_subgroup - ps36
sim./sech./mel. group - ps37
Drosophila_melanogaster - ps38
Phylostratum

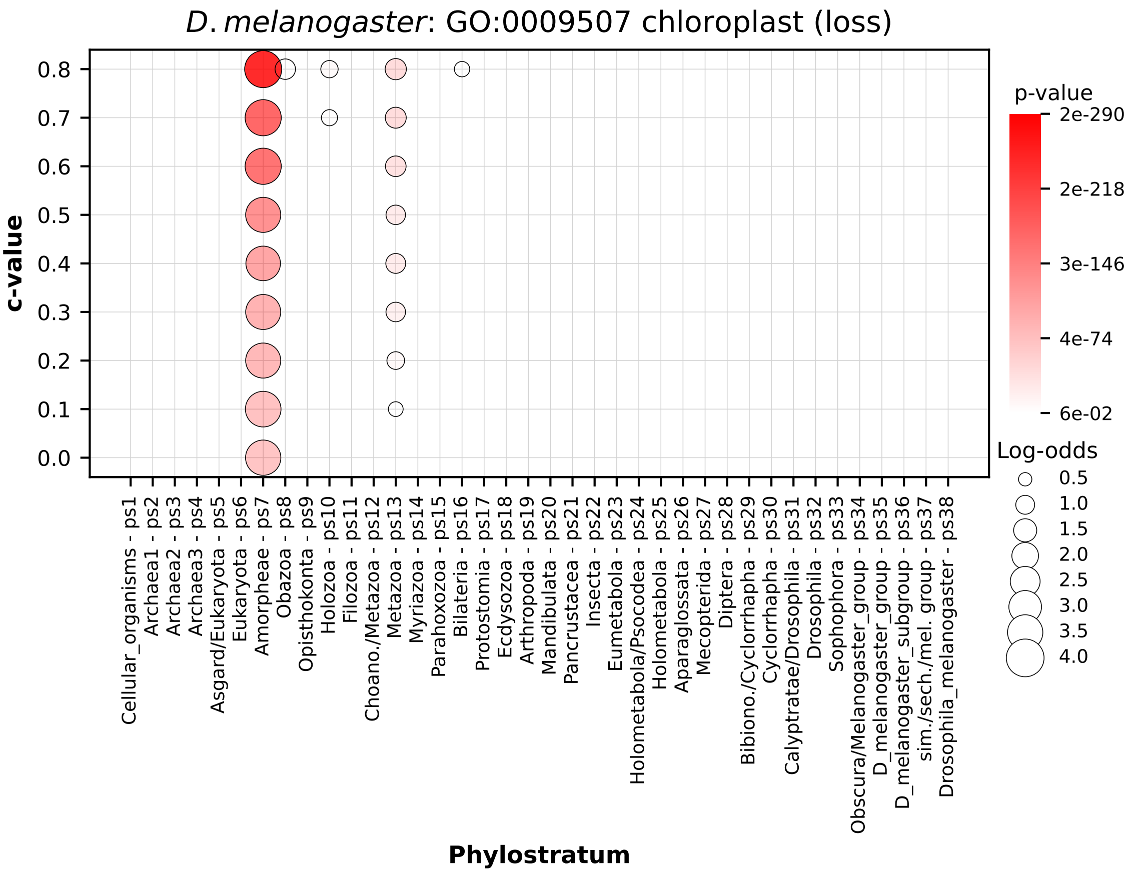

D. melanogaster: GO:0009507 chloroplast (loss)
c-value
0.8
0.7
0.6
0.5
0.4
0.3
0.2
0.1
0.0
Phylostratum
Cellular_organisms - ps1
Archaea1 - ps2
Archaea2 - ps3
Archaea3 - ps4
Asgard/Eukaryota - ps5
Eukaryota - ps6
Amorpheae - ps7
Obazoa - ps8
Opisthokonta - ps9
Holozoa - ps10
Filozoa - ps11
Choano./Metazoa - ps12
Metazoa - ps13
Myriazoa - ps14
Parahoxozoa - ps15
Bilateria - ps16
Protostomia - ps17
Ecdysozoa - ps18
Arthropoda - ps19
Mandibulata - ps20
Pancrustacea - ps21
Insecta - ps22
Eumetabola - ps23
Holometabola/Psocodea - ps24
Holometabola - ps25
Aparaglossata - ps26
Mecopterida - ps27
Diptera - ps28
Bibiono./Cyclorrhapha - ps29
Cyclorrhapha - ps30
Calyptratae/Drosophila - ps31
Drosophila - ps32
Sophophora - ps33
Obscura/Melanogaster_group - ps34
D_melanogaster_group - ps35
D_melanogaster_subgroup - ps36
sim./sech./mel. group - ps37
Drosophila_melanogaster - ps38
p-value
2e-290
2e-218
3e-146
4e-74
6e-02
Log-odds
0.5
1.0
1.5
2.0
2.5
3.0
3.5
4.0

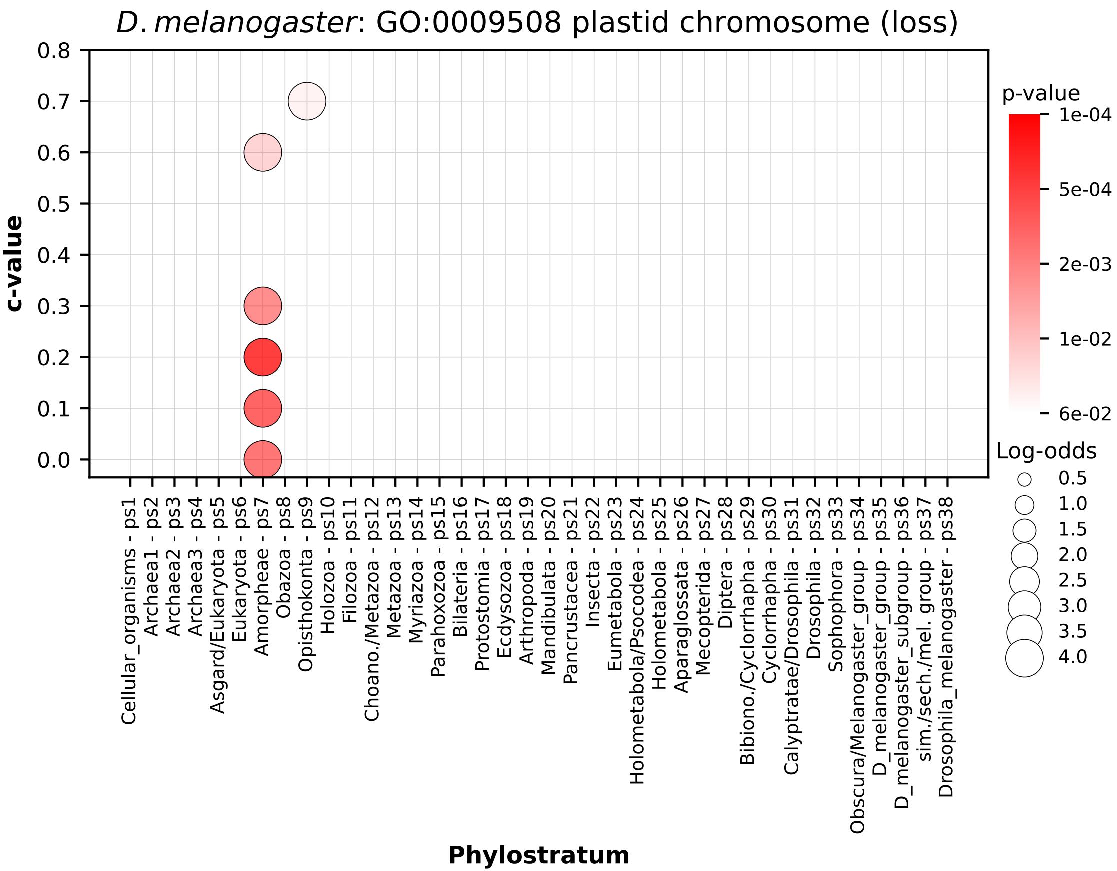

D. melanogaster: GO:0009508 plastid chromosome (loss)
c-value
0.8
0.7
0.6
0.5
0.4
0.3
0.2
0.1
0.0
Phylostratum
Cellular_organisms - ps1
Archaea1 - ps2
Archaea2 - ps3
Archaea3 - ps4
Asgard/Eukaryota - ps5
Eukaryota - ps6
Amorpheae - ps7
Obazoa - ps8
Opisthokonta - ps9
Holozoa - ps10
Filozoa - ps11
Choano./Metazoa - ps12
Metazoa - ps13
Myriazoa - ps14
Parahoxozoa - ps15
Bilateria - ps16
Protostomia - ps17
Ecdysozoa - ps18
Arthropoda - ps19
Mandibulata - ps20
Pancrustacea - ps21
Insecta - ps22
Eumetabola - ps23
Holometabola/Psocodea - ps24
Holometabola - ps25
Aparaglossata - ps26
Mecopterida - ps27
Diptera - ps28
Bibiono./Cyclorrhapha - ps29
Cyclorrhapha - ps30
Calyptratae/Drosophila - ps31
Drosophila - ps32
Sophophora - ps33
Obscura/Melanogaster_group - ps34
D_melanogaster_group - ps35
D_melanogaster_subgroup - ps36
sim./sech./mel. group - ps37
Drosophila_melanogaster - ps38
p-value
1e-04
5e-04
2e-03
1e-02
6e-02
Log-odds
0.5
1.0
1.5
2.0
2.5
3.0
3.5
4.0

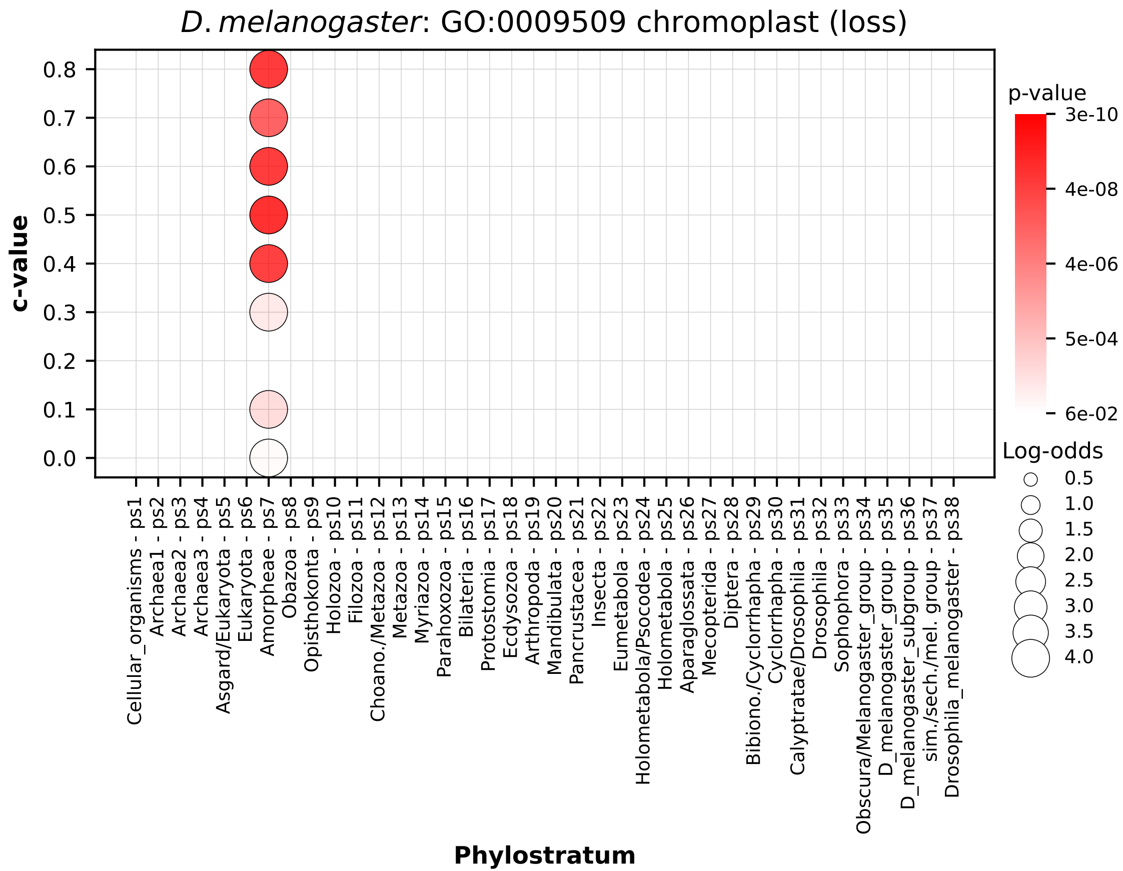

D. melanogaster: GO:0009509 chromoplast (loss)
c-value
0.0
0.1
0.2
0.3
0.4
0.5
0.6
0.7
0.8
Phylostratum
Cellular_organisms - ps1
Archaea1 - ps2
Archaea2 - ps3
Archaea3 - ps4
Asgard/Eukaryota - ps5
Eukaryota - ps6
Amorpheae - ps7
Obazoa - ps8
Opisthokonta - ps9
Holozoa - ps10
Filozoa - ps11
Choano./Metazoa - ps12
Metazoa - ps13
Myriazoa - ps14
Parahoxozoa - ps15
Bilateria - ps16
Protostomia - ps17
Ecdysozoa - ps18
Arthropoda - ps19
Mandibulata - ps20
Pancrustacea - ps21
Insecta - ps22
Eumetabola - ps23
Holometabola/Psocodea - ps24
Holometabola - ps25
Aparaglossata - ps26
Mecopterida - ps27
Diptera - ps28
Bibiono./Cyclorrhapha - ps29
Cyclorrhapha - ps30
Calyptratae/Drosophila - ps31
Drosophila - ps32
Sophophora - ps33
Obscura/Melanogaster_group - ps34
D_melanogaster_group - ps35
D_melanogaster_subgroup - ps36
sim./sech./mel. group - ps37
Drosophila_melanogaster - ps38
p-value
3e-10
4e-08
4e-06
5e-04
6e-02
Log-odds
0.5
1.0
1.5
2.0
2.5
3.0
3.5
4.0

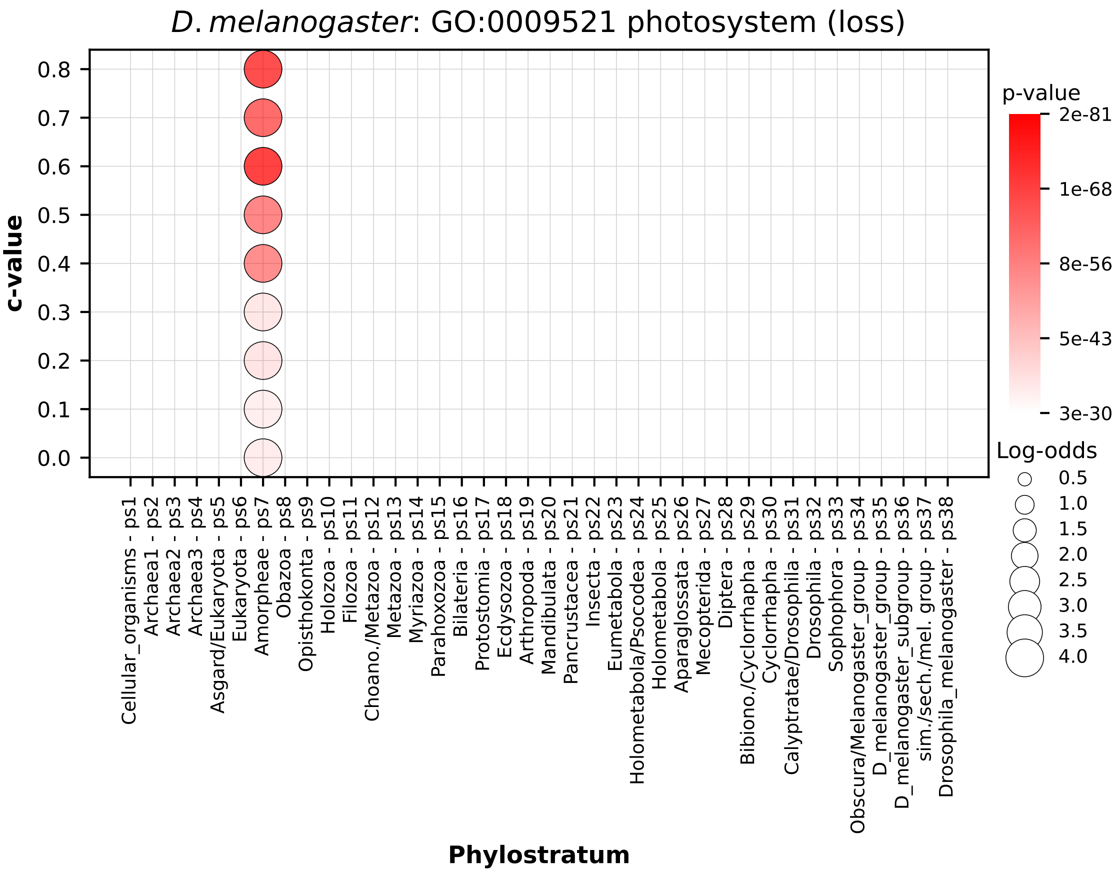

D. melanogaster: GO:0009521 photosystem (loss)
c-value
0.8
0.7
0.6
0.5
0.4
0.3
0.2
0.1
0.0
p-value
2e-81
1e-68
8e-56
5e-43
3e-30
Log-odds
0.5
1.0
1.5
2.0
2.5
3.0
3.5
4.0
Cellular_organisms - ps1
Archaea1 - ps2
Archaea2 - ps3
Archaea3 - ps4
Asgard/Eukaryota - ps5
Eukaryota - ps6
Amorpheae - ps7
Obazoa - ps8
Opisthokonta - ps9
Holozoa - ps10
Filozoa - ps11
Choano./Metazoa - ps12
Metazoa - ps13
Myriazoa - ps14
Parahoxozoa - ps15
Bilateria - ps16
Protostomia - ps17
Ecdysozoa - ps18
Arthropoda - ps19
Mandibulata - ps20
Pancrustacea - ps21
Insecta - ps22
Eumetabola - ps23
Holometabola/Psocodea - ps24
Holometabola - ps25
Aparaglossata - ps26
Mecopterida - ps27
Diptera - ps28
Bibiono./Cyclorrhapha - ps29
Cyclorrhapha - ps30
Calyptratae/Drosophila - ps31
Drosophila - ps32
Sophophora - ps33
Obscura/Melanogaster_group - ps34
D_melanogaster_group - ps35
D_melanogaster_subgroup - ps36
sim./sech./mel. group - ps37
Drosophila_melanogaster - ps38
Phylostratum

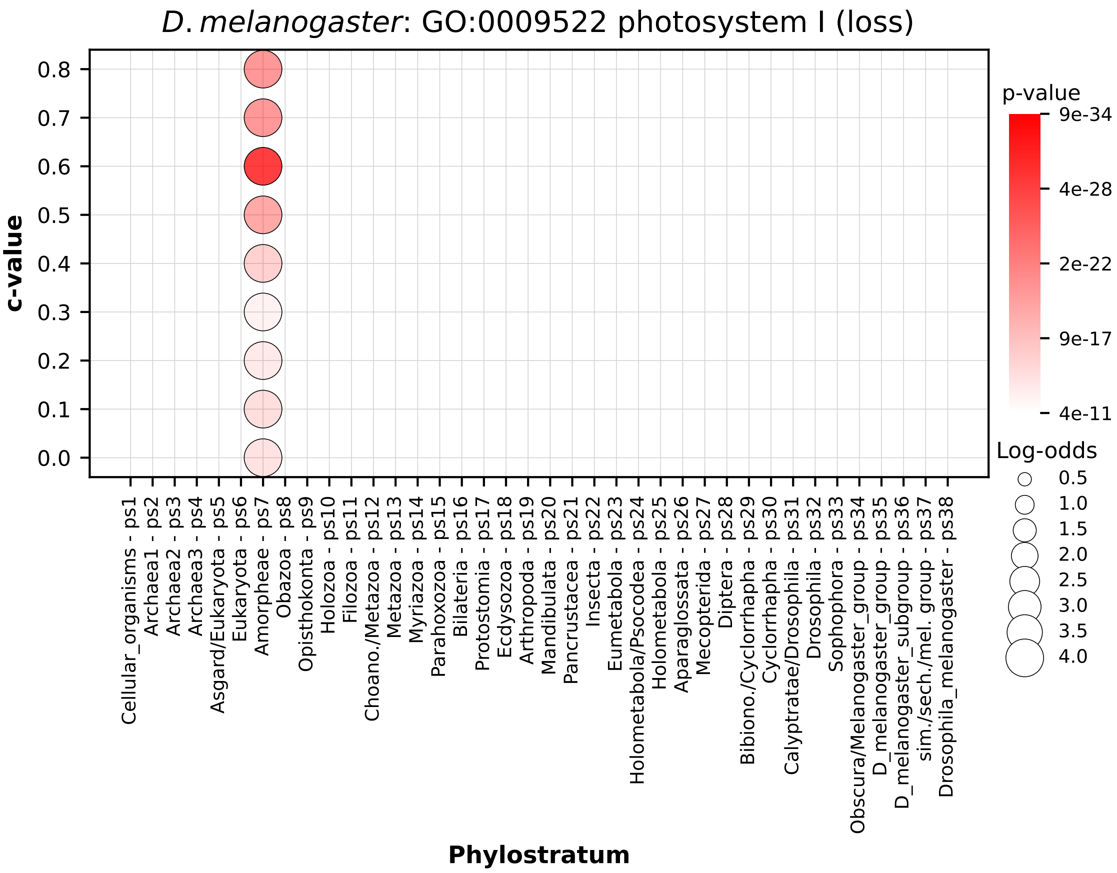

D. melanogaster: GO:0009522 photosystem I (loss)
c-value
0.0
0.1
0.2
0.3
0.4
0.5
0.6
0.7
0.8
Phylostratum
Cellular_organisms - ps1
Archaea1 - ps2
Archaea2 - ps3
Archaea3 - ps4
Asgard/Eukaryota - ps5
Eukaryota - ps6
Amorpheae - ps7
Obazoa - ps8
Opisthokonta - ps9
Holozoa - ps10
Filozoa - ps11
Choano./Metazoa - ps12
Metazoa - ps13
Myriazoa - ps14
Parahoxozoa - ps15
Bilateria - ps16
Protostomia - ps17
Ecdysozoa - ps18
Arthropoda - ps19
Mandibulata - ps20
Pancrustacea - ps21
Insecta - ps22
Eumetabola - ps23
Holometabola/Psocodea - ps24
Holometabola - ps25
Aparaglossata - ps26
Mecopterida - ps27
Diptera - ps28
Bibiono./Cyclorrhapha - ps29
Cyclorrhapha - ps30
Calyptratae/Drosophila - ps31
Drosophila - ps32
Sophophora - ps33
Obscura/Melanogaster_group - ps34
D_melanogaster_group - ps35
D_melanogaster_subgroup - ps36
sim./sech./mel. group - ps37
Drosophila_melanogaster - ps38
p-value
9e-34
4e-28
2e-22
9e-17
4e-11
Log-odds
0.5
1.0
1.5
2.0
2.5
3.0
3.5
4.0

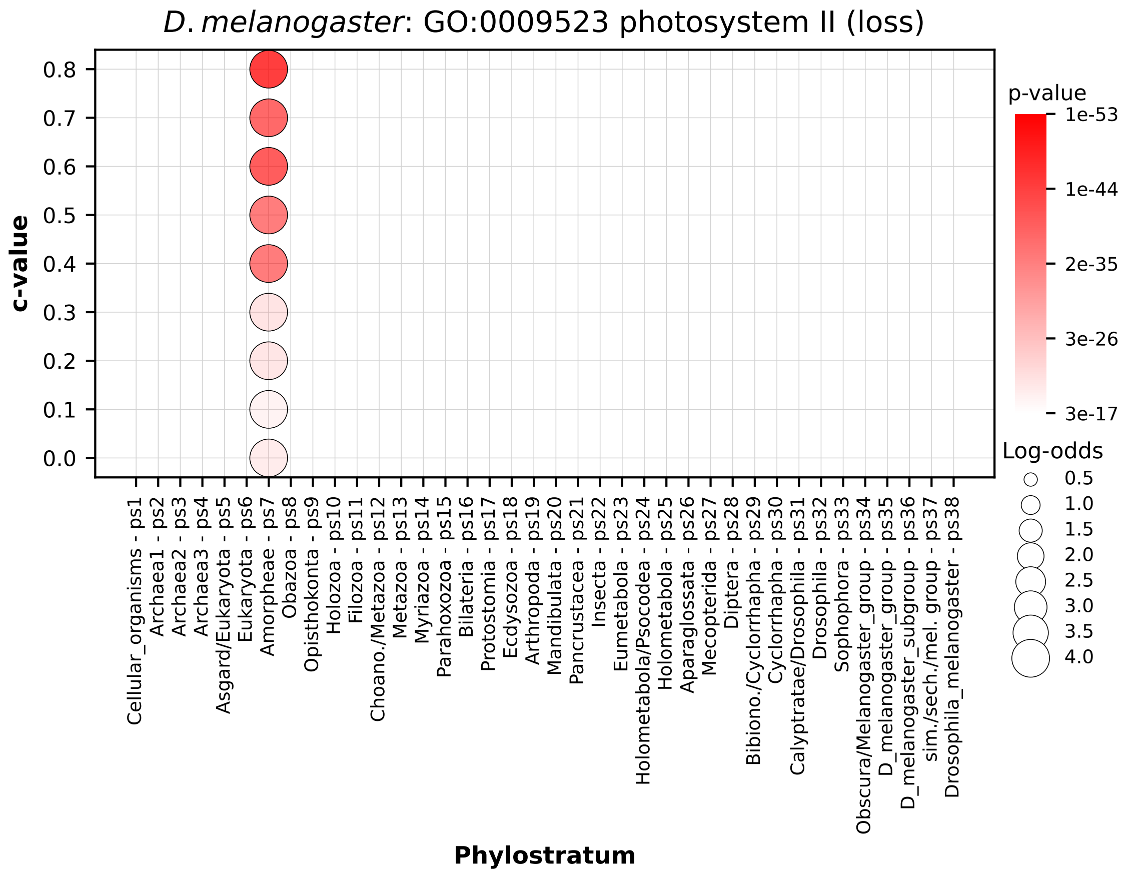

D. melanogaster: GO:0009523 photosystem II (loss)
c-value
0.0
0.1
0.2
0.3
0.4
0.5
0.6
0.7
0.8
Phylostratum
Cellular_organisms - ps1
Archaea1 - ps2
Archaea2 - ps3
Archaea3 - ps4
Asgard/Eukaryota - ps5
Eukaryota - ps6
Amorpheae - ps7
Obazoa - ps8
Opisthokonta - ps9
Holozoa - ps10
Filozoa - ps11
Choano./Metazoa - ps12
Metazoa - ps13
Myriazoa - ps14
Parahoxozoa - ps15
Bilateria - ps16
Protostomia - ps17
Ecdysozoa - ps18
Arthropoda - ps19
Mandibulata - ps20
Pancrustacea - ps21
Insecta - ps22
Eumetabola - ps23
Holometabola/Psocodea - ps24
Holometabola - ps25
Aparaglossata - ps26
Mecopterida - ps27
Diptera - ps28
Bibiono./Cyclorrhapha - ps29
Cyclorrhapha - ps30
Calyptratae/Drosophila - ps31
Drosophila - ps32
Sophophora - ps33
Obscura/Melanogaster_group - ps34
D_melanogaster_group - ps35
D_melanogaster_subgroup - ps36
sim./sech./mel. group - ps37
Drosophila_melanogaster - ps38
p-value
1e-53
1e-44
2e-35
3e-26
3e-17
Log-odds
0.5
1.0
1.5
2.0
2.5
3.0
3.5
4.0

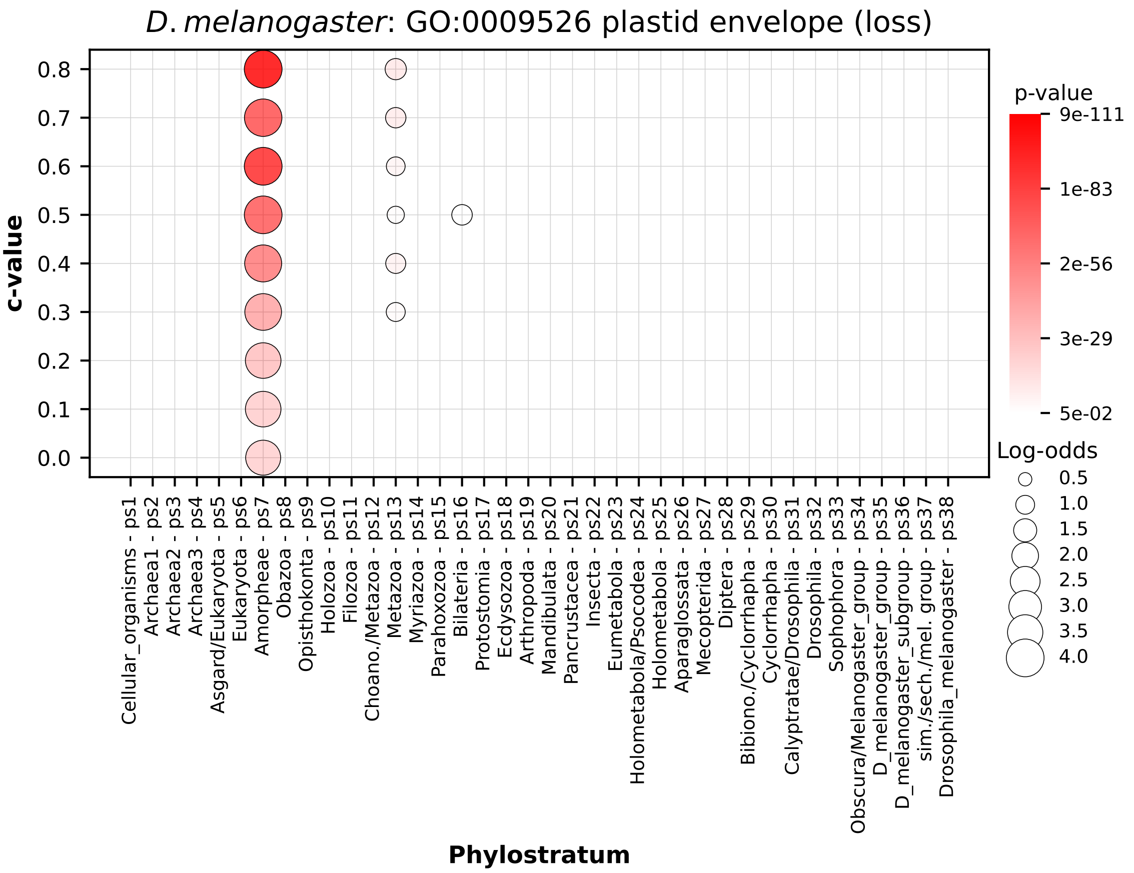

D. melanogaster: GO:0009526 plastid envelope (loss)
c-value
0.8
0.7
0.6
0.5
0.4
0.3
0.2
0.1
0.0
Phylostratum
Cellular_organisms - ps1
Archaea1 - ps2
Archaea2 - ps3
Archaea3 - ps4
Asgard/Eukaryota - ps5
Eukaryota - ps6
Amorpheae - ps7
Obazoa - ps8
Opisthokonta - ps9
Holozoa - ps10
Filozoa - ps11
Choano./Metazoa - ps12
Metazoa - ps13
Myriazoa - ps14
Parahoxozoa - ps15
Bilateria - ps16
Protostomia - ps17
Ecdysozoa - ps18
Arthropoda - ps19
Mandibulata - ps20
Pancrustacea - ps21
Insecta - ps22
Eumetabola - ps23
Holometabola/Psocodea - ps24
Holometabola - ps25
Aparaglossata - ps26
Mecopterida - ps27
Diptera - ps28
Bibiono./Cyclorrhapha - ps29
Cyclorrhapha - ps30
Calyptratae/Drosophila - ps31
Drosophila - ps32
Sophophora - ps33
Obscura/Melanogaster_group - ps34
D_melanogaster_group - ps35
D_melanogaster_subgroup - ps36
sim./sech./mel. group - ps37
Drosophila_melanogaster - ps38
p-value
9e-111
1e-83
2e-56
3e-29
5e-02
Log-odds
0.5
1.0
1.5
2.0
2.5
3.0
3.5
4.0

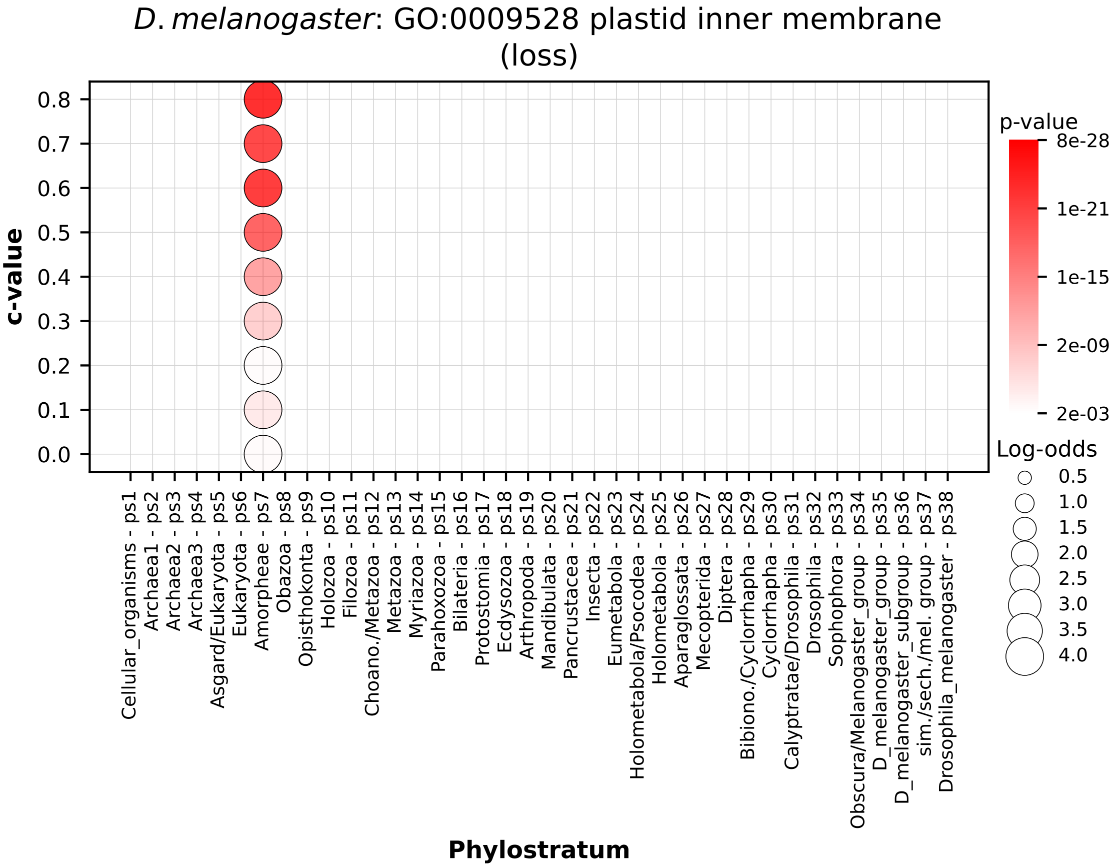

D. melanogaster: GO:0009528 plastid inner membrane (loss)
c-value
Phylostratum
p-value
8e-28
1e-21
1e-15
2e-09
2e-03
Log-odds
0.5
1.0
1.5
2.0
2.5
3.0
3.5
4.0
Cellular_organisms - ps1
Archaea1 - ps2
Archaea2 - ps3
Archaea3 - ps4
Asgard/Eukaryota - ps5
Eukaryota - ps6
Amorpheae - ps7
Obazoa - ps8
Opisthokonta - ps9
Holozoa - ps10
Filozoa - ps11
Choano./Metazoa - ps12
Metazoa - ps13
Myriazoa - ps14
Parahoxozoa - ps15
Bilateria - ps16
Protostomia - ps17
Ecdysozoa - ps18
Arthropoda - ps19
Mandibulata - ps20
Pancrustacea - ps21
Insecta - ps22
Eumetabola - ps23
Holometabola/Psocodea - ps24
Holometabola - ps25
Aparaglossata - ps26
Mecopterida - ps27
Diptera - ps28
Bibiono./Cyclorrhapha - ps29
Cyclorrhapha - ps30
Calyptratae/Drosophila - ps31
Drosophila - ps32
Sophophora - ps33
Obscura/Melanogaster_group - ps34
D_melanogaster_group - ps35
D_melanogaster_subgroup - ps36
sim./sech./mel. group - ps37
Drosophila_melanogaster - ps38

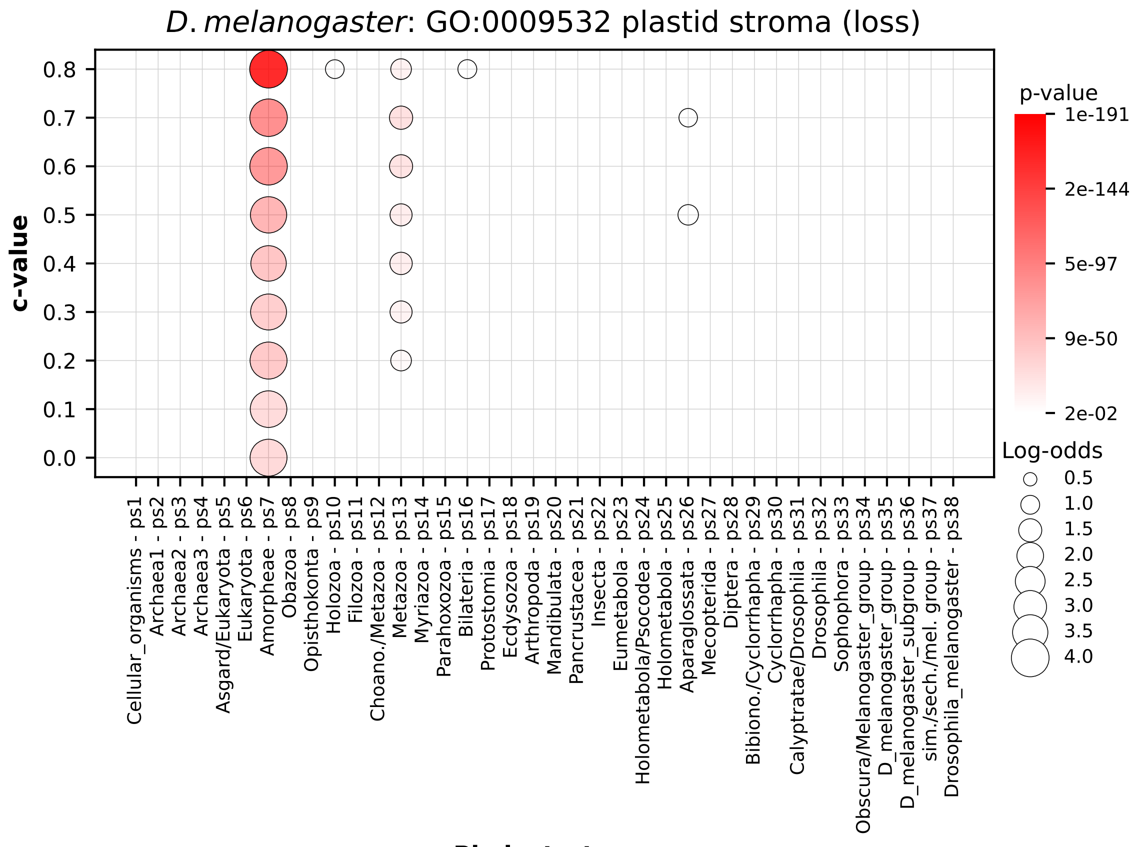

D. melanogaster: GO:0009532 plastid stroma (loss)
c-value
0.8
0.7
0.6
0.5
0.4
0.3
0.2
0.1
0.0
p-value
1e-191
2e-144
5e-97
9e-50
2e-02
Log-odds
0.5
1.0
1.5
2.0
2.5
3.0
3.5
4.0
Cellular_organisms - ps1
Archaea1 - ps2
Archaea2 - ps3
Archaea3 - ps4
Asgard/Eukaryota - ps5
Eukaryota - ps6
Amorpheae - ps7
Obazoa - ps8
Opisthokonta - ps9
Holozoa - ps10
Filozoa - ps11
Choano./Metazoa - ps12
Metazoa - ps13
Myriazoa - ps14
Parahoxozoa - ps15
Bilateria - ps16
Protostomia - ps17
Ecdysozoa - ps18
Arthropoda - ps19
Mandibulata - ps20
Pancrustacea - ps21
Insecta - ps22
Eumetabola - ps23
Holometabola/Psocodea - ps24
Holometabola - ps25
Aparaglossata - ps26
Mecopterida - ps27
Diptera - ps28
Bibiono./Cyclorrhapha - ps29
Cyclorrhapha - ps30
Calyptratae/Drosophila - ps31
Drosophila - ps32
Sophophora - ps33
Obscura/Melanogaster_group - ps34
D_melanogaster_group - ps35
D_melanogaster_subgroup - ps36
sim./sech./mel. group - ps37
Drosophila_melanogaster - ps38
Phylostratum

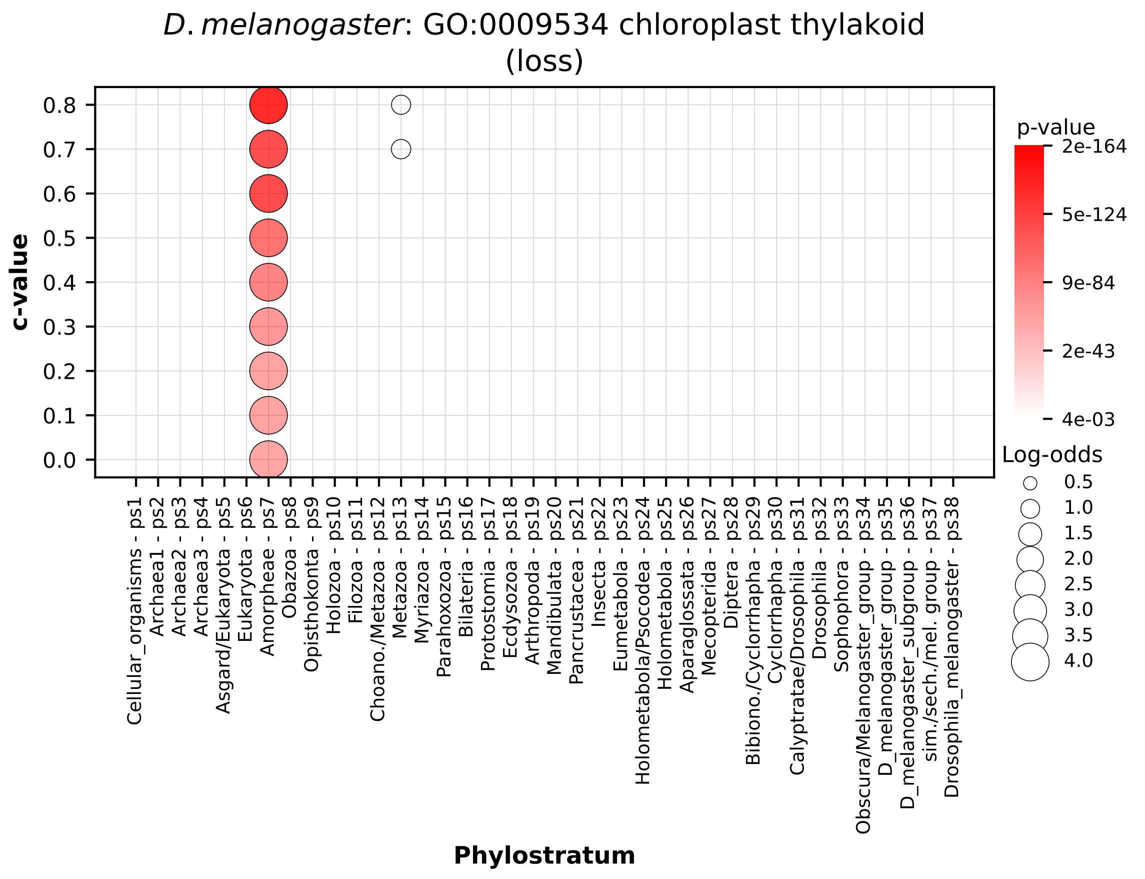
D. melanogaster: GO:0009534 chloroplast thylakoid
(loss)
c-value
0.8
0.7
0.6
0.5
0.4
0.3
0.2
0.1
0.0
p-value
2e-164
5e-124
9e-84
2e-43
4e-03
Log-odds
0.5
1.0
1.5
2.0
2.5
3.0
3.5
4.0
Cellular_organisms - ps1
Archaea1 - ps2
Archaea2 - ps3
Archaea3 - ps4
Asgard/Eukaryota - ps5
Eukaryota - ps6
Amorpheae - ps7
Obazoa - ps8
Opisthokonta - ps9
Holozoa - ps10
Filozoa - ps11
Choano./Metazoa - ps12
Metazoa - ps13
Myriazoa - ps14
Parahoxozoa - ps15
Bilateria - ps16
Protostomia - ps17
Ecdysozoa - ps18
Arthropoda - ps19
Mandibulata - ps20
Pancrustacea - ps21
Insecta - ps22
Eumetabola - ps23
Holometabola/Psocodea - ps24
Holometabola - ps25
Aparaglossata - ps26
Mecopterida - ps27
Diptera - ps28
Bibiono./Cyclorrhapha - ps29
Cyclorrhapha - ps30
Calyptratae/Drosophila - ps31
Drosophila - ps32
Sophophora - ps33
Obscura/Melanogaster_group - ps34
D_melanogaster_group - ps35
D_melanogaster_subgroup - ps36
sim./sech./mel. group - ps37
Drosophila_melanogaster - ps38
Phylostratum

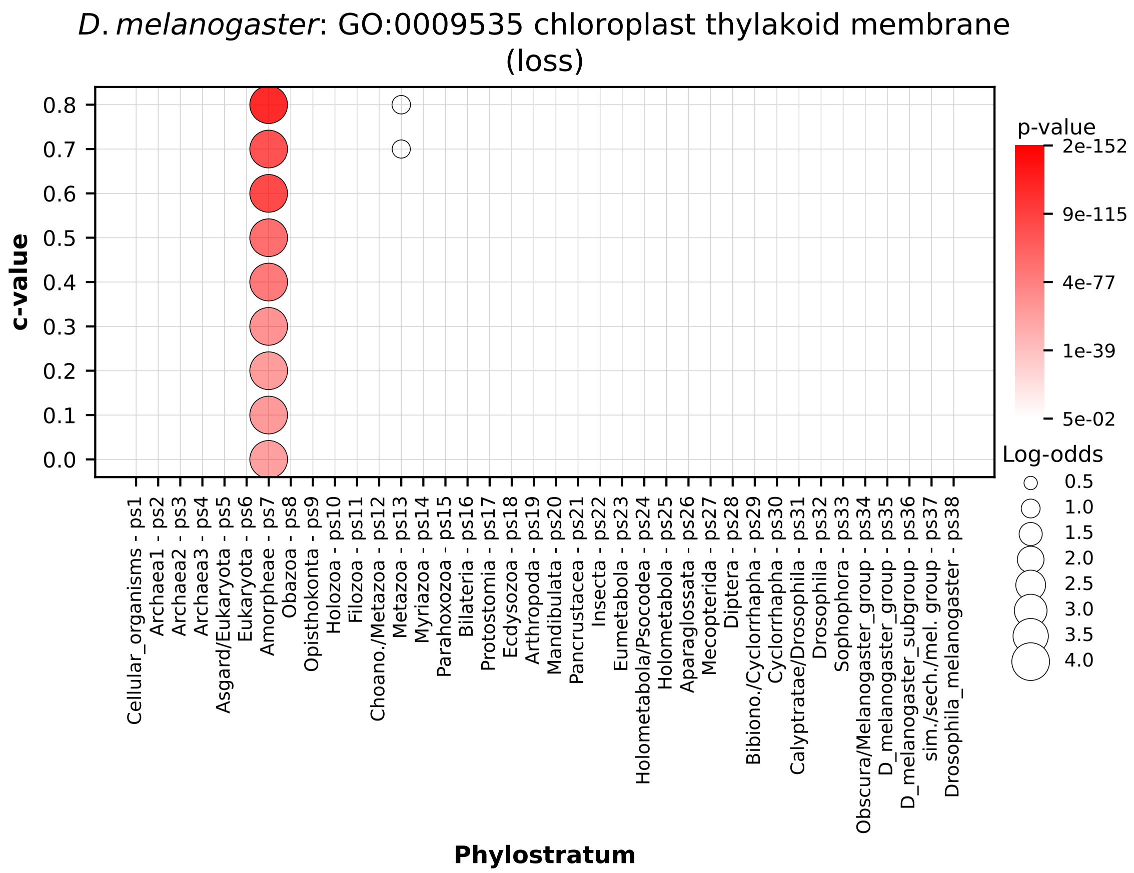

D. melanogaster: GO:0009535 chloroplast thylakoid membrane (loss)
c-value
0.8
0.7
0.6
0.5
0.4
0.3
0.2
0.1
0.0
p-value
2e-152
9e-115
4e-77
1e-39
5e-02
Log-odds
0.5
1.0
1.5
2.0
2.5
3.0
3.5
4.0
Cellular_organisms - ps1
Archaea1 - ps2
Archaea2 - ps3
Archaea3 - ps4
Asgard/Eukaryota - ps5
Eukaryota - ps6
Amorpheae - ps7
Obazoa - ps8
Opisthokonta - ps9
Holozoa - ps10
Filozoa - ps11
Choano./Metazoa - ps12
Metazoa - ps13
Myriazoa - ps14
Parahoxozoa - ps15
Bilateria - ps16
Protostomia - ps17
Ecdysozoa - ps18
Arthropoda - ps19
Mandibulata - ps20
Pancrustacea - ps21
Insecta - ps22
Eumetabola - ps23
Holometabola/Psocodea - ps24
Holometabola - ps25
Aparaglossata - ps26
Mecopterida - ps27
Diptera - ps28
Bibiono./Cyclorrhapha - ps29
Cyclorrhapha - ps30
Calyptratae/Drosophila - ps31
Drosophila - ps32
Sophophora - ps33
Obscura/Melanogaster_group - ps34
D_melanogaster_group - ps35
D_melanogaster_subgroup - ps36
sim./sech./mel. group - ps37
Drosophila_melanogaster - ps38
Phylostratum

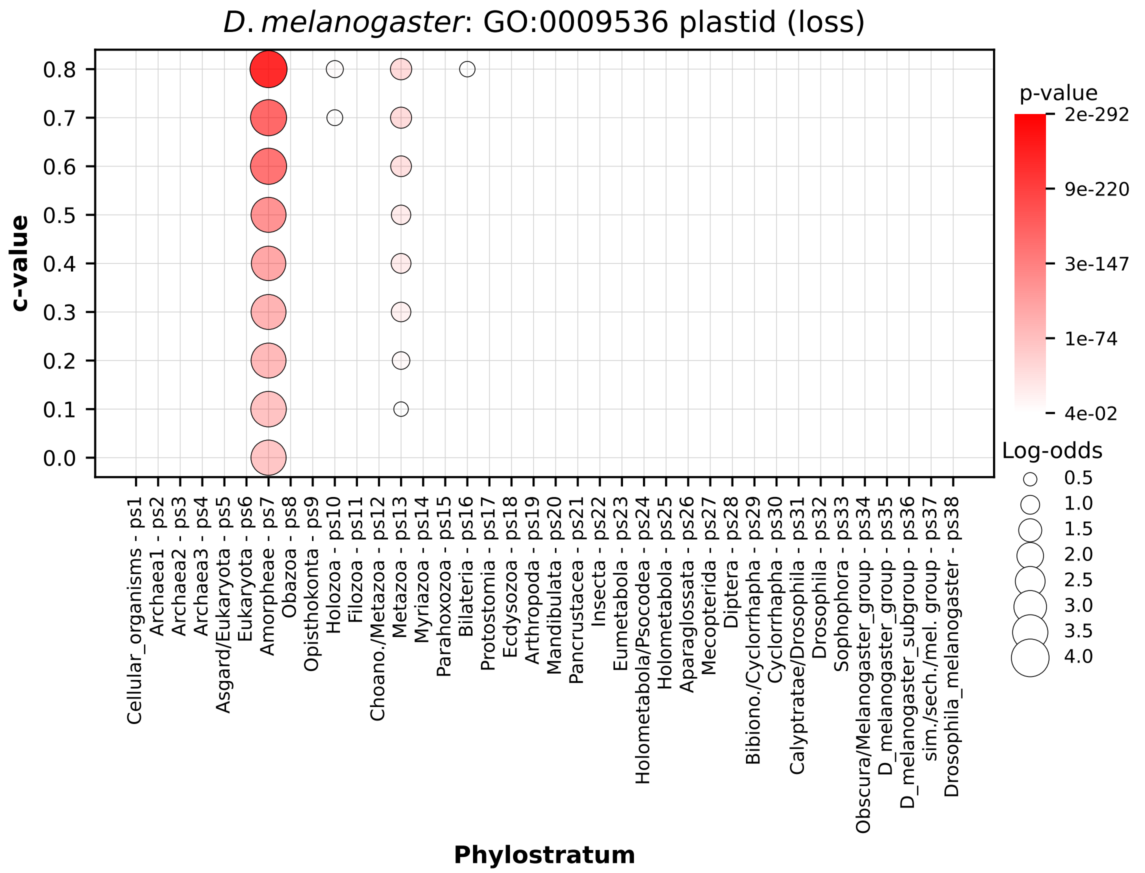

D. melanogaster: GO:0009536 plastid (loss)
c-value
0.8
0.7
0.6
0.5
0.4
0.3
0.2
0.1
0.0
Phylostratum
Cellular_organisms - ps1
Archaea1 - ps2
Archaea2 - ps3
Archaea3 - ps4
Asgard/Eukaryota - ps5
Eukaryota - ps6
Amorpheae - ps7
Obazoa - ps8
Opisthokonta - ps9
Holozoa - ps10
Filozoa - ps11
Choano./Metazoa - ps12
Metazoa - ps13
Myriazoa - ps14
Parahoxozoa - ps15
Bilateria - ps16
Protostomia - ps17
Ecdysozoa - ps18
Arthropoda - ps19
Mandibulata - ps20
Pancrustacea - ps21
Insecta - ps22
Eumetabola - ps23
Holometabola/Psocodea - ps24
Holometabola - ps25
Aparaglossata - ps26
Mecopterida - ps27
Diptera - ps28
Bibiono./Cyclorrhapha - ps29
Cyclorrhapha - ps30
Calyptratae/Drosophila - ps31
Drosophila - ps32
Sophophora - ps33
Obscura/Melanogaster_group - ps34
D_melanogaster_group - ps35
D_melanogaster_subgroup - ps36
sim./sech./mel. group - ps37
Drosophila_melanogaster - ps38
p-value
2e-292
9e-220
3e-147
1e-74
4e-02
Log-odds
0.5
1.0
1.5
2.0
2.5
3.0
3.5
4.0

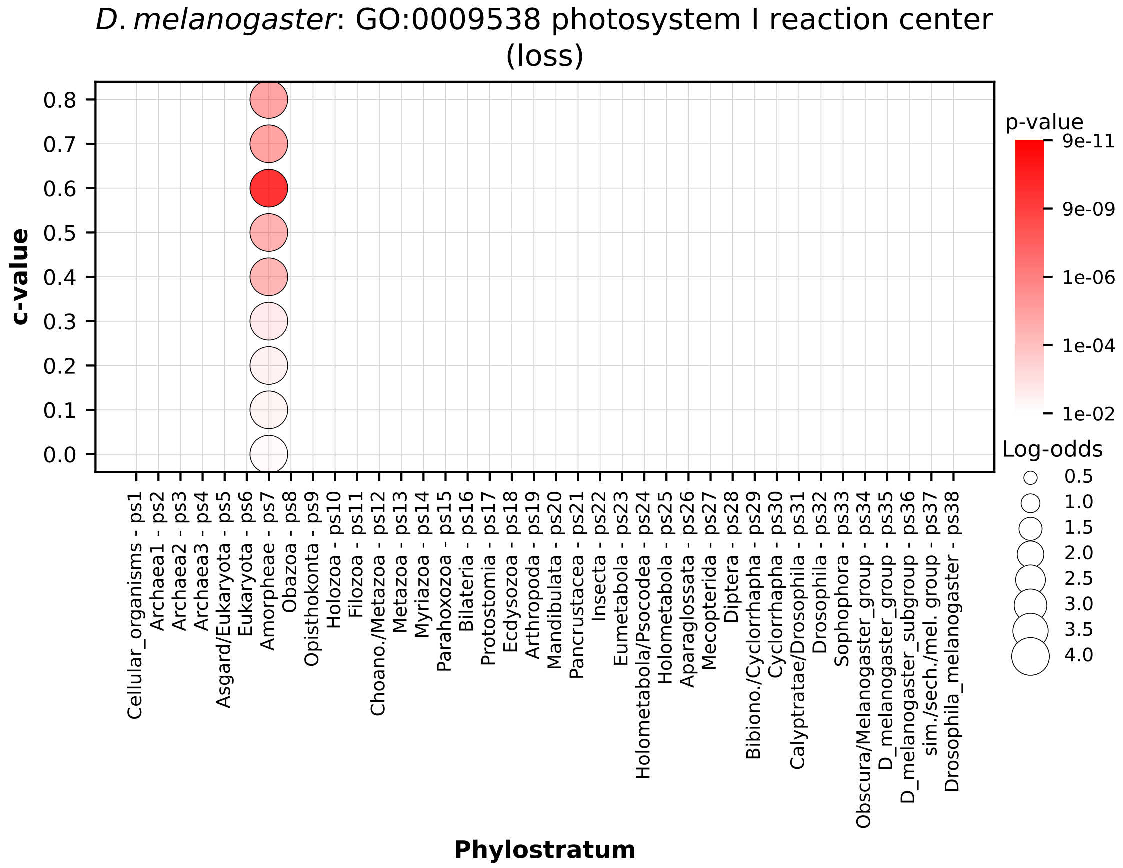

D. melanogaster: GO:0009538 photosystem I reaction center (loss)
c-value
0.8
0.7
0.6
0.5
0.4
0.3
0.2
0.1
0.0
Phylostratum
Cellular_organisms - ps1
Archaea1 - ps2
Archaea2 - ps3
Archaea3 - ps4
Asgard/Eukaryota - ps5
Eukaryota - ps6
Amorpheae - ps7
Obazoa - ps8
Opisthokonta - ps9
Holozoa - ps10
Filozoa - ps11
Choano./Metazoa - ps12
Metazoa - ps13
Myriazoa - ps14
Parahoxozoa - ps15
Bilateria - ps16
Protostomia - ps17
Ecdysozoa - ps18
Arthropoda - ps19
Mandibulata - ps20
Pancrustacea - ps21
Insecta - ps22
Eumetabola - ps23
Holometabola/Psocodea - ps24
Holometabola - ps25
Aparaglossata - ps26
Mecopterida - ps27
Diptera - ps28
Bibiono./Cyclorrhapha - ps29
Cyclorrhapha - ps30
Calyptratae/Drosophila - ps31
Drosophila - ps32
Sophophora - ps33
Obscura/Melanogaster_group - ps34
D_melanogaster_group - ps35
D_melanogaster_subgroup - ps36
sim./sech./mel. group - ps37
Drosophila_melanogaster - ps38
p-value
9e-11
9e-09
1e-06
1e-04
1e-02
Log-odds
0.5
1.0
1.5
2.0
2.5
3.0
3.5
4.0

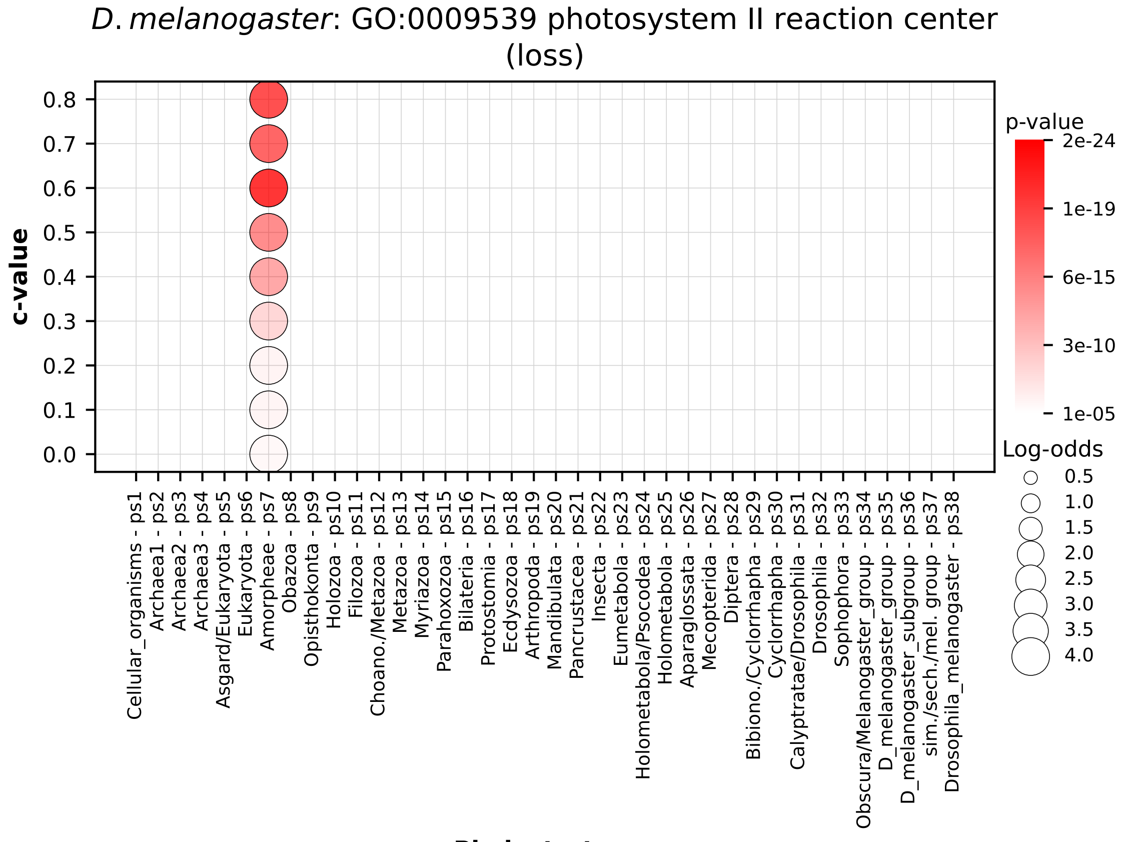

D. melanogaster: GO:0009539 photosystem II reaction center (loss)
c-value
0.8
0.7
0.6
0.5
0.4
0.3
0.2
0.1
0.0
p-value
2e-24
1e-19
6e-15
3e-10
1e-05
Log-odds
0.5
1.0
1.5
2.0
2.5
3.0
3.5
4.0
Cellular_organisms - ps1
Archaea1 - ps2
Archaea2 - ps3
Archaea3 - ps4
Asgard/Eukaryota - ps5
Eukaryota - ps6
Amorpheae - ps7
Obazoa - ps8
Opisthokonta - ps9
Holozoa - ps10
Filozoa - ps11
Choano./Metazoa - ps12
Metazoa - ps13
Myriazoa - ps14
Parahoxozoa - ps15
Bilateria - ps16
Protostomia - ps17
Ecdysozoa - ps18
Arthropoda - ps19
Mandibulata - ps20
Pancrustacea - ps21
Insecta - ps22
Eumetabola - ps23
Holometabola/Psocodea - ps24
Holometabola - ps25
Aparaglossata - ps26
Mecopterida - ps27
Diptera - ps28
Bibiono./Cyclorrhapha - ps29
Cyclorrhapha - ps30
Calyptratae/Drosophila - ps31
Drosophila - ps32
Sophophora - ps33
Obscura/Melanogaster_group - ps34
D_melanogaster_group - ps35
D_melanogaster_subgroup - ps36
sim./sech./mel. group - ps37
Drosophila_melanogaster - ps38
Phylostratum

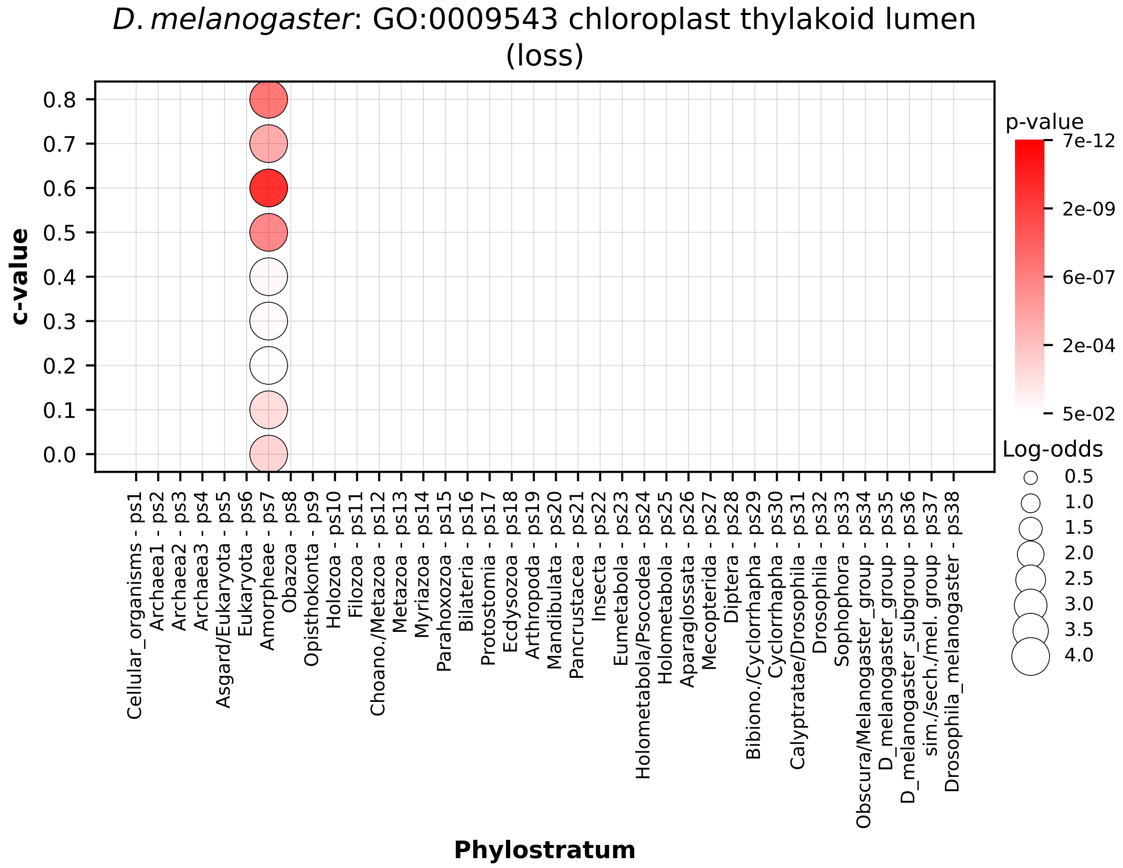

D. melanogaster: GO:0009543 chloroplast thylakoid lumen (loss)
c-value
0.8
0.7
0.6
0.5
0.4
0.3
0.2
0.1
0.0
p-value
7e-12
2e-09
6e-07
2e-04
5e-02
Log-odds
0.5
1.0
1.5
2.0
2.5
3.0
3.5
4.0
Cellular_organisms - ps1
Archaea1 - ps2
Archaea2 - ps3
Archaea3 - ps4
Asgard/Eukaryota - ps5
Eukaryota - ps6
Amorpheae - ps7
Obazoa - ps8
Opisthokonta - ps9
Holozoa - ps10
Filozoa - ps11
Choano./Metazoa - ps12
Metazoa - ps13
Myriazoa - ps14
Parahoxozoa - ps15
Bilateria - ps16
Protostomia - ps17
Ecdysozoa - ps18
Arthropoda - ps19
Mandibulata - ps20
Pancrustacea - ps21
Insecta - ps22
Eumetabola - ps23
Holometabola/Psocodea - ps24
Holometabola - ps25
Aparaglossata - ps26
Mecopterida - ps27
Diptera - ps28
Bibiono./Cyclorrhapha - ps29
Cyclorrhapha - ps30
Calyptratae/Drosophila - ps31
Drosophila - ps32
Sophophora - ps33
Obscura/Melanogaster_group - ps34
D_melanogaster_group - ps35
D_melanogaster_subgroup - ps36
sim./sech./mel. group - ps37
Drosophila_melanogaster - ps38
Phylostratum

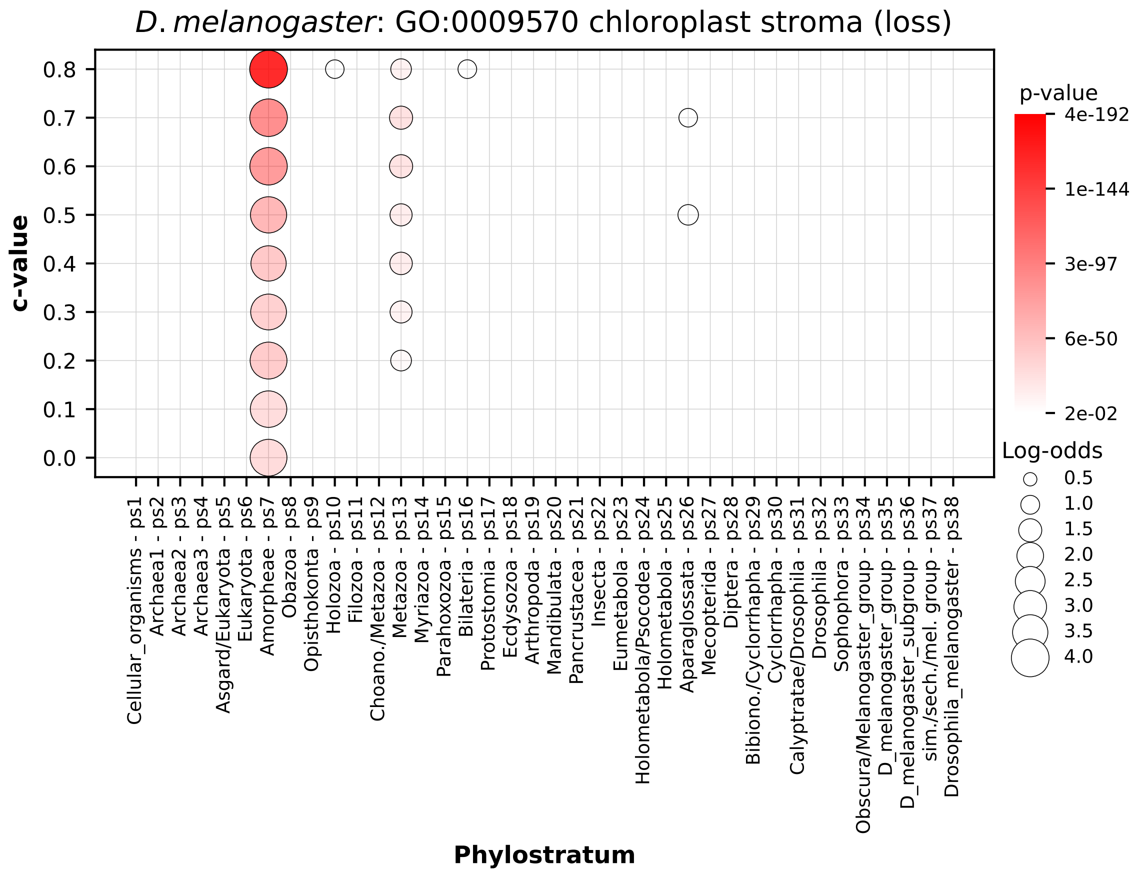

D. melanogaster: GO:0009570 chloroplast stroma (loss)
c-value
0.8
0.7
0.6
0.5
0.4
0.3
0.2
0.1
0.0
p-value
4e-192
1e-144
3e-97
6e-50
2e-02
Log-odds
0.5
1.0
1.5
2.0
2.5
3.0
3.5
4.0
Cellular_organisms - ps1
Archaea1 - ps2
Archaea2 - ps3
Archaea3 - ps4
Asgard/Eukaryota - ps5
Eukaryota - ps6
Amorpheae - ps7
Obazoa - ps8
Opisthokonta - ps9
Holozoa - ps10
Filozoa - ps11
Choano./Metazoa - ps12
Metazoa - ps13
Myriazoa - ps14
Parahoxozoa - ps15
Bilateria - ps16
Protostomia - ps17
Ecdysozoa - ps18
Arthropoda - ps19
Mandibulata - ps20
Pancrustacea - ps21
Insecta - ps22
Eumetabola - ps23
Holometabola/Psocodea - ps24
Holometabola - ps25
Aparaglossata - ps26
Mecopterida - ps27
Diptera - ps28
Bibiono./Cyclorrhapha - ps29
Cyclorrhapha - ps30
Calyptratae/Drosophila - ps31
Drosophila - ps32
Sophophora - ps33
Obscura/Melanogaster_group - ps34
D_melanogaster_group - ps35
D_melanogaster_subgroup - ps36
sim./sech./mel. group - ps37
Drosophila_melanogaster - ps38
Phylostratum

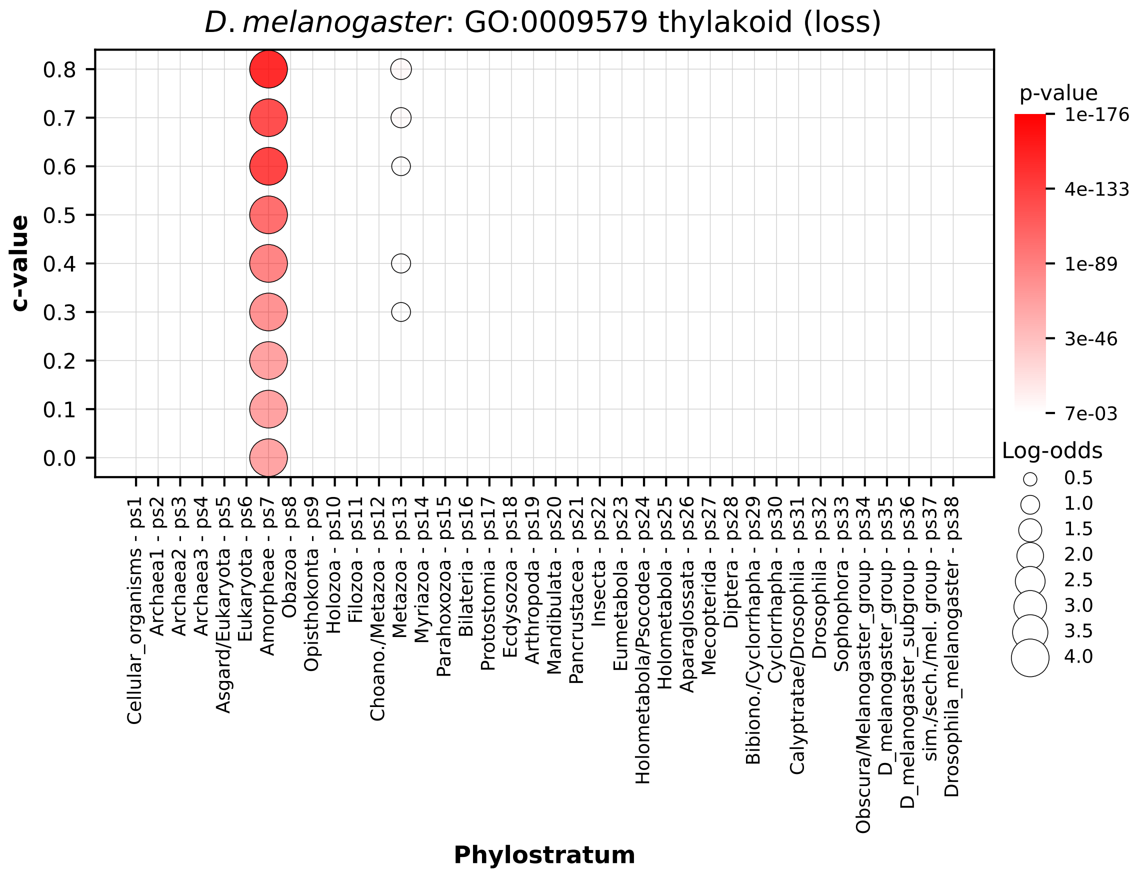
D. melanogaster: GO:0009579 thylakoid (loss)
c-value
0.8
0.7
0.6
0.5
0.4
0.3
0.2
0.1
0.0
p-value
1e-176
4e-133
1e-89
3e-46
7e-03
Log-odds
0.5
1.0
1.5
2.0
2.5
3.0
3.5
4.0
Cellular_organisms - ps1
Archaea1 - ps2
Archaea2 - ps3
Archaea3 - ps4
Asgard/Eukaryota - ps5
Eukaryota - ps6
Amorpheae - ps7
Obazoa - ps8
Opisthokonta - ps9
Holozoa - ps10
Filozoa - ps11
Choano./Metazoa - ps12
Metazoa - ps13
Myriazoa - ps14
Parahoxozoa - ps15
Bilateria - ps16
Protostomia - ps17
Ecdysozoa - ps18
Arthropoda - ps19
Mandibulata - ps20
Pancrustacea - ps21
Insecta - ps22
Eumetabola - ps23
Holometabola/Psocodea - ps24
Holometabola - ps25
Aparaglossata - ps26
Mecopterida - ps27
Diptera - ps28
Bibiono./Cyclorrhapha - ps29
Cyclorrhapha - ps30
Calyptratae/Drosophila - ps31
Drosophila - ps32
Sophophora - ps33
Obscura/Melanogaster_group - ps34
D_melanogaster_group - ps35
D_melanogaster_subgroup - ps36
sim./sech./mel. group - ps37
Drosophila_melanogaster - ps38
Phylostratum

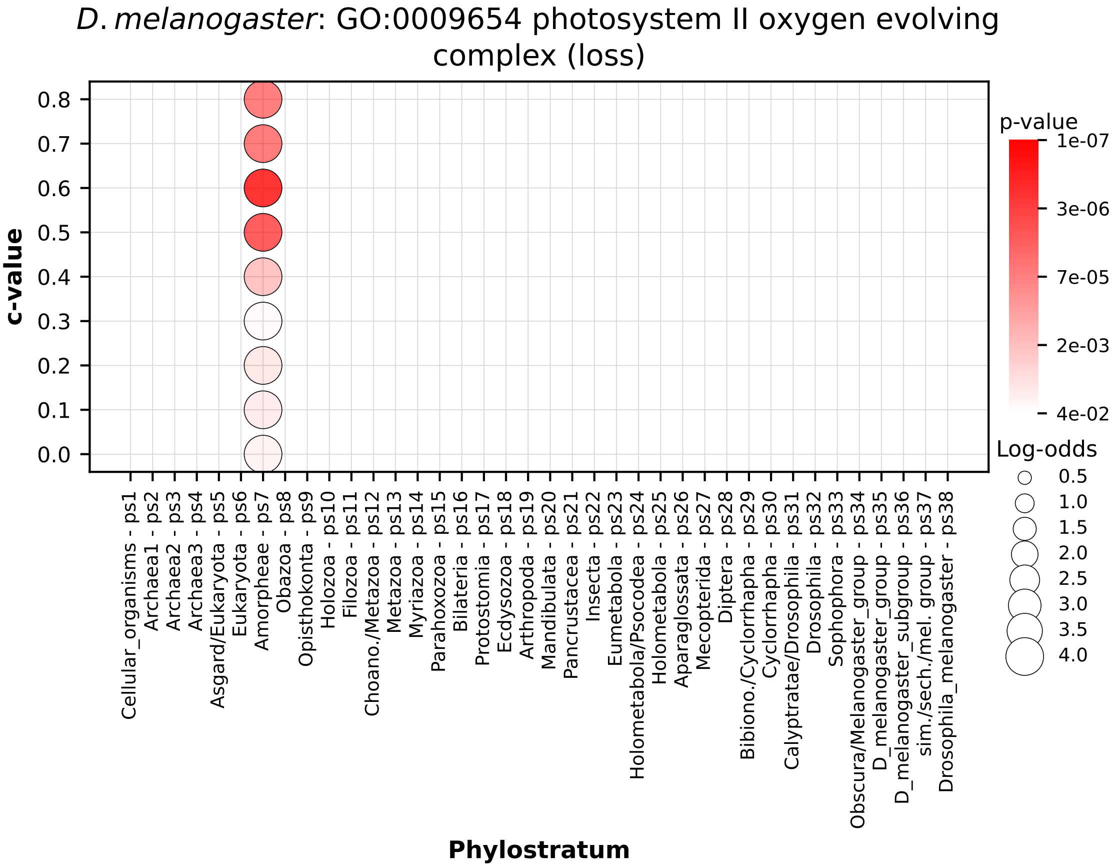

$D. melanogaster$: GO:0009654 photosystem II oxygen evolving complex (loss)
c-value
0.8
0.7
0.6
0.5
0.4
0.3
0.2
0.1
0.0
Phylostratum
Cellular_organisms - ps1
Archaea1 - ps2
Archaea2 - ps3
Archaea3 - ps4
Asgard/Eukaryota - ps5
Eukaryota - ps6
Amorpheae - ps7
Obazoa - ps8
Opisthokonta - ps9
Holozoa - ps10
Filozoa - ps11
Choano./Metazoa - ps12
Metazoa - ps13
Myriazoa - ps14
Parahoxozoa - ps15
Bilateria - ps16
Protostomia - ps17
Ecdysozoa - ps18
Arthropoda - ps19
Mandibulata - ps20
Pancrustacea - ps21
Insecta - ps22
Eumetabola - ps23
Holometabola/Psocodea - ps24
Holometabola - ps25
Aparaglossata - ps26
Mecopterida - ps27
Diptera - ps28
Bibiono./Cyclorrhapha - ps29
Cyclorrhapha - ps30
Calyptratae/Drosophila - ps31
Drosophila - ps32
Sophophora - ps33
Obscura/Melanogaster_group - ps34
D_melanogaster_group - ps35
D_melanogaster_subgroup - ps36
sim./sech./mel. group - ps37
Drosophila_melanogaster - ps38
p-value
1e-07
3e-06
7e-05
2e-03
4e-02
Log-odds
0.5
1.0
1.5
2.0
2.5
3.0
3.5
4.0

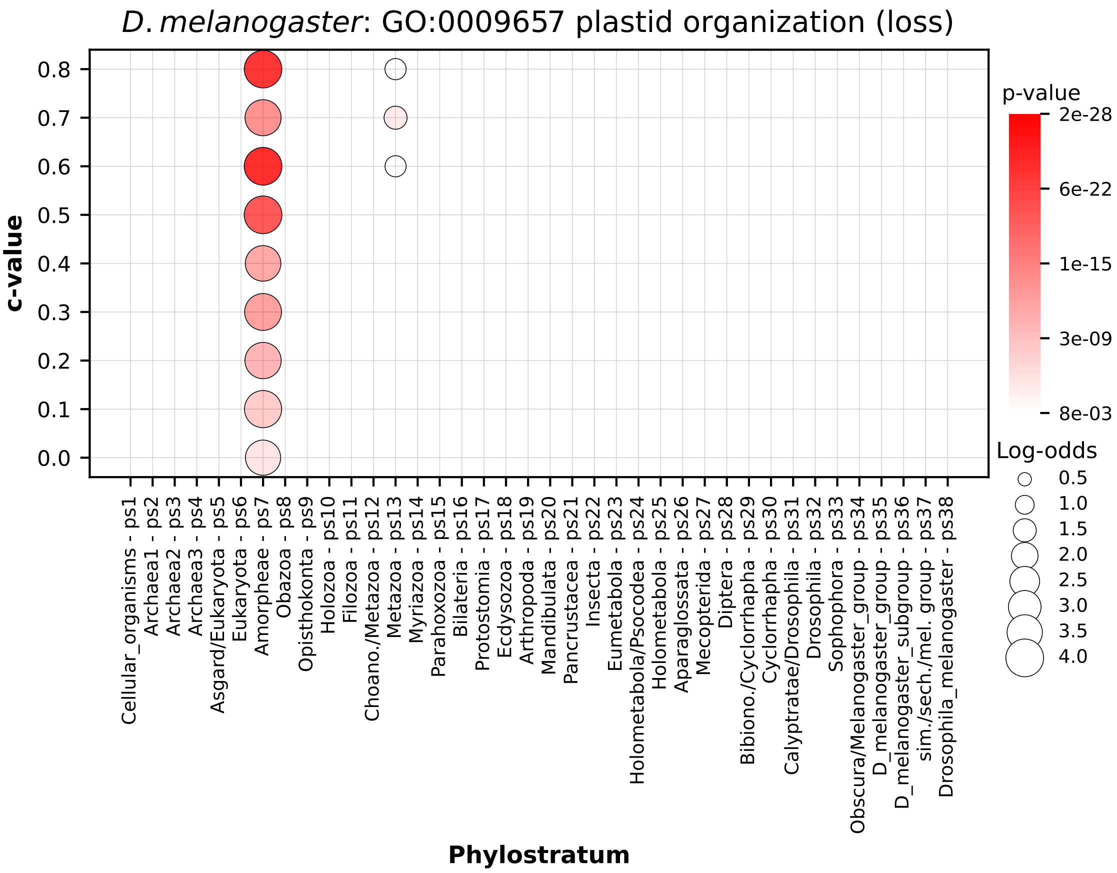

D. melanogaster: GO:0009657 plastid organization (loss)
c-value
0.8
0.7
0.6
0.5
0.4
0.3
0.2
0.1
0.0
Phylostratum
Cellular_organisms - ps1
Archaea1 - ps2
Archaea2 - ps3
Archaea3 - ps4
Asgard/Eukaryota - ps5
Eukaryota - ps6
Amorpheae - ps7
Obazoa - ps8
Opisthokonta - ps9
Holozoa - ps10
Filozoa - ps11
Choano./Metazoa - ps12
Metazoa - ps13
Myriazoa - ps14
Parahoxozoa - ps15
Bilateria - ps16
Protostomia - ps17
Ecdysozoa - ps18
Arthropoda - ps19
Mandibulata - ps20
Pancrustacea - ps21
Insecta - ps22
Eumetabola - ps23
Holometabola/Psocodea - ps24
Holometabola - ps25
Aparaglossata - ps26
Mecopterida - ps27
Diptera - ps28
Bibiono./Cyclorrhapha - ps29
Cyclorrhapha - ps30
Calyptratae/Drosophila - ps31
Drosophila - ps32
Sophophora - ps33
Obscura/Melanogaster_group - ps34
D_melanogaster_group - ps35
D_melanogaster_subgroup - ps36
sim./sech./mel. group - ps37
Drosophila_melanogaster - ps38
p-value
2e-28
6e-22
1e-15
3e-09
8e-03
Log-odds
0.5
1.0
1.5
2.0
2.5
3.0
3.5
4.0

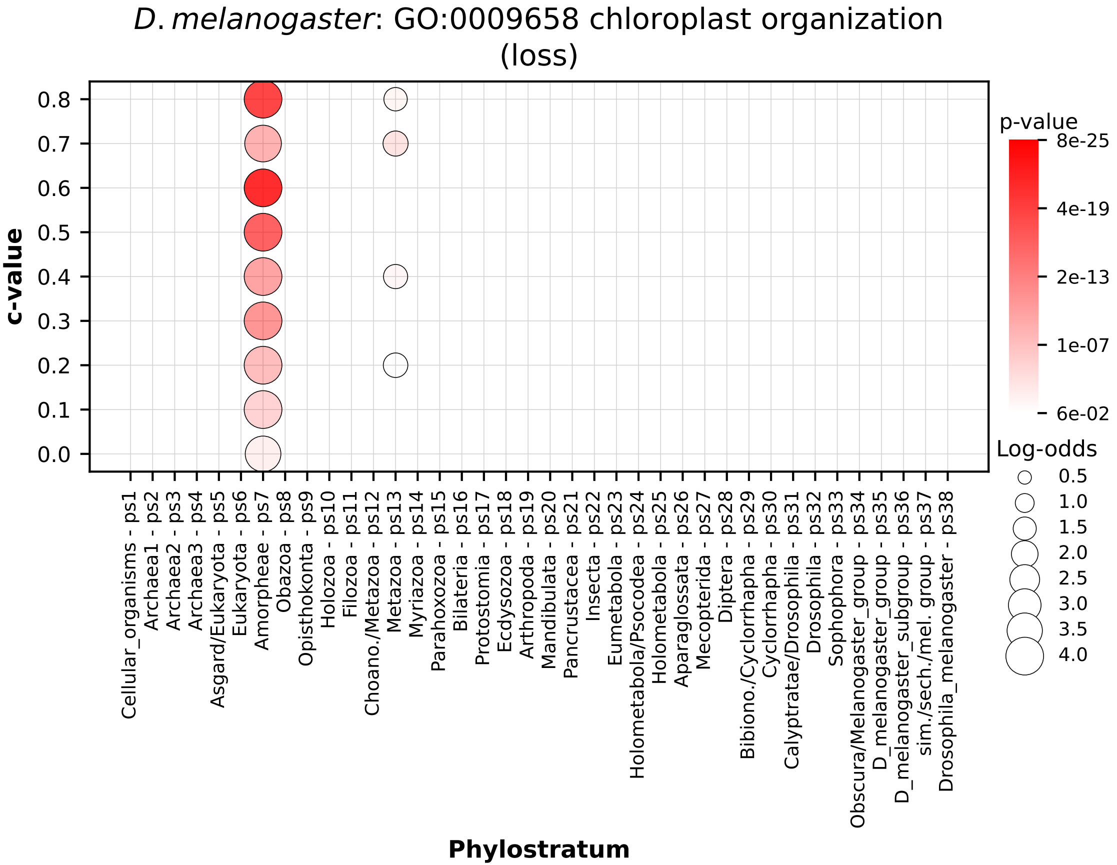

D. melanogaster: GO:0009658 chloroplast organization (loss)
c-value
0.8
0.7
0.6
0.5
0.4
0.3
0.2
0.1
0.0
p-value
8e-25
4e-19
2e-13
1e-07
6e-02
Log-odds
0.5
1.0
1.5
2.0
2.5
3.0
3.5
4.0
Cellular_organisms - ps1
Archaea1 - ps2
Archaea2 - ps3
Archaea3 - ps4
Asgard/Eukaryota - ps5
Eukaryota - ps6
Amorpheae - ps7
Obazoa - ps8
Opisthokonta - ps9
Holozoa - ps10
Filozoa - ps11
Choano./Metazoa - ps12
Metazoa - ps13
Myriazoa - ps14
Parahoxozoa - ps15
Bilateria - ps16
Protostomia - ps17
Ecdysozoa - ps18
Arthropoda - ps19
Mandibulata - ps20
Pancrustacea - ps21
Insecta - ps22
Eumetabola - ps23
Holometabola/Psocodea - ps24
Holometabola - ps25
Aparaglossata - ps26
Mecopterida - ps27
Diptera - ps28
Bibiono./Cyclorrhapha - ps29
Cyclorrhapha - ps30
Calyptratae/Drosophila - ps31
Drosophila - ps32
Sophophora - ps33
Obscura/Melanogaster_group - ps34
D_melanogaster_group - ps35
D_melanogaster_subgroup - ps36
sim./sech./mel. group - ps37
Drosophila_melanogaster - ps38
Phylostratum

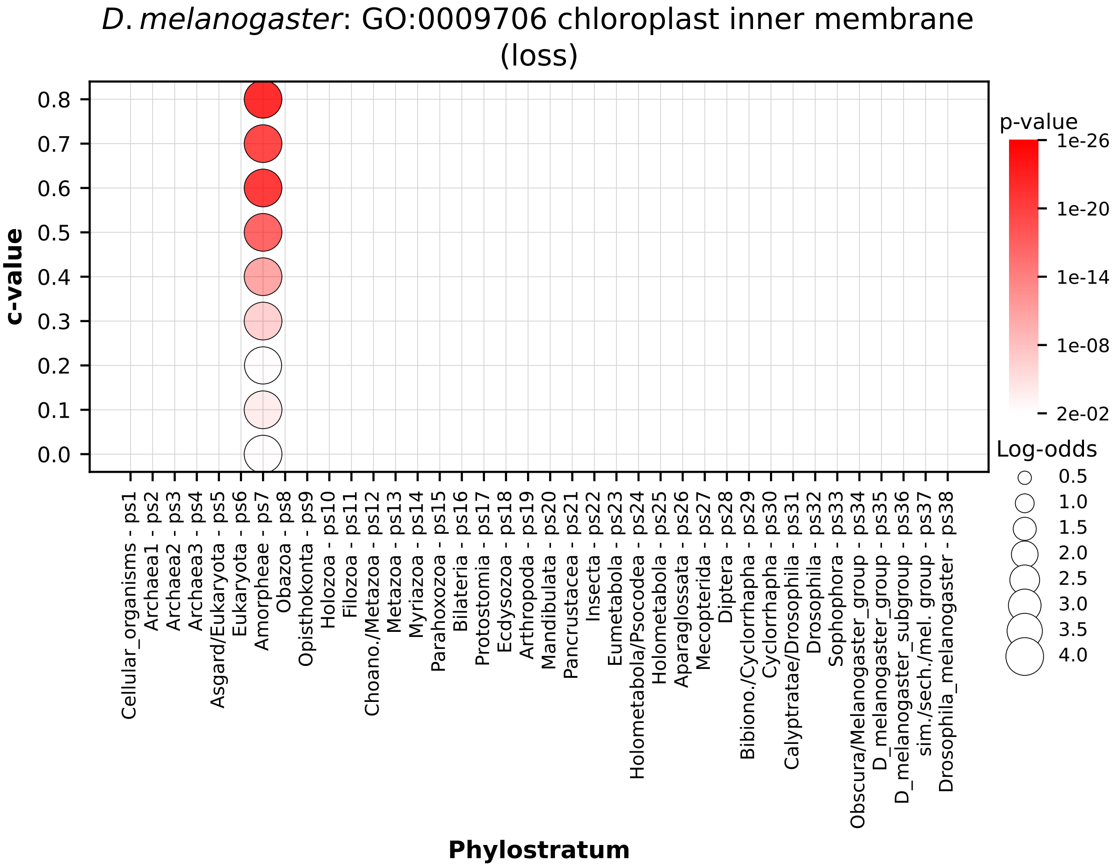
D. melanogaster: GO:0009706 chloroplast inner membrane (loss)
c-value
0.8
0.7
0.6
0.5
0.4
0.3
0.2
0.1
0.0
p-value
1e-26
1e-20
1e-14
1e-08
2e-02
Log-odds
0.5
1.0
1.5
2.0
2.5
3.0
3.5
4.0
Cellular_organisms - ps1
Archaea1 - ps2
Archaea2 - ps3
Archaea3 - ps4
Asgard/Eukaryota - ps5
Eukaryota - ps6
Amorpheae - ps7
Obazoa - ps8
Opisthokonta - ps9
Holozoa - ps10
Filozoa - ps11
Choano./Metazoa - ps12
Metazoa - ps13
Myriazoa - ps14
Parahoxozoa - ps15
Bilateria - ps16
Protostomia - ps17
Ecdysozoa - ps18
Arthropoda - ps19
Mandibulata - ps20
Pancrustacea - ps21
Insecta - ps22
Eumetabola - ps23
Holometabola/Psocodea - ps24
Holometabola - ps25
Aparaglossata - ps26
Mecopterida - ps27
Diptera - ps28
Bibiono./Cyclorrhapha - ps29
Cyclorrhapha - ps30
Calyptratae/Drosophila - ps31
Drosophila - ps32
Sophophora - ps33
Obscura/Melanogaster_group - ps34
D_melanogaster_group - ps35
D_melanogaster_subgroup - ps36
sim./sech./mel. group - ps37
Drosophila_melanogaster - ps38
Phylostratum

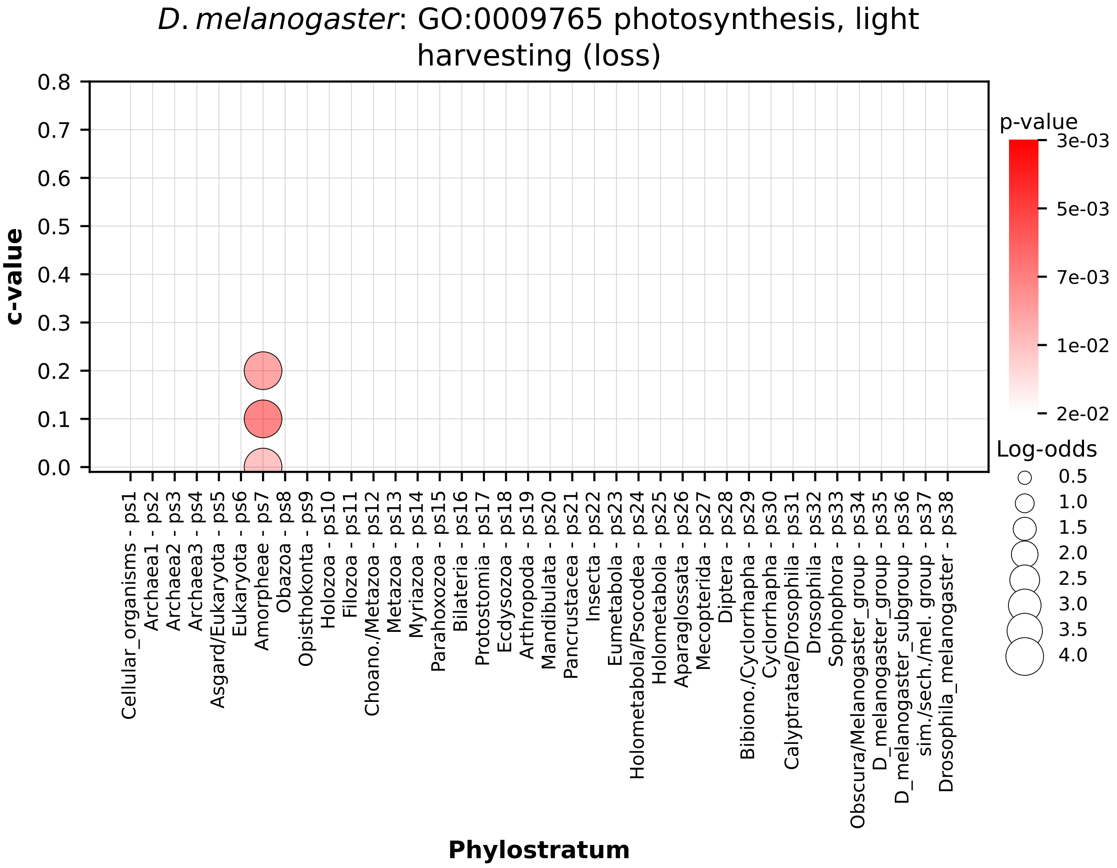

D. melanogaster: GO:0009765 photosynthesis, light harvesting (loss)
c-value
0.8
0.7
0.6
0.5
0.4
0.3
0.2
0.1
0.0
p-value
3e-03
5e-03
7e-03
1e-02
2e-02
Log-odds
0.5
1.0
1.5
2.0
2.5
3.0
3.5
4.0
Cellular_organisms - ps1
Archaea1 - ps2
Archaea2 - ps3
Archaea3 - ps4
Asgard/Eukaryota - ps5
Eukaryota - ps6
Amorpheae - ps7
Obazoa - ps8
Opisthokonta - ps9
Holozoa - ps10
Filozoa - ps11
Choano./Metazoa - ps12
Metazoa - ps13
Myriazoa - ps14
Parahoxozoa - ps15
Bilateria - ps16
Protostomia - ps17
Ecdysozoa - ps18
Arthropoda - ps19
Mandibulata - ps20
Pancrustacea - ps21
Insecta - ps22
Eumetabola - ps23
Holometabola/Psocodea - ps24
Holometabola - ps25
Aparaglossata - ps26
Mecopterida - ps27
Diptera - ps28
Bibiono./Cyclorrhapha - ps29
Cyclorrhapha - ps30
Calyptratae/Drosophila - ps31
Drosophila - ps32
Sophophora - ps33
Obscura/Melanogaster_group - ps34
D_melanogaster_group - ps35
D_melanogaster_subgroup - ps36
sim./sech./mel. group - ps37
Drosophila_melanogaster - ps38
Phylostratum

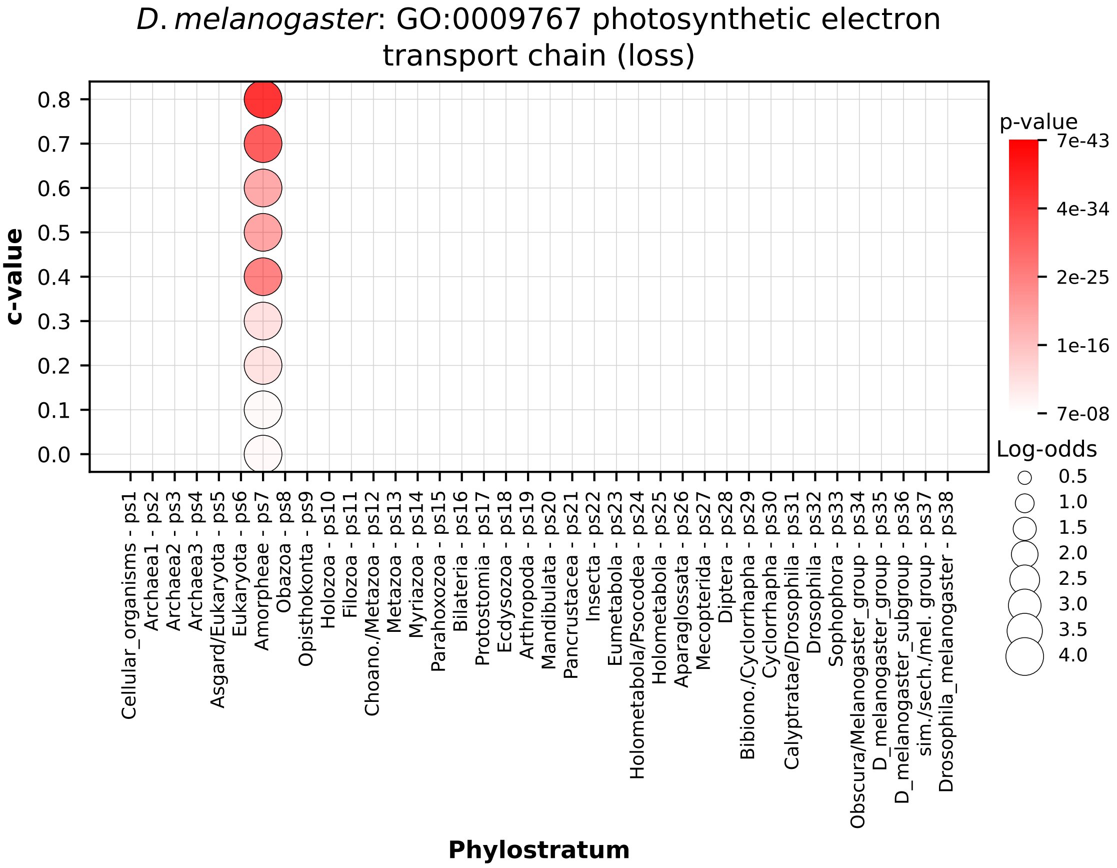

D. melanogaster: GO:0009767 photosynthetic electron transport chain (loss)
c-value
0.8
0.7
0.6
0.5
0.4
0.3
0.2
0.1
0.0
p-value
7e-43
4e-34
2e-25
1e-16
7e-08
Log-odds
0.5
1.0
1.5
2.0
2.5
3.0
3.5
4.0
Cellular_organisms - ps1
Archaea1 - ps2
Archaea2 - ps3
Archaea3 - ps4
Asgard/Eukaryota - ps5
Eukaryota - ps6
Amorpheae - ps7
Obazoa - ps8
Opisthokonta - ps9
Holozoa - ps10
Filozoa - ps11
Choano./Metazoa - ps12
Metazoa - ps13
Myriazoa - ps14
Parahoxozoa - ps15
Bilateria - ps16
Protostomia - ps17
Ecdysozoa - ps18
Arthropoda - ps19
Mandibulata - ps20
Pancrustacea - ps21
Insecta - ps22
Eumetabola - ps23
Holometabola/Psocodea - ps24
Holometabola - ps25
Aparaglossata - ps26
Mecopterida - ps27
Diptera - ps28
Bibiono./Cyclorrhapha - ps29
Cyclorrhapha - ps30
Calyptratae/Drosophila - ps31
Drosophila - ps32
Sophophora - ps33
Obscura/Melanogaster_group - ps34
D_melanogaster_group - ps35
D_melanogaster_subgroup - ps36
sim./sech./mel. group - ps37
Drosophila_melanogaster - ps38
Phylostratum

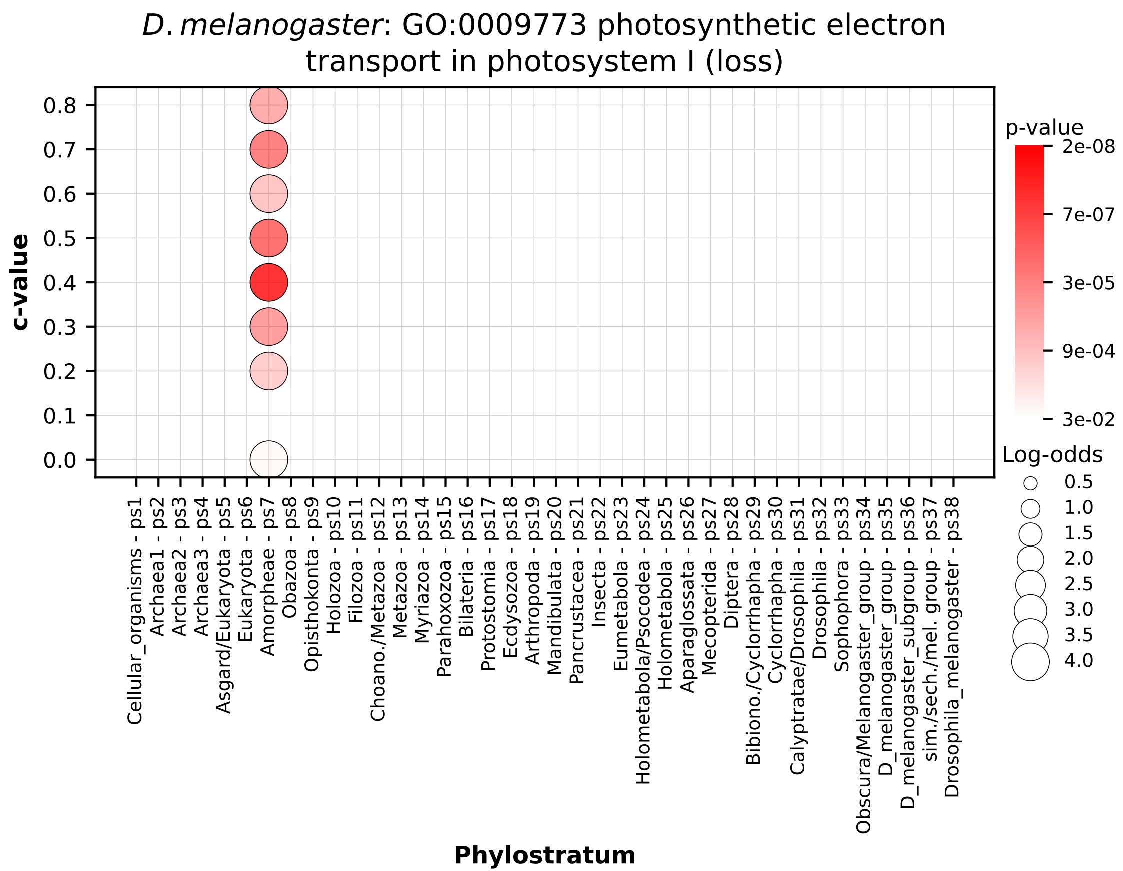

D. melanogaster: GO:0009773 photosynthetic electron transport in photosystem I (loss)
c-value
0.8
0.7
0.6
0.5
0.4
0.3
0.2
0.1
0.0
Phylostratum
Cellular_organisms - ps1
Archaea1 - ps2
Archaea2 - ps3
Archaea3 - ps4
Asgard/Eukaryota - ps5
Eukaryota - ps6
Amorpheae - ps7
Obazoa - ps8
Opisthokonta - ps9
Holozoa - ps10
Filozoa - ps11
Choano./Metazoa - ps12
Metazoa - ps13
Myriazoa - ps14
Parahoxozoa - ps15
Bilateria - ps16
Protostomia - ps17
Ecdysozoa - ps18
Arthropoda - ps19
Mandibulata - ps20
Pancrustacea - ps21
Insecta - ps22
Eumetabola - ps23
Holometabola/Psocodea - ps24
Holometabola - ps25
Aparaglossata - ps26
Mecopterida - ps27
Diptera - ps28
Bibiono./Cyclorrhapha - ps29
Cyclorrhapha - ps30
Calyptratae/Drosophila - ps31
Drosophila - ps32
Sophophora - ps33
Obscura/Melanogaster_group - ps34
D_melanogaster_group - ps35
D_melanogaster_subgroup - ps36
sim./sech./mel. group - ps37
Drosophila_melanogaster - ps38
p-value
2e-08
7e-07
3e-05
9e-04
3e-02
Log-odds
0.5
1.0
1.5
2.0
2.5
3.0
3.5
4.0

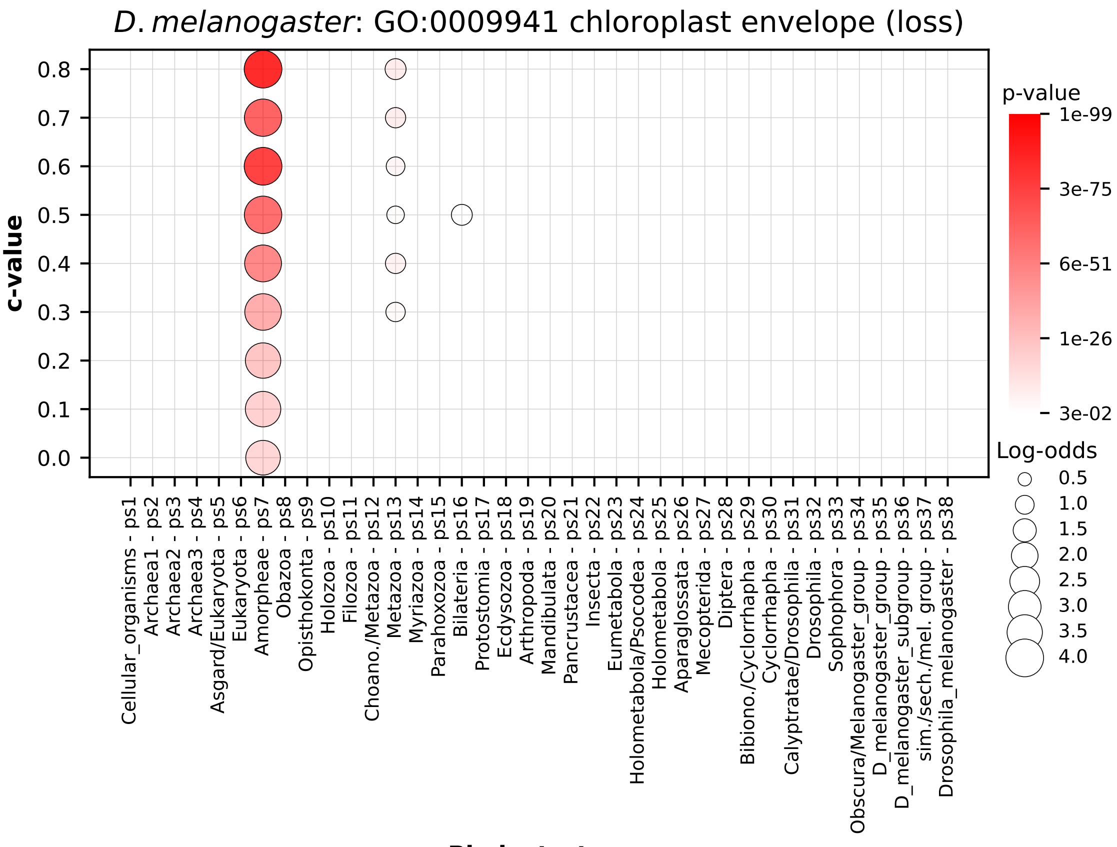

D. melanogaster: GO:0009941 chloroplast envelope (loss)
c-value
0.8
0.7
0.6
0.5
0.4
0.3
0.2
0.1
0.0
p-value
1e-99
3e-75
6e-51
1e-26
3e-02
Log-odds
0.5
1.0
1.5
2.0
2.5
3.0
3.5
4.0
Cellular_organisms - ps1
Archaea1 - ps2
Archaea2 - ps3
Archaea3 - ps4
Asgard/Eukaryota - ps5
Eukaryota - ps6
Amorpheae - ps7
Obazoa - ps8
Opisthokonta - ps9
Holozoa - ps10
Filozoa - ps11
Choano./Metazoa - ps12
Metazoa - ps13
Myriazoa - ps14
Parahoxozoa - ps15
Bilateria - ps16
Protostomia - ps17
Ecdysozoa - ps18
Arthropoda - ps19
Mandibulata - ps20
Pancrustacea - ps21
Insecta - ps22
Eumetabola - ps23
Holometabola/Psocodea - ps24
Holometabola - ps25
Aparaglossata - ps26
Mecopterida - ps27
Diptera - ps28
Bibiono./Cyclorrhapha - ps29
Cyclorrhapha - ps30
Calyptratae/Drosophila - ps31
Drosophila - ps32
Sophophora - ps33
Obscura/Melanogaster_group - ps34
D_melanogaster_group - ps35
D_melanogaster_subgroup - ps36
sim./sech./mel. group - ps37
Drosophila_melanogaster - ps38
Phylostratum

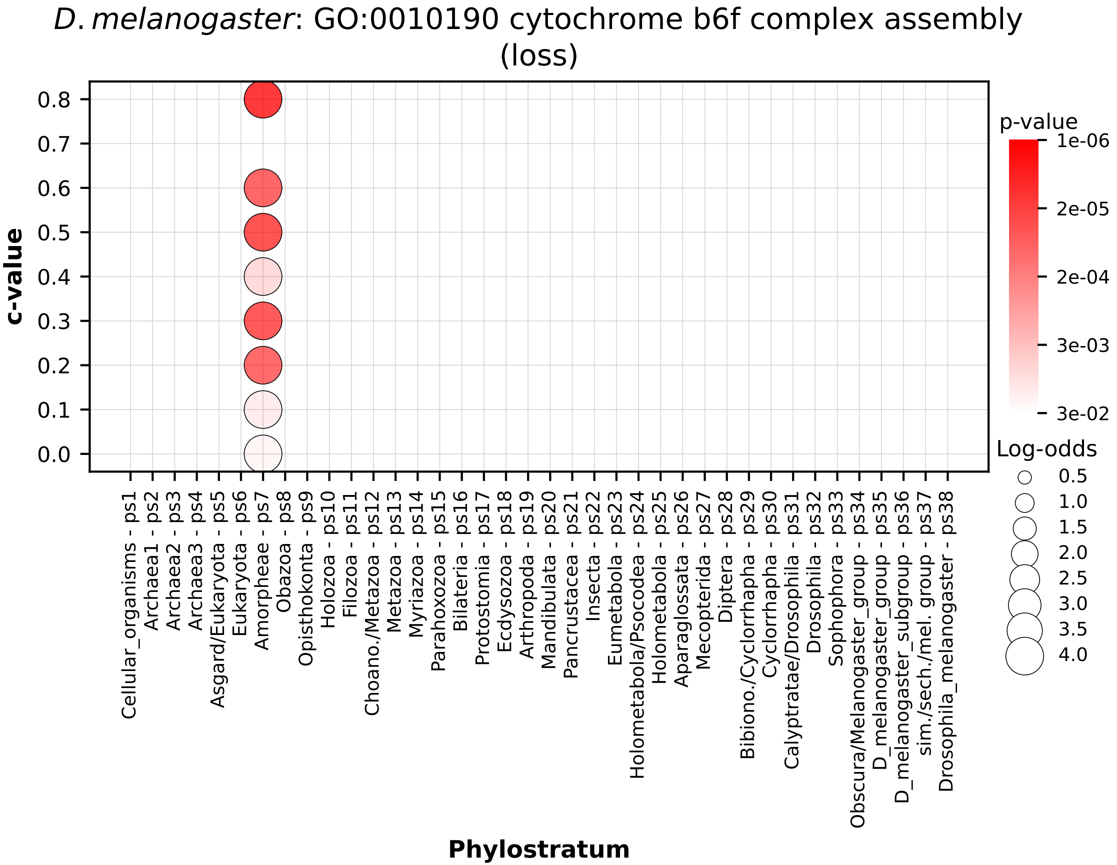
D. melanogaster: GO:0010190 cytochrome b6f complex assembly (loss)
c-value
0.8
0.7
0.6
0.5
0.4
0.3
0.2
0.1
0.0
p-value
1e-06
2e-05
2e-04
3e-03
3e-02
Log-odds
0.5
1.0
1.5
2.0
2.5
3.0
3.5
4.0
Cellular_organisms - ps1
Archaea1 - ps2
Archaea2 - ps3
Archaea3 - ps4
Asgard/Eukaryota - ps5
Eukaryota - ps6
Amorpheae - ps7
Obazoa - ps8
Opisthokonta - ps9
Holozoa - ps10
Filozoa - ps11
Choano./Metazoa - ps12
Metazoa - ps13
Myriazoa - ps14
Parahoxozoa - ps15
Bilateria - ps16
Protostomia - ps17
Ecdysozoa - ps18
Arthropoda - ps19
Mandibulata - ps20
Pancrustacea - ps21
Insecta - ps22
Eumetabola - ps23
Holometabola/Psocodea - ps24
Holometabola - ps25
Aparaglossata - ps26
Mecopterida - ps27
Diptera - ps28
Bibiono./Cyclorrhapha - ps29
Cyclorrhapha - ps30
Calyptratae/Drosophila - ps31
Drosophila - ps32
Sophophora - ps33
Obscura/Melanogaster_group - ps34
D_melanogaster_group - ps35
D_melanogaster_subgroup - ps36
sim./sech./mel. group - ps37
Drosophila_melanogaster - ps38
Phylostratum

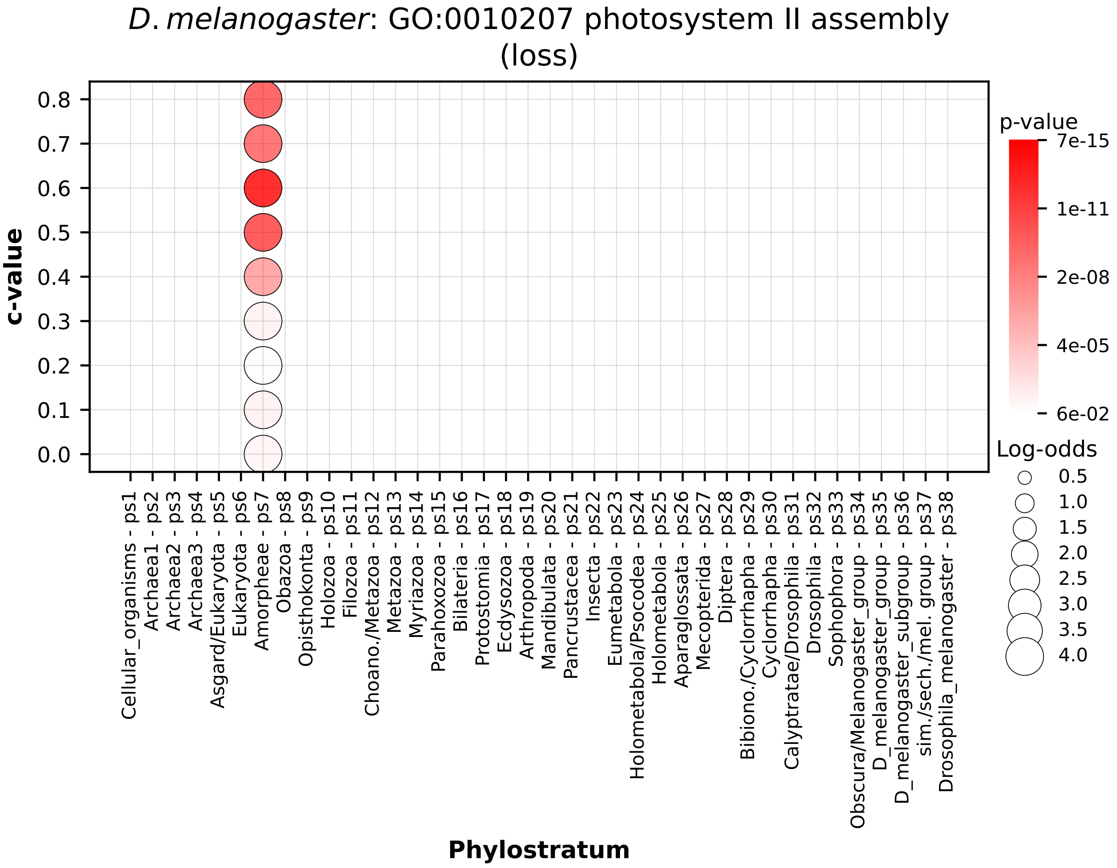
D. melanogaster: GO:0010207 photosystem II assembly (loss)
c-value
0.8
0.7
0.6
0.5
0.4
0.3
0.2
0.1
0.0
Phylostratum
Cellular_organisms - ps1
Archaea1 - ps2
Archaea2 - ps3
Archaea3 - ps4
Asgard/Eukaryota - ps5
Eukaryota - ps6
Amorpheae - ps7
Obazoa - ps8
Opisthokonta - ps9
Holozoa - ps10
Filozoa - ps11
Choano./Metazoa - ps12
Metazoa - ps13
Myriazoa - ps14
Parahoxozoa - ps15
Bilateria - ps16
Protostomia - ps17
Ecdysozoa - ps18
Arthropoda - ps19
Mandibulata - ps20
Pancrustacea - ps21
Insecta - ps22
Eumetabola - ps23
Holometabola/Psocodea - ps24
Holometabola - ps25
Aparaglossata - ps26
Mecopterida - ps27
Diptera - ps28
Bibiono./Cyclorrhapha - ps29
Cyclorrhapha - ps30
Calyptratae/Drosophila - ps31
Drosophila - ps32
Sophophora - ps33
Obscura/Melanogaster_group - ps34
D_melanogaster_group - ps35
D_melanogaster_subgroup - ps36
sim./sech./mel. group - ps37
Drosophila_melanogaster - ps38
p-value
7e-15
1e-11
2e-08
4e-05
6e-02
Log-odds
0.5
1.0
1.5
2.0
2.5
3.0
3.5
4.0

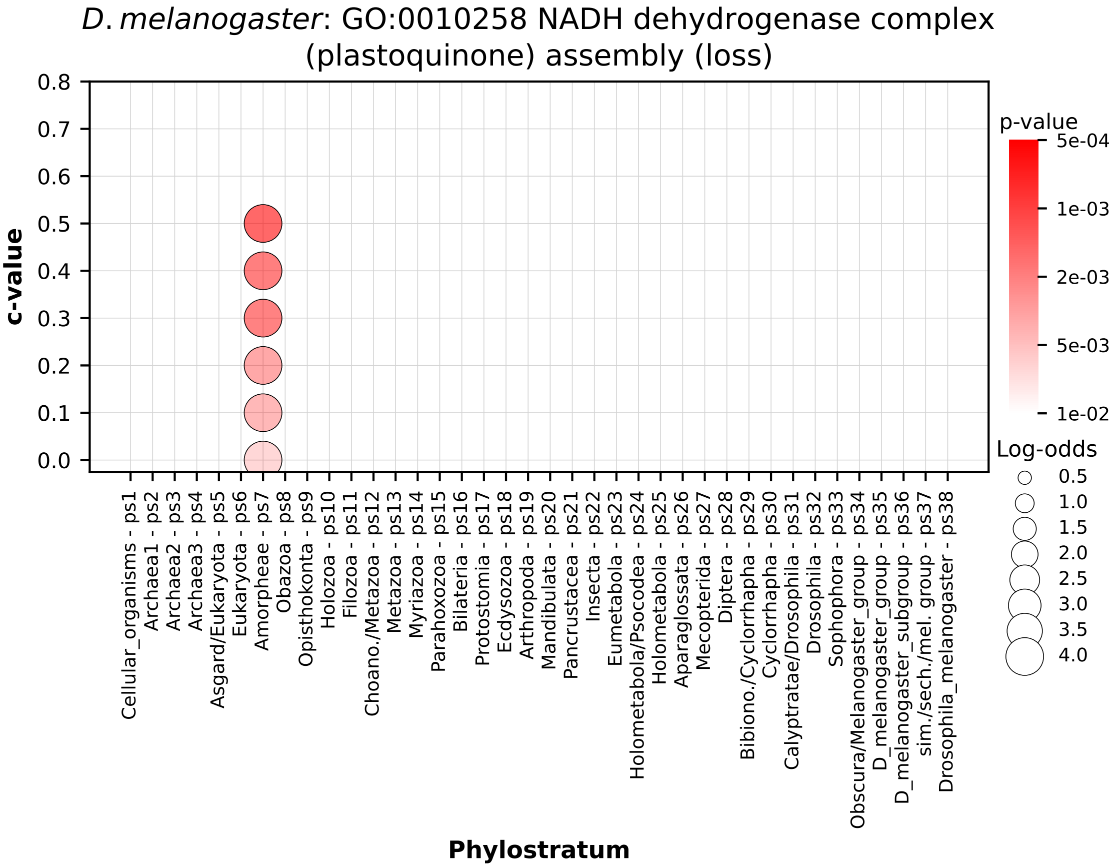

D. melanogaster: GO:0010258 NADH dehydrogenase complex (plastoquinone) assembly (loss)
c-value
0.8
0.7
0.6
0.5
0.4
0.3
0.2
0.1
0.0
p-value
5e-04
1e-03
2e-03
5e-03
1e-02
Log-odds
0.5
1.0
1.5
2.0
2.5
3.0
3.5
4.0
Cellular_organisms - ps1
Archaea1 - ps2
Archaea2 - ps3
Archaea3 - ps4
Asgard/Eukaryota - ps5
Eukaryota - ps6
Amorpheae - ps7
Obazoa - ps8
Opisthokonta - ps9
Holozoa - ps10
Filozoa - ps11
Choano./Metazoa - ps12
Metazoa - ps13
Myriazoa - ps14
Parahoxozoa - ps15
Bilateria - ps16
Protostomia - ps17
Ecdysozoa - ps18
Arthropoda - ps19
Mandibulata - ps20
Pancrustacea - ps21
Insecta - ps22
Eumetabola - ps23
Holometabola/Psocodea - ps24
Holometabola - ps25
Aparaglossata - ps26
Mecopterida - ps27
Diptera - ps28
Bibiono./Cyclorrhapha - ps29
Cyclorrhapha - ps30
Calyptratae/Drosophila - ps31
Drosophila - ps32
Sophophora - ps33
Obscura/Melanogaster_group - ps34
D_melanogaster_group - ps35
D_melanogaster_subgroup - ps36
sim./sech./mel. group - ps37
Drosophila_melanogaster - ps38
Phylostratum

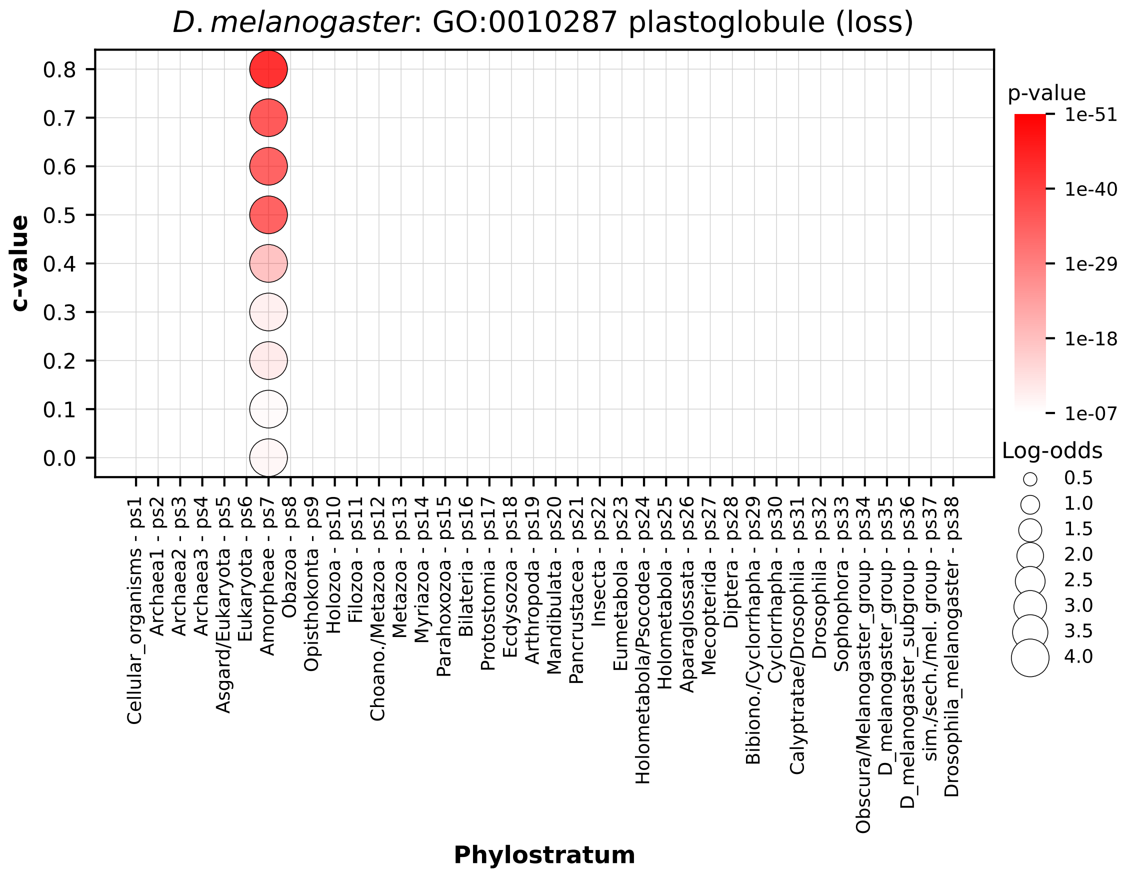

D. melanogaster: GO:0010287 plastoglobule (loss)
c-value
0.8
0.7
0.6
0.5
0.4
0.3
0.2
0.1
0.0
p-value
1e-51
1e-40
1e-29
1e-18
1e-07
Log-odds
0.5
1.0
1.5
2.0
2.5
3.0
3.5
4.0
Cellular_organisms - ps1
Archaea1 - ps2
Archaea2 - ps3
Archaea3 - ps4
Asgard/Eukaryota - ps5
Eukaryota - ps6
Amorpheae - ps7
Obazoa - ps8
Opisthokonta - ps9
Holozoa - ps10
Filozoa - ps11
Choano./Metazoa - ps12
Metazoa - ps13
Myriazoa - ps14
Parahoxozoa - ps15
Bilateria - ps16
Protostomia - ps17
Ecdysozoa - ps18
Arthropoda - ps19
Mandibulata - ps20
Pancrustacea - ps21
Insecta - ps22
Eumetabola - ps23
Holometabola/Psocodea - ps24
Holometabola - ps25
Aparaglossata - ps26
Mecopterida - ps27
Diptera - ps28
Bibiono./Cyclorrhapha - ps29
Cyclorrhapha - ps30
Calyptratae/Drosophila - ps31
Drosophila - ps32
Sophophora - ps33
Obscura/Melanogaster_group - ps34
D_melanogaster_group - ps35
D_melanogaster_subgroup - ps36
sim./sech./mel. group - ps37
Drosophila_melanogaster - ps38
Phylostratum

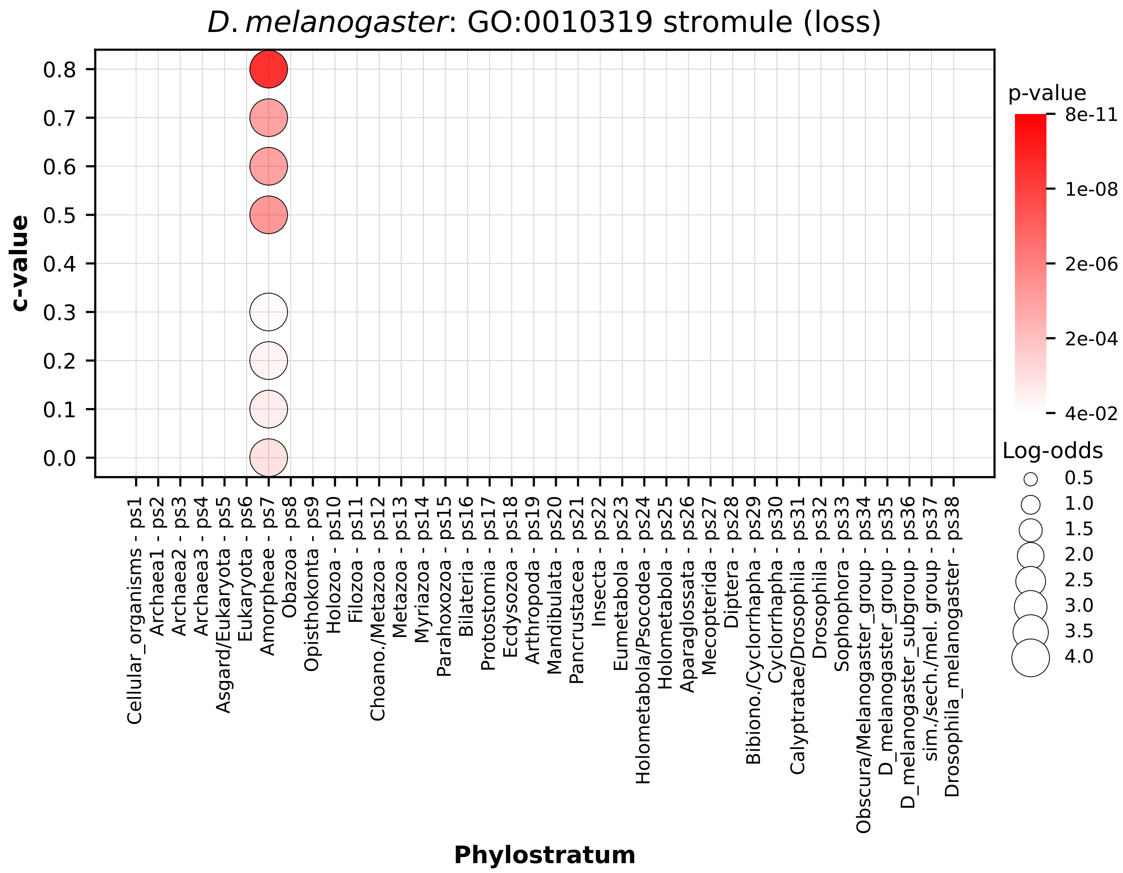

D. melanogaster: GO:0010319 stromule (loss)
c-value
0.0
0.1
0.2
0.3
0.4
0.5
0.6
0.7
0.8
p-value
8e-11
1e-08
2e-06
2e-04
4e-02
Log-odds
0.5
1.0
1.5
2.0
2.5
3.0
3.5
4.0
Cellular_organisms - ps1
Archaea1 - ps2
Archaea2 - ps3
Archaea3 - ps4
Asgard/Eukaryota - ps5
Eukaryota - ps6
Amorpheae - ps7
Obazoa - ps8
Opisthokonta - ps9
Holozoa - ps10
Filozoa - ps11
Choano./Metazoa - ps12
Metazoa - ps13
Myriazoa - ps14
Parahoxozoa - ps15
Bilateria - ps16
Protostomia - ps17
Ecdysozoa - ps18
Arthropoda - ps19
Mandibulata - ps20
Pancrustacea - ps21
Insecta - ps22
Eumetabola - ps23
Holometabola/Psocodea - ps24
Holometabola - ps25
Aparaglossata - ps26
Mecopterida - ps27
Diptera - ps28
Bibiono./Cyclorrhapha - ps29
Cyclorrhapha - ps30
Calyptratae/Drosophila - ps31
Drosophila - ps32
Sophophora - ps33
Obscura/Melanogaster_group - ps34
D_melanogaster_group - ps35
D_melanogaster_subgroup - ps36
sim./sech./mel. group - ps37
Drosophila_melanogaster - ps38
Phylostratum

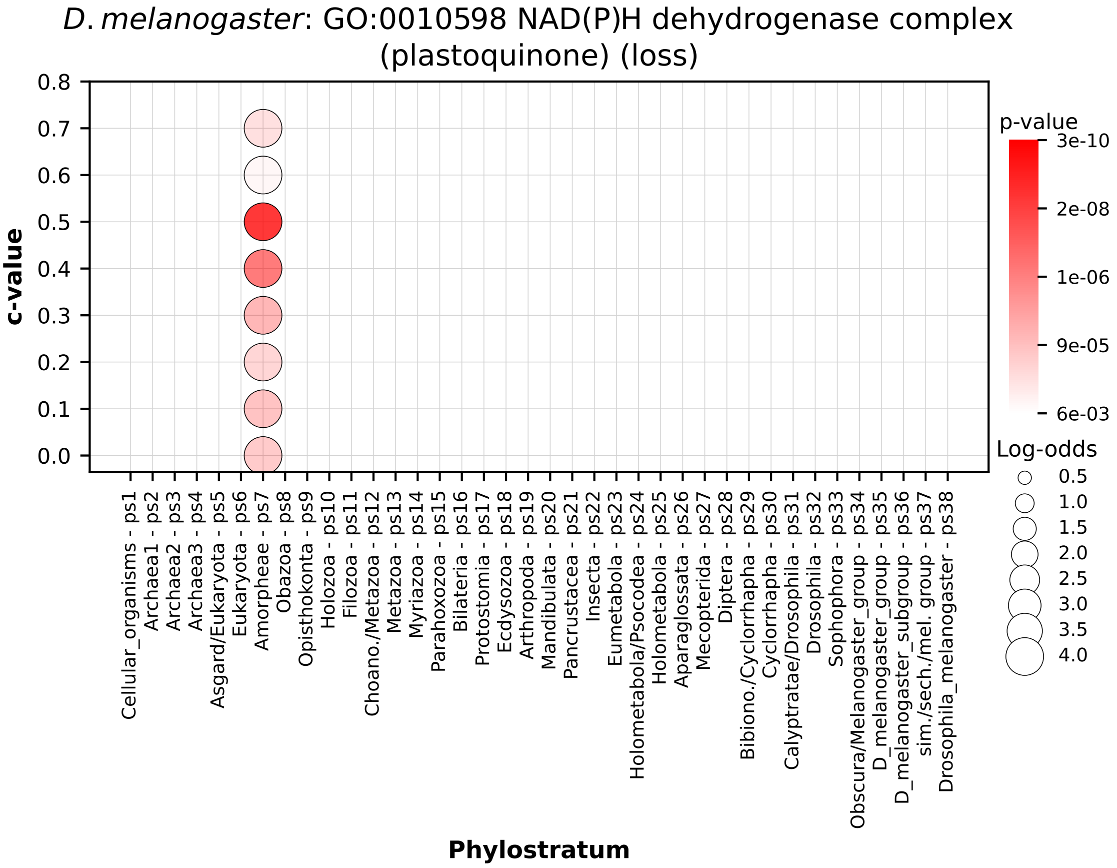
D. melanogaster: GO:0010598 NAD(P)H dehydrogenase complex (plastoquinone) (loss)
c-value
0.8
0.7
0.6
0.5
0.4
0.3
0.2
0.1
0.0
p-value
3e-10
2e-08
1e-06
9e-05
6e-03
Log-odds
0.5
1.0
1.5
2.0
2.5
3.0
3.5
4.0
Cellular_organisms - ps1
Archaea1 - ps2
Archaea2 - ps3
Archaea3 - ps4
Asgard/Eukaryota - ps5
Eukaryota - ps6
Amorpheae - ps7
Obazoa - ps8
Opisthokonta - ps9
Holozoa - ps10
Filozoa - ps11
Choano./Metazoa - ps12
Metazoa - ps13
Myriazoa - ps14
Parahoxozoa - ps15
Bilateria - ps16
Protostomia - ps17
Ecdysozoa - ps18
Arthropoda - ps19
Mandibulata - ps20
Pancrustacea - ps21
Insecta - ps22
Eumetabola - ps23
Holometabola/Psocodea - ps24
Holometabola - ps25
Aparaglossata - ps26
Mecopterida - ps27
Diptera - ps28
Bibiono./Cyclorrhapha - ps29
Cyclorrhapha - ps30
Calyptratae/Drosophila - ps31
Drosophila - ps32
Sophophora - ps33
Obscura/Melanogaster_group - ps34
D_melanogaster_group - ps35
D_melanogaster_subgroup - ps36
sim./sech./mel. group - ps37
Drosophila_melanogaster - ps38
Phylostratum

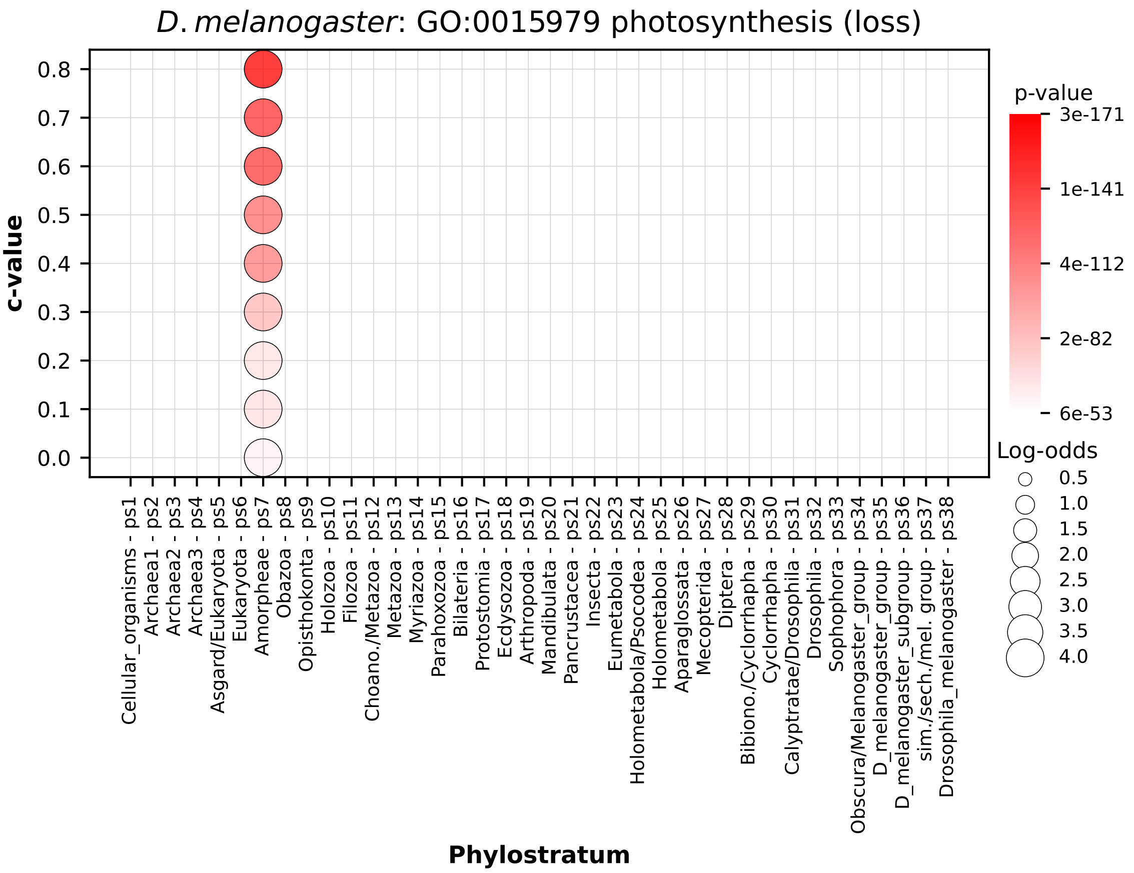
D. melanogaster: GO:0015979 photosynthesis (loss)
c-value
0.8
0.7
0.6
0.5
0.4
0.3
0.2
0.1
0.0
p-value
3e-171
1e-141
4e-112
2e-82
6e-53
Log-odds
0.5
1.0
1.5
2.0
2.5
3.0
3.5
4.0
Cellular_organisms - ps1
Archaea1 - ps2
Archaea2 - ps3
Archaea3 - ps4
Asgard/Eukaryota - ps5
Eukaryota - ps6
Amorpheae - ps7
Obazoa - ps8
Opisthokonta - ps9
Holozoa - ps10
Filozoa - ps11
Choano./Metazoa - ps12
Metazoa - ps13
Myriazoa - ps14
Parahoxozoa - ps15
Bilateria - ps16
Protostomia - ps17
Ecdysozoa - ps18
Arthropoda - ps19
Mandibulata - ps20
Pancrustacea - ps21
Insecta - ps22
Eumetabola - ps23
Holometabola/Psocodea - ps24
Holometabola - ps25
Aparaglossata - ps26
Mecopterida - ps27
Diptera - ps28
Bibiono./Cyclorrhapha - ps29
Cyclorrhapha - ps30
Calyptratae/Drosophila - ps31
Drosophila - ps32
Sophophora - ps33
Obscura/Melanogaster_group - ps34
D_melanogaster_group - ps35
D_melanogaster_subgroup - ps36
sim./sech./mel. group - ps37
Drosophila_melanogaster - ps38
Phylostratum

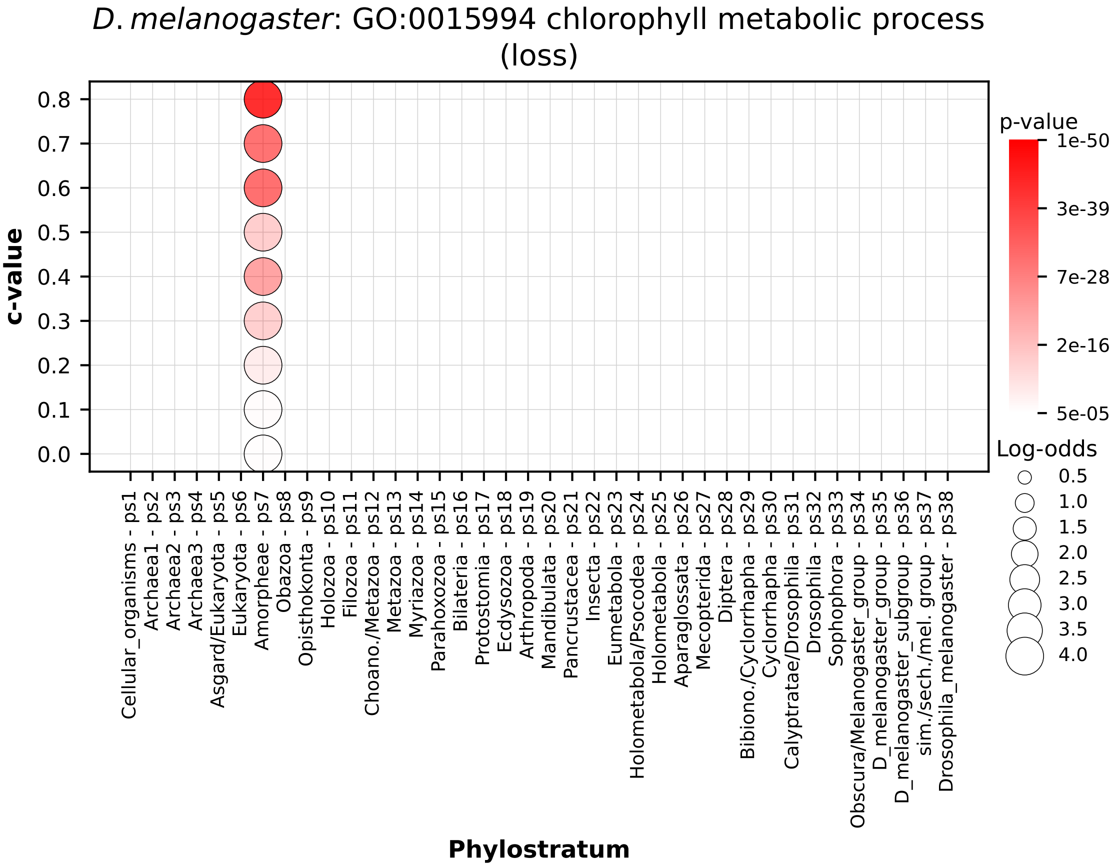

D. melanogaster: GO:0015994 chlorophyll metabolic process
(loss)
c-value
0.8
0.7
0.6
0.5
0.4
0.3
0.2
0.1
0.0
Phylostratum
Cellular_organisms - ps1
Archaea1 - ps2
Archaea2 - ps3
Archaea3 - ps4
Asgard/Eukaryota - ps5
Eukaryota - ps6
Amorpheae - ps7
Obazoa - ps8
Opisthokonta - ps9
Holozoa - ps10
Filozoa - ps11
Choano./Metazoa - ps12
Metazoa - ps13
Myriazoa - ps14
Parahoxozoa - ps15
Bilateria - ps16
Protostomia - ps17
Ecdysozoa - ps18
Arthropoda - ps19
Mandibulata - ps20
Pancrustacea - ps21
Insecta - ps22
Eumetabola - ps23
Holometabola/Psocodea - ps24
Holometabola - ps25
Aparaglossata - ps26
Mecopterida - ps27
Diptera - ps28
Bibiono./Cyclorrhapha - ps29
Cyclorrhapha - ps30
Calyptratae/Drosophila - ps31
Drosophila - ps32
Sophophora - ps33
Obscura/Melanogaster_group - ps34
D_melanogaster_group - ps35
D_melanogaster_subgroup - ps36
sim./sech./mel. group - ps37
Drosophila_melanogaster - ps38
p-value
1e-50
3e-39
7e-28
2e-16
5e-05
Log-odds
0.5
1.0
1.5
2.0
2.5
3.0
3.5
4.0

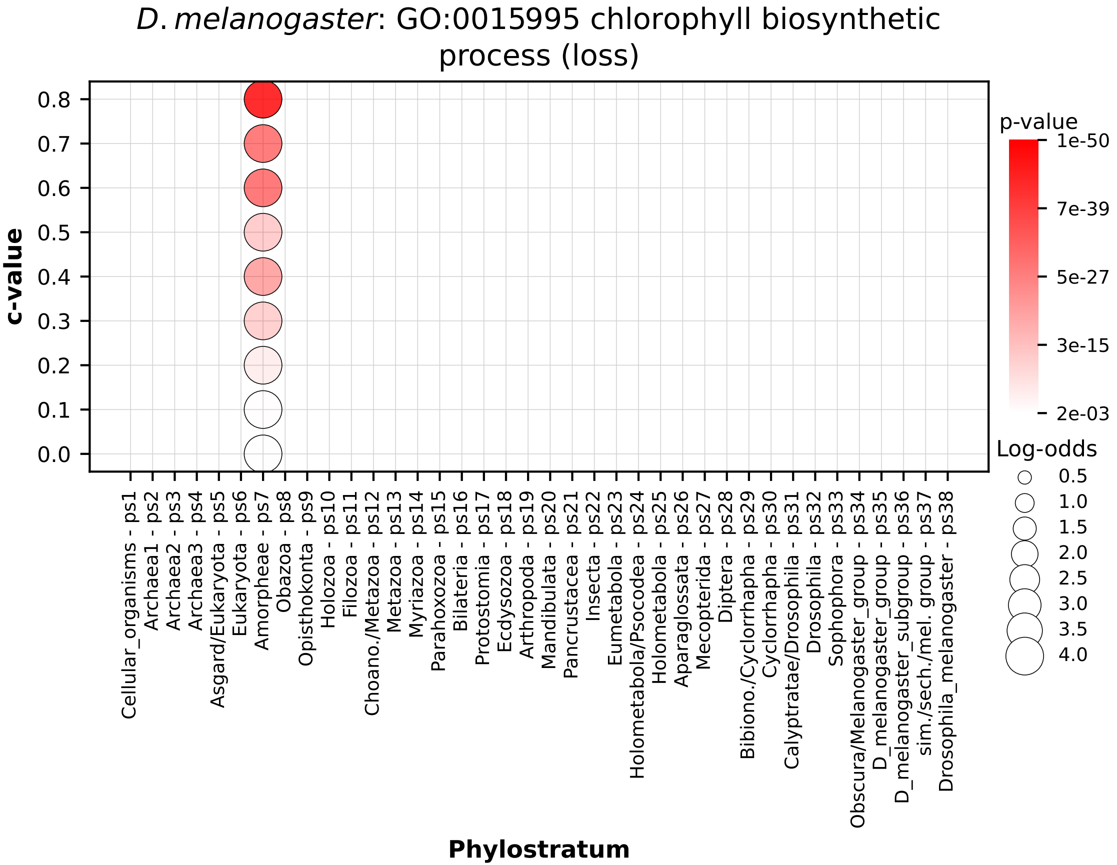

D. melanogaster: GO:0015995 chlorophyll biosynthetic process (loss)
c-value
0.8
0.7
0.6
0.5
0.4
0.3
0.2
0.1
0.0
p-value
1e-50
7e-39
5e-27
3e-15
2e-03
Log-odds
0.5
1.0
1.5
2.0
2.5
3.0
3.5
4.0
Cellular_organisms - ps1
Archaea1 - ps2
Archaea2 - ps3
Archaea3 - ps4
Asgard/Eukaryota - ps5
Eukaryota - ps6
Amorpheae - ps7
Obazoa - ps8
Opisthokonta - ps9
Holozoa - ps10
Filozoa - ps11
Choano./Metazoa - ps12
Metazoa - ps13
Myriazoa - ps14
Parahoxozoa - ps15
Bilateria - ps16
Protostomia - ps17
Ecdysozoa - ps18
Arthropoda - ps19
Mandibulata - ps20
Pancrustacea - ps21
Insecta - ps22
Eumetabola - ps23
Holometabola/Psocodea - ps24
Holometabola - ps25
Aparaglossata - ps26
Mecopterida - ps27
Diptera - ps28
Bibiono./Cyclorrhapha - ps29
Cyclorrhapha - ps30
Calyptratae/Drosophila - ps31
Drosophila - ps32
Sophophora - ps33
Obscura/Melanogaster_group - ps34
D_melanogaster_group - ps35
D_melanogaster_subgroup - ps36
sim./sech./mel. group - ps37
Drosophila_melanogaster - ps38
Phylostratum

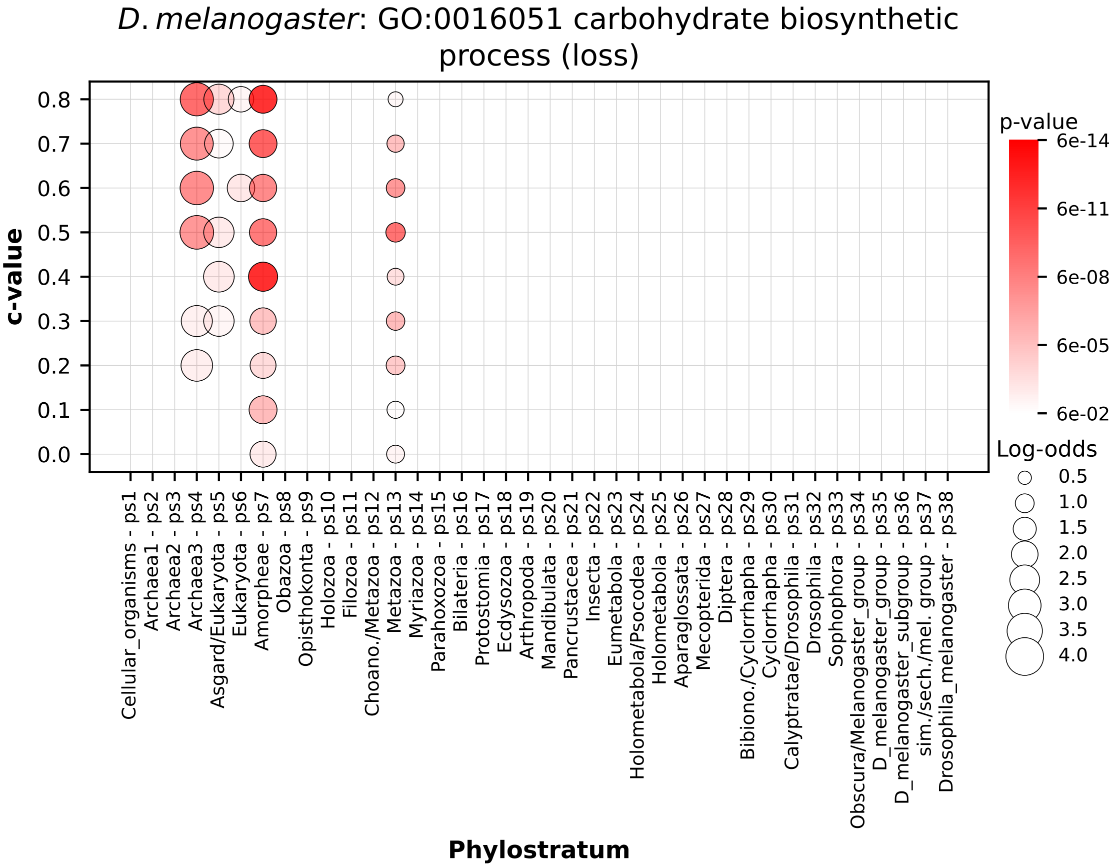

D. melanogaster: GO:0016051 carbohydrate biosynthetic process (loss)
c-value
0.8
0.7
0.6
0.5
0.4
0.3
0.2
0.1
0.0
p-value
6e-14
6e-11
6e-08
6e-05
6e-02
Log-odds
0.5
1.0
1.5
2.0
2.5
3.0
3.5
4.0
Cellular_organisms - ps1
Archaea1 - ps2
Archaea2 - ps3
Archaea3 - ps4
Asgard/Eukaryota - ps5
Eukaryota - ps6
Amorpheae - ps7
Obazoa - ps8
Opisthokonta - ps9
Holozoa - ps10
Filozoa - ps11
Choano./Metazoa - ps12
Metazoa - ps13
Myriazoa - ps14
Parahoxozoa - ps15
Bilateria - ps16
Protostomia - ps17
Ecdysozoa - ps18
Arthropoda - ps19
Mandibulata - ps20
Pancrustacea - ps21
Insecta - ps22
Eumetabola - ps23
Holometabola/Psocodea - ps24
Holometabola - ps25
Aparaglossata - ps26
Mecopterida - ps27
Diptera - ps28
Bibiono./Cyclorrhapha - ps29
Cyclorrhapha - ps30
Calyptratae/Drosophila - ps31
Drosophila - ps32
Sophophora - ps33
Obscura/Melanogaster_group - ps34
D_melanogaster_group - ps35
D_melanogaster_subgroup - ps36
sim./sech./mel. group - ps37
Drosophila_melanogaster - ps38
Phylostratum

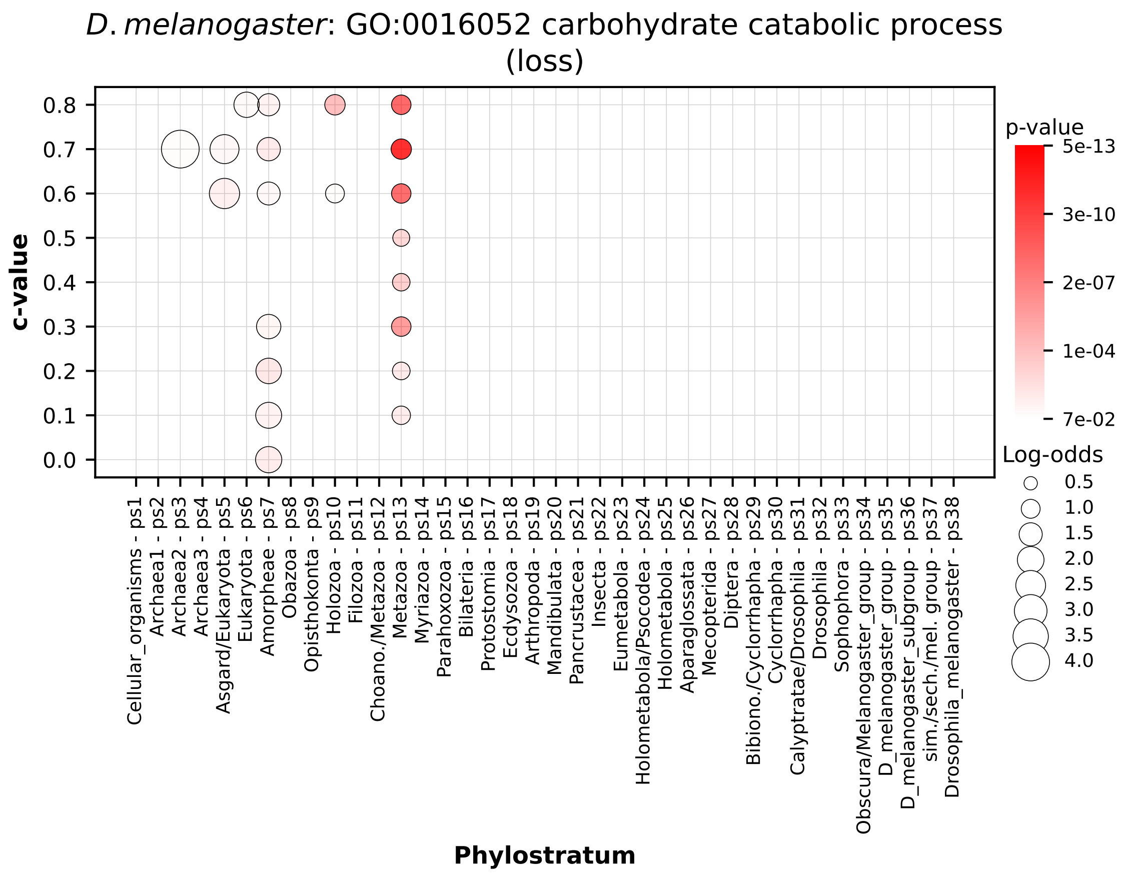

D. melanogaster: GO:0016052 carbohydrate catabolic process (loss)
c-value
0.8
0.7
0.6
0.5
0.4
0.3
0.2
0.1
0.0
p-value
5e-13
3e-10
2e-07
1e-04
7e-02
Log-odds
0.5
1.0
1.5
2.0
2.5
3.0
3.5
4.0
Cellular_organisms - ps1
Archaea1 - ps2
Archaea2 - ps3
Archaea3 - ps4
Asgard/Eukaryota - ps5
Eukaryota - ps6
Amorpheae - ps7
Obazoa - ps8
Opisthokonta - ps9
Holozoa - ps10
Filozoa - ps11
Choano./Metazoa - ps12
Metazoa - ps13
Myriazoa - ps14
Parahoxozoa - ps15
Bilateria - ps16
Protostomia - ps17
Ecdysozoa - ps18
Arthropoda - ps19
Mandibulata - ps20
Pancrustacea - ps21
Insecta - ps22
Eumetabola - ps23
Holometabola/Psocodea - ps24
Holometabola - ps25
Aparaglossata - ps26
Mecopterida - ps27
Diptera - ps28
Bibiono./Cyclorrhapha - ps29
Cyclorrhapha - ps30
Calyptratae/Drosophila - ps31
Drosophila - ps32
Sophophora - ps33
Obscura/Melanogaster_group - ps34
D_melanogaster_group - ps35
D_melanogaster_subgroup - ps36
sim./sech./mel. group - ps37
Drosophila_melanogaster - ps38
Phylostratum

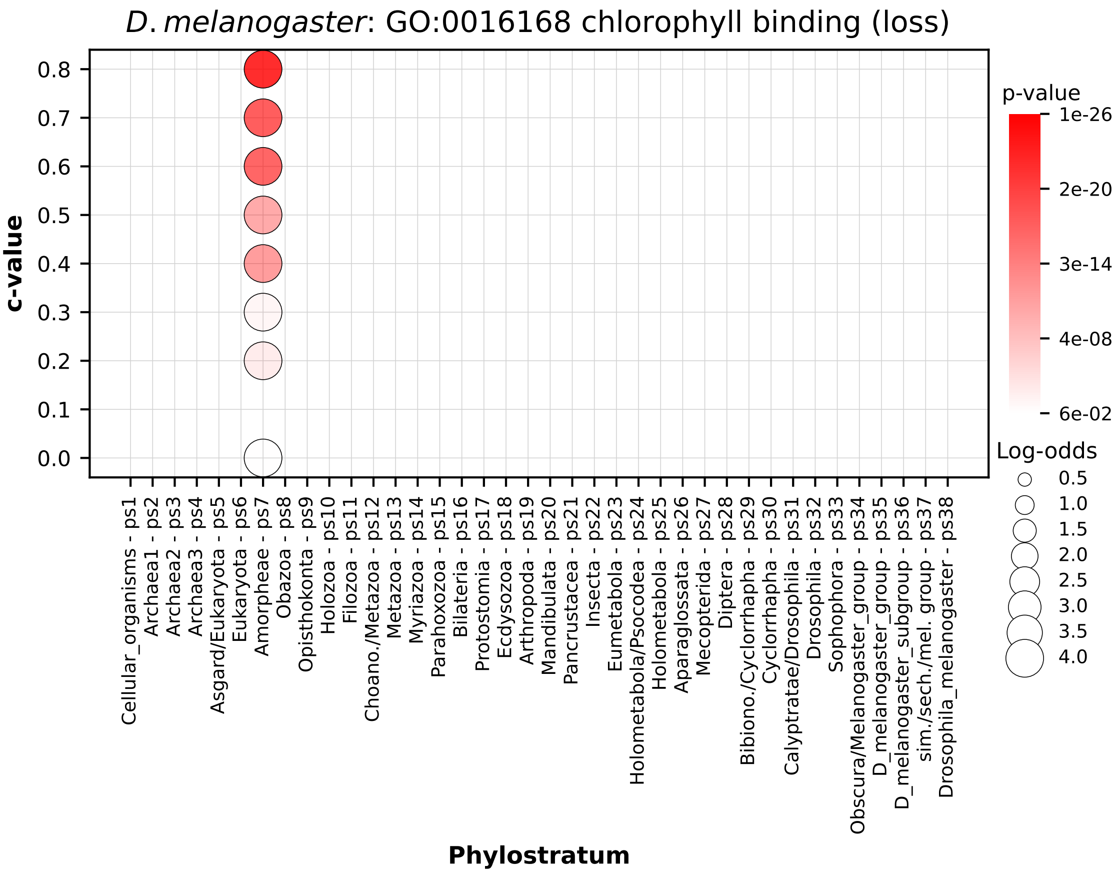

D. melanogaster: GO:0016168 chlorophyll binding (loss)
c-value
0.8
0.7
0.6
0.5
0.4
0.3
0.2
0.1
0.0
Phylostratum
Cellular_organisms - ps1
Archaea1 - ps2
Archaea2 - ps3
Archaea3 - ps4
Asgard/Eukaryota - ps5
Eukaryota - ps6
Amorpheae - ps7
Obazoa - ps8
Opisthokonta - ps9
Holozoa - ps10
Filozoa - ps11
Choano./Metazoa - ps12
Metazoa - ps13
Myriazoa - ps14
Parahoxozoa - ps15
Bilateria - ps16
Protostomia - ps17
Ecdysozoa - ps18
Arthropoda - ps19
Mandibulata - ps20
Pancrustacea - ps21
Insecta - ps22
Eumetabola - ps23
Holometabola/Psocodea - ps24
Holometabola - ps25
Aparaglossata - ps26
Mecopterida - ps27
Diptera - ps28
Bibiono./Cyclorrhapha - ps29
Cyclorrhapha - ps30
Calyptratae/Drosophila - ps31
Drosophila - ps32
Sophophora - ps33
Obscura/Melanogaster_group - ps34
D_melanogaster_group - ps35
D_melanogaster_subgroup - ps36
sim./sech./mel. group - ps37
Drosophila_melanogaster - ps38
p-value
1e-26
2e-20
3e-14
4e-08
6e-02
Log-odds
0.5
1.0
1.5
2.0
2.5
3.0
3.5
4.0

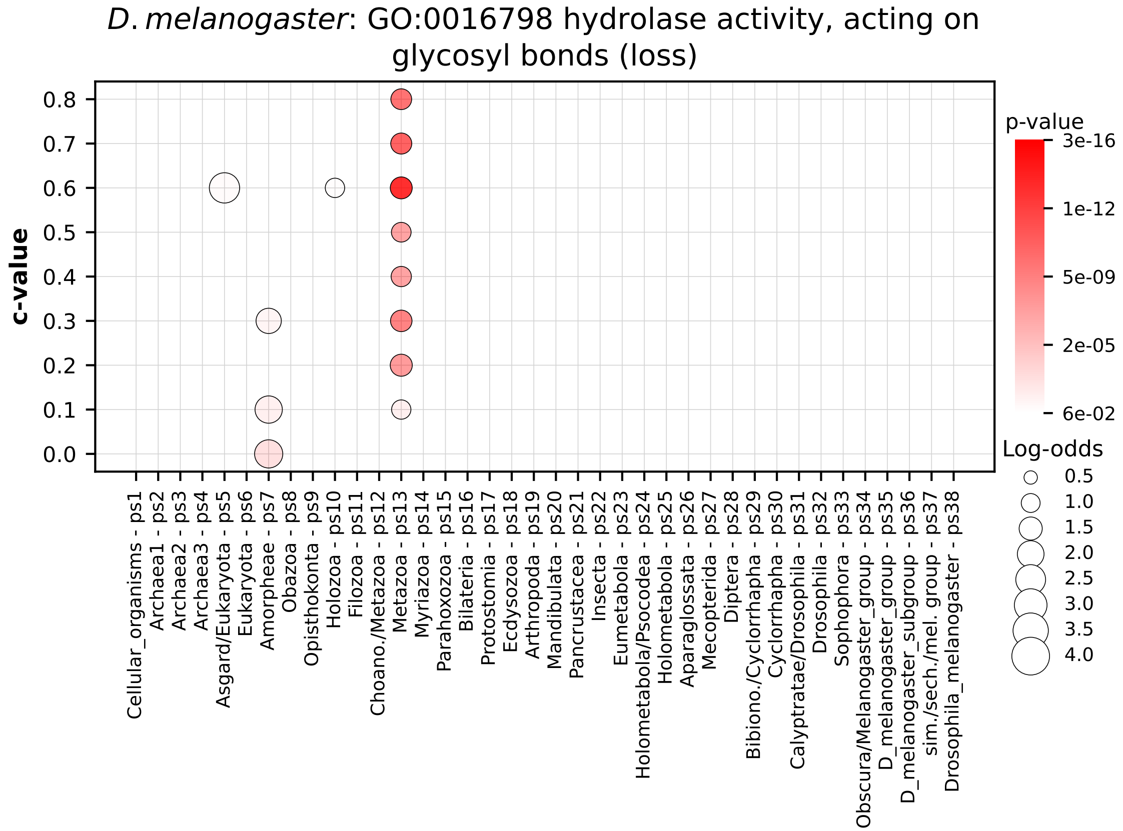
D. melanogaster: GO:0016798 hydrolase activity, acting on glycosyl bonds (loss)
c-value
0.8
0.7
0.6
0.5
0.4
0.3
0.2
0.1
0.0
Phylostratum
Cellular_organisms - ps1
Archaea1 - ps2
Archaea2 - ps3
Archaea3 - ps4
Asgard/Eukaryota - ps5
Eukaryota - ps6
Amorpheae - ps7
Obazoa - ps8
Opisthokonta - ps9
Holozoa - ps10
Filozoa - ps11
Choano./Metazoa - ps12
Metazoa - ps13
Myriazoa - ps14
Parahoxozoa - ps15
Bilateria - ps16
Protostomia - ps17
Ecdysozoa - ps18
Arthropoda - ps19
Mandibulata - ps20
Pancrustacea - ps21
Insecta - ps22
Eumetabola - ps23
Holometabola/Psocodea - ps24
Holometabola - ps25
Aparaglossata - ps26
Mecopterida - ps27
Diptera - ps28
Bibiono./Cyclorrhapha - ps29
Cyclorrhapha - ps30
Calyptratae/Drosophila - ps31
Drosophila - ps32
Sophophora - ps33
Obscura/Melanogaster_group - ps34
D_melanogaster_group - ps35
D_melanogaster_subgroup - ps36
sim./sech./mel. group - ps37
Drosophila_melanogaster - ps38
p-value
3e-16
1e-12
5e-09
2e-05
6e-02
Log-odds
0.5
1.0
1.5
2.0
2.5
3.0
3.5
4.0

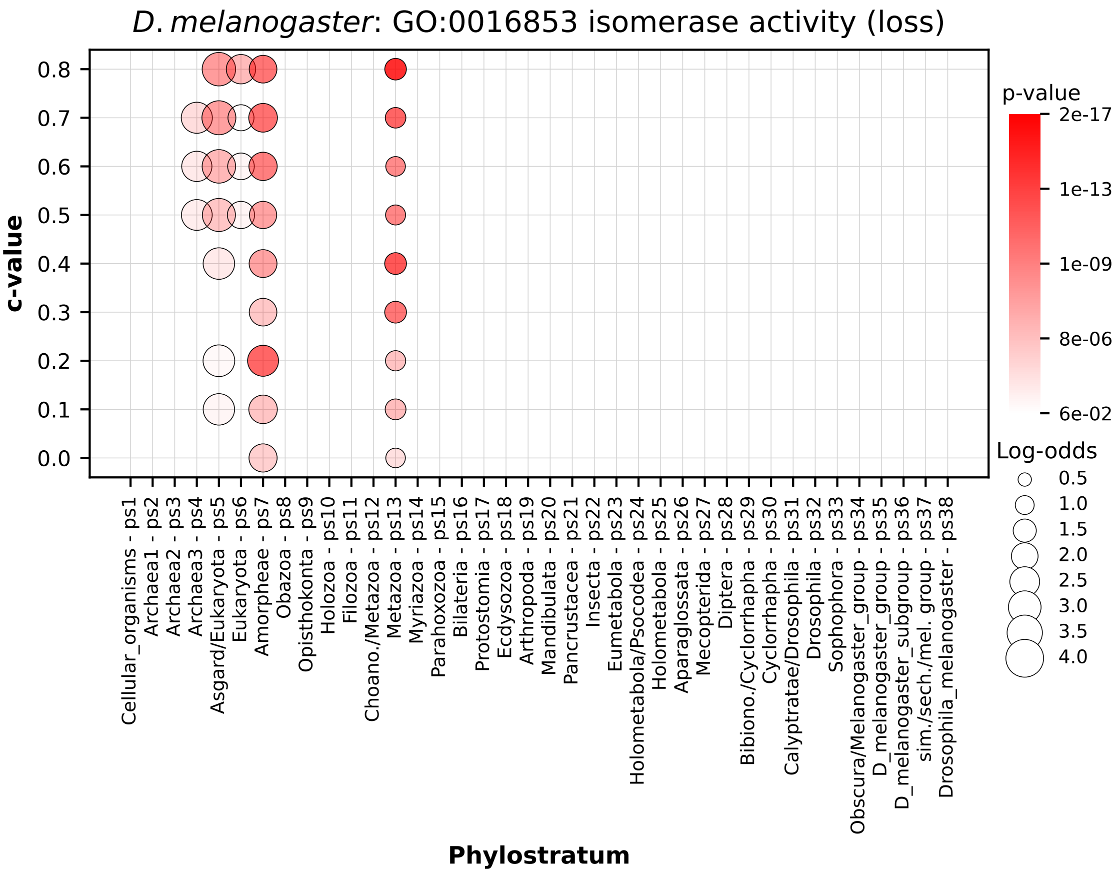

D. melanogaster: GO:0016853 isomerase activity (loss)
c-value
0.8
0.7
0.6
0.5
0.4
0.3
0.2
0.1
0.0
Phylostratum
Cellular_organisms - ps1
Archaea1 - ps2
Archaea2 - ps3
Archaea3 - ps4
Asgard/Eukaryota - ps5
Eukaryota - ps6
Amorpheae - ps7
Obazoa - ps8
Opisthokonta - ps9
Holozoa - ps10
Filozoa - ps11
Choano./Metazoa - ps12
Metazoa - ps13
Myriazoa - ps14
Parahoxozoa - ps15
Bilateria - ps16
Protostomia - ps17
Ecdysozoa - ps18
Arthropoda - ps19
Mandibulata - ps20
Pancrustacea - ps21
Insecta - ps22
Eumetabola - ps23
Holometabola/Psocodea - ps24
Holometabola - ps25
Aparaglossata - ps26
Mecopterida - ps27
Diptera - ps28
Bibiono./Cyclorrhapha - ps29
Cyclorrhapha - ps30
Calyptratae/Drosophila - ps31
Drosophila - ps32
Sophophora - ps33
Obscura/Melanogaster_group - ps34
D_melanogaster_group - ps35
D_melanogaster_subgroup - ps36
sim./sech./mel. group - ps37
Drosophila_melanogaster - ps38
p-value
2e-17
1e-13
1e-09
8e-06
6e-02
Log-odds
0.5
1.0
1.5
2.0
2.5
3.0
3.5
4.0

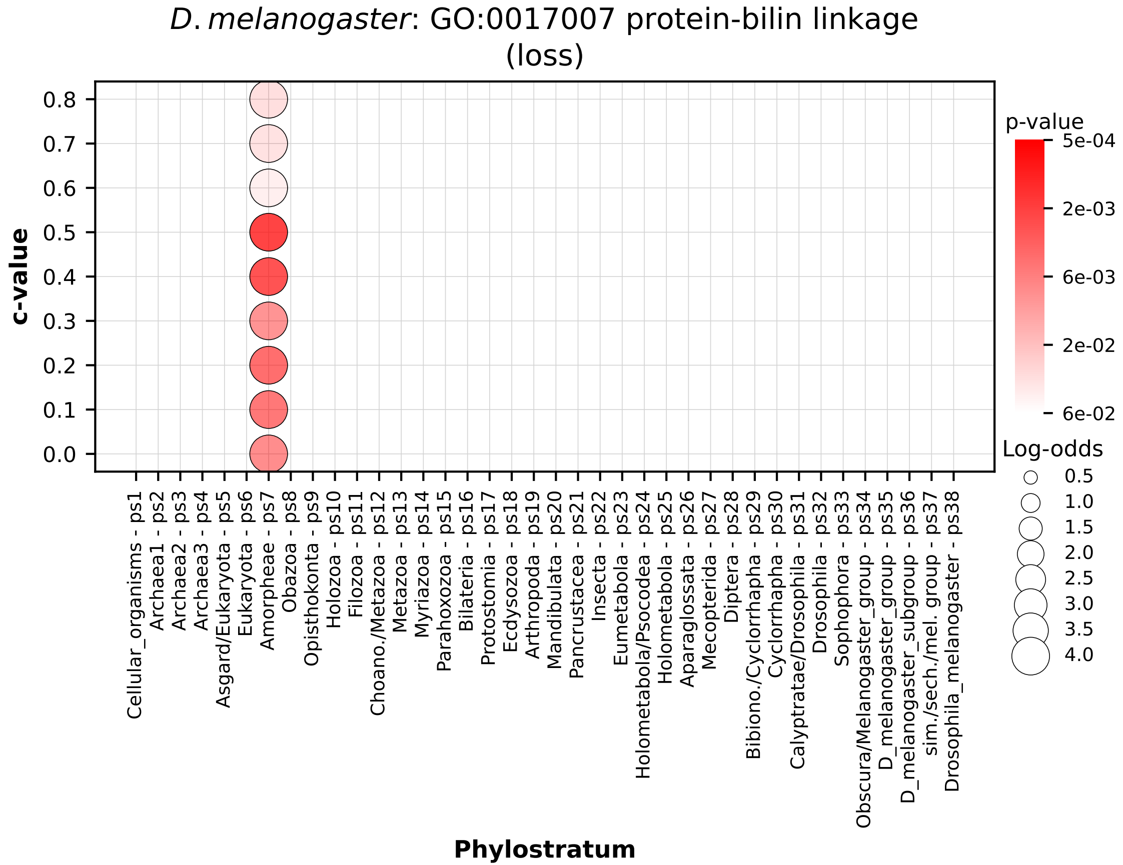

D. melanogaster: GO:0017007 protein-bilin linkage (loss)
c-value
p-value
5e-04
2e-03
6e-03
2e-02
6e-02
Log-odds
0.5
1.0
1.5
2.0
2.5
3.0
3.5
4.0
Phylostratum
Cellular_organisms - ps1
Archaea1 - ps2
Archaea2 - ps3
Archaea3 - ps4
Asgard/Eukaryota - ps5
Eukaryota - ps6
Amorpheae - ps7
Obazoa - ps8
Opisthokonta - ps9
Holozoa - ps10
Filozoa - ps11
Choano./Metazoa - ps12
Metazoa - ps13
Myriazoa - ps14
Parahoxozoa - ps15
Bilateria - ps16
Protostomia - ps17
Ecdysozoa - ps18
Arthropoda - ps19
Mandibulata - ps20
Pancrustacea - ps21
Insecta - ps22
Eumetabola - ps23
Holometabola/Psocodea - ps24
Holometabola - ps25
Aparaglossata - ps26
Mecopterida - ps27
Diptera - ps28
Bibiono./Cyclorrhapha - ps29
Cyclorrhapha - ps30
Calyptratae/Drosophila - ps31
Drosophila - ps32
Sophophora - ps33
Obscura/Melanogaster_group - ps34
D_melanogaster_group - ps35
D_melanogaster_subgroup - ps36
sim./sech./mel. group - ps37
Drosophila_melanogaster - ps38

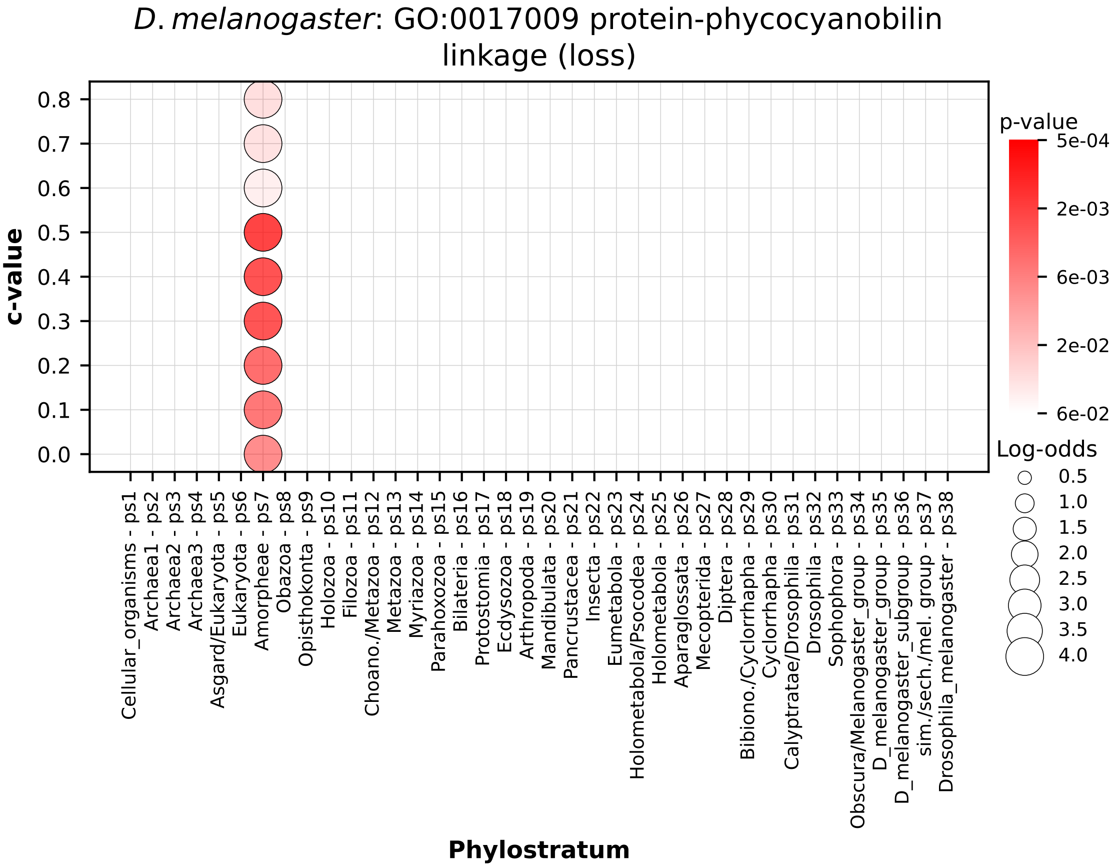
D. melanogaster: GO:0017009 protein-phycocyanobilin linkage (loss)
c-value
0.8
0.7
0.6
0.5
0.4
0.3
0.2
0.1
0.0
p-value
5e-04
2e-03
6e-03
2e-02
6e-02
Log-odds
0.5
1.0
1.5
2.0
2.5
3.0
3.5
4.0
Cellular_organisms - ps1
Archaea1 - ps2
Archaea2 - ps3
Archaea3 - ps4
Asgard/Eukaryota - ps5
Eukaryota - ps6
Amorpheae - ps7
Obazoa - ps8
Opisthokonta - ps9
Holozoa - ps10
Filozoa - ps11
Choano./Metazoa - ps12
Metazoa - ps13
Myriazoa - ps14
Parahoxozoa - ps15
Bilateria - ps16
Protostomia - ps17
Ecdysozoa - ps18
Arthropoda - ps19
Mandibulata - ps20
Pancrustacea - ps21
Insecta - ps22
Eumetabola - ps23
Holometabola/Psocodea - ps24
Holometabola - ps25
Aparaglossata - ps26
Mecopterida - ps27
Diptera - ps28
Bibiono./Cyclorrhapha - ps29
Cyclorrhapha - ps30
Calyptratae/Drosophila - ps31
Drosophila - ps32
Sophophora - ps33
Obscura/Melanogaster_group - ps34
D_melanogaster_group - ps35
D_melanogaster_subgroup - ps36
sim./sech./mel. group - ps37
Drosophila_melanogaster - ps38
Phylostratum

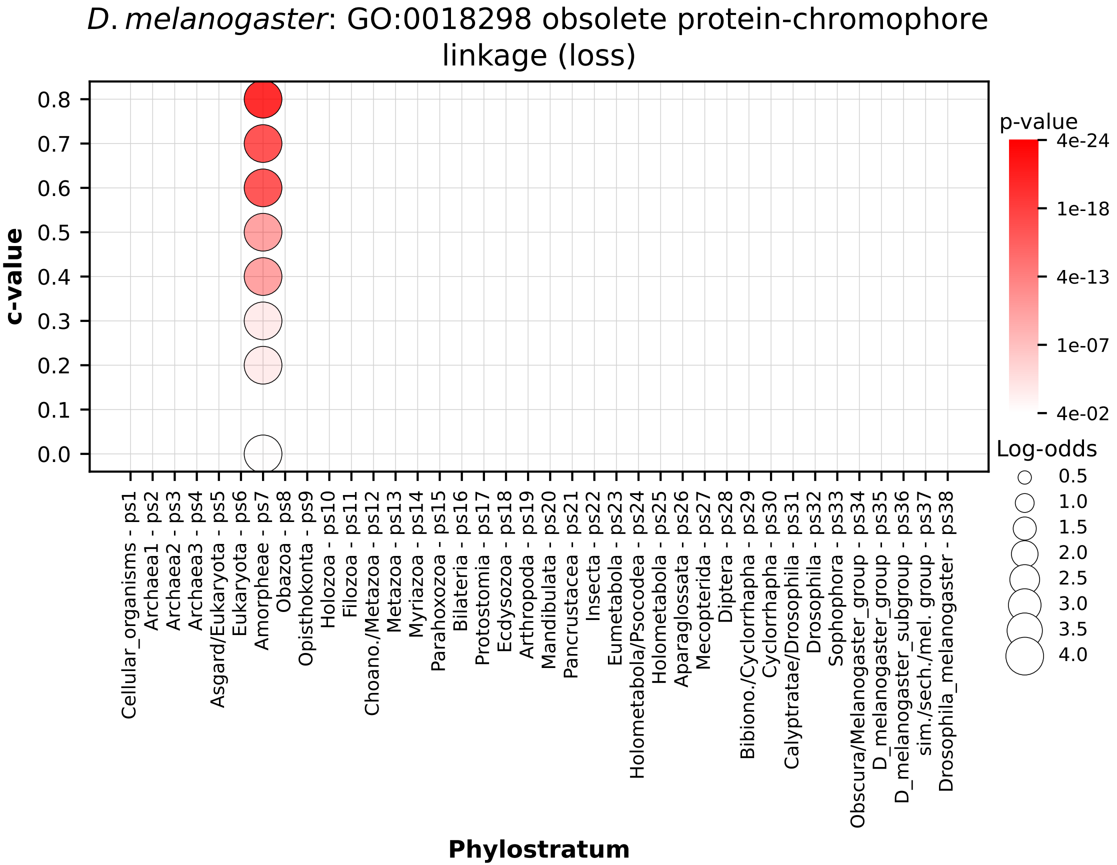

D. melanogaster: GO:0018298 obsolete protein-chromophore linkage (loss)
c-value
0.8
0.7
0.6
0.5
0.4
0.3
0.2
0.1
0.0
Phylostratum
Cellular_organisms - ps1
Archaea1 - ps2
Archaea2 - ps3
Archaea3 - ps4
Asgard/Eukaryota - ps5
Eukaryota - ps6
Amorpheae - ps7
Obazoa - ps8
Opisthokonta - ps9
Holozoa - ps10
Filozoa - ps11
Choano./Metazoa - ps12
Metazoa - ps13
Myriazoa - ps14
Parahoxozoa - ps15
Bilateria - ps16
Protostomia - ps17
Ecdysozoa - ps18
Arthropoda - ps19
Mandibulata - ps20
Pancrustacea - ps21
Insecta - ps22
Eumetabola - ps23
Holometabola/Psocodea - ps24
Holometabola - ps25
Aparaglossata - ps26
Mecopterida - ps27
Diptera - ps28
Bibiono./Cyclorrhapha - ps29
Cyclorrhapha - ps30
Calyptratae/Drosophila - ps31
Drosophila - ps32
Sophophora - ps33
Obscura/Melanogaster_group - ps34
D_melanogaster_group - ps35
D_melanogaster_subgroup - ps36
sim./sech./mel. group - ps37
Drosophila_melanogaster - ps38
p-value
4e-24
1e-18
4e-13
1e-07
4e-02
Log-odds
0.5
1.0
1.5
2.0
2.5
3.0
3.5
4.0

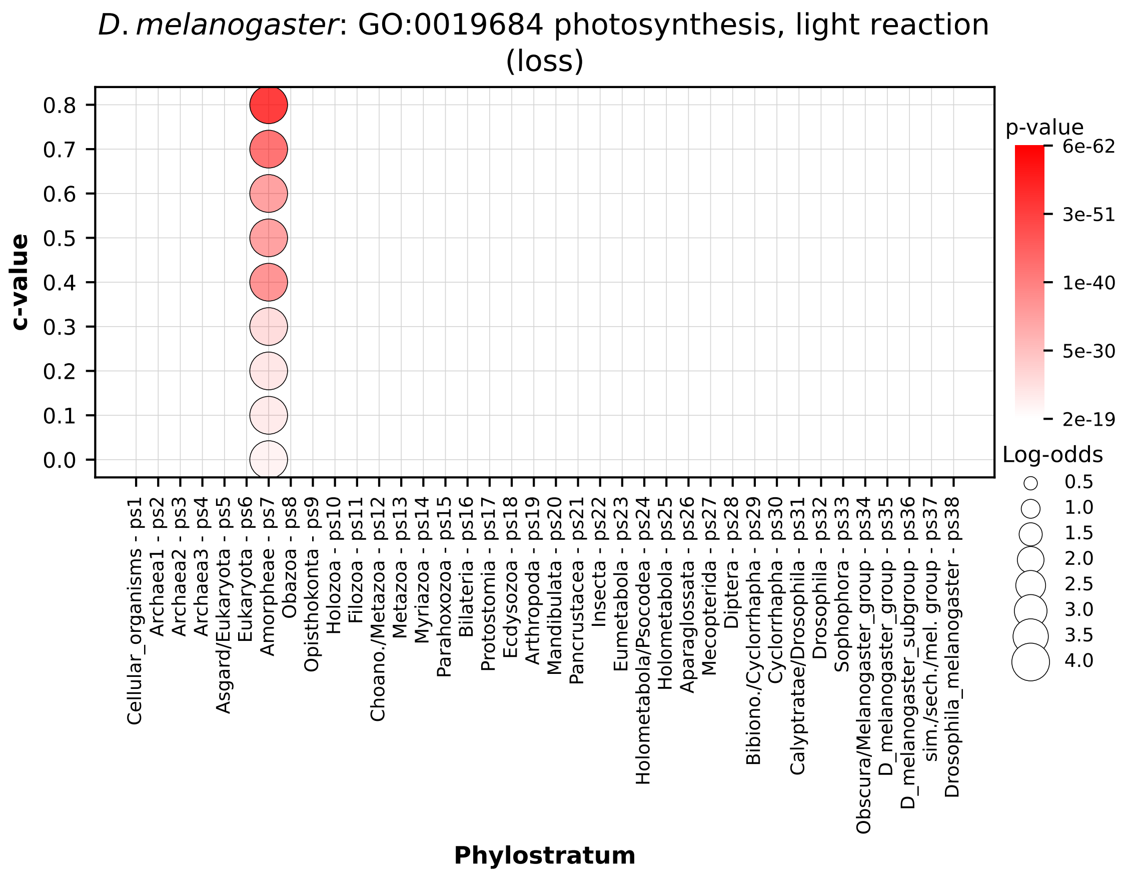

D. melanogaster: GO:0019684 photosynthesis, light reaction
(loss)
c-value
0.8
0.7
0.6
0.5
0.4
0.3
0.2
0.1
0.0
p-value
6e-62
3e-51
1e-40
5e-30
2e-19
Log-odds
0.5
1.0
1.5
2.0
2.5
3.0
3.5
4.0
Cellular_organisms - ps1
Archaea1 - ps2
Archaea2 - ps3
Archaea3 - ps4
Asgard/Eukaryota - ps5
Eukaryota - ps6
Amorpheae - ps7
Obazoa - ps8
Opisthokonta - ps9
Holozoa - ps10
Filozoa - ps11
Choano./Metazoa - ps12
Metazoa - ps13
Myriazoa - ps14
Parahoxozoa - ps15
Bilateria - ps16
Protostomia - ps17
Ecdysozoa - ps18
Arthropoda - ps19
Mandibulata - ps20
Pancrustacea - ps21
Insecta - ps22
Eumetabola - ps23
Holometabola/Psocodea - ps24
Holometabola - ps25
Aparaglossata - ps26
Mecopterida - ps27
Diptera - ps28
Bibiono./Cyclorrhapha - ps29
Cyclorrhapha - ps30
Calyptratae/Drosophila - ps31
Drosophila - ps32
Sophophora - ps33
Obscura/Melanogaster_group - ps34
D_melanogaster_group - ps35
D_melanogaster_subgroup - ps36
sim./sech./mel. group - ps37
Drosophila_melanogaster - ps38
Phylostratum

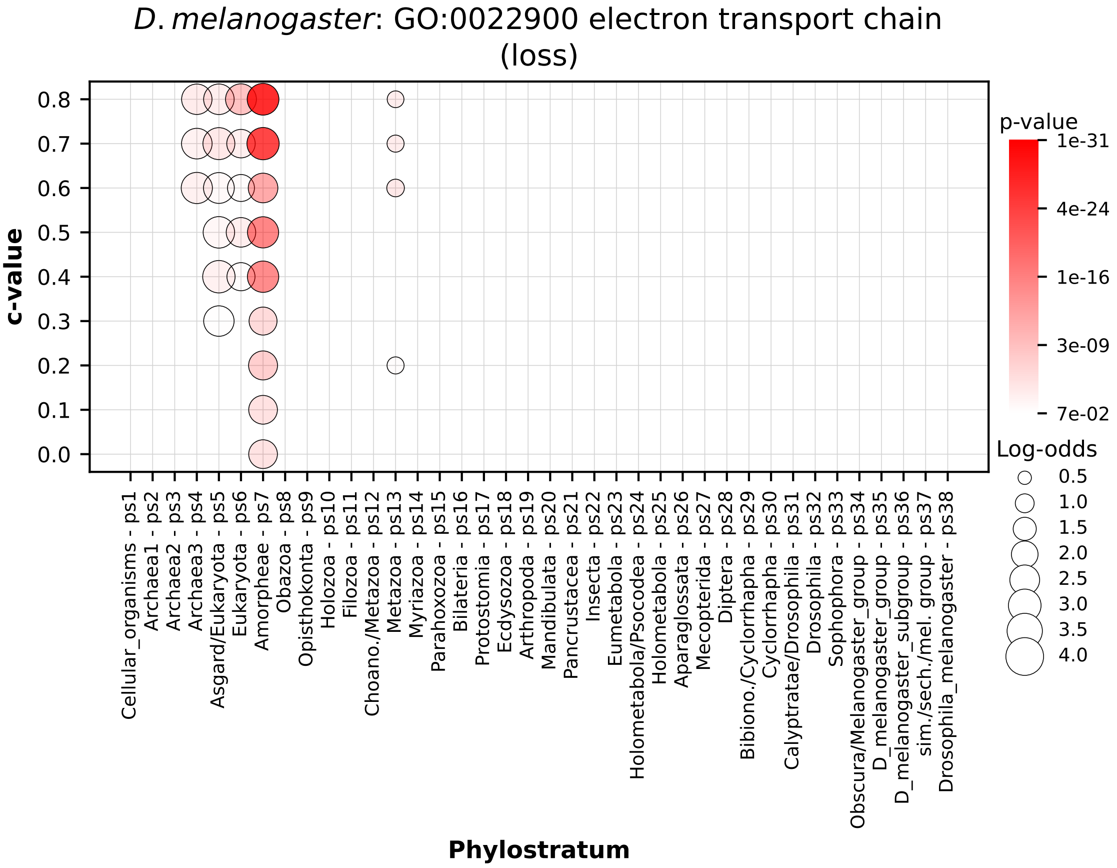

D. melanogaster: GO:0022900 electron transport chain
(loss)
c-value
0.8
0.7
0.6
0.5
0.4
0.3
0.2
0.1
0.0
Phylostratum
Cellular_organisms - ps1
Archaea1 - ps2
Archaea2 - ps3
Archaea3 - ps4
Asgard/Eukaryota - ps5
Eukaryota - ps6
Amorpheae - ps7
Obazoa - ps8
Opisthokonta - ps9
Holozoa - ps10
Filozoa - ps11
Choano./Metazoa - ps12
Metazoa - ps13
Myriazoa - ps14
Parahoxozoa - ps15
Bilateria - ps16
Protostomia - ps17
Ecdysozoa - ps18
Arthropoda - ps19
Mandibulata - ps20
Pancrustacea - ps21
Insecta - ps22
Eumetabola - ps23
Holometabola/Psocodea - ps24
Holometabola - ps25
Aparaglossata - ps26
Mecopterida - ps27
Diptera - ps28
Bibiono./Cyclorrhapha - ps29
Cyclorrhapha - ps30
Calyptratae/Drosophila - ps31
Drosophila - ps32
Sophophora - ps33
Obscura/Melanogaster_group - ps34
D_melanogaster_group - ps35
D_melanogaster_subgroup - ps36
sim./sech./mel. group - ps37
Drosophila_melanogaster - ps38
p-value
1e-31
4e-24
1e-16
3e-09
7e-02
Log-odds
0.5
1.0
1.5
2.0
2.5
3.0
3.5
4.0

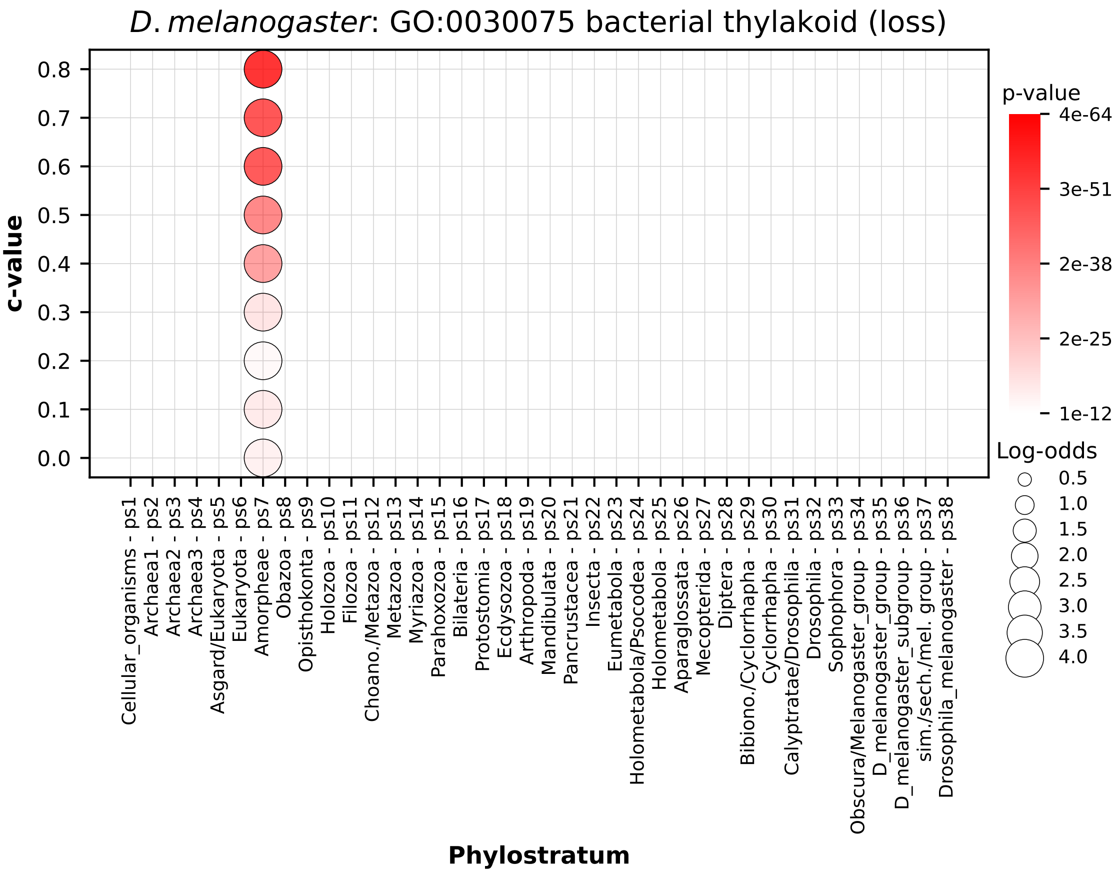

D. melanogaster: GO:0030075 bacterial thylakoid (loss)
c-value
0.8
0.7
0.6
0.5
0.4
0.3
0.2
0.1
0.0
p-value
4e-64
3e-51
2e-38
2e-25
1e-12
Log-odds
0.5
1.0
1.5
2.0
2.5
3.0
3.5
4.0
Cellular_organisms - ps1
Archaea1 - ps2
Archaea2 - ps3
Archaea3 - ps4
Asgard/Eukaryota - ps5
Eukaryota - ps6
Amorpheae - ps7
Obazoa - ps8
Opisthokonta - ps9
Holozoa - ps10
Filozoa - ps11
Choano./Metazoa - ps12
Metazoa - ps13
Myriazoa - ps14
Parahoxozoa - ps15
Bilateria - ps16
Protostomia - ps17
Ecdysozoa - ps18
Arthropoda - ps19
Mandibulata - ps20
Pancrustacea - ps21
Insecta - ps22
Eumetabola - ps23
Holometabola/Psocodea - ps24
Holometabola - ps25
Aparaglossata - ps26
Mecopterida - ps27
Diptera - ps28
Bibiono./Cyclorrhapha - ps29
Cyclorrhapha - ps30
Calyptratae/Drosophila - ps31
Drosophila - ps32
Sophophora - ps33
Obscura/Melanogaster_group - ps34
D_melanogaster_group - ps35
D_melanogaster_subgroup - ps36
sim./sech./mel. group - ps37
Drosophila_melanogaster - ps38
Phylostratum

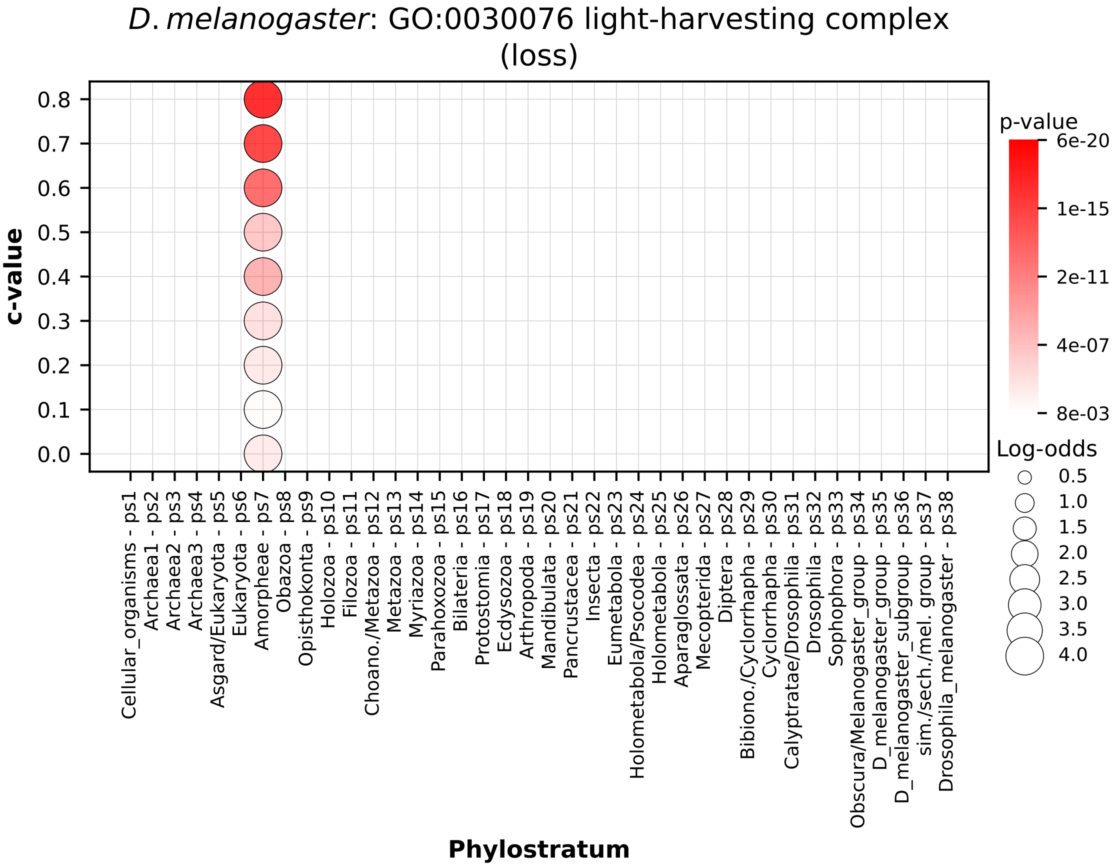

D. melanogaster: GO:0030076 light-harvesting complex
(loss)
c-value
0.8
0.7
0.6
0.5
0.4
0.3
0.2
0.1
0.0
p-value
6e-20
1e-15
2e-11
4e-07
8e-03
Log-odds
0.5
1.0
1.5
2.0
2.5
3.0
3.5
4.0
Cellular_organisms - ps1
Archaea1 - ps2
Archaea2 - ps3
Archaea3 - ps4
Asgard/Eukaryota - ps5
Eukaryota - ps6
Amorpheae - ps7
Obazoa - ps8
Opisthokonta - ps9
Holozoa - ps10
Filozoa - ps11
Choano./Metazoa - ps12
Metazoa - ps13
Myriazoa - ps14
Parahoxozoa - ps15
Bilateria - ps16
Protostomia - ps17
Ecdysozoa - ps18
Arthropoda - ps19
Mandibulata - ps20
Pancrustacea - ps21
Insecta - ps22
Eumetabola - ps23
Holometabola/Psocodea - ps24
Holometabola - ps25
Aparaglossata - ps26
Mecopterida - ps27
Diptera - ps28
Bibiono./Cyclorrhapha - ps29
Cyclorrhapha - ps30
Calyptratae/Drosophila - ps31
Drosophila - ps32
Sophophora - ps33
Obscura/Melanogaster_group - ps34
D_melanogaster_group - ps35
D_melanogaster_subgroup - ps36
sim./sech./mel. group - ps37
Drosophila_melanogaster - ps38
Phylostratum

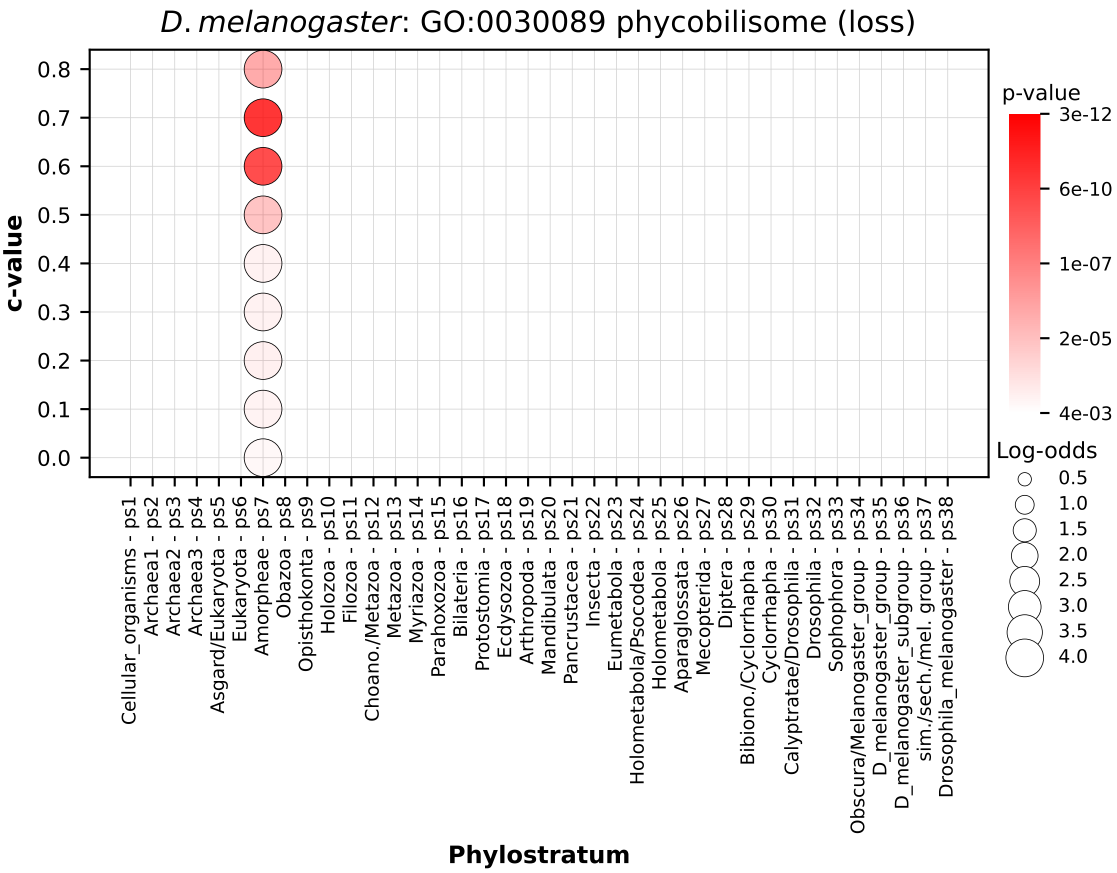

D. melanogaster: GO:0030089 phycobilisome (loss)
c-value
0.8
0.7
0.6
0.5
0.4
0.3
0.2
0.1
0.0
p-value
3e-12
6e-10
1e-07
2e-05
4e-03
Log-odds
0.5
1.0
1.5
2.0
2.5
3.0
3.5
4.0
Cellular_organisms - ps1
Archaea1 - ps2
Archaea2 - ps3
Archaea3 - ps4
Asgard/Eukaryota - ps5
Eukaryota - ps6
Amorpheae - ps7
Obazoa - ps8
Opisthokonta - ps9
Holozoa - ps10
Filozoa - ps11
Choano./Metazoa - ps12
Metazoa - ps13
Myriazoa - ps14
Parahoxozoa - ps15
Bilateria - ps16
Protostomia - ps17
Ecdysozoa - ps18
Arthropoda - ps19
Mandibulata - ps20
Pancrustacea - ps21
Insecta - ps22
Eumetabola - ps23
Holometabola/Psocodea - ps24
Holometabola - ps25
Aparaglossata - ps26
Mecopterida - ps27
Diptera - ps28
Bibiono./Cyclorrhapha - ps29
Cyclorrhapha - ps30
Calyptratae/Drosophila - ps31
Drosophila - ps32
Sophophora - ps33
Obscura/Melanogaster_group - ps34
D_melanogaster_group - ps35
D_melanogaster_subgroup - ps36
sim./sech./mel. group - ps37
Drosophila_melanogaster - ps38
Phylostratum

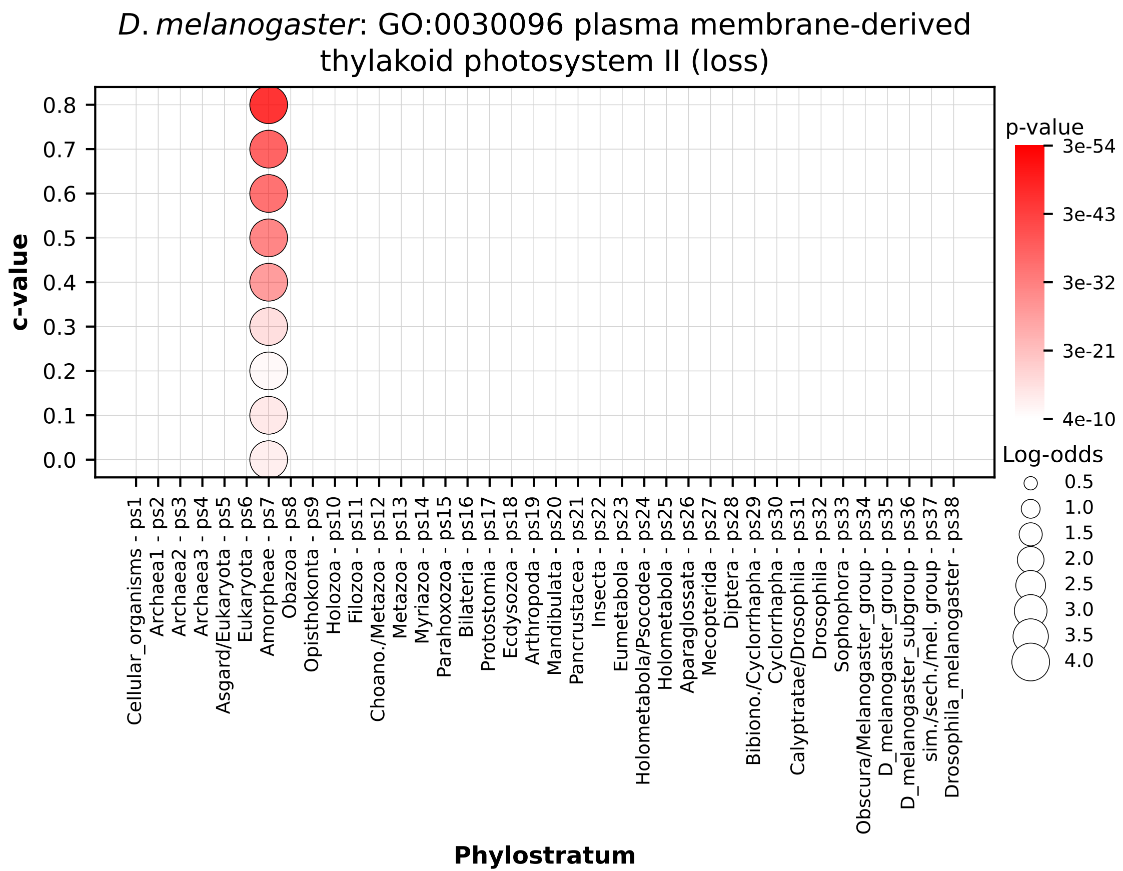

D. melanogaster: GO:0030096 plasma membrane-derived thylakoid photosystem II (loss)
c-value
0.8
0.7
0.6
0.5
0.4
0.3
0.2
0.1
0.0
p-value
3e-54
3e-43
3e-32
3e-21
4e-10
Log-odds
0.5
1.0
1.5
2.0
2.5
3.0
3.5
4.0
Cellular_organisms - ps1
Archaea1 - ps2
Archaea2 - ps3
Archaea3 - ps4
Asgard/Eukaryota - ps5
Eukaryota - ps6
Amorpheae - ps7
Obazoa - ps8
Opisthokonta - ps9
Holozoa - ps10
Filozoa - ps11
Choano./Metazoa - ps12
Metazoa - ps13
Myriazoa - ps14
Parahoxozoa - ps15
Bilateria - ps16
Protostomia - ps17
Ecdysozoa - ps18
Arthropoda - ps19
Mandibulata - ps20
Pancrustacea - ps21
Insecta - ps22
Eumetabola - ps23
Holometabola/Psocodea - ps24
Holometabola - ps25
Aparaglossata - ps26
Mecopterida - ps27
Diptera - ps28
Bibiono./Cyclorrhapha - ps29
Cyclorrhapha - ps30
Calyptratae/Drosophila - ps31
Drosophila - ps32
Sophophora - ps33
Obscura/Melanogaster_group - ps34
D_melanogaster_group - ps35
D_melanogaster_subgroup - ps36
sim./sech./mel. group - ps37
Drosophila_melanogaster - ps38
Phylostratum

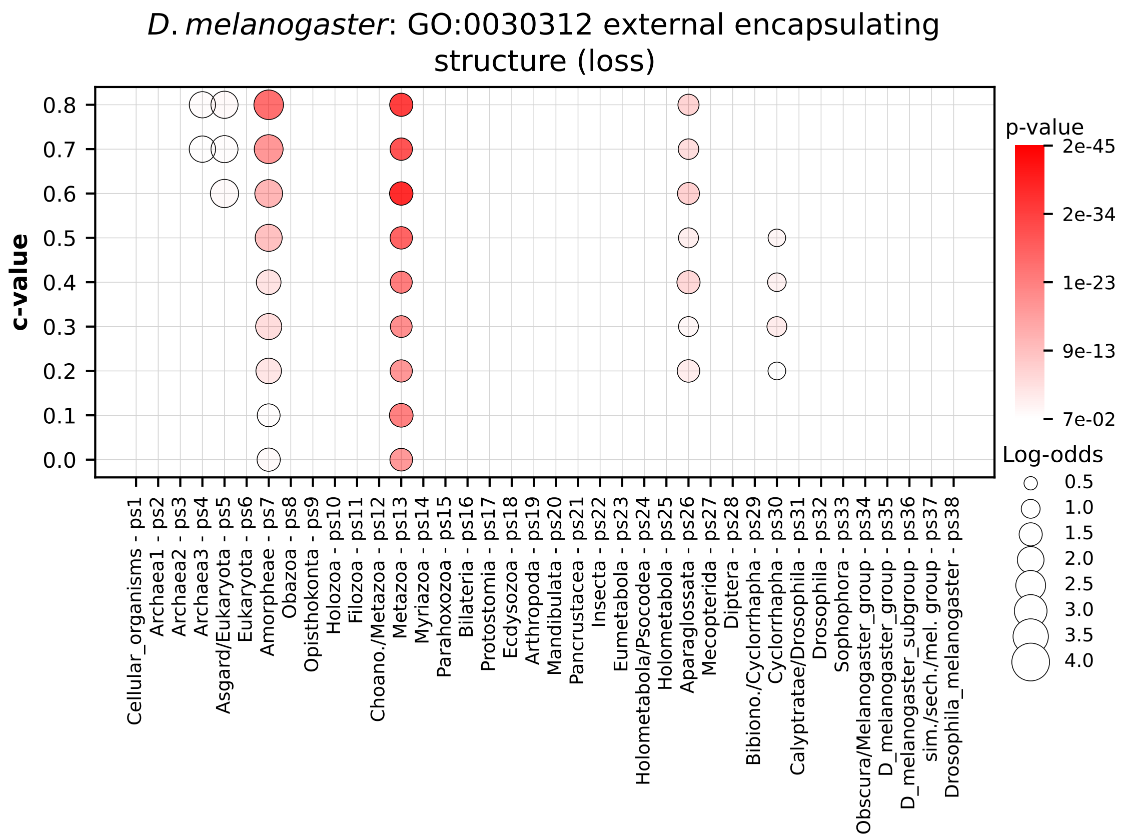
D. melanogaster: GO:0030312 external encapsulating structure (loss)
c-value
Phylostratum
p-value
2e-45
2e-34
1e-23
9e-13
7e-02
Log-odds
0.5
1.0
1.5
2.0
2.5
3.0
3.5
4.0
Cellular_organisms - ps1
Archaea1 - ps2
Archaea2 - ps3
Archaea3 - ps4
Asgard/Eukaryota - ps5
Eukaryota - ps6
Amorpheae - ps7
Obazoa - ps8
Opisthokonta - ps9
Holozoa - ps10
Filozoa - ps11
Choano./Metazoa - ps12
Metazoa - ps13
Myriazoa - ps14
Parahoxozoa - ps15
Bilateria - ps16
Protostomia - ps17
Ecdysozoa - ps18
Arthropoda - ps19
Mandibulata - ps20
Pancrustacea - ps21
Insecta - ps22
Eumetabola - ps23
Holometabola/Psocodea - ps24
Holometabola - ps25
Aparaglossata - ps26
Mecopterida - ps27
Diptera - ps28
Bibiono./Cyclorrhapha - ps29
Cyclorrhapha - ps30
Calyptratae/Drosophila - ps31
Drosophila - ps32
Sophophora - ps33
Obscura/Melanogaster_group - ps34
D_melanogaster_group - ps35
D_melanogaster_subgroup - ps36
sim./sech./mel. group - ps37
Drosophila_melanogaster - ps38

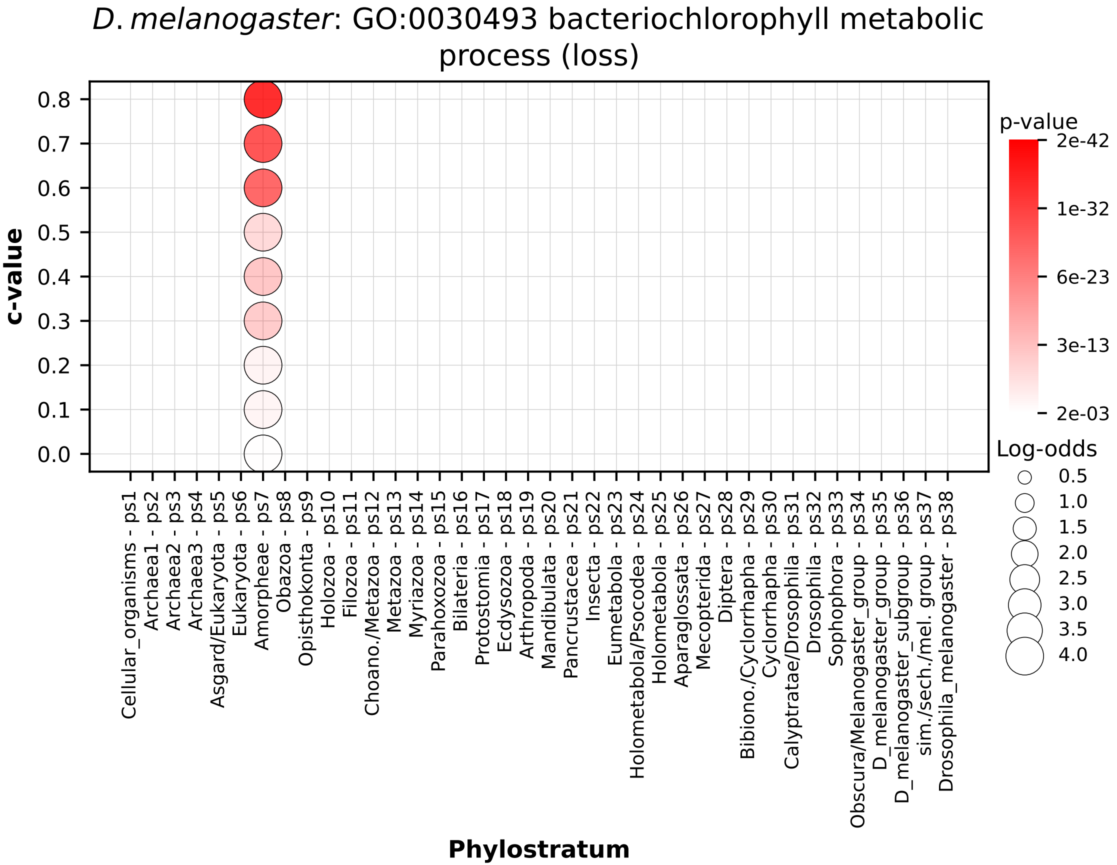
D. melanogaster: GO:0030493 bacteriochlorophyll metabolic process (loss)
c-value
0.8
0.7
0.6
0.5
0.4
0.3
0.2
0.1
0.0
p-value
2e-42
1e-32
6e-23
3e-13
2e-03
Log-odds
0.5
1.0
1.5
2.0
2.5
3.0
3.5
4.0
Cellular_organisms - ps1
Archaea1 - ps2
Archaea2 - ps3
Archaea3 - ps4
Asgard/Eukaryota - ps5
Eukaryota - ps6
Amorpheae - ps7
Obazoa - ps8
Opisthokonta - ps9
Holozoa - ps10
Filozoa - ps11
Choano./Metazoa - ps12
Metazoa - ps13
Myriazoa - ps14
Parahoxozoa - ps15
Bilateria - ps16
Protostomia - ps17
Ecdysozoa - ps18
Arthropoda - ps19
Mandibulata - ps20
Pancrustacea - ps21
Insecta - ps22
Eumetabola - ps23
Holometabola/Psocodea - ps24
Holometabola - ps25
Aparaglossata - ps26
Mecopterida - ps27
Diptera - ps28
Bibiono./Cyclorrhapha - ps29
Cyclorrhapha - ps30
Calyptratae/Drosophila - ps31
Drosophila - ps32
Sophophora - ps33
Obscura/Melanogaster_group - ps34
D_melanogaster_group - ps35
D_melanogaster_subgroup - ps36
sim./sech./mel. group - ps37
Drosophila_melanogaster - ps38
Phylostratum

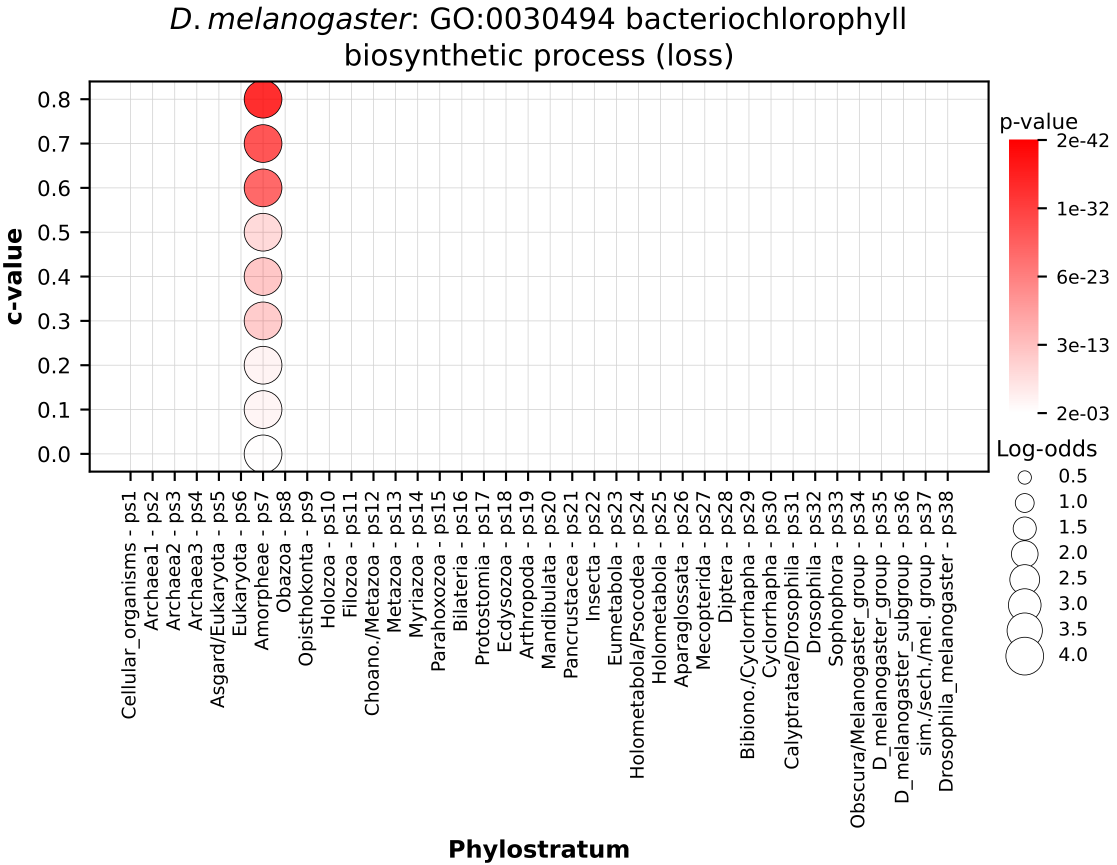

D. melanogaster: GO:0030494 bacteriochlorophyll biosynthetic process (loss)
c-value
0.8
0.7
0.6
0.5
0.4
0.3
0.2
0.1
0.0
p-value
2e-42
1e-32
6e-23
3e-13
2e-03
Log-odds
0.5
1.0
1.5
2.0
2.5
3.0
3.5
4.0
Cellular_organisms - ps1
Archaea1 - ps2
Archaea2 - ps3
Archaea3 - ps4
Asgard/Eukaryota - ps5
Eukaryota - ps6
Amorpheae - ps7
Obazoa - ps8
Opisthokonta - ps9
Holozoa - ps10
Filozoa - ps11
Choano./Metazoa - ps12
Metazoa - ps13
Myriazoa - ps14
Parahoxozoa - ps15
Bilateria - ps16
Protostomia - ps17
Ecdysozoa - ps18
Arthropoda - ps19
Mandibulata - ps20
Pancrustacea - ps21
Insecta - ps22
Eumetabola - ps23
Holometabola/Psocodea - ps24
Holometabola - ps25
Aparaglossata - ps26
Mecopterida - ps27
Diptera - ps28
Bibiono./Cyclorrhapha - ps29
Cyclorrhapha - ps30
Calyptratae/Drosophila - ps31
Drosophila - ps32
Sophophora - ps33
Obscura/Melanogaster_group - ps34
D_melanogaster_group - ps35
D_melanogaster_subgroup - ps36
sim./sech./mel. group - ps37
Drosophila_melanogaster - ps38
Phylostratum

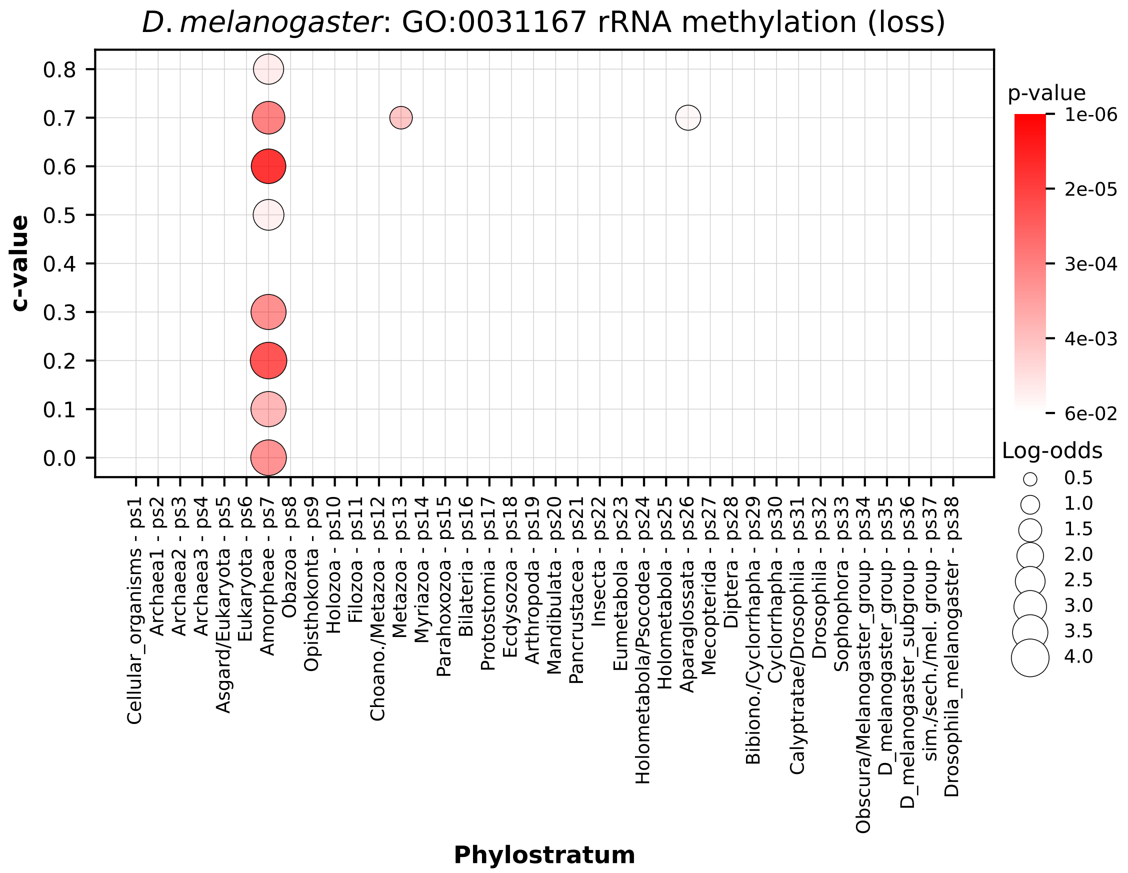
D. melanogaster: GO:0031167 rRNA methylation (loss)
c-value
0.0
0.1
0.2
0.3
0.4
0.5
0.6
0.7
0.8
Phylostratum
Cellular_organisms - ps1
Archaea1 - ps2
Archaea2 - ps3
Archaea3 - ps4
Asgard/Eukaryota - ps5
Eukaryota - ps6
Amorpheae - ps7
Obazoa - ps8
Opisthokonta - ps9
Holozoa - ps10
Filozoa - ps11
Choano./Metazoa - ps12
Metazoa - ps13
Myriazoa - ps14
Parahoxozoa - ps15
Bilateria - ps16
Protostomia - ps17
Ecdysozoa - ps18
Arthropoda - ps19
Mandibulata - ps20
Pancrustacea - ps21
Insecta - ps22
Eumetabola - ps23
Holometabola/Psocodea - ps24
Holometabola - ps25
Aparaglossata - ps26
Mecopterida - ps27
Diptera - ps28
Bibiono./Cyclorrhapha - ps29
Cyclorrhapha - ps30
Calyptratae/Drosophila - ps31
Drosophila - ps32
Sophophora - ps33
Obscura/Melanogaster_group - ps34
D_melanogaster_group - ps35
D_melanogaster_subgroup - ps36
sim./sech./mel. group - ps37
Drosophila_melanogaster - ps38
p-value
1e-06
2e-05
3e-04
4e-03
6e-02
Log-odds
0.5
1.0
1.5
2.0
2.5
3.0
3.5
4.0

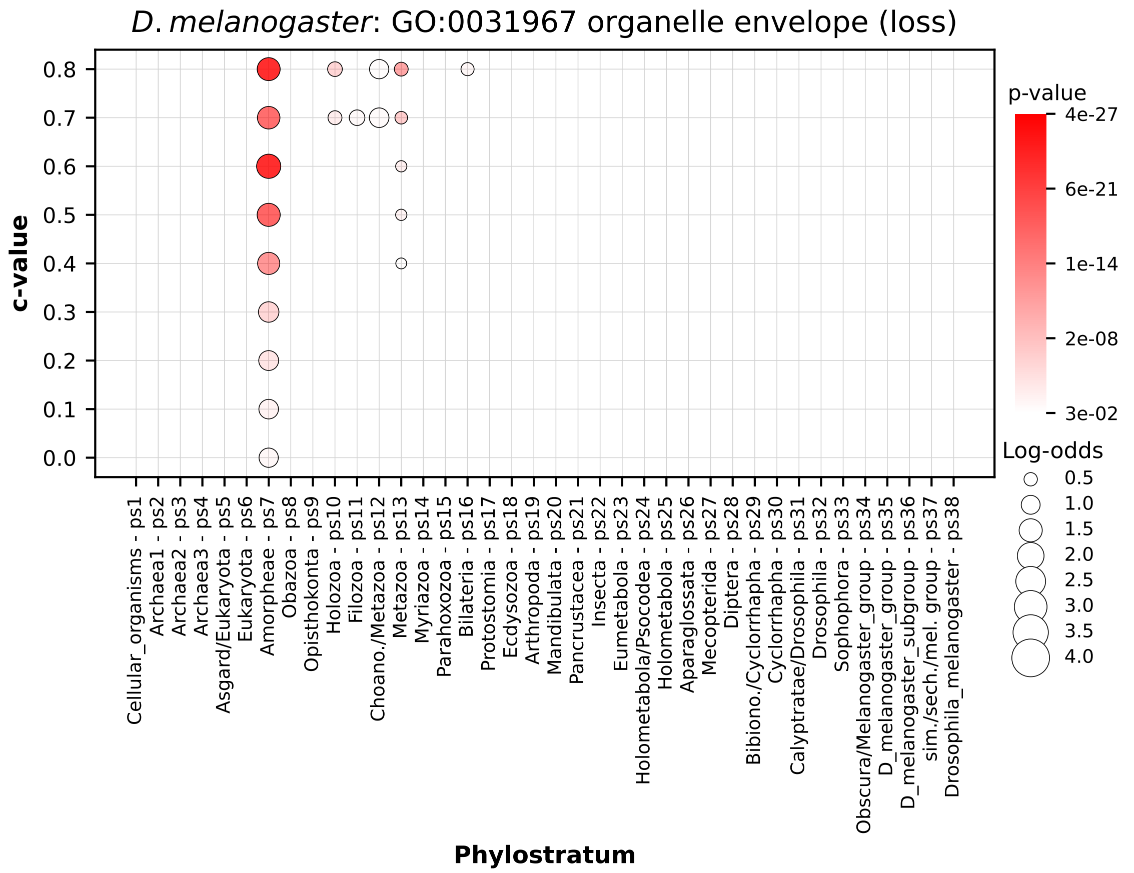
D. melanogaster: GO:0031967 organelle envelope (loss)
c-value
0.0
0.1
0.2
0.3
0.4
0.5
0.6
0.7
0.8
Phylostratum
Cellular_organisms - ps1
Archaea1 - ps2
Archaea2 - ps3
Archaea3 - ps4
Asgard/Eukaryota - ps5
Eukaryota - ps6
Amorpheae - ps7
Obazoa - ps8
Opisthokonta - ps9
Holozoa - ps10
Filozoa - ps11
Choano./Metazoa - ps12
Metazoa - ps13
Myriazoa - ps14
Parahoxozoa - ps15
Bilateria - ps16
Protostomia - ps17
Ecdysozoa - ps18
Arthropoda - ps19
Mandibulata - ps20
Pancrustacea - ps21
Insecta - ps22
Eumetabola - ps23
Holometabola/Psocodea - ps24
Holometabola - ps25
Aparaglossata - ps26
Mecopterida - ps27
Diptera - ps28
Bibiono./Cyclorrhapha - ps29
Cyclorrhapha - ps30
Calyptratae/Drosophila - ps31
Drosophila - ps32
Sophophora - ps33
Obscura/Melanogaster_group - ps34
D_melanogaster_group - ps35
D_melanogaster_subgroup - ps36
sim./sech./mel. group - ps37
Drosophila_melanogaster - ps38
p-value
4e-27
6e-21
1e-14
2e-08
3e-02
Log-odds
0.5
1.0
1.5
2.0
2.5
3.0
3.5
4.0

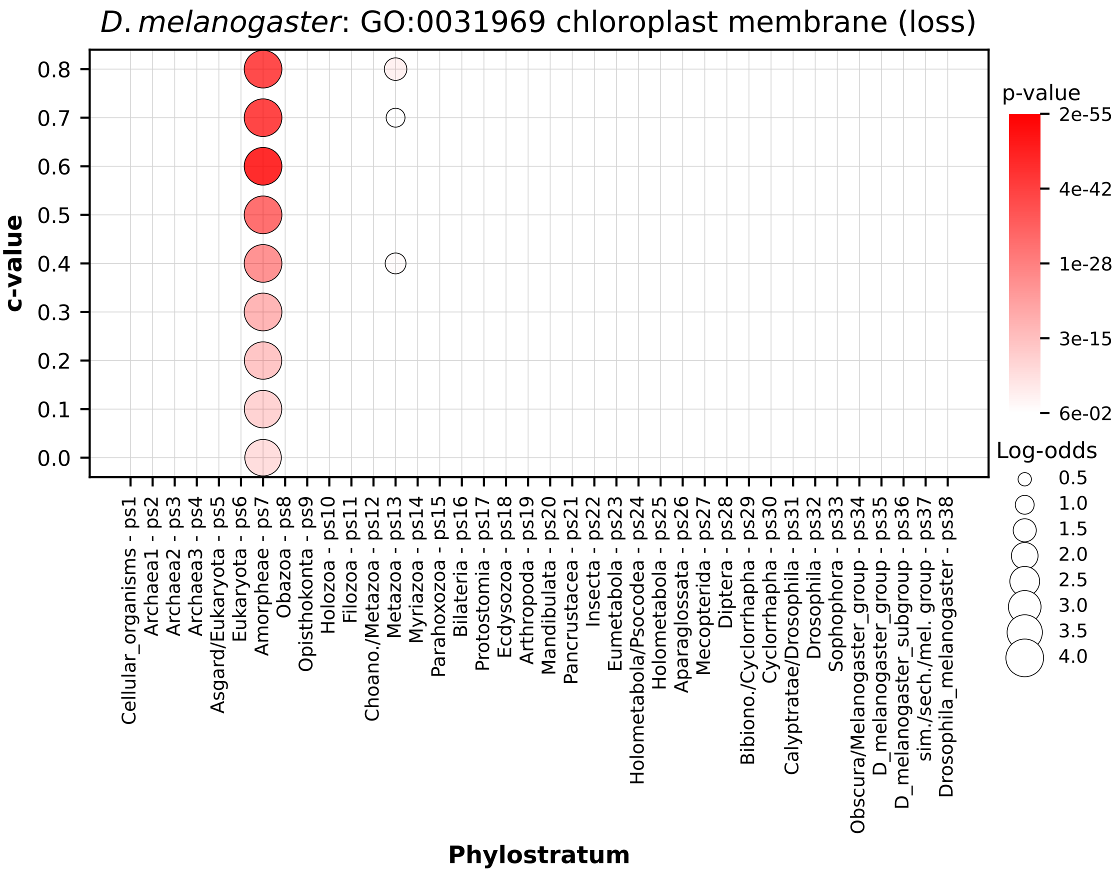

D. melanogaster: GO:0031969 chloroplast membrane (loss)
c-value
0.8
0.7
0.6
0.5
0.4
0.3
0.2
0.1
0.0
p-value
2e-55
4e-42
1e-28
3e-15
6e-02
Log-odds
0.5
1.0
1.5
2.0
2.5
3.0
3.5
4.0
Cellular_organisms - ps1
Archaea1 - ps2
Archaea2 - ps3
Archaea3 - ps4
Asgard/Eukaryota - ps5
Eukaryota - ps6
Amorpheae - ps7
Obazoa - ps8
Opisthokonta - ps9
Holozoa - ps10
Filozoa - ps11
Choano./Metazoa - ps12
Metazoa - ps13
Myriazoa - ps14
Parahoxozoa - ps15
Bilateria - ps16
Protostomia - ps17
Ecdysozoa - ps18
Arthropoda - ps19
Mandibulata - ps20
Pancrustacea - ps21
Insecta - ps22
Eumetabola - ps23
Holometabola/Psocodea - ps24
Holometabola - ps25
Aparaglossata - ps26
Mecopterida - ps27
Diptera - ps28
Bibiono./Cyclorrhapha - ps29
Cyclorrhapha - ps30
Calyptratae/Drosophila - ps31
Drosophila - ps32
Sophophora - ps33
Obscura/Melanogaster_group - ps34
D_melanogaster_group - ps35
D_melanogaster_subgroup - ps36
sim./sech./mel. group - ps37
Drosophila_melanogaster - ps38
Phylostratum

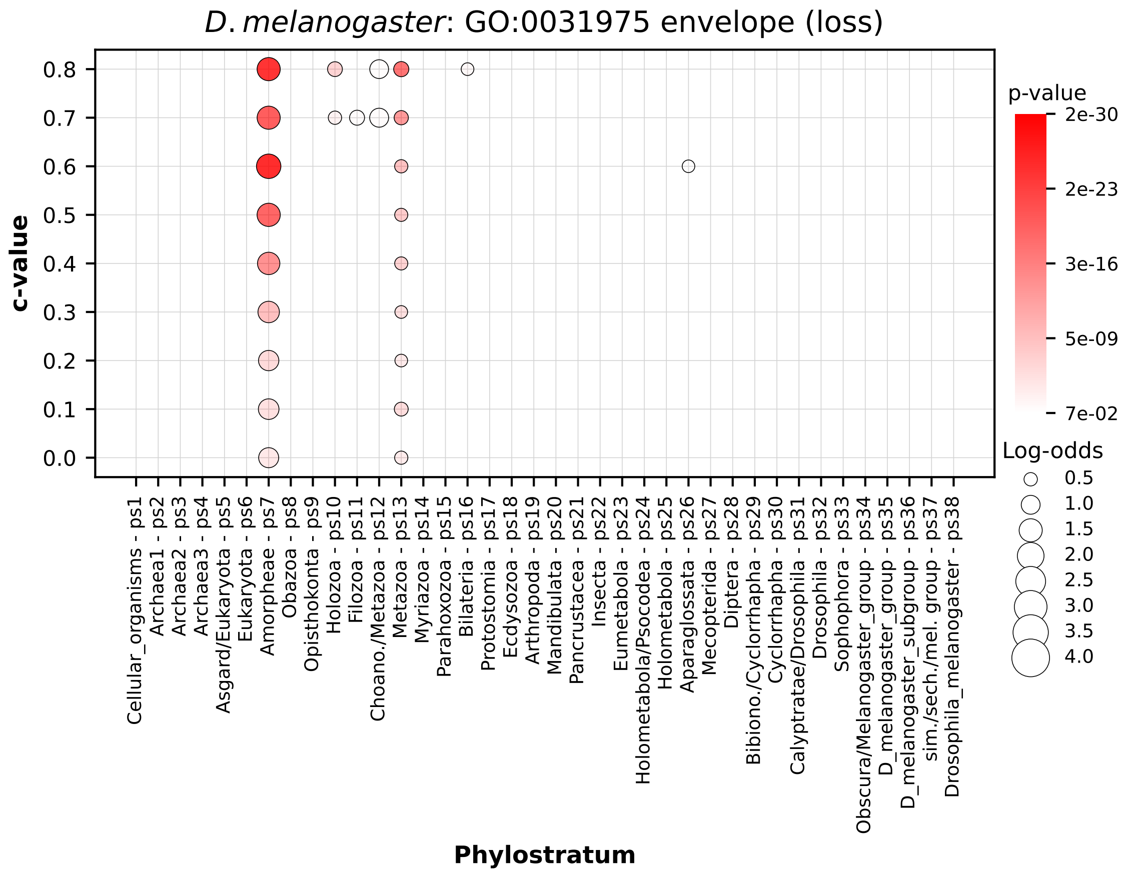

D. melanogaster: GO:0031975 envelope (loss)
c-value
0.8
0.7
0.6
0.5
0.4
0.3
0.2
0.1
0.0
Phylostratum
Cellular_organisms - ps1
Archaea1 - ps2
Archaea2 - ps3
Archaea3 - ps4
Asgard/Eukaryota - ps5
Eukaryota - ps6
Amorpheae - ps7
Obazoa - ps8
Opisthokonta - ps9
Holozoa - ps10
Filozoa - ps11
Choano./Metazoa - ps12
Metazoa - ps13
Myriazoa - ps14
Parahoxozoa - ps15
Bilateria - ps16
Protostomia - ps17
Ecdysozoa - ps18
Arthropoda - ps19
Mandibulata - ps20
Pancrustacea - ps21
Insecta - ps22
Eumetabola - ps23
Holometabola/Psocodea - ps24
Holometabola - ps25
Aparaglossata - ps26
Mecopterida - ps27
Diptera - ps28
Bibiono./Cyclorrhapha - ps29
Cyclorrhapha - ps30
Calyptratae/Drosophila - ps31
Drosophila - ps32
Sophophora - ps33
Obscura/Melanogaster_group - ps34
D_melanogaster_group - ps35
D_melanogaster_subgroup - ps36
sim./sech./mel. group - ps37
Drosophila_melanogaster - ps38
p-value
2e-30
2e-23
3e-16
5e-09
7e-02
Log-odds
0.5
1.0
1.5
2.0
2.5
3.0
3.5
4.0

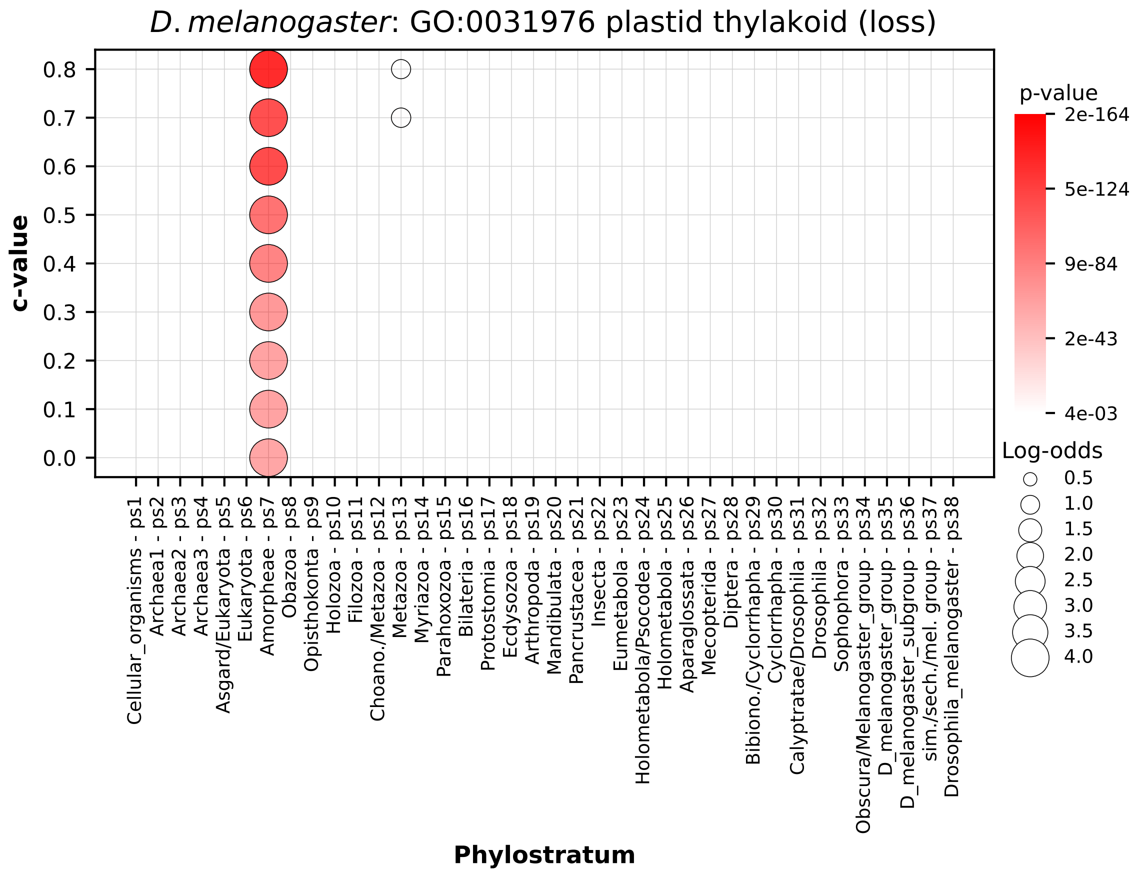
D. melanogaster: GO:0031976 plastid thylakoid (loss)
c-value
0.8
0.7
0.6
0.5
0.4
0.3
0.2
0.1
0.0
Phylostratum
Cellular_organisms - ps1
Archaea1 - ps2
Archaea2 - ps3
Archaea3 - ps4
Asgard/Eukaryota - ps5
Eukaryota - ps6
Amorpheae - ps7
Obazoa - ps8
Opisthokonta - ps9
Holozoa - ps10
Filozoa - ps11
Choano./Metazoa - ps12
Metazoa - ps13
Myriazoa - ps14
Parahoxozoa - ps15
Bilateria - ps16
Protostomia - ps17
Ecdysozoa - ps18
Arthropoda - ps19
Mandibulata - ps20
Pancrustacea - ps21
Insecta - ps22
Eumetabola - ps23
Holometabola/Psocodea - ps24
Holometabola - ps25
Aparaglossata - ps26
Mecopterida - ps27
Diptera - ps28
Bibiono./Cyclorrhapha - ps29
Cyclorrhapha - ps30
Calyptratae/Drosophila - ps31
Drosophila - ps32
Sophophora - ps33
Obscura/Melanogaster_group - ps34
D_melanogaster_group - ps35
D_melanogaster_subgroup - ps36
sim./sech./mel. group - ps37
Drosophila_melanogaster - ps38
p-value
2e-164
5e-124
9e-84
2e-43
4e-03
Log-odds
0.5
1.0
1.5
2.0
2.5
3.0
3.5
4.0

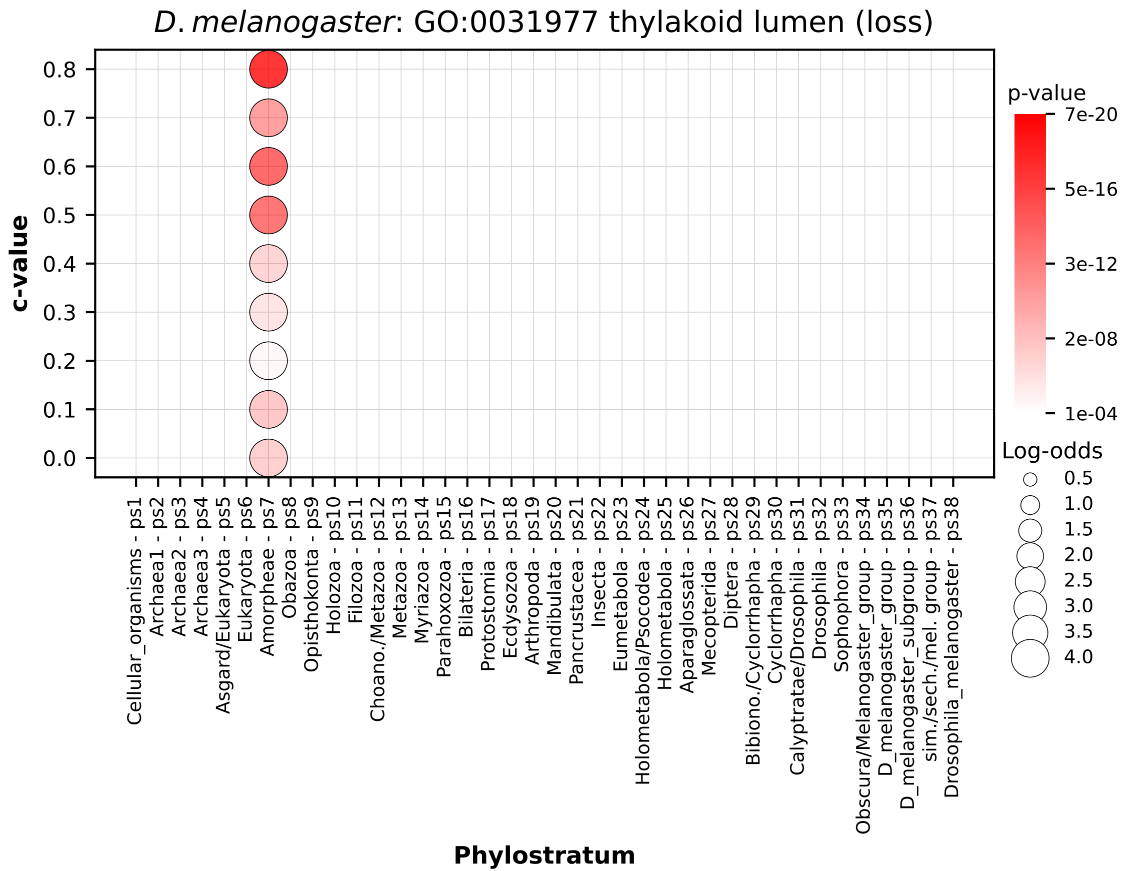
D. melanogaster: GO:0031977 thylakoid lumen (loss)
c-value
0.8
0.7
0.6
0.5
0.4
0.3
0.2
0.1
0.0
p-value
7e-20
5e-16
3e-12
2e-08
1e-04
Log-odds
0.5
1.0
1.5
2.0
2.5
3.0
3.5
4.0
Cellular_organisms - ps1
Archaea1 - ps2
Archaea2 - ps3
Archaea3 - ps4
Asgard/Eukaryota - ps5
Eukaryota - ps6
Amorpheae - ps7
Obazoa - ps8
Opisthokonta - ps9
Holozoa - ps10
Filozoa - ps11
Choano./Metazoa - ps12
Metazoa - ps13
Myriazoa - ps14
Parahoxozoa - ps15
Bilateria - ps16
Protostomia - ps17
Ecdysozoa - ps18
Arthropoda - ps19
Mandibulata - ps20
Pancrustacea - ps21
Insecta - ps22
Eumetabola - ps23
Holometabola/Psocodea - ps24
Holometabola - ps25
Aparaglossata - ps26
Mecopterida - ps27
Diptera - ps28
Bibiono./Cyclorrhapha - ps29
Cyclorrhapha - ps30
Calyptratae/Drosophila - ps31
Drosophila - ps32
Sophophora - ps33
Obscura/Melanogaster_group - ps34
D_melanogaster_group - ps35
D_melanogaster_subgroup - ps36
sim./sech./mel. group - ps37
Drosophila_melanogaster - ps38
Phylostratum

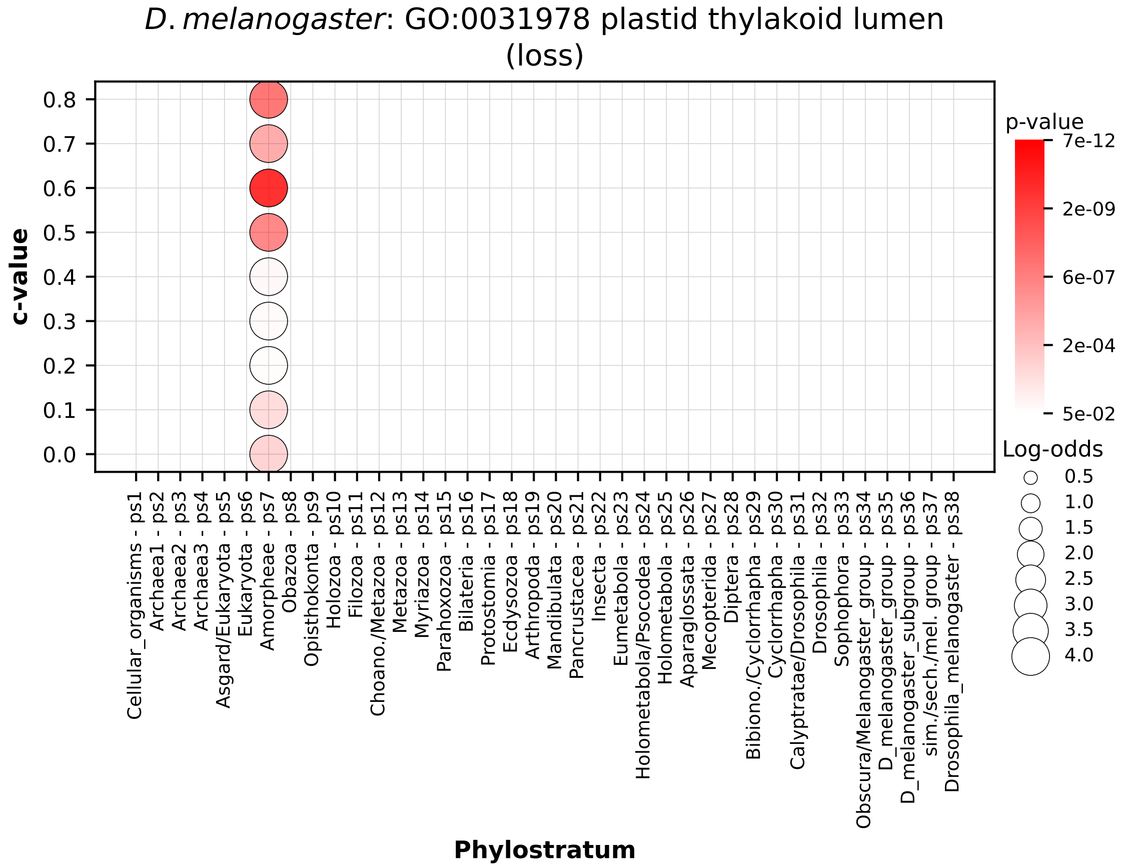

D. melanogaster: GO:0031978 plastid thylakoid lumen
(loss)
c-value
0.8
0.7
0.6
0.5
0.4
0.3
0.2
0.1
0.0
p-value
7e-12
2e-09
6e-07
2e-04
5e-02
Log-odds
0.5
1.0
1.5
2.0
2.5
3.0
3.5
4.0
Cellular_organisms - ps1
Archaea1 - ps2
Archaea2 - ps3
Archaea3 - ps4
Asgard/Eukaryota - ps5
Eukaryota - ps6
Amorpheae - ps7
Obazoa - ps8
Opisthokonta - ps9
Holozoa - ps10
Filozoa - ps11
Choano./Metazoa - ps12
Metazoa - ps13
Myriazoa - ps14
Parahoxozoa - ps15
Bilateria - ps16
Protostomia - ps17
Ecdysozoa - ps18
Arthropoda - ps19
Mandibulata - ps20
Pancrustacea - ps21
Insecta - ps22
Eumetabola - ps23
Holometabola/Psocodea - ps24
Holometabola - ps25
Aparaglossata - ps26
Mecopterida - ps27
Diptera - ps28
Bibiono./Cyclorrhapha - ps29
Cyclorrhapha - ps30
Calyptratae/Drosophila - ps31
Drosophila - ps32
Sophophora - ps33
Obscura/Melanogaster_group - ps34
D_melanogaster_group - ps35
D_melanogaster_subgroup - ps36
sim./sech./mel. group - ps37
Drosophila_melanogaster - ps38
Phylostratum

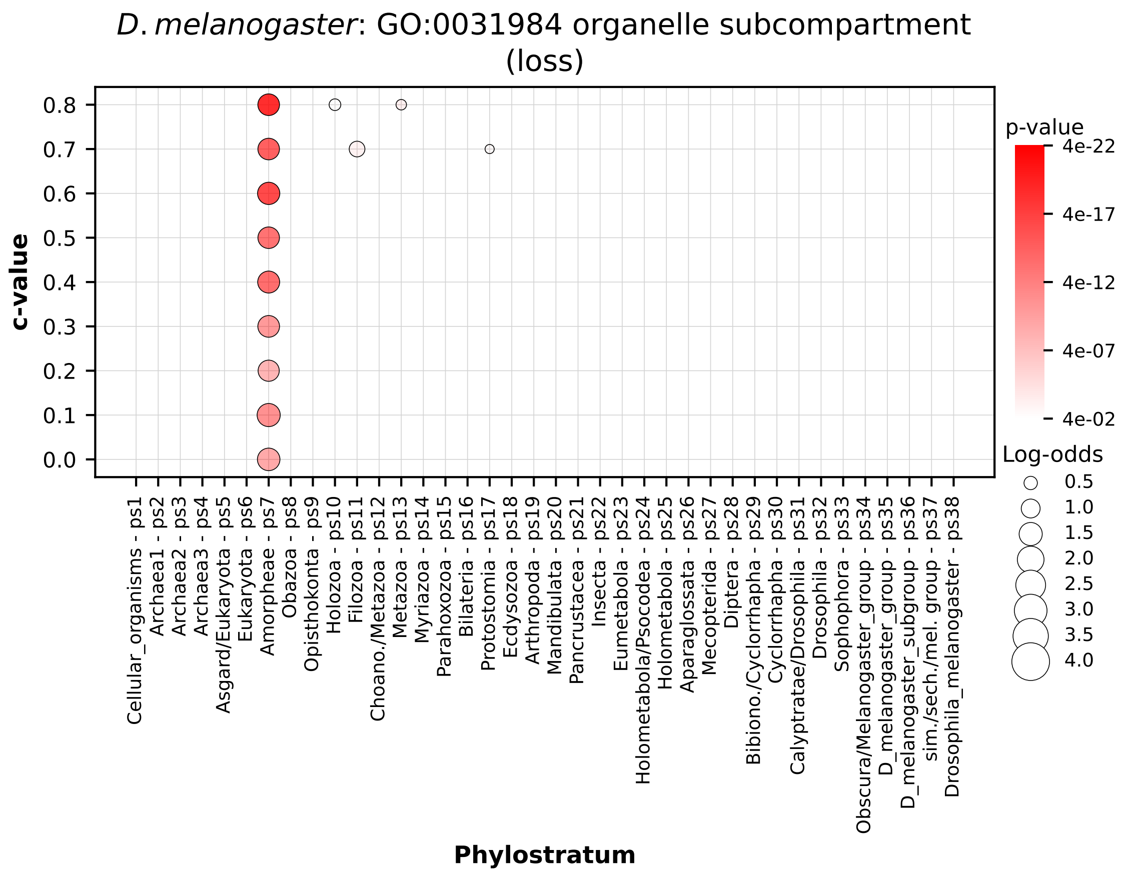

D. melanogaster: GO:0031984 organelle subcompartment (loss)
c-value
0.8
0.7
0.6
0.5
0.4
0.3
0.2
0.1
0.0
p-value
4e-22
4e-17
4e-12
4e-07
4e-02
Log-odds
0.5
1.0
1.5
2.0
2.5
3.0
3.5
4.0
Cellular_organisms - ps1
Archaea1 - ps2
Archaea2 - ps3
Archaea3 - ps4
Asgard/Eukaryota - ps5
Eukaryota - ps6
Amorpheae - ps7
Obazoa - ps8
Opisthokonta - ps9
Holozoa - ps10
Filozoa - ps11
Choano./Metazoa - ps12
Metazoa - ps13
Myriazoa - ps14
Parahoxozoa - ps15
Bilateria - ps16
Protostomia - ps17
Ecdysozoa - ps18
Arthropoda - ps19
Mandibulata - ps20
Pancrustacea - ps21
Insecta - ps22
Eumetabola - ps23
Holometabola/Psocodea - ps24
Holometabola - ps25
Aparaglossata - ps26
Mecopterida - ps27
Diptera - ps28
Bibiono./Cyclorrhapha - ps29
Cyclorrhapha - ps30
Calyptratae/Drosophila - ps31
Drosophila - ps32
Sophophora - ps33
Obscura/Melanogaster_group - ps34
D_melanogaster_group - ps35
D_melanogaster_subgroup - ps36
sim./sech./mel. group - ps37
Drosophila_melanogaster - ps38
Phylostratum

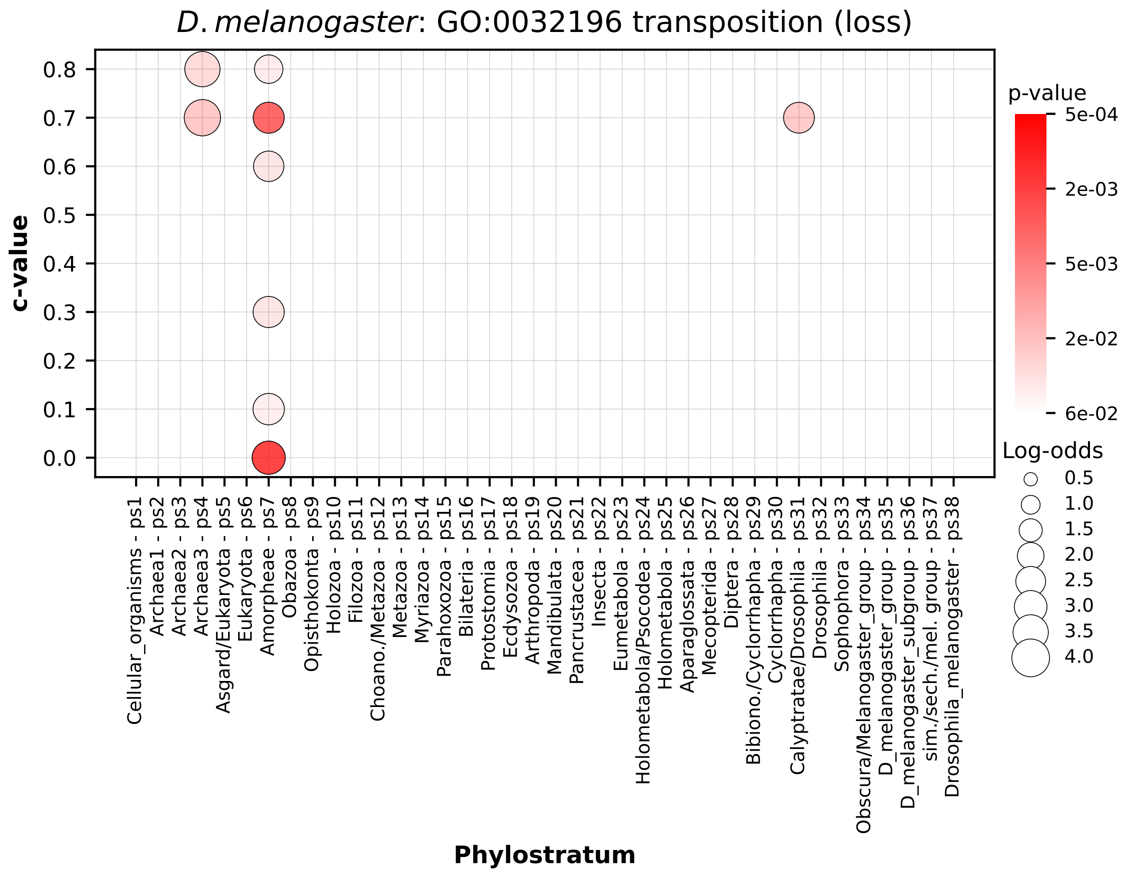

D. melanogaster: GO:0032196 transposition (loss)
c-value
0.0
0.1
0.2
0.3
0.4
0.5
0.6
0.7
0.8
Phylostratum
Cellular_organisms - ps1
Archaea1 - ps2
Archaea2 - ps3
Archaea3 - ps4
Asgard/Eukaryota - ps5
Eukaryota - ps6
Amorpheae - ps7
Obazoa - ps8
Opisthokonta - ps9
Holozoa - ps10
Filozoa - ps11
Choano./Metazoa - ps12
Metazoa - ps13
Myriazoa - ps14
Parahoxozoa - ps15
Bilateria - ps16
Protostomia - ps17
Ecdysozoa - ps18
Arthropoda - ps19
Mandibulata - ps20
Pancrustacea - ps21
Insecta - ps22
Eumetabola - ps23
Holometabola/Psocodea - ps24
Holometabola - ps25
Aparaglossata - ps26
Mecopterida - ps27
Diptera - ps28
Bibiono./Cyclorrhapha - ps29
Cyclorrhapha - ps30
Calyptratae/Drosophila - ps31
Drosophila - ps32
Sophophora - ps33
Obscura/Melanogaster_group - ps34
D_melanogaster_group - ps35
D_melanogaster_subgroup - ps36
sim./sech./mel. group - ps37
Drosophila_melanogaster - ps38
p-value
5e-04
2e-03
5e-03
2e-02
6e-02
Log-odds
0.5
1.0
1.5
2.0
2.5
3.0
3.5
4.0

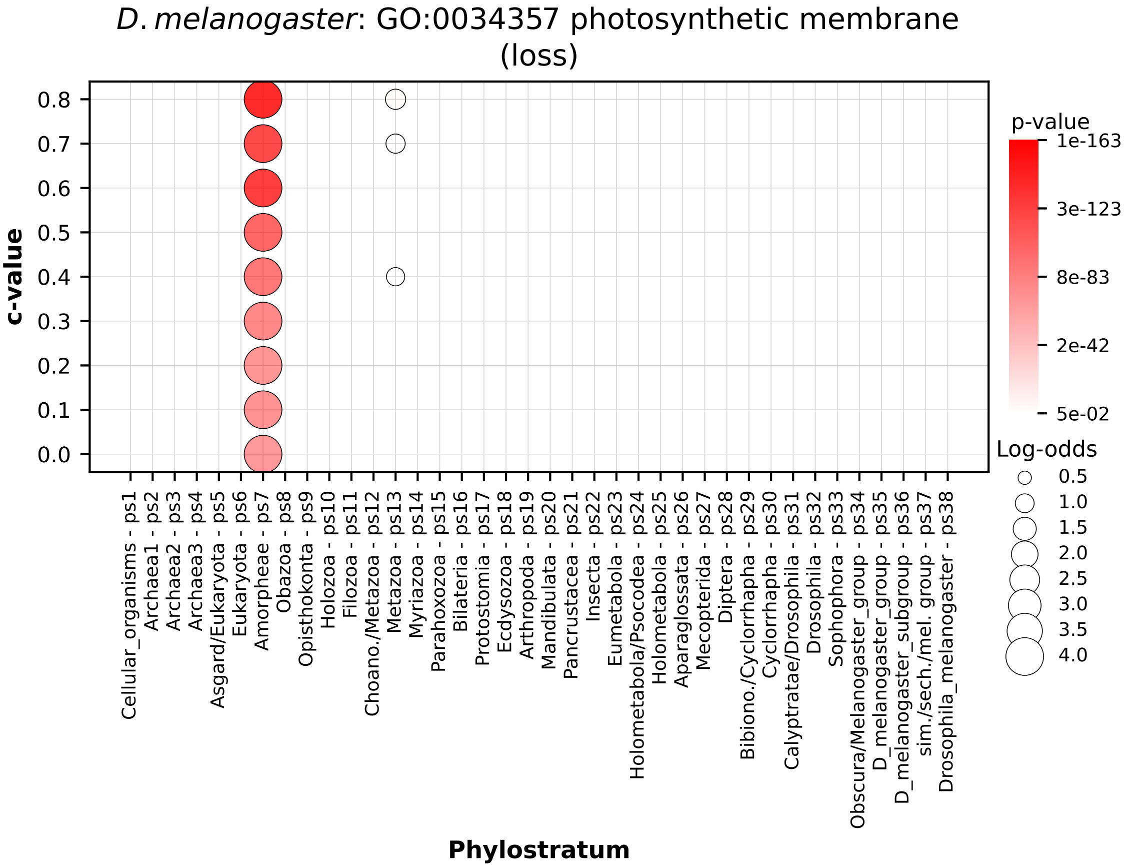
D. melanogaster: GO:0034357 photosynthetic membrane (loss)
c-value
0.8
0.7
0.6
0.5
0.4
0.3
0.2
0.1
0.0
p-value
1e-163
3e-123
8e-83
2e-42
5e-02
Log-odds
0.5
1.0
1.5
2.0
2.5
3.0
3.5
4.0
Cellular_organisms - ps1
Archaea1 - ps2
Archaea2 - ps3
Archaea3 - ps4
Asgard/Eukaryota - ps5
Eukaryota - ps6
Amorpheae - ps7
Obazoa - ps8
Opisthokonta - ps9
Holozoa - ps10
Filozoa - ps11
Choano./Metazoa - ps12
Metazoa - ps13
Myriazoa - ps14
Parahoxozoa - ps15
Bilateria - ps16
Protostomia - ps17
Ecdysozoa - ps18
Arthropoda - ps19
Mandibulata - ps20
Pancrustacea - ps21
Insecta - ps22
Eumetabola - ps23
Holometabola/Psocodea - ps24
Holometabola - ps25
Aparaglossata - ps26
Mecopterida - ps27
Diptera - ps28
Bibiono./Cyclorrhapha - ps29
Cyclorrhapha - ps30
Calyptratae/Drosophila - ps31
Drosophila - ps32
Sophophora - ps33
Obscura/Melanogaster_group - ps34
D_melanogaster_group - ps35
D_melanogaster_subgroup - ps36
sim./sech./mel. group - ps37
Drosophila_melanogaster - ps38
Phylostratum

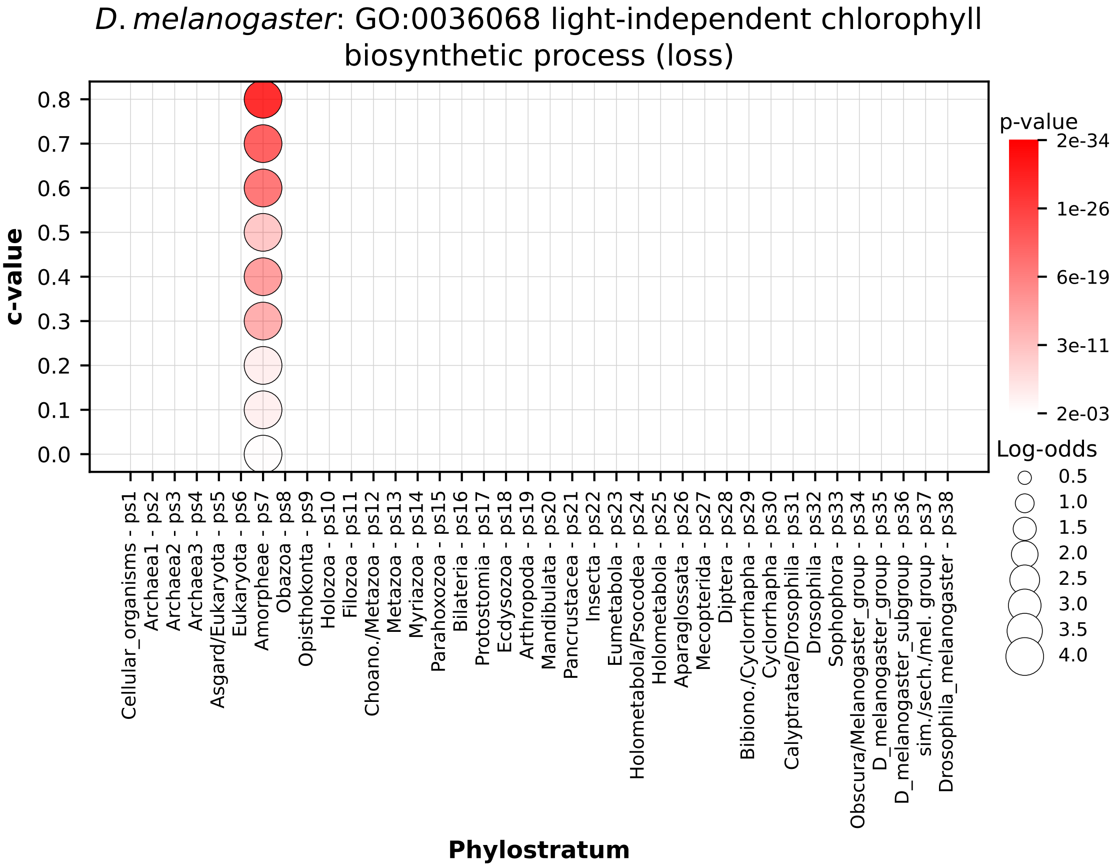

D. melanogaster: GO:0036068 light-independent chlorophyll biosynthetic process (loss)
c-value
0.8
0.7
0.6
0.5
0.4
0.3
0.2
0.1
0.0
p-value
2e-34
1e-26
6e-19
3e-11
2e-03
Log-odds
0.5
1.0
1.5
2.0
2.5
3.0
3.5
4.0
Cellular_organisms - ps1
Archaea1 - ps2
Archaea2 - ps3
Archaea3 - ps4
Asgard/Eukaryota - ps5
Eukaryota - ps6
Amorpheae - ps7
Obazoa - ps8
Opisthokonta - ps9
Holozoa - ps10
Filozoa - ps11
Choano./Metazoa - ps12
Metazoa - ps13
Myriazoa - ps14
Parahoxozoa - ps15
Bilateria - ps16
Protostomia - ps17
Ecdysozoa - ps18
Arthropoda - ps19
Mandibulata - ps20
Pancrustacea - ps21
Insecta - ps22
Eumetabola - ps23
Holometabola/Psocodea - ps24
Holometabola - ps25
Aparaglossata - ps26
Mecopterida - ps27
Diptera - ps28
Bibiono./Cyclorrhapha - ps29
Cyclorrhapha - ps30
Calyptratae/Drosophila - ps31
Drosophila - ps32
Sophophora - ps33
Obscura/Melanogaster_group - ps34
D_melanogaster_group - ps35
D_melanogaster_subgroup - ps36
sim./sech./mel. group - ps37
Drosophila_melanogaster - ps38
Phylostratum

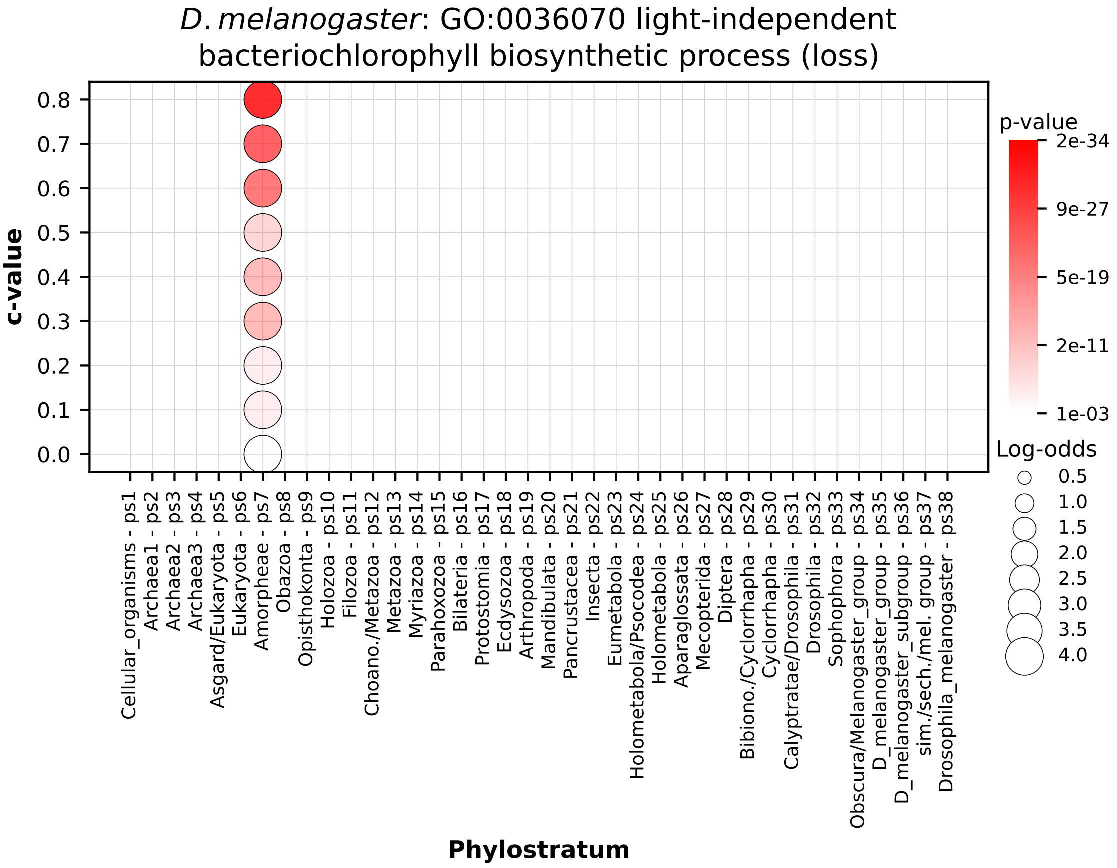
D. melanogaster: GO:0036070 light-independent bacteriochlorophyll biosynthetic process (loss)
c-value
0.8
0.7
0.6
0.5
0.4
0.3
0.2
0.1
0.0
p-value
2e-34
9e-27
5e-19
2e-11
1e-03
Log-odds
0.5
1.0
1.5
2.0
2.5
3.0
3.5
4.0
Cellular_organisms - ps1
Archaea1 - ps2
Archaea2 - ps3
Archaea3 - ps4
Asgard/Eukaryota - ps5
Eukaryota - ps6
Amorpheae - ps7
Obazoa - ps8
Opisthokonta - ps9
Holozoa - ps10
Filozoa - ps11
Choano./Metazoa - ps12
Metazoa - ps13
Myriazoa - ps14
Parahoxozoa - ps15
Bilateria - ps16
Protostomia - ps17
Ecdysozoa - ps18
Arthropoda - ps19
Mandibulata - ps20
Pancrustacea - ps21
Insecta - ps22
Eumetabola - ps23
Holometabola/Psocodea - ps24
Holometabola - ps25
Aparaglossata - ps26
Mecopterida - ps27
Diptera - ps28
Bibiono./Cyclorrhapha - ps29
Cyclorrhapha - ps30
Calyptratae/Drosophila - ps31
Drosophila - ps32
Sophophora - ps33
Obscura/Melanogaster_group - ps34
D_melanogaster_group - ps35
D_melanogaster_subgroup - ps36
sim./sech./mel. group - ps37
Drosophila_melanogaster - ps38
Phylostratum

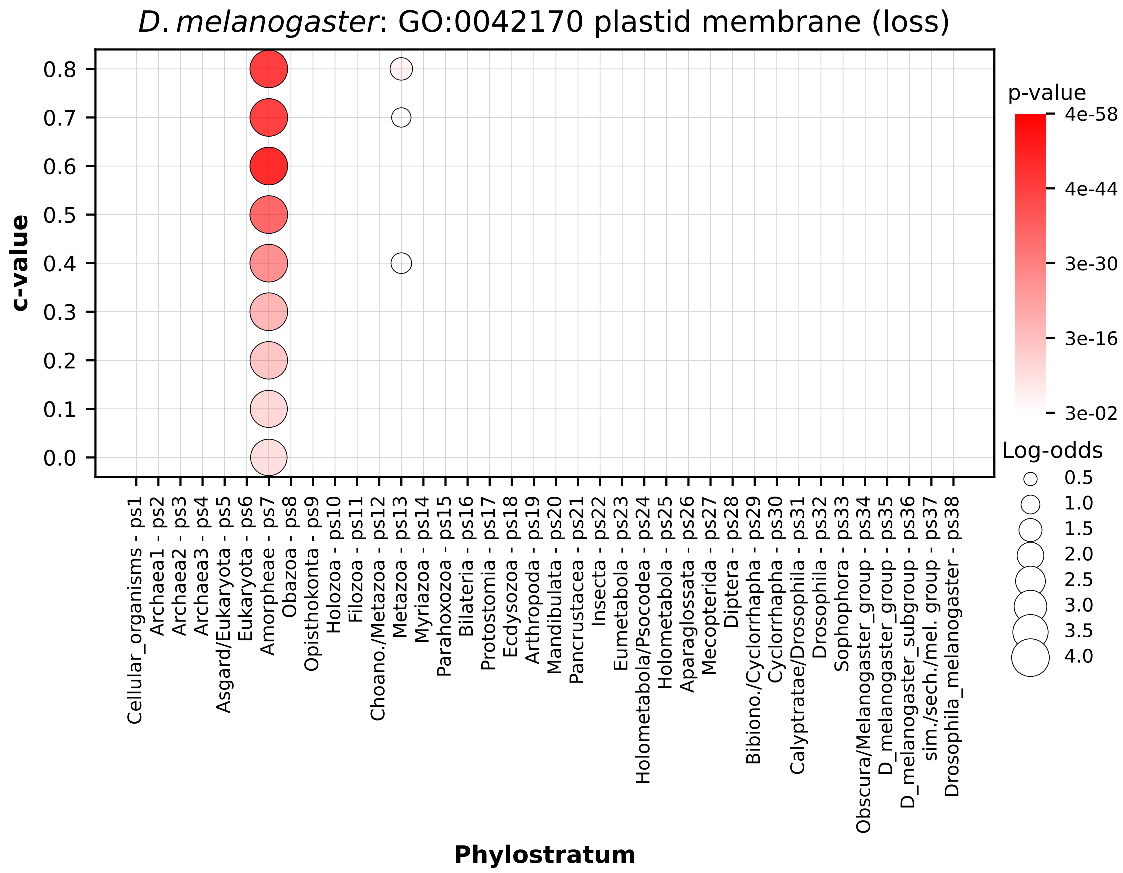

D. melanogaster: GO:0042170 plastid membrane (loss)
c-value
0.8
0.7
0.6
0.5
0.4
0.3
0.2
0.1
0.0
Phylostratum
Cellular_organisms - ps1
Archaea1 - ps2
Archaea2 - ps3
Archaea3 - ps4
Asgard/Eukaryota - ps5
Eukaryota - ps6
Amorpheae - ps7
Obazoa - ps8
Opisthokonta - ps9
Holozoa - ps10
Filozoa - ps11
Choano./Metazoa - ps12
Metazoa - ps13
Myriazoa - ps14
Parahoxozoa - ps15
Bilateria - ps16
Protostomia - ps17
Ecdysozoa - ps18
Arthropoda - ps19
Mandibulata - ps20
Pancrustacea - ps21
Insecta - ps22
Eumetabola - ps23
Holometabola/Psocodea - ps24
Holometabola - ps25
Aparaglossata - ps26
Mecopterida - ps27
Diptera - ps28
Bibiono./Cyclorrhapha - ps29
Cyclorrhapha - ps30
Calyptratae/Drosophila - ps31
Drosophila - ps32
Sophophora - ps33
Obscura/Melanogaster_group - ps34
D_melanogaster_group - ps35
D_melanogaster_subgroup - ps36
sim./sech./mel. group - ps37
Drosophila_melanogaster - ps38
p-value
4e-58
4e-44
3e-30
3e-16
3e-02
Log-odds
0.5
1.0
1.5
2.0
2.5
3.0
3.5
4.0

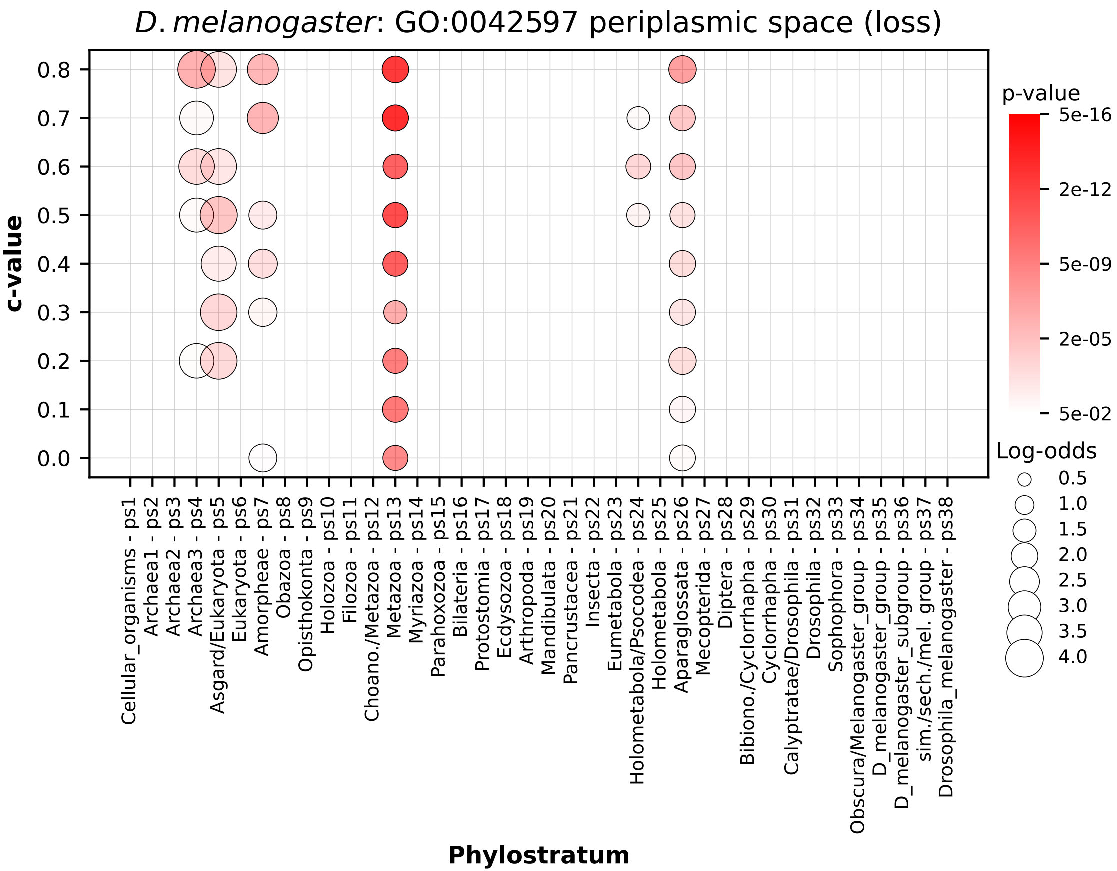

D. melanogaster: GO:0042597 periplasmic space (loss)
c-value
0.0
0.1
0.2
0.3
0.4
0.5
0.6
0.7
0.8
Phylostratum
Cellular_organisms - ps1
Archaea1 - ps2
Archaea2 - ps3
Archaea3 - ps4
Asgard/Eukaryota - ps5
Eukaryota - ps6
Amorpheae - ps7
Obazoa - ps8
Opisthokonta - ps9
Holozoa - ps10
Filozoa - ps11
Choano./Metazoa - ps12
Metazoa - ps13
Myriazoa - ps14
Parahoxozoa - ps15
Bilateria - ps16
Protostomia - ps17
Ecdysozoa - ps18
Arthropoda - ps19
Mandibulata - ps20
Pancrustacea - ps21
Insecta - ps22
Eumetabola - ps23
Holometabola/Psocodea - ps24
Holometabola - ps25
Aparaglossata - ps26
Mecopterida - ps27
Diptera - ps28
Bibiono./Cyclorrhapha - ps29
Cyclorrhapha - ps30
Calyptratae/Drosophila - ps31
Drosophila - ps32
Sophophora - ps33
Obscura/Melanogaster_group - ps34
D_melanogaster_group - ps35
D_melanogaster_subgroup - ps36
sim./sech./mel. group - ps37
Drosophila_melanogaster - ps38
p-value
5e-16
2e-12
5e-09
2e-05
5e-02
Log-odds
0.5
1.0
1.5
2.0
2.5
3.0
3.5
4.0

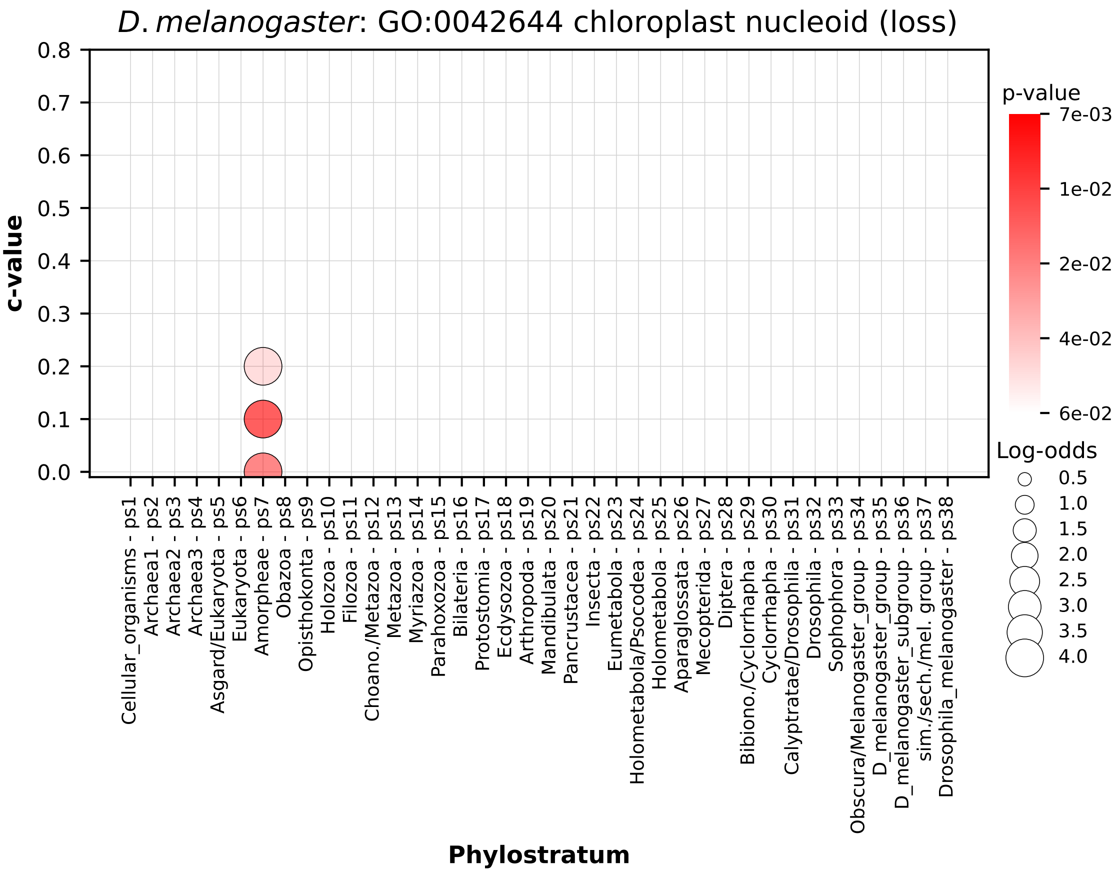

D. melanogaster: GO:0042644 chloroplast nucleoid (loss)
c-value
0.0
0.1
0.2
0.3
0.4
0.5
0.6
0.7
0.8
p-value
7e-03
1e-02
2e-02
4e-02
6e-02
Log-odds
0.5
1.0
1.5
2.0
2.5
3.0
3.5
4.0
Cellular_organisms - ps1
Archaea1 - ps2
Archaea2 - ps3
Archaea3 - ps4
Asgard/Eukaryota - ps5
Eukaryota - ps6
Amorpheae - ps7
Obazoa - ps8
Opisthokonta - ps9
Holozoa - ps10
Filozoa - ps11
Choano./Metazoa - ps12
Metazoa - ps13
Myriazoa - ps14
Parahoxozoa - ps15
Bilateria - ps16
Protostomia - ps17
Ecdysozoa - ps18
Arthropoda - ps19
Mandibulata - ps20
Pancrustacea - ps21
Insecta - ps22
Eumetabola - ps23
Holometabola/Psocodea - ps24
Holometabola - ps25
Aparaglossata - ps26
Mecopterida - ps27
Diptera - ps28
Bibiono./Cyclorrhapha - ps29
Cyclorrhapha - ps30
Calyptratae/Drosophila - ps31
Drosophila - ps32
Sophophora - ps33
Obscura/Melanogaster_group - ps34
D_melanogaster_group - ps35
D_melanogaster_subgroup - ps36
sim./sech./mel. group - ps37
Drosophila_melanogaster - ps38
Phylostratum

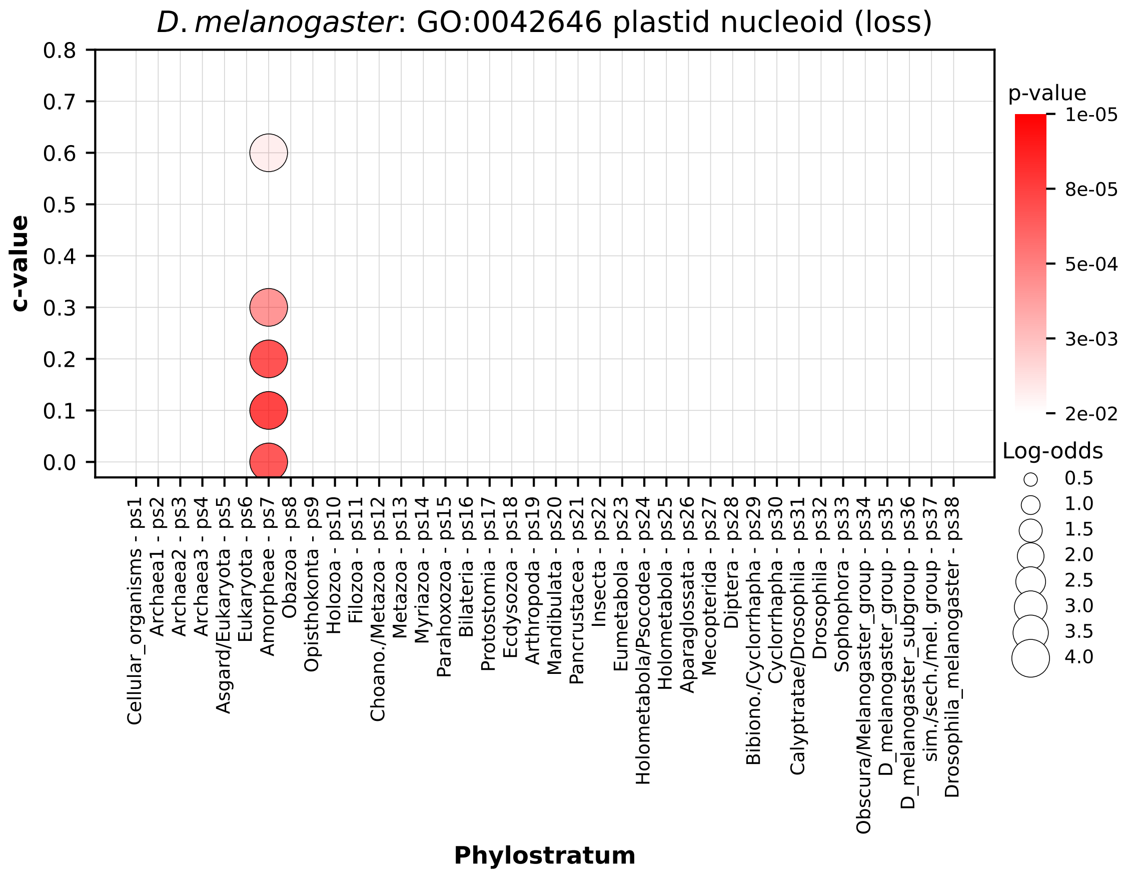

D. melanogaster: GO:0042646 plastid nucleoid (loss)
c-value
0.8
0.7
0.6
0.5
0.4
0.3
0.2
0.1
0.0
Phylostratum
Cellular_organisms - ps1
Archaea1 - ps2
Archaea2 - ps3
Archaea3 - ps4
Asgard/Eukaryota - ps5
Eukaryota - ps6
Amorpheae - ps7
Obazoa - ps8
Opisthokonta - ps9
Holozoa - ps10
Filozoa - ps11
Choano./Metazoa - ps12
Metazoa - ps13
Myriazoa - ps14
Parahoxozoa - ps15
Bilateria - ps16
Protostomia - ps17
Ecdysozoa - ps18
Arthropoda - ps19
Mandibulata - ps20
Pancrustacea - ps21
Insecta - ps22
Eumetabola - ps23
Holometabola/Psocodea - ps24
Holometabola - ps25
Aparaglossata - ps26
Mecopterida - ps27
Diptera - ps28
Bibiono./Cyclorrhapha - ps29
Cyclorrhapha - ps30
Calyptratae/Drosophila - ps31
Drosophila - ps32
Sophophora - ps33
Obscura/Melanogaster_group - ps34
D_melanogaster_group - ps35
D_melanogaster_subgroup - ps36
sim./sech./mel. group - ps37
Drosophila_melanogaster - ps38
p-value
1e-05
8e-05
5e-04
3e-03
2e-02
Log-odds
0.5
1.0
1.5
2.0
2.5
3.0
3.5
4.0

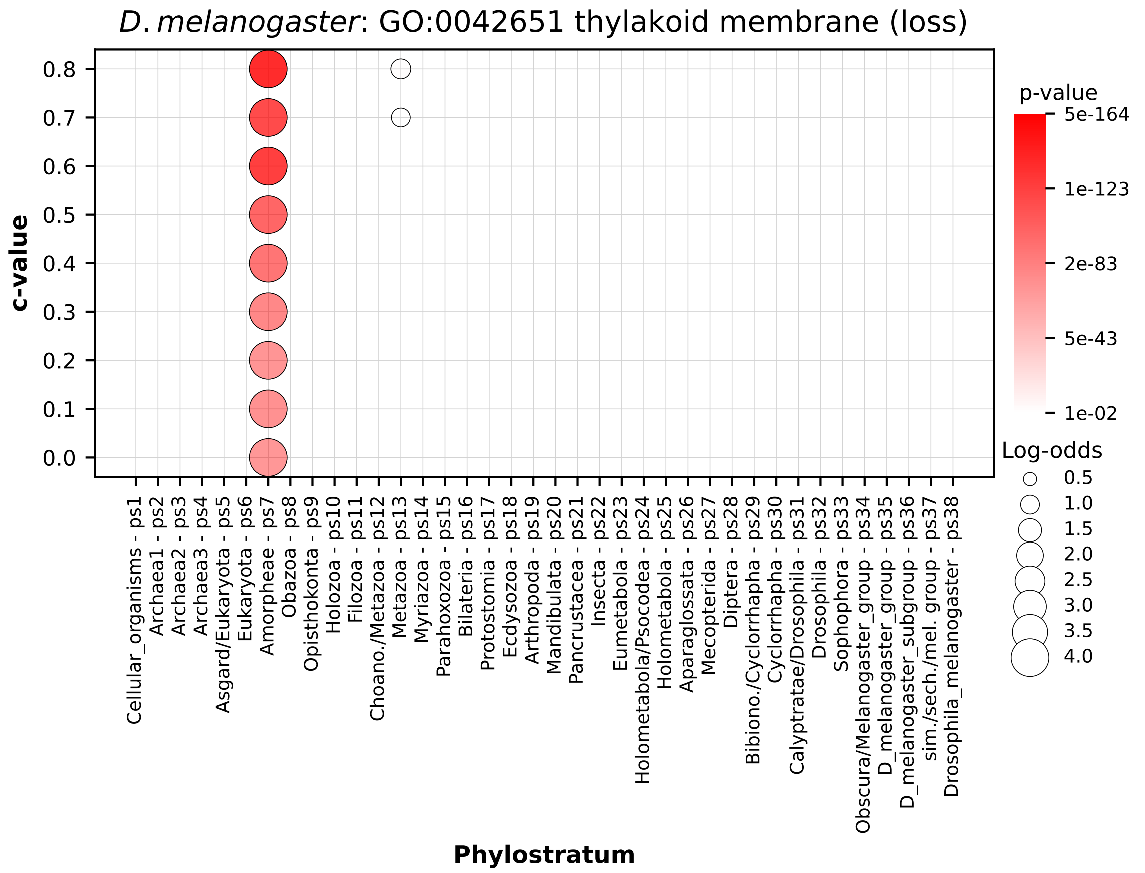
D. melanogaster: GO:0042651 thylakoid membrane (loss)
c-value
0.8
0.7
0.6
0.5
0.4
0.3
0.2
0.1
0.0
p-value
5e-164
1e-123
2e-83
5e-43
1e-02
Log-odds
0.5
1.0
1.5
2.0
2.5
3.0
3.5
4.0
Cellular_organisms - ps1
Archaea1 - ps2
Archaea2 - ps3
Archaea3 - ps4
Asgard/Eukaryota - ps5
Eukaryota - ps6
Amorpheae - ps7
Obazoa - ps8
Opisthokonta - ps9
Holozoa - ps10
Filozoa - ps11
Choano./Metazoa - ps12
Metazoa - ps13
Myriazoa - ps14
Parahoxozoa - ps15
Bilateria - ps16
Protostomia - ps17
Ecdysozoa - ps18
Arthropoda - ps19
Mandibulata - ps20
Pancrustacea - ps21
Insecta - ps22
Eumetabola - ps23
Holometabola/Psocodea - ps24
Holometabola - ps25
Aparaglossata - ps26
Mecopterida - ps27
Diptera - ps28
Bibiono./Cyclorrhapha - ps29
Cyclorrhapha - ps30
Calyptratae/Drosophila - ps31
Drosophila - ps32
Sophophora - ps33
Obscura/Melanogaster_group - ps34
D_melanogaster_group - ps35
D_melanogaster_subgroup - ps36
sim./sech./mel. group - ps37
Drosophila_melanogaster - ps38
Phylostratum

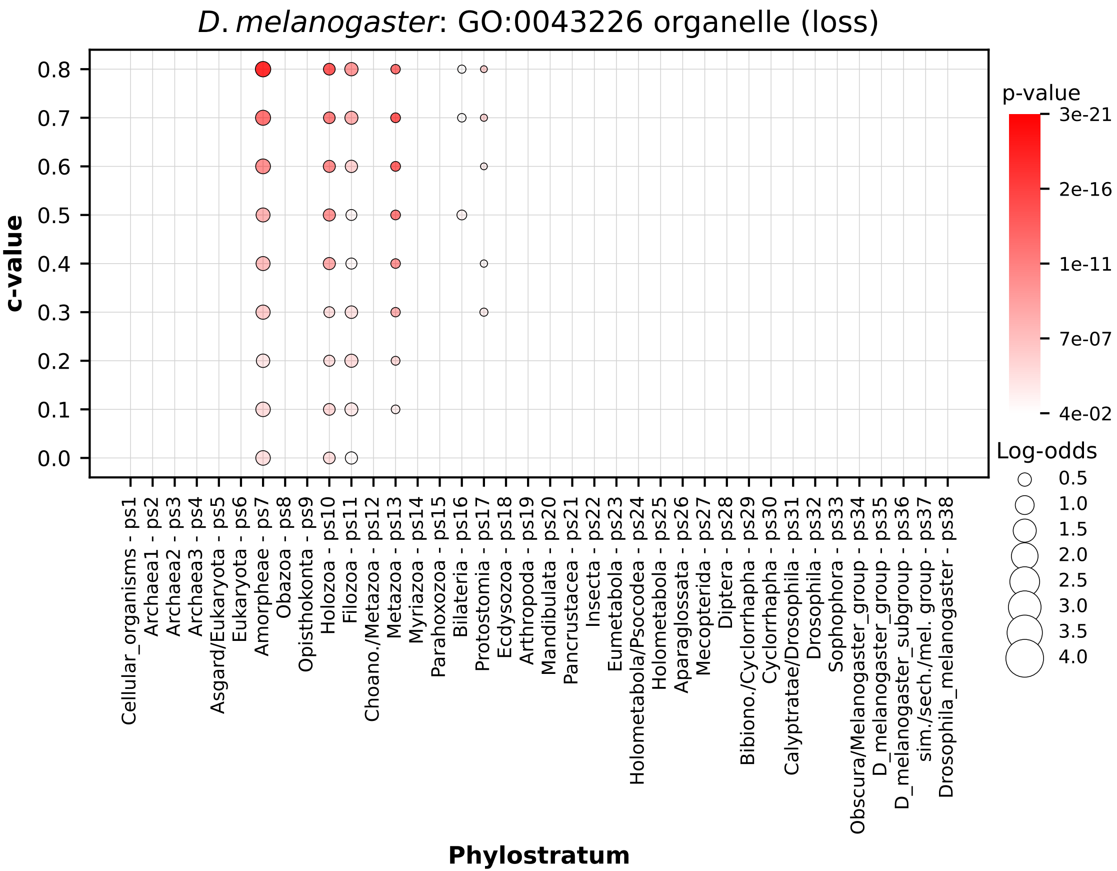

D. melanogaster: GO:0043226 organelle (loss)
c-value
0.8
0.7
0.6
0.5
0.4
0.3
0.2
0.1
0.0
Phylostratum
Cellular_organisms - ps1
Archaea1 - ps2
Archaea2 - ps3
Archaea3 - ps4
Asgard/Eukaryota - ps5
Eukaryota - ps6
Amorpheae - ps7
Obazoa - ps8
Opisthokonta - ps9
Holozoa - ps10
Filozoa - ps11
Choano./Metazoa - ps12
Metazoa - ps13
Myriazoa - ps14
Parahoxozoa - ps15
Bilateria - ps16
Protostomia - ps17
Ecdysozoa - ps18
Arthropoda - ps19
Mandibulata - ps20
Pancrustacea - ps21
Insecta - ps22
Eumetabola - ps23
Holometabola/Psocodea - ps24
Holometabola - ps25
Aparaglossata - ps26
Mecopterida - ps27
Diptera - ps28
Bibiono./Cyclorrhapha - ps29
Cyclorrhapha - ps30
Calyptratae/Drosophila - ps31
Drosophila - ps32
Sophophora - ps33
Obscura/Melanogaster_group - ps34
D_melanogaster_group - ps35
D_melanogaster_subgroup - ps36
sim./sech./mel. group - ps37
Drosophila_melanogaster - ps38
p-value
3e-21
2e-16
1e-11
7e-07
4e-02
Log-odds
0.5
1.0
1.5
2.0
2.5
3.0
3.5
4.0

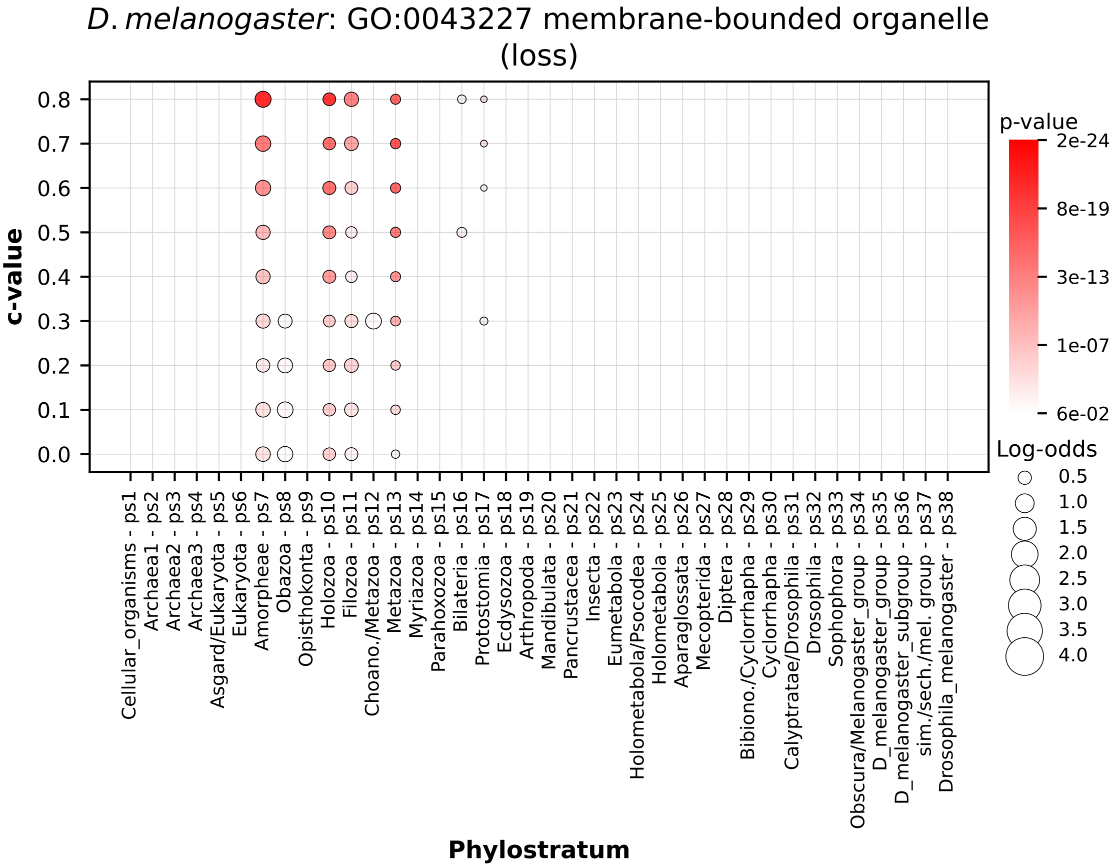

D. melanogaster: GO:0043227 membrane-bounded organelle (loss)
c-value
0.8
0.7
0.6
0.5
0.4
0.3
0.2
0.1
0.0
Phylostratum
Cellular_organisms - ps1
Archaea1 - ps2
Archaea2 - ps3
Archaea3 - ps4
Asgard/Eukaryota - ps5
Eukaryota - ps6
Amorpheae - ps7
Obazoa - ps8
Opisthokonta - ps9
Holozoa - ps10
Filozoa - ps11
Choano./Metazoa - ps12
Metazoa - ps13
Myriazoa - ps14
Parahoxozoa - ps15
Bilateria - ps16
Protostomia - ps17
Ecdysozoa - ps18
Arthropoda - ps19
Mandibulata - ps20
Pancrustacea - ps21
Insecta - ps22
Eumetabola - ps23
Holometabola/Psocodea - ps24
Holometabola - ps25
Aparaglossata - ps26
Mecopterida - ps27
Diptera - ps28
Bibiono./Cyclorrhapha - ps29
Cyclorrhapha - ps30
Calyptratae/Drosophila - ps31
Drosophila - ps32
Sophophora - ps33
Obscura/Melanogaster_group - ps34
D_melanogaster_group - ps35
D_melanogaster_subgroup - ps36
sim./sech./mel. group - ps37
Drosophila_melanogaster - ps38
p-value
2e-24
8e-19
3e-13
1e-07
6e-02
Log-odds
0.5
1.0
1.5
2.0
2.5
3.0
3.5
4.0

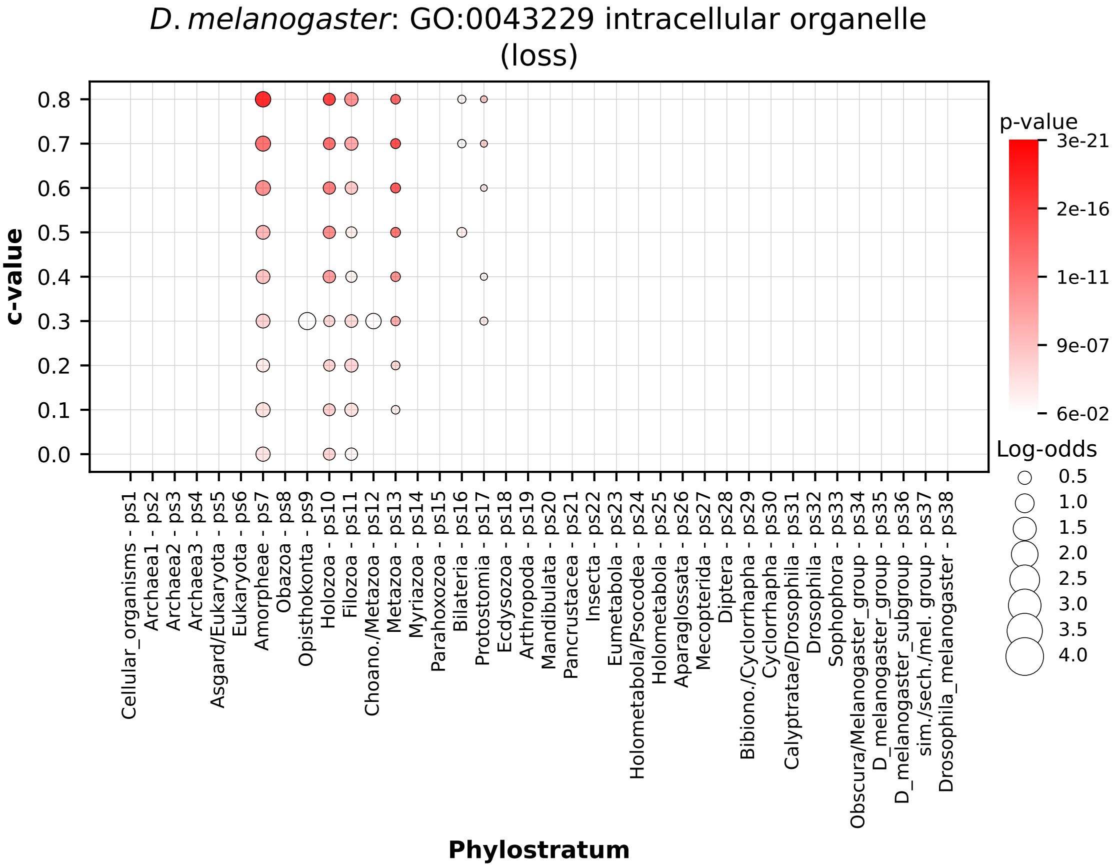

D. melanogaster: GO:0043229 intracellular organelle
(loss)
c-value
0.8
0.7
0.6
0.5
0.4
0.3
0.2
0.1
0.0
Phylostratum
Cellular_organisms - ps1
Archaea1 - ps2
Archaea2 - ps3
Archaea3 - ps4
Asgard/Eukaryota - ps5
Eukaryota - ps6
Amorpheae - ps7
Obazoa - ps8
Opisthokonta - ps9
Holozoa - ps10
Filozoa - ps11
Choano./Metazoa - ps12
Metazoa - ps13
Myriazoa - ps14
Parahoxozoa - ps15
Bilateria - ps16
Protostomia - ps17
Ecdysozoa - ps18
Arthropoda - ps19
Mandibulata - ps20
Pancrustacea - ps21
Insecta - ps22
Eumetabola - ps23
Holometabola/Psocodea - ps24
Holometabola - ps25
Aparaglossata - ps26
Mecopterida - ps27
Diptera - ps28
Bibiono./Cyclorrhapha - ps29
Cyclorrhapha - ps30
Calyptratae/Drosophila - ps31
Drosophila - ps32
Sophophora - ps33
Obscura/Melanogaster_group - ps34
D_melanogaster_group - ps35
D_melanogaster_subgroup - ps36
sim./sech./mel. group - ps37
Drosophila_melanogaster - ps38
p-value
3e-21
2e-16
1e-11
9e-07
6e-02
Log-odds
0.5
1.0
1.5
2.0
2.5
3.0
3.5
4.0

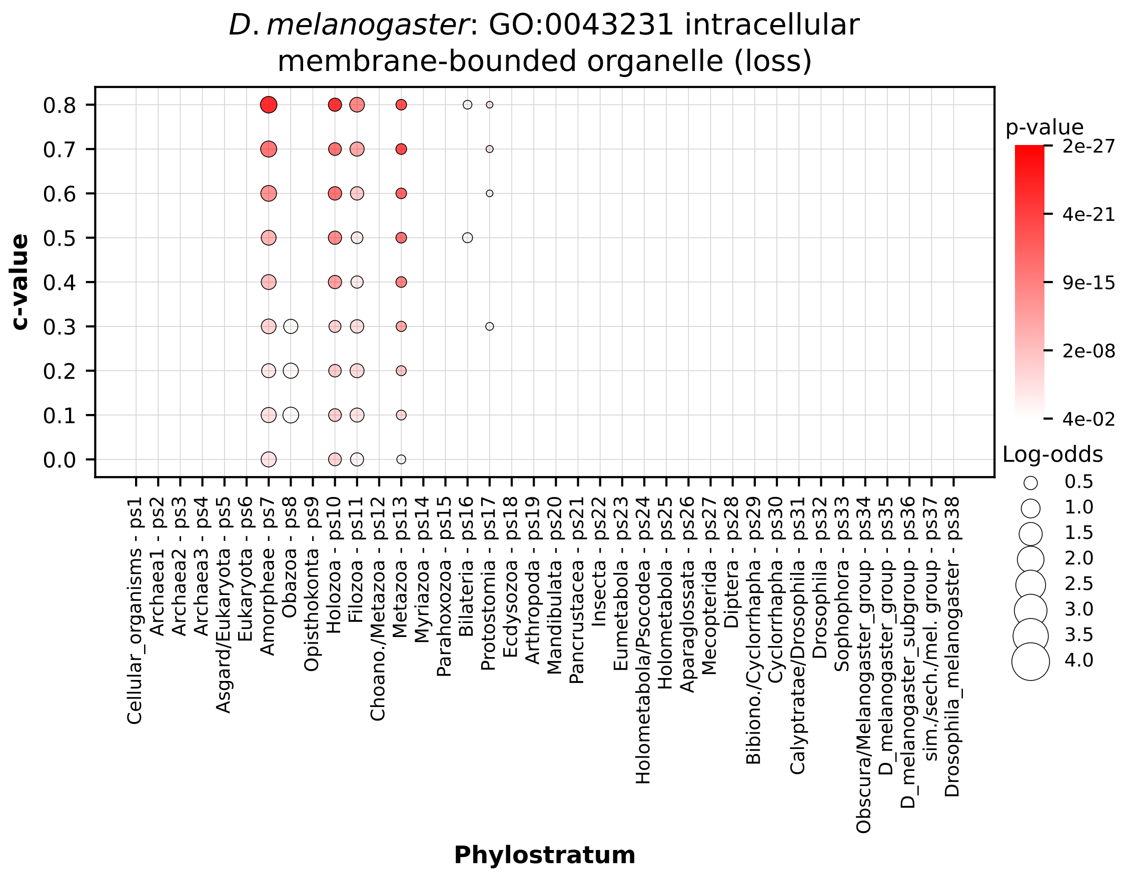

D. melanogaster: GO:0043231 intracellular membrane-bounded organelle (loss)
c-value
0.8
0.7
0.6
0.5
0.4
0.3
0.2
0.1
0.0
Phylostratum
Cellular_organisms - ps1
Archaea1 - ps2
Archaea2 - ps3
Archaea3 - ps4
Asgard/Eukaryota - ps5
Eukaryota - ps6
Amorpheae - ps7
Obazoa - ps8
Opisthokonta - ps9
Holozoa - ps10
Filozoa - ps11
Choano./Metazoa - ps12
Metazoa - ps13
Myriazoa - ps14
Parahoxozoa - ps15
Bilateria - ps16
Protostomia - ps17
Ecdysozoa - ps18
Arthropoda - ps19
Mandibulata - ps20
Pancrustacea - ps21
Insecta - ps22
Eumetabola - ps23
Holometabola/Psocodea - ps24
Holometabola - ps25
Aparaglossata - ps26
Mecopterida - ps27
Diptera - ps28
Bibiono./Cyclorrhapha - ps29
Cyclorrhapha - ps30
Calyptratae/Drosophila - ps31
Drosophila - ps32
Sophophora - ps33
Obscura/Melanogaster_group - ps34
D_melanogaster_group - ps35
D_melanogaster_subgroup - ps36
sim./sech./mel. group - ps37
Drosophila_melanogaster - ps38
p-value
2e-27
4e-21
9e-15
2e-08
4e-02
Log-odds
0.5
1.0
1.5
2.0
2.5
3.0
3.5
4.0

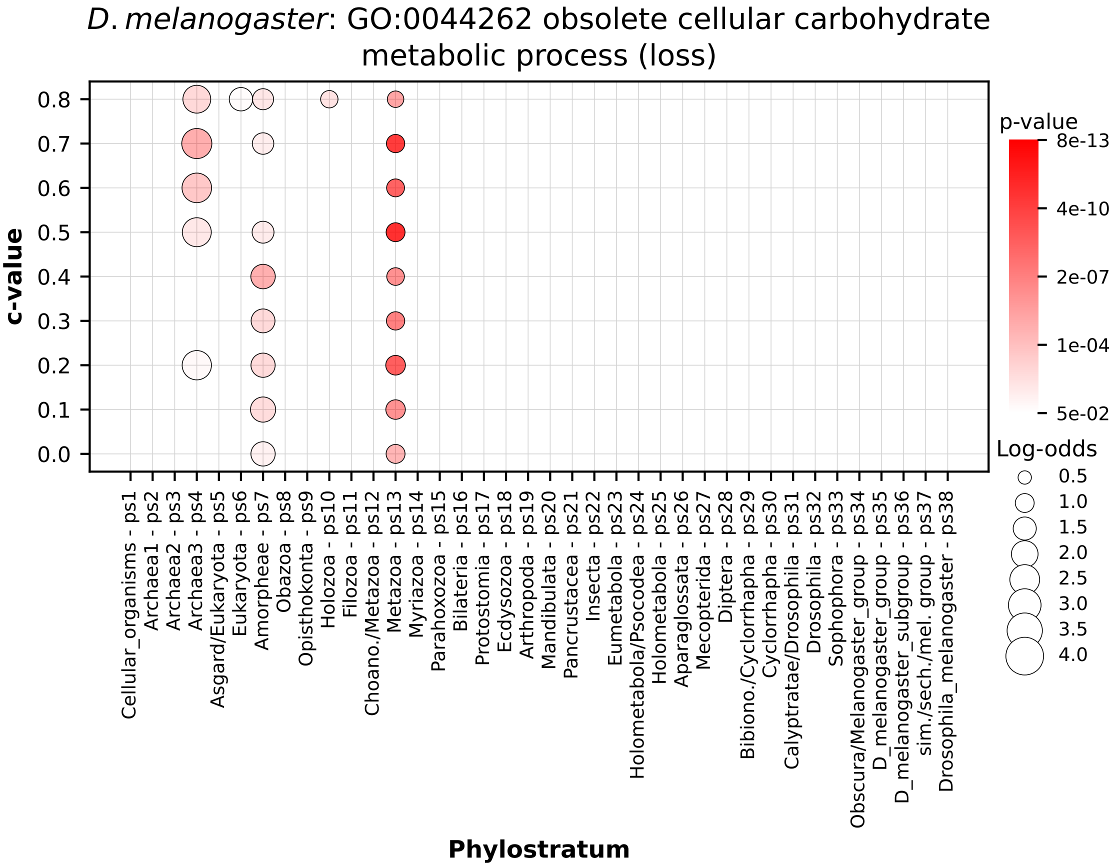

D. melanogaster: GO:0044262 obsolete cellular carbohydrate metabolic process (loss)
c-value
0.8
0.7
0.6
0.5
0.4
0.3
0.2
0.1
0.0
p-value
8e-13
4e-10
2e-07
1e-04
5e-02
Log-odds
0.5
1.0
1.5
2.0
2.5
3.0
3.5
4.0
Cellular_organisms - ps1
Archaea1 - ps2
Archaea2 - ps3
Archaea3 - ps4
Asgard/Eukaryota - ps5
Eukaryota - ps6
Amorpheae - ps7
Obazoa - ps8
Opisthokonta - ps9
Holozoa - ps10
Filozoa - ps11
Choano./Metazoa - ps12
Metazoa - ps13
Myriazoa - ps14
Parahoxozoa - ps15
Bilateria - ps16
Protostomia - ps17
Ecdysozoa - ps18
Arthropoda - ps19
Mandibulata - ps20
Pancrustacea - ps21
Insecta - ps22
Eumetabola - ps23
Holometabola/Psocodea - ps24
Holometabola - ps25
Aparaglossata - ps26
Mecopterida - ps27
Diptera - ps28
Bibiono./Cyclorrhapha - ps29
Cyclorrhapha - ps30
Calyptratae/Drosophila - ps31
Drosophila - ps32
Sophophora - ps33
Obscura/Melanogaster_group - ps34
D_melanogaster_group - ps35
D_melanogaster_subgroup - ps36
sim./sech./mel. group - ps37
Drosophila_melanogaster - ps38
Phylostratum

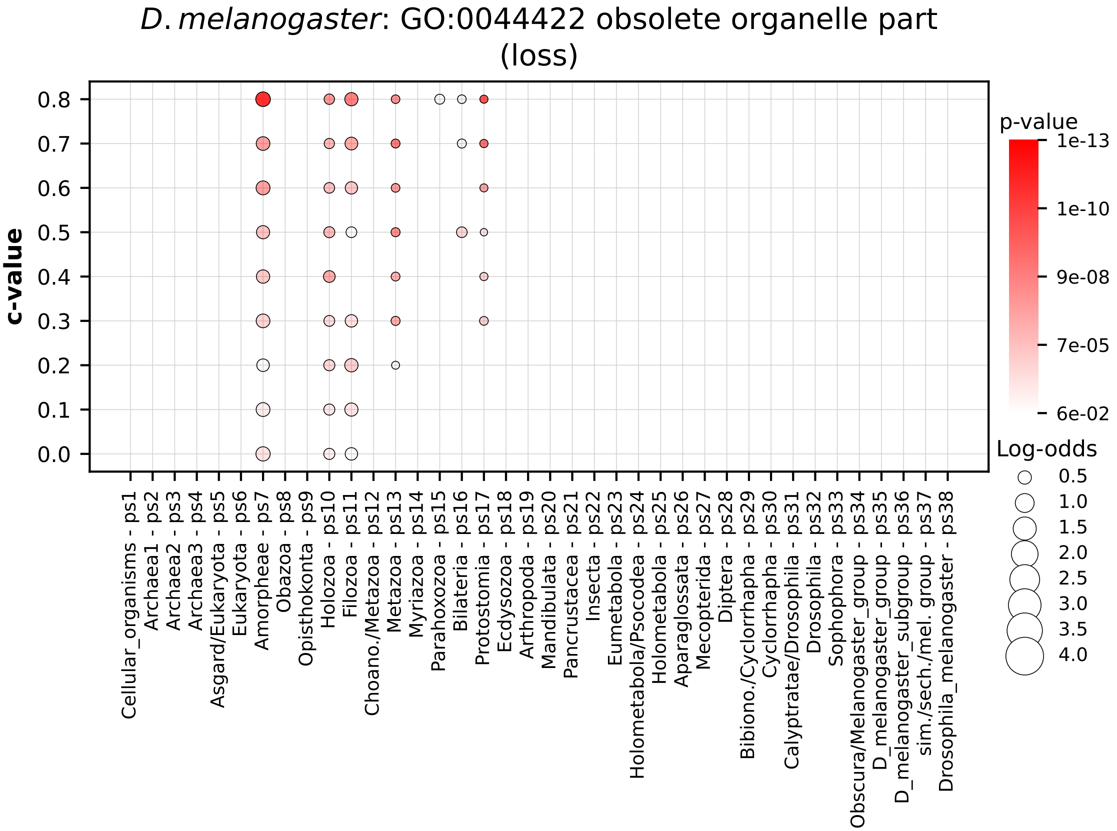

D. melanogaster: GO:0044422 obsolete organelle part (loss)
c-value
0.8
0.7
0.6
0.5
0.4
0.3
0.2
0.1
0.0
p-value
1e-13
1e-10
9e-08
7e-05
6e-02
Log-odds
0.5
1.0
1.5
2.0
2.5
3.0
3.5
4.0
Cellular_organisms - ps1
Archaea1 - ps2
Archaea2 - ps3
Archaea3 - ps4
Asgard/Eukaryota - ps5
Eukaryota - ps6
Amorpheae - ps7
Obazoa - ps8
Opisthokonta - ps9
Holozoa - ps10
Filozoa - ps11
Choano./Metazoa - ps12
Metazoa - ps13
Myriazoa - ps14
Parahoxozoa - ps15
Bilateria - ps16
Protostomia - ps17
Ecdysozoa - ps18
Arthropoda - ps19
Mandibulata - ps20
Pancrustacea - ps21
Insecta - ps22
Eumetabola - ps23
Holometabola/Psocodea - ps24
Holometabola - ps25
Aparaglossata - ps26
Mecopterida - ps27
Diptera - ps28
Bibiono./Cyclorrhapha - ps29
Cyclorrhapha - ps30
Calyptratae/Drosophila - ps31
Drosophila - ps32
Sophophora - ps33
Obscura/Melanogaster_group - ps34
D_melanogaster_group - ps35
D_melanogaster_subgroup - ps36
sim./sech./mel. group - ps37
Drosophila_melanogaster - ps38
Phylostratum

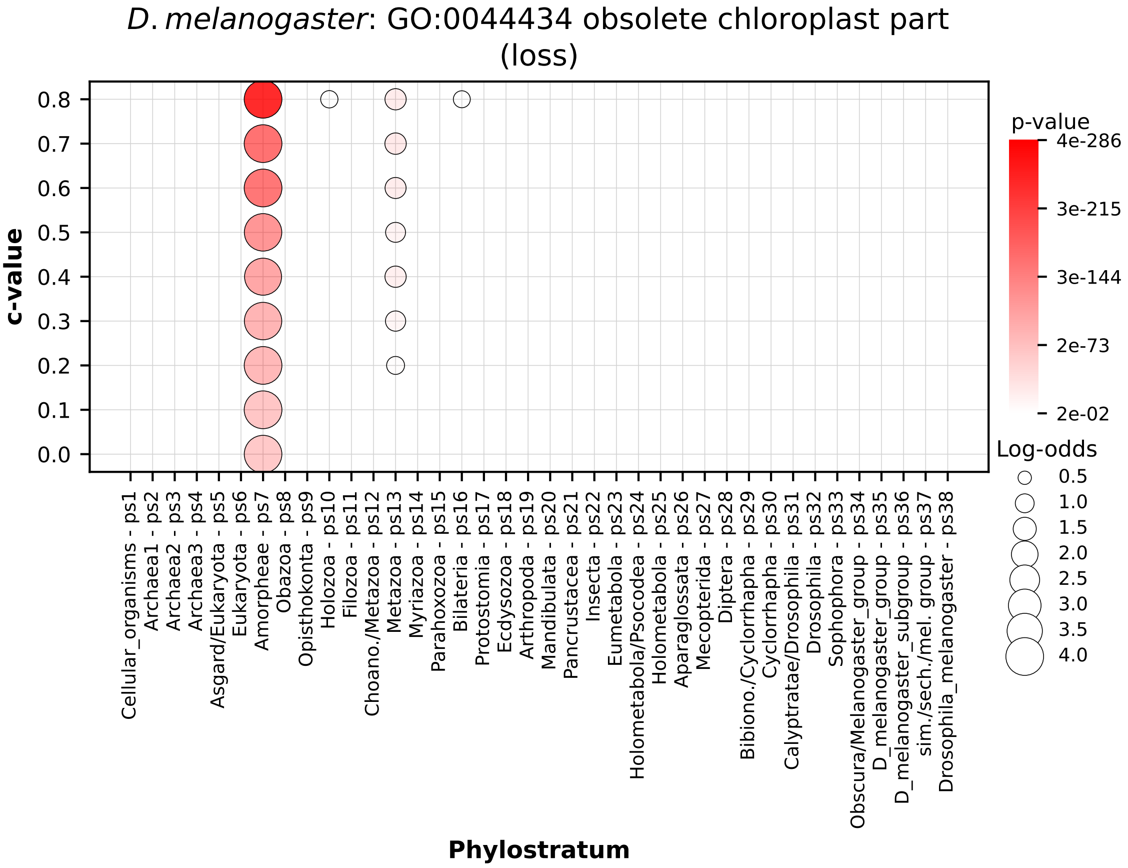

D. melanogaster: GO:0044434 obsolete chloroplast part
(loss)
c-value
0.8
0.7
0.6
0.5
0.4
0.3
0.2
0.1
0.0
p-value
4e-286
3e-215
3e-144
2e-73
2e-02
Log-odds
0.5
1.0
1.5
2.0
2.5
3.0
3.5
4.0
Cellular_organisms - ps1
Archaea1 - ps2
Archaea2 - ps3
Archaea3 - ps4
Asgard/Eukaryota - ps5
Eukaryota - ps6
Amorpheae - ps7
Obazoa - ps8
Opisthokonta - ps9
Holozoa - ps10
Filozoa - ps11
Choano./Metazoa - ps12
Metazoa - ps13
Myriazoa - ps14
Parahoxozoa - ps15
Bilateria - ps16
Protostomia - ps17
Ecdysozoa - ps18
Arthropoda - ps19
Mandibulata - ps20
Pancrustacea - ps21
Insecta - ps22
Eumetabola - ps23
Holometabola/Psocodea - ps24
Holometabola - ps25
Aparaglossata - ps26
Mecopterida - ps27
Diptera - ps28
Bibiono./Cyclorrhapha - ps29
Cyclorrhapha - ps30
Calyptratae/Drosophila - ps31
Drosophila - ps32
Sophophora - ps33
Obscura/Melanogaster_group - ps34
D_melanogaster_group - ps35
D_melanogaster_subgroup - ps36
sim./sech./mel. group - ps37
Drosophila_melanogaster - ps38
Phylostratum

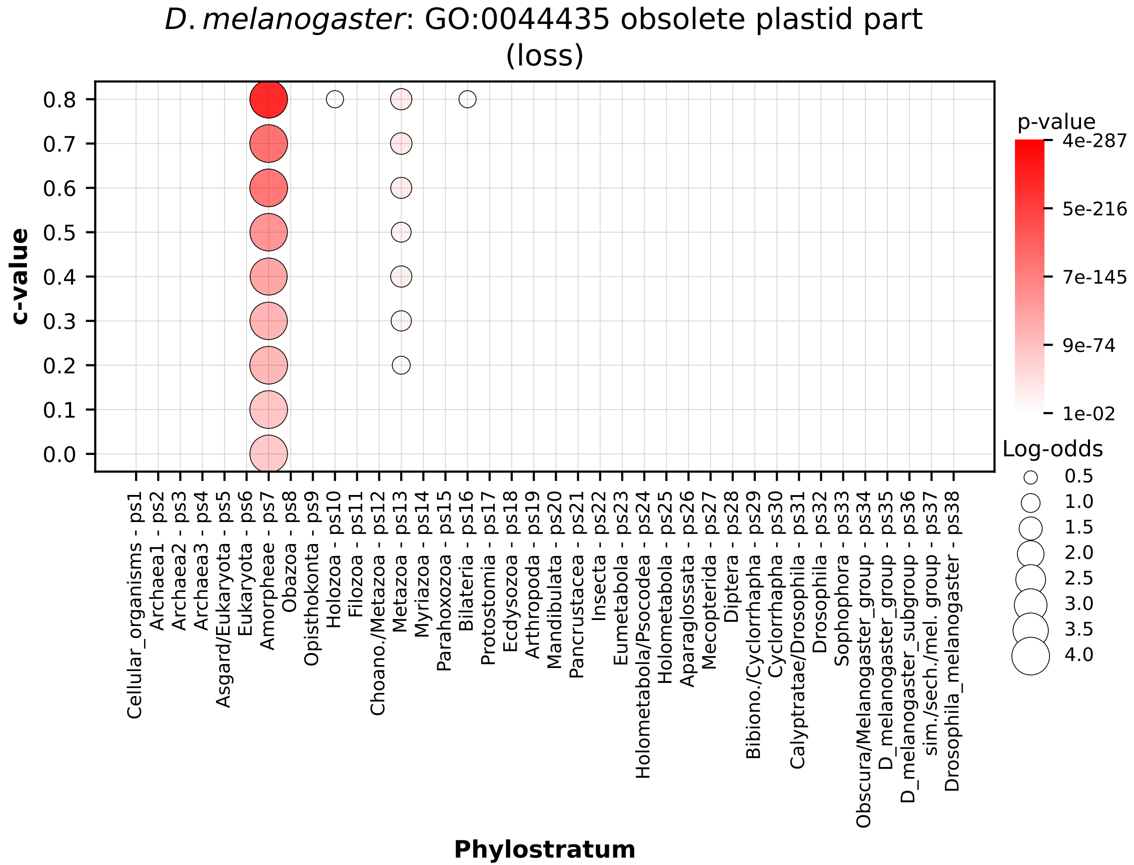

D. melanogaster: GO:0044435 obsolete plastid part
(loss)
c-value
0.8
0.7
0.6
0.5
0.4
0.3
0.2
0.1
0.0
p-value
4e-287
5e-216
7e-145
9e-74
1e-02
Log-odds
0.5
1.0
1.5
2.0
2.5
3.0
3.5
4.0
Cellular_organisms - ps1
Archaea1 - ps2
Archaea2 - ps3
Archaea3 - ps4
Asgard/Eukaryota - ps5
Eukaryota - ps6
Amorpheae - ps7
Obazoa - ps8
Opisthokonta - ps9
Holozoa - ps10
Filozoa - ps11
Choano./Metazoa - ps12
Metazoa - ps13
Myriazoa - ps14
Parahoxozoa - ps15
Bilateria - ps16
Protostomia - ps17
Ecdysozoa - ps18
Arthropoda - ps19
Mandibulata - ps20
Pancrustacea - ps21
Insecta - ps22
Eumetabola - ps23
Holometabola/Psocodea - ps24
Holometabola - ps25
Aparaglossata - ps26
Mecopterida - ps27
Diptera - ps28
Bibiono./Cyclorrhapha - ps29
Cyclorrhapha - ps30
Calyptratae/Drosophila - ps31
Drosophila - ps32
Sophophora - ps33
Obscura/Melanogaster_group - ps34
D_melanogaster_group - ps35
D_melanogaster_subgroup - ps36
sim./sech./mel. group - ps37
Drosophila_melanogaster - ps38
Phylostratum

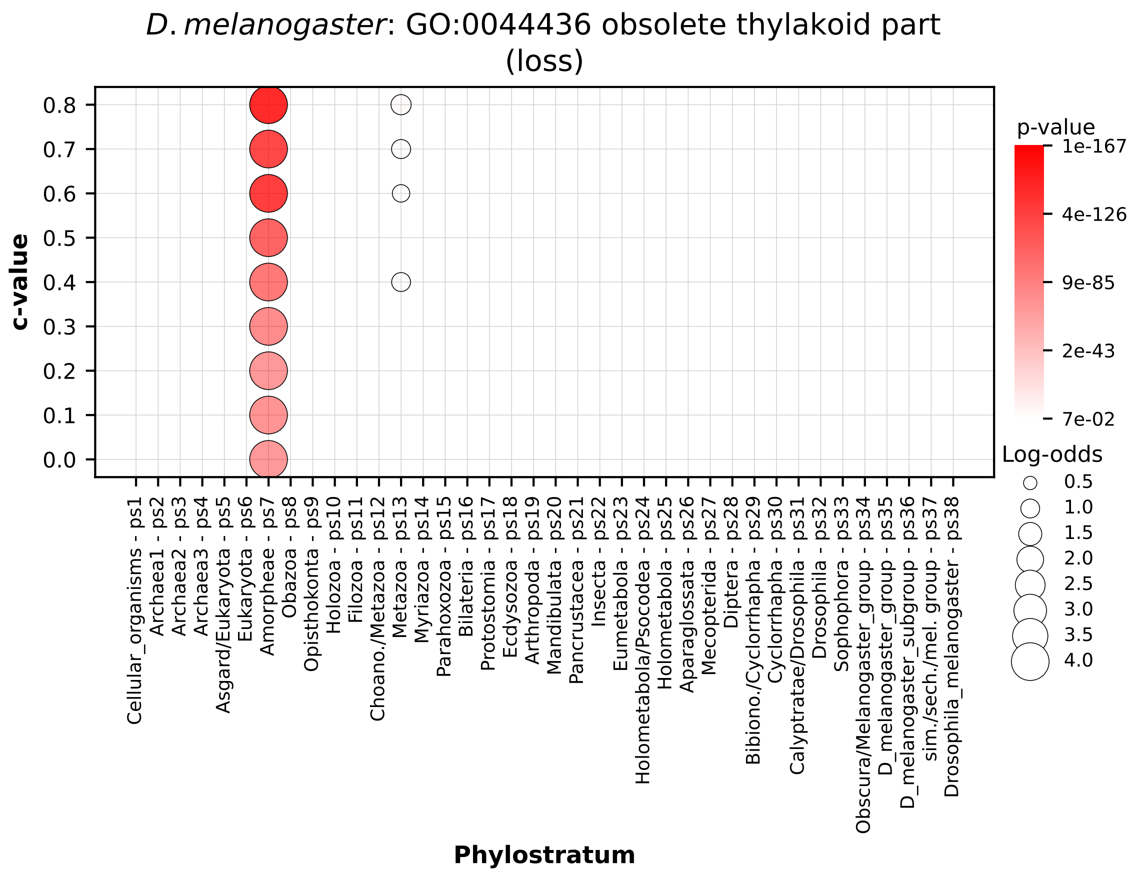

D. melanogaster: GO:0044436 obsolete thylakoid part
(loss)
c-value
0.8
0.7
0.6
0.5
0.4
0.3
0.2
0.1
0.0
p-value
1e-167
4e-126
9e-85
2e-43
7e-02
Log-odds
0.5
1.0
1.5
2.0
2.5
3.0
3.5
4.0
Cellular_organisms - ps1
Archaea1 - ps2
Archaea2 - ps3
Archaea3 - ps4
Asgard/Eukaryota - ps5
Eukaryota - ps6
Amorpheae - ps7
Obazoa - ps8
Opisthokonta - ps9
Holozoa - ps10
Filozoa - ps11
Choano./Metazoa - ps12
Metazoa - ps13
Myriazoa - ps14
Parahoxozoa - ps15
Bilateria - ps16
Protostomia - ps17
Ecdysozoa - ps18
Arthropoda - ps19
Mandibulata - ps20
Pancrustacea - ps21
Insecta - ps22
Eumetabola - ps23
Holometabola/Psocodea - ps24
Holometabola - ps25
Aparaglossata - ps26
Mecopterida - ps27
Diptera - ps28
Bibiono./Cyclorrhapha - ps29
Cyclorrhapha - ps30
Calyptratae/Drosophila - ps31
Drosophila - ps32
Sophophora - ps33
Obscura/Melanogaster_group - ps34
D_melanogaster_group - ps35
D_melanogaster_subgroup - ps36
sim./sech./mel. group - ps37
Drosophila_melanogaster - ps38
Phylostratum

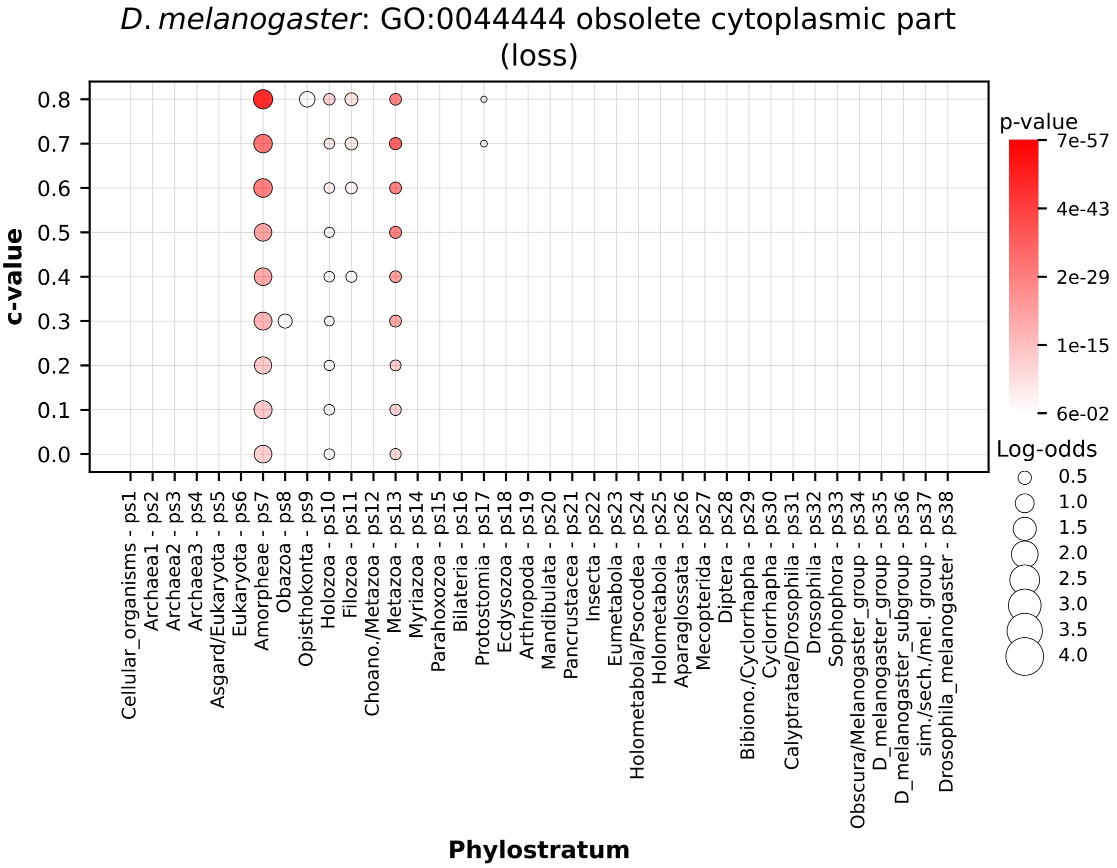

D. melanogaster: GO:0044444 obsolete cytoplasmic part
(loss)
c-value
0.8
0.7
0.6
0.5
0.4
0.3
0.2
0.1
0.0
Phylostratum
Cellular_organisms - ps1
Archaea1 - ps2
Archaea2 - ps3
Archaea3 - ps4
Asgard/Eukaryota - ps5
Eukaryota - ps6
Amorpheae - ps7
Obazoa - ps8
Opisthokonta - ps9
Holozoa - ps10
Filozoa - ps11
Choano./Metazoa - ps12
Metazoa - ps13
Myriazoa - ps14
Parahoxozoa - ps15
Bilateria - ps16
Protostomia - ps17
Ecdysozoa - ps18
Arthropoda - ps19
Mandibulata - ps20
Pancrustacea - ps21
Insecta - ps22
Eumetabola - ps23
Holometabola/Psocodea - ps24
Holometabola - ps25
Aparaglossata - ps26
Mecopterida - ps27
Diptera - ps28
Bibiono./Cyclorrhapha - ps29
Cyclorrhapha - ps30
Calyptratae/Drosophila - ps31
Drosophila - ps32
Sophophora - ps33
Obscura/Melanogaster_group - ps34
D_melanogaster_group - ps35
D_melanogaster_subgroup - ps36
sim./sech./mel. group - ps37
Drosophila_melanogaster - ps38
p-value
7e-57
4e-43
2e-29
1e-15
6e-02
Log-odds
0.5
1.0
1.5
2.0
2.5
3.0
3.5
4.0

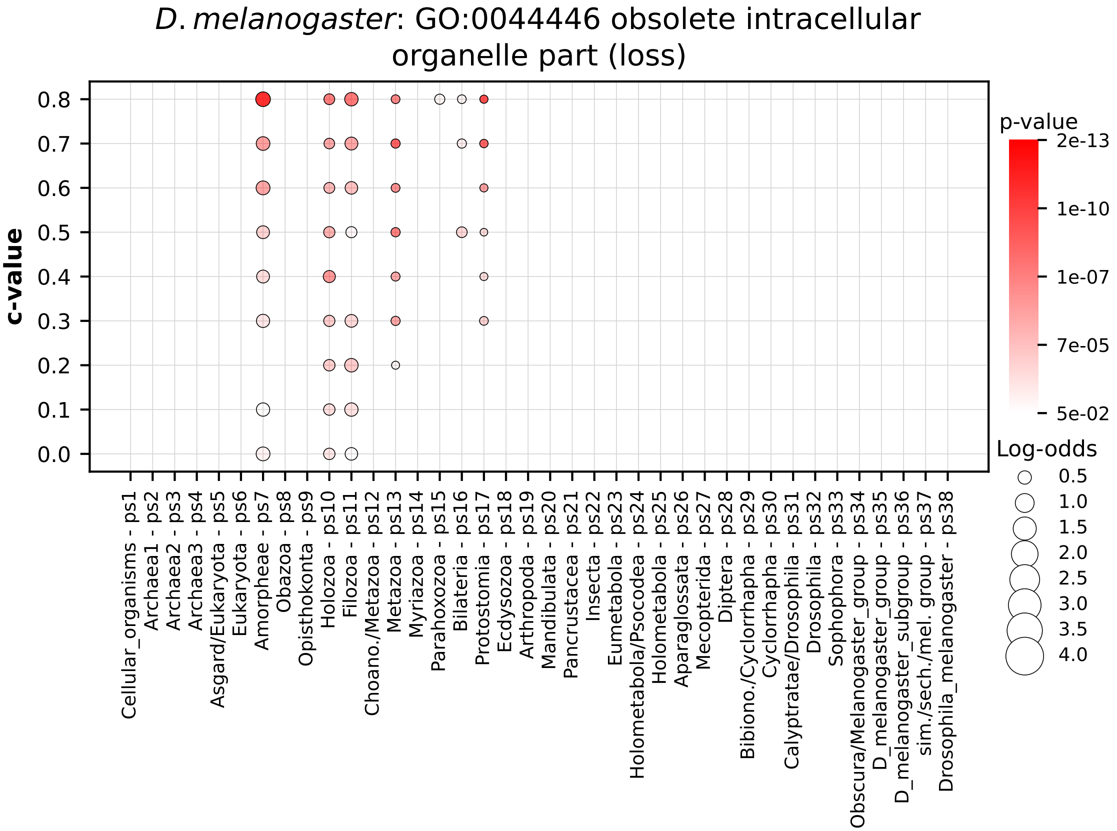

$D. melanogaster$: GO:0044446 obsolete intracellular organelle part (loss)
c-value
0.8
0.7
0.6
0.5
0.4
0.3
0.2
0.1
0.0
Phylostratum
Cellular_organisms - ps1
Archaea1 - ps2
Archaea2 - ps3
Archaea3 - ps4
Asgard/Eukaryota - ps5
Eukaryota - ps6
Amorpheae - ps7
Obazoa - ps8
Opisthokonta - ps9
Holozoa - ps10
Filozoa - ps11
Choano./Metazoa - ps12
Metazoa - ps13
Myriazoa - ps14
Parahoxozoa - ps15
Bilateria - ps16
Protostomia - ps17
Ecdysozoa - ps18
Arthropoda - ps19
Mandibulata - ps20
Pancrustacea - ps21
Insecta - ps22
Eumetabola - ps23
Holometabola/Psocodea - ps24
Holometabola - ps25
Aparaglossata - ps26
Mecopterida - ps27
Diptera - ps28
Bibiono./Cyclorrhapha - ps29
Cyclorrhapha - ps30
Calyptratae/Drosophila - ps31
Drosophila - ps32
Sophophora - ps33
Obscura/Melanogaster_group - ps34
D_melanogaster_group - ps35
D_melanogaster_subgroup - ps36
sim./sech./mel. group - ps37
Drosophila_melanogaster - ps38
p-value
2e-13
1e-10
1e-07
7e-05
5e-02
Log-odds
0.5
1.0
1.5
2.0
2.5
3.0
3.5
4.0

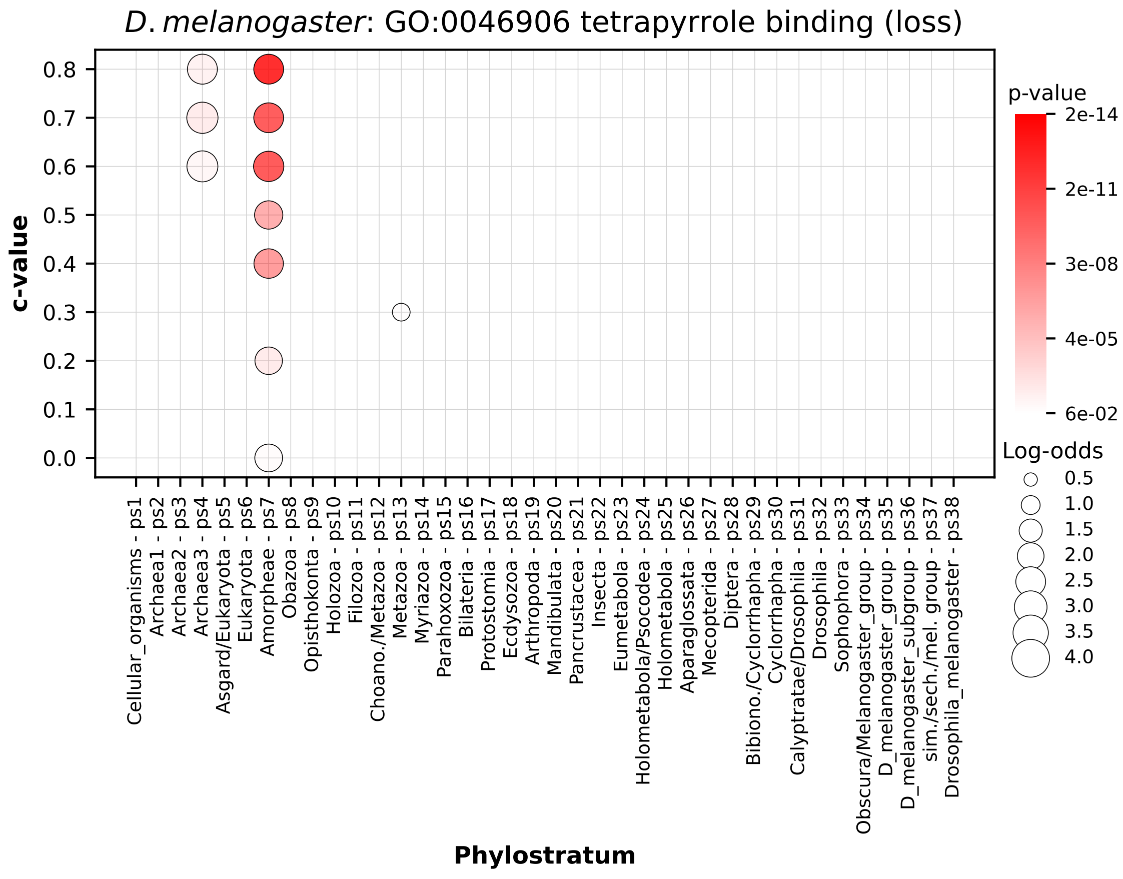

D. melanogaster: GO:0046906 tetrapyrrole binding (loss)
c-value
0.8
0.7
0.6
0.5
0.4
0.3
0.2
0.1
0.0
Phylostratum
Cellular_organisms - ps1
Archaea1 - ps2
Archaea2 - ps3
Archaea3 - ps4
Asgard/Eukaryota - ps5
Eukaryota - ps6
Amorpheae - ps7
Obazoa - ps8
Opisthokonta - ps9
Holozoa - ps10
Filozoa - ps11
Choano./Metazoa - ps12
Metazoa - ps13
Myriazoa - ps14
Parahoxozoa - ps15
Bilateria - ps16
Protostomia - ps17
Ecdysozoa - ps18
Arthropoda - ps19
Mandibulata - ps20
Pancrustacea - ps21
Insecta - ps22
Eumetabola - ps23
Holometabola/Psocodea - ps24
Holometabola - ps25
Aparaglossata - ps26
Mecopterida - ps27
Diptera - ps28
Bibiono./Cyclorrhapha - ps29
Cyclorrhapha - ps30
Calyptratae/Drosophila - ps31
Drosophila - ps32
Sophophora - ps33
Obscura/Melanogaster_group - ps34
D_melanogaster_group - ps35
D_melanogaster_subgroup - ps36
sim./sech./mel. group - ps37
Drosophila_melanogaster - ps38
p-value
2e-14
2e-11
3e-08
4e-05
6e-02
Log-odds
0.5
1.0
1.5
2.0
2.5
3.0
3.5
4.0

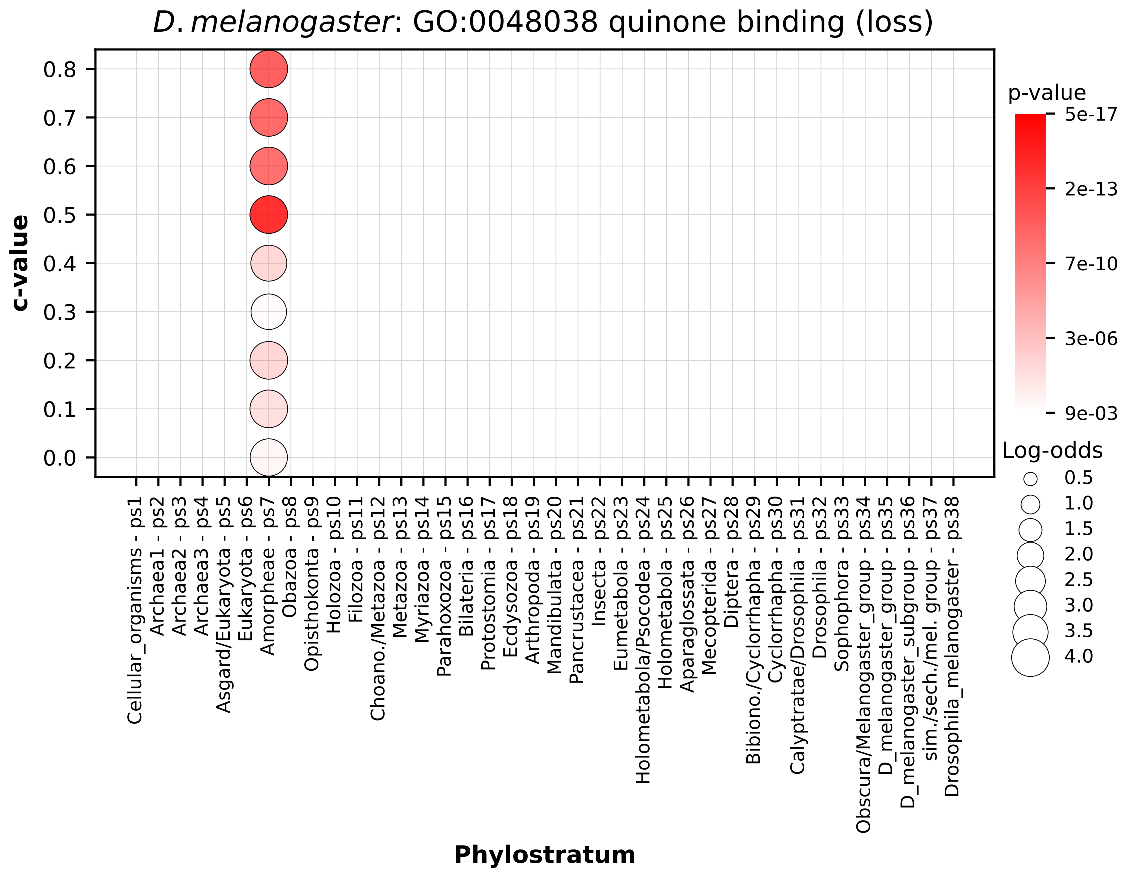

D. melanogaster: GO:0048038 quinone binding (loss)
c-value
0.8
0.7
0.6
0.5
0.4
0.3
0.2
0.1
0.0
p-value
5e-17
2e-13
7e-10
3e-06
9e-03
Log-odds
0.5
1.0
1.5
2.0
2.5
3.0
3.5
4.0
Cellular_organisms - ps1
Archaea1 - ps2
Archaea2 - ps3
Archaea3 - ps4
Asgard/Eukaryota - ps5
Eukaryota - ps6
Amorpheae - ps7
Obazoa - ps8
Opisthokonta - ps9
Holozoa - ps10
Filozoa - ps11
Choano./Metazoa - ps12
Metazoa - ps13
Myriazoa - ps14
Parahoxozoa - ps15
Bilateria - ps16
Protostomia - ps17
Ecdysozoa - ps18
Arthropoda - ps19
Mandibulata - ps20
Pancrustacea - ps21
Insecta - ps22
Eumetabola - ps23
Holometabola/Psocodea - ps24
Holometabola - ps25
Aparaglossata - ps26
Mecopterida - ps27
Diptera - ps28
Bibiono./Cyclorrhapha - ps29
Cyclorrhapha - ps30
Calyptratae/Drosophila - ps31
Drosophila - ps32
Sophophora - ps33
Obscura/Melanogaster_group - ps34
D_melanogaster_group - ps35
D_melanogaster_subgroup - ps36
sim./sech./mel. group - ps37
Drosophila_melanogaster - ps38
Phylostratum

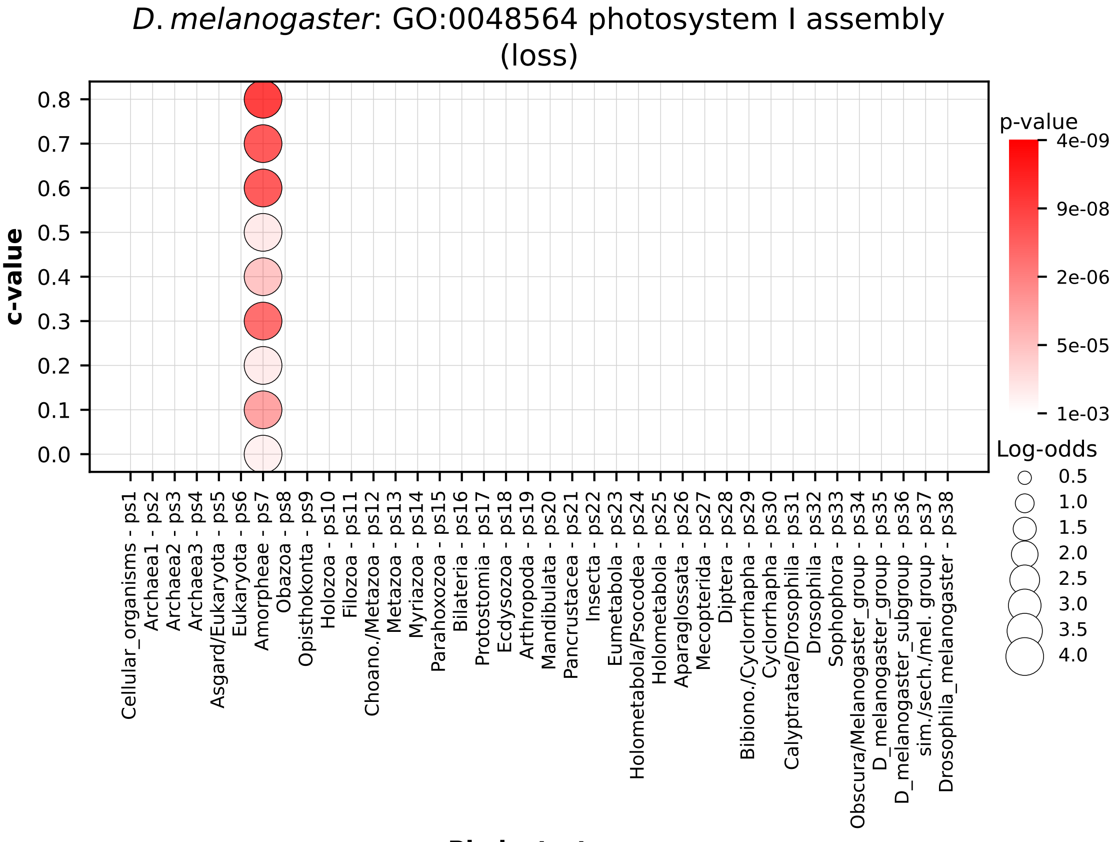

D. melanogaster: GO:0048564 photosystem I assembly (loss)
c-value
0.8
0.7
0.6
0.5
0.4
0.3
0.2
0.1
0.0
Phylostratum
Cellular_organisms - ps1
Archaea1 - ps2
Archaea2 - ps3
Archaea3 - ps4
Asgard/Eukaryota - ps5
Eukaryota - ps6
Amorpheae - ps7
Obazoa - ps8
Opisthokonta - ps9
Holozoa - ps10
Filozoa - ps11
Choano./Metazoa - ps12
Metazoa - ps13
Myriazoa - ps14
Parahoxozoa - ps15
Bilateria - ps16
Protostomia - ps17
Ecdysozoa - ps18
Arthropoda - ps19
Mandibulata - ps20
Pancrustacea - ps21
Insecta - ps22
Eumetabola - ps23
Holometabola/Psocodea - ps24
Holometabola - ps25
Aparaglossata - ps26
Mecopterida - ps27
Diptera - ps28
Bibiono./Cyclorrhapha - ps29
Cyclorrhapha - ps30
Calyptratae/Drosophila - ps31
Drosophila - ps32
Sophophora - ps33
Obscura/Melanogaster_group - ps34
D_melanogaster_group - ps35
D_melanogaster_subgroup - ps36
sim./sech./mel. group - ps37
Drosophila_melanogaster - ps38
p-value
4e-09
9e-08
2e-06
5e-05
1e-03
Log-odds
0.5
1.0
1.5
2.0
2.5
3.0
3.5
4.0

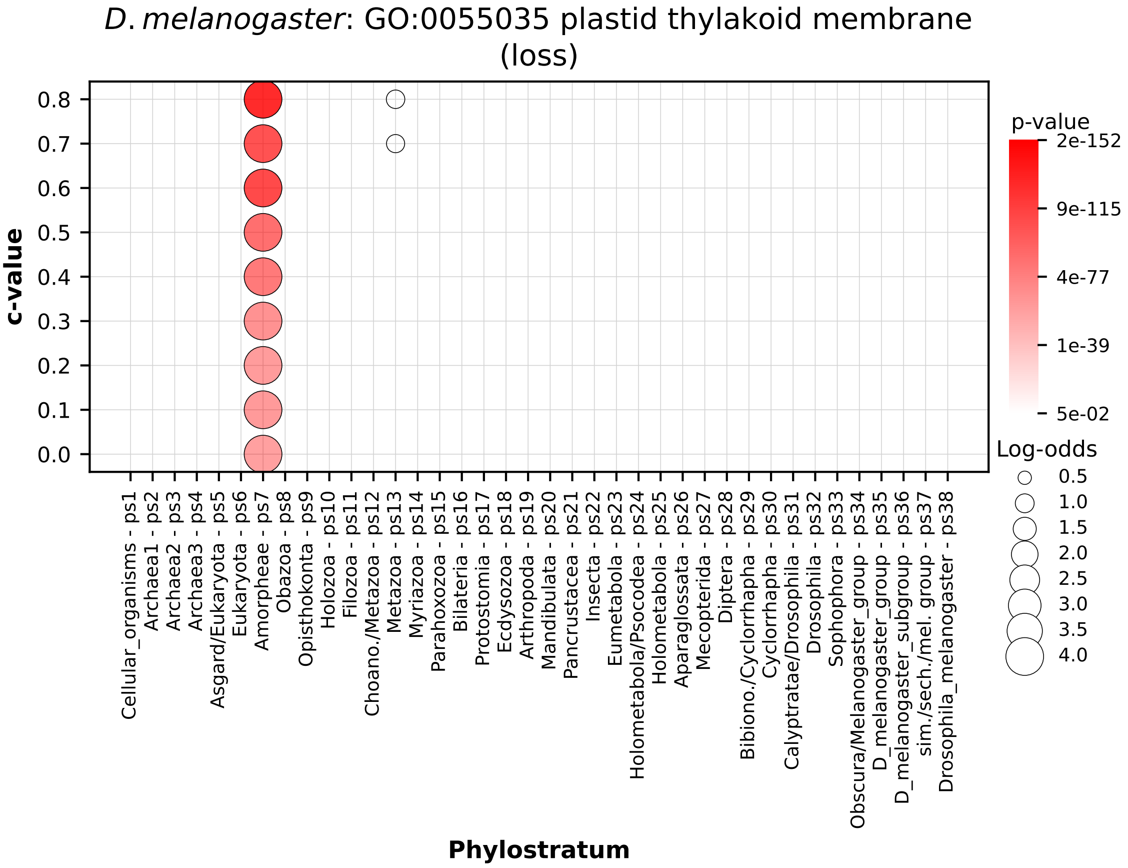

D. melanogaster: GO:0055035 plastid thylakoid membrane (loss)
c-value
0.8
0.7
0.6
0.5
0.4
0.3
0.2
0.1
0.0
Phylostratum
Cellular_organisms - ps1
Archaea1 - ps2
Archaea2 - ps3
Archaea3 - ps4
Asgard/Eukaryota - ps5
Eukaryota - ps6
Amorpheae - ps7
Obazoa - ps8
Opisthokonta - ps9
Holozoa - ps10
Filozoa - ps11
Choano./Metazoa - ps12
Metazoa - ps13
Myriazoa - ps14
Parahoxozoa - ps15
Bilateria - ps16
Protostomia - ps17
Ecdysozoa - ps18
Arthropoda - ps19
Mandibulata - ps20
Pancrustacea - ps21
Insecta - ps22
Eumetabola - ps23
Holometabola/Psocodea - ps24
Holometabola - ps25
Aparaglossata - ps26
Mecopterida - ps27
Diptera - ps28
Bibiono./Cyclorrhapha - ps29
Cyclorrhapha - ps30
Calyptratae/Drosophila - ps31
Drosophila - ps32
Sophophora - ps33
Obscura/Melanogaster_group - ps34
D_melanogaster_group - ps35
D_melanogaster_subgroup - ps36
sim./sech./mel. group - ps37
Drosophila_melanogaster - ps38
p-value
2e-152
9e-115
4e-77
1e-39
5e-02
Log-odds
0.5
1.0
1.5
2.0
2.5
3.0
3.5
4.0

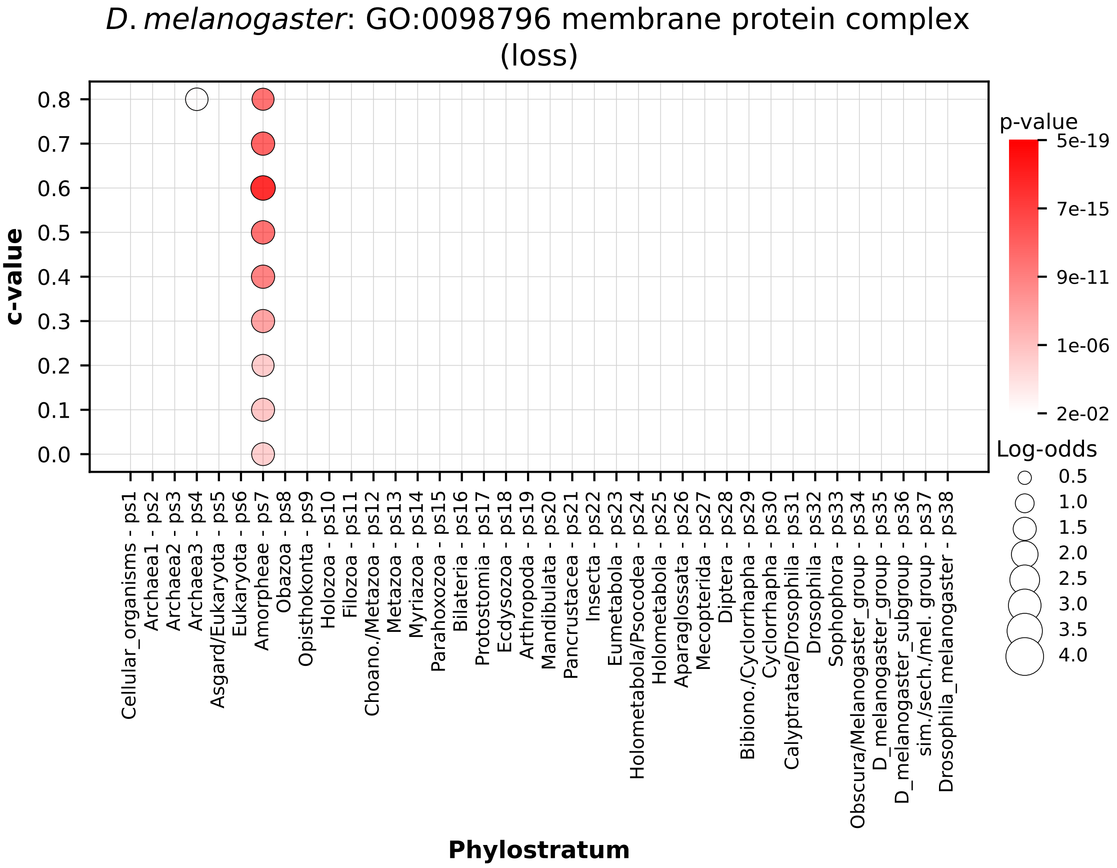

$D. melanogaster$: GO:0098796 membrane protein complex (loss)
c-value
0.8
0.7
0.6
0.5
0.4
0.3
0.2
0.1
0.0
p-value
5e-19
7e-15
9e-11
1e-06
2e-02
Log-odds
0.5
1.0
1.5
2.0
2.5
3.0
3.5
4.0
Cellular_organisms - ps1
Archaea1 - ps2
Archaea2 - ps3
Archaea3 - ps4
Asgard/Eukaryota - ps5
Eukaryota - ps6
Amorpheae - ps7
Obazoa - ps8
Opisthokonta - ps9
Holozoa - ps10
Filozoa - ps11
Choano./Metazoa - ps12
Metazoa - ps13
Myriazoa - ps14
Parahoxozoa - ps15
Bilateria - ps16
Protostomia - ps17
Ecdysozoa - ps18
Arthropoda - ps19
Mandibulata - ps20
Pancrustacea - ps21
Insecta - ps22
Eumetabola - ps23
Holometabola/Psocodea - ps24
Holometabola - ps25
Aparaglossata - ps26
Mecopterida - ps27
Diptera - ps28
Bibiono./Cyclorrhapha - ps29
Cyclorrhapha - ps30
Calyptratae/Drosophila - ps31
Drosophila - ps32
Sophophora - ps33
Obscura/Melanogaster_group - ps34
D_melanogaster_group - ps35
D_melanogaster_subgroup - ps36
sim./sech./mel. group - ps37
Drosophila_melanogaster - ps38
Phylostratum

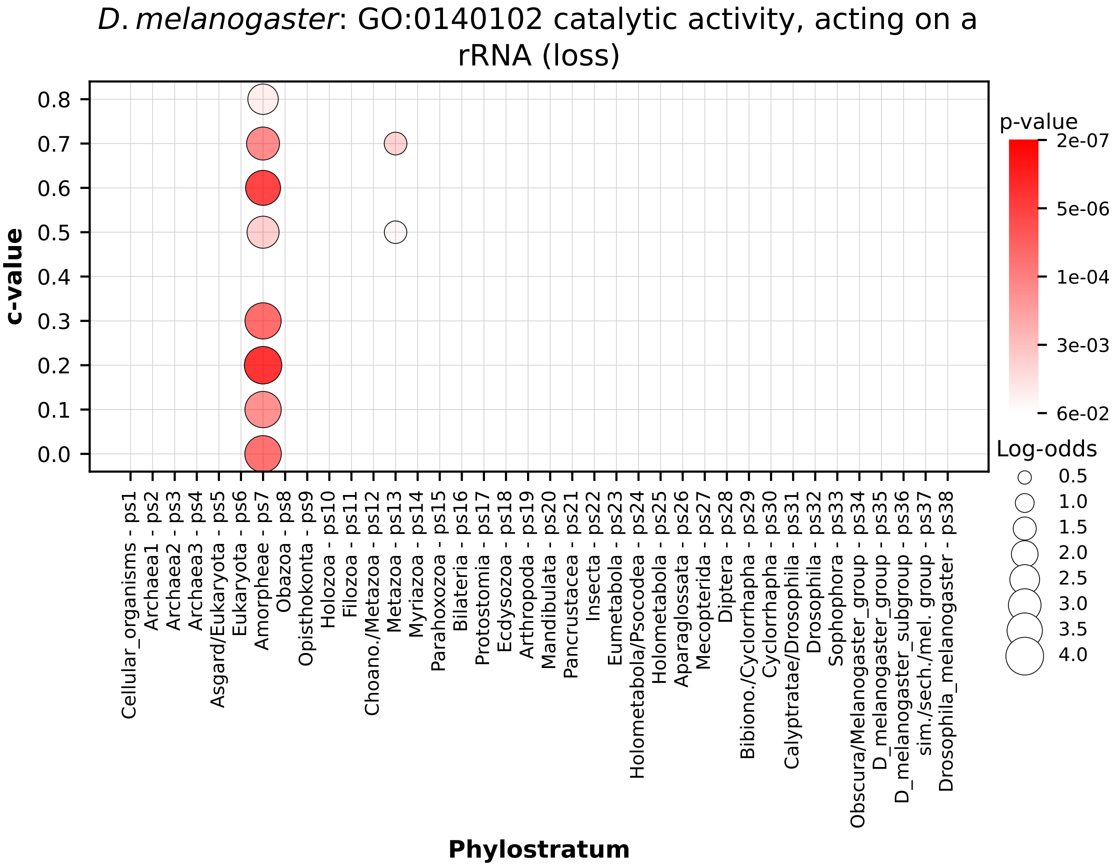
D. melanogaster: GO:0140102 catalytic activity, acting on a rRNA (loss)
c-value
0.8
0.7
0.6
0.5
0.4
0.3
0.2
0.1
0.0
Phylostratum
Cellular_organisms - ps1
Archaea1 - ps2
Archaea2 - ps3
Archaea3 - ps4
Asgard/Eukaryota - ps5
Eukaryota - ps6
Amorpheae - ps7
Obazoa - ps8
Opisthokonta - ps9
Holozoa - ps10
Filozoa - ps11
Choano./Metazoa - ps12
Metazoa - ps13
Myriazoa - ps14
Parahoxozoa - ps15
Bilateria - ps16
Protostomia - ps17
Ecdysozoa - ps18
Arthropoda - ps19
Mandibulata - ps20
Pancrustacea - ps21
Insecta - ps22
Eumetabola - ps23
Holometabola/Psocodea - ps24
Holometabola - ps25
Aparaglossata - ps26
Mecopterida - ps27
Diptera - ps28
Bibiono./Cyclorrhapha - ps29
Cyclorrhapha - ps30
Calyptratae/Drosophila - ps31
Drosophila - ps32
Sophophora - ps33
Obscura/Melanogaster_group - ps34
D_melanogaster_group - ps35
D_melanogaster_subgroup - ps36
sim./sech./mel. group - ps37
Drosophila_melanogaster - ps38
p-value
2e-07
5e-06
1e-04
3e-03
6e-02
Log-odds
0.5
1.0
1.5
2.0
2.5
3.0
3.5
4.0

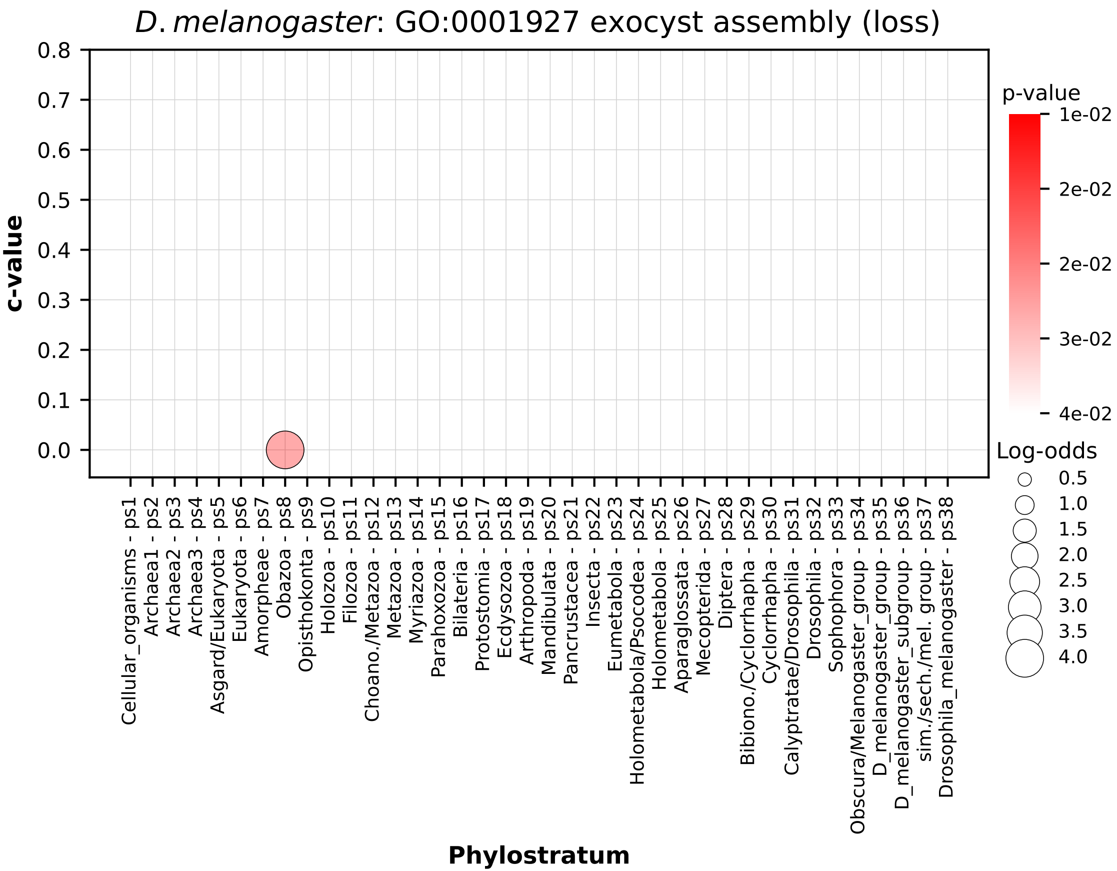

D. melanogaster: GO:0001927 exocyst assembly (loss)
c-value
0.8
0.7
0.6
0.5
0.4
0.3
0.2
0.1
0.0
Phylostratum
Cellular_organisms - ps1
Archaea1 - ps2
Archaea2 - ps3
Archaea3 - ps4
Asgard/Eukaryota - ps5
Eukaryota - ps6
Amorpheae - ps7
Obazoa - ps8
Opisthokonta - ps9
Holozoa - ps10
Filozoa - ps11
Choano./Metazoa - ps12
Metazoa - ps13
Myriazoa - ps14
Parahoxozoa - ps15
Bilateria - ps16
Protostomia - ps17
Ecdysozoa - ps18
Arthropoda - ps19
Mandibulata - ps20
Pancrustacea - ps21
Insecta - ps22
Eumetabola - ps23
Holometabola/Psocodea - ps24
Holometabola - ps25
Aparaglossata - ps26
Mecopterida - ps27
Diptera - ps28
Bibiono./Cyclorrhapha - ps29
Cyclorrhapha - ps30
Calyptratae/Drosophila - ps31
Drosophila - ps32
Sophophora - ps33
Obscura/Melanogaster_group - ps34
D_melanogaster_group - ps35
D_melanogaster_subgroup - ps36
sim./sech./mel. group - ps37
Drosophila_melanogaster - ps38
p-value
1e-02
2e-02
2e-02
3e-02
4e-02
Log-odds
0.5
1.0
1.5
2.0
2.5
3.0
3.5
4.0

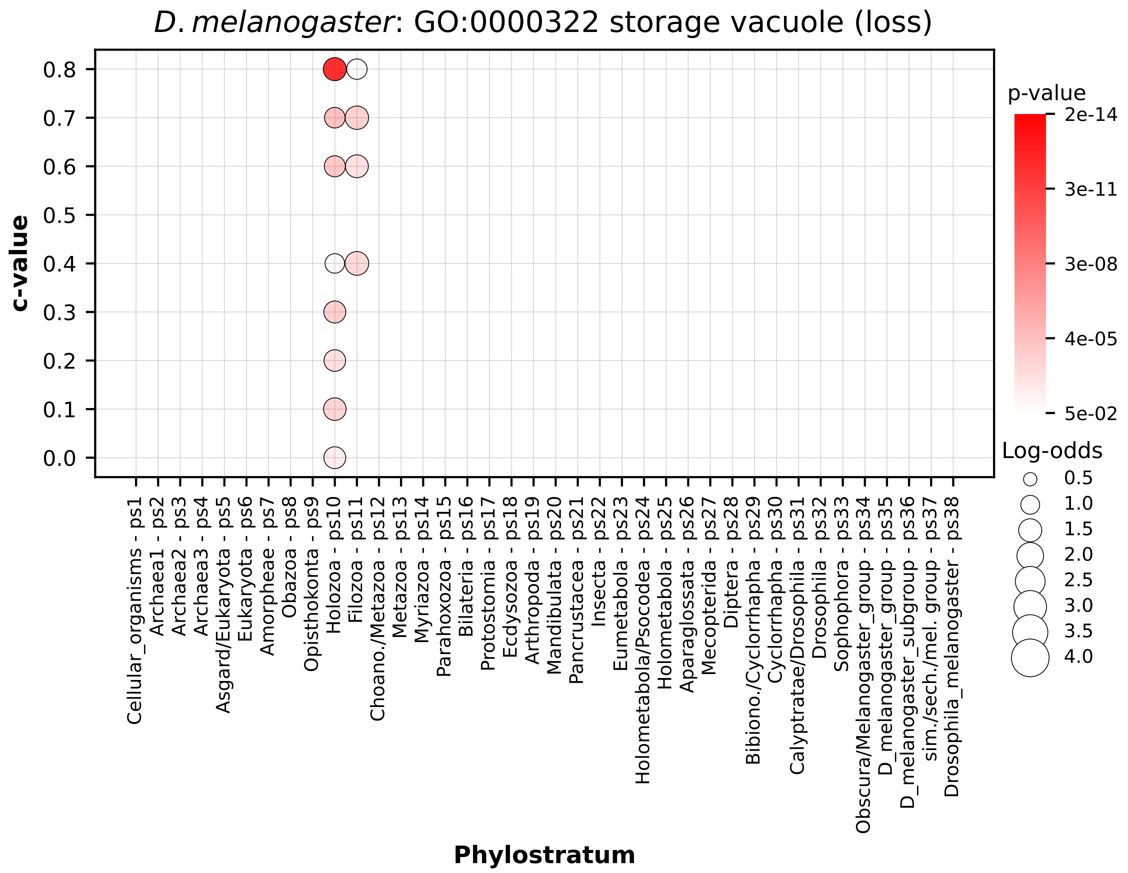
D. melanogaster: GO:0000322 storage vacuole (loss)
c-value
0.8
0.7
0.6
0.5
0.4
0.3
0.2
0.1
0.0
p-value
2e-14
3e-11
3e-08
4e-05
5e-02
Log-odds
0.5
1.0
1.5
2.0
2.5
3.0
3.5
4.0
Cellular_organisms - ps1
Archaea1 - ps2
Archaea2 - ps3
Archaea3 - ps4
Asgard/Eukaryota - ps5
Eukaryota - ps6
Amorpheae - ps7
Obazoa - ps8
Opisthokonta - ps9
Holozoa - ps10
Filozoa - ps11
Choano./Metazoa - ps12
Metazoa - ps13
Myriazoa - ps14
Parahoxozoa - ps15
Bilateria - ps16
Protostomia - ps17
Ecdysozoa - ps18
Arthropoda - ps19
Mandibulata - ps20
Pancrustacea - ps21
Insecta - ps22
Eumetabola - ps23
Holometabola/Psocodea - ps24
Holometabola - ps25
Aparaglossata - ps26
Mecopterida - ps27
Diptera - ps28
Bibiono./Cyclorrhapha - ps29
Cyclorrhapha - ps30
Calyptratae/Drosophila - ps31
Drosophila - ps32
Sophophora - ps33
Obscura/Melanogaster_group - ps34
D_melanogaster_group - ps35
D_melanogaster_subgroup - ps36
sim./sech./mel. group - ps37
Drosophila_melanogaster - ps38
Phylostratum

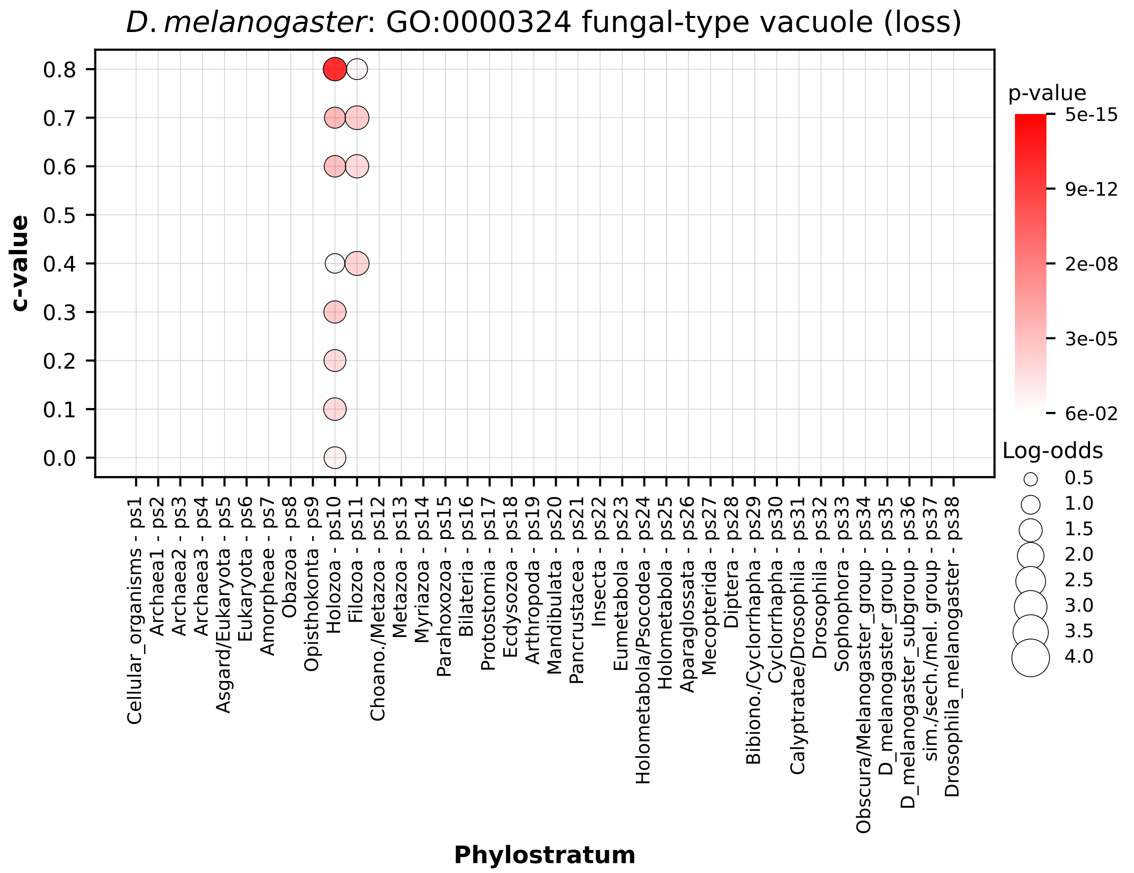
D. melanogaster: GO:0000324 fungal-type vacuole (loss)
c-value
0.0
0.1
0.2
0.3
0.4
0.5
0.6
0.7
0.8
Phylostratum
Cellular_organisms - ps1
Archaea1 - ps2
Archaea2 - ps3
Archaea3 - ps4
Asgard/Eukaryota - ps5
Eukaryota - ps6
Amorpheae - ps7
Obazoa - ps8
Opisthokonta - ps9
Holozoa - ps10
Filozoa - ps11
Choano./Metazoa - ps12
Metazoa - ps13
Myriazoa - ps14
Parahoxozoa - ps15
Bilateria - ps16
Protostomia - ps17
Ecdysozoa - ps18
Arthropoda - ps19
Mandibulata - ps20
Pancrustacea - ps21
Insecta - ps22
Eumetabola - ps23
Holometabola/Psocodea - ps24
Holometabola - ps25
Aparaglossata - ps26
Mecopterida - ps27
Diptera - ps28
Bibiono./Cyclorrhapha - ps29
Cyclorrhapha - ps30
Calyptratae/Drosophila - ps31
Drosophila - ps32
Sophophora - ps33
Obscura/Melanogaster_group - ps34
D_melanogaster_group - ps35
D_melanogaster_subgroup - ps36
sim./sech./mel. group - ps37
Drosophila_melanogaster - ps38
p-value
5e-15
9e-12
2e-08
3e-05
6e-02
Log-odds
0.5
1.0
1.5
2.0
2.5
3.0
3.5
4.0

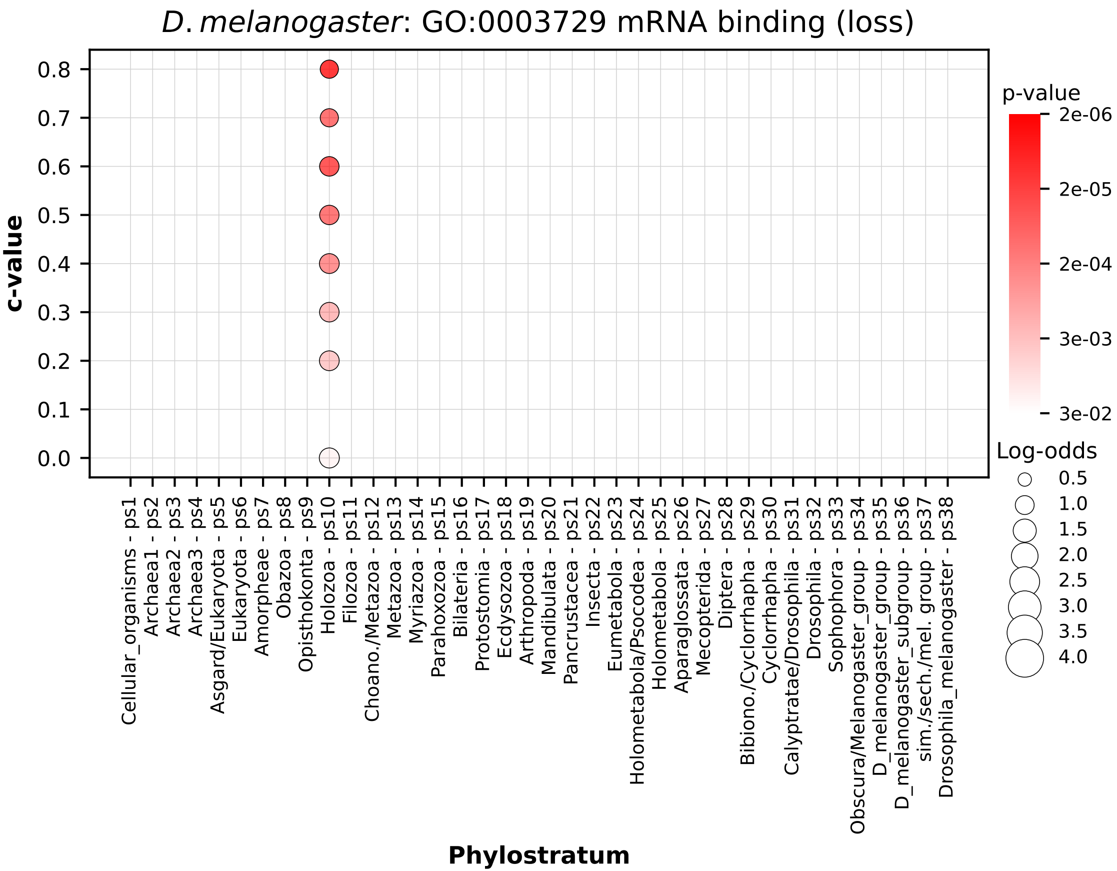

D. melanogaster: GO:0003729 mRNA binding (loss)
c-value
0.8
0.7
0.6
0.5
0.4
0.3
0.2
0.1
0.0
p-value
2e-06
2e-05
2e-04
3e-03
3e-02
Log-odds
0.5
1.0
1.5
2.0
2.5
3.0
3.5
4.0
Cellular_organisms - ps1
Archaea1 - ps2
Archaea2 - ps3
Archaea3 - ps4
Asgard/Eukaryota - ps5
Eukaryota - ps6
Amorpheae - ps7
Obazoa - ps8
Opisthokonta - ps9
Holozoa - ps10
Filozoa - ps11
Choano./Metazoa - ps12
Metazoa - ps13
Myriazoa - ps14
Parahoxozoa - ps15
Bilateria - ps16
Protostomia - ps17
Ecdysozoa - ps18
Arthropoda - ps19
Mandibulata - ps20
Pancrustacea - ps21
Insecta - ps22
Eumetabola - ps23
Holometabola/Psocodea - ps24
Holometabola - ps25
Aparaglossata - ps26
Mecopterida - ps27
Diptera - ps28
Bibiono./Cyclorrhapha - ps29
Cyclorrhapha - ps30
Calyptratae/Drosophila - ps31
Drosophila - ps32
Sophophora - ps33
Obscura/Melanogaster_group - ps34
D_melanogaster_group - ps35
D_melanogaster_subgroup - ps36
sim./sech./mel. group - ps37
Drosophila_melanogaster - ps38
Phylostratum

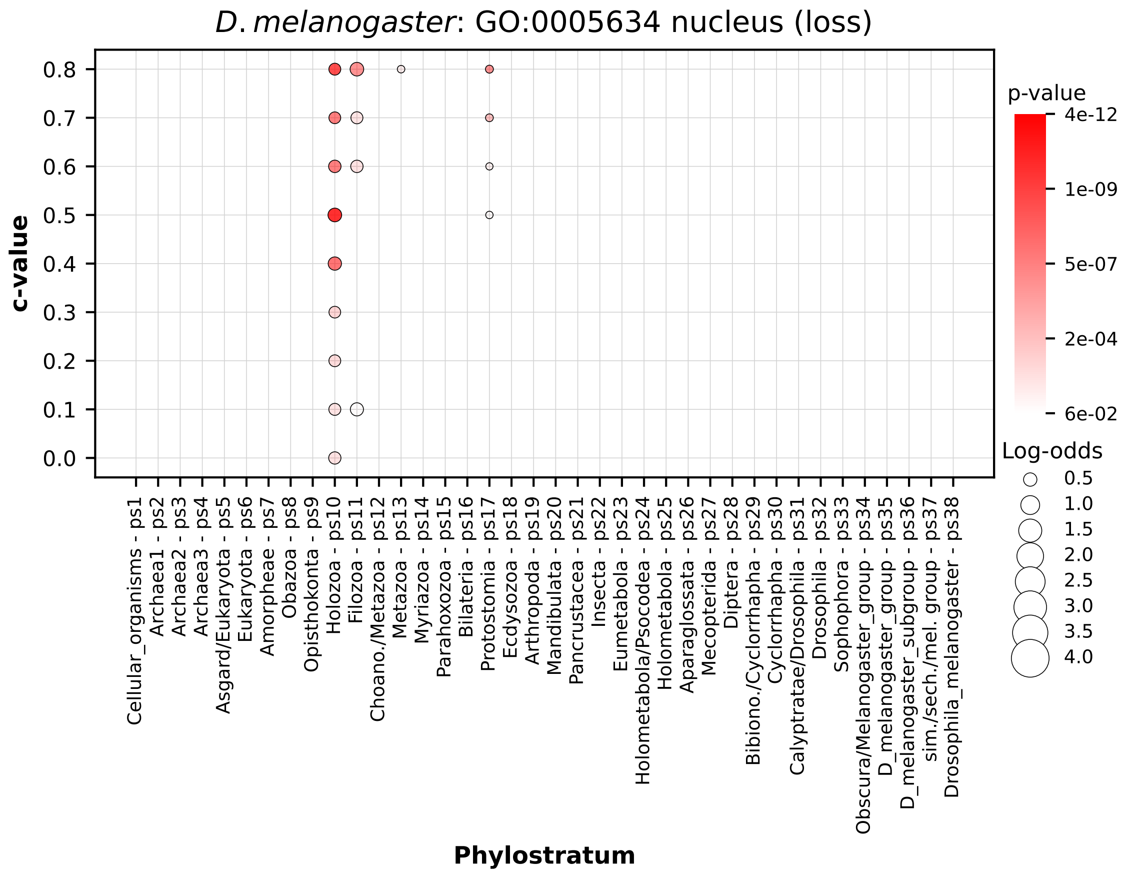
D. melanogaster: GO:0005634 nucleus (loss)
c-value
0.8
0.7
0.6
0.5
0.4
0.3
0.2
0.1
0.0
Phylostratum
Cellular_organisms - ps1
Archaea1 - ps2
Archaea2 - ps3
Archaea3 - ps4
Asgard/Eukaryota - ps5
Eukaryota - ps6
Amorpheae - ps7
Obazoa - ps8
Opisthokonta - ps9
Holozoa - ps10
Filozoa - ps11
Choano./Metazoa - ps12
Metazoa - ps13
Myriazoa - ps14
Parahoxozoa - ps15
Bilateria - ps16
Protostomia - ps17
Ecdysozoa - ps18
Arthropoda - ps19
Mandibulata - ps20
Pancrustacea - ps21
Insecta - ps22
Eumetabola - ps23
Holometabola/Psocodea - ps24
Holometabola - ps25
Aparaglossata - ps26
Mecopterida - ps27
Diptera - ps28
Bibiono./Cyclorrhapha - ps29
Cyclorrhapha - ps30
Calyptratae/Drosophila - ps31
Drosophila - ps32
Sophophora - ps33
Obscura/Melanogaster_group - ps34
D_melanogaster_group - ps35
D_melanogaster_subgroup - ps36
sim./sech./mel. group - ps37
Drosophila_melanogaster - ps38
p-value
4e-12
1e-09
5e-07
2e-04
6e-02
Log-odds
0.5
1.0
1.5
2.0
2.5
3.0
3.5
4.0

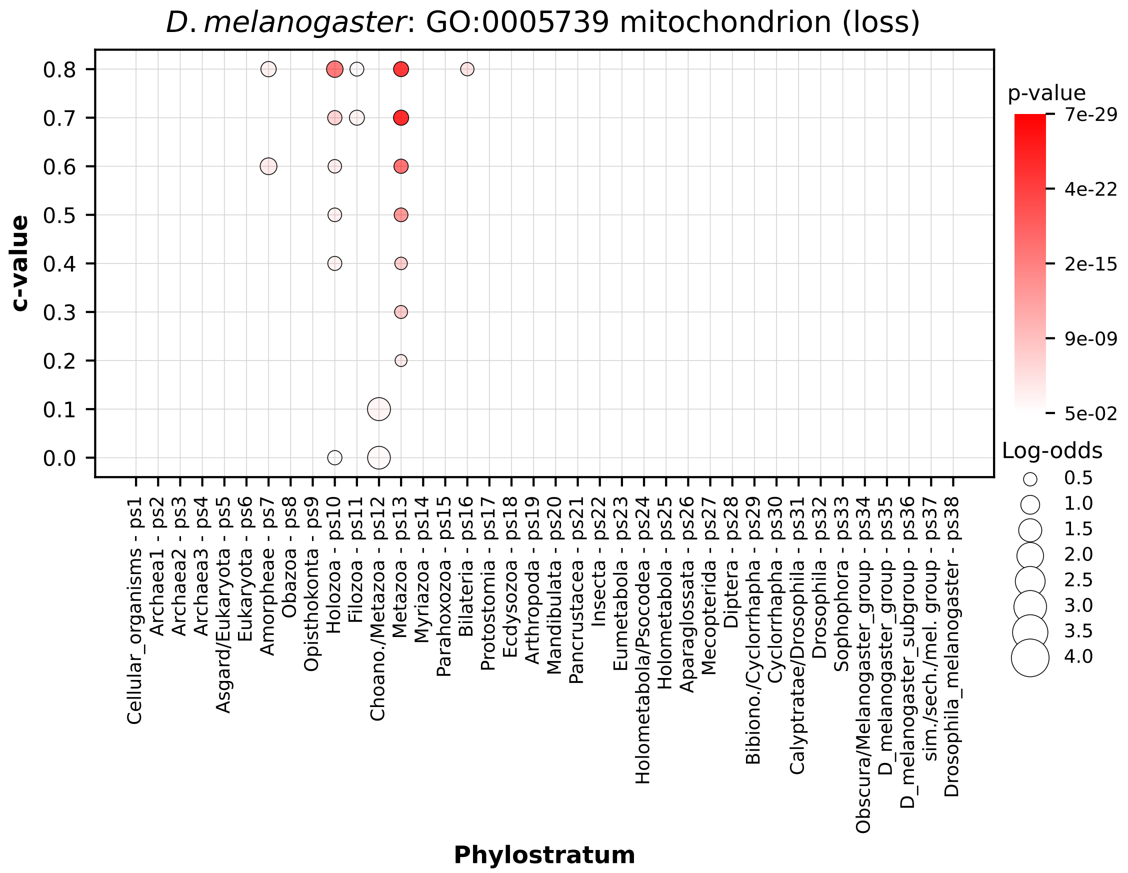

D. melanogaster: GO:0005739 mitochondrion (loss)
c-value
0.8
0.7
0.6
0.5
0.4
0.3
0.2
0.1
0.0
Phylostratum
Cellular_organisms - ps1
Archaea1 - ps2
Archaea2 - ps3
Archaea3 - ps4
Asgard/Eukaryota - ps5
Eukaryota - ps6
Amorpheae - ps7
Obazoa - ps8
Opisthokonta - ps9
Holozoa - ps10
Filozoa - ps11
Choano./Metazoa - ps12
Metazoa - ps13
Myriazoa - ps14
Parahoxozoa - ps15
Bilateria - ps16
Protostomia - ps17
Ecdysozoa - ps18
Arthropoda - ps19
Mandibulata - ps20
Pancrustacea - ps21
Insecta - ps22
Eumetabola - ps23
Holometabola/Psocodea - ps24
Holometabola - ps25
Aparaglossata - ps26
Mecopterida - ps27
Diptera - ps28
Bibiono./Cyclorrhapha - ps29
Cyclorrhapha - ps30
Calyptratae/Drosophila - ps31
Drosophila - ps32
Sophophora - ps33
Obscura/Melanogaster_group - ps34
D_melanogaster_group - ps35
D_melanogaster_subgroup - ps36
sim./sech./mel. group - ps37
Drosophila_melanogaster - ps38
p-value
7e-29
4e-22
2e-15
9e-09
5e-02
Log-odds
0.5
1.0
1.5
2.0
2.5
3.0
3.5
4.0

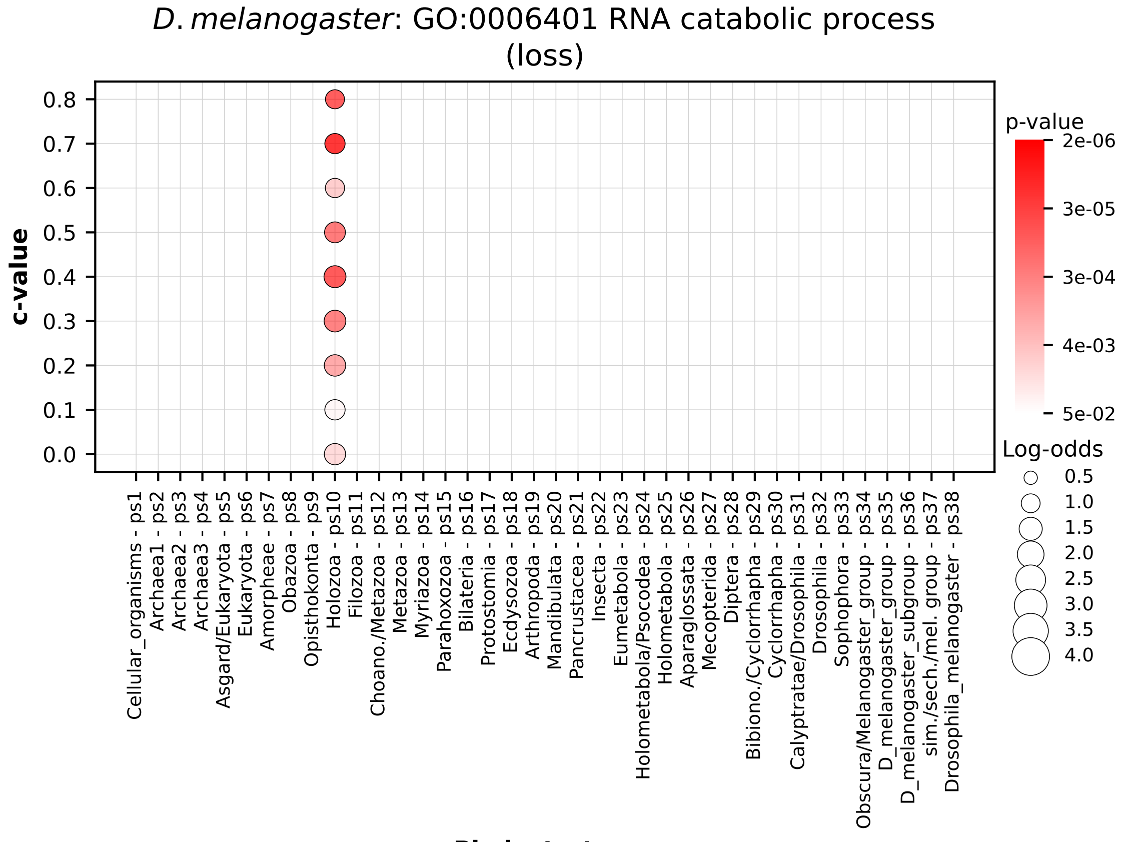

D. melanogaster: GO:0006401 RNA catabolic process
(loss)
c-value
0.8
0.7
0.6
0.5
0.4
0.3
0.2
0.1
0.0
Phylostratum
Cellular_organisms - ps1
Archaea1 - ps2
Archaea2 - ps3
Archaea3 - ps4
Asgard/Eukaryota - ps5
Eukaryota - ps6
Amorpheae - ps7
Obazoa - ps8
Opisthokonta - ps9
Holozoa - ps10
Filozoa - ps11
Choano./Metazoa - ps12
Metazoa - ps13
Myriazoa - ps14
Parahoxozoa - ps15
Bilateria - ps16
Protostomia - ps17
Ecdysozoa - ps18
Arthropoda - ps19
Mandibulata - ps20
Pancrustacea - ps21
Insecta - ps22
Eumetabola - ps23
Holometabola/Psocodea - ps24
Holometabola - ps25
Aparaglossata - ps26
Mecopterida - ps27
Diptera - ps28
Bibiono./Cyclorrhapha - ps29
Cyclorrhapha - ps30
Calyptratae/Drosophila - ps31
Drosophila - ps32
Sophophora - ps33
Obscura/Melanogaster_group - ps34
D_melanogaster_group - ps35
D_melanogaster_subgroup - ps36
sim./sech./mel. group - ps37
Drosophila_melanogaster - ps38
p-value
2e-06
3e-05
3e-04
4e-03
5e-02
Log-odds
0.5
1.0
1.5
2.0
2.5
3.0
3.5
4.0

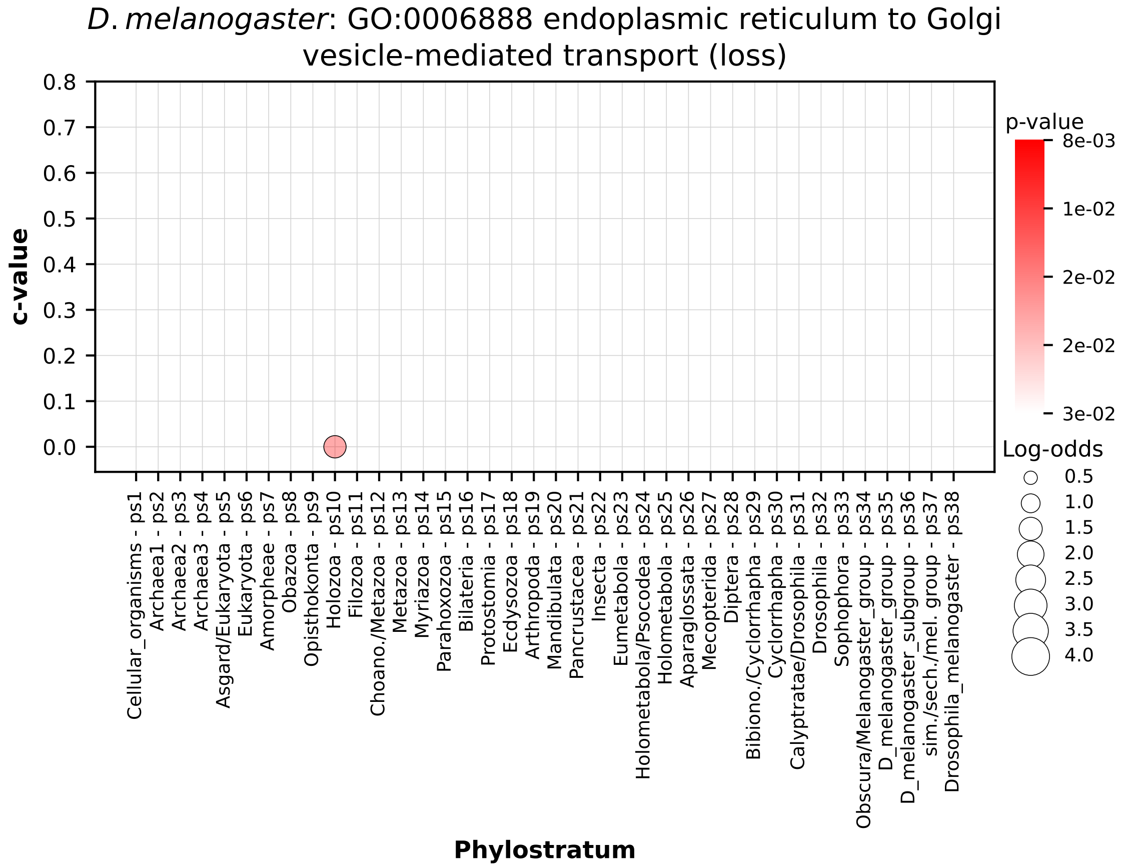

D. melanogaster: GO:0006888 endoplasmic reticulum to Golgi vesicle-mediated transport (loss)
c-value
0.8
0.7
0.6
0.5
0.4
0.3
0.2
0.1
0.0
Phylostratum
Cellular_organisms - ps1
Archaea1 - ps2
Archaea2 - ps3
Archaea3 - ps4
Asgard/Eukaryota - ps5
Eukaryota - ps6
Amorpheae - ps7
Obazoa - ps8
Opisthokonta - ps9
Holozoa - ps10
Filozoa - ps11
Choano./Metazoa - ps12
Metazoa - ps13
Myriazoa - ps14
Parahoxozoa - ps15
Bilateria - ps16
Protostomia - ps17
Ecdysozoa - ps18
Arthropoda - ps19
Mandibulata - ps20
Pancrustacea - ps21
Insecta - ps22
Eumetabola - ps23
Holometabola/Psocodea - ps24
Holometabola - ps25
Aparaglossata - ps26
Mecopterida - ps27
Diptera - ps28
Bibiono./Cyclorrhapha - ps29
Cyclorrhapha - ps30
Calyptratae/Drosophila - ps31
Drosophila - ps32
Sophophora - ps33
Obscura/Melanogaster_group - ps34
D_melanogaster_group - ps35
D_melanogaster_subgroup - ps36
sim./sech./mel. group - ps37
Drosophila_melanogaster - ps38
p-value
8e-03
1e-02
2e-02
2e-02
3e-02
Log-odds
0.5
1.0
1.5
2.0
2.5
3.0
3.5
4.0

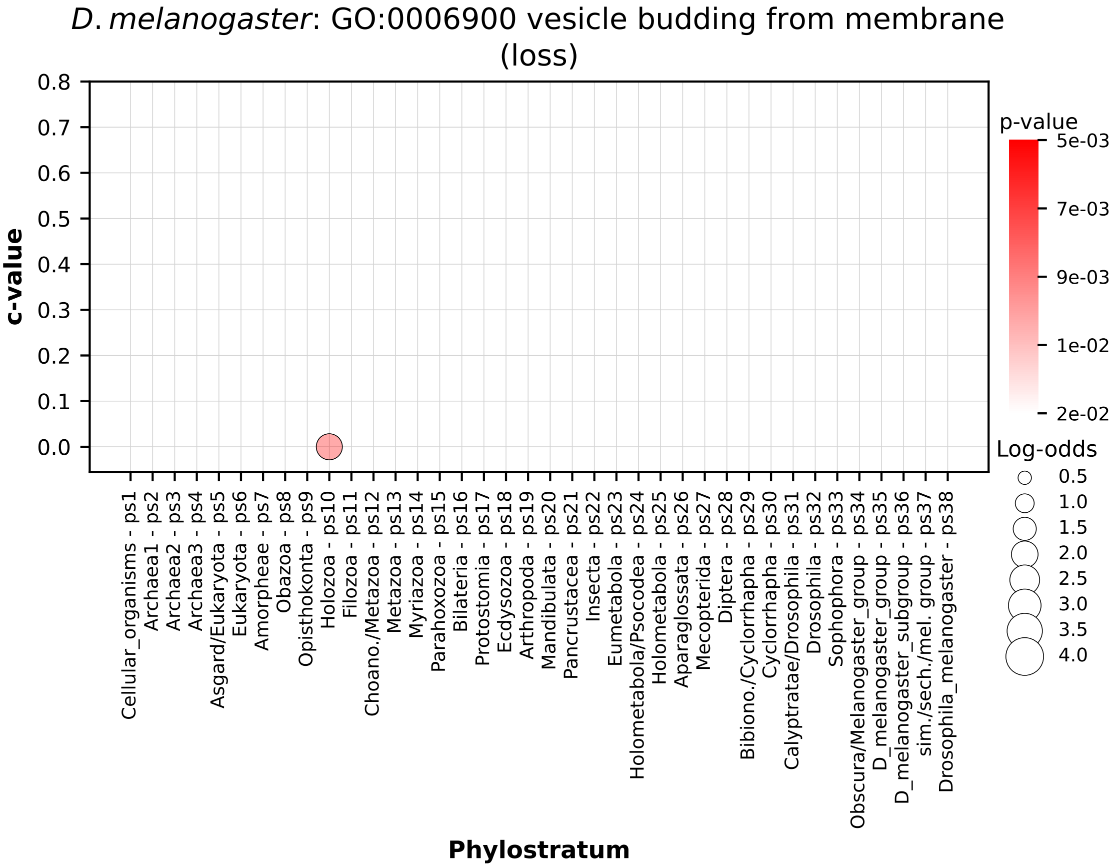

D. melanogaster: GO:0006900 vesicle budding from membrane (loss)
c-value
0.8
0.7
0.6
0.5
0.4
0.3
0.2
0.1
0.0
p-value
5e-03
7e-03
9e-03
1e-02
2e-02
Log-odds
0.5
1.0
1.5
2.0
2.5
3.0
3.5
4.0
Cellular_organisms - ps1
Archaea1 - ps2
Archaea2 - ps3
Archaea3 - ps4
Asgard/Eukaryota - ps5
Eukaryota - ps6
Amorpheae - ps7
Obazoa - ps8
Opisthokonta - ps9
Holozoa - ps10
Filozoa - ps11
Choano./Metazoa - ps12
Metazoa - ps13
Myriazoa - ps14
Parahoxozoa - ps15
Bilateria - ps16
Protostomia - ps17
Ecdysozoa - ps18
Arthropoda - ps19
Mandibulata - ps20
Pancrustacea - ps21
Insecta - ps22
Eumetabola - ps23
Holometabola/Psocodea - ps24
Holometabola - ps25
Aparaglossata - ps26
Mecopterida - ps27
Diptera - ps28
Bibiono./Cyclorrhapha - ps29
Cyclorrhapha - ps30
Calyptratae/Drosophila - ps31
Drosophila - ps32
Sophophora - ps33
Obscura/Melanogaster_group - ps34
D_melanogaster_group - ps35
D_melanogaster_subgroup - ps36
sim./sech./mel. group - ps37
Drosophila_melanogaster - ps38
Phylostratum

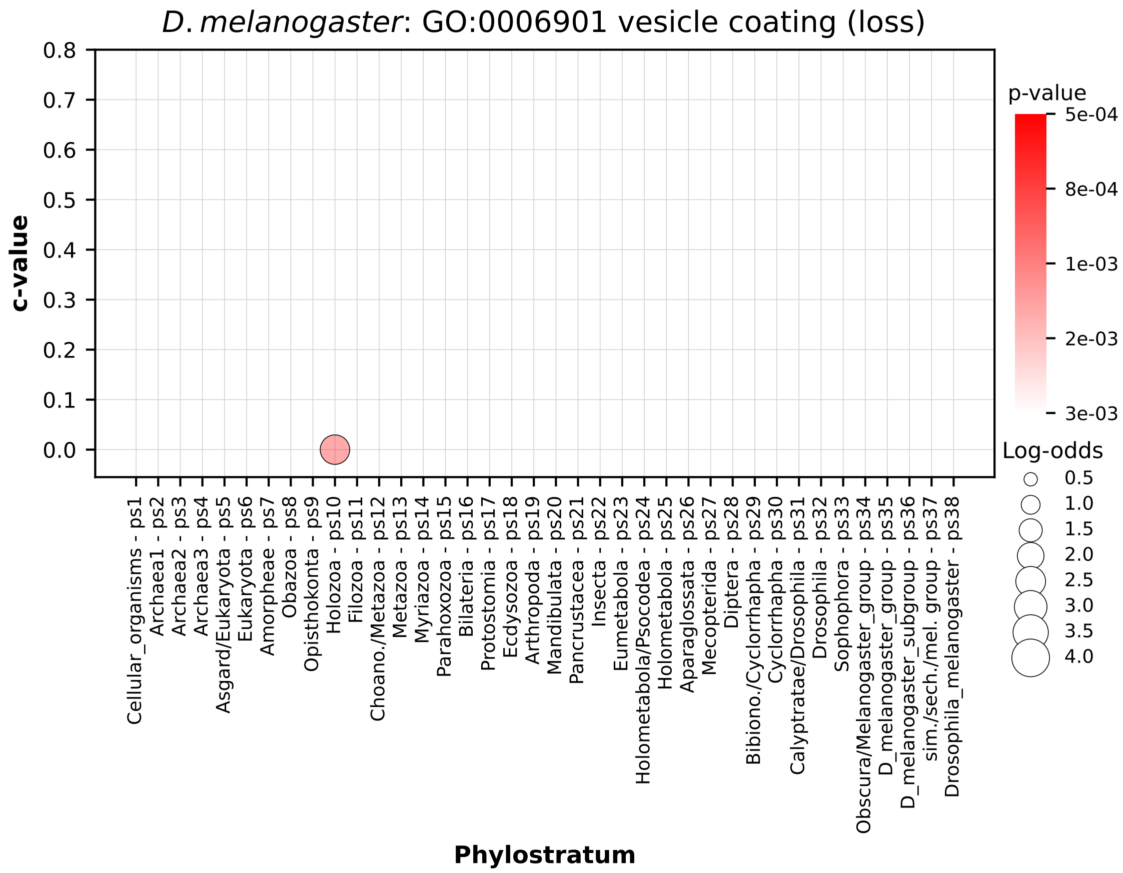

D. melanogaster: GO:0006901 vesicle coating (loss)
c-value
0.8
0.7
0.6
0.5
0.4
0.3
0.2
0.1
0.0
Phylostratum
Cellular_organisms - ps1
Archaea1 - ps2
Archaea2 - ps3
Archaea3 - ps4
Asgard/Eukaryota - ps5
Eukaryota - ps6
Amorpheae - ps7
Obazoa - ps8
Opisthokonta - ps9
Holozoa - ps10
Filozoa - ps11
Choano./Metazoa - ps12
Metazoa - ps13
Myriazoa - ps14
Parahoxozoa - ps15
Bilateria - ps16
Protostomia - ps17
Ecdysozoa - ps18
Arthropoda - ps19
Mandibulata - ps20
Pancrustacea - ps21
Insecta - ps22
Eumetabola - ps23
Holometabola/Psocodea - ps24
Holometabola - ps25
Aparaglossata - ps26
Mecopterida - ps27
Diptera - ps28
Bibiono./Cyclorrhapha - ps29
Cyclorrhapha - ps30
Calyptratae/Drosophila - ps31
Drosophila - ps32
Sophophora - ps33
Obscura/Melanogaster_group - ps34
D_melanogaster_group - ps35
D_melanogaster_subgroup - ps36
sim./sech./mel. group - ps37
Drosophila_melanogaster - ps38
p-value
5e-04
8e-04
1e-03
2e-03
3e-03
Log-odds
0.5
1.0
1.5
2.0
2.5
3.0
3.5
4.0

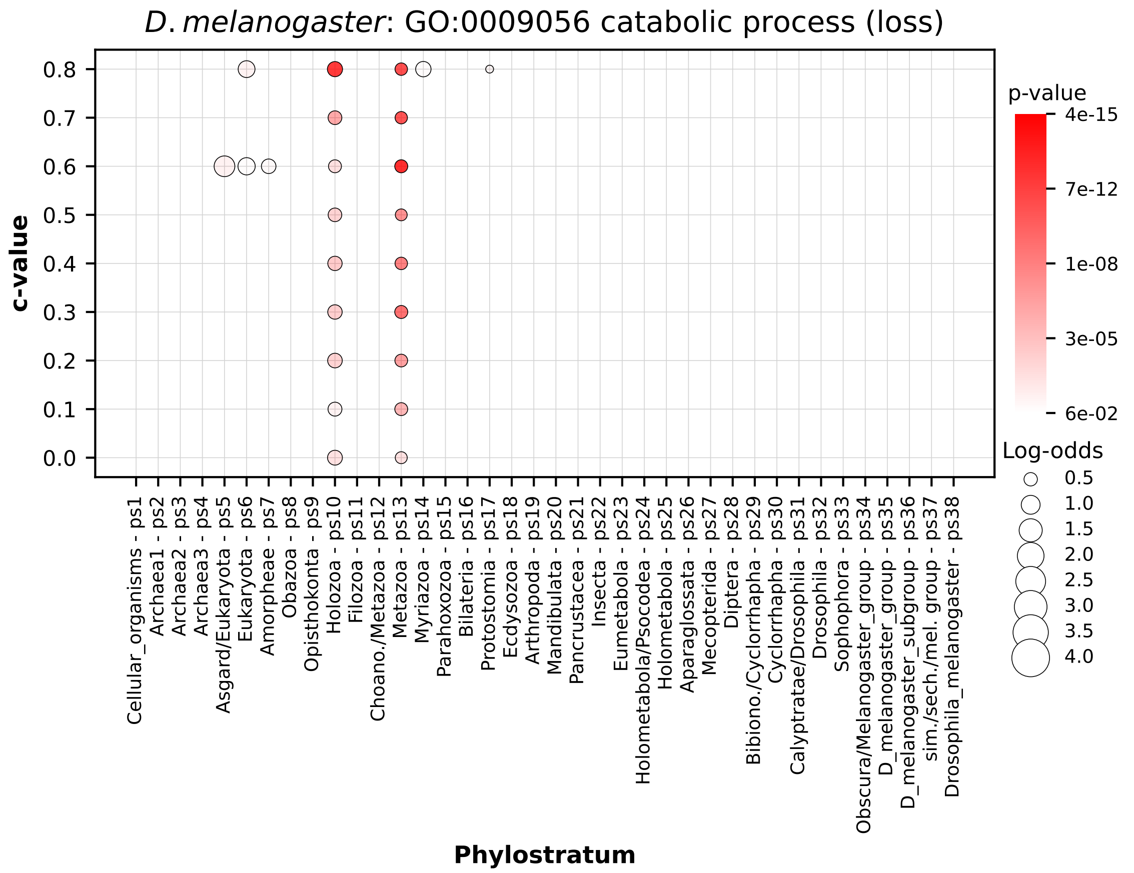
D. melanogaster: GO:0009056 catabolic process (loss)
c-value
0.8
0.7
0.6
0.5
0.4
0.3
0.2
0.1
0.0
p-value
4e-15
7e-12
1e-08
3e-05
6e-02
Log-odds
0.5
1.0
1.5
2.0
2.5
3.0
3.5
4.0
Cellular_organisms - ps1
Archaea1 - ps2
Archaea2 - ps3
Archaea3 - ps4
Asgard/Eukaryota - ps5
Eukaryota - ps6
Amorpheae - ps7
Obazoa - ps8
Opisthokonta - ps9
Holozoa - ps10
Filozoa - ps11
Choano./Metazoa - ps12
Metazoa - ps13
Myriazoa - ps14
Parahoxozoa - ps15
Bilateria - ps16
Protostomia - ps17
Ecdysozoa - ps18
Arthropoda - ps19
Mandibulata - ps20
Pancrustacea - ps21
Insecta - ps22
Eumetabola - ps23
Holometabola/Psocodea - ps24
Holometabola - ps25
Aparaglossata - ps26
Mecopterida - ps27
Diptera - ps28
Bibiono./Cyclorrhapha - ps29
Cyclorrhapha - ps30
Calyptratae/Drosophila - ps31
Drosophila - ps32
Sophophora - ps33
Obscura/Melanogaster_group - ps34
D_melanogaster_group - ps35
D_melanogaster_subgroup - ps36
sim./sech./mel. group - ps37
Drosophila_melanogaster - ps38
Phylostratum

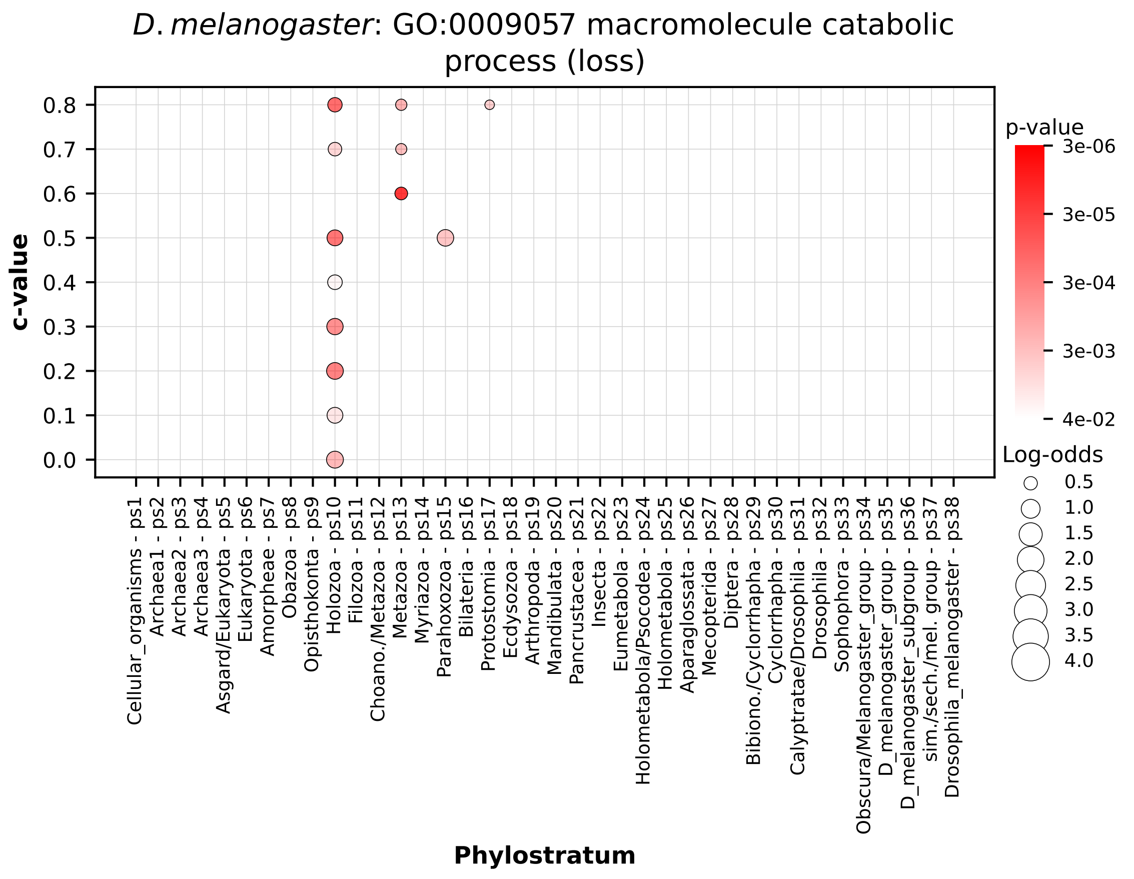

D. melanogaster: GO:0009057 macromolecule catabolic process (loss)
c-value
0.0
0.1
0.2
0.3
0.4
0.5
0.6
0.7
0.8
Phylostratum
Cellular_organisms - ps1
Archaea1 - ps2
Archaea2 - ps3
Archaea3 - ps4
Asgard/Eukaryota - ps5
Eukaryota - ps6
Amorpheae - ps7
Obazoa - ps8
Opisthokonta - ps9
Holozoa - ps10
Filozoa - ps11
Choano./Metazoa - ps12
Metazoa - ps13
Myriazoa - ps14
Parahoxozoa - ps15
Bilateria - ps16
Protostomia - ps17
Ecdysozoa - ps18
Arthropoda - ps19
Mandibulata - ps20
Pancrustacea - ps21
Insecta - ps22
Eumetabola - ps23
Holometabola/Psocodea - ps24
Holometabola - ps25
Aparaglossata - ps26
Mecopterida - ps27
Diptera - ps28
Bibiono./Cyclorrhapha - ps29
Cyclorrhapha - ps30
Calyptratae/Drosophila - ps31
Drosophila - ps32
Sophophora - ps33
Obscura/Melanogaster_group - ps34
D_melanogaster_group - ps35
D_melanogaster_subgroup - ps36
sim./sech./mel. group - ps37
Drosophila_melanogaster - ps38
p-value
3e-06
3e-05
3e-04
3e-03
4e-02
Log-odds
0.5
1.0
1.5
2.0
2.5
3.0
3.5
4.0

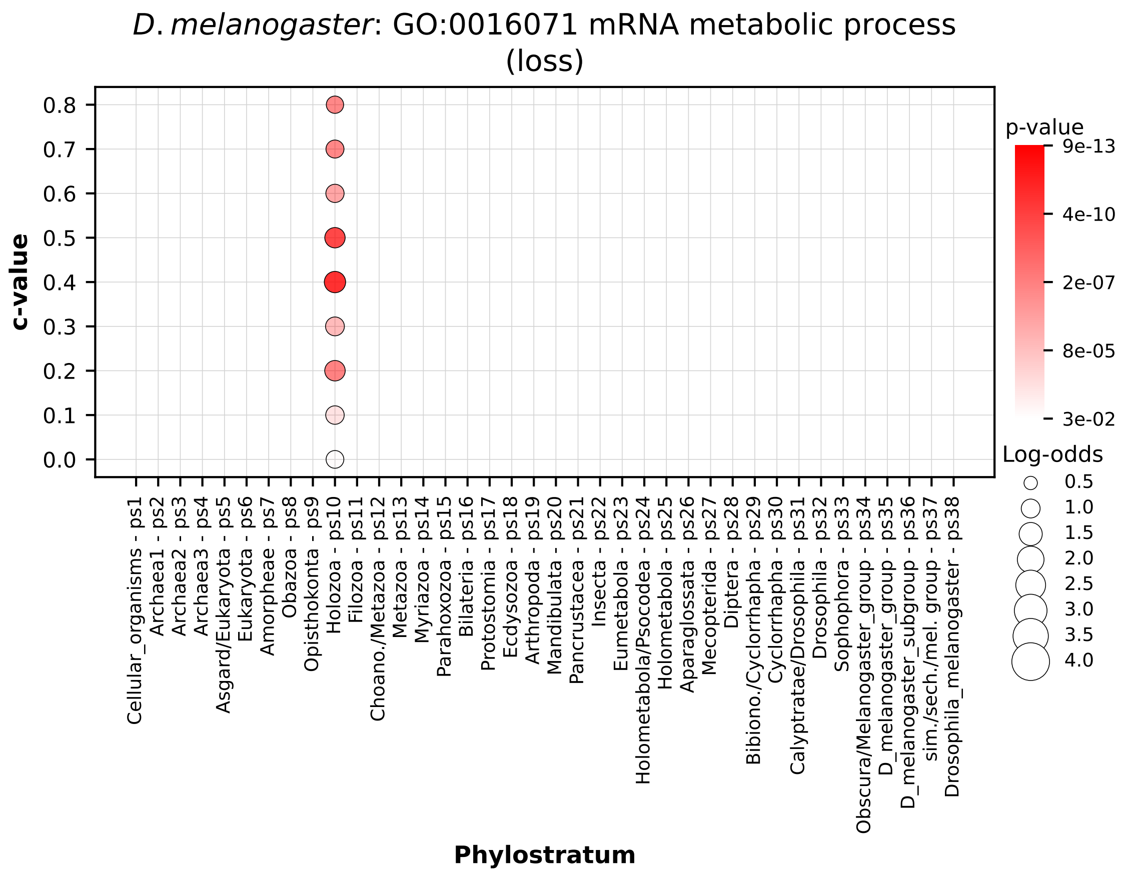

D. melanogaster: GO:0016071 mRNA metabolic process
(loss)
c-value
Phylostratum
p-value
9e-13
4e-10
2e-07
8e-05
3e-02
Log-odds
0.5
1.0
1.5
2.0
2.5
3.0
3.5
4.0
Cellular_organisms - ps1
Archaea1 - ps2
Archaea2 - ps3
Archaea3 - ps4
Asgard/Eukaryota - ps5
Eukaryota - ps6
Amorpheae - ps7
Obazoa - ps8
Opisthokonta - ps9
Holozoa - ps10
Filozoa - ps11
Choano./Metazoa - ps12
Metazoa - ps13
Myriazoa - ps14
Parahoxozoa - ps15
Bilateria - ps16
Protostomia - ps17
Ecdysozoa - ps18
Arthropoda - ps19
Mandibulata - ps20
Pancrustacea - ps21
Insecta - ps22
Eumetabola - ps23
Holometabola/Psocodea - ps24
Holometabola - ps25
Aparaglossata - ps26
Mecopterida - ps27
Diptera - ps28
Bibiono./Cyclorrhapha - ps29
Cyclorrhapha - ps30
Calyptratae/Drosophila - ps31
Drosophila - ps32
Sophophora - ps33
Obscura/Melanogaster_group - ps34
D_melanogaster_group - ps35
D_melanogaster_subgroup - ps36
sim./sech./mel. group - ps37
Drosophila_melanogaster - ps38

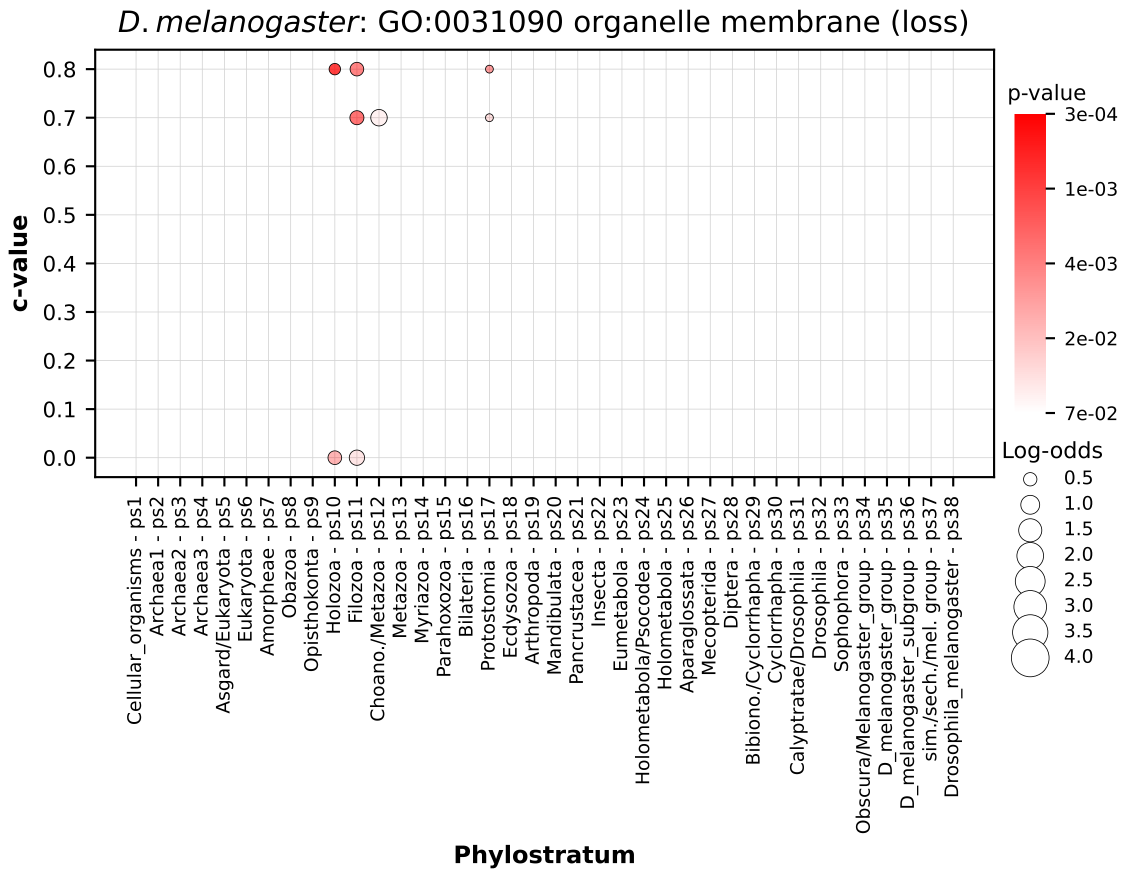
D. melanogaster: GO:0031090 organelle membrane (loss)
c-value
0.8
0.7
0.6
0.5
0.4
0.3
0.2
0.1
0.0
Phylostratum
Cellular_organisms - ps1
Archaea1 - ps2
Archaea2 - ps3
Archaea3 - ps4
Asgard/Eukaryota - ps5
Eukaryota - ps6
Amorpheae - ps7
Obazoa - ps8
Opisthokonta - ps9
Holozoa - ps10
Filozoa - ps11
Choano./Metazoa - ps12
Metazoa - ps13
Myriazoa - ps14
Parahoxozoa - ps15
Bilateria - ps16
Protostomia - ps17
Ecdysozoa - ps18
Arthropoda - ps19
Mandibulata - ps20
Pancrustacea - ps21
Insecta - ps22
Eumetabola - ps23
Holometabola/Psocodea - ps24
Holometabola - ps25
Aparaglossata - ps26
Mecopterida - ps27
Diptera - ps28
Bibiono./Cyclorrhapha - ps29
Cyclorrhapha - ps30
Calyptratae/Drosophila - ps31
Drosophila - ps32
Sophophora - ps33
Obscura/Melanogaster_group - ps34
D_melanogaster_group - ps35
D_melanogaster_subgroup - ps36
sim./sech./mel. group - ps37
Drosophila_melanogaster - ps38
p-value
3e-04
1e-03
4e-03
2e-02
7e-02
Log-odds
0.5
1.0
1.5
2.0
2.5
3.0
3.5
4.0

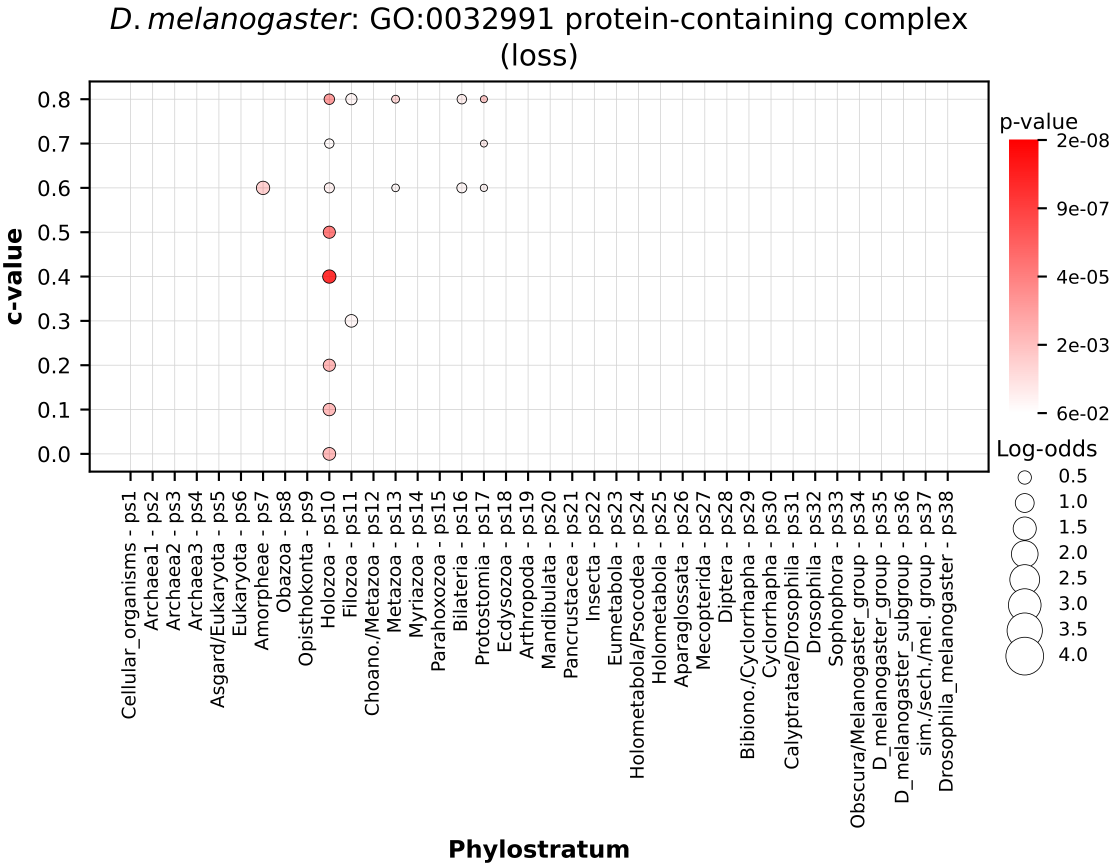

D. melanogaster: GO:0032991 protein-containing complex (loss)
c-value
0.8
0.7
0.6
0.5
0.4
0.3
0.2
0.1
0.0
p-value
2e-08
9e-07
4e-05
2e-03
6e-02
Log-odds
0.5
1.0
1.5
2.0
2.5
3.0
3.5
4.0
Cellular_organisms - ps1
Archaea1 - ps2
Archaea2 - ps3
Archaea3 - ps4
Asgard/Eukaryota - ps5
Eukaryota - ps6
Amorpheae - ps7
Obazoa - ps8
Opisthokonta - ps9
Holozoa - ps10
Filozoa - ps11
Choano./Metazoa - ps12
Metazoa - ps13
Myriazoa - ps14
Parahoxozoa - ps15
Bilateria - ps16
Protostomia - ps17
Ecdysozoa - ps18
Arthropoda - ps19
Mandibulata - ps20
Pancrustacea - ps21
Insecta - ps22
Eumetabola - ps23
Holometabola/Psocodea - ps24
Holometabola - ps25
Aparaglossata - ps26
Mecopterida - ps27
Diptera - ps28
Bibiono./Cyclorrhapha - ps29
Cyclorrhapha - ps30
Calyptratae/Drosophila - ps31
Drosophila - ps32
Sophophora - ps33
Obscura/Melanogaster_group - ps34
D_melanogaster_group - ps35
D_melanogaster_subgroup - ps36
sim./sech./mel. group - ps37
Drosophila_melanogaster - ps38
Phylostratum

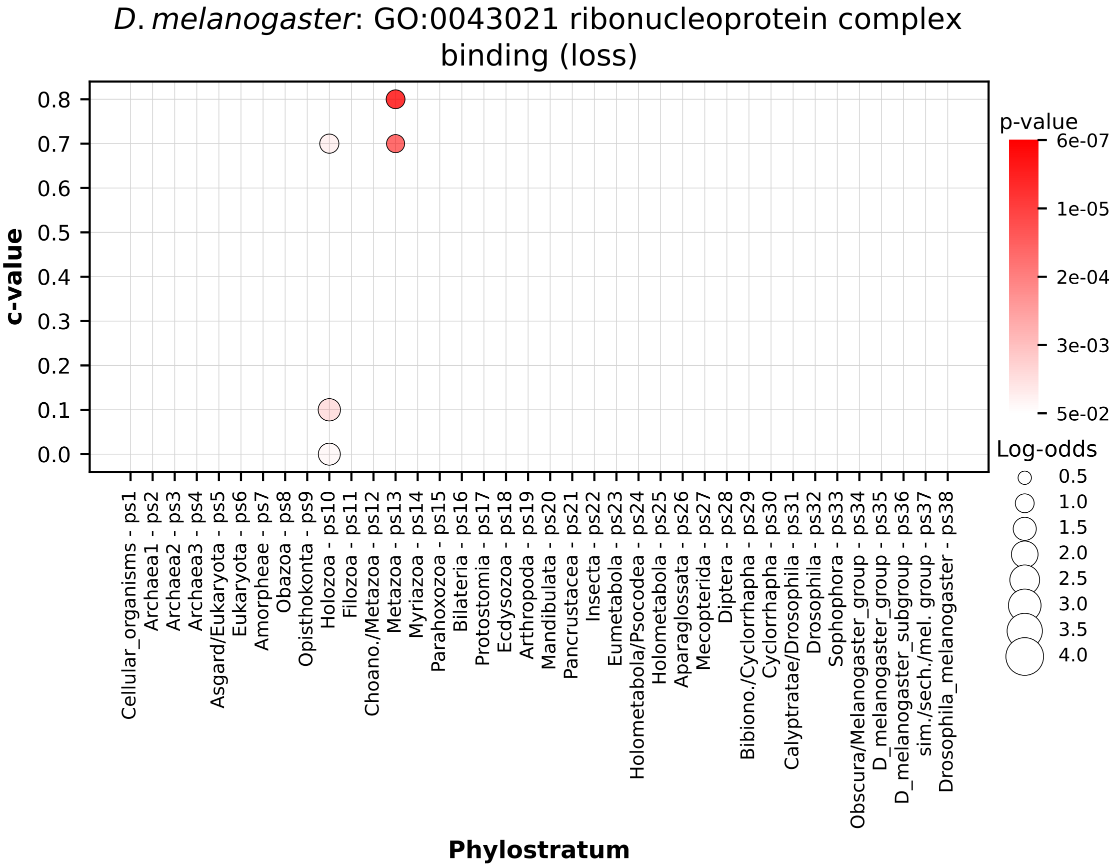

D. melanogaster: GO:0043021 ribonucleoprotein complex binding (loss)
c-value
0.8
0.7
0.6
0.5
0.4
0.3
0.2
0.1
0.0
p-value
6e-07
1e-05
2e-04
3e-03
5e-02
Log-odds
0.5
1.0
1.5
2.0
2.5
3.0
3.5
4.0
Cellular_organisms - ps1
Archaea1 - ps2
Archaea2 - ps3
Archaea3 - ps4
Asgard/Eukaryota - ps5
Eukaryota - ps6
Amorpheae - ps7
Obazoa - ps8
Opisthokonta - ps9
Holozoa - ps10
Filozoa - ps11
Choano./Metazoa - ps12
Metazoa - ps13
Myriazoa - ps14
Parahoxozoa - ps15
Bilateria - ps16
Protostomia - ps17
Ecdysozoa - ps18
Arthropoda - ps19
Mandibulata - ps20
Pancrustacea - ps21
Insecta - ps22
Eumetabola - ps23
Holometabola/Psocodea - ps24
Holometabola - ps25
Aparaglossata - ps26
Mecopterida - ps27
Diptera - ps28
Bibiono./Cyclorrhapha - ps29
Cyclorrhapha - ps30
Calyptratae/Drosophila - ps31
Drosophila - ps32
Sophophora - ps33
Obscura/Melanogaster_group - ps34
D_melanogaster_group - ps35
D_melanogaster_subgroup - ps36
sim./sech./mel. group - ps37
Drosophila_melanogaster - ps38
Phylostratum

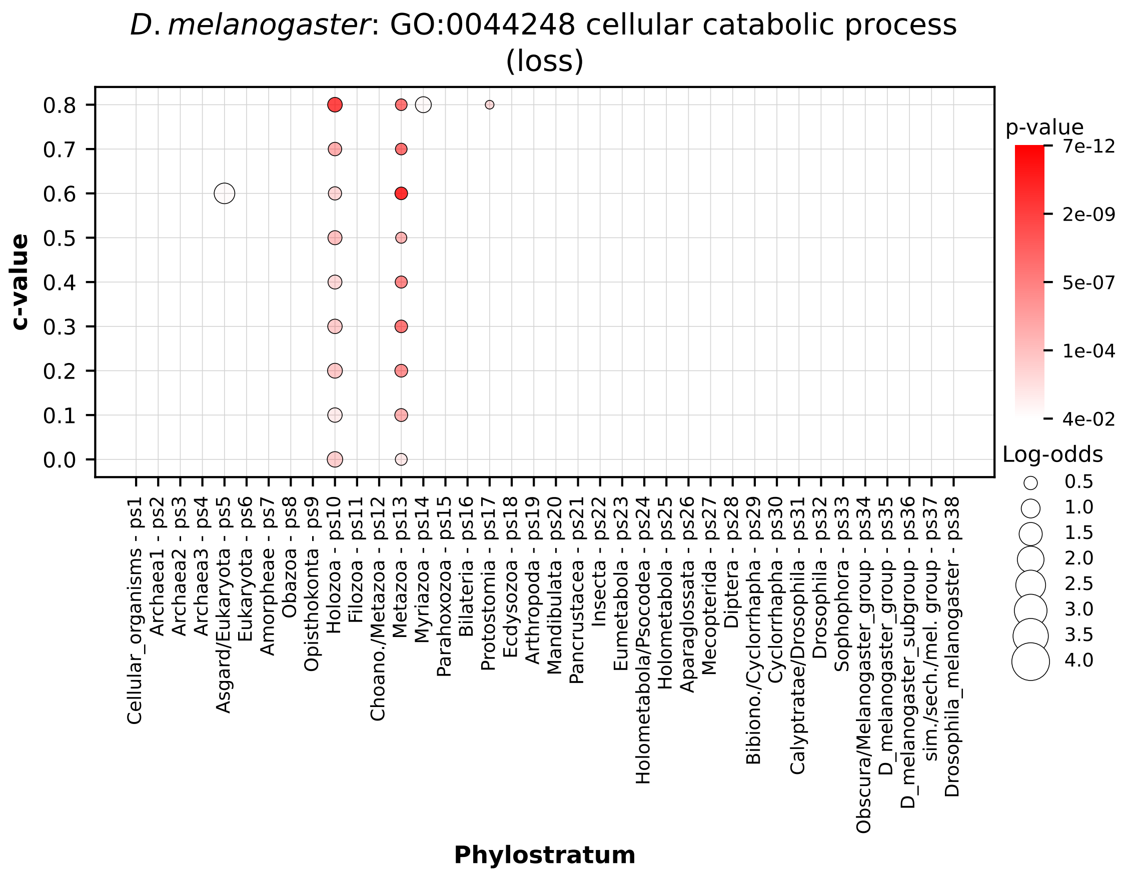

D. melanogaster: GO:0044248 cellular catabolic process
(loss)
c-value
0.0
0.1
0.2
0.3
0.4
0.5
0.6
0.7
0.8
Phylostratum
Cellular_organisms - ps1
Archaea1 - ps2
Archaea2 - ps3
Archaea3 - ps4
Asgard/Eukaryota - ps5
Eukaryota - ps6
Amorpheae - ps7
Obazoa - ps8
Opisthokonta - ps9
Holozoa - ps10
Filozoa - ps11
Choano./Metazoa - ps12
Metazoa - ps13
Myriazoa - ps14
Parahoxozoa - ps15
Bilateria - ps16
Protostomia - ps17
Ecdysozoa - ps18
Arthropoda - ps19
Mandibulata - ps20
Pancrustacea - ps21
Insecta - ps22
Eumetabola - ps23
Holometabola/Psocodea - ps24
Holometabola - ps25
Aparaglossata - ps26
Mecopterida - ps27
Diptera - ps28
Bibiono./Cyclorrhapha - ps29
Cyclorrhapha - ps30
Calyptratae/Drosophila - ps31
Drosophila - ps32
Sophophora - ps33
Obscura/Melanogaster_group - ps34
D_melanogaster_group - ps35
D_melanogaster_subgroup - ps36
sim./sech./mel. group - ps37
Drosophila_melanogaster - ps38
p-value
7e-12
2e-09
5e-07
1e-04
4e-02
Log-odds
0.5
1.0
1.5
2.0
2.5
3.0
3.5
4.0

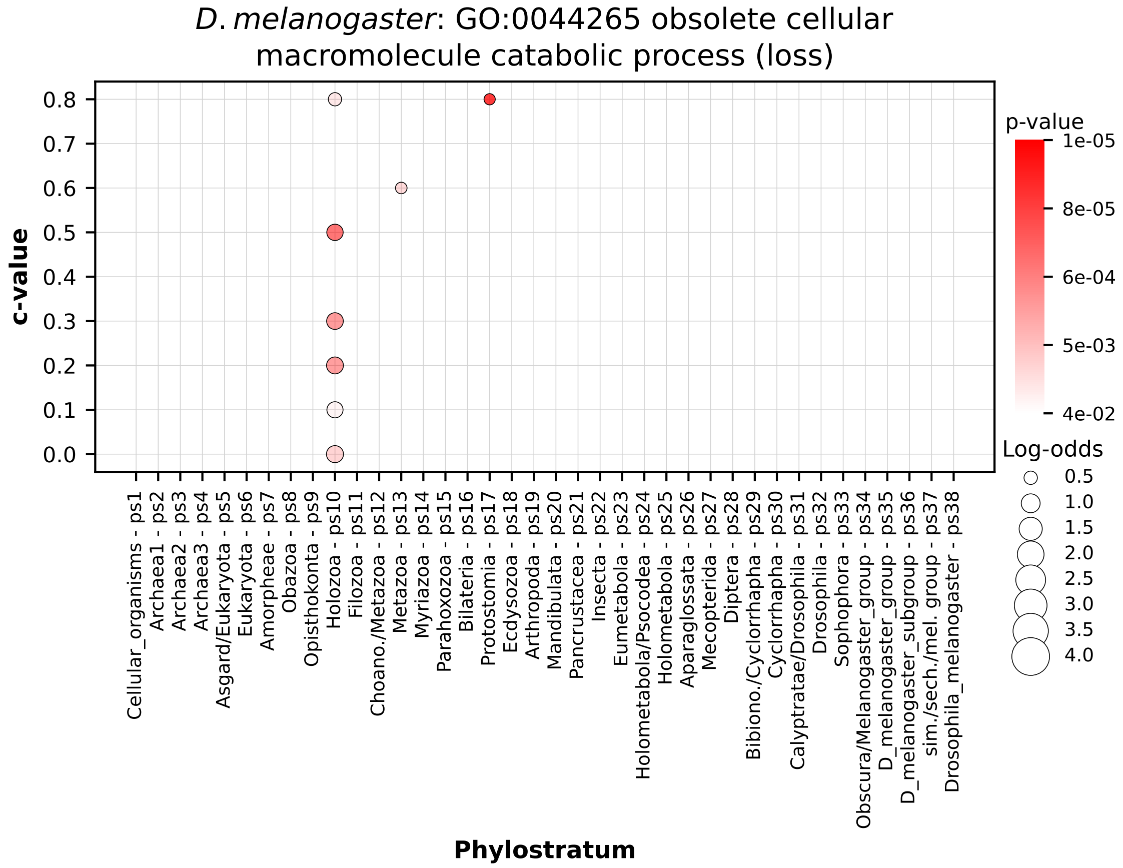

D. melanogaster: GO:0044265 obsolete cellular macromolecule catabolic process (loss)
c-value
0.0
0.1
0.2
0.3
0.4
0.5
0.6
0.7
0.8
Phylostratum
Cellular_organisms - ps1
Archaea1 - ps2
Archaea2 - ps3
Archaea3 - ps4
Asgard/Eukaryota - ps5
Eukaryota - ps6
Amorpheae - ps7
Obazoa - ps8
Opisthokonta - ps9
Holozoa - ps10
Filozoa - ps11
Choano./Metazoa - ps12
Metazoa - ps13
Myriazoa - ps14
Parahoxozoa - ps15
Bilateria - ps16
Protostomia - ps17
Ecdysozoa - ps18
Arthropoda - ps19
Mandibulata - ps20
Pancrustacea - ps21
Insecta - ps22
Eumetabola - ps23
Holometabola/Psocodea - ps24
Holometabola - ps25
Aparaglossata - ps26
Mecopterida - ps27
Diptera - ps28
Bibiono./Cyclorrhapha - ps29
Cyclorrhapha - ps30
Calyptratae/Drosophila - ps31
Drosophila - ps32
Sophophora - ps33
Obscura/Melanogaster_group - ps34
D_melanogaster_group - ps35
D_melanogaster_subgroup - ps36
sim./sech./mel. group - ps37
Drosophila_melanogaster - ps38
p-value
1e-05
8e-05
6e-04
5e-03
4e-02
Log-odds
0.5
1.0
1.5
2.0
2.5
3.0
3.5
4.0

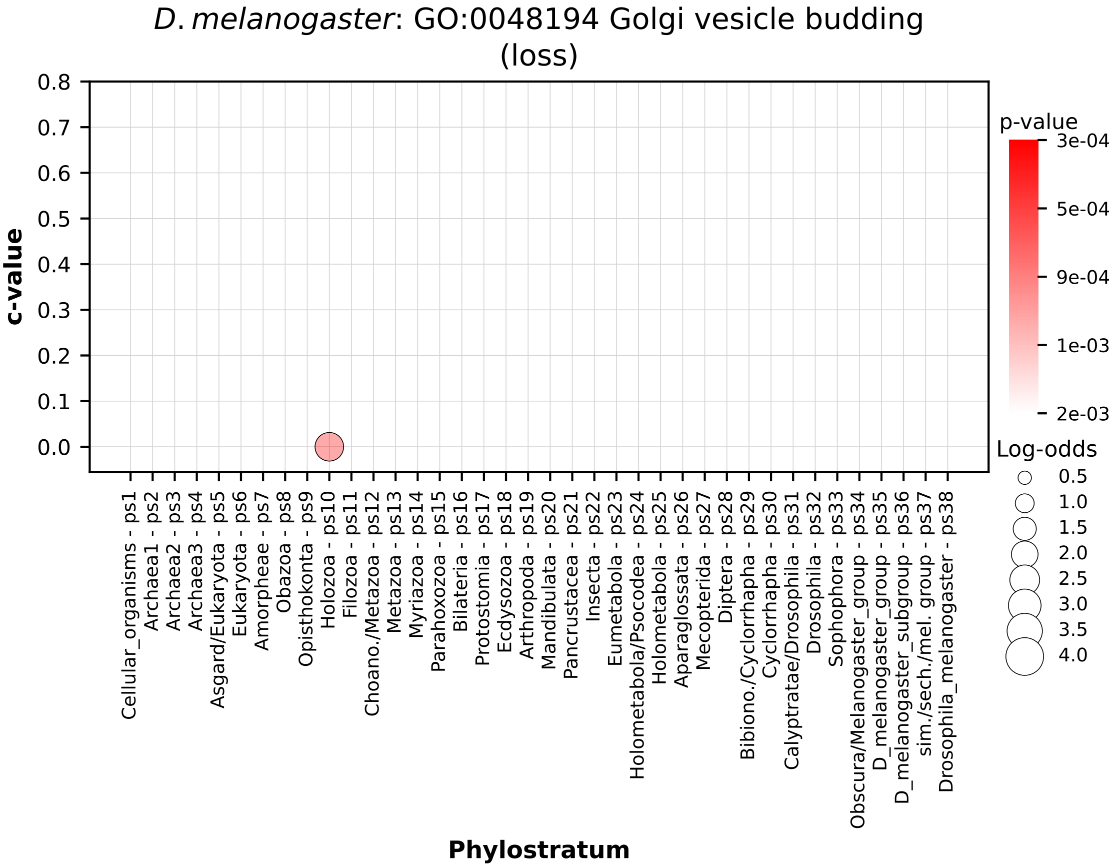

D. melanogaster: GO:0048194 Golgi vesicle budding
(loss)
c-value
0.8
0.7
0.6
0.5
0.4
0.3
0.2
0.1
0.0
p-value
3e-04
5e-04
9e-04
1e-03
2e-03
Log-odds
0.5
1.0
1.5
2.0
2.5
3.0
3.5
4.0
Cellular_organisms - ps1
Archaea1 - ps2
Archaea2 - ps3
Archaea3 - ps4
Asgard/Eukaryota - ps5
Eukaryota - ps6
Amorpheae - ps7
Obazoa - ps8
Opisthokonta - ps9
Holozoa - ps10
Filozoa - ps11
Choano./Metazoa - ps12
Metazoa - ps13
Myriazoa - ps14
Parahoxozoa - ps15
Bilateria - ps16
Protostomia - ps17
Ecdysozoa - ps18
Arthropoda - ps19
Mandibulata - ps20
Pancrustacea - ps21
Insecta - ps22
Eumetabola - ps23
Holometabola/Psocodea - ps24
Holometabola - ps25
Aparaglossata - ps26
Mecopterida - ps27
Diptera - ps28
Bibiono./Cyclorrhapha - ps29
Cyclorrhapha - ps30
Calyptratae/Drosophila - ps31
Drosophila - ps32
Sophophora - ps33
Obscura/Melanogaster_group - ps34
D_melanogaster_group - ps35
D_melanogaster_subgroup - ps36
sim./sech./mel. group - ps37
Drosophila_melanogaster - ps38
Phylostratum

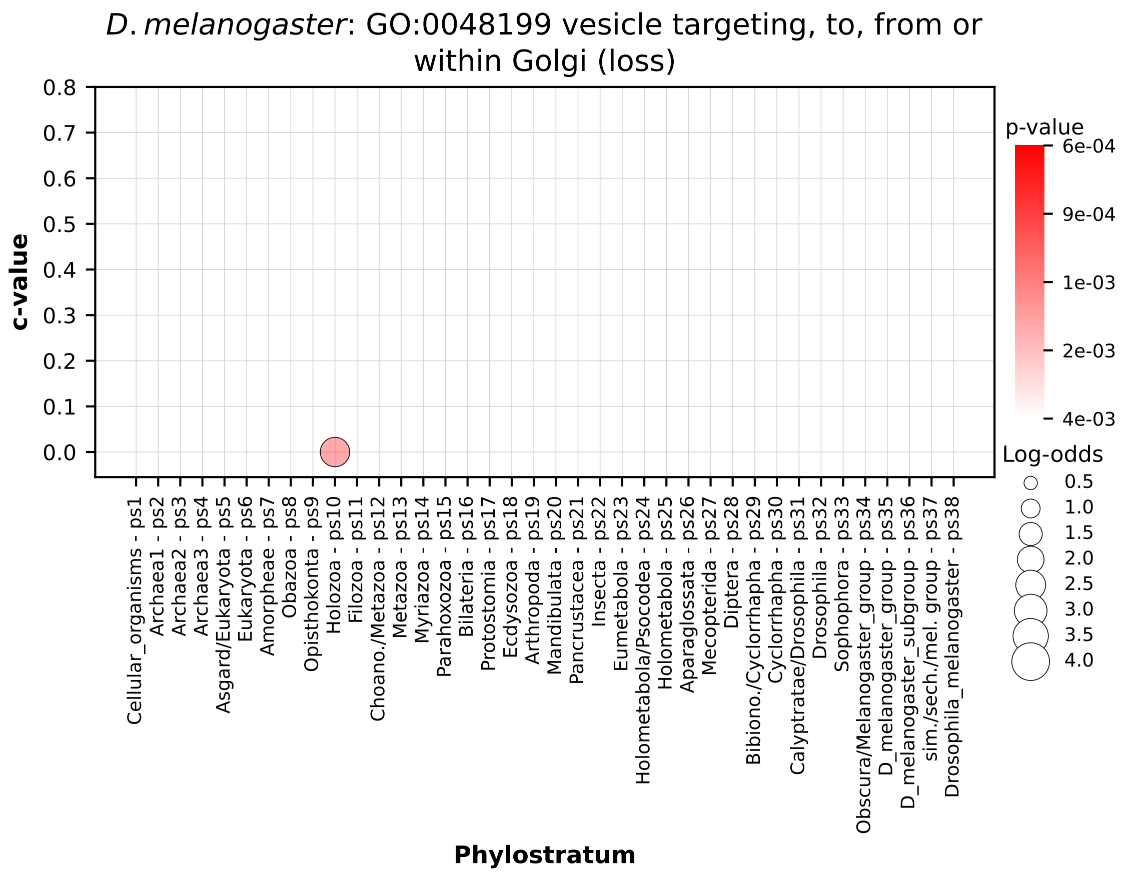

D. melanogaster: GO:0048199 vesicle targeting, to, from or within Golgi (loss)
c-value
0.8
0.7
0.6
0.5
0.4
0.3
0.2
0.1
0.0
Phylostratum
Cellular_organisms - ps1
Archaea1 - ps2
Archaea2 - ps3
Archaea3 - ps4
Asgard/Eukaryota - ps5
Eukaryota - ps6
Amorpheae - ps7
Obazoa - ps8
Opisthokonta - ps9
Holozoa - ps10
Filozoa - ps11
Choano./Metazoa - ps12
Metazoa - ps13
Myriazoa - ps14
Parahoxozoa - ps15
Bilateria - ps16
Protostomia - ps17
Ecdysozoa - ps18
Arthropoda - ps19
Mandibulata - ps20
Pancrustacea - ps21
Insecta - ps22
Eumetabola - ps23
Holometabola/Psocodea - ps24
Holometabola - ps25
Aparaglossata - ps26
Mecopterida - ps27
Diptera - ps28
Bibiono./Cyclorrhapha - ps29
Cyclorrhapha - ps30
Calyptratae/Drosophila - ps31
Drosophila - ps32
Sophophora - ps33
Obscura/Melanogaster_group - ps34
D_melanogaster_group - ps35
D_melanogaster_subgroup - ps36
sim./sech./mel. group - ps37
Drosophila_melanogaster - ps38
p-value
6e-04
9e-04
1e-03
2e-03
4e-03
Log-odds
0.5
1.0
1.5
2.0
2.5
3.0
3.5
4.0

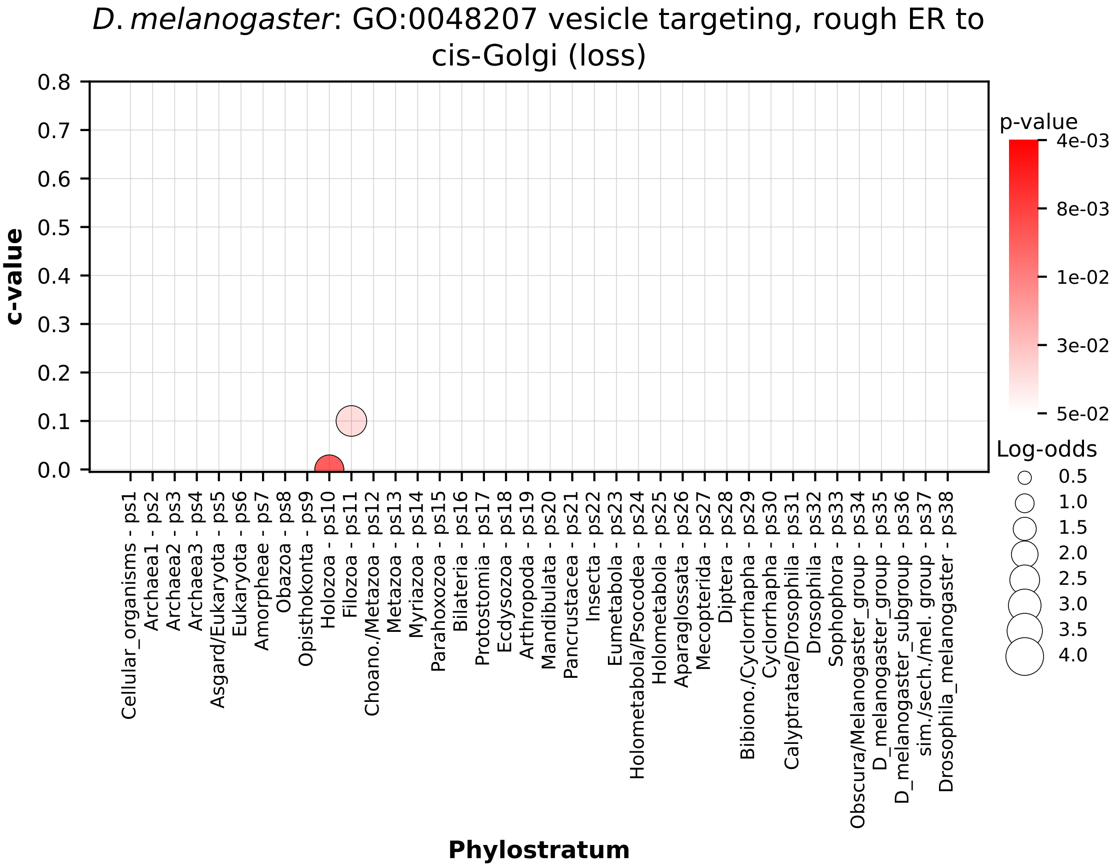

D. melanogaster: GO:0048207 vesicle targeting, rough ER to cis-Golgi (loss)
c-value
0.8
0.7
0.6
0.5
0.4
0.3
0.2
0.1
0.0
Phylostratum
Cellular_organisms - ps1
Archaea1 - ps2
Archaea2 - ps3
Archaea3 - ps4
Asgard/Eukaryota - ps5
Eukaryota - ps6
Amorpheae - ps7
Obazoa - ps8
Opisthokonta - ps9
Holozoa - ps10
Filozoa - ps11
Choano./Metazoa - ps12
Metazoa - ps13
Myriazoa - ps14
Parahoxozoa - ps15
Bilateria - ps16
Protostomia - ps17
Ecdysozoa - ps18
Arthropoda - ps19
Mandibulata - ps20
Pancrustacea - ps21
Insecta - ps22
Eumetabola - ps23
Holometabola/Psocodea - ps24
Holometabola - ps25
Aparaglossata - ps26
Mecopterida - ps27
Diptera - ps28
Bibiono./Cyclorrhapha - ps29
Cyclorrhapha - ps30
Calyptratae/Drosophila - ps31
Drosophila - ps32
Sophophora - ps33
Obscura/Melanogaster_group - ps34
D_melanogaster_group - ps35
D_melanogaster_subgroup - ps36
sim./sech./mel. group - ps37
Drosophila_melanogaster - ps38
p-value
4e-03
8e-03
1e-02
3e-02
5e-02
Log-odds
0.5
1.0
1.5
2.0
2.5
3.0
3.5
4.0

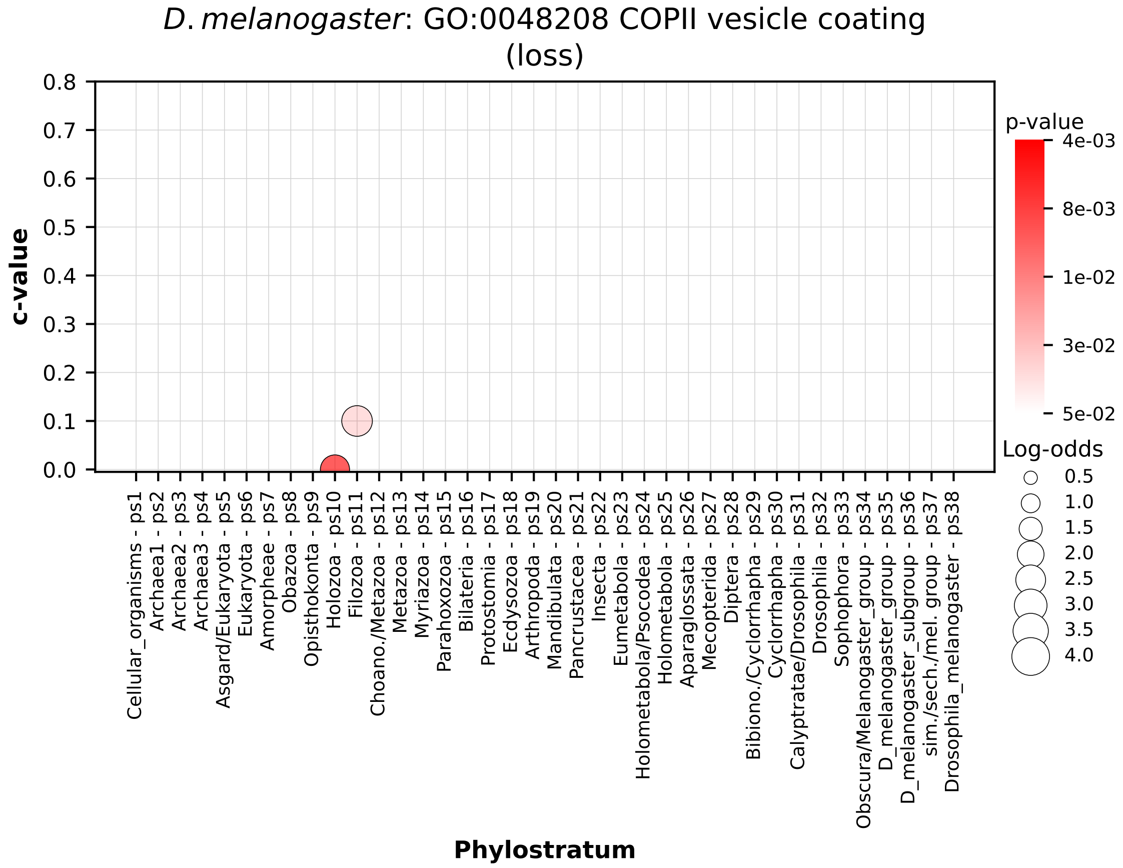
D. melanogaster: GO:0048208 COPII vesicle coating
(loss)
c-value
0.8
0.7
0.6
0.5
0.4
0.3
0.2
0.1
0.0
p-value
4e-03
8e-03
1e-02
3e-02
5e-02
Log-odds
0.5
1.0
1.5
2.0
2.5
3.0
3.5
4.0
Cellular_organisms - ps1
Archaea1 - ps2
Archaea2 - ps3
Archaea3 - ps4
Asgard/Eukaryota - ps5
Eukaryota - ps6
Amorpheae - ps7
Obazoa - ps8
Opisthokonta - ps9
Holozoa - ps10
Filozoa - ps11
Choano./Metazoa - ps12
Metazoa - ps13
Myriazoa - ps14
Parahoxozoa - ps15
Bilateria - ps16
Protostomia - ps17
Ecdysozoa - ps18
Arthropoda - ps19
Mandibulata - ps20
Pancrustacea - ps21
Insecta - ps22
Eumetabola - ps23
Holometabola/Psocodea - ps24
Holometabola - ps25
Aparaglossata - ps26
Mecopterida - ps27
Diptera - ps28
Bibiono./Cyclorrhapha - ps29
Cyclorrhapha - ps30
Calyptratae/Drosophila - ps31
Drosophila - ps32
Sophophora - ps33
Obscura/Melanogaster_group - ps34
D_melanogaster_group - ps35
D_melanogaster_subgroup - ps36
sim./sech./mel. group - ps37
Drosophila_melanogaster - ps38
Phylostratum

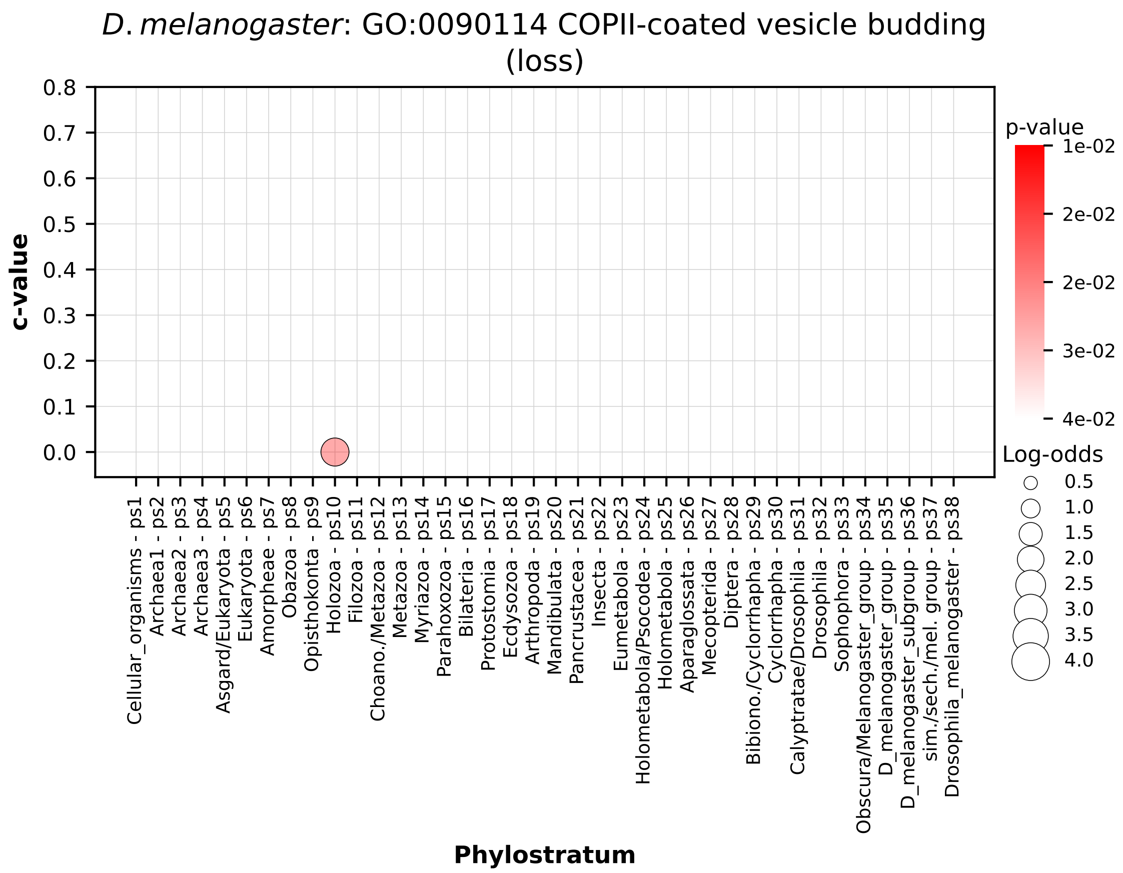

D. melanogaster: GO:0090114 COPII-coated vesicle budding (loss)
c-value
0.8
0.7
0.6
0.5
0.4
0.3
0.2
0.1
0.0
p-value
1e-02
2e-02
2e-02
3e-02
4e-02
Log-odds
0.5
1.0
1.5
2.0
2.5
3.0
3.5
4.0
Cellular_organisms - ps1
Archaea1 - ps2
Archaea2 - ps3
Archaea3 - ps4
Asgard/Eukaryota - ps5
Eukaryota - ps6
Amorpheae - ps7
Obazoa - ps8
Opisthokonta - ps9
Holozoa - ps10
Filozoa - ps11
Choano./Metazoa - ps12
Metazoa - ps13
Myriazoa - ps14
Parahoxozoa - ps15
Bilateria - ps16
Protostomia - ps17
Ecdysozoa - ps18
Arthropoda - ps19
Mandibulata - ps20
Pancrustacea - ps21
Insecta - ps22
Eumetabola - ps23
Holometabola/Psocodea - ps24
Holometabola - ps25
Aparaglossata - ps26
Mecopterida - ps27
Diptera - ps28
Bibiono./Cyclorrhapha - ps29
Cyclorrhapha - ps30
Calyptratae/Drosophila - ps31
Drosophila - ps32
Sophophora - ps33
Obscura/Melanogaster_group - ps34
D_melanogaster_group - ps35
D_melanogaster_subgroup - ps36
sim./sech./mel. group - ps37
Drosophila_melanogaster - ps38
Phylostratum

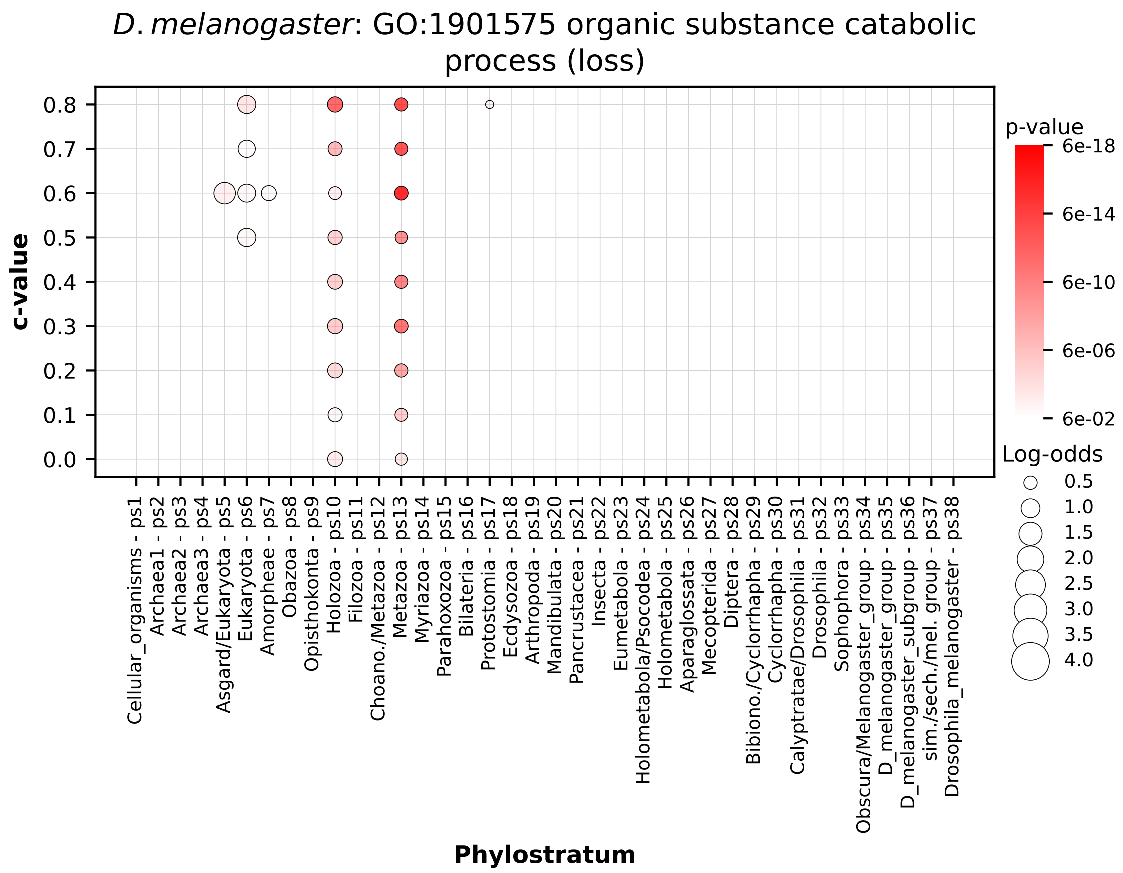

D. melanogaster: GO:1901575 organic substance catabolic process (loss)
c-value
0.8
0.7
0.6
0.5
0.4
0.3
0.2
0.1
0.0
p-value
6e-18
6e-14
6e-10
6e-06
6e-02
Log-odds
0.5
1.0
1.5
2.0
2.5
3.0
3.5
4.0
Cellular_organisms - ps1
Archaea1 - ps2
Archaea2 - ps3
Archaea3 - ps4
Asgard/Eukaryota - ps5
Eukaryota - ps6
Amorpheae - ps7
Obazoa - ps8
Opisthokonta - ps9
Holozoa - ps10
Filozoa - ps11
Choano./Metazoa - ps12
Metazoa - ps13
Myriazoa - ps14
Parahoxozoa - ps15
Bilateria - ps16
Protostomia - ps17
Ecdysozoa - ps18
Arthropoda - ps19
Mandibulata - ps20
Pancrustacea - ps21
Insecta - ps22
Eumetabola - ps23
Holometabola/Psocodea - ps24
Holometabola - ps25
Aparaglossata - ps26
Mecopterida - ps27
Diptera - ps28
Bibiono./Cyclorrhapha - ps29
Cyclorrhapha - ps30
Calyptratae/Drosophila - ps31
Drosophila - ps32
Sophophora - ps33
Obscura/Melanogaster_group - ps34
D_melanogaster_group - ps35
D_melanogaster_subgroup - ps36
sim./sech./mel. group - ps37
Drosophila_melanogaster - ps38
Phylostratum

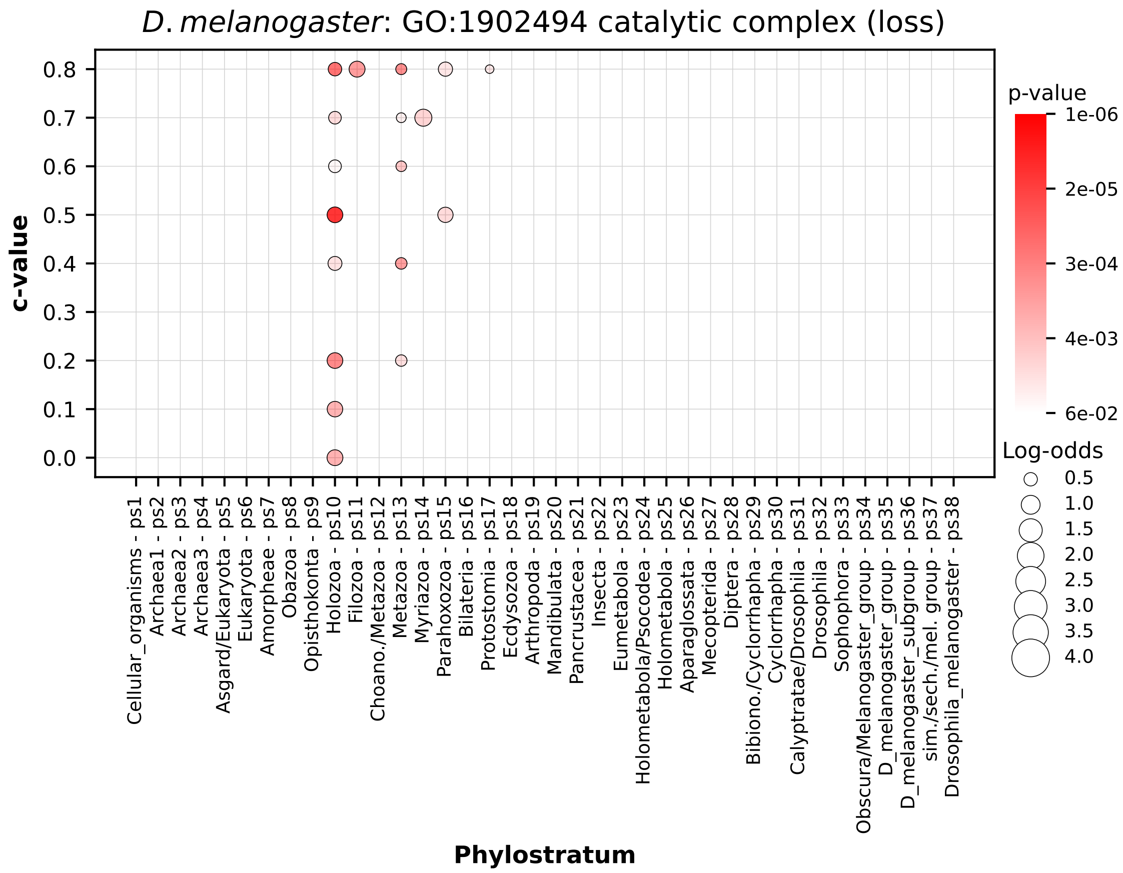
D. melanogaster: GO:1902494 catalytic complex (loss)
c-value
0.8
0.7
0.6
0.5
0.4
0.3
0.2
0.1
0.0
Phylostratum
Cellular_organisms - ps1
Archaea1 - ps2
Archaea2 - ps3
Archaea3 - ps4
Asgard/Eukaryota - ps5
Eukaryota - ps6
Amorpheae - ps7
Obazoa - ps8
Opisthokonta - ps9
Holozoa - ps10
Filozoa - ps11
Choano./Metazoa - ps12
Metazoa - ps13
Myriazoa - ps14
Parahoxozoa - ps15
Bilateria - ps16
Protostomia - ps17
Ecdysozoa - ps18
Arthropoda - ps19
Mandibulata - ps20
Pancrustacea - ps21
Insecta - ps22
Eumetabola - ps23
Holometabola/Psocodea - ps24
Holometabola - ps25
Aparaglossata - ps26
Mecopterida - ps27
Diptera - ps28
Bibiono./Cyclorrhapha - ps29
Cyclorrhapha - ps30
Calyptratae/Drosophila - ps31
Drosophila - ps32
Sophophora - ps33
Obscura/Melanogaster_group - ps34
D_melanogaster_group - ps35
D_melanogaster_subgroup - ps36
sim./sech./mel. group - ps37
Drosophila_melanogaster - ps38
p-value
1e-06
2e-05
3e-04
4e-03
6e-02
Log-odds
0.5
1.0
1.5
2.0
2.5
3.0
3.5
4.0

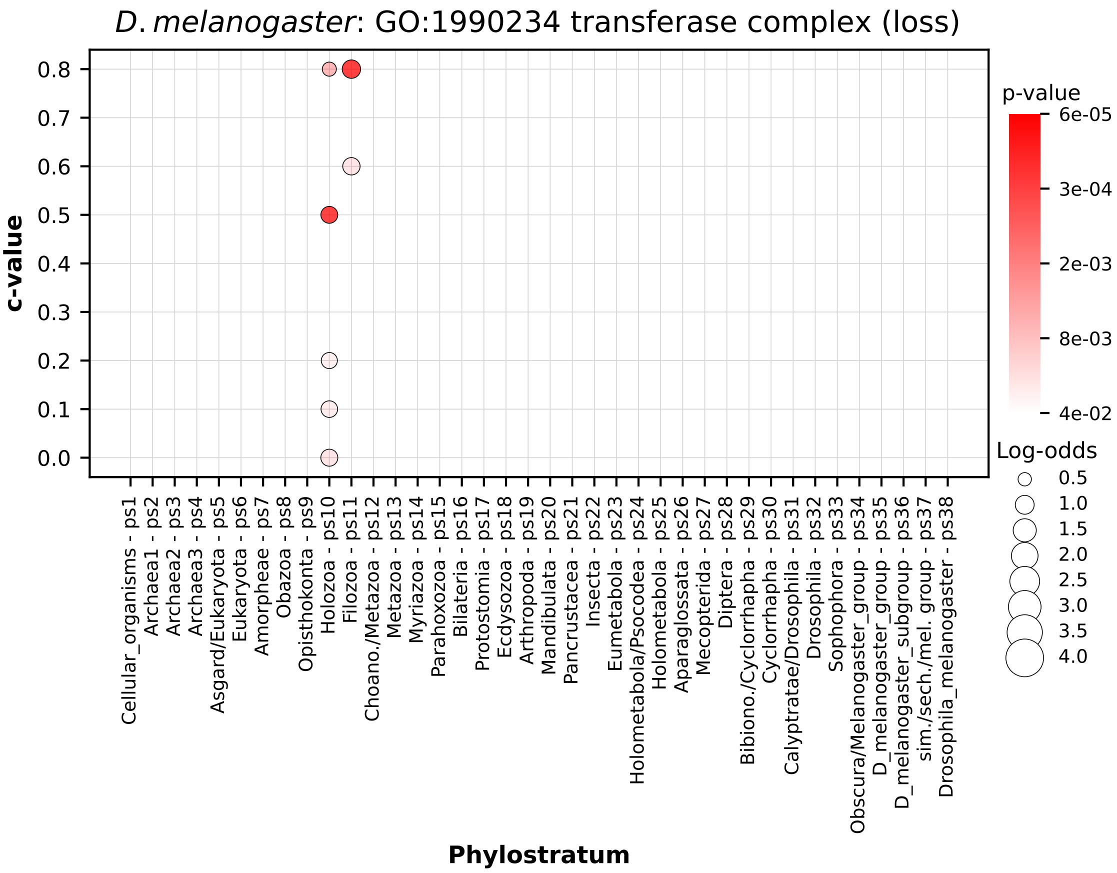

D. melanogaster: GO:1990234 transferase complex (loss)
c-value
0.8
0.7
0.6
0.5
0.4
0.3
0.2
0.1
0.0
Phylostratum
Cellular_organisms - ps1
Archaea1 - ps2
Archaea2 - ps3
Archaea3 - ps4
Asgard/Eukaryota - ps5
Eukaryota - ps6
Amorpheae - ps7
Obazoa - ps8
Opisthokonta - ps9
Holozoa - ps10
Filozoa - ps11
Choano./Metazoa - ps12
Metazoa - ps13
Myriazoa - ps14
Parahoxozoa - ps15
Bilateria - ps16
Protostomia - ps17
Ecdysozoa - ps18
Arthropoda - ps19
Mandibulata - ps20
Pancrustacea - ps21
Insecta - ps22
Eumetabola - ps23
Holometabola/Psocodea - ps24
Holometabola - ps25
Aparaglossata - ps26
Mecopterida - ps27
Diptera - ps28
Bibiono./Cyclorrhapha - ps29
Cyclorrhapha - ps30
Calyptratae/Drosophila - ps31
Drosophila - ps32
Sophophora - ps33
Obscura/Melanogaster_group - ps34
D_melanogaster_group - ps35
D_melanogaster_subgroup - ps36
sim./sech./mel. group - ps37
Drosophila_melanogaster - ps38
p-value
6e-05
3e-04
2e-03
8e-03
4e-02
Log-odds
0.5
1.0
1.5
2.0
2.5
3.0
3.5
4.0

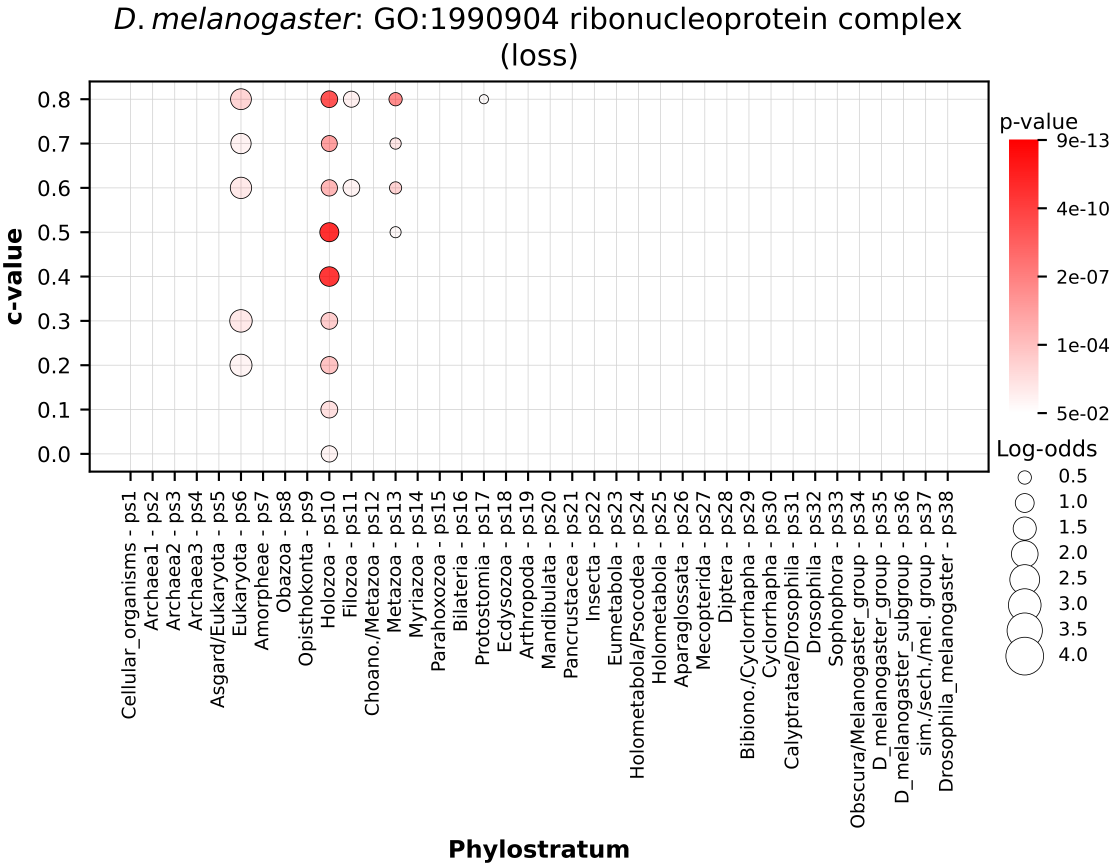

D. melanogaster: GO:1990904 ribonucleoprotein complex
(loss)
c-value
0.8
0.7
0.6
0.5
0.4
0.3
0.2
0.1
0.0
Phylostratum
Cellular_organisms - ps1
Archaea1 - ps2
Archaea2 - ps3
Archaea3 - ps4
Asgard/Eukaryota - ps5
Eukaryota - ps6
Amorpheae - ps7
Obazoa - ps8
Opisthokonta - ps9
Holozoa - ps10
Filozoa - ps11
Choano./Metazoa - ps12
Metazoa - ps13
Myriazoa - ps14
Parahoxozoa - ps15
Bilateria - ps16
Protostomia - ps17
Ecdysozoa - ps18
Arthropoda - ps19
Mandibulata - ps20
Pancrustacea - ps21
Insecta - ps22
Eumetabola - ps23
Holometabola/Psocodea - ps24
Holometabola - ps25
Aparaglossata - ps26
Mecopterida - ps27
Diptera - ps28
Bibiono./Cyclorrhapha - ps29
Cyclorrhapha - ps30
Calyptratae/Drosophila - ps31
Drosophila - ps32
Sophophora - ps33
Obscura/Melanogaster_group - ps34
D_melanogaster_group - ps35
D_melanogaster_subgroup - ps36
sim./sech./mel. group - ps37
Drosophila_melanogaster - ps38
p-value
9e-13
4e-10
2e-07
1e-04
5e-02
Log-odds
0.5
1.0
1.5
2.0
2.5
3.0
3.5
4.0

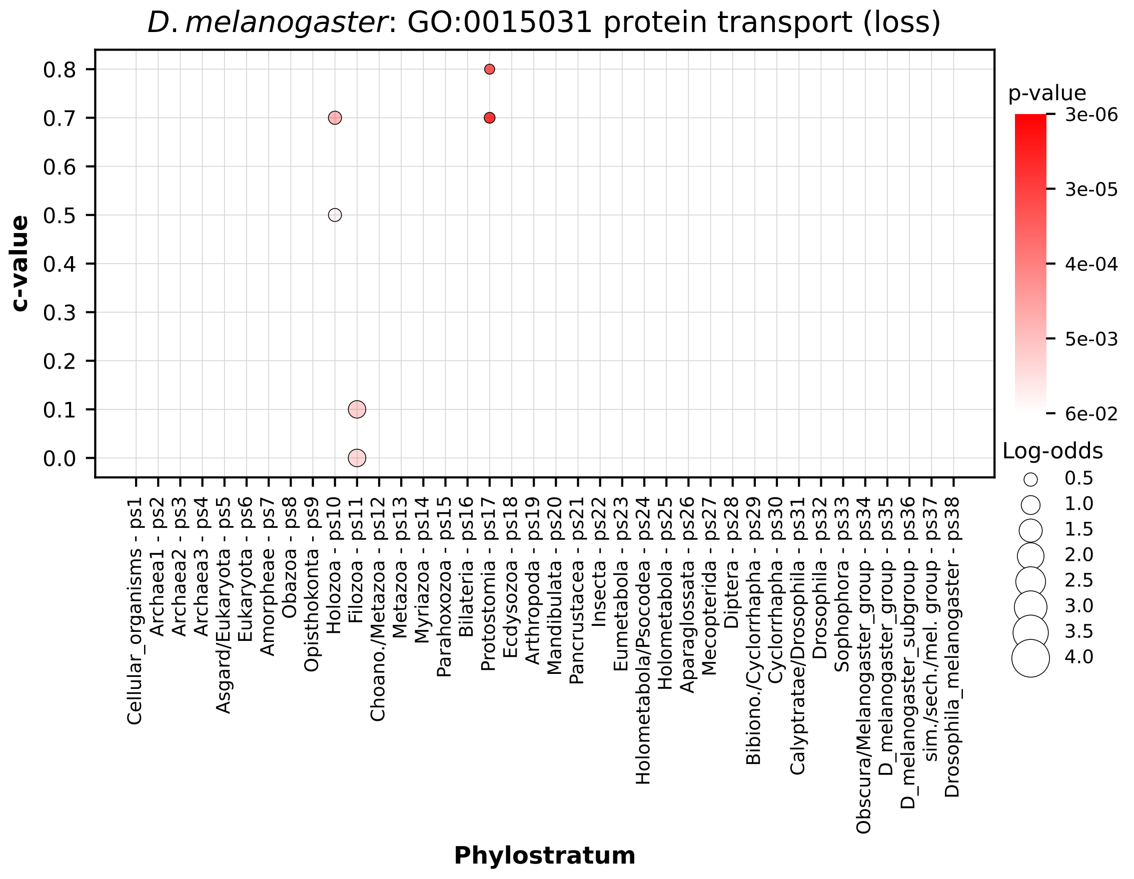

D. melanogaster: GO:0015031 protein transport (loss)
c-value
0.8
0.7
0.6
0.5
0.4
0.3
0.2
0.1
0.0
p-value
3e-06
3e-05
4e-04
5e-03
6e-02
Log-odds
0.5
1.0
1.5
2.0
2.5
3.0
3.5
4.0
Cellular_organisms - ps1
Archaea1 - ps2
Archaea2 - ps3
Archaea3 - ps4
Asgard/Eukaryota - ps5
Eukaryota - ps6
Amorpheae - ps7
Obazoa - ps8
Opisthokonta - ps9
Holozoa - ps10
Filozoa - ps11
Choano./Metazoa - ps12
Metazoa - ps13
Myriazoa - ps14
Parahoxozoa - ps15
Bilateria - ps16
Protostomia - ps17
Ecdysozoa - ps18
Arthropoda - ps19
Mandibulata - ps20
Pancrustacea - ps21
Insecta - ps22
Eumetabola - ps23
Holometabola/Psocodea - ps24
Holometabola - ps25
Aparaglossata - ps26
Mecopterida - ps27
Diptera - ps28
Bibiono./Cyclorrhapha - ps29
Cyclorrhapha - ps30
Calyptratae/Drosophila - ps31
Drosophila - ps32
Sophophora - ps33
Obscura/Melanogaster_group - ps34
D_melanogaster_group - ps35
D_melanogaster_subgroup - ps36
sim./sech./mel. group - ps37
Drosophila_melanogaster - ps38
Phylostratum

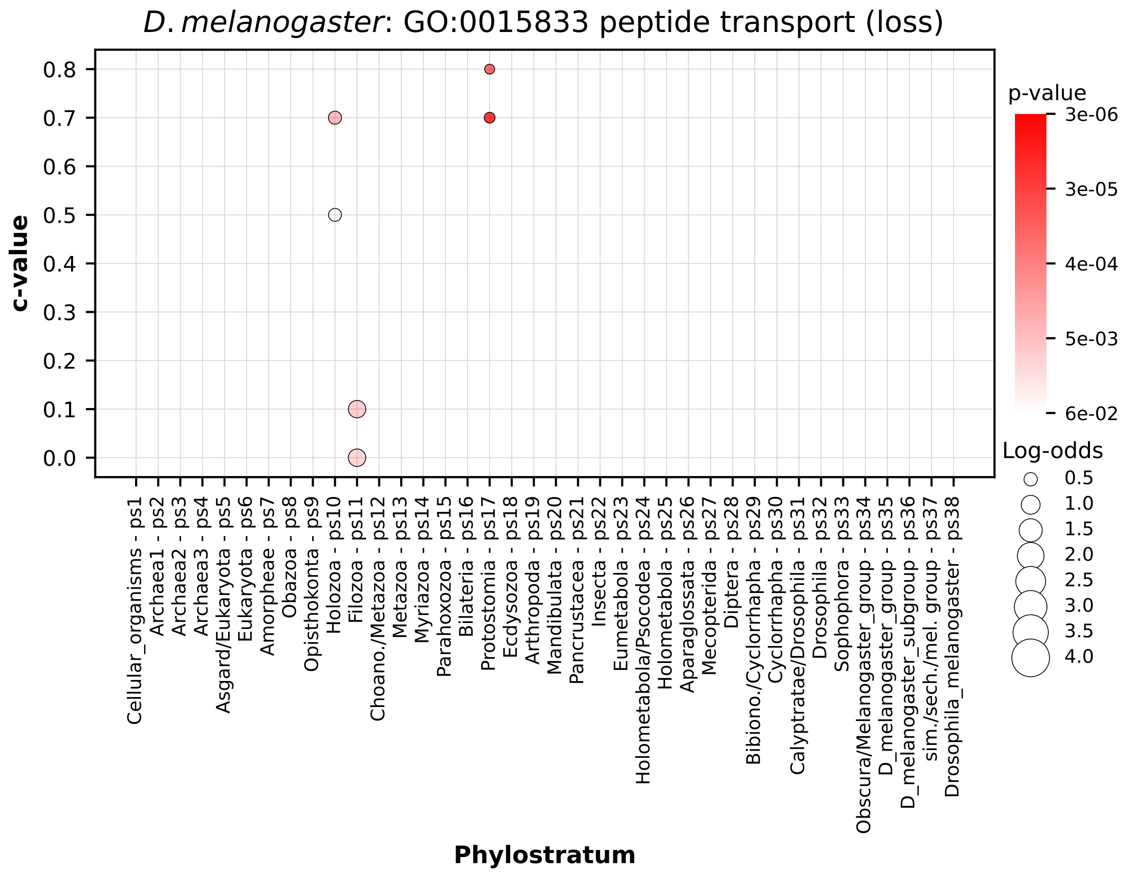
D. melanogaster: GO:0015833 peptide transport (loss)
c-value
0.8
0.7
0.6
0.5
0.4
0.3
0.2
0.1
0.0
Phylostratum
Cellular_organisms - ps1
Archaea1 - ps2
Archaea2 - ps3
Archaea3 - ps4
Asgard/Eukaryota - ps5
Eukaryota - ps6
Amorpheae - ps7
Obazoa - ps8
Opisthokonta - ps9
Holozoa - ps10
Filozoa - ps11
Choano./Metazoa - ps12
Metazoa - ps13
Myriazoa - ps14
Parahoxozoa - ps15
Bilateria - ps16
Protostomia - ps17
Ecdysozoa - ps18
Arthropoda - ps19
Mandibulata - ps20
Pancrustacea - ps21
Insecta - ps22
Eumetabola - ps23
Holometabola/Psocodea - ps24
Holometabola - ps25
Aparaglossata - ps26
Mecopterida - ps27
Diptera - ps28
Bibiono./Cyclorrhapha - ps29
Cyclorrhapha - ps30
Calyptratae/Drosophila - ps31
Drosophila - ps32
Sophophora - ps33
Obscura/Melanogaster_group - ps34
D_melanogaster_group - ps35
D_melanogaster_subgroup - ps36
sim./sech./mel. group - ps37
Drosophila_melanogaster - ps38
p-value
3e-06
3e-05
4e-04
5e-03
6e-02
Log-odds
0.5
1.0
1.5
2.0
2.5
3.0
3.5
4.0

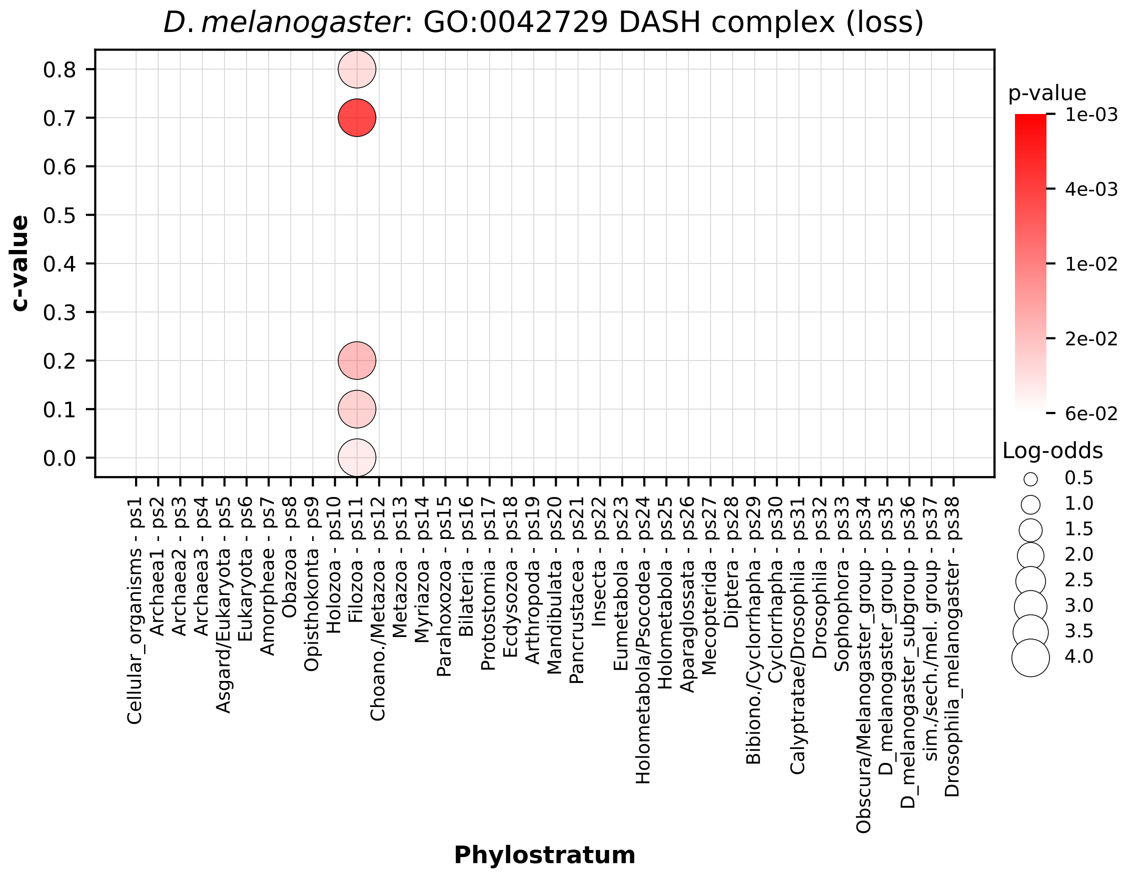

D. melanogaster: GO:0042729 DASH complex (loss)
c-value
0.0
0.1
0.2
0.3
0.4
0.5
0.6
0.7
0.8
Phylostratum
Cellular_organisms - ps1
Archaea1 - ps2
Archaea2 - ps3
Archaea3 - ps4
Asgard/Eukaryota - ps5
Eukaryota - ps6
Amorpheae - ps7
Obazoa - ps8
Opisthokonta - ps9
Holozoa - ps10
Filozoa - ps11
Choano./Metazoa - ps12
Metazoa - ps13
Myriazoa - ps14
Parahoxozoa - ps15
Bilateria - ps16
Protostomia - ps17
Ecdysozoa - ps18
Arthropoda - ps19
Mandibulata - ps20
Pancrustacea - ps21
Insecta - ps22
Eumetabola - ps23
Holometabola/Psocodea - ps24
Holometabola - ps25
Aparaglossata - ps26
Mecopterida - ps27
Diptera - ps28
Bibiono./Cyclorrhapha - ps29
Cyclorrhapha - ps30
Calyptratae/Drosophila - ps31
Drosophila - ps32
Sophophora - ps33
Obscura/Melanogaster_group - ps34
D_melanogaster_group - ps35
D_melanogaster_subgroup - ps36
sim./sech./mel. group - ps37
Drosophila_melanogaster - ps38
p-value
1e-03
4e-03
1e-02
2e-02
6e-02
Log-odds
0.5
1.0
1.5
2.0
2.5
3.0
3.5
4.0

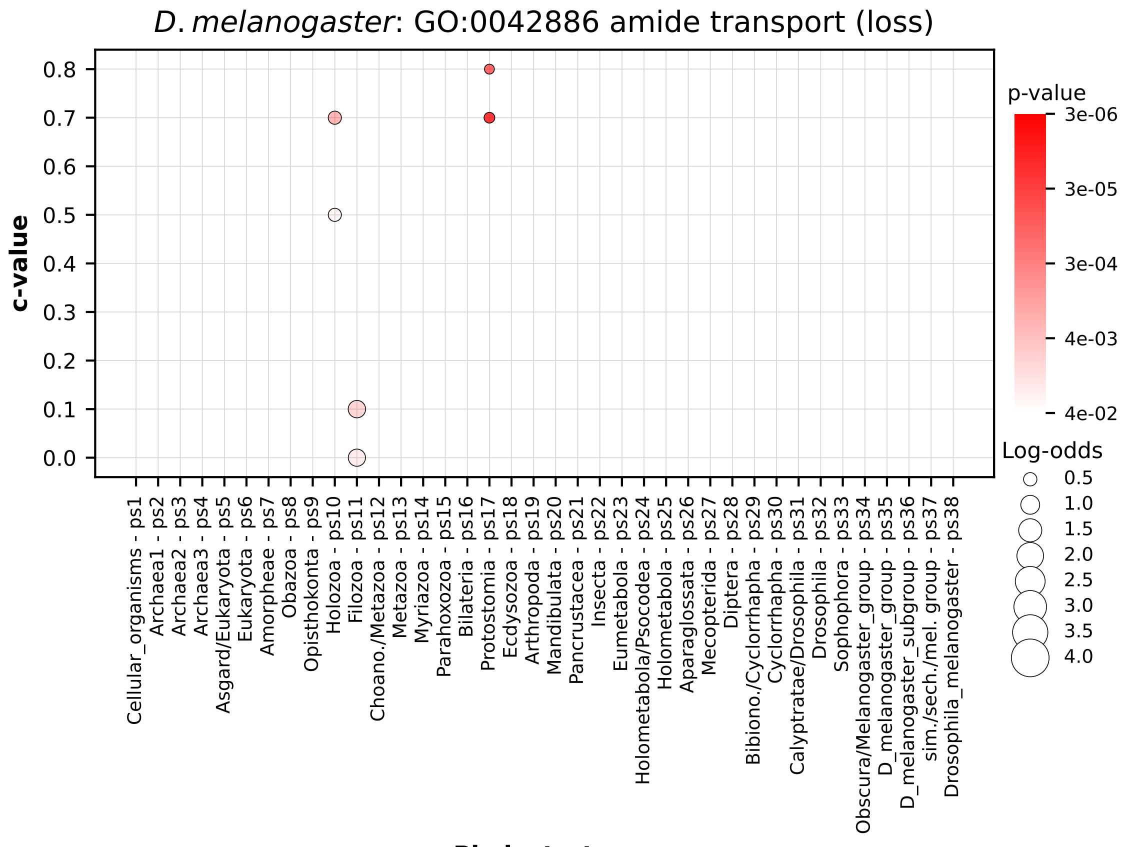

D. melanogaster: GO:0042886 amide transport (loss)
c-value
0.8
0.7
0.6
0.5
0.4
0.3
0.2
0.1
0.0
Phylostratum
Cellular_organisms - ps1
Archaea1 - ps2
Archaea2 - ps3
Archaea3 - ps4
Asgard/Eukaryota - ps5
Eukaryota - ps6
Amorpheae - ps7
Obazoa - ps8
Opisthokonta - ps9
Holozoa - ps10
Filozoa - ps11
Choano./Metazoa - ps12
Metazoa - ps13
Myriazoa - ps14
Parahoxozoa - ps15
Bilateria - ps16
Protostomia - ps17
Ecdysozoa - ps18
Arthropoda - ps19
Mandibulata - ps20
Pancrustacea - ps21
Insecta - ps22
Eumetabola - ps23
Holometabola/Psocodea - ps24
Holometabola - ps25
Aparaglossata - ps26
Mecopterida - ps27
Diptera - ps28
Bibiono./Cyclorrhapha - ps29
Cyclorrhapha - ps30
Calyptratae/Drosophila - ps31
Drosophila - ps32
Sophophora - ps33
Obscura/Melanogaster_group - ps34
D_melanogaster_group - ps35
D_melanogaster_subgroup - ps36
sim./sech./mel. group - ps37
Drosophila_melanogaster - ps38
p-value
3e-06
3e-05
3e-04
4e-03
4e-02
Log-odds
0.5
1.0
1.5
2.0
2.5
3.0
3.5
4.0

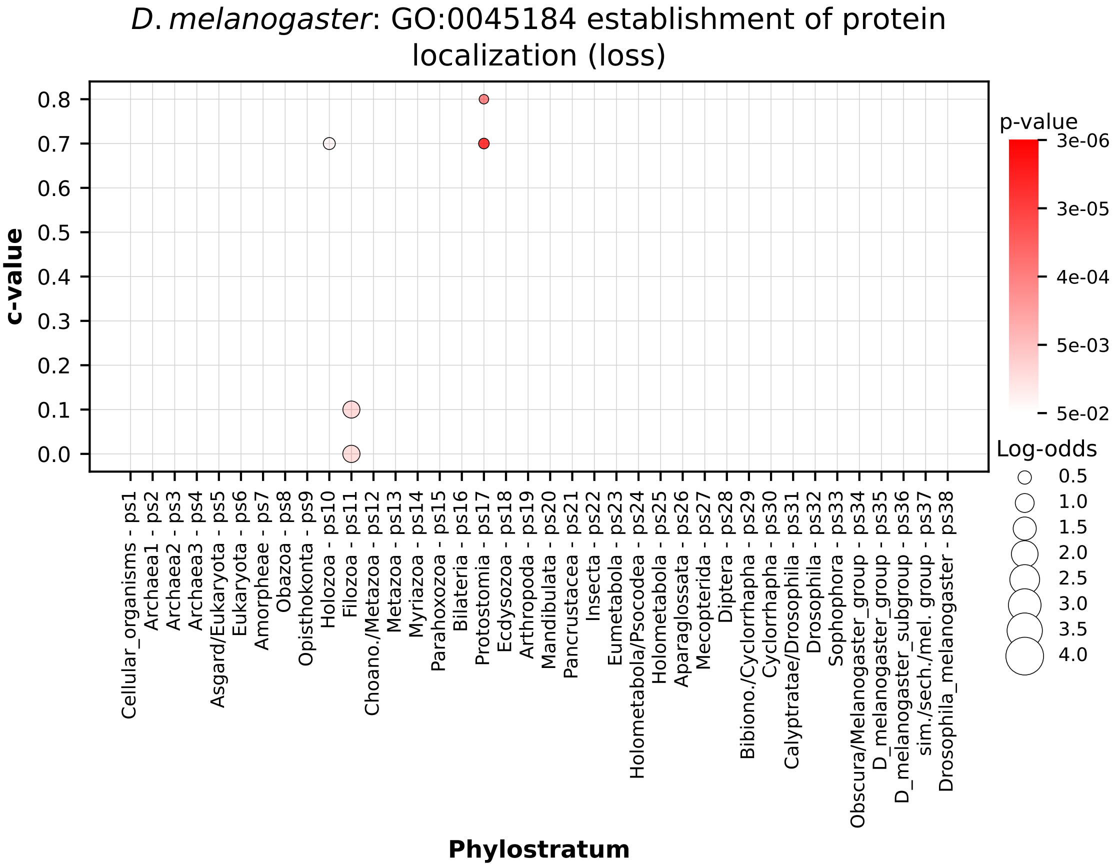

D. melanogaster: GO:0045184 establishment of protein localization (loss)
c-value
0.8
0.7
0.6
0.5
0.4
0.3
0.2
0.1
0.0
p-value
3e-06
3e-05
4e-04
5e-03
5e-02
Log-odds
0.5
1.0
1.5
2.0
2.5
3.0
3.5
4.0
Cellular_organisms - ps1
Archaea1 - ps2
Archaea2 - ps3
Archaea3 - ps4
Asgard/Eukaryota - ps5
Eukaryota - ps6
Amorpheae - ps7
Obazoa - ps8
Opisthokonta - ps9
Holozoa - ps10
Filozoa - ps11
Choano./Metazoa - ps12
Metazoa - ps13
Myriazoa - ps14
Parahoxozoa - ps15
Bilateria - ps16
Protostomia - ps17
Ecdysozoa - ps18
Arthropoda - ps19
Mandibulata - ps20
Pancrustacea - ps21
Insecta - ps22
Eumetabola - ps23
Holometabola/Psocodea - ps24
Holometabola - ps25
Aparaglossata - ps26
Mecopterida - ps27
Diptera - ps28
Bibiono./Cyclorrhapha - ps29
Cyclorrhapha - ps30
Calyptratae/Drosophila - ps31
Drosophila - ps32
Sophophora - ps33
Obscura/Melanogaster_group - ps34
D_melanogaster_group - ps35
D_melanogaster_subgroup - ps36
sim./sech./mel. group - ps37
Drosophila_melanogaster - ps38
Phylostratum

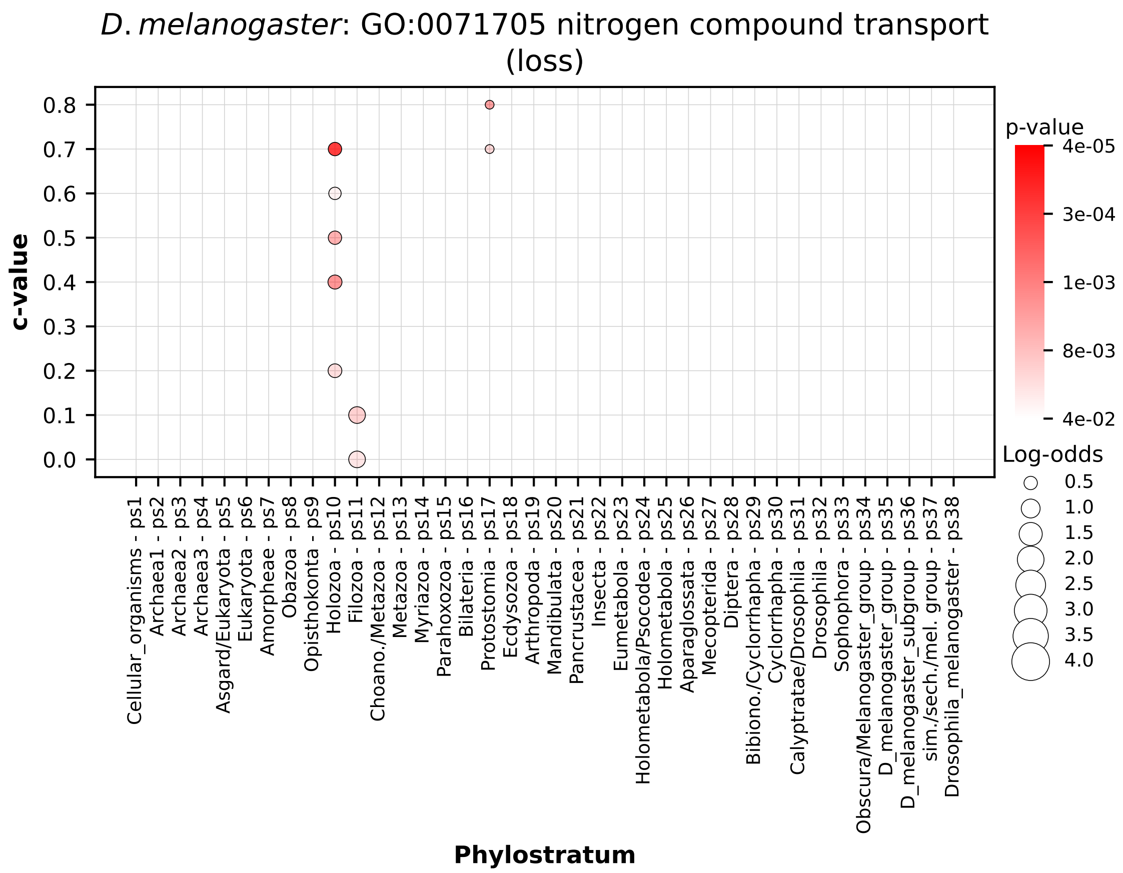

D. melanogaster: GO:0071705 nitrogen compound transport (loss)
c-value
0.0
0.1
0.2
0.3
0.4
0.5
0.6
0.7
0.8
Phylostratum
Cellular_organisms - ps1
Archaea1 - ps2
Archaea2 - ps3
Archaea3 - ps4
Asgard/Eukaryota - ps5
Eukaryota - ps6
Amorpheae - ps7
Obazoa - ps8
Opisthokonta - ps9
Holozoa - ps10
Filozoa - ps11
Choano./Metazoa - ps12
Metazoa - ps13
Myriazoa - ps14
Parahoxozoa - ps15
Bilateria - ps16
Protostomia - ps17
Ecdysozoa - ps18
Arthropoda - ps19
Mandibulata - ps20
Pancrustacea - ps21
Insecta - ps22
Eumetabola - ps23
Holometabola/Psocodea - ps24
Holometabola - ps25
Aparaglossata - ps26
Mecopterida - ps27
Diptera - ps28
Bibiono./Cyclorrhapha - ps29
Cyclorrhapha - ps30
Calyptratae/Drosophila - ps31
Drosophila - ps32
Sophophora - ps33
Obscura/Melanogaster_group - ps34
D_melanogaster_group - ps35
D_melanogaster_subgroup - ps36
sim./sech./mel. group - ps37
Drosophila_melanogaster - ps38
p-value
4e-05
3e-04
1e-03
8e-03
4e-02
Log-odds
0.5
1.0
1.5
2.0
2.5
3.0
3.5
4.0

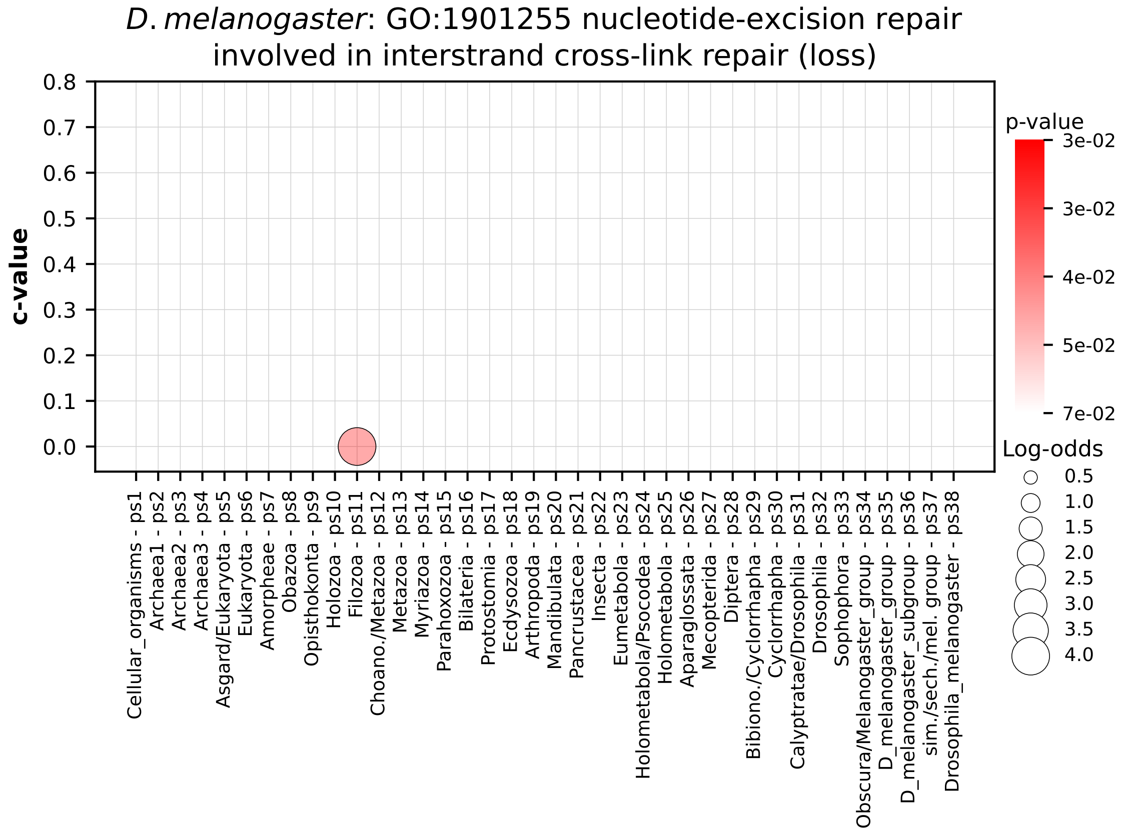

D. melanogaster: GO:1901255 nucleotide-excision repair involved in interstrand cross-link repair (loss)
c-value
0.8
0.7
0.6
0.5
0.4
0.3
0.2
0.1
0.0
p-value
3e-02
3e-02
4e-02
5e-02
7e-02
Log-odds
0.5
1.0
1.5
2.0
2.5
3.0
3.5
4.0
Cellular_organisms - ps1
Archaea1 - ps2
Archaea2 - ps3
Archaea3 - ps4
Asgard/Eukaryota - ps5
Eukaryota - ps6
Amorpheae - ps7
Obazoa - ps8
Opisthokonta - ps9
Holozoa - ps10
Filozoa - ps11
Choano./Metazoa - ps12
Metazoa - ps13
Myriazoa - ps14
Parahoxozoa - ps15
Bilateria - ps16
Protostomia - ps17
Ecdysozoa - ps18
Arthropoda - ps19
Mandibulata - ps20
Pancrustacea - ps21
Insecta - ps22
Eumetabola - ps23
Holometabola/Psocodea - ps24
Holometabola - ps25
Aparaglossata - ps26
Mecopterida - ps27
Diptera - ps28
Bibiono./Cyclorrhapha - ps29
Cyclorrhapha - ps30
Calyptratae/Drosophila - ps31
Drosophila - ps32
Sophophora - ps33
Obscura/Melanogaster_group - ps34
D_melanogaster_group - ps35
D_melanogaster_subgroup - ps36
sim./sech./mel. group - ps37
Drosophila_melanogaster - ps38
Phylostratum

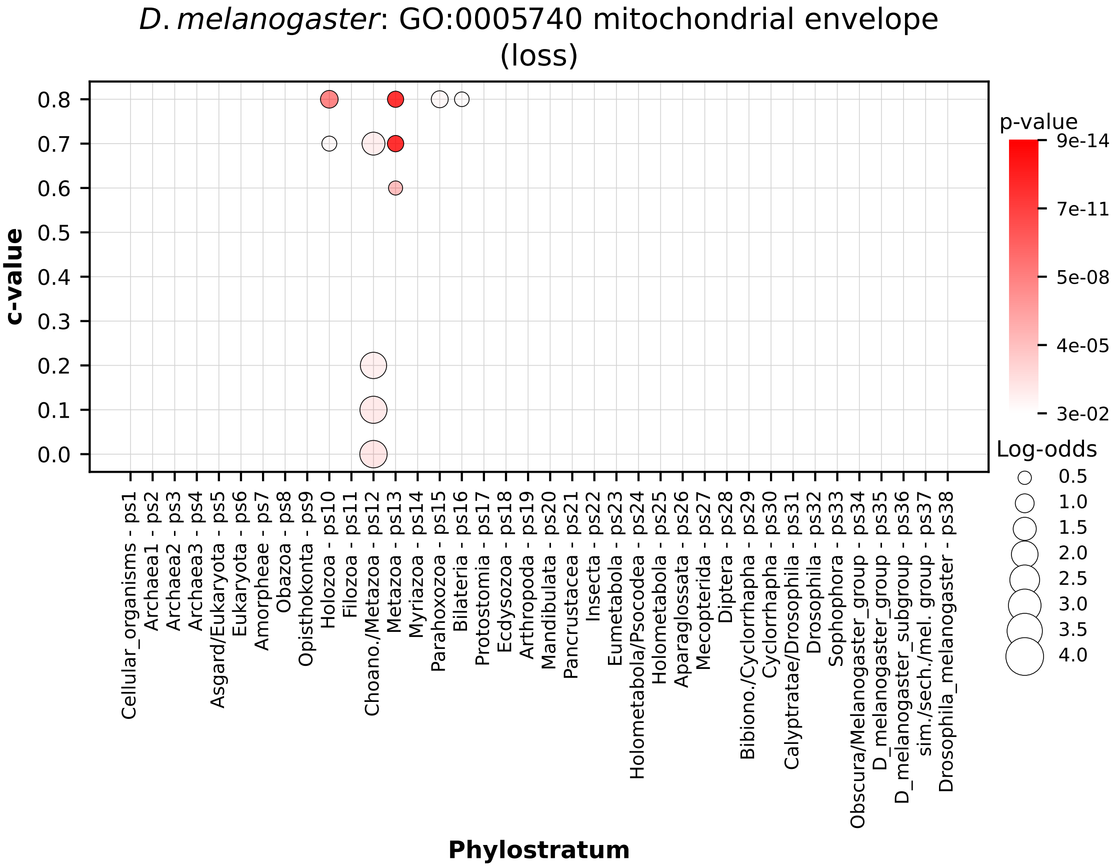

D. melanogaster: GO:0005740 mitochondrial envelope
(loss)
c-value
0.8
0.7
0.6
0.5
0.4
0.3
0.2
0.1
0.0
p-value
9e-14
7e-11
5e-08
4e-05
3e-02
Log-odds
0.5
1.0
1.5
2.0
2.5
3.0
3.5
4.0
Cellular_organisms - ps1
Archaea1 - ps2
Archaea2 - ps3
Archaea3 - ps4
Asgard/Eukaryota - ps5
Eukaryota - ps6
Amorpheae - ps7
Obazoa - ps8
Opisthokonta - ps9
Holozoa - ps10
Filozoa - ps11
Choano./Metazoa - ps12
Metazoa - ps13
Myriazoa - ps14
Parahoxozoa - ps15
Bilateria - ps16
Protostomia - ps17
Ecdysozoa - ps18
Arthropoda - ps19
Mandibulata - ps20
Pancrustacea - ps21
Insecta - ps22
Eumetabola - ps23
Holometabola/Psocodea - ps24
Holometabola - ps25
Aparaglossata - ps26
Mecopterida - ps27
Diptera - ps28
Bibiono./Cyclorrhapha - ps29
Cyclorrhapha - ps30
Calyptratae/Drosophila - ps31
Drosophila - ps32
Sophophora - ps33
Obscura/Melanogaster_group - ps34
D_melanogaster_group - ps35
D_melanogaster_subgroup - ps36
sim./sech./mel. group - ps37
Drosophila_melanogaster - ps38
Phylostratum

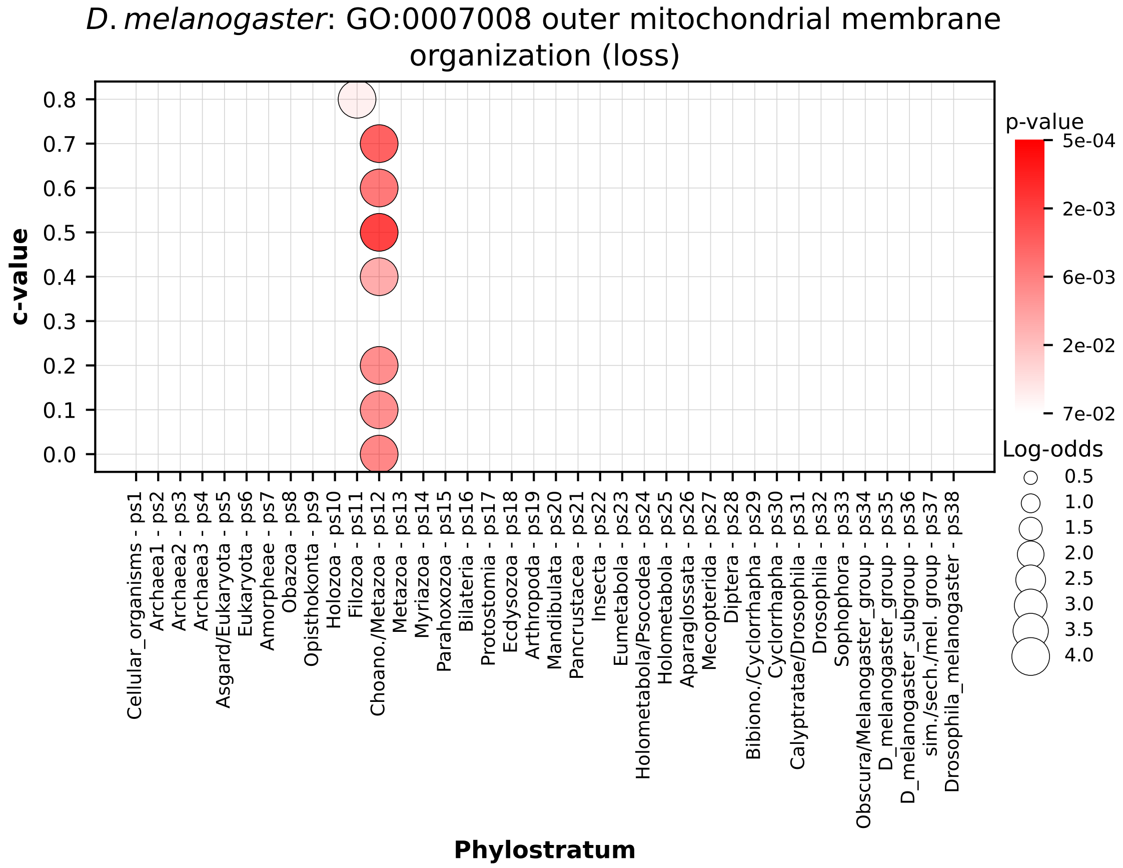
D. melanogaster: GO:0007008 outer mitochondrial membrane organization (loss)
c-value
0.8
0.7
0.6
0.5
0.4
0.3
0.2
0.1
0.0
p-value
5e-04
2e-03
6e-03
2e-02
7e-02
Log-odds
0.5
1.0
1.5
2.0
2.5
3.0
3.5
4.0
Cellular_organisms - ps1
Archaea1 - ps2
Archaea2 - ps3
Archaea3 - ps4
Asgard/Eukaryota - ps5
Eukaryota - ps6
Amorpheae - ps7
Obazoa - ps8
Opisthokonta - ps9
Holozoa - ps10
Filozoa - ps11
Choano./Metazoa - ps12
Metazoa - ps13
Myriazoa - ps14
Parahoxozoa - ps15
Bilateria - ps16
Protostomia - ps17
Ecdysozoa - ps18
Arthropoda - ps19
Mandibulata - ps20
Pancrustacea - ps21
Insecta - ps22
Eumetabola - ps23
Holometabola/Psocodea - ps24
Holometabola - ps25
Aparaglossata - ps26
Mecopterida - ps27
Diptera - ps28
Bibiono./Cyclorrhapha - ps29
Cyclorrhapha - ps30
Calyptratae/Drosophila - ps31
Drosophila - ps32
Sophophora - ps33
Obscura/Melanogaster_group - ps34
D_melanogaster_group - ps35
D_melanogaster_subgroup - ps36
sim./sech./mel. group - ps37
Drosophila_melanogaster - ps38
Phylostratum

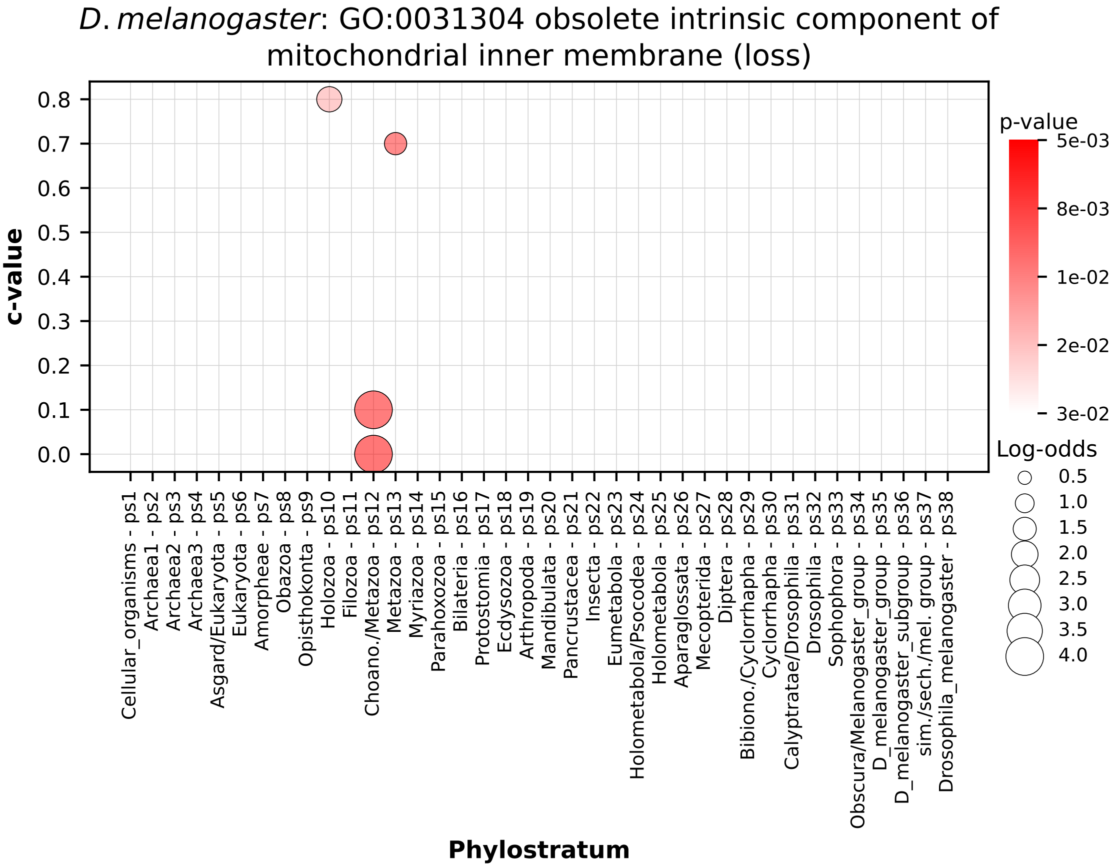

D. melanogaster: GO:0031304 obsolete intrinsic component of mitochondrial inner membrane (loss)
c-value
0.8
0.7
0.6
0.5
0.4
0.3
0.2
0.1
0.0
p-value
5e-03
8e-03
1e-02
2e-02
3e-02
Log-odds
0.5
1.0
1.5
2.0
2.5
3.0
3.5
4.0
Cellular_organisms - ps1
Archaea1 - ps2
Archaea2 - ps3
Archaea3 - ps4
Asgard/Eukaryota - ps5
Eukaryota - ps6
Amorpheae - ps7
Obazoa - ps8
Opisthokonta - ps9
Holozoa - ps10
Filozoa - ps11
Choano./Metazoa - ps12
Metazoa - ps13
Myriazoa - ps14
Parahoxozoa - ps15
Bilateria - ps16
Protostomia - ps17
Ecdysozoa - ps18
Arthropoda - ps19
Mandibulata - ps20
Pancrustacea - ps21
Insecta - ps22
Eumetabola - ps23
Holometabola/Psocodea - ps24
Holometabola - ps25
Aparaglossata - ps26
Mecopterida - ps27
Diptera - ps28
Bibiono./Cyclorrhapha - ps29
Cyclorrhapha - ps30
Calyptratae/Drosophila - ps31
Drosophila - ps32
Sophophora - ps33
Obscura/Melanogaster_group - ps34
D_melanogaster_group - ps35
D_melanogaster_subgroup - ps36
sim./sech./mel. group - ps37
Drosophila_melanogaster - ps38
Phylostratum

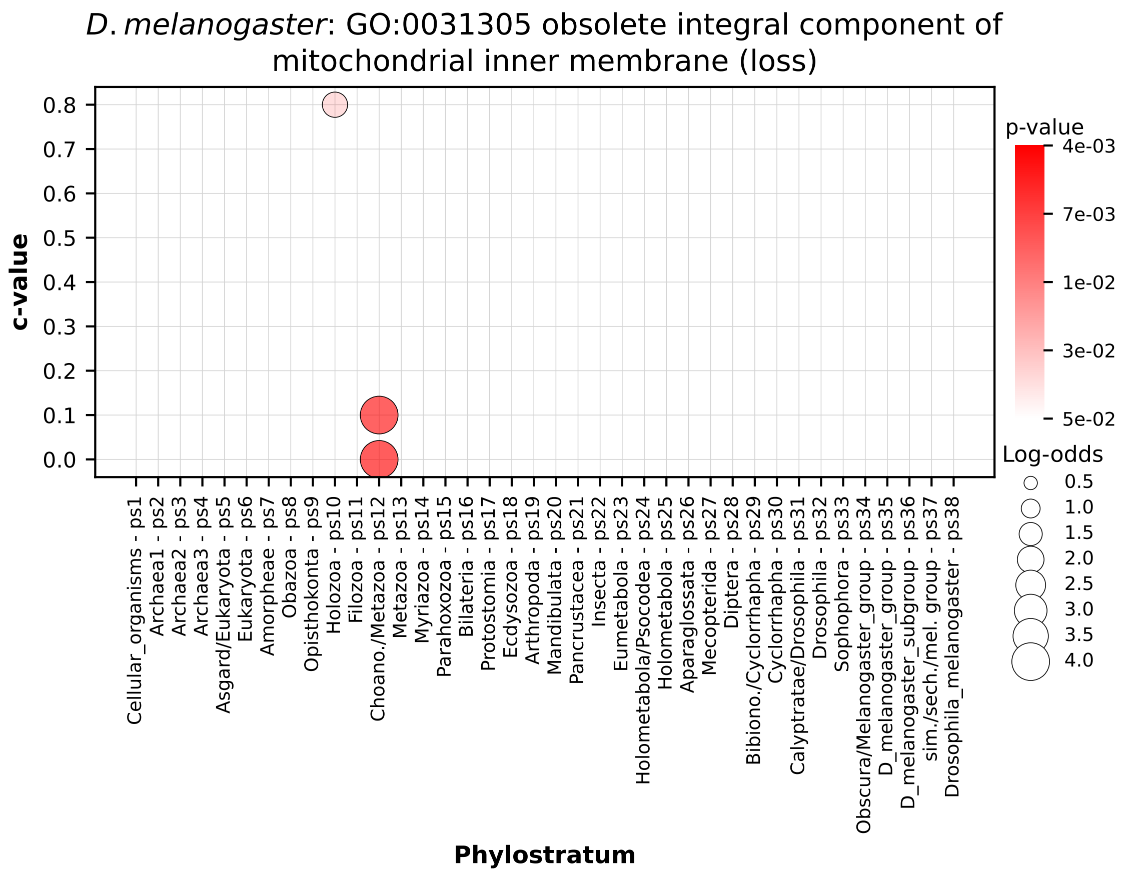
D. melanogaster: GO:0031305 obsolete integral component of mitochondrial inner membrane (loss)
c-value
Phylostratum
p-value
4e-03
7e-03
1e-02
3e-02
5e-02
Log-odds
0.5
1.0
1.5
2.0
2.5
3.0
3.5
4.0
Cellular_organisms - ps1
Archaea1 - ps2
Archaea2 - ps3
Archaea3 - ps4
Asgard/Eukaryota - ps5
Eukaryota - ps6
Amorpheae - ps7
Obazoa - ps8
Opisthokonta - ps9
Holozoa - ps10
Filozoa - ps11
Choano./Metazoa - ps12
Metazoa - ps13
Myriazoa - ps14
Parahoxozoa - ps15
Bilateria - ps16
Protostomia - ps17
Ecdysozoa - ps18
Arthropoda - ps19
Mandibulata - ps20
Pancrustacea - ps21
Insecta - ps22
Eumetabola - ps23
Holometabola/Psocodea - ps24
Holometabola - ps25
Aparaglossata - ps26
Mecopterida - ps27
Diptera - ps28
Bibiono./Cyclorrhapha - ps29
Cyclorrhapha - ps30
Calyptratae/Drosophila - ps31
Drosophila - ps32
Sophophora - ps33
Obscura/Melanogaster_group - ps34
D_melanogaster_group - ps35
D_melanogaster_subgroup - ps36
sim./sech./mel. group - ps37
Drosophila_melanogaster - ps38

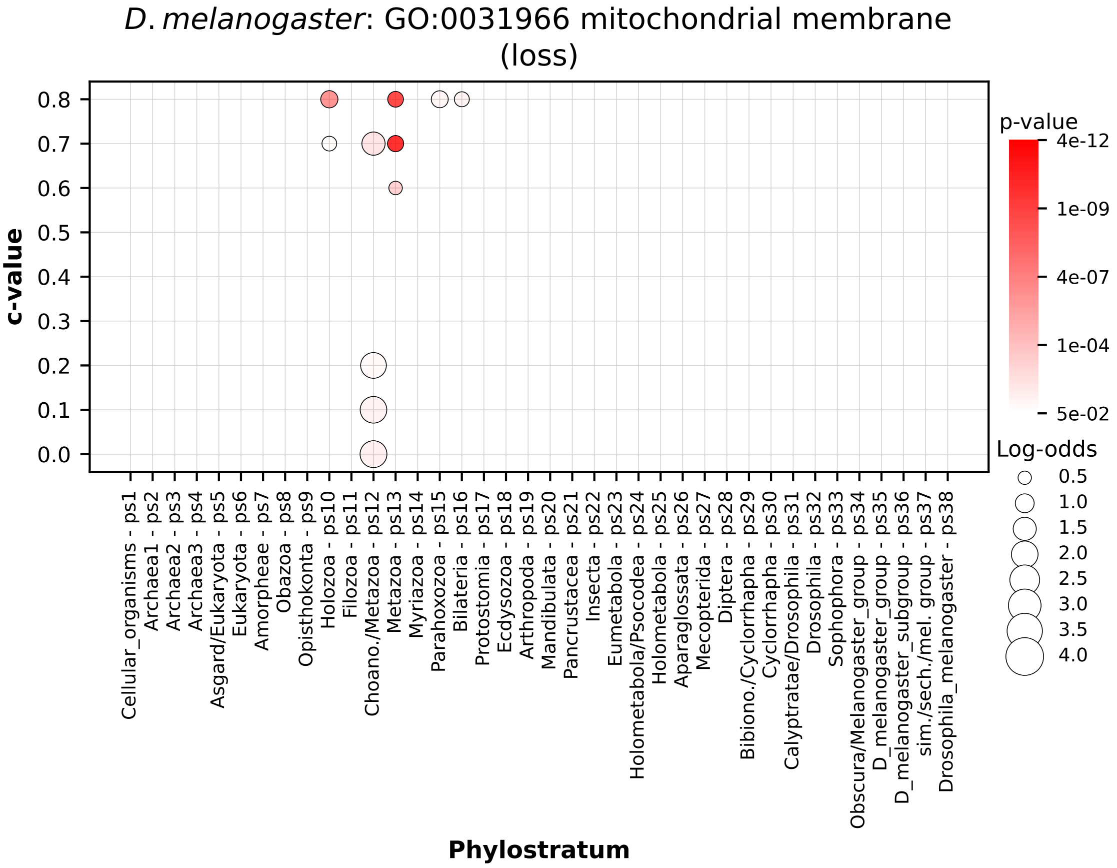

D. melanogaster: GO:0031966 mitochondrial membrane (loss)
c-value
0.8
0.7
0.6
0.5
0.4
0.3
0.2
0.1
0.0
Phylostratum
Cellular_organisms - ps1
Archaea1 - ps2
Archaea2 - ps3
Archaea3 - ps4
Asgard/Eukaryota - ps5
Eukaryota - ps6
Amorpheae - ps7
Obazoa - ps8
Opisthokonta - ps9
Holozoa - ps10
Filozoa - ps11
Choano./Metazoa - ps12
Metazoa - ps13
Myriazoa - ps14
Parahoxozoa - ps15
Bilateria - ps16
Protostomia - ps17
Ecdysozoa - ps18
Arthropoda - ps19
Mandibulata - ps20
Pancrustacea - ps21
Insecta - ps22
Eumetabola - ps23
Holometabola/Psocodea - ps24
Holometabola - ps25
Aparaglossata - ps26
Mecopterida - ps27
Diptera - ps28
Bibiono./Cyclorrhapha - ps29
Cyclorrhapha - ps30
Calyptratae/Drosophila - ps31
Drosophila - ps32
Sophophora - ps33
Obscura/Melanogaster_group - ps34
D_melanogaster_group - ps35
D_melanogaster_subgroup - ps36
sim./sech./mel. group - ps37
Drosophila_melanogaster - ps38
p-value
4e-12
1e-09
4e-07
1e-04
5e-02
Log-odds
0.5
1.0
1.5
2.0
2.5
3.0
3.5
4.0

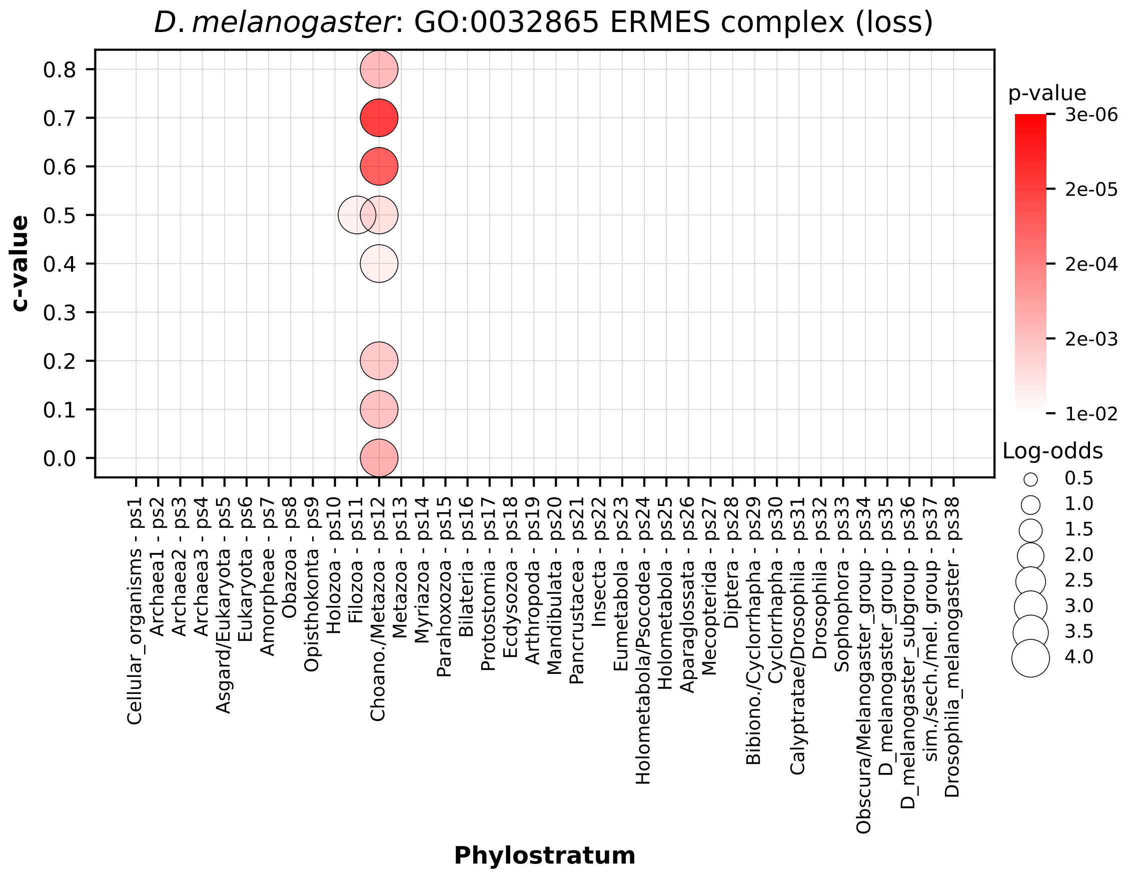

D. melanogaster: GO:0032865 ERMES complex (loss)
c-value
0.8
0.7
0.6
0.5
0.4
0.3
0.2
0.1
0.0
Phylostratum
Cellular_organisms - ps1
Archaea1 - ps2
Archaea2 - ps3
Archaea3 - ps4
Asgard/Eukaryota - ps5
Eukaryota - ps6
Amorpheae - ps7
Obazoa - ps8
Opisthokonta - ps9
Holozoa - ps10
Filozoa - ps11
Choano./Metazoa - ps12
Metazoa - ps13
Myriazoa - ps14
Parahoxozoa - ps15
Bilateria - ps16
Protostomia - ps17
Ecdysozoa - ps18
Arthropoda - ps19
Mandibulata - ps20
Pancrustacea - ps21
Insecta - ps22
Eumetabola - ps23
Holometabola/Psocodea - ps24
Holometabola - ps25
Aparaglossata - ps26
Mecopterida - ps27
Diptera - ps28
Bibiono./Cyclorrhapha - ps29
Cyclorrhapha - ps30
Calyptratae/Drosophila - ps31
Drosophila - ps32
Sophophora - ps33
Obscura/Melanogaster_group - ps34
D_melanogaster_group - ps35
D_melanogaster_subgroup - ps36
sim./sech./mel. group - ps37
Drosophila_melanogaster - ps38
p-value
3e-06
2e-05
2e-04
2e-03
1e-02
Log-odds
0.5
1.0
1.5
2.0
2.5
3.0
3.5
4.0

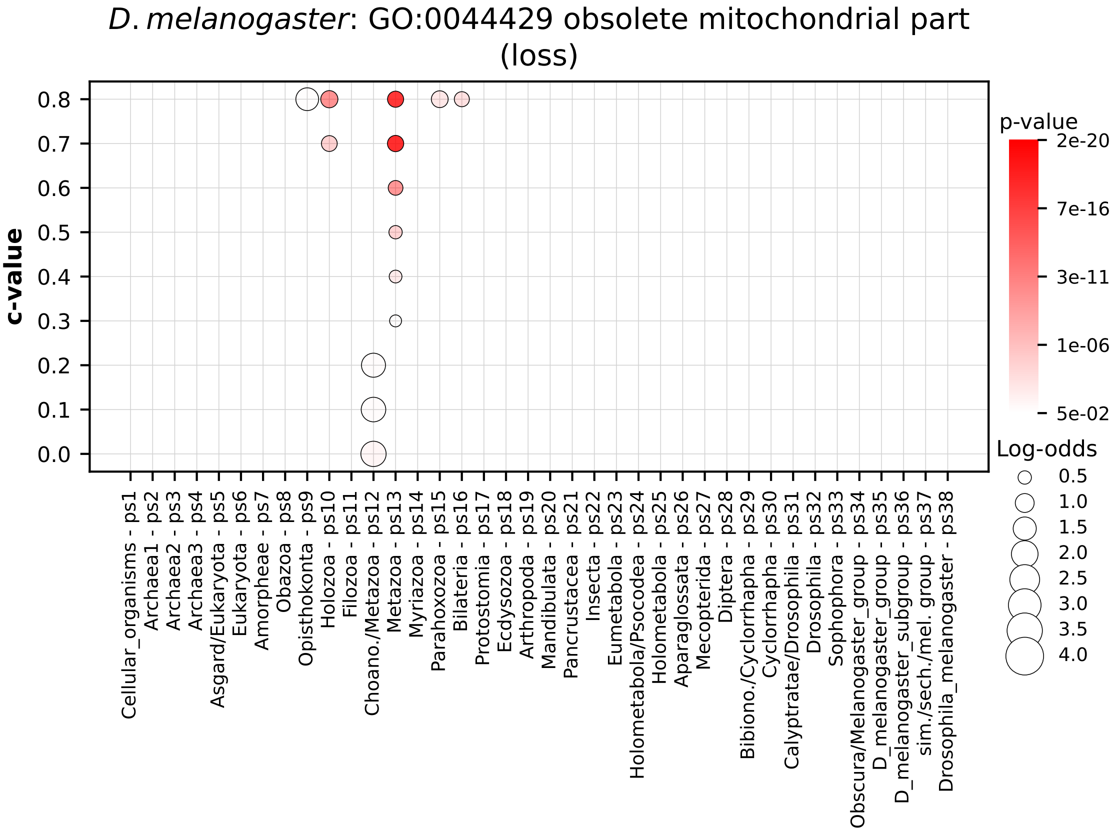

D. melanogaster: GO:0044429 obsolete mitochondrial part (loss)
c-value
0.8
0.7
0.6
0.5
0.4
0.3
0.2
0.1
0.0
p-value
2e-20
7e-16
3e-11
1e-06
5e-02
Log-odds
0.5
1.0
1.5
2.0
2.5
3.0
3.5
4.0
Cellular_organisms - ps1
Archaea1 - ps2
Archaea2 - ps3
Archaea3 - ps4
Asgard/Eukaryota - ps5
Eukaryota - ps6
Amorpheae - ps7
Obazoa - ps8
Opisthokonta - ps9
Holozoa - ps10
Filozoa - ps11
Choano./Metazoa - ps12
Metazoa - ps13
Myriazoa - ps14
Parahoxozoa - ps15
Bilateria - ps16
Protostomia - ps17
Ecdysozoa - ps18
Arthropoda - ps19
Mandibulata - ps20
Pancrustacea - ps21
Insecta - ps22
Eumetabola - ps23
Holometabola/Psocodea - ps24
Holometabola - ps25
Aparaglossata - ps26
Mecopterida - ps27
Diptera - ps28
Bibiono./Cyclorrhapha - ps29
Cyclorrhapha - ps30
Calyptratae/Drosophila - ps31
Drosophila - ps32
Sophophora - ps33
Obscura/Melanogaster_group - ps34
D_melanogaster_group - ps35
D_melanogaster_subgroup - ps36
sim./sech./mel. group - ps37
Drosophila_melanogaster - ps38
Phylostratum

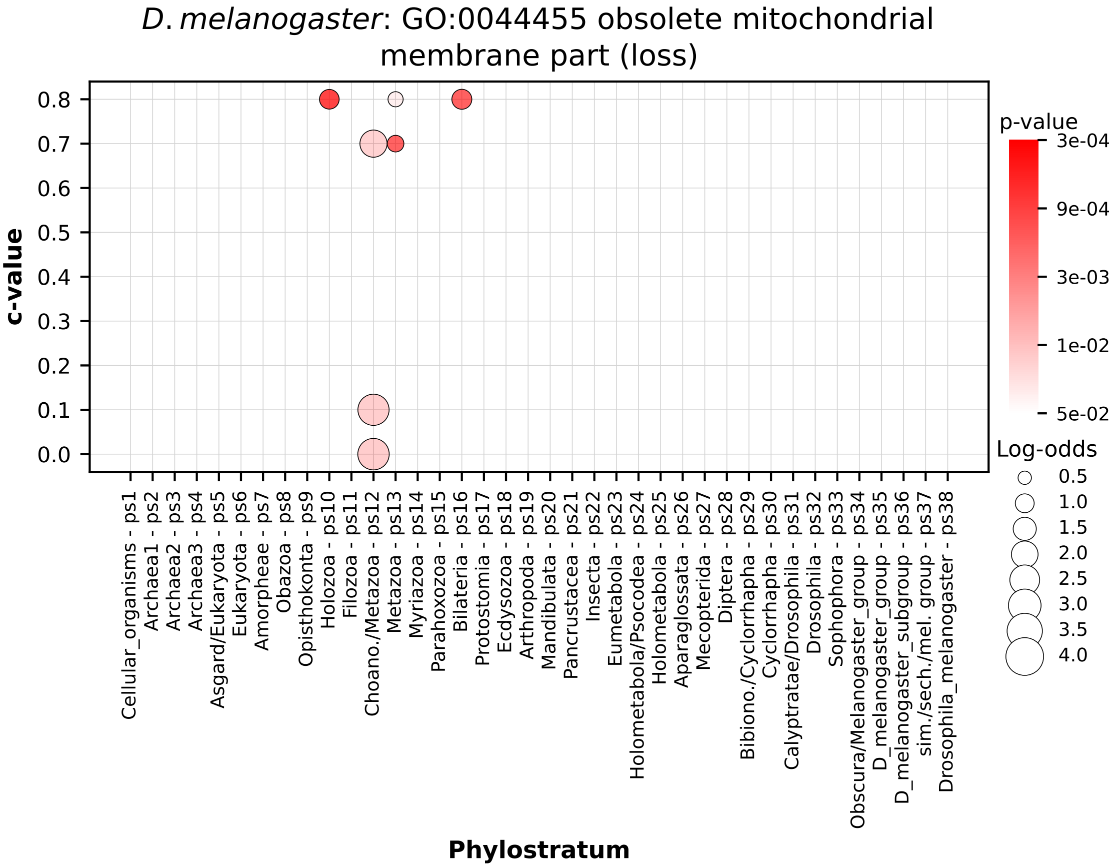

D. melanogaster: GO:0044455 obsolete mitochondrial membrane part (loss)
c-value
0.0
0.1
0.2
0.3
0.4
0.5
0.6
0.7
0.8
Phylostratum
Cellular_organisms - ps1
Archaea1 - ps2
Archaea2 - ps3
Archaea3 - ps4
Asgard/Eukaryota - ps5
Eukaryota - ps6
Amorpheae - ps7
Obazoa - ps8
Opisthokonta - ps9
Holozoa - ps10
Filozoa - ps11
Choano./Metazoa - ps12
Metazoa - ps13
Myriazoa - ps14
Parahoxozoa - ps15
Bilateria - ps16
Protostomia - ps17
Ecdysozoa - ps18
Arthropoda - ps19
Mandibulata - ps20
Pancrustacea - ps21
Insecta - ps22
Eumetabola - ps23
Holometabola/Psocodea - ps24
Holometabola - ps25
Aparaglossata - ps26
Mecopterida - ps27
Diptera - ps28
Bibiono./Cyclorrhapha - ps29
Cyclorrhapha - ps30
Calyptratae/Drosophila - ps31
Drosophila - ps32
Sophophora - ps33
Obscura/Melanogaster_group - ps34
D_melanogaster_group - ps35
D_melanogaster_subgroup - ps36
sim./sech./mel. group - ps37
Drosophila_melanogaster - ps38
p-value
3e-04
9e-04
3e-03
1e-02
5e-02
Log-odds
0.5
1.0
1.5
2.0
2.5
3.0
3.5
4.0

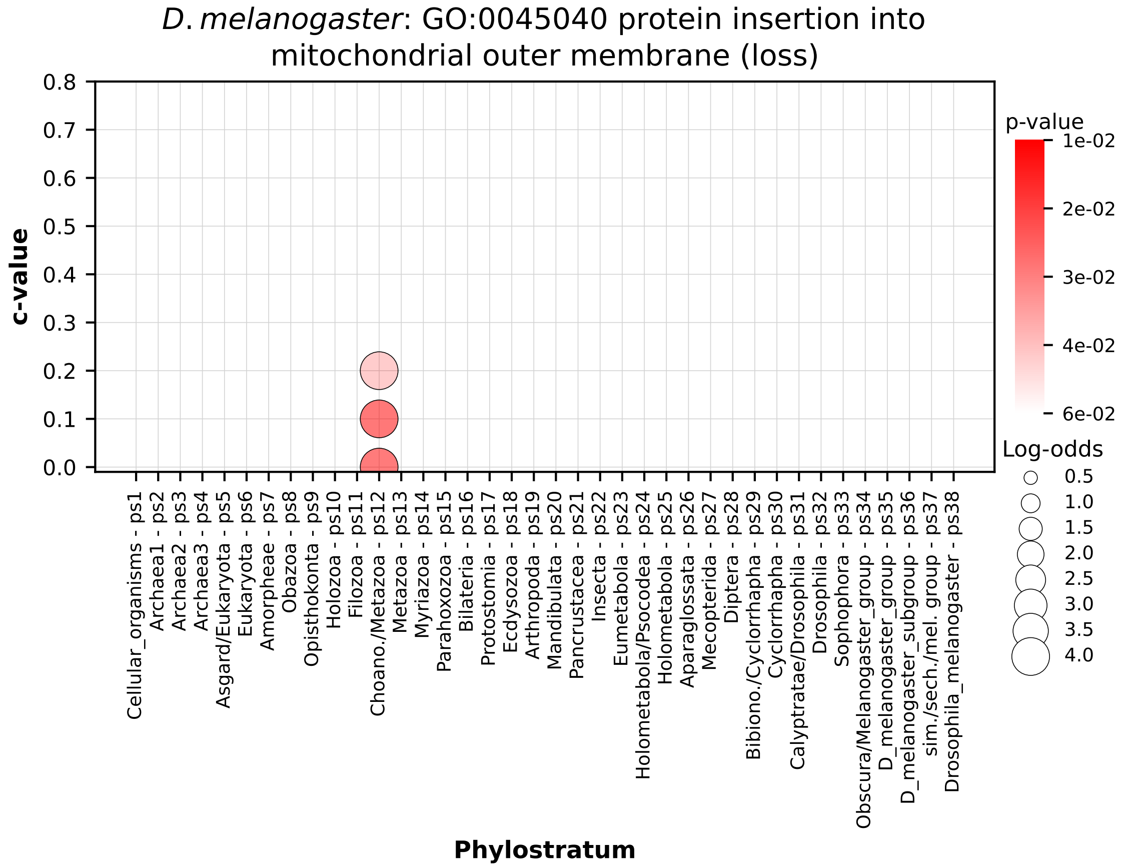

D. melanogaster: GO:0045040 protein insertion into mitochondrial outer membrane (loss)
c-value
0.8
0.7
0.6
0.5
0.4
0.3
0.2
0.1
0.0
p-value
1e-02
2e-02
3e-02
4e-02
6e-02
Log-odds
0.5
1.0
1.5
2.0
2.5
3.0
3.5
4.0
Cellular_organisms - ps1
Archaea1 - ps2
Archaea2 - ps3
Archaea3 - ps4
Asgard/Eukaryota - ps5
Eukaryota - ps6
Amorpheae - ps7
Obazoa - ps8
Opisthokonta - ps9
Holozoa - ps10
Filozoa - ps11
Choano./Metazoa - ps12
Metazoa - ps13
Myriazoa - ps14
Parahoxozoa - ps15
Bilateria - ps16
Protostomia - ps17
Ecdysozoa - ps18
Arthropoda - ps19
Mandibulata - ps20
Pancrustacea - ps21
Insecta - ps22
Eumetabola - ps23
Holometabola/Psocodea - ps24
Holometabola - ps25
Aparaglossata - ps26
Mecopterida - ps27
Diptera - ps28
Bibiono./Cyclorrhapha - ps29
Cyclorrhapha - ps30
Calyptratae/Drosophila - ps31
Drosophila - ps32
Sophophora - ps33
Obscura/Melanogaster_group - ps34
D_melanogaster_group - ps35
D_melanogaster_subgroup - ps36
sim./sech./mel. group - ps37
Drosophila_melanogaster - ps38
Phylostratum

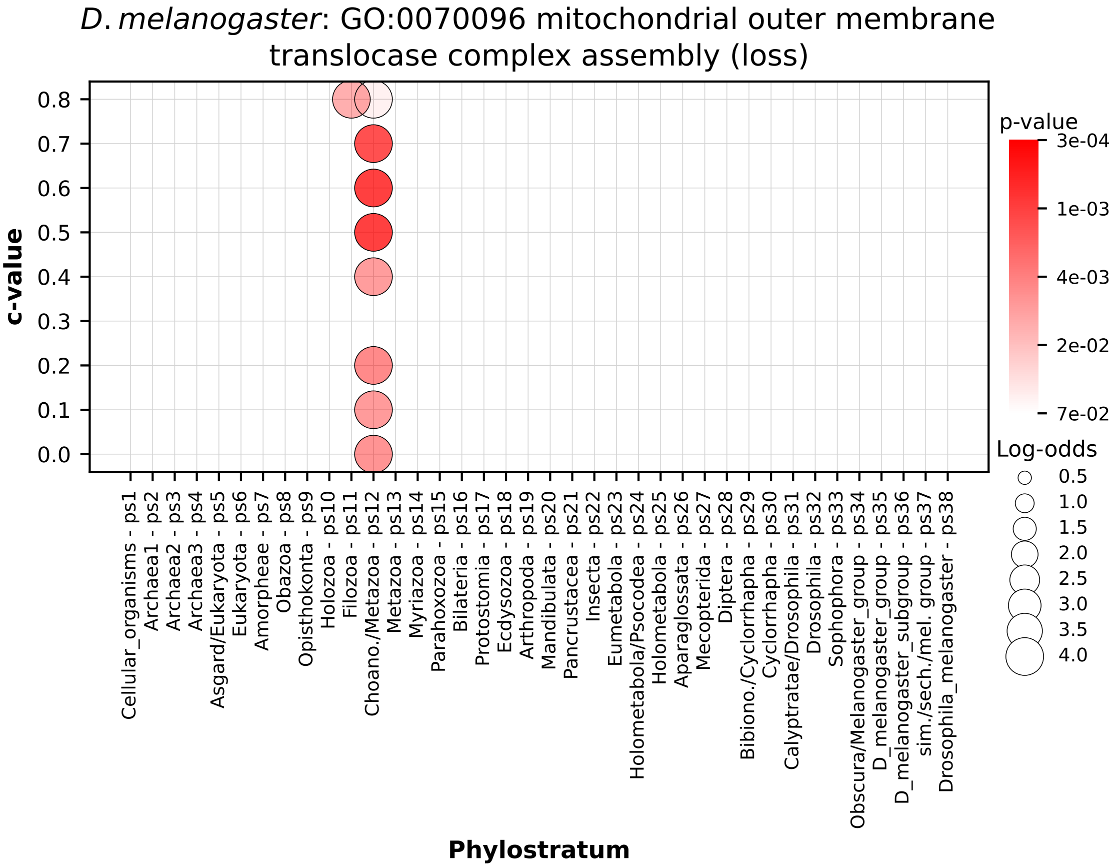

D. melanogaster: GO:0070096 mitochondrial outer membrane translocase complex assembly (loss)
c-value
0.8
0.7
0.6
0.5
0.4
0.3
0.2
0.1
0.0
p-value
3e-04
1e-03
4e-03
2e-02
7e-02
Log-odds
0.5
1.0
1.5
2.0
2.5
3.0
3.5
4.0
Cellular_organisms - ps1
Archaea1 - ps2
Archaea2 - ps3
Archaea3 - ps4
Asgard/Eukaryota - ps5
Eukaryota - ps6
Amorpheae - ps7
Obazoa - ps8
Opisthokonta - ps9
Holozoa - ps10
Filozoa - ps11
Choano./Metazoa - ps12
Metazoa - ps13
Myriazoa - ps14
Parahoxozoa - ps15
Bilateria - ps16
Protostomia - ps17
Ecdysozoa - ps18
Arthropoda - ps19
Mandibulata - ps20
Pancrustacea - ps21
Insecta - ps22
Eumetabola - ps23
Holometabola/Psocodea - ps24
Holometabola - ps25
Aparaglossata - ps26
Mecopterida - ps27
Diptera - ps28
Bibiono./Cyclorrhapha - ps29
Cyclorrhapha - ps30
Calyptratae/Drosophila - ps31
Drosophila - ps32
Sophophora - ps33
Obscura/Melanogaster_group - ps34
D_melanogaster_group - ps35
D_melanogaster_subgroup - ps36
sim./sech./mel. group - ps37
Drosophila_melanogaster - ps38
Phylostratum

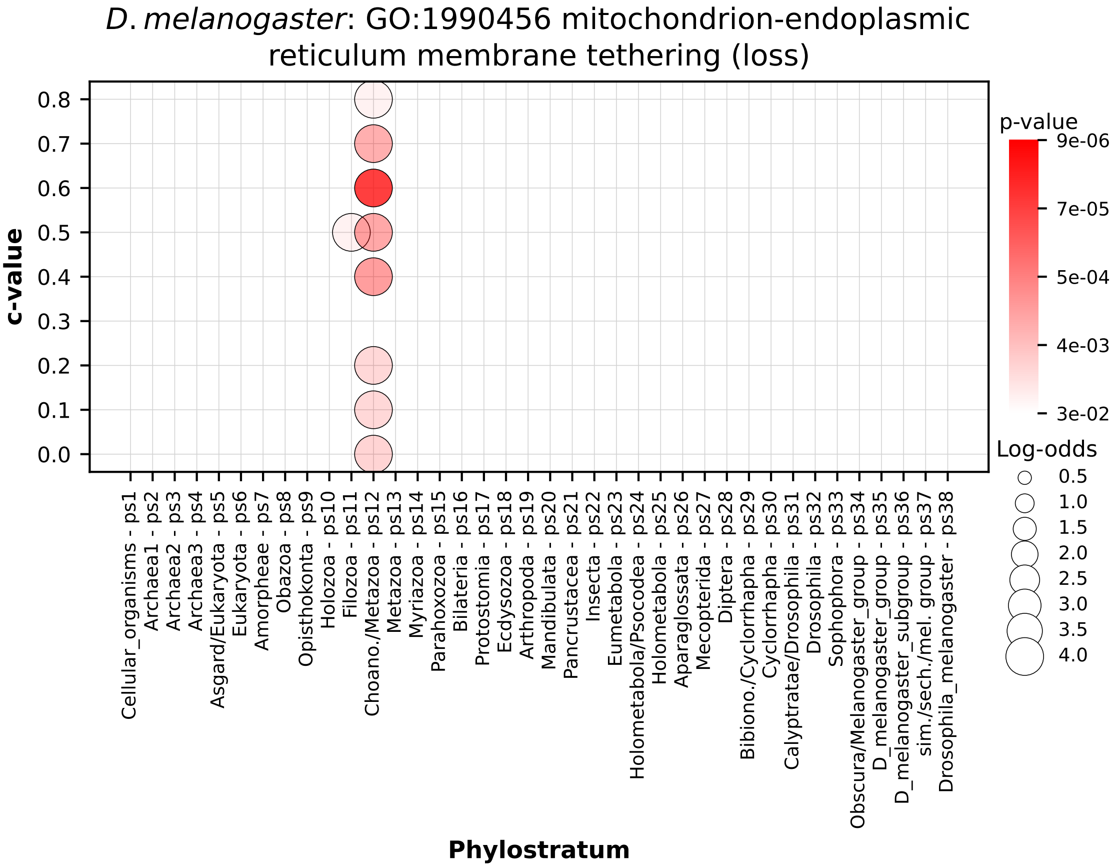

D. melanogaster: GO:1990456 mitochondrion-endoplasmic reticulum membrane tethering (loss)
c-value
0.0
0.1
0.2
0.3
0.4
0.5
0.6
0.7
0.8
p-value
9e-06
7e-05
5e-04
4e-03
3e-02
Log-odds
0.5
1.0
1.5
2.0
2.5
3.0
3.5
4.0
Cellular_organisms - ps1
Archaea1 - ps2
Archaea2 - ps3
Archaea3 - ps4
Asgard/Eukaryota - ps5
Eukaryota - ps6
Amorpheae - ps7
Obazoa - ps8
Opisthokonta - ps9
Holozoa - ps10
Filozoa - ps11
Choano./Metazoa - ps12
Metazoa - ps13
Myriazoa - ps14
Parahoxozoa - ps15
Bilateria - ps16
Protostomia - ps17
Ecdysozoa - ps18
Arthropoda - ps19
Mandibulata - ps20
Pancrustacea - ps21
Insecta - ps22
Eumetabola - ps23
Holometabola/Psocodea - ps24
Holometabola - ps25
Aparaglossata - ps26
Mecopterida - ps27
Diptera - ps28
Bibiono./Cyclorrhapha - ps29
Cyclorrhapha - ps30
Calyptratae/Drosophila - ps31
Drosophila - ps32
Sophophora - ps33
Obscura/Melanogaster_group - ps34
D_melanogaster_group - ps35
D_melanogaster_subgroup - ps36
sim./sech./mel. group - ps37
Drosophila_melanogaster - ps38
Phylostratum

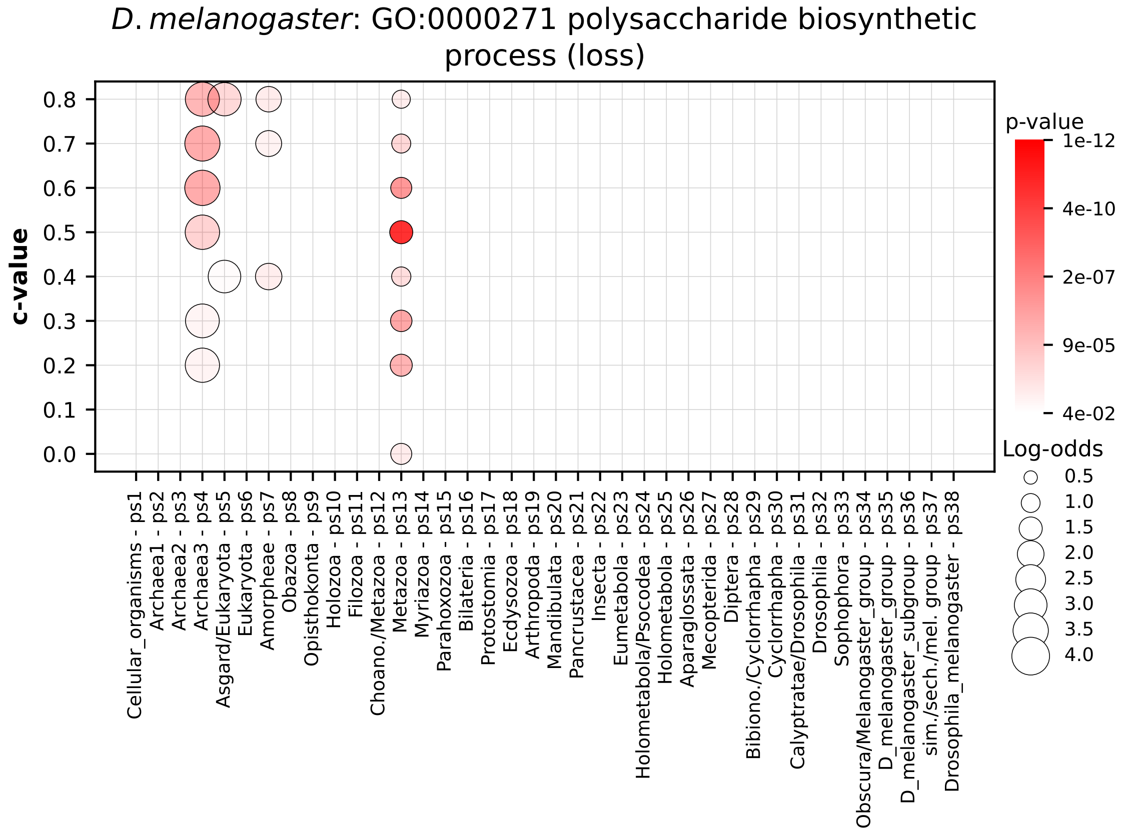

D. melanogaster: GO:0000271 polysaccharide biosynthetic process (loss)
c-value
0.8
0.7
0.6
0.5
0.4
0.3
0.2
0.1
0.0
p-value
1e-12
4e-10
2e-07
9e-05
4e-02
Log-odds
0.5
1.0
1.5
2.0
2.5
3.0
3.5
4.0
Cellular_organisms - ps1
Archaea1 - ps2
Archaea2 - ps3
Archaea3 - ps4
Asgard/Eukaryota - ps5
Eukaryota - ps6
Amorpheae - ps7
Obazoa - ps8
Opisthokonta - ps9
Holozoa - ps10
Filozoa - ps11
Choano./Metazoa - ps12
Metazoa - ps13
Myriazoa - ps14
Parahoxozoa - ps15
Bilateria - ps16
Protostomia - ps17
Ecdysozoa - ps18
Arthropoda - ps19
Mandibulata - ps20
Pancrustacea - ps21
Insecta - ps22
Eumetabola - ps23
Holometabola/Psocodea - ps24
Holometabola - ps25
Aparaglossata - ps26
Mecopterida - ps27
Diptera - ps28
Bibiono./Cyclorrhapha - ps29
Cyclorrhapha - ps30
Calyptratae/Drosophila - ps31
Drosophila - ps32
Sophophora - ps33
Obscura/Melanogaster_group - ps34
D_melanogaster_group - ps35
D_melanogaster_subgroup - ps36
sim./sech./mel. group - ps37
Drosophila_melanogaster - ps38
Phylostratum

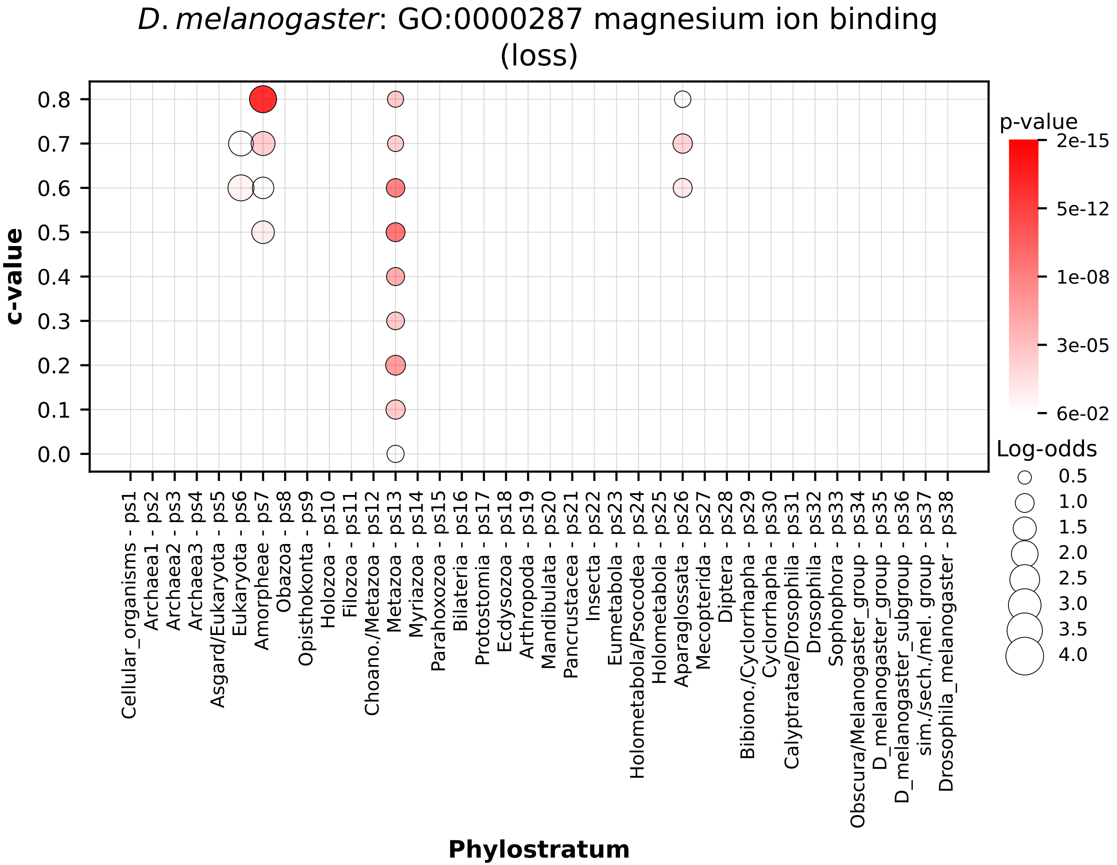

D. melanogaster: GO:0000287 magnesium ion binding (loss)
c-value
0.8
0.7
0.6
0.5
0.4
0.3
0.2
0.1
0.0
Phylostratum
Cellular_organisms - ps1
Archaea1 - ps2
Archaea2 - ps3
Archaea3 - ps4
Asgard/Eukaryota - ps5
Eukaryota - ps6
Amorpheae - ps7
Obazoa - ps8
Opisthokonta - ps9
Holozoa - ps10
Filozoa - ps11
Choano./Metazoa - ps12
Metazoa - ps13
Myriazoa - ps14
Parahoxozoa - ps15
Bilateria - ps16
Protostomia - ps17
Ecdysozoa - ps18
Arthropoda - ps19
Mandibulata - ps20
Pancrustacea - ps21
Insecta - ps22
Eumetabola - ps23
Holometabola/Psocodea - ps24
Holometabola - ps25
Aparaglossata - ps26
Mecopterida - ps27
Diptera - ps28
Bibiono./Cyclorrhapha - ps29
Cyclorrhapha - ps30
Calyptratae/Drosophila - ps31
Drosophila - ps32
Sophophora - ps33
Obscura/Melanogaster_group - ps34
D_melanogaster_group - ps35
D_melanogaster_subgroup - ps36
sim./sech./mel. group - ps37
Drosophila_melanogaster - ps38
p-value
2e-15
5e-12
1e-08
3e-05
6e-02
Log-odds
0.5
1.0
1.5
2.0
2.5
3.0
3.5
4.0

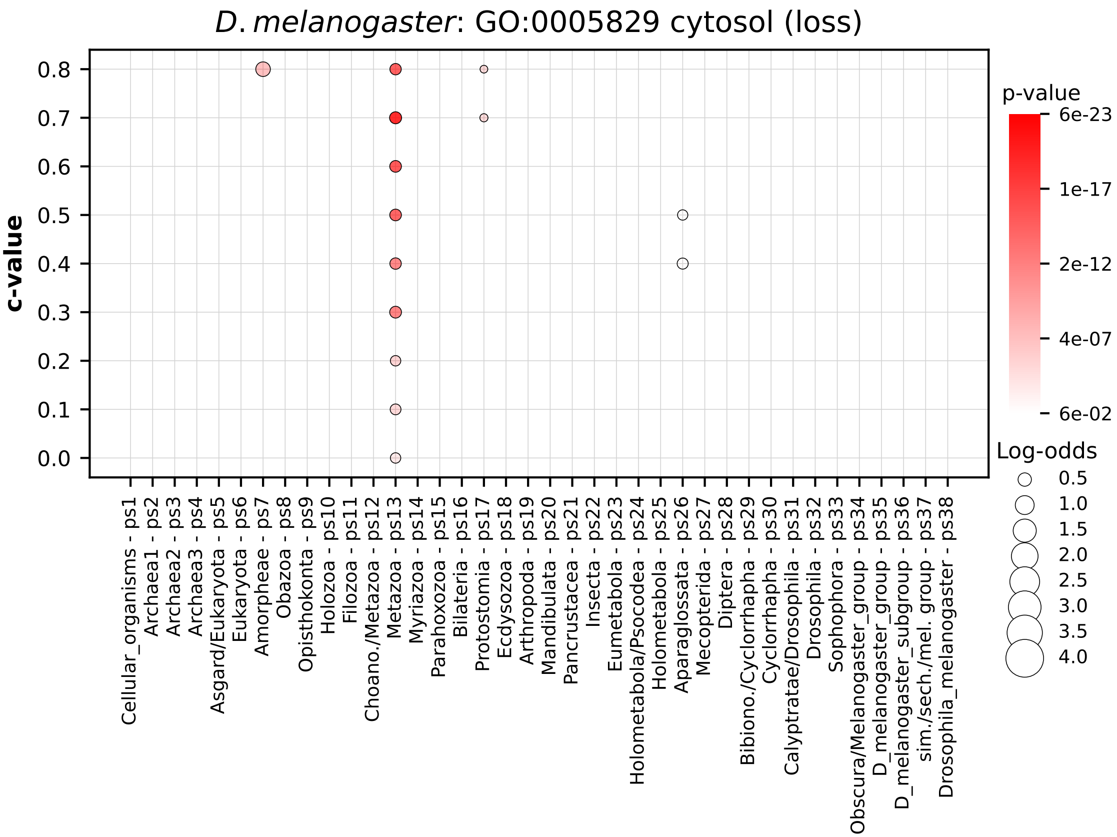

D. melanogaster: GO:0005829 cytosol (loss)
c-value
0.0
0.1
0.2
0.3
0.4
0.5
0.6
0.7
0.8
Phylostratum
Cellular_organisms - ps1
Archaea1 - ps2
Archaea2 - ps3
Archaea3 - ps4
Asgard/Eukaryota - ps5
Eukaryota - ps6
Amorpheae - ps7
Obazoa - ps8
Opisthokonta - ps9
Holozoa - ps10
Filozoa - ps11
Choano./Metazoa - ps12
Metazoa - ps13
Myriazoa - ps14
Parahoxozoa - ps15
Bilateria - ps16
Protostomia - ps17
Ecdysozoa - ps18
Arthropoda - ps19
Mandibulata - ps20
Pancrustacea - ps21
Insecta - ps22
Eumetabola - ps23
Holometabola/Psocodea - ps24
Holometabola - ps25
Aparaglossata - ps26
Mecopterida - ps27
Diptera - ps28
Bibiono./Cyclorrhapha - ps29
Cyclorrhapha - ps30
Calyptratae/Drosophila - ps31
Drosophila - ps32
Sophophora - ps33
Obscura/Melanogaster_group - ps34
D_melanogaster_group - ps35
D_melanogaster_subgroup - ps36
sim./sech./mel. group - ps37
Drosophila_melanogaster - ps38
p-value
6e-23
1e-17
2e-12
4e-07
6e-02
Log-odds
0.5
1.0
1.5
2.0
2.5
3.0
3.5
4.0

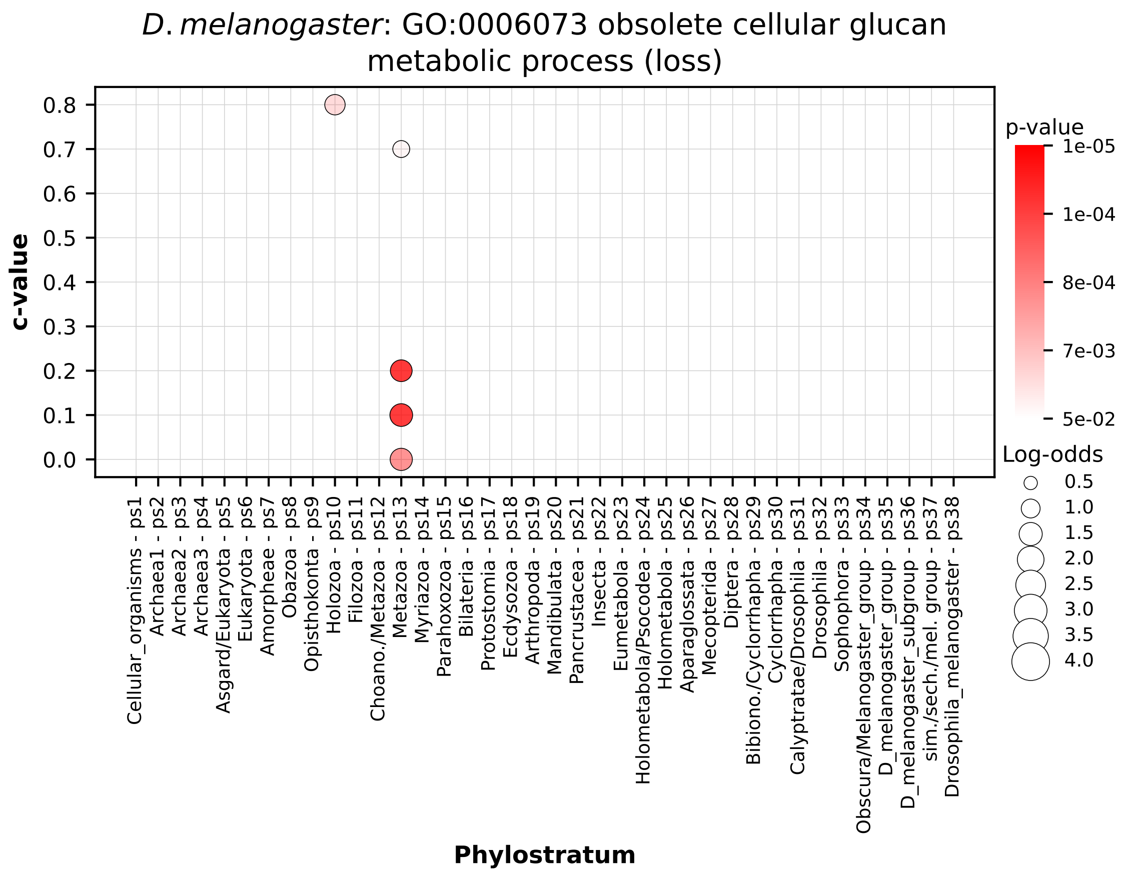

D. melanogaster: GO:0006073 obsolete cellular glucan metabolic process (loss)
c-value
0.8
0.7
0.6
0.5
0.4
0.3
0.2
0.1
0.0
p-value
1e-05
1e-04
8e-04
7e-03
5e-02
Log-odds
0.5
1.0
1.5
2.0
2.5
3.0
3.5
4.0
Cellular_organisms - ps1
Archaea1 - ps2
Archaea2 - ps3
Archaea3 - ps4
Asgard/Eukaryota - ps5
Eukaryota - ps6
Amorpheae - ps7
Obazoa - ps8
Opisthokonta - ps9
Holozoa - ps10
Filozoa - ps11
Choano./Metazoa - ps12
Metazoa - ps13
Myriazoa - ps14
Parahoxozoa - ps15
Bilateria - ps16
Protostomia - ps17
Ecdysozoa - ps18
Arthropoda - ps19
Mandibulata - ps20
Pancrustacea - ps21
Insecta - ps22
Eumetabola - ps23
Holometabola/Psocodea - ps24
Holometabola - ps25
Aparaglossata - ps26
Mecopterida - ps27
Diptera - ps28
Bibiono./Cyclorrhapha - ps29
Cyclorrhapha - ps30
Calyptratae/Drosophila - ps31
Drosophila - ps32
Sophophora - ps33
Obscura/Melanogaster_group - ps34
D_melanogaster_group - ps35
D_melanogaster_subgroup - ps36
sim./sech./mel. group - ps37
Drosophila_melanogaster - ps38
Phylostratum

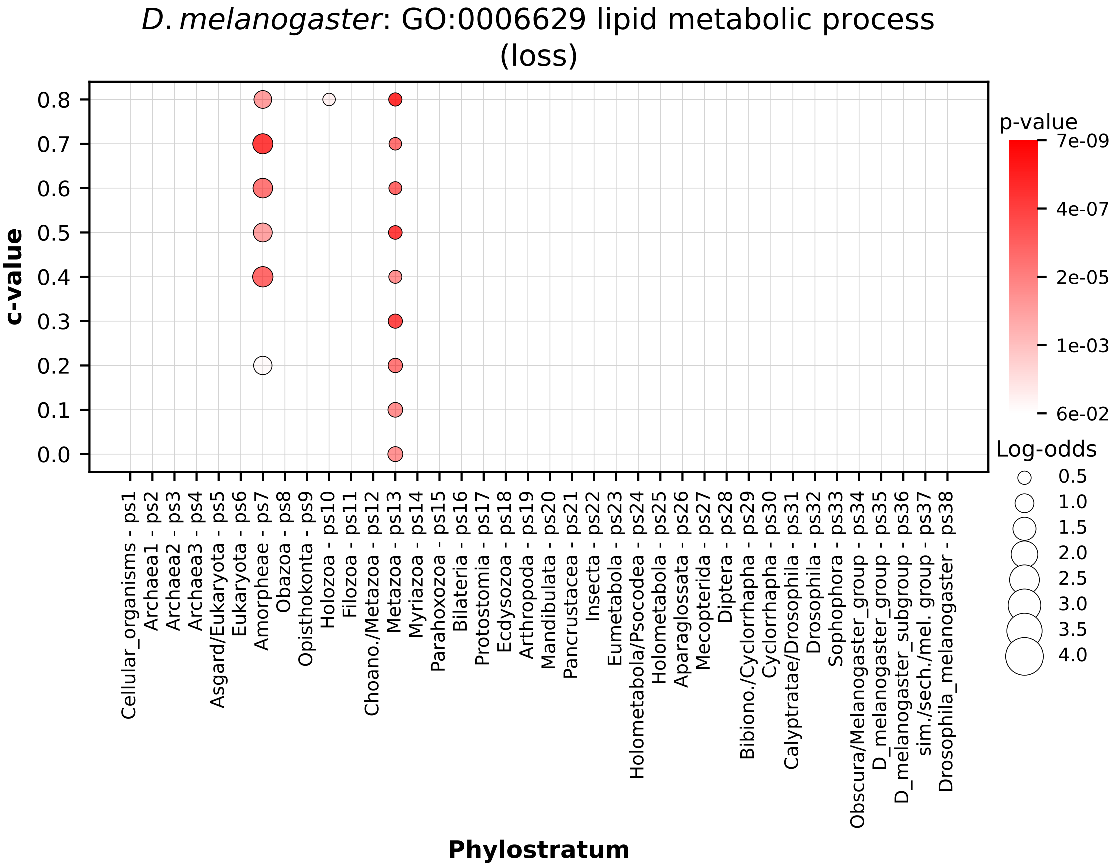
D. melanogaster: GO:0006629 lipid metabolic process
(loss)
c-value
0.8
0.7
0.6
0.5
0.4
0.3
0.2
0.1
0.0
p-value
7e-09
4e-07
2e-05
1e-03
6e-02
Log-odds
0.5
1.0
1.5
2.0
2.5
3.0
3.5
4.0
Cellular_organisms - ps1
Archaea1 - ps2
Archaea2 - ps3
Archaea3 - ps4
Asgard/Eukaryota - ps5
Eukaryota - ps6
Amorpheae - ps7
Obazoa - ps8
Opisthokonta - ps9
Holozoa - ps10
Filozoa - ps11
Choano./Metazoa - ps12
Metazoa - ps13
Myriazoa - ps14
Parahoxozoa - ps15
Bilateria - ps16
Protostomia - ps17
Ecdysozoa - ps18
Arthropoda - ps19
Mandibulata - ps20
Pancrustacea - ps21
Insecta - ps22
Eumetabola - ps23
Holometabola/Psocodea - ps24
Holometabola - ps25
Aparaglossata - ps26
Mecopterida - ps27
Diptera - ps28
Bibiono./Cyclorrhapha - ps29
Cyclorrhapha - ps30
Calyptratae/Drosophila - ps31
Drosophila - ps32
Sophophora - ps33
Obscura/Melanogaster_group - ps34
D_melanogaster_group - ps35
D_melanogaster_subgroup - ps36
sim./sech./mel. group - ps37
Drosophila_melanogaster - ps38
Phylostratum

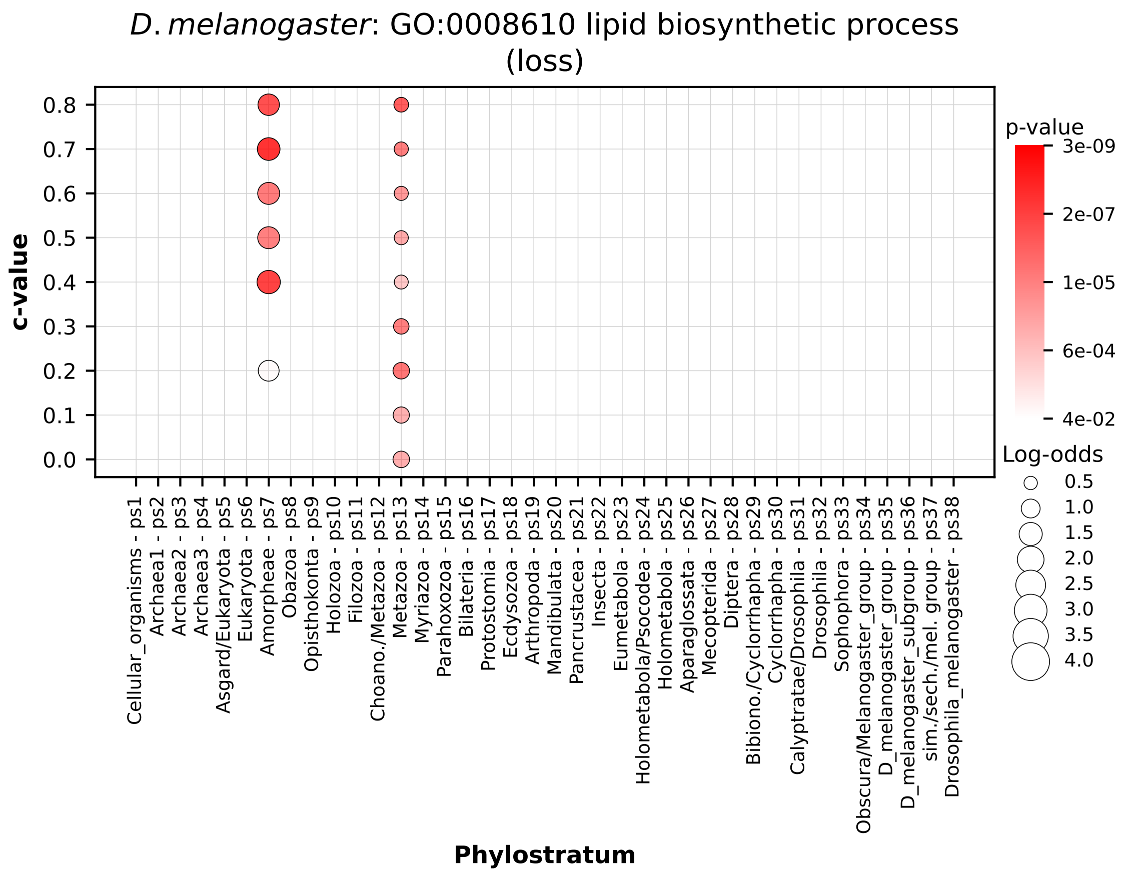

D. melanogaster: GO:0008610 lipid biosynthetic process (loss)
c-value
0.8
0.7
0.6
0.5
0.4
0.3
0.2
0.1
0.0
p-value
3e-09
2e-07
1e-05
6e-04
4e-02
Log-odds
0.5
1.0
1.5
2.0
2.5
3.0
3.5
4.0
Cellular_organisms - ps1
Archaea1 - ps2
Archaea2 - ps3
Archaea3 - ps4
Asgard/Eukaryota - ps5
Eukaryota - ps6
Amorpheae - ps7
Obazoa - ps8
Opisthokonta - ps9
Holozoa - ps10
Filozoa - ps11
Choano./Metazoa - ps12
Metazoa - ps13
Myriazoa - ps14
Parahoxozoa - ps15
Bilateria - ps16
Protostomia - ps17
Ecdysozoa - ps18
Arthropoda - ps19
Mandibulata - ps20
Pancrustacea - ps21
Insecta - ps22
Eumetabola - ps23
Holometabola/Psocodea - ps24
Holometabola - ps25
Aparaglossata - ps26
Mecopterida - ps27
Diptera - ps28
Bibiono./Cyclorrhapha - ps29
Cyclorrhapha - ps30
Calyptratae/Drosophila - ps31
Drosophila - ps32
Sophophora - ps33
Obscura/Melanogaster_group - ps34
D_melanogaster_group - ps35
D_melanogaster_subgroup - ps36
sim./sech./mel. group - ps37
Drosophila_melanogaster - ps38
Phylostratum

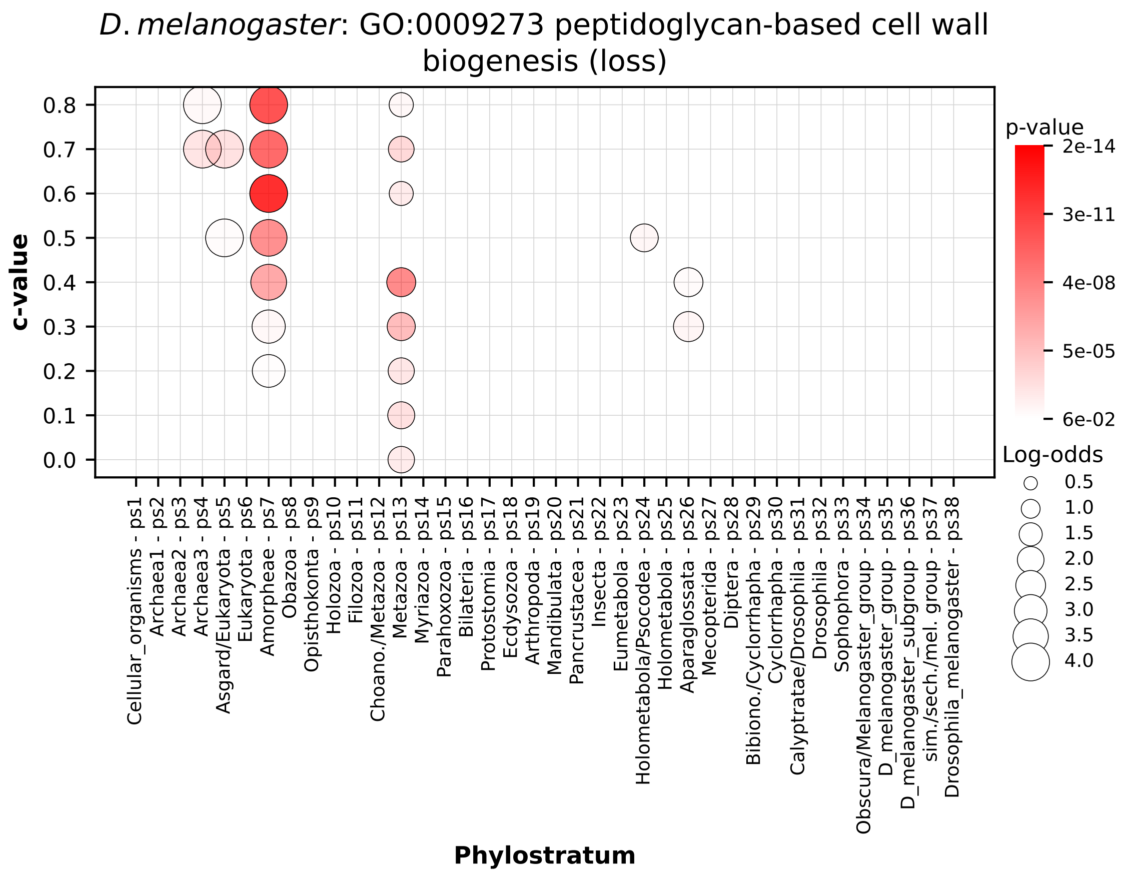
D. melanogaster: GO:0009273 peptidoglycan-based cell wall biogenesis (loss)
c-value
0.8
0.7
0.6
0.5
0.4
0.3
0.2
0.1
0.0
Phylostratum
Cellular_organisms - ps1
Archaea1 - ps2
Archaea2 - ps3
Archaea3 - ps4
Asgard/Eukaryota - ps5
Eukaryota - ps6
Amorpheae - ps7
Obazoa - ps8
Opisthokonta - ps9
Holozoa - ps10
Filozoa - ps11
Choano./Metazoa - ps12
Metazoa - ps13
Myriazoa - ps14
Parahoxozoa - ps15
Bilateria - ps16
Protostomia - ps17
Ecdysozoa - ps18
Arthropoda - ps19
Mandibulata - ps20
Pancrustacea - ps21
Insecta - ps22
Eumetabola - ps23
Holometabola/Psocodea - ps24
Holometabola - ps25
Aparaglossata - ps26
Mecopterida - ps27
Diptera - ps28
Bibiono./Cyclorrhapha - ps29
Cyclorrhapha - ps30
Calyptratae/Drosophila - ps31
Drosophila - ps32
Sophophora - ps33
Obscura/Melanogaster_group - ps34
D_melanogaster_group - ps35
D_melanogaster_subgroup - ps36
sim./sech./mel. group - ps37
Drosophila_melanogaster - ps38
p-value
2e-14
3e-11
4e-08
5e-05
6e-02
Log-odds
0.5
1.0
1.5
2.0
2.5
3.0
3.5
4.0

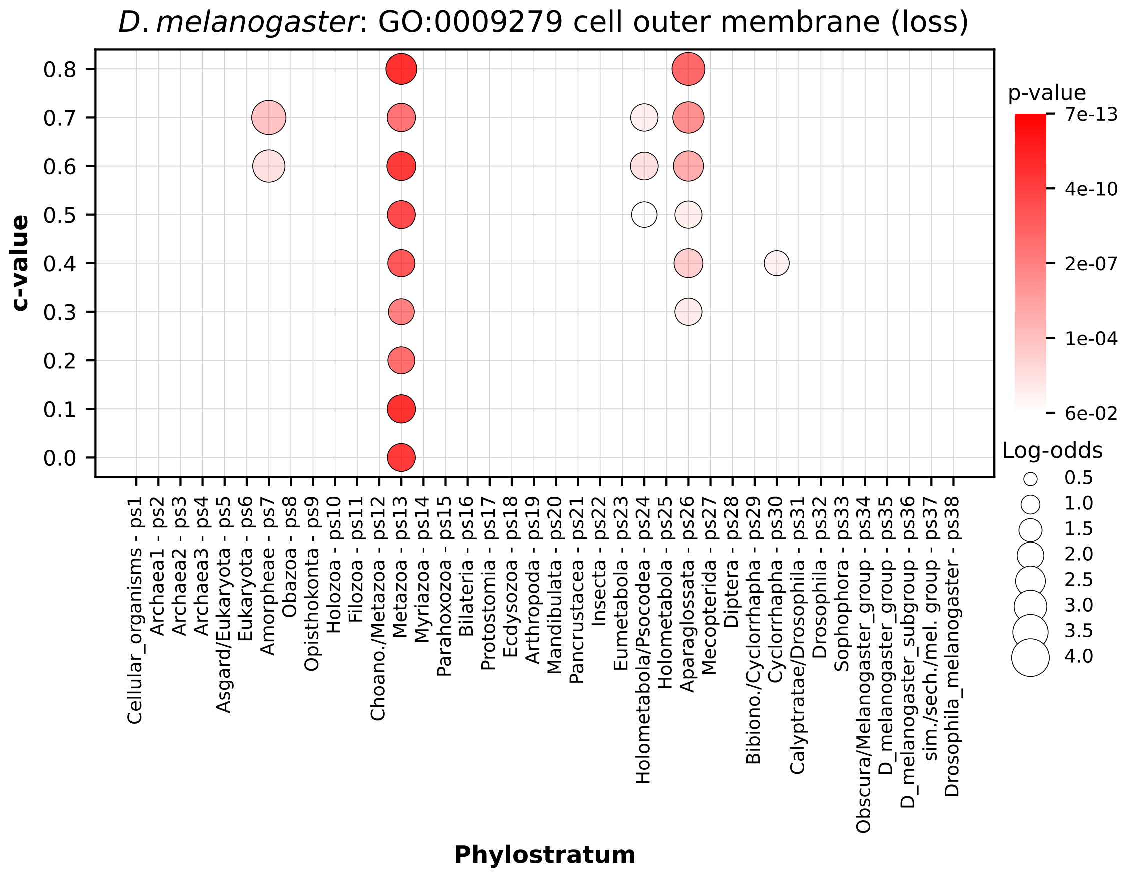
D. melanogaster: GO:0009279 cell outer membrane (loss)
c-value
0.8
0.7
0.6
0.5
0.4
0.3
0.2
0.1
0.0
Phylostratum
Cellular_organisms - ps1
Archaea1 - ps2
Archaea2 - ps3
Archaea3 - ps4
Asgard/Eukaryota - ps5
Eukaryota - ps6
Amorpheae - ps7
Obazoa - ps8
Opisthokonta - ps9
Holozoa - ps10
Filozoa - ps11
Choano./Metazoa - ps12
Metazoa - ps13
Myriazoa - ps14
Parahoxozoa - ps15
Bilateria - ps16
Protostomia - ps17
Ecdysozoa - ps18
Arthropoda - ps19
Mandibulata - ps20
Pancrustacea - ps21
Insecta - ps22
Eumetabola - ps23
Holometabola/Psocodea - ps24
Holometabola - ps25
Aparaglossata - ps26
Mecopterida - ps27
Diptera - ps28
Bibiono./Cyclorrhapha - ps29
Cyclorrhapha - ps30
Calyptratae/Drosophila - ps31
Drosophila - ps32
Sophophora - ps33
Obscura/Melanogaster_group - ps34
D_melanogaster_group - ps35
D_melanogaster_subgroup - ps36
sim./sech./mel. group - ps37
Drosophila_melanogaster - ps38
p-value
7e-13
4e-10
2e-07
1e-04
6e-02
Log-odds
0.5
1.0
1.5
2.0
2.5
3.0
3.5
4.0

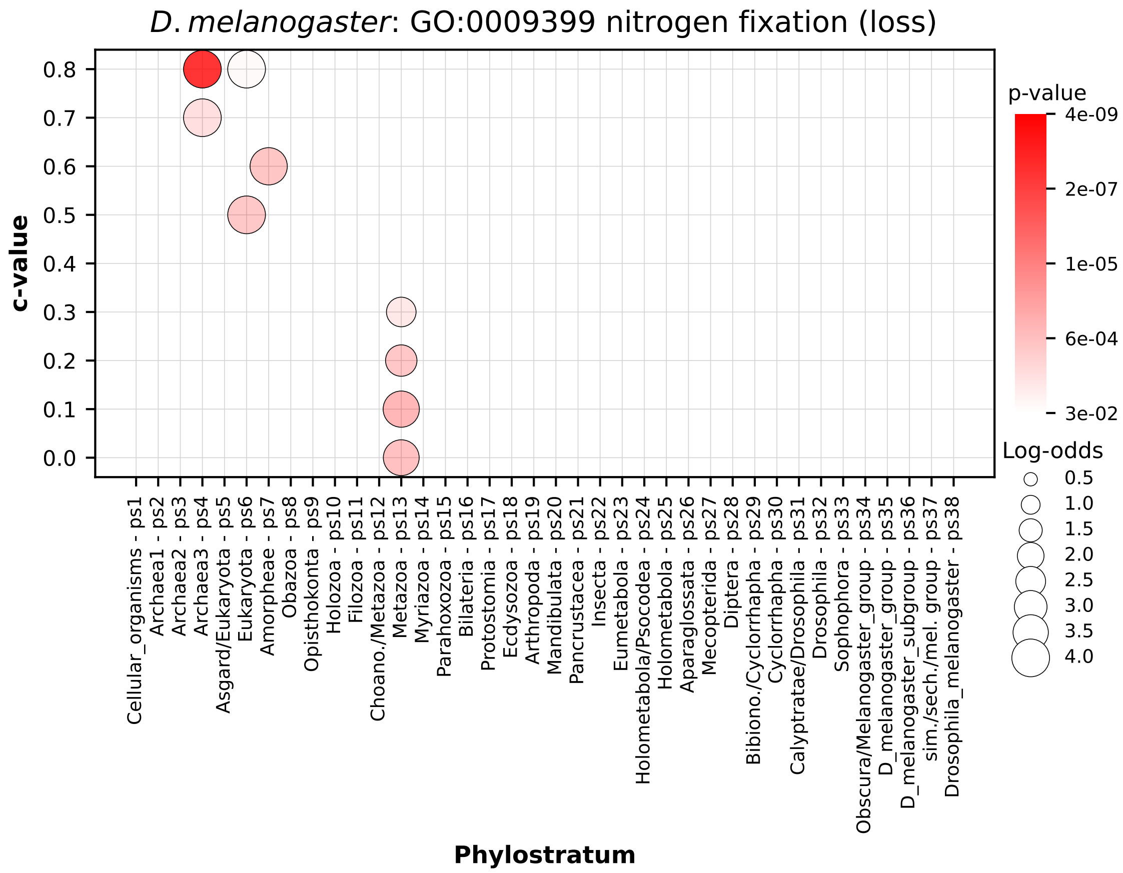
D. melanogaster: GO:0009399 nitrogen fixation (loss)
c-value
0.8
0.7
0.6
0.5
0.4
0.3
0.2
0.1
0.0
p-value
4e-09
2e-07
1e-05
6e-04
3e-02
Log-odds
0.5
1.0
1.5
2.0
2.5
3.0
3.5
4.0
Cellular_organisms - ps1
Archaea1 - ps2
Archaea2 - ps3
Archaea3 - ps4
Asgard/Eukaryota - ps5
Eukaryota - ps6
Amorpheae - ps7
Obazoa - ps8
Opisthokonta - ps9
Holozoa - ps10
Filozoa - ps11
Choano./Metazoa - ps12
Metazoa - ps13
Myriazoa - ps14
Parahoxozoa - ps15
Bilateria - ps16
Protostomia - ps17
Ecdysozoa - ps18
Arthropoda - ps19
Mandibulata - ps20
Pancrustacea - ps21
Insecta - ps22
Eumetabola - ps23
Holometabola/Psocodea - ps24
Holometabola - ps25
Aparaglossata - ps26
Mecopterida - ps27
Diptera - ps28
Bibiono./Cyclorrhapha - ps29
Cyclorrhapha - ps30
Calyptratae/Drosophila - ps31
Drosophila - ps32
Sophophora - ps33
Obscura/Melanogaster_group - ps34
D_melanogaster_group - ps35
D_melanogaster_subgroup - ps36
sim./sech./mel. group - ps37
Drosophila_melanogaster - ps38
Phylostratum

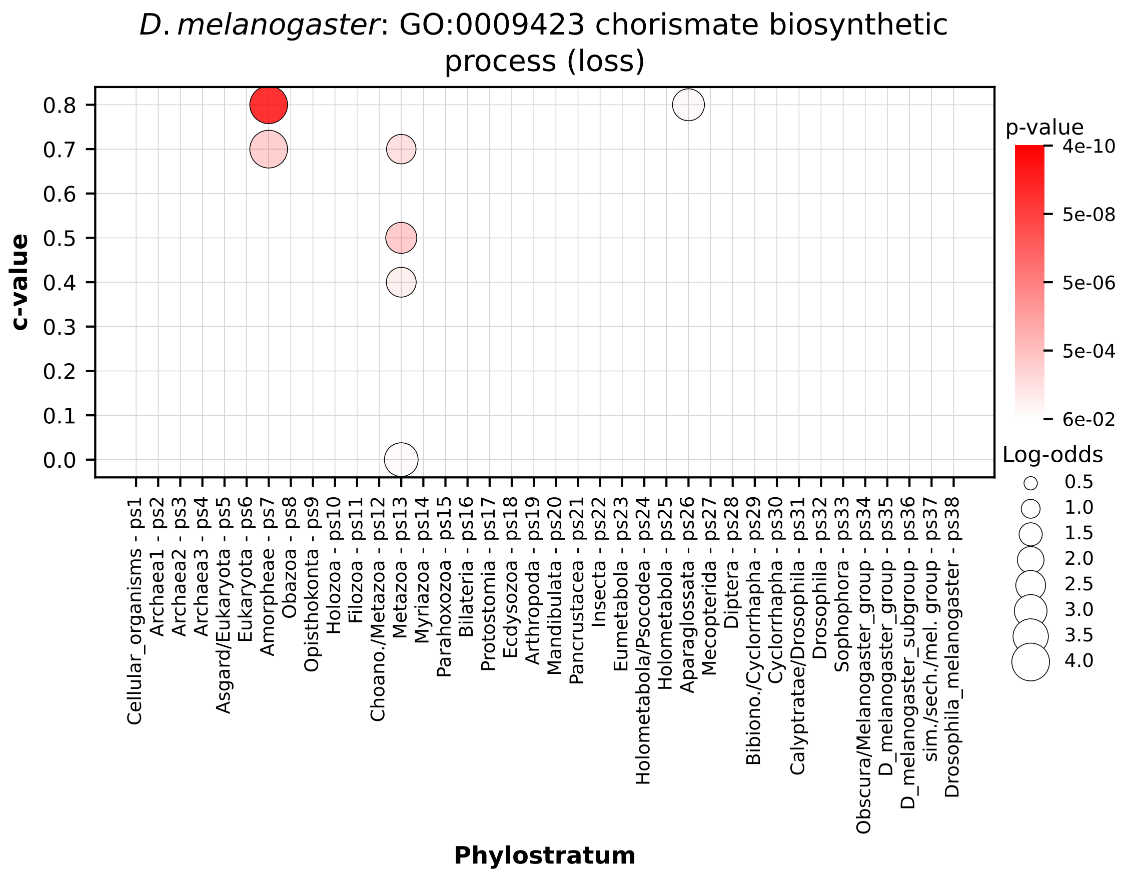

D. melanogaster: GO:0009423 chorismate biosynthetic process (loss)
c-value
0.0
0.1
0.2
0.3
0.4
0.5
0.6
0.7
0.8
p-value
4e-10
5e-08
5e-06
5e-04
6e-02
Log-odds
0.5
1.0
1.5
2.0
2.5
3.0
3.5
4.0
Cellular_organisms - ps1
Archaea1 - ps2
Archaea2 - ps3
Archaea3 - ps4
Asgard/Eukaryota - ps5
Eukaryota - ps6
Amorpheae - ps7
Obazoa - ps8
Opisthokonta - ps9
Holozoa - ps10
Filozoa - ps11
Choano./Metazoa - ps12
Metazoa - ps13
Myriazoa - ps14
Parahoxozoa - ps15
Bilateria - ps16
Protostomia - ps17
Ecdysozoa - ps18
Arthropoda - ps19
Mandibulata - ps20
Pancrustacea - ps21
Insecta - ps22
Eumetabola - ps23
Holometabola/Psocodea - ps24
Holometabola - ps25
Aparaglossata - ps26
Mecopterida - ps27
Diptera - ps28
Bibiono./Cyclorrhapha - ps29
Cyclorrhapha - ps30
Calyptratae/Drosophila - ps31
Drosophila - ps32
Sophophora - ps33
Obscura/Melanogaster_group - ps34
D_melanogaster_group - ps35
D_melanogaster_subgroup - ps36
sim./sech./mel. group - ps37
Drosophila_melanogaster - ps38
Phylostratum

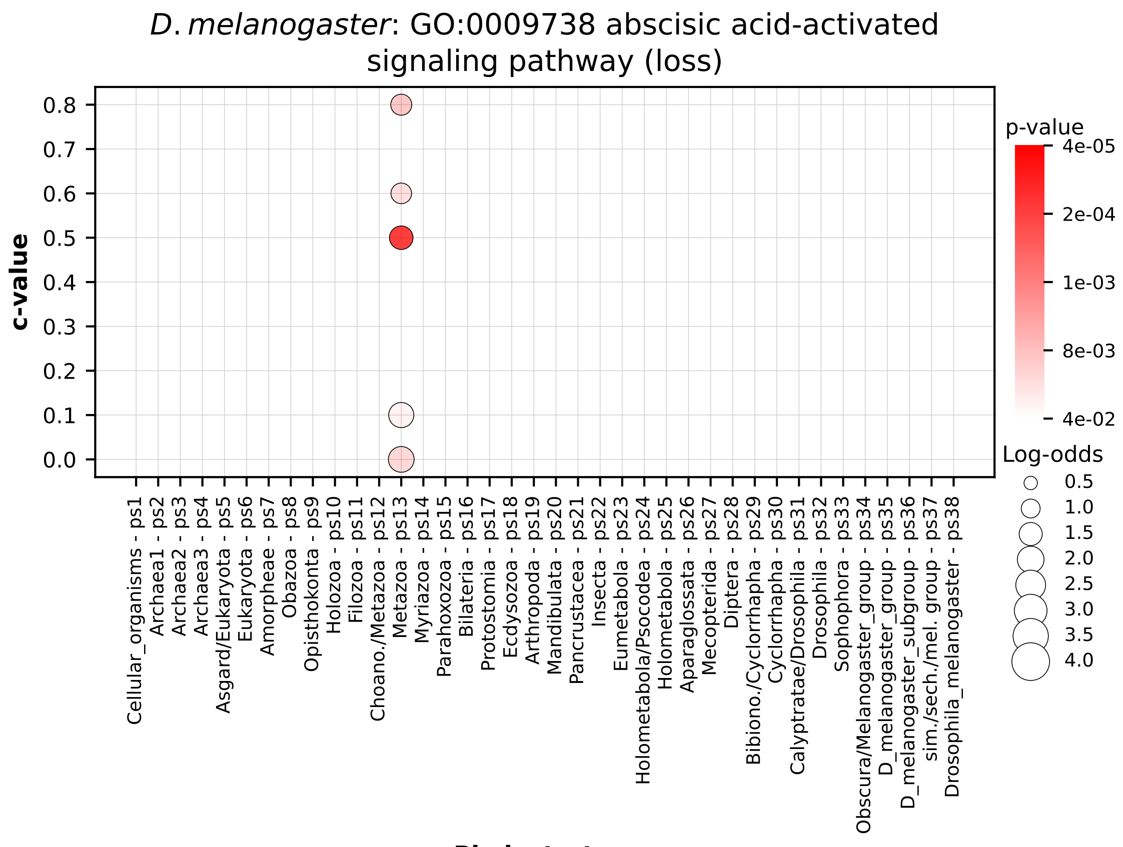

D. melanogaster: GO:0009738 abscisic acid-activated signaling pathway (loss)
c-value
0.0
0.1
0.2
0.3
0.4
0.5
0.6
0.7
0.8
Phylostratum
Cellular_organisms - ps1
Archaea1 - ps2
Archaea2 - ps3
Archaea3 - ps4
Asgard/Eukaryota - ps5
Eukaryota - ps6
Amorpheae - ps7
Obazoa - ps8
Opisthokonta - ps9
Holozoa - ps10
Filozoa - ps11
Choano./Metazoa - ps12
Metazoa - ps13
Myriazoa - ps14
Parahoxozoa - ps15
Bilateria - ps16
Protostomia - ps17
Ecdysozoa - ps18
Arthropoda - ps19
Mandibulata - ps20
Pancrustacea - ps21
Insecta - ps22
Eumetabola - ps23
Holometabola/Psocodea - ps24
Holometabola - ps25
Aparaglossata - ps26
Mecopterida - ps27
Diptera - ps28
Bibiono./Cyclorrhapha - ps29
Cyclorrhapha - ps30
Calyptratae/Drosophila - ps31
Drosophila - ps32
Sophophora - ps33
Obscura/Melanogaster_group - ps34
D_melanogaster_group - ps35
D_melanogaster_subgroup - ps36
sim./sech./mel. group - ps37
Drosophila_melanogaster - ps38
p-value
4e-05
2e-04
1e-03
8e-03
4e-02
Log-odds
0.5
1.0
1.5
2.0
2.5
3.0
3.5
4.0

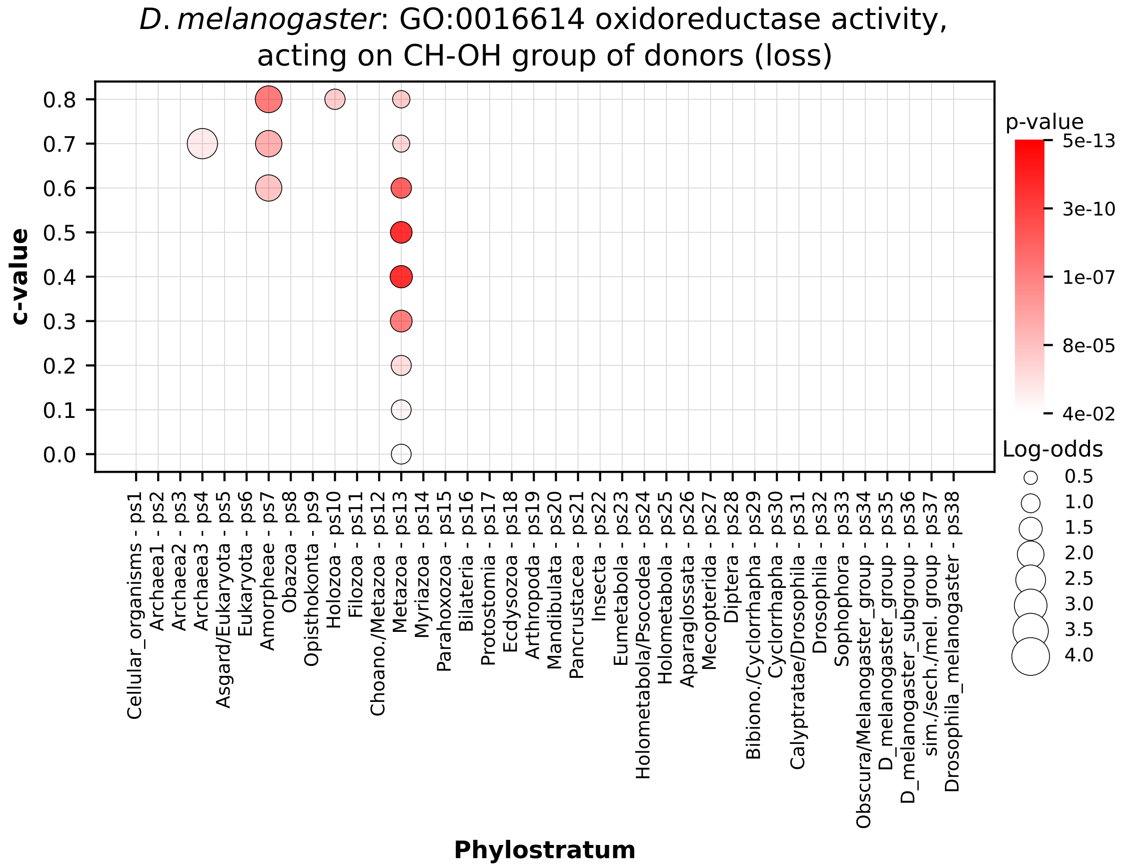

D. melanogaster: GO:0016614 oxidoreductase activity, acting on CH-OH group of donors (loss)
c-value
0.8
0.7
0.6
0.5
0.4
0.3
0.2
0.1
0.0
p-value
5e-13
3e-10
1e-07
8e-05
4e-02
Log-odds
0.5
1.0
1.5
2.0
2.5
3.0
3.5
4.0
Cellular_organisms - ps1
Archaea1 - ps2
Archaea2 - ps3
Archaea3 - ps4
Asgard/Eukaryota - ps5
Eukaryota - ps6
Amorpheae - ps7
Obazoa - ps8
Opisthokonta - ps9
Holozoa - ps10
Filozoa - ps11
Choano./Metazoa - ps12
Metazoa - ps13
Myriazoa - ps14
Parahoxozoa - ps15
Bilateria - ps16
Protostomia - ps17
Ecdysozoa - ps18
Arthropoda - ps19
Mandibulata - ps20
Pancrustacea - ps21
Insecta - ps22
Eumetabola - ps23
Holometabola/Psocodea - ps24
Holometabola - ps25
Aparaglossata - ps26
Mecopterida - ps27
Diptera - ps28
Bibiono./Cyclorrhapha - ps29
Cyclorrhapha - ps30
Calyptratae/Drosophila - ps31
Drosophila - ps32
Sophophora - ps33
Obscura/Melanogaster_group - ps34
D_melanogaster_group - ps35
D_melanogaster_subgroup - ps36
sim./sech./mel. group - ps37
Drosophila_melanogaster - ps38
Phylostratum

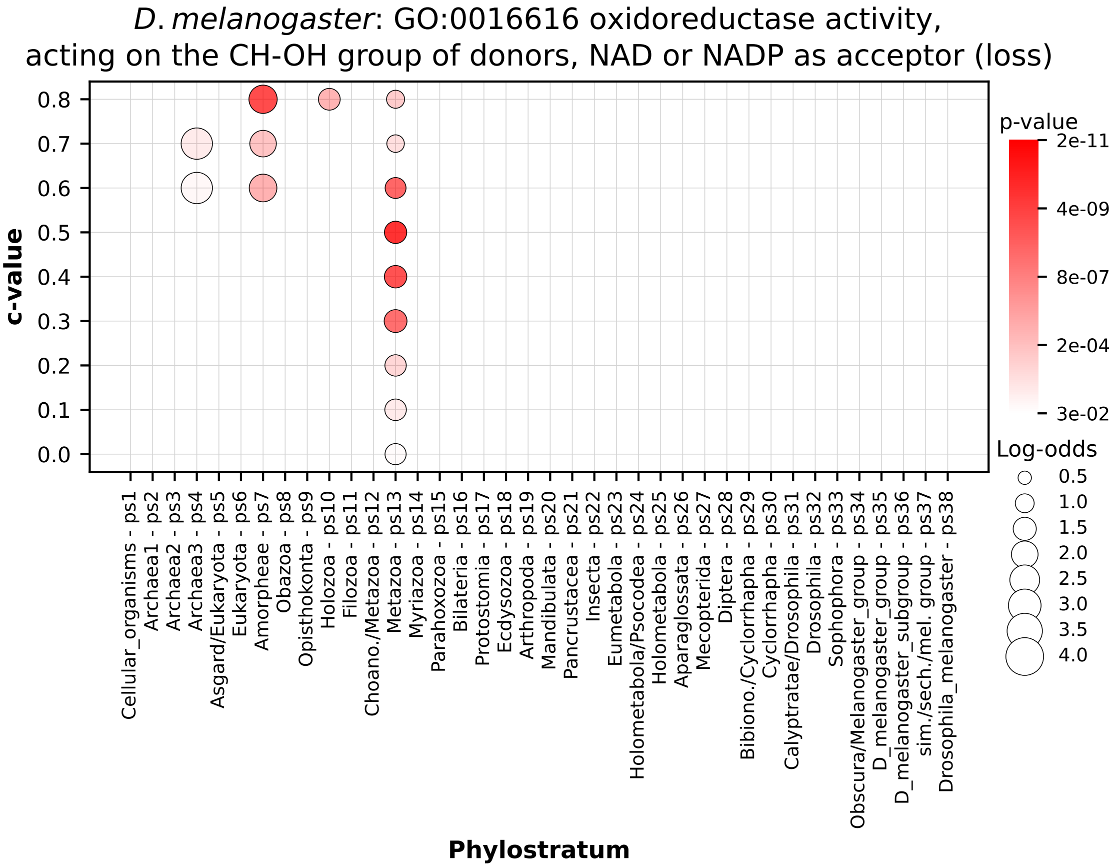

D. melanogaster: GO:0016616 oxidoreductase activity, acting on the CH-OH group of donors, NAD or NADP as acceptor (loss)
c-value
0.8
0.7
0.6
0.5
0.4
0.3
0.2
0.1
0.0
Phylostratum
Cellular_organisms - ps1
Archaea1 - ps2
Archaea2 - ps3
Archaea3 - ps4
Asgard/Eukaryota - ps5
Eukaryota - ps6
Amorpheae - ps7
Obazoa - ps8
Opisthokonta - ps9
Holozoa - ps10
Filozoa - ps11
Choano./Metazoa - ps12
Metazoa - ps13
Myriazoa - ps14
Parahoxozoa - ps15
Bilateria - ps16
Protostomia - ps17
Ecdysozoa - ps18
Arthropoda - ps19
Mandibulata - ps20
Pancrustacea - ps21
Insecta - ps22
Eumetabola - ps23
Holometabola/Psocodea - ps24
Holometabola - ps25
Aparaglossata - ps26
Mecopterida - ps27
Diptera - ps28
Bibiono./Cyclorrhapha - ps29
Cyclorrhapha - ps30
Calyptratae/Drosophila - ps31
Drosophila - ps32
Sophophora - ps33
Obscura/Melanogaster_group - ps34
D_melanogaster_group - ps35
D_melanogaster_subgroup - ps36
sim./sech./mel. group - ps37
Drosophila_melanogaster - ps38
p-value
2e-11
4e-09
8e-07
2e-04
3e-02
Log-odds
0.5
1.0
1.5
2.0
2.5
3.0
3.5
4.0

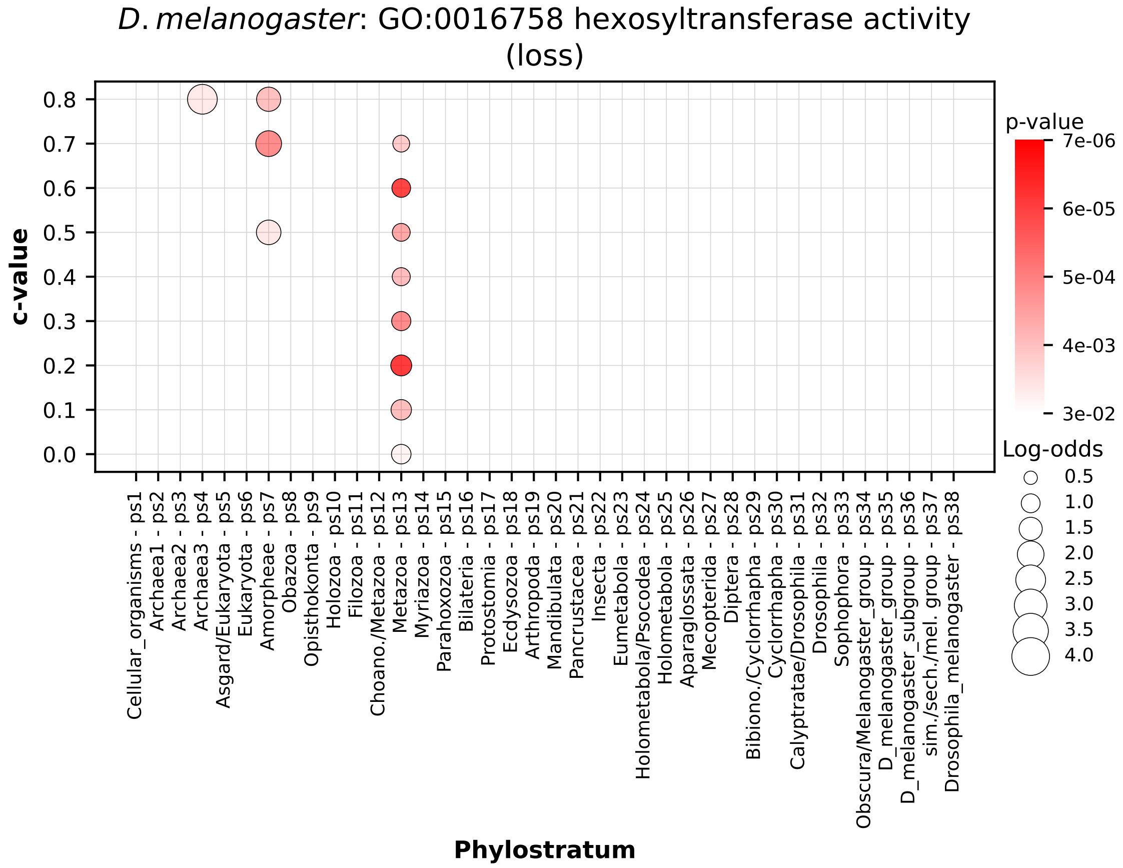

D. melanogaster: GO:0016758 hexosyltransferase activity
(loss)
c-value
0.8
0.7
0.6
0.5
0.4
0.3
0.2
0.1
0.0
p-value
7e-06
6e-05
5e-04
4e-03
3e-02
Log-odds
0.5
1.0
1.5
2.0
2.5
3.0
3.5
4.0
Cellular_organisms - ps1
Archaea1 - ps2
Archaea2 - ps3
Archaea3 - ps4
Asgard/Eukaryota - ps5
Eukaryota - ps6
Amorpheae - ps7
Obazoa - ps8
Opisthokonta - ps9
Holozoa - ps10
Filozoa - ps11
Choano./Metazoa - ps12
Metazoa - ps13
Myriazoa - ps14
Parahoxozoa - ps15
Bilateria - ps16
Protostomia - ps17
Ecdysozoa - ps18
Arthropoda - ps19
Mandibulata - ps20
Pancrustacea - ps21
Insecta - ps22
Eumetabola - ps23
Holometabola/Psocodea - ps24
Holometabola - ps25
Aparaglossata - ps26
Mecopterida - ps27
Diptera - ps28
Bibiono./Cyclorrhapha - ps29
Cyclorrhapha - ps30
Calyptratae/Drosophila - ps31
Drosophila - ps32
Sophophora - ps33
Obscura/Melanogaster_group - ps34
D_melanogaster_group - ps35
D_melanogaster_subgroup - ps36
sim./sech./mel. group - ps37
Drosophila_melanogaster - ps38
Phylostratum

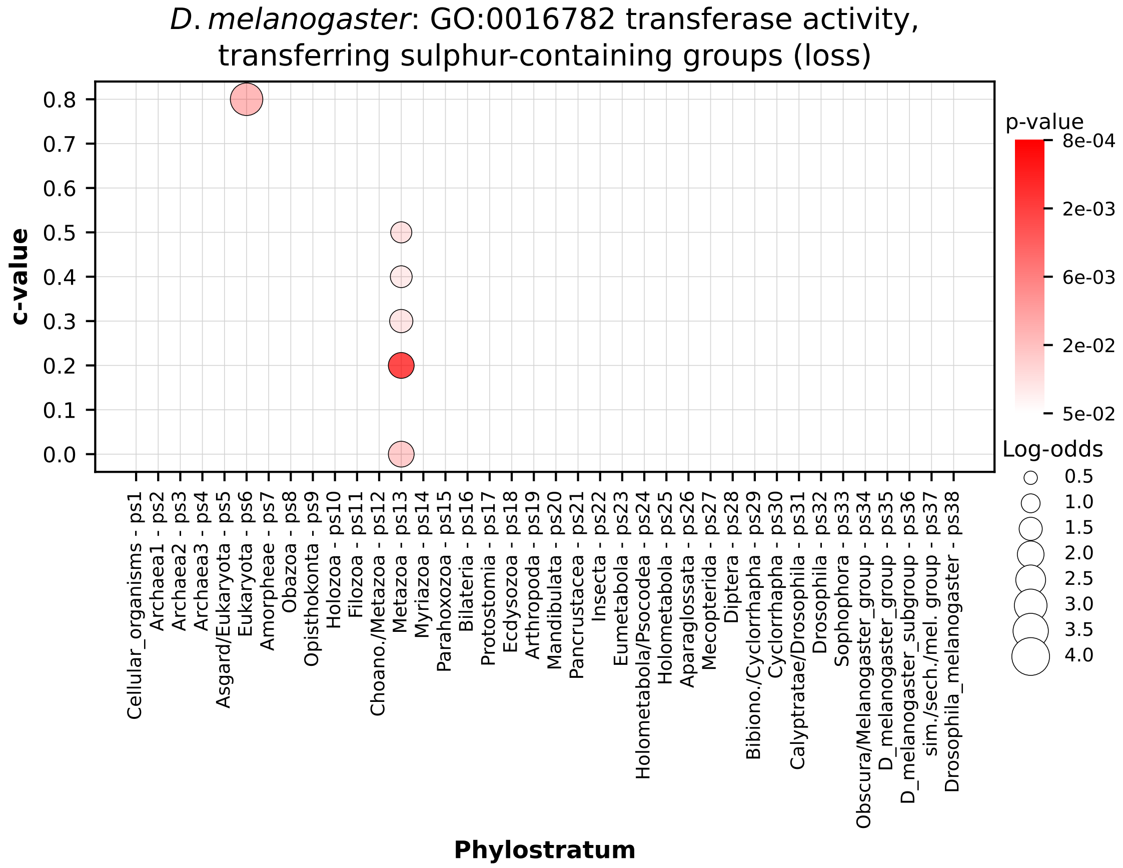

D. melanogaster: GO:0016782 transferase activity, transferring sulphur-containing groups (loss)
c-value
0.8
0.7
0.6
0.5
0.4
0.3
0.2
0.1
0.0
p-value
8e-04
2e-03
6e-03
2e-02
5e-02
Log-odds
0.5
1.0
1.5
2.0
2.5
3.0
3.5
4.0
Cellular_organisms - ps1
Archaea1 - ps2
Archaea2 - ps3
Archaea3 - ps4
Asgard/Eukaryota - ps5
Eukaryota - ps6
Amorpheae - ps7
Obazoa - ps8
Opisthokonta - ps9
Holozoa - ps10
Filozoa - ps11
Choano./Metazoa - ps12
Metazoa - ps13
Myriazoa - ps14
Parahoxozoa - ps15
Bilateria - ps16
Protostomia - ps17
Ecdysozoa - ps18
Arthropoda - ps19
Mandibulata - ps20
Pancrustacea - ps21
Insecta - ps22
Eumetabola - ps23
Holometabola/Psocodea - ps24
Holometabola - ps25
Aparaglossata - ps26
Mecopterida - ps27
Diptera - ps28
Bibiono./Cyclorrhapha - ps29
Cyclorrhapha - ps30
Calyptratae/Drosophila - ps31
Drosophila - ps32
Sophophora - ps33
Obscura/Melanogaster_group - ps34
D_melanogaster_group - ps35
D_melanogaster_subgroup - ps36
sim./sech./mel. group - ps37
Drosophila_melanogaster - ps38
Phylostratum

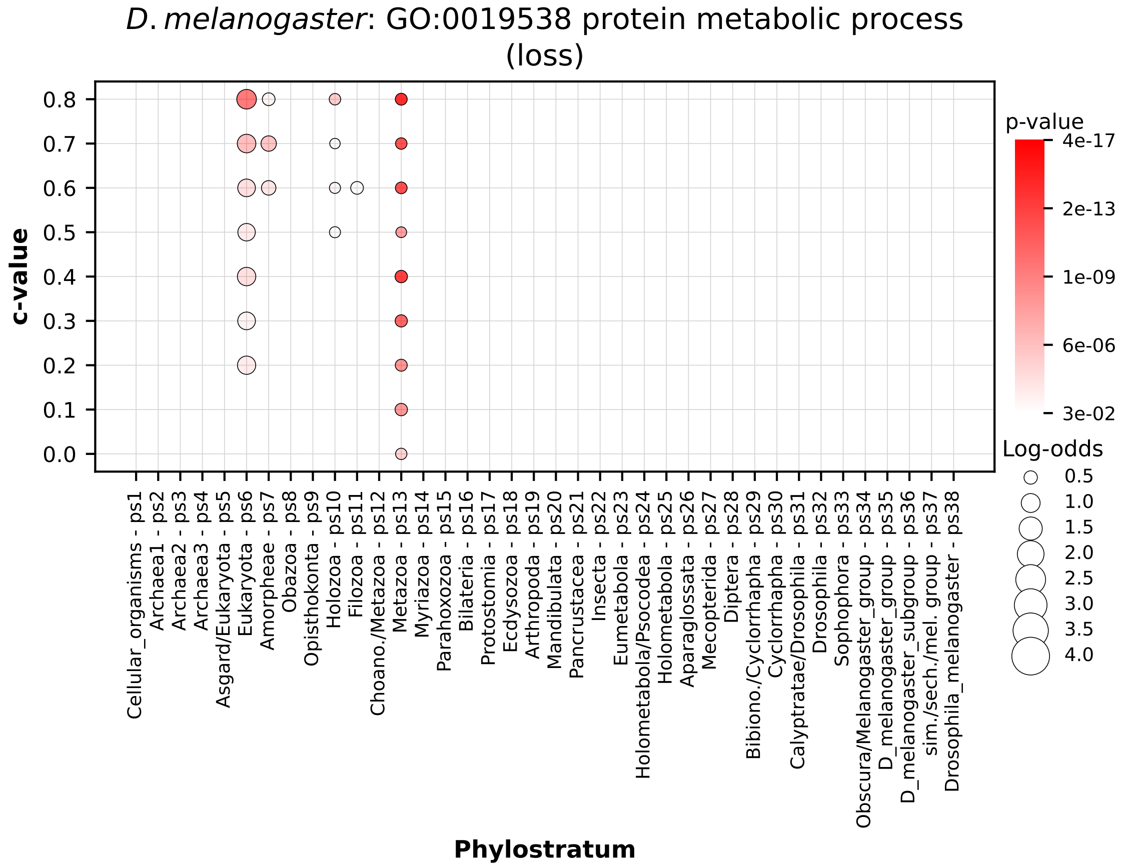

D. melanogaster: GO:0019538 protein metabolic process
(loss)
c-value
0.8
0.7
0.6
0.5
0.4
0.3
0.2
0.1
0.0
Phylostratum
Cellular_organisms - ps1
Archaea1 - ps2
Archaea2 - ps3
Archaea3 - ps4
Asgard/Eukaryota - ps5
Eukaryota - ps6
Amorpheae - ps7
Obazoa - ps8
Opisthokonta - ps9
Holozoa - ps10
Filozoa - ps11
Choano./Metazoa - ps12
Metazoa - ps13
Myriazoa - ps14
Parahoxozoa - ps15
Bilateria - ps16
Protostomia - ps17
Ecdysozoa - ps18
Arthropoda - ps19
Mandibulata - ps20
Pancrustacea - ps21
Insecta - ps22
Eumetabola - ps23
Holometabola/Psocodea - ps24
Holometabola - ps25
Aparaglossata - ps26
Mecopterida - ps27
Diptera - ps28
Bibiono./Cyclorrhapha - ps29
Cyclorrhapha - ps30
Calyptratae/Drosophila - ps31
Drosophila - ps32
Sophophora - ps33
Obscura/Melanogaster_group - ps34
D_melanogaster_group - ps35
D_melanogaster_subgroup - ps36
sim./sech./mel. group - ps37
Drosophila_melanogaster - ps38
p-value
4e-17
2e-13
1e-09
6e-06
3e-02
Log-odds
0.5
1.0
1.5
2.0
2.5
3.0
3.5
4.0

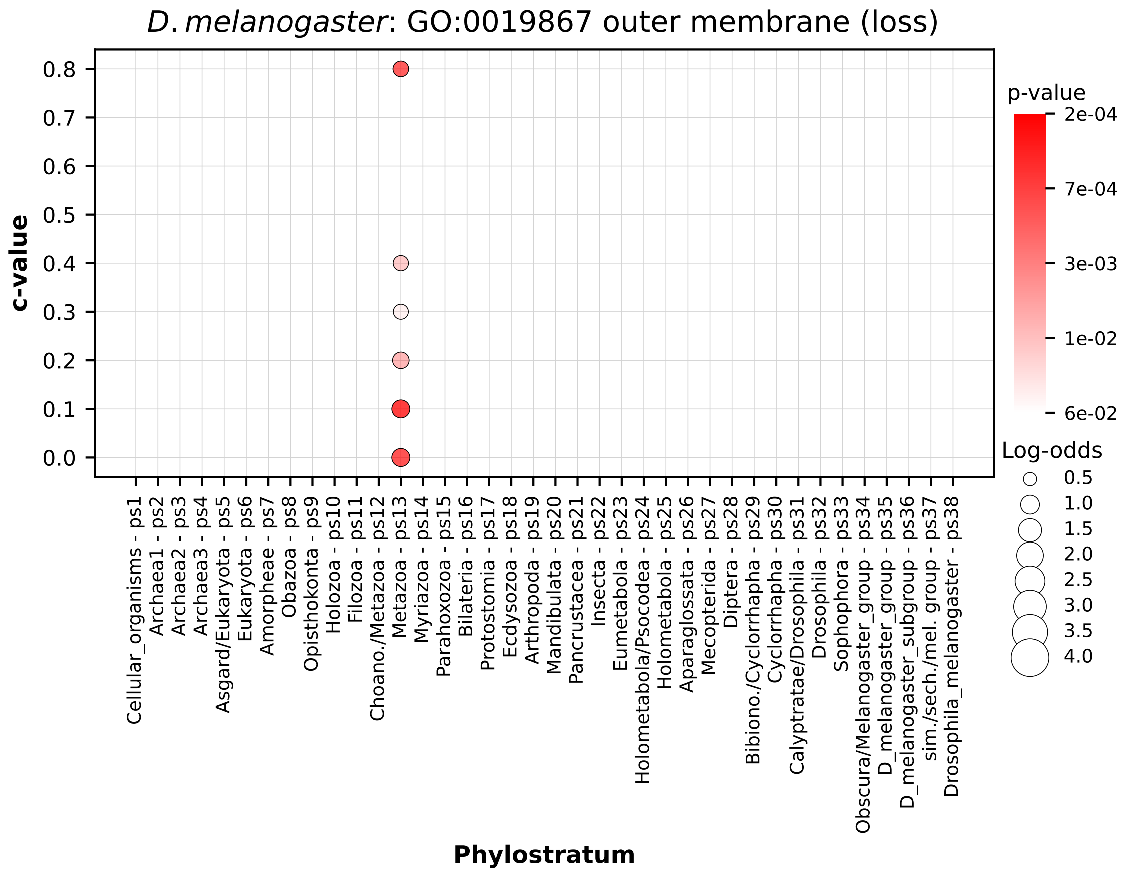

D. melanogaster: GO:0019867 outer membrane (loss)
c-value
0.8
0.7
0.6
0.5
0.4
0.3
0.2
0.1
0.0
Phylostratum
Cellular_organisms - ps1
Archaea1 - ps2
Archaea2 - ps3
Archaea3 - ps4
Asgard/Eukaryota - ps5
Eukaryota - ps6
Amorpheae - ps7
Obazoa - ps8
Opisthokonta - ps9
Holozoa - ps10
Filozoa - ps11
Choano./Metazoa - ps12
Metazoa - ps13
Myriazoa - ps14
Parahoxozoa - ps15
Bilateria - ps16
Protostomia - ps17
Ecdysozoa - ps18
Arthropoda - ps19
Mandibulata - ps20
Pancrustacea - ps21
Insecta - ps22
Eumetabola - ps23
Holometabola/Psocodea - ps24
Holometabola - ps25
Aparaglossata - ps26
Mecopterida - ps27
Diptera - ps28
Bibiono./Cyclorrhapha - ps29
Cyclorrhapha - ps30
Calyptratae/Drosophila - ps31
Drosophila - ps32
Sophophora - ps33
Obscura/Melanogaster_group - ps34
D_melanogaster_group - ps35
D_melanogaster_subgroup - ps36
sim./sech./mel. group - ps37
Drosophila_melanogaster - ps38
p-value
2e-04
7e-04
3e-03
1e-02
6e-02
Log-odds
0.5
1.0
1.5
2.0
2.5
3.0
3.5
4.0

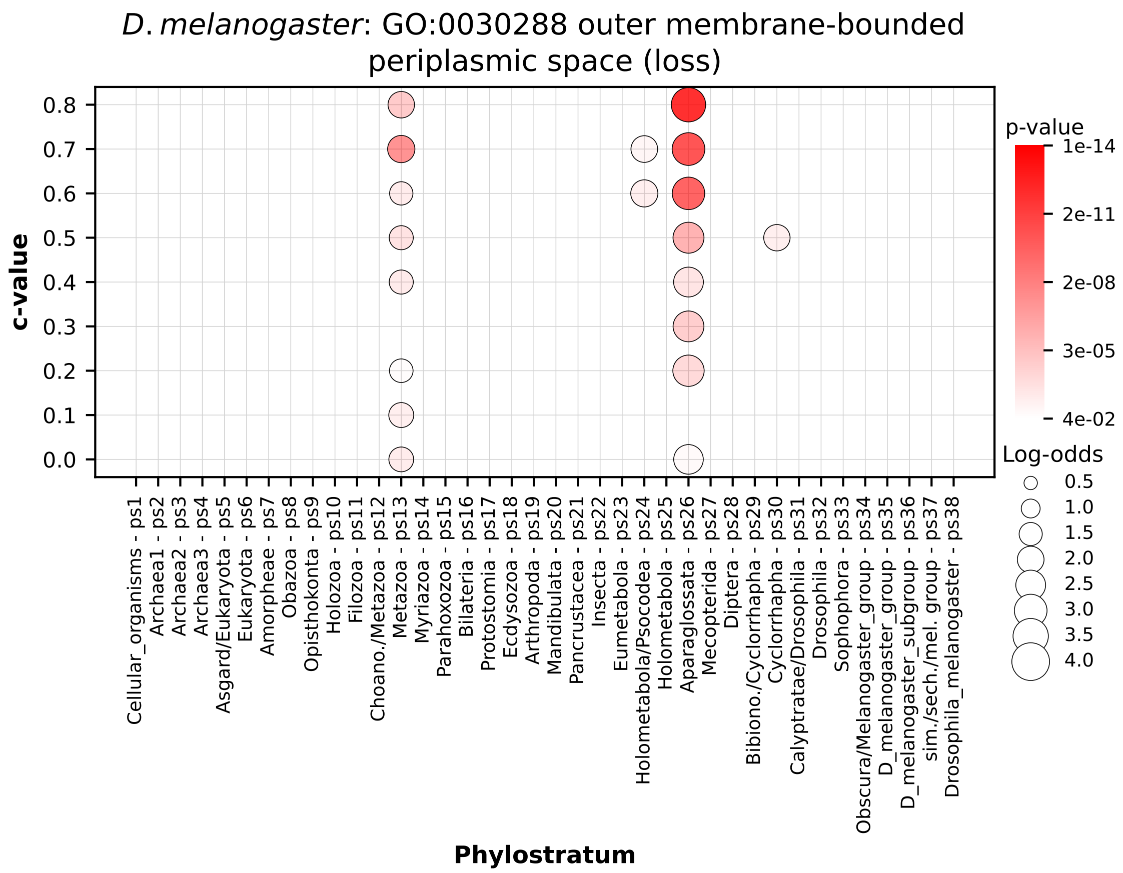

D. melanogaster: GO:0030288 outer membrane-bounded periplasmic space (loss)
c-value
0.8
0.7
0.6
0.5
0.4
0.3
0.2
0.1
0.0
p-value
1e-14
2e-11
2e-08
3e-05
4e-02
Log-odds
0.5
1.0
1.5
2.0
2.5
3.0
3.5
4.0
Cellular_organisms - ps1
Archaea1 - ps2
Archaea2 - ps3
Archaea3 - ps4
Asgard/Eukaryota - ps5
Eukaryota - ps6
Amorpheae - ps7
Obazoa - ps8
Opisthokonta - ps9
Holozoa - ps10
Filozoa - ps11
Choano./Metazoa - ps12
Metazoa - ps13
Myriazoa - ps14
Parahoxozoa - ps15
Bilateria - ps16
Protostomia - ps17
Ecdysozoa - ps18
Arthropoda - ps19
Mandibulata - ps20
Pancrustacea - ps21
Insecta - ps22
Eumetabola - ps23
Holometabola/Psocodea - ps24
Holometabola - ps25
Aparaglossata - ps26
Mecopterida - ps27
Diptera - ps28
Bibiono./Cyclorrhapha - ps29
Cyclorrhapha - ps30
Calyptratae/Drosophila - ps31
Drosophila - ps32
Sophophora - ps33
Obscura/Melanogaster_group - ps34
D_melanogaster_group - ps35
D_melanogaster_subgroup - ps36
sim./sech./mel. group - ps37
Drosophila_melanogaster - ps38
Phylostratum

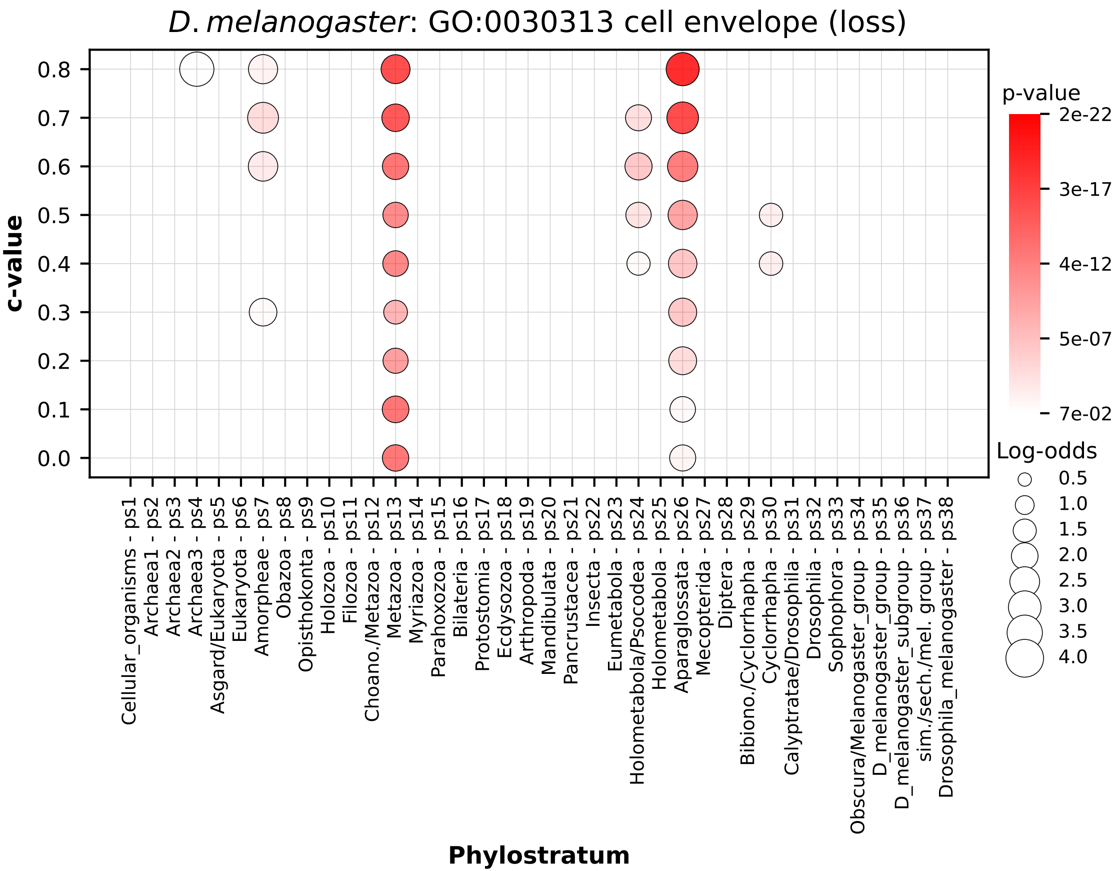

D. melanogaster: GO:0030313 cell envelope (loss)
c-value
0.8
0.7
0.6
0.5
0.4
0.3
0.2
0.1
0.0
Phylostratum
Cellular_organisms - ps1
Archaea1 - ps2
Archaea2 - ps3
Archaea3 - ps4
Asgard/Eukaryota - ps5
Eukaryota - ps6
Amorpheae - ps7
Obazoa - ps8
Opisthokonta - ps9
Holozoa - ps10
Filozoa - ps11
Choano./Metazoa - ps12
Metazoa - ps13
Myriazoa - ps14
Parahoxozoa - ps15
Bilateria - ps16
Protostomia - ps17
Ecdysozoa - ps18
Arthropoda - ps19
Mandibulata - ps20
Pancrustacea - ps21
Insecta - ps22
Eumetabola - ps23
Holometabola/Psocodea - ps24
Holometabola - ps25
Aparaglossata - ps26
Mecopterida - ps27
Diptera - ps28
Bibiono./Cyclorrhapha - ps29
Cyclorrhapha - ps30
Calyptratae/Drosophila - ps31
Drosophila - ps32
Sophophora - ps33
Obscura/Melanogaster_group - ps34
D_melanogaster_group - ps35
D_melanogaster_subgroup - ps36
sim./sech./mel. group - ps37
Drosophila_melanogaster - ps38
p-value
2e-22
3e-17
4e-12
5e-07
7e-02
Log-odds
0.5
1.0
1.5
2.0
2.5
3.0
3.5
4.0

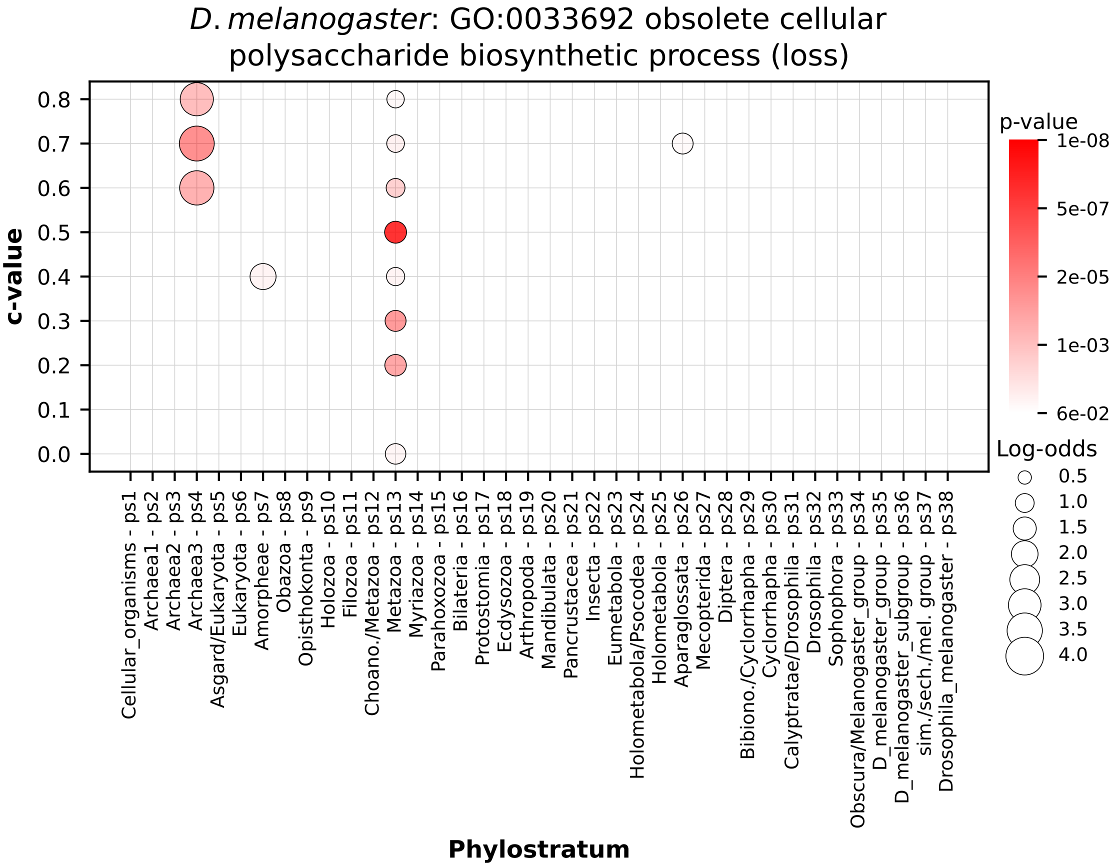

D. melanogaster: GO:0033692 obsolete cellular polysaccharide biosynthetic process (loss)
c-value
0.8
0.7
0.6
0.5
0.4
0.3
0.2
0.1
0.0
p-value
1e-08
5e-07
2e-05
1e-03
6e-02
Log-odds
0.5
1.0
1.5
2.0
2.5
3.0
3.5
4.0
Cellular_organisms - ps1
Archaea1 - ps2
Archaea2 - ps3
Archaea3 - ps4
Asgard/Eukaryota - ps5
Eukaryota - ps6
Amorpheae - ps7
Obazoa - ps8
Opisthokonta - ps9
Holozoa - ps10
Filozoa - ps11
Choano./Metazoa - ps12
Metazoa - ps13
Myriazoa - ps14
Parahoxozoa - ps15
Bilateria - ps16
Protostomia - ps17
Ecdysozoa - ps18
Arthropoda - ps19
Mandibulata - ps20
Pancrustacea - ps21
Insecta - ps22
Eumetabola - ps23
Holometabola/Psocodea - ps24
Holometabola - ps25
Aparaglossata - ps26
Mecopterida - ps27
Diptera - ps28
Bibiono./Cyclorrhapha - ps29
Cyclorrhapha - ps30
Calyptratae/Drosophila - ps31
Drosophila - ps32
Sophophora - ps33
Obscura/Melanogaster_group - ps34
D_melanogaster_group - ps35
D_melanogaster_subgroup - ps36
sim./sech./mel. group - ps37
Drosophila_melanogaster - ps38
Phylostratum

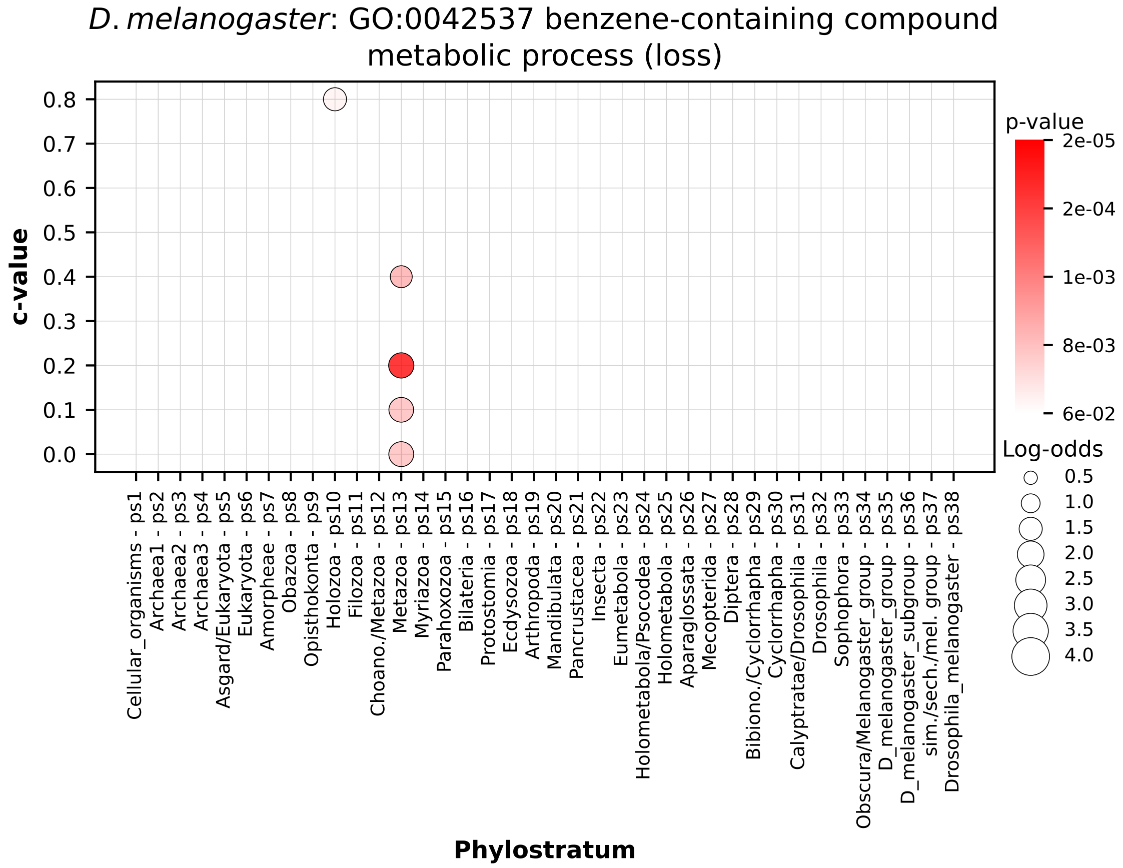

D. melanogaster: GO:0042537 benzene-containing compound metabolic process (loss)
c-value
0.8
0.7
0.6
0.5
0.4
0.3
0.2
0.1
0.0
p-value
2e-05
2e-04
1e-03
8e-03
6e-02
Log-odds
0.5
1.0
1.5
2.0
2.5
3.0
3.5
4.0
Cellular_organisms - ps1
Archaea1 - ps2
Archaea2 - ps3
Archaea3 - ps4
Asgard/Eukaryota - ps5
Eukaryota - ps6
Amorpheae - ps7
Obazoa - ps8
Opisthokonta - ps9
Holozoa - ps10
Filozoa - ps11
Choano./Metazoa - ps12
Metazoa - ps13
Myriazoa - ps14
Parahoxozoa - ps15
Bilateria - ps16
Protostomia - ps17
Ecdysozoa - ps18
Arthropoda - ps19
Mandibulata - ps20
Pancrustacea - ps21
Insecta - ps22
Eumetabola - ps23
Holometabola/Psocodea - ps24
Holometabola - ps25
Aparaglossata - ps26
Mecopterida - ps27
Diptera - ps28
Bibiono./Cyclorrhapha - ps29
Cyclorrhapha - ps30
Calyptratae/Drosophila - ps31
Drosophila - ps32
Sophophora - ps33
Obscura/Melanogaster_group - ps34
D_melanogaster_group - ps35
D_melanogaster_subgroup - ps36
sim./sech./mel. group - ps37
Drosophila_melanogaster - ps38
Phylostratum

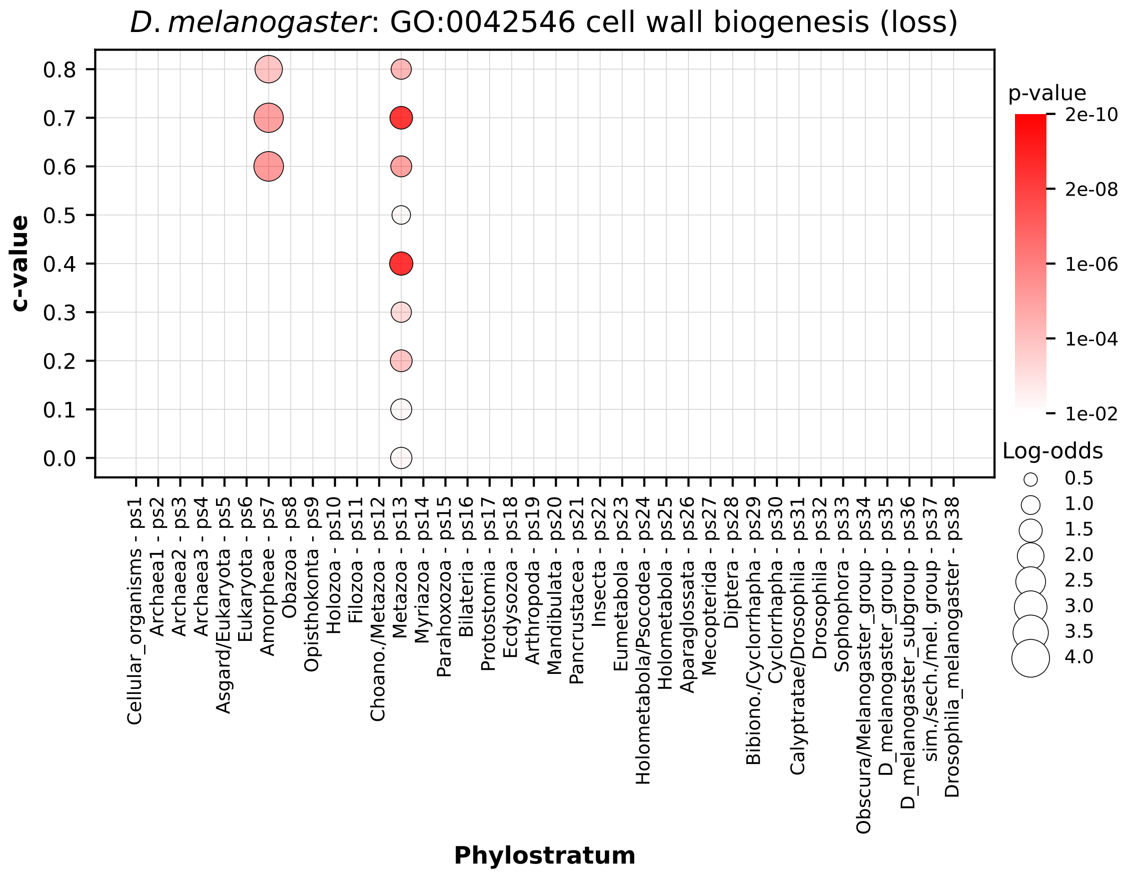

D. melanogaster: GO:0042546 cell wall biogenesis (loss)
c-value
0.8
0.7
0.6
0.5
0.4
0.3
0.2
0.1
0.0
Phylostratum
Cellular_organisms - ps1
Archaea1 - ps2
Archaea2 - ps3
Archaea3 - ps4
Asgard/Eukaryota - ps5
Eukaryota - ps6
Amorpheae - ps7
Obazoa - ps8
Opisthokonta - ps9
Holozoa - ps10
Filozoa - ps11
Choano./Metazoa - ps12
Metazoa - ps13
Myriazoa - ps14
Parahoxozoa - ps15
Bilateria - ps16
Protostomia - ps17
Ecdysozoa - ps18
Arthropoda - ps19
Mandibulata - ps20
Pancrustacea - ps21
Insecta - ps22
Eumetabola - ps23
Holometabola/Psocodea - ps24
Holometabola - ps25
Aparaglossata - ps26
Mecopterida - ps27
Diptera - ps28
Bibiono./Cyclorrhapha - ps29
Cyclorrhapha - ps30
Calyptratae/Drosophila - ps31
Drosophila - ps32
Sophophora - ps33
Obscura/Melanogaster_group - ps34
D_melanogaster_group - ps35
D_melanogaster_subgroup - ps36
sim./sech./mel. group - ps37
Drosophila_melanogaster - ps38
p-value
2e-10
2e-08
1e-06
1e-04
1e-02
Log-odds
0.5
1.0
1.5
2.0
2.5
3.0
3.5
4.0

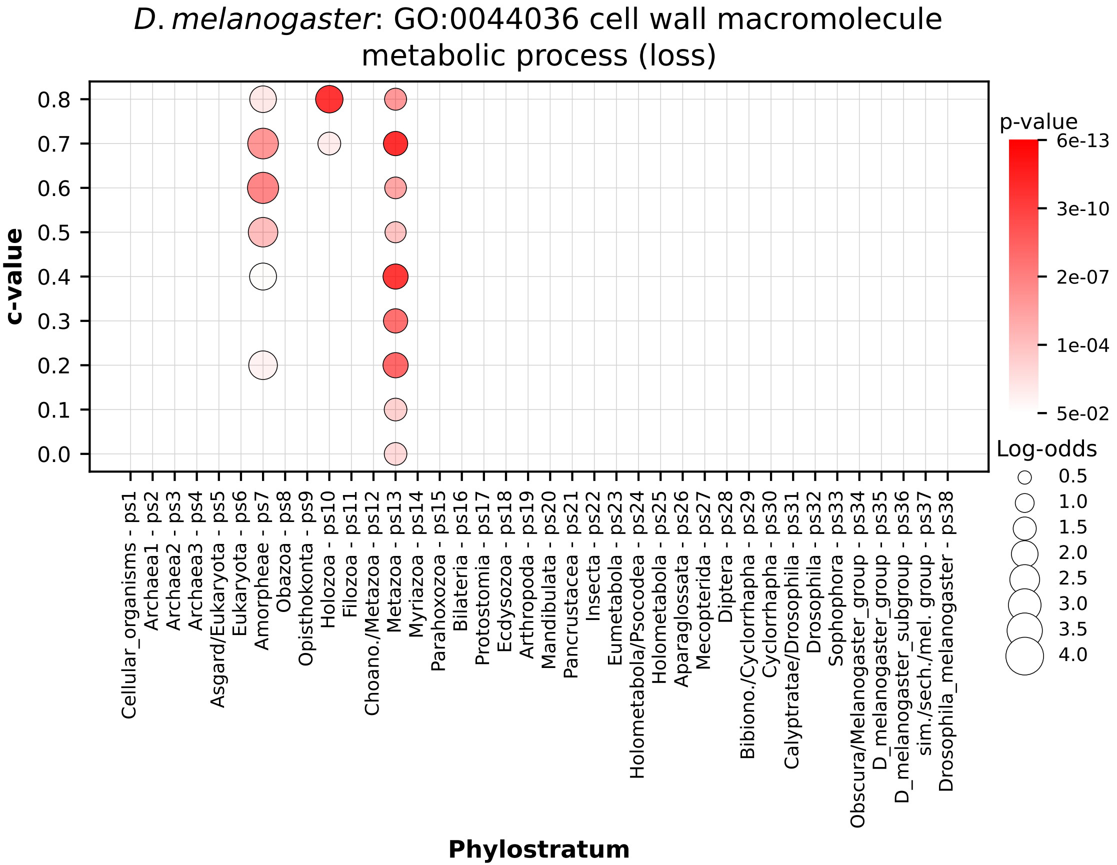
D. melanogaster: GO:0044036 cell wall macromolecule metabolic process (loss)
c-value
0.8
0.7
0.6
0.5
0.4
0.3
0.2
0.1
0.0
p-value
6e-13
3e-10
2e-07
1e-04
5e-02
Log-odds
0.5
1.0
1.5
2.0
2.5
3.0
3.5
4.0
Cellular_organisms - ps1
Archaea1 - ps2
Archaea2 - ps3
Archaea3 - ps4
Asgard/Eukaryota - ps5
Eukaryota - ps6
Amorpheae - ps7
Obazoa - ps8
Opisthokonta - ps9
Holozoa - ps10
Filozoa - ps11
Choano./Metazoa - ps12
Metazoa - ps13
Myriazoa - ps14
Parahoxozoa - ps15
Bilateria - ps16
Protostomia - ps17
Ecdysozoa - ps18
Arthropoda - ps19
Mandibulata - ps20
Pancrustacea - ps21
Insecta - ps22
Eumetabola - ps23
Holometabola/Psocodea - ps24
Holometabola - ps25
Aparaglossata - ps26
Mecopterida - ps27
Diptera - ps28
Bibiono./Cyclorrhapha - ps29
Cyclorrhapha - ps30
Calyptratae/Drosophila - ps31
Drosophila - ps32
Sophophora - ps33
Obscura/Melanogaster_group - ps34
D_melanogaster_group - ps35
D_melanogaster_subgroup - ps36
sim./sech./mel. group - ps37
Drosophila_melanogaster - ps38
Phylostratum

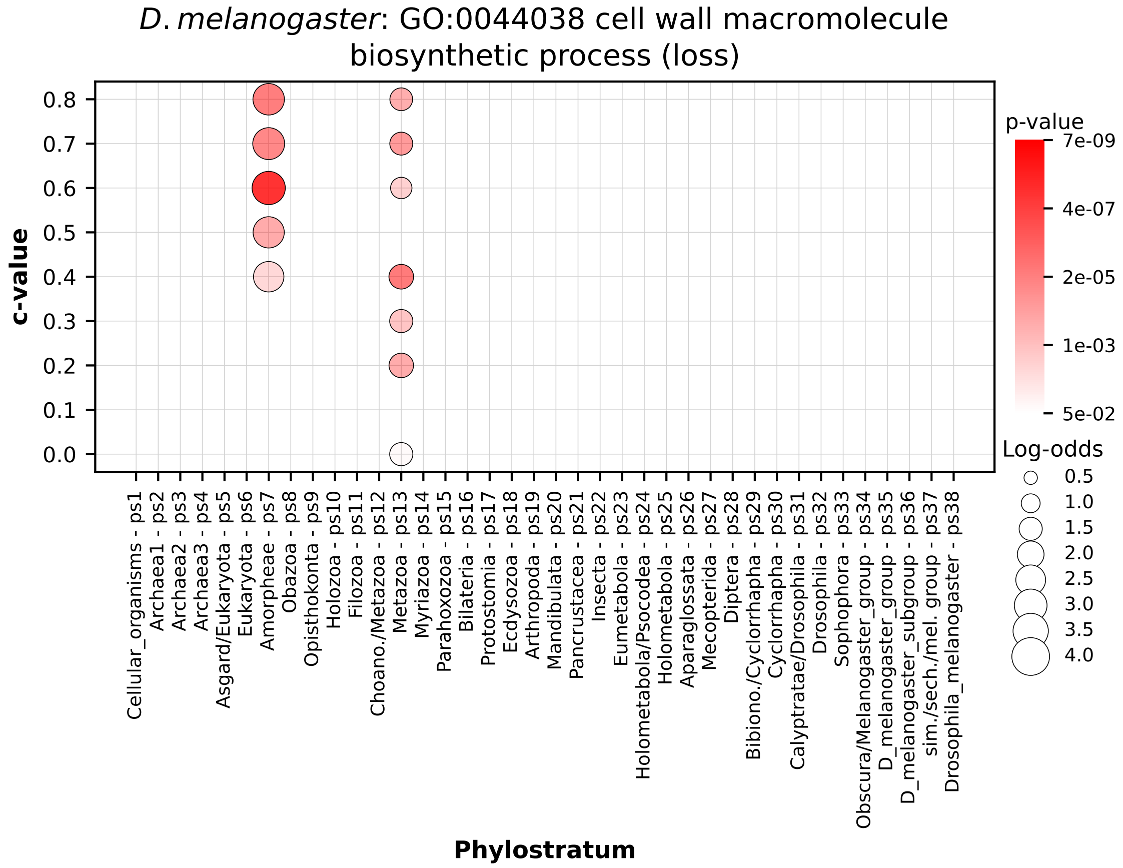

D. melanogaster: GO:0044038 cell wall macromolecule biosynthetic process (loss)
c-value
0.8
0.7
0.6
0.5
0.4
0.3
0.2
0.1
0.0
Phylostratum
Cellular_organisms - ps1
Archaea1 - ps2
Archaea2 - ps3
Archaea3 - ps4
Asgard/Eukaryota - ps5
Eukaryota - ps6
Amorpheae - ps7
Obazoa - ps8
Opisthokonta - ps9
Holozoa - ps10
Filozoa - ps11
Choano./Metazoa - ps12
Metazoa - ps13
Myriazoa - ps14
Parahoxozoa - ps15
Bilateria - ps16
Protostomia - ps17
Ecdysozoa - ps18
Arthropoda - ps19
Mandibulata - ps20
Pancrustacea - ps21
Insecta - ps22
Eumetabola - ps23
Holometabola/Psocodea - ps24
Holometabola - ps25
Aparaglossata - ps26
Mecopterida - ps27
Diptera - ps28
Bibiono./Cyclorrhapha - ps29
Cyclorrhapha - ps30
Calyptratae/Drosophila - ps31
Drosophila - ps32
Sophophora - ps33
Obscura/Melanogaster_group - ps34
D_melanogaster_group - ps35
D_melanogaster_subgroup - ps36
sim./sech./mel. group - ps37
Drosophila_melanogaster - ps38
p-value
7e-09
4e-07
2e-05
1e-03
5e-02
Log-odds
0.5
1.0
1.5
2.0
2.5
3.0
3.5
4.0

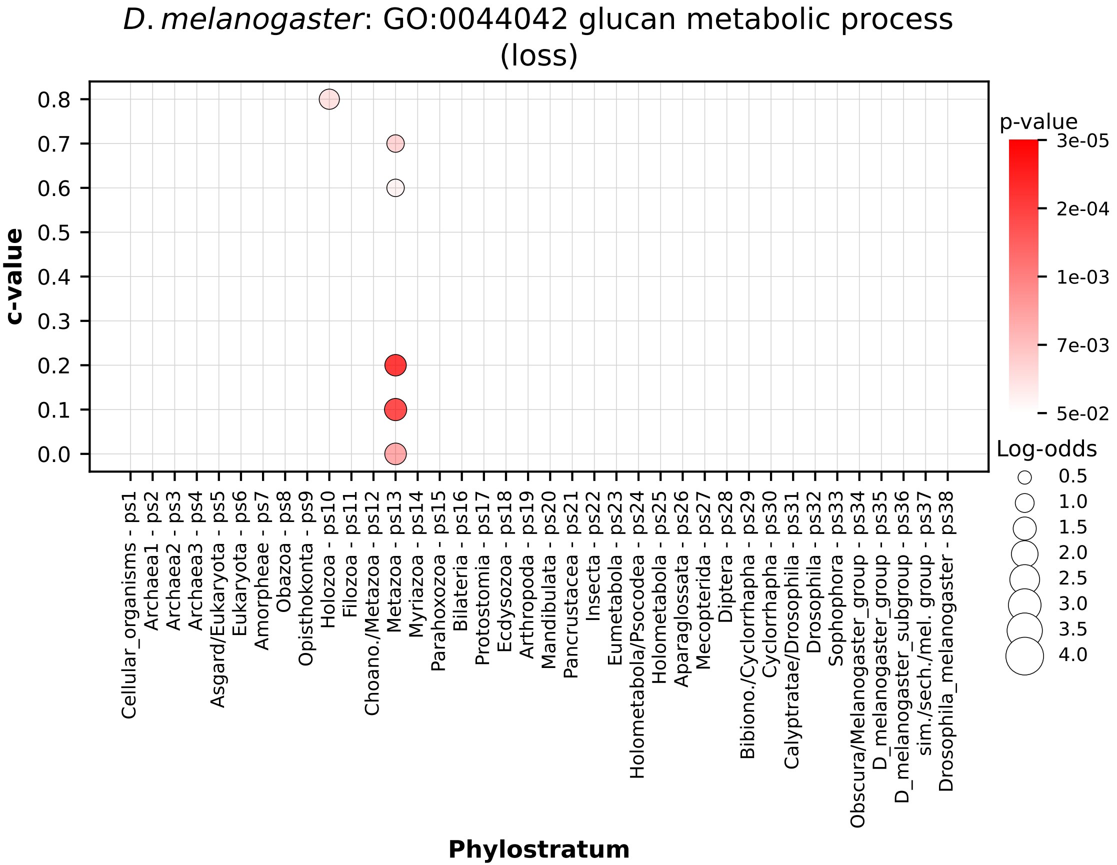

D. melanogaster: GO:0044042 glucan metabolic process
(loss)
c-value
0.8
0.7
0.6
0.5
0.4
0.3
0.2
0.1
0.0
p-value
3e-05
2e-04
1e-03
7e-03
5e-02
Log-odds
0.5
1.0
1.5
2.0
2.5
3.0
3.5
4.0
Cellular_organisms - ps1
Archaea1 - ps2
Archaea2 - ps3
Archaea3 - ps4
Asgard/Eukaryota - ps5
Eukaryota - ps6
Amorpheae - ps7
Obazoa - ps8
Opisthokonta - ps9
Holozoa - ps10
Filozoa - ps11
Choano./Metazoa - ps12
Metazoa - ps13
Myriazoa - ps14
Parahoxozoa - ps15
Bilateria - ps16
Protostomia - ps17
Ecdysozoa - ps18
Arthropoda - ps19
Mandibulata - ps20
Pancrustacea - ps21
Insecta - ps22
Eumetabola - ps23
Holometabola/Psocodea - ps24
Holometabola - ps25
Aparaglossata - ps26
Mecopterida - ps27
Diptera - ps28
Bibiono./Cyclorrhapha - ps29
Cyclorrhapha - ps30
Calyptratae/Drosophila - ps31
Drosophila - ps32
Sophophora - ps33
Obscura/Melanogaster_group - ps34
D_melanogaster_group - ps35
D_melanogaster_subgroup - ps36
sim./sech./mel. group - ps37
Drosophila_melanogaster - ps38
Phylostratum

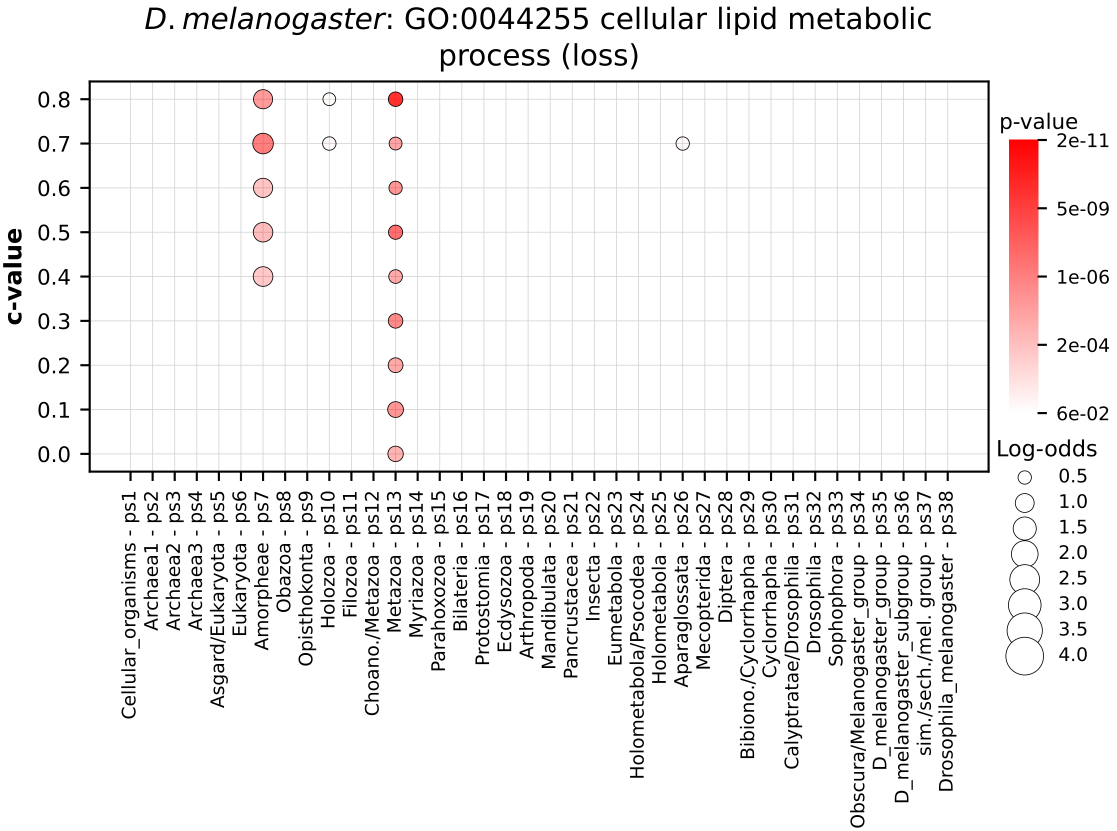

D. melanogaster: GO:0044255 cellular lipid metabolic process (loss)
c-value
Phylostratum
p-value
Log-odds
Cellular_organisms - ps1
Archaea1 - ps2
Archaea2 - ps3
Archaea3 - ps4
Asgard/Eukaryota - ps5
Eukaryota - ps6
Amorpheae - ps7
Obazoa - ps8
Opisthokonta - ps9
Holozoa - ps10
Filozoa - ps11
Choano./Metazoa - ps12
Metazoa - ps13
Myriazoa - ps14
Parahoxozoa - ps15
Bilateria - ps16
Protostomia - ps17
Ecdysozoa - ps18
Arthropoda - ps19
Mandibulata - ps20
Pancrustacea - ps21
Insecta - ps22
Eumetabola - ps23
Holometabola/Psocodea - ps24
Holometabola - ps25
Aparaglossata - ps26
Mecopterida - ps27
Diptera - ps28
Bibiono./Cyclorrhapha - ps29
Cyclorrhapha - ps30
Calyptratae/Drosophila - ps31
Drosophila - ps32
Sophophora - ps33
Obscura/Melanogaster_group - ps34
D_melanogaster_group - ps35
D_melanogaster_subgroup - ps36
sim./sech./mel. group - ps37
Drosophila_melanogaster - ps38

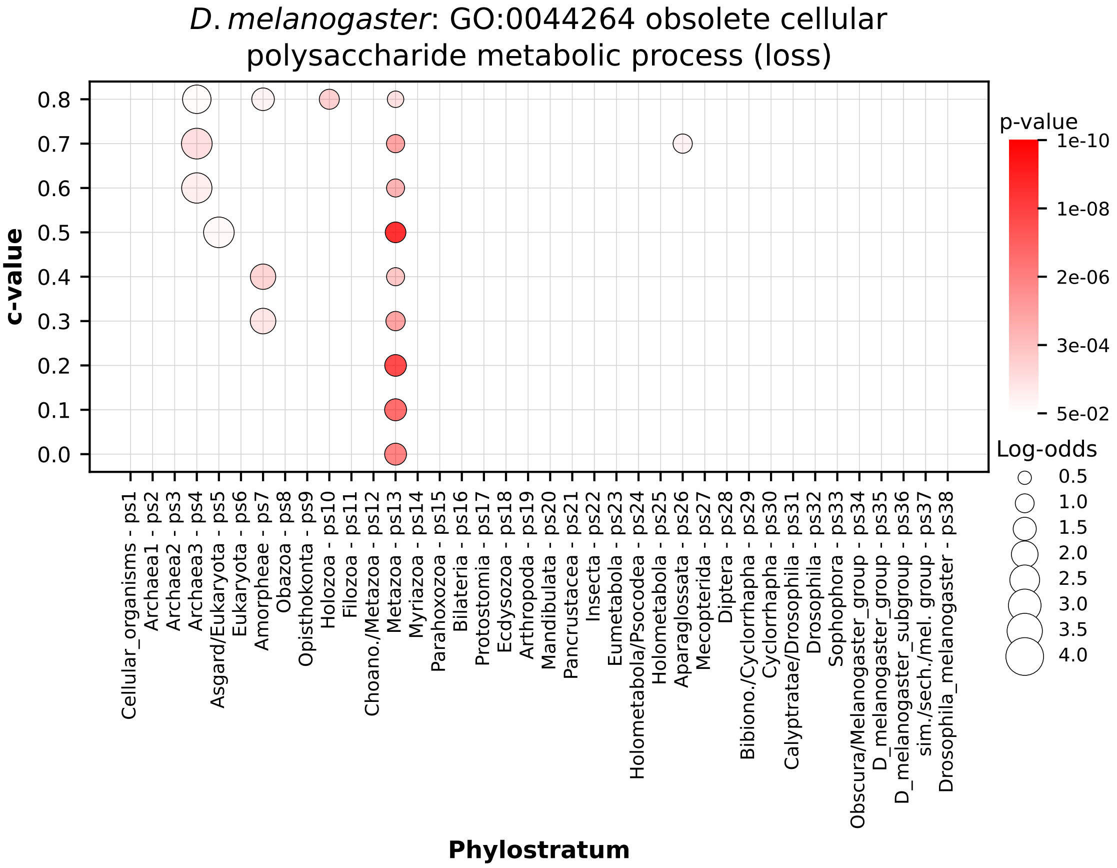
D. melanogaster: GO:0044264 obsolete cellular polysaccharide metabolic process (loss)
c-value
0.8
0.7
0.6
0.5
0.4
0.3
0.2
0.1
0.0
p-value
1e-10
1e-08
2e-06
3e-04
5e-02
Log-odds
0.5
1.0
1.5
2.0
2.5
3.0
3.5
4.0
Cellular_organisms - ps1
Archaea1 - ps2
Archaea2 - ps3
Archaea3 - ps4
Asgard/Eukaryota - ps5
Eukaryota - ps6
Amorpheae - ps7
Obazoa - ps8
Opisthokonta - ps9
Holozoa - ps10
Filozoa - ps11
Choano./Metazoa - ps12
Metazoa - ps13
Myriazoa - ps14
Parahoxozoa - ps15
Bilateria - ps16
Protostomia - ps17
Ecdysozoa - ps18
Arthropoda - ps19
Mandibulata - ps20
Pancrustacea - ps21
Insecta - ps22
Eumetabola - ps23
Holometabola/Psocodea - ps24
Holometabola - ps25
Aparaglossata - ps26
Mecopterida - ps27
Diptera - ps28
Bibiono./Cyclorrhapha - ps29
Cyclorrhapha - ps30
Calyptratae/Drosophila - ps31
Drosophila - ps32
Sophophora - ps33
Obscura/Melanogaster_group - ps34
D_melanogaster_group - ps35
D_melanogaster_subgroup - ps36
sim./sech./mel. group - ps37
Drosophila_melanogaster - ps38
Phylostratum

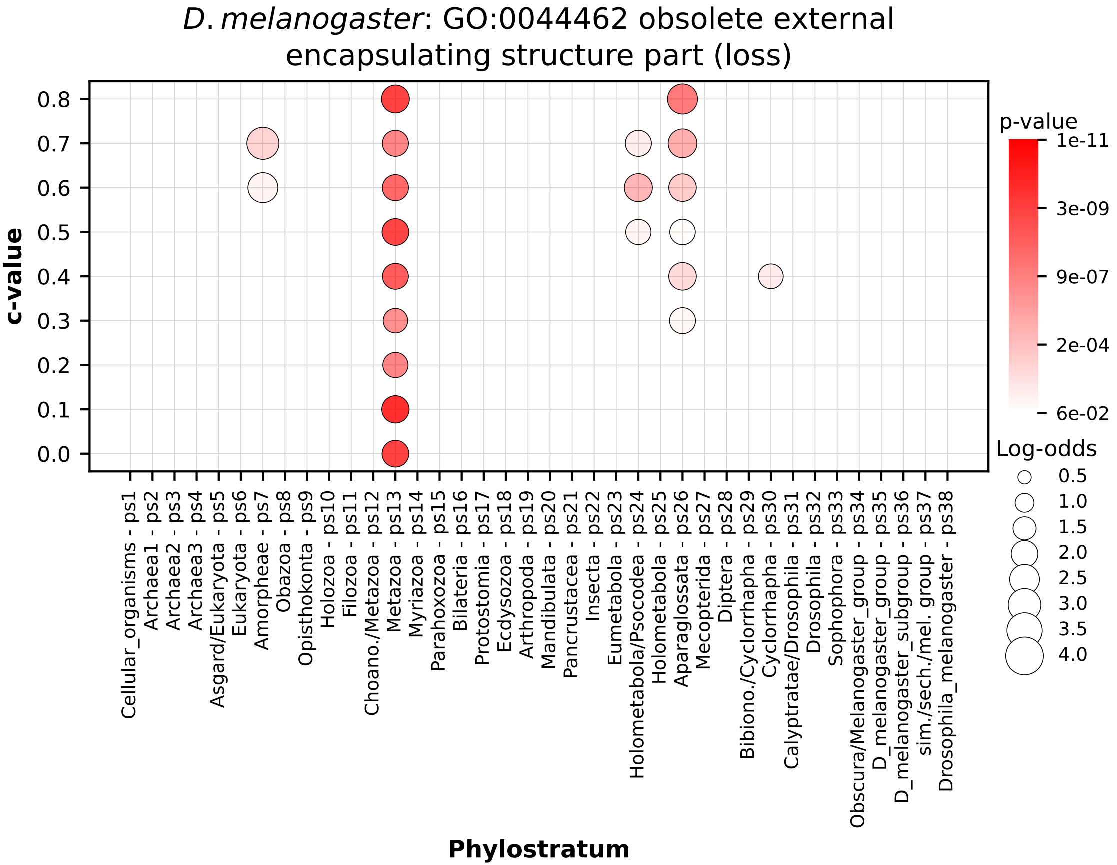
D. melanogaster: GO:0044462 obsolete external encapsulating structure part (loss)
c-value
Phylostratum
p-value
1e-11
3e-09
9e-07
2e-04
6e-02
Log-odds
0.5
1.0
1.5
2.0
2.5
3.0
3.5
4.0
Cellular_organisms - ps1
Archaea1 - ps2
Archaea2 - ps3
Archaea3 - ps4
Asgard/Eukaryota - ps5
Eukaryota - ps6
Amorpheae - ps7
Obazoa - ps8
Opisthokonta - ps9
Holozoa - ps10
Filozoa - ps11
Choano./Metazoa - ps12
Metazoa - ps13
Myriazoa - ps14
Parahoxozoa - ps15
Bilateria - ps16
Protostomia - ps17
Ecdysozoa - ps18
Arthropoda - ps19
Mandibulata - ps20
Pancrustacea - ps21
Insecta - ps22
Eumetabola - ps23
Holometabola/Psocodea - ps24
Holometabola - ps25
Aparaglossata - ps26
Mecopterida - ps27
Diptera - ps28
Bibiono./Cyclorrhapha - ps29
Cyclorrhapha - ps30
Calyptratae/Drosophila - ps31
Drosophila - ps32
Sophophora - ps33
Obscura/Melanogaster_group - ps34
D_melanogaster_group - ps35
D_melanogaster_subgroup - ps36
sim./sech./mel. group - ps37
Drosophila_melanogaster - ps38

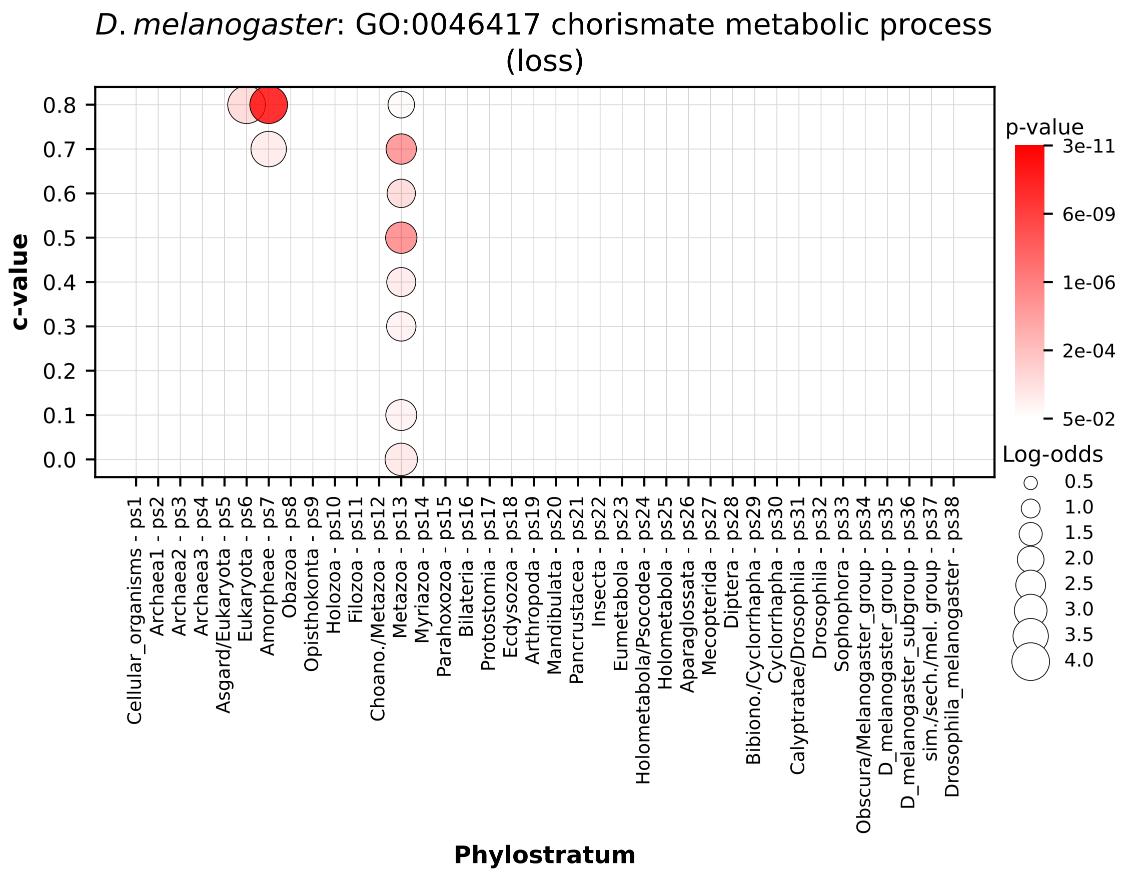

D. melanogaster: GO:0046417 chorismate metabolic process (loss)
c-value
0.8
0.7
0.6
0.5
0.4
0.3
0.2
0.1
0.0
p-value
3e-11
6e-09
1e-06
2e-04
5e-02
Log-odds
0.5
1.0
1.5
2.0
2.5
3.0
3.5
4.0
Cellular_organisms - ps1
Archaea1 - ps2
Archaea2 - ps3
Archaea3 - ps4
Asgard/Eukaryota - ps5
Eukaryota - ps6
Amorpheae - ps7
Obazoa - ps8
Opisthokonta - ps9
Holozoa - ps10
Filozoa - ps11
Choano./Metazoa - ps12
Metazoa - ps13
Myriazoa - ps14
Parahoxozoa - ps15
Bilateria - ps16
Protostomia - ps17
Ecdysozoa - ps18
Arthropoda - ps19
Mandibulata - ps20
Pancrustacea - ps21
Insecta - ps22
Eumetabola - ps23
Holometabola/Psocodea - ps24
Holometabola - ps25
Aparaglossata - ps26
Mecopterida - ps27
Diptera - ps28
Bibiono./Cyclorrhapha - ps29
Cyclorrhapha - ps30
Calyptratae/Drosophila - ps31
Drosophila - ps32
Sophophora - ps33
Obscura/Melanogaster_group - ps34
D_melanogaster_group - ps35
D_melanogaster_subgroup - ps36
sim./sech./mel. group - ps37
Drosophila_melanogaster - ps38
Phylostratum

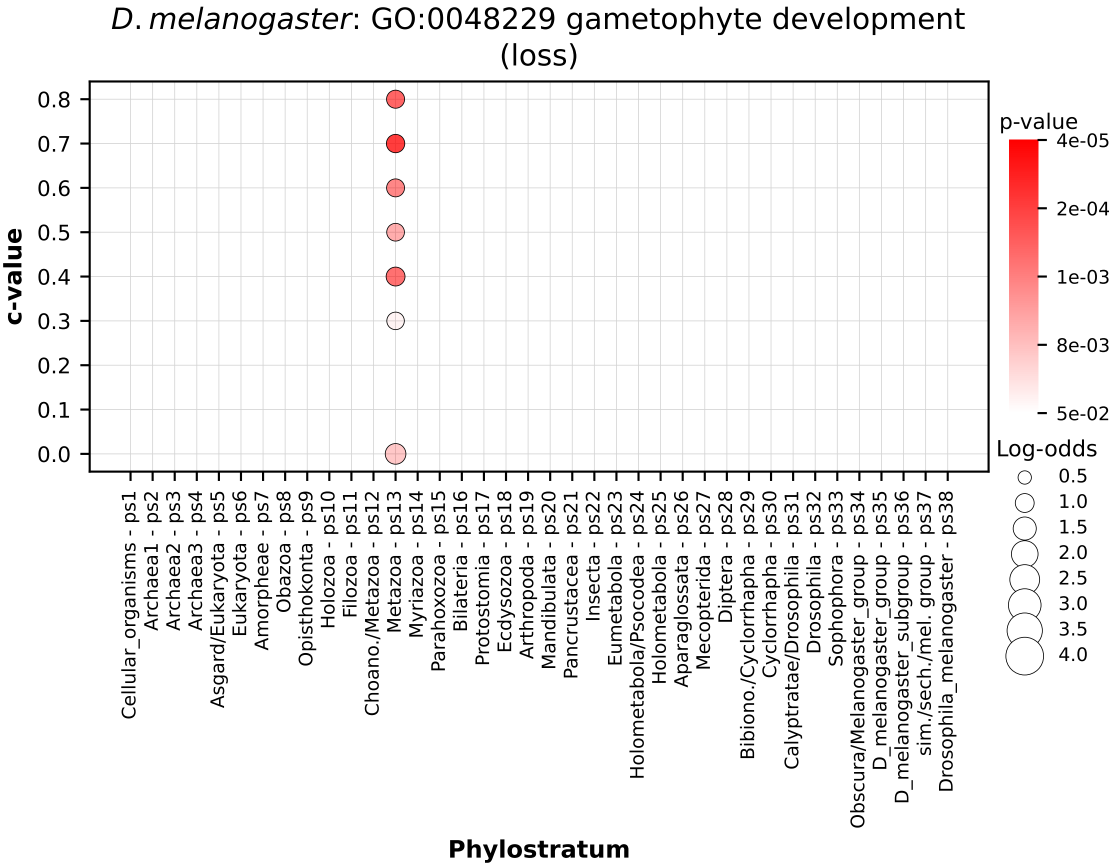

D. melanogaster: GO:0048229 gametophyte development
(loss)
c-value
0.8
0.7
0.6
0.5
0.4
0.3
0.2
0.1
0.0
p-value
4e-05
2e-04
1e-03
8e-03
5e-02
Log-odds
0.5
1.0
1.5
2.0
2.5
3.0
3.5
4.0
Cellular_organisms - ps1
Archaea1 - ps2
Archaea2 - ps3
Archaea3 - ps4
Asgard/Eukaryota - ps5
Eukaryota - ps6
Amorpheae - ps7
Obazoa - ps8
Opisthokonta - ps9
Holozoa - ps10
Filozoa - ps11
Choano./Metazoa - ps12
Metazoa - ps13
Myriazoa - ps14
Parahoxozoa - ps15
Bilateria - ps16
Protostomia - ps17
Ecdysozoa - ps18
Arthropoda - ps19
Mandibulata - ps20
Pancrustacea - ps21
Insecta - ps22
Eumetabola - ps23
Holometabola/Psocodea - ps24
Holometabola - ps25
Aparaglossata - ps26
Mecopterida - ps27
Diptera - ps28
Bibiono./Cyclorrhapha - ps29
Cyclorrhapha - ps30
Calyptratae/Drosophila - ps31
Drosophila - ps32
Sophophora - ps33
Obscura/Melanogaster_group - ps34
D_melanogaster_group - ps35
D_melanogaster_subgroup - ps36
sim./sech./mel. group - ps37
Drosophila_melanogaster - ps38
Phylostratum

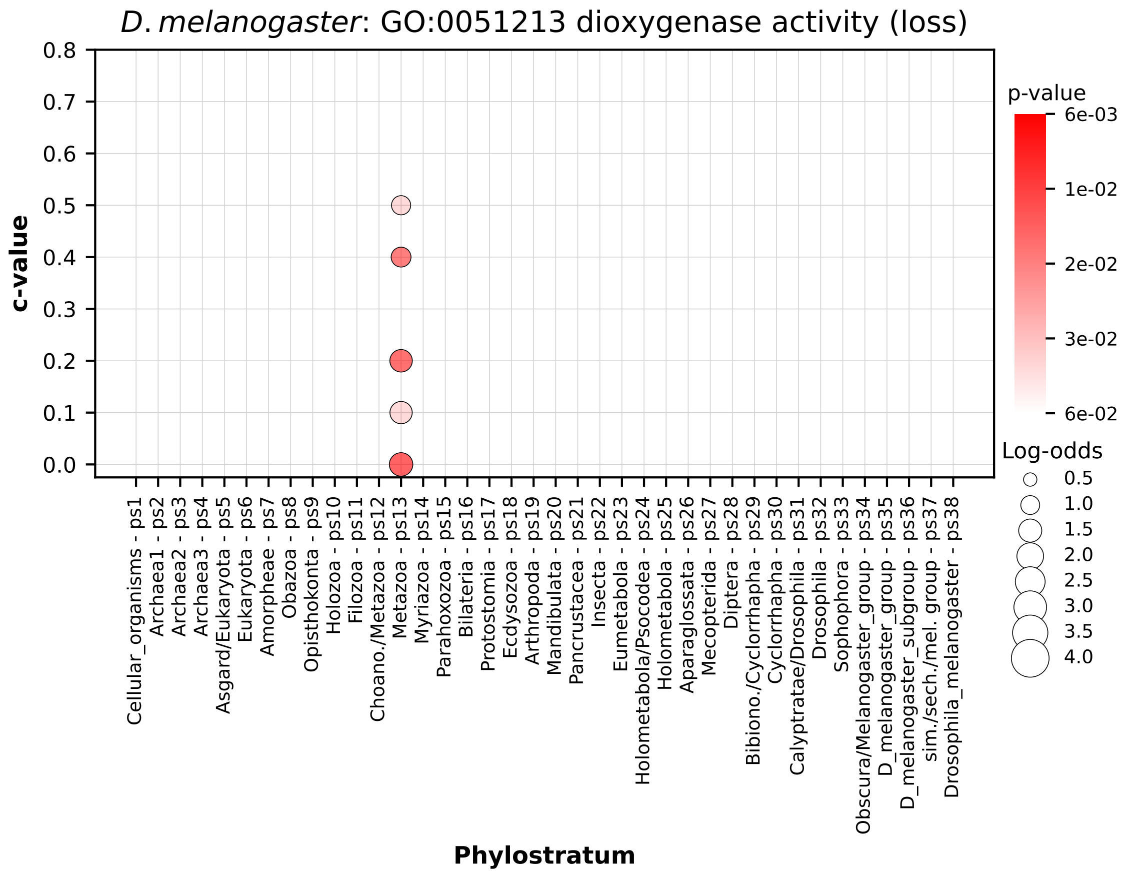
D. melanogaster: GO:0051213 dioxygenase activity (loss)
c-value
0.8
0.7
0.6
0.5
0.4
0.3
0.2
0.1
0.0
p-value
6e-03
1e-02
2e-02
3e-02
6e-02
Log-odds
0.5
1.0
1.5
2.0
2.5
3.0
3.5
4.0
Cellular_organisms - ps1
Archaea1 - ps2
Archaea2 - ps3
Archaea3 - ps4
Asgard/Eukaryota - ps5
Eukaryota - ps6
Amorpheae - ps7
Obazoa - ps8
Opisthokonta - ps9
Holozoa - ps10
Filozoa - ps11
Choano./Metazoa - ps12
Metazoa - ps13
Myriazoa - ps14
Parahoxozoa - ps15
Bilateria - ps16
Protostomia - ps17
Ecdysozoa - ps18
Arthropoda - ps19
Mandibulata - ps20
Pancrustacea - ps21
Insecta - ps22
Eumetabola - ps23
Holometabola/Psocodea - ps24
Holometabola - ps25
Aparaglossata - ps26
Mecopterida - ps27
Diptera - ps28
Bibiono./Cyclorrhapha - ps29
Cyclorrhapha - ps30
Calyptratae/Drosophila - ps31
Drosophila - ps32
Sophophora - ps33
Obscura/Melanogaster_group - ps34
D_melanogaster_group - ps35
D_melanogaster_subgroup - ps36
sim./sech./mel. group - ps37
Drosophila_melanogaster - ps38
Phylostratum

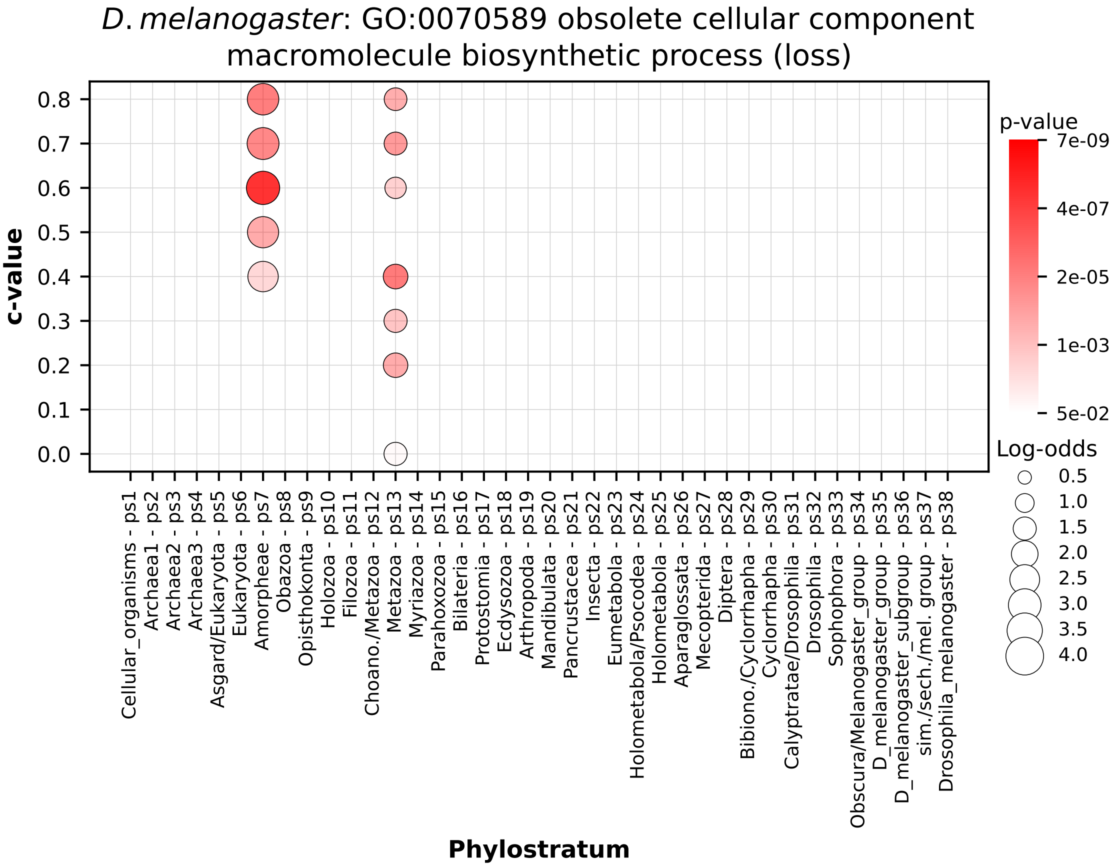

D. melanogaster: GO:0070589 obsolete cellular component macromolecule biosynthetic process (loss)
c-value
0.8
0.7
0.6
0.5
0.4
0.3
0.2
0.1
0.0
Phylostratum
Cellular_organisms - ps1
Archaea1 - ps2
Archaea2 - ps3
Archaea3 - ps4
Asgard/Eukaryota - ps5
Eukaryota - ps6
Amorpheae - ps7
Obazoa - ps8
Opisthokonta - ps9
Holozoa - ps10
Filozoa - ps11
Choano./Metazoa - ps12
Metazoa - ps13
Myriazoa - ps14
Parahoxozoa - ps15
Bilateria - ps16
Protostomia - ps17
Ecdysozoa - ps18
Arthropoda - ps19
Mandibulata - ps20
Pancrustacea - ps21
Insecta - ps22
Eumetabola - ps23
Holometabola/Psocodea - ps24
Holometabola - ps25
Aparaglossata - ps26
Mecopterida - ps27
Diptera - ps28
Bibiono./Cyclorrhapha - ps29
Cyclorrhapha - ps30
Calyptratae/Drosophila - ps31
Drosophila - ps32
Sophophora - ps33
Obscura/Melanogaster_group - ps34
D_melanogaster_group - ps35
D_melanogaster_subgroup - ps36
sim./sech./mel. group - ps37
Drosophila_melanogaster - ps38
p-value
7e-09
4e-07
2e-05
1e-03
5e-02
Log-odds
0.5
1.0
1.5
2.0
2.5
3.0
3.5
4.0

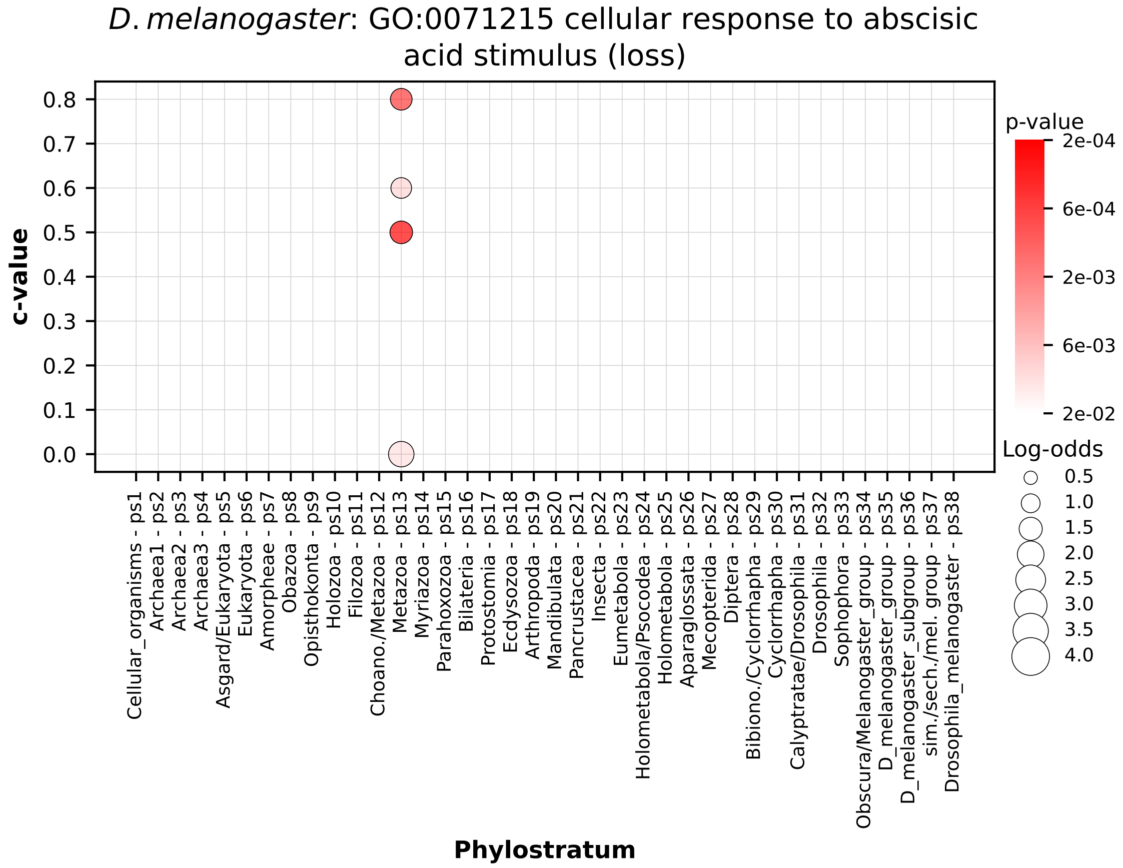

D. melanogaster: GO:0071215 cellular response to abscisic acid stimulus (loss)
c-value
0.8
0.7
0.6
0.5
0.4
0.3
0.2
0.1
0.0
Phylostratum
Cellular_organisms - ps1
Archaea1 - ps2
Archaea2 - ps3
Archaea3 - ps4
Asgard/Eukaryota - ps5
Eukaryota - ps6
Amorpheae - ps7
Obazoa - ps8
Opisthokonta - ps9
Holozoa - ps10
Filozoa - ps11
Choano./Metazoa - ps12
Metazoa - ps13
Myriazoa - ps14
Parahoxozoa - ps15
Bilateria - ps16
Protostomia - ps17
Ecdysozoa - ps18
Arthropoda - ps19
Mandibulata - ps20
Pancrustacea - ps21
Insecta - ps22
Eumetabola - ps23
Holometabola/Psocodea - ps24
Holometabola - ps25
Aparaglossata - ps26
Mecopterida - ps27
Diptera - ps28
Bibiono./Cyclorrhapha - ps29
Cyclorrhapha - ps30
Calyptratae/Drosophila - ps31
Drosophila - ps32
Sophophora - ps33
Obscura/Melanogaster_group - ps34
D_melanogaster_group - ps35
D_melanogaster_subgroup - ps36
sim./sech./mel. group - ps37
Drosophila_melanogaster - ps38
p-value
2e-04
6e-04
2e-03
6e-03
2e-02
Log-odds
0.5
1.0
1.5
2.0
2.5
3.0
3.5
4.0

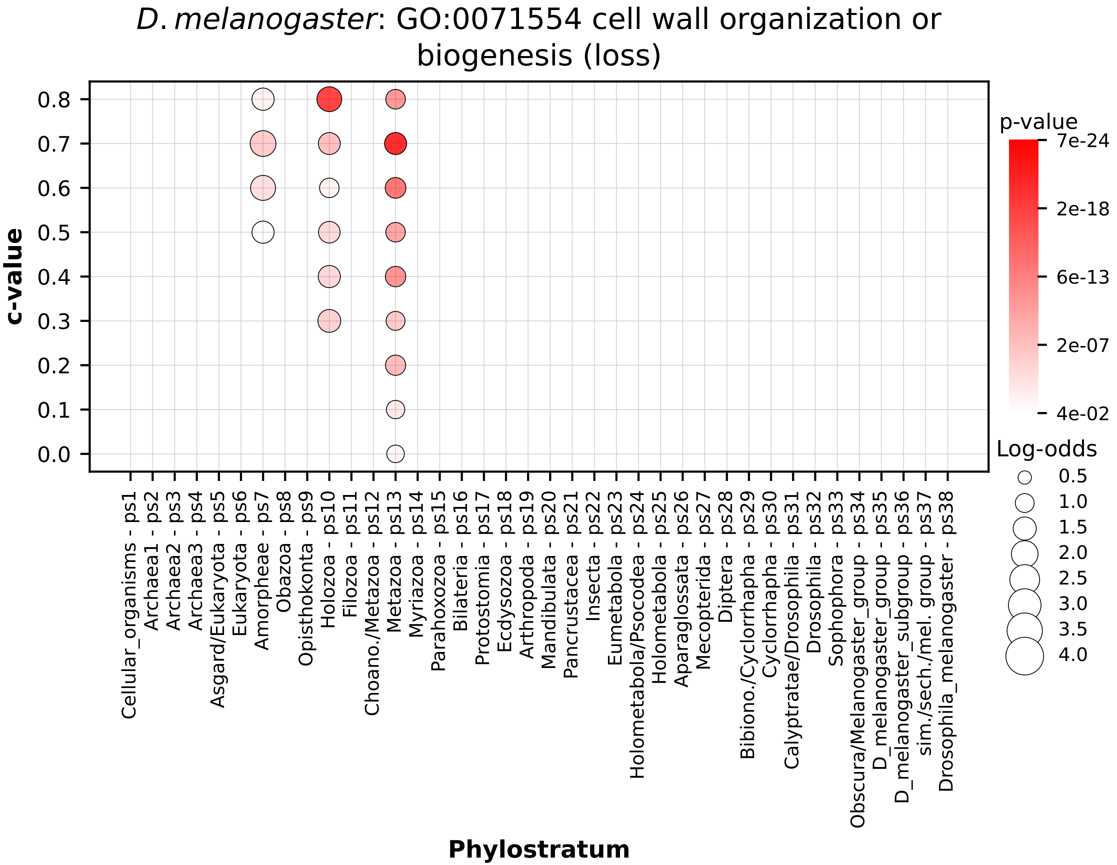

D. melanogaster: GO:0071554 cell wall organization or biogenesis (loss)
c-value
0.8
0.7
0.6
0.5
0.4
0.3
0.2
0.1
0.0
p-value
7e-24
2e-18
6e-13
2e-07
4e-02
Log-odds
0.5
1.0
1.5
2.0
2.5
3.0
3.5
4.0
Cellular_organisms - ps1
Archaea1 - ps2
Archaea2 - ps3
Archaea3 - ps4
Asgard/Eukaryota - ps5
Eukaryota - ps6
Amorpheae - ps7
Obazoa - ps8
Opisthokonta - ps9
Holozoa - ps10
Filozoa - ps11
Choano./Metazoa - ps12
Metazoa - ps13
Myriazoa - ps14
Parahoxozoa - ps15
Bilateria - ps16
Protostomia - ps17
Ecdysozoa - ps18
Arthropoda - ps19
Mandibulata - ps20
Pancrustacea - ps21
Insecta - ps22
Eumetabola - ps23
Holometabola/Psocodea - ps24
Holometabola - ps25
Aparaglossata - ps26
Mecopterida - ps27
Diptera - ps28
Bibiono./Cyclorrhapha - ps29
Cyclorrhapha - ps30
Calyptratae/Drosophila - ps31
Drosophila - ps32
Sophophora - ps33
Obscura/Melanogaster_group - ps34
D_melanogaster_group - ps35
D_melanogaster_subgroup - ps36
sim./sech./mel. group - ps37
Drosophila_melanogaster - ps38
Phylostratum

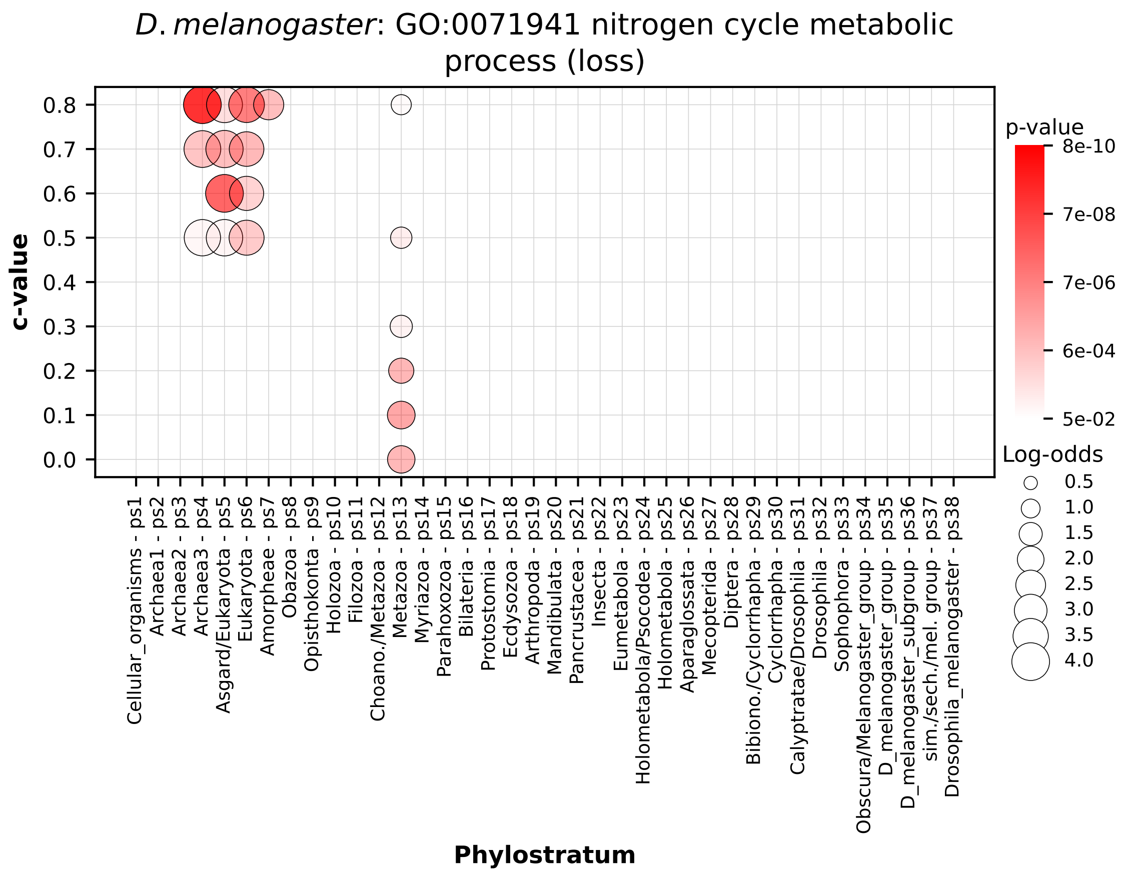

D. melanogaster: GO:0071941 nitrogen cycle metabolic process (loss)
c-value
0.8
0.7
0.6
0.5
0.4
0.3
0.2
0.1
0.0
Phylostratum
Cellular_organisms - ps1
Archaea1 - ps2
Archaea2 - ps3
Archaea3 - ps4
Asgard/Eukaryota - ps5
Eukaryota - ps6
Amorpheae - ps7
Obazoa - ps8
Opisthokonta - ps9
Holozoa - ps10
Filozoa - ps11
Choano./Metazoa - ps12
Metazoa - ps13
Myriazoa - ps14
Parahoxozoa - ps15
Bilateria - ps16
Protostomia - ps17
Ecdysozoa - ps18
Arthropoda - ps19
Mandibulata - ps20
Pancrustacea - ps21
Insecta - ps22
Eumetabola - ps23
Holometabola/Psocodea - ps24
Holometabola - ps25
Aparaglossata - ps26
Mecopterida - ps27
Diptera - ps28
Bibiono./Cyclorrhapha - ps29
Cyclorrhapha - ps30
Calyptratae/Drosophila - ps31
Drosophila - ps32
Sophophora - ps33
Obscura/Melanogaster_group - ps34
D_melanogaster_group - ps35
D_melanogaster_subgroup - ps36
sim./sech./mel. group - ps37
Drosophila_melanogaster - ps38
p-value
8e-10
7e-08
7e-06
6e-04
5e-02
Log-odds
0.5
1.0
1.5
2.0
2.5
3.0
3.5
4.0

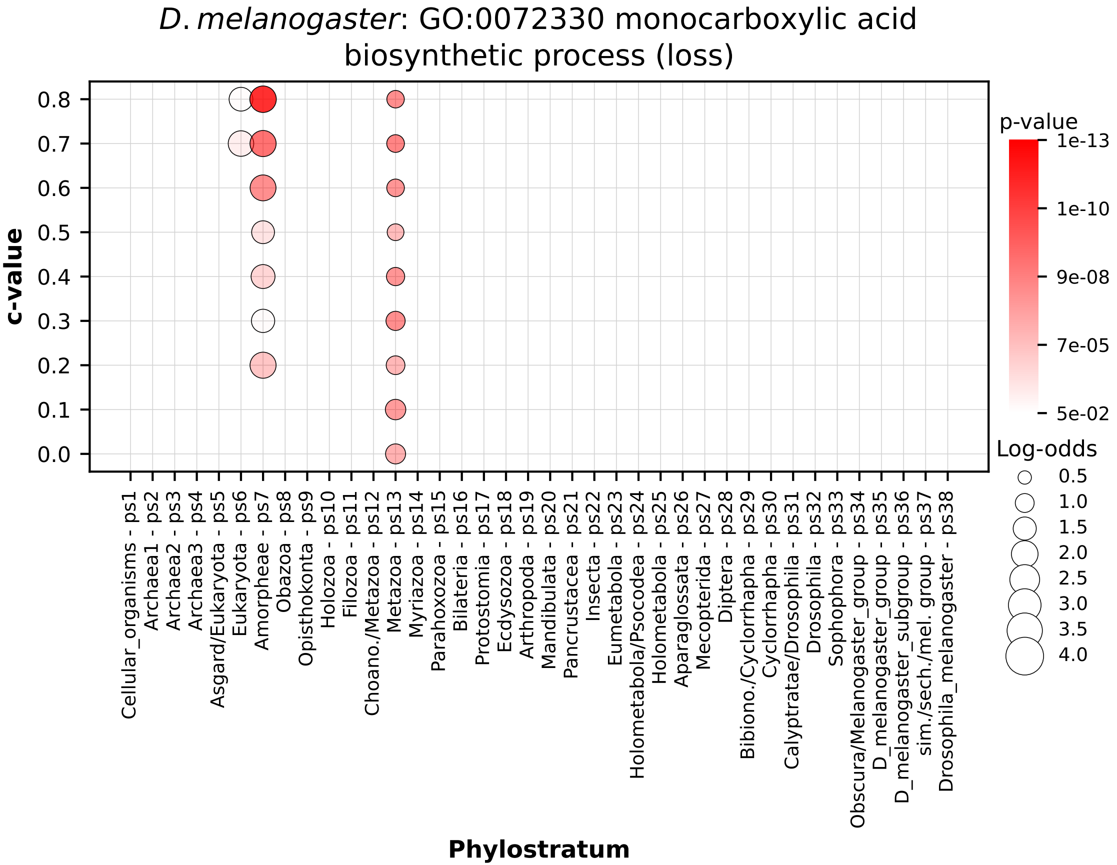

D. melanogaster: GO:0072330 monocarboxylic acid biosynthetic process (loss)
c-value
0.8
0.7
0.6
0.5
0.4
0.3
0.2
0.1
0.0
p-value
1e-13
1e-10
9e-08
7e-05
5e-02
Log-odds
0.5
1.0
1.5
2.0
2.5
3.0
3.5
4.0
Cellular_organisms - ps1
Archaea1 - ps2
Archaea2 - ps3
Archaea3 - ps4
Asgard/Eukaryota - ps5
Eukaryota - ps6
Amorpheae - ps7
Obazoa - ps8
Opisthokonta - ps9
Holozoa - ps10
Filozoa - ps11
Choano./Metazoa - ps12
Metazoa - ps13
Myriazoa - ps14
Parahoxozoa - ps15
Bilateria - ps16
Protostomia - ps17
Ecdysozoa - ps18
Arthropoda - ps19
Mandibulata - ps20
Pancrustacea - ps21
Insecta - ps22
Eumetabola - ps23
Holometabola/Psocodea - ps24
Holometabola - ps25
Aparaglossata - ps26
Mecopterida - ps27
Diptera - ps28
Bibiono./Cyclorrhapha - ps29
Cyclorrhapha - ps30
Calyptratae/Drosophila - ps31
Drosophila - ps32
Sophophora - ps33
Obscura/Melanogaster_group - ps34
D_melanogaster_group - ps35
D_melanogaster_subgroup - ps36
sim./sech./mel. group - ps37
Drosophila_melanogaster - ps38
Phylostratum

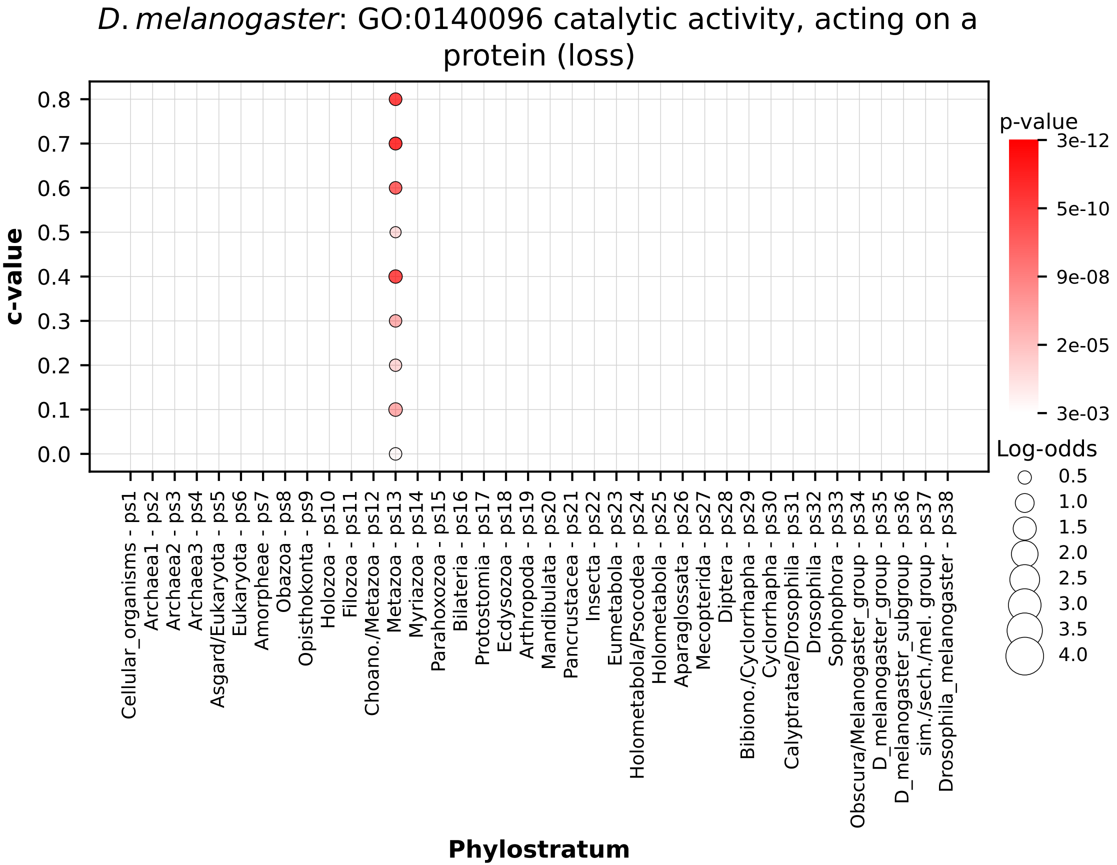

D. melanogaster: GO:0140096 catalytic activity, acting on a protein (loss)
c-value
0.8
0.7
0.6
0.5
0.4
0.3
0.2
0.1
0.0
p-value
3e-12
5e-10
9e-08
2e-05
3e-03
Log-odds
0.5
1.0
1.5
2.0
2.5
3.0
3.5
4.0
Cellular_organisms - ps1
Archaea1 - ps2
Archaea2 - ps3
Archaea3 - ps4
Asgard/Eukaryota - ps5
Eukaryota - ps6
Amorpheae - ps7
Obazoa - ps8
Opisthokonta - ps9
Holozoa - ps10
Filozoa - ps11
Choano./Metazoa - ps12
Metazoa - ps13
Myriazoa - ps14
Parahoxozoa - ps15
Bilateria - ps16
Protostomia - ps17
Ecdysozoa - ps18
Arthropoda - ps19
Mandibulata - ps20
Pancrustacea - ps21
Insecta - ps22
Eumetabola - ps23
Holometabola/Psocodea - ps24
Holometabola - ps25
Aparaglossata - ps26
Mecopterida - ps27
Diptera - ps28
Bibiono./Cyclorrhapha - ps29
Cyclorrhapha - ps30
Calyptratae/Drosophila - ps31
Drosophila - ps32
Sophophora - ps33
Obscura/Melanogaster_group - ps34
D_melanogaster_group - ps35
D_melanogaster_subgroup - ps36
sim./sech./mel. group - ps37
Drosophila_melanogaster - ps38
Phylostratum

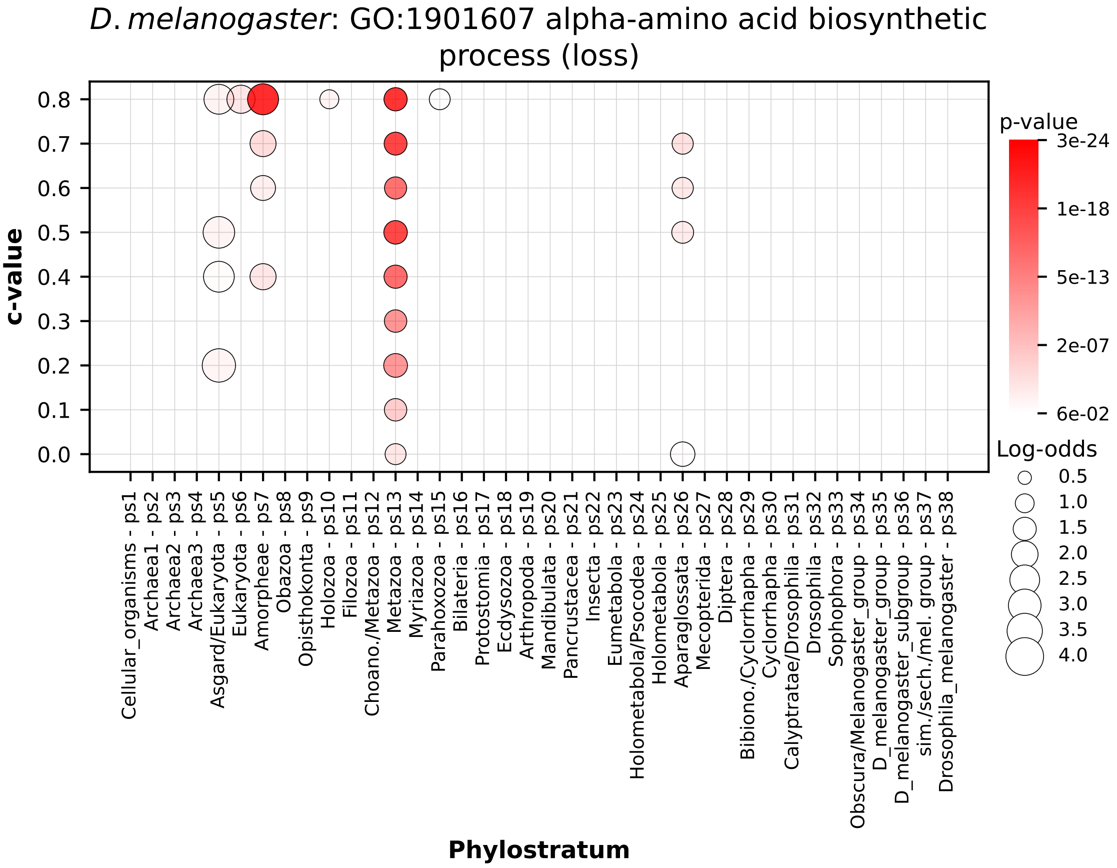

D. melanogaster: GO:1901607 alpha-amino acid biosynthetic process (loss)
c-value
0.8
0.7
0.6
0.5
0.4
0.3
0.2
0.1
0.0
p-value
3e-24
1e-18
5e-13
2e-07
6e-02
Log-odds
0.5
1.0
1.5
2.0
2.5
3.0
3.5
4.0
Cellular_organisms - ps1
Archaea1 - ps2
Archaea2 - ps3
Archaea3 - ps4
Asgard/Eukaryota - ps5
Eukaryota - ps6
Amorpheae - ps7
Obazoa - ps8
Opisthokonta - ps9
Holozoa - ps10
Filozoa - ps11
Choano./Metazoa - ps12
Metazoa - ps13
Myriazoa - ps14
Parahoxozoa - ps15
Bilateria - ps16
Protostomia - ps17
Ecdysozoa - ps18
Arthropoda - ps19
Mandibulata - ps20
Pancrustacea - ps21
Insecta - ps22
Eumetabola - ps23
Holometabola/Psocodea - ps24
Holometabola - ps25
Aparaglossata - ps26
Mecopterida - ps27
Diptera - ps28
Bibiono./Cyclorrhapha - ps29
Cyclorrhapha - ps30
Calyptratae/Drosophila - ps31
Drosophila - ps32
Sophophora - ps33
Obscura/Melanogaster_group - ps34
D_melanogaster_group - ps35
D_melanogaster_subgroup - ps36
sim./sech./mel. group - ps37
Drosophila_melanogaster - ps38
Phylostratum

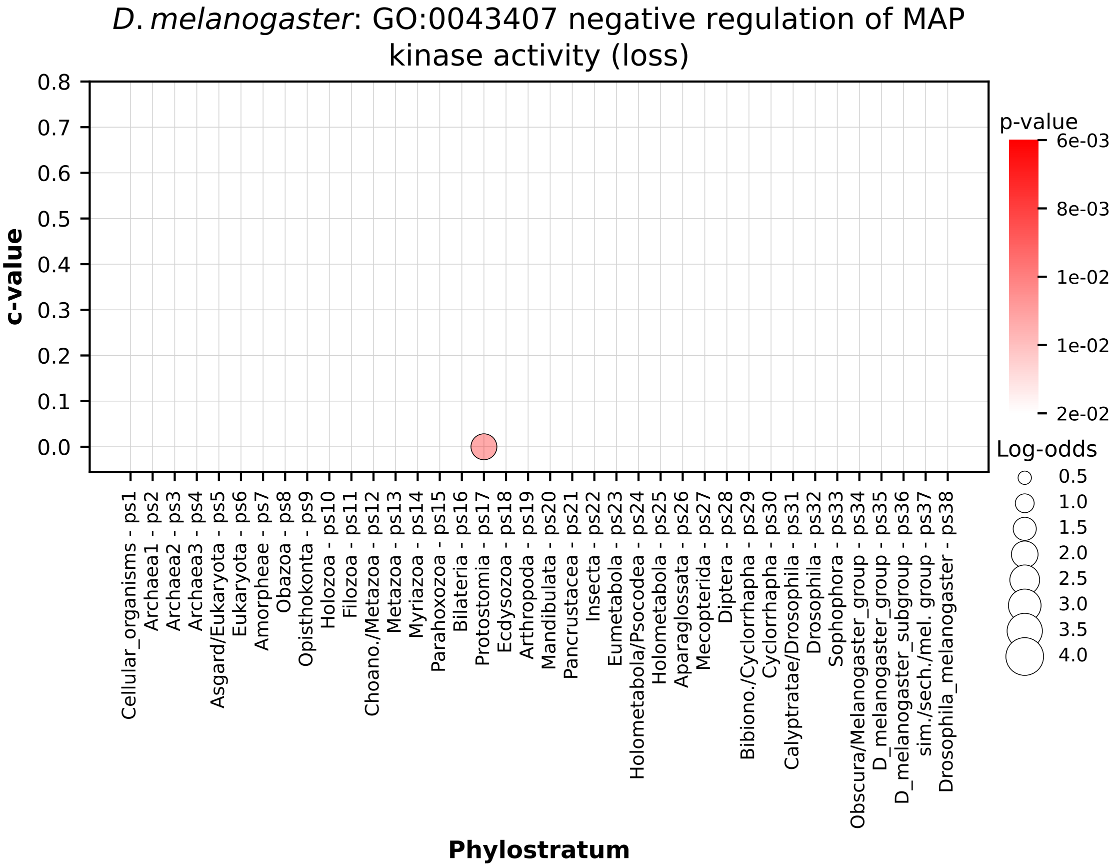
D. melanogaster: GO:0043407 negative regulation of MAP kinase activity (loss)
c-value
0.8
0.7
0.6
0.5
0.4
0.3
0.2
0.1
0.0
p-value
6e-03
8e-03
1e-02
1e-02
2e-02
Log-odds
0.5
1.0
1.5
2.0
2.5
3.0
3.5
4.0
Cellular_organisms - ps1
Archaea1 - ps2
Archaea2 - ps3
Archaea3 - ps4
Asgard/Eukaryota - ps5
Eukaryota - ps6
Amorpheae - ps7
Obazoa - ps8
Opisthokonta - ps9
Holozoa - ps10
Filozoa - ps11
Choano./Metazoa - ps12
Metazoa - ps13
Myriazoa - ps14
Parahoxozoa - ps15
Bilateria - ps16
Protostomia - ps17
Ecdysozoa - ps18
Arthropoda - ps19
Mandibulata - ps20
Pancrustacea - ps21
Insecta - ps22
Eumetabola - ps23
Holometabola/Psocodea - ps24
Holometabola - ps25
Aparaglossata - ps26
Mecopterida - ps27
Diptera - ps28
Bibiono./Cyclorrhapha - ps29
Cyclorrhapha - ps30
Calyptratae/Drosophila - ps31
Drosophila - ps32
Sophophora - ps33
Obscura/Melanogaster_group - ps34
D_melanogaster_group - ps35
D_melanogaster_subgroup - ps36
sim./sech./mel. group - ps37
Drosophila_melanogaster - ps38
Phylostratum

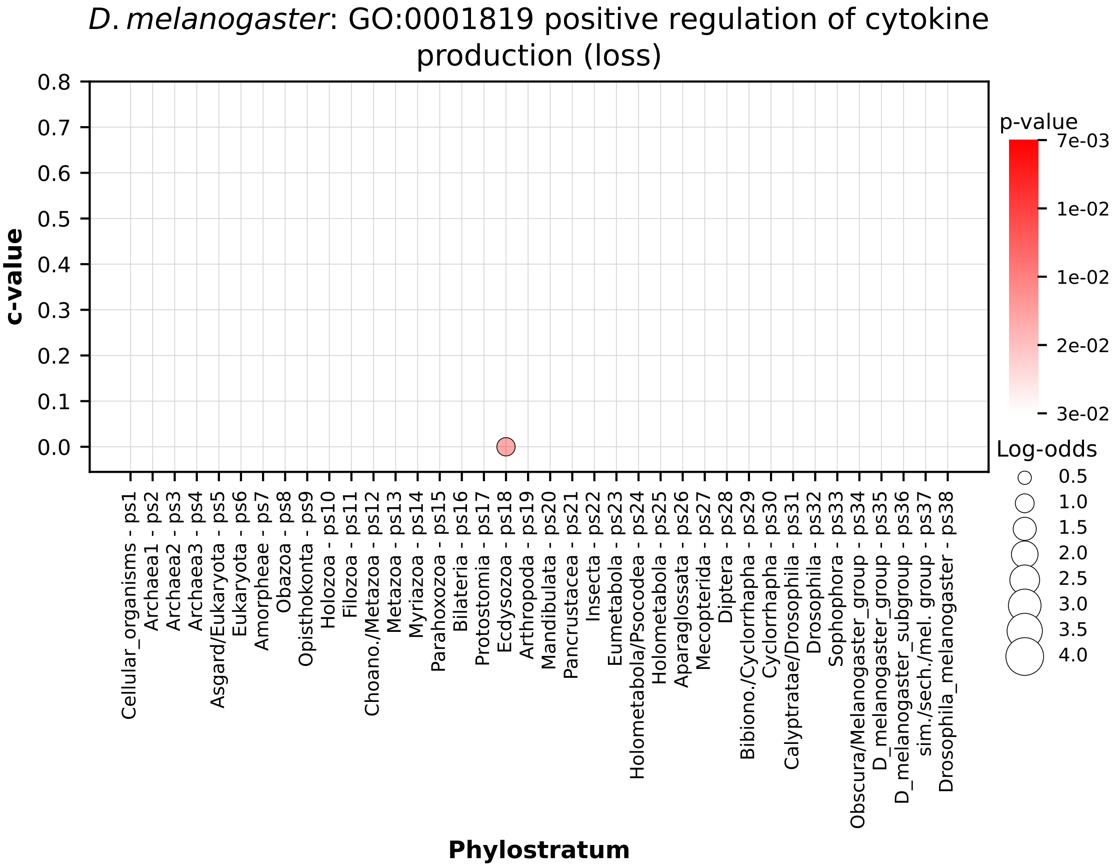

D. melanogaster: GO:0001819 positive regulation of cytokine production (loss)
c-value
0.8
0.7
0.6
0.5
0.4
0.3
0.2
0.1
0.0
p-value
7e-03
1e-02
1e-02
2e-02
3e-02
Log-odds
0.5
1.0
1.5
2.0
2.5
3.0
3.5
4.0
Cellular_organisms - ps1
Archaea1 - ps2
Archaea2 - ps3
Archaea3 - ps4
Asgard/Eukaryota - ps5
Eukaryota - ps6
Amorpheae - ps7
Obazoa - ps8
Opisthokonta - ps9
Holozoa - ps10
Filozoa - ps11
Choano./Metazoa - ps12
Metazoa - ps13
Myriazoa - ps14
Parahoxozoa - ps15
Bilateria - ps16
Protostomia - ps17
Ecdysozoa - ps18
Arthropoda - ps19
Mandibulata - ps20
Pancrustacea - ps21
Insecta - ps22
Eumetabola - ps23
Holometabola/Psocodea - ps24
Holometabola - ps25
Aparaglossata - ps26
Mecopterida - ps27
Diptera - ps28
Bibiono./Cyclorrhapha - ps29
Cyclorrhapha - ps30
Calyptratae/Drosophila - ps31
Drosophila - ps32
Sophophora - ps33
Obscura/Melanogaster_group - ps34
D_melanogaster_group - ps35
D_melanogaster_subgroup - ps36
sim./sech./mel. group - ps37
Drosophila_melanogaster - ps38
Phylostratum

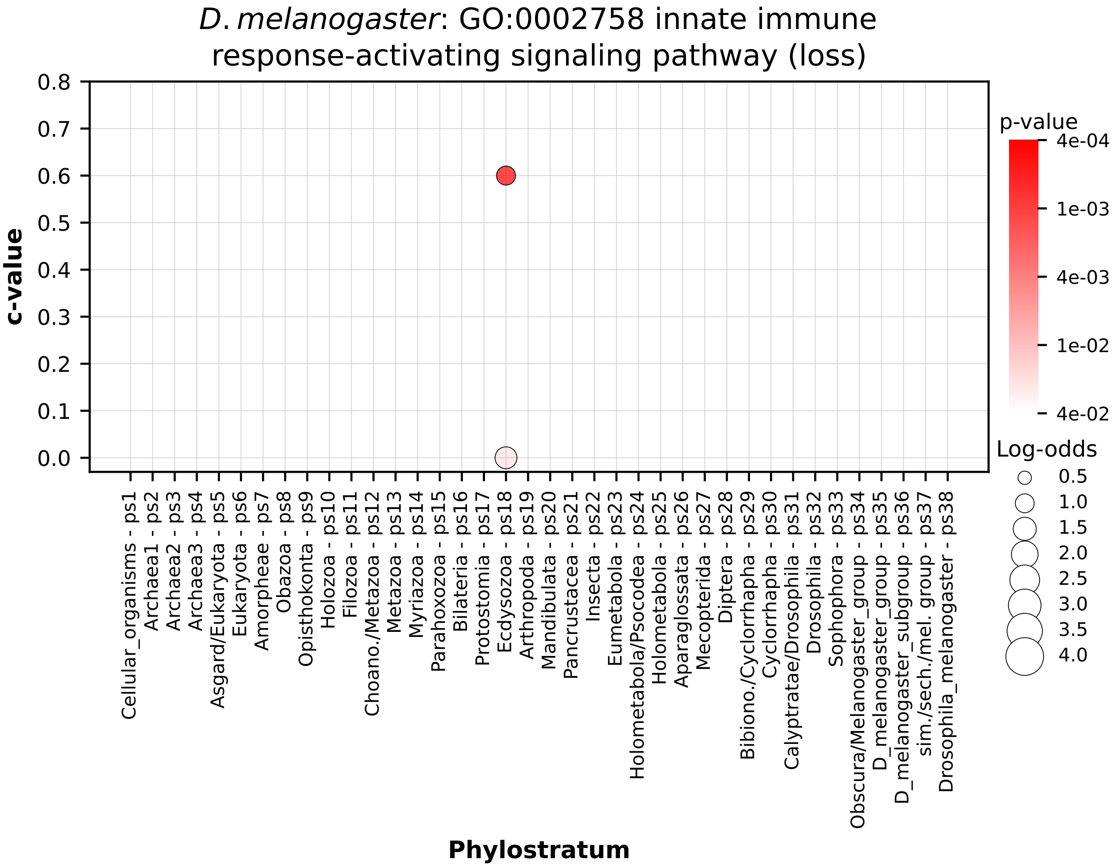

D. melanogaster: GO:0002758 innate immune response-activating signaling pathway (loss)
c-value
0.8
0.7
0.6
0.5
0.4
0.3
0.2
0.1
0.0
Phylostratum
Cellular_organisms - ps1
Archaea1 - ps2
Archaea2 - ps3
Archaea3 - ps4
Asgard/Eukaryota - ps5
Eukaryota - ps6
Amorpheae - ps7
Obazoa - ps8
Opisthokonta - ps9
Holozoa - ps10
Filozoa - ps11
Choano./Metazoa - ps12
Metazoa - ps13
Myriazoa - ps14
Parahoxozoa - ps15
Bilateria - ps16
Protostomia - ps17
Ecdysozoa - ps18
Arthropoda - ps19
Mandibulata - ps20
Pancrustacea - ps21
Insecta - ps22
Eumetabola - ps23
Holometabola/Psocodea - ps24
Holometabola - ps25
Aparaglossata - ps26
Mecopterida - ps27
Diptera - ps28
Bibiono./Cyclorrhapha - ps29
Cyclorrhapha - ps30
Calyptratae/Drosophila - ps31
Drosophila - ps32
Sophophora - ps33
Obscura/Melanogaster_group - ps34
D_melanogaster_group - ps35
D_melanogaster_subgroup - ps36
sim./sech./mel. group - ps37
Drosophila_melanogaster - ps38
p-value
4e-04
1e-03
4e-03
1e-02
4e-02
Log-odds
0.5
1.0
1.5
2.0
2.5
3.0
3.5
4.0

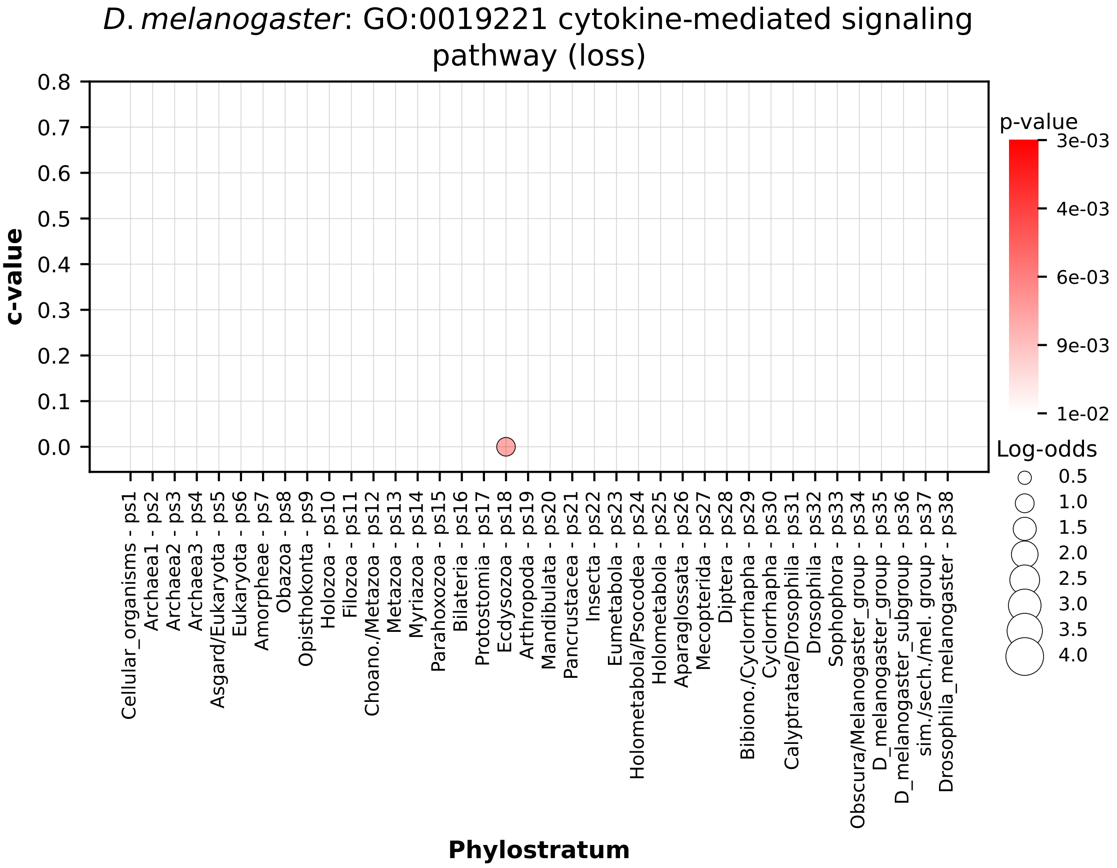

D. melanogaster: GO:0019221 cytokine-mediated signaling pathway (loss)
c-value
0.8
0.7
0.6
0.5
0.4
0.3
0.2
0.1
0.0
Phylostratum
Cellular_organisms - ps1
Archaea1 - ps2
Archaea2 - ps3
Archaea3 - ps4
Asgard/Eukaryota - ps5
Eukaryota - ps6
Amorpheae - ps7
Obazoa - ps8
Opisthokonta - ps9
Holozoa - ps10
Filozoa - ps11
Choano./Metazoa - ps12
Metazoa - ps13
Myriazoa - ps14
Parahoxozoa - ps15
Bilateria - ps16
Protostomia - ps17
Ecdysozoa - ps18
Arthropoda - ps19
Mandibulata - ps20
Pancrustacea - ps21
Insecta - ps22
Eumetabola - ps23
Holometabola/Psocodea - ps24
Holometabola - ps25
Aparaglossata - ps26
Mecopterida - ps27
Diptera - ps28
Bibiono./Cyclorrhapha - ps29
Cyclorrhapha - ps30
Calyptratae/Drosophila - ps31
Drosophila - ps32
Sophophora - ps33
Obscura/Melanogaster_group - ps34
D_melanogaster_group - ps35
D_melanogaster_subgroup - ps36
sim./sech./mel. group - ps37
Drosophila_melanogaster - ps38
p-value
3e-03
4e-03
6e-03
9e-03
1e-02
Log-odds
0.5
1.0
1.5
2.0
2.5
3.0
3.5
4.0

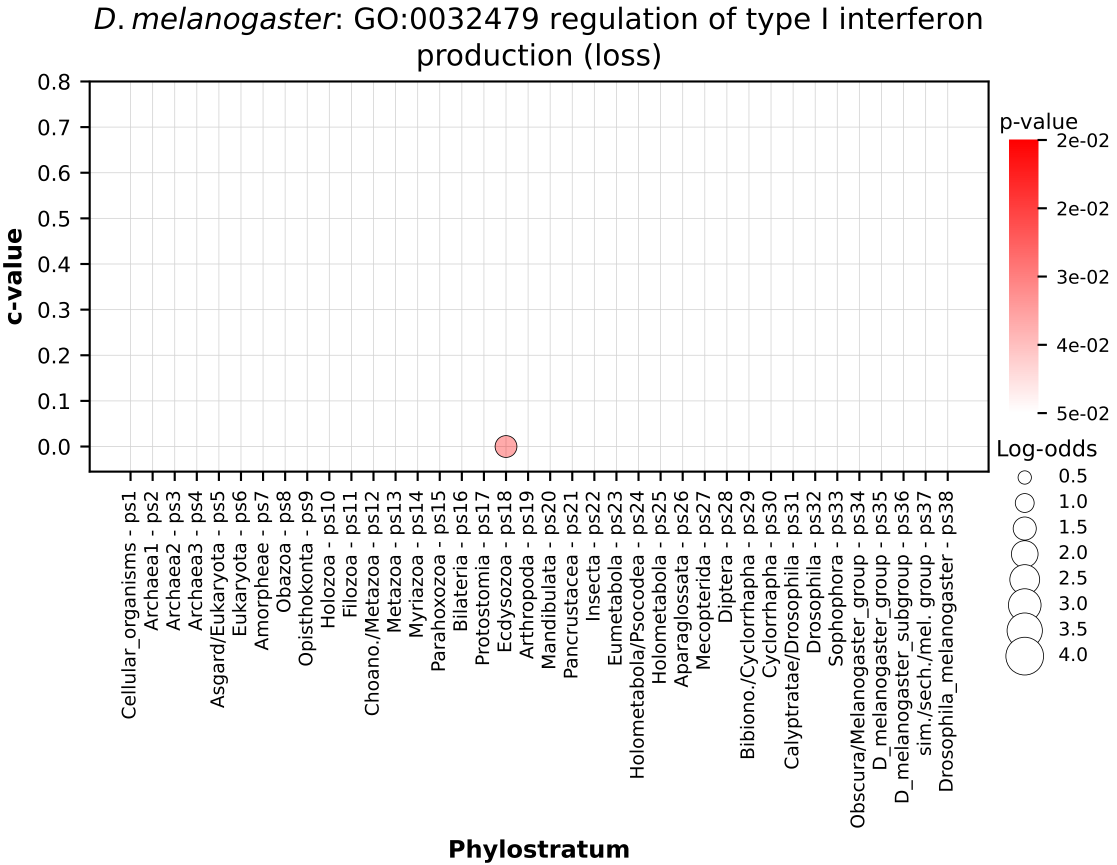

D. melanogaster: GO:0032479 regulation of type I interferon production (loss)
c-value
0.8
0.7
0.6
0.5
0.4
0.3
0.2
0.1
0.0
p-value
2e-02
2e-02
3e-02
4e-02
5e-02
Log-odds
0.5
1.0
1.5
2.0
2.5
3.0
3.5
4.0
Cellular_organisms - ps1
Archaea1 - ps2
Archaea2 - ps3
Archaea3 - ps4
Asgard/Eukaryota - ps5
Eukaryota - ps6
Amorpheae - ps7
Obazoa - ps8
Opisthokonta - ps9
Holozoa - ps10
Filozoa - ps11
Choano./Metazoa - ps12
Metazoa - ps13
Myriazoa - ps14
Parahoxozoa - ps15
Bilateria - ps16
Protostomia - ps17
Ecdysozoa - ps18
Arthropoda - ps19
Mandibulata - ps20
Pancrustacea - ps21
Insecta - ps22
Eumetabola - ps23
Holometabola/Psocodea - ps24
Holometabola - ps25
Aparaglossata - ps26
Mecopterida - ps27
Diptera - ps28
Bibiono./Cyclorrhapha - ps29
Cyclorrhapha - ps30
Calyptratae/Drosophila - ps31
Drosophila - ps32
Sophophora - ps33
Obscura/Melanogaster_group - ps34
D_melanogaster_group - ps35
D_melanogaster_subgroup - ps36
sim./sech./mel. group - ps37
Drosophila_melanogaster - ps38
Phylostratum

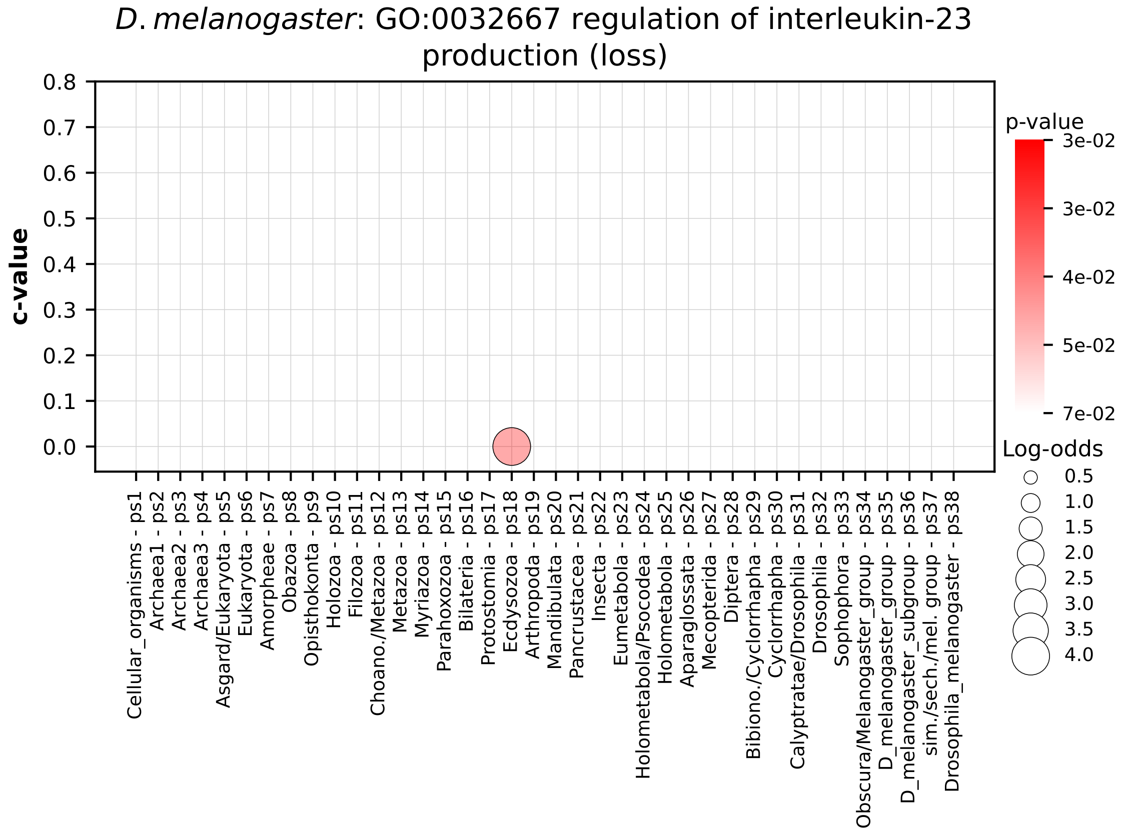

D. melanogaster: GO:0032667 regulation of interleukin-23 production (loss)
c-value
0.8
0.7
0.6
0.5
0.4
0.3
0.2
0.1
0.0
Phylostratum
Cellular_organisms - ps1
Archaea1 - ps2
Archaea2 - ps3
Archaea3 - ps4
Asgard/Eukaryota - ps5
Eukaryota - ps6
Amorpheae - ps7
Obazoa - ps8
Opisthokonta - ps9
Holozoa - ps10
Filozoa - ps11
Choano./Metazoa - ps12
Metazoa - ps13
Myriazoa - ps14
Parahoxozoa - ps15
Bilateria - ps16
Protostomia - ps17
Ecdysozoa - ps18
Arthropoda - ps19
Mandibulata - ps20
Pancrustacea - ps21
Insecta - ps22
Eumetabola - ps23
Holometabola/Psocodea - ps24
Holometabola - ps25
Aparaglossata - ps26
Mecopterida - ps27
Diptera - ps28
Bibiono./Cyclorrhapha - ps29
Cyclorrhapha - ps30
Calyptratae/Drosophila - ps31
Drosophila - ps32
Sophophora - ps33
Obscura/Melanogaster_group - ps34
D_melanogaster_group - ps35
D_melanogaster_subgroup - ps36
sim./sech./mel. group - ps37
Drosophila_melanogaster - ps38
p-value
3e-02
3e-02
4e-02
5e-02
7e-02
Log-odds
0.5
1.0
1.5
2.0
2.5
3.0
3.5
4.0

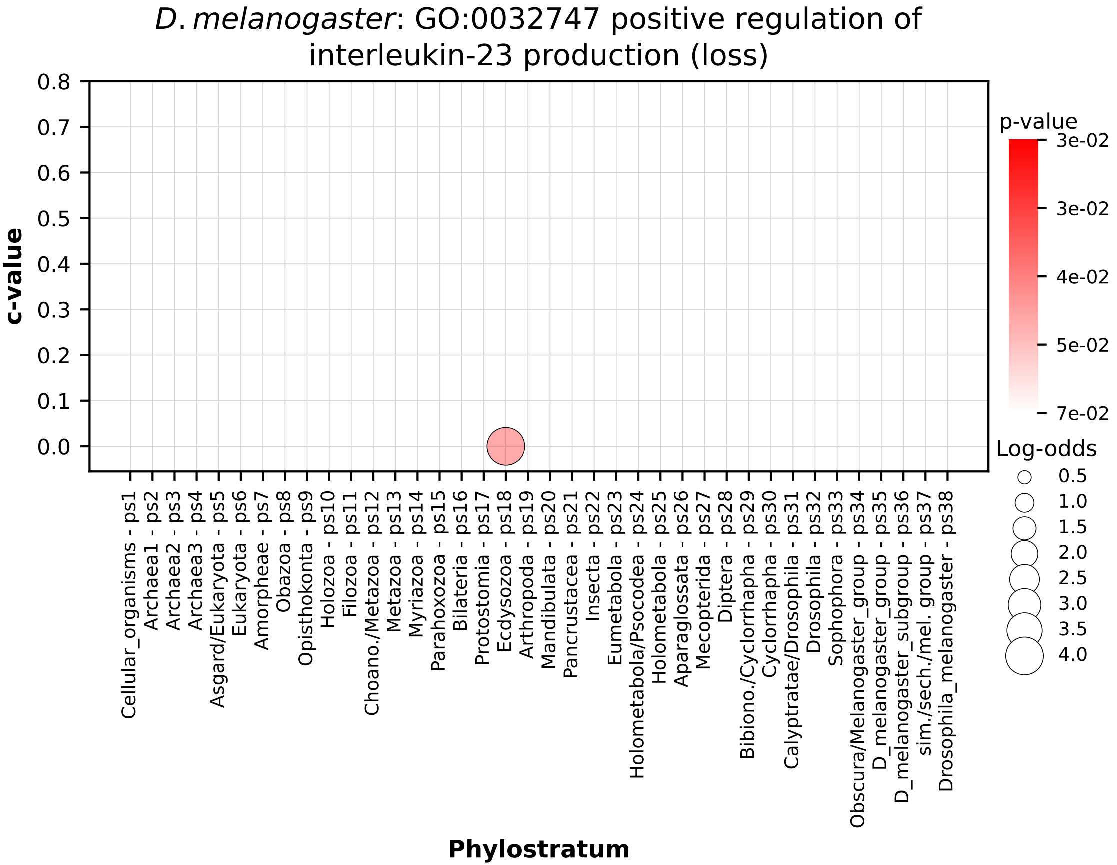

D. melanogaster: GO:0032747 positive regulation of interleukin-23 production (loss)
c-value
0.8
0.7
0.6
0.5
0.4
0.3
0.2
0.1
0.0
Phylostratum
Cellular_organisms - ps1
Archaea1 - ps2
Archaea2 - ps3
Archaea3 - ps4
Asgard/Eukaryota - ps5
Eukaryota - ps6
Amorpheae - ps7
Obazoa - ps8
Opisthokonta - ps9
Holozoa - ps10
Filozoa - ps11
Choano./Metazoa - ps12
Metazoa - ps13
Myriazoa - ps14
Parahoxozoa - ps15
Bilateria - ps16
Protostomia - ps17
Ecdysozoa - ps18
Arthropoda - ps19
Mandibulata - ps20
Pancrustacea - ps21
Insecta - ps22
Eumetabola - ps23
Holometabola/Psocodea - ps24
Holometabola - ps25
Aparaglossata - ps26
Mecopterida - ps27
Diptera - ps28
Bibiono./Cyclorrhapha - ps29
Cyclorrhapha - ps30
Calyptratae/Drosophila - ps31
Drosophila - ps32
Sophophora - ps33
Obscura/Melanogaster_group - ps34
D_melanogaster_group - ps35
D_melanogaster_subgroup - ps36
sim./sech./mel. group - ps37
Drosophila_melanogaster - ps38
p-value
3e-02
3e-02
4e-02
5e-02
7e-02
Log-odds
0.5
1.0
1.5
2.0
2.5
3.0
3.5
4.0

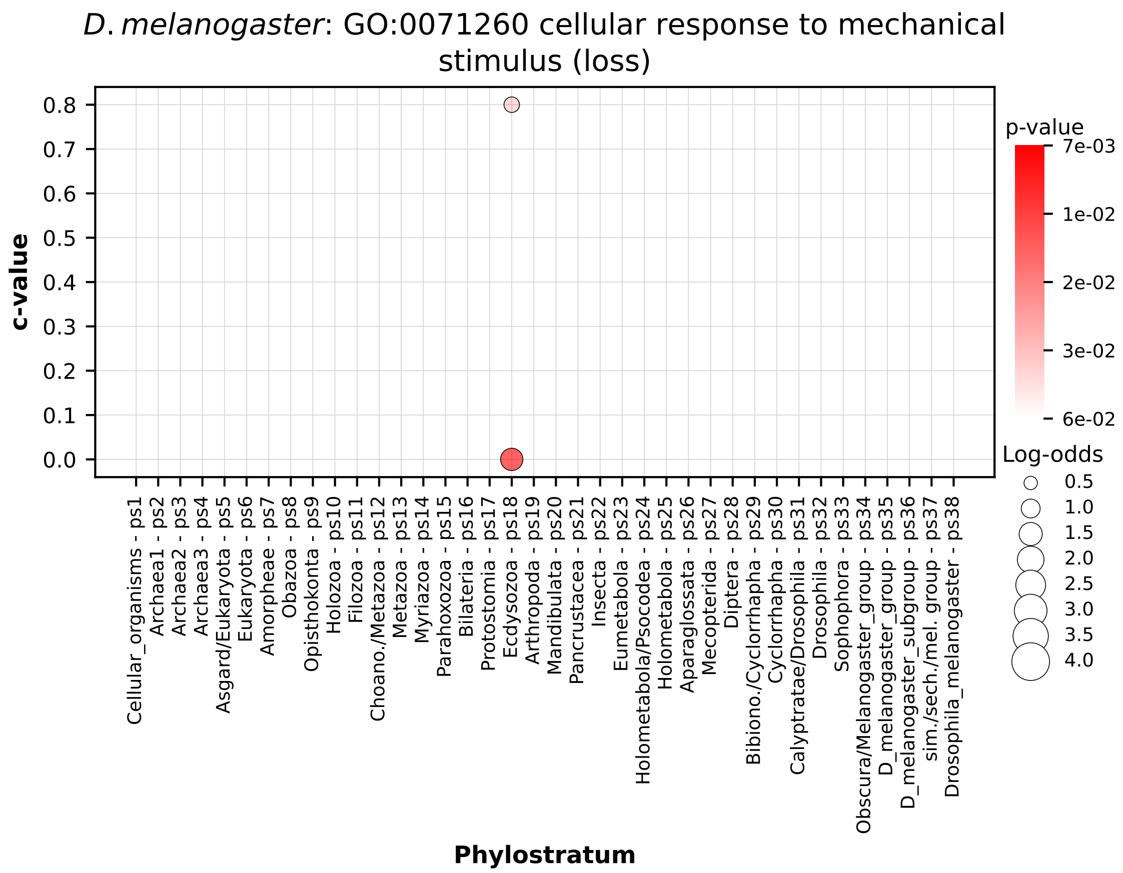

D. melanogaster: GO:0071260 cellular response to mechanical stimulus (loss)
c-value
0.0
0.1
0.2
0.3
0.4
0.5
0.6
0.7
0.8
Phylostratum
Cellular_organisms - ps1
Archaea1 - ps2
Archaea2 - ps3
Archaea3 - ps4
Asgard/Eukaryota - ps5
Eukaryota - ps6
Amorpheae - ps7
Obazoa - ps8
Opisthokonta - ps9
Holozoa - ps10
Filozoa - ps11
Choano./Metazoa - ps12
Metazoa - ps13
Myriazoa - ps14
Parahoxozoa - ps15
Bilateria - ps16
Protostomia - ps17
Ecdysozoa - ps18
Arthropoda - ps19
Mandibulata - ps20
Pancrustacea - ps21
Insecta - ps22
Eumetabola - ps23
Holometabola/Psocodea - ps24
Holometabola - ps25
Aparaglossata - ps26
Mecopterida - ps27
Diptera - ps28
Bibiono./Cyclorrhapha - ps29
Cyclorrhapha - ps30
Calyptratae/Drosophila - ps31
Drosophila - ps32
Sophophora - ps33
Obscura/Melanogaster_group - ps34
D_melanogaster_group - ps35
D_melanogaster_subgroup - ps36
sim./sech./mel. group - ps37
Drosophila_melanogaster - ps38
p-value
7e-03
1e-02
2e-02
3e-02
6e-02
Log-odds
0.5
1.0
1.5
2.0
2.5
3.0
3.5
4.0

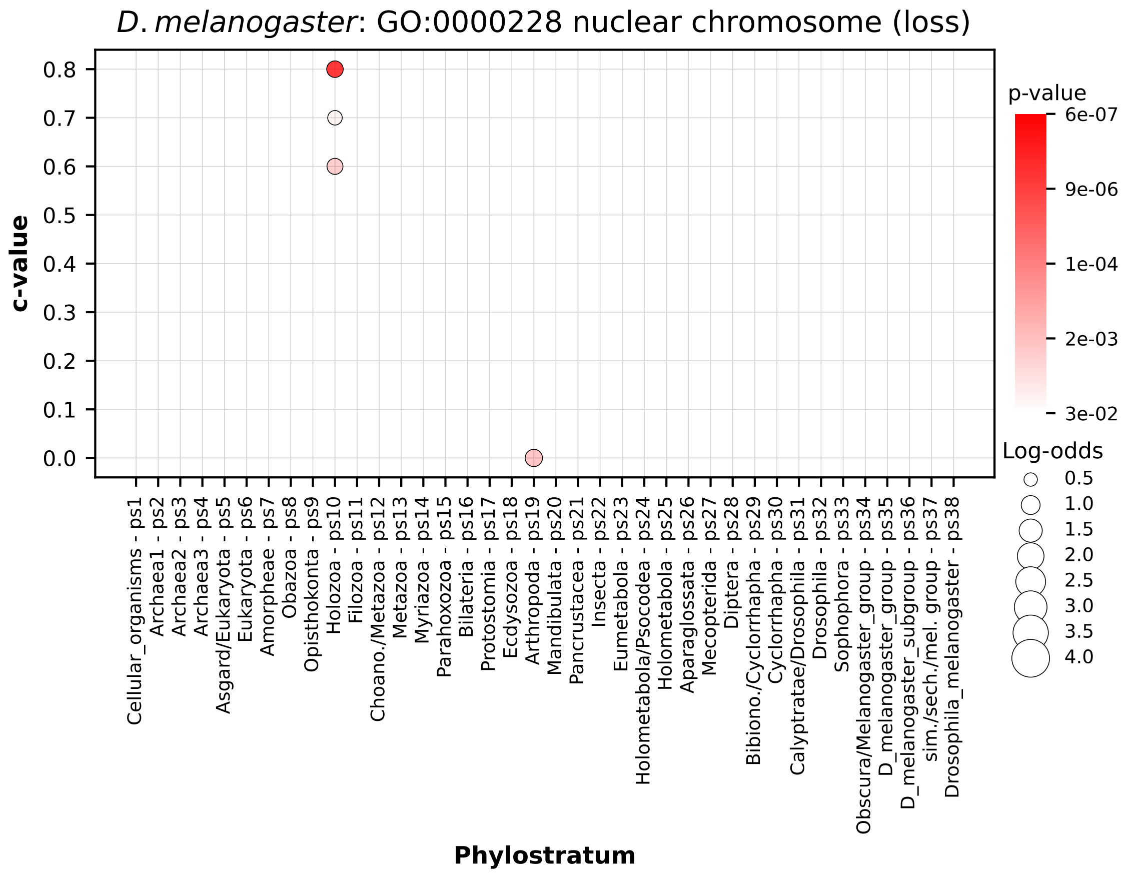

D. melanogaster: GO:0000228 nuclear chromosome (loss)
c-value
0.8
0.7
0.6
0.5
0.4
0.3
0.2
0.1
0.0
p-value
6e-07
9e-06
1e-04
2e-03
3e-02
Log-odds
0.5
1.0
1.5
2.0
2.5
3.0
3.5
4.0
Cellular_organisms - ps1
Archaea1 - ps2
Archaea2 - ps3
Archaea3 - ps4
Asgard/Eukaryota - ps5
Eukaryota - ps6
Amorpheae - ps7
Obazoa - ps8
Opisthokonta - ps9
Holozoa - ps10
Filozoa - ps11
Choano./Metazoa - ps12
Metazoa - ps13
Myriazoa - ps14
Parahoxozoa - ps15
Bilateria - ps16
Protostomia - ps17
Ecdysozoa - ps18
Arthropoda - ps19
Mandibulata - ps20
Pancrustacea - ps21
Insecta - ps22
Eumetabola - ps23
Holometabola/Psocodea - ps24
Holometabola - ps25
Aparaglossata - ps26
Mecopterida - ps27
Diptera - ps28
Bibiono./Cyclorrhapha - ps29
Cyclorrhapha - ps30
Calyptratae/Drosophila - ps31
Drosophila - ps32
Sophophora - ps33
Obscura/Melanogaster_group - ps34
D_melanogaster_group - ps35
D_melanogaster_subgroup - ps36
sim./sech./mel. group - ps37
Drosophila_melanogaster - ps38
Phylostratum

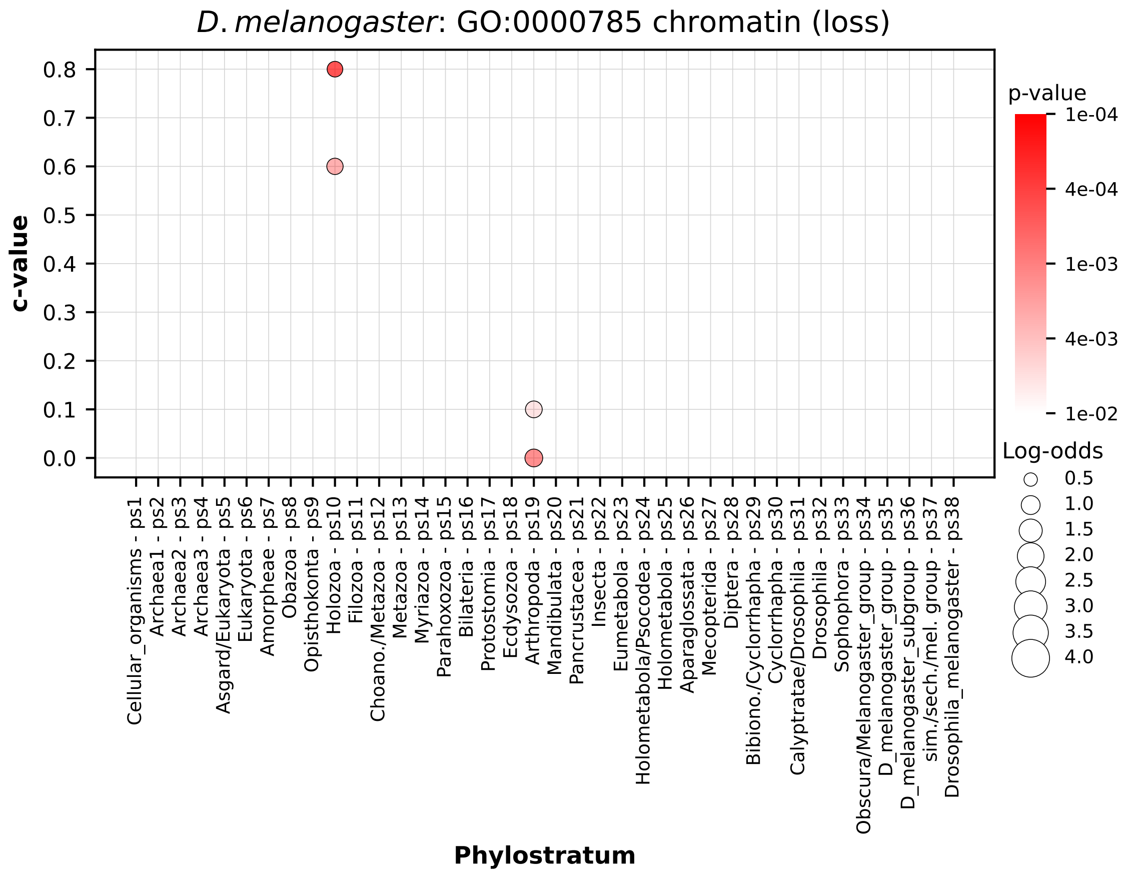
D. melanogaster: GO:0000785 chromatin (loss)
c-value
0.8
0.7
0.6
0.5
0.4
0.3
0.2
0.1
0.0
Phylostratum
Cellular_organisms - ps1
Archaea1 - ps2
Archaea2 - ps3
Archaea3 - ps4
Asgard/Eukaryota - ps5
Eukaryota - ps6
Amorpheae - ps7
Obazoa - ps8
Opisthokonta - ps9
Holozoa - ps10
Filozoa - ps11
Choano./Metazoa - ps12
Metazoa - ps13
Myriazoa - ps14
Parahoxozoa - ps15
Bilateria - ps16
Protostomia - ps17
Ecdysozoa - ps18
Arthropoda - ps19
Mandibulata - ps20
Pancrustacea - ps21
Insecta - ps22
Eumetabola - ps23
Holometabola/Psocodea - ps24
Holometabola - ps25
Aparaglossata - ps26
Mecopterida - ps27
Diptera - ps28
Bibiono./Cyclorrhapha - ps29
Cyclorrhapha - ps30
Calyptratae/Drosophila - ps31
Drosophila - ps32
Sophophora - ps33
Obscura/Melanogaster_group - ps34
D_melanogaster_group - ps35
D_melanogaster_subgroup - ps36
sim./sech./mel. group - ps37
Drosophila_melanogaster - ps38
p-value
1e-04
4e-04
1e-03
4e-03
1e-02
Log-odds
0.5
1.0
1.5
2.0
2.5
3.0
3.5
4.0

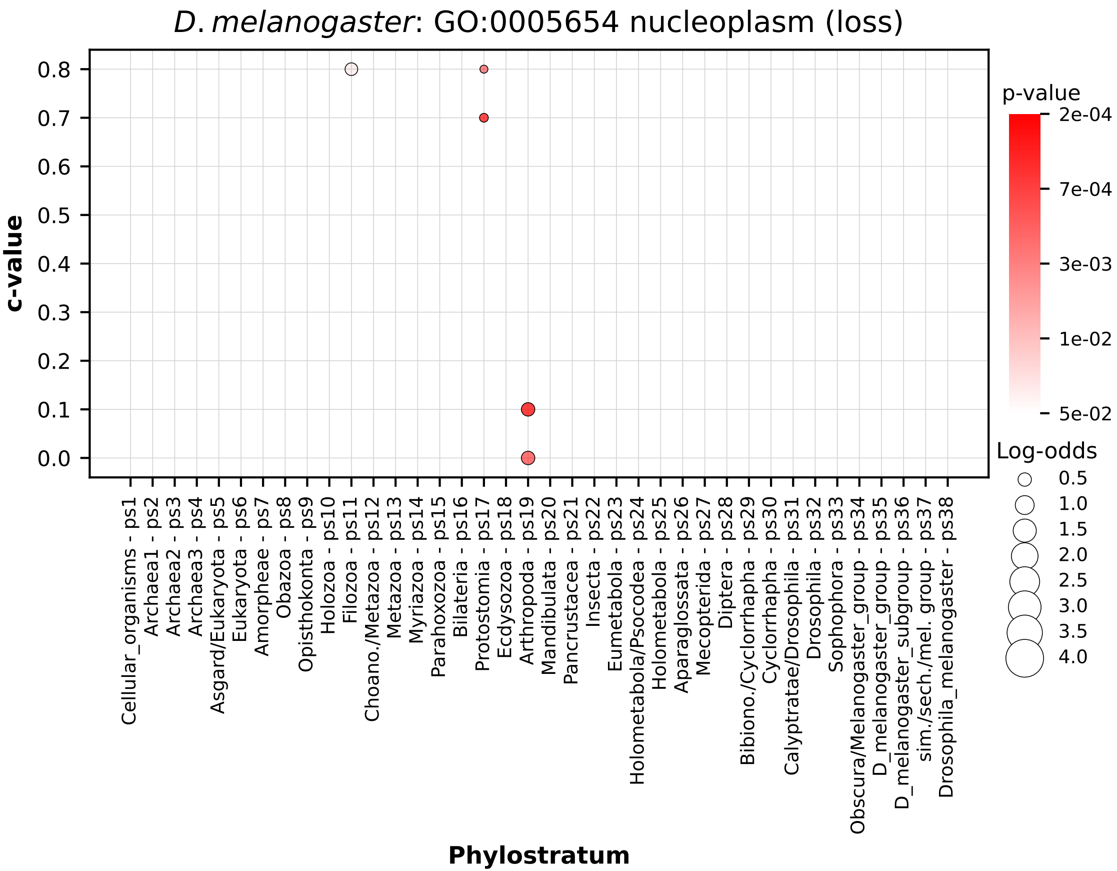

D. melanogaster: GO:0005654 nucleoplasm (loss)
c-value
0.8
0.7
0.6
0.5
0.4
0.3
0.2
0.1
0.0
p-value
2e-04
7e-04
3e-03
1e-02
5e-02
Log-odds
0.5
1.0
1.5
2.0
2.5
3.0
3.5
4.0
Cellular_organisms - ps1
Archaea1 - ps2
Archaea2 - ps3
Archaea3 - ps4
Asgard/Eukaryota - ps5
Eukaryota - ps6
Amorpheae - ps7
Obazoa - ps8
Opisthokonta - ps9
Holozoa - ps10
Filozoa - ps11
Choano./Metazoa - ps12
Metazoa - ps13
Myriazoa - ps14
Parahoxozoa - ps15
Bilateria - ps16
Protostomia - ps17
Ecdysozoa - ps18
Arthropoda - ps19
Mandibulata - ps20
Pancrustacea - ps21
Insecta - ps22
Eumetabola - ps23
Holometabola/Psocodea - ps24
Holometabola - ps25
Aparaglossata - ps26
Mecopterida - ps27
Diptera - ps28
Bibiono./Cyclorrhapha - ps29
Cyclorrhapha - ps30
Calyptratae/Drosophila - ps31
Drosophila - ps32
Sophophora - ps33
Obscura/Melanogaster_group - ps34
D_melanogaster_group - ps35
D_melanogaster_subgroup - ps36
sim./sech./mel. group - ps37
Drosophila_melanogaster - ps38
Phylostratum

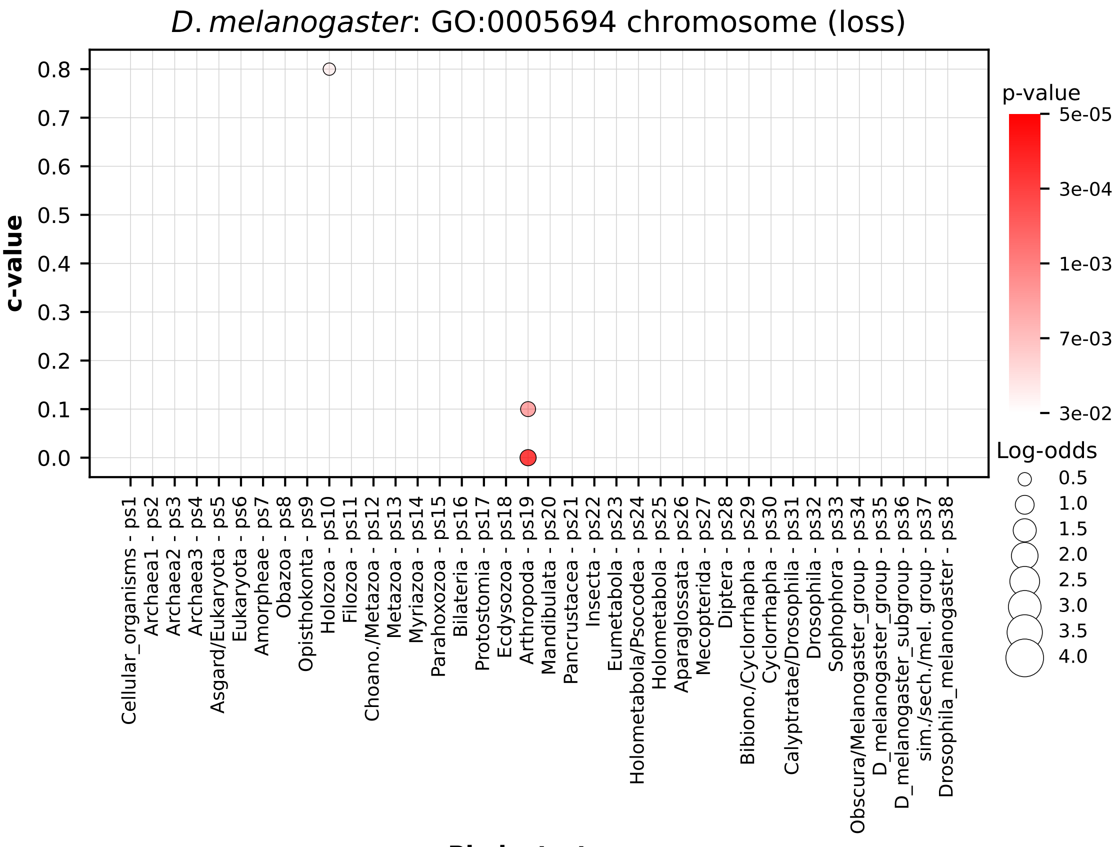

D. melanogaster: GO:0005694 chromosome (loss)
c-value
0.8
0.7
0.6
0.5
0.4
0.3
0.2
0.1
0.0
Phylostratum
Cellular_organisms - ps1
Archaea1 - ps2
Archaea2 - ps3
Archaea3 - ps4
Asgard/Eukaryota - ps5
Eukaryota - ps6
Amorpheae - ps7
Obazoa - ps8
Opisthokonta - ps9
Holozoa - ps10
Filozoa - ps11
Choano./Metazoa - ps12
Metazoa - ps13
Myriazoa - ps14
Parahoxozoa - ps15
Bilateria - ps16
Protostomia - ps17
Ecdysozoa - ps18
Arthropoda - ps19
Mandibulata - ps20
Pancrustacea - ps21
Insecta - ps22
Eumetabola - ps23
Holometabola/Psocodea - ps24
Holometabola - ps25
Aparaglossata - ps26
Mecopterida - ps27
Diptera - ps28
Bibiono./Cyclorrhapha - ps29
Cyclorrhapha - ps30
Calyptratae/Drosophila - ps31
Drosophila - ps32
Sophophora - ps33
Obscura/Melanogaster_group - ps34
D_melanogaster_group - ps35
D_melanogaster_subgroup - ps36
sim./sech./mel. group - ps37
Drosophila_melanogaster - ps38
p-value
5e-05
3e-04
1e-03
7e-03
3e-02
Log-odds
0.5
1.0
1.5
2.0
2.5
3.0
3.5
4.0

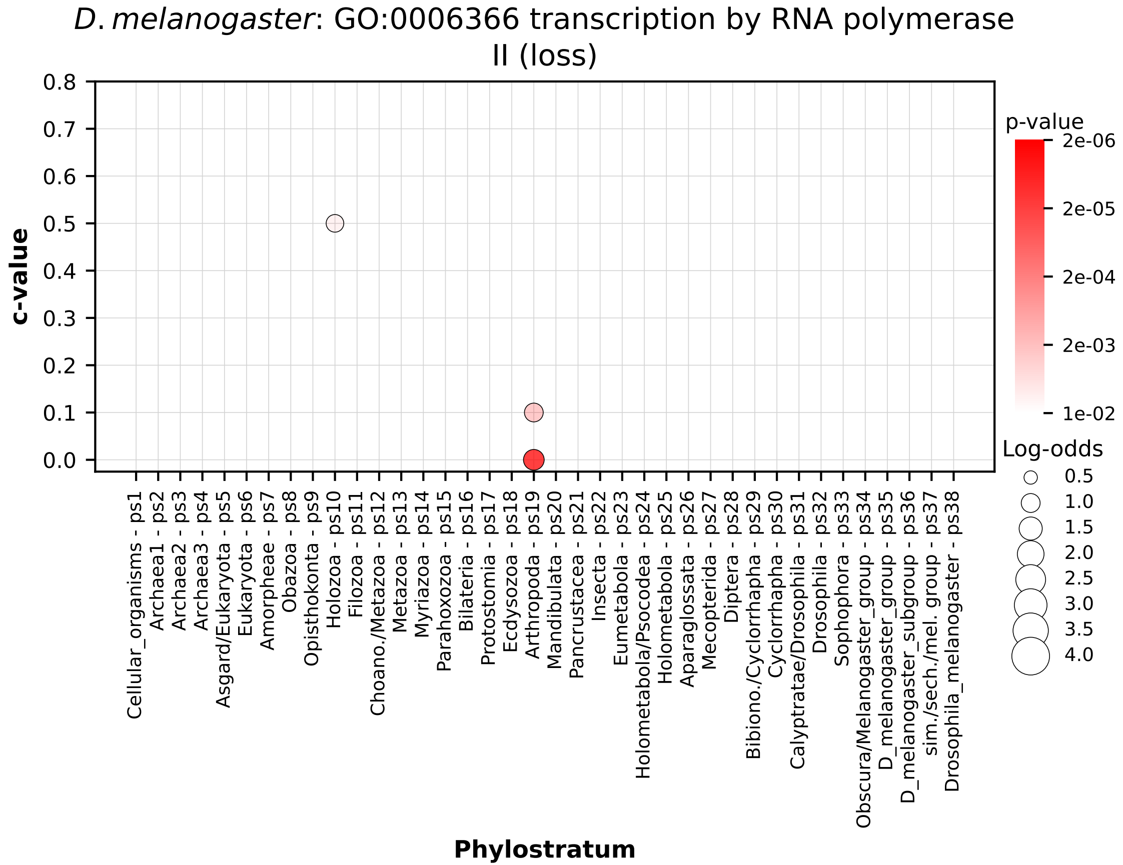

D. melanogaster: GO:0006366 transcription by RNA polymerase II (loss)
c-value
0.8
0.7
0.6
0.5
0.4
0.3
0.2
0.1
0.0
Phylostratum
Cellular_organisms - ps1
Archaea1 - ps2
Archaea2 - ps3
Archaea3 - ps4
Asgard/Eukaryota - ps5
Eukaryota - ps6
Amorpheae - ps7
Obazoa - ps8
Opisthokonta - ps9
Holozoa - ps10
Filozoa - ps11
Choano./Metazoa - ps12
Metazoa - ps13
Myriazoa - ps14
Parahoxozoa - ps15
Bilateria - ps16
Protostomia - ps17
Ecdysozoa - ps18
Arthropoda - ps19
Mandibulata - ps20
Pancrustacea - ps21
Insecta - ps22
Eumetabola - ps23
Holometabola/Psocodea - ps24
Holometabola - ps25
Aparaglossata - ps26
Mecopterida - ps27
Diptera - ps28
Bibiono./Cyclorrhapha - ps29
Cyclorrhapha - ps30
Calyptratae/Drosophila - ps31
Drosophila - ps32
Sophophora - ps33
Obscura/Melanogaster_group - ps34
D_melanogaster_group - ps35
D_melanogaster_subgroup - ps36
sim./sech./mel. group - ps37
Drosophila_melanogaster - ps38
p-value
2e-06
2e-05
2e-04
2e-03
1e-02
Log-odds
0.5
1.0
1.5
2.0
2.5
3.0
3.5
4.0

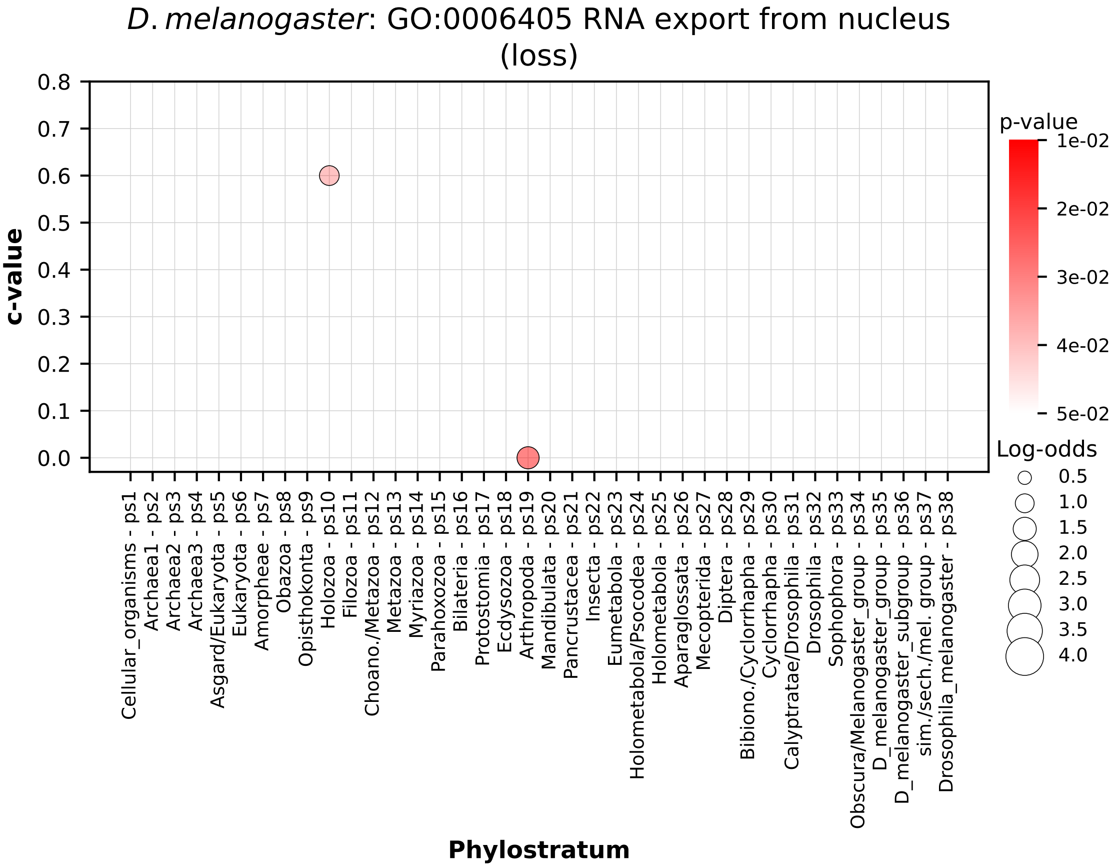
D. melanogaster: GO:0006405 RNA export from nucleus
(loss)
c-value
Phylostratum
p-value
1e-02
2e-02
3e-02
4e-02
5e-02
Log-odds
0.5
1.0
1.5
2.0
2.5
3.0
3.5
4.0
0.0
0.1
0.2
0.3
0.4
0.5
0.6
0.7
0.8
Cellular_organisms - ps1
Archaea1 - ps2
Archaea2 - ps3
Archaea3 - ps4
Asgard/Eukaryota - ps5
Eukaryota - ps6
Amorpheae - ps7
Obazoa - ps8
Opisthokonta - ps9
Holozoa - ps10
Filozoa - ps11
Choano./Metazoa - ps12
Metazoa - ps13
Myriazoa - ps14
Parahoxozoa - ps15
Bilateria - ps16
Protostomia - ps17
Ecdysozoa - ps18
Arthropoda - ps19
Mandibulata - ps20
Pancrustacea - ps21
Insecta - ps22
Eumetabola - ps23
Holometabola/Psocodea - ps24
Holometabola - ps25
Aparaglossata - ps26
Mecopterida - ps27
Diptera - ps28
Bibiono./Cyclorrhapha - ps29
Cyclorrhapha - ps30
Calyptratae/Drosophila - ps31
Drosophila - ps32
Sophophora - ps33
Obscura/Melanogaster_group - ps34
D_melanogaster_group - ps35
D_melanogaster_subgroup - ps36
sim./sech./mel. group - ps37
Drosophila_melanogaster - ps38

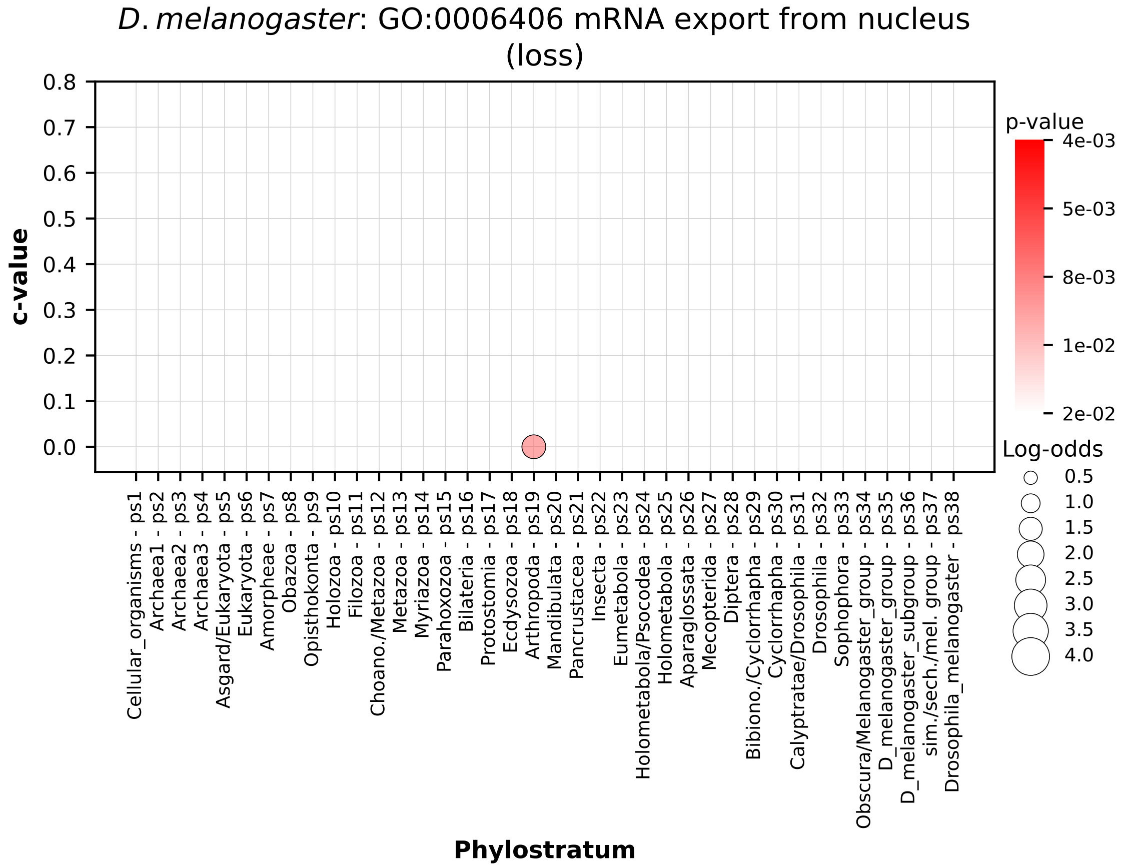

D. melanogaster: GO:0006406 mRNA export from nucleus (loss)
c-value
0.8
0.7
0.6
0.5
0.4
0.3
0.2
0.1
0.0
p-value
4e-03
5e-03
8e-03
1e-02
2e-02
Log-odds
0.5
1.0
1.5
2.0
2.5
3.0
3.5
4.0
Cellular_organisms - ps1
Archaea1 - ps2
Archaea2 - ps3
Archaea3 - ps4
Asgard/Eukaryota - ps5
Eukaryota - ps6
Amorpheae - ps7
Obazoa - ps8
Opisthokonta - ps9
Holozoa - ps10
Filozoa - ps11
Choano./Metazoa - ps12
Metazoa - ps13
Myriazoa - ps14
Parahoxozoa - ps15
Bilateria - ps16
Protostomia - ps17
Ecdysozoa - ps18
Arthropoda - ps19
Mandibulata - ps20
Pancrustacea - ps21
Insecta - ps22
Eumetabola - ps23
Holometabola/Psocodea - ps24
Holometabola - ps25
Aparaglossata - ps26
Mecopterida - ps27
Diptera - ps28
Bibiono./Cyclorrhapha - ps29
Cyclorrhapha - ps30
Calyptratae/Drosophila - ps31
Drosophila - ps32
Sophophora - ps33
Obscura/Melanogaster_group - ps34
D_melanogaster_group - ps35
D_melanogaster_subgroup - ps36
sim./sech./mel. group - ps37
Drosophila_melanogaster - ps38
Phylostratum

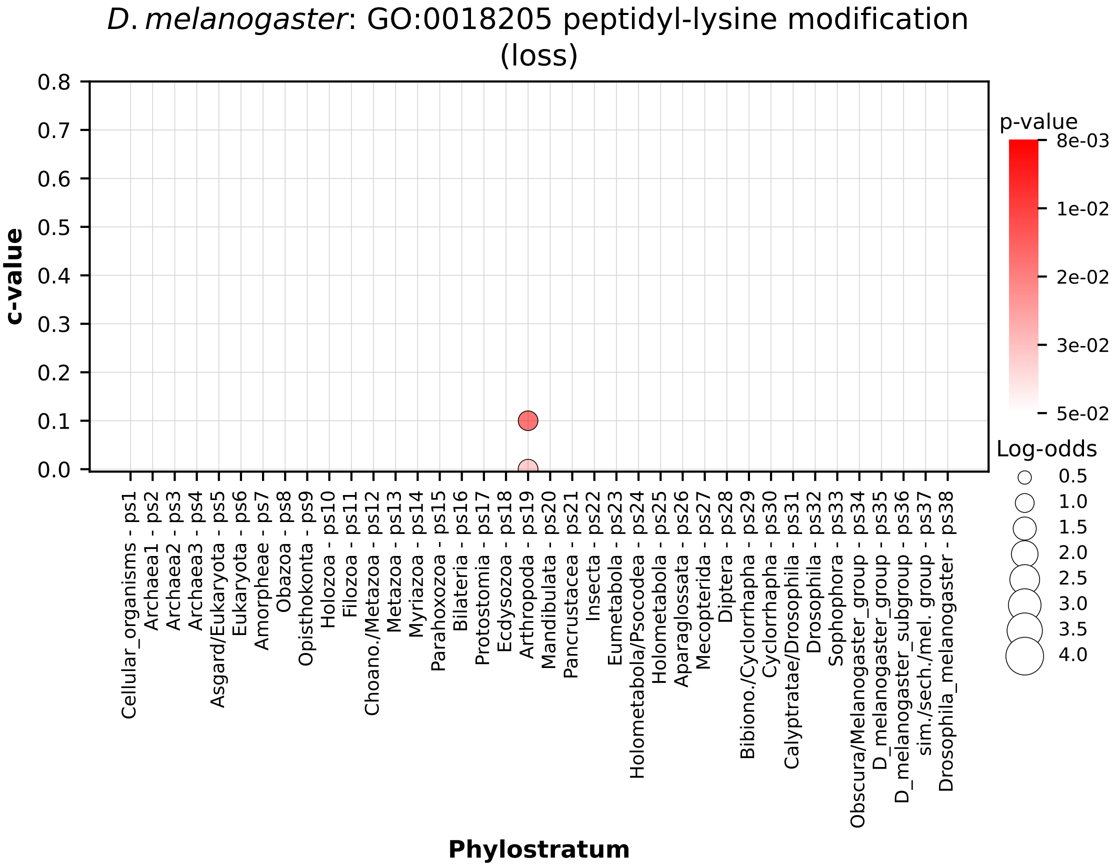

D. melanogaster: GO:0018205 peptidyl-lysine modification (loss)
c-value
0.8
0.7
0.6
0.5
0.4
0.3
0.2
0.1
0.0
Phylostratum
Cellular_organisms - ps1
Archaea1 - ps2
Archaea2 - ps3
Archaea3 - ps4
Asgard/Eukaryota - ps5
Eukaryota - ps6
Amorpheae - ps7
Obazoa - ps8
Opisthokonta - ps9
Holozoa - ps10
Filozoa - ps11
Choano./Metazoa - ps12
Metazoa - ps13
Myriazoa - ps14
Parahoxozoa - ps15
Bilateria - ps16
Protostomia - ps17
Ecdysozoa - ps18
Arthropoda - ps19
Mandibulata - ps20
Pancrustacea - ps21
Insecta - ps22
Eumetabola - ps23
Holometabola/Psocodea - ps24
Holometabola - ps25
Aparaglossata - ps26
Mecopterida - ps27
Diptera - ps28
Bibiono./Cyclorrhapha - ps29
Cyclorrhapha - ps30
Calyptratae/Drosophila - ps31
Drosophila - ps32
Sophophora - ps33
Obscura/Melanogaster_group - ps34
D_melanogaster_group - ps35
D_melanogaster_subgroup - ps36
sim./sech./mel. group - ps37
Drosophila_melanogaster - ps38
p-value
8e-03
1e-02
2e-02
3e-02
5e-02
Log-odds
0.5
1.0
1.5
2.0
2.5
3.0
3.5
4.0

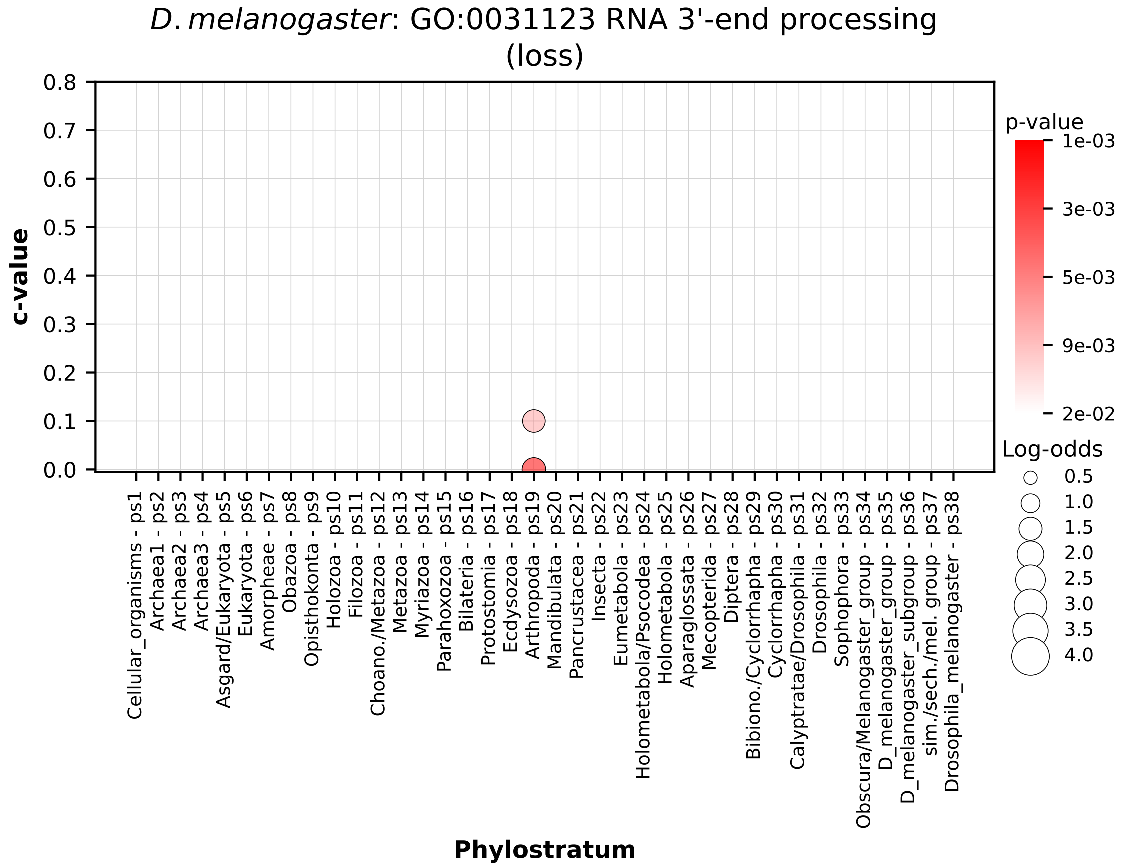

D. melanogaster: GO:0031123 RNA 3'-end processing
(loss)
c-value
0.8
0.7
0.6
0.5
0.4
0.3
0.2
0.1
0.0
p-value
1e-03
3e-03
5e-03
9e-03
2e-02
Log-odds
0.5
1.0
1.5
2.0
2.5
3.0
3.5
4.0
Cellular_organisms - ps1
Archaea1 - ps2
Archaea2 - ps3
Archaea3 - ps4
Asgard/Eukaryota - ps5
Eukaryota - ps6
Amorpheae - ps7
Obazoa - ps8
Opisthokonta - ps9
Holozoa - ps10
Filozoa - ps11
Choano./Metazoa - ps12
Metazoa - ps13
Myriazoa - ps14
Parahoxozoa - ps15
Bilateria - ps16
Protostomia - ps17
Ecdysozoa - ps18
Arthropoda - ps19
Mandibulata - ps20
Pancrustacea - ps21
Insecta - ps22
Eumetabola - ps23
Holometabola/Psocodea - ps24
Holometabola - ps25
Aparaglossata - ps26
Mecopterida - ps27
Diptera - ps28
Bibiono./Cyclorrhapha - ps29
Cyclorrhapha - ps30
Calyptratae/Drosophila - ps31
Drosophila - ps32
Sophophora - ps33
Obscura/Melanogaster_group - ps34
D_melanogaster_group - ps35
D_melanogaster_subgroup - ps36
sim./sech./mel. group - ps37
Drosophila_melanogaster - ps38
Phylostratum

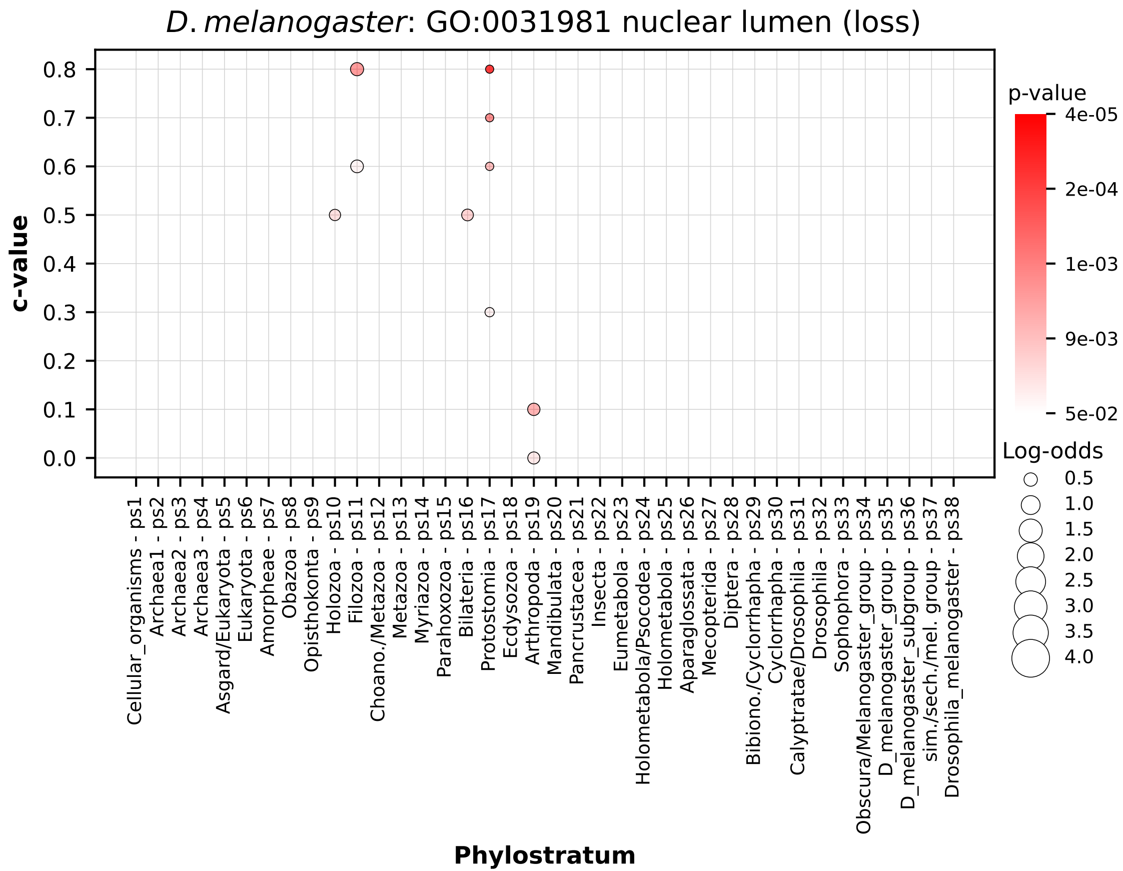

D. melanogaster: GO:0031981 nuclear lumen (loss)
c-value
0.8
0.7
0.6
0.5
0.4
0.3
0.2
0.1
0.0
p-value
4e-05
2e-04
1e-03
9e-03
5e-02
Log-odds
0.5
1.0
1.5
2.0
2.5
3.0
3.5
4.0
Cellular_organisms - ps1
Archaea1 - ps2
Archaea2 - ps3
Archaea3 - ps4
Asgard/Eukaryota - ps5
Eukaryota - ps6
Amorpheae - ps7
Obazoa - ps8
Opisthokonta - ps9
Holozoa - ps10
Filozoa - ps11
Choano./Metazoa - ps12
Metazoa - ps13
Myriazoa - ps14
Parahoxozoa - ps15
Bilateria - ps16
Protostomia - ps17
Ecdysozoa - ps18
Arthropoda - ps19
Mandibulata - ps20
Pancrustacea - ps21
Insecta - ps22
Eumetabola - ps23
Holometabola/Psocodea - ps24
Holometabola - ps25
Aparaglossata - ps26
Mecopterida - ps27
Diptera - ps28
Bibiono./Cyclorrhapha - ps29
Cyclorrhapha - ps30
Calyptratae/Drosophila - ps31
Drosophila - ps32
Sophophora - ps33
Obscura/Melanogaster_group - ps34
D_melanogaster_group - ps35
D_melanogaster_subgroup - ps36
sim./sech./mel. group - ps37
Drosophila_melanogaster - ps38
Phylostratum

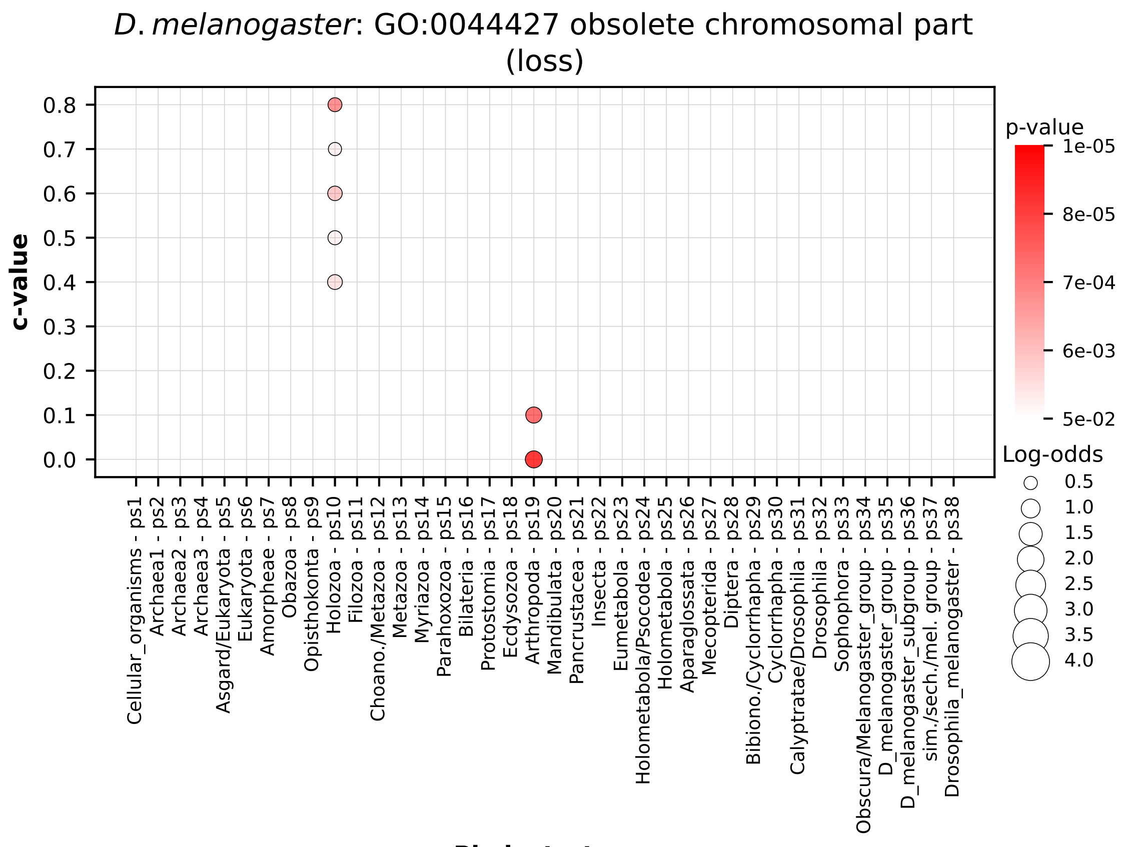

D. melanogaster: GO:0044427 obsolete chromosomal part
(loss)
c-value
0.8
0.7
0.6
0.5
0.4
0.3
0.2
0.1
0.0
Phylostratum
Cellular_organisms - ps1
Archaea1 - ps2
Archaea2 - ps3
Archaea3 - ps4
Asgard/Eukaryota - ps5
Eukaryota - ps6
Amorpheae - ps7
Obazoa - ps8
Opisthokonta - ps9
Holozoa - ps10
Filozoa - ps11
Choano./Metazoa - ps12
Metazoa - ps13
Myriazoa - ps14
Parahoxozoa - ps15
Bilateria - ps16
Protostomia - ps17
Ecdysozoa - ps18
Arthropoda - ps19
Mandibulata - ps20
Pancrustacea - ps21
Insecta - ps22
Eumetabola - ps23
Holometabola/Psocodea - ps24
Holometabola - ps25
Aparaglossata - ps26
Mecopterida - ps27
Diptera - ps28
Bibiono./Cyclorrhapha - ps29
Cyclorrhapha - ps30
Calyptratae/Drosophila - ps31
Drosophila - ps32
Sophophora - ps33
Obscura/Melanogaster_group - ps34
D_melanogaster_group - ps35
D_melanogaster_subgroup - ps36
sim./sech./mel. group - ps37
Drosophila_melanogaster - ps38
p-value
1e-05
8e-05
7e-04
6e-03
5e-02
Log-odds
0.5
1.0
1.5
2.0
2.5
3.0
3.5
4.0

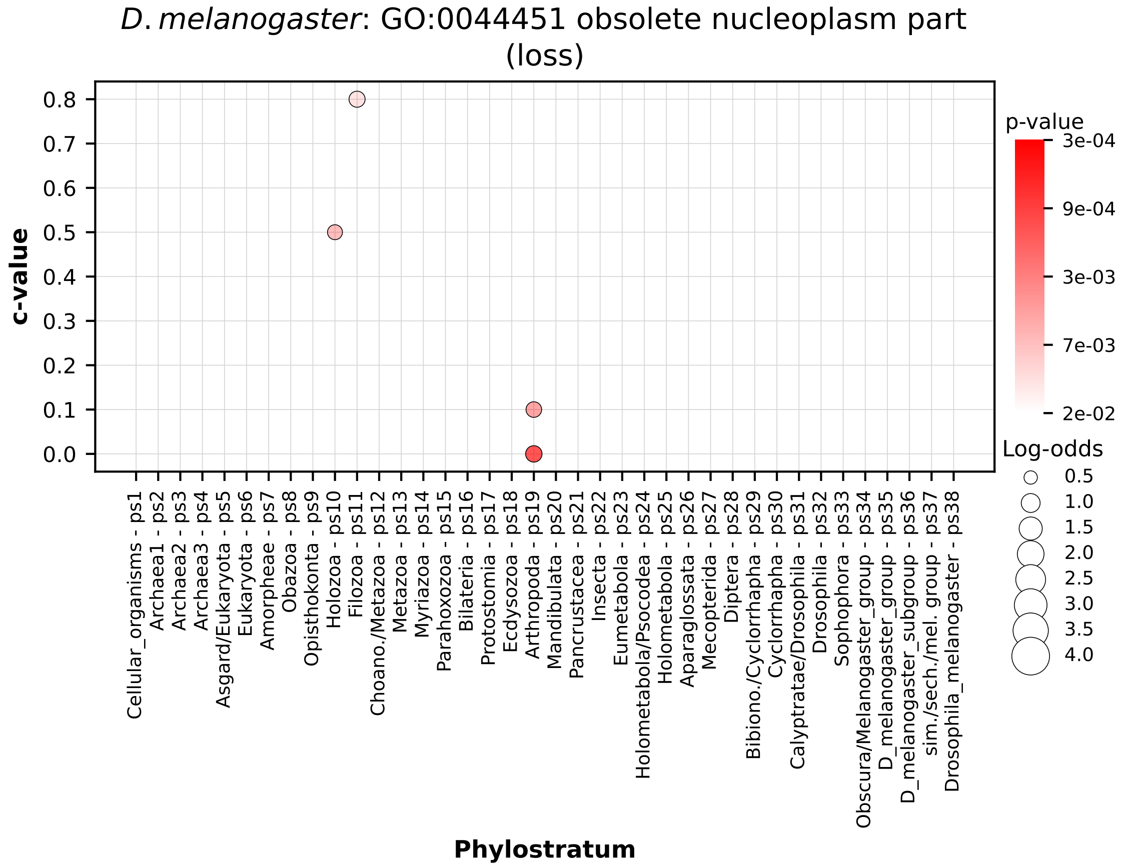

D. melanogaster: GO:0044451 obsolete nucleoplasm part (loss)
c-value
0.0
0.1
0.2
0.3
0.4
0.5
0.6
0.7
0.8
p-value
3e-04
9e-04
3e-03
7e-03
2e-02
Log-odds
0.5
1.0
1.5
2.0
2.5
3.0
3.5
4.0
Cellular_organisms - ps1
Archaea1 - ps2
Archaea2 - ps3
Archaea3 - ps4
Asgard/Eukaryota - ps5
Eukaryota - ps6
Amorpheae - ps7
Obazoa - ps8
Opisthokonta - ps9
Holozoa - ps10
Filozoa - ps11
Choano./Metazoa - ps12
Metazoa - ps13
Myriazoa - ps14
Parahoxozoa - ps15
Bilateria - ps16
Protostomia - ps17
Ecdysozoa - ps18
Arthropoda - ps19
Mandibulata - ps20
Pancrustacea - ps21
Insecta - ps22
Eumetabola - ps23
Holometabola/Psocodea - ps24
Holometabola - ps25
Aparaglossata - ps26
Mecopterida - ps27
Diptera - ps28
Bibiono./Cyclorrhapha - ps29
Cyclorrhapha - ps30
Calyptratae/Drosophila - ps31
Drosophila - ps32
Sophophora - ps33
Obscura/Melanogaster_group - ps34
D_melanogaster_group - ps35
D_melanogaster_subgroup - ps36
sim./sech./mel. group - ps37
Drosophila_melanogaster - ps38
Phylostratum

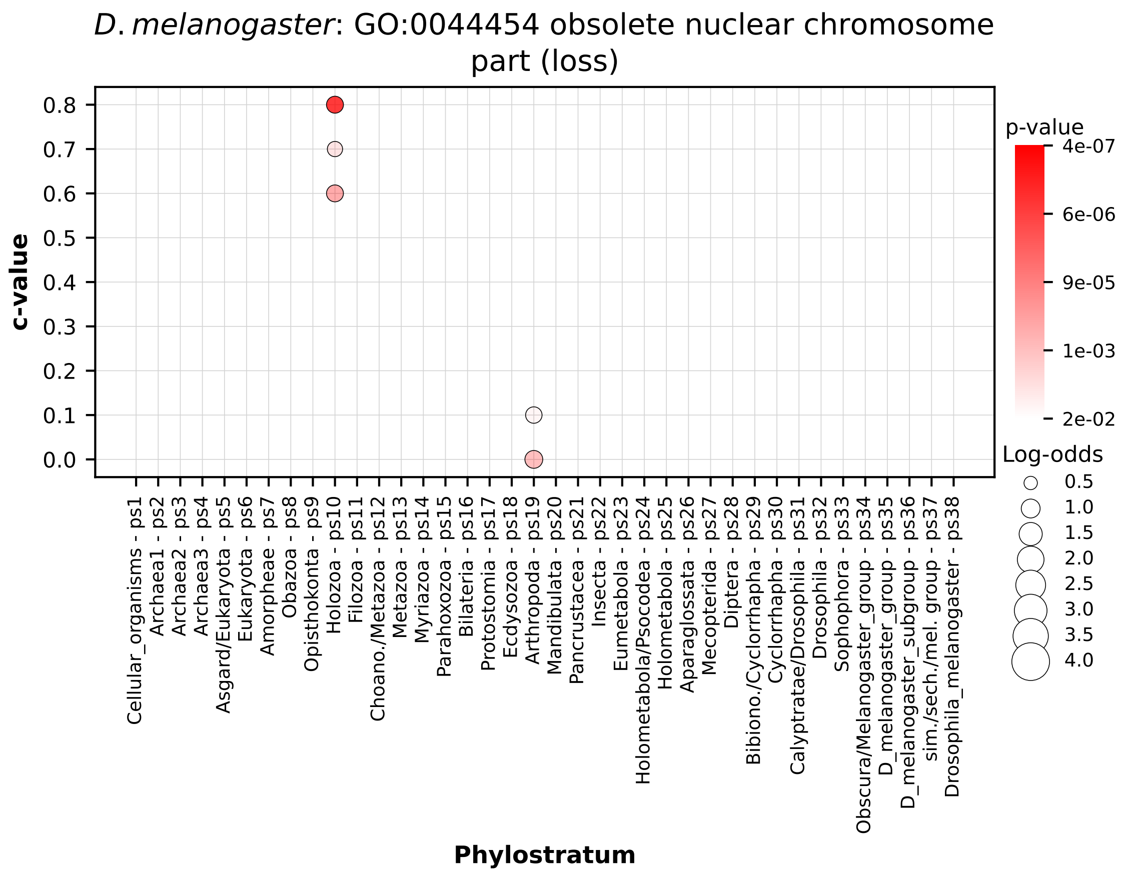

D. melanogaster: GO:0044454 obsolete nuclear chromosome part (loss)
c-value
0.8
0.7
0.6
0.5
0.4
0.3
0.2
0.1
0.0
Phylostratum
Cellular_organisms - ps1
Archaea1 - ps2
Archaea2 - ps3
Archaea3 - ps4
Asgard/Eukaryota - ps5
Eukaryota - ps6
Amorpheae - ps7
Obazoa - ps8
Opisthokonta - ps9
Holozoa - ps10
Filozoa - ps11
Choano./Metazoa - ps12
Metazoa - ps13
Myriazoa - ps14
Parahoxozoa - ps15
Bilateria - ps16
Protostomia - ps17
Ecdysozoa - ps18
Arthropoda - ps19
Mandibulata - ps20
Pancrustacea - ps21
Insecta - ps22
Eumetabola - ps23
Holometabola/Psocodea - ps24
Holometabola - ps25
Aparaglossata - ps26
Mecopterida - ps27
Diptera - ps28
Bibiono./Cyclorrhapha - ps29
Cyclorrhapha - ps30
Calyptratae/Drosophila - ps31
Drosophila - ps32
Sophophora - ps33
Obscura/Melanogaster_group - ps34
D_melanogaster_group - ps35
D_melanogaster_subgroup - ps36
sim./sech./mel. group - ps37
Drosophila_melanogaster - ps38
p-value
4e-07
6e-06
9e-05
1e-03
2e-02
Log-odds
0.5
1.0
1.5
2.0
2.5
3.0
3.5
4.0

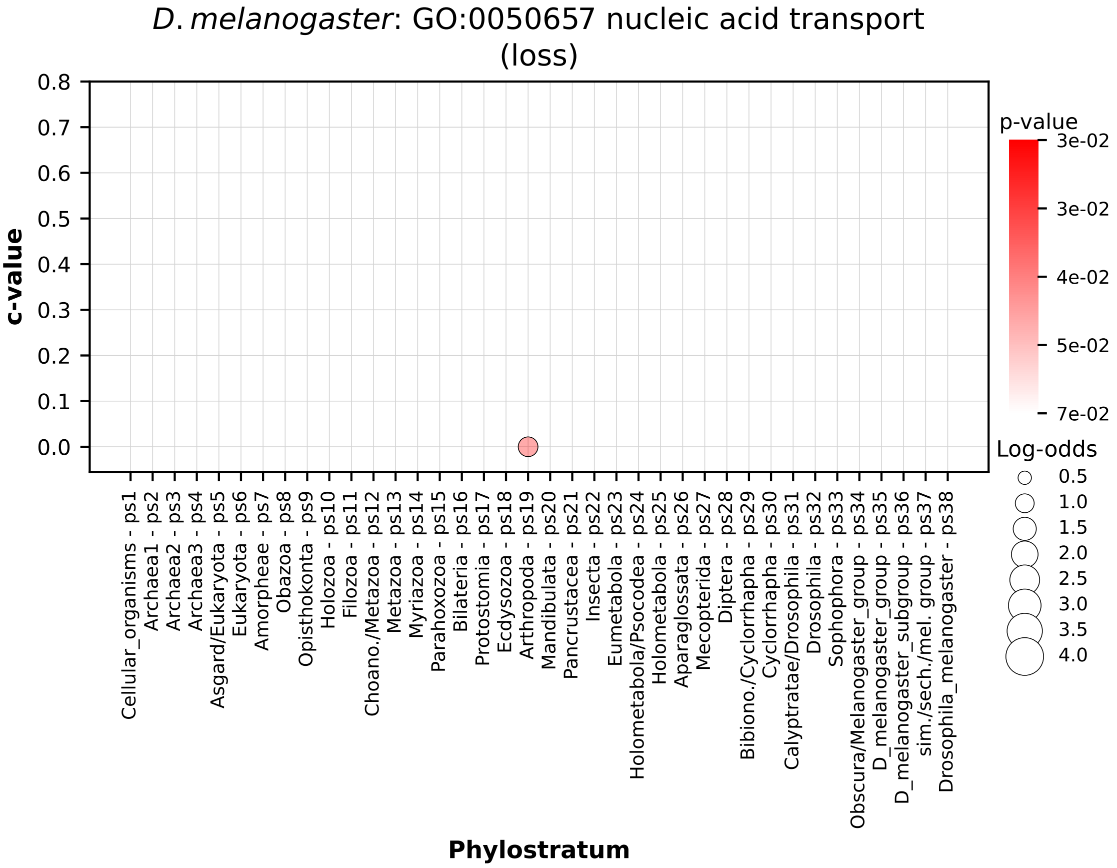

D. melanogaster: GO:0050657 nucleic acid transport
(loss)
c-value
0.8
0.7
0.6
0.5
0.4
0.3
0.2
0.1
0.0
Phylostratum
Cellular_organisms - ps1
Archaea1 - ps2
Archaea2 - ps3
Archaea3 - ps4
Asgard/Eukaryota - ps5
Eukaryota - ps6
Amorpheae - ps7
Obazoa - ps8
Opisthokonta - ps9
Holozoa - ps10
Filozoa - ps11
Choano./Metazoa - ps12
Metazoa - ps13
Myriazoa - ps14
Parahoxozoa - ps15
Bilateria - ps16
Protostomia - ps17
Ecdysozoa - ps18
Arthropoda - ps19
Mandibulata - ps20
Pancrustacea - ps21
Insecta - ps22
Eumetabola - ps23
Holometabola/Psocodea - ps24
Holometabola - ps25
Aparaglossata - ps26
Mecopterida - ps27
Diptera - ps28
Bibiono./Cyclorrhapha - ps29
Cyclorrhapha - ps30
Calyptratae/Drosophila - ps31
Drosophila - ps32
Sophophora - ps33
Obscura/Melanogaster_group - ps34
D_melanogaster_group - ps35
D_melanogaster_subgroup - ps36
sim./sech./mel. group - ps37
Drosophila_melanogaster - ps38
p-value
3e-02
3e-02
4e-02
5e-02
7e-02
Log-odds
0.5
1.0
1.5
2.0
2.5
3.0
3.5
4.0

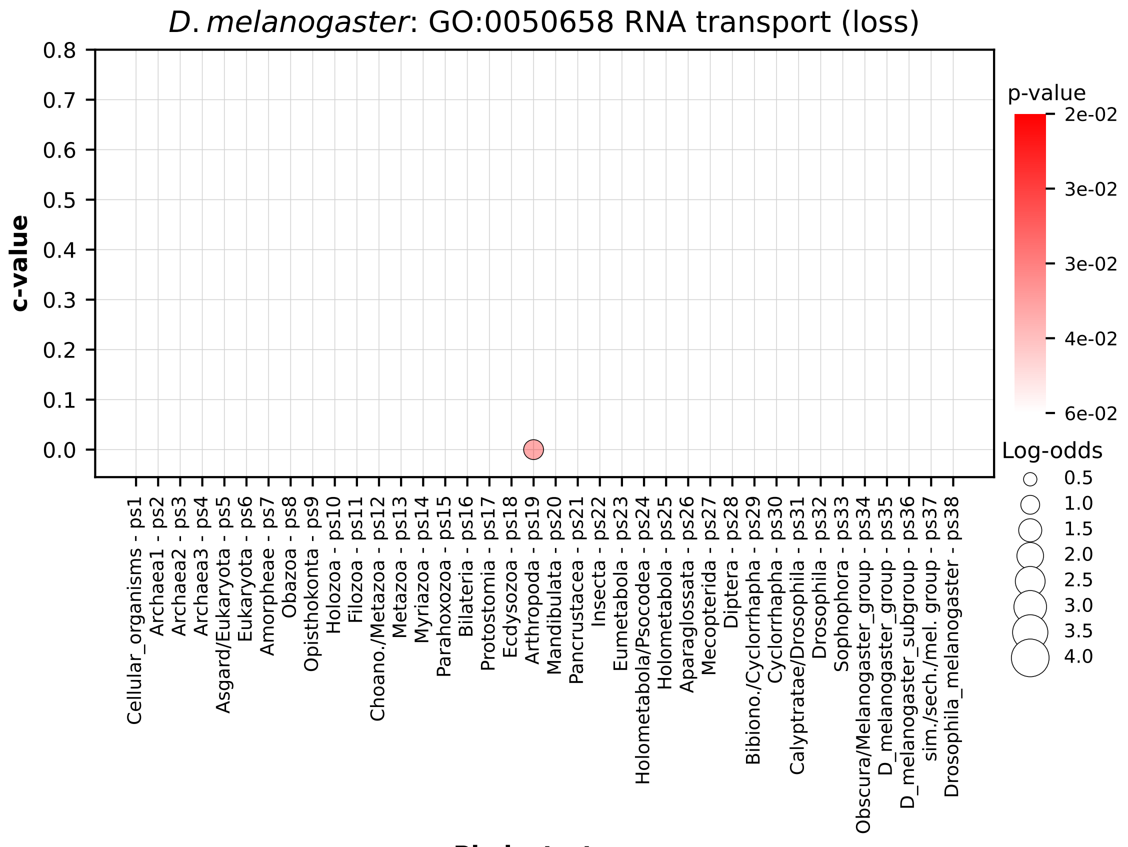

D. melanogaster: GO:0050658 RNA transport (loss)
c-value
0.8
0.7
0.6
0.5
0.4
0.3
0.2
0.1
0.0
p-value
2e-02
3e-02
3e-02
4e-02
6e-02
Log-odds
0.5
1.0
1.5
2.0
2.5
3.0
3.5
4.0
Cellular_organisms - ps1
Archaea1 - ps2
Archaea2 - ps3
Archaea3 - ps4
Asgard/Eukaryota - ps5
Eukaryota - ps6
Amorpheae - ps7
Obazoa - ps8
Opisthokonta - ps9
Holozoa - ps10
Filozoa - ps11
Choano./Metazoa - ps12
Metazoa - ps13
Myriazoa - ps14
Parahoxozoa - ps15
Bilateria - ps16
Protostomia - ps17
Ecdysozoa - ps18
Arthropoda - ps19
Mandibulata - ps20
Pancrustacea - ps21
Insecta - ps22
Eumetabola - ps23
Holometabola/Psocodea - ps24
Holometabola - ps25
Aparaglossata - ps26
Mecopterida - ps27
Diptera - ps28
Bibiono./Cyclorrhapha - ps29
Cyclorrhapha - ps30
Calyptratae/Drosophila - ps31
Drosophila - ps32
Sophophora - ps33
Obscura/Melanogaster_group - ps34
D_melanogaster_group - ps35
D_melanogaster_subgroup - ps36
sim./sech./mel. group - ps37
Drosophila_melanogaster - ps38
Phylostratum

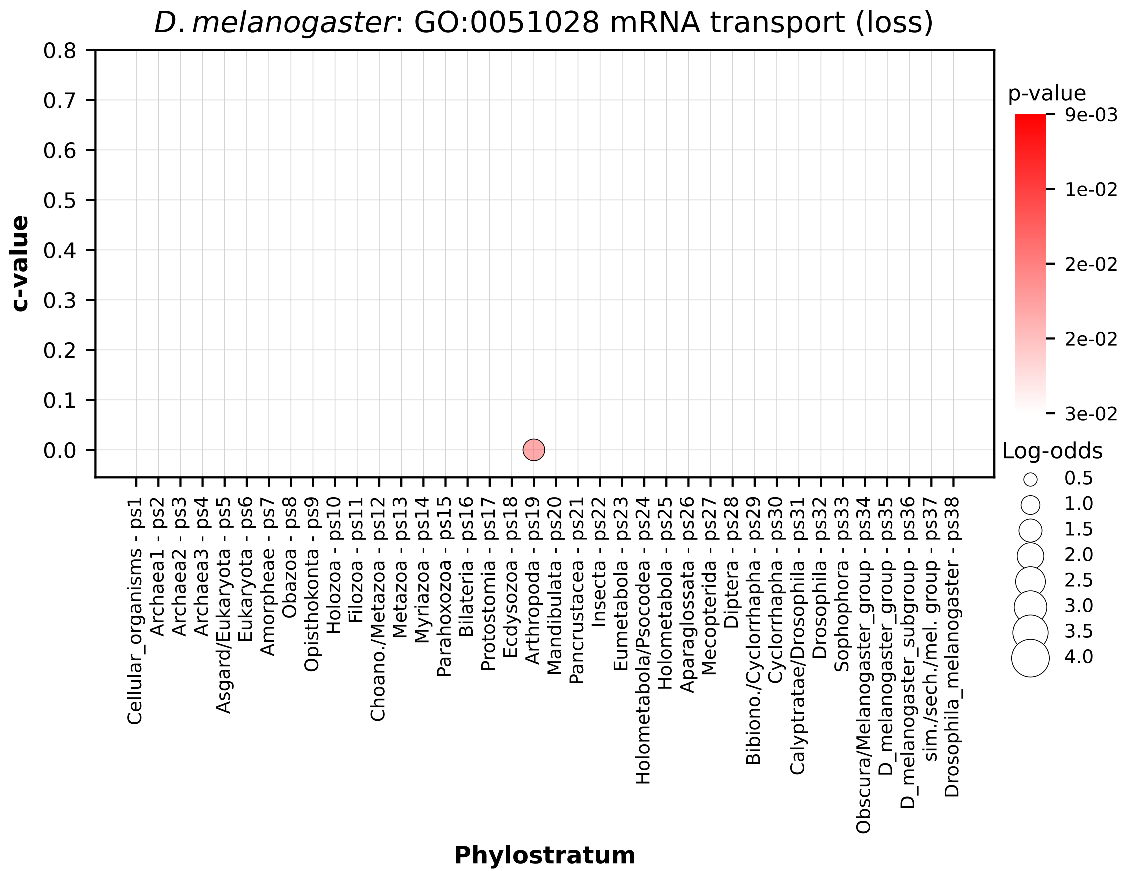

D. melanogaster: GO:0051028 mRNA transport (loss)
c-value
0.8
0.7
0.6
0.5
0.4
0.3
0.2
0.1
0.0
Phylostratum
Cellular_organisms - ps1
Archaea1 - ps2
Archaea2 - ps3
Archaea3 - ps4
Asgard/Eukaryota - ps5
Eukaryota - ps6
Amorpheae - ps7
Obazoa - ps8
Opisthokonta - ps9
Holozoa - ps10
Filozoa - ps11
Choano./Metazoa - ps12
Metazoa - ps13
Myriazoa - ps14
Parahoxozoa - ps15
Bilateria - ps16
Protostomia - ps17
Ecdysozoa - ps18
Arthropoda - ps19
Mandibulata - ps20
Pancrustacea - ps21
Insecta - ps22
Eumetabola - ps23
Holometabola/Psocodea - ps24
Holometabola - ps25
Aparaglossata - ps26
Mecopterida - ps27
Diptera - ps28
Bibiono./Cyclorrhapha - ps29
Cyclorrhapha - ps30
Calyptratae/Drosophila - ps31
Drosophila - ps32
Sophophora - ps33
Obscura/Melanogaster_group - ps34
D_melanogaster_group - ps35
D_melanogaster_subgroup - ps36
sim./sech./mel. group - ps37
Drosophila_melanogaster - ps38
p-value
9e-03
1e-02
2e-02
2e-02
3e-02
Log-odds
0.5
1.0
1.5
2.0
2.5
3.0
3.5
4.0

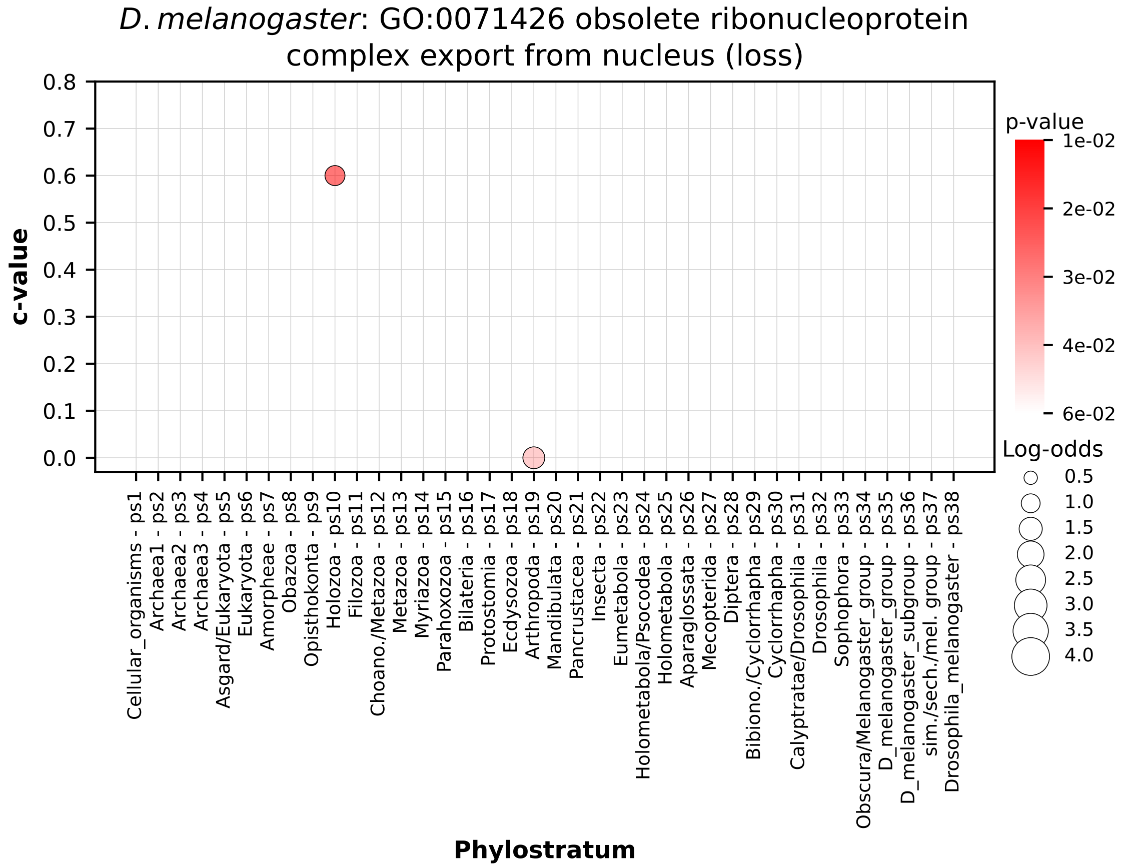

D. melanogaster: GO:0071426 obsolete ribonucleoprotein complex export from nucleus (loss)
c-value
0.0
0.1
0.2
0.3
0.4
0.5
0.6
0.7
0.8
Phylostratum
Cellular_organisms - ps1
Archaea1 - ps2
Archaea2 - ps3
Archaea3 - ps4
Asgard/Eukaryota - ps5
Eukaryota - ps6
Amorpheae - ps7
Obazoa - ps8
Opisthokonta - ps9
Holozoa - ps10
Filozoa - ps11
Choano./Metazoa - ps12
Metazoa - ps13
Myriazoa - ps14
Parahoxozoa - ps15
Bilateria - ps16
Protostomia - ps17
Ecdysozoa - ps18
Arthropoda - ps19
Mandibulata - ps20
Pancrustacea - ps21
Insecta - ps22
Eumetabola - ps23
Holometabola/Psocodea - ps24
Holometabola - ps25
Aparaglossata - ps26
Mecopterida - ps27
Diptera - ps28
Bibiono./Cyclorrhapha - ps29
Cyclorrhapha - ps30
Calyptratae/Drosophila - ps31
Drosophila - ps32
Sophophora - ps33
Obscura/Melanogaster_group - ps34
D_melanogaster_group - ps35
D_melanogaster_subgroup - ps36
sim./sech./mel. group - ps37
Drosophila_melanogaster - ps38
p-value
1e-02
2e-02
3e-02
4e-02
6e-02
Log-odds
0.5
1.0
1.5
2.0
2.5
3.0
3.5
4.0

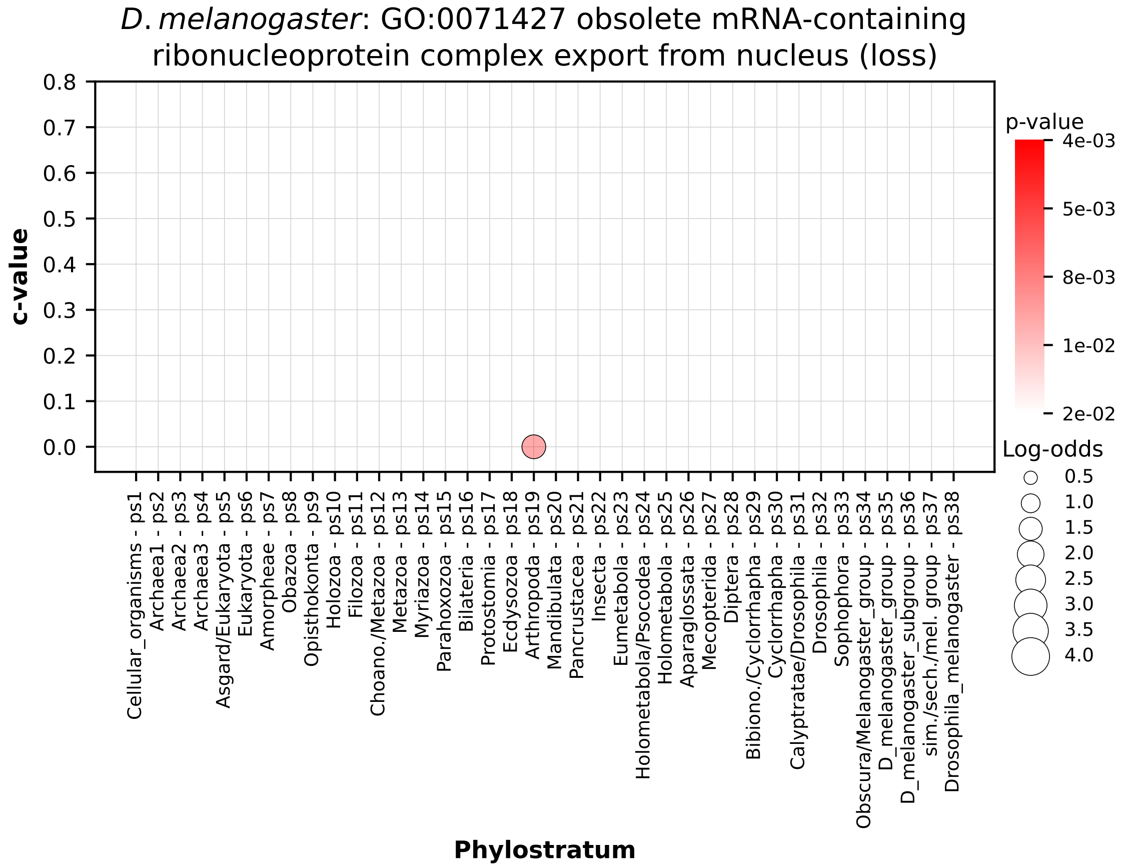

D. melanogaster: GO:0071427 obsolete mRNA-containing ribonucleoprotein complex export from nucleus (loss)
c-value
0.8
0.7
0.6
0.5
0.4
0.3
0.2
0.1
0.0
p-value
4e-03
5e-03
8e-03
1e-02
2e-02
Log-odds
0.5
1.0
1.5
2.0
2.5
3.0
3.5
4.0
Cellular_organisms - ps1
Archaea1 - ps2
Archaea2 - ps3
Archaea3 - ps4
Asgard/Eukaryota - ps5
Eukaryota - ps6
Amorpheae - ps7
Obazoa - ps8
Opisthokonta - ps9
Holozoa - ps10
Filozoa - ps11
Choano./Metazoa - ps12
Metazoa - ps13
Myriazoa - ps14
Parahoxozoa - ps15
Bilateria - ps16
Protostomia - ps17
Ecdysozoa - ps18
Arthropoda - ps19
Mandibulata - ps20
Pancrustacea - ps21
Insecta - ps22
Eumetabola - ps23
Holometabola/Psocodea - ps24
Holometabola - ps25
Aparaglossata - ps26
Mecopterida - ps27
Diptera - ps28
Bibiono./Cyclorrhapha - ps29
Cyclorrhapha - ps30
Calyptratae/Drosophila - ps31
Drosophila - ps32
Sophophora - ps33
Obscura/Melanogaster_group - ps34
D_melanogaster_group - ps35
D_melanogaster_subgroup - ps36
sim./sech./mel. group - ps37
Drosophila_melanogaster - ps38
Phylostratum

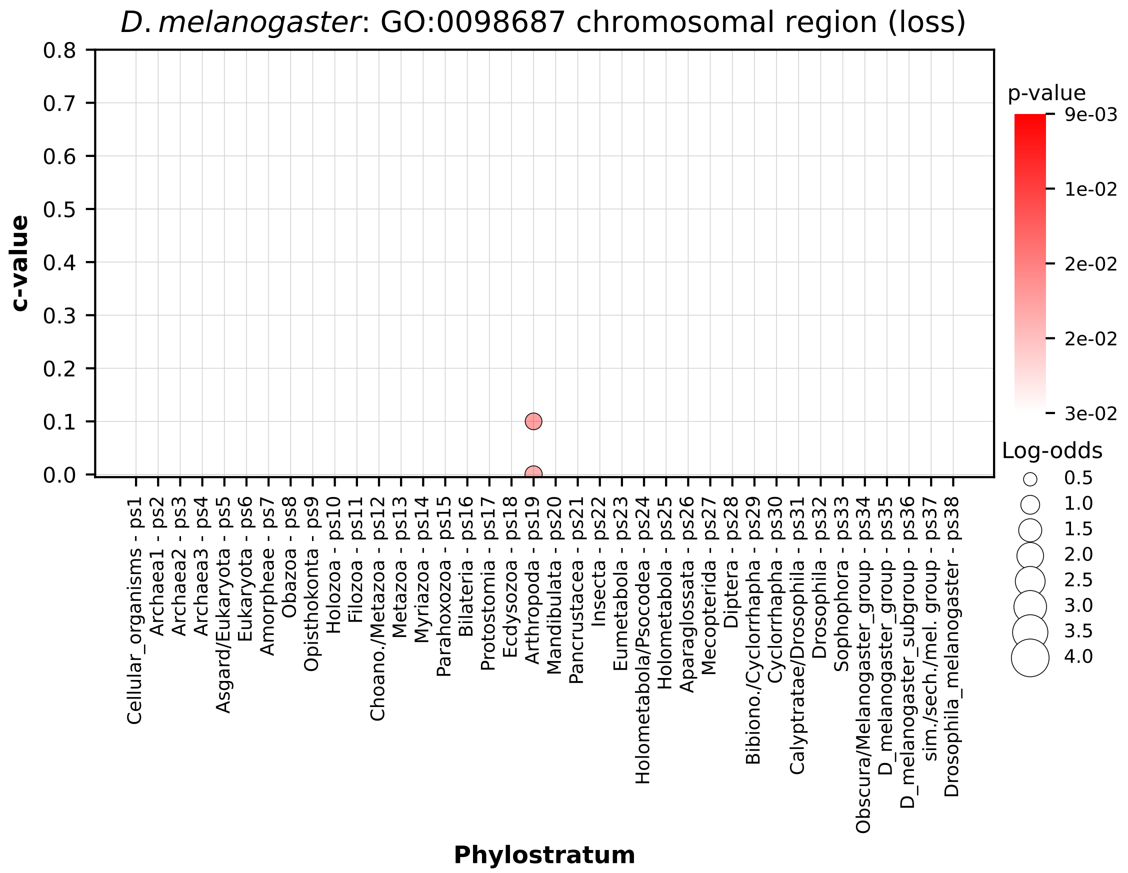
D. melanogaster: GO:0098687 chromosomal region (loss)
c-value
0.0
0.1
0.2
0.3
0.4
0.5
0.6
0.7
0.8
Phylostratum
Cellular_organisms - ps1
Archaea1 - ps2
Archaea2 - ps3
Archaea3 - ps4
Asgard/Eukaryota - ps5
Eukaryota - ps6
Amorpheae - ps7
Obazoa - ps8
Opisthokonta - ps9
Holozoa - ps10
Filozoa - ps11
Choano./Metazoa - ps12
Metazoa - ps13
Myriazoa - ps14
Parahoxozoa - ps15
Bilateria - ps16
Protostomia - ps17
Ecdysozoa - ps18
Arthropoda - ps19
Mandibulata - ps20
Pancrustacea - ps21
Insecta - ps22
Eumetabola - ps23
Holometabola/Psocodea - ps24
Holometabola - ps25
Aparaglossata - ps26
Mecopterida - ps27
Diptera - ps28
Bibiono./Cyclorrhapha - ps29
Cyclorrhapha - ps30
Calyptratae/Drosophila - ps31
Drosophila - ps32
Sophophora - ps33
Obscura/Melanogaster_group - ps34
D_melanogaster_group - ps35
D_melanogaster_subgroup - ps36
sim./sech./mel. group - ps37
Drosophila_melanogaster - ps38
p-value
9e-03
1e-02
2e-02
2e-02
3e-02
Log-odds
0.5
1.0
1.5
2.0
2.5
3.0
3.5
4.0

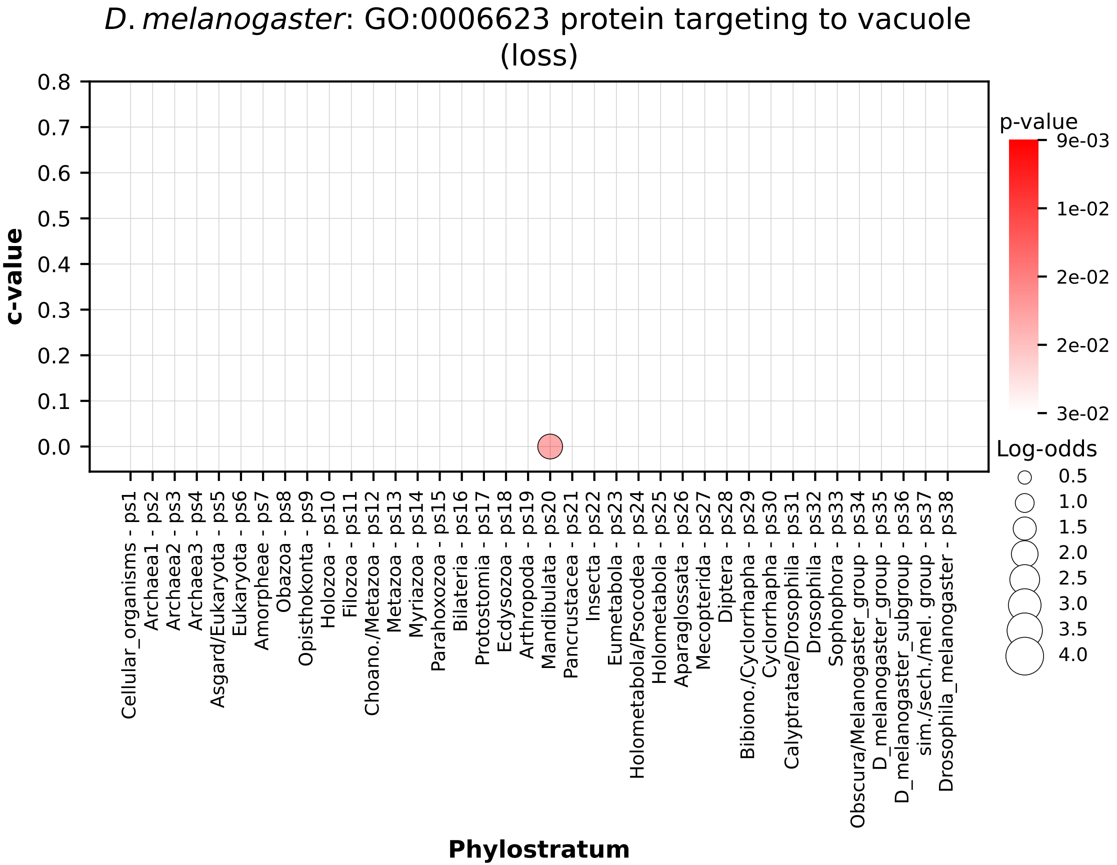

D. melanogaster: GO:0006623 protein targeting to vacuole (loss)
c-value
0.8
0.7
0.6
0.5
0.4
0.3
0.2
0.1
0.0
p-value
9e-03
1e-02
2e-02
2e-02
3e-02
Log-odds
0.5
1.0
1.5
2.0
2.5
3.0
3.5
4.0
Cellular_organisms - ps1
Archaea1 - ps2
Archaea2 - ps3
Archaea3 - ps4
Asgard/Eukaryota - ps5
Eukaryota - ps6
Amorpheae - ps7
Obazoa - ps8
Opisthokonta - ps9
Holozoa - ps10
Filozoa - ps11
Choano./Metazoa - ps12
Metazoa - ps13
Myriazoa - ps14
Parahoxozoa - ps15
Bilateria - ps16
Protostomia - ps17
Ecdysozoa - ps18
Arthropoda - ps19
Mandibulata - ps20
Pancrustacea - ps21
Insecta - ps22
Eumetabola - ps23
Holometabola/Psocodea - ps24
Holometabola - ps25
Aparaglossata - ps26
Mecopterida - ps27
Diptera - ps28
Bibiono./Cyclorrhapha - ps29
Cyclorrhapha - ps30
Calyptratae/Drosophila - ps31
Drosophila - ps32
Sophophora - ps33
Obscura/Melanogaster_group - ps34
D_melanogaster_group - ps35
D_melanogaster_subgroup - ps36
sim./sech./mel. group - ps37
Drosophila_melanogaster - ps38
Phylostratum

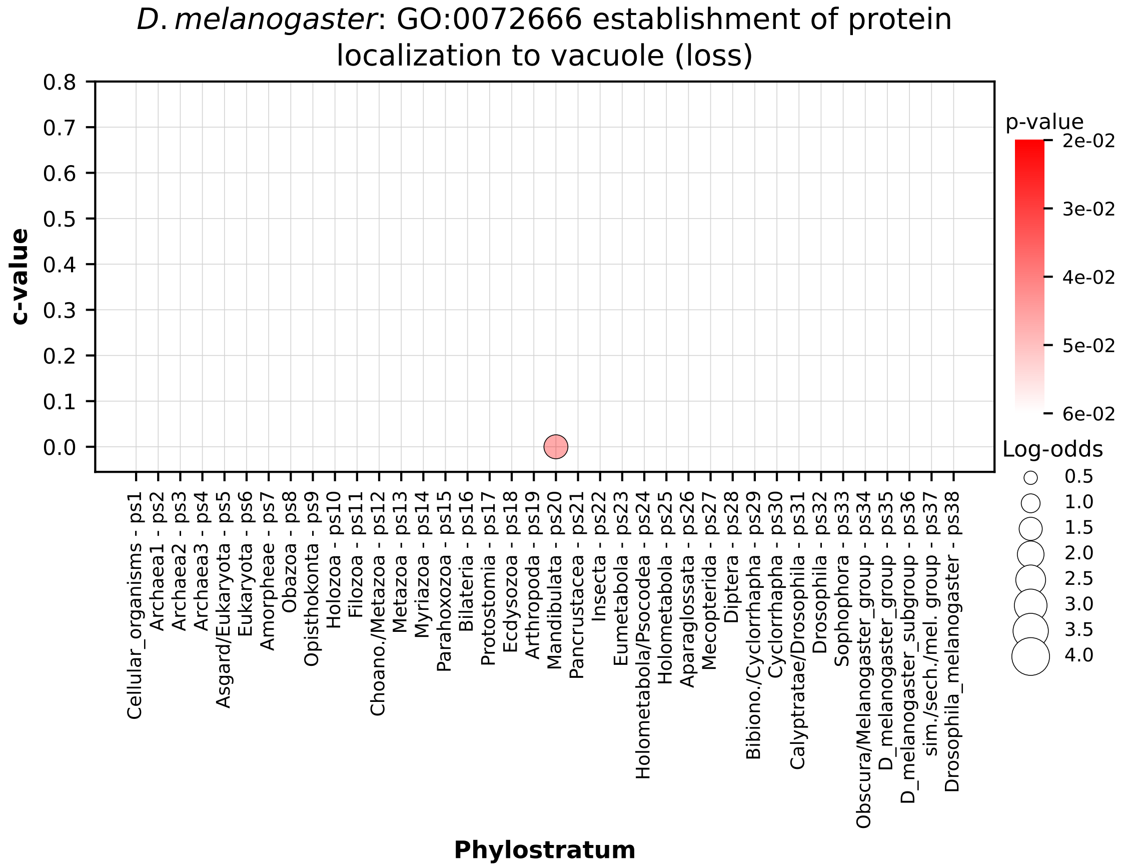

D. melanogaster: GO:0072666 establishment of protein localization to vacuole (loss)
c-value
0.8
0.7
0.6
0.5
0.4
0.3
0.2
0.1
0.0
p-value
2e-02
3e-02
4e-02
5e-02
6e-02
Log-odds
0.5
1.0
1.5
2.0
2.5
3.0
3.5
4.0
Cellular_organisms - ps1
Archaea1 - ps2
Archaea2 - ps3
Archaea3 - ps4
Asgard/Eukaryota - ps5
Eukaryota - ps6
Amorpheae - ps7
Obazoa - ps8
Opisthokonta - ps9
Holozoa - ps10
Filozoa - ps11
Choano./Metazoa - ps12
Metazoa - ps13
Myriazoa - ps14
Parahoxozoa - ps15
Bilateria - ps16
Protostomia - ps17
Ecdysozoa - ps18
Arthropoda - ps19
Mandibulata - ps20
Pancrustacea - ps21
Insecta - ps22
Eumetabola - ps23
Holometabola/Psocodea - ps24
Holometabola - ps25
Aparaglossata - ps26
Mecopterida - ps27
Diptera - ps28
Bibiono./Cyclorrhapha - ps29
Cyclorrhapha - ps30
Calyptratae/Drosophila - ps31
Drosophila - ps32
Sophophora - ps33
Obscura/Melanogaster_group - ps34
D_melanogaster_group - ps35
D_melanogaster_subgroup - ps36
sim./sech./mel. group - ps37
Drosophila_melanogaster - ps38
Phylostratum

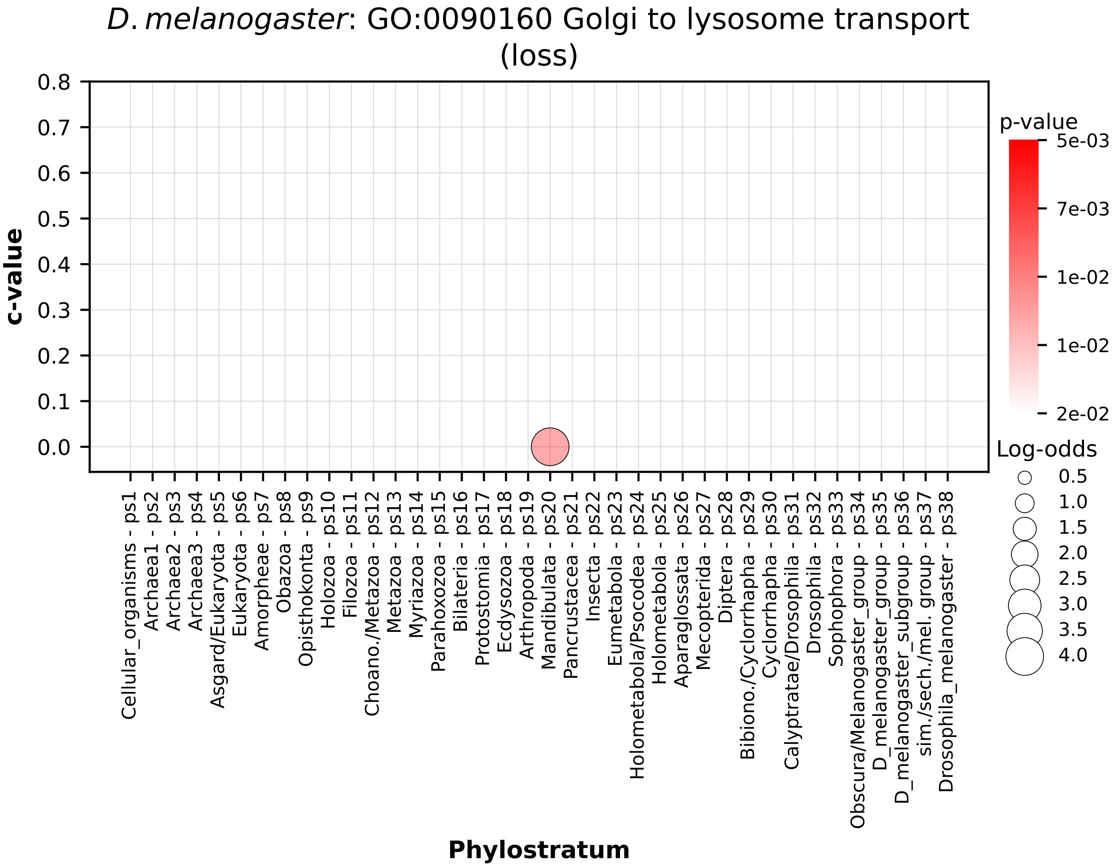

D. melanogaster: GO:0090160 Golgi to lysosome transport (loss)
c-value
0.8
0.7
0.6
0.5
0.4
0.3
0.2
0.1
0.0
Phylostratum
Cellular_organisms - ps1
Archaea1 - ps2
Archaea2 - ps3
Archaea3 - ps4
Asgard/Eukaryota - ps5
Eukaryota - ps6
Amorpheae - ps7
Obazoa - ps8
Opisthokonta - ps9
Holozoa - ps10
Filozoa - ps11
Choano./Metazoa - ps12
Metazoa - ps13
Myriazoa - ps14
Parahoxozoa - ps15
Bilateria - ps16
Protostomia - ps17
Ecdysozoa - ps18
Arthropoda - ps19
Mandibulata - ps20
Pancrustacea - ps21
Insecta - ps22
Eumetabola - ps23
Holometabola/Psocodea - ps24
Holometabola - ps25
Aparaglossata - ps26
Mecopterida - ps27
Diptera - ps28
Bibiono./Cyclorrhapha - ps29
Cyclorrhapha - ps30
Calyptratae/Drosophila - ps31
Drosophila - ps32
Sophophora - ps33
Obscura/Melanogaster_group - ps34
D_melanogaster_group - ps35
D_melanogaster_subgroup - ps36
sim./sech./mel. group - ps37
Drosophila_melanogaster - ps38
p-value
5e-03
7e-03
1e-02
1e-02
2e-02
Log-odds
0.5
1.0
1.5
2.0
2.5
3.0
3.5
4.0

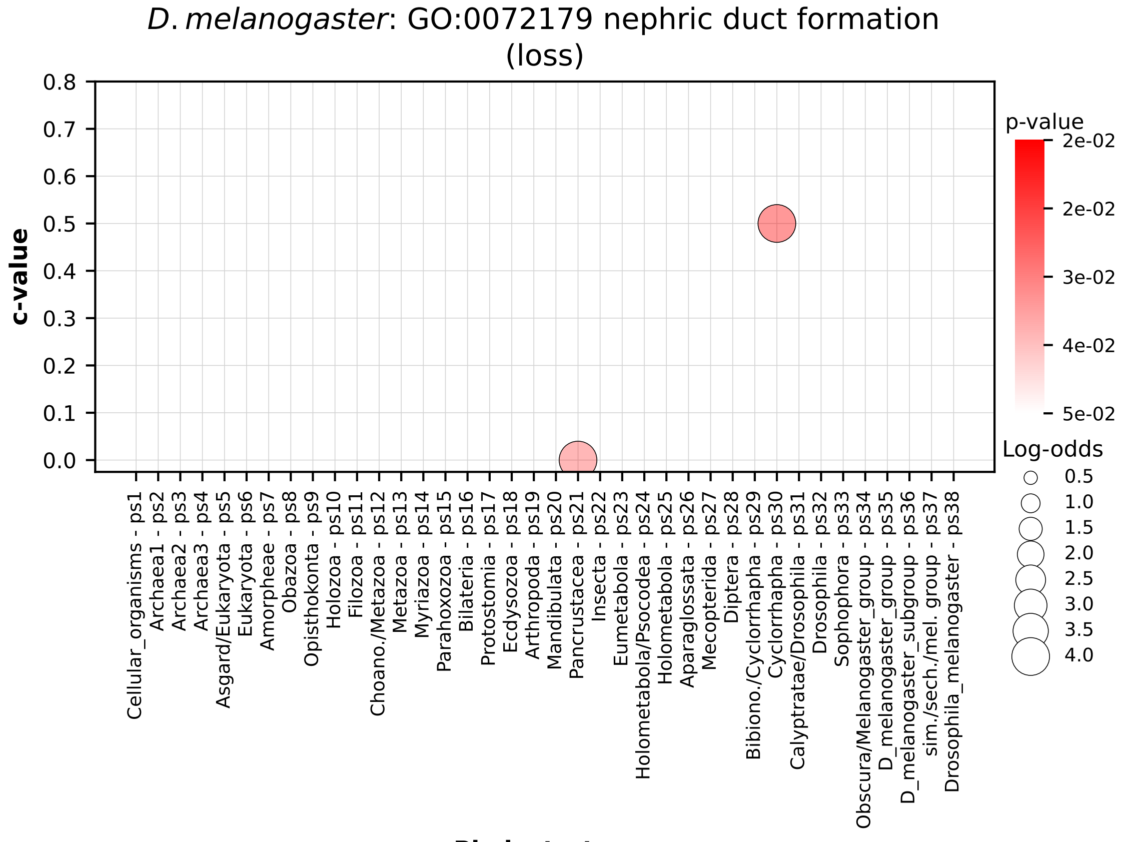
D. melanogaster: GO:0072179 nephric duct formation
(loss)
c-value
0.8
0.7
0.6
0.5
0.4
0.3
0.2
0.1
0.0
p-value
2e-02
2e-02
3e-02
4e-02
5e-02
Log-odds
0.5
1.0
1.5
2.0
2.5
3.0
3.5
4.0
Cellular_organisms - ps1
Archaea1 - ps2
Archaea2 - ps3
Archaea3 - ps4
Asgard/Eukaryota - ps5
Eukaryota - ps6
Amorpheae - ps7
Obazoa - ps8
Opisthokonta - ps9
Holozoa - ps10
Filozoa - ps11
Choano./Metazoa - ps12
Metazoa - ps13
Myriazoa - ps14
Parahoxozoa - ps15
Bilateria - ps16
Protostomia - ps17
Ecdysozoa - ps18
Arthropoda - ps19
Mandibulata - ps20
Pancrustacea - ps21
Insecta - ps22
Eumetabola - ps23
Holometabola/Psocodea - ps24
Holometabola - ps25
Aparaglossata - ps26
Mecopterida - ps27
Diptera - ps28
Bibiono./Cyclorrhapha - ps29
Cyclorrhapha - ps30
Calyptratae/Drosophila - ps31
Drosophila - ps32
Sophophora - ps33
Obscura/Melanogaster_group - ps34
D_melanogaster_group - ps35
D_melanogaster_subgroup - ps36
sim./sech./mel. group - ps37
Drosophila_melanogaster - ps38
Phylostratum

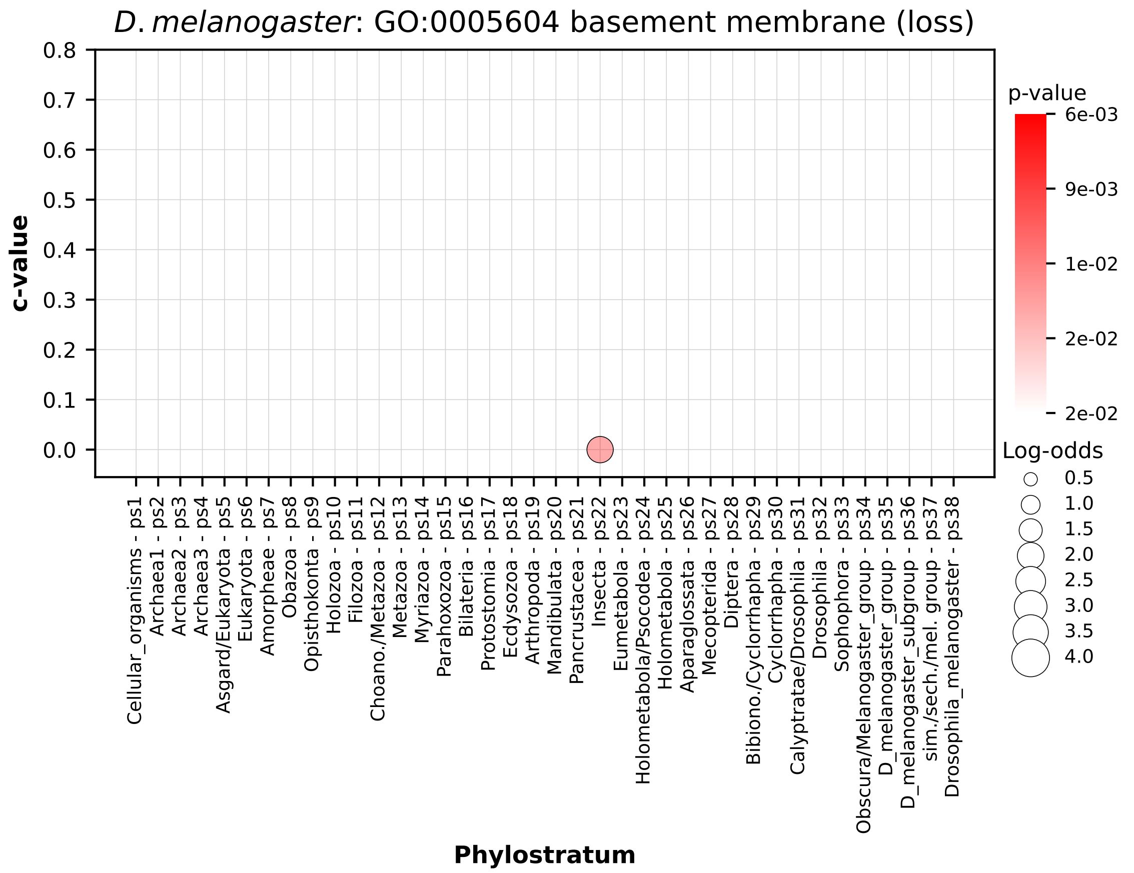

D. melanogaster: GO:0005604 basement membrane (loss)
c-value
0.8
0.7
0.6
0.5
0.4
0.3
0.2
0.1
0.0
Phylostratum
Cellular_organisms - ps1
Archaea1 - ps2
Archaea2 - ps3
Archaea3 - ps4
Asgard/Eukaryota - ps5
Eukaryota - ps6
Amorpheae - ps7
Obazoa - ps8
Opisthokonta - ps9
Holozoa - ps10
Filozoa - ps11
Choano./Metazoa - ps12
Metazoa - ps13
Myriazoa - ps14
Parahoxozoa - ps15
Bilateria - ps16
Protostomia - ps17
Ecdysozoa - ps18
Arthropoda - ps19
Mandibulata - ps20
Pancrustacea - ps21
Insecta - ps22
Eumetabola - ps23
Holometabola/Psocodea - ps24
Holometabola - ps25
Aparaglossata - ps26
Mecopterida - ps27
Diptera - ps28
Bibiono./Cyclorrhapha - ps29
Cyclorrhapha - ps30
Calyptratae/Drosophila - ps31
Drosophila - ps32
Sophophora - ps33
Obscura/Melanogaster_group - ps34
D_melanogaster_group - ps35
D_melanogaster_subgroup - ps36
sim./sech./mel. group - ps37
Drosophila_melanogaster - ps38
p-value
6e-03
9e-03
1e-02
2e-02
2e-02
Log-odds
0.5
1.0
1.5
2.0
2.5
3.0
3.5
4.0

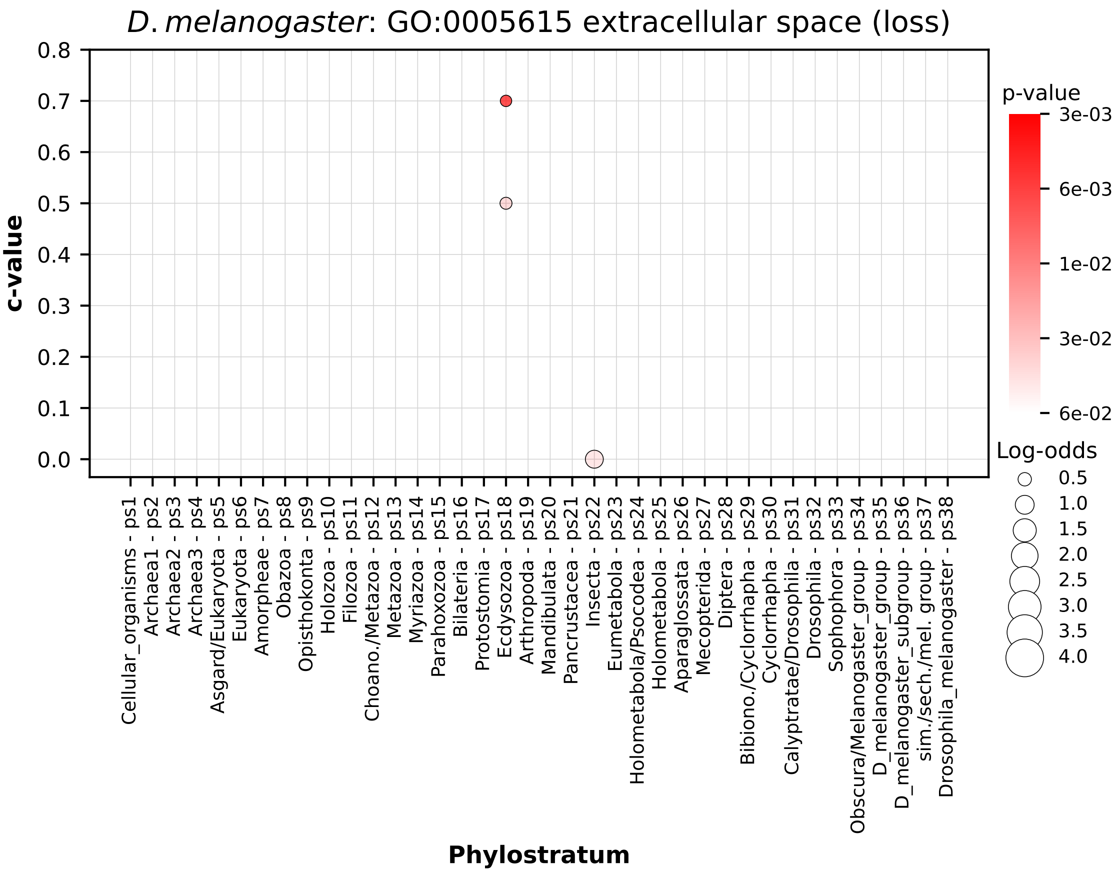

D. melanogaster: GO:0005615 extracellular space (loss)
c-value
0.8
0.7
0.6
0.5
0.4
0.3
0.2
0.1
0.0
Phylostratum
Cellular_organisms - ps1
Archaea1 - ps2
Archaea2 - ps3
Archaea3 - ps4
Asgard/Eukaryota - ps5
Eukaryota - ps6
Amorpheae - ps7
Obazoa - ps8
Opisthokonta - ps9
Holozoa - ps10
Filozoa - ps11
Choano./Metazoa - ps12
Metazoa - ps13
Myriazoa - ps14
Parahoxozoa - ps15
Bilateria - ps16
Protostomia - ps17
Ecdysozoa - ps18
Arthropoda - ps19
Mandibulata - ps20
Pancrustacea - ps21
Insecta - ps22
Eumetabola - ps23
Holometabola/Psocodea - ps24
Holometabola - ps25
Aparaglossata - ps26
Mecopterida - ps27
Diptera - ps28
Bibiono./Cyclorrhapha - ps29
Cyclorrhapha - ps30
Calyptratae/Drosophila - ps31
Drosophila - ps32
Sophophora - ps33
Obscura/Melanogaster_group - ps34
D_melanogaster_group - ps35
D_melanogaster_subgroup - ps36
sim./sech./mel. group - ps37
Drosophila_melanogaster - ps38
p-value
3e-03
6e-03
1e-02
3e-02
6e-02
Log-odds
0.5
1.0
1.5
2.0
2.5
3.0
3.5
4.0

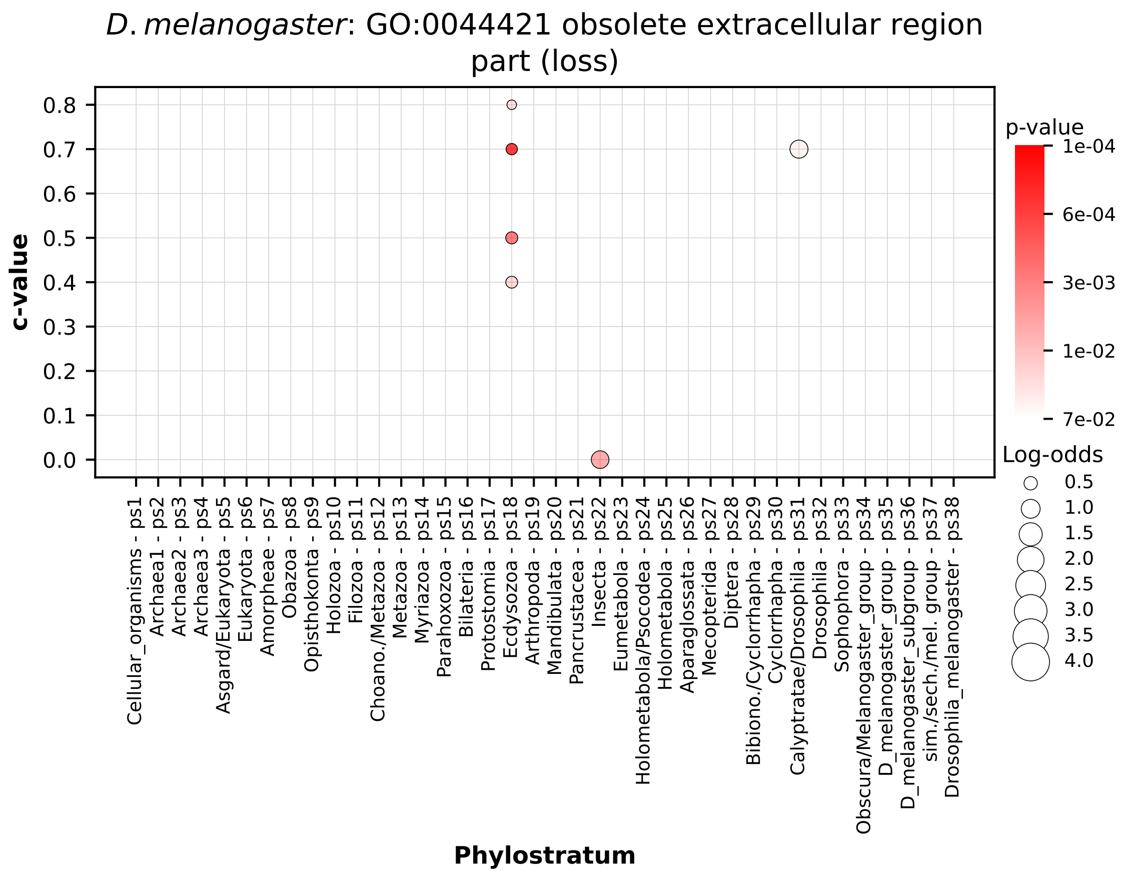

D. melanogaster: GO:0044421 obsolete extracellular region part (loss)
c-value
0.0
0.1
0.2
0.3
0.4
0.5
0.6
0.7
0.8
Phylostratum
Cellular_organisms - ps1
Archaea1 - ps2
Archaea2 - ps3
Archaea3 - ps4
Asgard/Eukaryota - ps5
Eukaryota - ps6
Amorpheae - ps7
Obazoa - ps8
Opisthokonta - ps9
Holozoa - ps10
Filozoa - ps11
Choano./Metazoa - ps12
Metazoa - ps13
Myriazoa - ps14
Parahoxozoa - ps15
Bilateria - ps16
Protostomia - ps17
Ecdysozoa - ps18
Arthropoda - ps19
Mandibulata - ps20
Pancrustacea - ps21
Insecta - ps22
Eumetabola - ps23
Holometabola/Psocodea - ps24
Holometabola - ps25
Aparaglossata - ps26
Mecopterida - ps27
Diptera - ps28
Bibiono./Cyclorrhapha - ps29
Cyclorrhapha - ps30
Calyptratae/Drosophila - ps31
Drosophila - ps32
Sophophora - ps33
Obscura/Melanogaster_group - ps34
D_melanogaster_group - ps35
D_melanogaster_subgroup - ps36
sim./sech./mel. group - ps37
Drosophila_melanogaster - ps38
p-value
1e-04
6e-04
3e-03
1e-02
7e-02
Log-odds
0.5
1.0
1.5
2.0
2.5
3.0
3.5
4.0

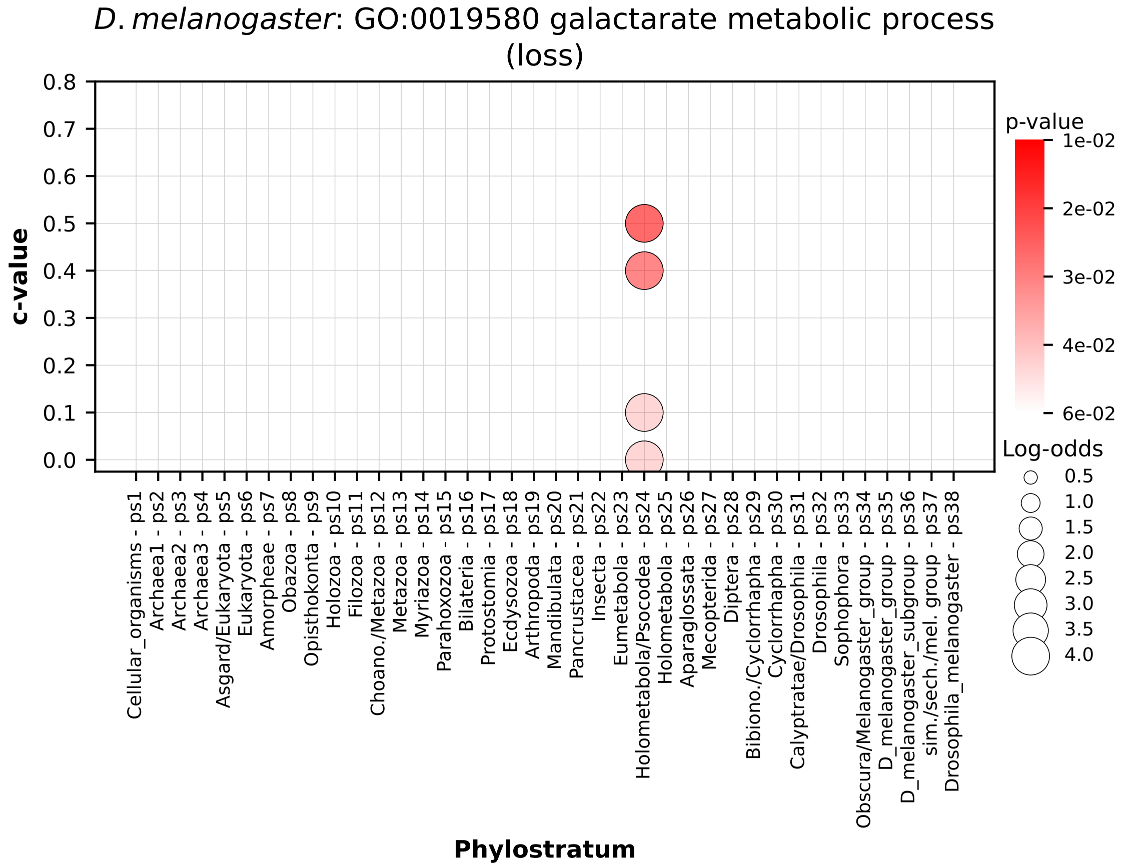

D. melanogaster: GO:0019580 galactarate metabolic process
(loss)
c-value
0.8
0.7
0.6
0.5
0.4
0.3
0.2
0.1
0.0
p-value
1e-02
2e-02
3e-02
4e-02
6e-02
Log-odds
0.5
1.0
1.5
2.0
2.5
3.0
3.5
4.0
Cellular_organisms - ps1
Archaea1 - ps2
Archaea2 - ps3
Archaea3 - ps4
Asgard/Eukaryota - ps5
Eukaryota - ps6
Amorpheae - ps7
Obazoa - ps8
Opisthokonta - ps9
Holozoa - ps10
Filozoa - ps11
Choano./Metazoa - ps12
Metazoa - ps13
Myriazoa - ps14
Parahoxozoa - ps15
Bilateria - ps16
Protostomia - ps17
Ecdysozoa - ps18
Arthropoda - ps19
Mandibulata - ps20
Pancrustacea - ps21
Insecta - ps22
Eumetabola - ps23
Holometabola/Psocodea - ps24
Holometabola - ps25
Aparaglossata - ps26
Mecopterida - ps27
Diptera - ps28
Bibiono./Cyclorrhapha - ps29
Cyclorrhapha - ps30
Calyptratae/Drosophila - ps31
Drosophila - ps32
Sophophora - ps33
Obscura/Melanogaster_group - ps34
D_melanogaster_group - ps35
D_melanogaster_subgroup - ps36
sim./sech./mel. group - ps37
Drosophila_melanogaster - ps38
Phylostratum

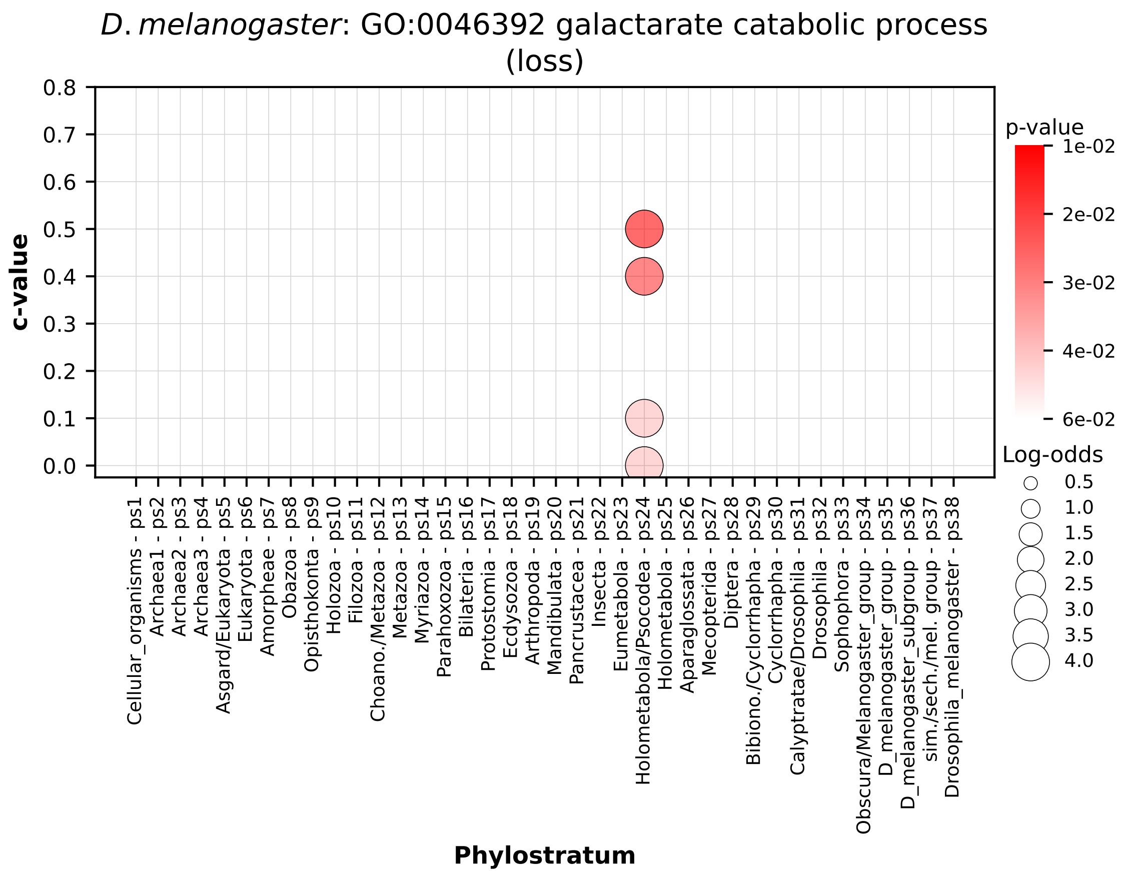

D. melanogaster: GO:0046392 galactarate catabolic process (loss)
c-value
0.8
0.7
0.6
0.5
0.4
0.3
0.2
0.1
0.0
p-value
1e-02
2e-02
3e-02
4e-02
6e-02
Log-odds
0.5
1.0
1.5
2.0
2.5
3.0
3.5
4.0
Cellular_organisms - ps1
Archaea1 - ps2
Archaea2 - ps3
Archaea3 - ps4
Asgard/Eukaryota - ps5
Eukaryota - ps6
Amorpheae - ps7
Obazoa - ps8
Opisthokonta - ps9
Holozoa - ps10
Filozoa - ps11
Choano./Metazoa - ps12
Metazoa - ps13
Myriazoa - ps14
Parahoxozoa - ps15
Bilateria - ps16
Protostomia - ps17
Ecdysozoa - ps18
Arthropoda - ps19
Mandibulata - ps20
Pancrustacea - ps21
Insecta - ps22
Eumetabola - ps23
Holometabola/Psocodea - ps24
Holometabola - ps25
Aparaglossata - ps26
Mecopterida - ps27
Diptera - ps28
Bibiono./Cyclorrhapha - ps29
Cyclorrhapha - ps30
Calyptratae/Drosophila - ps31
Drosophila - ps32
Sophophora - ps33
Obscura/Melanogaster_group - ps34
D_melanogaster_group - ps35
D_melanogaster_subgroup - ps36
sim./sech./mel. group - ps37
Drosophila_melanogaster - ps38
Phylostratum

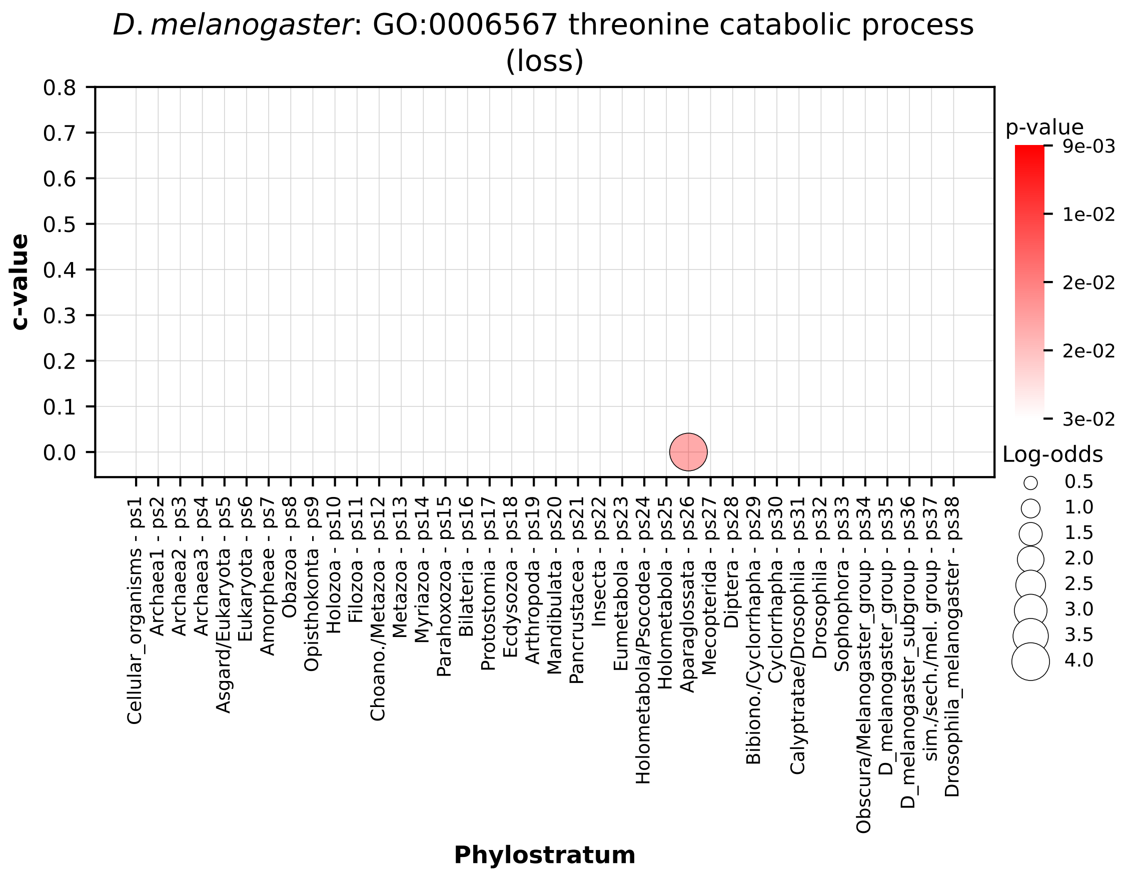

D. melanogaster: GO:0006567 threonine catabolic process (loss)
c-value
0.8
0.7
0.6
0.5
0.4
0.3
0.2
0.1
0.0
p-value
9e-03
1e-02
2e-02
2e-02
3e-02
Log-odds
0.5
1.0
1.5
2.0
2.5
3.0
3.5
4.0
Cellular_organisms - ps1
Archaea1 - ps2
Archaea2 - ps3
Archaea3 - ps4
Asgard/Eukaryota - ps5
Eukaryota - ps6
Amorpheae - ps7
Obazoa - ps8
Opisthokonta - ps9
Holozoa - ps10
Filozoa - ps11
Choano./Metazoa - ps12
Metazoa - ps13
Myriazoa - ps14
Parahoxozoa - ps15
Bilateria - ps16
Protostomia - ps17
Ecdysozoa - ps18
Arthropoda - ps19
Mandibulata - ps20
Pancrustacea - ps21
Insecta - ps22
Eumetabola - ps23
Holometabola/Psocodea - ps24
Holometabola - ps25
Aparaglossata - ps26
Mecopterida - ps27
Diptera - ps28
Bibiono./Cyclorrhapha - ps29
Cyclorrhapha - ps30
Calyptratae/Drosophila - ps31
Drosophila - ps32
Sophophora - ps33
Obscura/Melanogaster_group - ps34
D_melanogaster_group - ps35
D_melanogaster_subgroup - ps36
sim./sech./mel. group - ps37
Drosophila_melanogaster - ps38
Phylostratum

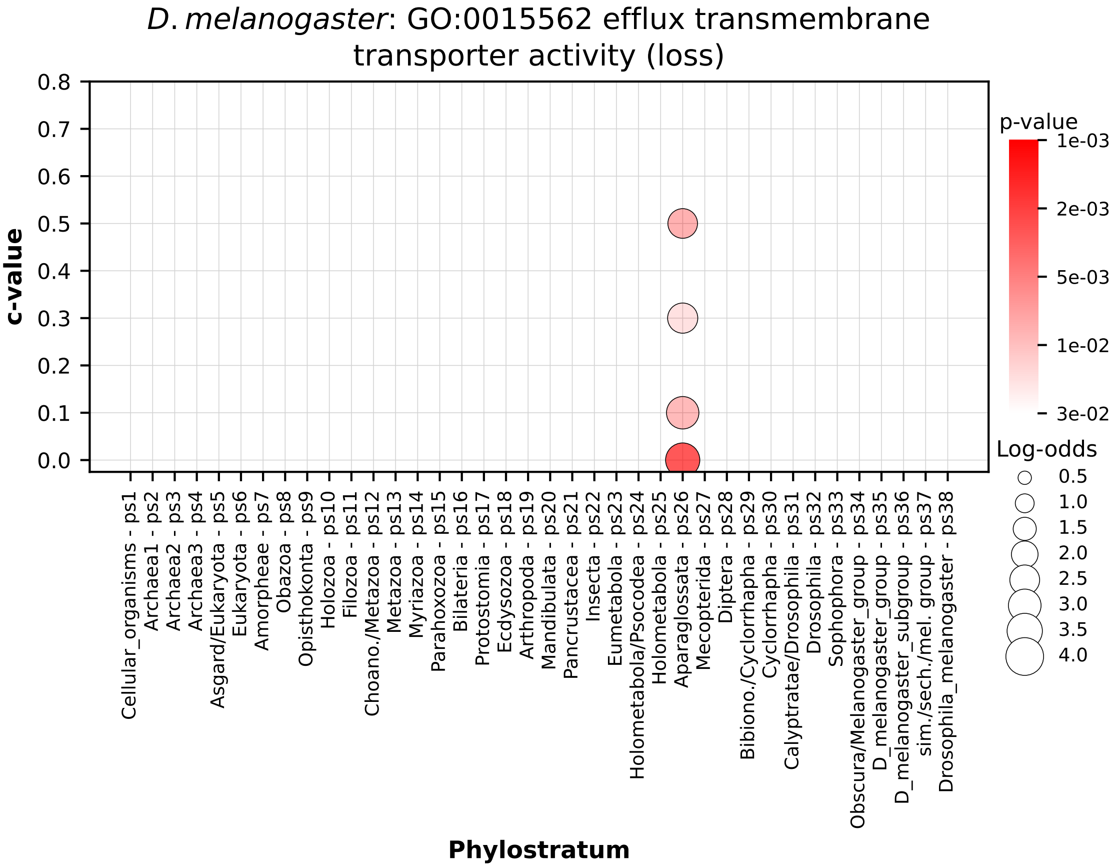

D. melanogaster: GO:0015562 efflux transmembrane transporter activity (loss)
c-value
0.8
0.7
0.6
0.5
0.4
0.3
0.2
0.1
0.0
Phylostratum
Cellular_organisms - ps1
Archaea1 - ps2
Archaea2 - ps3
Archaea3 - ps4
Asgard/Eukaryota - ps5
Eukaryota - ps6
Amorpheae - ps7
Obazoa - ps8
Opisthokonta - ps9
Holozoa - ps10
Filozoa - ps11
Choano./Metazoa - ps12
Metazoa - ps13
Myriazoa - ps14
Parahoxozoa - ps15
Bilateria - ps16
Protostomia - ps17
Ecdysozoa - ps18
Arthropoda - ps19
Mandibulata - ps20
Pancrustacea - ps21
Insecta - ps22
Eumetabola - ps23
Holometabola/Psocodea - ps24
Holometabola - ps25
Aparaglossata - ps26
Mecopterida - ps27
Diptera - ps28
Bibiono./Cyclorrhapha - ps29
Cyclorrhapha - ps30
Calyptratae/Drosophila - ps31
Drosophila - ps32
Sophophora - ps33
Obscura/Melanogaster_group - ps34
D_melanogaster_group - ps35
D_melanogaster_subgroup - ps36
sim./sech./mel. group - ps37
Drosophila_melanogaster - ps38
p-value
1e-03
2e-03
5e-03
1e-02
3e-02
Log-odds
0.5
1.0
1.5
2.0
2.5
3.0
3.5
4.0

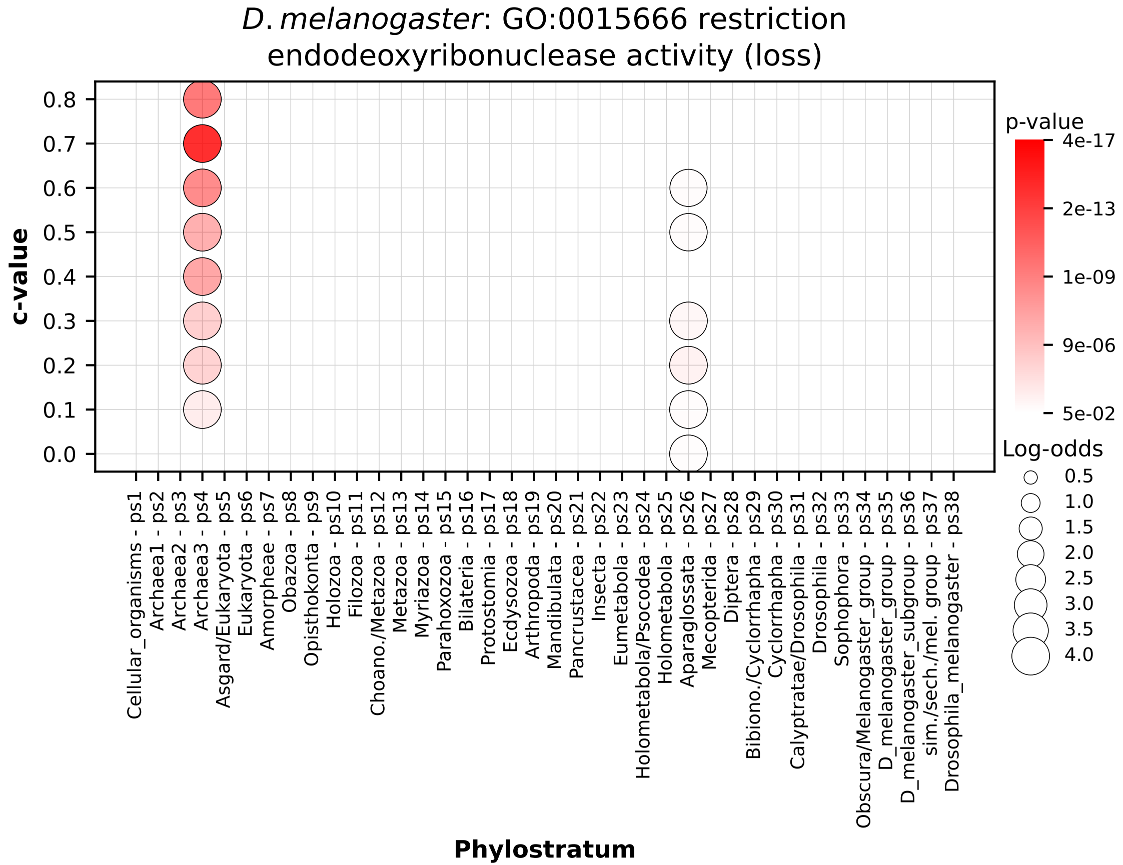

D. melanogaster: GO:0015666 restriction endodeoxyribonuclease activity (loss)
c-value
0.8
0.7
0.6
0.5
0.4
0.3
0.2
0.1
0.0
p-value
4e-17
2e-13
1e-09
9e-06
5e-02
Log-odds
0.5
1.0
1.5
2.0
2.5
3.0
3.5
4.0
Cellular_organisms - ps1
Archaea1 - ps2
Archaea2 - ps3
Archaea3 - ps4
Asgard/Eukaryota - ps5
Eukaryota - ps6
Amorpheae - ps7
Obazoa - ps8
Opisthokonta - ps9
Holozoa - ps10
Filozoa - ps11
Choano./Metazoa - ps12
Metazoa - ps13
Myriazoa - ps14
Parahoxozoa - ps15
Bilateria - ps16
Protostomia - ps17
Ecdysozoa - ps18
Arthropoda - ps19
Mandibulata - ps20
Pancrustacea - ps21
Insecta - ps22
Eumetabola - ps23
Holometabola/Psocodea - ps24
Holometabola - ps25
Aparaglossata - ps26
Mecopterida - ps27
Diptera - ps28
Bibiono./Cyclorrhapha - ps29
Cyclorrhapha - ps30
Calyptratae/Drosophila - ps31
Drosophila - ps32
Sophophora - ps33
Obscura/Melanogaster_group - ps34
D_melanogaster_group - ps35
D_melanogaster_subgroup - ps36
sim./sech./mel. group - ps37
Drosophila_melanogaster - ps38
Phylostratum

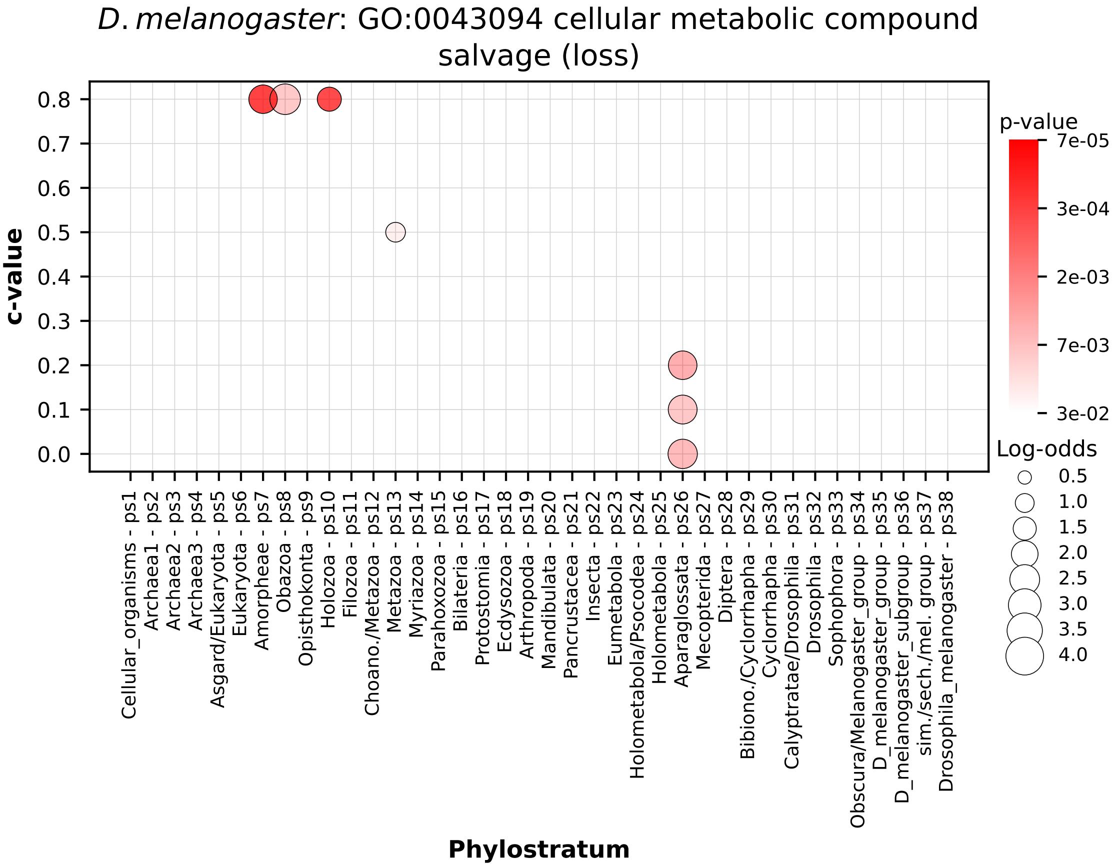

D. melanogaster: GO:0043094 cellular metabolic compound salvage (loss)
c-value
0.0
0.1
0.2
0.3
0.4
0.5
0.6
0.7
0.8
p-value
7e-05
3e-04
2e-03
7e-03
3e-02
Log-odds
0.5
1.0
1.5
2.0
2.5
3.0
3.5
4.0
Cellular_organisms - ps1
Archaea1 - ps2
Archaea2 - ps3
Archaea3 - ps4
Asgard/Eukaryota - ps5
Eukaryota - ps6
Amorpheae - ps7
Obazoa - ps8
Opisthokonta - ps9
Holozoa - ps10
Filozoa - ps11
Choano./Metazoa - ps12
Metazoa - ps13
Myriazoa - ps14
Parahoxozoa - ps15
Bilateria - ps16
Protostomia - ps17
Ecdysozoa - ps18
Arthropoda - ps19
Mandibulata - ps20
Pancrustacea - ps21
Insecta - ps22
Eumetabola - ps23
Holometabola/Psocodea - ps24
Holometabola - ps25
Aparaglossata - ps26
Mecopterida - ps27
Diptera - ps28
Bibiono./Cyclorrhapha - ps29
Cyclorrhapha - ps30
Calyptratae/Drosophila - ps31
Drosophila - ps32
Sophophora - ps33
Obscura/Melanogaster_group - ps34
D_melanogaster_group - ps35
D_melanogaster_subgroup - ps36
sim./sech./mel. group - ps37
Drosophila_melanogaster - ps38
Phylostratum

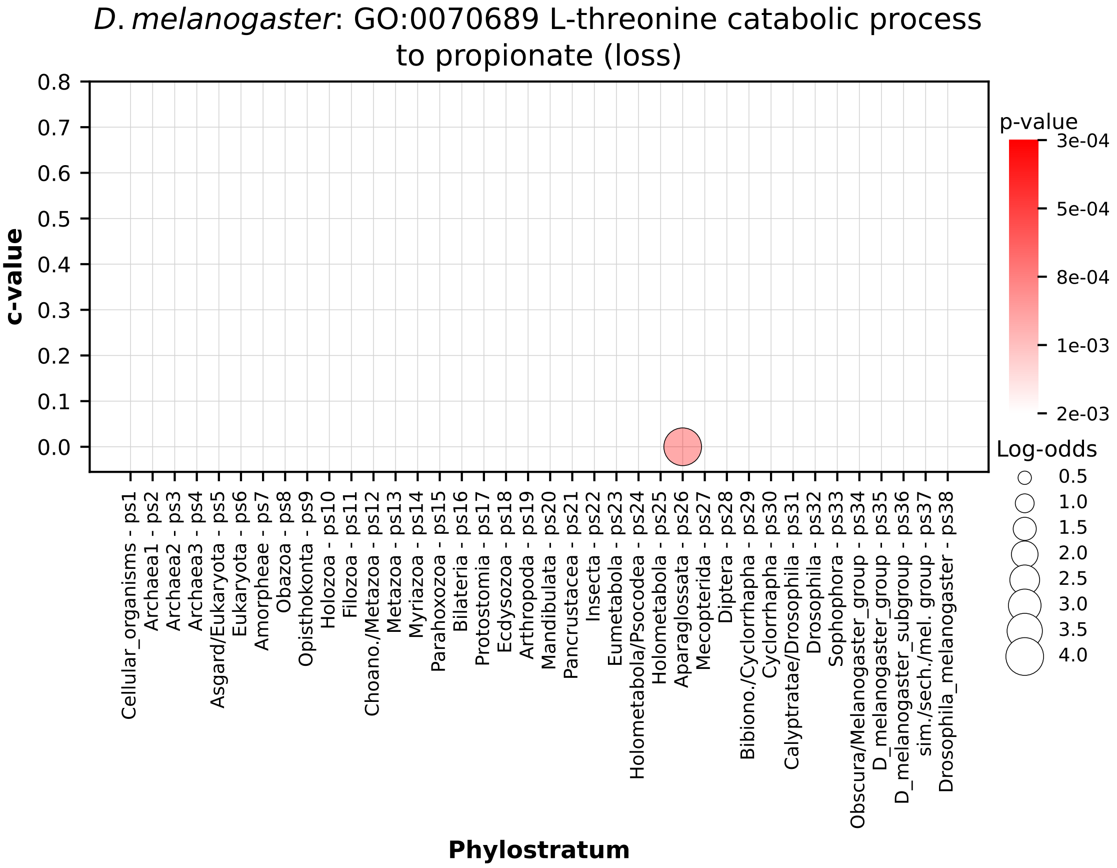
D. melanogaster: GO:0070689 L-threonine catabolic process to propionate (loss)
c-value
0.8
0.7
0.6
0.5
0.4
0.3
0.2
0.1
0.0
p-value
3e-04
5e-04
8e-04
1e-03
2e-03
Log-odds
0.5
1.0
1.5
2.0
2.5
3.0
3.5
4.0
Cellular_organisms - ps1
Archaea1 - ps2
Archaea2 - ps3
Archaea3 - ps4
Asgard/Eukaryota - ps5
Eukaryota - ps6
Amorpheae - ps7
Obazoa - ps8
Opisthokonta - ps9
Holozoa - ps10
Filozoa - ps11
Choano./Metazoa - ps12
Metazoa - ps13
Myriazoa - ps14
Parahoxozoa - ps15
Bilateria - ps16
Protostomia - ps17
Ecdysozoa - ps18
Arthropoda - ps19
Mandibulata - ps20
Pancrustacea - ps21
Insecta - ps22
Eumetabola - ps23
Holometabola/Psocodea - ps24
Holometabola - ps25
Aparaglossata - ps26
Mecopterida - ps27
Diptera - ps28
Bibiono./Cyclorrhapha - ps29
Cyclorrhapha - ps30
Calyptratae/Drosophila - ps31
Drosophila - ps32
Sophophora - ps33
Obscura/Melanogaster_group - ps34
D_melanogaster_group - ps35
D_melanogaster_subgroup - ps36
sim./sech./mel. group - ps37
Drosophila_melanogaster - ps38
Phylostratum

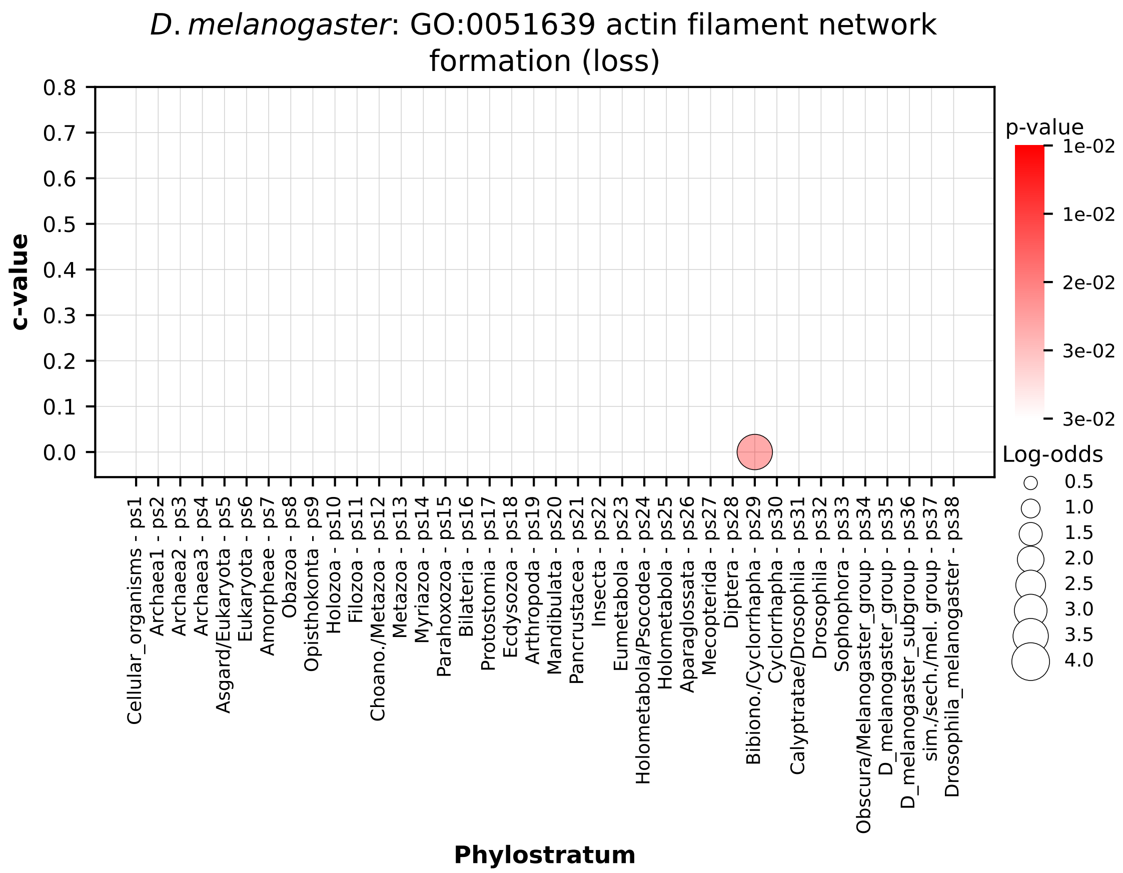

D. melanogaster: GO:0051639 actin filament network formation (loss)
c-value
0.8
0.7
0.6
0.5
0.4
0.3
0.2
0.1
0.0
p-value
1e-02
1e-02
2e-02
3e-02
3e-02
Log-odds
0.5
1.0
1.5
2.0
2.5
3.0
3.5
4.0
Cellular_organisms - ps1
Archaea1 - ps2
Archaea2 - ps3
Archaea3 - ps4
Asgard/Eukaryota - ps5
Eukaryota - ps6
Amorpheae - ps7
Obazoa - ps8
Opisthokonta - ps9
Holozoa - ps10
Filozoa - ps11
Choano./Metazoa - ps12
Metazoa - ps13
Myriazoa - ps14
Parahoxozoa - ps15
Bilateria - ps16
Protostomia - ps17
Ecdysozoa - ps18
Arthropoda - ps19
Mandibulata - ps20
Pancrustacea - ps21
Insecta - ps22
Eumetabola - ps23
Holometabola/Psocodea - ps24
Holometabola - ps25
Aparaglossata - ps26
Mecopterida - ps27
Diptera - ps28
Bibiono./Cyclorrhapha - ps29
Cyclorrhapha - ps30
Calyptratae/Drosophila - ps31
Drosophila - ps32
Sophophora - ps33
Obscura/Melanogaster_group - ps34
D_melanogaster_group - ps35
D_melanogaster_subgroup - ps36
sim./sech./mel. group - ps37
Drosophila_melanogaster - ps38
Phylostratum

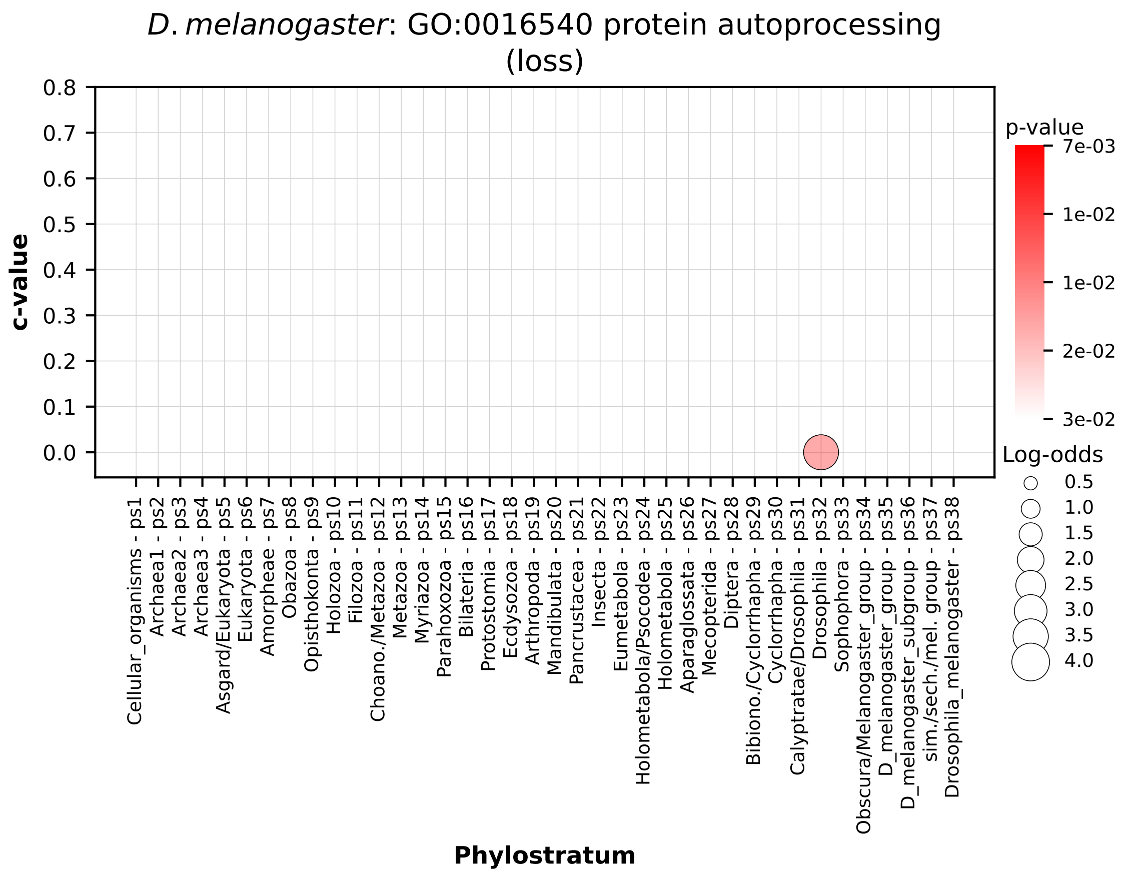

$D. melanogaster$: GO:0016540 protein autoprocessing
(loss)
c-value
0.8
0.7
0.6
0.5
0.4
0.3
0.2
0.1
0.0
p-value
7e-03
1e-02
1e-02
2e-02
3e-02
Log-odds
0.5
1.0
1.5
2.0
2.5
3.0
3.5
4.0
Cellular_organisms - ps1
Archaea1 - ps2
Archaea2 - ps3
Archaea3 - ps4
Asgard/Eukaryota - ps5
Eukaryota - ps6
Amorpheae - ps7
Obazoa - ps8
Opisthokonta - ps9
Holozoa - ps10
Filozoa - ps11
Choano./Metazoa - ps12
Metazoa - ps13
Myriazoa - ps14
Parahoxozoa - ps15
Bilateria - ps16
Protostomia - ps17
Ecdysozoa - ps18
Arthropoda - ps19
Mandibulata - ps20
Pancrustacea - ps21
Insecta - ps22
Eumetabola - ps23
Holometabola/Psocodea - ps24
Holometabola - ps25
Aparaglossata - ps26
Mecopterida - ps27
Diptera - ps28
Bibiono./Cyclorrhapha - ps29
Cyclorrhapha - ps30
Calyptratae/Drosophila - ps31
Drosophila - ps32
Sophophora - ps33
Obscura/Melanogaster_group - ps34
D_melanogaster_group - ps35
D_melanogaster_subgroup - ps36
sim./sech./mel. group - ps37
Drosophila_melanogaster - ps38
Phylostratum

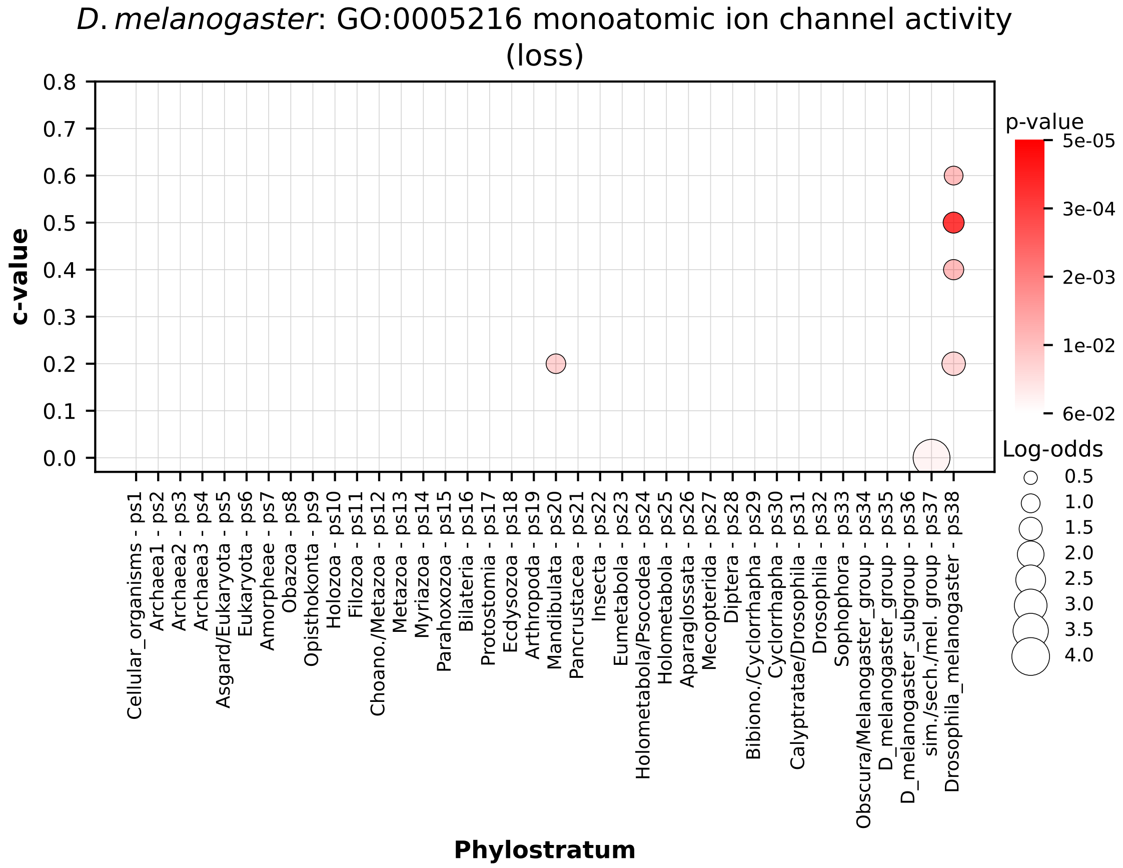
D. melanogaster: GO:0005216 monoatomic ion channel activity
(loss)
c-value
0.8
0.7
0.6
0.5
0.4
0.3
0.2
0.1
0.0
Phylostratum
Cellular_organisms - ps1
Archaea1 - ps2
Archaea2 - ps3
Archaea3 - ps4
Asgard/Eukaryota - ps5
Eukaryota - ps6
Amorpheae - ps7
Obazoa - ps8
Opisthokonta - ps9
Holozoa - ps10
Filozoa - ps11
Choano./Metazoa - ps12
Metazoa - ps13
Myriazoa - ps14
Parahoxozoa - ps15
Bilateria - ps16
Protostomia - ps17
Ecdysozoa - ps18
Arthropoda - ps19
Mandibulata - ps20
Pancrustacea - ps21
Insecta - ps22
Eumetabola - ps23
Holometabola/Psocodea - ps24
Holometabola - ps25
Aparaglossata - ps26
Mecopterida - ps27
Diptera - ps28
Bibiono./Cyclorrhapha - ps29
Cyclorrhapha - ps30
Calyptratae/Drosophila - ps31
Drosophila - ps32
Sophophora - ps33
Obscura/Melanogaster_group - ps34
D_melanogaster_group - ps35
D_melanogaster_subgroup - ps36
sim./sech./mel. group - ps37
Drosophila_melanogaster - ps38
p-value
5e-05
3e-04
2e-03
1e-02
6e-02
Log-odds
0.5
1.0
1.5
2.0
2.5
3.0
3.5
4.0

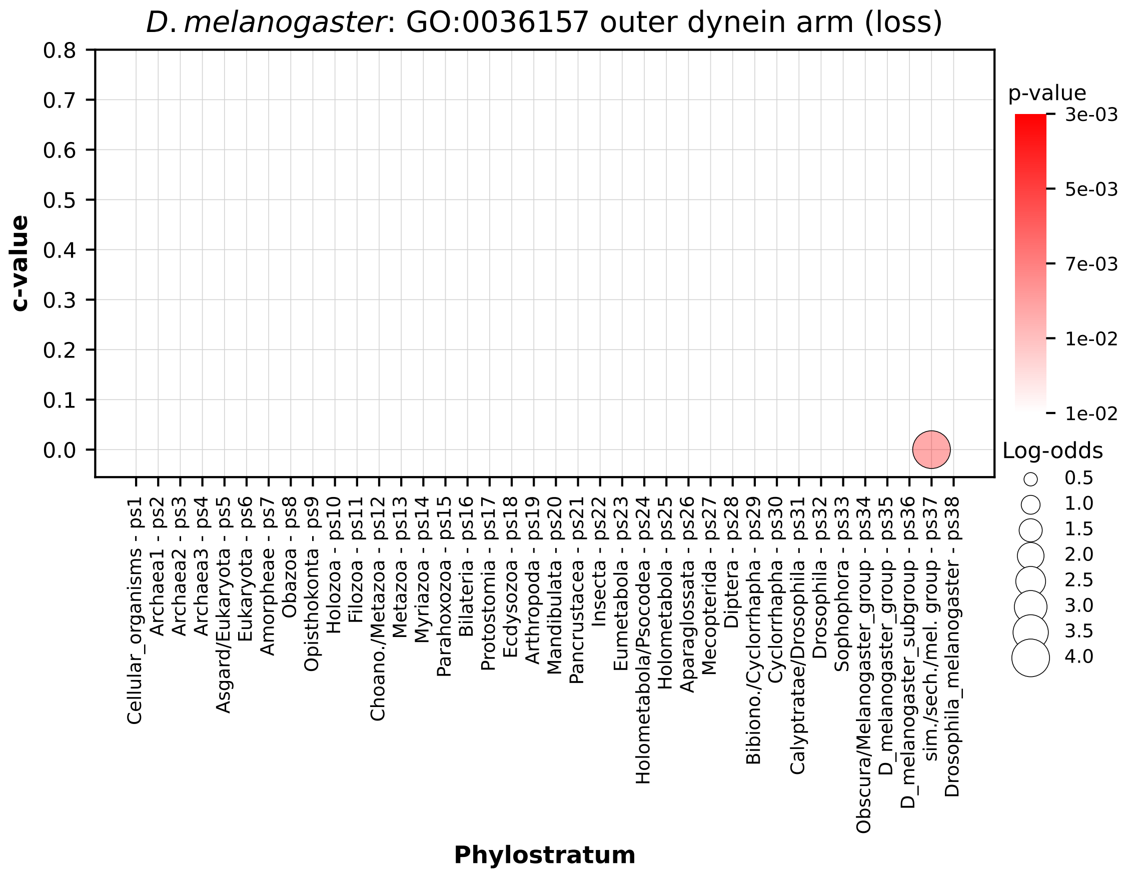
D. melanogaster: GO:0036157 outer dynein arm (loss)
c-value
0.8
0.7
0.6
0.5
0.4
0.3
0.2
0.1
0.0
p-value
3e-03
5e-03
7e-03
1e-02
1e-02
Log-odds
0.5
1.0
1.5
2.0
2.5
3.0
3.5
4.0
Cellular_organisms - ps1
Archaea1 - ps2
Archaea2 - ps3
Archaea3 - ps4
Asgard/Eukaryota - ps5
Eukaryota - ps6
Amorpheae - ps7
Obazoa - ps8
Opisthokonta - ps9
Holozoa - ps10
Filozoa - ps11
Choano./Metazoa - ps12
Metazoa - ps13
Myriazoa - ps14
Parahoxozoa - ps15
Bilateria - ps16
Protostomia - ps17
Ecdysozoa - ps18
Arthropoda - ps19
Mandibulata - ps20
Pancrustacea - ps21
Insecta - ps22
Eumetabola - ps23
Holometabola/Psocodea - ps24
Holometabola - ps25
Aparaglossata - ps26
Mecopterida - ps27
Diptera - ps28
Bibiono./Cyclorrhapha - ps29
Cyclorrhapha - ps30
Calyptratae/Drosophila - ps31
Drosophila - ps32
Sophophora - ps33
Obscura/Melanogaster_group - ps34
D_melanogaster_group - ps35
D_melanogaster_subgroup - ps36
sim./sech./mel. group - ps37
Drosophila_melanogaster - ps38
Phylostratum

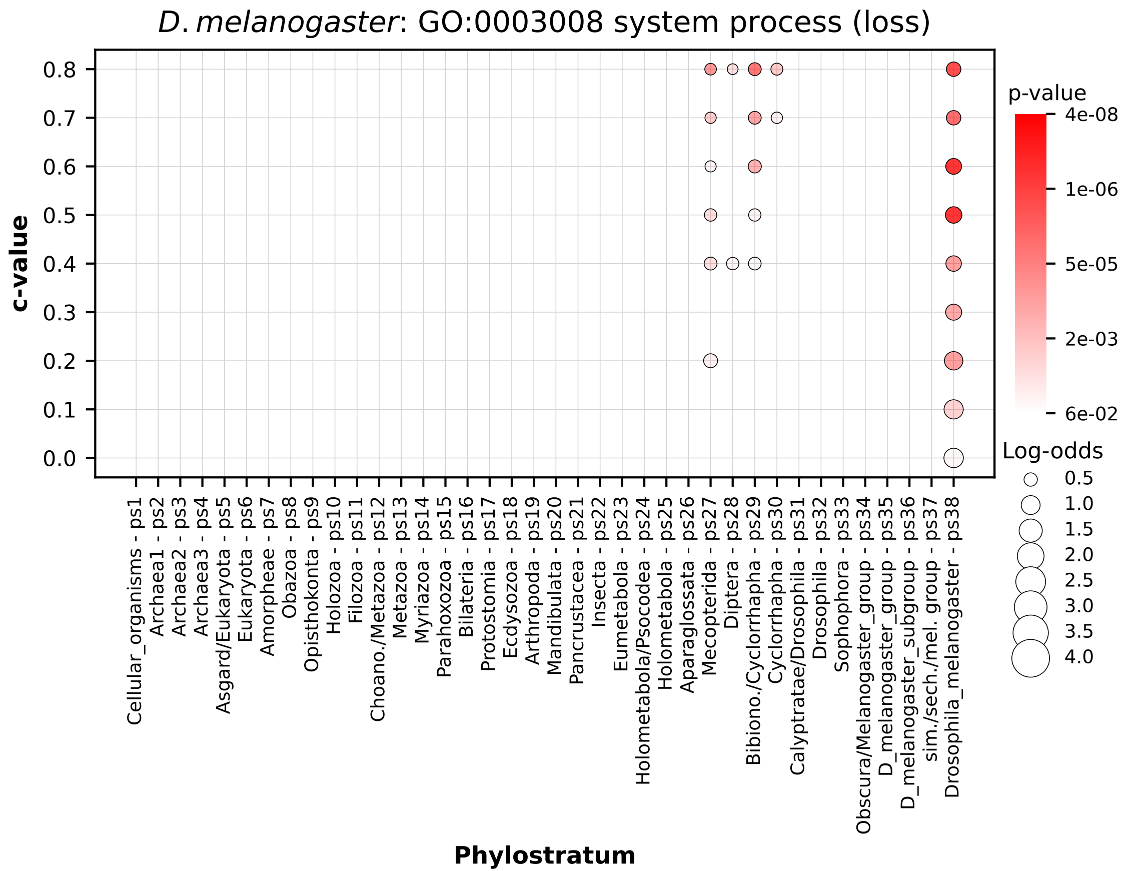
D. melanogaster: GO:0003008 system process (loss)
c-value
0.8
0.7
0.6
0.5
0.4
0.3
0.2
0.1
0.0
p-value
4e-08
1e-06
5e-05
2e-03
6e-02
Log-odds
0.5
1.0
1.5
2.0
2.5
3.0
3.5
4.0
Cellular_organisms - ps1
Archaea1 - ps2
Archaea2 - ps3
Archaea3 - ps4
Asgard/Eukaryota - ps5
Eukaryota - ps6
Amorpheae - ps7
Obazoa - ps8
Opisthokonta - ps9
Holozoa - ps10
Filozoa - ps11
Choano./Metazoa - ps12
Metazoa - ps13
Myriazoa - ps14
Parahoxozoa - ps15
Bilateria - ps16
Protostomia - ps17
Ecdysozoa - ps18
Arthropoda - ps19
Mandibulata - ps20
Pancrustacea - ps21
Insecta - ps22
Eumetabola - ps23
Holometabola/Psocodea - ps24
Holometabola - ps25
Aparaglossata - ps26
Mecopterida - ps27
Diptera - ps28
Bibiono./Cyclorrhapha - ps29
Cyclorrhapha - ps30
Calyptratae/Drosophila - ps31
Drosophila - ps32
Sophophora - ps33
Obscura/Melanogaster_group - ps34
D_melanogaster_group - ps35
D_melanogaster_subgroup - ps36
sim./sech./mel. group - ps37
Drosophila_melanogaster - ps38
Phylostratum

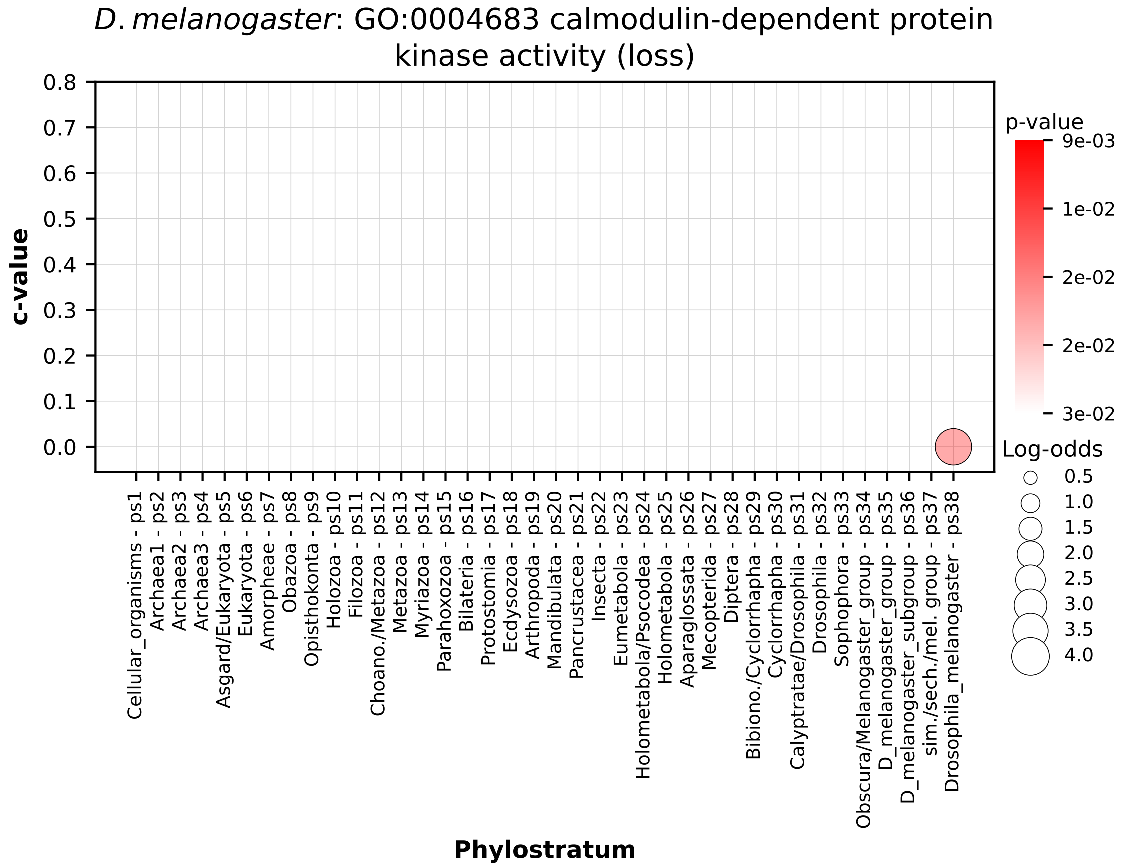

D. melanogaster: GO:0004683 calmodulin-dependent protein kinase activity (loss)
c-value
0.8
0.7
0.6
0.5
0.4
0.3
0.2
0.1
0.0
Phylostratum
Cellular_organisms - ps1
Archaea1 - ps2
Archaea2 - ps3
Archaea3 - ps4
Asgard/Eukaryota - ps5
Eukaryota - ps6
Amorpheae - ps7
Obazoa - ps8
Opisthokonta - ps9
Holozoa - ps10
Filozoa - ps11
Choano./Metazoa - ps12
Metazoa - ps13
Myriazoa - ps14
Parahoxozoa - ps15
Bilateria - ps16
Protostomia - ps17
Ecdysozoa - ps18
Arthropoda - ps19
Mandibulata - ps20
Pancrustacea - ps21
Insecta - ps22
Eumetabola - ps23
Holometabola/Psocodea - ps24
Holometabola - ps25
Aparaglossata - ps26
Mecopterida - ps27
Diptera - ps28
Bibiono./Cyclorrhapha - ps29
Cyclorrhapha - ps30
Calyptratae/Drosophila - ps31
Drosophila - ps32
Sophophora - ps33
Obscura/Melanogaster_group - ps34
D_melanogaster_group - ps35
D_melanogaster_subgroup - ps36
sim./sech./mel. group - ps37
Drosophila_melanogaster - ps38
p-value
9e-03
1e-02
2e-02
2e-02
3e-02
Log-odds
0.5
1.0
1.5
2.0
2.5
3.0
3.5
4.0

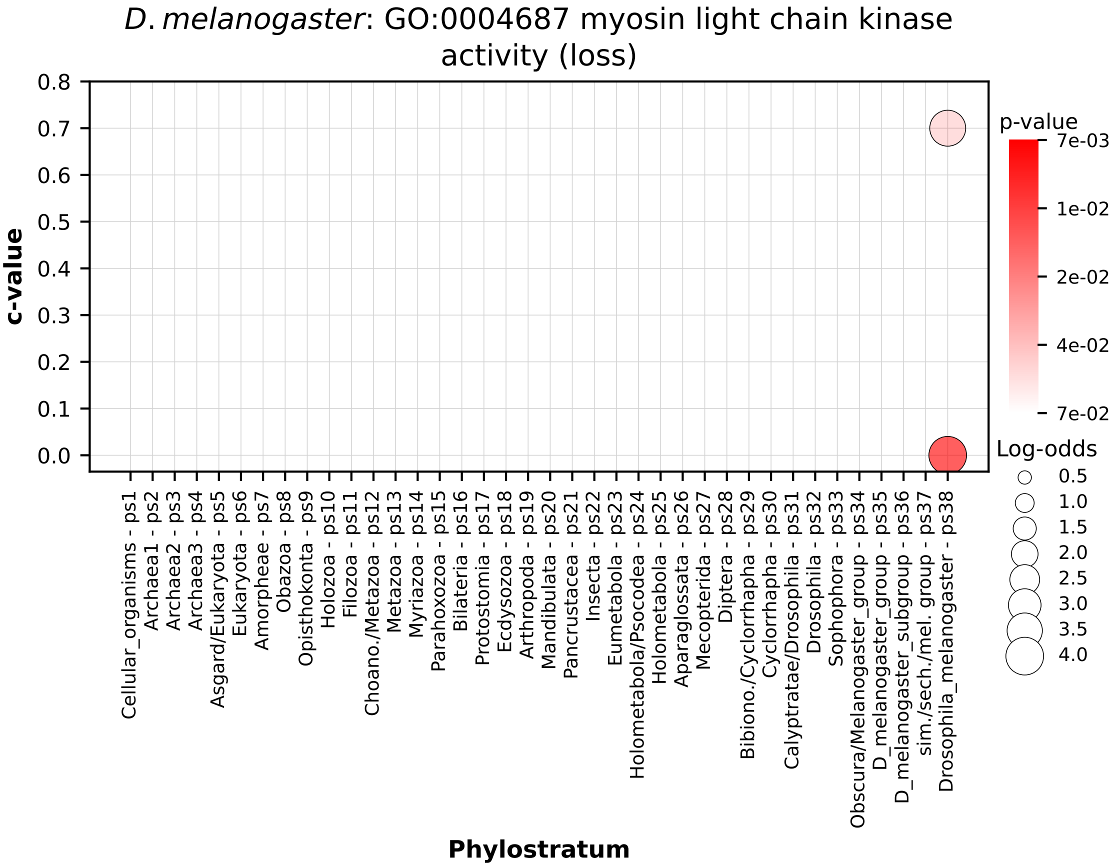
D. melanogaster: GO:0004687 myosin light chain kinase activity (loss)
c-value
0.8
0.7
0.6
0.5
0.4
0.3
0.2
0.1
0.0
p-value
7e-03
1e-02
2e-02
4e-02
7e-02
Log-odds
0.5
1.0
1.5
2.0
2.5
3.0
3.5
4.0
Cellular_organisms - ps1
Archaea1 - ps2
Archaea2 - ps3
Archaea3 - ps4
Asgard/Eukaryota - ps5
Eukaryota - ps6
Amorpheae - ps7
Obazoa - ps8
Opisthokonta - ps9
Holozoa - ps10
Filozoa - ps11
Choano./Metazoa - ps12
Metazoa - ps13
Myriazoa - ps14
Parahoxozoa - ps15
Bilateria - ps16
Protostomia - ps17
Ecdysozoa - ps18
Arthropoda - ps19
Mandibulata - ps20
Pancrustacea - ps21
Insecta - ps22
Eumetabola - ps23
Holometabola/Psocodea - ps24
Holometabola - ps25
Aparaglossata - ps26
Mecopterida - ps27
Diptera - ps28
Bibiono./Cyclorrhapha - ps29
Cyclorrhapha - ps30
Calyptratae/Drosophila - ps31
Drosophila - ps32
Sophophora - ps33
Obscura/Melanogaster_group - ps34
D_melanogaster_group - ps35
D_melanogaster_subgroup - ps36
sim./sech./mel. group - ps37
Drosophila_melanogaster - ps38
Phylostratum

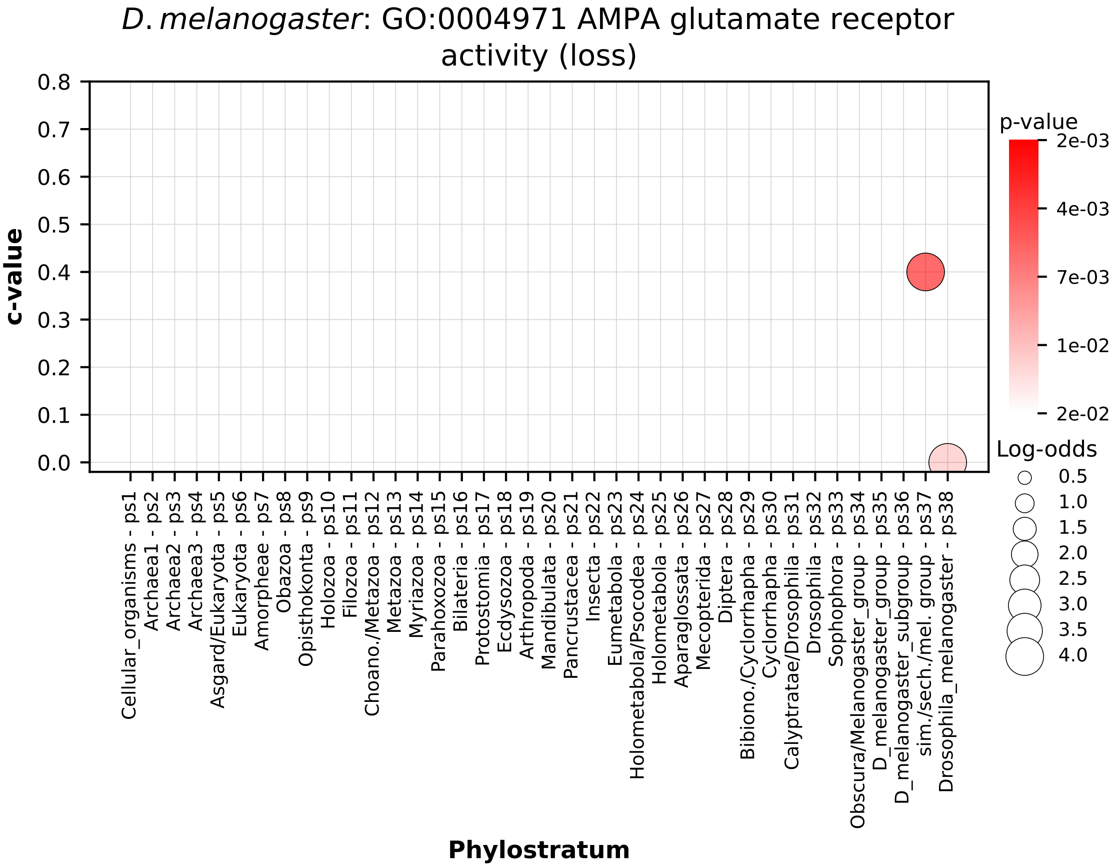

D. melanogaster: GO:0004971 AMPA glutamate receptor activity (loss)
c-value
0.8
0.7
0.6
0.5
0.4
0.3
0.2
0.1
0.0
p-value
2e-03
4e-03
7e-03
1e-02
2e-02
Log-odds
0.5
1.0
1.5
2.0
2.5
3.0
3.5
4.0
Cellular_organisms - ps1
Archaea1 - ps2
Archaea2 - ps3
Archaea3 - ps4
Asgard/Eukaryota - ps5
Eukaryota - ps6
Amorpheae - ps7
Obazoa - ps8
Opisthokonta - ps9
Holozoa - ps10
Filozoa - ps11
Choano./Metazoa - ps12
Metazoa - ps13
Myriazoa - ps14
Parahoxozoa - ps15
Bilateria - ps16
Protostomia - ps17
Ecdysozoa - ps18
Arthropoda - ps19
Mandibulata - ps20
Pancrustacea - ps21
Insecta - ps22
Eumetabola - ps23
Holometabola/Psocodea - ps24
Holometabola - ps25
Aparaglossata - ps26
Mecopterida - ps27
Diptera - ps28
Bibiono./Cyclorrhapha - ps29
Cyclorrhapha - ps30
Calyptratae/Drosophila - ps31
Drosophila - ps32
Sophophora - ps33
Obscura/Melanogaster_group - ps34
D_melanogaster_group - ps35
D_melanogaster_subgroup - ps36
sim./sech./mel. group - ps37
Drosophila_melanogaster - ps38
Phylostratum

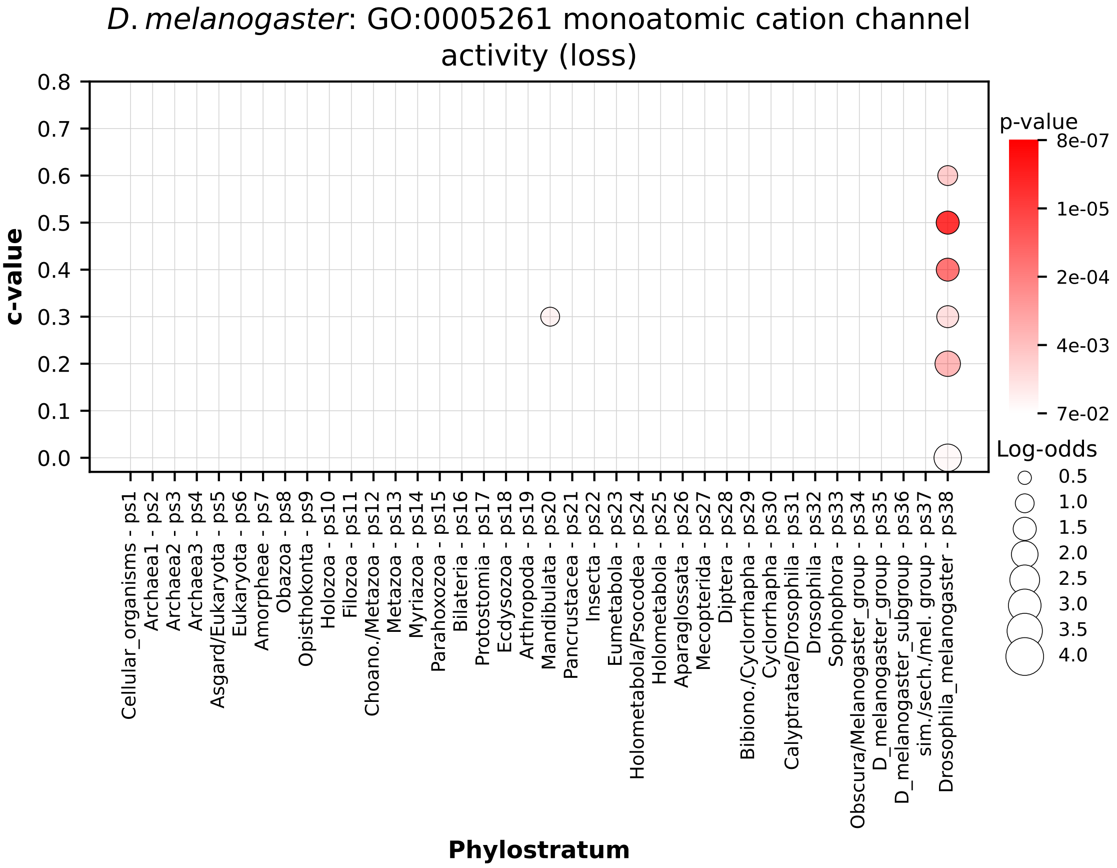
D. melanogaster: GO:0005261 monoatomic cation channel activity (loss)
c-value
0.8
0.7
0.6
0.5
0.4
0.3
0.2
0.1
0.0
p-value
8e-07
1e-05
2e-04
4e-03
7e-02
Log-odds
0.5
1.0
1.5
2.0
2.5
3.0
3.5
4.0
Cellular_organisms - ps1
Archaea1 - ps2
Archaea2 - ps3
Archaea3 - ps4
Asgard/Eukaryota - ps5
Eukaryota - ps6
Amorpheae - ps7
Obazoa - ps8
Opisthokonta - ps9
Holozoa - ps10
Filozoa - ps11
Choano./Metazoa - ps12
Metazoa - ps13
Myriazoa - ps14
Parahoxozoa - ps15
Bilateria - ps16
Protostomia - ps17
Ecdysozoa - ps18
Arthropoda - ps19
Mandibulata - ps20
Pancrustacea - ps21
Insecta - ps22
Eumetabola - ps23
Holometabola/Psocodea - ps24
Holometabola - ps25
Aparaglossata - ps26
Mecopterida - ps27
Diptera - ps28
Bibiono./Cyclorrhapha - ps29
Cyclorrhapha - ps30
Calyptratae/Drosophila - ps31
Drosophila - ps32
Sophophora - ps33
Obscura/Melanogaster_group - ps34
D_melanogaster_group - ps35
D_melanogaster_subgroup - ps36
sim./sech./mel. group - ps37
Drosophila_melanogaster - ps38
Phylostratum

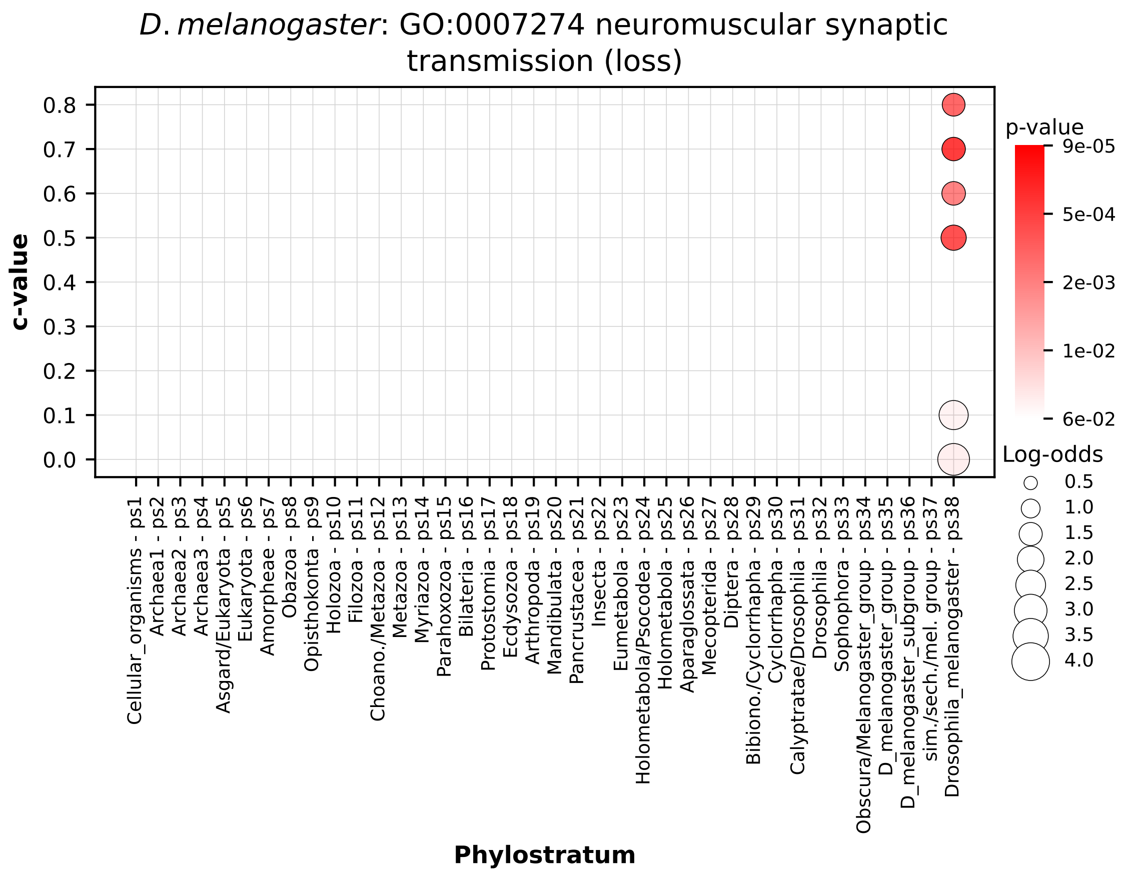

D. melanogaster: GO:0007274 neuromuscular synaptic transmission (loss)
c-value
0.0
0.1
0.2
0.3
0.4
0.5
0.6
0.7
0.8
Phylostratum
Cellular_organisms - ps1
Archaea1 - ps2
Archaea2 - ps3
Archaea3 - ps4
Asgard/Eukaryota - ps5
Eukaryota - ps6
Amorpheae - ps7
Obazoa - ps8
Opisthokonta - ps9
Holozoa - ps10
Filozoa - ps11
Choano./Metazoa - ps12
Metazoa - ps13
Myriazoa - ps14
Parahoxozoa - ps15
Bilateria - ps16
Protostomia - ps17
Ecdysozoa - ps18
Arthropoda - ps19
Mandibulata - ps20
Pancrustacea - ps21
Insecta - ps22
Eumetabola - ps23
Holometabola/Psocodea - ps24
Holometabola - ps25
Aparaglossata - ps26
Mecopterida - ps27
Diptera - ps28
Bibiono./Cyclorrhapha - ps29
Cyclorrhapha - ps30
Calyptratae/Drosophila - ps31
Drosophila - ps32
Sophophora - ps33
Obscura/Melanogaster_group - ps34
D_melanogaster_group - ps35
D_melanogaster_subgroup - ps36
sim./sech./mel. group - ps37
Drosophila_melanogaster - ps38
p-value
9e-05
5e-04
2e-03
1e-02
6e-02
Log-odds
0.5
1.0
1.5
2.0
2.5
3.0
3.5
4.0

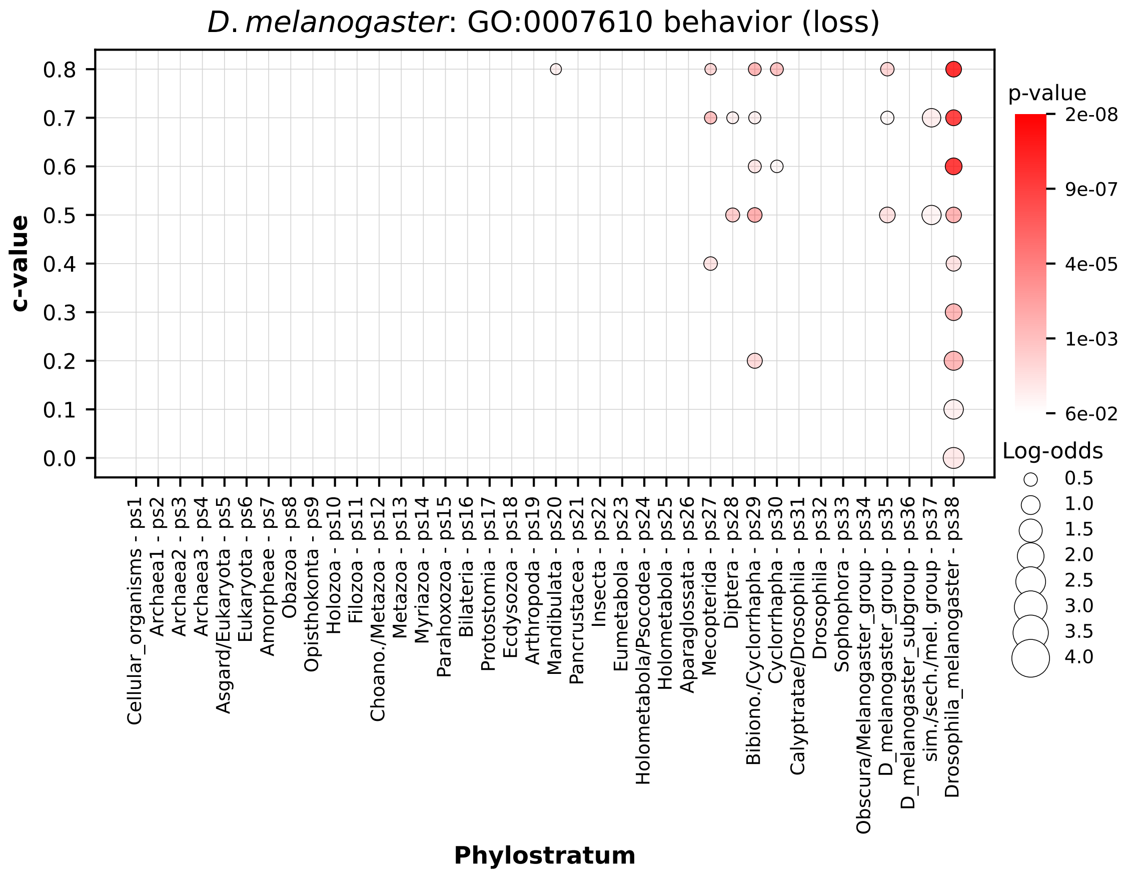

D. melanogaster: GO:0007610 behavior (loss)
c-value
0.8
0.7
0.6
0.5
0.4
0.3
0.2
0.1
0.0
p-value
2e-08
9e-07
4e-05
1e-03
6e-02
Log-odds
0.5
1.0
1.5
2.0
2.5
3.0
3.5
4.0
Cellular_organisms - ps1
Archaea1 - ps2
Archaea2 - ps3
Archaea3 - ps4
Asgard/Eukaryota - ps5
Eukaryota - ps6
Amorpheae - ps7
Obazoa - ps8
Opisthokonta - ps9
Holozoa - ps10
Filozoa - ps11
Choano./Metazoa - ps12
Metazoa - ps13
Myriazoa - ps14
Parahoxozoa - ps15
Bilateria - ps16
Protostomia - ps17
Ecdysozoa - ps18
Arthropoda - ps19
Mandibulata - ps20
Pancrustacea - ps21
Insecta - ps22
Eumetabola - ps23
Holometabola/Psocodea - ps24
Holometabola - ps25
Aparaglossata - ps26
Mecopterida - ps27
Diptera - ps28
Bibiono./Cyclorrhapha - ps29
Cyclorrhapha - ps30
Calyptratae/Drosophila - ps31
Drosophila - ps32
Sophophora - ps33
Obscura/Melanogaster_group - ps34
D_melanogaster_group - ps35
D_melanogaster_subgroup - ps36
sim./sech./mel. group - ps37
Drosophila_melanogaster - ps38
Phylostratum

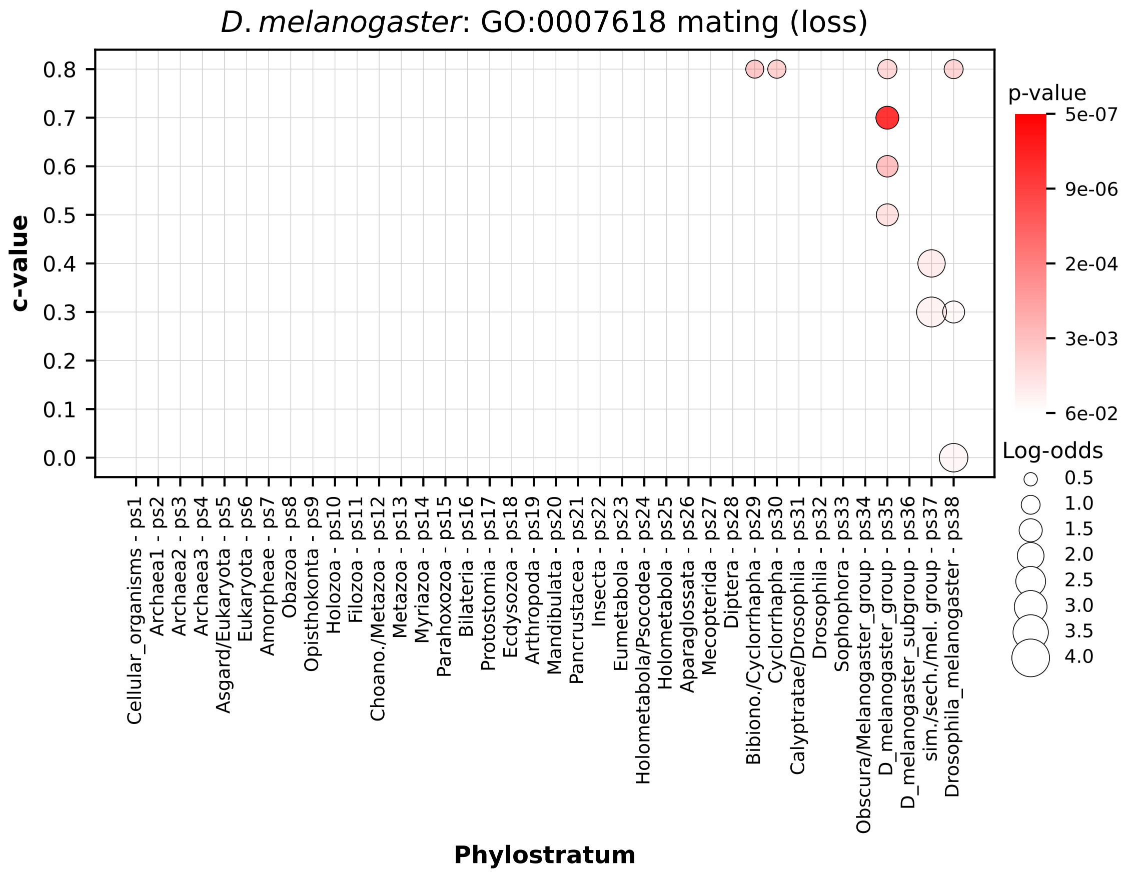
D. melanogaster: GO:0007618 mating (loss)
c-value
0.8
0.7
0.6
0.5
0.4
0.3
0.2
0.1
0.0
Phylostratum
Cellular_organisms - ps1
Archaea1 - ps2
Archaea2 - ps3
Archaea3 - ps4
Asgard/Eukaryota - ps5
Eukaryota - ps6
Amorpheae - ps7
Obazoa - ps8
Opisthokonta - ps9
Holozoa - ps10
Filozoa - ps11
Choano./Metazoa - ps12
Metazoa - ps13
Myriazoa - ps14
Parahoxozoa - ps15
Bilateria - ps16
Protostomia - ps17
Ecdysozoa - ps18
Arthropoda - ps19
Mandibulata - ps20
Pancrustacea - ps21
Insecta - ps22
Eumetabola - ps23
Holometabola/Psocodea - ps24
Holometabola - ps25
Aparaglossata - ps26
Mecopterida - ps27
Diptera - ps28
Bibiono./Cyclorrhapha - ps29
Cyclorrhapha - ps30
Calyptratae/Drosophila - ps31
Drosophila - ps32
Sophophora - ps33
Obscura/Melanogaster_group - ps34
D_melanogaster_group - ps35
D_melanogaster_subgroup - ps36
sim./sech./mel. group - ps37
Drosophila_melanogaster - ps38
p-value
5e-07
9e-06
2e-04
3e-03
6e-02
Log-odds
0.5
1.0
1.5
2.0
2.5
3.0
3.5
4.0

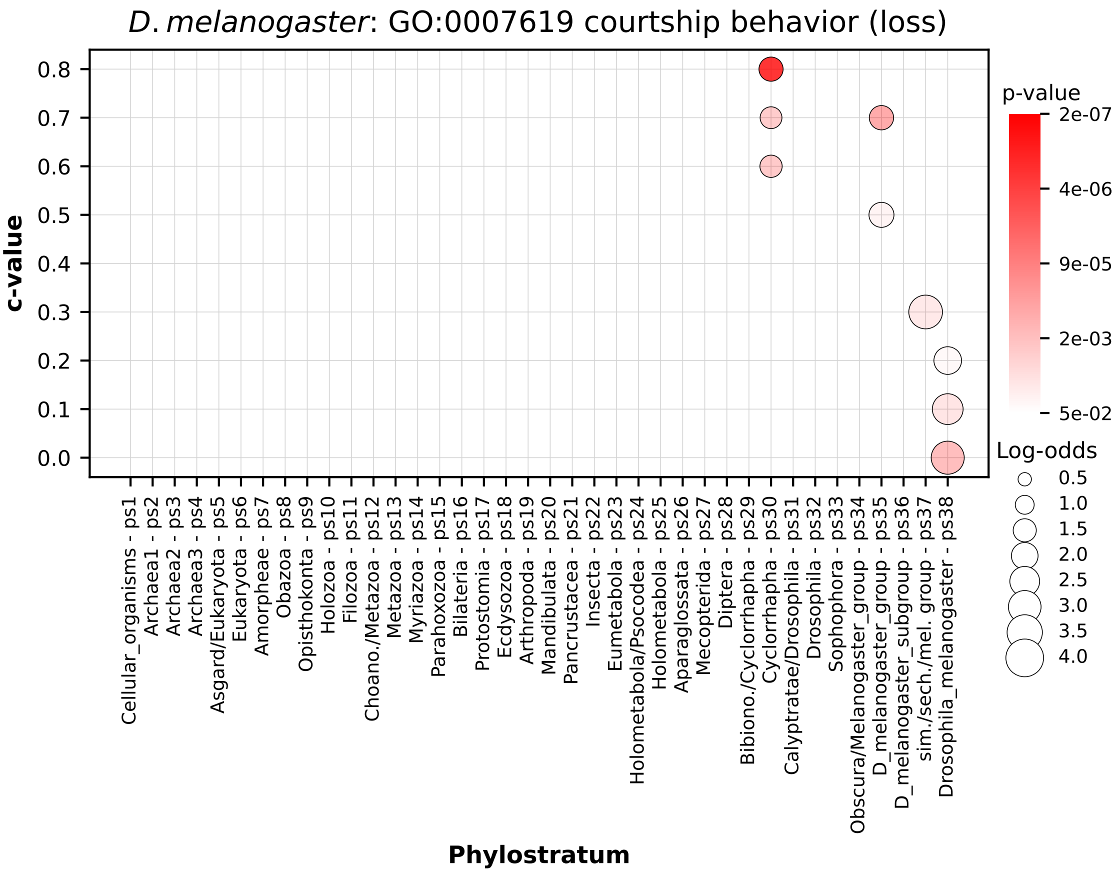

D. melanogaster: GO:0007619 courtship behavior (loss)
c-value
0.0
0.1
0.2
0.3
0.4
0.5
0.6
0.7
0.8
Phylostratum
Cellular_organisms - ps1
Archaea1 - ps2
Archaea2 - ps3
Archaea3 - ps4
Asgard/Eukaryota - ps5
Eukaryota - ps6
Amorpheae - ps7
Obazoa - ps8
Opisthokonta - ps9
Holozoa - ps10
Filozoa - ps11
Choano./Metazoa - ps12
Metazoa - ps13
Myriazoa - ps14
Parahoxozoa - ps15
Bilateria - ps16
Protostomia - ps17
Ecdysozoa - ps18
Arthropoda - ps19
Mandibulata - ps20
Pancrustacea - ps21
Insecta - ps22
Eumetabola - ps23
Holometabola/Psocodea - ps24
Holometabola - ps25
Aparaglossata - ps26
Mecopterida - ps27
Diptera - ps28
Bibiono./Cyclorrhapha - ps29
Cyclorrhapha - ps30
Calyptratae/Drosophila - ps31
Drosophila - ps32
Sophophora - ps33
Obscura/Melanogaster_group - ps34
D_melanogaster_group - ps35
D_melanogaster_subgroup - ps36
sim./sech./mel. group - ps37
Drosophila_melanogaster - ps38
p-value
2e-07
4e-06
9e-05
2e-03
5e-02
Log-odds
0.5
1.0
1.5
2.0
2.5
3.0
3.5
4.0

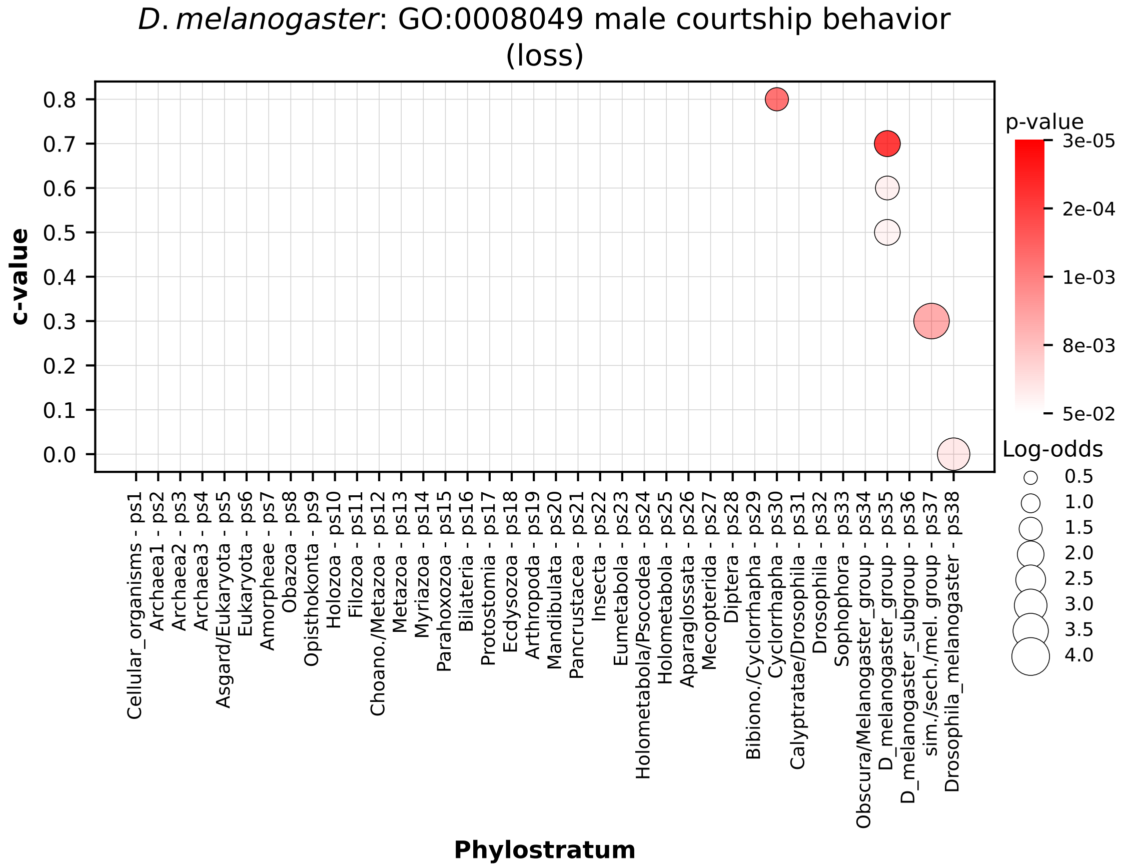

D. melanogaster: GO:0008049 male courtship behavior
(loss)
c-value
0.8
0.7
0.6
0.5
0.4
0.3
0.2
0.1
0.0
p-value
3e-05
2e-04
1e-03
8e-03
5e-02
Log-odds
0.5
1.0
1.5
2.0
2.5
3.0
3.5
4.0
Cellular_organisms - ps1
Archaea1 - ps2
Archaea2 - ps3
Archaea3 - ps4
Asgard/Eukaryota - ps5
Eukaryota - ps6
Amorpheae - ps7
Obazoa - ps8
Opisthokonta - ps9
Holozoa - ps10
Filozoa - ps11
Choano./Metazoa - ps12
Metazoa - ps13
Myriazoa - ps14
Parahoxozoa - ps15
Bilateria - ps16
Protostomia - ps17
Ecdysozoa - ps18
Arthropoda - ps19
Mandibulata - ps20
Pancrustacea - ps21
Insecta - ps22
Eumetabola - ps23
Holometabola/Psocodea - ps24
Holometabola - ps25
Aparaglossata - ps26
Mecopterida - ps27
Diptera - ps28
Bibiono./Cyclorrhapha - ps29
Cyclorrhapha - ps30
Calyptratae/Drosophila - ps31
Drosophila - ps32
Sophophora - ps33
Obscura/Melanogaster_group - ps34
D_melanogaster_group - ps35
D_melanogaster_subgroup - ps36
sim./sech./mel. group - ps37
Drosophila_melanogaster - ps38
Phylostratum

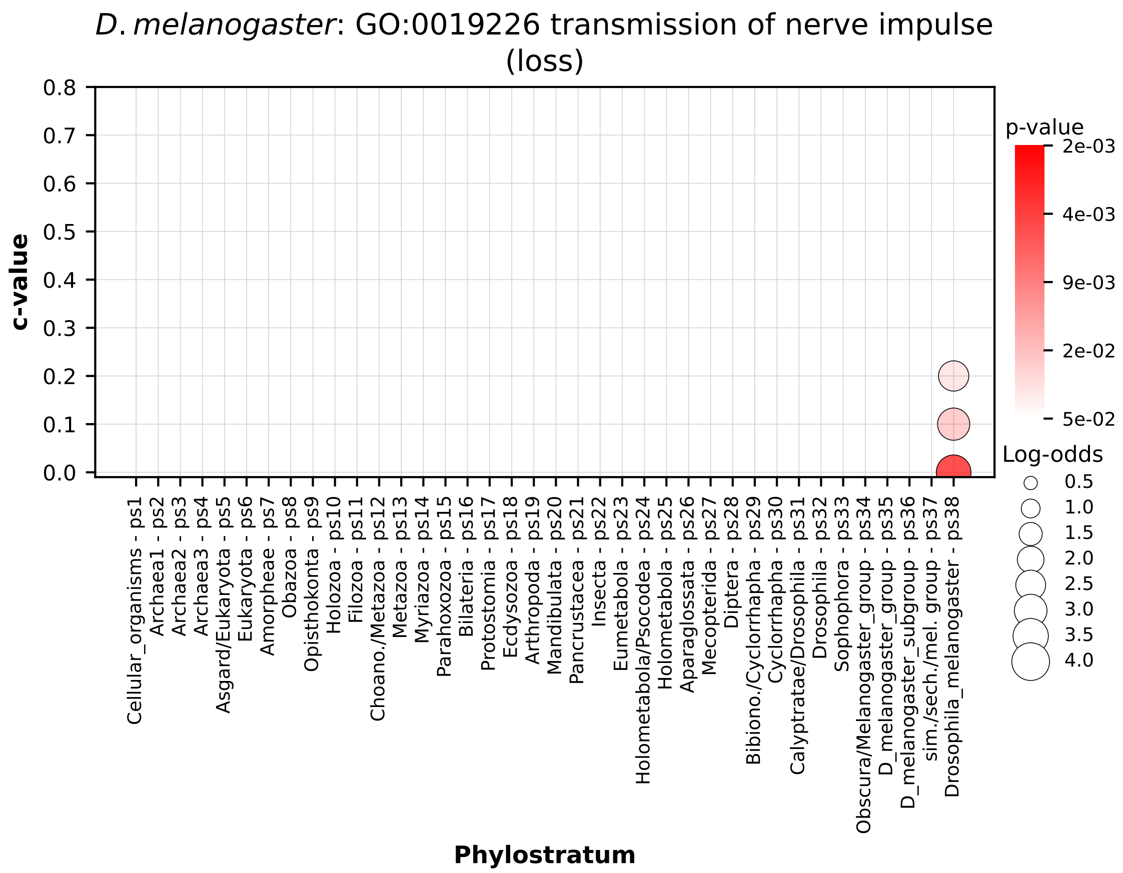
D. melanogaster: GO:0019226 transmission of nerve impulse
(loss)
c-value
0.0
0.1
0.2
0.3
0.4
0.5
0.6
0.7
0.8
p-value
2e-03
4e-03
9e-03
2e-02
5e-02
Log-odds
0.5
1.0
1.5
2.0
2.5
3.0
3.5
4.0
Cellular_organisms - ps1
Archaea1 - ps2
Archaea2 - ps3
Archaea3 - ps4
Asgard/Eukaryota - ps5
Eukaryota - ps6
Amorpheae - ps7
Obazoa - ps8
Opisthokonta - ps9
Holozoa - ps10
Filozoa - ps11
Choano./Metazoa - ps12
Metazoa - ps13
Myriazoa - ps14
Parahoxozoa - ps15
Bilateria - ps16
Protostomia - ps17
Ecdysozoa - ps18
Arthropoda - ps19
Mandibulata - ps20
Pancrustacea - ps21
Insecta - ps22
Eumetabola - ps23
Holometabola/Psocodea - ps24
Holometabola - ps25
Aparaglossata - ps26
Mecopterida - ps27
Diptera - ps28
Bibiono./Cyclorrhapha - ps29
Cyclorrhapha - ps30
Calyptratae/Drosophila - ps31
Drosophila - ps32
Sophophora - ps33
Obscura/Melanogaster_group - ps34
D_melanogaster_group - ps35
D_melanogaster_subgroup - ps36
sim./sech./mel. group - ps37
Drosophila_melanogaster - ps38
Phylostratum

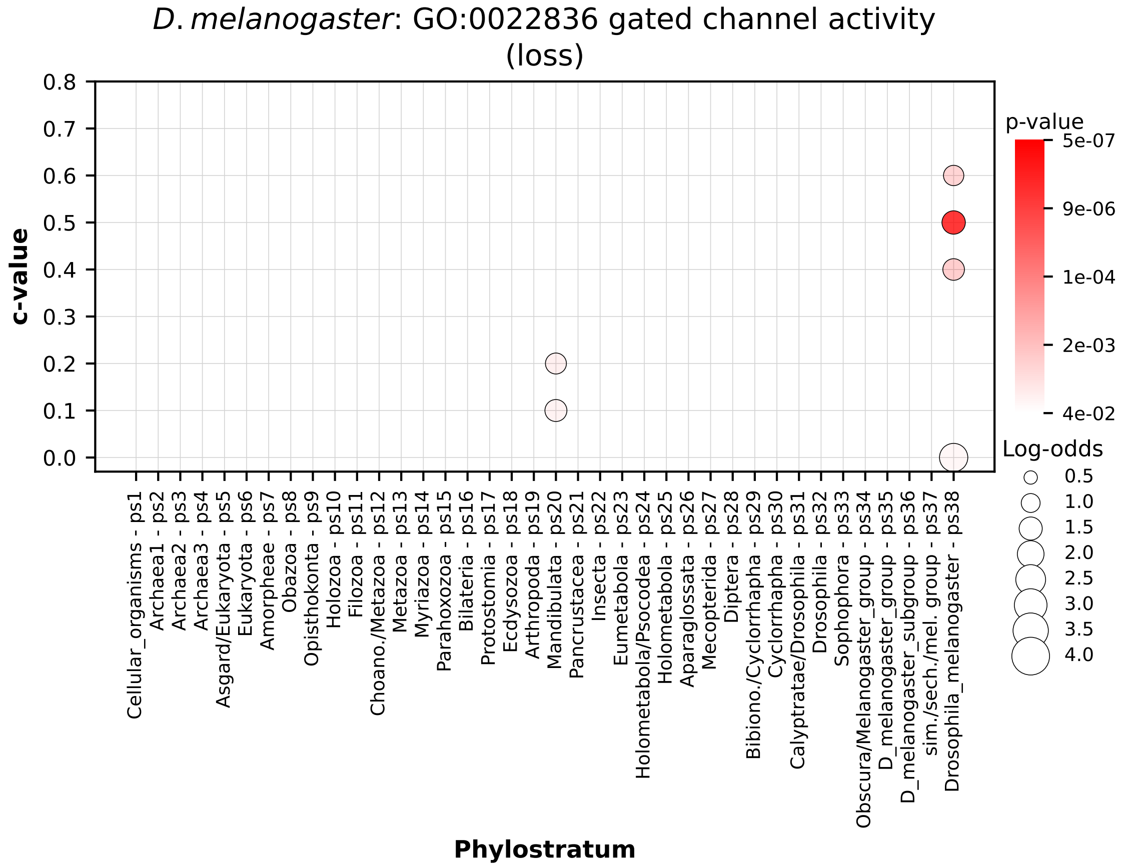

D. melanogaster: GO:0022836 gated channel activity
(loss)
c-value
0.8
0.7
0.6
0.5
0.4
0.3
0.2
0.1
0.0
p-value
5e-07
9e-06
1e-04
2e-03
4e-02
Log-odds
0.5
1.0
1.5
2.0
2.5
3.0
3.5
4.0
Cellular_organisms - ps1
Archaea1 - ps2
Archaea2 - ps3
Archaea3 - ps4
Asgard/Eukaryota - ps5
Eukaryota - ps6
Amorpheae - ps7
Obazoa - ps8
Opisthokonta - ps9
Holozoa - ps10
Filozoa - ps11
Choano./Metazoa - ps12
Metazoa - ps13
Myriazoa - ps14
Parahoxozoa - ps15
Bilateria - ps16
Protostomia - ps17
Ecdysozoa - ps18
Arthropoda - ps19
Mandibulata - ps20
Pancrustacea - ps21
Insecta - ps22
Eumetabola - ps23
Holometabola/Psocodea - ps24
Holometabola - ps25
Aparaglossata - ps26
Mecopterida - ps27
Diptera - ps28
Bibiono./Cyclorrhapha - ps29
Cyclorrhapha - ps30
Calyptratae/Drosophila - ps31
Drosophila - ps32
Sophophora - ps33
Obscura/Melanogaster_group - ps34
D_melanogaster_group - ps35
D_melanogaster_subgroup - ps36
sim./sech./mel. group - ps37
Drosophila_melanogaster - ps38
Phylostratum

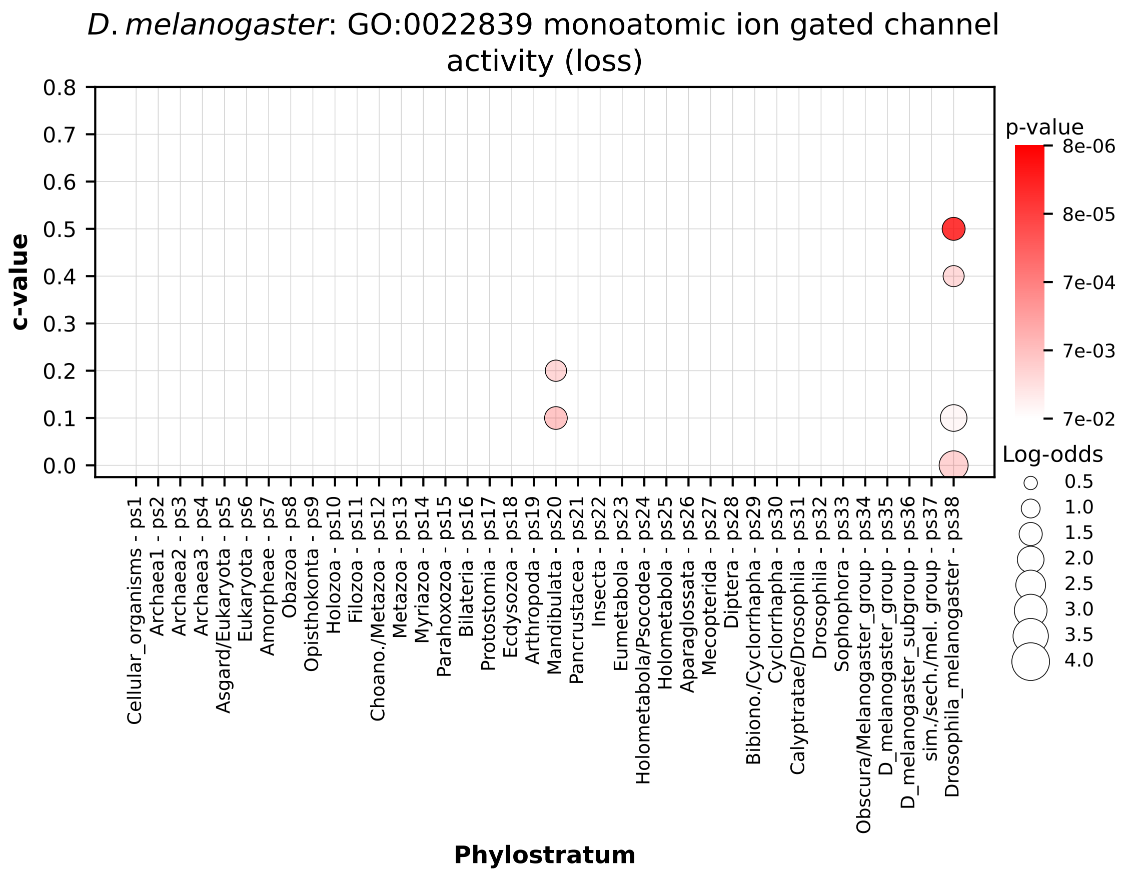
D. melanogaster: GO:0022839 monoatomic ion gated channel activity (loss)
c-value
0.8
0.7
0.6
0.5
0.4
0.3
0.2
0.1
0.0
p-value
8e-06
8e-05
7e-04
7e-03
7e-02
Log-odds
0.5
1.0
1.5
2.0
2.5
3.0
3.5
4.0
Cellular_organisms - ps1
Archaea1 - ps2
Archaea2 - ps3
Archaea3 - ps4
Asgard/Eukaryota - ps5
Eukaryota - ps6
Amorpheae - ps7
Obazoa - ps8
Opisthokonta - ps9
Holozoa - ps10
Filozoa - ps11
Choano./Metazoa - ps12
Metazoa - ps13
Myriazoa - ps14
Parahoxozoa - ps15
Bilateria - ps16
Protostomia - ps17
Ecdysozoa - ps18
Arthropoda - ps19
Mandibulata - ps20
Pancrustacea - ps21
Insecta - ps22
Eumetabola - ps23
Holometabola/Psocodea - ps24
Holometabola - ps25
Aparaglossata - ps26
Mecopterida - ps27
Diptera - ps28
Bibiono./Cyclorrhapha - ps29
Cyclorrhapha - ps30
Calyptratae/Drosophila - ps31
Drosophila - ps32
Sophophora - ps33
Obscura/Melanogaster_group - ps34
D_melanogaster_group - ps35
D_melanogaster_subgroup - ps36
sim./sech./mel. group - ps37
Drosophila_melanogaster - ps38
Phylostratum

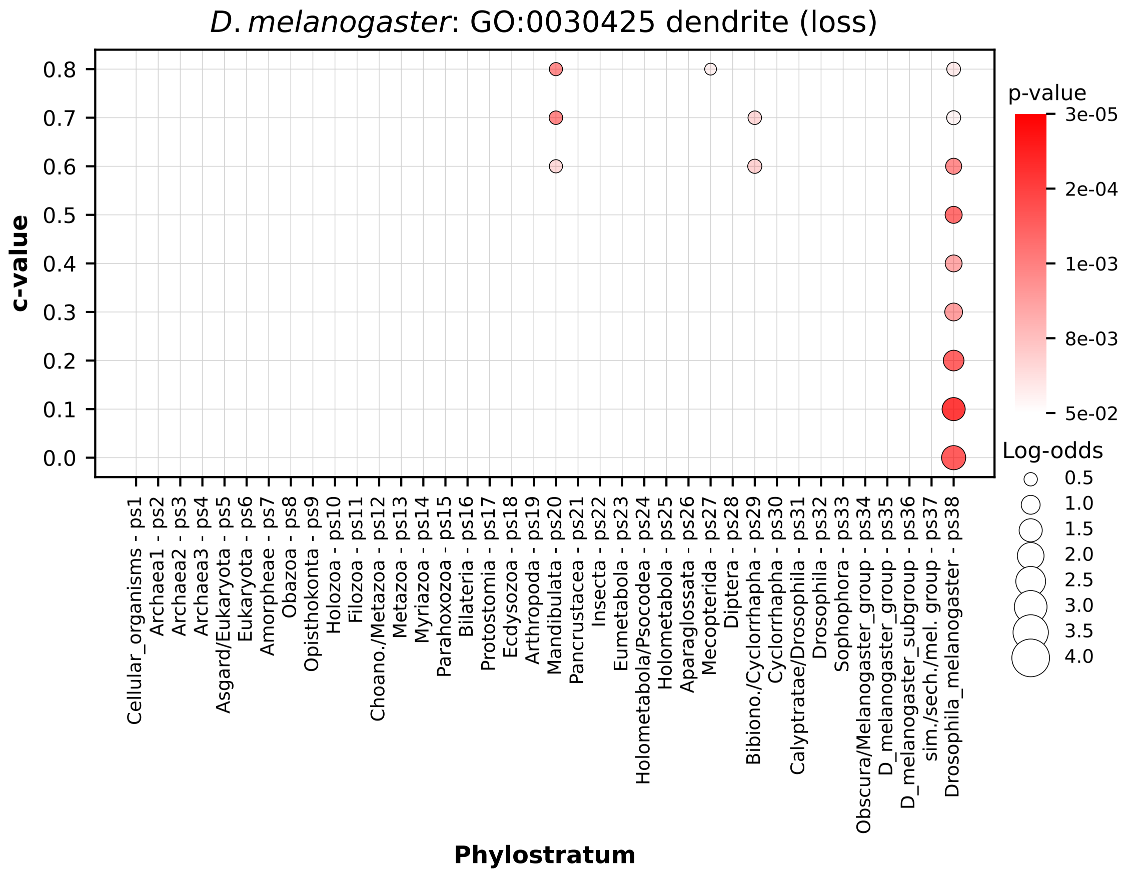
D. melanogaster: GO:0030425 dendrite (loss)
c-value
0.8
0.7
0.6
0.5
0.4
0.3
0.2
0.1
0.0
Phylostratum
Cellular_organisms - ps1
Archaea1 - ps2
Archaea2 - ps3
Archaea3 - ps4
Asgard/Eukaryota - ps5
Eukaryota - ps6
Amorpheae - ps7
Obazoa - ps8
Opisthokonta - ps9
Holozoa - ps10
Filozoa - ps11
Choano./Metazoa - ps12
Metazoa - ps13
Myriazoa - ps14
Parahoxozoa - ps15
Bilateria - ps16
Protostomia - ps17
Ecdysozoa - ps18
Arthropoda - ps19
Mandibulata - ps20
Pancrustacea - ps21
Insecta - ps22
Eumetabola - ps23
Holometabola/Psocodea - ps24
Holometabola - ps25
Aparaglossata - ps26
Mecopterida - ps27
Diptera - ps28
Bibiono./Cyclorrhapha - ps29
Cyclorrhapha - ps30
Calyptratae/Drosophila - ps31
Drosophila - ps32
Sophophora - ps33
Obscura/Melanogaster_group - ps34
D_melanogaster_group - ps35
D_melanogaster_subgroup - ps36
sim./sech./mel. group - ps37
Drosophila_melanogaster - ps38
p-value
3e-05
2e-04
1e-03
8e-03
5e-02
Log-odds
0.5
1.0
1.5
2.0
2.5
3.0
3.5
4.0

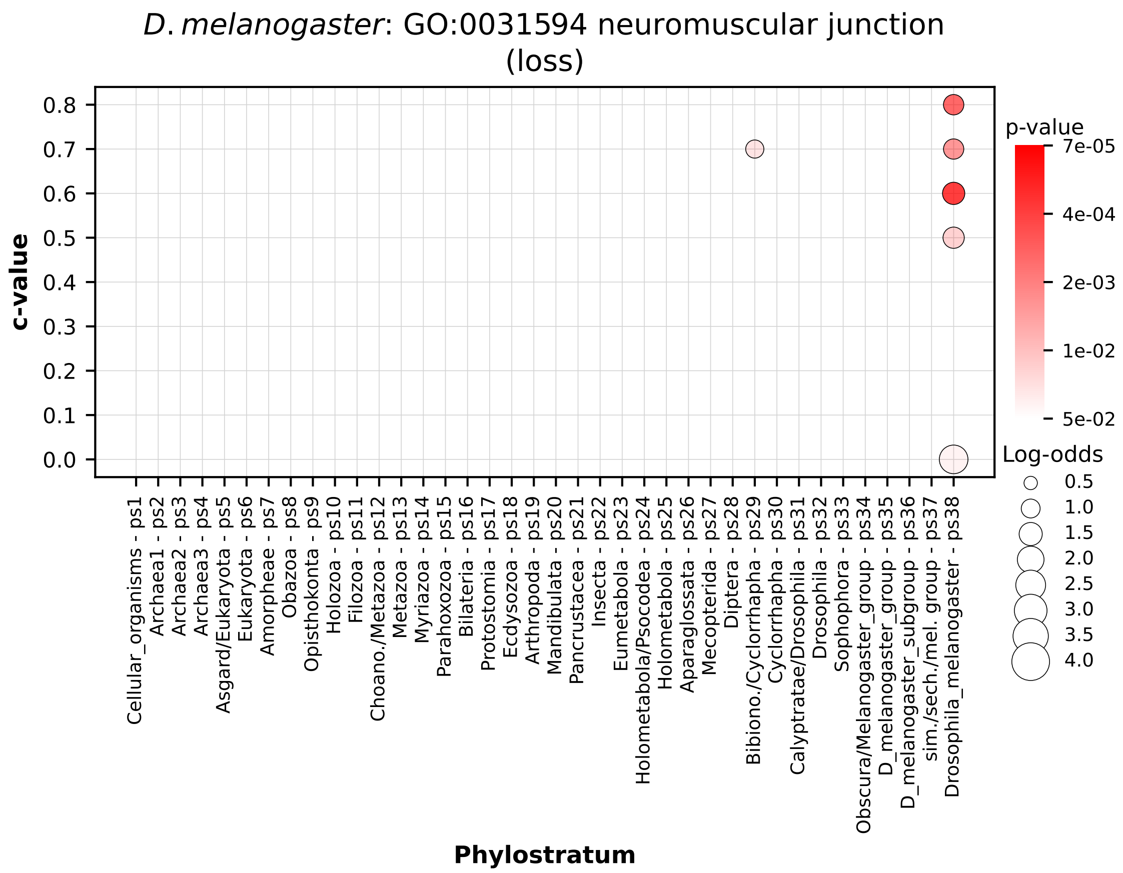

D. melanogaster: GO:0031594 neuromuscular junction
(loss)
c-value
0.8
0.7
0.6
0.5
0.4
0.3
0.2
0.1
0.0
p-value
7e-05
4e-04
2e-03
1e-02
5e-02
Log-odds
0.5
1.0
1.5
2.0
2.5
3.0
3.5
4.0
Cellular_organisms - ps1
Archaea1 - ps2
Archaea2 - ps3
Archaea3 - ps4
Asgard/Eukaryota - ps5
Eukaryota - ps6
Amorpheae - ps7
Obazoa - ps8
Opisthokonta - ps9
Holozoa - ps10
Filozoa - ps11
Choano./Metazoa - ps12
Metazoa - ps13
Myriazoa - ps14
Parahoxozoa - ps15
Bilateria - ps16
Protostomia - ps17
Ecdysozoa - ps18
Arthropoda - ps19
Mandibulata - ps20
Pancrustacea - ps21
Insecta - ps22
Eumetabola - ps23
Holometabola/Psocodea - ps24
Holometabola - ps25
Aparaglossata - ps26
Mecopterida - ps27
Diptera - ps28
Bibiono./Cyclorrhapha - ps29
Cyclorrhapha - ps30
Calyptratae/Drosophila - ps31
Drosophila - ps32
Sophophora - ps33
Obscura/Melanogaster_group - ps34
D_melanogaster_group - ps35
D_melanogaster_subgroup - ps36
sim./sech./mel. group - ps37
Drosophila_melanogaster - ps38
Phylostratum

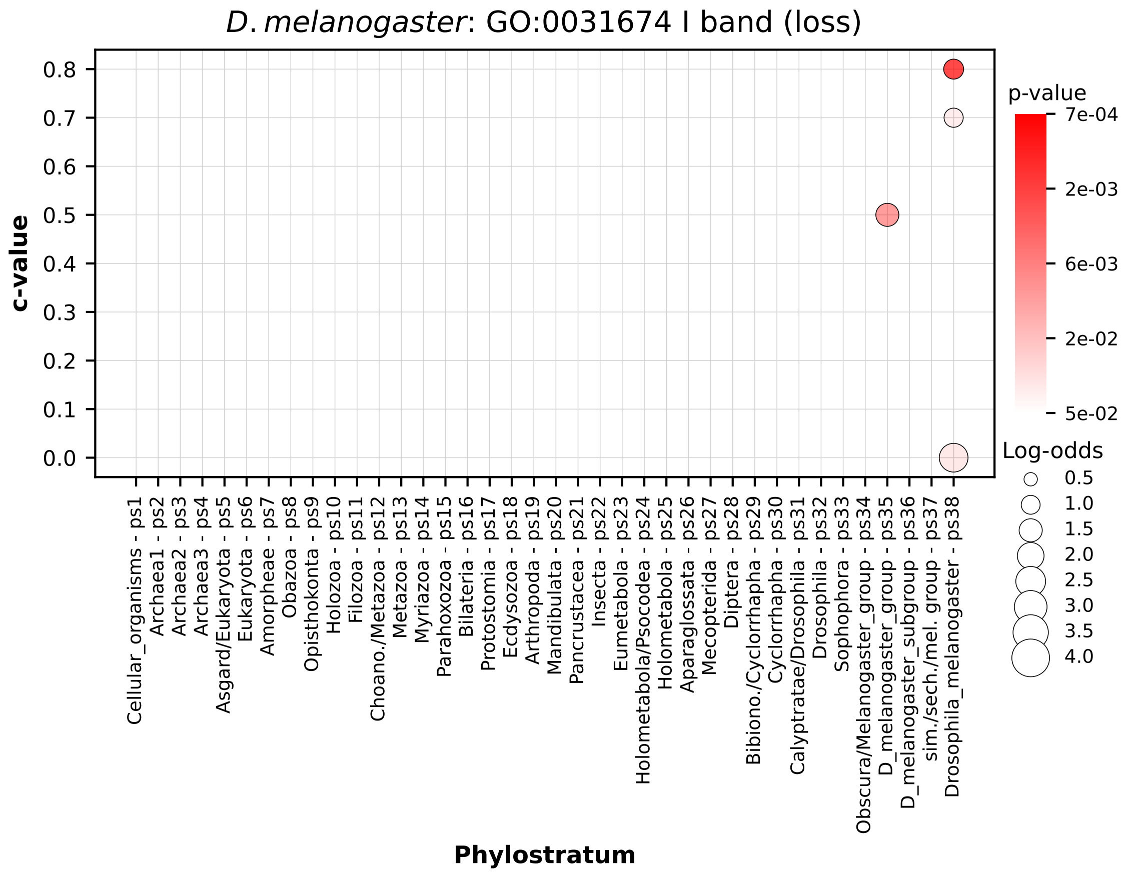

D. melanogaster: GO:0031674 I band (loss)
c-value
0.0
0.1
0.2
0.3
0.4
0.5
0.6
0.7
0.8
Phylostratum
Cellular_organisms - ps1
Archaea1 - ps2
Archaea2 - ps3
Archaea3 - ps4
Asgard/Eukaryota - ps5
Eukaryota - ps6
Amorpheae - ps7
Obazoa - ps8
Opisthokonta - ps9
Holozoa - ps10
Filozoa - ps11
Choano./Metazoa - ps12
Metazoa - ps13
Myriazoa - ps14
Parahoxozoa - ps15
Bilateria - ps16
Protostomia - ps17
Ecdysozoa - ps18
Arthropoda - ps19
Mandibulata - ps20
Pancrustacea - ps21
Insecta - ps22
Eumetabola - ps23
Holometabola/Psocodea - ps24
Holometabola - ps25
Aparaglossata - ps26
Mecopterida - ps27
Diptera - ps28
Bibiono./Cyclorrhapha - ps29
Cyclorrhapha - ps30
Calyptratae/Drosophila - ps31
Drosophila - ps32
Sophophora - ps33
Obscura/Melanogaster_group - ps34
D_melanogaster_group - ps35
D_melanogaster_subgroup - ps36
sim./sech./mel. group - ps37
Drosophila_melanogaster - ps38
p-value
7e-04
2e-03
6e-03
2e-02
5e-02
Log-odds
0.5
1.0
1.5
2.0
2.5
3.0
3.5
4.0

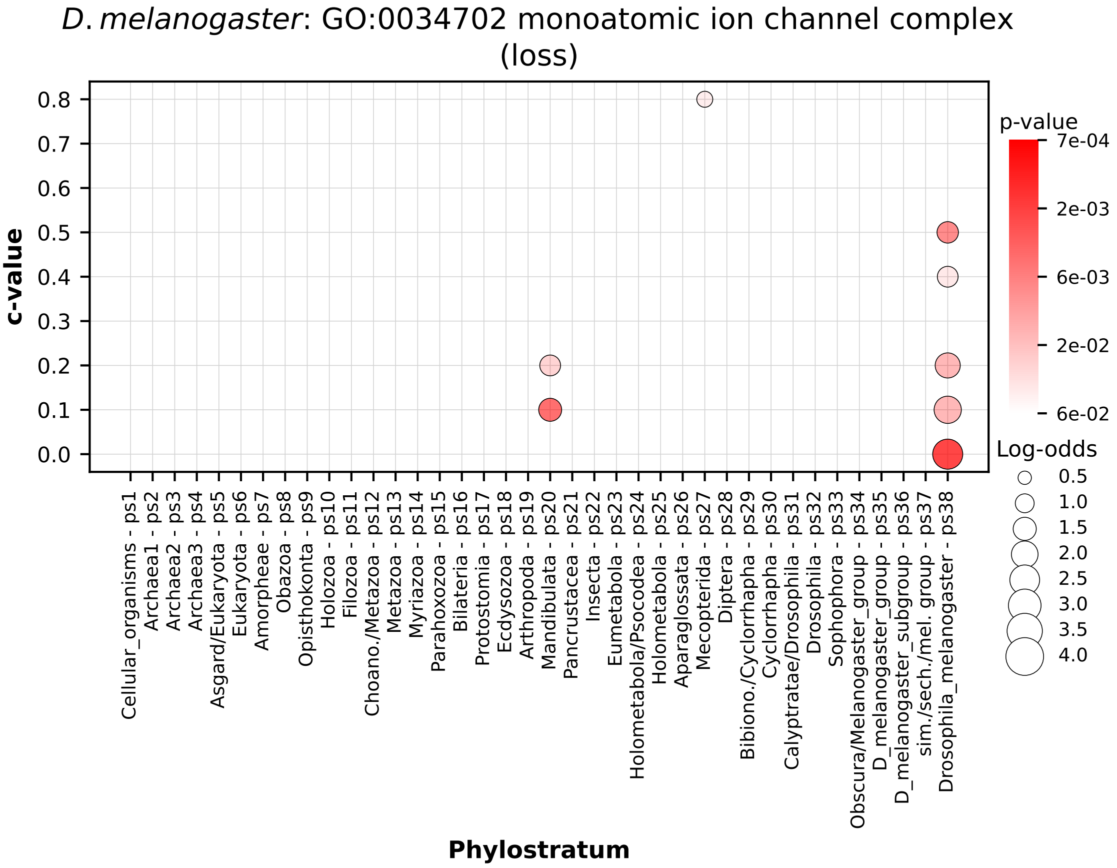

D. melanogaster: GO:0034702 monoatomic ion channel complex
(loss)
c-value
0.8
0.7
0.6
0.5
0.4
0.3
0.2
0.1
0.0
Phylostratum
Cellular_organisms - ps1
Archaea1 - ps2
Archaea2 - ps3
Archaea3 - ps4
Asgard/Eukaryota - ps5
Eukaryota - ps6
Amorpheae - ps7
Obazoa - ps8
Opisthokonta - ps9
Holozoa - ps10
Filozoa - ps11
Choano./Metazoa - ps12
Metazoa - ps13
Myriazoa - ps14
Parahoxozoa - ps15
Bilateria - ps16
Protostomia - ps17
Ecdysozoa - ps18
Arthropoda - ps19
Mandibulata - ps20
Pancrustacea - ps21
Insecta - ps22
Eumetabola - ps23
Holometabola/Psocodea - ps24
Holometabola - ps25
Aparaglossata - ps26
Mecopterida - ps27
Diptera - ps28
Bibiono./Cyclorrhapha - ps29
Cyclorrhapha - ps30
Calyptratae/Drosophila - ps31
Drosophila - ps32
Sophophora - ps33
Obscura/Melanogaster_group - ps34
D_melanogaster_group - ps35
D_melanogaster_subgroup - ps36
sim./sech./mel. group - ps37
Drosophila_melanogaster - ps38
p-value
7e-04
2e-03
6e-03
2e-02
6e-02
Log-odds
0.5
1.0
1.5
2.0
2.5
3.0
3.5
4.0

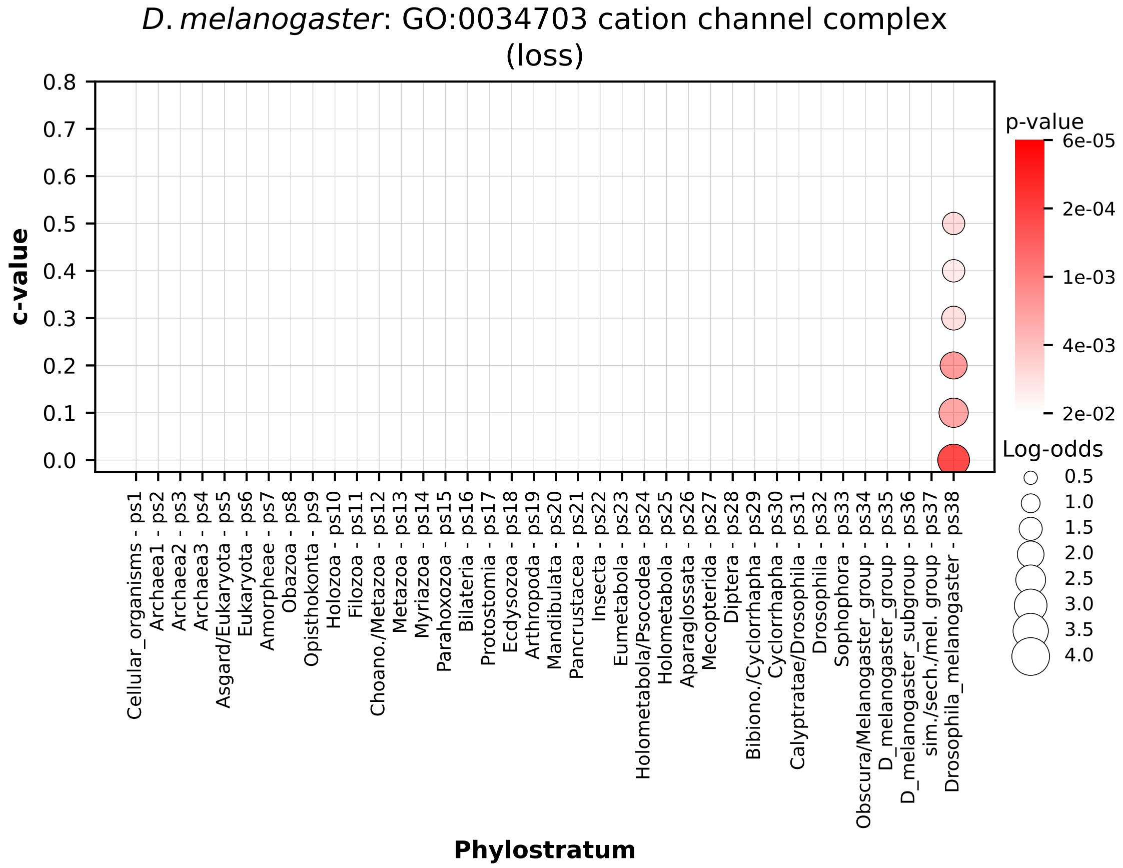
D. melanogaster: GO:0034703 cation channel complex
(loss)
c-value
0.8
0.7
0.6
0.5
0.4
0.3
0.2
0.1
0.0
p-value
6e-05
2e-04
1e-03
4e-03
2e-02
Log-odds
0.5
1.0
1.5
2.0
2.5
3.0
3.5
4.0
Cellular_organisms - ps1
Archaea1 - ps2
Archaea2 - ps3
Archaea3 - ps4
Asgard/Eukaryota - ps5
Eukaryota - ps6
Amorpheae - ps7
Obazoa - ps8
Opisthokonta - ps9
Holozoa - ps10
Filozoa - ps11
Choano./Metazoa - ps12
Metazoa - ps13
Myriazoa - ps14
Parahoxozoa - ps15
Bilateria - ps16
Protostomia - ps17
Ecdysozoa - ps18
Arthropoda - ps19
Mandibulata - ps20
Pancrustacea - ps21
Insecta - ps22
Eumetabola - ps23
Holometabola/Psocodea - ps24
Holometabola - ps25
Aparaglossata - ps26
Mecopterida - ps27
Diptera - ps28
Bibiono./Cyclorrhapha - ps29
Cyclorrhapha - ps30
Calyptratae/Drosophila - ps31
Drosophila - ps32
Sophophora - ps33
Obscura/Melanogaster_group - ps34
D_melanogaster_group - ps35
D_melanogaster_subgroup - ps36
sim./sech./mel. group - ps37
Drosophila_melanogaster - ps38
Phylostratum

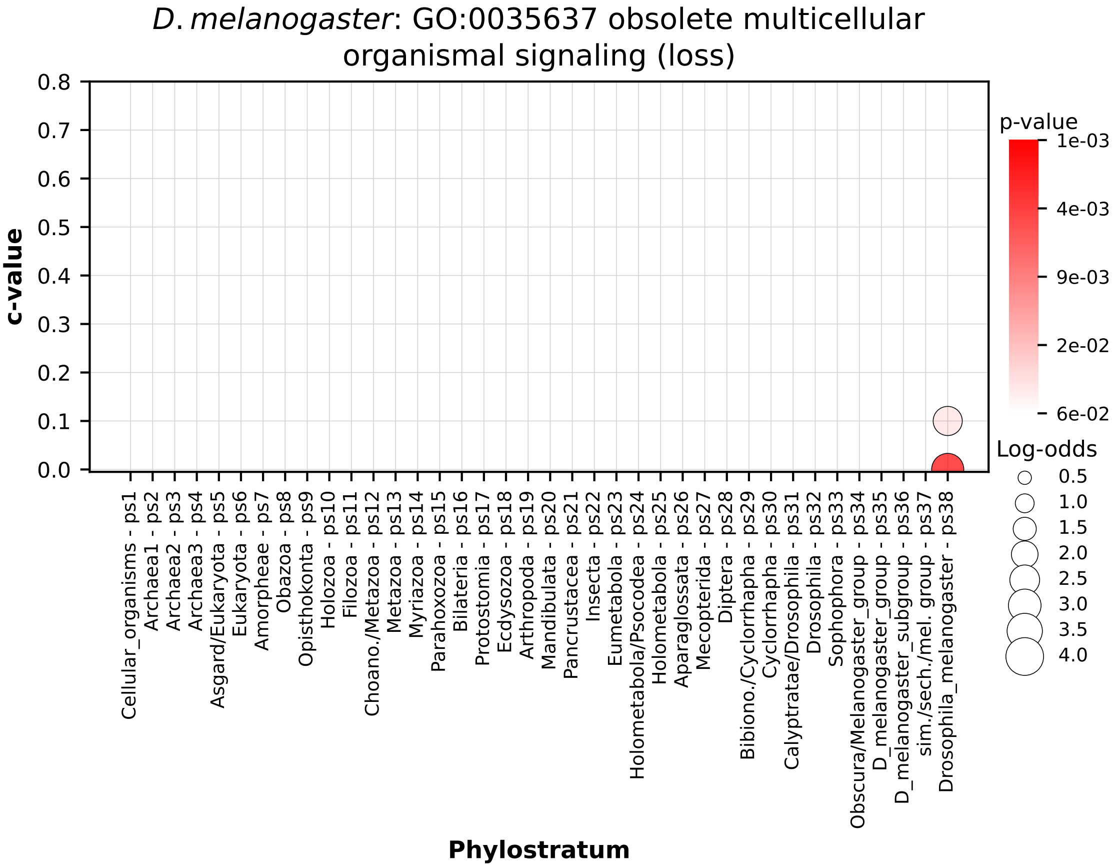
D. melanogaster: GO:0035637 obsolete multicellular organismal signaling (loss)
c-value
0.8
0.7
0.6
0.5
0.4
0.3
0.2
0.1
0.0
Phylostratum
Cellular_organisms - ps1
Archaea1 - ps2
Archaea2 - ps3
Archaea3 - ps4
Asgard/Eukaryota - ps5
Eukaryota - ps6
Amorpheae - ps7
Obazoa - ps8
Opisthokonta - ps9
Holozoa - ps10
Filozoa - ps11
Choano./Metazoa - ps12
Metazoa - ps13
Myriazoa - ps14
Parahoxozoa - ps15
Bilateria - ps16
Protostomia - ps17
Ecdysozoa - ps18
Arthropoda - ps19
Mandibulata - ps20
Pancrustacea - ps21
Insecta - ps22
Eumetabola - ps23
Holometabola/Psocodea - ps24
Holometabola - ps25
Aparaglossata - ps26
Mecopterida - ps27
Diptera - ps28
Bibiono./Cyclorrhapha - ps29
Cyclorrhapha - ps30
Calyptratae/Drosophila - ps31
Drosophila - ps32
Sophophora - ps33
Obscura/Melanogaster_group - ps34
D_melanogaster_group - ps35
D_melanogaster_subgroup - ps36
sim./sech./mel. group - ps37
Drosophila_melanogaster - ps38
p-value
1e-03
4e-03
9e-03
2e-02
6e-02
Log-odds
0.5
1.0
1.5
2.0
2.5
3.0
3.5
4.0

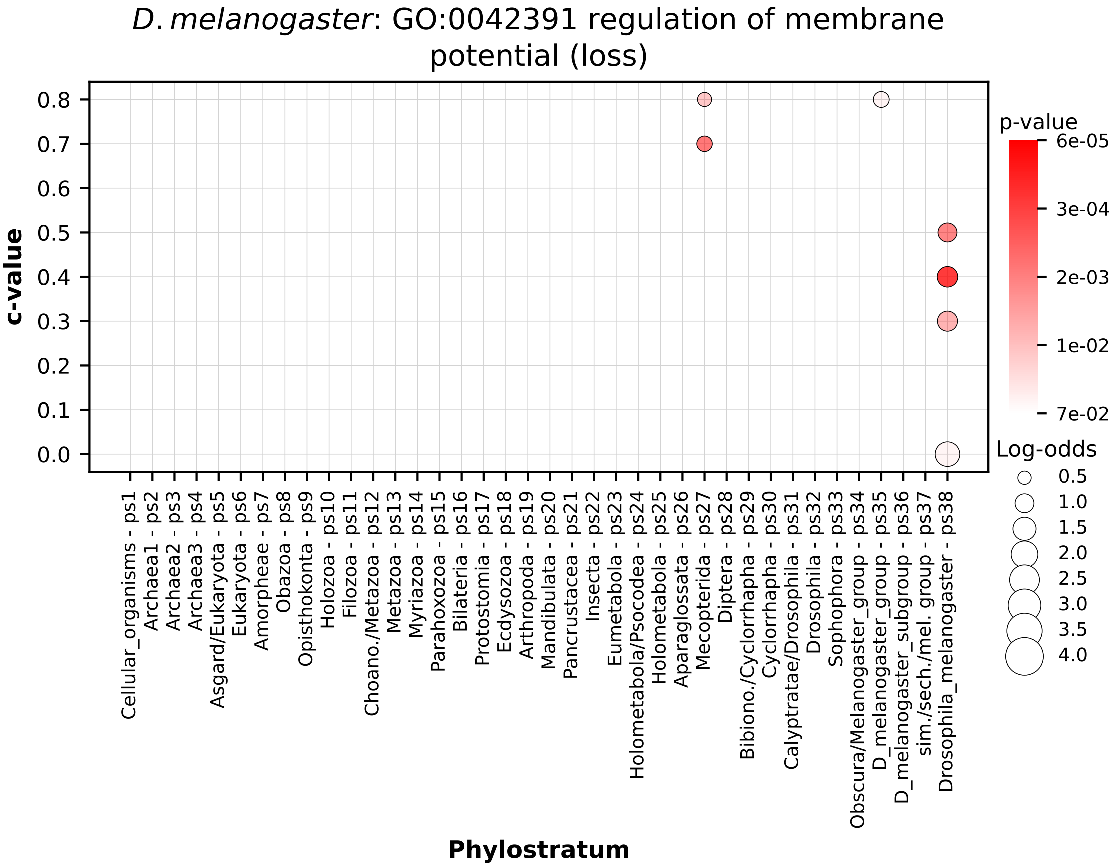
D. melanogaster: GO:0042391 regulation of membrane potential (loss)
c-value
0.8
0.7
0.6
0.5
0.4
0.3
0.2
0.1
0.0
Phylostratum
Cellular_organisms - ps1
Archaea1 - ps2
Archaea2 - ps3
Archaea3 - ps4
Asgard/Eukaryota - ps5
Eukaryota - ps6
Amorpheae - ps7
Obazoa - ps8
Opisthokonta - ps9
Holozoa - ps10
Filozoa - ps11
Choano./Metazoa - ps12
Metazoa - ps13
Myriazoa - ps14
Parahoxozoa - ps15
Bilateria - ps16
Protostomia - ps17
Ecdysozoa - ps18
Arthropoda - ps19
Mandibulata - ps20
Pancrustacea - ps21
Insecta - ps22
Eumetabola - ps23
Holometabola/Psocodea - ps24
Holometabola - ps25
Aparaglossata - ps26
Mecopterida - ps27
Diptera - ps28
Bibiono./Cyclorrhapha - ps29
Cyclorrhapha - ps30
Calyptratae/Drosophila - ps31
Drosophila - ps32
Sophophora - ps33
Obscura/Melanogaster_group - ps34
D_melanogaster_group - ps35
D_melanogaster_subgroup - ps36
sim./sech./mel. group - ps37
Drosophila_melanogaster - ps38
p-value
6e-05
3e-04
2e-03
1e-02
7e-02
Log-odds
0.5
1.0
1.5
2.0
2.5
3.0
3.5
4.0

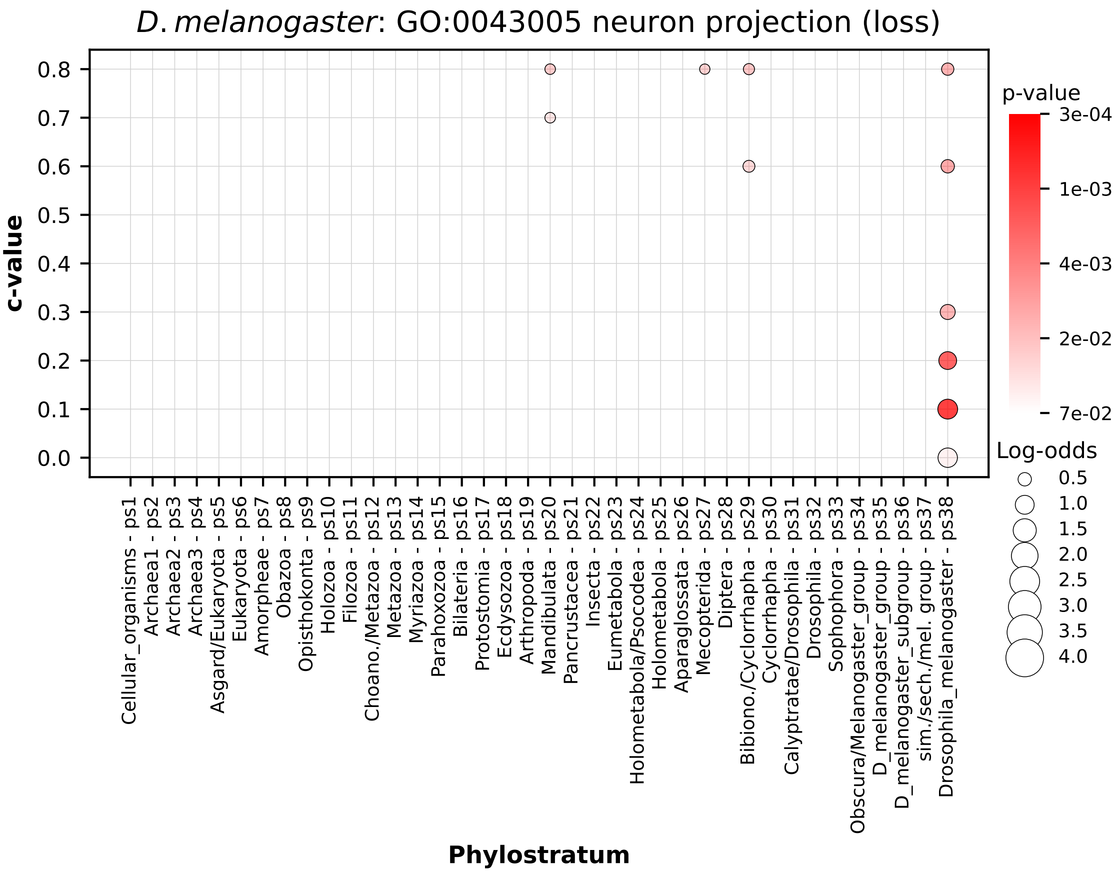

D. melanogaster: GO:0043005 neuron projection (loss)
c-value
0.0
0.1
0.2
0.3
0.4
0.5
0.6
0.7
0.8
Phylostratum
Cellular_organisms - ps1
Archaea1 - ps2
Archaea2 - ps3
Archaea3 - ps4
Asgard/Eukaryota - ps5
Eukaryota - ps6
Amorpheae - ps7
Obazoa - ps8
Opisthokonta - ps9
Holozoa - ps10
Filozoa - ps11
Choano./Metazoa - ps12
Metazoa - ps13
Myriazoa - ps14
Parahoxozoa - ps15
Bilateria - ps16
Protostomia - ps17
Ecdysozoa - ps18
Arthropoda - ps19
Mandibulata - ps20
Pancrustacea - ps21
Insecta - ps22
Eumetabola - ps23
Holometabola/Psocodea - ps24
Holometabola - ps25
Aparaglossata - ps26
Mecopterida - ps27
Diptera - ps28
Bibiono./Cyclorrhapha - ps29
Cyclorrhapha - ps30
Calyptratae/Drosophila - ps31
Drosophila - ps32
Sophophora - ps33
Obscura/Melanogaster_group - ps34
D_melanogaster_group - ps35
D_melanogaster_subgroup - ps36
sim./sech./mel. group - ps37
Drosophila_melanogaster - ps38
p-value
3e-04
1e-03
4e-03
2e-02
7e-02
Log-odds
0.5
1.0
1.5
2.0
2.5
3.0
3.5
4.0

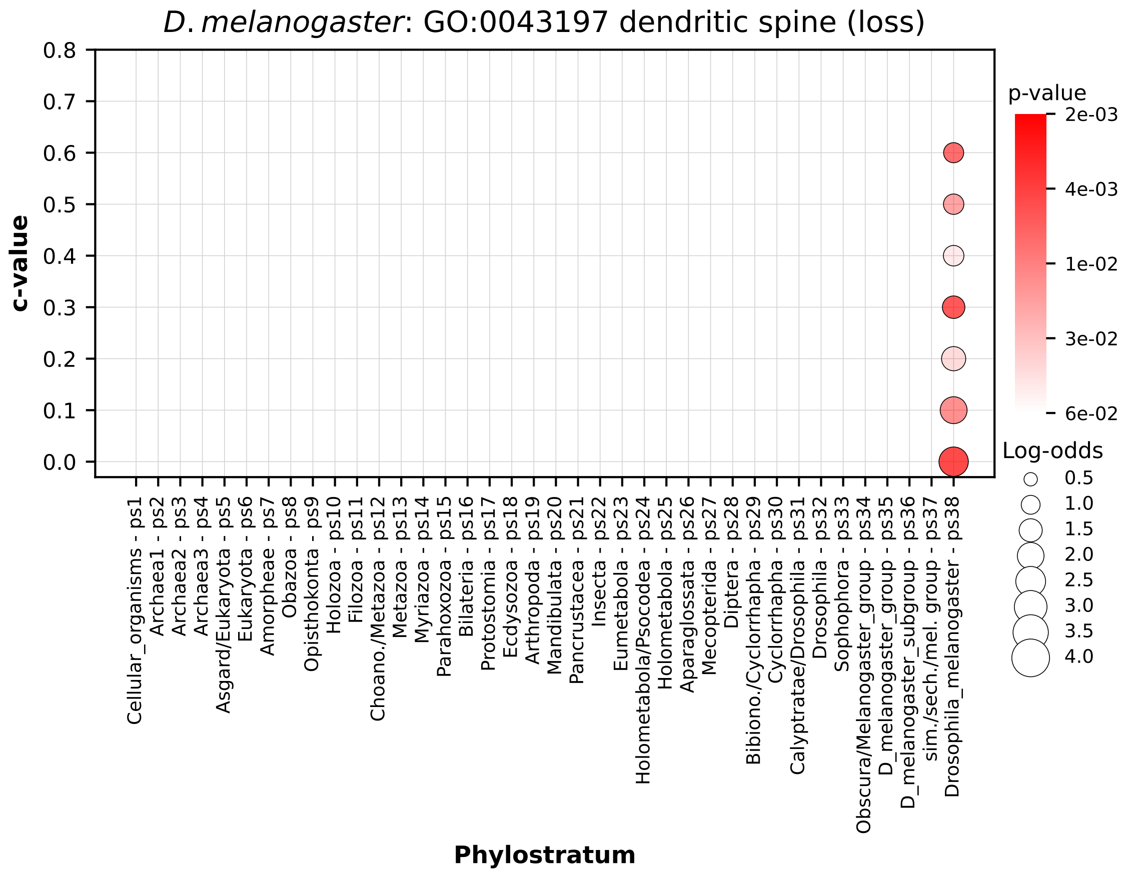

D. melanogaster: GO:0043197 dendritic spine (loss)
c-value
0.8
0.7
0.6
0.5
0.4
0.3
0.2
0.1
0.0
Phylostratum
Cellular_organisms - ps1
Archaea1 - ps2
Archaea2 - ps3
Archaea3 - ps4
Asgard/Eukaryota - ps5
Eukaryota - ps6
Amorpheae - ps7
Obazoa - ps8
Opisthokonta - ps9
Holozoa - ps10
Filozoa - ps11
Choano./Metazoa - ps12
Metazoa - ps13
Myriazoa - ps14
Parahoxozoa - ps15
Bilateria - ps16
Protostomia - ps17
Ecdysozoa - ps18
Arthropoda - ps19
Mandibulata - ps20
Pancrustacea - ps21
Insecta - ps22
Eumetabola - ps23
Holometabola/Psocodea - ps24
Holometabola - ps25
Aparaglossata - ps26
Mecopterida - ps27
Diptera - ps28
Bibiono./Cyclorrhapha - ps29
Cyclorrhapha - ps30
Calyptratae/Drosophila - ps31
Drosophila - ps32
Sophophora - ps33
Obscura/Melanogaster_group - ps34
D_melanogaster_group - ps35
D_melanogaster_subgroup - ps36
sim./sech./mel. group - ps37
Drosophila_melanogaster - ps38
p-value
2e-03
4e-03
1e-02
3e-02
6e-02
Log-odds
0.5
1.0
1.5
2.0
2.5
3.0
3.5
4.0

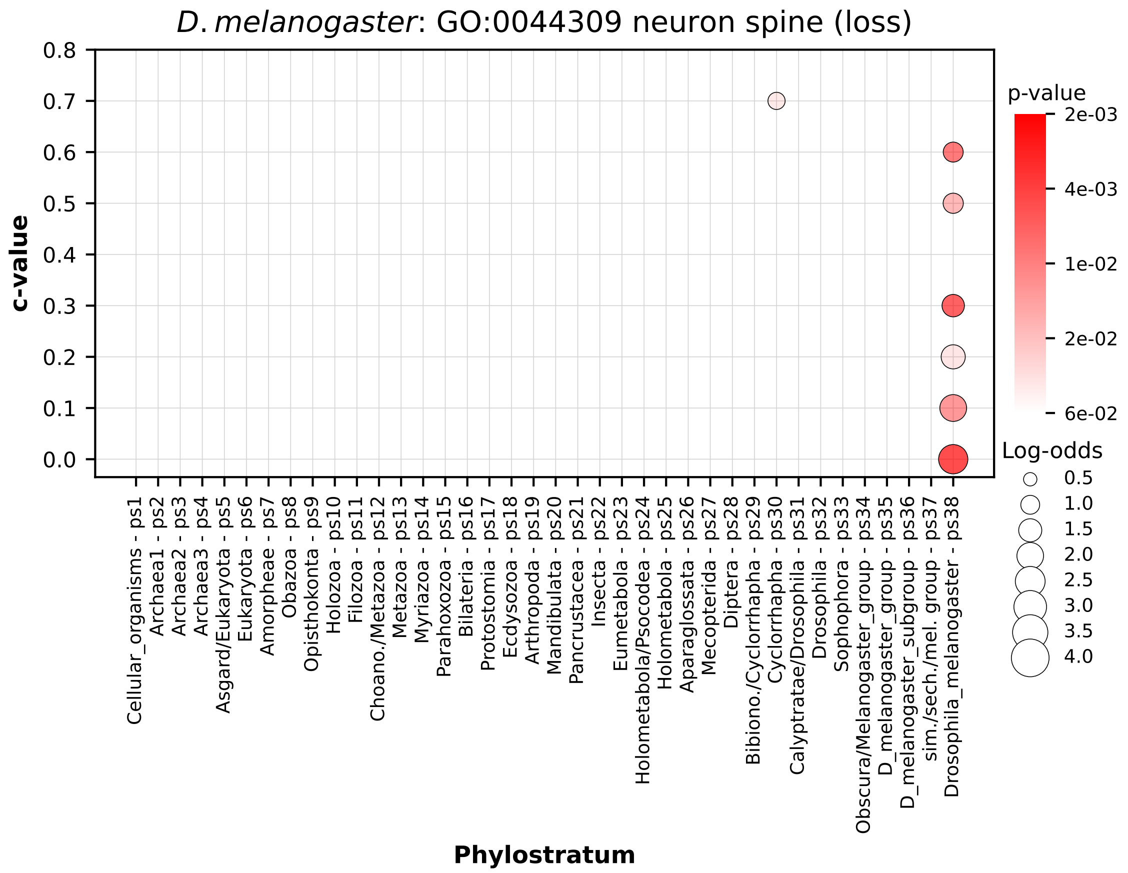

D. melanogaster: GO:0044309 neuron spine (loss)
c-value
0.0
0.1
0.2
0.3
0.4
0.5
0.6
0.7
0.8
Phylostratum
Cellular_organisms - ps1
Archaea1 - ps2
Archaea2 - ps3
Archaea3 - ps4
Asgard/Eukaryota - ps5
Eukaryota - ps6
Amorpheae - ps7
Obazoa - ps8
Opisthokonta - ps9
Holozoa - ps10
Filozoa - ps11
Choano./Metazoa - ps12
Metazoa - ps13
Myriazoa - ps14
Parahoxozoa - ps15
Bilateria - ps16
Protostomia - ps17
Ecdysozoa - ps18
Arthropoda - ps19
Mandibulata - ps20
Pancrustacea - ps21
Insecta - ps22
Eumetabola - ps23
Holometabola/Psocodea - ps24
Holometabola - ps25
Aparaglossata - ps26
Mecopterida - ps27
Diptera - ps28
Bibiono./Cyclorrhapha - ps29
Cyclorrhapha - ps30
Calyptratae/Drosophila - ps31
Drosophila - ps32
Sophophora - ps33
Obscura/Melanogaster_group - ps34
D_melanogaster_group - ps35
D_melanogaster_subgroup - ps36
sim./sech./mel. group - ps37
Drosophila_melanogaster - ps38
p-value
2e-03
4e-03
1e-02
2e-02
6e-02
Log-odds
0.5
1.0
1.5
2.0
2.5
3.0
3.5
4.0

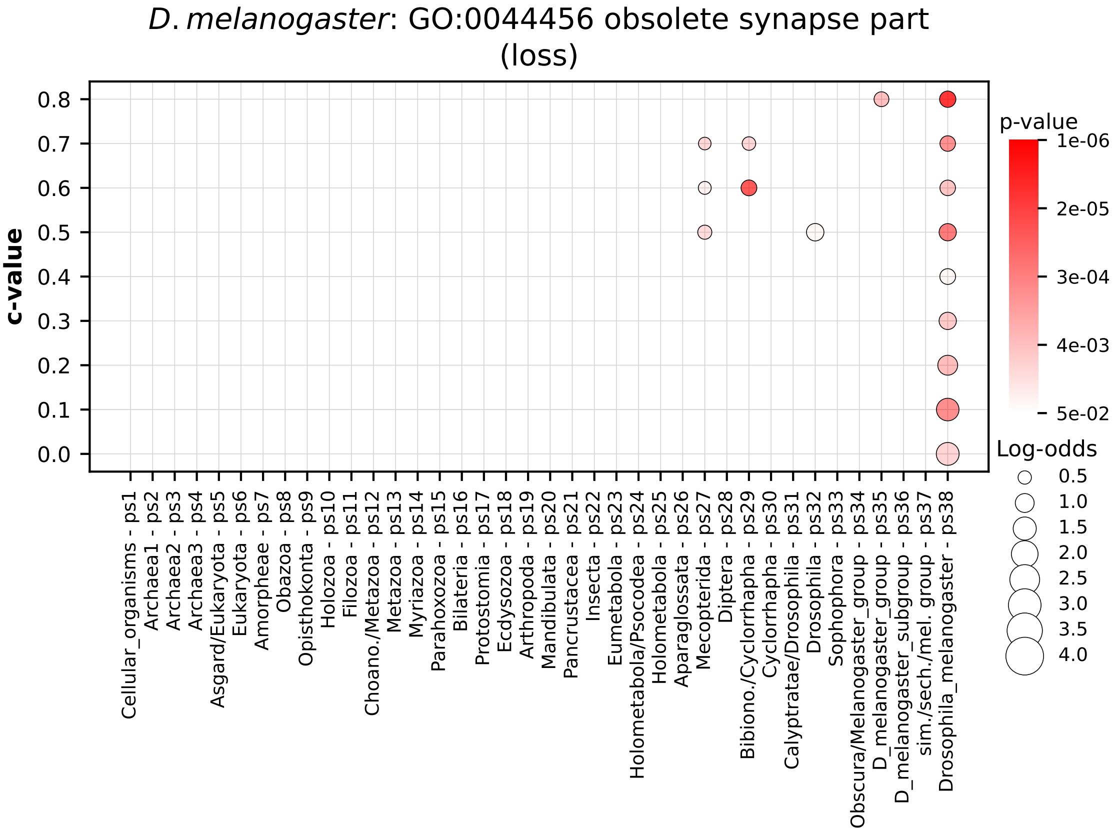

D. melanogaster: GO:0044456 obsolete synapse part
(loss)
c-value
0.8
0.7
0.6
0.5
0.4
0.3
0.2
0.1
0.0
p-value
1e-06
2e-05
3e-04
4e-03
5e-02
Log-odds
0.5
1.0
1.5
2.0
2.5
3.0
3.5
4.0
Cellular_organisms - ps1
Archaea1 - ps2
Archaea2 - ps3
Archaea3 - ps4
Asgard/Eukaryota - ps5
Eukaryota - ps6
Amorpheae - ps7
Obazoa - ps8
Opisthokonta - ps9
Holozoa - ps10
Filozoa - ps11
Choano./Metazoa - ps12
Metazoa - ps13
Myriazoa - ps14
Parahoxozoa - ps15
Bilateria - ps16
Protostomia - ps17
Ecdysozoa - ps18
Arthropoda - ps19
Mandibulata - ps20
Pancrustacea - ps21
Insecta - ps22
Eumetabola - ps23
Holometabola/Psocodea - ps24
Holometabola - ps25
Aparaglossata - ps26
Mecopterida - ps27
Diptera - ps28
Bibiono./Cyclorrhapha - ps29
Cyclorrhapha - ps30
Calyptratae/Drosophila - ps31
Drosophila - ps32
Sophophora - ps33
Obscura/Melanogaster_group - ps34
D_melanogaster_group - ps35
D_melanogaster_subgroup - ps36
sim./sech./mel. group - ps37
Drosophila_melanogaster - ps38
Phylostratum

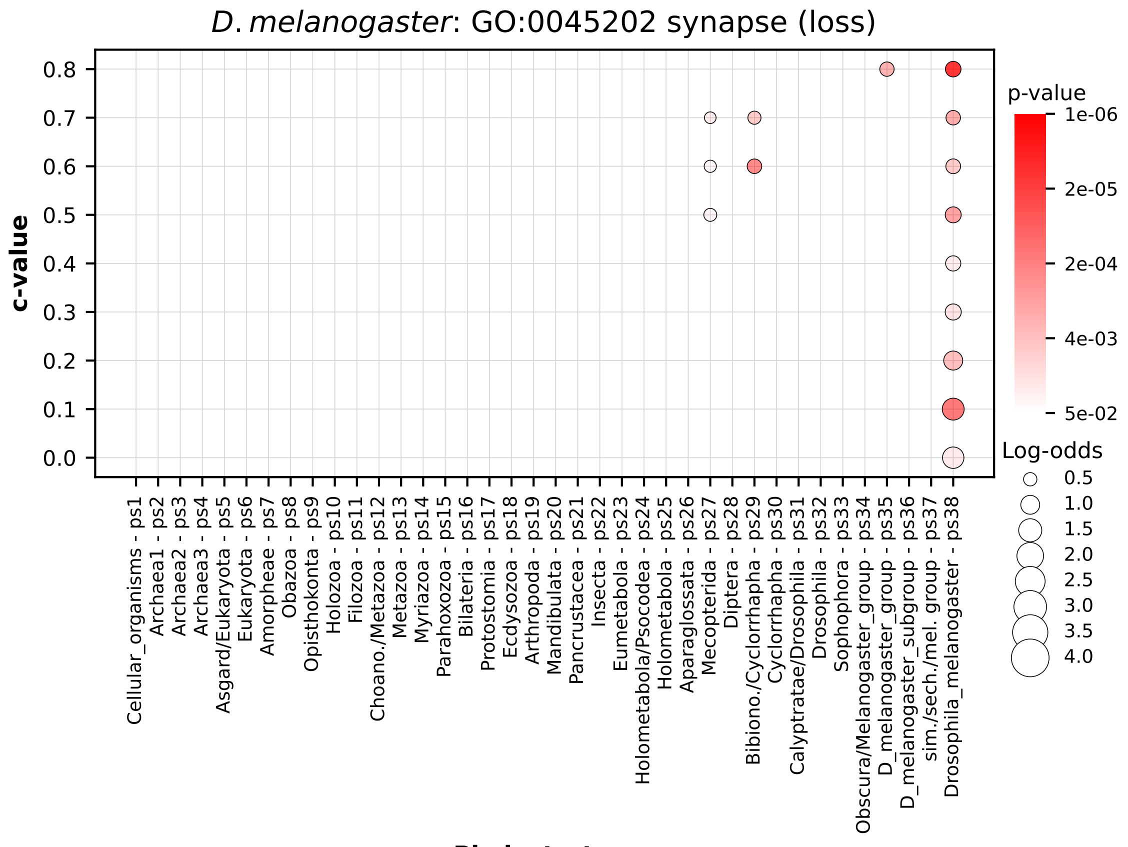

D. melanogaster: GO:0045202 synapse (loss)
c-value
0.8
0.7
0.6
0.5
0.4
0.3
0.2
0.1
0.0
p-value
1e-06
2e-05
2e-04
4e-03
5e-02
Log-odds
0.5
1.0
1.5
2.0
2.5
3.0
3.5
4.0
Cellular_organisms - ps1
Archaea1 - ps2
Archaea2 - ps3
Archaea3 - ps4
Asgard/Eukaryota - ps5
Eukaryota - ps6
Amorpheae - ps7
Obazoa - ps8
Opisthokonta - ps9
Holozoa - ps10
Filozoa - ps11
Choano./Metazoa - ps12
Metazoa - ps13
Myriazoa - ps14
Parahoxozoa - ps15
Bilateria - ps16
Protostomia - ps17
Ecdysozoa - ps18
Arthropoda - ps19
Mandibulata - ps20
Pancrustacea - ps21
Insecta - ps22
Eumetabola - ps23
Holometabola/Psocodea - ps24
Holometabola - ps25
Aparaglossata - ps26
Mecopterida - ps27
Diptera - ps28
Bibiono./Cyclorrhapha - ps29
Cyclorrhapha - ps30
Calyptratae/Drosophila - ps31
Drosophila - ps32
Sophophora - ps33
Obscura/Melanogaster_group - ps34
D_melanogaster_group - ps35
D_melanogaster_subgroup - ps36
sim./sech./mel. group - ps37
Drosophila_melanogaster - ps38
Phylostratum

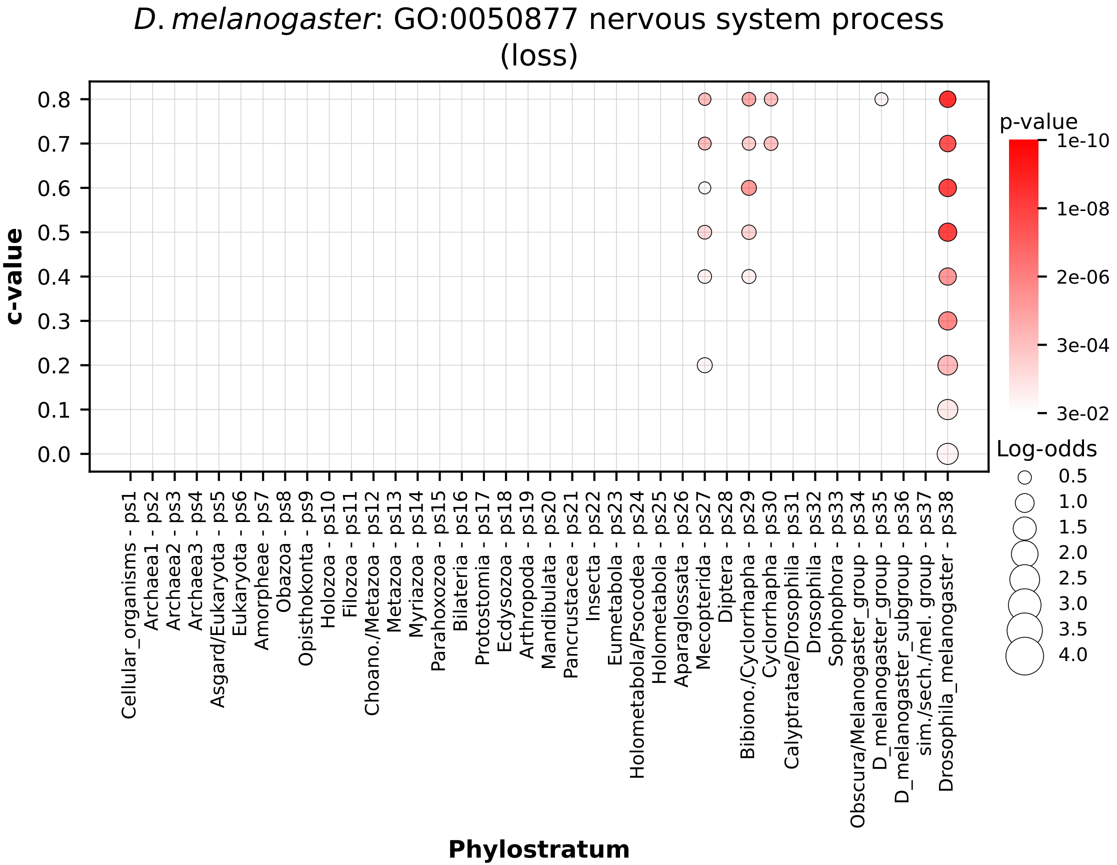

D. melanogaster: GO:0050877 nervous system process
(loss)
c-value
0.8
0.7
0.6
0.5
0.4
0.3
0.2
0.1
0.0
p-value
1e-10
1e-08
2e-06
3e-04
3e-02
Log-odds
0.5
1.0
1.5
2.0
2.5
3.0
3.5
4.0
Cellular_organisms - ps1
Archaea1 - ps2
Archaea2 - ps3
Archaea3 - ps4
Asgard/Eukaryota - ps5
Eukaryota - ps6
Amorpheae - ps7
Obazoa - ps8
Opisthokonta - ps9
Holozoa - ps10
Filozoa - ps11
Choano./Metazoa - ps12
Metazoa - ps13
Myriazoa - ps14
Parahoxozoa - ps15
Bilateria - ps16
Protostomia - ps17
Ecdysozoa - ps18
Arthropoda - ps19
Mandibulata - ps20
Pancrustacea - ps21
Insecta - ps22
Eumetabola - ps23
Holometabola/Psocodea - ps24
Holometabola - ps25
Aparaglossata - ps26
Mecopterida - ps27
Diptera - ps28
Bibiono./Cyclorrhapha - ps29
Cyclorrhapha - ps30
Calyptratae/Drosophila - ps31
Drosophila - ps32
Sophophora - ps33
Obscura/Melanogaster_group - ps34
D_melanogaster_group - ps35
D_melanogaster_subgroup - ps36
sim./sech./mel. group - ps37
Drosophila_melanogaster - ps38
Phylostratum

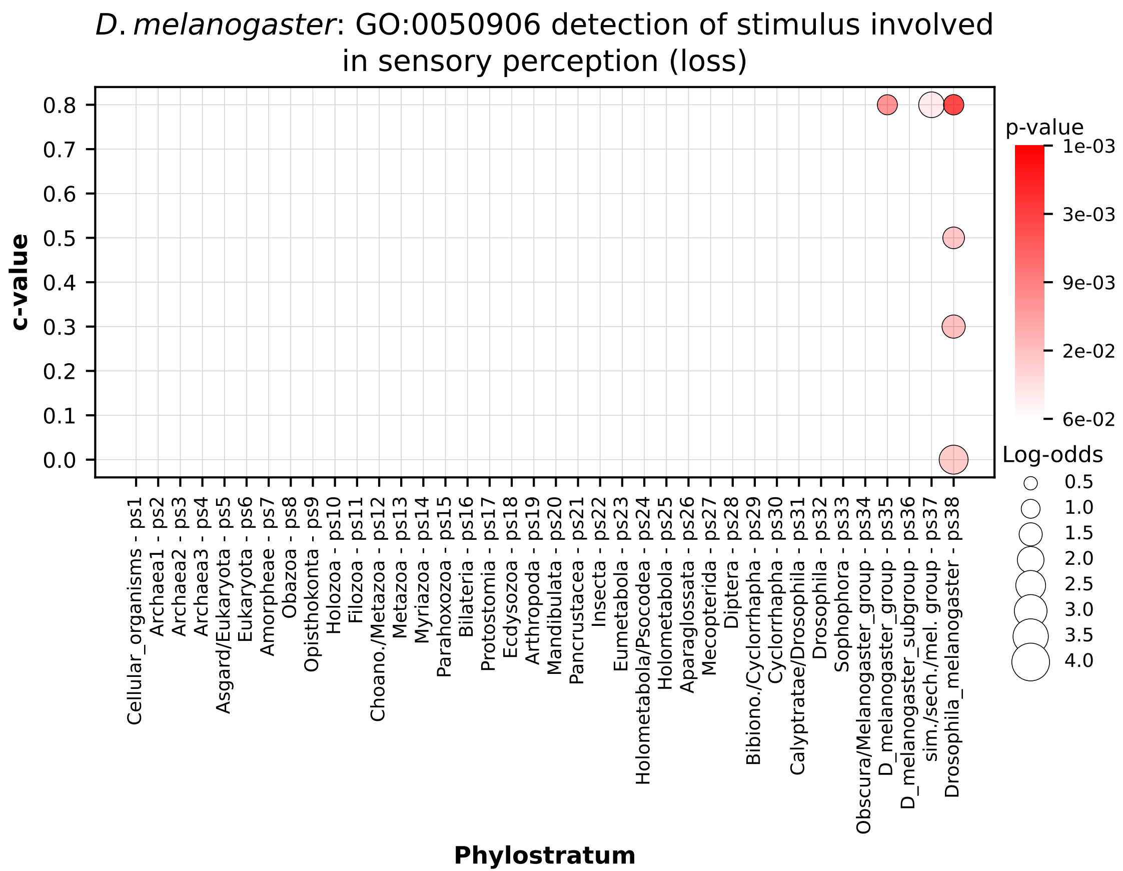

D. melanogaster: GO:0050906 detection of stimulus involved in sensory perception (loss)
c-value
0.8
0.7
0.6
0.5
0.4
0.3
0.2
0.1
0.0
p-value
1e-03
3e-03
9e-03
2e-02
6e-02
Log-odds
0.5
1.0
1.5
2.0
2.5
3.0
3.5
4.0
Cellular_organisms - ps1
Archaea1 - ps2
Archaea2 - ps3
Archaea3 - ps4
Asgard/Eukaryota - ps5
Eukaryota - ps6
Amorpheae - ps7
Obazoa - ps8
Opisthokonta - ps9
Holozoa - ps10
Filozoa - ps11
Choano./Metazoa - ps12
Metazoa - ps13
Myriazoa - ps14
Parahoxozoa - ps15
Bilateria - ps16
Protostomia - ps17
Ecdysozoa - ps18
Arthropoda - ps19
Mandibulata - ps20
Pancrustacea - ps21
Insecta - ps22
Eumetabola - ps23
Holometabola/Psocodea - ps24
Holometabola - ps25
Aparaglossata - ps26
Mecopterida - ps27
Diptera - ps28
Bibiono./Cyclorrhapha - ps29
Cyclorrhapha - ps30
Calyptratae/Drosophila - ps31
Drosophila - ps32
Sophophora - ps33
Obscura/Melanogaster_group - ps34
D_melanogaster_group - ps35
D_melanogaster_subgroup - ps36
sim./sech./mel. group - ps37
Drosophila_melanogaster - ps38
Phylostratum

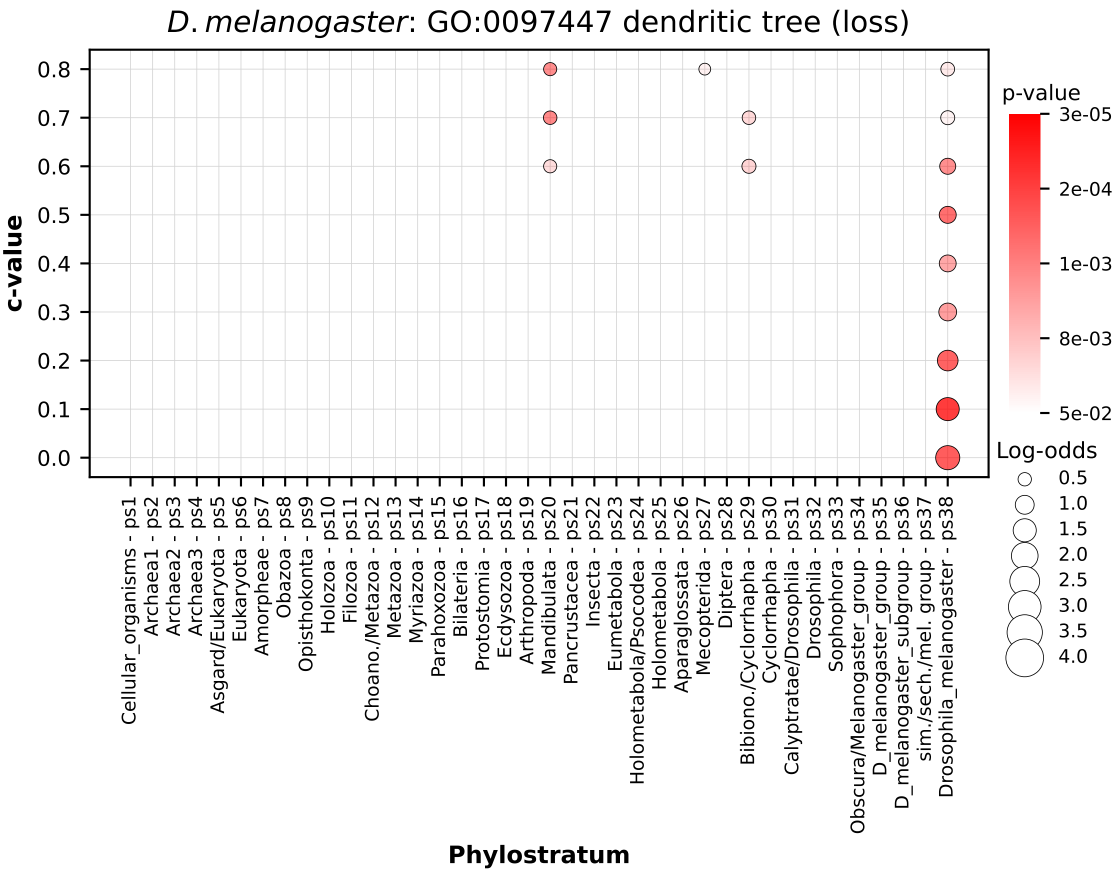

D. melanogaster: GO:0097447 dendritic tree (loss)
c-value
0.8
0.7
0.6
0.5
0.4
0.3
0.2
0.1
0.0
p-value
3e-05
2e-04
1e-03
8e-03
5e-02
Log-odds
0.5
1.0
1.5
2.0
2.5
3.0
3.5
4.0
Cellular_organisms - ps1
Archaea1 - ps2
Archaea2 - ps3
Archaea3 - ps4
Asgard/Eukaryota - ps5
Eukaryota - ps6
Amorpheae - ps7
Obazoa - ps8
Opisthokonta - ps9
Holozoa - ps10
Filozoa - ps11
Choano./Metazoa - ps12
Metazoa - ps13
Myriazoa - ps14
Parahoxozoa - ps15
Bilateria - ps16
Protostomia - ps17
Ecdysozoa - ps18
Arthropoda - ps19
Mandibulata - ps20
Pancrustacea - ps21
Insecta - ps22
Eumetabola - ps23
Holometabola/Psocodea - ps24
Holometabola - ps25
Aparaglossata - ps26
Mecopterida - ps27
Diptera - ps28
Bibiono./Cyclorrhapha - ps29
Cyclorrhapha - ps30
Calyptratae/Drosophila - ps31
Drosophila - ps32
Sophophora - ps33
Obscura/Melanogaster_group - ps34
D_melanogaster_group - ps35
D_melanogaster_subgroup - ps36
sim./sech./mel. group - ps37
Drosophila_melanogaster - ps38
Phylostratum

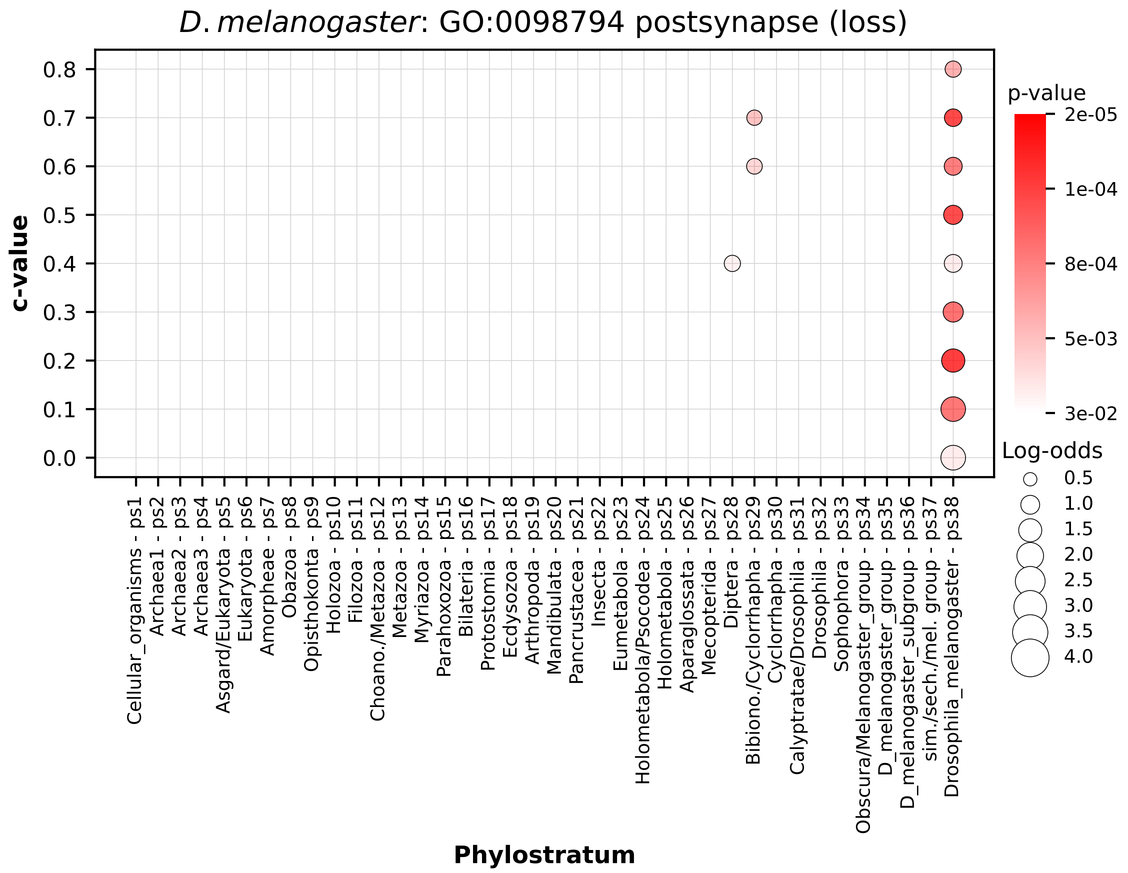

D. melanogaster: GO:0098794 postsynapse (loss)
c-value
0.0
0.1
0.2
0.3
0.4
0.5
0.6
0.7
0.8
Phylostratum
Cellular_organisms - ps1
Archaea1 - ps2
Archaea2 - ps3
Archaea3 - ps4
Asgard/Eukaryota - ps5
Eukaryota - ps6
Amorpheae - ps7
Obazoa - ps8
Opisthokonta - ps9
Holozoa - ps10
Filozoa - ps11
Choano./Metazoa - ps12
Metazoa - ps13
Myriazoa - ps14
Parahoxozoa - ps15
Bilateria - ps16
Protostomia - ps17
Ecdysozoa - ps18
Arthropoda - ps19
Mandibulata - ps20
Pancrustacea - ps21
Insecta - ps22
Eumetabola - ps23
Holometabola/Psocodea - ps24
Holometabola - ps25
Aparaglossata - ps26
Mecopterida - ps27
Diptera - ps28
Bibiono./Cyclorrhapha - ps29
Cyclorrhapha - ps30
Calyptratae/Drosophila - ps31
Drosophila - ps32
Sophophora - ps33
Obscura/Melanogaster_group - ps34
D_melanogaster_group - ps35
D_melanogaster_subgroup - ps36
sim./sech./mel. group - ps37
Drosophila_melanogaster - ps38
p-value
2e-05
1e-04
8e-04
5e-03
3e-02
Log-odds
0.5
1.0
1.5
2.0
2.5
3.0
3.5
4.0

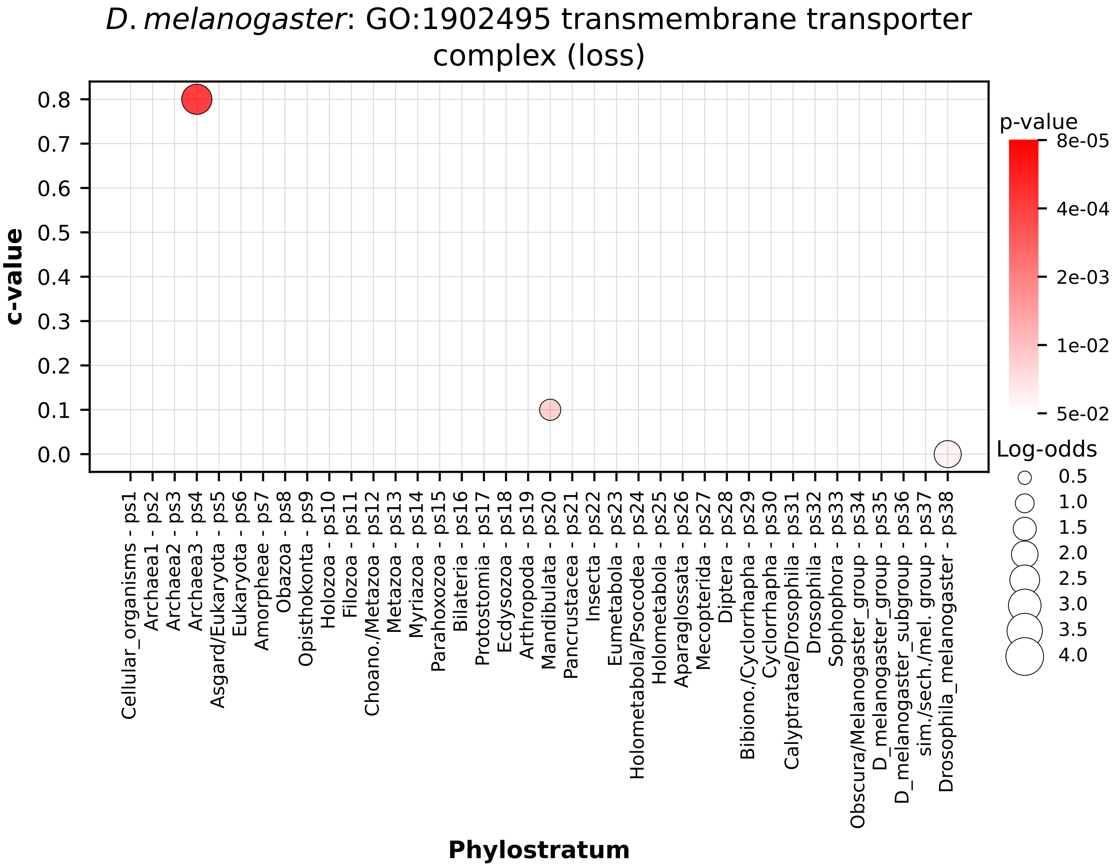
D. melanogaster: GO:1902495 transmembrane transporter complex (loss)
c-value
Phylostratum
p-value
8e-05
4e-04
2e-03
1e-02
5e-02
Log-odds
0.5
1.0
1.5
2.0
2.5
3.0
3.5
4.0
Cellular_organisms - ps1
Archaea1 - ps2
Archaea2 - ps3
Archaea3 - ps4
Asgard/Eukaryota - ps5
Eukaryota - ps6
Amorpheae - ps7
Obazoa - ps8
Opisthokonta - ps9
Holozoa - ps10
Filozoa - ps11
Choano./Metazoa - ps12
Metazoa - ps13
Myriazoa - ps14
Parahoxozoa - ps15
Bilateria - ps16
Protostomia - ps17
Ecdysozoa - ps18
Arthropoda - ps19
Mandibulata - ps20
Pancrustacea - ps21
Insecta - ps22
Eumetabola - ps23
Holometabola/Psocodea - ps24
Holometabola - ps25
Aparaglossata - ps26
Mecopterida - ps27
Diptera - ps28
Bibiono./Cyclorrhapha - ps29
Cyclorrhapha - ps30
Calyptratae/Drosophila - ps31
Drosophila - ps32
Sophophora - ps33
Obscura/Melanogaster_group - ps34
D_melanogaster_group - ps35
D_melanogaster_subgroup - ps36
sim./sech./mel. group - ps37
Drosophila_melanogaster - ps38

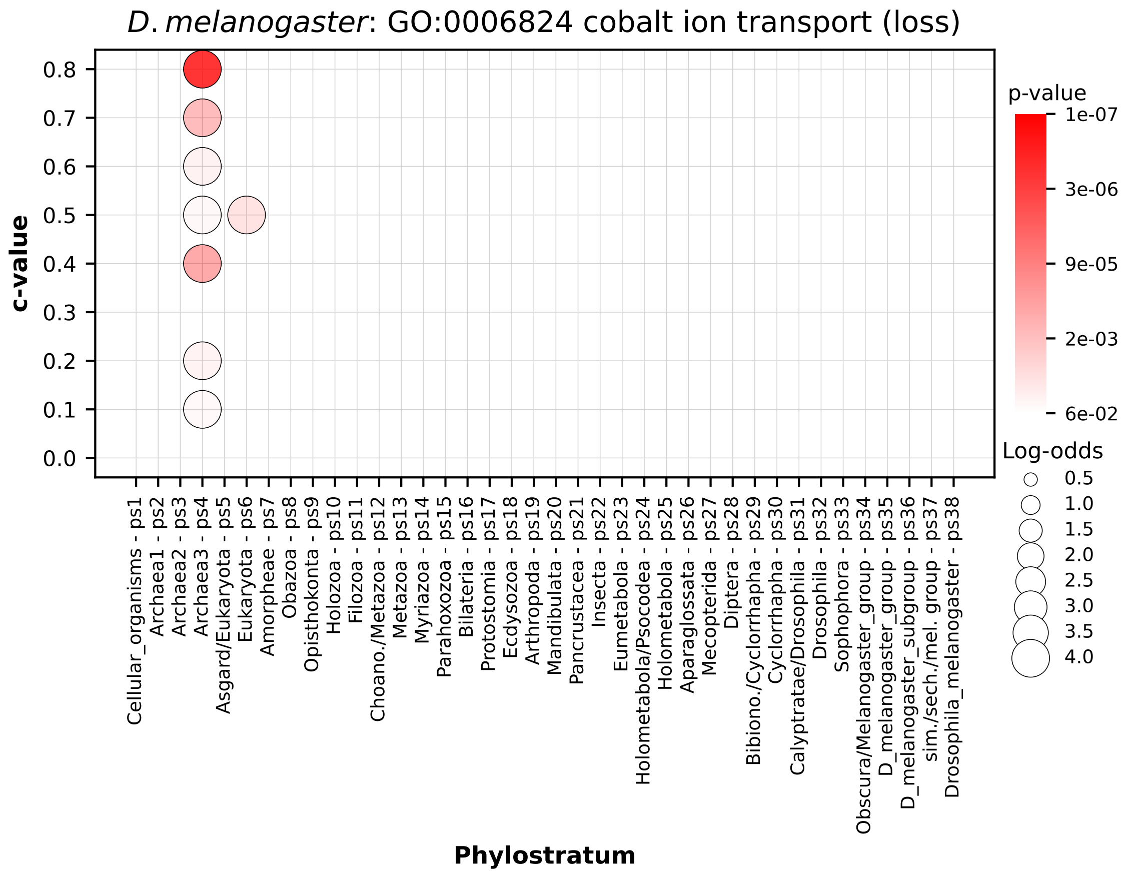

D. melanogaster: GO:0006824 cobalt ion transport (loss)
c-value
0.0
0.1
0.2
0.3
0.4
0.5
0.6
0.7
0.8
p-value
1e-07
3e-06
9e-05
2e-03
6e-02
Log-odds
0.5
1.0
1.5
2.0
2.5
3.0
3.5
4.0
Cellular_organisms - ps1
Archaea1 - ps2
Archaea2 - ps3
Archaea3 - ps4
Asgard/Eukaryota - ps5
Eukaryota - ps6
Amorpheae - ps7
Obazoa - ps8
Opisthokonta - ps9
Holozoa - ps10
Filozoa - ps11
Choano./Metazoa - ps12
Metazoa - ps13
Myriazoa - ps14
Parahoxozoa - ps15
Bilateria - ps16
Protostomia - ps17
Ecdysozoa - ps18
Arthropoda - ps19
Mandibulata - ps20
Pancrustacea - ps21
Insecta - ps22
Eumetabola - ps23
Holometabola/Psocodea - ps24
Holometabola - ps25
Aparaglossata - ps26
Mecopterida - ps27
Diptera - ps28
Bibiono./Cyclorrhapha - ps29
Cyclorrhapha - ps30
Calyptratae/Drosophila - ps31
Drosophila - ps32
Sophophora - ps33
Obscura/Melanogaster_group - ps34
D_melanogaster_group - ps35
D_melanogaster_subgroup - ps36
sim./sech./mel. group - ps37
Drosophila_melanogaster - ps38
Phylostratum

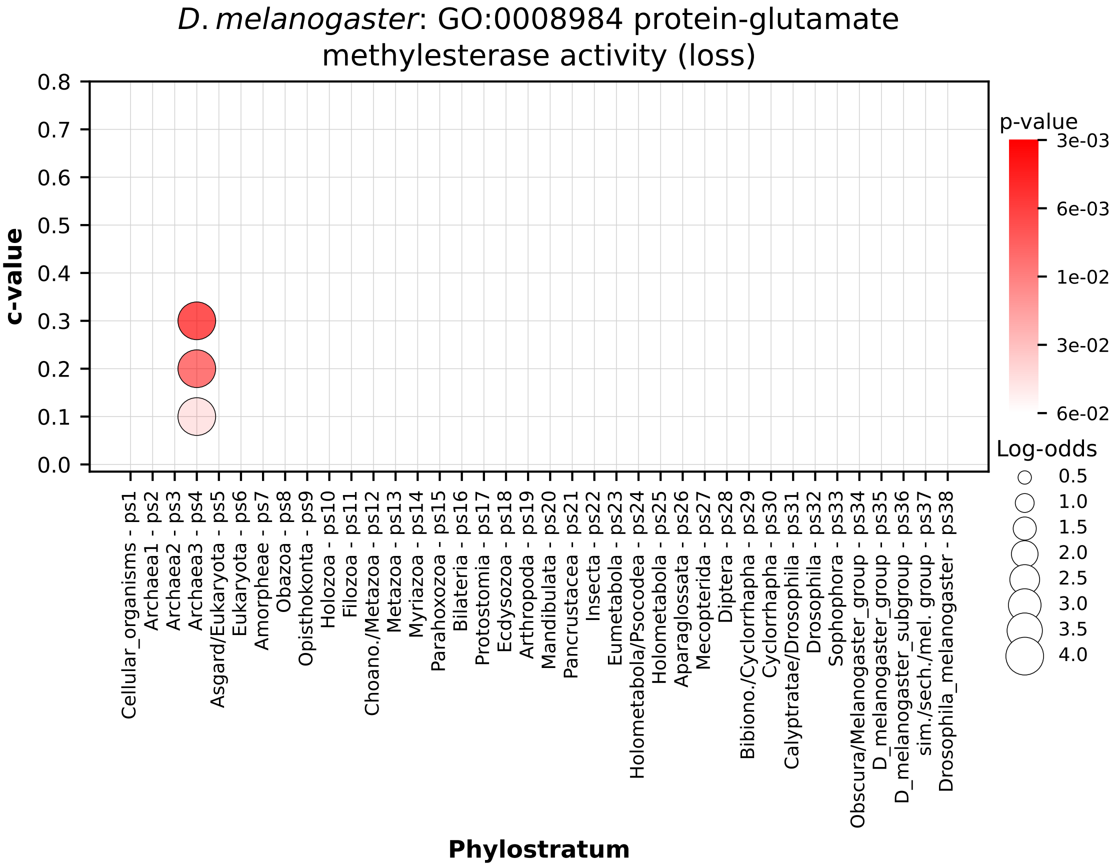
D. melanogaster: GO:0008984 protein-glutamate methylesterase activity (loss)
c-value
0.8
0.7
0.6
0.5
0.4
0.3
0.2
0.1
0.0
p-value
3e-03
6e-03
1e-02
3e-02
6e-02
Log-odds
0.5
1.0
1.5
2.0
2.5
3.0
3.5
4.0
Cellular_organisms - ps1
Archaea1 - ps2
Archaea2 - ps3
Archaea3 - ps4
Asgard/Eukaryota - ps5
Eukaryota - ps6
Amorpheae - ps7
Obazoa - ps8
Opisthokonta - ps9
Holozoa - ps10
Filozoa - ps11
Choano./Metazoa - ps12
Metazoa - ps13
Myriazoa - ps14
Parahoxozoa - ps15
Bilateria - ps16
Protostomia - ps17
Ecdysozoa - ps18
Arthropoda - ps19
Mandibulata - ps20
Pancrustacea - ps21
Insecta - ps22
Eumetabola - ps23
Holometabola/Psocodea - ps24
Holometabola - ps25
Aparaglossata - ps26
Mecopterida - ps27
Diptera - ps28
Bibiono./Cyclorrhapha - ps29
Cyclorrhapha - ps30
Calyptratae/Drosophila - ps31
Drosophila - ps32
Sophophora - ps33
Obscura/Melanogaster_group - ps34
D_melanogaster_group - ps35
D_melanogaster_subgroup - ps36
sim./sech./mel. group - ps37
Drosophila_melanogaster - ps38
Phylostratum

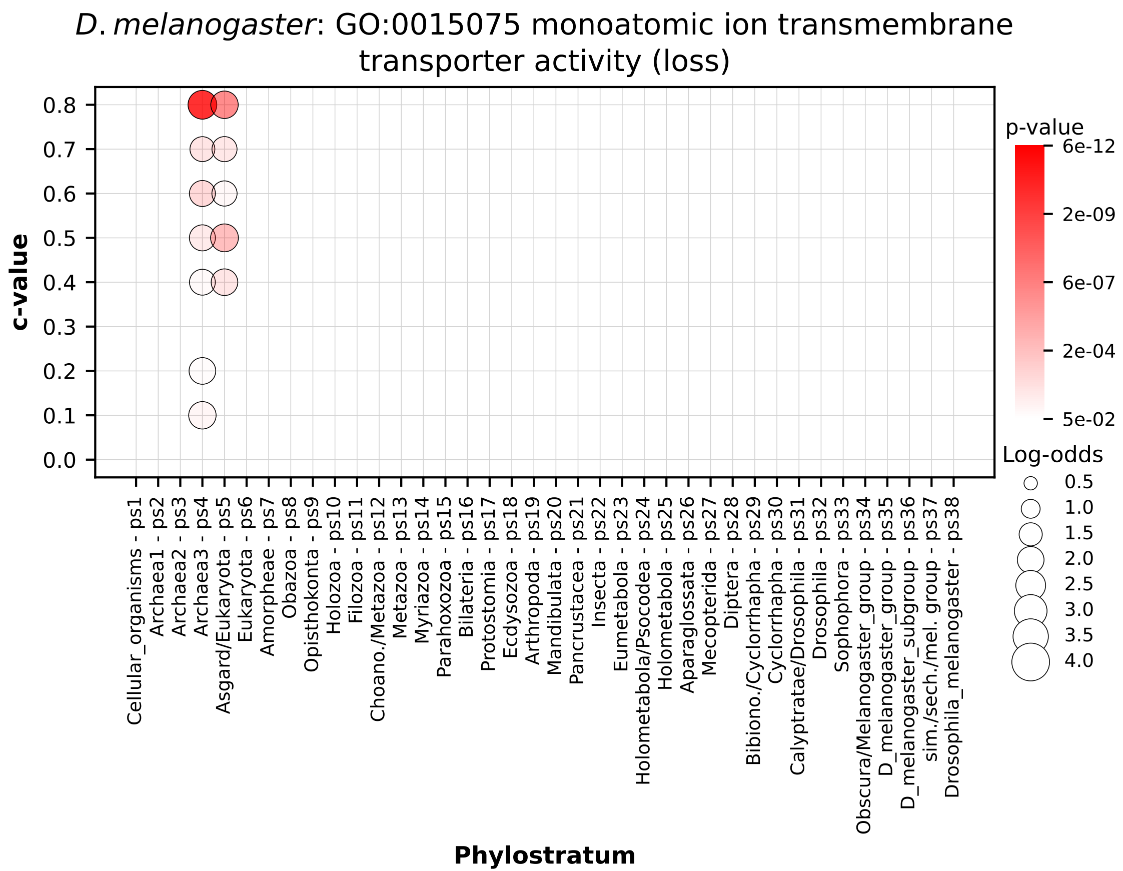

D. melanogaster: GO:0015075 monoatomic ion transmembrane transporter activity (loss)
c-value
0.8
0.7
0.6
0.5
0.4
0.3
0.2
0.1
0.0
p-value
6e-12
2e-09
6e-07
2e-04
5e-02
Log-odds
0.5
1.0
1.5
2.0
2.5
3.0
3.5
4.0
Cellular_organisms - ps1
Archaea1 - ps2
Archaea2 - ps3
Archaea3 - ps4
Asgard/Eukaryota - ps5
Eukaryota - ps6
Amorpheae - ps7
Obazoa - ps8
Opisthokonta - ps9
Holozoa - ps10
Filozoa - ps11
Choano./Metazoa - ps12
Metazoa - ps13
Myriazoa - ps14
Parahoxozoa - ps15
Bilateria - ps16
Protostomia - ps17
Ecdysozoa - ps18
Arthropoda - ps19
Mandibulata - ps20
Pancrustacea - ps21
Insecta - ps22
Eumetabola - ps23
Holometabola/Psocodea - ps24
Holometabola - ps25
Aparaglossata - ps26
Mecopterida - ps27
Diptera - ps28
Bibiono./Cyclorrhapha - ps29
Cyclorrhapha - ps30
Calyptratae/Drosophila - ps31
Drosophila - ps32
Sophophora - ps33
Obscura/Melanogaster_group - ps34
D_melanogaster_group - ps35
D_melanogaster_subgroup - ps36
sim./sech./mel. group - ps37
Drosophila_melanogaster - ps38
Phylostratum

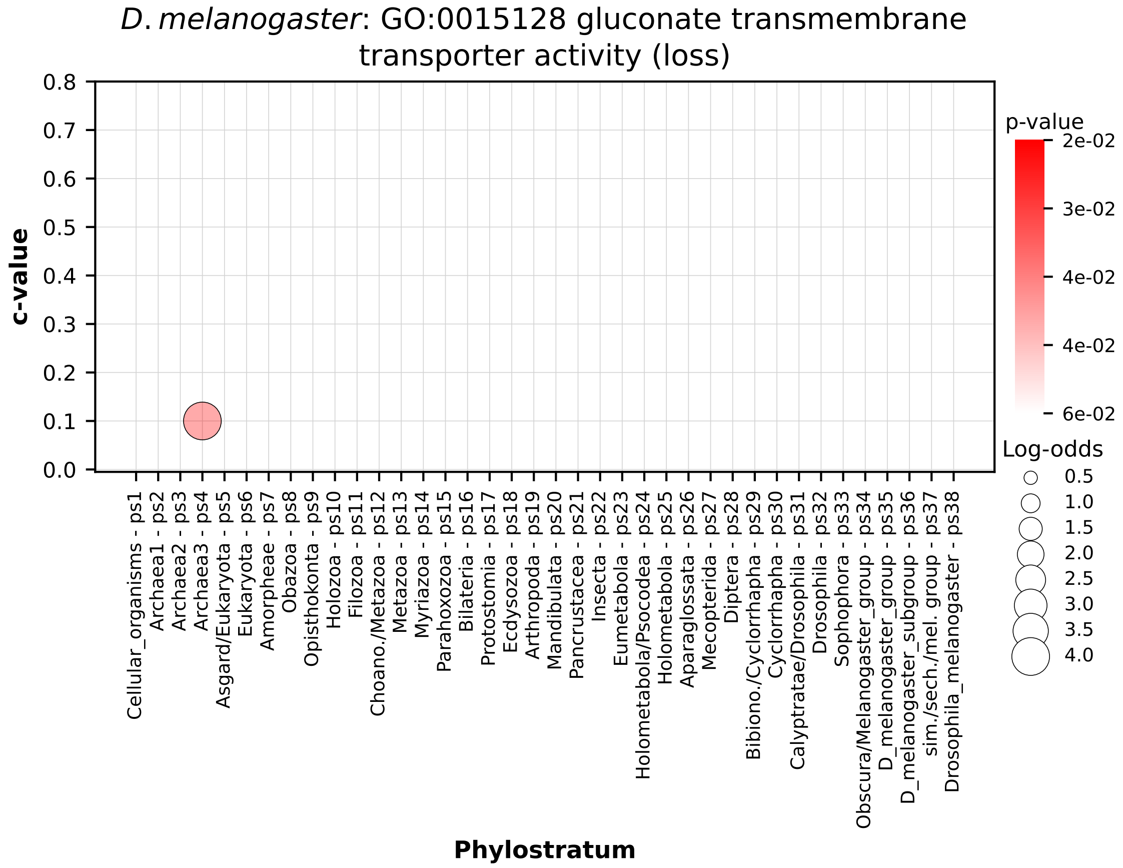
D. melanogaster: GO:0015128 gluconate transmembrane transporter activity (loss)
c-value
0.8
0.7
0.6
0.5
0.4
0.3
0.2
0.1
0.0
Phylostratum
Cellular_organisms - ps1
Archaea1 - ps2
Archaea2 - ps3
Archaea3 - ps4
Asgard/Eukaryota - ps5
Eukaryota - ps6
Amorpheae - ps7
Obazoa - ps8
Opisthokonta - ps9
Holozoa - ps10
Filozoa - ps11
Choano./Metazoa - ps12
Metazoa - ps13
Myriazoa - ps14
Parahoxozoa - ps15
Bilateria - ps16
Protostomia - ps17
Ecdysozoa - ps18
Arthropoda - ps19
Mandibulata - ps20
Pancrustacea - ps21
Insecta - ps22
Eumetabola - ps23
Holometabola/Psocodea - ps24
Holometabola - ps25
Aparaglossata - ps26
Mecopterida - ps27
Diptera - ps28
Bibiono./Cyclorrhapha - ps29
Cyclorrhapha - ps30
Calyptratae/Drosophila - ps31
Drosophila - ps32
Sophophora - ps33
Obscura/Melanogaster_group - ps34
D_melanogaster_group - ps35
D_melanogaster_subgroup - ps36
sim./sech./mel. group - ps37
Drosophila_melanogaster - ps38
p-value
2e-02
3e-02
4e-02
4e-02
6e-02
Log-odds
0.5
1.0
1.5
2.0
2.5
3.0
3.5
4.0

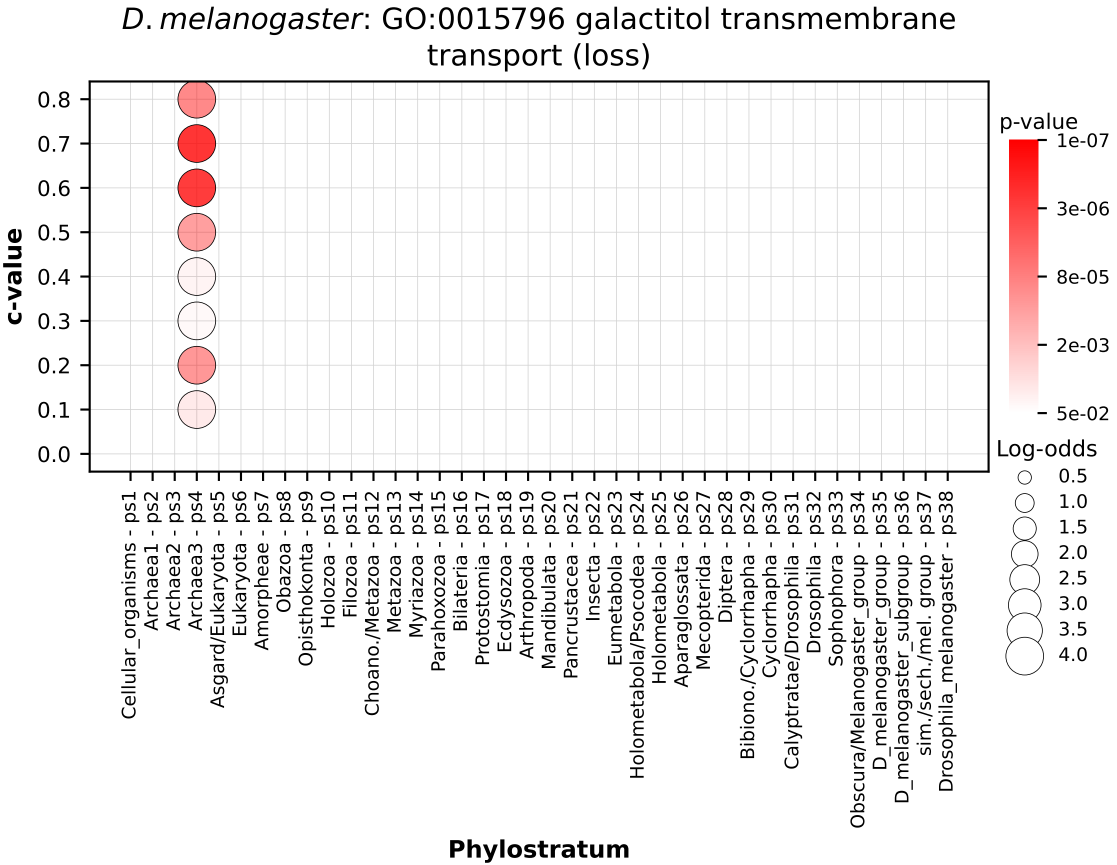

D. melanogaster: GO:0015796 galactitol transmembrane transport (loss)
c-value
0.0
0.1
0.2
0.3
0.4
0.5
0.6
0.7
0.8
p-value
1e-07
3e-06
8e-05
2e-03
5e-02
Log-odds
0.5
1.0
1.5
2.0
2.5
3.0
3.5
4.0
Cellular_organisms - ps1
Archaea1 - ps2
Archaea2 - ps3
Archaea3 - ps4
Asgard/Eukaryota - ps5
Eukaryota - ps6
Amorpheae - ps7
Obazoa - ps8
Opisthokonta - ps9
Holozoa - ps10
Filozoa - ps11
Choano./Metazoa - ps12
Metazoa - ps13
Myriazoa - ps14
Parahoxozoa - ps15
Bilateria - ps16
Protostomia - ps17
Ecdysozoa - ps18
Arthropoda - ps19
Mandibulata - ps20
Pancrustacea - ps21
Insecta - ps22
Eumetabola - ps23
Holometabola/Psocodea - ps24
Holometabola - ps25
Aparaglossata - ps26
Mecopterida - ps27
Diptera - ps28
Bibiono./Cyclorrhapha - ps29
Cyclorrhapha - ps30
Calyptratae/Drosophila - ps31
Drosophila - ps32
Sophophora - ps33
Obscura/Melanogaster_group - ps34
D_melanogaster_group - ps35
D_melanogaster_subgroup - ps36
sim./sech./mel. group - ps37
Drosophila_melanogaster - ps38
Phylostratum

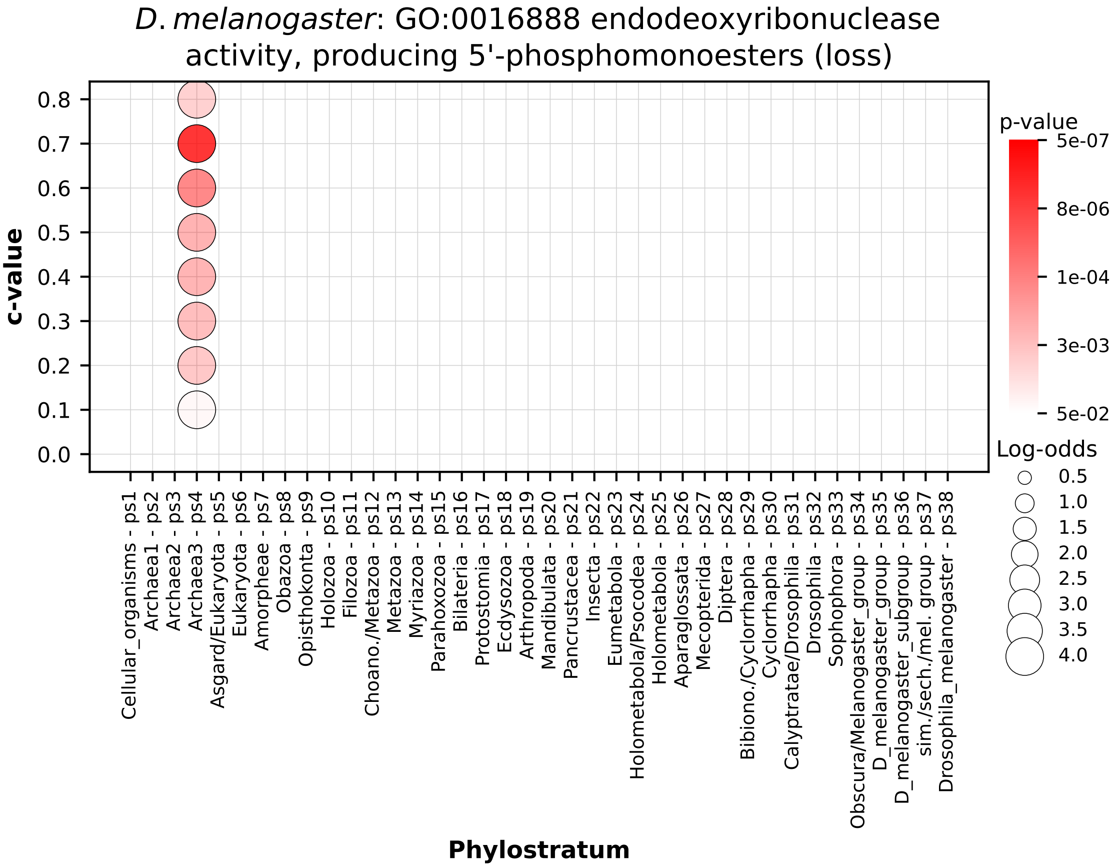
D. melanogaster: GO:0016888 endodeoxyribonuclease activity, producing 5'-phosphomonoesters (loss)
c-value
0.8
0.7
0.6
0.5
0.4
0.3
0.2
0.1
0.0
p-value
5e-07
8e-06
1e-04
3e-03
5e-02
Log-odds
0.5
1.0
1.5
2.0
2.5
3.0
3.5
4.0
Cellular_organisms - ps1
Archaea1 - ps2
Archaea2 - ps3
Archaea3 - ps4
Asgard/Eukaryota - ps5
Eukaryota - ps6
Amorpheae - ps7
Obazoa - ps8
Opisthokonta - ps9
Holozoa - ps10
Filozoa - ps11
Choano./Metazoa - ps12
Metazoa - ps13
Myriazoa - ps14
Parahoxozoa - ps15
Bilateria - ps16
Protostomia - ps17
Ecdysozoa - ps18
Arthropoda - ps19
Mandibulata - ps20
Pancrustacea - ps21
Insecta - ps22
Eumetabola - ps23
Holometabola/Psocodea - ps24
Holometabola - ps25
Aparaglossata - ps26
Mecopterida - ps27
Diptera - ps28
Bibiono./Cyclorrhapha - ps29
Cyclorrhapha - ps30
Calyptratae/Drosophila - ps31
Drosophila - ps32
Sophophora - ps33
Obscura/Melanogaster_group - ps34
D_melanogaster_group - ps35
D_melanogaster_subgroup - ps36
sim./sech./mel. group - ps37
Drosophila_melanogaster - ps38
Phylostratum

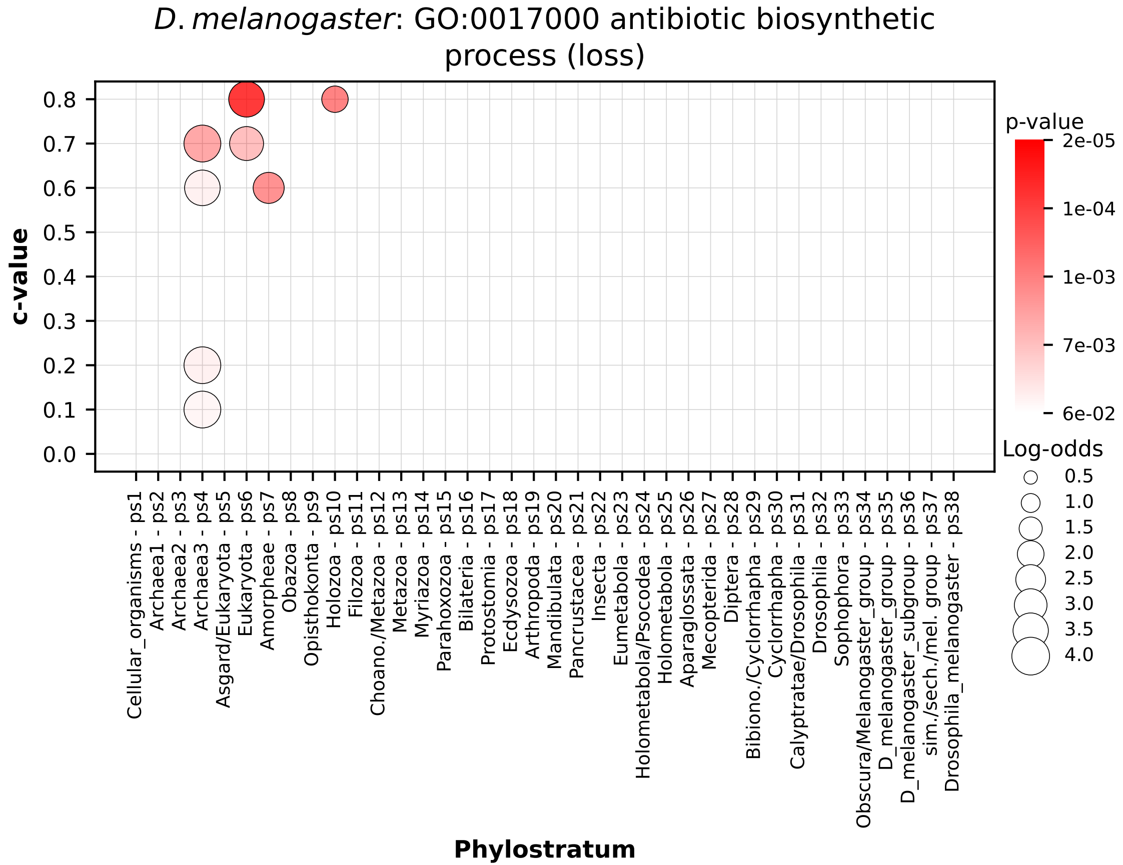
D. melanogaster: GO:0017000 antibiotic biosynthetic process (loss)
c-value
0.8
0.7
0.6
0.5
0.4
0.3
0.2
0.1
0.0
Phylostratum
Cellular_organisms - ps1
Archaea1 - ps2
Archaea2 - ps3
Archaea3 - ps4
Asgard/Eukaryota - ps5
Eukaryota - ps6
Amorpheae - ps7
Obazoa - ps8
Opisthokonta - ps9
Holozoa - ps10
Filozoa - ps11
Choano./Metazoa - ps12
Metazoa - ps13
Myriazoa - ps14
Parahoxozoa - ps15
Bilateria - ps16
Protostomia - ps17
Ecdysozoa - ps18
Arthropoda - ps19
Mandibulata - ps20
Pancrustacea - ps21
Insecta - ps22
Eumetabola - ps23
Holometabola/Psocodea - ps24
Holometabola - ps25
Aparaglossata - ps26
Mecopterida - ps27
Diptera - ps28
Bibiono./Cyclorrhapha - ps29
Cyclorrhapha - ps30
Calyptratae/Drosophila - ps31
Drosophila - ps32
Sophophora - ps33
Obscura/Melanogaster_group - ps34
D_melanogaster_group - ps35
D_melanogaster_subgroup - ps36
sim./sech./mel. group - ps37
Drosophila_melanogaster - ps38
p-value
2e-05
1e-04
1e-03
7e-03
6e-02
Log-odds
0.5
1.0
1.5
2.0
2.5
3.0
3.5
4.0

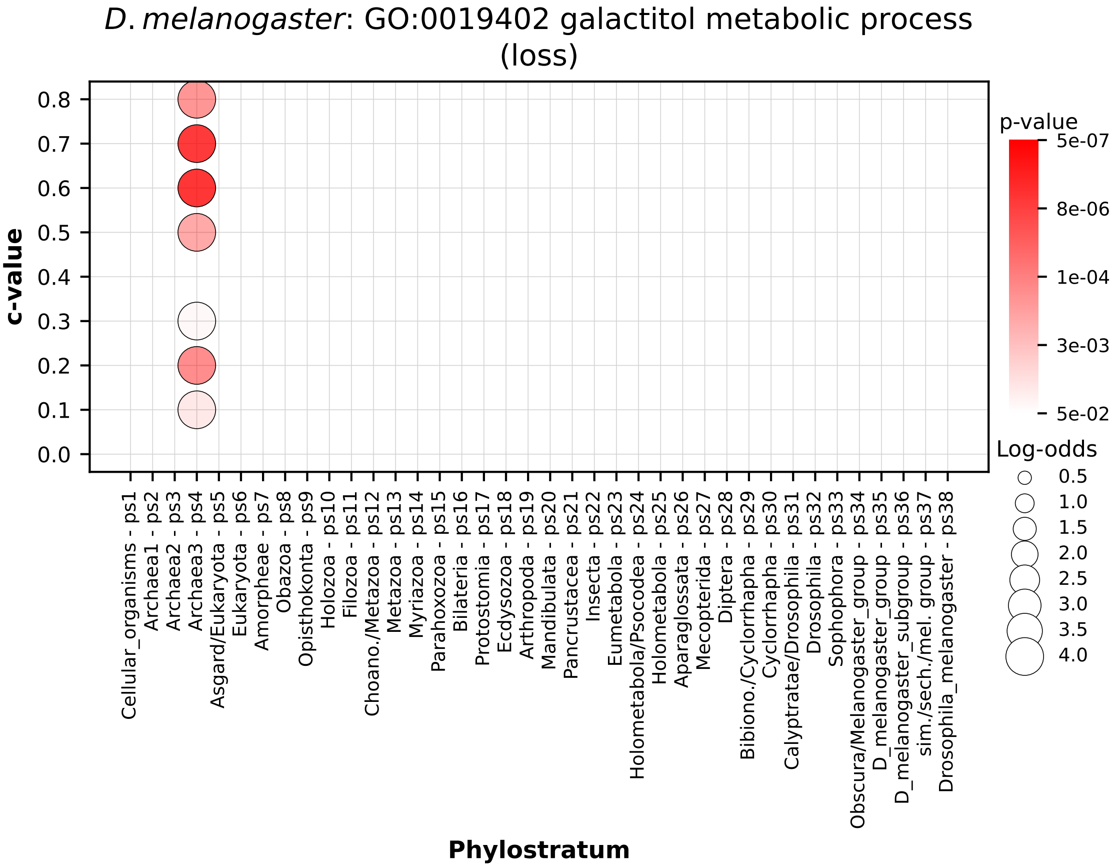

D. melanogaster: GO:0019402 galactitol metabolic process
(loss)
c-value
0.0
0.1
0.2
0.3
0.4
0.5
0.6
0.7
0.8
Phylostratum
Cellular_organisms - ps1
Archaea1 - ps2
Archaea2 - ps3
Archaea3 - ps4
Asgard/Eukaryota - ps5
Eukaryota - ps6
Amorpheae - ps7
Obazoa - ps8
Opisthokonta - ps9
Holozoa - ps10
Filozoa - ps11
Choano./Metazoa - ps12
Metazoa - ps13
Myriazoa - ps14
Parahoxozoa - ps15
Bilateria - ps16
Protostomia - ps17
Ecdysozoa - ps18
Arthropoda - ps19
Mandibulata - ps20
Pancrustacea - ps21
Insecta - ps22
Eumetabola - ps23
Holometabola/Psocodea - ps24
Holometabola - ps25
Aparaglossata - ps26
Mecopterida - ps27
Diptera - ps28
Bibiono./Cyclorrhapha - ps29
Cyclorrhapha - ps30
Calyptratae/Drosophila - ps31
Drosophila - ps32
Sophophora - ps33
Obscura/Melanogaster_group - ps34
D_melanogaster_group - ps35
D_melanogaster_subgroup - ps36
sim./sech./mel. group - ps37
Drosophila_melanogaster - ps38
p-value
5e-07
8e-06
1e-04
3e-03
5e-02
Log-odds
0.5
1.0
1.5
2.0
2.5
3.0
3.5
4.0

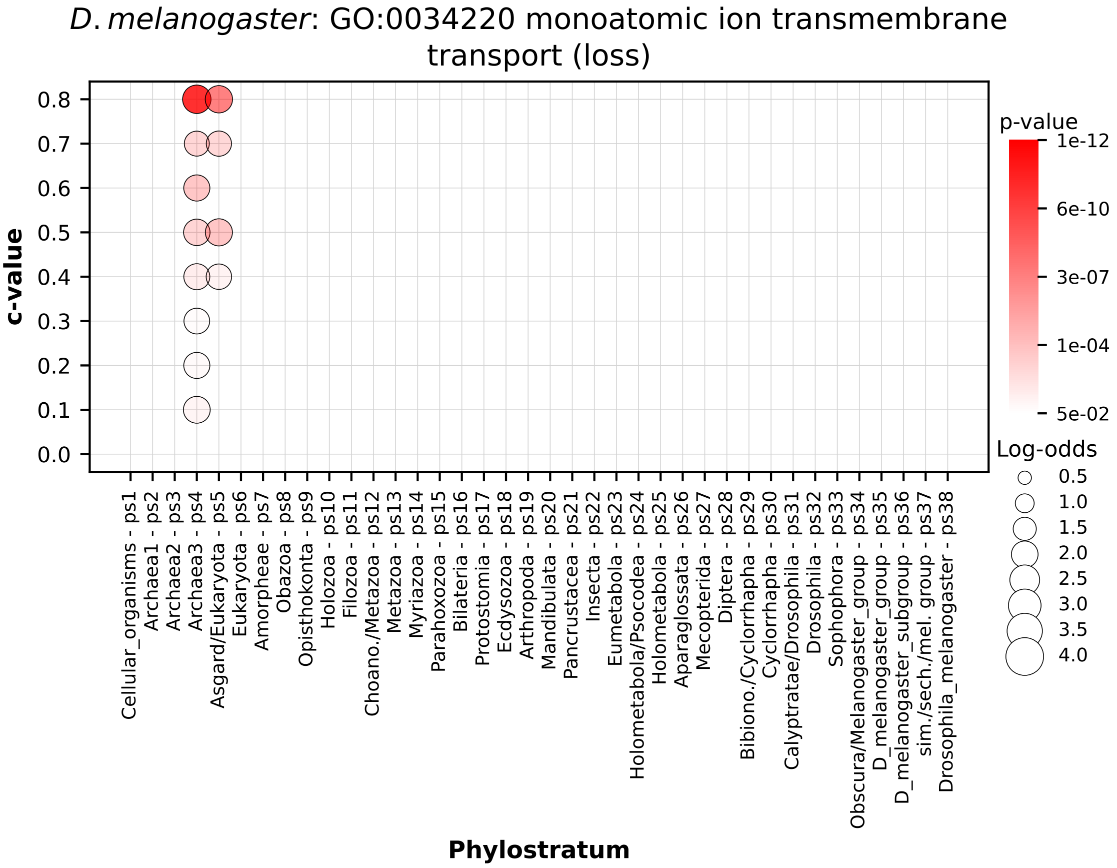

D. melanogaster: GO:0034220 monoatomic ion transmembrane transport (loss)
c-value
0.8
0.7
0.6
0.5
0.4
0.3
0.2
0.1
0.0
p-value
1e-12
6e-10
3e-07
1e-04
5e-02
Log-odds
0.5
1.0
1.5
2.0
2.5
3.0
3.5
4.0
Cellular_organisms - ps1
Archaea1 - ps2
Archaea2 - ps3
Archaea3 - ps4
Asgard/Eukaryota - ps5
Eukaryota - ps6
Amorpheae - ps7
Obazoa - ps8
Opisthokonta - ps9
Holozoa - ps10
Filozoa - ps11
Choano./Metazoa - ps12
Metazoa - ps13
Myriazoa - ps14
Parahoxozoa - ps15
Bilateria - ps16
Protostomia - ps17
Ecdysozoa - ps18
Arthropoda - ps19
Mandibulata - ps20
Pancrustacea - ps21
Insecta - ps22
Eumetabola - ps23
Holometabola/Psocodea - ps24
Holometabola - ps25
Aparaglossata - ps26
Mecopterida - ps27
Diptera - ps28
Bibiono./Cyclorrhapha - ps29
Cyclorrhapha - ps30
Calyptratae/Drosophila - ps31
Drosophila - ps32
Sophophora - ps33
Obscura/Melanogaster_group - ps34
D_melanogaster_group - ps35
D_melanogaster_subgroup - ps36
sim./sech./mel. group - ps37
Drosophila_melanogaster - ps38
Phylostratum

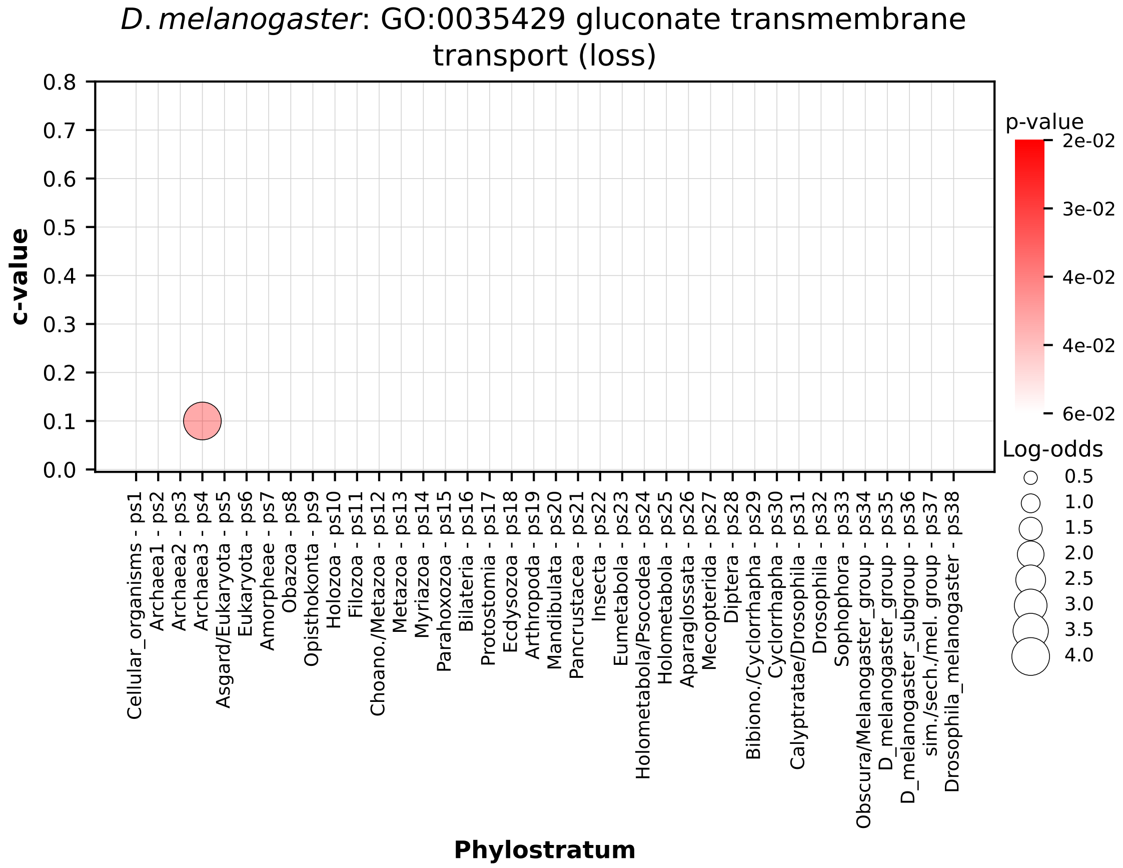

D. melanogaster: GO:0035429 gluconate transmembrane transport (loss)
c-value
0.8
0.7
0.6
0.5
0.4
0.3
0.2
0.1
0.0
Phylostratum
Cellular_organisms - ps1
Archaea1 - ps2
Archaea2 - ps3
Archaea3 - ps4
Asgard/Eukaryota - ps5
Eukaryota - ps6
Amorpheae - ps7
Obazoa - ps8
Opisthokonta - ps9
Holozoa - ps10
Filozoa - ps11
Choano./Metazoa - ps12
Metazoa - ps13
Myriazoa - ps14
Parahoxozoa - ps15
Bilateria - ps16
Protostomia - ps17
Ecdysozoa - ps18
Arthropoda - ps19
Mandibulata - ps20
Pancrustacea - ps21
Insecta - ps22
Eumetabola - ps23
Holometabola/Psocodea - ps24
Holometabola - ps25
Aparaglossata - ps26
Mecopterida - ps27
Diptera - ps28
Bibiono./Cyclorrhapha - ps29
Cyclorrhapha - ps30
Calyptratae/Drosophila - ps31
Drosophila - ps32
Sophophora - ps33
Obscura/Melanogaster_group - ps34
D_melanogaster_group - ps35
D_melanogaster_subgroup - ps36
sim./sech./mel. group - ps37
Drosophila_melanogaster - ps38
p-value
2e-02
3e-02
4e-02
4e-02
6e-02
Log-odds
0.5
1.0
1.5
2.0
2.5
3.0
3.5
4.0

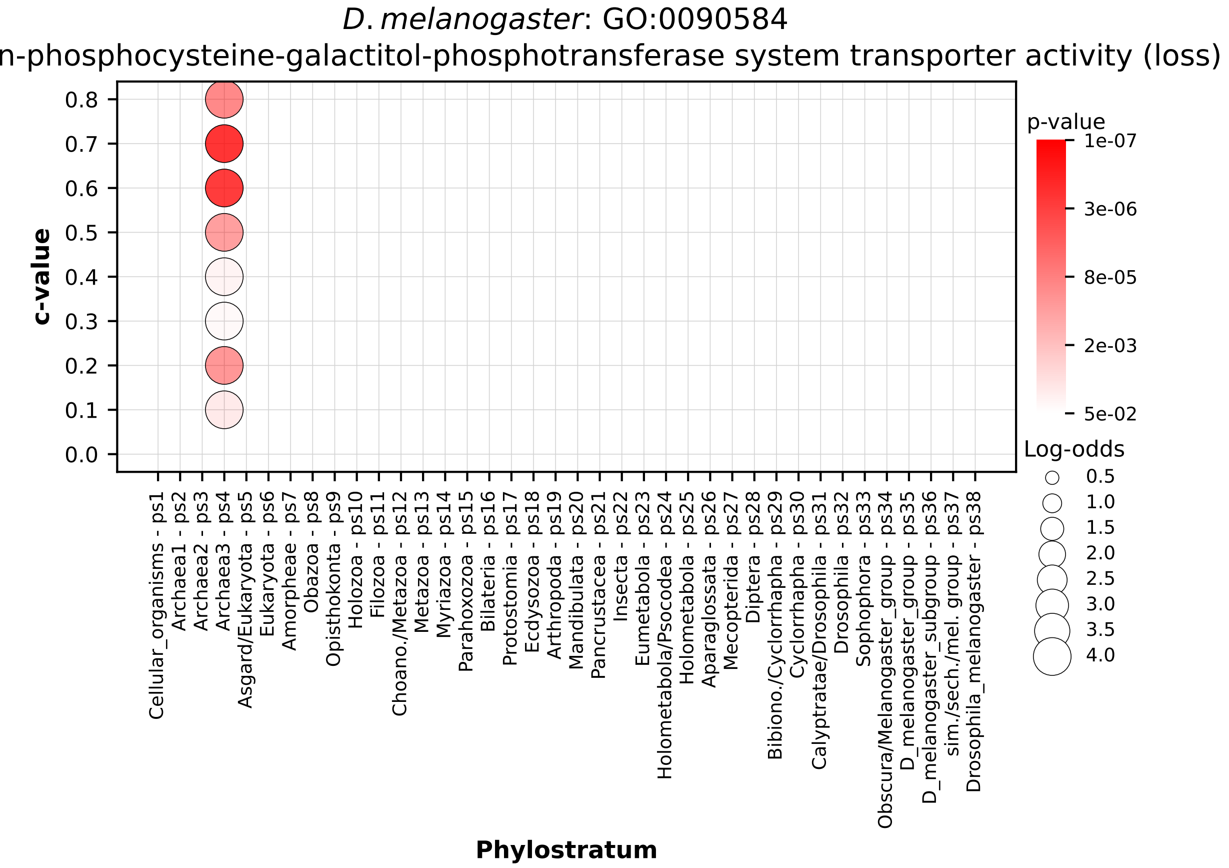

D. melanogaster: GO:0090584
n-phosphocysteine-galactitol-phosphotransferase system transporter activity (loss)
c-value
0.8
0.7
0.6
0.5
0.4
0.3
0.2
0.1
0.0
p-value
1e-07
3e-06
8e-05
2e-03
5e-02
Log-odds
0.5
1.0
1.5
2.0
2.5
3.0
3.5
4.0
Cellular_organisms - ps1
Archaea1 - ps2
Archaea2 - ps3
Archaea3 - ps4
Asgard/Eukaryota - ps5
Eukaryota - ps6
Amorpheae - ps7
Obazoa - ps8
Opisthokonta - ps9
Holozoa - ps10
Filozoa - ps11
Choano./Metazoa - ps12
Metazoa - ps13
Myriazoa - ps14
Parahoxozoa - ps15
Bilateria - ps16
Protostomia - ps17
Ecdysozoa - ps18
Arthropoda - ps19
Mandibulata - ps20
Pancrustacea - ps21
Insecta - ps22
Eumetabola - ps23
Holometabola/Psocodea - ps24
Holometabola - ps25
Aparaglossata - ps26
Mecopterida - ps27
Diptera - ps28
Bibiono./Cyclorrhapha - ps29
Cyclorrhapha - ps30
Calyptratae/Drosophila - ps31
Drosophila - ps32
Sophophora - ps33
Obscura/Melanogaster_group - ps34
D_melanogaster_group - ps35
D_melanogaster_subgroup - ps36
sim./sech./mel. group - ps37
Drosophila_melanogaster - ps38
Phylostratum

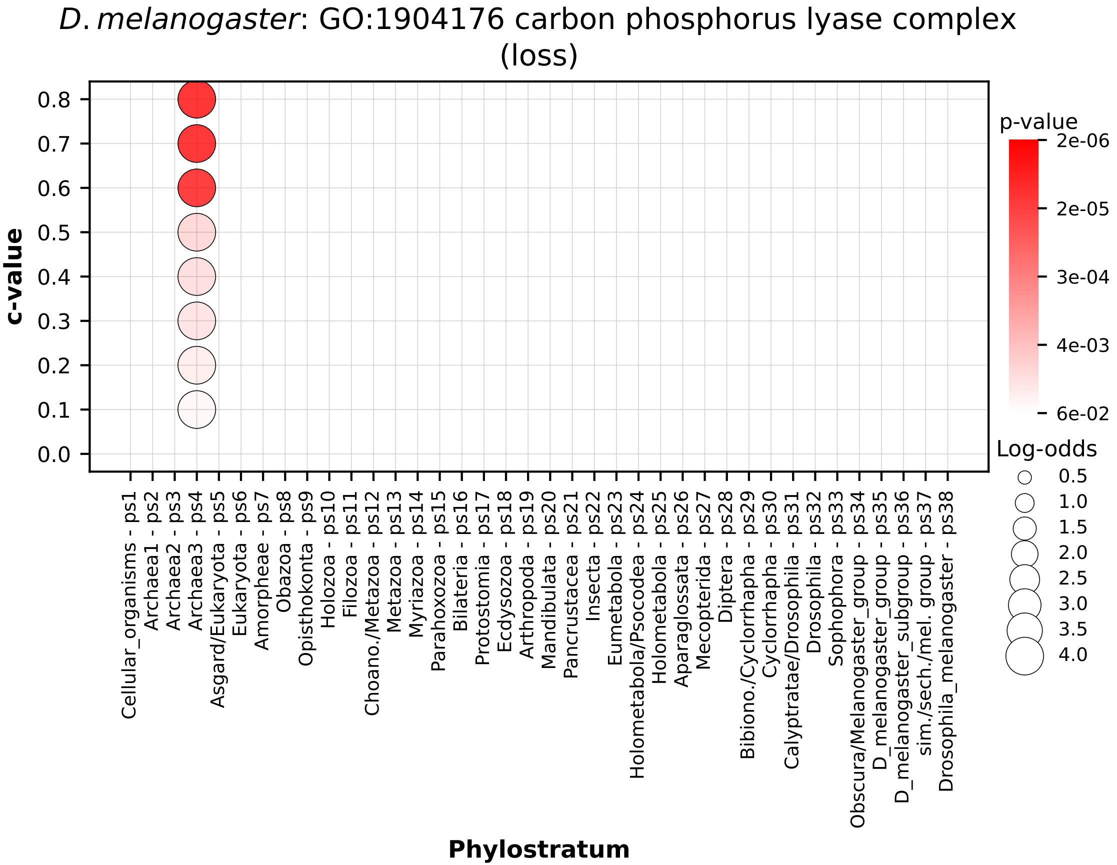

D. melanogaster: GO:1904176 carbon phosphorus lyase complex (loss)
c-value
0.8
0.7
0.6
0.5
0.4
0.3
0.2
0.1
0.0
Phylostratum
Cellular_organisms - ps1
Archaea1 - ps2
Archaea2 - ps3
Archaea3 - ps4
Asgard/Eukaryota - ps5
Eukaryota - ps6
Amorpheae - ps7
Obazoa - ps8
Opisthokonta - ps9
Holozoa - ps10
Filozoa - ps11
Choano./Metazoa - ps12
Metazoa - ps13
Myriazoa - ps14
Parahoxozoa - ps15
Bilateria - ps16
Protostomia - ps17
Ecdysozoa - ps18
Arthropoda - ps19
Mandibulata - ps20
Pancrustacea - ps21
Insecta - ps22
Eumetabola - ps23
Holometabola/Psocodea - ps24
Holometabola - ps25
Aparaglossata - ps26
Mecopterida - ps27
Diptera - ps28
Bibiono./Cyclorrhapha - ps29
Cyclorrhapha - ps30
Calyptratae/Drosophila - ps31
Drosophila - ps32
Sophophora - ps33
Obscura/Melanogaster_group - ps34
D_melanogaster_group - ps35
D_melanogaster_subgroup - ps36
sim./sech./mel. group - ps37
Drosophila_melanogaster - ps38
p-value
2e-06
2e-05
3e-04
4e-03
6e-02
Log-odds
0.5
1.0
1.5
2.0
2.5
3.0
3.5
4.0

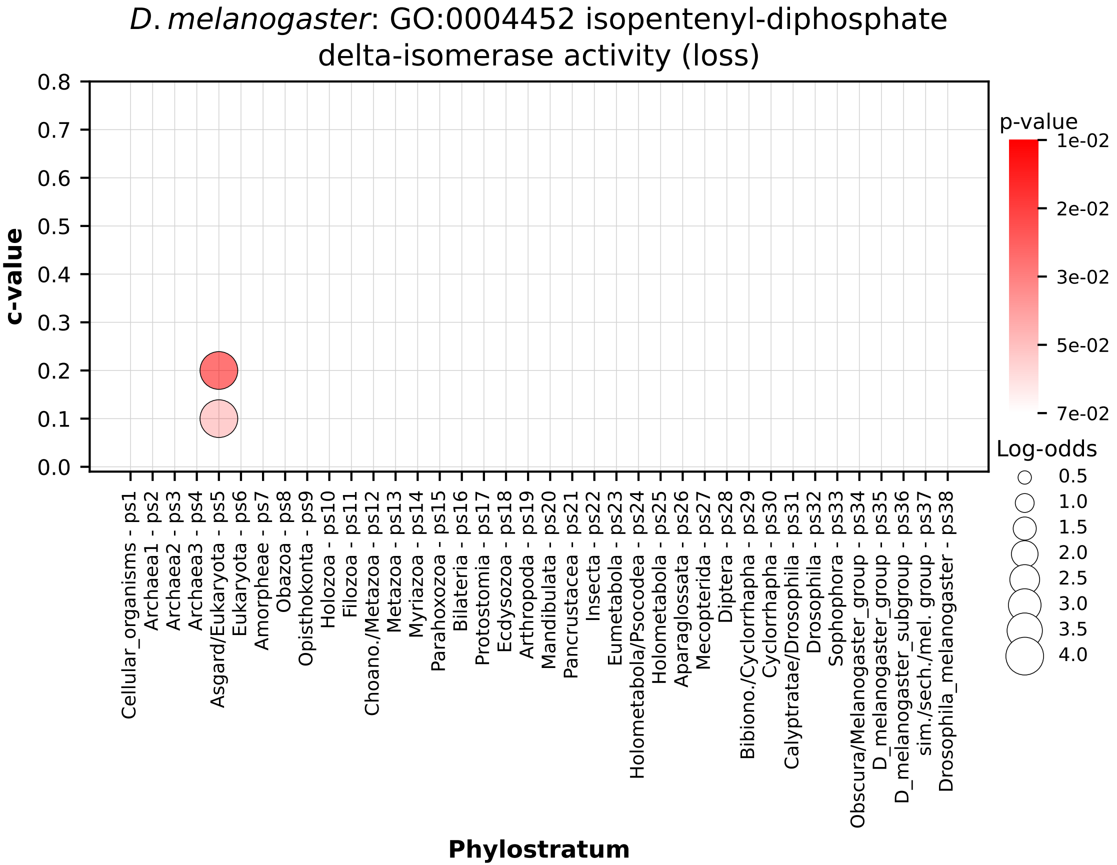

D. melanogaster: GO:0004452 isopentenyl-diphosphate delta-isomerase activity (loss)
c-value
0.0
0.1
0.2
0.3
0.4
0.5
0.6
0.7
0.8
Phylostratum
Cellular_organisms - ps1
Archaea1 - ps2
Archaea2 - ps3
Archaea3 - ps4
Asgard/Eukaryota - ps5
Eukaryota - ps6
Amorpheae - ps7
Obazoa - ps8
Opisthokonta - ps9
Holozoa - ps10
Filozoa - ps11
Choano./Metazoa - ps12
Metazoa - ps13
Myriazoa - ps14
Parahoxozoa - ps15
Bilateria - ps16
Protostomia - ps17
Ecdysozoa - ps18
Arthropoda - ps19
Mandibulata - ps20
Pancrustacea - ps21
Insecta - ps22
Eumetabola - ps23
Holometabola/Psocodea - ps24
Holometabola - ps25
Aparaglossata - ps26
Mecopterida - ps27
Diptera - ps28
Bibiono./Cyclorrhapha - ps29
Cyclorrhapha - ps30
Calyptratae/Drosophila - ps31
Drosophila - ps32
Sophophora - ps33
Obscura/Melanogaster_group - ps34
D_melanogaster_group - ps35
D_melanogaster_subgroup - ps36
sim./sech./mel. group - ps37
Drosophila_melanogaster - ps38
p-value
1e-02
2e-02
3e-02
5e-02
7e-02
Log-odds
0.5
1.0
1.5
2.0
2.5
3.0
3.5
4.0

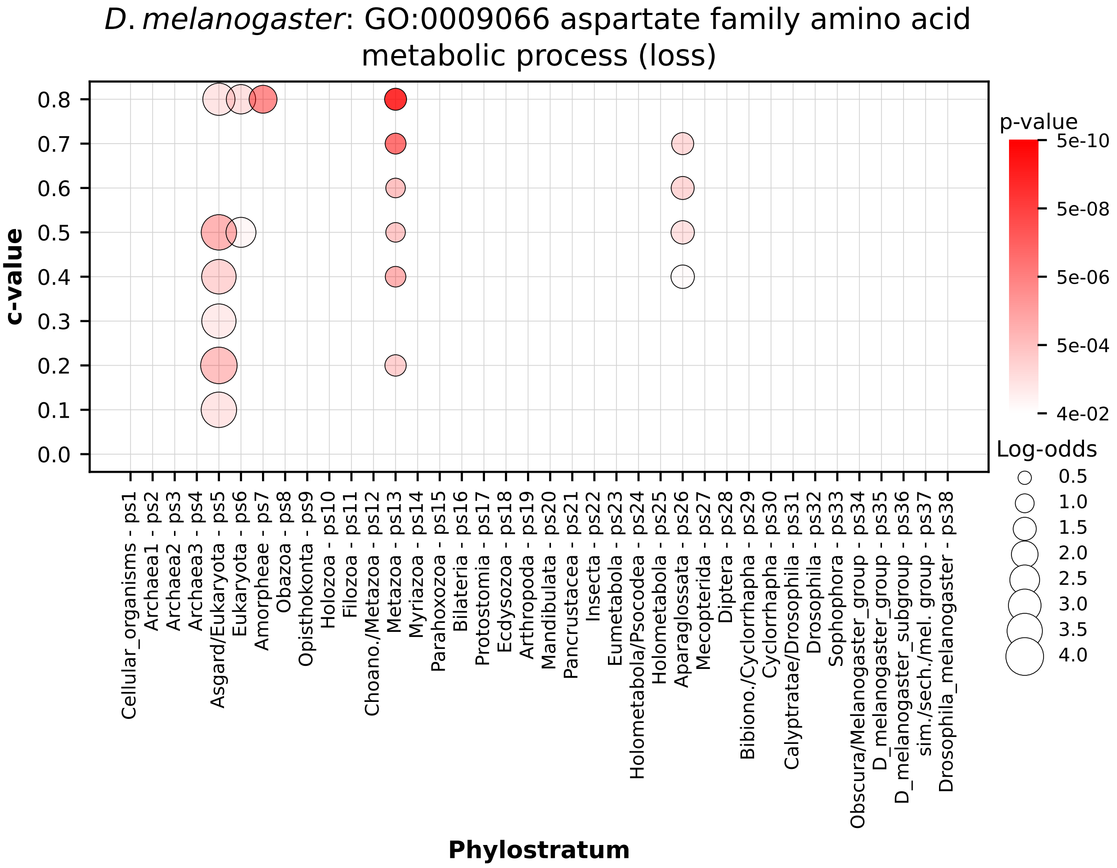

D. melanogaster: GO:0009066 aspartate family amino acid metabolic process (loss)
c-value
0.0
0.1
0.2
0.3
0.4
0.5
0.6
0.7
0.8
Phylostratum
Cellular_organisms - ps1
Archaea1 - ps2
Archaea2 - ps3
Archaea3 - ps4
Asgard/Eukaryota - ps5
Eukaryota - ps6
Amorpheae - ps7
Obazoa - ps8
Opisthokonta - ps9
Holozoa - ps10
Filozoa - ps11
Choano./Metazoa - ps12
Metazoa - ps13
Myriazoa - ps14
Parahoxozoa - ps15
Bilateria - ps16
Protostomia - ps17
Ecdysozoa - ps18
Arthropoda - ps19
Mandibulata - ps20
Pancrustacea - ps21
Insecta - ps22
Eumetabola - ps23
Holometabola/Psocodea - ps24
Holometabola - ps25
Aparaglossata - ps26
Mecopterida - ps27
Diptera - ps28
Bibiono./Cyclorrhapha - ps29
Cyclorrhapha - ps30
Calyptratae/Drosophila - ps31
Drosophila - ps32
Sophophora - ps33
Obscura/Melanogaster_group - ps34
D_melanogaster_group - ps35
D_melanogaster_subgroup - ps36
sim./sech./mel. group - ps37
Drosophila_melanogaster - ps38
p-value
5e-10
5e-08
5e-06
5e-04
4e-02
Log-odds
0.5
1.0
1.5
2.0
2.5
3.0
3.5
4.0

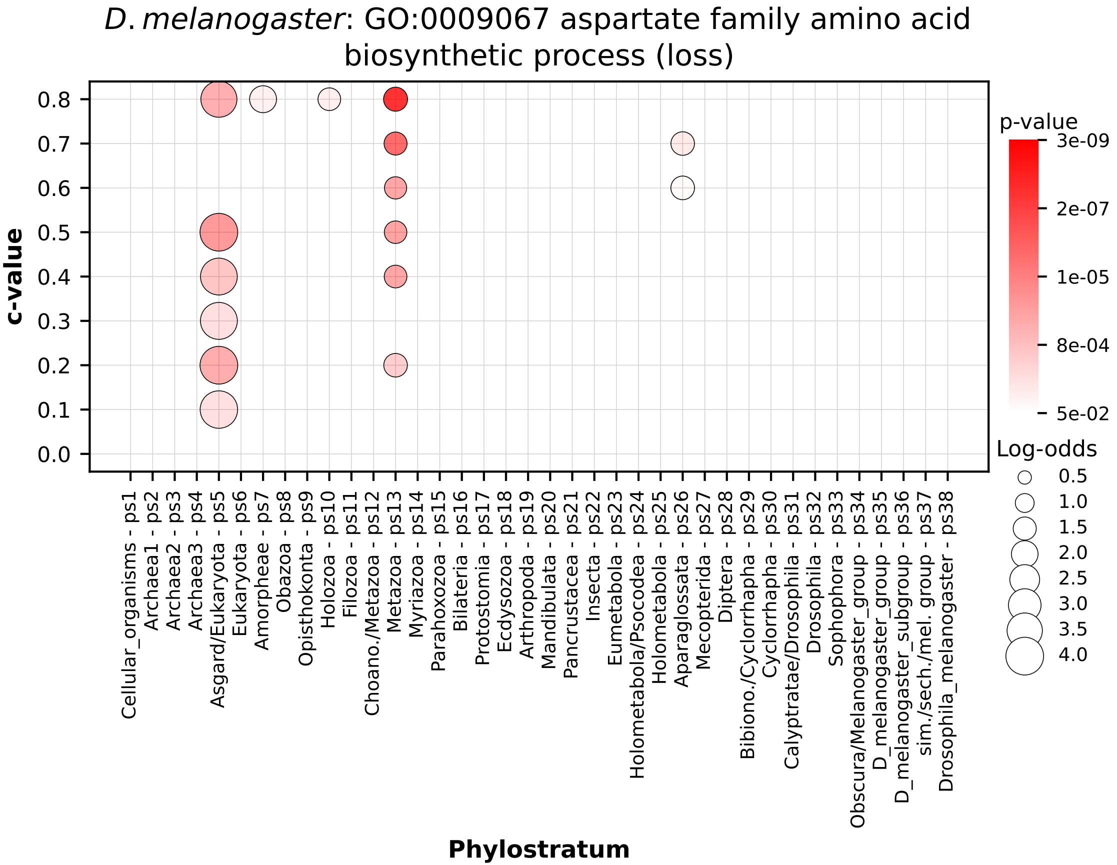

D. melanogaster: GO:0009067 aspartate family amino acid biosynthetic process (loss)
c-value
0.8
0.7
0.6
0.5
0.4
0.3
0.2
0.1
0.0
p-value
3e-09
2e-07
1e-05
8e-04
5e-02
Log-odds
0.5
1.0
1.5
2.0
2.5
3.0
3.5
4.0
Cellular_organisms - ps1
Archaea1 - ps2
Archaea2 - ps3
Archaea3 - ps4
Asgard/Eukaryota - ps5
Eukaryota - ps6
Amorpheae - ps7
Obazoa - ps8
Opisthokonta - ps9
Holozoa - ps10
Filozoa - ps11
Choano./Metazoa - ps12
Metazoa - ps13
Myriazoa - ps14
Parahoxozoa - ps15
Bilateria - ps16
Protostomia - ps17
Ecdysozoa - ps18
Arthropoda - ps19
Mandibulata - ps20
Pancrustacea - ps21
Insecta - ps22
Eumetabola - ps23
Holometabola/Psocodea - ps24
Holometabola - ps25
Aparaglossata - ps26
Mecopterida - ps27
Diptera - ps28
Bibiono./Cyclorrhapha - ps29
Cyclorrhapha - ps30
Calyptratae/Drosophila - ps31
Drosophila - ps32
Sophophora - ps33
Obscura/Melanogaster_group - ps34
D_melanogaster_group - ps35
D_melanogaster_subgroup - ps36
sim./sech./mel. group - ps37
Drosophila_melanogaster - ps38
Phylostratum

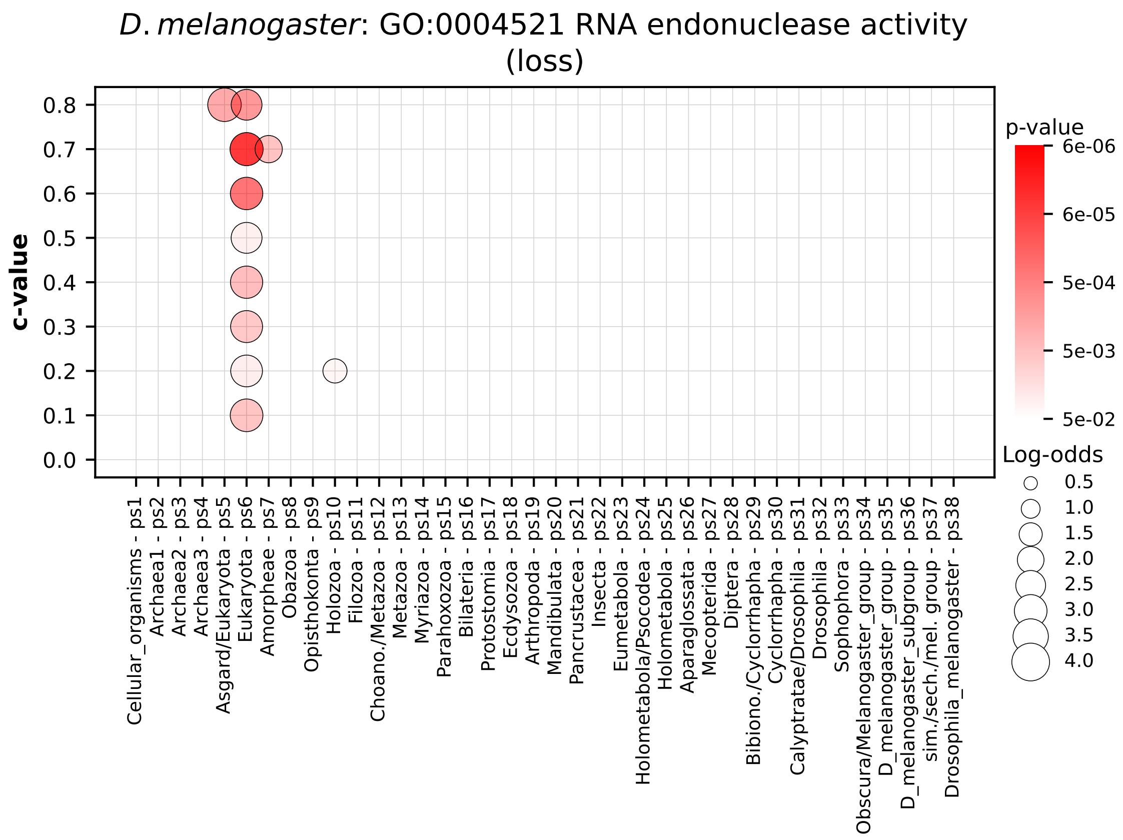

D. melanogaster: GO:0004521 RNA endonuclease activity
(loss)
c-value
0.0
0.1
0.2
0.3
0.4
0.5
0.6
0.7
0.8
Phylostratum
Cellular_organisms - ps1
Archaea1 - ps2
Archaea2 - ps3
Archaea3 - ps4
Asgard/Eukaryota - ps5
Eukaryota - ps6
Amorpheae - ps7
Obazoa - ps8
Opisthokonta - ps9
Holozoa - ps10
Filozoa - ps11
Choano./Metazoa - ps12
Metazoa - ps13
Myriazoa - ps14
Parahoxozoa - ps15
Bilateria - ps16
Protostomia - ps17
Ecdysozoa - ps18
Arthropoda - ps19
Mandibulata - ps20
Pancrustacea - ps21
Insecta - ps22
Eumetabola - ps23
Holometabola/Psocodea - ps24
Holometabola - ps25
Aparaglossata - ps26
Mecopterida - ps27
Diptera - ps28
Bibiono./Cyclorrhapha - ps29
Cyclorrhapha - ps30
Calyptratae/Drosophila - ps31
Drosophila - ps32
Sophophora - ps33
Obscura/Melanogaster_group - ps34
D_melanogaster_group - ps35
D_melanogaster_subgroup - ps36
sim./sech./mel. group - ps37
Drosophila_melanogaster - ps38
p-value
6e-06
6e-05
5e-04
5e-03
5e-02
Log-odds
0.5
1.0
1.5
2.0
2.5
3.0
3.5
4.0

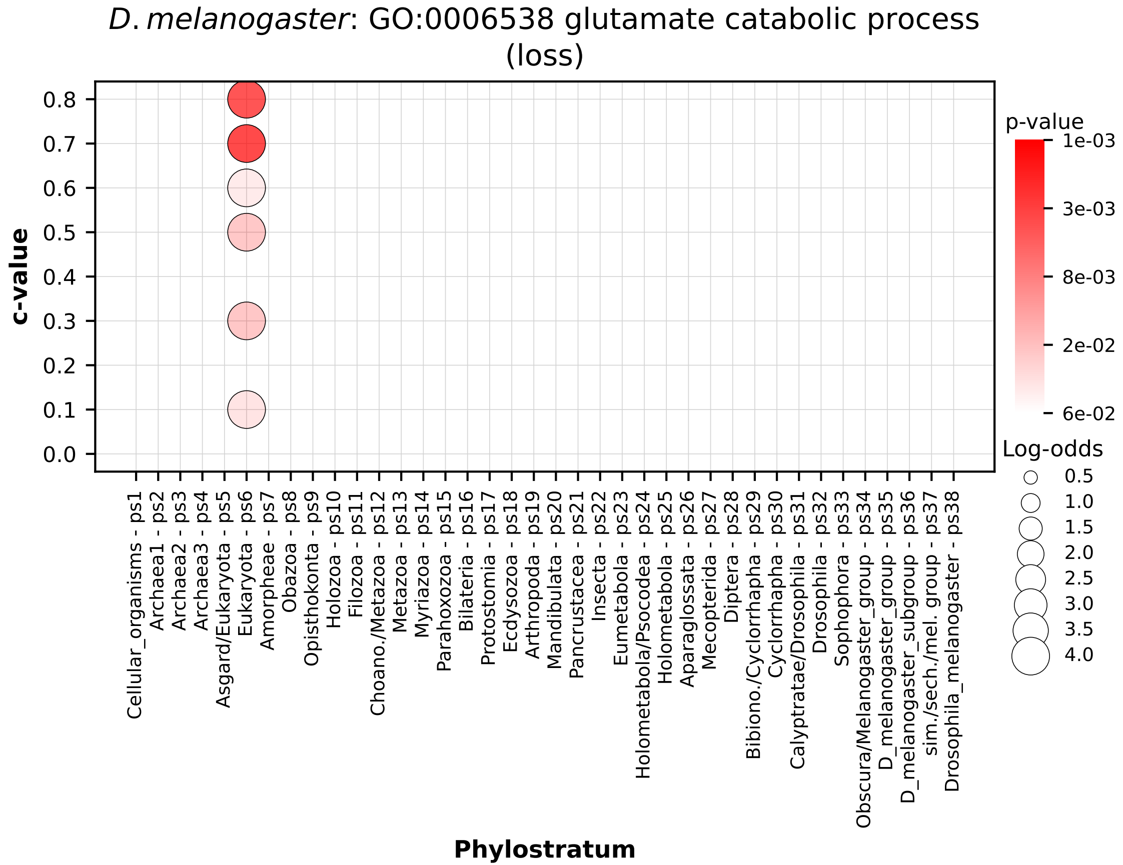

D. melanogaster: GO:0006538 glutamate catabolic process (loss)
c-value
0.8
0.7
0.6
0.5
0.4
0.3
0.2
0.1
0.0
p-value
1e-03
3e-03
8e-03
2e-02
6e-02
Log-odds
0.5
1.0
1.5
2.0
2.5
3.0
3.5
4.0
Cellular_organisms - ps1
Archaea1 - ps2
Archaea2 - ps3
Archaea3 - ps4
Asgard/Eukaryota - ps5
Eukaryota - ps6
Amorpheae - ps7
Obazoa - ps8
Opisthokonta - ps9
Holozoa - ps10
Filozoa - ps11
Choano./Metazoa - ps12
Metazoa - ps13
Myriazoa - ps14
Parahoxozoa - ps15
Bilateria - ps16
Protostomia - ps17
Ecdysozoa - ps18
Arthropoda - ps19
Mandibulata - ps20
Pancrustacea - ps21
Insecta - ps22
Eumetabola - ps23
Holometabola/Psocodea - ps24
Holometabola - ps25
Aparaglossata - ps26
Mecopterida - ps27
Diptera - ps28
Bibiono./Cyclorrhapha - ps29
Cyclorrhapha - ps30
Calyptratae/Drosophila - ps31
Drosophila - ps32
Sophophora - ps33
Obscura/Melanogaster_group - ps34
D_melanogaster_group - ps35
D_melanogaster_subgroup - ps36
sim./sech./mel. group - ps37
Drosophila_melanogaster - ps38
Phylostratum

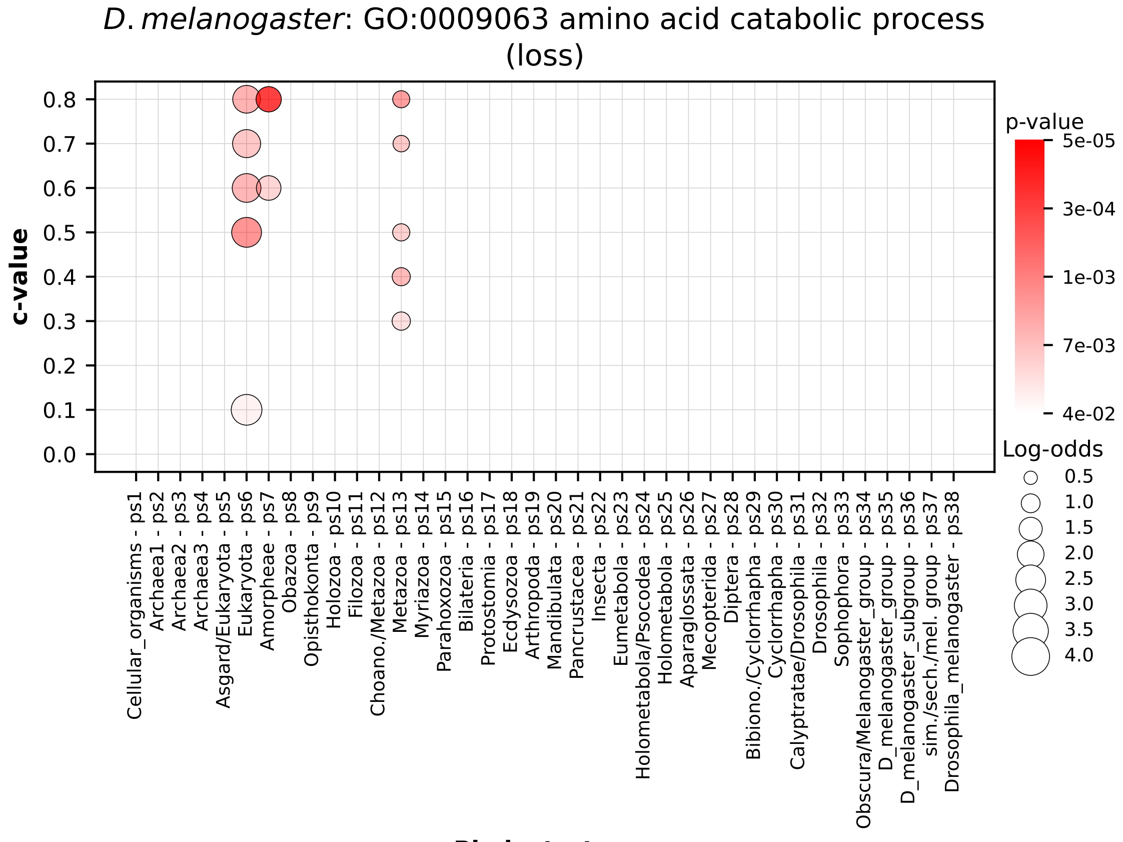

D. melanogaster: GO:0009063 amino acid catabolic process (loss)
c-value
0.0
0.1
0.2
0.3
0.4
0.5
0.6
0.7
0.8
Phylostratum
Cellular_organisms - ps1
Archaea1 - ps2
Archaea2 - ps3
Archaea3 - ps4
Asgard/Eukaryota - ps5
Eukaryota - ps6
Amorpheae - ps7
Obazoa - ps8
Opisthokonta - ps9
Holozoa - ps10
Filozoa - ps11
Choano./Metazoa - ps12
Metazoa - ps13
Myriazoa - ps14
Parahoxozoa - ps15
Bilateria - ps16
Protostomia - ps17
Ecdysozoa - ps18
Arthropoda - ps19
Mandibulata - ps20
Pancrustacea - ps21
Insecta - ps22
Eumetabola - ps23
Holometabola/Psocodea - ps24
Holometabola - ps25
Aparaglossata - ps26
Mecopterida - ps27
Diptera - ps28
Bibiono./Cyclorrhapha - ps29
Cyclorrhapha - ps30
Calyptratae/Drosophila - ps31
Drosophila - ps32
Sophophora - ps33
Obscura/Melanogaster_group - ps34
D_melanogaster_group - ps35
D_melanogaster_subgroup - ps36
sim./sech./mel. group - ps37
Drosophila_melanogaster - ps38
p-value
5e-05
3e-04
1e-03
7e-03
4e-02
Log-odds
0.5
1.0
1.5
2.0
2.5
3.0
3.5
4.0

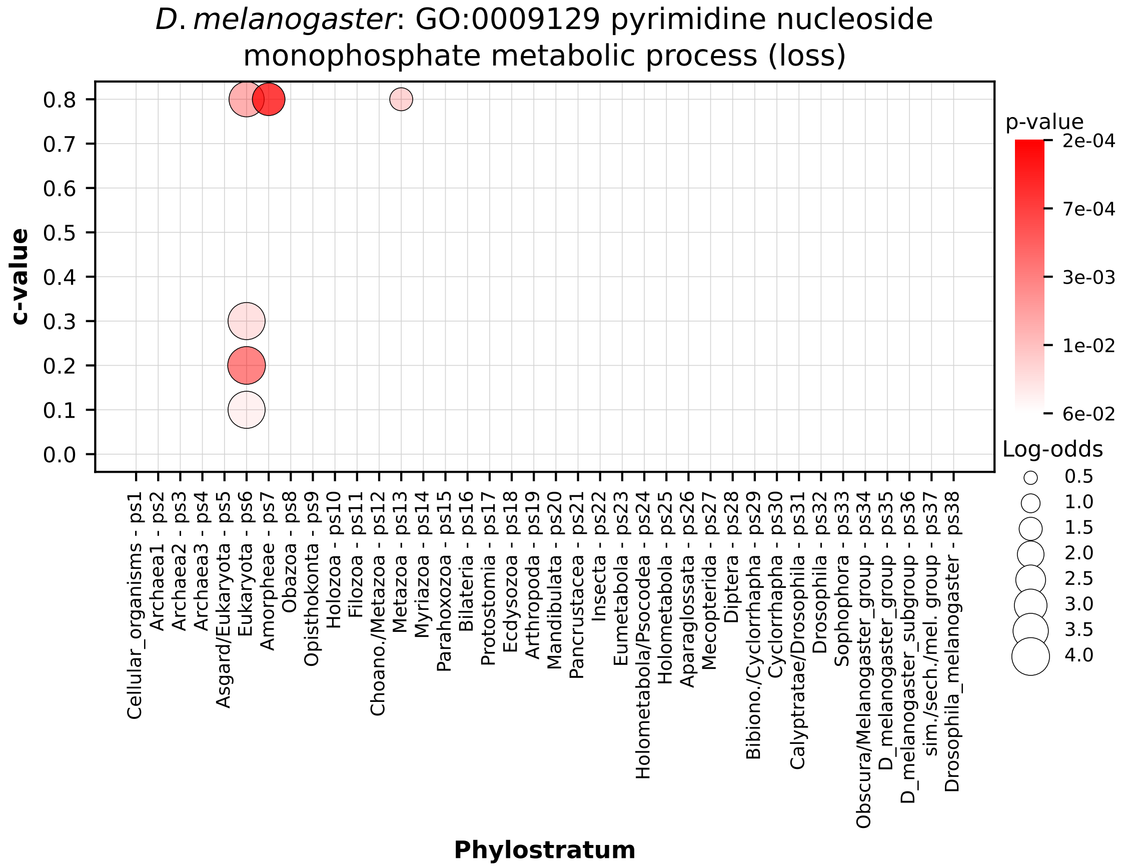

D. melanogaster: GO:0009129 pyrimidine nucleoside monophosphate metabolic process (loss)
c-value
0.8
0.7
0.6
0.5
0.4
0.3
0.2
0.1
0.0
p-value
2e-04
7e-04
3e-03
1e-02
6e-02
Log-odds
0.5
1.0
1.5
2.0
2.5
3.0
3.5
4.0
Cellular_organisms - ps1
Archaea1 - ps2
Archaea2 - ps3
Archaea3 - ps4
Asgard/Eukaryota - ps5
Eukaryota - ps6
Amorpheae - ps7
Obazoa - ps8
Opisthokonta - ps9
Holozoa - ps10
Filozoa - ps11
Choano./Metazoa - ps12
Metazoa - ps13
Myriazoa - ps14
Parahoxozoa - ps15
Bilateria - ps16
Protostomia - ps17
Ecdysozoa - ps18
Arthropoda - ps19
Mandibulata - ps20
Pancrustacea - ps21
Insecta - ps22
Eumetabola - ps23
Holometabola/Psocodea - ps24
Holometabola - ps25
Aparaglossata - ps26
Mecopterida - ps27
Diptera - ps28
Bibiono./Cyclorrhapha - ps29
Cyclorrhapha - ps30
Calyptratae/Drosophila - ps31
Drosophila - ps32
Sophophora - ps33
Obscura/Melanogaster_group - ps34
D_melanogaster_group - ps35
D_melanogaster_subgroup - ps36
sim./sech./mel. group - ps37
Drosophila_melanogaster - ps38
Phylostratum

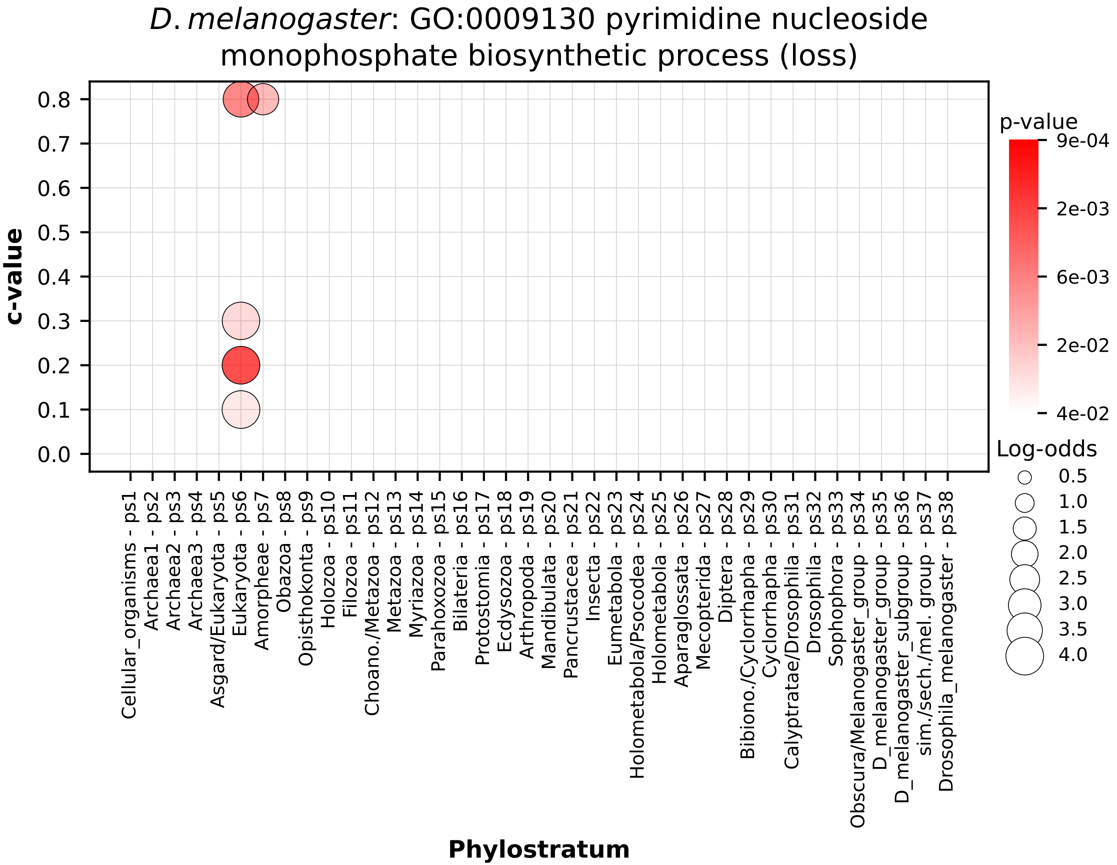

D. melanogaster: GO:0009130 pyrimidine nucleoside monophosphate biosynthetic process (loss)
c-value
0.0
0.1
0.2
0.3
0.4
0.5
0.6
0.7
0.8
p-value
9e-04
2e-03
6e-03
2e-02
4e-02
Log-odds
0.5
1.0
1.5
2.0
2.5
3.0
3.5
4.0
Cellular_organisms - ps1
Archaea1 - ps2
Archaea2 - ps3
Archaea3 - ps4
Asgard/Eukaryota - ps5
Eukaryota - ps6
Amorpheae - ps7
Obazoa - ps8
Opisthokonta - ps9
Holozoa - ps10
Filozoa - ps11
Choano./Metazoa - ps12
Metazoa - ps13
Myriazoa - ps14
Parahoxozoa - ps15
Bilateria - ps16
Protostomia - ps17
Ecdysozoa - ps18
Arthropoda - ps19
Mandibulata - ps20
Pancrustacea - ps21
Insecta - ps22
Eumetabola - ps23
Holometabola/Psocodea - ps24
Holometabola - ps25
Aparaglossata - ps26
Mecopterida - ps27
Diptera - ps28
Bibiono./Cyclorrhapha - ps29
Cyclorrhapha - ps30
Calyptratae/Drosophila - ps31
Drosophila - ps32
Sophophora - ps33
Obscura/Melanogaster_group - ps34
D_melanogaster_group - ps35
D_melanogaster_subgroup - ps36
sim./sech./mel. group - ps37
Drosophila_melanogaster - ps38
Phylostratum

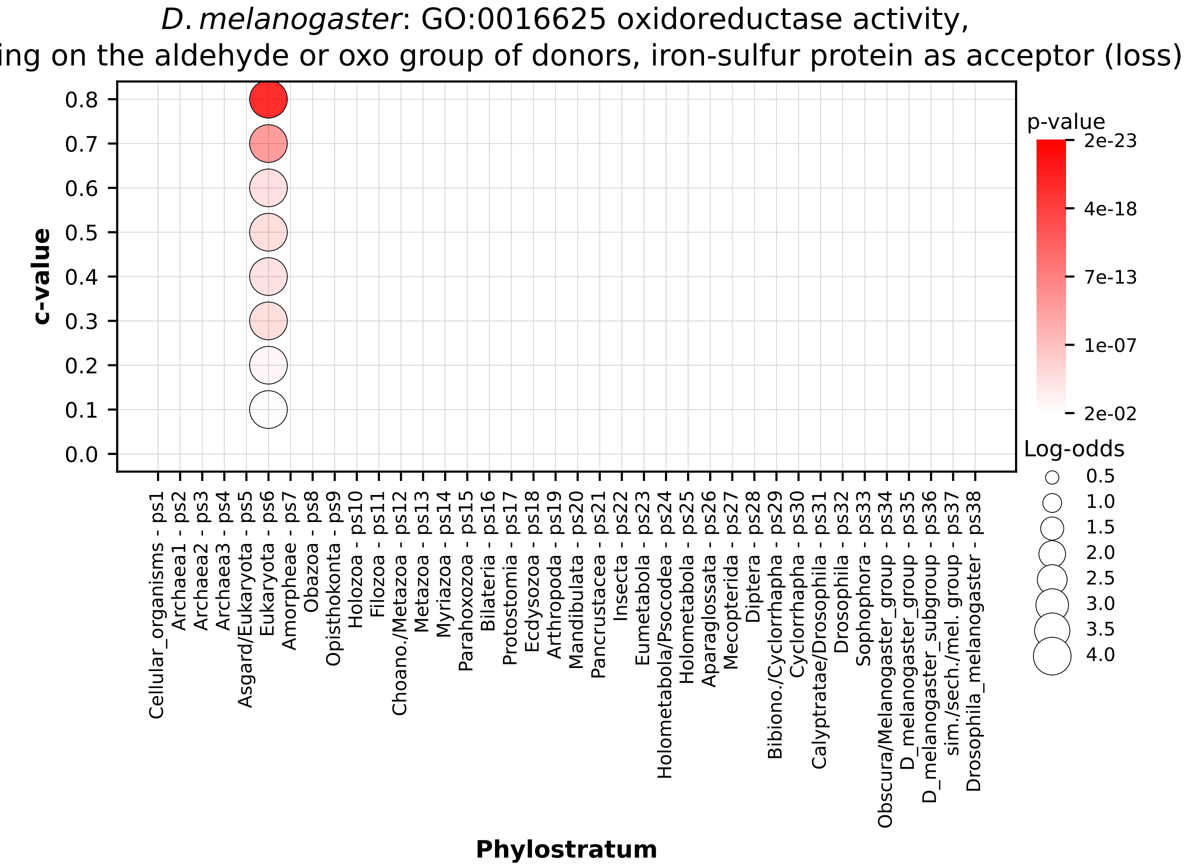
D. melanogaster: GO:0016625 oxidoreductase activity,
ing on the aldehyde or oxo group of donors, iron-sulfur protein as acceptor (loss)
c-value
0.0
0.1
0.2
0.3
0.4
0.5
0.6
0.7
0.8
p-value
2e-23
4e-18
7e-13
1e-07
2e-02
Log-odds
0.5
1.0
1.5
2.0
2.5
3.0
3.5
4.0
Phylostratum
Cellular_organisms - ps1
Archaea1 - ps2
Archaea2 - ps3
Archaea3 - ps4
Asgard/Eukaryota - ps5
Eukaryota - ps6
Amorpheae - ps7
Obazoa - ps8
Opisthokonta - ps9
Holozoa - ps10
Filozoa - ps11
Choano./Metazoa - ps12
Metazoa - ps13
Myriazoa - ps14
Parahoxozoa - ps15
Bilateria - ps16
Protostomia - ps17
Ecdysozoa - ps18
Arthropoda - ps19
Mandibulata - ps20
Pancrustacea - ps21
Insecta - ps22
Eumetabola - ps23
Holometabola/Psocodea - ps24
Holometabola - ps25
Aparaglossata - ps26
Mecopterida - ps27
Diptera - ps28
Bibiono./Cyclorrhapha - ps29
Cyclorrhapha - ps30
Calyptratae/Drosophila - ps31
Drosophila - ps32
Sophophora - ps33
Obscura/Melanogaster_group - ps34
D_melanogaster_group - ps35
D_melanogaster_subgroup - ps36
sim./sech./mel. group - ps37
Drosophila_melanogaster - ps38

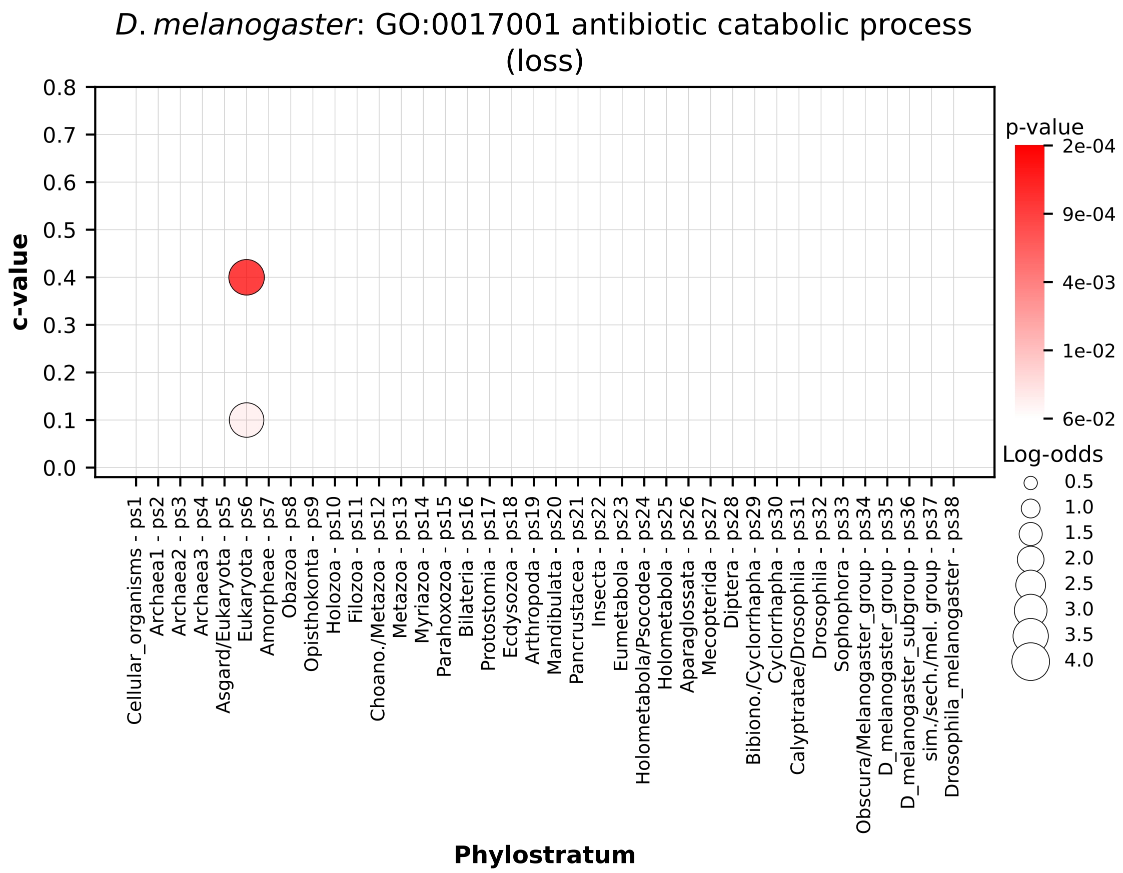

D. melanogaster: GO:0017001 antibiotic catabolic process (loss)
c-value
0.0
0.1
0.2
0.3
0.4
0.5
0.6
0.7
0.8
Phylostratum
Cellular_organisms - ps1
Archaea1 - ps2
Archaea2 - ps3
Archaea3 - ps4
Asgard/Eukaryota - ps5
Eukaryota - ps6
Amorpheae - ps7
Obazoa - ps8
Opisthokonta - ps9
Holozoa - ps10
Filozoa - ps11
Choano./Metazoa - ps12
Metazoa - ps13
Myriazoa - ps14
Parahoxozoa - ps15
Bilateria - ps16
Protostomia - ps17
Ecdysozoa - ps18
Arthropoda - ps19
Mandibulata - ps20
Pancrustacea - ps21
Insecta - ps22
Eumetabola - ps23
Holometabola/Psocodea - ps24
Holometabola - ps25
Aparaglossata - ps26
Mecopterida - ps27
Diptera - ps28
Bibiono./Cyclorrhapha - ps29
Cyclorrhapha - ps30
Calyptratae/Drosophila - ps31
Drosophila - ps32
Sophophora - ps33
Obscura/Melanogaster_group - ps34
D_melanogaster_group - ps35
D_melanogaster_subgroup - ps36
sim./sech./mel. group - ps37
Drosophila_melanogaster - ps38
p-value
2e-04
9e-04
4e-03
1e-02
6e-02
Log-odds
0.5
1.0
1.5
2.0
2.5
3.0
3.5
4.0

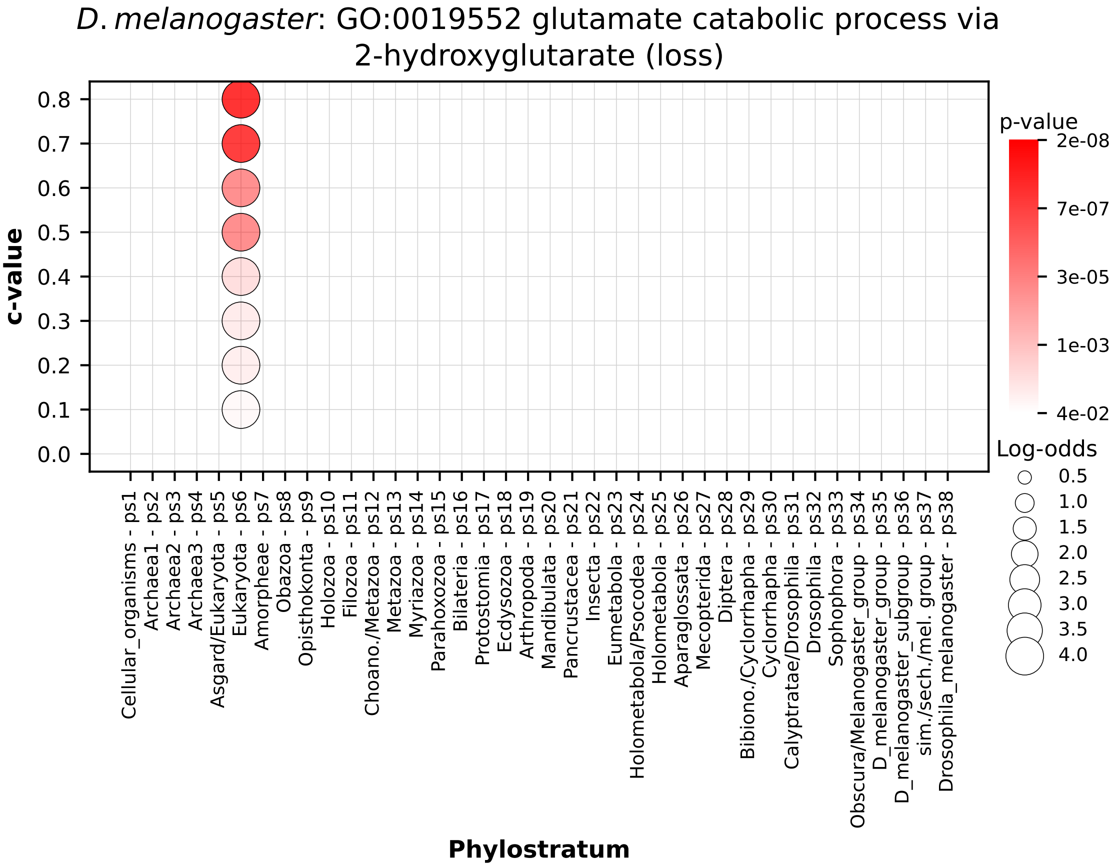

D. melanogaster: GO:0019552 glutamate catabolic process via 2-hydroxyglutarate (loss)
c-value
0.8
0.7
0.6
0.5
0.4
0.3
0.2
0.1
0.0
Phylostratum
Cellular_organisms - ps1
Archaea1 - ps2
Archaea2 - ps3
Archaea3 - ps4
Asgard/Eukaryota - ps5
Eukaryota - ps6
Amorpheae - ps7
Obazoa - ps8
Opisthokonta - ps9
Holozoa - ps10
Filozoa - ps11
Choano./Metazoa - ps12
Metazoa - ps13
Myriazoa - ps14
Parahoxozoa - ps15
Bilateria - ps16
Protostomia - ps17
Ecdysozoa - ps18
Arthropoda - ps19
Mandibulata - ps20
Pancrustacea - ps21
Insecta - ps22
Eumetabola - ps23
Holometabola/Psocodea - ps24
Holometabola - ps25
Aparaglossata - ps26
Mecopterida - ps27
Diptera - ps28
Bibiono./Cyclorrhapha - ps29
Cyclorrhapha - ps30
Calyptratae/Drosophila - ps31
Drosophila - ps32
Sophophora - ps33
Obscura/Melanogaster_group - ps34
D_melanogaster_group - ps35
D_melanogaster_subgroup - ps36
sim./sech./mel. group - ps37
Drosophila_melanogaster - ps38
p-value
2e-08
7e-07
3e-05
1e-03
4e-02
Log-odds
0.5
1.0
1.5
2.0
2.5
3.0
3.5
4.0

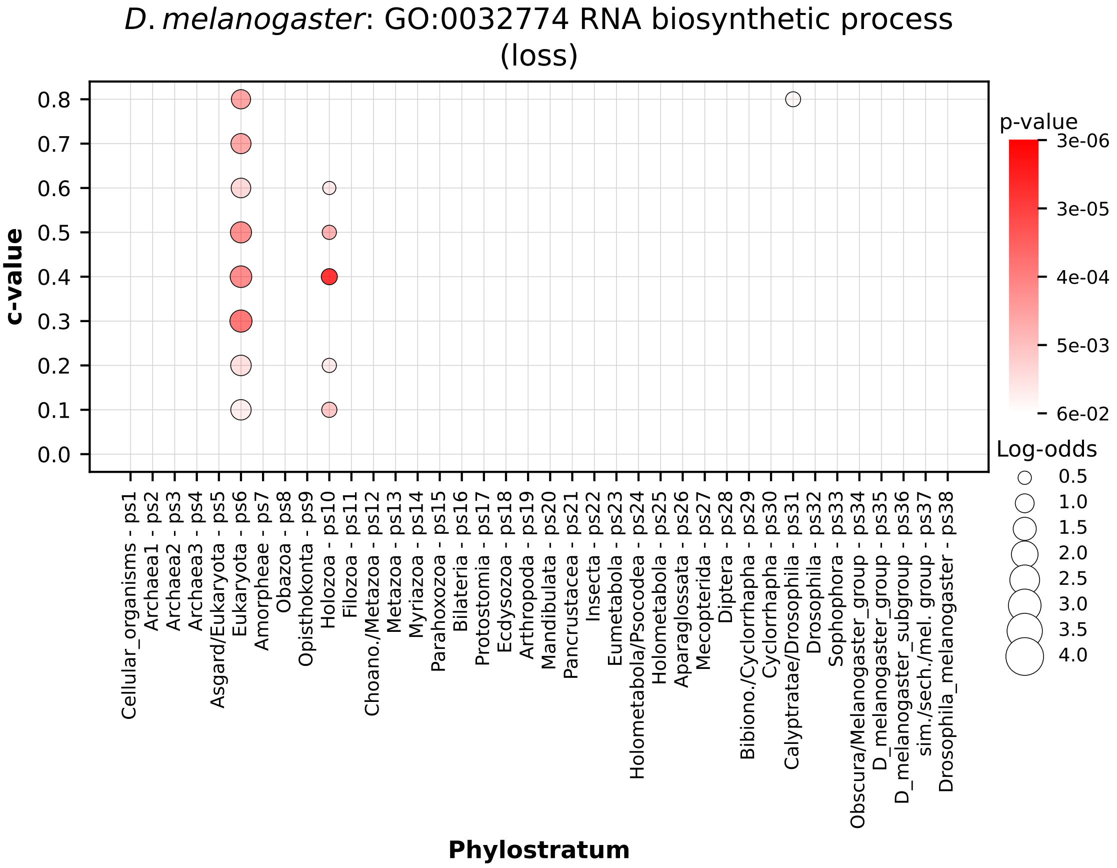

$D. melanogaster$: GO:0032774 RNA biosynthetic process (loss)
c-value
0.0
0.1
0.2
0.3
0.4
0.5
0.6
0.7
0.8
Phylostratum
Cellular_organisms - ps1
Archaea1 - ps2
Archaea2 - ps3
Archaea3 - ps4
Asgard/Eukaryota - ps5
Eukaryota - ps6
Amorpheae - ps7
Obazoa - ps8
Opisthokonta - ps9
Holozoa - ps10
Filozoa - ps11
Choano./Metazoa - ps12
Metazoa - ps13
Myriazoa - ps14
Parahoxozoa - ps15
Bilateria - ps16
Protostomia - ps17
Ecdysozoa - ps18
Arthropoda - ps19
Mandibulata - ps20
Pancrustacea - ps21
Insecta - ps22
Eumetabola - ps23
Holometabola/Psocodea - ps24
Holometabola - ps25
Aparaglossata - ps26
Mecopterida - ps27
Diptera - ps28
Bibiono./Cyclorrhapha - ps29
Cyclorrhapha - ps30
Calyptratae/Drosophila - ps31
Drosophila - ps32
Sophophora - ps33
Obscura/Melanogaster_group - ps34
D_melanogaster_group - ps35
D_melanogaster_subgroup - ps36
sim./sech./mel. group - ps37
Drosophila_melanogaster - ps38
p-value
3e-06
3e-05
4e-04
5e-03
6e-02
Log-odds
0.5
1.0
1.5
2.0
2.5
3.0
3.5
4.0

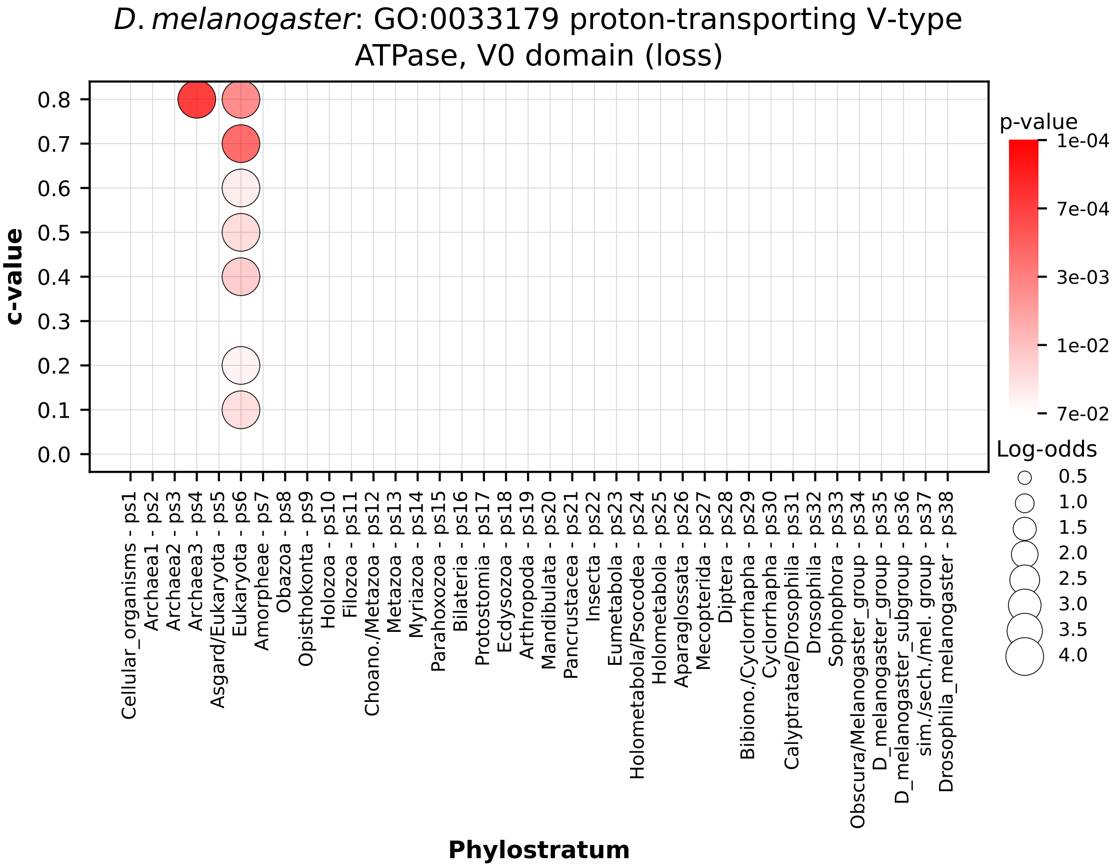

D. melanogaster: GO:0033179 proton-transporting V-type ATPase, V0 domain (loss)
c-value
0.0
0.1
0.2
0.3
0.4
0.5
0.6
0.7
0.8
Phylostratum
Cellular_organisms - ps1
Archaea1 - ps2
Archaea2 - ps3
Archaea3 - ps4
Asgard/Eukaryota - ps5
Eukaryota - ps6
Amorpheae - ps7
Obazoa - ps8
Opisthokonta - ps9
Holozoa - ps10
Filozoa - ps11
Choano./Metazoa - ps12
Metazoa - ps13
Myriazoa - ps14
Parahoxozoa - ps15
Bilateria - ps16
Protostomia - ps17
Ecdysozoa - ps18
Arthropoda - ps19
Mandibulata - ps20
Pancrustacea - ps21
Insecta - ps22
Eumetabola - ps23
Holometabola/Psocodea - ps24
Holometabola - ps25
Aparaglossata - ps26
Mecopterida - ps27
Diptera - ps28
Bibiono./Cyclorrhapha - ps29
Cyclorrhapha - ps30
Calyptratae/Drosophila - ps31
Drosophila - ps32
Sophophora - ps33
Obscura/Melanogaster_group - ps34
D_melanogaster_group - ps35
D_melanogaster_subgroup - ps36
sim./sech./mel. group - ps37
Drosophila_melanogaster - ps38
p-value
1e-04
7e-04
3e-03
1e-02
7e-02
Log-odds
0.5
1.0
1.5
2.0
2.5
3.0
3.5
4.0

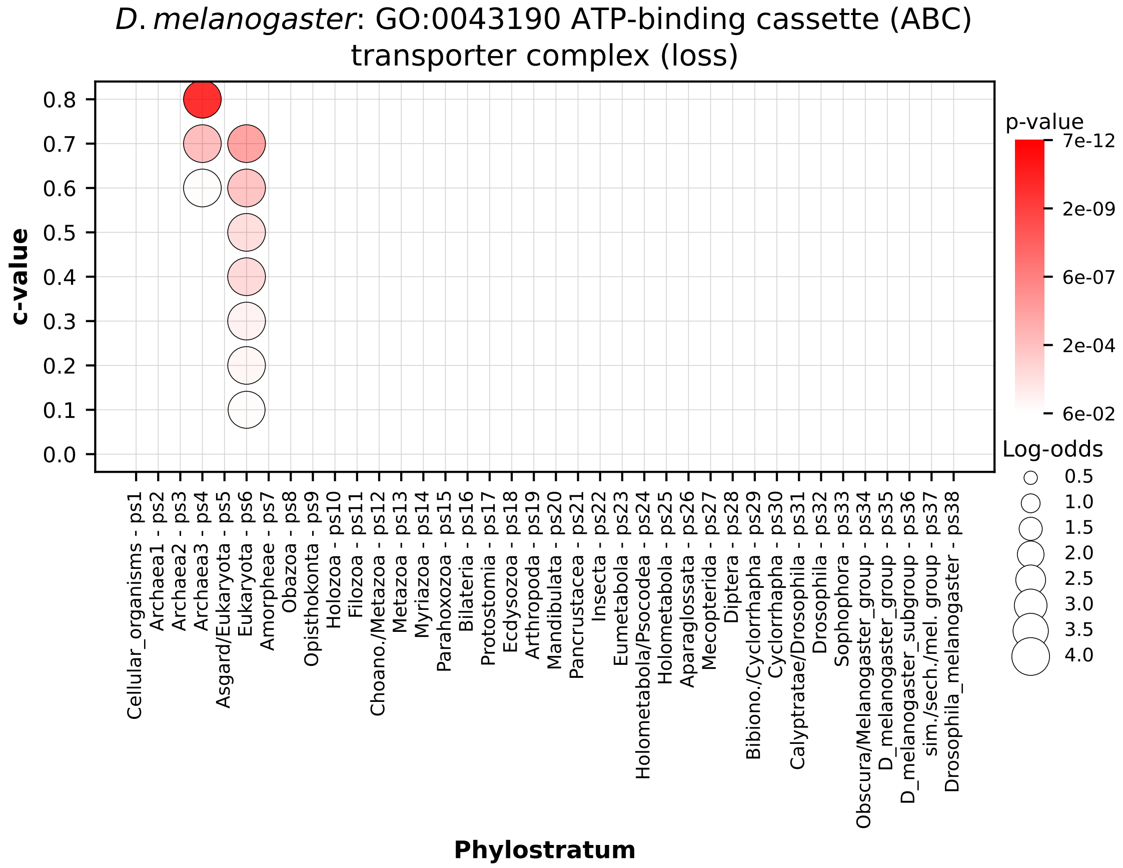

D. melanogaster: GO:0043190 ATP-binding cassette (ABC) transporter complex (loss)
c-value
0.8
0.7
0.6
0.5
0.4
0.3
0.2
0.1
0.0
p-value
7e-12
2e-09
6e-07
2e-04
6e-02
Log-odds
0.5
1.0
1.5
2.0
2.5
3.0
3.5
4.0
Cellular_organisms - ps1
Archaea1 - ps2
Archaea2 - ps3
Archaea3 - ps4
Asgard/Eukaryota - ps5
Eukaryota - ps6
Amorpheae - ps7
Obazoa - ps8
Opisthokonta - ps9
Holozoa - ps10
Filozoa - ps11
Choano./Metazoa - ps12
Metazoa - ps13
Myriazoa - ps14
Parahoxozoa - ps15
Bilateria - ps16
Protostomia - ps17
Ecdysozoa - ps18
Arthropoda - ps19
Mandibulata - ps20
Pancrustacea - ps21
Insecta - ps22
Eumetabola - ps23
Holometabola/Psocodea - ps24
Holometabola - ps25
Aparaglossata - ps26
Mecopterida - ps27
Diptera - ps28
Bibiono./Cyclorrhapha - ps29
Cyclorrhapha - ps30
Calyptratae/Drosophila - ps31
Drosophila - ps32
Sophophora - ps33
Obscura/Melanogaster_group - ps34
D_melanogaster_group - ps35
D_melanogaster_subgroup - ps36
sim./sech./mel. group - ps37
Drosophila_melanogaster - ps38
Phylostratum

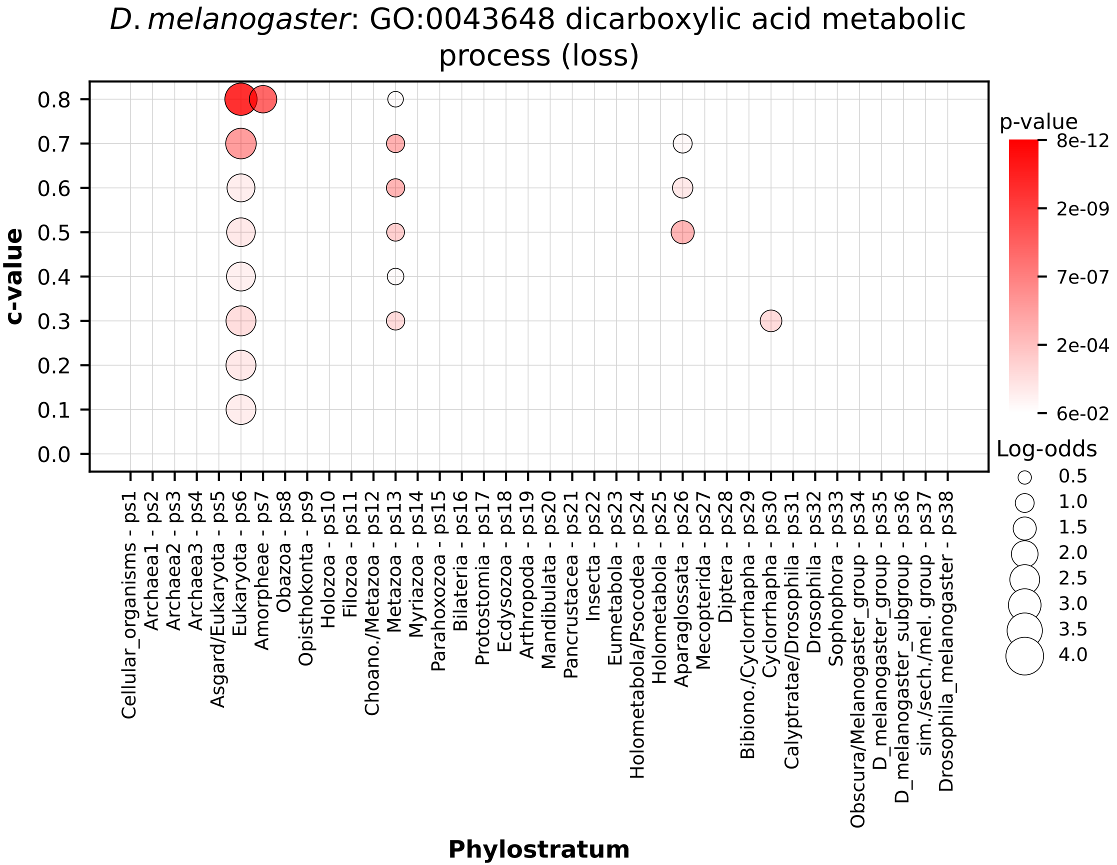

D. melanogaster: GO:0043648 dicarboxylic acid metabolic process (loss)
c-value
0.8
0.7
0.6
0.5
0.4
0.3
0.2
0.1
0.0
Phylostratum
Cellular_organisms - ps1
Archaea1 - ps2
Archaea2 - ps3
Archaea3 - ps4
Asgard/Eukaryota - ps5
Eukaryota - ps6
Amorpheae - ps7
Obazoa - ps8
Opisthokonta - ps9
Holozoa - ps10
Filozoa - ps11
Choano./Metazoa - ps12
Metazoa - ps13
Myriazoa - ps14
Parahoxozoa - ps15
Bilateria - ps16
Protostomia - ps17
Ecdysozoa - ps18
Arthropoda - ps19
Mandibulata - ps20
Pancrustacea - ps21
Insecta - ps22
Eumetabola - ps23
Holometabola/Psocodea - ps24
Holometabola - ps25
Aparaglossata - ps26
Mecopterida - ps27
Diptera - ps28
Bibiono./Cyclorrhapha - ps29
Cyclorrhapha - ps30
Calyptratae/Drosophila - ps31
Drosophila - ps32
Sophophora - ps33
Obscura/Melanogaster_group - ps34
D_melanogaster_group - ps35
D_melanogaster_subgroup - ps36
sim./sech./mel. group - ps37
Drosophila_melanogaster - ps38
p-value
8e-12
2e-09
7e-07
2e-04
6e-02
Log-odds
0.5
1.0
1.5
2.0
2.5
3.0
3.5
4.0

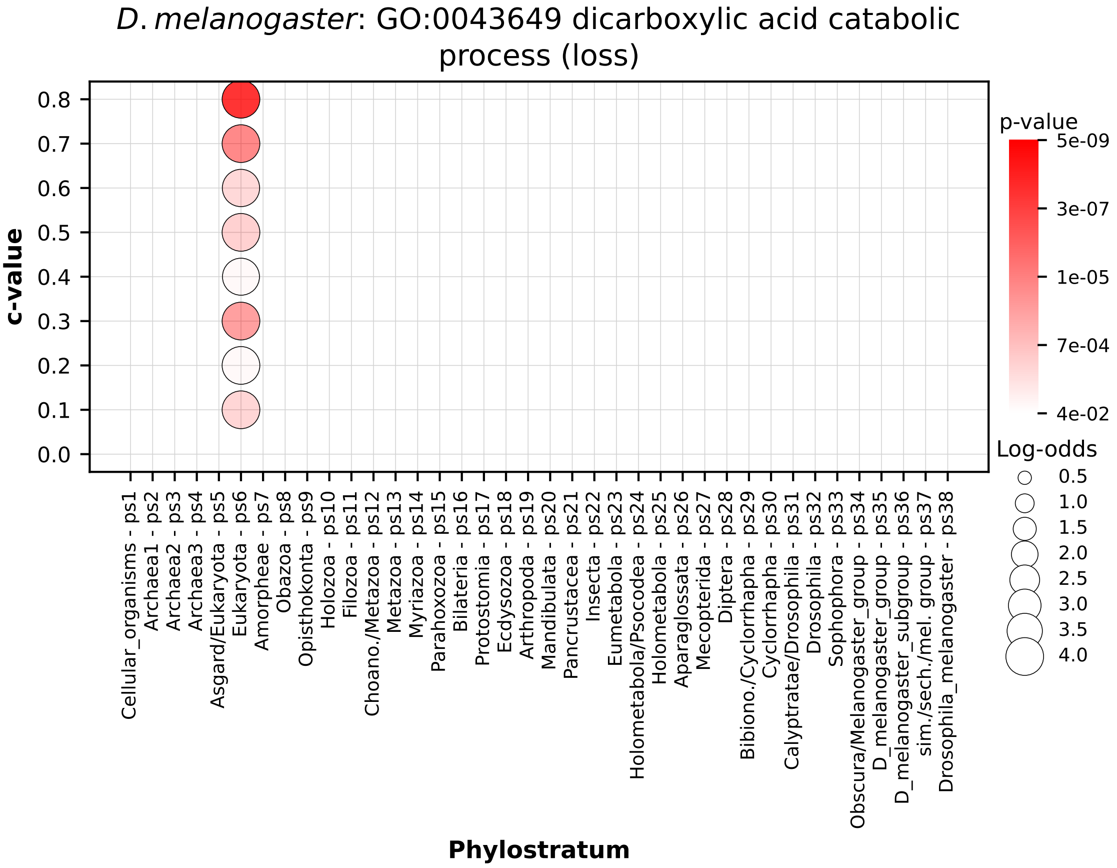

D. melanogaster: GO:0043649 dicarboxylic acid catabolic process (loss)
c-value
0.0
0.1
0.2
0.3
0.4
0.5
0.6
0.7
0.8
Phylostratum
Cellular_organisms - ps1
Archaea1 - ps2
Archaea2 - ps3
Archaea3 - ps4
Asgard/Eukaryota - ps5
Eukaryota - ps6
Amorpheae - ps7
Obazoa - ps8
Opisthokonta - ps9
Holozoa - ps10
Filozoa - ps11
Choano./Metazoa - ps12
Metazoa - ps13
Myriazoa - ps14
Parahoxozoa - ps15
Bilateria - ps16
Protostomia - ps17
Ecdysozoa - ps18
Arthropoda - ps19
Mandibulata - ps20
Pancrustacea - ps21
Insecta - ps22
Eumetabola - ps23
Holometabola/Psocodea - ps24
Holometabola - ps25
Aparaglossata - ps26
Mecopterida - ps27
Diptera - ps28
Bibiono./Cyclorrhapha - ps29
Cyclorrhapha - ps30
Calyptratae/Drosophila - ps31
Drosophila - ps32
Sophophora - ps33
Obscura/Melanogaster_group - ps34
D_melanogaster_group - ps35
D_melanogaster_subgroup - ps36
sim./sech./mel. group - ps37
Drosophila_melanogaster - ps38
p-value
5e-09
3e-07
1e-05
7e-04
4e-02
Log-odds
0.5
1.0
1.5
2.0
2.5
3.0
3.5
4.0

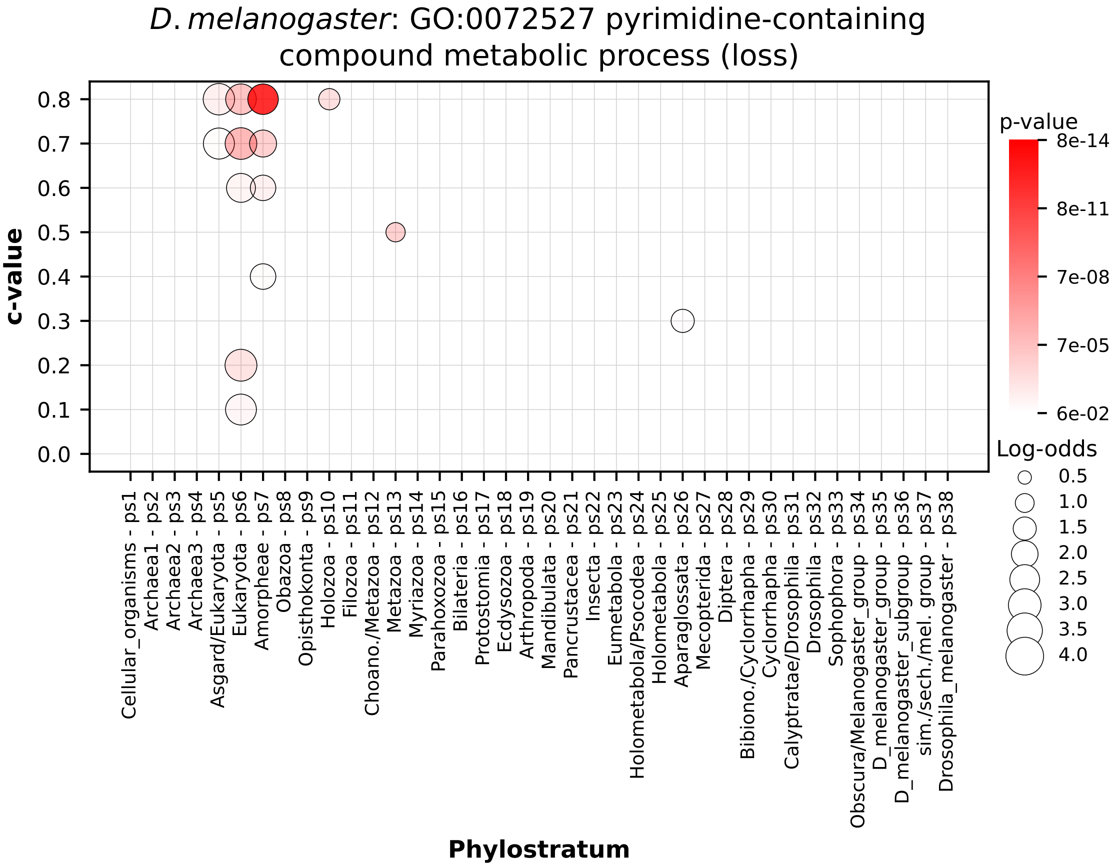

D. melanogaster: GO:0072527 pyrimidine-containing compound metabolic process (loss)
c-value
0.8
0.7
0.6
0.5
0.4
0.3
0.2
0.1
0.0
p-value
8e-14
8e-11
7e-08
7e-05
6e-02
Log-odds
0.5
1.0
1.5
2.0
2.5
3.0
3.5
4.0
Cellular_organisms - ps1
Archaea1 - ps2
Archaea2 - ps3
Archaea3 - ps4
Asgard/Eukaryota - ps5
Eukaryota - ps6
Amorpheae - ps7
Obazoa - ps8
Opisthokonta - ps9
Holozoa - ps10
Filozoa - ps11
Choano./Metazoa - ps12
Metazoa - ps13
Myriazoa - ps14
Parahoxozoa - ps15
Bilateria - ps16
Protostomia - ps17
Ecdysozoa - ps18
Arthropoda - ps19
Mandibulata - ps20
Pancrustacea - ps21
Insecta - ps22
Eumetabola - ps23
Holometabola/Psocodea - ps24
Holometabola - ps25
Aparaglossata - ps26
Mecopterida - ps27
Diptera - ps28
Bibiono./Cyclorrhapha - ps29
Cyclorrhapha - ps30
Calyptratae/Drosophila - ps31
Drosophila - ps32
Sophophora - ps33
Obscura/Melanogaster_group - ps34
D_melanogaster_group - ps35
D_melanogaster_subgroup - ps36
sim./sech./mel. group - ps37
Drosophila_melanogaster - ps38
Phylostratum

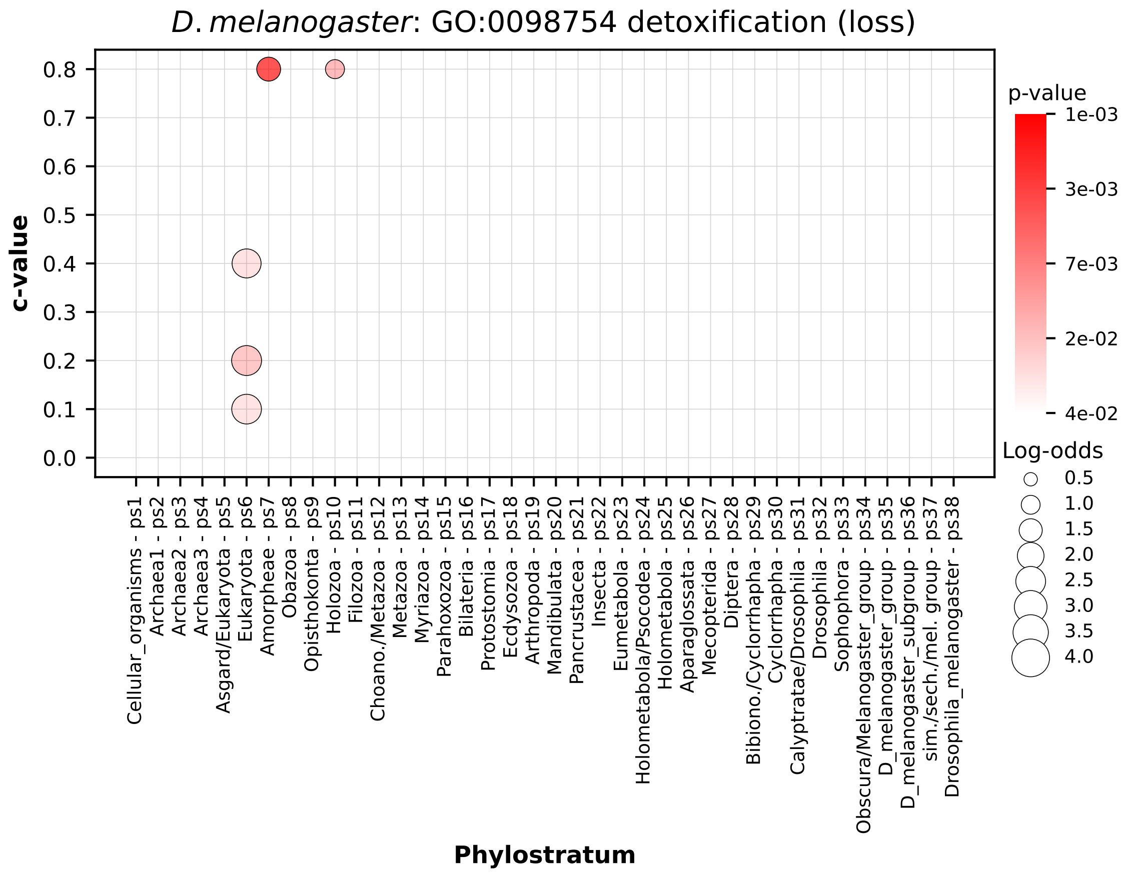
D. melanogaster: GO:0098754 detoxification (loss)
c-value
0.0
0.1
0.2
0.3
0.4
0.5
0.6
0.7
0.8
Phylostratum
Cellular_organisms - ps1
Archaea1 - ps2
Archaea2 - ps3
Archaea3 - ps4
Asgard/Eukaryota - ps5
Eukaryota - ps6
Amorpheae - ps7
Obazoa - ps8
Opisthokonta - ps9
Holozoa - ps10
Filozoa - ps11
Choano./Metazoa - ps12
Metazoa - ps13
Myriazoa - ps14
Parahoxozoa - ps15
Bilateria - ps16
Protostomia - ps17
Ecdysozoa - ps18
Arthropoda - ps19
Mandibulata - ps20
Pancrustacea - ps21
Insecta - ps22
Eumetabola - ps23
Holometabola/Psocodea - ps24
Holometabola - ps25
Aparaglossata - ps26
Mecopterida - ps27
Diptera - ps28
Bibiono./Cyclorrhapha - ps29
Cyclorrhapha - ps30
Calyptratae/Drosophila - ps31
Drosophila - ps32
Sophophora - ps33
Obscura/Melanogaster_group - ps34
D_melanogaster_group - ps35
D_melanogaster_subgroup - ps36
sim./sech./mel. group - ps37
Drosophila_melanogaster - ps38
p-value
1e-03
3e-03
7e-03
2e-02
4e-02
Log-odds
0.5
1.0
1.5
2.0
2.5
3.0
3.5
4.0

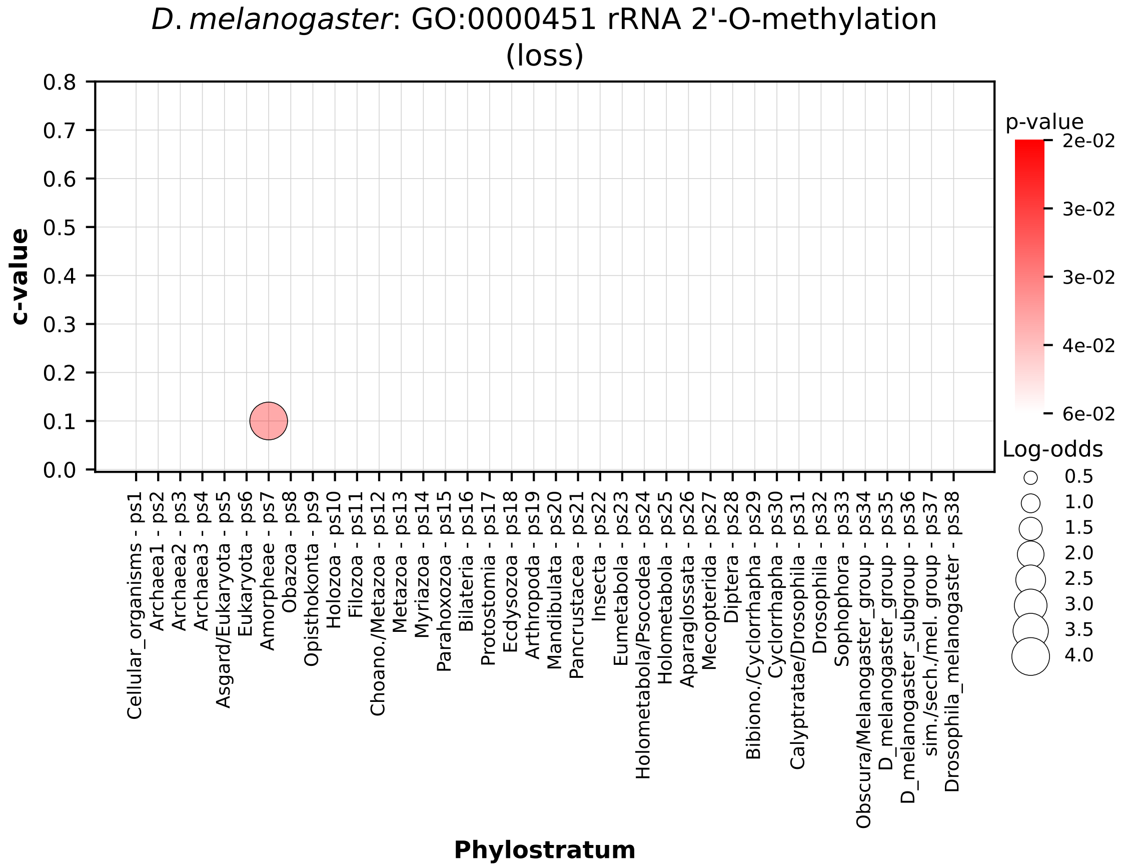

D. melanogaster: GO:0000451 rRNA 2'-O-methylation (loss)
c-value
Phylostratum
p-value
2e-02
3e-02
3e-02
4e-02
6e-02
Log-odds
0.5
1.0
1.5
2.0
2.5
3.0
3.5
4.0
Cellular_organisms - ps1
Archaea1 - ps2
Archaea2 - ps3
Archaea3 - ps4
Asgard/Eukaryota - ps5
Eukaryota - ps6
Amorpheae - ps7
Obazoa - ps8
Opisthokonta - ps9
Holozoa - ps10
Filozoa - ps11
Choano./Metazoa - ps12
Metazoa - ps13
Myriazoa - ps14
Parahoxozoa - ps15
Bilateria - ps16
Protostomia - ps17
Ecdysozoa - ps18
Arthropoda - ps19
Mandibulata - ps20
Pancrustacea - ps21
Insecta - ps22
Eumetabola - ps23
Holometabola/Psocodea - ps24
Holometabola - ps25
Aparaglossata - ps26
Mecopterida - ps27
Diptera - ps28
Bibiono./Cyclorrhapha - ps29
Cyclorrhapha - ps30
Calyptratae/Drosophila - ps31
Drosophila - ps32
Sophophora - ps33
Obscura/Melanogaster_group - ps34
D_melanogaster_group - ps35
D_melanogaster_subgroup - ps36
sim./sech./mel. group - ps37
Drosophila_melanogaster - ps38

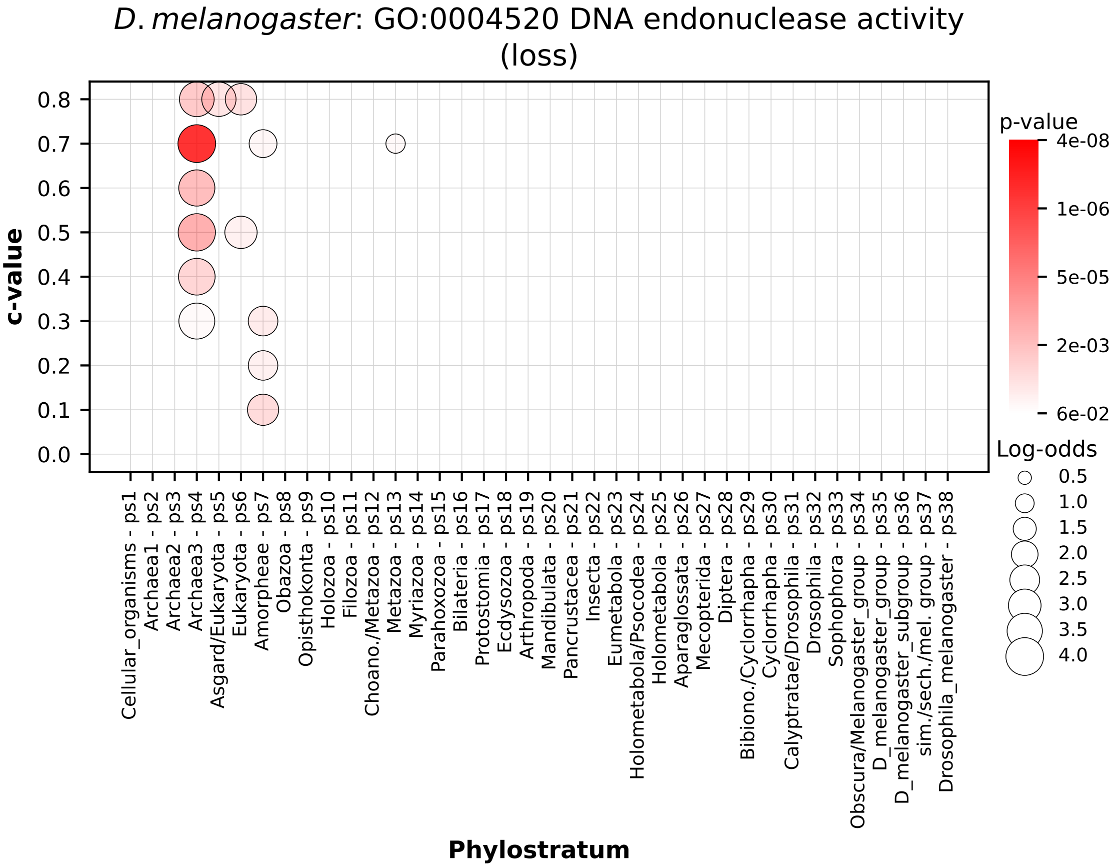

D. melanogaster: GO:0004520 DNA endonuclease activity
(loss)
c-value
0.8
0.7
0.6
0.5
0.4
0.3
0.2
0.1
0.0
p-value
4e-08
1e-06
5e-05
2e-03
6e-02
Log-odds
0.5
1.0
1.5
2.0
2.5
3.0
3.5
4.0
Cellular_organisms - ps1
Archaea1 - ps2
Archaea2 - ps3
Archaea3 - ps4
Asgard/Eukaryota - ps5
Eukaryota - ps6
Amorpheae - ps7
Obazoa - ps8
Opisthokonta - ps9
Holozoa - ps10
Filozoa - ps11
Choano./Metazoa - ps12
Metazoa - ps13
Myriazoa - ps14
Parahoxozoa - ps15
Bilateria - ps16
Protostomia - ps17
Ecdysozoa - ps18
Arthropoda - ps19
Mandibulata - ps20
Pancrustacea - ps21
Insecta - ps22
Eumetabola - ps23
Holometabola/Psocodea - ps24
Holometabola - ps25
Aparaglossata - ps26
Mecopterida - ps27
Diptera - ps28
Bibiono./Cyclorrhapha - ps29
Cyclorrhapha - ps30
Calyptratae/Drosophila - ps31
Drosophila - ps32
Sophophora - ps33
Obscura/Melanogaster_group - ps34
D_melanogaster_group - ps35
D_melanogaster_subgroup - ps36
sim./sech./mel. group - ps37
Drosophila_melanogaster - ps38
Phylostratum

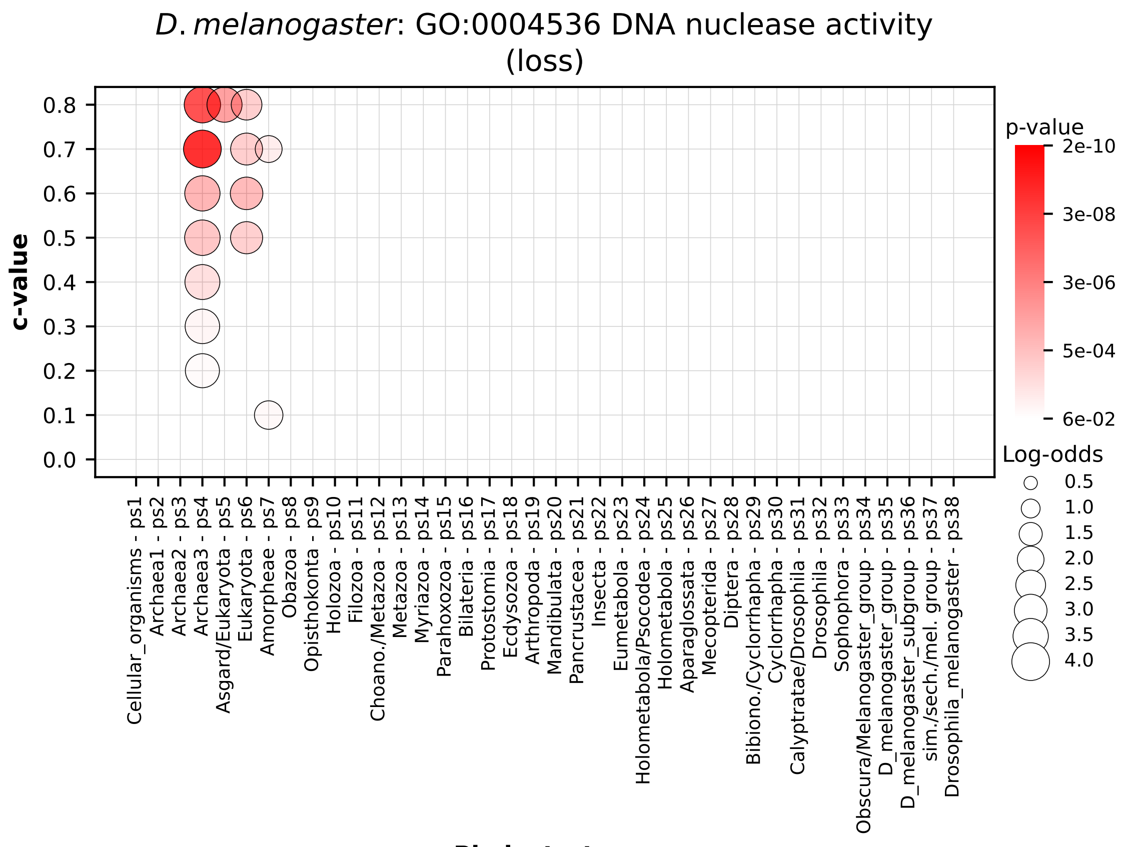

D. melanogaster: GO:0004536 DNA nuclease activity
(loss)
c-value
0.8
0.7
0.6
0.5
0.4
0.3
0.2
0.1
0.0
p-value
2e-10
3e-08
3e-06
5e-04
6e-02
Log-odds
0.5
1.0
1.5
2.0
2.5
3.0
3.5
4.0
Cellular_organisms - ps1
Archaea1 - ps2
Archaea2 - ps3
Archaea3 - ps4
Asgard/Eukaryota - ps5
Eukaryota - ps6
Amorpheae - ps7
Obazoa - ps8
Opisthokonta - ps9
Holozoa - ps10
Filozoa - ps11
Choano./Metazoa - ps12
Metazoa - ps13
Myriazoa - ps14
Parahoxozoa - ps15
Bilateria - ps16
Protostomia - ps17
Ecdysozoa - ps18
Arthropoda - ps19
Mandibulata - ps20
Pancrustacea - ps21
Insecta - ps22
Eumetabola - ps23
Holometabola/Psocodea - ps24
Holometabola - ps25
Aparaglossata - ps26
Mecopterida - ps27
Diptera - ps28
Bibiono./Cyclorrhapha - ps29
Cyclorrhapha - ps30
Calyptratae/Drosophila - ps31
Drosophila - ps32
Sophophora - ps33
Obscura/Melanogaster_group - ps34
D_melanogaster_group - ps35
D_melanogaster_subgroup - ps36
sim./sech./mel. group - ps37
Drosophila_melanogaster - ps38
Phylostratum

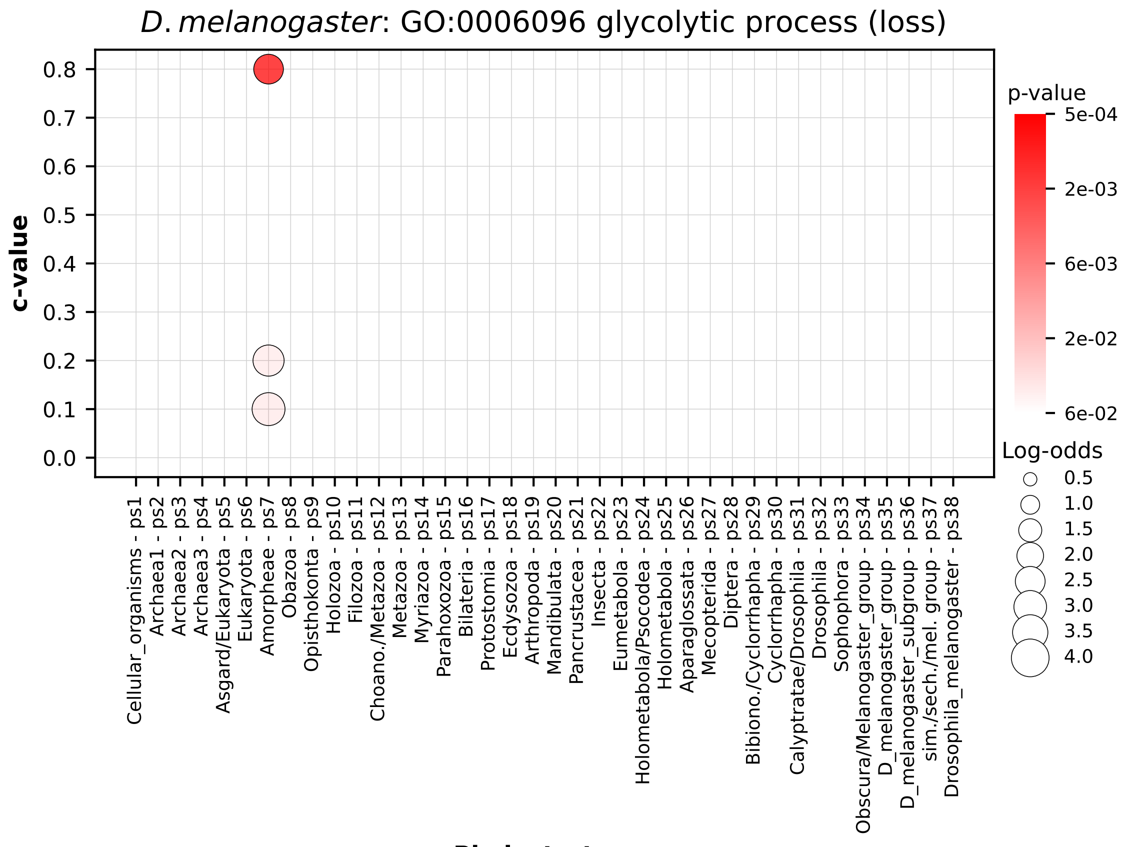

D. melanogaster: GO:0006096 glycolytic process (loss)
c-value
0.0
0.1
0.2
0.3
0.4
0.5
0.6
0.7
0.8
Phylostratum
Cellular_organisms - ps1
Archaea1 - ps2
Archaea2 - ps3
Archaea3 - ps4
Asgard/Eukaryota - ps5
Eukaryota - ps6
Amorpheae - ps7
Obazoa - ps8
Opisthokonta - ps9
Holozoa - ps10
Filozoa - ps11
Choano./Metazoa - ps12
Metazoa - ps13
Myriazoa - ps14
Parahoxozoa - ps15
Bilateria - ps16
Protostomia - ps17
Ecdysozoa - ps18
Arthropoda - ps19
Mandibulata - ps20
Pancrustacea - ps21
Insecta - ps22
Eumetabola - ps23
Holometabola/Psocodea - ps24
Holometabola - ps25
Aparaglossata - ps26
Mecopterida - ps27
Diptera - ps28
Bibiono./Cyclorrhapha - ps29
Cyclorrhapha - ps30
Calyptratae/Drosophila - ps31
Drosophila - ps32
Sophophora - ps33
Obscura/Melanogaster_group - ps34
D_melanogaster_group - ps35
D_melanogaster_subgroup - ps36
sim./sech./mel. group - ps37
Drosophila_melanogaster - ps38
p-value
5e-04
2e-03
6e-03
2e-02
6e-02
Log-odds
0.5
1.0
1.5
2.0
2.5
3.0
3.5
4.0

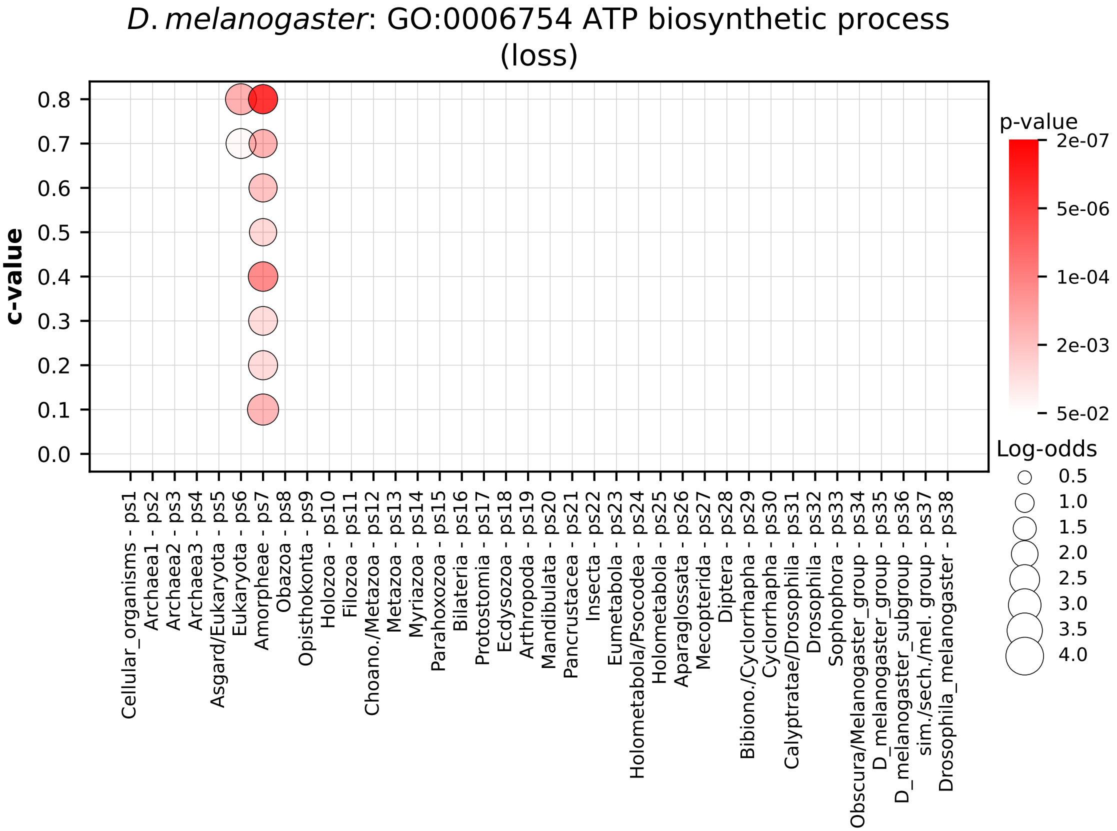
D. melanogaster: GO:0006754 ATP biosynthetic process
(loss)
c-value
0.0
0.1
0.2
0.3
0.4
0.5
0.6
0.7
0.8
Phylostratum
Cellular_organisms - ps1
Archaea1 - ps2
Archaea2 - ps3
Archaea3 - ps4
Asgard/Eukaryota - ps5
Eukaryota - ps6
Amorpheae - ps7
Obazoa - ps8
Opisthokonta - ps9
Holozoa - ps10
Filozoa - ps11
Choano./Metazoa - ps12
Metazoa - ps13
Myriazoa - ps14
Parahoxozoa - ps15
Bilateria - ps16
Protostomia - ps17
Ecdysozoa - ps18
Arthropoda - ps19
Mandibulata - ps20
Pancrustacea - ps21
Insecta - ps22
Eumetabola - ps23
Holometabola/Psocodea - ps24
Holometabola - ps25
Aparaglossata - ps26
Mecopterida - ps27
Diptera - ps28
Bibiono./Cyclorrhapha - ps29
Cyclorrhapha - ps30
Calyptratae/Drosophila - ps31
Drosophila - ps32
Sophophora - ps33
Obscura/Melanogaster_group - ps34
D_melanogaster_group - ps35
D_melanogaster_subgroup - ps36
sim./sech./mel. group - ps37
Drosophila_melanogaster - ps38
p-value
2e-07
5e-06
1e-04
2e-03
5e-02
Log-odds
0.5
1.0
1.5
2.0
2.5
3.0
3.5
4.0

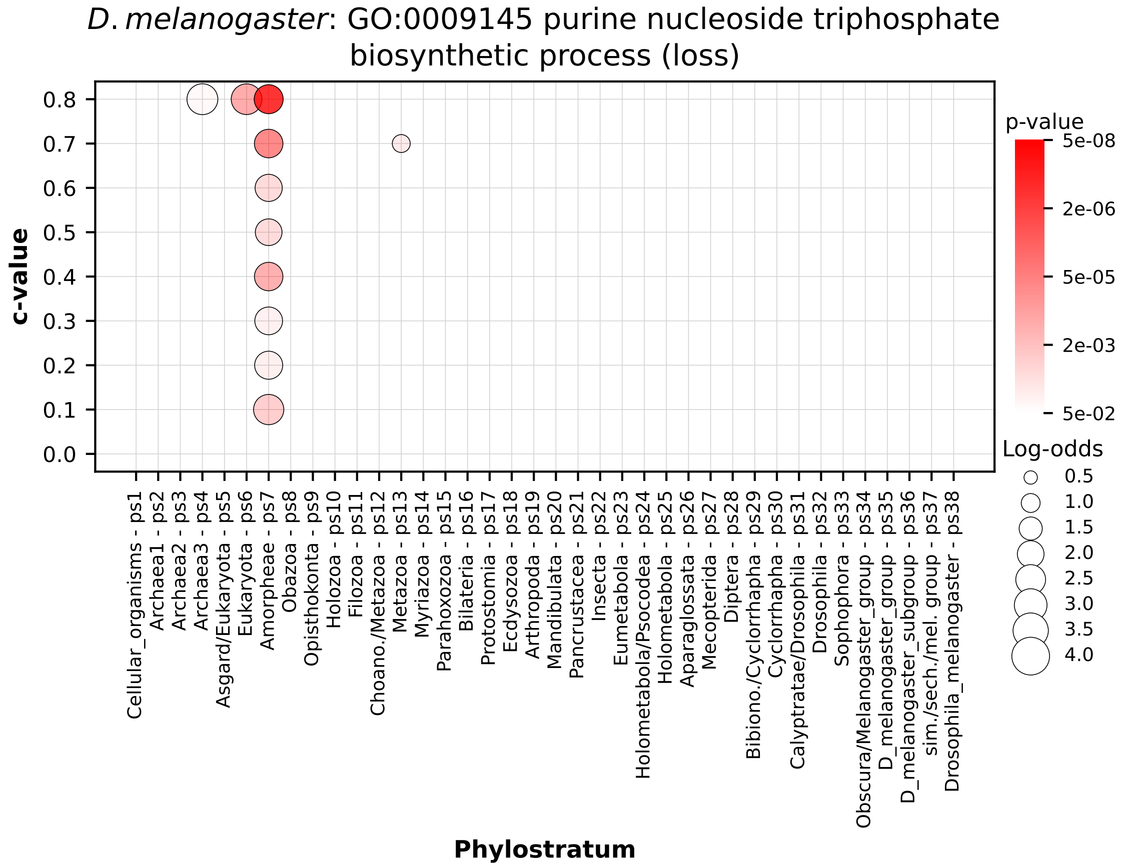
D. melanogaster: GO:0009145 purine nucleoside triphosphate biosynthetic process (loss)
c-value
0.8
0.7
0.6
0.5
0.4
0.3
0.2
0.1
0.0
p-value
5e-08
2e-06
5e-05
2e-03
5e-02
Log-odds
0.5
1.0
1.5
2.0
2.5
3.0
3.5
4.0
Cellular_organisms - ps1
Archaea1 - ps2
Archaea2 - ps3
Archaea3 - ps4
Asgard/Eukaryota - ps5
Eukaryota - ps6
Amorpheae - ps7
Obazoa - ps8
Opisthokonta - ps9
Holozoa - ps10
Filozoa - ps11
Choano./Metazoa - ps12
Metazoa - ps13
Myriazoa - ps14
Parahoxozoa - ps15
Bilateria - ps16
Protostomia - ps17
Ecdysozoa - ps18
Arthropoda - ps19
Mandibulata - ps20
Pancrustacea - ps21
Insecta - ps22
Eumetabola - ps23
Holometabola/Psocodea - ps24
Holometabola - ps25
Aparaglossata - ps26
Mecopterida - ps27
Diptera - ps28
Bibiono./Cyclorrhapha - ps29
Cyclorrhapha - ps30
Calyptratae/Drosophila - ps31
Drosophila - ps32
Sophophora - ps33
Obscura/Melanogaster_group - ps34
D_melanogaster_group - ps35
D_melanogaster_subgroup - ps36
sim./sech./mel. group - ps37
Drosophila_melanogaster - ps38
Phylostratum

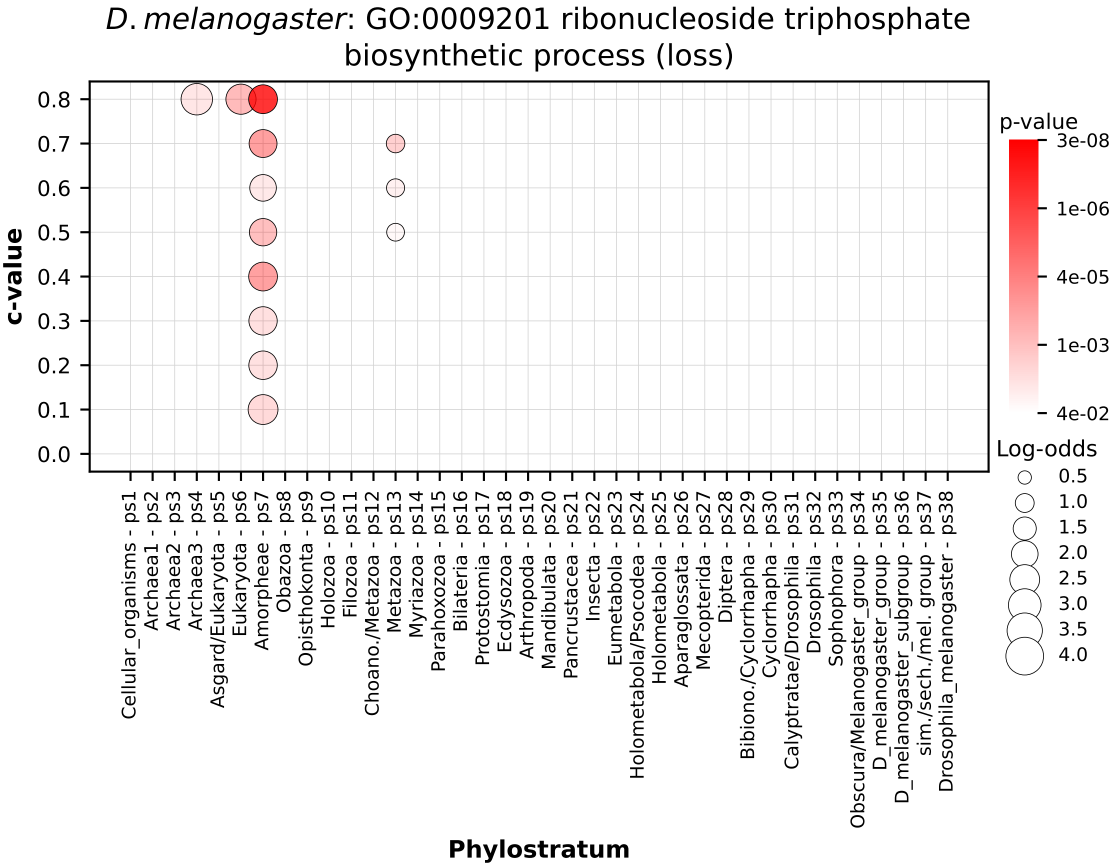
D. melanogaster: GO:0009201 ribonucleoside triphosphate biosynthetic process (loss)
c-value
0.0
0.1
0.2
0.3
0.4
0.5
0.6
0.7
0.8
p-value
3e-08
1e-06
4e-05
1e-03
4e-02
Log-odds
0.5
1.0
1.5
2.0
2.5
3.0
3.5
4.0
Cellular_organisms - ps1
Archaea1 - ps2
Archaea2 - ps3
Archaea3 - ps4
Asgard/Eukaryota - ps5
Eukaryota - ps6
Amorpheae - ps7
Obazoa - ps8
Opisthokonta - ps9
Holozoa - ps10
Filozoa - ps11
Choano./Metazoa - ps12
Metazoa - ps13
Myriazoa - ps14
Parahoxozoa - ps15
Bilateria - ps16
Protostomia - ps17
Ecdysozoa - ps18
Arthropoda - ps19
Mandibulata - ps20
Pancrustacea - ps21
Insecta - ps22
Eumetabola - ps23
Holometabola/Psocodea - ps24
Holometabola - ps25
Aparaglossata - ps26
Mecopterida - ps27
Diptera - ps28
Bibiono./Cyclorrhapha - ps29
Cyclorrhapha - ps30
Calyptratae/Drosophila - ps31
Drosophila - ps32
Sophophora - ps33
Obscura/Melanogaster_group - ps34
D_melanogaster_group - ps35
D_melanogaster_subgroup - ps36
sim./sech./mel. group - ps37
Drosophila_melanogaster - ps38
Phylostratum

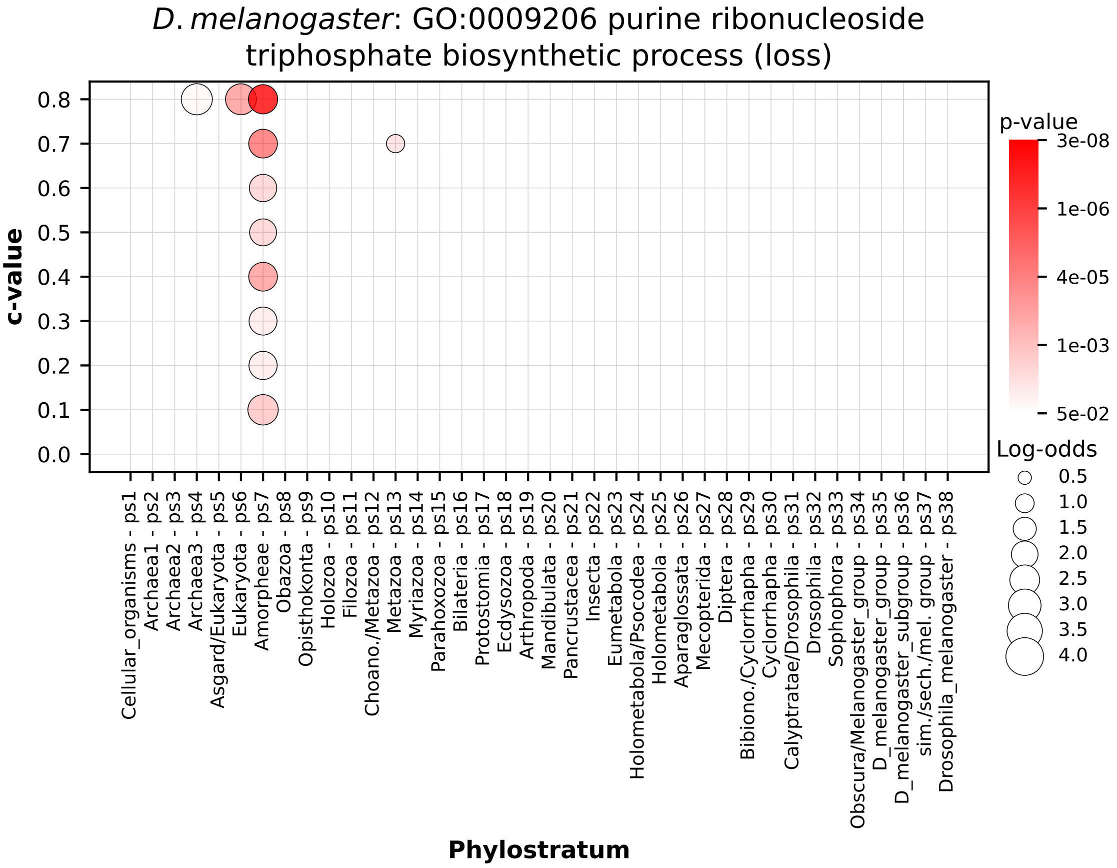

D. melanogaster: GO:0009206 purine ribonucleoside triphosphate biosynthetic process (loss)
c-value
0.0
0.1
0.2
0.3
0.4
0.5
0.6
0.7
0.8
Phylostratum
Cellular_organisms - ps1
Archaea1 - ps2
Archaea2 - ps3
Archaea3 - ps4
Asgard/Eukaryota - ps5
Eukaryota - ps6
Amorpheae - ps7
Obazoa - ps8
Opisthokonta - ps9
Holozoa - ps10
Filozoa - ps11
Choano./Metazoa - ps12
Metazoa - ps13
Myriazoa - ps14
Parahoxozoa - ps15
Bilateria - ps16
Protostomia - ps17
Ecdysozoa - ps18
Arthropoda - ps19
Mandibulata - ps20
Pancrustacea - ps21
Insecta - ps22
Eumetabola - ps23
Holometabola/Psocodea - ps24
Holometabola - ps25
Aparaglossata - ps26
Mecopterida - ps27
Diptera - ps28
Bibiono./Cyclorrhapha - ps29
Cyclorrhapha - ps30
Calyptratae/Drosophila - ps31
Drosophila - ps32
Sophophora - ps33
Obscura/Melanogaster_group - ps34
D_melanogaster_group - ps35
D_melanogaster_subgroup - ps36
sim./sech./mel. group - ps37
Drosophila_melanogaster - ps38
p-value
3e-08
1e-06
4e-05
1e-03
5e-02
Log-odds
0.5
1.0
1.5
2.0
2.5
3.0
3.5
4.0

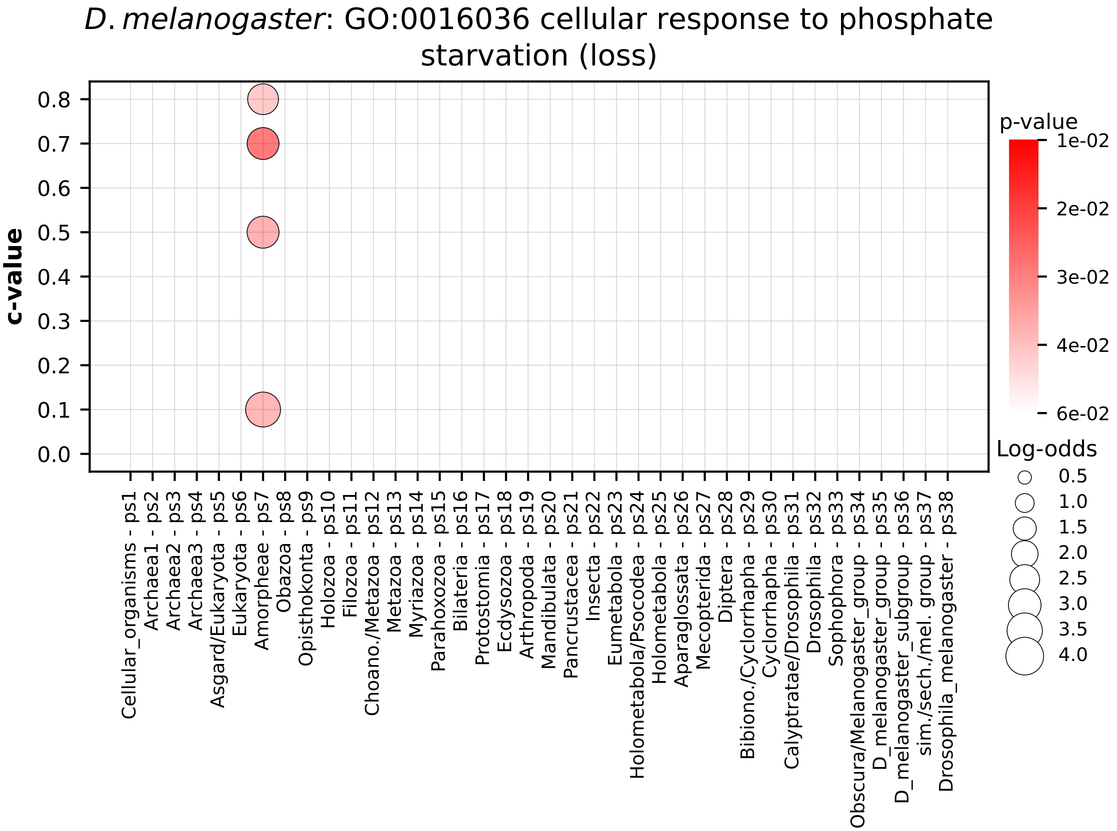
D. melanogaster: GO:0016036 cellular response to phosphate starvation (loss)
c-value
Phylostratum
p-value
1e-02
2e-02
3e-02
4e-02
6e-02
Log-odds
0.5
1.0
1.5
2.0
2.5
3.0
3.5
4.0
Cellular_organisms - ps1
Archaea1 - ps2
Archaea2 - ps3
Archaea3 - ps4
Asgard/Eukaryota - ps5
Eukaryota - ps6
Amorpheae - ps7
Obazoa - ps8
Opisthokonta - ps9
Holozoa - ps10
Filozoa - ps11
Choano./Metazoa - ps12
Metazoa - ps13
Myriazoa - ps14
Parahoxozoa - ps15
Bilateria - ps16
Protostomia - ps17
Ecdysozoa - ps18
Arthropoda - ps19
Mandibulata - ps20
Pancrustacea - ps21
Insecta - ps22
Eumetabola - ps23
Holometabola/Psocodea - ps24
Holometabola - ps25
Aparaglossata - ps26
Mecopterida - ps27
Diptera - ps28
Bibiono./Cyclorrhapha - ps29
Cyclorrhapha - ps30
Calyptratae/Drosophila - ps31
Drosophila - ps32
Sophophora - ps33
Obscura/Melanogaster_group - ps34
D_melanogaster_group - ps35
D_melanogaster_subgroup - ps36
sim./sech./mel. group - ps37
Drosophila_melanogaster - ps38

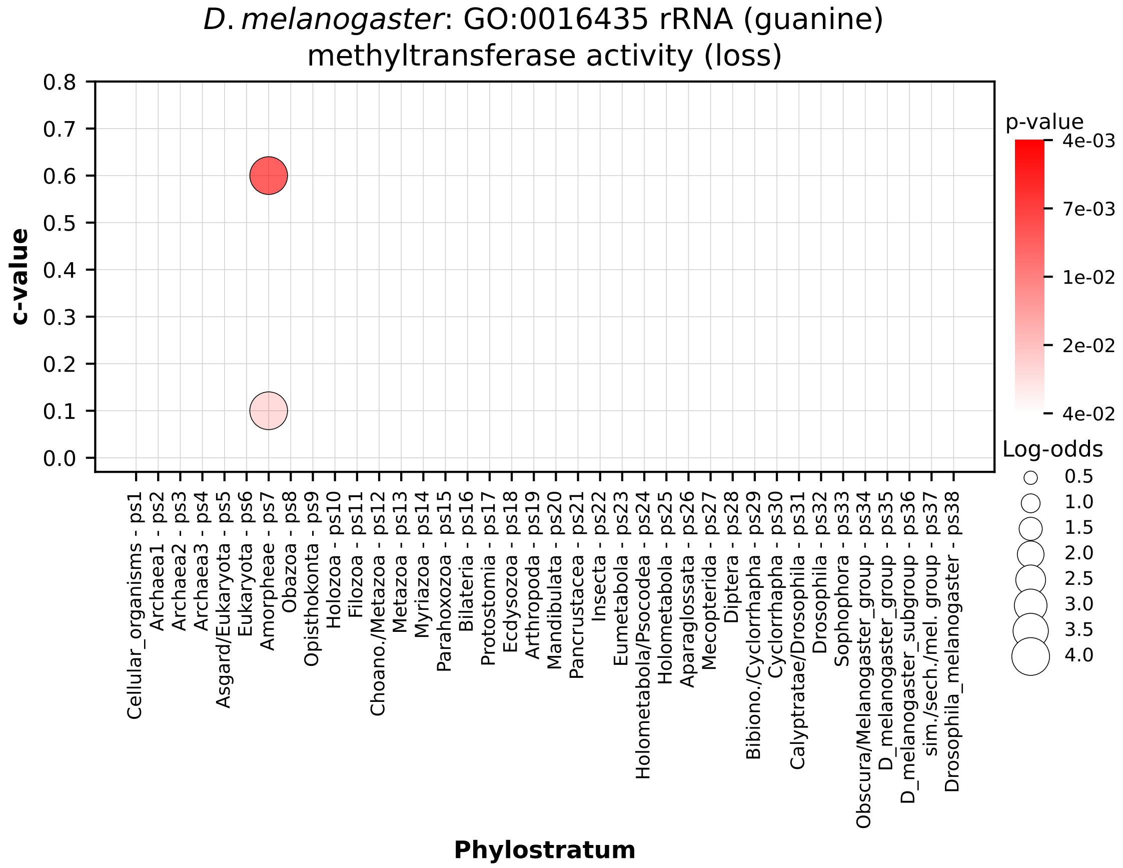
D. melanogaster: GO:0016435 rRNA (guanine) methyltransferase activity (loss)
c-value
Phylostratum
p-value
4e-03
7e-03
1e-02
2e-02
4e-02
Log-odds
0.5
1.0
1.5
2.0
2.5
3.0
3.5
4.0
Cellular_organisms - ps1
Archaea1 - ps2
Archaea2 - ps3
Archaea3 - ps4
Asgard/Eukaryota - ps5
Eukaryota - ps6
Amorpheae - ps7
Obazoa - ps8
Opisthokonta - ps9
Holozoa - ps10
Filozoa - ps11
Choano./Metazoa - ps12
Metazoa - ps13
Myriazoa - ps14
Parahoxozoa - ps15
Bilateria - ps16
Protostomia - ps17
Ecdysozoa - ps18
Arthropoda - ps19
Mandibulata - ps20
Pancrustacea - ps21
Insecta - ps22
Eumetabola - ps23
Holometabola/Psocodea - ps24
Holometabola - ps25
Aparaglossata - ps26
Mecopterida - ps27
Diptera - ps28
Bibiono./Cyclorrhapha - ps29
Cyclorrhapha - ps30
Calyptratae/Drosophila - ps31
Drosophila - ps32
Sophophora - ps33
Obscura/Melanogaster_group - ps34
D_melanogaster_group - ps35
D_melanogaster_subgroup - ps36
sim./sech./mel. group - ps37
Drosophila_melanogaster - ps38

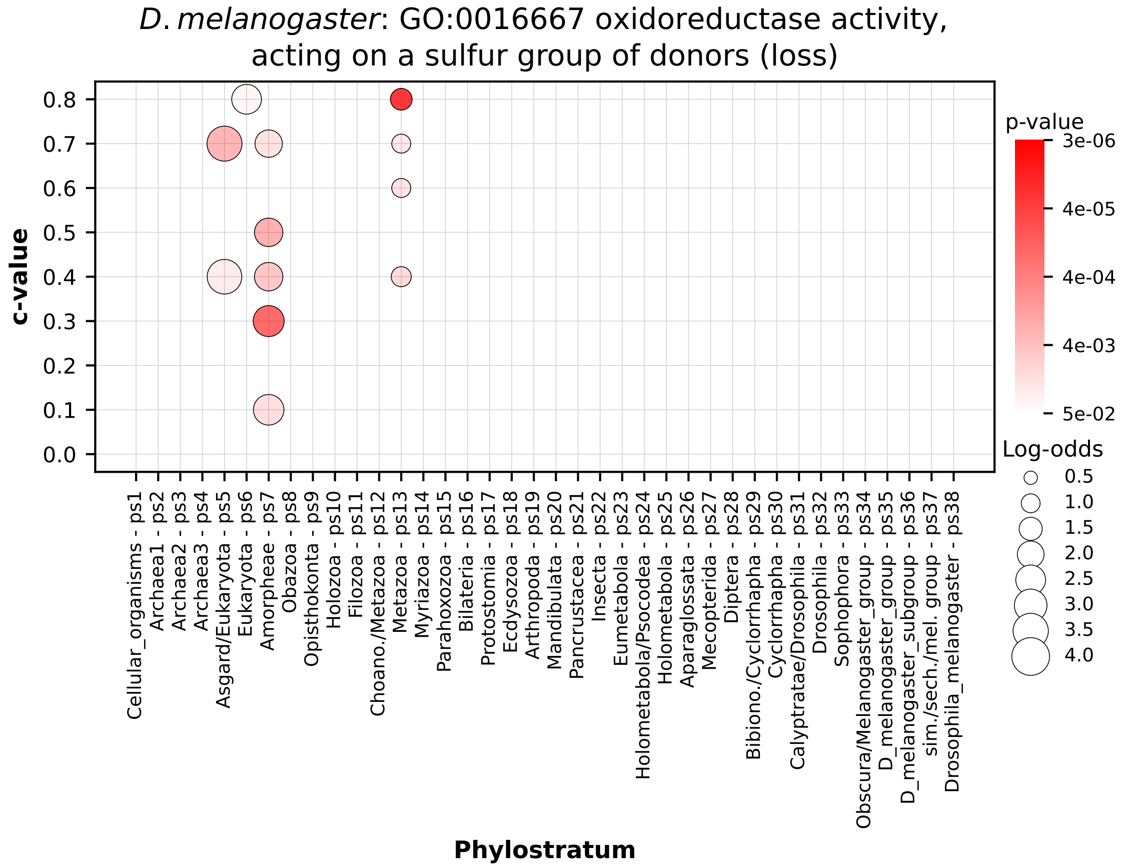

D. melanogaster: GO:0016667 oxidoreductase activity, acting on a sulfur group of donors (loss)
c-value
0.8
0.7
0.6
0.5
0.4
0.3
0.2
0.1
0.0
p-value
3e-06
4e-05
4e-04
4e-03
5e-02
Log-odds
0.5
1.0
1.5
2.0
2.5
3.0
3.5
4.0
Cellular_organisms - ps1
Archaea1 - ps2
Archaea2 - ps3
Archaea3 - ps4
Asgard/Eukaryota - ps5
Eukaryota - ps6
Amorpheae - ps7
Obazoa - ps8
Opisthokonta - ps9
Holozoa - ps10
Filozoa - ps11
Choano./Metazoa - ps12
Metazoa - ps13
Myriazoa - ps14
Parahoxozoa - ps15
Bilateria - ps16
Protostomia - ps17
Ecdysozoa - ps18
Arthropoda - ps19
Mandibulata - ps20
Pancrustacea - ps21
Insecta - ps22
Eumetabola - ps23
Holometabola/Psocodea - ps24
Holometabola - ps25
Aparaglossata - ps26
Mecopterida - ps27
Diptera - ps28
Bibiono./Cyclorrhapha - ps29
Cyclorrhapha - ps30
Calyptratae/Drosophila - ps31
Drosophila - ps32
Sophophora - ps33
Obscura/Melanogaster_group - ps34
D_melanogaster_group - ps35
D_melanogaster_subgroup - ps36
sim./sech./mel. group - ps37
Drosophila_melanogaster - ps38
Phylostratum

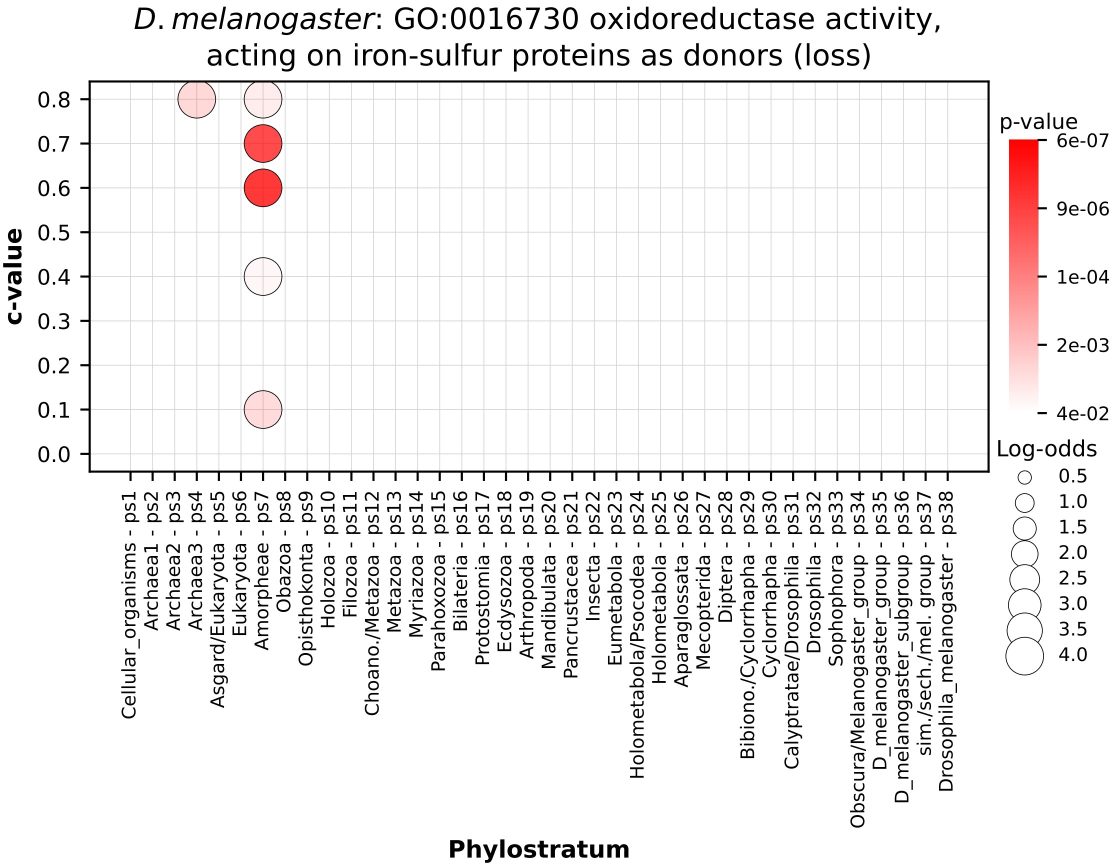

D. melanogaster: GO:0016730 oxidoreductase activity, acting on iron-sulfur proteins as donors (loss)
c-value
0.8
0.7
0.6
0.5
0.4
0.3
0.2
0.1
0.0
Phylostratum
Cellular_organisms - ps1
Archaea1 - ps2
Archaea2 - ps3
Archaea3 - ps4
Asgard/Eukaryota - ps5
Eukaryota - ps6
Amorpheae - ps7
Obazoa - ps8
Opisthokonta - ps9
Holozoa - ps10
Filozoa - ps11
Choano./Metazoa - ps12
Metazoa - ps13
Myriazoa - ps14
Parahoxozoa - ps15
Bilateria - ps16
Protostomia - ps17
Ecdysozoa - ps18
Arthropoda - ps19
Mandibulata - ps20
Pancrustacea - ps21
Insecta - ps22
Eumetabola - ps23
Holometabola/Psocodea - ps24
Holometabola - ps25
Aparaglossata - ps26
Mecopterida - ps27
Diptera - ps28
Bibiono./Cyclorrhapha - ps29
Cyclorrhapha - ps30
Calyptratae/Drosophila - ps31
Drosophila - ps32
Sophophora - ps33
Obscura/Melanogaster_group - ps34
D_melanogaster_group - ps35
D_melanogaster_subgroup - ps36
sim./sech./mel. group - ps37
Drosophila_melanogaster - ps38
p-value
6e-07
9e-06
1e-04
2e-03
4e-02
Log-odds
0.5
1.0
1.5
2.0
2.5
3.0
3.5
4.0

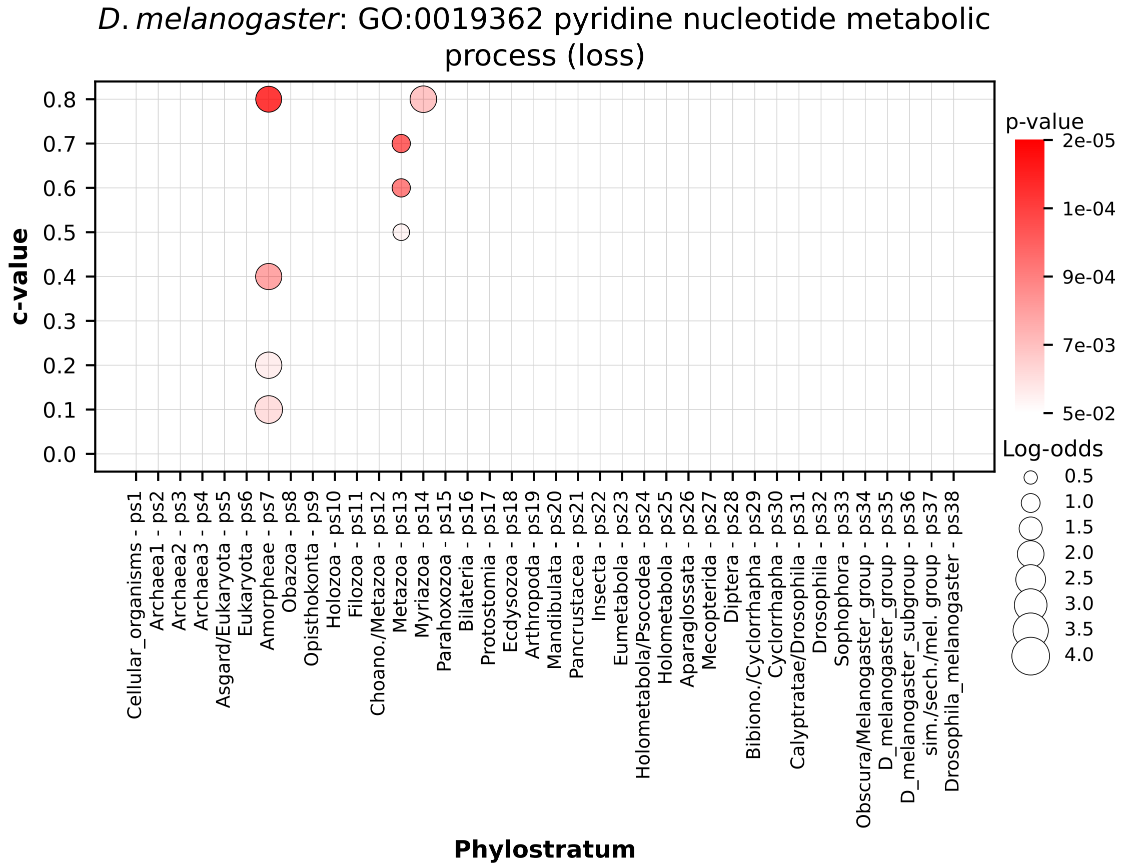

D. melanogaster: GO:0019362 pyridine nucleotide metabolic process (loss)
c-value
0.8
0.7
0.6
0.5
0.4
0.3
0.2
0.1
0.0
p-value
2e-05
1e-04
9e-04
7e-03
5e-02
Log-odds
0.5
1.0
1.5
2.0
2.5
3.0
3.5
4.0
Cellular_organisms - ps1
Archaea1 - ps2
Archaea2 - ps3
Archaea3 - ps4
Asgard/Eukaryota - ps5
Eukaryota - ps6
Amorpheae - ps7
Obazoa - ps8
Opisthokonta - ps9
Holozoa - ps10
Filozoa - ps11
Choano./Metazoa - ps12
Metazoa - ps13
Myriazoa - ps14
Parahoxozoa - ps15
Bilateria - ps16
Protostomia - ps17
Ecdysozoa - ps18
Arthropoda - ps19
Mandibulata - ps20
Pancrustacea - ps21
Insecta - ps22
Eumetabola - ps23
Holometabola/Psocodea - ps24
Holometabola - ps25
Aparaglossata - ps26
Mecopterida - ps27
Diptera - ps28
Bibiono./Cyclorrhapha - ps29
Cyclorrhapha - ps30
Calyptratae/Drosophila - ps31
Drosophila - ps32
Sophophora - ps33
Obscura/Melanogaster_group - ps34
D_melanogaster_group - ps35
D_melanogaster_subgroup - ps36
sim./sech./mel. group - ps37
Drosophila_melanogaster - ps38
Phylostratum

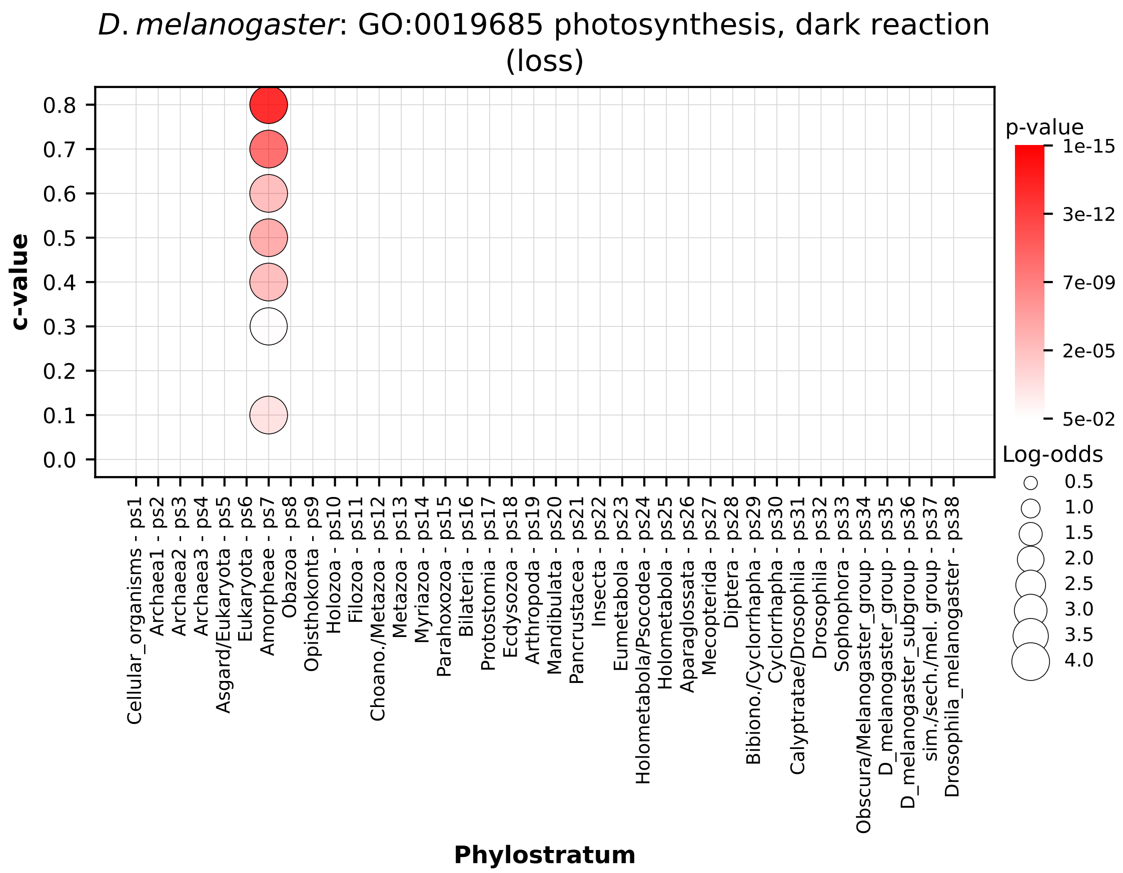

D. melanogaster: GO:0019685 photosynthesis, dark reaction
(loss)
c-value
0.8
0.7
0.6
0.5
0.4
0.3
0.2
0.1
0.0
p-value
1e-15
3e-12
7e-09
2e-05
5e-02
Log-odds
0.5
1.0
1.5
2.0
2.5
3.0
3.5
4.0
Cellular_organisms - ps1
Archaea1 - ps2
Archaea2 - ps3
Archaea3 - ps4
Asgard/Eukaryota - ps5
Eukaryota - ps6
Amorpheae - ps7
Obazoa - ps8
Opisthokonta - ps9
Holozoa - ps10
Filozoa - ps11
Choano./Metazoa - ps12
Metazoa - ps13
Myriazoa - ps14
Parahoxozoa - ps15
Bilateria - ps16
Protostomia - ps17
Ecdysozoa - ps18
Arthropoda - ps19
Mandibulata - ps20
Pancrustacea - ps21
Insecta - ps22
Eumetabola - ps23
Holometabola/Psocodea - ps24
Holometabola - ps25
Aparaglossata - ps26
Mecopterida - ps27
Diptera - ps28
Bibiono./Cyclorrhapha - ps29
Cyclorrhapha - ps30
Calyptratae/Drosophila - ps31
Drosophila - ps32
Sophophora - ps33
Obscura/Melanogaster_group - ps34
D_melanogaster_group - ps35
D_melanogaster_subgroup - ps36
sim./sech./mel. group - ps37
Drosophila_melanogaster - ps38
Phylostratum

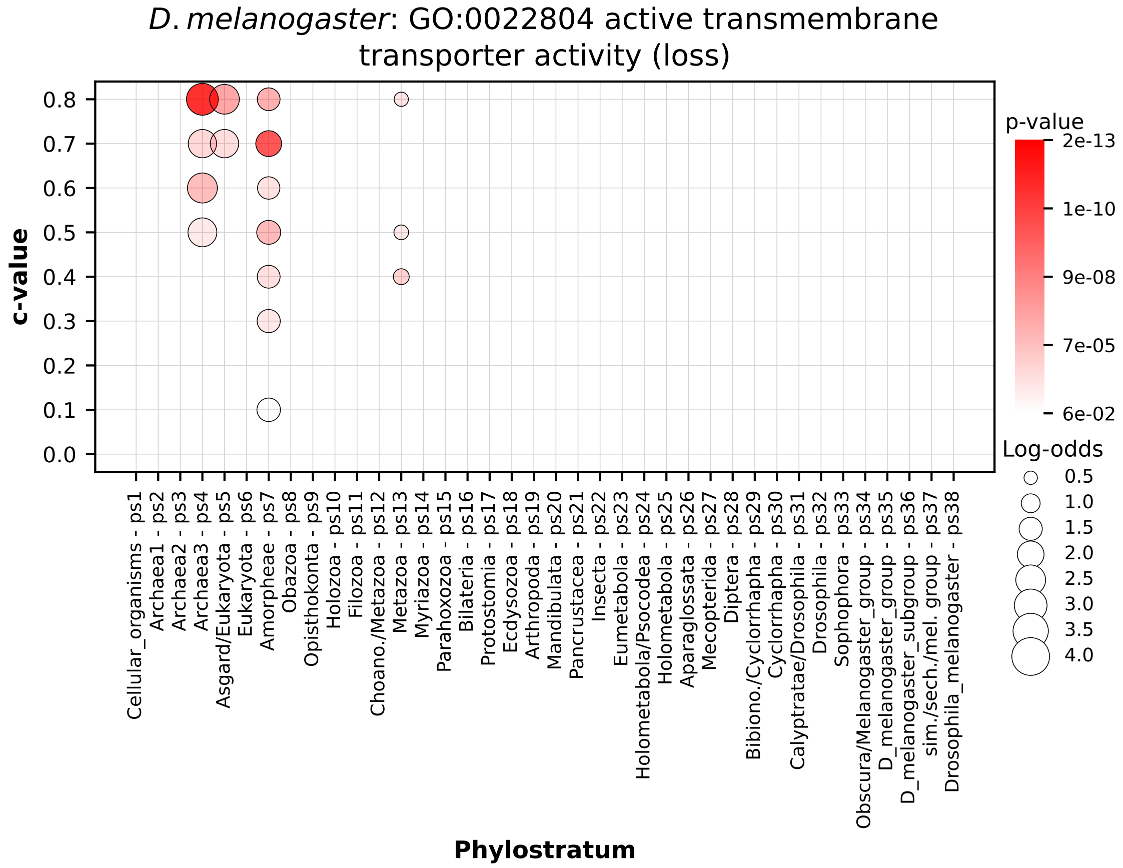

D. melanogaster: GO:0022804 active transmembrane transporter activity (loss)
c-value
0.8
0.7
0.6
0.5
0.4
0.3
0.2
0.1
0.0
Phylostratum
Cellular_organisms - ps1
Archaea1 - ps2
Archaea2 - ps3
Archaea3 - ps4
Asgard/Eukaryota - ps5
Eukaryota - ps6
Amorpheae - ps7
Obazoa - ps8
Opisthokonta - ps9
Holozoa - ps10
Filozoa - ps11
Choano./Metazoa - ps12
Metazoa - ps13
Myriazoa - ps14
Parahoxozoa - ps15
Bilateria - ps16
Protostomia - ps17
Ecdysozoa - ps18
Arthropoda - ps19
Mandibulata - ps20
Pancrustacea - ps21
Insecta - ps22
Eumetabola - ps23
Holometabola/Psocodea - ps24
Holometabola - ps25
Aparaglossata - ps26
Mecopterida - ps27
Diptera - ps28
Bibiono./Cyclorrhapha - ps29
Cyclorrhapha - ps30
Calyptratae/Drosophila - ps31
Drosophila - ps32
Sophophora - ps33
Obscura/Melanogaster_group - ps34
D_melanogaster_group - ps35
D_melanogaster_subgroup - ps36
sim./sech./mel. group - ps37
Drosophila_melanogaster - ps38
p-value
2e-13
1e-10
9e-08
7e-05
6e-02
Log-odds
0.5
1.0
1.5
2.0
2.5
3.0
3.5
4.0

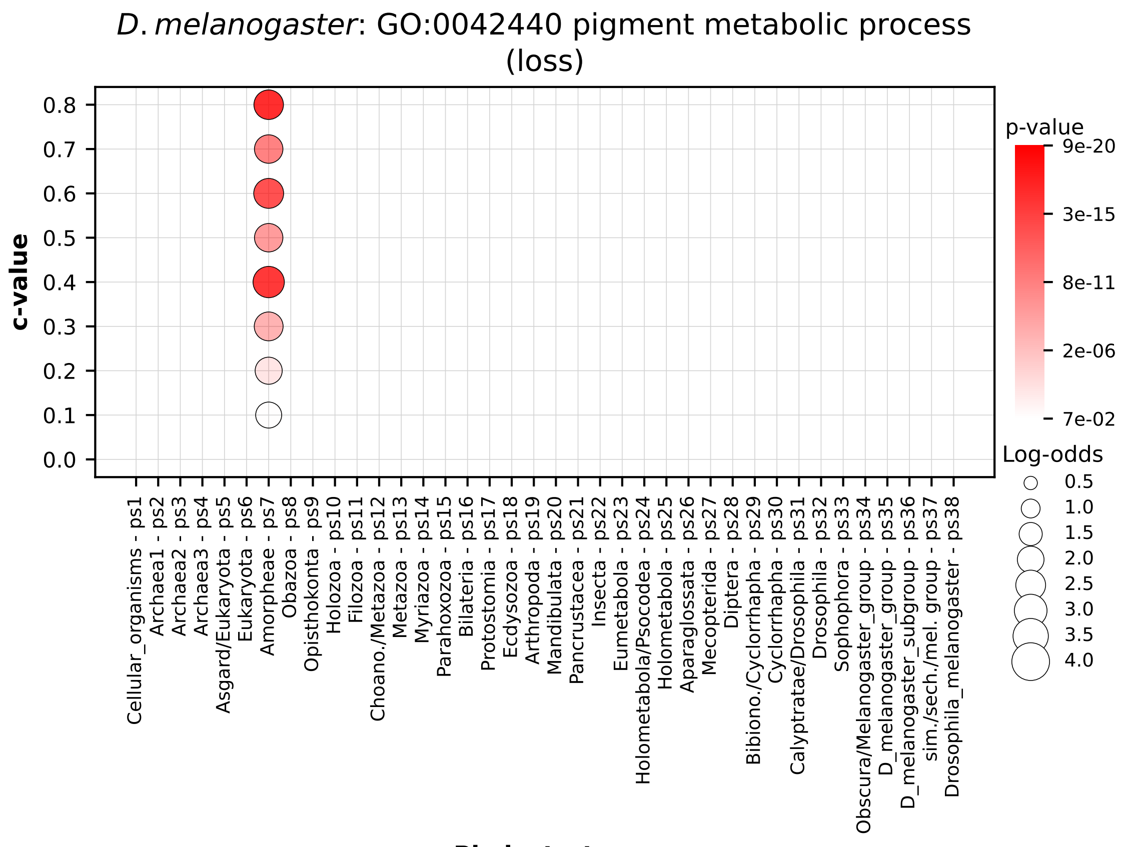

D. melanogaster: GO:0042440 pigment metabolic process
(loss)
c-value
0.8
0.7
0.6
0.5
0.4
0.3
0.2
0.1
0.0
Phylostratum
Cellular_organisms - ps1
Archaea1 - ps2
Archaea2 - ps3
Archaea3 - ps4
Asgard/Eukaryota - ps5
Eukaryota - ps6
Amorpheae - ps7
Obazoa - ps8
Opisthokonta - ps9
Holozoa - ps10
Filozoa - ps11
Choano./Metazoa - ps12
Metazoa - ps13
Myriazoa - ps14
Parahoxozoa - ps15
Bilateria - ps16
Protostomia - ps17
Ecdysozoa - ps18
Arthropoda - ps19
Mandibulata - ps20
Pancrustacea - ps21
Insecta - ps22
Eumetabola - ps23
Holometabola/Psocodea - ps24
Holometabola - ps25
Aparaglossata - ps26
Mecopterida - ps27
Diptera - ps28
Bibiono./Cyclorrhapha - ps29
Cyclorrhapha - ps30
Calyptratae/Drosophila - ps31
Drosophila - ps32
Sophophora - ps33
Obscura/Melanogaster_group - ps34
D_melanogaster_group - ps35
D_melanogaster_subgroup - ps36
sim./sech./mel. group - ps37
Drosophila_melanogaster - ps38
p-value
9e-20
3e-15
8e-11
2e-06
7e-02
Log-odds
0.5
1.0
1.5
2.0
2.5
3.0
3.5
4.0

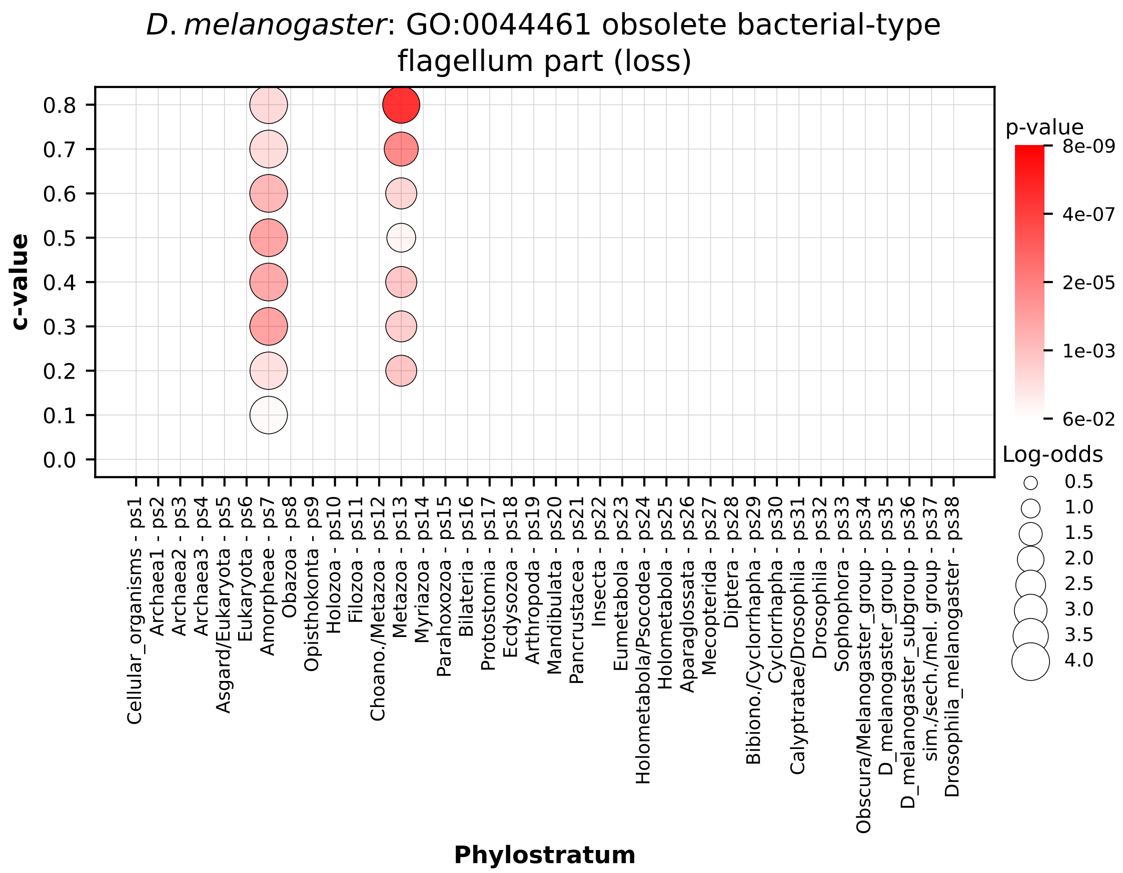

D. melanogaster: GO:0044461 obsolete bacterial-type flagellum part (loss)
c-value
0.8
0.7
0.6
0.5
0.4
0.3
0.2
0.1
0.0
p-value
8e-09
4e-07
2e-05
1e-03
6e-02
Log-odds
0.5
1.0
1.5
2.0
2.5
3.0
3.5
4.0
Cellular_organisms - ps1
Archaea1 - ps2
Archaea2 - ps3
Archaea3 - ps4
Asgard/Eukaryota - ps5
Eukaryota - ps6
Amorpheae - ps7
Obazoa - ps8
Opisthokonta - ps9
Holozoa - ps10
Filozoa - ps11
Choano./Metazoa - ps12
Metazoa - ps13
Myriazoa - ps14
Parahoxozoa - ps15
Bilateria - ps16
Protostomia - ps17
Ecdysozoa - ps18
Arthropoda - ps19
Mandibulata - ps20
Pancrustacea - ps21
Insecta - ps22
Eumetabola - ps23
Holometabola/Psocodea - ps24
Holometabola - ps25
Aparaglossata - ps26
Mecopterida - ps27
Diptera - ps28
Bibiono./Cyclorrhapha - ps29
Cyclorrhapha - ps30
Calyptratae/Drosophila - ps31
Drosophila - ps32
Sophophora - ps33
Obscura/Melanogaster_group - ps34
D_melanogaster_group - ps35
D_melanogaster_subgroup - ps36
sim./sech./mel. group - ps37
Drosophila_melanogaster - ps38
Phylostratum

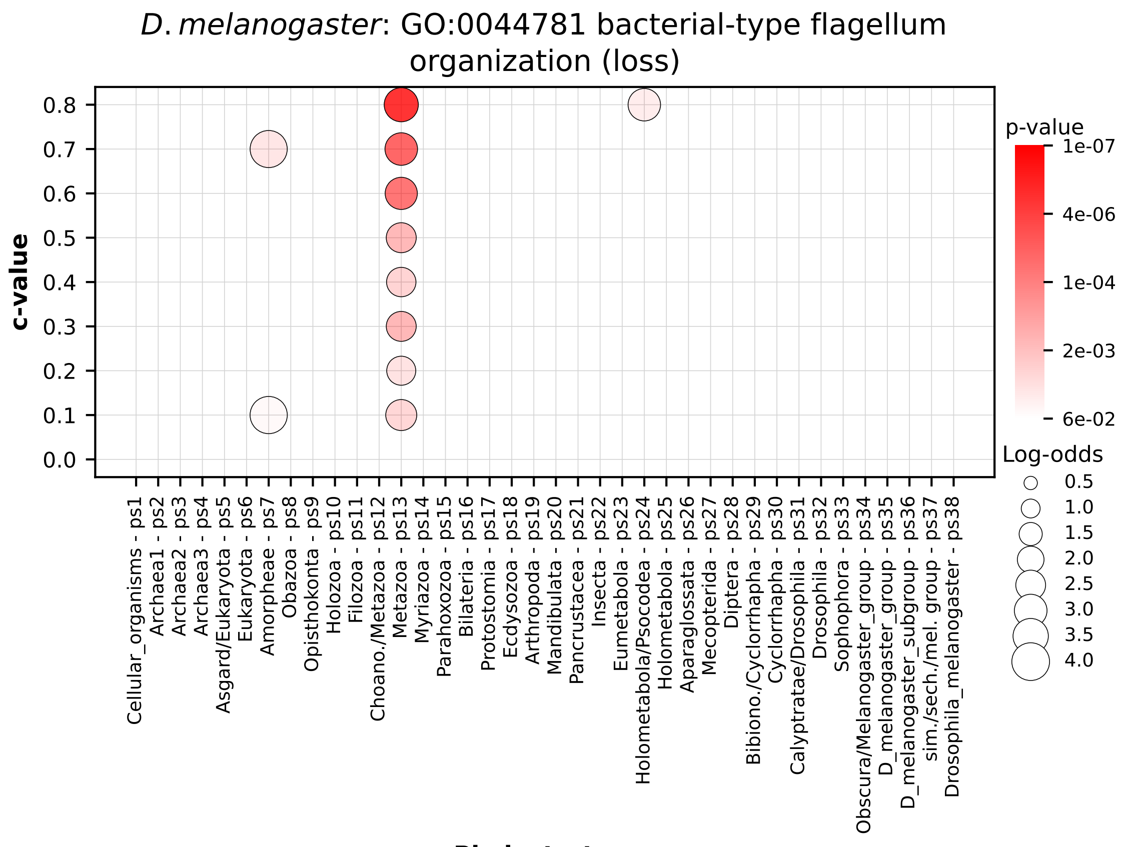

D. melanogaster: GO:0044781 bacterial-type flagellum organization (loss)
c-value
0.8
0.7
0.6
0.5
0.4
0.3
0.2
0.1
0.0
p-value
1e-07
4e-06
1e-04
2e-03
6e-02
Log-odds
0.5
1.0
1.5
2.0
2.5
3.0
3.5
4.0
Cellular_organisms - ps1
Archaea1 - ps2
Archaea2 - ps3
Archaea3 - ps4
Asgard/Eukaryota - ps5
Eukaryota - ps6
Amorpheae - ps7
Obazoa - ps8
Opisthokonta - ps9
Holozoa - ps10
Filozoa - ps11
Choano./Metazoa - ps12
Metazoa - ps13
Myriazoa - ps14
Parahoxozoa - ps15
Bilateria - ps16
Protostomia - ps17
Ecdysozoa - ps18
Arthropoda - ps19
Mandibulata - ps20
Pancrustacea - ps21
Insecta - ps22
Eumetabola - ps23
Holometabola/Psocodea - ps24
Holometabola - ps25
Aparaglossata - ps26
Mecopterida - ps27
Diptera - ps28
Bibiono./Cyclorrhapha - ps29
Cyclorrhapha - ps30
Calyptratae/Drosophila - ps31
Drosophila - ps32
Sophophora - ps33
Obscura/Melanogaster_group - ps34
D_melanogaster_group - ps35
D_melanogaster_subgroup - ps36
sim./sech./mel. group - ps37
Drosophila_melanogaster - ps38
Phylostratum

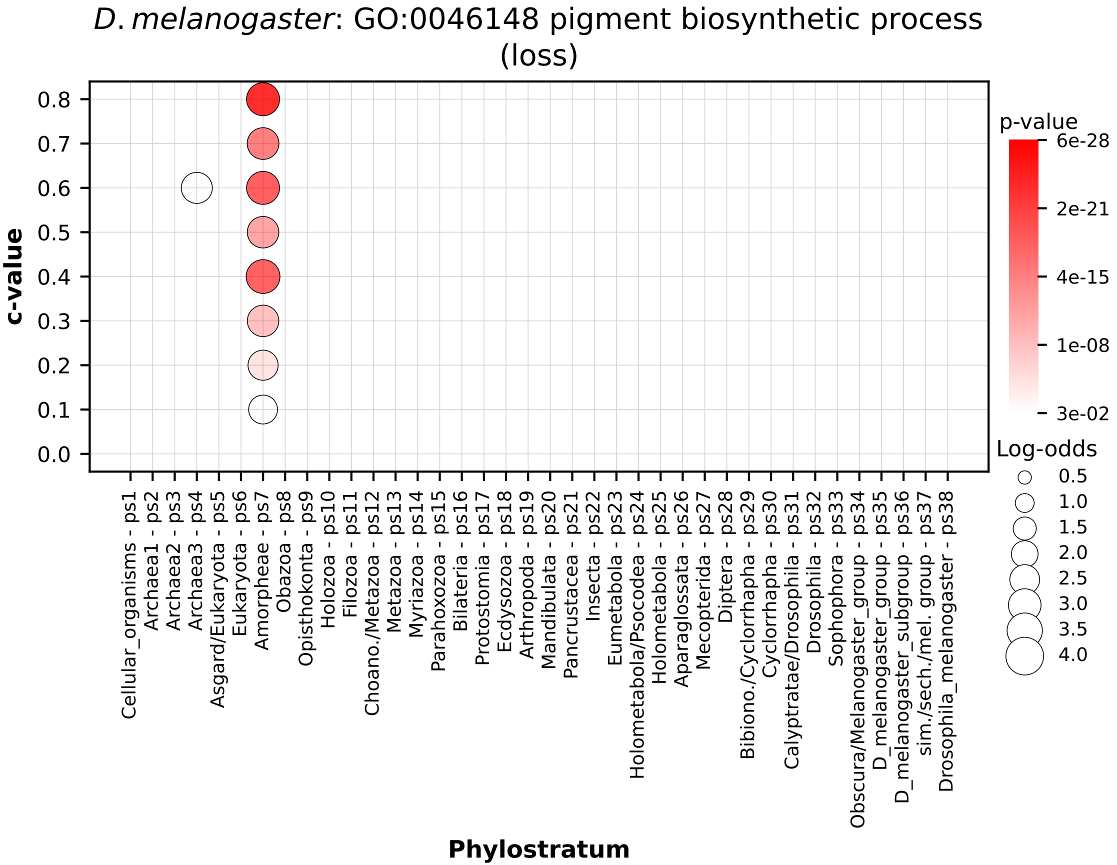

$D. melanogaster$: GO:0046148 pigment biosynthetic process
(loss)
c-value
0.8
0.7
0.6
0.5
0.4
0.3
0.2
0.1
0.0
p-value
6e-28
2e-21
4e-15
1e-08
3e-02
Log-odds
0.5
1.0
1.5
2.0
2.5
3.0
3.5
4.0
Cellular_organisms - ps1
Archaea1 - ps2
Archaea2 - ps3
Archaea3 - ps4
Asgard/Eukaryota - ps5
Eukaryota - ps6
Amorpheae - ps7
Obazoa - ps8
Opisthokonta - ps9
Holozoa - ps10
Filozoa - ps11
Choano./Metazoa - ps12
Metazoa - ps13
Myriazoa - ps14
Parahoxozoa - ps15
Bilateria - ps16
Protostomia - ps17
Ecdysozoa - ps18
Arthropoda - ps19
Mandibulata - ps20
Pancrustacea - ps21
Insecta - ps22
Eumetabola - ps23
Holometabola/Psocodea - ps24
Holometabola - ps25
Aparaglossata - ps26
Mecopterida - ps27
Diptera - ps28
Bibiono./Cyclorrhapha - ps29
Cyclorrhapha - ps30
Calyptratae/Drosophila - ps31
Drosophila - ps32
Sophophora - ps33
Obscura/Melanogaster_group - ps34
D_melanogaster_group - ps35
D_melanogaster_subgroup - ps36
sim./sech./mel. group - ps37
Drosophila_melanogaster - ps38
Phylostratum

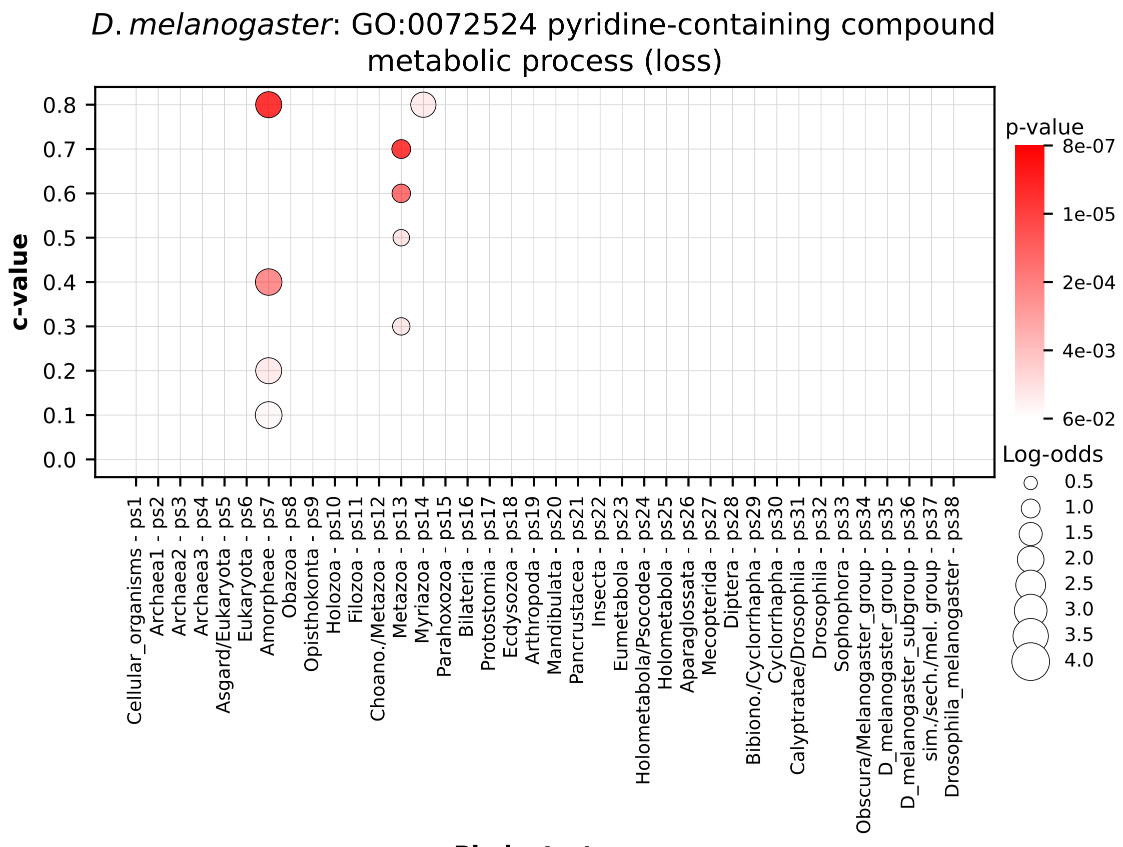

D. melanogaster: GO:0072524 pyridine-containing compound metabolic process (loss)
c-value
0.8
0.7
0.6
0.5
0.4
0.3
0.2
0.1
0.0
p-value
8e-07
1e-05
2e-04
4e-03
6e-02
Log-odds
0.5
1.0
1.5
2.0
2.5
3.0
3.5
4.0
Cellular_organisms - ps1
Archaea1 - ps2
Archaea2 - ps3
Archaea3 - ps4
Asgard/Eukaryota - ps5
Eukaryota - ps6
Amorpheae - ps7
Obazoa - ps8
Opisthokonta - ps9
Holozoa - ps10
Filozoa - ps11
Choano./Metazoa - ps12
Metazoa - ps13
Myriazoa - ps14
Parahoxozoa - ps15
Bilateria - ps16
Protostomia - ps17
Ecdysozoa - ps18
Arthropoda - ps19
Mandibulata - ps20
Pancrustacea - ps21
Insecta - ps22
Eumetabola - ps23
Holometabola/Psocodea - ps24
Holometabola - ps25
Aparaglossata - ps26
Mecopterida - ps27
Diptera - ps28
Bibiono./Cyclorrhapha - ps29
Cyclorrhapha - ps30
Calyptratae/Drosophila - ps31
Drosophila - ps32
Sophophora - ps33
Obscura/Melanogaster_group - ps34
D_melanogaster_group - ps35
D_melanogaster_subgroup - ps36
sim./sech./mel. group - ps37
Drosophila_melanogaster - ps38
Phylostratum

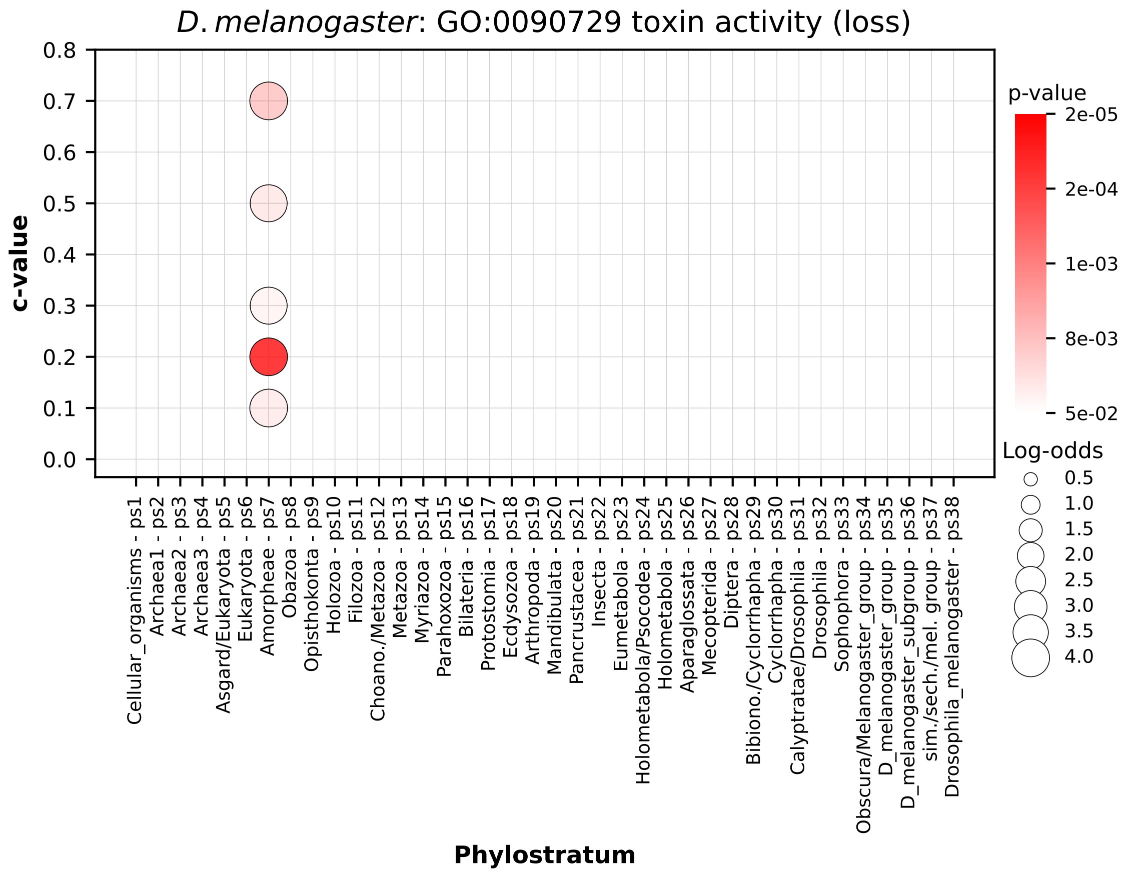

D. melanogaster: GO:0090729 toxin activity (loss)
c-value
0.8
0.7
0.6
0.5
0.4
0.3
0.2
0.1
0.0
Phylostratum
Cellular_organisms - ps1
Archaea1 - ps2
Archaea2 - ps3
Archaea3 - ps4
Asgard/Eukaryota - ps5
Eukaryota - ps6
Amorpheae - ps7
Obazoa - ps8
Opisthokonta - ps9
Holozoa - ps10
Filozoa - ps11
Choano./Metazoa - ps12
Metazoa - ps13
Myriazoa - ps14
Parahoxozoa - ps15
Bilateria - ps16
Protostomia - ps17
Ecdysozoa - ps18
Arthropoda - ps19
Mandibulata - ps20
Pancrustacea - ps21
Insecta - ps22
Eumetabola - ps23
Holometabola/Psocodea - ps24
Holometabola - ps25
Aparaglossata - ps26
Mecopterida - ps27
Diptera - ps28
Bibiono./Cyclorrhapha - ps29
Cyclorrhapha - ps30
Calyptratae/Drosophila - ps31
Drosophila - ps32
Sophophora - ps33
Obscura/Melanogaster_group - ps34
D_melanogaster_group - ps35
D_melanogaster_subgroup - ps36
sim./sech./mel. group - ps37
Drosophila_melanogaster - ps38
p-value
2e-05
2e-04
1e-03
8e-03
5e-02
Log-odds
0.5
1.0
1.5
2.0
2.5
3.0
3.5
4.0

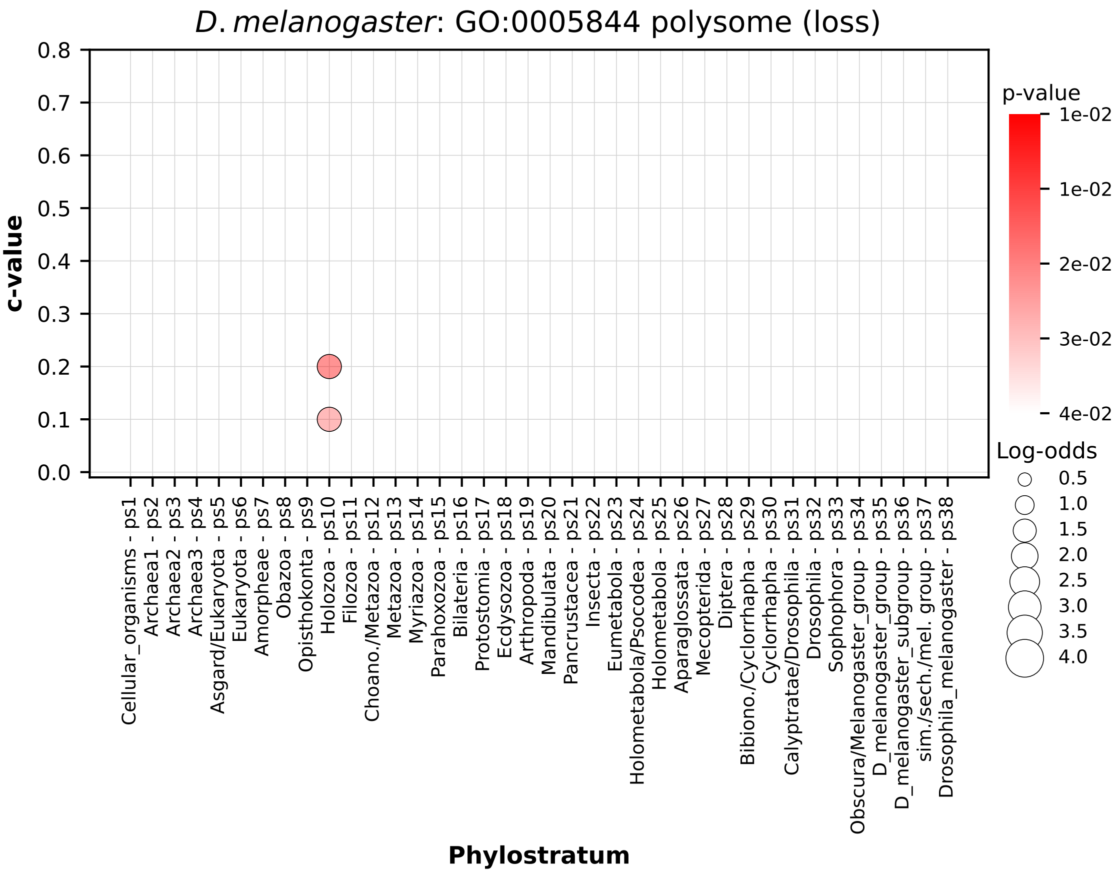

D. melanogaster: GO:0005844 polysome (loss)
c-value
0.0
0.1
0.2
0.3
0.4
0.5
0.6
0.7
0.8
Phylostratum
Cellular_organisms - ps1
Archaea1 - ps2
Archaea2 - ps3
Archaea3 - ps4
Asgard/Eukaryota - ps5
Eukaryota - ps6
Amorpheae - ps7
Obazoa - ps8
Opisthokonta - ps9
Holozoa - ps10
Filozoa - ps11
Choano./Metazoa - ps12
Metazoa - ps13
Myriazoa - ps14
Parahoxozoa - ps15
Bilateria - ps16
Protostomia - ps17
Ecdysozoa - ps18
Arthropoda - ps19
Mandibulata - ps20
Pancrustacea - ps21
Insecta - ps22
Eumetabola - ps23
Holometabola/Psocodea - ps24
Holometabola - ps25
Aparaglossata - ps26
Mecopterida - ps27
Diptera - ps28
Bibiono./Cyclorrhapha - ps29
Cyclorrhapha - ps30
Calyptratae/Drosophila - ps31
Drosophila - ps32
Sophophora - ps33
Obscura/Melanogaster_group - ps34
D_melanogaster_group - ps35
D_melanogaster_subgroup - ps36
sim./sech./mel. group - ps37
Drosophila_melanogaster - ps38
p-value
1e-02
1e-02
2e-02
3e-02
4e-02
Log-odds
0.5
1.0
1.5
2.0
2.5
3.0
3.5
4.0

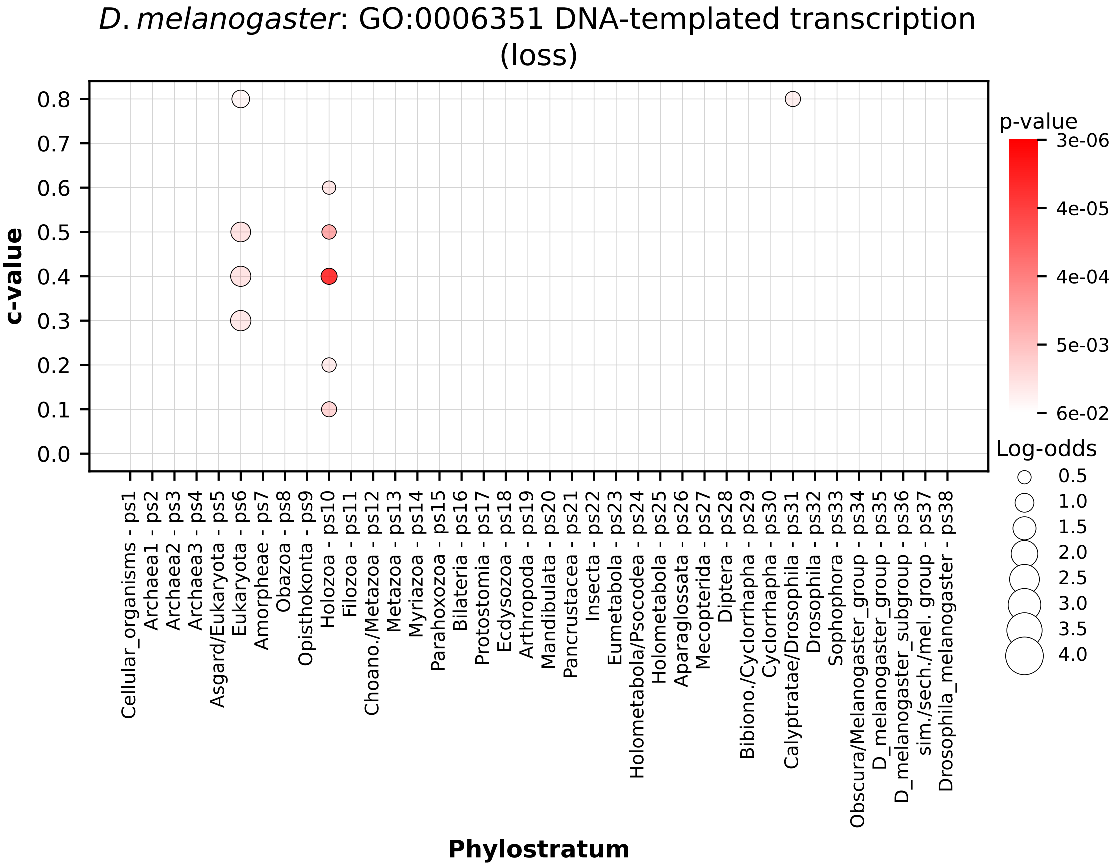
D. melanogaster: GO:0006351 DNA-templated transcription (loss)
c-value
Phylostratum
p-value
3e-06
4e-05
4e-04
5e-03
6e-02
Log-odds
0.5
1.0
1.5
2.0
2.5
3.0
3.5
4.0
Cellular_organisms - ps1
Archaea1 - ps2
Archaea2 - ps3
Archaea3 - ps4
Asgard/Eukaryota - ps5
Eukaryota - ps6
Amorpheae - ps7
Obazoa - ps8
Opisthokonta - ps9
Holozoa - ps10
Filozoa - ps11
Choano./Metazoa - ps12
Metazoa - ps13
Myriazoa - ps14
Parahoxozoa - ps15
Bilateria - ps16
Protostomia - ps17
Ecdysozoa - ps18
Arthropoda - ps19
Mandibulata - ps20
Pancrustacea - ps21
Insecta - ps22
Eumetabola - ps23
Holometabola/Psocodea - ps24
Holometabola - ps25
Aparaglossata - ps26
Mecopterida - ps27
Diptera - ps28
Bibiono./Cyclorrhapha - ps29
Cyclorrhapha - ps30
Calyptratae/Drosophila - ps31
Drosophila - ps32
Sophophora - ps33
Obscura/Melanogaster_group - ps34
D_melanogaster_group - ps35
D_melanogaster_subgroup - ps36
sim./sech./mel. group - ps37
Drosophila_melanogaster - ps38

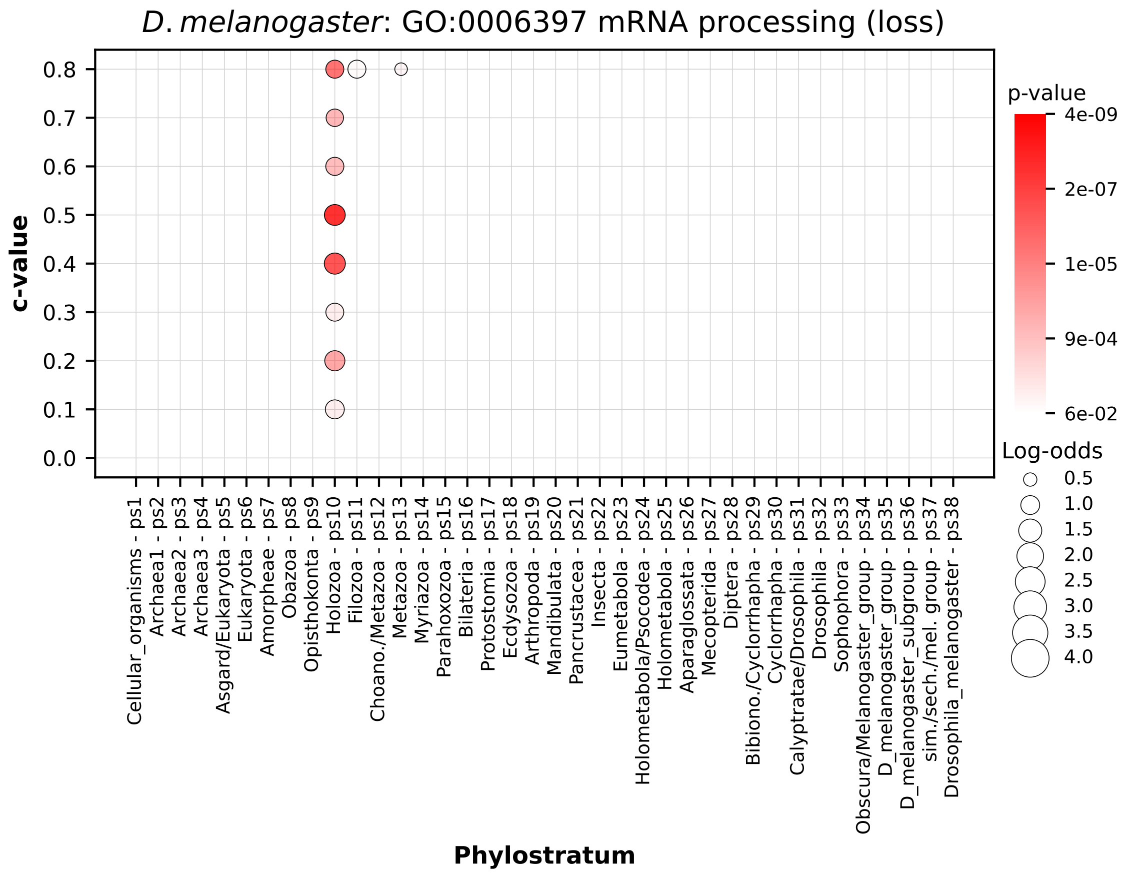

D. melanogaster: GO:0006397 mRNA processing (loss)
c-value
0.8
0.7
0.6
0.5
0.4
0.3
0.2
0.1
0.0
p-value
4e-09
2e-07
1e-05
9e-04
6e-02
Log-odds
0.5
1.0
1.5
2.0
2.5
3.0
3.5
4.0
Cellular_organisms - ps1
Archaea1 - ps2
Archaea2 - ps3
Archaea3 - ps4
Asgard/Eukaryota - ps5
Eukaryota - ps6
Amorpheae - ps7
Obazoa - ps8
Opisthokonta - ps9
Holozoa - ps10
Filozoa - ps11
Choano./Metazoa - ps12
Metazoa - ps13
Myriazoa - ps14
Parahoxozoa - ps15
Bilateria - ps16
Protostomia - ps17
Ecdysozoa - ps18
Arthropoda - ps19
Mandibulata - ps20
Pancrustacea - ps21
Insecta - ps22
Eumetabola - ps23
Holometabola/Psocodea - ps24
Holometabola - ps25
Aparaglossata - ps26
Mecopterida - ps27
Diptera - ps28
Bibiono./Cyclorrhapha - ps29
Cyclorrhapha - ps30
Calyptratae/Drosophila - ps31
Drosophila - ps32
Sophophora - ps33
Obscura/Melanogaster_group - ps34
D_melanogaster_group - ps35
D_melanogaster_subgroup - ps36
sim./sech./mel. group - ps37
Drosophila_melanogaster - ps38
Phylostratum

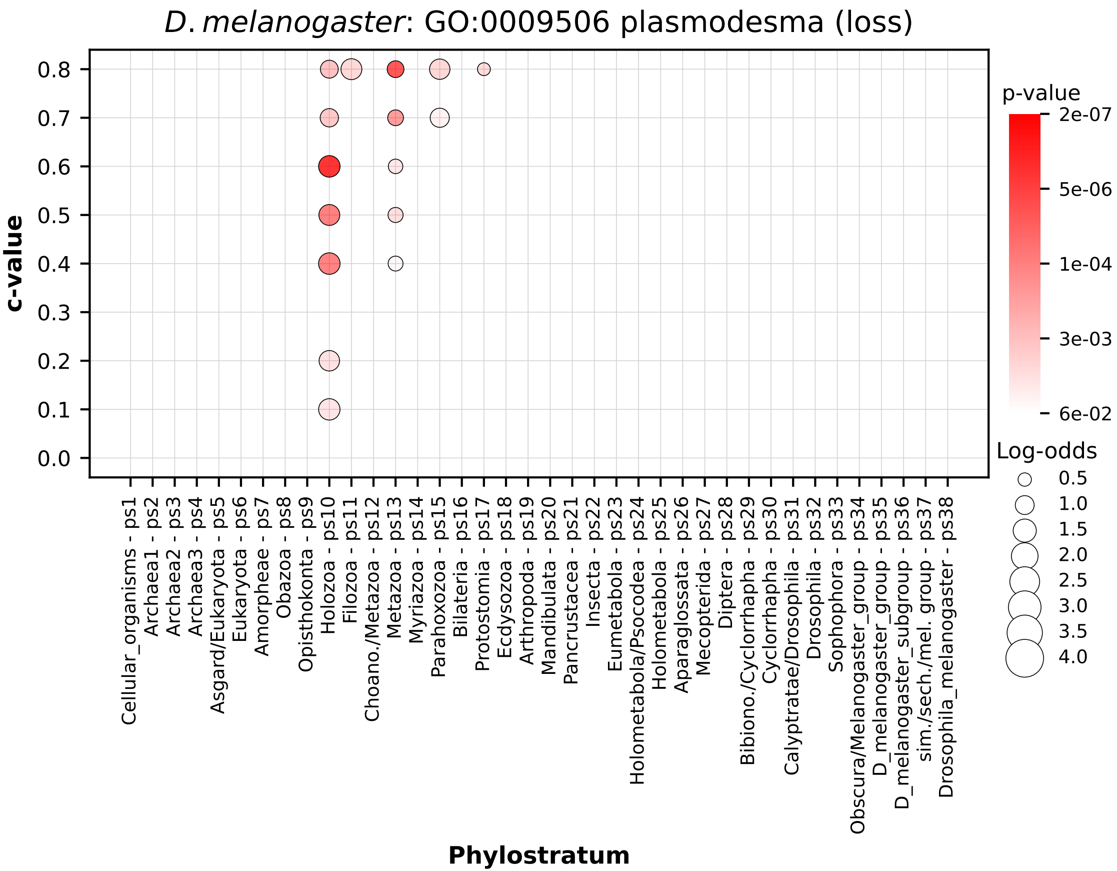

D. melanogaster: GO:0009506 plasmodesma (loss)
c-value
0.8
0.7
0.6
0.5
0.4
0.3
0.2
0.1
0.0
Phylostratum
Cellular_organisms - ps1
Archaea1 - ps2
Archaea2 - ps3
Archaea3 - ps4
Asgard/Eukaryota - ps5
Eukaryota - ps6
Amorpheae - ps7
Obazoa - ps8
Opisthokonta - ps9
Holozoa - ps10
Filozoa - ps11
Choano./Metazoa - ps12
Metazoa - ps13
Myriazoa - ps14
Parahoxozoa - ps15
Bilateria - ps16
Protostomia - ps17
Ecdysozoa - ps18
Arthropoda - ps19
Mandibulata - ps20
Pancrustacea - ps21
Insecta - ps22
Eumetabola - ps23
Holometabola/Psocodea - ps24
Holometabola - ps25
Aparaglossata - ps26
Mecopterida - ps27
Diptera - ps28
Bibiono./Cyclorrhapha - ps29
Cyclorrhapha - ps30
Calyptratae/Drosophila - ps31
Drosophila - ps32
Sophophora - ps33
Obscura/Melanogaster_group - ps34
D_melanogaster_group - ps35
D_melanogaster_subgroup - ps36
sim./sech./mel. group - ps37
Drosophila_melanogaster - ps38
p-value
2e-07
5e-06
1e-04
3e-03
6e-02
Log-odds
0.5
1.0
1.5
2.0
2.5
3.0
3.5
4.0

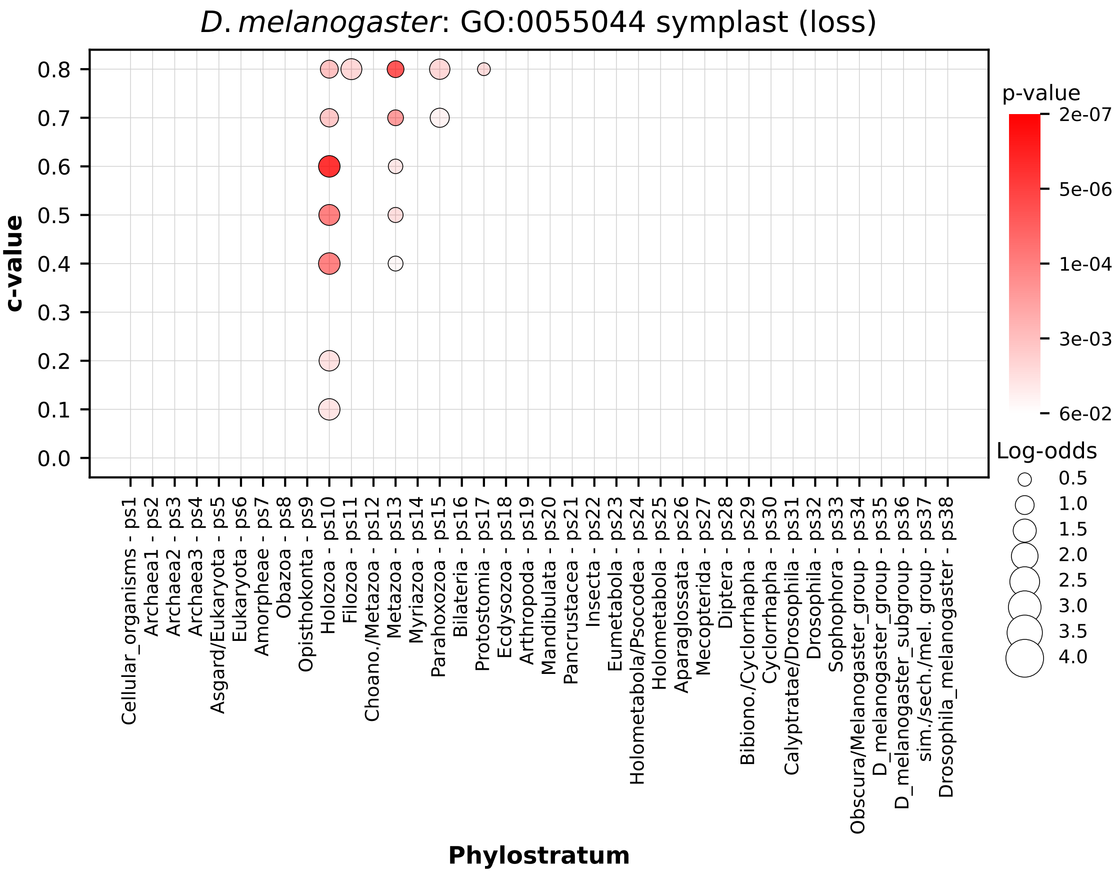

D. melanogaster: GO:0055044 symplast (loss)
c-value
0.8
0.7
0.6
0.5
0.4
0.3
0.2
0.1
0.0
Phylostratum
Cellular_organisms - ps1
Archaea1 - ps2
Archaea2 - ps3
Archaea3 - ps4
Asgard/Eukaryota - ps5
Eukaryota - ps6
Amorpheae - ps7
Obazoa - ps8
Opisthokonta - ps9
Holozoa - ps10
Filozoa - ps11
Choano./Metazoa - ps12
Metazoa - ps13
Myriazoa - ps14
Parahoxozoa - ps15
Bilateria - ps16
Protostomia - ps17
Ecdysozoa - ps18
Arthropoda - ps19
Mandibulata - ps20
Pancrustacea - ps21
Insecta - ps22
Eumetabola - ps23
Holometabola/Psocodea - ps24
Holometabola - ps25
Aparaglossata - ps26
Mecopterida - ps27
Diptera - ps28
Bibiono./Cyclorrhapha - ps29
Cyclorrhapha - ps30
Calyptratae/Drosophila - ps31
Drosophila - ps32
Sophophora - ps33
Obscura/Melanogaster_group - ps34
D_melanogaster_group - ps35
D_melanogaster_subgroup - ps36
sim./sech./mel. group - ps37
Drosophila_melanogaster - ps38
p-value
2e-07
5e-06
1e-04
3e-03
6e-02
Log-odds
0.5
1.0
1.5
2.0
2.5
3.0
3.5
4.0

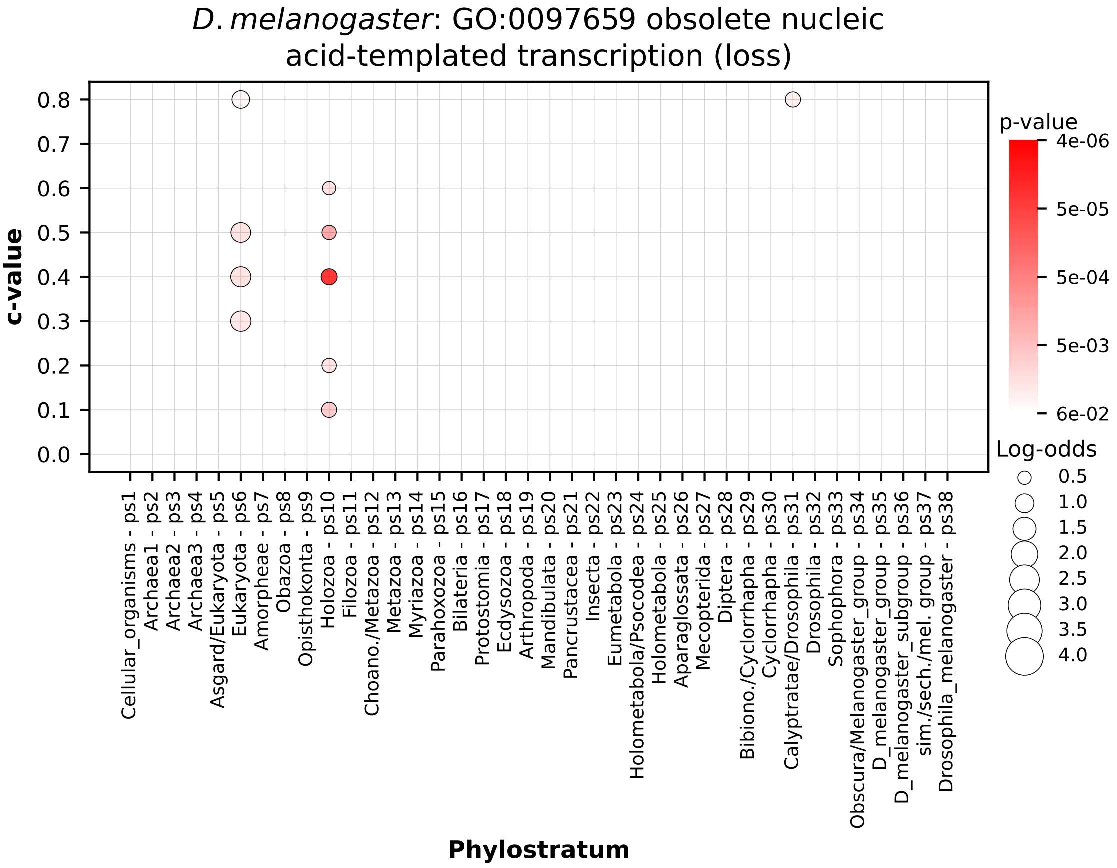
D. melanogaster: GO:0097659 obsolete nucleic acid-templated transcription (loss)
c-value
0.0
0.1
0.2
0.3
0.4
0.5
0.6
0.7
0.8
Phylostratum
Cellular_organisms - ps1
Archaea1 - ps2
Archaea2 - ps3
Archaea3 - ps4
Asgard/Eukaryota - ps5
Eukaryota - ps6
Amorpheae - ps7
Obazoa - ps8
Opisthokonta - ps9
Holozoa - ps10
Filozoa - ps11
Choano./Metazoa - ps12
Metazoa - ps13
Myriazoa - ps14
Parahoxozoa - ps15
Bilateria - ps16
Protostomia - ps17
Ecdysozoa - ps18
Arthropoda - ps19
Mandibulata - ps20
Pancrustacea - ps21
Insecta - ps22
Eumetabola - ps23
Holometabola/Psocodea - ps24
Holometabola - ps25
Aparaglossata - ps26
Mecopterida - ps27
Diptera - ps28
Bibiono./Cyclorrhapha - ps29
Cyclorrhapha - ps30
Calyptratae/Drosophila - ps31
Drosophila - ps32
Sophophora - ps33
Obscura/Melanogaster_group - ps34
D_melanogaster_group - ps35
D_melanogaster_subgroup - ps36
sim./sech./mel. group - ps37
Drosophila_melanogaster - ps38
p-value
4e-06
5e-05
5e-04
5e-03
6e-02
Log-odds
0.5
1.0
1.5
2.0
2.5
3.0
3.5
4.0

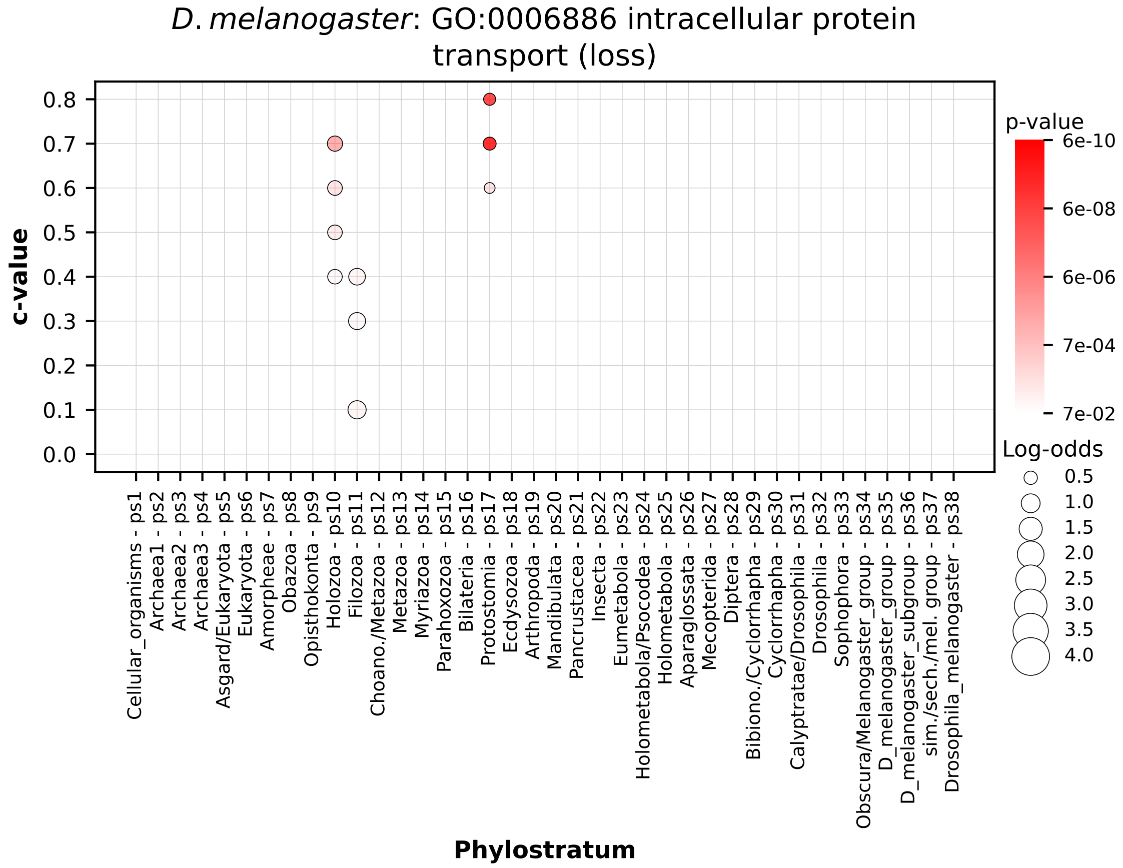
D. melanogaster: GO:0006886 intracellular protein transport (loss)
c-value
0.8
0.7
0.6
0.5
0.4
0.3
0.2
0.1
0.0
Phylostratum
Cellular_organisms - ps1
Archaea1 - ps2
Archaea2 - ps3
Archaea3 - ps4
Asgard/Eukaryota - ps5
Eukaryota - ps6
Amorpheae - ps7
Obazoa - ps8
Opisthokonta - ps9
Holozoa - ps10
Filozoa - ps11
Choano./Metazoa - ps12
Metazoa - ps13
Myriazoa - ps14
Parahoxozoa - ps15
Bilateria - ps16
Protostomia - ps17
Ecdysozoa - ps18
Arthropoda - ps19
Mandibulata - ps20
Pancrustacea - ps21
Insecta - ps22
Eumetabola - ps23
Holometabola/Psocodea - ps24
Holometabola - ps25
Aparaglossata - ps26
Mecopterida - ps27
Diptera - ps28
Bibiono./Cyclorrhapha - ps29
Cyclorrhapha - ps30
Calyptratae/Drosophila - ps31
Drosophila - ps32
Sophophora - ps33
Obscura/Melanogaster_group - ps34
D_melanogaster_group - ps35
D_melanogaster_subgroup - ps36
sim./sech./mel. group - ps37
Drosophila_melanogaster - ps38
p-value
6e-10
6e-08
6e-06
7e-04
7e-02
Log-odds
0.5
1.0
1.5
2.0
2.5
3.0
3.5
4.0

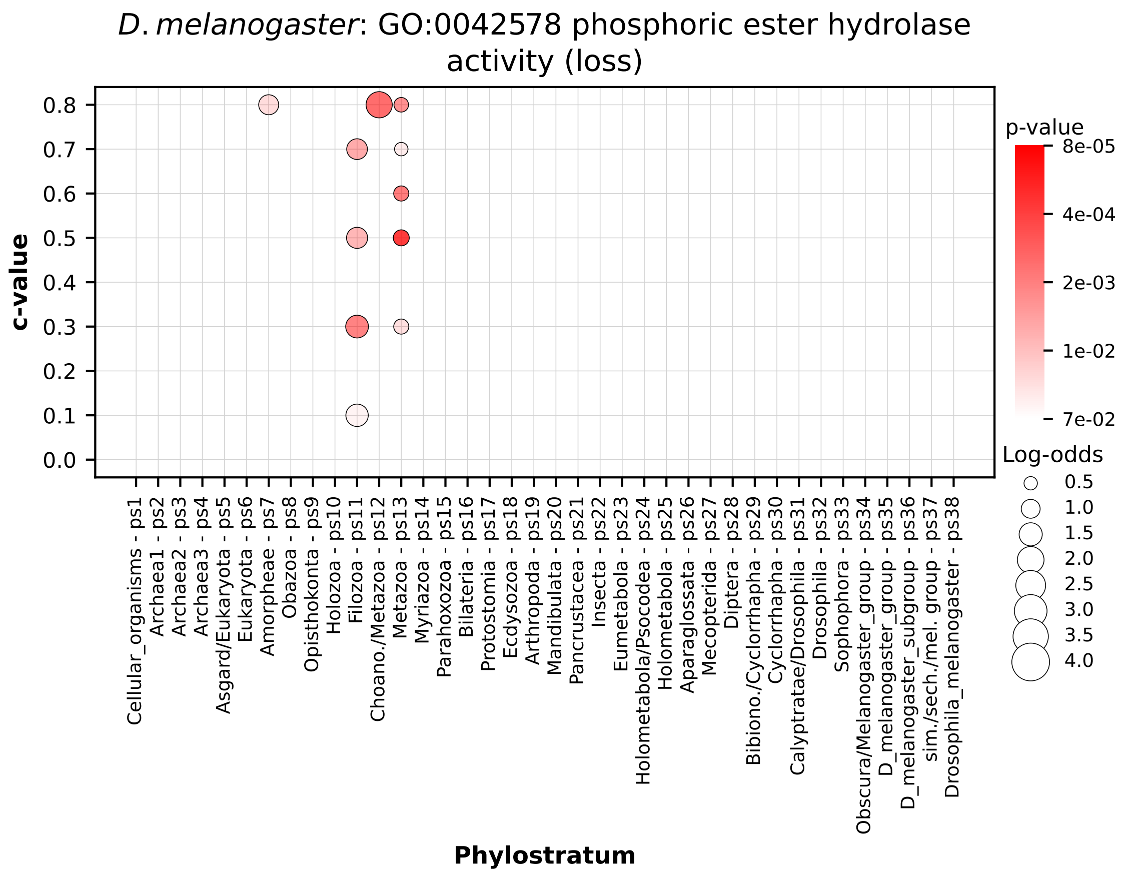

D. melanogaster: GO:0042578 phosphoric ester hydrolase activity (loss)
c-value
0.0
0.1
0.2
0.3
0.4
0.5
0.6
0.7
0.8
p-value
8e-05
4e-04
2e-03
1e-02
7e-02
Log-odds
0.5
1.0
1.5
2.0
2.5
3.0
3.5
4.0
Cellular_organisms - ps1
Archaea1 - ps2
Archaea2 - ps3
Archaea3 - ps4
Asgard/Eukaryota - ps5
Eukaryota - ps6
Amorpheae - ps7
Obazoa - ps8
Opisthokonta - ps9
Holozoa - ps10
Filozoa - ps11
Choano./Metazoa - ps12
Metazoa - ps13
Myriazoa - ps14
Parahoxozoa - ps15
Bilateria - ps16
Protostomia - ps17
Ecdysozoa - ps18
Arthropoda - ps19
Mandibulata - ps20
Pancrustacea - ps21
Insecta - ps22
Eumetabola - ps23
Holometabola/Psocodea - ps24
Holometabola - ps25
Aparaglossata - ps26
Mecopterida - ps27
Diptera - ps28
Bibiono./Cyclorrhapha - ps29
Cyclorrhapha - ps30
Calyptratae/Drosophila - ps31
Drosophila - ps32
Sophophora - ps33
Obscura/Melanogaster_group - ps34
D_melanogaster_group - ps35
D_melanogaster_subgroup - ps36
sim./sech./mel. group - ps37
Drosophila_melanogaster - ps38
Phylostratum

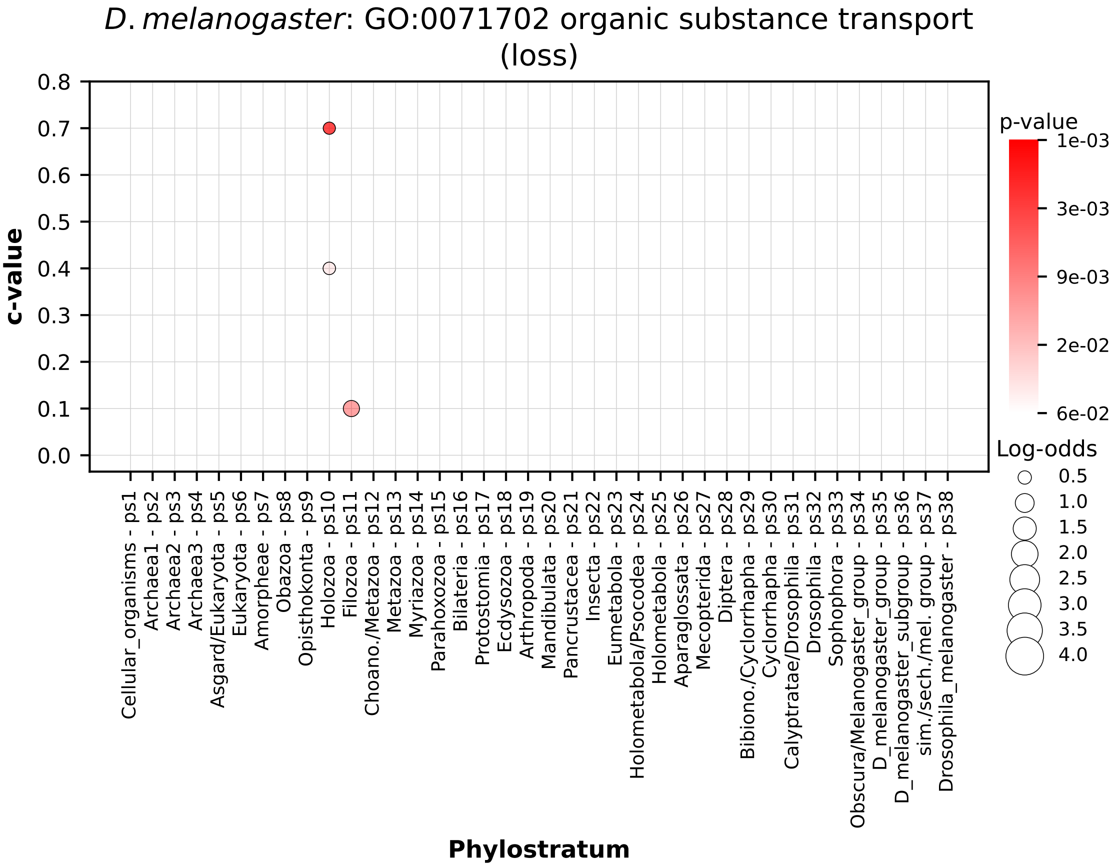

D. melanogaster: GO:0071702 organic substance transport (loss)
c-value
0.8
0.7
0.6
0.5
0.4
0.3
0.2
0.1
0.0
Phylostratum
Cellular_organisms - ps1
Archaea1 - ps2
Archaea2 - ps3
Archaea3 - ps4
Asgard/Eukaryota - ps5
Eukaryota - ps6
Amorpheae - ps7
Obazoa - ps8
Opisthokonta - ps9
Holozoa - ps10
Filozoa - ps11
Choano./Metazoa - ps12
Metazoa - ps13
Myriazoa - ps14
Parahoxozoa - ps15
Bilateria - ps16
Protostomia - ps17
Ecdysozoa - ps18
Arthropoda - ps19
Mandibulata - ps20
Pancrustacea - ps21
Insecta - ps22
Eumetabola - ps23
Holometabola/Psocodea - ps24
Holometabola - ps25
Aparaglossata - ps26
Mecopterida - ps27
Diptera - ps28
Bibiono./Cyclorrhapha - ps29
Cyclorrhapha - ps30
Calyptratae/Drosophila - ps31
Drosophila - ps32
Sophophora - ps33
Obscura/Melanogaster_group - ps34
D_melanogaster_group - ps35
D_melanogaster_subgroup - ps36
sim./sech./mel. group - ps37
Drosophila_melanogaster - ps38
p-value
1e-03
3e-03
9e-03
2e-02
6e-02
Log-odds
0.5
1.0
1.5
2.0
2.5
3.0
3.5
4.0

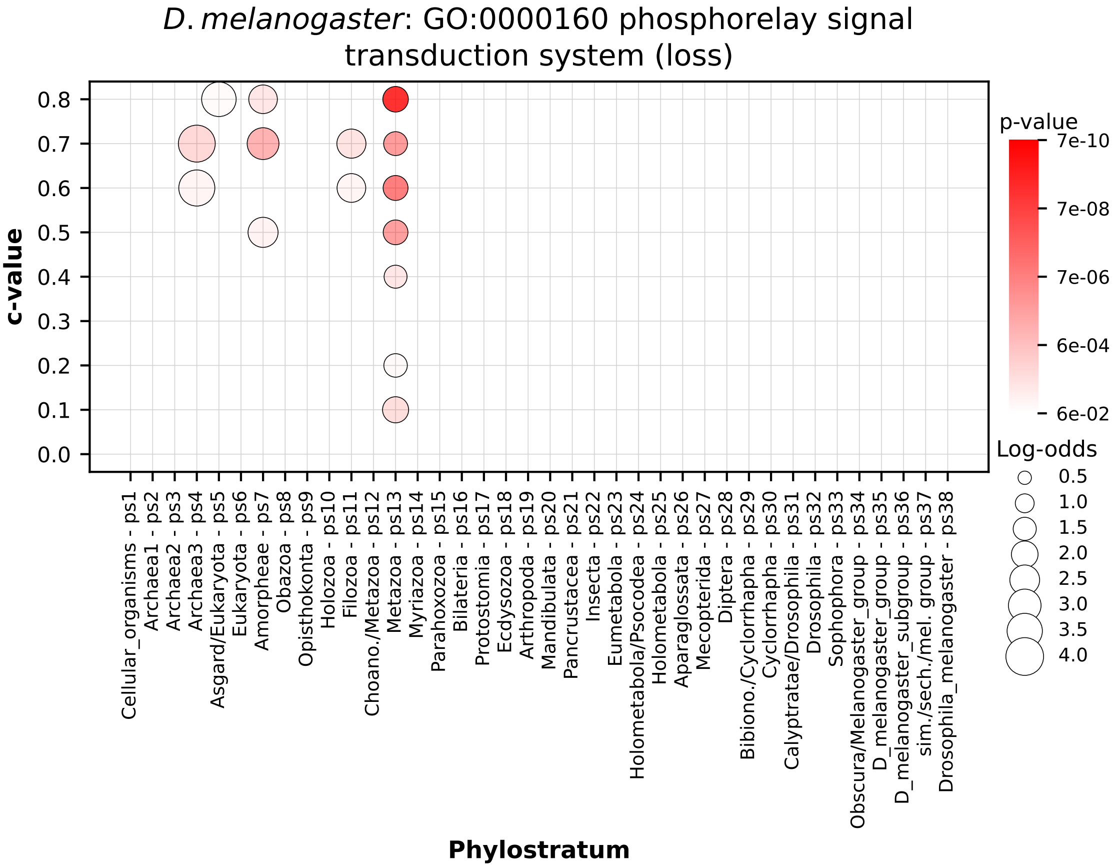

D. melanogaster: GO:0000160 phosphorelay signal transduction system (loss)
c-value
0.8
0.7
0.6
0.5
0.4
0.3
0.2
0.1
0.0
Phylostratum
Cellular_organisms - ps1
Archaea1 - ps2
Archaea2 - ps3
Archaea3 - ps4
Asgard/Eukaryota - ps5
Eukaryota - ps6
Amorpheae - ps7
Obazoa - ps8
Opisthokonta - ps9
Holozoa - ps10
Filozoa - ps11
Choano./Metazoa - ps12
Metazoa - ps13
Myriazoa - ps14
Parahoxozoa - ps15
Bilateria - ps16
Protostomia - ps17
Ecdysozoa - ps18
Arthropoda - ps19
Mandibulata - ps20
Pancrustacea - ps21
Insecta - ps22
Eumetabola - ps23
Holometabola/Psocodea - ps24
Holometabola - ps25
Aparaglossata - ps26
Mecopterida - ps27
Diptera - ps28
Bibiono./Cyclorrhapha - ps29
Cyclorrhapha - ps30
Calyptratae/Drosophila - ps31
Drosophila - ps32
Sophophora - ps33
Obscura/Melanogaster_group - ps34
D_melanogaster_group - ps35
D_melanogaster_subgroup - ps36
sim./sech./mel. group - ps37
Drosophila_melanogaster - ps38
p-value
7e-10
7e-08
7e-06
6e-04
6e-02
Log-odds
0.5
1.0
1.5
2.0
2.5
3.0
3.5
4.0

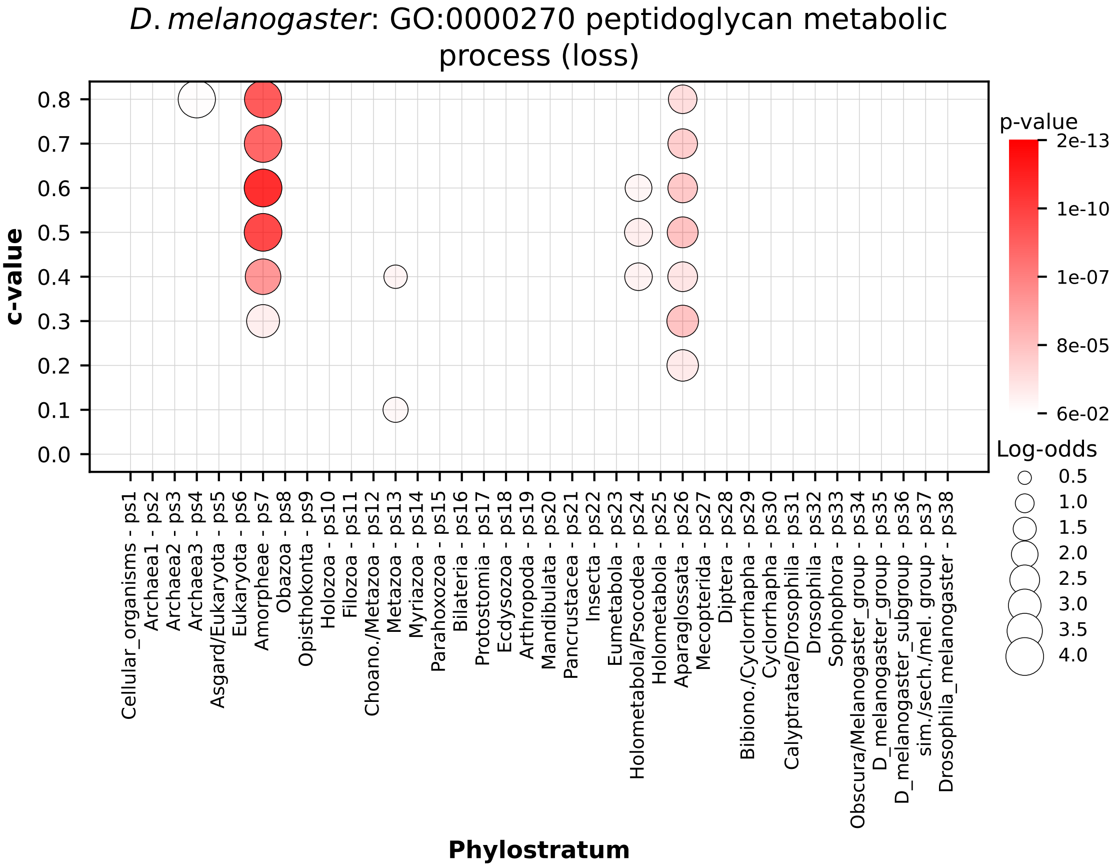

D. melanogaster: GO:0000270 peptidoglycan metabolic process (loss)
c-value
0.0
0.1
0.2
0.3
0.4
0.5
0.6
0.7
0.8
Phylostratum
Cellular_organisms - ps1
Archaea1 - ps2
Archaea2 - ps3
Archaea3 - ps4
Asgard/Eukaryota - ps5
Eukaryota - ps6
Amorpheae - ps7
Obazoa - ps8
Opisthokonta - ps9
Holozoa - ps10
Filozoa - ps11
Choano./Metazoa - ps12
Metazoa - ps13
Myriazoa - ps14
Parahoxozoa - ps15
Bilateria - ps16
Protostomia - ps17
Ecdysozoa - ps18
Arthropoda - ps19
Mandibulata - ps20
Pancrustacea - ps21
Insecta - ps22
Eumetabola - ps23
Holometabola/Psocodea - ps24
Holometabola - ps25
Aparaglossata - ps26
Mecopterida - ps27
Diptera - ps28
Bibiono./Cyclorrhapha - ps29
Cyclorrhapha - ps30
Calyptratae/Drosophila - ps31
Drosophila - ps32
Sophophora - ps33
Obscura/Melanogaster_group - ps34
D_melanogaster_group - ps35
D_melanogaster_subgroup - ps36
sim./sech./mel. group - ps37
Drosophila_melanogaster - ps38
p-value
2e-13
1e-10
1e-07
8e-05
6e-02
Log-odds
0.5
1.0
1.5
2.0
2.5
3.0
3.5
4.0

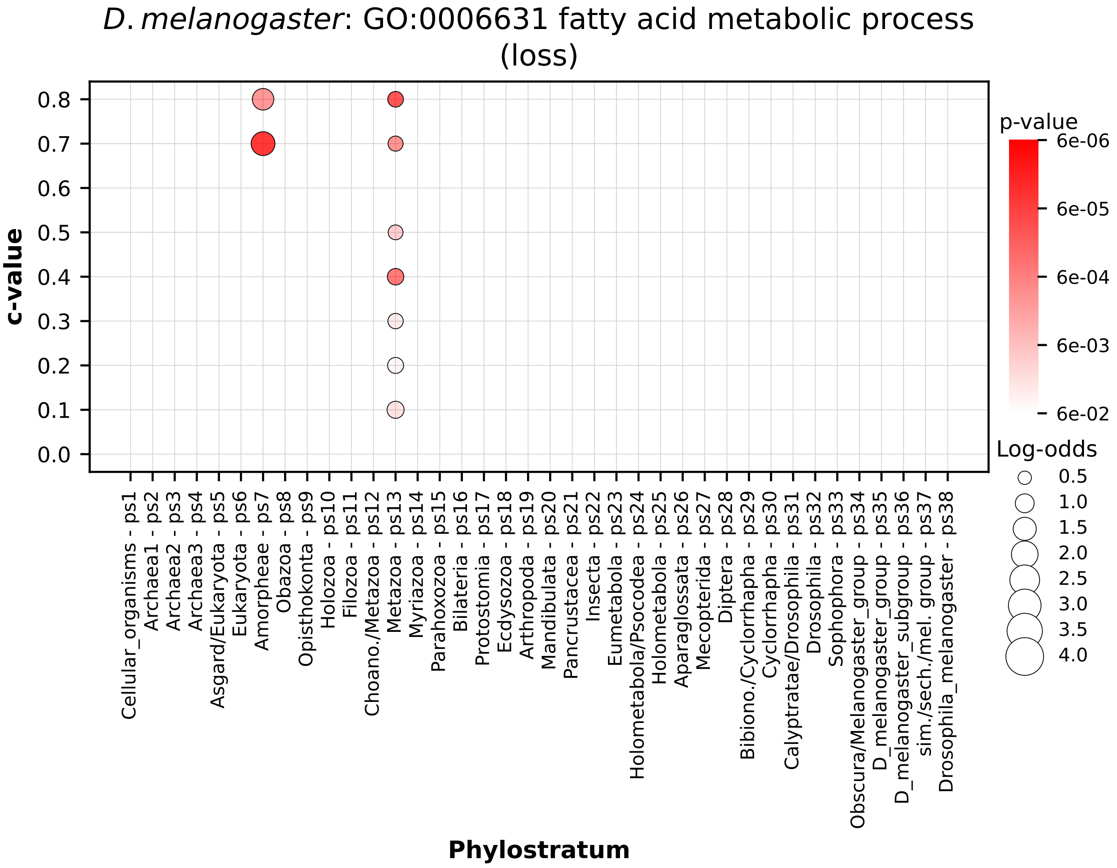

D. melanogaster: GO:0006631 fatty acid metabolic process (loss)
c-value
0.0
0.1
0.2
0.3
0.4
0.5
0.6
0.7
0.8
Phylostratum
Cellular_organisms - ps1
Archaea1 - ps2
Archaea2 - ps3
Archaea3 - ps4
Asgard/Eukaryota - ps5
Eukaryota - ps6
Amorpheae - ps7
Obazoa - ps8
Opisthokonta - ps9
Holozoa - ps10
Filozoa - ps11
Choano./Metazoa - ps12
Metazoa - ps13
Myriazoa - ps14
Parahoxozoa - ps15
Bilateria - ps16
Protostomia - ps17
Ecdysozoa - ps18
Arthropoda - ps19
Mandibulata - ps20
Pancrustacea - ps21
Insecta - ps22
Eumetabola - ps23
Holometabola/Psocodea - ps24
Holometabola - ps25
Aparaglossata - ps26
Mecopterida - ps27
Diptera - ps28
Bibiono./Cyclorrhapha - ps29
Cyclorrhapha - ps30
Calyptratae/Drosophila - ps31
Drosophila - ps32
Sophophora - ps33
Obscura/Melanogaster_group - ps34
D_melanogaster_group - ps35
D_melanogaster_subgroup - ps36
sim./sech./mel. group - ps37
Drosophila_melanogaster - ps38
p-value
6e-06
6e-05
6e-04
6e-03
6e-02
Log-odds
0.5
1.0
1.5
2.0
2.5
3.0
3.5
4.0

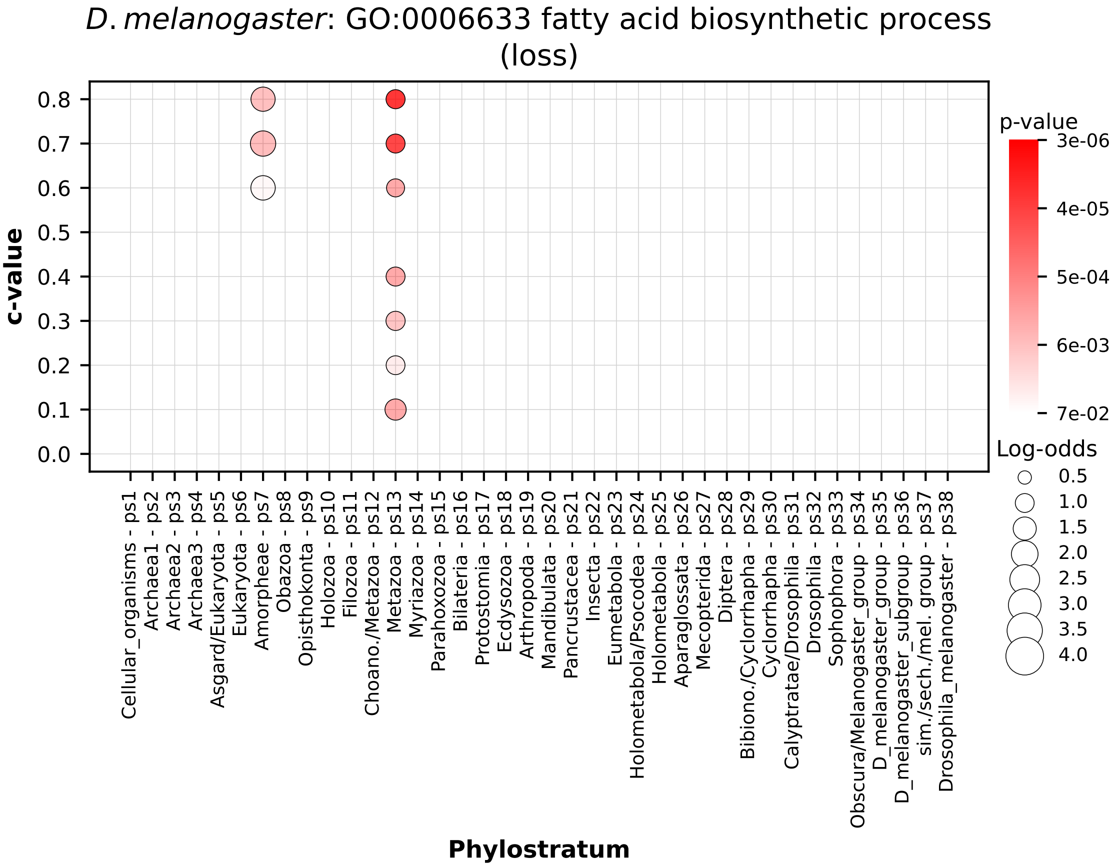

D. melanogaster: GO:0006633 fatty acid biosynthetic process (loss)
c-value
0.0
0.1
0.2
0.3
0.4
0.5
0.6
0.7
0.8
Phylostratum
Cellular_organisms - ps1
Archaea1 - ps2
Archaea2 - ps3
Archaea3 - ps4
Asgard/Eukaryota - ps5
Eukaryota - ps6
Amorpheae - ps7
Obazoa - ps8
Opisthokonta - ps9
Holozoa - ps10
Filozoa - ps11
Choano./Metazoa - ps12
Metazoa - ps13
Myriazoa - ps14
Parahoxozoa - ps15
Bilateria - ps16
Protostomia - ps17
Ecdysozoa - ps18
Arthropoda - ps19
Mandibulata - ps20
Pancrustacea - ps21
Insecta - ps22
Eumetabola - ps23
Holometabola/Psocodea - ps24
Holometabola - ps25
Aparaglossata - ps26
Mecopterida - ps27
Diptera - ps28
Bibiono./Cyclorrhapha - ps29
Cyclorrhapha - ps30
Calyptratae/Drosophila - ps31
Drosophila - ps32
Sophophora - ps33
Obscura/Melanogaster_group - ps34
D_melanogaster_group - ps35
D_melanogaster_subgroup - ps36
sim./sech./mel. group - ps37
Drosophila_melanogaster - ps38
p-value
3e-06
4e-05
5e-04
6e-03
7e-02
Log-odds
0.5
1.0
1.5
2.0
2.5
3.0
3.5
4.0

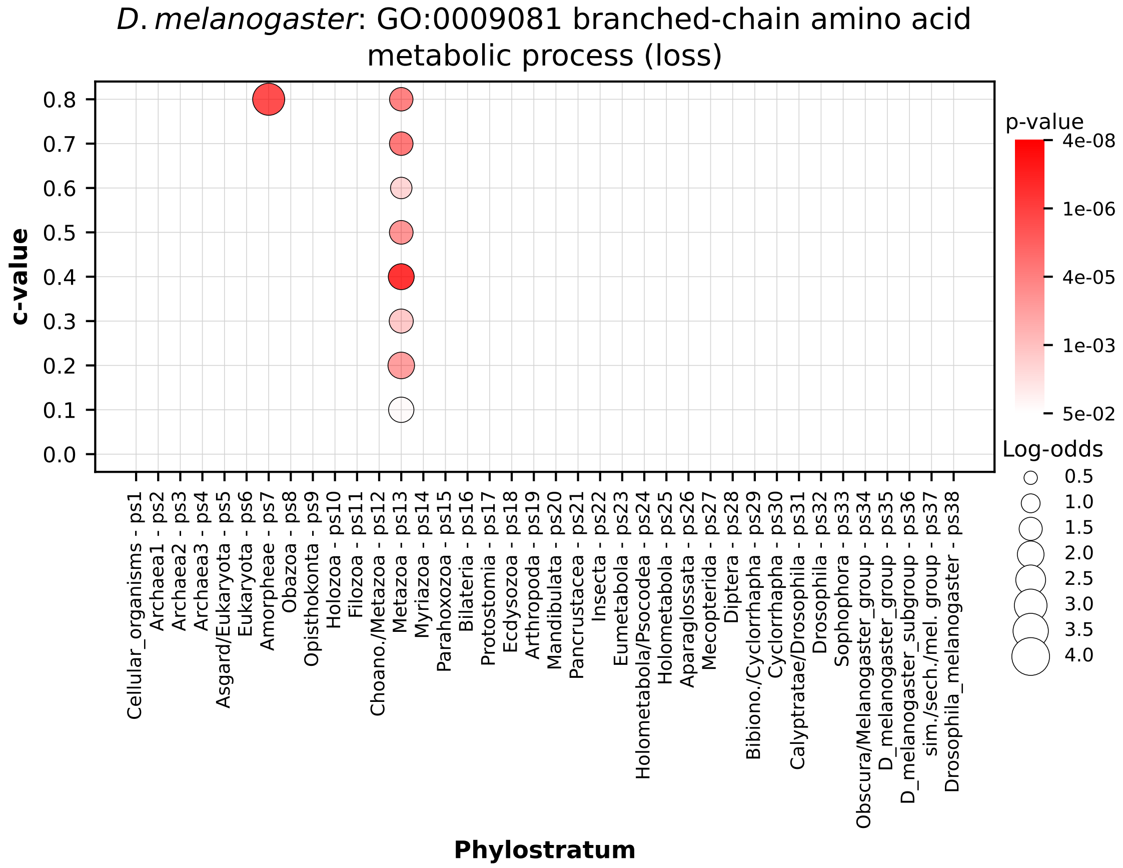

D. melanogaster: GO:0009081 branched-chain amino acid metabolic process (loss)
c-value
0.8
0.7
0.6
0.5
0.4
0.3
0.2
0.1
0.0
p-value
4e-08
1e-06
4e-05
1e-03
5e-02
Log-odds
0.5
1.0
1.5
2.0
2.5
3.0
3.5
4.0
Cellular_organisms - ps1
Archaea1 - ps2
Archaea2 - ps3
Archaea3 - ps4
Asgard/Eukaryota - ps5
Eukaryota - ps6
Amorpheae - ps7
Obazoa - ps8
Opisthokonta - ps9
Holozoa - ps10
Filozoa - ps11
Choano./Metazoa - ps12
Metazoa - ps13
Myriazoa - ps14
Parahoxozoa - ps15
Bilateria - ps16
Protostomia - ps17
Ecdysozoa - ps18
Arthropoda - ps19
Mandibulata - ps20
Pancrustacea - ps21
Insecta - ps22
Eumetabola - ps23
Holometabola/Psocodea - ps24
Holometabola - ps25
Aparaglossata - ps26
Mecopterida - ps27
Diptera - ps28
Bibiono./Cyclorrhapha - ps29
Cyclorrhapha - ps30
Calyptratae/Drosophila - ps31
Drosophila - ps32
Sophophora - ps33
Obscura/Melanogaster_group - ps34
D_melanogaster_group - ps35
D_melanogaster_subgroup - ps36
sim./sech./mel. group - ps37
Drosophila_melanogaster - ps38
Phylostratum

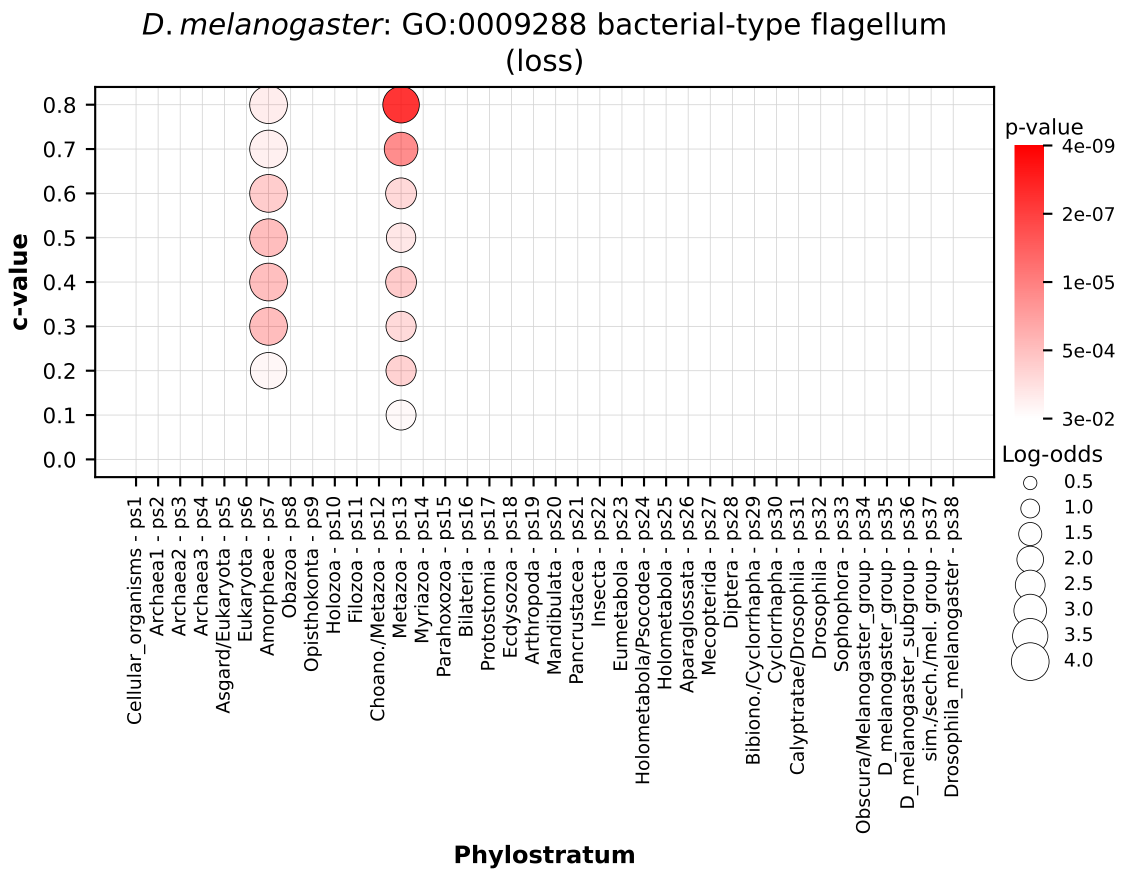

D. melanogaster: GO:0009288 bacterial-type flagellum
(loss)
c-value
0.8
0.7
0.6
0.5
0.4
0.3
0.2
0.1
0.0
p-value
4e-09
2e-07
1e-05
5e-04
3e-02
Log-odds
0.5
1.0
1.5
2.0
2.5
3.0
3.5
4.0
Cellular_organisms - ps1
Archaea1 - ps2
Archaea2 - ps3
Archaea3 - ps4
Asgard/Eukaryota - ps5
Eukaryota - ps6
Amorpheae - ps7
Obazoa - ps8
Opisthokonta - ps9
Holozoa - ps10
Filozoa - ps11
Choano./Metazoa - ps12
Metazoa - ps13
Myriazoa - ps14
Parahoxozoa - ps15
Bilateria - ps16
Protostomia - ps17
Ecdysozoa - ps18
Arthropoda - ps19
Mandibulata - ps20
Pancrustacea - ps21
Insecta - ps22
Eumetabola - ps23
Holometabola/Psocodea - ps24
Holometabola - ps25
Aparaglossata - ps26
Mecopterida - ps27
Diptera - ps28
Bibiono./Cyclorrhapha - ps29
Cyclorrhapha - ps30
Calyptratae/Drosophila - ps31
Drosophila - ps32
Sophophora - ps33
Obscura/Melanogaster_group - ps34
D_melanogaster_group - ps35
D_melanogaster_subgroup - ps36
sim./sech./mel. group - ps37
Drosophila_melanogaster - ps38
Phylostratum

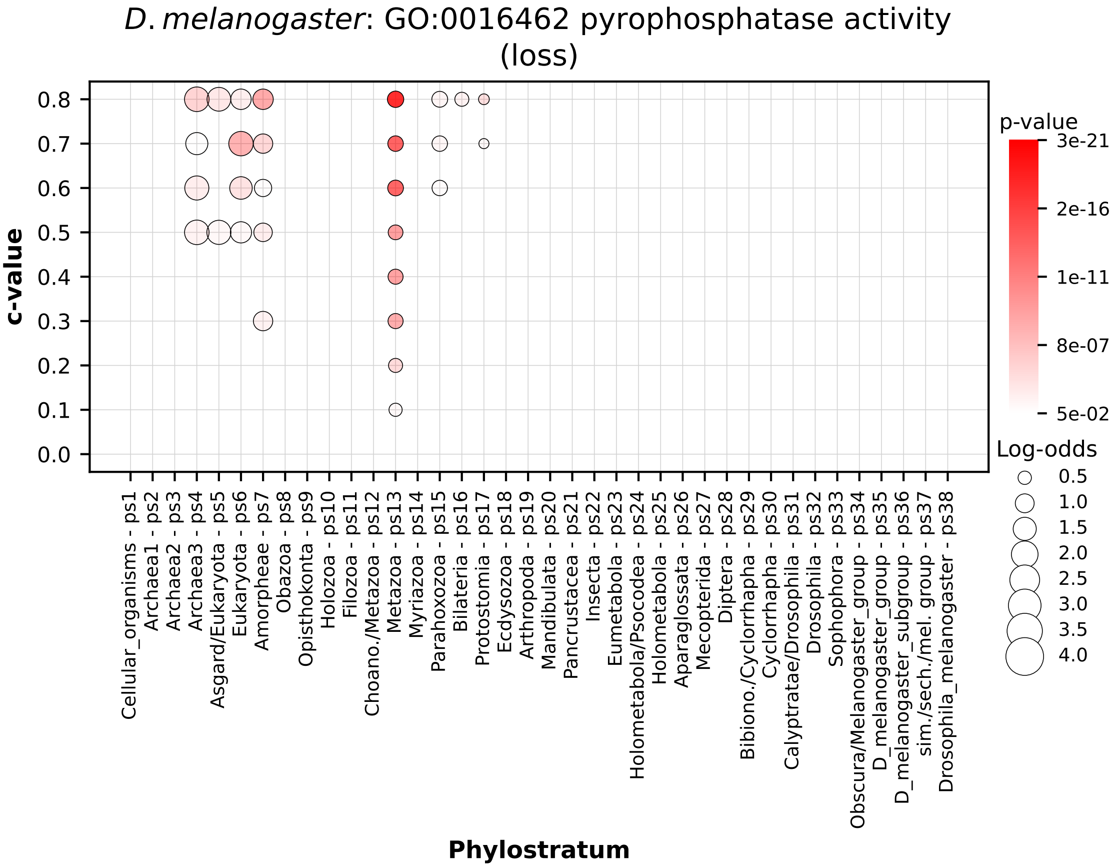
D. melanogaster: GO:0016462 pyrophosphatase activity (loss)
c-value
0.0
0.1
0.2
0.3
0.4
0.5
0.6
0.7
0.8
Phylostratum
Cellular_organisms - ps1
Archaea1 - ps2
Archaea2 - ps3
Archaea3 - ps4
Asgard/Eukaryota - ps5
Eukaryota - ps6
Amorpheae - ps7
Obazoa - ps8
Opisthokonta - ps9
Holozoa - ps10
Filozoa - ps11
Choano./Metazoa - ps12
Metazoa - ps13
Myriazoa - ps14
Parahoxozoa - ps15
Bilateria - ps16
Protostomia - ps17
Ecdysozoa - ps18
Arthropoda - ps19
Mandibulata - ps20
Pancrustacea - ps21
Insecta - ps22
Eumetabola - ps23
Holometabola/Psocodea - ps24
Holometabola - ps25
Aparaglossata - ps26
Mecopterida - ps27
Diptera - ps28
Bibiono./Cyclorrhapha - ps29
Cyclorrhapha - ps30
Calyptratae/Drosophila - ps31
Drosophila - ps32
Sophophora - ps33
Obscura/Melanogaster_group - ps34
D_melanogaster_group - ps35
D_melanogaster_subgroup - ps36
sim./sech./mel. group - ps37
Drosophila_melanogaster - ps38
p-value
3e-21
2e-16
1e-11
8e-07
5e-02
Log-odds
0.5
1.0
1.5
2.0
2.5
3.0
3.5
4.0

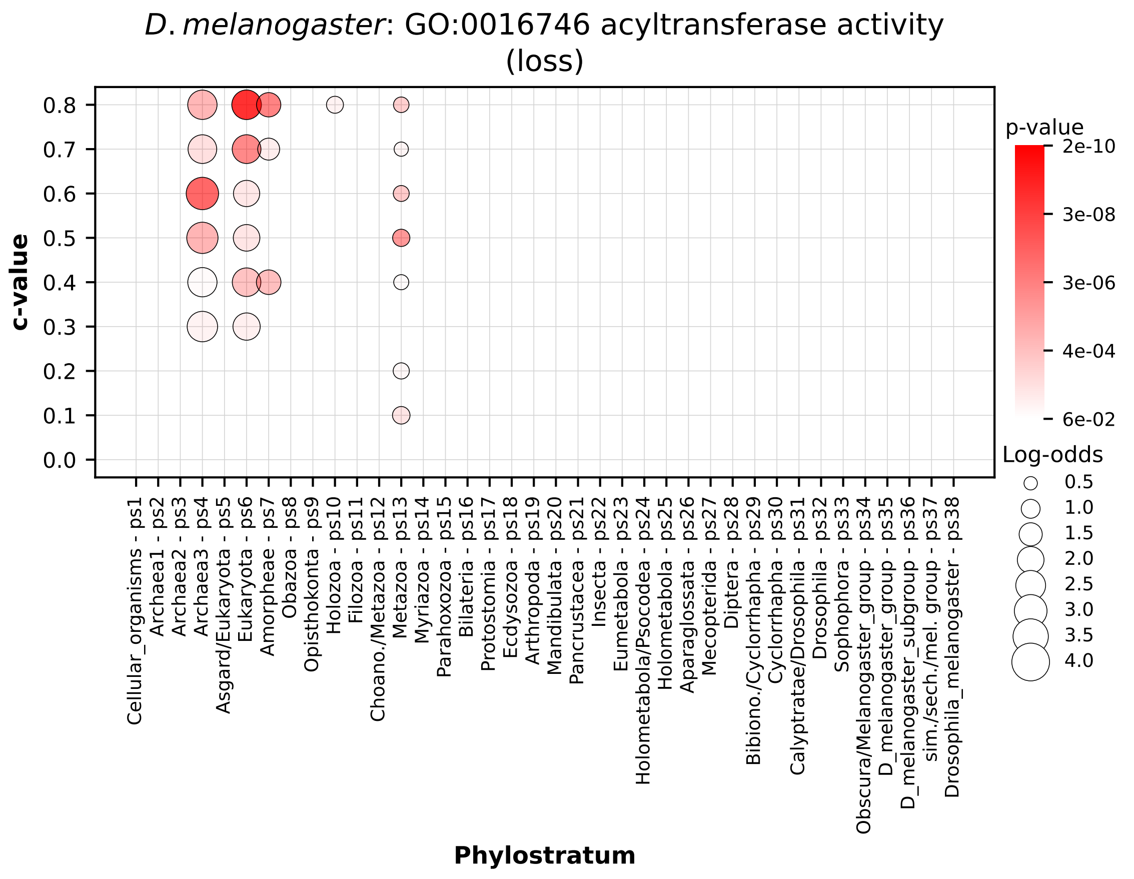

D. melanogaster: GO:0016746 acyltransferase activity
(loss)
c-value
0.8
0.7
0.6
0.5
0.4
0.3
0.2
0.1
0.0
Phylostratum
Cellular_organisms - ps1
Archaea1 - ps2
Archaea2 - ps3
Archaea3 - ps4
Asgard/Eukaryota - ps5
Eukaryota - ps6
Amorpheae - ps7
Obazoa - ps8
Opisthokonta - ps9
Holozoa - ps10
Filozoa - ps11
Choano./Metazoa - ps12
Metazoa - ps13
Myriazoa - ps14
Parahoxozoa - ps15
Bilateria - ps16
Protostomia - ps17
Ecdysozoa - ps18
Arthropoda - ps19
Mandibulata - ps20
Pancrustacea - ps21
Insecta - ps22
Eumetabola - ps23
Holometabola/Psocodea - ps24
Holometabola - ps25
Aparaglossata - ps26
Mecopterida - ps27
Diptera - ps28
Bibiono./Cyclorrhapha - ps29
Cyclorrhapha - ps30
Calyptratae/Drosophila - ps31
Drosophila - ps32
Sophophora - ps33
Obscura/Melanogaster_group - ps34
D_melanogaster_group - ps35
D_melanogaster_subgroup - ps36
sim./sech./mel. group - ps37
Drosophila_melanogaster - ps38
p-value
2e-10
3e-08
3e-06
4e-04
6e-02
Log-odds
0.5
1.0
1.5
2.0
2.5
3.0
3.5
4.0

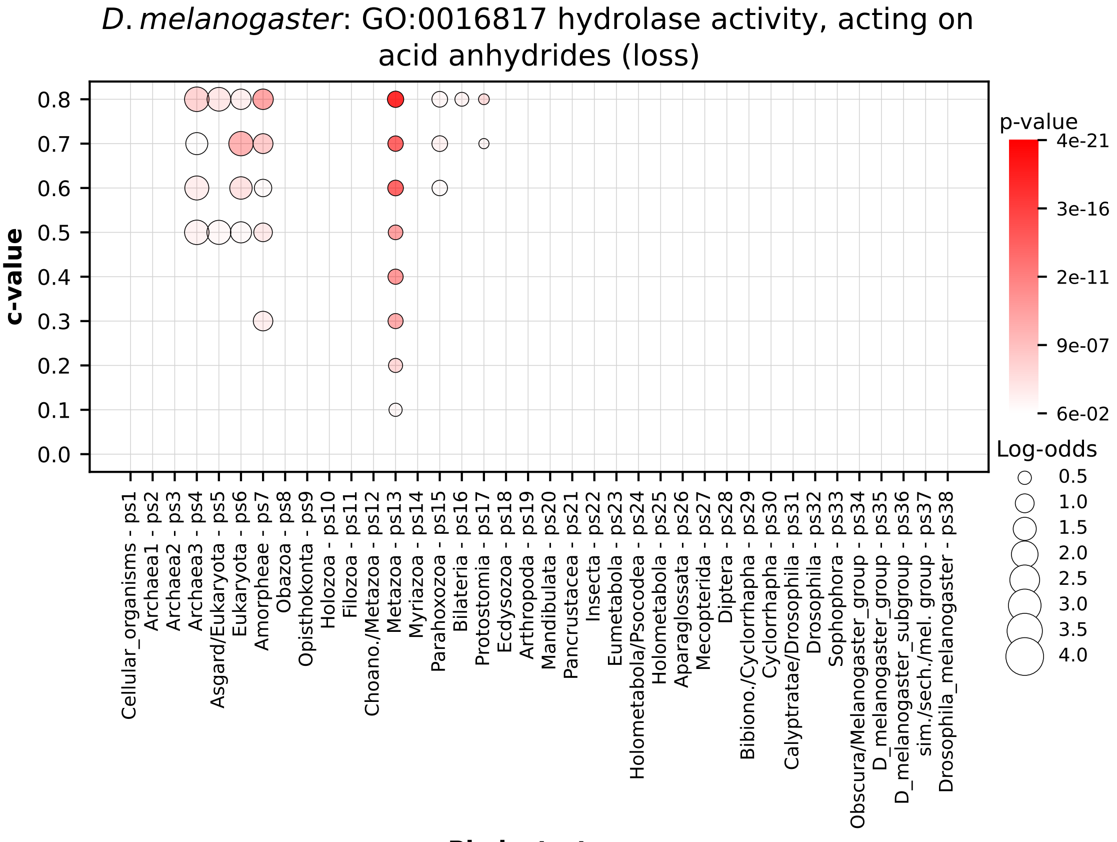

D. melanogaster: GO:0016817 hydrolase activity, acting on acid anhydrides (loss)
c-value
0.0
0.1
0.2
0.3
0.4
0.5
0.6
0.7
0.8
p-value
4e-21
3e-16
2e-11
9e-07
6e-02
Log-odds
0.5
1.0
1.5
2.0
2.5
3.0
3.5
4.0
Cellular_organisms - ps1
Archaea1 - ps2
Archaea2 - ps3
Archaea3 - ps4
Asgard/Eukaryota - ps5
Eukaryota - ps6
Amorpheae - ps7
Obazoa - ps8
Opisthokonta - ps9
Holozoa - ps10
Filozoa - ps11
Choano./Metazoa - ps12
Metazoa - ps13
Myriazoa - ps14
Parahoxozoa - ps15
Bilateria - ps16
Protostomia - ps17
Ecdysozoa - ps18
Arthropoda - ps19
Mandibulata - ps20
Pancrustacea - ps21
Insecta - ps22
Eumetabola - ps23
Holometabola/Psocodea - ps24
Holometabola - ps25
Aparaglossata - ps26
Mecopterida - ps27
Diptera - ps28
Bibiono./Cyclorrhapha - ps29
Cyclorrhapha - ps30
Calyptratae/Drosophila - ps31
Drosophila - ps32
Sophophora - ps33
Obscura/Melanogaster_group - ps34
D_melanogaster_group - ps35
D_melanogaster_subgroup - ps36
sim./sech./mel. group - ps37
Drosophila_melanogaster - ps38
Phylostratum

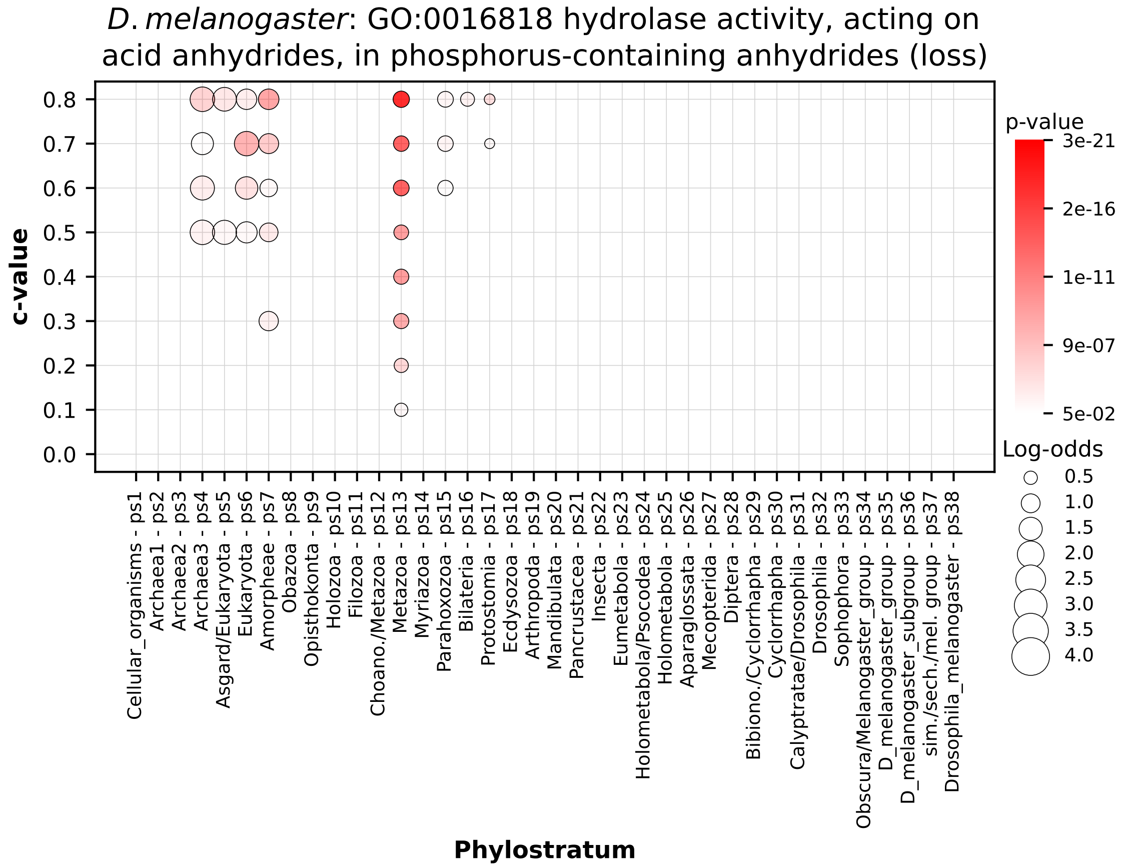

D. melanogaster: GO:0016818 hydrolase activity, acting on acid anhydrides, in phosphorus-containing anhydrides (loss)
c-value
0.8
0.7
0.6
0.5
0.4
0.3
0.2
0.1
0.0
p-value
3e-21
2e-16
1e-11
9e-07
5e-02
Log-odds
0.5
1.0
1.5
2.0
2.5
3.0
3.5
4.0
Cellular_organisms - ps1
Archaea1 - ps2
Archaea2 - ps3
Archaea3 - ps4
Asgard/Eukaryota - ps5
Eukaryota - ps6
Amorpheae - ps7
Obazoa - ps8
Opisthokonta - ps9
Holozoa - ps10
Filozoa - ps11
Choano./Metazoa - ps12
Metazoa - ps13
Myriazoa - ps14
Parahoxozoa - ps15
Bilateria - ps16
Protostomia - ps17
Ecdysozoa - ps18
Arthropoda - ps19
Mandibulata - ps20
Pancrustacea - ps21
Insecta - ps22
Eumetabola - ps23
Holometabola/Psocodea - ps24
Holometabola - ps25
Aparaglossata - ps26
Mecopterida - ps27
Diptera - ps28
Bibiono./Cyclorrhapha - ps29
Cyclorrhapha - ps30
Calyptratae/Drosophila - ps31
Drosophila - ps32
Sophophora - ps33
Obscura/Melanogaster_group - ps34
D_melanogaster_group - ps35
D_melanogaster_subgroup - ps36
sim./sech./mel. group - ps37
Drosophila_melanogaster - ps38
Phylostratum

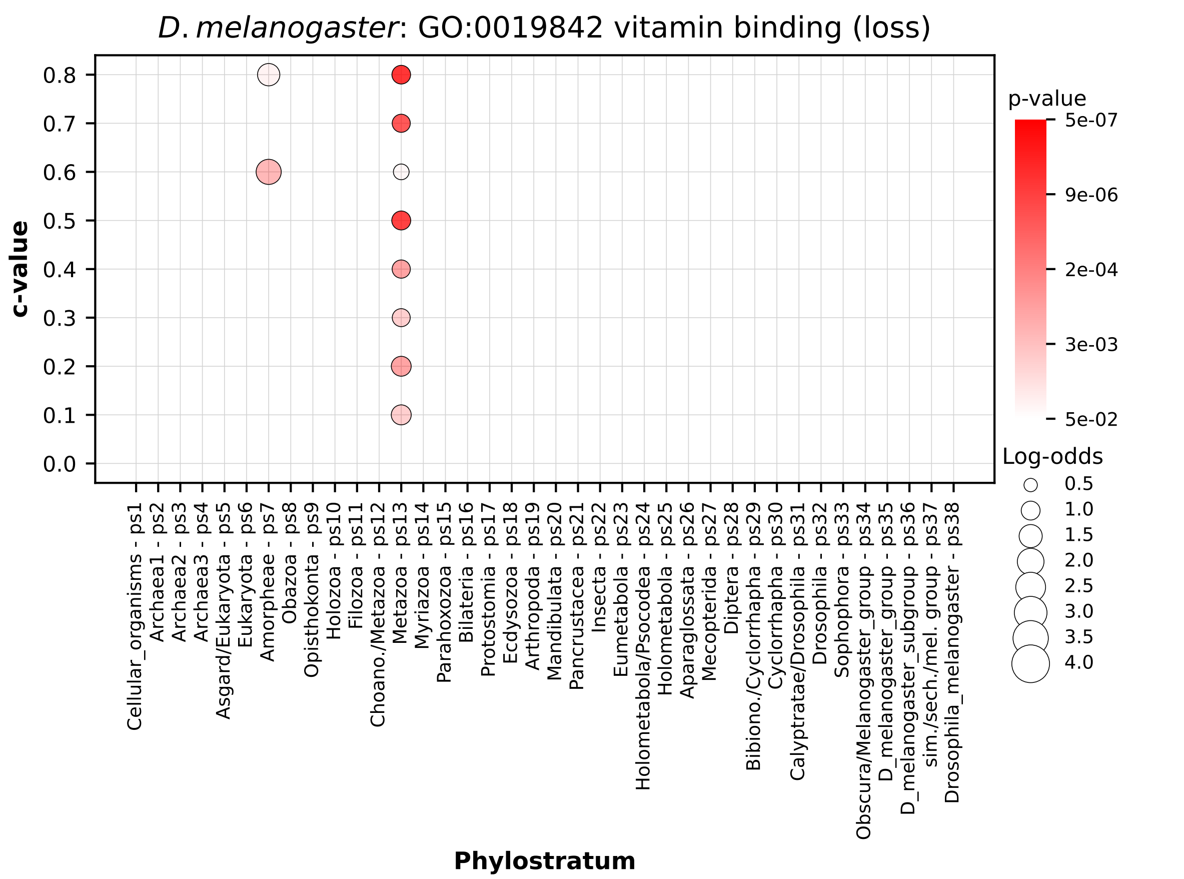

D. melanogaster: GO:0019842 vitamin binding (loss)
c-value
0.0
0.1
0.2
0.3
0.4
0.5
0.6
0.7
0.8
Phylostratum
Cellular_organisms - ps1
Archaea1 - ps2
Archaea2 - ps3
Archaea3 - ps4
Asgard/Eukaryota - ps5
Eukaryota - ps6
Amorpheae - ps7
Obazoa - ps8
Opisthokonta - ps9
Holozoa - ps10
Filozoa - ps11
Choano./Metazoa - ps12
Metazoa - ps13
Myriazoa - ps14
Parahoxozoa - ps15
Bilateria - ps16
Protostomia - ps17
Ecdysozoa - ps18
Arthropoda - ps19
Mandibulata - ps20
Pancrustacea - ps21
Insecta - ps22
Eumetabola - ps23
Holometabola/Psocodea - ps24
Holometabola - ps25
Aparaglossata - ps26
Mecopterida - ps27
Diptera - ps28
Bibiono./Cyclorrhapha - ps29
Cyclorrhapha - ps30
Calyptratae/Drosophila - ps31
Drosophila - ps32
Sophophora - ps33
Obscura/Melanogaster_group - ps34
D_melanogaster_group - ps35
D_melanogaster_subgroup - ps36
sim./sech./mel. group - ps37
Drosophila_melanogaster - ps38
p-value
5e-07
9e-06
2e-04
3e-03
5e-02
Log-odds
0.5
1.0
1.5
2.0
2.5
3.0
3.5
4.0

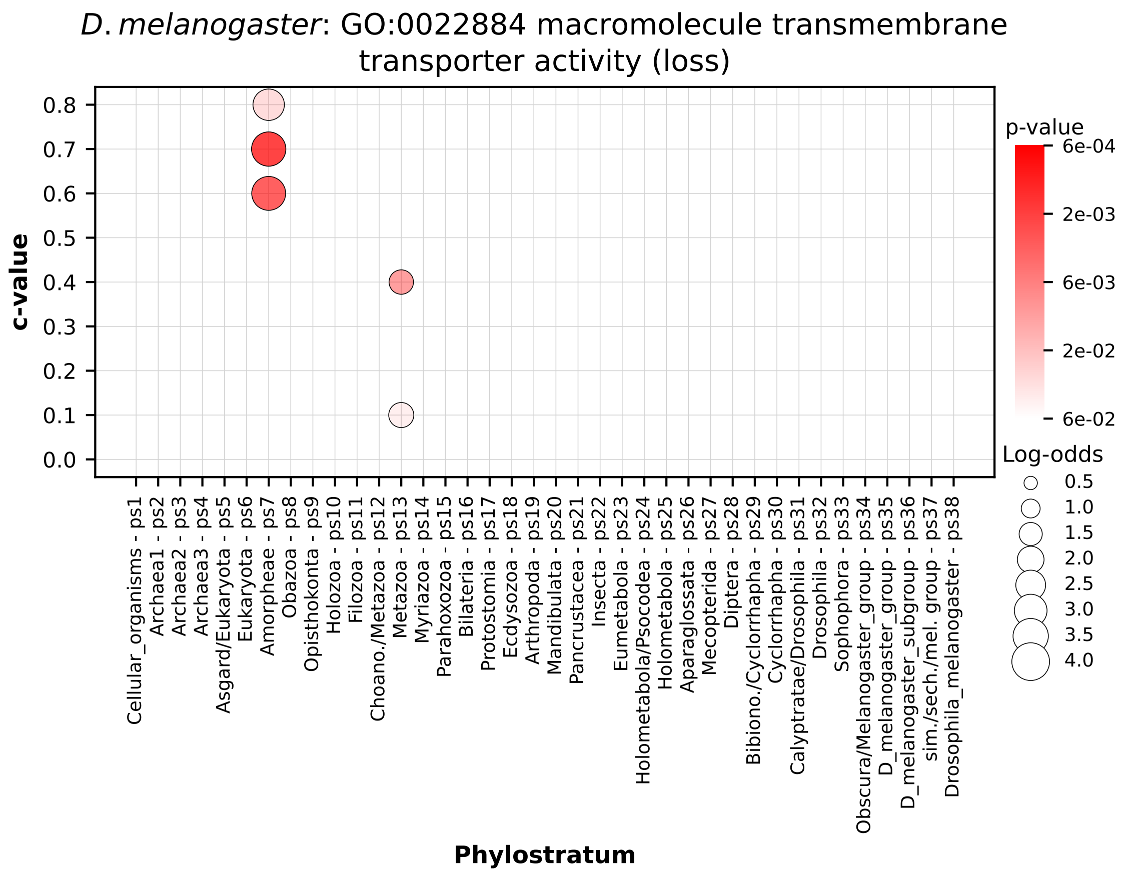
D. melanogaster: GO:0022884 macromolecule transmembrane transporter activity (loss)
c-value
0.0
0.1
0.2
0.3
0.4
0.5
0.6
0.7
0.8
p-value
6e-04
2e-03
6e-03
2e-02
6e-02
Log-odds
0.5
1.0
1.5
2.0
2.5
3.0
3.5
4.0
Cellular_organisms - ps1
Archaea1 - ps2
Archaea2 - ps3
Archaea3 - ps4
Asgard/Eukaryota - ps5
Eukaryota - ps6
Amorpheae - ps7
Obazoa - ps8
Opisthokonta - ps9
Holozoa - ps10
Filozoa - ps11
Choano./Metazoa - ps12
Metazoa - ps13
Myriazoa - ps14
Parahoxozoa - ps15
Bilateria - ps16
Protostomia - ps17
Ecdysozoa - ps18
Arthropoda - ps19
Mandibulata - ps20
Pancrustacea - ps21
Insecta - ps22
Eumetabola - ps23
Holometabola/Psocodea - ps24
Holometabola - ps25
Aparaglossata - ps26
Mecopterida - ps27
Diptera - ps28
Bibiono./Cyclorrhapha - ps29
Cyclorrhapha - ps30
Calyptratae/Drosophila - ps31
Drosophila - ps32
Sophophora - ps33
Obscura/Melanogaster_group - ps34
D_melanogaster_group - ps35
D_melanogaster_subgroup - ps36
sim./sech./mel. group - ps37
Drosophila_melanogaster - ps38
Phylostratum

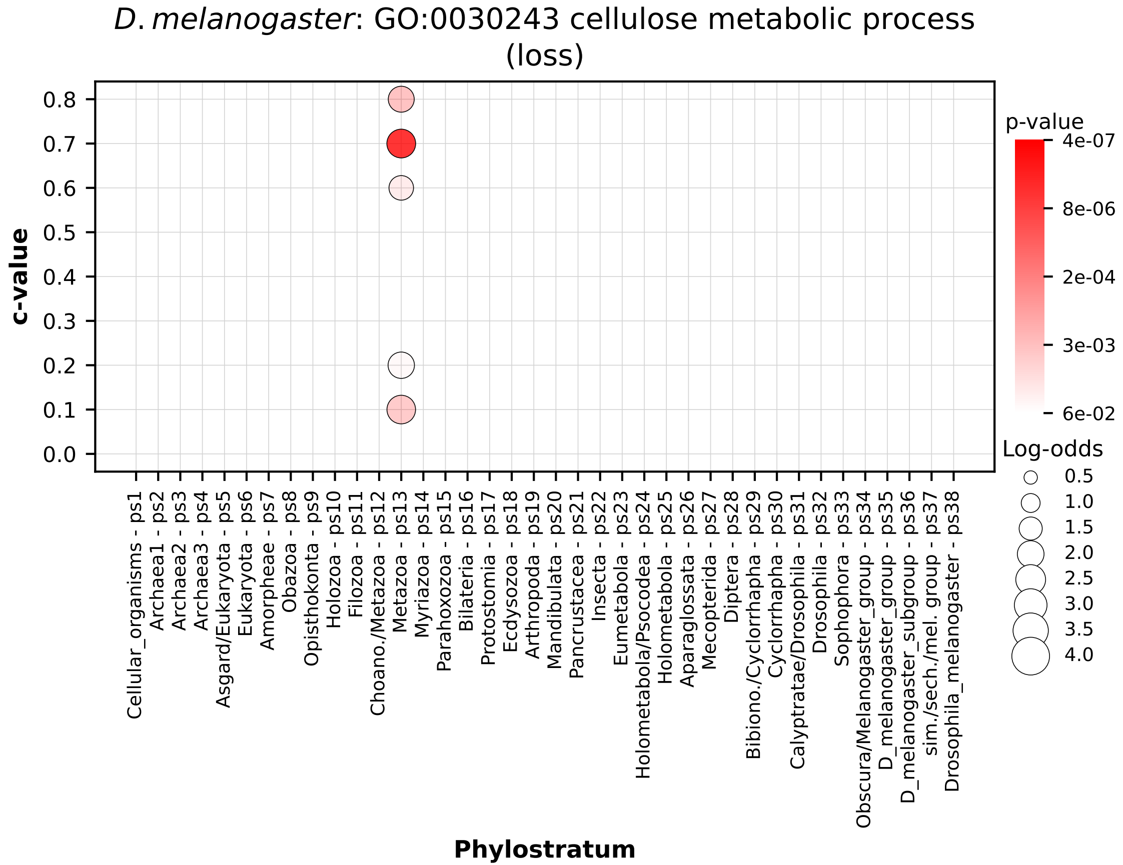

D. melanogaster: GO:0030243 cellulose metabolic process (loss)
c-value
0.8
0.7
0.6
0.5
0.4
0.3
0.2
0.1
0.0
p-value
4e-07
8e-06
2e-04
3e-03
6e-02
Log-odds
0.5
1.0
1.5
2.0
2.5
3.0
3.5
4.0
Cellular_organisms - ps1
Archaea1 - ps2
Archaea2 - ps3
Archaea3 - ps4
Asgard/Eukaryota - ps5
Eukaryota - ps6
Amorpheae - ps7
Obazoa - ps8
Opisthokonta - ps9
Holozoa - ps10
Filozoa - ps11
Choano./Metazoa - ps12
Metazoa - ps13
Myriazoa - ps14
Parahoxozoa - ps15
Bilateria - ps16
Protostomia - ps17
Ecdysozoa - ps18
Arthropoda - ps19
Mandibulata - ps20
Pancrustacea - ps21
Insecta - ps22
Eumetabola - ps23
Holometabola/Psocodea - ps24
Holometabola - ps25
Aparaglossata - ps26
Mecopterida - ps27
Diptera - ps28
Bibiono./Cyclorrhapha - ps29
Cyclorrhapha - ps30
Calyptratae/Drosophila - ps31
Drosophila - ps32
Sophophora - ps33
Obscura/Melanogaster_group - ps34
D_melanogaster_group - ps35
D_melanogaster_subgroup - ps36
sim./sech./mel. group - ps37
Drosophila_melanogaster - ps38
Phylostratum

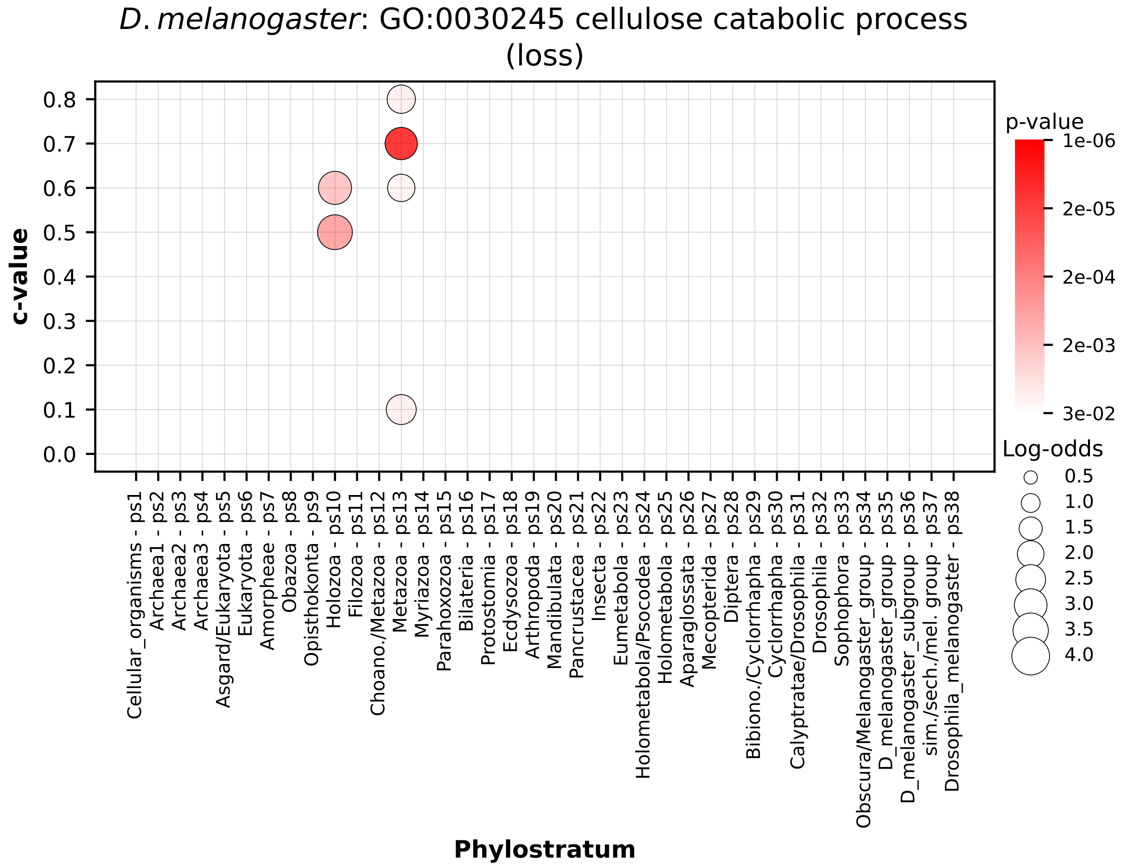
D. melanogaster: GO:0030245 cellulose catabolic process
(loss)
c-value
0.8
0.7
0.6
0.5
0.4
0.3
0.2
0.1
0.0
Phylostratum
Cellular_organisms - ps1
Archaea1 - ps2
Archaea2 - ps3
Archaea3 - ps4
Asgard/Eukaryota - ps5
Eukaryota - ps6
Amorpheae - ps7
Obazoa - ps8
Opisthokonta - ps9
Holozoa - ps10
Filozoa - ps11
Choano./Metazoa - ps12
Metazoa - ps13
Myriazoa - ps14
Parahoxozoa - ps15
Bilateria - ps16
Protostomia - ps17
Ecdysozoa - ps18
Arthropoda - ps19
Mandibulata - ps20
Pancrustacea - ps21
Insecta - ps22
Eumetabola - ps23
Holometabola/Psocodea - ps24
Holometabola - ps25
Aparaglossata - ps26
Mecopterida - ps27
Diptera - ps28
Bibiono./Cyclorrhapha - ps29
Cyclorrhapha - ps30
Calyptratae/Drosophila - ps31
Drosophila - ps32
Sophophora - ps33
Obscura/Melanogaster_group - ps34
D_melanogaster_group - ps35
D_melanogaster_subgroup - ps36
sim./sech./mel. group - ps37
Drosophila_melanogaster - ps38
p-value
1e-06
2e-05
2e-04
2e-03
3e-02
Log-odds
0.5
1.0
1.5
2.0
2.5
3.0
3.5
4.0

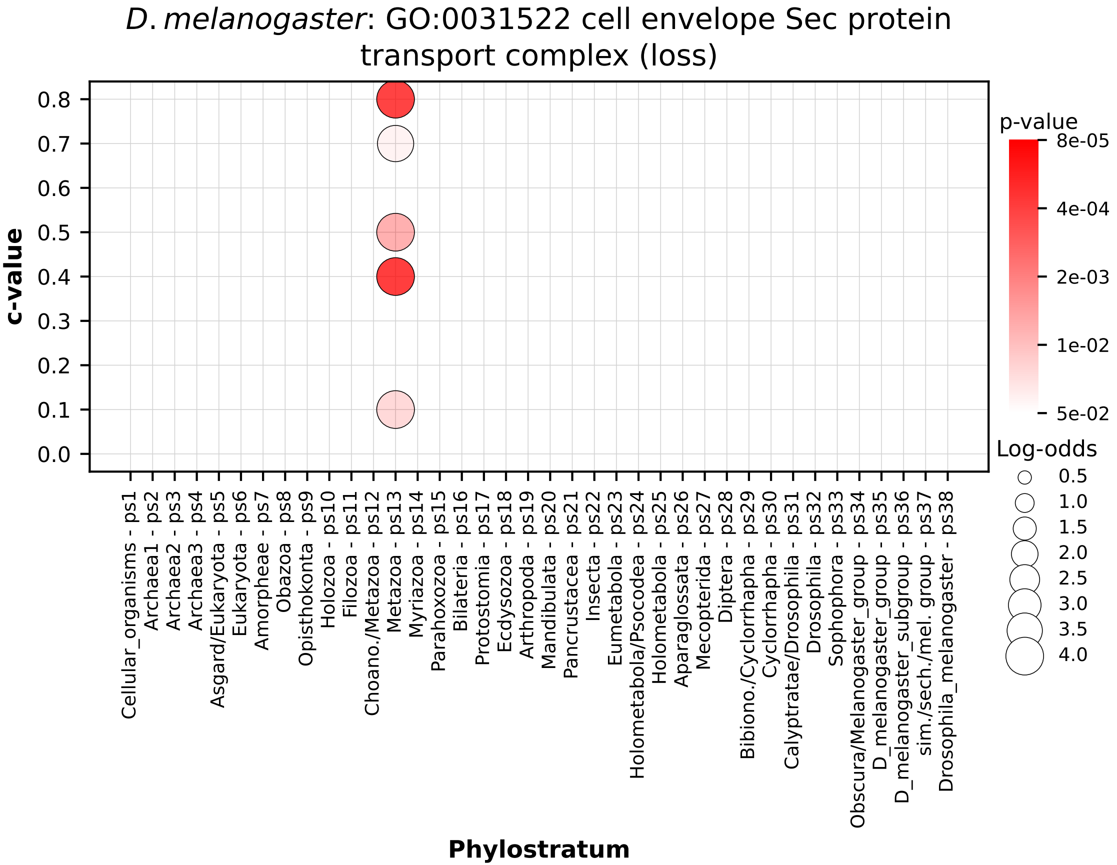

D. melanogaster: GO:0031522 cell envelope Sec protein transport complex (loss)
c-value
0.8
0.7
0.6
0.5
0.4
0.3
0.2
0.1
0.0
Phylostratum
Cellular_organisms - ps1
Archaea1 - ps2
Archaea2 - ps3
Archaea3 - ps4
Asgard/Eukaryota - ps5
Eukaryota - ps6
Amorpheae - ps7
Obazoa - ps8
Opisthokonta - ps9
Holozoa - ps10
Filozoa - ps11
Choano./Metazoa - ps12
Metazoa - ps13
Myriazoa - ps14
Parahoxozoa - ps15
Bilateria - ps16
Protostomia - ps17
Ecdysozoa - ps18
Arthropoda - ps19
Mandibulata - ps20
Pancrustacea - ps21
Insecta - ps22
Eumetabola - ps23
Holometabola/Psocodea - ps24
Holometabola - ps25
Aparaglossata - ps26
Mecopterida - ps27
Diptera - ps28
Bibiono./Cyclorrhapha - ps29
Cyclorrhapha - ps30
Calyptratae/Drosophila - ps31
Drosophila - ps32
Sophophora - ps33
Obscura/Melanogaster_group - ps34
D_melanogaster_group - ps35
D_melanogaster_subgroup - ps36
sim./sech./mel. group - ps37
Drosophila_melanogaster - ps38
p-value
8e-05
4e-04
2e-03
1e-02
5e-02
Log-odds
0.5
1.0
1.5
2.0
2.5
3.0
3.5
4.0

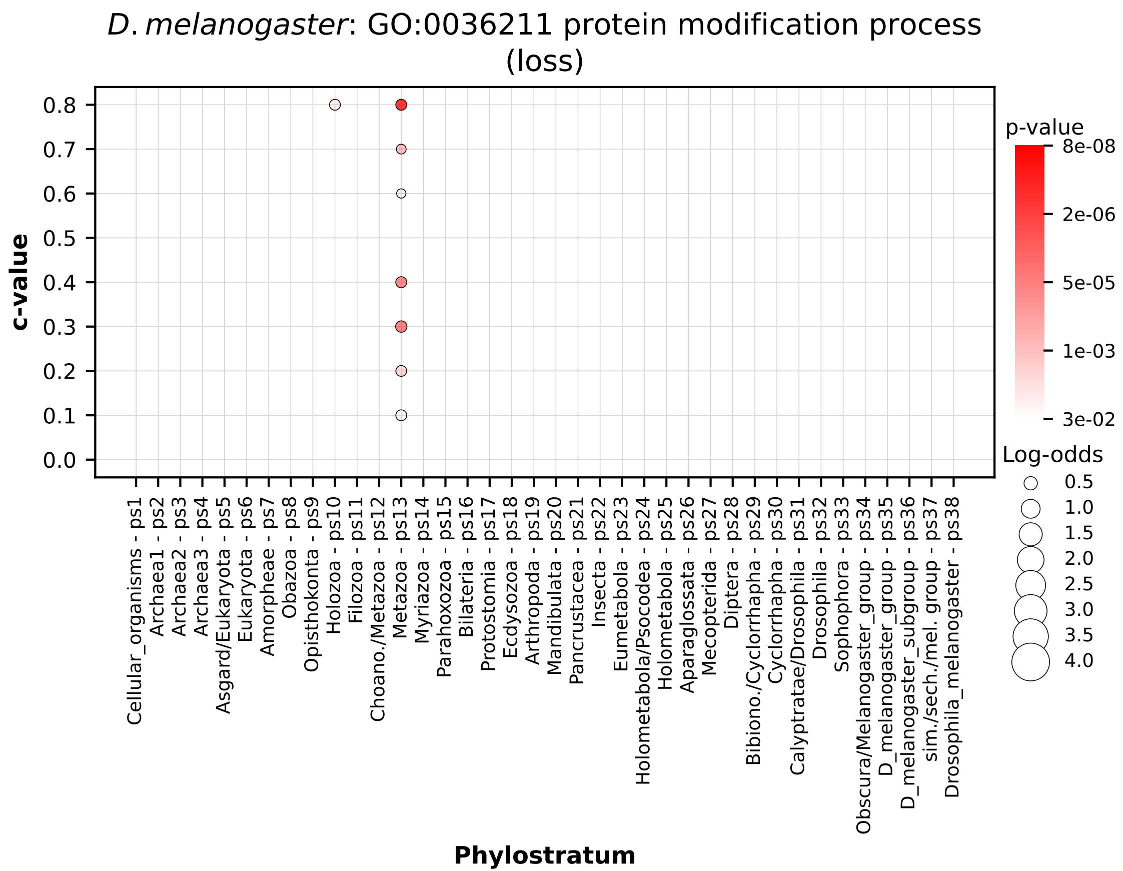

D. melanogaster: GO:0036211 protein modification process
(loss)
c-value
0.8
0.7
0.6
0.5
0.4
0.3
0.2
0.1
0.0
p-value
8e-08
2e-06
5e-05
1e-03
3e-02
Log-odds
0.5
1.0
1.5
2.0
2.5
3.0
3.5
4.0
Cellular_organisms - ps1
Archaea1 - ps2
Archaea2 - ps3
Archaea3 - ps4
Asgard/Eukaryota - ps5
Eukaryota - ps6
Amorpheae - ps7
Obazoa - ps8
Opisthokonta - ps9
Holozoa - ps10
Filozoa - ps11
Choano./Metazoa - ps12
Metazoa - ps13
Myriazoa - ps14
Parahoxozoa - ps15
Bilateria - ps16
Protostomia - ps17
Ecdysozoa - ps18
Arthropoda - ps19
Mandibulata - ps20
Pancrustacea - ps21
Insecta - ps22
Eumetabola - ps23
Holometabola/Psocodea - ps24
Holometabola - ps25
Aparaglossata - ps26
Mecopterida - ps27
Diptera - ps28
Bibiono./Cyclorrhapha - ps29
Cyclorrhapha - ps30
Calyptratae/Drosophila - ps31
Drosophila - ps32
Sophophora - ps33
Obscura/Melanogaster_group - ps34
D_melanogaster_group - ps35
D_melanogaster_subgroup - ps36
sim./sech./mel. group - ps37
Drosophila_melanogaster - ps38
Phylostratum

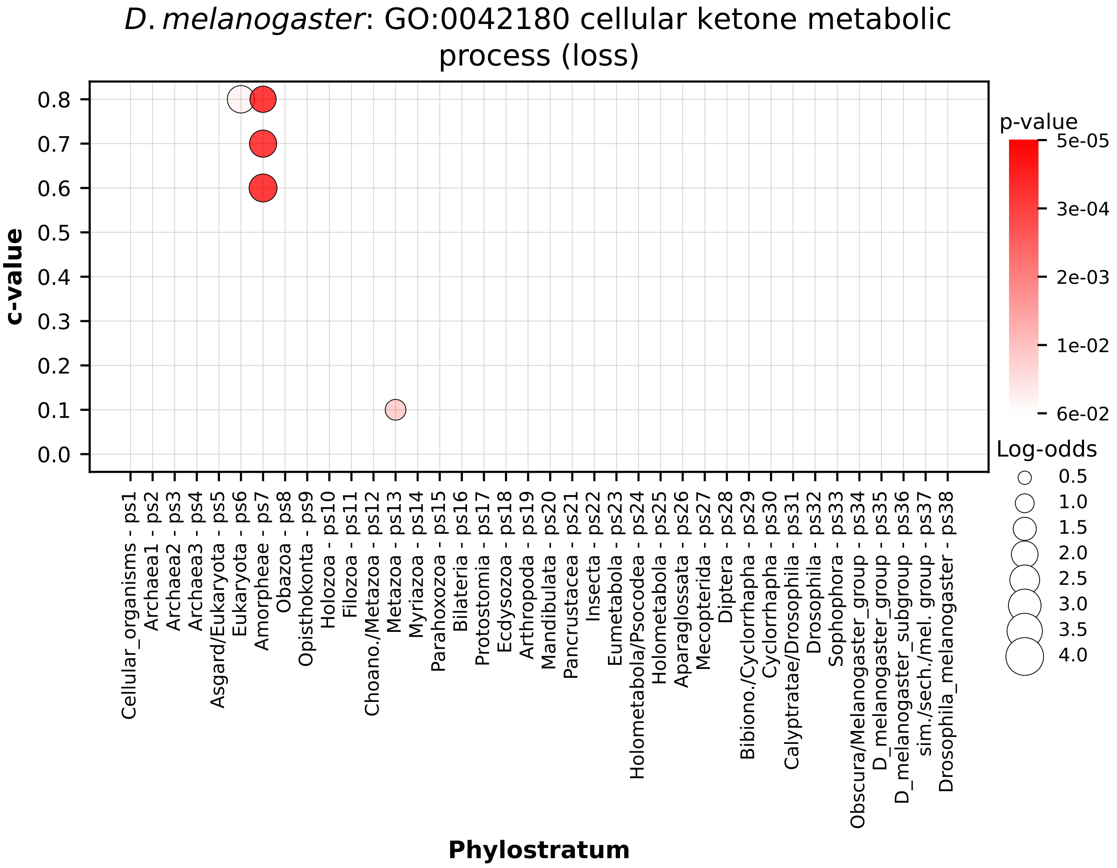

D. melanogaster: GO:0042180 cellular ketone metabolic process (loss)
c-value
0.0
0.1
0.2
0.3
0.4
0.5
0.6
0.7
0.8
p-value
5e-05
3e-04
2e-03
1e-02
6e-02
Log-odds
0.5
1.0
1.5
2.0
2.5
3.0
3.5
4.0
Cellular_organisms - ps1
Archaea1 - ps2
Archaea2 - ps3
Archaea3 - ps4
Asgard/Eukaryota - ps5
Eukaryota - ps6
Amorpheae - ps7
Obazoa - ps8
Opisthokonta - ps9
Holozoa - ps10
Filozoa - ps11
Choano./Metazoa - ps12
Metazoa - ps13
Myriazoa - ps14
Parahoxozoa - ps15
Bilateria - ps16
Protostomia - ps17
Ecdysozoa - ps18
Arthropoda - ps19
Mandibulata - ps20
Pancrustacea - ps21
Insecta - ps22
Eumetabola - ps23
Holometabola/Psocodea - ps24
Holometabola - ps25
Aparaglossata - ps26
Mecopterida - ps27
Diptera - ps28
Bibiono./Cyclorrhapha - ps29
Cyclorrhapha - ps30
Calyptratae/Drosophila - ps31
Drosophila - ps32
Sophophora - ps33
Obscura/Melanogaster_group - ps34
D_melanogaster_group - ps35
D_melanogaster_subgroup - ps36
sim./sech./mel. group - ps37
Drosophila_melanogaster - ps38
Phylostratum

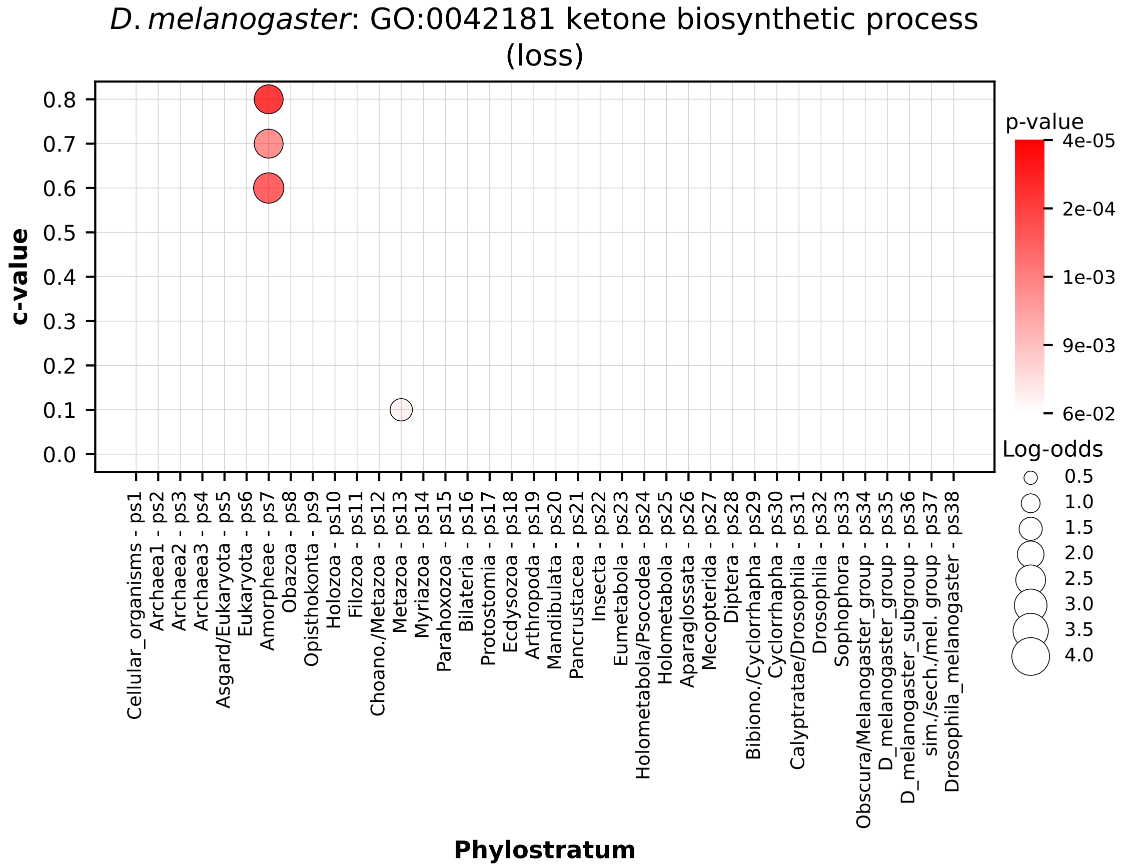
D. melanogaster: GO:0042181 ketone biosynthetic process
(loss)
c-value
0.0
0.1
0.2
0.3
0.4
0.5
0.6
0.7
0.8
Phylostratum
Cellular_organisms - ps1
Archaea1 - ps2
Archaea2 - ps3
Archaea3 - ps4
Asgard/Eukaryota - ps5
Eukaryota - ps6
Amorpheae - ps7
Obazoa - ps8
Opisthokonta - ps9
Holozoa - ps10
Filozoa - ps11
Choano./Metazoa - ps12
Metazoa - ps13
Myriazoa - ps14
Parahoxozoa - ps15
Bilateria - ps16
Protostomia - ps17
Ecdysozoa - ps18
Arthropoda - ps19
Mandibulata - ps20
Pancrustacea - ps21
Insecta - ps22
Eumetabola - ps23
Holometabola/Psocodea - ps24
Holometabola - ps25
Aparaglossata - ps26
Mecopterida - ps27
Diptera - ps28
Bibiono./Cyclorrhapha - ps29
Cyclorrhapha - ps30
Calyptratae/Drosophila - ps31
Drosophila - ps32
Sophophora - ps33
Obscura/Melanogaster_group - ps34
D_melanogaster_group - ps35
D_melanogaster_subgroup - ps36
sim./sech./mel. group - ps37
Drosophila_melanogaster - ps38
p-value
4e-05
2e-04
1e-03
9e-03
6e-02
Log-odds
0.5
1.0
1.5
2.0
2.5
3.0
3.5
4.0

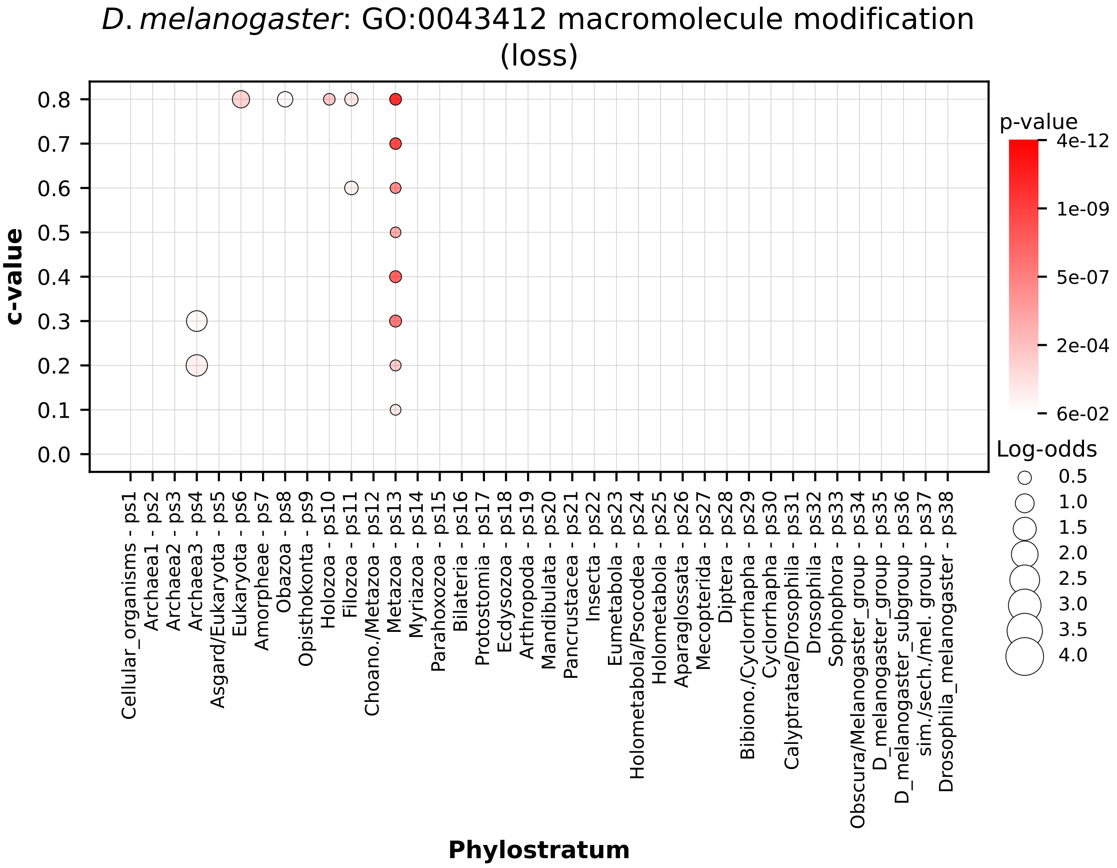

D. melanogaster: GO:0043412 macromolecule modification (loss)
c-value
0.0
0.1
0.2
0.3
0.4
0.5
0.6
0.7
0.8
Phylostratum
Cellular_organisms - ps1
Archaea1 - ps2
Archaea2 - ps3
Archaea3 - ps4
Asgard/Eukaryota - ps5
Eukaryota - ps6
Amorpheae - ps7
Obazoa - ps8
Opisthokonta - ps9
Holozoa - ps10
Filozoa - ps11
Choano./Metazoa - ps12
Metazoa - ps13
Myriazoa - ps14
Parahoxozoa - ps15
Bilateria - ps16
Protostomia - ps17
Ecdysozoa - ps18
Arthropoda - ps19
Mandibulata - ps20
Pancrustacea - ps21
Insecta - ps22
Eumetabola - ps23
Holometabola/Psocodea - ps24
Holometabola - ps25
Aparaglossata - ps26
Mecopterida - ps27
Diptera - ps28
Bibiono./Cyclorrhapha - ps29
Cyclorrhapha - ps30
Calyptratae/Drosophila - ps31
Drosophila - ps32
Sophophora - ps33
Obscura/Melanogaster_group - ps34
D_melanogaster_group - ps35
D_melanogaster_subgroup - ps36
sim./sech./mel. group - ps37
Drosophila_melanogaster - ps38
p-value
4e-12
1e-09
5e-07
2e-04
6e-02
Log-odds
0.5
1.0
1.5
2.0
2.5
3.0
3.5
4.0

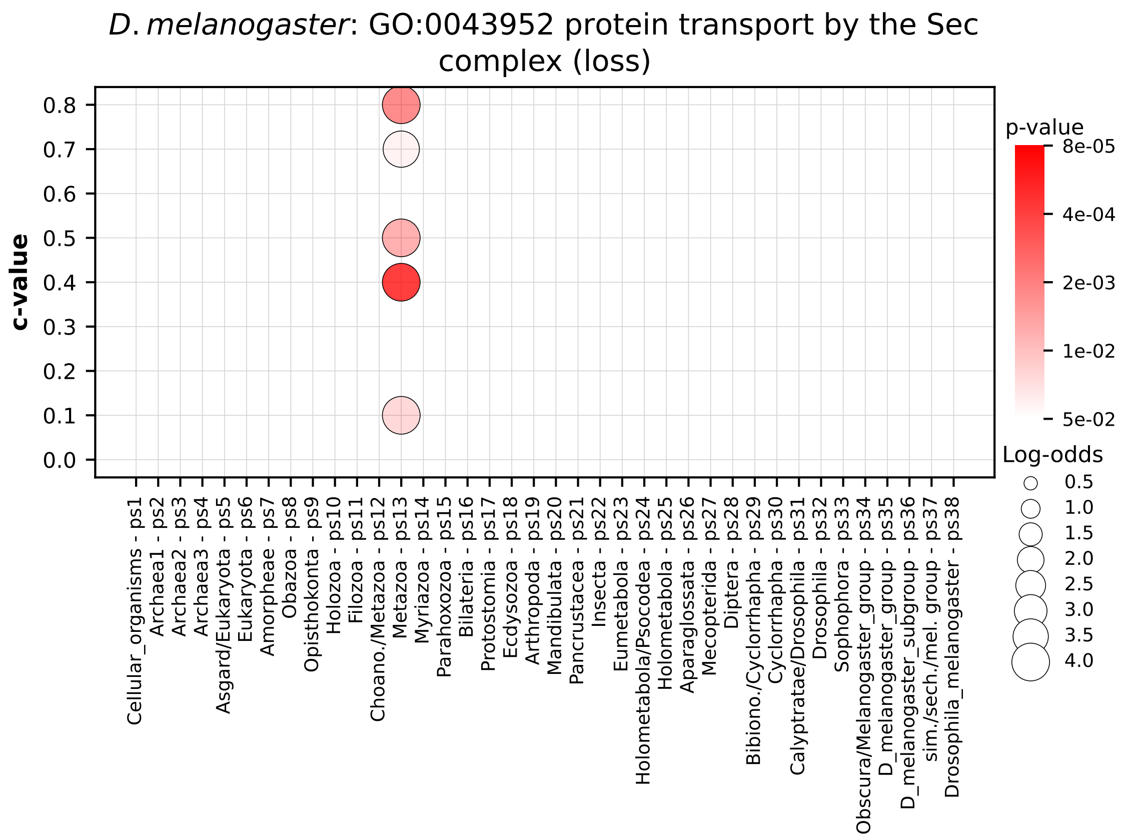

D. melanogaster: GO:0043952 protein transport by the Sec complex (loss)
c-value
0.8
0.7
0.6
0.5
0.4
0.3
0.2
0.1
0.0
Phylostratum
Cellular_organisms - ps1
Archaea1 - ps2
Archaea2 - ps3
Archaea3 - ps4
Asgard/Eukaryota - ps5
Eukaryota - ps6
Amorpheae - ps7
Obazoa - ps8
Opisthokonta - ps9
Holozoa - ps10
Filozoa - ps11
Choano./Metazoa - ps12
Metazoa - ps13
Myriazoa - ps14
Parahoxozoa - ps15
Bilateria - ps16
Protostomia - ps17
Ecdysozoa - ps18
Arthropoda - ps19
Mandibulata - ps20
Pancrustacea - ps21
Insecta - ps22
Eumetabola - ps23
Holometabola/Psocodea - ps24
Holometabola - ps25
Aparaglossata - ps26
Mecopterida - ps27
Diptera - ps28
Bibiono./Cyclorrhapha - ps29
Cyclorrhapha - ps30
Calyptratae/Drosophila - ps31
Drosophila - ps32
Sophophora - ps33
Obscura/Melanogaster_group - ps34
D_melanogaster_group - ps35
D_melanogaster_subgroup - ps36
sim./sech./mel. group - ps37
Drosophila_melanogaster - ps38
p-value
8e-05
4e-04
2e-03
1e-02
5e-02
Log-odds
0.5
1.0
1.5
2.0
2.5
3.0
3.5
4.0

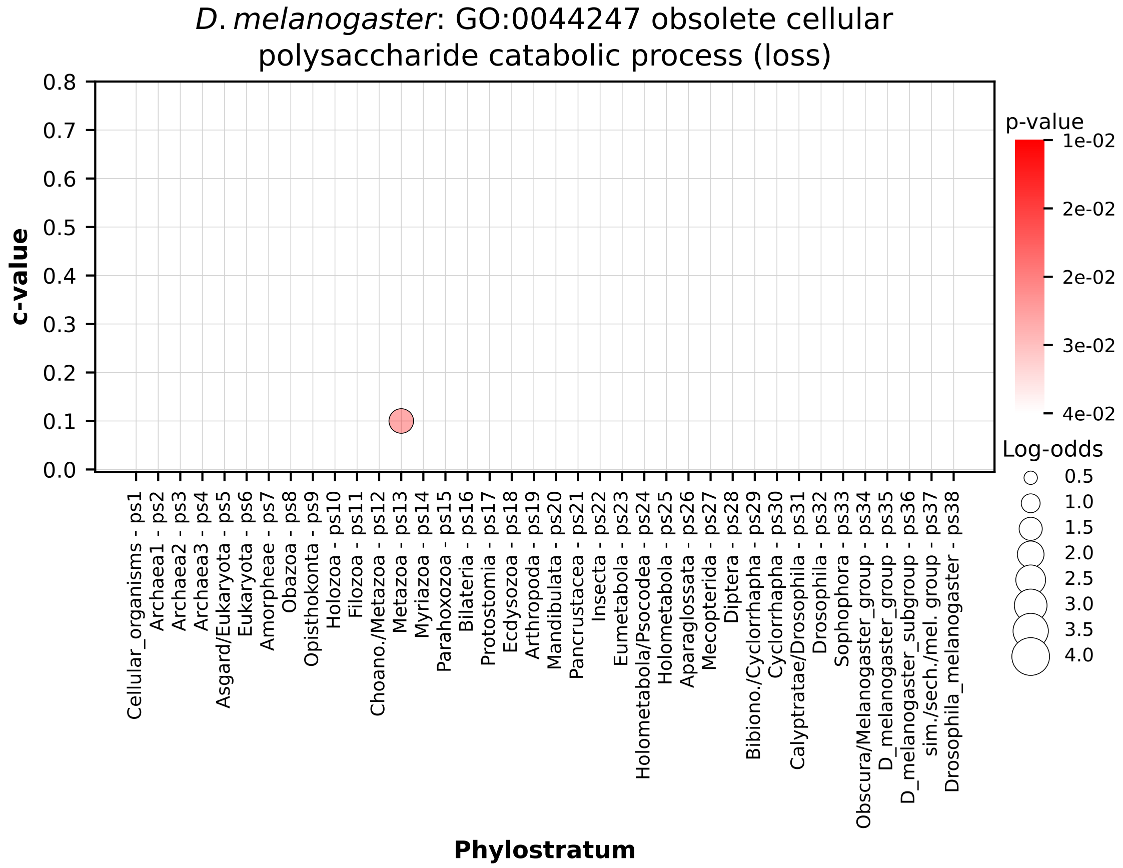

D. melanogaster: GO:0044247 obsolete cellular polysaccharide catabolic process (loss)
c-value
0.8
0.7
0.6
0.5
0.4
0.3
0.2
0.1
0.0
p-value
1e-02
2e-02
2e-02
3e-02
4e-02
Log-odds
0.5
1.0
1.5
2.0
2.5
3.0
3.5
4.0
Cellular_organisms - ps1
Archaea1 - ps2
Archaea2 - ps3
Archaea3 - ps4
Asgard/Eukaryota - ps5
Eukaryota - ps6
Amorpheae - ps7
Obazoa - ps8
Opisthokonta - ps9
Holozoa - ps10
Filozoa - ps11
Choano./Metazoa - ps12
Metazoa - ps13
Myriazoa - ps14
Parahoxozoa - ps15
Bilateria - ps16
Protostomia - ps17
Ecdysozoa - ps18
Arthropoda - ps19
Mandibulata - ps20
Pancrustacea - ps21
Insecta - ps22
Eumetabola - ps23
Holometabola/Psocodea - ps24
Holometabola - ps25
Aparaglossata - ps26
Mecopterida - ps27
Diptera - ps28
Bibiono./Cyclorrhapha - ps29
Cyclorrhapha - ps30
Calyptratae/Drosophila - ps31
Drosophila - ps32
Sophophora - ps33
Obscura/Melanogaster_group - ps34
D_melanogaster_group - ps35
D_melanogaster_subgroup - ps36
sim./sech./mel. group - ps37
Drosophila_melanogaster - ps38
Phylostratum

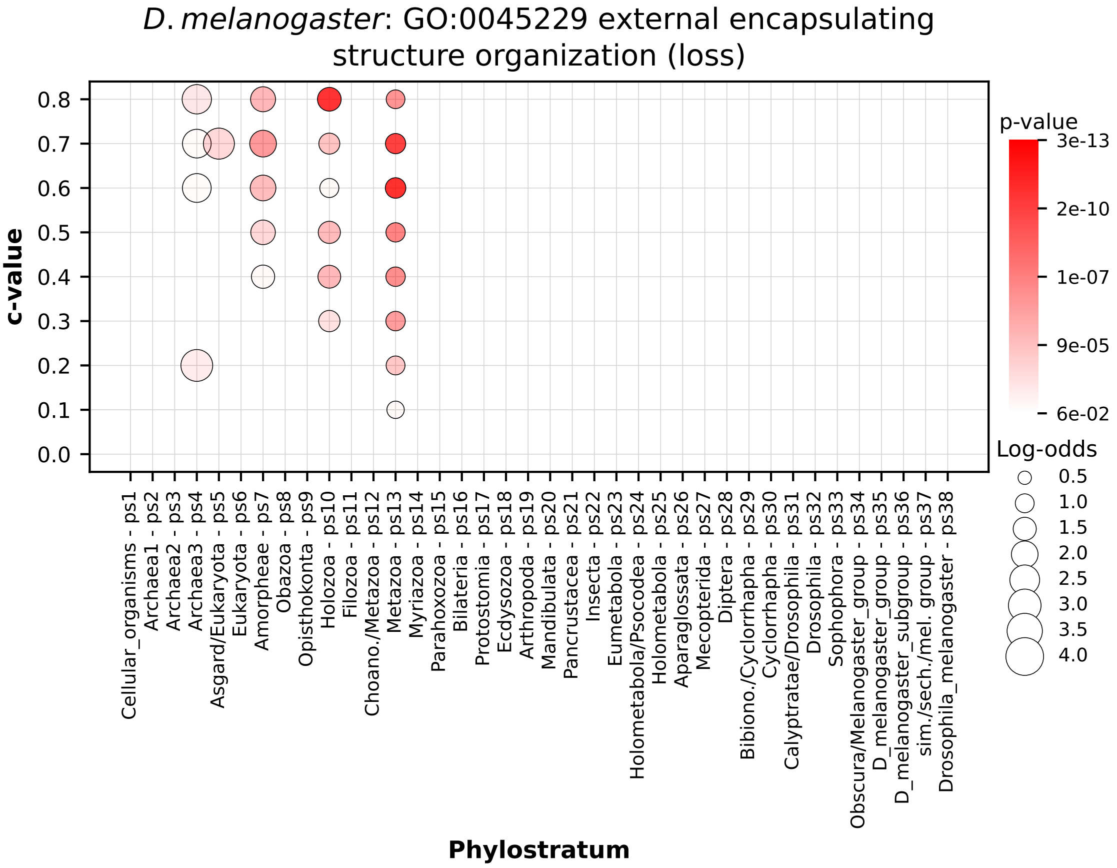

D. melanogaster: GO:0045229 external encapsulating structure organization (loss)
c-value
0.8
0.7
0.6
0.5
0.4
0.3
0.2
0.1
0.0
Phylostratum
Cellular_organisms - ps1
Archaea1 - ps2
Archaea2 - ps3
Archaea3 - ps4
Asgard/Eukaryota - ps5
Eukaryota - ps6
Amorpheae - ps7
Obazoa - ps8
Opisthokonta - ps9
Holozoa - ps10
Filozoa - ps11
Choano./Metazoa - ps12
Metazoa - ps13
Myriazoa - ps14
Parahoxozoa - ps15
Bilateria - ps16
Protostomia - ps17
Ecdysozoa - ps18
Arthropoda - ps19
Mandibulata - ps20
Pancrustacea - ps21
Insecta - ps22
Eumetabola - ps23
Holometabola/Psocodea - ps24
Holometabola - ps25
Aparaglossata - ps26
Mecopterida - ps27
Diptera - ps28
Bibiono./Cyclorrhapha - ps29
Cyclorrhapha - ps30
Calyptratae/Drosophila - ps31
Drosophila - ps32
Sophophora - ps33
Obscura/Melanogaster_group - ps34
D_melanogaster_group - ps35
D_melanogaster_subgroup - ps36
sim./sech./mel. group - ps37
Drosophila_melanogaster - ps38
p-value
3e-13
2e-10
1e-07
9e-05
6e-02
Log-odds
0.5
1.0
1.5
2.0
2.5
3.0
3.5
4.0

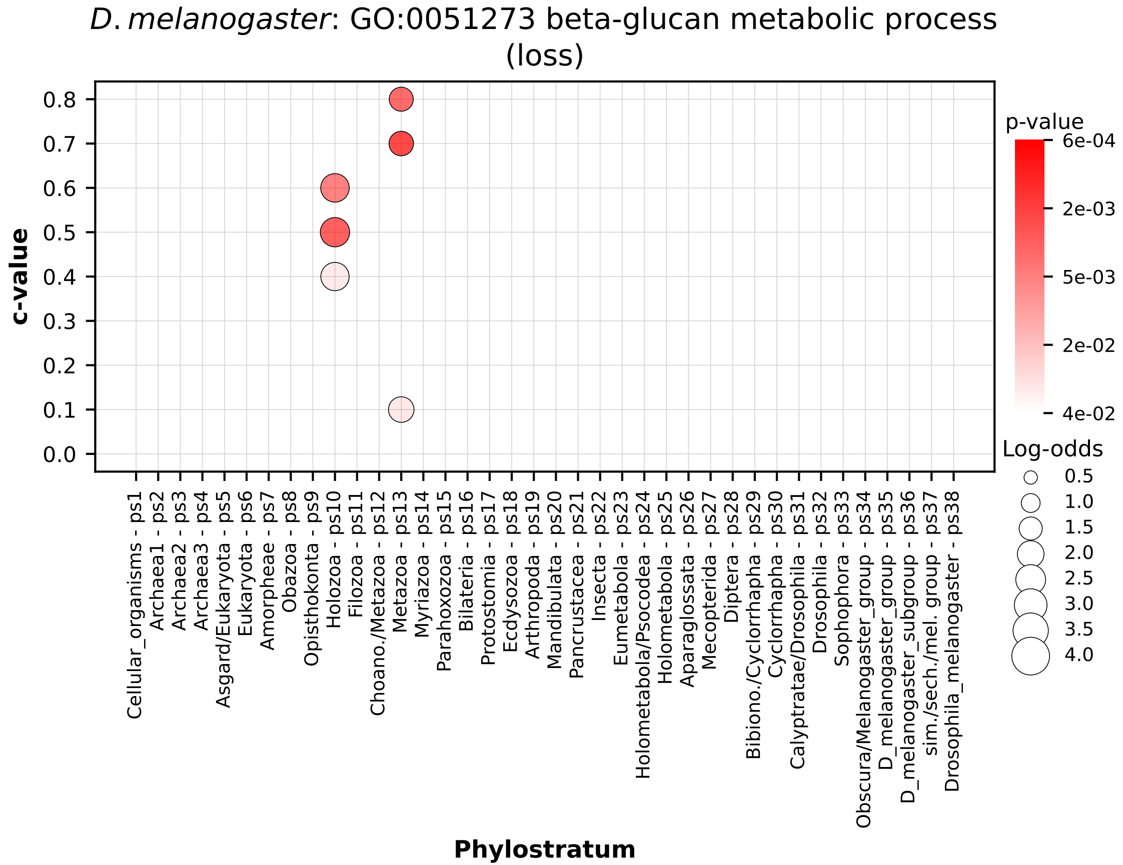

D. melanogaster: GO:0051273 beta-glucan metabolic process (loss)
c-value
0.8
0.7
0.6
0.5
0.4
0.3
0.2
0.1
0.0
p-value
6e-04
2e-03
5e-03
2e-02
4e-02
Log-odds
0.5
1.0
1.5
2.0
2.5
3.0
3.5
4.0
Cellular_organisms - ps1
Archaea1 - ps2
Archaea2 - ps3
Archaea3 - ps4
Asgard/Eukaryota - ps5
Eukaryota - ps6
Amorpheae - ps7
Obazoa - ps8
Opisthokonta - ps9
Holozoa - ps10
Filozoa - ps11
Choano./Metazoa - ps12
Metazoa - ps13
Myriazoa - ps14
Parahoxozoa - ps15
Bilateria - ps16
Protostomia - ps17
Ecdysozoa - ps18
Arthropoda - ps19
Mandibulata - ps20
Pancrustacea - ps21
Insecta - ps22
Eumetabola - ps23
Holometabola/Psocodea - ps24
Holometabola - ps25
Aparaglossata - ps26
Mecopterida - ps27
Diptera - ps28
Bibiono./Cyclorrhapha - ps29
Cyclorrhapha - ps30
Calyptratae/Drosophila - ps31
Drosophila - ps32
Sophophora - ps33
Obscura/Melanogaster_group - ps34
D_melanogaster_group - ps35
D_melanogaster_subgroup - ps36
sim./sech./mel. group - ps37
Drosophila_melanogaster - ps38
Phylostratum

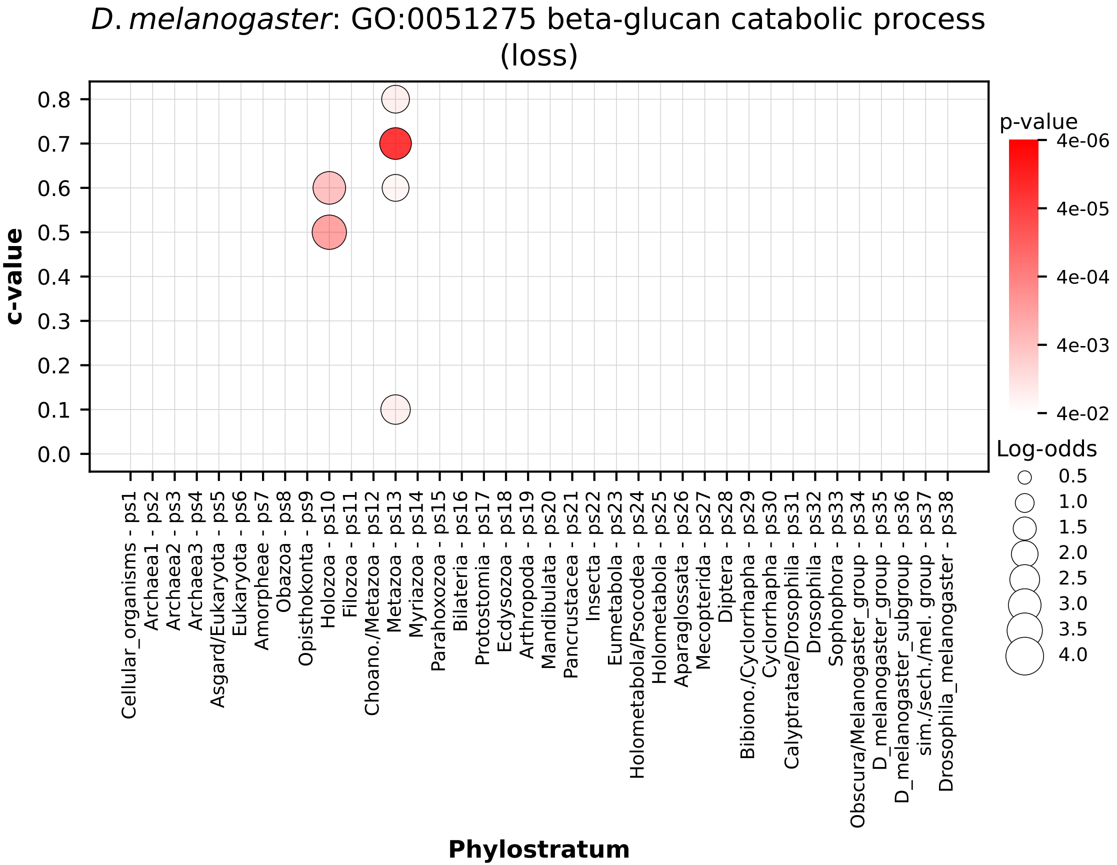

D. melanogaster: GO:0051275 beta-glucan catabolic process (loss)
c-value
Phylostratum
p-value
4e-06
4e-05
4e-04
4e-03
4e-02
Log-odds
0.5
1.0
1.5
2.0
2.5
3.0
3.5
4.0
Cellular_organisms - ps1
Archaea1 - ps2
Archaea2 - ps3
Archaea3 - ps4
Asgard/Eukaryota - ps5
Eukaryota - ps6
Amorpheae - ps7
Obazoa - ps8
Opisthokonta - ps9
Holozoa - ps10
Filozoa - ps11
Choano./Metazoa - ps12
Metazoa - ps13
Myriazoa - ps14
Parahoxozoa - ps15
Bilateria - ps16
Protostomia - ps17
Ecdysozoa - ps18
Arthropoda - ps19
Mandibulata - ps20
Pancrustacea - ps21
Insecta - ps22
Eumetabola - ps23
Holometabola/Psocodea - ps24
Holometabola - ps25
Aparaglossata - ps26
Mecopterida - ps27
Diptera - ps28
Bibiono./Cyclorrhapha - ps29
Cyclorrhapha - ps30
Calyptratae/Drosophila - ps31
Drosophila - ps32
Sophophora - ps33
Obscura/Melanogaster_group - ps34
D_melanogaster_group - ps35
D_melanogaster_subgroup - ps36
sim./sech./mel. group - ps37
Drosophila_melanogaster - ps38

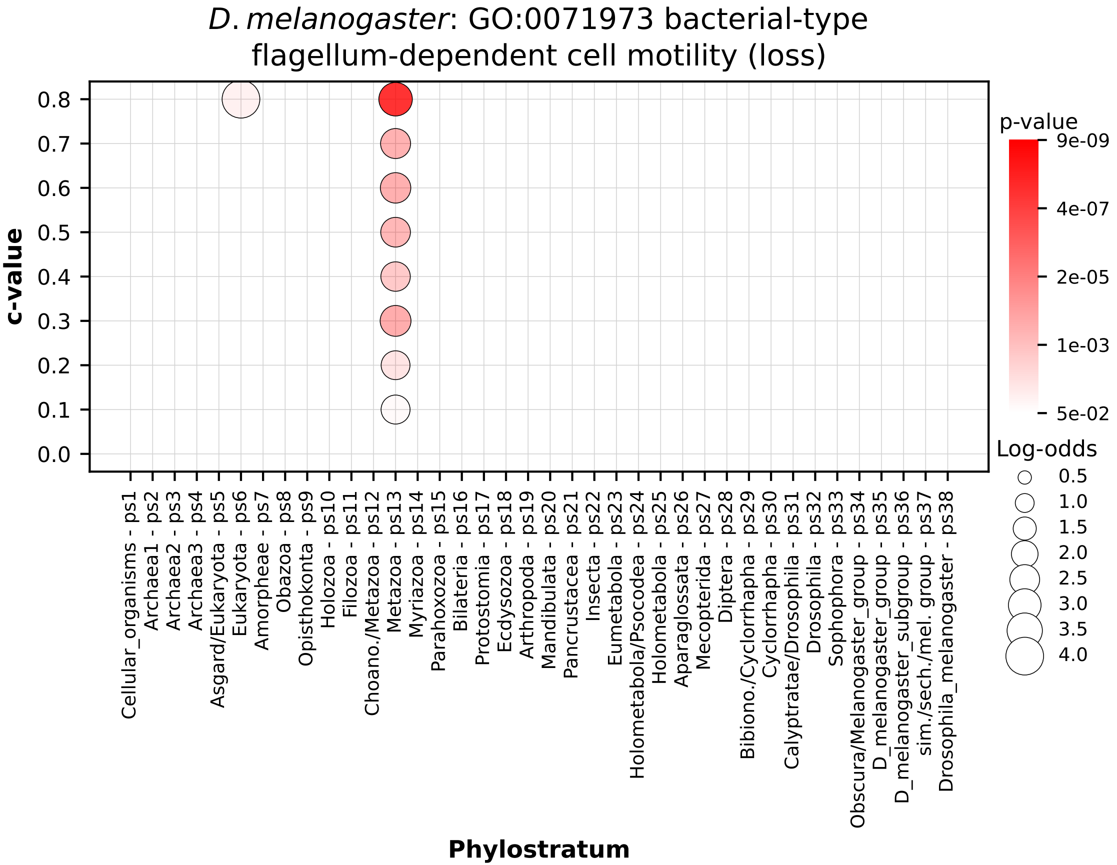

$D. melanogaster$: GO:0071973 bacterial-type flagellum-dependent cell motility (loss)
c-value
0.8
0.7
0.6
0.5
0.4
0.3
0.2
0.1
0.0
p-value
9e-09
4e-07
2e-05
1e-03
5e-02
Log-odds
0.5
1.0
1.5
2.0
2.5
3.0
3.5
4.0
Cellular_organisms - ps1
Archaea1 - ps2
Archaea2 - ps3
Archaea3 - ps4
Asgard/Eukaryota - ps5
Eukaryota - ps6
Amorpheae - ps7
Obazoa - ps8
Opisthokonta - ps9
Holozoa - ps10
Filozoa - ps11
Choano./Metazoa - ps12
Metazoa - ps13
Myriazoa - ps14
Parahoxozoa - ps15
Bilateria - ps16
Protostomia - ps17
Ecdysozoa - ps18
Arthropoda - ps19
Mandibulata - ps20
Pancrustacea - ps21
Insecta - ps22
Eumetabola - ps23
Holometabola/Psocodea - ps24
Holometabola - ps25
Aparaglossata - ps26
Mecopterida - ps27
Diptera - ps28
Bibiono./Cyclorrhapha - ps29
Cyclorrhapha - ps30
Calyptratae/Drosophila - ps31
Drosophila - ps32
Sophophora - ps33
Obscura/Melanogaster_group - ps34
D_melanogaster_group - ps35
D_melanogaster_subgroup - ps36
sim./sech./mel. group - ps37
Drosophila_melanogaster - ps38
Phylostratum

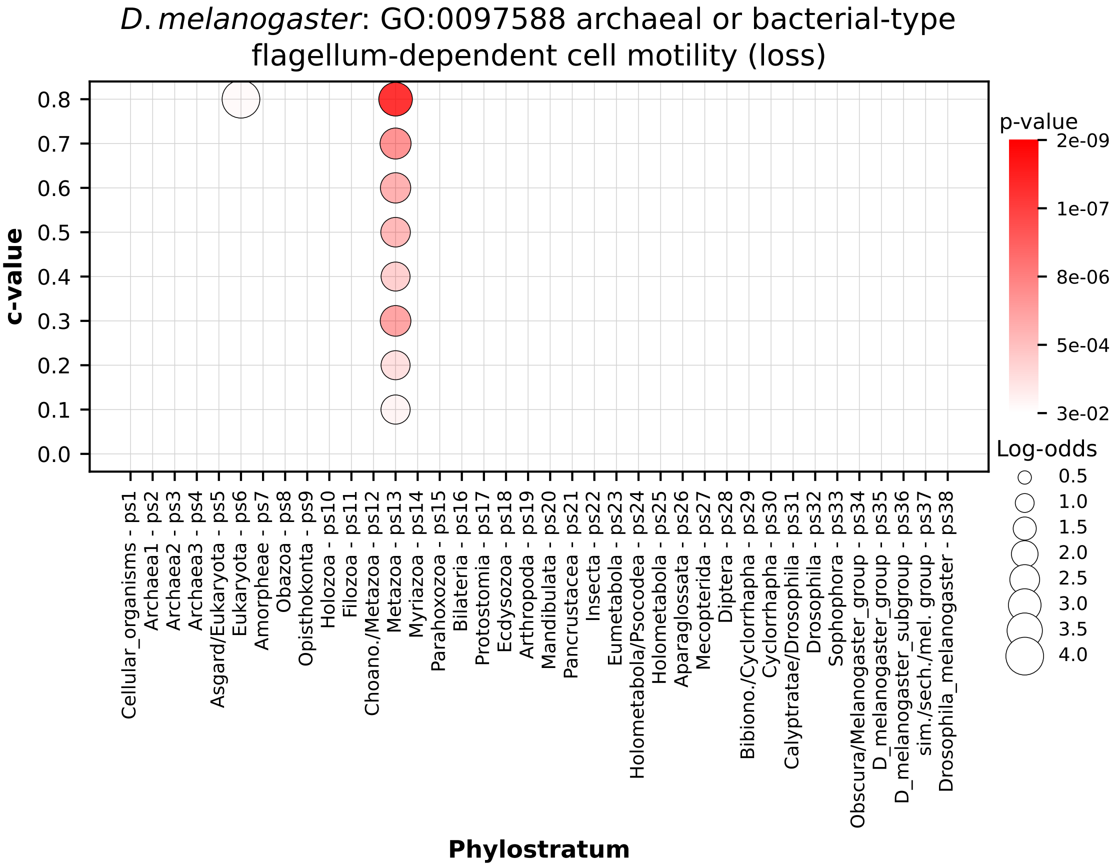

D. melanogaster: GO:0097588 archaeal or bacterial-type flagellum-dependent cell motility (loss)
c-value
0.8
0.7
0.6
0.5
0.4
0.3
0.2
0.1
0.0
p-value
2e-09
1e-07
8e-06
5e-04
3e-02
Log-odds
0.5
1.0
1.5
2.0
2.5
3.0
3.5
4.0
Cellular_organisms - ps1
Archaea1 - ps2
Archaea2 - ps3
Archaea3 - ps4
Asgard/Eukaryota - ps5
Eukaryota - ps6
Amorpheae - ps7
Obazoa - ps8
Opisthokonta - ps9
Holozoa - ps10
Filozoa - ps11
Choano./Metazoa - ps12
Metazoa - ps13
Myriazoa - ps14
Parahoxozoa - ps15
Bilateria - ps16
Protostomia - ps17
Ecdysozoa - ps18
Arthropoda - ps19
Mandibulata - ps20
Pancrustacea - ps21
Insecta - ps22
Eumetabola - ps23
Holometabola/Psocodea - ps24
Holometabola - ps25
Aparaglossata - ps26
Mecopterida - ps27
Diptera - ps28
Bibiono./Cyclorrhapha - ps29
Cyclorrhapha - ps30
Calyptratae/Drosophila - ps31
Drosophila - ps32
Sophophora - ps33
Obscura/Melanogaster_group - ps34
D_melanogaster_group - ps35
D_melanogaster_subgroup - ps36
sim./sech./mel. group - ps37
Drosophila_melanogaster - ps38
Phylostratum

D. melanogaster: GO:1901663 quinone biosynthetic process (loss)

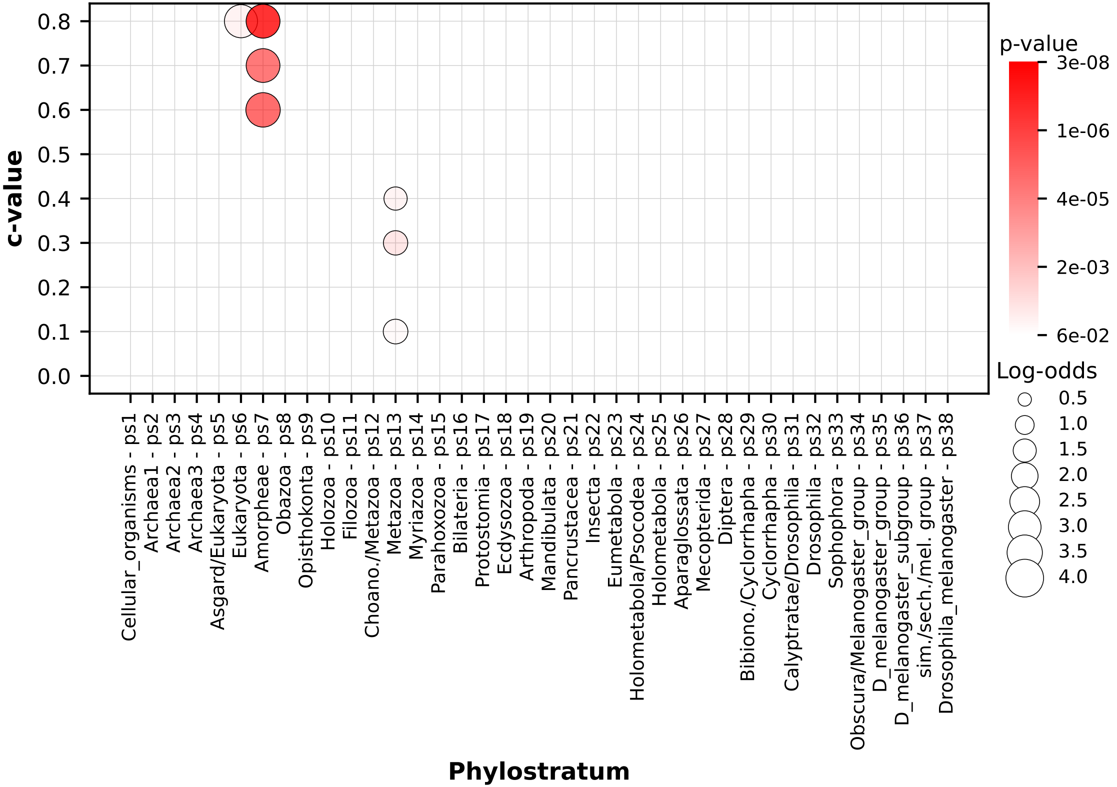

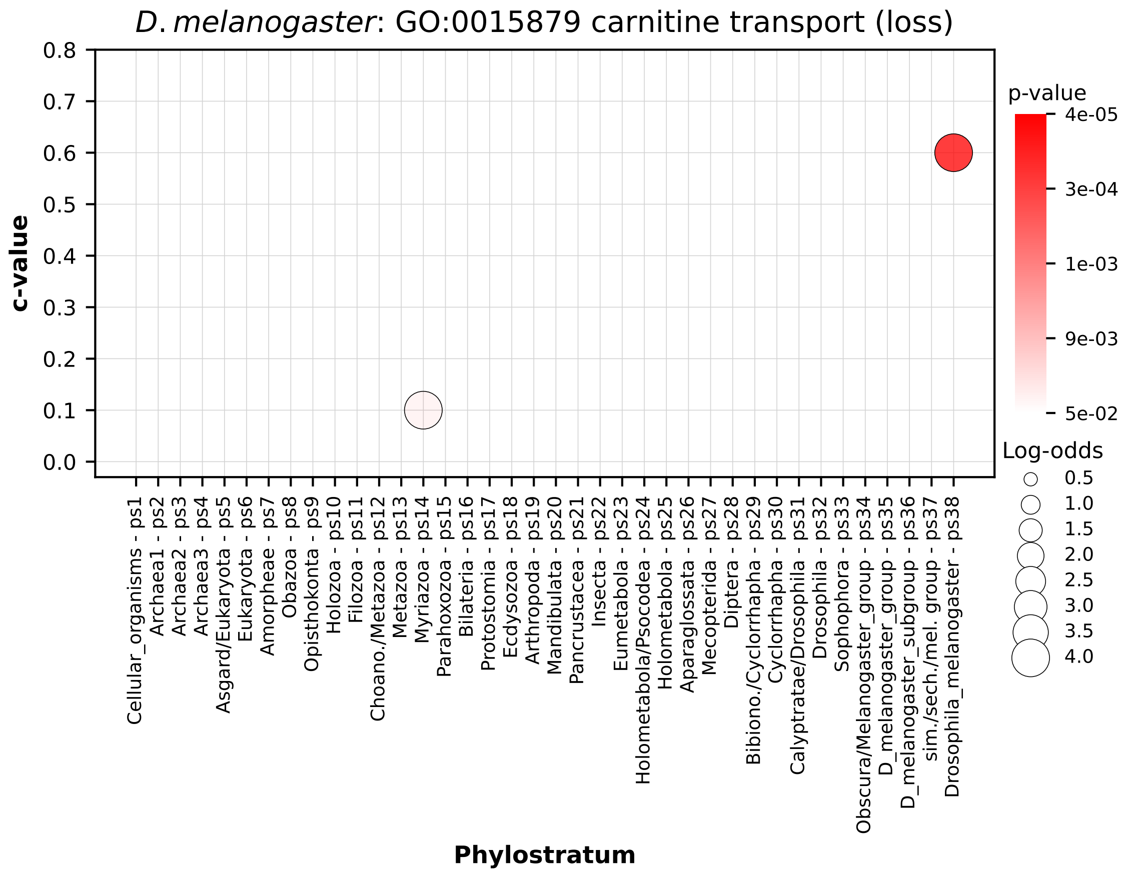

D. melanogaster: GO:0015879 carnitine transport (loss)
c-value
0.0
0.1
0.2
0.3
0.4
0.5
0.6
0.7
0.8
Phylostratum
Cellular_organisms - ps1
Archaea1 - ps2
Archaea2 - ps3
Archaea3 - ps4
Asgard/Eukaryota - ps5
Eukaryota - ps6
Amorpheae - ps7
Obazoa - ps8
Opisthokonta - ps9
Holozoa - ps10
Filozoa - ps11
Choano./Metazoa - ps12
Metazoa - ps13
Myriazoa - ps14
Parahoxozoa - ps15
Bilateria - ps16
Protostomia - ps17
Ecdysozoa - ps18
Arthropoda - ps19
Mandibulata - ps20
Pancrustacea - ps21
Insecta - ps22
Eumetabola - ps23
Holometabola/Psocodea - ps24
Holometabola - ps25
Aparaglossata - ps26
Mecopterida - ps27
Diptera - ps28
Bibiono./Cyclorrhapha - ps29
Cyclorrhapha - ps30
Calyptratae/Drosophila - ps31
Drosophila - ps32
Sophophora - ps33
Obscura/Melanogaster_group - ps34
D_melanogaster_group - ps35
D_melanogaster_subgroup - ps36
sim./sech./mel. group - ps37
Drosophila_melanogaster - ps38
p-value
4e-05
3e-04
1e-03
9e-03
5e-02
Log-odds
0.5
1.0
1.5
2.0
2.5
3.0
3.5
4.0

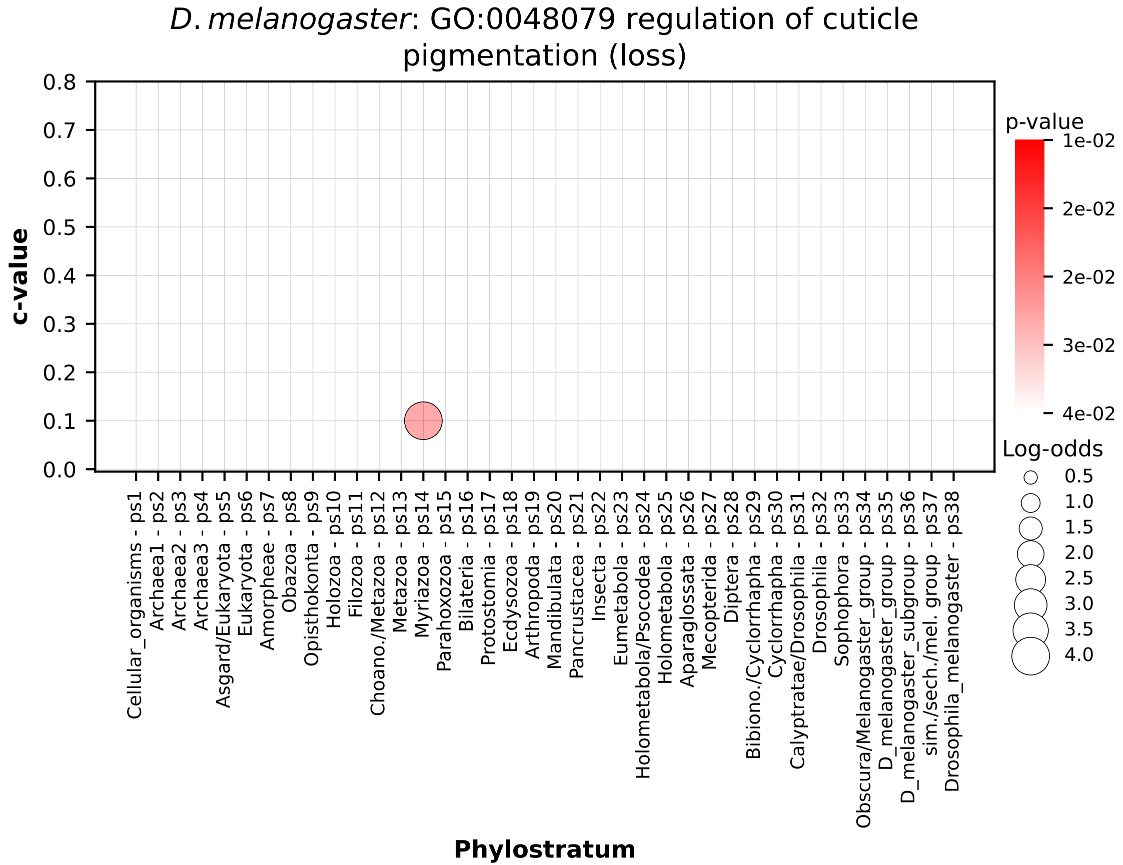

D. melanogaster: GO:0048079 regulation of cuticle pigmentation (loss)
c-value
0.8
0.7
0.6
0.5
0.4
0.3
0.2
0.1
0.0
p-value
1e-02
2e-02
2e-02
3e-02
4e-02
Log-odds
0.5
1.0
1.5
2.0
2.5
3.0
3.5
4.0
Cellular_organisms - ps1
Archaea1 - ps2
Archaea2 - ps3
Archaea3 - ps4
Asgard/Eukaryota - ps5
Eukaryota - ps6
Amorpheae - ps7
Obazoa - ps8
Opisthokonta - ps9
Holozoa - ps10
Filozoa - ps11
Choano./Metazoa - ps12
Metazoa - ps13
Myriazoa - ps14
Parahoxozoa - ps15
Bilateria - ps16
Protostomia - ps17
Ecdysozoa - ps18
Arthropoda - ps19
Mandibulata - ps20
Pancrustacea - ps21
Insecta - ps22
Eumetabola - ps23
Holometabola/Psocodea - ps24
Holometabola - ps25
Aparaglossata - ps26
Mecopterida - ps27
Diptera - ps28
Bibiono./Cyclorrhapha - ps29
Cyclorrhapha - ps30
Calyptratae/Drosophila - ps31
Drosophila - ps32
Sophophora - ps33
Obscura/Melanogaster_group - ps34
D_melanogaster_group - ps35
D_melanogaster_subgroup - ps36
sim./sech./mel. group - ps37
Drosophila_melanogaster - ps38
Phylostratum

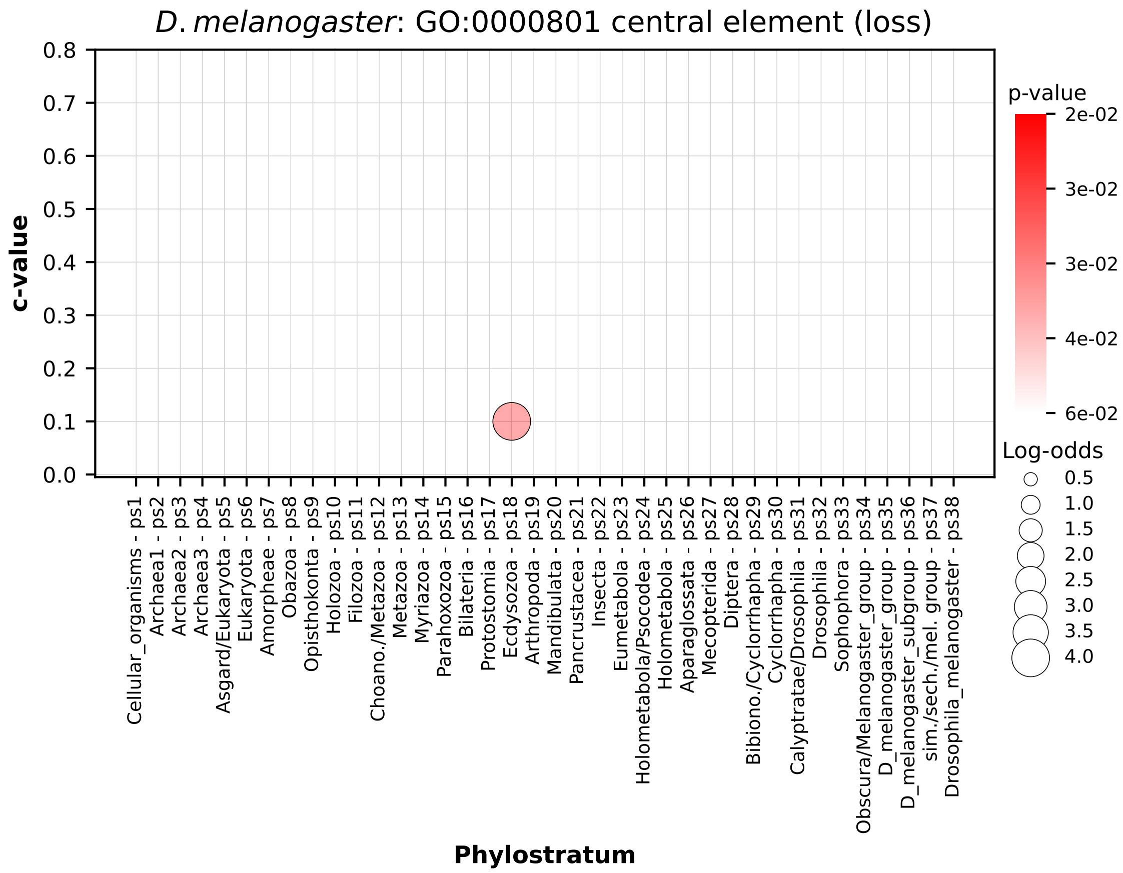

D. melanogaster: GO:0000801 central element (loss)
c-value
0.0
0.1
0.2
0.3
0.4
0.5
0.6
0.7
0.8
Phylostratum
Cellular_organisms - ps1
Archaea1 - ps2
Archaea2 - ps3
Archaea3 - ps4
Asgard/Eukaryota - ps5
Eukaryota - ps6
Amorpheae - ps7
Obazoa - ps8
Opisthokonta - ps9
Holozoa - ps10
Filozoa - ps11
Choano./Metazoa - ps12
Metazoa - ps13
Myriazoa - ps14
Parahoxozoa - ps15
Bilateria - ps16
Protostomia - ps17
Ecdysozoa - ps18
Arthropoda - ps19
Mandibulata - ps20
Pancrustacea - ps21
Insecta - ps22
Eumetabola - ps23
Holometabola/Psocodea - ps24
Holometabola - ps25
Aparaglossata - ps26
Mecopterida - ps27
Diptera - ps28
Bibiono./Cyclorrhapha - ps29
Cyclorrhapha - ps30
Calyptratae/Drosophila - ps31
Drosophila - ps32
Sophophora - ps33
Obscura/Melanogaster_group - ps34
D_melanogaster_group - ps35
D_melanogaster_subgroup - ps36
sim./sech./mel. group - ps37
Drosophila_melanogaster - ps38
p-value
2e-02
3e-02
3e-02
4e-02
6e-02
Log-odds
0.5
1.0
1.5
2.0
2.5
3.0
3.5
4.0

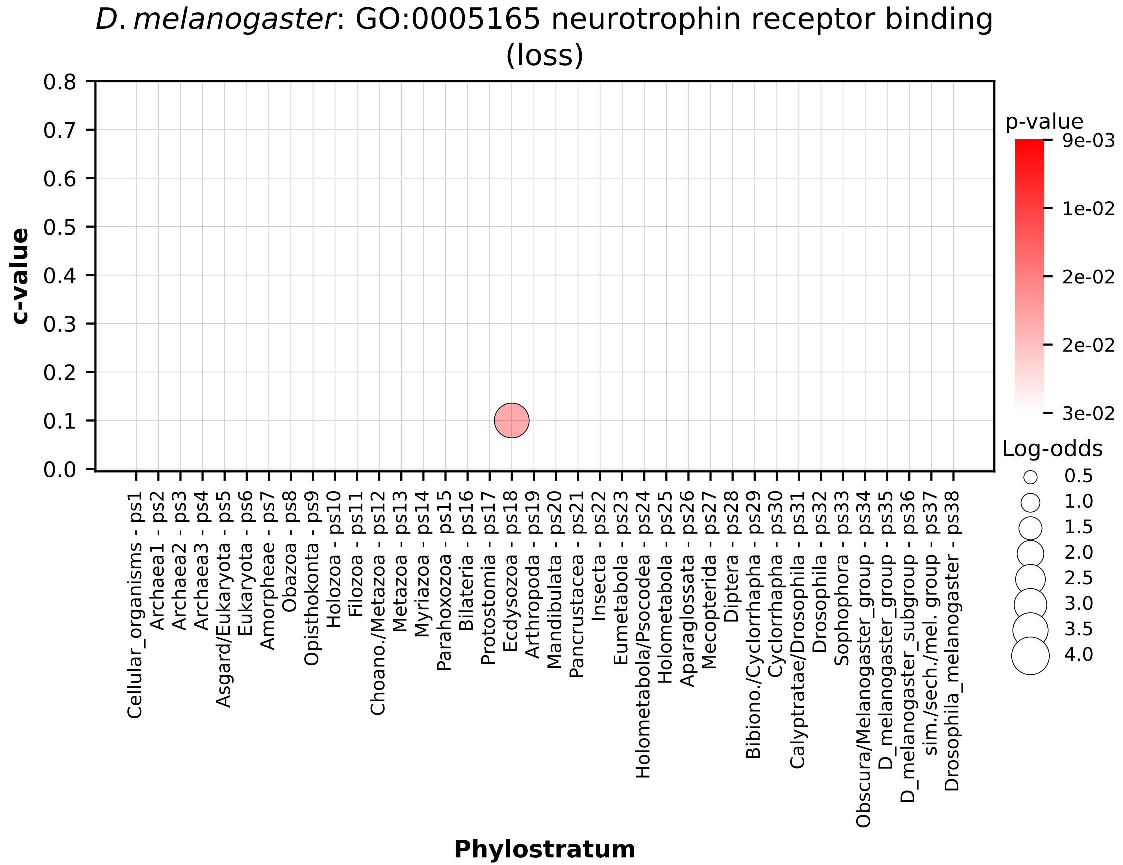
D. melanogaster: GO:0005165 neurotrophin receptor binding
(loss)
c-value
0.0
0.1
0.2
0.3
0.4
0.5
0.6
0.7
0.8
Phylostratum
Cellular_organisms - ps1
Archaea1 - ps2
Archaea2 - ps3
Archaea3 - ps4
Asgard/Eukaryota - ps5
Eukaryota - ps6
Amorpheae - ps7
Obazoa - ps8
Opisthokonta - ps9
Holozoa - ps10
Filozoa - ps11
Choano./Metazoa - ps12
Metazoa - ps13
Myriazoa - ps14
Parahoxozoa - ps15
Bilateria - ps16
Protostomia - ps17
Ecdysozoa - ps18
Arthropoda - ps19
Mandibulata - ps20
Pancrustacea - ps21
Insecta - ps22
Eumetabola - ps23
Holometabola/Psocodea - ps24
Holometabola - ps25
Aparaglossata - ps26
Mecopterida - ps27
Diptera - ps28
Bibiono./Cyclorrhapha - ps29
Cyclorrhapha - ps30
Calyptratae/Drosophila - ps31
Drosophila - ps32
Sophophora - ps33
Obscura/Melanogaster_group - ps34
D_melanogaster_group - ps35
D_melanogaster_subgroup - ps36
sim./sech./mel. group - ps37
Drosophila_melanogaster - ps38
p-value
9e-03
1e-02
2e-02
2e-02
3e-02
Log-odds
0.5
1.0
1.5
2.0
2.5
3.0
3.5
4.0

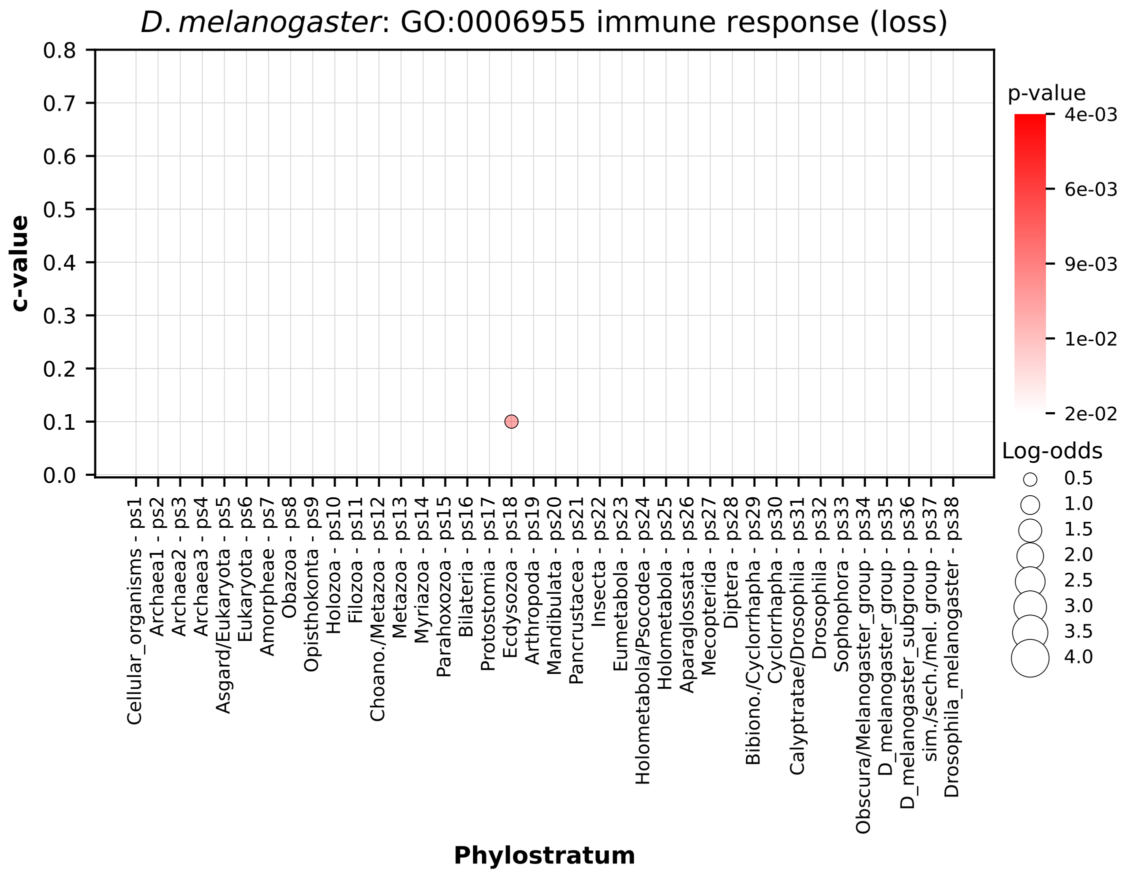

D. melanogaster: GO:0006955 immune response (loss)
c-value
0.8
0.7
0.6
0.5
0.4
0.3
0.2
0.1
0.0
p-value
4e-03
6e-03
9e-03
1e-02
2e-02
Log-odds
0.5
1.0
1.5
2.0
2.5
3.0
3.5
4.0
Cellular_organisms - ps1
Archaea1 - ps2
Archaea2 - ps3
Archaea3 - ps4
Asgard/Eukaryota - ps5
Eukaryota - ps6
Amorpheae - ps7
Obazoa - ps8
Opisthokonta - ps9
Holozoa - ps10
Filozoa - ps11
Choano./Metazoa - ps12
Metazoa - ps13
Myriazoa - ps14
Parahoxozoa - ps15
Bilateria - ps16
Protostomia - ps17
Ecdysozoa - ps18
Arthropoda - ps19
Mandibulata - ps20
Pancrustacea - ps21
Insecta - ps22
Eumetabola - ps23
Holometabola/Psocodea - ps24
Holometabola - ps25
Aparaglossata - ps26
Mecopterida - ps27
Diptera - ps28
Bibiono./Cyclorrhapha - ps29
Cyclorrhapha - ps30
Calyptratae/Drosophila - ps31
Drosophila - ps32
Sophophora - ps33
Obscura/Melanogaster_group - ps34
D_melanogaster_group - ps35
D_melanogaster_subgroup - ps36
sim./sech./mel. group - ps37
Drosophila_melanogaster - ps38
Phylostratum

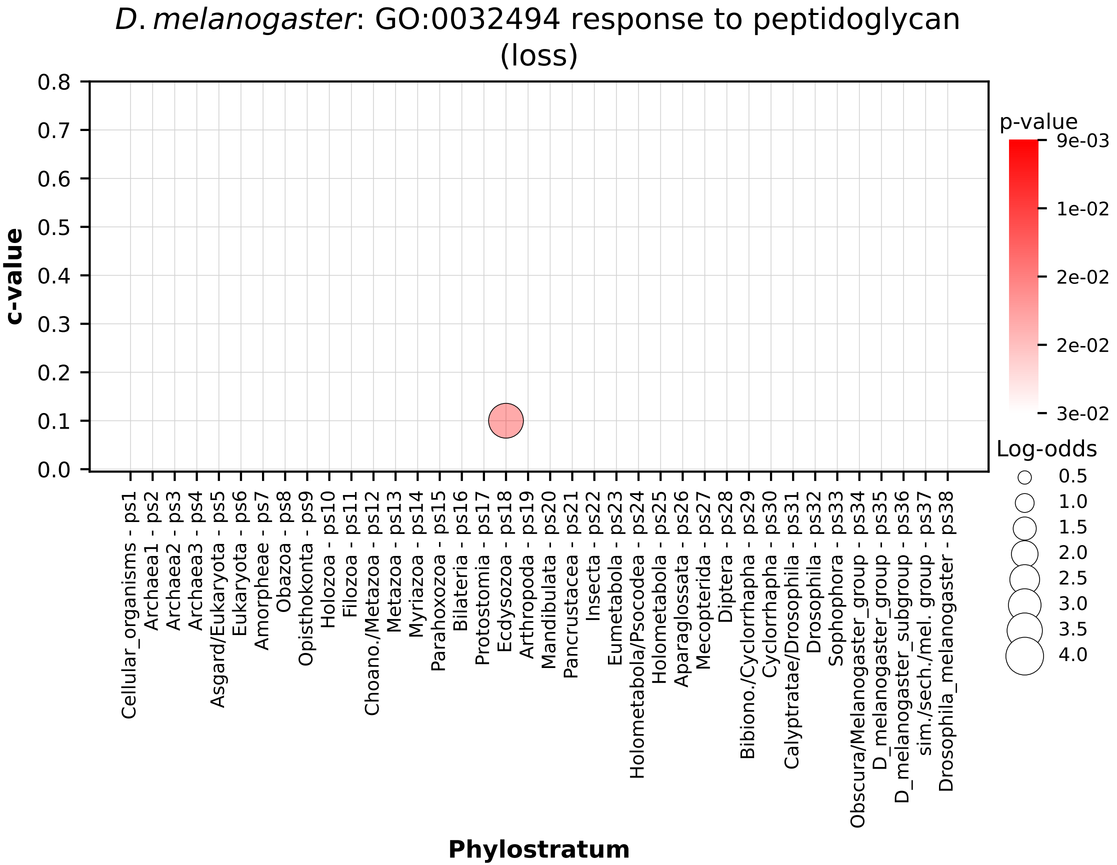
D. melanogaster: GO:0032494 response to peptidoglycan
(loss)
c-value
0.8
0.7
0.6
0.5
0.4
0.3
0.2
0.1
0.0
p-value
9e-03
1e-02
2e-02
2e-02
3e-02
Log-odds
0.5
1.0
1.5
2.0
2.5
3.0
3.5
4.0
Cellular_organisms - ps1
Archaea1 - ps2
Archaea2 - ps3
Archaea3 - ps4
Asgard/Eukaryota - ps5
Eukaryota - ps6
Amorpheae - ps7
Obazoa - ps8
Opisthokonta - ps9
Holozoa - ps10
Filozoa - ps11
Choano./Metazoa - ps12
Metazoa - ps13
Myriazoa - ps14
Parahoxozoa - ps15
Bilateria - ps16
Protostomia - ps17
Ecdysozoa - ps18
Arthropoda - ps19
Mandibulata - ps20
Pancrustacea - ps21
Insecta - ps22
Eumetabola - ps23
Holometabola/Psocodea - ps24
Holometabola - ps25
Aparaglossata - ps26
Mecopterida - ps27
Diptera - ps28
Bibiono./Cyclorrhapha - ps29
Cyclorrhapha - ps30
Calyptratae/Drosophila - ps31
Drosophila - ps32
Sophophora - ps33
Obscura/Melanogaster_group - ps34
D_melanogaster_group - ps35
D_melanogaster_subgroup - ps36
sim./sech./mel. group - ps37
Drosophila_melanogaster - ps38
Phylostratum

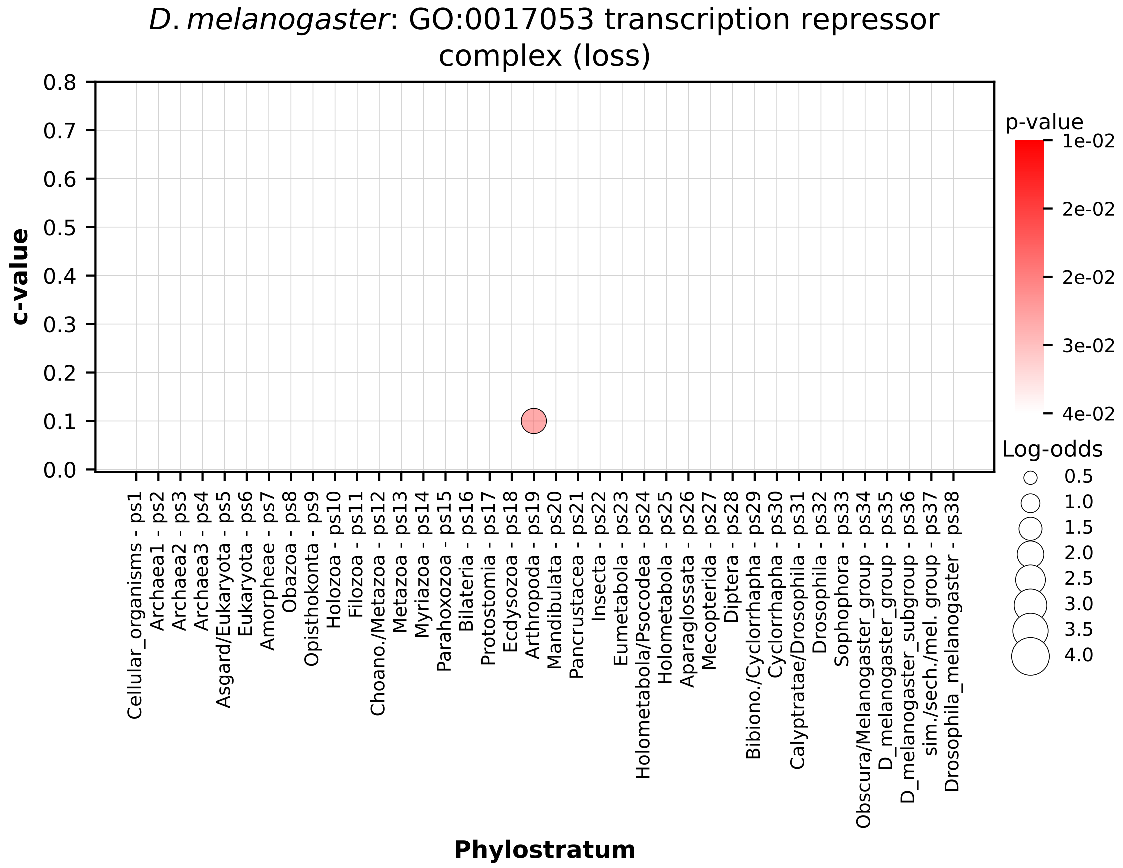
D. melanogaster: GO:0017053 transcription repressor complex (loss)
c-value
0.8
0.7
0.6
0.5
0.4
0.3
0.2
0.1
0.0
Phylostratum
Cellular_organisms - ps1
Archaea1 - ps2
Archaea2 - ps3
Archaea3 - ps4
Asgard/Eukaryota - ps5
Eukaryota - ps6
Amorpheae - ps7
Obazoa - ps8
Opisthokonta - ps9
Holozoa - ps10
Filozoa - ps11
Choano./Metazoa - ps12
Metazoa - ps13
Myriazoa - ps14
Parahoxozoa - ps15
Bilateria - ps16
Protostomia - ps17
Ecdysozoa - ps18
Arthropoda - ps19
Mandibulata - ps20
Pancrustacea - ps21
Insecta - ps22
Eumetabola - ps23
Holometabola/Psocodea - ps24
Holometabola - ps25
Aparaglossata - ps26
Mecopterida - ps27
Diptera - ps28
Bibiono./Cyclorrhapha - ps29
Cyclorrhapha - ps30
Calyptratae/Drosophila - ps31
Drosophila - ps32
Sophophora - ps33
Obscura/Melanogaster_group - ps34
D_melanogaster_group - ps35
D_melanogaster_subgroup - ps36
sim./sech./mel. group - ps37
Drosophila_melanogaster - ps38
p-value
1e-02
2e-02
2e-02
3e-02
4e-02
Log-odds
0.5
1.0
1.5
2.0
2.5
3.0
3.5
4.0

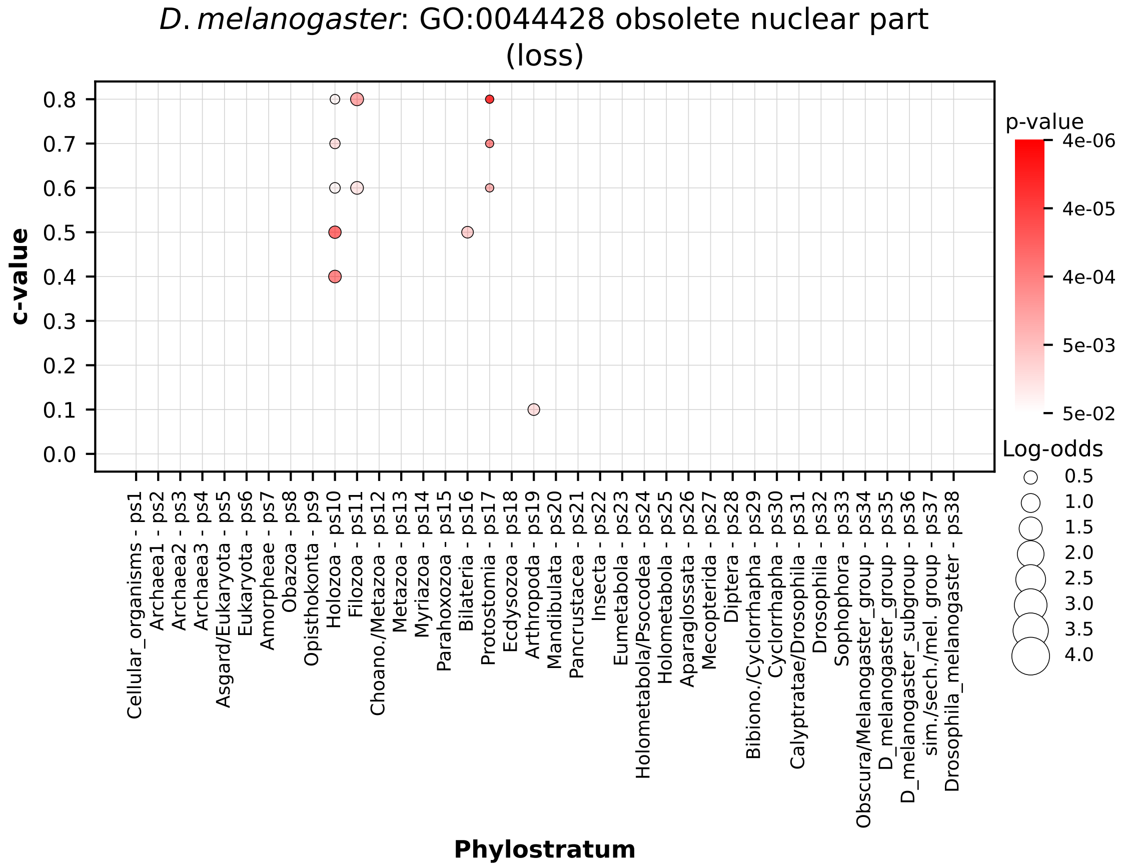

D. melanogaster: GO:0044428 obsolete nuclear part
(loss)
c-value
0.8
0.7
0.6
0.5
0.4
0.3
0.2
0.1
0.0
Phylostratum
Cellular_organisms - ps1
Archaea1 - ps2
Archaea2 - ps3
Archaea3 - ps4
Asgard/Eukaryota - ps5
Eukaryota - ps6
Amorpheae - ps7
Obazoa - ps8
Opisthokonta - ps9
Holozoa - ps10
Filozoa - ps11
Choano./Metazoa - ps12
Metazoa - ps13
Myriazoa - ps14
Parahoxozoa - ps15
Bilateria - ps16
Protostomia - ps17
Ecdysozoa - ps18
Arthropoda - ps19
Mandibulata - ps20
Pancrustacea - ps21
Insecta - ps22
Eumetabola - ps23
Holometabola/Psocodea - ps24
Holometabola - ps25
Aparaglossata - ps26
Mecopterida - ps27
Diptera - ps28
Bibiono./Cyclorrhapha - ps29
Cyclorrhapha - ps30
Calyptratae/Drosophila - ps31
Drosophila - ps32
Sophophora - ps33
Obscura/Melanogaster_group - ps34
D_melanogaster_group - ps35
D_melanogaster_subgroup - ps36
sim./sech./mel. group - ps37
Drosophila_melanogaster - ps38
p-value
4e-06
4e-05
4e-04
5e-03
5e-02
Log-odds
0.5
1.0
1.5
2.0
2.5
3.0
3.5
4.0

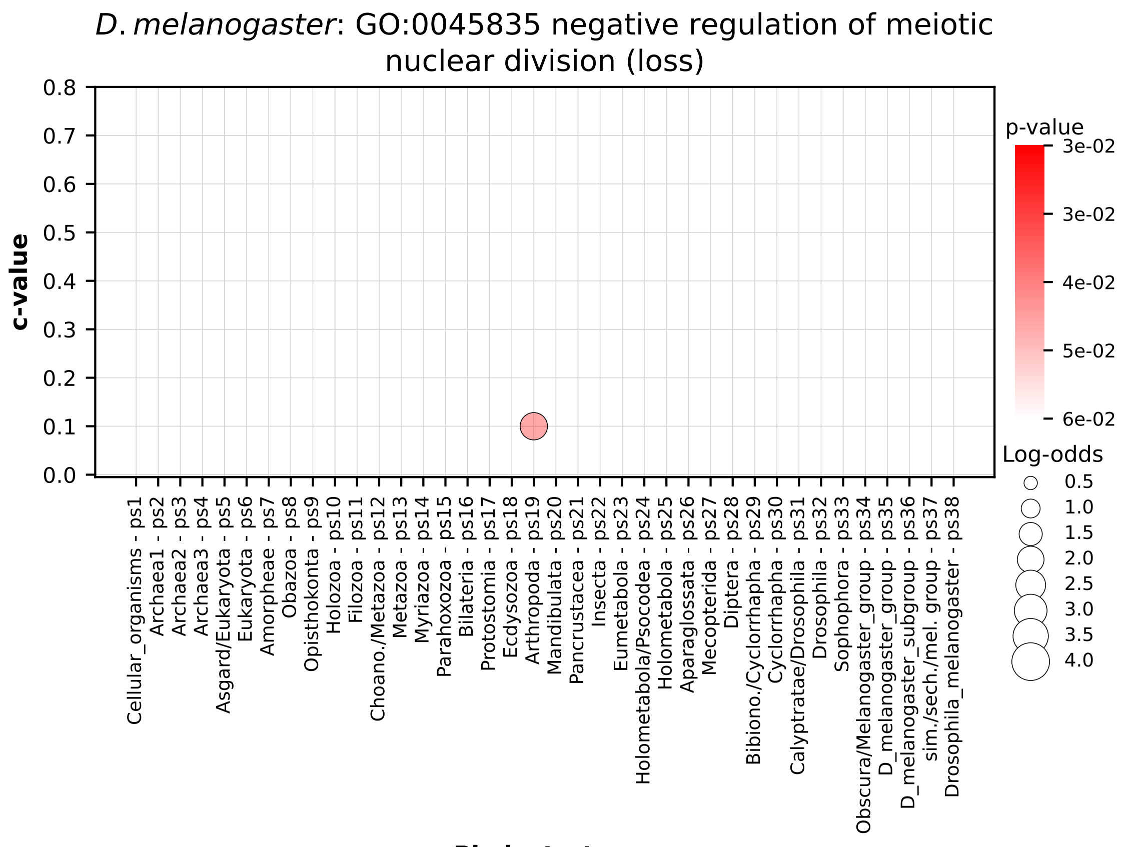

D. melanogaster: GO:0045835 negative regulation of meiotic nuclear division (loss)
c-value
0.8
0.7
0.6
0.5
0.4
0.3
0.2
0.1
0.0
Phylostratum
Cellular_organisms - ps1
Archaea1 - ps2
Archaea2 - ps3
Archaea3 - ps4
Asgard/Eukaryota - ps5
Eukaryota - ps6
Amorpheae - ps7
Obazoa - ps8
Opisthokonta - ps9
Holozoa - ps10
Filozoa - ps11
Choano./Metazoa - ps12
Metazoa - ps13
Myriazoa - ps14
Parahoxozoa - ps15
Bilateria - ps16
Protostomia - ps17
Ecdysozoa - ps18
Arthropoda - ps19
Mandibulata - ps20
Pancrustacea - ps21
Insecta - ps22
Eumetabola - ps23
Holometabola/Psocodea - ps24
Holometabola - ps25
Aparaglossata - ps26
Mecopterida - ps27
Diptera - ps28
Bibiono./Cyclorrhapha - ps29
Cyclorrhapha - ps30
Calyptratae/Drosophila - ps31
Drosophila - ps32
Sophophora - ps33
Obscura/Melanogaster_group - ps34
D_melanogaster_group - ps35
D_melanogaster_subgroup - ps36
sim./sech./mel. group - ps37
Drosophila_melanogaster - ps38
p-value
3e-02
3e-02
4e-02
5e-02
6e-02
Log-odds
0.5
1.0
1.5
2.0
2.5
3.0
3.5
4.0

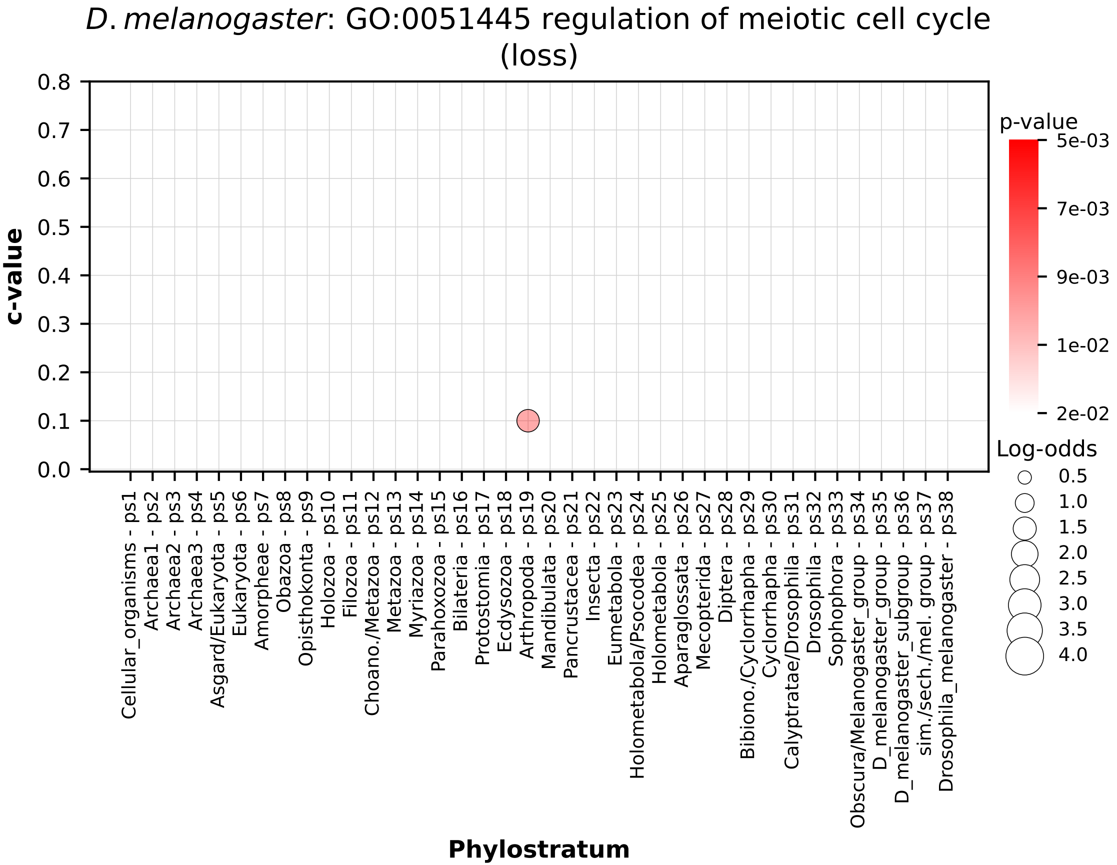
D. melanogaster: GO:0051445 regulation of meiotic cell cycle
(loss)
c-value
0.8
0.7
0.6
0.5
0.4
0.3
0.2
0.1
0.0
Phylostratum
Cellular_organisms - ps1
Archaea1 - ps2
Archaea2 - ps3
Archaea3 - ps4
Asgard/Eukaryota - ps5
Eukaryota - ps6
Amorpheae - ps7
Obazoa - ps8
Opisthokonta - ps9
Holozoa - ps10
Filozoa - ps11
Choano./Metazoa - ps12
Metazoa - ps13
Myriazoa - ps14
Parahoxozoa - ps15
Bilateria - ps16
Protostomia - ps17
Ecdysozoa - ps18
Arthropoda - ps19
Mandibulata - ps20
Pancrustacea - ps21
Insecta - ps22
Eumetabola - ps23
Holometabola/Psocodea - ps24
Holometabola - ps25
Aparaglossata - ps26
Mecopterida - ps27
Diptera - ps28
Bibiono./Cyclorrhapha - ps29
Cyclorrhapha - ps30
Calyptratae/Drosophila - ps31
Drosophila - ps32
Sophophora - ps33
Obscura/Melanogaster_group - ps34
D_melanogaster_group - ps35
D_melanogaster_subgroup - ps36
sim./sech./mel. group - ps37
Drosophila_melanogaster - ps38
p-value
5e-03
7e-03
9e-03
1e-02
2e-02
Log-odds
0.5
1.0
1.5
2.0
2.5
3.0
3.5
4.0

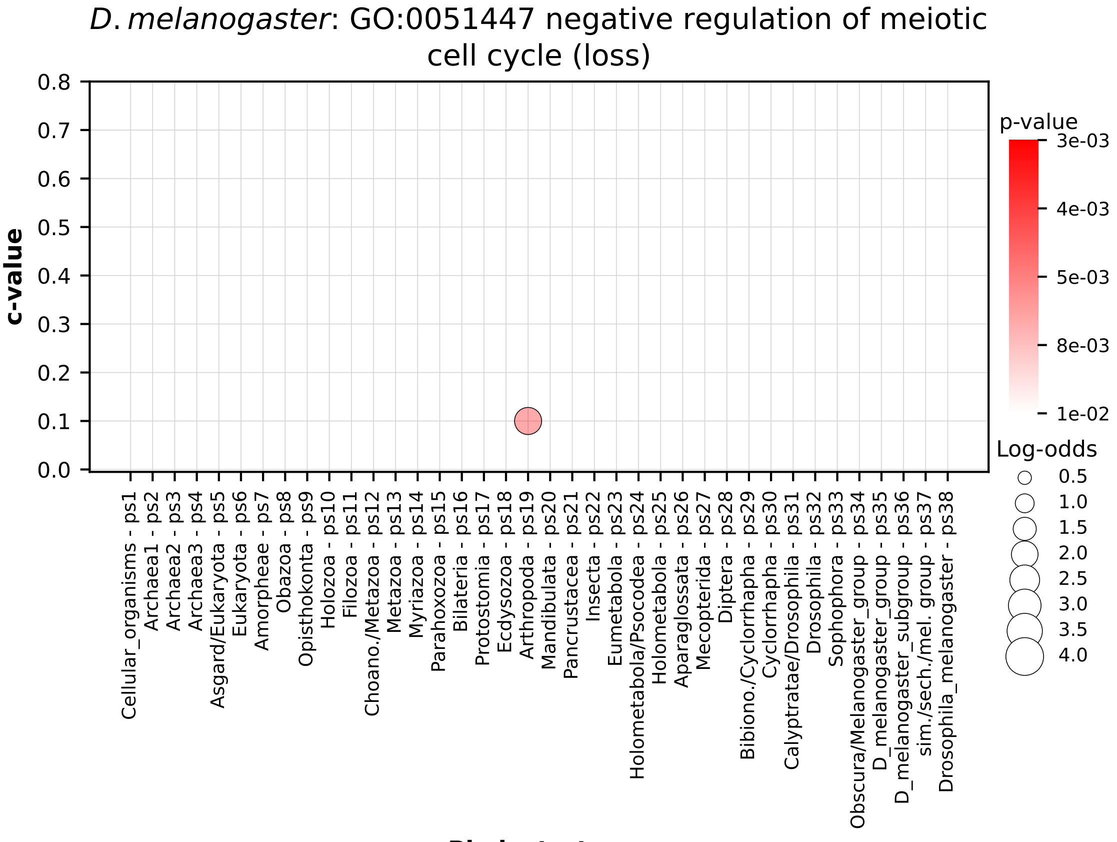

D. melanogaster: GO:0051447 negative regulation of meiotic cell cycle (loss)
c-value
0.0
0.1
0.2
0.3
0.4
0.5
0.6
0.7
0.8
Phylostratum
Cellular_organisms - ps1
Archaea1 - ps2
Archaea2 - ps3
Archaea3 - ps4
Asgard/Eukaryota - ps5
Eukaryota - ps6
Amorpheae - ps7
Obazoa - ps8
Opisthokonta - ps9
Holozoa - ps10
Filozoa - ps11
Choano./Metazoa - ps12
Metazoa - ps13
Myriazoa - ps14
Parahoxozoa - ps15
Bilateria - ps16
Protostomia - ps17
Ecdysozoa - ps18
Arthropoda - ps19
Mandibulata - ps20
Pancrustacea - ps21
Insecta - ps22
Eumetabola - ps23
Holometabola/Psocodea - ps24
Holometabola - ps25
Aparaglossata - ps26
Mecopterida - ps27
Diptera - ps28
Bibiono./Cyclorrhapha - ps29
Cyclorrhapha - ps30
Calyptratae/Drosophila - ps31
Drosophila - ps32
Sophophora - ps33
Obscura/Melanogaster_group - ps34
D_melanogaster_group - ps35
D_melanogaster_subgroup - ps36
sim./sech./mel. group - ps37
Drosophila_melanogaster - ps38
p-value
3e-03
4e-03
5e-03
8e-03
1e-02
Log-odds
0.5
1.0
1.5
2.0
2.5
3.0
3.5
4.0

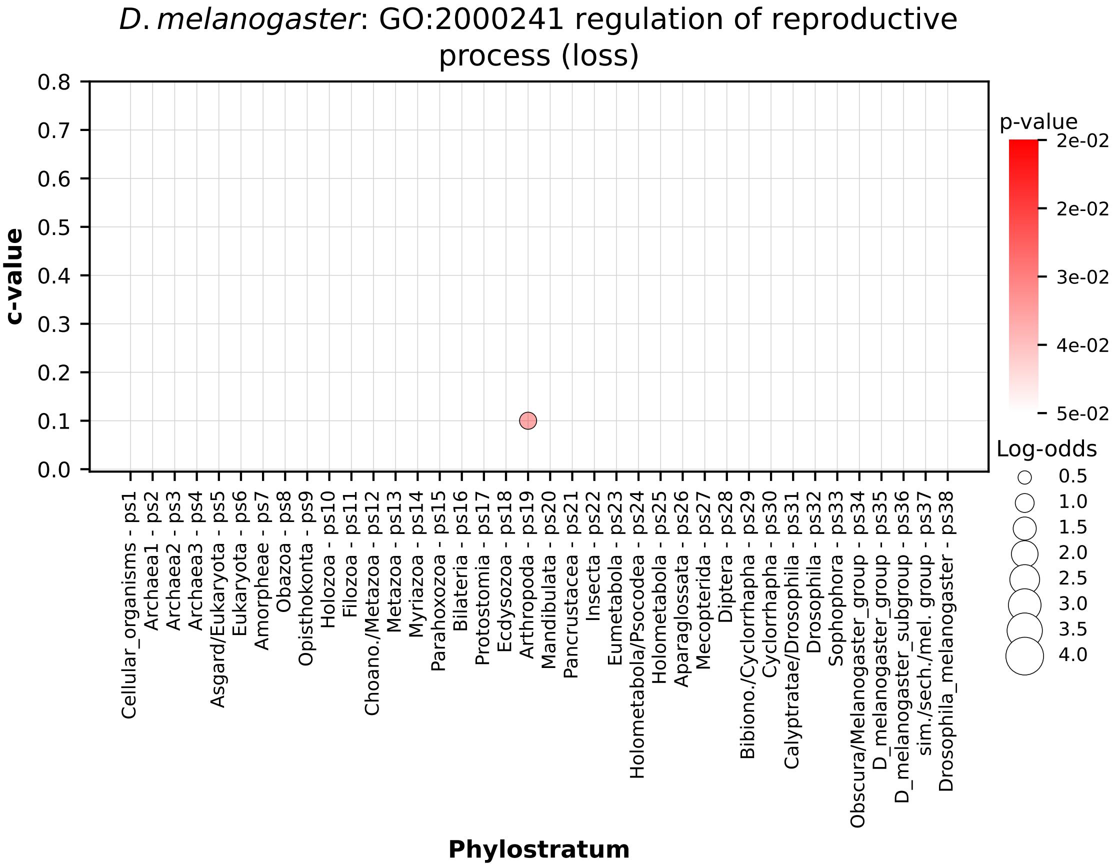

D. melanogaster: GO:2000241 regulation of reproductive process (loss)
c-value
0.0
0.1
0.2
0.3
0.4
0.5
0.6
0.7
0.8
Phylostratum
Cellular_organisms - ps1
Archaea1 - ps2
Archaea2 - ps3
Archaea3 - ps4
Asgard/Eukaryota - ps5
Eukaryota - ps6
Amorpheae - ps7
Obazoa - ps8
Opisthokonta - ps9
Holozoa - ps10
Filozoa - ps11
Choano./Metazoa - ps12
Metazoa - ps13
Myriazoa - ps14
Parahoxozoa - ps15
Bilateria - ps16
Protostomia - ps17
Ecdysozoa - ps18
Arthropoda - ps19
Mandibulata - ps20
Pancrustacea - ps21
Insecta - ps22
Eumetabola - ps23
Holometabola/Psocodea - ps24
Holometabola - ps25
Aparaglossata - ps26
Mecopterida - ps27
Diptera - ps28
Bibiono./Cyclorrhapha - ps29
Cyclorrhapha - ps30
Calyptratae/Drosophila - ps31
Drosophila - ps32
Sophophora - ps33
Obscura/Melanogaster_group - ps34
D_melanogaster_group - ps35
D_melanogaster_subgroup - ps36
sim./sech./mel. group - ps37
Drosophila_melanogaster - ps38
p-value
2e-02
2e-02
3e-02
4e-02
5e-02
Log-odds
0.5
1.0
1.5
2.0
2.5
3.0
3.5
4.0

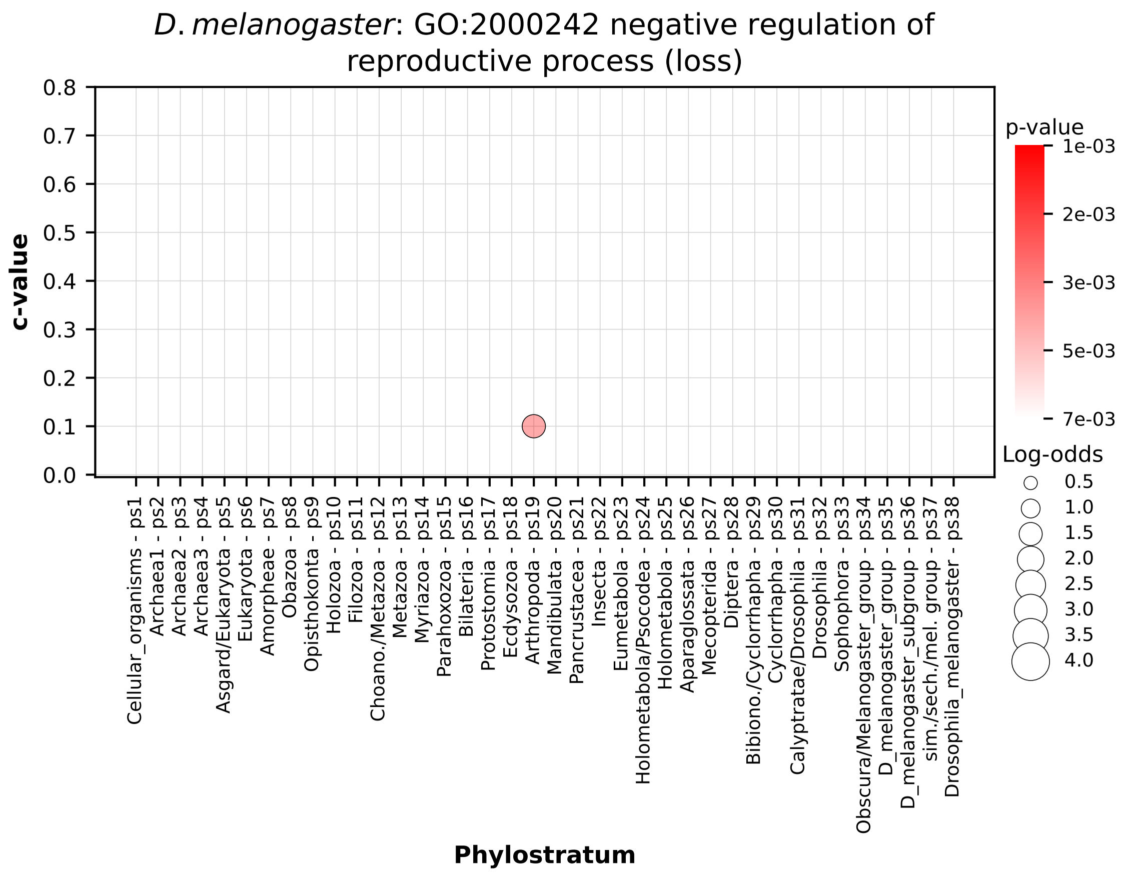

D. melanogaster: GO:2000242 negative regulation of reproductive process (loss)
c-value
0.8
0.7
0.6
0.5
0.4
0.3
0.2
0.1
0.0
p-value
1e-03
2e-03
3e-03
5e-03
7e-03
Log-odds
0.5
1.0
1.5
2.0
2.5
3.0
3.5
4.0
Cellular_organisms - ps1
Archaea1 - ps2
Archaea2 - ps3
Archaea3 - ps4
Asgard/Eukaryota - ps5
Eukaryota - ps6
Amorpheae - ps7
Obazoa - ps8
Opisthokonta - ps9
Holozoa - ps10
Filozoa - ps11
Choano./Metazoa - ps12
Metazoa - ps13
Myriazoa - ps14
Parahoxozoa - ps15
Bilateria - ps16
Protostomia - ps17
Ecdysozoa - ps18
Arthropoda - ps19
Mandibulata - ps20
Pancrustacea - ps21
Insecta - ps22
Eumetabola - ps23
Holometabola/Psocodea - ps24
Holometabola - ps25
Aparaglossata - ps26
Mecopterida - ps27
Diptera - ps28
Bibiono./Cyclorrhapha - ps29
Cyclorrhapha - ps30
Calyptratae/Drosophila - ps31
Drosophila - ps32
Sophophora - ps33
Obscura/Melanogaster_group - ps34
D_melanogaster_group - ps35
D_melanogaster_subgroup - ps36
sim./sech./mel. group - ps37
Drosophila_melanogaster - ps38
Phylostratum

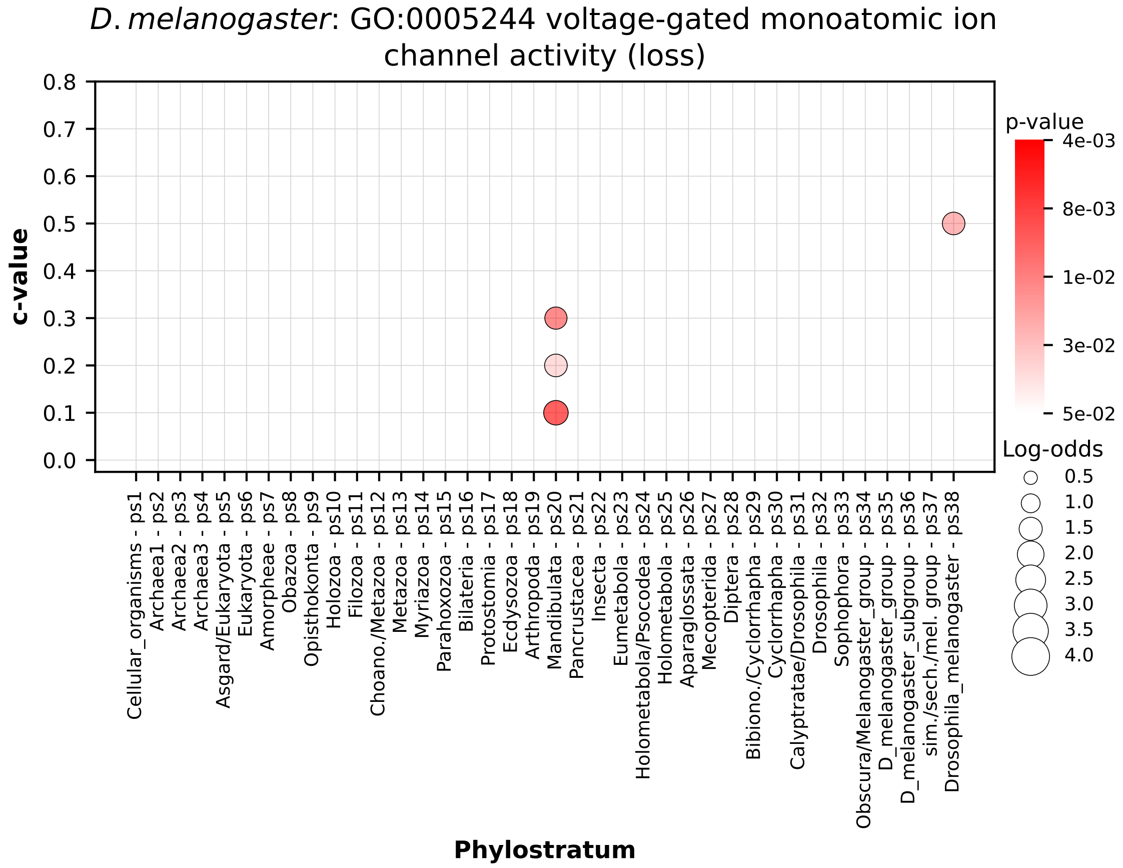

D. melanogaster: GO:0005244 voltage-gated monoatomic ion channel activity (loss)
c-value
0.8
0.7
0.6
0.5
0.4
0.3
0.2
0.1
0.0
p-value
4e-03
8e-03
1e-02
3e-02
5e-02
Log-odds
0.5
1.0
1.5
2.0
2.5
3.0
3.5
4.0
Cellular_organisms - ps1
Archaea1 - ps2
Archaea2 - ps3
Archaea3 - ps4
Asgard/Eukaryota - ps5
Eukaryota - ps6
Amorpheae - ps7
Obazoa - ps8
Opisthokonta - ps9
Holozoa - ps10
Filozoa - ps11
Choano./Metazoa - ps12
Metazoa - ps13
Myriazoa - ps14
Parahoxozoa - ps15
Bilateria - ps16
Protostomia - ps17
Ecdysozoa - ps18
Arthropoda - ps19
Mandibulata - ps20
Pancrustacea - ps21
Insecta - ps22
Eumetabola - ps23
Holometabola/Psocodea - ps24
Holometabola - ps25
Aparaglossata - ps26
Mecopterida - ps27
Diptera - ps28
Bibiono./Cyclorrhapha - ps29
Cyclorrhapha - ps30
Calyptratae/Drosophila - ps31
Drosophila - ps32
Sophophora - ps33
Obscura/Melanogaster_group - ps34
D_melanogaster_group - ps35
D_melanogaster_subgroup - ps36
sim./sech./mel. group - ps37
Drosophila_melanogaster - ps38
Phylostratum

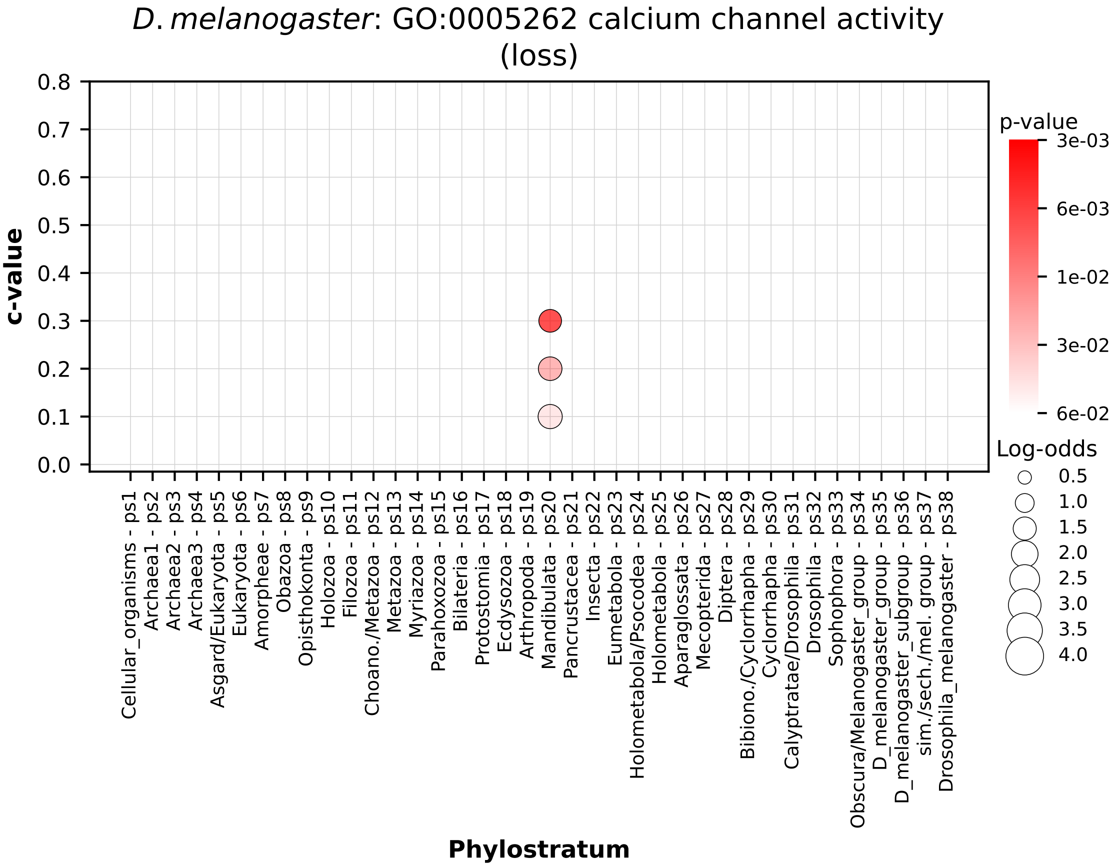

D. melanogaster: GO:0005262 calcium channel activity
(loss)
c-value
0.8
0.7
0.6
0.5
0.4
0.3
0.2
0.1
0.0
p-value
3e-03
6e-03
1e-02
3e-02
6e-02
Log-odds
0.5
1.0
1.5
2.0
2.5
3.0
3.5
4.0
Cellular_organisms - ps1
Archaea1 - ps2
Archaea2 - ps3
Archaea3 - ps4
Asgard/Eukaryota - ps5
Eukaryota - ps6
Amorpheae - ps7
Obazoa - ps8
Opisthokonta - ps9
Holozoa - ps10
Filozoa - ps11
Choano./Metazoa - ps12
Metazoa - ps13
Myriazoa - ps14
Parahoxozoa - ps15
Bilateria - ps16
Protostomia - ps17
Ecdysozoa - ps18
Arthropoda - ps19
Mandibulata - ps20
Pancrustacea - ps21
Insecta - ps22
Eumetabola - ps23
Holometabola/Psocodea - ps24
Holometabola - ps25
Aparaglossata - ps26
Mecopterida - ps27
Diptera - ps28
Bibiono./Cyclorrhapha - ps29
Cyclorrhapha - ps30
Calyptratae/Drosophila - ps31
Drosophila - ps32
Sophophora - ps33
Obscura/Melanogaster_group - ps34
D_melanogaster_group - ps35
D_melanogaster_subgroup - ps36
sim./sech./mel. group - ps37
Drosophila_melanogaster - ps38
Phylostratum

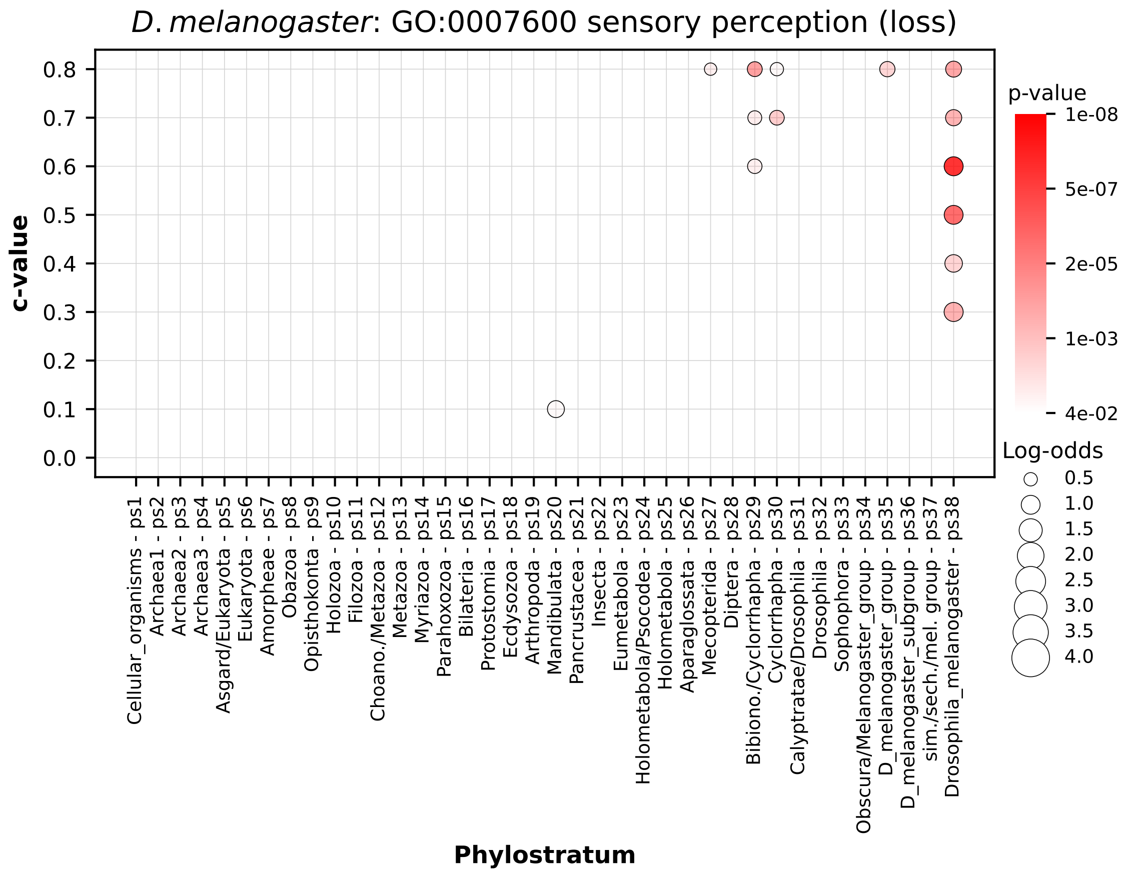

D. melanogaster: GO:0007600 sensory perception (loss)
c-value
0.0
0.1
0.2
0.3
0.4
0.5
0.6
0.7
0.8
Phylostratum
Cellular_organisms - ps1
Archaea1 - ps2
Archaea2 - ps3
Archaea3 - ps4
Asgard/Eukaryota - ps5
Eukaryota - ps6
Amorpheae - ps7
Obazoa - ps8
Opisthokonta - ps9
Holozoa - ps10
Filozoa - ps11
Choano./Metazoa - ps12
Metazoa - ps13
Myriazoa - ps14
Parahoxozoa - ps15
Bilateria - ps16
Protostomia - ps17
Ecdysozoa - ps18
Arthropoda - ps19
Mandibulata - ps20
Pancrustacea - ps21
Insecta - ps22
Eumetabola - ps23
Holometabola/Psocodea - ps24
Holometabola - ps25
Aparaglossata - ps26
Mecopterida - ps27
Diptera - ps28
Bibiono./Cyclorrhapha - ps29
Cyclorrhapha - ps30
Calyptratae/Drosophila - ps31
Drosophila - ps32
Sophophora - ps33
Obscura/Melanogaster_group - ps34
D_melanogaster_group - ps35
D_melanogaster_subgroup - ps36
sim./sech./mel. group - ps37
Drosophila_melanogaster - ps38
p-value
1e-08
5e-07
2e-05
1e-03
4e-02
Log-odds
0.5
1.0
1.5
2.0
2.5
3.0
3.5
4.0

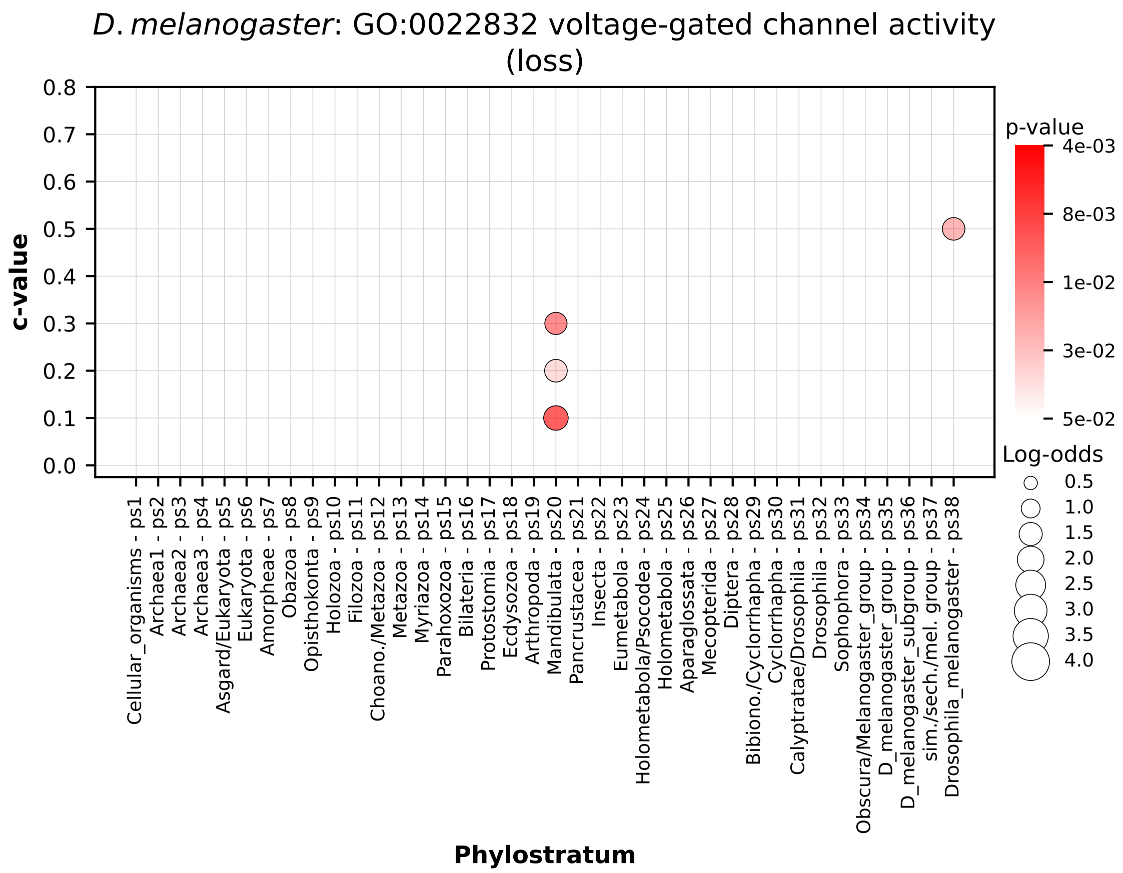

D. melanogaster: GO:0022832 voltage-gated channel activity (loss)
c-value
0.0
0.1
0.2
0.3
0.4
0.5
0.6
0.7
0.8
Phylostratum
Cellular_organisms - ps1
Archaea1 - ps2
Archaea2 - ps3
Archaea3 - ps4
Asgard/Eukaryota - ps5
Eukaryota - ps6
Amorpheae - ps7
Obazoa - ps8
Opisthokonta - ps9
Holozoa - ps10
Filozoa - ps11
Choano./Metazoa - ps12
Metazoa - ps13
Myriazoa - ps14
Parahoxozoa - ps15
Bilateria - ps16
Protostomia - ps17
Ecdysozoa - ps18
Arthropoda - ps19
Mandibulata - ps20
Pancrustacea - ps21
Insecta - ps22
Eumetabola - ps23
Holometabola/Psocodea - ps24
Holometabola - ps25
Aparaglossata - ps26
Mecopterida - ps27
Diptera - ps28
Bibiono./Cyclorrhapha - ps29
Cyclorrhapha - ps30
Calyptratae/Drosophila - ps31
Drosophila - ps32
Sophophora - ps33
Obscura/Melanogaster_group - ps34
D_melanogaster_group - ps35
D_melanogaster_subgroup - ps36
sim./sech./mel. group - ps37
Drosophila_melanogaster - ps38
p-value
4e-03
8e-03
1e-02
3e-02
5e-02
Log-odds
0.5
1.0
1.5
2.0
2.5
3.0
3.5
4.0

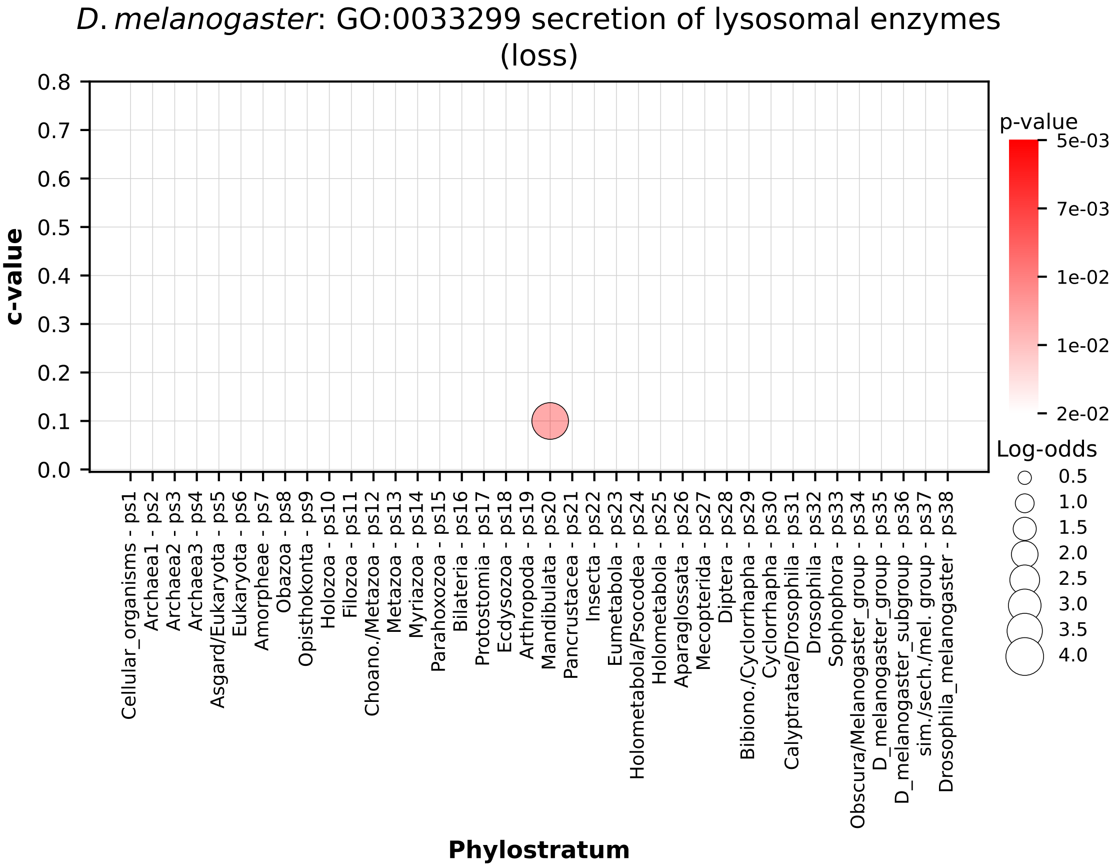

D. melanogaster: GO:0033299 secretion of lysosomal enzymes
(loss)
c-value
0.0
0.1
0.2
0.3
0.4
0.5
0.6
0.7
0.8
Phylostratum
Cellular_organisms - ps1
Archaea1 - ps2
Archaea2 - ps3
Archaea3 - ps4
Asgard/Eukaryota - ps5
Eukaryota - ps6
Amorpheae - ps7
Obazoa - ps8
Opisthokonta - ps9
Holozoa - ps10
Filozoa - ps11
Choano./Metazoa - ps12
Metazoa - ps13
Myriazoa - ps14
Parahoxozoa - ps15
Bilateria - ps16
Protostomia - ps17
Ecdysozoa - ps18
Arthropoda - ps19
Mandibulata - ps20
Pancrustacea - ps21
Insecta - ps22
Eumetabola - ps23
Holometabola/Psocodea - ps24
Holometabola - ps25
Aparaglossata - ps26
Mecopterida - ps27
Diptera - ps28
Bibiono./Cyclorrhapha - ps29
Cyclorrhapha - ps30
Calyptratae/Drosophila - ps31
Drosophila - ps32
Sophophora - ps33
Obscura/Melanogaster_group - ps34
D_melanogaster_group - ps35
D_melanogaster_subgroup - ps36
sim./sech./mel. group - ps37
Drosophila_melanogaster - ps38
p-value
5e-03
7e-03
1e-02
1e-02
2e-02
Log-odds
0.5
1.0
1.5
2.0
2.5
3.0
3.5
4.0

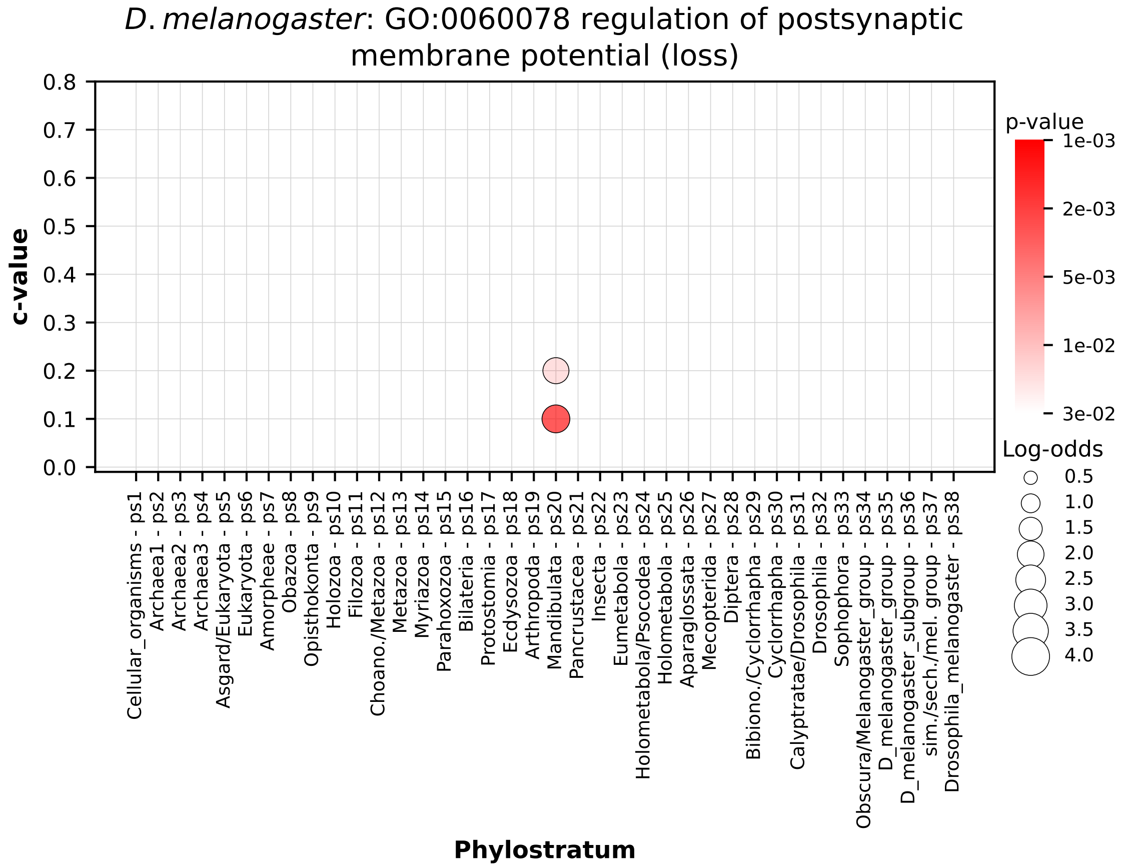

D. melanogaster: GO:0060078 regulation of postsynaptic membrane potential (loss)
c-value
0.0
0.1
0.2
0.3
0.4
0.5
0.6
0.7
0.8
Phylostratum
Cellular_organisms - ps1
Archaea1 - ps2
Archaea2 - ps3
Archaea3 - ps4
Asgard/Eukaryota - ps5
Eukaryota - ps6
Amorpheae - ps7
Obazoa - ps8
Opisthokonta - ps9
Holozoa - ps10
Filozoa - ps11
Choano./Metazoa - ps12
Metazoa - ps13
Myriazoa - ps14
Parahoxozoa - ps15
Bilateria - ps16
Protostomia - ps17
Ecdysozoa - ps18
Arthropoda - ps19
Mandibulata - ps20
Pancrustacea - ps21
Insecta - ps22
Eumetabola - ps23
Holometabola/Psocodea - ps24
Holometabola - ps25
Aparaglossata - ps26
Mecopterida - ps27
Diptera - ps28
Bibiono./Cyclorrhapha - ps29
Cyclorrhapha - ps30
Calyptratae/Drosophila - ps31
Drosophila - ps32
Sophophora - ps33
Obscura/Melanogaster_group - ps34
D_melanogaster_group - ps35
D_melanogaster_subgroup - ps36
sim./sech./mel. group - ps37
Drosophila_melanogaster - ps38
p-value
1e-03
2e-03
5e-03
1e-02
3e-02
Log-odds
0.5
1.0
1.5
2.0
2.5
3.0
3.5
4.0

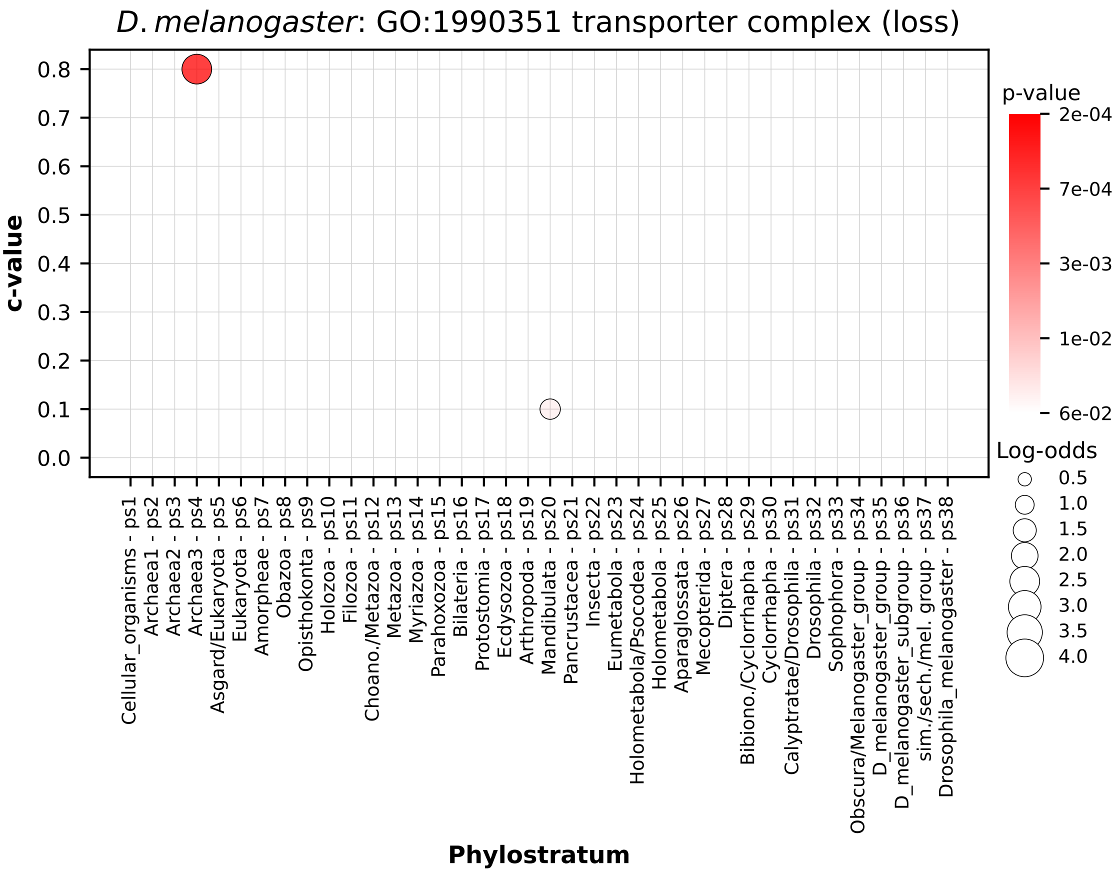

D. melanogaster: GO:1990351 transporter complex (loss)
c-value
0.8
0.7
0.6
0.5
0.4
0.3
0.2
0.1
0.0
p-value
2e-04
7e-04
3e-03
1e-02
6e-02
Log-odds
0.5
1.0
1.5
2.0
2.5
3.0
3.5
4.0
Cellular_organisms - ps1
Archaea1 - ps2
Archaea2 - ps3
Archaea3 - ps4
Asgard/Eukaryota - ps5
Eukaryota - ps6
Amorpheae - ps7
Obazoa - ps8
Opisthokonta - ps9
Holozoa - ps10
Filozoa - ps11
Choano./Metazoa - ps12
Metazoa - ps13
Myriazoa - ps14
Parahoxozoa - ps15
Bilateria - ps16
Protostomia - ps17
Ecdysozoa - ps18
Arthropoda - ps19
Mandibulata - ps20
Pancrustacea - ps21
Insecta - ps22
Eumetabola - ps23
Holometabola/Psocodea - ps24
Holometabola - ps25
Aparaglossata - ps26
Mecopterida - ps27
Diptera - ps28
Bibiono./Cyclorrhapha - ps29
Cyclorrhapha - ps30
Calyptratae/Drosophila - ps31
Drosophila - ps32
Sophophora - ps33
Obscura/Melanogaster_group - ps34
D_melanogaster_group - ps35
D_melanogaster_subgroup - ps36
sim./sech./mel. group - ps37
Drosophila_melanogaster - ps38
Phylostratum

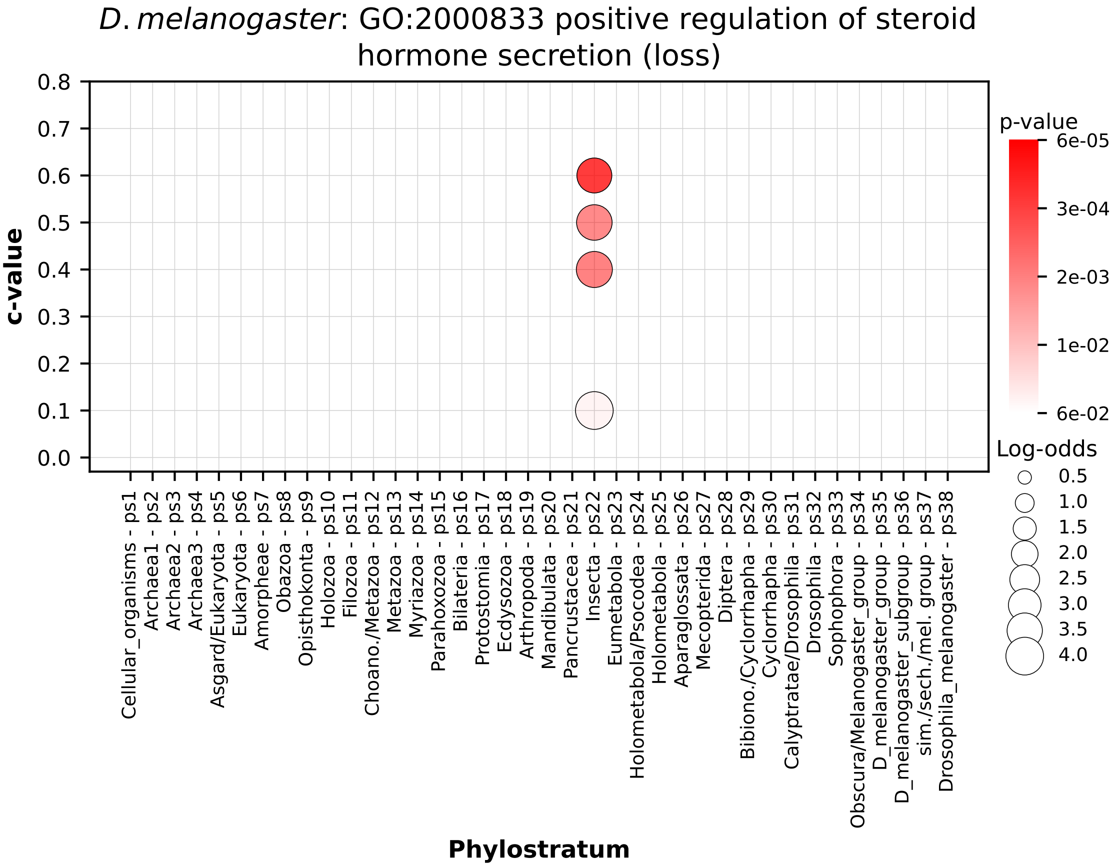

D. melanogaster: GO:2000833 positive regulation of steroid hormone secretion (loss)
c-value
0.8
0.7
0.6
0.5
0.4
0.3
0.2
0.1
0.0
Phylostratum
Cellular_organisms - ps1
Archaea1 - ps2
Archaea2 - ps3
Archaea3 - ps4
Asgard/Eukaryota - ps5
Eukaryota - ps6
Amorpheae - ps7
Obazoa - ps8
Opisthokonta - ps9
Holozoa - ps10
Filozoa - ps11
Choano./Metazoa - ps12
Metazoa - ps13
Myriazoa - ps14
Parahoxozoa - ps15
Bilateria - ps16
Protostomia - ps17
Ecdysozoa - ps18
Arthropoda - ps19
Mandibulata - ps20
Pancrustacea - ps21
Insecta - ps22
Eumetabola - ps23
Holometabola/Psocodea - ps24
Holometabola - ps25
Aparaglossata - ps26
Mecopterida - ps27
Diptera - ps28
Bibiono./Cyclorrhapha - ps29
Cyclorrhapha - ps30
Calyptratae/Drosophila - ps31
Drosophila - ps32
Sophophora - ps33
Obscura/Melanogaster_group - ps34
D_melanogaster_group - ps35
D_melanogaster_subgroup - ps36
sim./sech./mel. group - ps37
Drosophila_melanogaster - ps38
p-value
6e-05
3e-04
2e-03
1e-02
6e-02
Log-odds
0.5
1.0
1.5
2.0
2.5
3.0
3.5
4.0

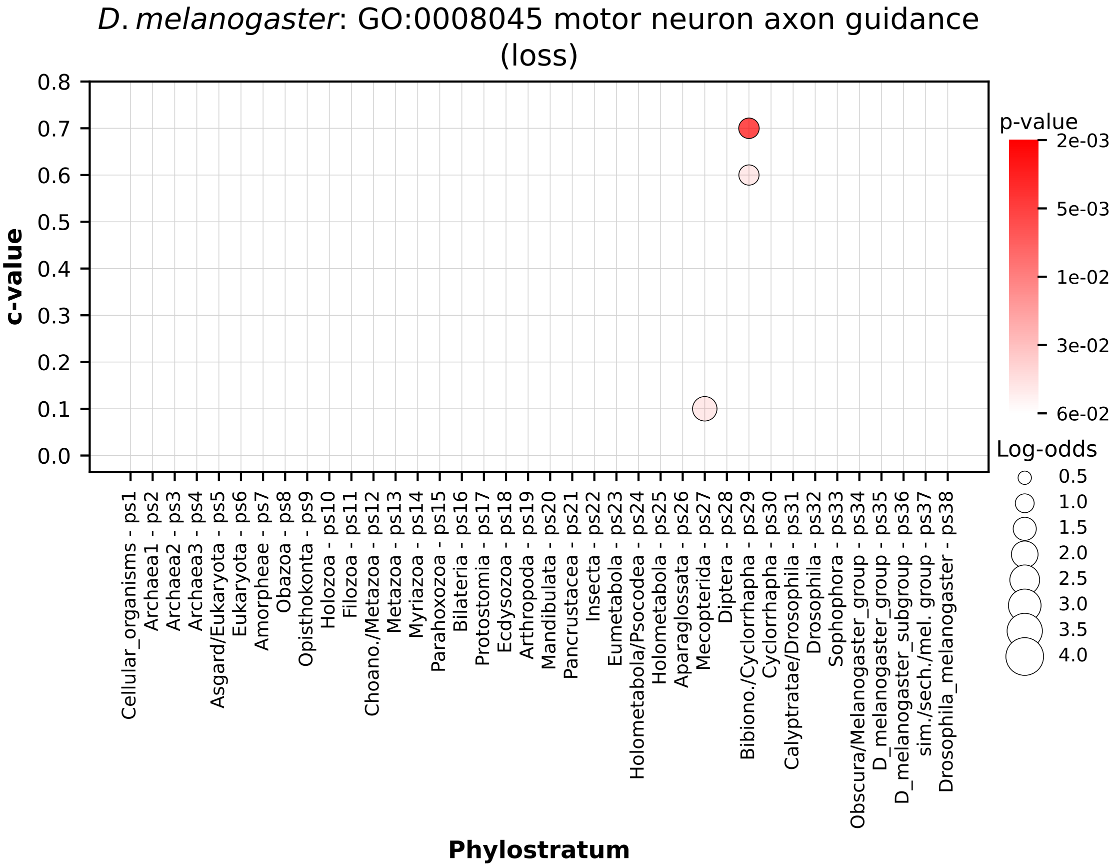
D. melanogaster: GO:0008045 motor neuron axon guidance
(loss)
c-value
0.8
0.7
0.6
0.5
0.4
0.3
0.2
0.1
0.0
Phylostratum
Cellular_organisms - ps1
Archaea1 - ps2
Archaea2 - ps3
Archaea3 - ps4
Asgard/Eukaryota - ps5
Eukaryota - ps6
Amorpheae - ps7
Obazoa - ps8
Opisthokonta - ps9
Holozoa - ps10
Filozoa - ps11
Choano./Metazoa - ps12
Metazoa - ps13
Myriazoa - ps14
Parahoxozoa - ps15
Bilateria - ps16
Protostomia - ps17
Ecdysozoa - ps18
Arthropoda - ps19
Mandibulata - ps20
Pancrustacea - ps21
Insecta - ps22
Eumetabola - ps23
Holometabola/Psocodea - ps24
Holometabola - ps25
Aparaglossata - ps26
Mecopterida - ps27
Diptera - ps28
Bibiono./Cyclorrhapha - ps29
Cyclorrhapha - ps30
Calyptratae/Drosophila - ps31
Drosophila - ps32
Sophophora - ps33
Obscura/Melanogaster_group - ps34
D_melanogaster_group - ps35
D_melanogaster_subgroup - ps36
sim./sech./mel. group - ps37
Drosophila_melanogaster - ps38
p-value
2e-03
5e-03
1e-02
3e-02
6e-02
Log-odds
0.5
1.0
1.5
2.0
2.5
3.0
3.5
4.0

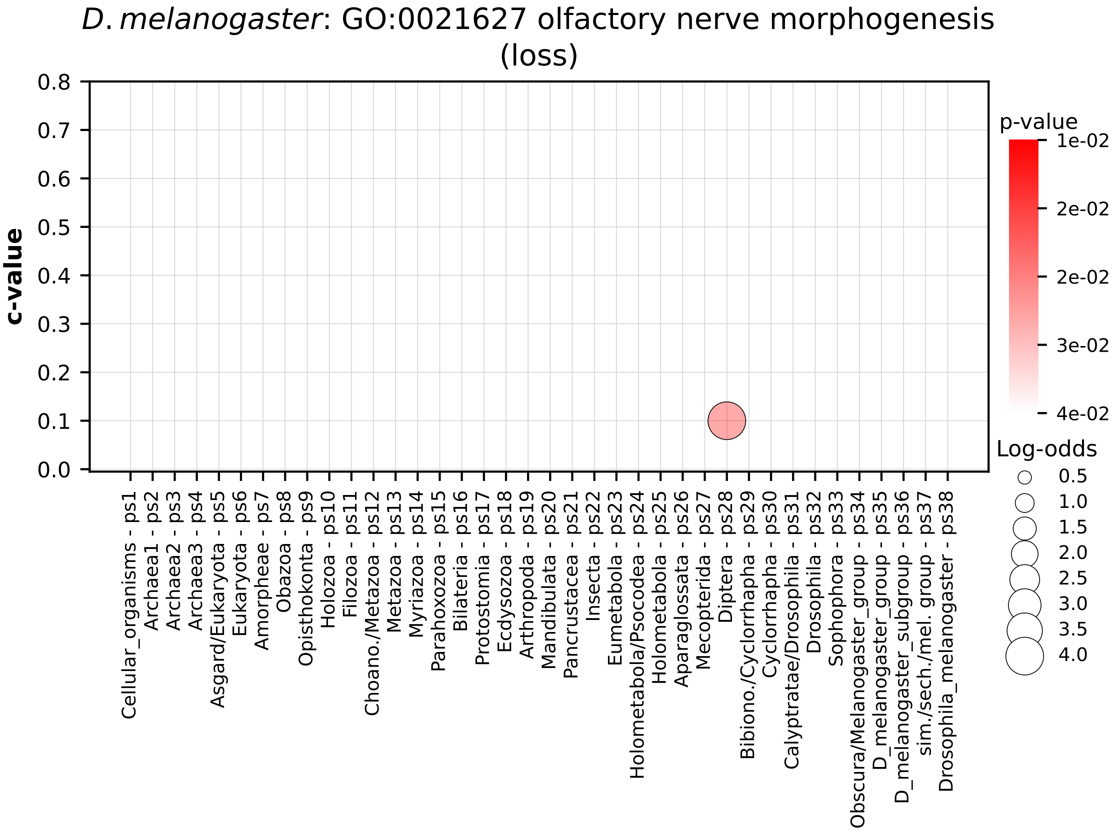

D. melanogaster: GO:0021627 olfactory nerve morphogenesis (loss)
c-value
0.0
0.1
0.2
0.3
0.4
0.5
0.6
0.7
0.8
Phylostratum
Cellular_organisms - ps1
Archaea1 - ps2
Archaea2 - ps3
Archaea3 - ps4
Asgard/Eukaryota - ps5
Eukaryota - ps6
Amorpheae - ps7
Obazoa - ps8
Opisthokonta - ps9
Holozoa - ps10
Filozoa - ps11
Choano./Metazoa - ps12
Metazoa - ps13
Myriazoa - ps14
Parahoxozoa - ps15
Bilateria - ps16
Protostomia - ps17
Ecdysozoa - ps18
Arthropoda - ps19
Mandibulata - ps20
Pancrustacea - ps21
Insecta - ps22
Eumetabola - ps23
Holometabola/Psocodea - ps24
Holometabola - ps25
Aparaglossata - ps26
Mecopterida - ps27
Diptera - ps28
Bibiono./Cyclorrhapha - ps29
Cyclorrhapha - ps30
Calyptratae/Drosophila - ps31
Drosophila - ps32
Sophophora - ps33
Obscura/Melanogaster_group - ps34
D_melanogaster_group - ps35
D_melanogaster_subgroup - ps36
sim./sech./mel. group - ps37
Drosophila_melanogaster - ps38
p-value
1e-02
2e-02
2e-02
3e-02
4e-02
Log-odds
0.5
1.0
1.5
2.0
2.5
3.0
3.5
4.0

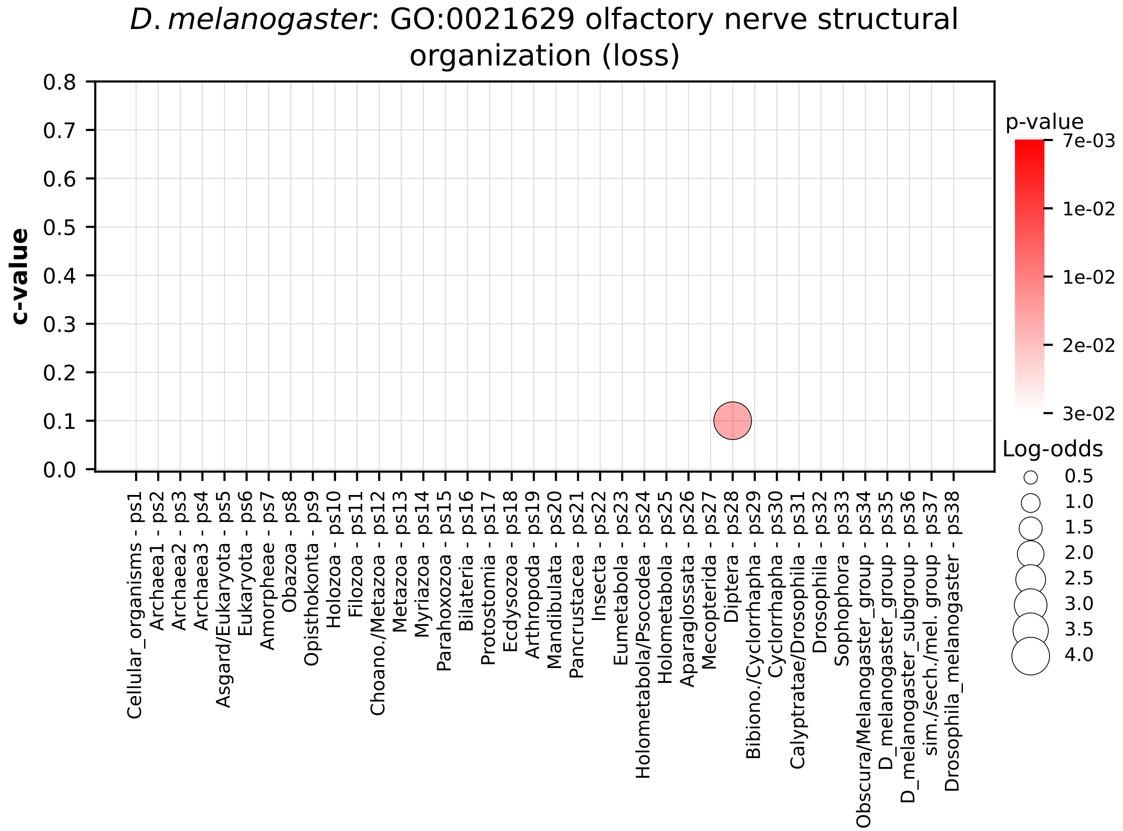

D. melanogaster: GO:0021629 olfactory nerve structural organization (loss)
c-value
0.8
0.7
0.6
0.5
0.4
0.3
0.2
0.1
0.0
p-value
7e-03
1e-02
1e-02
2e-02
3e-02
Log-odds
0.5
1.0
1.5
2.0
2.5
3.0
3.5
4.0
Cellular_organisms - ps1
Archaea1 - ps2
Archaea2 - ps3
Archaea3 - ps4
Asgard/Eukaryota - ps5
Eukaryota - ps6
Amorpheae - ps7
Obazoa - ps8
Opisthokonta - ps9
Holozoa - ps10
Filozoa - ps11
Choano./Metazoa - ps12
Metazoa - ps13
Myriazoa - ps14
Parahoxozoa - ps15
Bilateria - ps16
Protostomia - ps17
Ecdysozoa - ps18
Arthropoda - ps19
Mandibulata - ps20
Pancrustacea - ps21
Insecta - ps22
Eumetabola - ps23
Holometabola/Psocodea - ps24
Holometabola - ps25
Aparaglossata - ps26
Mecopterida - ps27
Diptera - ps28
Bibiono./Cyclorrhapha - ps29
Cyclorrhapha - ps30
Calyptratae/Drosophila - ps31
Drosophila - ps32
Sophophora - ps33
Obscura/Melanogaster_group - ps34
D_melanogaster_group - ps35
D_melanogaster_subgroup - ps36
sim./sech./mel. group - ps37
Drosophila_melanogaster - ps38
Phylostratum

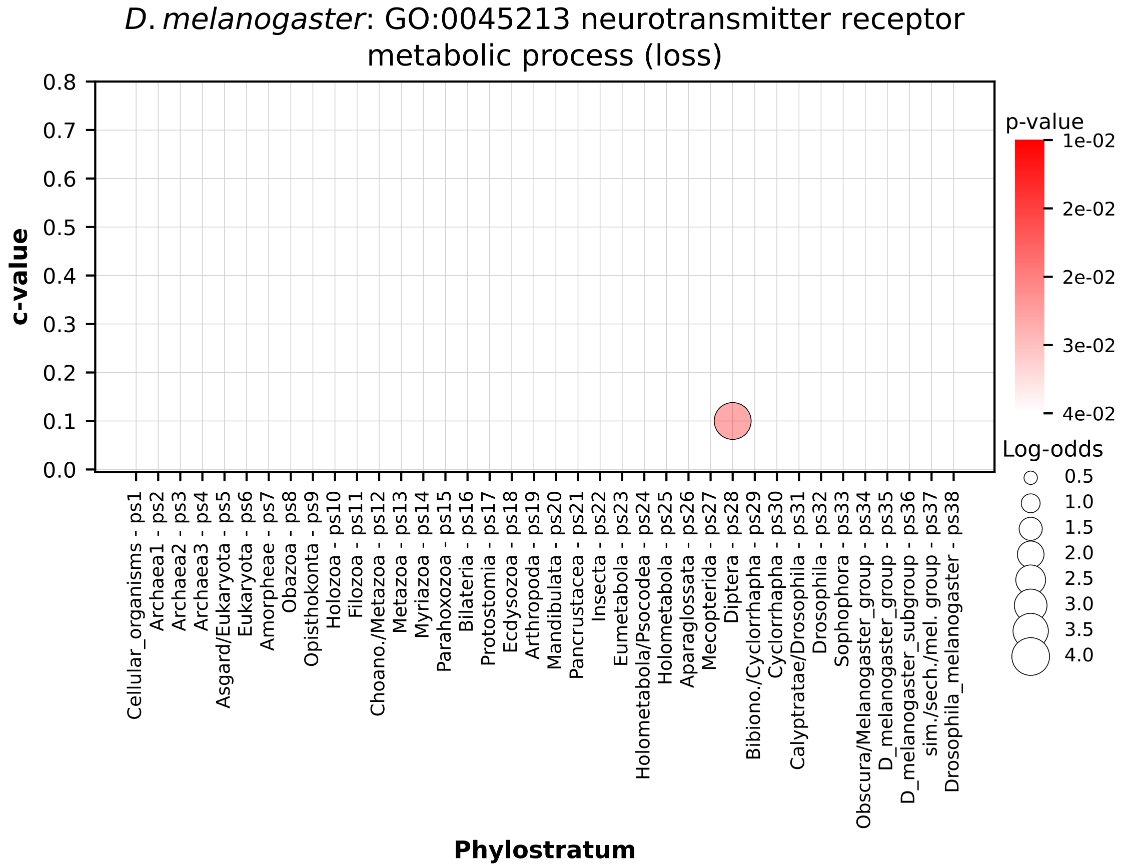

D. melanogaster: GO:0045213 neurotransmitter receptor metabolic process (loss)
c-value
0.0
0.1
0.2
0.3
0.4
0.5
0.6
0.7
0.8
Phylostratum
Cellular_organisms - ps1
Archaea1 - ps2
Archaea2 - ps3
Archaea3 - ps4
Asgard/Eukaryota - ps5
Eukaryota - ps6
Amorpheae - ps7
Obazoa - ps8
Opisthokonta - ps9
Holozoa - ps10
Filozoa - ps11
Choano./Metazoa - ps12
Metazoa - ps13
Myriazoa - ps14
Parahoxozoa - ps15
Bilateria - ps16
Protostomia - ps17
Ecdysozoa - ps18
Arthropoda - ps19
Mandibulata - ps20
Pancrustacea - ps21
Insecta - ps22
Eumetabola - ps23
Holometabola/Psocodea - ps24
Holometabola - ps25
Aparaglossata - ps26
Mecopterida - ps27
Diptera - ps28
Bibiono./Cyclorrhapha - ps29
Cyclorrhapha - ps30
Calyptratae/Drosophila - ps31
Drosophila - ps32
Sophophora - ps33
Obscura/Melanogaster_group - ps34
D_melanogaster_group - ps35
D_melanogaster_subgroup - ps36
sim./sech./mel. group - ps37
Drosophila_melanogaster - ps38
p-value
1e-02
2e-02
2e-02
3e-02
4e-02
Log-odds
0.5
1.0
1.5
2.0
2.5
3.0
3.5
4.0

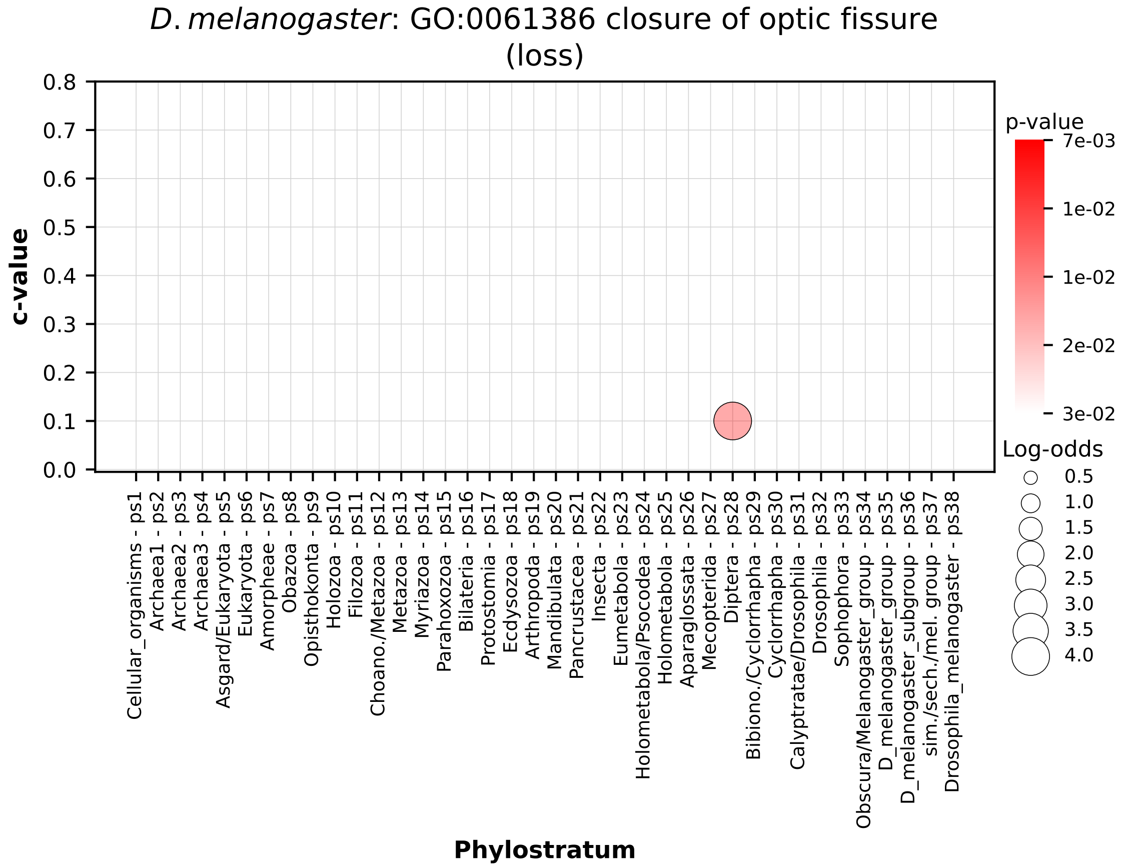

D. melanogaster: GO:0061386 closure of optic fissure (loss)
c-value
0.0
0.1
0.2
0.3
0.4
0.5
0.6
0.7
0.8
p-value
7e-03
1e-02
1e-02
2e-02
3e-02
Log-odds
0.5
1.0
1.5
2.0
2.5
3.0
3.5
4.0
Cellular_organisms - ps1
Archaea1 - ps2
Archaea2 - ps3
Archaea3 - ps4
Asgard/Eukaryota - ps5
Eukaryota - ps6
Amorpheae - ps7
Obazoa - ps8
Opisthokonta - ps9
Holozoa - ps10
Filozoa - ps11
Choano./Metazoa - ps12
Metazoa - ps13
Myriazoa - ps14
Parahoxozoa - ps15
Bilateria - ps16
Protostomia - ps17
Ecdysozoa - ps18
Arthropoda - ps19
Mandibulata - ps20
Pancrustacea - ps21
Insecta - ps22
Eumetabola - ps23
Holometabola/Psocodea - ps24
Holometabola - ps25
Aparaglossata - ps26
Mecopterida - ps27
Diptera - ps28
Bibiono./Cyclorrhapha - ps29
Cyclorrhapha - ps30
Calyptratae/Drosophila - ps31
Drosophila - ps32
Sophophora - ps33
Obscura/Melanogaster_group - ps34
D_melanogaster_group - ps35
D_melanogaster_subgroup - ps36
sim./sech./mel. group - ps37
Drosophila_melanogaster - ps38
Phylostratum

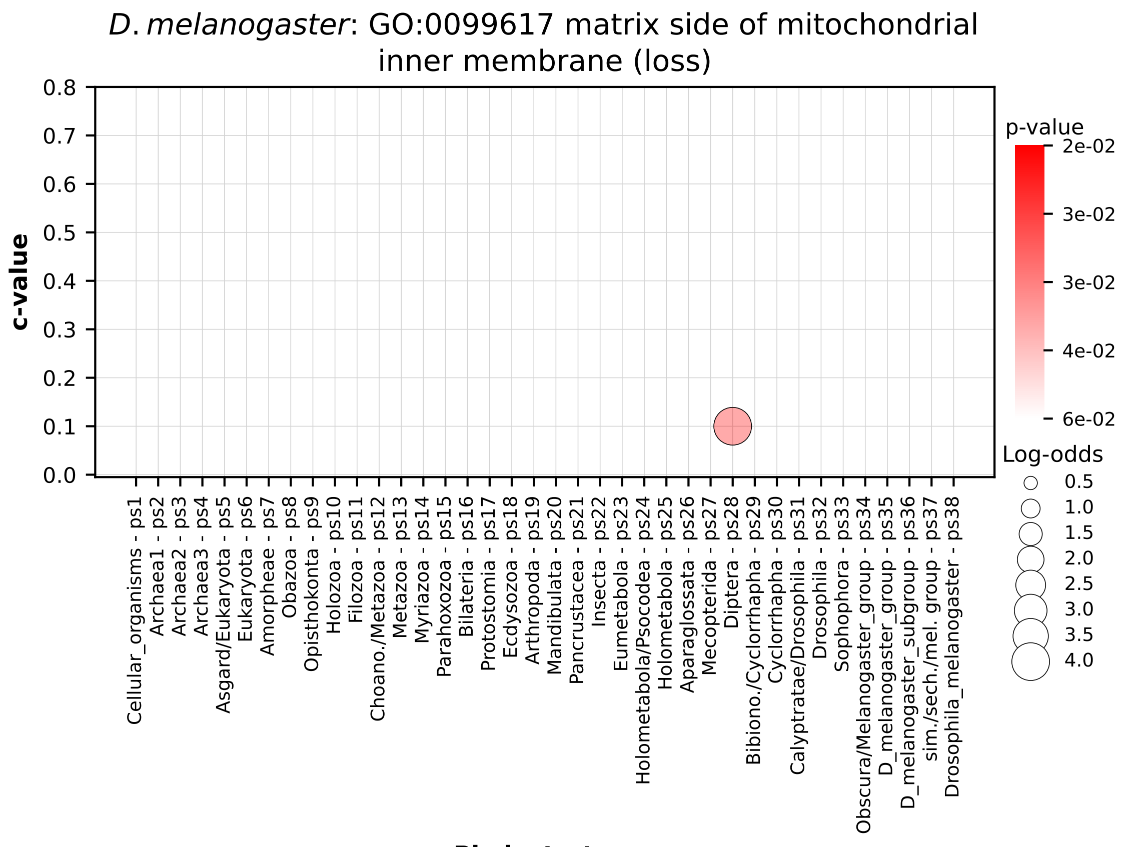

D. melanogaster: GO:0099617 matrix side of mitochondrial inner membrane (loss)
c-value
0.0
0.1
0.2
0.3
0.4
0.5
0.6
0.7
0.8
Phylostratum
Cellular_organisms - ps1
Archaea1 - ps2
Archaea2 - ps3
Archaea3 - ps4
Asgard/Eukaryota - ps5
Eukaryota - ps6
Amorpheae - ps7
Obazoa - ps8
Opisthokonta - ps9
Holozoa - ps10
Filozoa - ps11
Choano./Metazoa - ps12
Metazoa - ps13
Myriazoa - ps14
Parahoxozoa - ps15
Bilateria - ps16
Protostomia - ps17
Ecdysozoa - ps18
Arthropoda - ps19
Mandibulata - ps20
Pancrustacea - ps21
Insecta - ps22
Eumetabola - ps23
Holometabola/Psocodea - ps24
Holometabola - ps25
Aparaglossata - ps26
Mecopterida - ps27
Diptera - ps28
Bibiono./Cyclorrhapha - ps29
Cyclorrhapha - ps30
Calyptratae/Drosophila - ps31
Drosophila - ps32
Sophophora - ps33
Obscura/Melanogaster_group - ps34
D_melanogaster_group - ps35
D_melanogaster_subgroup - ps36
sim./sech./mel. group - ps37
Drosophila_melanogaster - ps38
p-value
2e-02
3e-02
3e-02
4e-02
6e-02
Log-odds
0.5
1.0
1.5
2.0
2.5
3.0
3.5
4.0

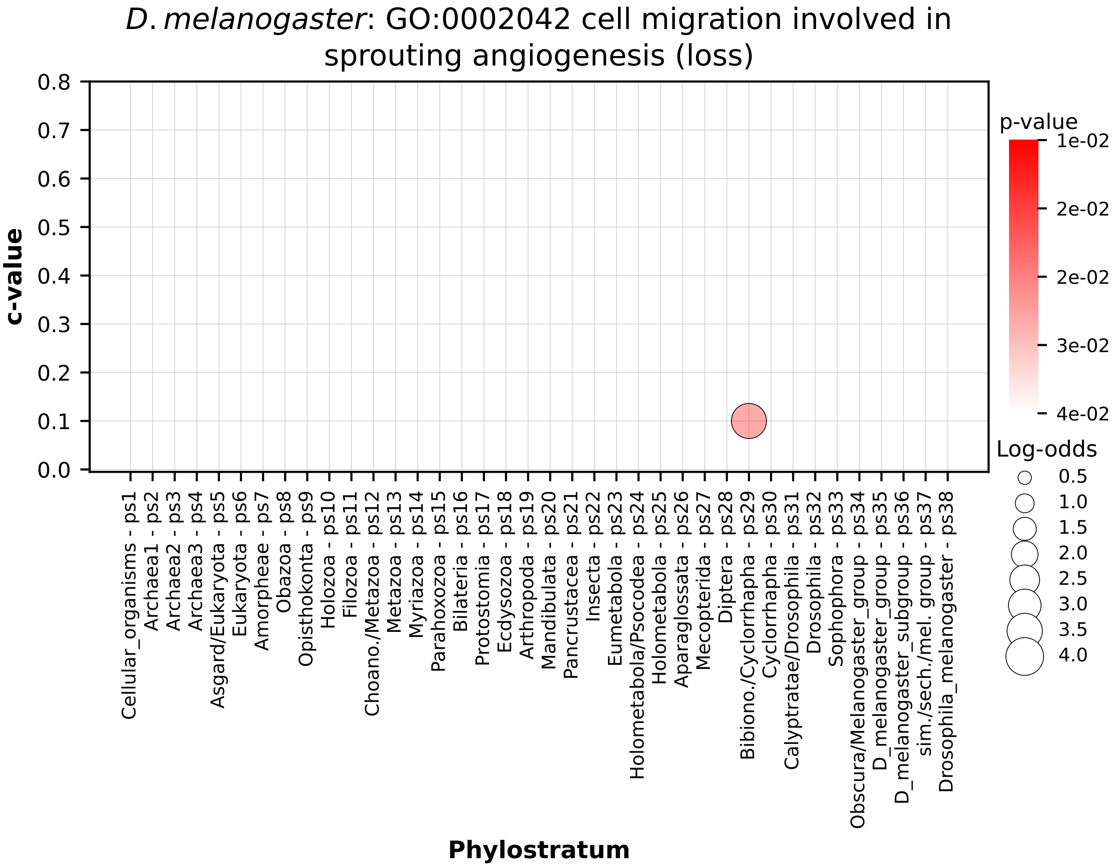

D. melanogaster: GO:0002042 cell migration involved in sprouting angiogenesis (loss)
c-value
0.0
0.1
0.2
0.3
0.4
0.5
0.6
0.7
0.8
p-value
1e-02
2e-02
2e-02
3e-02
4e-02
Log-odds
0.5
1.0
1.5
2.0
2.5
3.0
3.5
4.0
Cellular_organisms - ps1
Archaea1 - ps2
Archaea2 - ps3
Archaea3 - ps4
Asgard/Eukaryota - ps5
Eukaryota - ps6
Amorpheae - ps7
Obazoa - ps8
Opisthokonta - ps9
Holozoa - ps10
Filozoa - ps11
Choano./Metazoa - ps12
Metazoa - ps13
Myriazoa - ps14
Parahoxozoa - ps15
Bilateria - ps16
Protostomia - ps17
Ecdysozoa - ps18
Arthropoda - ps19
Mandibulata - ps20
Pancrustacea - ps21
Insecta - ps22
Eumetabola - ps23
Holometabola/Psocodea - ps24
Holometabola - ps25
Aparaglossata - ps26
Mecopterida - ps27
Diptera - ps28
Bibiono./Cyclorrhapha - ps29
Cyclorrhapha - ps30
Calyptratae/Drosophila - ps31
Drosophila - ps32
Sophophora - ps33
Obscura/Melanogaster_group - ps34
D_melanogaster_group - ps35
D_melanogaster_subgroup - ps36
sim./sech./mel. group - ps37
Drosophila_melanogaster - ps38
Phylostratum

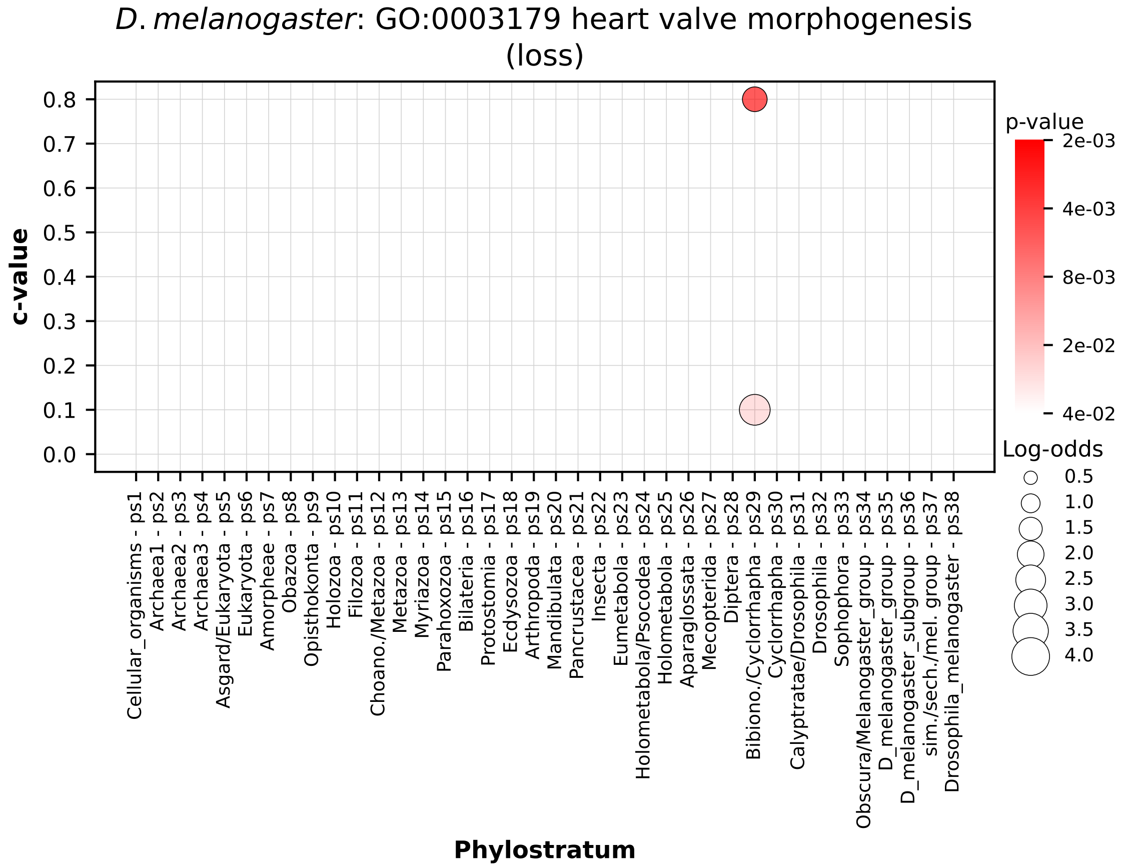

D. melanogaster: GO:0003179 heart valve morphogenesis
(loss)
c-value
0.0
0.1
0.2
0.3
0.4
0.5
0.6
0.7
0.8
p-value
2e-03
4e-03
8e-03
2e-02
4e-02
Log-odds
0.5
1.0
1.5
2.0
2.5
3.0
3.5
4.0
Cellular_organisms - ps1
Archaea1 - ps2
Archaea2 - ps3
Archaea3 - ps4
Asgard/Eukaryota - ps5
Eukaryota - ps6
Amorpheae - ps7
Obazoa - ps8
Opisthokonta - ps9
Holozoa - ps10
Filozoa - ps11
Choano./Metazoa - ps12
Metazoa - ps13
Myriazoa - ps14
Parahoxozoa - ps15
Bilateria - ps16
Protostomia - ps17
Ecdysozoa - ps18
Arthropoda - ps19
Mandibulata - ps20
Pancrustacea - ps21
Insecta - ps22
Eumetabola - ps23
Holometabola/Psocodea - ps24
Holometabola - ps25
Aparaglossata - ps26
Mecopterida - ps27
Diptera - ps28
Bibiono./Cyclorrhapha - ps29
Cyclorrhapha - ps30
Calyptratae/Drosophila - ps31
Drosophila - ps32
Sophophora - ps33
Obscura/Melanogaster_group - ps34
D_melanogaster_group - ps35
D_melanogaster_subgroup - ps36
sim./sech./mel. group - ps37
Drosophila_melanogaster - ps38
Phylostratum

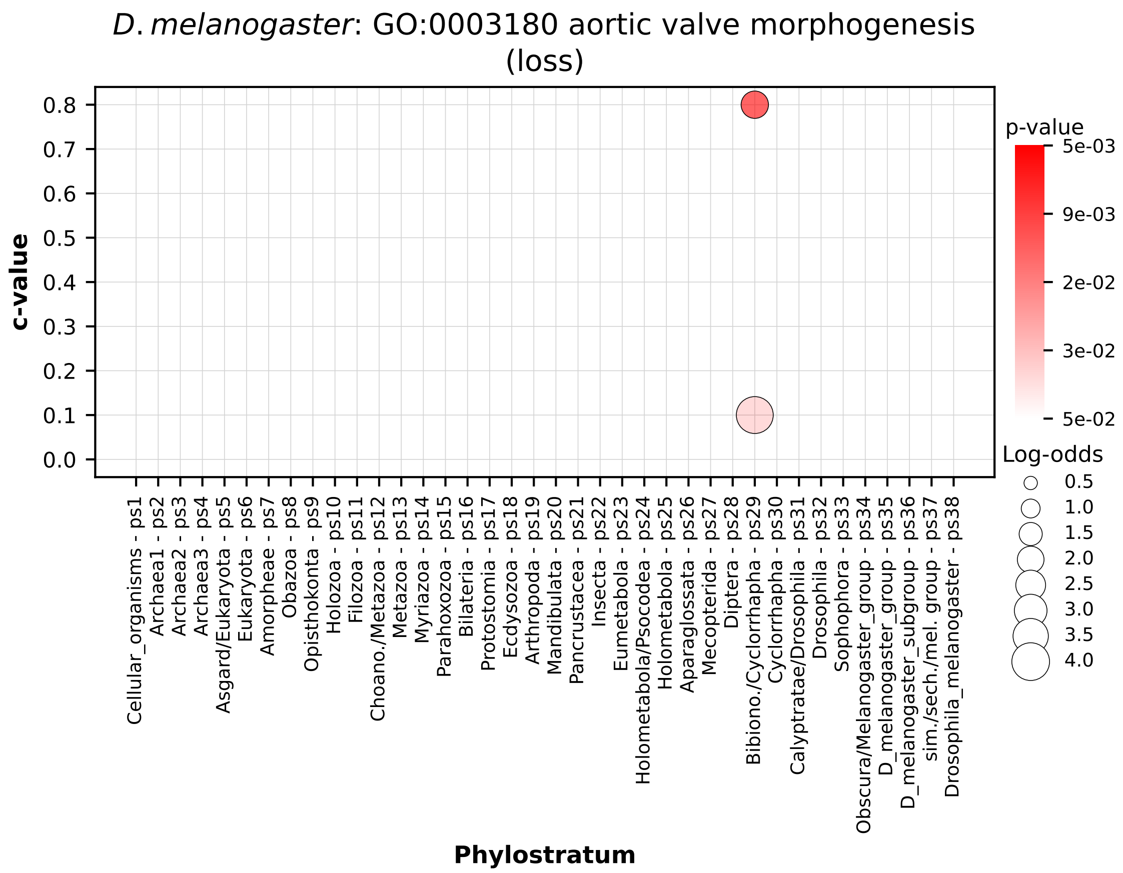

D. melanogaster: GO:0003180 aortic valve morphogenesis
(loss)
c-value
0.8
0.7
0.6
0.5
0.4
0.3
0.2
0.1
0.0
p-value
5e-03
9e-03
2e-02
3e-02
5e-02
Log-odds
0.5
1.0
1.5
2.0
2.5
3.0
3.5
4.0
Cellular_organisms - ps1
Archaea1 - ps2
Archaea2 - ps3
Archaea3 - ps4
Asgard/Eukaryota - ps5
Eukaryota - ps6
Amorpheae - ps7
Obazoa - ps8
Opisthokonta - ps9
Holozoa - ps10
Filozoa - ps11
Choano./Metazoa - ps12
Metazoa - ps13
Myriazoa - ps14
Parahoxozoa - ps15
Bilateria - ps16
Protostomia - ps17
Ecdysozoa - ps18
Arthropoda - ps19
Mandibulata - ps20
Pancrustacea - ps21
Insecta - ps22
Eumetabola - ps23
Holometabola/Psocodea - ps24
Holometabola - ps25
Aparaglossata - ps26
Mecopterida - ps27
Diptera - ps28
Bibiono./Cyclorrhapha - ps29
Cyclorrhapha - ps30
Calyptratae/Drosophila - ps31
Drosophila - ps32
Sophophora - ps33
Obscura/Melanogaster_group - ps34
D_melanogaster_group - ps35
D_melanogaster_subgroup - ps36
sim./sech./mel. group - ps37
Drosophila_melanogaster - ps38
Phylostratum

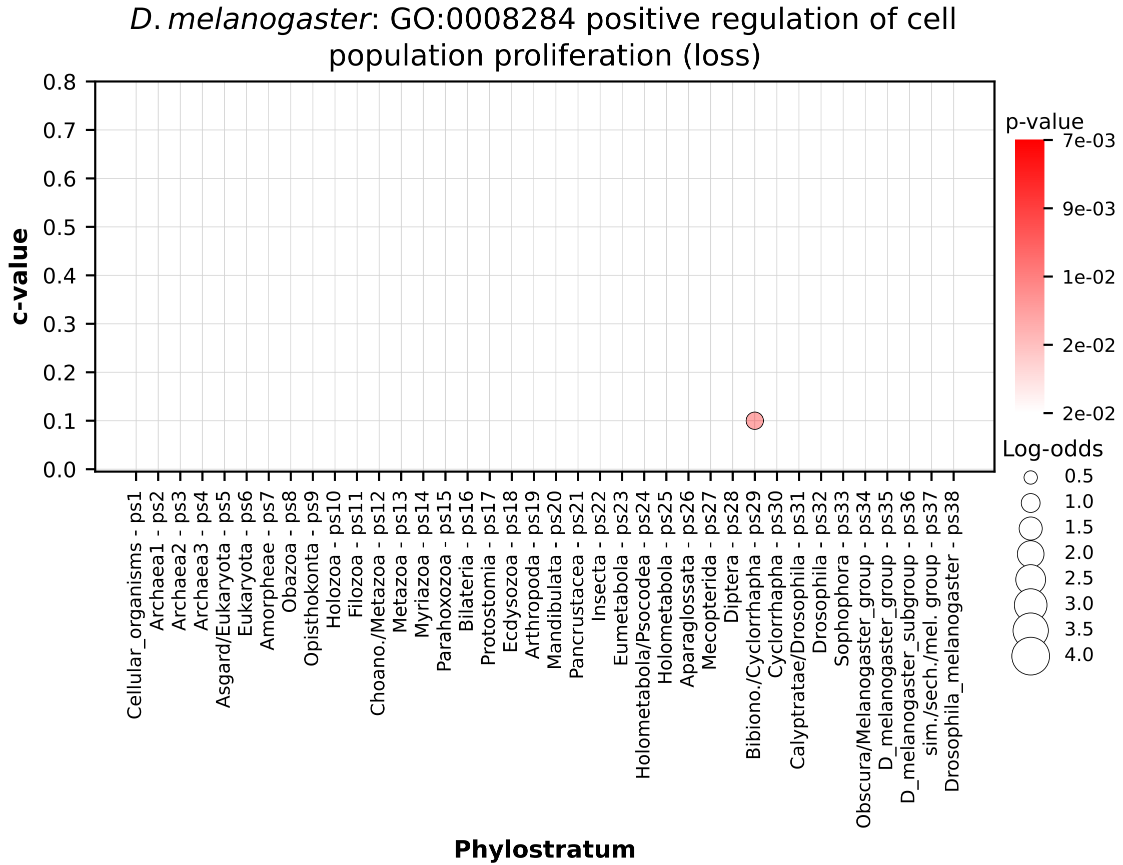

$D. melanogaster$: GO:0008284 positive regulation of cell population proliferation (loss)
c-value
0.8
0.7
0.6
0.5
0.4
0.3
0.2
0.1
0.0
p-value
7e-03
9e-03
1e-02
2e-02
2e-02
Log-odds
0.5
1.0
1.5
2.0
2.5
3.0
3.5
4.0
Cellular_organisms - ps1
Archaea1 - ps2
Archaea2 - ps3
Archaea3 - ps4
Asgard/Eukaryota - ps5
Eukaryota - ps6
Amorpheae - ps7
Obazoa - ps8
Opisthokonta - ps9
Holozoa - ps10
Filozoa - ps11
Choano./Metazoa - ps12
Metazoa - ps13
Myriazoa - ps14
Parahoxozoa - ps15
Bilateria - ps16
Protostomia - ps17
Ecdysozoa - ps18
Arthropoda - ps19
Mandibulata - ps20
Pancrustacea - ps21
Insecta - ps22
Eumetabola - ps23
Holometabola/Psocodea - ps24
Holometabola - ps25
Aparaglossata - ps26
Mecopterida - ps27
Diptera - ps28
Bibiono./Cyclorrhapha - ps29
Cyclorrhapha - ps30
Calyptratae/Drosophila - ps31
Drosophila - ps32
Sophophora - ps33
Obscura/Melanogaster_group - ps34
D_melanogaster_group - ps35
D_melanogaster_subgroup - ps36
sim./sech./mel. group - ps37
Drosophila_melanogaster - ps38
Phylostratum

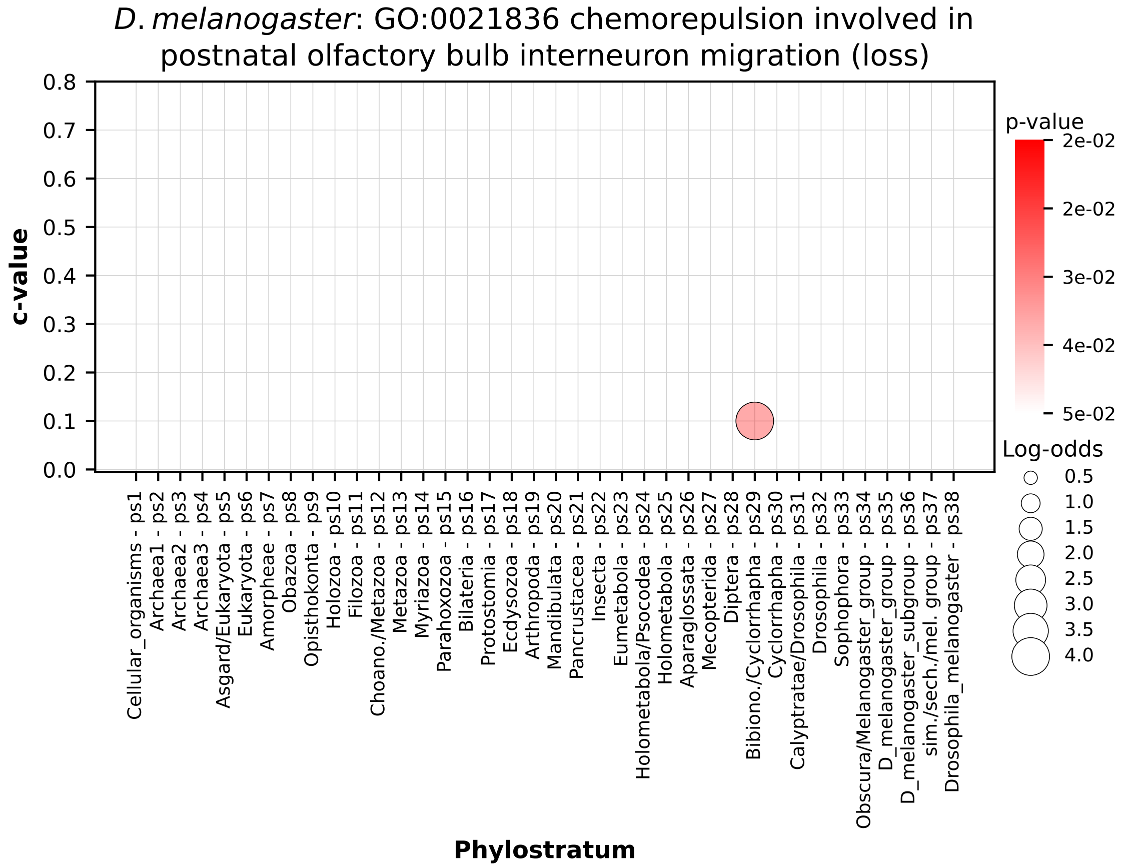

D. melanogaster: GO:0021836 chemorepulsion involved in postnatal olfactory bulb interneuron migration (loss)
c-value
0.0
0.1
0.2
0.3
0.4
0.5
0.6
0.7
0.8
Phylostratum
Cellular_organisms - ps1
Archaea1 - ps2
Archaea2 - ps3
Archaea3 - ps4
Asgard/Eukaryota - ps5
Eukaryota - ps6
Amorpheae - ps7
Obazoa - ps8
Opisthokonta - ps9
Holozoa - ps10
Filozoa - ps11
Choano./Metazoa - ps12
Metazoa - ps13
Myriazoa - ps14
Parahoxozoa - ps15
Bilateria - ps16
Protostomia - ps17
Ecdysozoa - ps18
Arthropoda - ps19
Mandibulata - ps20
Pancrustacea - ps21
Insecta - ps22
Eumetabola - ps23
Holometabola/Psocodea - ps24
Holometabola - ps25
Aparaglossata - ps26
Mecopterida - ps27
Diptera - ps28
Bibiono./Cyclorrhapha - ps29
Cyclorrhapha - ps30
Calyptratae/Drosophila - ps31
Drosophila - ps32
Sophophora - ps33
Obscura/Melanogaster_group - ps34
D_melanogaster_group - ps35
D_melanogaster_subgroup - ps36
sim./sech./mel. group - ps37
Drosophila_melanogaster - ps38
p-value
2e-02
2e-02
3e-02
4e-02
5e-02
Log-odds
0.5
1.0
1.5
2.0
2.5
3.0
3.5
4.0

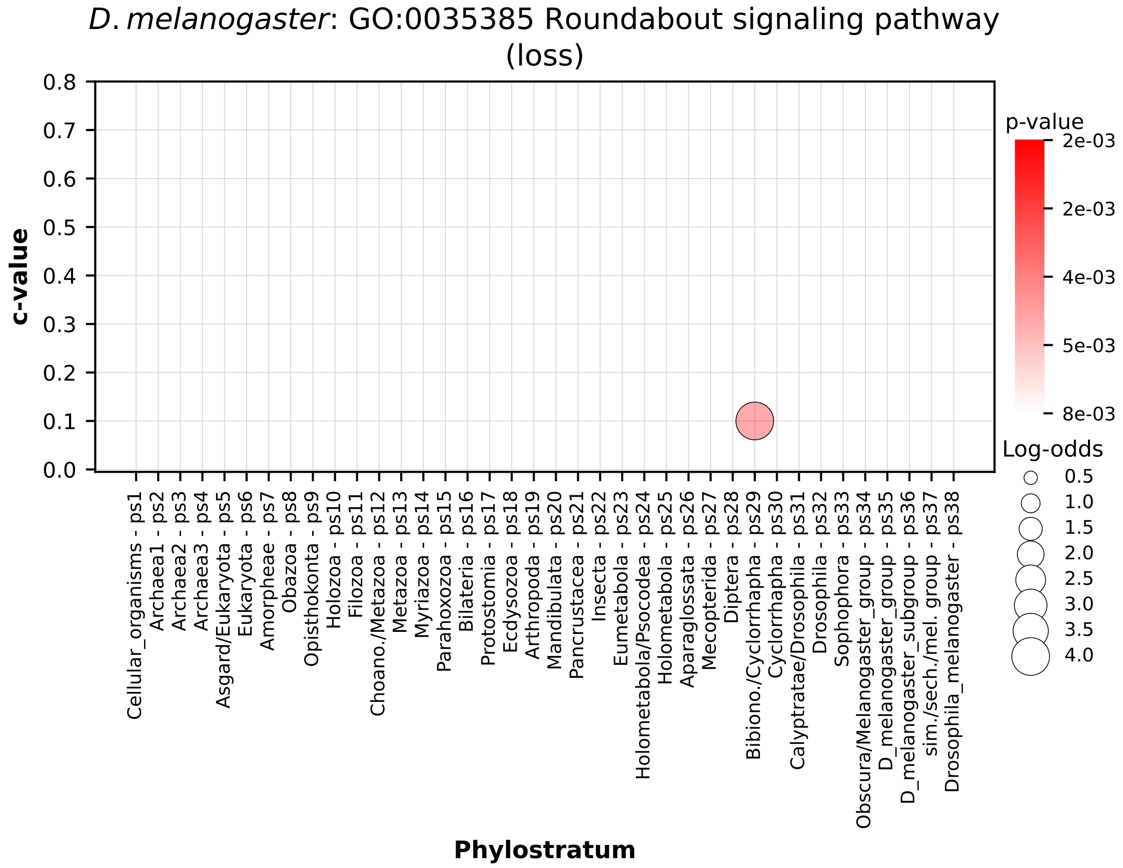
D. melanogaster: GO:0035385 Roundabout signaling pathway (loss)
c-value
0.0
0.1
0.2
0.3
0.4
0.5
0.6
0.7
0.8
Phylostratum
Cellular_organisms - ps1
Archaea1 - ps2
Archaea2 - ps3
Archaea3 - ps4
Asgard/Eukaryota - ps5
Eukaryota - ps6
Amorpheae - ps7
Obazoa - ps8
Opisthokonta - ps9
Holozoa - ps10
Filozoa - ps11
Choano./Metazoa - ps12
Metazoa - ps13
Myriazoa - ps14
Parahoxozoa - ps15
Bilateria - ps16
Protostomia - ps17
Ecdysozoa - ps18
Arthropoda - ps19
Mandibulata - ps20
Pancrustacea - ps21
Insecta - ps22
Eumetabola - ps23
Holometabola/Psocodea - ps24
Holometabola - ps25
Aparaglossata - ps26
Mecopterida - ps27
Diptera - ps28
Bibiono./Cyclorrhapha - ps29
Cyclorrhapha - ps30
Calyptratae/Drosophila - ps31
Drosophila - ps32
Sophophora - ps33
Obscura/Melanogaster_group - ps34
D_melanogaster_group - ps35
D_melanogaster_subgroup - ps36
sim./sech./mel. group - ps37
Drosophila_melanogaster - ps38
p-value
2e-03
2e-03
4e-03
5e-03
8e-03
Log-odds
0.5
1.0
1.5
2.0
2.5
3.0
3.5
4.0

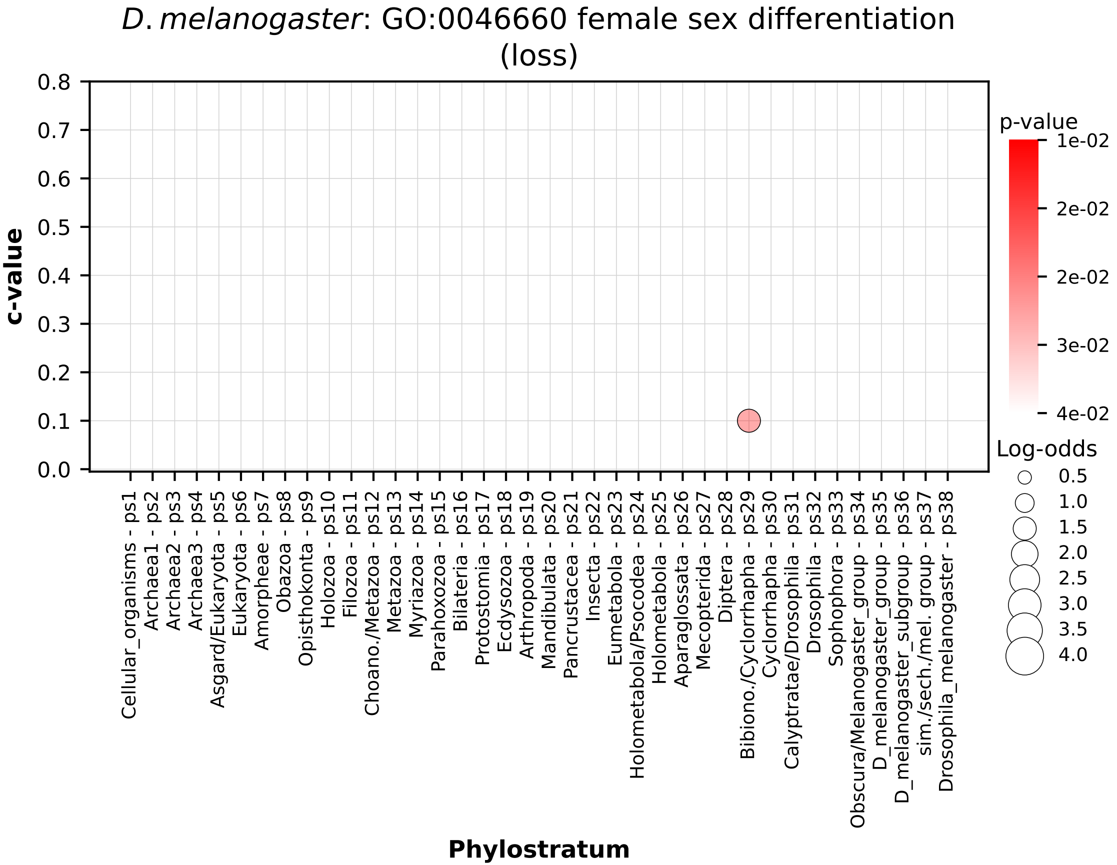

D. melanogaster: GO:0046660 female sex differentiation
(loss)
c-value
0.0
0.1
0.2
0.3
0.4
0.5
0.6
0.7
0.8
Phylostratum
Cellular_organisms - ps1
Archaea1 - ps2
Archaea2 - ps3
Archaea3 - ps4
Asgard/Eukaryota - ps5
Eukaryota - ps6
Amorpheae - ps7
Obazoa - ps8
Opisthokonta - ps9
Holozoa - ps10
Filozoa - ps11
Choano./Metazoa - ps12
Metazoa - ps13
Myriazoa - ps14
Parahoxozoa - ps15
Bilateria - ps16
Protostomia - ps17
Ecdysozoa - ps18
Arthropoda - ps19
Mandibulata - ps20
Pancrustacea - ps21
Insecta - ps22
Eumetabola - ps23
Holometabola/Psocodea - ps24
Holometabola - ps25
Aparaglossata - ps26
Mecopterida - ps27
Diptera - ps28
Bibiono./Cyclorrhapha - ps29
Cyclorrhapha - ps30
Calyptratae/Drosophila - ps31
Drosophila - ps32
Sophophora - ps33
Obscura/Melanogaster_group - ps34
D_melanogaster_group - ps35
D_melanogaster_subgroup - ps36
sim./sech./mel. group - ps37
Drosophila_melanogaster - ps38
p-value
1e-02
2e-02
2e-02
3e-02
4e-02
Log-odds
0.5
1.0
1.5
2.0
2.5
3.0
3.5
4.0

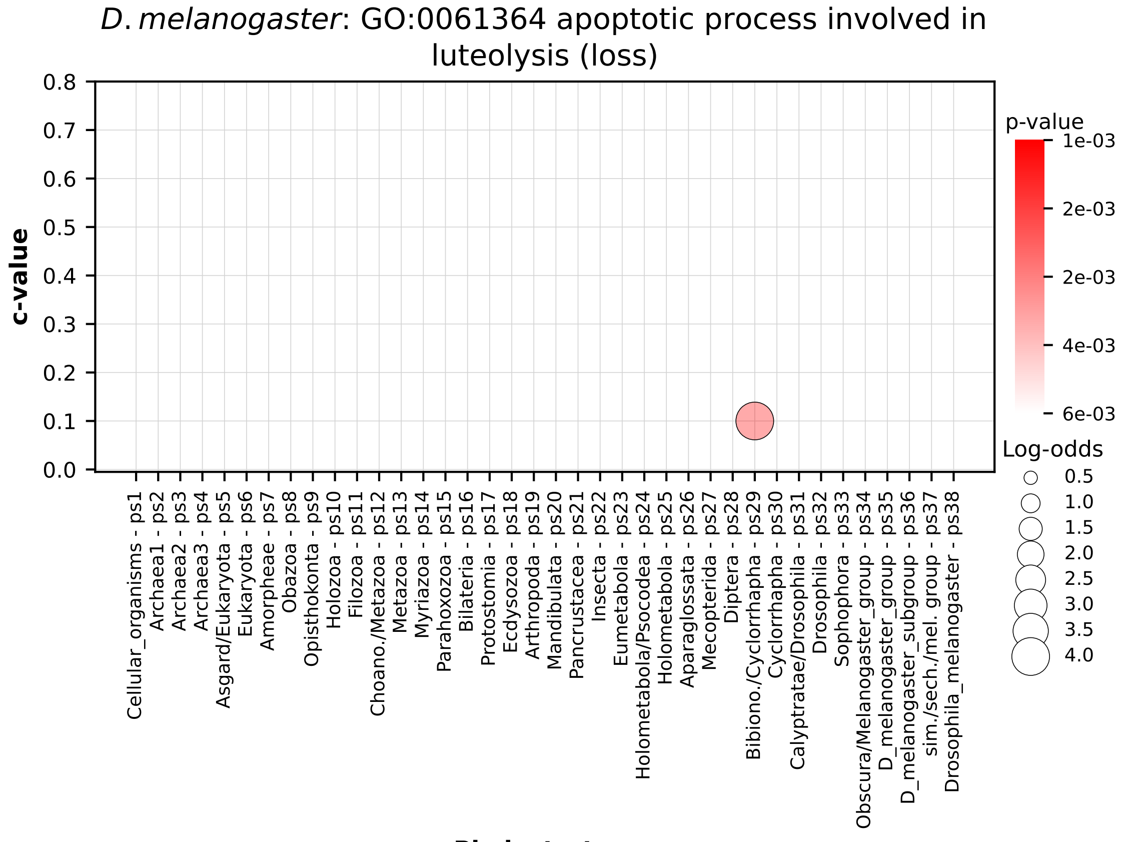
D. melanogaster: GO:0061364 apoptotic process involved in luteolysis (loss)
c-value
0.0
0.1
0.2
0.3
0.4
0.5
0.6
0.7
0.8
Phylostratum
Cellular_organisms - ps1
Archaea1 - ps2
Archaea2 - ps3
Archaea3 - ps4
Asgard/Eukaryota - ps5
Eukaryota - ps6
Amorpheae - ps7
Obazoa - ps8
Opisthokonta - ps9
Holozoa - ps10
Filozoa - ps11
Choano./Metazoa - ps12
Metazoa - ps13
Myriazoa - ps14
Parahoxozoa - ps15
Bilateria - ps16
Protostomia - ps17
Ecdysozoa - ps18
Arthropoda - ps19
Mandibulata - ps20
Pancrustacea - ps21
Insecta - ps22
Eumetabola - ps23
Holometabola/Psocodea - ps24
Holometabola - ps25
Aparaglossata - ps26
Mecopterida - ps27
Diptera - ps28
Bibiono./Cyclorrhapha - ps29
Cyclorrhapha - ps30
Calyptratae/Drosophila - ps31
Drosophila - ps32
Sophophora - ps33
Obscura/Melanogaster_group - ps34
D_melanogaster_group - ps35
D_melanogaster_subgroup - ps36
sim./sech./mel. group - ps37
Drosophila_melanogaster - ps38
p-value
1e-03
2e-03
2e-03
4e-03
6e-03
Log-odds
0.5
1.0
1.5
2.0
2.5
3.0
3.5
4.0

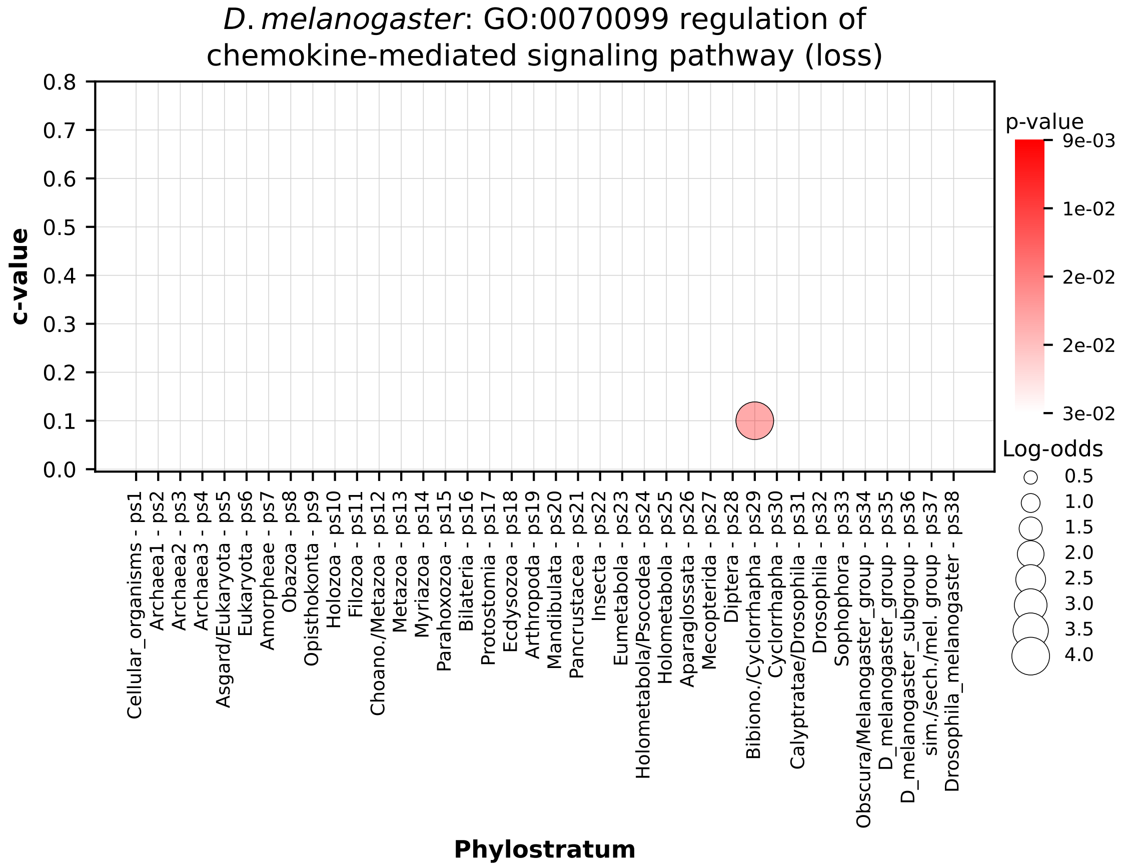

D. melanogaster: GO:0070099 regulation of chemokine-mediated signaling pathway (loss)
c-value
0.8
0.7
0.6
0.5
0.4
0.3
0.2
0.1
0.0
Phylostratum
Cellular_organisms - ps1
Archaea1 - ps2
Archaea2 - ps3
Archaea3 - ps4
Asgard/Eukaryota - ps5
Eukaryota - ps6
Amorpheae - ps7
Obazoa - ps8
Opisthokonta - ps9
Holozoa - ps10
Filozoa - ps11
Choano./Metazoa - ps12
Metazoa - ps13
Myriazoa - ps14
Parahoxozoa - ps15
Bilateria - ps16
Protostomia - ps17
Ecdysozoa - ps18
Arthropoda - ps19
Mandibulata - ps20
Pancrustacea - ps21
Insecta - ps22
Eumetabola - ps23
Holometabola/Psocodea - ps24
Holometabola - ps25
Aparaglossata - ps26
Mecopterida - ps27
Diptera - ps28
Bibiono./Cyclorrhapha - ps29
Cyclorrhapha - ps30
Calyptratae/Drosophila - ps31
Drosophila - ps32
Sophophora - ps33
Obscura/Melanogaster_group - ps34
D_melanogaster_group - ps35
D_melanogaster_subgroup - ps36
sim./sech./mel. group - ps37
Drosophila_melanogaster - ps38
p-value
9e-03
1e-02
2e-02
2e-02
3e-02
Log-odds
0.5
1.0
1.5
2.0
2.5
3.0
3.5
4.0

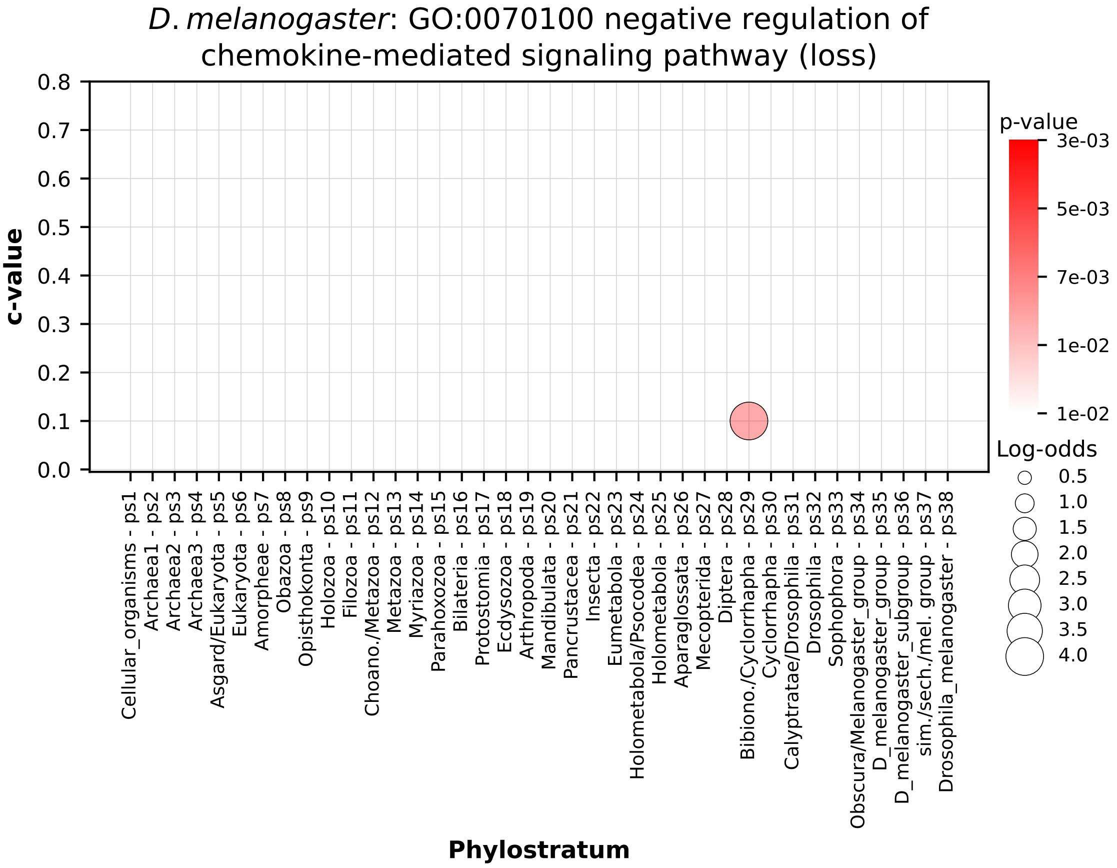

D. melanogaster: GO:0070100 negative regulation of chemokine-mediated signaling pathway (loss)
c-value
0.8
0.7
0.6
0.5
0.4
0.3
0.2
0.1
0.0
p-value
3e-03
5e-03
7e-03
1e-02
1e-02
Log-odds
0.5
1.0
1.5
2.0
2.5
3.0
3.5
4.0
Cellular_organisms - ps1
Archaea1 - ps2
Archaea2 - ps3
Archaea3 - ps4
Asgard/Eukaryota - ps5
Eukaryota - ps6
Amorpheae - ps7
Obazoa - ps8
Opisthokonta - ps9
Holozoa - ps10
Filozoa - ps11
Choano./Metazoa - ps12
Metazoa - ps13
Myriazoa - ps14
Parahoxozoa - ps15
Bilateria - ps16
Protostomia - ps17
Ecdysozoa - ps18
Arthropoda - ps19
Mandibulata - ps20
Pancrustacea - ps21
Insecta - ps22
Eumetabola - ps23
Holometabola/Psocodea - ps24
Holometabola - ps25
Aparaglossata - ps26
Mecopterida - ps27
Diptera - ps28
Bibiono./Cyclorrhapha - ps29
Cyclorrhapha - ps30
Calyptratae/Drosophila - ps31
Drosophila - ps32
Sophophora - ps33
Obscura/Melanogaster_group - ps34
D_melanogaster_group - ps35
D_melanogaster_subgroup - ps36
sim./sech./mel. group - ps37
Drosophila_melanogaster - ps38
Phylostratum

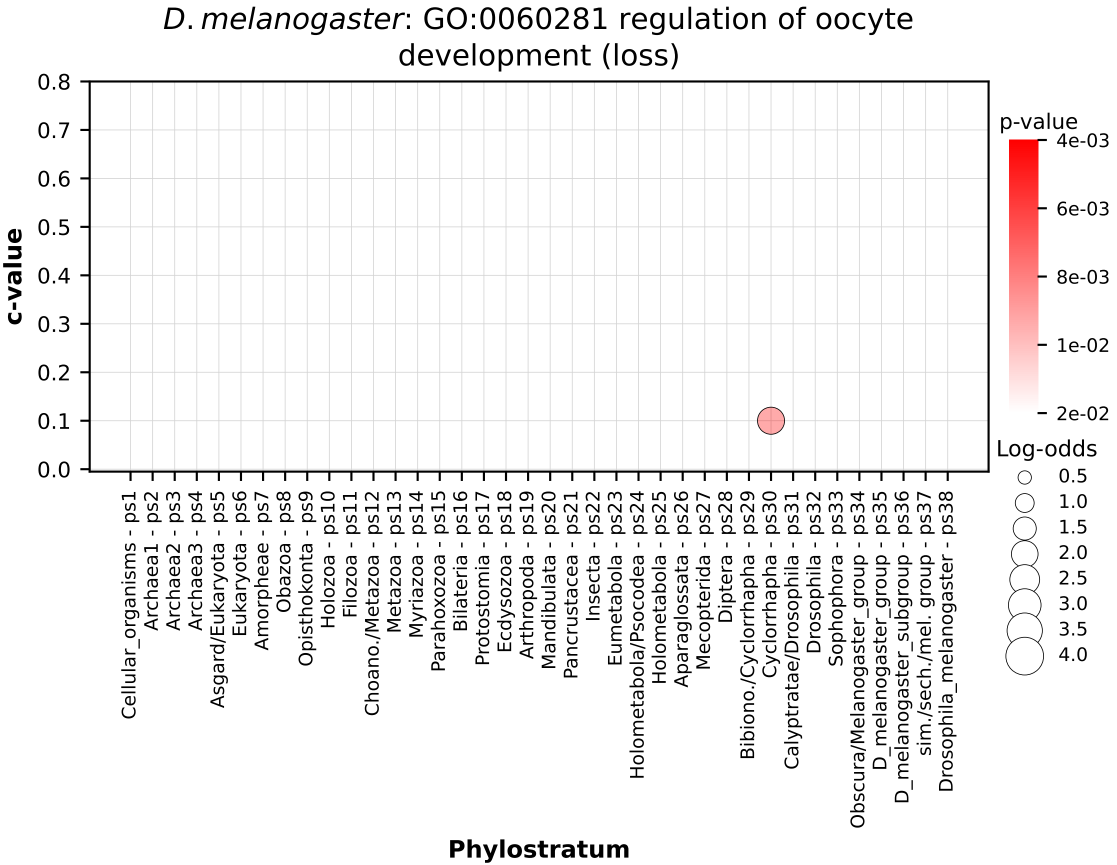

D. melanogaster: GO:0060281 regulation of oocyte development (loss)
c-value
0.0
0.1
0.2
0.3
0.4
0.5
0.6
0.7
0.8
Phylostratum
Cellular_organisms - ps1
Archaea1 - ps2
Archaea2 - ps3
Archaea3 - ps4
Asgard/Eukaryota - ps5
Eukaryota - ps6
Amorpheae - ps7
Obazoa - ps8
Opisthokonta - ps9
Holozoa - ps10
Filozoa - ps11
Choano./Metazoa - ps12
Metazoa - ps13
Myriazoa - ps14
Parahoxozoa - ps15
Bilateria - ps16
Protostomia - ps17
Ecdysozoa - ps18
Arthropoda - ps19
Mandibulata - ps20
Pancrustacea - ps21
Insecta - ps22
Eumetabola - ps23
Holometabola/Psocodea - ps24
Holometabola - ps25
Aparaglossata - ps26
Mecopterida - ps27
Diptera - ps28
Bibiono./Cyclorrhapha - ps29
Cyclorrhapha - ps30
Calyptratae/Drosophila - ps31
Drosophila - ps32
Sophophora - ps33
Obscura/Melanogaster_group - ps34
D_melanogaster_group - ps35
D_melanogaster_subgroup - ps36
sim./sech./mel. group - ps37
Drosophila_melanogaster - ps38
p-value
4e-03
6e-03
8e-03
1e-02
2e-02
Log-odds
0.5
1.0
1.5
2.0
2.5
3.0
3.5
4.0

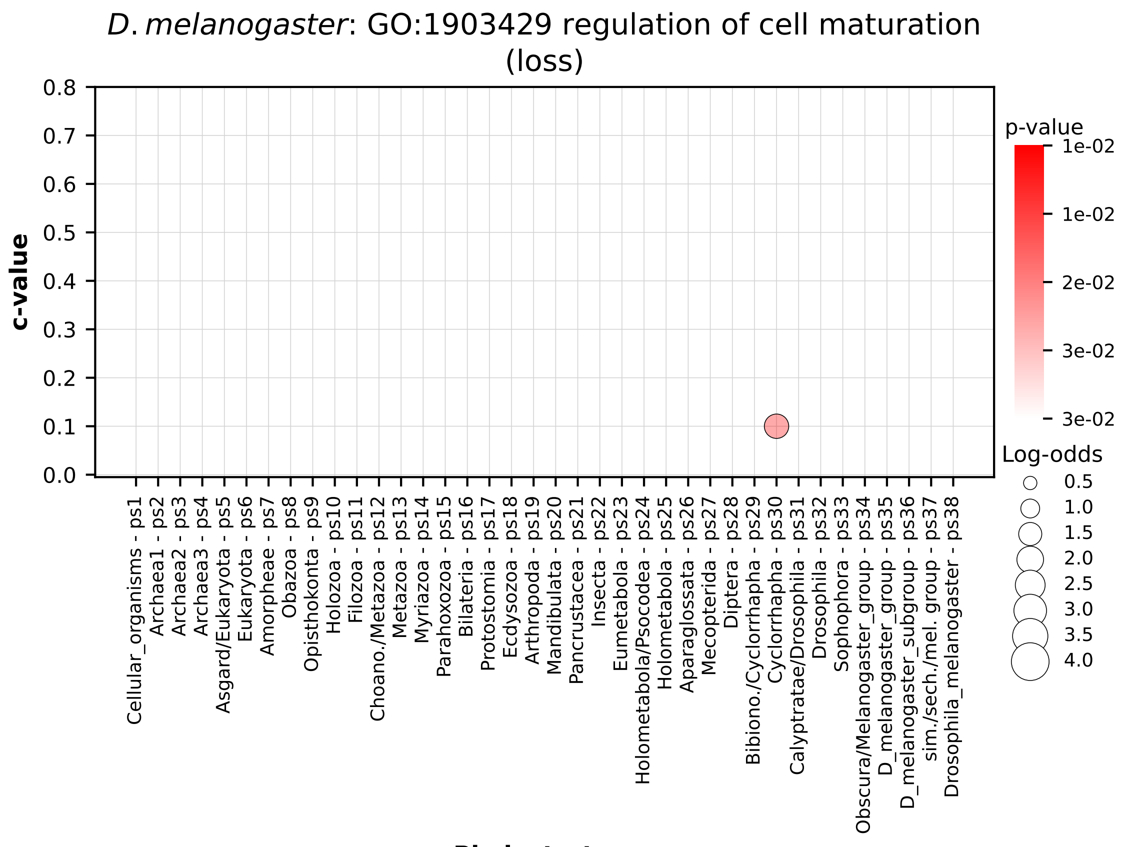

D. melanogaster: GO:1903429 regulation of cell maturation (loss)
c-value
0.8
0.7
0.6
0.5
0.4
0.3
0.2
0.1
0.0
Phylostratum
Cellular_organisms - ps1
Archaea1 - ps2
Archaea2 - ps3
Archaea3 - ps4
Asgard/Eukaryota - ps5
Eukaryota - ps6
Amorpheae - ps7
Obazoa - ps8
Opisthokonta - ps9
Holozoa - ps10
Filozoa - ps11
Choano./Metazoa - ps12
Metazoa - ps13
Myriazoa - ps14
Parahoxozoa - ps15
Bilateria - ps16
Protostomia - ps17
Ecdysozoa - ps18
Arthropoda - ps19
Mandibulata - ps20
Pancrustacea - ps21
Insecta - ps22
Eumetabola - ps23
Holometabola/Psocodea - ps24
Holometabola - ps25
Aparaglossata - ps26
Mecopterida - ps27
Diptera - ps28
Bibiono./Cyclorrhapha - ps29
Cyclorrhapha - ps30
Calyptratae/Drosophila - ps31
Drosophila - ps32
Sophophora - ps33
Obscura/Melanogaster_group - ps34
D_melanogaster_group - ps35
D_melanogaster_subgroup - ps36
sim./sech./mel. group - ps37
Drosophila_melanogaster - ps38
p-value
1e-02
1e-02
2e-02
3e-02
3e-02
Log-odds
0.5
1.0
1.5
2.0
2.5
3.0
3.5
4.0

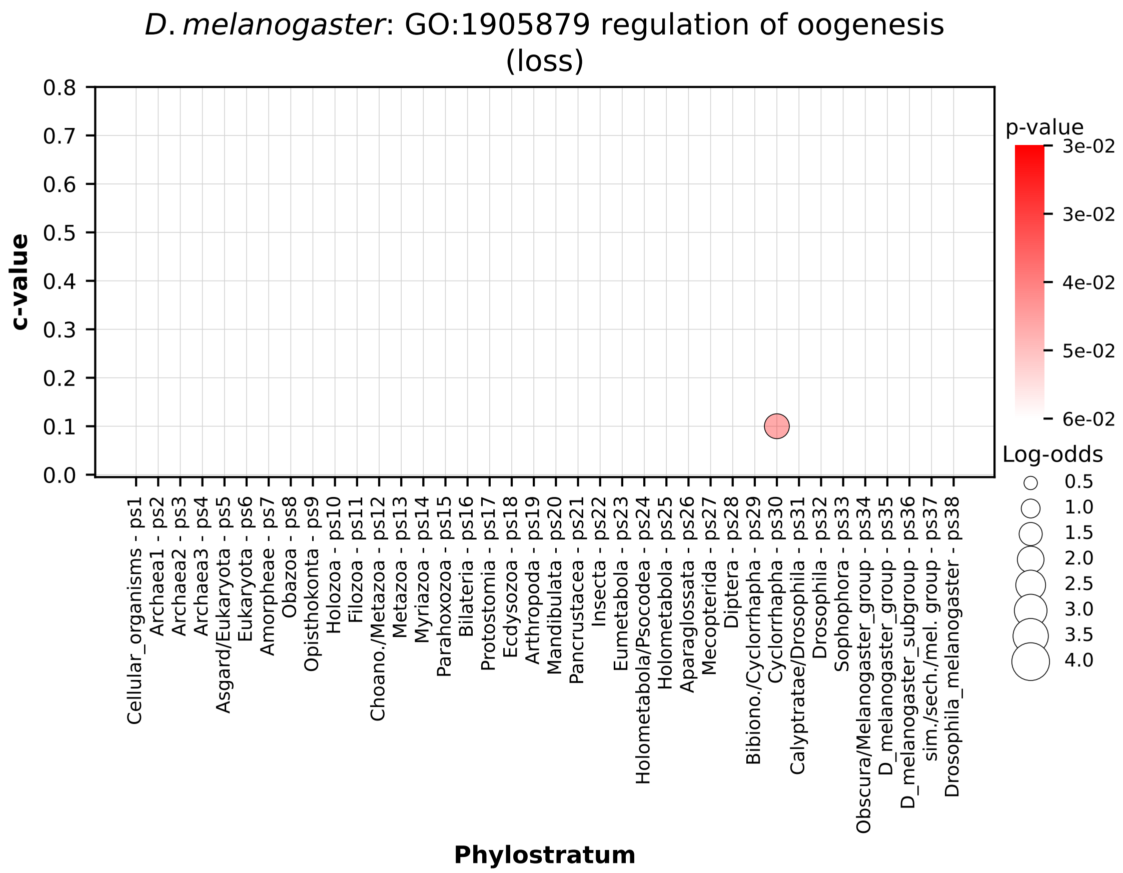
D. melanogaster: GO:1905879 regulation of oogenesis
(loss)
c-value
0.8
0.7
0.6
0.5
0.4
0.3
0.2
0.1
0.0
p-value
3e-02
3e-02
4e-02
5e-02
6e-02
Log-odds
0.5
1.0
1.5
2.0
2.5
3.0
3.5
4.0
Cellular_organisms - ps1
Archaea1 - ps2
Archaea2 - ps3
Archaea3 - ps4
Asgard/Eukaryota - ps5
Eukaryota - ps6
Amorpheae - ps7
Obazoa - ps8
Opisthokonta - ps9
Holozoa - ps10
Filozoa - ps11
Choano./Metazoa - ps12
Metazoa - ps13
Myriazoa - ps14
Parahoxozoa - ps15
Bilateria - ps16
Protostomia - ps17
Ecdysozoa - ps18
Arthropoda - ps19
Mandibulata - ps20
Pancrustacea - ps21
Insecta - ps22
Eumetabola - ps23
Holometabola/Psocodea - ps24
Holometabola - ps25
Aparaglossata - ps26
Mecopterida - ps27
Diptera - ps28
Bibiono./Cyclorrhapha - ps29
Cyclorrhapha - ps30
Calyptratae/Drosophila - ps31
Drosophila - ps32
Sophophora - ps33
Obscura/Melanogaster_group - ps34
D_melanogaster_group - ps35
D_melanogaster_subgroup - ps36
sim./sech./mel. group - ps37
Drosophila_melanogaster - ps38
Phylostratum

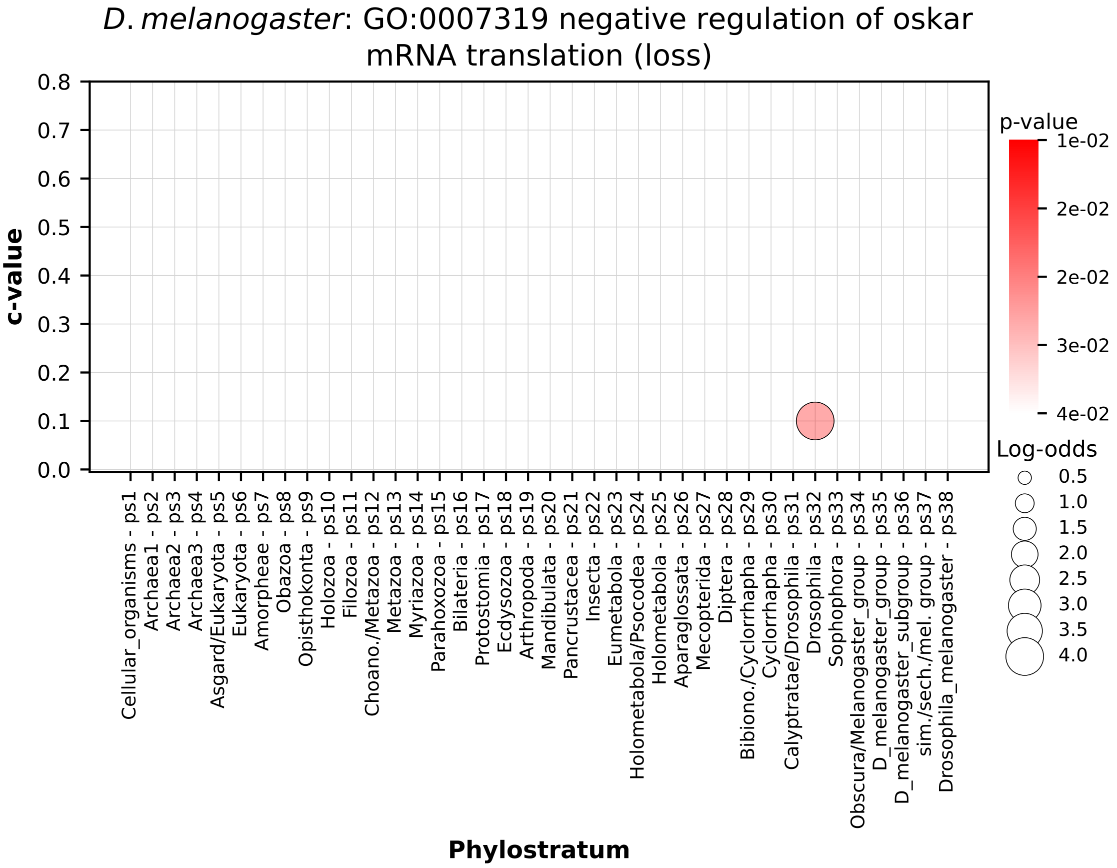
D. melanogaster: GO:0007319 negative regulation of oskar mRNA translation (loss)
c-value
0.8
0.7
0.6
0.5
0.4
0.3
0.2
0.1
0.0
p-value
1e-02
2e-02
2e-02
3e-02
4e-02
Log-odds
0.5
1.0
1.5
2.0
2.5
3.0
3.5
4.0
Cellular_organisms - ps1
Archaea1 - ps2
Archaea2 - ps3
Archaea3 - ps4
Asgard/Eukaryota - ps5
Eukaryota - ps6
Amorpheae - ps7
Obazoa - ps8
Opisthokonta - ps9
Holozoa - ps10
Filozoa - ps11
Choano./Metazoa - ps12
Metazoa - ps13
Myriazoa - ps14
Parahoxozoa - ps15
Bilateria - ps16
Protostomia - ps17
Ecdysozoa - ps18
Arthropoda - ps19
Mandibulata - ps20
Pancrustacea - ps21
Insecta - ps22
Eumetabola - ps23
Holometabola/Psocodea - ps24
Holometabola - ps25
Aparaglossata - ps26
Mecopterida - ps27
Diptera - ps28
Bibiono./Cyclorrhapha - ps29
Cyclorrhapha - ps30
Calyptratae/Drosophila - ps31
Drosophila - ps32
Sophophora - ps33
Obscura/Melanogaster_group - ps34
D_melanogaster_group - ps35
D_melanogaster_subgroup - ps36
sim./sech./mel. group - ps37
Drosophila_melanogaster - ps38
Phylostratum

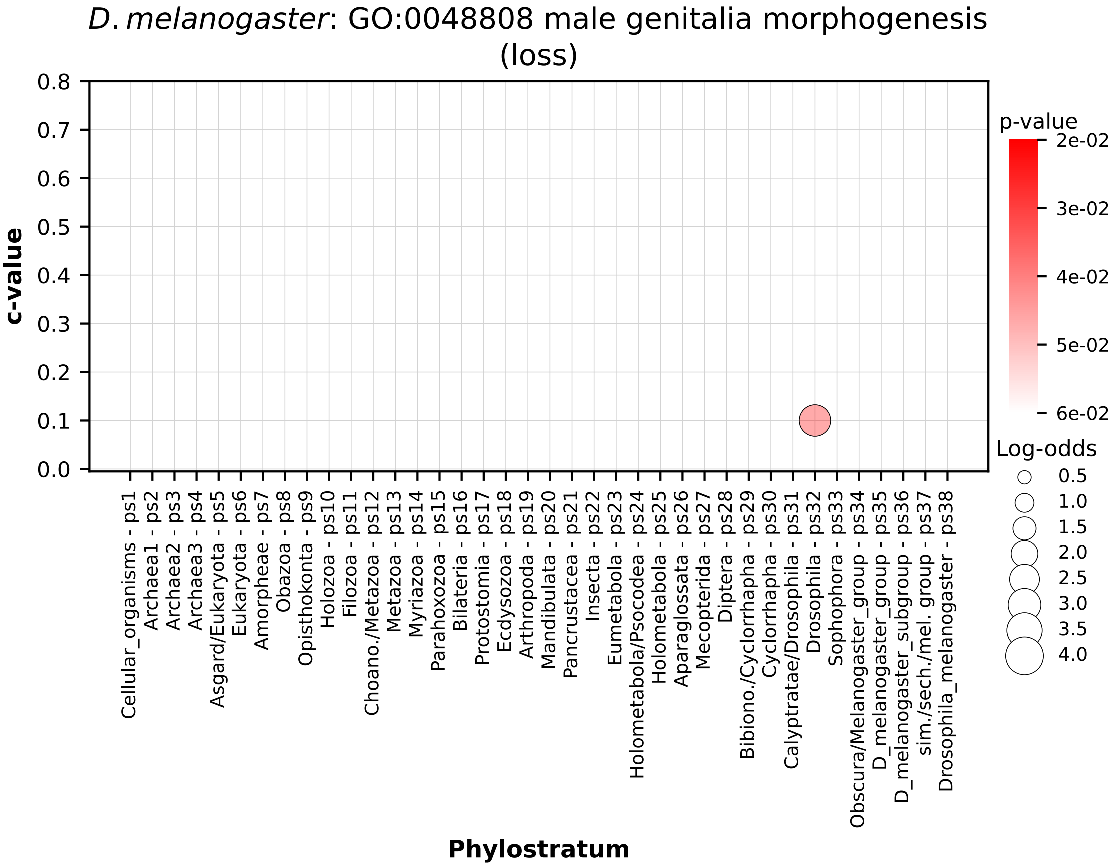

D. melanogaster: GO:0048808 male genitalia morphogenesis (loss)
c-value
0.0
0.1
0.2
0.3
0.4
0.5
0.6
0.7
0.8
Phylostratum
Cellular_organisms - ps1
Archaea1 - ps2
Archaea2 - ps3
Archaea3 - ps4
Asgard/Eukaryota - ps5
Eukaryota - ps6
Amorpheae - ps7
Obazoa - ps8
Opisthokonta - ps9
Holozoa - ps10
Filozoa - ps11
Choano./Metazoa - ps12
Metazoa - ps13
Myriazoa - ps14
Parahoxozoa - ps15
Bilateria - ps16
Protostomia - ps17
Ecdysozoa - ps18
Arthropoda - ps19
Mandibulata - ps20
Pancrustacea - ps21
Insecta - ps22
Eumetabola - ps23
Holometabola/Psocodea - ps24
Holometabola - ps25
Aparaglossata - ps26
Mecopterida - ps27
Diptera - ps28
Bibiono./Cyclorrhapha - ps29
Cyclorrhapha - ps30
Calyptratae/Drosophila - ps31
Drosophila - ps32
Sophophora - ps33
Obscura/Melanogaster_group - ps34
D_melanogaster_group - ps35
D_melanogaster_subgroup - ps36
sim./sech./mel. group - ps37
Drosophila_melanogaster - ps38
p-value
2e-02
3e-02
4e-02
5e-02
6e-02
Log-odds
0.5
1.0
1.5
2.0
2.5
3.0
3.5
4.0

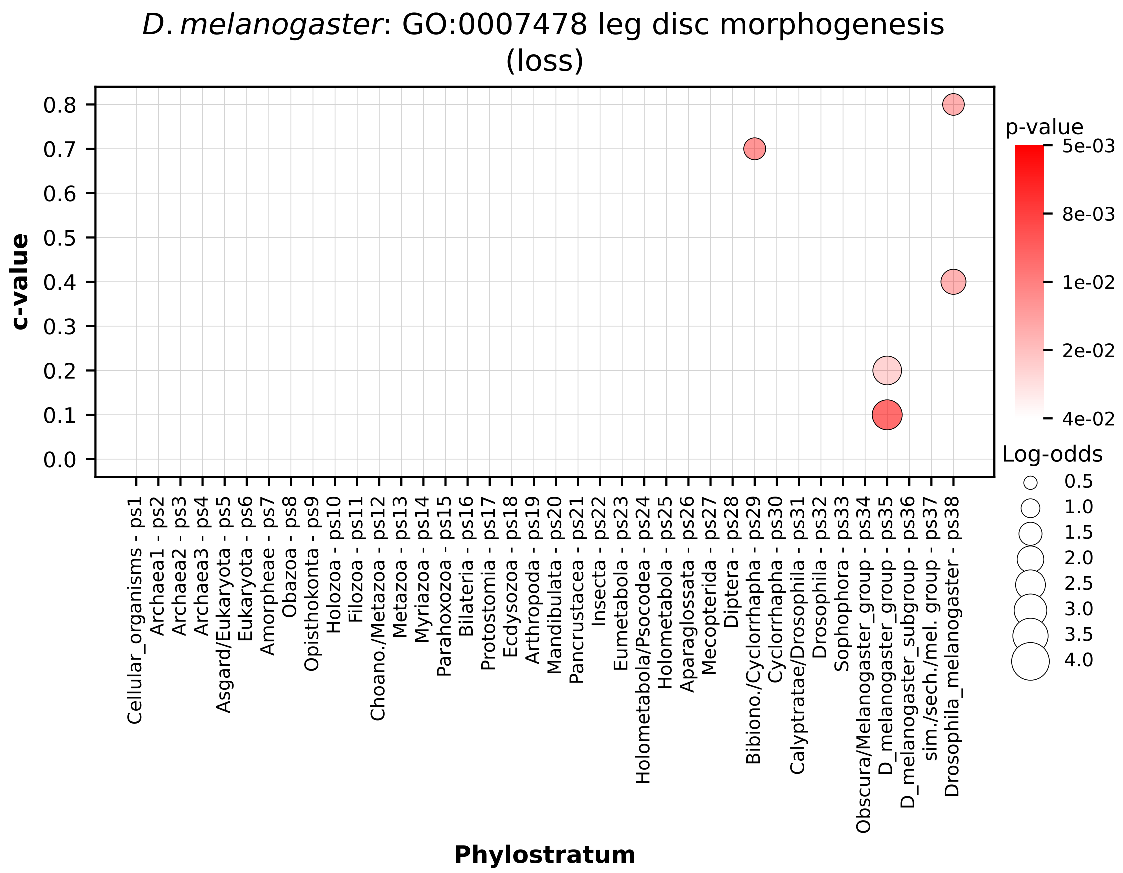

D. melanogaster: GO:0007478 leg disc morphogenesis
(loss)
c-value
0.8
0.7
0.6
0.5
0.4
0.3
0.2
0.1
0.0
p-value
5e-03
8e-03
1e-02
2e-02
4e-02
Log-odds
0.5
1.0
1.5
2.0
2.5
3.0
3.5
4.0
Cellular_organisms - ps1
Archaea1 - ps2
Archaea2 - ps3
Archaea3 - ps4
Asgard/Eukaryota - ps5
Eukaryota - ps6
Amorpheae - ps7
Obazoa - ps8
Opisthokonta - ps9
Holozoa - ps10
Filozoa - ps11
Choano./Metazoa - ps12
Metazoa - ps13
Myriazoa - ps14
Parahoxozoa - ps15
Bilateria - ps16
Protostomia - ps17
Ecdysozoa - ps18
Arthropoda - ps19
Mandibulata - ps20
Pancrustacea - ps21
Insecta - ps22
Eumetabola - ps23
Holometabola/Psocodea - ps24
Holometabola - ps25
Aparaglossata - ps26
Mecopterida - ps27
Diptera - ps28
Bibiono./Cyclorrhapha - ps29
Cyclorrhapha - ps30
Calyptratae/Drosophila - ps31
Drosophila - ps32
Sophophora - ps33
Obscura/Melanogaster_group - ps34
D_melanogaster_group - ps35
D_melanogaster_subgroup - ps36
sim./sech./mel. group - ps37
Drosophila_melanogaster - ps38
Phylostratum

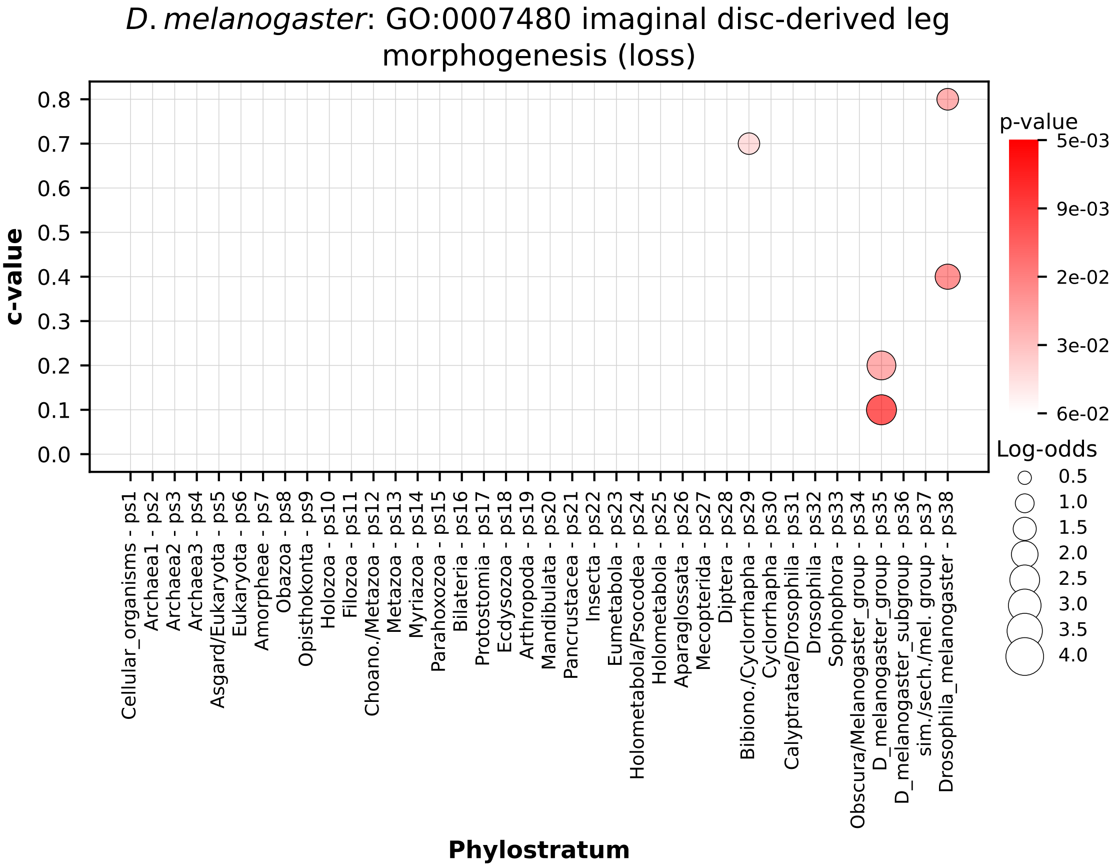

D. melanogaster: GO:0007480 imaginal disc-derived leg morphogenesis (loss)
c-value
0.8
0.7
0.6
0.5
0.4
0.3
0.2
0.1
0.0
p-value
5e-03
9e-03
2e-02
3e-02
6e-02
Log-odds
0.5
1.0
1.5
2.0
2.5
3.0
3.5
4.0
Cellular_organisms - ps1
Archaea1 - ps2
Archaea2 - ps3
Archaea3 - ps4
Asgard/Eukaryota - ps5
Eukaryota - ps6
Amorpheae - ps7
Obazoa - ps8
Opisthokonta - ps9
Holozoa - ps10
Filozoa - ps11
Choano./Metazoa - ps12
Metazoa - ps13
Myriazoa - ps14
Parahoxozoa - ps15
Bilateria - ps16
Protostomia - ps17
Ecdysozoa - ps18
Arthropoda - ps19
Mandibulata - ps20
Pancrustacea - ps21
Insecta - ps22
Eumetabola - ps23
Holometabola/Psocodea - ps24
Holometabola - ps25
Aparaglossata - ps26
Mecopterida - ps27
Diptera - ps28
Bibiono./Cyclorrhapha - ps29
Cyclorrhapha - ps30
Calyptratae/Drosophila - ps31
Drosophila - ps32
Sophophora - ps33
Obscura/Melanogaster_group - ps34
D_melanogaster_group - ps35
D_melanogaster_subgroup - ps36
sim./sech./mel. group - ps37
Drosophila_melanogaster - ps38
Phylostratum

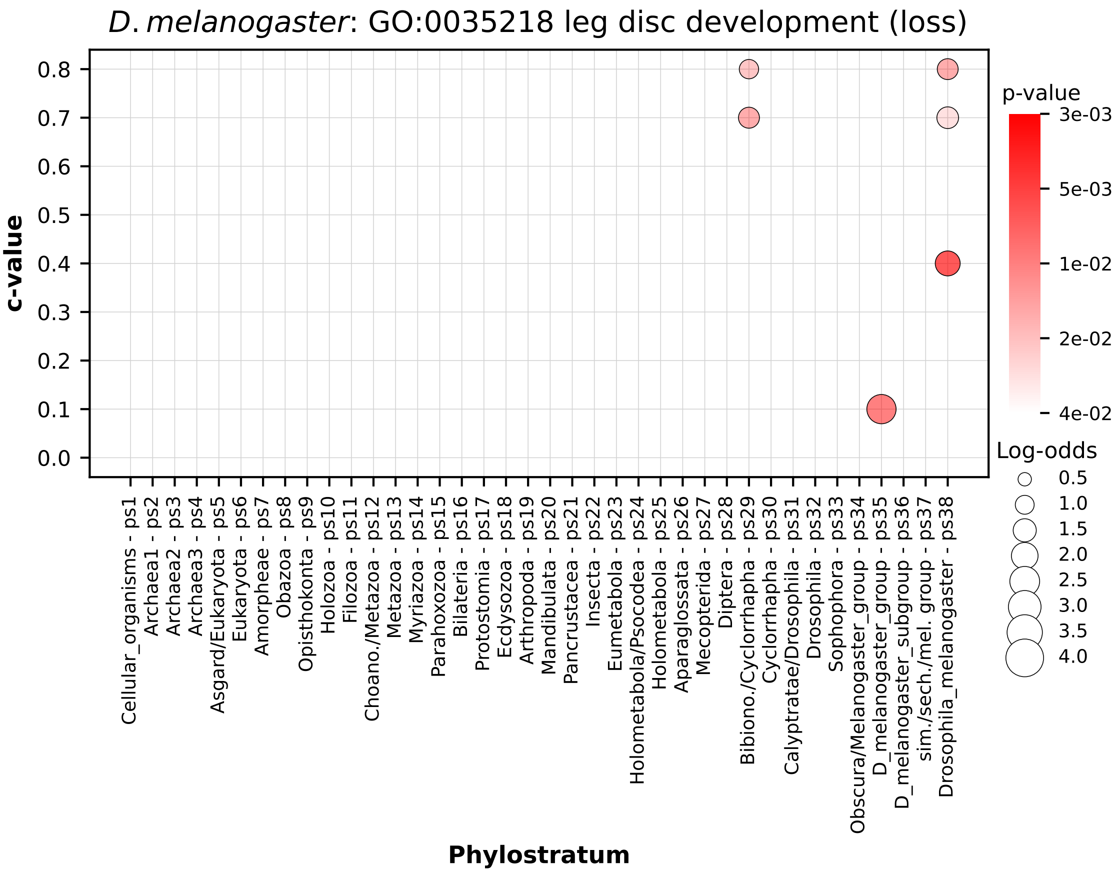

D. melanogaster: GO:0035218 leg disc development (loss)
c-value
0.0
0.1
0.2
0.3
0.4
0.5
0.6
0.7
0.8
p-value
3e-03
5e-03
1e-02
2e-02
4e-02
Log-odds
0.5
1.0
1.5
2.0
2.5
3.0
3.5
4.0
Cellular_organisms - ps1
Archaea1 - ps2
Archaea2 - ps3
Archaea3 - ps4
Asgard/Eukaryota - ps5
Eukaryota - ps6
Amorpheae - ps7
Obazoa - ps8
Opisthokonta - ps9
Holozoa - ps10
Filozoa - ps11
Choano./Metazoa - ps12
Metazoa - ps13
Myriazoa - ps14
Parahoxozoa - ps15
Bilateria - ps16
Protostomia - ps17
Ecdysozoa - ps18
Arthropoda - ps19
Mandibulata - ps20
Pancrustacea - ps21
Insecta - ps22
Eumetabola - ps23
Holometabola/Psocodea - ps24
Holometabola - ps25
Aparaglossata - ps26
Mecopterida - ps27
Diptera - ps28
Bibiono./Cyclorrhapha - ps29
Cyclorrhapha - ps30
Calyptratae/Drosophila - ps31
Drosophila - ps32
Sophophora - ps33
Obscura/Melanogaster_group - ps34
D_melanogaster_group - ps35
D_melanogaster_subgroup - ps36
sim./sech./mel. group - ps37
Drosophila_melanogaster - ps38
Phylostratum

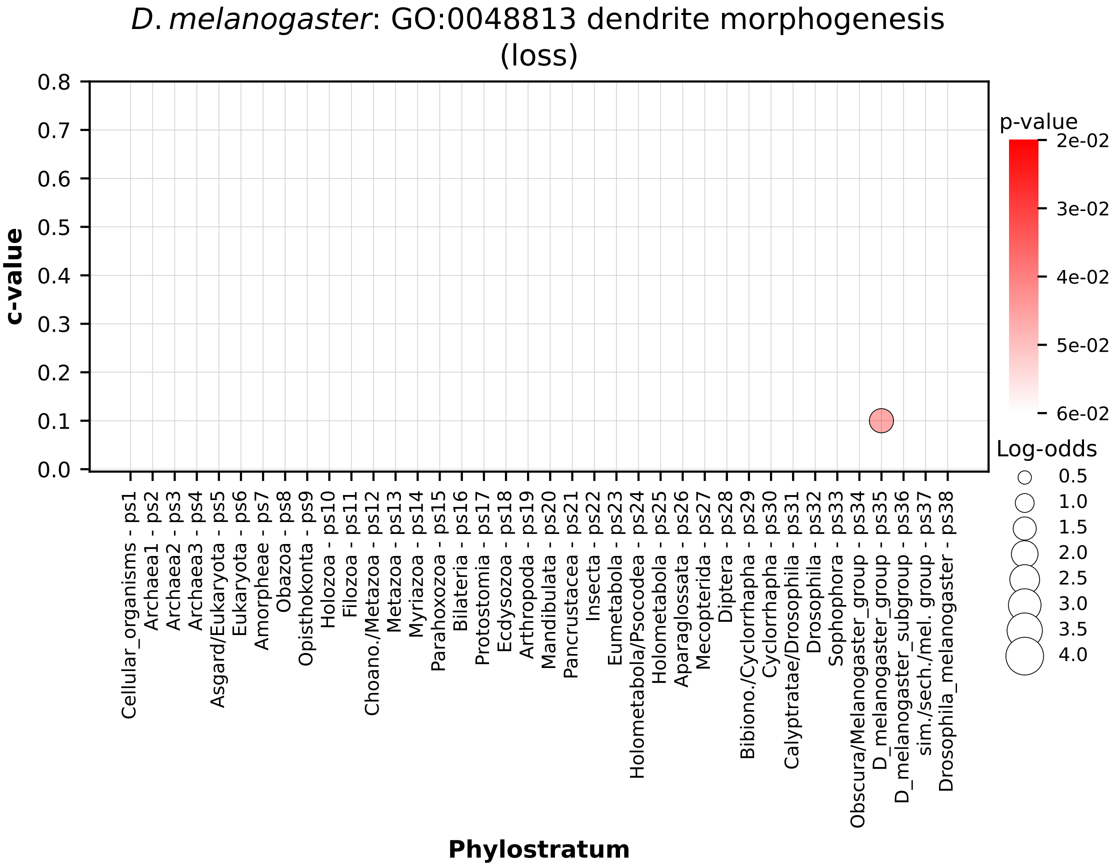
D. melanogaster: GO:0048813 dendrite morphogenesis
(loss)
c-value
0.0
0.1
0.2
0.3
0.4
0.5
0.6
0.7
0.8
p-value
2e-02
3e-02
4e-02
5e-02
6e-02
Log-odds
0.5
1.0
1.5
2.0
2.5
3.0
3.5
4.0
Cellular_organisms - ps1
Archaea1 - ps2
Archaea2 - ps3
Archaea3 - ps4
Asgard/Eukaryota - ps5
Eukaryota - ps6
Amorpheae - ps7
Obazoa - ps8
Opisthokonta - ps9
Holozoa - ps10
Filozoa - ps11
Choano./Metazoa - ps12
Metazoa - ps13
Myriazoa - ps14
Parahoxozoa - ps15
Bilateria - ps16
Protostomia - ps17
Ecdysozoa - ps18
Arthropoda - ps19
Mandibulata - ps20
Pancrustacea - ps21
Insecta - ps22
Eumetabola - ps23
Holometabola/Psocodea - ps24
Holometabola - ps25
Aparaglossata - ps26
Mecopterida - ps27
Diptera - ps28
Bibiono./Cyclorrhapha - ps29
Cyclorrhapha - ps30
Calyptratae/Drosophila - ps31
Drosophila - ps32
Sophophora - ps33
Obscura/Melanogaster_group - ps34
D_melanogaster_group - ps35
D_melanogaster_subgroup - ps36
sim./sech./mel. group - ps37
Drosophila_melanogaster - ps38
Phylostratum

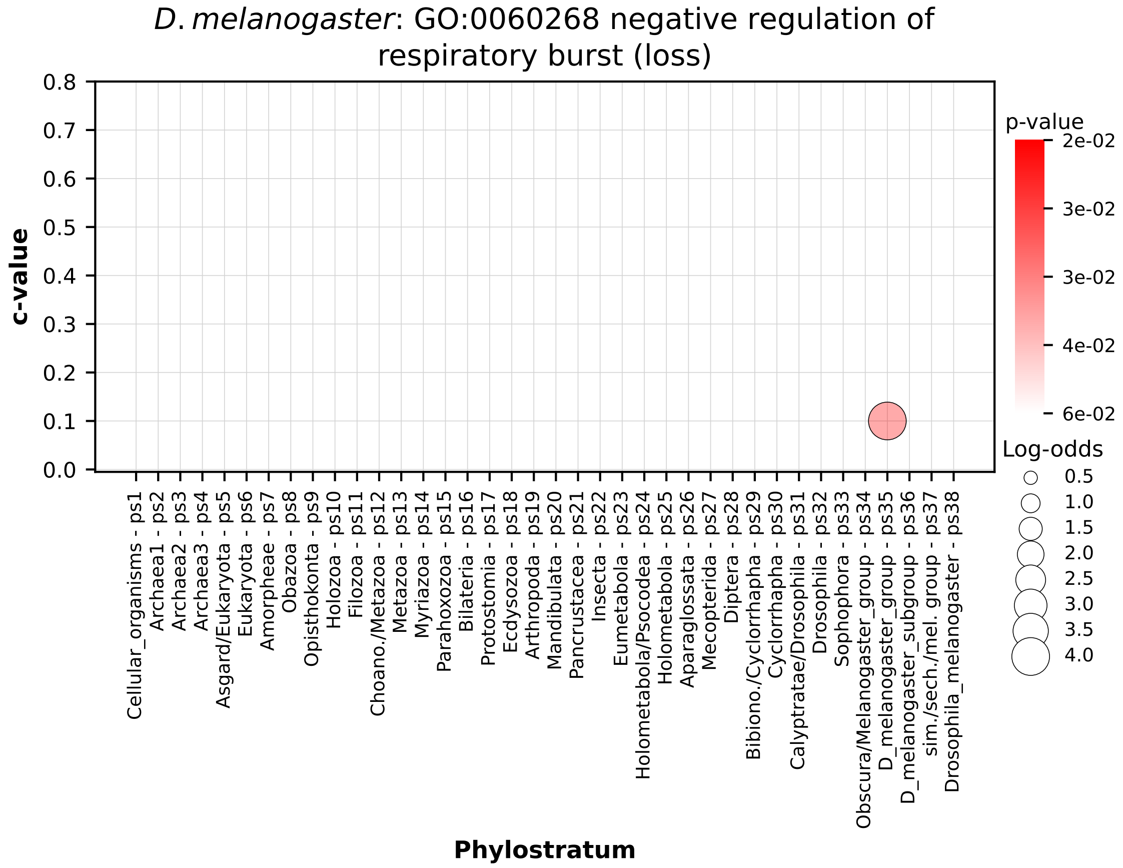

D. melanogaster: GO:0060268 negative regulation of respiratory burst (loss)
c-value
0.0
0.1
0.2
0.3
0.4
0.5
0.6
0.7
0.8
p-value
2e-02
3e-02
3e-02
4e-02
6e-02
Log-odds
0.5
1.0
1.5
2.0
2.5
3.0
3.5
4.0
Cellular_organisms - ps1
Archaea1 - ps2
Archaea2 - ps3
Archaea3 - ps4
Asgard/Eukaryota - ps5
Eukaryota - ps6
Amorpheae - ps7
Obazoa - ps8
Opisthokonta - ps9
Holozoa - ps10
Filozoa - ps11
Choano./Metazoa - ps12
Metazoa - ps13
Myriazoa - ps14
Parahoxozoa - ps15
Bilateria - ps16
Protostomia - ps17
Ecdysozoa - ps18
Arthropoda - ps19
Mandibulata - ps20
Pancrustacea - ps21
Insecta - ps22
Eumetabola - ps23
Holometabola/Psocodea - ps24
Holometabola - ps25
Aparaglossata - ps26
Mecopterida - ps27
Diptera - ps28
Bibiono./Cyclorrhapha - ps29
Cyclorrhapha - ps30
Calyptratae/Drosophila - ps31
Drosophila - ps32
Sophophora - ps33
Obscura/Melanogaster_group - ps34
D_melanogaster_group - ps35
D_melanogaster_subgroup - ps36
sim./sech./mel. group - ps37
Drosophila_melanogaster - ps38
Phylostratum

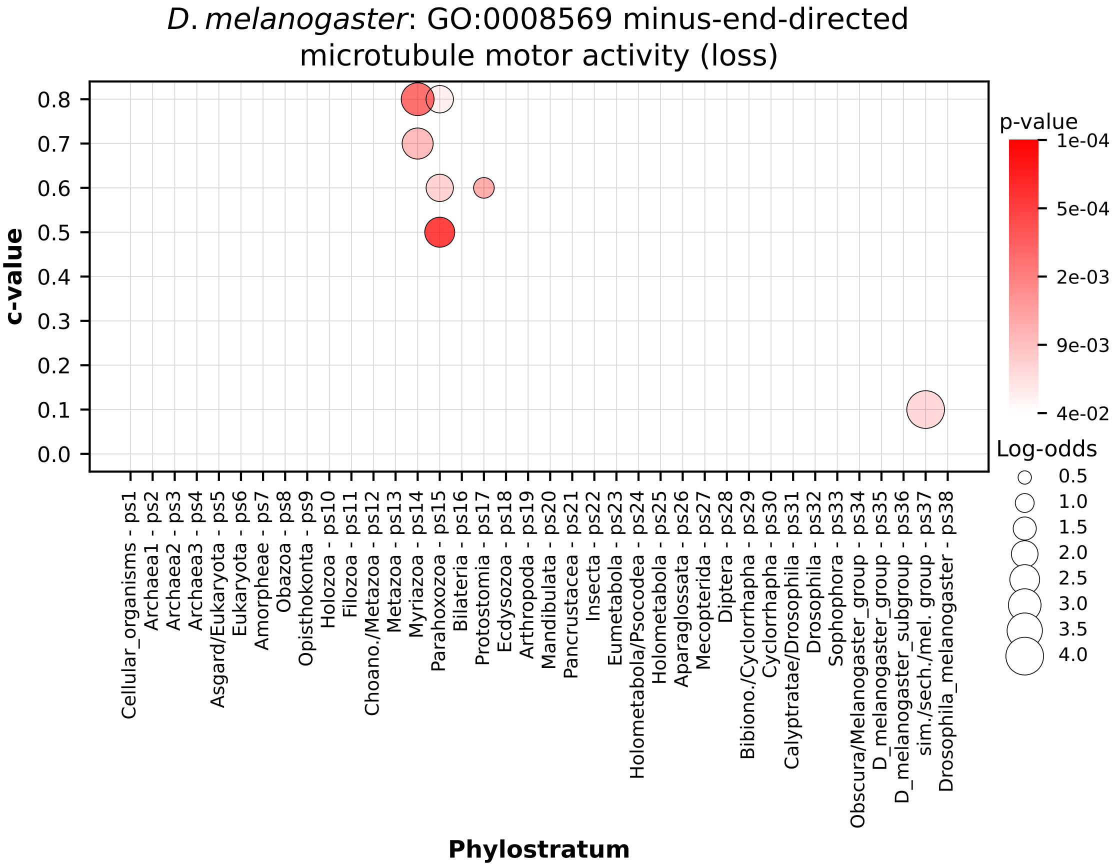
D. melanogaster: GO:0008569 minus-end-directed microtubule motor activity (loss)
c-value
0.0
0.1
0.2
0.3
0.4
0.5
0.6
0.7
0.8
Phylostratum
Cellular_organisms - ps1
Archaea1 - ps2
Archaea2 - ps3
Archaea3 - ps4
Asgard/Eukaryota - ps5
Eukaryota - ps6
Amorpheae - ps7
Obazoa - ps8
Opisthokonta - ps9
Holozoa - ps10
Filozoa - ps11
Choano./Metazoa - ps12
Metazoa - ps13
Myriazoa - ps14
Parahoxozoa - ps15
Bilateria - ps16
Protostomia - ps17
Ecdysozoa - ps18
Arthropoda - ps19
Mandibulata - ps20
Pancrustacea - ps21
Insecta - ps22
Eumetabola - ps23
Holometabola/Psocodea - ps24
Holometabola - ps25
Aparaglossata - ps26
Mecopterida - ps27
Diptera - ps28
Bibiono./Cyclorrhapha - ps29
Cyclorrhapha - ps30
Calyptratae/Drosophila - ps31
Drosophila - ps32
Sophophora - ps33
Obscura/Melanogaster_group - ps34
D_melanogaster_group - ps35
D_melanogaster_subgroup - ps36
sim./sech./mel. group - ps37
Drosophila_melanogaster - ps38
p-value
1e-04
5e-04
2e-03
9e-03
4e-02
Log-odds
0.5
1.0
1.5
2.0
2.5
3.0
3.5
4.0

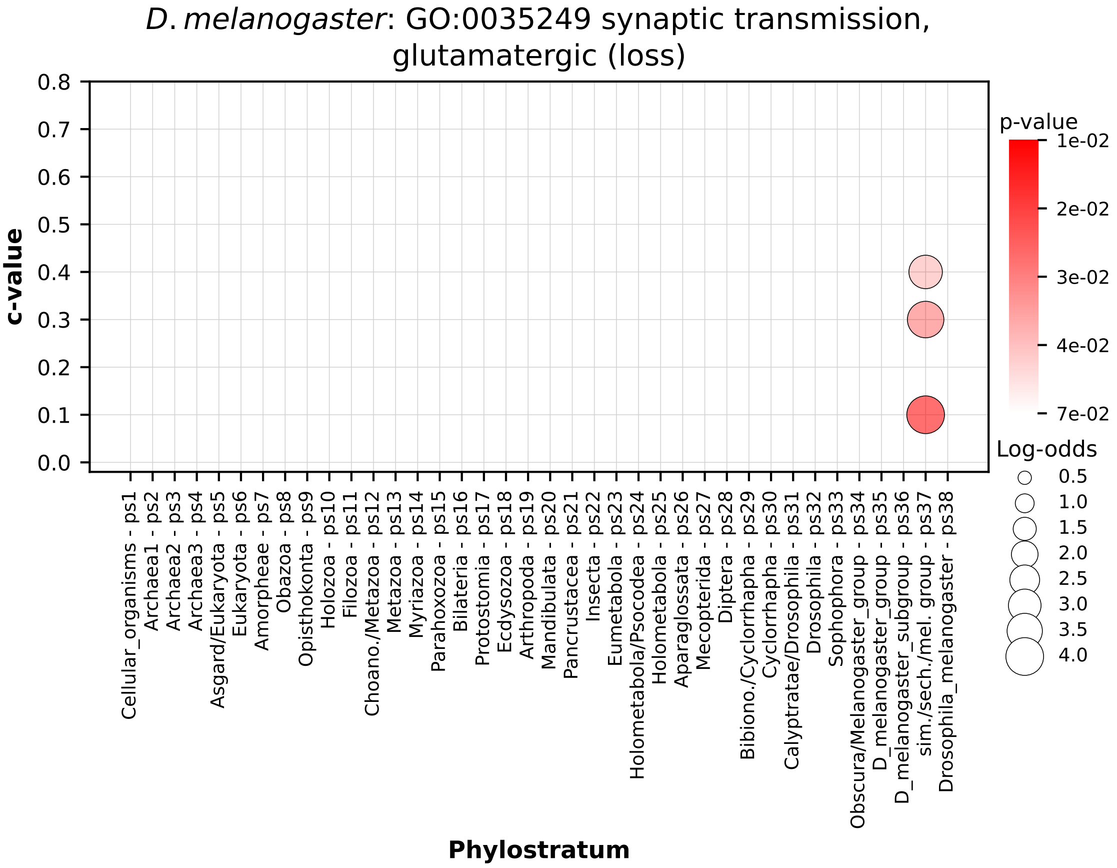

D. melanogaster: GO:0035249 synaptic transmission, glutamatergic (loss)
c-value
0.8
0.7
0.6
0.5
0.4
0.3
0.2
0.1
0.0
p-value
1e-02
2e-02
3e-02
4e-02
7e-02
Log-odds
0.5
1.0
1.5
2.0
2.5
3.0
3.5
4.0
Cellular_organisms - ps1
Archaea1 - ps2
Archaea2 - ps3
Archaea3 - ps4
Asgard/Eukaryota - ps5
Eukaryota - ps6
Amorpheae - ps7
Obazoa - ps8
Opisthokonta - ps9
Holozoa - ps10
Filozoa - ps11
Choano./Metazoa - ps12
Metazoa - ps13
Myriazoa - ps14
Parahoxozoa - ps15
Bilateria - ps16
Protostomia - ps17
Ecdysozoa - ps18
Arthropoda - ps19
Mandibulata - ps20
Pancrustacea - ps21
Insecta - ps22
Eumetabola - ps23
Holometabola/Psocodea - ps24
Holometabola - ps25
Aparaglossata - ps26
Mecopterida - ps27
Diptera - ps28
Bibiono./Cyclorrhapha - ps29
Cyclorrhapha - ps30
Calyptratae/Drosophila - ps31
Drosophila - ps32
Sophophora - ps33
Obscura/Melanogaster_group - ps34
D_melanogaster_group - ps35
D_melanogaster_subgroup - ps36
sim./sech./mel. group - ps37
Drosophila_melanogaster - ps38
Phylostratum

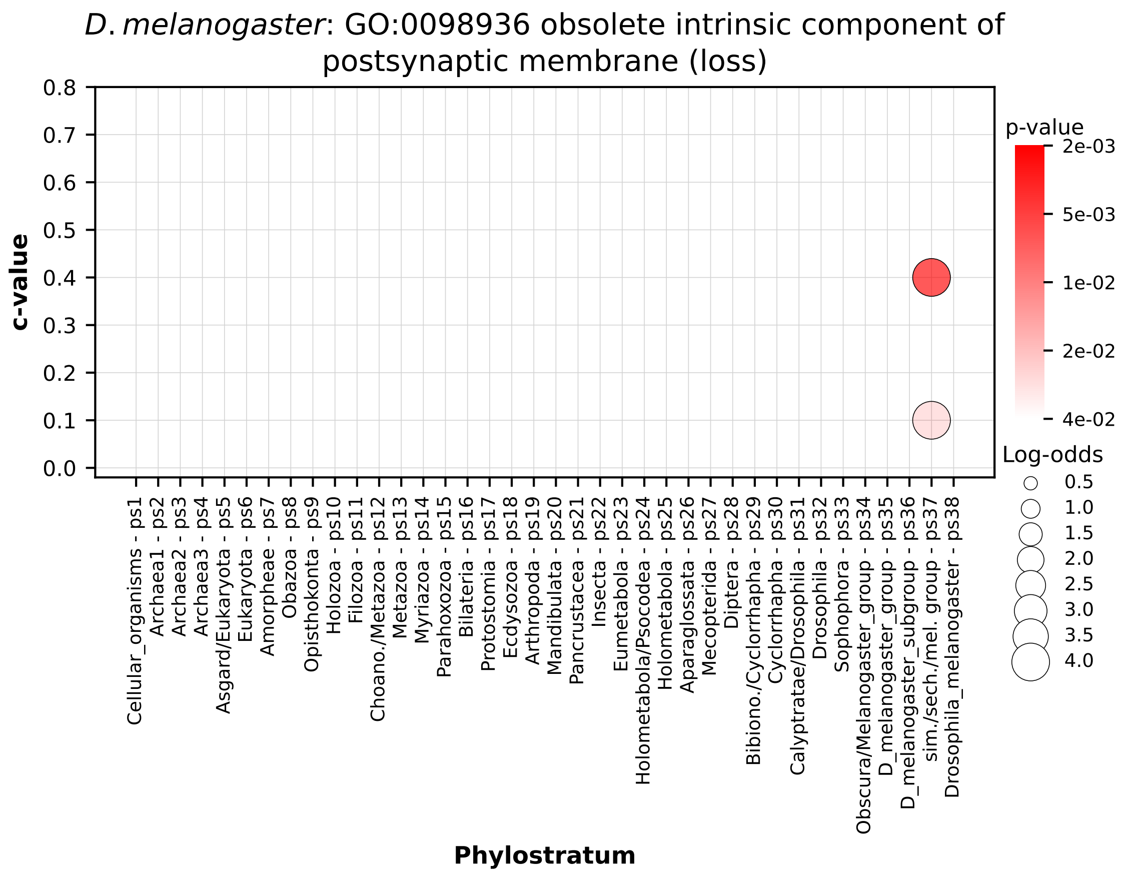

D. melanogaster: GO:0098936 obsolete intrinsic component of postsynaptic membrane (loss)
c-value
0.8
0.7
0.6
0.5
0.4
0.3
0.2
0.1
0.0
p-value
2e-03
5e-03
1e-02
2e-02
4e-02
Log-odds
0.5
1.0
1.5
2.0
2.5
3.0
3.5
4.0
Cellular_organisms - ps1
Archaea1 - ps2
Archaea2 - ps3
Archaea3 - ps4
Asgard/Eukaryota - ps5
Eukaryota - ps6
Amorpheae - ps7
Obazoa - ps8
Opisthokonta - ps9
Holozoa - ps10
Filozoa - ps11
Choano./Metazoa - ps12
Metazoa - ps13
Myriazoa - ps14
Parahoxozoa - ps15
Bilateria - ps16
Protostomia - ps17
Ecdysozoa - ps18
Arthropoda - ps19
Mandibulata - ps20
Pancrustacea - ps21
Insecta - ps22
Eumetabola - ps23
Holometabola/Psocodea - ps24
Holometabola - ps25
Aparaglossata - ps26
Mecopterida - ps27
Diptera - ps28
Bibiono./Cyclorrhapha - ps29
Cyclorrhapha - ps30
Calyptratae/Drosophila - ps31
Drosophila - ps32
Sophophora - ps33
Obscura/Melanogaster_group - ps34
D_melanogaster_group - ps35
D_melanogaster_subgroup - ps36
sim./sech./mel. group - ps37
Drosophila_melanogaster - ps38
Phylostratum

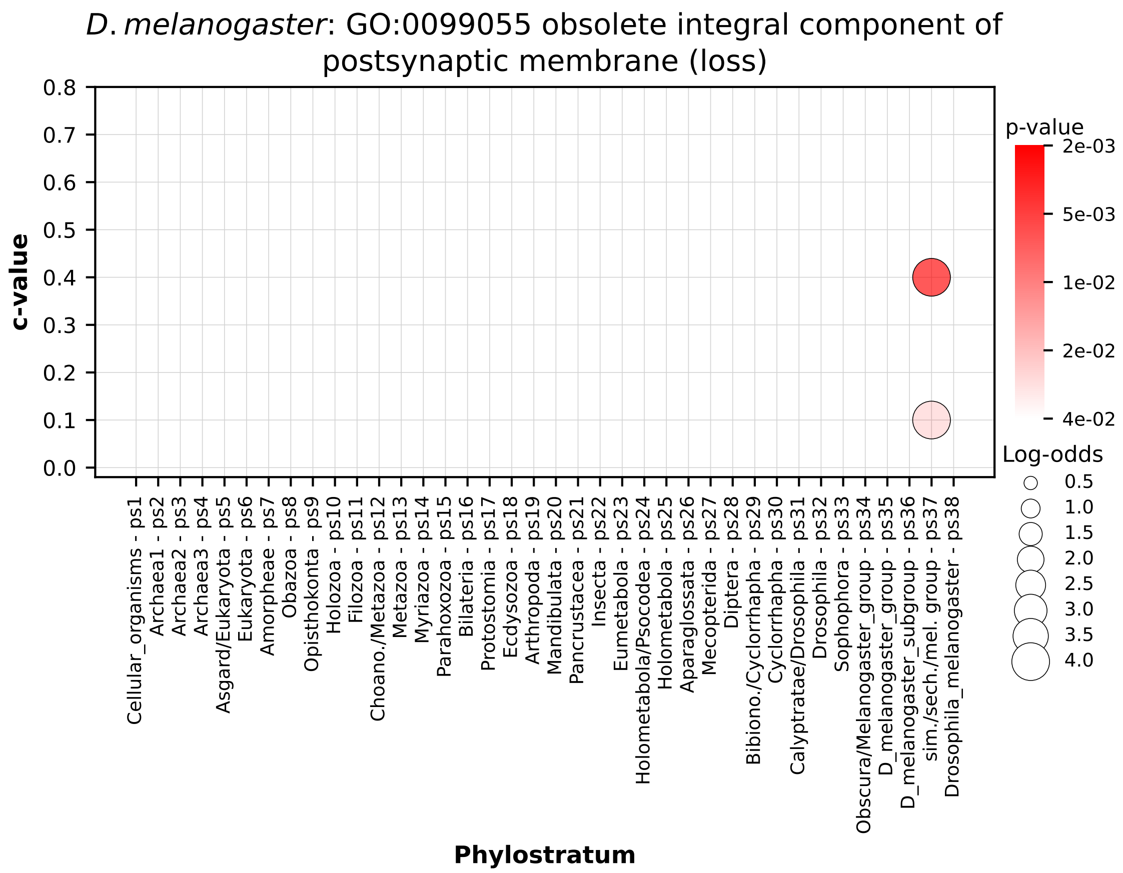

D. melanogaster: GO:0099055 obsolete integral component of postsynaptic membrane (loss)
c-value
0.8
0.7
0.6
0.5
0.4
0.3
0.2
0.1
0.0
Phylostratum
Cellular_organisms - ps1
Archaea1 - ps2
Archaea2 - ps3
Archaea3 - ps4
Asgard/Eukaryota - ps5
Eukaryota - ps6
Amorpheae - ps7
Obazoa - ps8
Opisthokonta - ps9
Holozoa - ps10
Filozoa - ps11
Choano./Metazoa - ps12
Metazoa - ps13
Myriazoa - ps14
Parahoxozoa - ps15
Bilateria - ps16
Protostomia - ps17
Ecdysozoa - ps18
Arthropoda - ps19
Mandibulata - ps20
Pancrustacea - ps21
Insecta - ps22
Eumetabola - ps23
Holometabola/Psocodea - ps24
Holometabola - ps25
Aparaglossata - ps26
Mecopterida - ps27
Diptera - ps28
Bibiono./Cyclorrhapha - ps29
Cyclorrhapha - ps30
Calyptratae/Drosophila - ps31
Drosophila - ps32
Sophophora - ps33
Obscura/Melanogaster_group - ps34
D_melanogaster_group - ps35
D_melanogaster_subgroup - ps36
sim./sech./mel. group - ps37
Drosophila_melanogaster - ps38
p-value
2e-03
5e-03
1e-02
2e-02
4e-02
Log-odds
0.5
1.0
1.5
2.0
2.5
3.0
3.5
4.0

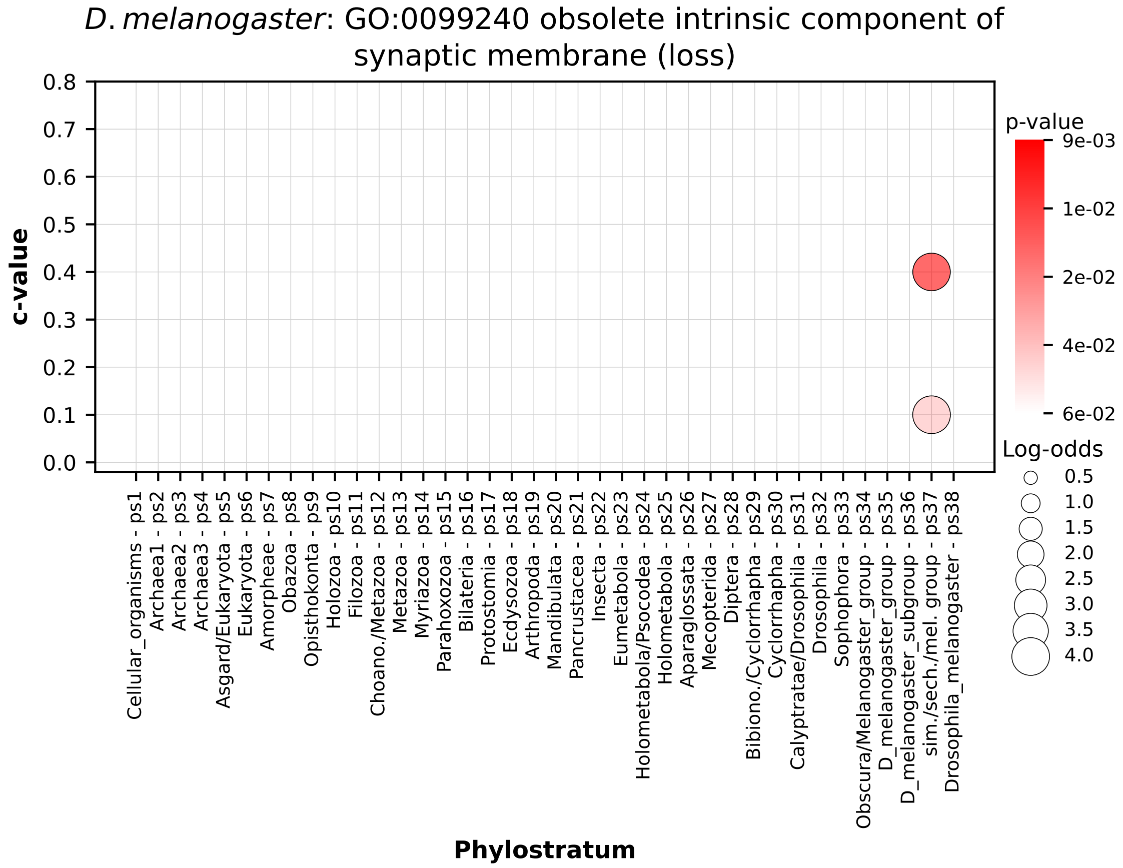
D. melanogaster: GO:0099240 obsolete intrinsic component of synaptic membrane (loss)
c-value
0.8
0.7
0.6
0.5
0.4
0.3
0.2
0.1
0.0
p-value
9e-03
1e-02
2e-02
4e-02
6e-02
Log-odds
0.5
1.0
1.5
2.0
2.5
3.0
3.5
4.0
Cellular_organisms - ps1
Archaea1 - ps2
Archaea2 - ps3
Archaea3 - ps4
Asgard/Eukaryota - ps5
Eukaryota - ps6
Amorpheae - ps7
Obazoa - ps8
Opisthokonta - ps9
Holozoa - ps10
Filozoa - ps11
Choano./Metazoa - ps12
Metazoa - ps13
Myriazoa - ps14
Parahoxozoa - ps15
Bilateria - ps16
Protostomia - ps17
Ecdysozoa - ps18
Arthropoda - ps19
Mandibulata - ps20
Pancrustacea - ps21
Insecta - ps22
Eumetabola - ps23
Holometabola/Psocodea - ps24
Holometabola - ps25
Aparaglossata - ps26
Mecopterida - ps27
Diptera - ps28
Bibiono./Cyclorrhapha - ps29
Cyclorrhapha - ps30
Calyptratae/Drosophila - ps31
Drosophila - ps32
Sophophora - ps33
Obscura/Melanogaster_group - ps34
D_melanogaster_group - ps35
D_melanogaster_subgroup - ps36
sim./sech./mel. group - ps37
Drosophila_melanogaster - ps38
Phylostratum

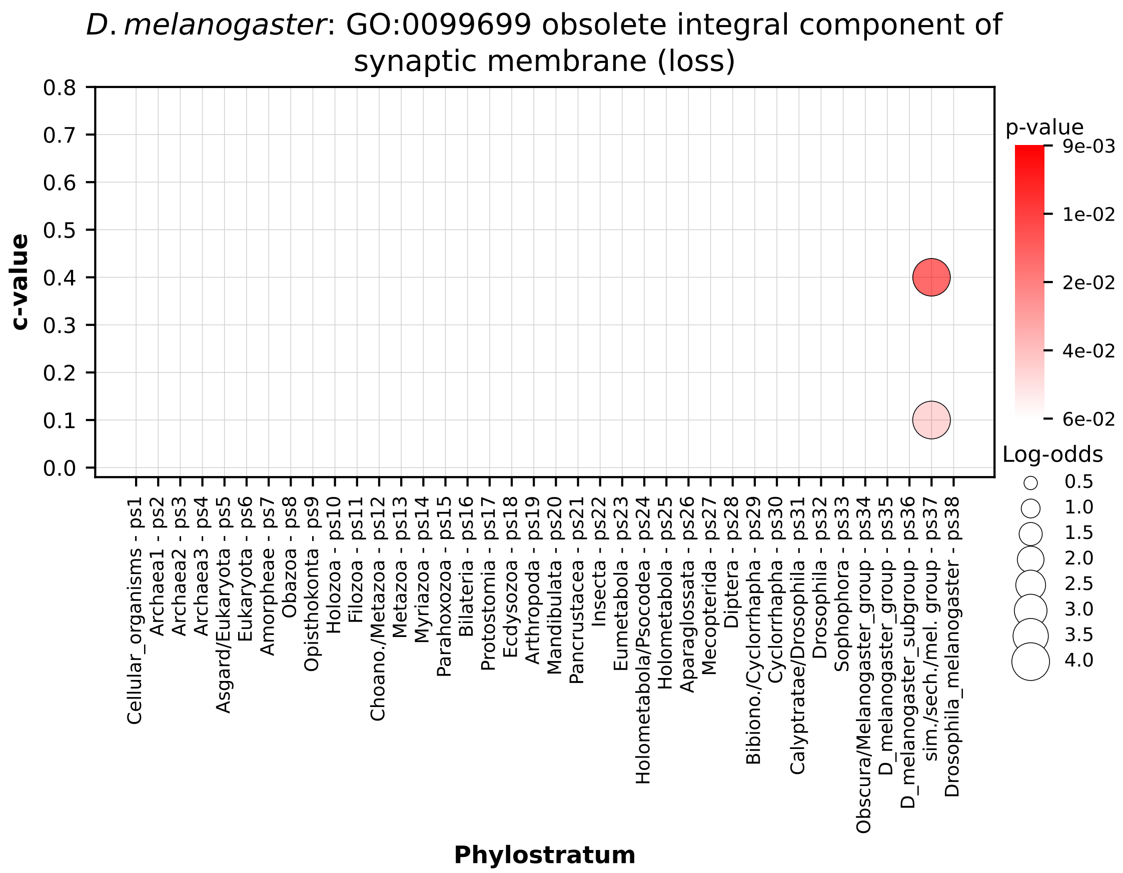

D. melanogaster: GO:0099699 obsolete integral component of synaptic membrane (loss)
c-value
0.8
0.7
0.6
0.5
0.4
0.3
0.2
0.1
0.0
p-value
9e-03
1e-02
2e-02
4e-02
6e-02
Log-odds
0.5
1.0
1.5
2.0
2.5
3.0
3.5
4.0
Cellular_organisms - ps1
Archaea1 - ps2
Archaea2 - ps3
Archaea3 - ps4
Asgard/Eukaryota - ps5
Eukaryota - ps6
Amorpheae - ps7
Obazoa - ps8
Opisthokonta - ps9
Holozoa - ps10
Filozoa - ps11
Choano./Metazoa - ps12
Metazoa - ps13
Myriazoa - ps14
Parahoxozoa - ps15
Bilateria - ps16
Protostomia - ps17
Ecdysozoa - ps18
Arthropoda - ps19
Mandibulata - ps20
Pancrustacea - ps21
Insecta - ps22
Eumetabola - ps23
Holometabola/Psocodea - ps24
Holometabola - ps25
Aparaglossata - ps26
Mecopterida - ps27
Diptera - ps28
Bibiono./Cyclorrhapha - ps29
Cyclorrhapha - ps30
Calyptratae/Drosophila - ps31
Drosophila - ps32
Sophophora - ps33
Obscura/Melanogaster_group - ps34
D_melanogaster_group - ps35
D_melanogaster_subgroup - ps36
sim./sech./mel. group - ps37
Drosophila_melanogaster - ps38
Phylostratum

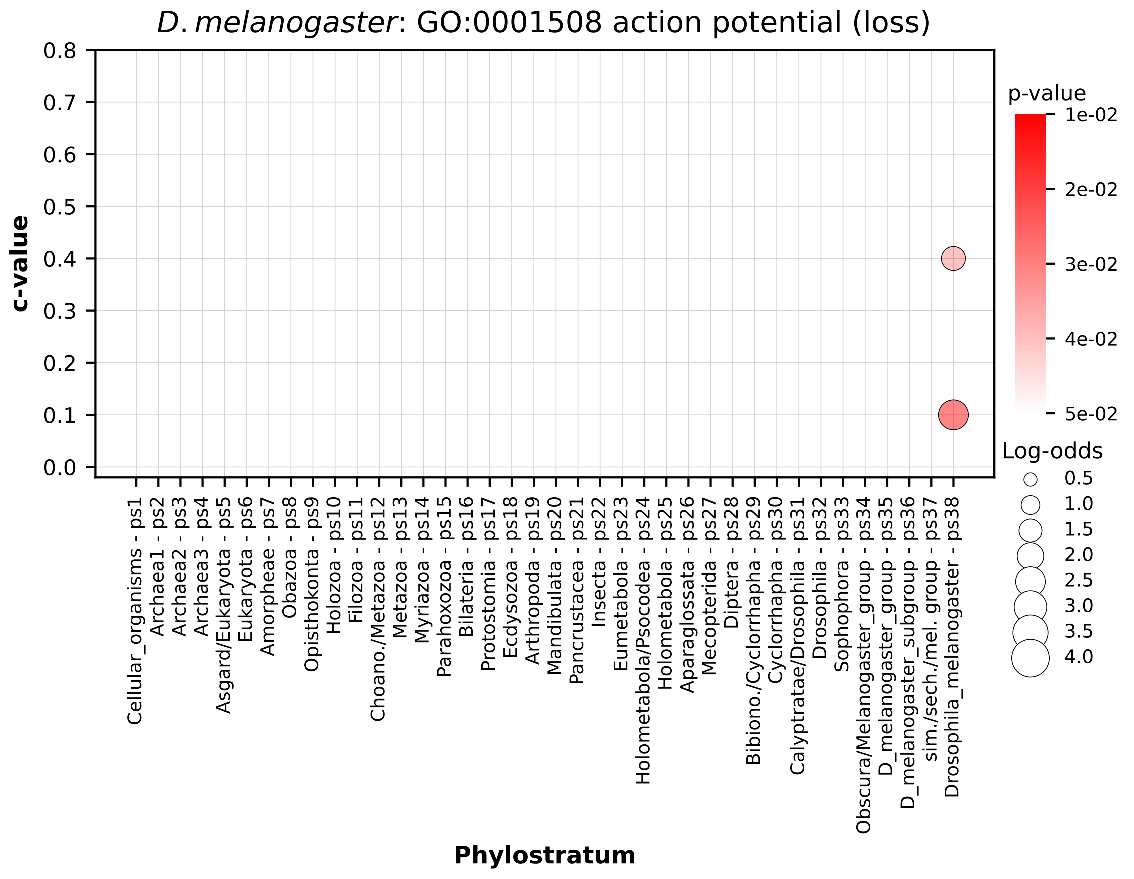

D. melanogaster: GO:0001508 action potential (loss)
c-value
0.0
0.1
0.2
0.3
0.4
0.5
0.6
0.7
0.8
Phylostratum
Cellular_organisms - ps1
Archaea1 - ps2
Archaea2 - ps3
Archaea3 - ps4
Asgard/Eukaryota - ps5
Eukaryota - ps6
Amorpheae - ps7
Obazoa - ps8
Opisthokonta - ps9
Holozoa - ps10
Filozoa - ps11
Choano./Metazoa - ps12
Metazoa - ps13
Myriazoa - ps14
Parahoxozoa - ps15
Bilateria - ps16
Protostomia - ps17
Ecdysozoa - ps18
Arthropoda - ps19
Mandibulata - ps20
Pancrustacea - ps21
Insecta - ps22
Eumetabola - ps23
Holometabola/Psocodea - ps24
Holometabola - ps25
Aparaglossata - ps26
Mecopterida - ps27
Diptera - ps28
Bibiono./Cyclorrhapha - ps29
Cyclorrhapha - ps30
Calyptratae/Drosophila - ps31
Drosophila - ps32
Sophophora - ps33
Obscura/Melanogaster_group - ps34
D_melanogaster_group - ps35
D_melanogaster_subgroup - ps36
sim./sech./mel. group - ps37
Drosophila_melanogaster - ps38
p-value
1e-02
2e-02
3e-02
4e-02
5e-02
Log-odds
0.5
1.0
1.5
2.0
2.5
3.0
3.5
4.0

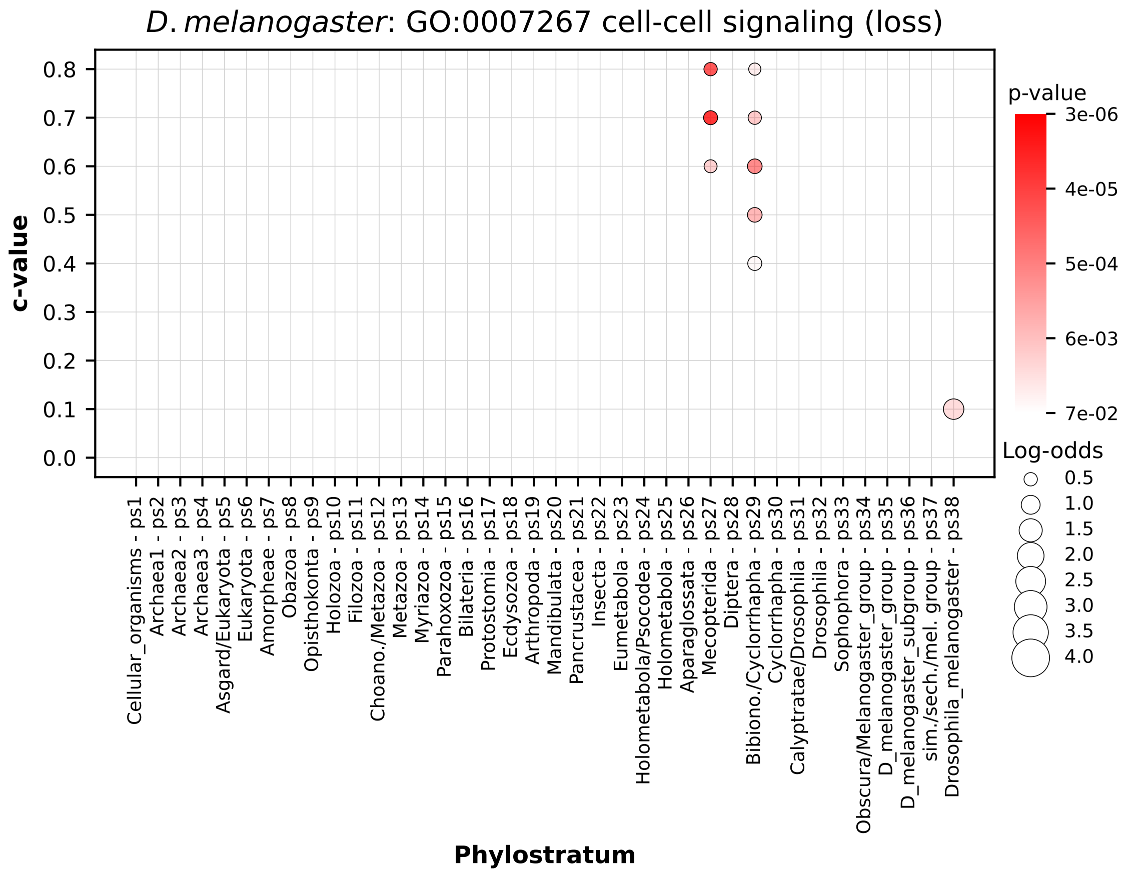

D. melanogaster: GO:0007267 cell-cell signaling (loss)
c-value
0.8
0.7
0.6
0.5
0.4
0.3
0.2
0.1
0.0
p-value
3e-06
4e-05
5e-04
6e-03
7e-02
Log-odds
0.5
1.0
1.5
2.0
2.5
3.0
3.5
4.0
Cellular_organisms - ps1
Archaea1 - ps2
Archaea2 - ps3
Archaea3 - ps4
Asgard/Eukaryota - ps5
Eukaryota - ps6
Amorpheae - ps7
Obazoa - ps8
Opisthokonta - ps9
Holozoa - ps10
Filozoa - ps11
Choano./Metazoa - ps12
Metazoa - ps13
Myriazoa - ps14
Parahoxozoa - ps15
Bilateria - ps16
Protostomia - ps17
Ecdysozoa - ps18
Arthropoda - ps19
Mandibulata - ps20
Pancrustacea - ps21
Insecta - ps22
Eumetabola - ps23
Holometabola/Psocodea - ps24
Holometabola - ps25
Aparaglossata - ps26
Mecopterida - ps27
Diptera - ps28
Bibiono./Cyclorrhapha - ps29
Cyclorrhapha - ps30
Calyptratae/Drosophila - ps31
Drosophila - ps32
Sophophora - ps33
Obscura/Melanogaster_group - ps34
D_melanogaster_group - ps35
D_melanogaster_subgroup - ps36
sim./sech./mel. group - ps37
Drosophila_melanogaster - ps38
Phylostratum

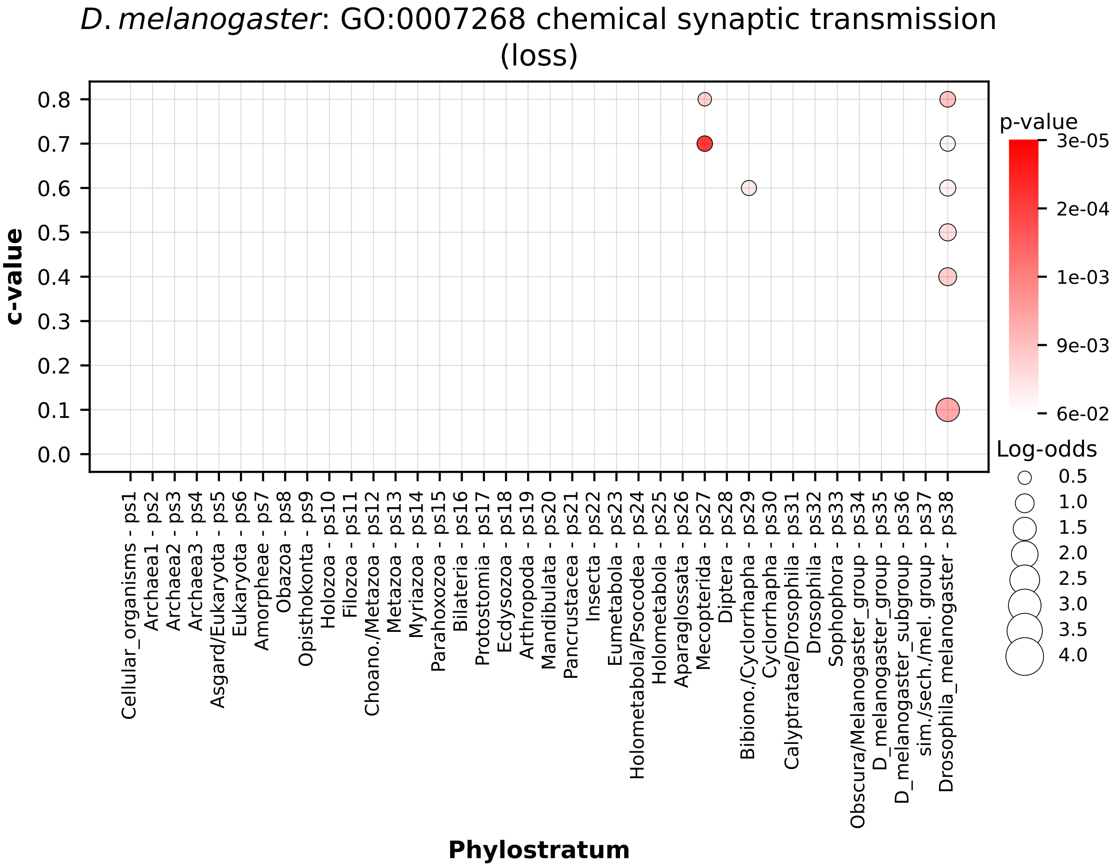

D. melanogaster: GO:0007268 chemical synaptic transmission (loss)
c-value
0.8
0.7
0.6
0.5
0.4
0.3
0.2
0.1
0.0
Phylostratum
Cellular_organisms - ps1
Archaea1 - ps2
Archaea2 - ps3
Archaea3 - ps4
Asgard/Eukaryota - ps5
Eukaryota - ps6
Amorpheae - ps7
Obazoa - ps8
Opisthokonta - ps9
Holozoa - ps10
Filozoa - ps11
Choano./Metazoa - ps12
Metazoa - ps13
Myriazoa - ps14
Parahoxozoa - ps15
Bilateria - ps16
Protostomia - ps17
Ecdysozoa - ps18
Arthropoda - ps19
Mandibulata - ps20
Pancrustacea - ps21
Insecta - ps22
Eumetabola - ps23
Holometabola/Psocodea - ps24
Holometabola - ps25
Aparaglossata - ps26
Mecopterida - ps27
Diptera - ps28
Bibiono./Cyclorrhapha - ps29
Cyclorrhapha - ps30
Calyptratae/Drosophila - ps31
Drosophila - ps32
Sophophora - ps33
Obscura/Melanogaster_group - ps34
D_melanogaster_group - ps35
D_melanogaster_subgroup - ps36
sim./sech./mel. group - ps37
Drosophila_melanogaster - ps38
p-value
3e-05
2e-04
1e-03
9e-03
6e-02
Log-odds
0.5
1.0
1.5
2.0
2.5
3.0
3.5
4.0

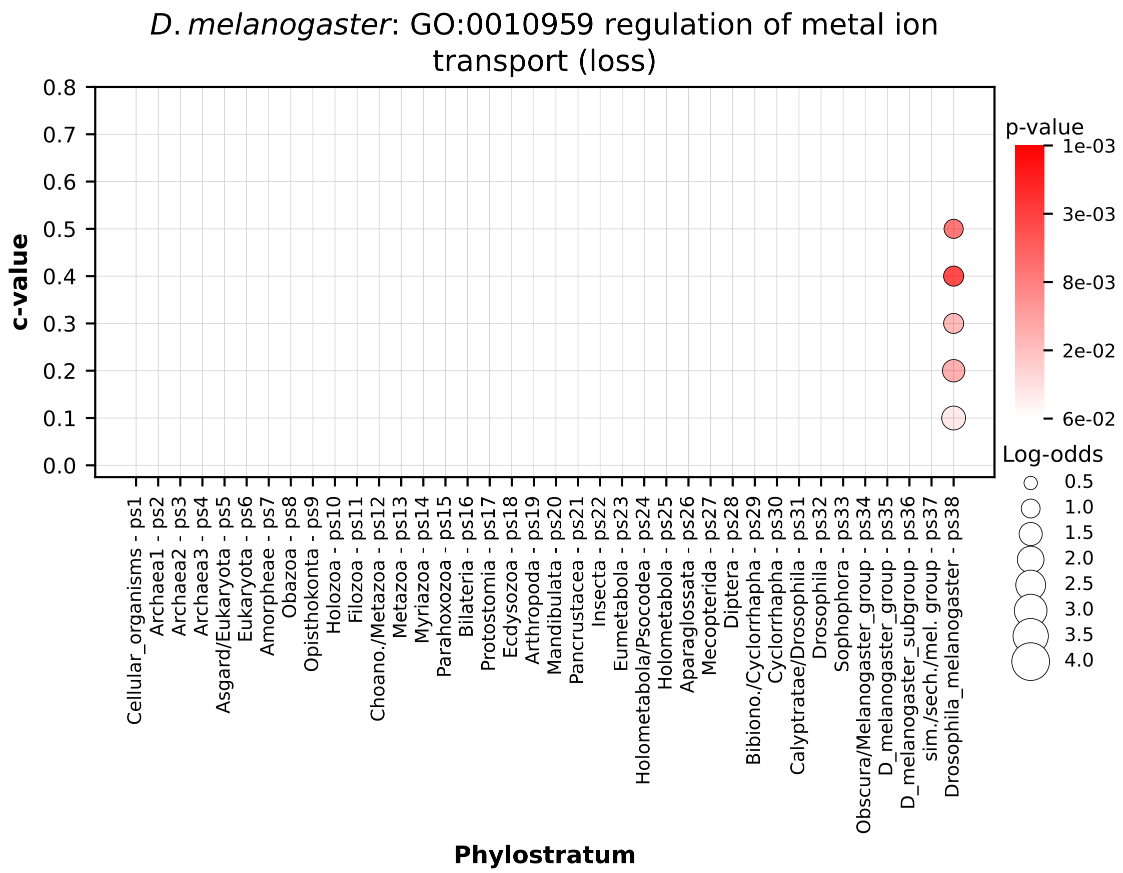
D. melanogaster: GO:0010959 regulation of metal ion transport (loss)
c-value
0.0
0.1
0.2
0.3
0.4
0.5
0.6
0.7
0.8
Phylostratum
Cellular_organisms - ps1
Archaea1 - ps2
Archaea2 - ps3
Archaea3 - ps4
Asgard/Eukaryota - ps5
Eukaryota - ps6
Amorpheae - ps7
Obazoa - ps8
Opisthokonta - ps9
Holozoa - ps10
Filozoa - ps11
Choano./Metazoa - ps12
Metazoa - ps13
Myriazoa - ps14
Parahoxozoa - ps15
Bilateria - ps16
Protostomia - ps17
Ecdysozoa - ps18
Arthropoda - ps19
Mandibulata - ps20
Pancrustacea - ps21
Insecta - ps22
Eumetabola - ps23
Holometabola/Psocodea - ps24
Holometabola - ps25
Aparaglossata - ps26
Mecopterida - ps27
Diptera - ps28
Bibiono./Cyclorrhapha - ps29
Cyclorrhapha - ps30
Calyptratae/Drosophila - ps31
Drosophila - ps32
Sophophora - ps33
Obscura/Melanogaster_group - ps34
D_melanogaster_group - ps35
D_melanogaster_subgroup - ps36
sim./sech./mel. group - ps37
Drosophila_melanogaster - ps38
p-value
1e-03
3e-03
8e-03
2e-02
6e-02
Log-odds
0.5
1.0
1.5
2.0
2.5
3.0
3.5
4.0

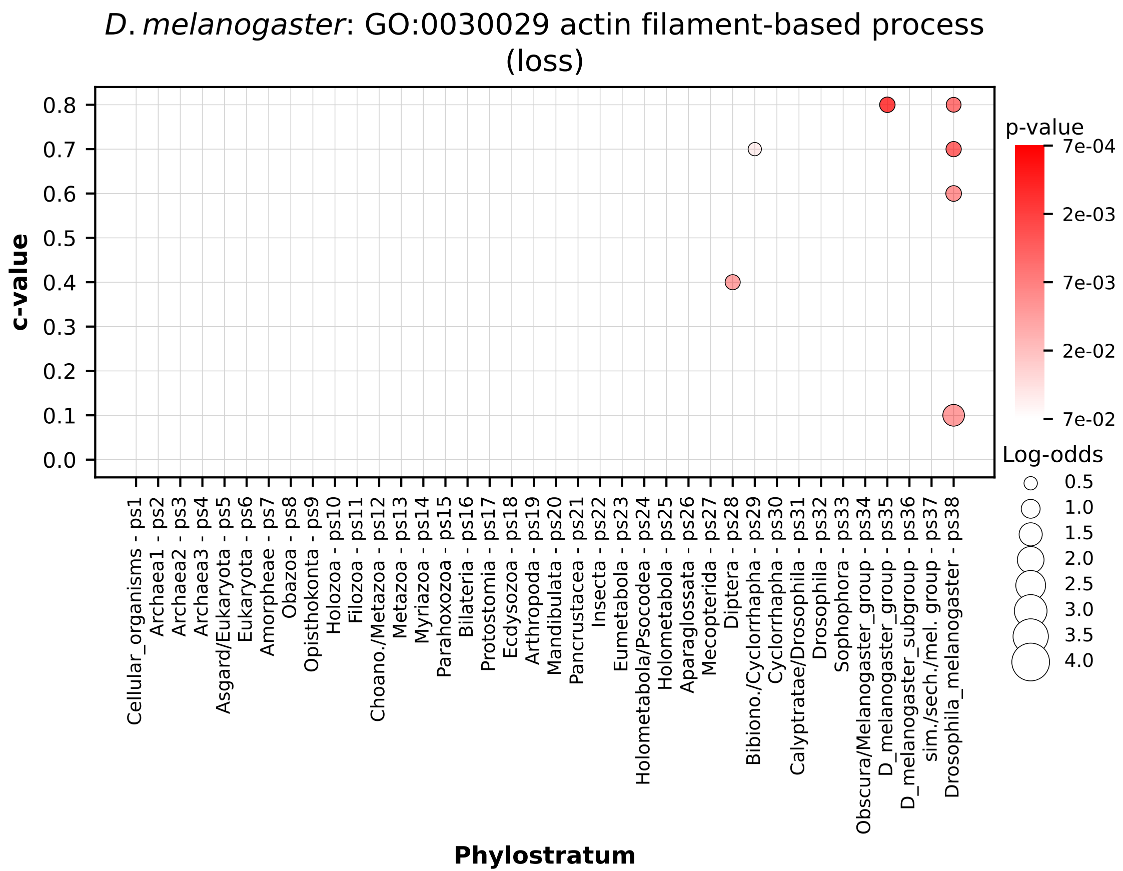
D. melanogaster: GO:0030029 actin filament-based process
(loss)
c-value
0.8
0.7
0.6
0.5
0.4
0.3
0.2
0.1
0.0
p-value
7e-04
2e-03
7e-03
2e-02
7e-02
Log-odds
0.5
1.0
1.5
2.0
2.5
3.0
3.5
4.0
Cellular_organisms - ps1
Archaea1 - ps2
Archaea2 - ps3
Archaea3 - ps4
Asgard/Eukaryota - ps5
Eukaryota - ps6
Amorpheae - ps7
Obazoa - ps8
Opisthokonta - ps9
Holozoa - ps10
Filozoa - ps11
Choano./Metazoa - ps12
Metazoa - ps13
Myriazoa - ps14
Parahoxozoa - ps15
Bilateria - ps16
Protostomia - ps17
Ecdysozoa - ps18
Arthropoda - ps19
Mandibulata - ps20
Pancrustacea - ps21
Insecta - ps22
Eumetabola - ps23
Holometabola/Psocodea - ps24
Holometabola - ps25
Aparaglossata - ps26
Mecopterida - ps27
Diptera - ps28
Bibiono./Cyclorrhapha - ps29
Cyclorrhapha - ps30
Calyptratae/Drosophila - ps31
Drosophila - ps32
Sophophora - ps33
Obscura/Melanogaster_group - ps34
D_melanogaster_group - ps35
D_melanogaster_subgroup - ps36
sim./sech./mel. group - ps37
Drosophila_melanogaster - ps38
Phylostratum

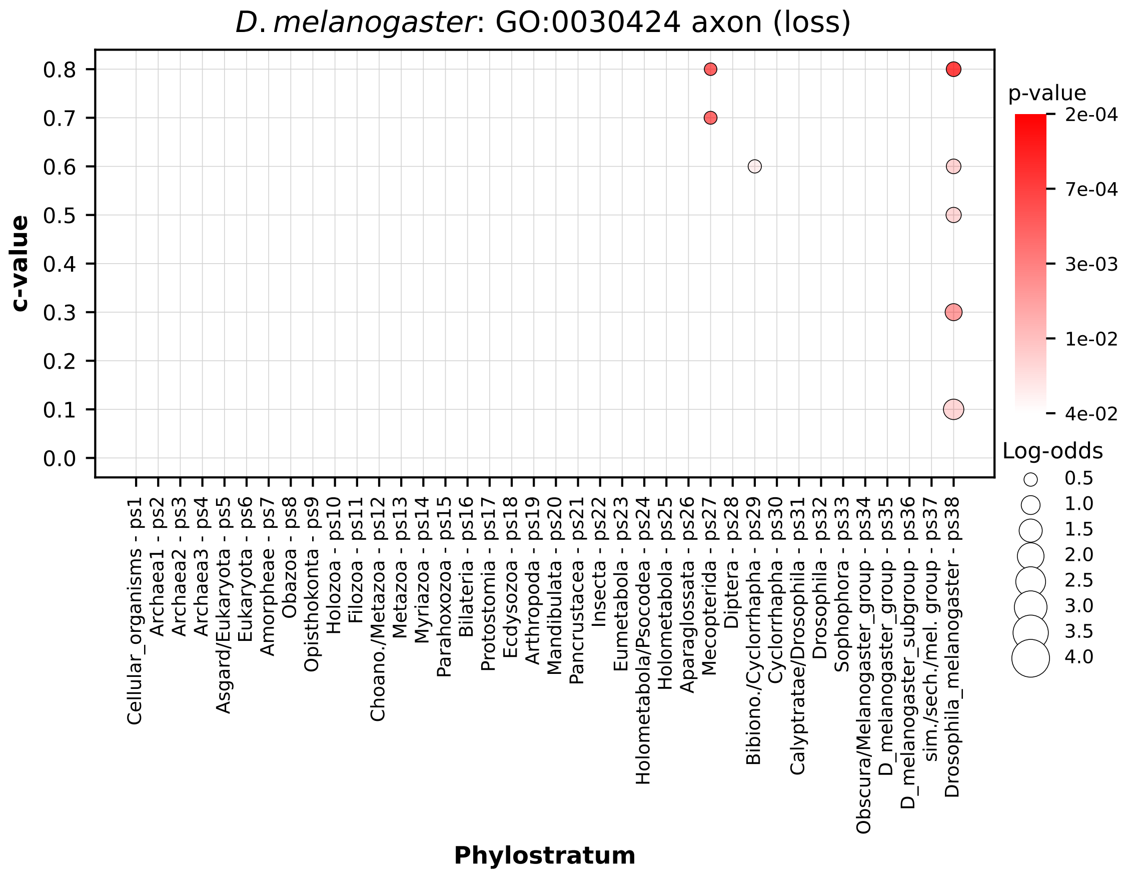

D. melanogaster: GO:0030424 axon (loss)
c-value
0.8
0.7
0.6
0.5
0.4
0.3
0.2
0.1
0.0
Phylostratum
Cellular_organisms - ps1
Archaea1 - ps2
Archaea2 - ps3
Archaea3 - ps4
Asgard/Eukaryota - ps5
Eukaryota - ps6
Amorpheae - ps7
Obazoa - ps8
Opisthokonta - ps9
Holozoa - ps10
Filozoa - ps11
Choano./Metazoa - ps12
Metazoa - ps13
Myriazoa - ps14
Parahoxozoa - ps15
Bilateria - ps16
Protostomia - ps17
Ecdysozoa - ps18
Arthropoda - ps19
Mandibulata - ps20
Pancrustacea - ps21
Insecta - ps22
Eumetabola - ps23
Holometabola/Psocodea - ps24
Holometabola - ps25
Aparaglossata - ps26
Mecopterida - ps27
Diptera - ps28
Bibiono./Cyclorrhapha - ps29
Cyclorrhapha - ps30
Calyptratae/Drosophila - ps31
Drosophila - ps32
Sophophora - ps33
Obscura/Melanogaster_group - ps34
D_melanogaster_group - ps35
D_melanogaster_subgroup - ps36
sim./sech./mel. group - ps37
Drosophila_melanogaster - ps38
p-value
2e-04
7e-04
3e-03
1e-02
4e-02
Log-odds
0.5
1.0
1.5
2.0
2.5
3.0
3.5
4.0

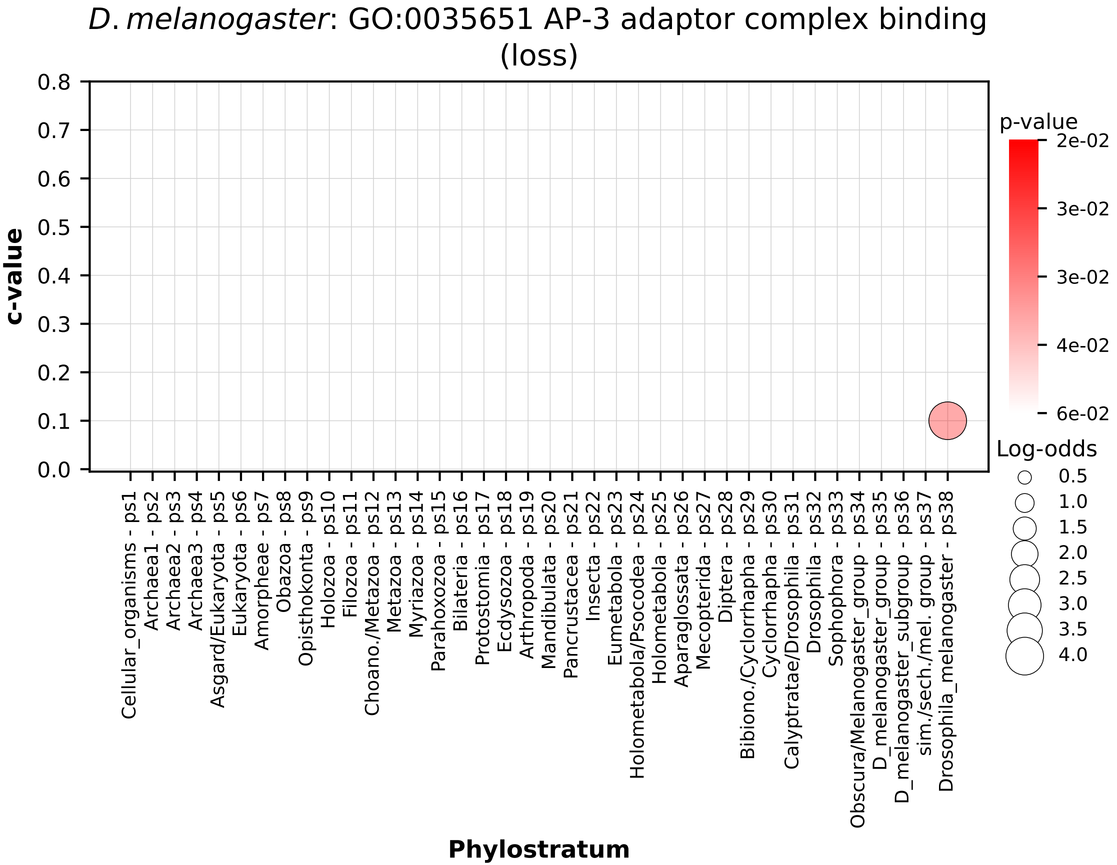
D. melanogaster: GO:0035651 AP-3 adaptor complex binding
(loss)
c-value
0.8
0.7
0.6
0.5
0.4
0.3
0.2
0.1
0.0
Phylostratum
Cellular_organisms - ps1
Archaea1 - ps2
Archaea2 - ps3
Archaea3 - ps4
Asgard/Eukaryota - ps5
Eukaryota - ps6
Amorpheae - ps7
Obazoa - ps8
Opisthokonta - ps9
Holozoa - ps10
Filozoa - ps11
Choano./Metazoa - ps12
Metazoa - ps13
Myriazoa - ps14
Parahoxozoa - ps15
Bilateria - ps16
Protostomia - ps17
Ecdysozoa - ps18
Arthropoda - ps19
Mandibulata - ps20
Pancrustacea - ps21
Insecta - ps22
Eumetabola - ps23
Holometabola/Psocodea - ps24
Holometabola - ps25
Aparaglossata - ps26
Mecopterida - ps27
Diptera - ps28
Bibiono./Cyclorrhapha - ps29
Cyclorrhapha - ps30
Calyptratae/Drosophila - ps31
Drosophila - ps32
Sophophora - ps33
Obscura/Melanogaster_group - ps34
D_melanogaster_group - ps35
D_melanogaster_subgroup - ps36
sim./sech./mel. group - ps37
Drosophila_melanogaster - ps38
p-value
2e-02
3e-02
3e-02
4e-02
6e-02
Log-odds
0.5
1.0
1.5
2.0
2.5
3.0
3.5
4.0

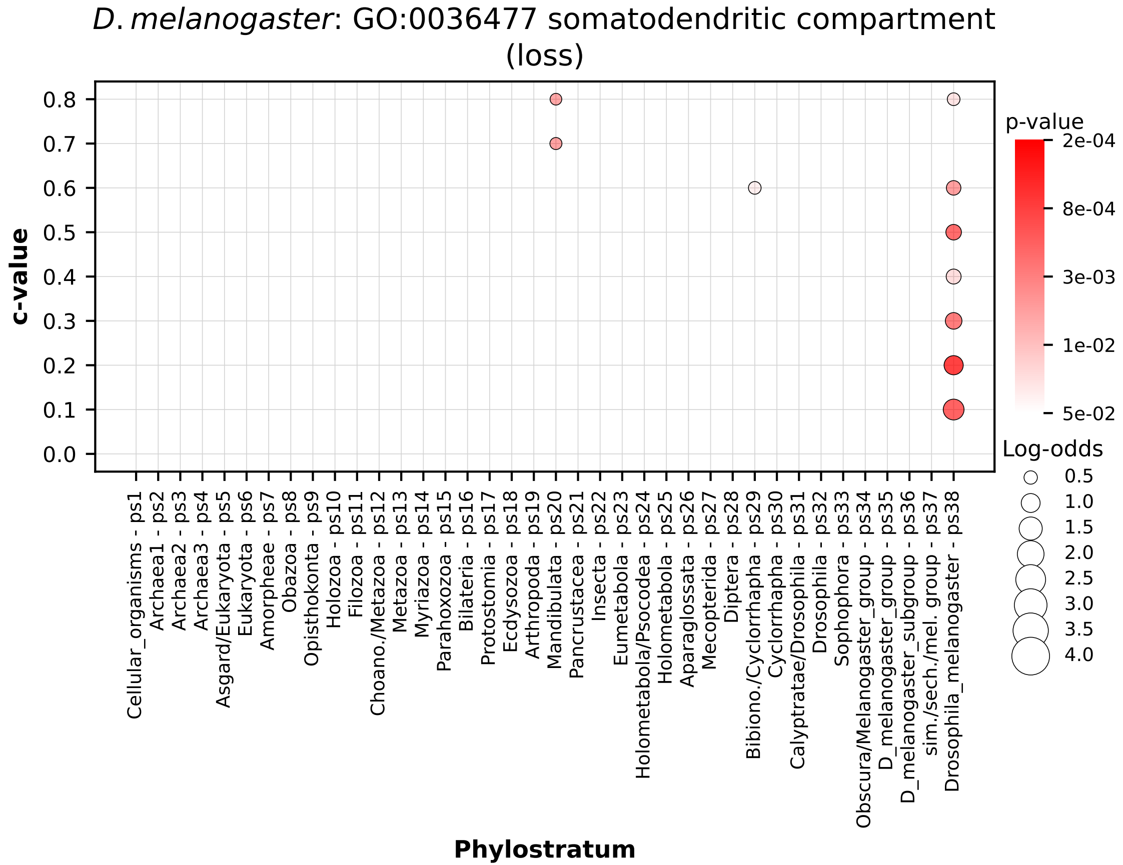
D. melanogaster: GO:0036477 somatodendritic compartment
(loss)
c-value
0.8
0.7
0.6
0.5
0.4
0.3
0.2
0.1
0.0
p-value
2e-04
8e-04
3e-03
1e-02
5e-02
Log-odds
0.5
1.0
1.5
2.0
2.5
3.0
3.5
4.0
Cellular_organisms - ps1
Archaea1 - ps2
Archaea2 - ps3
Archaea3 - ps4
Asgard/Eukaryota - ps5
Eukaryota - ps6
Amorpheae - ps7
Obazoa - ps8
Opisthokonta - ps9
Holozoa - ps10
Filozoa - ps11
Choano./Metazoa - ps12
Metazoa - ps13
Myriazoa - ps14
Parahoxozoa - ps15
Bilateria - ps16
Protostomia - ps17
Ecdysozoa - ps18
Arthropoda - ps19
Mandibulata - ps20
Pancrustacea - ps21
Insecta - ps22
Eumetabola - ps23
Holometabola/Psocodea - ps24
Holometabola - ps25
Aparaglossata - ps26
Mecopterida - ps27
Diptera - ps28
Bibiono./Cyclorrhapha - ps29
Cyclorrhapha - ps30
Calyptratae/Drosophila - ps31
Drosophila - ps32
Sophophora - ps33
Obscura/Melanogaster_group - ps34
D_melanogaster_group - ps35
D_melanogaster_subgroup - ps36
sim./sech./mel. group - ps37
Drosophila_melanogaster - ps38
Phylostratum

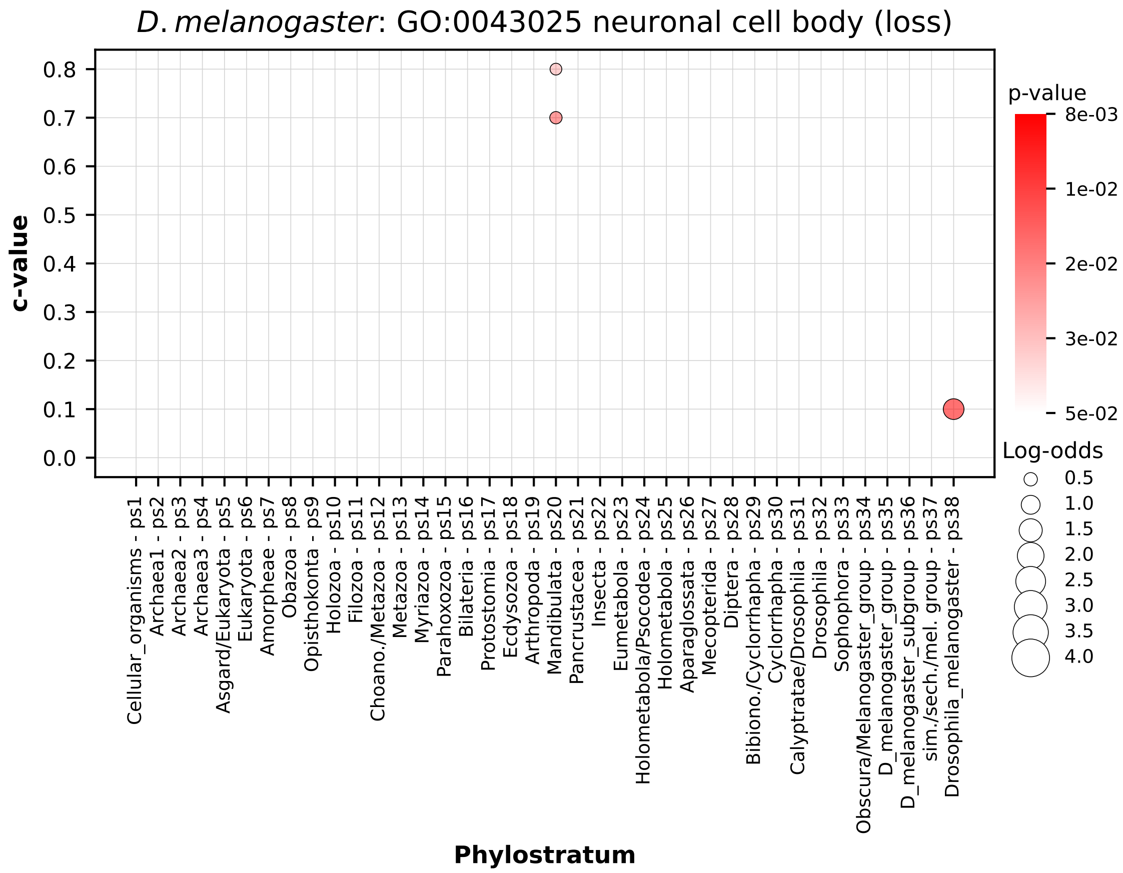
D. melanogaster: GO:0043025 neuronal cell body (loss)
c-value
0.8
0.7
0.6
0.5
0.4
0.3
0.2
0.1
0.0
Phylostratum
Cellular_organisms - ps1
Archaea1 - ps2
Archaea2 - ps3
Archaea3 - ps4
Asgard/Eukaryota - ps5
Eukaryota - ps6
Amorpheae - ps7
Obazoa - ps8
Opisthokonta - ps9
Holozoa - ps10
Filozoa - ps11
Choano./Metazoa - ps12
Metazoa - ps13
Myriazoa - ps14
Parahoxozoa - ps15
Bilateria - ps16
Protostomia - ps17
Ecdysozoa - ps18
Arthropoda - ps19
Mandibulata - ps20
Pancrustacea - ps21
Insecta - ps22
Eumetabola - ps23
Holometabola/Psocodea - ps24
Holometabola - ps25
Aparaglossata - ps26
Mecopterida - ps27
Diptera - ps28
Bibiono./Cyclorrhapha - ps29
Cyclorrhapha - ps30
Calyptratae/Drosophila - ps31
Drosophila - ps32
Sophophora - ps33
Obscura/Melanogaster_group - ps34
D_melanogaster_group - ps35
D_melanogaster_subgroup - ps36
sim./sech./mel. group - ps37
Drosophila_melanogaster - ps38
p-value
8e-03
1e-02
2e-02
3e-02
5e-02
Log-odds
0.5
1.0
1.5
2.0
2.5
3.0
3.5
4.0

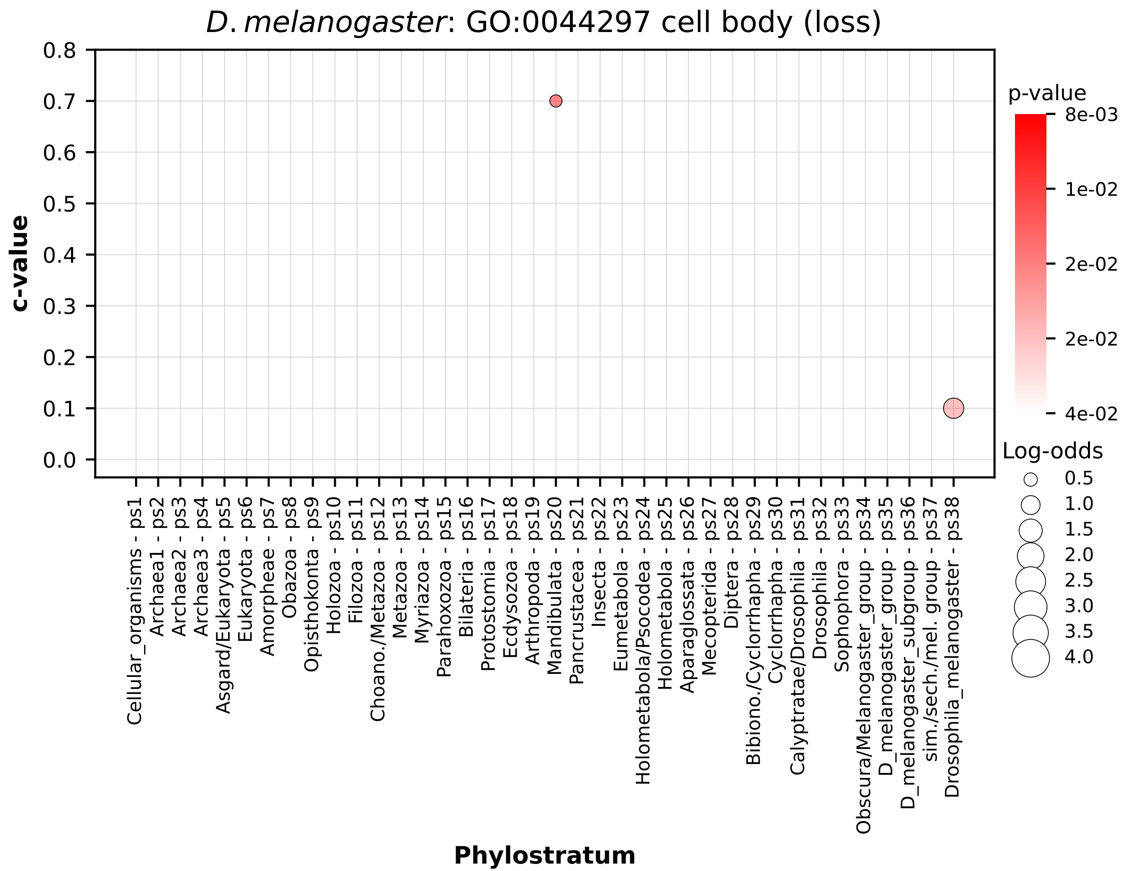

D. melanogaster: GO:0044297 cell body (loss)
c-value
0.0
0.1
0.2
0.3
0.4
0.5
0.6
0.7
0.8
Phylostratum
Cellular_organisms - ps1
Archaea1 - ps2
Archaea2 - ps3
Archaea3 - ps4
Asgard/Eukaryota - ps5
Eukaryota - ps6
Amorpheae - ps7
Obazoa - ps8
Opisthokonta - ps9
Holozoa - ps10
Filozoa - ps11
Choano./Metazoa - ps12
Metazoa - ps13
Myriazoa - ps14
Parahoxozoa - ps15
Bilateria - ps16
Protostomia - ps17
Ecdysozoa - ps18
Arthropoda - ps19
Mandibulata - ps20
Pancrustacea - ps21
Insecta - ps22
Eumetabola - ps23
Holometabola/Psocodea - ps24
Holometabola - ps25
Aparaglossata - ps26
Mecopterida - ps27
Diptera - ps28
Bibiono./Cyclorrhapha - ps29
Cyclorrhapha - ps30
Calyptratae/Drosophila - ps31
Drosophila - ps32
Sophophora - ps33
Obscura/Melanogaster_group - ps34
D_melanogaster_group - ps35
D_melanogaster_subgroup - ps36
sim./sech./mel. group - ps37
Drosophila_melanogaster - ps38
p-value
8e-03
1e-02
2e-02
2e-02
4e-02
Log-odds
0.5
1.0
1.5
2.0
2.5
3.0
3.5
4.0

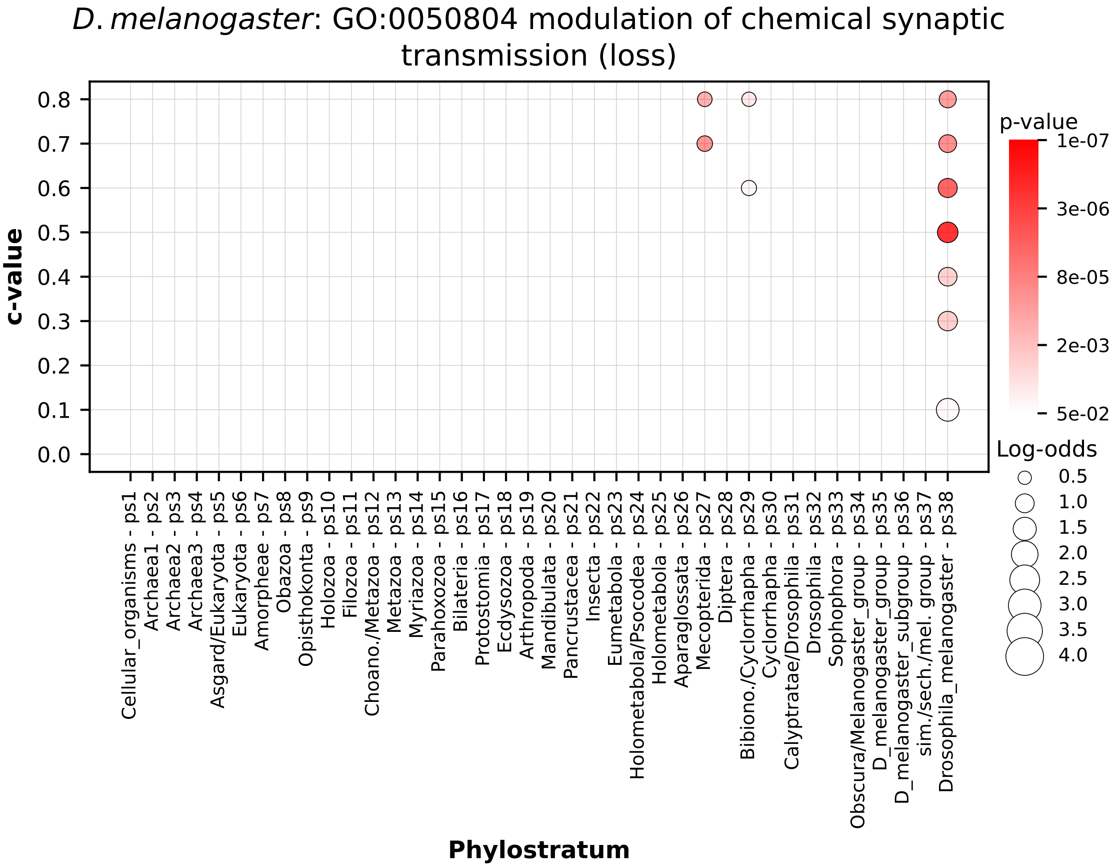

D. melanogaster: GO:0050804 modulation of chemical synaptic transmission (loss)
c-value
0.0
0.1
0.2
0.3
0.4
0.5
0.6
0.7
0.8
p-value
1e-07
3e-06
8e-05
2e-03
5e-02
Log-odds
0.5
1.0
1.5
2.0
2.5
3.0
3.5
4.0
Cellular_organisms - ps1
Archaea1 - ps2
Archaea2 - ps3
Archaea3 - ps4
Asgard/Eukaryota - ps5
Eukaryota - ps6
Amorpheae - ps7
Obazoa - ps8
Opisthokonta - ps9
Holozoa - ps10
Filozoa - ps11
Choano./Metazoa - ps12
Metazoa - ps13
Myriazoa - ps14
Parahoxozoa - ps15
Bilateria - ps16
Protostomia - ps17
Ecdysozoa - ps18
Arthropoda - ps19
Mandibulata - ps20
Pancrustacea - ps21
Insecta - ps22
Eumetabola - ps23
Holometabola/Psocodea - ps24
Holometabola - ps25
Aparaglossata - ps26
Mecopterida - ps27
Diptera - ps28
Bibiono./Cyclorrhapha - ps29
Cyclorrhapha - ps30
Calyptratae/Drosophila - ps31
Drosophila - ps32
Sophophora - ps33
Obscura/Melanogaster_group - ps34
D_melanogaster_group - ps35
D_melanogaster_subgroup - ps36
sim./sech./mel. group - ps37
Drosophila_melanogaster - ps38
Phylostratum

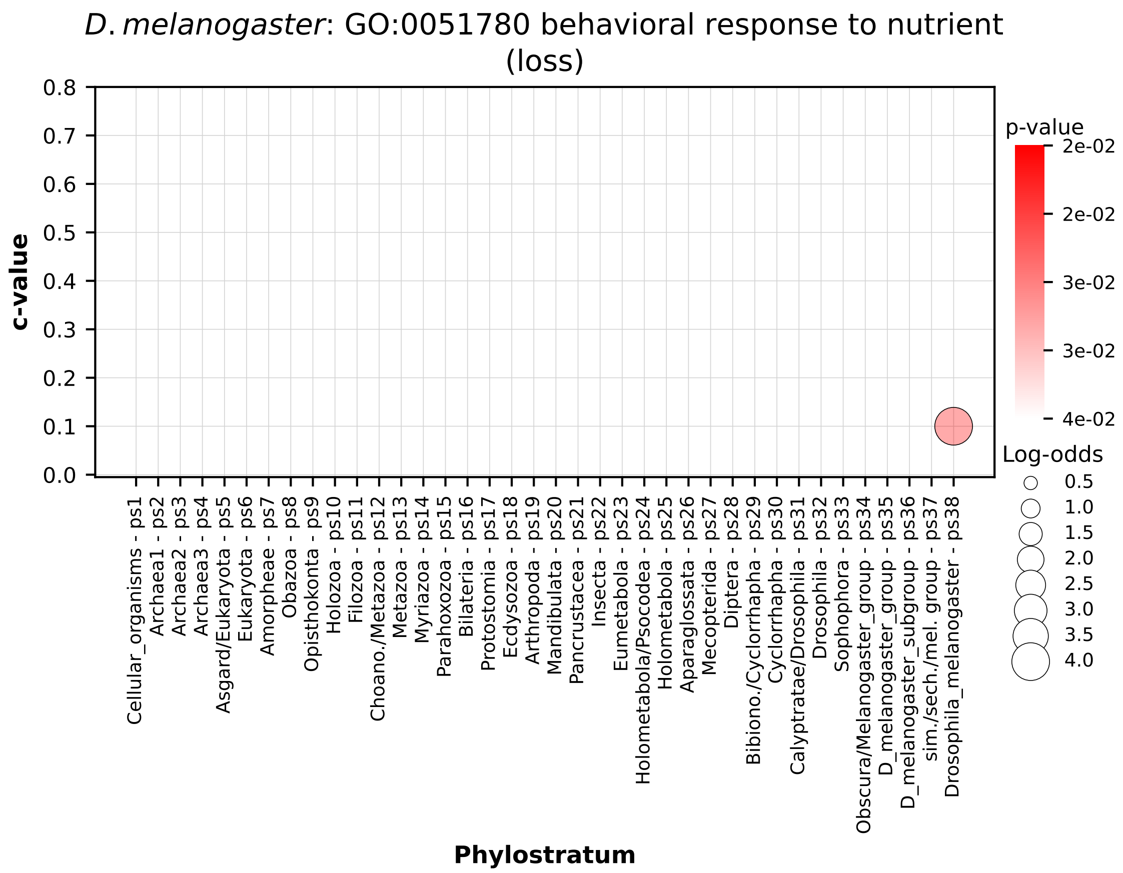

D. melanogaster: GO:0051780 behavioral response to nutrient (loss)
c-value
0.8
0.7
0.6
0.5
0.4
0.3
0.2
0.1
0.0
Phylostratum
Cellular_organisms - ps1
Archaea1 - ps2
Archaea2 - ps3
Archaea3 - ps4
Asgard/Eukaryota - ps5
Eukaryota - ps6
Amorpheae - ps7
Obazoa - ps8
Opisthokonta - ps9
Holozoa - ps10
Filozoa - ps11
Choano./Metazoa - ps12
Metazoa - ps13
Myriazoa - ps14
Parahoxozoa - ps15
Bilateria - ps16
Protostomia - ps17
Ecdysozoa - ps18
Arthropoda - ps19
Mandibulata - ps20
Pancrustacea - ps21
Insecta - ps22
Eumetabola - ps23
Holometabola/Psocodea - ps24
Holometabola - ps25
Aparaglossata - ps26
Mecopterida - ps27
Diptera - ps28
Bibiono./Cyclorrhapha - ps29
Cyclorrhapha - ps30
Calyptratae/Drosophila - ps31
Drosophila - ps32
Sophophora - ps33
Obscura/Melanogaster_group - ps34
D_melanogaster_group - ps35
D_melanogaster_subgroup - ps36
sim./sech./mel. group - ps37
Drosophila_melanogaster - ps38
p-value
2e-02
2e-02
3e-02
3e-02
4e-02
Log-odds
0.5
1.0
1.5
2.0
2.5
3.0
3.5
4.0

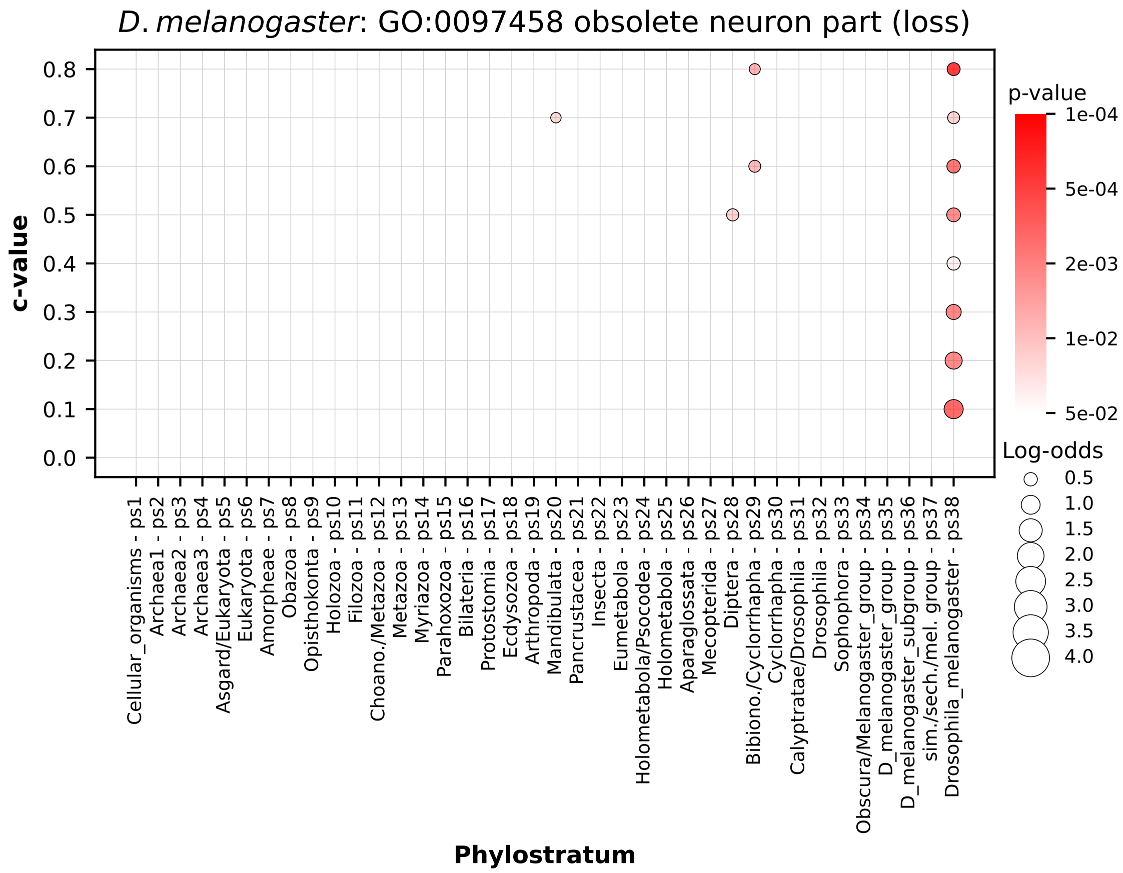

D. melanogaster: GO:0097458 obsolete neuron part (loss)
c-value
0.0
0.1
0.2
0.3
0.4
0.5
0.6
0.7
0.8
p-value
1e-04
5e-04
2e-03
1e-02
5e-02
Log-odds
0.5
1.0
1.5
2.0
2.5
3.0
3.5
4.0
Cellular_organisms - ps1
Archaea1 - ps2
Archaea2 - ps3
Archaea3 - ps4
Asgard/Eukaryota - ps5
Eukaryota - ps6
Amorpheae - ps7
Obazoa - ps8
Opisthokonta - ps9
Holozoa - ps10
Filozoa - ps11
Choano./Metazoa - ps12
Metazoa - ps13
Myriazoa - ps14
Parahoxozoa - ps15
Bilateria - ps16
Protostomia - ps17
Ecdysozoa - ps18
Arthropoda - ps19
Mandibulata - ps20
Pancrustacea - ps21
Insecta - ps22
Eumetabola - ps23
Holometabola/Psocodea - ps24
Holometabola - ps25
Aparaglossata - ps26
Mecopterida - ps27
Diptera - ps28
Bibiono./Cyclorrhapha - ps29
Cyclorrhapha - ps30
Calyptratae/Drosophila - ps31
Drosophila - ps32
Sophophora - ps33
Obscura/Melanogaster_group - ps34
D_melanogaster_group - ps35
D_melanogaster_subgroup - ps36
sim./sech./mel. group - ps37
Drosophila_melanogaster - ps38
Phylostratum

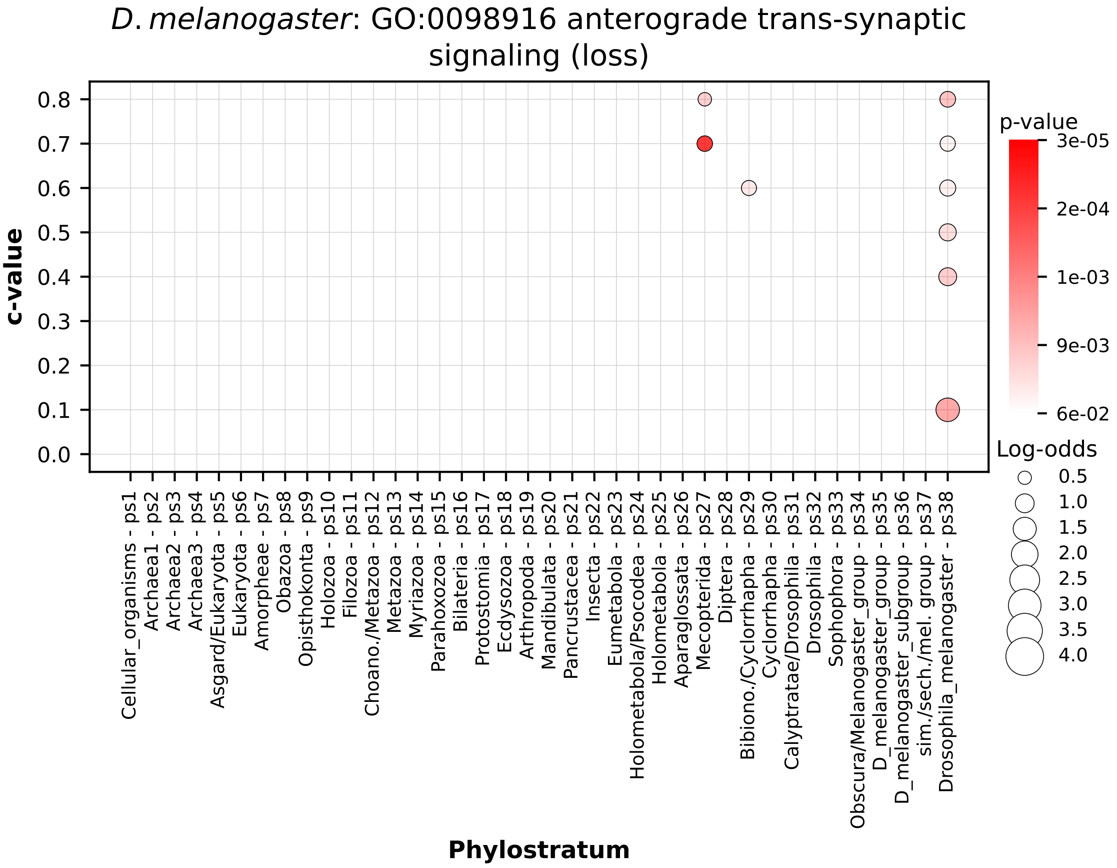
D. melanogaster: GO:0098916 anterograde trans-synaptic signaling (loss)
c-value
0.8
0.7
0.6
0.5
0.4
0.3
0.2
0.1
0.0
Phylostratum
Cellular_organisms - ps1
Archaea1 - ps2
Archaea2 - ps3
Archaea3 - ps4
Asgard/Eukaryota - ps5
Eukaryota - ps6
Amorpheae - ps7
Obazoa - ps8
Opisthokonta - ps9
Holozoa - ps10
Filozoa - ps11
Choano./Metazoa - ps12
Metazoa - ps13
Myriazoa - ps14
Parahoxozoa - ps15
Bilateria - ps16
Protostomia - ps17
Ecdysozoa - ps18
Arthropoda - ps19
Mandibulata - ps20
Pancrustacea - ps21
Insecta - ps22
Eumetabola - ps23
Holometabola/Psocodea - ps24
Holometabola - ps25
Aparaglossata - ps26
Mecopterida - ps27
Diptera - ps28
Bibiono./Cyclorrhapha - ps29
Cyclorrhapha - ps30
Calyptratae/Drosophila - ps31
Drosophila - ps32
Sophophora - ps33
Obscura/Melanogaster_group - ps34
D_melanogaster_group - ps35
D_melanogaster_subgroup - ps36
sim./sech./mel. group - ps37
Drosophila_melanogaster - ps38
p-value
3e-05
2e-04
1e-03
9e-03
6e-02
Log-odds
0.5
1.0
1.5
2.0
2.5
3.0
3.5
4.0

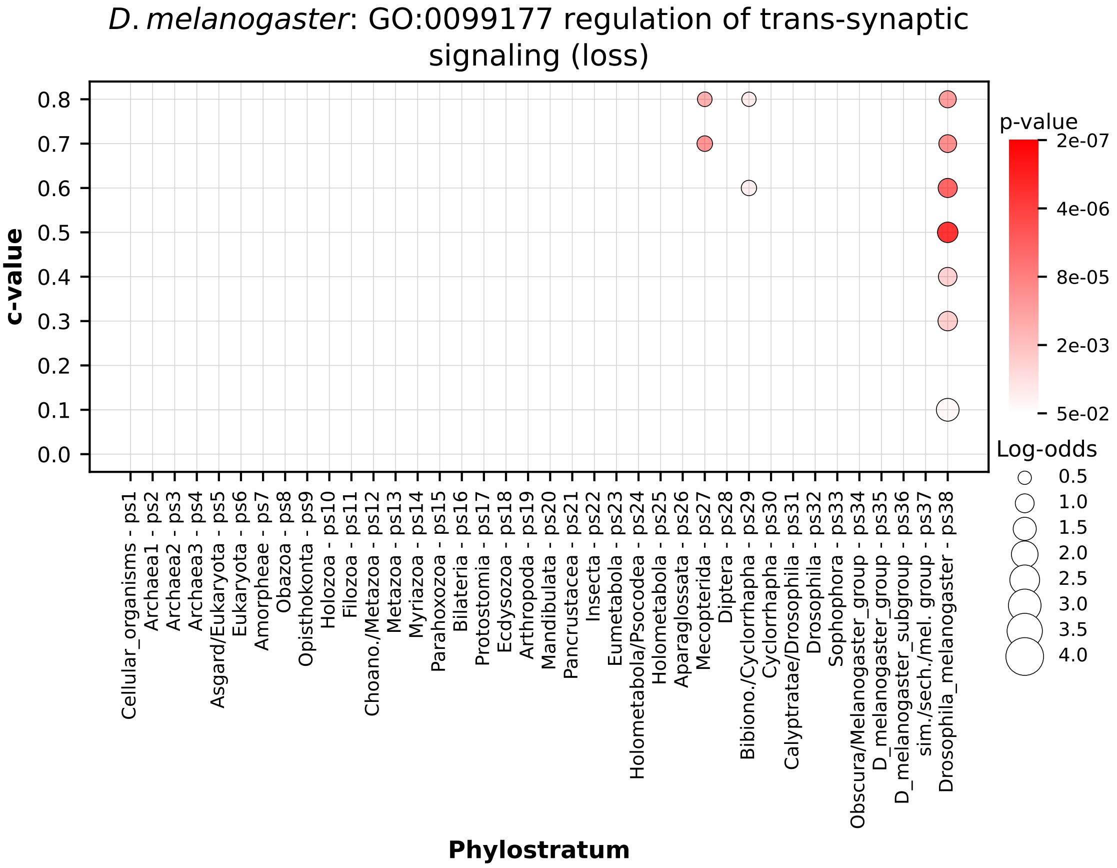

D. melanogaster: GO:0099177 regulation of trans-synaptic signaling (loss)
c-value
0.0
0.1
0.2
0.3
0.4
0.5
0.6
0.7
0.8
Phylostratum
Cellular_organisms - ps1
Archaea1 - ps2
Archaea2 - ps3
Archaea3 - ps4
Asgard/Eukaryota - ps5
Eukaryota - ps6
Amorpheae - ps7
Obazoa - ps8
Opisthokonta - ps9
Holozoa - ps10
Filozoa - ps11
Choano./Metazoa - ps12
Metazoa - ps13
Myriazoa - ps14
Parahoxozoa - ps15
Bilateria - ps16
Protostomia - ps17
Ecdysozoa - ps18
Arthropoda - ps19
Mandibulata - ps20
Pancrustacea - ps21
Insecta - ps22
Eumetabola - ps23
Holometabola/Psocodea - ps24
Holometabola - ps25
Aparaglossata - ps26
Mecopterida - ps27
Diptera - ps28
Bibiono./Cyclorrhapha - ps29
Cyclorrhapha - ps30
Calyptratae/Drosophila - ps31
Drosophila - ps32
Sophophora - ps33
Obscura/Melanogaster_group - ps34
D_melanogaster_group - ps35
D_melanogaster_subgroup - ps36
sim./sech./mel. group - ps37
Drosophila_melanogaster - ps38
p-value
2e-07
4e-06
8e-05
2e-03
5e-02
Log-odds
0.5
1.0
1.5
2.0
2.5
3.0
3.5
4.0

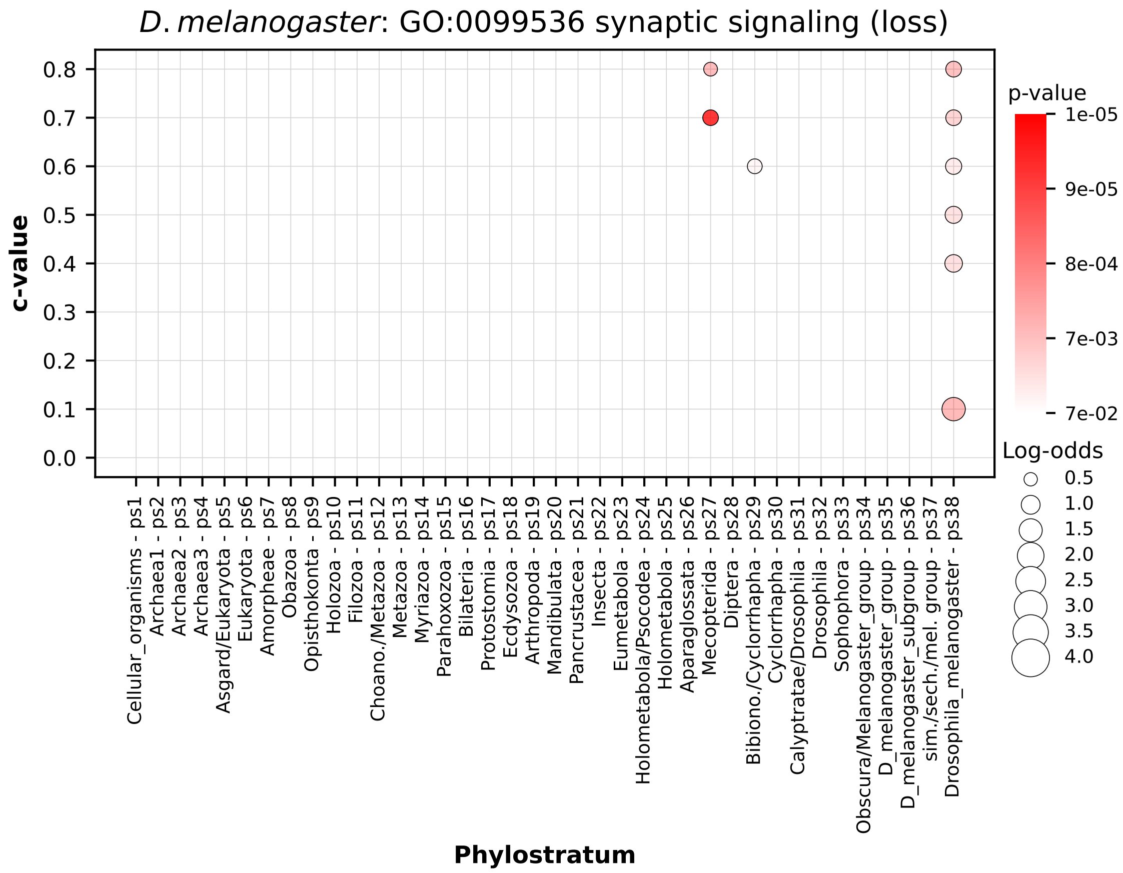

D. melanogaster: GO:0099536 synaptic signaling (loss)
c-value
0.8
0.7
0.6
0.5
0.4
0.3
0.2
0.1
0.0
p-value
1e-05
9e-05
8e-04
7e-03
7e-02
Log-odds
0.5
1.0
1.5
2.0
2.5
3.0
3.5
4.0
Cellular_organisms - ps1
Archaea1 - ps2
Archaea2 - ps3
Archaea3 - ps4
Asgard/Eukaryota - ps5
Eukaryota - ps6
Amorpheae - ps7
Obazoa - ps8
Opisthokonta - ps9
Holozoa - ps10
Filozoa - ps11
Choano./Metazoa - ps12
Metazoa - ps13
Myriazoa - ps14
Parahoxozoa - ps15
Bilateria - ps16
Protostomia - ps17
Ecdysozoa - ps18
Arthropoda - ps19
Mandibulata - ps20
Pancrustacea - ps21
Insecta - ps22
Eumetabola - ps23
Holometabola/Psocodea - ps24
Holometabola - ps25
Aparaglossata - ps26
Mecopterida - ps27
Diptera - ps28
Bibiono./Cyclorrhapha - ps29
Cyclorrhapha - ps30
Calyptratae/Drosophila - ps31
Drosophila - ps32
Sophophora - ps33
Obscura/Melanogaster_group - ps34
D_melanogaster_group - ps35
D_melanogaster_subgroup - ps36
sim./sech./mel. group - ps37
Drosophila_melanogaster - ps38
Phylostratum

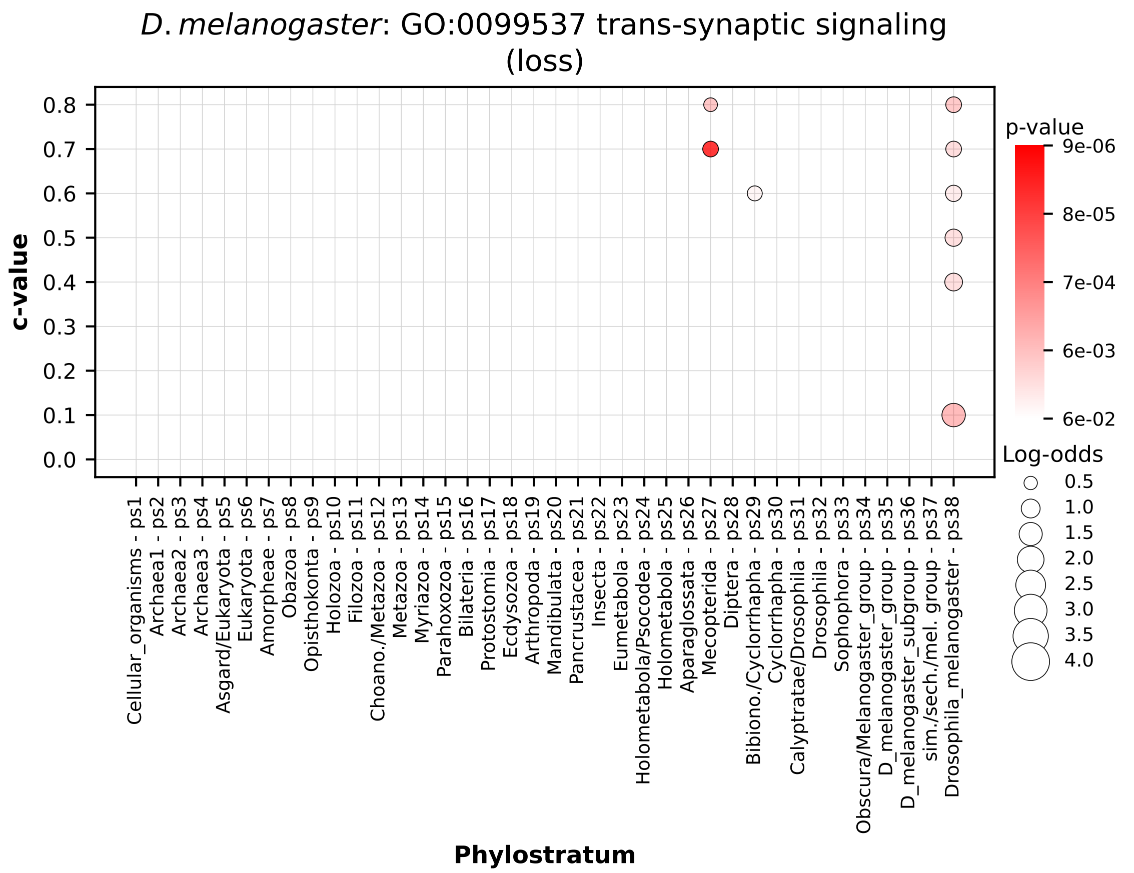

D. melanogaster: GO:0099537 trans-synaptic signaling (loss)
c-value
Phylostratum
p-value
9e-06
8e-05
7e-04
6e-03
6e-02
Log-odds
0.5
1.0
1.5
2.0
2.5
3.0
3.5
4.0
0.0
0.1
0.2
0.3
0.4
0.5
0.6
0.7
0.8
Cellular_organisms - ps1
Archaea1 - ps2
Archaea2 - ps3
Archaea3 - ps4
Asgard/Eukaryota - ps5
Eukaryota - ps6
Amorpheae - ps7
Obazoa - ps8
Opisthokonta - ps9
Holozoa - ps10
Filozoa - ps11
Choano./Metazoa - ps12
Metazoa - ps13
Myriazoa - ps14
Parahoxozoa - ps15
Bilateria - ps16
Protostomia - ps17
Ecdysozoa - ps18
Arthropoda - ps19
Mandibulata - ps20
Pancrustacea - ps21
Insecta - ps22
Eumetabola - ps23
Holometabola/Psocodea - ps24
Holometabola - ps25
Aparaglossata - ps26
Mecopterida - ps27
Diptera - ps28
Bibiono./Cyclorrhapha - ps29
Cyclorrhapha - ps30
Calyptratae/Drosophila - ps31
Drosophila - ps32
Sophophora - ps33
Obscura/Melanogaster_group - ps34
D_melanogaster_group - ps35
D_melanogaster_subgroup - ps36
sim./sech./mel. group - ps37
Drosophila_melanogaster - ps38

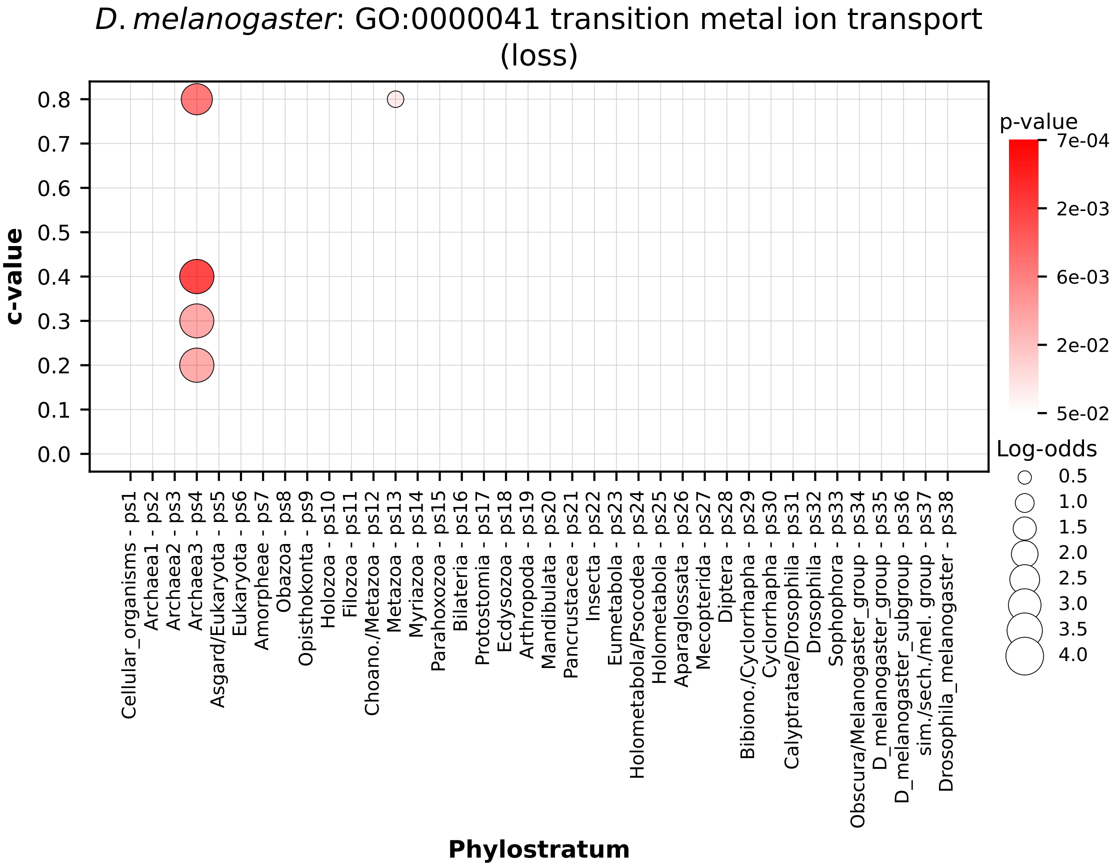

D. melanogaster: GO:0000041 transition metal ion transport (loss)
c-value
0.8
0.7
0.6
0.5
0.4
0.3
0.2
0.1
0.0
Cellular_organisms - ps1
Archaea1 - ps2
Archaea2 - ps3
Archaea3 - ps4
Asgard/Eukaryota - ps5
Eukaryota - ps6
Amorpheae - ps7
Obazoa - ps8
Opisthokonta - ps9
Holozoa - ps10
Filozoa - ps11
Choano./Metazoa - ps12
Metazoa - ps13
Myriazoa - ps14
Parahoxozoa - ps15
Bilateria - ps16
Protostomia - ps17
Ecdysozoa - ps18
Arthropoda - ps19
Mandibulata - ps20
Pancrustacea - ps21
Insecta - ps22
Eumetabola - ps23
Holometabola/Psocodea - ps24
Holometabola - ps25
Aparaglossata - ps26
Mecopterida - ps27
Diptera - ps28
Bibiono./Cyclorrhapha - ps29
Cyclorrhapha - ps30
Calyptratae/Drosophila - ps31
Drosophila - ps32
Sophophora - ps33
Obscura/Melanogaster_group - ps34
D_melanogaster_group - ps35
D_melanogaster_subgroup - ps36
sim./sech./mel. group - ps37
Drosophila_melanogaster - ps38
Phylostratum
p-value
7e-04
2e-03
6e-03
2e-02
5e-02
Log-odds
0.5
1.0
1.5
2.0
2.5
3.0
3.5
4.0

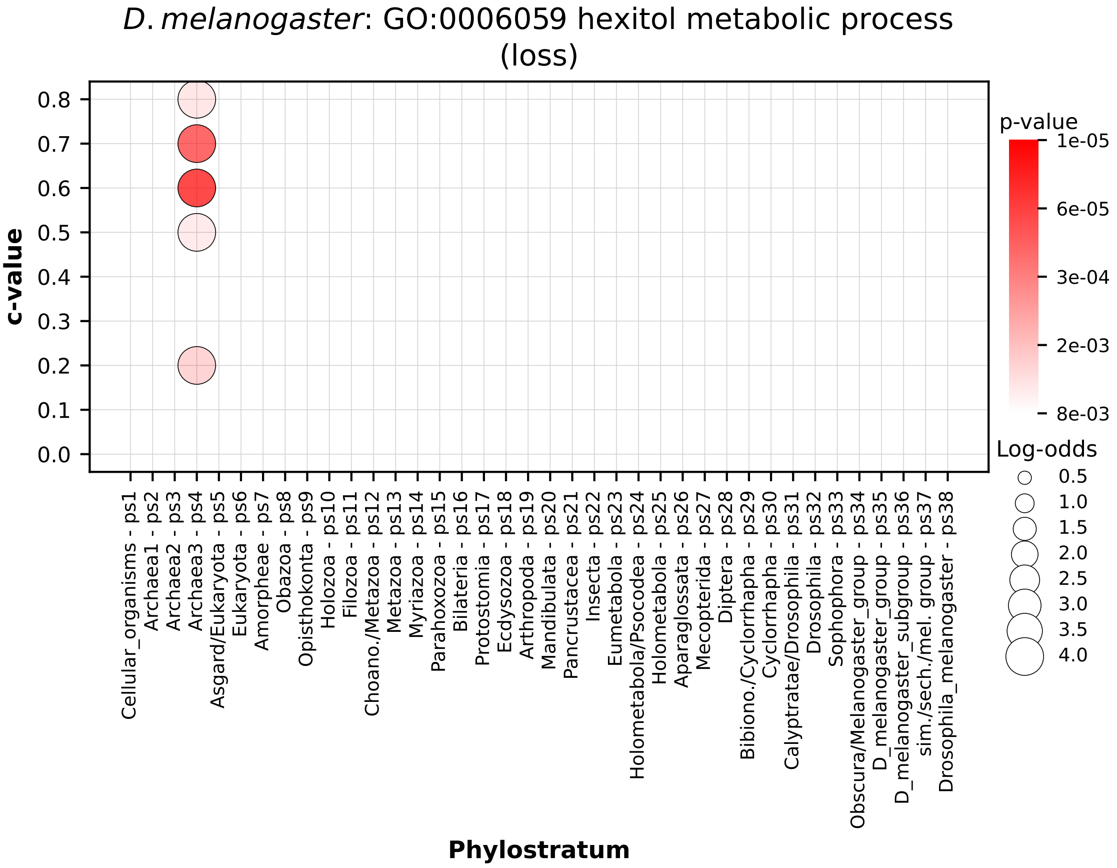

D. melanogaster: GO:0006059 hexitol metabolic process
(loss)
c-value
0.0
0.1
0.2
0.3
0.4
0.5
0.6
0.7
0.8
Phylostratum
Cellular_organisms - ps1
Archaea1 - ps2
Archaea2 - ps3
Archaea3 - ps4
Asgard/Eukaryota - ps5
Eukaryota - ps6
Amorpheae - ps7
Obazoa - ps8
Opisthokonta - ps9
Holozoa - ps10
Filozoa - ps11
Choano./Metazoa - ps12
Metazoa - ps13
Myriazoa - ps14
Parahoxozoa - ps15
Bilateria - ps16
Protostomia - ps17
Ecdysozoa - ps18
Arthropoda - ps19
Mandibulata - ps20
Pancrustacea - ps21
Insecta - ps22
Eumetabola - ps23
Holometabola/Psocodea - ps24
Holometabola - ps25
Aparaglossata - ps26
Mecopterida - ps27
Diptera - ps28
Bibiono./Cyclorrhapha - ps29
Cyclorrhapha - ps30
Calyptratae/Drosophila - ps31
Drosophila - ps32
Sophophora - ps33
Obscura/Melanogaster_group - ps34
D_melanogaster_group - ps35
D_melanogaster_subgroup - ps36
sim./sech./mel. group - ps37
Drosophila_melanogaster - ps38
p-value
1e-05
6e-05
3e-04
2e-03
8e-03
Log-odds
0.5
1.0
1.5
2.0
2.5
3.0
3.5
4.0

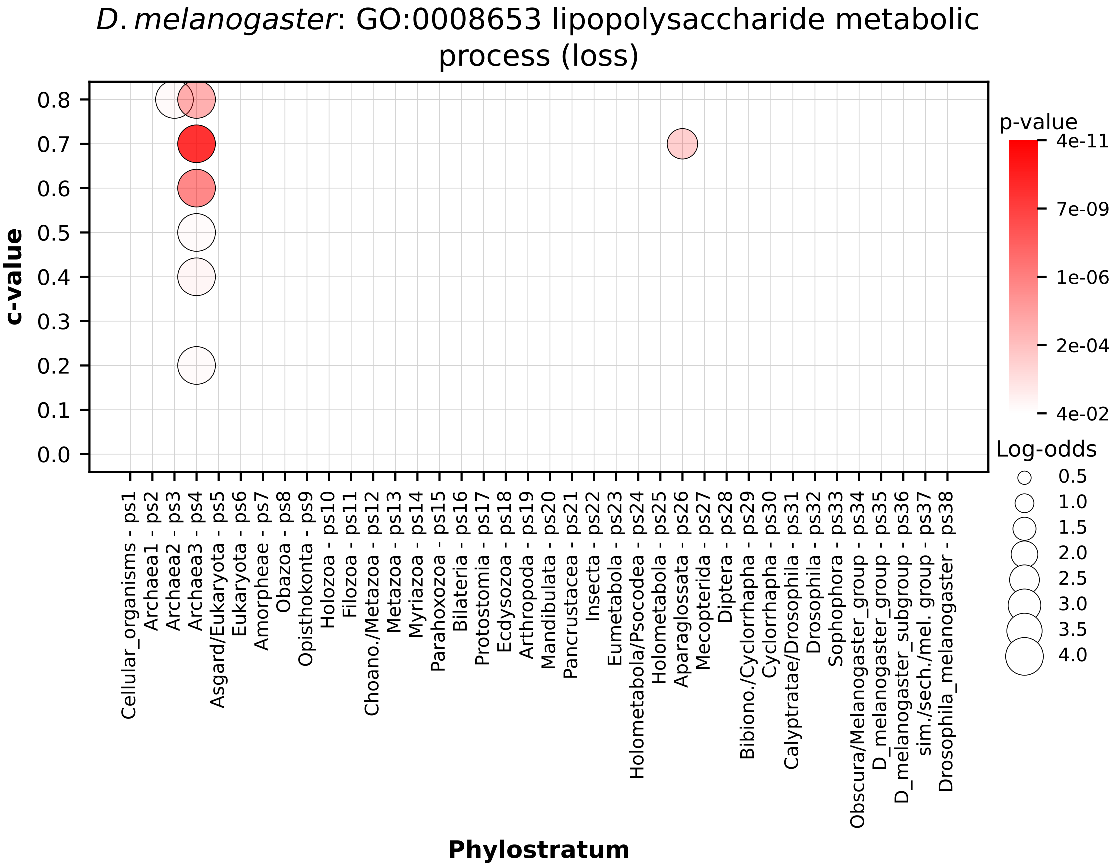
D. melanogaster: GO:0008653 lipopolysaccharide metabolic process (loss)
c-value
0.0
0.1
0.2
0.3
0.4
0.5
0.6
0.7
0.8
Phylostratum
Cellular_organisms - ps1
Archaea1 - ps2
Archaea2 - ps3
Archaea3 - ps4
Asgard/Eukaryota - ps5
Eukaryota - ps6
Amorpheae - ps7
Obazoa - ps8
Opisthokonta - ps9
Holozoa - ps10
Filozoa - ps11
Choano./Metazoa - ps12
Metazoa - ps13
Myriazoa - ps14
Parahoxozoa - ps15
Bilateria - ps16
Protostomia - ps17
Ecdysozoa - ps18
Arthropoda - ps19
Mandibulata - ps20
Pancrustacea - ps21
Insecta - ps22
Eumetabola - ps23
Holometabola/Psocodea - ps24
Holometabola - ps25
Aparaglossata - ps26
Mecopterida - ps27
Diptera - ps28
Bibiono./Cyclorrhapha - ps29
Cyclorrhapha - ps30
Calyptratae/Drosophila - ps31
Drosophila - ps32
Sophophora - ps33
Obscura/Melanogaster_group - ps34
D_melanogaster_group - ps35
D_melanogaster_subgroup - ps36
sim./sech./mel. group - ps37
Drosophila_melanogaster - ps38
p-value
4e-11
7e-09
1e-06
2e-04
4e-02
Log-odds
0.5
1.0
1.5
2.0
2.5
3.0
3.5
4.0

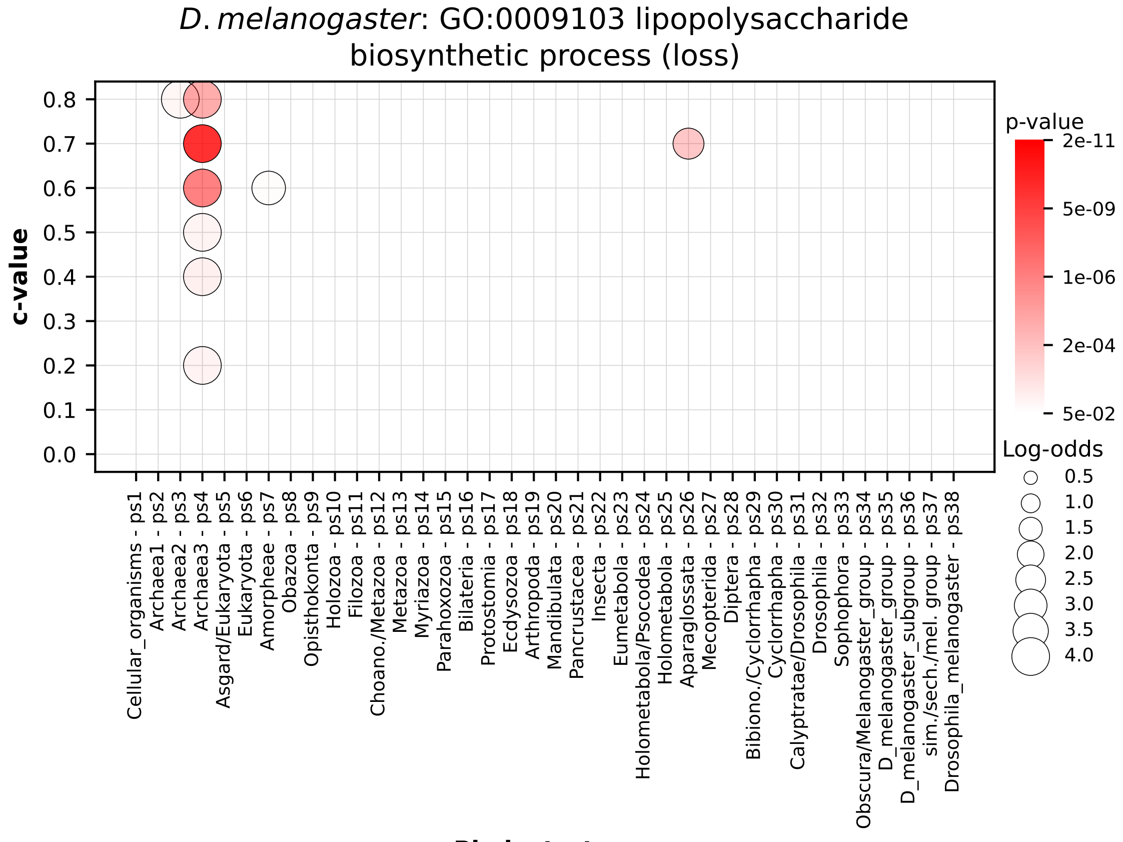

D. melanogaster: GO:0009103 lipopolysaccharide biosynthetic process (loss)
c-value
0.0
0.1
0.2
0.3
0.4
0.5
0.6
0.7
0.8
Phylostratum
Cellular_organisms - ps1
Archaea1 - ps2
Archaea2 - ps3
Archaea3 - ps4
Asgard/Eukaryota - ps5
Eukaryota - ps6
Amorpheae - ps7
Obazoa - ps8
Opisthokonta - ps9
Holozoa - ps10
Filozoa - ps11
Choano./Metazoa - ps12
Metazoa - ps13
Myriazoa - ps14
Parahoxozoa - ps15
Bilateria - ps16
Protostomia - ps17
Ecdysozoa - ps18
Arthropoda - ps19
Mandibulata - ps20
Pancrustacea - ps21
Insecta - ps22
Eumetabola - ps23
Holometabola/Psocodea - ps24
Holometabola - ps25
Aparaglossata - ps26
Mecopterida - ps27
Diptera - ps28
Bibiono./Cyclorrhapha - ps29
Cyclorrhapha - ps30
Calyptratae/Drosophila - ps31
Drosophila - ps32
Sophophora - ps33
Obscura/Melanogaster_group - ps34
D_melanogaster_group - ps35
D_melanogaster_subgroup - ps36
sim./sech./mel. group - ps37
Drosophila_melanogaster - ps38
p-value
2e-11
5e-09
1e-06
2e-04
5e-02
Log-odds
0.5
1.0
1.5
2.0
2.5
3.0
3.5
4.0

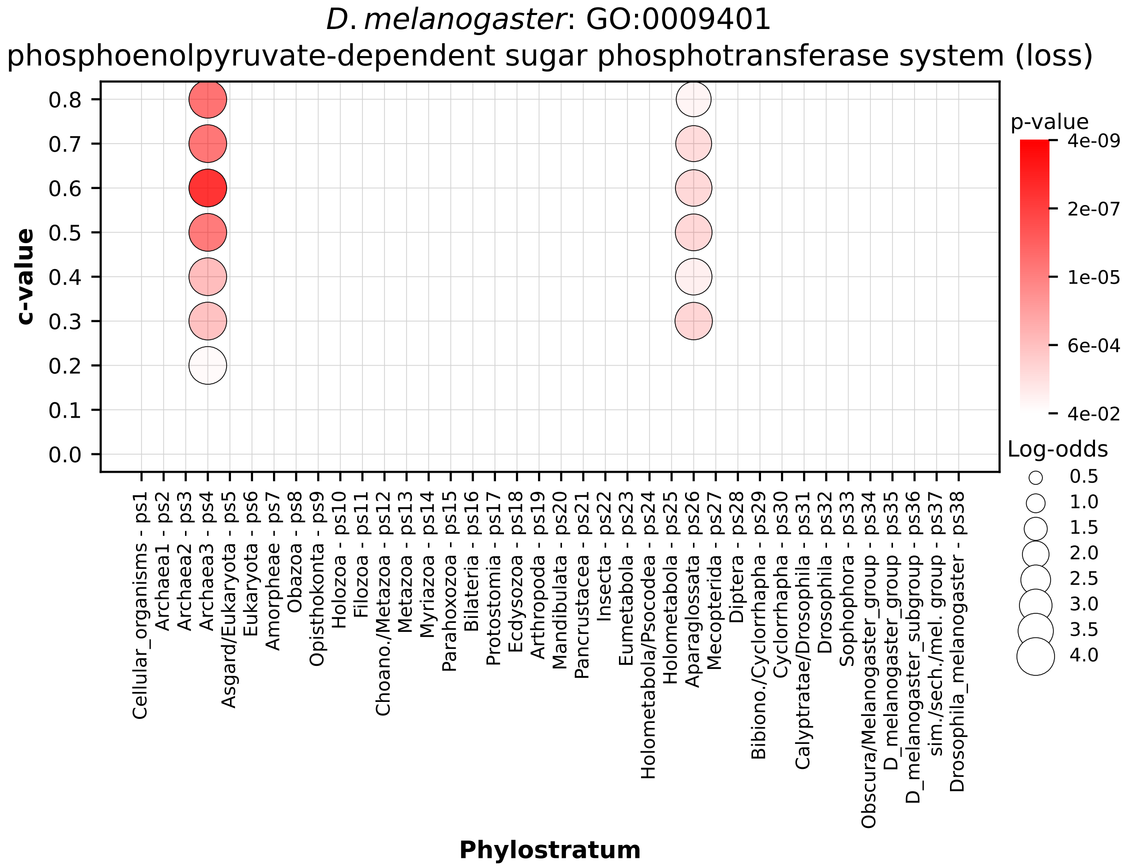

D. melanogaster: GO:0009401
phosphoenolpyruvate-dependent sugar phosphotransferase system (loss)
c-value
0.8
0.7
0.6
0.5
0.4
0.3
0.2
0.1
0.0
p-value
4e-09
2e-07
1e-05
6e-04
4e-02
Log-odds
0.5
1.0
1.5
2.0
2.5
3.0
3.5
4.0
Cellular_organisms - ps1
Archaea1 - ps2
Archaea2 - ps3
Archaea3 - ps4
Asgard/Eukaryota - ps5
Eukaryota - ps6
Amorpheae - ps7
Obazoa - ps8
Opisthokonta - ps9
Holozoa - ps10
Filozoa - ps11
Choano./Metazoa - ps12
Metazoa - ps13
Myriazoa - ps14
Parahoxozoa - ps15
Bilateria - ps16
Protostomia - ps17
Ecdysozoa - ps18
Arthropoda - ps19
Mandibulata - ps20
Pancrustacea - ps21
Insecta - ps22
Eumetabola - ps23
Holometabola/Psocodea - ps24
Holometabola - ps25
Aparaglossata - ps26
Mecopterida - ps27
Diptera - ps28
Bibiono./Cyclorrhapha - ps29
Cyclorrhapha - ps30
Calyptratae/Drosophila - ps31
Drosophila - ps32
Sophophora - ps33
Obscura/Melanogaster_group - ps34
D_melanogaster_group - ps35
D_melanogaster_subgroup - ps36
sim./sech./mel. group - ps37
Drosophila_melanogaster - ps38
Phylostratum

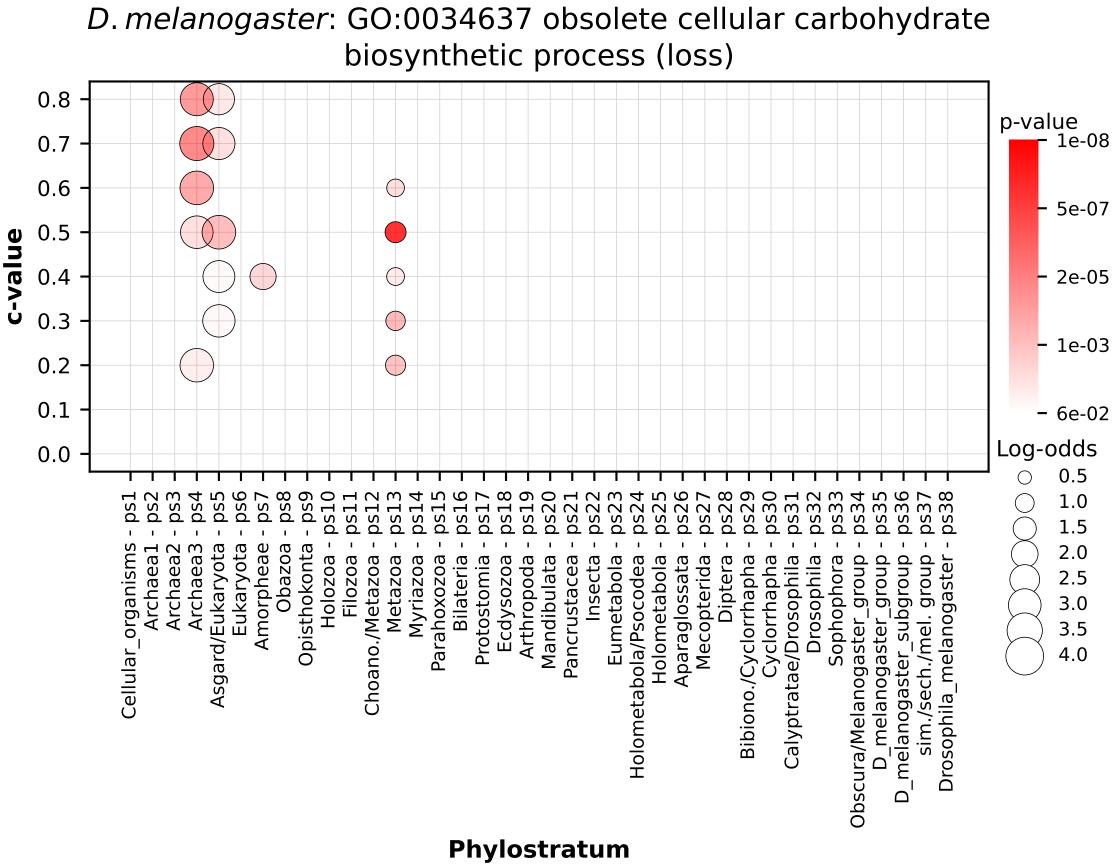

D. melanogaster: GO:0034637 obsolete cellular carbohydrate biosynthetic process (loss)
c-value
0.0
0.1
0.2
0.3
0.4
0.5
0.6
0.7
0.8
p-value
1e-08
5e-07
2e-05
1e-03
6e-02
Log-odds
0.5
1.0
1.5
2.0
2.5
3.0
3.5
4.0
Cellular_organisms - ps1
Archaea1 - ps2
Archaea2 - ps3
Archaea3 - ps4
Asgard/Eukaryota - ps5
Eukaryota - ps6
Amorpheae - ps7
Obazoa - ps8
Opisthokonta - ps9
Holozoa - ps10
Filozoa - ps11
Choano./Metazoa - ps12
Metazoa - ps13
Myriazoa - ps14
Parahoxozoa - ps15
Bilateria - ps16
Protostomia - ps17
Ecdysozoa - ps18
Arthropoda - ps19
Mandibulata - ps20
Pancrustacea - ps21
Insecta - ps22
Eumetabola - ps23
Holometabola/Psocodea - ps24
Holometabola - ps25
Aparaglossata - ps26
Mecopterida - ps27
Diptera - ps28
Bibiono./Cyclorrhapha - ps29
Cyclorrhapha - ps30
Calyptratae/Drosophila - ps31
Drosophila - ps32
Sophophora - ps33
Obscura/Melanogaster_group - ps34
D_melanogaster_group - ps35
D_melanogaster_subgroup - ps36
sim./sech./mel. group - ps37
Drosophila_melanogaster - ps38
Phylostratum

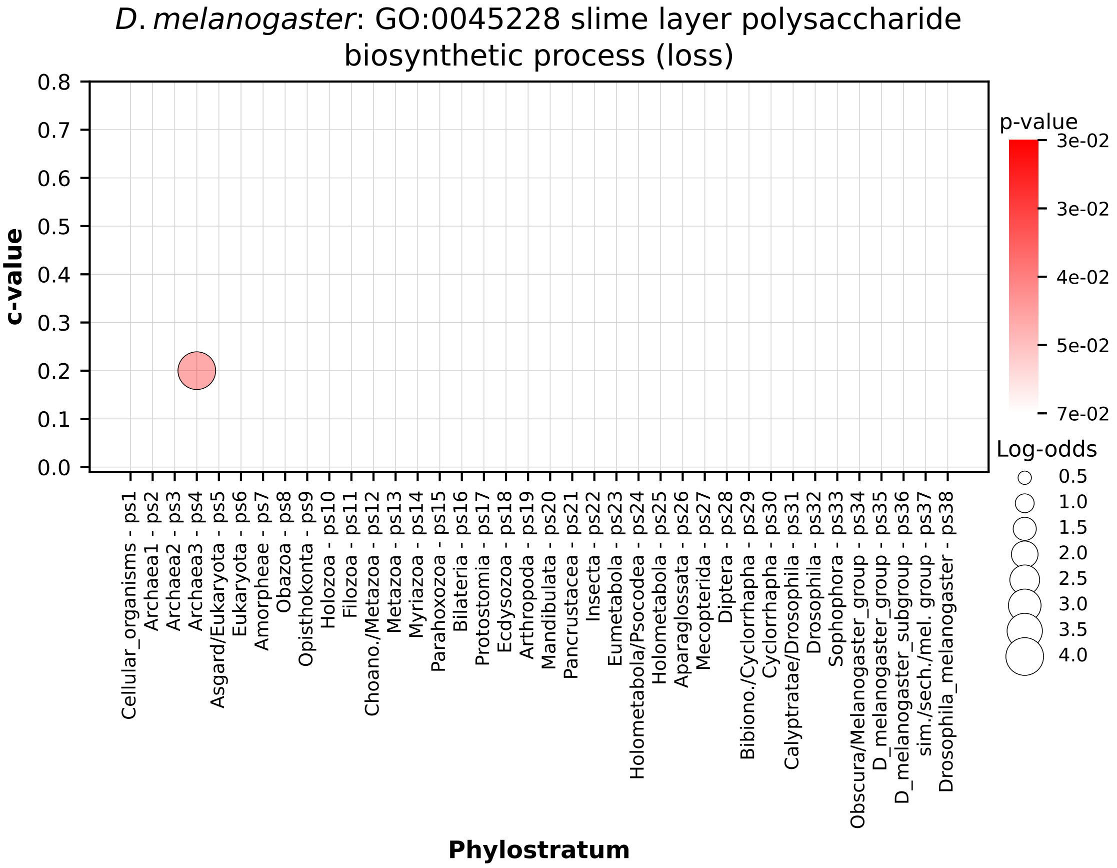

D. melanogaster: GO:0045228 slime layer polysaccharide biosynthetic process (loss)
c-value
0.8
0.7
0.6
0.5
0.4
0.3
0.2
0.1
0.0
p-value
3e-02
3e-02
4e-02
5e-02
7e-02
Log-odds
0.5
1.0
1.5
2.0
2.5
3.0
3.5
4.0
Cellular_organisms - ps1
Archaea1 - ps2
Archaea2 - ps3
Archaea3 - ps4
Asgard/Eukaryota - ps5
Eukaryota - ps6
Amorpheae - ps7
Obazoa - ps8
Opisthokonta - ps9
Holozoa - ps10
Filozoa - ps11
Choano./Metazoa - ps12
Metazoa - ps13
Myriazoa - ps14
Parahoxozoa - ps15
Bilateria - ps16
Protostomia - ps17
Ecdysozoa - ps18
Arthropoda - ps19
Mandibulata - ps20
Pancrustacea - ps21
Insecta - ps22
Eumetabola - ps23
Holometabola/Psocodea - ps24
Holometabola - ps25
Aparaglossata - ps26
Mecopterida - ps27
Diptera - ps28
Bibiono./Cyclorrhapha - ps29
Cyclorrhapha - ps30
Calyptratae/Drosophila - ps31
Drosophila - ps32
Sophophora - ps33
Obscura/Melanogaster_group - ps34
D_melanogaster_group - ps35
D_melanogaster_subgroup - ps36
sim./sech./mel. group - ps37
Drosophila_melanogaster - ps38
Phylostratum

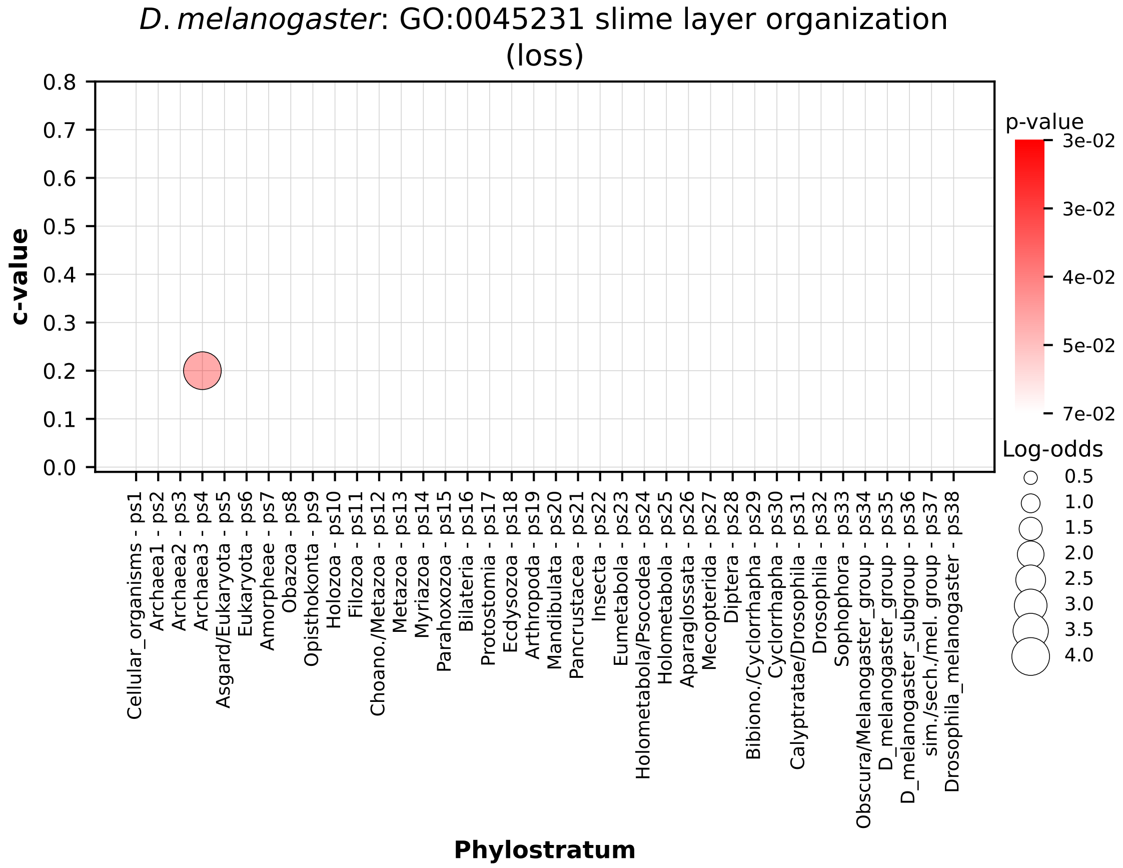

D. melanogaster: GO:0045231 slime layer organization
(loss)
c-value
0.8
0.7
0.6
0.5
0.4
0.3
0.2
0.1
0.0
Phylostratum
Cellular_organisms - ps1
Archaea1 - ps2
Archaea2 - ps3
Archaea3 - ps4
Asgard/Eukaryota - ps5
Eukaryota - ps6
Amorpheae - ps7
Obazoa - ps8
Opisthokonta - ps9
Holozoa - ps10
Filozoa - ps11
Choano./Metazoa - ps12
Metazoa - ps13
Myriazoa - ps14
Parahoxozoa - ps15
Bilateria - ps16
Protostomia - ps17
Ecdysozoa - ps18
Arthropoda - ps19
Mandibulata - ps20
Pancrustacea - ps21
Insecta - ps22
Eumetabola - ps23
Holometabola/Psocodea - ps24
Holometabola - ps25
Aparaglossata - ps26
Mecopterida - ps27
Diptera - ps28
Bibiono./Cyclorrhapha - ps29
Cyclorrhapha - ps30
Calyptratae/Drosophila - ps31
Drosophila - ps32
Sophophora - ps33
Obscura/Melanogaster_group - ps34
D_melanogaster_group - ps35
D_melanogaster_subgroup - ps36
sim./sech./mel. group - ps37
Drosophila_melanogaster - ps38
p-value
3e-02
3e-02
4e-02
5e-02
7e-02
Log-odds
0.5
1.0
1.5
2.0
2.5
3.0
3.5
4.0

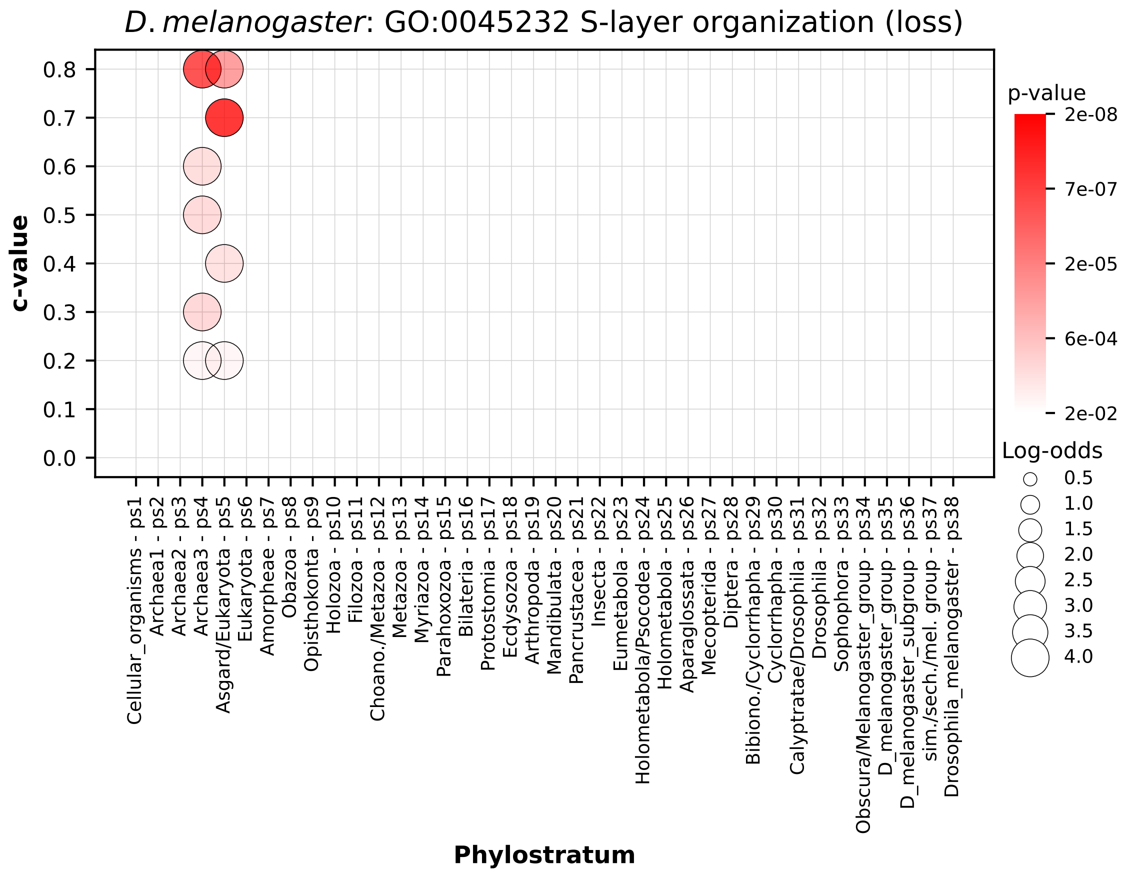
D. melanogaster: GO:0045232 S-layer organization (loss)
c-value
0.0
0.1
0.2
0.3
0.4
0.5
0.6
0.7
0.8
Phylostratum
Cellular_organisms - ps1
Archaea1 - ps2
Archaea2 - ps3
Archaea3 - ps4
Asgard/Eukaryota - ps5
Eukaryota - ps6
Amorpheae - ps7
Obazoa - ps8
Opisthokonta - ps9
Holozoa - ps10
Filozoa - ps11
Choano./Metazoa - ps12
Metazoa - ps13
Myriazoa - ps14
Parahoxozoa - ps15
Bilateria - ps16
Protostomia - ps17
Ecdysozoa - ps18
Arthropoda - ps19
Mandibulata - ps20
Pancrustacea - ps21
Insecta - ps22
Eumetabola - ps23
Holometabola/Psocodea - ps24
Holometabola - ps25
Aparaglossata - ps26
Mecopterida - ps27
Diptera - ps28
Bibiono./Cyclorrhapha - ps29
Cyclorrhapha - ps30
Calyptratae/Drosophila - ps31
Drosophila - ps32
Sophophora - ps33
Obscura/Melanogaster_group - ps34
D_melanogaster_group - ps35
D_melanogaster_subgroup - ps36
sim./sech./mel. group - ps37
Drosophila_melanogaster - ps38
p-value
2e-08
7e-07
2e-05
6e-04
2e-02
Log-odds
0.5
1.0
1.5
2.0
2.5
3.0
3.5
4.0

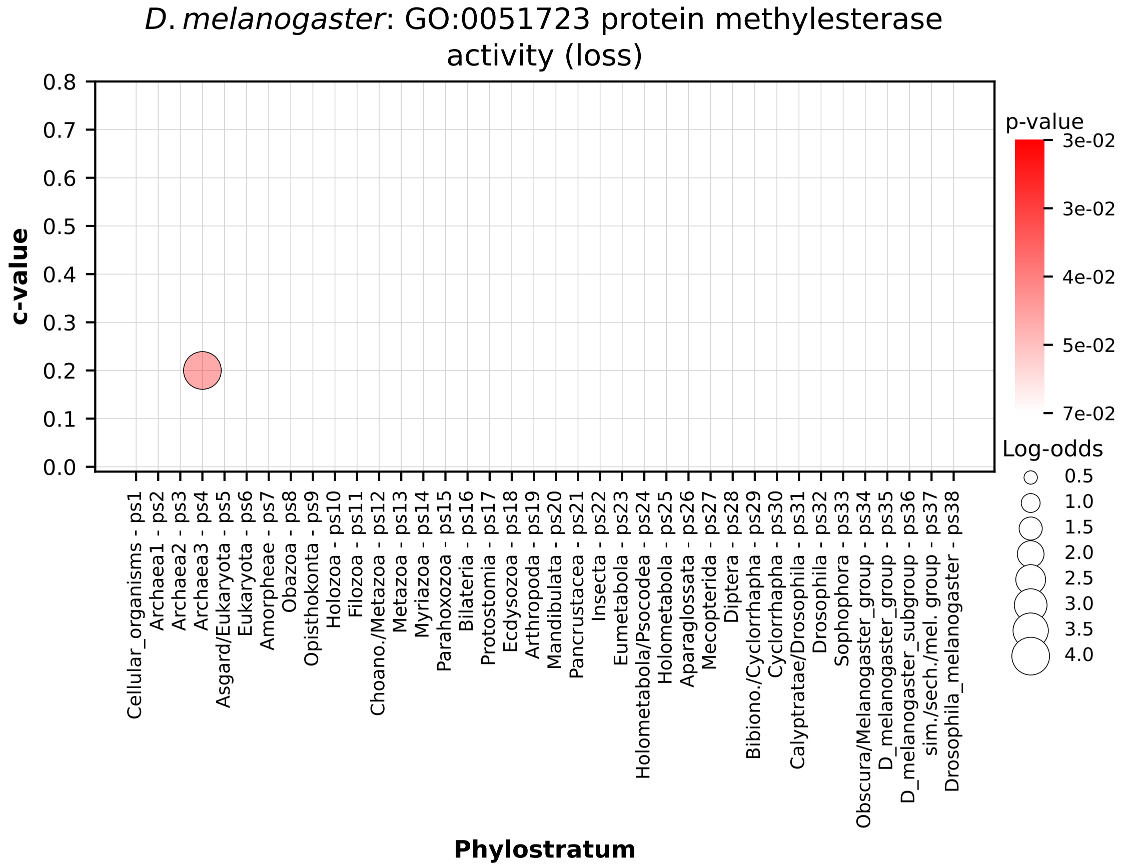
D. melanogaster: GO:0051723 protein methylesterase activity (loss)
c-value
0.8
0.7
0.6
0.5
0.4
0.3
0.2
0.1
0.0
Phylostratum
Cellular_organisms - ps1
Archaea1 - ps2
Archaea2 - ps3
Archaea3 - ps4
Asgard/Eukaryota - ps5
Eukaryota - ps6
Amorpheae - ps7
Obazoa - ps8
Opisthokonta - ps9
Holozoa - ps10
Filozoa - ps11
Choano./Metazoa - ps12
Metazoa - ps13
Myriazoa - ps14
Parahoxozoa - ps15
Bilateria - ps16
Protostomia - ps17
Ecdysozoa - ps18
Arthropoda - ps19
Mandibulata - ps20
Pancrustacea - ps21
Insecta - ps22
Eumetabola - ps23
Holometabola/Psocodea - ps24
Holometabola - ps25
Aparaglossata - ps26
Mecopterida - ps27
Diptera - ps28
Bibiono./Cyclorrhapha - ps29
Cyclorrhapha - ps30
Calyptratae/Drosophila - ps31
Drosophila - ps32
Sophophora - ps33
Obscura/Melanogaster_group - ps34
D_melanogaster_group - ps35
D_melanogaster_subgroup - ps36
sim./sech./mel. group - ps37
Drosophila_melanogaster - ps38
p-value
3e-02
3e-02
4e-02
5e-02
7e-02
Log-odds
0.5
1.0
1.5
2.0
2.5
3.0
3.5
4.0

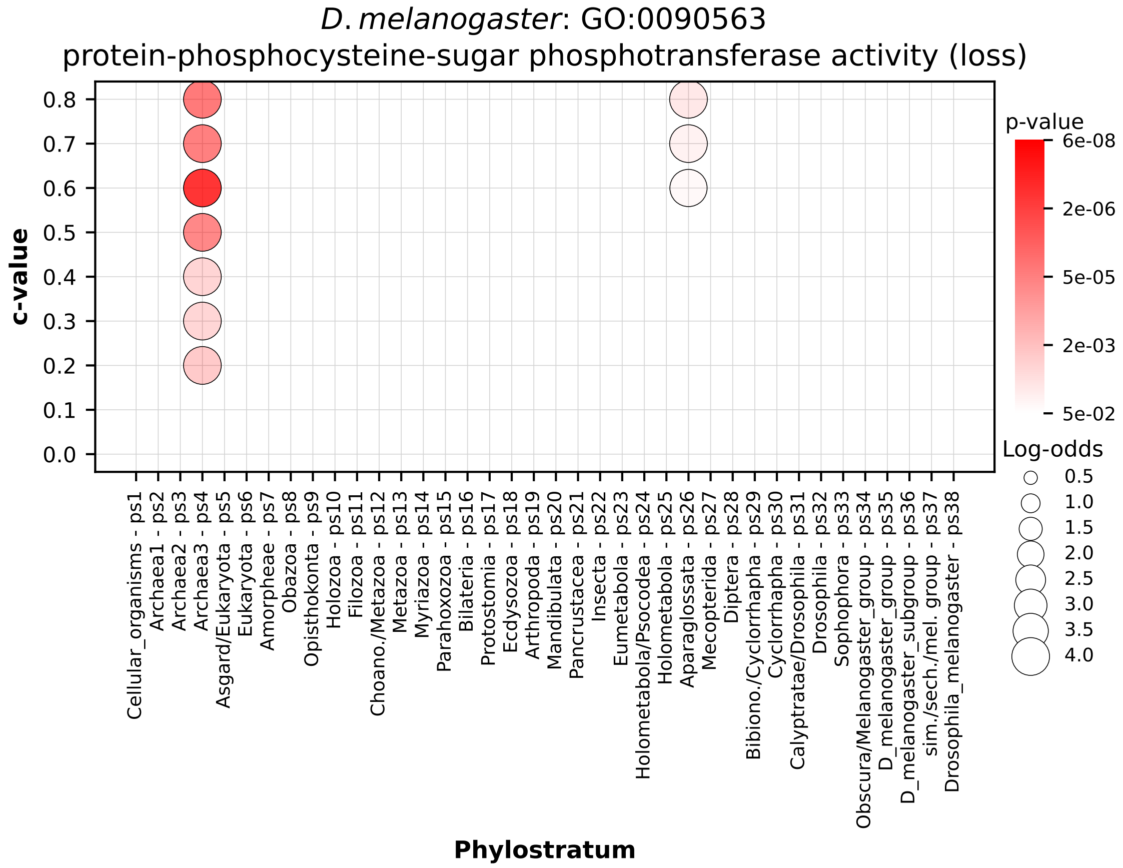

D. melanogaster: GO:0090563
protein-phosphocysteine-sugar phosphotransferase activity (loss)
c-value
0.0
0.1
0.2
0.3
0.4
0.5
0.6
0.7
0.8
p-value
6e-08
2e-06
5e-05
2e-03
5e-02
Log-odds
0.5
1.0
1.5
2.0
2.5
3.0
3.5
4.0
Cellular_organisms - ps1
Archaea1 - ps2
Archaea2 - ps3
Archaea3 - ps4
Asgard/Eukaryota - ps5
Eukaryota - ps6
Amorpheae - ps7
Obazoa - ps8
Opisthokonta - ps9
Holozoa - ps10
Filozoa - ps11
Choano./Metazoa - ps12
Metazoa - ps13
Myriazoa - ps14
Parahoxozoa - ps15
Bilateria - ps16
Protostomia - ps17
Ecdysozoa - ps18
Arthropoda - ps19
Mandibulata - ps20
Pancrustacea - ps21
Insecta - ps22
Eumetabola - ps23
Holometabola/Psocodea - ps24
Holometabola - ps25
Aparaglossata - ps26
Mecopterida - ps27
Diptera - ps28
Bibiono./Cyclorrhapha - ps29
Cyclorrhapha - ps30
Calyptratae/Drosophila - ps31
Drosophila - ps32
Sophophora - ps33
Obscura/Melanogaster_group - ps34
D_melanogaster_group - ps35
D_melanogaster_subgroup - ps36
sim./sech./mel. group - ps37
Drosophila_melanogaster - ps38
Phylostratum

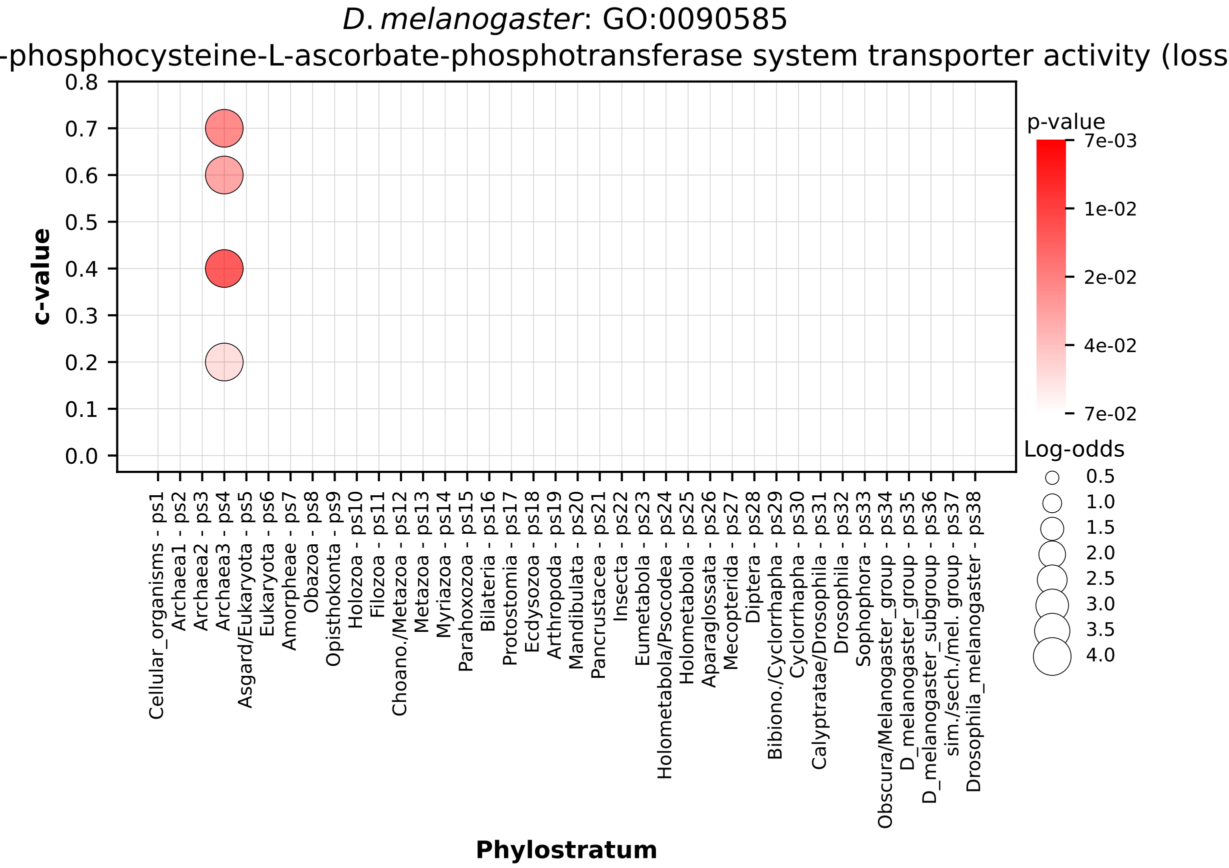

D. melanogaster: GO:0090585
-phosphocysteine-L-ascorbate-phosphotransferase system transporter activity (loss
c-value
0.8
0.7
0.6
0.5
0.4
0.3
0.2
0.1
0.0
p-value
7e-03
1e-02
2e-02
4e-02
7e-02
Log-odds
0.5
1.0
1.5
2.0
2.5
3.0
3.5
4.0
Cellular_organisms - ps1
Archaea1 - ps2
Archaea2 - ps3
Archaea3 - ps4
Asgard/Eukaryota - ps5
Eukaryota - ps6
Amorpheae - ps7
Obazoa - ps8
Opisthokonta - ps9
Holozoa - ps10
Filozoa - ps11
Choano./Metazoa - ps12
Metazoa - ps13
Myriazoa - ps14
Parahoxozoa - ps15
Bilateria - ps16
Protostomia - ps17
Ecdysozoa - ps18
Arthropoda - ps19
Mandibulata - ps20
Pancrustacea - ps21
Insecta - ps22
Eumetabola - ps23
Holometabola/Psocodea - ps24
Holometabola - ps25
Aparaglossata - ps26
Mecopterida - ps27
Diptera - ps28
Bibiono./Cyclorrhapha - ps29
Cyclorrhapha - ps30
Calyptratae/Drosophila - ps31
Drosophila - ps32
Sophophora - ps33
Obscura/Melanogaster_group - ps34
D_melanogaster_group - ps35
D_melanogaster_subgroup - ps36
sim./sech./mel. group - ps37
Drosophila_melanogaster - ps38
Phylostratum

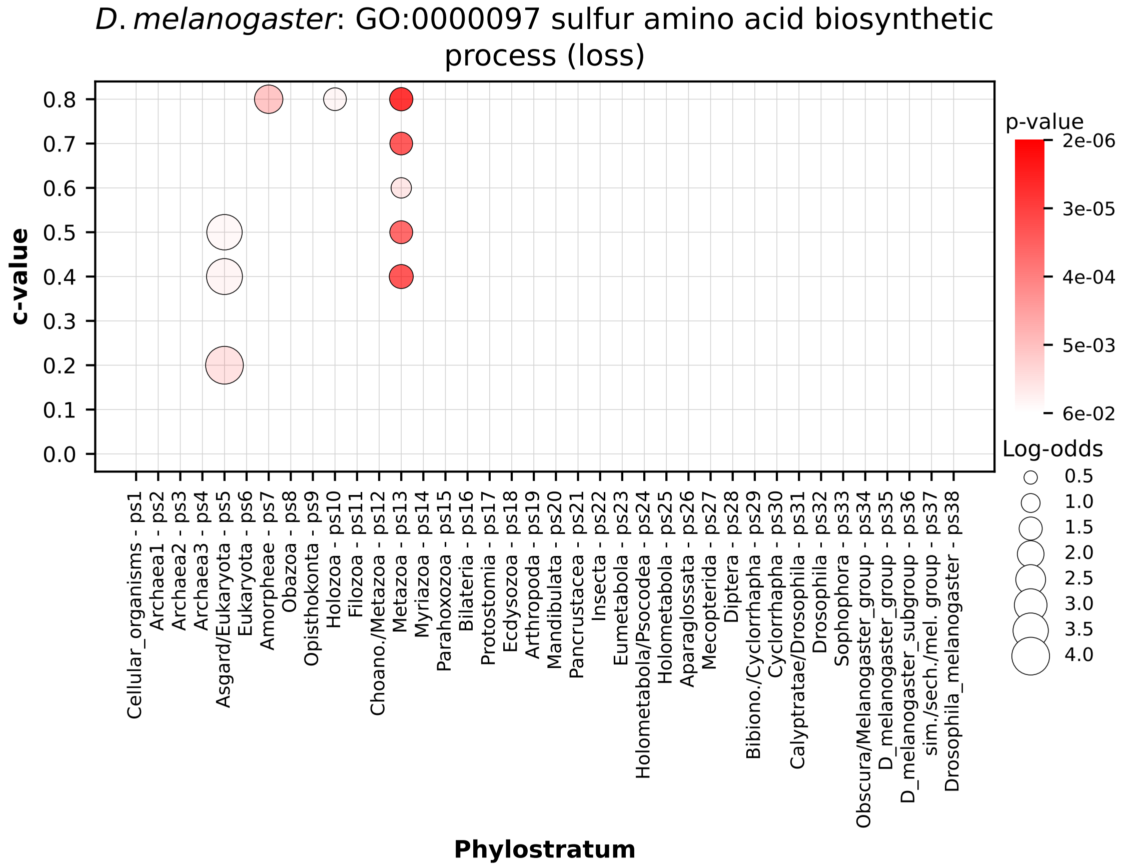

D. melanogaster: GO:0000097 sulfur amino acid biosynthetic process (loss)
c-value
0.8
0.7
0.6
0.5
0.4
0.3
0.2
0.1
0.0
Phylostratum
Cellular_organisms - ps1
Archaea1 - ps2
Archaea2 - ps3
Archaea3 - ps4
Asgard/Eukaryota - ps5
Eukaryota - ps6
Amorpheae - ps7
Obazoa - ps8
Opisthokonta - ps9
Holozoa - ps10
Filozoa - ps11
Choano./Metazoa - ps12
Metazoa - ps13
Myriazoa - ps14
Parahoxozoa - ps15
Bilateria - ps16
Protostomia - ps17
Ecdysozoa - ps18
Arthropoda - ps19
Mandibulata - ps20
Pancrustacea - ps21
Insecta - ps22
Eumetabola - ps23
Holometabola/Psocodea - ps24
Holometabola - ps25
Aparaglossata - ps26
Mecopterida - ps27
Diptera - ps28
Bibiono./Cyclorrhapha - ps29
Cyclorrhapha - ps30
Calyptratae/Drosophila - ps31
Drosophila - ps32
Sophophora - ps33
Obscura/Melanogaster_group - ps34
D_melanogaster_group - ps35
D_melanogaster_subgroup - ps36
sim./sech./mel. group - ps37
Drosophila_melanogaster - ps38
p-value
2e-06
3e-05
4e-04
5e-03
6e-02
Log-odds
0.5
1.0
1.5
2.0
2.5
3.0
3.5
4.0

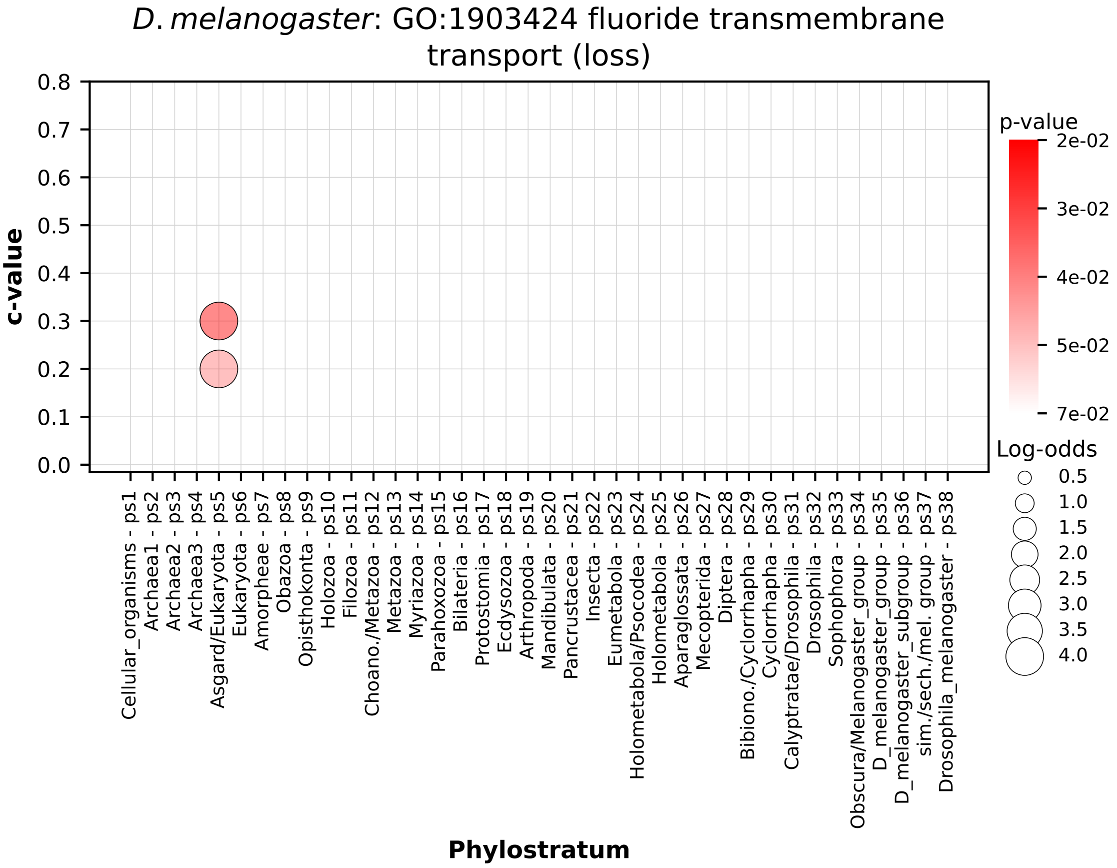

D. melanogaster: GO:1903424 fluoride transmembrane transport (loss)
c-value
0.0
0.1
0.2
0.3
0.4
0.5
0.6
0.7
0.8
Phylostratum
Cellular_organisms - ps1
Archaea1 - ps2
Archaea2 - ps3
Archaea3 - ps4
Asgard/Eukaryota - ps5
Eukaryota - ps6
Amorpheae - ps7
Obazoa - ps8
Opisthokonta - ps9
Holozoa - ps10
Filozoa - ps11
Choano./Metazoa - ps12
Metazoa - ps13
Myriazoa - ps14
Parahoxozoa - ps15
Bilateria - ps16
Protostomia - ps17
Ecdysozoa - ps18
Arthropoda - ps19
Mandibulata - ps20
Pancrustacea - ps21
Insecta - ps22
Eumetabola - ps23
Holometabola/Psocodea - ps24
Holometabola - ps25
Aparaglossata - ps26
Mecopterida - ps27
Diptera - ps28
Bibiono./Cyclorrhapha - ps29
Cyclorrhapha - ps30
Calyptratae/Drosophila - ps31
Drosophila - ps32
Sophophora - ps33
Obscura/Melanogaster_group - ps34
D_melanogaster_group - ps35
D_melanogaster_subgroup - ps36
sim./sech./mel. group - ps37
Drosophila_melanogaster - ps38
p-value
2e-02
3e-02
4e-02
5e-02
7e-02
Log-odds
0.5
1.0
1.5
2.0
2.5
3.0
3.5
4.0

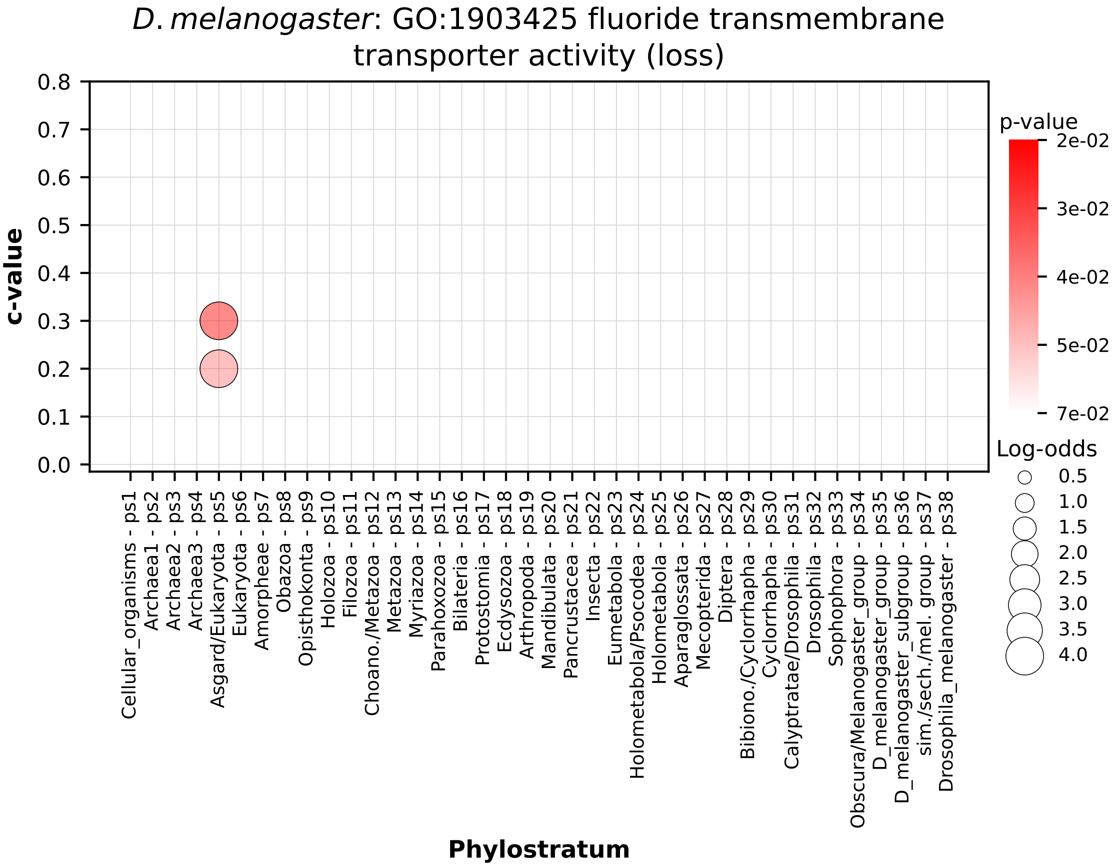

D. melanogaster: GO:1903425 fluoride transmembrane transporter activity (loss)
c-value
0.0
0.1
0.2
0.3
0.4
0.5
0.6
0.7
0.8
p-value
2e-02
3e-02
4e-02
5e-02
7e-02
Log-odds
0.5
1.0
1.5
2.0
2.5
3.0
3.5
4.0
Cellular_organisms - ps1
Archaea1 - ps2
Archaea2 - ps3
Archaea3 - ps4
Asgard/Eukaryota - ps5
Eukaryota - ps6
Amorpheae - ps7
Obazoa - ps8
Opisthokonta - ps9
Holozoa - ps10
Filozoa - ps11
Choano./Metazoa - ps12
Metazoa - ps13
Myriazoa - ps14
Parahoxozoa - ps15
Bilateria - ps16
Protostomia - ps17
Ecdysozoa - ps18
Arthropoda - ps19
Mandibulata - ps20
Pancrustacea - ps21
Insecta - ps22
Eumetabola - ps23
Holometabola/Psocodea - ps24
Holometabola - ps25
Aparaglossata - ps26
Mecopterida - ps27
Diptera - ps28
Bibiono./Cyclorrhapha - ps29
Cyclorrhapha - ps30
Calyptratae/Drosophila - ps31
Drosophila - ps32
Sophophora - ps33
Obscura/Melanogaster_group - ps34
D_melanogaster_group - ps35
D_melanogaster_subgroup - ps36
sim./sech./mel. group - ps37
Drosophila_melanogaster - ps38
Phylostratum

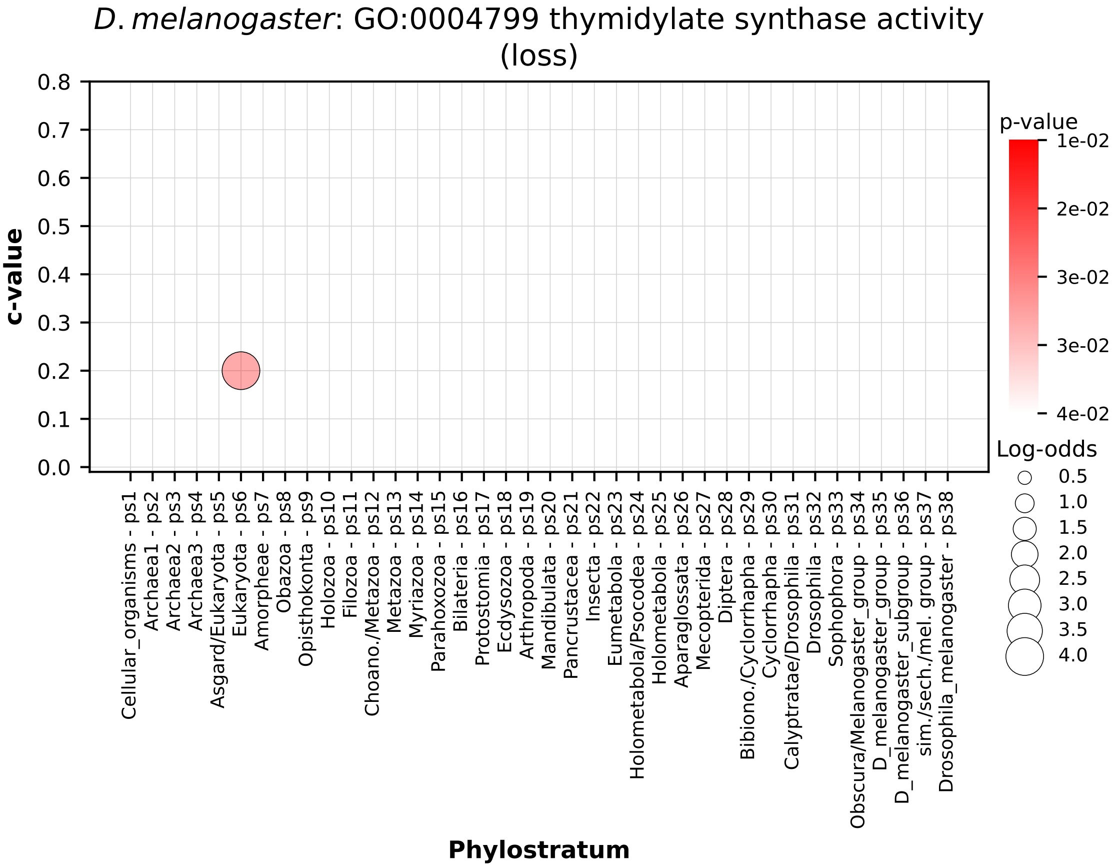

D. melanogaster: GO:0004799 thymidylate synthase activity (loss)
c-value
0.8
0.7
0.6
0.5
0.4
0.3
0.2
0.1
0.0
p-value
1e-02
2e-02
3e-02
3e-02
4e-02
Log-odds
0.5
1.0
1.5
2.0
2.5
3.0
3.5
4.0
Cellular_organisms - ps1
Archaea1 - ps2
Archaea2 - ps3
Archaea3 - ps4
Asgard/Eukaryota - ps5
Eukaryota - ps6
Amorpheae - ps7
Obazoa - ps8
Opisthokonta - ps9
Holozoa - ps10
Filozoa - ps11
Choano./Metazoa - ps12
Metazoa - ps13
Myriazoa - ps14
Parahoxozoa - ps15
Bilateria - ps16
Protostomia - ps17
Ecdysozoa - ps18
Arthropoda - ps19
Mandibulata - ps20
Pancrustacea - ps21
Insecta - ps22
Eumetabola - ps23
Holometabola/Psocodea - ps24
Holometabola - ps25
Aparaglossata - ps26
Mecopterida - ps27
Diptera - ps28
Bibiono./Cyclorrhapha - ps29
Cyclorrhapha - ps30
Calyptratae/Drosophila - ps31
Drosophila - ps32
Sophophora - ps33
Obscura/Melanogaster_group - ps34
D_melanogaster_group - ps35
D_melanogaster_subgroup - ps36
sim./sech./mel. group - ps37
Drosophila_melanogaster - ps38
Phylostratum

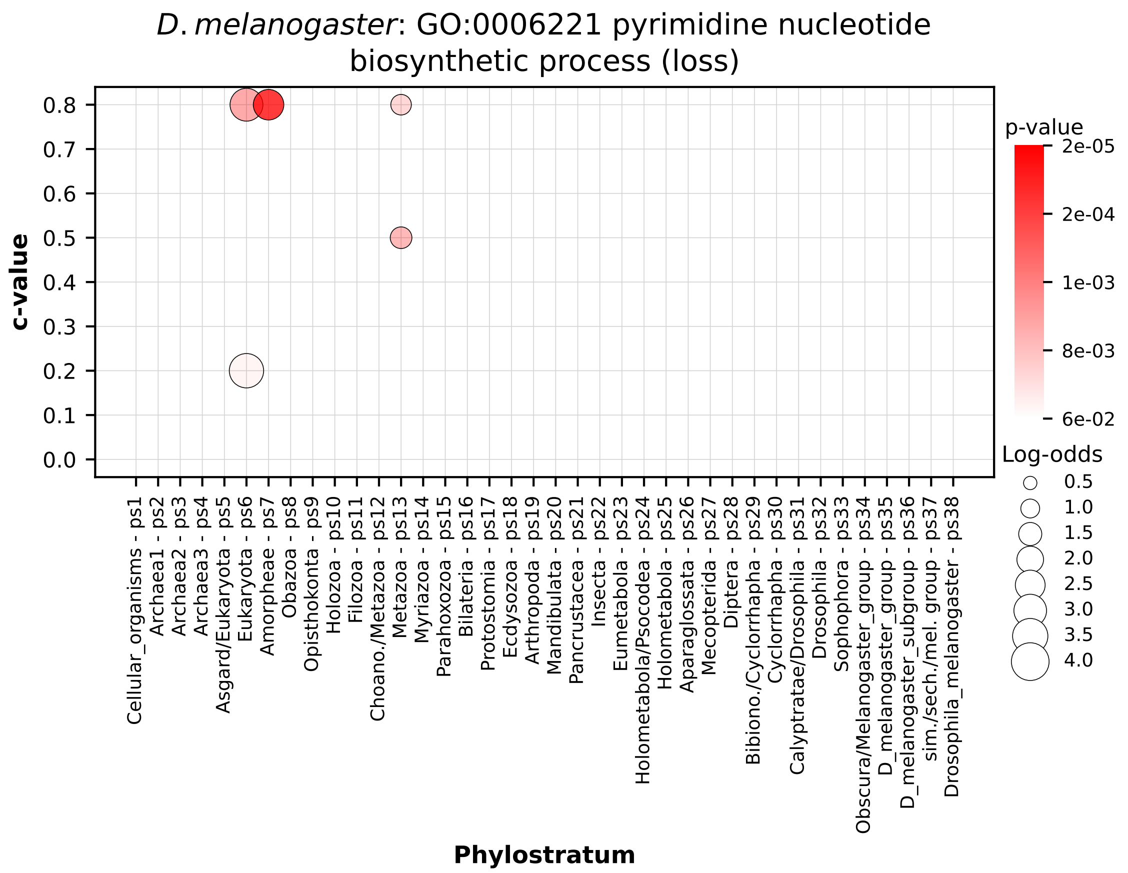

D. melanogaster: GO:0006221 pyrimidine nucleotide biosynthetic process (loss)
c-value
0.0
0.1
0.2
0.3
0.4
0.5
0.6
0.7
0.8
Phylostratum
Cellular_organisms - ps1
Archaea1 - ps2
Archaea2 - ps3
Archaea3 - ps4
Asgard/Eukaryota - ps5
Eukaryota - ps6
Amorpheae - ps7
Obazoa - ps8
Opisthokonta - ps9
Holozoa - ps10
Filozoa - ps11
Choano./Metazoa - ps12
Metazoa - ps13
Myriazoa - ps14
Parahoxozoa - ps15
Bilateria - ps16
Protostomia - ps17
Ecdysozoa - ps18
Arthropoda - ps19
Mandibulata - ps20
Pancrustacea - ps21
Insecta - ps22
Eumetabola - ps23
Holometabola/Psocodea - ps24
Holometabola - ps25
Aparaglossata - ps26
Mecopterida - ps27
Diptera - ps28
Bibiono./Cyclorrhapha - ps29
Cyclorrhapha - ps30
Calyptratae/Drosophila - ps31
Drosophila - ps32
Sophophora - ps33
Obscura/Melanogaster_group - ps34
D_melanogaster_group - ps35
D_melanogaster_subgroup - ps36
sim./sech./mel. group - ps37
Drosophila_melanogaster - ps38
p-value
2e-05
2e-04
1e-03
8e-03
6e-02
Log-odds
0.5
1.0
1.5
2.0
2.5
3.0
3.5
4.0

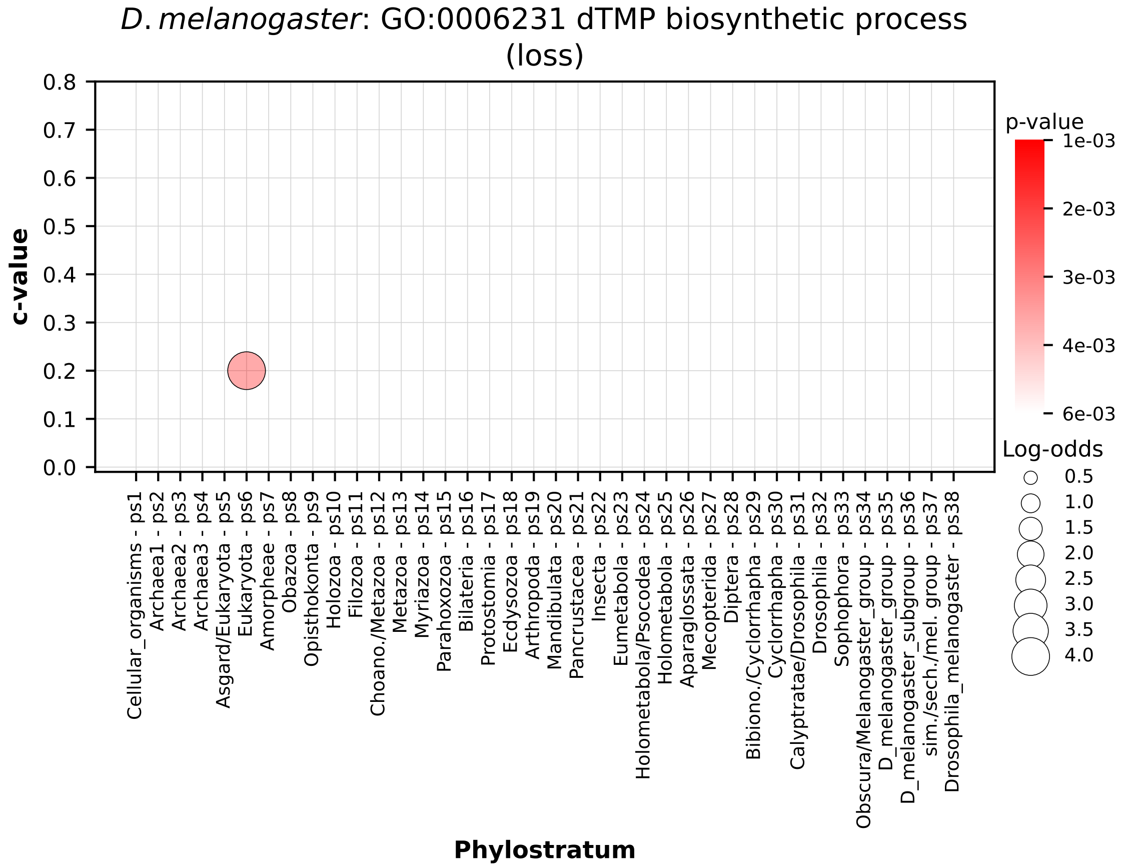

D. melanogaster: GO:0006231 dTMP biosynthetic process
(loss)
c-value
0.8
0.7
0.6
0.5
0.4
0.3
0.2
0.1
0.0
p-value
1e-03
2e-03
3e-03
4e-03
6e-03
Log-odds
0.5
1.0
1.5
2.0
2.5
3.0
3.5
4.0
Cellular_organisms - ps1
Archaea1 - ps2
Archaea2 - ps3
Archaea3 - ps4
Asgard/Eukaryota - ps5
Eukaryota - ps6
Amorpheae - ps7
Obazoa - ps8
Opisthokonta - ps9
Holozoa - ps10
Filozoa - ps11
Choano./Metazoa - ps12
Metazoa - ps13
Myriazoa - ps14
Parahoxozoa - ps15
Bilateria - ps16
Protostomia - ps17
Ecdysozoa - ps18
Arthropoda - ps19
Mandibulata - ps20
Pancrustacea - ps21
Insecta - ps22
Eumetabola - ps23
Holometabola/Psocodea - ps24
Holometabola - ps25
Aparaglossata - ps26
Mecopterida - ps27
Diptera - ps28
Bibiono./Cyclorrhapha - ps29
Cyclorrhapha - ps30
Calyptratae/Drosophila - ps31
Drosophila - ps32
Sophophora - ps33
Obscura/Melanogaster_group - ps34
D_melanogaster_group - ps35
D_melanogaster_subgroup - ps36
sim./sech./mel. group - ps37
Drosophila_melanogaster - ps38
Phylostratum

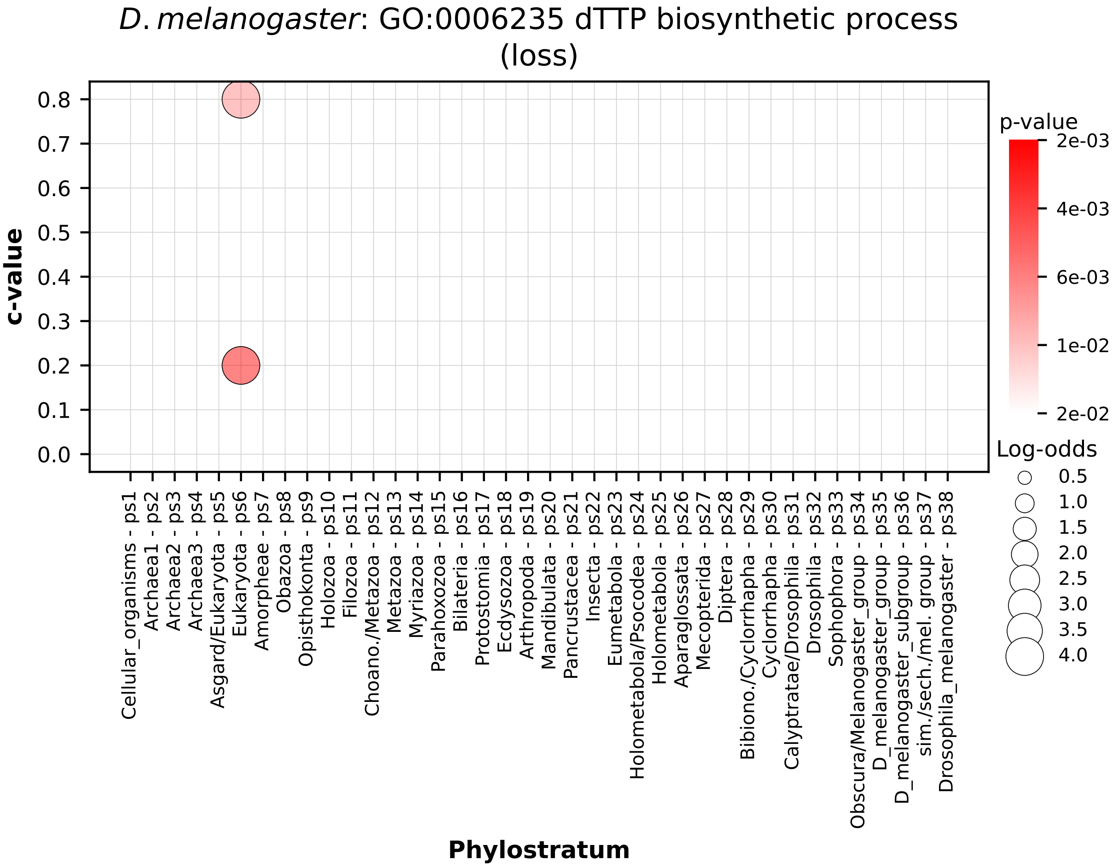
D. melanogaster: GO:0006235 dTTP biosynthetic process
(loss)
c-value
0.0
0.1
0.2
0.3
0.4
0.5
0.6
0.7
0.8
Phylostratum
Cellular_organisms - ps1
Archaea1 - ps2
Archaea2 - ps3
Archaea3 - ps4
Asgard/Eukaryota - ps5
Eukaryota - ps6
Amorpheae - ps7
Obazoa - ps8
Opisthokonta - ps9
Holozoa - ps10
Filozoa - ps11
Choano./Metazoa - ps12
Metazoa - ps13
Myriazoa - ps14
Parahoxozoa - ps15
Bilateria - ps16
Protostomia - ps17
Ecdysozoa - ps18
Arthropoda - ps19
Mandibulata - ps20
Pancrustacea - ps21
Insecta - ps22
Eumetabola - ps23
Holometabola/Psocodea - ps24
Holometabola - ps25
Aparaglossata - ps26
Mecopterida - ps27
Diptera - ps28
Bibiono./Cyclorrhapha - ps29
Cyclorrhapha - ps30
Calyptratae/Drosophila - ps31
Drosophila - ps32
Sophophora - ps33
Obscura/Melanogaster_group - ps34
D_melanogaster_group - ps35
D_melanogaster_subgroup - ps36
sim./sech./mel. group - ps37
Drosophila_melanogaster - ps38
p-value
2e-03
4e-03
6e-03
1e-02
2e-02
Log-odds
0.5
1.0
1.5
2.0
2.5
3.0
3.5
4.0

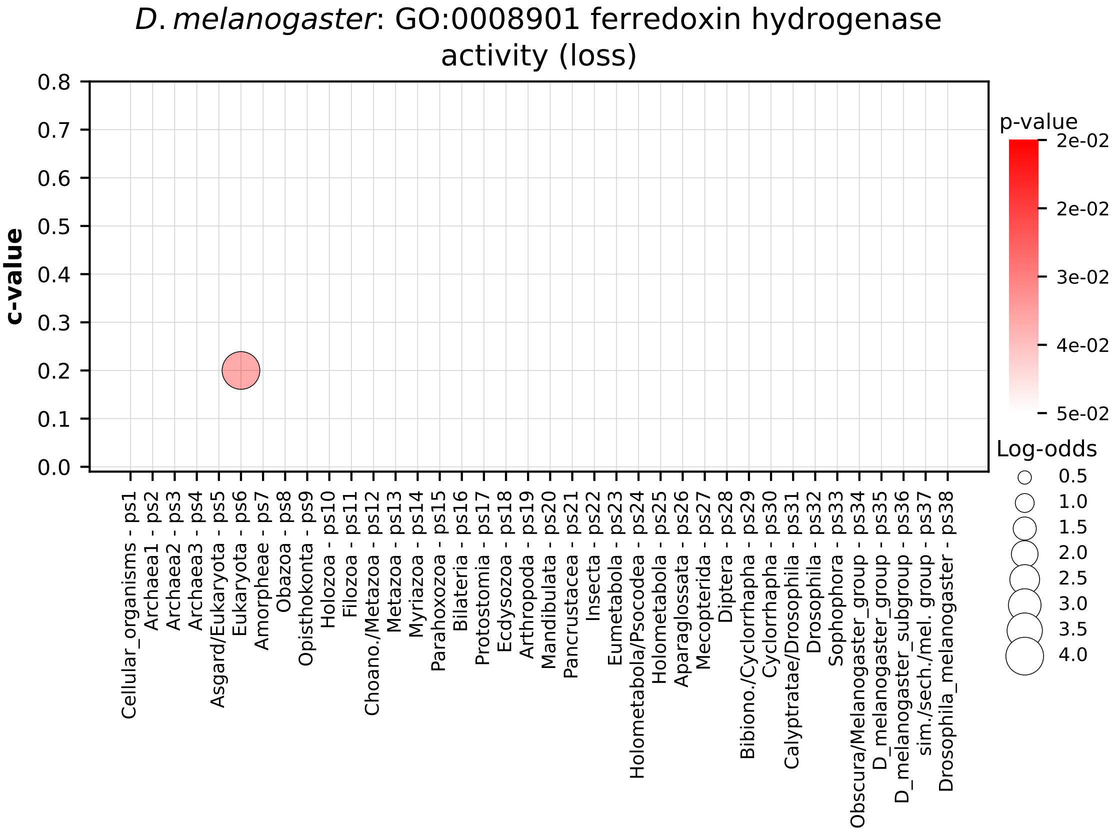

D. melanogaster: GO:0008901 ferredoxin hydrogenase activity (loss)
c-value
0.0
0.1
0.2
0.3
0.4
0.5
0.6
0.7
0.8
Phylostratum
Cellular_organisms - ps1
Archaea1 - ps2
Archaea2 - ps3
Archaea3 - ps4
Asgard/Eukaryota - ps5
Eukaryota - ps6
Amorpheae - ps7
Obazoa - ps8
Opisthokonta - ps9
Holozoa - ps10
Filozoa - ps11
Choano./Metazoa - ps12
Metazoa - ps13
Myriazoa - ps14
Parahoxozoa - ps15
Bilateria - ps16
Protostomia - ps17
Ecdysozoa - ps18
Arthropoda - ps19
Mandibulata - ps20
Pancrustacea - ps21
Insecta - ps22
Eumetabola - ps23
Holometabola/Psocodea - ps24
Holometabola - ps25
Aparaglossata - ps26
Mecopterida - ps27
Diptera - ps28
Bibiono./Cyclorrhapha - ps29
Cyclorrhapha - ps30
Calyptratae/Drosophila - ps31
Drosophila - ps32
Sophophora - ps33
Obscura/Melanogaster_group - ps34
D_melanogaster_group - ps35
D_melanogaster_subgroup - ps36
sim./sech./mel. group - ps37
Drosophila_melanogaster - ps38
p-value
2e-02
2e-02
3e-02
4e-02
5e-02
Log-odds
0.5
1.0
1.5
2.0
2.5
3.0
3.5
4.0

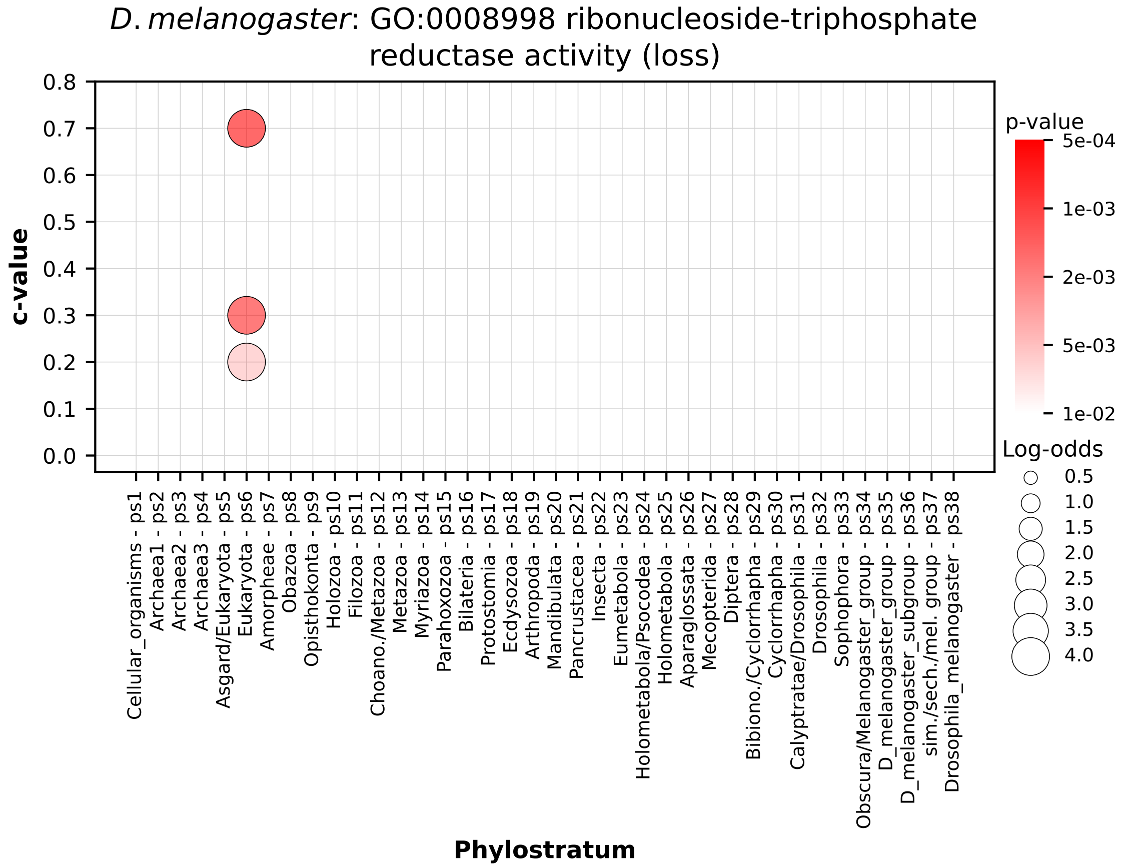

D. melanogaster: GO:0008998 ribonucleoside-triphosphate reductase activity (loss)
c-value
0.8
0.7
0.6
0.5
0.4
0.3
0.2
0.1
0.0
p-value
5e-04
1e-03
2e-03
5e-03
1e-02
Log-odds
0.5
1.0
1.5
2.0
2.5
3.0
3.5
4.0
Cellular_organisms - ps1
Archaea1 - ps2
Archaea2 - ps3
Archaea3 - ps4
Asgard/Eukaryota - ps5
Eukaryota - ps6
Amorpheae - ps7
Obazoa - ps8
Opisthokonta - ps9
Holozoa - ps10
Filozoa - ps11
Choano./Metazoa - ps12
Metazoa - ps13
Myriazoa - ps14
Parahoxozoa - ps15
Bilateria - ps16
Protostomia - ps17
Ecdysozoa - ps18
Arthropoda - ps19
Mandibulata - ps20
Pancrustacea - ps21
Insecta - ps22
Eumetabola - ps23
Holometabola/Psocodea - ps24
Holometabola - ps25
Aparaglossata - ps26
Mecopterida - ps27
Diptera - ps28
Bibiono./Cyclorrhapha - ps29
Cyclorrhapha - ps30
Calyptratae/Drosophila - ps31
Drosophila - ps32
Sophophora - ps33
Obscura/Melanogaster_group - ps34
D_melanogaster_group - ps35
D_melanogaster_subgroup - ps36
sim./sech./mel. group - ps37
Drosophila_melanogaster - ps38
Phylostratum

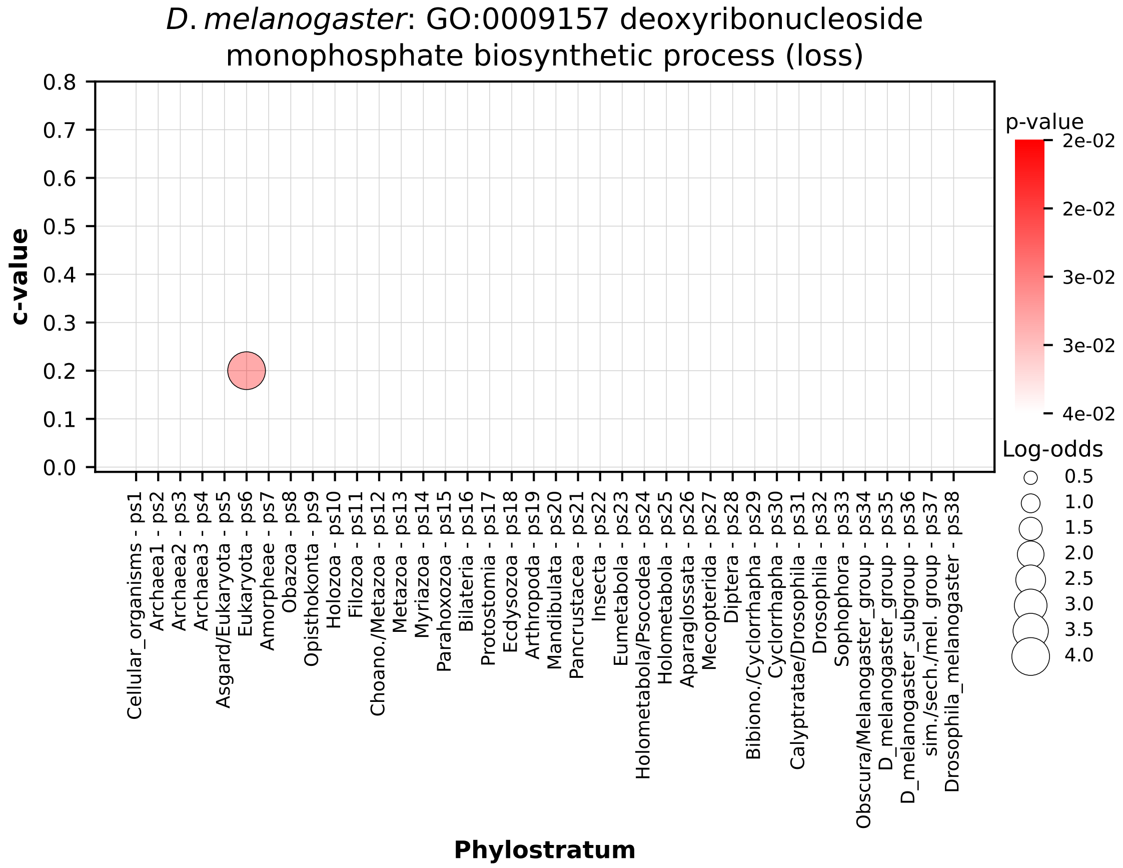

D. melanogaster: GO:0009157 deoxyribonucleoside monophosphate biosynthetic process (loss)
c-value
0.8
0.7
0.6
0.5
0.4
0.3
0.2
0.1
0.0
p-value
2e-02
2e-02
3e-02
3e-02
4e-02
Log-odds
0.5
1.0
1.5
2.0
2.5
3.0
3.5
4.0
Cellular_organisms - ps1
Archaea1 - ps2
Archaea2 - ps3
Archaea3 - ps4
Asgard/Eukaryota - ps5
Eukaryota - ps6
Amorpheae - ps7
Obazoa - ps8
Opisthokonta - ps9
Holozoa - ps10
Filozoa - ps11
Choano./Metazoa - ps12
Metazoa - ps13
Myriazoa - ps14
Parahoxozoa - ps15
Bilateria - ps16
Protostomia - ps17
Ecdysozoa - ps18
Arthropoda - ps19
Mandibulata - ps20
Pancrustacea - ps21
Insecta - ps22
Eumetabola - ps23
Holometabola/Psocodea - ps24
Holometabola - ps25
Aparaglossata - ps26
Mecopterida - ps27
Diptera - ps28
Bibiono./Cyclorrhapha - ps29
Cyclorrhapha - ps30
Calyptratae/Drosophila - ps31
Drosophila - ps32
Sophophora - ps33
Obscura/Melanogaster_group - ps34
D_melanogaster_group - ps35
D_melanogaster_subgroup - ps36
sim./sech./mel. group - ps37
Drosophila_melanogaster - ps38
Phylostratum

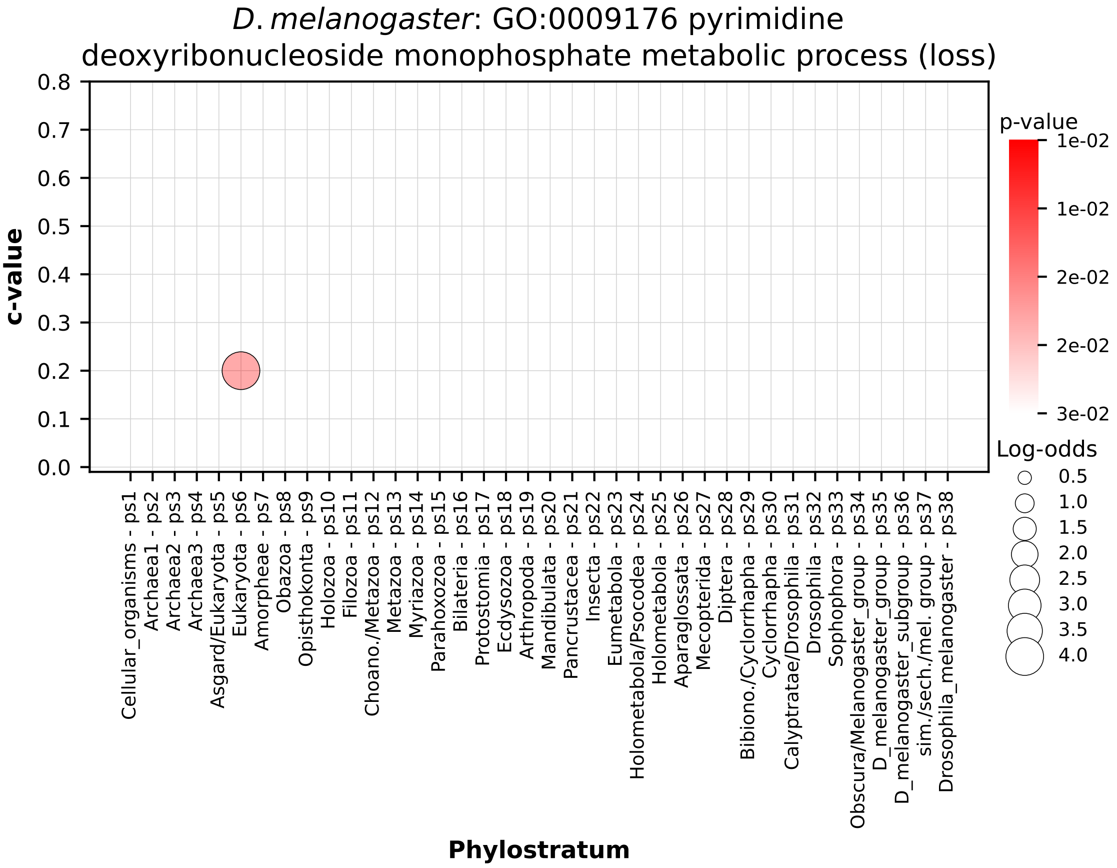

D. melanogaster: GO:0009176 pyrimidine deoxyribonucleoside monophosphate metabolic process (loss)
c-value
0.0
0.1
0.2
0.3
0.4
0.5
0.6
0.7
0.8
Phylostratum
Cellular_organisms - ps1
Archaea1 - ps2
Archaea2 - ps3
Archaea3 - ps4
Asgard/Eukaryota - ps5
Eukaryota - ps6
Amorpheae - ps7
Obazoa - ps8
Opisthokonta - ps9
Holozoa - ps10
Filozoa - ps11
Choano./Metazoa - ps12
Metazoa - ps13
Myriazoa - ps14
Parahoxozoa - ps15
Bilateria - ps16
Protostomia - ps17
Ecdysozoa - ps18
Arthropoda - ps19
Mandibulata - ps20
Pancrustacea - ps21
Insecta - ps22
Eumetabola - ps23
Holometabola/Psocodea - ps24
Holometabola - ps25
Aparaglossata - ps26
Mecopterida - ps27
Diptera - ps28
Bibiono./Cyclorrhapha - ps29
Cyclorrhapha - ps30
Calyptratae/Drosophila - ps31
Drosophila - ps32
Sophophora - ps33
Obscura/Melanogaster_group - ps34
D_melanogaster_group - ps35
D_melanogaster_subgroup - ps36
sim./sech./mel. group - ps37
Drosophila_melanogaster - ps38
p-value
1e-02
1e-02
2e-02
2e-02
3e-02
Log-odds
0.5
1.0
1.5
2.0
2.5
3.0
3.5
4.0

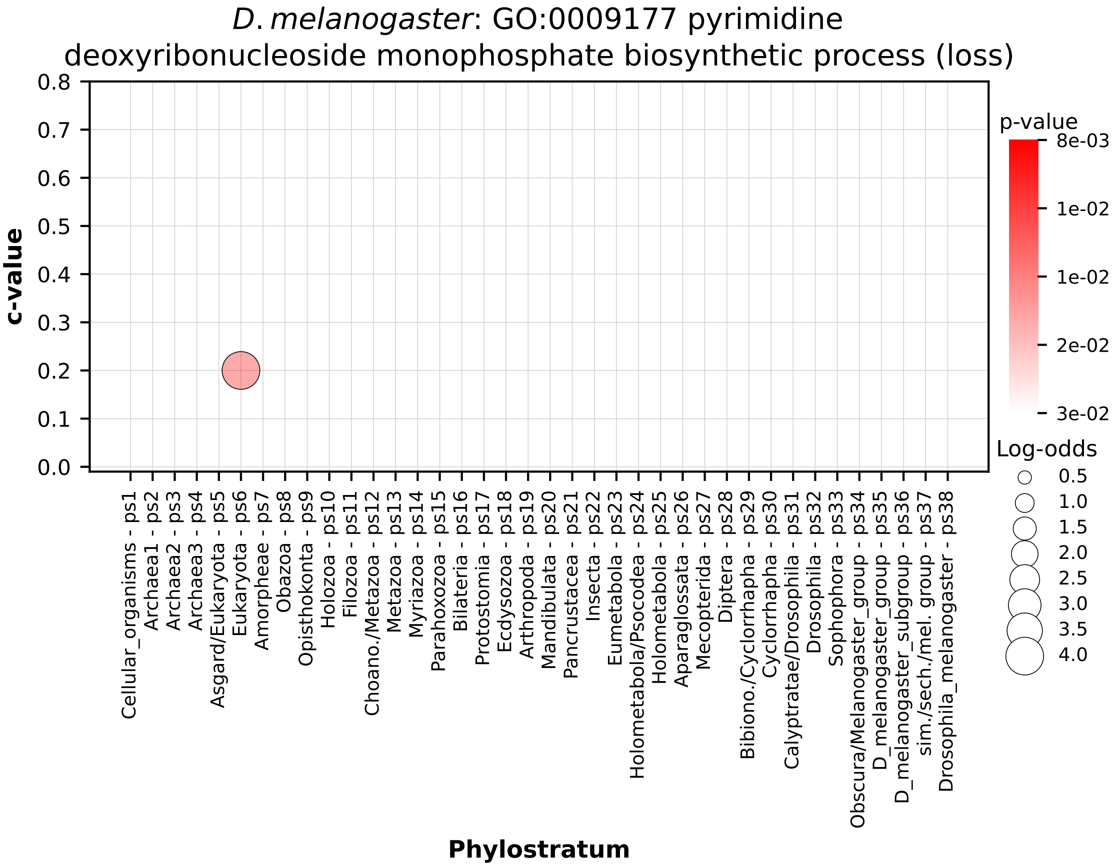

D. melanogaster: GO:0009177 pyrimidine deoxyribonucleoside monophosphate biosynthetic process (loss)
c-value
0.0
0.1
0.2
0.3
0.4
0.5
0.6
0.7
0.8
Phylostratum
Cellular_organisms - ps1
Archaea1 - ps2
Archaea2 - ps3
Archaea3 - ps4
Asgard/Eukaryota - ps5
Eukaryota - ps6
Amorpheae - ps7
Obazoa - ps8
Opisthokonta - ps9
Holozoa - ps10
Filozoa - ps11
Choano./Metazoa - ps12
Metazoa - ps13
Myriazoa - ps14
Parahoxozoa - ps15
Bilateria - ps16
Protostomia - ps17
Ecdysozoa - ps18
Arthropoda - ps19
Mandibulata - ps20
Pancrustacea - ps21
Insecta - ps22
Eumetabola - ps23
Holometabola/Psocodea - ps24
Holometabola - ps25
Aparaglossata - ps26
Mecopterida - ps27
Diptera - ps28
Bibiono./Cyclorrhapha - ps29
Cyclorrhapha - ps30
Calyptratae/Drosophila - ps31
Drosophila - ps32
Sophophora - ps33
Obscura/Melanogaster_group - ps34
D_melanogaster_group - ps35
D_melanogaster_subgroup - ps36
sim./sech./mel. group - ps37
Drosophila_melanogaster - ps38
p-value
8e-03
1e-02
1e-02
2e-02
3e-02
Log-odds
0.5
1.0
1.5
2.0
2.5
3.0
3.5
4.0

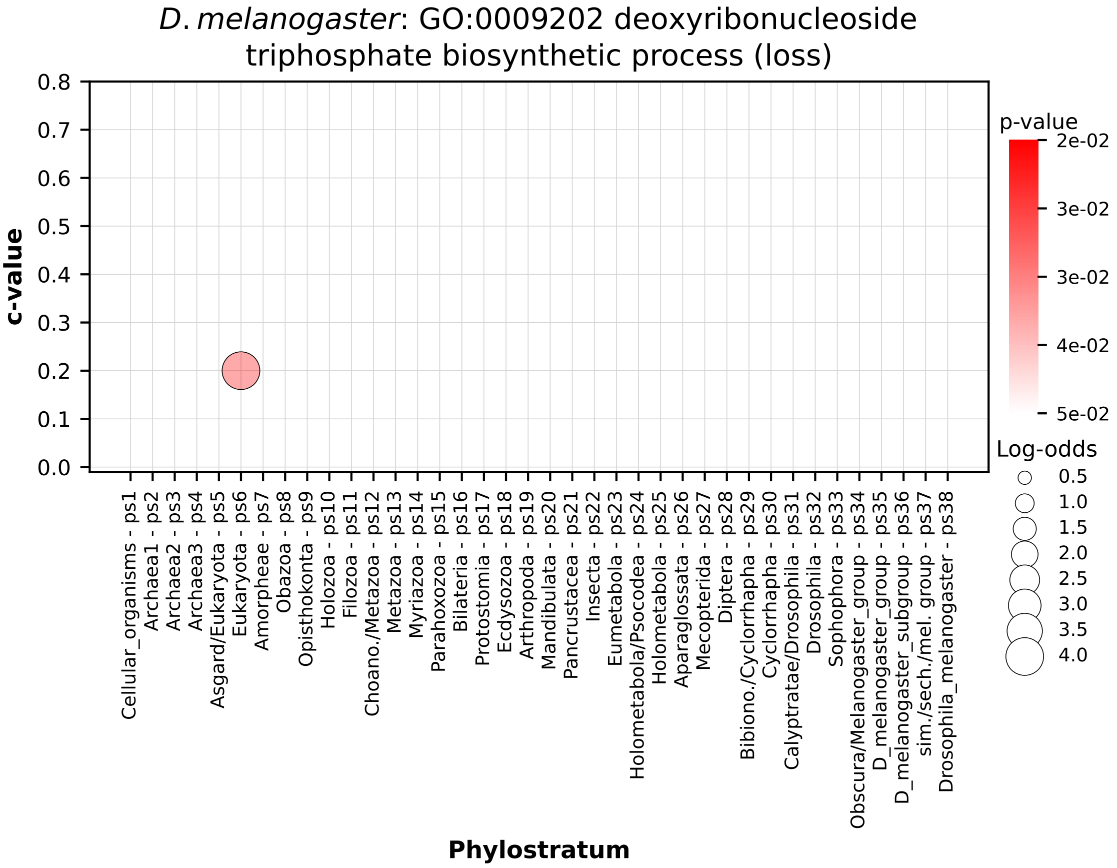

D. melanogaster: GO:0009202 deoxyribonucleoside triphosphate biosynthetic process (loss)
c-value
0.8
0.7
0.6
0.5
0.4
0.3
0.2
0.1
0.0
p-value
2e-02
3e-02
3e-02
4e-02
5e-02
Log-odds
0.5
1.0
1.5
2.0
2.5
3.0
3.5
4.0
Cellular_organisms - ps1
Archaea1 - ps2
Archaea2 - ps3
Archaea3 - ps4
Asgard/Eukaryota - ps5
Eukaryota - ps6
Amorpheae - ps7
Obazoa - ps8
Opisthokonta - ps9
Holozoa - ps10
Filozoa - ps11
Choano./Metazoa - ps12
Metazoa - ps13
Myriazoa - ps14
Parahoxozoa - ps15
Bilateria - ps16
Protostomia - ps17
Ecdysozoa - ps18
Arthropoda - ps19
Mandibulata - ps20
Pancrustacea - ps21
Insecta - ps22
Eumetabola - ps23
Holometabola/Psocodea - ps24
Holometabola - ps25
Aparaglossata - ps26
Mecopterida - ps27
Diptera - ps28
Bibiono./Cyclorrhapha - ps29
Cyclorrhapha - ps30
Calyptratae/Drosophila - ps31
Drosophila - ps32
Sophophora - ps33
Obscura/Melanogaster_group - ps34
D_melanogaster_group - ps35
D_melanogaster_subgroup - ps36
sim./sech./mel. group - ps37
Drosophila_melanogaster - ps38
Phylostratum

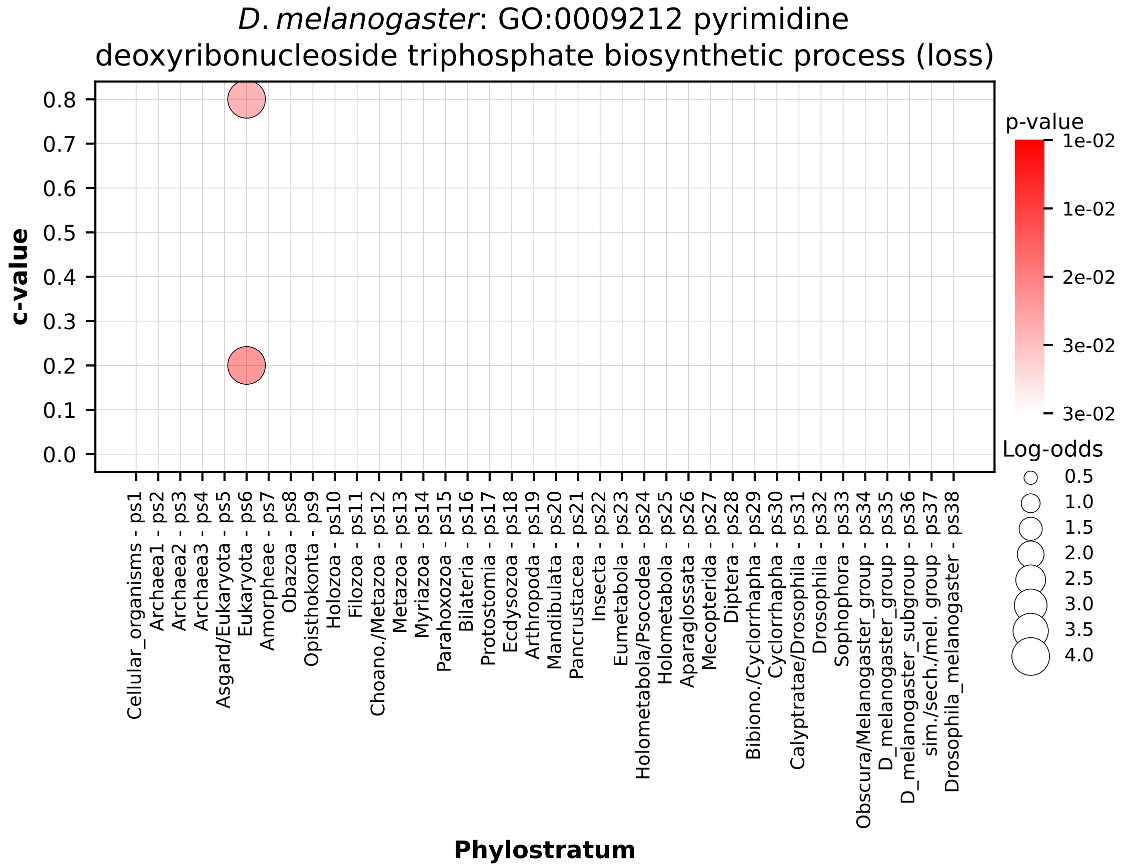

D. melanogaster: GO:0009212 pyrimidine deoxyribonucleoside triphosphate biosynthetic process (loss)
c-value
0.8
0.7
0.6
0.5
0.4
0.3
0.2
0.1
0.0
p-value
1e-02
1e-02
2e-02
3e-02
3e-02
Log-odds
0.5
1.0
1.5
2.0
2.5
3.0
3.5
4.0
Cellular_organisms - ps1
Archaea1 - ps2
Archaea2 - ps3
Archaea3 - ps4
Asgard/Eukaryota - ps5
Eukaryota - ps6
Amorpheae - ps7
Obazoa - ps8
Opisthokonta - ps9
Holozoa - ps10
Filozoa - ps11
Choano./Metazoa - ps12
Metazoa - ps13
Myriazoa - ps14
Parahoxozoa - ps15
Bilateria - ps16
Protostomia - ps17
Ecdysozoa - ps18
Arthropoda - ps19
Mandibulata - ps20
Pancrustacea - ps21
Insecta - ps22
Eumetabola - ps23
Holometabola/Psocodea - ps24
Holometabola - ps25
Aparaglossata - ps26
Mecopterida - ps27
Diptera - ps28
Bibiono./Cyclorrhapha - ps29
Cyclorrhapha - ps30
Calyptratae/Drosophila - ps31
Drosophila - ps32
Sophophora - ps33
Obscura/Melanogaster_group - ps34
D_melanogaster_group - ps35
D_melanogaster_subgroup - ps36
sim./sech./mel. group - ps37
Drosophila_melanogaster - ps38
Phylostratum

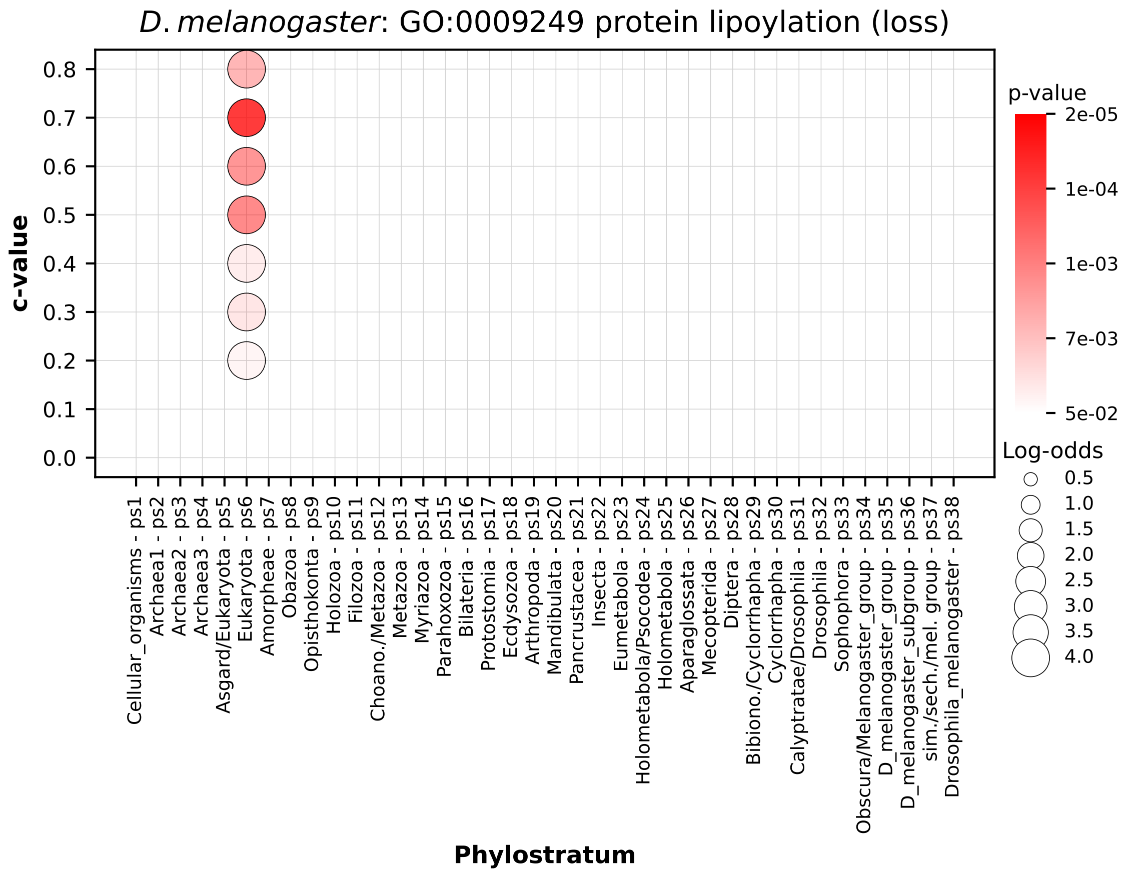

D. melanogaster: GO:0009249 protein lipoylation (loss)
c-value
0.8
0.7
0.6
0.5
0.4
0.3
0.2
0.1
0.0
Phylostratum
Cellular_organisms - ps1
Archaea1 - ps2
Archaea2 - ps3
Archaea3 - ps4
Asgard/Eukaryota - ps5
Eukaryota - ps6
Amorpheae - ps7
Obazoa - ps8
Opisthokonta - ps9
Holozoa - ps10
Filozoa - ps11
Choano./Metazoa - ps12
Metazoa - ps13
Myriazoa - ps14
Parahoxozoa - ps15
Bilateria - ps16
Protostomia - ps17
Ecdysozoa - ps18
Arthropoda - ps19
Mandibulata - ps20
Pancrustacea - ps21
Insecta - ps22
Eumetabola - ps23
Holometabola/Psocodea - ps24
Holometabola - ps25
Aparaglossata - ps26
Mecopterida - ps27
Diptera - ps28
Bibiono./Cyclorrhapha - ps29
Cyclorrhapha - ps30
Calyptratae/Drosophila - ps31
Drosophila - ps32
Sophophora - ps33
Obscura/Melanogaster_group - ps34
D_melanogaster_group - ps35
D_melanogaster_subgroup - ps36
sim./sech./mel. group - ps37
Drosophila_melanogaster - ps38
p-value
2e-05
1e-04
1e-03
7e-03
5e-02
Log-odds
0.5
1.0
1.5
2.0
2.5
3.0
3.5
4.0

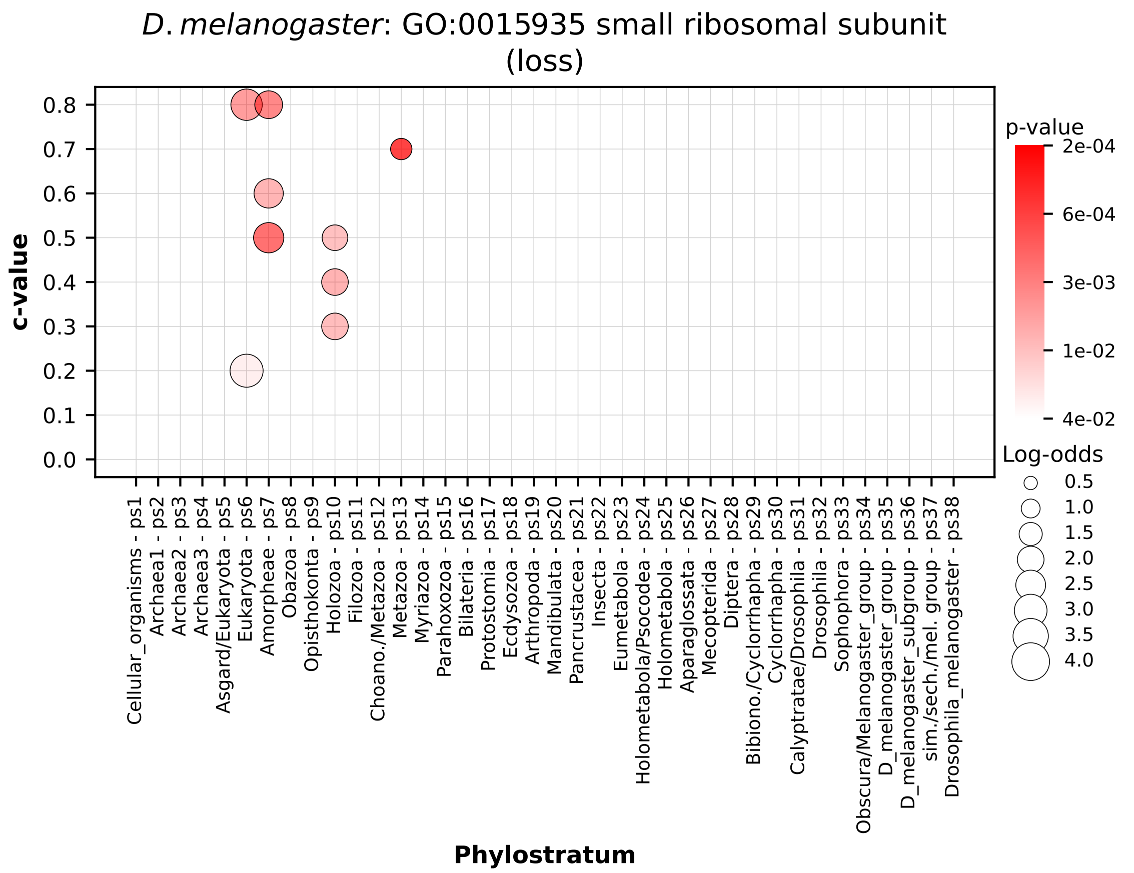

D. melanogaster: GO:0015935 small ribosomal subunit (loss)
c-value
0.0
0.1
0.2
0.3
0.4
0.5
0.6
0.7
0.8
Phylostratum
Cellular_organisms - ps1
Archaea1 - ps2
Archaea2 - ps3
Archaea3 - ps4
Asgard/Eukaryota - ps5
Eukaryota - ps6
Amorpheae - ps7
Obazoa - ps8
Opisthokonta - ps9
Holozoa - ps10
Filozoa - ps11
Choano./Metazoa - ps12
Metazoa - ps13
Myriazoa - ps14
Parahoxozoa - ps15
Bilateria - ps16
Protostomia - ps17
Ecdysozoa - ps18
Arthropoda - ps19
Mandibulata - ps20
Pancrustacea - ps21
Insecta - ps22
Eumetabola - ps23
Holometabola/Psocodea - ps24
Holometabola - ps25
Aparaglossata - ps26
Mecopterida - ps27
Diptera - ps28
Bibiono./Cyclorrhapha - ps29
Cyclorrhapha - ps30
Calyptratae/Drosophila - ps31
Drosophila - ps32
Sophophora - ps33
Obscura/Melanogaster_group - ps34
D_melanogaster_group - ps35
D_melanogaster_subgroup - ps36
sim./sech./mel. group - ps37
Drosophila_melanogaster - ps38
p-value
2e-04
6e-04
3e-03
1e-02
4e-02
Log-odds
0.5
1.0
1.5
2.0
2.5
3.0
3.5
4.0

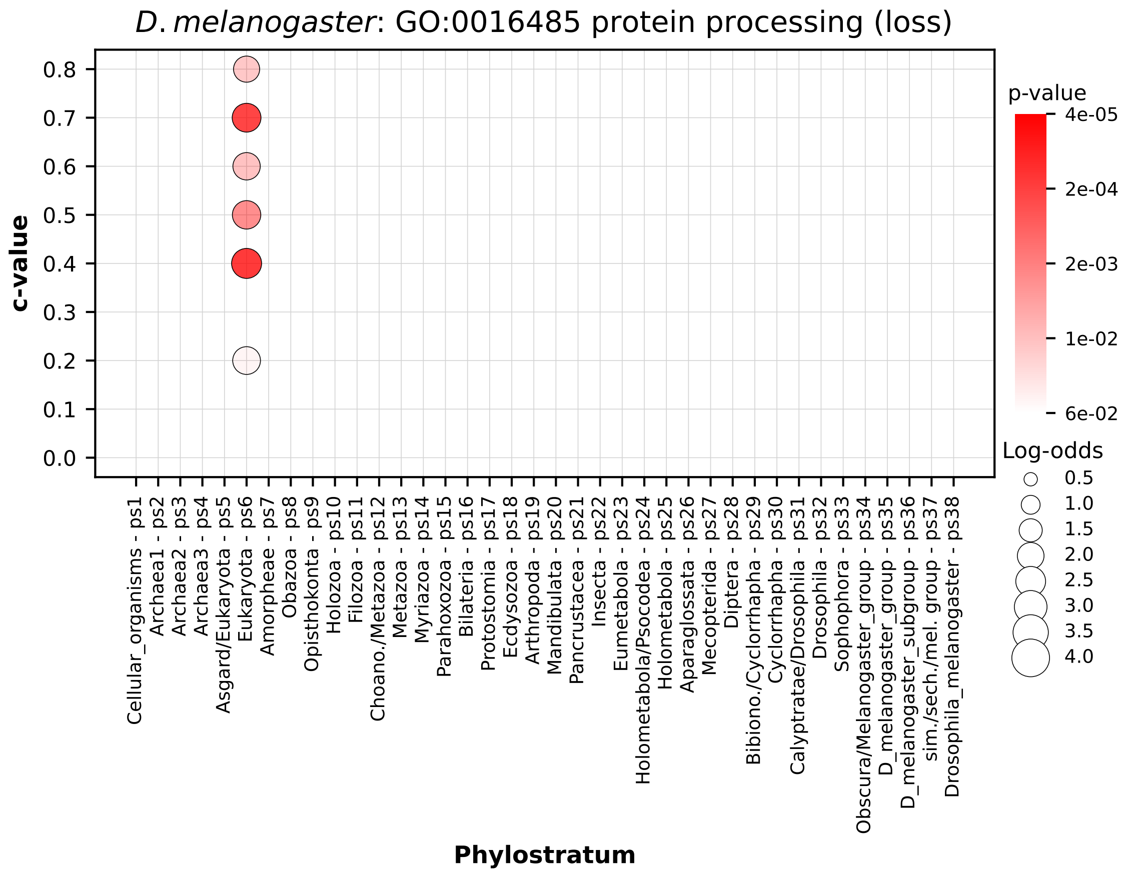

D. melanogaster: GO:0016485 protein processing (loss)
c-value
0.8
0.7
0.6
0.5
0.4
0.3
0.2
0.1
0.0
Phylostratum
Cellular_organisms - ps1
Archaea1 - ps2
Archaea2 - ps3
Archaea3 - ps4
Asgard/Eukaryota - ps5
Eukaryota - ps6
Amorpheae - ps7
Obazoa - ps8
Opisthokonta - ps9
Holozoa - ps10
Filozoa - ps11
Choano./Metazoa - ps12
Metazoa - ps13
Myriazoa - ps14
Parahoxozoa - ps15
Bilateria - ps16
Protostomia - ps17
Ecdysozoa - ps18
Arthropoda - ps19
Mandibulata - ps20
Pancrustacea - ps21
Insecta - ps22
Eumetabola - ps23
Holometabola/Psocodea - ps24
Holometabola - ps25
Aparaglossata - ps26
Mecopterida - ps27
Diptera - ps28
Bibiono./Cyclorrhapha - ps29
Cyclorrhapha - ps30
Calyptratae/Drosophila - ps31
Drosophila - ps32
Sophophora - ps33
Obscura/Melanogaster_group - ps34
D_melanogaster_group - ps35
D_melanogaster_subgroup - ps36
sim./sech./mel. group - ps37
Drosophila_melanogaster - ps38
p-value
4e-05
2e-04
2e-03
1e-02
6e-02
Log-odds
0.5
1.0
1.5
2.0
2.5
3.0
3.5
4.0

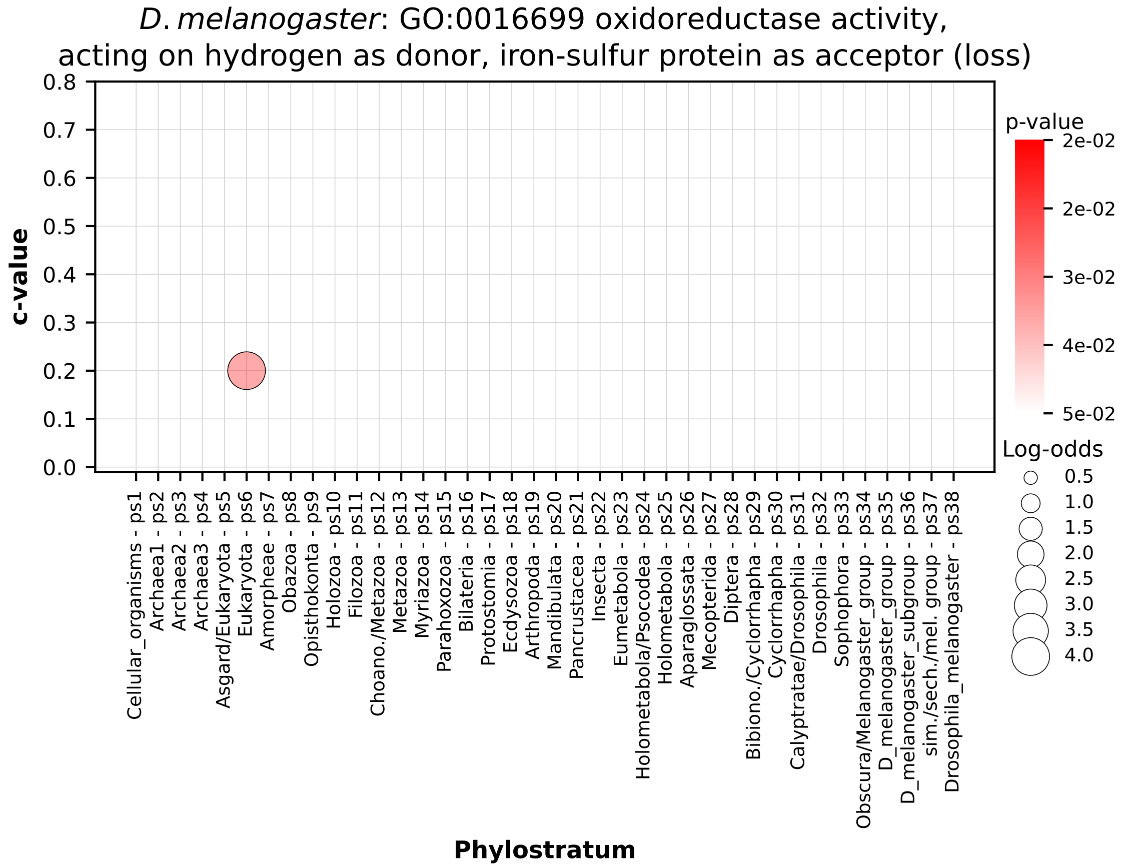

D. melanogaster: GO:0016699 oxidoreductase activity,
acting on hydrogen as donor, iron-sulfur protein as acceptor (loss)
c-value
0.8
0.7
0.6
0.5
0.4
0.3
0.2
0.1
0.0
p-value
2e-02
2e-02
3e-02
4e-02
5e-02
Log-odds
0.5
1.0
1.5
2.0
2.5
3.0
3.5
4.0
Cellular_organisms - ps1
Archaea1 - ps2
Archaea2 - ps3
Archaea3 - ps4
Asgard/Eukaryota - ps5
Eukaryota - ps6
Amorpheae - ps7
Obazoa - ps8
Opisthokonta - ps9
Holozoa - ps10
Filozoa - ps11
Choano./Metazoa - ps12
Metazoa - ps13
Myriazoa - ps14
Parahoxozoa - ps15
Bilateria - ps16
Protostomia - ps17
Ecdysozoa - ps18
Arthropoda - ps19
Mandibulata - ps20
Pancrustacea - ps21
Insecta - ps22
Eumetabola - ps23
Holometabola/Psocodea - ps24
Holometabola - ps25
Aparaglossata - ps26
Mecopterida - ps27
Diptera - ps28
Bibiono./Cyclorrhapha - ps29
Cyclorrhapha - ps30
Calyptratae/Drosophila - ps31
Drosophila - ps32
Sophophora - ps33
Obscura/Melanogaster_group - ps34
D_melanogaster_group - ps35
D_melanogaster_subgroup - ps36
sim./sech./mel. group - ps37
Drosophila_melanogaster - ps38
Phylostratum

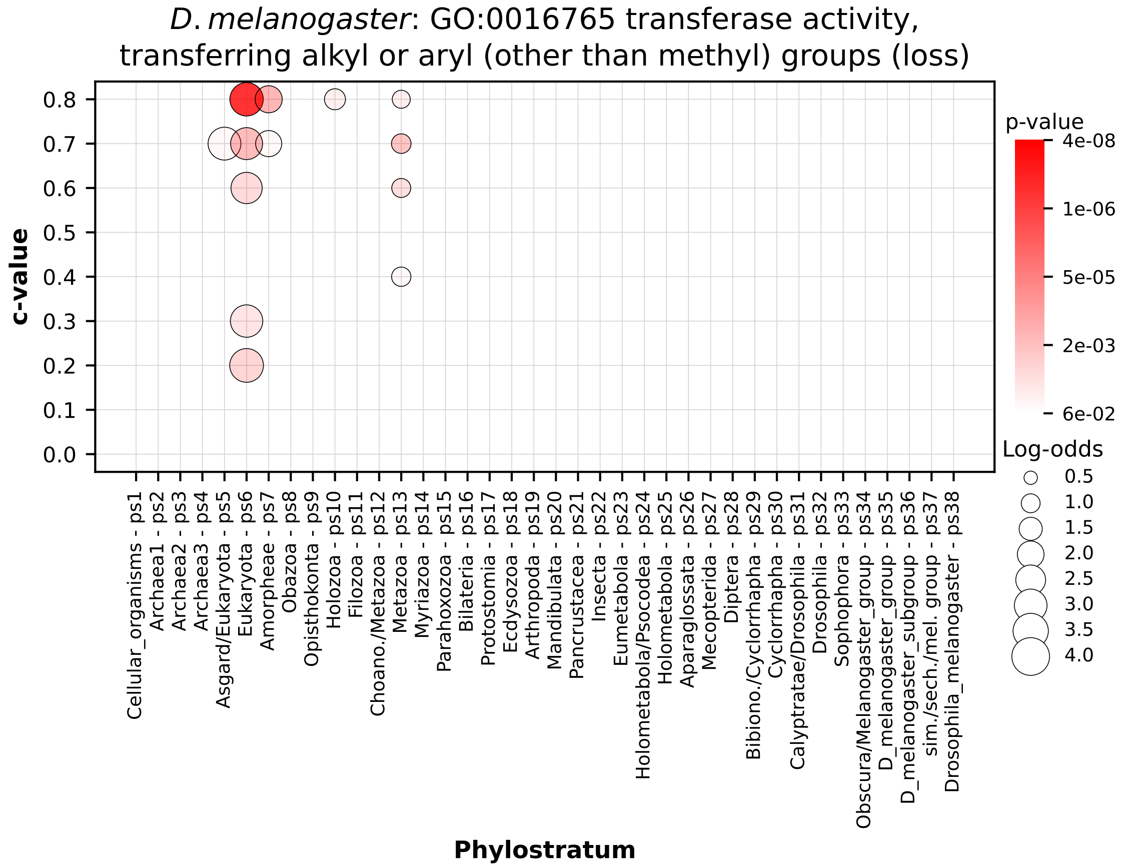

D. melanogaster: GO:0016765 transferase activity, transferring alkyl or aryl (other than methyl) groups (loss)
c-value
Phylostratum
p-value
4e-08
1e-06
5e-05
2e-03
6e-02
Log-odds
0.5
1.0
1.5
2.0
2.5
3.0
3.5
4.0
Cellular_organisms - ps1
Archaea1 - ps2
Archaea2 - ps3
Archaea3 - ps4
Asgard/Eukaryota - ps5
Eukaryota - ps6
Amorpheae - ps7
Obazoa - ps8
Opisthokonta - ps9
Holozoa - ps10
Filozoa - ps11
Choano./Metazoa - ps12
Metazoa - ps13
Myriazoa - ps14
Parahoxozoa - ps15
Bilateria - ps16
Protostomia - ps17
Ecdysozoa - ps18
Arthropoda - ps19
Mandibulata - ps20
Pancrustacea - ps21
Insecta - ps22
Eumetabola - ps23
Holometabola/Psocodea - ps24
Holometabola - ps25
Aparaglossata - ps26
Mecopterida - ps27
Diptera - ps28
Bibiono./Cyclorrhapha - ps29
Cyclorrhapha - ps30
Calyptratae/Drosophila - ps31
Drosophila - ps32
Sophophora - ps33
Obscura/Melanogaster_group - ps34
D_melanogaster_group - ps35
D_melanogaster_subgroup - ps36
sim./sech./mel. group - ps37
Drosophila_melanogaster - ps38

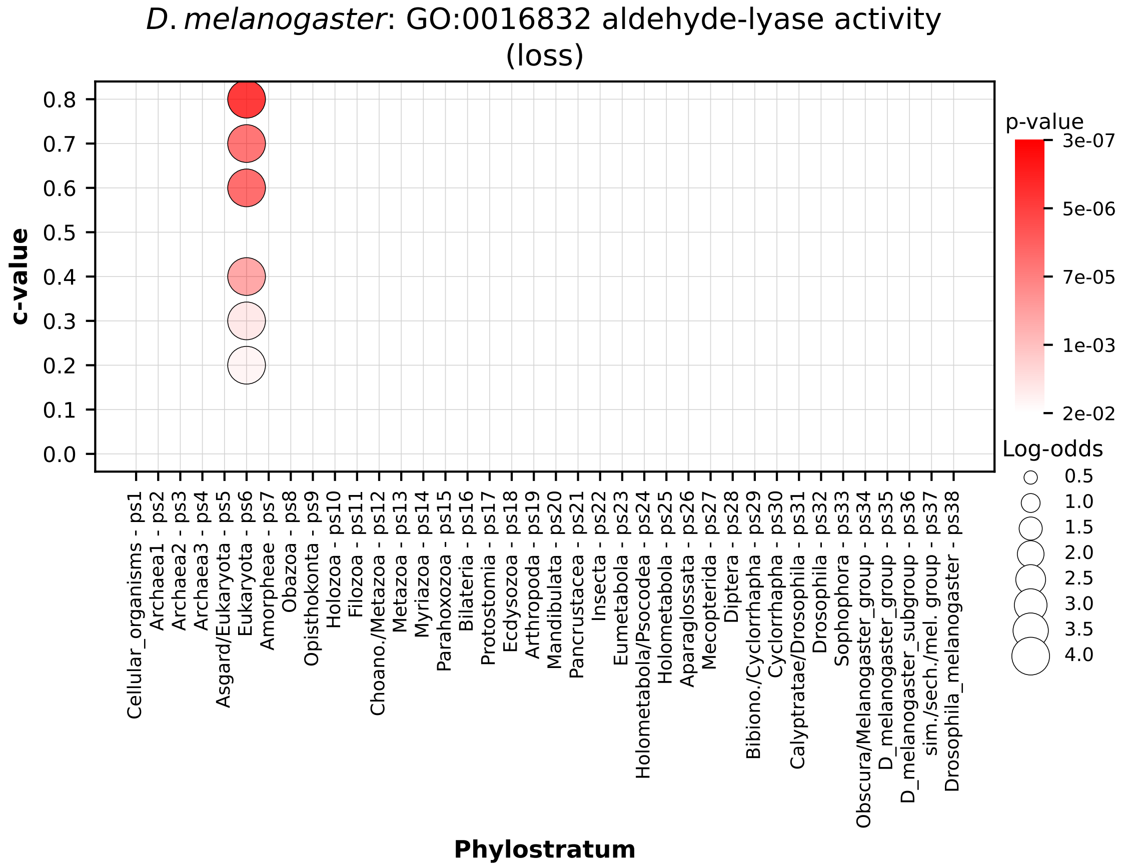
D. melanogaster: GO:0016832 aldehyde-lyase activity
(loss)
c-value
0.8
0.7
0.6
0.5
0.4
0.3
0.2
0.1
0.0
p-value
3e-07
5e-06
7e-05
1e-03
2e-02
Log-odds
0.5
1.0
1.5
2.0
2.5
3.0
3.5
4.0
Cellular_organisms - ps1
Archaea1 - ps2
Archaea2 - ps3
Archaea3 - ps4
Asgard/Eukaryota - ps5
Eukaryota - ps6
Amorpheae - ps7
Obazoa - ps8
Opisthokonta - ps9
Holozoa - ps10
Filozoa - ps11
Choano./Metazoa - ps12
Metazoa - ps13
Myriazoa - ps14
Parahoxozoa - ps15
Bilateria - ps16
Protostomia - ps17
Ecdysozoa - ps18
Arthropoda - ps19
Mandibulata - ps20
Pancrustacea - ps21
Insecta - ps22
Eumetabola - ps23
Holometabola/Psocodea - ps24
Holometabola - ps25
Aparaglossata - ps26
Mecopterida - ps27
Diptera - ps28
Bibiono./Cyclorrhapha - ps29
Cyclorrhapha - ps30
Calyptratae/Drosophila - ps31
Drosophila - ps32
Sophophora - ps33
Obscura/Melanogaster_group - ps34
D_melanogaster_group - ps35
D_melanogaster_subgroup - ps36
sim./sech./mel. group - ps37
Drosophila_melanogaster - ps38
Phylostratum

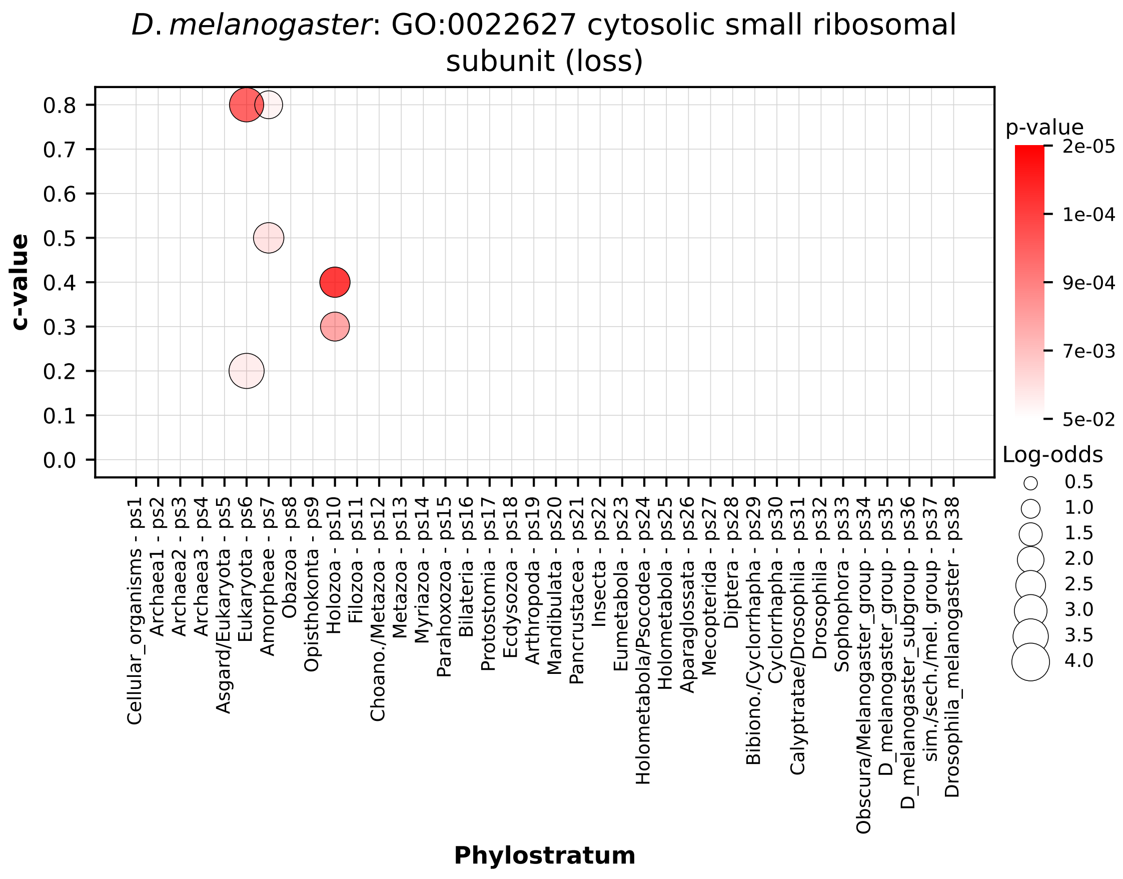

D. melanogaster: GO:0022627 cytosolic small ribosomal subunit (loss)
c-value
0.0
0.1
0.2
0.3
0.4
0.5
0.6
0.7
0.8
Phylostratum
Cellular_organisms - ps1
Archaea1 - ps2
Archaea2 - ps3
Archaea3 - ps4
Asgard/Eukaryota - ps5
Eukaryota - ps6
Amorpheae - ps7
Obazoa - ps8
Opisthokonta - ps9
Holozoa - ps10
Filozoa - ps11
Choano./Metazoa - ps12
Metazoa - ps13
Myriazoa - ps14
Parahoxozoa - ps15
Bilateria - ps16
Protostomia - ps17
Ecdysozoa - ps18
Arthropoda - ps19
Mandibulata - ps20
Pancrustacea - ps21
Insecta - ps22
Eumetabola - ps23
Holometabola/Psocodea - ps24
Holometabola - ps25
Aparaglossata - ps26
Mecopterida - ps27
Diptera - ps28
Bibiono./Cyclorrhapha - ps29
Cyclorrhapha - ps30
Calyptratae/Drosophila - ps31
Drosophila - ps32
Sophophora - ps33
Obscura/Melanogaster_group - ps34
D_melanogaster_group - ps35
D_melanogaster_subgroup - ps36
sim./sech./mel. group - ps37
Drosophila_melanogaster - ps38
p-value
2e-05
1e-04
9e-04
7e-03
5e-02
Log-odds
0.5
1.0
1.5
2.0
2.5
3.0
3.5
4.0

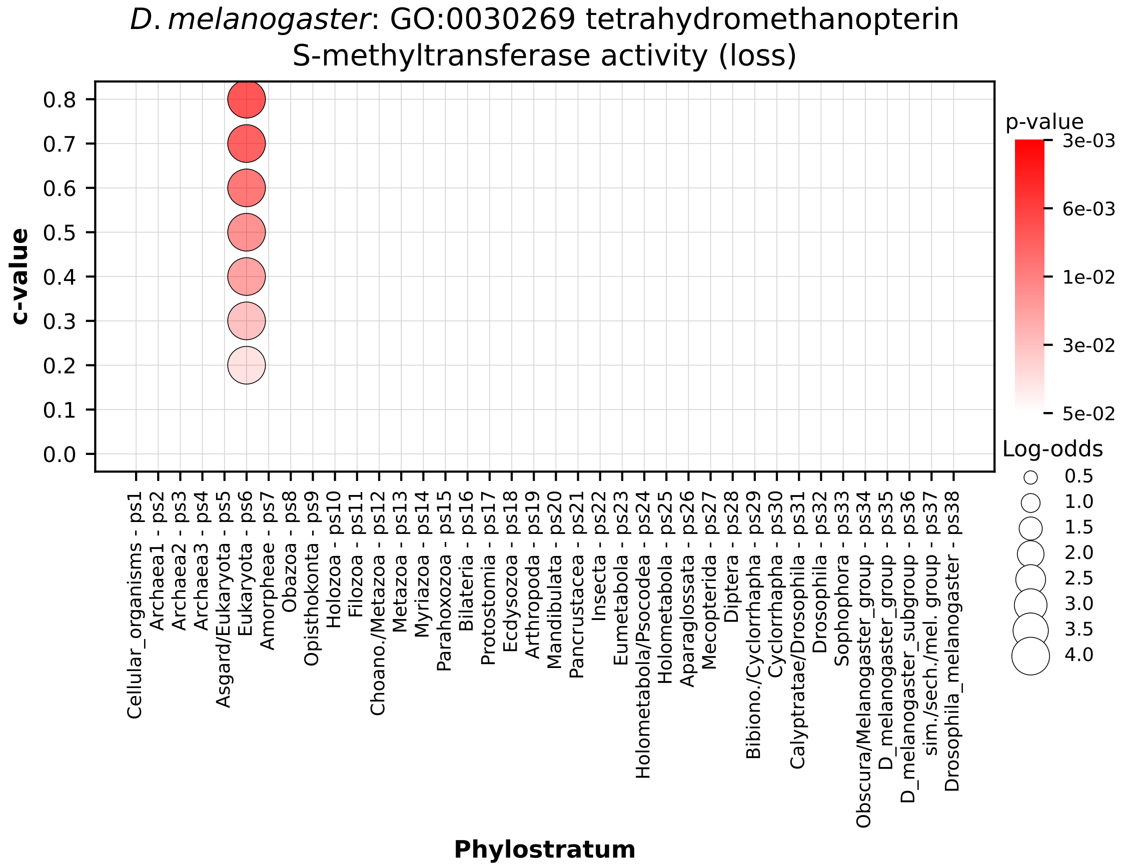

D. melanogaster: GO:0030269 tetrahydromethanopterin S-methyltransferase activity (loss)
c-value
Phylostratum
p-value
3e-03
6e-03
1e-02
3e-02
5e-02
Log-odds
0.5
1.0
1.5
2.0
2.5
3.0
3.5
4.0
0.0
0.1
0.2
0.3
0.4
0.5
0.6
0.7
0.8
Cellular_organisms - ps1
Archaea1 - ps2
Archaea2 - ps3
Archaea3 - ps4
Asgard/Eukaryota - ps5
Eukaryota - ps6
Amorpheae - ps7
Obazoa - ps8
Opisthokonta - ps9
Holozoa - ps10
Filozoa - ps11
Choano./Metazoa - ps12
Metazoa - ps13
Myriazoa - ps14
Parahoxozoa - ps15
Bilateria - ps16
Protostomia - ps17
Ecdysozoa - ps18
Arthropoda - ps19
Mandibulata - ps20
Pancrustacea - ps21
Insecta - ps22
Eumetabola - ps23
Holometabola/Psocodea - ps24
Holometabola - ps25
Aparaglossata - ps26
Mecopterida - ps27
Diptera - ps28
Bibiono./Cyclorrhapha - ps29
Cyclorrhapha - ps30
Calyptratae/Drosophila - ps31
Drosophila - ps32
Sophophora - ps33
Obscura/Melanogaster_group - ps34
D_melanogaster_group - ps35
D_melanogaster_subgroup - ps36
sim./sech./mel. group - ps37
Drosophila_melanogaster - ps38

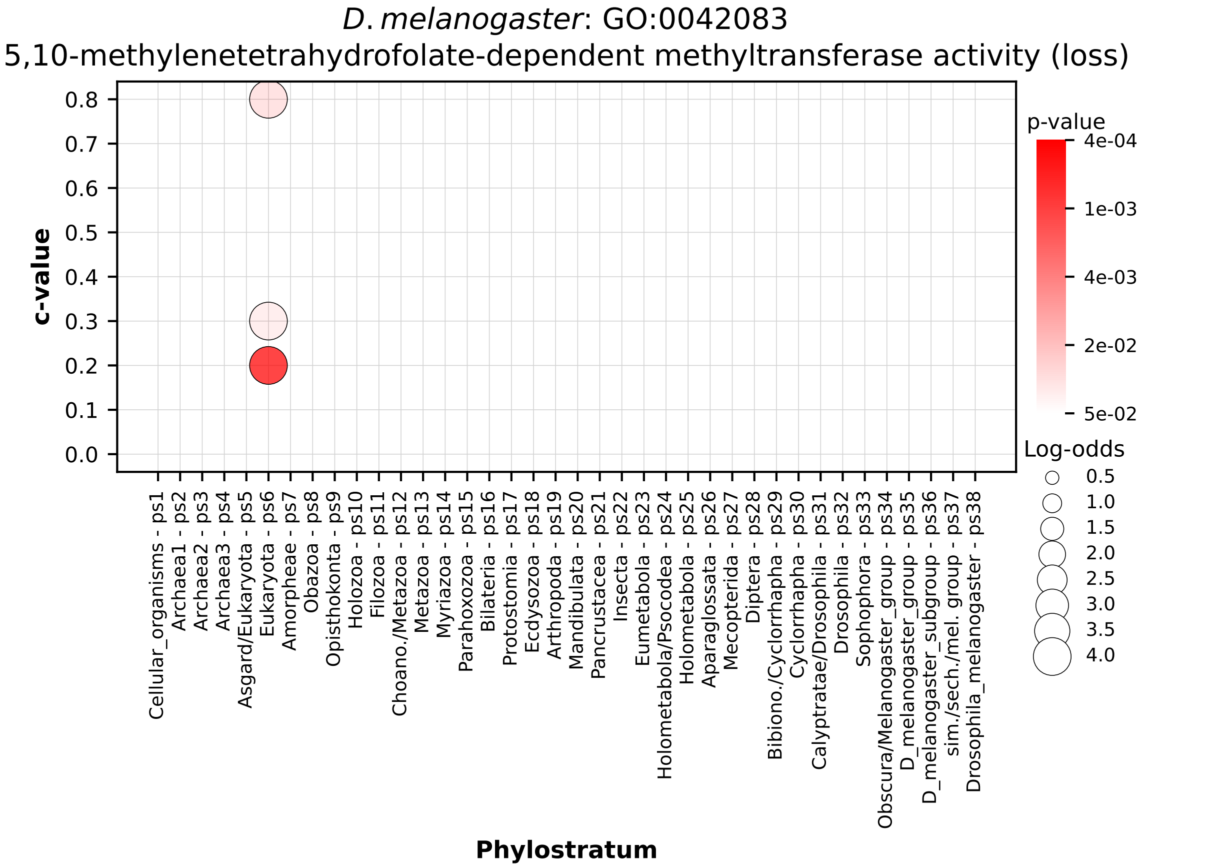

D. melanogaster: GO:0042083
5,10-methylenetetrahydrofolate-dependent methyltransferase activity (loss)
c-value
0.8
0.7
0.6
0.5
0.4
0.3
0.2
0.1
0.0
p-value
4e-04
1e-03
4e-03
2e-02
5e-02
Log-odds
0.5
1.0
1.5
2.0
2.5
3.0
3.5
4.0
Cellular_organisms - ps1
Archaea1 - ps2
Archaea2 - ps3
Archaea3 - ps4
Asgard/Eukaryota - ps5
Eukaryota - ps6
Amorpheae - ps7
Obazoa - ps8
Opisthokonta - ps9
Holozoa - ps10
Filozoa - ps11
Choano./Metazoa - ps12
Metazoa - ps13
Myriazoa - ps14
Parahoxozoa - ps15
Bilateria - ps16
Protostomia - ps17
Ecdysozoa - ps18
Arthropoda - ps19
Mandibulata - ps20
Pancrustacea - ps21
Insecta - ps22
Eumetabola - ps23
Holometabola/Psocodea - ps24
Holometabola - ps25
Aparaglossata - ps26
Mecopterida - ps27
Diptera - ps28
Bibiono./Cyclorrhapha - ps29
Cyclorrhapha - ps30
Calyptratae/Drosophila - ps31
Drosophila - ps32
Sophophora - ps33
Obscura/Melanogaster_group - ps34
D_melanogaster_group - ps35
D_melanogaster_subgroup - ps36
sim./sech./mel. group - ps37
Drosophila_melanogaster - ps38
Phylostratum

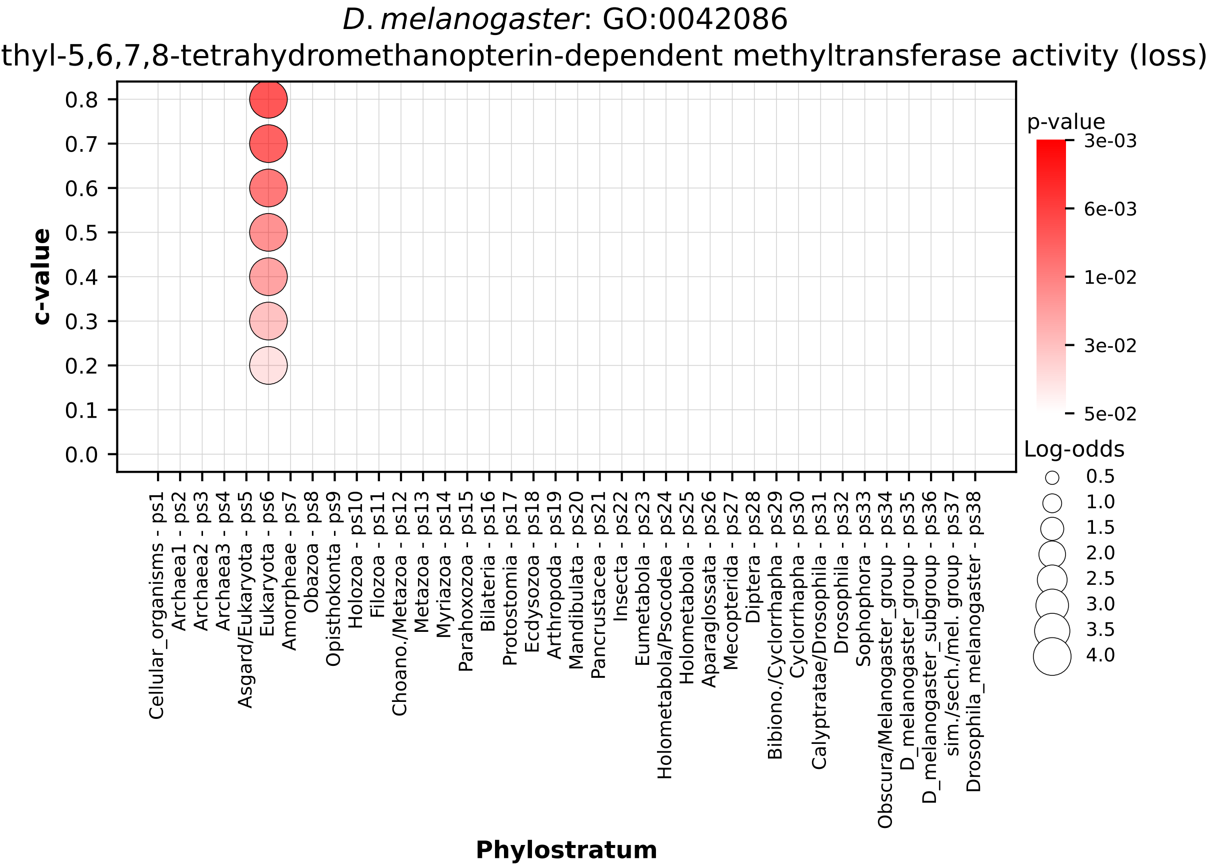

D. melanogaster: GO:0042086
thyl-5,6,7,8-tetrahydromethanopterin-dependent methyltransferase activity (loss)
c-value
0.8
0.7
0.6
0.5
0.4
0.3
0.2
0.1
0.0
p-value
3e-03
6e-03
1e-02
3e-02
5e-02
Log-odds
0.5
1.0
1.5
2.0
2.5
3.0
3.5
4.0
Cellular_organisms - ps1
Archaea1 - ps2
Archaea2 - ps3
Archaea3 - ps4
Asgard/Eukaryota - ps5
Eukaryota - ps6
Amorpheae - ps7
Obazoa - ps8
Opisthokonta - ps9
Holozoa - ps10
Filozoa - ps11
Choano./Metazoa - ps12
Metazoa - ps13
Myriazoa - ps14
Parahoxozoa - ps15
Bilateria - ps16
Protostomia - ps17
Ecdysozoa - ps18
Arthropoda - ps19
Mandibulata - ps20
Pancrustacea - ps21
Insecta - ps22
Eumetabola - ps23
Holometabola/Psocodea - ps24
Holometabola - ps25
Aparaglossata - ps26
Mecopterida - ps27
Diptera - ps28
Bibiono./Cyclorrhapha - ps29
Cyclorrhapha - ps30
Calyptratae/Drosophila - ps31
Drosophila - ps32
Sophophora - ps33
Obscura/Melanogaster_group - ps34
D_melanogaster_group - ps35
D_melanogaster_subgroup - ps36
sim./sech./mel. group - ps37
Drosophila_melanogaster - ps38
Phylostratum

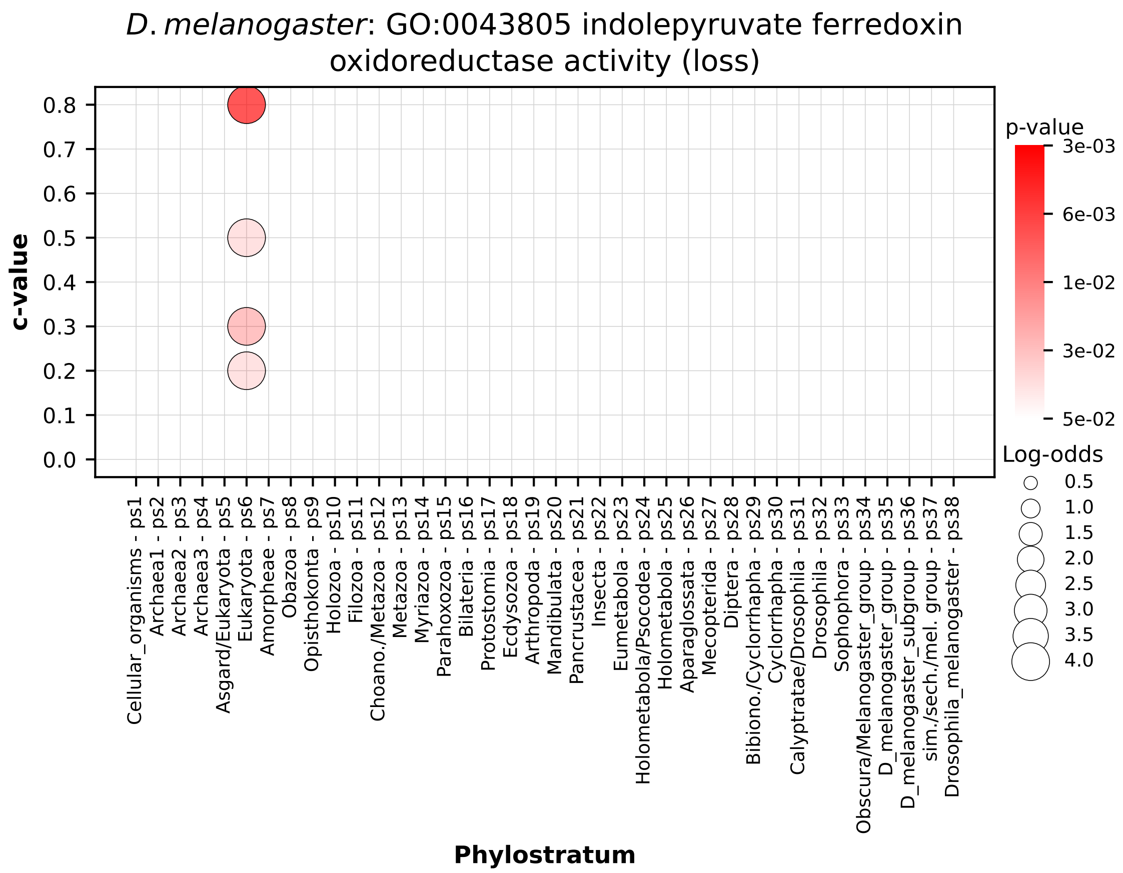

D. melanogaster: GO:0043805 indolepyruvate ferredoxin oxidoreductase activity (loss)
c-value
0.8
0.7
0.6
0.5
0.4
0.3
0.2
0.1
0.0
p-value
3e-03
6e-03
1e-02
3e-02
5e-02
Log-odds
0.5
1.0
1.5
2.0
2.5
3.0
3.5
4.0
Cellular_organisms - ps1
Archaea1 - ps2
Archaea2 - ps3
Archaea3 - ps4
Asgard/Eukaryota - ps5
Eukaryota - ps6
Amorpheae - ps7
Obazoa - ps8
Opisthokonta - ps9
Holozoa - ps10
Filozoa - ps11
Choano./Metazoa - ps12
Metazoa - ps13
Myriazoa - ps14
Parahoxozoa - ps15
Bilateria - ps16
Protostomia - ps17
Ecdysozoa - ps18
Arthropoda - ps19
Mandibulata - ps20
Pancrustacea - ps21
Insecta - ps22
Eumetabola - ps23
Holometabola/Psocodea - ps24
Holometabola - ps25
Aparaglossata - ps26
Mecopterida - ps27
Diptera - ps28
Bibiono./Cyclorrhapha - ps29
Cyclorrhapha - ps30
Calyptratae/Drosophila - ps31
Drosophila - ps32
Sophophora - ps33
Obscura/Melanogaster_group - ps34
D_melanogaster_group - ps35
D_melanogaster_subgroup - ps36
sim./sech./mel. group - ps37
Drosophila_melanogaster - ps38
Phylostratum

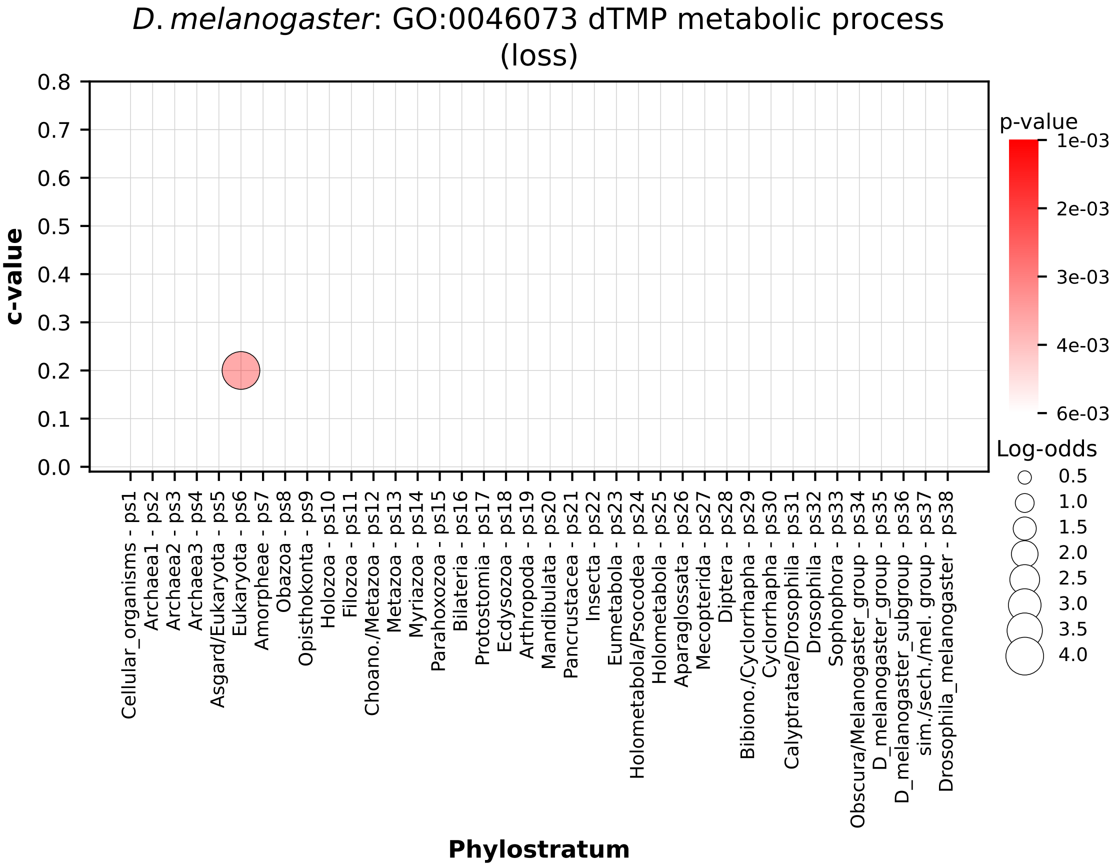

D. melanogaster: GO:0046073 dTMP metabolic process (loss)
c-value
0.0
0.1
0.2
0.3
0.4
0.5
0.6
0.7
0.8
Phylostratum
Cellular_organisms - ps1
Archaea1 - ps2
Archaea2 - ps3
Archaea3 - ps4
Asgard/Eukaryota - ps5
Eukaryota - ps6
Amorpheae - ps7
Obazoa - ps8
Opisthokonta - ps9
Holozoa - ps10
Filozoa - ps11
Choano./Metazoa - ps12
Metazoa - ps13
Myriazoa - ps14
Parahoxozoa - ps15
Bilateria - ps16
Protostomia - ps17
Ecdysozoa - ps18
Arthropoda - ps19
Mandibulata - ps20
Pancrustacea - ps21
Insecta - ps22
Eumetabola - ps23
Holometabola/Psocodea - ps24
Holometabola - ps25
Aparaglossata - ps26
Mecopterida - ps27
Diptera - ps28
Bibiono./Cyclorrhapha - ps29
Cyclorrhapha - ps30
Calyptratae/Drosophila - ps31
Drosophila - ps32
Sophophora - ps33
Obscura/Melanogaster_group - ps34
D_melanogaster_group - ps35
D_melanogaster_subgroup - ps36
sim./sech./mel. group - ps37
Drosophila_melanogaster - ps38
p-value
1e-03
2e-03
3e-03
4e-03
6e-03
Log-odds
0.5
1.0
1.5
2.0
2.5
3.0
3.5
4.0

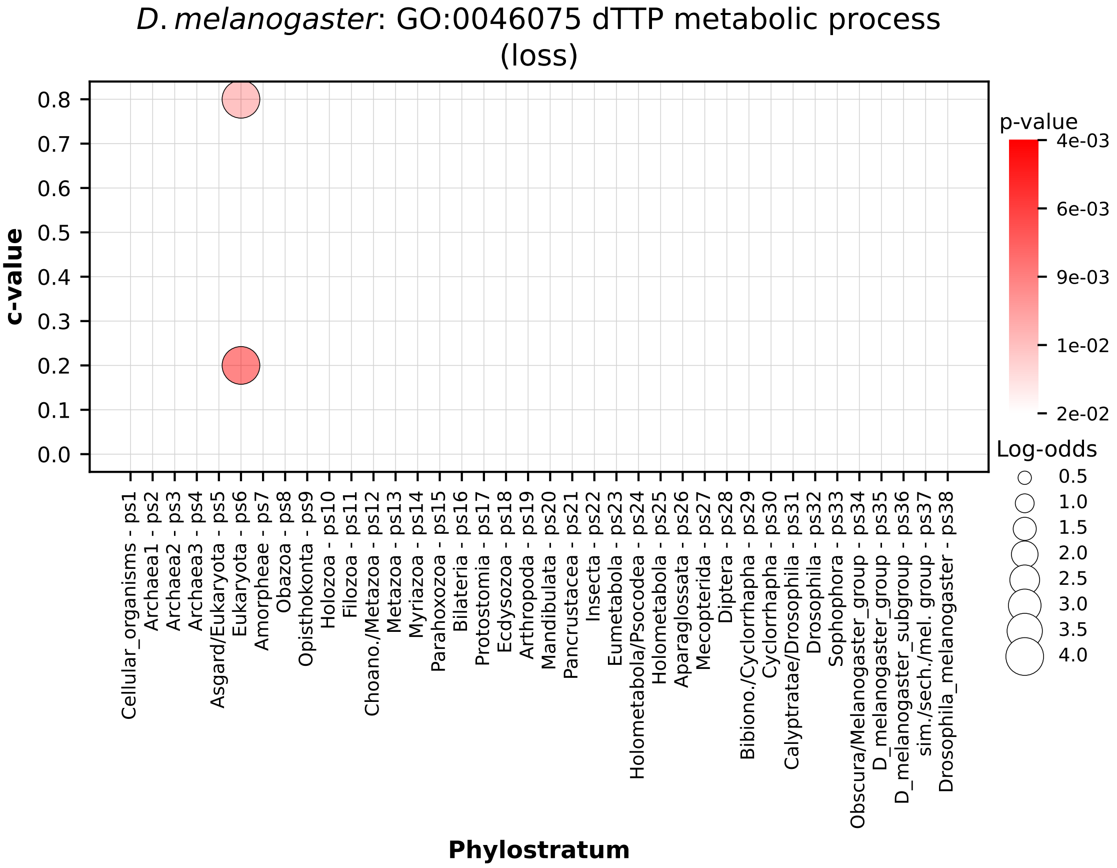

D. melanogaster: GO:0046075 dTTP metabolic process
(loss)
c-value
0.0
0.1
0.2
0.3
0.4
0.5
0.6
0.7
0.8
p-value
4e-03
6e-03
9e-03
1e-02
2e-02
Log-odds
0.5
1.0
1.5
2.0
2.5
3.0
3.5
4.0
Cellular_organisms - ps1
Archaea1 - ps2
Archaea2 - ps3
Archaea3 - ps4
Asgard/Eukaryota - ps5
Eukaryota - ps6
Amorpheae - ps7
Obazoa - ps8
Opisthokonta - ps9
Holozoa - ps10
Filozoa - ps11
Choano./Metazoa - ps12
Metazoa - ps13
Myriazoa - ps14
Parahoxozoa - ps15
Bilateria - ps16
Protostomia - ps17
Ecdysozoa - ps18
Arthropoda - ps19
Mandibulata - ps20
Pancrustacea - ps21
Insecta - ps22
Eumetabola - ps23
Holometabola/Psocodea - ps24
Holometabola - ps25
Aparaglossata - ps26
Mecopterida - ps27
Diptera - ps28
Bibiono./Cyclorrhapha - ps29
Cyclorrhapha - ps30
Calyptratae/Drosophila - ps31
Drosophila - ps32
Sophophora - ps33
Obscura/Melanogaster_group - ps34
D_melanogaster_group - ps35
D_melanogaster_subgroup - ps36
sim./sech./mel. group - ps37
Drosophila_melanogaster - ps38
Phylostratum

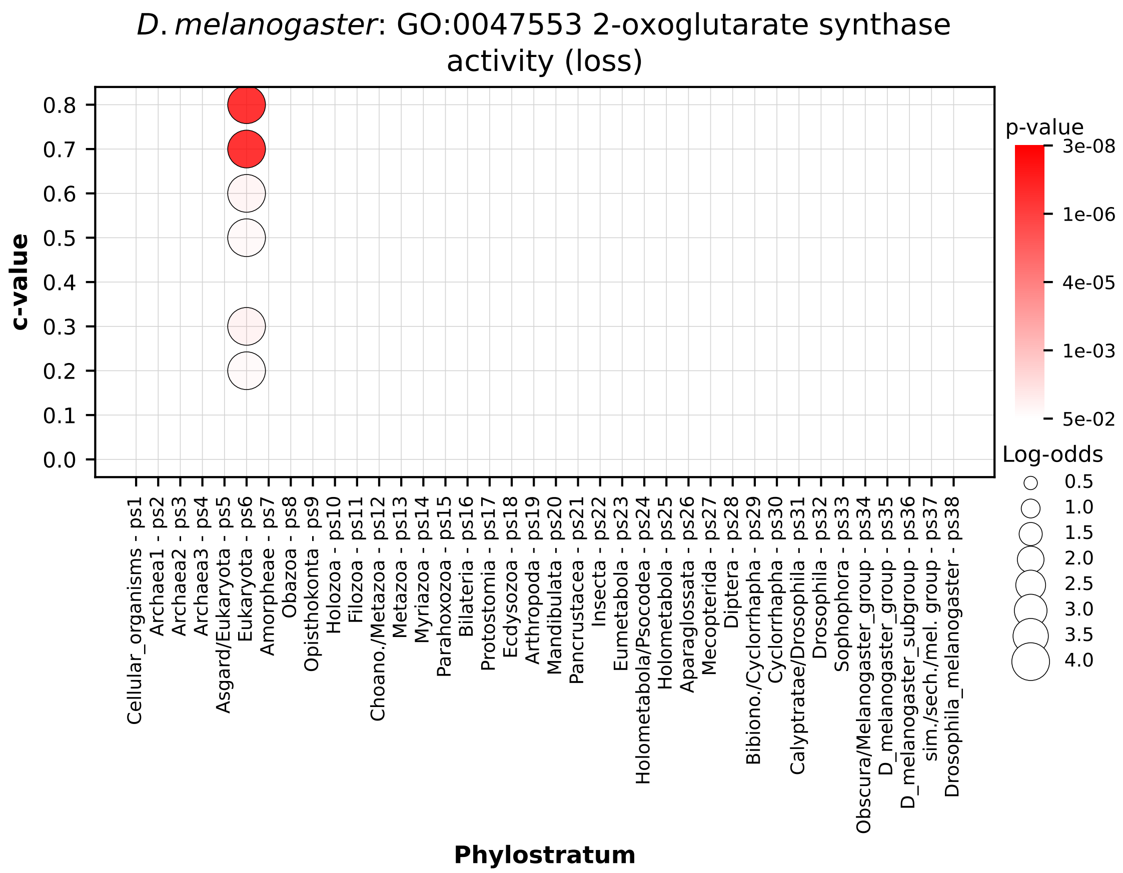
D. melanogaster: GO:0047553 2-oxoglutarate synthase activity (loss)
c-value
0.8
0.7
0.6
0.5
0.4
0.3
0.2
0.1
0.0
Phylostratum
Cellular_organisms - ps1
Archaea1 - ps2
Archaea2 - ps3
Archaea3 - ps4
Asgard/Eukaryota - ps5
Eukaryota - ps6
Amorpheae - ps7
Obazoa - ps8
Opisthokonta - ps9
Holozoa - ps10
Filozoa - ps11
Choano./Metazoa - ps12
Metazoa - ps13
Myriazoa - ps14
Parahoxozoa - ps15
Bilateria - ps16
Protostomia - ps17
Ecdysozoa - ps18
Arthropoda - ps19
Mandibulata - ps20
Pancrustacea - ps21
Insecta - ps22
Eumetabola - ps23
Holometabola/Psocodea - ps24
Holometabola - ps25
Aparaglossata - ps26
Mecopterida - ps27
Diptera - ps28
Bibiono./Cyclorrhapha - ps29
Cyclorrhapha - ps30
Calyptratae/Drosophila - ps31
Drosophila - ps32
Sophophora - ps33
Obscura/Melanogaster_group - ps34
D_melanogaster_group - ps35
D_melanogaster_subgroup - ps36
sim./sech./mel. group - ps37
Drosophila_melanogaster - ps38
p-value
3e-08
1e-06
4e-05
1e-03
5e-02
Log-odds
0.5
1.0
1.5
2.0
2.5
3.0
3.5
4.0

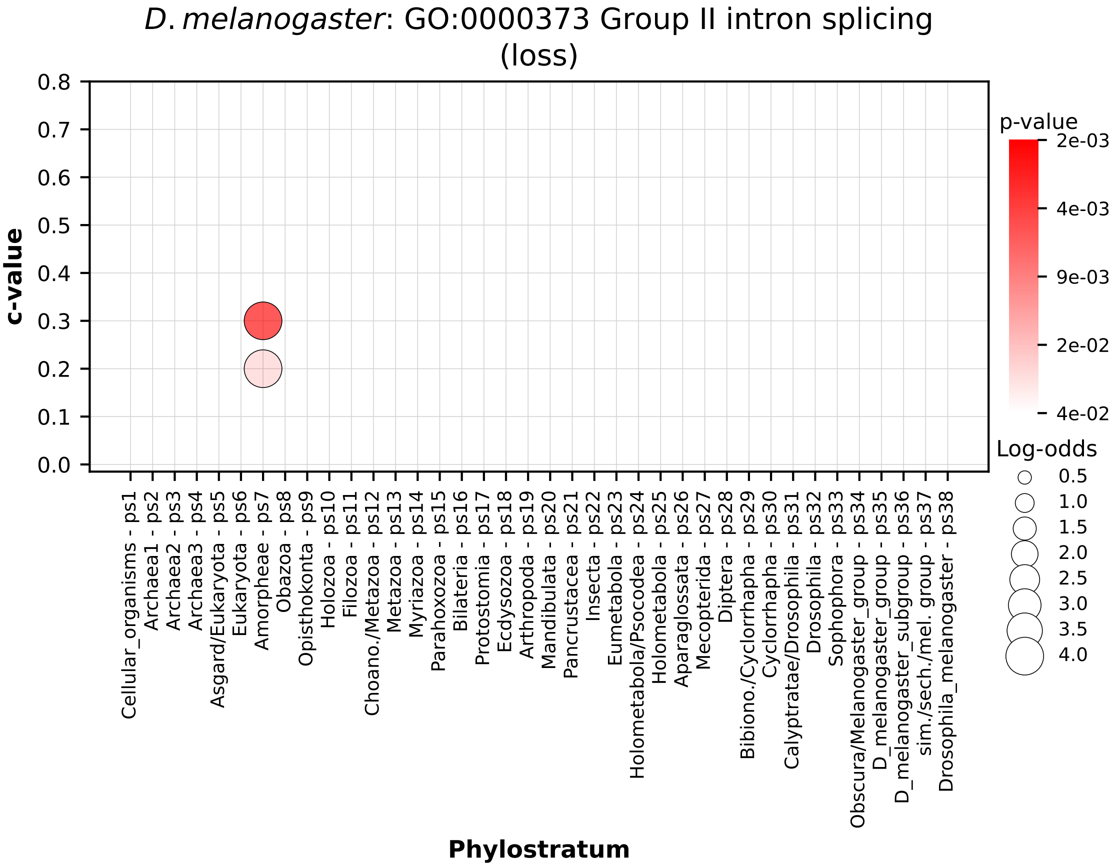

D. melanogaster: GO:0000373 Group II intron splicing
(loss)
c-value
0.8
0.7
0.6
0.5
0.4
0.3
0.2
0.1
0.0
p-value
2e-03
4e-03
9e-03
2e-02
4e-02
Log-odds
0.5
1.0
1.5
2.0
2.5
3.0
3.5
4.0
Cellular_organisms - ps1
Archaea1 - ps2
Archaea2 - ps3
Archaea3 - ps4
Asgard/Eukaryota - ps5
Eukaryota - ps6
Amorpheae - ps7
Obazoa - ps8
Opisthokonta - ps9
Holozoa - ps10
Filozoa - ps11
Choano./Metazoa - ps12
Metazoa - ps13
Myriazoa - ps14
Parahoxozoa - ps15
Bilateria - ps16
Protostomia - ps17
Ecdysozoa - ps18
Arthropoda - ps19
Mandibulata - ps20
Pancrustacea - ps21
Insecta - ps22
Eumetabola - ps23
Holometabola/Psocodea - ps24
Holometabola - ps25
Aparaglossata - ps26
Mecopterida - ps27
Diptera - ps28
Bibiono./Cyclorrhapha - ps29
Cyclorrhapha - ps30
Calyptratae/Drosophila - ps31
Drosophila - ps32
Sophophora - ps33
Obscura/Melanogaster_group - ps34
D_melanogaster_group - ps35
D_melanogaster_subgroup - ps36
sim./sech./mel. group - ps37
Drosophila_melanogaster - ps38
Phylostratum

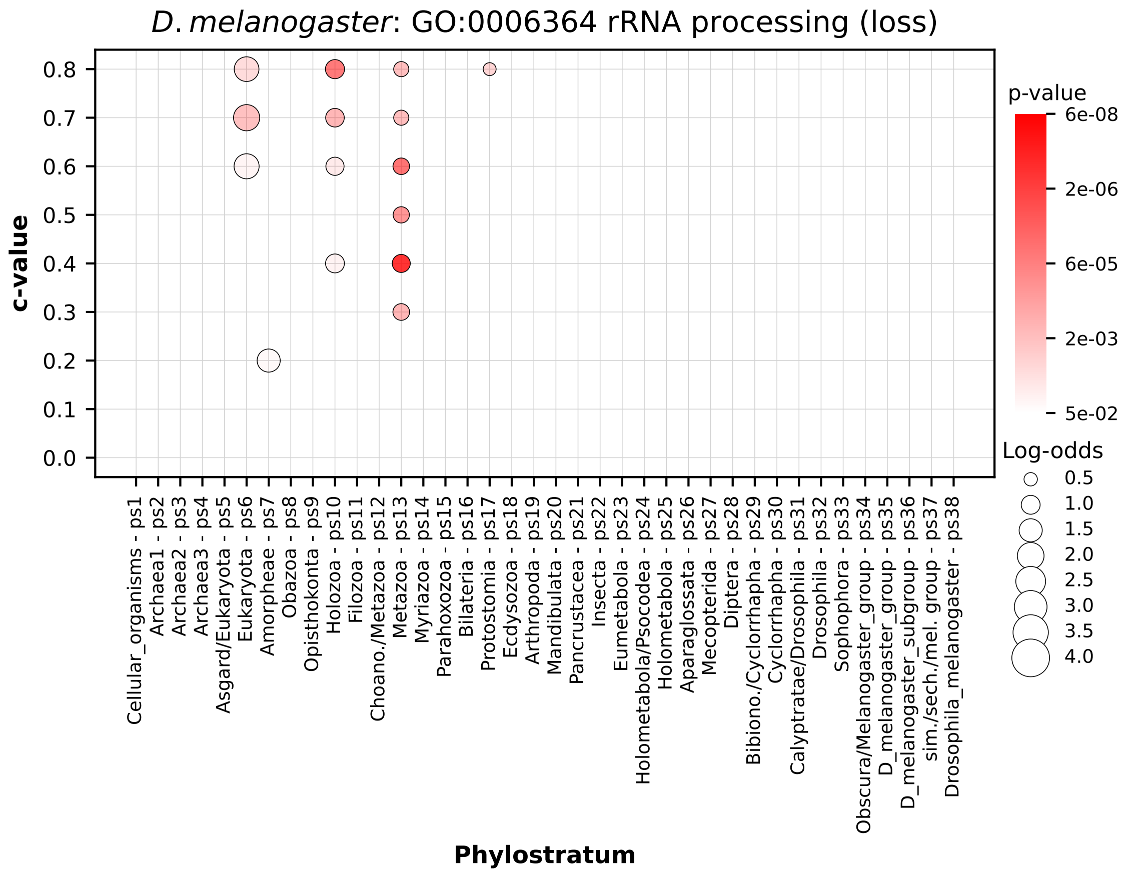
D. melanogaster: GO:0006364 rRNA processing (loss)
c-value
0.0
0.1
0.2
0.3
0.4
0.5
0.6
0.7
0.8
Phylostratum
Cellular_organisms - ps1
Archaea1 - ps2
Archaea2 - ps3
Archaea3 - ps4
Asgard/Eukaryota - ps5
Eukaryota - ps6
Amorpheae - ps7
Obazoa - ps8
Opisthokonta - ps9
Holozoa - ps10
Filozoa - ps11
Choano./Metazoa - ps12
Metazoa - ps13
Myriazoa - ps14
Parahoxozoa - ps15
Bilateria - ps16
Protostomia - ps17
Ecdysozoa - ps18
Arthropoda - ps19
Mandibulata - ps20
Pancrustacea - ps21
Insecta - ps22
Eumetabola - ps23
Holometabola/Psocodea - ps24
Holometabola - ps25
Aparaglossata - ps26
Mecopterida - ps27
Diptera - ps28
Bibiono./Cyclorrhapha - ps29
Cyclorrhapha - ps30
Calyptratae/Drosophila - ps31
Drosophila - ps32
Sophophora - ps33
Obscura/Melanogaster_group - ps34
D_melanogaster_group - ps35
D_melanogaster_subgroup - ps36
sim./sech./mel. group - ps37
Drosophila_melanogaster - ps38
p-value
6e-08
2e-06
6e-05
2e-03
5e-02
Log-odds
0.5
1.0
1.5
2.0
2.5
3.0
3.5
4.0

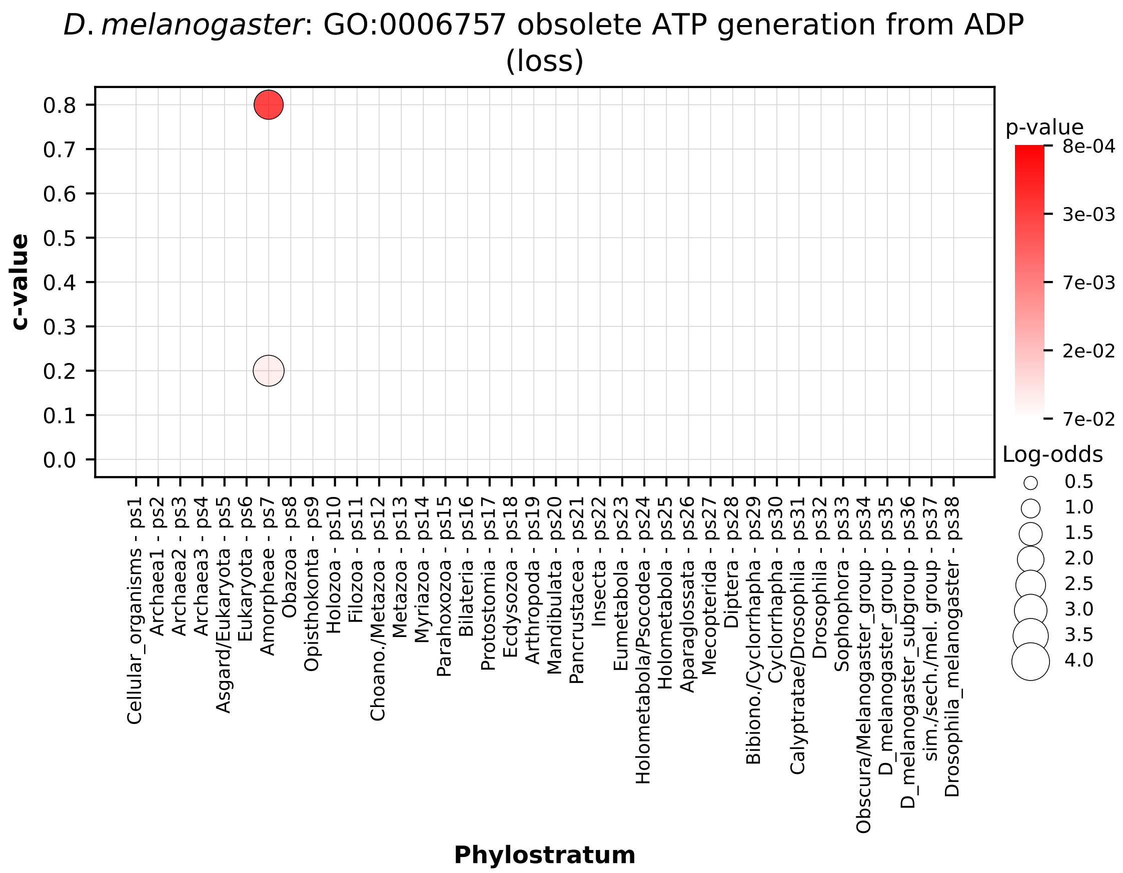

D. melanogaster: GO:0006757 obsolete ATP generation from ADP (loss)
c-value
0.8
0.7
0.6
0.5
0.4
0.3
0.2
0.1
0.0
p-value
8e-04
3e-03
7e-03
2e-02
7e-02
Log-odds
0.5
1.0
1.5
2.0
2.5
3.0
3.5
4.0
Cellular_organisms - ps1
Archaea1 - ps2
Archaea2 - ps3
Archaea3 - ps4
Asgard/Eukaryota - ps5
Eukaryota - ps6
Amorpheae - ps7
Obazoa - ps8
Opisthokonta - ps9
Holozoa - ps10
Filozoa - ps11
Choano./Metazoa - ps12
Metazoa - ps13
Myriazoa - ps14
Parahoxozoa - ps15
Bilateria - ps16
Protostomia - ps17
Ecdysozoa - ps18
Arthropoda - ps19
Mandibulata - ps20
Pancrustacea - ps21
Insecta - ps22
Eumetabola - ps23
Holometabola/Psocodea - ps24
Holometabola - ps25
Aparaglossata - ps26
Mecopterida - ps27
Diptera - ps28
Bibiono./Cyclorrhapha - ps29
Cyclorrhapha - ps30
Calyptratae/Drosophila - ps31
Drosophila - ps32
Sophophora - ps33
Obscura/Melanogaster_group - ps34
D_melanogaster_group - ps35
D_melanogaster_subgroup - ps36
sim./sech./mel. group - ps37
Drosophila_melanogaster - ps38
Phylostratum

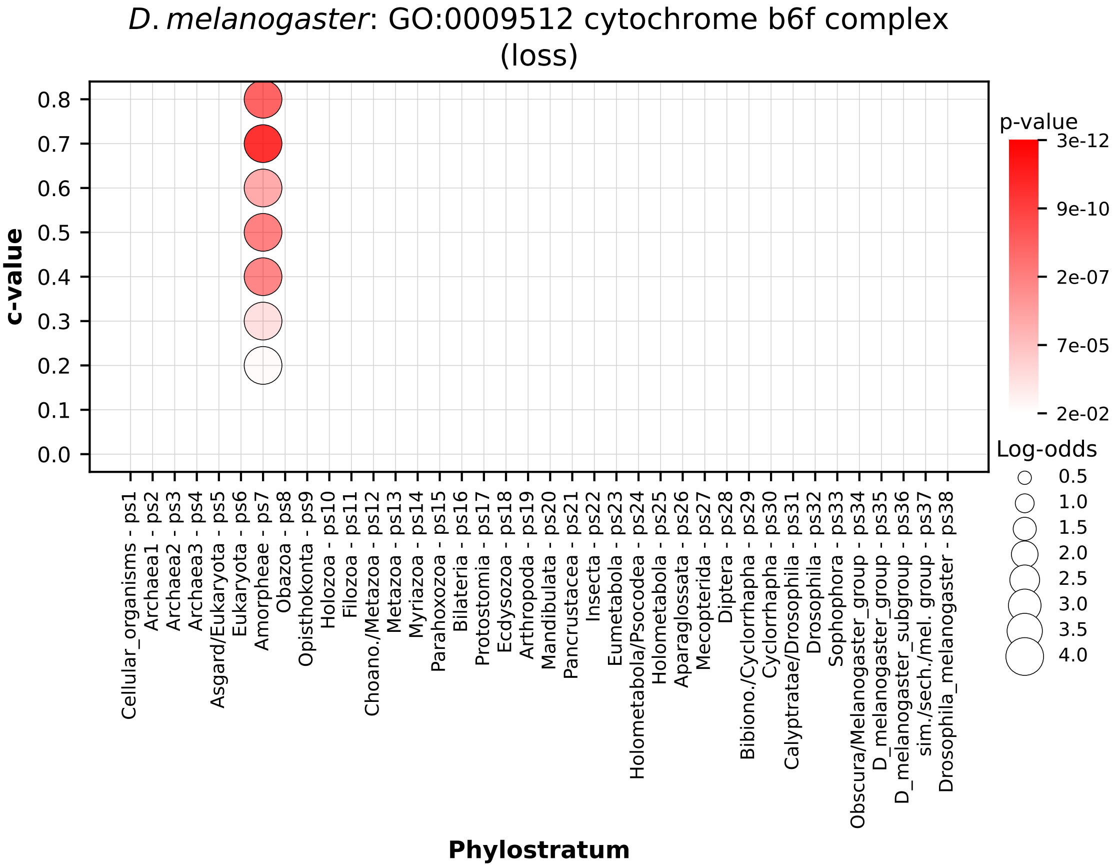

D. melanogaster: GO:0009512 cytochrome b6f complex (loss)
c-value
0.8
0.7
0.6
0.5
0.4
0.3
0.2
0.1
0.0
p-value
3e-12
9e-10
2e-07
7e-05
2e-02
Log-odds
0.5
1.0
1.5
2.0
2.5
3.0
3.5
4.0
Cellular_organisms - ps1
Archaea1 - ps2
Archaea2 - ps3
Archaea3 - ps4
Asgard/Eukaryota - ps5
Eukaryota - ps6
Amorpheae - ps7
Obazoa - ps8
Opisthokonta - ps9
Holozoa - ps10
Filozoa - ps11
Choano./Metazoa - ps12
Metazoa - ps13
Myriazoa - ps14
Parahoxozoa - ps15
Bilateria - ps16
Protostomia - ps17
Ecdysozoa - ps18
Arthropoda - ps19
Mandibulata - ps20
Pancrustacea - ps21
Insecta - ps22
Eumetabola - ps23
Holometabola/Psocodea - ps24
Holometabola - ps25
Aparaglossata - ps26
Mecopterida - ps27
Diptera - ps28
Bibiono./Cyclorrhapha - ps29
Cyclorrhapha - ps30
Calyptratae/Drosophila - ps31
Drosophila - ps32
Sophophora - ps33
Obscura/Melanogaster_group - ps34
D_melanogaster_group - ps35
D_melanogaster_subgroup - ps36
sim./sech./mel. group - ps37
Drosophila_melanogaster - ps38
Phylostratum

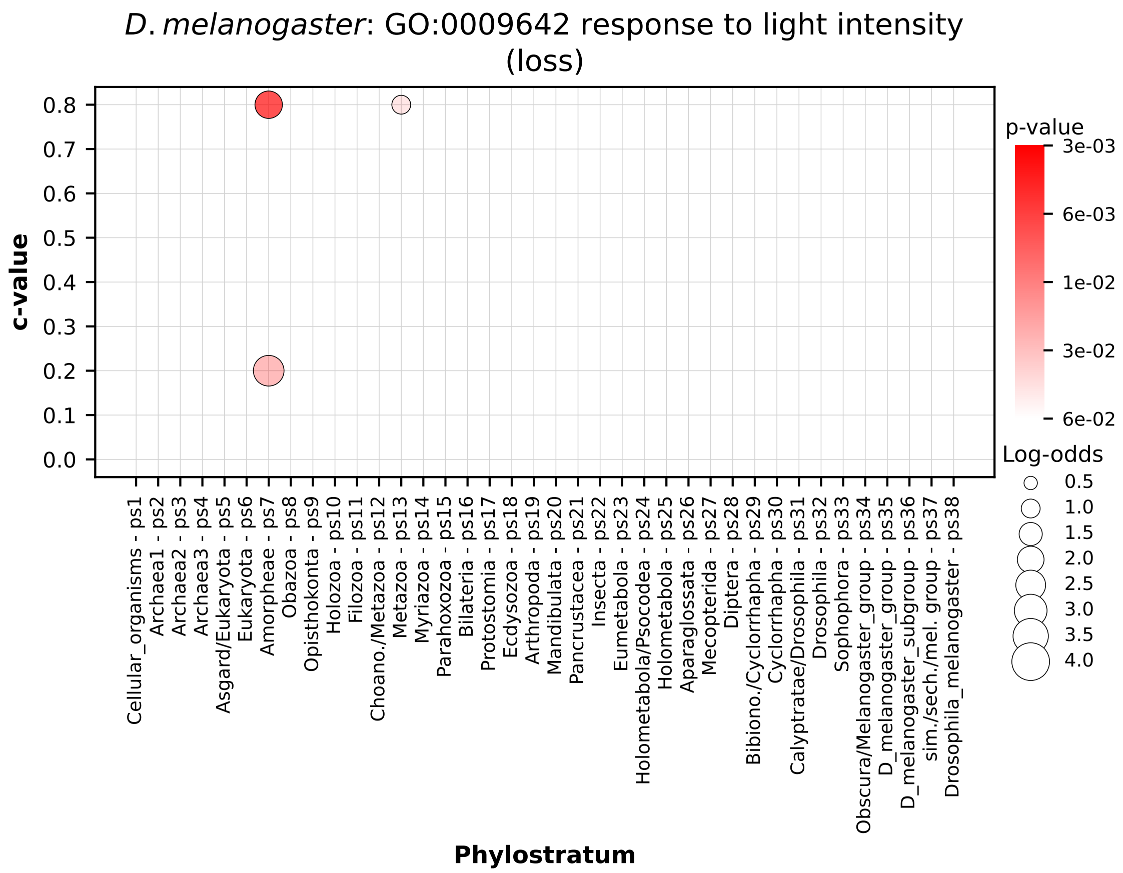

D. melanogaster: GO:0009642 response to light intensity (loss)
c-value
0.0
0.1
0.2
0.3
0.4
0.5
0.6
0.7
0.8
Phylostratum
Cellular_organisms - ps1
Archaea1 - ps2
Archaea2 - ps3
Archaea3 - ps4
Asgard/Eukaryota - ps5
Eukaryota - ps6
Amorpheae - ps7
Obazoa - ps8
Opisthokonta - ps9
Holozoa - ps10
Filozoa - ps11
Choano./Metazoa - ps12
Metazoa - ps13
Myriazoa - ps14
Parahoxozoa - ps15
Bilateria - ps16
Protostomia - ps17
Ecdysozoa - ps18
Arthropoda - ps19
Mandibulata - ps20
Pancrustacea - ps21
Insecta - ps22
Eumetabola - ps23
Holometabola/Psocodea - ps24
Holometabola - ps25
Aparaglossata - ps26
Mecopterida - ps27
Diptera - ps28
Bibiono./Cyclorrhapha - ps29
Cyclorrhapha - ps30
Calyptratae/Drosophila - ps31
Drosophila - ps32
Sophophora - ps33
Obscura/Melanogaster_group - ps34
D_melanogaster_group - ps35
D_melanogaster_subgroup - ps36
sim./sech./mel. group - ps37
Drosophila_melanogaster - ps38
p-value
3e-03
6e-03
1e-02
3e-02
6e-02
Log-odds
0.5
1.0
1.5
2.0
2.5
3.0
3.5
4.0

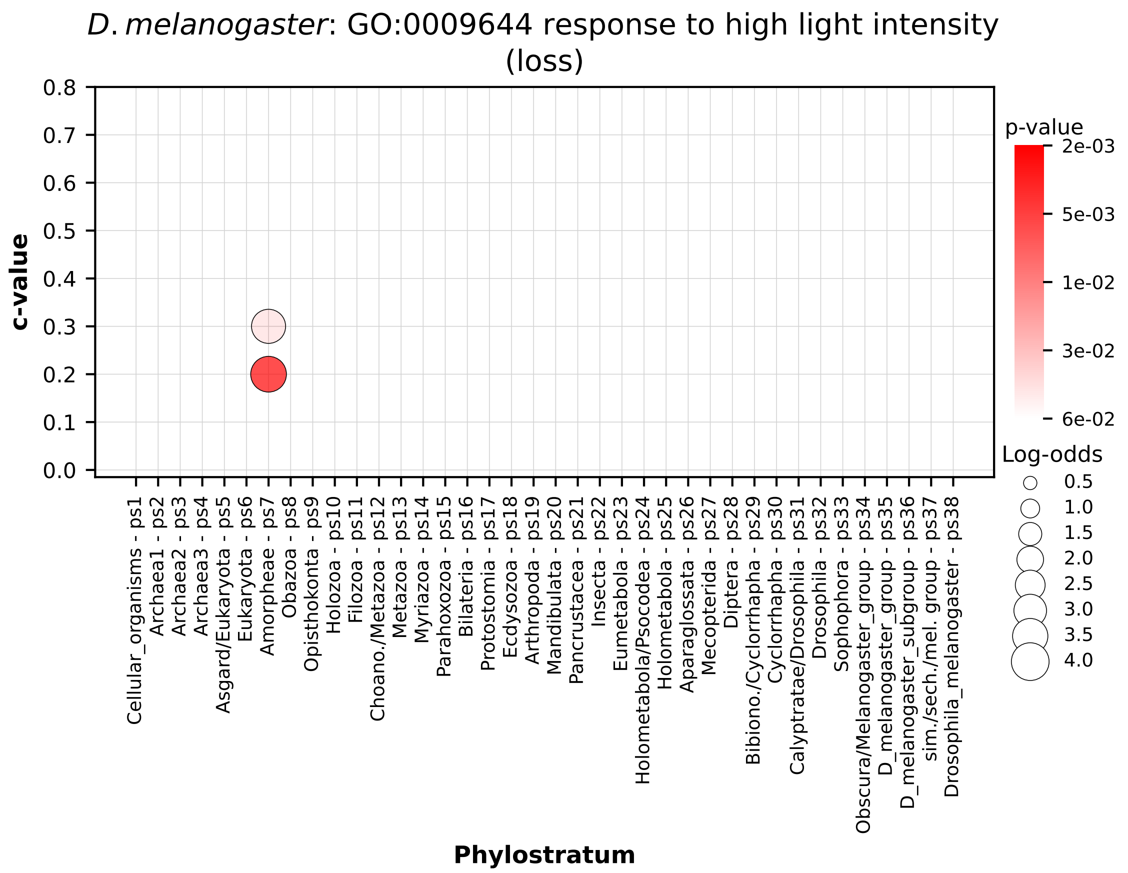

D. melanogaster: GO:0009644 response to high light intensity
(loss)
c-value
0.8
0.7
0.6
0.5
0.4
0.3
0.2
0.1
0.0
Phylostratum
Cellular_organisms - ps1
Archaea1 - ps2
Archaea2 - ps3
Archaea3 - ps4
Asgard/Eukaryota - ps5
Eukaryota - ps6
Amorpheae - ps7
Obazoa - ps8
Opisthokonta - ps9
Holozoa - ps10
Filozoa - ps11
Choano./Metazoa - ps12
Metazoa - ps13
Myriazoa - ps14
Parahoxozoa - ps15
Bilateria - ps16
Protostomia - ps17
Ecdysozoa - ps18
Arthropoda - ps19
Mandibulata - ps20
Pancrustacea - ps21
Insecta - ps22
Eumetabola - ps23
Holometabola/Psocodea - ps24
Holometabola - ps25
Aparaglossata - ps26
Mecopterida - ps27
Diptera - ps28
Bibiono./Cyclorrhapha - ps29
Cyclorrhapha - ps30
Calyptratae/Drosophila - ps31
Drosophila - ps32
Sophophora - ps33
Obscura/Melanogaster_group - ps34
D_melanogaster_group - ps35
D_melanogaster_subgroup - ps36
sim./sech./mel. group - ps37
Drosophila_melanogaster - ps38
p-value
2e-03
5e-03
1e-02
3e-02
6e-02
Log-odds
0.5
1.0
1.5
2.0
2.5
3.0
3.5
4.0

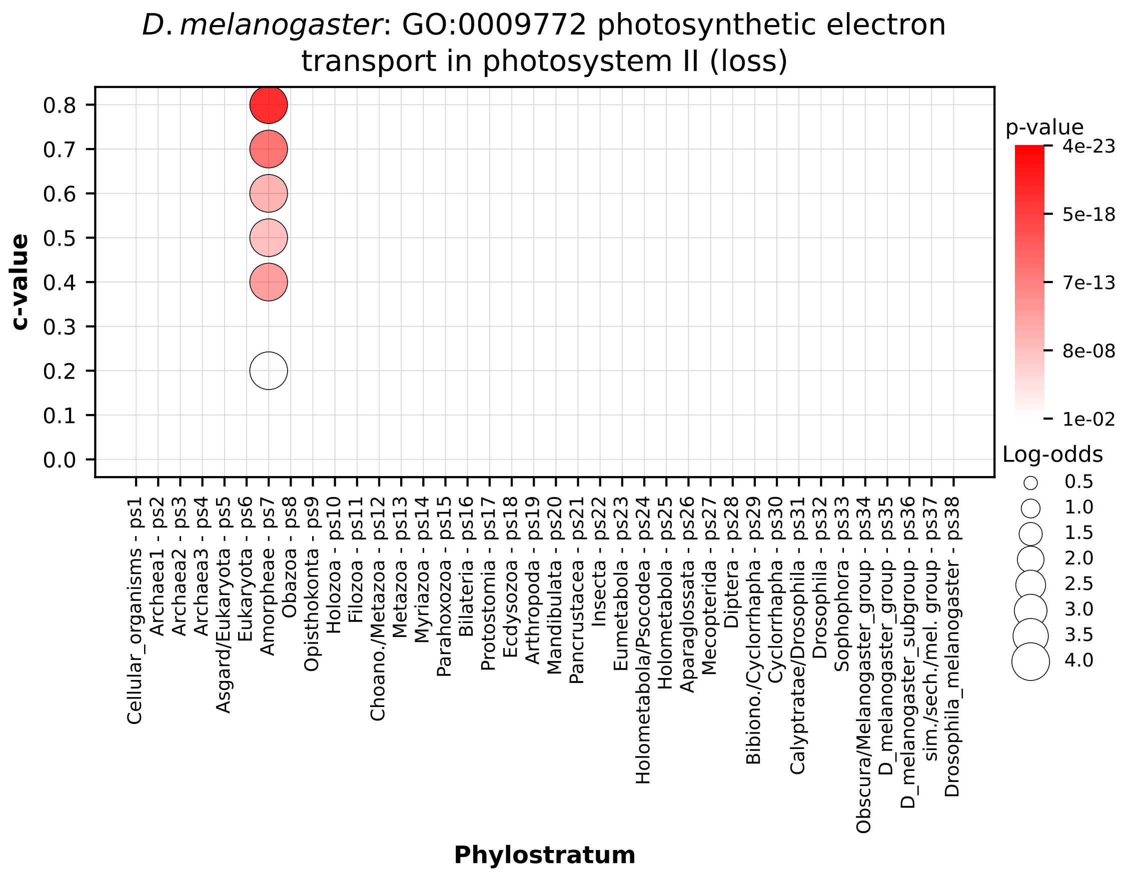
D. melanogaster: GO:0009772 photosynthetic electron transport in photosystem II (loss)
c-value
0.8
0.7
0.6
0.5
0.4
0.3
0.2
0.1
0.0
Phylostratum
Cellular_organisms - ps1
Archaea1 - ps2
Archaea2 - ps3
Archaea3 - ps4
Asgard/Eukaryota - ps5
Eukaryota - ps6
Amorpheae - ps7
Obazoa - ps8
Opisthokonta - ps9
Holozoa - ps10
Filozoa - ps11
Choano./Metazoa - ps12
Metazoa - ps13
Myriazoa - ps14
Parahoxozoa - ps15
Bilateria - ps16
Protostomia - ps17
Ecdysozoa - ps18
Arthropoda - ps19
Mandibulata - ps20
Pancrustacea - ps21
Insecta - ps22
Eumetabola - ps23
Holometabola/Psocodea - ps24
Holometabola - ps25
Aparaglossata - ps26
Mecopterida - ps27
Diptera - ps28
Bibiono./Cyclorrhapha - ps29
Cyclorrhapha - ps30
Calyptratae/Drosophila - ps31
Drosophila - ps32
Sophophora - ps33
Obscura/Melanogaster_group - ps34
D_melanogaster_group - ps35
D_melanogaster_subgroup - ps36
sim./sech./mel. group - ps37
Drosophila_melanogaster - ps38
p-value
4e-23
5e-18
7e-13
8e-08
1e-02
Log-odds
0.5
1.0
1.5
2.0
2.5
3.0
3.5
4.0

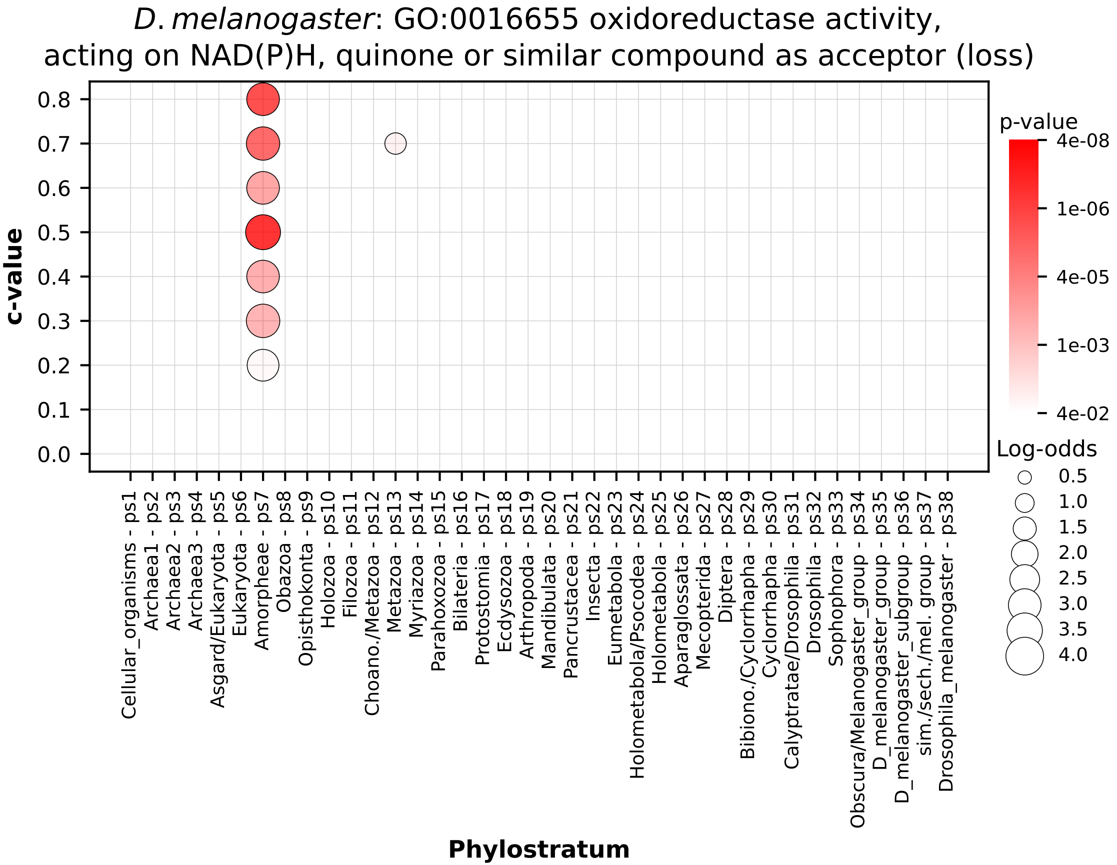

D. melanogaster: GO:0016655 oxidoreductase activity,
acting on NAD(P)H, quinone or similar compound as acceptor (loss)
c-value
0.0
0.1
0.2
0.3
0.4
0.5
0.6
0.7
0.8
p-value
4e-08
1e-06
4e-05
1e-03
4e-02
Log-odds
0.5
1.0
1.5
2.0
2.5
3.0
3.5
4.0
Cellular_organisms - ps1
Archaea1 - ps2
Archaea2 - ps3
Archaea3 - ps4
Asgard/Eukaryota - ps5
Eukaryota - ps6
Amorpheae - ps7
Obazoa - ps8
Opisthokonta - ps9
Holozoa - ps10
Filozoa - ps11
Choano./Metazoa - ps12
Metazoa - ps13
Myriazoa - ps14
Parahoxozoa - ps15
Bilateria - ps16
Protostomia - ps17
Ecdysozoa - ps18
Arthropoda - ps19
Mandibulata - ps20
Pancrustacea - ps21
Insecta - ps22
Eumetabola - ps23
Holometabola/Psocodea - ps24
Holometabola - ps25
Aparaglossata - ps26
Mecopterida - ps27
Diptera - ps28
Bibiono./Cyclorrhapha - ps29
Cyclorrhapha - ps30
Calyptratae/Drosophila - ps31
Drosophila - ps32
Sophophora - ps33
Obscura/Melanogaster_group - ps34
D_melanogaster_group - ps35
D_melanogaster_subgroup - ps36
sim./sech./mel. group - ps37
Drosophila_melanogaster - ps38
Phylostratum

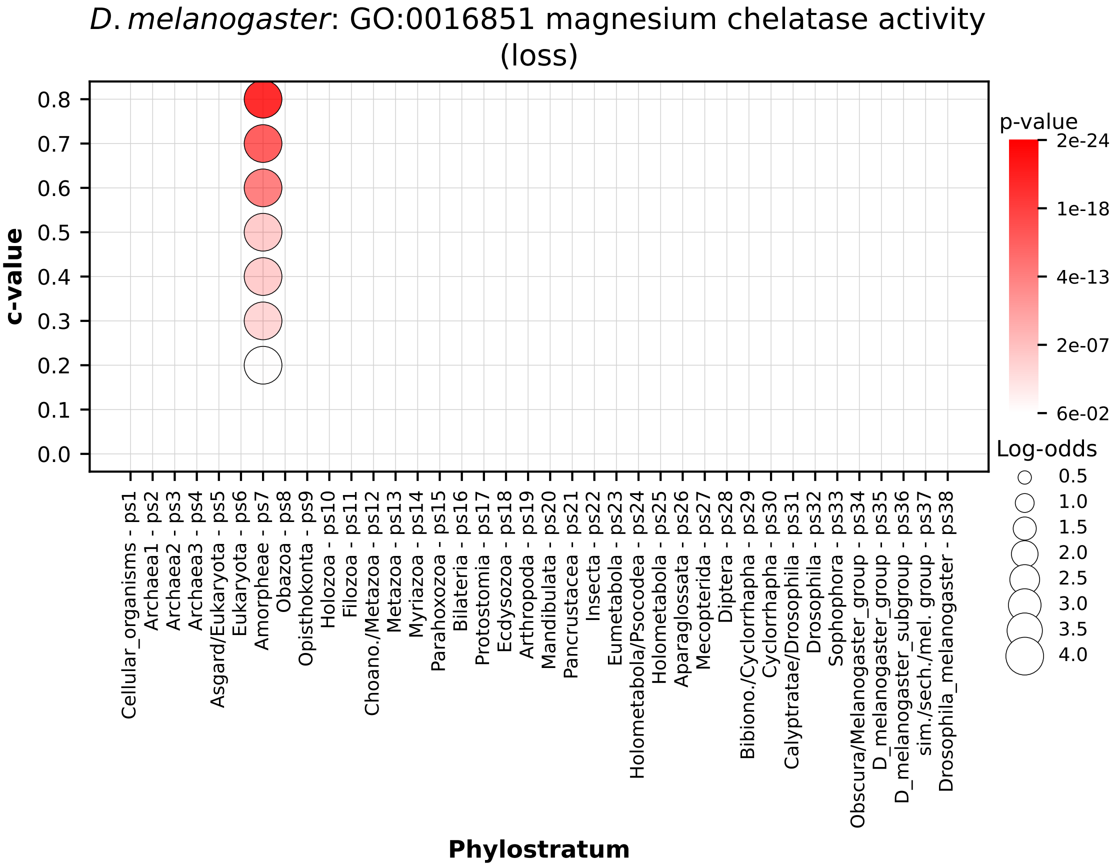

D. melanogaster: GO:0016851 magnesium chelatase activity (loss)
c-value
0.0
0.1
0.2
0.3
0.4
0.5
0.6
0.7
0.8
Phylostratum
Cellular_organisms - ps1
Archaea1 - ps2
Archaea2 - ps3
Archaea3 - ps4
Asgard/Eukaryota - ps5
Eukaryota - ps6
Amorpheae - ps7
Obazoa - ps8
Opisthokonta - ps9
Holozoa - ps10
Filozoa - ps11
Choano./Metazoa - ps12
Metazoa - ps13
Myriazoa - ps14
Parahoxozoa - ps15
Bilateria - ps16
Protostomia - ps17
Ecdysozoa - ps18
Arthropoda - ps19
Mandibulata - ps20
Pancrustacea - ps21
Insecta - ps22
Eumetabola - ps23
Holometabola/Psocodea - ps24
Holometabola - ps25
Aparaglossata - ps26
Mecopterida - ps27
Diptera - ps28
Bibiono./Cyclorrhapha - ps29
Cyclorrhapha - ps30
Calyptratae/Drosophila - ps31
Drosophila - ps32
Sophophora - ps33
Obscura/Melanogaster_group - ps34
D_melanogaster_group - ps35
D_melanogaster_subgroup - ps36
sim./sech./mel. group - ps37
Drosophila_melanogaster - ps38
p-value
2e-24
1e-18
4e-13
2e-07
6e-02
Log-odds
0.5
1.0
1.5
2.0
2.5
3.0
3.5
4.0

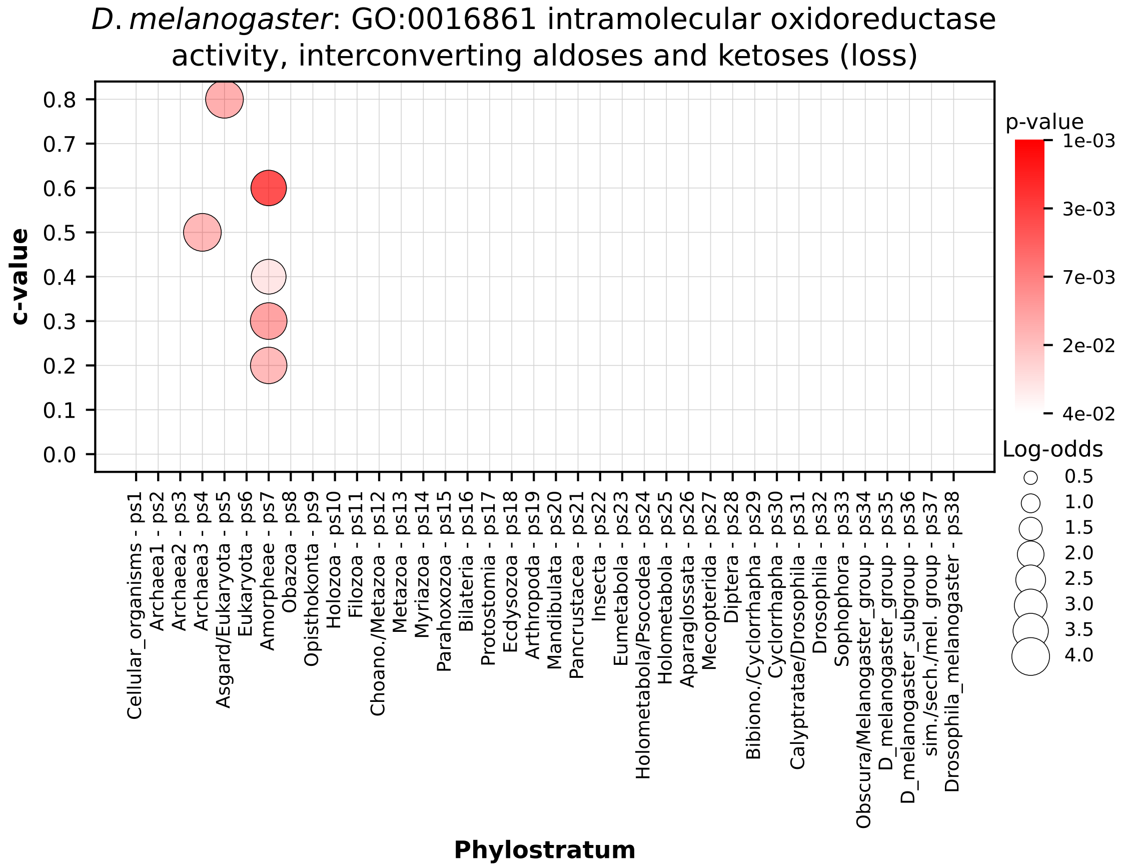
D. melanogaster: GO:0016861 intramolecular oxidoreductase activity, interconverting aldoses and ketoses (loss)
c-value
0.0
0.1
0.2
0.3
0.4
0.5
0.6
0.7
0.8
Phylostratum
Cellular_organisms - ps1
Archaea1 - ps2
Archaea2 - ps3
Archaea3 - ps4
Asgard/Eukaryota - ps5
Eukaryota - ps6
Amorpheae - ps7
Obazoa - ps8
Opisthokonta - ps9
Holozoa - ps10
Filozoa - ps11
Choano./Metazoa - ps12
Metazoa - ps13
Myriazoa - ps14
Parahoxozoa - ps15
Bilateria - ps16
Protostomia - ps17
Ecdysozoa - ps18
Arthropoda - ps19
Mandibulata - ps20
Pancrustacea - ps21
Insecta - ps22
Eumetabola - ps23
Holometabola/Psocodea - ps24
Holometabola - ps25
Aparaglossata - ps26
Mecopterida - ps27
Diptera - ps28
Bibiono./Cyclorrhapha - ps29
Cyclorrhapha - ps30
Calyptratae/Drosophila - ps31
Drosophila - ps32
Sophophora - ps33
Obscura/Melanogaster_group - ps34
D_melanogaster_group - ps35
D_melanogaster_subgroup - ps36
sim./sech./mel. group - ps37
Drosophila_melanogaster - ps38
p-value
1e-03
3e-03
7e-03
2e-02
4e-02
Log-odds
0.5
1.0
1.5
2.0
2.5
3.0
3.5
4.0

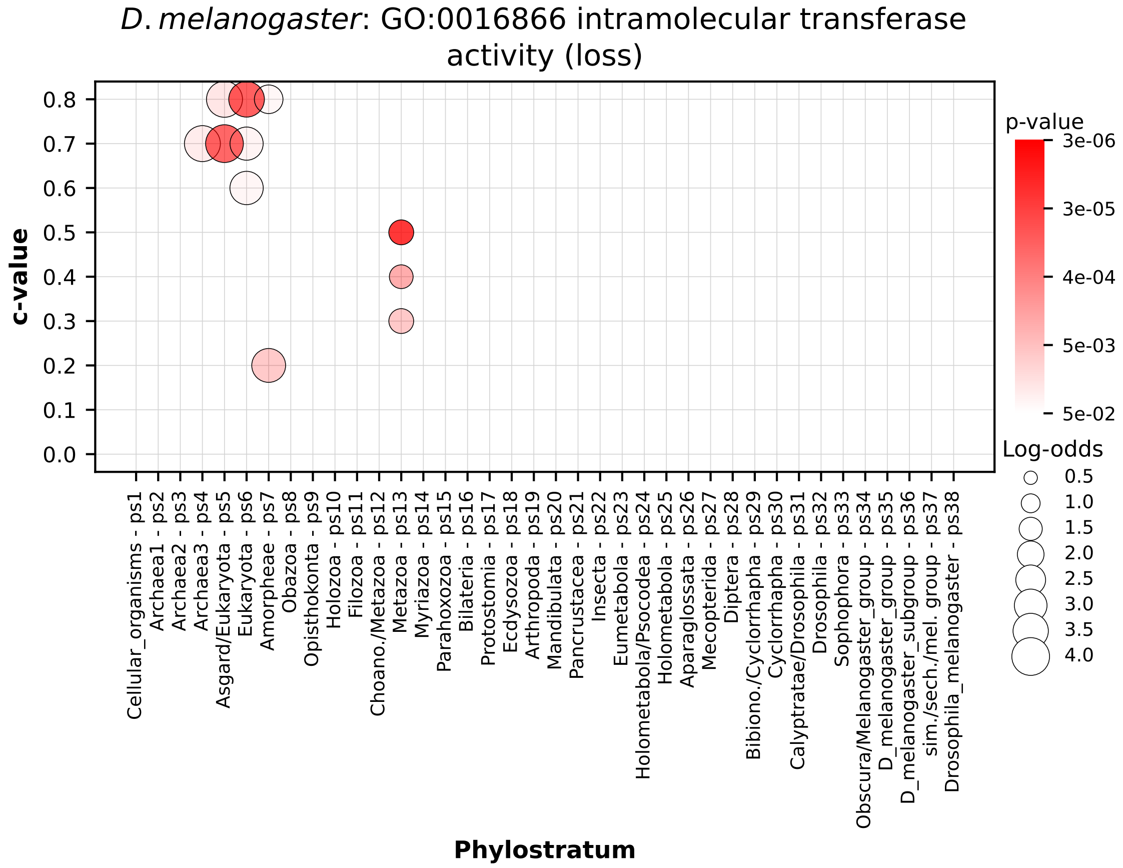

D. melanogaster: GO:0016866 intramolecular transferase activity (loss)
c-value
0.8
0.7
0.6
0.5
0.4
0.3
0.2
0.1
0.0
p-value
3e-06
3e-05
4e-04
5e-03
5e-02
Log-odds
0.5
1.0
1.5
2.0
2.5
3.0
3.5
4.0
Cellular_organisms - ps1
Archaea1 - ps2
Archaea2 - ps3
Archaea3 - ps4
Asgard/Eukaryota - ps5
Eukaryota - ps6
Amorpheae - ps7
Obazoa - ps8
Opisthokonta - ps9
Holozoa - ps10
Filozoa - ps11
Choano./Metazoa - ps12
Metazoa - ps13
Myriazoa - ps14
Parahoxozoa - ps15
Bilateria - ps16
Protostomia - ps17
Ecdysozoa - ps18
Arthropoda - ps19
Mandibulata - ps20
Pancrustacea - ps21
Insecta - ps22
Eumetabola - ps23
Holometabola/Psocodea - ps24
Holometabola - ps25
Aparaglossata - ps26
Mecopterida - ps27
Diptera - ps28
Bibiono./Cyclorrhapha - ps29
Cyclorrhapha - ps30
Calyptratae/Drosophila - ps31
Drosophila - ps32
Sophophora - ps33
Obscura/Melanogaster_group - ps34
D_melanogaster_group - ps35
D_melanogaster_subgroup - ps36
sim./sech./mel. group - ps37
Drosophila_melanogaster - ps38
Phylostratum

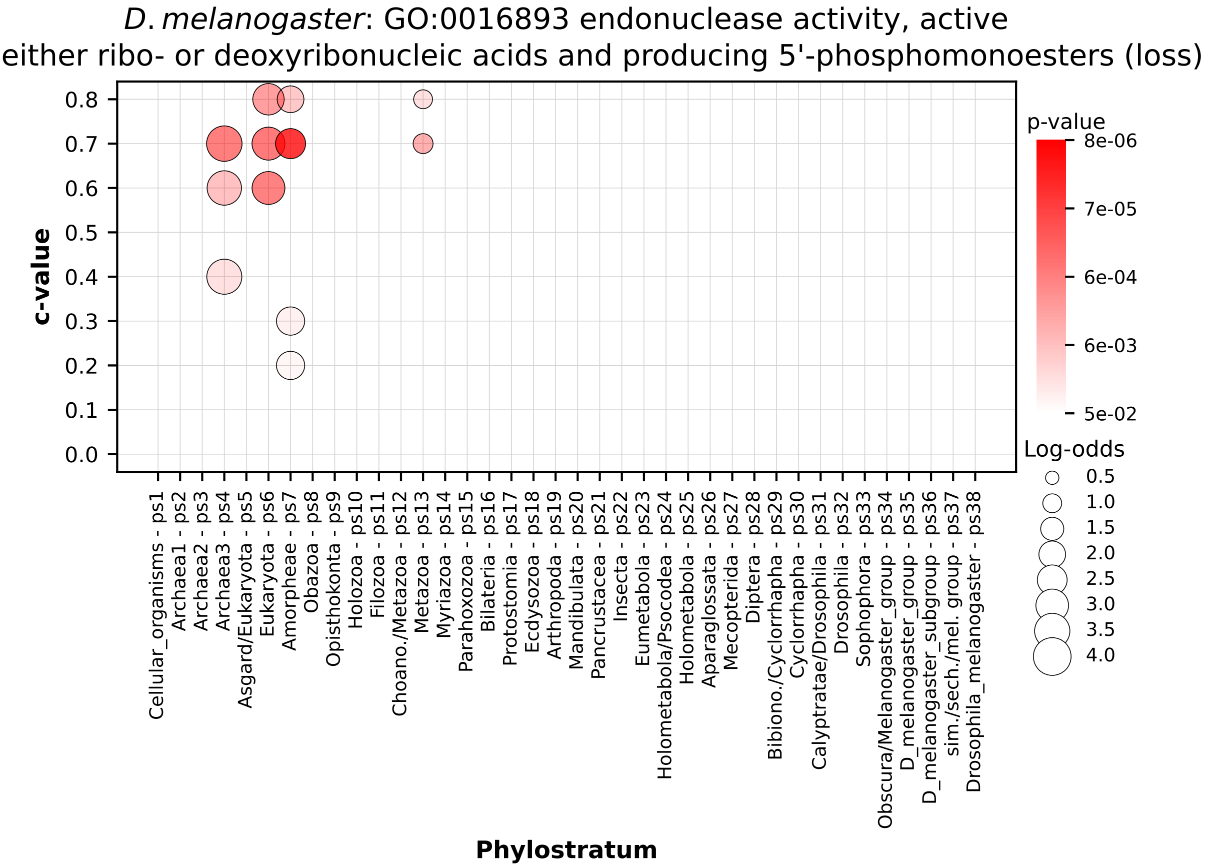

D. melanogaster: GO:0016893 endonuclease activity, active
either ribo- or deoxyribonucleic acids and producing 5'-phosphomonoesters (loss)
c-value
0.8
0.7
0.6
0.5
0.4
0.3
0.2
0.1
0.0
Phylostratum
Cellular_organisms - ps1
Archaea1 - ps2
Archaea2 - ps3
Archaea3 - ps4
Asgard/Eukaryota - ps5
Eukaryota - ps6
Amorpheae - ps7
Obazoa - ps8
Opisthokonta - ps9
Holozoa - ps10
Filozoa - ps11
Choano./Metazoa - ps12
Metazoa - ps13
Myriazoa - ps14
Parahoxozoa - ps15
Bilateria - ps16
Protostomia - ps17
Ecdysozoa - ps18
Arthropoda - ps19
Mandibulata - ps20
Pancrustacea - ps21
Insecta - ps22
Eumetabola - ps23
Holometabola/Psocodea - ps24
Holometabola - ps25
Aparaglossata - ps26
Mecopterida - ps27
Diptera - ps28
Bibiono./Cyclorrhapha - ps29
Cyclorrhapha - ps30
Calyptratae/Drosophila - ps31
Drosophila - ps32
Sophophora - ps33
Obscura/Melanogaster_group - ps34
D_melanogaster_group - ps35
D_melanogaster_subgroup - ps36
sim./sech./mel. group - ps37
Drosophila_melanogaster - ps38
p-value
8e-06
7e-05
6e-04
6e-03
5e-02
Log-odds
0.5
1.0
1.5
2.0
2.5
3.0
3.5
4.0

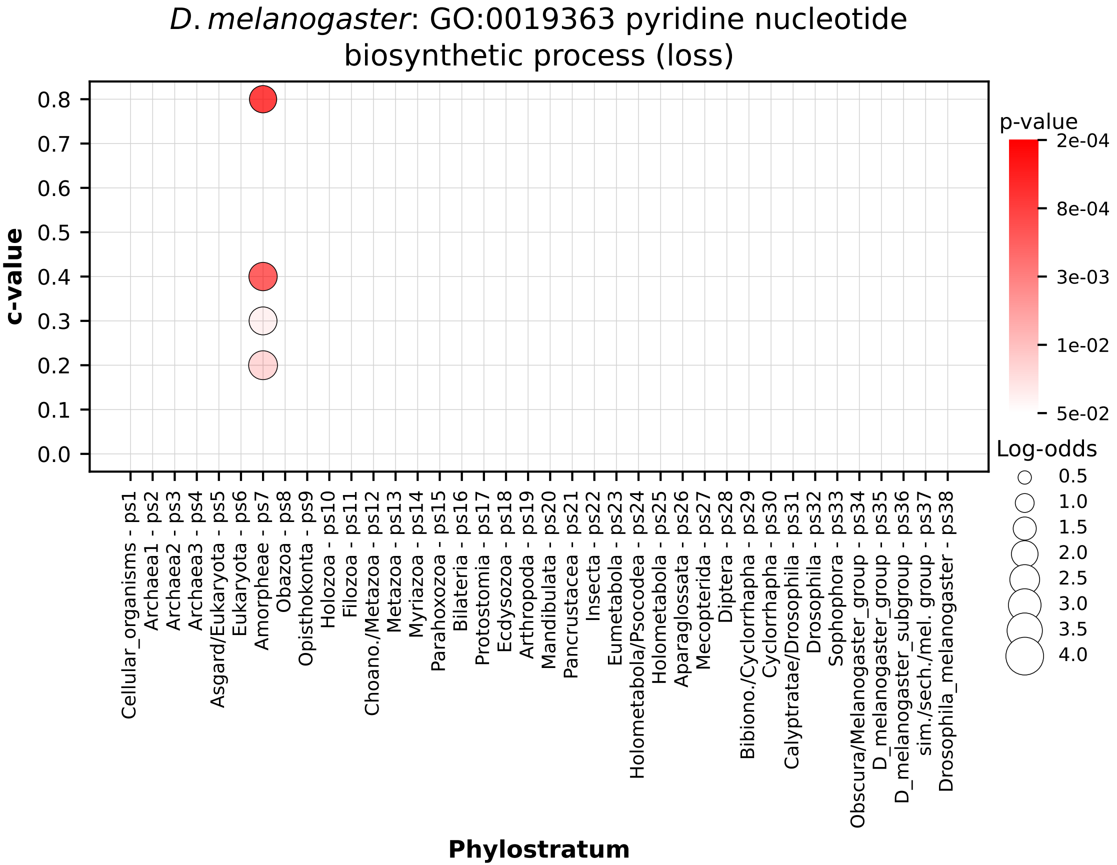

D. melanogaster: GO:0019363 pyridine nucleotide biosynthetic process (loss)
c-value
0.0
0.1
0.2
0.3
0.4
0.5
0.6
0.7
0.8
Phylostratum
Cellular_organisms - ps1
Archaea1 - ps2
Archaea2 - ps3
Archaea3 - ps4
Asgard/Eukaryota - ps5
Eukaryota - ps6
Amorpheae - ps7
Obazoa - ps8
Opisthokonta - ps9
Holozoa - ps10
Filozoa - ps11
Choano./Metazoa - ps12
Metazoa - ps13
Myriazoa - ps14
Parahoxozoa - ps15
Bilateria - ps16
Protostomia - ps17
Ecdysozoa - ps18
Arthropoda - ps19
Mandibulata - ps20
Pancrustacea - ps21
Insecta - ps22
Eumetabola - ps23
Holometabola/Psocodea - ps24
Holometabola - ps25
Aparaglossata - ps26
Mecopterida - ps27
Diptera - ps28
Bibiono./Cyclorrhapha - ps29
Cyclorrhapha - ps30
Calyptratae/Drosophila - ps31
Drosophila - ps32
Sophophora - ps33
Obscura/Melanogaster_group - ps34
D_melanogaster_group - ps35
D_melanogaster_subgroup - ps36
sim./sech./mel. group - ps37
Drosophila_melanogaster - ps38
p-value
2e-04
8e-04
3e-03
1e-02
5e-02
Log-odds
0.5
1.0
1.5
2.0
2.5
3.0
3.5
4.0

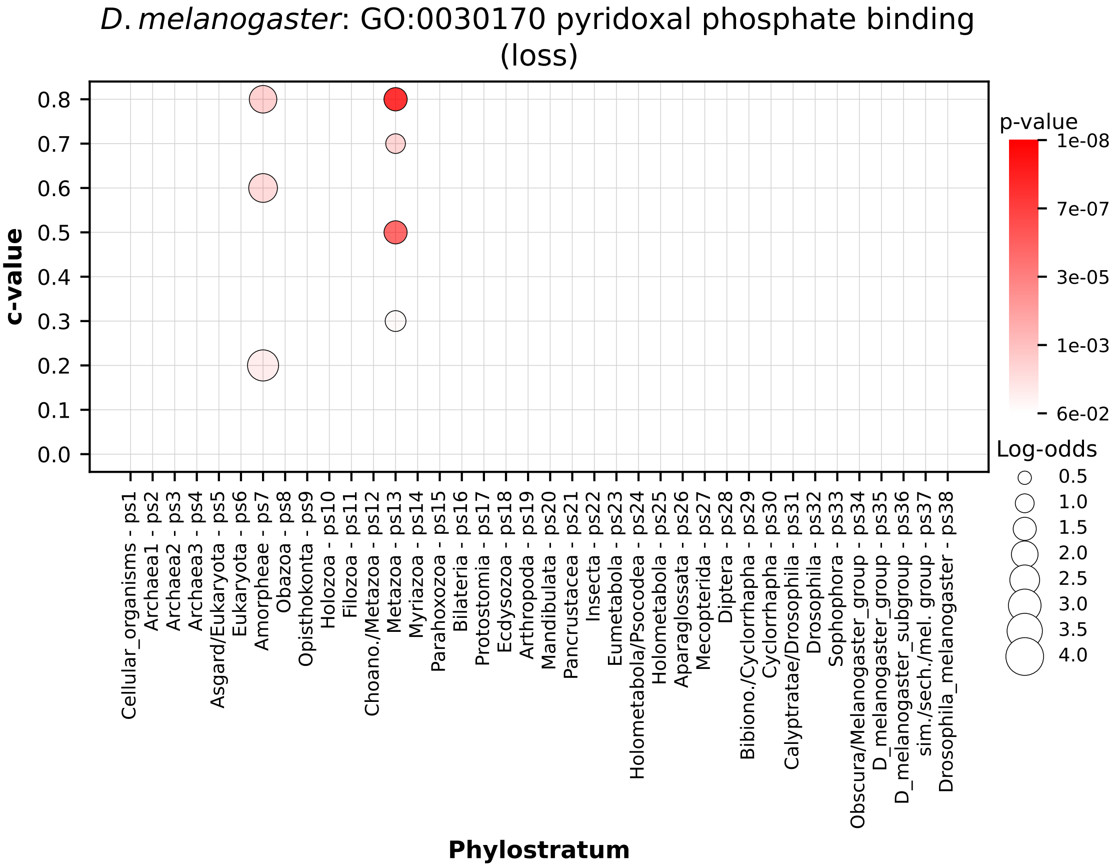

D. melanogaster: GO:0030170 pyridoxal phosphate binding (loss)
c-value
0.8
0.7
0.6
0.5
0.4
0.3
0.2
0.1
0.0
Phylostratum
Cellular_organisms - ps1
Archaea1 - ps2
Archaea2 - ps3
Archaea3 - ps4
Asgard/Eukaryota - ps5
Eukaryota - ps6
Amorpheae - ps7
Obazoa - ps8
Opisthokonta - ps9
Holozoa - ps10
Filozoa - ps11
Choano./Metazoa - ps12
Metazoa - ps13
Myriazoa - ps14
Parahoxozoa - ps15
Bilateria - ps16
Protostomia - ps17
Ecdysozoa - ps18
Arthropoda - ps19
Mandibulata - ps20
Pancrustacea - ps21
Insecta - ps22
Eumetabola - ps23
Holometabola/Psocodea - ps24
Holometabola - ps25
Aparaglossata - ps26
Mecopterida - ps27
Diptera - ps28
Bibiono./Cyclorrhapha - ps29
Cyclorrhapha - ps30
Calyptratae/Drosophila - ps31
Drosophila - ps32
Sophophora - ps33
Obscura/Melanogaster_group - ps34
D_melanogaster_group - ps35
D_melanogaster_subgroup - ps36
sim./sech./mel. group - ps37
Drosophila_melanogaster - ps38
p-value
1e-08
7e-07
3e-05
1e-03
6e-02
Log-odds
0.5
1.0
1.5
2.0
2.5
3.0
3.5
4.0

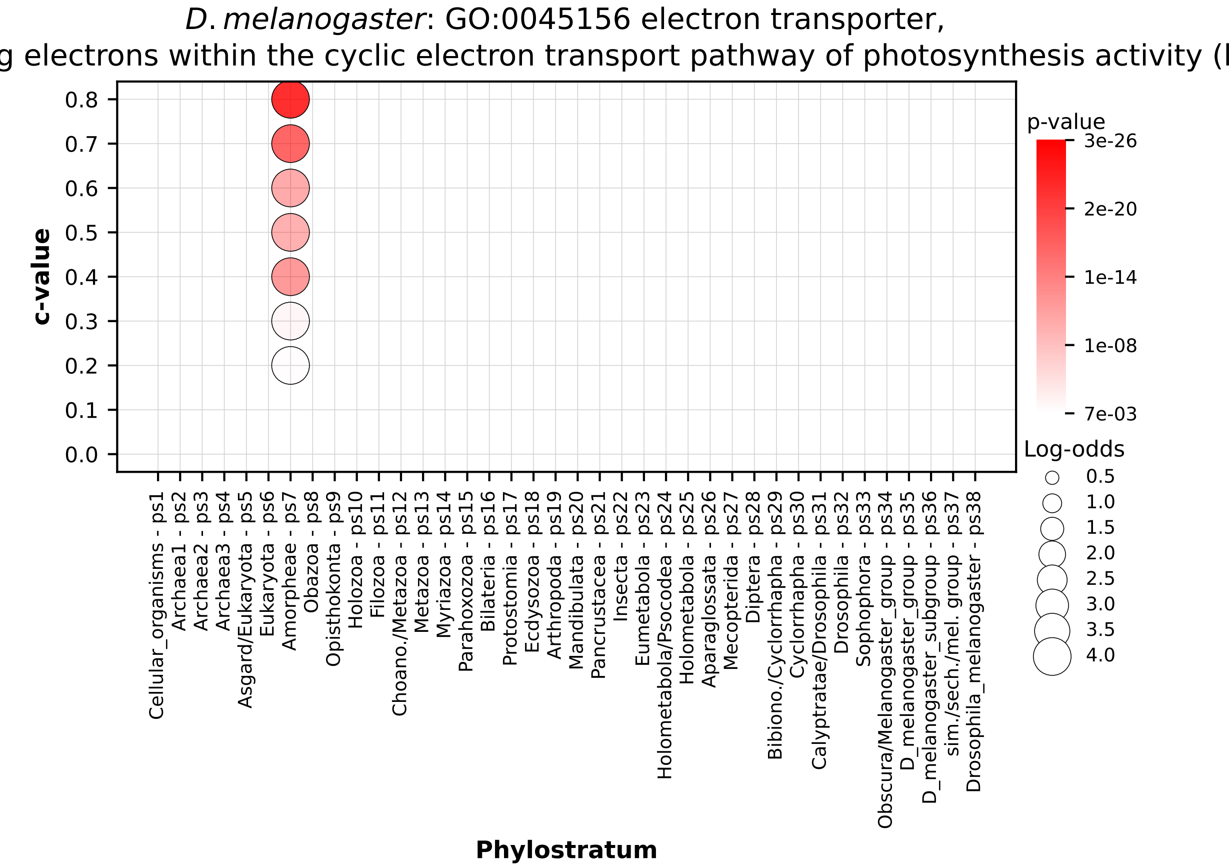
D. melanogaster: GO:0045156 electron transporter,
g electrons within the cyclic electron transport pathway of photosynthesis activity (I
c-value
0.8
0.7
0.6
0.5
0.4
0.3
0.2
0.1
0.0
p-value
3e-26
2e-20
1e-14
1e-08
7e-03
Log-odds
0.5
1.0
1.5
2.0
2.5
3.0
3.5
4.0
Cellular_organisms - ps1
Archaea1 - ps2
Archaea2 - ps3
Archaea3 - ps4
Asgard/Eukaryota - ps5
Eukaryota - ps6
Amorpheae - ps7
Obazoa - ps8
Opisthokonta - ps9
Holozoa - ps10
Filozoa - ps11
Choano./Metazoa - ps12
Metazoa - ps13
Myriazoa - ps14
Parahoxozoa - ps15
Bilateria - ps16
Protostomia - ps17
Ecdysozoa - ps18
Arthropoda - ps19
Mandibulata - ps20
Pancrustacea - ps21
Insecta - ps22
Eumetabola - ps23
Holometabola/Psocodea - ps24
Holometabola - ps25
Aparaglossata - ps26
Mecopterida - ps27
Diptera - ps28
Bibiono./Cyclorrhapha - ps29
Cyclorrhapha - ps30
Calyptratae/Drosophila - ps31
Drosophila - ps32
Sophophora - ps33
Obscura/Melanogaster_group - ps34
D_melanogaster_group - ps35
D_melanogaster_subgroup - ps36
sim./sech./mel. group - ps37
Drosophila_melanogaster - ps38
Phylostratum

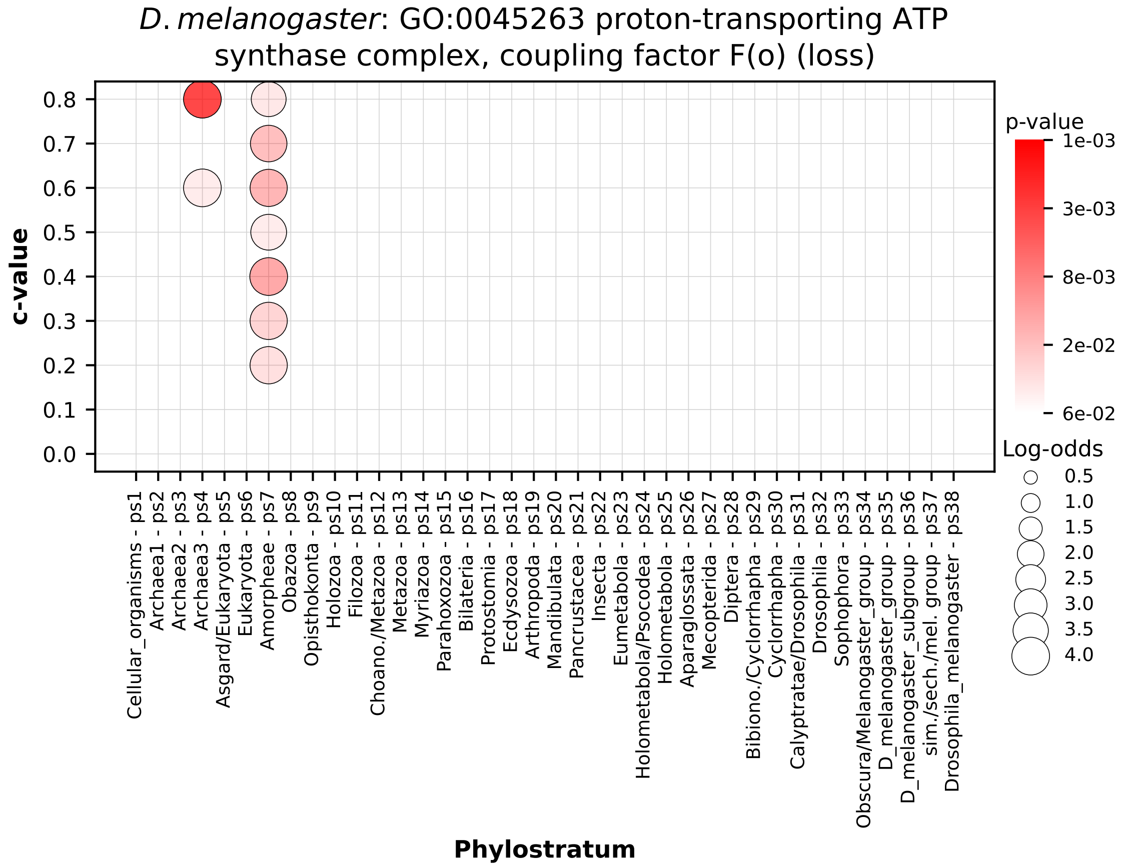
D. melanogaster: GO:0045263 proton-transporting ATP synthase complex, coupling factor F(o) (loss)
c-value
0.8
0.7
0.6
0.5
0.4
0.3
0.2
0.1
0.0
Phylostratum
Cellular_organisms - ps1
Archaea1 - ps2
Archaea2 - ps3
Archaea3 - ps4
Asgard/Eukaryota - ps5
Eukaryota - ps6
Amorpheae - ps7
Obazoa - ps8
Opisthokonta - ps9
Holozoa - ps10
Filozoa - ps11
Choano./Metazoa - ps12
Metazoa - ps13
Myriazoa - ps14
Parahoxozoa - ps15
Bilateria - ps16
Protostomia - ps17
Ecdysozoa - ps18
Arthropoda - ps19
Mandibulata - ps20
Pancrustacea - ps21
Insecta - ps22
Eumetabola - ps23
Holometabola/Psocodea - ps24
Holometabola - ps25
Aparaglossata - ps26
Mecopterida - ps27
Diptera - ps28
Bibiono./Cyclorrhapha - ps29
Cyclorrhapha - ps30
Calyptratae/Drosophila - ps31
Drosophila - ps32
Sophophora - ps33
Obscura/Melanogaster_group - ps34
D_melanogaster_group - ps35
D_melanogaster_subgroup - ps36
sim./sech./mel. group - ps37
Drosophila_melanogaster - ps38
p-value
1e-03
3e-03
8e-03
2e-02
6e-02
Log-odds
0.5
1.0
1.5
2.0
2.5
3.0
3.5
4.0

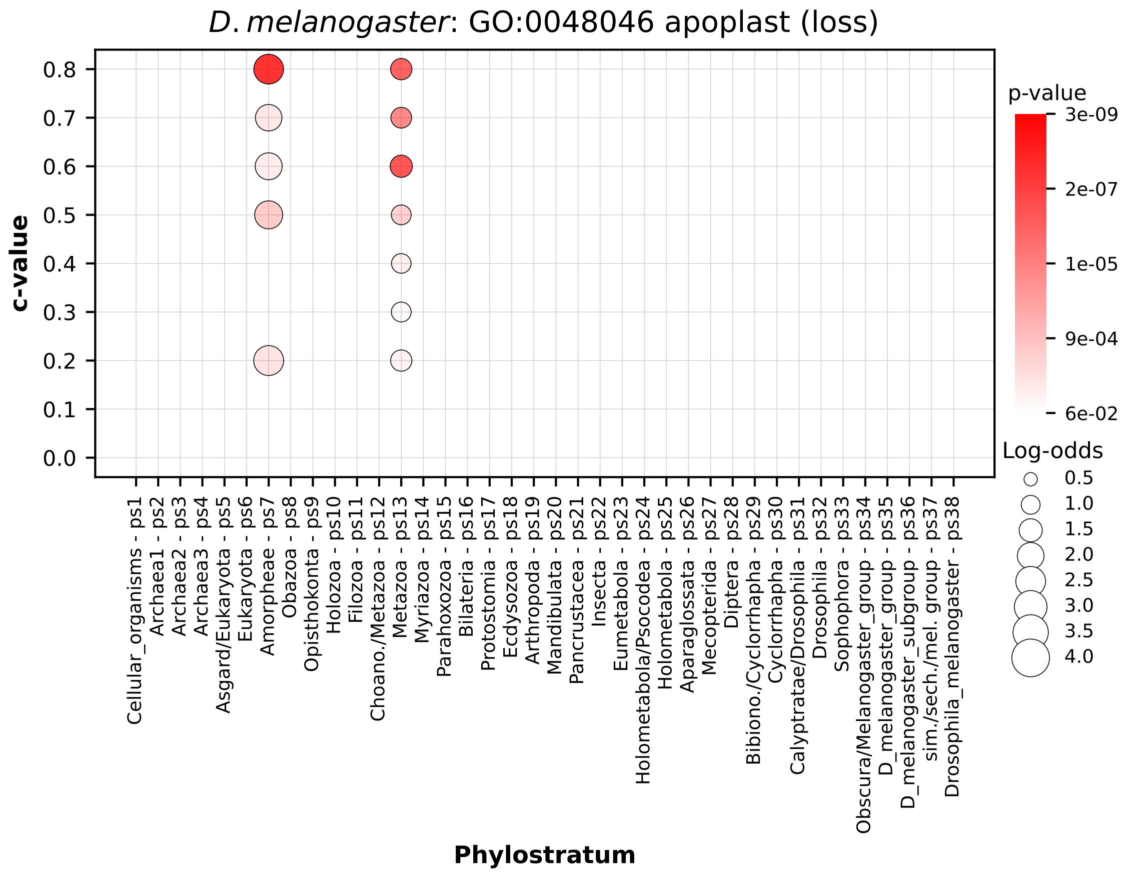

D. melanogaster: GO:0048046 apoplast (loss)
c-value
0.0
0.1
0.2
0.3
0.4
0.5
0.6
0.7
0.8
p-value
3e-09
2e-07
1e-05
9e-04
6e-02
Log-odds
0.5
1.0
1.5
2.0
2.5
3.0
3.5
4.0
Cellular_organisms - ps1
Archaea1 - ps2
Archaea2 - ps3
Archaea3 - ps4
Asgard/Eukaryota - ps5
Eukaryota - ps6
Amorpheae - ps7
Obazoa - ps8
Opisthokonta - ps9
Holozoa - ps10
Filozoa - ps11
Choano./Metazoa - ps12
Metazoa - ps13
Myriazoa - ps14
Parahoxozoa - ps15
Bilateria - ps16
Protostomia - ps17
Ecdysozoa - ps18
Arthropoda - ps19
Mandibulata - ps20
Pancrustacea - ps21
Insecta - ps22
Eumetabola - ps23
Holometabola/Psocodea - ps24
Holometabola - ps25
Aparaglossata - ps26
Mecopterida - ps27
Diptera - ps28
Bibiono./Cyclorrhapha - ps29
Cyclorrhapha - ps30
Calyptratae/Drosophila - ps31
Drosophila - ps32
Sophophora - ps33
Obscura/Melanogaster_group - ps34
D_melanogaster_group - ps35
D_melanogaster_subgroup - ps36
sim./sech./mel. group - ps37
Drosophila_melanogaster - ps38
Phylostratum

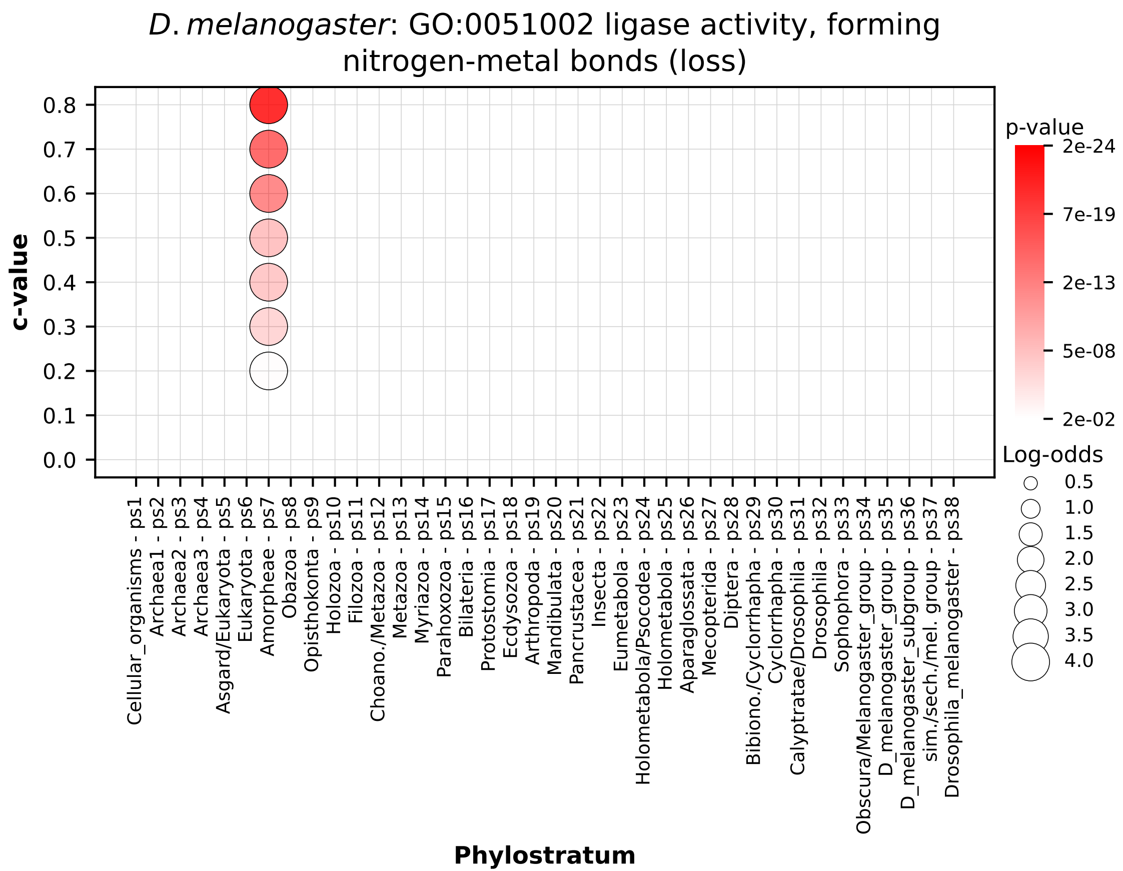
D. melanogaster: GO:0051002 ligase activity, forming nitrogen-metal bonds (loss)
c-value
0.0
0.1
0.2
0.3
0.4
0.5
0.6
0.7
0.8
p-value
2e-24
7e-19
2e-13
5e-08
2e-02
Log-odds
0.5
1.0
1.5
2.0
2.5
3.0
3.5
4.0
Cellular_organisms - ps1
Archaea1 - ps2
Archaea2 - ps3
Archaea3 - ps4
Asgard/Eukaryota - ps5
Eukaryota - ps6
Amorpheae - ps7
Obazoa - ps8
Opisthokonta - ps9
Holozoa - ps10
Filozoa - ps11
Choano./Metazoa - ps12
Metazoa - ps13
Myriazoa - ps14
Parahoxozoa - ps15
Bilateria - ps16
Protostomia - ps17
Ecdysozoa - ps18
Arthropoda - ps19
Mandibulata - ps20
Pancrustacea - ps21
Insecta - ps22
Eumetabola - ps23
Holometabola/Psocodea - ps24
Holometabola - ps25
Aparaglossata - ps26
Mecopterida - ps27
Diptera - ps28
Bibiono./Cyclorrhapha - ps29
Cyclorrhapha - ps30
Calyptratae/Drosophila - ps31
Drosophila - ps32
Sophophora - ps33
Obscura/Melanogaster_group - ps34
D_melanogaster_group - ps35
D_melanogaster_subgroup - ps36
sim./sech./mel. group - ps37
Drosophila_melanogaster - ps38
Phylostratum

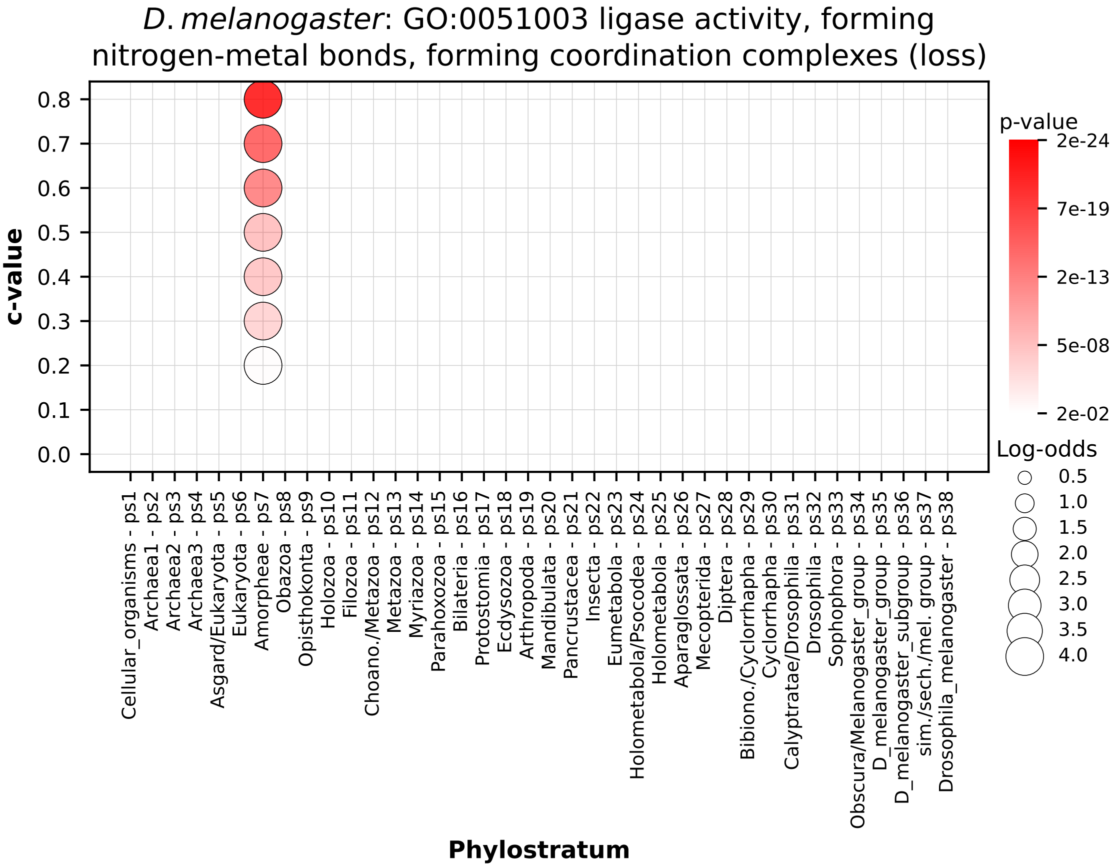

D. melanogaster: GO:0051003 ligase activity, forming nitrogen-metal bonds, forming coordination complexes (loss)
c-value
0.8
0.7
0.6
0.5
0.4
0.3
0.2
0.1
0.0
p-value
2e-24
7e-19
2e-13
5e-08
2e-02
Log-odds
0.5
1.0
1.5
2.0
2.5
3.0
3.5
4.0
Cellular_organisms - ps1
Archaea1 - ps2
Archaea2 - ps3
Archaea3 - ps4
Asgard/Eukaryota - ps5
Eukaryota - ps6
Amorpheae - ps7
Obazoa - ps8
Opisthokonta - ps9
Holozoa - ps10
Filozoa - ps11
Choano./Metazoa - ps12
Metazoa - ps13
Myriazoa - ps14
Parahoxozoa - ps15
Bilateria - ps16
Protostomia - ps17
Ecdysozoa - ps18
Arthropoda - ps19
Mandibulata - ps20
Pancrustacea - ps21
Insecta - ps22
Eumetabola - ps23
Holometabola/Psocodea - ps24
Holometabola - ps25
Aparaglossata - ps26
Mecopterida - ps27
Diptera - ps28
Bibiono./Cyclorrhapha - ps29
Cyclorrhapha - ps30
Calyptratae/Drosophila - ps31
Drosophila - ps32
Sophophora - ps33
Obscura/Melanogaster_group - ps34
D_melanogaster_group - ps35
D_melanogaster_subgroup - ps36
sim./sech./mel. group - ps37
Drosophila_melanogaster - ps38
Phylostratum

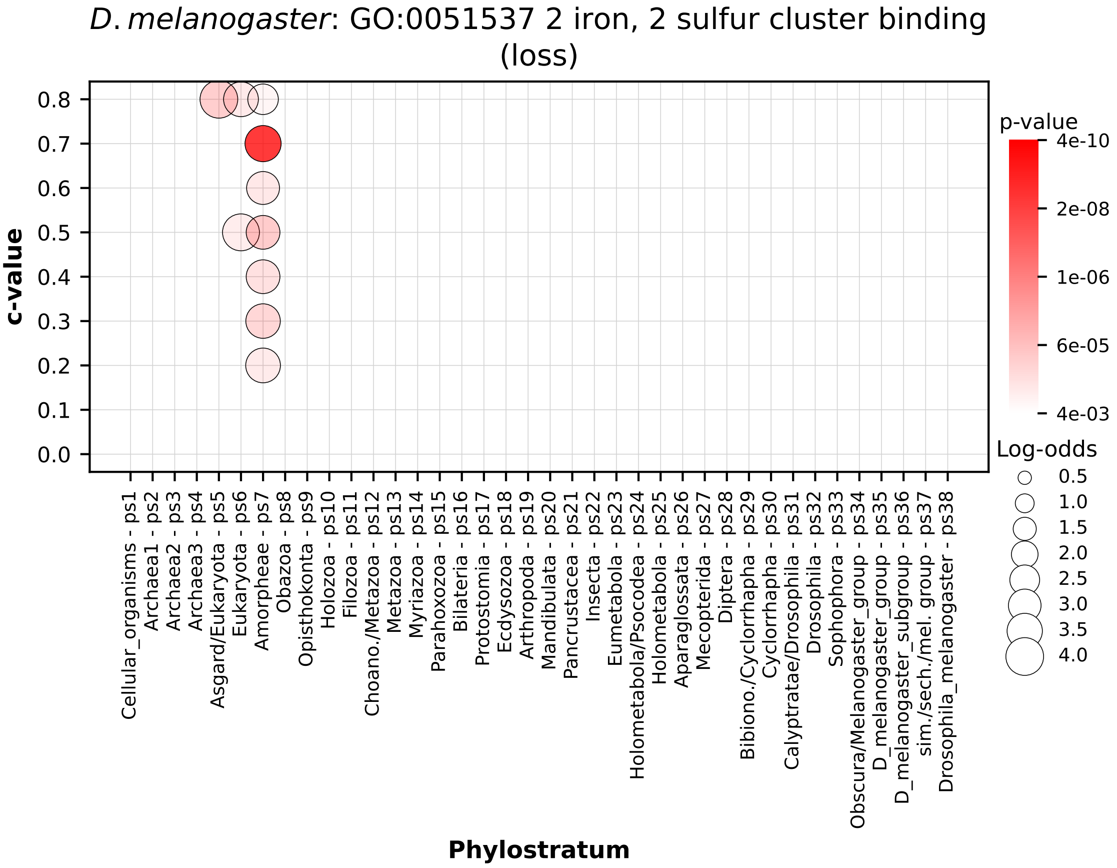

D. melanogaster: GO:0051537 2 iron, 2 sulfur cluster binding (loss)
c-value
0.0
0.1
0.2
0.3
0.4
0.5
0.6
0.7
0.8
Phylostratum
Cellular_organisms - ps1
Archaea1 - ps2
Archaea2 - ps3
Archaea3 - ps4
Asgard/Eukaryota - ps5
Eukaryota - ps6
Amorpheae - ps7
Obazoa - ps8
Opisthokonta - ps9
Holozoa - ps10
Filozoa - ps11
Choano./Metazoa - ps12
Metazoa - ps13
Myriazoa - ps14
Parahoxozoa - ps15
Bilateria - ps16
Protostomia - ps17
Ecdysozoa - ps18
Arthropoda - ps19
Mandibulata - ps20
Pancrustacea - ps21
Insecta - ps22
Eumetabola - ps23
Holometabola/Psocodea - ps24
Holometabola - ps25
Aparaglossata - ps26
Mecopterida - ps27
Diptera - ps28
Bibiono./Cyclorrhapha - ps29
Cyclorrhapha - ps30
Calyptratae/Drosophila - ps31
Drosophila - ps32
Sophophora - ps33
Obscura/Melanogaster_group - ps34
D_melanogaster_group - ps35
D_melanogaster_subgroup - ps36
sim./sech./mel. group - ps37
Drosophila_melanogaster - ps38
p-value
4e-10
2e-08
1e-06
6e-05
4e-03
Log-odds
0.5
1.0
1.5
2.0
2.5
3.0
3.5
4.0

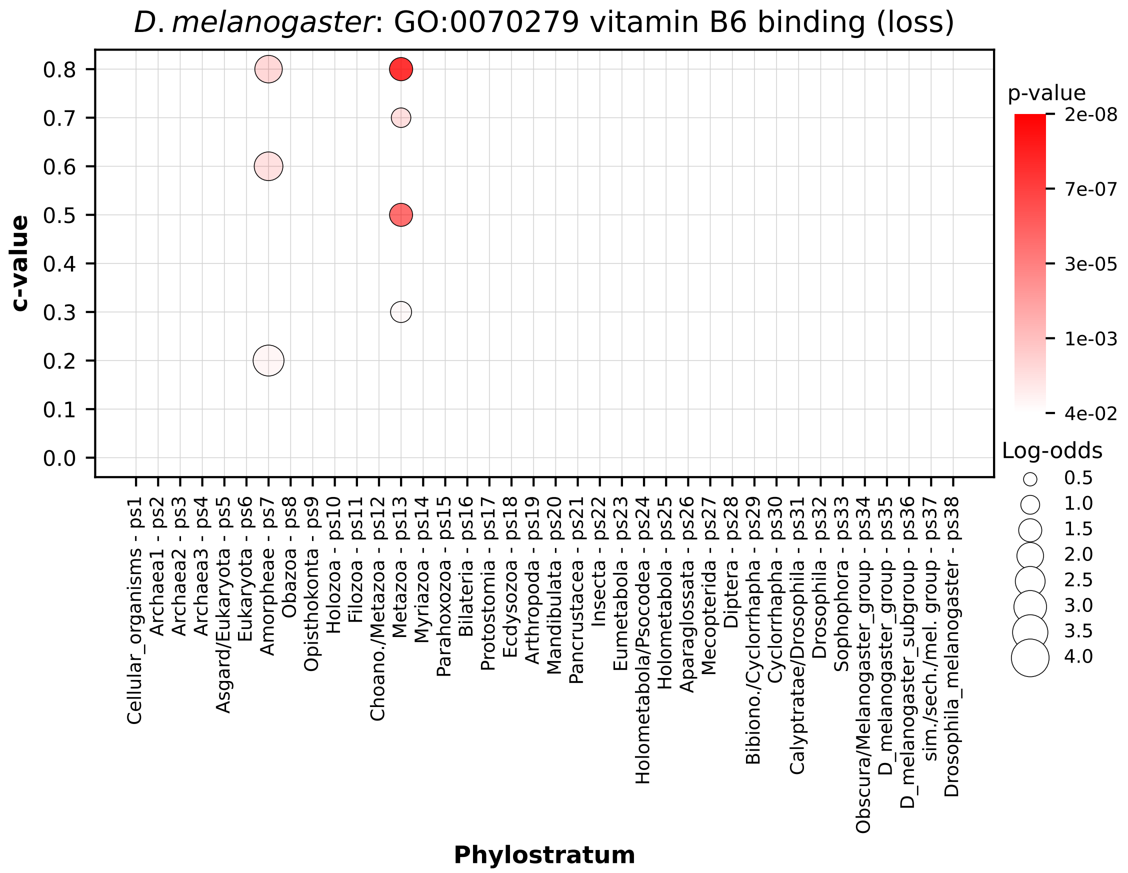

D. melanogaster: GO:0070279 vitamin B6 binding (loss)
c-value
0.0
0.1
0.2
0.3
0.4
0.5
0.6
0.7
0.8
Phylostratum
Cellular_organisms - ps1
Archaea1 - ps2
Archaea2 - ps3
Archaea3 - ps4
Asgard/Eukaryota - ps5
Eukaryota - ps6
Amorpheae - ps7
Obazoa - ps8
Opisthokonta - ps9
Holozoa - ps10
Filozoa - ps11
Choano./Metazoa - ps12
Metazoa - ps13
Myriazoa - ps14
Parahoxozoa - ps15
Bilateria - ps16
Protostomia - ps17
Ecdysozoa - ps18
Arthropoda - ps19
Mandibulata - ps20
Pancrustacea - ps21
Insecta - ps22
Eumetabola - ps23
Holometabola/Psocodea - ps24
Holometabola - ps25
Aparaglossata - ps26
Mecopterida - ps27
Diptera - ps28
Bibiono./Cyclorrhapha - ps29
Cyclorrhapha - ps30
Calyptratae/Drosophila - ps31
Drosophila - ps32
Sophophora - ps33
Obscura/Melanogaster_group - ps34
D_melanogaster_group - ps35
D_melanogaster_subgroup - ps36
sim./sech./mel. group - ps37
Drosophila_melanogaster - ps38
p-value
2e-08
7e-07
3e-05
1e-03
4e-02
Log-odds
0.5
1.0
1.5
2.0
2.5
3.0
3.5
4.0

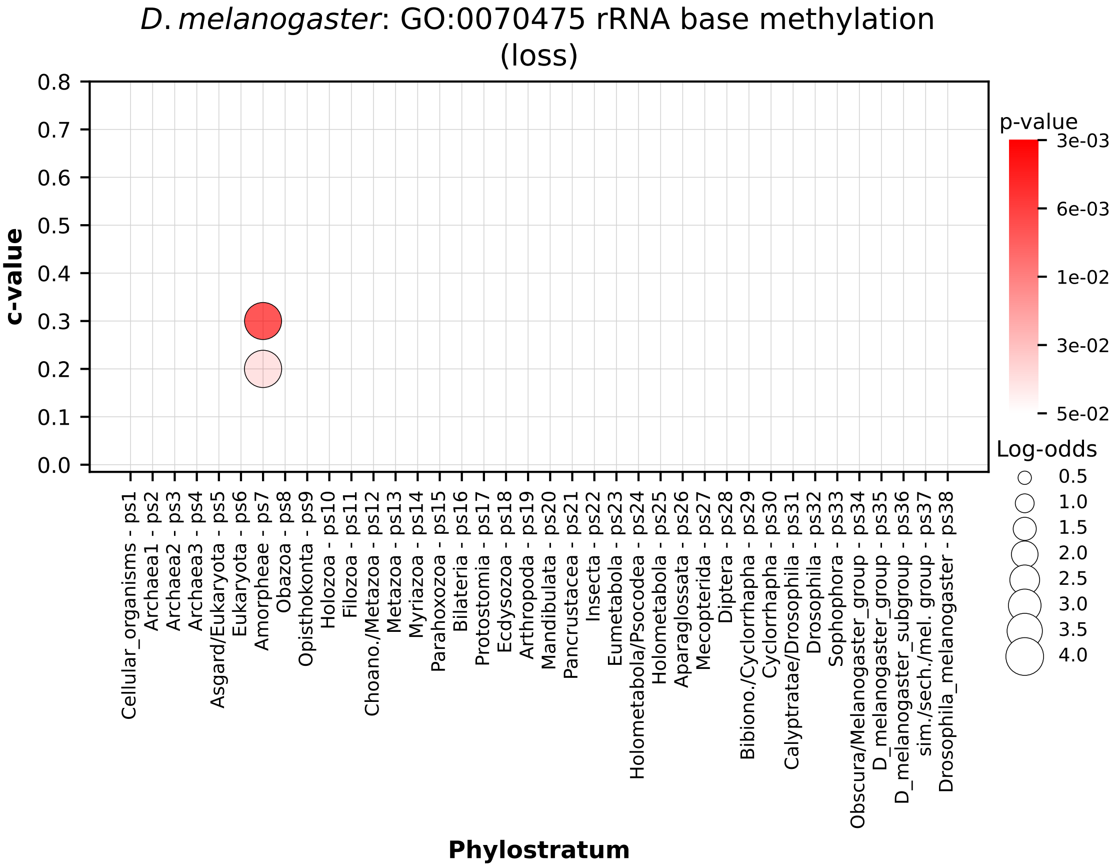
D. melanogaster: GO:0070475 rRNA base methylation
(loss)
c-value
0.8
0.7
0.6
0.5
0.4
0.3
0.2
0.1
0.0
p-value
3e-03
6e-03
1e-02
3e-02
5e-02
Log-odds
0.5
1.0
1.5
2.0
2.5
3.0
3.5
4.0
Cellular_organisms - ps1
Archaea1 - ps2
Archaea2 - ps3
Archaea3 - ps4
Asgard/Eukaryota - ps5
Eukaryota - ps6
Amorpheae - ps7
Obazoa - ps8
Opisthokonta - ps9
Holozoa - ps10
Filozoa - ps11
Choano./Metazoa - ps12
Metazoa - ps13
Myriazoa - ps14
Parahoxozoa - ps15
Bilateria - ps16
Protostomia - ps17
Ecdysozoa - ps18
Arthropoda - ps19
Mandibulata - ps20
Pancrustacea - ps21
Insecta - ps22
Eumetabola - ps23
Holometabola/Psocodea - ps24
Holometabola - ps25
Aparaglossata - ps26
Mecopterida - ps27
Diptera - ps28
Bibiono./Cyclorrhapha - ps29
Cyclorrhapha - ps30
Calyptratae/Drosophila - ps31
Drosophila - ps32
Sophophora - ps33
Obscura/Melanogaster_group - ps34
D_melanogaster_group - ps35
D_melanogaster_subgroup - ps36
sim./sech./mel. group - ps37
Drosophila_melanogaster - ps38
Phylostratum

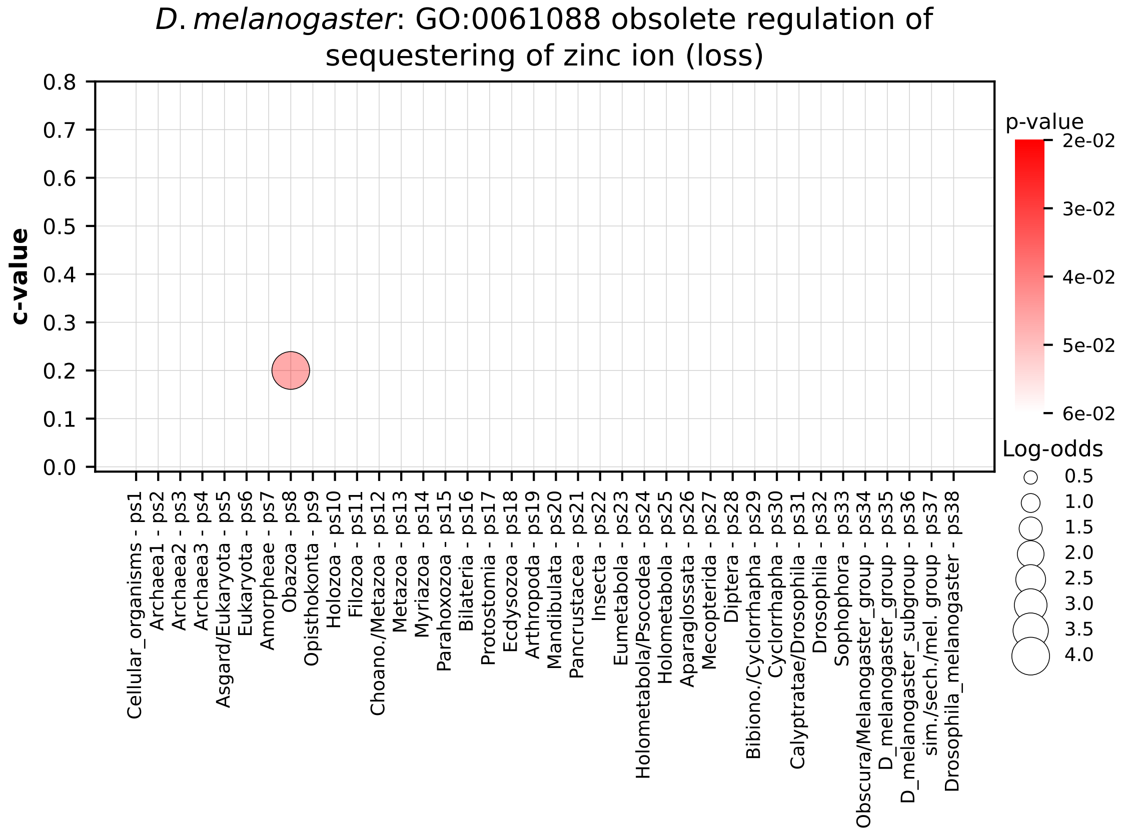

D. melanogaster: GO:0061088 obsolete regulation of sequestering of zinc ion (loss)
c-value
0.0
0.1
0.2
0.3
0.4
0.5
0.6
0.7
0.8
Phylostratum
Cellular_organisms - ps1
Archaea1 - ps2
Archaea2 - ps3
Archaea3 - ps4
Asgard/Eukaryota - ps5
Eukaryota - ps6
Amorpheae - ps7
Obazoa - ps8
Opisthokonta - ps9
Holozoa - ps10
Filozoa - ps11
Choano./Metazoa - ps12
Metazoa - ps13
Myriazoa - ps14
Parahoxozoa - ps15
Bilateria - ps16
Protostomia - ps17
Ecdysozoa - ps18
Arthropoda - ps19
Mandibulata - ps20
Pancrustacea - ps21
Insecta - ps22
Eumetabola - ps23
Holometabola/Psocodea - ps24
Holometabola - ps25
Aparaglossata - ps26
Mecopterida - ps27
Diptera - ps28
Bibiono./Cyclorrhapha - ps29
Cyclorrhapha - ps30
Calyptratae/Drosophila - ps31
Drosophila - ps32
Sophophora - ps33
Obscura/Melanogaster_group - ps34
D_melanogaster_group - ps35
D_melanogaster_subgroup - ps36
sim./sech./mel. group - ps37
Drosophila_melanogaster - ps38
p-value
2e-02
3e-02
4e-02
5e-02
6e-02
Log-odds
0.5
1.0
1.5
2.0
2.5
3.0
3.5
4.0

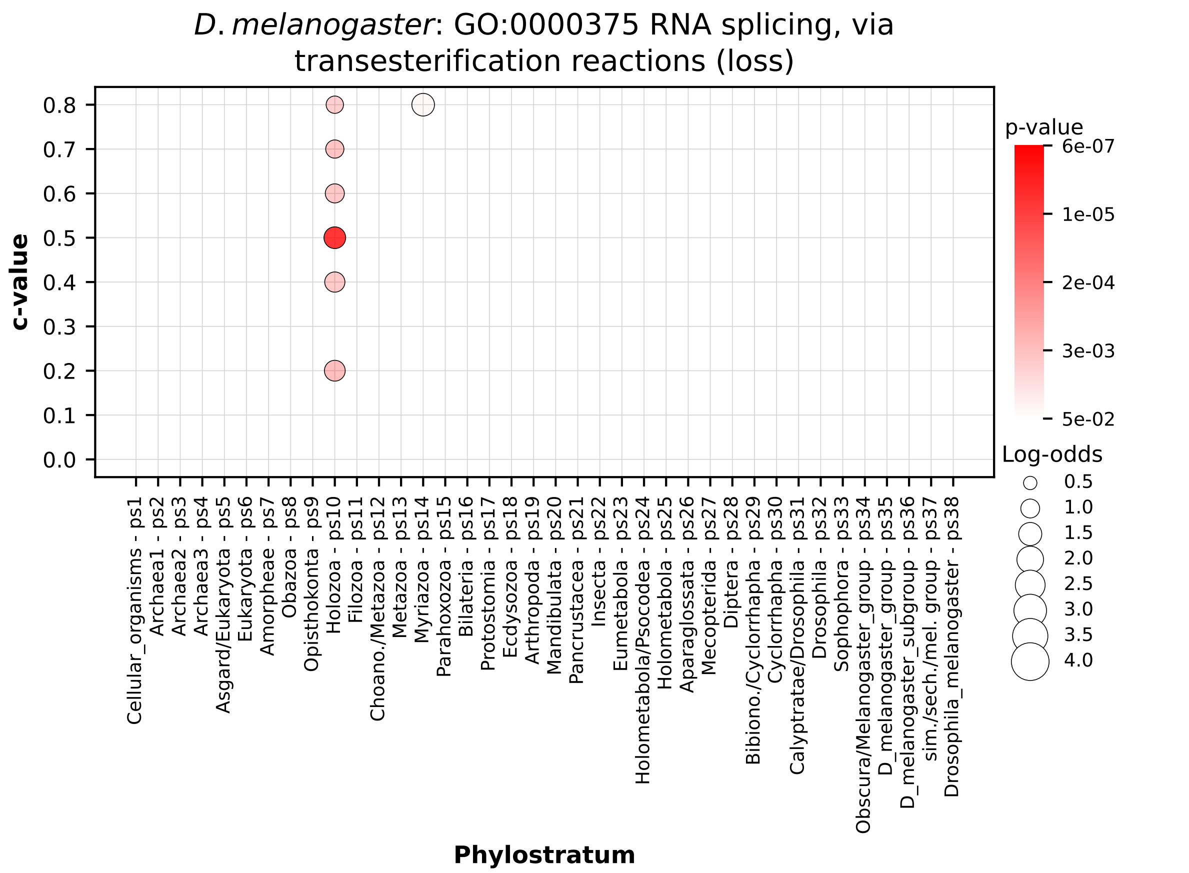

D. melanogaster: GO:0000375 RNA splicing, via transesterification reactions (loss)
c-value
Phylostratum
p-value
6e-07
1e-05
2e-04
3e-03
5e-02
Log-odds
0.5
1.0
1.5
2.0
2.5
3.0
3.5
4.0
Cellular_organisms - ps1
Archaea1 - ps2
Archaea2 - ps3
Archaea3 - ps4
Asgard/Eukaryota - ps5
Eukaryota - ps6
Amorpheae - ps7
Obazoa - ps8
Opisthokonta - ps9
Holozoa - ps10
Filozoa - ps11
Choano./Metazoa - ps12
Metazoa - ps13
Myriazoa - ps14
Parahoxozoa - ps15
Bilateria - ps16
Protostomia - ps17
Ecdysozoa - ps18
Arthropoda - ps19
Mandibulata - ps20
Pancrustacea - ps21
Insecta - ps22
Eumetabola - ps23
Holometabola/Psocodea - ps24
Holometabola - ps25
Aparaglossata - ps26
Mecopterida - ps27
Diptera - ps28
Bibiono./Cyclorrhapha - ps29
Cyclorrhapha - ps30
Calyptratae/Drosophila - ps31
Drosophila - ps32
Sophophora - ps33
Obscura/Melanogaster_group - ps34
D_melanogaster_group - ps35
D_melanogaster_subgroup - ps36
sim./sech./mel. group - ps37
Drosophila_melanogaster - ps38

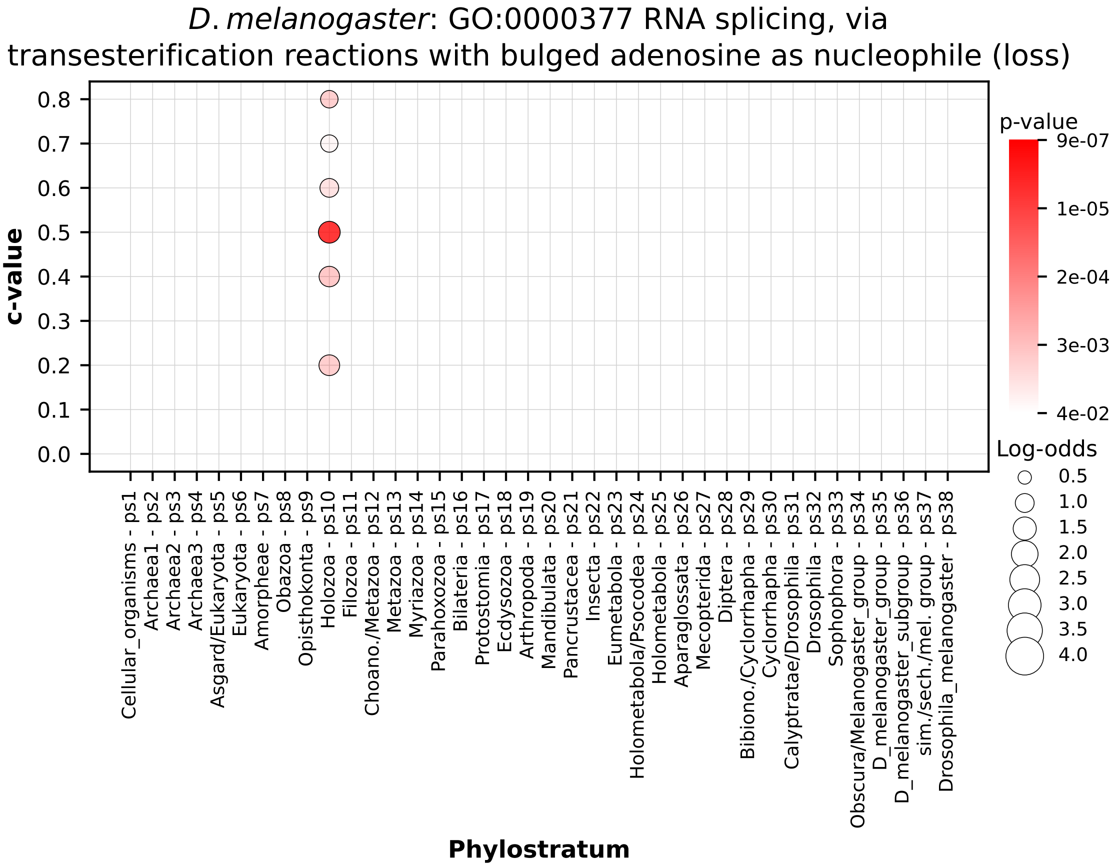

D. melanogaster: GO:0000377 RNA splicing, via transesterification reactions with bulged adenosine as nucleophile (loss)
c-value
0.0
0.1
0.2
0.3
0.4
0.5
0.6
0.7
0.8
p-value
9e-07
1e-05
2e-04
3e-03
4e-02
Log-odds
0.5
1.0
1.5
2.0
2.5
3.0
3.5
4.0
Cellular_organisms - ps1
Archaea1 - ps2
Archaea2 - ps3
Archaea3 - ps4
Asgard/Eukaryota - ps5
Eukaryota - ps6
Amorpheae - ps7
Obazoa - ps8
Opisthokonta - ps9
Holozoa - ps10
Filozoa - ps11
Choano./Metazoa - ps12
Metazoa - ps13
Myriazoa - ps14
Parahoxozoa - ps15
Bilateria - ps16
Protostomia - ps17
Ecdysozoa - ps18
Arthropoda - ps19
Mandibulata - ps20
Pancrustacea - ps21
Insecta - ps22
Eumetabola - ps23
Holometabola/Psocodea - ps24
Holometabola - ps25
Aparaglossata - ps26
Mecopterida - ps27
Diptera - ps28
Bibiono./Cyclorrhapha - ps29
Cyclorrhapha - ps30
Calyptratae/Drosophila - ps31
Drosophila - ps32
Sophophora - ps33
Obscura/Melanogaster_group - ps34
D_melanogaster_group - ps35
D_melanogaster_subgroup - ps36
sim./sech./mel. group - ps37
Drosophila_melanogaster - ps38
Phylostratum

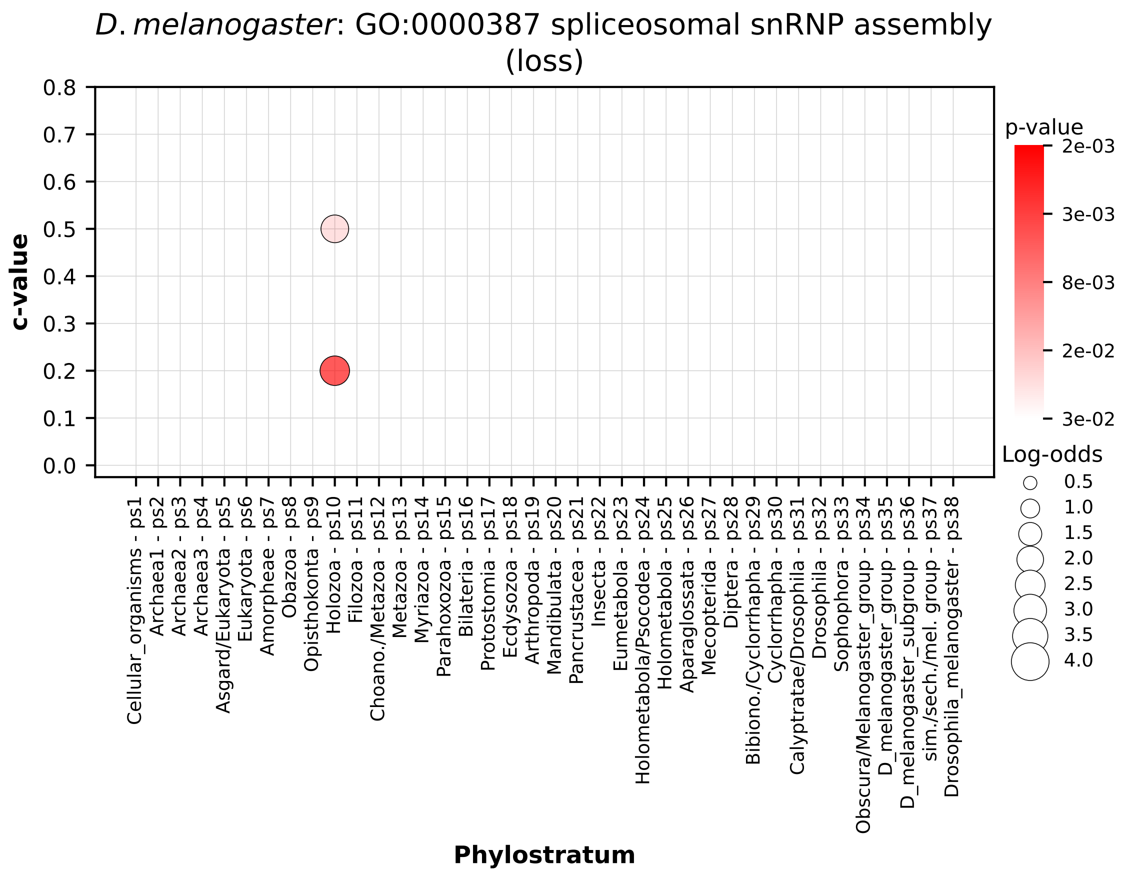
D. melanogaster: GO:0000387 spliceosomal snRNP assembly (loss)
c-value
0.0
0.1
0.2
0.3
0.4
0.5
0.6
0.7
0.8
Phylostratum
Cellular_organisms - ps1
Archaea1 - ps2
Archaea2 - ps3
Archaea3 - ps4
Asgard/Eukaryota - ps5
Eukaryota - ps6
Amorpheae - ps7
Obazoa - ps8
Opisthokonta - ps9
Holozoa - ps10
Filozoa - ps11
Choano./Metazoa - ps12
Metazoa - ps13
Myriazoa - ps14
Parahoxozoa - ps15
Bilateria - ps16
Protostomia - ps17
Ecdysozoa - ps18
Arthropoda - ps19
Mandibulata - ps20
Pancrustacea - ps21
Insecta - ps22
Eumetabola - ps23
Holometabola/Psocodea - ps24
Holometabola - ps25
Aparaglossata - ps26
Mecopterida - ps27
Diptera - ps28
Bibiono./Cyclorrhapha - ps29
Cyclorrhapha - ps30
Calyptratae/Drosophila - ps31
Drosophila - ps32
Sophophora - ps33
Obscura/Melanogaster_group - ps34
D_melanogaster_group - ps35
D_melanogaster_subgroup - ps36
sim./sech./mel. group - ps37
Drosophila_melanogaster - ps38
p-value
2e-03
3e-03
8e-03
2e-02
3e-02
Log-odds
0.5
1.0
1.5
2.0
2.5
3.0
3.5
4.0

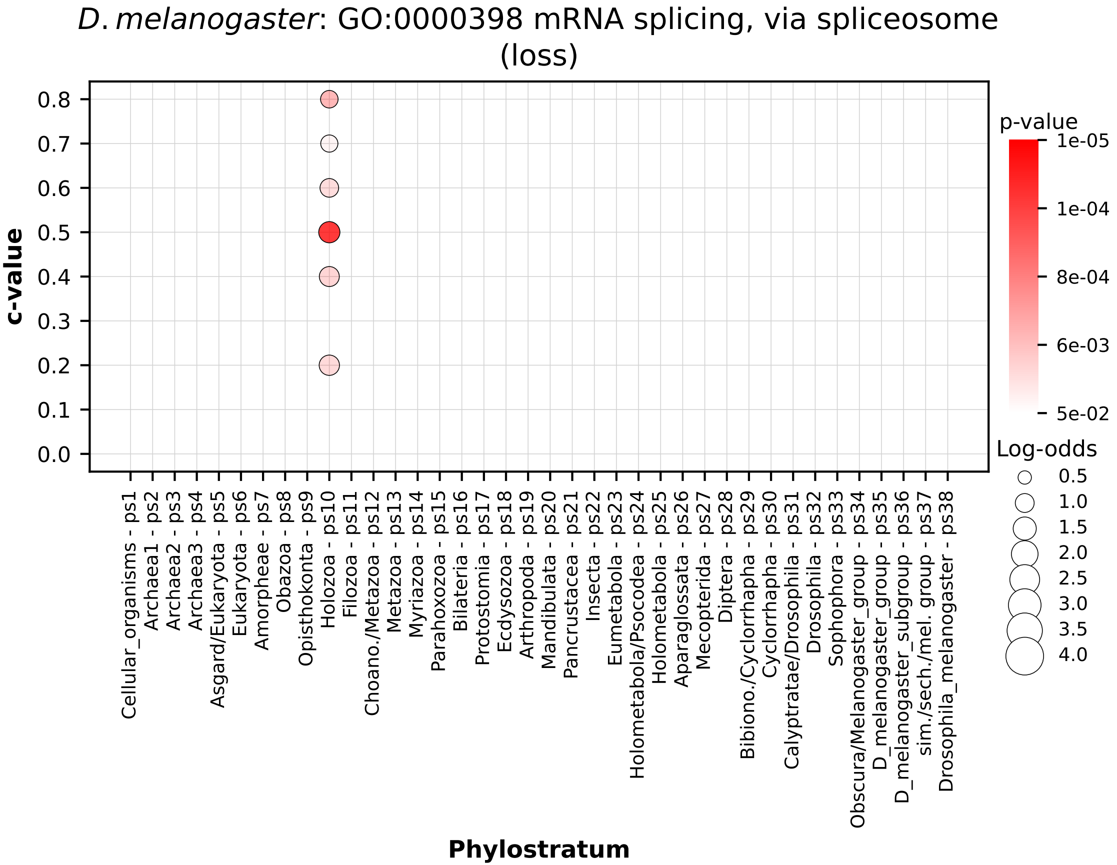

D. melanogaster: GO:0000398 mRNA splicing, via spliceosome
(loss)
c-value
0.0
0.1
0.2
0.3
0.4
0.5
0.6
0.7
0.8
Phylostratum
Cellular_organisms - ps1
Archaea1 - ps2
Archaea2 - ps3
Archaea3 - ps4
Asgard/Eukaryota - ps5
Eukaryota - ps6
Amorpheae - ps7
Obazoa - ps8
Opisthokonta - ps9
Holozoa - ps10
Filozoa - ps11
Choano./Metazoa - ps12
Metazoa - ps13
Myriazoa - ps14
Parahoxozoa - ps15
Bilateria - ps16
Protostomia - ps17
Ecdysozoa - ps18
Arthropoda - ps19
Mandibulata - ps20
Pancrustacea - ps21
Insecta - ps22
Eumetabola - ps23
Holometabola/Psocodea - ps24
Holometabola - ps25
Aparaglossata - ps26
Mecopterida - ps27
Diptera - ps28
Bibiono./Cyclorrhapha - ps29
Cyclorrhapha - ps30
Calyptratae/Drosophila - ps31
Drosophila - ps32
Sophophora - ps33
Obscura/Melanogaster_group - ps34
D_melanogaster_group - ps35
D_melanogaster_subgroup - ps36
sim./sech./mel. group - ps37
Drosophila_melanogaster - ps38
p-value
1e-05
1e-04
8e-04
6e-03
5e-02
Log-odds
0.5
1.0
1.5
2.0
2.5
3.0
3.5
4.0

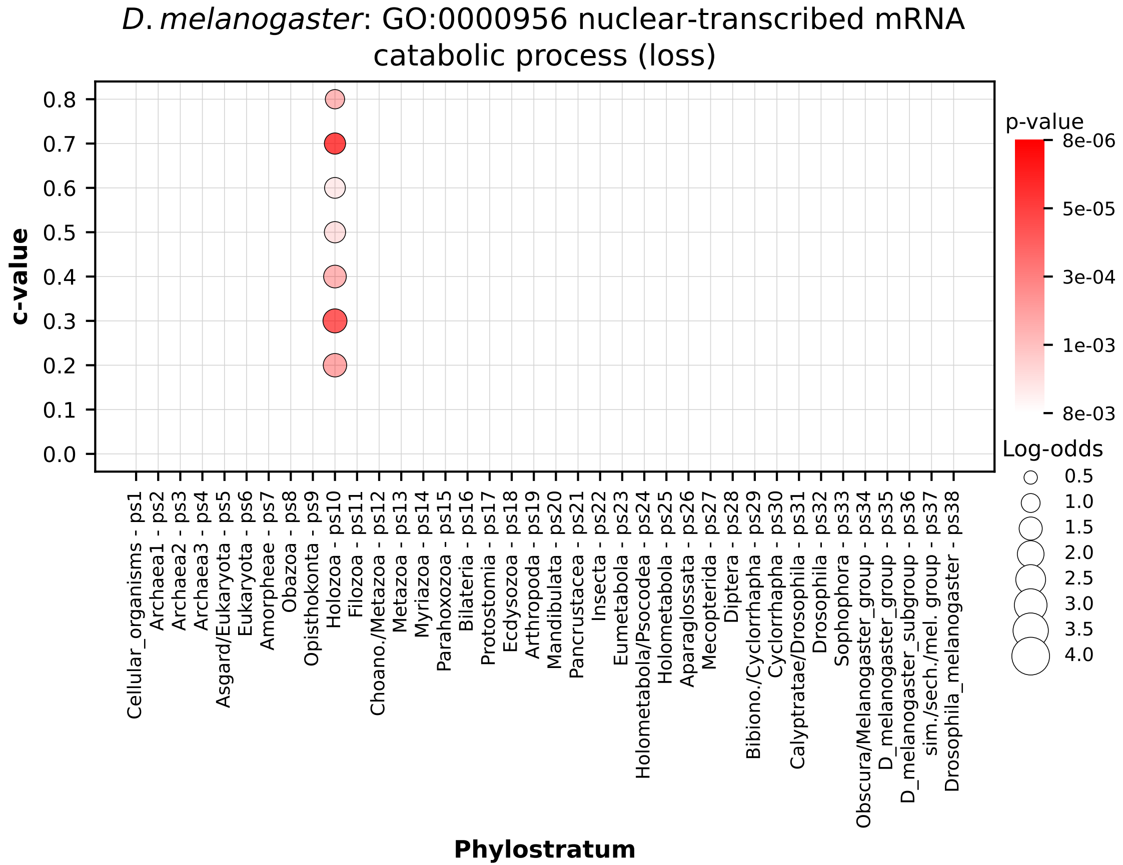

D. melanogaster: GO:0000956 nuclear-transcribed mRNA catabolic process (loss)
c-value
0.8
0.7
0.6
0.5
0.4
0.3
0.2
0.1
0.0
Phylostratum
Cellular_organisms - ps1
Archaea1 - ps2
Archaea2 - ps3
Archaea3 - ps4
Asgard/Eukaryota - ps5
Eukaryota - ps6
Amorpheae - ps7
Obazoa - ps8
Opisthokonta - ps9
Holozoa - ps10
Filozoa - ps11
Choano./Metazoa - ps12
Metazoa - ps13
Myriazoa - ps14
Parahoxozoa - ps15
Bilateria - ps16
Protostomia - ps17
Ecdysozoa - ps18
Arthropoda - ps19
Mandibulata - ps20
Pancrustacea - ps21
Insecta - ps22
Eumetabola - ps23
Holometabola/Psocodea - ps24
Holometabola - ps25
Aparaglossata - ps26
Mecopterida - ps27
Diptera - ps28
Bibiono./Cyclorrhapha - ps29
Cyclorrhapha - ps30
Calyptratae/Drosophila - ps31
Drosophila - ps32
Sophophora - ps33
Obscura/Melanogaster_group - ps34
D_melanogaster_group - ps35
D_melanogaster_subgroup - ps36
sim./sech./mel. group - ps37
Drosophila_melanogaster - ps38
p-value
8e-06
5e-05
3e-04
1e-03
8e-03
Log-odds
0.5
1.0
1.5
2.0
2.5
3.0
3.5
4.0

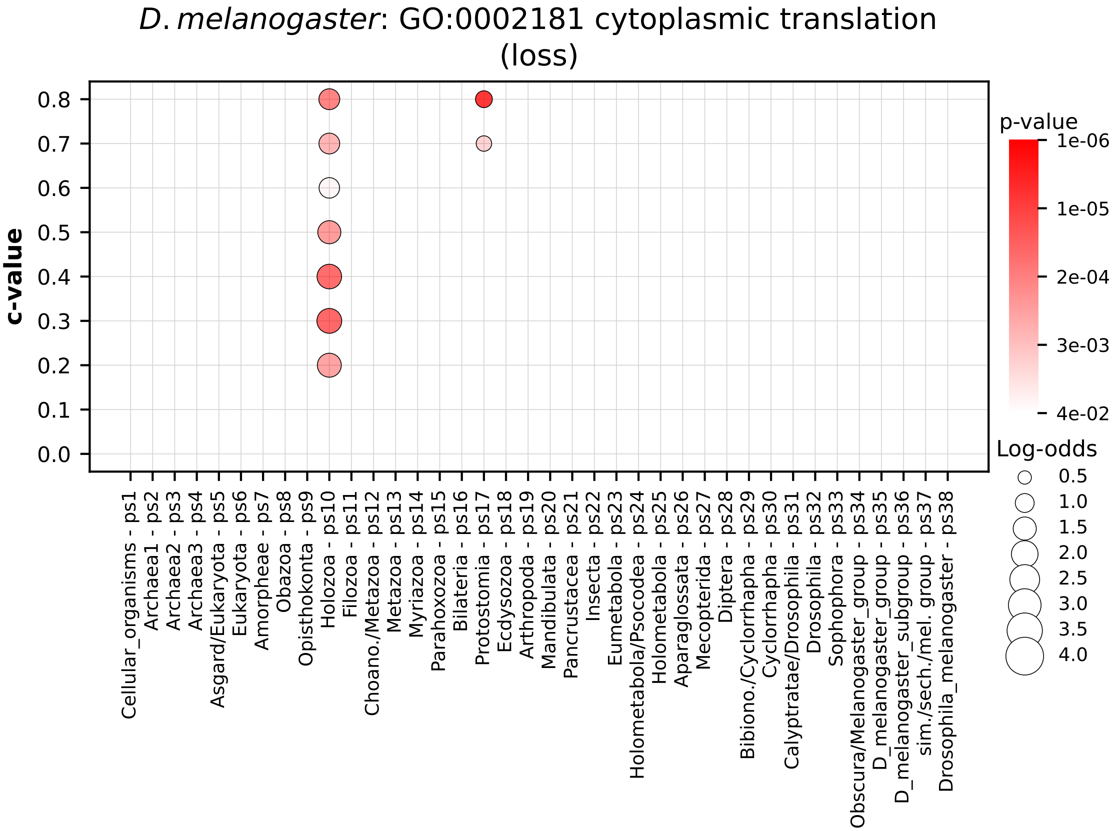

D. melanogaster: GO:0002181 cytoplasmic translation
(loss)
c-value
0.0
0.1
0.2
0.3
0.4
0.5
0.6
0.7
0.8
Phylostratum
Cellular_organisms - ps1
Archaea1 - ps2
Archaea2 - ps3
Archaea3 - ps4
Asgard/Eukaryota - ps5
Eukaryota - ps6
Amorpheae - ps7
Obazoa - ps8
Opisthokonta - ps9
Holozoa - ps10
Filozoa - ps11
Choano./Metazoa - ps12
Metazoa - ps13
Myriazoa - ps14
Parahoxozoa - ps15
Bilateria - ps16
Protostomia - ps17
Ecdysozoa - ps18
Arthropoda - ps19
Mandibulata - ps20
Pancrustacea - ps21
Insecta - ps22
Eumetabola - ps23
Holometabola/Psocodea - ps24
Holometabola - ps25
Aparaglossata - ps26
Mecopterida - ps27
Diptera - ps28
Bibiono./Cyclorrhapha - ps29
Cyclorrhapha - ps30
Calyptratae/Drosophila - ps31
Drosophila - ps32
Sophophora - ps33
Obscura/Melanogaster_group - ps34
D_melanogaster_group - ps35
D_melanogaster_subgroup - ps36
sim./sech./mel. group - ps37
Drosophila_melanogaster - ps38
p-value
1e-06
1e-05
2e-04
3e-03
4e-02
Log-odds
0.5
1.0
1.5
2.0
2.5
3.0
3.5
4.0

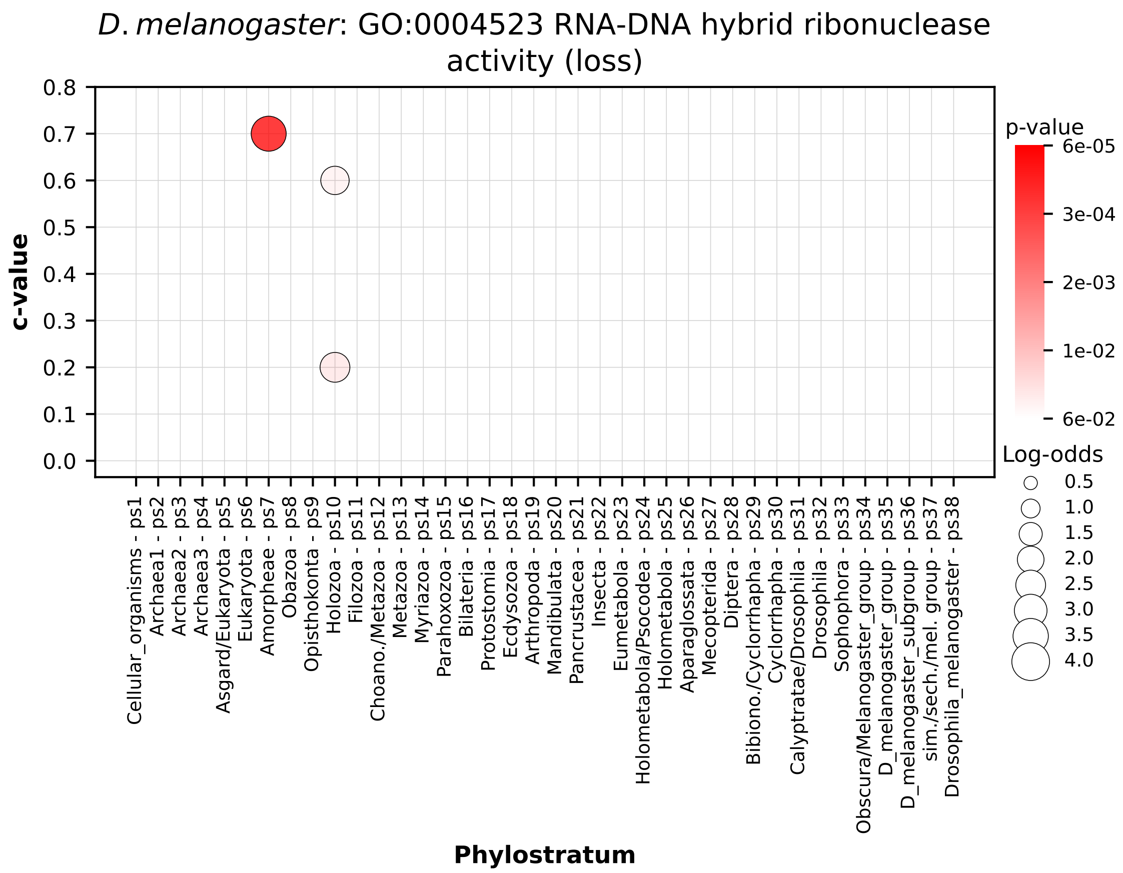

D. melanogaster: GO:0004523 RNA-DNA hybrid ribonuclease activity (loss)
c-value
0.0
0.1
0.2
0.3
0.4
0.5
0.6
0.7
0.8
Phylostratum
Cellular_organisms - ps1
Archaea1 - ps2
Archaea2 - ps3
Archaea3 - ps4
Asgard/Eukaryota - ps5
Eukaryota - ps6
Amorpheae - ps7
Obazoa - ps8
Opisthokonta - ps9
Holozoa - ps10
Filozoa - ps11
Choano./Metazoa - ps12
Metazoa - ps13
Myriazoa - ps14
Parahoxozoa - ps15
Bilateria - ps16
Protostomia - ps17
Ecdysozoa - ps18
Arthropoda - ps19
Mandibulata - ps20
Pancrustacea - ps21
Insecta - ps22
Eumetabola - ps23
Holometabola/Psocodea - ps24
Holometabola - ps25
Aparaglossata - ps26
Mecopterida - ps27
Diptera - ps28
Bibiono./Cyclorrhapha - ps29
Cyclorrhapha - ps30
Calyptratae/Drosophila - ps31
Drosophila - ps32
Sophophora - ps33
Obscura/Melanogaster_group - ps34
D_melanogaster_group - ps35
D_melanogaster_subgroup - ps36
sim./sech./mel. group - ps37
Drosophila_melanogaster - ps38
p-value
6e-05
3e-04
2e-03
1e-02
6e-02
Log-odds
0.5
1.0
1.5
2.0
2.5
3.0
3.5
4.0

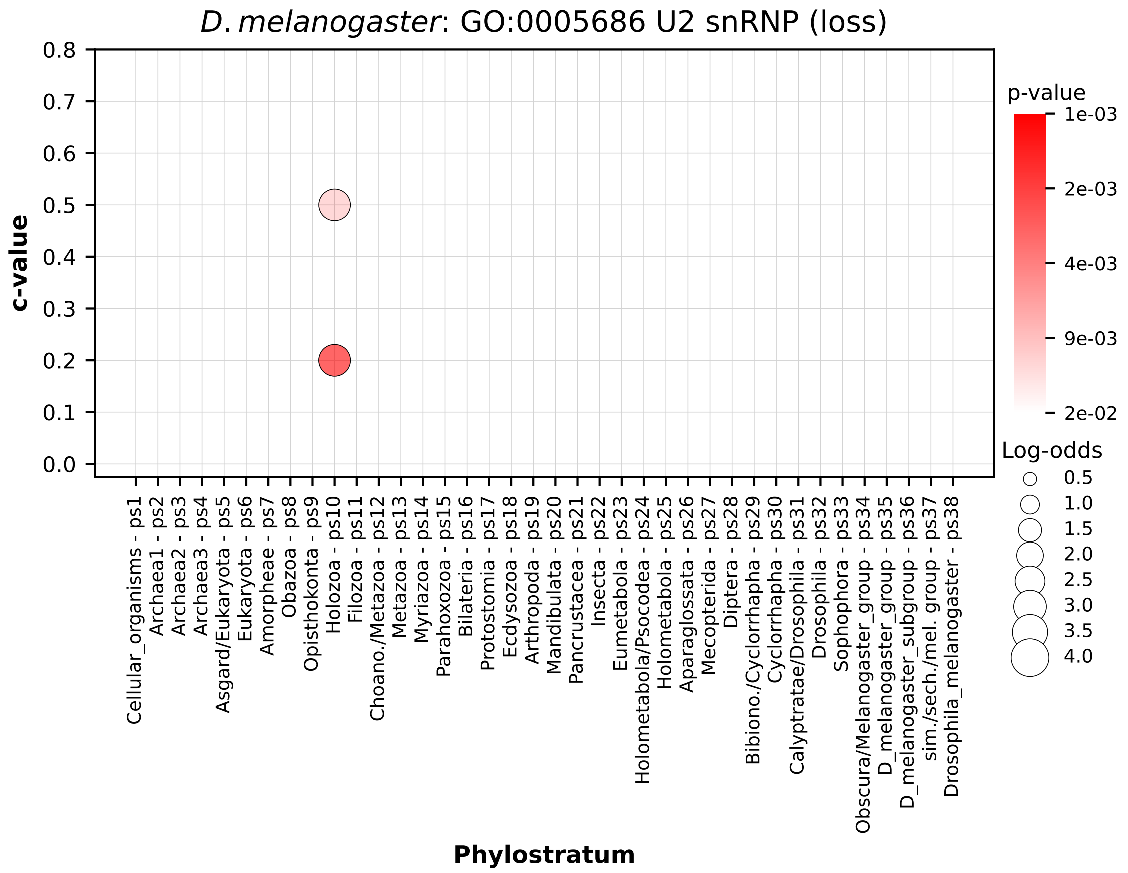
D. melanogaster: GO:0005686 U2 snRNP (loss)
c-value
0.0
0.1
0.2
0.3
0.4
0.5
0.6
0.7
0.8
Phylostratum
Cellular_organisms - ps1
Archaea1 - ps2
Archaea2 - ps3
Archaea3 - ps4
Asgard/Eukaryota - ps5
Eukaryota - ps6
Amorpheae - ps7
Obazoa - ps8
Opisthokonta - ps9
Holozoa - ps10
Filozoa - ps11
Choano./Metazoa - ps12
Metazoa - ps13
Myriazoa - ps14
Parahoxozoa - ps15
Bilateria - ps16
Protostomia - ps17
Ecdysozoa - ps18
Arthropoda - ps19
Mandibulata - ps20
Pancrustacea - ps21
Insecta - ps22
Eumetabola - ps23
Holometabola/Psocodea - ps24
Holometabola - ps25
Aparaglossata - ps26
Mecopterida - ps27
Diptera - ps28
Bibiono./Cyclorrhapha - ps29
Cyclorrhapha - ps30
Calyptratae/Drosophila - ps31
Drosophila - ps32
Sophophora - ps33
Obscura/Melanogaster_group - ps34
D_melanogaster_group - ps35
D_melanogaster_subgroup - ps36
sim./sech./mel. group - ps37
Drosophila_melanogaster - ps38
p-value
1e-03
2e-03
4e-03
9e-03
2e-02
Log-odds
0.5
1.0
1.5
2.0
2.5
3.0
3.5
4.0

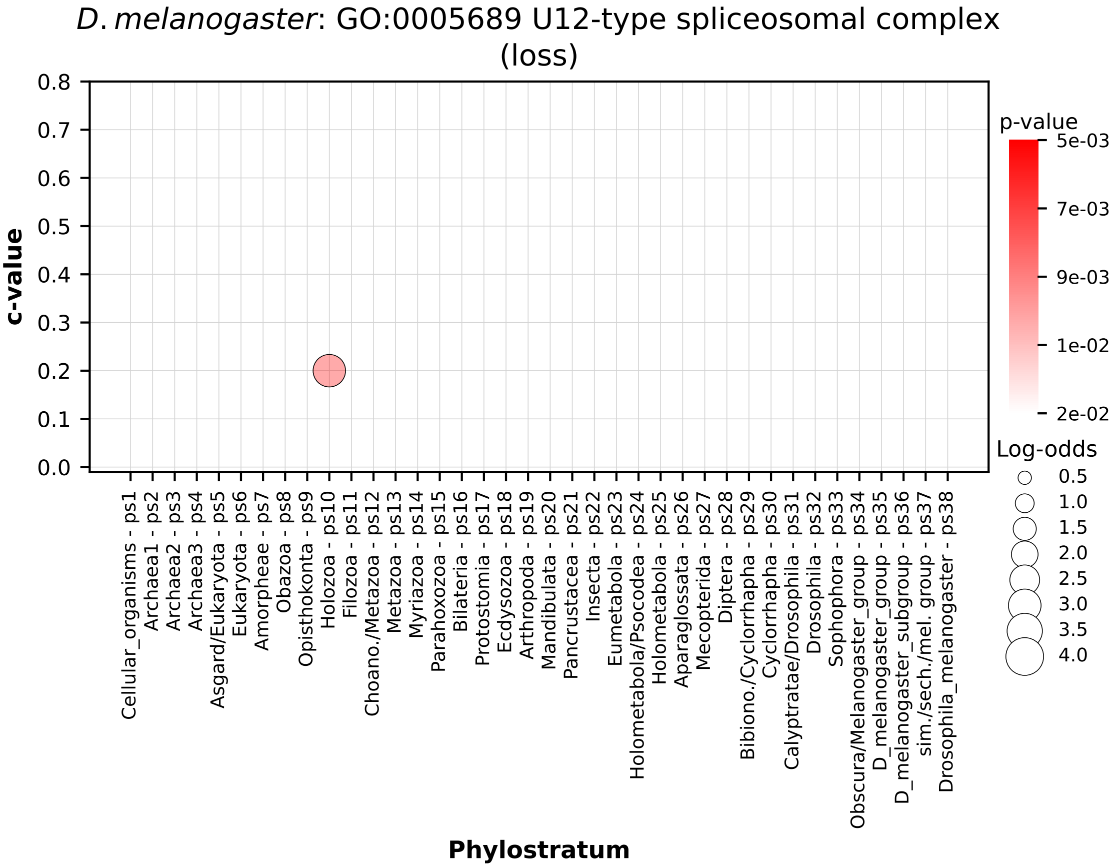

D. melanogaster: GO:0005689 U12-type spliceosomal complex (loss)
c-value
0.8
0.7
0.6
0.5
0.4
0.3
0.2
0.1
0.0
Phylostratum
Cellular_organisms - ps1
Archaea1 - ps2
Archaea2 - ps3
Archaea3 - ps4
Asgard/Eukaryota - ps5
Eukaryota - ps6
Amorpheae - ps7
Obazoa - ps8
Opisthokonta - ps9
Holozoa - ps10
Filozoa - ps11
Choano./Metazoa - ps12
Metazoa - ps13
Myriazoa - ps14
Parahoxozoa - ps15
Bilateria - ps16
Protostomia - ps17
Ecdysozoa - ps18
Arthropoda - ps19
Mandibulata - ps20
Pancrustacea - ps21
Insecta - ps22
Eumetabola - ps23
Holometabola/Psocodea - ps24
Holometabola - ps25
Aparaglossata - ps26
Mecopterida - ps27
Diptera - ps28
Bibiono./Cyclorrhapha - ps29
Cyclorrhapha - ps30
Calyptratae/Drosophila - ps31
Drosophila - ps32
Sophophora - ps33
Obscura/Melanogaster_group - ps34
D_melanogaster_group - ps35
D_melanogaster_subgroup - ps36
sim./sech./mel. group - ps37
Drosophila_melanogaster - ps38
p-value
5e-03
7e-03
9e-03
1e-02
2e-02
Log-odds
0.5
1.0
1.5
2.0
2.5
3.0
3.5
4.0

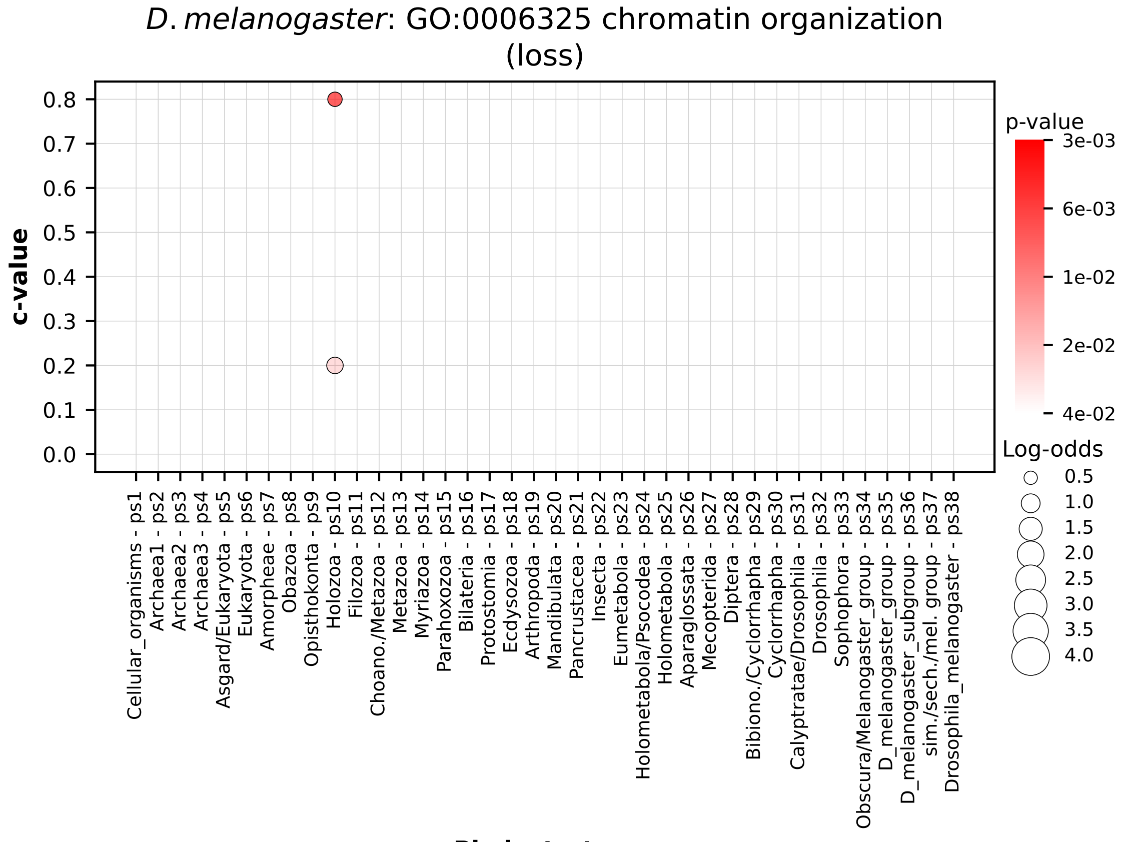

D. melanogaster: GO:0006325 chromatin organization (loss)
c-value
0.8
0.7
0.6
0.5
0.4
0.3
0.2
0.1
0.0
Phylostratum
Cellular_organisms - ps1
Archaea1 - ps2
Archaea2 - ps3
Archaea3 - ps4
Asgard/Eukaryota - ps5
Eukaryota - ps6
Amorpheae - ps7
Obazoa - ps8
Opisthokonta - ps9
Holozoa - ps10
Filozoa - ps11
Choano./Metazoa - ps12
Metazoa - ps13
Myriazoa - ps14
Parahoxozoa - ps15
Bilateria - ps16
Protostomia - ps17
Ecdysozoa - ps18
Arthropoda - ps19
Mandibulata - ps20
Pancrustacea - ps21
Insecta - ps22
Eumetabola - ps23
Holometabola/Psocodea - ps24
Holometabola - ps25
Aparaglossata - ps26
Mecopterida - ps27
Diptera - ps28
Bibiono./Cyclorrhapha - ps29
Cyclorrhapha - ps30
Calyptratae/Drosophila - ps31
Drosophila - ps32
Sophophora - ps33
Obscura/Melanogaster_group - ps34
D_melanogaster_group - ps35
D_melanogaster_subgroup - ps36
sim./sech./mel. group - ps37
Drosophila_melanogaster - ps38
p-value
3e-03
6e-03
1e-02
2e-02
4e-02
Log-odds
0.5
1.0
1.5
2.0
2.5
3.0
3.5
4.0

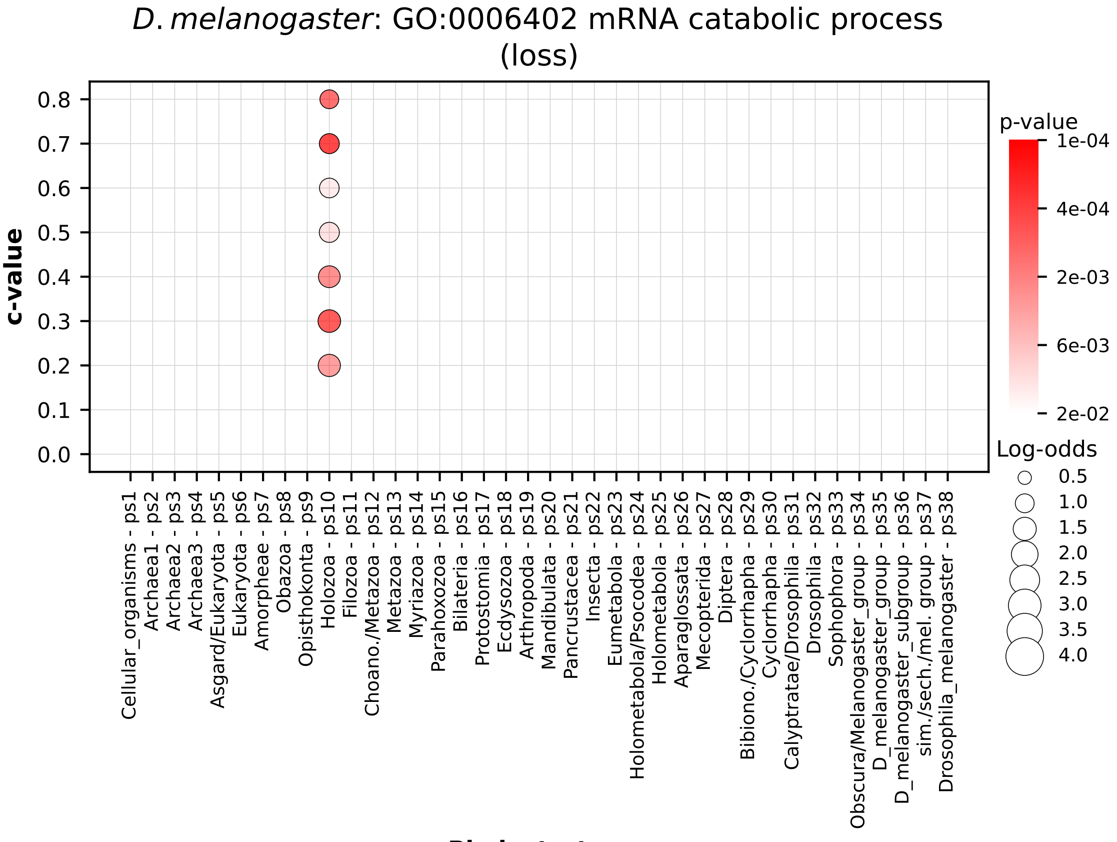

D. melanogaster: GO:0006402 mRNA catabolic process (loss)
c-value
0.0
0.1
0.2
0.3
0.4
0.5
0.6
0.7
0.8
Phylostratum
Cellular_organisms - ps1
Archaea1 - ps2
Archaea2 - ps3
Archaea3 - ps4
Asgard/Eukaryota - ps5
Eukaryota - ps6
Amorpheae - ps7
Obazoa - ps8
Opisthokonta - ps9
Holozoa - ps10
Filozoa - ps11
Choano./Metazoa - ps12
Metazoa - ps13
Myriazoa - ps14
Parahoxozoa - ps15
Bilateria - ps16
Protostomia - ps17
Ecdysozoa - ps18
Arthropoda - ps19
Mandibulata - ps20
Pancrustacea - ps21
Insecta - ps22
Eumetabola - ps23
Holometabola/Psocodea - ps24
Holometabola - ps25
Aparaglossata - ps26
Mecopterida - ps27
Diptera - ps28
Bibiono./Cyclorrhapha - ps29
Cyclorrhapha - ps30
Calyptratae/Drosophila - ps31
Drosophila - ps32
Sophophora - ps33
Obscura/Melanogaster_group - ps34
D_melanogaster_group - ps35
D_melanogaster_subgroup - ps36
sim./sech./mel. group - ps37
Drosophila_melanogaster - ps38
p-value
1e-04
4e-04
2e-03
6e-03
2e-02
Log-odds
0.5
1.0
1.5
2.0
2.5
3.0
3.5
4.0

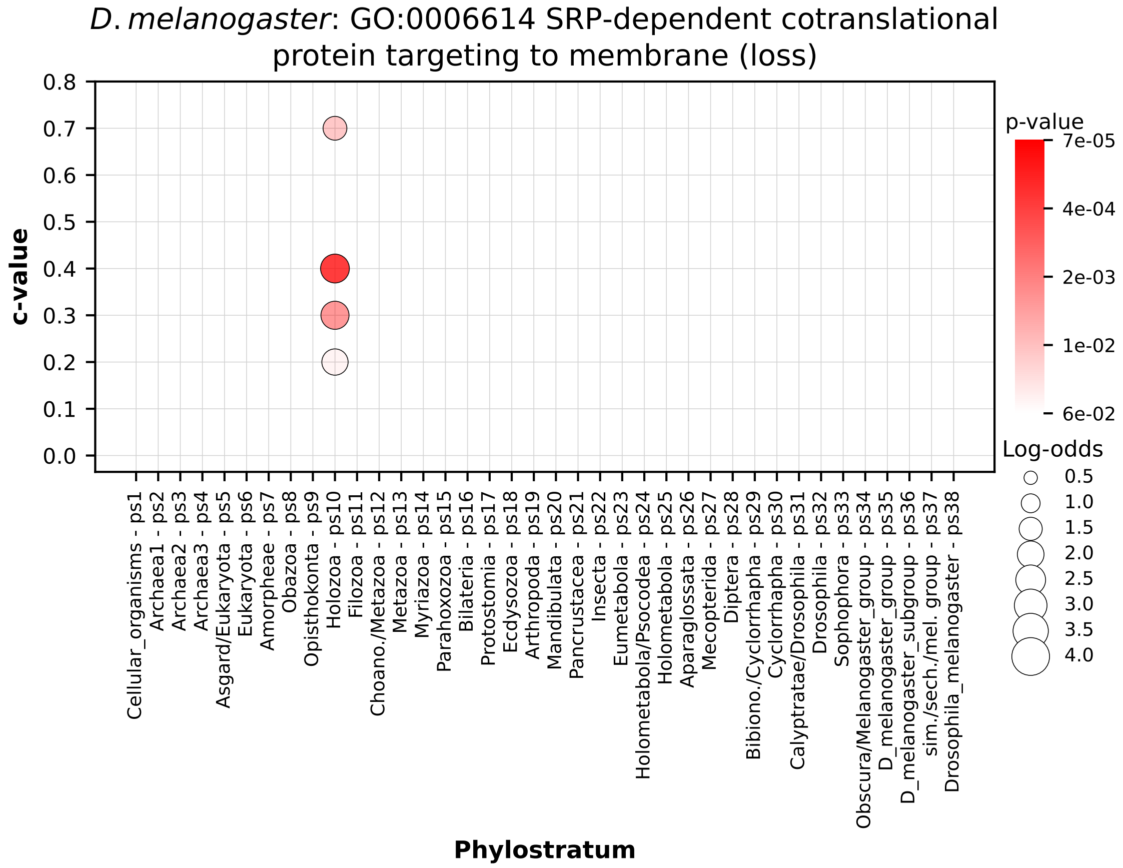

D. melanogaster: GO:0006614 SRP-dependent cotranslational protein targeting to membrane (loss)
c-value
0.8
0.7
0.6
0.5
0.4
0.3
0.2
0.1
0.0
p-value
7e-05
4e-04
2e-03
1e-02
6e-02
Log-odds
0.5
1.0
1.5
2.0
2.5
3.0
3.5
4.0
Cellular_organisms - ps1
Archaea1 - ps2
Archaea2 - ps3
Archaea3 - ps4
Asgard/Eukaryota - ps5
Eukaryota - ps6
Amorpheae - ps7
Obazoa - ps8
Opisthokonta - ps9
Holozoa - ps10
Filozoa - ps11
Choano./Metazoa - ps12
Metazoa - ps13
Myriazoa - ps14
Parahoxozoa - ps15
Bilateria - ps16
Protostomia - ps17
Ecdysozoa - ps18
Arthropoda - ps19
Mandibulata - ps20
Pancrustacea - ps21
Insecta - ps22
Eumetabola - ps23
Holometabola/Psocodea - ps24
Holometabola - ps25
Aparaglossata - ps26
Mecopterida - ps27
Diptera - ps28
Bibiono./Cyclorrhapha - ps29
Cyclorrhapha - ps30
Calyptratae/Drosophila - ps31
Drosophila - ps32
Sophophora - ps33
Obscura/Melanogaster_group - ps34
D_melanogaster_group - ps35
D_melanogaster_subgroup - ps36
sim./sech./mel. group - ps37
Drosophila_melanogaster - ps38
Phylostratum

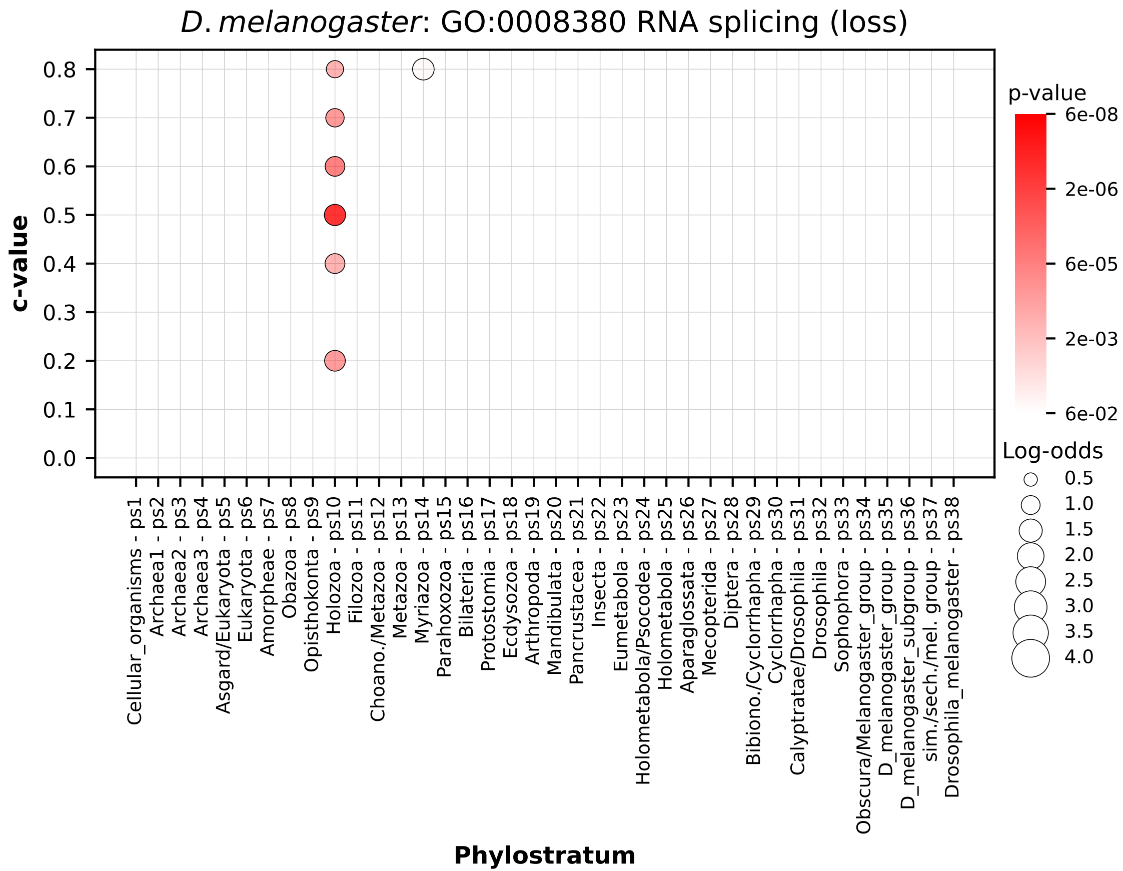

D. melanogaster: GO:0008380 RNA splicing (loss)
c-value
0.0
0.1
0.2
0.3
0.4
0.5
0.6
0.7
0.8
p-value
6e-08
2e-06
6e-05
2e-03
6e-02
Log-odds
0.5
1.0
1.5
2.0
2.5
3.0
3.5
4.0
Cellular_organisms - ps1
Archaea1 - ps2
Archaea2 - ps3
Archaea3 - ps4
Asgard/Eukaryota - ps5
Eukaryota - ps6
Amorpheae - ps7
Obazoa - ps8
Opisthokonta - ps9
Holozoa - ps10
Filozoa - ps11
Choano./Metazoa - ps12
Metazoa - ps13
Myriazoa - ps14
Parahoxozoa - ps15
Bilateria - ps16
Protostomia - ps17
Ecdysozoa - ps18
Arthropoda - ps19
Mandibulata - ps20
Pancrustacea - ps21
Insecta - ps22
Eumetabola - ps23
Holometabola/Psocodea - ps24
Holometabola - ps25
Aparaglossata - ps26
Mecopterida - ps27
Diptera - ps28
Bibiono./Cyclorrhapha - ps29
Cyclorrhapha - ps30
Calyptratae/Drosophila - ps31
Drosophila - ps32
Sophophora - ps33
Obscura/Melanogaster_group - ps34
D_melanogaster_group - ps35
D_melanogaster_subgroup - ps36
sim./sech./mel. group - ps37
Drosophila_melanogaster - ps38
Phylostratum

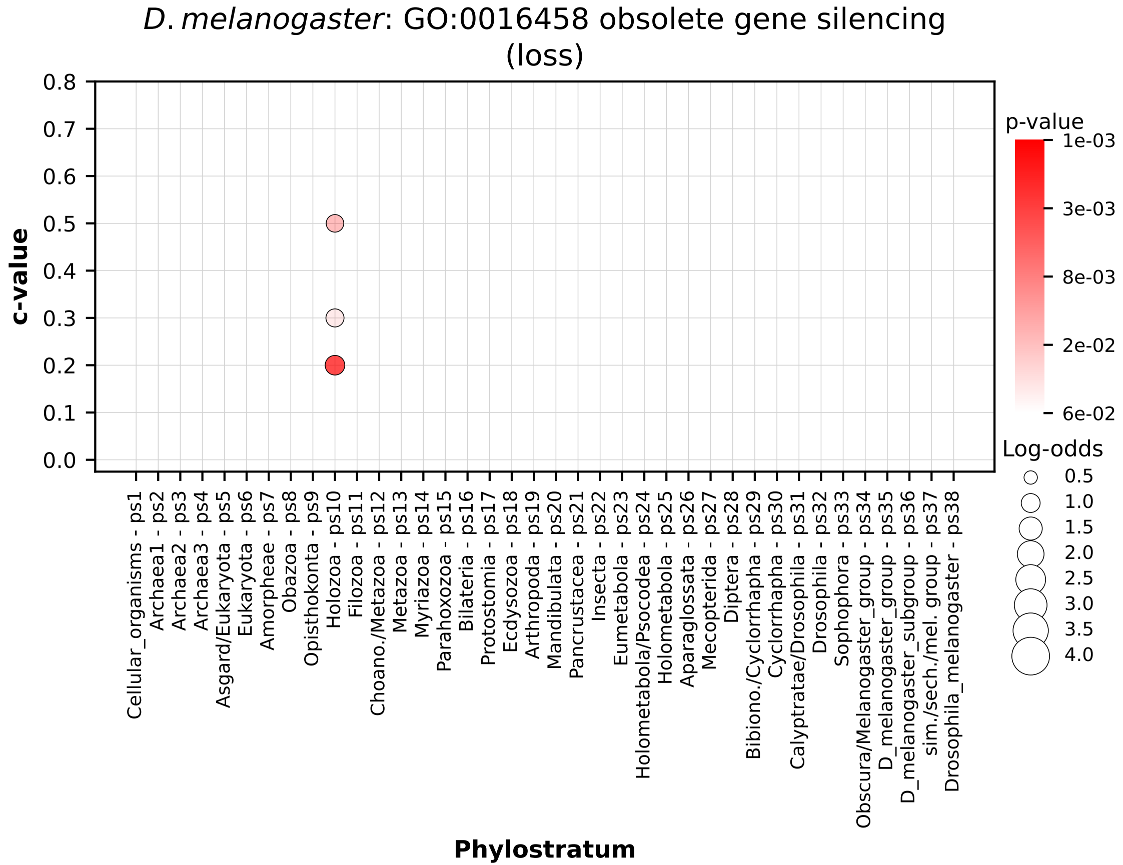

D. melanogaster: GO:0016458 obsolete gene silencing
(loss)
c-value
0.8
0.7
0.6
0.5
0.4
0.3
0.2
0.1
0.0
Phylostratum
Cellular_organisms - ps1
Archaea1 - ps2
Archaea2 - ps3
Archaea3 - ps4
Asgard/Eukaryota - ps5
Eukaryota - ps6
Amorpheae - ps7
Obazoa - ps8
Opisthokonta - ps9
Holozoa - ps10
Filozoa - ps11
Choano./Metazoa - ps12
Metazoa - ps13
Myriazoa - ps14
Parahoxozoa - ps15
Bilateria - ps16
Protostomia - ps17
Ecdysozoa - ps18
Arthropoda - ps19
Mandibulata - ps20
Pancrustacea - ps21
Insecta - ps22
Eumetabola - ps23
Holometabola/Psocodea - ps24
Holometabola - ps25
Aparaglossata - ps26
Mecopterida - ps27
Diptera - ps28
Bibiono./Cyclorrhapha - ps29
Cyclorrhapha - ps30
Calyptratae/Drosophila - ps31
Drosophila - ps32
Sophophora - ps33
Obscura/Melanogaster_group - ps34
D_melanogaster_group - ps35
D_melanogaster_subgroup - ps36
sim./sech./mel. group - ps37
Drosophila_melanogaster - ps38
p-value
1e-03
3e-03
8e-03
2e-02
6e-02
Log-odds
0.5
1.0
1.5
2.0
2.5
3.0
3.5
4.0

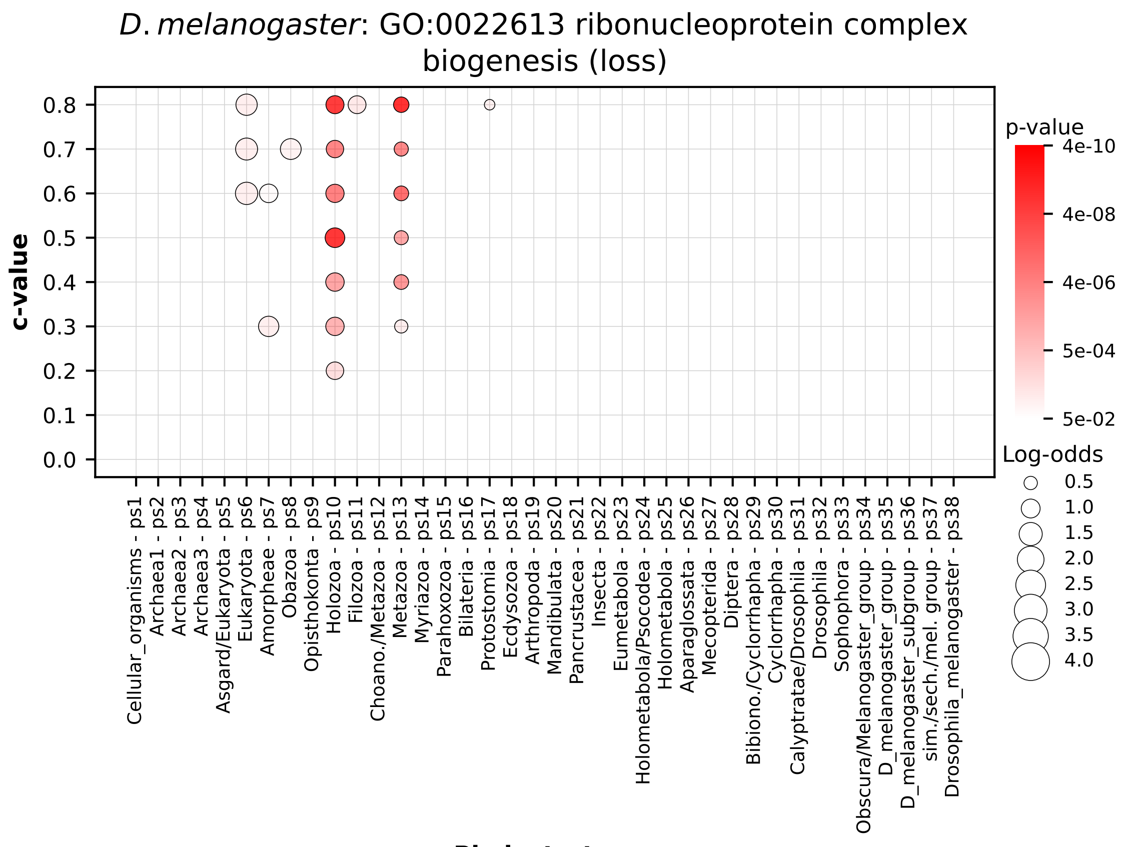

D. melanogaster: GO:0022613 ribonucleoprotein complex biogenesis (loss)
c-value
0.8
0.7
0.6
0.5
0.4
0.3
0.2
0.1
0.0
p-value
4e-10
4e-08
4e-06
5e-04
5e-02
Log-odds
0.5
1.0
1.5
2.0
2.5
3.0
3.5
4.0
Cellular_organisms - ps1
Archaea1 - ps2
Archaea2 - ps3
Archaea3 - ps4
Asgard/Eukaryota - ps5
Eukaryota - ps6
Amorpheae - ps7
Obazoa - ps8
Opisthokonta - ps9
Holozoa - ps10
Filozoa - ps11
Choano./Metazoa - ps12
Metazoa - ps13
Myriazoa - ps14
Parahoxozoa - ps15
Bilateria - ps16
Protostomia - ps17
Ecdysozoa - ps18
Arthropoda - ps19
Mandibulata - ps20
Pancrustacea - ps21
Insecta - ps22
Eumetabola - ps23
Holometabola/Psocodea - ps24
Holometabola - ps25
Aparaglossata - ps26
Mecopterida - ps27
Diptera - ps28
Bibiono./Cyclorrhapha - ps29
Cyclorrhapha - ps30
Calyptratae/Drosophila - ps31
Drosophila - ps32
Sophophora - ps33
Obscura/Melanogaster_group - ps34
D_melanogaster_group - ps35
D_melanogaster_subgroup - ps36
sim./sech./mel. group - ps37
Drosophila_melanogaster - ps38
Phylostratum

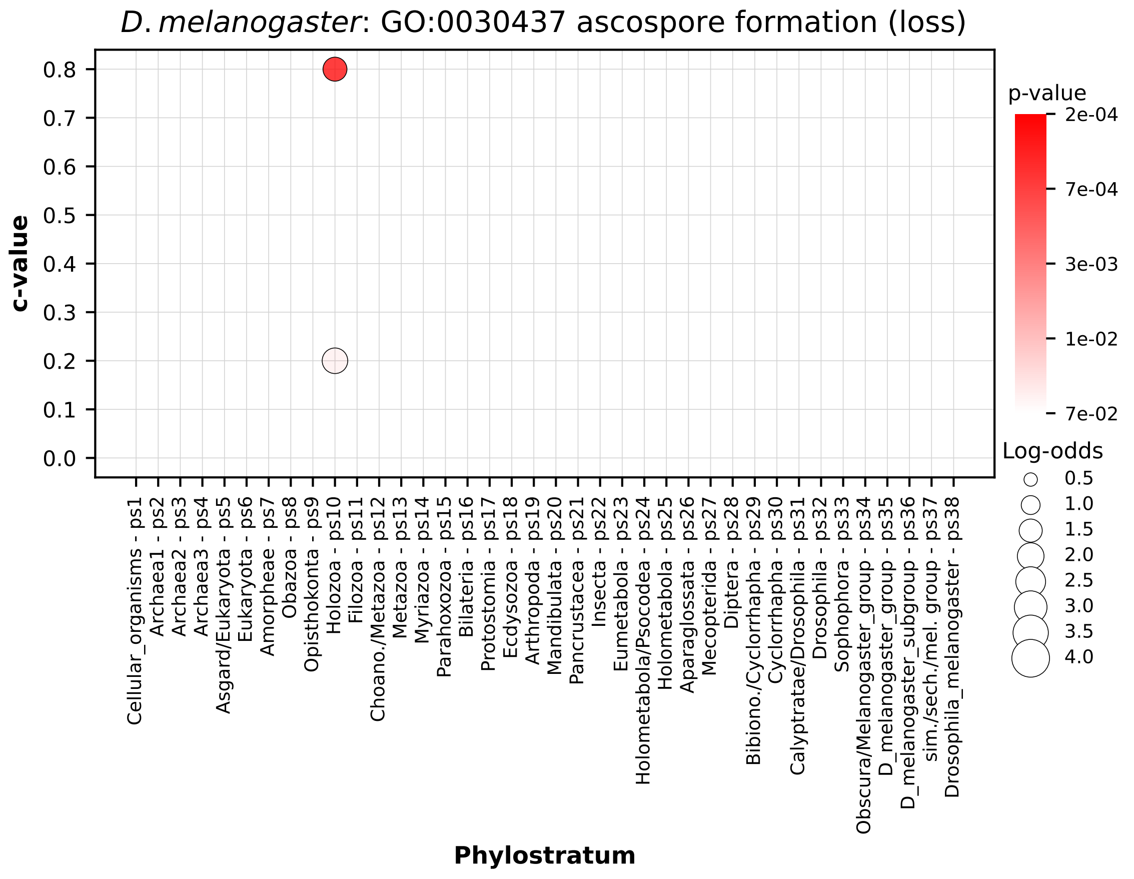

D. melanogaster: GO:0030437 ascospore formation (loss)
c-value
0.0
0.1
0.2
0.3
0.4
0.5
0.6
0.7
0.8
Phylostratum
Cellular_organisms - ps1
Archaea1 - ps2
Archaea2 - ps3
Archaea3 - ps4
Asgard/Eukaryota - ps5
Eukaryota - ps6
Amorpheae - ps7
Obazoa - ps8
Opisthokonta - ps9
Holozoa - ps10
Filozoa - ps11
Choano./Metazoa - ps12
Metazoa - ps13
Myriazoa - ps14
Parahoxozoa - ps15
Bilateria - ps16
Protostomia - ps17
Ecdysozoa - ps18
Arthropoda - ps19
Mandibulata - ps20
Pancrustacea - ps21
Insecta - ps22
Eumetabola - ps23
Holometabola/Psocodea - ps24
Holometabola - ps25
Aparaglossata - ps26
Mecopterida - ps27
Diptera - ps28
Bibiono./Cyclorrhapha - ps29
Cyclorrhapha - ps30
Calyptratae/Drosophila - ps31
Drosophila - ps32
Sophophora - ps33
Obscura/Melanogaster_group - ps34
D_melanogaster_group - ps35
D_melanogaster_subgroup - ps36
sim./sech./mel. group - ps37
Drosophila_melanogaster - ps38
p-value
2e-04
7e-04
3e-03
1e-02
7e-02
Log-odds
0.5
1.0
1.5
2.0
2.5
3.0
3.5
4.0

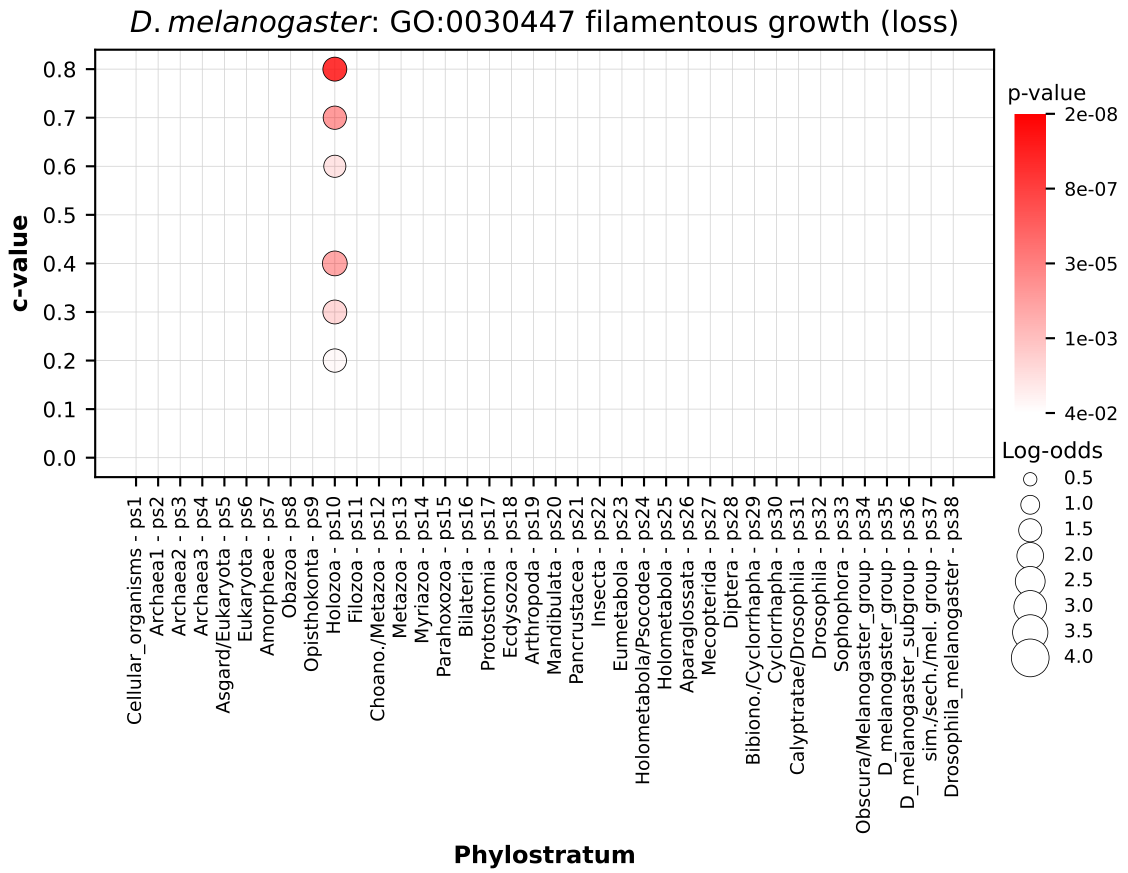

D. melanogaster: GO:0030447 filamentous growth (loss)
c-value
0.8
0.7
0.6
0.5
0.4
0.3
0.2
0.1
0.0
p-value
2e-08
8e-07
3e-05
1e-03
4e-02
Log-odds
0.5
1.0
1.5
2.0
2.5
3.0
3.5
4.0
Cellular_organisms - ps1
Archaea1 - ps2
Archaea2 - ps3
Archaea3 - ps4
Asgard/Eukaryota - ps5
Eukaryota - ps6
Amorpheae - ps7
Obazoa - ps8
Opisthokonta - ps9
Holozoa - ps10
Filozoa - ps11
Choano./Metazoa - ps12
Metazoa - ps13
Myriazoa - ps14
Parahoxozoa - ps15
Bilateria - ps16
Protostomia - ps17
Ecdysozoa - ps18
Arthropoda - ps19
Mandibulata - ps20
Pancrustacea - ps21
Insecta - ps22
Eumetabola - ps23
Holometabola/Psocodea - ps24
Holometabola - ps25
Aparaglossata - ps26
Mecopterida - ps27
Diptera - ps28
Bibiono./Cyclorrhapha - ps29
Cyclorrhapha - ps30
Calyptratae/Drosophila - ps31
Drosophila - ps32
Sophophora - ps33
Obscura/Melanogaster_group - ps34
D_melanogaster_group - ps35
D_melanogaster_subgroup - ps36
sim./sech./mel. group - ps37
Drosophila_melanogaster - ps38
Phylostratum

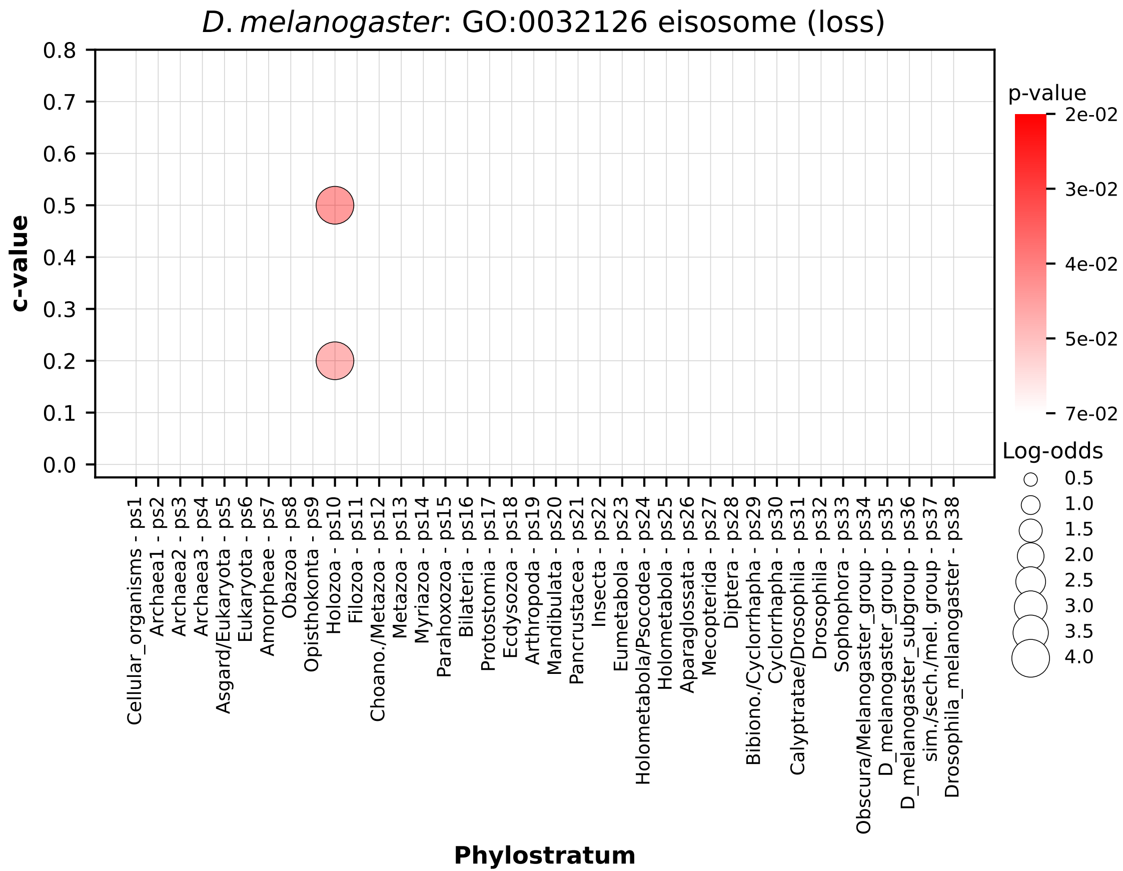
D. melanogaster: GO:0032126 eisosome (loss)
c-value
0.0
0.1
0.2
0.3
0.4
0.5
0.6
0.7
0.8
Phylostratum
Cellular_organisms - ps1
Archaea1 - ps2
Archaea2 - ps3
Archaea3 - ps4
Asgard/Eukaryota - ps5
Eukaryota - ps6
Amorpheae - ps7
Obazoa - ps8
Opisthokonta - ps9
Holozoa - ps10
Filozoa - ps11
Choano./Metazoa - ps12
Metazoa - ps13
Myriazoa - ps14
Parahoxozoa - ps15
Bilateria - ps16
Protostomia - ps17
Ecdysozoa - ps18
Arthropoda - ps19
Mandibulata - ps20
Pancrustacea - ps21
Insecta - ps22
Eumetabola - ps23
Holometabola/Psocodea - ps24
Holometabola - ps25
Aparaglossata - ps26
Mecopterida - ps27
Diptera - ps28
Bibiono./Cyclorrhapha - ps29
Cyclorrhapha - ps30
Calyptratae/Drosophila - ps31
Drosophila - ps32
Sophophora - ps33
Obscura/Melanogaster_group - ps34
D_melanogaster_group - ps35
D_melanogaster_subgroup - ps36
sim./sech./mel. group - ps37
Drosophila_melanogaster - ps38
p-value
2e-02
3e-02
4e-02
5e-02
7e-02
Log-odds
0.5
1.0
1.5
2.0
2.5
3.0
3.5
4.0

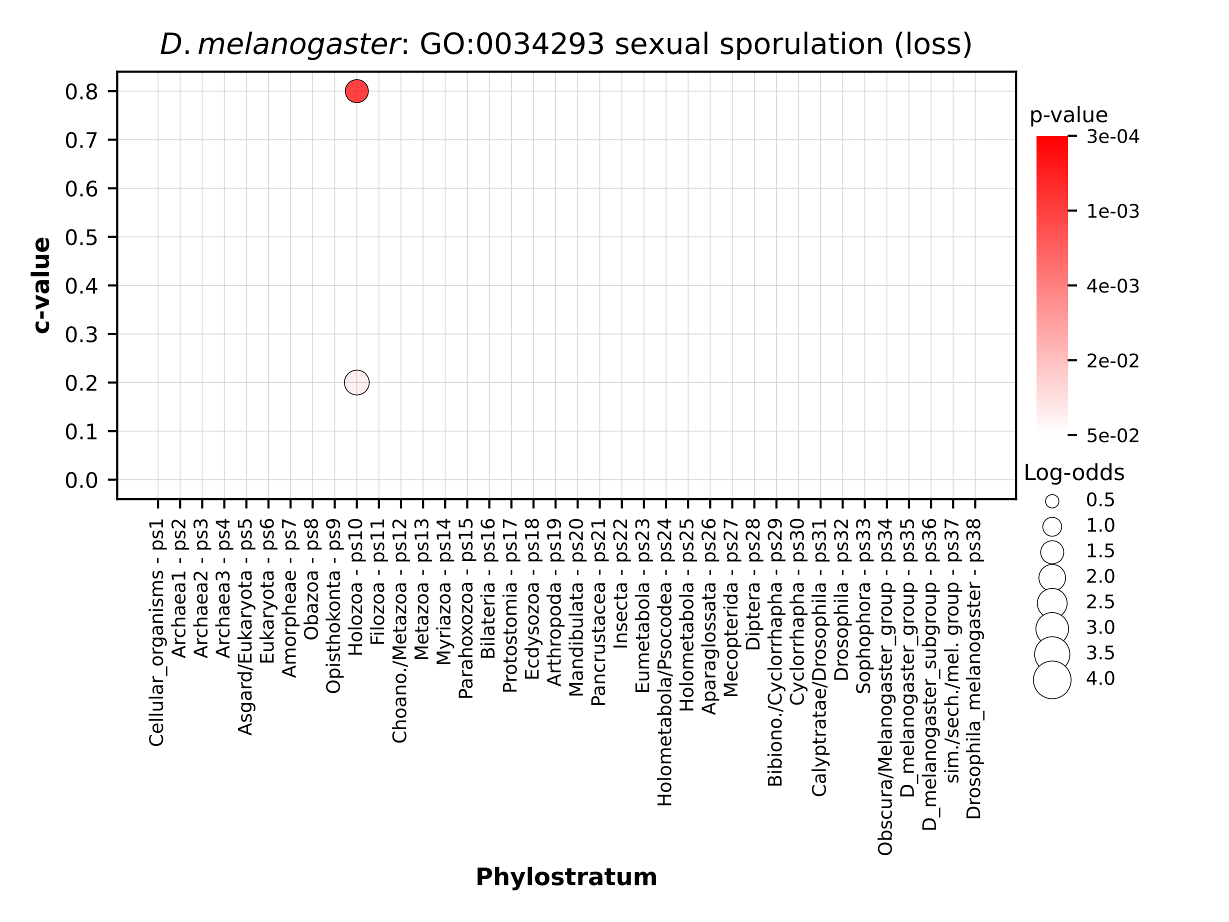

D. melanogaster: GO:0034293 sexual sporulation (loss)
c-value
0.0
0.1
0.2
0.3
0.4
0.5
0.6
0.7
0.8
Phylostratum
Cellular_organisms - ps1
Archaea1 - ps2
Archaea2 - ps3
Archaea3 - ps4
Asgard/Eukaryota - ps5
Eukaryota - ps6
Amorpheae - ps7
Obazoa - ps8
Opisthokonta - ps9
Holozoa - ps10
Filozoa - ps11
Choano./Metazoa - ps12
Metazoa - ps13
Myriazoa - ps14
Parahoxozoa - ps15
Bilateria - ps16
Protostomia - ps17
Ecdysozoa - ps18
Arthropoda - ps19
Mandibulata - ps20
Pancrustacea - ps21
Insecta - ps22
Eumetabola - ps23
Holometabola/Psocodea - ps24
Holometabola - ps25
Aparaglossata - ps26
Mecopterida - ps27
Diptera - ps28
Bibiono./Cyclorrhapha - ps29
Cyclorrhapha - ps30
Calyptratae/Drosophila - ps31
Drosophila - ps32
Sophophora - ps33
Obscura/Melanogaster_group - ps34
D_melanogaster_group - ps35
D_melanogaster_subgroup - ps36
sim./sech./mel. group - ps37
Drosophila_melanogaster - ps38
p-value
3e-04
1e-03
4e-03
2e-02
5e-02
Log-odds
0.5
1.0
1.5
2.0
2.5
3.0
3.5
4.0

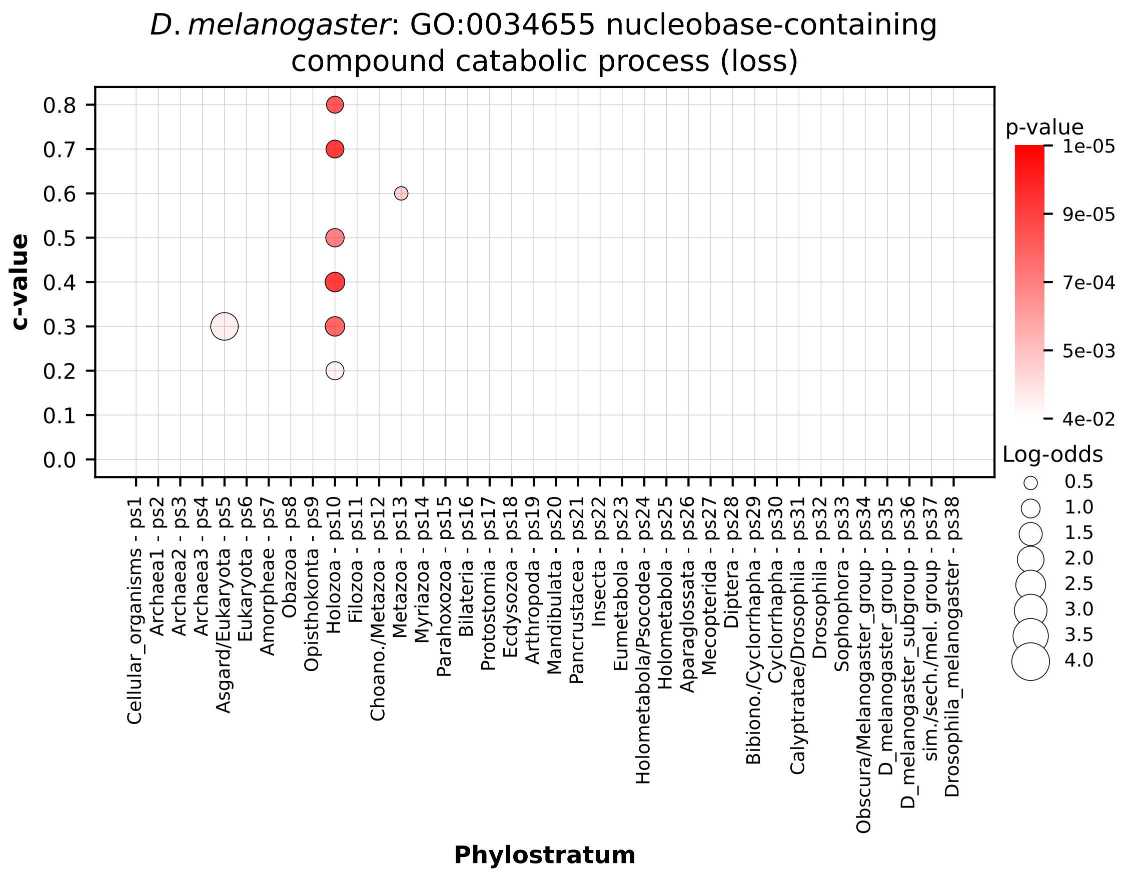
D. melanogaster: GO:0034655 nucleobase-containing compound catabolic process (loss)
c-value
0.0
0.1
0.2
0.3
0.4
0.5
0.6
0.7
0.8
Phylostratum
Cellular_organisms - ps1
Archaea1 - ps2
Archaea2 - ps3
Archaea3 - ps4
Asgard/Eukaryota - ps5
Eukaryota - ps6
Amorpheae - ps7
Obazoa - ps8
Opisthokonta - ps9
Holozoa - ps10
Filozoa - ps11
Choano./Metazoa - ps12
Metazoa - ps13
Myriazoa - ps14
Parahoxozoa - ps15
Bilateria - ps16
Protostomia - ps17
Ecdysozoa - ps18
Arthropoda - ps19
Mandibulata - ps20
Pancrustacea - ps21
Insecta - ps22
Eumetabola - ps23
Holometabola/Psocodea - ps24
Holometabola - ps25
Aparaglossata - ps26
Mecopterida - ps27
Diptera - ps28
Bibiono./Cyclorrhapha - ps29
Cyclorrhapha - ps30
Calyptratae/Drosophila - ps31
Drosophila - ps32
Sophophora - ps33
Obscura/Melanogaster_group - ps34
D_melanogaster_group - ps35
D_melanogaster_subgroup - ps36
sim./sech./mel. group - ps37
Drosophila_melanogaster - ps38
p-value
1e-05
9e-05
7e-04
5e-03
4e-02
Log-odds
0.5
1.0
1.5
2.0
2.5
3.0
3.5
4.0

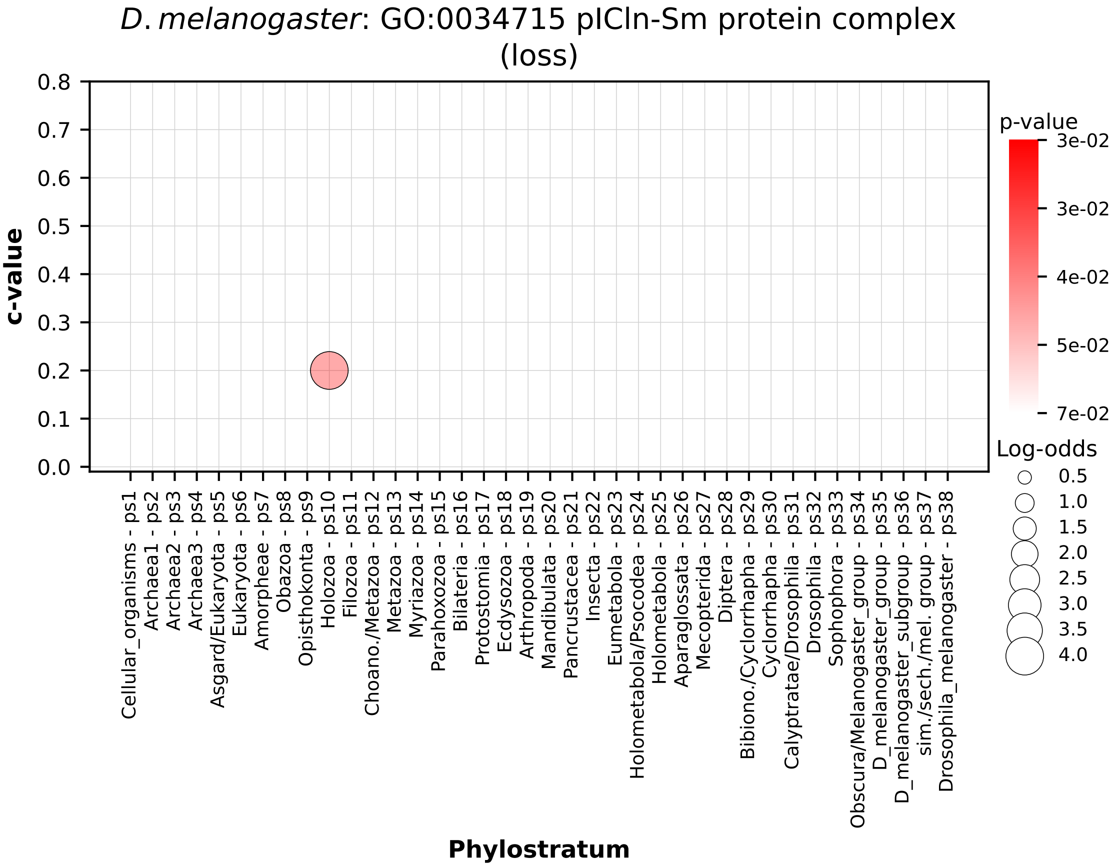
D. melanogaster: GO:0034715 pICln-Sm protein complex (loss)
c-value
0.8
0.7
0.6
0.5
0.4
0.3
0.2
0.1
0.0
Phylostratum
Cellular_organisms - ps1
Archaea1 - ps2
Archaea2 - ps3
Archaea3 - ps4
Asgard/Eukaryota - ps5
Eukaryota - ps6
Amorpheae - ps7
Obazoa - ps8
Opisthokonta - ps9
Holozoa - ps10
Filozoa - ps11
Choano./Metazoa - ps12
Metazoa - ps13
Myriazoa - ps14
Parahoxozoa - ps15
Bilateria - ps16
Protostomia - ps17
Ecdysozoa - ps18
Arthropoda - ps19
Mandibulata - ps20
Pancrustacea - ps21
Insecta - ps22
Eumetabola - ps23
Holometabola/Psocodea - ps24
Holometabola - ps25
Aparaglossata - ps26
Mecopterida - ps27
Diptera - ps28
Bibiono./Cyclorrhapha - ps29
Cyclorrhapha - ps30
Calyptratae/Drosophila - ps31
Drosophila - ps32
Sophophora - ps33
Obscura/Melanogaster_group - ps34
D_melanogaster_group - ps35
D_melanogaster_subgroup - ps36
sim./sech./mel. group - ps37
Drosophila_melanogaster - ps38
p-value
3e-02
3e-02
4e-02
5e-02
7e-02
Log-odds
0.5
1.0
1.5
2.0
2.5
3.0
3.5
4.0

D. melanogaster: GO:0040029 epigenetic regulation of gene expression (loss)

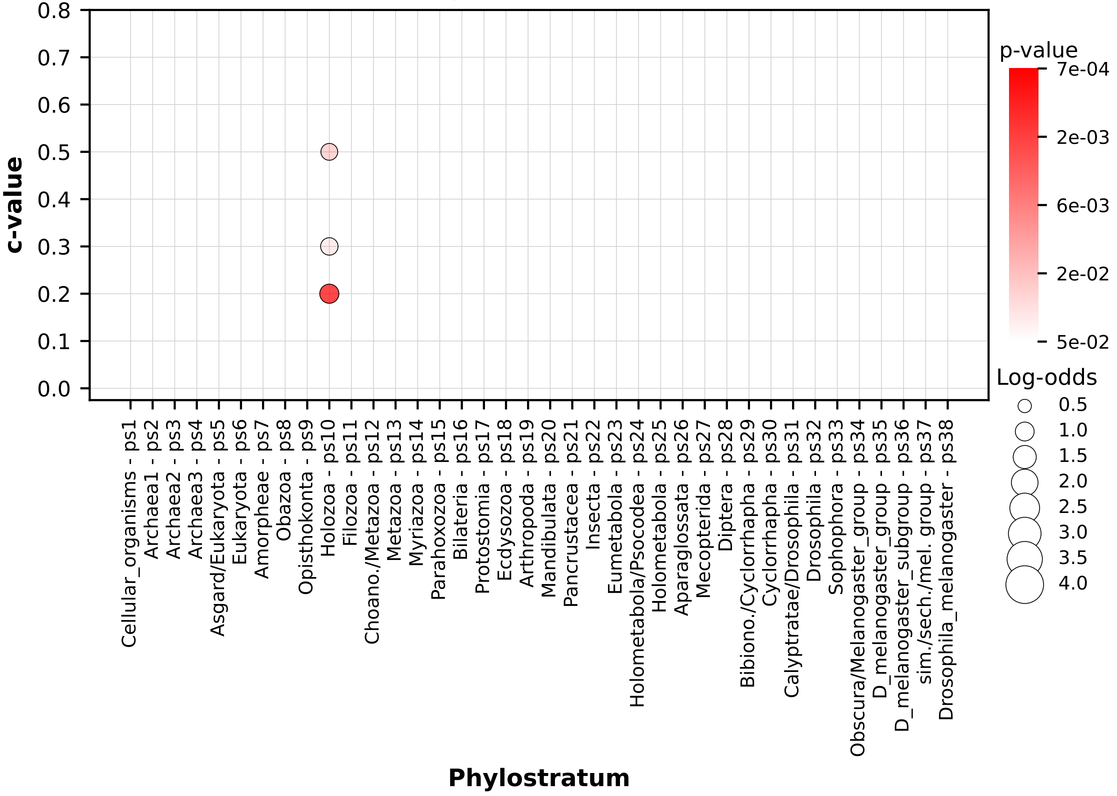

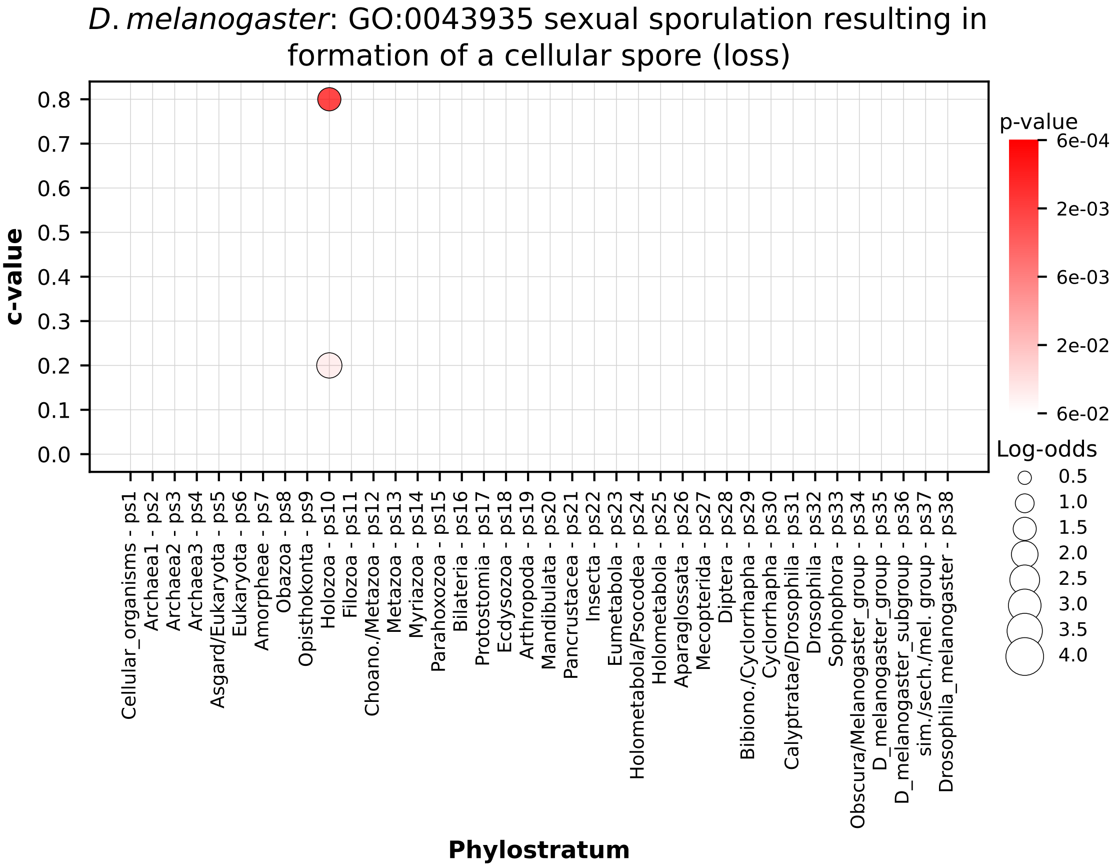

D. melanogaster: GO:0043935 sexual sporulation resulting in formation of a cellular spore (loss)
c-value
0.8
0.7
0.6
0.5
0.4
0.3
0.2
0.1
0.0
p-value
6e-04
2e-03
6e-03
2e-02
6e-02
Log-odds
0.5
1.0
1.5
2.0
2.5
3.0
3.5
4.0
Cellular_organisms - ps1
Archaea1 - ps2
Archaea2 - ps3
Archaea3 - ps4
Asgard/Eukaryota - ps5
Eukaryota - ps6
Amorpheae - ps7
Obazoa - ps8
Opisthokonta - ps9
Holozoa - ps10
Filozoa - ps11
Choano./Metazoa - ps12
Metazoa - ps13
Myriazoa - ps14
Parahoxozoa - ps15
Bilateria - ps16
Protostomia - ps17
Ecdysozoa - ps18
Arthropoda - ps19
Mandibulata - ps20
Pancrustacea - ps21
Insecta - ps22
Eumetabola - ps23
Holometabola/Psocodea - ps24
Holometabola - ps25
Aparaglossata - ps26
Mecopterida - ps27
Diptera - ps28
Bibiono./Cyclorrhapha - ps29
Cyclorrhapha - ps30
Calyptratae/Drosophila - ps31
Drosophila - ps32
Sophophora - ps33
Obscura/Melanogaster_group - ps34
D_melanogaster_group - ps35
D_melanogaster_subgroup - ps36
sim./sech./mel. group - ps37
Drosophila_melanogaster - ps38
Phylostratum

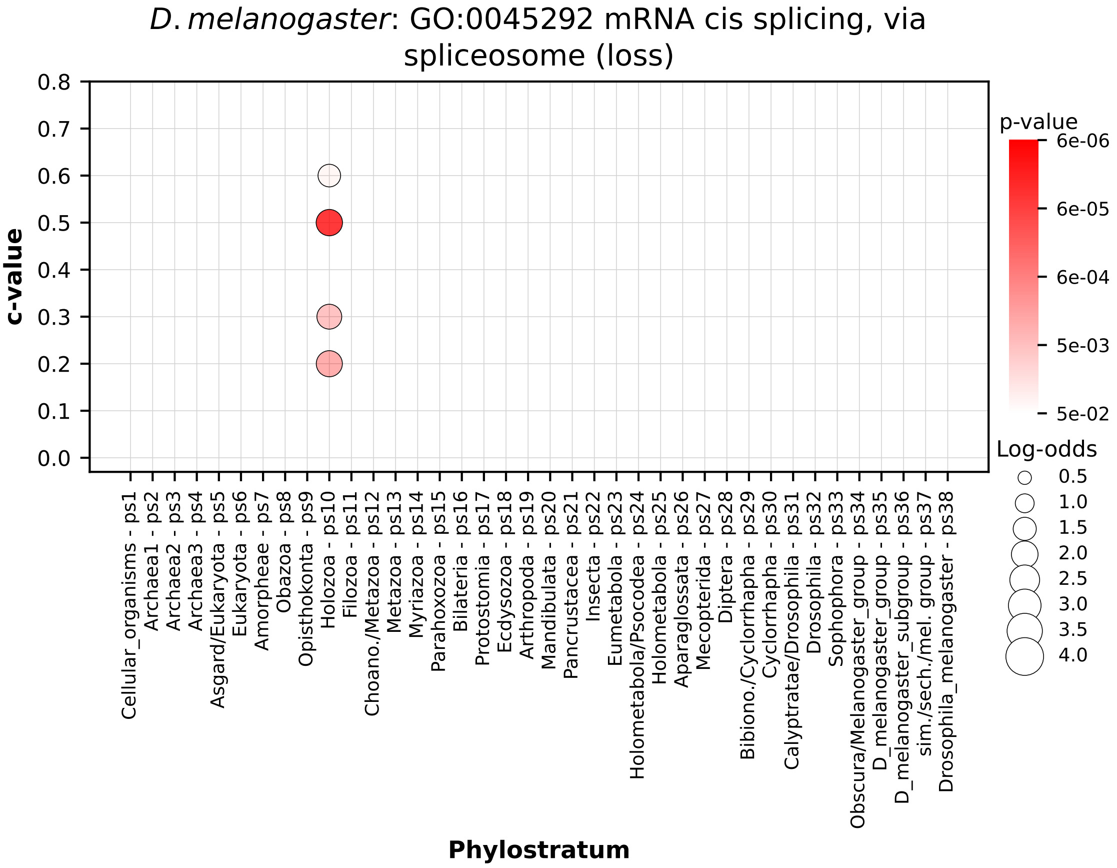

D. melanogaster: GO:0045292 mRNA cis splicing, via spliceosome (loss)
c-value
0.8
0.7
0.6
0.5
0.4
0.3
0.2
0.1
0.0
Phylostratum
Cellular_organisms - ps1
Archaea1 - ps2
Archaea2 - ps3
Archaea3 - ps4
Asgard/Eukaryota - ps5
Eukaryota - ps6
Amorpheae - ps7
Obazoa - ps8
Opisthokonta - ps9
Holozoa - ps10
Filozoa - ps11
Choano./Metazoa - ps12
Metazoa - ps13
Myriazoa - ps14
Parahoxozoa - ps15
Bilateria - ps16
Protostomia - ps17
Ecdysozoa - ps18
Arthropoda - ps19
Mandibulata - ps20
Pancrustacea - ps21
Insecta - ps22
Eumetabola - ps23
Holometabola/Psocodea - ps24
Holometabola - ps25
Aparaglossata - ps26
Mecopterida - ps27
Diptera - ps28
Bibiono./Cyclorrhapha - ps29
Cyclorrhapha - ps30
Calyptratae/Drosophila - ps31
Drosophila - ps32
Sophophora - ps33
Obscura/Melanogaster_group - ps34
D_melanogaster_group - ps35
D_melanogaster_subgroup - ps36
sim./sech./mel. group - ps37
Drosophila_melanogaster - ps38
p-value
6e-06
6e-05
6e-04
5e-03
5e-02
Log-odds
0.5
1.0
1.5
2.0
2.5
3.0
3.5
4.0

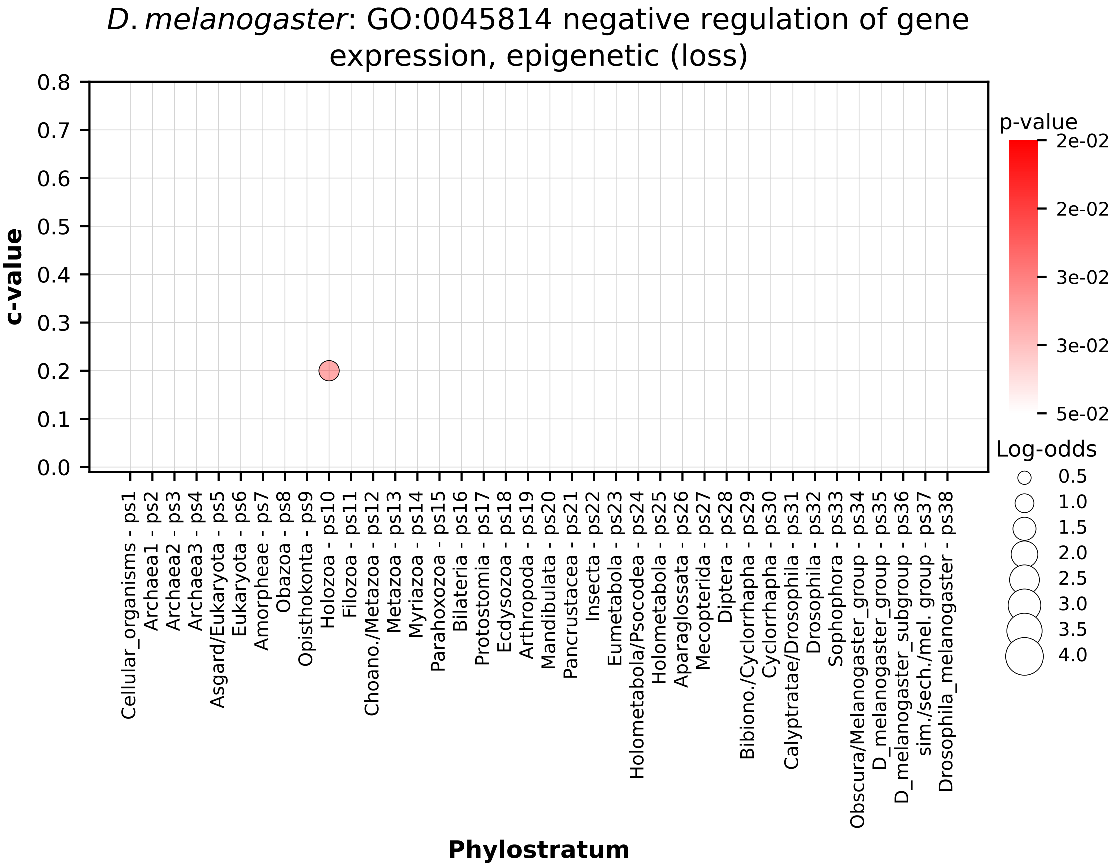

D. melanogaster: GO:0045814 negative regulation of gene expression, epigenetic (loss)
c-value
0.8
0.7
0.6
0.5
0.4
0.3
0.2
0.1
0.0
Phylostratum
Cellular_organisms - ps1
Archaea1 - ps2
Archaea2 - ps3
Archaea3 - ps4
Asgard/Eukaryota - ps5
Eukaryota - ps6
Amorpheae - ps7
Obazoa - ps8
Opisthokonta - ps9
Holozoa - ps10
Filozoa - ps11
Choano./Metazoa - ps12
Metazoa - ps13
Myriazoa - ps14
Parahoxozoa - ps15
Bilateria - ps16
Protostomia - ps17
Ecdysozoa - ps18
Arthropoda - ps19
Mandibulata - ps20
Pancrustacea - ps21
Insecta - ps22
Eumetabola - ps23
Holometabola/Psocodea - ps24
Holometabola - ps25
Aparaglossata - ps26
Mecopterida - ps27
Diptera - ps28
Bibiono./Cyclorrhapha - ps29
Cyclorrhapha - ps30
Calyptratae/Drosophila - ps31
Drosophila - ps32
Sophophora - ps33
Obscura/Melanogaster_group - ps34
D_melanogaster_group - ps35
D_melanogaster_subgroup - ps36
sim./sech./mel. group - ps37
Drosophila_melanogaster - ps38
p-value
2e-02
2e-02
3e-02
3e-02
5e-02
Log-odds
0.5
1.0
1.5
2.0
2.5
3.0
3.5
4.0

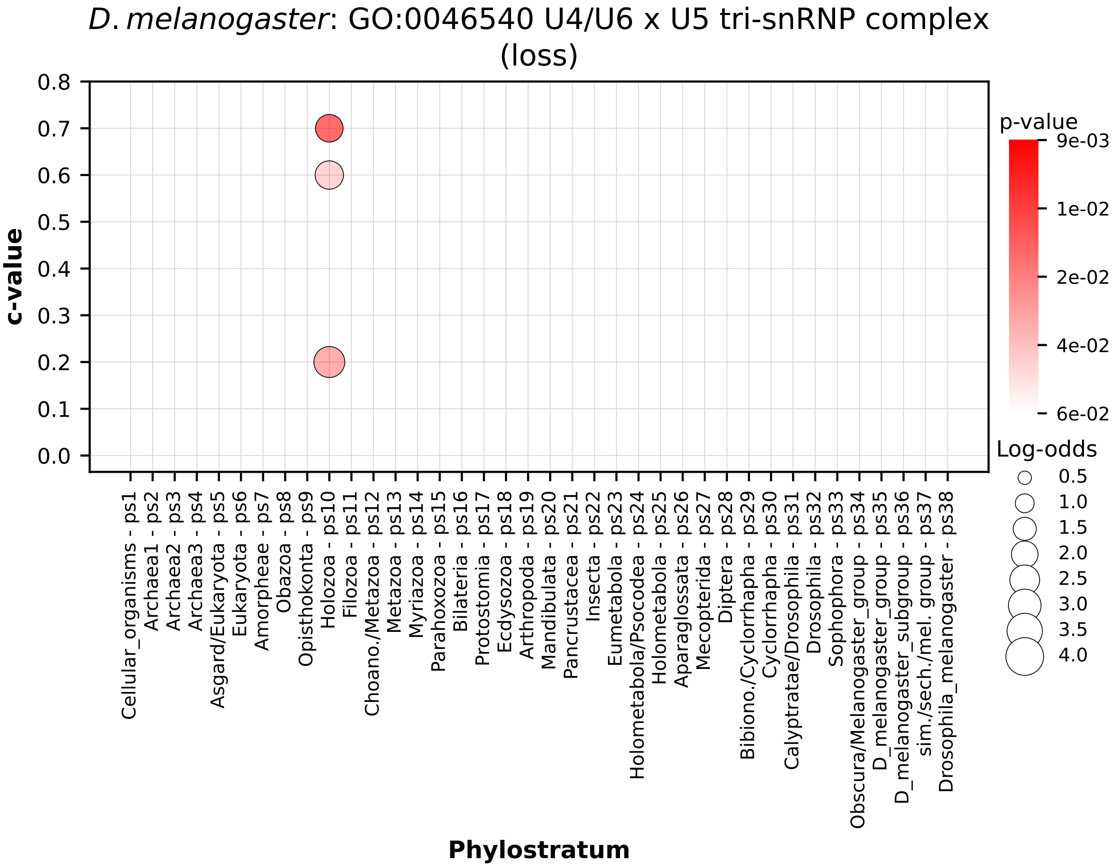
D. melanogaster: GO:0046540 U4/U6 x U5 tri-snRNP complex (loss)
c-value
0.8
0.7
0.6
0.5
0.4
0.3
0.2
0.1
0.0
p-value
9e-03
1e-02
2e-02
4e-02
6e-02
Log-odds
0.5
1.0
1.5
2.0
2.5
3.0
3.5
4.0
Cellular_organisms - ps1
Archaea1 - ps2
Archaea2 - ps3
Archaea3 - ps4
Asgard/Eukaryota - ps5
Eukaryota - ps6
Amorpheae - ps7
Obazoa - ps8
Opisthokonta - ps9
Holozoa - ps10
Filozoa - ps11
Choano./Metazoa - ps12
Metazoa - ps13
Myriazoa - ps14
Parahoxozoa - ps15
Bilateria - ps16
Protostomia - ps17
Ecdysozoa - ps18
Arthropoda - ps19
Mandibulata - ps20
Pancrustacea - ps21
Insecta - ps22
Eumetabola - ps23
Holometabola/Psocodea - ps24
Holometabola - ps25
Aparaglossata - ps26
Mecopterida - ps27
Diptera - ps28
Bibiono./Cyclorrhapha - ps29
Cyclorrhapha - ps30
Calyptratae/Drosophila - ps31
Drosophila - ps32
Sophophora - ps33
Obscura/Melanogaster_group - ps34
D_melanogaster_group - ps35
D_melanogaster_subgroup - ps36
sim./sech./mel. group - ps37
Drosophila_melanogaster - ps38
Phylostratum

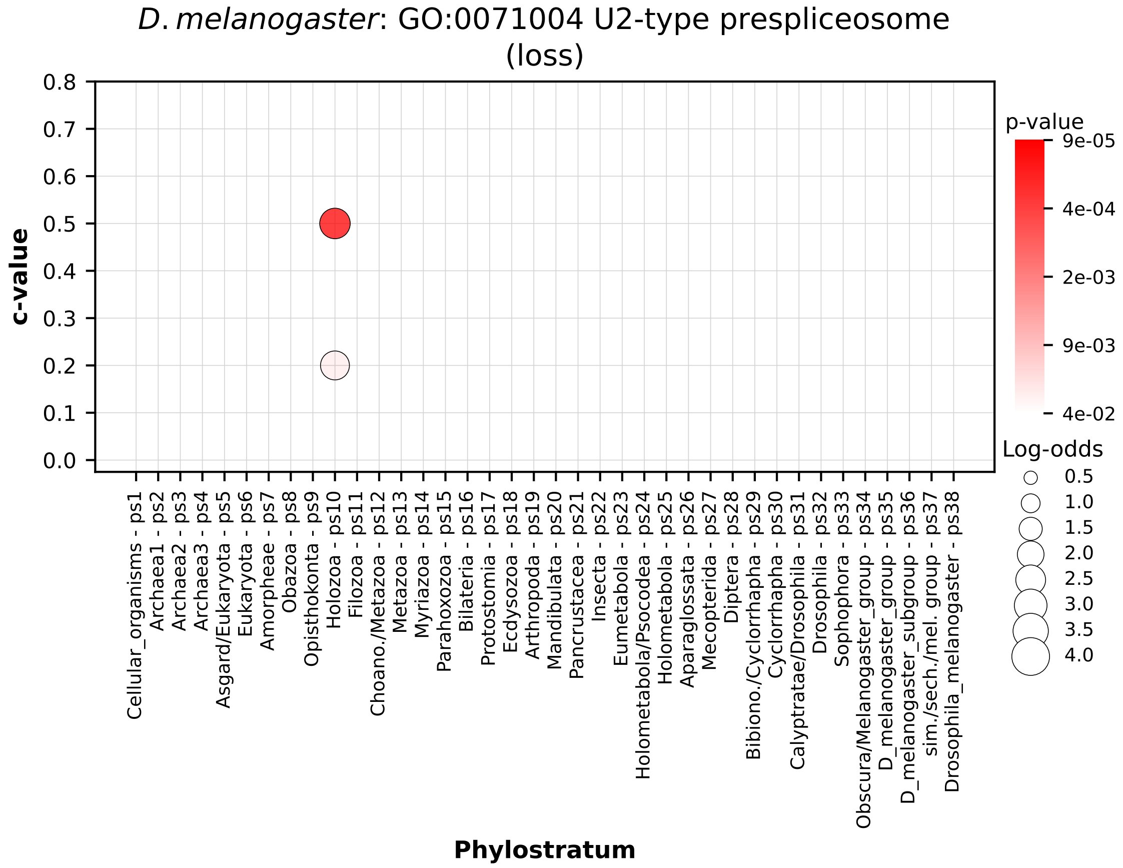

D. melanogaster: GO:0071004 U2-type prespliceosome
(loss)
c-value
0.8
0.7
0.6
0.5
0.4
0.3
0.2
0.1
0.0
p-value
9e-05
4e-04
2e-03
9e-03
4e-02
Log-odds
0.5
1.0
1.5
2.0
2.5
3.0
3.5
4.0
Cellular_organisms - ps1
Archaea1 - ps2
Archaea2 - ps3
Archaea3 - ps4
Asgard/Eukaryota - ps5
Eukaryota - ps6
Amorpheae - ps7
Obazoa - ps8
Opisthokonta - ps9
Holozoa - ps10
Filozoa - ps11
Choano./Metazoa - ps12
Metazoa - ps13
Myriazoa - ps14
Parahoxozoa - ps15
Bilateria - ps16
Protostomia - ps17
Ecdysozoa - ps18
Arthropoda - ps19
Mandibulata - ps20
Pancrustacea - ps21
Insecta - ps22
Eumetabola - ps23
Holometabola/Psocodea - ps24
Holometabola - ps25
Aparaglossata - ps26
Mecopterida - ps27
Diptera - ps28
Bibiono./Cyclorrhapha - ps29
Cyclorrhapha - ps30
Calyptratae/Drosophila - ps31
Drosophila - ps32
Sophophora - ps33
Obscura/Melanogaster_group - ps34
D_melanogaster_group - ps35
D_melanogaster_subgroup - ps36
sim./sech./mel. group - ps37
Drosophila_melanogaster - ps38
Phylostratum

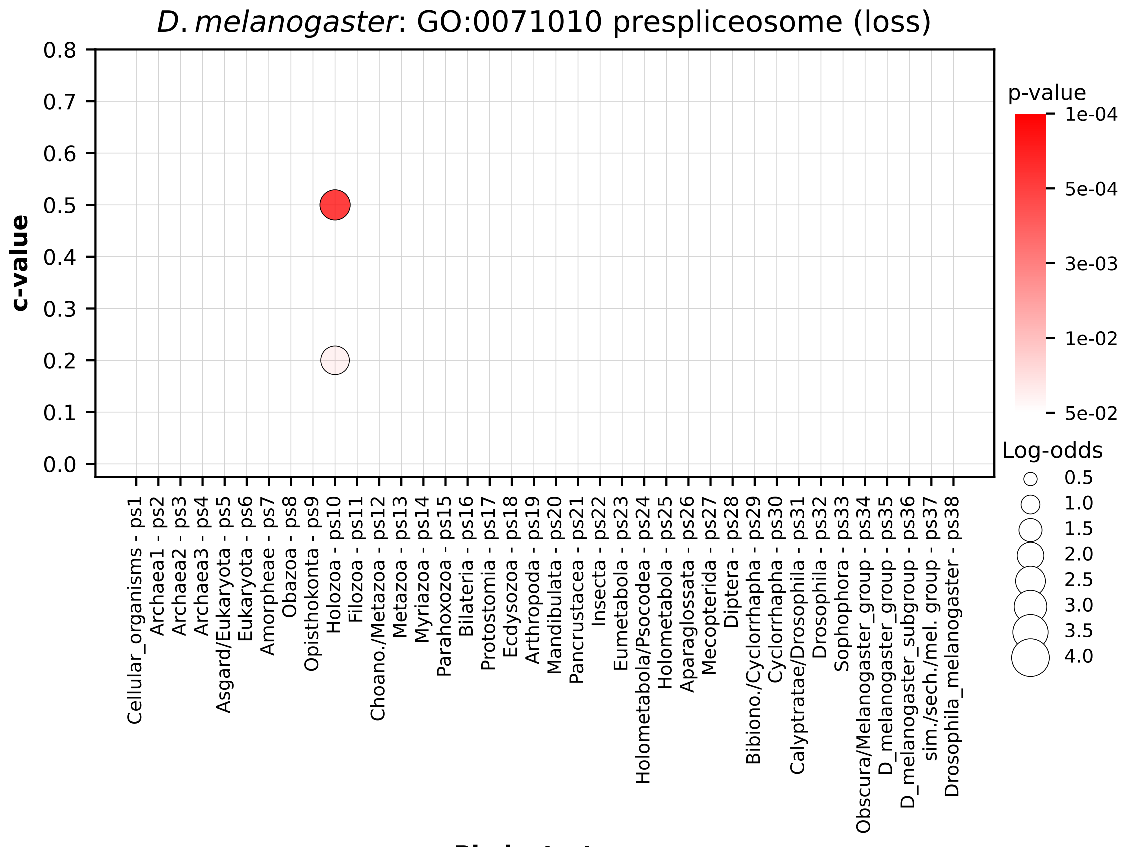

D. melanogaster: GO:0071010 prespliceosome (loss)
c-value
0.8
0.7
0.6
0.5
0.4
0.3
0.2
0.1
0.0
Phylostratum
Cellular_organisms - ps1
Archaea1 - ps2
Archaea2 - ps3
Archaea3 - ps4
Asgard/Eukaryota - ps5
Eukaryota - ps6
Amorpheae - ps7
Obazoa - ps8
Opisthokonta - ps9
Holozoa - ps10
Filozoa - ps11
Choano./Metazoa - ps12
Metazoa - ps13
Myriazoa - ps14
Parahoxozoa - ps15
Bilateria - ps16
Protostomia - ps17
Ecdysozoa - ps18
Arthropoda - ps19
Mandibulata - ps20
Pancrustacea - ps21
Insecta - ps22
Eumetabola - ps23
Holometabola/Psocodea - ps24
Holometabola - ps25
Aparaglossata - ps26
Mecopterida - ps27
Diptera - ps28
Bibiono./Cyclorrhapha - ps29
Cyclorrhapha - ps30
Calyptratae/Drosophila - ps31
Drosophila - ps32
Sophophora - ps33
Obscura/Melanogaster_group - ps34
D_melanogaster_group - ps35
D_melanogaster_subgroup - ps36
sim./sech./mel. group - ps37
Drosophila_melanogaster - ps38
p-value
1e-04
5e-04
3e-03
1e-02
5e-02
Log-odds
0.5
1.0
1.5
2.0
2.5
3.0
3.5
4.0

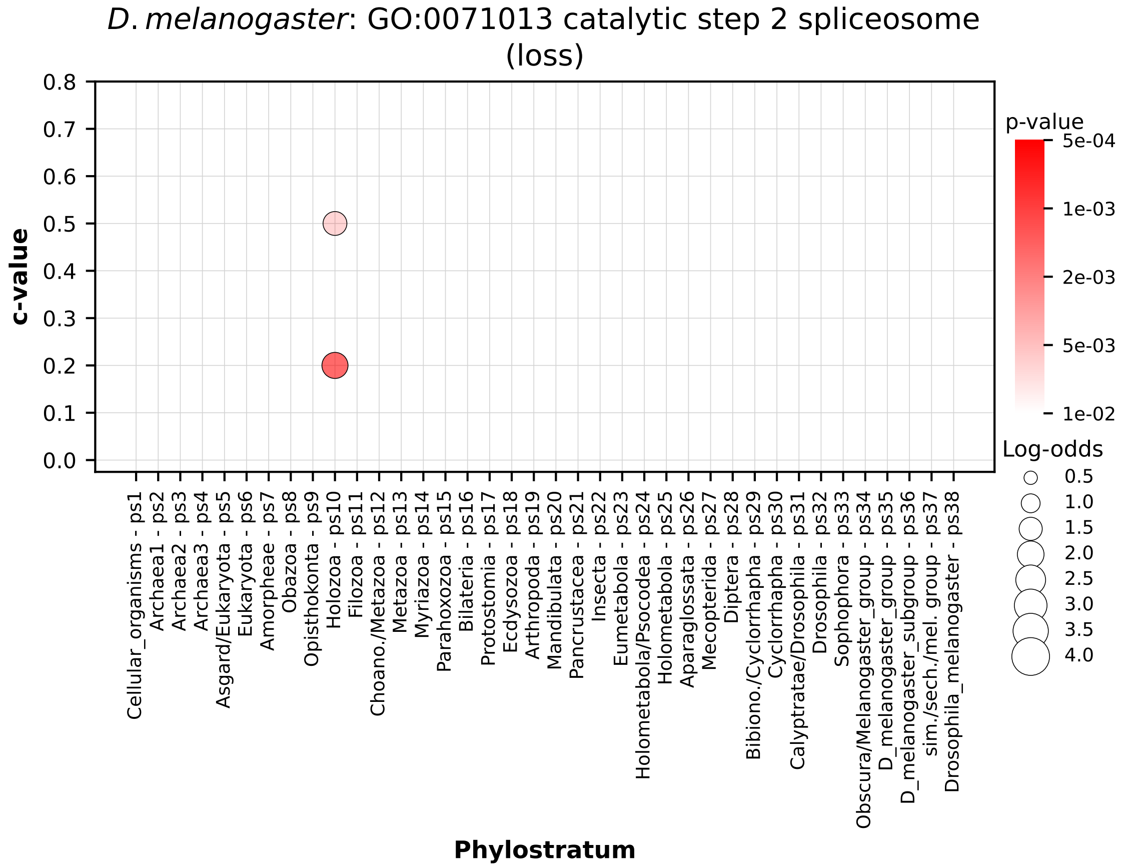

D. melanogaster: GO:0071013 catalytic step 2 spliceosome (loss)
c-value
0.0
0.1
0.2
0.3
0.4
0.5
0.6
0.7
0.8
p-value
5e-04
1e-03
2e-03
5e-03
1e-02
Log-odds
0.5
1.0
1.5
2.0
2.5
3.0
3.5
4.0
Cellular_organisms - ps1
Archaea1 - ps2
Archaea2 - ps3
Archaea3 - ps4
Asgard/Eukaryota - ps5
Eukaryota - ps6
Amorpheae - ps7
Obazoa - ps8
Opisthokonta - ps9
Holozoa - ps10
Filozoa - ps11
Choano./Metazoa - ps12
Metazoa - ps13
Myriazoa - ps14
Parahoxozoa - ps15
Bilateria - ps16
Protostomia - ps17
Ecdysozoa - ps18
Arthropoda - ps19
Mandibulata - ps20
Pancrustacea - ps21
Insecta - ps22
Eumetabola - ps23
Holometabola/Psocodea - ps24
Holometabola - ps25
Aparaglossata - ps26
Mecopterida - ps27
Diptera - ps28
Bibiono./Cyclorrhapha - ps29
Cyclorrhapha - ps30
Calyptratae/Drosophila - ps31
Drosophila - ps32
Sophophora - ps33
Obscura/Melanogaster_group - ps34
D_melanogaster_group - ps35
D_melanogaster_subgroup - ps36
sim./sech./mel. group - ps37
Drosophila_melanogaster - ps38
Phylostratum

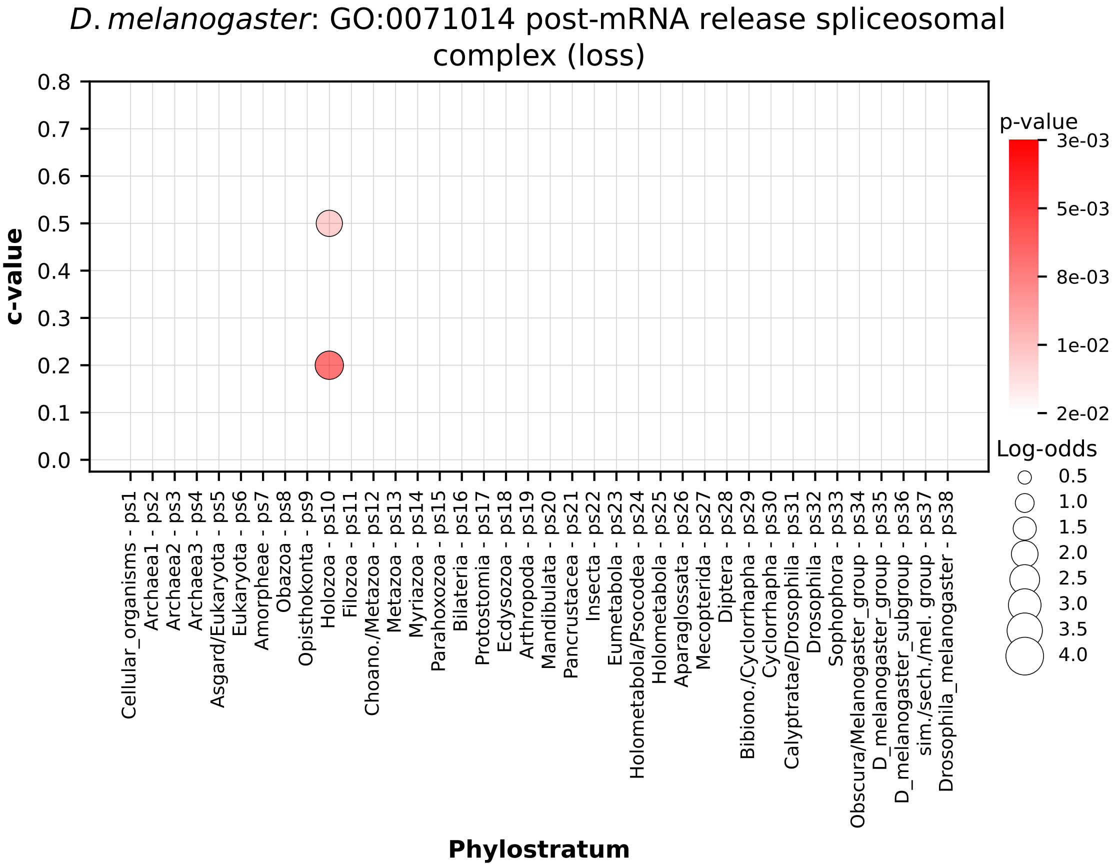
D. melanogaster: GO:0071014 post-mRNA release spliceosomal complex (loss)
c-value
0.0
0.1
0.2
0.3
0.4
0.5
0.6
0.7
0.8
Phylostratum
Cellular_organisms - ps1
Archaea1 - ps2
Archaea2 - ps3
Archaea3 - ps4
Asgard/Eukaryota - ps5
Eukaryota - ps6
Amorpheae - ps7
Obazoa - ps8
Opisthokonta - ps9
Holozoa - ps10
Filozoa - ps11
Choano./Metazoa - ps12
Metazoa - ps13
Myriazoa - ps14
Parahoxozoa - ps15
Bilateria - ps16
Protostomia - ps17
Ecdysozoa - ps18
Arthropoda - ps19
Mandibulata - ps20
Pancrustacea - ps21
Insecta - ps22
Eumetabola - ps23
Holometabola/Psocodea - ps24
Holometabola - ps25
Aparaglossata - ps26
Mecopterida - ps27
Diptera - ps28
Bibiono./Cyclorrhapha - ps29
Cyclorrhapha - ps30
Calyptratae/Drosophila - ps31
Drosophila - ps32
Sophophora - ps33
Obscura/Melanogaster_group - ps34
D_melanogaster_group - ps35
D_melanogaster_subgroup - ps36
sim./sech./mel. group - ps37
Drosophila_melanogaster - ps38
p-value
3e-03
5e-03
8e-03
1e-02
2e-02
Log-odds
0.5
1.0
1.5
2.0
2.5
3.0
3.5
4.0

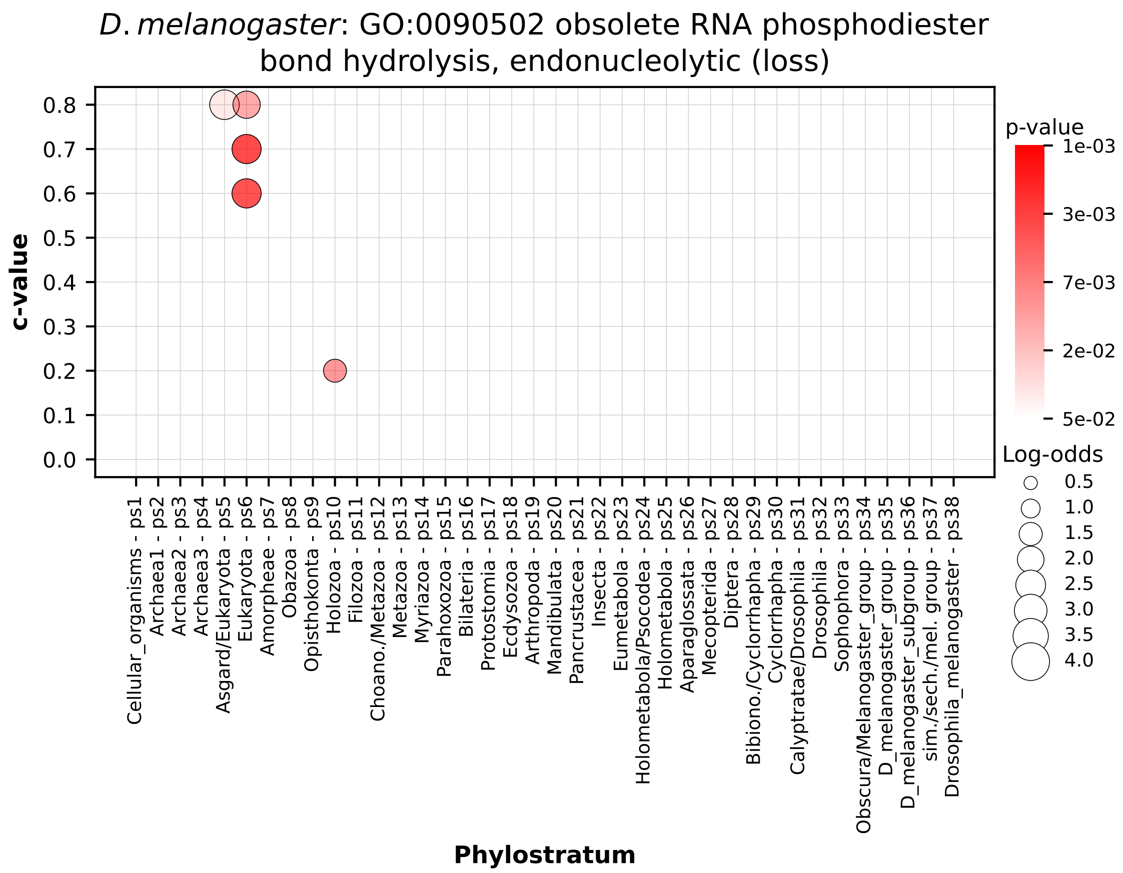

D. melanogaster: GO:0090502 obsolete RNA phosphodiester bond hydrolysis, endonucleolytic (loss)
c-value
0.8
0.7
0.6
0.5
0.4
0.3
0.2
0.1
0.0
p-value
1e-03
3e-03
7e-03
2e-02
5e-02
Log-odds
0.5
1.0
1.5
2.0
2.5
3.0
3.5
4.0
Cellular_organisms - ps1
Archaea1 - ps2
Archaea2 - ps3
Archaea3 - ps4
Asgard/Eukaryota - ps5
Eukaryota - ps6
Amorpheae - ps7
Obazoa - ps8
Opisthokonta - ps9
Holozoa - ps10
Filozoa - ps11
Choano./Metazoa - ps12
Metazoa - ps13
Myriazoa - ps14
Parahoxozoa - ps15
Bilateria - ps16
Protostomia - ps17
Ecdysozoa - ps18
Arthropoda - ps19
Mandibulata - ps20
Pancrustacea - ps21
Insecta - ps22
Eumetabola - ps23
Holometabola/Psocodea - ps24
Holometabola - ps25
Aparaglossata - ps26
Mecopterida - ps27
Diptera - ps28
Bibiono./Cyclorrhapha - ps29
Cyclorrhapha - ps30
Calyptratae/Drosophila - ps31
Drosophila - ps32
Sophophora - ps33
Obscura/Melanogaster_group - ps34
D_melanogaster_group - ps35
D_melanogaster_subgroup - ps36
sim./sech./mel. group - ps37
Drosophila_melanogaster - ps38
Phylostratum

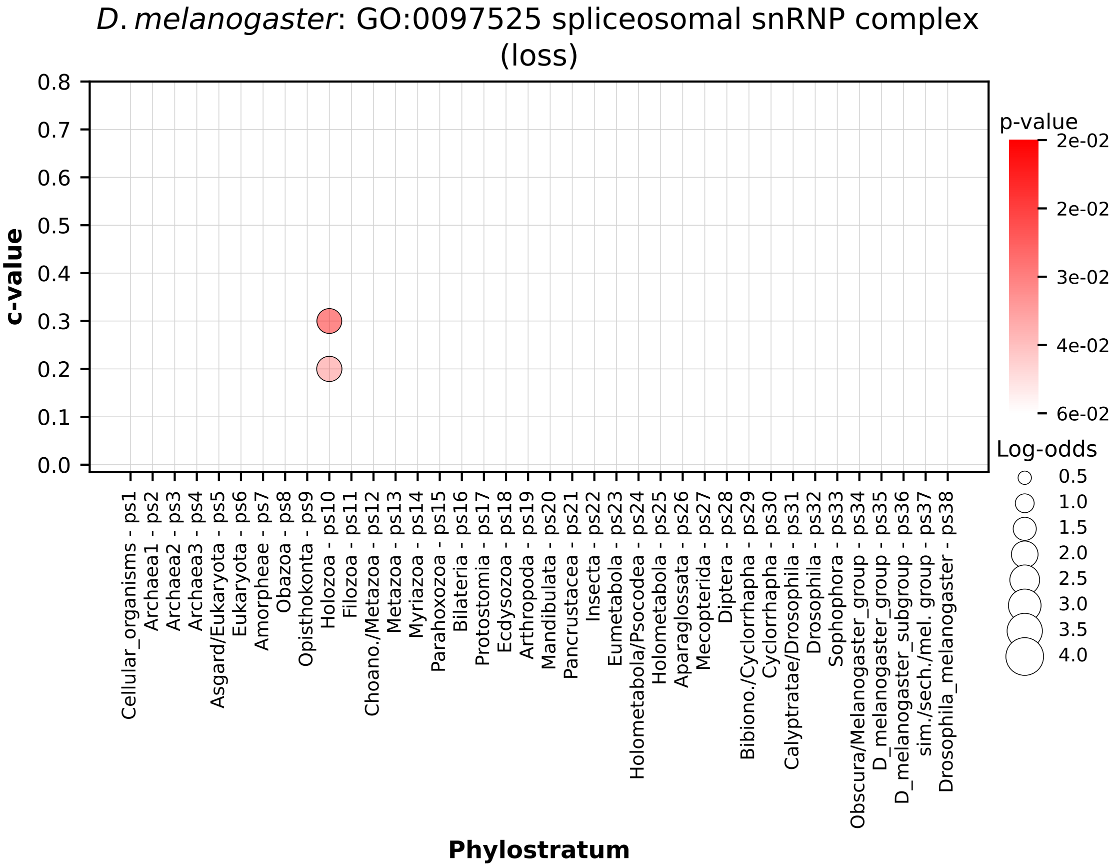

D. melanogaster: GO:0097525 spliceosomal snRNP complex (loss)
c-value
0.0
0.1
0.2
0.3
0.4
0.5
0.6
0.7
0.8
Phylostratum
Cellular_organisms - ps1
Archaea1 - ps2
Archaea2 - ps3
Archaea3 - ps4
Asgard/Eukaryota - ps5
Eukaryota - ps6
Amorpheae - ps7
Obazoa - ps8
Opisthokonta - ps9
Holozoa - ps10
Filozoa - ps11
Choano./Metazoa - ps12
Metazoa - ps13
Myriazoa - ps14
Parahoxozoa - ps15
Bilateria - ps16
Protostomia - ps17
Ecdysozoa - ps18
Arthropoda - ps19
Mandibulata - ps20
Pancrustacea - ps21
Insecta - ps22
Eumetabola - ps23
Holometabola/Psocodea - ps24
Holometabola - ps25
Aparaglossata - ps26
Mecopterida - ps27
Diptera - ps28
Bibiono./Cyclorrhapha - ps29
Cyclorrhapha - ps30
Calyptratae/Drosophila - ps31
Drosophila - ps32
Sophophora - ps33
Obscura/Melanogaster_group - ps34
D_melanogaster_group - ps35
D_melanogaster_subgroup - ps36
sim./sech./mel. group - ps37
Drosophila_melanogaster - ps38
p-value
2e-02
2e-02
3e-02
4e-02
6e-02
Log-odds
0.5
1.0
1.5
2.0
2.5
3.0
3.5
4.0

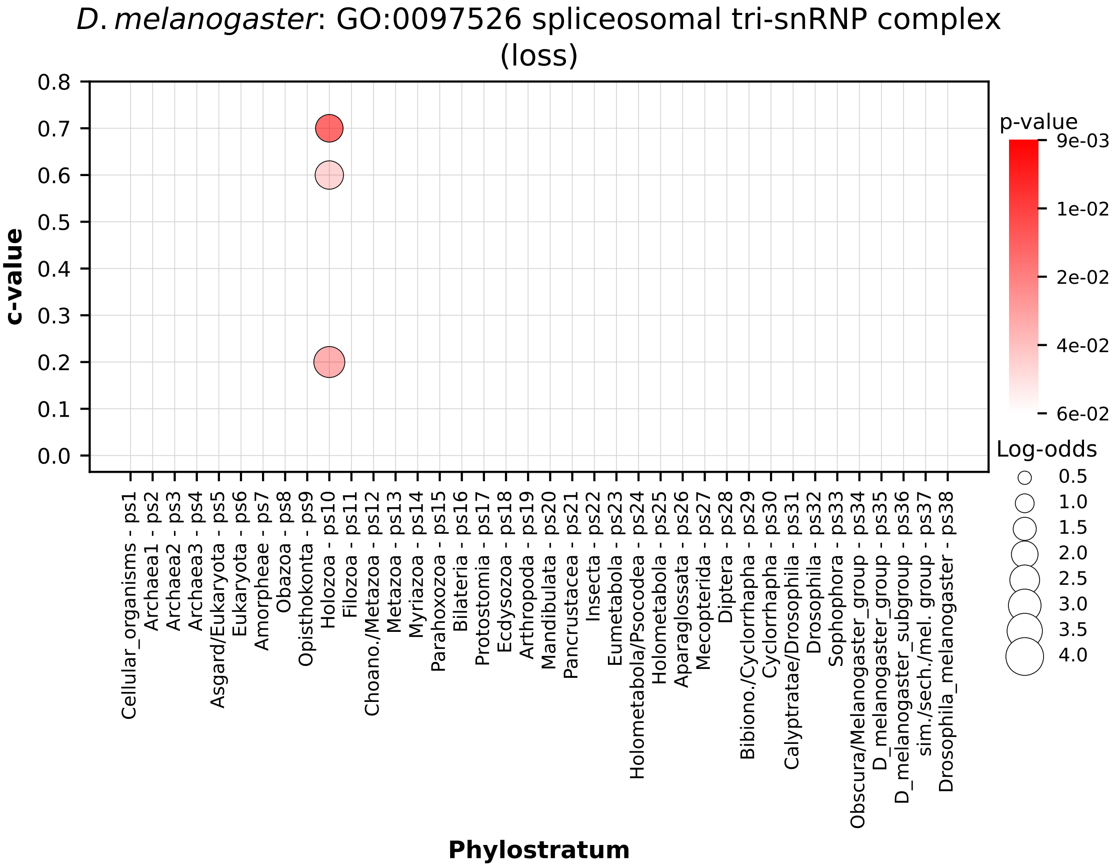
D. melanogaster: GO:0097526 spliceosomal tri-snRNP complex
(loss)
c-value
0.8
0.7
0.6
0.5
0.4
0.3
0.2
0.1
0.0
p-value
9e-03
1e-02
2e-02
4e-02
6e-02
Log-odds
0.5
1.0
1.5
2.0
2.5
3.0
3.5
4.0
Cellular_organisms - ps1
Archaea1 - ps2
Archaea2 - ps3
Archaea3 - ps4
Asgard/Eukaryota - ps5
Eukaryota - ps6
Amorpheae - ps7
Obazoa - ps8
Opisthokonta - ps9
Holozoa - ps10
Filozoa - ps11
Choano./Metazoa - ps12
Metazoa - ps13
Myriazoa - ps14
Parahoxozoa - ps15
Bilateria - ps16
Protostomia - ps17
Ecdysozoa - ps18
Arthropoda - ps19
Mandibulata - ps20
Pancrustacea - ps21
Insecta - ps22
Eumetabola - ps23
Holometabola/Psocodea - ps24
Holometabola - ps25
Aparaglossata - ps26
Mecopterida - ps27
Diptera - ps28
Bibiono./Cyclorrhapha - ps29
Cyclorrhapha - ps30
Calyptratae/Drosophila - ps31
Drosophila - ps32
Sophophora - ps33
Obscura/Melanogaster_group - ps34
D_melanogaster_group - ps35
D_melanogaster_subgroup - ps36
sim./sech./mel. group - ps37
Drosophila_melanogaster - ps38
Phylostratum

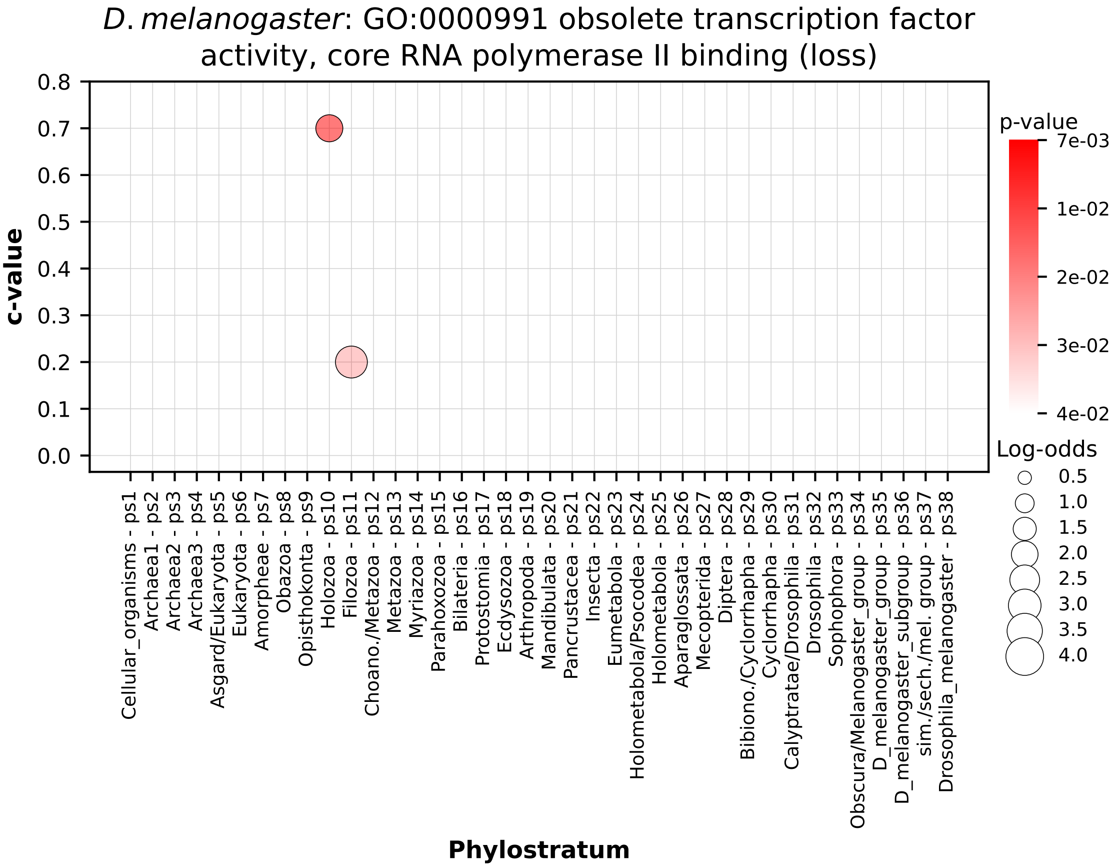
D. melanogaster: GO:0000991 obsolete transcription factor activity, core RNA polymerase II binding (loss)
c-value
0.8
0.7
0.6
0.5
0.4
0.3
0.2
0.1
0.0
Phylostratum
Cellular_organisms - ps1
Archaea1 - ps2
Archaea2 - ps3
Archaea3 - ps4
Asgard/Eukaryota - ps5
Eukaryota - ps6
Amorpheae - ps7
Obazoa - ps8
Opisthokonta - ps9
Holozoa - ps10
Filozoa - ps11
Choano./Metazoa - ps12
Metazoa - ps13
Myriazoa - ps14
Parahoxozoa - ps15
Bilateria - ps16
Protostomia - ps17
Ecdysozoa - ps18
Arthropoda - ps19
Mandibulata - ps20
Pancrustacea - ps21
Insecta - ps22
Eumetabola - ps23
Holometabola/Psocodea - ps24
Holometabola - ps25
Aparaglossata - ps26
Mecopterida - ps27
Diptera - ps28
Bibiono./Cyclorrhapha - ps29
Cyclorrhapha - ps30
Calyptratae/Drosophila - ps31
Drosophila - ps32
Sophophora - ps33
Obscura/Melanogaster_group - ps34
D_melanogaster_group - ps35
D_melanogaster_subgroup - ps36
sim./sech./mel. group - ps37
Drosophila_melanogaster - ps38
p-value
7e-03
1e-02
2e-02
3e-02
4e-02
Log-odds
0.5
1.0
1.5
2.0
2.5
3.0
3.5
4.0

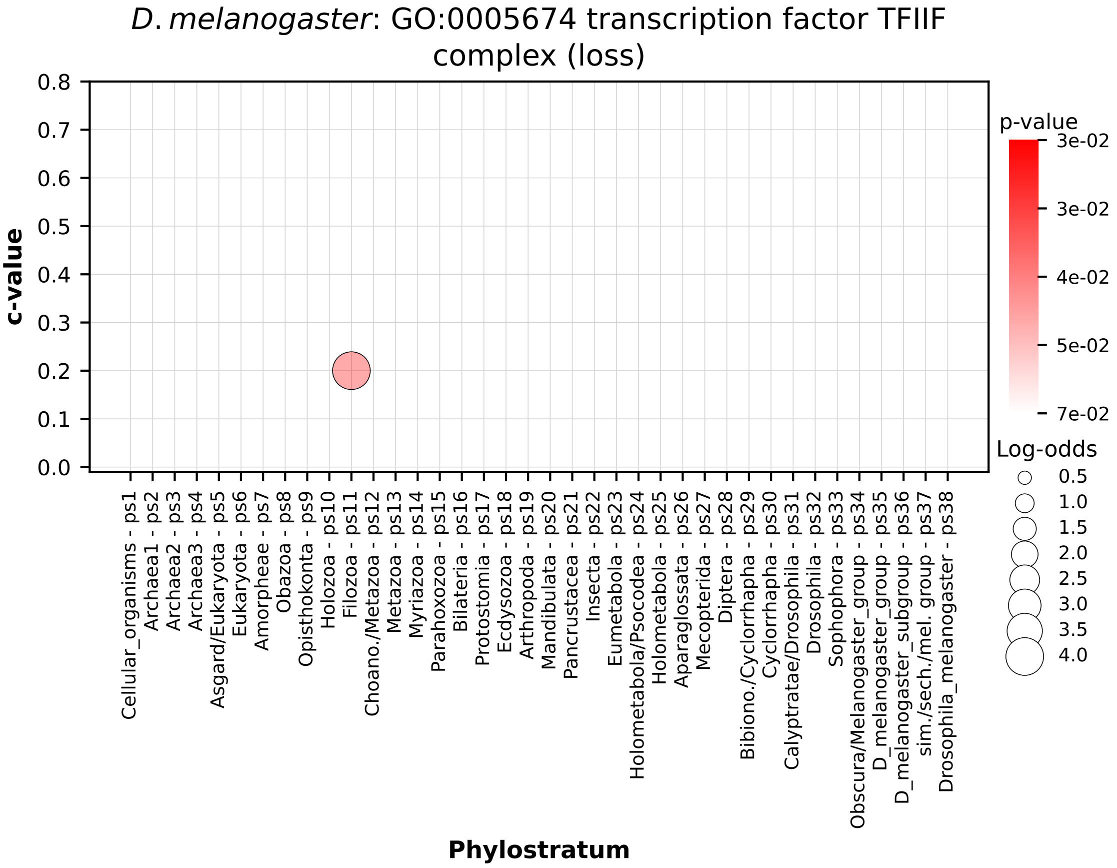

D. melanogaster: GO:0005674 transcription factor TFIIF complex (loss)
c-value
0.0
0.1
0.2
0.3
0.4
0.5
0.6
0.7
0.8
Phylostratum
Cellular_organisms - ps1
Archaea1 - ps2
Archaea2 - ps3
Archaea3 - ps4
Asgard/Eukaryota - ps5
Eukaryota - ps6
Amorpheae - ps7
Obazoa - ps8
Opisthokonta - ps9
Holozoa - ps10
Filozoa - ps11
Choano./Metazoa - ps12
Metazoa - ps13
Myriazoa - ps14
Parahoxozoa - ps15
Bilateria - ps16
Protostomia - ps17
Ecdysozoa - ps18
Arthropoda - ps19
Mandibulata - ps20
Pancrustacea - ps21
Insecta - ps22
Eumetabola - ps23
Holometabola/Psocodea - ps24
Holometabola - ps25
Aparaglossata - ps26
Mecopterida - ps27
Diptera - ps28
Bibiono./Cyclorrhapha - ps29
Cyclorrhapha - ps30
Calyptratae/Drosophila - ps31
Drosophila - ps32
Sophophora - ps33
Obscura/Melanogaster_group - ps34
D_melanogaster_group - ps35
D_melanogaster_subgroup - ps36
sim./sech./mel. group - ps37
Drosophila_melanogaster - ps38
p-value
3e-02
3e-02
4e-02
5e-02
7e-02
Log-odds
0.5
1.0
1.5
2.0
2.5
3.0
3.5
4.0

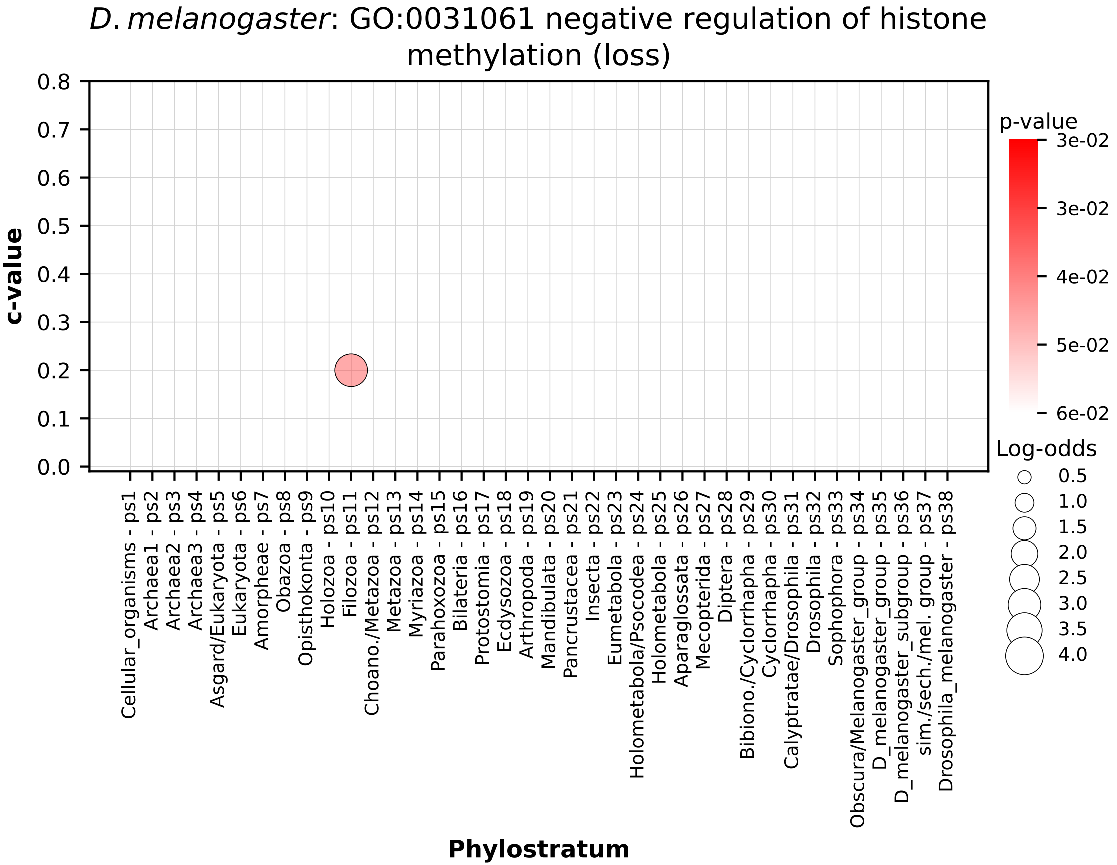

D. melanogaster: GO:0031061 negative regulation of histone methylation (loss)
c-value
0.0
0.1
0.2
0.3
0.4
0.5
0.6
0.7
0.8
Phylostratum
Cellular_organisms - ps1
Archaea1 - ps2
Archaea2 - ps3
Archaea3 - ps4
Asgard/Eukaryota - ps5
Eukaryota - ps6
Amorpheae - ps7
Obazoa - ps8
Opisthokonta - ps9
Holozoa - ps10
Filozoa - ps11
Choano./Metazoa - ps12
Metazoa - ps13
Myriazoa - ps14
Parahoxozoa - ps15
Bilateria - ps16
Protostomia - ps17
Ecdysozoa - ps18
Arthropoda - ps19
Mandibulata - ps20
Pancrustacea - ps21
Insecta - ps22
Eumetabola - ps23
Holometabola/Psocodea - ps24
Holometabola - ps25
Aparaglossata - ps26
Mecopterida - ps27
Diptera - ps28
Bibiono./Cyclorrhapha - ps29
Cyclorrhapha - ps30
Calyptratae/Drosophila - ps31
Drosophila - ps32
Sophophora - ps33
Obscura/Melanogaster_group - ps34
D_melanogaster_group - ps35
D_melanogaster_subgroup - ps36
sim./sech./mel. group - ps37
Drosophila_melanogaster - ps38
p-value
3e-02
3e-02
4e-02
5e-02
6e-02
Log-odds
0.5
1.0
1.5
2.0
2.5
3.0
3.5
4.0

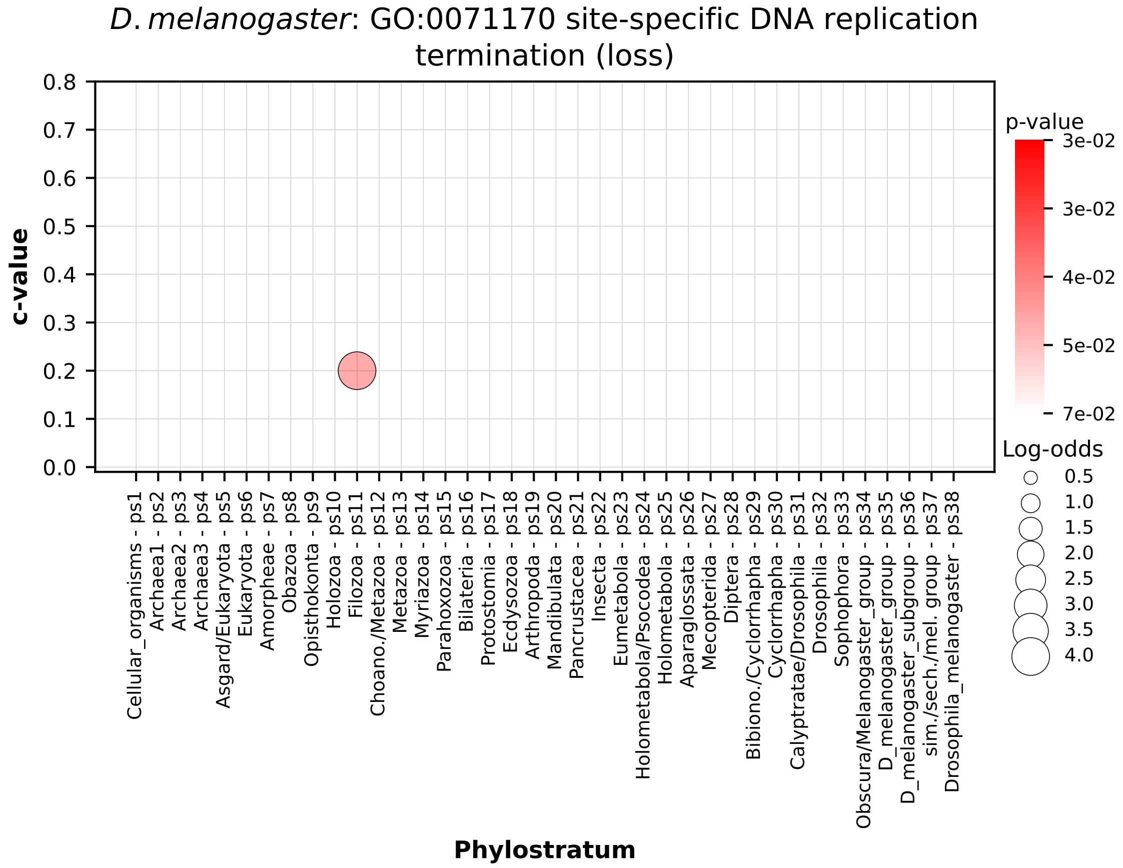

D. melanogaster: GO:0071170 site-specific DNA replication termination (loss)
c-value
0.8
0.7
0.6
0.5
0.4
0.3
0.2
0.1
0.0
p-value
3e-02
3e-02
4e-02
5e-02
7e-02
Log-odds
0.5
1.0
1.5
2.0
2.5
3.0
3.5
4.0
Cellular_organisms - ps1
Archaea1 - ps2
Archaea2 - ps3
Archaea3 - ps4
Asgard/Eukaryota - ps5
Eukaryota - ps6
Amorpheae - ps7
Obazoa - ps8
Opisthokonta - ps9
Holozoa - ps10
Filozoa - ps11
Choano./Metazoa - ps12
Metazoa - ps13
Myriazoa - ps14
Parahoxozoa - ps15
Bilateria - ps16
Protostomia - ps17
Ecdysozoa - ps18
Arthropoda - ps19
Mandibulata - ps20
Pancrustacea - ps21
Insecta - ps22
Eumetabola - ps23
Holometabola/Psocodea - ps24
Holometabola - ps25
Aparaglossata - ps26
Mecopterida - ps27
Diptera - ps28
Bibiono./Cyclorrhapha - ps29
Cyclorrhapha - ps30
Calyptratae/Drosophila - ps31
Drosophila - ps32
Sophophora - ps33
Obscura/Melanogaster_group - ps34
D_melanogaster_group - ps35
D_melanogaster_subgroup - ps36
sim./sech./mel. group - ps37
Drosophila_melanogaster - ps38
Phylostratum

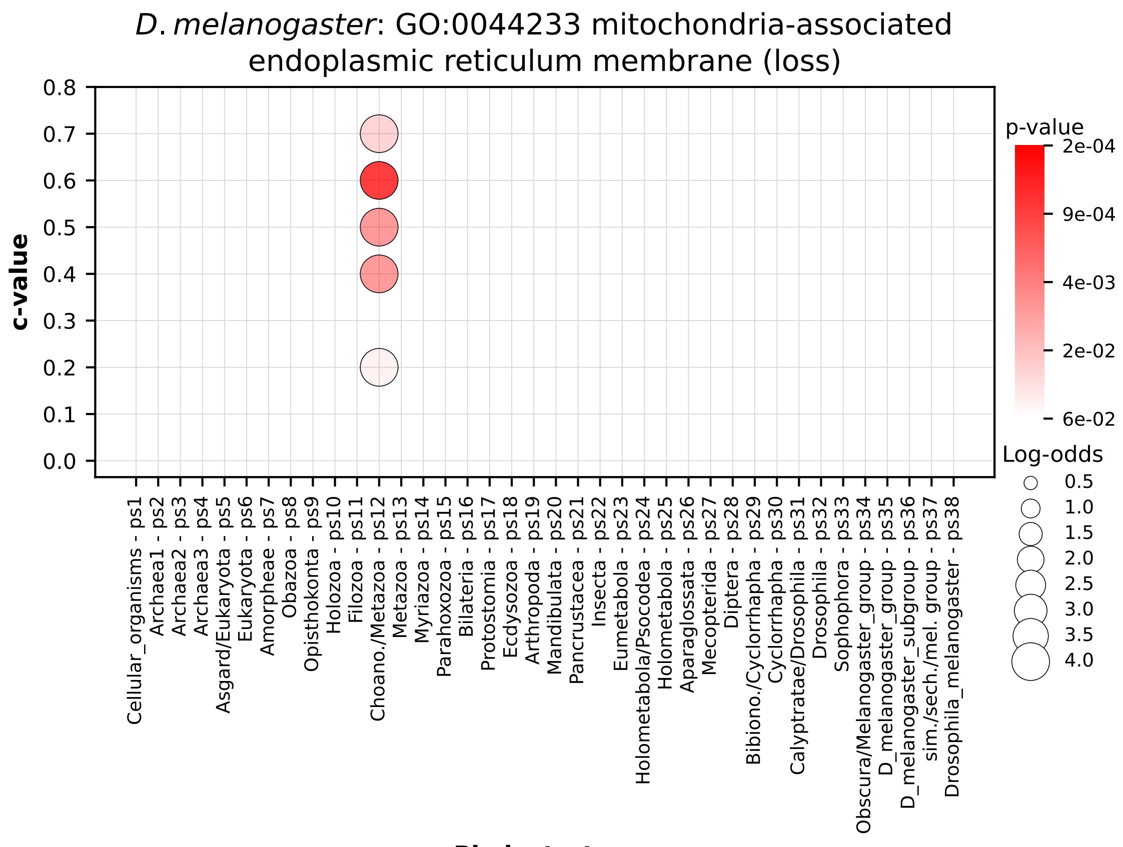
D. melanogaster: GO:0044233 mitochondria-associated endoplasmic reticulum membrane (loss)
c-value
0.0
0.1
0.2
0.3
0.4
0.5
0.6
0.7
0.8
Phylostratum
Cellular_organisms - ps1
Archaea1 - ps2
Archaea2 - ps3
Archaea3 - ps4
Asgard/Eukaryota - ps5
Eukaryota - ps6
Amorpheae - ps7
Obazoa - ps8
Opisthokonta - ps9
Holozoa - ps10
Filozoa - ps11
Choano./Metazoa - ps12
Metazoa - ps13
Myriazoa - ps14
Parahoxozoa - ps15
Bilateria - ps16
Protostomia - ps17
Ecdysozoa - ps18
Arthropoda - ps19
Mandibulata - ps20
Pancrustacea - ps21
Insecta - ps22
Eumetabola - ps23
Holometabola/Psocodea - ps24
Holometabola - ps25
Aparaglossata - ps26
Mecopterida - ps27
Diptera - ps28
Bibiono./Cyclorrhapha - ps29
Cyclorrhapha - ps30
Calyptratae/Drosophila - ps31
Drosophila - ps32
Sophophora - ps33
Obscura/Melanogaster_group - ps34
D_melanogaster_group - ps35
D_melanogaster_subgroup - ps36
sim./sech./mel. group - ps37
Drosophila_melanogaster - ps38
p-value
2e-04
9e-04
4e-03
2e-02
6e-02
Log-odds
0.5
1.0
1.5
2.0
2.5
3.0
3.5
4.0

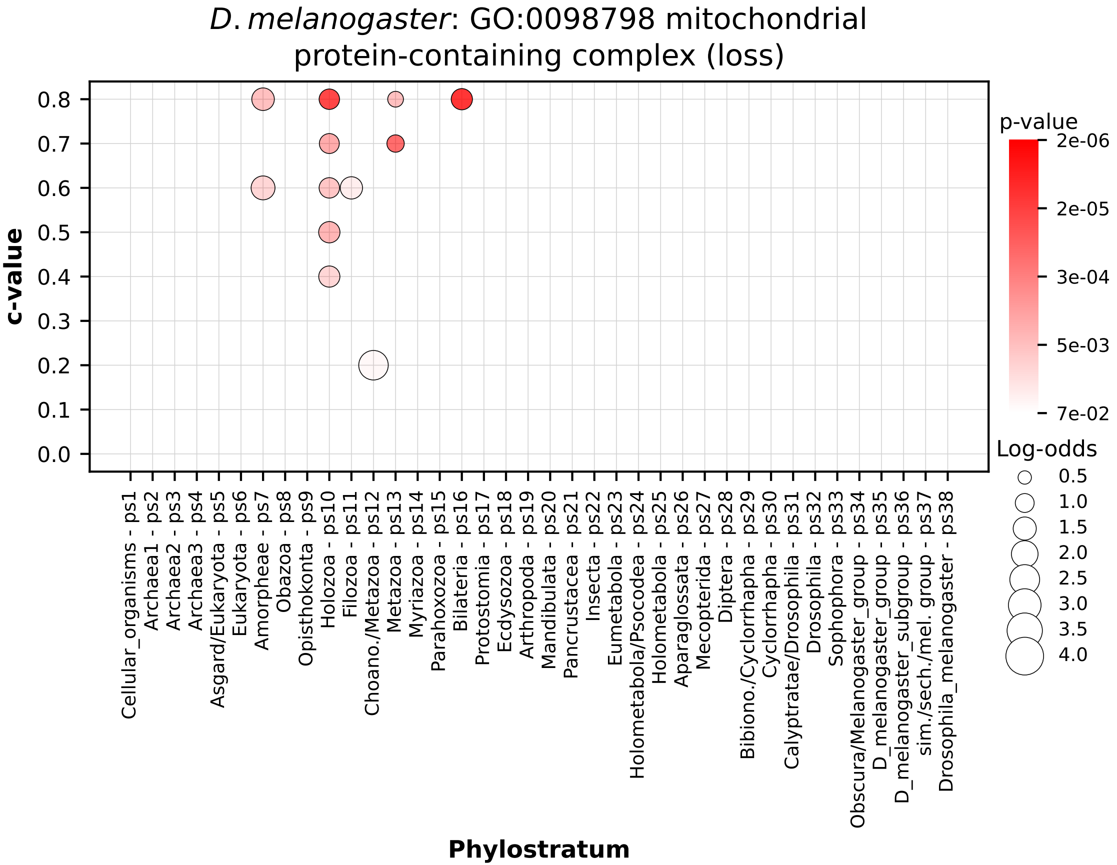

D. melanogaster: GO:0098798 mitochondrial protein-containing complex (loss)
c-value
0.8
0.7
0.6
0.5
0.4
0.3
0.2
0.1
0.0
Phylostratum
Cellular_organisms - ps1
Archaea1 - ps2
Archaea2 - ps3
Archaea3 - ps4
Asgard/Eukaryota - ps5
Eukaryota - ps6
Amorpheae - ps7
Obazoa - ps8
Opisthokonta - ps9
Holozoa - ps10
Filozoa - ps11
Choano./Metazoa - ps12
Metazoa - ps13
Myriazoa - ps14
Parahoxozoa - ps15
Bilateria - ps16
Protostomia - ps17
Ecdysozoa - ps18
Arthropoda - ps19
Mandibulata - ps20
Pancrustacea - ps21
Insecta - ps22
Eumetabola - ps23
Holometabola/Psocodea - ps24
Holometabola - ps25
Aparaglossata - ps26
Mecopterida - ps27
Diptera - ps28
Bibiono./Cyclorrhapha - ps29
Cyclorrhapha - ps30
Calyptratae/Drosophila - ps31
Drosophila - ps32
Sophophora - ps33
Obscura/Melanogaster_group - ps34
D_melanogaster_group - ps35
D_melanogaster_subgroup - ps36
sim./sech./mel. group - ps37
Drosophila_melanogaster - ps38
p-value
2e-06
2e-05
3e-04
5e-03
7e-02
Log-odds
0.5
1.0
1.5
2.0
2.5
3.0
3.5
4.0

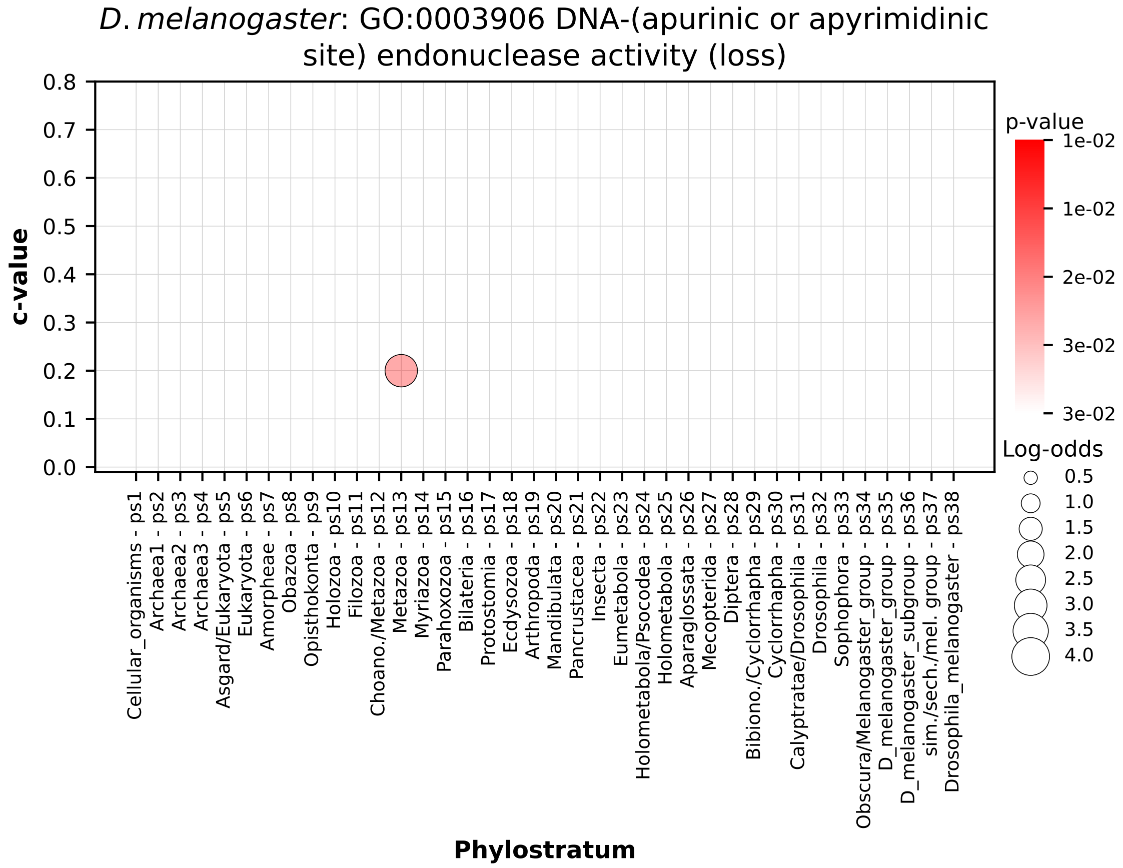

D. melanogaster: GO:0003906 DNA-(apurinic or apyrimidinic site) endonuclease activity (loss)
c-value
0.8
0.7
0.6
0.5
0.4
0.3
0.2
0.1
0.0
Phylostratum
Cellular_organisms - ps1
Archaea1 - ps2
Archaea2 - ps3
Archaea3 - ps4
Asgard/Eukaryota - ps5
Eukaryota - ps6
Amorpheae - ps7
Obazoa - ps8
Opisthokonta - ps9
Holozoa - ps10
Filozoa - ps11
Choano./Metazoa - ps12
Metazoa - ps13
Myriazoa - ps14
Parahoxozoa - ps15
Bilateria - ps16
Protostomia - ps17
Ecdysozoa - ps18
Arthropoda - ps19
Mandibulata - ps20
Pancrustacea - ps21
Insecta - ps22
Eumetabola - ps23
Holometabola/Psocodea - ps24
Holometabola - ps25
Aparaglossata - ps26
Mecopterida - ps27
Diptera - ps28
Bibiono./Cyclorrhapha - ps29
Cyclorrhapha - ps30
Calyptratae/Drosophila - ps31
Drosophila - ps32
Sophophora - ps33
Obscura/Melanogaster_group - ps34
D_melanogaster_group - ps35
D_melanogaster_subgroup - ps36
sim./sech./mel. group - ps37
Drosophila_melanogaster - ps38
p-value
1e-02
1e-02
2e-02
3e-02
3e-02
Log-odds
0.5
1.0
1.5
2.0
2.5
3.0
3.5
4.0

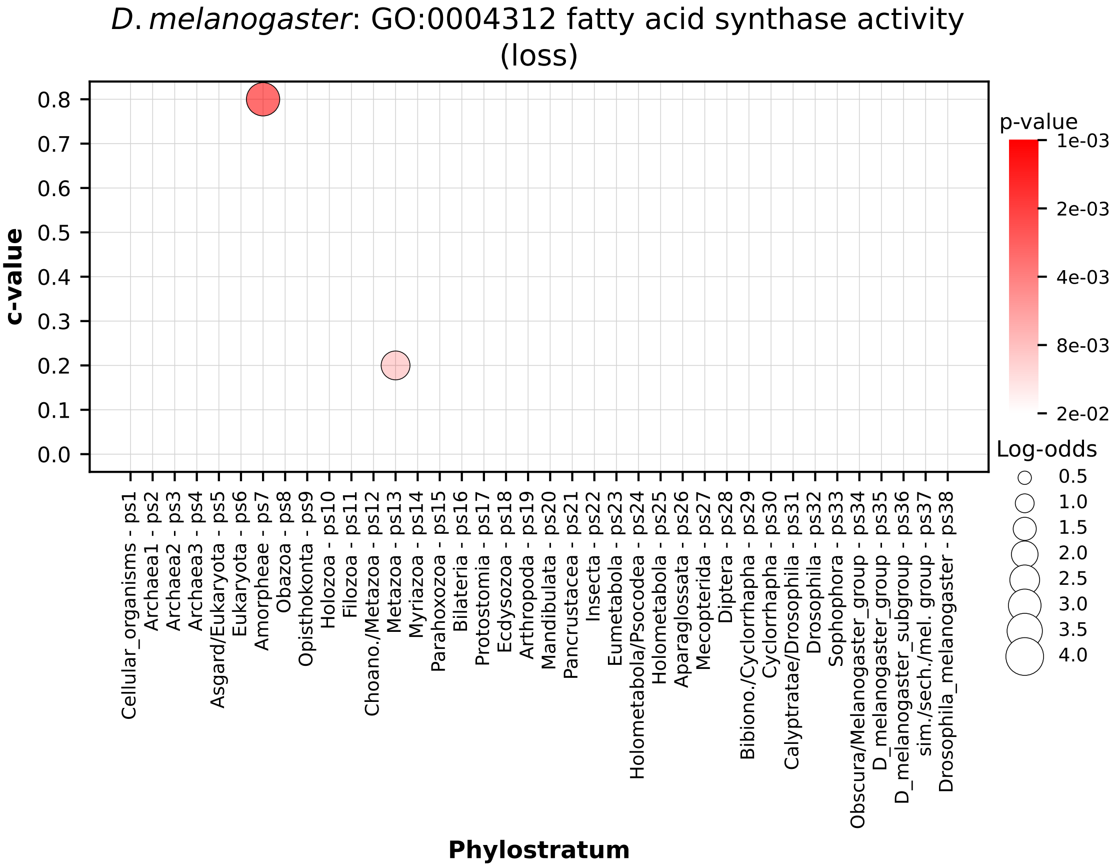
D. melanogaster: GO:0004312 fatty acid synthase activity
(loss)
c-value
0.8
0.7
0.6
0.5
0.4
0.3
0.2
0.1
0.0
Phylostratum
Cellular_organisms - ps1
Archaea1 - ps2
Archaea2 - ps3
Archaea3 - ps4
Asgard/Eukaryota - ps5
Eukaryota - ps6
Amorpheae - ps7
Obazoa - ps8
Opisthokonta - ps9
Holozoa - ps10
Filozoa - ps11
Choano./Metazoa - ps12
Metazoa - ps13
Myriazoa - ps14
Parahoxozoa - ps15
Bilateria - ps16
Protostomia - ps17
Ecdysozoa - ps18
Arthropoda - ps19
Mandibulata - ps20
Pancrustacea - ps21
Insecta - ps22
Eumetabola - ps23
Holometabola/Psocodea - ps24
Holometabola - ps25
Aparaglossata - ps26
Mecopterida - ps27
Diptera - ps28
Bibiono./Cyclorrhapha - ps29
Cyclorrhapha - ps30
Calyptratae/Drosophila - ps31
Drosophila - ps32
Sophophora - ps33
Obscura/Melanogaster_group - ps34
D_melanogaster_group - ps35
D_melanogaster_subgroup - ps36
sim./sech./mel. group - ps37
Drosophila_melanogaster - ps38
p-value
1e-03
2e-03
4e-03
8e-03
2e-02
Log-odds
0.5
1.0
1.5
2.0
2.5
3.0
3.5
4.0

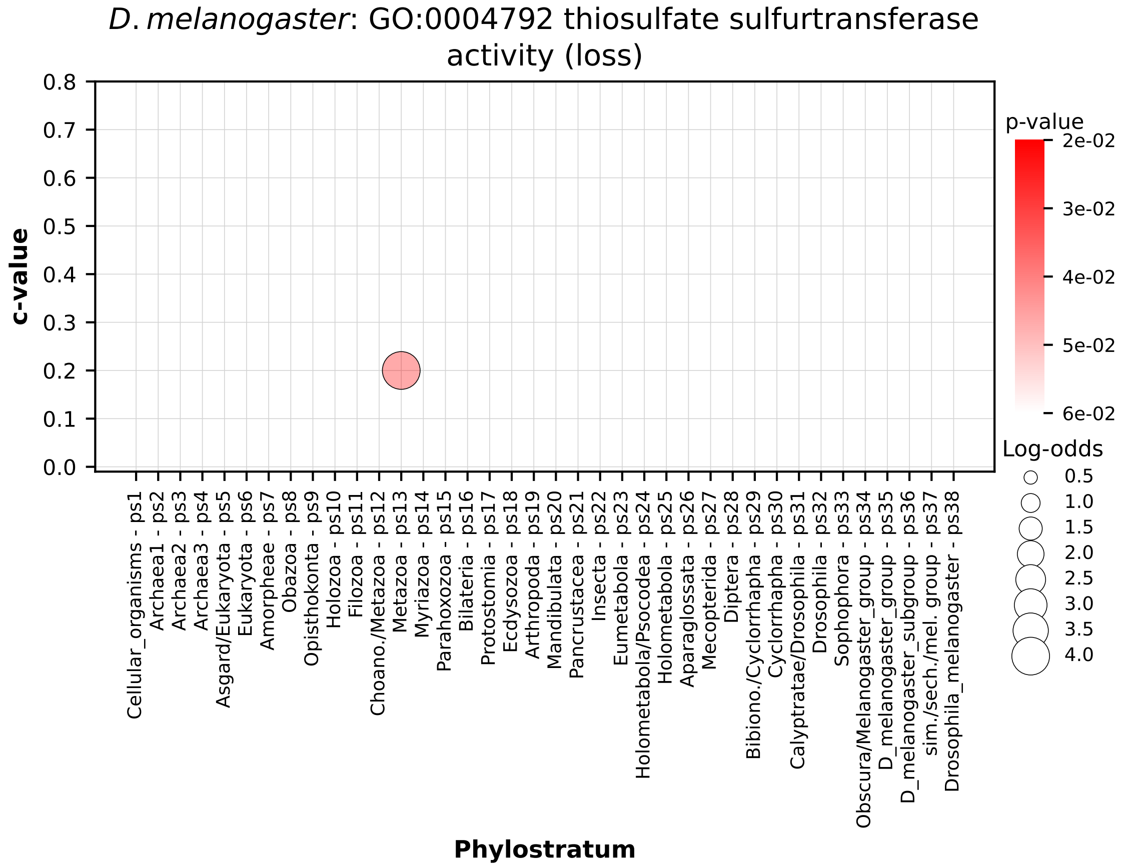

D. melanogaster: GO:0004792 thiosulfate sulfurtransferase activity (loss)
c-value
0.0
0.1
0.2
0.3
0.4
0.5
0.6
0.7
0.8
Phylostratum
Cellular_organisms - ps1
Archaea1 - ps2
Archaea2 - ps3
Archaea3 - ps4
Asgard/Eukaryota - ps5
Eukaryota - ps6
Amorpheae - ps7
Obazoa - ps8
Opisthokonta - ps9
Holozoa - ps10
Filozoa - ps11
Choano./Metazoa - ps12
Metazoa - ps13
Myriazoa - ps14
Parahoxozoa - ps15
Bilateria - ps16
Protostomia - ps17
Ecdysozoa - ps18
Arthropoda - ps19
Mandibulata - ps20
Pancrustacea - ps21
Insecta - ps22
Eumetabola - ps23
Holometabola/Psocodea - ps24
Holometabola - ps25
Aparaglossata - ps26
Mecopterida - ps27
Diptera - ps28
Bibiono./Cyclorrhapha - ps29
Cyclorrhapha - ps30
Calyptratae/Drosophila - ps31
Drosophila - ps32
Sophophora - ps33
Obscura/Melanogaster_group - ps34
D_melanogaster_group - ps35
D_melanogaster_subgroup - ps36
sim./sech./mel. group - ps37
Drosophila_melanogaster - ps38
p-value
2e-02
3e-02
4e-02
5e-02
6e-02
Log-odds
0.5
1.0
1.5
2.0
2.5
3.0
3.5
4.0

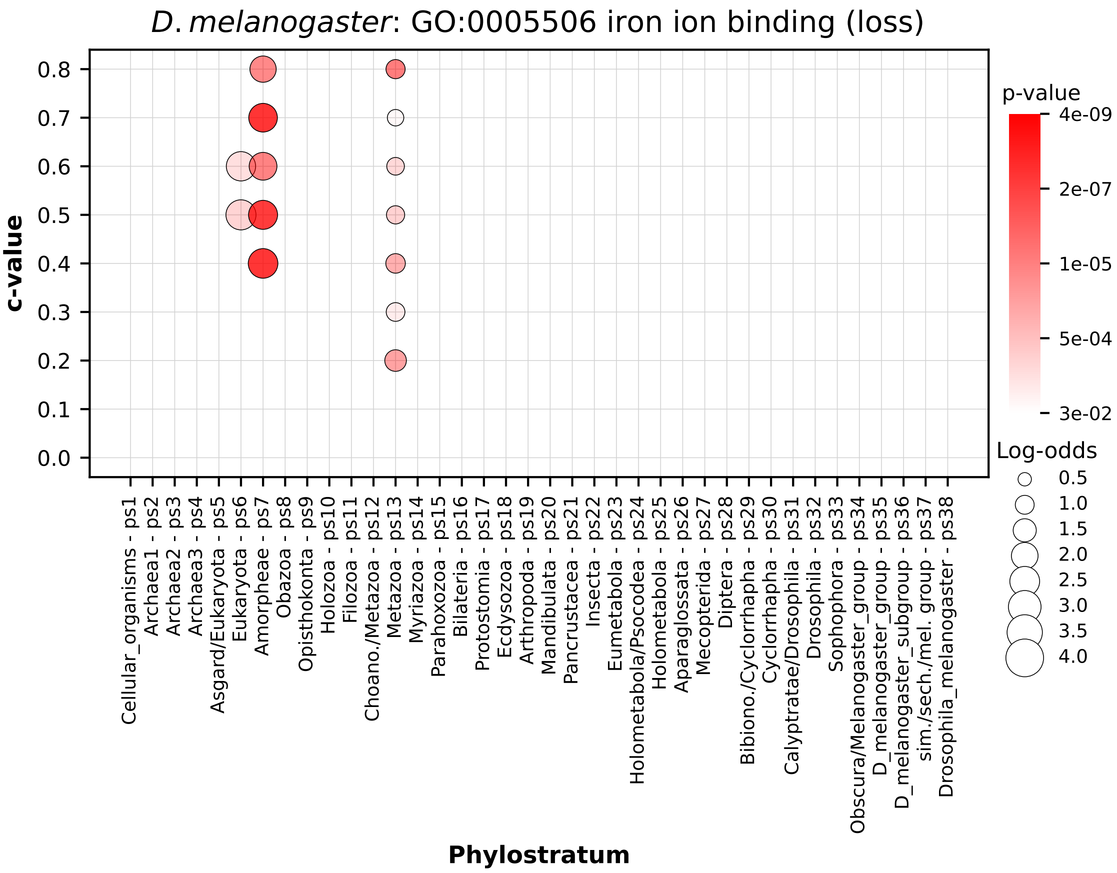

D. melanogaster: GO:0005506 iron ion binding (loss)
c-value
0.0
0.1
0.2
0.3
0.4
0.5
0.6
0.7
0.8
Phylostratum
Cellular_organisms - ps1
Archaea1 - ps2
Archaea2 - ps3
Archaea3 - ps4
Asgard/Eukaryota - ps5
Eukaryota - ps6
Amorpheae - ps7
Obazoa - ps8
Opisthokonta - ps9
Holozoa - ps10
Filozoa - ps11
Choano./Metazoa - ps12
Metazoa - ps13
Myriazoa - ps14
Parahoxozoa - ps15
Bilateria - ps16
Protostomia - ps17
Ecdysozoa - ps18
Arthropoda - ps19
Mandibulata - ps20
Pancrustacea - ps21
Insecta - ps22
Eumetabola - ps23
Holometabola/Psocodea - ps24
Holometabola - ps25
Aparaglossata - ps26
Mecopterida - ps27
Diptera - ps28
Bibiono./Cyclorrhapha - ps29
Cyclorrhapha - ps30
Calyptratae/Drosophila - ps31
Drosophila - ps32
Sophophora - ps33
Obscura/Melanogaster_group - ps34
D_melanogaster_group - ps35
D_melanogaster_subgroup - ps36
sim./sech./mel. group - ps37
Drosophila_melanogaster - ps38
p-value
4e-09
2e-07
1e-05
5e-04
3e-02
Log-odds
0.5
1.0
1.5
2.0
2.5
3.0
3.5
4.0

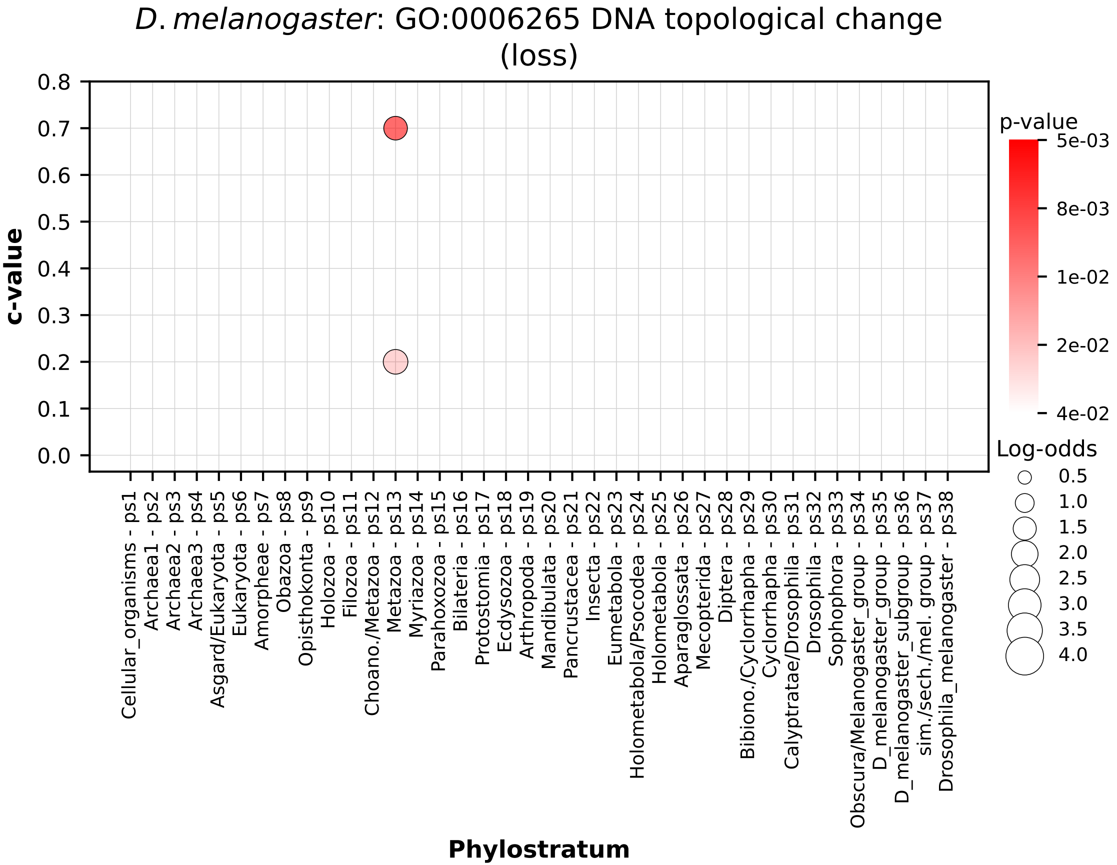

D. melanogaster: GO:0006265 DNA topological change
(loss)
c-value
0.8
0.7
0.6
0.5
0.4
0.3
0.2
0.1
0.0
p-value
5e-03
8e-03
1e-02
2e-02
4e-02
Log-odds
0.5
1.0
1.5
2.0
2.5
3.0
3.5
4.0
Cellular_organisms - ps1
Archaea1 - ps2
Archaea2 - ps3
Archaea3 - ps4
Asgard/Eukaryota - ps5
Eukaryota - ps6
Amorpheae - ps7
Obazoa - ps8
Opisthokonta - ps9
Holozoa - ps10
Filozoa - ps11
Choano./Metazoa - ps12
Metazoa - ps13
Myriazoa - ps14
Parahoxozoa - ps15
Bilateria - ps16
Protostomia - ps17
Ecdysozoa - ps18
Arthropoda - ps19
Mandibulata - ps20
Pancrustacea - ps21
Insecta - ps22
Eumetabola - ps23
Holometabola/Psocodea - ps24
Holometabola - ps25
Aparaglossata - ps26
Mecopterida - ps27
Diptera - ps28
Bibiono./Cyclorrhapha - ps29
Cyclorrhapha - ps30
Calyptratae/Drosophila - ps31
Drosophila - ps32
Sophophora - ps33
Obscura/Melanogaster_group - ps34
D_melanogaster_group - ps35
D_melanogaster_subgroup - ps36
sim./sech./mel. group - ps37
Drosophila_melanogaster - ps38
Phylostratum

D. melanogaster: GO:0006281 DNA repair (loss)

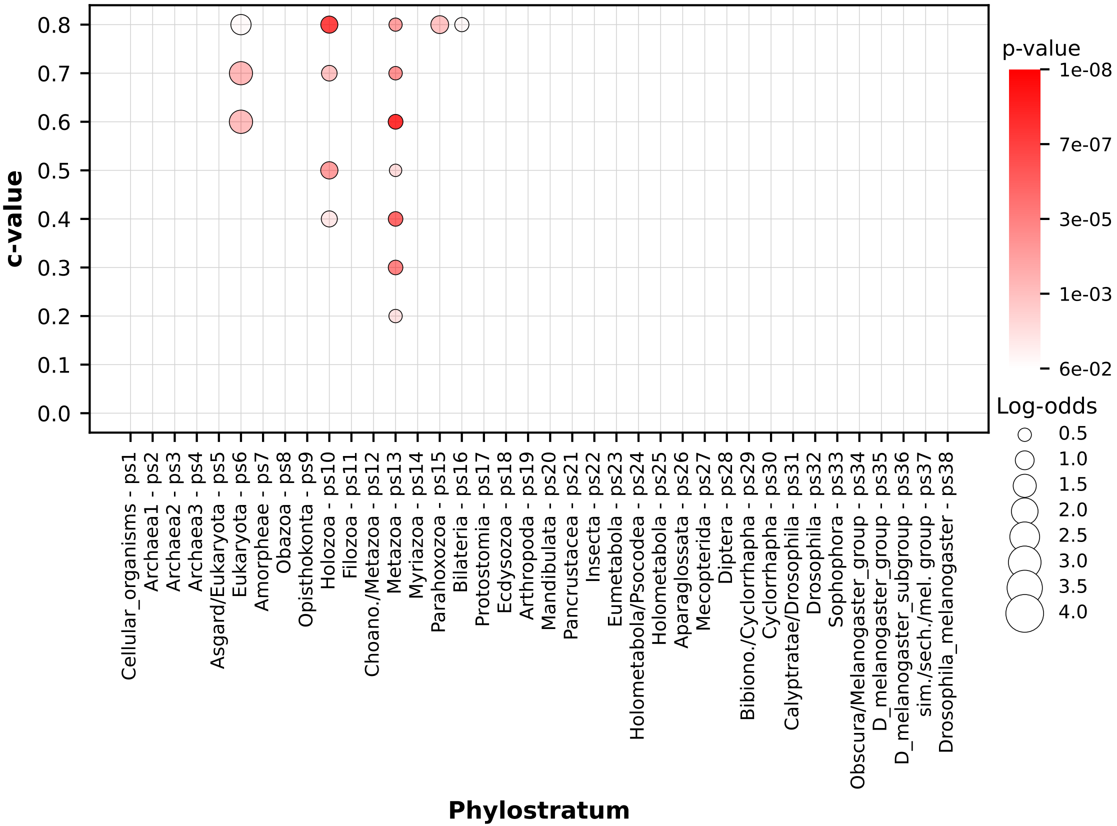

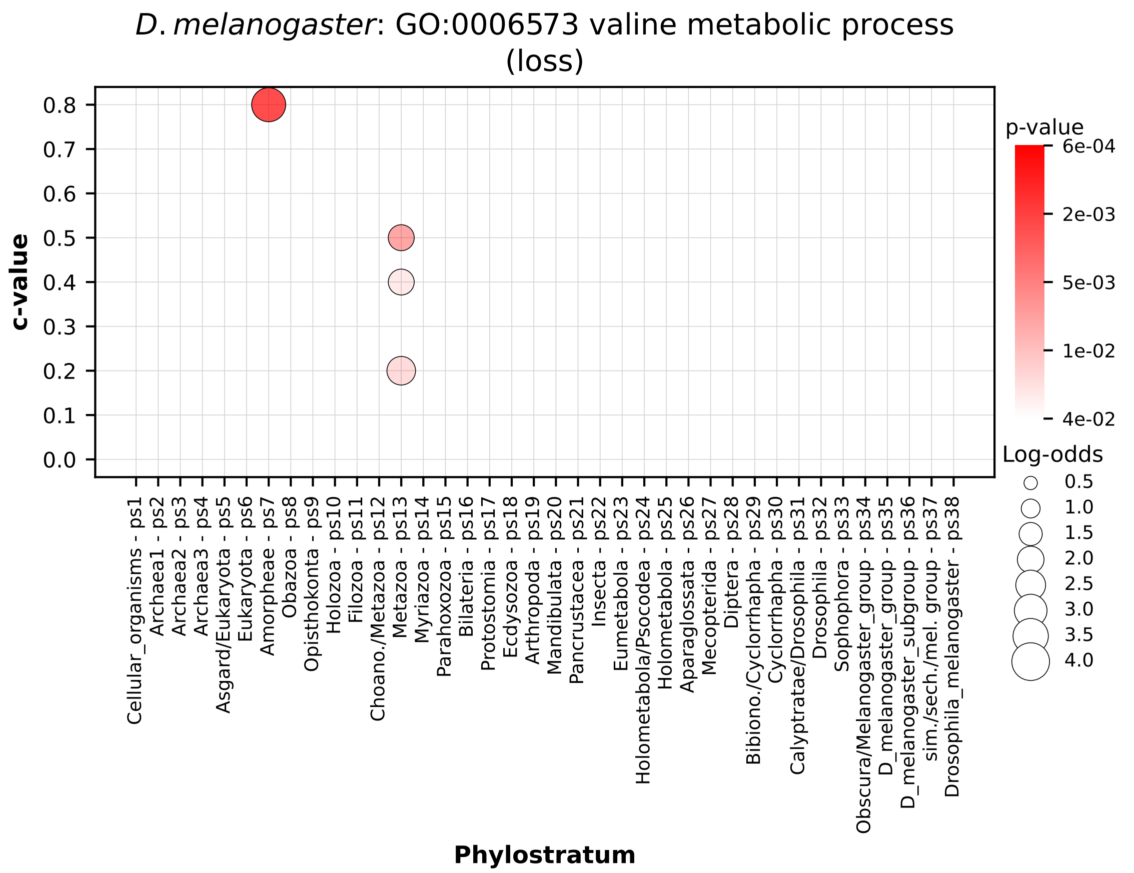

D. melanogaster: GO:0006573 valine metabolic process
(loss)
c-value
0.0
0.1
0.2
0.3
0.4
0.5
0.6
0.7
0.8
Phylostratum
Cellular_organisms - ps1
Archaea1 - ps2
Archaea2 - ps3
Archaea3 - ps4
Asgard/Eukaryota - ps5
Eukaryota - ps6
Amorpheae - ps7
Obazoa - ps8
Opisthokonta - ps9
Holozoa - ps10
Filozoa - ps11
Choano./Metazoa - ps12
Metazoa - ps13
Myriazoa - ps14
Parahoxozoa - ps15
Bilateria - ps16
Protostomia - ps17
Ecdysozoa - ps18
Arthropoda - ps19
Mandibulata - ps20
Pancrustacea - ps21
Insecta - ps22
Eumetabola - ps23
Holometabola/Psocodea - ps24
Holometabola - ps25
Aparaglossata - ps26
Mecopterida - ps27
Diptera - ps28
Bibiono./Cyclorrhapha - ps29
Cyclorrhapha - ps30
Calyptratae/Drosophila - ps31
Drosophila - ps32
Sophophora - ps33
Obscura/Melanogaster_group - ps34
D_melanogaster_group - ps35
D_melanogaster_subgroup - ps36
sim./sech./mel. group - ps37
Drosophila_melanogaster - ps38
p-value
6e-04
2e-03
5e-03
1e-02
4e-02
Log-odds
0.5
1.0
1.5
2.0
2.5
3.0
3.5
4.0

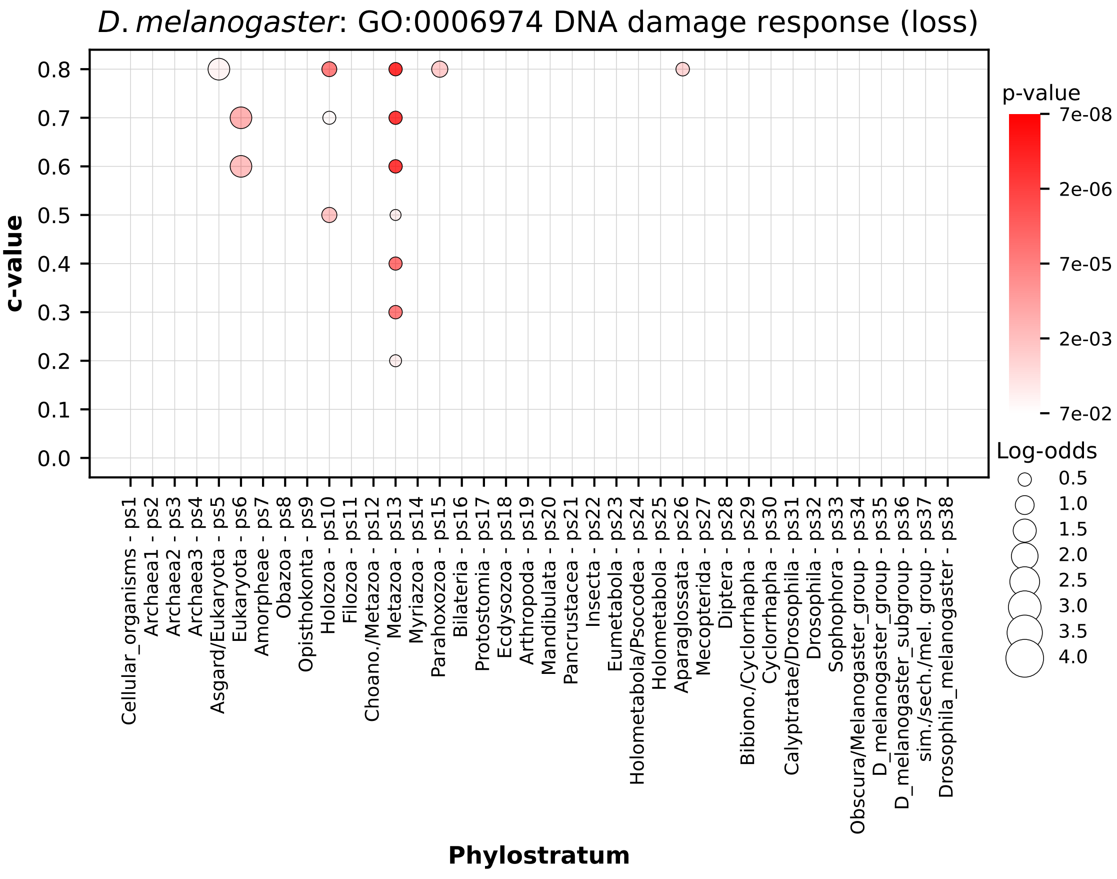

D. melanogaster: GO:0006974 DNA damage response (loss)
c-value
0.8
0.7
0.6
0.5
0.4
0.3
0.2
0.1
0.0
Phylostratum
Cellular_organisms - ps1
Archaea1 - ps2
Archaea2 - ps3
Archaea3 - ps4
Asgard/Eukaryota - ps5
Eukaryota - ps6
Amorpheae - ps7
Obazoa - ps8
Opisthokonta - ps9
Holozoa - ps10
Filozoa - ps11
Choano./Metazoa - ps12
Metazoa - ps13
Myriazoa - ps14
Parahoxozoa - ps15
Bilateria - ps16
Protostomia - ps17
Ecdysozoa - ps18
Arthropoda - ps19
Mandibulata - ps20
Pancrustacea - ps21
Insecta - ps22
Eumetabola - ps23
Holometabola/Psocodea - ps24
Holometabola - ps25
Aparaglossata - ps26
Mecopterida - ps27
Diptera - ps28
Bibiono./Cyclorrhapha - ps29
Cyclorrhapha - ps30
Calyptratae/Drosophila - ps31
Drosophila - ps32
Sophophora - ps33
Obscura/Melanogaster_group - ps34
D_melanogaster_group - ps35
D_melanogaster_subgroup - ps36
sim./sech./mel. group - ps37
Drosophila_melanogaster - ps38
p-value
7e-08
2e-06
7e-05
2e-03
7e-02
Log-odds
0.5
1.0
1.5
2.0
2.5
3.0
3.5
4.0

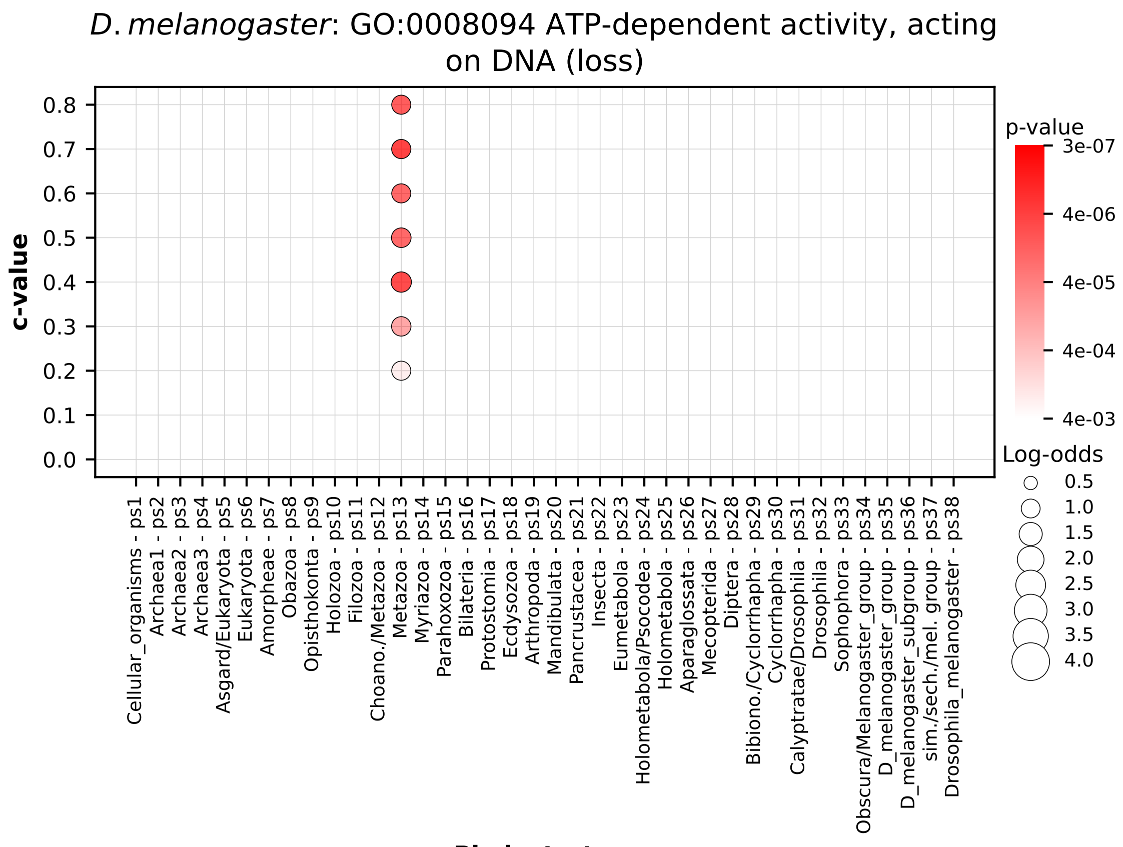

D. melanogaster: GO:0008094 ATP-dependent activity, acting on DNA (loss)
c-value
0.8
0.7
0.6
0.5
0.4
0.3
0.2
0.1
0.0
p-value
3e-07
4e-06
4e-05
4e-04
4e-03
Log-odds
0.5
1.0
1.5
2.0
2.5
3.0
3.5
4.0
Cellular_organisms - ps1
Archaea1 - ps2
Archaea2 - ps3
Archaea3 - ps4
Asgard/Eukaryota - ps5
Eukaryota - ps6
Amorpheae - ps7
Obazoa - ps8
Opisthokonta - ps9
Holozoa - ps10
Filozoa - ps11
Choano./Metazoa - ps12
Metazoa - ps13
Myriazoa - ps14
Parahoxozoa - ps15
Bilateria - ps16
Protostomia - ps17
Ecdysozoa - ps18
Arthropoda - ps19
Mandibulata - ps20
Pancrustacea - ps21
Insecta - ps22
Eumetabola - ps23
Holometabola/Psocodea - ps24
Holometabola - ps25
Aparaglossata - ps26
Mecopterida - ps27
Diptera - ps28
Bibiono./Cyclorrhapha - ps29
Cyclorrhapha - ps30
Calyptratae/Drosophila - ps31
Drosophila - ps32
Sophophora - ps33
Obscura/Melanogaster_group - ps34
D_melanogaster_group - ps35
D_melanogaster_subgroup - ps36
sim./sech./mel. group - ps37
Drosophila_melanogaster - ps38
Phylostratum

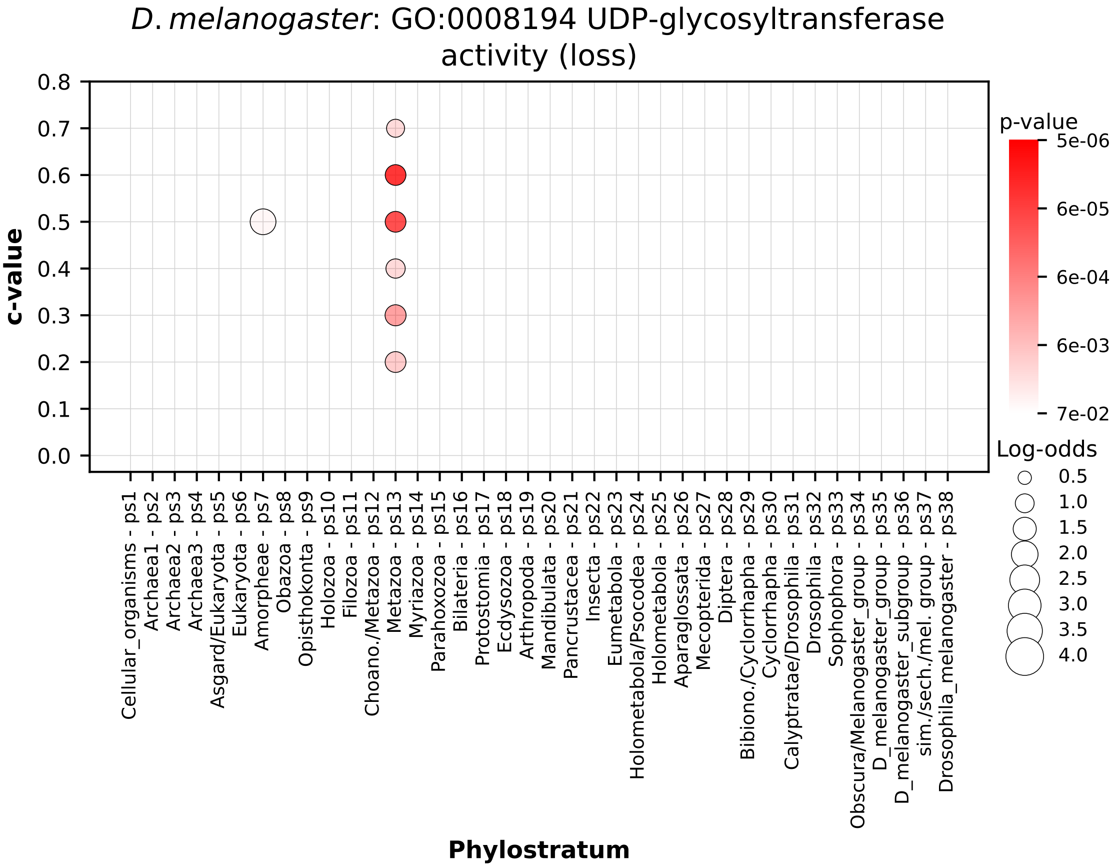
D. melanogaster: GO:0008194 UDP-glycosyltransferase activity (loss)
c-value
0.8
0.7
0.6
0.5
0.4
0.3
0.2
0.1
0.0
p-value
5e-06
6e-05
6e-04
6e-03
7e-02
Log-odds
0.5
1.0
1.5
2.0
2.5
3.0
3.5
4.0
Cellular_organisms - ps1
Archaea1 - ps2
Archaea2 - ps3
Archaea3 - ps4
Asgard/Eukaryota - ps5
Eukaryota - ps6
Amorpheae - ps7
Obazoa - ps8
Opisthokonta - ps9
Holozoa - ps10
Filozoa - ps11
Choano./Metazoa - ps12
Metazoa - ps13
Myriazoa - ps14
Parahoxozoa - ps15
Bilateria - ps16
Protostomia - ps17
Ecdysozoa - ps18
Arthropoda - ps19
Mandibulata - ps20
Pancrustacea - ps21
Insecta - ps22
Eumetabola - ps23
Holometabola/Psocodea - ps24
Holometabola - ps25
Aparaglossata - ps26
Mecopterida - ps27
Diptera - ps28
Bibiono./Cyclorrhapha - ps29
Cyclorrhapha - ps30
Calyptratae/Drosophila - ps31
Drosophila - ps32
Sophophora - ps33
Obscura/Melanogaster_group - ps34
D_melanogaster_group - ps35
D_melanogaster_subgroup - ps36
sim./sech./mel. group - ps37
Drosophila_melanogaster - ps38
Phylostratum

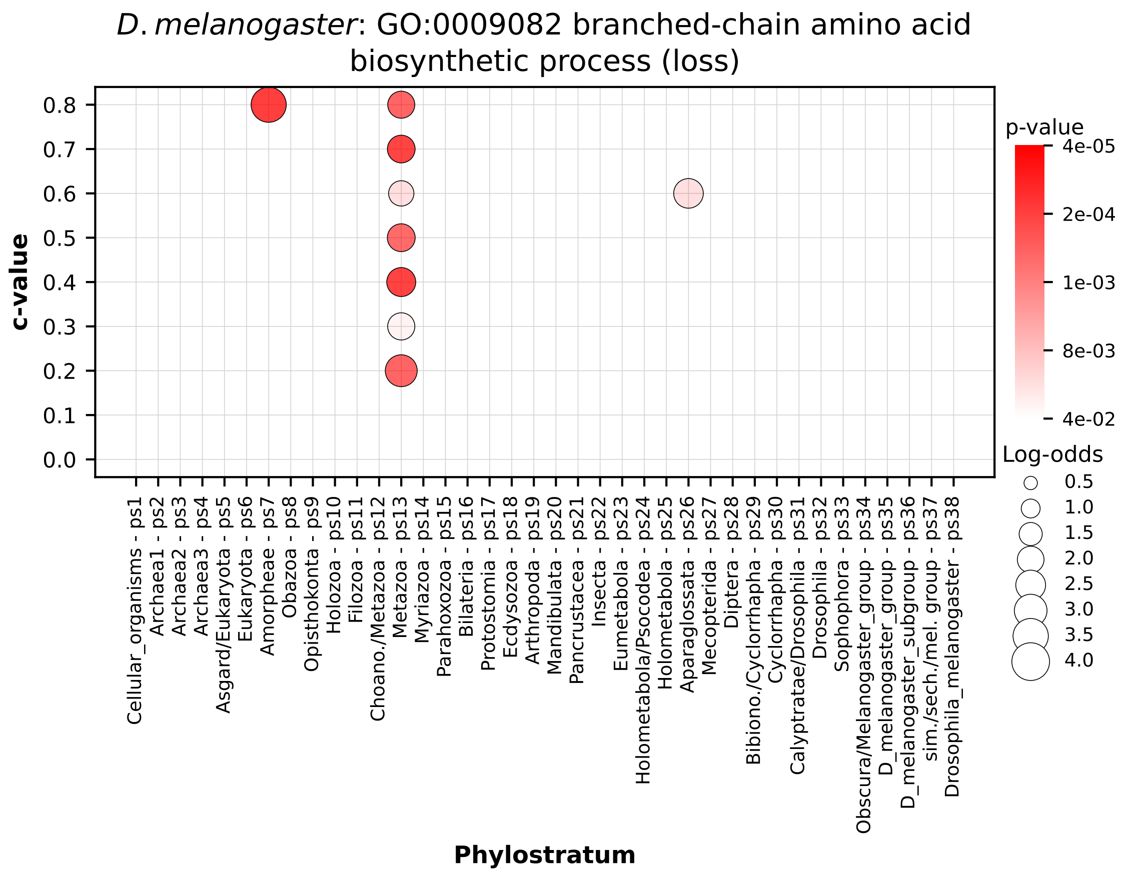

D. melanogaster: GO:0009082 branched-chain amino acid biosynthetic process (loss)
c-value
0.8
0.7
0.6
0.5
0.4
0.3
0.2
0.1
0.0
Phylostratum
Cellular_organisms - ps1
Archaea1 - ps2
Archaea2 - ps3
Archaea3 - ps4
Asgard/Eukaryota - ps5
Eukaryota - ps6
Amorpheae - ps7
Obazoa - ps8
Opisthokonta - ps9
Holozoa - ps10
Filozoa - ps11
Choano./Metazoa - ps12
Metazoa - ps13
Myriazoa - ps14
Parahoxozoa - ps15
Bilateria - ps16
Protostomia - ps17
Ecdysozoa - ps18
Arthropoda - ps19
Mandibulata - ps20
Pancrustacea - ps21
Insecta - ps22
Eumetabola - ps23
Holometabola/Psocodea - ps24
Holometabola - ps25
Aparaglossata - ps26
Mecopterida - ps27
Diptera - ps28
Bibiono./Cyclorrhapha - ps29
Cyclorrhapha - ps30
Calyptratae/Drosophila - ps31
Drosophila - ps32
Sophophora - ps33
Obscura/Melanogaster_group - ps34
D_melanogaster_group - ps35
D_melanogaster_subgroup - ps36
sim./sech./mel. group - ps37
Drosophila_melanogaster - ps38
p-value
4e-05
2e-04
1e-03
8e-03
4e-02
Log-odds
0.5
1.0
1.5
2.0
2.5
3.0
3.5
4.0

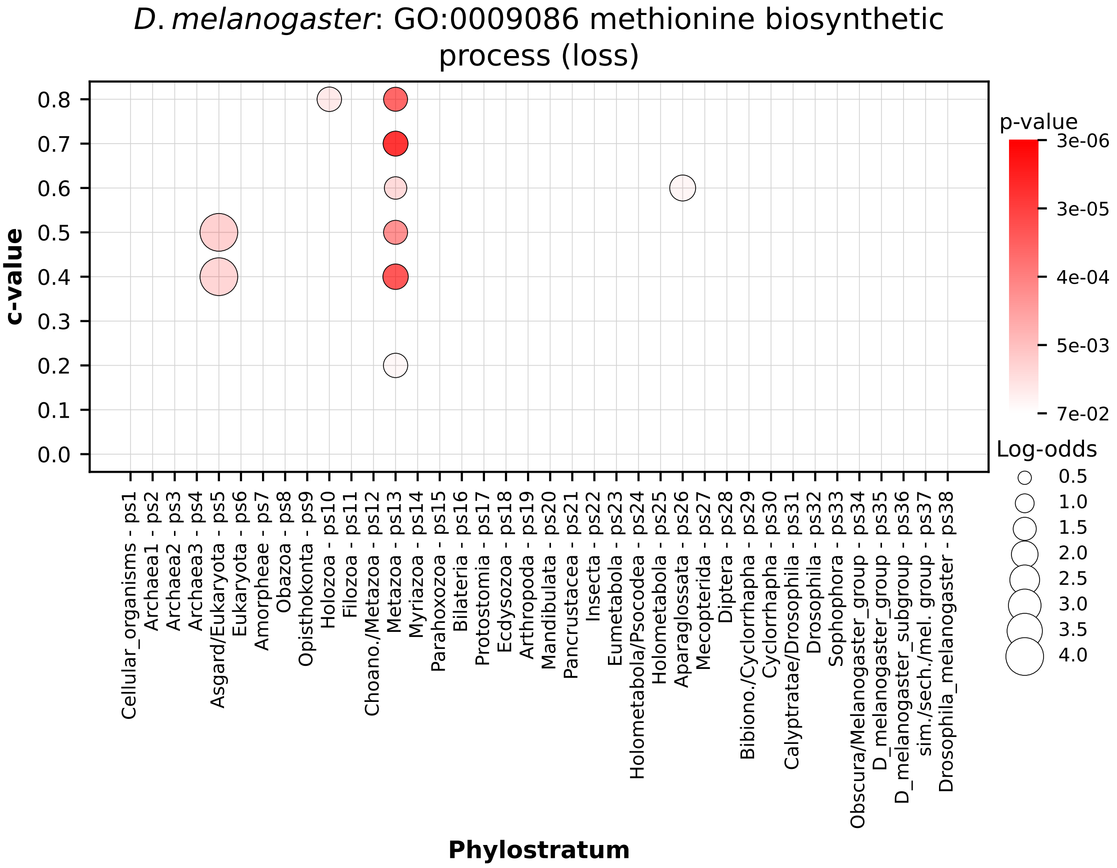

D. melanogaster: GO:0009086 methionine biosynthetic process (loss)
c-value
0.8
0.7
0.6
0.5
0.4
0.3
0.2
0.1
0.0
p-value
3e-06
3e-05
4e-04
5e-03
7e-02
Log-odds
0.5
1.0
1.5
2.0
2.5
3.0
3.5
4.0
Cellular_organisms - ps1
Archaea1 - ps2
Archaea2 - ps3
Archaea3 - ps4
Asgard/Eukaryota - ps5
Eukaryota - ps6
Amorpheae - ps7
Obazoa - ps8
Opisthokonta - ps9
Holozoa - ps10
Filozoa - ps11
Choano./Metazoa - ps12
Metazoa - ps13
Myriazoa - ps14
Parahoxozoa - ps15
Bilateria - ps16
Protostomia - ps17
Ecdysozoa - ps18
Arthropoda - ps19
Mandibulata - ps20
Pancrustacea - ps21
Insecta - ps22
Eumetabola - ps23
Holometabola/Psocodea - ps24
Holometabola - ps25
Aparaglossata - ps26
Mecopterida - ps27
Diptera - ps28
Bibiono./Cyclorrhapha - ps29
Cyclorrhapha - ps30
Calyptratae/Drosophila - ps31
Drosophila - ps32
Sophophora - ps33
Obscura/Melanogaster_group - ps34
D_melanogaster_group - ps35
D_melanogaster_subgroup - ps36
sim./sech./mel. group - ps37
Drosophila_melanogaster - ps38
Phylostratum

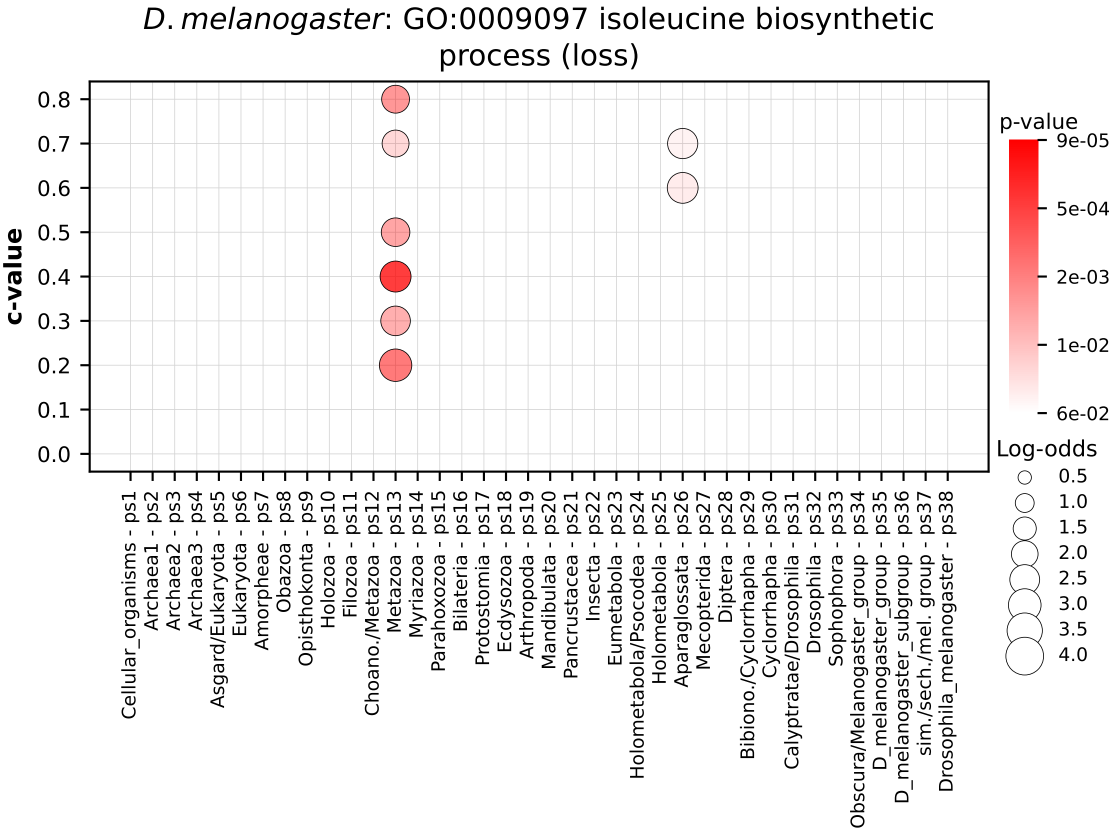
D. melanogaster: GO:0009097 isoleucine biosynthetic process (loss)
c-value
0.8
0.7
0.6
0.5
0.4
0.3
0.2
0.1
0.0
Phylostratum
Cellular_organisms - ps1
Archaea1 - ps2
Archaea2 - ps3
Archaea3 - ps4
Asgard/Eukaryota - ps5
Eukaryota - ps6
Amorpheae - ps7
Obazoa - ps8
Opisthokonta - ps9
Holozoa - ps10
Filozoa - ps11
Choano./Metazoa - ps12
Metazoa - ps13
Myriazoa - ps14
Parahoxozoa - ps15
Bilateria - ps16
Protostomia - ps17
Ecdysozoa - ps18
Arthropoda - ps19
Mandibulata - ps20
Pancrustacea - ps21
Insecta - ps22
Eumetabola - ps23
Holometabola/Psocodea - ps24
Holometabola - ps25
Aparaglossata - ps26
Mecopterida - ps27
Diptera - ps28
Bibiono./Cyclorrhapha - ps29
Cyclorrhapha - ps30
Calyptratae/Drosophila - ps31
Drosophila - ps32
Sophophora - ps33
Obscura/Melanogaster_group - ps34
D_melanogaster_group - ps35
D_melanogaster_subgroup - ps36
sim./sech./mel. group - ps37
Drosophila_melanogaster - ps38
p-value
9e-05
5e-04
2e-03
1e-02
6e-02
Log-odds
0.5
1.0
1.5
2.0
2.5
3.0
3.5
4.0

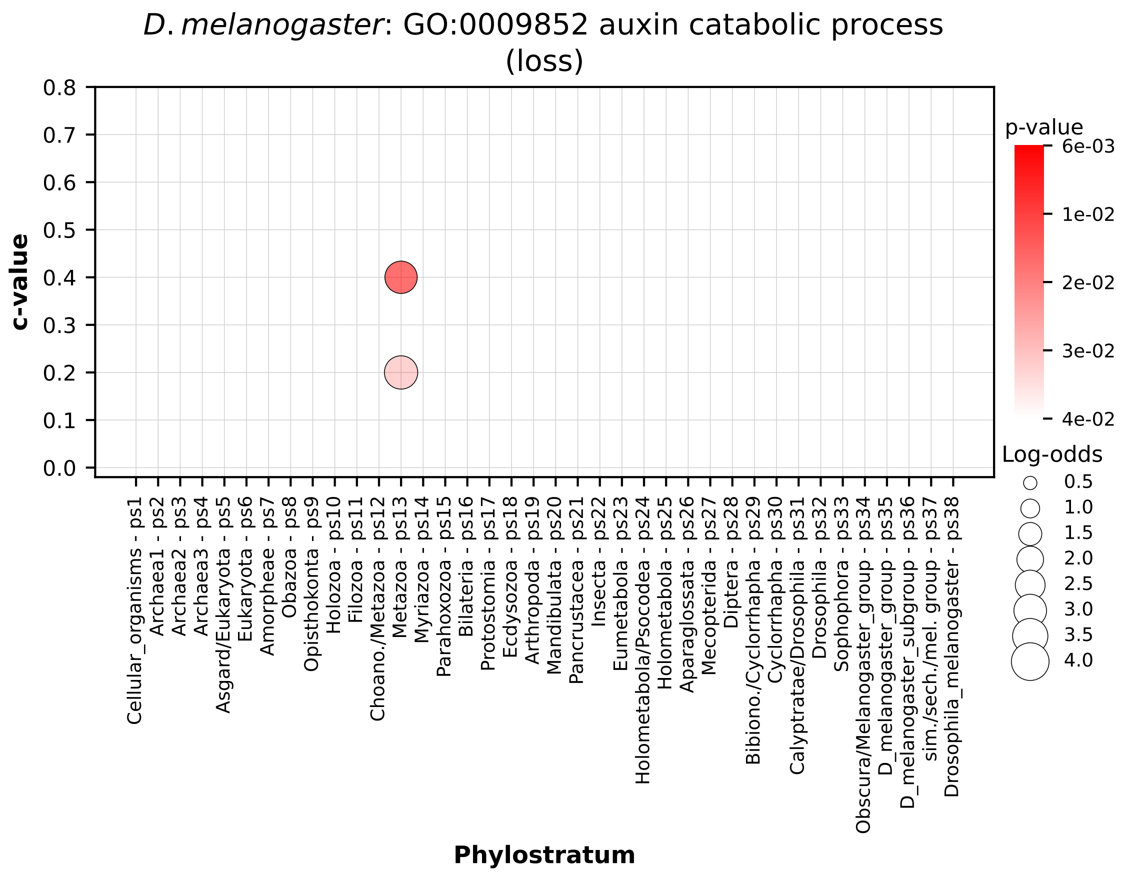

D. melanogaster: GO:0009852 auxin catabolic process
(loss)
c-value
0.8
0.7
0.6
0.5
0.4
0.3
0.2
0.1
0.0
p-value
6e-03
1e-02
2e-02
3e-02
4e-02
Log-odds
0.5
1.0
1.5
2.0
2.5
3.0
3.5
4.0
Cellular_organisms - ps1
Archaea1 - ps2
Archaea2 - ps3
Archaea3 - ps4
Asgard/Eukaryota - ps5
Eukaryota - ps6
Amorpheae - ps7
Obazoa - ps8
Opisthokonta - ps9
Holozoa - ps10
Filozoa - ps11
Choano./Metazoa - ps12
Metazoa - ps13
Myriazoa - ps14
Parahoxozoa - ps15
Bilateria - ps16
Protostomia - ps17
Ecdysozoa - ps18
Arthropoda - ps19
Mandibulata - ps20
Pancrustacea - ps21
Insecta - ps22
Eumetabola - ps23
Holometabola/Psocodea - ps24
Holometabola - ps25
Aparaglossata - ps26
Mecopterida - ps27
Diptera - ps28
Bibiono./Cyclorrhapha - ps29
Cyclorrhapha - ps30
Calyptratae/Drosophila - ps31
Drosophila - ps32
Sophophora - ps33
Obscura/Melanogaster_group - ps34
D_melanogaster_group - ps35
D_melanogaster_subgroup - ps36
sim./sech./mel. group - ps37
Drosophila_melanogaster - ps38
Phylostratum

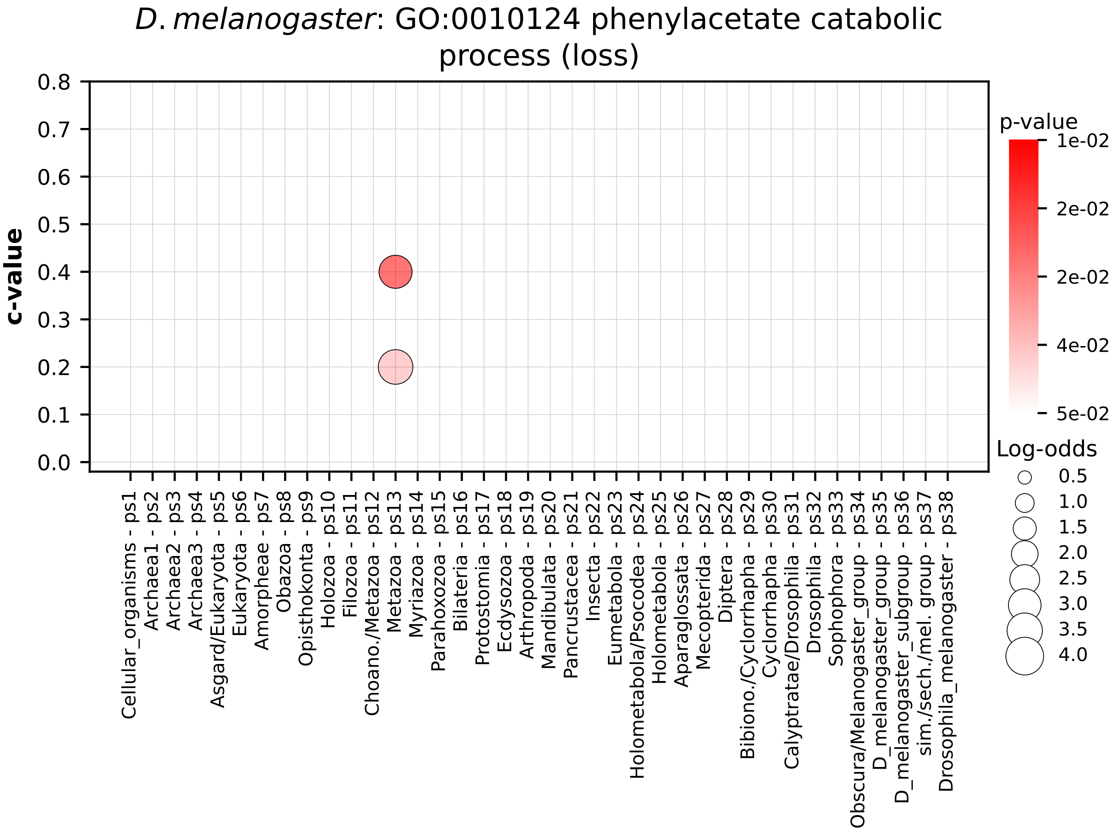

D. melanogaster: GO:0010124 phenylacetate catabolic process (loss)
c-value
0.8
0.7
0.6
0.5
0.4
0.3
0.2
0.1
0.0
p-value
1e-02
2e-02
2e-02
4e-02
5e-02
Log-odds
0.5
1.0
1.5
2.0
2.5
3.0
3.5
4.0
Cellular_organisms - ps1
Archaea1 - ps2
Archaea2 - ps3
Archaea3 - ps4
Asgard/Eukaryota - ps5
Eukaryota - ps6
Amorpheae - ps7
Obazoa - ps8
Opisthokonta - ps9
Holozoa - ps10
Filozoa - ps11
Choano./Metazoa - ps12
Metazoa - ps13
Myriazoa - ps14
Parahoxozoa - ps15
Bilateria - ps16
Protostomia - ps17
Ecdysozoa - ps18
Arthropoda - ps19
Mandibulata - ps20
Pancrustacea - ps21
Insecta - ps22
Eumetabola - ps23
Holometabola/Psocodea - ps24
Holometabola - ps25
Aparaglossata - ps26
Mecopterida - ps27
Diptera - ps28
Bibiono./Cyclorrhapha - ps29
Cyclorrhapha - ps30
Calyptratae/Drosophila - ps31
Drosophila - ps32
Sophophora - ps33
Obscura/Melanogaster_group - ps34
D_melanogaster_group - ps35
D_melanogaster_subgroup - ps36
sim./sech./mel. group - ps37
Drosophila_melanogaster - ps38
Phylostratum

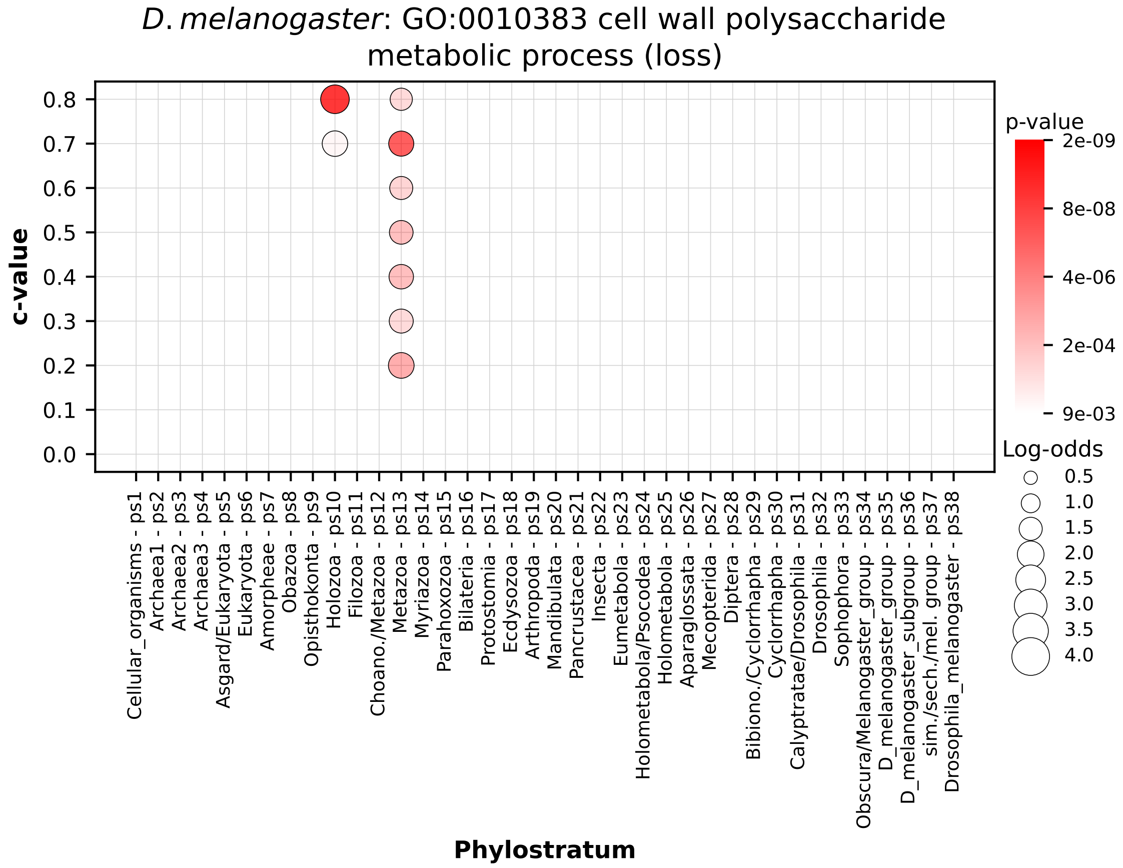

D. melanogaster: GO:0010383 cell wall polysaccharide metabolic process (loss)
c-value
0.8
0.7
0.6
0.5
0.4
0.3
0.2
0.1
0.0
p-value
2e-09
8e-08
4e-06
2e-04
9e-03
Log-odds
0.5
1.0
1.5
2.0
2.5
3.0
3.5
4.0
Cellular_organisms - ps1
Archaea1 - ps2
Archaea2 - ps3
Archaea3 - ps4
Asgard/Eukaryota - ps5
Eukaryota - ps6
Amorpheae - ps7
Obazoa - ps8
Opisthokonta - ps9
Holozoa - ps10
Filozoa - ps11
Choano./Metazoa - ps12
Metazoa - ps13
Myriazoa - ps14
Parahoxozoa - ps15
Bilateria - ps16
Protostomia - ps17
Ecdysozoa - ps18
Arthropoda - ps19
Mandibulata - ps20
Pancrustacea - ps21
Insecta - ps22
Eumetabola - ps23
Holometabola/Psocodea - ps24
Holometabola - ps25
Aparaglossata - ps26
Mecopterida - ps27
Diptera - ps28
Bibiono./Cyclorrhapha - ps29
Cyclorrhapha - ps30
Calyptratae/Drosophila - ps31
Drosophila - ps32
Sophophora - ps33
Obscura/Melanogaster_group - ps34
D_melanogaster_group - ps35
D_melanogaster_subgroup - ps36
sim./sech./mel. group - ps37
Drosophila_melanogaster - ps38
Phylostratum

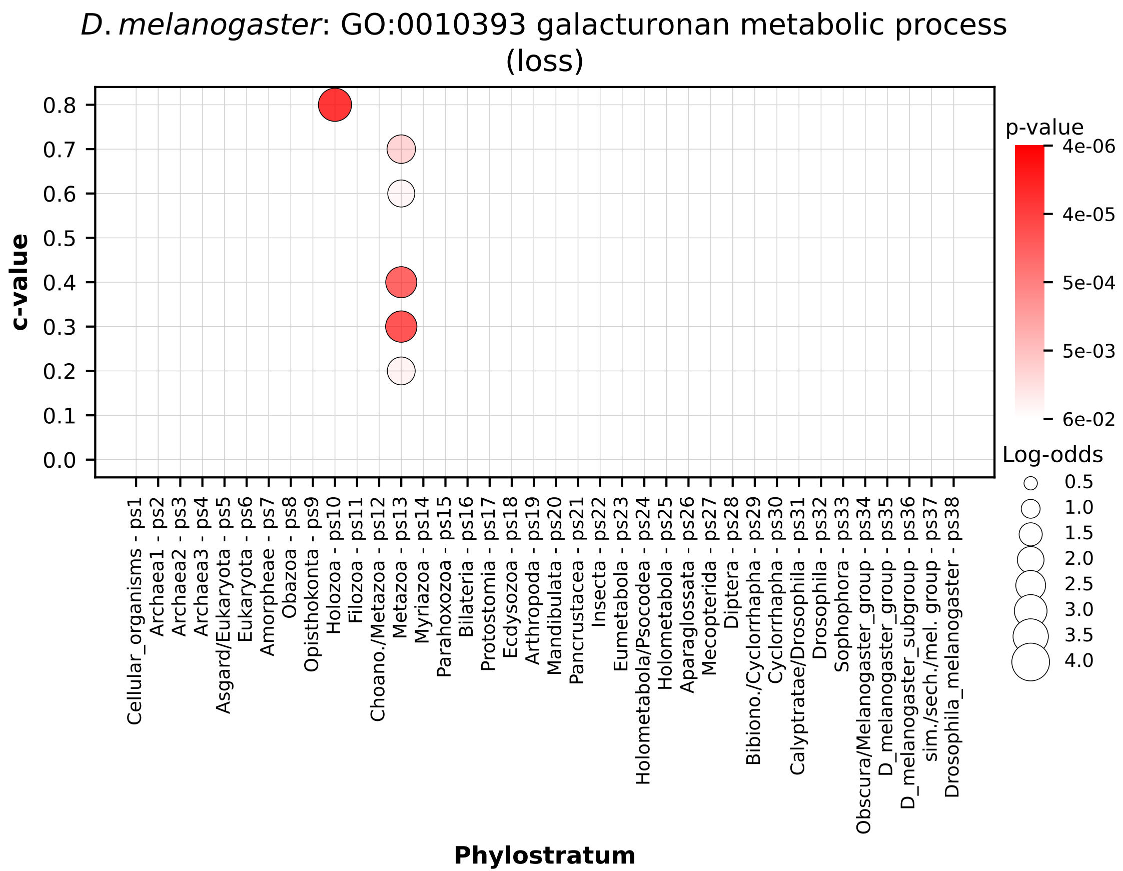

D. melanogaster: GO:0010393 galacturonan metabolic process (loss)
c-value
0.8
0.7
0.6
0.5
0.4
0.3
0.2
0.1
0.0
p-value
4e-06
4e-05
5e-04
5e-03
6e-02
Log-odds
0.5
1.0
1.5
2.0
2.5
3.0
3.5
4.0
Cellular_organisms - ps1
Archaea1 - ps2
Archaea2 - ps3
Archaea3 - ps4
Asgard/Eukaryota - ps5
Eukaryota - ps6
Amorpheae - ps7
Obazoa - ps8
Opisthokonta - ps9
Holozoa - ps10
Filozoa - ps11
Choano./Metazoa - ps12
Metazoa - ps13
Myriazoa - ps14
Parahoxozoa - ps15
Bilateria - ps16
Protostomia - ps17
Ecdysozoa - ps18
Arthropoda - ps19
Mandibulata - ps20
Pancrustacea - ps21
Insecta - ps22
Eumetabola - ps23
Holometabola/Psocodea - ps24
Holometabola - ps25
Aparaglossata - ps26
Mecopterida - ps27
Diptera - ps28
Bibiono./Cyclorrhapha - ps29
Cyclorrhapha - ps30
Calyptratae/Drosophila - ps31
Drosophila - ps32
Sophophora - ps33
Obscura/Melanogaster_group - ps34
D_melanogaster_group - ps35
D_melanogaster_subgroup - ps36
sim./sech./mel. group - ps37
Drosophila_melanogaster - ps38
Phylostratum

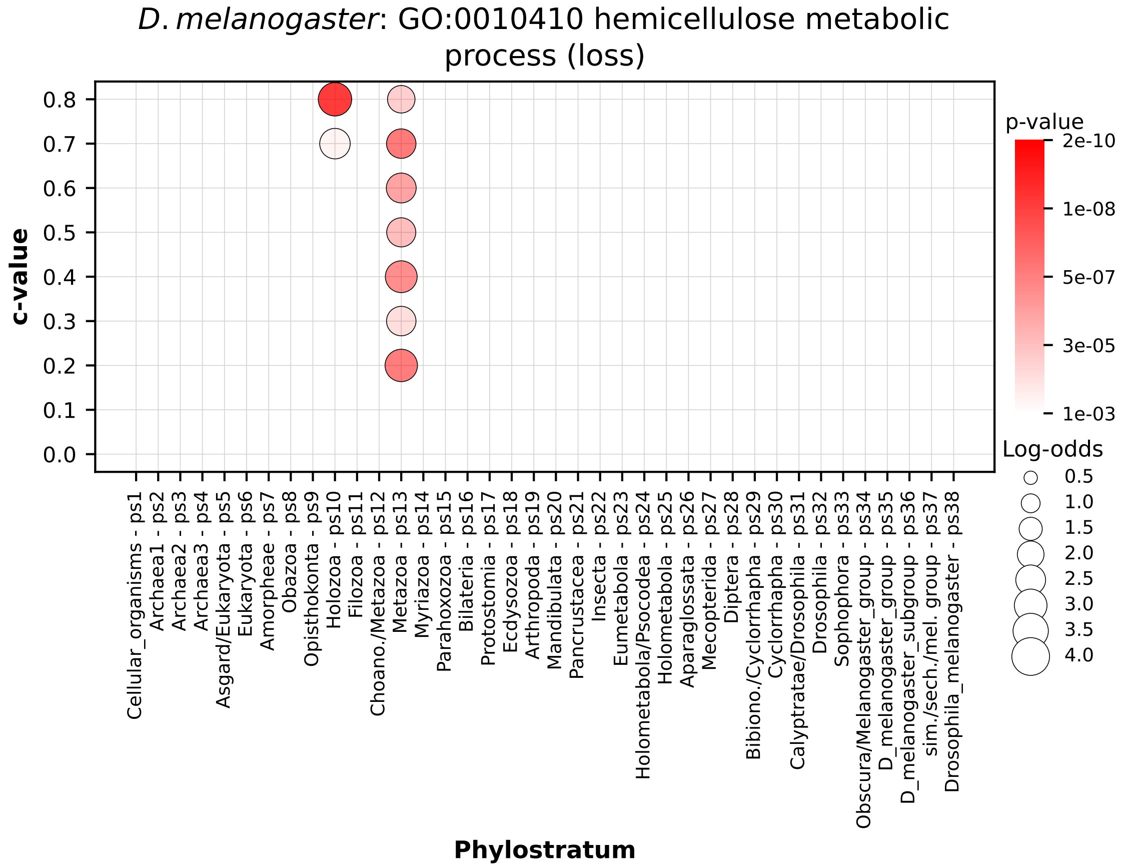
D. melanogaster: GO:0010410 hemicellulose metabolic process (loss)
c-value
0.8
0.7
0.6
0.5
0.4
0.3
0.2
0.1
0.0
Phylostratum
Cellular_organisms - ps1
Archaea1 - ps2
Archaea2 - ps3
Archaea3 - ps4
Asgard/Eukaryota - ps5
Eukaryota - ps6
Amorpheae - ps7
Obazoa - ps8
Opisthokonta - ps9
Holozoa - ps10
Filozoa - ps11
Choano./Metazoa - ps12
Metazoa - ps13
Myriazoa - ps14
Parahoxozoa - ps15
Bilateria - ps16
Protostomia - ps17
Ecdysozoa - ps18
Arthropoda - ps19
Mandibulata - ps20
Pancrustacea - ps21
Insecta - ps22
Eumetabola - ps23
Holometabola/Psocodea - ps24
Holometabola - ps25
Aparaglossata - ps26
Mecopterida - ps27
Diptera - ps28
Bibiono./Cyclorrhapha - ps29
Cyclorrhapha - ps30
Calyptratae/Drosophila - ps31
Drosophila - ps32
Sophophora - ps33
Obscura/Melanogaster_group - ps34
D_melanogaster_group - ps35
D_melanogaster_subgroup - ps36
sim./sech./mel. group - ps37
Drosophila_melanogaster - ps38
p-value
2e-10
1e-08
5e-07
3e-05
1e-03
Log-odds
0.5
1.0
1.5
2.0
2.5
3.0
3.5
4.0

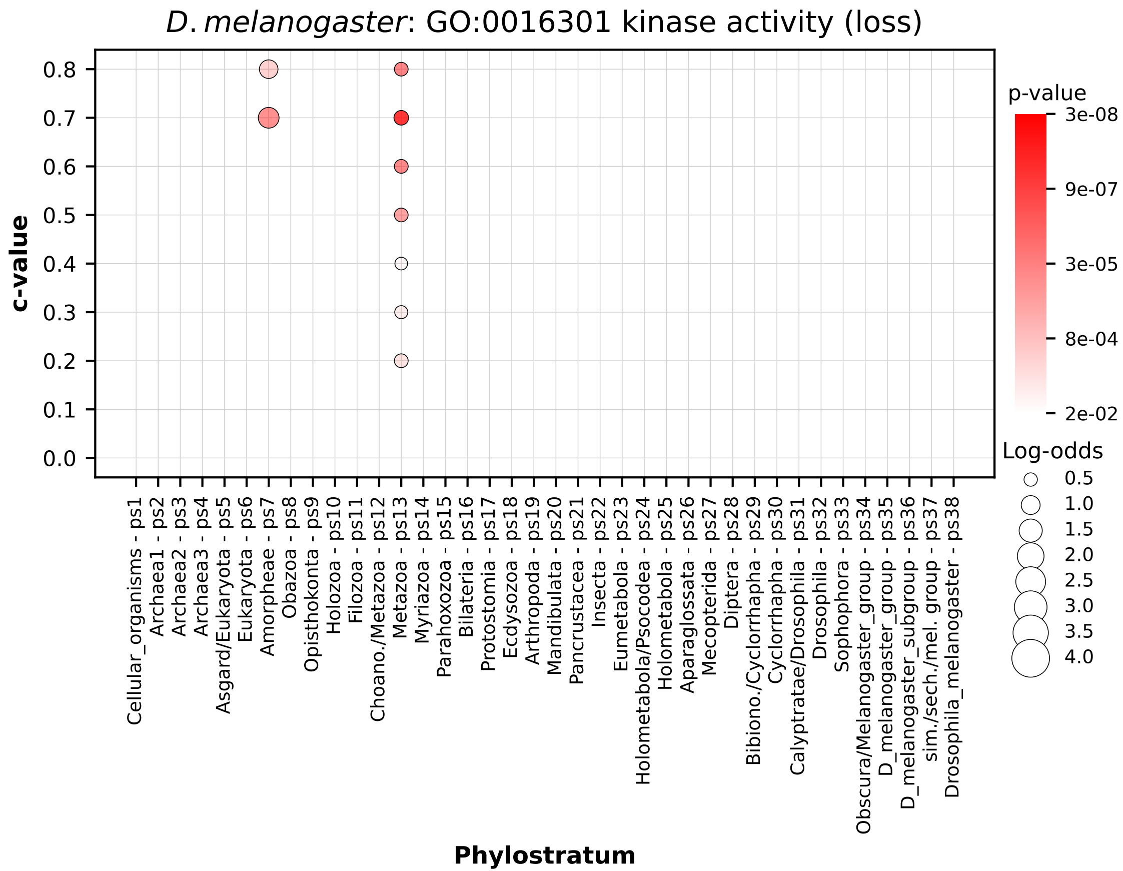
D. melanogaster: GO:0016301 kinase activity (loss)
c-value
0.8
0.7
0.6
0.5
0.4
0.3
0.2
0.1
0.0
p-value
3e-08
9e-07
3e-05
8e-04
2e-02
Log-odds
0.5
1.0
1.5
2.0
2.5
3.0
3.5
4.0
Cellular_organisms - ps1
Archaea1 - ps2
Archaea2 - ps3
Archaea3 - ps4
Asgard/Eukaryota - ps5
Eukaryota - ps6
Amorpheae - ps7
Obazoa - ps8
Opisthokonta - ps9
Holozoa - ps10
Filozoa - ps11
Choano./Metazoa - ps12
Metazoa - ps13
Myriazoa - ps14
Parahoxozoa - ps15
Bilateria - ps16
Protostomia - ps17
Ecdysozoa - ps18
Arthropoda - ps19
Mandibulata - ps20
Pancrustacea - ps21
Insecta - ps22
Eumetabola - ps23
Holometabola/Psocodea - ps24
Holometabola - ps25
Aparaglossata - ps26
Mecopterida - ps27
Diptera - ps28
Bibiono./Cyclorrhapha - ps29
Cyclorrhapha - ps30
Calyptratae/Drosophila - ps31
Drosophila - ps32
Sophophora - ps33
Obscura/Melanogaster_group - ps34
D_melanogaster_group - ps35
D_melanogaster_subgroup - ps36
sim./sech./mel. group - ps37
Drosophila_melanogaster - ps38
Phylostratum

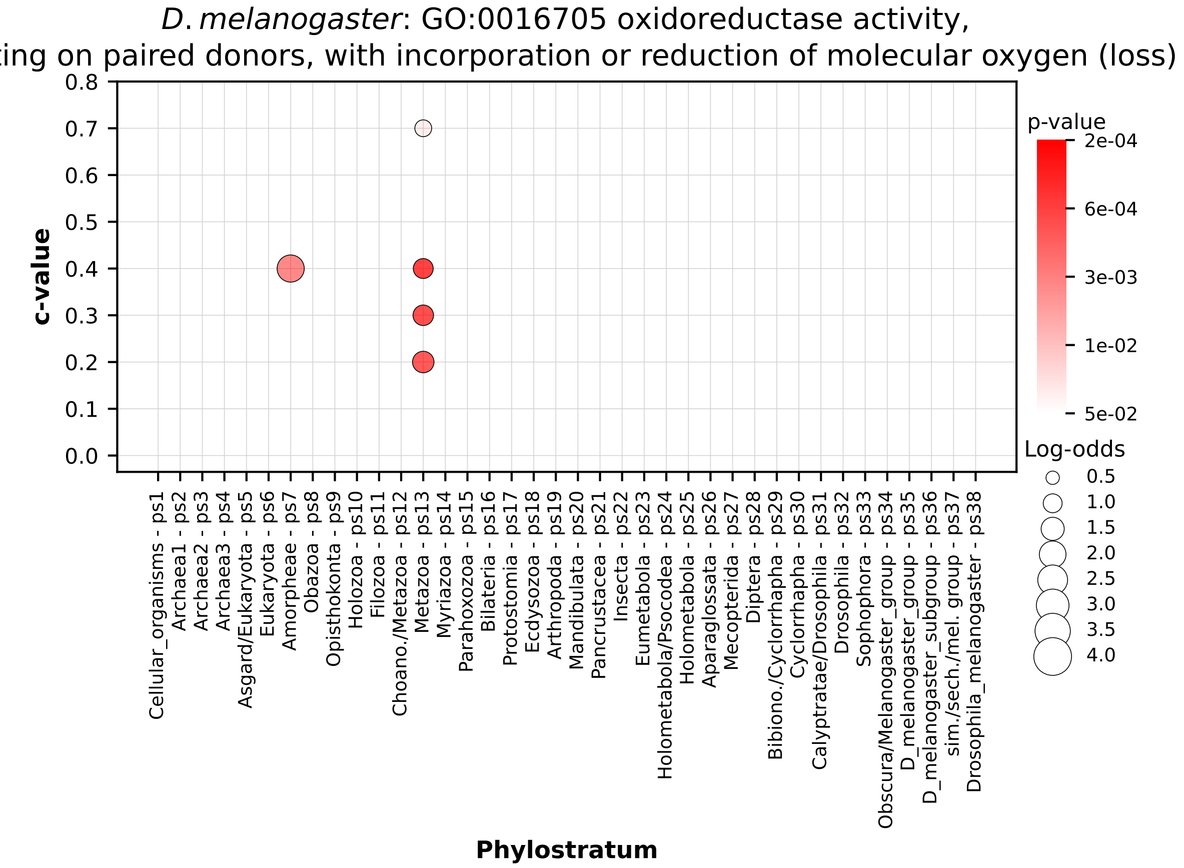

D. melanogaster: GO:0016705 oxidoreductase activity,
ting on paired donors, with incorporation or reduction of molecular oxygen (loss)
c-value
0.8
0.7
0.6
0.5
0.4
0.3
0.2
0.1
0.0
p-value
2e-04
6e-04
3e-03
1e-02
5e-02
Log-odds
0.5
1.0
1.5
2.0
2.5
3.0
3.5
4.0
Cellular_organisms - ps1
Archaea1 - ps2
Archaea2 - ps3
Archaea3 - ps4
Asgard/Eukaryota - ps5
Eukaryota - ps6
Amorpheae - ps7
Obazoa - ps8
Opisthokonta - ps9
Holozoa - ps10
Filozoa - ps11
Choano./Metazoa - ps12
Metazoa - ps13
Myriazoa - ps14
Parahoxozoa - ps15
Bilateria - ps16
Protostomia - ps17
Ecdysozoa - ps18
Arthropoda - ps19
Mandibulata - ps20
Pancrustacea - ps21
Insecta - ps22
Eumetabola - ps23
Holometabola/Psocodea - ps24
Holometabola - ps25
Aparaglossata - ps26
Mecopterida - ps27
Diptera - ps28
Bibiono./Cyclorrhapha - ps29
Cyclorrhapha - ps30
Calyptratae/Drosophila - ps31
Drosophila - ps32
Sophophora - ps33
Obscura/Melanogaster_group - ps34
D_melanogaster_group - ps35
D_melanogaster_subgroup - ps36
sim./sech./mel. group - ps37
Drosophila_melanogaster - ps38
Phylostratum

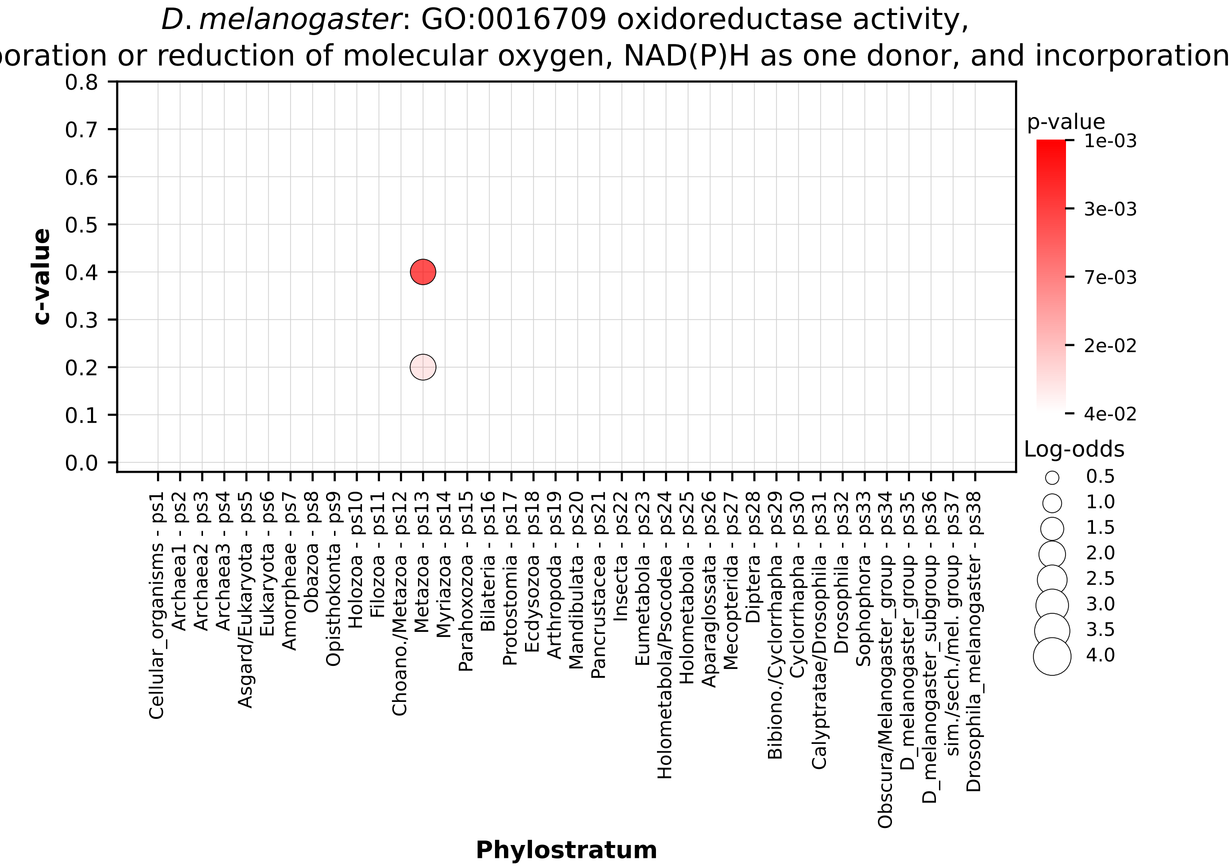
D. melanogaster: GO:0016709 oxidoreductase activity,
poration or reduction of molecular oxygen, NAD(P)H as one donor, and incorporation
c-value
0.8
0.7
0.6
0.5
0.4
0.3
0.2
0.1
0.0
p-value
1e-03
3e-03
7e-03
2e-02
4e-02
Log-odds
0.5
1.0
1.5
2.0
2.5
3.0
3.5
4.0
Cellular_organisms - ps1
Archaea1 - ps2
Archaea2 - ps3
Archaea3 - ps4
Asgard/Eukaryota - ps5
Eukaryota - ps6
Amorpheae - ps7
Obazoa - ps8
Opisthokonta - ps9
Holozoa - ps10
Filozoa - ps11
Choano./Metazoa - ps12
Metazoa - ps13
Myriazoa - ps14
Parahoxozoa - ps15
Bilateria - ps16
Protostomia - ps17
Ecdysozoa - ps18
Arthropoda - ps19
Mandibulata - ps20
Pancrustacea - ps21
Insecta - ps22
Eumetabola - ps23
Holometabola/Psocodea - ps24
Holometabola - ps25
Aparaglossata - ps26
Mecopterida - ps27
Diptera - ps28
Bibiono./Cyclorrhapha - ps29
Cyclorrhapha - ps30
Calyptratae/Drosophila - ps31
Drosophila - ps32
Sophophora - ps33
Obscura/Melanogaster_group - ps34
D_melanogaster_group - ps35
D_melanogaster_subgroup - ps36
sim./sech./mel. group - ps37
Drosophila_melanogaster - ps38
Phylostratum

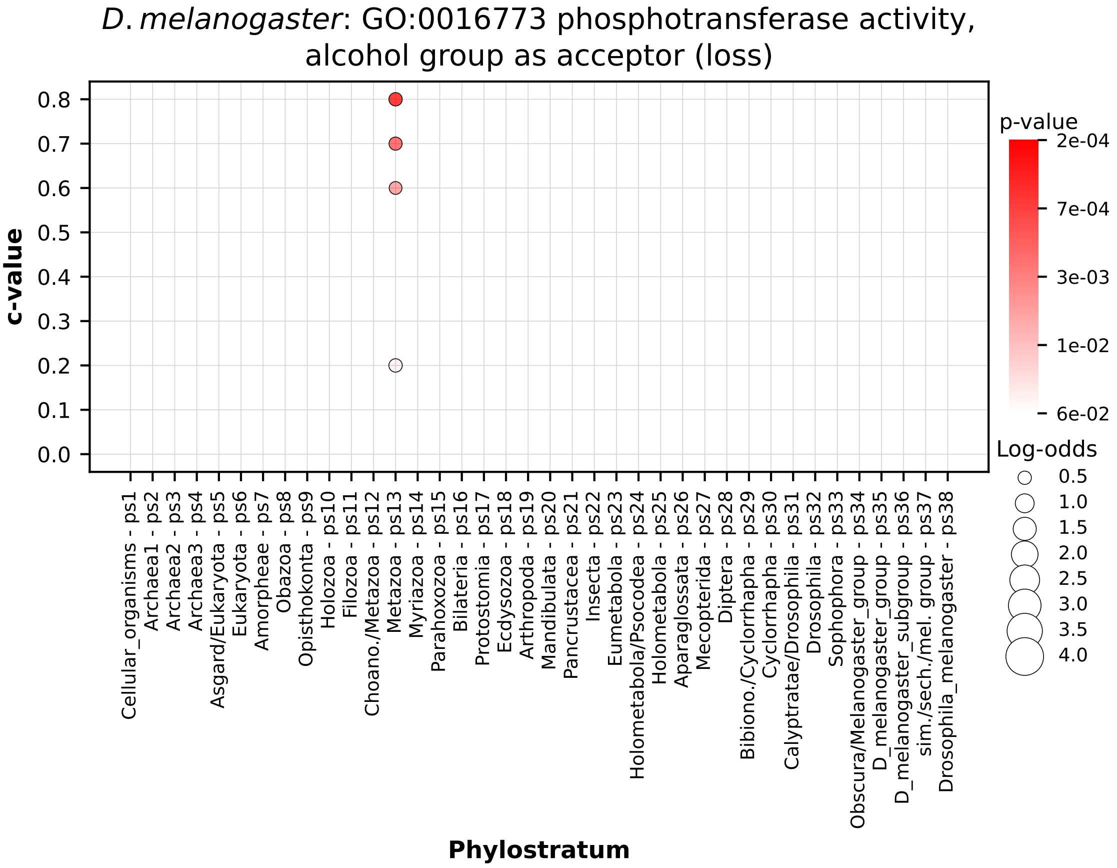

D. melanogaster: GO:0016773 phosphotransferase activity, alcohol group as acceptor (loss)
c-value
0.8
0.7
0.6
0.5
0.4
0.3
0.2
0.1
0.0
p-value
2e-04
7e-04
3e-03
1e-02
6e-02
Log-odds
0.5
1.0
1.5
2.0
2.5
3.0
3.5
4.0
Cellular_organisms - ps1
Archaea1 - ps2
Archaea2 - ps3
Archaea3 - ps4
Asgard/Eukaryota - ps5
Eukaryota - ps6
Amorpheae - ps7
Obazoa - ps8
Opisthokonta - ps9
Holozoa - ps10
Filozoa - ps11
Choano./Metazoa - ps12
Metazoa - ps13
Myriazoa - ps14
Parahoxozoa - ps15
Bilateria - ps16
Protostomia - ps17
Ecdysozoa - ps18
Arthropoda - ps19
Mandibulata - ps20
Pancrustacea - ps21
Insecta - ps22
Eumetabola - ps23
Holometabola/Psocodea - ps24
Holometabola - ps25
Aparaglossata - ps26
Mecopterida - ps27
Diptera - ps28
Bibiono./Cyclorrhapha - ps29
Cyclorrhapha - ps30
Calyptratae/Drosophila - ps31
Drosophila - ps32
Sophophora - ps33
Obscura/Melanogaster_group - ps34
D_melanogaster_group - ps35
D_melanogaster_subgroup - ps36
sim./sech./mel. group - ps37
Drosophila_melanogaster - ps38
Phylostratum

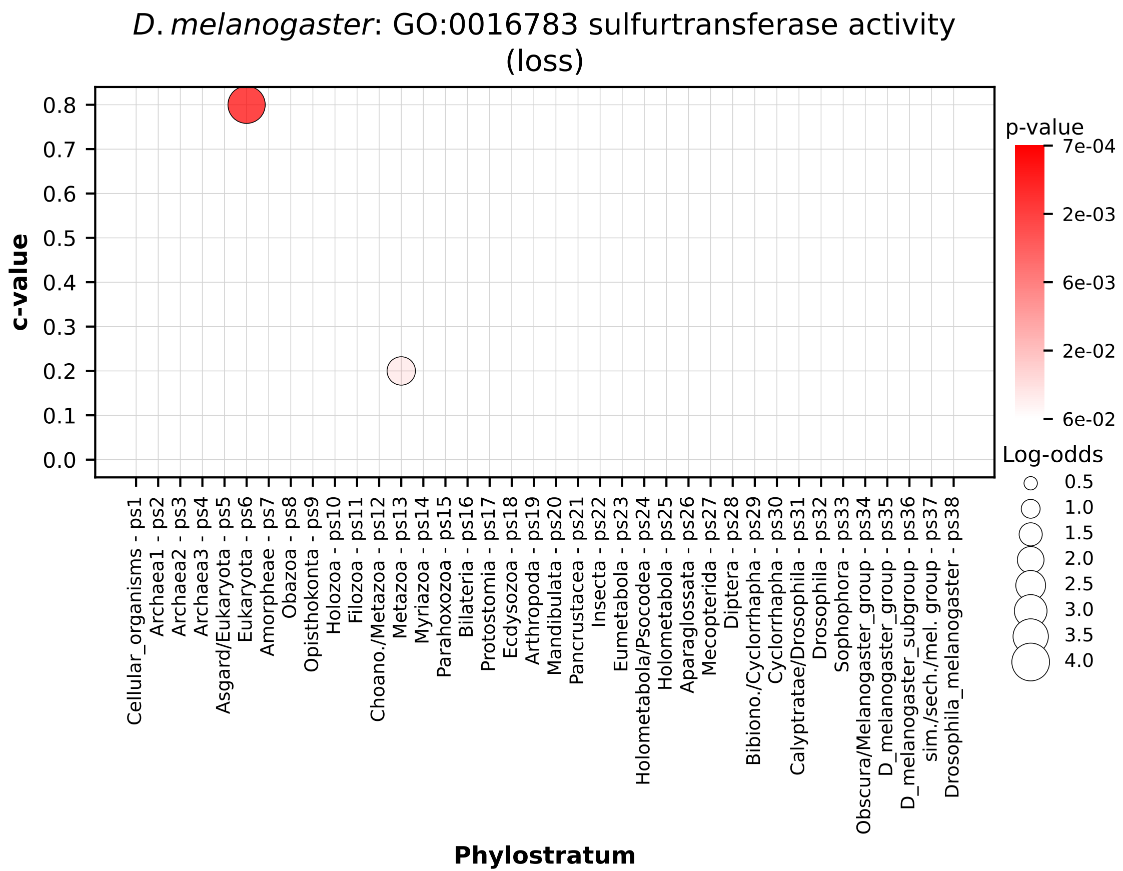

D. melanogaster: GO:0016783 sulfurtransferase activity (loss)
c-value
0.8
0.7
0.6
0.5
0.4
0.3
0.2
0.1
0.0
Cellular_organisms - ps1
Archaea1 - ps2
Archaea2 - ps3
Archaea3 - ps4
Asgard/Eukaryota - ps5
Eukaryota - ps6
Amorpheae - ps7
Obazoa - ps8
Opisthokonta - ps9
Holozoa - ps10
Filozoa - ps11
Choano./Metazoa - ps12
Metazoa - ps13
Myriazoa - ps14
Parahoxozoa - ps15
Bilateria - ps16
Protostomia - ps17
Ecdysozoa - ps18
Arthropoda - ps19
Mandibulata - ps20
Pancrustacea - ps21
Insecta - ps22
Eumetabola - ps23
Holometabola/Psocodea - ps24
Holometabola - ps25
Aparaglossata - ps26
Mecopterida - ps27
Diptera - ps28
Bibiono./Cyclorrhapha - ps29
Cyclorrhapha - ps30
Calyptratae/Drosophila - ps31
Drosophila - ps32
Sophophora - ps33
Obscura/Melanogaster_group - ps34
D_melanogaster_group - ps35
D_melanogaster_subgroup - ps36
sim./sech./mel. group - ps37
Drosophila_melanogaster - ps38
Phylostratum
p-value
7e-04
2e-03
6e-03
2e-02
6e-02
Log-odds
0.5
1.0
1.5
2.0
2.5
3.0
3.5
4.0

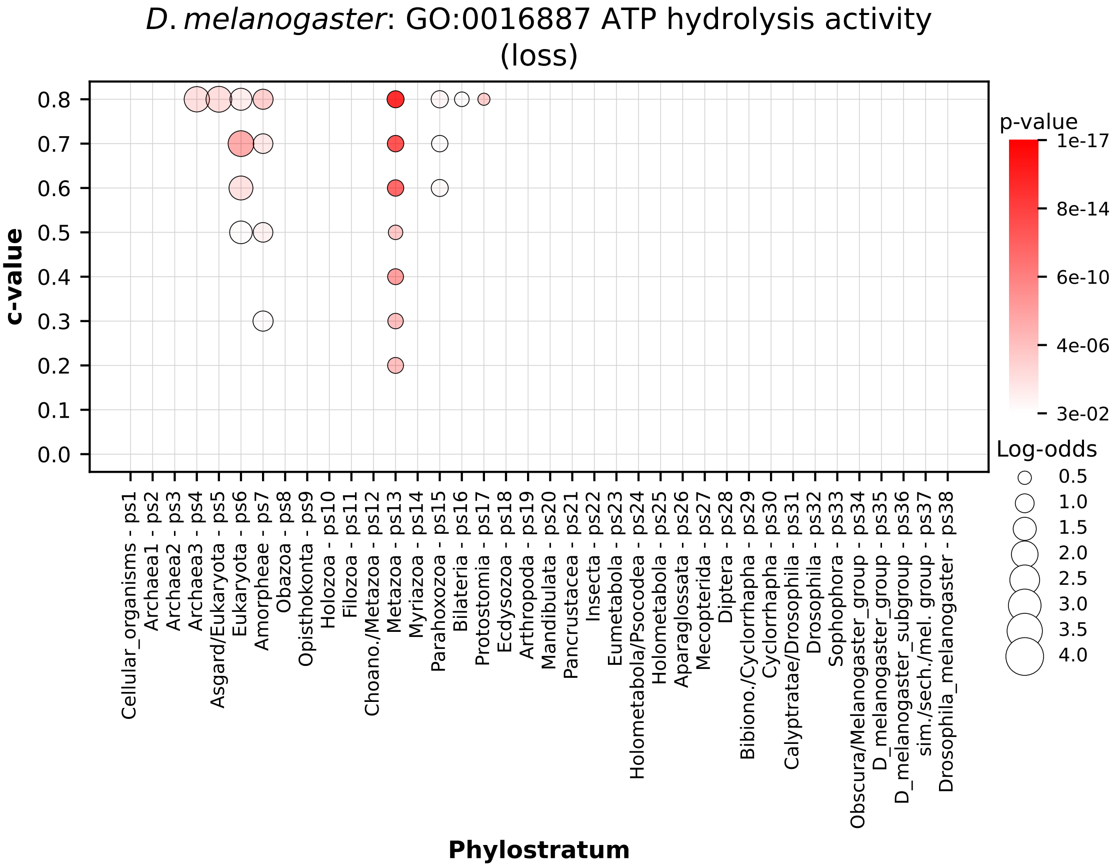

D. melanogaster: GO:0016887 ATP hydrolysis activity (loss)
c-value
0.8
0.7
0.6
0.5
0.4
0.3
0.2
0.1
0.0
Phylostratum
Cellular_organisms - ps1
Archaea1 - ps2
Archaea2 - ps3
Archaea3 - ps4
Asgard/Eukaryota - ps5
Eukaryota - ps6
Amorpheae - ps7
Obazoa - ps8
Opisthokonta - ps9
Holozoa - ps10
Filozoa - ps11
Choano./Metazoa - ps12
Metazoa - ps13
Myriazoa - ps14
Parahoxozoa - ps15
Bilateria - ps16
Protostomia - ps17
Ecdysozoa - ps18
Arthropoda - ps19
Mandibulata - ps20
Pancrustacea - ps21
Insecta - ps22
Eumetabola - ps23
Holometabola/Psocodea - ps24
Holometabola - ps25
Aparaglossata - ps26
Mecopterida - ps27
Diptera - ps28
Bibiono./Cyclorrhapha - ps29
Cyclorrhapha - ps30
Calyptratae/Drosophila - ps31
Drosophila - ps32
Sophophora - ps33
Obscura/Melanogaster_group - ps34
D_melanogaster_group - ps35
D_melanogaster_subgroup - ps36
sim./sech./mel. group - ps37
Drosophila_melanogaster - ps38
p-value
1e-17
8e-14
6e-10
4e-06
3e-02
Log-odds
0.5
1.0
1.5
2.0
2.5
3.0
3.5
4.0

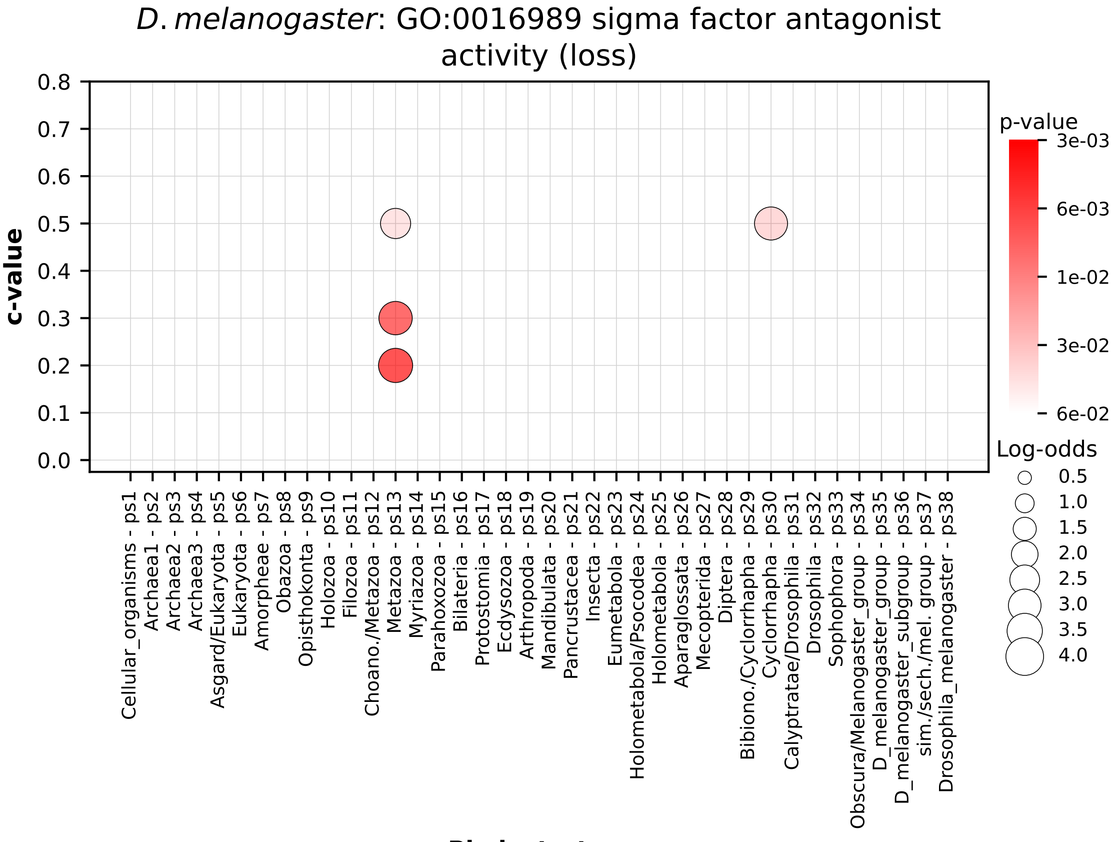

D. melanogaster: GO:0016989 sigma factor antagonist activity (loss)
c-value
0.0
0.1
0.2
0.3
0.4
0.5
0.6
0.7
0.8
Phylostratum
Cellular_organisms - ps1
Archaea1 - ps2
Archaea2 - ps3
Archaea3 - ps4
Asgard/Eukaryota - ps5
Eukaryota - ps6
Amorpheae - ps7
Obazoa - ps8
Opisthokonta - ps9
Holozoa - ps10
Filozoa - ps11
Choano./Metazoa - ps12
Metazoa - ps13
Myriazoa - ps14
Parahoxozoa - ps15
Bilateria - ps16
Protostomia - ps17
Ecdysozoa - ps18
Arthropoda - ps19
Mandibulata - ps20
Pancrustacea - ps21
Insecta - ps22
Eumetabola - ps23
Holometabola/Psocodea - ps24
Holometabola - ps25
Aparaglossata - ps26
Mecopterida - ps27
Diptera - ps28
Bibiono./Cyclorrhapha - ps29
Cyclorrhapha - ps30
Calyptratae/Drosophila - ps31
Drosophila - ps32
Sophophora - ps33
Obscura/Melanogaster_group - ps34
D_melanogaster_group - ps35
D_melanogaster_subgroup - ps36
sim./sech./mel. group - ps37
Drosophila_melanogaster - ps38
p-value
3e-03
6e-03
1e-02
3e-02
6e-02
Log-odds
0.5
1.0
1.5
2.0
2.5
3.0
3.5
4.0

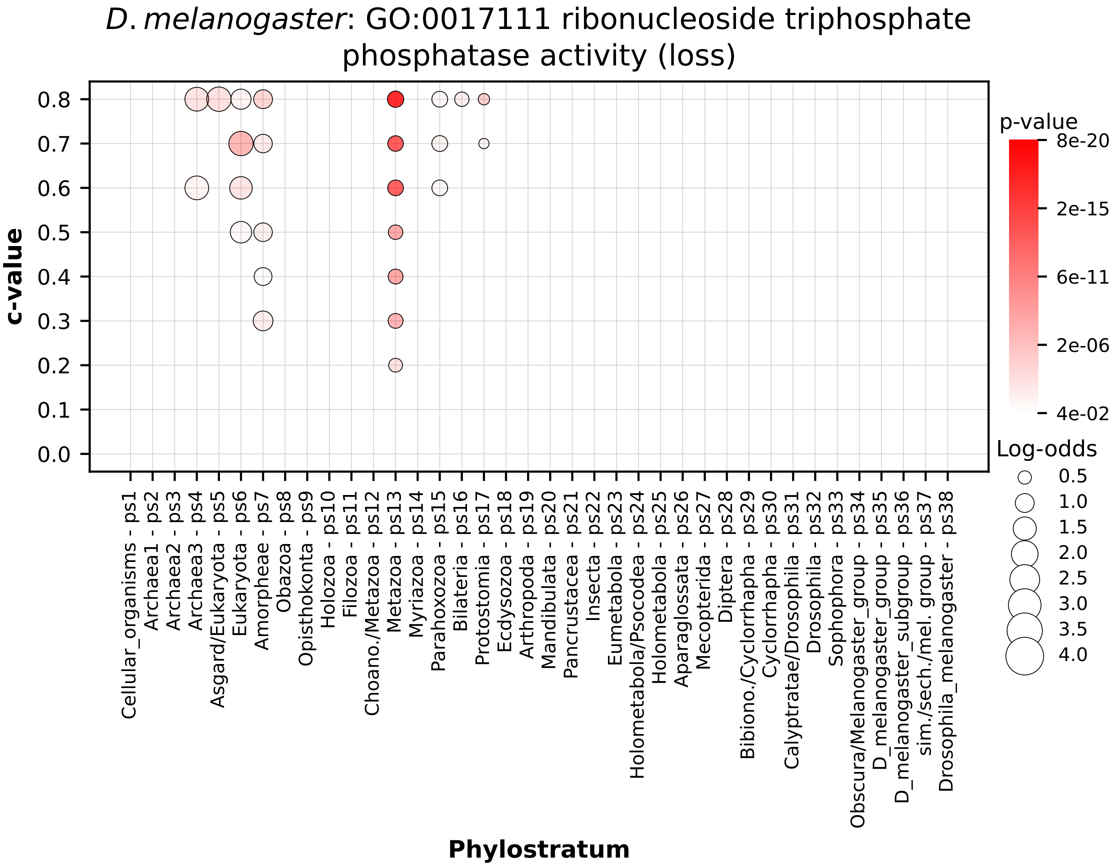

D. melanogaster: GO:0017111 ribonucleoside triphosphate phosphatase activity (loss)
c-value
0.8
0.7
0.6
0.5
0.4
0.3
0.2
0.1
0.0
p-value
8e-20
2e-15
6e-11
2e-06
4e-02
Log-odds
0.5
1.0
1.5
2.0
2.5
3.0
3.5
4.0
Cellular_organisms - ps1
Archaea1 - ps2
Archaea2 - ps3
Archaea3 - ps4
Asgard/Eukaryota - ps5
Eukaryota - ps6
Amorpheae - ps7
Obazoa - ps8
Opisthokonta - ps9
Holozoa - ps10
Filozoa - ps11
Choano./Metazoa - ps12
Metazoa - ps13
Myriazoa - ps14
Parahoxozoa - ps15
Bilateria - ps16
Protostomia - ps17
Ecdysozoa - ps18
Arthropoda - ps19
Mandibulata - ps20
Pancrustacea - ps21
Insecta - ps22
Eumetabola - ps23
Holometabola/Psocodea - ps24
Holometabola - ps25
Aparaglossata - ps26
Mecopterida - ps27
Diptera - ps28
Bibiono./Cyclorrhapha - ps29
Cyclorrhapha - ps30
Calyptratae/Drosophila - ps31
Drosophila - ps32
Sophophora - ps33
Obscura/Melanogaster_group - ps34
D_melanogaster_group - ps35
D_melanogaster_subgroup - ps36
sim./sech./mel. group - ps37
Drosophila_melanogaster - ps38
Phylostratum

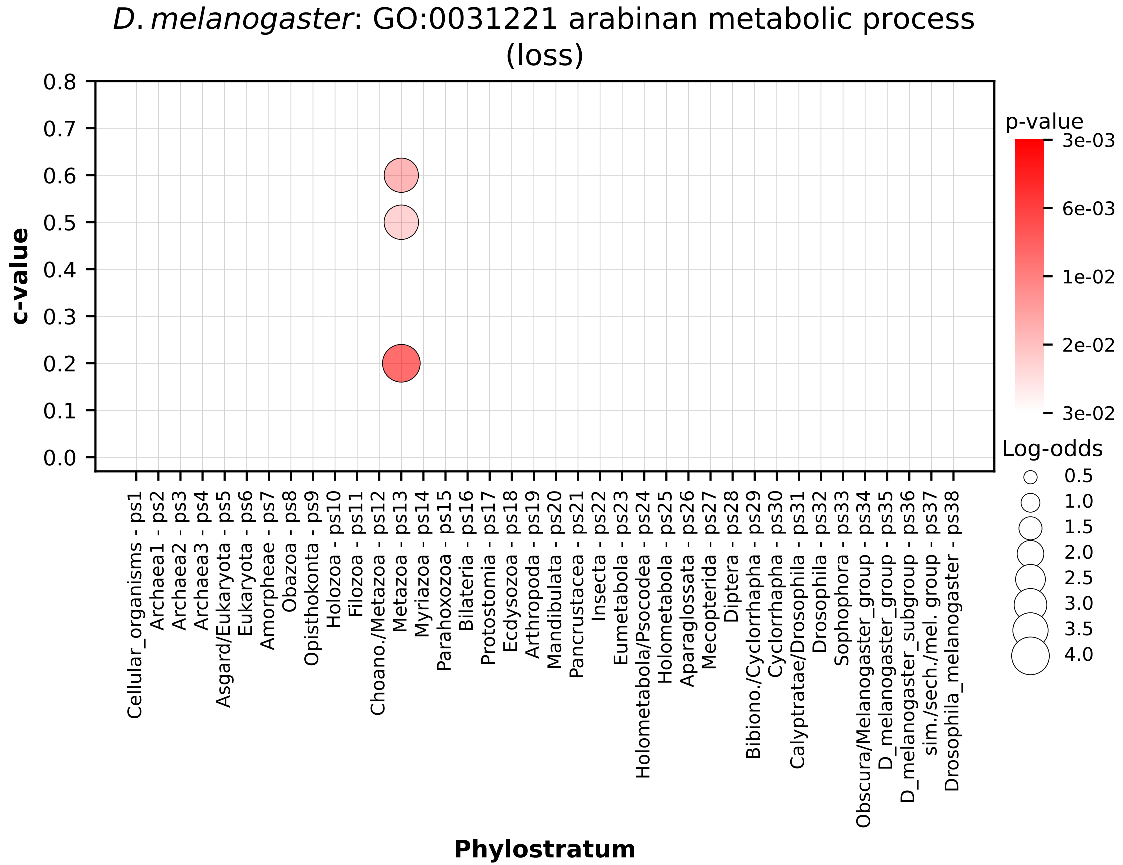

D. melanogaster: GO:0031221 arabinan metabolic process (loss)
c-value
0.8
0.7
0.6
0.5
0.4
0.3
0.2
0.1
0.0
Phylostratum
Cellular_organisms - ps1
Archaea1 - ps2
Archaea2 - ps3
Archaea3 - ps4
Asgard/Eukaryota - ps5
Eukaryota - ps6
Amorpheae - ps7
Obazoa - ps8
Opisthokonta - ps9
Holozoa - ps10
Filozoa - ps11
Choano./Metazoa - ps12
Metazoa - ps13
Myriazoa - ps14
Parahoxozoa - ps15
Bilateria - ps16
Protostomia - ps17
Ecdysozoa - ps18
Arthropoda - ps19
Mandibulata - ps20
Pancrustacea - ps21
Insecta - ps22
Eumetabola - ps23
Holometabola/Psocodea - ps24
Holometabola - ps25
Aparaglossata - ps26
Mecopterida - ps27
Diptera - ps28
Bibiono./Cyclorrhapha - ps29
Cyclorrhapha - ps30
Calyptratae/Drosophila - ps31
Drosophila - ps32
Sophophora - ps33
Obscura/Melanogaster_group - ps34
D_melanogaster_group - ps35
D_melanogaster_subgroup - ps36
sim./sech./mel. group - ps37
Drosophila_melanogaster - ps38
p-value
3e-03
6e-03
1e-02
2e-02
3e-02
Log-odds
0.5
1.0
1.5
2.0
2.5
3.0
3.5
4.0

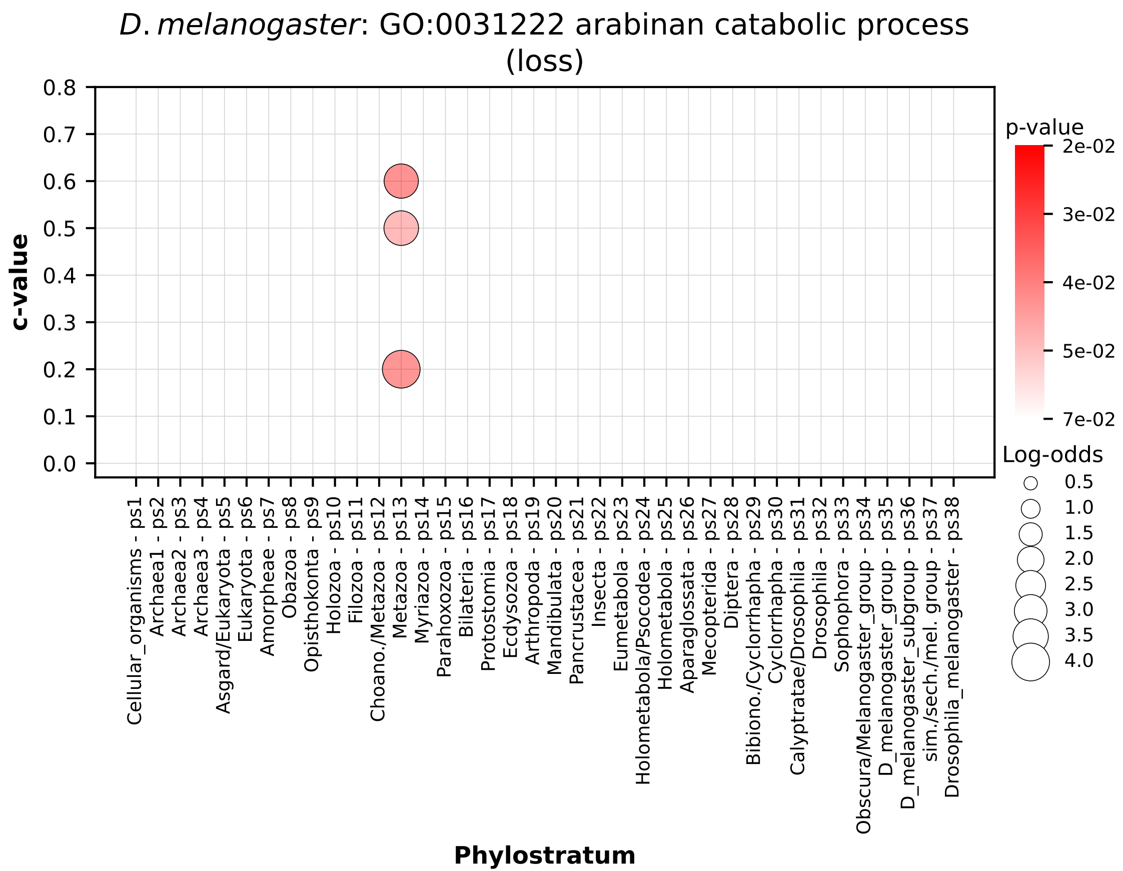

D. melanogaster: GO:0031222 arabinan catabolic process
(loss)
c-value
0.8
0.7
0.6
0.5
0.4
0.3
0.2
0.1
0.0
Phylostratum
Cellular_organisms - ps1
Archaea1 - ps2
Archaea2 - ps3
Archaea3 - ps4
Asgard/Eukaryota - ps5
Eukaryota - ps6
Amorpheae - ps7
Obazoa - ps8
Opisthokonta - ps9
Holozoa - ps10
Filozoa - ps11
Choano./Metazoa - ps12
Metazoa - ps13
Myriazoa - ps14
Parahoxozoa - ps15
Bilateria - ps16
Protostomia - ps17
Ecdysozoa - ps18
Arthropoda - ps19
Mandibulata - ps20
Pancrustacea - ps21
Insecta - ps22
Eumetabola - ps23
Holometabola/Psocodea - ps24
Holometabola - ps25
Aparaglossata - ps26
Mecopterida - ps27
Diptera - ps28
Bibiono./Cyclorrhapha - ps29
Cyclorrhapha - ps30
Calyptratae/Drosophila - ps31
Drosophila - ps32
Sophophora - ps33
Obscura/Melanogaster_group - ps34
D_melanogaster_group - ps35
D_melanogaster_subgroup - ps36
sim./sech./mel. group - ps37
Drosophila_melanogaster - ps38
p-value
2e-02
3e-02
4e-02
5e-02
7e-02
Log-odds
0.5
1.0
1.5
2.0
2.5
3.0
3.5
4.0

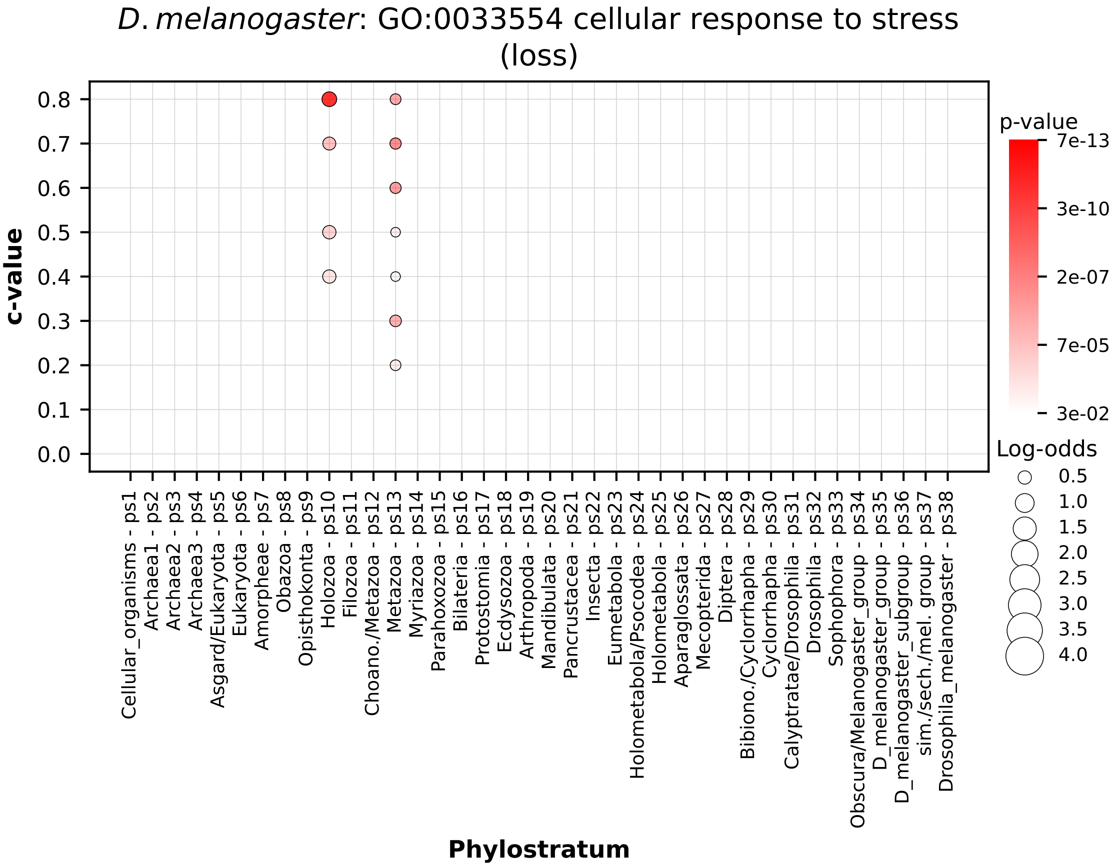
D. melanogaster: GO:0033554 cellular response to stress (loss)
c-value
0.8
0.7
0.6
0.5
0.4
0.3
0.2
0.1
0.0
p-value
7e-13
3e-10
2e-07
7e-05
3e-02
Log-odds
0.5
1.0
1.5
2.0
2.5
3.0
3.5
4.0
Cellular_organisms - ps1
Archaea1 - ps2
Archaea2 - ps3
Archaea3 - ps4
Asgard/Eukaryota - ps5
Eukaryota - ps6
Amorpheae - ps7
Obazoa - ps8
Opisthokonta - ps9
Holozoa - ps10
Filozoa - ps11
Choano./Metazoa - ps12
Metazoa - ps13
Myriazoa - ps14
Parahoxozoa - ps15
Bilateria - ps16
Protostomia - ps17
Ecdysozoa - ps18
Arthropoda - ps19
Mandibulata - ps20
Pancrustacea - ps21
Insecta - ps22
Eumetabola - ps23
Holometabola/Psocodea - ps24
Holometabola - ps25
Aparaglossata - ps26
Mecopterida - ps27
Diptera - ps28
Bibiono./Cyclorrhapha - ps29
Cyclorrhapha - ps30
Calyptratae/Drosophila - ps31
Drosophila - ps32
Sophophora - ps33
Obscura/Melanogaster_group - ps34
D_melanogaster_group - ps35
D_melanogaster_subgroup - ps36
sim./sech./mel. group - ps37
Drosophila_melanogaster - ps38
Phylostratum

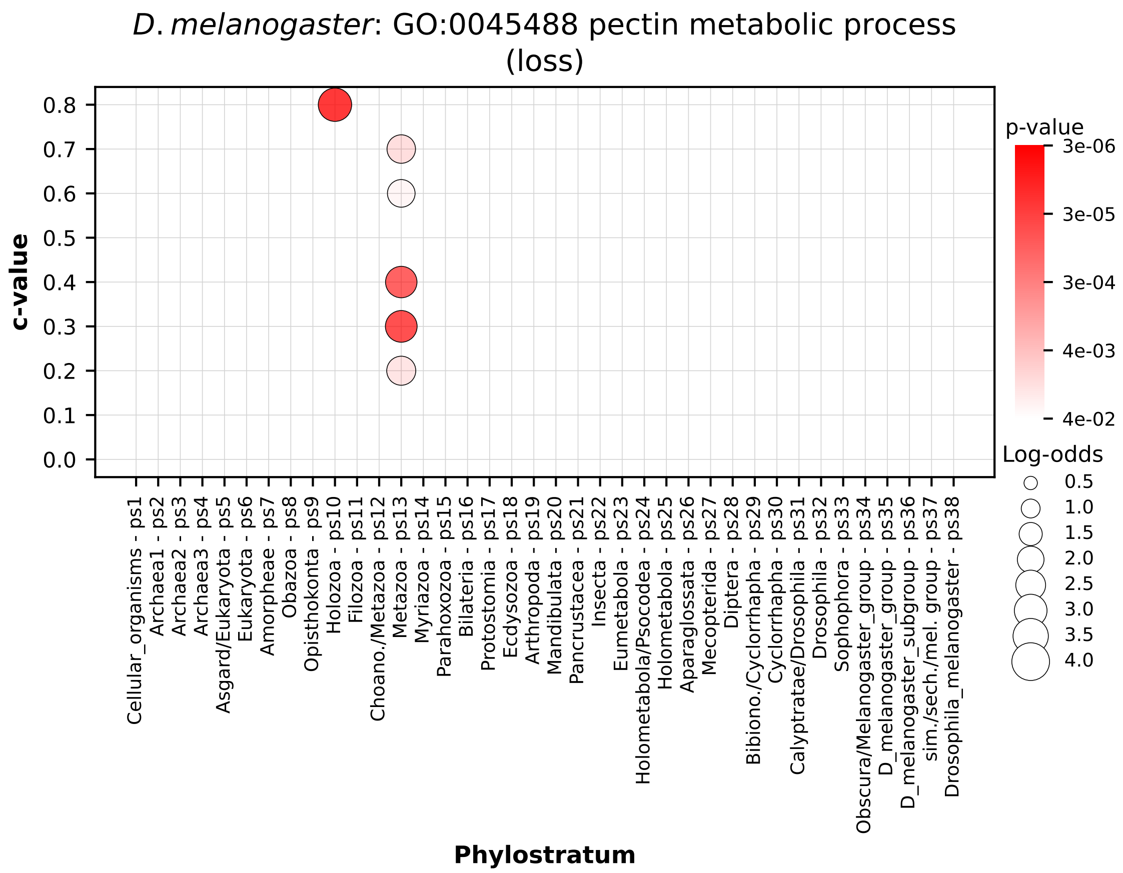
D. melanogaster: GO:0045488 pectin metabolic process
(loss)
c-value
0.8
0.7
0.6
0.5
0.4
0.3
0.2
0.1
0.0
p-value
3e-06
3e-05
3e-04
4e-03
4e-02
Log-odds
0.5
1.0
1.5
2.0
2.5
3.0
3.5
4.0
Cellular_organisms - ps1
Archaea1 - ps2
Archaea2 - ps3
Archaea3 - ps4
Asgard/Eukaryota - ps5
Eukaryota - ps6
Amorpheae - ps7
Obazoa - ps8
Opisthokonta - ps9
Holozoa - ps10
Filozoa - ps11
Choano./Metazoa - ps12
Metazoa - ps13
Myriazoa - ps14
Parahoxozoa - ps15
Bilateria - ps16
Protostomia - ps17
Ecdysozoa - ps18
Arthropoda - ps19
Mandibulata - ps20
Pancrustacea - ps21
Insecta - ps22
Eumetabola - ps23
Holometabola/Psocodea - ps24
Holometabola - ps25
Aparaglossata - ps26
Mecopterida - ps27
Diptera - ps28
Bibiono./Cyclorrhapha - ps29
Cyclorrhapha - ps30
Calyptratae/Drosophila - ps31
Drosophila - ps32
Sophophora - ps33
Obscura/Melanogaster_group - ps34
D_melanogaster_group - ps35
D_melanogaster_subgroup - ps36
sim./sech./mel. group - ps37
Drosophila_melanogaster - ps38
Phylostratum

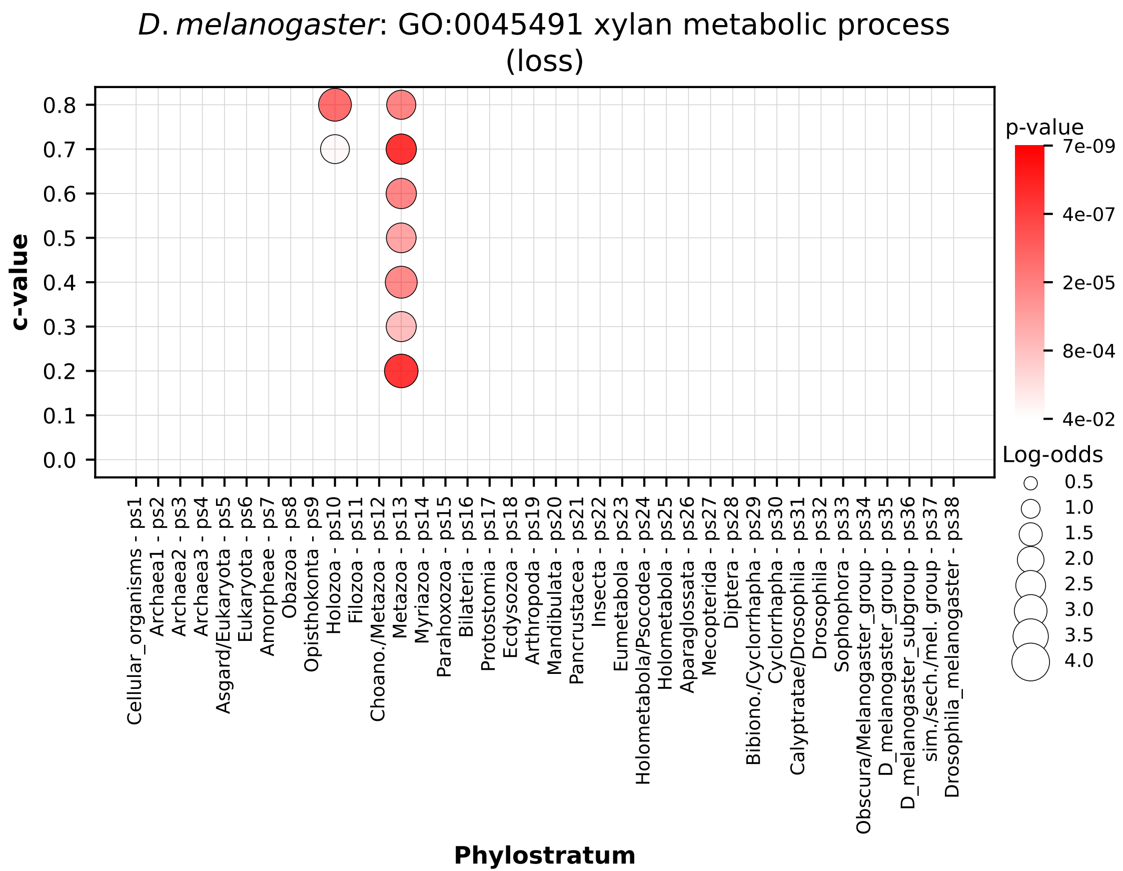

D. melanogaster: GO:0045491 xylan metabolic process (loss)
c-value
0.8
0.7
0.6
0.5
0.4
0.3
0.2
0.1
0.0
Phylostratum
Cellular_organisms - ps1
Archaea1 - ps2
Archaea2 - ps3
Archaea3 - ps4
Asgard/Eukaryota - ps5
Eukaryota - ps6
Amorpheae - ps7
Obazoa - ps8
Opisthokonta - ps9
Holozoa - ps10
Filozoa - ps11
Choano./Metazoa - ps12
Metazoa - ps13
Myriazoa - ps14
Parahoxozoa - ps15
Bilateria - ps16
Protostomia - ps17
Ecdysozoa - ps18
Arthropoda - ps19
Mandibulata - ps20
Pancrustacea - ps21
Insecta - ps22
Eumetabola - ps23
Holometabola/Psocodea - ps24
Holometabola - ps25
Aparaglossata - ps26
Mecopterida - ps27
Diptera - ps28
Bibiono./Cyclorrhapha - ps29
Cyclorrhapha - ps30
Calyptratae/Drosophila - ps31
Drosophila - ps32
Sophophora - ps33
Obscura/Melanogaster_group - ps34
D_melanogaster_group - ps35
D_melanogaster_subgroup - ps36
sim./sech./mel. group - ps37
Drosophila_melanogaster - ps38
p-value
7e-09
4e-07
2e-05
8e-04
4e-02
Log-odds
0.5
1.0
1.5
2.0
2.5
3.0
3.5
4.0

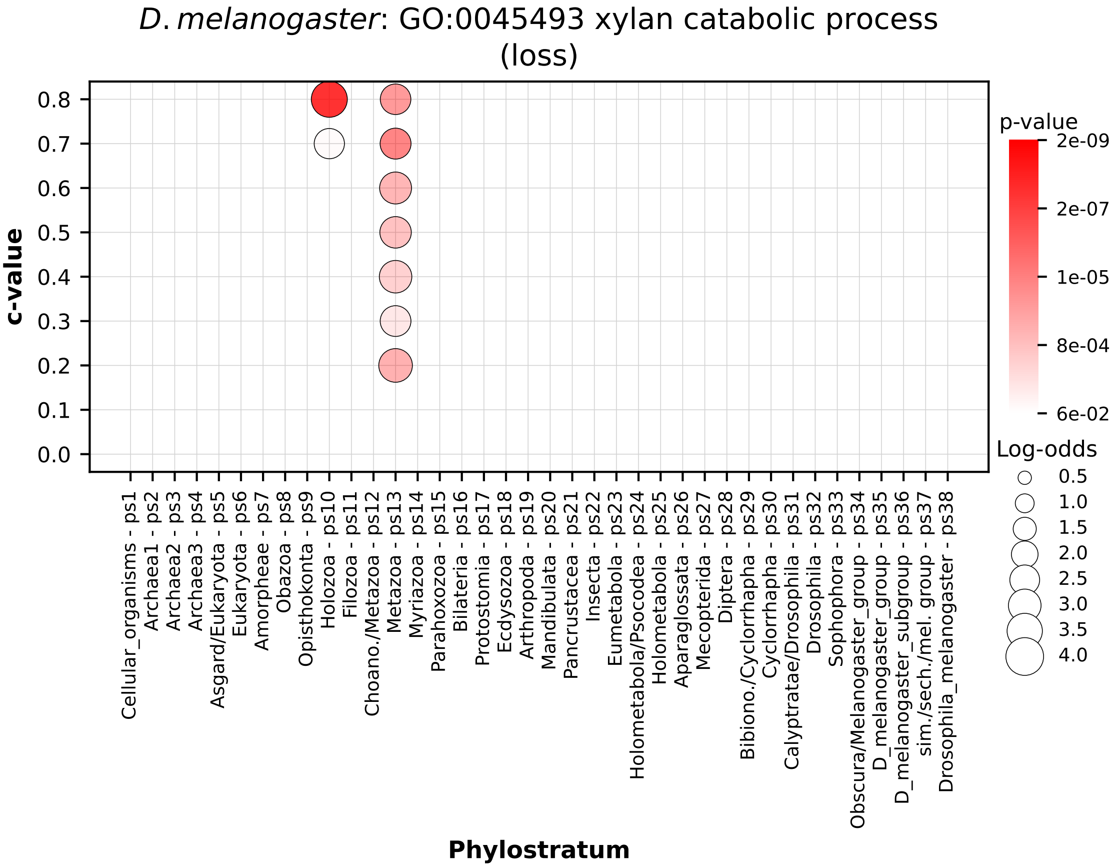

D. melanogaster: GO:0045493 xylan catabolic process (loss)
c-value
0.8
0.7
0.6
0.5
0.4
0.3
0.2
0.1
0.0
Phylostratum
Cellular_organisms - ps1
Archaea1 - ps2
Archaea2 - ps3
Archaea3 - ps4
Asgard/Eukaryota - ps5
Eukaryota - ps6
Amorpheae - ps7
Obazoa - ps8
Opisthokonta - ps9
Holozoa - ps10
Filozoa - ps11
Choano./Metazoa - ps12
Metazoa - ps13
Myriazoa - ps14
Parahoxozoa - ps15
Bilateria - ps16
Protostomia - ps17
Ecdysozoa - ps18
Arthropoda - ps19
Mandibulata - ps20
Pancrustacea - ps21
Insecta - ps22
Eumetabola - ps23
Holometabola/Psocodea - ps24
Holometabola - ps25
Aparaglossata - ps26
Mecopterida - ps27
Diptera - ps28
Bibiono./Cyclorrhapha - ps29
Cyclorrhapha - ps30
Calyptratae/Drosophila - ps31
Drosophila - ps32
Sophophora - ps33
Obscura/Melanogaster_group - ps34
D_melanogaster_group - ps35
D_melanogaster_subgroup - ps36
sim./sech./mel. group - ps37
Drosophila_melanogaster - ps38
p-value
2e-09
2e-07
1e-05
8e-04
6e-02
Log-odds
0.5
1.0
1.5
2.0
2.5
3.0
3.5
4.0

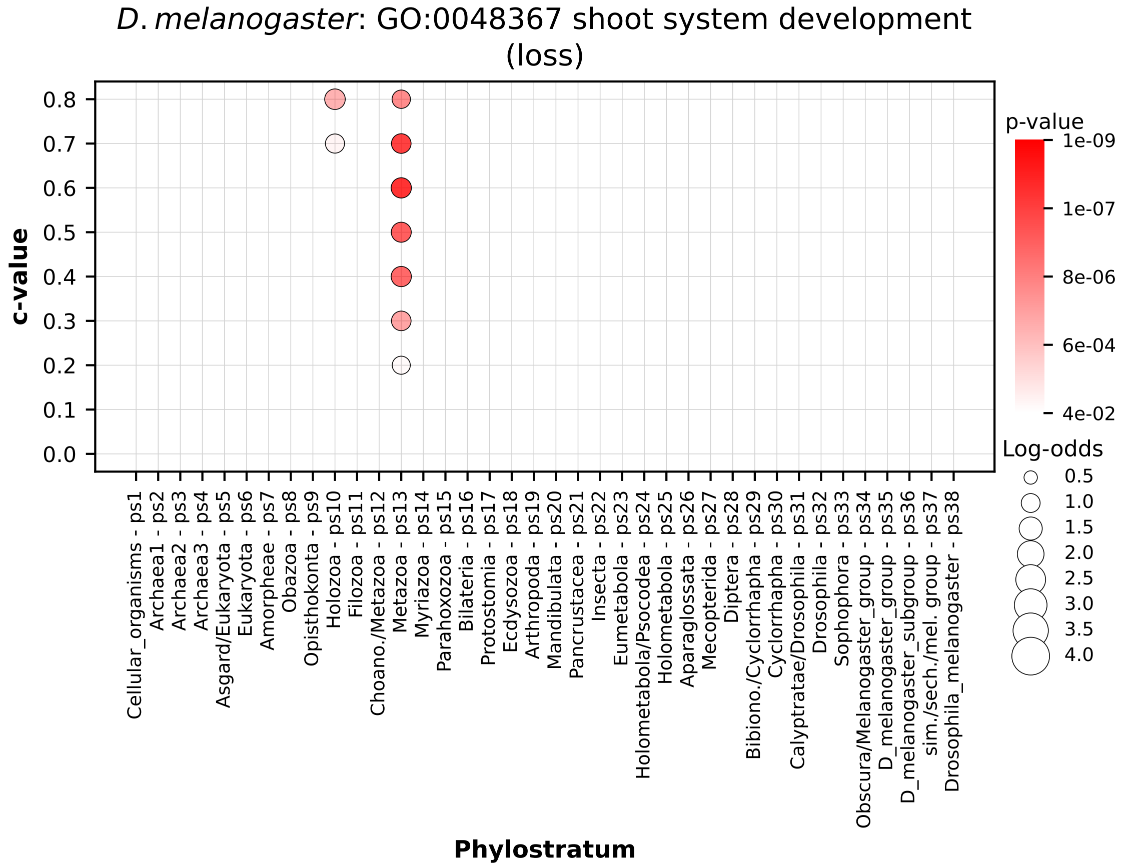

D. melanogaster: GO:0048367 shoot system development
(loss)
c-value
0.8
0.7
0.6
0.5
0.4
0.3
0.2
0.1
0.0
p-value
1e-09
1e-07
8e-06
6e-04
4e-02
Log-odds
0.5
1.0
1.5
2.0
2.5
3.0
3.5
4.0
Cellular_organisms - ps1
Archaea1 - ps2
Archaea2 - ps3
Archaea3 - ps4
Asgard/Eukaryota - ps5
Eukaryota - ps6
Amorpheae - ps7
Obazoa - ps8
Opisthokonta - ps9
Holozoa - ps10
Filozoa - ps11
Choano./Metazoa - ps12
Metazoa - ps13
Myriazoa - ps14
Parahoxozoa - ps15
Bilateria - ps16
Protostomia - ps17
Ecdysozoa - ps18
Arthropoda - ps19
Mandibulata - ps20
Pancrustacea - ps21
Insecta - ps22
Eumetabola - ps23
Holometabola/Psocodea - ps24
Holometabola - ps25
Aparaglossata - ps26
Mecopterida - ps27
Diptera - ps28
Bibiono./Cyclorrhapha - ps29
Cyclorrhapha - ps30
Calyptratae/Drosophila - ps31
Drosophila - ps32
Sophophora - ps33
Obscura/Melanogaster_group - ps34
D_melanogaster_group - ps35
D_melanogaster_subgroup - ps36
sim./sech./mel. group - ps37
Drosophila_melanogaster - ps38
Phylostratum

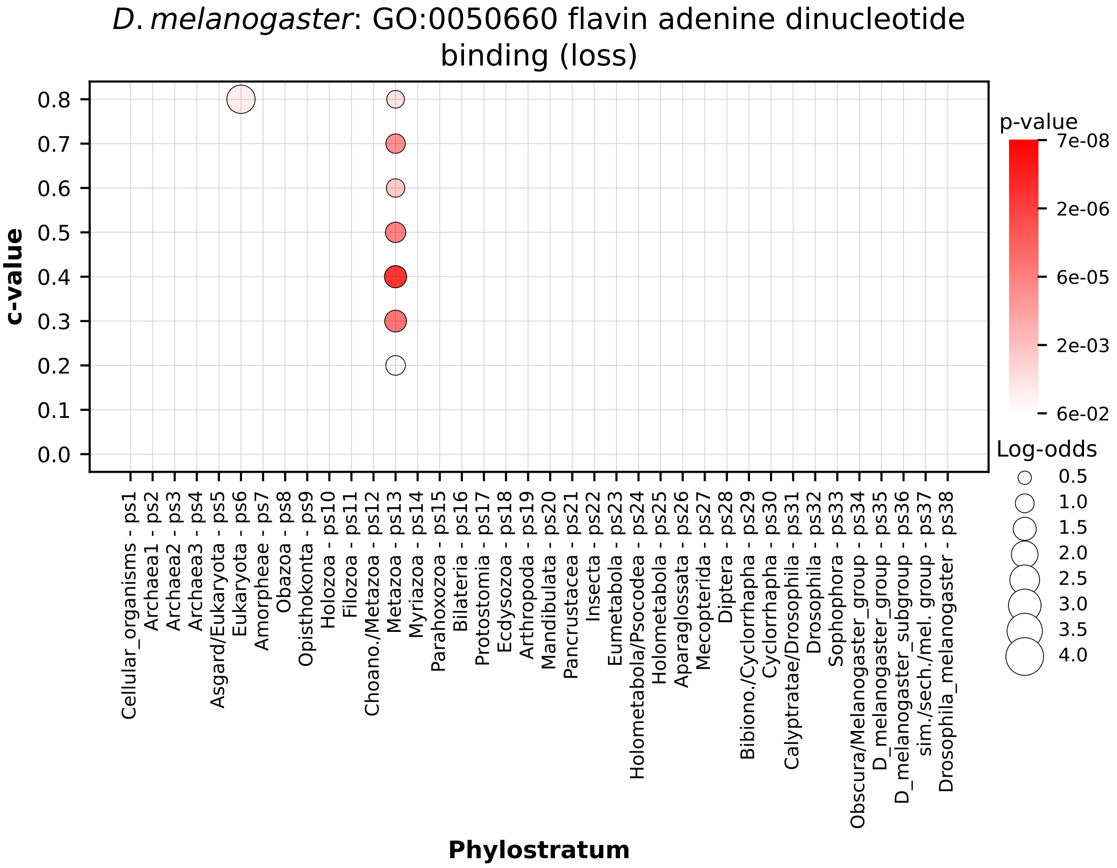

D. melanogaster: GO:0050660 flavin adenine dinucleotide binding (loss)
c-value
0.0
0.1
0.2
0.3
0.4
0.5
0.6
0.7
0.8
Phylostratum
Cellular_organisms - ps1
Archaea1 - ps2
Archaea2 - ps3
Archaea3 - ps4
Asgard/Eukaryota - ps5
Eukaryota - ps6
Amorpheae - ps7
Obazoa - ps8
Opisthokonta - ps9
Holozoa - ps10
Filozoa - ps11
Choano./Metazoa - ps12
Metazoa - ps13
Myriazoa - ps14
Parahoxozoa - ps15
Bilateria - ps16
Protostomia - ps17
Ecdysozoa - ps18
Arthropoda - ps19
Mandibulata - ps20
Pancrustacea - ps21
Insecta - ps22
Eumetabola - ps23
Holometabola/Psocodea - ps24
Holometabola - ps25
Aparaglossata - ps26
Mecopterida - ps27
Diptera - ps28
Bibiono./Cyclorrhapha - ps29
Cyclorrhapha - ps30
Calyptratae/Drosophila - ps31
Drosophila - ps32
Sophophora - ps33
Obscura/Melanogaster_group - ps34
D_melanogaster_group - ps35
D_melanogaster_subgroup - ps36
sim./sech./mel. group - ps37
Drosophila_melanogaster - ps38
p-value
7e-08
2e-06
6e-05
2e-03
6e-02
Log-odds
0.5
1.0
1.5
2.0
2.5
3.0
3.5
4.0

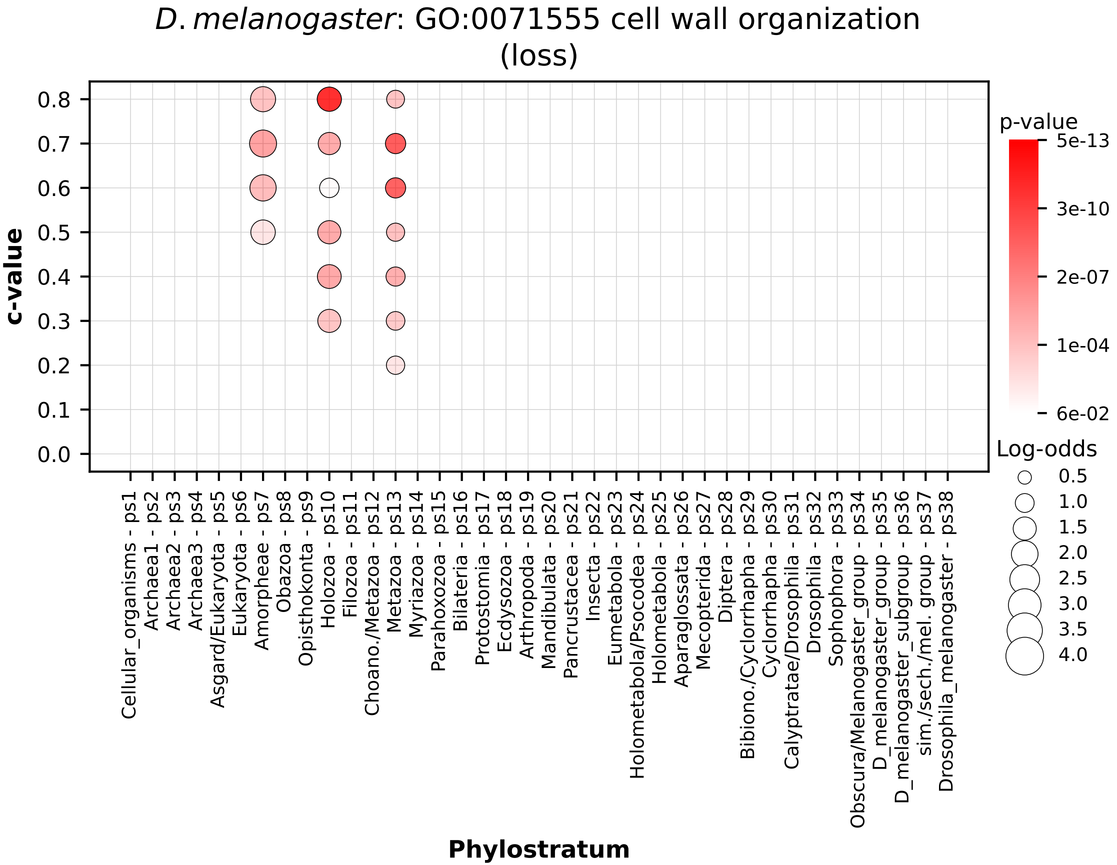

D. melanogaster: GO:0071555 cell wall organization
(loss)
c-value
0.8
0.7
0.6
0.5
0.4
0.3
0.2
0.1
0.0
Phylostratum
Cellular_organisms - ps1
Archaea1 - ps2
Archaea2 - ps3
Archaea3 - ps4
Asgard/Eukaryota - ps5
Eukaryota - ps6
Amorpheae - ps7
Obazoa - ps8
Opisthokonta - ps9
Holozoa - ps10
Filozoa - ps11
Choano./Metazoa - ps12
Metazoa - ps13
Myriazoa - ps14
Parahoxozoa - ps15
Bilateria - ps16
Protostomia - ps17
Ecdysozoa - ps18
Arthropoda - ps19
Mandibulata - ps20
Pancrustacea - ps21
Insecta - ps22
Eumetabola - ps23
Holometabola/Psocodea - ps24
Holometabola - ps25
Aparaglossata - ps26
Mecopterida - ps27
Diptera - ps28
Bibiono./Cyclorrhapha - ps29
Cyclorrhapha - ps30
Calyptratae/Drosophila - ps31
Drosophila - ps32
Sophophora - ps33
Obscura/Melanogaster_group - ps34
D_melanogaster_group - ps35
D_melanogaster_subgroup - ps36
sim./sech./mel. group - ps37
Drosophila_melanogaster - ps38
p-value
5e-13
3e-10
2e-07
1e-04
6e-02
Log-odds
0.5
1.0
1.5
2.0
2.5
3.0
3.5
4.0

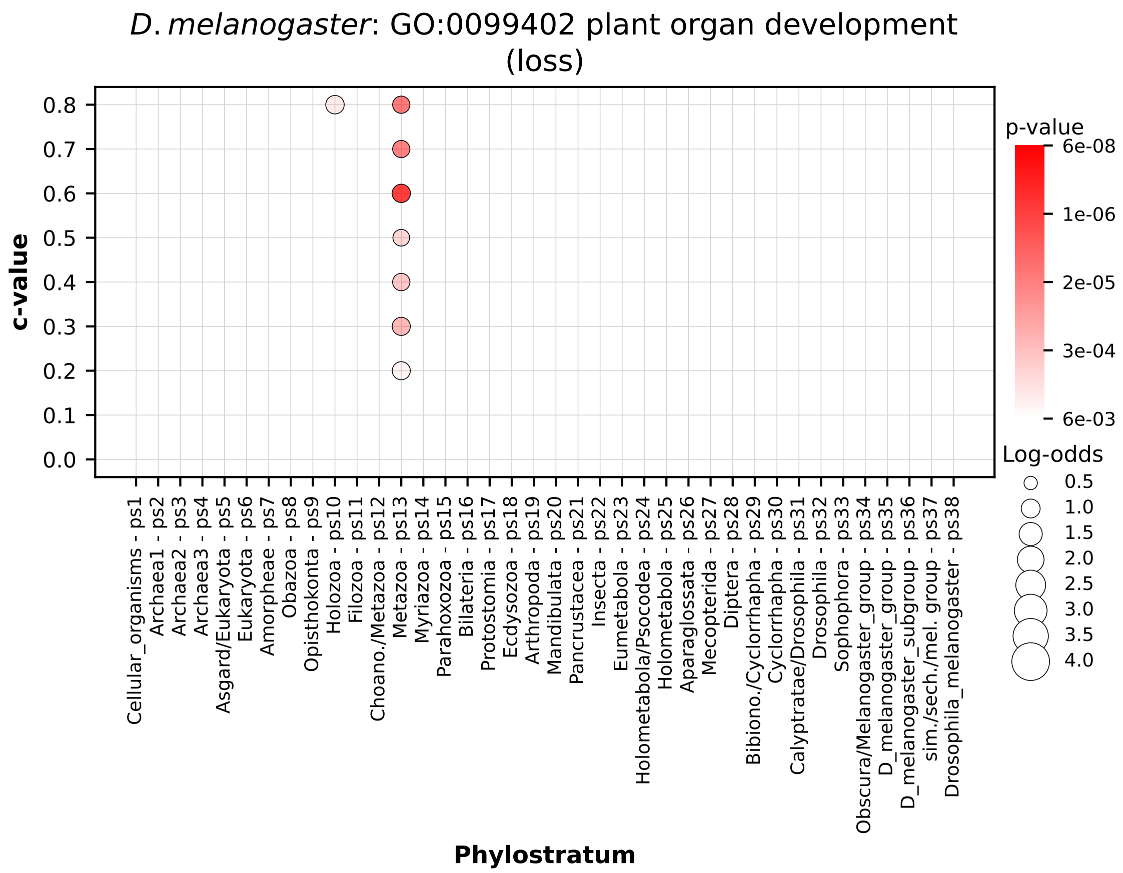

$D. melanogaster$: GO:0099402 plant organ development (loss)
c-value
0.8
0.7
0.6
0.5
0.4
0.3
0.2
0.1
0.0
Phylostratum
Cellular_organisms - ps1
Archaea1 - ps2
Archaea2 - ps3
Archaea3 - ps4
Asgard/Eukaryota - ps5
Eukaryota - ps6
Amorpheae - ps7
Obazoa - ps8
Opisthokonta - ps9
Holozoa - ps10
Filozoa - ps11
Choano./Metazoa - ps12
Metazoa - ps13
Myriazoa - ps14
Parahoxozoa - ps15
Bilateria - ps16
Protostomia - ps17
Ecdysozoa - ps18
Arthropoda - ps19
Mandibulata - ps20
Pancrustacea - ps21
Insecta - ps22
Eumetabola - ps23
Holometabola/Psocodea - ps24
Holometabola - ps25
Aparaglossata - ps26
Mecopterida - ps27
Diptera - ps28
Bibiono./Cyclorrhapha - ps29
Cyclorrhapha - ps30
Calyptratae/Drosophila - ps31
Drosophila - ps32
Sophophora - ps33
Obscura/Melanogaster_group - ps34
D_melanogaster_group - ps35
D_melanogaster_subgroup - ps36
sim./sech./mel. group - ps37
Drosophila_melanogaster - ps38
p-value
6e-08
1e-06
2e-05
3e-04
6e-03
Log-odds
0.5
1.0
1.5
2.0
2.5
3.0
3.5
4.0

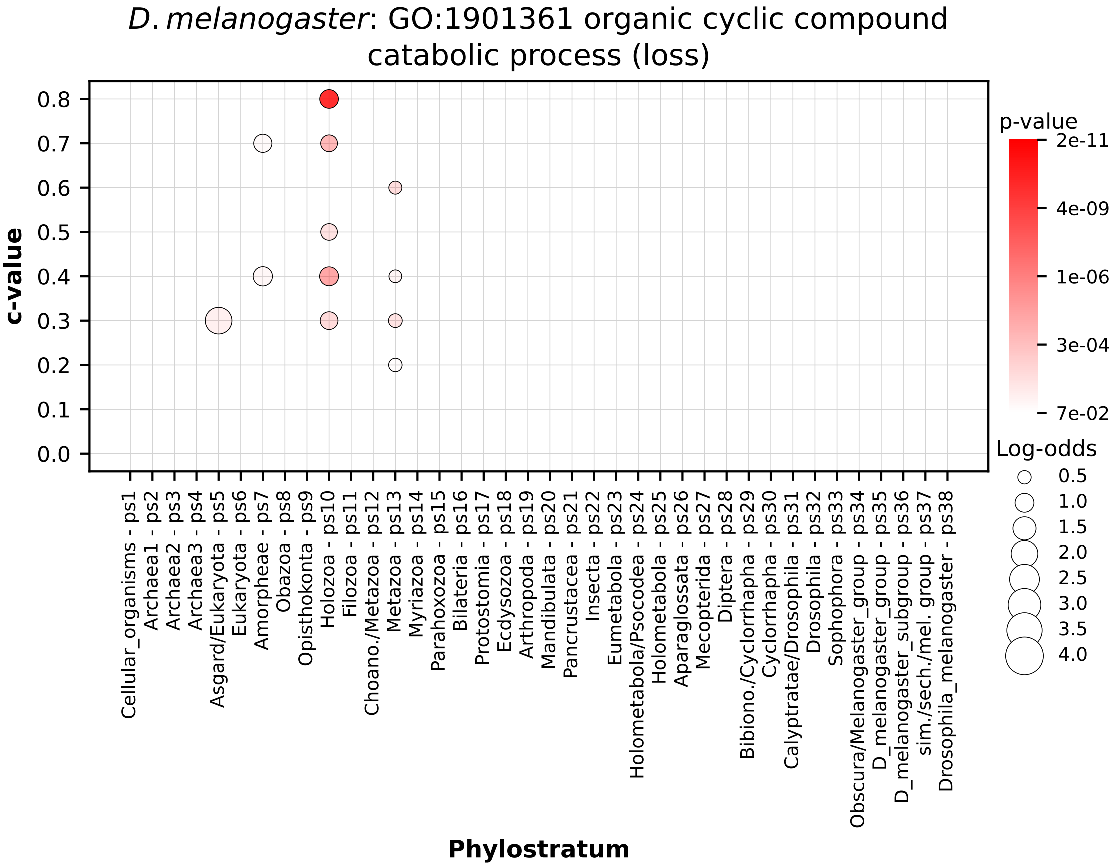
D. melanogaster: GO:1901361 organic cyclic compound catabolic process (loss)
c-value
0.0
0.1
0.2
0.3
0.4
0.5
0.6
0.7
0.8
Phylostratum
Cellular_organisms - ps1
Archaea1 - ps2
Archaea2 - ps3
Archaea3 - ps4
Asgard/Eukaryota - ps5
Eukaryota - ps6
Amorpheae - ps7
Obazoa - ps8
Opisthokonta - ps9
Holozoa - ps10
Filozoa - ps11
Choano./Metazoa - ps12
Metazoa - ps13
Myriazoa - ps14
Parahoxozoa - ps15
Bilateria - ps16
Protostomia - ps17
Ecdysozoa - ps18
Arthropoda - ps19
Mandibulata - ps20
Pancrustacea - ps21
Insecta - ps22
Eumetabola - ps23
Holometabola/Psocodea - ps24
Holometabola - ps25
Aparaglossata - ps26
Mecopterida - ps27
Diptera - ps28
Bibiono./Cyclorrhapha - ps29
Cyclorrhapha - ps30
Calyptratae/Drosophila - ps31
Drosophila - ps32
Sophophora - ps33
Obscura/Melanogaster_group - ps34
D_melanogaster_group - ps35
D_melanogaster_subgroup - ps36
sim./sech./mel. group - ps37
Drosophila_melanogaster - ps38
p-value
2e-11
4e-09
1e-06
3e-04
7e-02
Log-odds
0.5
1.0
1.5
2.0
2.5
3.0
3.5
4.0

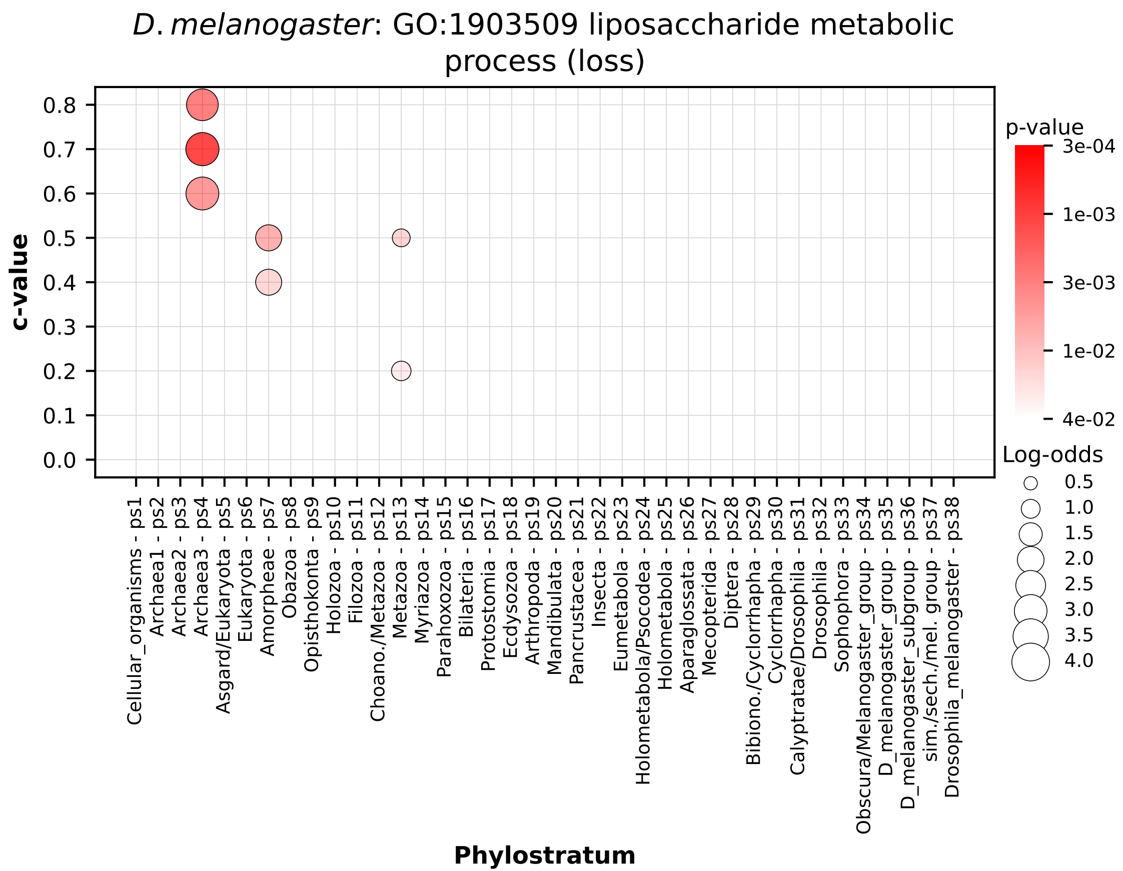

D. melanogaster: GO:1903509 liposaccharide metabolic process (loss)
c-value
0.8
0.7
0.6
0.5
0.4
0.3
0.2
0.1
0.0
Phylostratum
Cellular_organisms - ps1
Archaea1 - ps2
Archaea2 - ps3
Archaea3 - ps4
Asgard/Eukaryota - ps5
Eukaryota - ps6
Amorpheae - ps7
Obazoa - ps8
Opisthokonta - ps9
Holozoa - ps10
Filozoa - ps11
Choano./Metazoa - ps12
Metazoa - ps13
Myriazoa - ps14
Parahoxozoa - ps15
Bilateria - ps16
Protostomia - ps17
Ecdysozoa - ps18
Arthropoda - ps19
Mandibulata - ps20
Pancrustacea - ps21
Insecta - ps22
Eumetabola - ps23
Holometabola/Psocodea - ps24
Holometabola - ps25
Aparaglossata - ps26
Mecopterida - ps27
Diptera - ps28
Bibiono./Cyclorrhapha - ps29
Cyclorrhapha - ps30
Calyptratae/Drosophila - ps31
Drosophila - ps32
Sophophora - ps33
Obscura/Melanogaster_group - ps34
D_melanogaster_group - ps35
D_melanogaster_subgroup - ps36
sim./sech./mel. group - ps37
Drosophila_melanogaster - ps38
p-value
3e-04
1e-03
3e-03
1e-02
4e-02
Log-odds
0.5
1.0
1.5
2.0
2.5
3.0
3.5
4.0

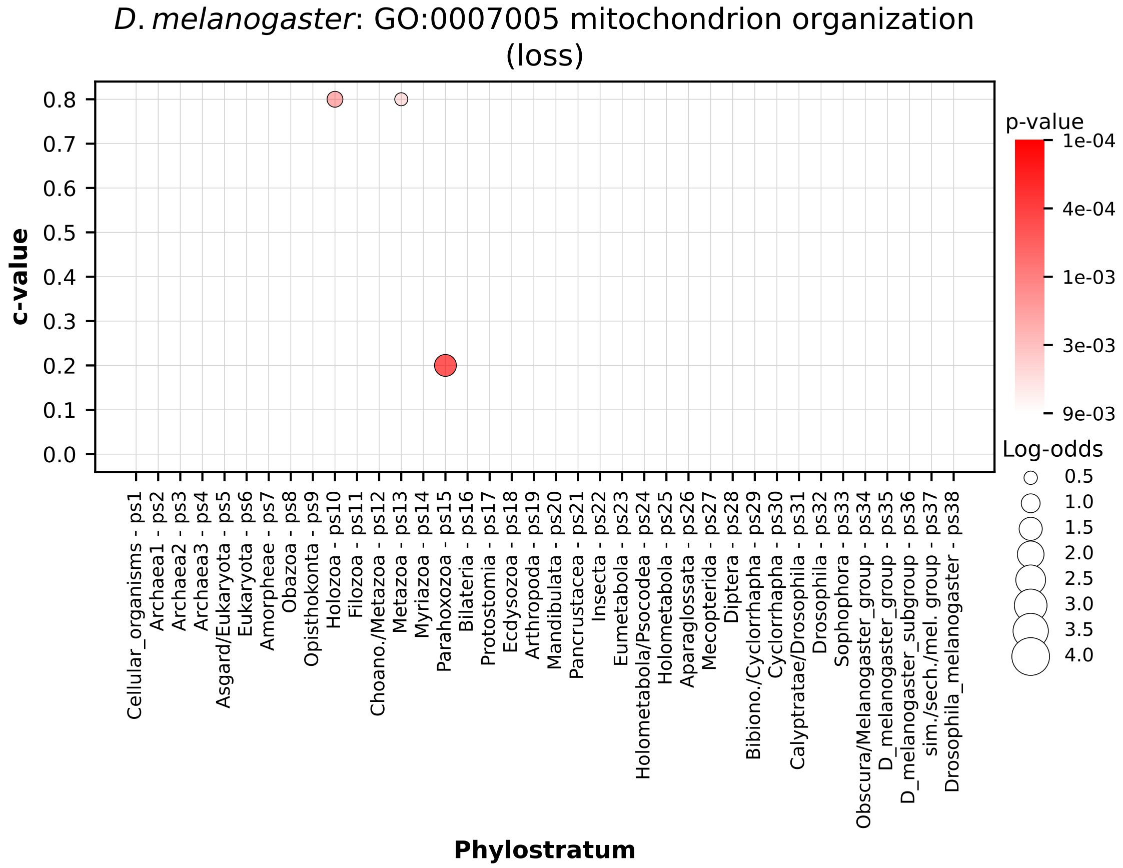

D. melanogaster: GO:0007005 mitochondrion organization (loss)
c-value
0.0
0.1
0.2
0.3
0.4
0.5
0.6
0.7
0.8
p-value
1e-04
4e-04
1e-03
3e-03
9e-03
Log-odds
0.5
1.0
1.5
2.0
2.5
3.0
3.5
4.0
Cellular_organisms - ps1
Archaea1 - ps2
Archaea2 - ps3
Archaea3 - ps4
Asgard/Eukaryota - ps5
Eukaryota - ps6
Amorpheae - ps7
Obazoa - ps8
Opisthokonta - ps9
Holozoa - ps10
Filozoa - ps11
Choano./Metazoa - ps12
Metazoa - ps13
Myriazoa - ps14
Parahoxozoa - ps15
Bilateria - ps16
Protostomia - ps17
Ecdysozoa - ps18
Arthropoda - ps19
Mandibulata - ps20
Pancrustacea - ps21
Insecta - ps22
Eumetabola - ps23
Holometabola/Psocodea - ps24
Holometabola - ps25
Aparaglossata - ps26
Mecopterida - ps27
Diptera - ps28
Bibiono./Cyclorrhapha - ps29
Cyclorrhapha - ps30
Calyptratae/Drosophila - ps31
Drosophila - ps32
Sophophora - ps33
Obscura/Melanogaster_group - ps34
D_melanogaster_group - ps35
D_melanogaster_subgroup - ps36
sim./sech./mel. group - ps37
Drosophila_melanogaster - ps38
Phylostratum

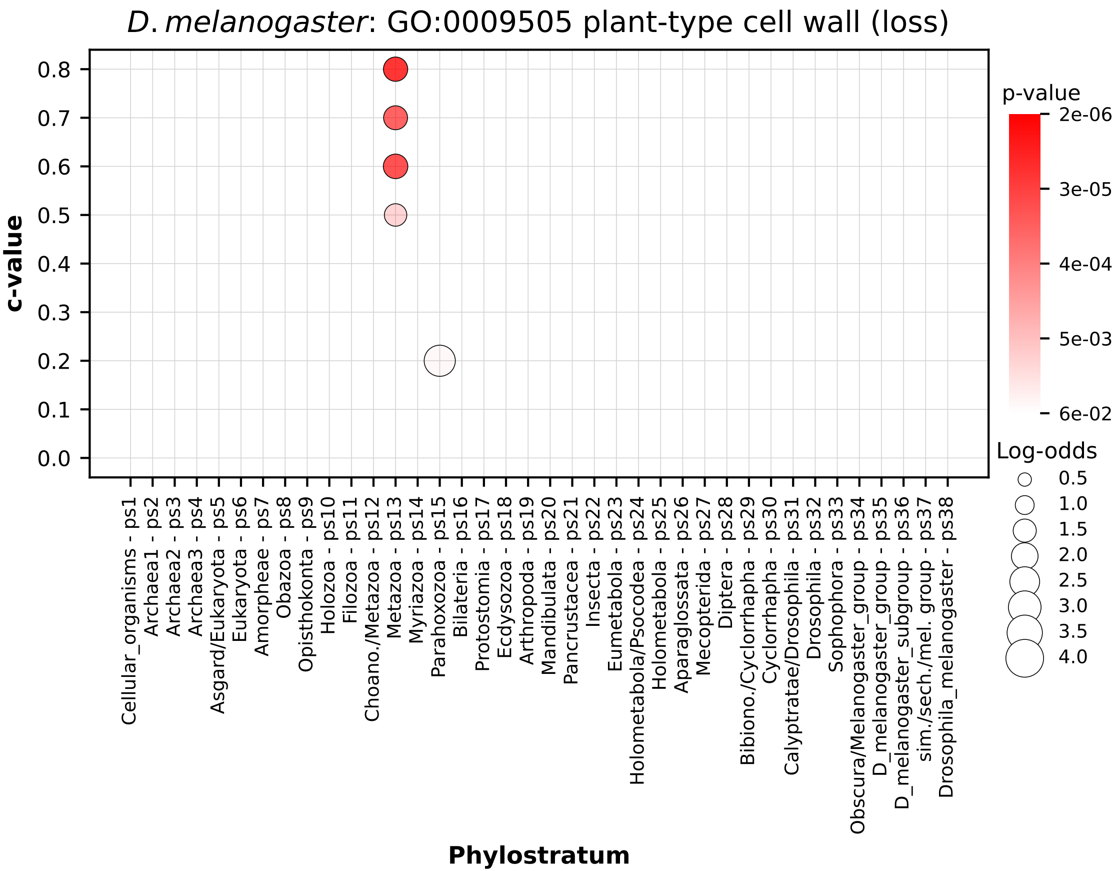

D. melanogaster: GO:0009505 plant-type cell wall (loss)
c-value
0.0
0.1
0.2
0.3
0.4
0.5
0.6
0.7
0.8
Phylostratum
Cellular_organisms - ps1
Archaea1 - ps2
Archaea2 - ps3
Archaea3 - ps4
Asgard/Eukaryota - ps5
Eukaryota - ps6
Amorpheae - ps7
Obazoa - ps8
Opisthokonta - ps9
Holozoa - ps10
Filozoa - ps11
Choano./Metazoa - ps12
Metazoa - ps13
Myriazoa - ps14
Parahoxozoa - ps15
Bilateria - ps16
Protostomia - ps17
Ecdysozoa - ps18
Arthropoda - ps19
Mandibulata - ps20
Pancrustacea - ps21
Insecta - ps22
Eumetabola - ps23
Holometabola/Psocodea - ps24
Holometabola - ps25
Aparaglossata - ps26
Mecopterida - ps27
Diptera - ps28
Bibiono./Cyclorrhapha - ps29
Cyclorrhapha - ps30
Calyptratae/Drosophila - ps31
Drosophila - ps32
Sophophora - ps33
Obscura/Melanogaster_group - ps34
D_melanogaster_group - ps35
D_melanogaster_subgroup - ps36
sim./sech./mel. group - ps37
Drosophila_melanogaster - ps38
p-value
2e-06
3e-05
4e-04
5e-03
6e-02
Log-odds
0.5
1.0
1.5
2.0
2.5
3.0
3.5
4.0

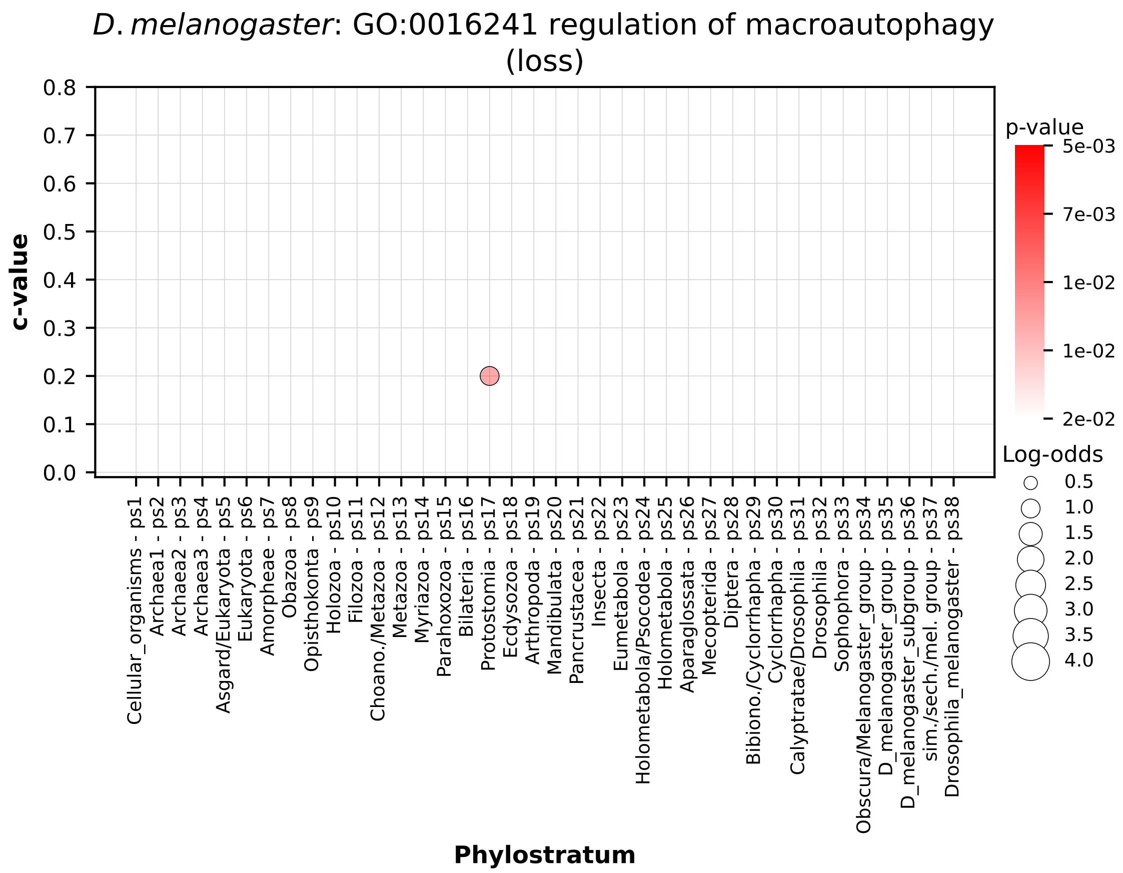
D. melanogaster: GO:0016241 regulation of macroautophagy
(loss)
c-value
0.8
0.7
0.6
0.5
0.4
0.3
0.2
0.1
0.0
p-value
5e-03
7e-03
1e-02
1e-02
2e-02
Log-odds
0.5
1.0
1.5
2.0
2.5
3.0
3.5
4.0
Cellular_organisms - ps1
Archaea1 - ps2
Archaea2 - ps3
Archaea3 - ps4
Asgard/Eukaryota - ps5
Eukaryota - ps6
Amorpheae - ps7
Obazoa - ps8
Opisthokonta - ps9
Holozoa - ps10
Filozoa - ps11
Choano./Metazoa - ps12
Metazoa - ps13
Myriazoa - ps14
Parahoxozoa - ps15
Bilateria - ps16
Protostomia - ps17
Ecdysozoa - ps18
Arthropoda - ps19
Mandibulata - ps20
Pancrustacea - ps21
Insecta - ps22
Eumetabola - ps23
Holometabola/Psocodea - ps24
Holometabola - ps25
Aparaglossata - ps26
Mecopterida - ps27
Diptera - ps28
Bibiono./Cyclorrhapha - ps29
Cyclorrhapha - ps30
Calyptratae/Drosophila - ps31
Drosophila - ps32
Sophophora - ps33
Obscura/Melanogaster_group - ps34
D_melanogaster_group - ps35
D_melanogaster_subgroup - ps36
sim./sech./mel. group - ps37
Drosophila_melanogaster - ps38
Phylostratum

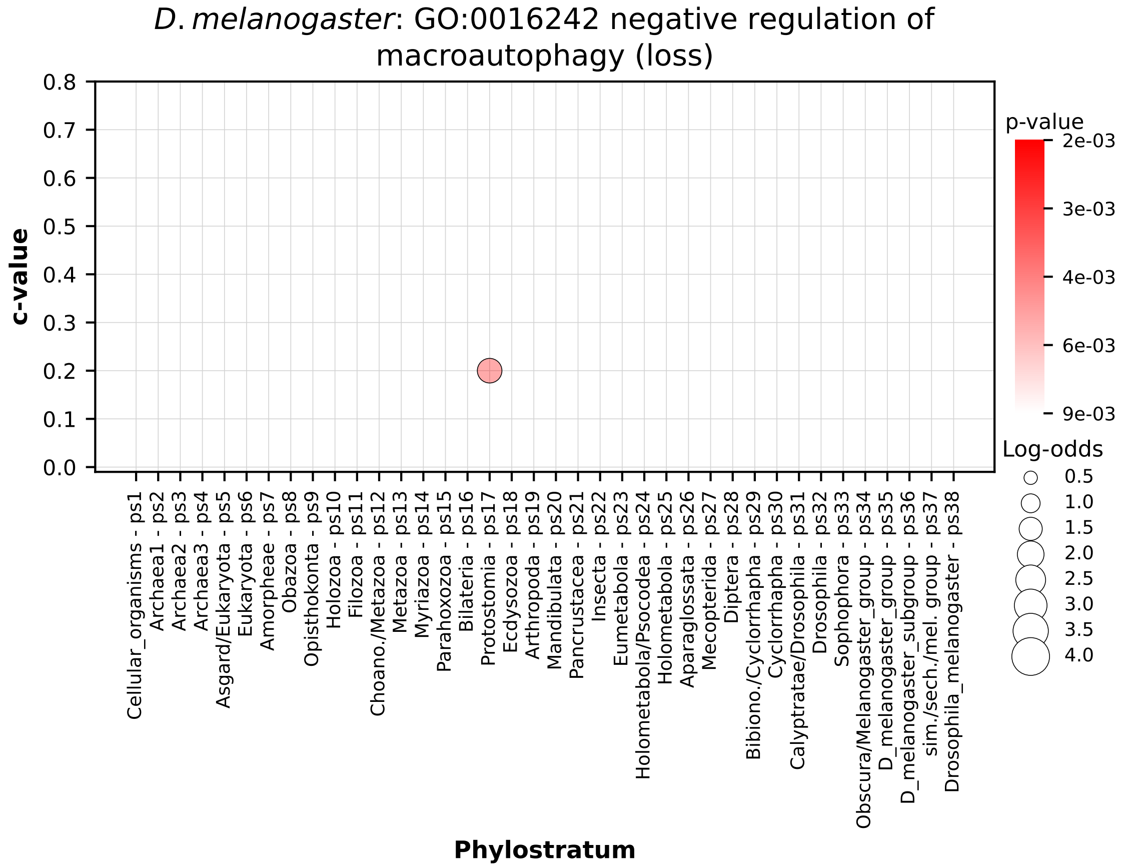

D. melanogaster: GO:0016242 negative regulation of macroautophagy (loss)
c-value
0.8
0.7
0.6
0.5
0.4
0.3
0.2
0.1
0.0
Phylostratum
Cellular_organisms - ps1
Archaea1 - ps2
Archaea2 - ps3
Archaea3 - ps4
Asgard/Eukaryota - ps5
Eukaryota - ps6
Amorpheae - ps7
Obazoa - ps8
Opisthokonta - ps9
Holozoa - ps10
Filozoa - ps11
Choano./Metazoa - ps12
Metazoa - ps13
Myriazoa - ps14
Parahoxozoa - ps15
Bilateria - ps16
Protostomia - ps17
Ecdysozoa - ps18
Arthropoda - ps19
Mandibulata - ps20
Pancrustacea - ps21
Insecta - ps22
Eumetabola - ps23
Holometabola/Psocodea - ps24
Holometabola - ps25
Aparaglossata - ps26
Mecopterida - ps27
Diptera - ps28
Bibiono./Cyclorrhapha - ps29
Cyclorrhapha - ps30
Calyptratae/Drosophila - ps31
Drosophila - ps32
Sophophora - ps33
Obscura/Melanogaster_group - ps34
D_melanogaster_group - ps35
D_melanogaster_subgroup - ps36
sim./sech./mel. group - ps37
Drosophila_melanogaster - ps38
p-value
2e-03
3e-03
4e-03
6e-03
9e-03
Log-odds
0.5
1.0
1.5
2.0
2.5
3.0
3.5
4.0

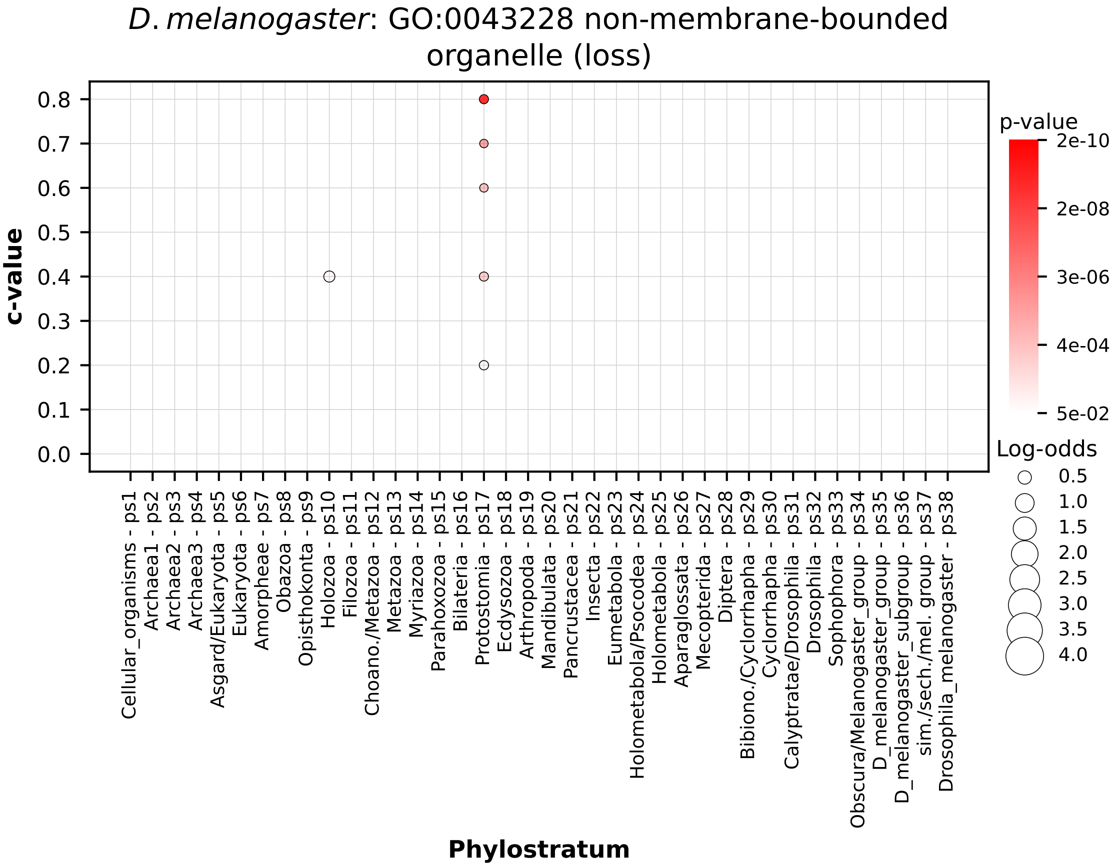
D. melanogaster: GO:0043228 non-membrane-bounded organelle (loss)
c-value
0.8
0.7
0.6
0.5
0.4
0.3
0.2
0.1
0.0
Phylostratum
Cellular_organisms - ps1
Archaea1 - ps2
Archaea2 - ps3
Archaea3 - ps4
Asgard/Eukaryota - ps5
Eukaryota - ps6
Amorpheae - ps7
Obazoa - ps8
Opisthokonta - ps9
Holozoa - ps10
Filozoa - ps11
Choano./Metazoa - ps12
Metazoa - ps13
Myriazoa - ps14
Parahoxozoa - ps15
Bilateria - ps16
Protostomia - ps17
Ecdysozoa - ps18
Arthropoda - ps19
Mandibulata - ps20
Pancrustacea - ps21
Insecta - ps22
Eumetabola - ps23
Holometabola/Psocodea - ps24
Holometabola - ps25
Aparaglossata - ps26
Mecopterida - ps27
Diptera - ps28
Bibiono./Cyclorrhapha - ps29
Cyclorrhapha - ps30
Calyptratae/Drosophila - ps31
Drosophila - ps32
Sophophora - ps33
Obscura/Melanogaster_group - ps34
D_melanogaster_group - ps35
D_melanogaster_subgroup - ps36
sim./sech./mel. group - ps37
Drosophila_melanogaster - ps38
p-value
2e-10
2e-08
3e-06
4e-04
5e-02
Log-odds
0.5
1.0
1.5
2.0
2.5
3.0
3.5
4.0

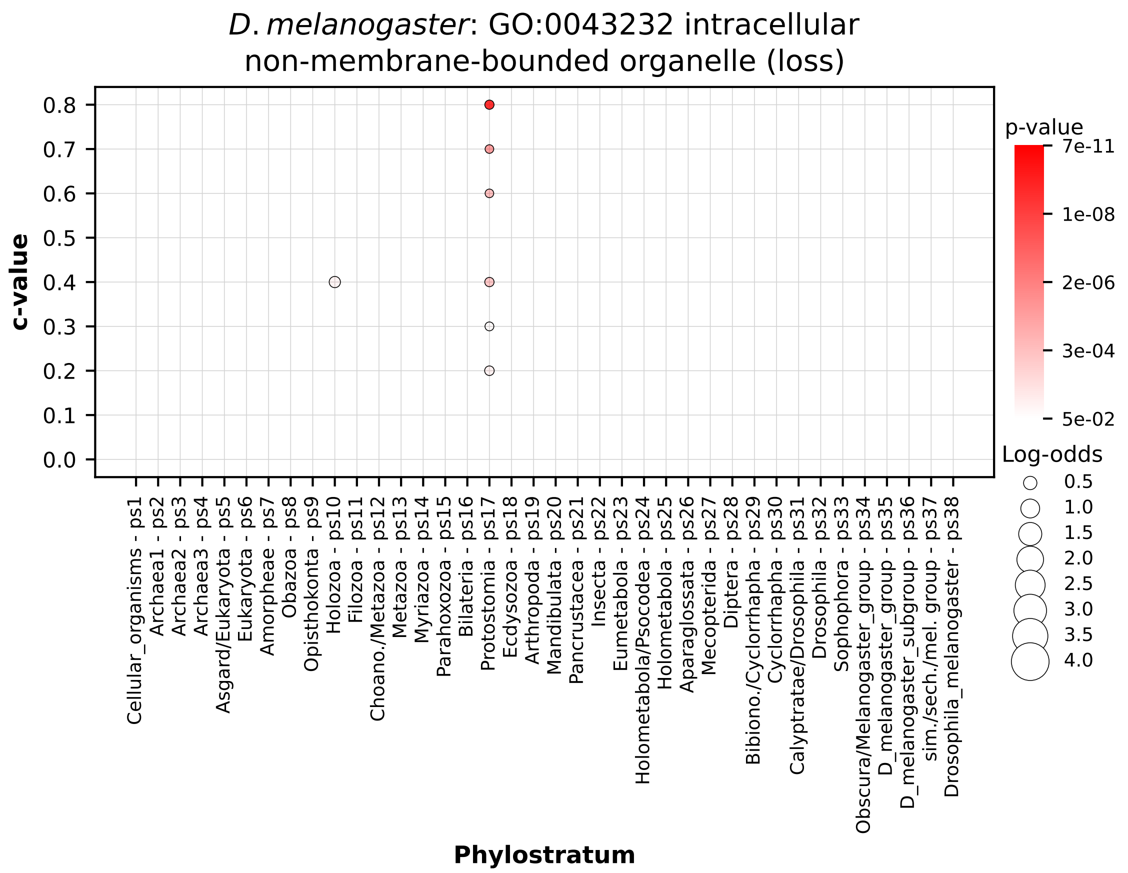

D. melanogaster: GO:0043232 intracellular non-membrane-bounded organelle (loss)
c-value
0.8
0.7
0.6
0.5
0.4
0.3
0.2
0.1
0.0
p-value
7e-11
1e-08
2e-06
3e-04
5e-02
Log-odds
0.5
1.0
1.5
2.0
2.5
3.0
3.5
4.0
Cellular_organisms - ps1
Archaea1 - ps2
Archaea2 - ps3
Archaea3 - ps4
Asgard/Eukaryota - ps5
Eukaryota - ps6
Amorpheae - ps7
Obazoa - ps8
Opisthokonta - ps9
Holozoa - ps10
Filozoa - ps11
Choano./Metazoa - ps12
Metazoa - ps13
Myriazoa - ps14
Parahoxozoa - ps15
Bilateria - ps16
Protostomia - ps17
Ecdysozoa - ps18
Arthropoda - ps19
Mandibulata - ps20
Pancrustacea - ps21
Insecta - ps22
Eumetabola - ps23
Holometabola/Psocodea - ps24
Holometabola - ps25
Aparaglossata - ps26
Mecopterida - ps27
Diptera - ps28
Bibiono./Cyclorrhapha - ps29
Cyclorrhapha - ps30
Calyptratae/Drosophila - ps31
Drosophila - ps32
Sophophora - ps33
Obscura/Melanogaster_group - ps34
D_melanogaster_group - ps35
D_melanogaster_subgroup - ps36
sim./sech./mel. group - ps37
Drosophila_melanogaster - ps38
Phylostratum

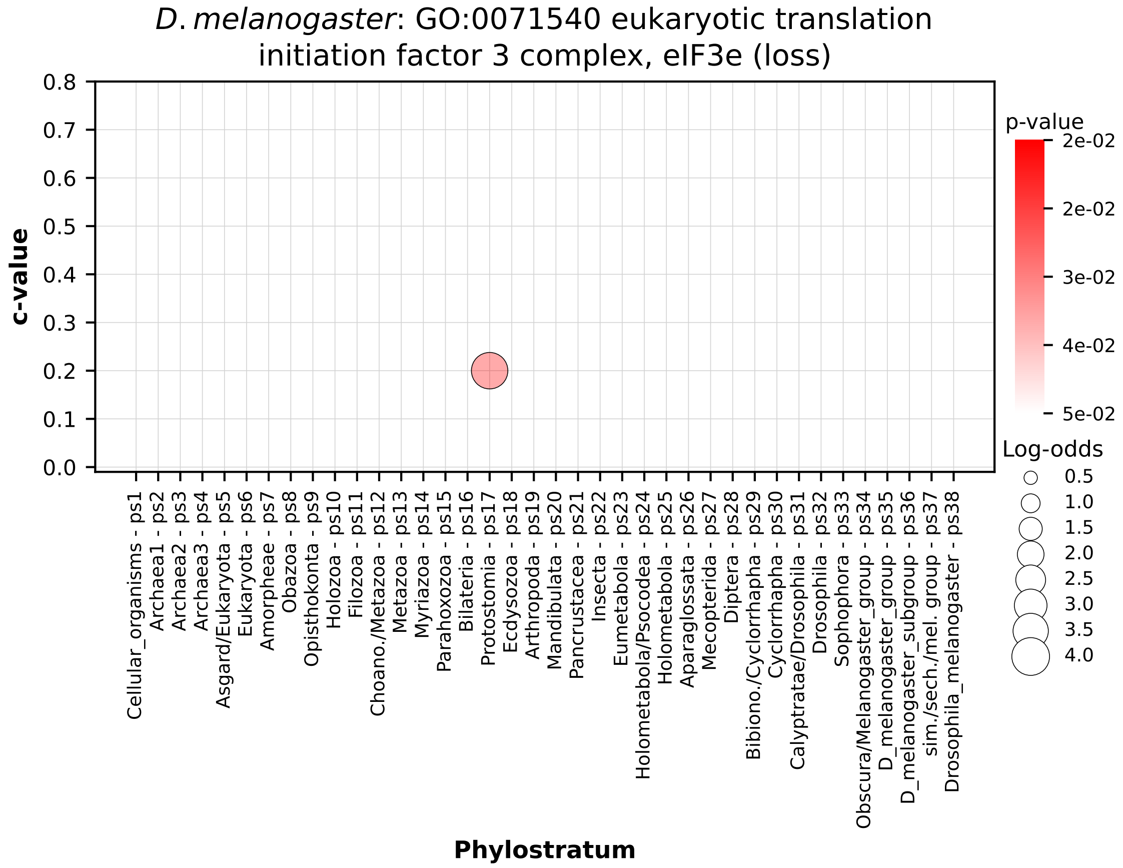

D. melanogaster: GO:0071540 eukaryotic translation initiation factor 3 complex, eIF3e (loss)
c-value
0.8
0.7
0.6
0.5
0.4
0.3
0.2
0.1
0.0
Phylostratum
Cellular_organisms - ps1
Archaea1 - ps2
Archaea2 - ps3
Archaea3 - ps4
Asgard/Eukaryota - ps5
Eukaryota - ps6
Amorpheae - ps7
Obazoa - ps8
Opisthokonta - ps9
Holozoa - ps10
Filozoa - ps11
Choano./Metazoa - ps12
Metazoa - ps13
Myriazoa - ps14
Parahoxozoa - ps15
Bilateria - ps16
Protostomia - ps17
Ecdysozoa - ps18
Arthropoda - ps19
Mandibulata - ps20
Pancrustacea - ps21
Insecta - ps22
Eumetabola - ps23
Holometabola/Psocodea - ps24
Holometabola - ps25
Aparaglossata - ps26
Mecopterida - ps27
Diptera - ps28
Bibiono./Cyclorrhapha - ps29
Cyclorrhapha - ps30
Calyptratae/Drosophila - ps31
Drosophila - ps32
Sophophora - ps33
Obscura/Melanogaster_group - ps34
D_melanogaster_group - ps35
D_melanogaster_subgroup - ps36
sim./sech./mel. group - ps37
Drosophila_melanogaster - ps38
p-value
2e-02
2e-02
3e-02
4e-02
5e-02
Log-odds
0.5
1.0
1.5
2.0
2.5
3.0
3.5
4.0

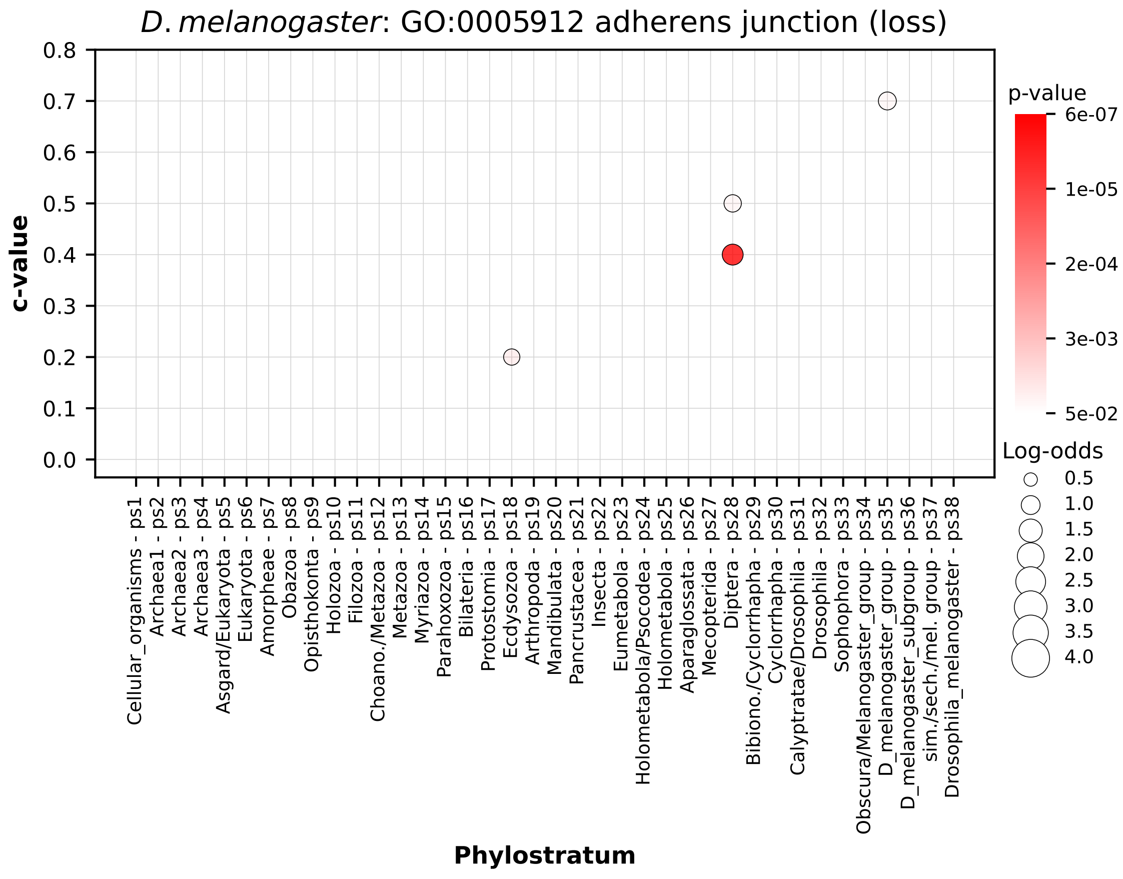

D. melanogaster: GO:0005912 adherens junction (loss)
c-value
0.0
0.1
0.2
0.3
0.4
0.5
0.6
0.7
0.8
Phylostratum
Cellular_organisms - ps1
Archaea1 - ps2
Archaea2 - ps3
Archaea3 - ps4
Asgard/Eukaryota - ps5
Eukaryota - ps6
Amorpheae - ps7
Obazoa - ps8
Opisthokonta - ps9
Holozoa - ps10
Filozoa - ps11
Choano./Metazoa - ps12
Metazoa - ps13
Myriazoa - ps14
Parahoxozoa - ps15
Bilateria - ps16
Protostomia - ps17
Ecdysozoa - ps18
Arthropoda - ps19
Mandibulata - ps20
Pancrustacea - ps21
Insecta - ps22
Eumetabola - ps23
Holometabola/Psocodea - ps24
Holometabola - ps25
Aparaglossata - ps26
Mecopterida - ps27
Diptera - ps28
Bibiono./Cyclorrhapha - ps29
Cyclorrhapha - ps30
Calyptratae/Drosophila - ps31
Drosophila - ps32
Sophophora - ps33
Obscura/Melanogaster_group - ps34
D_melanogaster_group - ps35
D_melanogaster_subgroup - ps36
sim./sech./mel. group - ps37
Drosophila_melanogaster - ps38
p-value
6e-07
1e-05
2e-04
3e-03
5e-02
Log-odds
0.5
1.0
1.5
2.0
2.5
3.0
3.5
4.0

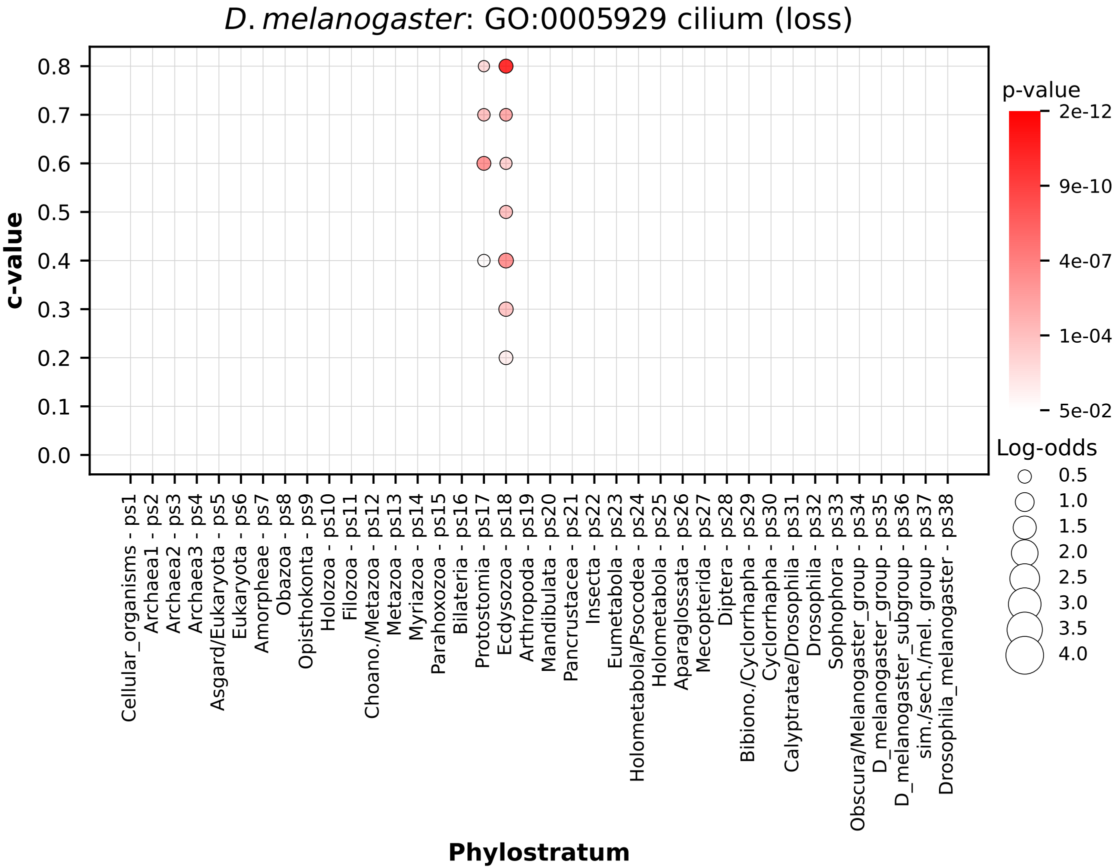

D. melanogaster: GO:0005929 cilium (loss)
c-value
0.8
0.7
0.6
0.5
0.4
0.3
0.2
0.1
0.0
p-value
2e-12
9e-10
4e-07
1e-04
5e-02
Log-odds
0.5
1.0
1.5
2.0
2.5
3.0
3.5
4.0
Cellular_organisms - ps1
Archaea1 - ps2
Archaea2 - ps3
Archaea3 - ps4
Asgard/Eukaryota - ps5
Eukaryota - ps6
Amorpheae - ps7
Obazoa - ps8
Opisthokonta - ps9
Holozoa - ps10
Filozoa - ps11
Choano./Metazoa - ps12
Metazoa - ps13
Myriazoa - ps14
Parahoxozoa - ps15
Bilateria - ps16
Protostomia - ps17
Ecdysozoa - ps18
Arthropoda - ps19
Mandibulata - ps20
Pancrustacea - ps21
Insecta - ps22
Eumetabola - ps23
Holometabola/Psocodea - ps24
Holometabola - ps25
Aparaglossata - ps26
Mecopterida - ps27
Diptera - ps28
Bibiono./Cyclorrhapha - ps29
Cyclorrhapha - ps30
Calyptratae/Drosophila - ps31
Drosophila - ps32
Sophophora - ps33
Obscura/Melanogaster_group - ps34
D_melanogaster_group - ps35
D_melanogaster_subgroup - ps36
sim./sech./mel. group - ps37
Drosophila_melanogaster - ps38
Phylostratum

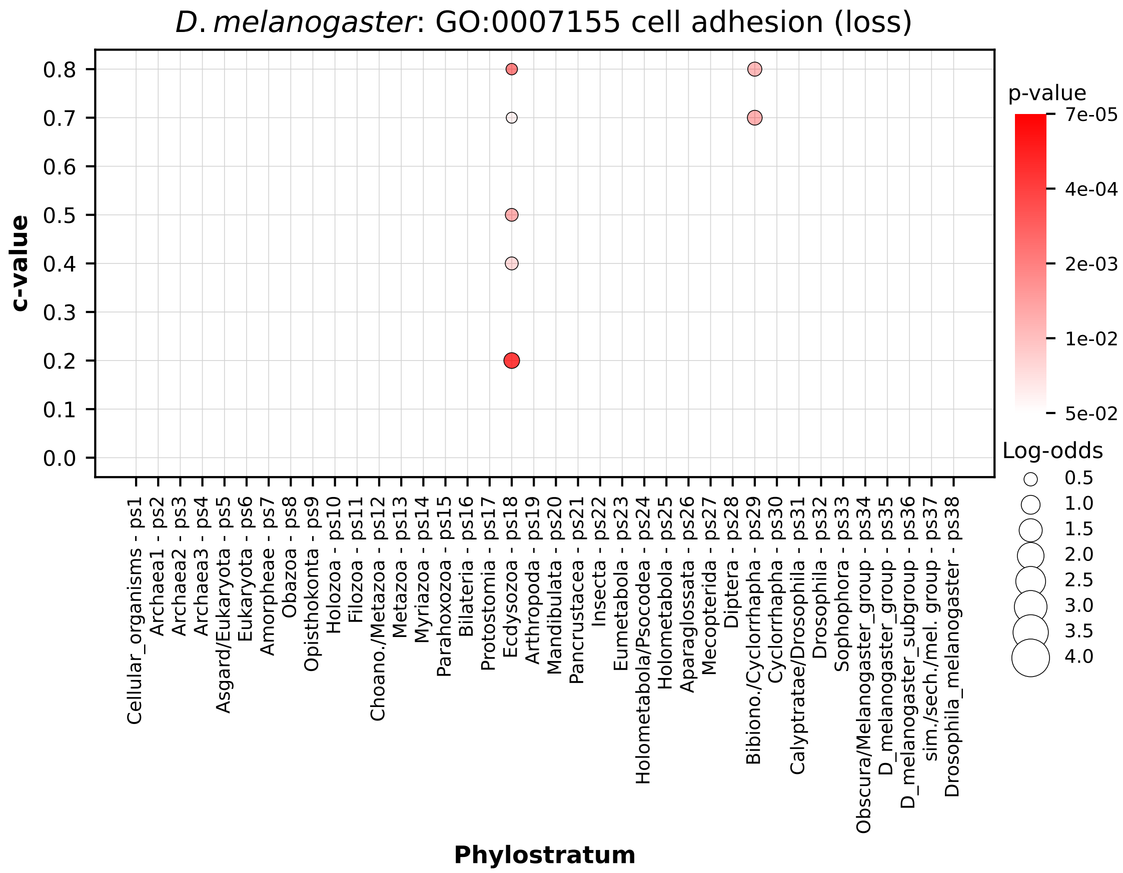

D. melanogaster: GO:0007155 cell adhesion (loss)
c-value
0.8
0.7
0.6
0.5
0.4
0.3
0.2
0.1
0.0
p-value
7e-05
4e-04
2e-03
1e-02
5e-02
Log-odds
0.5
1.0
1.5
2.0
2.5
3.0
3.5
4.0
Cellular_organisms - ps1
Archaea1 - ps2
Archaea2 - ps3
Archaea3 - ps4
Asgard/Eukaryota - ps5
Eukaryota - ps6
Amorpheae - ps7
Obazoa - ps8
Opisthokonta - ps9
Holozoa - ps10
Filozoa - ps11
Choano./Metazoa - ps12
Metazoa - ps13
Myriazoa - ps14
Parahoxozoa - ps15
Bilateria - ps16
Protostomia - ps17
Ecdysozoa - ps18
Arthropoda - ps19
Mandibulata - ps20
Pancrustacea - ps21
Insecta - ps22
Eumetabola - ps23
Holometabola/Psocodea - ps24
Holometabola - ps25
Aparaglossata - ps26
Mecopterida - ps27
Diptera - ps28
Bibiono./Cyclorrhapha - ps29
Cyclorrhapha - ps30
Calyptratae/Drosophila - ps31
Drosophila - ps32
Sophophora - ps33
Obscura/Melanogaster_group - ps34
D_melanogaster_group - ps35
D_melanogaster_subgroup - ps36
sim./sech./mel. group - ps37
Drosophila_melanogaster - ps38
Phylostratum

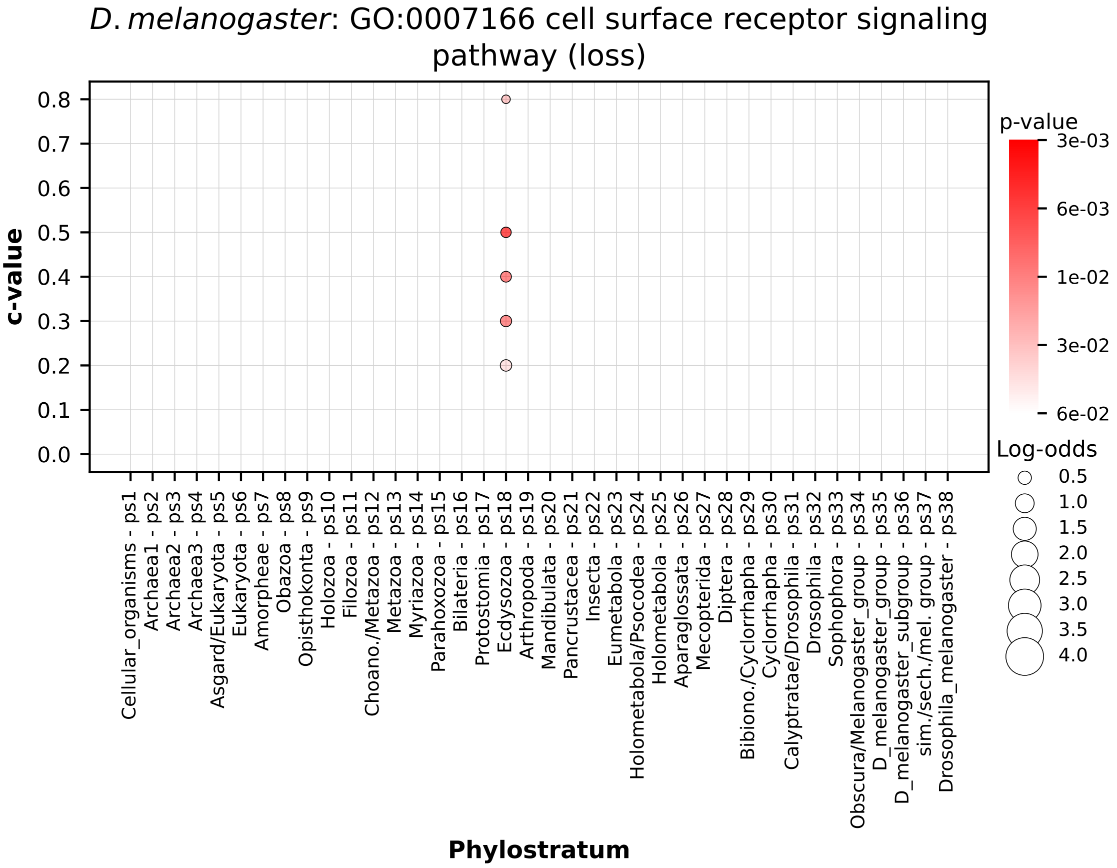
D. melanogaster: GO:0007166 cell surface receptor signaling pathway (loss)
c-value
0.8
0.7
0.6
0.5
0.4
0.3
0.2
0.1
0.0
p-value
3e-03
6e-03
1e-02
3e-02
6e-02
Log-odds
0.5
1.0
1.5
2.0
2.5
3.0
3.5
4.0
Cellular_organisms - ps1
Archaea1 - ps2
Archaea2 - ps3
Archaea3 - ps4
Asgard/Eukaryota - ps5
Eukaryota - ps6
Amorpheae - ps7
Obazoa - ps8
Opisthokonta - ps9
Holozoa - ps10
Filozoa - ps11
Choano./Metazoa - ps12
Metazoa - ps13
Myriazoa - ps14
Parahoxozoa - ps15
Bilateria - ps16
Protostomia - ps17
Ecdysozoa - ps18
Arthropoda - ps19
Mandibulata - ps20
Pancrustacea - ps21
Insecta - ps22
Eumetabola - ps23
Holometabola/Psocodea - ps24
Holometabola - ps25
Aparaglossata - ps26
Mecopterida - ps27
Diptera - ps28
Bibiono./Cyclorrhapha - ps29
Cyclorrhapha - ps30
Calyptratae/Drosophila - ps31
Drosophila - ps32
Sophophora - ps33
Obscura/Melanogaster_group - ps34
D_melanogaster_group - ps35
D_melanogaster_subgroup - ps36
sim./sech./mel. group - ps37
Drosophila_melanogaster - ps38
Phylostratum

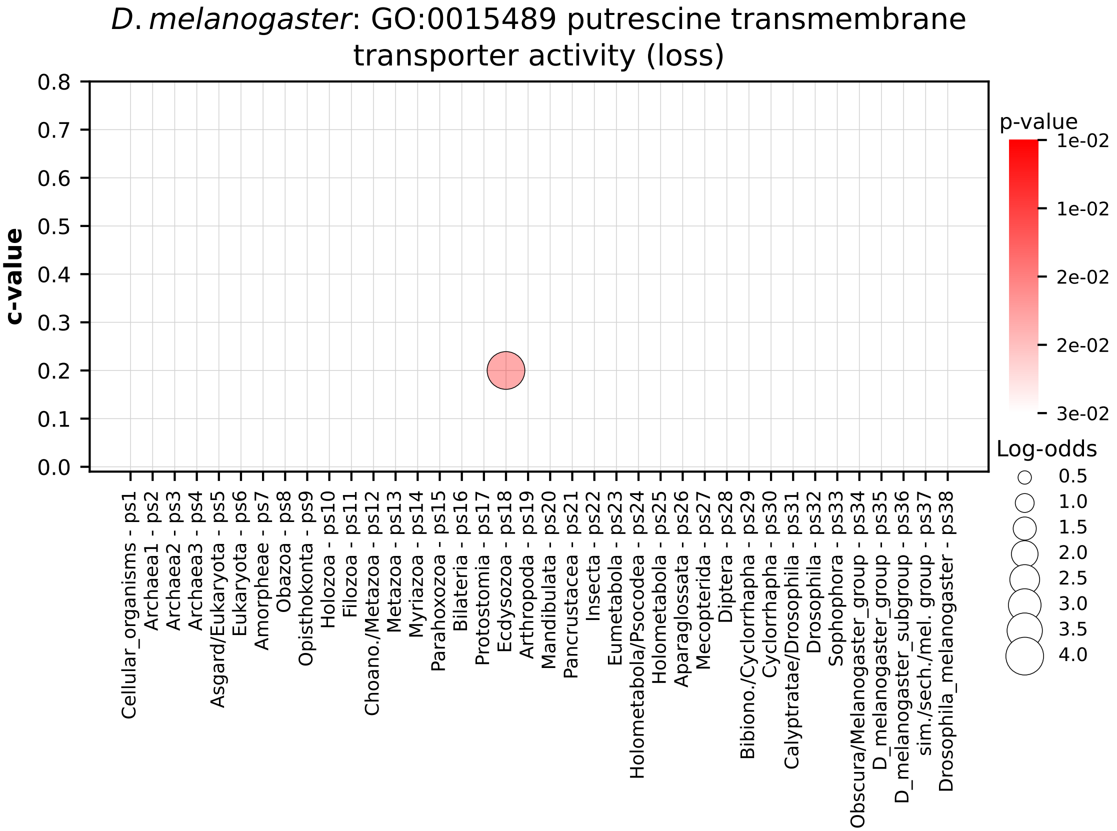

D. melanogaster: GO:0015489 putrescine transmembrane transporter activity (loss)
c-value
0.8
0.7
0.6
0.5
0.4
0.3
0.2
0.1
0.0
p-value
1e-02
1e-02
2e-02
2e-02
3e-02
Log-odds
0.5
1.0
1.5
2.0
2.5
3.0
3.5
4.0
Cellular_organisms - ps1
Archaea1 - ps2
Archaea2 - ps3
Archaea3 - ps4
Asgard/Eukaryota - ps5
Eukaryota - ps6
Amorpheae - ps7
Obazoa - ps8
Opisthokonta - ps9
Holozoa - ps10
Filozoa - ps11
Choano./Metazoa - ps12
Metazoa - ps13
Myriazoa - ps14
Parahoxozoa - ps15
Bilateria - ps16
Protostomia - ps17
Ecdysozoa - ps18
Arthropoda - ps19
Mandibulata - ps20
Pancrustacea - ps21
Insecta - ps22
Eumetabola - ps23
Holometabola/Psocodea - ps24
Holometabola - ps25
Aparaglossata - ps26
Mecopterida - ps27
Diptera - ps28
Bibiono./Cyclorrhapha - ps29
Cyclorrhapha - ps30
Calyptratae/Drosophila - ps31
Drosophila - ps32
Sophophora - ps33
Obscura/Melanogaster_group - ps34
D_melanogaster_group - ps35
D_melanogaster_subgroup - ps36
sim./sech./mel. group - ps37
Drosophila_melanogaster - ps38
Phylostratum

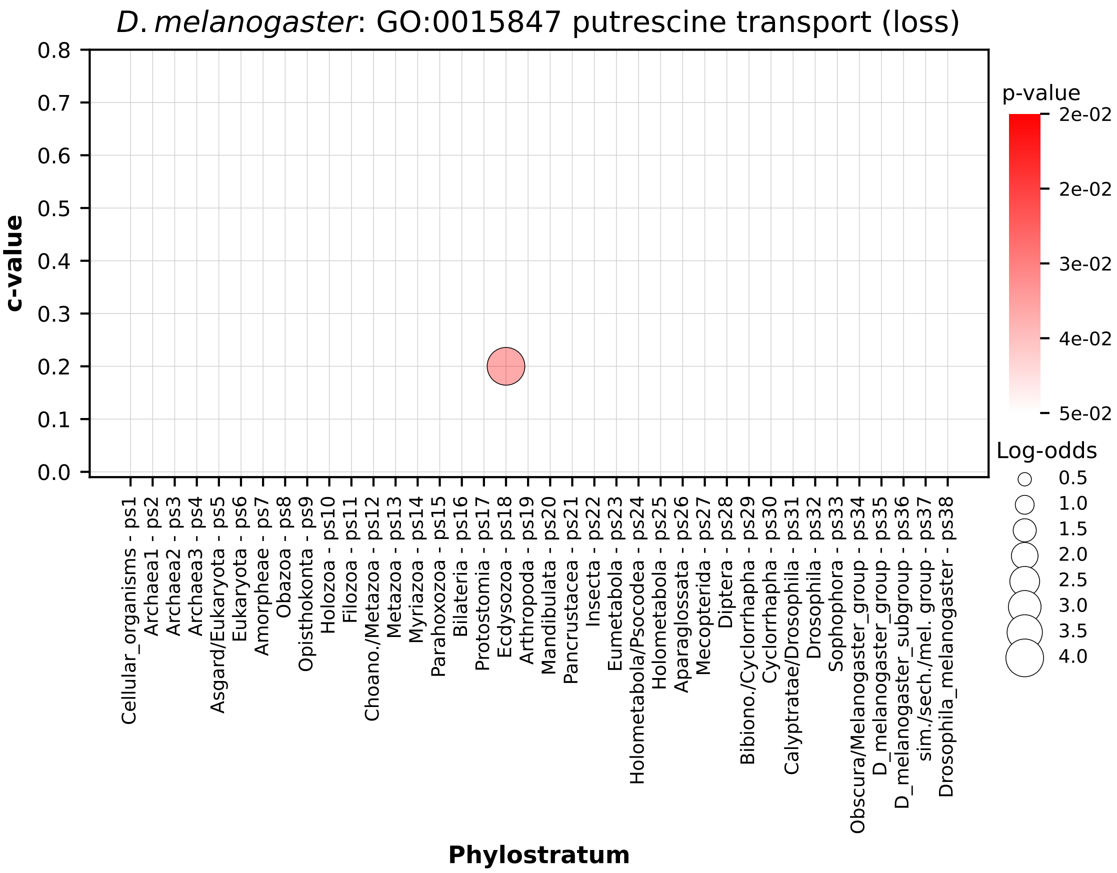

D. melanogaster: GO:0015847 putrescine transport (loss)
c-value
0.8
0.7
0.6
0.5
0.4
0.3
0.2
0.1
0.0
Phylostratum
Cellular_organisms - ps1
Archaea1 - ps2
Archaea2 - ps3
Archaea3 - ps4
Asgard/Eukaryota - ps5
Eukaryota - ps6
Amorpheae - ps7
Obazoa - ps8
Opisthokonta - ps9
Holozoa - ps10
Filozoa - ps11
Choano./Metazoa - ps12
Metazoa - ps13
Myriazoa - ps14
Parahoxozoa - ps15
Bilateria - ps16
Protostomia - ps17
Ecdysozoa - ps18
Arthropoda - ps19
Mandibulata - ps20
Pancrustacea - ps21
Insecta - ps22
Eumetabola - ps23
Holometabola/Psocodea - ps24
Holometabola - ps25
Aparaglossata - ps26
Mecopterida - ps27
Diptera - ps28
Bibiono./Cyclorrhapha - ps29
Cyclorrhapha - ps30
Calyptratae/Drosophila - ps31
Drosophila - ps32
Sophophora - ps33
Obscura/Melanogaster_group - ps34
D_melanogaster_group - ps35
D_melanogaster_subgroup - ps36
sim./sech./mel. group - ps37
Drosophila_melanogaster - ps38
p-value
2e-02
2e-02
3e-02
4e-02
5e-02
Log-odds
0.5
1.0
1.5
2.0
2.5
3.0
3.5
4.0

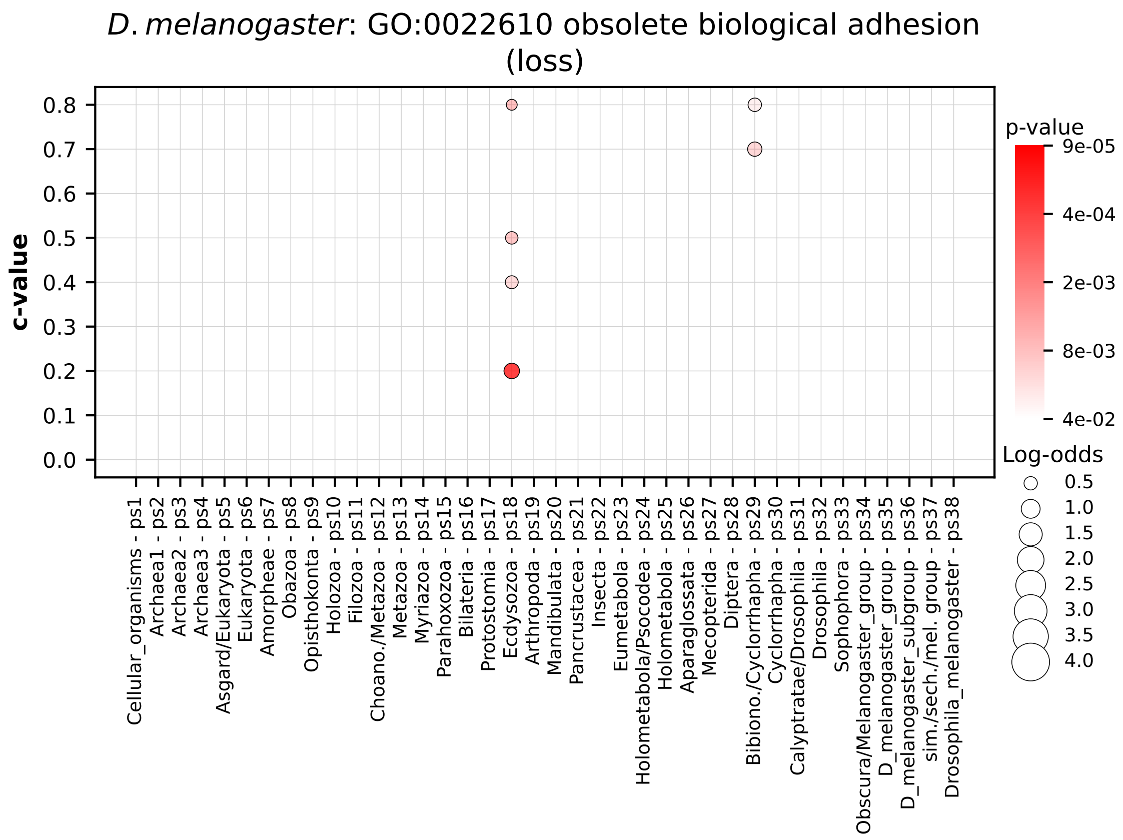

D. melanogaster: GO:0022610 obsolete biological adhesion (loss)
c-value
0.8
0.7
0.6
0.5
0.4
0.3
0.2
0.1
0.0
Phylostratum
Cellular_organisms - ps1
Archaea1 - ps2
Archaea2 - ps3
Archaea3 - ps4
Asgard/Eukaryota - ps5
Eukaryota - ps6
Amorpheae - ps7
Obazoa - ps8
Opisthokonta - ps9
Holozoa - ps10
Filozoa - ps11
Choano./Metazoa - ps12
Metazoa - ps13
Myriazoa - ps14
Parahoxozoa - ps15
Bilateria - ps16
Protostomia - ps17
Ecdysozoa - ps18
Arthropoda - ps19
Mandibulata - ps20
Pancrustacea - ps21
Insecta - ps22
Eumetabola - ps23
Holometabola/Psocodea - ps24
Holometabola - ps25
Aparaglossata - ps26
Mecopterida - ps27
Diptera - ps28
Bibiono./Cyclorrhapha - ps29
Cyclorrhapha - ps30
Calyptratae/Drosophila - ps31
Drosophila - ps32
Sophophora - ps33
Obscura/Melanogaster_group - ps34
D_melanogaster_group - ps35
D_melanogaster_subgroup - ps36
sim./sech./mel. group - ps37
Drosophila_melanogaster - ps38
p-value
9e-05
4e-04
2e-03
8e-03
4e-02
Log-odds
0.5
1.0
1.5
2.0
2.5
3.0
3.5
4.0

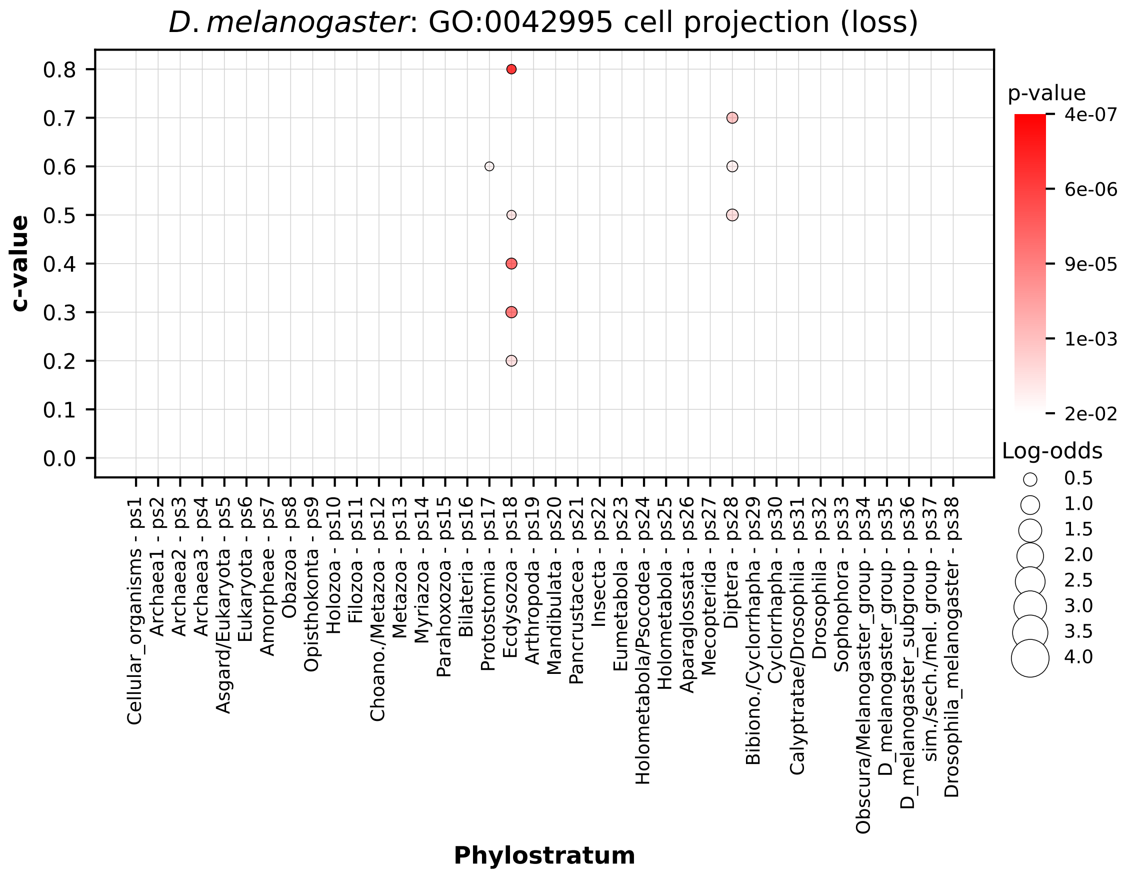

D. melanogaster: GO:0042995 cell projection (loss)
c-value
0.8
0.7
0.6
0.5
0.4
0.3
0.2
0.1
0.0
p-value
4e-07
6e-06
9e-05
1e-03
2e-02
Log-odds
0.5
1.0
1.5
2.0
2.5
3.0
3.5
4.0
Cellular_organisms - ps1
Archaea1 - ps2
Archaea2 - ps3
Archaea3 - ps4
Asgard/Eukaryota - ps5
Eukaryota - ps6
Amorpheae - ps7
Obazoa - ps8
Opisthokonta - ps9
Holozoa - ps10
Filozoa - ps11
Choano./Metazoa - ps12
Metazoa - ps13
Myriazoa - ps14
Parahoxozoa - ps15
Bilateria - ps16
Protostomia - ps17
Ecdysozoa - ps18
Arthropoda - ps19
Mandibulata - ps20
Pancrustacea - ps21
Insecta - ps22
Eumetabola - ps23
Holometabola/Psocodea - ps24
Holometabola - ps25
Aparaglossata - ps26
Mecopterida - ps27
Diptera - ps28
Bibiono./Cyclorrhapha - ps29
Cyclorrhapha - ps30
Calyptratae/Drosophila - ps31
Drosophila - ps32
Sophophora - ps33
Obscura/Melanogaster_group - ps34
D_melanogaster_group - ps35
D_melanogaster_subgroup - ps36
sim./sech./mel. group - ps37
Drosophila_melanogaster - ps38
Phylostratum

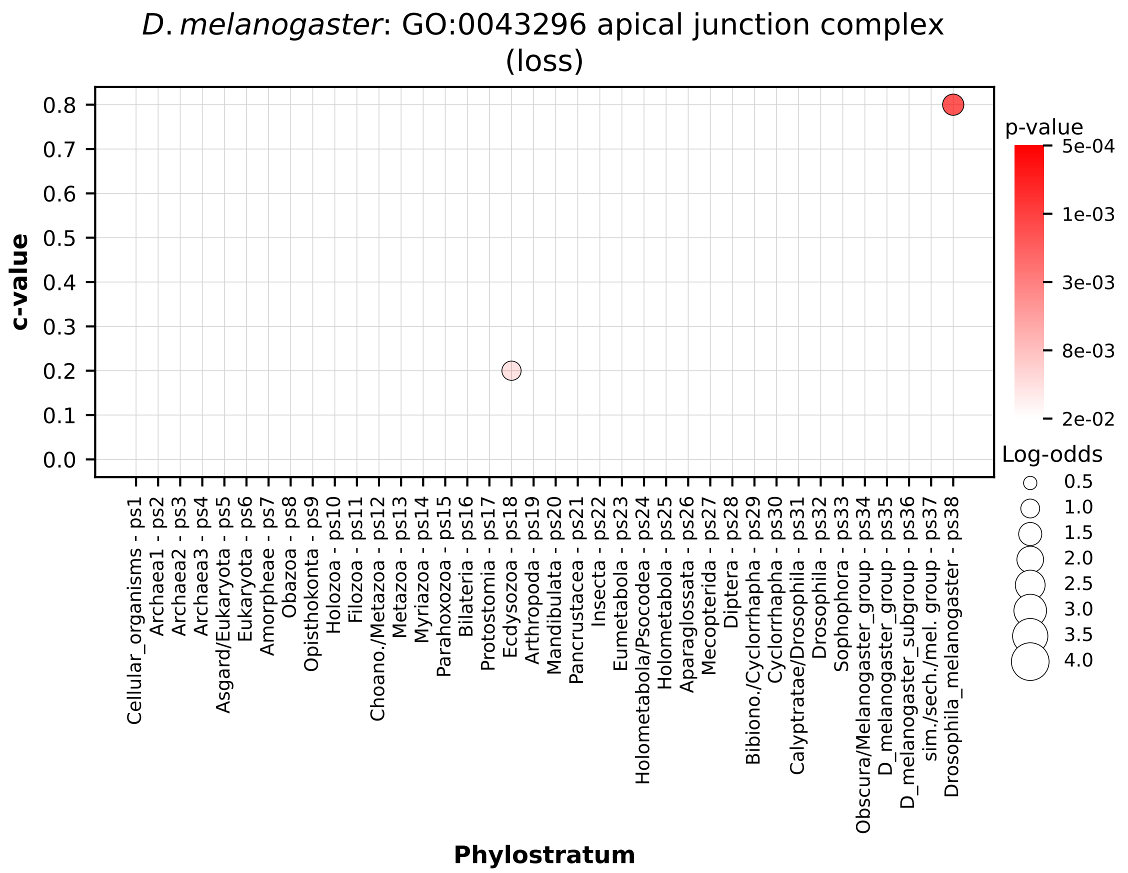
D. melanogaster: GO:0043296 apical junction complex
(loss)
c-value
0.0
0.1
0.2
0.3
0.4
0.5
0.6
0.7
0.8
Phylostratum
Cellular_organisms - ps1
Archaea1 - ps2
Archaea2 - ps3
Archaea3 - ps4
Asgard/Eukaryota - ps5
Eukaryota - ps6
Amorpheae - ps7
Obazoa - ps8
Opisthokonta - ps9
Holozoa - ps10
Filozoa - ps11
Choano./Metazoa - ps12
Metazoa - ps13
Myriazoa - ps14
Parahoxozoa - ps15
Bilateria - ps16
Protostomia - ps17
Ecdysozoa - ps18
Arthropoda - ps19
Mandibulata - ps20
Pancrustacea - ps21
Insecta - ps22
Eumetabola - ps23
Holometabola/Psocodea - ps24
Holometabola - ps25
Aparaglossata - ps26
Mecopterida - ps27
Diptera - ps28
Bibiono./Cyclorrhapha - ps29
Cyclorrhapha - ps30
Calyptratae/Drosophila - ps31
Drosophila - ps32
Sophophora - ps33
Obscura/Melanogaster_group - ps34
D_melanogaster_group - ps35
D_melanogaster_subgroup - ps36
sim./sech./mel. group - ps37
Drosophila_melanogaster - ps38
p-value
5e-04
1e-03
3e-03
8e-03
2e-02
Log-odds
0.5
1.0
1.5
2.0
2.5
3.0
3.5
4.0

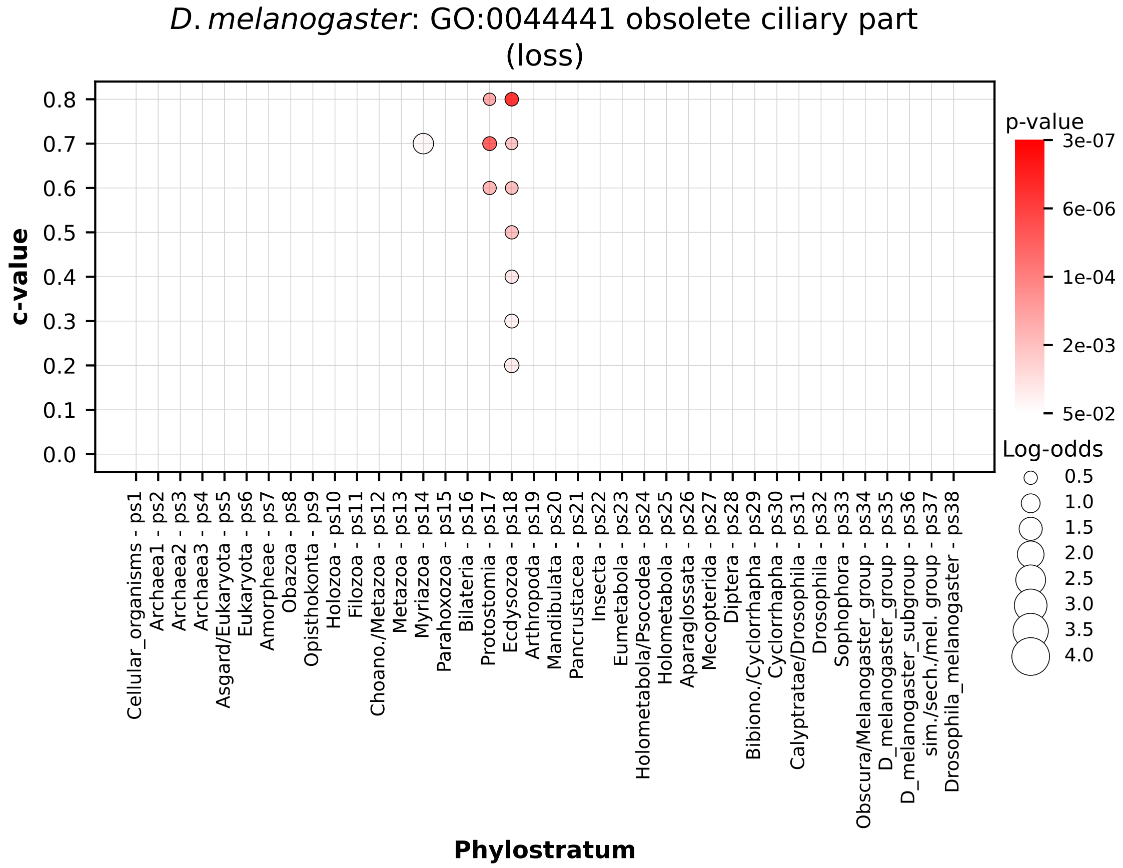

D. melanogaster: GO:0044441 obsolete ciliary part
(loss)
c-value
0.8
0.7
0.6
0.5
0.4
0.3
0.2
0.1
0.0
p-value
3e-07
6e-06
1e-04
2e-03
5e-02
Log-odds
0.5
1.0
1.5
2.0
2.5
3.0
3.5
4.0
Cellular_organisms - ps1
Archaea1 - ps2
Archaea2 - ps3
Archaea3 - ps4
Asgard/Eukaryota - ps5
Eukaryota - ps6
Amorpheae - ps7
Obazoa - ps8
Opisthokonta - ps9
Holozoa - ps10
Filozoa - ps11
Choano./Metazoa - ps12
Metazoa - ps13
Myriazoa - ps14
Parahoxozoa - ps15
Bilateria - ps16
Protostomia - ps17
Ecdysozoa - ps18
Arthropoda - ps19
Mandibulata - ps20
Pancrustacea - ps21
Insecta - ps22
Eumetabola - ps23
Holometabola/Psocodea - ps24
Holometabola - ps25
Aparaglossata - ps26
Mecopterida - ps27
Diptera - ps28
Bibiono./Cyclorrhapha - ps29
Cyclorrhapha - ps30
Calyptratae/Drosophila - ps31
Drosophila - ps32
Sophophora - ps33
Obscura/Melanogaster_group - ps34
D_melanogaster_group - ps35
D_melanogaster_subgroup - ps36
sim./sech./mel. group - ps37
Drosophila_melanogaster - ps38
Phylostratum

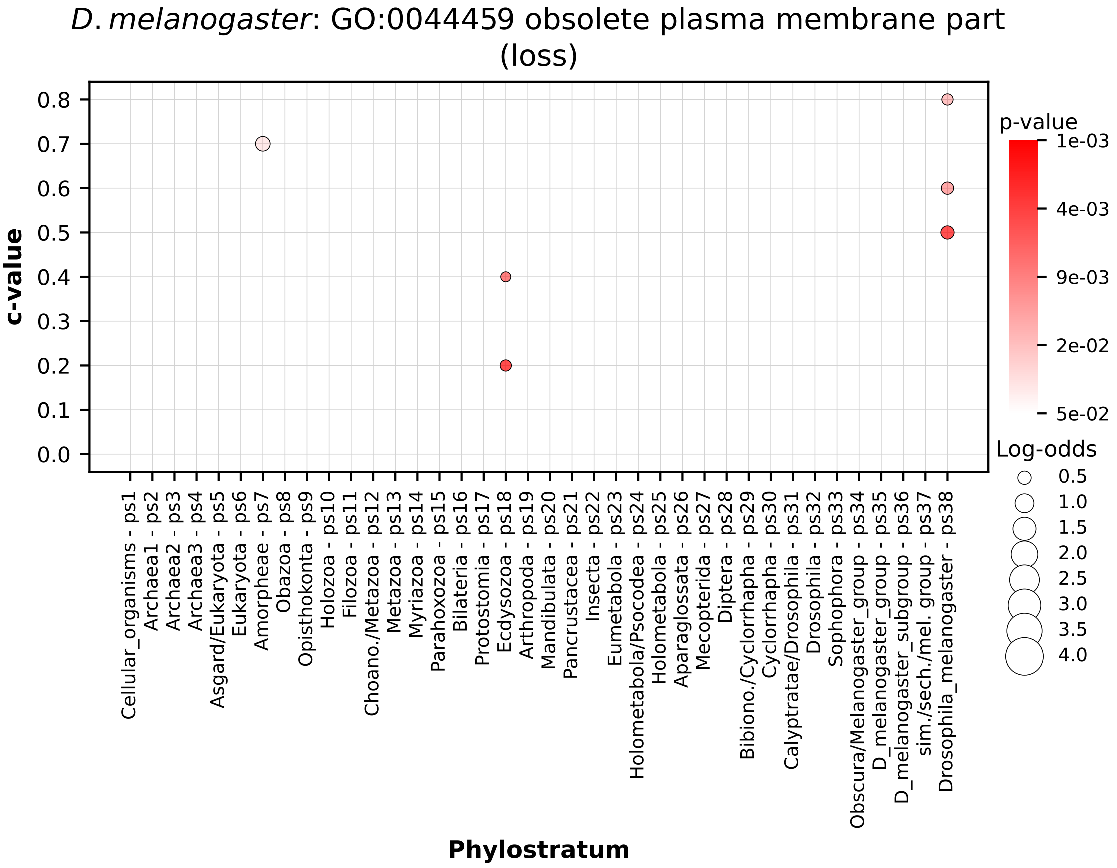

D. melanogaster: GO:0044459 obsolete plasma membrane part (loss)
c-value
0.8
0.7
0.6
0.5
0.4
0.3
0.2
0.1
0.0
p-value
1e-03
4e-03
9e-03
2e-02
5e-02
Log-odds
0.5
1.0
1.5
2.0
2.5
3.0
3.5
4.0
Cellular_organisms - ps1
Archaea1 - ps2
Archaea2 - ps3
Archaea3 - ps4
Asgard/Eukaryota - ps5
Eukaryota - ps6
Amorpheae - ps7
Obazoa - ps8
Opisthokonta - ps9
Holozoa - ps10
Filozoa - ps11
Choano./Metazoa - ps12
Metazoa - ps13
Myriazoa - ps14
Parahoxozoa - ps15
Bilateria - ps16
Protostomia - ps17
Ecdysozoa - ps18
Arthropoda - ps19
Mandibulata - ps20
Pancrustacea - ps21
Insecta - ps22
Eumetabola - ps23
Holometabola/Psocodea - ps24
Holometabola - ps25
Aparaglossata - ps26
Mecopterida - ps27
Diptera - ps28
Bibiono./Cyclorrhapha - ps29
Cyclorrhapha - ps30
Calyptratae/Drosophila - ps31
Drosophila - ps32
Sophophora - ps33
Obscura/Melanogaster_group - ps34
D_melanogaster_group - ps35
D_melanogaster_subgroup - ps36
sim./sech./mel. group - ps37
Drosophila_melanogaster - ps38
Phylostratum

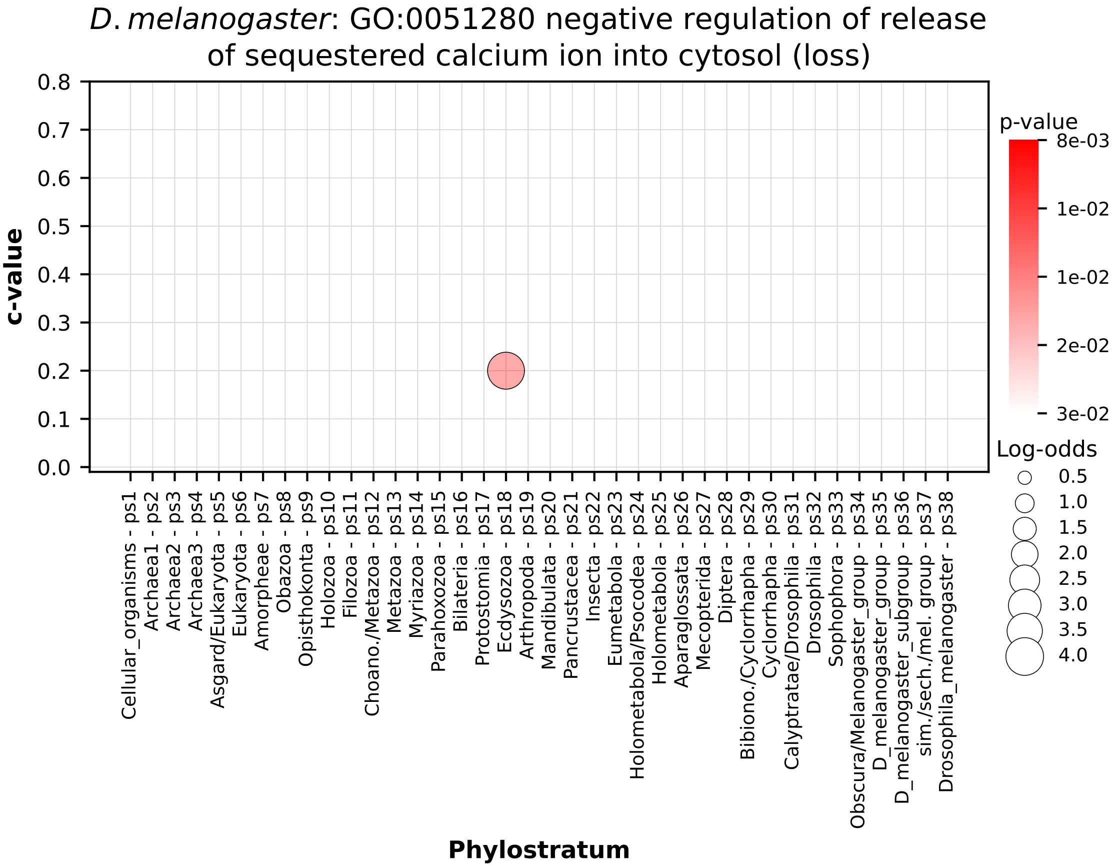

D. melanogaster: GO:0051280 negative regulation of release of sequestered calcium ion into cytosol (loss)
c-value
0.0
0.1
0.2
0.3
0.4
0.5
0.6
0.7
0.8
Phylostratum
Cellular_organisms - ps1
Archaea1 - ps2
Archaea2 - ps3
Archaea3 - ps4
Asgard/Eukaryota - ps5
Eukaryota - ps6
Amorpheae - ps7
Obazoa - ps8
Opisthokonta - ps9
Holozoa - ps10
Filozoa - ps11
Choano./Metazoa - ps12
Metazoa - ps13
Myriazoa - ps14
Parahoxozoa - ps15
Bilateria - ps16
Protostomia - ps17
Ecdysozoa - ps18
Arthropoda - ps19
Mandibulata - ps20
Pancrustacea - ps21
Insecta - ps22
Eumetabola - ps23
Holometabola/Psocodea - ps24
Holometabola - ps25
Aparaglossata - ps26
Mecopterida - ps27
Diptera - ps28
Bibiono./Cyclorrhapha - ps29
Cyclorrhapha - ps30
Calyptratae/Drosophila - ps31
Drosophila - ps32
Sophophora - ps33
Obscura/Melanogaster_group - ps34
D_melanogaster_group - ps35
D_melanogaster_subgroup - ps36
sim./sech./mel. group - ps37
Drosophila_melanogaster - ps38
p-value
8e-03
1e-02
1e-02
2e-02
3e-02
Log-odds
0.5
1.0
1.5
2.0
2.5
3.0
3.5
4.0

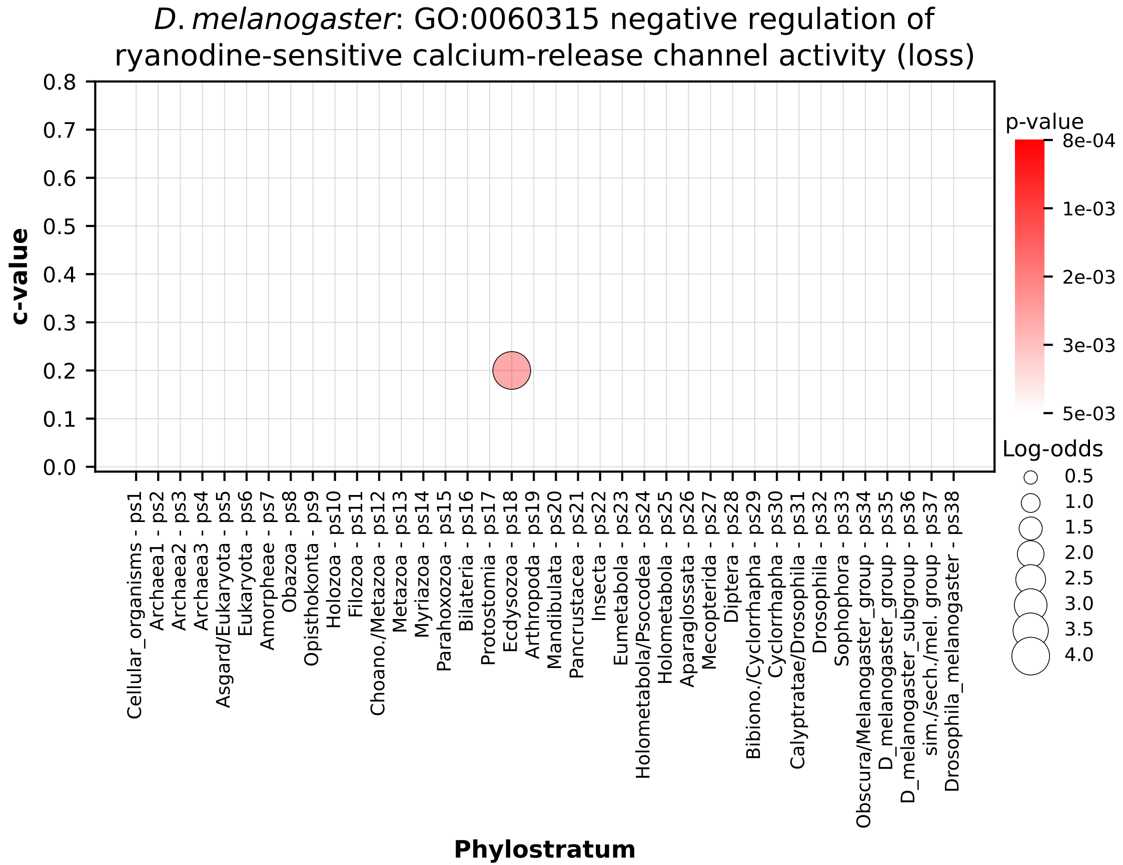

D. melanogaster: GO:0060315 negative regulation of ryanodine-sensitive calcium-release channel activity (loss)
c-value
0.0
0.1
0.2
0.3
0.4
0.5
0.6
0.7
0.8
Phylostratum
Cellular_organisms - ps1
Archaea1 - ps2
Archaea2 - ps3
Archaea3 - ps4
Asgard/Eukaryota - ps5
Eukaryota - ps6
Amorpheae - ps7
Obazoa - ps8
Opisthokonta - ps9
Holozoa - ps10
Filozoa - ps11
Choano./Metazoa - ps12
Metazoa - ps13
Myriazoa - ps14
Parahoxozoa - ps15
Bilateria - ps16
Protostomia - ps17
Ecdysozoa - ps18
Arthropoda - ps19
Mandibulata - ps20
Pancrustacea - ps21
Insecta - ps22
Eumetabola - ps23
Holometabola/Psocodea - ps24
Holometabola - ps25
Aparaglossata - ps26
Mecopterida - ps27
Diptera - ps28
Bibiono./Cyclorrhapha - ps29
Cyclorrhapha - ps30
Calyptratae/Drosophila - ps31
Drosophila - ps32
Sophophora - ps33
Obscura/Melanogaster_group - ps34
D_melanogaster_group - ps35
D_melanogaster_subgroup - ps36
sim./sech./mel. group - ps37
Drosophila_melanogaster - ps38
p-value
8e-04
1e-03
2e-03
3e-03
5e-03
Log-odds
0.5
1.0
1.5
2.0
2.5
3.0
3.5
4.0

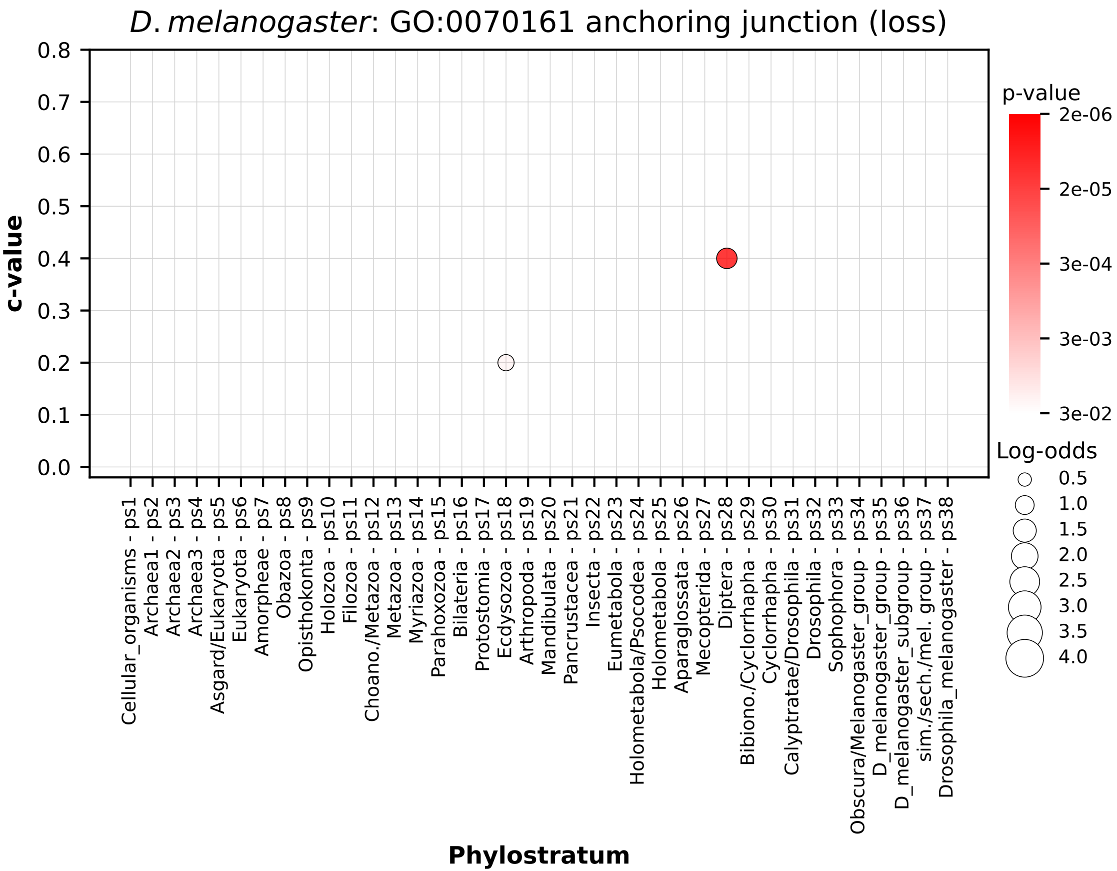

D. melanogaster: GO:0070161 anchoring junction (loss)
c-value
0.0
0.1
0.2
0.3
0.4
0.5
0.6
0.7
0.8
p-value
2e-06
2e-05
3e-04
3e-03
3e-02
Log-odds
0.5
1.0
1.5
2.0
2.5
3.0
3.5
4.0
Cellular_organisms - ps1
Archaea1 - ps2
Archaea2 - ps3
Archaea3 - ps4
Asgard/Eukaryota - ps5
Eukaryota - ps6
Amorpheae - ps7
Obazoa - ps8
Opisthokonta - ps9
Holozoa - ps10
Filozoa - ps11
Choano./Metazoa - ps12
Metazoa - ps13
Myriazoa - ps14
Parahoxozoa - ps15
Bilateria - ps16
Protostomia - ps17
Ecdysozoa - ps18
Arthropoda - ps19
Mandibulata - ps20
Pancrustacea - ps21
Insecta - ps22
Eumetabola - ps23
Holometabola/Psocodea - ps24
Holometabola - ps25
Aparaglossata - ps26
Mecopterida - ps27
Diptera - ps28
Bibiono./Cyclorrhapha - ps29
Cyclorrhapha - ps30
Calyptratae/Drosophila - ps31
Drosophila - ps32
Sophophora - ps33
Obscura/Melanogaster_group - ps34
D_melanogaster_group - ps35
D_melanogaster_subgroup - ps36
sim./sech./mel. group - ps37
Drosophila_melanogaster - ps38
Phylostratum

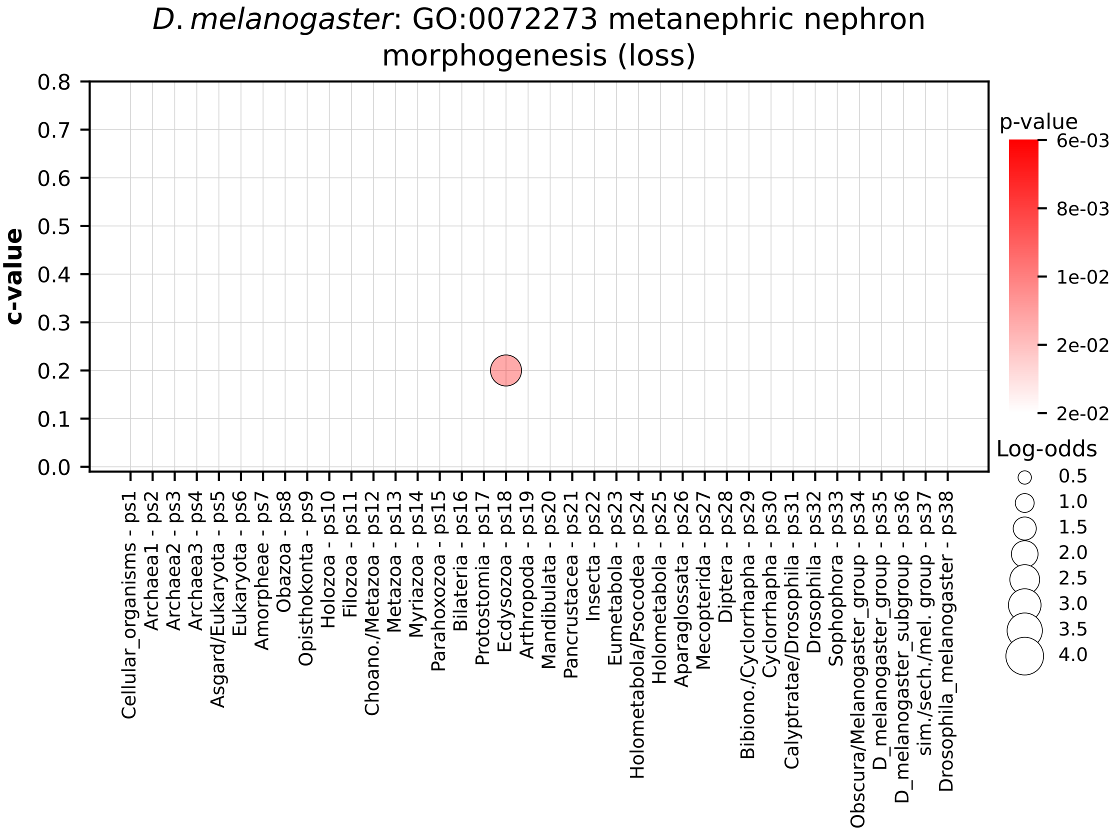
D. melanogaster: GO:0072273 metanephric nephron morphogenesis (loss)
c-value
0.0
0.1
0.2
0.3
0.4
0.5
0.6
0.7
0.8
Phylostratum
Cellular_organisms - ps1
Archaea1 - ps2
Archaea2 - ps3
Archaea3 - ps4
Asgard/Eukaryota - ps5
Eukaryota - ps6
Amorpheae - ps7
Obazoa - ps8
Opisthokonta - ps9
Holozoa - ps10
Filozoa - ps11
Choano./Metazoa - ps12
Metazoa - ps13
Myriazoa - ps14
Parahoxozoa - ps15
Bilateria - ps16
Protostomia - ps17
Ecdysozoa - ps18
Arthropoda - ps19
Mandibulata - ps20
Pancrustacea - ps21
Insecta - ps22
Eumetabola - ps23
Holometabola/Psocodea - ps24
Holometabola - ps25
Aparaglossata - ps26
Mecopterida - ps27
Diptera - ps28
Bibiono./Cyclorrhapha - ps29
Cyclorrhapha - ps30
Calyptratae/Drosophila - ps31
Drosophila - ps32
Sophophora - ps33
Obscura/Melanogaster_group - ps34
D_melanogaster_group - ps35
D_melanogaster_subgroup - ps36
sim./sech./mel. group - ps37
Drosophila_melanogaster - ps38
p-value
6e-03
8e-03
1e-02
2e-02
2e-02
Log-odds
0.5
1.0
1.5
2.0
2.5
3.0
3.5
4.0

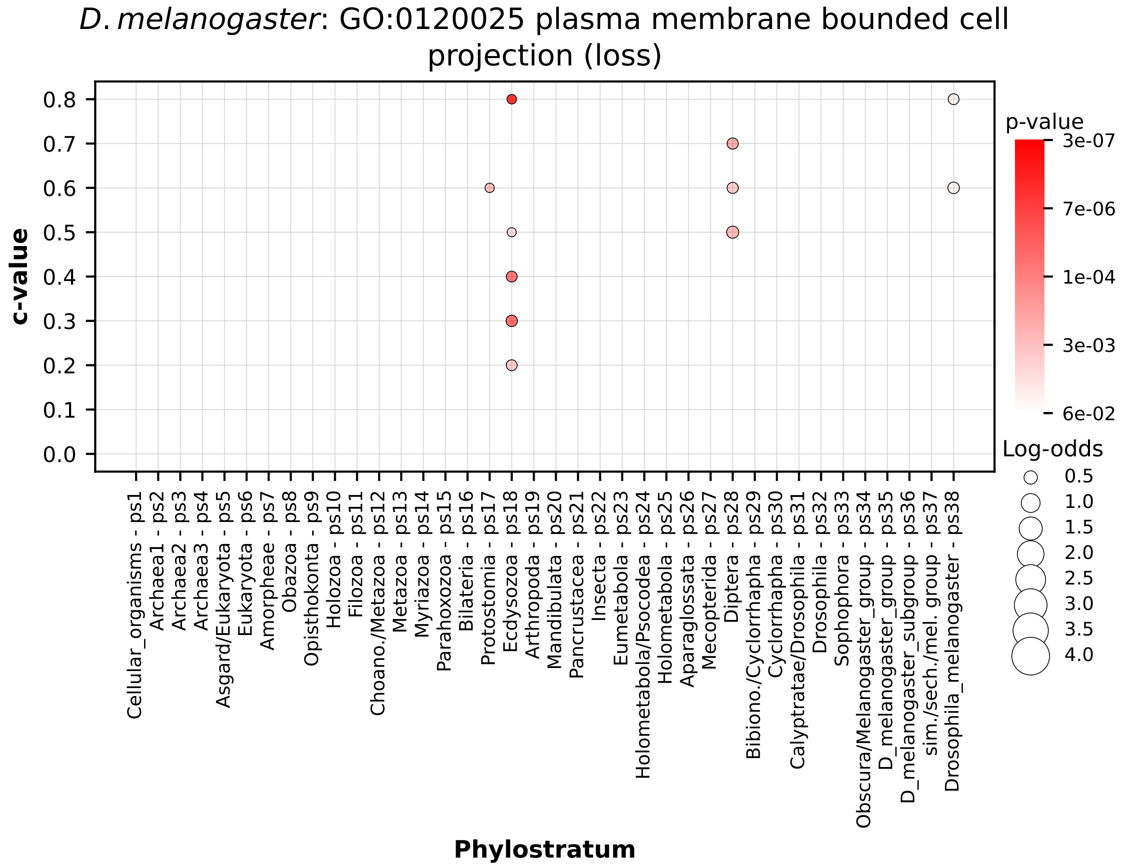

D. melanogaster: GO:0120025 plasma membrane bounded cell projection (loss)
c-value
Phylostratum
p-value
3e-07
7e-06
1e-04
3e-03
6e-02
Log-odds
0.5
1.0
1.5
2.0
2.5
3.0
3.5
4.0
Cellular_organisms - ps1
Archaea1 - ps2
Archaea2 - ps3
Archaea3 - ps4
Asgard/Eukaryota - ps5
Eukaryota - ps6
Amorpheae - ps7
Obazoa - ps8
Opisthokonta - ps9
Holozoa - ps10
Filozoa - ps11
Choano./Metazoa - ps12
Metazoa - ps13
Myriazoa - ps14
Parahoxozoa - ps15
Bilateria - ps16
Protostomia - ps17
Ecdysozoa - ps18
Arthropoda - ps19
Mandibulata - ps20
Pancrustacea - ps21
Insecta - ps22
Eumetabola - ps23
Holometabola/Psocodea - ps24
Holometabola - ps25
Aparaglossata - ps26
Mecopterida - ps27
Diptera - ps28
Bibiono./Cyclorrhapha - ps29
Cyclorrhapha - ps30
Calyptratae/Drosophila - ps31
Drosophila - ps32
Sophophora - ps33
Obscura/Melanogaster_group - ps34
D_melanogaster_group - ps35
D_melanogaster_subgroup - ps36
sim./sech./mel. group - ps37
Drosophila_melanogaster - ps38

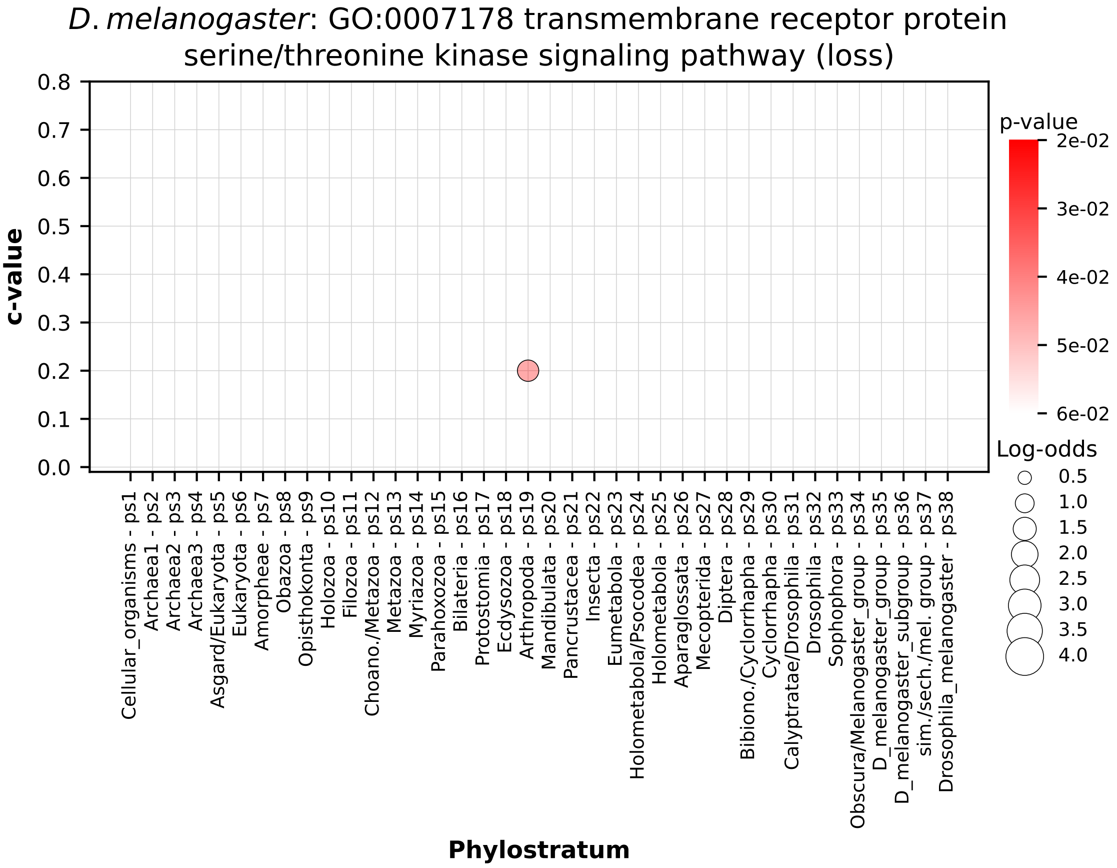

D. melanogaster: GO:0007178 transmembrane receptor protein serine/threonine kinase signaling pathway (loss)
c-value
0.8
0.7
0.6
0.5
0.4
0.3
0.2
0.1
0.0
Phylostratum
Cellular_organisms - ps1
Archaea1 - ps2
Archaea2 - ps3
Archaea3 - ps4
Asgard/Eukaryota - ps5
Eukaryota - ps6
Amorpheae - ps7
Obazoa - ps8
Opisthokonta - ps9
Holozoa - ps10
Filozoa - ps11
Choano./Metazoa - ps12
Metazoa - ps13
Myriazoa - ps14
Parahoxozoa - ps15
Bilateria - ps16
Protostomia - ps17
Ecdysozoa - ps18
Arthropoda - ps19
Mandibulata - ps20
Pancrustacea - ps21
Insecta - ps22
Eumetabola - ps23
Holometabola/Psocodea - ps24
Holometabola - ps25
Aparaglossata - ps26
Mecopterida - ps27
Diptera - ps28
Bibiono./Cyclorrhapha - ps29
Cyclorrhapha - ps30
Calyptratae/Drosophila - ps31
Drosophila - ps32
Sophophora - ps33
Obscura/Melanogaster_group - ps34
D_melanogaster_group - ps35
D_melanogaster_subgroup - ps36
sim./sech./mel. group - ps37
Drosophila_melanogaster - ps38
p-value
2e-02
3e-02
4e-02
5e-02
6e-02
Log-odds
0.5
1.0
1.5
2.0
2.5
3.0
3.5
4.0

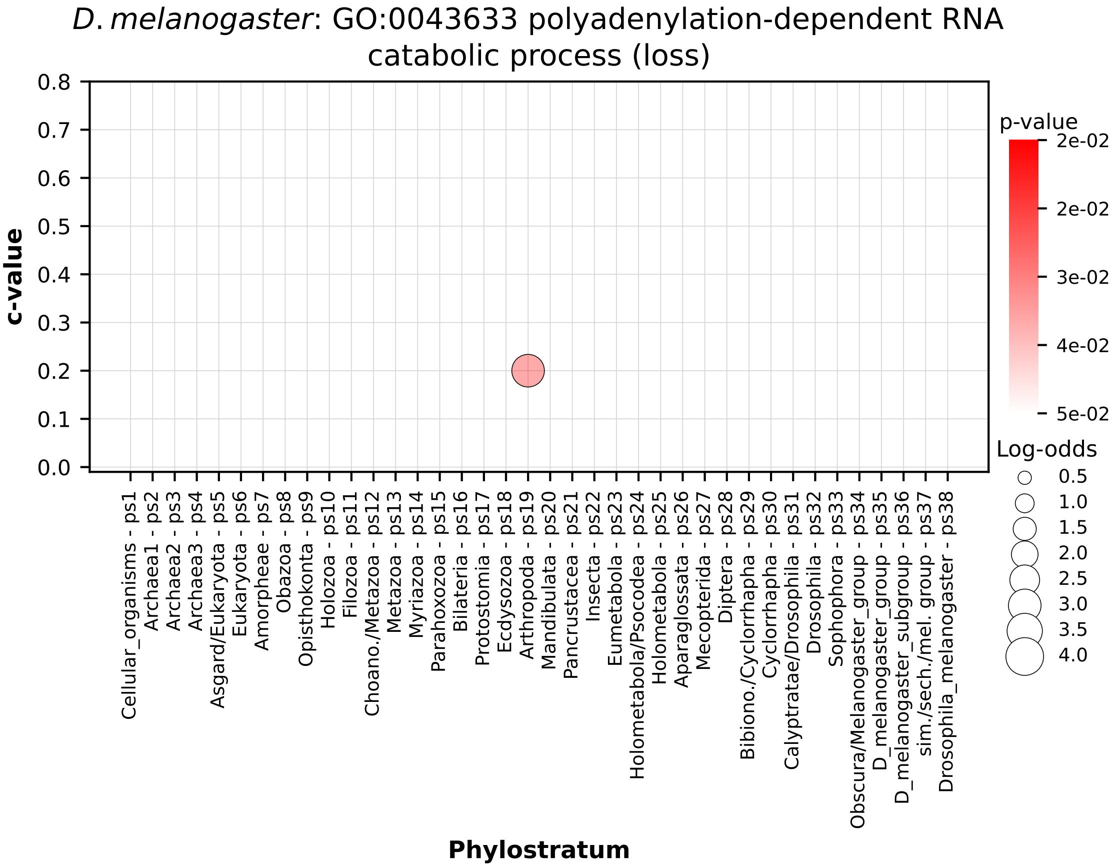

D. melanogaster: GO:0043633 polyadenylation-dependent RNA catabolic process (loss)
c-value
0.8
0.7
0.6
0.5
0.4
0.3
0.2
0.1
0.0
Phylostratum
Cellular_organisms - ps1
Archaea1 - ps2
Archaea2 - ps3
Archaea3 - ps4
Asgard/Eukaryota - ps5
Eukaryota - ps6
Amorpheae - ps7
Obazoa - ps8
Opisthokonta - ps9
Holozoa - ps10
Filozoa - ps11
Choano./Metazoa - ps12
Metazoa - ps13
Myriazoa - ps14
Parahoxozoa - ps15
Bilateria - ps16
Protostomia - ps17
Ecdysozoa - ps18
Arthropoda - ps19
Mandibulata - ps20
Pancrustacea - ps21
Insecta - ps22
Eumetabola - ps23
Holometabola/Psocodea - ps24
Holometabola - ps25
Aparaglossata - ps26
Mecopterida - ps27
Diptera - ps28
Bibiono./Cyclorrhapha - ps29
Cyclorrhapha - ps30
Calyptratae/Drosophila - ps31
Drosophila - ps32
Sophophora - ps33
Obscura/Melanogaster_group - ps34
D_melanogaster_group - ps35
D_melanogaster_subgroup - ps36
sim./sech./mel. group - ps37
Drosophila_melanogaster - ps38
p-value
2e-02
2e-02
3e-02
4e-02
5e-02
Log-odds
0.5
1.0
1.5
2.0
2.5
3.0
3.5
4.0

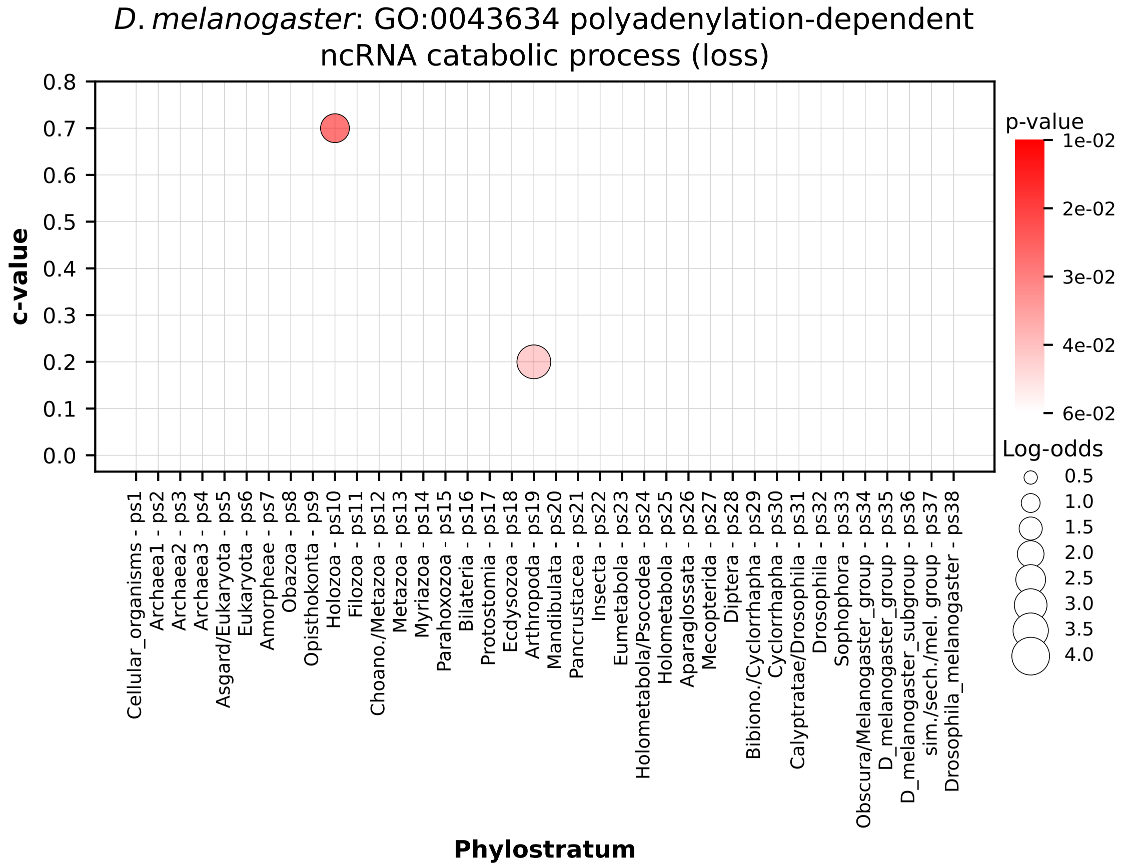

$D. melanogaster$: GO:0043634 polyadenylation-dependent ncRNA catabolic process (loss)
c-value
0.8
0.7
0.6
0.5
0.4
0.3
0.2
0.1
0.0
Phylostratum
Cellular_organisms - ps1
Archaea1 - ps2
Archaea2 - ps3
Archaea3 - ps4
Asgard/Eukaryota - ps5
Eukaryota - ps6
Amorpheae - ps7
Obazoa - ps8
Opisthokonta - ps9
Holozoa - ps10
Filozoa - ps11
Choano./Metazoa - ps12
Metazoa - ps13
Myriazoa - ps14
Parahoxozoa - ps15
Bilateria - ps16
Protostomia - ps17
Ecdysozoa - ps18
Arthropoda - ps19
Mandibulata - ps20
Pancrustacea - ps21
Insecta - ps22
Eumetabola - ps23
Holometabola/Psocodea - ps24
Holometabola - ps25
Aparaglossata - ps26
Mecopterida - ps27
Diptera - ps28
Bibiono./Cyclorrhapha - ps29
Cyclorrhapha - ps30
Calyptratae/Drosophila - ps31
Drosophila - ps32
Sophophora - ps33
Obscura/Melanogaster_group - ps34
D_melanogaster_group - ps35
D_melanogaster_subgroup - ps36
sim./sech./mel. group - ps37
Drosophila_melanogaster - ps38
p-value
1e-02
2e-02
3e-02
4e-02
6e-02
Log-odds
0.5
1.0
1.5
2.0
2.5
3.0
3.5
4.0

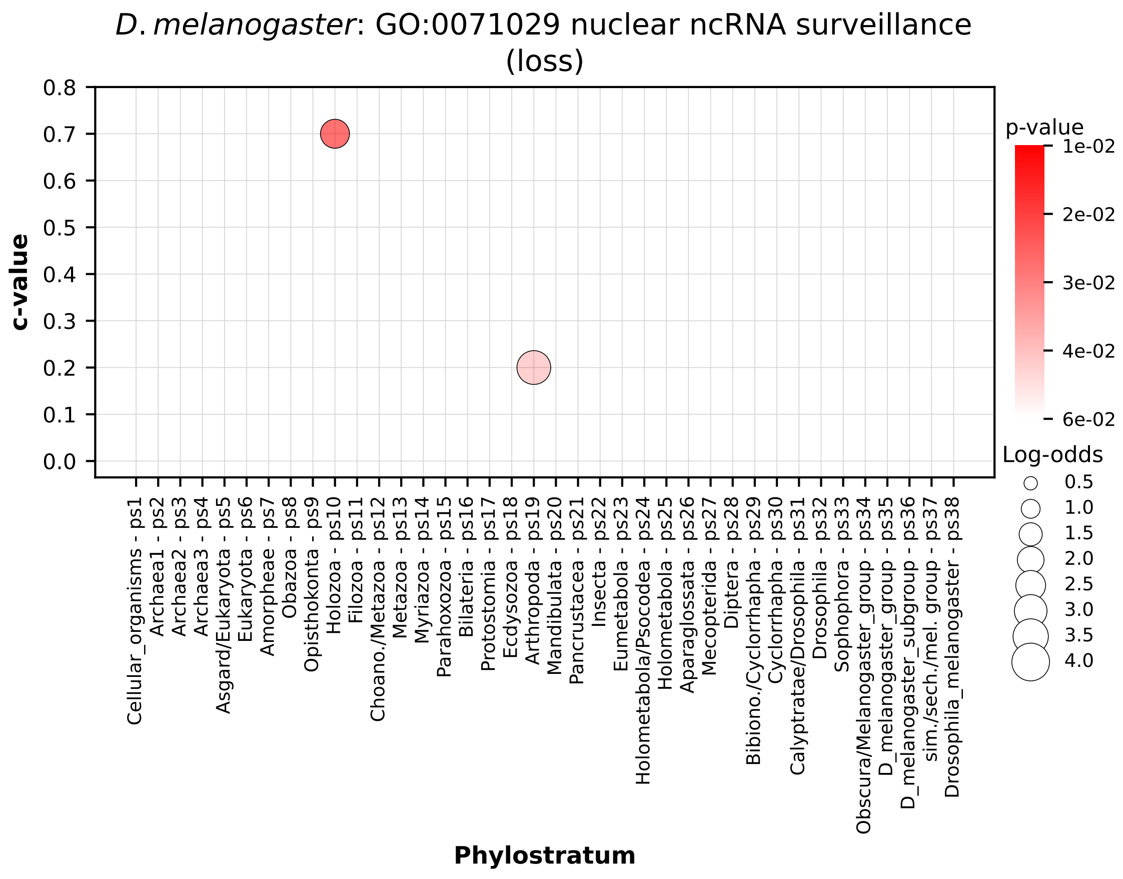

D. melanogaster: GO:0071029 nuclear ncRNA surveillance (loss)
c-value
0.8
0.7
0.6
0.5
0.4
0.3
0.2
0.1
0.0
Phylostratum
Cellular_organisms - ps1
Archaea1 - ps2
Archaea2 - ps3
Archaea3 - ps4
Asgard/Eukaryota - ps5
Eukaryota - ps6
Amorpheae - ps7
Obazoa - ps8
Opisthokonta - ps9
Holozoa - ps10
Filozoa - ps11
Choano./Metazoa - ps12
Metazoa - ps13
Myriazoa - ps14
Parahoxozoa - ps15
Bilateria - ps16
Protostomia - ps17
Ecdysozoa - ps18
Arthropoda - ps19
Mandibulata - ps20
Pancrustacea - ps21
Insecta - ps22
Eumetabola - ps23
Holometabola/Psocodea - ps24
Holometabola - ps25
Aparaglossata - ps26
Mecopterida - ps27
Diptera - ps28
Bibiono./Cyclorrhapha - ps29
Cyclorrhapha - ps30
Calyptratae/Drosophila - ps31
Drosophila - ps32
Sophophora - ps33
Obscura/Melanogaster_group - ps34
D_melanogaster_group - ps35
D_melanogaster_subgroup - ps36
sim./sech./mel. group - ps37
Drosophila_melanogaster - ps38
p-value
1e-02
2e-02
3e-02
4e-02
6e-02
Log-odds
0.5
1.0
1.5
2.0
2.5
3.0
3.5
4.0

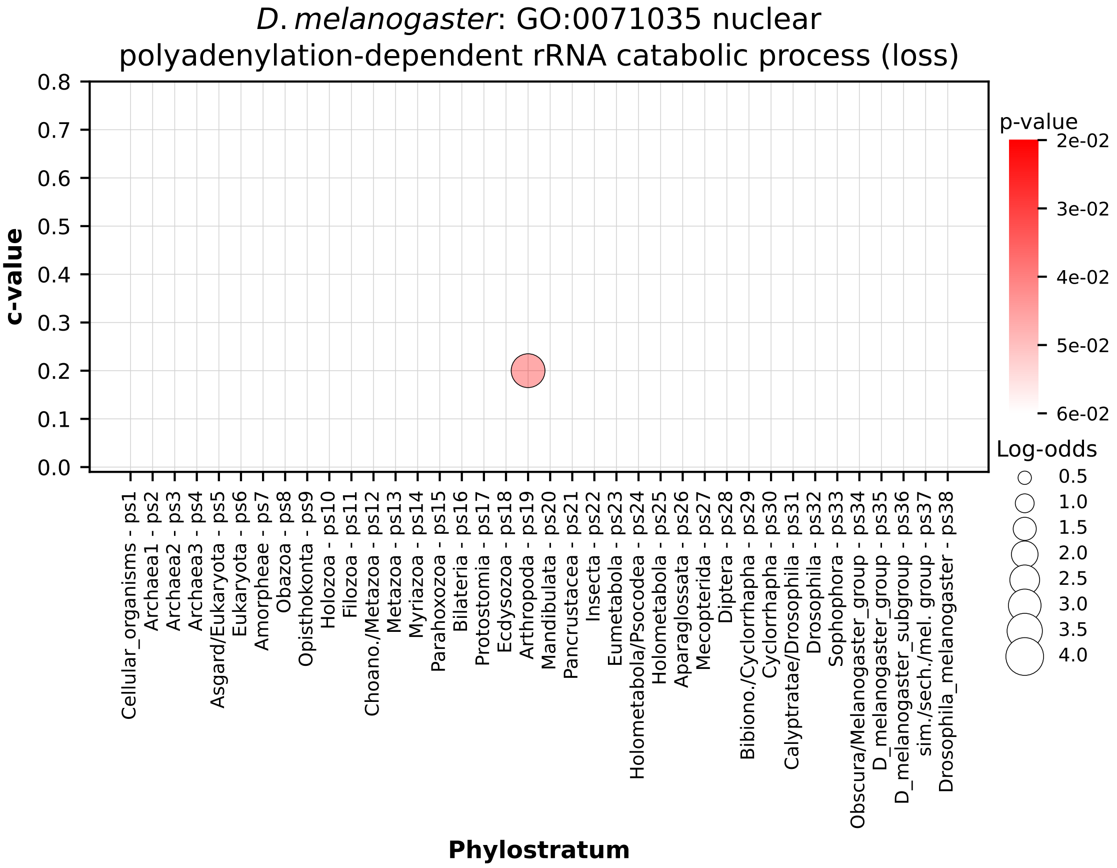

D. melanogaster: GO:0071035 nuclear polyadenylation-dependent rRNA catabolic process (loss)
c-value
0.8
0.7
0.6
0.5
0.4
0.3
0.2
0.1
0.0
p-value
2e-02
3e-02
4e-02
5e-02
6e-02
Log-odds
0.5
1.0
1.5
2.0
2.5
3.0
3.5
4.0
Cellular_organisms - ps1
Archaea1 - ps2
Archaea2 - ps3
Archaea3 - ps4
Asgard/Eukaryota - ps5
Eukaryota - ps6
Amorpheae - ps7
Obazoa - ps8
Opisthokonta - ps9
Holozoa - ps10
Filozoa - ps11
Choano./Metazoa - ps12
Metazoa - ps13
Myriazoa - ps14
Parahoxozoa - ps15
Bilateria - ps16
Protostomia - ps17
Ecdysozoa - ps18
Arthropoda - ps19
Mandibulata - ps20
Pancrustacea - ps21
Insecta - ps22
Eumetabola - ps23
Holometabola/Psocodea - ps24
Holometabola - ps25
Aparaglossata - ps26
Mecopterida - ps27
Diptera - ps28
Bibiono./Cyclorrhapha - ps29
Cyclorrhapha - ps30
Calyptratae/Drosophila - ps31
Drosophila - ps32
Sophophora - ps33
Obscura/Melanogaster_group - ps34
D_melanogaster_group - ps35
D_melanogaster_subgroup - ps36
sim./sech./mel. group - ps37
Drosophila_melanogaster - ps38
Phylostratum

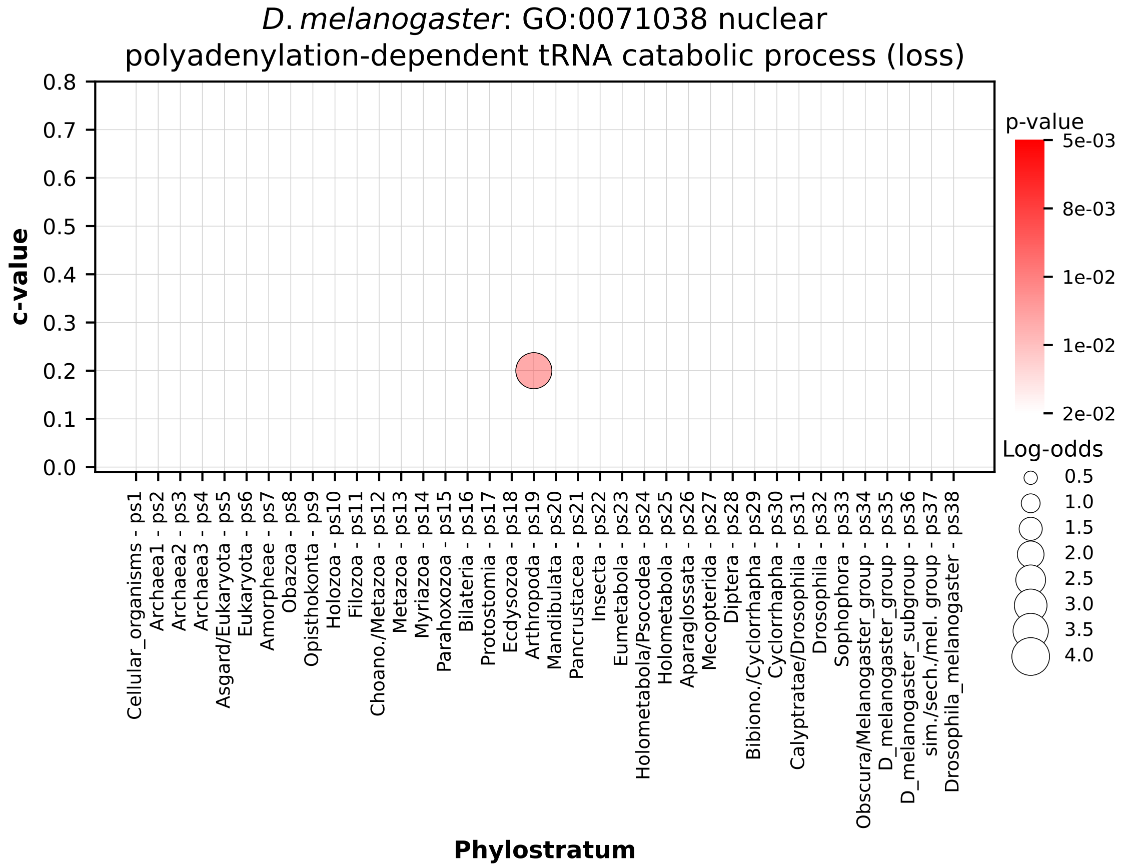

D. melanogaster: GO:0071038 nuclear polyadenylation-dependent tRNA catabolic process (loss)
c-value
0.8
0.7
0.6
0.5
0.4
0.3
0.2
0.1
0.0
Phylostratum
Cellular_organisms - ps1
Archaea1 - ps2
Archaea2 - ps3
Archaea3 - ps4
Asgard/Eukaryota - ps5
Eukaryota - ps6
Amorpheae - ps7
Obazoa - ps8
Opisthokonta - ps9
Holozoa - ps10
Filozoa - ps11
Choano./Metazoa - ps12
Metazoa - ps13
Myriazoa - ps14
Parahoxozoa - ps15
Bilateria - ps16
Protostomia - ps17
Ecdysozoa - ps18
Arthropoda - ps19
Mandibulata - ps20
Pancrustacea - ps21
Insecta - ps22
Eumetabola - ps23
Holometabola/Psocodea - ps24
Holometabola - ps25
Aparaglossata - ps26
Mecopterida - ps27
Diptera - ps28
Bibiono./Cyclorrhapha - ps29
Cyclorrhapha - ps30
Calyptratae/Drosophila - ps31
Drosophila - ps32
Sophophora - ps33
Obscura/Melanogaster_group - ps34
D_melanogaster_group - ps35
D_melanogaster_subgroup - ps36
sim./sech./mel. group - ps37
Drosophila_melanogaster - ps38
p-value
5e-03
8e-03
1e-02
1e-02
2e-02
Log-odds
0.5
1.0
1.5
2.0
2.5
3.0
3.5
4.0

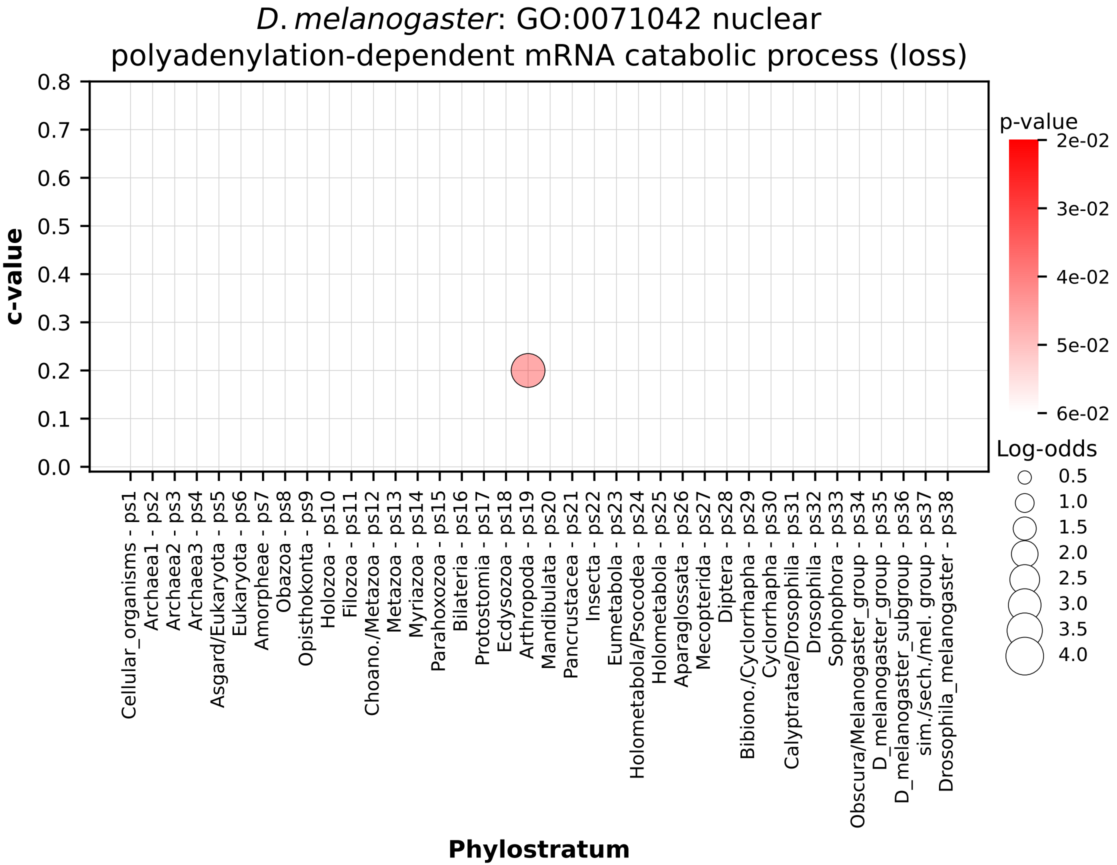

D. melanogaster: GO:0071042 nuclear polyadenylation-dependent mRNA catabolic process (loss)
c-value
p-value
2e-02
3e-02
4e-02
5e-02
6e-02
Log-odds
0.5
1.0
1.5
2.0
2.5
3.0
3.5
4.0
Cellular_organisms - ps1
Archaea1 - ps2
Archaea2 - ps3
Archaea3 - ps4
Asgard/Eukaryota - ps5
Eukaryota - ps6
Amorpheae - ps7
Obazoa - ps8
Opisthokonta - ps9
Holozoa - ps10
Filozoa - ps11
Choano./Metazoa - ps12
Metazoa - ps13
Myriazoa - ps14
Parahoxozoa - ps15
Bilateria - ps16
Protostomia - ps17
Ecdysozoa - ps18
Arthropoda - ps19
Mandibulata - ps20
Pancrustacea - ps21
Insecta - ps22
Eumetabola - ps23
Holometabola/Psocodea - ps24
Holometabola - ps25
Aparaglossata - ps26
Mecopterida - ps27
Diptera - ps28
Bibiono./Cyclorrhapha - ps29
Cyclorrhapha - ps30
Calyptratae/Drosophila - ps31
Drosophila - ps32
Sophophora - ps33
Obscura/Melanogaster_group - ps34
D_melanogaster_group - ps35
D_melanogaster_subgroup - ps36
sim./sech./mel. group - ps37
Drosophila_melanogaster - ps38
Phylostratum

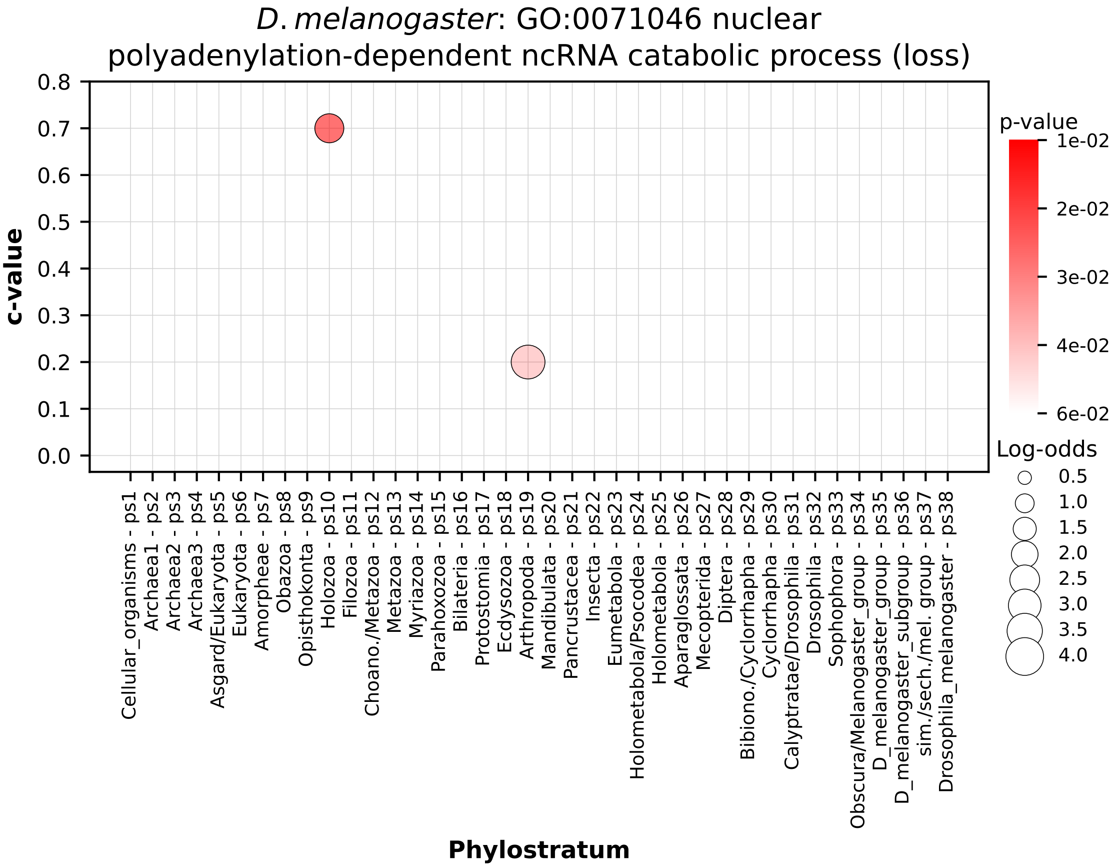
D. melanogaster: GO:0071046 nuclear polyadenylation-dependent ncRNA catabolic process (loss)
c-value
0.8
0.7
0.6
0.5
0.4
0.3
0.2
0.1
0.0
Phylostratum
Cellular_organisms - ps1
Archaea1 - ps2
Archaea2 - ps3
Archaea3 - ps4
Asgard/Eukaryota - ps5
Eukaryota - ps6
Amorpheae - ps7
Obazoa - ps8
Opisthokonta - ps9
Holozoa - ps10
Filozoa - ps11
Choano./Metazoa - ps12
Metazoa - ps13
Myriazoa - ps14
Parahoxozoa - ps15
Bilateria - ps16
Protostomia - ps17
Ecdysozoa - ps18
Arthropoda - ps19
Mandibulata - ps20
Pancrustacea - ps21
Insecta - ps22
Eumetabola - ps23
Holometabola/Psocodea - ps24
Holometabola - ps25
Aparaglossata - ps26
Mecopterida - ps27
Diptera - ps28
Bibiono./Cyclorrhapha - ps29
Cyclorrhapha - ps30
Calyptratae/Drosophila - ps31
Drosophila - ps32
Sophophora - ps33
Obscura/Melanogaster_group - ps34
D_melanogaster_group - ps35
D_melanogaster_subgroup - ps36
sim./sech./mel. group - ps37
Drosophila_melanogaster - ps38
p-value
1e-02
2e-02
3e-02
4e-02
6e-02
Log-odds
0.5
1.0
1.5
2.0
2.5
3.0
3.5
4.0

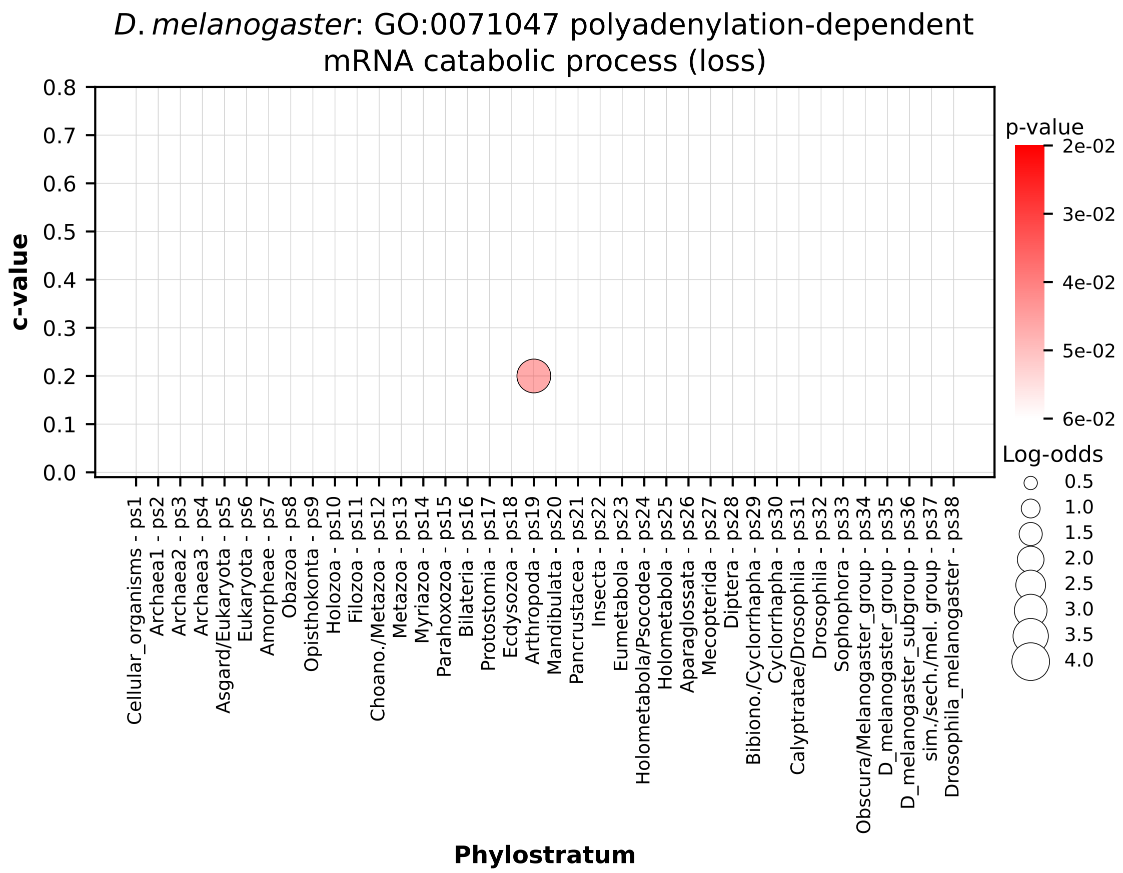
D. melanogaster: GO:0071047 polyadenylation-dependent mRNA catabolic process (loss)
c-value
0.0
0.1
0.2
0.3
0.4
0.5
0.6
0.7
0.8
Phylostratum
Cellular_organisms - ps1
Archaea1 - ps2
Archaea2 - ps3
Archaea3 - ps4
Asgard/Eukaryota - ps5
Eukaryota - ps6
Amorpheae - ps7
Obazoa - ps8
Opisthokonta - ps9
Holozoa - ps10
Filozoa - ps11
Choano./Metazoa - ps12
Metazoa - ps13
Myriazoa - ps14
Parahoxozoa - ps15
Bilateria - ps16
Protostomia - ps17
Ecdysozoa - ps18
Arthropoda - ps19
Mandibulata - ps20
Pancrustacea - ps21
Insecta - ps22
Eumetabola - ps23
Holometabola/Psocodea - ps24
Holometabola - ps25
Aparaglossata - ps26
Mecopterida - ps27
Diptera - ps28
Bibiono./Cyclorrhapha - ps29
Cyclorrhapha - ps30
Calyptratae/Drosophila - ps31
Drosophila - ps32
Sophophora - ps33
Obscura/Melanogaster_group - ps34
D_melanogaster_group - ps35
D_melanogaster_subgroup - ps36
sim./sech./mel. group - ps37
Drosophila_melanogaster - ps38
p-value
2e-02
3e-02
4e-02
5e-02
6e-02
Log-odds
0.5
1.0
1.5
2.0
2.5
3.0
3.5
4.0

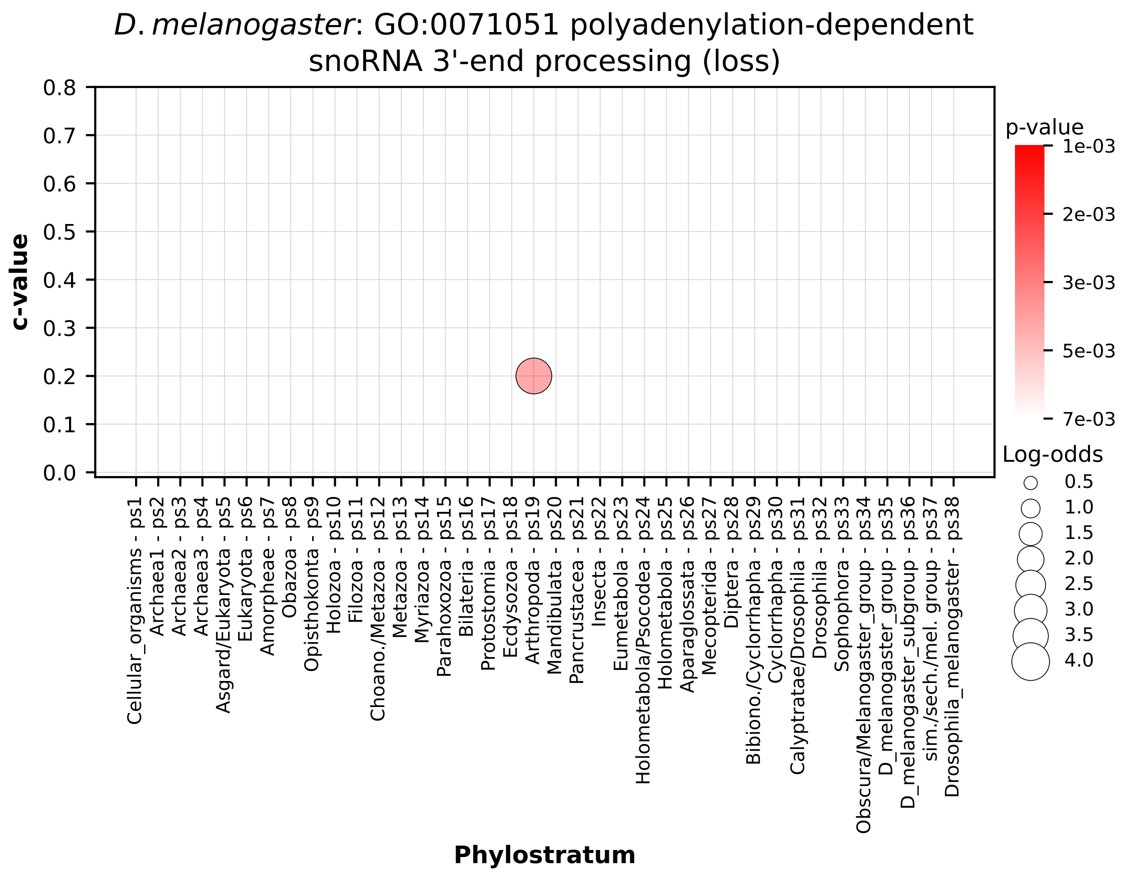

D. melanogaster: GO:0071051 polyadenylation-dependent snoRNA 3'-end processing (loss)
c-value
0.0
0.1
0.2
0.3
0.4
0.5
0.6
0.7
0.8
p-value
1e-03
2e-03
3e-03
5e-03
7e-03
Log-odds
0.5
1.0
1.5
2.0
2.5
3.0
3.5
4.0
Cellular_organisms - ps1
Archaea1 - ps2
Archaea2 - ps3
Archaea3 - ps4
Asgard/Eukaryota - ps5
Eukaryota - ps6
Amorpheae - ps7
Obazoa - ps8
Opisthokonta - ps9
Holozoa - ps10
Filozoa - ps11
Choano./Metazoa - ps12
Metazoa - ps13
Myriazoa - ps14
Parahoxozoa - ps15
Bilateria - ps16
Protostomia - ps17
Ecdysozoa - ps18
Arthropoda - ps19
Mandibulata - ps20
Pancrustacea - ps21
Insecta - ps22
Eumetabola - ps23
Holometabola/Psocodea - ps24
Holometabola - ps25
Aparaglossata - ps26
Mecopterida - ps27
Diptera - ps28
Bibiono./Cyclorrhapha - ps29
Cyclorrhapha - ps30
Calyptratae/Drosophila - ps31
Drosophila - ps32
Sophophora - ps33
Obscura/Melanogaster_group - ps34
D_melanogaster_group - ps35
D_melanogaster_subgroup - ps36
sim./sech./mel. group - ps37
Drosophila_melanogaster - ps38
Phylostratum

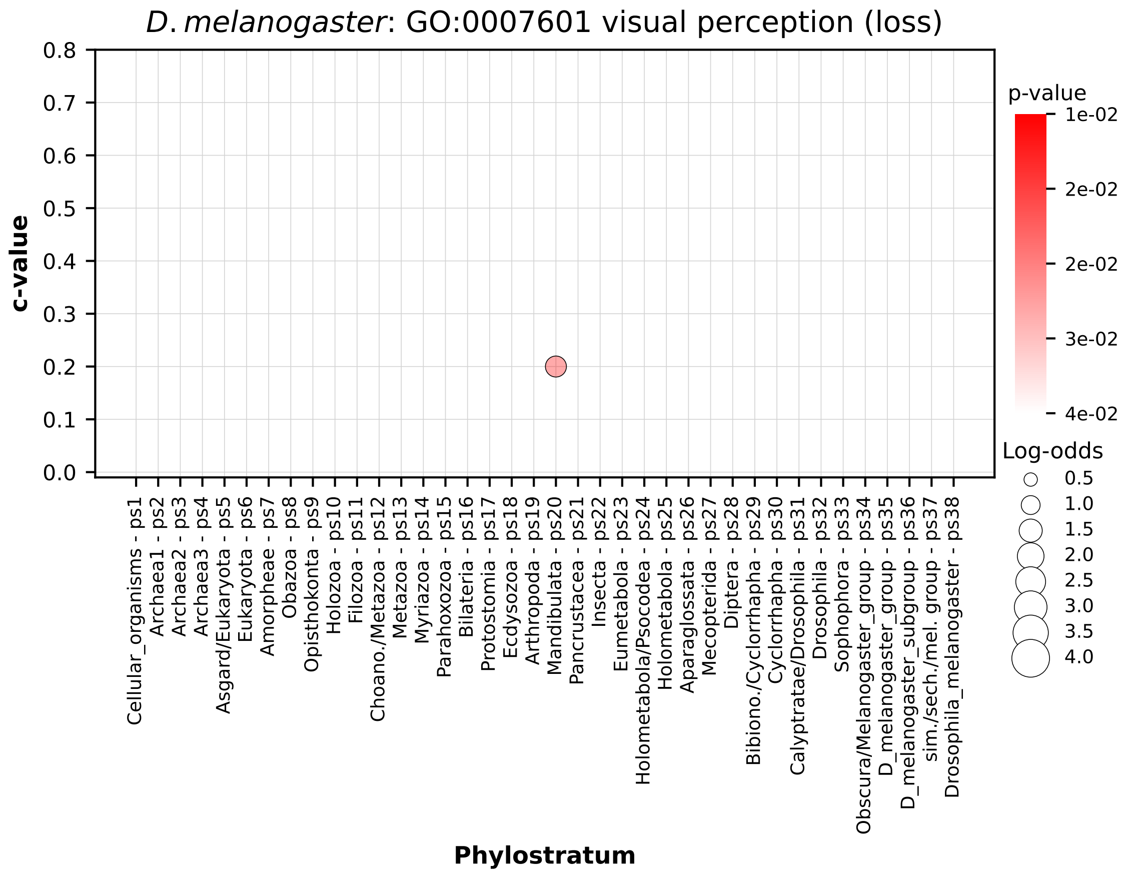

D. melanogaster: GO:0007601 visual perception (loss)
c-value
0.8
0.7
0.6
0.5
0.4
0.3
0.2
0.1
0.0
p-value
1e-02
2e-02
2e-02
3e-02
4e-02
Log-odds
0.5
1.0
1.5
2.0
2.5
3.0
3.5
4.0
Cellular_organisms - ps1
Archaea1 - ps2
Archaea2 - ps3
Archaea3 - ps4
Asgard/Eukaryota - ps5
Eukaryota - ps6
Amorpheae - ps7
Obazoa - ps8
Opisthokonta - ps9
Holozoa - ps10
Filozoa - ps11
Choano./Metazoa - ps12
Metazoa - ps13
Myriazoa - ps14
Parahoxozoa - ps15
Bilateria - ps16
Protostomia - ps17
Ecdysozoa - ps18
Arthropoda - ps19
Mandibulata - ps20
Pancrustacea - ps21
Insecta - ps22
Eumetabola - ps23
Holometabola/Psocodea - ps24
Holometabola - ps25
Aparaglossata - ps26
Mecopterida - ps27
Diptera - ps28
Bibiono./Cyclorrhapha - ps29
Cyclorrhapha - ps30
Calyptratae/Drosophila - ps31
Drosophila - ps32
Sophophora - ps33
Obscura/Melanogaster_group - ps34
D_melanogaster_group - ps35
D_melanogaster_subgroup - ps36
sim./sech./mel. group - ps37
Drosophila_melanogaster - ps38
Phylostratum

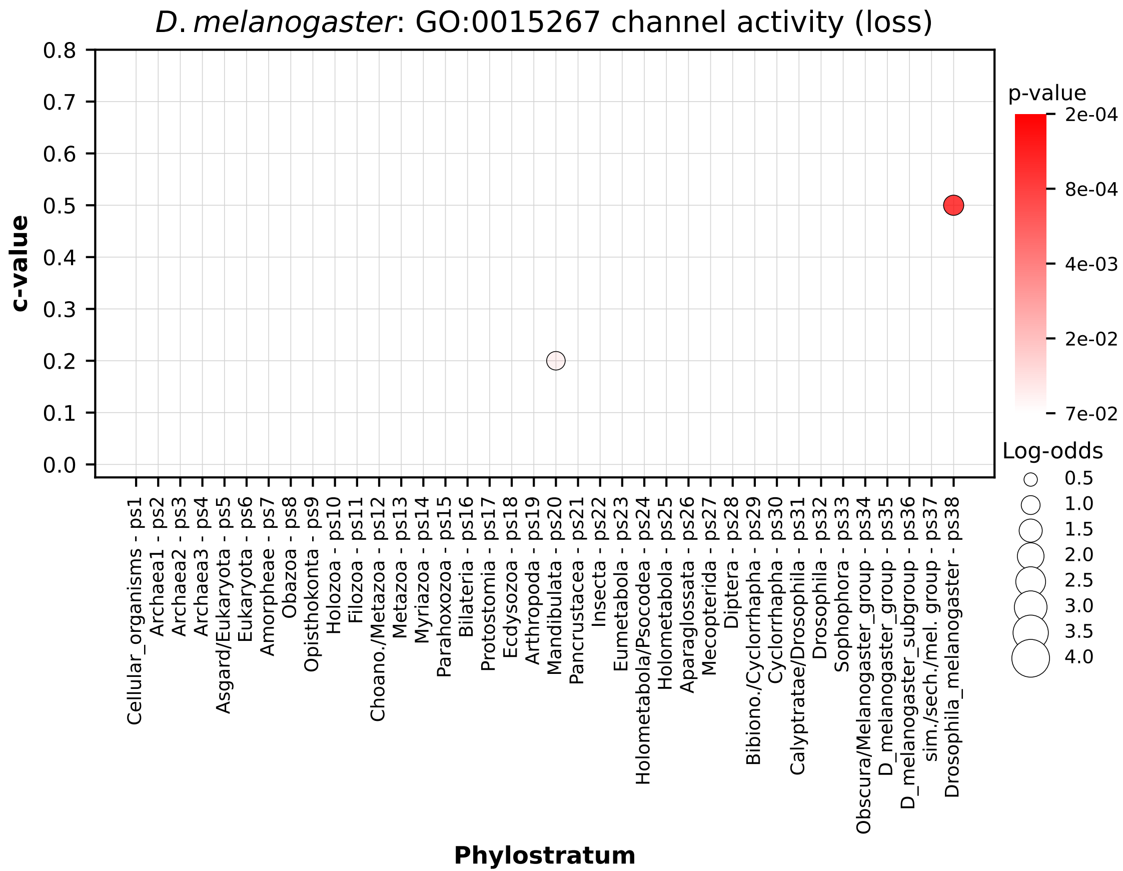

D. melanogaster: GO:0015267 channel activity (loss)
c-value
0.8
0.7
0.6
0.5
0.4
0.3
0.2
0.1
0.0
Phylostratum
Cellular_organisms - ps1
Archaea1 - ps2
Archaea2 - ps3
Archaea3 - ps4
Asgard/Eukaryota - ps5
Eukaryota - ps6
Amorpheae - ps7
Obazoa - ps8
Opisthokonta - ps9
Holozoa - ps10
Filozoa - ps11
Choano./Metazoa - ps12
Metazoa - ps13
Myriazoa - ps14
Parahoxozoa - ps15
Bilateria - ps16
Protostomia - ps17
Ecdysozoa - ps18
Arthropoda - ps19
Mandibulata - ps20
Pancrustacea - ps21
Insecta - ps22
Eumetabola - ps23
Holometabola/Psocodea - ps24
Holometabola - ps25
Aparaglossata - ps26
Mecopterida - ps27
Diptera - ps28
Bibiono./Cyclorrhapha - ps29
Cyclorrhapha - ps30
Calyptratae/Drosophila - ps31
Drosophila - ps32
Sophophora - ps33
Obscura/Melanogaster_group - ps34
D_melanogaster_group - ps35
D_melanogaster_subgroup - ps36
sim./sech./mel. group - ps37
Drosophila_melanogaster - ps38
p-value
2e-04
8e-04
4e-03
2e-02
7e-02
Log-odds
0.5
1.0
1.5
2.0
2.5
3.0
3.5
4.0

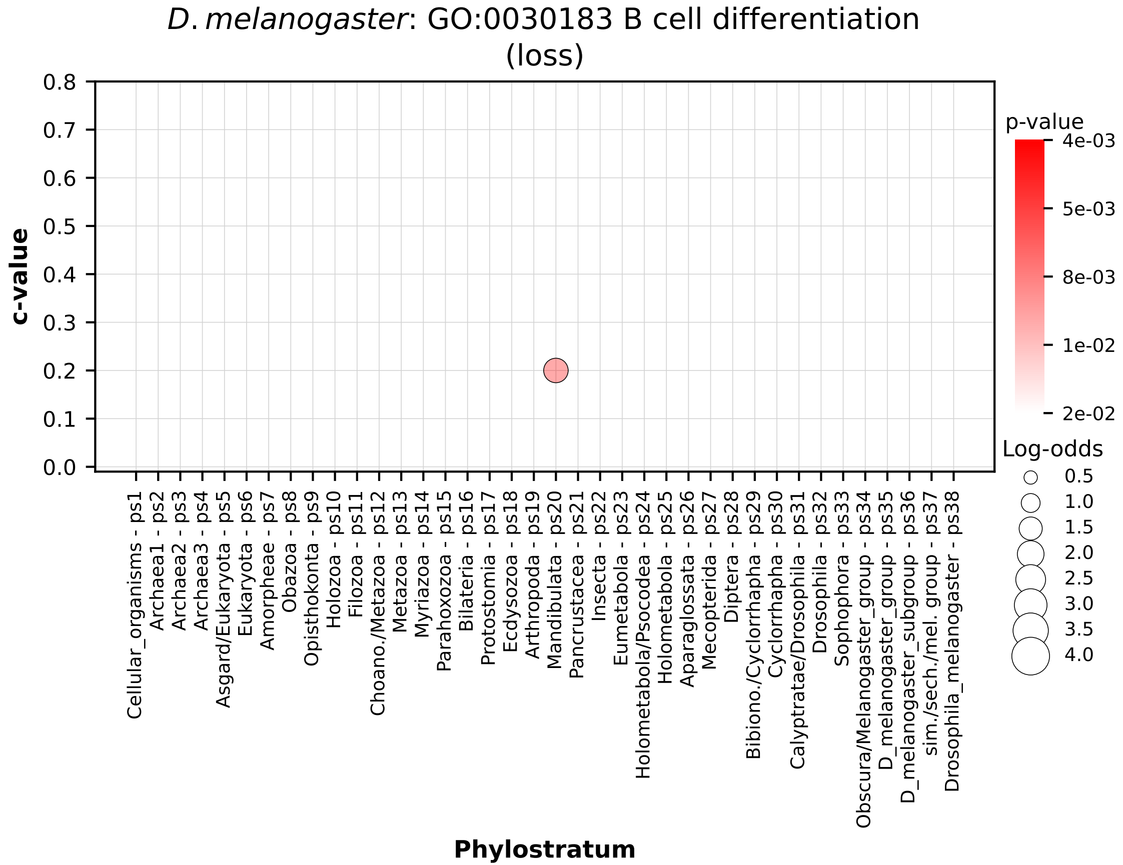

D. melanogaster: GO:0030183 B cell differentiation
(loss)
c-value
0.8
0.7
0.6
0.5
0.4
0.3
0.2
0.1
0.0
Phylostratum
Cellular_organisms - ps1
Archaea1 - ps2
Archaea2 - ps3
Archaea3 - ps4
Asgard/Eukaryota - ps5
Eukaryota - ps6
Amorpheae - ps7
Obazoa - ps8
Opisthokonta - ps9
Holozoa - ps10
Filozoa - ps11
Choano./Metazoa - ps12
Metazoa - ps13
Myriazoa - ps14
Parahoxozoa - ps15
Bilateria - ps16
Protostomia - ps17
Ecdysozoa - ps18
Arthropoda - ps19
Mandibulata - ps20
Pancrustacea - ps21
Insecta - ps22
Eumetabola - ps23
Holometabola/Psocodea - ps24
Holometabola - ps25
Aparaglossata - ps26
Mecopterida - ps27
Diptera - ps28
Bibiono./Cyclorrhapha - ps29
Cyclorrhapha - ps30
Calyptratae/Drosophila - ps31
Drosophila - ps32
Sophophora - ps33
Obscura/Melanogaster_group - ps34
D_melanogaster_group - ps35
D_melanogaster_subgroup - ps36
sim./sech./mel. group - ps37
Drosophila_melanogaster - ps38
p-value
4e-03
5e-03
8e-03
1e-02
2e-02
Log-odds
0.5
1.0
1.5
2.0
2.5
3.0
3.5
4.0

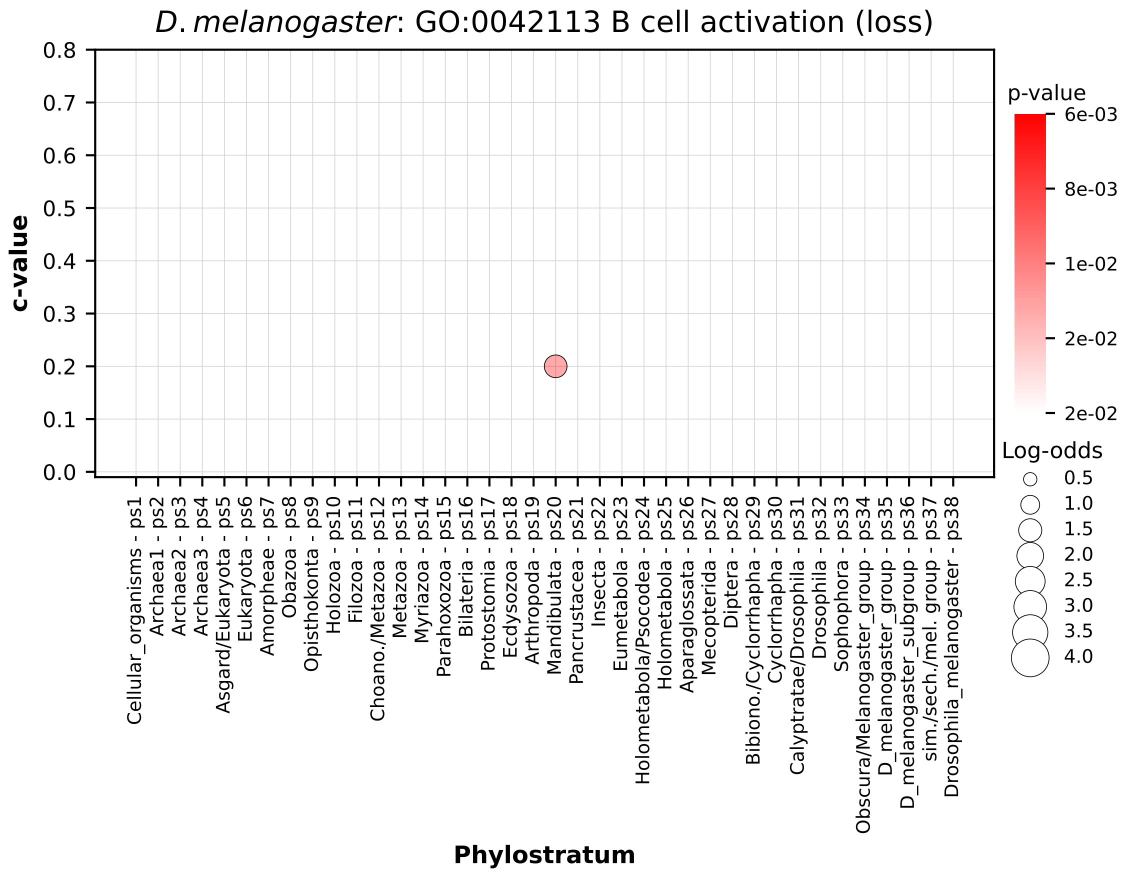
D. melanogaster: GO:0042113 B cell activation (loss)
c-value
0.0
0.1
0.2
0.3
0.4
0.5
0.6
0.7
0.8
p-value
6e-03
8e-03
1e-02
2e-02
2e-02
Log-odds
0.5
1.0
1.5
2.0
2.5
3.0
3.5
4.0
Cellular_organisms - ps1
Archaea1 - ps2
Archaea2 - ps3
Archaea3 - ps4
Asgard/Eukaryota - ps5
Eukaryota - ps6
Amorpheae - ps7
Obazoa - ps8
Opisthokonta - ps9
Holozoa - ps10
Filozoa - ps11
Choano./Metazoa - ps12
Metazoa - ps13
Myriazoa - ps14
Parahoxozoa - ps15
Bilateria - ps16
Protostomia - ps17
Ecdysozoa - ps18
Arthropoda - ps19
Mandibulata - ps20
Pancrustacea - ps21
Insecta - ps22
Eumetabola - ps23
Holometabola/Psocodea - ps24
Holometabola - ps25
Aparaglossata - ps26
Mecopterida - ps27
Diptera - ps28
Bibiono./Cyclorrhapha - ps29
Cyclorrhapha - ps30
Calyptratae/Drosophila - ps31
Drosophila - ps32
Sophophora - ps33
Obscura/Melanogaster_group - ps34
D_melanogaster_group - ps35
D_melanogaster_subgroup - ps36
sim./sech./mel. group - ps37
Drosophila_melanogaster - ps38
Phylostratum

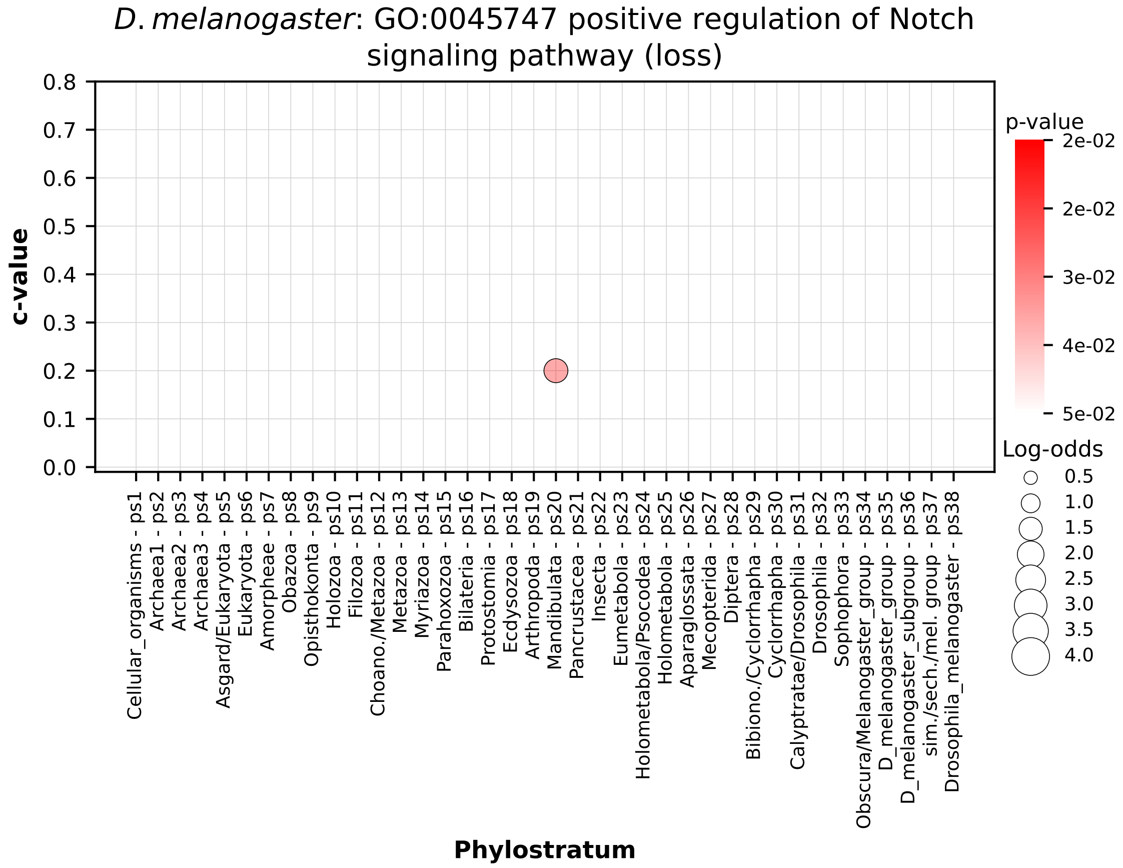

D. melanogaster: GO:0045747 positive regulation of Notch signaling pathway (loss)
c-value
0.0
0.1
0.2
0.3
0.4
0.5
0.6
0.7
0.8
Phylostratum
Cellular_organisms - ps1
Archaea1 - ps2
Archaea2 - ps3
Archaea3 - ps4
Asgard/Eukaryota - ps5
Eukaryota - ps6
Amorpheae - ps7
Obazoa - ps8
Opisthokonta - ps9
Holozoa - ps10
Filozoa - ps11
Choano./Metazoa - ps12
Metazoa - ps13
Myriazoa - ps14
Parahoxozoa - ps15
Bilateria - ps16
Protostomia - ps17
Ecdysozoa - ps18
Arthropoda - ps19
Mandibulata - ps20
Pancrustacea - ps21
Insecta - ps22
Eumetabola - ps23
Holometabola/Psocodea - ps24
Holometabola - ps25
Aparaglossata - ps26
Mecopterida - ps27
Diptera - ps28
Bibiono./Cyclorrhapha - ps29
Cyclorrhapha - ps30
Calyptratae/Drosophila - ps31
Drosophila - ps32
Sophophora - ps33
Obscura/Melanogaster_group - ps34
D_melanogaster_group - ps35
D_melanogaster_subgroup - ps36
sim./sech./mel. group - ps37
Drosophila_melanogaster - ps38
p-value
2e-02
2e-02
3e-02
4e-02
5e-02
Log-odds
0.5
1.0
1.5
2.0
2.5
3.0
3.5
4.0

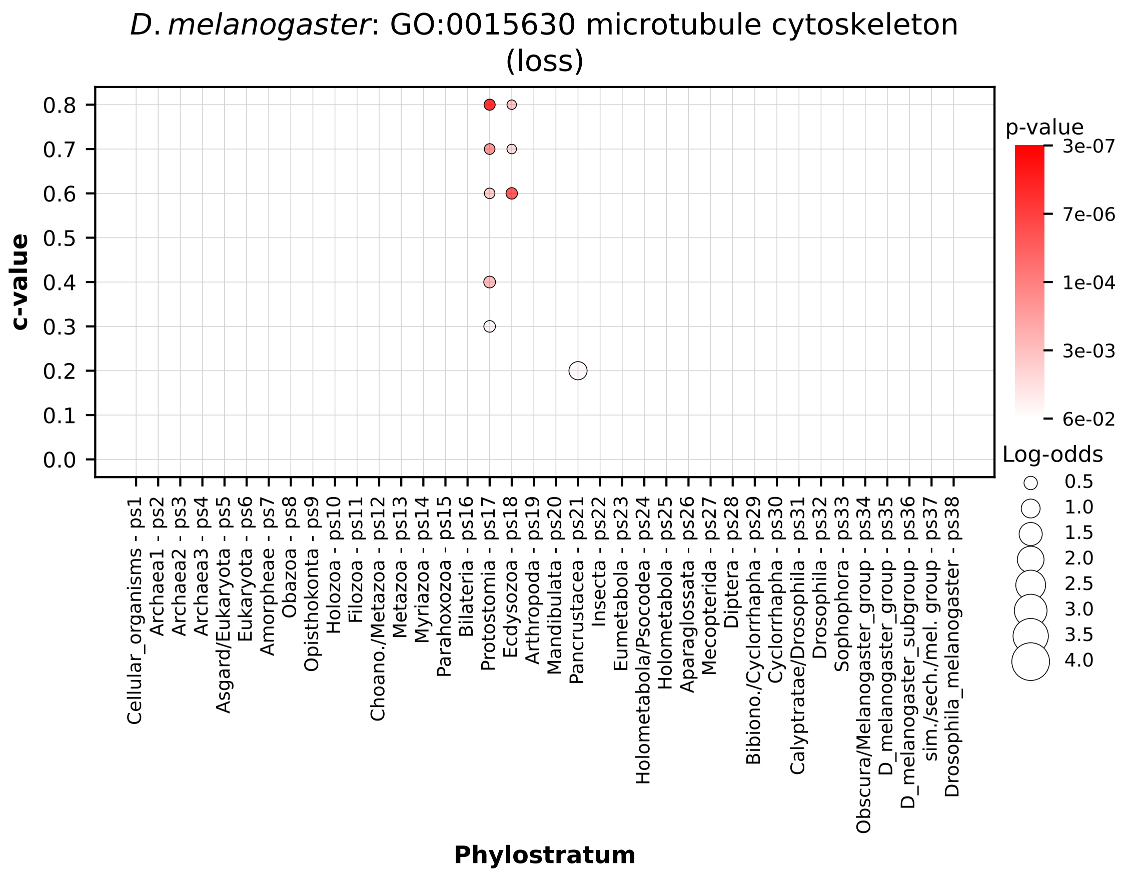

D. melanogaster: GO:0015630 microtubule cytoskeleton
(loss)
c-value
0.8
0.7
0.6
0.5
0.4
0.3
0.2
0.1
0.0
Phylostratum
Cellular_organisms - ps1
Archaea1 - ps2
Archaea2 - ps3
Archaea3 - ps4
Asgard/Eukaryota - ps5
Eukaryota - ps6
Amorpheae - ps7
Obazoa - ps8
Opisthokonta - ps9
Holozoa - ps10
Filozoa - ps11
Choano./Metazoa - ps12
Metazoa - ps13
Myriazoa - ps14
Parahoxozoa - ps15
Bilateria - ps16
Protostomia - ps17
Ecdysozoa - ps18
Arthropoda - ps19
Mandibulata - ps20
Pancrustacea - ps21
Insecta - ps22
Eumetabola - ps23
Holometabola/Psocodea - ps24
Holometabola - ps25
Aparaglossata - ps26
Mecopterida - ps27
Diptera - ps28
Bibiono./Cyclorrhapha - ps29
Cyclorrhapha - ps30
Calyptratae/Drosophila - ps31
Drosophila - ps32
Sophophora - ps33
Obscura/Melanogaster_group - ps34
D_melanogaster_group - ps35
D_melanogaster_subgroup - ps36
sim./sech./mel. group - ps37
Drosophila_melanogaster - ps38
p-value
3e-07
7e-06
1e-04
3e-03
6e-02
Log-odds
0.5
1.0
1.5
2.0
2.5
3.0
3.5
4.0

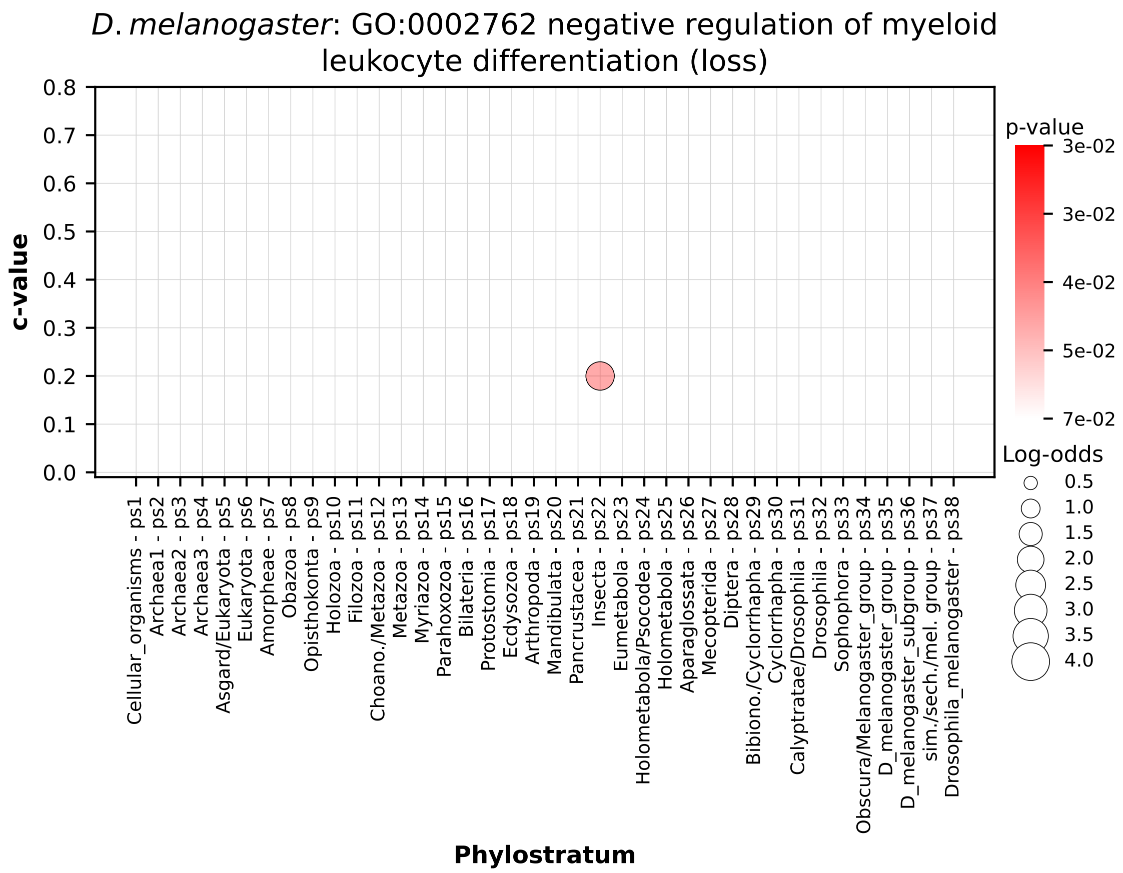

D. melanogaster: GO:0002762 negative regulation of myeloid leukocyte differentiation (loss)
c-value
0.8
0.7
0.6
0.5
0.4
0.3
0.2
0.1
0.0
Phylostratum
Cellular_organisms - ps1
Archaea1 - ps2
Archaea2 - ps3
Archaea3 - ps4
Asgard/Eukaryota - ps5
Eukaryota - ps6
Amorpheae - ps7
Obazoa - ps8
Opisthokonta - ps9
Holozoa - ps10
Filozoa - ps11
Choano./Metazoa - ps12
Metazoa - ps13
Myriazoa - ps14
Parahoxozoa - ps15
Bilateria - ps16
Protostomia - ps17
Ecdysozoa - ps18
Arthropoda - ps19
Mandibulata - ps20
Pancrustacea - ps21
Insecta - ps22
Eumetabola - ps23
Holometabola/Psocodea - ps24
Holometabola - ps25
Aparaglossata - ps26
Mecopterida - ps27
Diptera - ps28
Bibiono./Cyclorrhapha - ps29
Cyclorrhapha - ps30
Calyptratae/Drosophila - ps31
Drosophila - ps32
Sophophora - ps33
Obscura/Melanogaster_group - ps34
D_melanogaster_group - ps35
D_melanogaster_subgroup - ps36
sim./sech./mel. group - ps37
Drosophila_melanogaster - ps38
p-value
3e-02
3e-02
4e-02
5e-02
7e-02
Log-odds
0.5
1.0
1.5
2.0
2.5
3.0
3.5
4.0

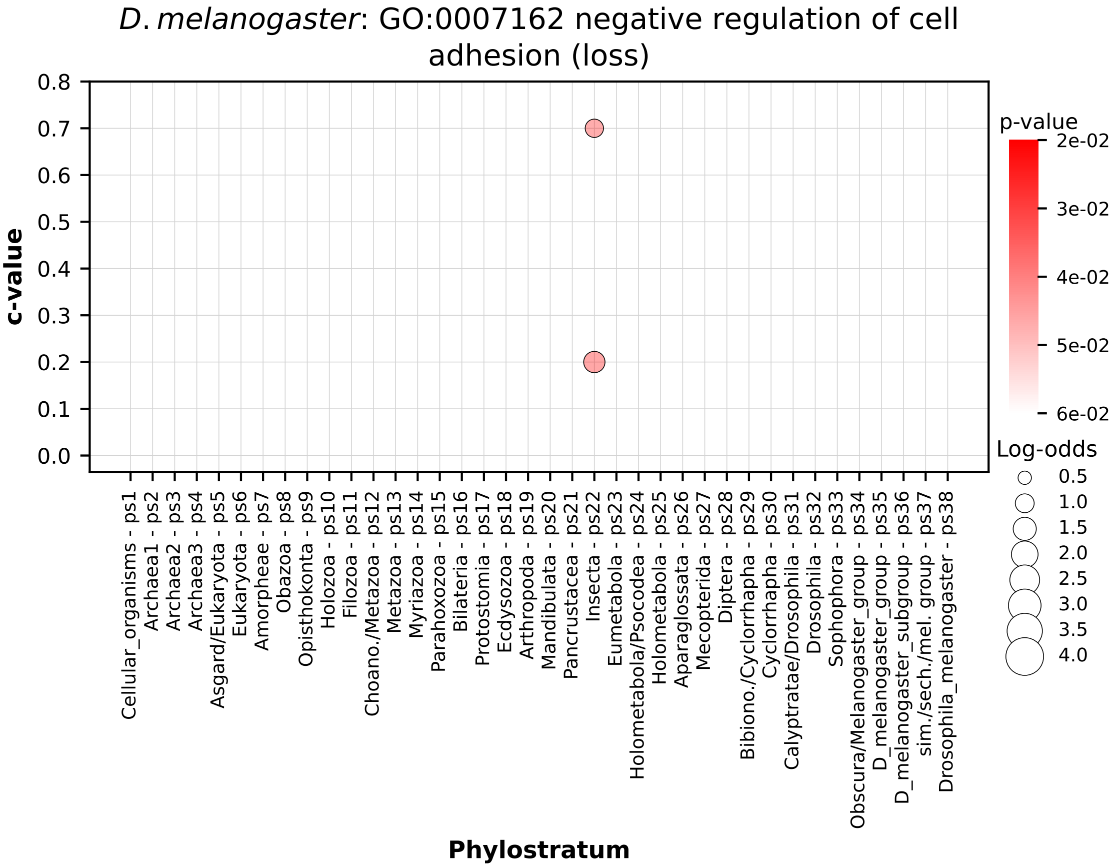

D. melanogaster: GO:0007162 negative regulation of cell adhesion (loss)
c-value
0.8
0.7
0.6
0.5
0.4
0.3
0.2
0.1
0.0
p-value
2e-02
3e-02
4e-02
5e-02
6e-02
Log-odds
0.5
1.0
1.5
2.0
2.5
3.0
3.5
4.0
Cellular_organisms - ps1
Archaea1 - ps2
Archaea2 - ps3
Archaea3 - ps4
Asgard/Eukaryota - ps5
Eukaryota - ps6
Amorpheae - ps7
Obazoa - ps8
Opisthokonta - ps9
Holozoa - ps10
Filozoa - ps11
Choano./Metazoa - ps12
Metazoa - ps13
Myriazoa - ps14
Parahoxozoa - ps15
Bilateria - ps16
Protostomia - ps17
Ecdysozoa - ps18
Arthropoda - ps19
Mandibulata - ps20
Pancrustacea - ps21
Insecta - ps22
Eumetabola - ps23
Holometabola/Psocodea - ps24
Holometabola - ps25
Aparaglossata - ps26
Mecopterida - ps27
Diptera - ps28
Bibiono./Cyclorrhapha - ps29
Cyclorrhapha - ps30
Calyptratae/Drosophila - ps31
Drosophila - ps32
Sophophora - ps33
Obscura/Melanogaster_group - ps34
D_melanogaster_group - ps35
D_melanogaster_subgroup - ps36
sim./sech./mel. group - ps37
Drosophila_melanogaster - ps38
Phylostratum

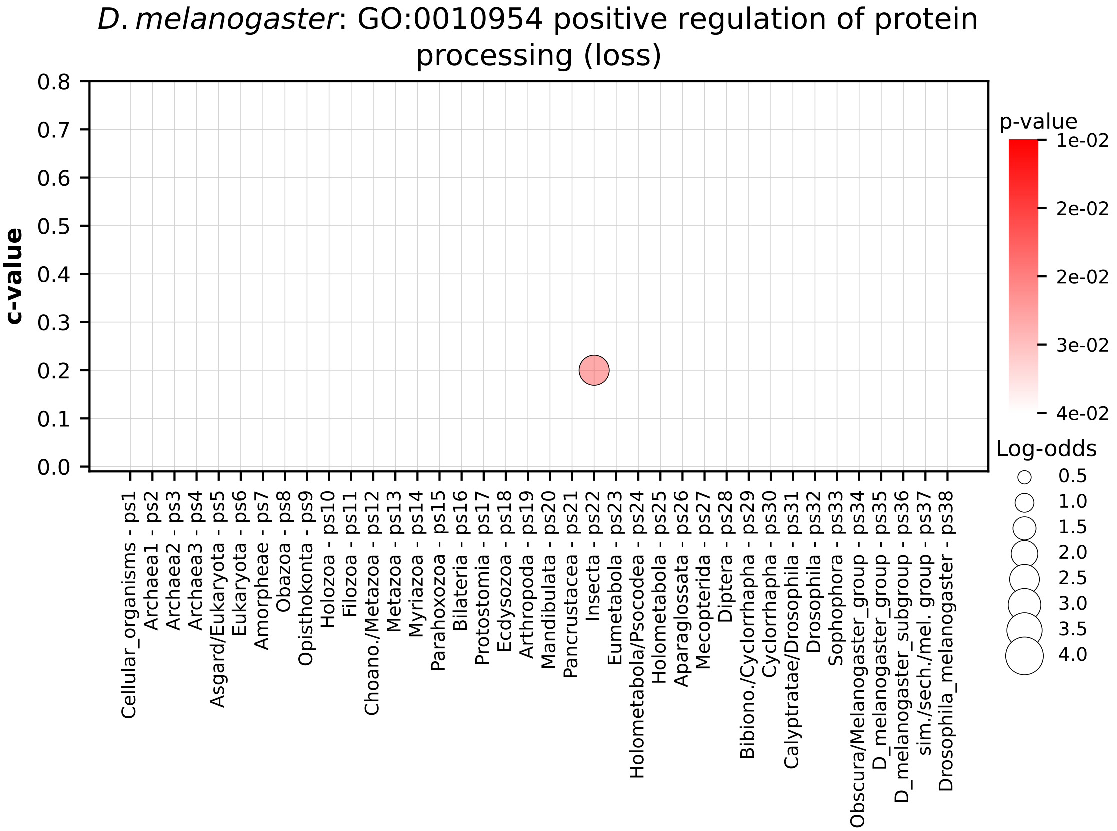

D. melanogaster: GO:0010954 positive regulation of protein processing (loss)
c-value
0.8
0.7
0.6
0.5
0.4
0.3
0.2
0.1
0.0
Phylostratum
Cellular_organisms - ps1
Archaea1 - ps2
Archaea2 - ps3
Archaea3 - ps4
Asgard/Eukaryota - ps5
Eukaryota - ps6
Amorpheae - ps7
Obazoa - ps8
Opisthokonta - ps9
Holozoa - ps10
Filozoa - ps11
Choano./Metazoa - ps12
Metazoa - ps13
Myriazoa - ps14
Parahoxozoa - ps15
Bilateria - ps16
Protostomia - ps17
Ecdysozoa - ps18
Arthropoda - ps19
Mandibulata - ps20
Pancrustacea - ps21
Insecta - ps22
Eumetabola - ps23
Holometabola/Psocodea - ps24
Holometabola - ps25
Aparaglossata - ps26
Mecopterida - ps27
Diptera - ps28
Bibiono./Cyclorrhapha - ps29
Cyclorrhapha - ps30
Calyptratae/Drosophila - ps31
Drosophila - ps32
Sophophora - ps33
Obscura/Melanogaster_group - ps34
D_melanogaster_group - ps35
D_melanogaster_subgroup - ps36
sim./sech./mel. group - ps37
Drosophila_melanogaster - ps38
p-value
1e-02
2e-02
2e-02
3e-02
4e-02
Log-odds
0.5
1.0
1.5
2.0
2.5
3.0
3.5
4.0

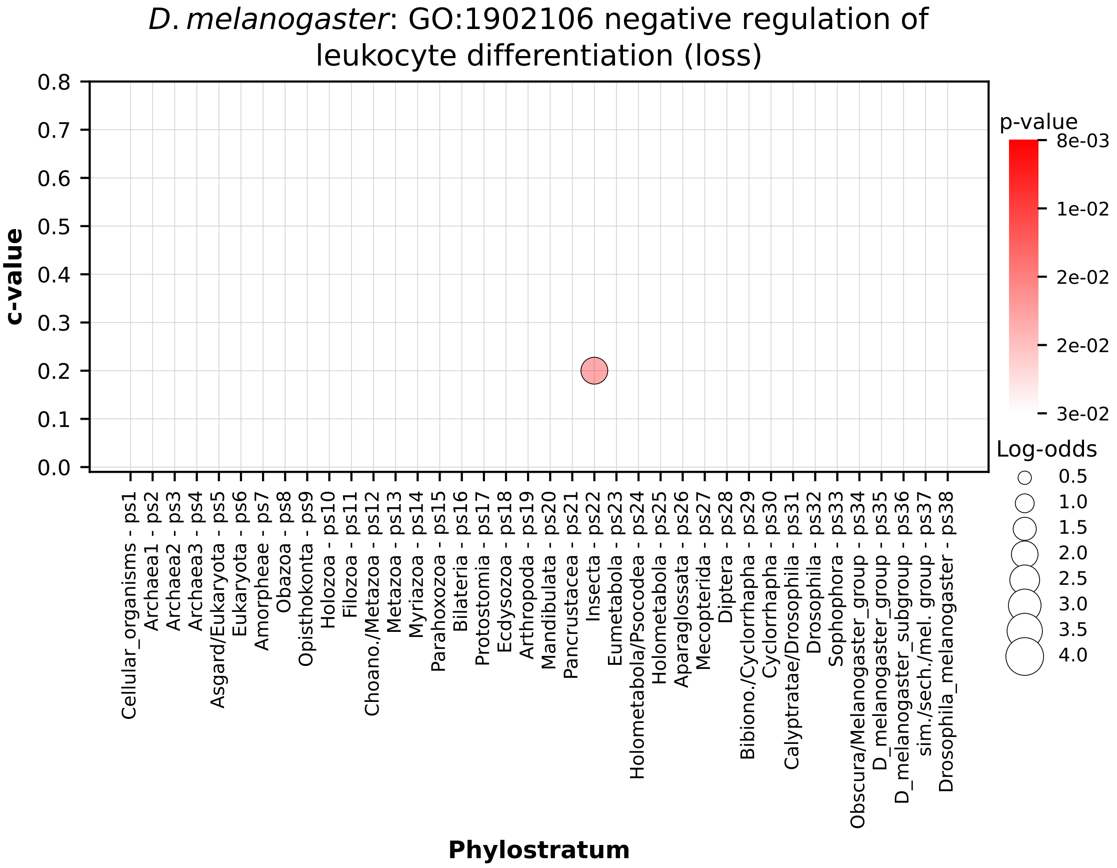

D. melanogaster: GO:1902106 negative regulation of leukocyte differentiation (loss)
c-value
0.8
0.7
0.6
0.5
0.4
0.3
0.2
0.1
0.0
p-value
8e-03
1e-02
2e-02
2e-02
3e-02
Log-odds
0.5
1.0
1.5
2.0
2.5
3.0
3.5
4.0
Cellular_organisms - ps1
Archaea1 - ps2
Archaea2 - ps3
Archaea3 - ps4
Asgard/Eukaryota - ps5
Eukaryota - ps6
Amorpheae - ps7
Obazoa - ps8
Opisthokonta - ps9
Holozoa - ps10
Filozoa - ps11
Choano./Metazoa - ps12
Metazoa - ps13
Myriazoa - ps14
Parahoxozoa - ps15
Bilateria - ps16
Protostomia - ps17
Ecdysozoa - ps18
Arthropoda - ps19
Mandibulata - ps20
Pancrustacea - ps21
Insecta - ps22
Eumetabola - ps23
Holometabola/Psocodea - ps24
Holometabola - ps25
Aparaglossata - ps26
Mecopterida - ps27
Diptera - ps28
Bibiono./Cyclorrhapha - ps29
Cyclorrhapha - ps30
Calyptratae/Drosophila - ps31
Drosophila - ps32
Sophophora - ps33
Obscura/Melanogaster_group - ps34
D_melanogaster_group - ps35
D_melanogaster_subgroup - ps36
sim./sech./mel. group - ps37
Drosophila_melanogaster - ps38
Phylostratum

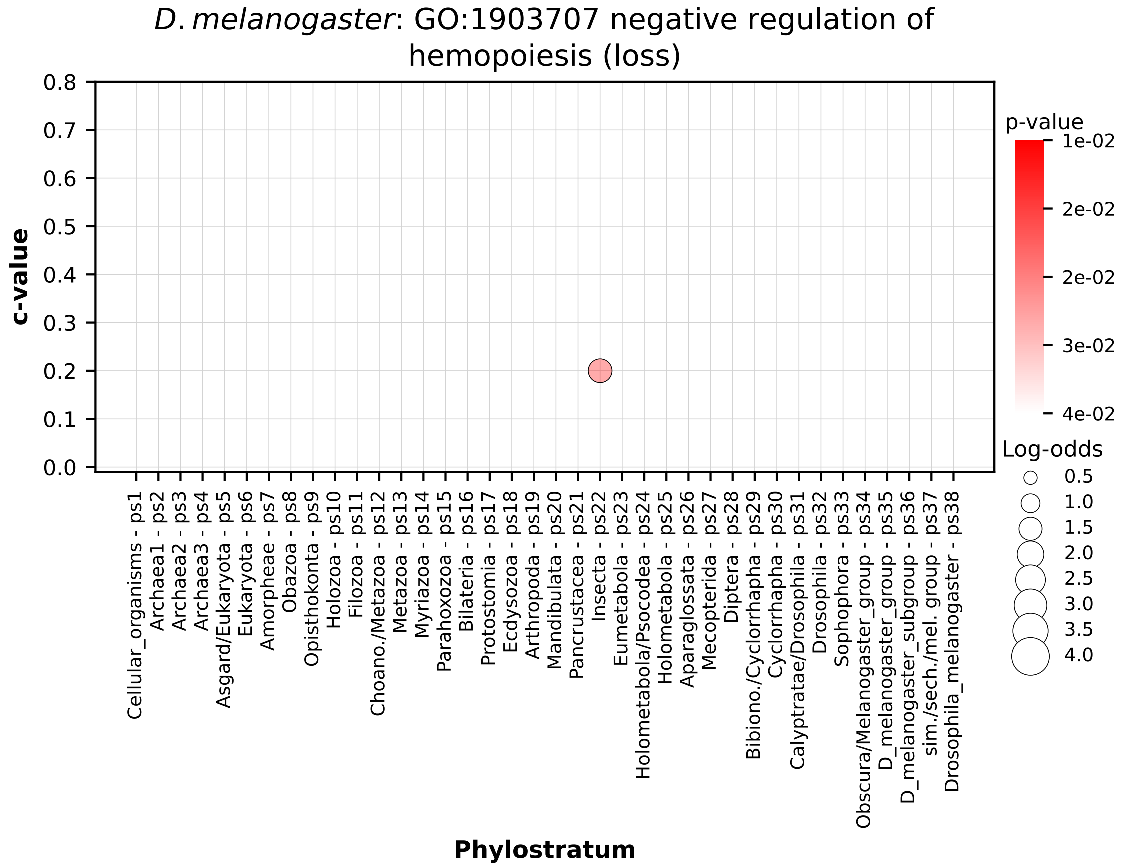
D. melanogaster: GO:1903707 negative regulation of hemopoiesis (loss)
c-value
0.0
0.1
0.2
0.3
0.4
0.5
0.6
0.7
0.8
p-value
1e-02
2e-02
2e-02
3e-02
4e-02
Log-odds
0.5
1.0
1.5
2.0
2.5
3.0
3.5
4.0
Cellular_organisms - ps1
Archaea1 - ps2
Archaea2 - ps3
Archaea3 - ps4
Asgard/Eukaryota - ps5
Eukaryota - ps6
Amorpheae - ps7
Obazoa - ps8
Opisthokonta - ps9
Holozoa - ps10
Filozoa - ps11
Choano./Metazoa - ps12
Metazoa - ps13
Myriazoa - ps14
Parahoxozoa - ps15
Bilateria - ps16
Protostomia - ps17
Ecdysozoa - ps18
Arthropoda - ps19
Mandibulata - ps20
Pancrustacea - ps21
Insecta - ps22
Eumetabola - ps23
Holometabola/Psocodea - ps24
Holometabola - ps25
Aparaglossata - ps26
Mecopterida - ps27
Diptera - ps28
Bibiono./Cyclorrhapha - ps29
Cyclorrhapha - ps30
Calyptratae/Drosophila - ps31
Drosophila - ps32
Sophophora - ps33
Obscura/Melanogaster_group - ps34
D_melanogaster_group - ps35
D_melanogaster_subgroup - ps36
sim./sech./mel. group - ps37
Drosophila_melanogaster - ps38
Phylostratum

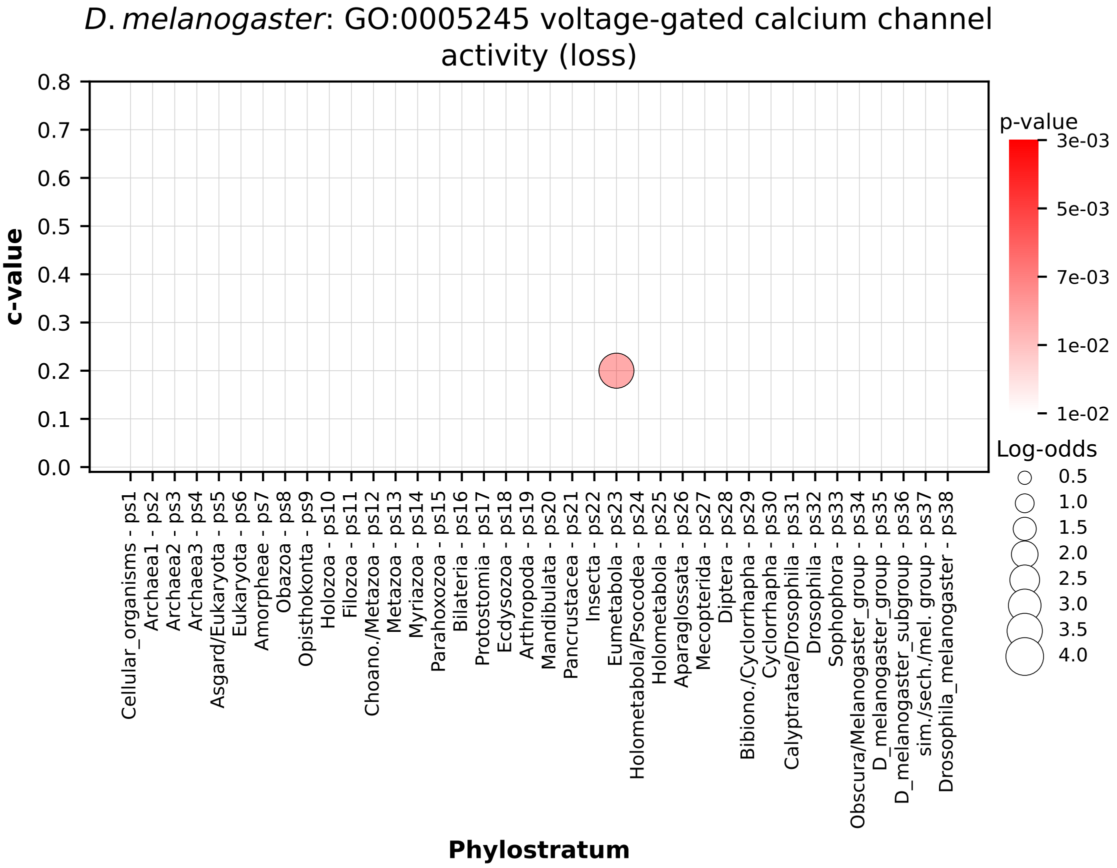

D. melanogaster: GO:0005245 voltage-gated calcium channel activity (loss)
c-value
0.8
0.7
0.6
0.5
0.4
0.3
0.2
0.1
0.0
p-value
3e-03
5e-03
7e-03
1e-02
1e-02
Log-odds
0.5
1.0
1.5
2.0
2.5
3.0
3.5
4.0
Cellular_organisms - ps1
Archaea1 - ps2
Archaea2 - ps3
Archaea3 - ps4
Asgard/Eukaryota - ps5
Eukaryota - ps6
Amorpheae - ps7
Obazoa - ps8
Opisthokonta - ps9
Holozoa - ps10
Filozoa - ps11
Choano./Metazoa - ps12
Metazoa - ps13
Myriazoa - ps14
Parahoxozoa - ps15
Bilateria - ps16
Protostomia - ps17
Ecdysozoa - ps18
Arthropoda - ps19
Mandibulata - ps20
Pancrustacea - ps21
Insecta - ps22
Eumetabola - ps23
Holometabola/Psocodea - ps24
Holometabola - ps25
Aparaglossata - ps26
Mecopterida - ps27
Diptera - ps28
Bibiono./Cyclorrhapha - ps29
Cyclorrhapha - ps30
Calyptratae/Drosophila - ps31
Drosophila - ps32
Sophophora - ps33
Obscura/Melanogaster_group - ps34
D_melanogaster_group - ps35
D_melanogaster_subgroup - ps36
sim./sech./mel. group - ps37
Drosophila_melanogaster - ps38
Phylostratum

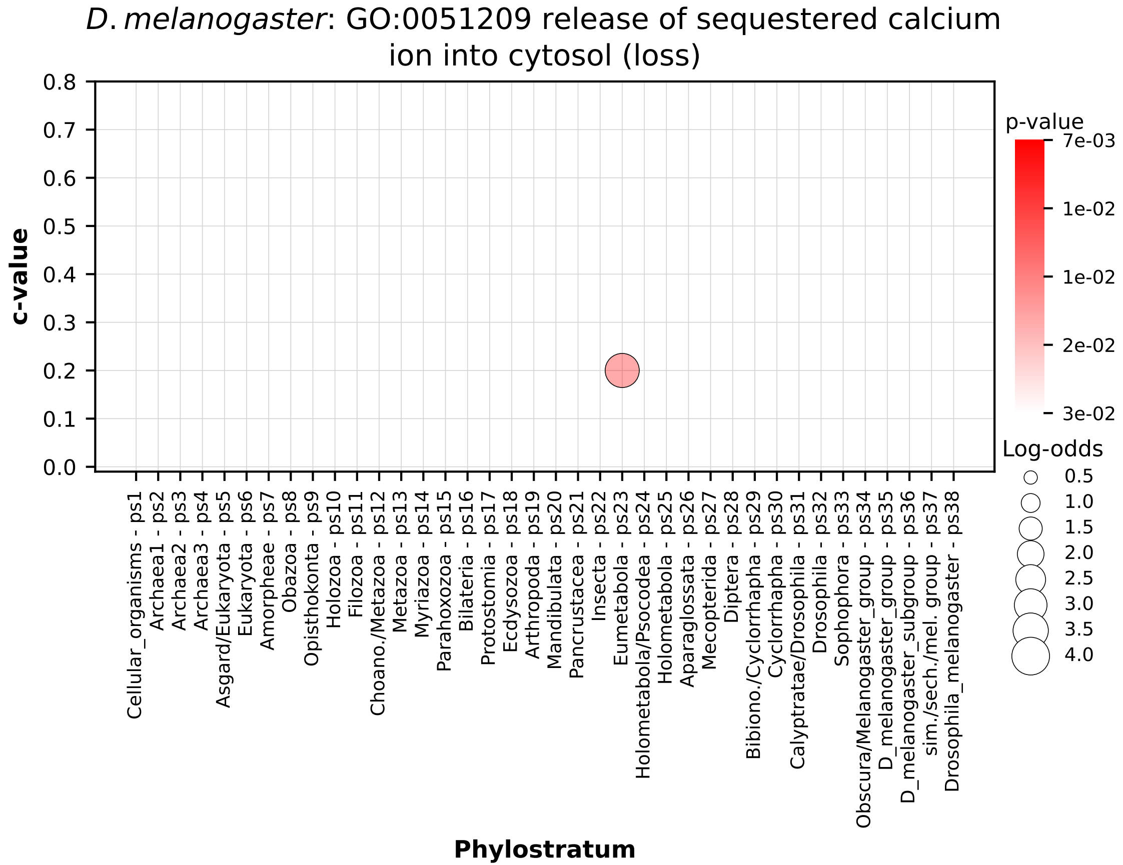

D. melanogaster: GO:0051209 release of sequestered calcium ion into cytosol (loss)
c-value
0.8
0.7
0.6
0.5
0.4
0.3
0.2
0.1
0.0
Phylostratum
Cellular_organisms - ps1
Archaea1 - ps2
Archaea2 - ps3
Archaea3 - ps4
Asgard/Eukaryota - ps5
Eukaryota - ps6
Amorpheae - ps7
Obazoa - ps8
Opisthokonta - ps9
Holozoa - ps10
Filozoa - ps11
Choano./Metazoa - ps12
Metazoa - ps13
Myriazoa - ps14
Parahoxozoa - ps15
Bilateria - ps16
Protostomia - ps17
Ecdysozoa - ps18
Arthropoda - ps19
Mandibulata - ps20
Pancrustacea - ps21
Insecta - ps22
Eumetabola - ps23
Holometabola/Psocodea - ps24
Holometabola - ps25
Aparaglossata - ps26
Mecopterida - ps27
Diptera - ps28
Bibiono./Cyclorrhapha - ps29
Cyclorrhapha - ps30
Calyptratae/Drosophila - ps31
Drosophila - ps32
Sophophora - ps33
Obscura/Melanogaster_group - ps34
D_melanogaster_group - ps35
D_melanogaster_subgroup - ps36
sim./sech./mel. group - ps37
Drosophila_melanogaster - ps38
p-value
7e-03
1e-02
1e-02
2e-02
3e-02
Log-odds
0.5
1.0
1.5
2.0
2.5
3.0
3.5
4.0

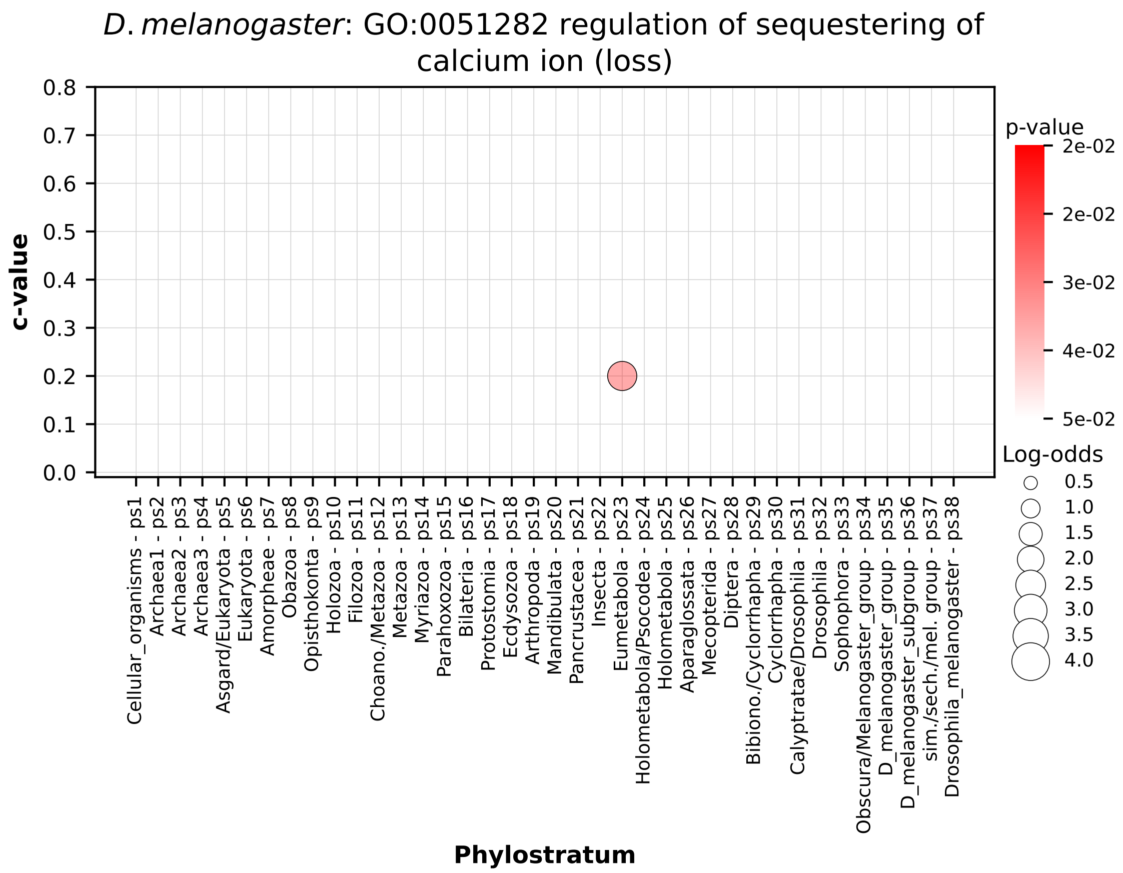
D. melanogaster: GO:0051282 regulation of sequestering of calcium ion (loss)
c-value
0.8
0.7
0.6
0.5
0.4
0.3
0.2
0.1
0.0
Phylostratum
Cellular_organisms - ps1
Archaea1 - ps2
Archaea2 - ps3
Archaea3 - ps4
Asgard/Eukaryota - ps5
Eukaryota - ps6
Amorpheae - ps7
Obazoa - ps8
Opisthokonta - ps9
Holozoa - ps10
Filozoa - ps11
Choano./Metazoa - ps12
Metazoa - ps13
Myriazoa - ps14
Parahoxozoa - ps15
Bilateria - ps16
Protostomia - ps17
Ecdysozoa - ps18
Arthropoda - ps19
Mandibulata - ps20
Pancrustacea - ps21
Insecta - ps22
Eumetabola - ps23
Holometabola/Psocodea - ps24
Holometabola - ps25
Aparaglossata - ps26
Mecopterida - ps27
Diptera - ps28
Bibiono./Cyclorrhapha - ps29
Cyclorrhapha - ps30
Calyptratae/Drosophila - ps31
Drosophila - ps32
Sophophora - ps33
Obscura/Melanogaster_group - ps34
D_melanogaster_group - ps35
D_melanogaster_subgroup - ps36
sim./sech./mel. group - ps37
Drosophila_melanogaster - ps38
p-value
2e-02
2e-02
3e-02
4e-02
5e-02
Log-odds
0.5
1.0
1.5
2.0
2.5
3.0
3.5
4.0

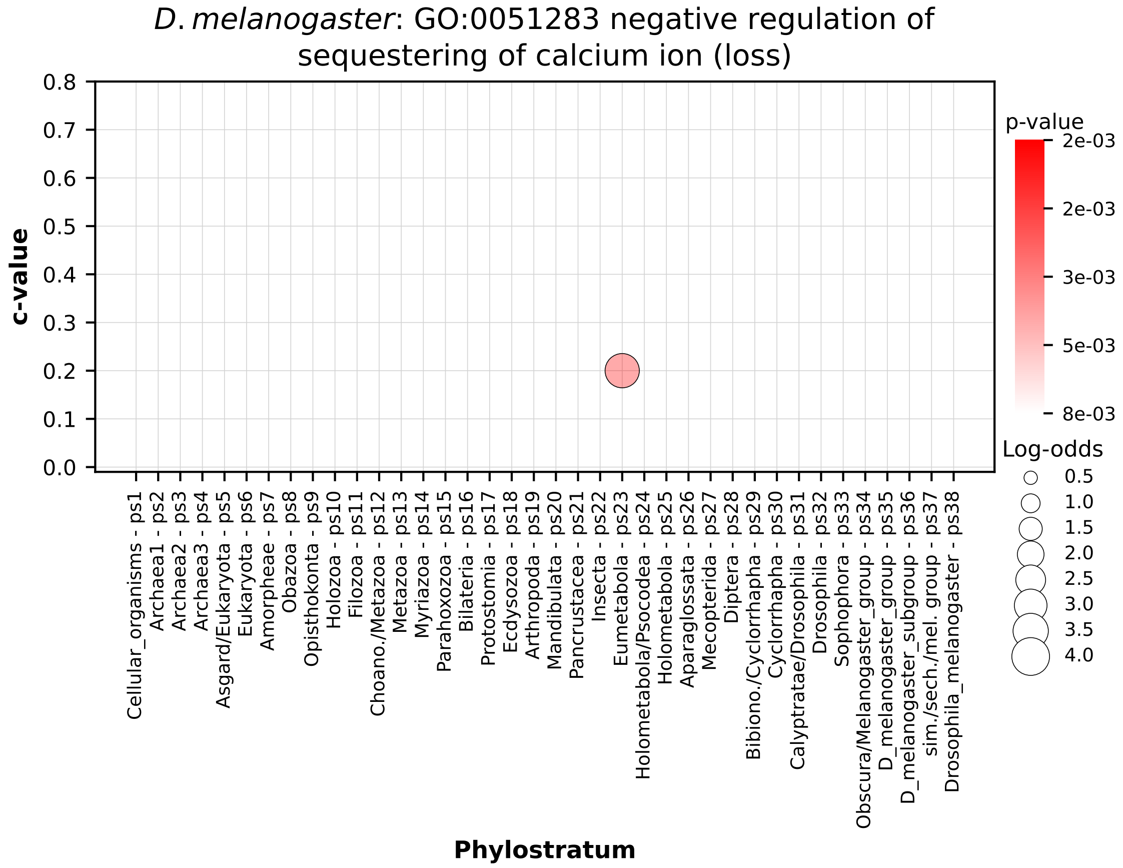

D. melanogaster: GO:0051283 negative regulation of sequestering of calcium ion (loss)
c-value
0.8
0.7
0.6
0.5
0.4
0.3
0.2
0.1
0.0
Phylostratum
Cellular_organisms - ps1
Archaea1 - ps2
Archaea2 - ps3
Archaea3 - ps4
Asgard/Eukaryota - ps5
Eukaryota - ps6
Amorpheae - ps7
Obazoa - ps8
Opisthokonta - ps9
Holozoa - ps10
Filozoa - ps11
Choano./Metazoa - ps12
Metazoa - ps13
Myriazoa - ps14
Parahoxozoa - ps15
Bilateria - ps16
Protostomia - ps17
Ecdysozoa - ps18
Arthropoda - ps19
Mandibulata - ps20
Pancrustacea - ps21
Insecta - ps22
Eumetabola - ps23
Holometabola/Psocodea - ps24
Holometabola - ps25
Aparaglossata - ps26
Mecopterida - ps27
Diptera - ps28
Bibiono./Cyclorrhapha - ps29
Cyclorrhapha - ps30
Calyptratae/Drosophila - ps31
Drosophila - ps32
Sophophora - ps33
Obscura/Melanogaster_group - ps34
D_melanogaster_group - ps35
D_melanogaster_subgroup - ps36
sim./sech./mel. group - ps37
Drosophila_melanogaster - ps38
p-value
2e-03
2e-03
3e-03
5e-03
8e-03
Log-odds
0.5
1.0
1.5
2.0
2.5
3.0
3.5
4.0

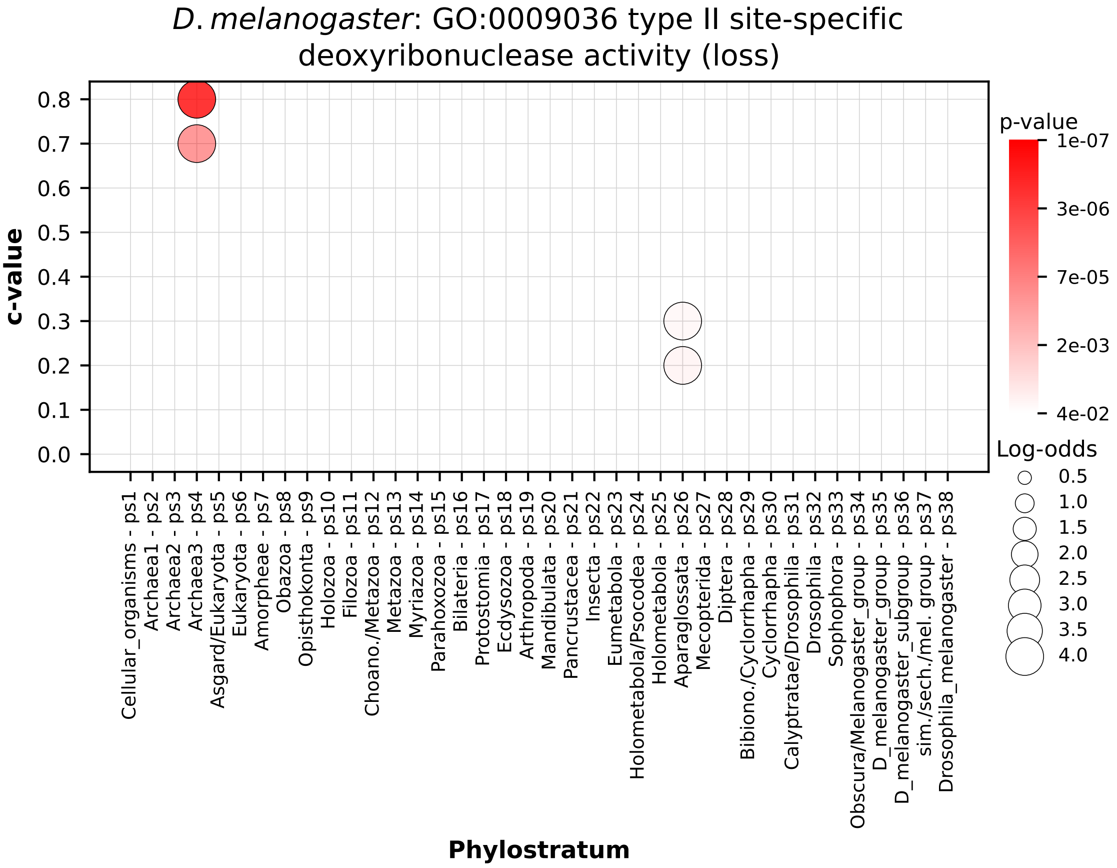

D. melanogaster: GO:0009036 type II site-specific deoxyribonuclease activity (loss)
c-value
0.0
0.1
0.2
0.3
0.4
0.5
0.6
0.7
0.8
Phylostratum
Cellular_organisms - ps1
Archaea1 - ps2
Archaea2 - ps3
Archaea3 - ps4
Asgard/Eukaryota - ps5
Eukaryota - ps6
Amorpheae - ps7
Obazoa - ps8
Opisthokonta - ps9
Holozoa - ps10
Filozoa - ps11
Choano./Metazoa - ps12
Metazoa - ps13
Myriazoa - ps14
Parahoxozoa - ps15
Bilateria - ps16
Protostomia - ps17
Ecdysozoa - ps18
Arthropoda - ps19
Mandibulata - ps20
Pancrustacea - ps21
Insecta - ps22
Eumetabola - ps23
Holometabola/Psocodea - ps24
Holometabola - ps25
Aparaglossata - ps26
Mecopterida - ps27
Diptera - ps28
Bibiono./Cyclorrhapha - ps29
Cyclorrhapha - ps30
Calyptratae/Drosophila - ps31
Drosophila - ps32
Sophophora - ps33
Obscura/Melanogaster_group - ps34
D_melanogaster_group - ps35
D_melanogaster_subgroup - ps36
sim./sech./mel. group - ps37
Drosophila_melanogaster - ps38
p-value
1e-07
3e-06
7e-05
2e-03
4e-02
Log-odds
0.5
1.0
1.5
2.0
2.5
3.0
3.5
4.0

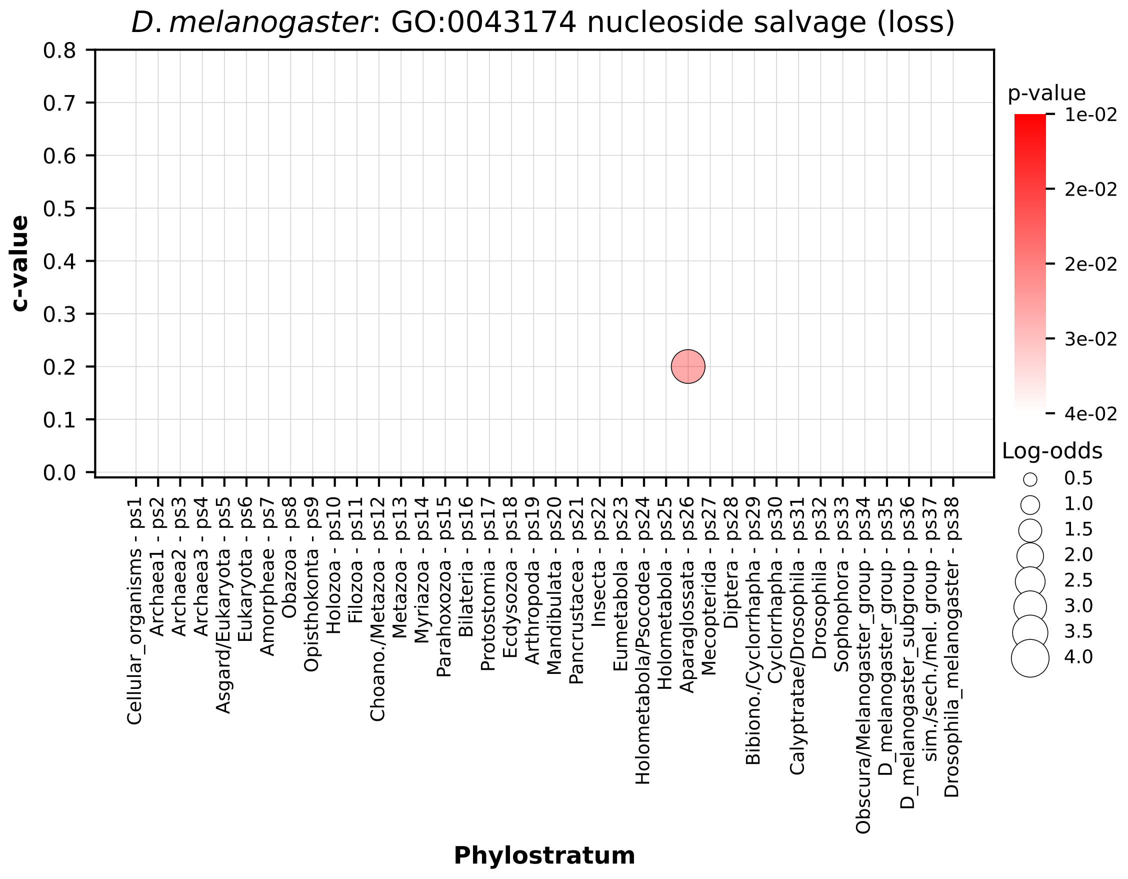

D. melanogaster: GO:0043174 nucleoside salvage (loss)
c-value
0.8
0.7
0.6
0.5
0.4
0.3
0.2
0.1
0.0
p-value
1e-02
2e-02
2e-02
3e-02
4e-02
Log-odds
0.5
1.0
1.5
2.0
2.5
3.0
3.5
4.0
Cellular_organisms - ps1
Archaea1 - ps2
Archaea2 - ps3
Archaea3 - ps4
Asgard/Eukaryota - ps5
Eukaryota - ps6
Amorpheae - ps7
Obazoa - ps8
Opisthokonta - ps9
Holozoa - ps10
Filozoa - ps11
Choano./Metazoa - ps12
Metazoa - ps13
Myriazoa - ps14
Parahoxozoa - ps15
Bilateria - ps16
Protostomia - ps17
Ecdysozoa - ps18
Arthropoda - ps19
Mandibulata - ps20
Pancrustacea - ps21
Insecta - ps22
Eumetabola - ps23
Holometabola/Psocodea - ps24
Holometabola - ps25
Aparaglossata - ps26
Mecopterida - ps27
Diptera - ps28
Bibiono./Cyclorrhapha - ps29
Cyclorrhapha - ps30
Calyptratae/Drosophila - ps31
Drosophila - ps32
Sophophora - ps33
Obscura/Melanogaster_group - ps34
D_melanogaster_group - ps35
D_melanogaster_subgroup - ps36
sim./sech./mel. group - ps37
Drosophila_melanogaster - ps38
Phylostratum

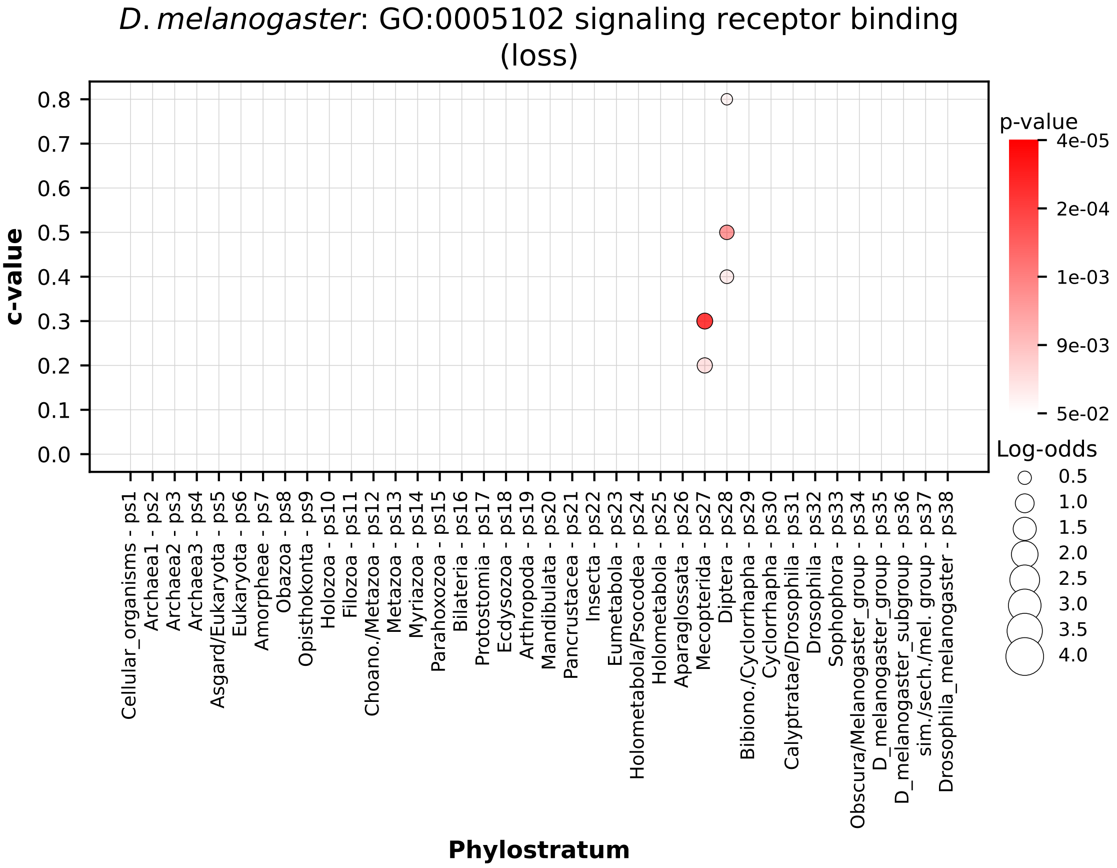

D. melanogaster: GO:0005102 signaling receptor binding
(loss)
c-value
0.0
0.1
0.2
0.3
0.4
0.5
0.6
0.7
0.8
p-value
4e-05
2e-04
1e-03
9e-03
5e-02
Log-odds
0.5
1.0
1.5
2.0
2.5
3.0
3.5
4.0
Cellular_organisms - ps1
Archaea1 - ps2
Archaea2 - ps3
Archaea3 - ps4
Asgard/Eukaryota - ps5
Eukaryota - ps6
Amorpheae - ps7
Obazoa - ps8
Opisthokonta - ps9
Holozoa - ps10
Filozoa - ps11
Choano./Metazoa - ps12
Metazoa - ps13
Myriazoa - ps14
Parahoxozoa - ps15
Bilateria - ps16
Protostomia - ps17
Ecdysozoa - ps18
Arthropoda - ps19
Mandibulata - ps20
Pancrustacea - ps21
Insecta - ps22
Eumetabola - ps23
Holometabola/Psocodea - ps24
Holometabola - ps25
Aparaglossata - ps26
Mecopterida - ps27
Diptera - ps28
Bibiono./Cyclorrhapha - ps29
Cyclorrhapha - ps30
Calyptratae/Drosophila - ps31
Drosophila - ps32
Sophophora - ps33
Obscura/Melanogaster_group - ps34
D_melanogaster_group - ps35
D_melanogaster_subgroup - ps36
sim./sech./mel. group - ps37
Drosophila_melanogaster - ps38
Phylostratum

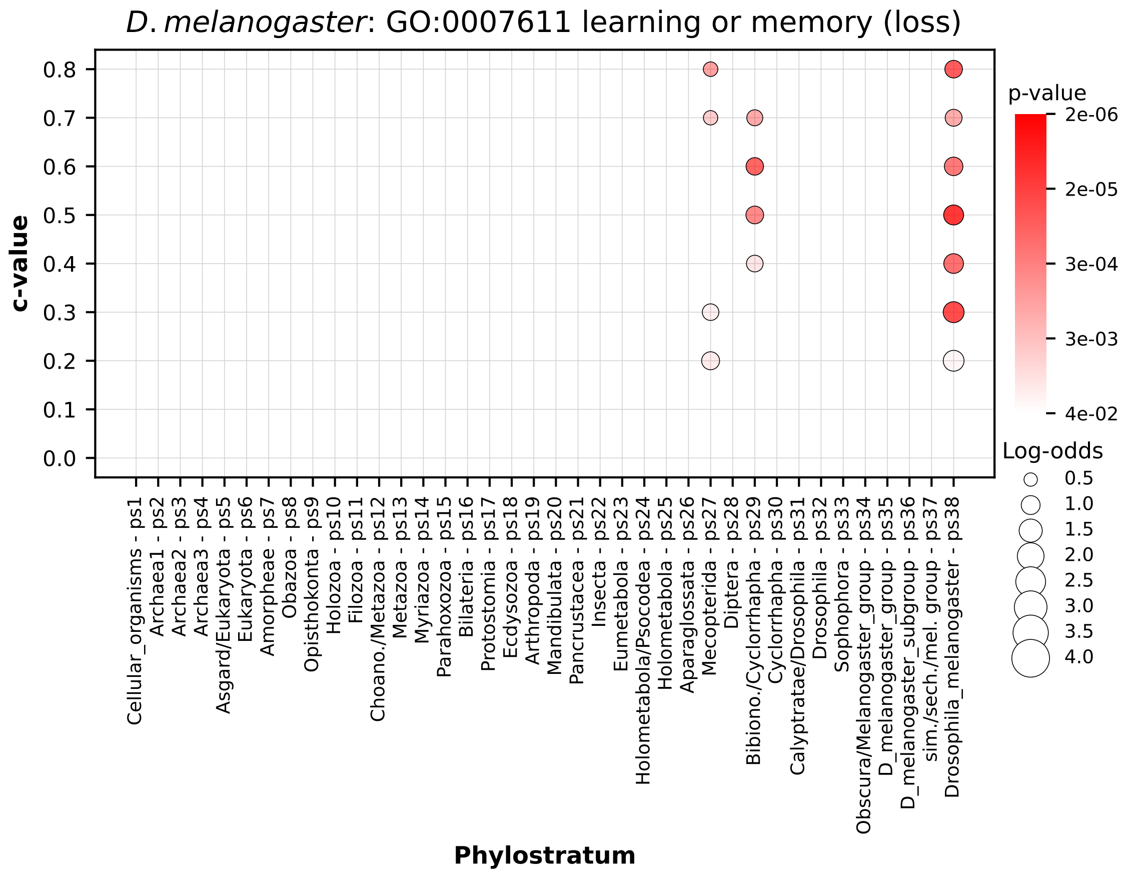

D. melanogaster: GO:0007611 learning or memory (loss)
c-value
0.0
0.1
0.2
0.3
0.4
0.5
0.6
0.7
0.8
Phylostratum
Cellular_organisms - ps1
Archaea1 - ps2
Archaea2 - ps3
Archaea3 - ps4
Asgard/Eukaryota - ps5
Eukaryota - ps6
Amorpheae - ps7
Obazoa - ps8
Opisthokonta - ps9
Holozoa - ps10
Filozoa - ps11
Choano./Metazoa - ps12
Metazoa - ps13
Myriazoa - ps14
Parahoxozoa - ps15
Bilateria - ps16
Protostomia - ps17
Ecdysozoa - ps18
Arthropoda - ps19
Mandibulata - ps20
Pancrustacea - ps21
Insecta - ps22
Eumetabola - ps23
Holometabola/Psocodea - ps24
Holometabola - ps25
Aparaglossata - ps26
Mecopterida - ps27
Diptera - ps28
Bibiono./Cyclorrhapha - ps29
Cyclorrhapha - ps30
Calyptratae/Drosophila - ps31
Drosophila - ps32
Sophophora - ps33
Obscura/Melanogaster_group - ps34
D_melanogaster_group - ps35
D_melanogaster_subgroup - ps36
sim./sech./mel. group - ps37
Drosophila_melanogaster - ps38
p-value
2e-06
2e-05
3e-04
3e-03
4e-02
Log-odds
0.5
1.0
1.5
2.0
2.5
3.0
3.5
4.0

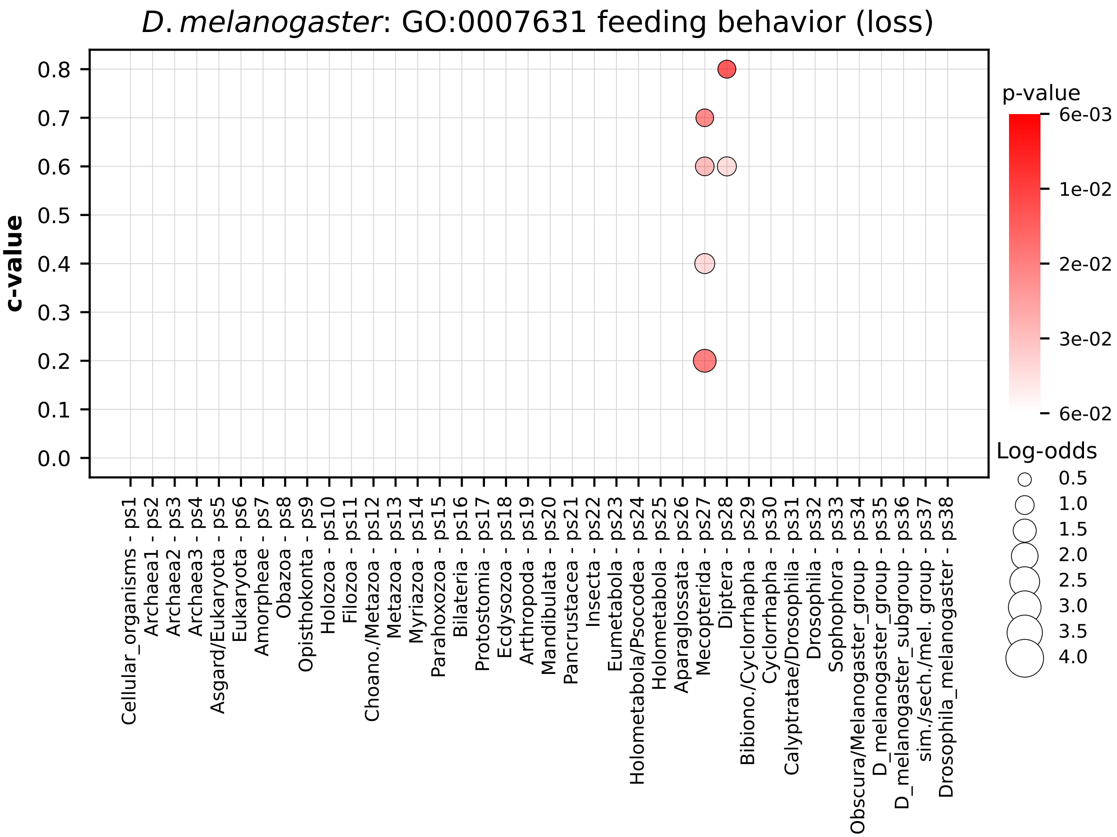

D. melanogaster: GO:0007631 feeding behavior (loss)
c-value
0.0
0.1
0.2
0.3
0.4
0.5
0.6
0.7
0.8
Phylostratum
Cellular_organisms - ps1
Archaea1 - ps2
Archaea2 - ps3
Archaea3 - ps4
Asgard/Eukaryota - ps5
Eukaryota - ps6
Amorpheae - ps7
Obazoa - ps8
Opisthokonta - ps9
Holozoa - ps10
Filozoa - ps11
Choano./Metazoa - ps12
Metazoa - ps13
Myriazoa - ps14
Parahoxozoa - ps15
Bilateria - ps16
Protostomia - ps17
Ecdysozoa - ps18
Arthropoda - ps19
Mandibulata - ps20
Pancrustacea - ps21
Insecta - ps22
Eumetabola - ps23
Holometabola/Psocodea - ps24
Holometabola - ps25
Aparaglossata - ps26
Mecopterida - ps27
Diptera - ps28
Bibiono./Cyclorrhapha - ps29
Cyclorrhapha - ps30
Calyptratae/Drosophila - ps31
Drosophila - ps32
Sophophora - ps33
Obscura/Melanogaster_group - ps34
D_melanogaster_group - ps35
D_melanogaster_subgroup - ps36
sim./sech./mel. group - ps37
Drosophila_melanogaster - ps38
p-value
6e-03
1e-02
2e-02
3e-02
6e-02
Log-odds
0.5
1.0
1.5
2.0
2.5
3.0
3.5
4.0

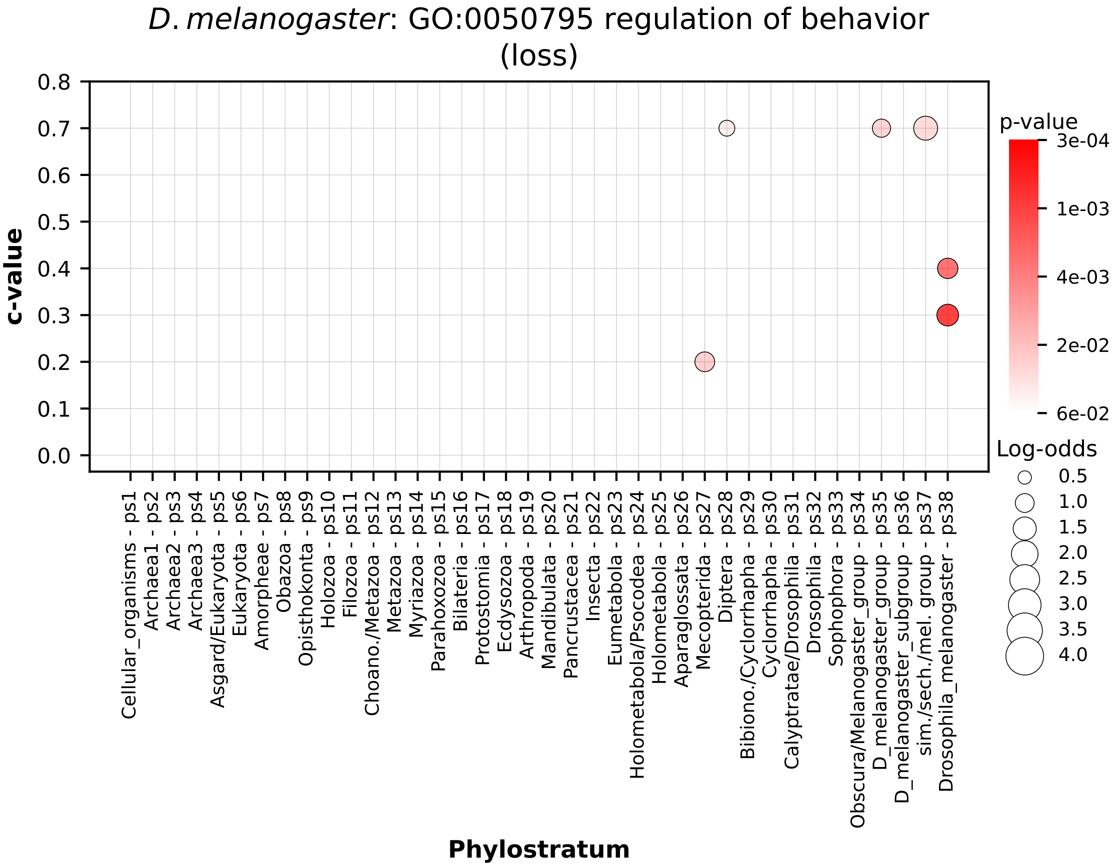

D. melanogaster: GO:0050795 regulation of behavior
(loss)
c-value
0.8
0.7
0.6
0.5
0.4
0.3
0.2
0.1
0.0
Phylostratum
Cellular_organisms - ps1
Archaea1 - ps2
Archaea2 - ps3
Archaea3 - ps4
Asgard/Eukaryota - ps5
Eukaryota - ps6
Amorpheae - ps7
Obazoa - ps8
Opisthokonta - ps9
Holozoa - ps10
Filozoa - ps11
Choano./Metazoa - ps12
Metazoa - ps13
Myriazoa - ps14
Parahoxozoa - ps15
Bilateria - ps16
Protostomia - ps17
Ecdysozoa - ps18
Arthropoda - ps19
Mandibulata - ps20
Pancrustacea - ps21
Insecta - ps22
Eumetabola - ps23
Holometabola/Psocodea - ps24
Holometabola - ps25
Aparaglossata - ps26
Mecopterida - ps27
Diptera - ps28
Bibiono./Cyclorrhapha - ps29
Cyclorrhapha - ps30
Calyptratae/Drosophila - ps31
Drosophila - ps32
Sophophora - ps33
Obscura/Melanogaster_group - ps34
D_melanogaster_group - ps35
D_melanogaster_subgroup - ps36
sim./sech./mel. group - ps37
Drosophila_melanogaster - ps38
p-value
3e-04
1e-03
4e-03
2e-02
6e-02
Log-odds
0.5
1.0
1.5
2.0
2.5
3.0
3.5
4.0

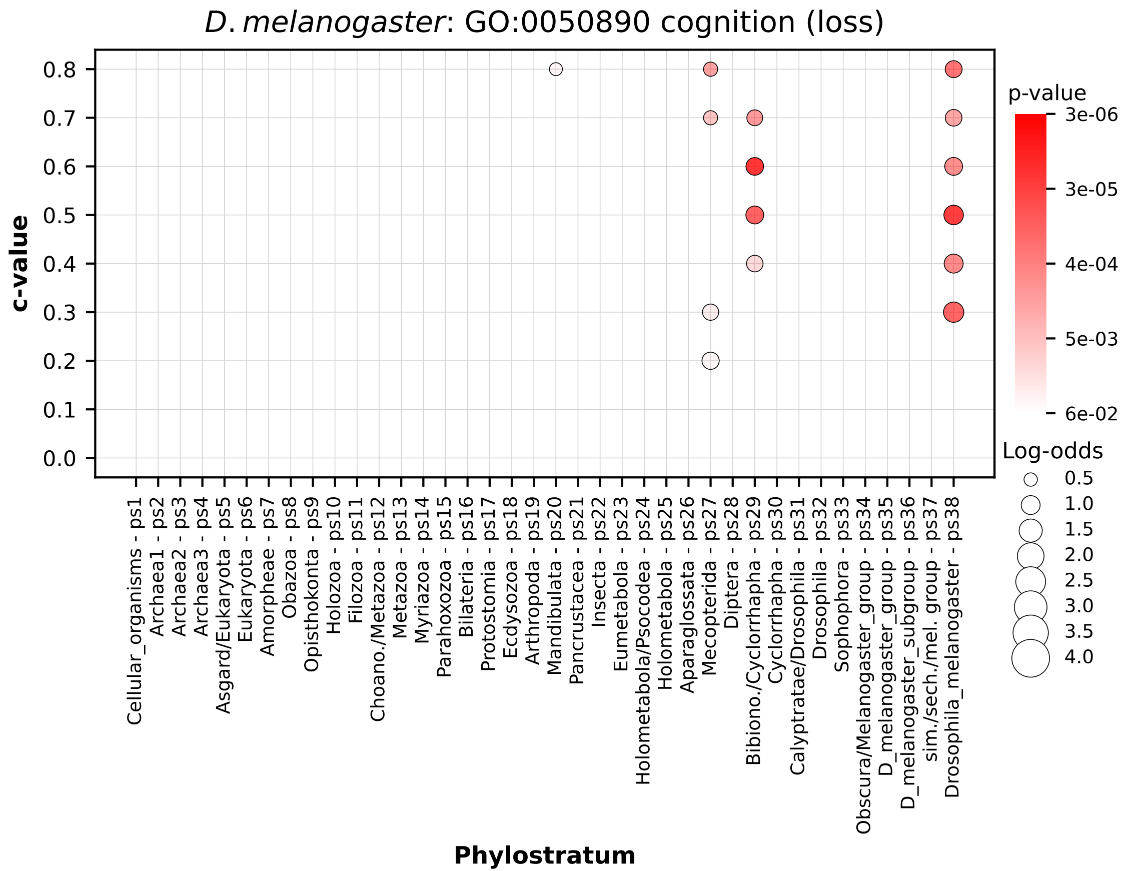
D. melanogaster: GO:0050890 cognition (loss)
c-value
0.0
0.1
0.2
0.3
0.4
0.5
0.6
0.7
0.8
Phylostratum
Cellular_organisms - ps1
Archaea1 - ps2
Archaea2 - ps3
Archaea3 - ps4
Asgard/Eukaryota - ps5
Eukaryota - ps6
Amorpheae - ps7
Obazoa - ps8
Opisthokonta - ps9
Holozoa - ps10
Filozoa - ps11
Choano./Metazoa - ps12
Metazoa - ps13
Myriazoa - ps14
Parahoxozoa - ps15
Bilateria - ps16
Protostomia - ps17
Ecdysozoa - ps18
Arthropoda - ps19
Mandibulata - ps20
Pancrustacea - ps21
Insecta - ps22
Eumetabola - ps23
Holometabola/Psocodea - ps24
Holometabola - ps25
Aparaglossata - ps26
Mecopterida - ps27
Diptera - ps28
Bibiono./Cyclorrhapha - ps29
Cyclorrhapha - ps30
Calyptratae/Drosophila - ps31
Drosophila - ps32
Sophophora - ps33
Obscura/Melanogaster_group - ps34
D_melanogaster_group - ps35
D_melanogaster_subgroup - ps36
sim./sech./mel. group - ps37
Drosophila_melanogaster - ps38
p-value
3e-06
3e-05
4e-04
5e-03
6e-02
Log-odds
0.5
1.0
1.5
2.0
2.5
3.0
3.5
4.0

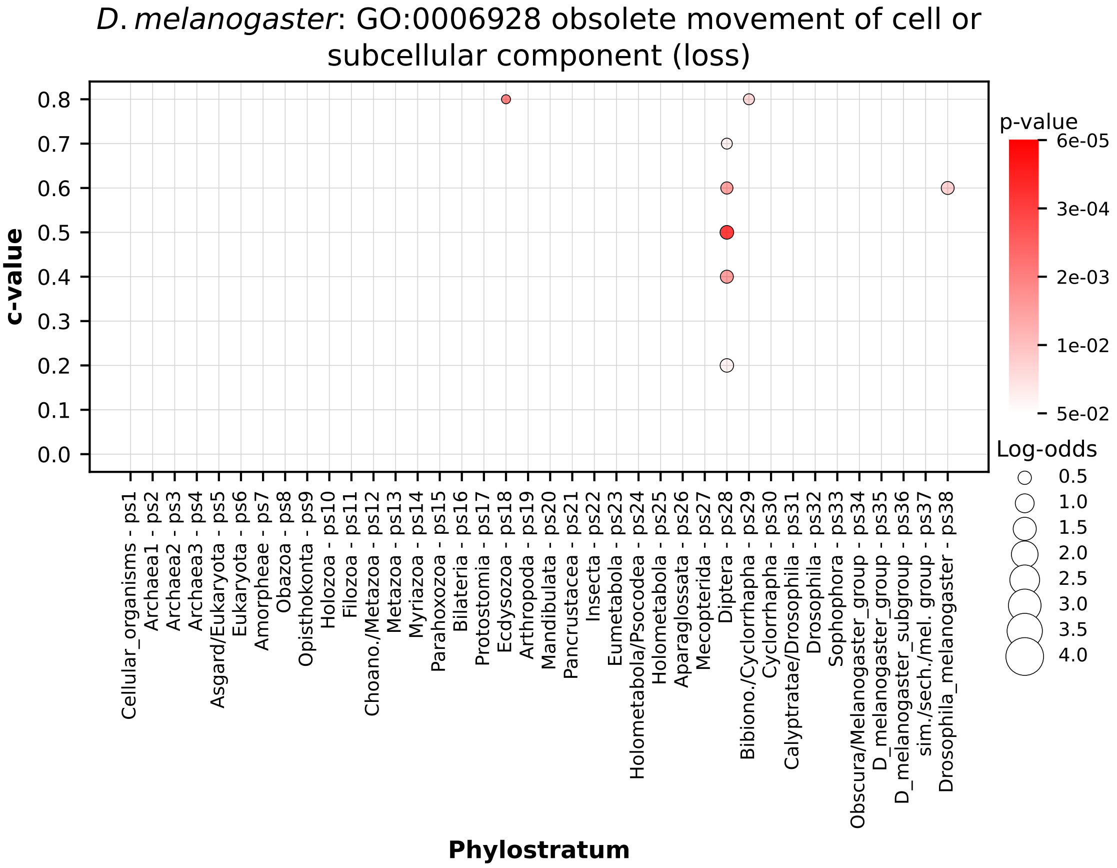

D. melanogaster: GO:0006928 obsolete movement of cell or subcellular component (loss)
c-value
0.0
0.1
0.2
0.3
0.4
0.5
0.6
0.7
0.8
p-value
6e-05
3e-04
2e-03
1e-02
5e-02
Log-odds
0.5
1.0
1.5
2.0
2.5
3.0
3.5
4.0
Cellular_organisms - ps1
Archaea1 - ps2
Archaea2 - ps3
Archaea3 - ps4
Asgard/Eukaryota - ps5
Eukaryota - ps6
Amorpheae - ps7
Obazoa - ps8
Opisthokonta - ps9
Holozoa - ps10
Filozoa - ps11
Choano./Metazoa - ps12
Metazoa - ps13
Myriazoa - ps14
Parahoxozoa - ps15
Bilateria - ps16
Protostomia - ps17
Ecdysozoa - ps18
Arthropoda - ps19
Mandibulata - ps20
Pancrustacea - ps21
Insecta - ps22
Eumetabola - ps23
Holometabola/Psocodea - ps24
Holometabola - ps25
Aparaglossata - ps26
Mecopterida - ps27
Diptera - ps28
Bibiono./Cyclorrhapha - ps29
Cyclorrhapha - ps30
Calyptratae/Drosophila - ps31
Drosophila - ps32
Sophophora - ps33
Obscura/Melanogaster_group - ps34
D_melanogaster_group - ps35
D_melanogaster_subgroup - ps36
sim./sech./mel. group - ps37
Drosophila_melanogaster - ps38
Phylostratum

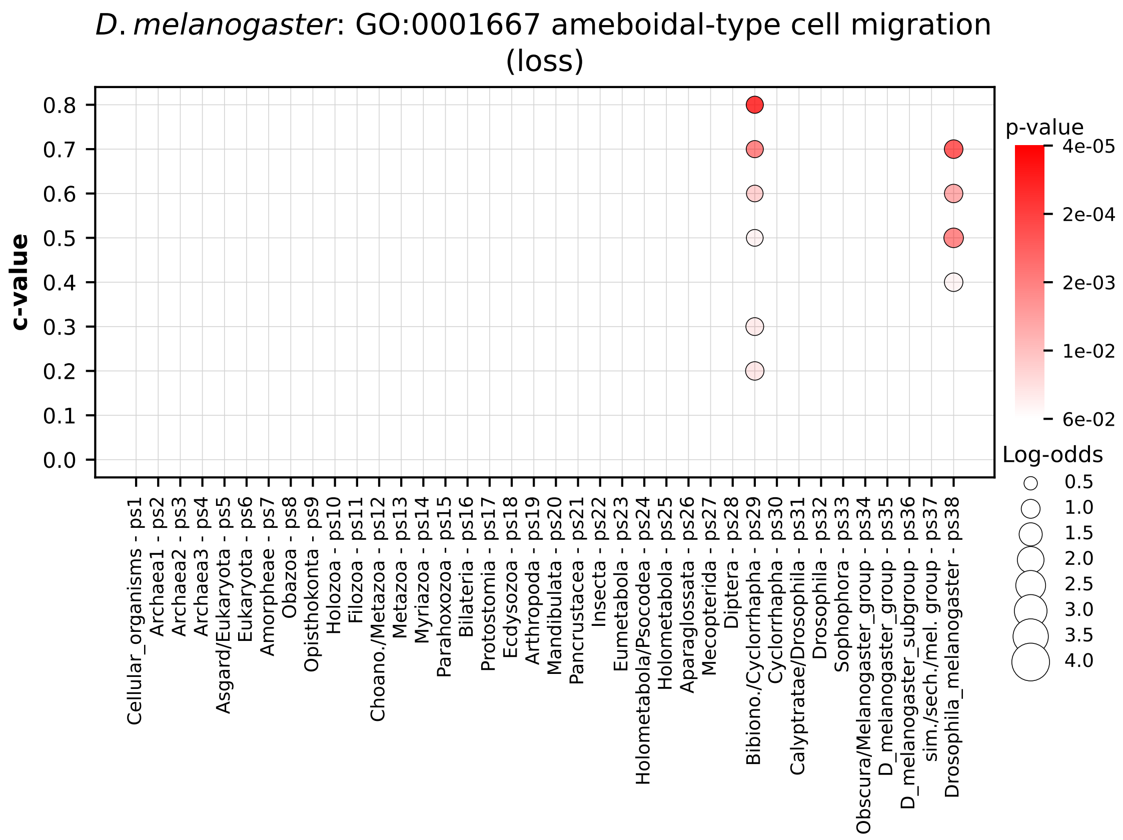
D. melanogaster: GO:0001667 ameboidal-type cell migration
(loss)
c-value
0.8
0.7
0.6
0.5
0.4
0.3
0.2
0.1
0.0
Phylostratum
Cellular_organisms - ps1
Archaea1 - ps2
Archaea2 - ps3
Archaea3 - ps4
Asgard/Eukaryota - ps5
Eukaryota - ps6
Amorpheae - ps7
Obazoa - ps8
Opisthokonta - ps9
Holozoa - ps10
Filozoa - ps11
Choano./Metazoa - ps12
Metazoa - ps13
Myriazoa - ps14
Parahoxozoa - ps15
Bilateria - ps16
Protostomia - ps17
Ecdysozoa - ps18
Arthropoda - ps19
Mandibulata - ps20
Pancrustacea - ps21
Insecta - ps22
Eumetabola - ps23
Holometabola/Psocodea - ps24
Holometabola - ps25
Aparaglossata - ps26
Mecopterida - ps27
Diptera - ps28
Bibiono./Cyclorrhapha - ps29
Cyclorrhapha - ps30
Calyptratae/Drosophila - ps31
Drosophila - ps32
Sophophora - ps33
Obscura/Melanogaster_group - ps34
D_melanogaster_group - ps35
D_melanogaster_subgroup - ps36
sim./sech./mel. group - ps37
Drosophila_melanogaster - ps38
p-value
4e-05
2e-04
2e-03
1e-02
6e-02
Log-odds
0.5
1.0
1.5
2.0
2.5
3.0
3.5
4.0

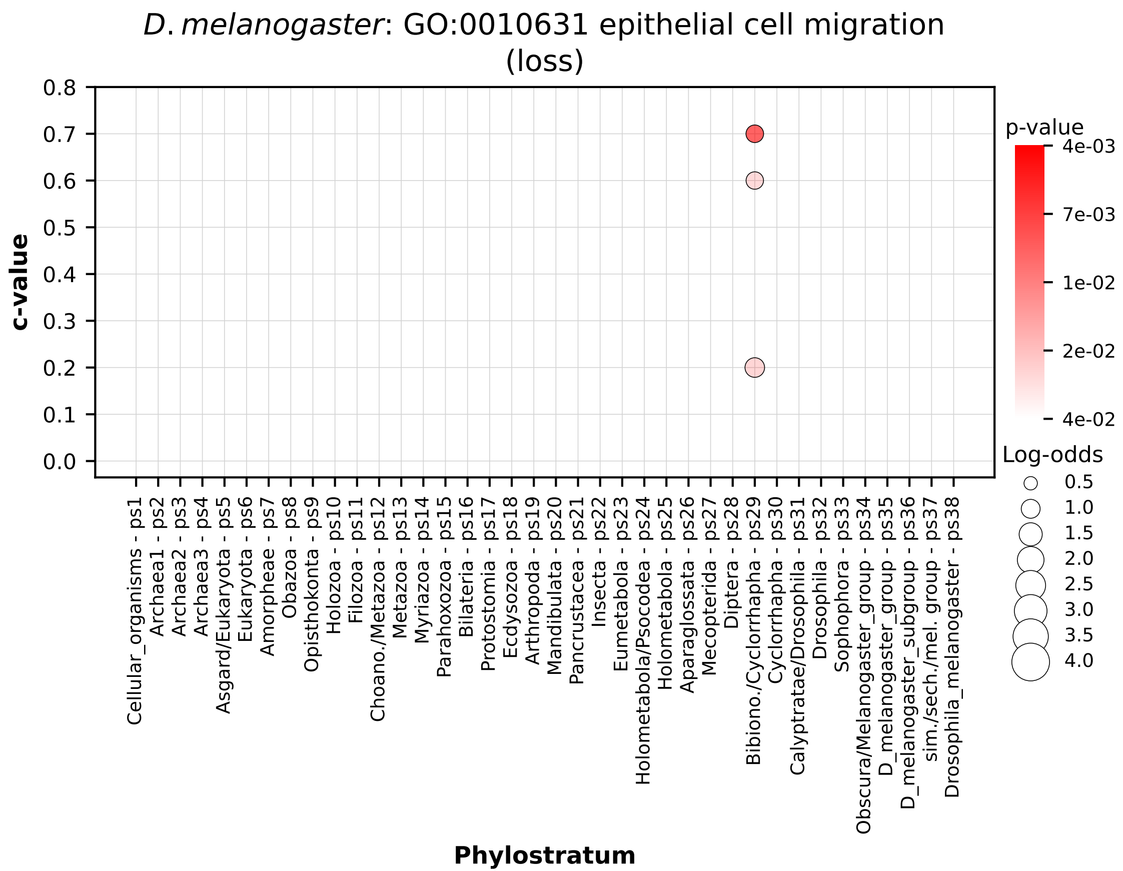

D. melanogaster: GO:0010631 epithelial cell migration
(loss)
c-value
0.0
0.1
0.2
0.3
0.4
0.5
0.6
0.7
0.8
p-value
4e-03
7e-03
1e-02
2e-02
4e-02
Log-odds
0.5
1.0
1.5
2.0
2.5
3.0
3.5
4.0
Cellular_organisms - ps1
Archaea1 - ps2
Archaea2 - ps3
Archaea3 - ps4
Asgard/Eukaryota - ps5
Eukaryota - ps6
Amorpheae - ps7
Obazoa - ps8
Opisthokonta - ps9
Holozoa - ps10
Filozoa - ps11
Choano./Metazoa - ps12
Metazoa - ps13
Myriazoa - ps14
Parahoxozoa - ps15
Bilateria - ps16
Protostomia - ps17
Ecdysozoa - ps18
Arthropoda - ps19
Mandibulata - ps20
Pancrustacea - ps21
Insecta - ps22
Eumetabola - ps23
Holometabola/Psocodea - ps24
Holometabola - ps25
Aparaglossata - ps26
Mecopterida - ps27
Diptera - ps28
Bibiono./Cyclorrhapha - ps29
Cyclorrhapha - ps30
Calyptratae/Drosophila - ps31
Drosophila - ps32
Sophophora - ps33
Obscura/Melanogaster_group - ps34
D_melanogaster_group - ps35
D_melanogaster_subgroup - ps36
sim./sech./mel. group - ps37
Drosophila_melanogaster - ps38
Phylostratum

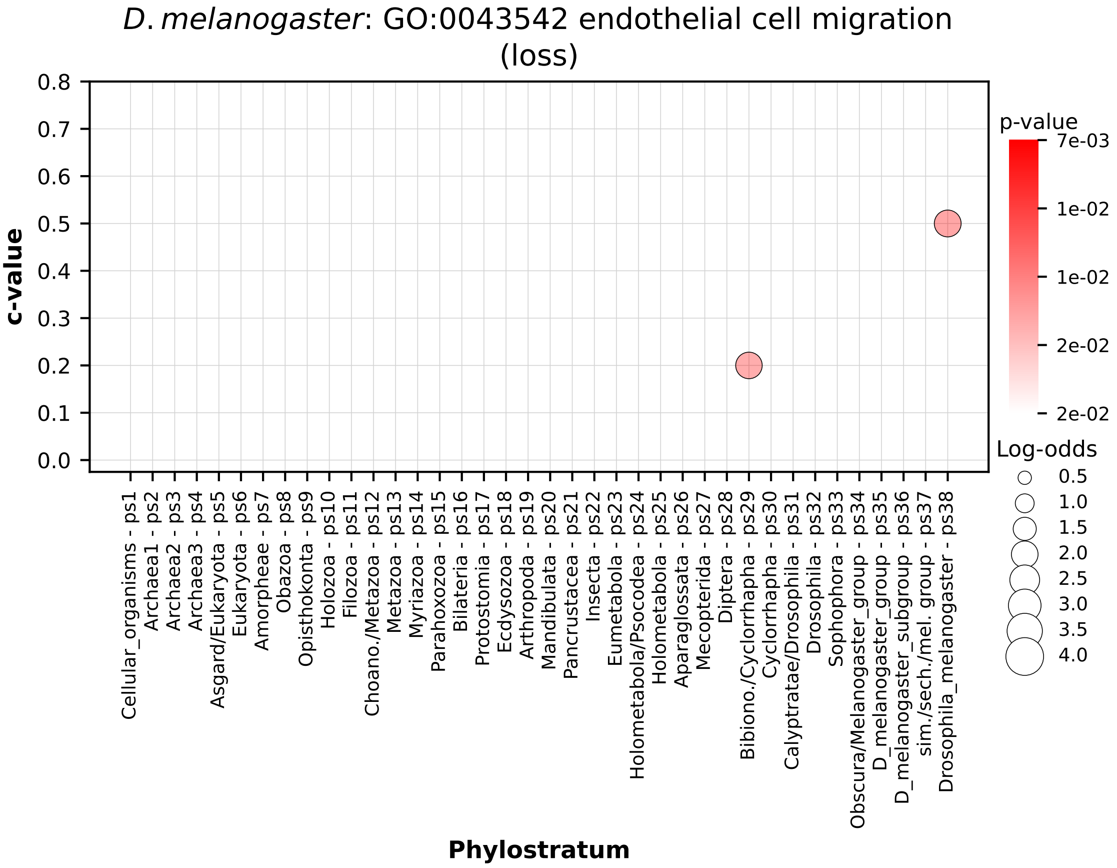

D. melanogaster: GO:0043542 endothelial cell migration (loss)
c-value
0.8
0.7
0.6
0.5
0.4
0.3
0.2
0.1
0.0
p-value
7e-03
1e-02
1e-02
2e-02
2e-02
Log-odds
0.5
1.0
1.5
2.0
2.5
3.0
3.5
4.0
Cellular_organisms - ps1
Archaea1 - ps2
Archaea2 - ps3
Archaea3 - ps4
Asgard/Eukaryota - ps5
Eukaryota - ps6
Amorpheae - ps7
Obazoa - ps8
Opisthokonta - ps9
Holozoa - ps10
Filozoa - ps11
Choano./Metazoa - ps12
Metazoa - ps13
Myriazoa - ps14
Parahoxozoa - ps15
Bilateria - ps16
Protostomia - ps17
Ecdysozoa - ps18
Arthropoda - ps19
Mandibulata - ps20
Pancrustacea - ps21
Insecta - ps22
Eumetabola - ps23
Holometabola/Psocodea - ps24
Holometabola - ps25
Aparaglossata - ps26
Mecopterida - ps27
Diptera - ps28
Bibiono./Cyclorrhapha - ps29
Cyclorrhapha - ps30
Calyptratae/Drosophila - ps31
Drosophila - ps32
Sophophora - ps33
Obscura/Melanogaster_group - ps34
D_melanogaster_group - ps35
D_melanogaster_subgroup - ps36
sim./sech./mel. group - ps37
Drosophila_melanogaster - ps38
Phylostratum

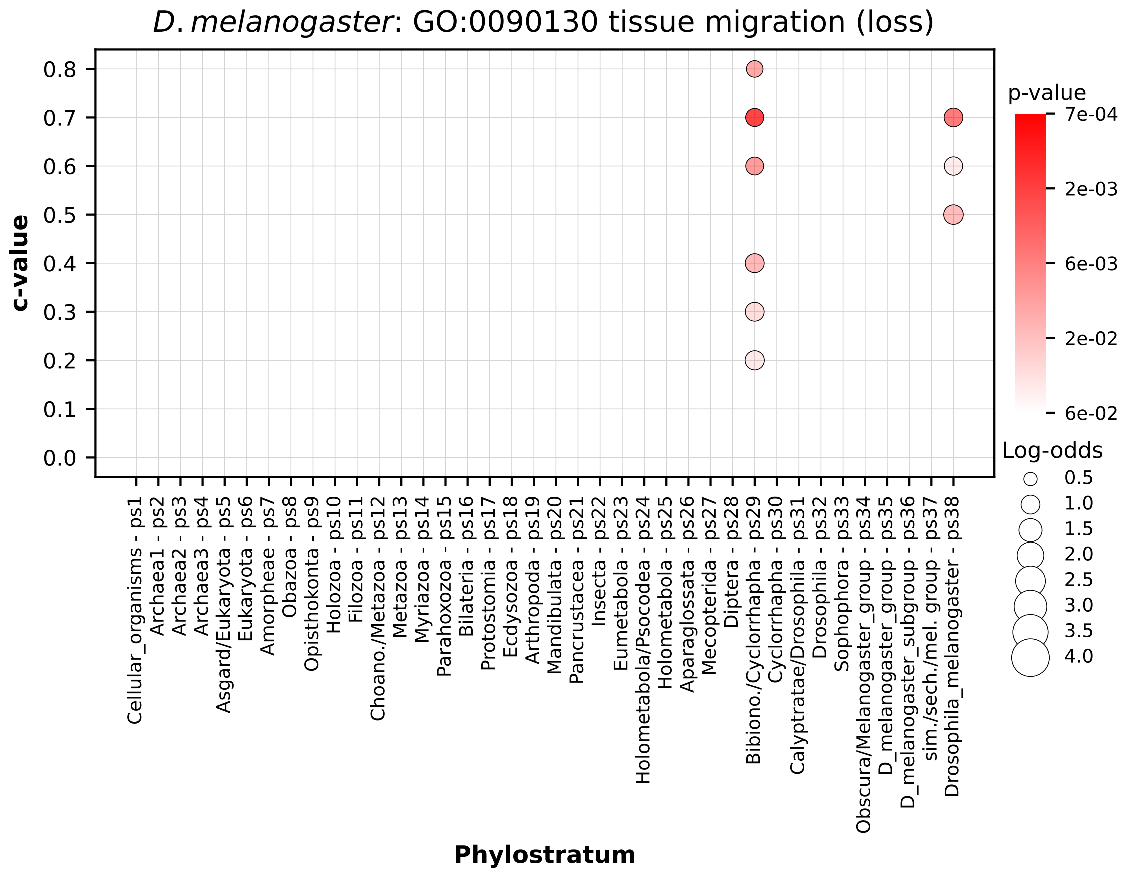
D. melanogaster: GO:0090130 tissue migration (loss)
c-value
0.0
0.1
0.2
0.3
0.4
0.5
0.6
0.7
0.8
Phylostratum
Cellular_organisms - ps1
Archaea1 - ps2
Archaea2 - ps3
Archaea3 - ps4
Asgard/Eukaryota - ps5
Eukaryota - ps6
Amorpheae - ps7
Obazoa - ps8
Opisthokonta - ps9
Holozoa - ps10
Filozoa - ps11
Choano./Metazoa - ps12
Metazoa - ps13
Myriazoa - ps14
Parahoxozoa - ps15
Bilateria - ps16
Protostomia - ps17
Ecdysozoa - ps18
Arthropoda - ps19
Mandibulata - ps20
Pancrustacea - ps21
Insecta - ps22
Eumetabola - ps23
Holometabola/Psocodea - ps24
Holometabola - ps25
Aparaglossata - ps26
Mecopterida - ps27
Diptera - ps28
Bibiono./Cyclorrhapha - ps29
Cyclorrhapha - ps30
Calyptratae/Drosophila - ps31
Drosophila - ps32
Sophophora - ps33
Obscura/Melanogaster_group - ps34
D_melanogaster_group - ps35
D_melanogaster_subgroup - ps36
sim./sech./mel. group - ps37
Drosophila_melanogaster - ps38
p-value
7e-04
2e-03
6e-03
2e-02
6e-02
Log-odds
0.5
1.0
1.5
2.0
2.5
3.0
3.5
4.0

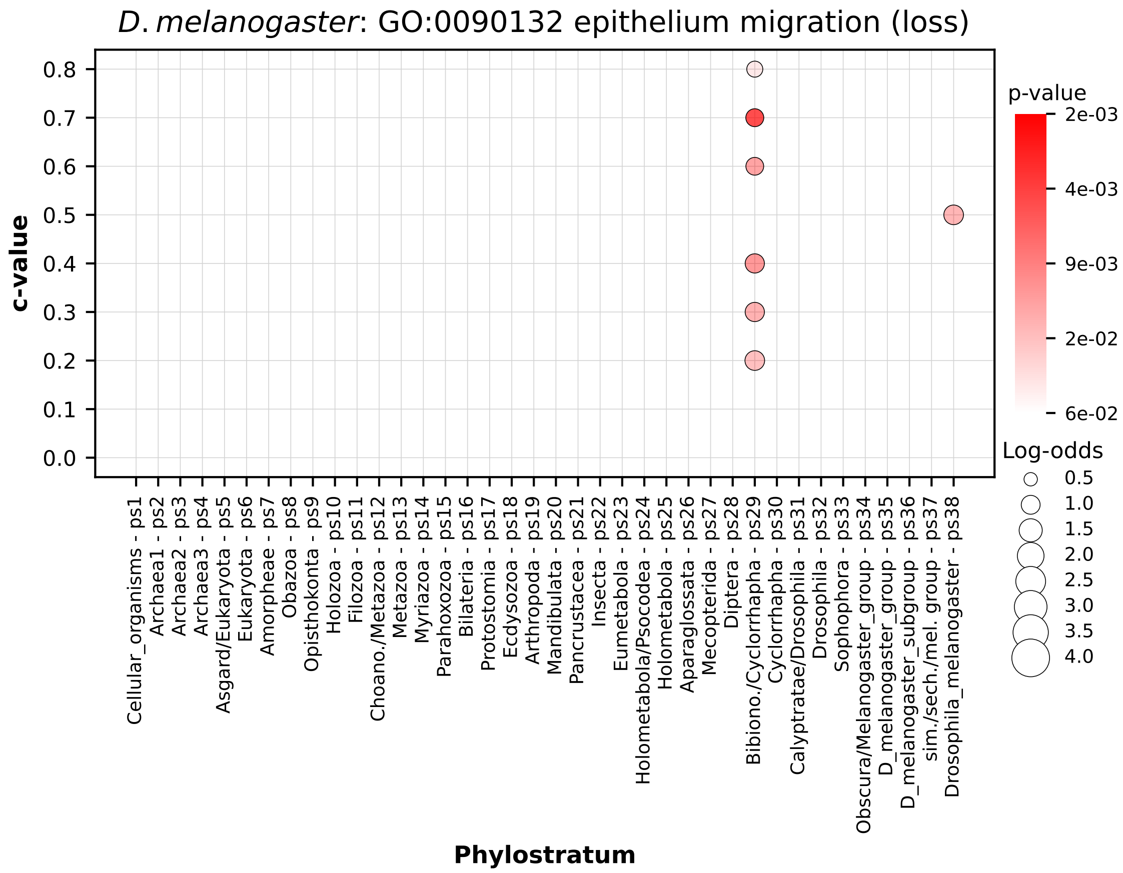

D. melanogaster: GO:0090132 epithelium migration (loss)
c-value
0.0
0.1
0.2
0.3
0.4
0.5
0.6
0.7
0.8
Phylostratum
Cellular_organisms - ps1
Archaea1 - ps2
Archaea2 - ps3
Archaea3 - ps4
Asgard/Eukaryota - ps5
Eukaryota - ps6
Amorpheae - ps7
Obazoa - ps8
Opisthokonta - ps9
Holozoa - ps10
Filozoa - ps11
Choano./Metazoa - ps12
Metazoa - ps13
Myriazoa - ps14
Parahoxozoa - ps15
Bilateria - ps16
Protostomia - ps17
Ecdysozoa - ps18
Arthropoda - ps19
Mandibulata - ps20
Pancrustacea - ps21
Insecta - ps22
Eumetabola - ps23
Holometabola/Psocodea - ps24
Holometabola - ps25
Aparaglossata - ps26
Mecopterida - ps27
Diptera - ps28
Bibiono./Cyclorrhapha - ps29
Cyclorrhapha - ps30
Calyptratae/Drosophila - ps31
Drosophila - ps32
Sophophora - ps33
Obscura/Melanogaster_group - ps34
D_melanogaster_group - ps35
D_melanogaster_subgroup - ps36
sim./sech./mel. group - ps37
Drosophila_melanogaster - ps38
p-value
2e-03
4e-03
9e-03
2e-02
6e-02
Log-odds
0.5
1.0
1.5
2.0
2.5
3.0
3.5
4.0

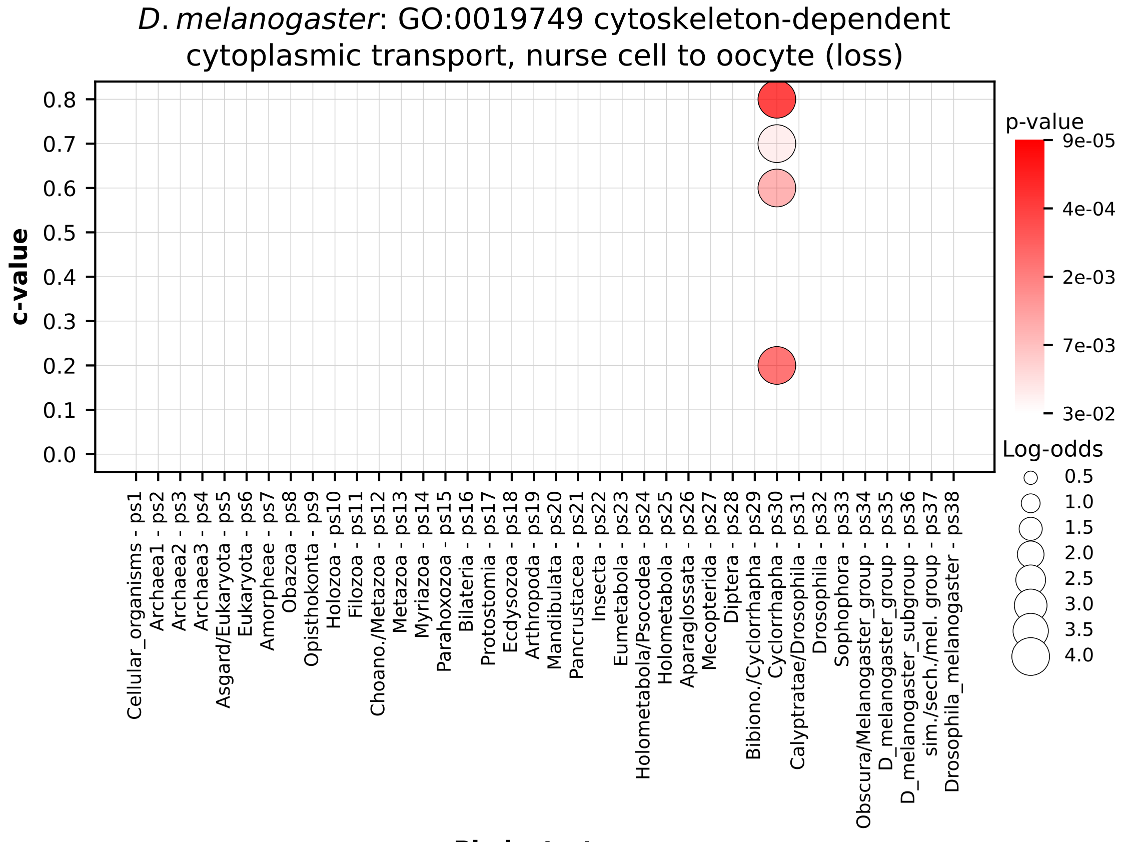

D. melanogaster: GO:0019749 cytoskeleton-dependent cytoplasmic transport, nurse cell to oocyte (loss)
c-value
0.0
0.1
0.2
0.3
0.4
0.5
0.6
0.7
0.8
p-value
9e-05
4e-04
2e-03
7e-03
3e-02
Log-odds
0.5
1.0
1.5
2.0
2.5
3.0
3.5
4.0
Cellular_organisms - ps1
Archaea1 - ps2
Archaea2 - ps3
Archaea3 - ps4
Asgard/Eukaryota - ps5
Eukaryota - ps6
Amorpheae - ps7
Obazoa - ps8
Opisthokonta - ps9
Holozoa - ps10
Filozoa - ps11
Choano./Metazoa - ps12
Metazoa - ps13
Myriazoa - ps14
Parahoxozoa - ps15
Bilateria - ps16
Protostomia - ps17
Ecdysozoa - ps18
Arthropoda - ps19
Mandibulata - ps20
Pancrustacea - ps21
Insecta - ps22
Eumetabola - ps23
Holometabola/Psocodea - ps24
Holometabola - ps25
Aparaglossata - ps26
Mecopterida - ps27
Diptera - ps28
Bibiono./Cyclorrhapha - ps29
Cyclorrhapha - ps30
Calyptratae/Drosophila - ps31
Drosophila - ps32
Sophophora - ps33
Obscura/Melanogaster_group - ps34
D_melanogaster_group - ps35
D_melanogaster_subgroup - ps36
sim./sech./mel. group - ps37
Drosophila_melanogaster - ps38
Phylostratum

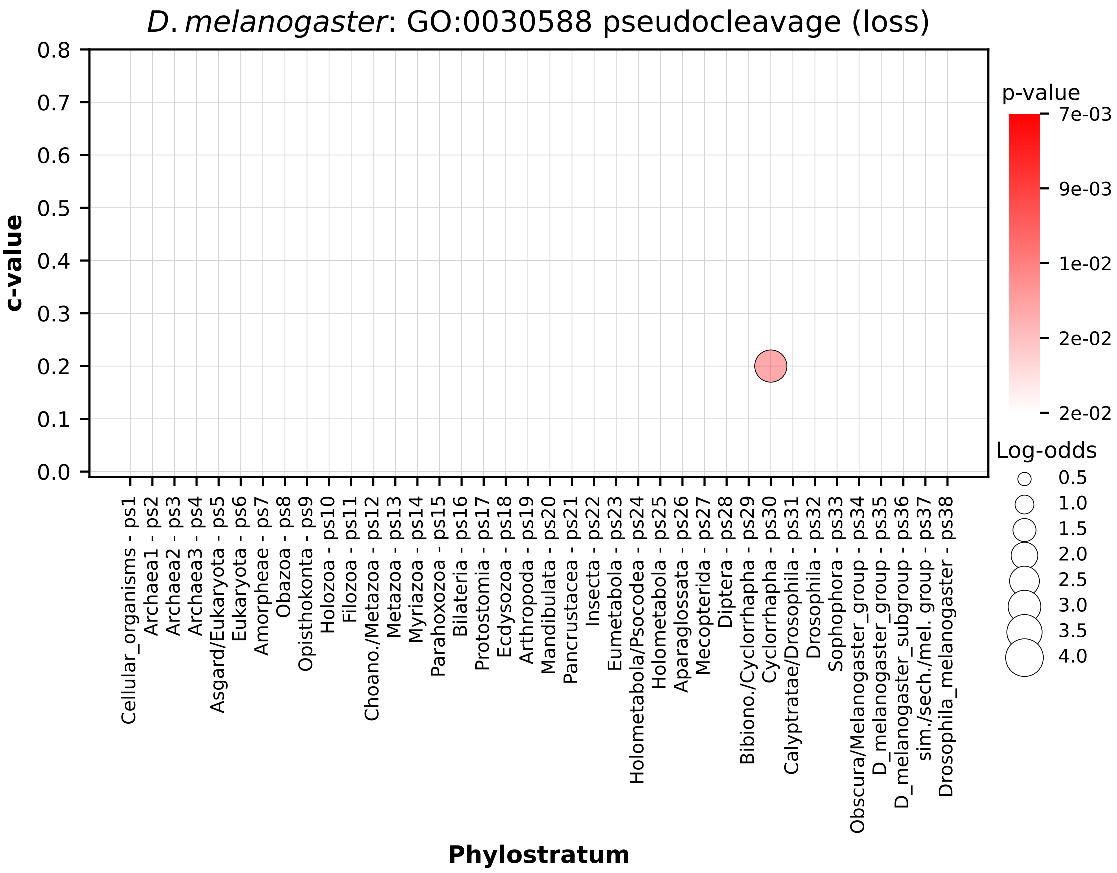
D. melanogaster: GO:0030588 pseudocleavage (loss)
c-value
0.0
0.1
0.2
0.3
0.4
0.5
0.6
0.7
0.8
Phylostratum
Cellular_organisms - ps1
Archaea1 - ps2
Archaea2 - ps3
Archaea3 - ps4
Asgard/Eukaryota - ps5
Eukaryota - ps6
Amorpheae - ps7
Obazoa - ps8
Opisthokonta - ps9
Holozoa - ps10
Filozoa - ps11
Choano./Metazoa - ps12
Metazoa - ps13
Myriazoa - ps14
Parahoxozoa - ps15
Bilateria - ps16
Protostomia - ps17
Ecdysozoa - ps18
Arthropoda - ps19
Mandibulata - ps20
Pancrustacea - ps21
Insecta - ps22
Eumetabola - ps23
Holometabola/Psocodea - ps24
Holometabola - ps25
Aparaglossata - ps26
Mecopterida - ps27
Diptera - ps28
Bibiono./Cyclorrhapha - ps29
Cyclorrhapha - ps30
Calyptratae/Drosophila - ps31
Drosophila - ps32
Sophophora - ps33
Obscura/Melanogaster_group - ps34
D_melanogaster_group - ps35
D_melanogaster_subgroup - ps36
sim./sech./mel. group - ps37
Drosophila_melanogaster - ps38
p-value
7e-03
9e-03
1e-02
2e-02
2e-02
Log-odds
0.5
1.0
1.5
2.0
2.5
3.0
3.5
4.0

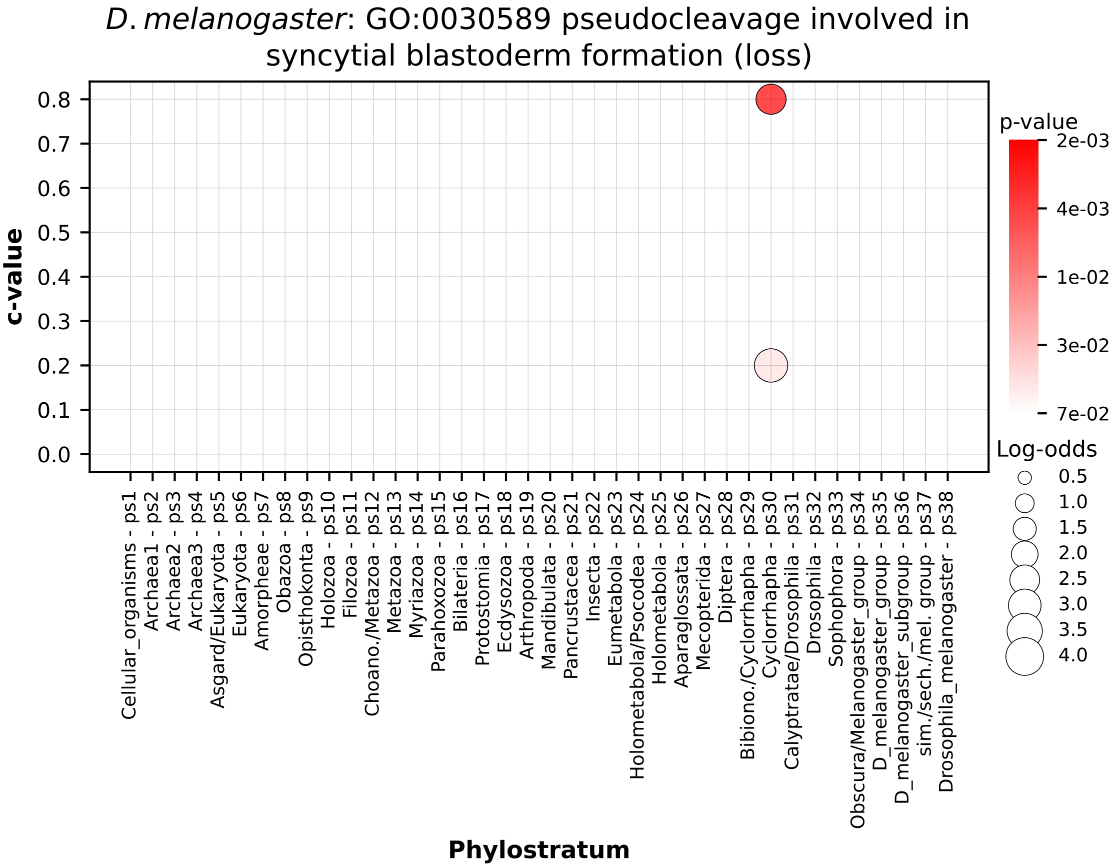

D. melanogaster: GO:0030589 pseudocleavage involved in syncytial blastoderm formation (loss)
c-value
0.8
0.7
0.6
0.5
0.4
0.3
0.2
0.1
0.0
p-value
2e-03
4e-03
1e-02
3e-02
7e-02
Log-odds
0.5
1.0
1.5
2.0
2.5
3.0
3.5
4.0
Cellular_organisms - ps1
Archaea1 - ps2
Archaea2 - ps3
Archaea3 - ps4
Asgard/Eukaryota - ps5
Eukaryota - ps6
Amorpheae - ps7
Obazoa - ps8
Opisthokonta - ps9
Holozoa - ps10
Filozoa - ps11
Choano./Metazoa - ps12
Metazoa - ps13
Myriazoa - ps14
Parahoxozoa - ps15
Bilateria - ps16
Protostomia - ps17
Ecdysozoa - ps18
Arthropoda - ps19
Mandibulata - ps20
Pancrustacea - ps21
Insecta - ps22
Eumetabola - ps23
Holometabola/Psocodea - ps24
Holometabola - ps25
Aparaglossata - ps26
Mecopterida - ps27
Diptera - ps28
Bibiono./Cyclorrhapha - ps29
Cyclorrhapha - ps30
Calyptratae/Drosophila - ps31
Drosophila - ps32
Sophophora - ps33
Obscura/Melanogaster_group - ps34
D_melanogaster_group - ps35
D_melanogaster_subgroup - ps36
sim./sech./mel. group - ps37
Drosophila_melanogaster - ps38
Phylostratum

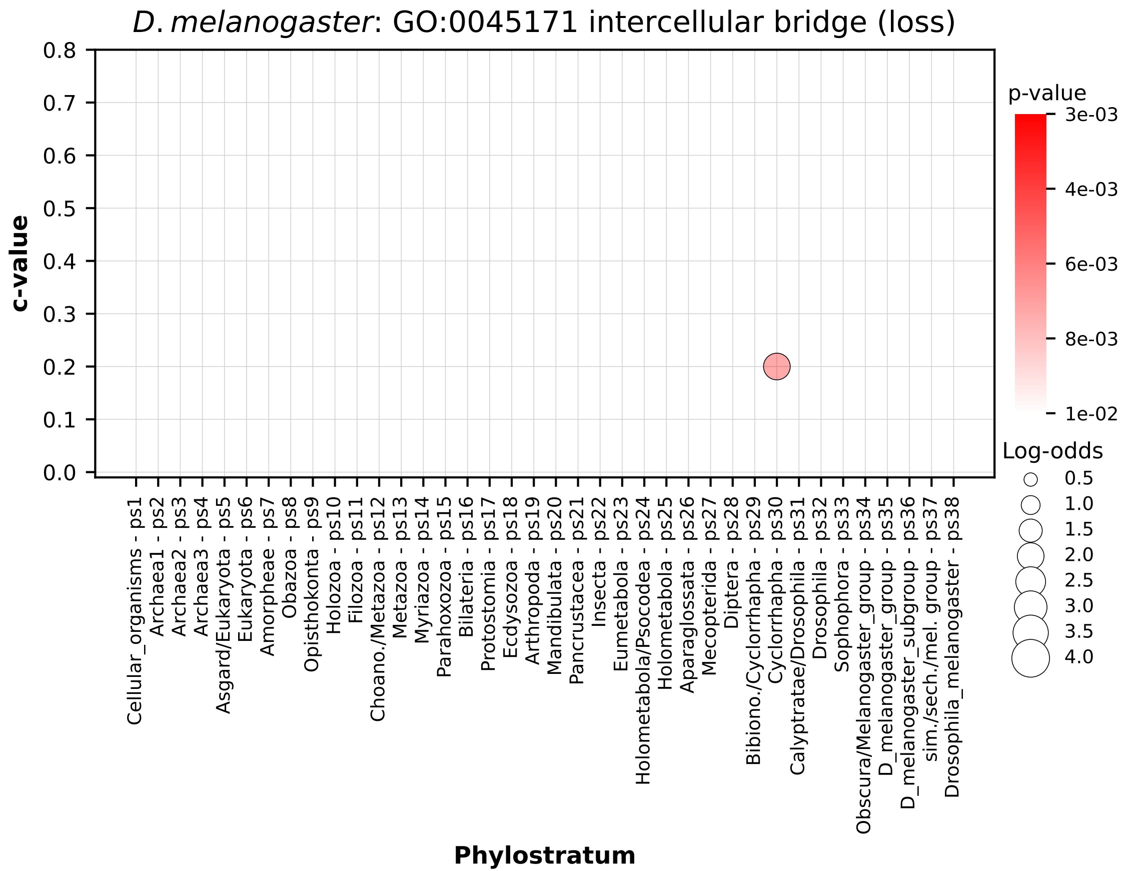

D. melanogaster: GO:0045171 intercellular bridge (loss)
c-value
0.0
0.1
0.2
0.3
0.4
0.5
0.6
0.7
0.8
Phylostratum
Cellular_organisms - ps1
Archaea1 - ps2
Archaea2 - ps3
Archaea3 - ps4
Asgard/Eukaryota - ps5
Eukaryota - ps6
Amorpheae - ps7
Obazoa - ps8
Opisthokonta - ps9
Holozoa - ps10
Filozoa - ps11
Choano./Metazoa - ps12
Metazoa - ps13
Myriazoa - ps14
Parahoxozoa - ps15
Bilateria - ps16
Protostomia - ps17
Ecdysozoa - ps18
Arthropoda - ps19
Mandibulata - ps20
Pancrustacea - ps21
Insecta - ps22
Eumetabola - ps23
Holometabola/Psocodea - ps24
Holometabola - ps25
Aparaglossata - ps26
Mecopterida - ps27
Diptera - ps28
Bibiono./Cyclorrhapha - ps29
Cyclorrhapha - ps30
Calyptratae/Drosophila - ps31
Drosophila - ps32
Sophophora - ps33
Obscura/Melanogaster_group - ps34
D_melanogaster_group - ps35
D_melanogaster_subgroup - ps36
sim./sech./mel. group - ps37
Drosophila_melanogaster - ps38
p-value
3e-03
4e-03
6e-03
8e-03
1e-02
Log-odds
0.5
1.0
1.5
2.0
2.5
3.0
3.5
4.0

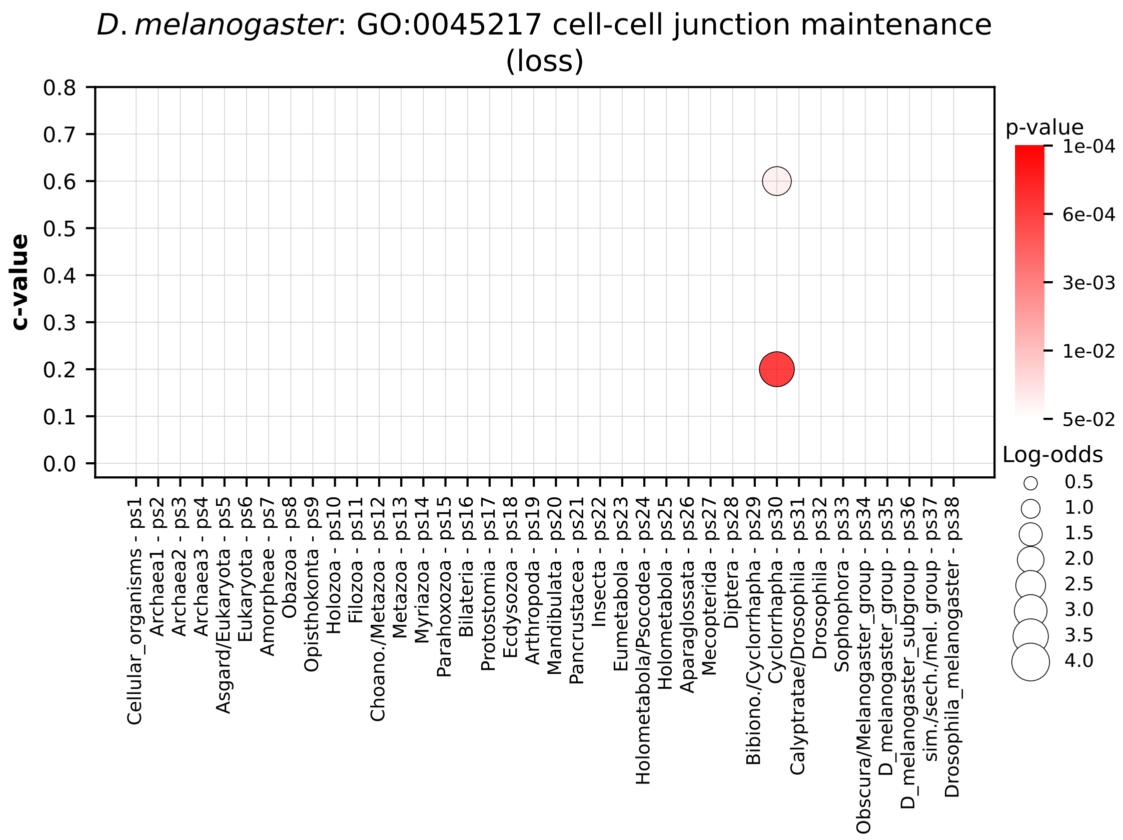

D. melanogaster: GO:0045217 cell-cell junction maintenance (loss)
c-value
0.8
0.7
0.6
0.5
0.4
0.3
0.2
0.1
0.0
Phylostratum
Cellular_organisms - ps1
Archaea1 - ps2
Archaea2 - ps3
Archaea3 - ps4
Asgard/Eukaryota - ps5
Eukaryota - ps6
Amorpheae - ps7
Obazoa - ps8
Opisthokonta - ps9
Holozoa - ps10
Filozoa - ps11
Choano./Metazoa - ps12
Metazoa - ps13
Myriazoa - ps14
Parahoxozoa - ps15
Bilateria - ps16
Protostomia - ps17
Ecdysozoa - ps18
Arthropoda - ps19
Mandibulata - ps20
Pancrustacea - ps21
Insecta - ps22
Eumetabola - ps23
Holometabola/Psocodea - ps24
Holometabola - ps25
Aparaglossata - ps26
Mecopterida - ps27
Diptera - ps28
Bibiono./Cyclorrhapha - ps29
Cyclorrhapha - ps30
Calyptratae/Drosophila - ps31
Drosophila - ps32
Sophophora - ps33
Obscura/Melanogaster_group - ps34
D_melanogaster_group - ps35
D_melanogaster_subgroup - ps36
sim./sech./mel. group - ps37
Drosophila_melanogaster - ps38
p-value
1e-04
6e-04
3e-03
1e-02
5e-02
Log-odds
0.5
1.0
1.5
2.0
2.5
3.0
3.5
4.0

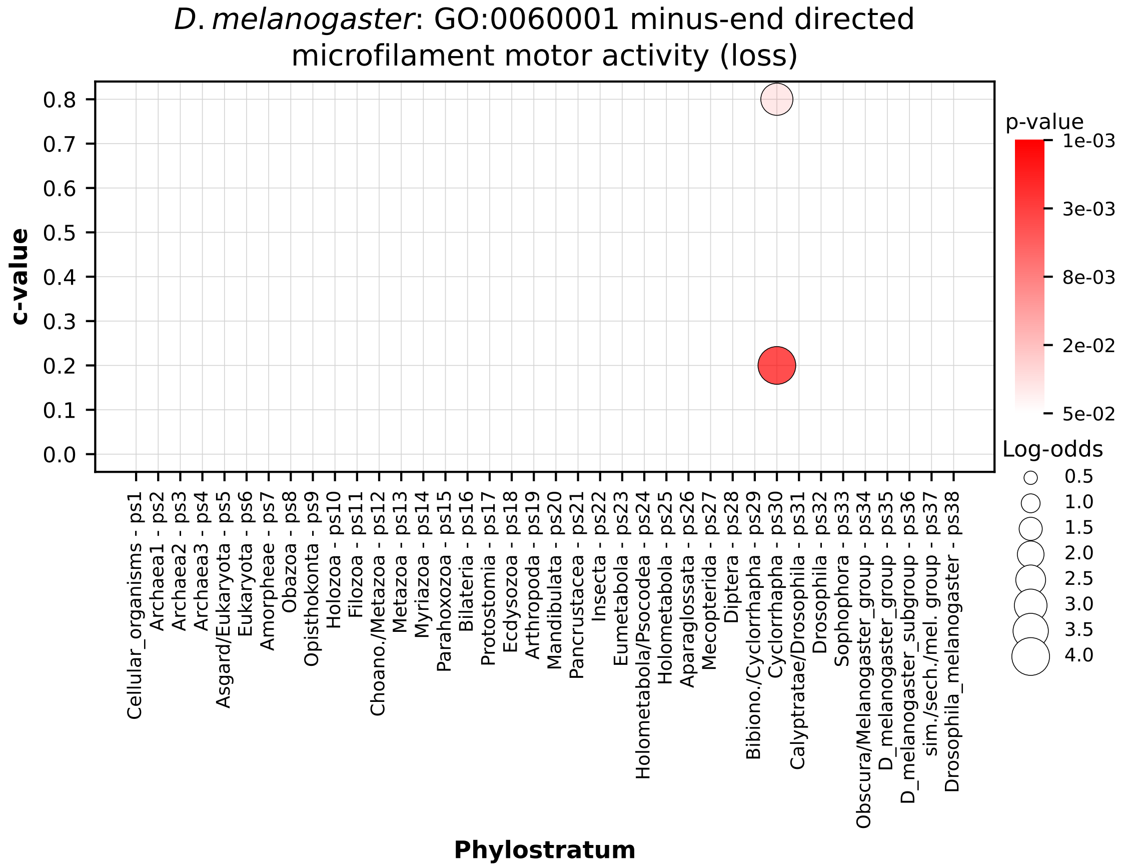

D. melanogaster: GO:0060001 minus-end directed microfilament motor activity (loss)
c-value
0.0
0.1
0.2
0.3
0.4
0.5
0.6
0.7
0.8
Phylostratum
Cellular_organisms - ps1
Archaea1 - ps2
Archaea2 - ps3
Archaea3 - ps4
Asgard/Eukaryota - ps5
Eukaryota - ps6
Amorpheae - ps7
Obazoa - ps8
Opisthokonta - ps9
Holozoa - ps10
Filozoa - ps11
Choano./Metazoa - ps12
Metazoa - ps13
Myriazoa - ps14
Parahoxozoa - ps15
Bilateria - ps16
Protostomia - ps17
Ecdysozoa - ps18
Arthropoda - ps19
Mandibulata - ps20
Pancrustacea - ps21
Insecta - ps22
Eumetabola - ps23
Holometabola/Psocodea - ps24
Holometabola - ps25
Aparaglossata - ps26
Mecopterida - ps27
Diptera - ps28
Bibiono./Cyclorrhapha - ps29
Cyclorrhapha - ps30
Calyptratae/Drosophila - ps31
Drosophila - ps32
Sophophora - ps33
Obscura/Melanogaster_group - ps34
D_melanogaster_group - ps35
D_melanogaster_subgroup - ps36
sim./sech./mel. group - ps37
Drosophila_melanogaster - ps38
p-value
1e-03
3e-03
8e-03
2e-02
5e-02
Log-odds
0.5
1.0
1.5
2.0
2.5
3.0
3.5
4.0

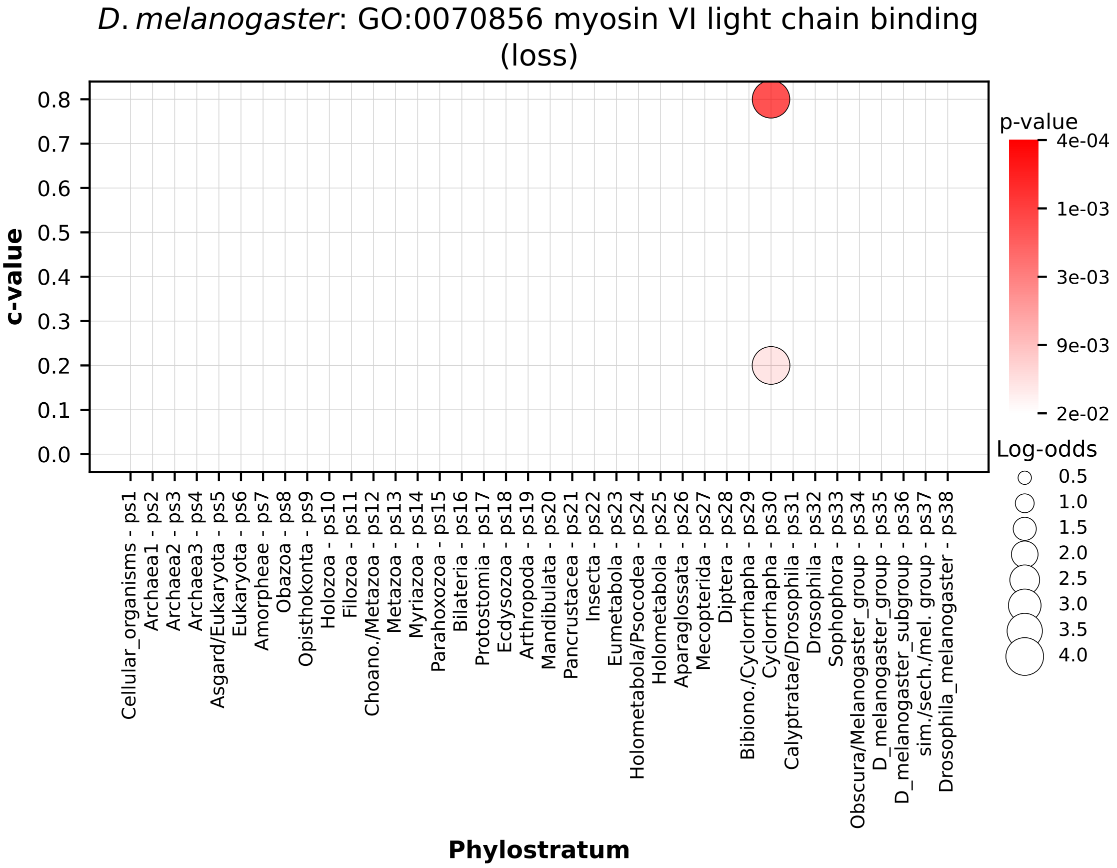

D. melanogaster: GO:0070856 myosin VI light chain binding
(loss)
c-value
0.8
0.7
0.6
0.5
0.4
0.3
0.2
0.1
0.0
p-value
4e-04
1e-03
3e-03
9e-03
2e-02
Log-odds
0.5
1.0
1.5
2.0
2.5
3.0
3.5
4.0
Cellular_organisms - ps1
Archaea1 - ps2
Archaea2 - ps3
Archaea3 - ps4
Asgard/Eukaryota - ps5
Eukaryota - ps6
Amorpheae - ps7
Obazoa - ps8
Opisthokonta - ps9
Holozoa - ps10
Filozoa - ps11
Choano./Metazoa - ps12
Metazoa - ps13
Myriazoa - ps14
Parahoxozoa - ps15
Bilateria - ps16
Protostomia - ps17
Ecdysozoa - ps18
Arthropoda - ps19
Mandibulata - ps20
Pancrustacea - ps21
Insecta - ps22
Eumetabola - ps23
Holometabola/Psocodea - ps24
Holometabola - ps25
Aparaglossata - ps26
Mecopterida - ps27
Diptera - ps28
Bibiono./Cyclorrhapha - ps29
Cyclorrhapha - ps30
Calyptratae/Drosophila - ps31
Drosophila - ps32
Sophophora - ps33
Obscura/Melanogaster_group - ps34
D_melanogaster_group - ps35
D_melanogaster_subgroup - ps36
sim./sech./mel. group - ps37
Drosophila_melanogaster - ps38
Phylostratum

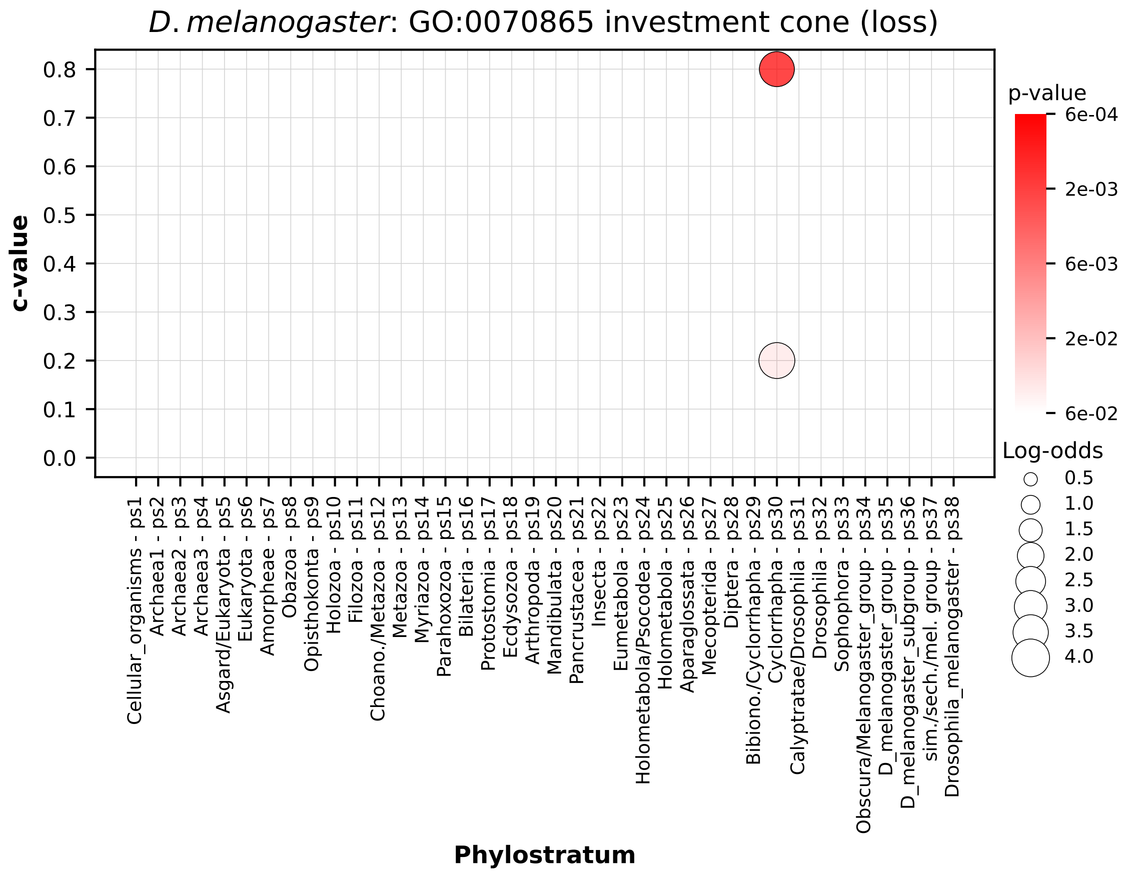
D. melanogaster: GO:0070865 investment cone (loss)
c-value
Phylostratum
p-value
6e-04
2e-03
6e-03
2e-02
6e-02
Log-odds
0.5
1.0
1.5
2.0
2.5
3.0
3.5
4.0
Cellular_organisms - ps1
Archaea1 - ps2
Archaea2 - ps3
Archaea3 - ps4
Asgard/Eukaryota - ps5
Eukaryota - ps6
Amorpheae - ps7
Obazoa - ps8
Opisthokonta - ps9
Holozoa - ps10
Filozoa - ps11
Choano./Metazoa - ps12
Metazoa - ps13
Myriazoa - ps14
Parahoxozoa - ps15
Bilateria - ps16
Protostomia - ps17
Ecdysozoa - ps18
Arthropoda - ps19
Mandibulata - ps20
Pancrustacea - ps21
Insecta - ps22
Eumetabola - ps23
Holometabola/Psocodea - ps24
Holometabola - ps25
Aparaglossata - ps26
Mecopterida - ps27
Diptera - ps28
Bibiono./Cyclorrhapha - ps29
Cyclorrhapha - ps30
Calyptratae/Drosophila - ps31
Drosophila - ps32
Sophophora - ps33
Obscura/Melanogaster_group - ps34
D_melanogaster_group - ps35
D_melanogaster_subgroup - ps36
sim./sech./mel. group - ps37
Drosophila_melanogaster - ps38

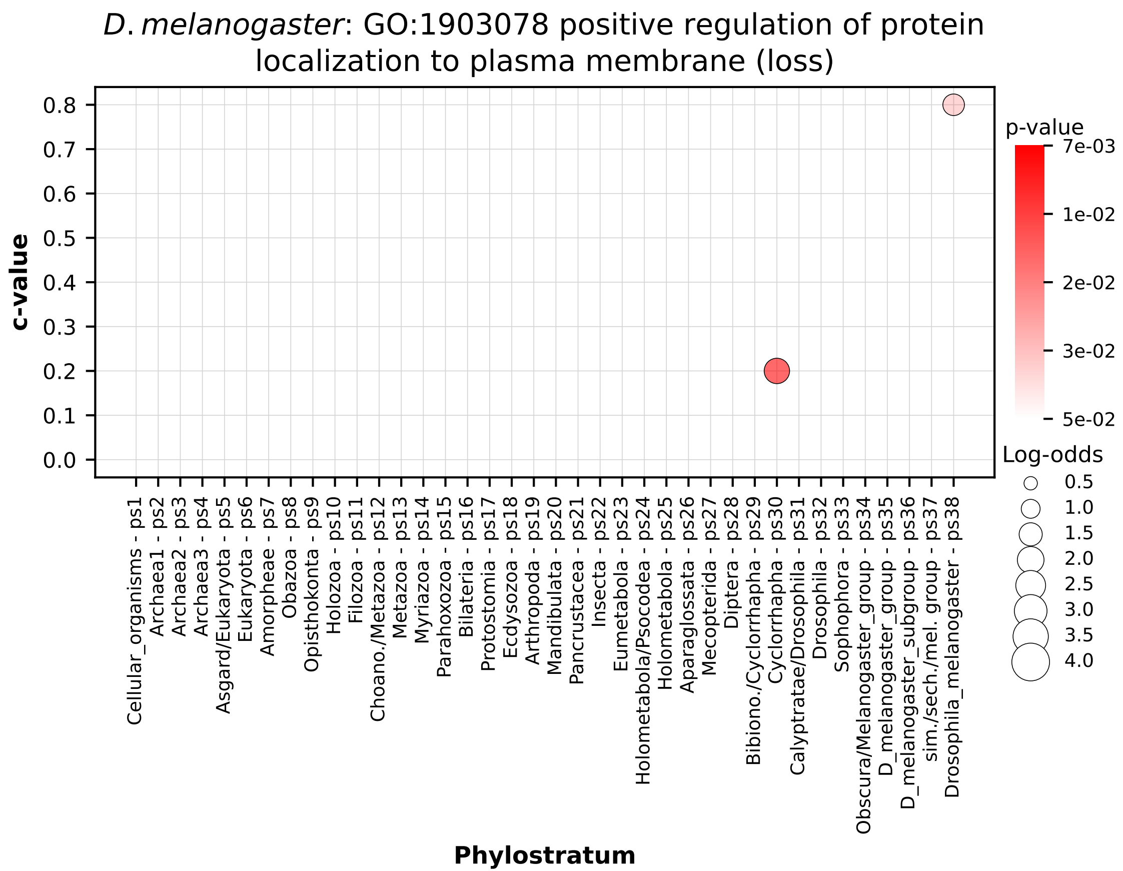

D. melanogaster: GO:1903078 positive regulation of protein localization to plasma membrane (loss)
c-value
0.8
0.7
0.6
0.5
0.4
0.3
0.2
0.1
0.0
p-value
7e-03
1e-02
2e-02
3e-02
5e-02
Log-odds
0.5
1.0
1.5
2.0
2.5
3.0
3.5
4.0
Cellular_organisms - ps1
Archaea1 - ps2
Archaea2 - ps3
Archaea3 - ps4
Asgard/Eukaryota - ps5
Eukaryota - ps6
Amorpheae - ps7
Obazoa - ps8
Opisthokonta - ps9
Holozoa - ps10
Filozoa - ps11
Choano./Metazoa - ps12
Metazoa - ps13
Myriazoa - ps14
Parahoxozoa - ps15
Bilateria - ps16
Protostomia - ps17
Ecdysozoa - ps18
Arthropoda - ps19
Mandibulata - ps20
Pancrustacea - ps21
Insecta - ps22
Eumetabola - ps23
Holometabola/Psocodea - ps24
Holometabola - ps25
Aparaglossata - ps26
Mecopterida - ps27
Diptera - ps28
Bibiono./Cyclorrhapha - ps29
Cyclorrhapha - ps30
Calyptratae/Drosophila - ps31
Drosophila - ps32
Sophophora - ps33
Obscura/Melanogaster_group - ps34
D_melanogaster_group - ps35
D_melanogaster_subgroup - ps36
sim./sech./mel. group - ps37
Drosophila_melanogaster - ps38
Phylostratum

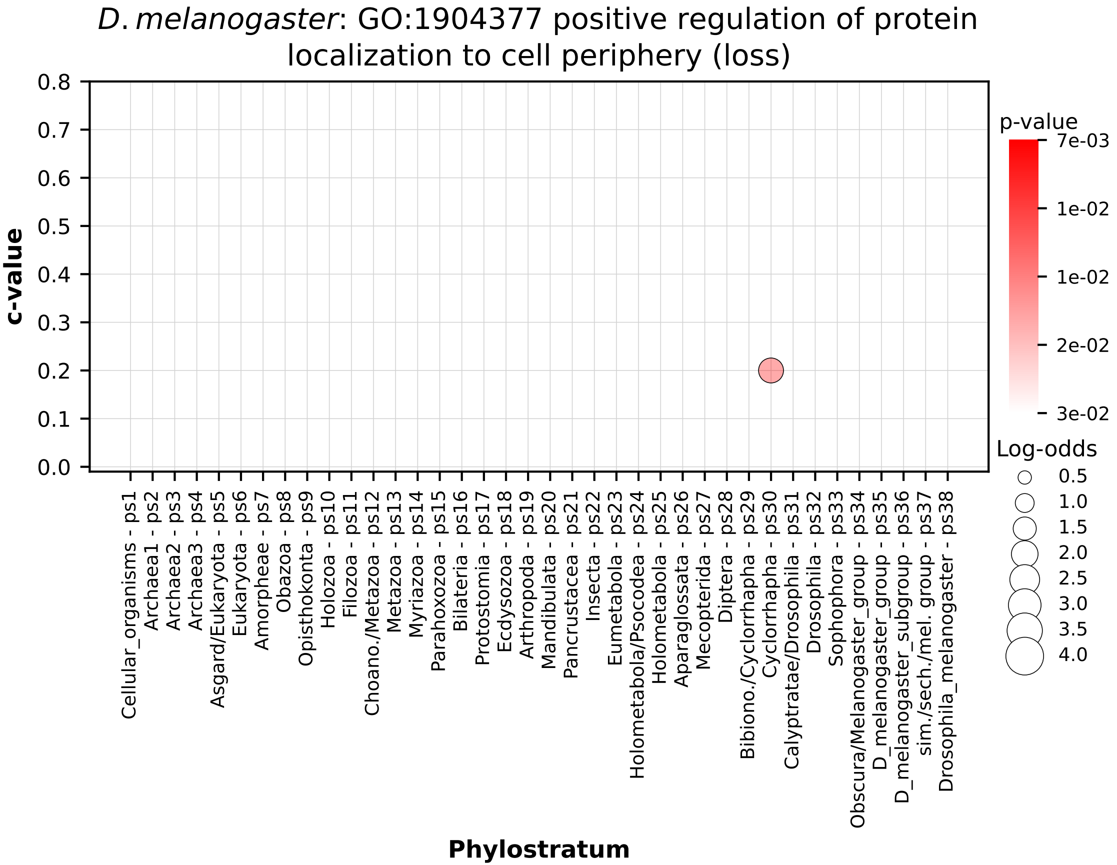

D. melanogaster: GO:1904377 positive regulation of protein localization to cell periphery (loss)
c-value
0.8
0.7
0.6
0.5
0.4
0.3
0.2
0.1
0.0
p-value
7e-03
1e-02
1e-02
2e-02
3e-02
Log-odds
0.5
1.0
1.5
2.0
2.5
3.0
3.5
4.0
Cellular_organisms - ps1
Archaea1 - ps2
Archaea2 - ps3
Archaea3 - ps4
Asgard/Eukaryota - ps5
Eukaryota - ps6
Amorpheae - ps7
Obazoa - ps8
Opisthokonta - ps9
Holozoa - ps10
Filozoa - ps11
Choano./Metazoa - ps12
Metazoa - ps13
Myriazoa - ps14
Parahoxozoa - ps15
Bilateria - ps16
Protostomia - ps17
Ecdysozoa - ps18
Arthropoda - ps19
Mandibulata - ps20
Pancrustacea - ps21
Insecta - ps22
Eumetabola - ps23
Holometabola/Psocodea - ps24
Holometabola - ps25
Aparaglossata - ps26
Mecopterida - ps27
Diptera - ps28
Bibiono./Cyclorrhapha - ps29
Cyclorrhapha - ps30
Calyptratae/Drosophila - ps31
Drosophila - ps32
Sophophora - ps33
Obscura/Melanogaster_group - ps34
D_melanogaster_group - ps35
D_melanogaster_subgroup - ps36
sim./sech./mel. group - ps37
Drosophila_melanogaster - ps38
Phylostratum

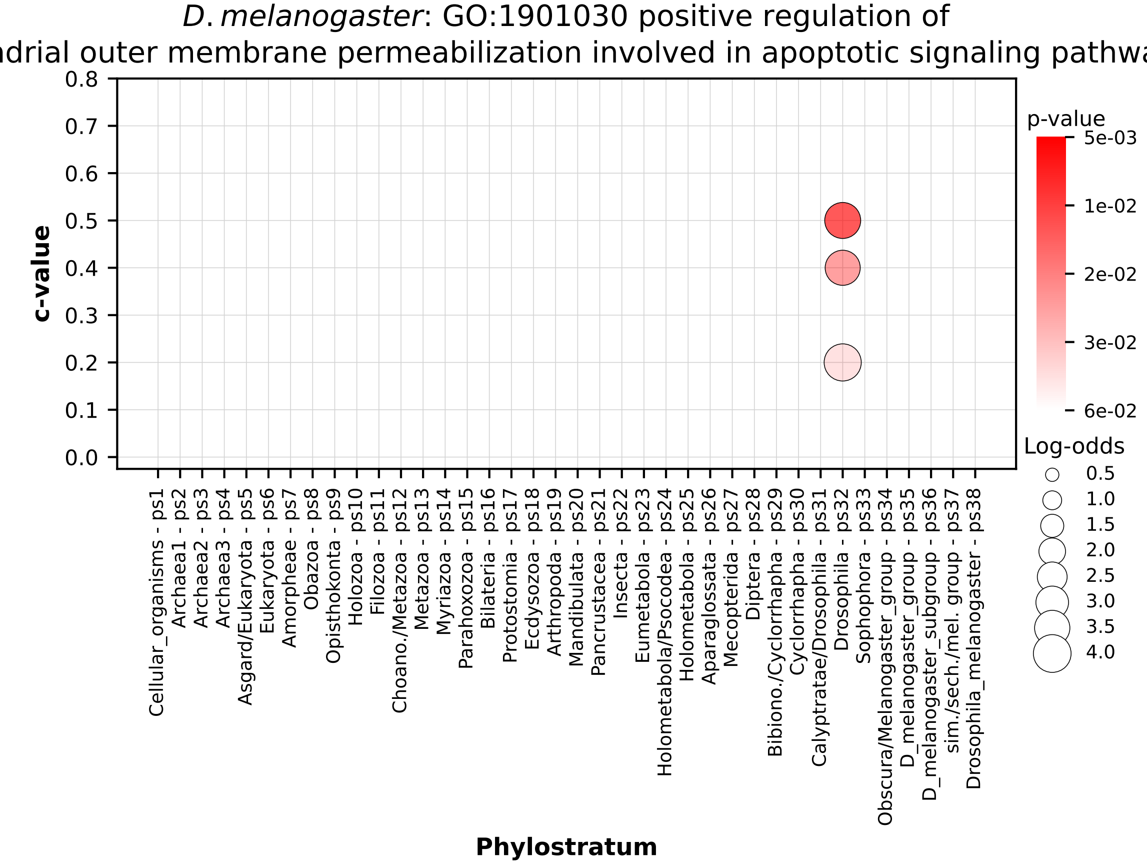

D. melanogaster: GO:1901030 positive regulation of
ndrial outer membrane permeabilization involved in apoptotic signaling pathway (los
c-value
0.8
0.7
0.6
0.5
0.4
0.3
0.2
0.1
0.0
p-value
5e-03
1e-02
2e-02
3e-02
6e-02
Log-odds
0.5
1.0
1.5
2.0
2.5
3.0
3.5
4.0
Cellular_organisms - ps1
Archaea1 - ps2
Archaea2 - ps3
Archaea3 - ps4
Asgard/Eukaryota - ps5
Eukaryota - ps6
Amorpheae - ps7
Obazoa - ps8
Opisthokonta - ps9
Holozoa - ps10
Filozoa - ps11
Choano./Metazoa - ps12
Metazoa - ps13
Myriazoa - ps14
Parahoxozoa - ps15
Bilateria - ps16
Protostomia - ps17
Ecdysozoa - ps18
Arthropoda - ps19
Mandibulata - ps20
Pancrustacea - ps21
Insecta - ps22
Eumetabola - ps23
Holometabola/Psocodea - ps24
Holometabola - ps25
Aparaglossata - ps26
Mecopterida - ps27
Diptera - ps28
Bibiono./Cyclorrhapha - ps29
Cyclorrhapha - ps30
Calyptratae/Drosophila - ps31
Drosophila - ps32
Sophophora - ps33
Obscura/Melanogaster_group - ps34
D_melanogaster_group - ps35
D_melanogaster_subgroup - ps36
sim./sech./mel. group - ps37
Drosophila_melanogaster - ps38
Phylostratum

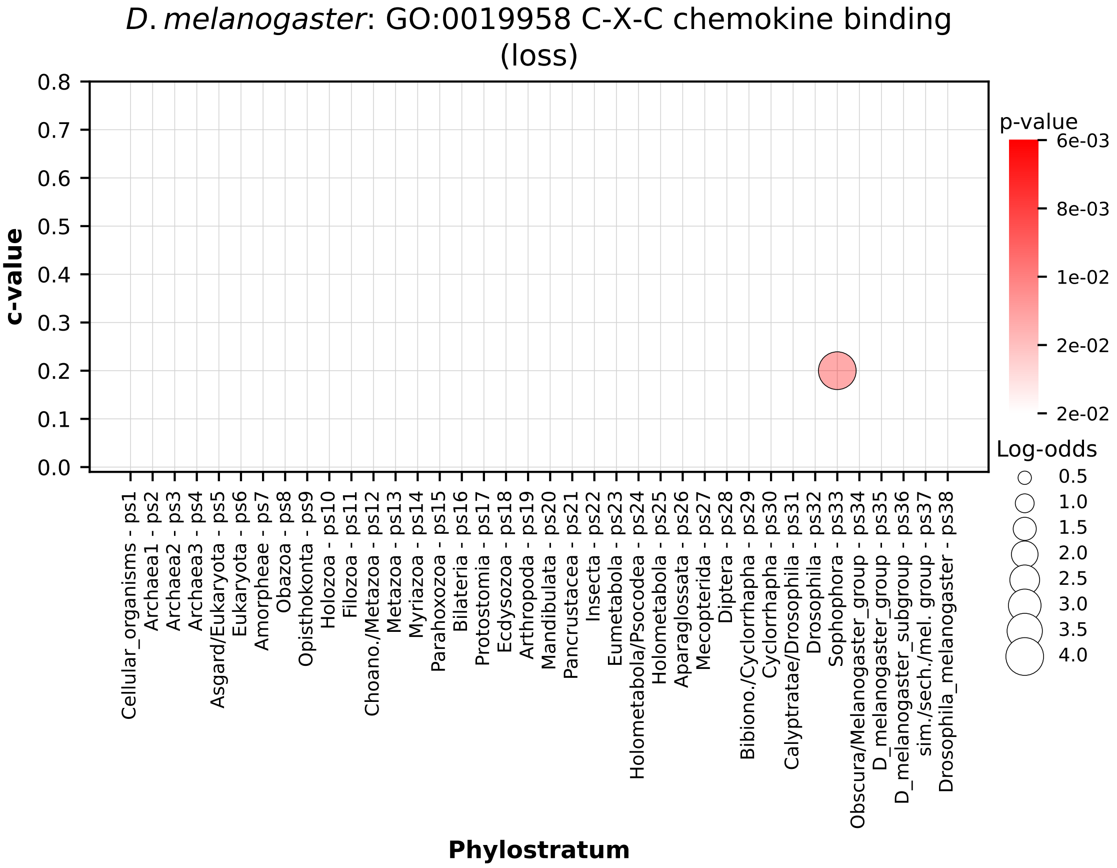
D. melanogaster: GO:0019958 C-X-C chemokine binding
(loss)
c-value
0.0
0.1
0.2
0.3
0.4
0.5
0.6
0.7
0.8
Phylostratum
Cellular_organisms - ps1
Archaea1 - ps2
Archaea2 - ps3
Archaea3 - ps4
Asgard/Eukaryota - ps5
Eukaryota - ps6
Amorpheae - ps7
Obazoa - ps8
Opisthokonta - ps9
Holozoa - ps10
Filozoa - ps11
Choano./Metazoa - ps12
Metazoa - ps13
Myriazoa - ps14
Parahoxozoa - ps15
Bilateria - ps16
Protostomia - ps17
Ecdysozoa - ps18
Arthropoda - ps19
Mandibulata - ps20
Pancrustacea - ps21
Insecta - ps22
Eumetabola - ps23
Holometabola/Psocodea - ps24
Holometabola - ps25
Aparaglossata - ps26
Mecopterida - ps27
Diptera - ps28
Bibiono./Cyclorrhapha - ps29
Cyclorrhapha - ps30
Calyptratae/Drosophila - ps31
Drosophila - ps32
Sophophora - ps33
Obscura/Melanogaster_group - ps34
D_melanogaster_group - ps35
D_melanogaster_subgroup - ps36
sim./sech./mel. group - ps37
Drosophila_melanogaster - ps38
p-value
6e-03
8e-03
1e-02
2e-02
2e-02
Log-odds
0.5
1.0
1.5
2.0
2.5
3.0
3.5
4.0

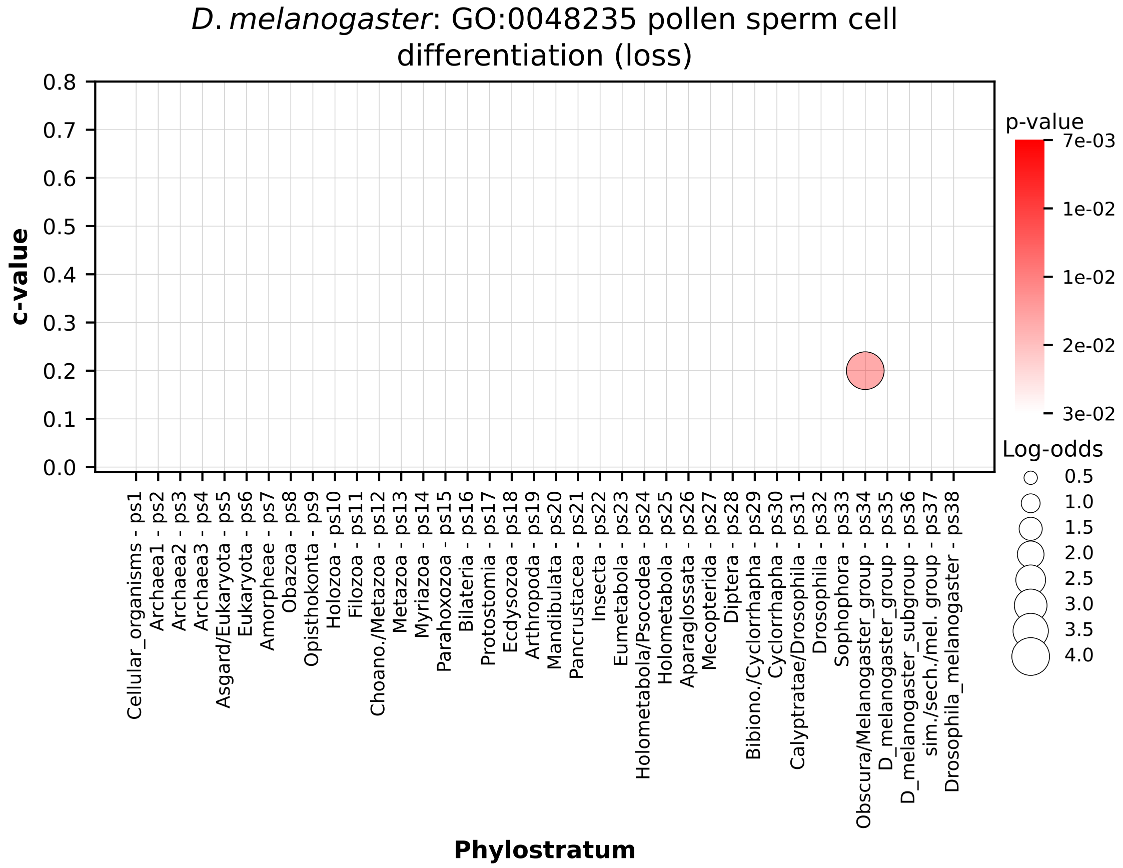

D. melanogaster: GO:0048235 pollen sperm cell differentiation (loss)
c-value
0.8
0.7
0.6
0.5
0.4
0.3
0.2
0.1
0.0
p-value
7e-03
1e-02
1e-02
2e-02
3e-02
Log-odds
0.5
1.0
1.5
2.0
2.5
3.0
3.5
4.0
Cellular_organisms - ps1
Archaea1 - ps2
Archaea2 - ps3
Archaea3 - ps4
Asgard/Eukaryota - ps5
Eukaryota - ps6
Amorpheae - ps7
Obazoa - ps8
Opisthokonta - ps9
Holozoa - ps10
Filozoa - ps11
Choano./Metazoa - ps12
Metazoa - ps13
Myriazoa - ps14
Parahoxozoa - ps15
Bilateria - ps16
Protostomia - ps17
Ecdysozoa - ps18
Arthropoda - ps19
Mandibulata - ps20
Pancrustacea - ps21
Insecta - ps22
Eumetabola - ps23
Holometabola/Psocodea - ps24
Holometabola - ps25
Aparaglossata - ps26
Mecopterida - ps27
Diptera - ps28
Bibiono./Cyclorrhapha - ps29
Cyclorrhapha - ps30
Calyptratae/Drosophila - ps31
Drosophila - ps32
Sophophora - ps33
Obscura/Melanogaster_group - ps34
D_melanogaster_group - ps35
D_melanogaster_subgroup - ps36
sim./sech./mel. group - ps37
Drosophila_melanogaster - ps38
Phylostratum

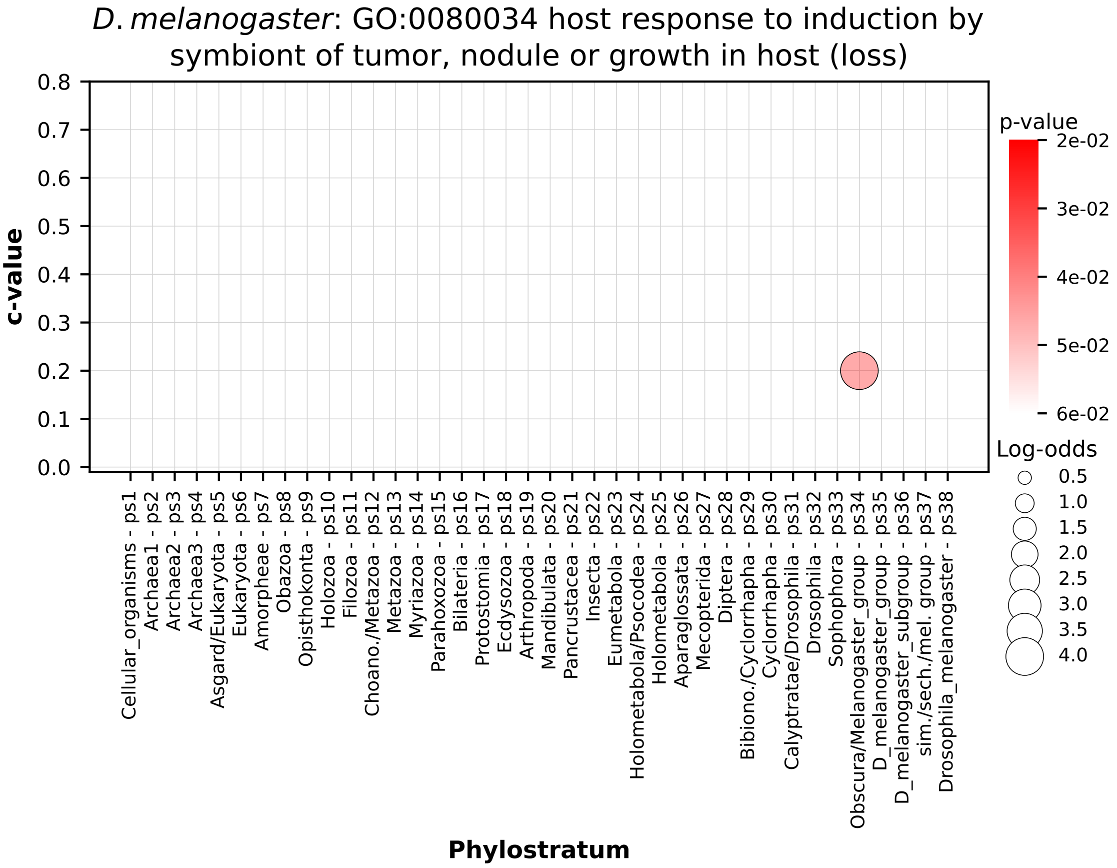
D. melanogaster: GO:0080034 host response to induction by symbiont of tumor, nodule or growth in host (loss)
c-value
0.8
0.7
0.6
0.5
0.4
0.3
0.2
0.1
0.0
p-value
2e-02
3e-02
4e-02
5e-02
6e-02
Log-odds
0.5
1.0
1.5
2.0
2.5
3.0
3.5
4.0
Cellular_organisms - ps1
Archaea1 - ps2
Archaea2 - ps3
Archaea3 - ps4
Asgard/Eukaryota - ps5
Eukaryota - ps6
Amorpheae - ps7
Obazoa - ps8
Opisthokonta - ps9
Holozoa - ps10
Filozoa - ps11
Choano./Metazoa - ps12
Metazoa - ps13
Myriazoa - ps14
Parahoxozoa - ps15
Bilateria - ps16
Protostomia - ps17
Ecdysozoa - ps18
Arthropoda - ps19
Mandibulata - ps20
Pancrustacea - ps21
Insecta - ps22
Eumetabola - ps23
Holometabola/Psocodea - ps24
Holometabola - ps25
Aparaglossata - ps26
Mecopterida - ps27
Diptera - ps28
Bibiono./Cyclorrhapha - ps29
Cyclorrhapha - ps30
Calyptratae/Drosophila - ps31
Drosophila - ps32
Sophophora - ps33
Obscura/Melanogaster_group - ps34
D_melanogaster_group - ps35
D_melanogaster_subgroup - ps36
sim./sech./mel. group - ps37
Drosophila_melanogaster - ps38
Phylostratum

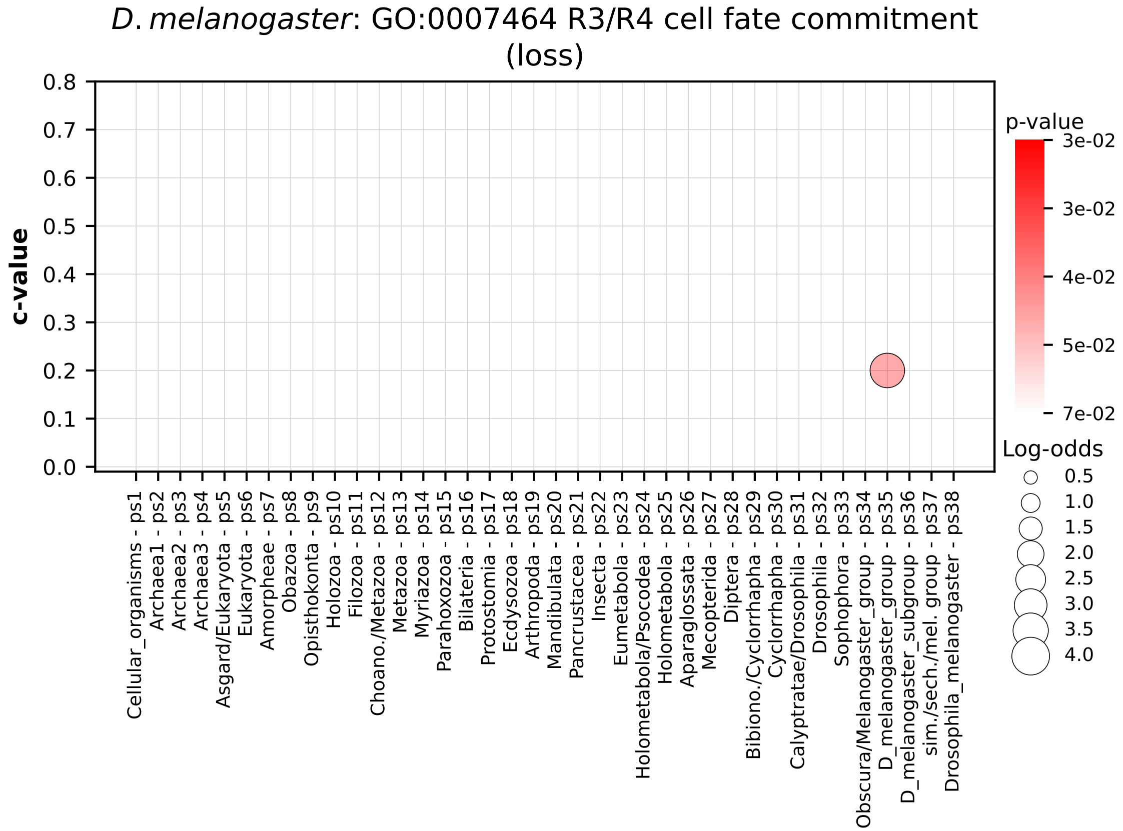

D. melanogaster: GO:0007464 R3/R4 cell fate commitment (loss)
c-value
0.0
0.1
0.2
0.3
0.4
0.5
0.6
0.7
0.8
Phylostratum
Cellular_organisms - ps1
Archaea1 - ps2
Archaea2 - ps3
Archaea3 - ps4
Asgard/Eukaryota - ps5
Eukaryota - ps6
Amorpheae - ps7
Obazoa - ps8
Opisthokonta - ps9
Holozoa - ps10
Filozoa - ps11
Choano./Metazoa - ps12
Metazoa - ps13
Myriazoa - ps14
Parahoxozoa - ps15
Bilateria - ps16
Protostomia - ps17
Ecdysozoa - ps18
Arthropoda - ps19
Mandibulata - ps20
Pancrustacea - ps21
Insecta - ps22
Eumetabola - ps23
Holometabola/Psocodea - ps24
Holometabola - ps25
Aparaglossata - ps26
Mecopterida - ps27
Diptera - ps28
Bibiono./Cyclorrhapha - ps29
Cyclorrhapha - ps30
Calyptratae/Drosophila - ps31
Drosophila - ps32
Sophophora - ps33
Obscura/Melanogaster_group - ps34
D_melanogaster_group - ps35
D_melanogaster_subgroup - ps36
sim./sech./mel. group - ps37
Drosophila_melanogaster - ps38
p-value
3e-02
3e-02
4e-02
5e-02
7e-02
Log-odds
0.5
1.0
1.5
2.0
2.5
3.0
3.5
4.0

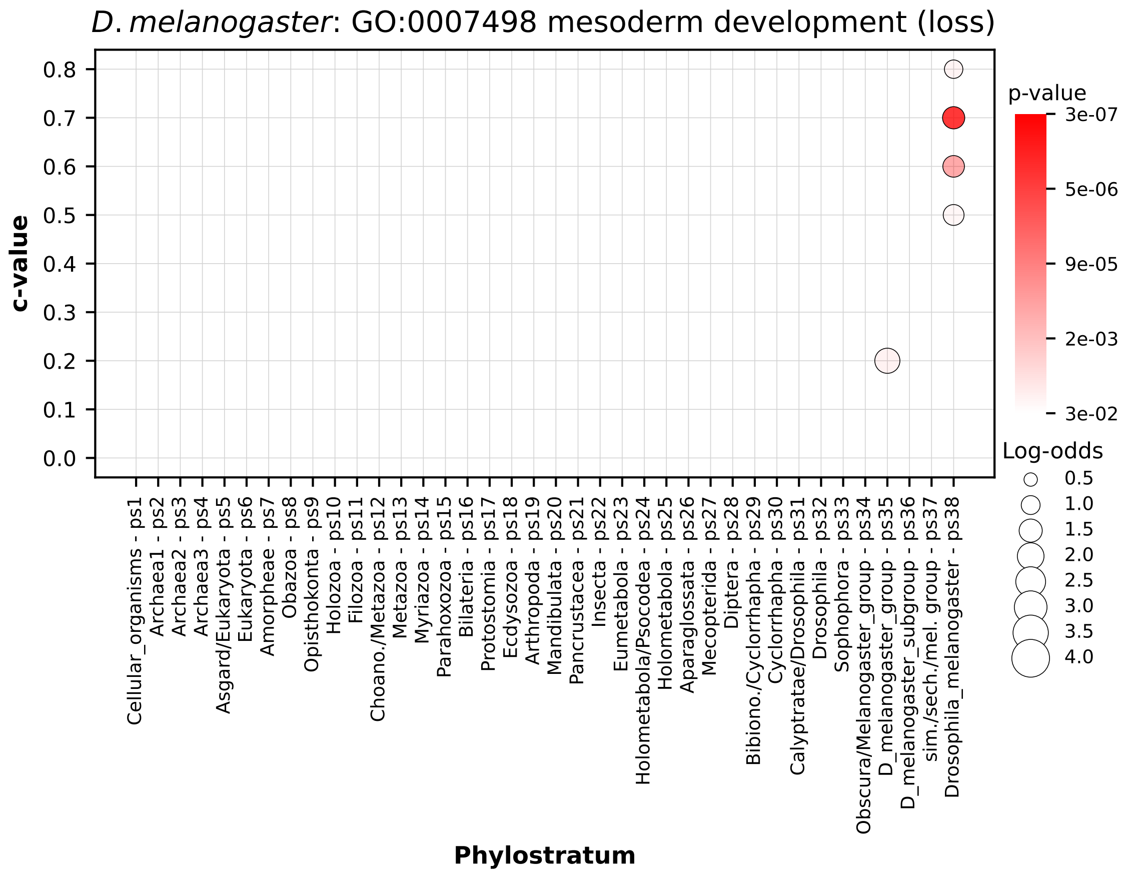

D. melanogaster: GO:0007498 mesoderm development (loss)
c-value
0.8
0.7
0.6
0.5
0.4
0.3
0.2
0.1
0.0
p-value
3e-07
5e-06
9e-05
2e-03
3e-02
Log-odds
0.5
1.0
1.5
2.0
2.5
3.0
3.5
4.0
Cellular_organisms - ps1
Archaea1 - ps2
Archaea2 - ps3
Archaea3 - ps4
Asgard/Eukaryota - ps5
Eukaryota - ps6
Amorpheae - ps7
Obazoa - ps8
Opisthokonta - ps9
Holozoa - ps10
Filozoa - ps11
Choano./Metazoa - ps12
Metazoa - ps13
Myriazoa - ps14
Parahoxozoa - ps15
Bilateria - ps16
Protostomia - ps17
Ecdysozoa - ps18
Arthropoda - ps19
Mandibulata - ps20
Pancrustacea - ps21
Insecta - ps22
Eumetabola - ps23
Holometabola/Psocodea - ps24
Holometabola - ps25
Aparaglossata - ps26
Mecopterida - ps27
Diptera - ps28
Bibiono./Cyclorrhapha - ps29
Cyclorrhapha - ps30
Calyptratae/Drosophila - ps31
Drosophila - ps32
Sophophora - ps33
Obscura/Melanogaster_group - ps34
D_melanogaster_group - ps35
D_melanogaster_subgroup - ps36
sim./sech./mel. group - ps37
Drosophila_melanogaster - ps38
Phylostratum

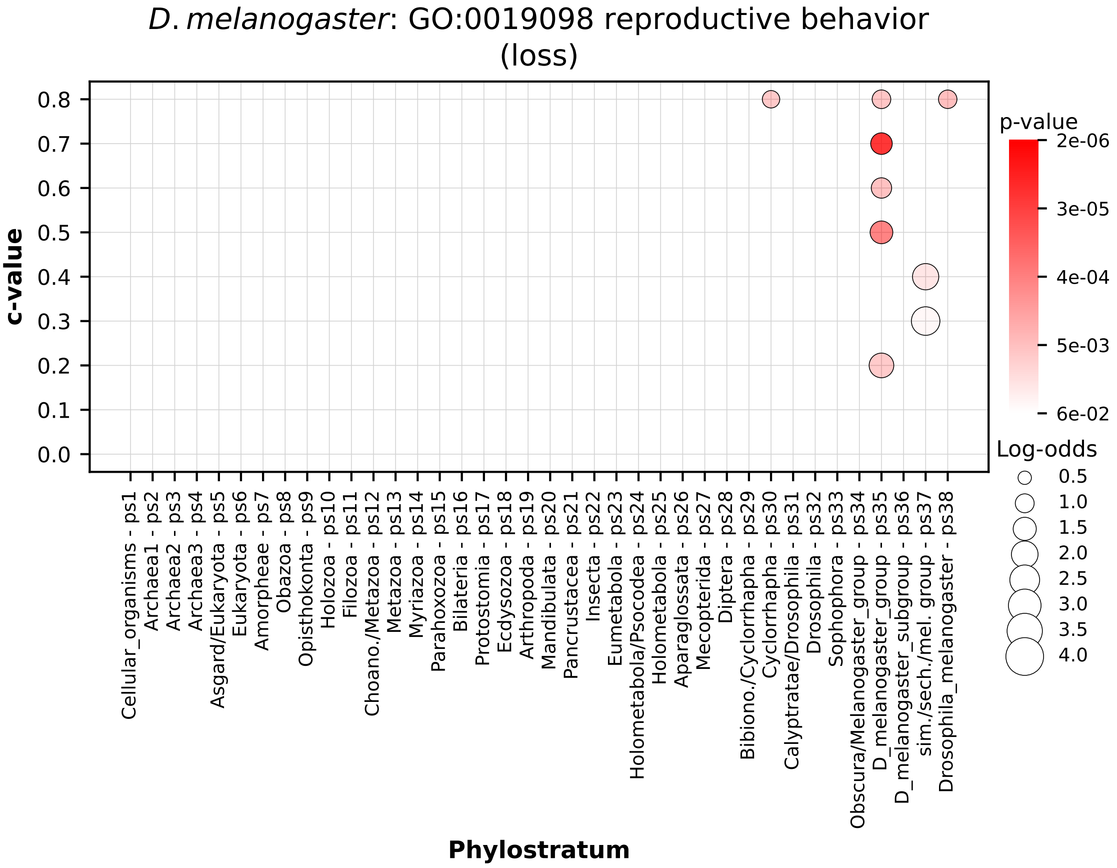

D. melanogaster: GO:0019098 reproductive behavior
(loss)
c-value
0.8
0.7
0.6
0.5
0.4
0.3
0.2
0.1
0.0
p-value
2e-06
3e-05
4e-04
5e-03
6e-02
Log-odds
0.5
1.0
1.5
2.0
2.5
3.0
3.5
4.0
Cellular_organisms - ps1
Archaea1 - ps2
Archaea2 - ps3
Archaea3 - ps4
Asgard/Eukaryota - ps5
Eukaryota - ps6
Amorpheae - ps7
Obazoa - ps8
Opisthokonta - ps9
Holozoa - ps10
Filozoa - ps11
Choano./Metazoa - ps12
Metazoa - ps13
Myriazoa - ps14
Parahoxozoa - ps15
Bilateria - ps16
Protostomia - ps17
Ecdysozoa - ps18
Arthropoda - ps19
Mandibulata - ps20
Pancrustacea - ps21
Insecta - ps22
Eumetabola - ps23
Holometabola/Psocodea - ps24
Holometabola - ps25
Aparaglossata - ps26
Mecopterida - ps27
Diptera - ps28
Bibiono./Cyclorrhapha - ps29
Cyclorrhapha - ps30
Calyptratae/Drosophila - ps31
Drosophila - ps32
Sophophora - ps33
Obscura/Melanogaster_group - ps34
D_melanogaster_group - ps35
D_melanogaster_subgroup - ps36
sim./sech./mel. group - ps37
Drosophila_melanogaster - ps38
Phylostratum

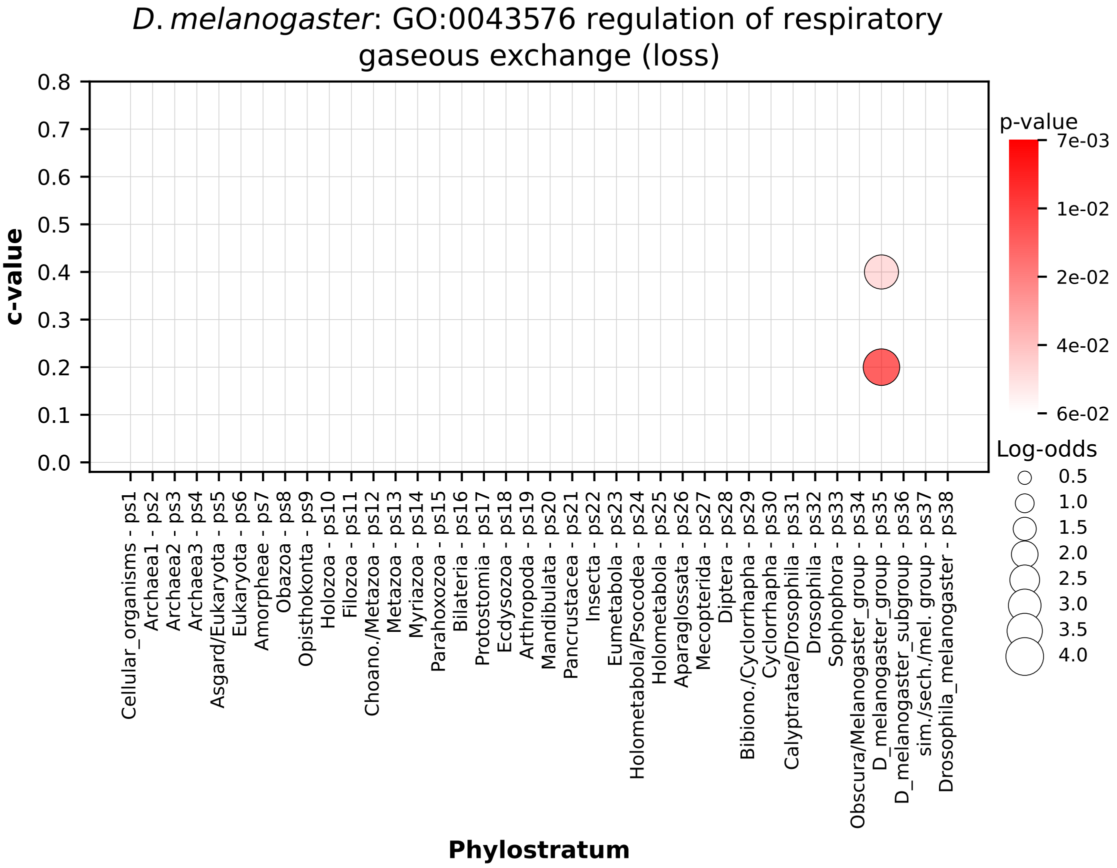

D. melanogaster: GO:0043576 regulation of respiratory gaseous exchange (loss)
c-value
0.0
0.1
0.2
0.3
0.4
0.5
0.6
0.7
0.8
Phylostratum
Cellular_organisms - ps1
Archaea1 - ps2
Archaea2 - ps3
Archaea3 - ps4
Asgard/Eukaryota - ps5
Eukaryota - ps6
Amorpheae - ps7
Obazoa - ps8
Opisthokonta - ps9
Holozoa - ps10
Filozoa - ps11
Choano./Metazoa - ps12
Metazoa - ps13
Myriazoa - ps14
Parahoxozoa - ps15
Bilateria - ps16
Protostomia - ps17
Ecdysozoa - ps18
Arthropoda - ps19
Mandibulata - ps20
Pancrustacea - ps21
Insecta - ps22
Eumetabola - ps23
Holometabola/Psocodea - ps24
Holometabola - ps25
Aparaglossata - ps26
Mecopterida - ps27
Diptera - ps28
Bibiono./Cyclorrhapha - ps29
Cyclorrhapha - ps30
Calyptratae/Drosophila - ps31
Drosophila - ps32
Sophophora - ps33
Obscura/Melanogaster_group - ps34
D_melanogaster_group - ps35
D_melanogaster_subgroup - ps36
sim./sech./mel. group - ps37
Drosophila_melanogaster - ps38
p-value
7e-03
1e-02
2e-02
4e-02
6e-02
Log-odds
0.5
1.0
1.5
2.0
2.5
3.0
3.5
4.0

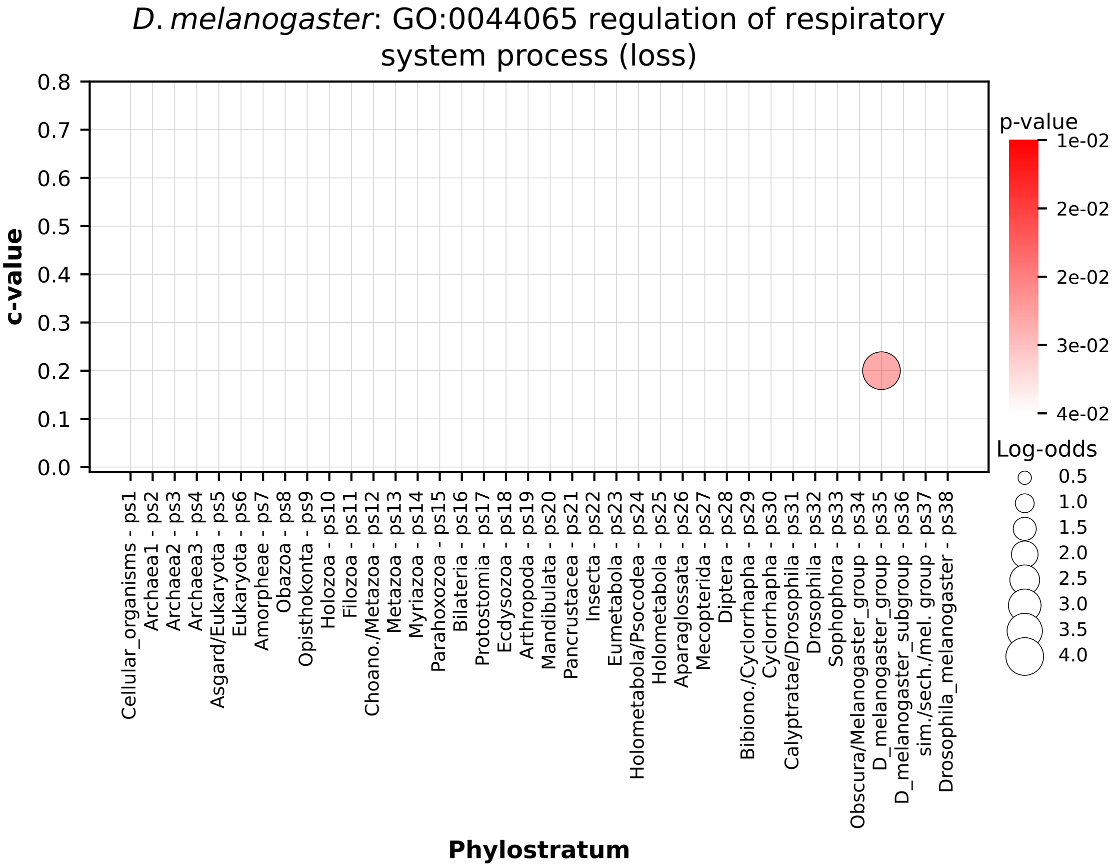

D. melanogaster: GO:0044065 regulation of respiratory system process (loss)
c-value
0.0
0.1
0.2
0.3
0.4
0.5
0.6
0.7
0.8
Phylostratum
Cellular_organisms - ps1
Archaea1 - ps2
Archaea2 - ps3
Archaea3 - ps4
Asgard/Eukaryota - ps5
Eukaryota - ps6
Amorpheae - ps7
Obazoa - ps8
Opisthokonta - ps9
Holozoa - ps10
Filozoa - ps11
Choano./Metazoa - ps12
Metazoa - ps13
Myriazoa - ps14
Parahoxozoa - ps15
Bilateria - ps16
Protostomia - ps17
Ecdysozoa - ps18
Arthropoda - ps19
Mandibulata - ps20
Pancrustacea - ps21
Insecta - ps22
Eumetabola - ps23
Holometabola/Psocodea - ps24
Holometabola - ps25
Aparaglossata - ps26
Mecopterida - ps27
Diptera - ps28
Bibiono./Cyclorrhapha - ps29
Cyclorrhapha - ps30
Calyptratae/Drosophila - ps31
Drosophila - ps32
Sophophora - ps33
Obscura/Melanogaster_group - ps34
D_melanogaster_group - ps35
D_melanogaster_subgroup - ps36
sim./sech./mel. group - ps37
Drosophila_melanogaster - ps38
p-value
1e-02
2e-02
2e-02
3e-02
4e-02
Log-odds
0.5
1.0
1.5
2.0
2.5
3.0
3.5
4.0

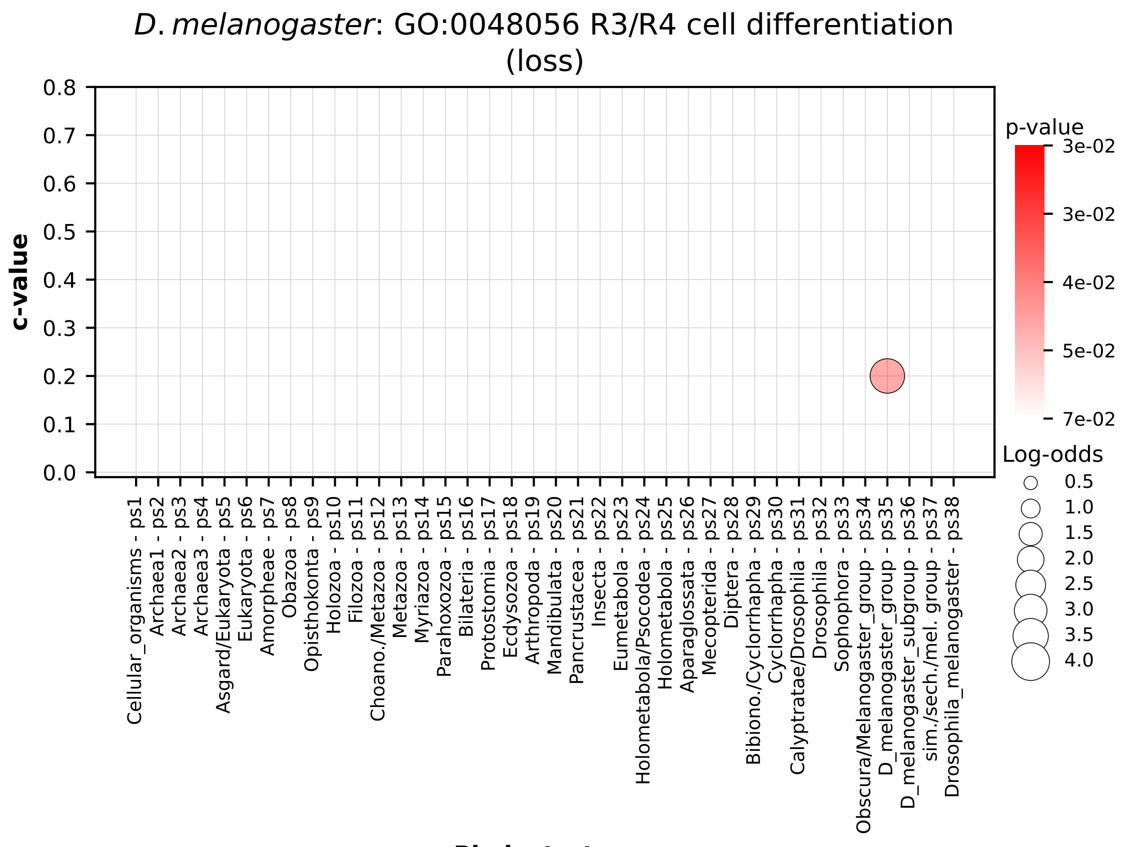

D. melanogaster: GO:0048056 R3/R4 cell differentiation (loss)
c-value
0.0
0.1
0.2
0.3
0.4
0.5
0.6
0.7
0.8
Phylostratum
Cellular_organisms - ps1
Archaea1 - ps2
Archaea2 - ps3
Archaea3 - ps4
Asgard/Eukaryota - ps5
Eukaryota - ps6
Amorpheae - ps7
Obazoa - ps8
Opisthokonta - ps9
Holozoa - ps10
Filozoa - ps11
Choano./Metazoa - ps12
Metazoa - ps13
Myriazoa - ps14
Parahoxozoa - ps15
Bilateria - ps16
Protostomia - ps17
Ecdysozoa - ps18
Arthropoda - ps19
Mandibulata - ps20
Pancrustacea - ps21
Insecta - ps22
Eumetabola - ps23
Holometabola/Psocodea - ps24
Holometabola - ps25
Aparaglossata - ps26
Mecopterida - ps27
Diptera - ps28
Bibiono./Cyclorrhapha - ps29
Cyclorrhapha - ps30
Calyptratae/Drosophila - ps31
Drosophila - ps32
Sophophora - ps33
Obscura/Melanogaster_group - ps34
D_melanogaster_group - ps35
D_melanogaster_subgroup - ps36
sim./sech./mel. group - ps37
Drosophila_melanogaster - ps38
p-value
3e-02
3e-02
4e-02
5e-02
7e-02
Log-odds
0.5
1.0
1.5
2.0
2.5
3.0
3.5
4.0

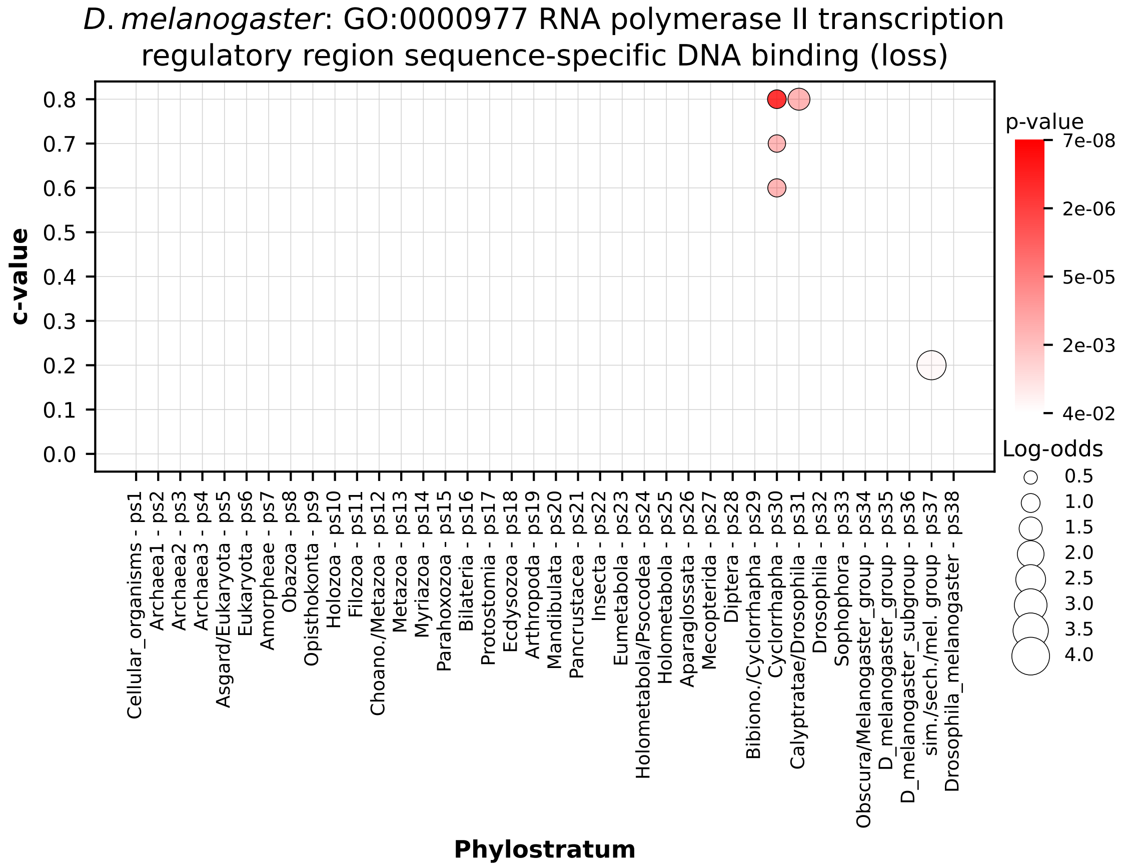

D. melanogaster: GO:0000977 RNA polymerase II transcription regulatory region sequence-specific DNA binding (loss)
c-value
0.8
0.7
0.6
0.5
0.4
0.3
0.2
0.1
0.0
p-value
7e-08
2e-06
5e-05
2e-03
4e-02
Log-odds
0.5
1.0
1.5
2.0
2.5
3.0
3.5
4.0
Cellular_organisms - ps1
Archaea1 - ps2
Archaea2 - ps3
Archaea3 - ps4
Asgard/Eukaryota - ps5
Eukaryota - ps6
Amorpheae - ps7
Obazoa - ps8
Opisthokonta - ps9
Holozoa - ps10
Filozoa - ps11
Choano./Metazoa - ps12
Metazoa - ps13
Myriazoa - ps14
Parahoxozoa - ps15
Bilateria - ps16
Protostomia - ps17
Ecdysozoa - ps18
Arthropoda - ps19
Mandibulata - ps20
Pancrustacea - ps21
Insecta - ps22
Eumetabola - ps23
Holometabola/Psocodea - ps24
Holometabola - ps25
Aparaglossata - ps26
Mecopterida - ps27
Diptera - ps28
Bibiono./Cyclorrhapha - ps29
Cyclorrhapha - ps30
Calyptratae/Drosophila - ps31
Drosophila - ps32
Sophophora - ps33
Obscura/Melanogaster_group - ps34
D_melanogaster_group - ps35
D_melanogaster_subgroup - ps36
sim./sech./mel. group - ps37
Drosophila_melanogaster - ps38
Phylostratum

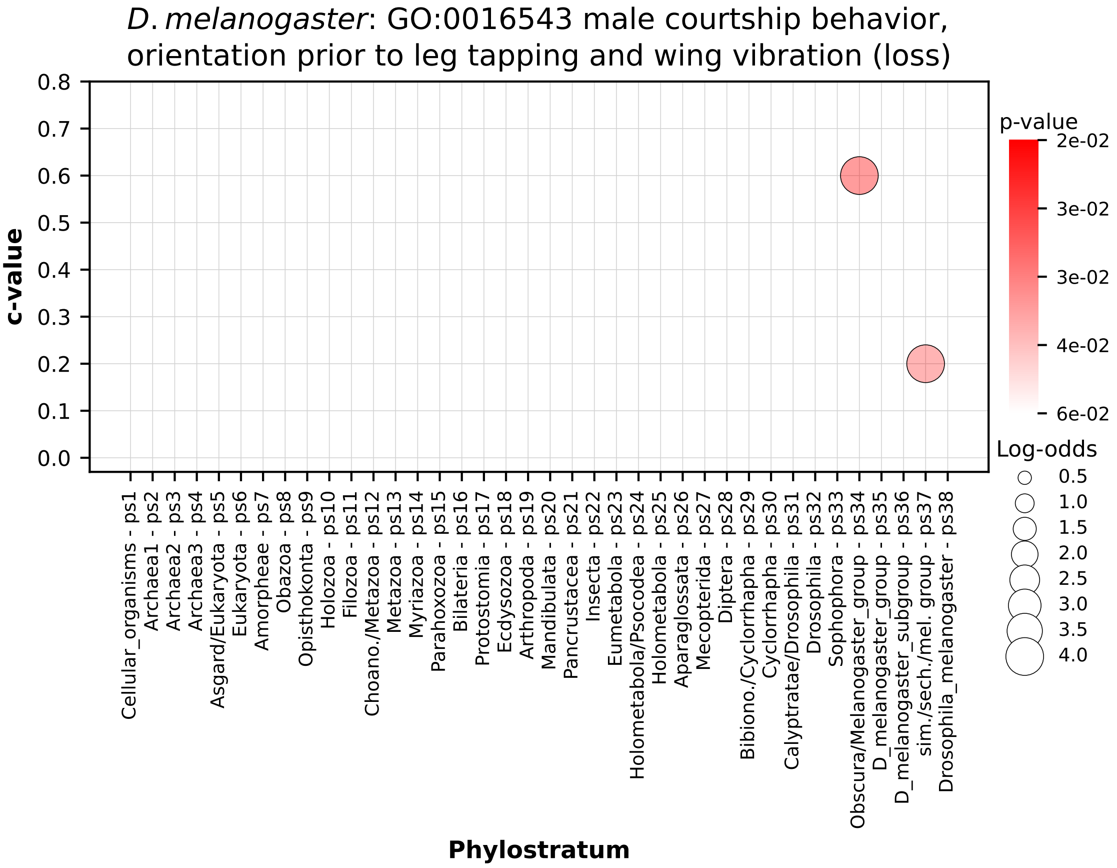
D. melanogaster: GO:0016543 male courtship behavior, orientation prior to leg tapping and wing vibration (loss)
c-value
0.8
0.7
0.6
0.5
0.4
0.3
0.2
0.1
0.0
Phylostratum
Cellular_organisms - ps1
Archaea1 - ps2
Archaea2 - ps3
Archaea3 - ps4
Asgard/Eukaryota - ps5
Eukaryota - ps6
Amorpheae - ps7
Obazoa - ps8
Opisthokonta - ps9
Holozoa - ps10
Filozoa - ps11
Choano./Metazoa - ps12
Metazoa - ps13
Myriazoa - ps14
Parahoxozoa - ps15
Bilateria - ps16
Protostomia - ps17
Ecdysozoa - ps18
Arthropoda - ps19
Mandibulata - ps20
Pancrustacea - ps21
Insecta - ps22
Eumetabola - ps23
Holometabola/Psocodea - ps24
Holometabola - ps25
Aparaglossata - ps26
Mecopterida - ps27
Diptera - ps28
Bibiono./Cyclorrhapha - ps29
Cyclorrhapha - ps30
Calyptratae/Drosophila - ps31
Drosophila - ps32
Sophophora - ps33
Obscura/Melanogaster_group - ps34
D_melanogaster_group - ps35
D_melanogaster_subgroup - ps36
sim./sech./mel. group - ps37
Drosophila_melanogaster - ps38
p-value
2e-02
3e-02
3e-02
4e-02
6e-02
Log-odds
0.5
1.0
1.5
2.0
2.5
3.0
3.5
4.0

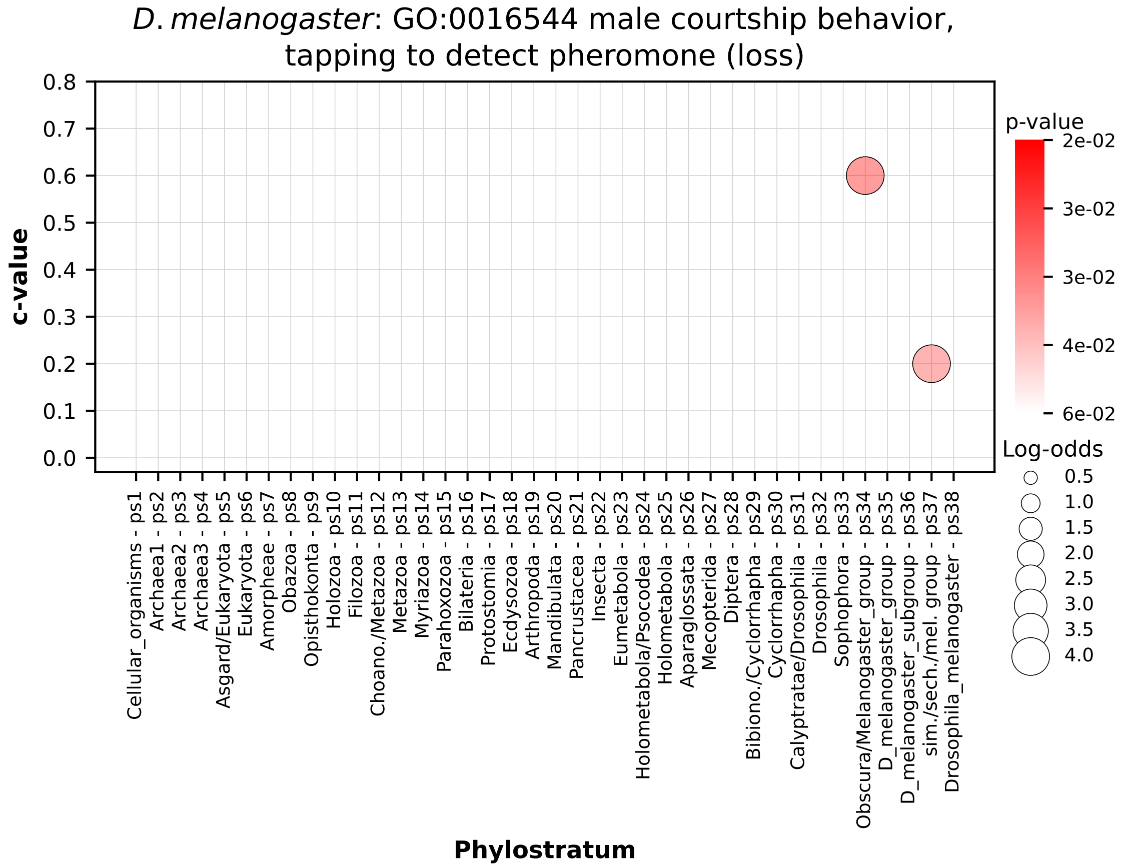

D. melanogaster: GO:0016544 male courtship behavior, tapping to detect pheromone (loss)
c-value
0.8
0.7
0.6
0.5
0.4
0.3
0.2
0.1
0.0
Phylostratum
Cellular_organisms - ps1
Archaea1 - ps2
Archaea2 - ps3
Archaea3 - ps4
Asgard/Eukaryota - ps5
Eukaryota - ps6
Amorpheae - ps7
Obazoa - ps8
Opisthokonta - ps9
Holozoa - ps10
Filozoa - ps11
Choano./Metazoa - ps12
Metazoa - ps13
Myriazoa - ps14
Parahoxozoa - ps15
Bilateria - ps16
Protostomia - ps17
Ecdysozoa - ps18
Arthropoda - ps19
Mandibulata - ps20
Pancrustacea - ps21
Insecta - ps22
Eumetabola - ps23
Holometabola/Psocodea - ps24
Holometabola - ps25
Aparaglossata - ps26
Mecopterida - ps27
Diptera - ps28
Bibiono./Cyclorrhapha - ps29
Cyclorrhapha - ps30
Calyptratae/Drosophila - ps31
Drosophila - ps32
Sophophora - ps33
Obscura/Melanogaster_group - ps34
D_melanogaster_group - ps35
D_melanogaster_subgroup - ps36
sim./sech./mel. group - ps37
Drosophila_melanogaster - ps38
p-value
2e-02
3e-02
3e-02
4e-02
6e-02
Log-odds
0.5
1.0
1.5
2.0
2.5
3.0
3.5
4.0

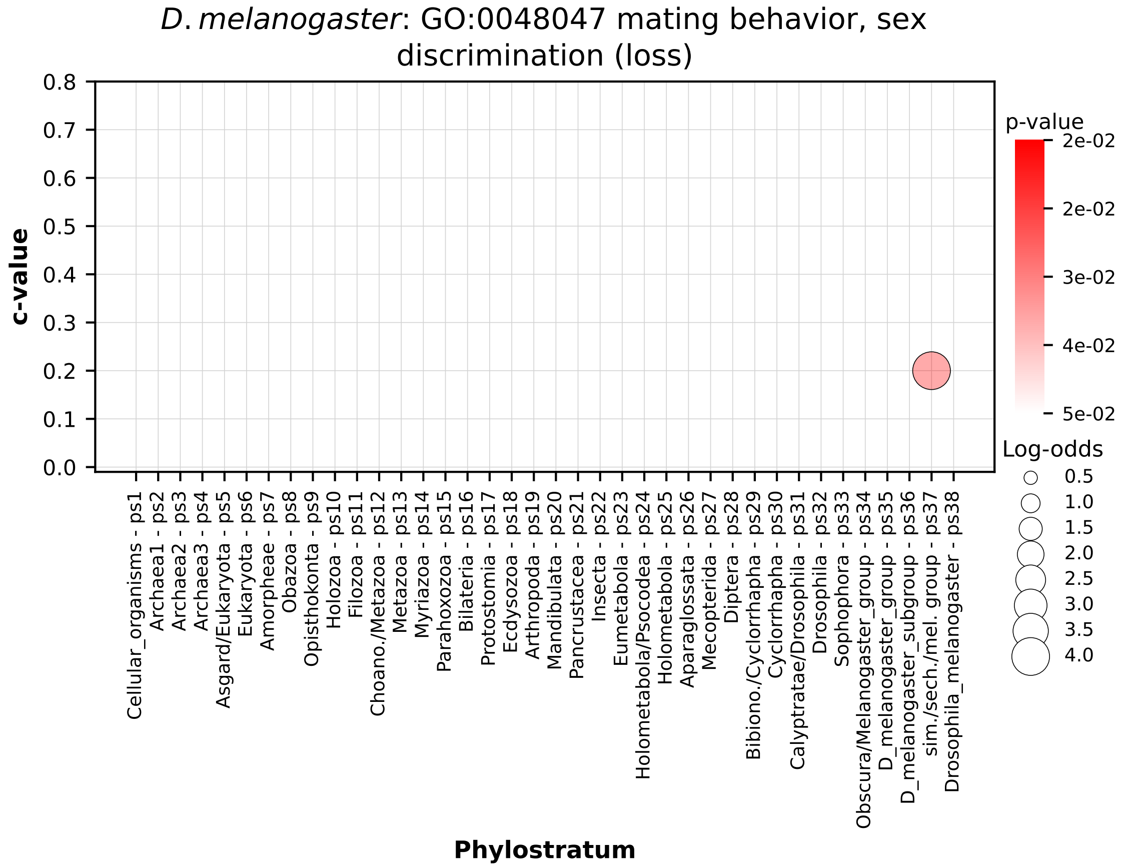

D. melanogaster: GO:0048047 mating behavior, sex discrimination (loss)
c-value
0.0
0.1
0.2
0.3
0.4
0.5
0.6
0.7
0.8
p-value
2e-02
2e-02
3e-02
4e-02
5e-02
Log-odds
0.5
1.0
1.5
2.0
2.5
3.0
3.5
4.0
Cellular_organisms - ps1
Archaea1 - ps2
Archaea2 - ps3
Archaea3 - ps4
Asgard/Eukaryota - ps5
Eukaryota - ps6
Amorpheae - ps7
Obazoa - ps8
Opisthokonta - ps9
Holozoa - ps10
Filozoa - ps11
Choano./Metazoa - ps12
Metazoa - ps13
Myriazoa - ps14
Parahoxozoa - ps15
Bilateria - ps16
Protostomia - ps17
Ecdysozoa - ps18
Arthropoda - ps19
Mandibulata - ps20
Pancrustacea - ps21
Insecta - ps22
Eumetabola - ps23
Holometabola/Psocodea - ps24
Holometabola - ps25
Aparaglossata - ps26
Mecopterida - ps27
Diptera - ps28
Bibiono./Cyclorrhapha - ps29
Cyclorrhapha - ps30
Calyptratae/Drosophila - ps31
Drosophila - ps32
Sophophora - ps33
Obscura/Melanogaster_group - ps34
D_melanogaster_group - ps35
D_melanogaster_subgroup - ps36
sim./sech./mel. group - ps37
Drosophila_melanogaster - ps38
Phylostratum

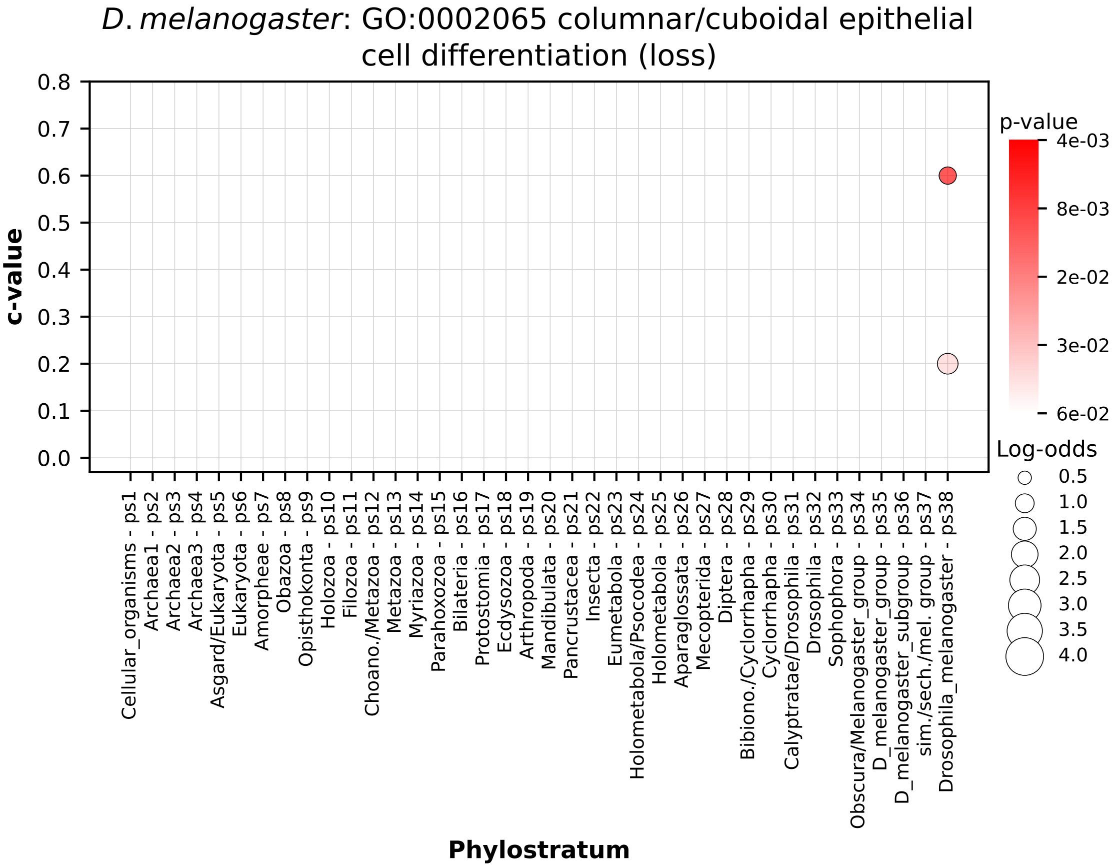
D. melanogaster: GO:0002065 columnar/cuboidal epithelial cell differentiation (loss)
c-value
0.8
0.7
0.6
0.5
0.4
0.3
0.2
0.1
0.0
p-value
4e-03
8e-03
2e-02
3e-02
6e-02
Log-odds
0.5
1.0
1.5
2.0
2.5
3.0
3.5
4.0
Cellular_organisms - ps1
Archaea1 - ps2
Archaea2 - ps3
Archaea3 - ps4
Asgard/Eukaryota - ps5
Eukaryota - ps6
Amorpheae - ps7
Obazoa - ps8
Opisthokonta - ps9
Holozoa - ps10
Filozoa - ps11
Choano./Metazoa - ps12
Metazoa - ps13
Myriazoa - ps14
Parahoxozoa - ps15
Bilateria - ps16
Protostomia - ps17
Ecdysozoa - ps18
Arthropoda - ps19
Mandibulata - ps20
Pancrustacea - ps21
Insecta - ps22
Eumetabola - ps23
Holometabola/Psocodea - ps24
Holometabola - ps25
Aparaglossata - ps26
Mecopterida - ps27
Diptera - ps28
Bibiono./Cyclorrhapha - ps29
Cyclorrhapha - ps30
Calyptratae/Drosophila - ps31
Drosophila - ps32
Sophophora - ps33
Obscura/Melanogaster_group - ps34
D_melanogaster_group - ps35
D_melanogaster_subgroup - ps36
sim./sech./mel. group - ps37
Drosophila_melanogaster - ps38
Phylostratum

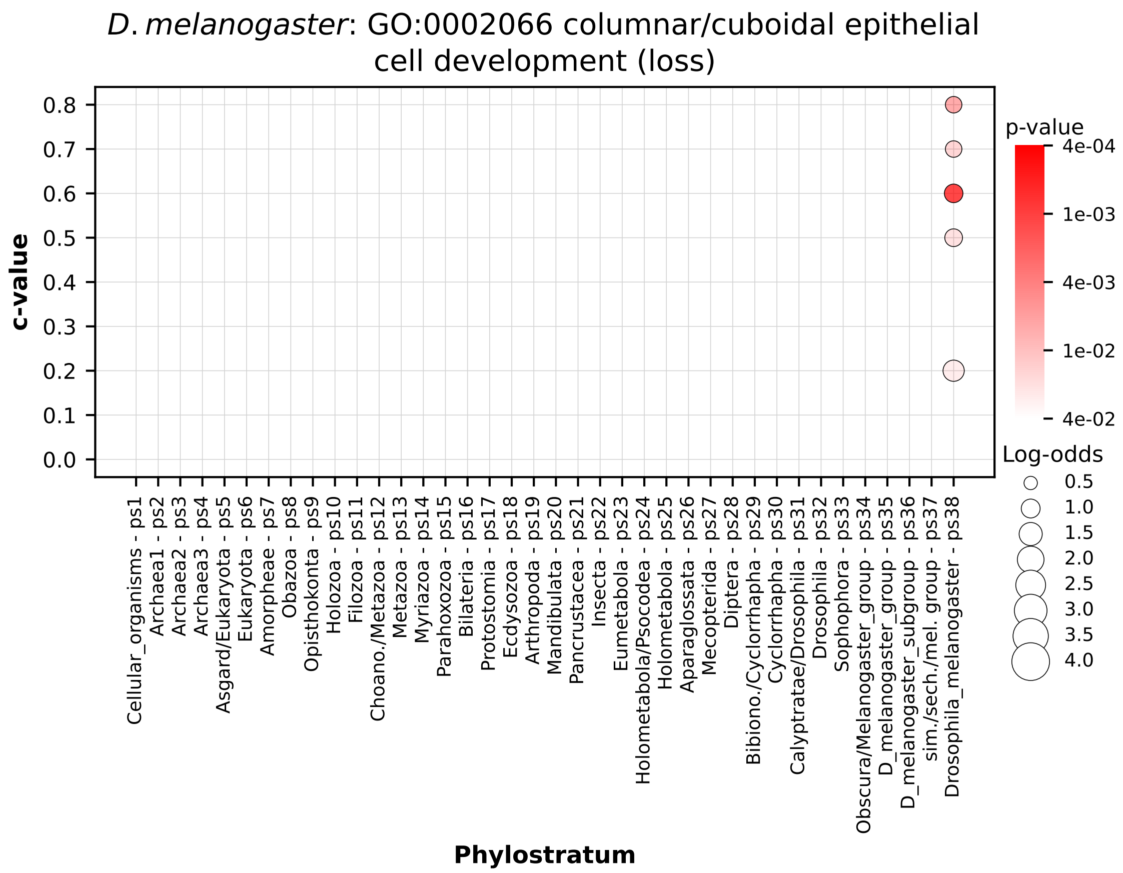

D. melanogaster: GO:0002066 columnar/cuboidal epithelial cell development (loss)
c-value
0.8
0.7
0.6
0.5
0.4
0.3
0.2
0.1
0.0
Phylostratum
Cellular_organisms - ps1
Archaea1 - ps2
Archaea2 - ps3
Archaea3 - ps4
Asgard/Eukaryota - ps5
Eukaryota - ps6
Amorpheae - ps7
Obazoa - ps8
Opisthokonta - ps9
Holozoa - ps10
Filozoa - ps11
Choano./Metazoa - ps12
Metazoa - ps13
Myriazoa - ps14
Parahoxozoa - ps15
Bilateria - ps16
Protostomia - ps17
Ecdysozoa - ps18
Arthropoda - ps19
Mandibulata - ps20
Pancrustacea - ps21
Insecta - ps22
Eumetabola - ps23
Holometabola/Psocodea - ps24
Holometabola - ps25
Aparaglossata - ps26
Mecopterida - ps27
Diptera - ps28
Bibiono./Cyclorrhapha - ps29
Cyclorrhapha - ps30
Calyptratae/Drosophila - ps31
Drosophila - ps32
Sophophora - ps33
Obscura/Melanogaster_group - ps34
D_melanogaster_group - ps35
D_melanogaster_subgroup - ps36
sim./sech./mel. group - ps37
Drosophila_melanogaster - ps38
p-value
4e-04
1e-03
4e-03
1e-02
4e-02
Log-odds
0.5
1.0
1.5
2.0
2.5
3.0
3.5
4.0

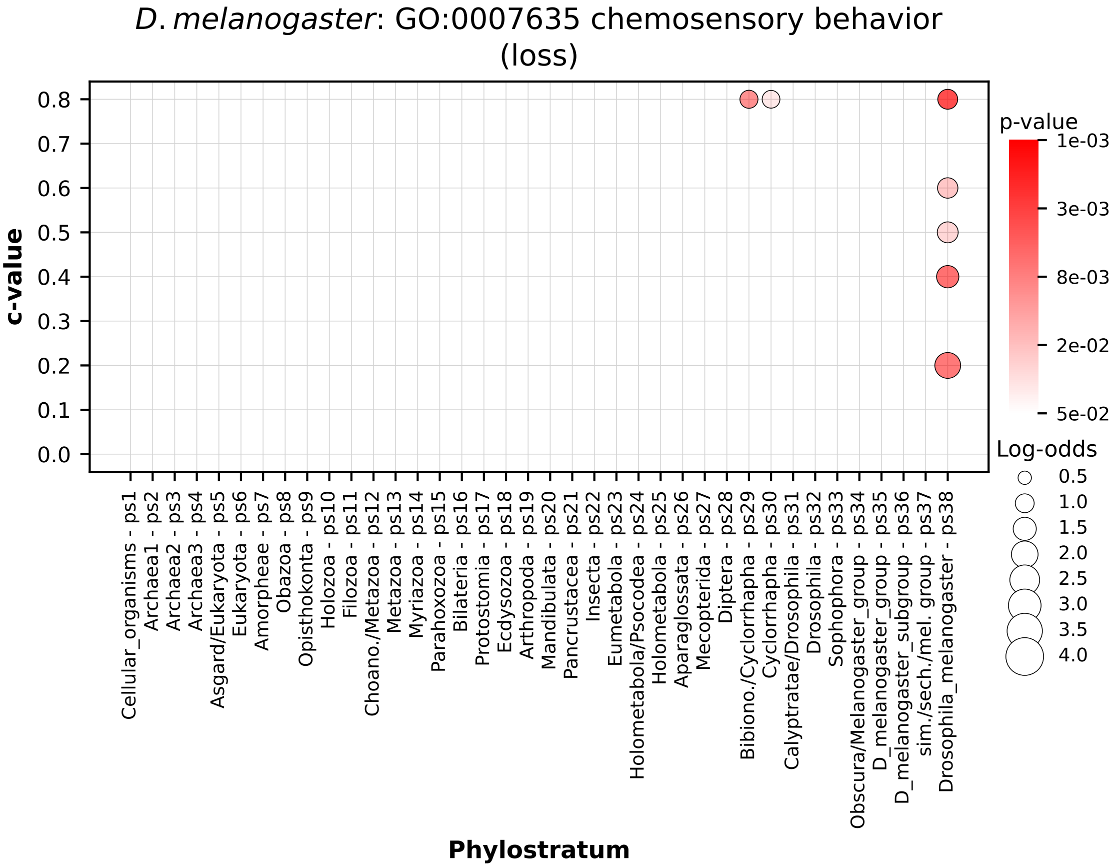
D. melanogaster: GO:0007635 chemosensory behavior
(loss)
c-value
0.8
0.7
0.6
0.5
0.4
0.3
0.2
0.1
0.0
p-value
1e-03
3e-03
8e-03
2e-02
5e-02
Log-odds
0.5
1.0
1.5
2.0
2.5
3.0
3.5
4.0
Cellular_organisms - ps1
Archaea1 - ps2
Archaea2 - ps3
Archaea3 - ps4
Asgard/Eukaryota - ps5
Eukaryota - ps6
Amorpheae - ps7
Obazoa - ps8
Opisthokonta - ps9
Holozoa - ps10
Filozoa - ps11
Choano./Metazoa - ps12
Metazoa - ps13
Myriazoa - ps14
Parahoxozoa - ps15
Bilateria - ps16
Protostomia - ps17
Ecdysozoa - ps18
Arthropoda - ps19
Mandibulata - ps20
Pancrustacea - ps21
Insecta - ps22
Eumetabola - ps23
Holometabola/Psocodea - ps24
Holometabola - ps25
Aparaglossata - ps26
Mecopterida - ps27
Diptera - ps28
Bibiono./Cyclorrhapha - ps29
Cyclorrhapha - ps30
Calyptratae/Drosophila - ps31
Drosophila - ps32
Sophophora - ps33
Obscura/Melanogaster_group - ps34
D_melanogaster_group - ps35
D_melanogaster_subgroup - ps36
sim./sech./mel. group - ps37
Drosophila_melanogaster - ps38
Phylostratum

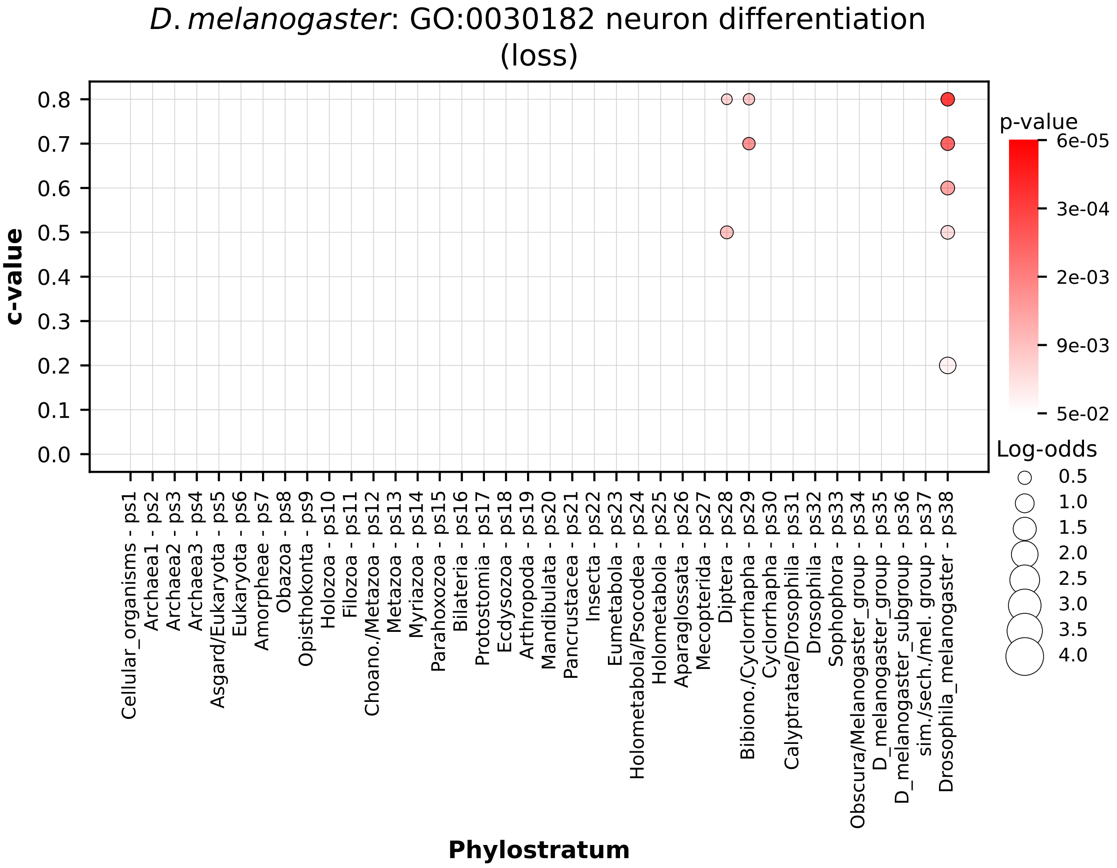

D. melanogaster: GO:0030182 neuron differentiation
(loss)
c-value
0.8
0.7
0.6
0.5
0.4
0.3
0.2
0.1
0.0
p-value
6e-05
3e-04
2e-03
9e-03
5e-02
Log-odds
0.5
1.0
1.5
2.0
2.5
3.0
3.5
4.0
Cellular_organisms - ps1
Archaea1 - ps2
Archaea2 - ps3
Archaea3 - ps4
Asgard/Eukaryota - ps5
Eukaryota - ps6
Amorpheae - ps7
Obazoa - ps8
Opisthokonta - ps9
Holozoa - ps10
Filozoa - ps11
Choano./Metazoa - ps12
Metazoa - ps13
Myriazoa - ps14
Parahoxozoa - ps15
Bilateria - ps16
Protostomia - ps17
Ecdysozoa - ps18
Arthropoda - ps19
Mandibulata - ps20
Pancrustacea - ps21
Insecta - ps22
Eumetabola - ps23
Holometabola/Psocodea - ps24
Holometabola - ps25
Aparaglossata - ps26
Mecopterida - ps27
Diptera - ps28
Bibiono./Cyclorrhapha - ps29
Cyclorrhapha - ps30
Calyptratae/Drosophila - ps31
Drosophila - ps32
Sophophora - ps33
Obscura/Melanogaster_group - ps34
D_melanogaster_group - ps35
D_melanogaster_subgroup - ps36
sim./sech./mel. group - ps37
Drosophila_melanogaster - ps38
Phylostratum

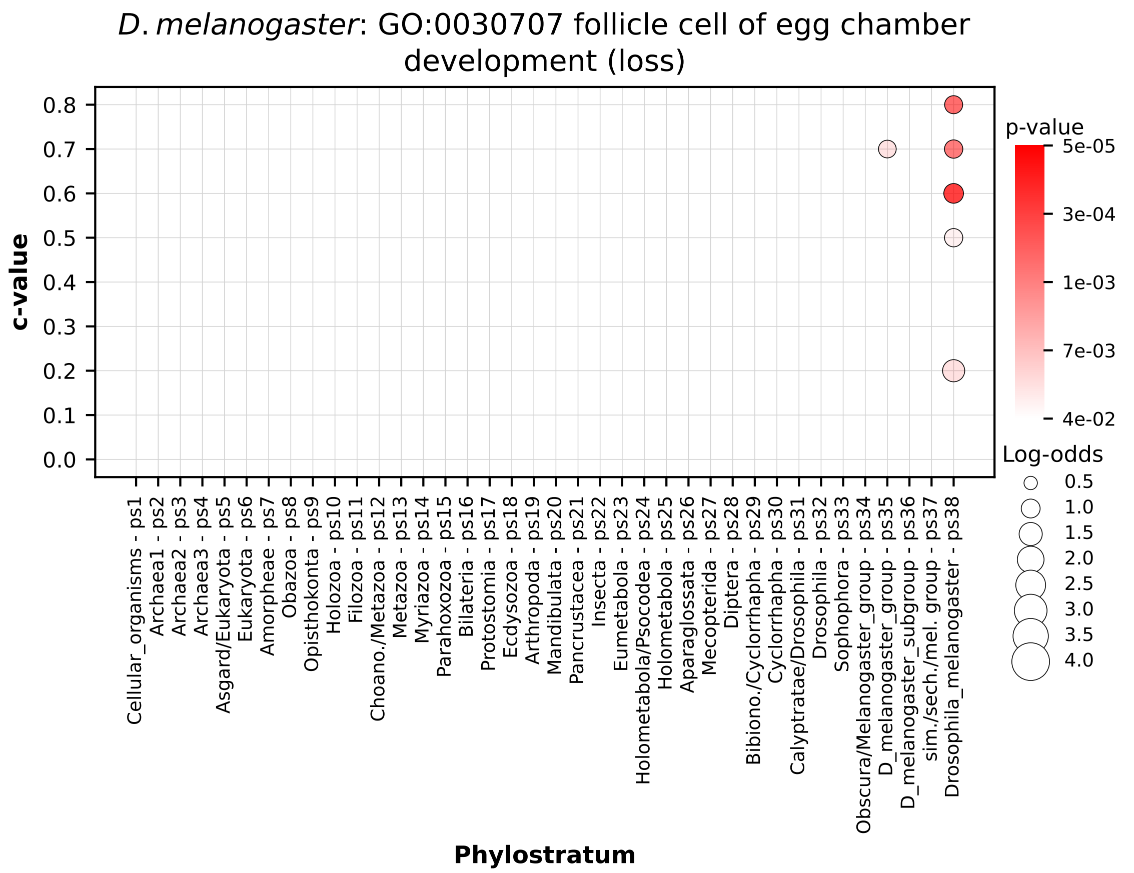
D. melanogaster: GO:0030707 follicle cell of egg chamber development (loss)
c-value
Phylostratum
p-value
5e-05
3e-04
1e-03
7e-03
4e-02
Log-odds
0.5
1.0
1.5
2.0
2.5
3.0
3.5
4.0
Cellular_organisms - ps1
Archaea1 - ps2
Archaea2 - ps3
Archaea3 - ps4
Asgard/Eukaryota - ps5
Eukaryota - ps6
Amorpheae - ps7
Obazoa - ps8
Opisthokonta - ps9
Holozoa - ps10
Filozoa - ps11
Choano./Metazoa - ps12
Metazoa - ps13
Myriazoa - ps14
Parahoxozoa - ps15
Bilateria - ps16
Protostomia - ps17
Ecdysozoa - ps18
Arthropoda - ps19
Mandibulata - ps20
Pancrustacea - ps21
Insecta - ps22
Eumetabola - ps23
Holometabola/Psocodea - ps24
Holometabola - ps25
Aparaglossata - ps26
Mecopterida - ps27
Diptera - ps28
Bibiono./Cyclorrhapha - ps29
Cyclorrhapha - ps30
Calyptratae/Drosophila - ps31
Drosophila - ps32
Sophophora - ps33
Obscura/Melanogaster_group - ps34
D_melanogaster_group - ps35
D_melanogaster_subgroup - ps36
sim./sech./mel. group - ps37
Drosophila_melanogaster - ps38

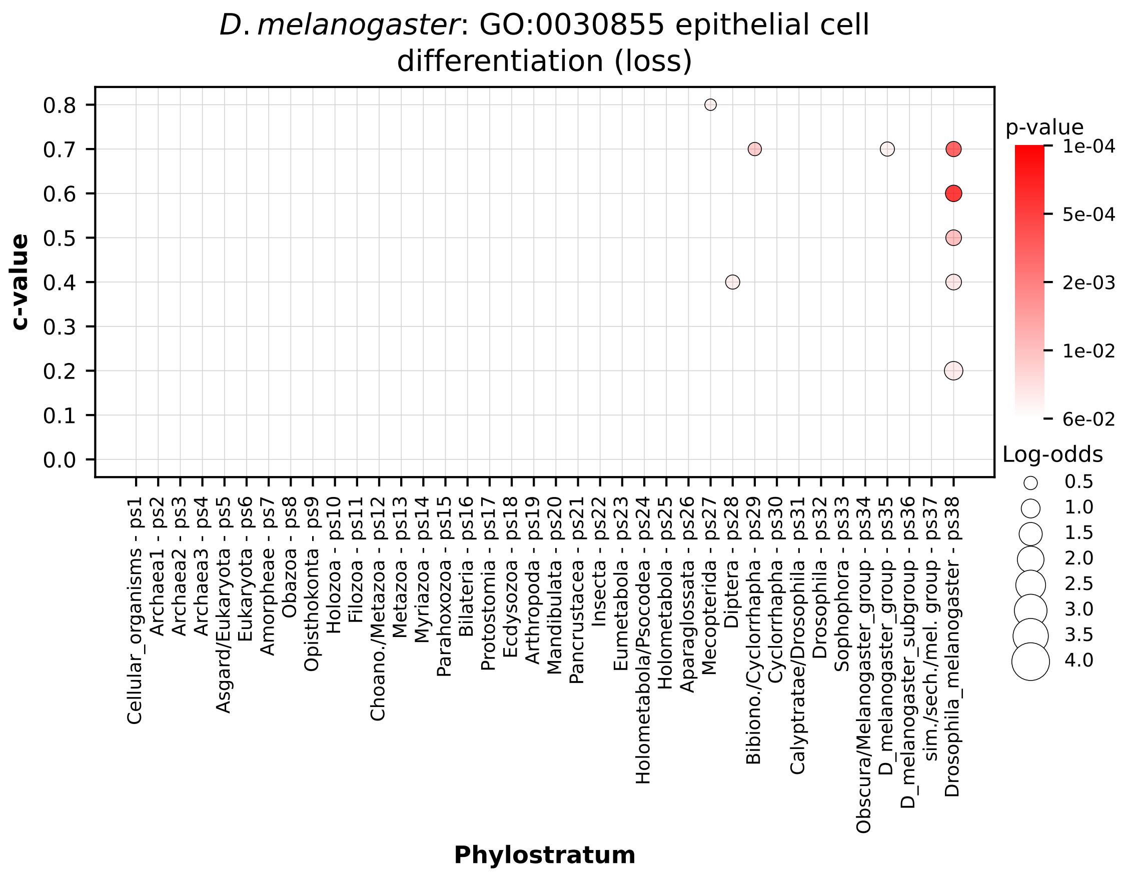

D. melanogaster: GO:0030855 epithelial cell differentiation (loss)
c-value
0.8
0.7
0.6
0.5
0.4
0.3
0.2
0.1
0.0
Phylostratum
Cellular_organisms - ps1
Archaea1 - ps2
Archaea2 - ps3
Archaea3 - ps4
Asgard/Eukaryota - ps5
Eukaryota - ps6
Amorpheae - ps7
Obazoa - ps8
Opisthokonta - ps9
Holozoa - ps10
Filozoa - ps11
Choano./Metazoa - ps12
Metazoa - ps13
Myriazoa - ps14
Parahoxozoa - ps15
Bilateria - ps16
Protostomia - ps17
Ecdysozoa - ps18
Arthropoda - ps19
Mandibulata - ps20
Pancrustacea - ps21
Insecta - ps22
Eumetabola - ps23
Holometabola/Psocodea - ps24
Holometabola - ps25
Aparaglossata - ps26
Mecopterida - ps27
Diptera - ps28
Bibiono./Cyclorrhapha - ps29
Cyclorrhapha - ps30
Calyptratae/Drosophila - ps31
Drosophila - ps32
Sophophora - ps33
Obscura/Melanogaster_group - ps34
D_melanogaster_group - ps35
D_melanogaster_subgroup - ps36
sim./sech./mel. group - ps37
Drosophila_melanogaster - ps38
p-value
1e-04
5e-04
2e-03
1e-02
6e-02
Log-odds
0.5
1.0
1.5
2.0
2.5
3.0
3.5
4.0

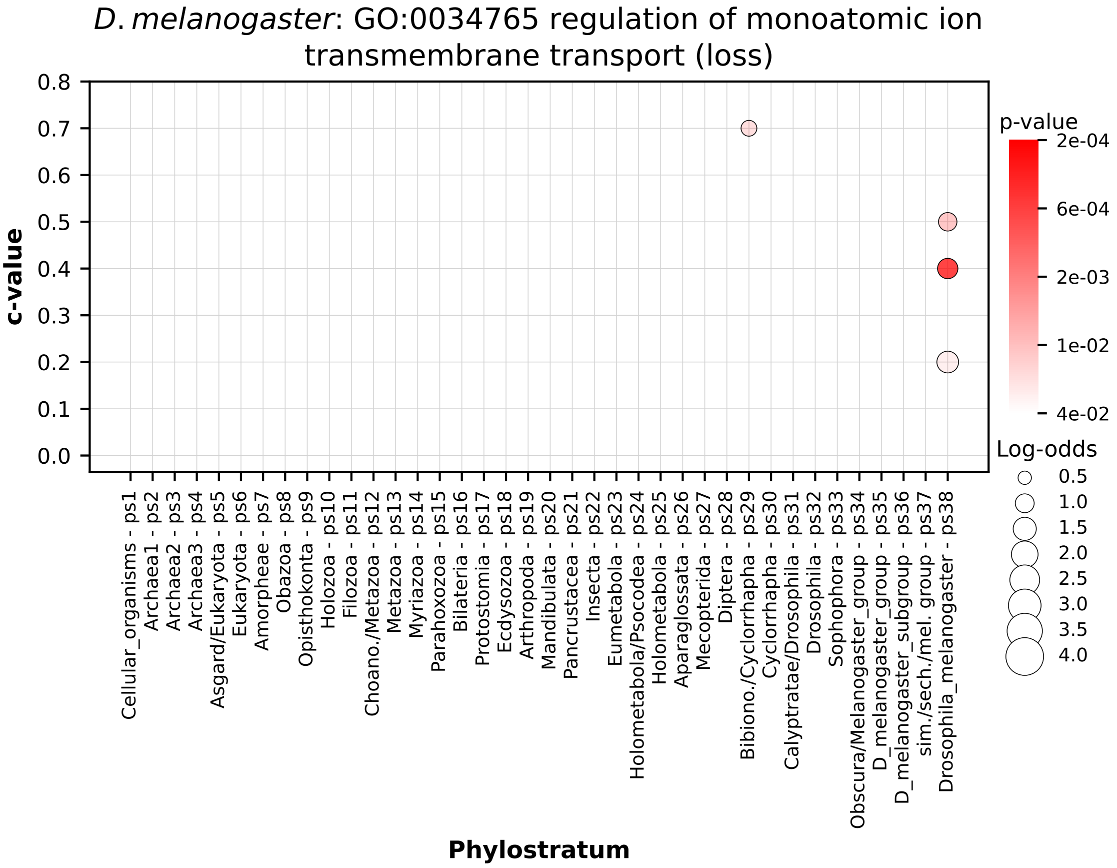
D. melanogaster: GO:0034765 regulation of monoatomic ion transmembrane transport (loss)
c-value
0.8
0.7
0.6
0.5
0.4
0.3
0.2
0.1
0.0
Phylostratum
Cellular_organisms - ps1
Archaea1 - ps2
Archaea2 - ps3
Archaea3 - ps4
Asgard/Eukaryota - ps5
Eukaryota - ps6
Amorpheae - ps7
Obazoa - ps8
Opisthokonta - ps9
Holozoa - ps10
Filozoa - ps11
Choano./Metazoa - ps12
Metazoa - ps13
Myriazoa - ps14
Parahoxozoa - ps15
Bilateria - ps16
Protostomia - ps17
Ecdysozoa - ps18
Arthropoda - ps19
Mandibulata - ps20
Pancrustacea - ps21
Insecta - ps22
Eumetabola - ps23
Holometabola/Psocodea - ps24
Holometabola - ps25
Aparaglossata - ps26
Mecopterida - ps27
Diptera - ps28
Bibiono./Cyclorrhapha - ps29
Cyclorrhapha - ps30
Calyptratae/Drosophila - ps31
Drosophila - ps32
Sophophora - ps33
Obscura/Melanogaster_group - ps34
D_melanogaster_group - ps35
D_melanogaster_subgroup - ps36
sim./sech./mel. group - ps37
Drosophila_melanogaster - ps38
p-value
2e-04
6e-04
2e-03
1e-02
4e-02
Log-odds
0.5
1.0
1.5
2.0
2.5
3.0
3.5
4.0

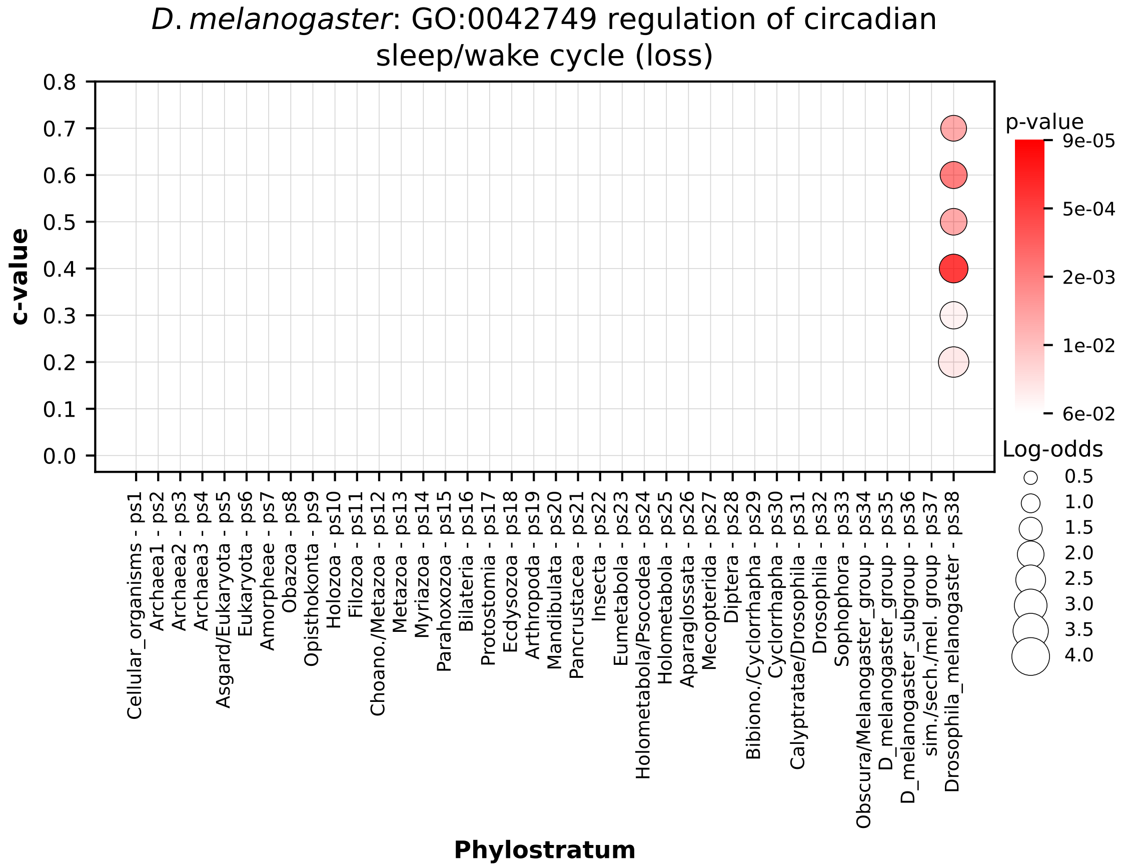
D. melanogaster: GO:0042749 regulation of circadian sleep/wake cycle (loss)
c-value
0.8
0.7
0.6
0.5
0.4
0.3
0.2
0.1
0.0
Phylostratum
Cellular_organisms - ps1
Archaea1 - ps2
Archaea2 - ps3
Archaea3 - ps4
Asgard/Eukaryota - ps5
Eukaryota - ps6
Amorpheae - ps7
Obazoa - ps8
Opisthokonta - ps9
Holozoa - ps10
Filozoa - ps11
Choano./Metazoa - ps12
Metazoa - ps13
Myriazoa - ps14
Parahoxozoa - ps15
Bilateria - ps16
Protostomia - ps17
Ecdysozoa - ps18
Arthropoda - ps19
Mandibulata - ps20
Pancrustacea - ps21
Insecta - ps22
Eumetabola - ps23
Holometabola/Psocodea - ps24
Holometabola - ps25
Aparaglossata - ps26
Mecopterida - ps27
Diptera - ps28
Bibiono./Cyclorrhapha - ps29
Cyclorrhapha - ps30
Calyptratae/Drosophila - ps31
Drosophila - ps32
Sophophora - ps33
Obscura/Melanogaster_group - ps34
D_melanogaster_group - ps35
D_melanogaster_subgroup - ps36
sim./sech./mel. group - ps37
Drosophila_melanogaster - ps38
p-value
9e-05
5e-04
2e-03
1e-02
6e-02
Log-odds
0.5
1.0
1.5
2.0
2.5
3.0
3.5
4.0

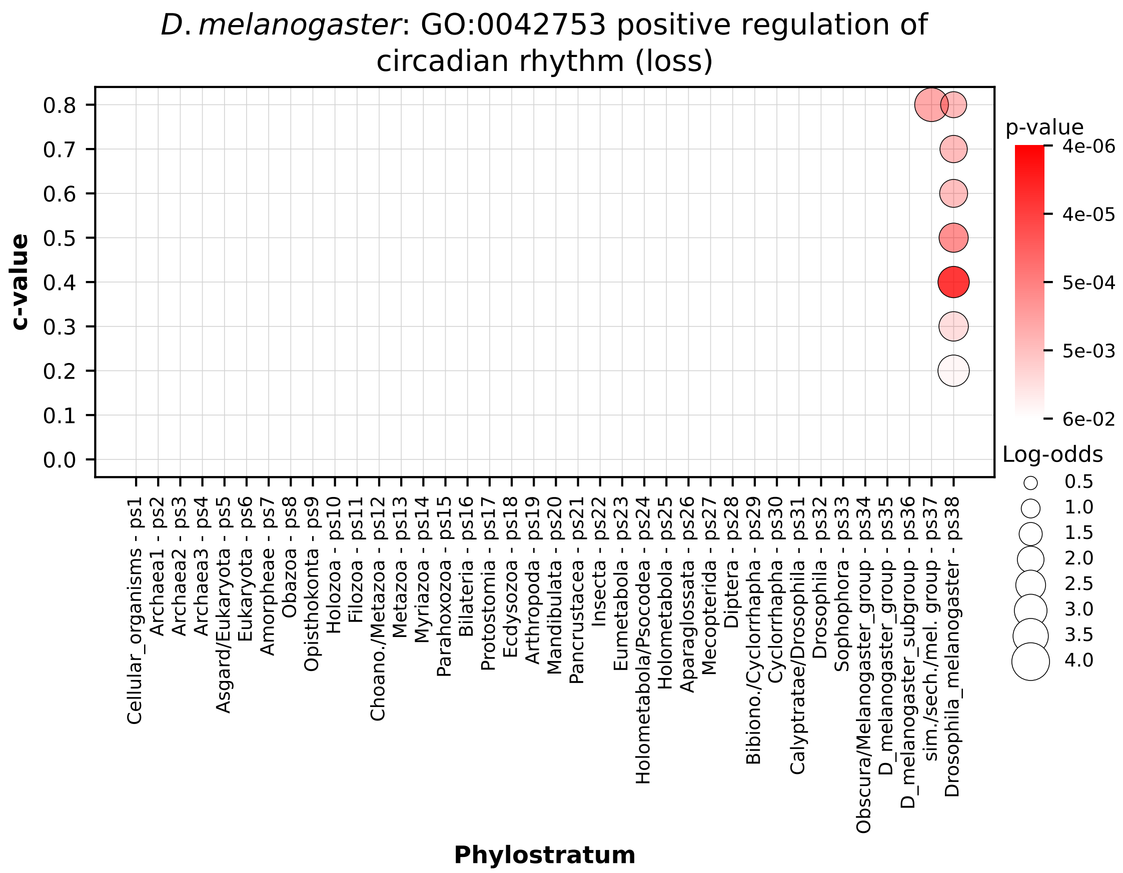

D. melanogaster: GO:0042753 positive regulation of circadian rhythm (loss)
c-value
0.0
0.1
0.2
0.3
0.4
0.5
0.6
0.7
0.8
Phylostratum
Cellular_organisms - ps1
Archaea1 - ps2
Archaea2 - ps3
Archaea3 - ps4
Asgard/Eukaryota - ps5
Eukaryota - ps6
Amorpheae - ps7
Obazoa - ps8
Opisthokonta - ps9
Holozoa - ps10
Filozoa - ps11
Choano./Metazoa - ps12
Metazoa - ps13
Myriazoa - ps14
Parahoxozoa - ps15
Bilateria - ps16
Protostomia - ps17
Ecdysozoa - ps18
Arthropoda - ps19
Mandibulata - ps20
Pancrustacea - ps21
Insecta - ps22
Eumetabola - ps23
Holometabola/Psocodea - ps24
Holometabola - ps25
Aparaglossata - ps26
Mecopterida - ps27
Diptera - ps28
Bibiono./Cyclorrhapha - ps29
Cyclorrhapha - ps30
Calyptratae/Drosophila - ps31
Drosophila - ps32
Sophophora - ps33
Obscura/Melanogaster_group - ps34
D_melanogaster_group - ps35
D_melanogaster_subgroup - ps36
sim./sech./mel. group - ps37
Drosophila_melanogaster - ps38
p-value
4e-06
4e-05
5e-04
5e-03
6e-02
Log-odds
0.5
1.0
1.5
2.0
2.5
3.0
3.5
4.0

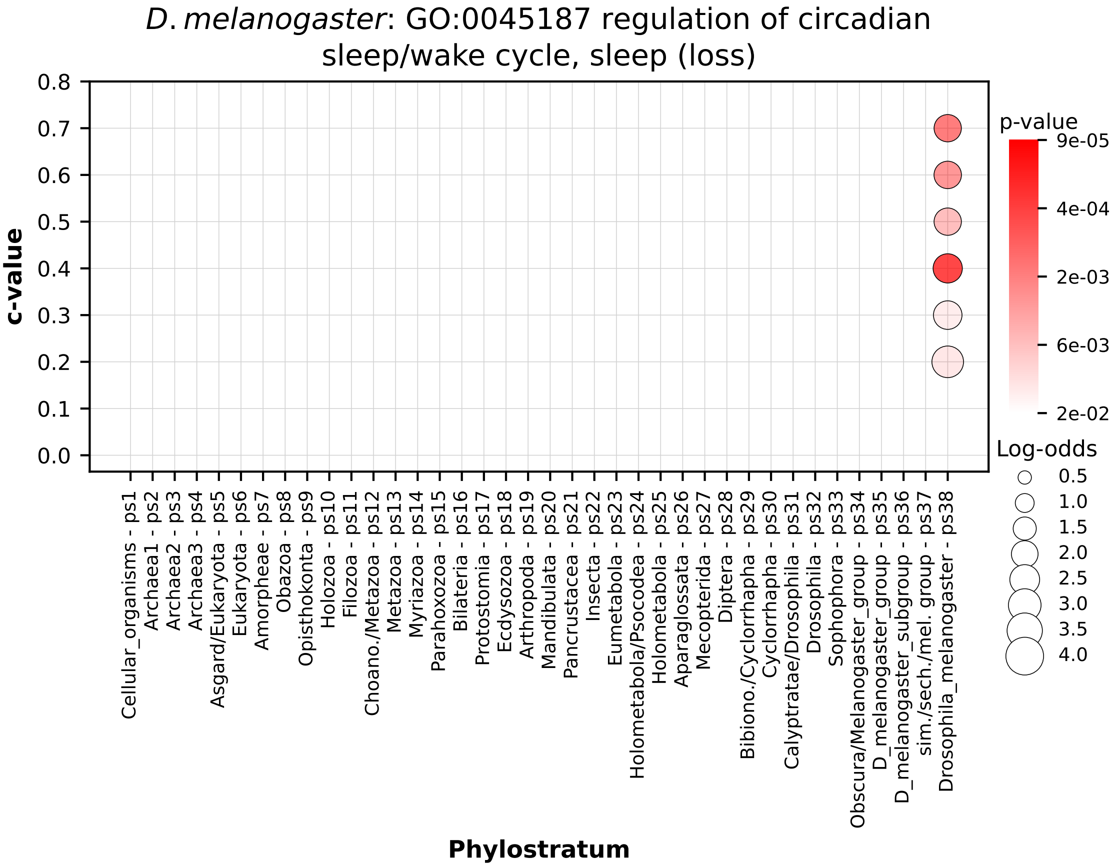

D. melanogaster: GO:0045187 regulation of circadian sleep/wake cycle, sleep (loss)
c-value
0.0
0.1
0.2
0.3
0.4
0.5
0.6
0.7
0.8
Phylostratum
Cellular_organisms - ps1
Archaea1 - ps2
Archaea2 - ps3
Archaea3 - ps4
Asgard/Eukaryota - ps5
Eukaryota - ps6
Amorpheae - ps7
Obazoa - ps8
Opisthokonta - ps9
Holozoa - ps10
Filozoa - ps11
Choano./Metazoa - ps12
Metazoa - ps13
Myriazoa - ps14
Parahoxozoa - ps15
Bilateria - ps16
Protostomia - ps17
Ecdysozoa - ps18
Arthropoda - ps19
Mandibulata - ps20
Pancrustacea - ps21
Insecta - ps22
Eumetabola - ps23
Holometabola/Psocodea - ps24
Holometabola - ps25
Aparaglossata - ps26
Mecopterida - ps27
Diptera - ps28
Bibiono./Cyclorrhapha - ps29
Cyclorrhapha - ps30
Calyptratae/Drosophila - ps31
Drosophila - ps32
Sophophora - ps33
Obscura/Melanogaster_group - ps34
D_melanogaster_group - ps35
D_melanogaster_subgroup - ps36
sim./sech./mel. group - ps37
Drosophila_melanogaster - ps38
p-value
9e-05
4e-04
2e-03
6e-03
2e-02
Log-odds
0.5
1.0
1.5
2.0
2.5
3.0
3.5
4.0

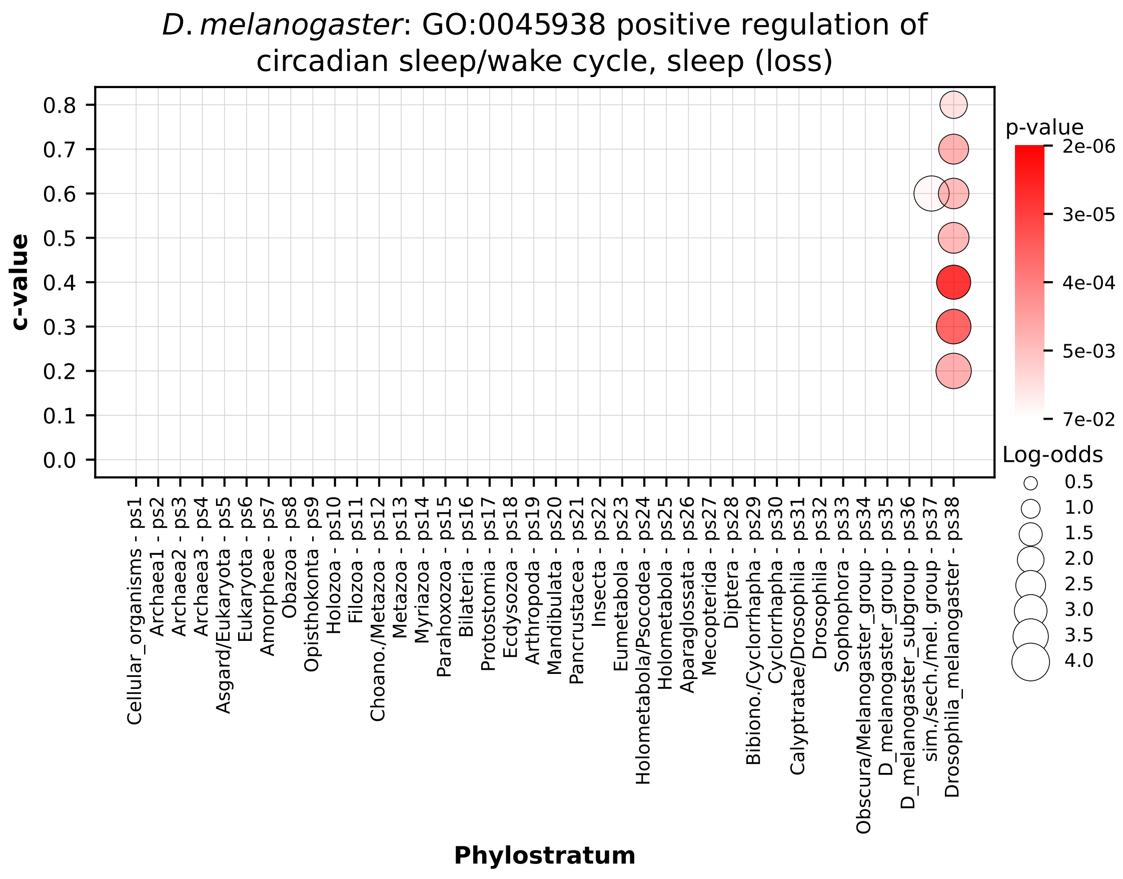

D. melanogaster: GO:0045938 positive regulation of circadian sleep/wake cycle, sleep (loss)
c-value
0.0
0.1
0.2
0.3
0.4
0.5
0.6
0.7
0.8
p-value
2e-06
3e-05
4e-04
5e-03
7e-02
Log-odds
0.5
1.0
1.5
2.0
2.5
3.0
3.5
4.0
Cellular_organisms - ps1
Archaea1 - ps2
Archaea2 - ps3
Archaea3 - ps4
Asgard/Eukaryota - ps5
Eukaryota - ps6
Amorpheae - ps7
Obazoa - ps8
Opisthokonta - ps9
Holozoa - ps10
Filozoa - ps11
Choano./Metazoa - ps12
Metazoa - ps13
Myriazoa - ps14
Parahoxozoa - ps15
Bilateria - ps16
Protostomia - ps17
Ecdysozoa - ps18
Arthropoda - ps19
Mandibulata - ps20
Pancrustacea - ps21
Insecta - ps22
Eumetabola - ps23
Holometabola/Psocodea - ps24
Holometabola - ps25
Aparaglossata - ps26
Mecopterida - ps27
Diptera - ps28
Bibiono./Cyclorrhapha - ps29
Cyclorrhapha - ps30
Calyptratae/Drosophila - ps31
Drosophila - ps32
Sophophora - ps33
Obscura/Melanogaster_group - ps34
D_melanogaster_group - ps35
D_melanogaster_subgroup - ps36
sim./sech./mel. group - ps37
Drosophila_melanogaster - ps38
Phylostratum

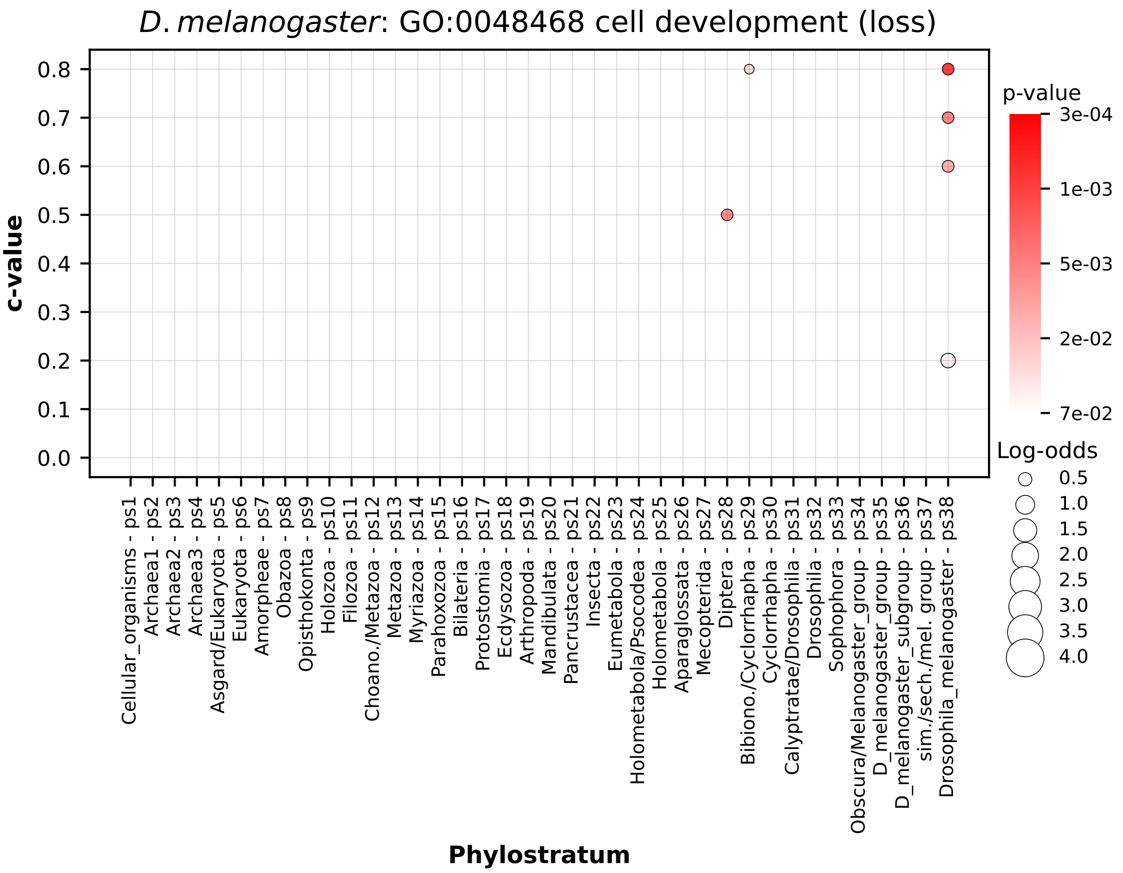

D. melanogaster: GO:0048468 cell development (loss)
c-value
0.0
0.1
0.2
0.3
0.4
0.5
0.6
0.7
0.8
Phylostratum
Cellular_organisms - ps1
Archaea1 - ps2
Archaea2 - ps3
Archaea3 - ps4
Asgard/Eukaryota - ps5
Eukaryota - ps6
Amorpheae - ps7
Obazoa - ps8
Opisthokonta - ps9
Holozoa - ps10
Filozoa - ps11
Choano./Metazoa - ps12
Metazoa - ps13
Myriazoa - ps14
Parahoxozoa - ps15
Bilateria - ps16
Protostomia - ps17
Ecdysozoa - ps18
Arthropoda - ps19
Mandibulata - ps20
Pancrustacea - ps21
Insecta - ps22
Eumetabola - ps23
Holometabola/Psocodea - ps24
Holometabola - ps25
Aparaglossata - ps26
Mecopterida - ps27
Diptera - ps28
Bibiono./Cyclorrhapha - ps29
Cyclorrhapha - ps30
Calyptratae/Drosophila - ps31
Drosophila - ps32
Sophophora - ps33
Obscura/Melanogaster_group - ps34
D_melanogaster_group - ps35
D_melanogaster_subgroup - ps36
sim./sech./mel. group - ps37
Drosophila_melanogaster - ps38
p-value
3e-04
1e-03
5e-03
2e-02
7e-02
Log-odds
0.5
1.0
1.5
2.0
2.5
3.0
3.5
4.0

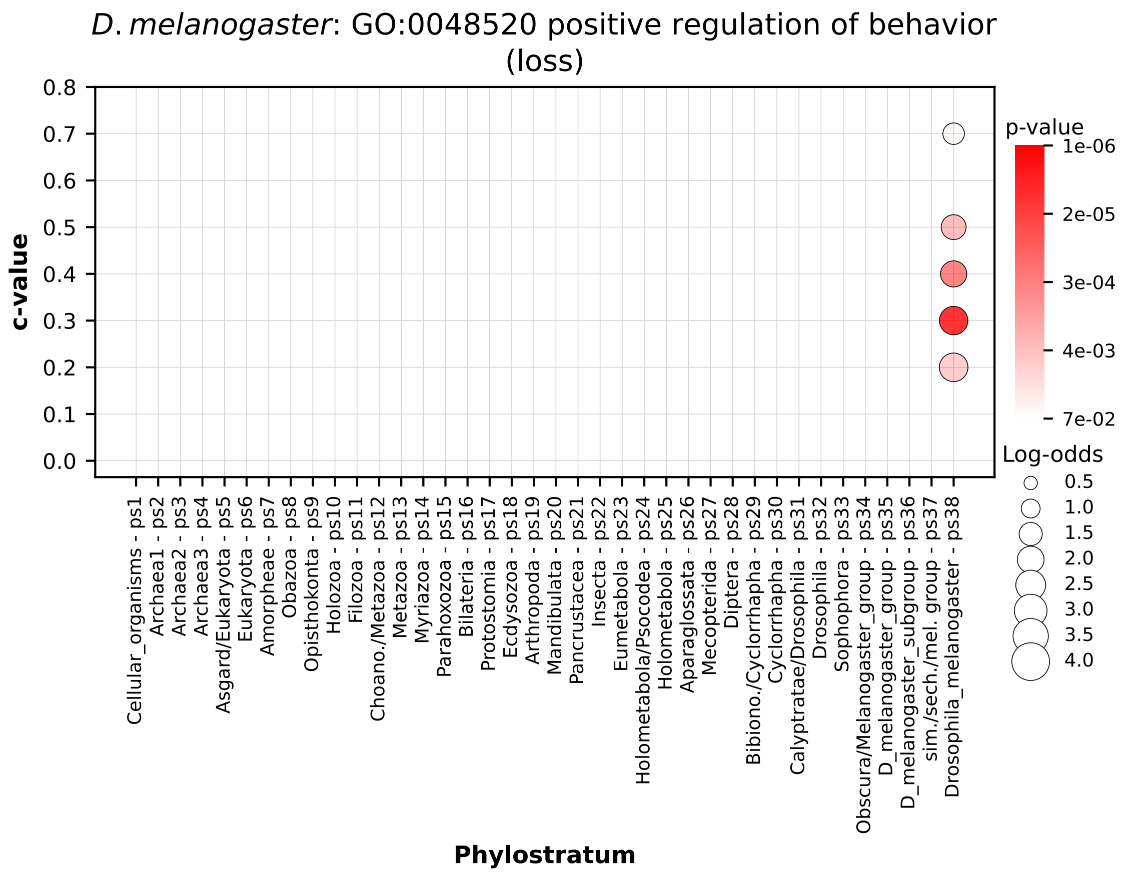

D. melanogaster: GO:0048520 positive regulation of behavior (loss)
c-value
0.0
0.1
0.2
0.3
0.4
0.5
0.6
0.7
0.8
Phylostratum
Cellular_organisms - ps1
Archaea1 - ps2
Archaea2 - ps3
Archaea3 - ps4
Asgard/Eukaryota - ps5
Eukaryota - ps6
Amorpheae - ps7
Obazoa - ps8
Opisthokonta - ps9
Holozoa - ps10
Filozoa - ps11
Choano./Metazoa - ps12
Metazoa - ps13
Myriazoa - ps14
Parahoxozoa - ps15
Bilateria - ps16
Protostomia - ps17
Ecdysozoa - ps18
Arthropoda - ps19
Mandibulata - ps20
Pancrustacea - ps21
Insecta - ps22
Eumetabola - ps23
Holometabola/Psocodea - ps24
Holometabola - ps25
Aparaglossata - ps26
Mecopterida - ps27
Diptera - ps28
Bibiono./Cyclorrhapha - ps29
Cyclorrhapha - ps30
Calyptratae/Drosophila - ps31
Drosophila - ps32
Sophophora - ps33
Obscura/Melanogaster_group - ps34
D_melanogaster_group - ps35
D_melanogaster_subgroup - ps36
sim./sech./mel. group - ps37
Drosophila_melanogaster - ps38
p-value
1e-06
2e-05
3e-04
4e-03
7e-02
Log-odds
0.5
1.0
1.5
2.0
2.5
3.0
3.5
4.0

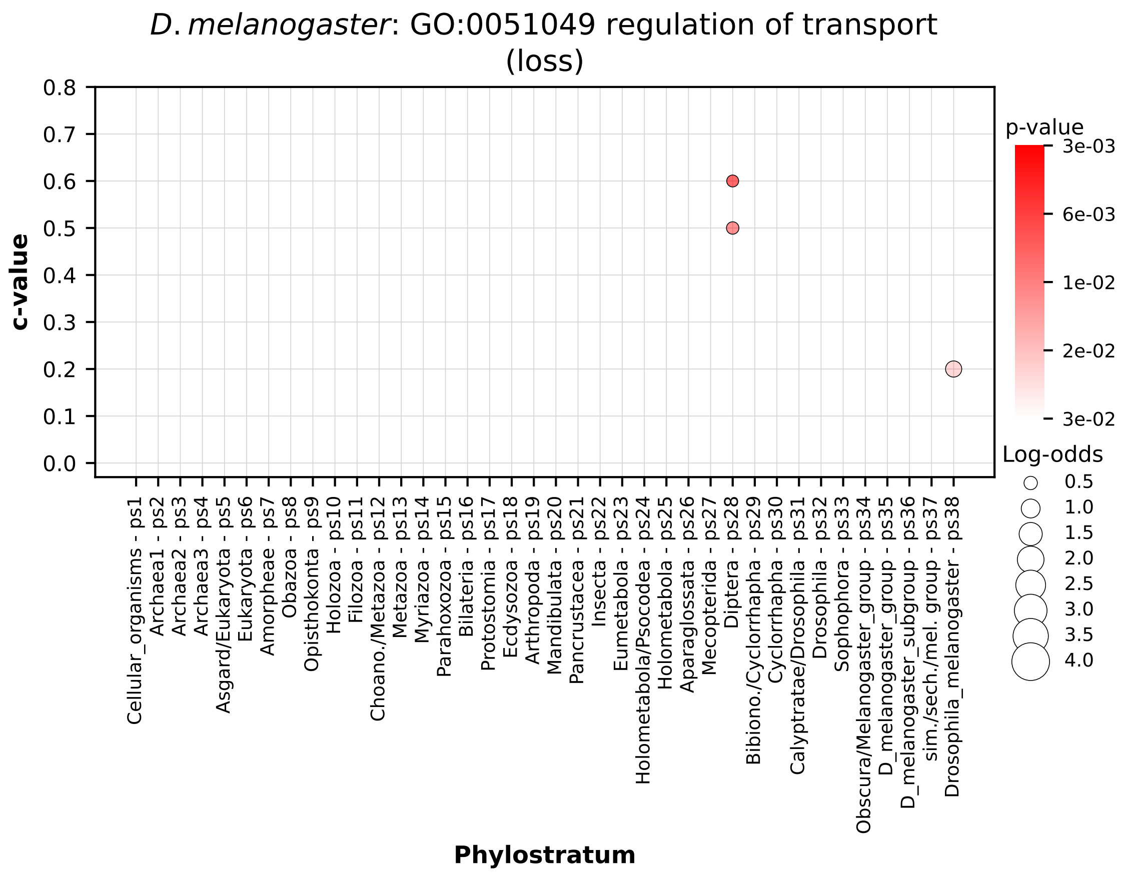

D. melanogaster: GO:0051049 regulation of transport (loss)
c-value
0.8
0.7
0.6
0.5
0.4
0.3
0.2
0.1
0.0
p-value
3e-03
6e-03
1e-02
2e-02
3e-02
Log-odds
0.5
1.0
1.5
2.0
2.5
3.0
3.5
4.0
Cellular_organisms - ps1
Archaea1 - ps2
Archaea2 - ps3
Archaea3 - ps4
Asgard/Eukaryota - ps5
Eukaryota - ps6
Amorpheae - ps7
Obazoa - ps8
Opisthokonta - ps9
Holozoa - ps10
Filozoa - ps11
Choano./Metazoa - ps12
Metazoa - ps13
Myriazoa - ps14
Parahoxozoa - ps15
Bilateria - ps16
Protostomia - ps17
Ecdysozoa - ps18
Arthropoda - ps19
Mandibulata - ps20
Pancrustacea - ps21
Insecta - ps22
Eumetabola - ps23
Holometabola/Psocodea - ps24
Holometabola - ps25
Aparaglossata - ps26
Mecopterida - ps27
Diptera - ps28
Bibiono./Cyclorrhapha - ps29
Cyclorrhapha - ps30
Calyptratae/Drosophila - ps31
Drosophila - ps32
Sophophora - ps33
Obscura/Melanogaster_group - ps34
D_melanogaster_group - ps35
D_melanogaster_subgroup - ps36
sim./sech./mel. group - ps37
Drosophila_melanogaster - ps38
Phylostratum

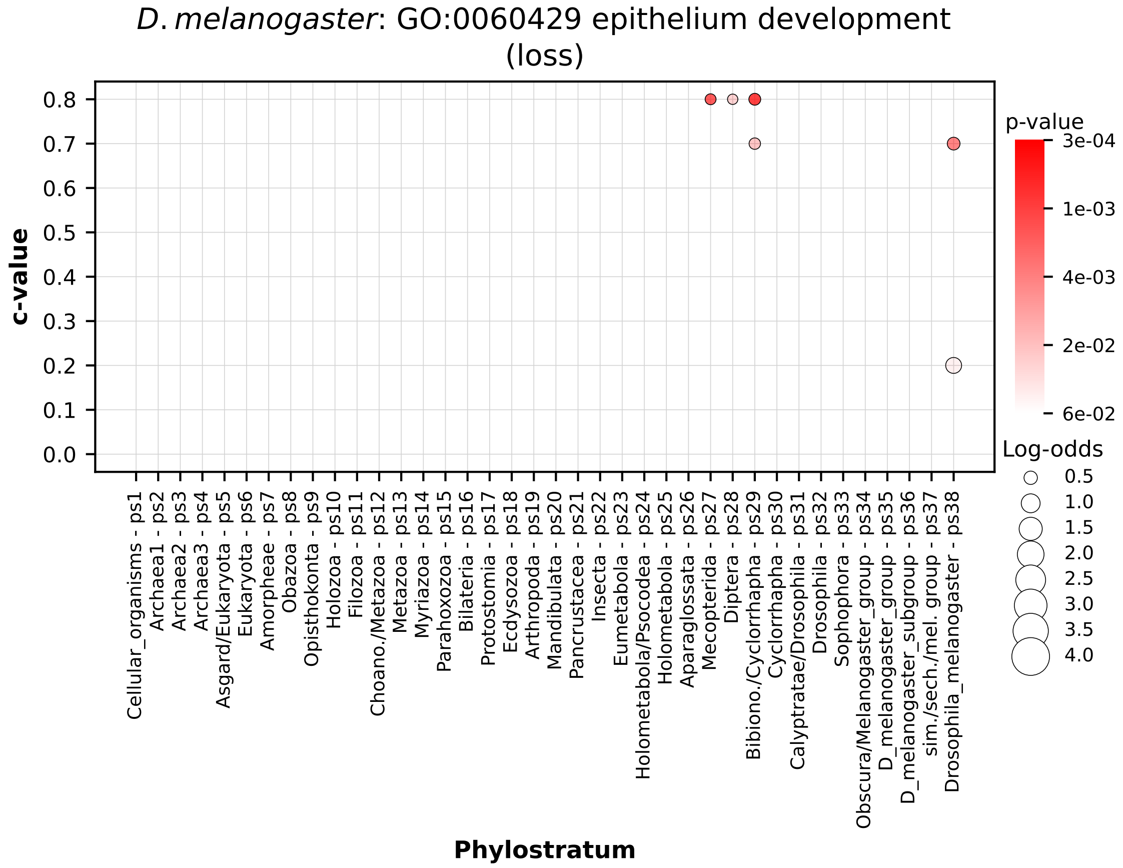

D. melanogaster: GO:0060429 epithelium development
(loss)
c-value
0.0
0.1
0.2
0.3
0.4
0.5
0.6
0.7
0.8
Phylostratum
Cellular_organisms - ps1
Archaea1 - ps2
Archaea2 - ps3
Archaea3 - ps4
Asgard/Eukaryota - ps5
Eukaryota - ps6
Amorpheae - ps7
Obazoa - ps8
Opisthokonta - ps9
Holozoa - ps10
Filozoa - ps11
Choano./Metazoa - ps12
Metazoa - ps13
Myriazoa - ps14
Parahoxozoa - ps15
Bilateria - ps16
Protostomia - ps17
Ecdysozoa - ps18
Arthropoda - ps19
Mandibulata - ps20
Pancrustacea - ps21
Insecta - ps22
Eumetabola - ps23
Holometabola/Psocodea - ps24
Holometabola - ps25
Aparaglossata - ps26
Mecopterida - ps27
Diptera - ps28
Bibiono./Cyclorrhapha - ps29
Cyclorrhapha - ps30
Calyptratae/Drosophila - ps31
Drosophila - ps32
Sophophora - ps33
Obscura/Melanogaster_group - ps34
D_melanogaster_group - ps35
D_melanogaster_subgroup - ps36
sim./sech./mel. group - ps37
Drosophila_melanogaster - ps38
p-value
3e-04
1e-03
4e-03
2e-02
6e-02
Log-odds
0.5
1.0
1.5
2.0
2.5
3.0
3.5
4.0

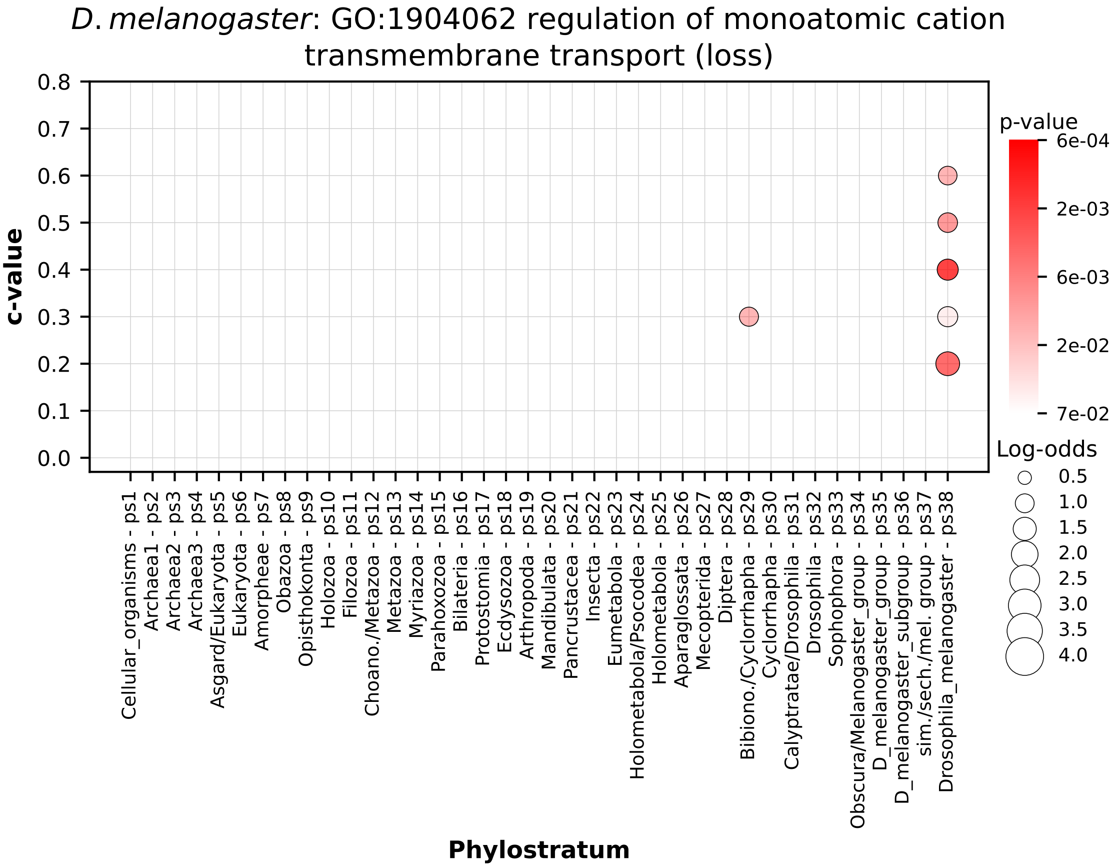

D. melanogaster: GO:1904062 regulation of monoatomic cation transmembrane transport (loss)
c-value
0.8
0.7
0.6
0.5
0.4
0.3
0.2
0.1
0.0
p-value
6e-04
2e-03
6e-03
2e-02
7e-02
Log-odds
0.5
1.0
1.5
2.0
2.5
3.0
3.5
4.0
Cellular_organisms - ps1
Archaea1 - ps2
Archaea2 - ps3
Archaea3 - ps4
Asgard/Eukaryota - ps5
Eukaryota - ps6
Amorpheae - ps7
Obazoa - ps8
Opisthokonta - ps9
Holozoa - ps10
Filozoa - ps11
Choano./Metazoa - ps12
Metazoa - ps13
Myriazoa - ps14
Parahoxozoa - ps15
Bilateria - ps16
Protostomia - ps17
Ecdysozoa - ps18
Arthropoda - ps19
Mandibulata - ps20
Pancrustacea - ps21
Insecta - ps22
Eumetabola - ps23
Holometabola/Psocodea - ps24
Holometabola - ps25
Aparaglossata - ps26
Mecopterida - ps27
Diptera - ps28
Bibiono./Cyclorrhapha - ps29
Cyclorrhapha - ps30
Calyptratae/Drosophila - ps31
Drosophila - ps32
Sophophora - ps33
Obscura/Melanogaster_group - ps34
D_melanogaster_group - ps35
D_melanogaster_subgroup - ps36
sim./sech./mel. group - ps37
Drosophila_melanogaster - ps38
Phylostratum

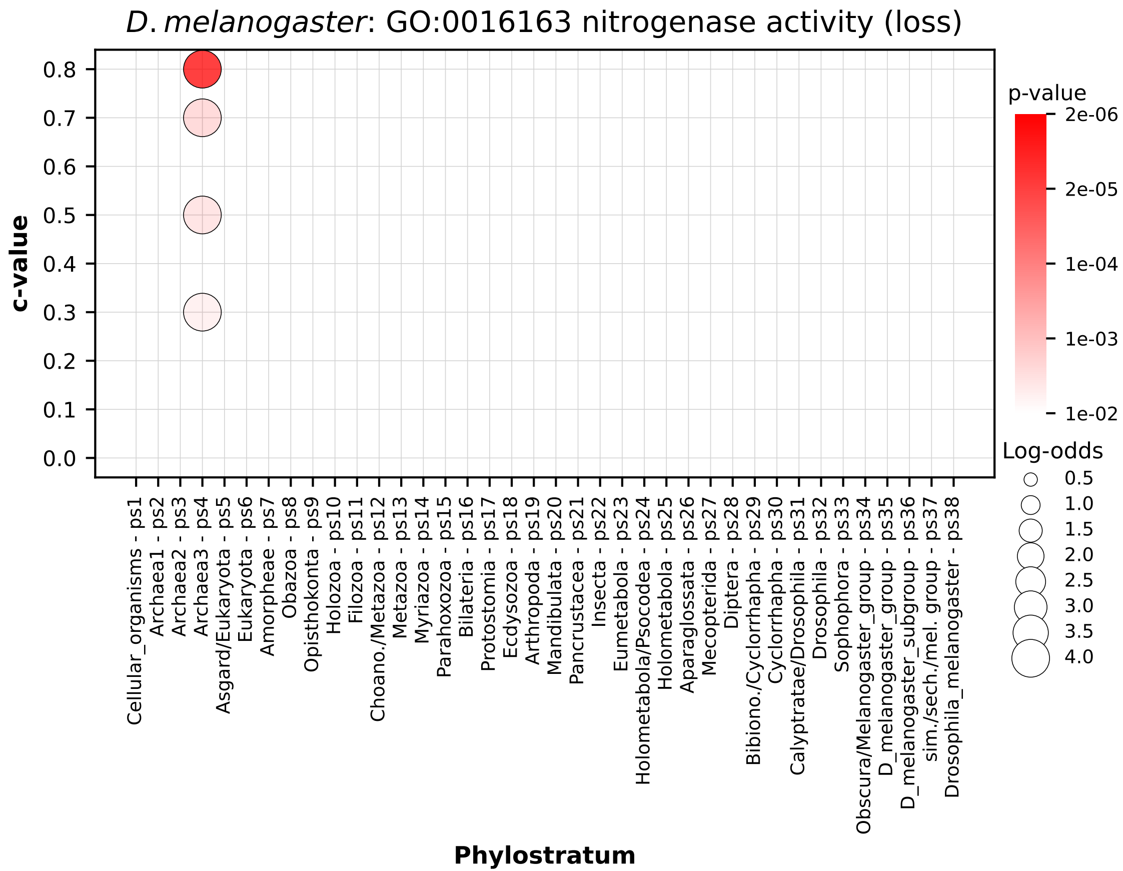

D. melanogaster: GO:0016163 nitrogenase activity (loss)
c-value
0.8
0.7
0.6
0.5
0.4
0.3
0.2
0.1
0.0
p-value
2e-06
2e-05
1e-04
1e-03
1e-02
Log-odds
0.5
1.0
1.5
2.0
2.5
3.0
3.5
4.0
Cellular_organisms - ps1
Archaea1 - ps2
Archaea2 - ps3
Archaea3 - ps4
Asgard/Eukaryota - ps5
Eukaryota - ps6
Amorpheae - ps7
Obazoa - ps8
Opisthokonta - ps9
Holozoa - ps10
Filozoa - ps11
Choano./Metazoa - ps12
Metazoa - ps13
Myriazoa - ps14
Parahoxozoa - ps15
Bilateria - ps16
Protostomia - ps17
Ecdysozoa - ps18
Arthropoda - ps19
Mandibulata - ps20
Pancrustacea - ps21
Insecta - ps22
Eumetabola - ps23
Holometabola/Psocodea - ps24
Holometabola - ps25
Aparaglossata - ps26
Mecopterida - ps27
Diptera - ps28
Bibiono./Cyclorrhapha - ps29
Cyclorrhapha - ps30
Calyptratae/Drosophila - ps31
Drosophila - ps32
Sophophora - ps33
Obscura/Melanogaster_group - ps34
D_melanogaster_group - ps35
D_melanogaster_subgroup - ps36
sim./sech./mel. group - ps37
Drosophila_melanogaster - ps38
Phylostratum

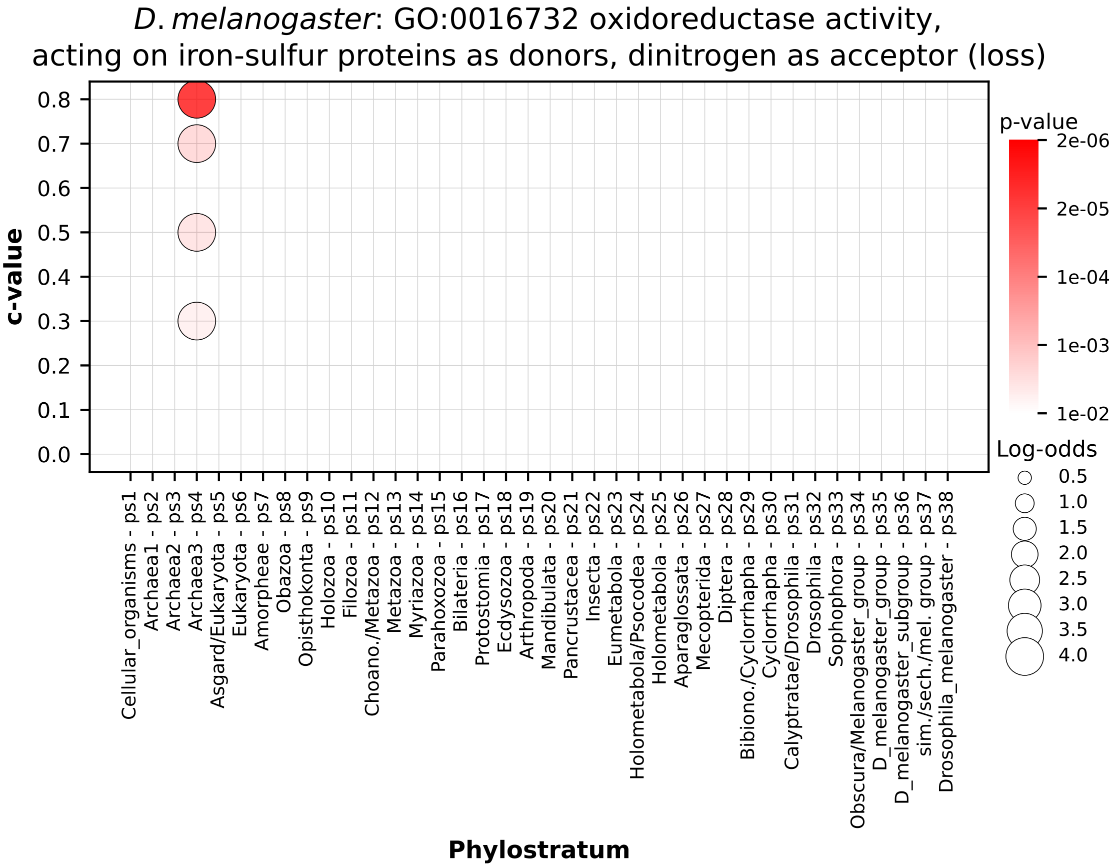

D. melanogaster: GO:0016732 oxidoreductase activity,
acting on iron-sulfur proteins as donors, dinitrogen as acceptor (loss)
c-value
0.0
0.1
0.2
0.3
0.4
0.5
0.6
0.7
0.8
Phylostratum
Cellular_organisms - ps1
Archaea1 - ps2
Archaea2 - ps3
Archaea3 - ps4
Asgard/Eukaryota - ps5
Eukaryota - ps6
Amorpheae - ps7
Obazoa - ps8
Opisthokonta - ps9
Holozoa - ps10
Filozoa - ps11
Choano./Metazoa - ps12
Metazoa - ps13
Myriazoa - ps14
Parahoxozoa - ps15
Bilateria - ps16
Protostomia - ps17
Ecdysozoa - ps18
Arthropoda - ps19
Mandibulata - ps20
Pancrustacea - ps21
Insecta - ps22
Eumetabola - ps23
Holometabola/Psocodea - ps24
Holometabola - ps25
Aparaglossata - ps26
Mecopterida - ps27
Diptera - ps28
Bibiono./Cyclorrhapha - ps29
Cyclorrhapha - ps30
Calyptratae/Drosophila - ps31
Drosophila - ps32
Sophophora - ps33
Obscura/Melanogaster_group - ps34
D_melanogaster_group - ps35
D_melanogaster_subgroup - ps36
sim./sech./mel. group - ps37
Drosophila_melanogaster - ps38
p-value
2e-06
2e-05
1e-04
1e-03
1e-02
Log-odds
0.5
1.0
1.5
2.0
2.5
3.0
3.5
4.0

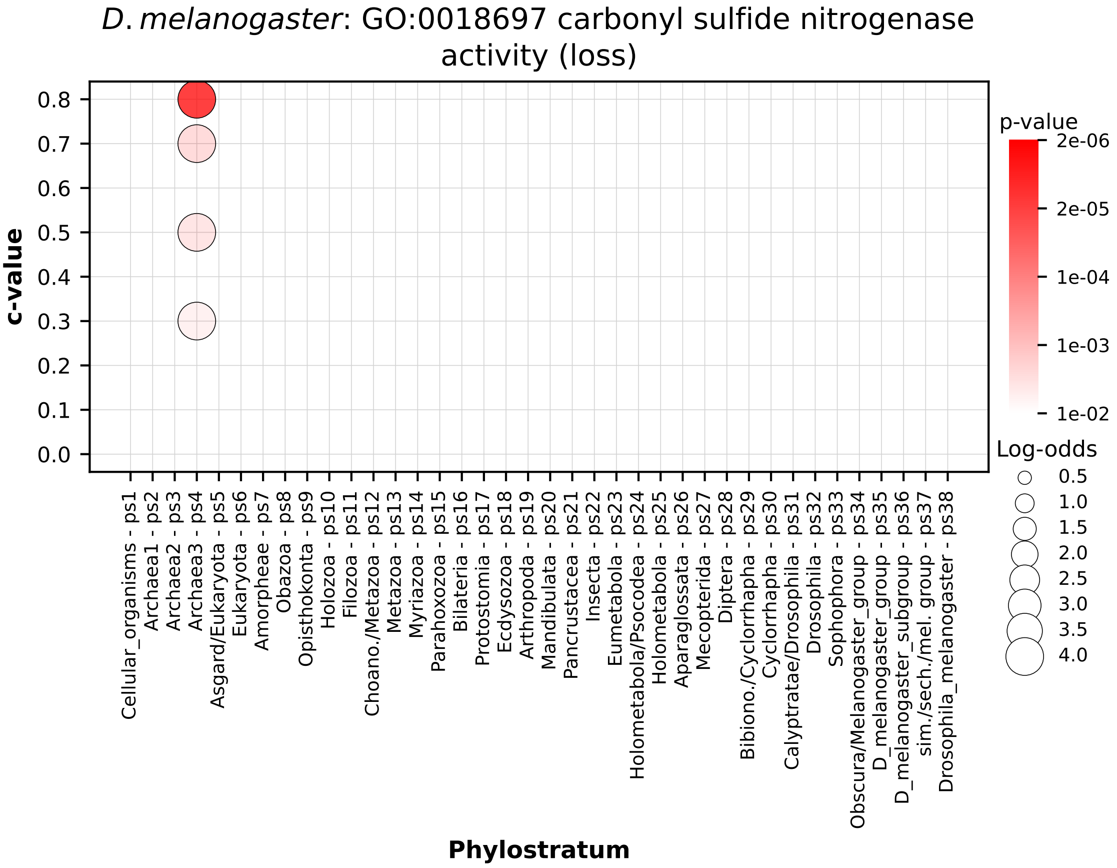

D. melanogaster: GO:0018697 carbonyl sulfide nitrogenase activity (loss)
c-value
0.0
0.1
0.2
0.3
0.4
0.5
0.6
0.7
0.8
Phylostratum
Cellular_organisms - ps1
Archaea1 - ps2
Archaea2 - ps3
Archaea3 - ps4
Asgard/Eukaryota - ps5
Eukaryota - ps6
Amorpheae - ps7
Obazoa - ps8
Opisthokonta - ps9
Holozoa - ps10
Filozoa - ps11
Choano./Metazoa - ps12
Metazoa - ps13
Myriazoa - ps14
Parahoxozoa - ps15
Bilateria - ps16
Protostomia - ps17
Ecdysozoa - ps18
Arthropoda - ps19
Mandibulata - ps20
Pancrustacea - ps21
Insecta - ps22
Eumetabola - ps23
Holometabola/Psocodea - ps24
Holometabola - ps25
Aparaglossata - ps26
Mecopterida - ps27
Diptera - ps28
Bibiono./Cyclorrhapha - ps29
Cyclorrhapha - ps30
Calyptratae/Drosophila - ps31
Drosophila - ps32
Sophophora - ps33
Obscura/Melanogaster_group - ps34
D_melanogaster_group - ps35
D_melanogaster_subgroup - ps36
sim./sech./mel. group - ps37
Drosophila_melanogaster - ps38
p-value
2e-06
2e-05
1e-04
1e-03
1e-02
Log-odds
0.5
1.0
1.5
2.0
2.5
3.0
3.5
4.0

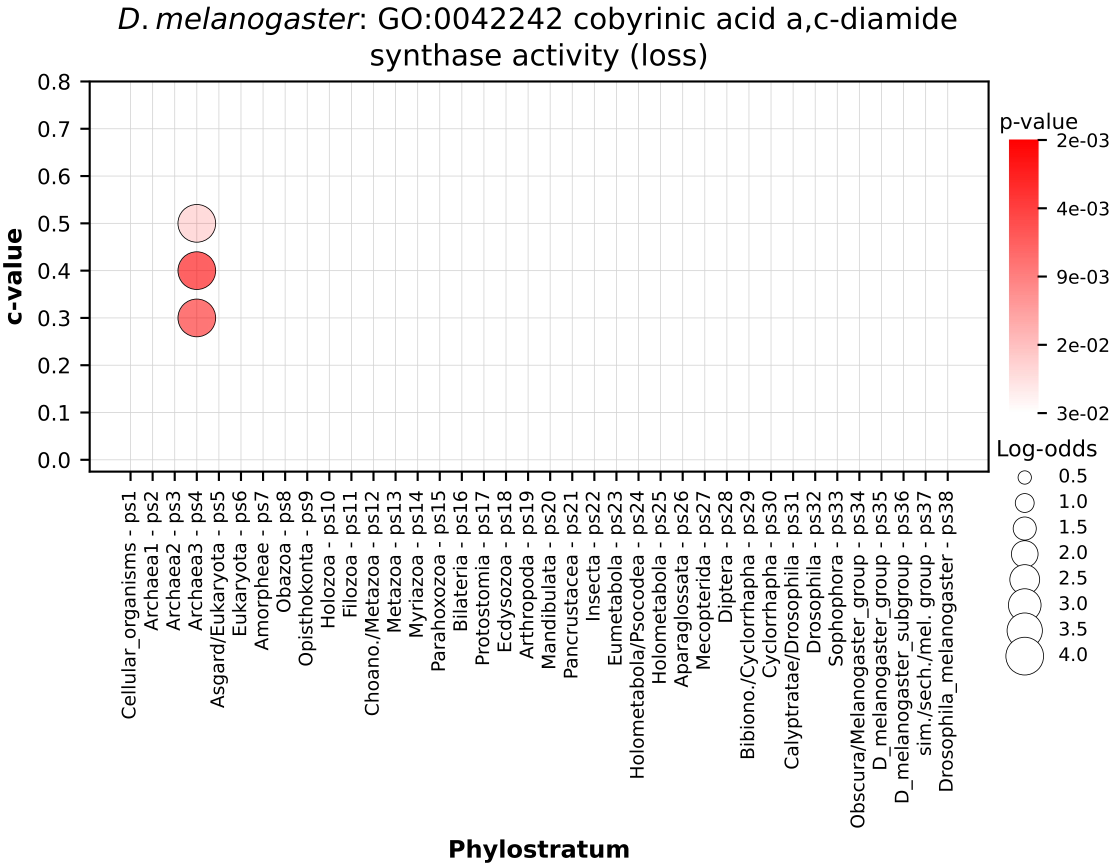

D. melanogaster: GO:0042242 cobyrinic acid a,c-diamide synthase activity (loss)
c-value
0.0
0.1
0.2
0.3
0.4
0.5
0.6
0.7
0.8
Phylostratum
Cellular_organisms - ps1
Archaea1 - ps2
Archaea2 - ps3
Archaea3 - ps4
Asgard/Eukaryota - ps5
Eukaryota - ps6
Amorpheae - ps7
Obazoa - ps8
Opisthokonta - ps9
Holozoa - ps10
Filozoa - ps11
Choano./Metazoa - ps12
Metazoa - ps13
Myriazoa - ps14
Parahoxozoa - ps15
Bilateria - ps16
Protostomia - ps17
Ecdysozoa - ps18
Arthropoda - ps19
Mandibulata - ps20
Pancrustacea - ps21
Insecta - ps22
Eumetabola - ps23
Holometabola/Psocodea - ps24
Holometabola - ps25
Aparaglossata - ps26
Mecopterida - ps27
Diptera - ps28
Bibiono./Cyclorrhapha - ps29
Cyclorrhapha - ps30
Calyptratae/Drosophila - ps31
Drosophila - ps32
Sophophora - ps33
Obscura/Melanogaster_group - ps34
D_melanogaster_group - ps35
D_melanogaster_subgroup - ps36
sim./sech./mel. group - ps37
Drosophila_melanogaster - ps38
p-value
2e-03
4e-03
9e-03
2e-02
3e-02
Log-odds
0.5
1.0
1.5
2.0
2.5
3.0
3.5
4.0

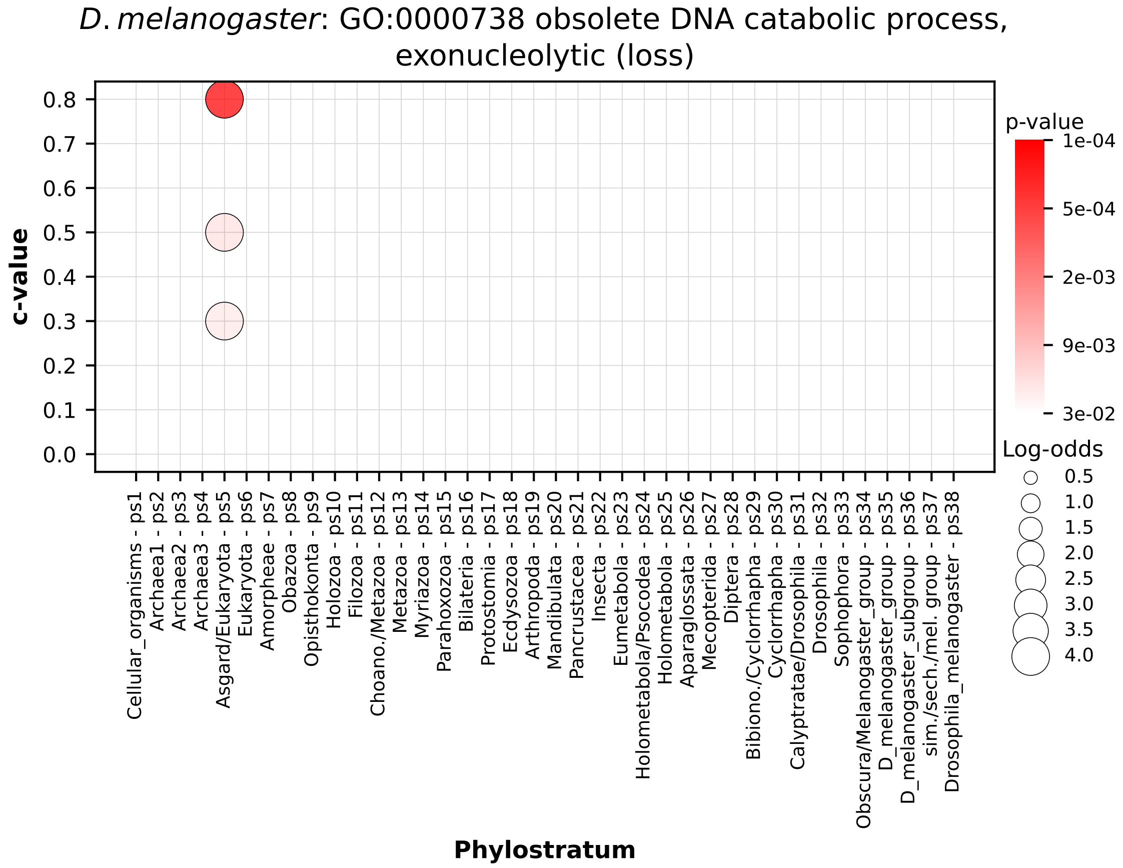

D. melanogaster: GO:0000738 obsolete DNA catabolic process, exonucleolytic (loss)
c-value
0.0
0.1
0.2
0.3
0.4
0.5
0.6
0.7
0.8
Phylostratum
Cellular_organisms - ps1
Archaea1 - ps2
Archaea2 - ps3
Archaea3 - ps4
Asgard/Eukaryota - ps5
Eukaryota - ps6
Amorpheae - ps7
Obazoa - ps8
Opisthokonta - ps9
Holozoa - ps10
Filozoa - ps11
Choano./Metazoa - ps12
Metazoa - ps13
Myriazoa - ps14
Parahoxozoa - ps15
Bilateria - ps16
Protostomia - ps17
Ecdysozoa - ps18
Arthropoda - ps19
Mandibulata - ps20
Pancrustacea - ps21
Insecta - ps22
Eumetabola - ps23
Holometabola/Psocodea - ps24
Holometabola - ps25
Aparaglossata - ps26
Mecopterida - ps27
Diptera - ps28
Bibiono./Cyclorrhapha - ps29
Cyclorrhapha - ps30
Calyptratae/Drosophila - ps31
Drosophila - ps32
Sophophora - ps33
Obscura/Melanogaster_group - ps34
D_melanogaster_group - ps35
D_melanogaster_subgroup - ps36
sim./sech./mel. group - ps37
Drosophila_melanogaster - ps38
p-value
1e-04
5e-04
2e-03
9e-03
3e-02
Log-odds
0.5
1.0
1.5
2.0
2.5
3.0
3.5
4.0

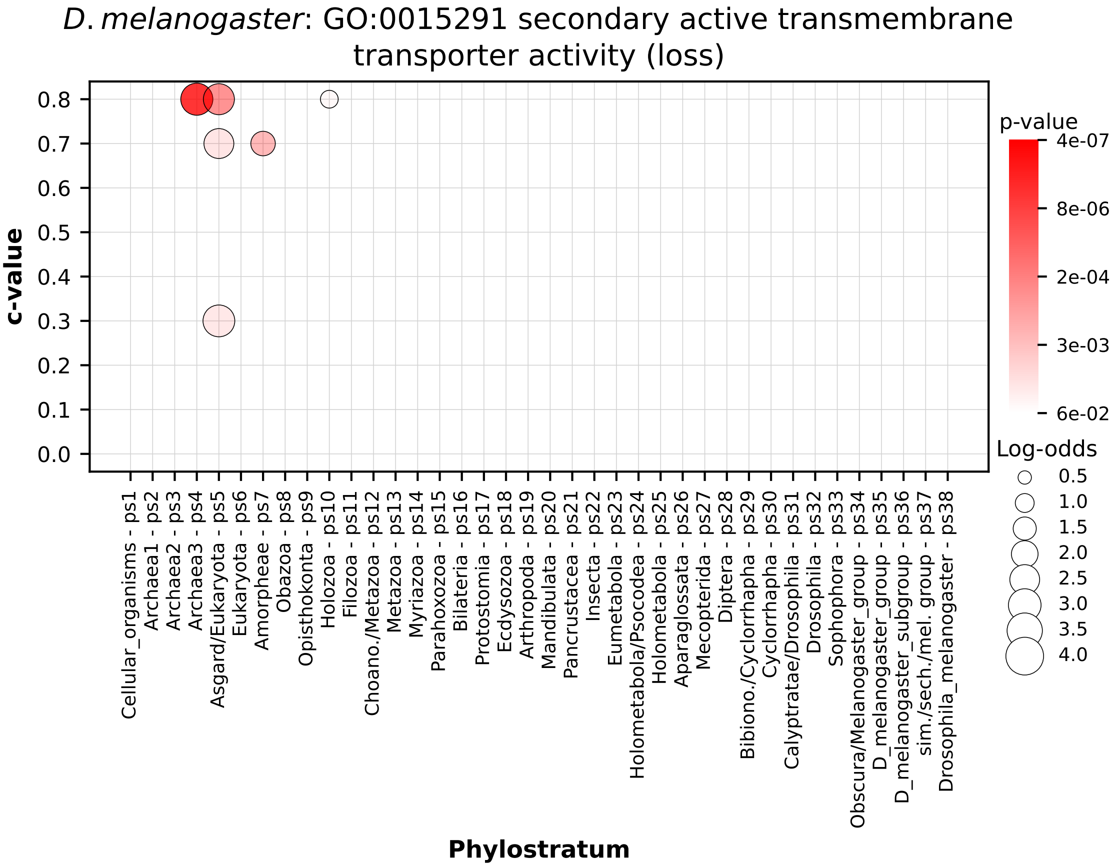

D. melanogaster: GO:0015291 secondary active transmembrane transporter activity (loss)
c-value
0.8
0.7
0.6
0.5
0.4
0.3
0.2
0.1
0.0
p-value
4e-07
8e-06
2e-04
3e-03
6e-02
Log-odds
0.5
1.0
1.5
2.0
2.5
3.0
3.5
4.0
Cellular_organisms - ps1
Archaea1 - ps2
Archaea2 - ps3
Archaea3 - ps4
Asgard/Eukaryota - ps5
Eukaryota - ps6
Amorpheae - ps7
Obazoa - ps8
Opisthokonta - ps9
Holozoa - ps10
Filozoa - ps11
Choano./Metazoa - ps12
Metazoa - ps13
Myriazoa - ps14
Parahoxozoa - ps15
Bilateria - ps16
Protostomia - ps17
Ecdysozoa - ps18
Arthropoda - ps19
Mandibulata - ps20
Pancrustacea - ps21
Insecta - ps22
Eumetabola - ps23
Holometabola/Psocodea - ps24
Holometabola - ps25
Aparaglossata - ps26
Mecopterida - ps27
Diptera - ps28
Bibiono./Cyclorrhapha - ps29
Cyclorrhapha - ps30
Calyptratae/Drosophila - ps31
Drosophila - ps32
Sophophora - ps33
Obscura/Melanogaster_group - ps34
D_melanogaster_group - ps35
D_melanogaster_subgroup - ps36
sim./sech./mel. group - ps37
Drosophila_melanogaster - ps38
Phylostratum

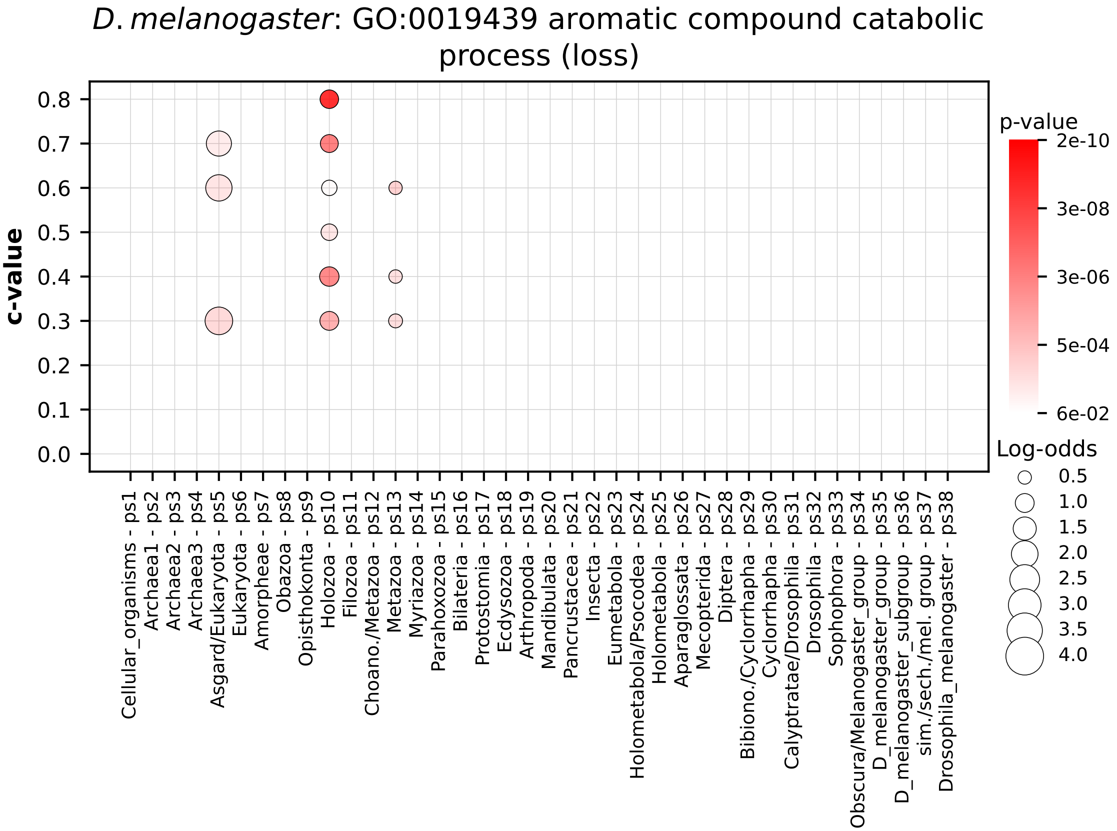

D. melanogaster: GO:0019439 aromatic compound catabolic process (loss)
c-value
0.8
0.7
0.6
0.5
0.4
0.3
0.2
0.1
0.0
Phylostratum
Cellular_organisms - ps1
Archaea1 - ps2
Archaea2 - ps3
Archaea3 - ps4
Asgard/Eukaryota - ps5
Eukaryota - ps6
Amorpheae - ps7
Obazoa - ps8
Opisthokonta - ps9
Holozoa - ps10
Filozoa - ps11
Choano./Metazoa - ps12
Metazoa - ps13
Myriazoa - ps14
Parahoxozoa - ps15
Bilateria - ps16
Protostomia - ps17
Ecdysozoa - ps18
Arthropoda - ps19
Mandibulata - ps20
Pancrustacea - ps21
Insecta - ps22
Eumetabola - ps23
Holometabola/Psocodea - ps24
Holometabola - ps25
Aparaglossata - ps26
Mecopterida - ps27
Diptera - ps28
Bibiono./Cyclorrhapha - ps29
Cyclorrhapha - ps30
Calyptratae/Drosophila - ps31
Drosophila - ps32
Sophophora - ps33
Obscura/Melanogaster_group - ps34
D_melanogaster_group - ps35
D_melanogaster_subgroup - ps36
sim./sech./mel. group - ps37
Drosophila_melanogaster - ps38
p-value
2e-10
3e-08
3e-06
5e-04
6e-02
Log-odds
0.5
1.0
1.5
2.0
2.5
3.0
3.5
4.0

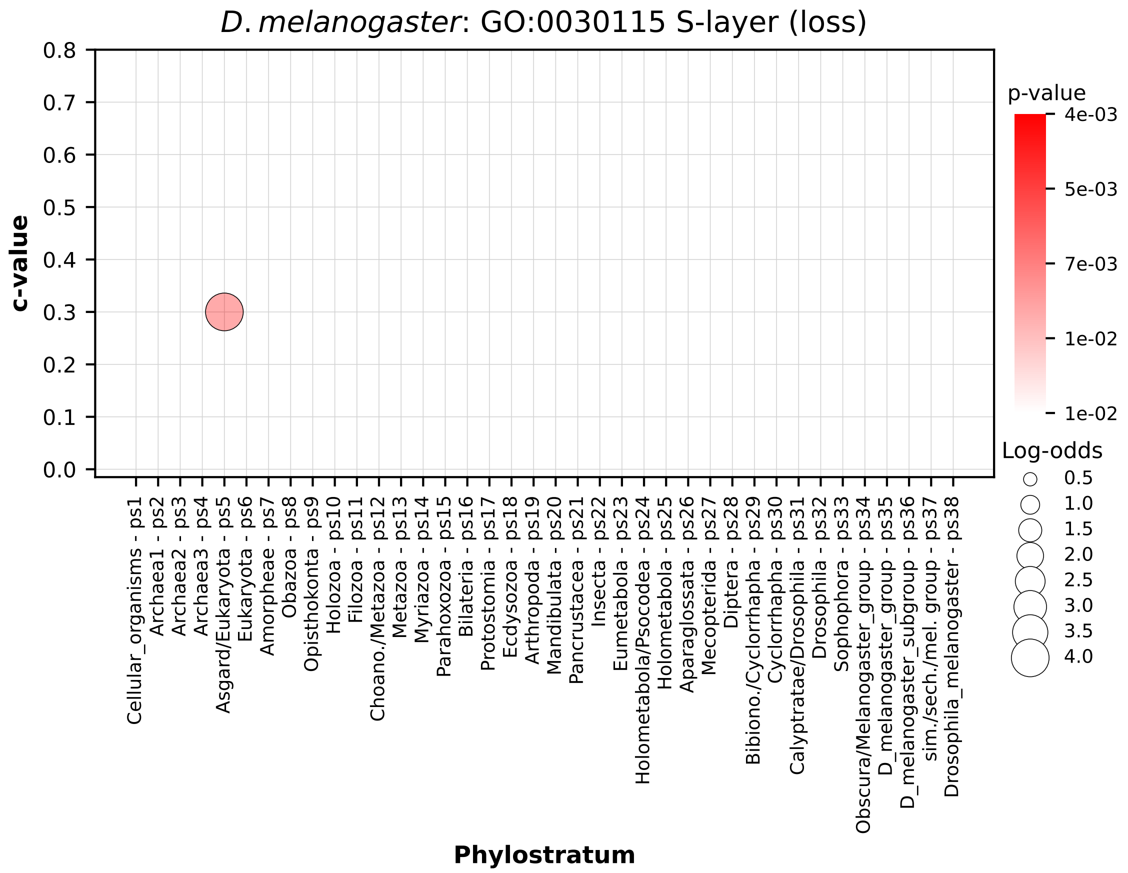

D. melanogaster: GO:0030115 S-layer (loss)
c-value
0.8
0.7
0.6
0.5
0.4
0.3
0.2
0.1
0.0
p-value
4e-03
5e-03
7e-03
1e-02
1e-02
Log-odds
0.5
1.0
1.5
2.0
2.5
3.0
3.5
4.0
Cellular_organisms - ps1
Archaea1 - ps2
Archaea2 - ps3
Archaea3 - ps4
Asgard/Eukaryota - ps5
Eukaryota - ps6
Amorpheae - ps7
Obazoa - ps8
Opisthokonta - ps9
Holozoa - ps10
Filozoa - ps11
Choano./Metazoa - ps12
Metazoa - ps13
Myriazoa - ps14
Parahoxozoa - ps15
Bilateria - ps16
Protostomia - ps17
Ecdysozoa - ps18
Arthropoda - ps19
Mandibulata - ps20
Pancrustacea - ps21
Insecta - ps22
Eumetabola - ps23
Holometabola/Psocodea - ps24
Holometabola - ps25
Aparaglossata - ps26
Mecopterida - ps27
Diptera - ps28
Bibiono./Cyclorrhapha - ps29
Cyclorrhapha - ps30
Calyptratae/Drosophila - ps31
Drosophila - ps32
Sophophora - ps33
Obscura/Melanogaster_group - ps34
D_melanogaster_group - ps35
D_melanogaster_subgroup - ps36
sim./sech./mel. group - ps37
Drosophila_melanogaster - ps38
Phylostratum

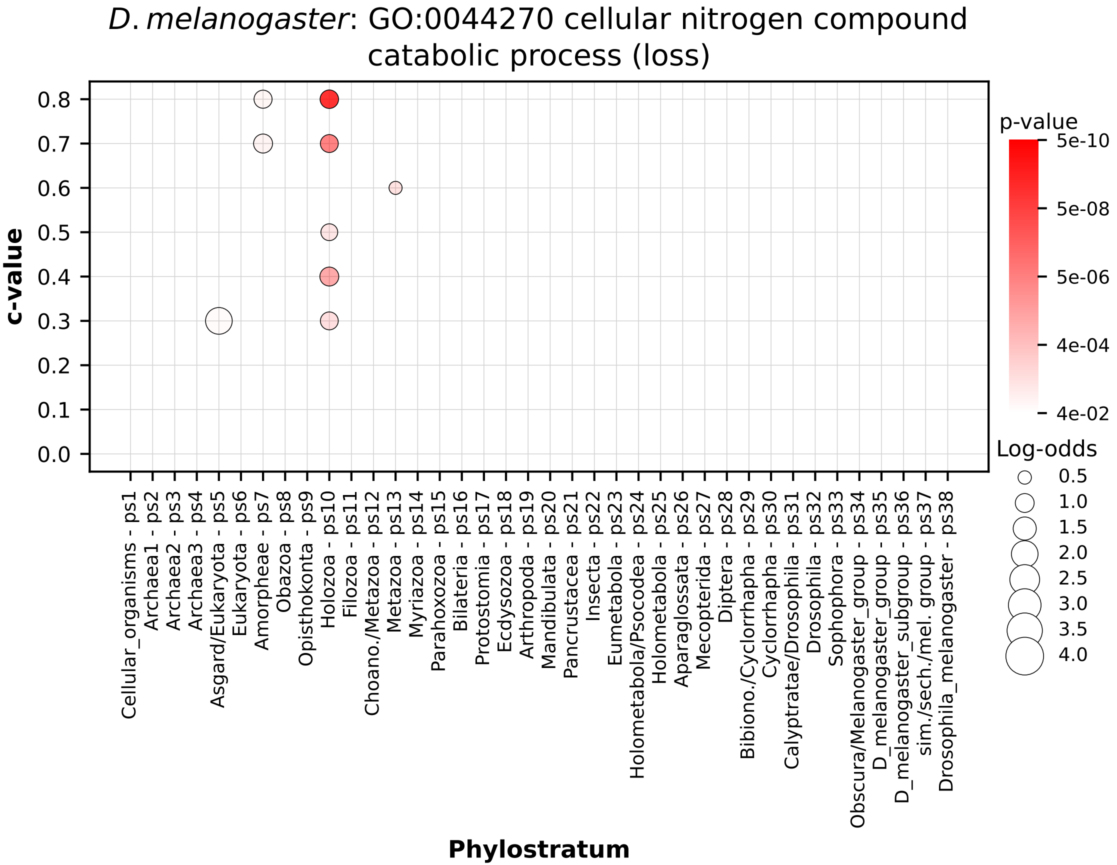

D. melanogaster: GO:0044270 cellular nitrogen compound catabolic process (loss)
c-value
0.8
0.7
0.6
0.5
0.4
0.3
0.2
0.1
0.0
p-value
5e-10
5e-08
5e-06
4e-04
4e-02
Log-odds
0.5
1.0
1.5
2.0
2.5
3.0
3.5
4.0
Cellular_organisms - ps1
Archaea1 - ps2
Archaea2 - ps3
Archaea3 - ps4
Asgard/Eukaryota - ps5
Eukaryota - ps6
Amorpheae - ps7
Obazoa - ps8
Opisthokonta - ps9
Holozoa - ps10
Filozoa - ps11
Choano./Metazoa - ps12
Metazoa - ps13
Myriazoa - ps14
Parahoxozoa - ps15
Bilateria - ps16
Protostomia - ps17
Ecdysozoa - ps18
Arthropoda - ps19
Mandibulata - ps20
Pancrustacea - ps21
Insecta - ps22
Eumetabola - ps23
Holometabola/Psocodea - ps24
Holometabola - ps25
Aparaglossata - ps26
Mecopterida - ps27
Diptera - ps28
Bibiono./Cyclorrhapha - ps29
Cyclorrhapha - ps30
Calyptratae/Drosophila - ps31
Drosophila - ps32
Sophophora - ps33
Obscura/Melanogaster_group - ps34
D_melanogaster_group - ps35
D_melanogaster_subgroup - ps36
sim./sech./mel. group - ps37
Drosophila_melanogaster - ps38
Phylostratum

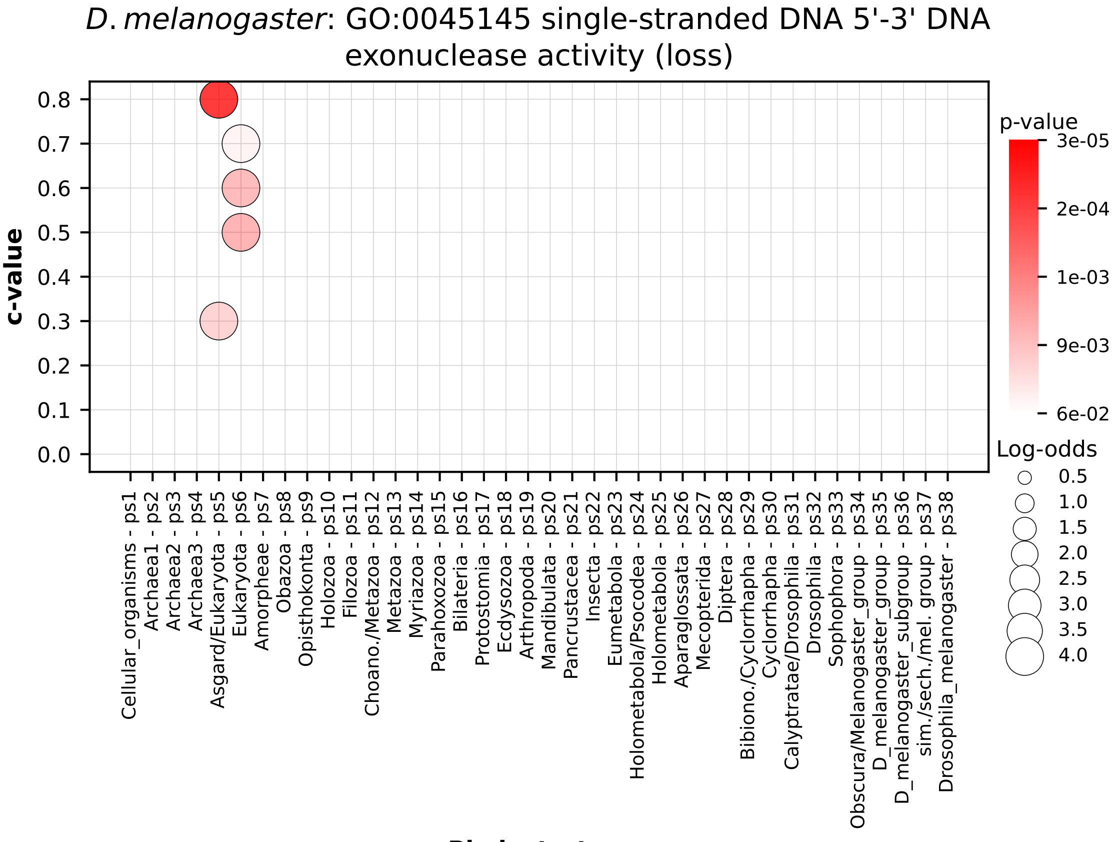

D. melanogaster: GO:0045145 single-stranded DNA 5'-3' DNA exonuclease activity (loss)
c-value
0.8
0.7
0.6
0.5
0.4
0.3
0.2
0.1
0.0
p-value
3e-05
2e-04
1e-03
9e-03
6e-02
Log-odds
0.5
1.0
1.5
2.0
2.5
3.0
3.5
4.0
Cellular_organisms - ps1
Archaea1 - ps2
Archaea2 - ps3
Archaea3 - ps4
Asgard/Eukaryota - ps5
Eukaryota - ps6
Amorpheae - ps7
Obazoa - ps8
Opisthokonta - ps9
Holozoa - ps10
Filozoa - ps11
Choano./Metazoa - ps12
Metazoa - ps13
Myriazoa - ps14
Parahoxozoa - ps15
Bilateria - ps16
Protostomia - ps17
Ecdysozoa - ps18
Arthropoda - ps19
Mandibulata - ps20
Pancrustacea - ps21
Insecta - ps22
Eumetabola - ps23
Holometabola/Psocodea - ps24
Holometabola - ps25
Aparaglossata - ps26
Mecopterida - ps27
Diptera - ps28
Bibiono./Cyclorrhapha - ps29
Cyclorrhapha - ps30
Calyptratae/Drosophila - ps31
Drosophila - ps32
Sophophora - ps33
Obscura/Melanogaster_group - ps34
D_melanogaster_group - ps35
D_melanogaster_subgroup - ps36
sim./sech./mel. group - ps37
Drosophila_melanogaster - ps38
Phylostratum

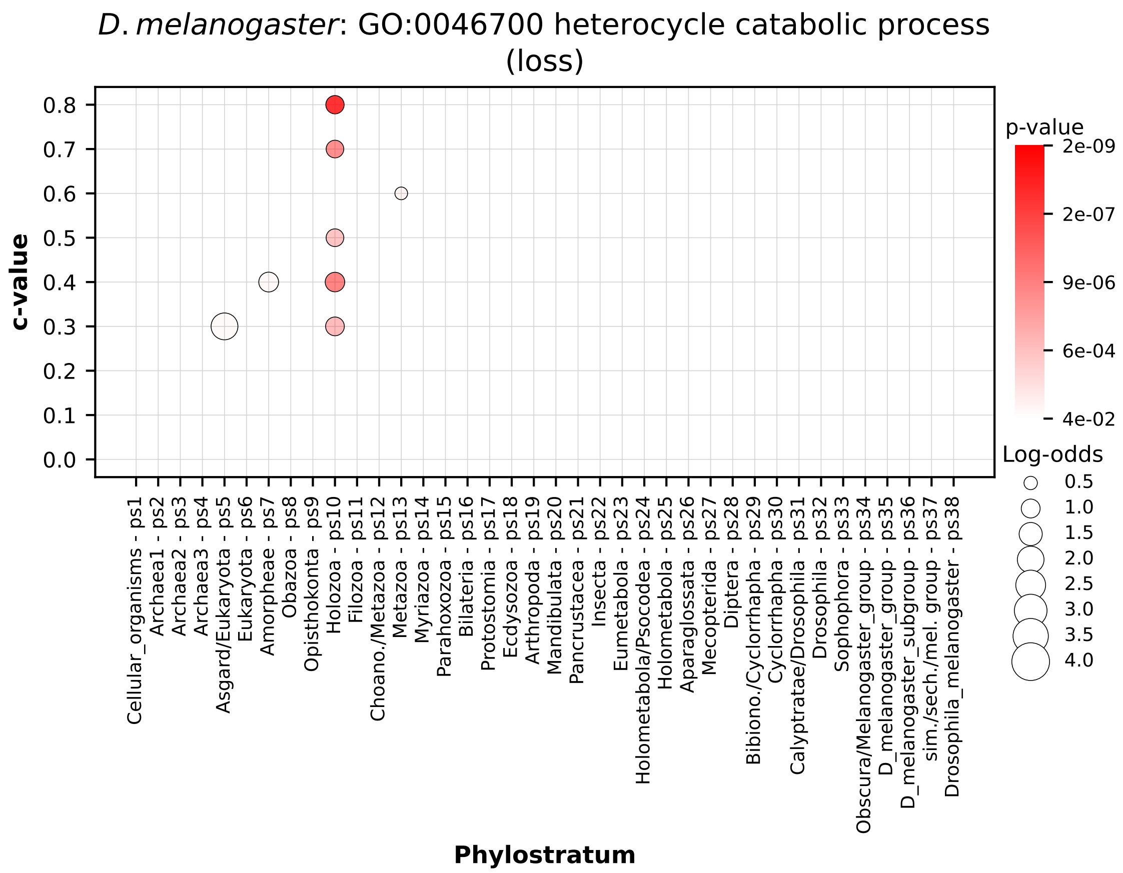
D. melanogaster: GO:0046700 heterocycle catabolic process
(loss)
c-value
0.0
0.1
0.2
0.3
0.4
0.5
0.6
0.7
0.8
p-value
2e-09
2e-07
9e-06
6e-04
4e-02
Log-odds
0.5
1.0
1.5
2.0
2.5
3.0
3.5
4.0
Cellular_organisms - ps1
Archaea1 - ps2
Archaea2 - ps3
Archaea3 - ps4
Asgard/Eukaryota - ps5
Eukaryota - ps6
Amorpheae - ps7
Obazoa - ps8
Opisthokonta - ps9
Holozoa - ps10
Filozoa - ps11
Choano./Metazoa - ps12
Metazoa - ps13
Myriazoa - ps14
Parahoxozoa - ps15
Bilateria - ps16
Protostomia - ps17
Ecdysozoa - ps18
Arthropoda - ps19
Mandibulata - ps20
Pancrustacea - ps21
Insecta - ps22
Eumetabola - ps23
Holometabola/Psocodea - ps24
Holometabola - ps25
Aparaglossata - ps26
Mecopterida - ps27
Diptera - ps28
Bibiono./Cyclorrhapha - ps29
Cyclorrhapha - ps30
Calyptratae/Drosophila - ps31
Drosophila - ps32
Sophophora - ps33
Obscura/Melanogaster_group - ps34
D_melanogaster_group - ps35
D_melanogaster_subgroup - ps36
sim./sech./mel. group - ps37
Drosophila_melanogaster - ps38
Phylostratum

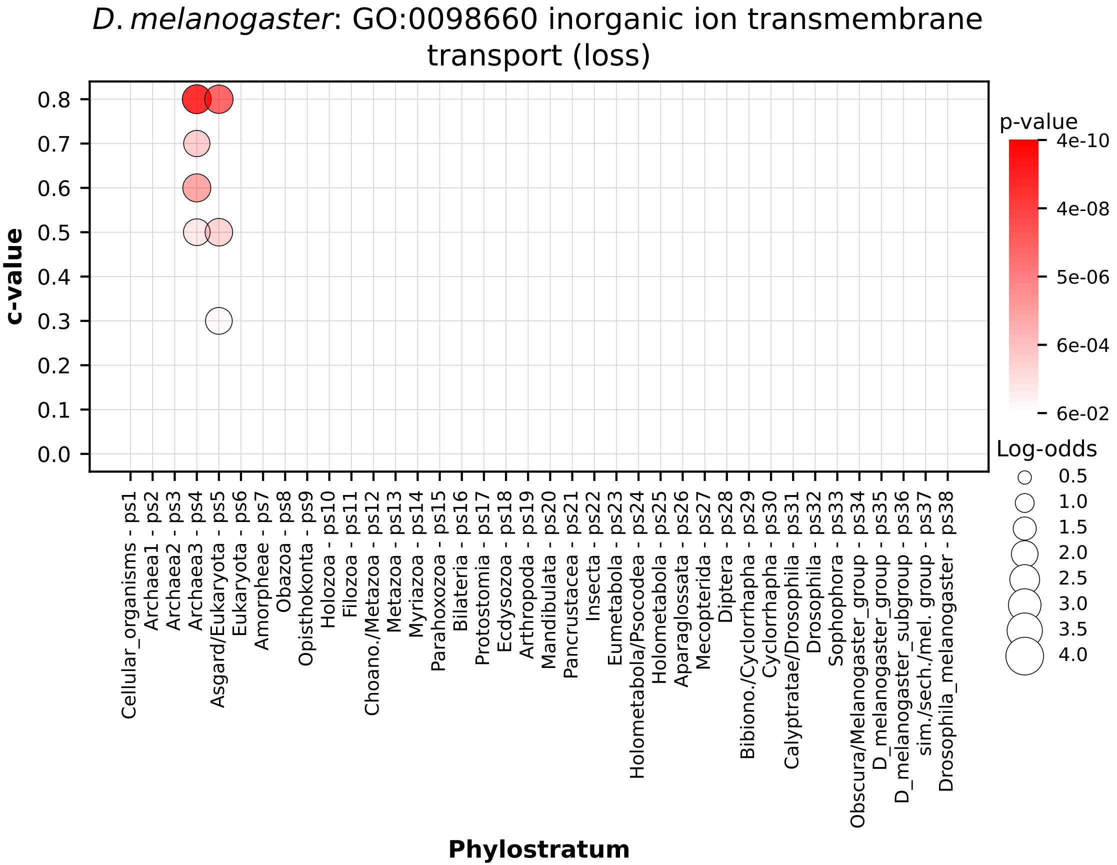
D. melanogaster: GO:0098660 inorganic ion transmembrane transport (loss)
c-value
0.8
0.7
0.6
0.5
0.4
0.3
0.2
0.1
0.0
p-value
4e-10
4e-08
5e-06
6e-04
6e-02
Log-odds
0.5
1.0
1.5
2.0
2.5
3.0
3.5
4.0
Cellular_organisms - ps1
Archaea1 - ps2
Archaea2 - ps3
Archaea3 - ps4
Asgard/Eukaryota - ps5
Eukaryota - ps6
Amorpheae - ps7
Obazoa - ps8
Opisthokonta - ps9
Holozoa - ps10
Filozoa - ps11
Choano./Metazoa - ps12
Metazoa - ps13
Myriazoa - ps14
Parahoxozoa - ps15
Bilateria - ps16
Protostomia - ps17
Ecdysozoa - ps18
Arthropoda - ps19
Mandibulata - ps20
Pancrustacea - ps21
Insecta - ps22
Eumetabola - ps23
Holometabola/Psocodea - ps24
Holometabola - ps25
Aparaglossata - ps26
Mecopterida - ps27
Diptera - ps28
Bibiono./Cyclorrhapha - ps29
Cyclorrhapha - ps30
Calyptratae/Drosophila - ps31
Drosophila - ps32
Sophophora - ps33
Obscura/Melanogaster_group - ps34
D_melanogaster_group - ps35
D_melanogaster_subgroup - ps36
sim./sech./mel. group - ps37
Drosophila_melanogaster - ps38
Phylostratum

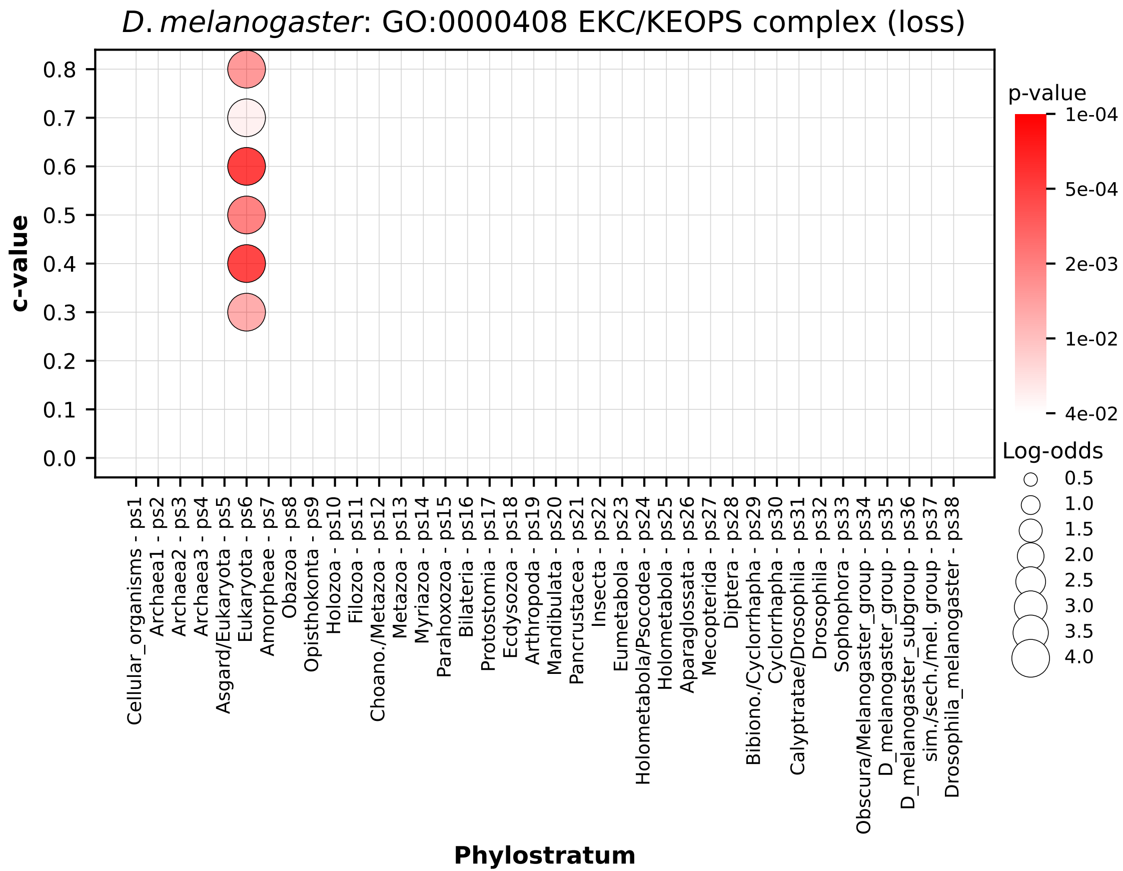

D. melanogaster: GO:0000408 EKC/KEOPS complex (loss)
c-value
0.8
0.7
0.6
0.5
0.4
0.3
0.2
0.1
0.0
Phylostratum
Cellular_organisms - ps1
Archaea1 - ps2
Archaea2 - ps3
Archaea3 - ps4
Asgard/Eukaryota - ps5
Eukaryota - ps6
Amorpheae - ps7
Obazoa - ps8
Opisthokonta - ps9
Holozoa - ps10
Filozoa - ps11
Choano./Metazoa - ps12
Metazoa - ps13
Myriazoa - ps14
Parahoxozoa - ps15
Bilateria - ps16
Protostomia - ps17
Ecdysozoa - ps18
Arthropoda - ps19
Mandibulata - ps20
Pancrustacea - ps21
Insecta - ps22
Eumetabola - ps23
Holometabola/Psocodea - ps24
Holometabola - ps25
Aparaglossata - ps26
Mecopterida - ps27
Diptera - ps28
Bibiono./Cyclorrhapha - ps29
Cyclorrhapha - ps30
Calyptratae/Drosophila - ps31
Drosophila - ps32
Sophophora - ps33
Obscura/Melanogaster_group - ps34
D_melanogaster_group - ps35
D_melanogaster_subgroup - ps36
sim./sech./mel. group - ps37
Drosophila_melanogaster - ps38
p-value
1e-04
5e-04
2e-03
1e-02
4e-02
Log-odds
0.5
1.0
1.5
2.0
2.5
3.0
3.5
4.0

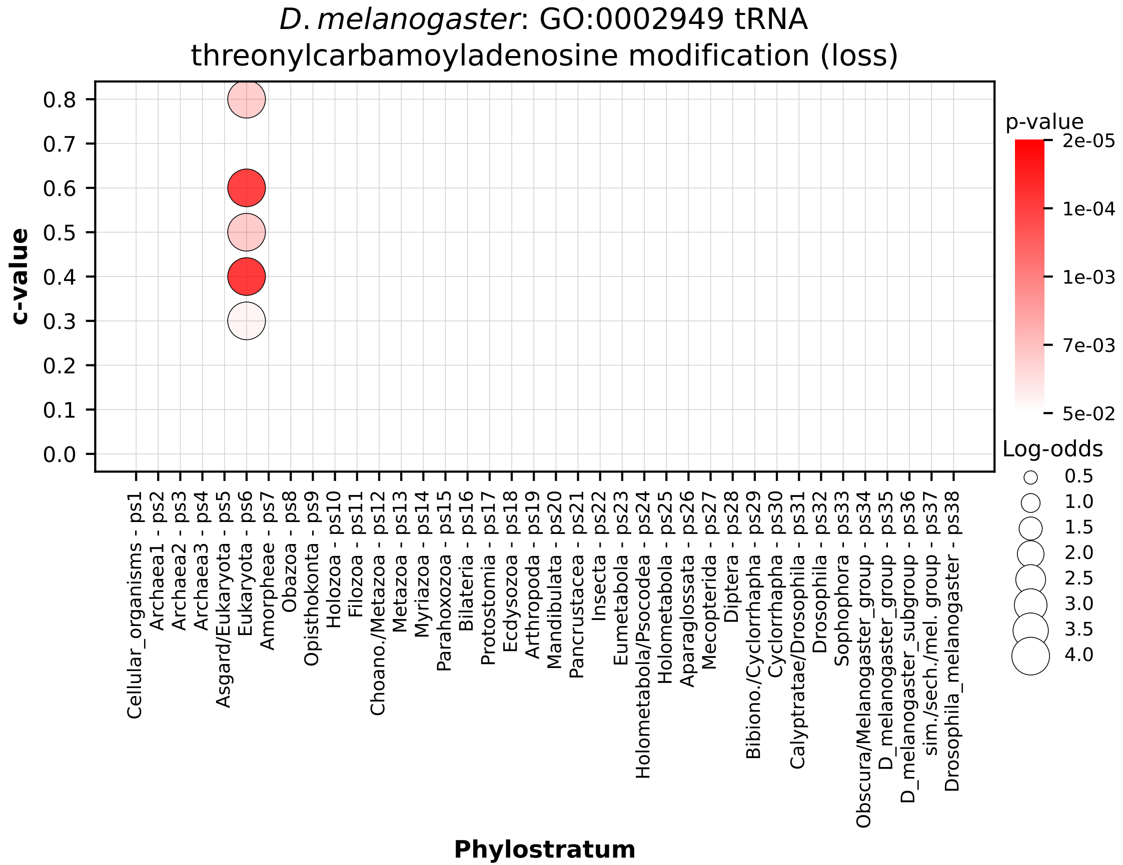

D. melanogaster: GO:0002949 tRNA threonylcarbamoyladenosine modification (loss)
c-value
0.8
0.7
0.6
0.5
0.4
0.3
0.2
0.1
0.0
Phylostratum
Cellular_organisms - ps1
Archaea1 - ps2
Archaea2 - ps3
Archaea3 - ps4
Asgard/Eukaryota - ps5
Eukaryota - ps6
Amorpheae - ps7
Obazoa - ps8
Opisthokonta - ps9
Holozoa - ps10
Filozoa - ps11
Choano./Metazoa - ps12
Metazoa - ps13
Myriazoa - ps14
Parahoxozoa - ps15
Bilateria - ps16
Protostomia - ps17
Ecdysozoa - ps18
Arthropoda - ps19
Mandibulata - ps20
Pancrustacea - ps21
Insecta - ps22
Eumetabola - ps23
Holometabola/Psocodea - ps24
Holometabola - ps25
Aparaglossata - ps26
Mecopterida - ps27
Diptera - ps28
Bibiono./Cyclorrhapha - ps29
Cyclorrhapha - ps30
Calyptratae/Drosophila - ps31
Drosophila - ps32
Sophophora - ps33
Obscura/Melanogaster_group - ps34
D_melanogaster_group - ps35
D_melanogaster_subgroup - ps36
sim./sech./mel. group - ps37
Drosophila_melanogaster - ps38
p-value
2e-05
1e-04
1e-03
7e-03
5e-02
Log-odds
0.5
1.0
1.5
2.0
2.5
3.0
3.5
4.0

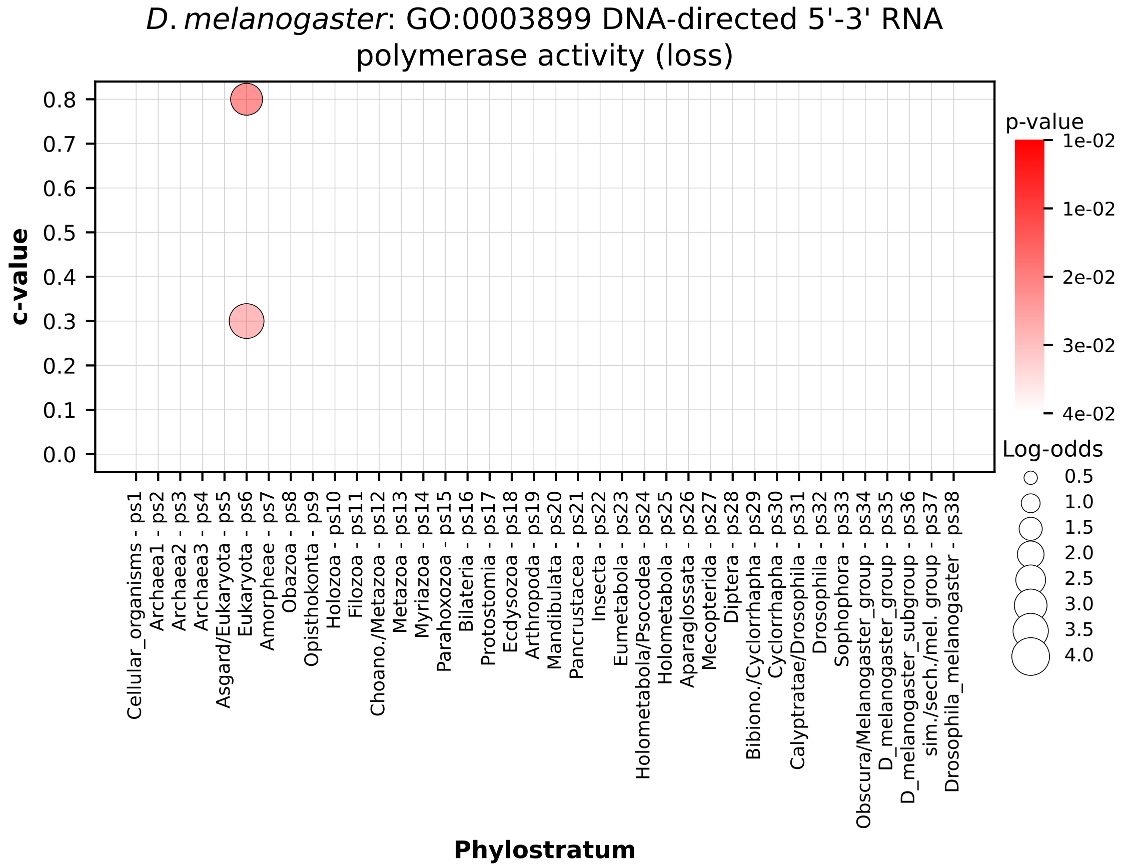
D. melanogaster: GO:0003899 DNA-directed 5'-3' RNA polymerase activity (loss)
c-value
0.8
0.7
0.6
0.5
0.4
0.3
0.2
0.1
0.0
p-value
1e-02
1e-02
2e-02
3e-02
4e-02
Log-odds
0.5
1.0
1.5
2.0
2.5
3.0
3.5
4.0
Cellular_organisms - ps1
Archaea1 - ps2
Archaea2 - ps3
Archaea3 - ps4
Asgard/Eukaryota - ps5
Eukaryota - ps6
Amorpheae - ps7
Obazoa - ps8
Opisthokonta - ps9
Holozoa - ps10
Filozoa - ps11
Choano./Metazoa - ps12
Metazoa - ps13
Myriazoa - ps14
Parahoxozoa - ps15
Bilateria - ps16
Protostomia - ps17
Ecdysozoa - ps18
Arthropoda - ps19
Mandibulata - ps20
Pancrustacea - ps21
Insecta - ps22
Eumetabola - ps23
Holometabola/Psocodea - ps24
Holometabola - ps25
Aparaglossata - ps26
Mecopterida - ps27
Diptera - ps28
Bibiono./Cyclorrhapha - ps29
Cyclorrhapha - ps30
Calyptratae/Drosophila - ps31
Drosophila - ps32
Sophophora - ps33
Obscura/Melanogaster_group - ps34
D_melanogaster_group - ps35
D_melanogaster_subgroup - ps36
sim./sech./mel. group - ps37
Drosophila_melanogaster - ps38
Phylostratum

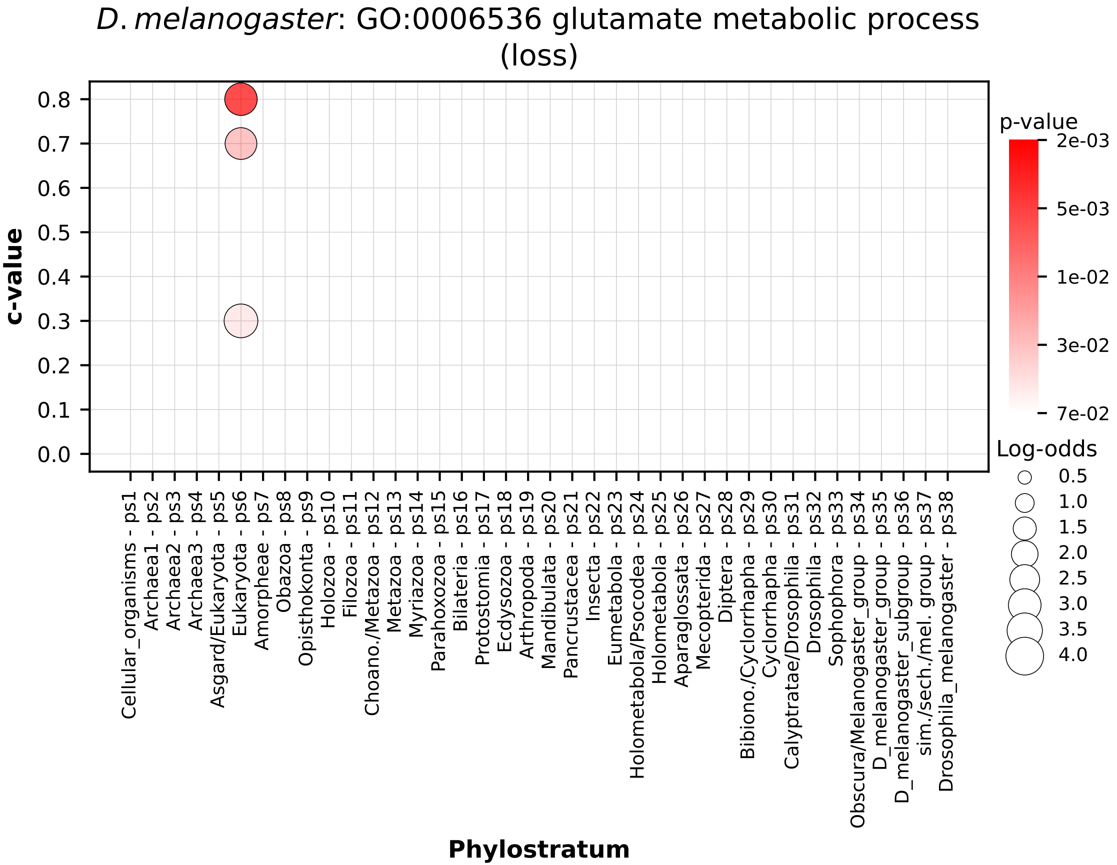
D. melanogaster: GO:0006536 glutamate metabolic process (loss)
c-value
0.8
0.7
0.6
0.5
0.4
0.3
0.2
0.1
0.0
p-value
2e-03
5e-03
1e-02
3e-02
7e-02
Log-odds
0.5
1.0
1.5
2.0
2.5
3.0
3.5
4.0
Cellular_organisms - ps1
Archaea1 - ps2
Archaea2 - ps3
Archaea3 - ps4
Asgard/Eukaryota - ps5
Eukaryota - ps6
Amorpheae - ps7
Obazoa - ps8
Opisthokonta - ps9
Holozoa - ps10
Filozoa - ps11
Choano./Metazoa - ps12
Metazoa - ps13
Myriazoa - ps14
Parahoxozoa - ps15
Bilateria - ps16
Protostomia - ps17
Ecdysozoa - ps18
Arthropoda - ps19
Mandibulata - ps20
Pancrustacea - ps21
Insecta - ps22
Eumetabola - ps23
Holometabola/Psocodea - ps24
Holometabola - ps25
Aparaglossata - ps26
Mecopterida - ps27
Diptera - ps28
Bibiono./Cyclorrhapha - ps29
Cyclorrhapha - ps30
Calyptratae/Drosophila - ps31
Drosophila - ps32
Sophophora - ps33
Obscura/Melanogaster_group - ps34
D_melanogaster_group - ps35
D_melanogaster_subgroup - ps36
sim./sech./mel. group - ps37
Drosophila_melanogaster - ps38
Phylostratum

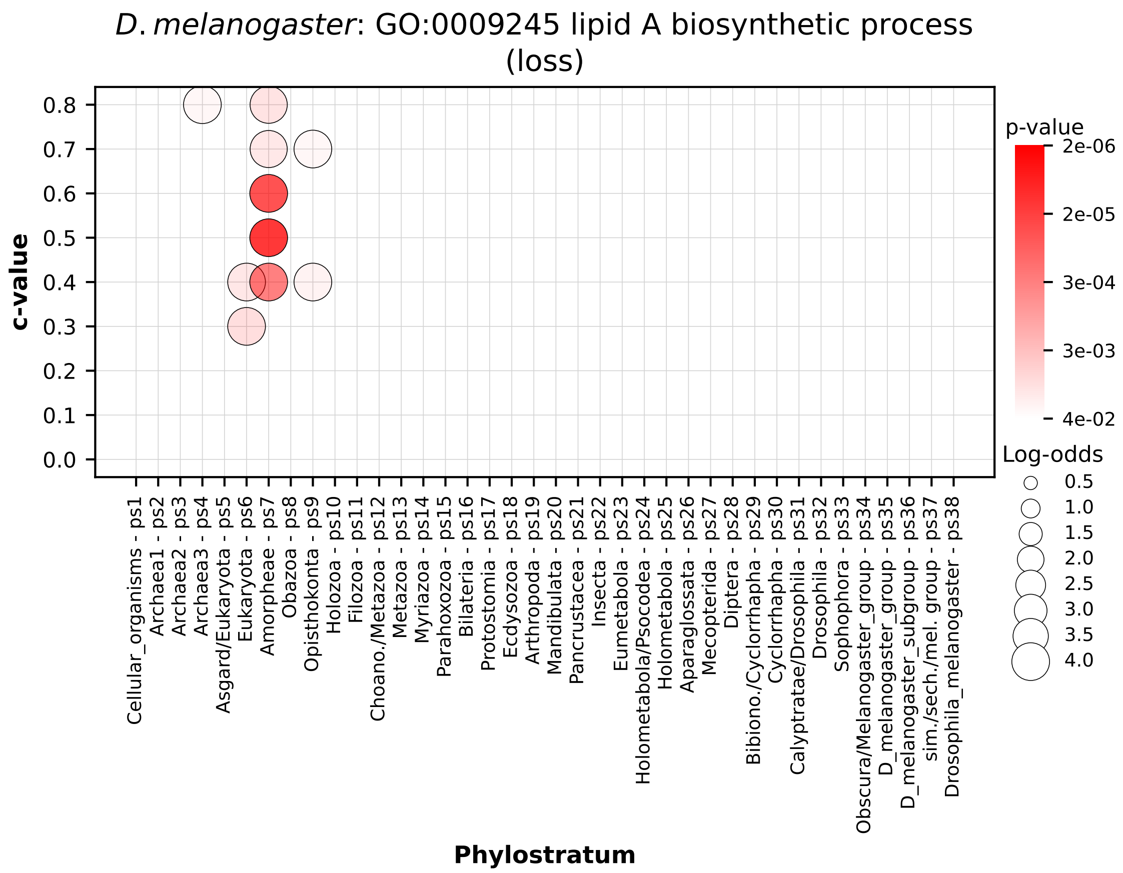

$D. melanogaster$: GO:0009245 lipid A biosynthetic process (loss)
c-value
0.8
0.7
0.6
0.5
0.4
0.3
0.2
0.1
0.0
Phylostratum
Cellular_organisms - ps1
Archaea1 - ps2
Archaea2 - ps3
Archaea3 - ps4
Asgard/Eukaryota - ps5
Eukaryota - ps6
Amorpheae - ps7
Obazoa - ps8
Opisthokonta - ps9
Holozoa - ps10
Filozoa - ps11
Choano./Metazoa - ps12
Metazoa - ps13
Myriazoa - ps14
Parahoxozoa - ps15
Bilateria - ps16
Protostomia - ps17
Ecdysozoa - ps18
Arthropoda - ps19
Mandibulata - ps20
Pancrustacea - ps21
Insecta - ps22
Eumetabola - ps23
Holometabola/Psocodea - ps24
Holometabola - ps25
Aparaglossata - ps26
Mecopterida - ps27
Diptera - ps28
Bibiono./Cyclorrhapha - ps29
Cyclorrhapha - ps30
Calyptratae/Drosophila - ps31
Drosophila - ps32
Sophophora - ps33
Obscura/Melanogaster_group - ps34
D_melanogaster_group - ps35
D_melanogaster_subgroup - ps36
sim./sech./mel. group - ps37
Drosophila_melanogaster - ps38
p-value
2e-06
2e-05
3e-04
3e-03
4e-02
Log-odds
0.5
1.0
1.5
2.0
2.5
3.0
3.5
4.0

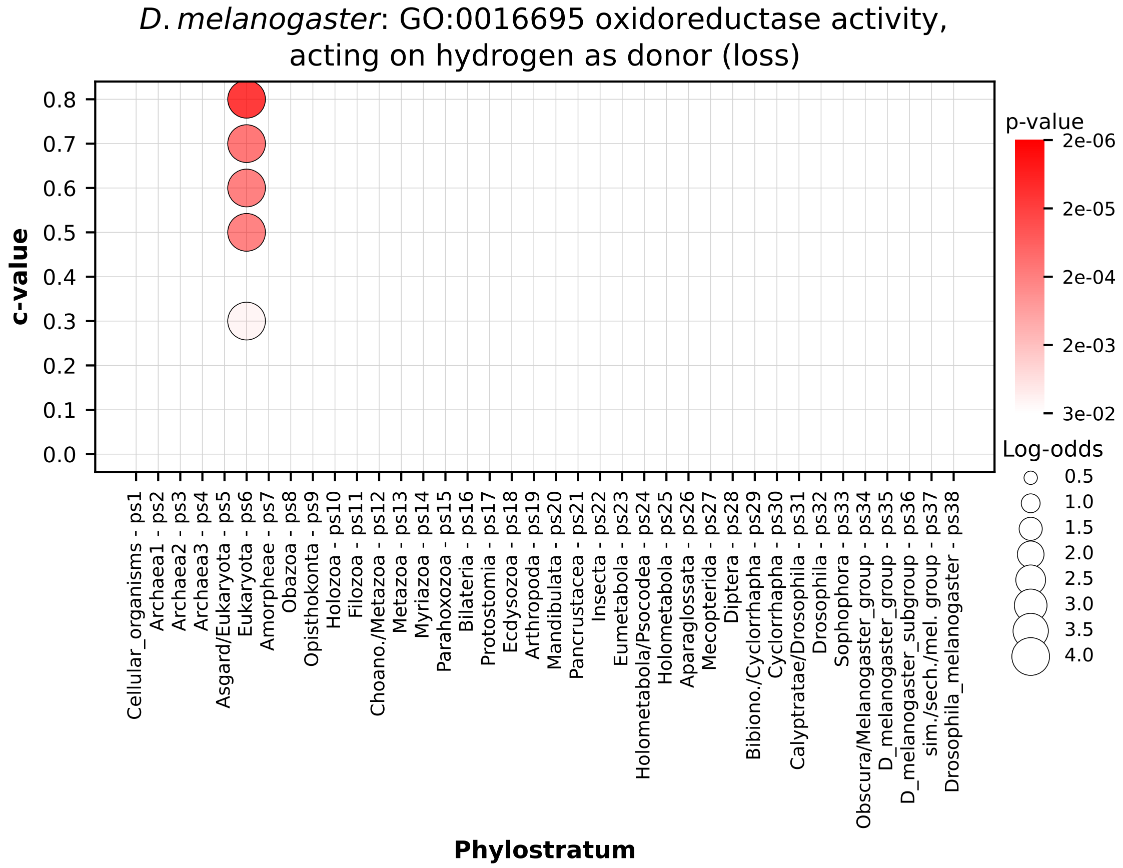

D. melanogaster: GO:0016695 oxidoreductase activity, acting on hydrogen as donor (loss)
c-value
0.8
0.7
0.6
0.5
0.4
0.3
0.2
0.1
0.0
Phylostratum
Cellular_organisms - ps1
Archaea1 - ps2
Archaea2 - ps3
Archaea3 - ps4
Asgard/Eukaryota - ps5
Eukaryota - ps6
Amorpheae - ps7
Obazoa - ps8
Opisthokonta - ps9
Holozoa - ps10
Filozoa - ps11
Choano./Metazoa - ps12
Metazoa - ps13
Myriazoa - ps14
Parahoxozoa - ps15
Bilateria - ps16
Protostomia - ps17
Ecdysozoa - ps18
Arthropoda - ps19
Mandibulata - ps20
Pancrustacea - ps21
Insecta - ps22
Eumetabola - ps23
Holometabola/Psocodea - ps24
Holometabola - ps25
Aparaglossata - ps26
Mecopterida - ps27
Diptera - ps28
Bibiono./Cyclorrhapha - ps29
Cyclorrhapha - ps30
Calyptratae/Drosophila - ps31
Drosophila - ps32
Sophophora - ps33
Obscura/Melanogaster_group - ps34
D_melanogaster_group - ps35
D_melanogaster_subgroup - ps36
sim./sech./mel. group - ps37
Drosophila_melanogaster - ps38
p-value
2e-06
2e-05
2e-04
2e-03
3e-02
Log-odds
0.5
1.0
1.5
2.0
2.5
3.0
3.5
4.0

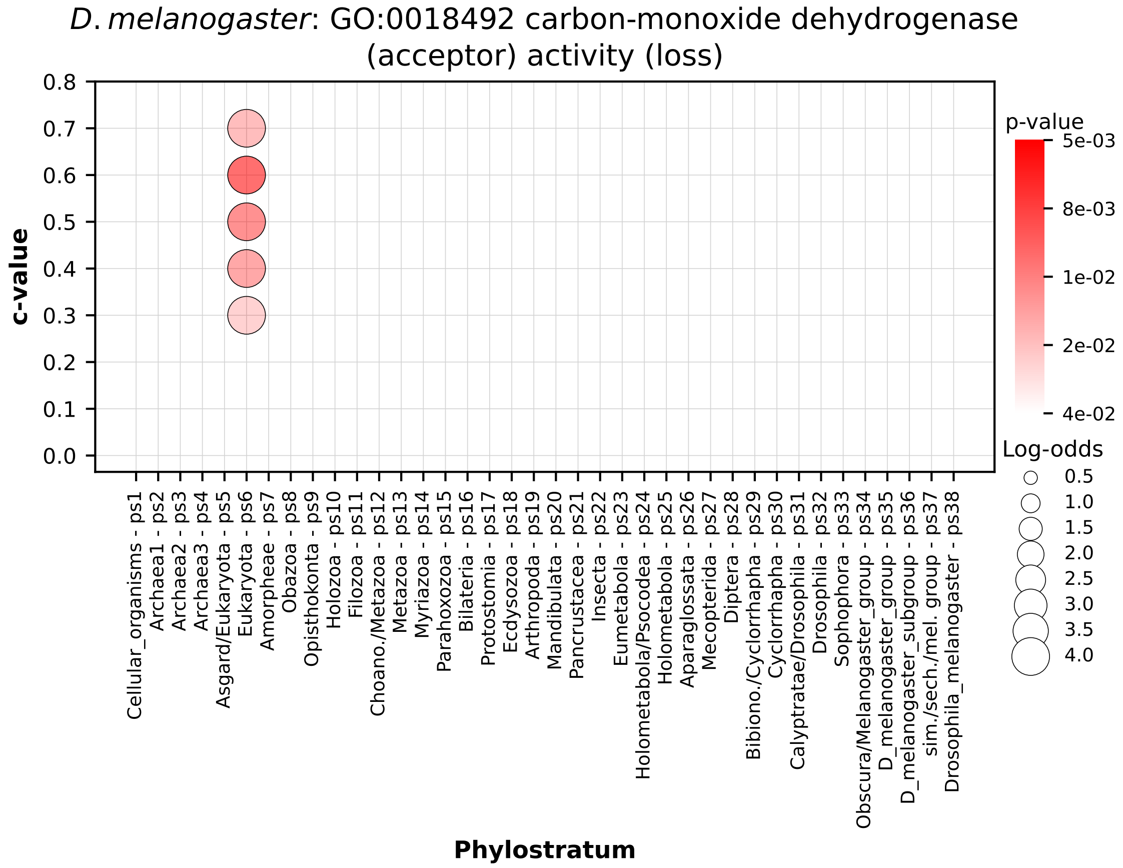

D. melanogaster: GO:0018492 carbon-monoxide dehydrogenase (acceptor) activity (loss)
c-value
0.8
0.7
0.6
0.5
0.4
0.3
0.2
0.1
0.0
Phylostratum
Cellular_organisms - ps1
Archaea1 - ps2
Archaea2 - ps3
Archaea3 - ps4
Asgard/Eukaryota - ps5
Eukaryota - ps6
Amorpheae - ps7
Obazoa - ps8
Opisthokonta - ps9
Holozoa - ps10
Filozoa - ps11
Choano./Metazoa - ps12
Metazoa - ps13
Myriazoa - ps14
Parahoxozoa - ps15
Bilateria - ps16
Protostomia - ps17
Ecdysozoa - ps18
Arthropoda - ps19
Mandibulata - ps20
Pancrustacea - ps21
Insecta - ps22
Eumetabola - ps23
Holometabola/Psocodea - ps24
Holometabola - ps25
Aparaglossata - ps26
Mecopterida - ps27
Diptera - ps28
Bibiono./Cyclorrhapha - ps29
Cyclorrhapha - ps30
Calyptratae/Drosophila - ps31
Drosophila - ps32
Sophophora - ps33
Obscura/Melanogaster_group - ps34
D_melanogaster_group - ps35
D_melanogaster_subgroup - ps36
sim./sech./mel. group - ps37
Drosophila_melanogaster - ps38
p-value
5e-03
8e-03
1e-02
2e-02
4e-02
Log-odds
0.5
1.0
1.5
2.0
2.5
3.0
3.5
4.0

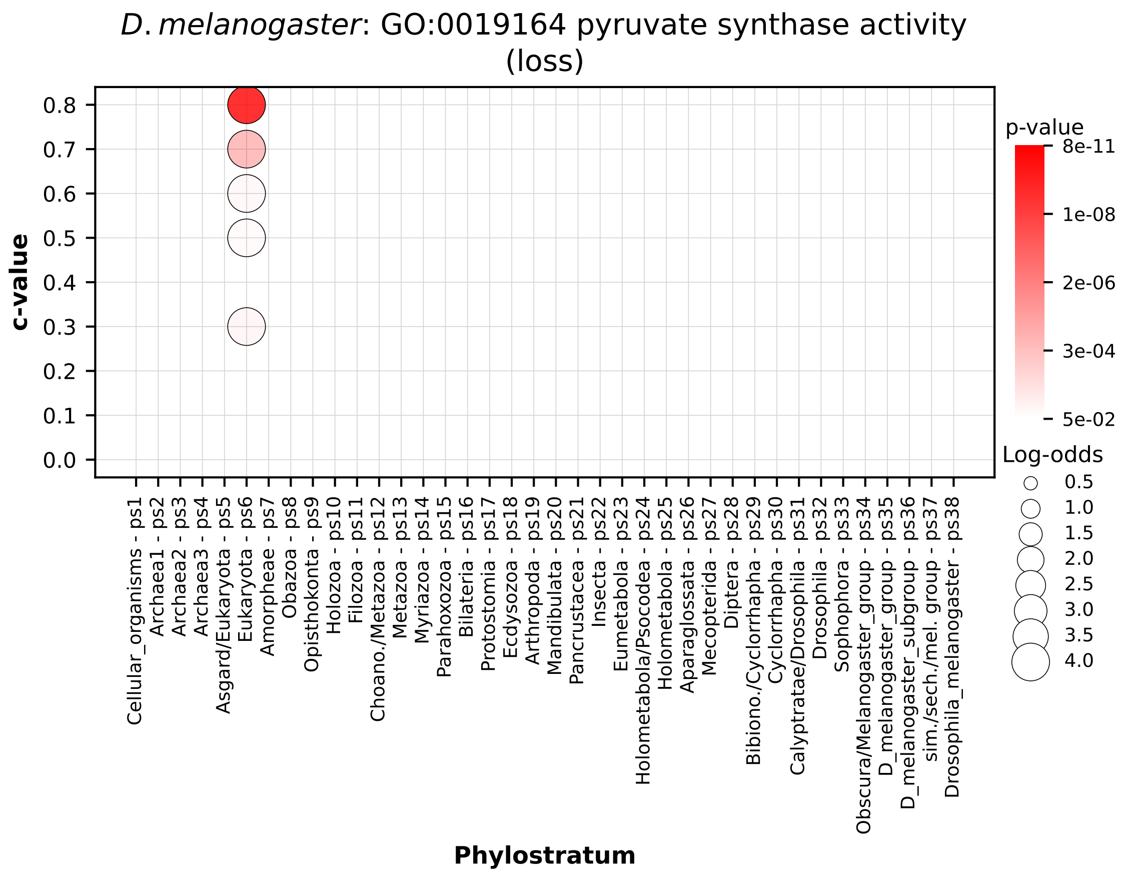
D. melanogaster: GO:0019164 pyruvate synthase activity
(loss)
c-value
0.8
0.7
0.6
0.5
0.4
0.3
0.2
0.1
0.0
p-value
8e-11
1e-08
2e-06
3e-04
5e-02
Log-odds
0.5
1.0
1.5
2.0
2.5
3.0
3.5
4.0
Cellular_organisms - ps1
Archaea1 - ps2
Archaea2 - ps3
Archaea3 - ps4
Asgard/Eukaryota - ps5
Eukaryota - ps6
Amorpheae - ps7
Obazoa - ps8
Opisthokonta - ps9
Holozoa - ps10
Filozoa - ps11
Choano./Metazoa - ps12
Metazoa - ps13
Myriazoa - ps14
Parahoxozoa - ps15
Bilateria - ps16
Protostomia - ps17
Ecdysozoa - ps18
Arthropoda - ps19
Mandibulata - ps20
Pancrustacea - ps21
Insecta - ps22
Eumetabola - ps23
Holometabola/Psocodea - ps24
Holometabola - ps25
Aparaglossata - ps26
Mecopterida - ps27
Diptera - ps28
Bibiono./Cyclorrhapha - ps29
Cyclorrhapha - ps30
Calyptratae/Drosophila - ps31
Drosophila - ps32
Sophophora - ps33
Obscura/Melanogaster_group - ps34
D_melanogaster_group - ps35
D_melanogaster_subgroup - ps36
sim./sech./mel. group - ps37
Drosophila_melanogaster - ps38
Phylostratum

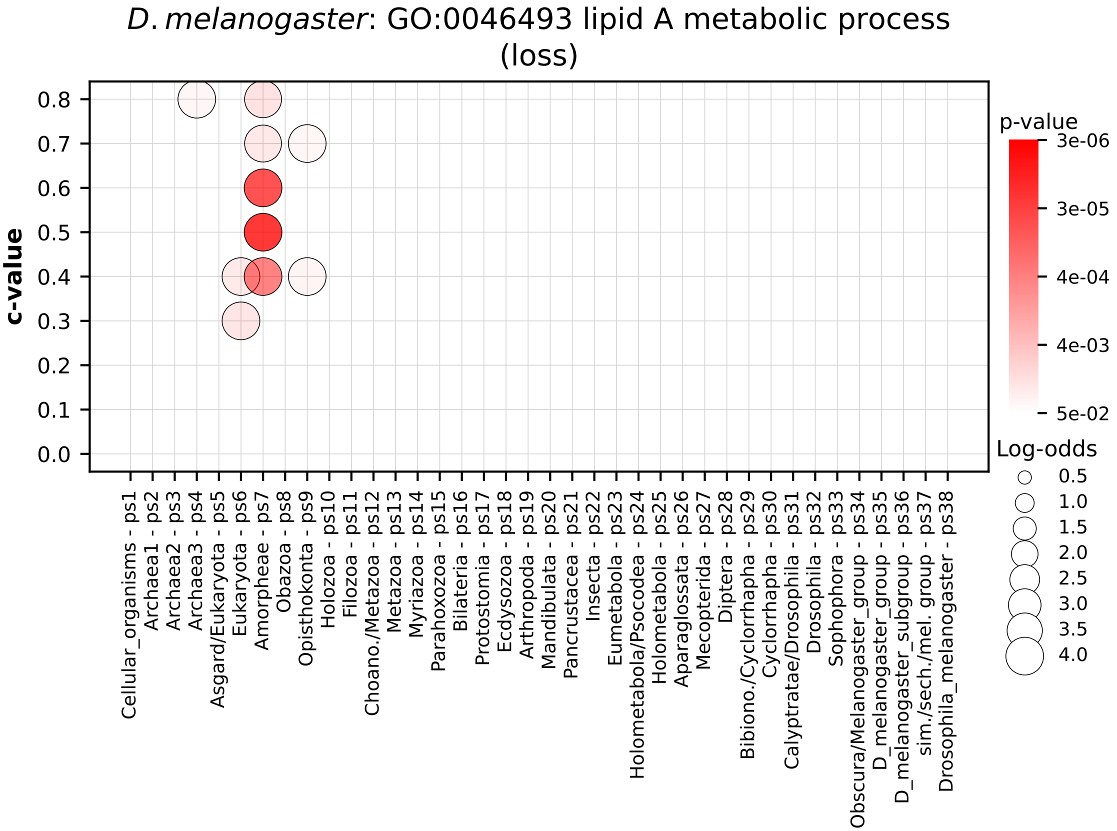
D. melanogaster: GO:0046493 lipid A metabolic process
(loss)
c-value
0.0
0.1
0.2
0.3
0.4
0.5
0.6
0.7
0.8
Phylostratum
Cellular_organisms - ps1
Archaea1 - ps2
Archaea2 - ps3
Archaea3 - ps4
Asgard/Eukaryota - ps5
Eukaryota - ps6
Amorpheae - ps7
Obazoa - ps8
Opisthokonta - ps9
Holozoa - ps10
Filozoa - ps11
Choano./Metazoa - ps12
Metazoa - ps13
Myriazoa - ps14
Parahoxozoa - ps15
Bilateria - ps16
Protostomia - ps17
Ecdysozoa - ps18
Arthropoda - ps19
Mandibulata - ps20
Pancrustacea - ps21
Insecta - ps22
Eumetabola - ps23
Holometabola/Psocodea - ps24
Holometabola - ps25
Aparaglossata - ps26
Mecopterida - ps27
Diptera - ps28
Bibiono./Cyclorrhapha - ps29
Cyclorrhapha - ps30
Calyptratae/Drosophila - ps31
Drosophila - ps32
Sophophora - ps33
Obscura/Melanogaster_group - ps34
D_melanogaster_group - ps35
D_melanogaster_subgroup - ps36
sim./sech./mel. group - ps37
Drosophila_melanogaster - ps38
p-value
3e-06
3e-05
4e-04
4e-03
5e-02
Log-odds
0.5
1.0
1.5
2.0
2.5
3.0
3.5
4.0

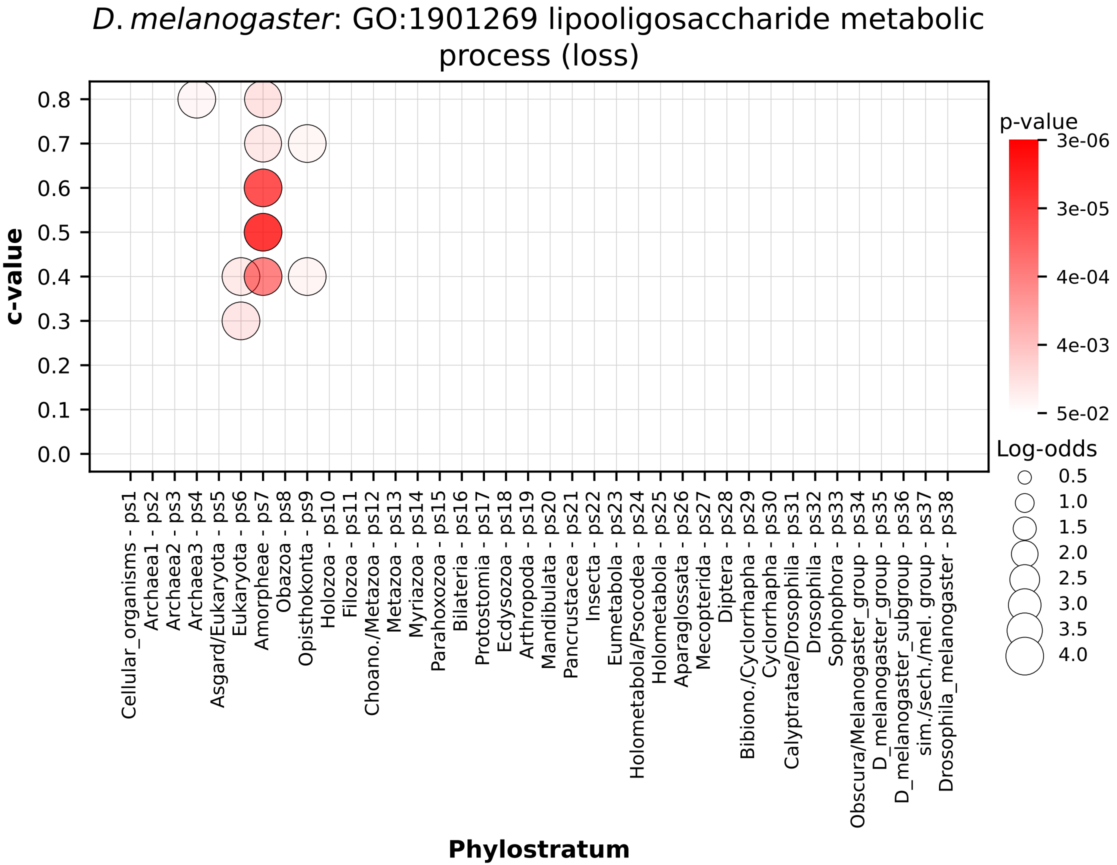

D. melanogaster: GO:1901269 lipooligosaccharide metabolic process (loss)
c-value
0.8
0.7
0.6
0.5
0.4
0.3
0.2
0.1
0.0
Phylostratum
Cellular_organisms - ps1
Archaea1 - ps2
Archaea2 - ps3
Archaea3 - ps4
Asgard/Eukaryota - ps5
Eukaryota - ps6
Amorpheae - ps7
Obazoa - ps8
Opisthokonta - ps9
Holozoa - ps10
Filozoa - ps11
Choano./Metazoa - ps12
Metazoa - ps13
Myriazoa - ps14
Parahoxozoa - ps15
Bilateria - ps16
Protostomia - ps17
Ecdysozoa - ps18
Arthropoda - ps19
Mandibulata - ps20
Pancrustacea - ps21
Insecta - ps22
Eumetabola - ps23
Holometabola/Psocodea - ps24
Holometabola - ps25
Aparaglossata - ps26
Mecopterida - ps27
Diptera - ps28
Bibiono./Cyclorrhapha - ps29
Cyclorrhapha - ps30
Calyptratae/Drosophila - ps31
Drosophila - ps32
Sophophora - ps33
Obscura/Melanogaster_group - ps34
D_melanogaster_group - ps35
D_melanogaster_subgroup - ps36
sim./sech./mel. group - ps37
Drosophila_melanogaster - ps38
p-value
3e-06
3e-05
4e-04
4e-03
5e-02
Log-odds
0.5
1.0
1.5
2.0
2.5
3.0
3.5
4.0

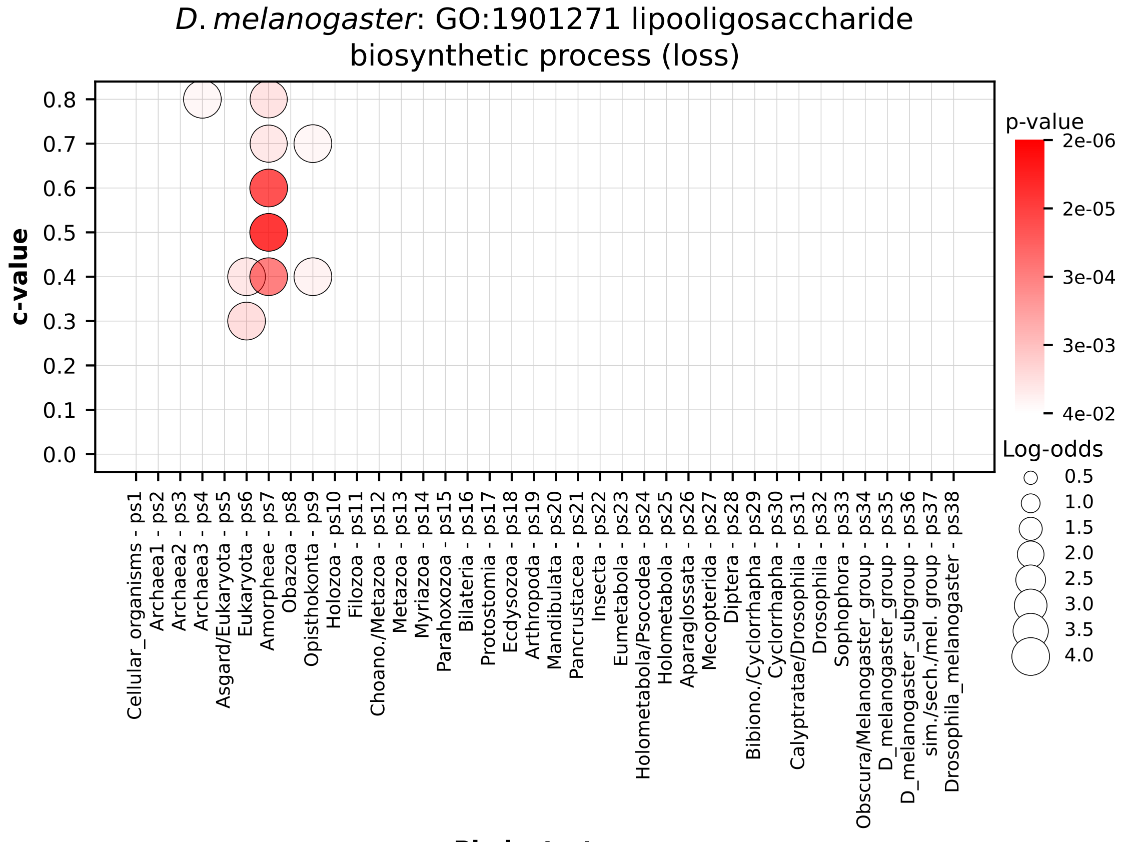

D. melanogaster: GO:1901271 lipooligosaccharide biosynthetic process (loss)
c-value
0.8
0.7
0.6
0.5
0.4
0.3
0.2
0.1
0.0
Phylostratum
Cellular_organisms - ps1
Archaea1 - ps2
Archaea2 - ps3
Archaea3 - ps4
Asgard/Eukaryota - ps5
Eukaryota - ps6
Amorpheae - ps7
Obazoa - ps8
Opisthokonta - ps9
Holozoa - ps10
Filozoa - ps11
Choano./Metazoa - ps12
Metazoa - ps13
Myriazoa - ps14
Parahoxozoa - ps15
Bilateria - ps16
Protostomia - ps17
Ecdysozoa - ps18
Arthropoda - ps19
Mandibulata - ps20
Pancrustacea - ps21
Insecta - ps22
Eumetabola - ps23
Holometabola/Psocodea - ps24
Holometabola - ps25
Aparaglossata - ps26
Mecopterida - ps27
Diptera - ps28
Bibiono./Cyclorrhapha - ps29
Cyclorrhapha - ps30
Calyptratae/Drosophila - ps31
Drosophila - ps32
Sophophora - ps33
Obscura/Melanogaster_group - ps34
D_melanogaster_group - ps35
D_melanogaster_subgroup - ps36
sim./sech./mel. group - ps37
Drosophila_melanogaster - ps38
p-value
2e-06
2e-05
3e-04
3e-03
4e-02
Log-odds
0.5
1.0
1.5
2.0
2.5
3.0
3.5
4.0

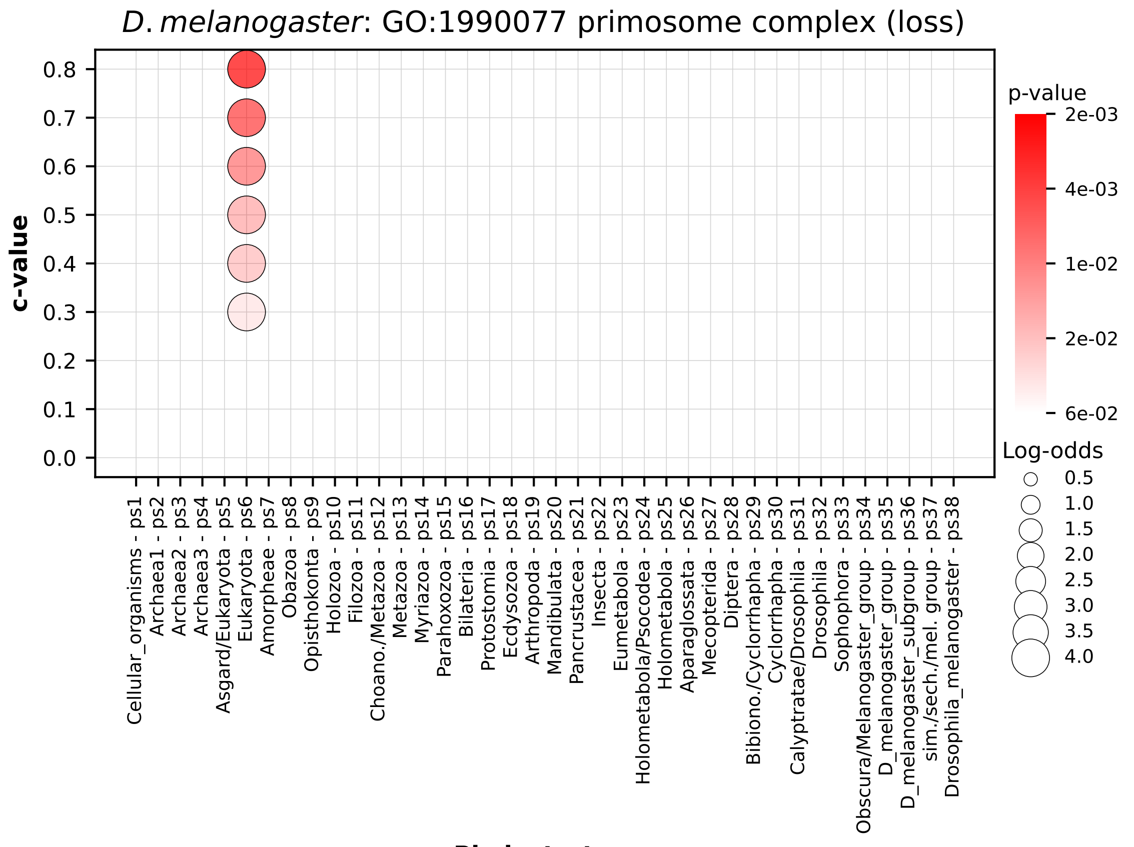

D. melanogaster: GO:1990077 primosome complex (loss)
c-value
0.8
0.7
0.6
0.5
0.4
0.3
0.2
0.1
0.0
Phylostratum
Cellular_organisms - ps1
Archaea1 - ps2
Archaea2 - ps3
Archaea3 - ps4
Asgard/Eukaryota - ps5
Eukaryota - ps6
Amorpheae - ps7
Obazoa - ps8
Opisthokonta - ps9
Holozoa - ps10
Filozoa - ps11
Choano./Metazoa - ps12
Metazoa - ps13
Myriazoa - ps14
Parahoxozoa - ps15
Bilateria - ps16
Protostomia - ps17
Ecdysozoa - ps18
Arthropoda - ps19
Mandibulata - ps20
Pancrustacea - ps21
Insecta - ps22
Eumetabola - ps23
Holometabola/Psocodea - ps24
Holometabola - ps25
Aparaglossata - ps26
Mecopterida - ps27
Diptera - ps28
Bibiono./Cyclorrhapha - ps29
Cyclorrhapha - ps30
Calyptratae/Drosophila - ps31
Drosophila - ps32
Sophophora - ps33
Obscura/Melanogaster_group - ps34
D_melanogaster_group - ps35
D_melanogaster_subgroup - ps36
sim./sech./mel. group - ps37
Drosophila_melanogaster - ps38
p-value
2e-03
4e-03
1e-02
2e-02
6e-02
Log-odds
0.5
1.0
1.5
2.0
2.5
3.0
3.5
4.0

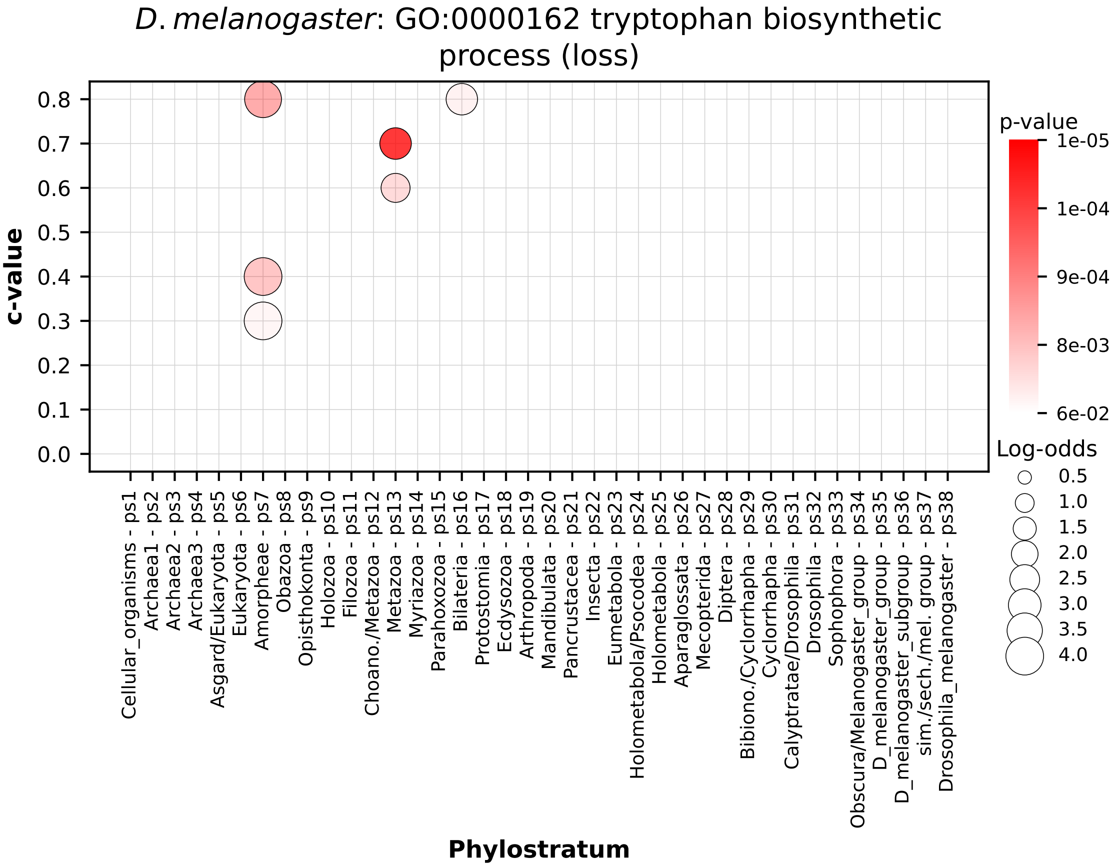
D. melanogaster: GO:0000162 tryptophan biosynthetic process (loss)
c-value
0.8
0.7
0.6
0.5
0.4
0.3
0.2
0.1
0.0
Phylostratum
Cellular_organisms - ps1
Archaea1 - ps2
Archaea2 - ps3
Archaea3 - ps4
Asgard/Eukaryota - ps5
Eukaryota - ps6
Amorpheae - ps7
Obazoa - ps8
Opisthokonta - ps9
Holozoa - ps10
Filozoa - ps11
Choano./Metazoa - ps12
Metazoa - ps13
Myriazoa - ps14
Parahoxozoa - ps15
Bilateria - ps16
Protostomia - ps17
Ecdysozoa - ps18
Arthropoda - ps19
Mandibulata - ps20
Pancrustacea - ps21
Insecta - ps22
Eumetabola - ps23
Holometabola/Psocodea - ps24
Holometabola - ps25
Aparaglossata - ps26
Mecopterida - ps27
Diptera - ps28
Bibiono./Cyclorrhapha - ps29
Cyclorrhapha - ps30
Calyptratae/Drosophila - ps31
Drosophila - ps32
Sophophora - ps33
Obscura/Melanogaster_group - ps34
D_melanogaster_group - ps35
D_melanogaster_subgroup - ps36
sim./sech./mel. group - ps37
Drosophila_melanogaster - ps38
p-value
1e-05
1e-04
9e-04
8e-03
6e-02
Log-odds
0.5
1.0
1.5
2.0
2.5
3.0
3.5
4.0

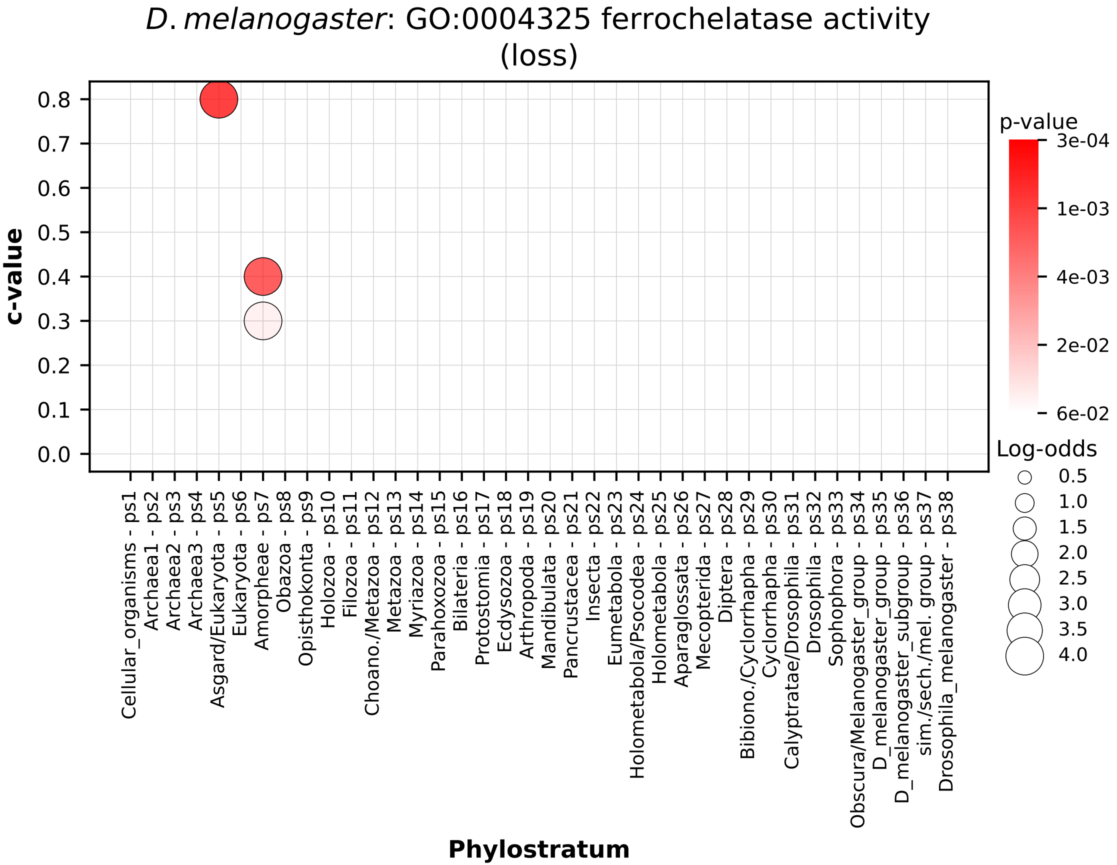

D. melanogaster: GO:0004325 ferrochelatase activity (loss)
c-value
Phylostratum
p-value
3e-04
1e-03
4e-03
2e-02
6e-02
Log-odds
0.5
1.0
1.5
2.0
2.5
3.0
3.5
4.0
Cellular_organisms - ps1
Archaea1 - ps2
Archaea2 - ps3
Archaea3 - ps4
Asgard/Eukaryota - ps5
Eukaryota - ps6
Amorpheae - ps7
Obazoa - ps8
Opisthokonta - ps9
Holozoa - ps10
Filozoa - ps11
Choano./Metazoa - ps12
Metazoa - ps13
Myriazoa - ps14
Parahoxozoa - ps15
Bilateria - ps16
Protostomia - ps17
Ecdysozoa - ps18
Arthropoda - ps19
Mandibulata - ps20
Pancrustacea - ps21
Insecta - ps22
Eumetabola - ps23
Holometabola/Psocodea - ps24
Holometabola - ps25
Aparaglossata - ps26
Mecopterida - ps27
Diptera - ps28
Bibiono./Cyclorrhapha - ps29
Cyclorrhapha - ps30
Calyptratae/Drosophila - ps31
Drosophila - ps32
Sophophora - ps33
Obscura/Melanogaster_group - ps34
D_melanogaster_group - ps35
D_melanogaster_subgroup - ps36
sim./sech./mel. group - ps37
Drosophila_melanogaster - ps38

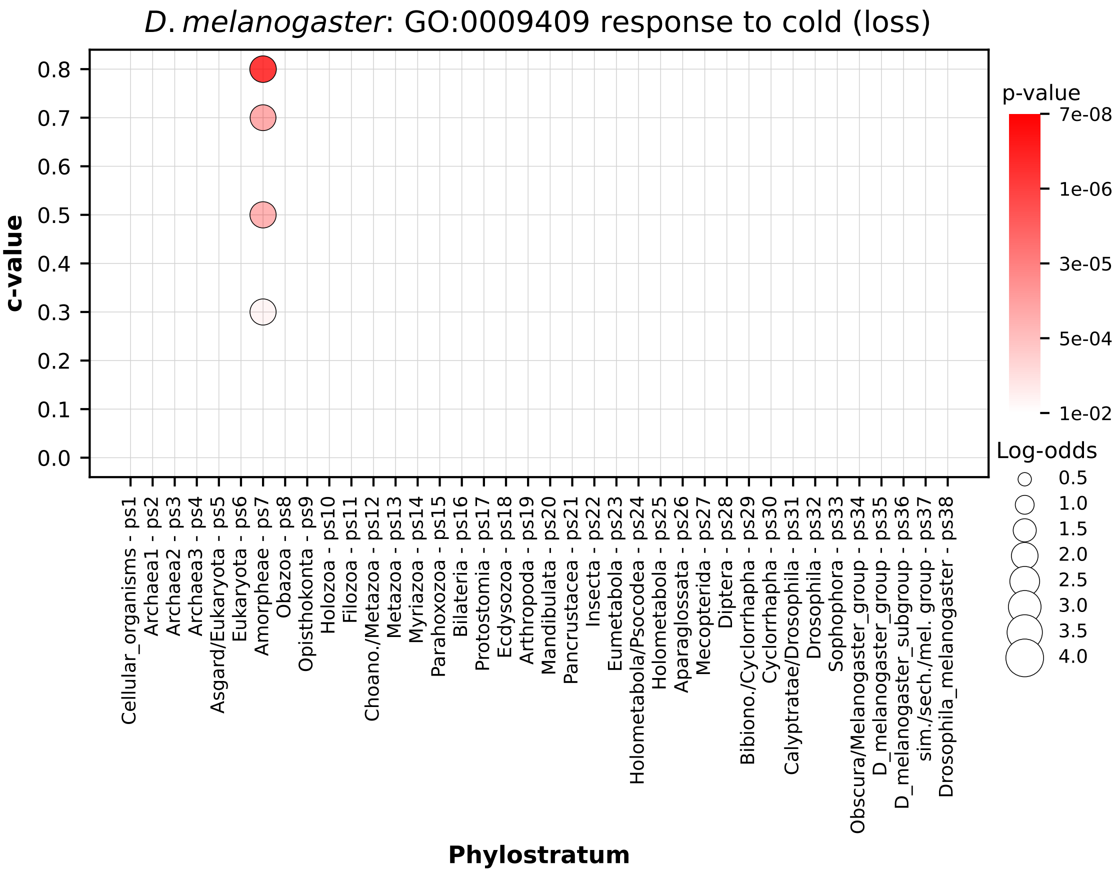
D. melanogaster: GO:0009409 response to cold (loss)
c-value
0.8
0.7
0.6
0.5
0.4
0.3
0.2
0.1
0.0
p-value
7e-08
1e-06
3e-05
5e-04
1e-02
Log-odds
0.5
1.0
1.5
2.0
2.5
3.0
3.5
4.0
Cellular_organisms - ps1
Archaea1 - ps2
Archaea2 - ps3
Archaea3 - ps4
Asgard/Eukaryota - ps5
Eukaryota - ps6
Amorpheae - ps7
Obazoa - ps8
Opisthokonta - ps9
Holozoa - ps10
Filozoa - ps11
Choano./Metazoa - ps12
Metazoa - ps13
Myriazoa - ps14
Parahoxozoa - ps15
Bilateria - ps16
Protostomia - ps17
Ecdysozoa - ps18
Arthropoda - ps19
Mandibulata - ps20
Pancrustacea - ps21
Insecta - ps22
Eumetabola - ps23
Holometabola/Psocodea - ps24
Holometabola - ps25
Aparaglossata - ps26
Mecopterida - ps27
Diptera - ps28
Bibiono./Cyclorrhapha - ps29
Cyclorrhapha - ps30
Calyptratae/Drosophila - ps31
Drosophila - ps32
Sophophora - ps33
Obscura/Melanogaster_group - ps34
D_melanogaster_group - ps35
D_melanogaster_subgroup - ps36
sim./sech./mel. group - ps37
Drosophila_melanogaster - ps38
Phylostratum

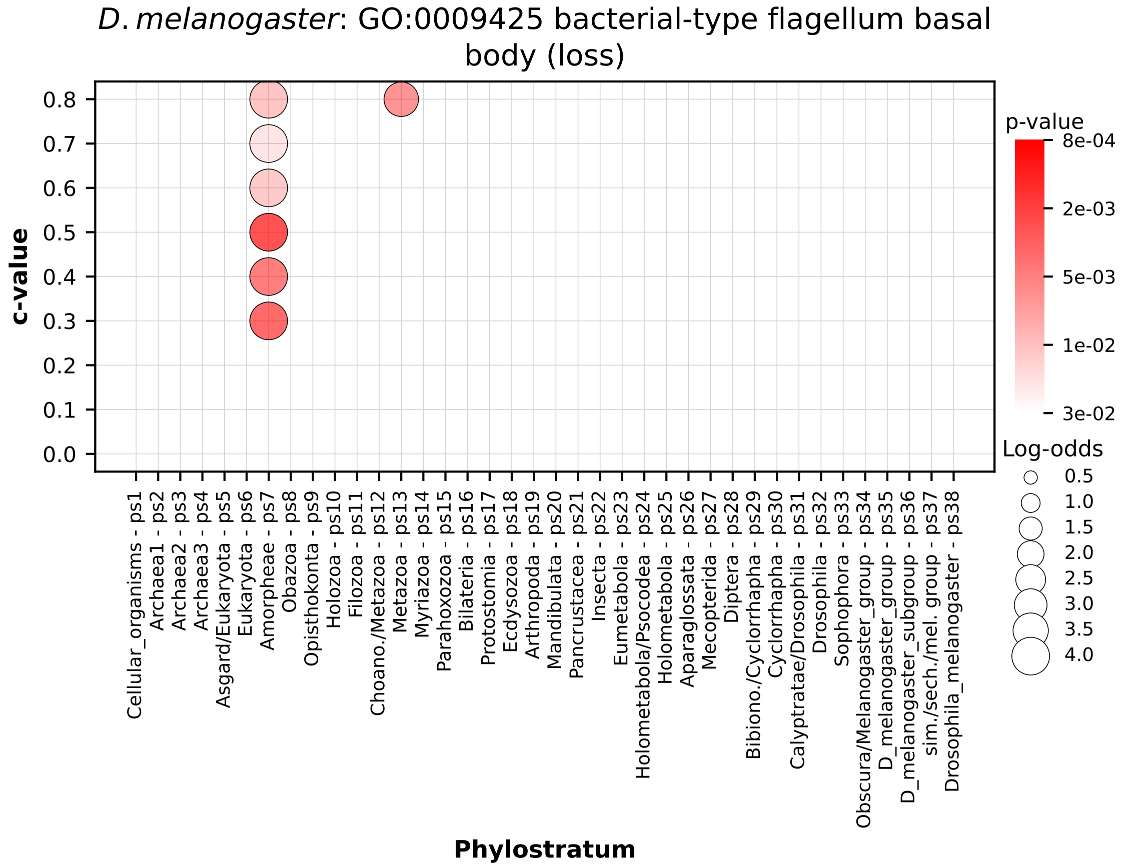

D. melanogaster: GO:0009425 bacterial-type flagellum basal body (loss)
c-value
0.8
0.7
0.6
0.5
0.4
0.3
0.2
0.1
0.0
Phylostratum
Cellular_organisms - ps1
Archaea1 - ps2
Archaea2 - ps3
Archaea3 - ps4
Asgard/Eukaryota - ps5
Eukaryota - ps6
Amorpheae - ps7
Obazoa - ps8
Opisthokonta - ps9
Holozoa - ps10
Filozoa - ps11
Choano./Metazoa - ps12
Metazoa - ps13
Myriazoa - ps14
Parahoxozoa - ps15
Bilateria - ps16
Protostomia - ps17
Ecdysozoa - ps18
Arthropoda - ps19
Mandibulata - ps20
Pancrustacea - ps21
Insecta - ps22
Eumetabola - ps23
Holometabola/Psocodea - ps24
Holometabola - ps25
Aparaglossata - ps26
Mecopterida - ps27
Diptera - ps28
Bibiono./Cyclorrhapha - ps29
Cyclorrhapha - ps30
Calyptratae/Drosophila - ps31
Drosophila - ps32
Sophophora - ps33
Obscura/Melanogaster_group - ps34
D_melanogaster_group - ps35
D_melanogaster_subgroup - ps36
sim./sech./mel. group - ps37
Drosophila_melanogaster - ps38
p-value
8e-04
2e-03
5e-03
1e-02
3e-02
Log-odds
0.5
1.0
1.5
2.0
2.5
3.0
3.5
4.0

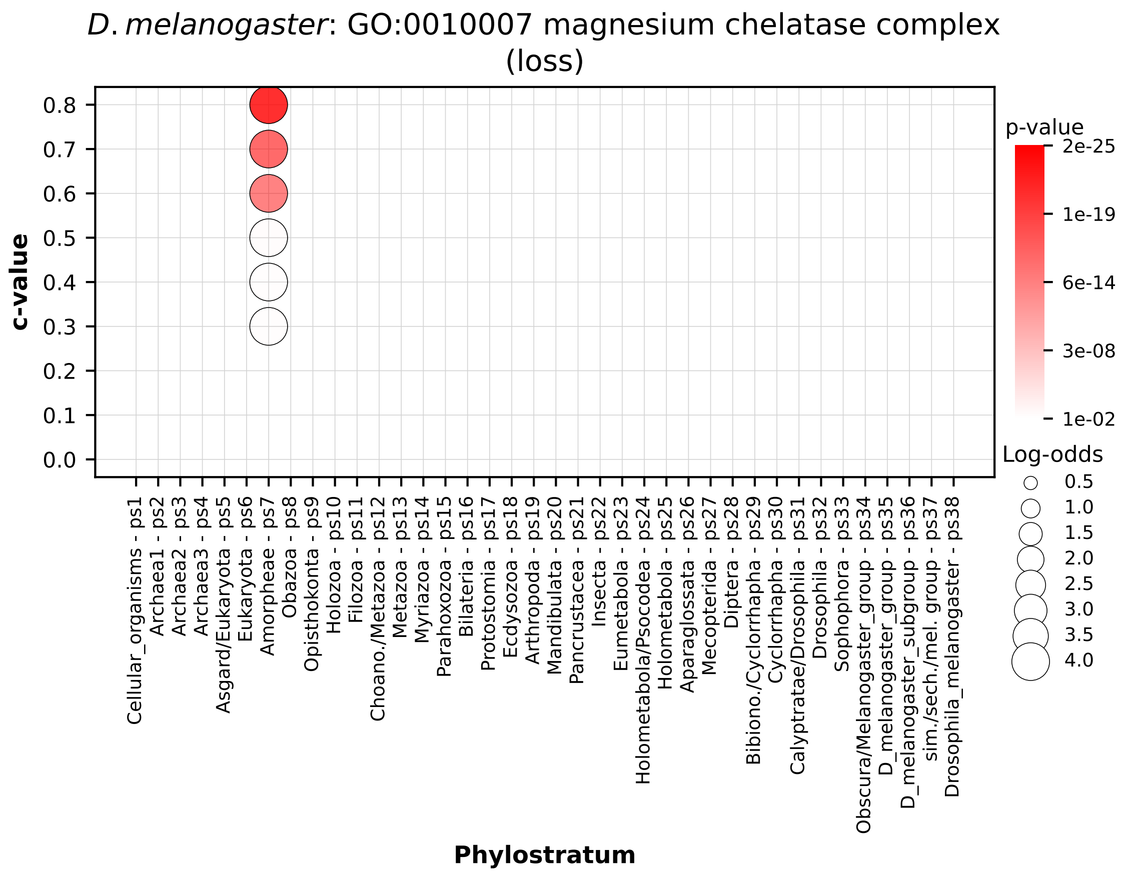

D. melanogaster: GO:0010007 magnesium chelatase complex
(loss)
c-value
0.8
0.7
0.6
0.5
0.4
0.3
0.2
0.1
0.0
Phylostratum
Cellular_organisms - ps1
Archaea1 - ps2
Archaea2 - ps3
Archaea3 - ps4
Asgard/Eukaryota - ps5
Eukaryota - ps6
Amorpheae - ps7
Obazoa - ps8
Opisthokonta - ps9
Holozoa - ps10
Filozoa - ps11
Choano./Metazoa - ps12
Metazoa - ps13
Myriazoa - ps14
Parahoxozoa - ps15
Bilateria - ps16
Protostomia - ps17
Ecdysozoa - ps18
Arthropoda - ps19
Mandibulata - ps20
Pancrustacea - ps21
Insecta - ps22
Eumetabola - ps23
Holometabola/Psocodea - ps24
Holometabola - ps25
Aparaglossata - ps26
Mecopterida - ps27
Diptera - ps28
Bibiono./Cyclorrhapha - ps29
Cyclorrhapha - ps30
Calyptratae/Drosophila - ps31
Drosophila - ps32
Sophophora - ps33
Obscura/Melanogaster_group - ps34
D_melanogaster_group - ps35
D_melanogaster_subgroup - ps36
sim./sech./mel. group - ps37
Drosophila_melanogaster - ps38
p-value
2e-25
1e-19
6e-14
3e-08
1e-02
Log-odds
0.5
1.0
1.5
2.0
2.5
3.0
3.5
4.0

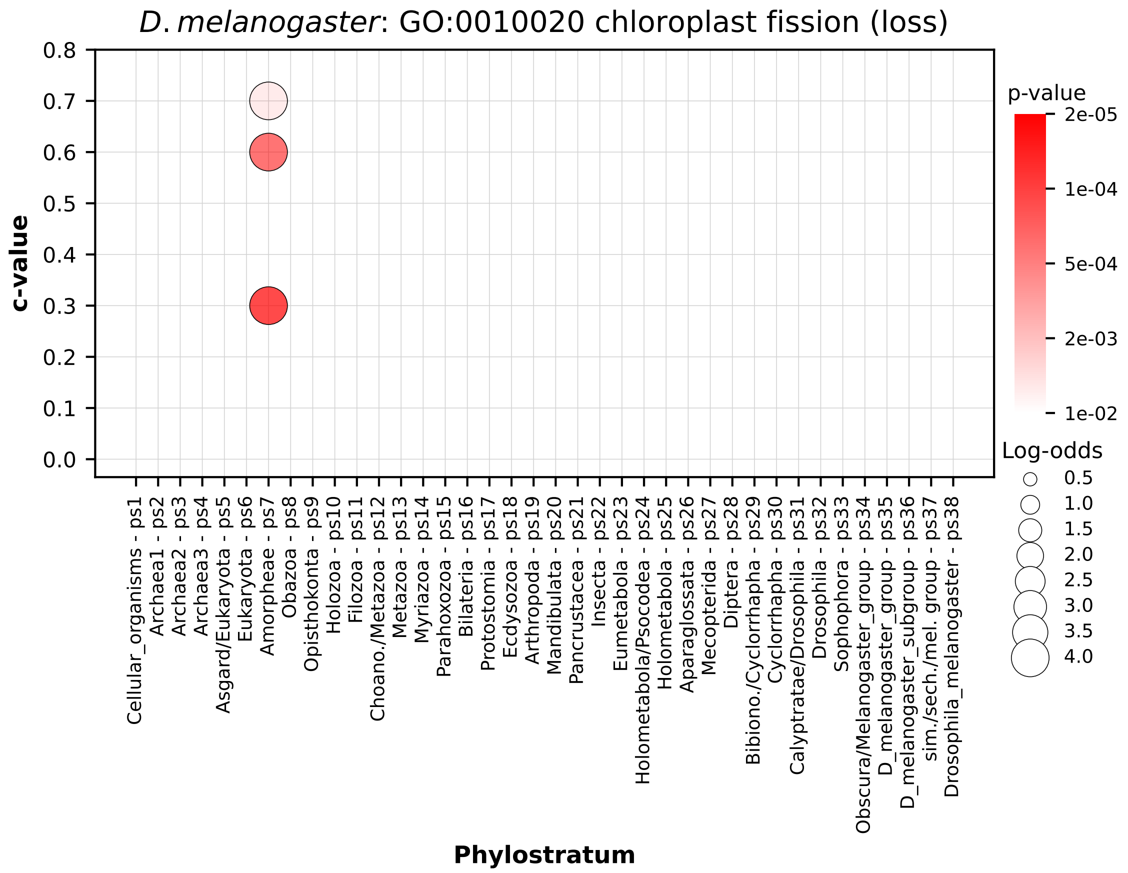
D. melanogaster: GO:0010020 chloroplast fission (loss)
c-value
0.8
0.7
0.6
0.5
0.4
0.3
0.2
0.1
0.0
Phylostratum
Cellular_organisms - ps1
Archaea1 - ps2
Archaea2 - ps3
Archaea3 - ps4
Asgard/Eukaryota - ps5
Eukaryota - ps6
Amorpheae - ps7
Obazoa - ps8
Opisthokonta - ps9
Holozoa - ps10
Filozoa - ps11
Choano./Metazoa - ps12
Metazoa - ps13
Myriazoa - ps14
Parahoxozoa - ps15
Bilateria - ps16
Protostomia - ps17
Ecdysozoa - ps18
Arthropoda - ps19
Mandibulata - ps20
Pancrustacea - ps21
Insecta - ps22
Eumetabola - ps23
Holometabola/Psocodea - ps24
Holometabola - ps25
Aparaglossata - ps26
Mecopterida - ps27
Diptera - ps28
Bibiono./Cyclorrhapha - ps29
Cyclorrhapha - ps30
Calyptratae/Drosophila - ps31
Drosophila - ps32
Sophophora - ps33
Obscura/Melanogaster_group - ps34
D_melanogaster_group - ps35
D_melanogaster_subgroup - ps36
sim./sech./mel. group - ps37
Drosophila_melanogaster - ps38
p-value
2e-05
1e-04
5e-04
2e-03
1e-02
Log-odds
0.5
1.0
1.5
2.0
2.5
3.0
3.5
4.0

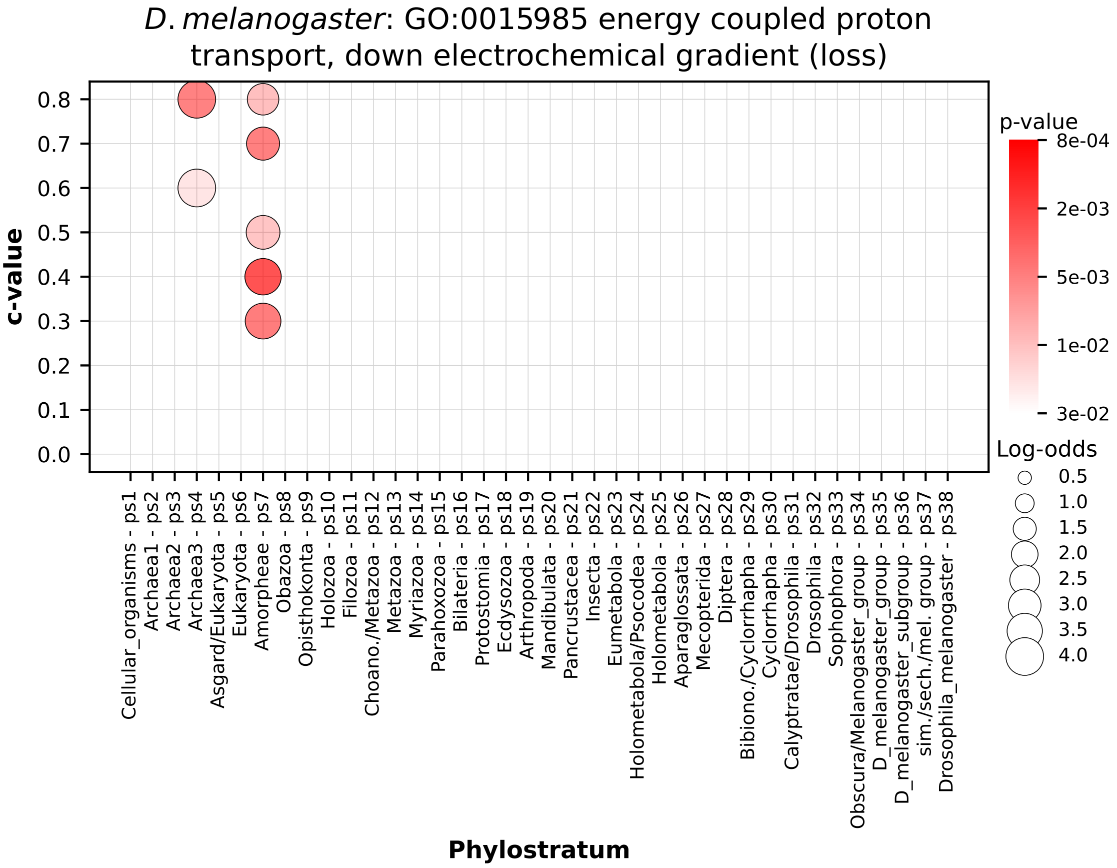
D. melanogaster: GO:0015985 energy coupled proton transport, down electrochemical gradient (loss)
c-value
0.0
0.1
0.2
0.3
0.4
0.5
0.6
0.7
0.8
Phylostratum
Cellular_organisms - ps1
Archaea1 - ps2
Archaea2 - ps3
Archaea3 - ps4
Asgard/Eukaryota - ps5
Eukaryota - ps6
Amorpheae - ps7
Obazoa - ps8
Opisthokonta - ps9
Holozoa - ps10
Filozoa - ps11
Choano./Metazoa - ps12
Metazoa - ps13
Myriazoa - ps14
Parahoxozoa - ps15
Bilateria - ps16
Protostomia - ps17
Ecdysozoa - ps18
Arthropoda - ps19
Mandibulata - ps20
Pancrustacea - ps21
Insecta - ps22
Eumetabola - ps23
Holometabola/Psocodea - ps24
Holometabola - ps25
Aparaglossata - ps26
Mecopterida - ps27
Diptera - ps28
Bibiono./Cyclorrhapha - ps29
Cyclorrhapha - ps30
Calyptratae/Drosophila - ps31
Drosophila - ps32
Sophophora - ps33
Obscura/Melanogaster_group - ps34
D_melanogaster_group - ps35
D_melanogaster_subgroup - ps36
sim./sech./mel. group - ps37
Drosophila_melanogaster - ps38
p-value
8e-04
2e-03
5e-03
1e-02
3e-02
Log-odds
0.5
1.0
1.5
2.0
2.5
3.0
3.5
4.0

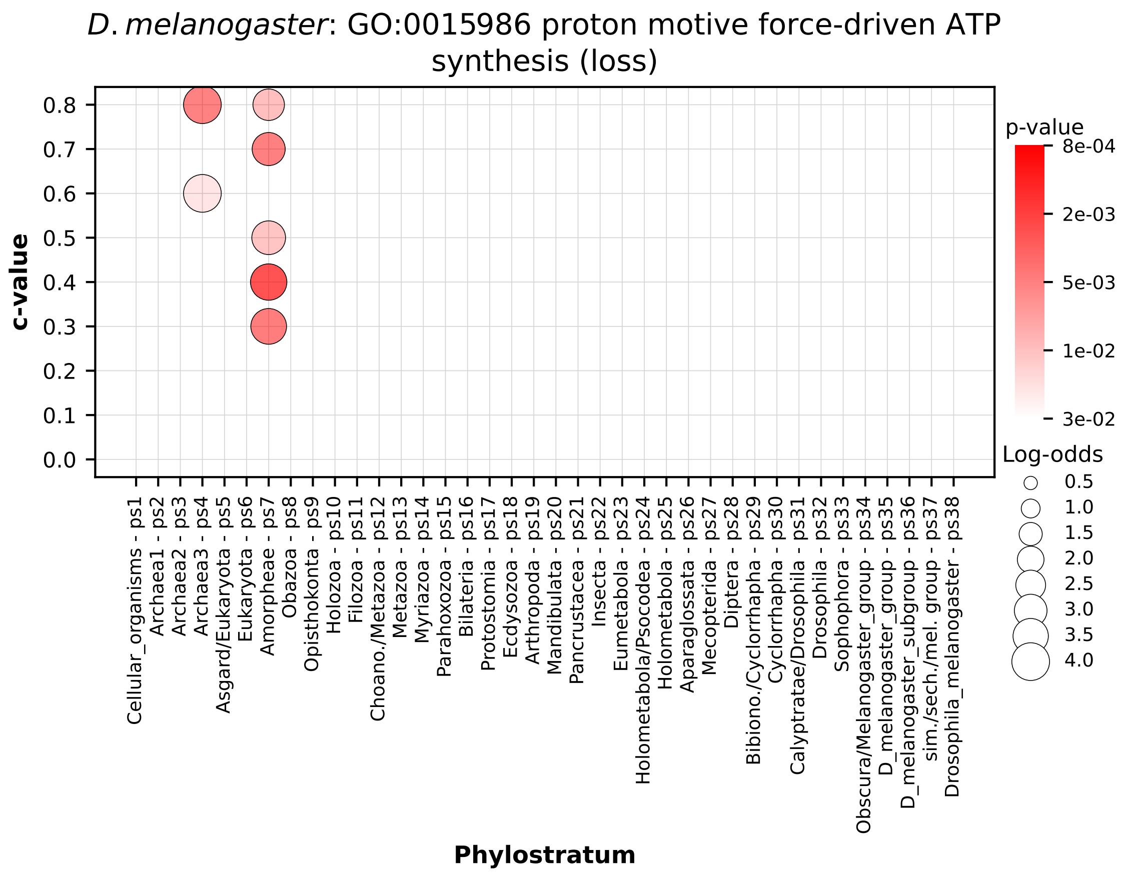

D. melanogaster: GO:0015986 proton motive force-driven ATP synthesis (loss)
c-value
0.8
0.7
0.6
0.5
0.4
0.3
0.2
0.1
0.0
Phylostratum
Cellular_organisms - ps1
Archaea1 - ps2
Archaea2 - ps3
Archaea3 - ps4
Asgard/Eukaryota - ps5
Eukaryota - ps6
Amorpheae - ps7
Obazoa - ps8
Opisthokonta - ps9
Holozoa - ps10
Filozoa - ps11
Choano./Metazoa - ps12
Metazoa - ps13
Myriazoa - ps14
Parahoxozoa - ps15
Bilateria - ps16
Protostomia - ps17
Ecdysozoa - ps18
Arthropoda - ps19
Mandibulata - ps20
Pancrustacea - ps21
Insecta - ps22
Eumetabola - ps23
Holometabola/Psocodea - ps24
Holometabola - ps25
Aparaglossata - ps26
Mecopterida - ps27
Diptera - ps28
Bibiono./Cyclorrhapha - ps29
Cyclorrhapha - ps30
Calyptratae/Drosophila - ps31
Drosophila - ps32
Sophophora - ps33
Obscura/Melanogaster_group - ps34
D_melanogaster_group - ps35
D_melanogaster_subgroup - ps36
sim./sech./mel. group - ps37
Drosophila_melanogaster - ps38
p-value
8e-04
2e-03
5e-03
1e-02
3e-02
Log-odds
0.5
1.0
1.5
2.0
2.5
3.0
3.5
4.0

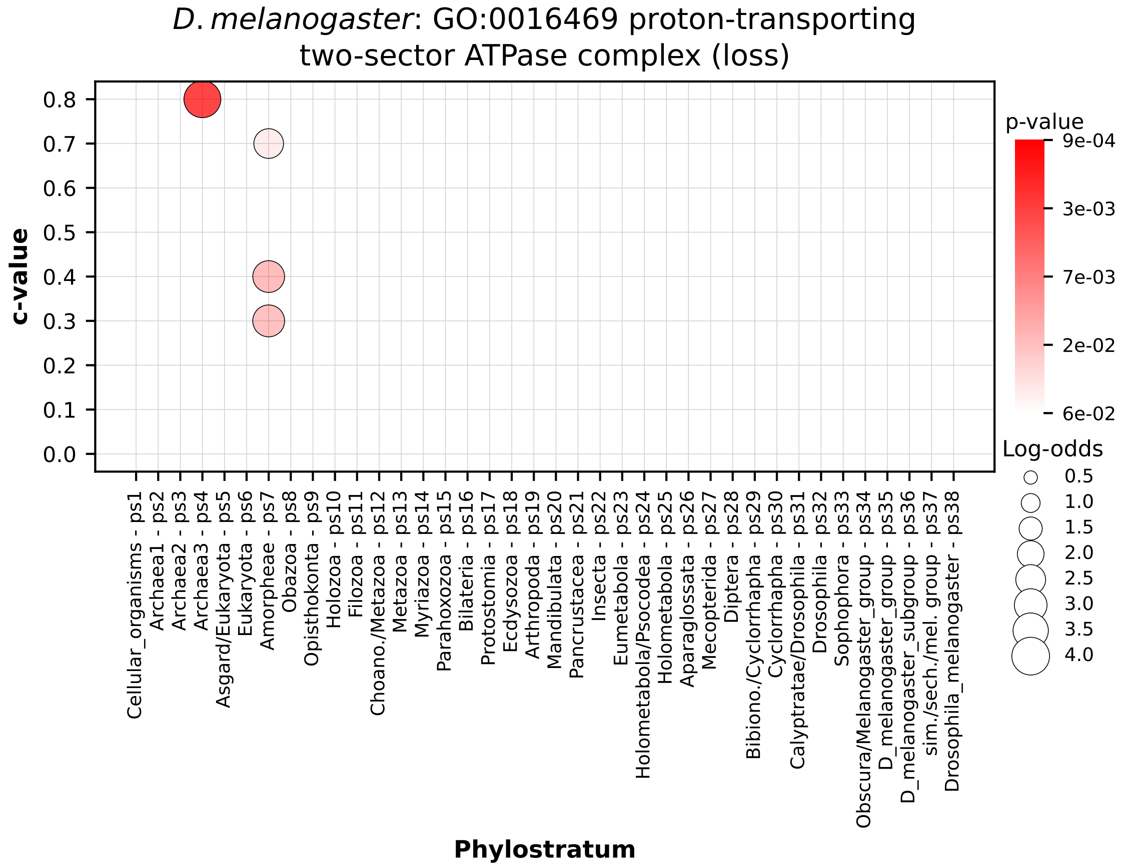
D. melanogaster: GO:0016469 proton-transporting two-sector ATPase complex (loss)
c-value
0.0
0.1
0.2
0.3
0.4
0.5
0.6
0.7
0.8
Phylostratum
Cellular_organisms - ps1
Archaea1 - ps2
Archaea2 - ps3
Archaea3 - ps4
Asgard/Eukaryota - ps5
Eukaryota - ps6
Amorpheae - ps7
Obazoa - ps8
Opisthokonta - ps9
Holozoa - ps10
Filozoa - ps11
Choano./Metazoa - ps12
Metazoa - ps13
Myriazoa - ps14
Parahoxozoa - ps15
Bilateria - ps16
Protostomia - ps17
Ecdysozoa - ps18
Arthropoda - ps19
Mandibulata - ps20
Pancrustacea - ps21
Insecta - ps22
Eumetabola - ps23
Holometabola/Psocodea - ps24
Holometabola - ps25
Aparaglossata - ps26
Mecopterida - ps27
Diptera - ps28
Bibiono./Cyclorrhapha - ps29
Cyclorrhapha - ps30
Calyptratae/Drosophila - ps31
Drosophila - ps32
Sophophora - ps33
Obscura/Melanogaster_group - ps34
D_melanogaster_group - ps35
D_melanogaster_subgroup - ps36
sim./sech./mel. group - ps37
Drosophila_melanogaster - ps38
p-value
9e-04
3e-03
7e-03
2e-02
6e-02
Log-odds
0.5
1.0
1.5
2.0
2.5
3.0
3.5
4.0

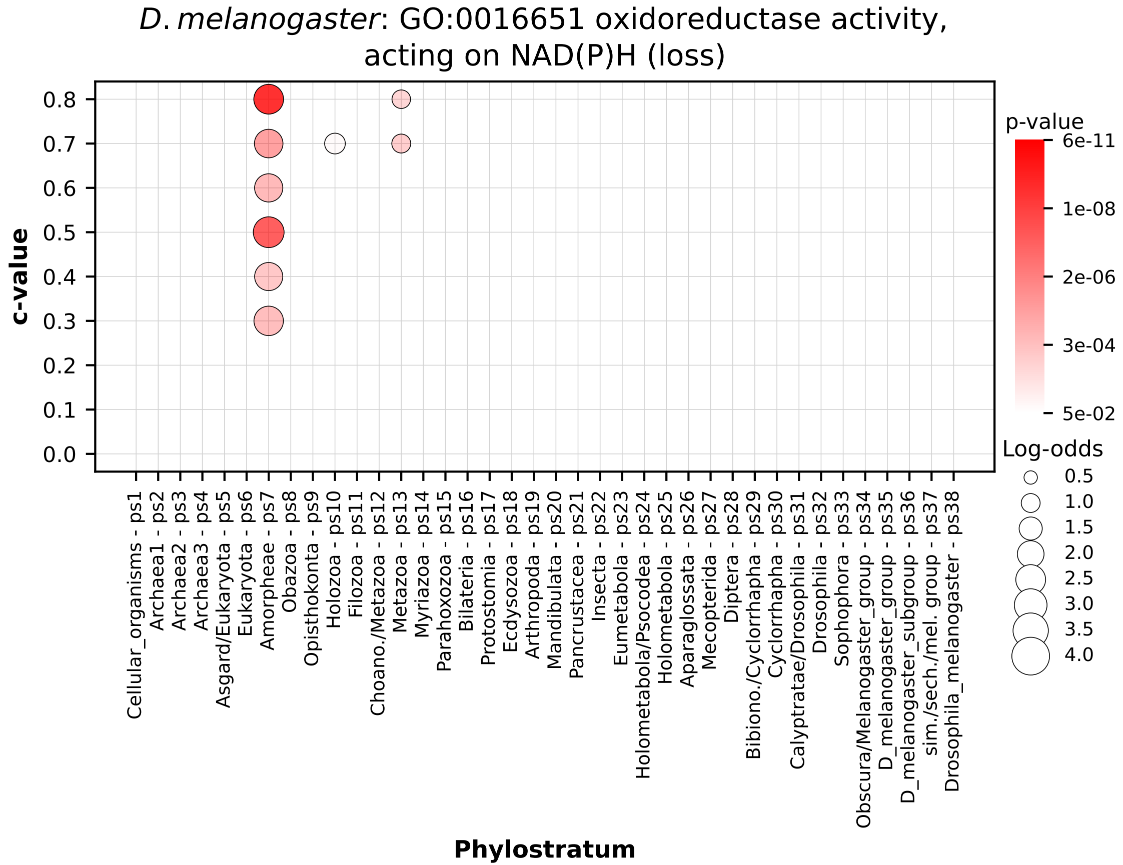

D. melanogaster: GO:0016651 oxidoreductase activity, acting on NAD(P)H (loss)
c-value
0.8
0.7
0.6
0.5
0.4
0.3
0.2
0.1
0.0
p-value
6e-11
1e-08
2e-06
3e-04
5e-02
Log-odds
0.5
1.0
1.5
2.0
2.5
3.0
3.5
4.0
Cellular_organisms - ps1
Archaea1 - ps2
Archaea2 - ps3
Archaea3 - ps4
Asgard/Eukaryota - ps5
Eukaryota - ps6
Amorpheae - ps7
Obazoa - ps8
Opisthokonta - ps9
Holozoa - ps10
Filozoa - ps11
Choano./Metazoa - ps12
Metazoa - ps13
Myriazoa - ps14
Parahoxozoa - ps15
Bilateria - ps16
Protostomia - ps17
Ecdysozoa - ps18
Arthropoda - ps19
Mandibulata - ps20
Pancrustacea - ps21
Insecta - ps22
Eumetabola - ps23
Holometabola/Psocodea - ps24
Holometabola - ps25
Aparaglossata - ps26
Mecopterida - ps27
Diptera - ps28
Bibiono./Cyclorrhapha - ps29
Cyclorrhapha - ps30
Calyptratae/Drosophila - ps31
Drosophila - ps32
Sophophora - ps33
Obscura/Melanogaster_group - ps34
D_melanogaster_group - ps35
D_melanogaster_subgroup - ps36
sim./sech./mel. group - ps37
Drosophila_melanogaster - ps38
Phylostratum

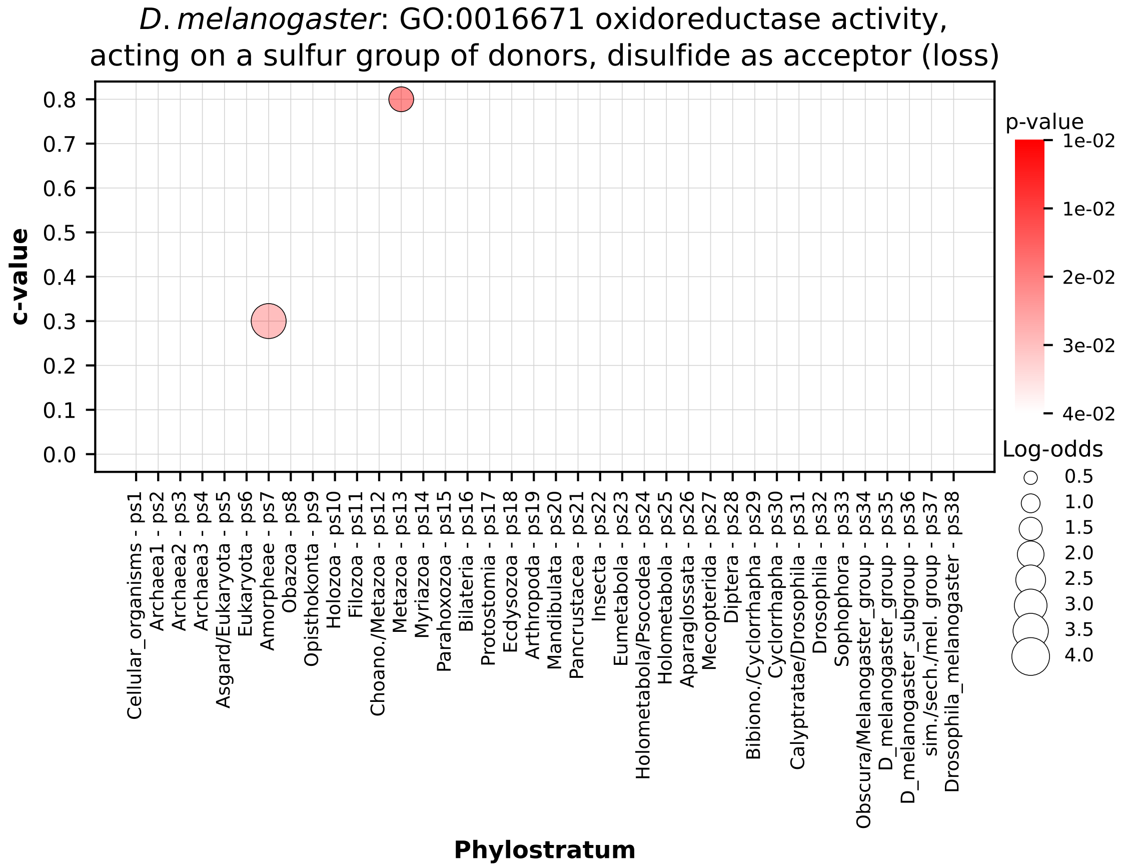

D. melanogaster: GO:0016671 oxidoreductase activity, acting on a sulfur group of donors, disulfide as acceptor (loss)
c-value
0.8
0.7
0.6
0.5
0.4
0.3
0.2
0.1
0.0
p-value
1e-02
1e-02
2e-02
3e-02
4e-02
Log-odds
0.5
1.0
1.5
2.0
2.5
3.0
3.5
4.0
Cellular_organisms - ps1
Archaea1 - ps2
Archaea2 - ps3
Archaea3 - ps4
Asgard/Eukaryota - ps5
Eukaryota - ps6
Amorpheae - ps7
Obazoa - ps8
Opisthokonta - ps9
Holozoa - ps10
Filozoa - ps11
Choano./Metazoa - ps12
Metazoa - ps13
Myriazoa - ps14
Parahoxozoa - ps15
Bilateria - ps16
Protostomia - ps17
Ecdysozoa - ps18
Arthropoda - ps19
Mandibulata - ps20
Pancrustacea - ps21
Insecta - ps22
Eumetabola - ps23
Holometabola/Psocodea - ps24
Holometabola - ps25
Aparaglossata - ps26
Mecopterida - ps27
Diptera - ps28
Bibiono./Cyclorrhapha - ps29
Cyclorrhapha - ps30
Calyptratae/Drosophila - ps31
Drosophila - ps32
Sophophora - ps33
Obscura/Melanogaster_group - ps34
D_melanogaster_group - ps35
D_melanogaster_subgroup - ps36
sim./sech./mel. group - ps37
Drosophila_melanogaster - ps38
Phylostratum

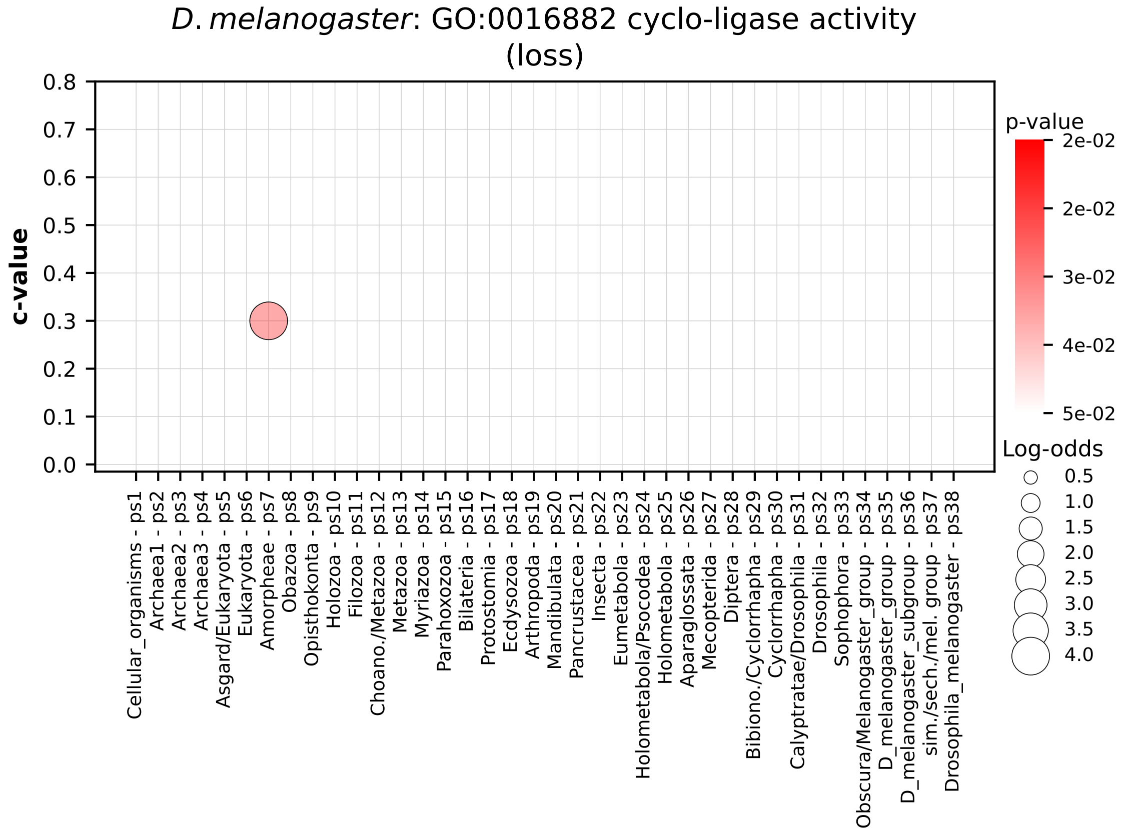

D. melanogaster: GO:0016882 cyclo-ligase activity
(loss)
c-value
Phylostratum
p-value
2e-02
2e-02
3e-02
4e-02
5e-02
Log-odds
0.5
1.0
1.5
2.0
2.5
3.0
3.5
4.0
0.0
0.1
0.2
0.3
0.4
0.5
0.6
0.7
0.8
Cellular_organisms - ps1
Archaea1 - ps2
Archaea2 - ps3
Archaea3 - ps4
Asgard/Eukaryota - ps5
Eukaryota - ps6
Amorpheae - ps7
Obazoa - ps8
Opisthokonta - ps9
Holozoa - ps10
Filozoa - ps11
Choano./Metazoa - ps12
Metazoa - ps13
Myriazoa - ps14
Parahoxozoa - ps15
Bilateria - ps16
Protostomia - ps17
Ecdysozoa - ps18
Arthropoda - ps19
Mandibulata - ps20
Pancrustacea - ps21
Insecta - ps22
Eumetabola - ps23
Holometabola/Psocodea - ps24
Holometabola - ps25
Aparaglossata - ps26
Mecopterida - ps27
Diptera - ps28
Bibiono./Cyclorrhapha - ps29
Cyclorrhapha - ps30
Calyptratae/Drosophila - ps31
Drosophila - ps32
Sophophora - ps33
Obscura/Melanogaster_group - ps34
D_melanogaster_group - ps35
D_melanogaster_subgroup - ps36
sim./sech./mel. group - ps37
Drosophila_melanogaster - ps38

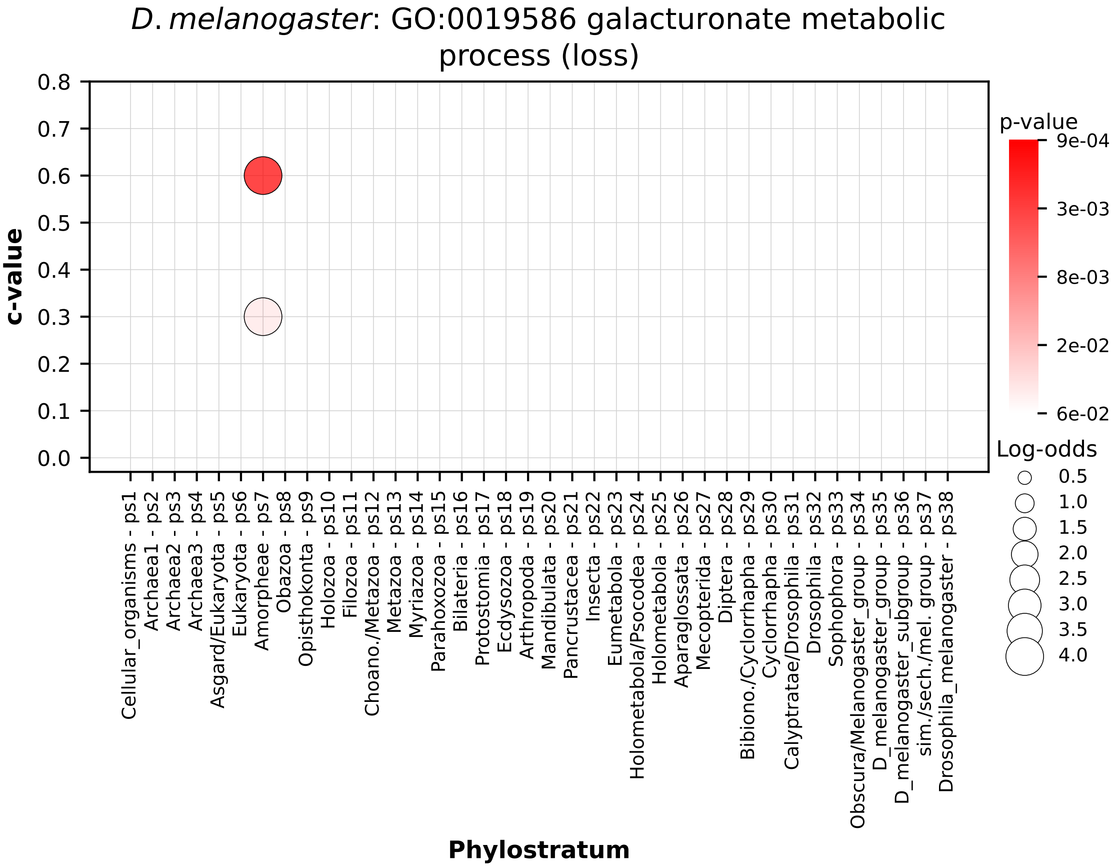

D. melanogaster: GO:0019586 galacturonate metabolic process (loss)
c-value
0.0
0.1
0.2
0.3
0.4
0.5
0.6
0.7
0.8
p-value
9e-04
3e-03
8e-03
2e-02
6e-02
Log-odds
0.5
1.0
1.5
2.0
2.5
3.0
3.5
4.0
Cellular_organisms - ps1
Archaea1 - ps2
Archaea2 - ps3
Archaea3 - ps4
Asgard/Eukaryota - ps5
Eukaryota - ps6
Amorpheae - ps7
Obazoa - ps8
Opisthokonta - ps9
Holozoa - ps10
Filozoa - ps11
Choano./Metazoa - ps12
Metazoa - ps13
Myriazoa - ps14
Parahoxozoa - ps15
Bilateria - ps16
Protostomia - ps17
Ecdysozoa - ps18
Arthropoda - ps19
Mandibulata - ps20
Pancrustacea - ps21
Insecta - ps22
Eumetabola - ps23
Holometabola/Psocodea - ps24
Holometabola - ps25
Aparaglossata - ps26
Mecopterida - ps27
Diptera - ps28
Bibiono./Cyclorrhapha - ps29
Cyclorrhapha - ps30
Calyptratae/Drosophila - ps31
Drosophila - ps32
Sophophora - ps33
Obscura/Melanogaster_group - ps34
D_melanogaster_group - ps35
D_melanogaster_subgroup - ps36
sim./sech./mel. group - ps37
Drosophila_melanogaster - ps38
Phylostratum

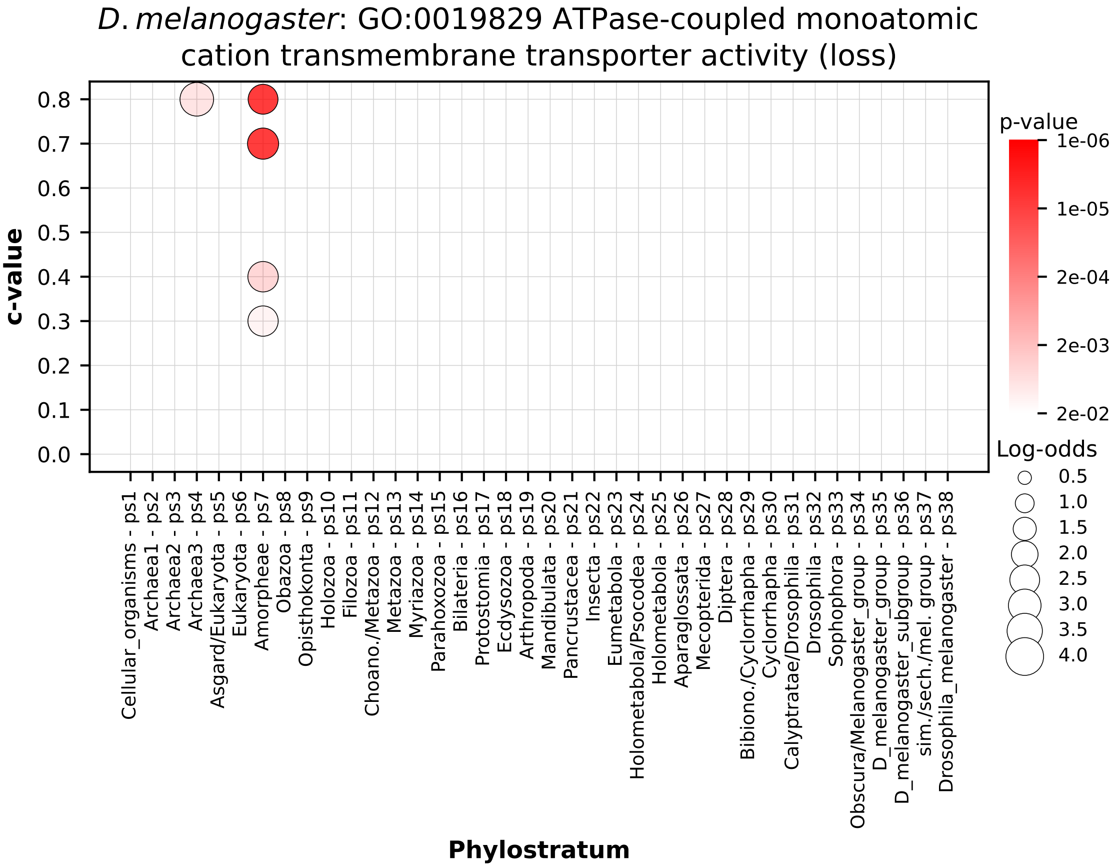

D. melanogaster: GO:0019829 ATPase-coupled monoatomic cation transmembrane transporter activity (loss)
c-value
0.8
0.7
0.6
0.5
0.4
0.3
0.2
0.1
0.0
Phylostratum
Cellular_organisms - ps1
Archaea1 - ps2
Archaea2 - ps3
Archaea3 - ps4
Asgard/Eukaryota - ps5
Eukaryota - ps6
Amorpheae - ps7
Obazoa - ps8
Opisthokonta - ps9
Holozoa - ps10
Filozoa - ps11
Choano./Metazoa - ps12
Metazoa - ps13
Myriazoa - ps14
Parahoxozoa - ps15
Bilateria - ps16
Protostomia - ps17
Ecdysozoa - ps18
Arthropoda - ps19
Mandibulata - ps20
Pancrustacea - ps21
Insecta - ps22
Eumetabola - ps23
Holometabola/Psocodea - ps24
Holometabola - ps25
Aparaglossata - ps26
Mecopterida - ps27
Diptera - ps28
Bibiono./Cyclorrhapha - ps29
Cyclorrhapha - ps30
Calyptratae/Drosophila - ps31
Drosophila - ps32
Sophophora - ps33
Obscura/Melanogaster_group - ps34
D_melanogaster_group - ps35
D_melanogaster_subgroup - ps36
sim./sech./mel. group - ps37
Drosophila_melanogaster - ps38
p-value
1e-06
1e-05
2e-04
2e-03
2e-02
Log-odds
0.5
1.0
1.5
2.0
2.5
3.0
3.5
4.0

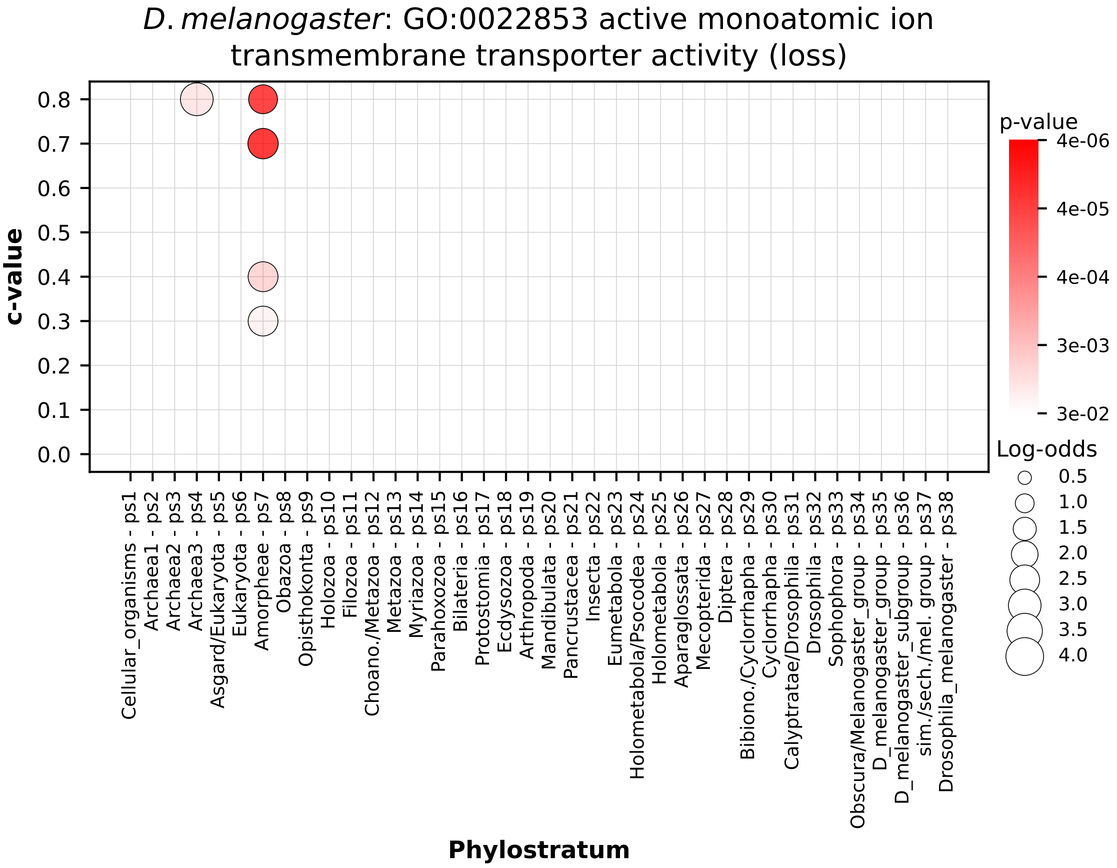

D. melanogaster: GO:0022853 active monoatomic ion transmembrane transporter activity (loss)
c-value
0.8
0.7
0.6
0.5
0.4
0.3
0.2
0.1
0.0
p-value
4e-06
4e-05
4e-04
3e-03
3e-02
Log-odds
0.5
1.0
1.5
2.0
2.5
3.0
3.5
4.0
Cellular_organisms - ps1
Archaea1 - ps2
Archaea2 - ps3
Archaea3 - ps4
Asgard/Eukaryota - ps5
Eukaryota - ps6
Amorpheae - ps7
Obazoa - ps8
Opisthokonta - ps9
Holozoa - ps10
Filozoa - ps11
Choano./Metazoa - ps12
Metazoa - ps13
Myriazoa - ps14
Parahoxozoa - ps15
Bilateria - ps16
Protostomia - ps17
Ecdysozoa - ps18
Arthropoda - ps19
Mandibulata - ps20
Pancrustacea - ps21
Insecta - ps22
Eumetabola - ps23
Holometabola/Psocodea - ps24
Holometabola - ps25
Aparaglossata - ps26
Mecopterida - ps27
Diptera - ps28
Bibiono./Cyclorrhapha - ps29
Cyclorrhapha - ps30
Calyptratae/Drosophila - ps31
Drosophila - ps32
Sophophora - ps33
Obscura/Melanogaster_group - ps34
D_melanogaster_group - ps35
D_melanogaster_subgroup - ps36
sim./sech./mel. group - ps37
Drosophila_melanogaster - ps38
Phylostratum

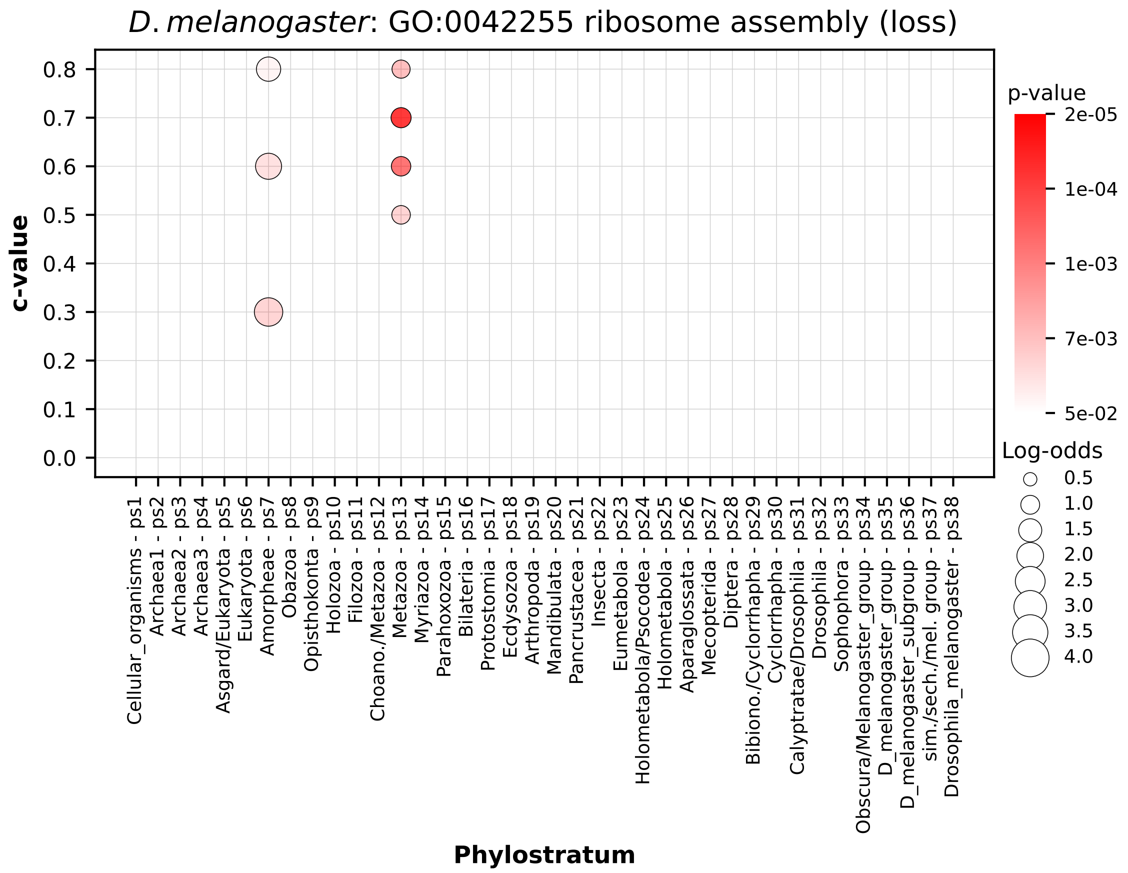

D. melanogaster: GO:0042255 ribosome assembly (loss)
c-value
0.8
0.7
0.6
0.5
0.4
0.3
0.2
0.1
0.0
Phylostratum
Cellular_organisms - ps1
Archaea1 - ps2
Archaea2 - ps3
Archaea3 - ps4
Asgard/Eukaryota - ps5
Eukaryota - ps6
Amorpheae - ps7
Obazoa - ps8
Opisthokonta - ps9
Holozoa - ps10
Filozoa - ps11
Choano./Metazoa - ps12
Metazoa - ps13
Myriazoa - ps14
Parahoxozoa - ps15
Bilateria - ps16
Protostomia - ps17
Ecdysozoa - ps18
Arthropoda - ps19
Mandibulata - ps20
Pancrustacea - ps21
Insecta - ps22
Eumetabola - ps23
Holometabola/Psocodea - ps24
Holometabola - ps25
Aparaglossata - ps26
Mecopterida - ps27
Diptera - ps28
Bibiono./Cyclorrhapha - ps29
Cyclorrhapha - ps30
Calyptratae/Drosophila - ps31
Drosophila - ps32
Sophophora - ps33
Obscura/Melanogaster_group - ps34
D_melanogaster_group - ps35
D_melanogaster_subgroup - ps36
sim./sech./mel. group - ps37
Drosophila_melanogaster - ps38
p-value
2e-05
1e-04
1e-03
7e-03
5e-02
Log-odds
0.5
1.0
1.5
2.0
2.5
3.0
3.5
4.0

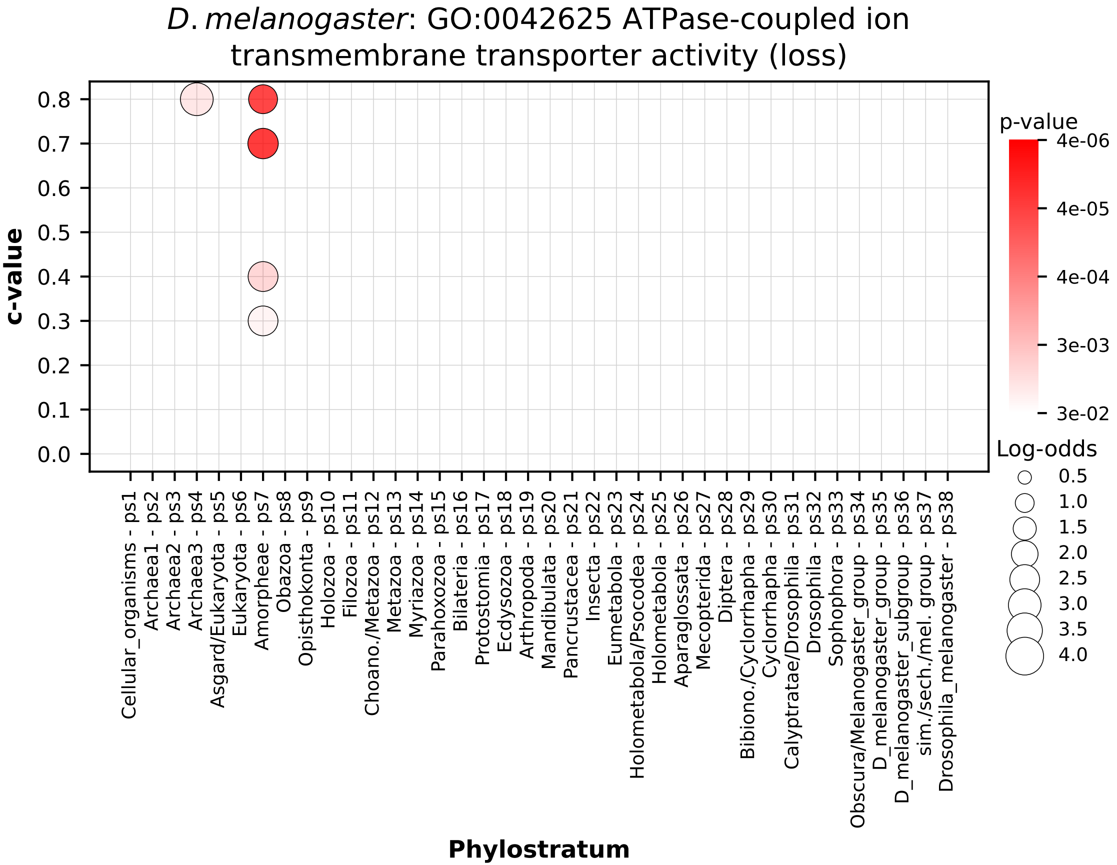

D. melanogaster: GO:0042625 ATPase-coupled ion transmembrane transporter activity (loss)
c-value
Phylostratum
p-value
4e-06
4e-05
4e-04
3e-03
3e-02
Log-odds
0.5
1.0
1.5
2.0
2.5
3.0
3.5
4.0
Cellular_organisms - ps1
Archaea1 - ps2
Archaea2 - ps3
Archaea3 - ps4
Asgard/Eukaryota - ps5
Eukaryota - ps6
Amorpheae - ps7
Obazoa - ps8
Opisthokonta - ps9
Holozoa - ps10
Filozoa - ps11
Choano./Metazoa - ps12
Metazoa - ps13
Myriazoa - ps14
Parahoxozoa - ps15
Bilateria - ps16
Protostomia - ps17
Ecdysozoa - ps18
Arthropoda - ps19
Mandibulata - ps20
Pancrustacea - ps21
Insecta - ps22
Eumetabola - ps23
Holometabola/Psocodea - ps24
Holometabola - ps25
Aparaglossata - ps26
Mecopterida - ps27
Diptera - ps28
Bibiono./Cyclorrhapha - ps29
Cyclorrhapha - ps30
Calyptratae/Drosophila - ps31
Drosophila - ps32
Sophophora - ps33
Obscura/Melanogaster_group - ps34
D_melanogaster_group - ps35
D_melanogaster_subgroup - ps36
sim./sech./mel. group - ps37
Drosophila_melanogaster - ps38

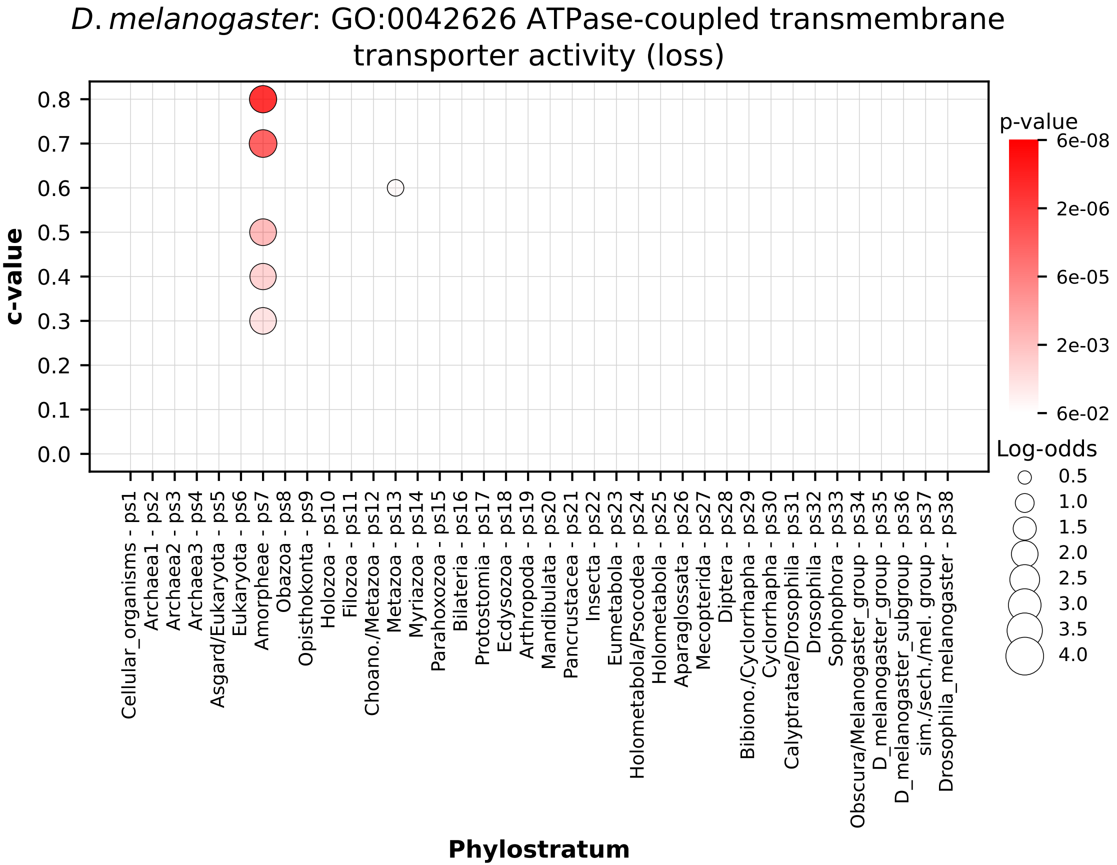
D. melanogaster: GO:0042626 ATPase-coupled transmembrane transporter activity (loss)
c-value
0.8
0.7
0.6
0.5
0.4
0.3
0.2
0.1
0.0
Phylostratum
Cellular_organisms - ps1
Archaea1 - ps2
Archaea2 - ps3
Archaea3 - ps4
Asgard/Eukaryota - ps5
Eukaryota - ps6
Amorpheae - ps7
Obazoa - ps8
Opisthokonta - ps9
Holozoa - ps10
Filozoa - ps11
Choano./Metazoa - ps12
Metazoa - ps13
Myriazoa - ps14
Parahoxozoa - ps15
Bilateria - ps16
Protostomia - ps17
Ecdysozoa - ps18
Arthropoda - ps19
Mandibulata - ps20
Pancrustacea - ps21
Insecta - ps22
Eumetabola - ps23
Holometabola/Psocodea - ps24
Holometabola - ps25
Aparaglossata - ps26
Mecopterida - ps27
Diptera - ps28
Bibiono./Cyclorrhapha - ps29
Cyclorrhapha - ps30
Calyptratae/Drosophila - ps31
Drosophila - ps32
Sophophora - ps33
Obscura/Melanogaster_group - ps34
D_melanogaster_group - ps35
D_melanogaster_subgroup - ps36
sim./sech./mel. group - ps37
Drosophila_melanogaster - ps38
p-value
6e-08
2e-06
6e-05
2e-03
6e-02
Log-odds
0.5
1.0
1.5
2.0
2.5
3.0
3.5
4.0

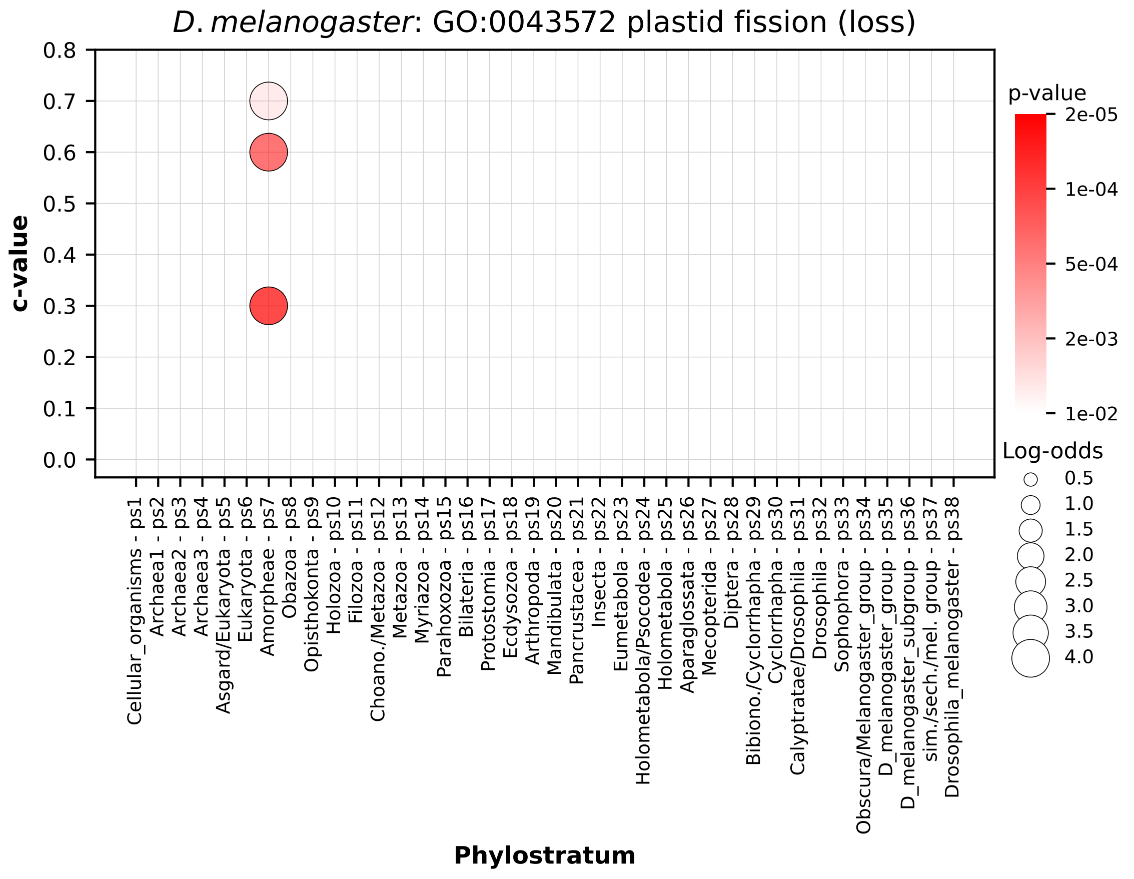

D. melanogaster: GO:0043572 plastid fission (loss)
c-value
0.8
0.7
0.6
0.5
0.4
0.3
0.2
0.1
0.0
Phylostratum
Cellular_organisms - ps1
Archaea1 - ps2
Archaea2 - ps3
Archaea3 - ps4
Asgard/Eukaryota - ps5
Eukaryota - ps6
Amorpheae - ps7
Obazoa - ps8
Opisthokonta - ps9
Holozoa - ps10
Filozoa - ps11
Choano./Metazoa - ps12
Metazoa - ps13
Myriazoa - ps14
Parahoxozoa - ps15
Bilateria - ps16
Protostomia - ps17
Ecdysozoa - ps18
Arthropoda - ps19
Mandibulata - ps20
Pancrustacea - ps21
Insecta - ps22
Eumetabola - ps23
Holometabola/Psocodea - ps24
Holometabola - ps25
Aparaglossata - ps26
Mecopterida - ps27
Diptera - ps28
Bibiono./Cyclorrhapha - ps29
Cyclorrhapha - ps30
Calyptratae/Drosophila - ps31
Drosophila - ps32
Sophophora - ps33
Obscura/Melanogaster_group - ps34
D_melanogaster_group - ps35
D_melanogaster_subgroup - ps36
sim./sech./mel. group - ps37
Drosophila_melanogaster - ps38
p-value
2e-05
1e-04
5e-04
2e-03
1e-02
Log-odds
0.5
1.0
1.5
2.0
2.5
3.0
3.5
4.0

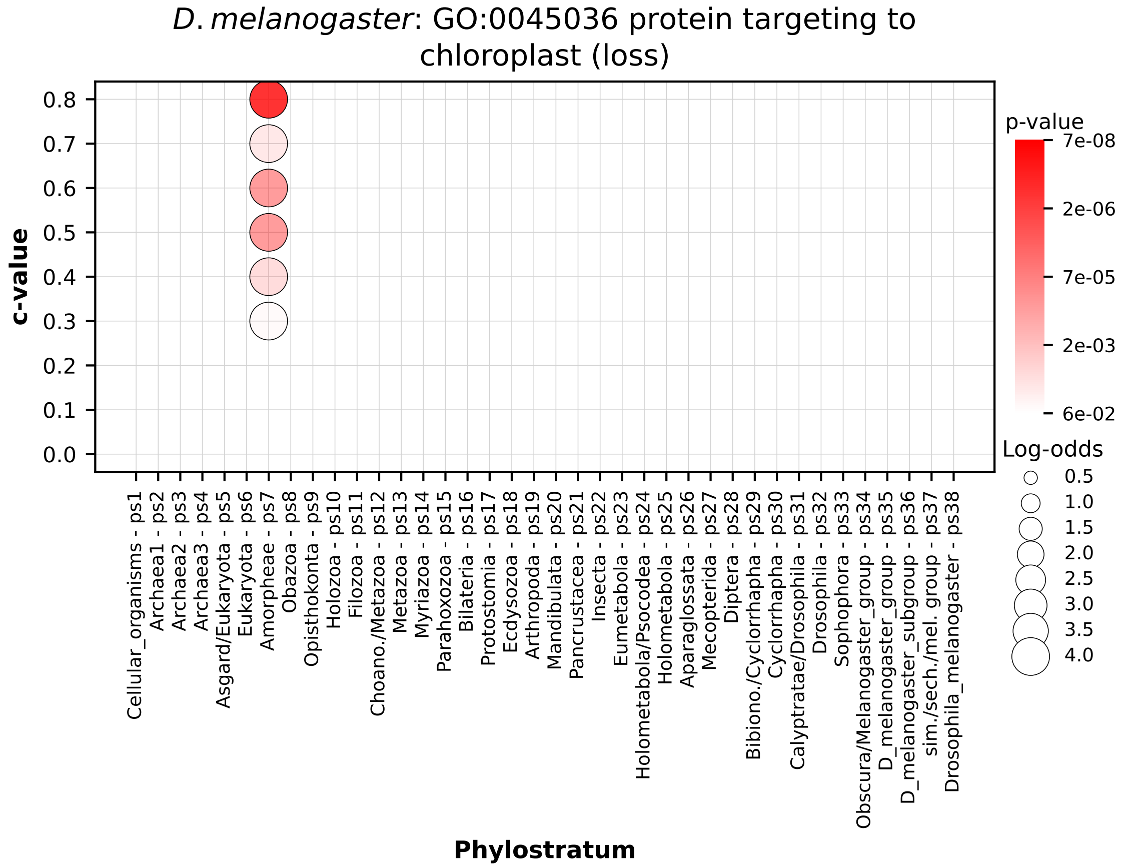

D. melanogaster: GO:0045036 protein targeting to chloroplast (loss)
c-value
0.0
0.1
0.2
0.3
0.4
0.5
0.6
0.7
0.8
p-value
7e-08
2e-06
7e-05
2e-03
6e-02
Log-odds
0.5
1.0
1.5
2.0
2.5
3.0
3.5
4.0
Cellular_organisms - ps1
Archaea1 - ps2
Archaea2 - ps3
Archaea3 - ps4
Asgard/Eukaryota - ps5
Eukaryota - ps6
Amorpheae - ps7
Obazoa - ps8
Opisthokonta - ps9
Holozoa - ps10
Filozoa - ps11
Choano./Metazoa - ps12
Metazoa - ps13
Myriazoa - ps14
Parahoxozoa - ps15
Bilateria - ps16
Protostomia - ps17
Ecdysozoa - ps18
Arthropoda - ps19
Mandibulata - ps20
Pancrustacea - ps21
Insecta - ps22
Eumetabola - ps23
Holometabola/Psocodea - ps24
Holometabola - ps25
Aparaglossata - ps26
Mecopterida - ps27
Diptera - ps28
Bibiono./Cyclorrhapha - ps29
Cyclorrhapha - ps30
Calyptratae/Drosophila - ps31
Drosophila - ps32
Sophophora - ps33
Obscura/Melanogaster_group - ps34
D_melanogaster_group - ps35
D_melanogaster_subgroup - ps36
sim./sech./mel. group - ps37
Drosophila_melanogaster - ps38
Phylostratum

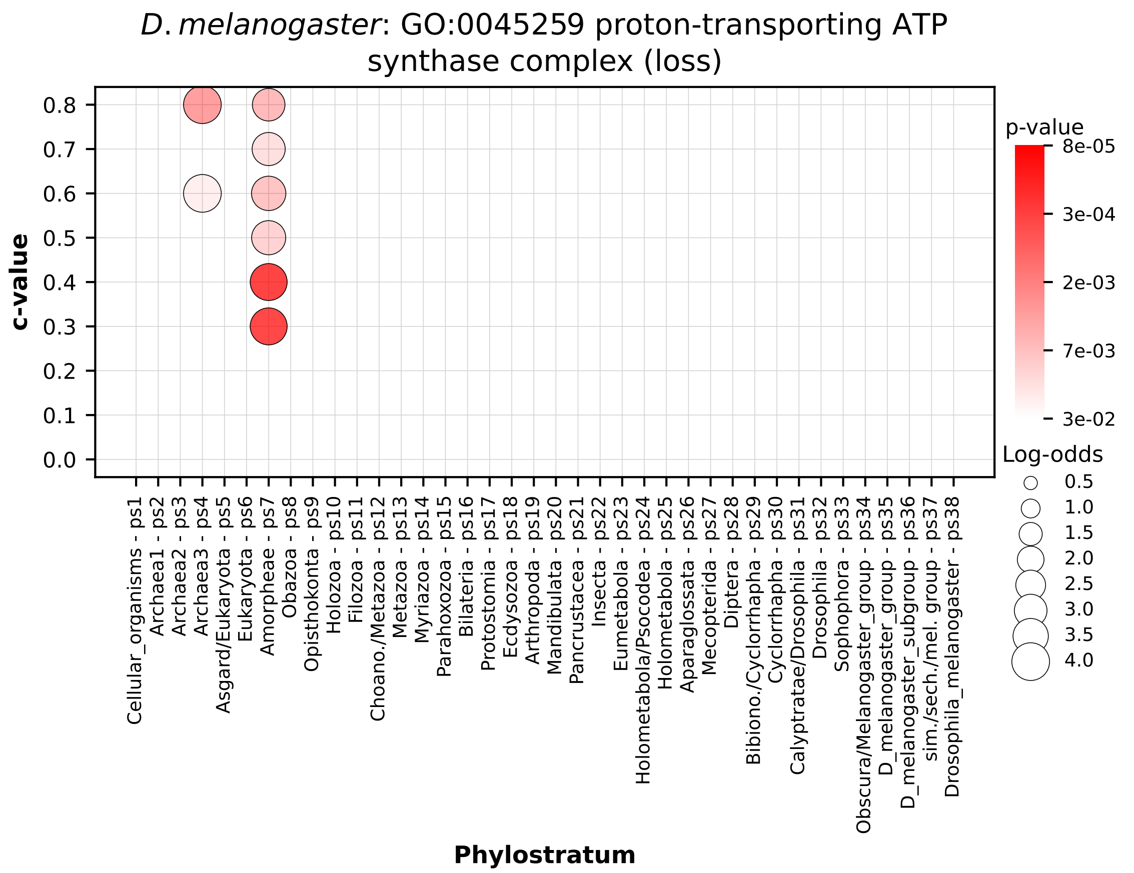

D. melanogaster: GO:0045259 proton-transporting ATP synthase complex (loss)
c-value
0.8
0.7
0.6
0.5
0.4
0.3
0.2
0.1
0.0
p-value
8e-05
3e-04
2e-03
7e-03
3e-02
Log-odds
0.5
1.0
1.5
2.0
2.5
3.0
3.5
4.0
Cellular_organisms - ps1
Archaea1 - ps2
Archaea2 - ps3
Archaea3 - ps4
Asgard/Eukaryota - ps5
Eukaryota - ps6
Amorpheae - ps7
Obazoa - ps8
Opisthokonta - ps9
Holozoa - ps10
Filozoa - ps11
Choano./Metazoa - ps12
Metazoa - ps13
Myriazoa - ps14
Parahoxozoa - ps15
Bilateria - ps16
Protostomia - ps17
Ecdysozoa - ps18
Arthropoda - ps19
Mandibulata - ps20
Pancrustacea - ps21
Insecta - ps22
Eumetabola - ps23
Holometabola/Psocodea - ps24
Holometabola - ps25
Aparaglossata - ps26
Mecopterida - ps27
Diptera - ps28
Bibiono./Cyclorrhapha - ps29
Cyclorrhapha - ps30
Calyptratae/Drosophila - ps31
Drosophila - ps32
Sophophora - ps33
Obscura/Melanogaster_group - ps34
D_melanogaster_group - ps35
D_melanogaster_subgroup - ps36
sim./sech./mel. group - ps37
Drosophila_melanogaster - ps38
Phylostratum

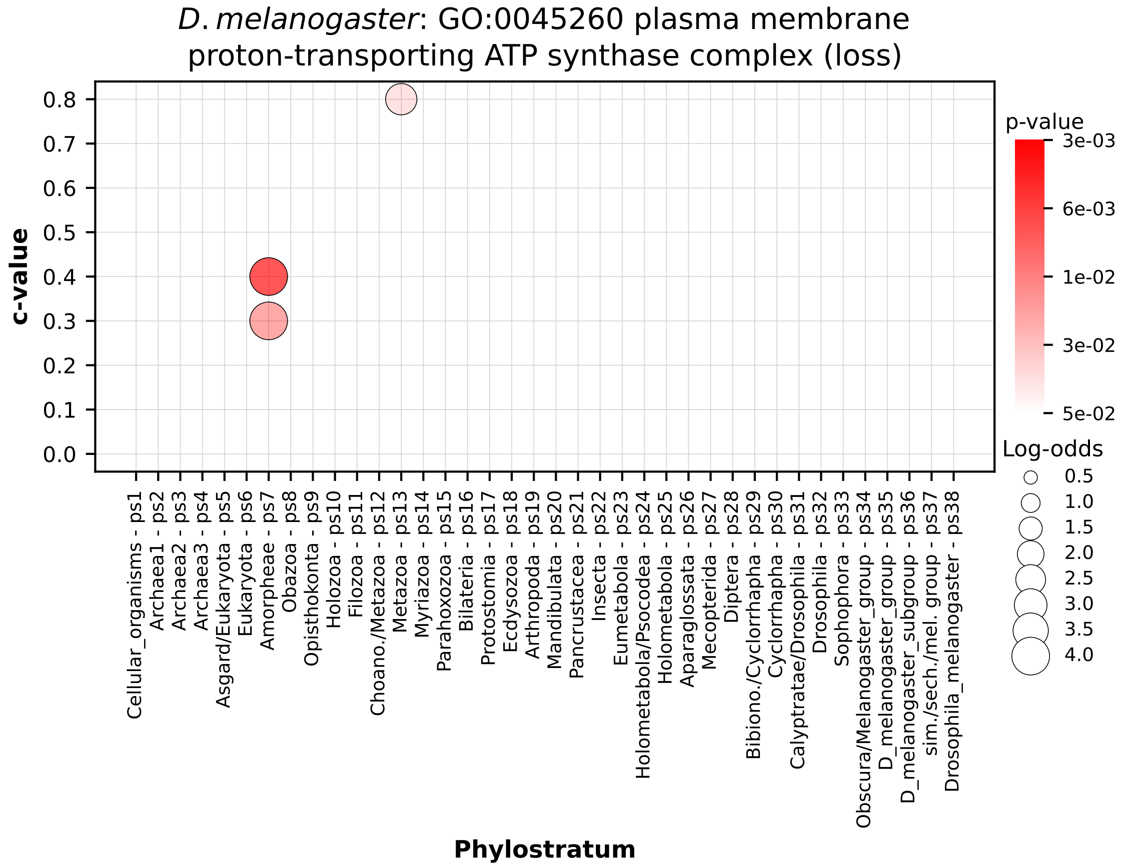
D. melanogaster: GO:0045260 plasma membrane proton-transporting ATP synthase complex (loss)
c-value
0.0
0.1
0.2
0.3
0.4
0.5
0.6
0.7
0.8
p-value
3e-03
6e-03
1e-02
3e-02
5e-02
Log-odds
0.5
1.0
1.5
2.0
2.5
3.0
3.5
4.0
Cellular_organisms - ps1
Archaea1 - ps2
Archaea2 - ps3
Archaea3 - ps4
Asgard/Eukaryota - ps5
Eukaryota - ps6
Amorpheae - ps7
Obazoa - ps8
Opisthokonta - ps9
Holozoa - ps10
Filozoa - ps11
Choano./Metazoa - ps12
Metazoa - ps13
Myriazoa - ps14
Parahoxozoa - ps15
Bilateria - ps16
Protostomia - ps17
Ecdysozoa - ps18
Arthropoda - ps19
Mandibulata - ps20
Pancrustacea - ps21
Insecta - ps22
Eumetabola - ps23
Holometabola/Psocodea - ps24
Holometabola - ps25
Aparaglossata - ps26
Mecopterida - ps27
Diptera - ps28
Bibiono./Cyclorrhapha - ps29
Cyclorrhapha - ps30
Calyptratae/Drosophila - ps31
Drosophila - ps32
Sophophora - ps33
Obscura/Melanogaster_group - ps34
D_melanogaster_group - ps35
D_melanogaster_subgroup - ps36
sim./sech./mel. group - ps37
Drosophila_melanogaster - ps38
Phylostratum

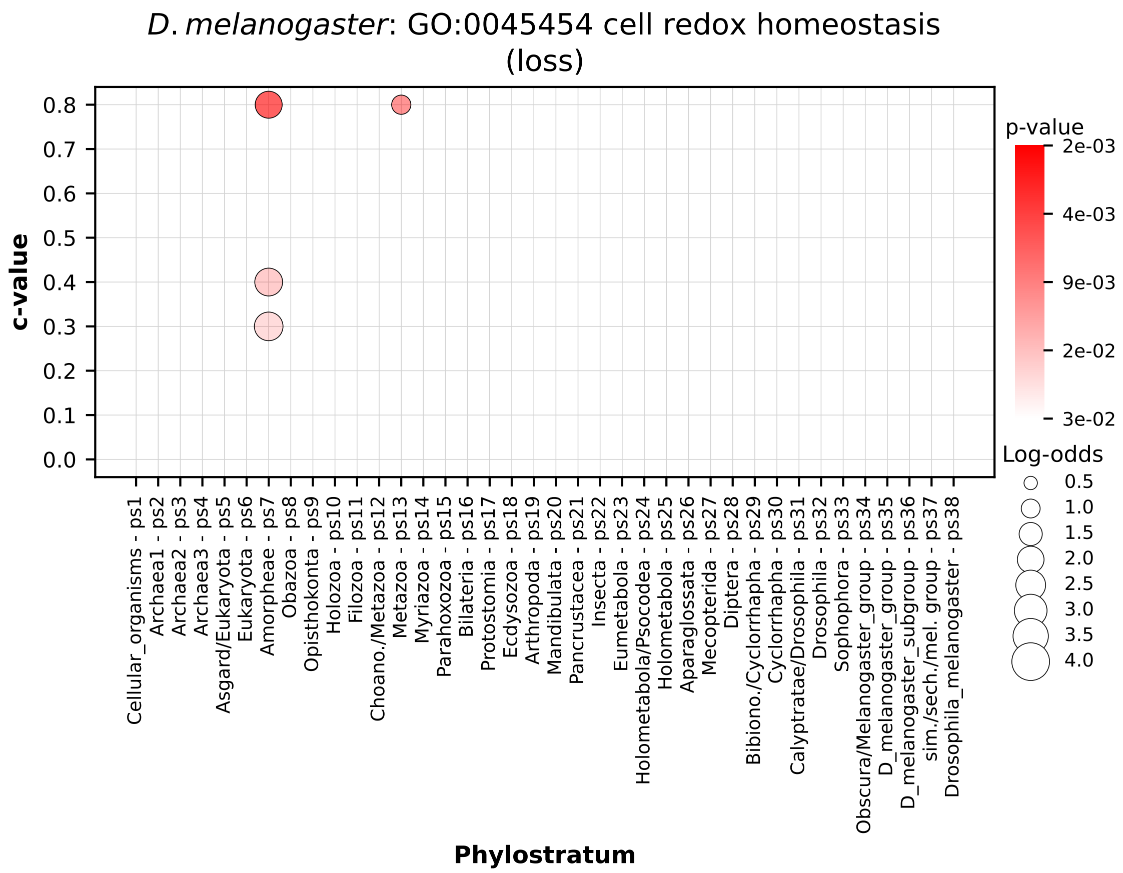

D. melanogaster: GO:0045454 cell redox homeostasis
(loss)
c-value
0.0
0.1
0.2
0.3
0.4
0.5
0.6
0.7
0.8
Phylostratum
Cellular_organisms - ps1
Archaea1 - ps2
Archaea2 - ps3
Archaea3 - ps4
Asgard/Eukaryota - ps5
Eukaryota - ps6
Amorpheae - ps7
Obazoa - ps8
Opisthokonta - ps9
Holozoa - ps10
Filozoa - ps11
Choano./Metazoa - ps12
Metazoa - ps13
Myriazoa - ps14
Parahoxozoa - ps15
Bilateria - ps16
Protostomia - ps17
Ecdysozoa - ps18
Arthropoda - ps19
Mandibulata - ps20
Pancrustacea - ps21
Insecta - ps22
Eumetabola - ps23
Holometabola/Psocodea - ps24
Holometabola - ps25
Aparaglossata - ps26
Mecopterida - ps27
Diptera - ps28
Bibiono./Cyclorrhapha - ps29
Cyclorrhapha - ps30
Calyptratae/Drosophila - ps31
Drosophila - ps32
Sophophora - ps33
Obscura/Melanogaster_group - ps34
D_melanogaster_group - ps35
D_melanogaster_subgroup - ps36
sim./sech./mel. group - ps37
Drosophila_melanogaster - ps38
p-value
2e-03
4e-03
9e-03
2e-02
3e-02
Log-odds
0.5
1.0
1.5
2.0
2.5
3.0
3.5
4.0

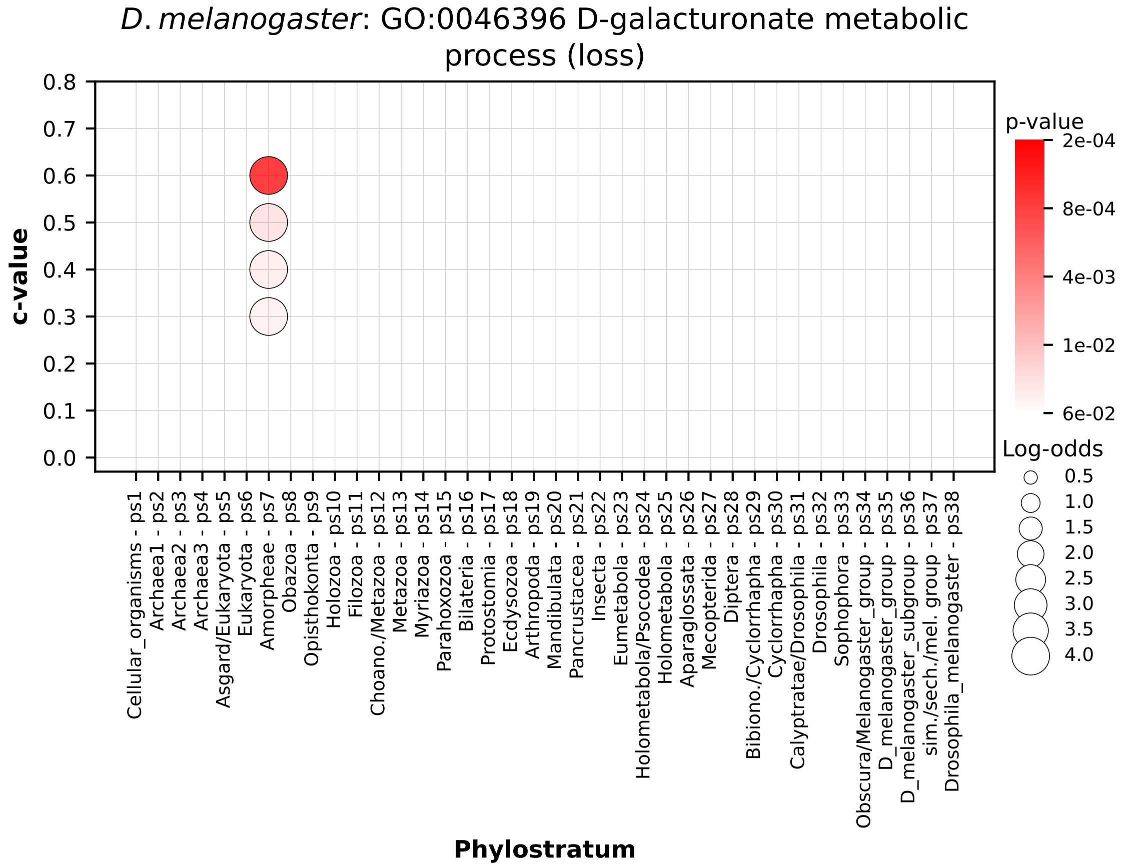

D. melanogaster: GO:0046396 D-galacturonate metabolic process (loss)
c-value
Phylostratum
p-value
2e-04
8e-04
4e-03
1e-02
6e-02
Log-odds
0.5
1.0
1.5
2.0
2.5
3.0
3.5
4.0
Cellular_organisms - ps1
Archaea1 - ps2
Archaea2 - ps3
Archaea3 - ps4
Asgard/Eukaryota - ps5
Eukaryota - ps6
Amorpheae - ps7
Obazoa - ps8
Opisthokonta - ps9
Holozoa - ps10
Filozoa - ps11
Choano./Metazoa - ps12
Metazoa - ps13
Myriazoa - ps14
Parahoxozoa - ps15
Bilateria - ps16
Protostomia - ps17
Ecdysozoa - ps18
Arthropoda - ps19
Mandibulata - ps20
Pancrustacea - ps21
Insecta - ps22
Eumetabola - ps23
Holometabola/Psocodea - ps24
Holometabola - ps25
Aparaglossata - ps26
Mecopterida - ps27
Diptera - ps28
Bibiono./Cyclorrhapha - ps29
Cyclorrhapha - ps30
Calyptratae/Drosophila - ps31
Drosophila - ps32
Sophophora - ps33
Obscura/Melanogaster_group - ps34
D_melanogaster_group - ps35
D_melanogaster_subgroup - ps36
sim./sech./mel. group - ps37
Drosophila_melanogaster - ps38

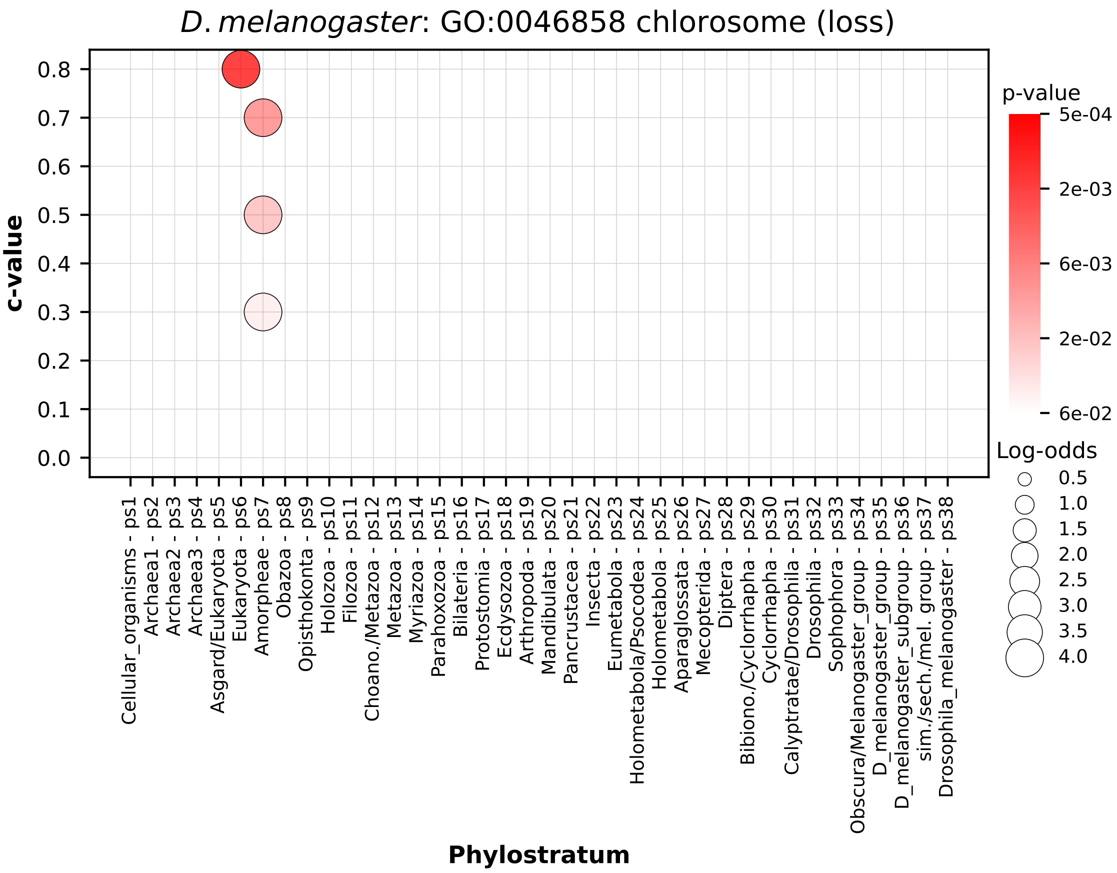

D. melanogaster: GO:0046858 chlorosome (loss)
c-value
0.8
0.7
0.6
0.5
0.4
0.3
0.2
0.1
0.0
Phylostratum
Cellular_organisms - ps1
Archaea1 - ps2
Archaea2 - ps3
Archaea3 - ps4
Asgard/Eukaryota - ps5
Eukaryota - ps6
Amorpheae - ps7
Obazoa - ps8
Opisthokonta - ps9
Holozoa - ps10
Filozoa - ps11
Choano./Metazoa - ps12
Metazoa - ps13
Myriazoa - ps14
Parahoxozoa - ps15
Bilateria - ps16
Protostomia - ps17
Ecdysozoa - ps18
Arthropoda - ps19
Mandibulata - ps20
Pancrustacea - ps21
Insecta - ps22
Eumetabola - ps23
Holometabola/Psocodea - ps24
Holometabola - ps25
Aparaglossata - ps26
Mecopterida - ps27
Diptera - ps28
Bibiono./Cyclorrhapha - ps29
Cyclorrhapha - ps30
Calyptratae/Drosophila - ps31
Drosophila - ps32
Sophophora - ps33
Obscura/Melanogaster_group - ps34
D_melanogaster_group - ps35
D_melanogaster_subgroup - ps36
sim./sech./mel. group - ps37
Drosophila_melanogaster - ps38
p-value
5e-04
2e-03
6e-03
2e-02
6e-02
Log-odds
0.5
1.0
1.5
2.0
2.5
3.0
3.5
4.0

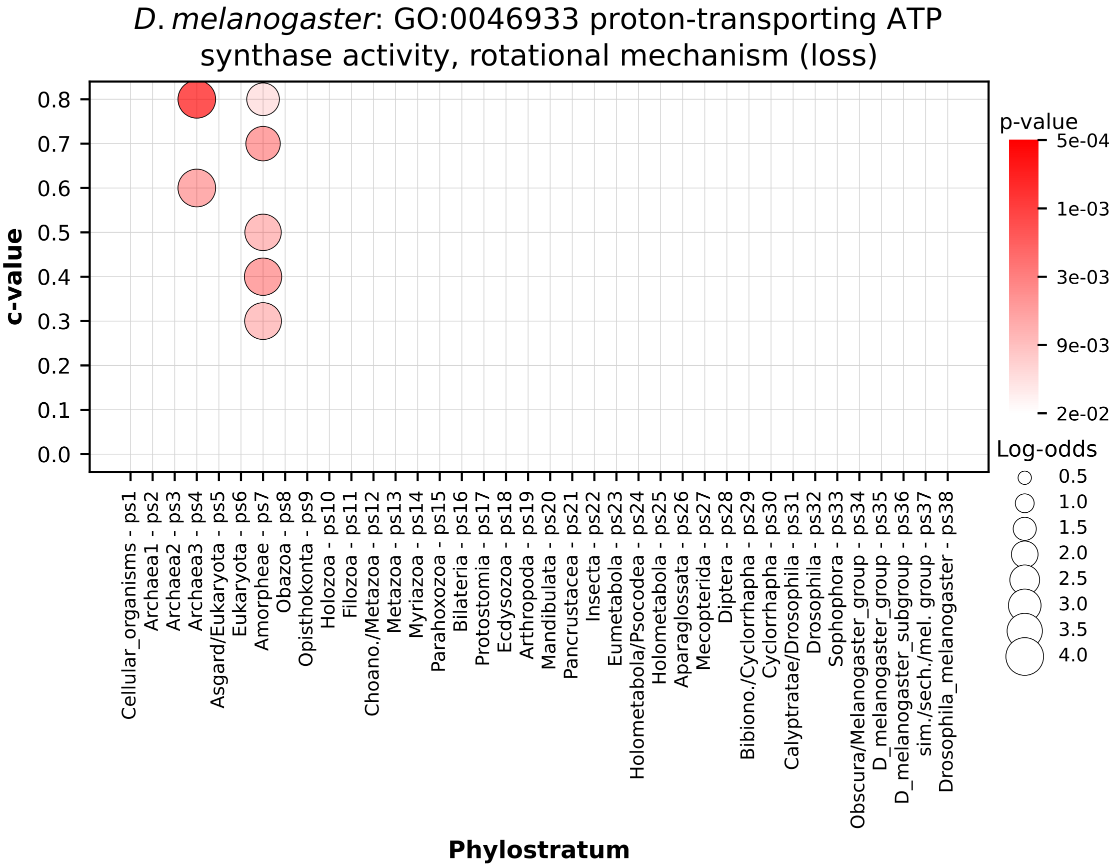

D. melanogaster: GO:0046933 proton-transporting ATP synthase activity, rotational mechanism (loss)
c-value
0.8
0.7
0.6
0.5
0.4
0.3
0.2
0.1
0.0
p-value
5e-04
1e-03
3e-03
9e-03
2e-02
Log-odds
0.5
1.0
1.5
2.0
2.5
3.0
3.5
4.0
Cellular_organisms - ps1
Archaea1 - ps2
Archaea2 - ps3
Archaea3 - ps4
Asgard/Eukaryota - ps5
Eukaryota - ps6
Amorpheae - ps7
Obazoa - ps8
Opisthokonta - ps9
Holozoa - ps10
Filozoa - ps11
Choano./Metazoa - ps12
Metazoa - ps13
Myriazoa - ps14
Parahoxozoa - ps15
Bilateria - ps16
Protostomia - ps17
Ecdysozoa - ps18
Arthropoda - ps19
Mandibulata - ps20
Pancrustacea - ps21
Insecta - ps22
Eumetabola - ps23
Holometabola/Psocodea - ps24
Holometabola - ps25
Aparaglossata - ps26
Mecopterida - ps27
Diptera - ps28
Bibiono./Cyclorrhapha - ps29
Cyclorrhapha - ps30
Calyptratae/Drosophila - ps31
Drosophila - ps32
Sophophora - ps33
Obscura/Melanogaster_group - ps34
D_melanogaster_group - ps35
D_melanogaster_subgroup - ps36
sim./sech./mel. group - ps37
Drosophila_melanogaster - ps38
Phylostratum

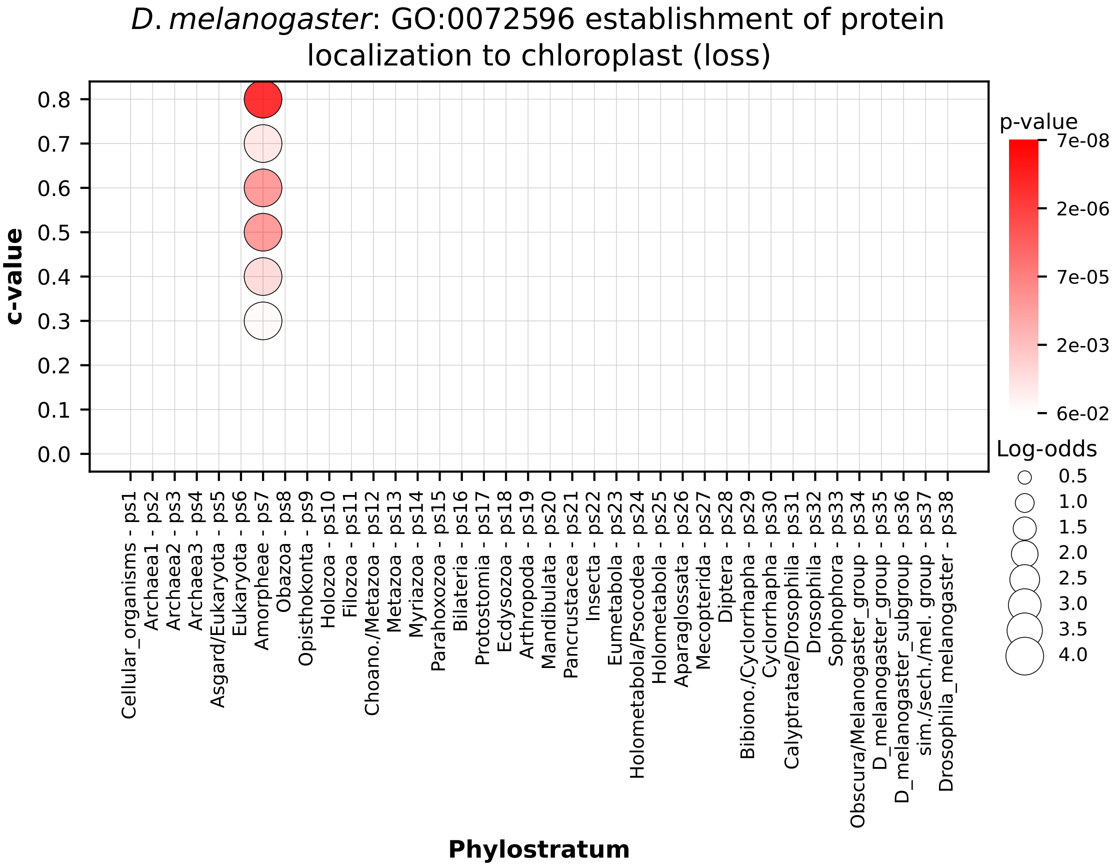

D. melanogaster: GO:0072596 establishment of protein localization to chloroplast (loss)
c-value
0.0
0.1
0.2
0.3
0.4
0.5
0.6
0.7
0.8
Phylostratum
Cellular_organisms - ps1
Archaea1 - ps2
Archaea2 - ps3
Archaea3 - ps4
Asgard/Eukaryota - ps5
Eukaryota - ps6
Amorpheae - ps7
Obazoa - ps8
Opisthokonta - ps9
Holozoa - ps10
Filozoa - ps11
Choano./Metazoa - ps12
Metazoa - ps13
Myriazoa - ps14
Parahoxozoa - ps15
Bilateria - ps16
Protostomia - ps17
Ecdysozoa - ps18
Arthropoda - ps19
Mandibulata - ps20
Pancrustacea - ps21
Insecta - ps22
Eumetabola - ps23
Holometabola/Psocodea - ps24
Holometabola - ps25
Aparaglossata - ps26
Mecopterida - ps27
Diptera - ps28
Bibiono./Cyclorrhapha - ps29
Cyclorrhapha - ps30
Calyptratae/Drosophila - ps31
Drosophila - ps32
Sophophora - ps33
Obscura/Melanogaster_group - ps34
D_melanogaster_group - ps35
D_melanogaster_subgroup - ps36
sim./sech./mel. group - ps37
Drosophila_melanogaster - ps38
p-value
7e-08
2e-06
7e-05
2e-03
6e-02
Log-odds
0.5
1.0
1.5
2.0
2.5
3.0
3.5
4.0

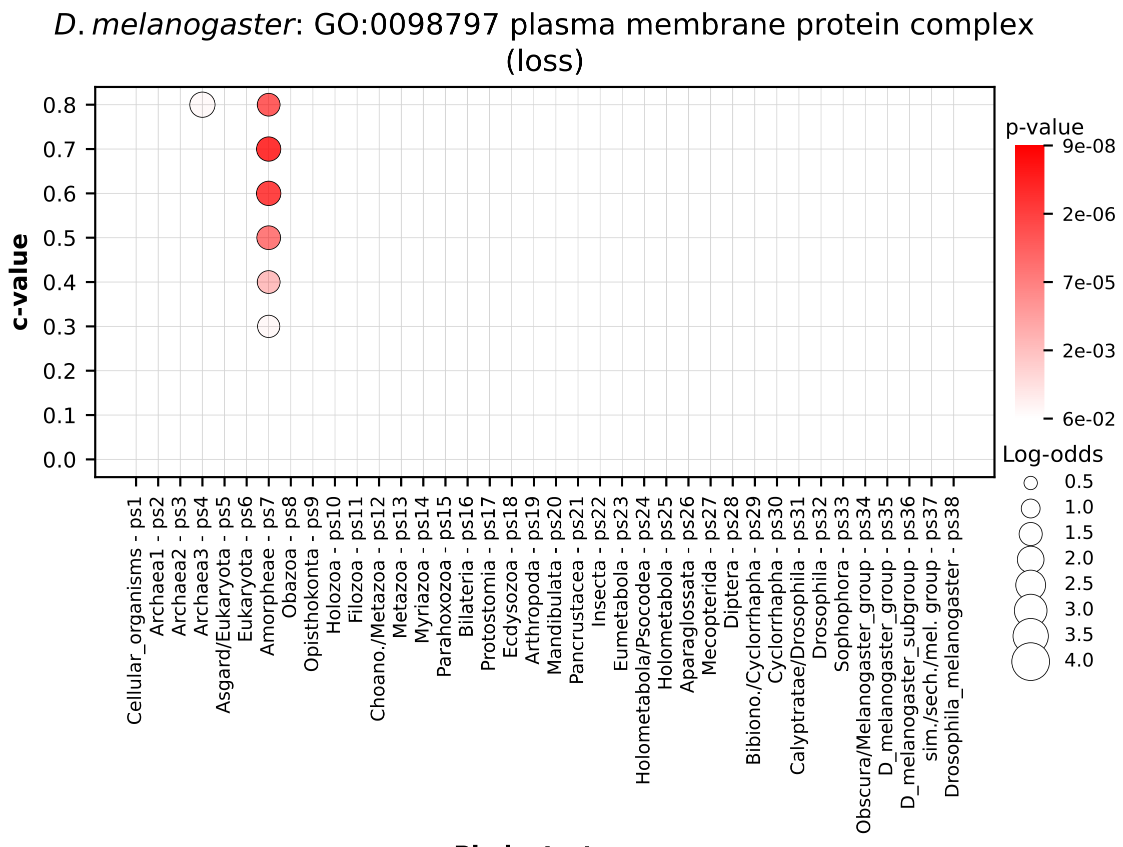

D. melanogaster: GO:0098797 plasma membrane protein complex (loss)
c-value
0.0
0.1
0.2
0.3
0.4
0.5
0.6
0.7
0.8
Phylostratum
Cellular_organisms - ps1
Archaea1 - ps2
Archaea2 - ps3
Archaea3 - ps4
Asgard/Eukaryota - ps5
Eukaryota - ps6
Amorpheae - ps7
Obazoa - ps8
Opisthokonta - ps9
Holozoa - ps10
Filozoa - ps11
Choano./Metazoa - ps12
Metazoa - ps13
Myriazoa - ps14
Parahoxozoa - ps15
Bilateria - ps16
Protostomia - ps17
Ecdysozoa - ps18
Arthropoda - ps19
Mandibulata - ps20
Pancrustacea - ps21
Insecta - ps22
Eumetabola - ps23
Holometabola/Psocodea - ps24
Holometabola - ps25
Aparaglossata - ps26
Mecopterida - ps27
Diptera - ps28
Bibiono./Cyclorrhapha - ps29
Cyclorrhapha - ps30
Calyptratae/Drosophila - ps31
Drosophila - ps32
Sophophora - ps33
Obscura/Melanogaster_group - ps34
D_melanogaster_group - ps35
D_melanogaster_subgroup - ps36
sim./sech./mel. group - ps37
Drosophila_melanogaster - ps38
p-value
9e-08
2e-06
7e-05
2e-03
6e-02
Log-odds
0.5
1.0
1.5
2.0
2.5
3.0
3.5
4.0

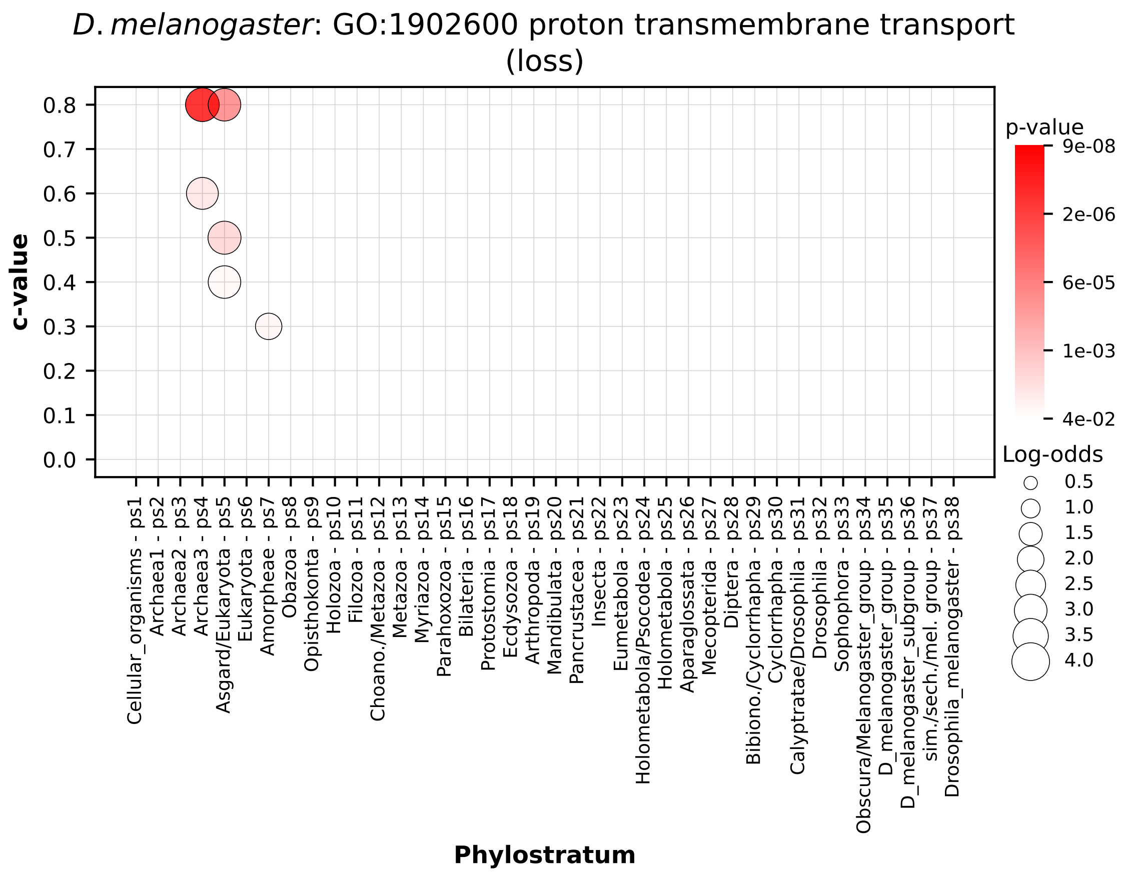
D. melanogaster: GO:1902600 proton transmembrane transport (loss)
c-value
Phylostratum
p-value
9e-08
2e-06
6e-05
1e-03
4e-02
Log-odds
0.5
1.0
1.5
2.0
2.5
3.0
3.5
4.0
Cellular_organisms - ps1
Archaea1 - ps2
Archaea2 - ps3
Archaea3 - ps4
Asgard/Eukaryota - ps5
Eukaryota - ps6
Amorpheae - ps7
Obazoa - ps8
Opisthokonta - ps9
Holozoa - ps10
Filozoa - ps11
Choano./Metazoa - ps12
Metazoa - ps13
Myriazoa - ps14
Parahoxozoa - ps15
Bilateria - ps16
Protostomia - ps17
Ecdysozoa - ps18
Arthropoda - ps19
Mandibulata - ps20
Pancrustacea - ps21
Insecta - ps22
Eumetabola - ps23
Holometabola/Psocodea - ps24
Holometabola - ps25
Aparaglossata - ps26
Mecopterida - ps27
Diptera - ps28
Bibiono./Cyclorrhapha - ps29
Cyclorrhapha - ps30
Calyptratae/Drosophila - ps31
Drosophila - ps32
Sophophora - ps33
Obscura/Melanogaster_group - ps34
D_melanogaster_group - ps35
D_melanogaster_subgroup - ps36
sim./sech./mel. group - ps37
Drosophila_melanogaster - ps38

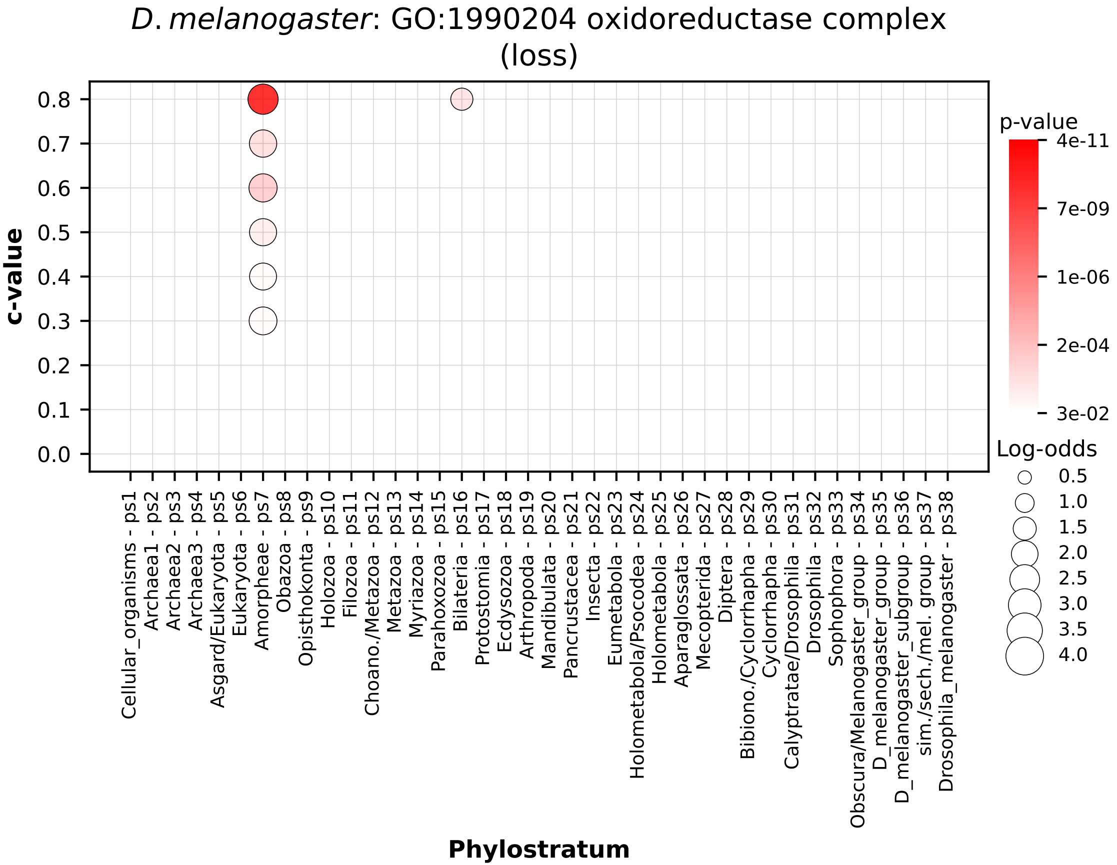

D. melanogaster: GO:1990204 oxidoreductase complex
(loss)
c-value
0.8
0.7
0.6
0.5
0.4
0.3
0.2
0.1
0.0
p-value
4e-11
7e-09
1e-06
2e-04
3e-02
Log-odds
0.5
1.0
1.5
2.0
2.5
3.0
3.5
4.0
Cellular_organisms - ps1
Archaea1 - ps2
Archaea2 - ps3
Archaea3 - ps4
Asgard/Eukaryota - ps5
Eukaryota - ps6
Amorpheae - ps7
Obazoa - ps8
Opisthokonta - ps9
Holozoa - ps10
Filozoa - ps11
Choano./Metazoa - ps12
Metazoa - ps13
Myriazoa - ps14
Parahoxozoa - ps15
Bilateria - ps16
Protostomia - ps17
Ecdysozoa - ps18
Arthropoda - ps19
Mandibulata - ps20
Pancrustacea - ps21
Insecta - ps22
Eumetabola - ps23
Holometabola/Psocodea - ps24
Holometabola - ps25
Aparaglossata - ps26
Mecopterida - ps27
Diptera - ps28
Bibiono./Cyclorrhapha - ps29
Cyclorrhapha - ps30
Calyptratae/Drosophila - ps31
Drosophila - ps32
Sophophora - ps33
Obscura/Melanogaster_group - ps34
D_melanogaster_group - ps35
D_melanogaster_subgroup - ps36
sim./sech./mel. group - ps37
Drosophila_melanogaster - ps38
Phylostratum

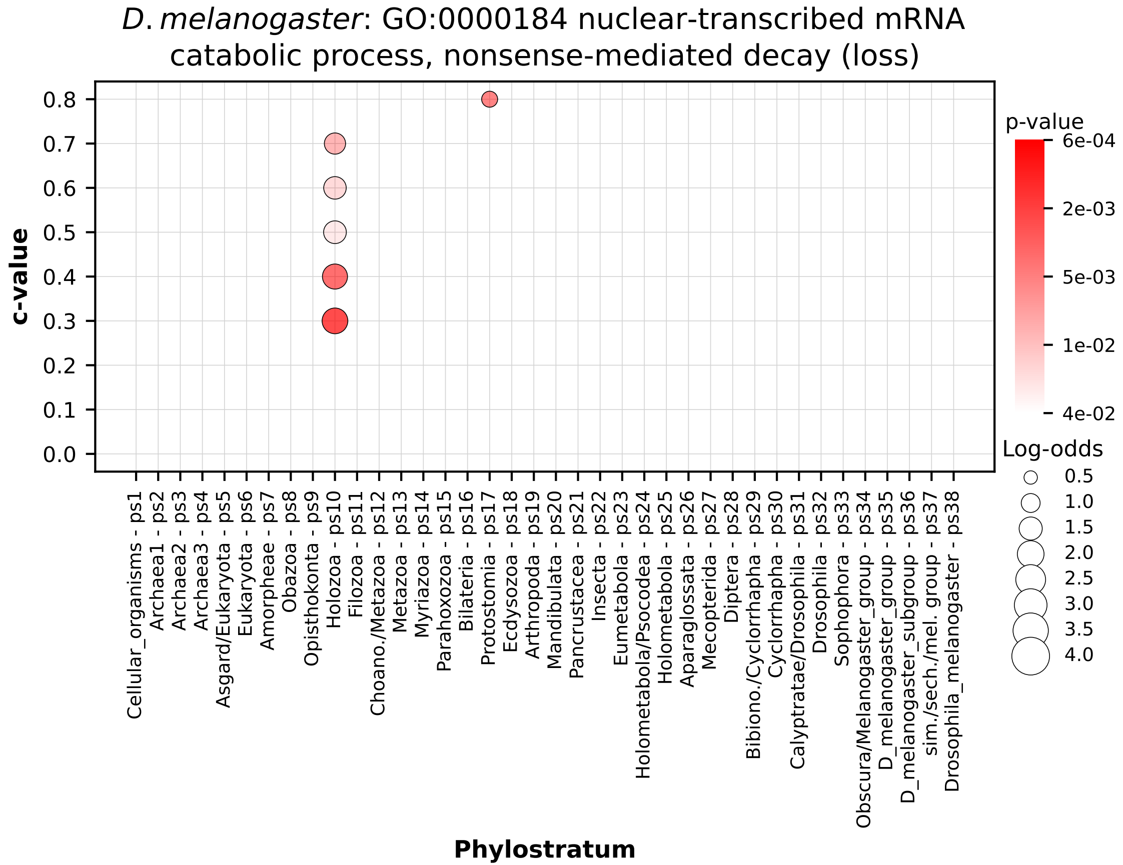

D. melanogaster: GO:0000184 nuclear-transcribed mRNA catabolic process, nonsense-mediated decay (loss)
c-value
0.8
0.7
0.6
0.5
0.4
0.3
0.2
0.1
0.0
Phylostratum
Cellular_organisms - ps1
Archaea1 - ps2
Archaea2 - ps3
Archaea3 - ps4
Asgard/Eukaryota - ps5
Eukaryota - ps6
Amorpheae - ps7
Obazoa - ps8
Opisthokonta - ps9
Holozoa - ps10
Filozoa - ps11
Choano./Metazoa - ps12
Metazoa - ps13
Myriazoa - ps14
Parahoxozoa - ps15
Bilateria - ps16
Protostomia - ps17
Ecdysozoa - ps18
Arthropoda - ps19
Mandibulata - ps20
Pancrustacea - ps21
Insecta - ps22
Eumetabola - ps23
Holometabola/Psocodea - ps24
Holometabola - ps25
Aparaglossata - ps26
Mecopterida - ps27
Diptera - ps28
Bibiono./Cyclorrhapha - ps29
Cyclorrhapha - ps30
Calyptratae/Drosophila - ps31
Drosophila - ps32
Sophophora - ps33
Obscura/Melanogaster_group - ps34
D_melanogaster_group - ps35
D_melanogaster_subgroup - ps36
sim./sech./mel. group - ps37
Drosophila_melanogaster - ps38
p-value
6e-04
2e-03
5e-03
1e-02
4e-02
Log-odds
0.5
1.0
1.5
2.0
2.5
3.0
3.5
4.0

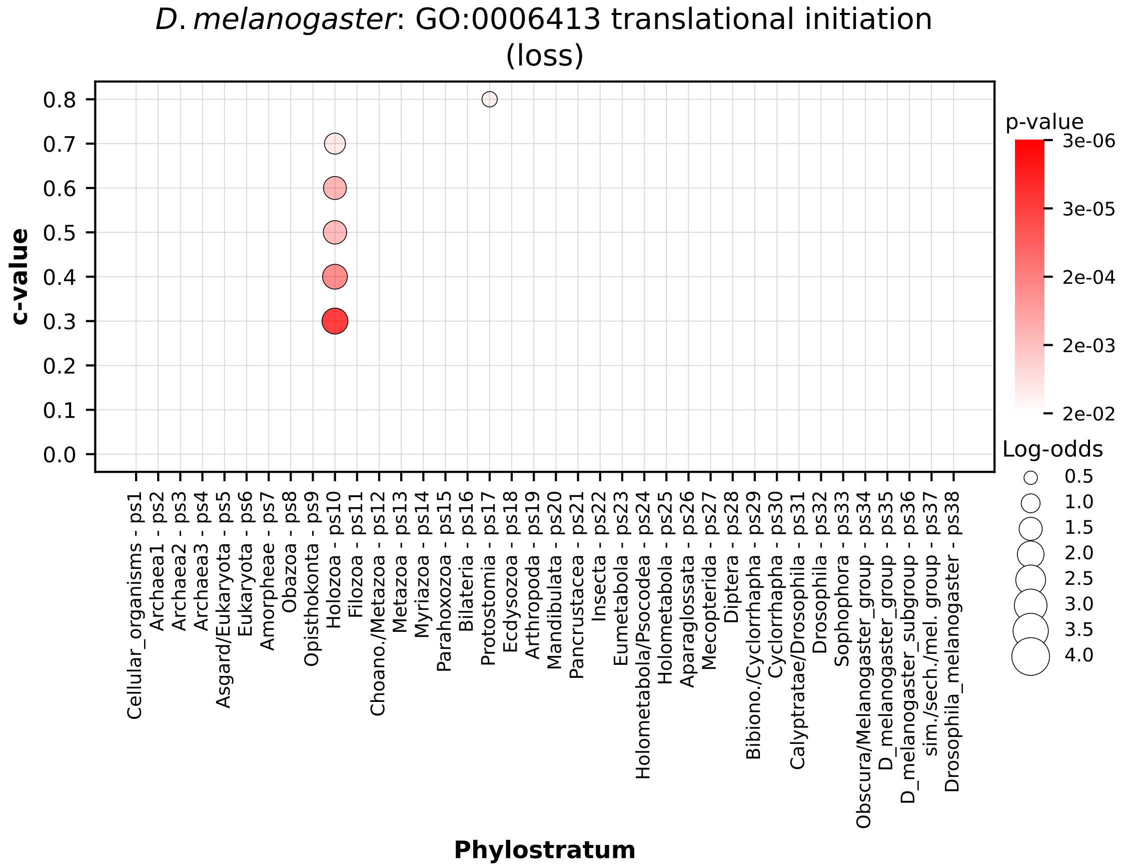

D. melanogaster: GO:0006413 translational initiation
(loss)
c-value
0.8
0.7
0.6
0.5
0.4
0.3
0.2
0.1
0.0
p-value
3e-06
3e-05
2e-04
2e-03
2e-02
Log-odds
0.5
1.0
1.5
2.0
2.5
3.0
3.5
4.0
Cellular_organisms - ps1
Archaea1 - ps2
Archaea2 - ps3
Archaea3 - ps4
Asgard/Eukaryota - ps5
Eukaryota - ps6
Amorpheae - ps7
Obazoa - ps8
Opisthokonta - ps9
Holozoa - ps10
Filozoa - ps11
Choano./Metazoa - ps12
Metazoa - ps13
Myriazoa - ps14
Parahoxozoa - ps15
Bilateria - ps16
Protostomia - ps17
Ecdysozoa - ps18
Arthropoda - ps19
Mandibulata - ps20
Pancrustacea - ps21
Insecta - ps22
Eumetabola - ps23
Holometabola/Psocodea - ps24
Holometabola - ps25
Aparaglossata - ps26
Mecopterida - ps27
Diptera - ps28
Bibiono./Cyclorrhapha - ps29
Cyclorrhapha - ps30
Calyptratae/Drosophila - ps31
Drosophila - ps32
Sophophora - ps33
Obscura/Melanogaster_group - ps34
D_melanogaster_group - ps35
D_melanogaster_subgroup - ps36
sim./sech./mel. group - ps37
Drosophila_melanogaster - ps38
Phylostratum

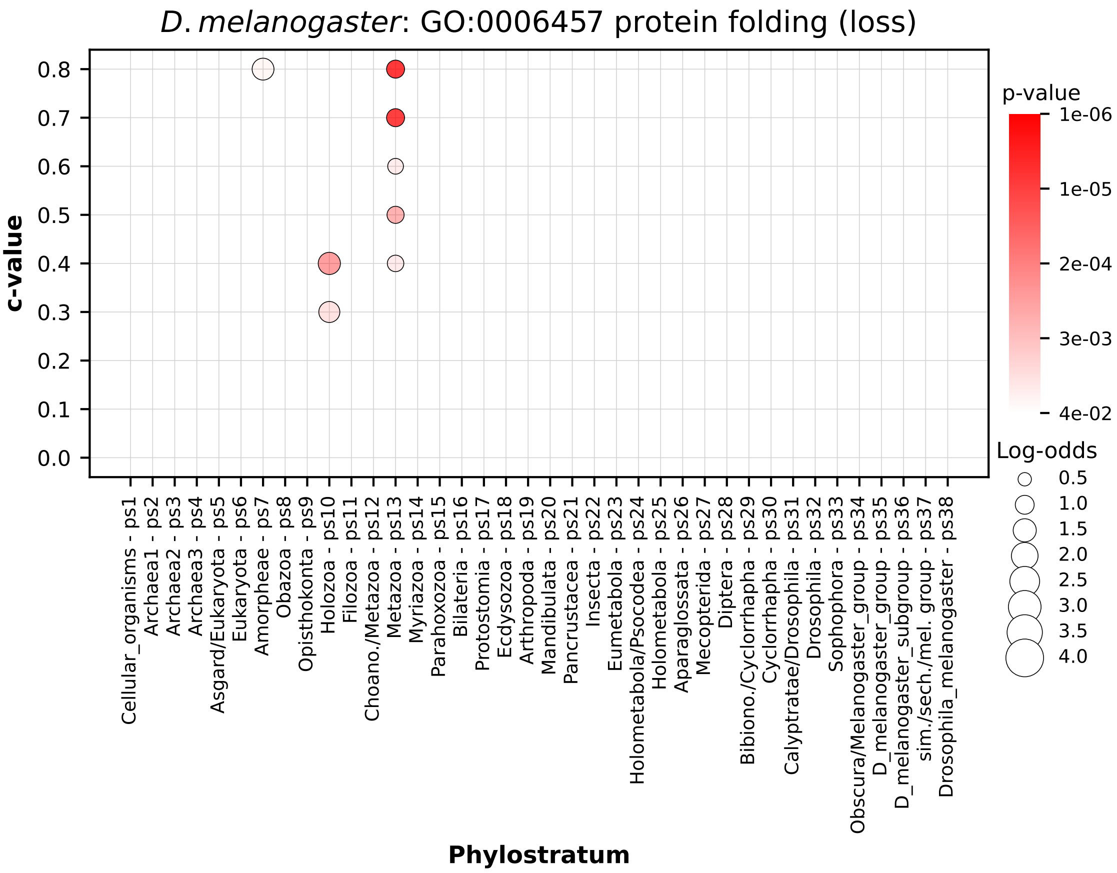

D. melanogaster: GO:0006457 protein folding (loss)
c-value
0.8
0.7
0.6
0.5
0.4
0.3
0.2
0.1
0.0
Phylostratum
Cellular_organisms - ps1
Archaea1 - ps2
Archaea2 - ps3
Archaea3 - ps4
Asgard/Eukaryota - ps5
Eukaryota - ps6
Amorpheae - ps7
Obazoa - ps8
Opisthokonta - ps9
Holozoa - ps10
Filozoa - ps11
Choano./Metazoa - ps12
Metazoa - ps13
Myriazoa - ps14
Parahoxozoa - ps15
Bilateria - ps16
Protostomia - ps17
Ecdysozoa - ps18
Arthropoda - ps19
Mandibulata - ps20
Pancrustacea - ps21
Insecta - ps22
Eumetabola - ps23
Holometabola/Psocodea - ps24
Holometabola - ps25
Aparaglossata - ps26
Mecopterida - ps27
Diptera - ps28
Bibiono./Cyclorrhapha - ps29
Cyclorrhapha - ps30
Calyptratae/Drosophila - ps31
Drosophila - ps32
Sophophora - ps33
Obscura/Melanogaster_group - ps34
D_melanogaster_group - ps35
D_melanogaster_subgroup - ps36
sim./sech./mel. group - ps37
Drosophila_melanogaster - ps38
p-value
1e-06
1e-05
2e-04
3e-03
4e-02
Log-odds
0.5
1.0
1.5
2.0
2.5
3.0
3.5
4.0

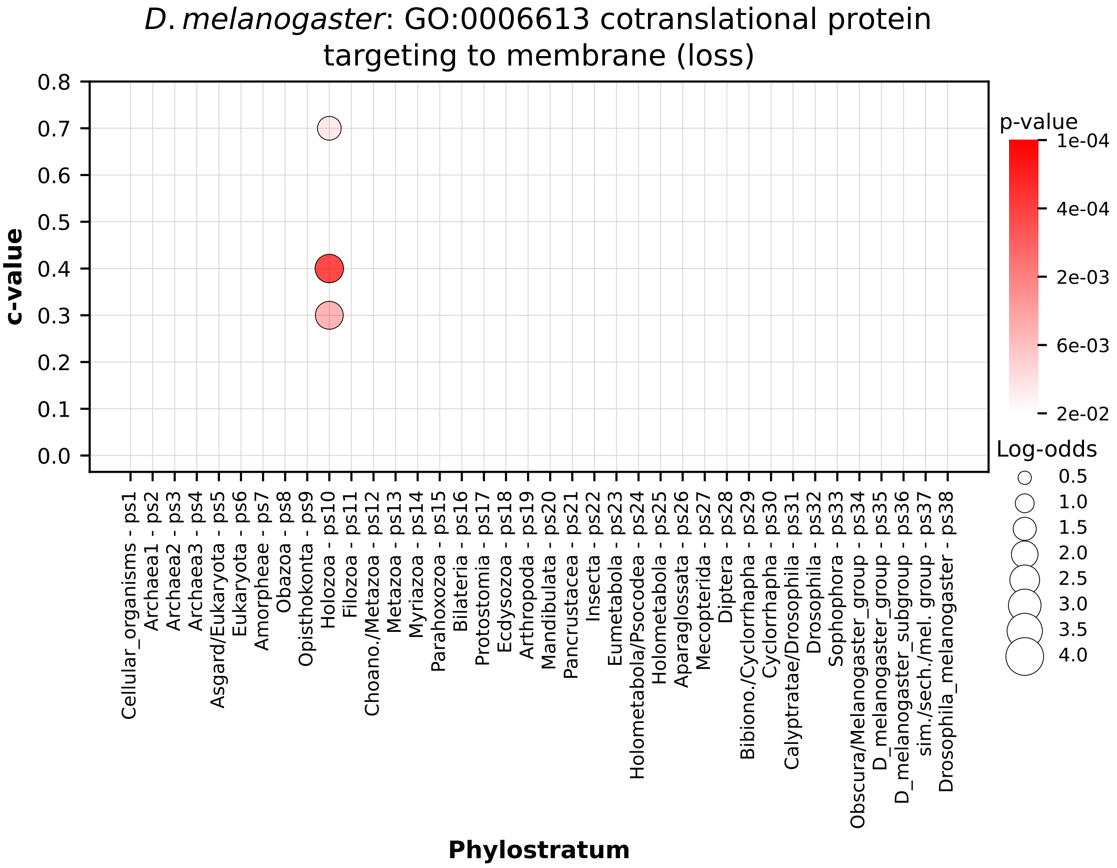

D. melanogaster: GO:0006613 cotranslational protein targeting to membrane (loss)
c-value
0.8
0.7
0.6
0.5
0.4
0.3
0.2
0.1
0.0
Phylostratum
Cellular_organisms - ps1
Archaea1 - ps2
Archaea2 - ps3
Archaea3 - ps4
Asgard/Eukaryota - ps5
Eukaryota - ps6
Amorpheae - ps7
Obazoa - ps8
Opisthokonta - ps9
Holozoa - ps10
Filozoa - ps11
Choano./Metazoa - ps12
Metazoa - ps13
Myriazoa - ps14
Parahoxozoa - ps15
Bilateria - ps16
Protostomia - ps17
Ecdysozoa - ps18
Arthropoda - ps19
Mandibulata - ps20
Pancrustacea - ps21
Insecta - ps22
Eumetabola - ps23
Holometabola/Psocodea - ps24
Holometabola - ps25
Aparaglossata - ps26
Mecopterida - ps27
Diptera - ps28
Bibiono./Cyclorrhapha - ps29
Cyclorrhapha - ps30
Calyptratae/Drosophila - ps31
Drosophila - ps32
Sophophora - ps33
Obscura/Melanogaster_group - ps34
D_melanogaster_group - ps35
D_melanogaster_subgroup - ps36
sim./sech./mel. group - ps37
Drosophila_melanogaster - ps38
p-value
1e-04
4e-04
2e-03
6e-03
2e-02
Log-odds
0.5
1.0
1.5
2.0
2.5
3.0
3.5
4.0

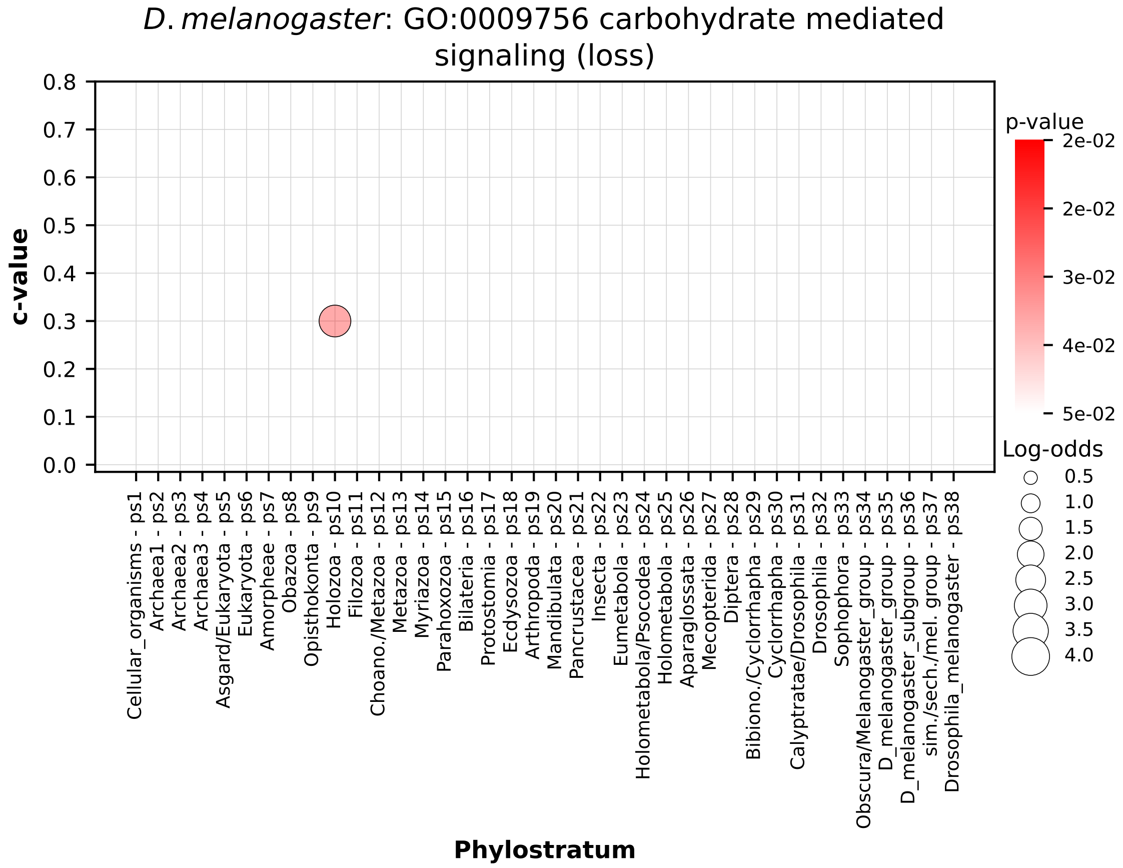

D. melanogaster: GO:0009756 carbohydrate mediated signaling (loss)
c-value
0.0
0.1
0.2
0.3
0.4
0.5
0.6
0.7
0.8
Phylostratum
Cellular_organisms - ps1
Archaea1 - ps2
Archaea2 - ps3
Archaea3 - ps4
Asgard/Eukaryota - ps5
Eukaryota - ps6
Amorpheae - ps7
Obazoa - ps8
Opisthokonta - ps9
Holozoa - ps10
Filozoa - ps11
Choano./Metazoa - ps12
Metazoa - ps13
Myriazoa - ps14
Parahoxozoa - ps15
Bilateria - ps16
Protostomia - ps17
Ecdysozoa - ps18
Arthropoda - ps19
Mandibulata - ps20
Pancrustacea - ps21
Insecta - ps22
Eumetabola - ps23
Holometabola/Psocodea - ps24
Holometabola - ps25
Aparaglossata - ps26
Mecopterida - ps27
Diptera - ps28
Bibiono./Cyclorrhapha - ps29
Cyclorrhapha - ps30
Calyptratae/Drosophila - ps31
Drosophila - ps32
Sophophora - ps33
Obscura/Melanogaster_group - ps34
D_melanogaster_group - ps35
D_melanogaster_subgroup - ps36
sim./sech./mel. group - ps37
Drosophila_melanogaster - ps38
p-value
2e-02
2e-02
3e-02
4e-02
5e-02
Log-odds
0.5
1.0
1.5
2.0
2.5
3.0
3.5
4.0

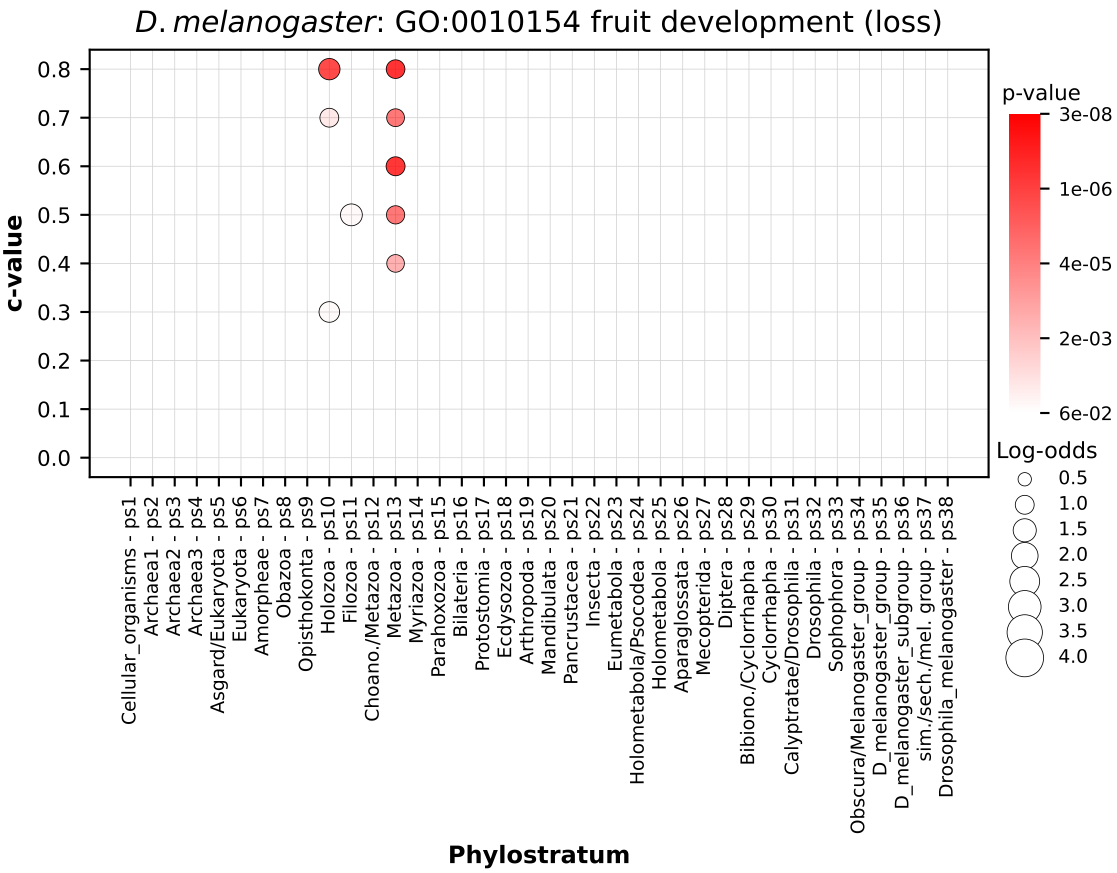

D. melanogaster: GO:0010154 fruit development (loss)
c-value
0.8
0.7
0.6
0.5
0.4
0.3
0.2
0.1
0.0
p-value
3e-08
1e-06
4e-05
2e-03
6e-02
Log-odds
0.5
1.0
1.5
2.0
2.5
3.0
3.5
4.0
Cellular_organisms - ps1
Archaea1 - ps2
Archaea2 - ps3
Archaea3 - ps4
Asgard/Eukaryota - ps5
Eukaryota - ps6
Amorpheae - ps7
Obazoa - ps8
Opisthokonta - ps9
Holozoa - ps10
Filozoa - ps11
Choano./Metazoa - ps12
Metazoa - ps13
Myriazoa - ps14
Parahoxozoa - ps15
Bilateria - ps16
Protostomia - ps17
Ecdysozoa - ps18
Arthropoda - ps19
Mandibulata - ps20
Pancrustacea - ps21
Insecta - ps22
Eumetabola - ps23
Holometabola/Psocodea - ps24
Holometabola - ps25
Aparaglossata - ps26
Mecopterida - ps27
Diptera - ps28
Bibiono./Cyclorrhapha - ps29
Cyclorrhapha - ps30
Calyptratae/Drosophila - ps31
Drosophila - ps32
Sophophora - ps33
Obscura/Melanogaster_group - ps34
D_melanogaster_group - ps35
D_melanogaster_subgroup - ps36
sim./sech./mel. group - ps37
Drosophila_melanogaster - ps38
Phylostratum

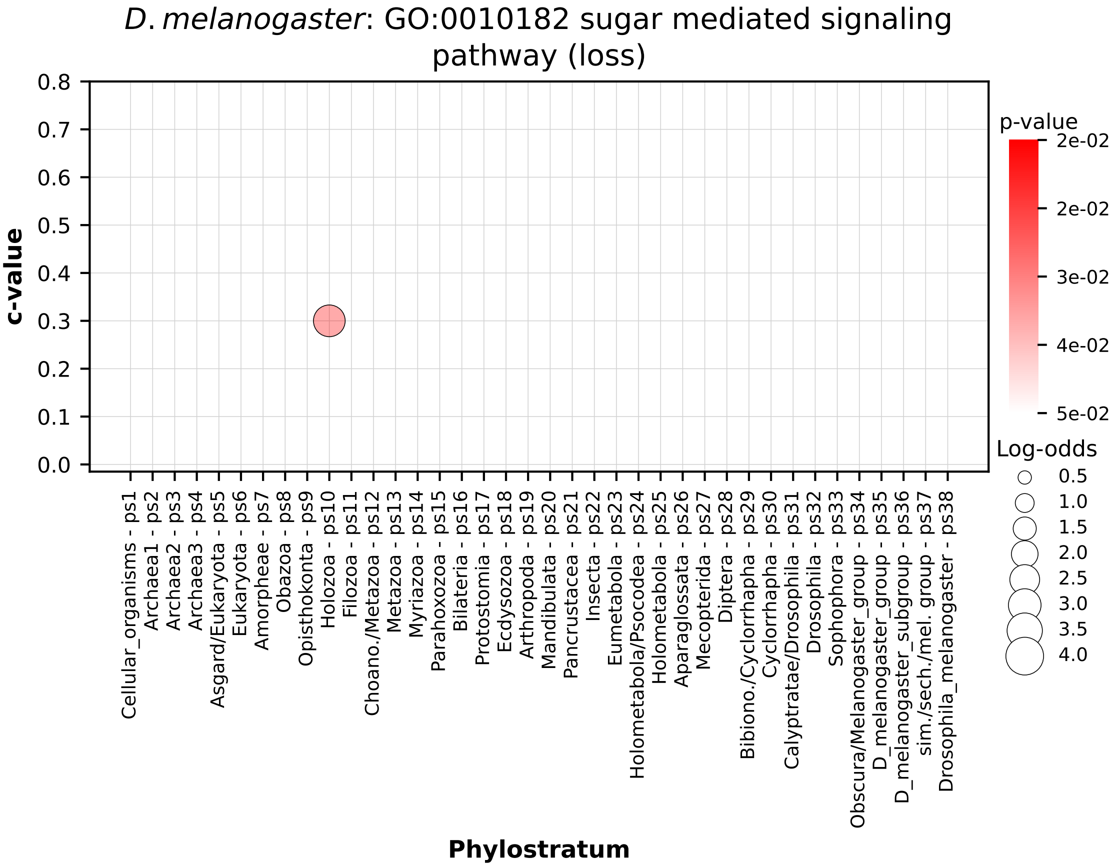
D. melanogaster: GO:0010182 sugar mediated signaling pathway (loss)
c-value
0.0
0.1
0.2
0.3
0.4
0.5
0.6
0.7
0.8
p-value
2e-02
2e-02
3e-02
4e-02
5e-02
Log-odds
0.5
1.0
1.5
2.0
2.5
3.0
3.5
4.0
Cellular_organisms - ps1
Archaea1 - ps2
Archaea2 - ps3
Archaea3 - ps4
Asgard/Eukaryota - ps5
Eukaryota - ps6
Amorpheae - ps7
Obazoa - ps8
Opisthokonta - ps9
Holozoa - ps10
Filozoa - ps11
Choano./Metazoa - ps12
Metazoa - ps13
Myriazoa - ps14
Parahoxozoa - ps15
Bilateria - ps16
Protostomia - ps17
Ecdysozoa - ps18
Arthropoda - ps19
Mandibulata - ps20
Pancrustacea - ps21
Insecta - ps22
Eumetabola - ps23
Holometabola/Psocodea - ps24
Holometabola - ps25
Aparaglossata - ps26
Mecopterida - ps27
Diptera - ps28
Bibiono./Cyclorrhapha - ps29
Cyclorrhapha - ps30
Calyptratae/Drosophila - ps31
Drosophila - ps32
Sophophora - ps33
Obscura/Melanogaster_group - ps34
D_melanogaster_group - ps35
D_melanogaster_subgroup - ps36
sim./sech./mel. group - ps37
Drosophila_melanogaster - ps38
Phylostratum

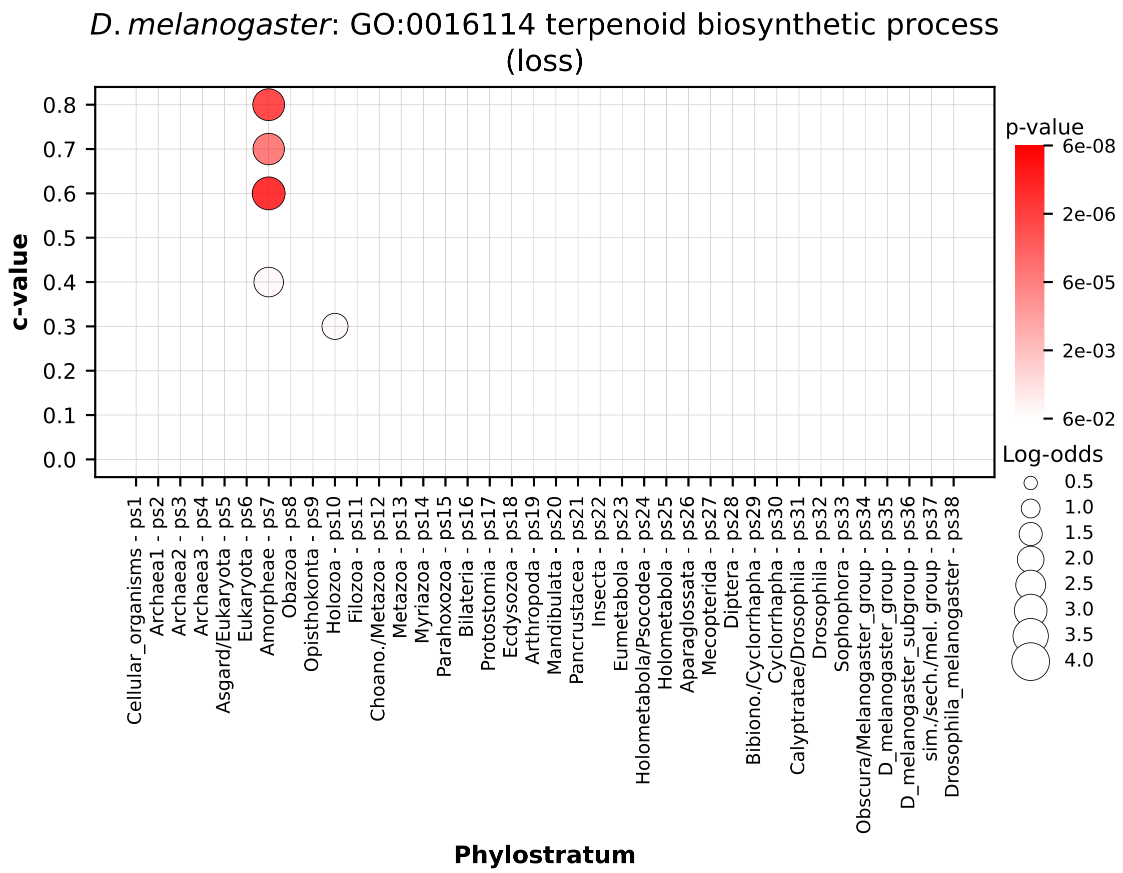

D. melanogaster: GO:0016114 terpenoid biosynthetic process (loss)
c-value
0.0
0.1
0.2
0.3
0.4
0.5
0.6
0.7
0.8
Phylostratum
Cellular_organisms - ps1
Archaea1 - ps2
Archaea2 - ps3
Archaea3 - ps4
Asgard/Eukaryota - ps5
Eukaryota - ps6
Amorpheae - ps7
Obazoa - ps8
Opisthokonta - ps9
Holozoa - ps10
Filozoa - ps11
Choano./Metazoa - ps12
Metazoa - ps13
Myriazoa - ps14
Parahoxozoa - ps15
Bilateria - ps16
Protostomia - ps17
Ecdysozoa - ps18
Arthropoda - ps19
Mandibulata - ps20
Pancrustacea - ps21
Insecta - ps22
Eumetabola - ps23
Holometabola/Psocodea - ps24
Holometabola - ps25
Aparaglossata - ps26
Mecopterida - ps27
Diptera - ps28
Bibiono./Cyclorrhapha - ps29
Cyclorrhapha - ps30
Calyptratae/Drosophila - ps31
Drosophila - ps32
Sophophora - ps33
Obscura/Melanogaster_group - ps34
D_melanogaster_group - ps35
D_melanogaster_subgroup - ps36
sim./sech./mel. group - ps37
Drosophila_melanogaster - ps38
p-value
6e-08
2e-06
6e-05
2e-03
6e-02
Log-odds
0.5
1.0
1.5
2.0
2.5
3.0
3.5
4.0

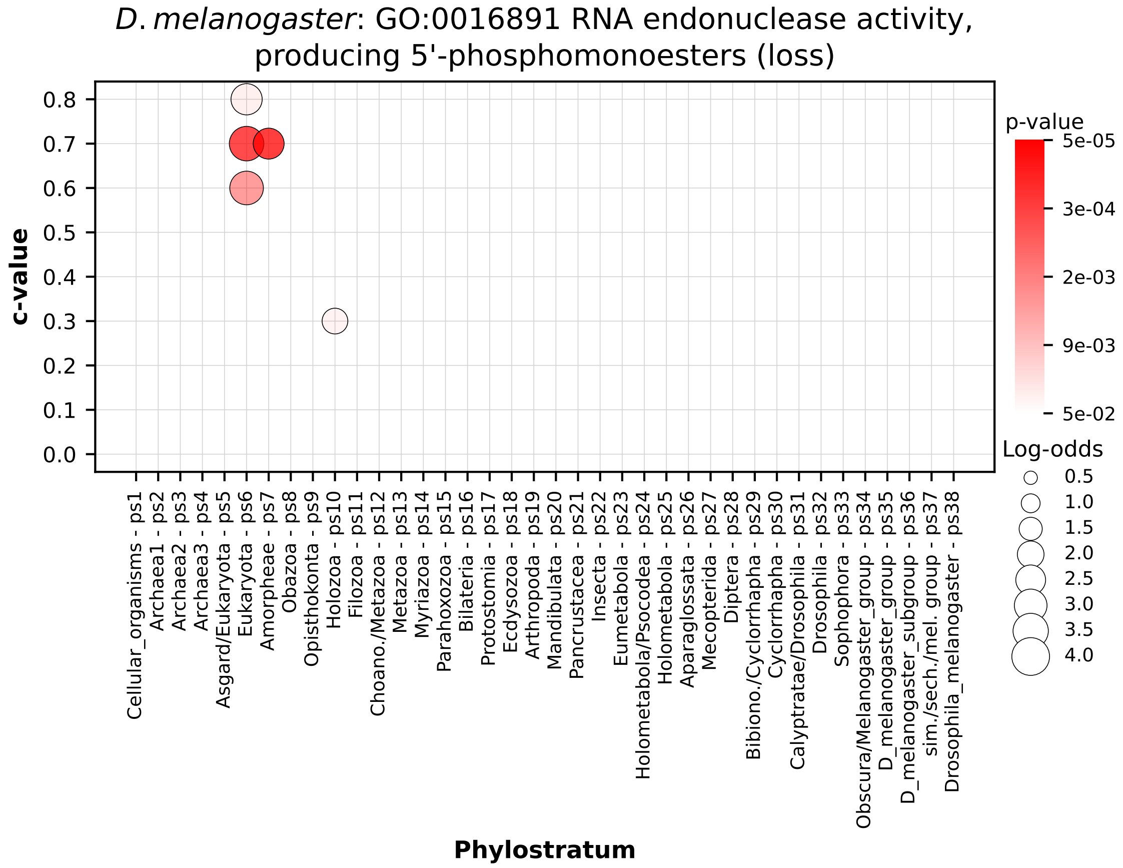

D. melanogaster: GO:0016891 RNA endonuclease activity, producing 5'-phosphomonoesters (loss)
c-value
0.8
0.7
0.6
0.5
0.4
0.3
0.2
0.1
0.0
p-value
5e-05
3e-04
2e-03
9e-03
5e-02
Log-odds
0.5
1.0
1.5
2.0
2.5
3.0
3.5
4.0
Cellular_organisms - ps1
Archaea1 - ps2
Archaea2 - ps3
Archaea3 - ps4
Asgard/Eukaryota - ps5
Eukaryota - ps6
Amorpheae - ps7
Obazoa - ps8
Opisthokonta - ps9
Holozoa - ps10
Filozoa - ps11
Choano./Metazoa - ps12
Metazoa - ps13
Myriazoa - ps14
Parahoxozoa - ps15
Bilateria - ps16
Protostomia - ps17
Ecdysozoa - ps18
Arthropoda - ps19
Mandibulata - ps20
Pancrustacea - ps21
Insecta - ps22
Eumetabola - ps23
Holometabola/Psocodea - ps24
Holometabola - ps25
Aparaglossata - ps26
Mecopterida - ps27
Diptera - ps28
Bibiono./Cyclorrhapha - ps29
Cyclorrhapha - ps30
Calyptratae/Drosophila - ps31
Drosophila - ps32
Sophophora - ps33
Obscura/Melanogaster_group - ps34
D_melanogaster_group - ps35
D_melanogaster_subgroup - ps36
sim./sech./mel. group - ps37
Drosophila_melanogaster - ps38
Phylostratum

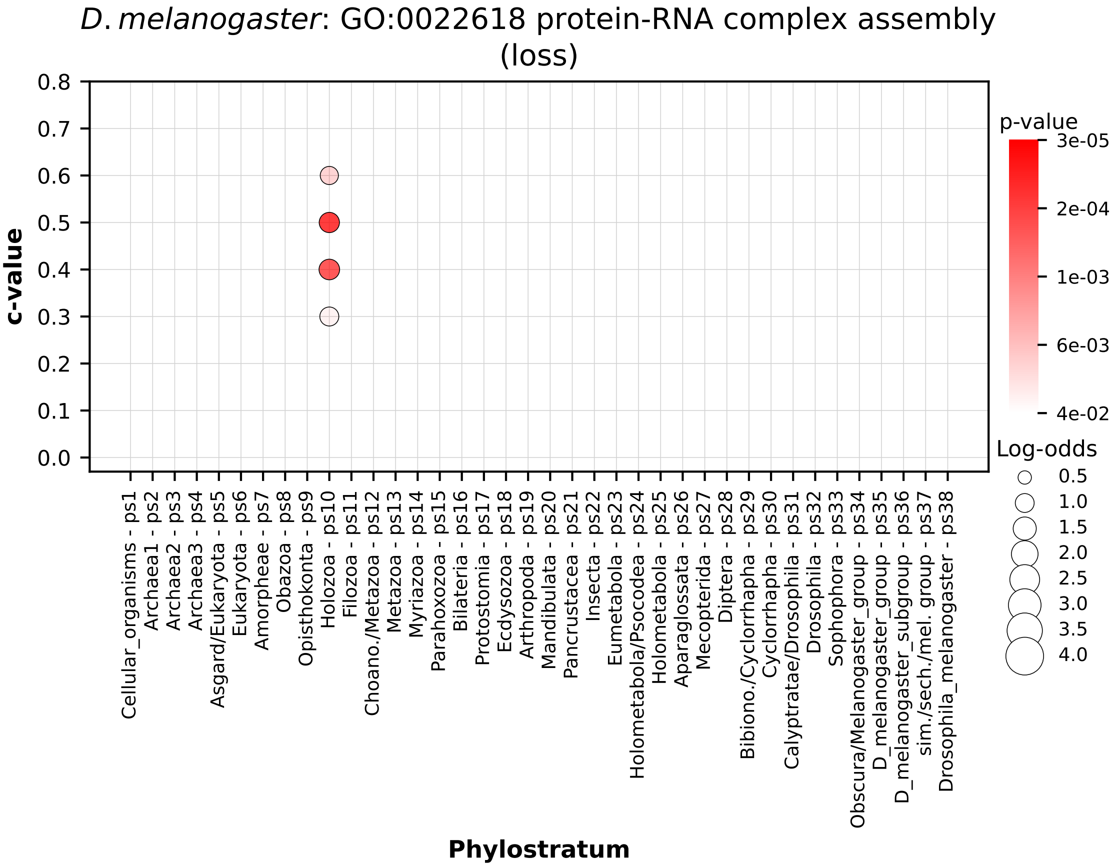

D. melanogaster: GO:0022618 protein-RNA complex assembly
(loss)
c-value
0.8
0.7
0.6
0.5
0.4
0.3
0.2
0.1
0.0
Phylostratum
Cellular_organisms - ps1
Archaea1 - ps2
Archaea2 - ps3
Archaea3 - ps4
Asgard/Eukaryota - ps5
Eukaryota - ps6
Amorpheae - ps7
Obazoa - ps8
Opisthokonta - ps9
Holozoa - ps10
Filozoa - ps11
Choano./Metazoa - ps12
Metazoa - ps13
Myriazoa - ps14
Parahoxozoa - ps15
Bilateria - ps16
Protostomia - ps17
Ecdysozoa - ps18
Arthropoda - ps19
Mandibulata - ps20
Pancrustacea - ps21
Insecta - ps22
Eumetabola - ps23
Holometabola/Psocodea - ps24
Holometabola - ps25
Aparaglossata - ps26
Mecopterida - ps27
Diptera - ps28
Bibiono./Cyclorrhapha - ps29
Cyclorrhapha - ps30
Calyptratae/Drosophila - ps31
Drosophila - ps32
Sophophora - ps33
Obscura/Melanogaster_group - ps34
D_melanogaster_group - ps35
D_melanogaster_subgroup - ps36
sim./sech./mel. group - ps37
Drosophila_melanogaster - ps38
p-value
3e-05
2e-04
1e-03
6e-03
4e-02
Log-odds
0.5
1.0
1.5
2.0
2.5
3.0
3.5
4.0

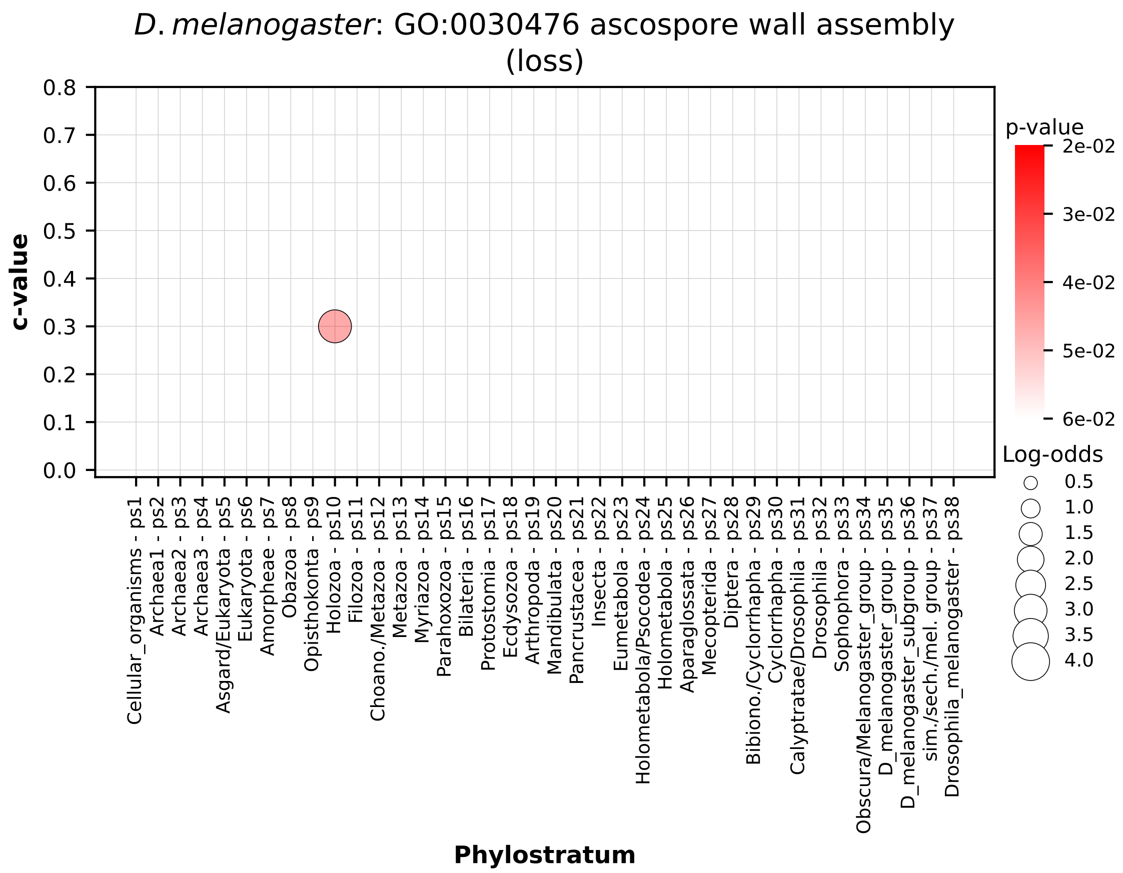
D. melanogaster: GO:0030476 ascospore wall assembly
(loss)
c-value
Phylostratum
p-value
2e-02
3e-02
4e-02
5e-02
6e-02
Log-odds
0.5
1.0
1.5
2.0
2.5
3.0
3.5
4.0
Cellular_organisms - ps1
Archaea1 - ps2
Archaea2 - ps3
Archaea3 - ps4
Asgard/Eukaryota - ps5
Eukaryota - ps6
Amorpheae - ps7
Obazoa - ps8
Opisthokonta - ps9
Holozoa - ps10
Filozoa - ps11
Choano./Metazoa - ps12
Metazoa - ps13
Myriazoa - ps14
Parahoxozoa - ps15
Bilateria - ps16
Protostomia - ps17
Ecdysozoa - ps18
Arthropoda - ps19
Mandibulata - ps20
Pancrustacea - ps21
Insecta - ps22
Eumetabola - ps23
Holometabola/Psocodea - ps24
Holometabola - ps25
Aparaglossata - ps26
Mecopterida - ps27
Diptera - ps28
Bibiono./Cyclorrhapha - ps29
Cyclorrhapha - ps30
Calyptratae/Drosophila - ps31
Drosophila - ps32
Sophophora - ps33
Obscura/Melanogaster_group - ps34
D_melanogaster_group - ps35
D_melanogaster_subgroup - ps36
sim./sech./mel. group - ps37
Drosophila_melanogaster - ps38

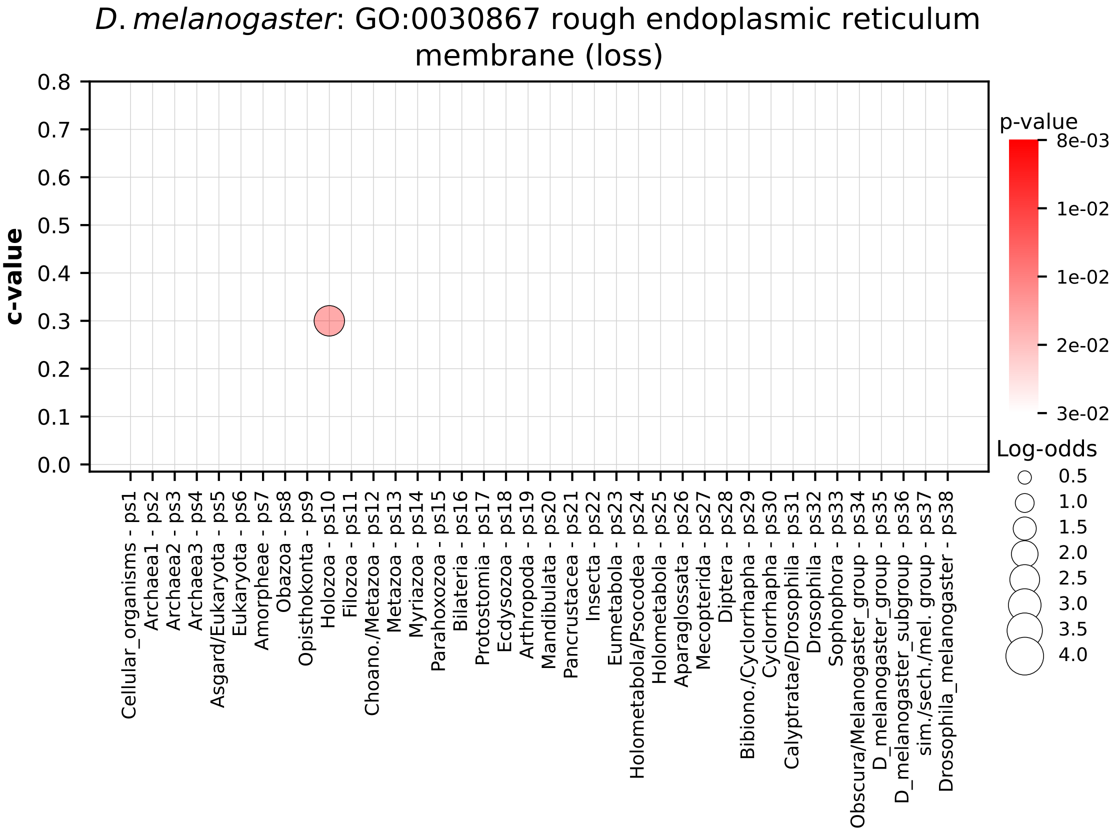
D. melanogaster: GO:0030867 rough endoplasmic reticulum membrane (loss)
c-value
0.8
0.7
0.6
0.5
0.4
0.3
0.2
0.1
0.0
Phylostratum
Cellular_organisms - ps1
Archaea1 - ps2
Archaea2 - ps3
Archaea3 - ps4
Asgard/Eukaryota - ps5
Eukaryota - ps6
Amorpheae - ps7
Obazoa - ps8
Opisthokonta - ps9
Holozoa - ps10
Filozoa - ps11
Choano./Metazoa - ps12
Metazoa - ps13
Myriazoa - ps14
Parahoxozoa - ps15
Bilateria - ps16
Protostomia - ps17
Ecdysozoa - ps18
Arthropoda - ps19
Mandibulata - ps20
Pancrustacea - ps21
Insecta - ps22
Eumetabola - ps23
Holometabola/Psocodea - ps24
Holometabola - ps25
Aparaglossata - ps26
Mecopterida - ps27
Diptera - ps28
Bibiono./Cyclorrhapha - ps29
Cyclorrhapha - ps30
Calyptratae/Drosophila - ps31
Drosophila - ps32
Sophophora - ps33
Obscura/Melanogaster_group - ps34
D_melanogaster_group - ps35
D_melanogaster_subgroup - ps36
sim./sech./mel. group - ps37
Drosophila_melanogaster - ps38
p-value
8e-03
1e-02
1e-02
2e-02
3e-02
Log-odds
0.5
1.0
1.5
2.0
2.5
3.0
3.5
4.0

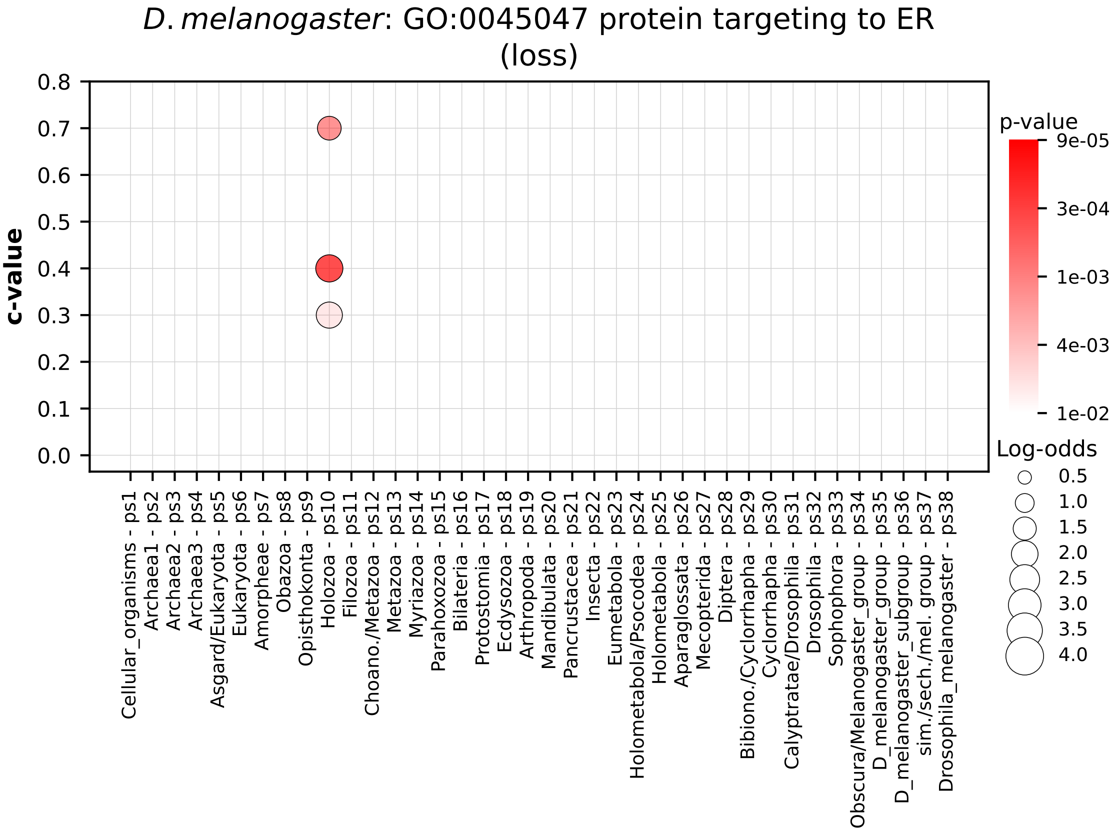
D. melanogaster: GO:0045047 protein targeting to ER
(loss)
c-value
0.8
0.7
0.6
0.5
0.4
0.3
0.2
0.1
0.0
p-value
9e-05
3e-04
1e-03
4e-03
1e-02
Log-odds
0.5
1.0
1.5
2.0
2.5
3.0
3.5
4.0
Cellular_organisms - ps1
Archaea1 - ps2
Archaea2 - ps3
Archaea3 - ps4
Asgard/Eukaryota - ps5
Eukaryota - ps6
Amorpheae - ps7
Obazoa - ps8
Opisthokonta - ps9
Holozoa - ps10
Filozoa - ps11
Choano./Metazoa - ps12
Metazoa - ps13
Myriazoa - ps14
Parahoxozoa - ps15
Bilateria - ps16
Protostomia - ps17
Ecdysozoa - ps18
Arthropoda - ps19
Mandibulata - ps20
Pancrustacea - ps21
Insecta - ps22
Eumetabola - ps23
Holometabola/Psocodea - ps24
Holometabola - ps25
Aparaglossata - ps26
Mecopterida - ps27
Diptera - ps28
Bibiono./Cyclorrhapha - ps29
Cyclorrhapha - ps30
Calyptratae/Drosophila - ps31
Drosophila - ps32
Sophophora - ps33
Obscura/Melanogaster_group - ps34
D_melanogaster_group - ps35
D_melanogaster_subgroup - ps36
sim./sech./mel. group - ps37
Drosophila_melanogaster - ps38
Phylostratum

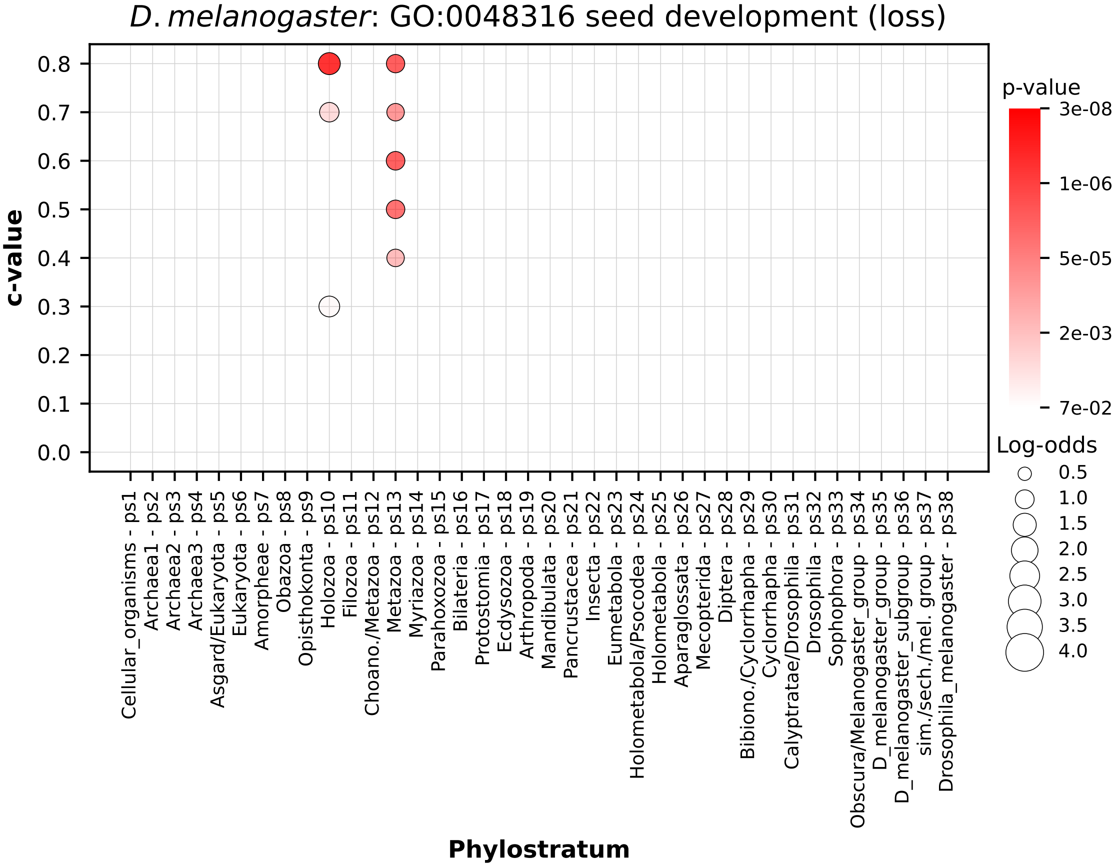
D. melanogaster: GO:0048316 seed development (loss)
c-value
0.0
0.1
0.2
0.3
0.4
0.5
0.6
0.7
0.8
p-value
3e-08
1e-06
5e-05
2e-03
7e-02
Log-odds
0.5
1.0
1.5
2.0
2.5
3.0
3.5
4.0
Cellular_organisms - ps1
Archaea1 - ps2
Archaea2 - ps3
Archaea3 - ps4
Asgard/Eukaryota - ps5
Eukaryota - ps6
Amorpheae - ps7
Obazoa - ps8
Opisthokonta - ps9
Holozoa - ps10
Filozoa - ps11
Choano./Metazoa - ps12
Metazoa - ps13
Myriazoa - ps14
Parahoxozoa - ps15
Bilateria - ps16
Protostomia - ps17
Ecdysozoa - ps18
Arthropoda - ps19
Mandibulata - ps20
Pancrustacea - ps21
Insecta - ps22
Eumetabola - ps23
Holometabola/Psocodea - ps24
Holometabola - ps25
Aparaglossata - ps26
Mecopterida - ps27
Diptera - ps28
Bibiono./Cyclorrhapha - ps29
Cyclorrhapha - ps30
Calyptratae/Drosophila - ps31
Drosophila - ps32
Sophophora - ps33
Obscura/Melanogaster_group - ps34
D_melanogaster_group - ps35
D_melanogaster_subgroup - ps36
sim./sech./mel. group - ps37
Drosophila_melanogaster - ps38
Phylostratum

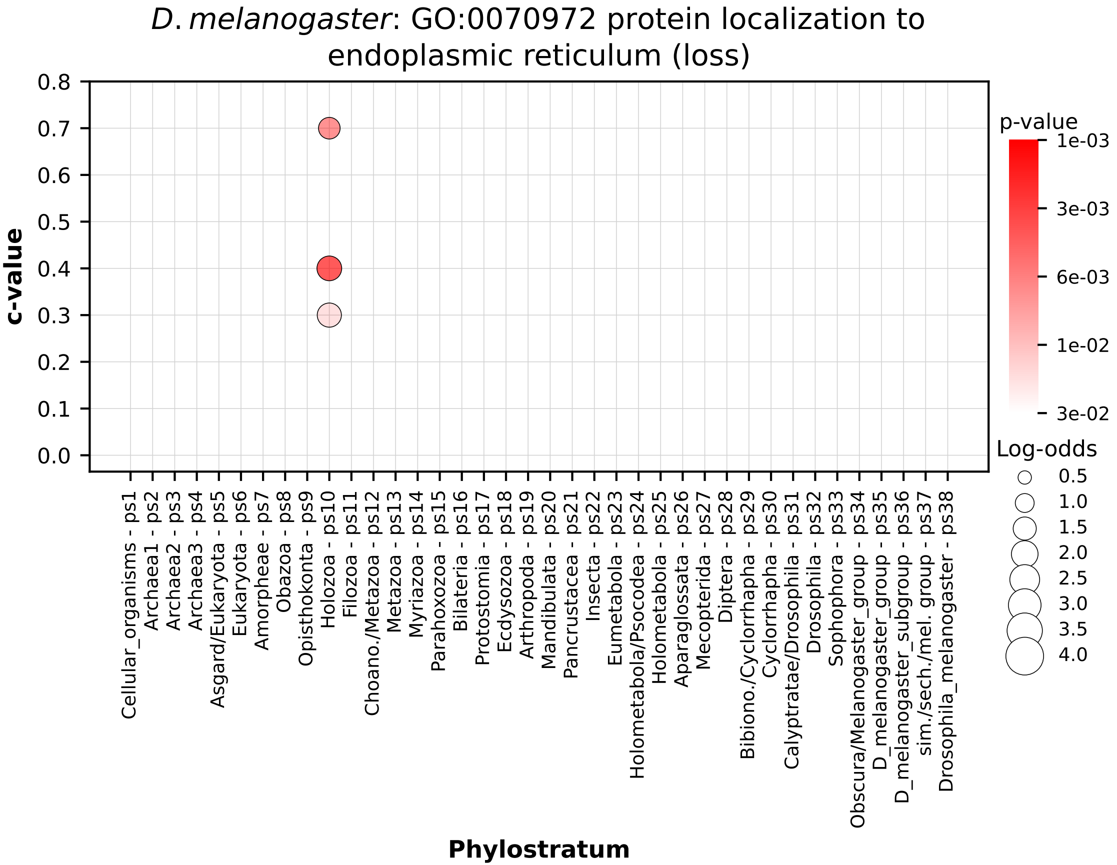

D. melanogaster: GO:0070972 protein localization to endoplasmic reticulum (loss)
c-value
0.8
0.7
0.6
0.5
0.4
0.3
0.2
0.1
0.0
Phylostratum
Cellular_organisms - ps1
Archaea1 - ps2
Archaea2 - ps3
Archaea3 - ps4
Asgard/Eukaryota - ps5
Eukaryota - ps6
Amorpheae - ps7
Obazoa - ps8
Opisthokonta - ps9
Holozoa - ps10
Filozoa - ps11
Choano./Metazoa - ps12
Metazoa - ps13
Myriazoa - ps14
Parahoxozoa - ps15
Bilateria - ps16
Protostomia - ps17
Ecdysozoa - ps18
Arthropoda - ps19
Mandibulata - ps20
Pancrustacea - ps21
Insecta - ps22
Eumetabola - ps23
Holometabola/Psocodea - ps24
Holometabola - ps25
Aparaglossata - ps26
Mecopterida - ps27
Diptera - ps28
Bibiono./Cyclorrhapha - ps29
Cyclorrhapha - ps30
Calyptratae/Drosophila - ps31
Drosophila - ps32
Sophophora - ps33
Obscura/Melanogaster_group - ps34
D_melanogaster_group - ps35
D_melanogaster_subgroup - ps36
sim./sech./mel. group - ps37
Drosophila_melanogaster - ps38
p-value
1e-03
3e-03
6e-03
1e-02
3e-02
Log-odds
0.5
1.0
1.5
2.0
2.5
3.0
3.5
4.0

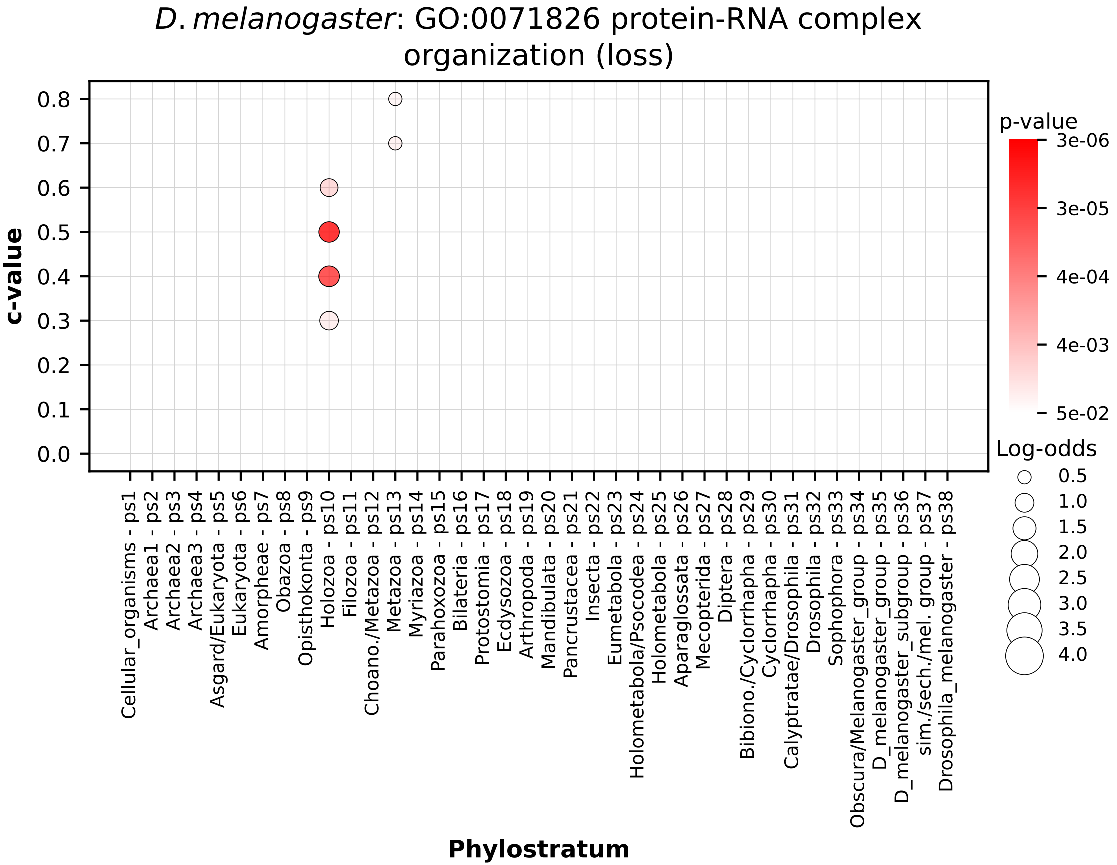

D. melanogaster: GO:0071826 protein-RNA complex organization (loss)
c-value
0.0
0.1
0.2
0.3
0.4
0.5
0.6
0.7
0.8
Phylostratum
Cellular_organisms - ps1
Archaea1 - ps2
Archaea2 - ps3
Archaea3 - ps4
Asgard/Eukaryota - ps5
Eukaryota - ps6
Amorpheae - ps7
Obazoa - ps8
Opisthokonta - ps9
Holozoa - ps10
Filozoa - ps11
Choano./Metazoa - ps12
Metazoa - ps13
Myriazoa - ps14
Parahoxozoa - ps15
Bilateria - ps16
Protostomia - ps17
Ecdysozoa - ps18
Arthropoda - ps19
Mandibulata - ps20
Pancrustacea - ps21
Insecta - ps22
Eumetabola - ps23
Holometabola/Psocodea - ps24
Holometabola - ps25
Aparaglossata - ps26
Mecopterida - ps27
Diptera - ps28
Bibiono./Cyclorrhapha - ps29
Cyclorrhapha - ps30
Calyptratae/Drosophila - ps31
Drosophila - ps32
Sophophora - ps33
Obscura/Melanogaster_group - ps34
D_melanogaster_group - ps35
D_melanogaster_subgroup - ps36
sim./sech./mel. group - ps37
Drosophila_melanogaster - ps38
p-value
3e-06
3e-05
4e-04
4e-03
5e-02
Log-odds
0.5
1.0
1.5
2.0
2.5
3.0
3.5
4.0

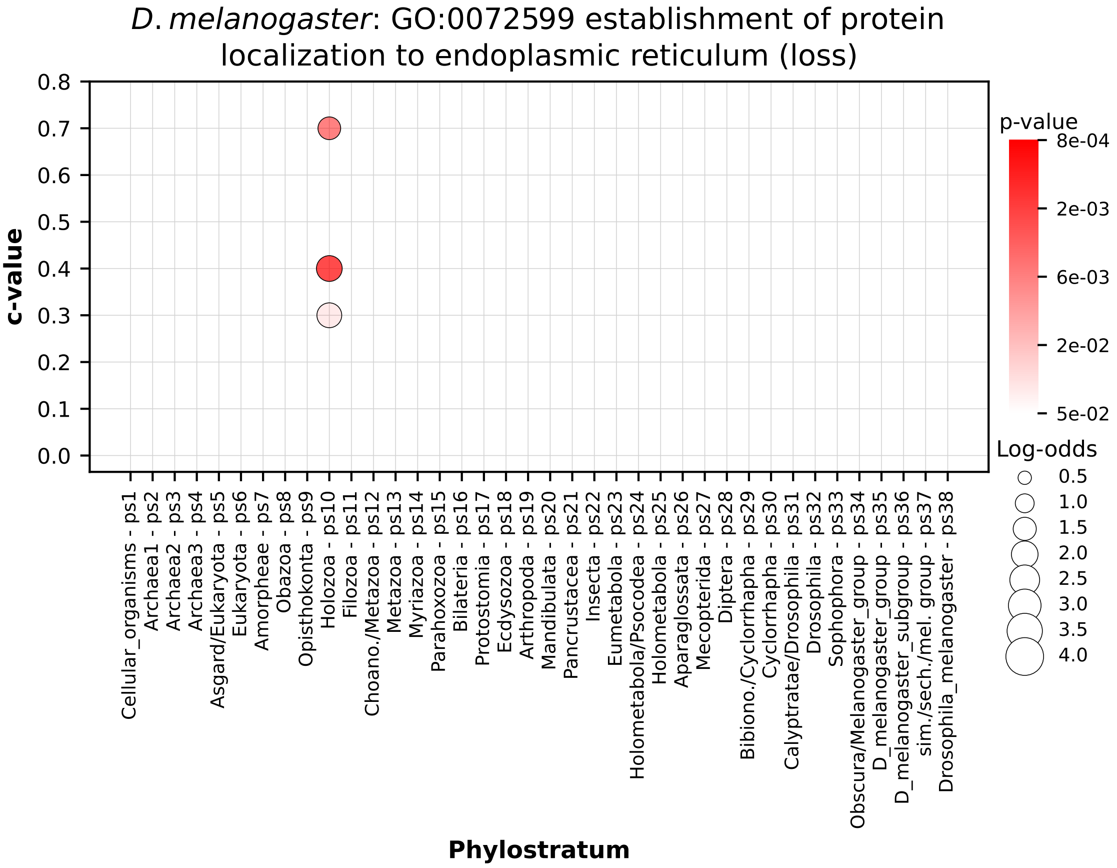

D. melanogaster: GO:0072599 establishment of protein localization to endoplasmic reticulum (loss)
c-value
0.8
0.7
0.6
0.5
0.4
0.3
0.2
0.1
0.0
Phylostratum
Cellular_organisms - ps1
Archaea1 - ps2
Archaea2 - ps3
Archaea3 - ps4
Asgard/Eukaryota - ps5
Eukaryota - ps6
Amorpheae - ps7
Obazoa - ps8
Opisthokonta - ps9
Holozoa - ps10
Filozoa - ps11
Choano./Metazoa - ps12
Metazoa - ps13
Myriazoa - ps14
Parahoxozoa - ps15
Bilateria - ps16
Protostomia - ps17
Ecdysozoa - ps18
Arthropoda - ps19
Mandibulata - ps20
Pancrustacea - ps21
Insecta - ps22
Eumetabola - ps23
Holometabola/Psocodea - ps24
Holometabola - ps25
Aparaglossata - ps26
Mecopterida - ps27
Diptera - ps28
Bibiono./Cyclorrhapha - ps29
Cyclorrhapha - ps30
Calyptratae/Drosophila - ps31
Drosophila - ps32
Sophophora - ps33
Obscura/Melanogaster_group - ps34
D_melanogaster_group - ps35
D_melanogaster_subgroup - ps36
sim./sech./mel. group - ps37
Drosophila_melanogaster - ps38
p-value
8e-04
2e-03
6e-03
2e-02
5e-02
Log-odds
0.5
1.0
1.5
2.0
2.5
3.0
3.5
4.0

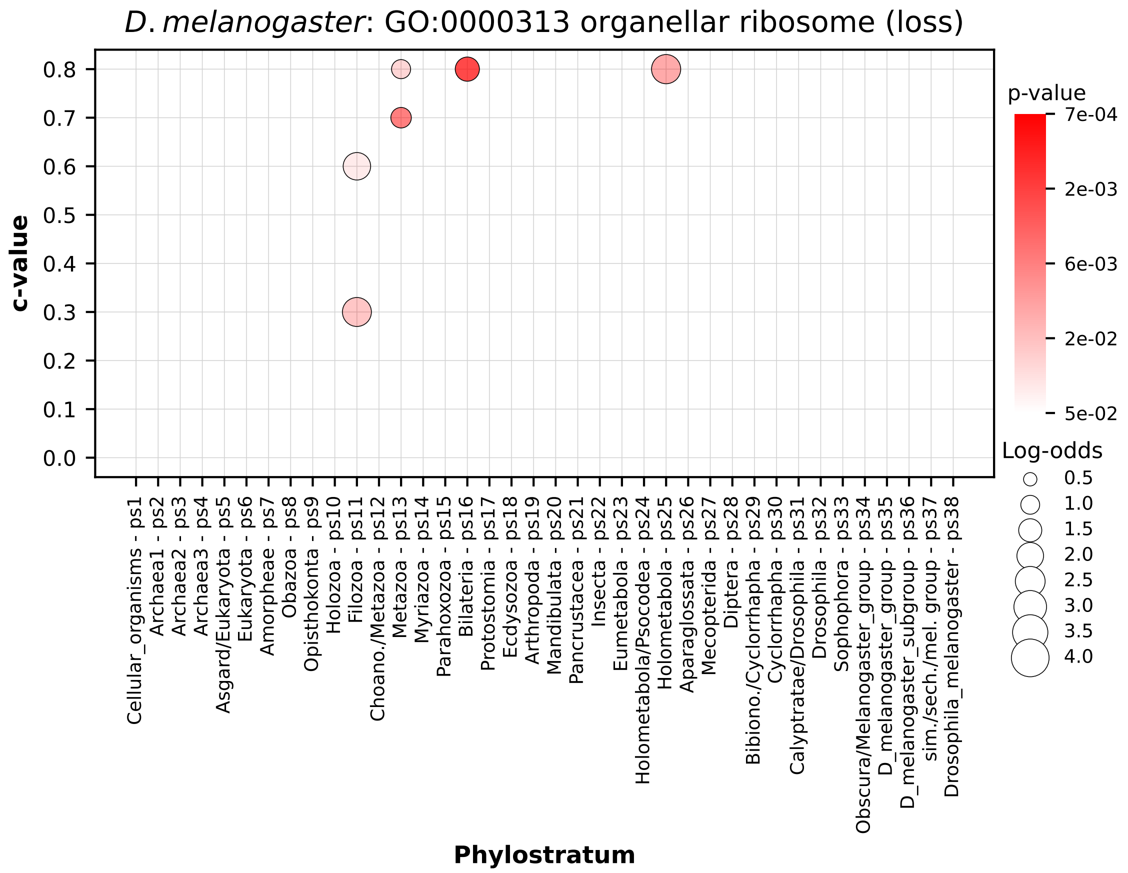

D. melanogaster: GO:0000313 organellar ribosome (loss)
c-value
0.0
0.1
0.2
0.3
0.4
0.5
0.6
0.7
0.8
Phylostratum
Cellular_organisms - ps1
Archaea1 - ps2
Archaea2 - ps3
Archaea3 - ps4
Asgard/Eukaryota - ps5
Eukaryota - ps6
Amorpheae - ps7
Obazoa - ps8
Opisthokonta - ps9
Holozoa - ps10
Filozoa - ps11
Choano./Metazoa - ps12
Metazoa - ps13
Myriazoa - ps14
Parahoxozoa - ps15
Bilateria - ps16
Protostomia - ps17
Ecdysozoa - ps18
Arthropoda - ps19
Mandibulata - ps20
Pancrustacea - ps21
Insecta - ps22
Eumetabola - ps23
Holometabola/Psocodea - ps24
Holometabola - ps25
Aparaglossata - ps26
Mecopterida - ps27
Diptera - ps28
Bibiono./Cyclorrhapha - ps29
Cyclorrhapha - ps30
Calyptratae/Drosophila - ps31
Drosophila - ps32
Sophophora - ps33
Obscura/Melanogaster_group - ps34
D_melanogaster_group - ps35
D_melanogaster_subgroup - ps36
sim./sech./mel. group - ps37
Drosophila_melanogaster - ps38
p-value
7e-04
2e-03
6e-03
2e-02
5e-02
Log-odds
0.5
1.0
1.5
2.0
2.5
3.0
3.5
4.0

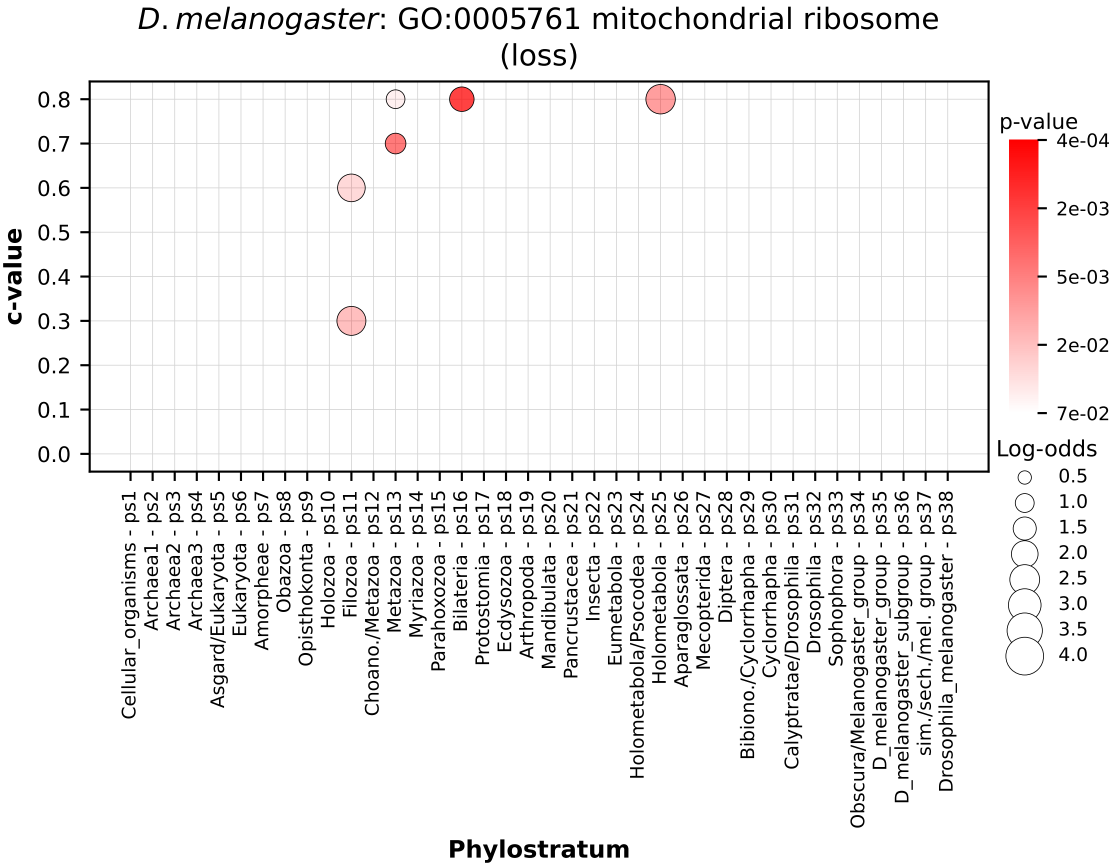

D. melanogaster: GO:0005761 mitochondrial ribosome (loss)
c-value
0.0
0.1
0.2
0.3
0.4
0.5
0.6
0.7
0.8
Phylostratum
Cellular_organisms - ps1
Archaea1 - ps2
Archaea2 - ps3
Archaea3 - ps4
Asgard/Eukaryota - ps5
Eukaryota - ps6
Amorpheae - ps7
Obazoa - ps8
Opisthokonta - ps9
Holozoa - ps10
Filozoa - ps11
Choano./Metazoa - ps12
Metazoa - ps13
Myriazoa - ps14
Parahoxozoa - ps15
Bilateria - ps16
Protostomia - ps17
Ecdysozoa - ps18
Arthropoda - ps19
Mandibulata - ps20
Pancrustacea - ps21
Insecta - ps22
Eumetabola - ps23
Holometabola/Psocodea - ps24
Holometabola - ps25
Aparaglossata - ps26
Mecopterida - ps27
Diptera - ps28
Bibiono./Cyclorrhapha - ps29
Cyclorrhapha - ps30
Calyptratae/Drosophila - ps31
Drosophila - ps32
Sophophora - ps33
Obscura/Melanogaster_group - ps34
D_melanogaster_group - ps35
D_melanogaster_subgroup - ps36
sim./sech./mel. group - ps37
Drosophila_melanogaster - ps38
p-value
4e-04
2e-03
5e-03
2e-02
7e-02
Log-odds
0.5
1.0
1.5
2.0
2.5
3.0
3.5
4.0

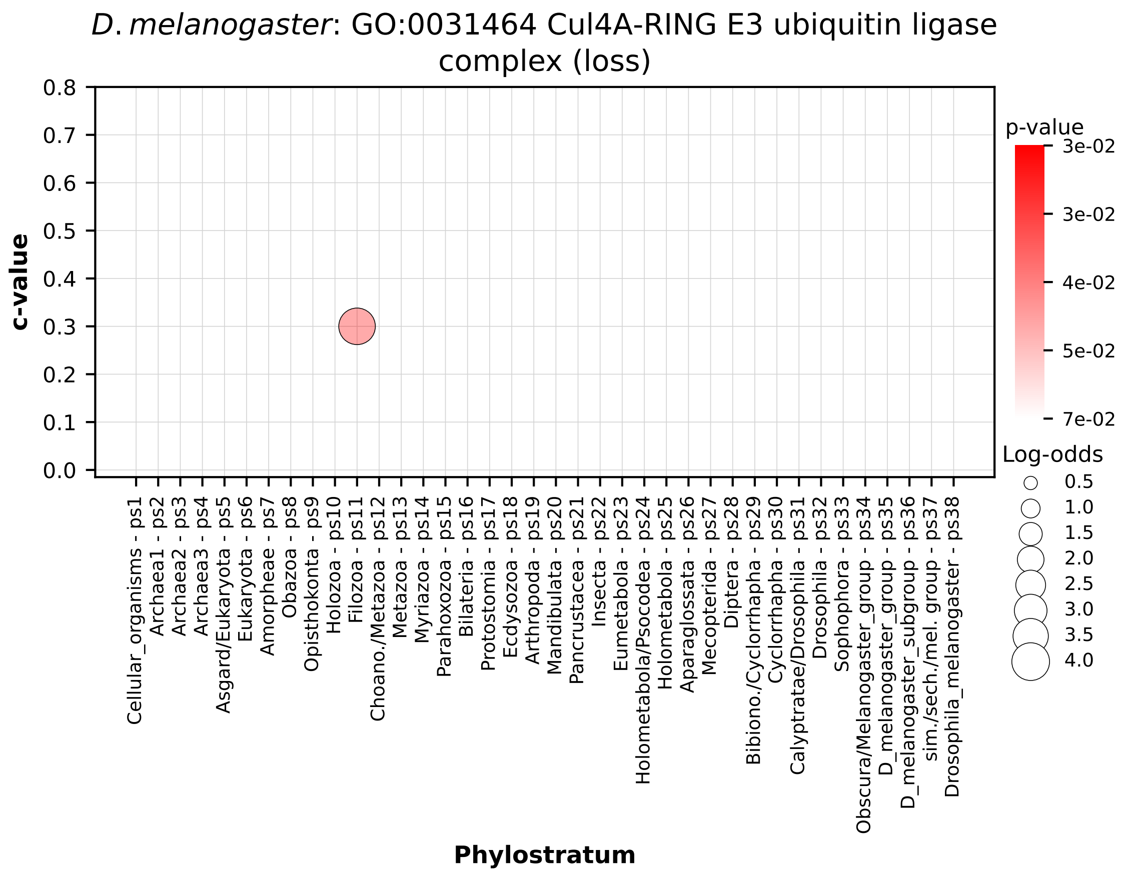

D. melanogaster: GO:0031464 Cul4A-RING E3 ubiquitin ligase complex (loss)
c-value
0.0
0.1
0.2
0.3
0.4
0.5
0.6
0.7
0.8
Phylostratum
Cellular_organisms - ps1
Archaea1 - ps2
Archaea2 - ps3
Archaea3 - ps4
Asgard/Eukaryota - ps5
Eukaryota - ps6
Amorpheae - ps7
Obazoa - ps8
Opisthokonta - ps9
Holozoa - ps10
Filozoa - ps11
Choano./Metazoa - ps12
Metazoa - ps13
Myriazoa - ps14
Parahoxozoa - ps15
Bilateria - ps16
Protostomia - ps17
Ecdysozoa - ps18
Arthropoda - ps19
Mandibulata - ps20
Pancrustacea - ps21
Insecta - ps22
Eumetabola - ps23
Holometabola/Psocodea - ps24
Holometabola - ps25
Aparaglossata - ps26
Mecopterida - ps27
Diptera - ps28
Bibiono./Cyclorrhapha - ps29
Cyclorrhapha - ps30
Calyptratae/Drosophila - ps31
Drosophila - ps32
Sophophora - ps33
Obscura/Melanogaster_group - ps34
D_melanogaster_group - ps35
D_melanogaster_subgroup - ps36
sim./sech./mel. group - ps37
Drosophila_melanogaster - ps38
p-value
3e-02
3e-02
4e-02
5e-02
7e-02
Log-odds
0.5
1.0
1.5
2.0
2.5
3.0
3.5
4.0

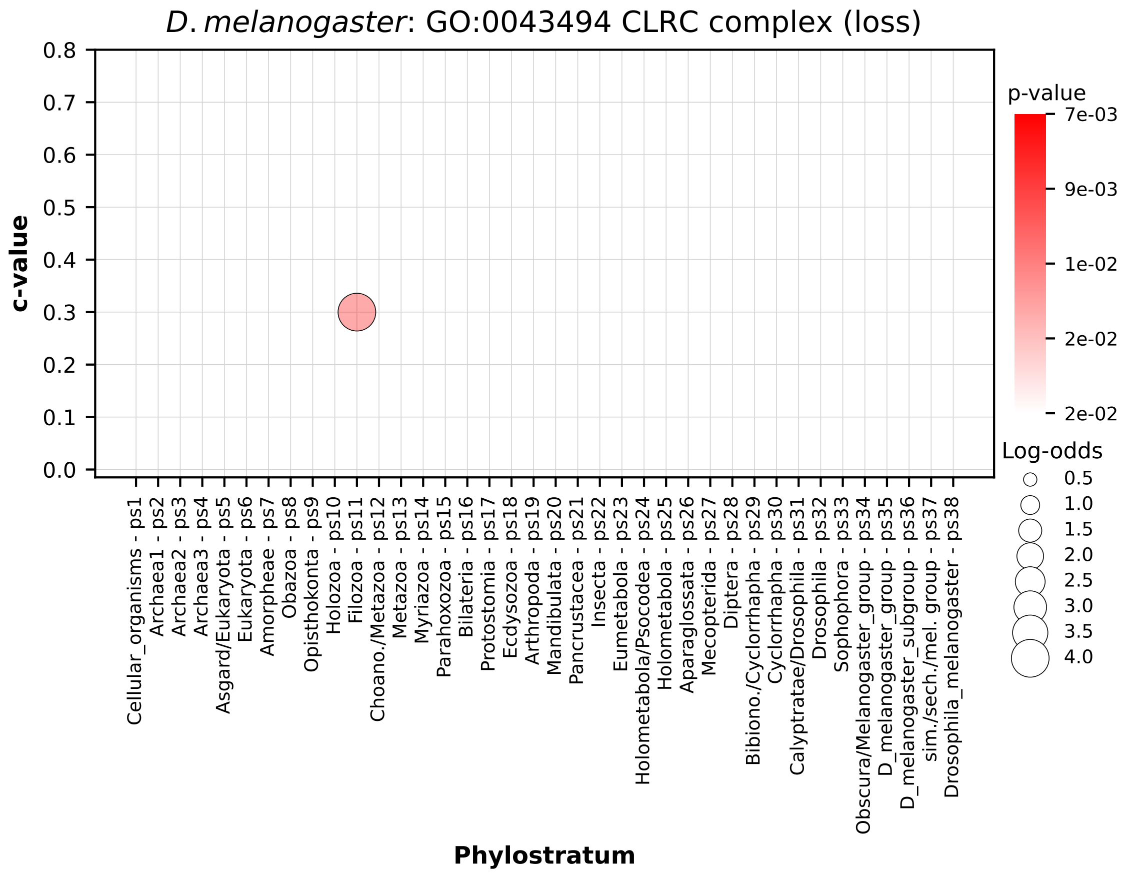

D. melanogaster: GO:0043494 CLRC complex (loss)
c-value
0.8
0.7
0.6
0.5
0.4
0.3
0.2
0.1
0.0
Phylostratum
Cellular_organisms - ps1
Archaea1 - ps2
Archaea2 - ps3
Archaea3 - ps4
Asgard/Eukaryota - ps5
Eukaryota - ps6
Amorpheae - ps7
Obazoa - ps8
Opisthokonta - ps9
Holozoa - ps10
Filozoa - ps11
Choano./Metazoa - ps12
Metazoa - ps13
Myriazoa - ps14
Parahoxozoa - ps15
Bilateria - ps16
Protostomia - ps17
Ecdysozoa - ps18
Arthropoda - ps19
Mandibulata - ps20
Pancrustacea - ps21
Insecta - ps22
Eumetabola - ps23
Holometabola/Psocodea - ps24
Holometabola - ps25
Aparaglossata - ps26
Mecopterida - ps27
Diptera - ps28
Bibiono./Cyclorrhapha - ps29
Cyclorrhapha - ps30
Calyptratae/Drosophila - ps31
Drosophila - ps32
Sophophora - ps33
Obscura/Melanogaster_group - ps34
D_melanogaster_group - ps35
D_melanogaster_subgroup - ps36
sim./sech./mel. group - ps37
Drosophila_melanogaster - ps38
p-value
7e-03
9e-03
1e-02
2e-02
2e-02
Log-odds
0.5
1.0
1.5
2.0
2.5
3.0
3.5
4.0

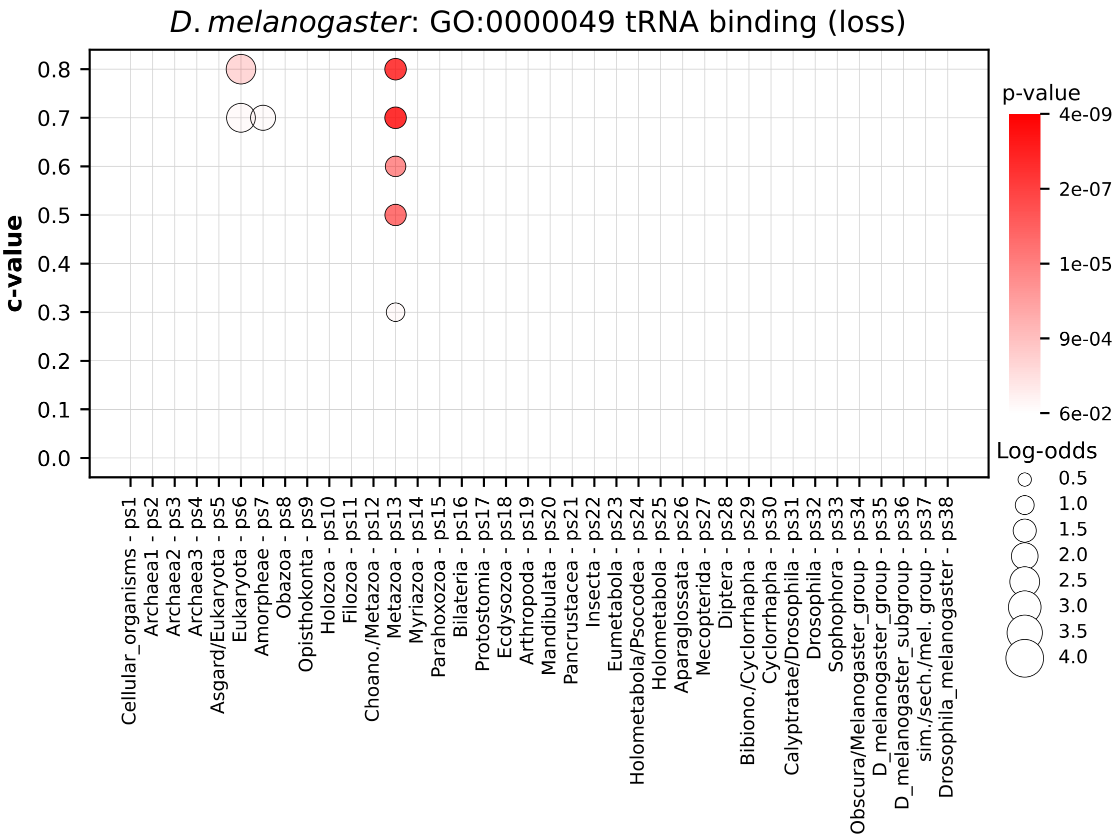

D. melanogaster: GO:0000049 tRNA binding (loss)
c-value
0.8
0.7
0.6
0.5
0.4
0.3
0.2
0.1
0.0
Phylostratum
Cellular_organisms - ps1
Archaea1 - ps2
Archaea2 - ps3
Archaea3 - ps4
Asgard/Eukaryota - ps5
Eukaryota - ps6
Amorpheae - ps7
Obazoa - ps8
Opisthokonta - ps9
Holozoa - ps10
Filozoa - ps11
Choano./Metazoa - ps12
Metazoa - ps13
Myriazoa - ps14
Parahoxozoa - ps15
Bilateria - ps16
Protostomia - ps17
Ecdysozoa - ps18
Arthropoda - ps19
Mandibulata - ps20
Pancrustacea - ps21
Insecta - ps22
Eumetabola - ps23
Holometabola/Psocodea - ps24
Holometabola - ps25
Aparaglossata - ps26
Mecopterida - ps27
Diptera - ps28
Bibiono./Cyclorrhapha - ps29
Cyclorrhapha - ps30
Calyptratae/Drosophila - ps31
Drosophila - ps32
Sophophora - ps33
Obscura/Melanogaster_group - ps34
D_melanogaster_group - ps35
D_melanogaster_subgroup - ps36
sim./sech./mel. group - ps37
Drosophila_melanogaster - ps38
p-value
4e-09
2e-07
1e-05
9e-04
6e-02
Log-odds
0.5
1.0
1.5
2.0
2.5
3.0
3.5
4.0

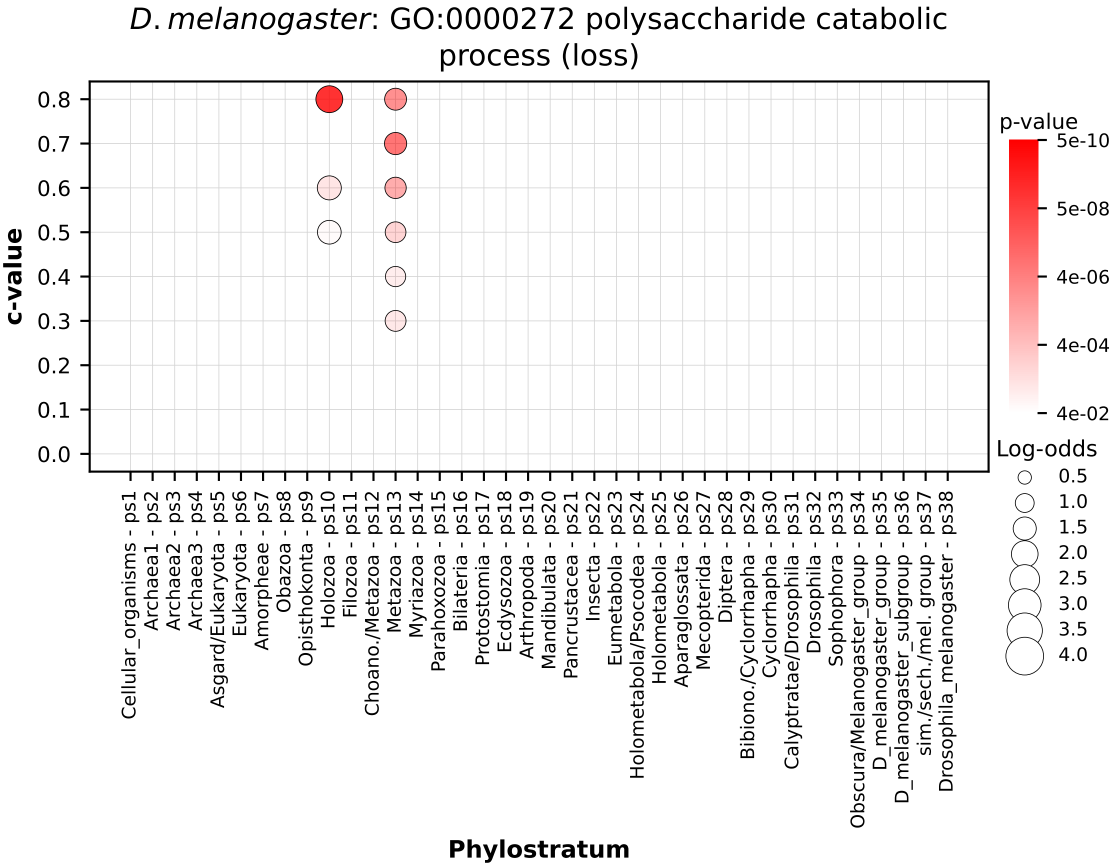

D. melanogaster: GO:0000272 polysaccharide catabolic process (loss)
c-value
0.8
0.7
0.6
0.5
0.4
0.3
0.2
0.1
0.0
Phylostratum
Cellular_organisms - ps1
Archaea1 - ps2
Archaea2 - ps3
Archaea3 - ps4
Asgard/Eukaryota - ps5
Eukaryota - ps6
Amorpheae - ps7
Obazoa - ps8
Opisthokonta - ps9
Holozoa - ps10
Filozoa - ps11
Choano./Metazoa - ps12
Metazoa - ps13
Myriazoa - ps14
Parahoxozoa - ps15
Bilateria - ps16
Protostomia - ps17
Ecdysozoa - ps18
Arthropoda - ps19
Mandibulata - ps20
Pancrustacea - ps21
Insecta - ps22
Eumetabola - ps23
Holometabola/Psocodea - ps24
Holometabola - ps25
Aparaglossata - ps26
Mecopterida - ps27
Diptera - ps28
Bibiono./Cyclorrhapha - ps29
Cyclorrhapha - ps30
Calyptratae/Drosophila - ps31
Drosophila - ps32
Sophophora - ps33
Obscura/Melanogaster_group - ps34
D_melanogaster_group - ps35
D_melanogaster_subgroup - ps36
sim./sech./mel. group - ps37
Drosophila_melanogaster - ps38
p-value
5e-10
5e-08
4e-06
4e-04
4e-02
Log-odds
0.5
1.0
1.5
2.0
2.5
3.0
3.5
4.0

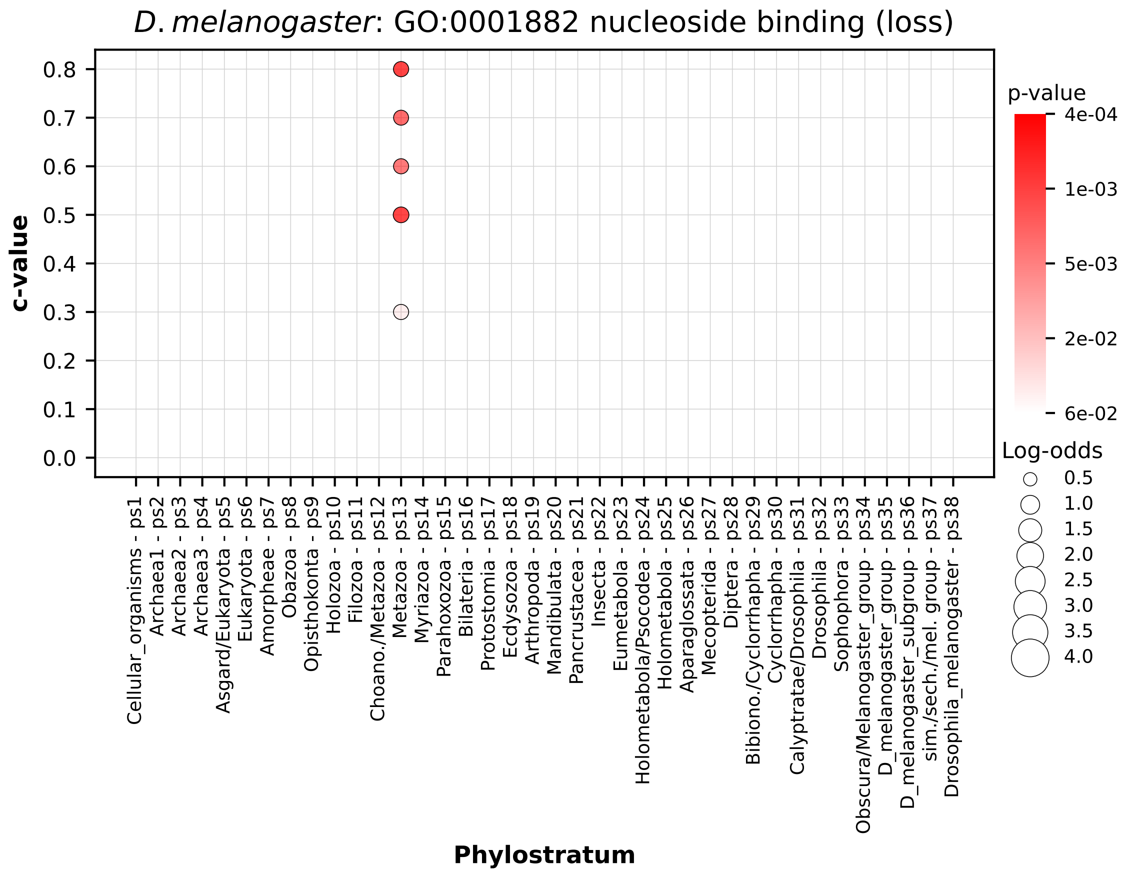

D. melanogaster: GO:0001882 nucleoside binding (loss)
c-value
0.0
0.1
0.2
0.3
0.4
0.5
0.6
0.7
0.8
Phylostratum
Cellular_organisms - ps1
Archaea1 - ps2
Archaea2 - ps3
Archaea3 - ps4
Asgard/Eukaryota - ps5
Eukaryota - ps6
Amorpheae - ps7
Obazoa - ps8
Opisthokonta - ps9
Holozoa - ps10
Filozoa - ps11
Choano./Metazoa - ps12
Metazoa - ps13
Myriazoa - ps14
Parahoxozoa - ps15
Bilateria - ps16
Protostomia - ps17
Ecdysozoa - ps18
Arthropoda - ps19
Mandibulata - ps20
Pancrustacea - ps21
Insecta - ps22
Eumetabola - ps23
Holometabola/Psocodea - ps24
Holometabola - ps25
Aparaglossata - ps26
Mecopterida - ps27
Diptera - ps28
Bibiono./Cyclorrhapha - ps29
Cyclorrhapha - ps30
Calyptratae/Drosophila - ps31
Drosophila - ps32
Sophophora - ps33
Obscura/Melanogaster_group - ps34
D_melanogaster_group - ps35
D_melanogaster_subgroup - ps36
sim./sech./mel. group - ps37
Drosophila_melanogaster - ps38
p-value
4e-04
1e-03
5e-03
2e-02
6e-02
Log-odds
0.5
1.0
1.5
2.0
2.5
3.0
3.5
4.0

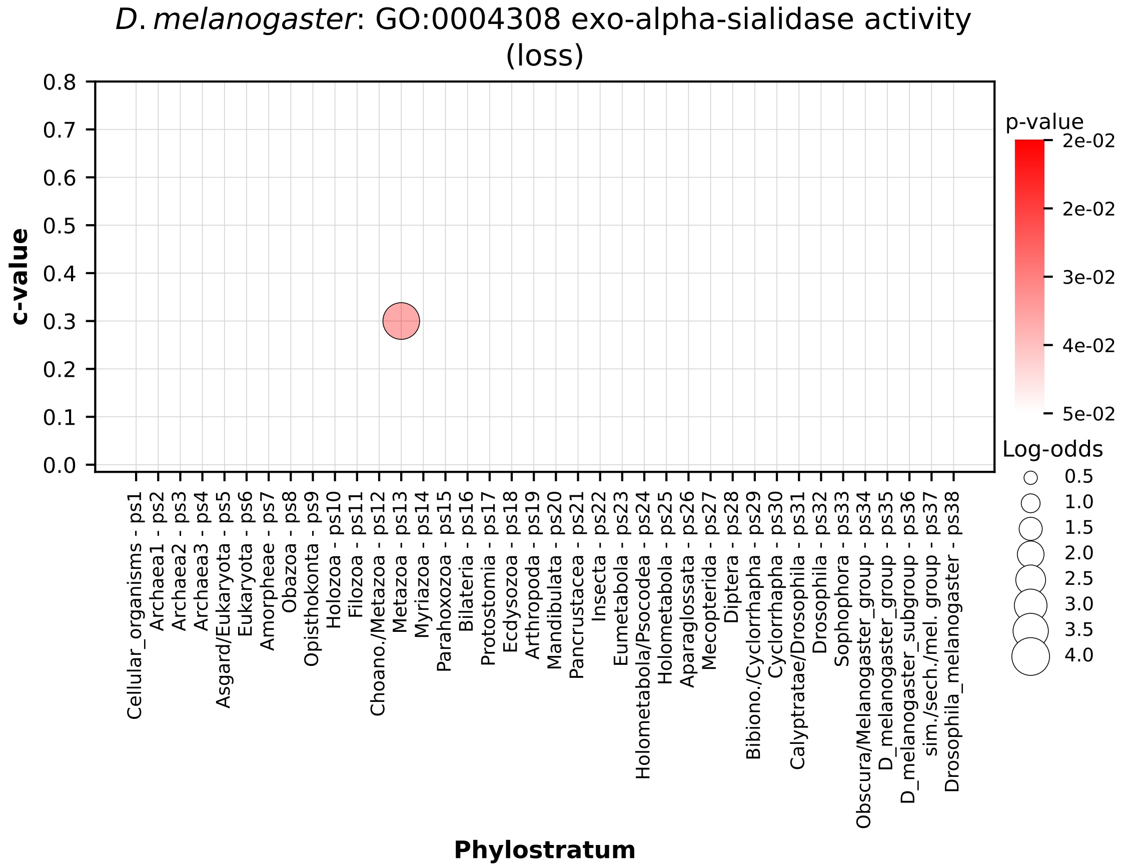
D. melanogaster: GO:0004308 exo-alpha-sialidase activity
(loss)
c-value
0.8
0.7
0.6
0.5
0.4
0.3
0.2
0.1
0.0
p-value
2e-02
2e-02
3e-02
4e-02
5e-02
Log-odds
0.5
1.0
1.5
2.0
2.5
3.0
3.5
4.0
Cellular_organisms - ps1
Archaea1 - ps2
Archaea2 - ps3
Archaea3 - ps4
Asgard/Eukaryota - ps5
Eukaryota - ps6
Amorpheae - ps7
Obazoa - ps8
Opisthokonta - ps9
Holozoa - ps10
Filozoa - ps11
Choano./Metazoa - ps12
Metazoa - ps13
Myriazoa - ps14
Parahoxozoa - ps15
Bilateria - ps16
Protostomia - ps17
Ecdysozoa - ps18
Arthropoda - ps19
Mandibulata - ps20
Pancrustacea - ps21
Insecta - ps22
Eumetabola - ps23
Holometabola/Psocodea - ps24
Holometabola - ps25
Aparaglossata - ps26
Mecopterida - ps27
Diptera - ps28
Bibiono./Cyclorrhapha - ps29
Cyclorrhapha - ps30
Calyptratae/Drosophila - ps31
Drosophila - ps32
Sophophora - ps33
Obscura/Melanogaster_group - ps34
D_melanogaster_group - ps35
D_melanogaster_subgroup - ps36
sim./sech./mel. group - ps37
Drosophila_melanogaster - ps38
Phylostratum

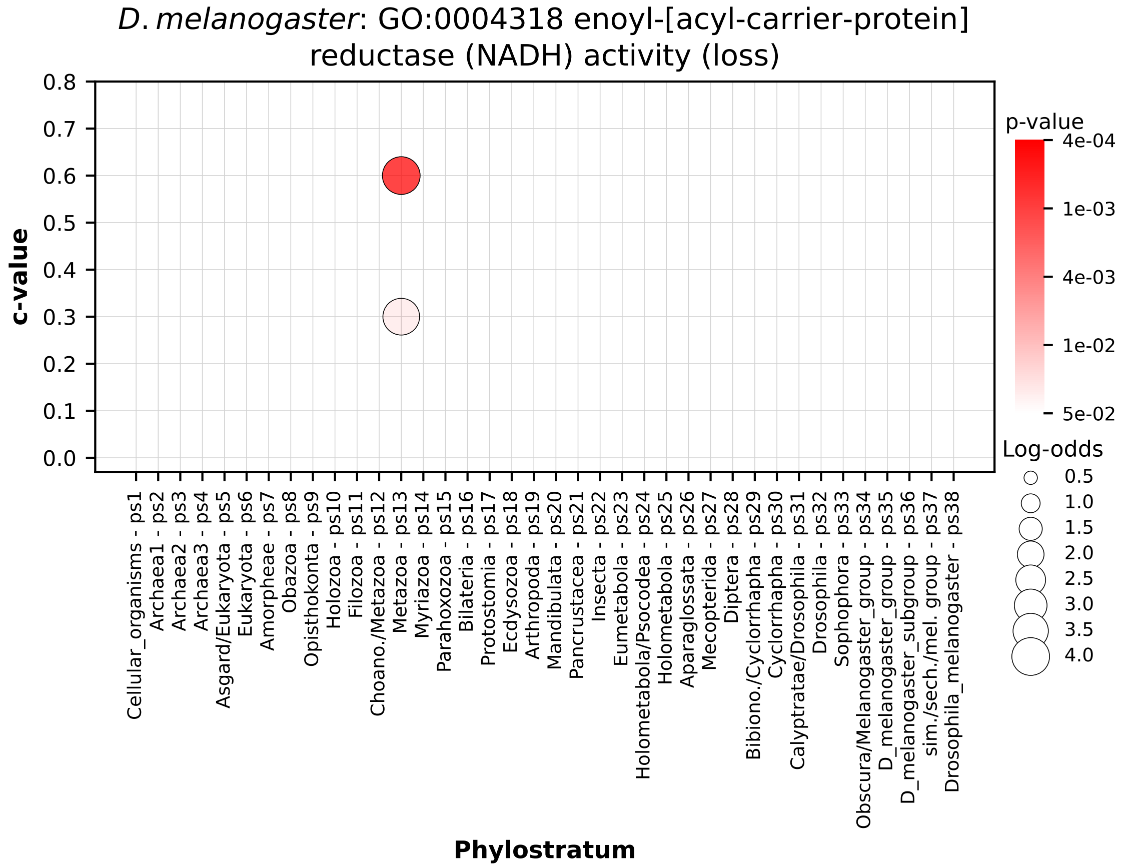

D. melanogaster: GO:0004318 enoyl-[acyl-carrier-protein] reductase (NADH) activity (loss)
c-value
0.8
0.7
0.6
0.5
0.4
0.3
0.2
0.1
0.0
p-value
4e-04
1e-03
4e-03
1e-02
5e-02
Log-odds
0.5
1.0
1.5
2.0
2.5
3.0
3.5
4.0
Cellular_organisms - ps1
Archaea1 - ps2
Archaea2 - ps3
Archaea3 - ps4
Asgard/Eukaryota - ps5
Eukaryota - ps6
Amorpheae - ps7
Obazoa - ps8
Opisthokonta - ps9
Holozoa - ps10
Filozoa - ps11
Choano./Metazoa - ps12
Metazoa - ps13
Myriazoa - ps14
Parahoxozoa - ps15
Bilateria - ps16
Protostomia - ps17
Ecdysozoa - ps18
Arthropoda - ps19
Mandibulata - ps20
Pancrustacea - ps21
Insecta - ps22
Eumetabola - ps23
Holometabola/Psocodea - ps24
Holometabola - ps25
Aparaglossata - ps26
Mecopterida - ps27
Diptera - ps28
Bibiono./Cyclorrhapha - ps29
Cyclorrhapha - ps30
Calyptratae/Drosophila - ps31
Drosophila - ps32
Sophophora - ps33
Obscura/Melanogaster_group - ps34
D_melanogaster_group - ps35
D_melanogaster_subgroup - ps36
sim./sech./mel. group - ps37
Drosophila_melanogaster - ps38
Phylostratum

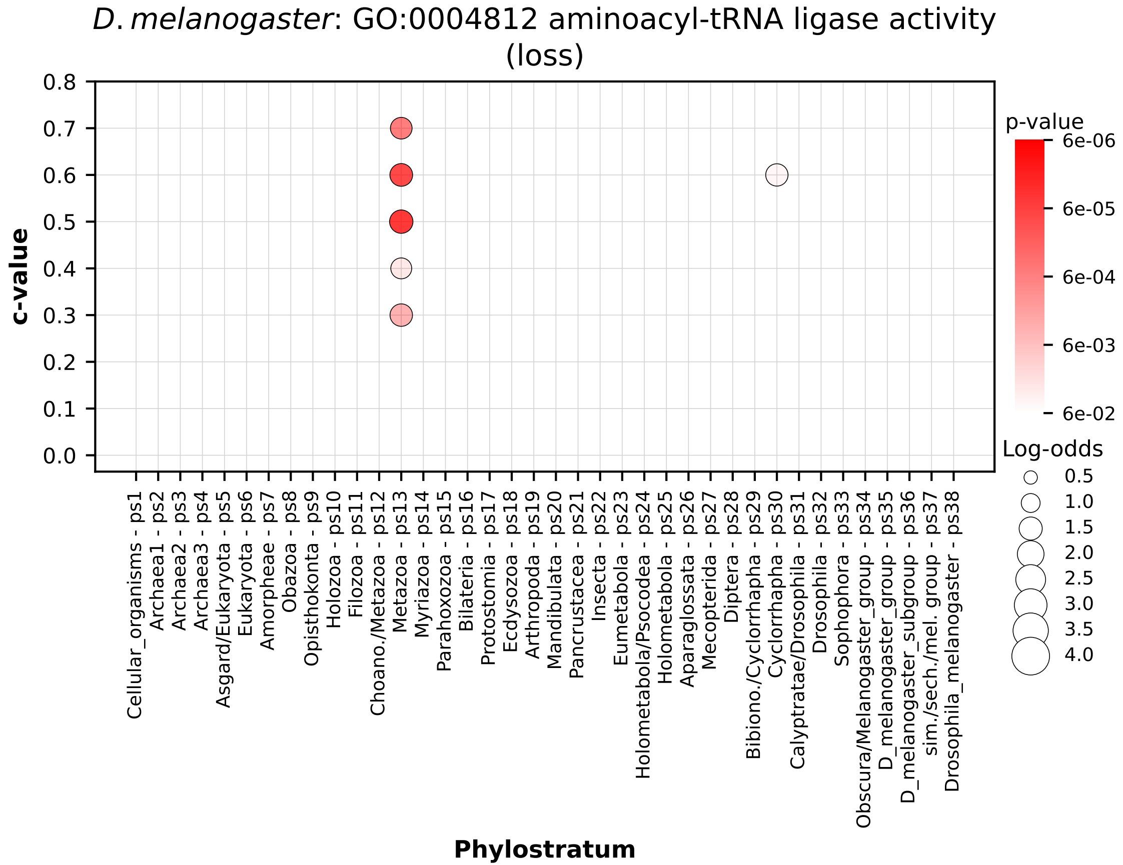

D. melanogaster: GO:0004812 aminoacyl-tRNA ligase activity (loss)
c-value
0.0
0.1
0.2
0.3
0.4
0.5
0.6
0.7
0.8
Phylostratum
Cellular_organisms - ps1
Archaea1 - ps2
Archaea2 - ps3
Archaea3 - ps4
Asgard/Eukaryota - ps5
Eukaryota - ps6
Amorpheae - ps7
Obazoa - ps8
Opisthokonta - ps9
Holozoa - ps10
Filozoa - ps11
Choano./Metazoa - ps12
Metazoa - ps13
Myriazoa - ps14
Parahoxozoa - ps15
Bilateria - ps16
Protostomia - ps17
Ecdysozoa - ps18
Arthropoda - ps19
Mandibulata - ps20
Pancrustacea - ps21
Insecta - ps22
Eumetabola - ps23
Holometabola/Psocodea - ps24
Holometabola - ps25
Aparaglossata - ps26
Mecopterida - ps27
Diptera - ps28
Bibiono./Cyclorrhapha - ps29
Cyclorrhapha - ps30
Calyptratae/Drosophila - ps31
Drosophila - ps32
Sophophora - ps33
Obscura/Melanogaster_group - ps34
D_melanogaster_group - ps35
D_melanogaster_subgroup - ps36
sim./sech./mel. group - ps37
Drosophila_melanogaster - ps38
p-value
6e-06
6e-05
6e-04
6e-03
6e-02
Log-odds
0.5
1.0
1.5
2.0
2.5
3.0
3.5
4.0

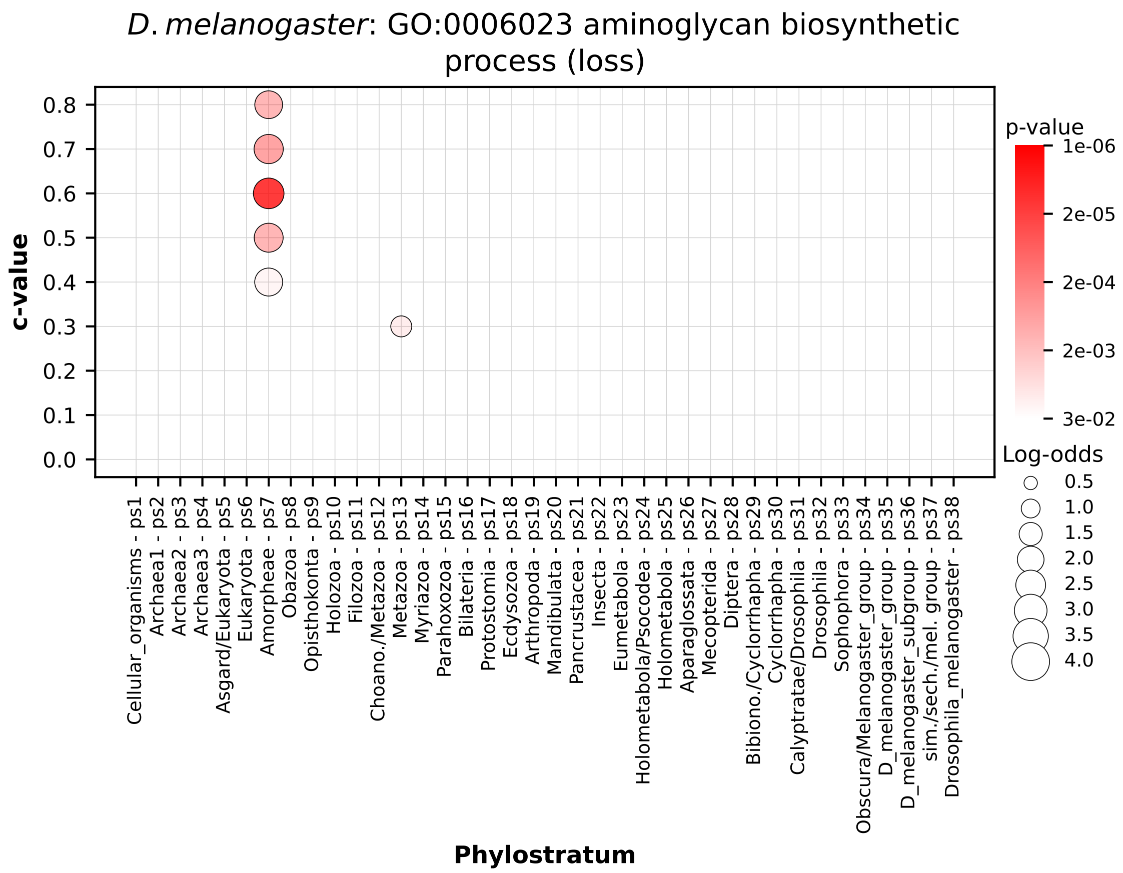

D. melanogaster: GO:0006023 aminoglycan biosynthetic process (loss)
c-value
0.8
0.7
0.6
0.5
0.4
0.3
0.2
0.1
0.0
Phylostratum
Cellular_organisms - ps1
Archaea1 - ps2
Archaea2 - ps3
Archaea3 - ps4
Asgard/Eukaryota - ps5
Eukaryota - ps6
Amorpheae - ps7
Obazoa - ps8
Opisthokonta - ps9
Holozoa - ps10
Filozoa - ps11
Choano./Metazoa - ps12
Metazoa - ps13
Myriazoa - ps14
Parahoxozoa - ps15
Bilateria - ps16
Protostomia - ps17
Ecdysozoa - ps18
Arthropoda - ps19
Mandibulata - ps20
Pancrustacea - ps21
Insecta - ps22
Eumetabola - ps23
Holometabola/Psocodea - ps24
Holometabola - ps25
Aparaglossata - ps26
Mecopterida - ps27
Diptera - ps28
Bibiono./Cyclorrhapha - ps29
Cyclorrhapha - ps30
Calyptratae/Drosophila - ps31
Drosophila - ps32
Sophophora - ps33
Obscura/Melanogaster_group - ps34
D_melanogaster_group - ps35
D_melanogaster_subgroup - ps36
sim./sech./mel. group - ps37
Drosophila_melanogaster - ps38
p-value
1e-06
2e-05
2e-04
2e-03
3e-02
Log-odds
0.5
1.0
1.5
2.0
2.5
3.0
3.5
4.0

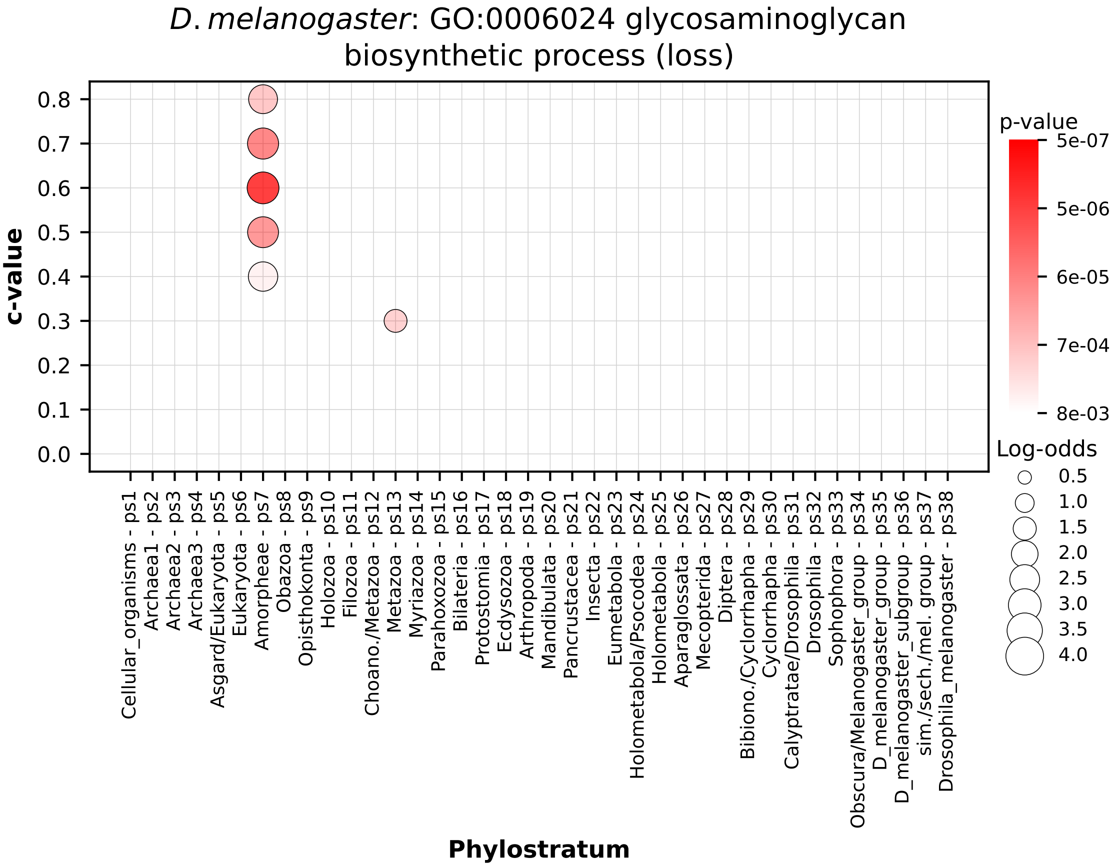

D. melanogaster: GO:0006024 glycosaminoglycan biosynthetic process (loss)
c-value
0.8
0.7
0.6
0.5
0.4
0.3
0.2
0.1
0.0
p-value
5e-07
5e-06
6e-05
7e-04
8e-03
Log-odds
0.5
1.0
1.5
2.0
2.5
3.0
3.5
4.0
Cellular_organisms - ps1
Archaea1 - ps2
Archaea2 - ps3
Archaea3 - ps4
Asgard/Eukaryota - ps5
Eukaryota - ps6
Amorpheae - ps7
Obazoa - ps8
Opisthokonta - ps9
Holozoa - ps10
Filozoa - ps11
Choano./Metazoa - ps12
Metazoa - ps13
Myriazoa - ps14
Parahoxozoa - ps15
Bilateria - ps16
Protostomia - ps17
Ecdysozoa - ps18
Arthropoda - ps19
Mandibulata - ps20
Pancrustacea - ps21
Insecta - ps22
Eumetabola - ps23
Holometabola/Psocodea - ps24
Holometabola - ps25
Aparaglossata - ps26
Mecopterida - ps27
Diptera - ps28
Bibiono./Cyclorrhapha - ps29
Cyclorrhapha - ps30
Calyptratae/Drosophila - ps31
Drosophila - ps32
Sophophora - ps33
Obscura/Melanogaster_group - ps34
D_melanogaster_group - ps35
D_melanogaster_subgroup - ps36
sim./sech./mel. group - ps37
Drosophila_melanogaster - ps38
Phylostratum

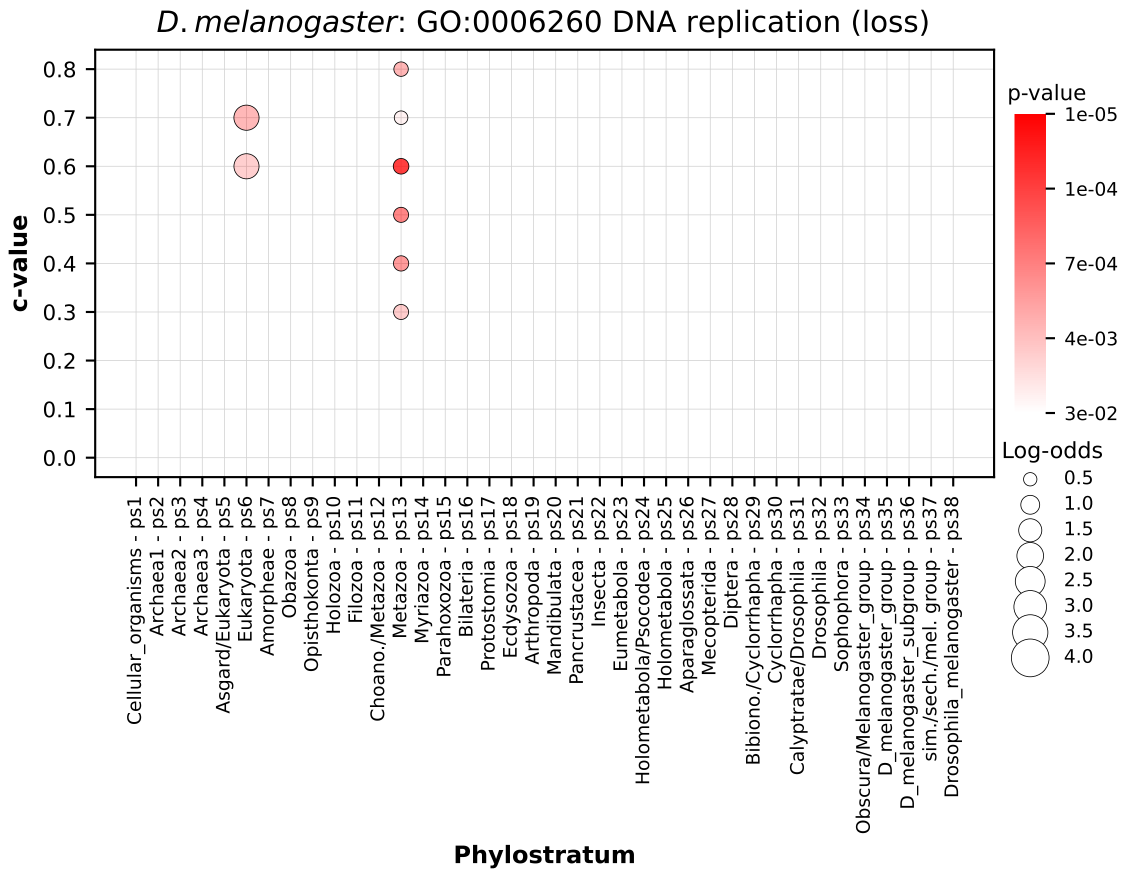
D. melanogaster: GO:0006260 DNA replication (loss)
c-value
0.0
0.1
0.2
0.3
0.4
0.5
0.6
0.7
0.8
Phylostratum
Cellular_organisms - ps1
Archaea1 - ps2
Archaea2 - ps3
Archaea3 - ps4
Asgard/Eukaryota - ps5
Eukaryota - ps6
Amorpheae - ps7
Obazoa - ps8
Opisthokonta - ps9
Holozoa - ps10
Filozoa - ps11
Choano./Metazoa - ps12
Metazoa - ps13
Myriazoa - ps14
Parahoxozoa - ps15
Bilateria - ps16
Protostomia - ps17
Ecdysozoa - ps18
Arthropoda - ps19
Mandibulata - ps20
Pancrustacea - ps21
Insecta - ps22
Eumetabola - ps23
Holometabola/Psocodea - ps24
Holometabola - ps25
Aparaglossata - ps26
Mecopterida - ps27
Diptera - ps28
Bibiono./Cyclorrhapha - ps29
Cyclorrhapha - ps30
Calyptratae/Drosophila - ps31
Drosophila - ps32
Sophophora - ps33
Obscura/Melanogaster_group - ps34
D_melanogaster_group - ps35
D_melanogaster_subgroup - ps36
sim./sech./mel. group - ps37
Drosophila_melanogaster - ps38
p-value
1e-05
1e-04
7e-04
4e-03
3e-02
Log-odds
0.5
1.0
1.5
2.0
2.5
3.0
3.5
4.0

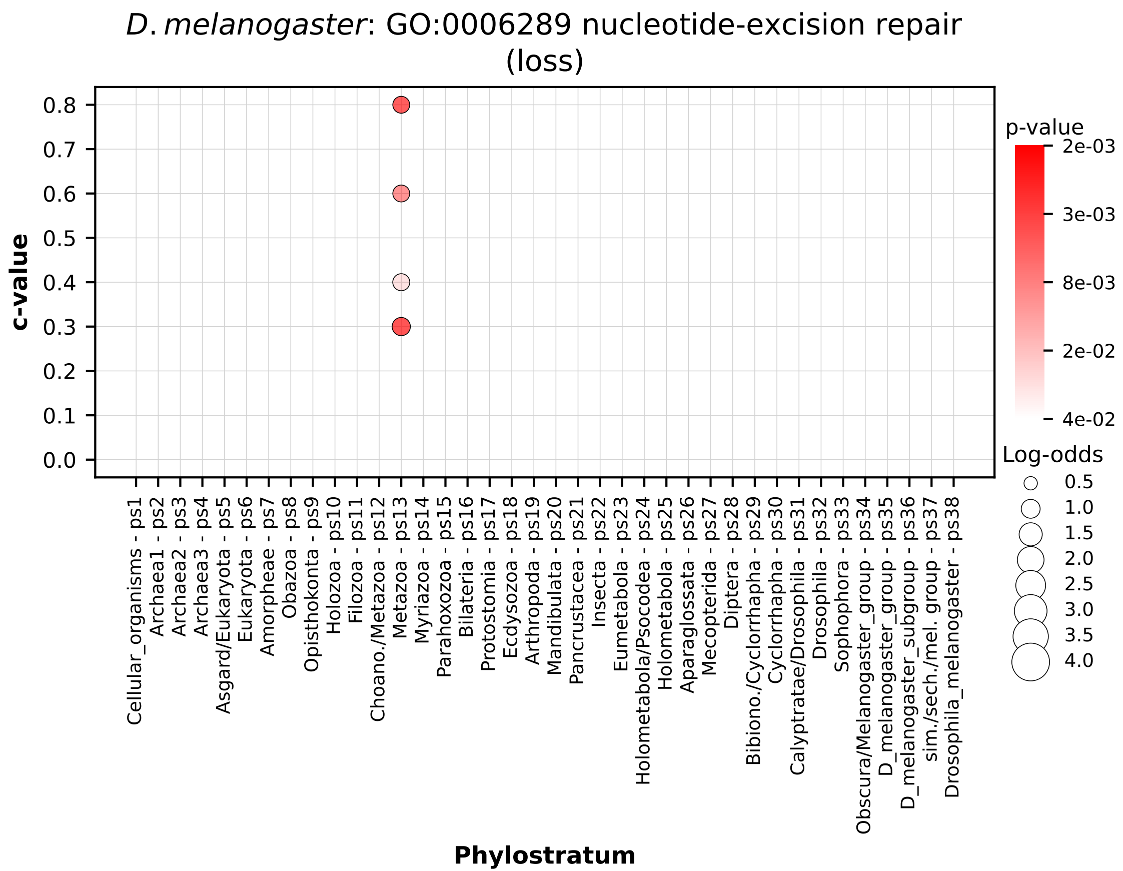

D. melanogaster: GO:0006289 nucleotide-excision repair (loss)
c-value
0.8
0.7
0.6
0.5
0.4
0.3
0.2
0.1
0.0
p-value
2e-03
3e-03
8e-03
2e-02
4e-02
Log-odds
0.5
1.0
1.5
2.0
2.5
3.0
3.5
4.0
Cellular_organisms - ps1
Archaea1 - ps2
Archaea2 - ps3
Archaea3 - ps4
Asgard/Eukaryota - ps5
Eukaryota - ps6
Amorpheae - ps7
Obazoa - ps8
Opisthokonta - ps9
Holozoa - ps10
Filozoa - ps11
Choano./Metazoa - ps12
Metazoa - ps13
Myriazoa - ps14
Parahoxozoa - ps15
Bilateria - ps16
Protostomia - ps17
Ecdysozoa - ps18
Arthropoda - ps19
Mandibulata - ps20
Pancrustacea - ps21
Insecta - ps22
Eumetabola - ps23
Holometabola/Psocodea - ps24
Holometabola - ps25
Aparaglossata - ps26
Mecopterida - ps27
Diptera - ps28
Bibiono./Cyclorrhapha - ps29
Cyclorrhapha - ps30
Calyptratae/Drosophila - ps31
Drosophila - ps32
Sophophora - ps33
Obscura/Melanogaster_group - ps34
D_melanogaster_group - ps35
D_melanogaster_subgroup - ps36
sim./sech./mel. group - ps37
Drosophila_melanogaster - ps38
Phylostratum

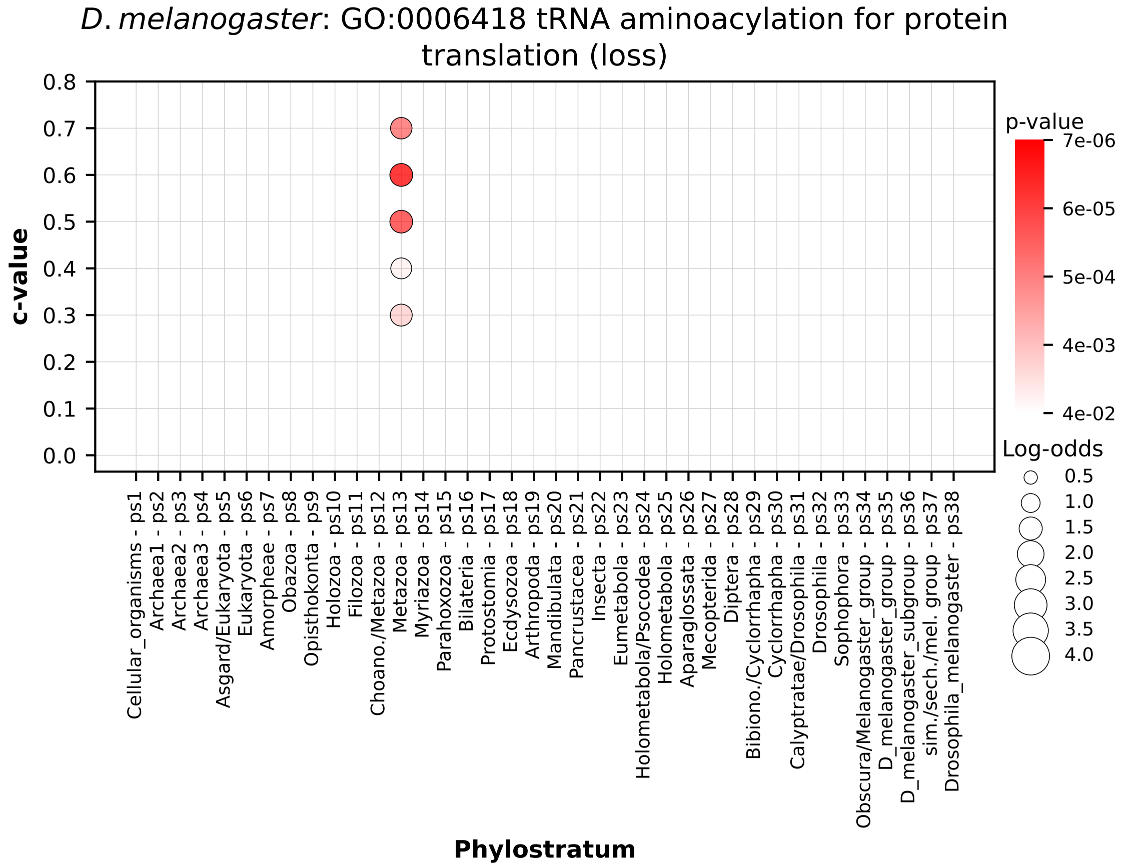
D. melanogaster: GO:0006418 tRNA aminoacylation for protein translation (loss)
c-value
0.8
0.7
0.6
0.5
0.4
0.3
0.2
0.1
0.0
p-value
7e-06
6e-05
5e-04
4e-03
4e-02
Log-odds
0.5
1.0
1.5
2.0
2.5
3.0
3.5
4.0
Cellular_organisms - ps1
Archaea1 - ps2
Archaea2 - ps3
Archaea3 - ps4
Asgard/Eukaryota - ps5
Eukaryota - ps6
Amorpheae - ps7
Obazoa - ps8
Opisthokonta - ps9
Holozoa - ps10
Filozoa - ps11
Choano./Metazoa - ps12
Metazoa - ps13
Myriazoa - ps14
Parahoxozoa - ps15
Bilateria - ps16
Protostomia - ps17
Ecdysozoa - ps18
Arthropoda - ps19
Mandibulata - ps20
Pancrustacea - ps21
Insecta - ps22
Eumetabola - ps23
Holometabola/Psocodea - ps24
Holometabola - ps25
Aparaglossata - ps26
Mecopterida - ps27
Diptera - ps28
Bibiono./Cyclorrhapha - ps29
Cyclorrhapha - ps30
Calyptratae/Drosophila - ps31
Drosophila - ps32
Sophophora - ps33
Obscura/Melanogaster_group - ps34
D_melanogaster_group - ps35
D_melanogaster_subgroup - ps36
sim./sech./mel. group - ps37
Drosophila_melanogaster - ps38
Phylostratum

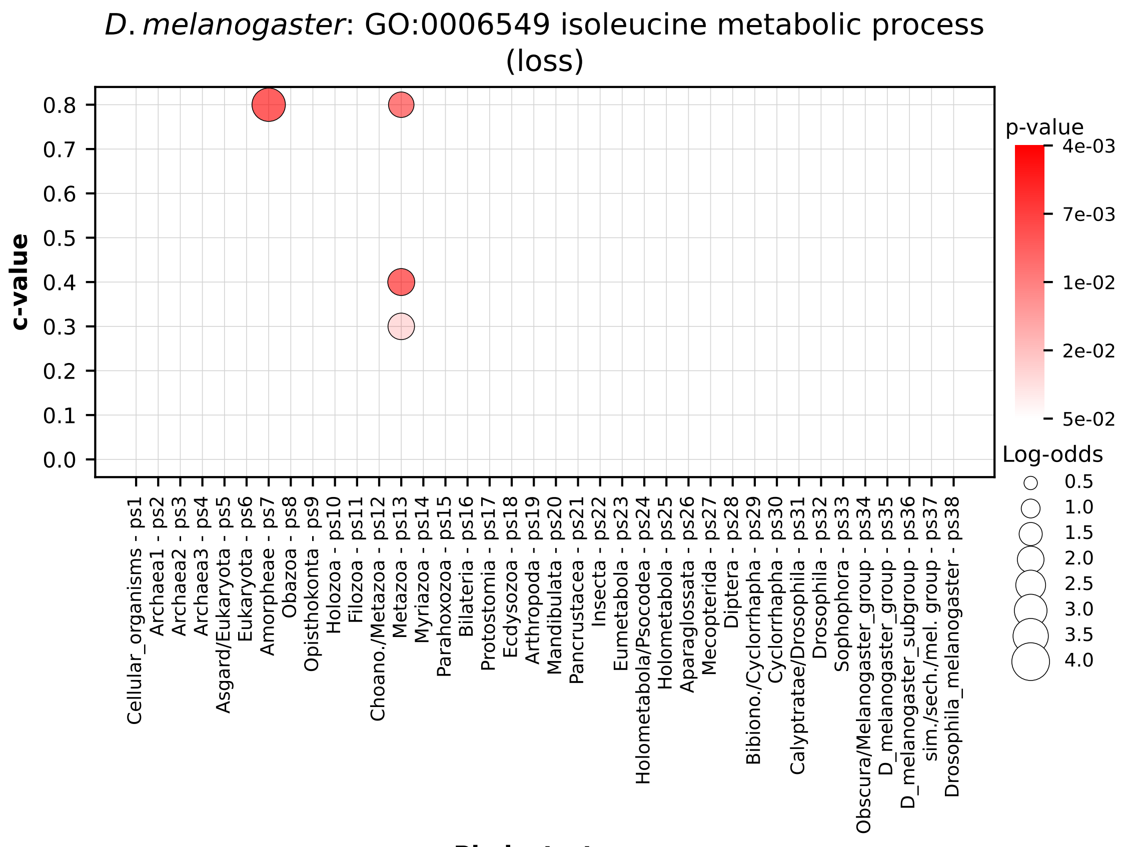

D. melanogaster: GO:0006549 isoleucine metabolic process (loss)
c-value
0.0
0.1
0.2
0.3
0.4
0.5
0.6
0.7
0.8
Phylostratum
Cellular_organisms - ps1
Archaea1 - ps2
Archaea2 - ps3
Archaea3 - ps4
Asgard/Eukaryota - ps5
Eukaryota - ps6
Amorpheae - ps7
Obazoa - ps8
Opisthokonta - ps9
Holozoa - ps10
Filozoa - ps11
Choano./Metazoa - ps12
Metazoa - ps13
Myriazoa - ps14
Parahoxozoa - ps15
Bilateria - ps16
Protostomia - ps17
Ecdysozoa - ps18
Arthropoda - ps19
Mandibulata - ps20
Pancrustacea - ps21
Insecta - ps22
Eumetabola - ps23
Holometabola/Psocodea - ps24
Holometabola - ps25
Aparaglossata - ps26
Mecopterida - ps27
Diptera - ps28
Bibiono./Cyclorrhapha - ps29
Cyclorrhapha - ps30
Calyptratae/Drosophila - ps31
Drosophila - ps32
Sophophora - ps33
Obscura/Melanogaster_group - ps34
D_melanogaster_group - ps35
D_melanogaster_subgroup - ps36
sim./sech./mel. group - ps37
Drosophila_melanogaster - ps38
p-value
4e-03
7e-03
1e-02
2e-02
5e-02
Log-odds
0.5
1.0
1.5
2.0
2.5
3.0
3.5
4.0

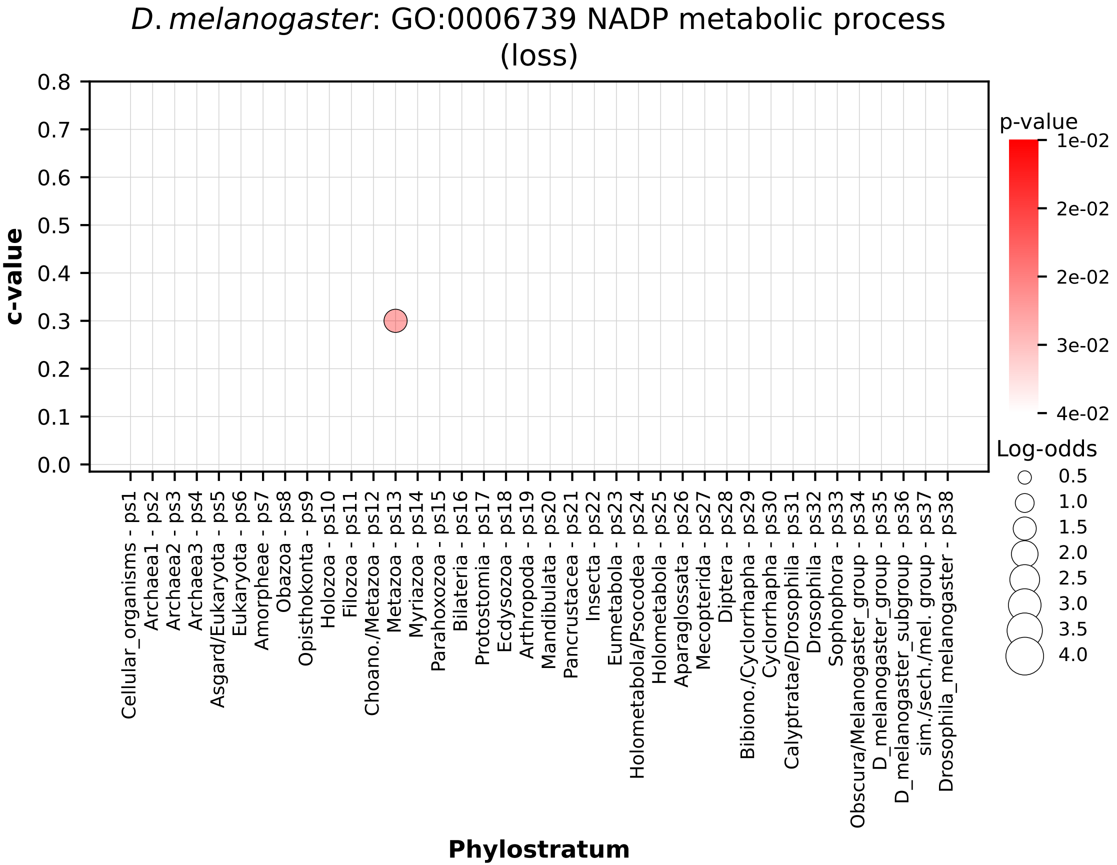
D. melanogaster: GO:0006739 NADP metabolic process
(loss)
c-value
0.0
0.1
0.2
0.3
0.4
0.5
0.6
0.7
0.8
Phylostratum
Cellular_organisms - ps1
Archaea1 - ps2
Archaea2 - ps3
Archaea3 - ps4
Asgard/Eukaryota - ps5
Eukaryota - ps6
Amorpheae - ps7
Obazoa - ps8
Opisthokonta - ps9
Holozoa - ps10
Filozoa - ps11
Choano./Metazoa - ps12
Metazoa - ps13
Myriazoa - ps14
Parahoxozoa - ps15
Bilateria - ps16
Protostomia - ps17
Ecdysozoa - ps18
Arthropoda - ps19
Mandibulata - ps20
Pancrustacea - ps21
Insecta - ps22
Eumetabola - ps23
Holometabola/Psocodea - ps24
Holometabola - ps25
Aparaglossata - ps26
Mecopterida - ps27
Diptera - ps28
Bibiono./Cyclorrhapha - ps29
Cyclorrhapha - ps30
Calyptratae/Drosophila - ps31
Drosophila - ps32
Sophophora - ps33
Obscura/Melanogaster_group - ps34
D_melanogaster_group - ps35
D_melanogaster_subgroup - ps36
sim./sech./mel. group - ps37
Drosophila_melanogaster - ps38
p-value
1e-02
2e-02
2e-02
3e-02
4e-02
Log-odds
0.5
1.0
1.5
2.0
2.5
3.0
3.5
4.0

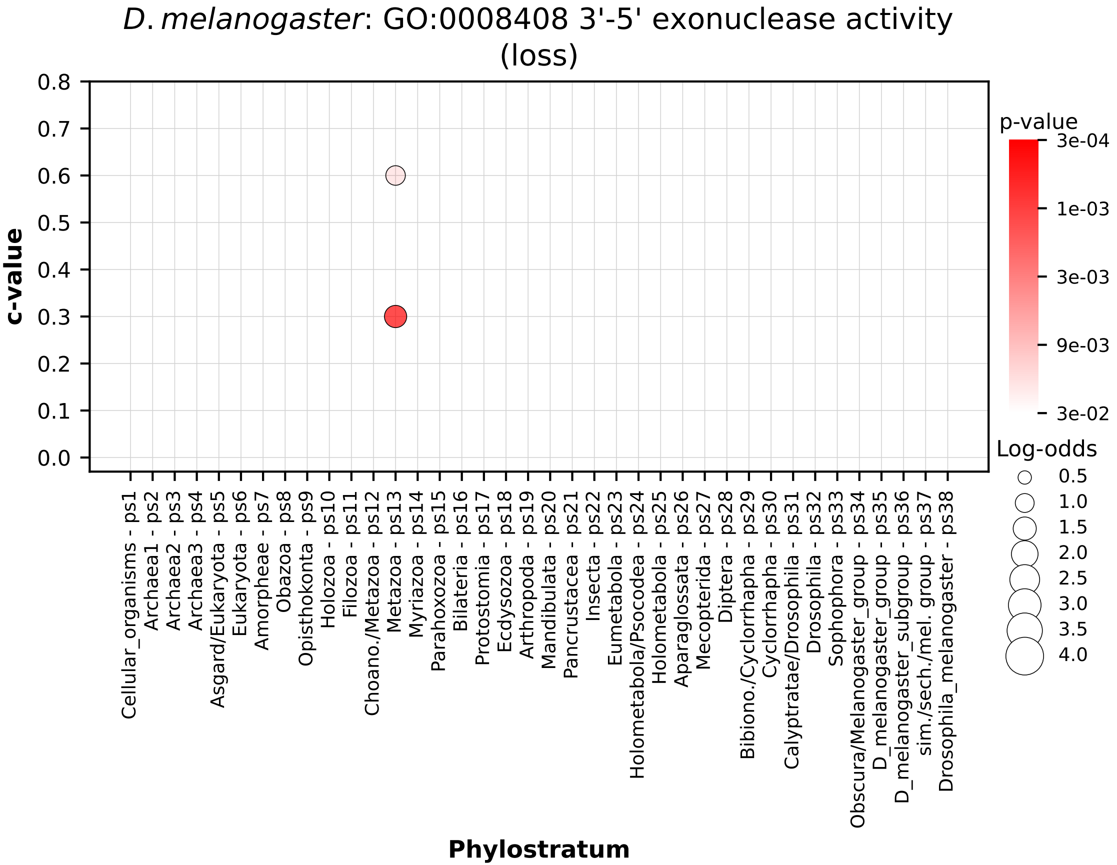

D. melanogaster: GO:0008408 3'-5' exonuclease activity
(loss)
c-value
0.8
0.7
0.6
0.5
0.4
0.3
0.2
0.1
0.0
p-value
3e-04
1e-03
3e-03
9e-03
3e-02
Log-odds
0.5
1.0
1.5
2.0
2.5
3.0
3.5
4.0
Cellular_organisms - ps1
Archaea1 - ps2
Archaea2 - ps3
Archaea3 - ps4
Asgard/Eukaryota - ps5
Eukaryota - ps6
Amorpheae - ps7
Obazoa - ps8
Opisthokonta - ps9
Holozoa - ps10
Filozoa - ps11
Choano./Metazoa - ps12
Metazoa - ps13
Myriazoa - ps14
Parahoxozoa - ps15
Bilateria - ps16
Protostomia - ps17
Ecdysozoa - ps18
Arthropoda - ps19
Mandibulata - ps20
Pancrustacea - ps21
Insecta - ps22
Eumetabola - ps23
Holometabola/Psocodea - ps24
Holometabola - ps25
Aparaglossata - ps26
Mecopterida - ps27
Diptera - ps28
Bibiono./Cyclorrhapha - ps29
Cyclorrhapha - ps30
Calyptratae/Drosophila - ps31
Drosophila - ps32
Sophophora - ps33
Obscura/Melanogaster_group - ps34
D_melanogaster_group - ps35
D_melanogaster_subgroup - ps36
sim./sech./mel. group - ps37
Drosophila_melanogaster - ps38
Phylostratum

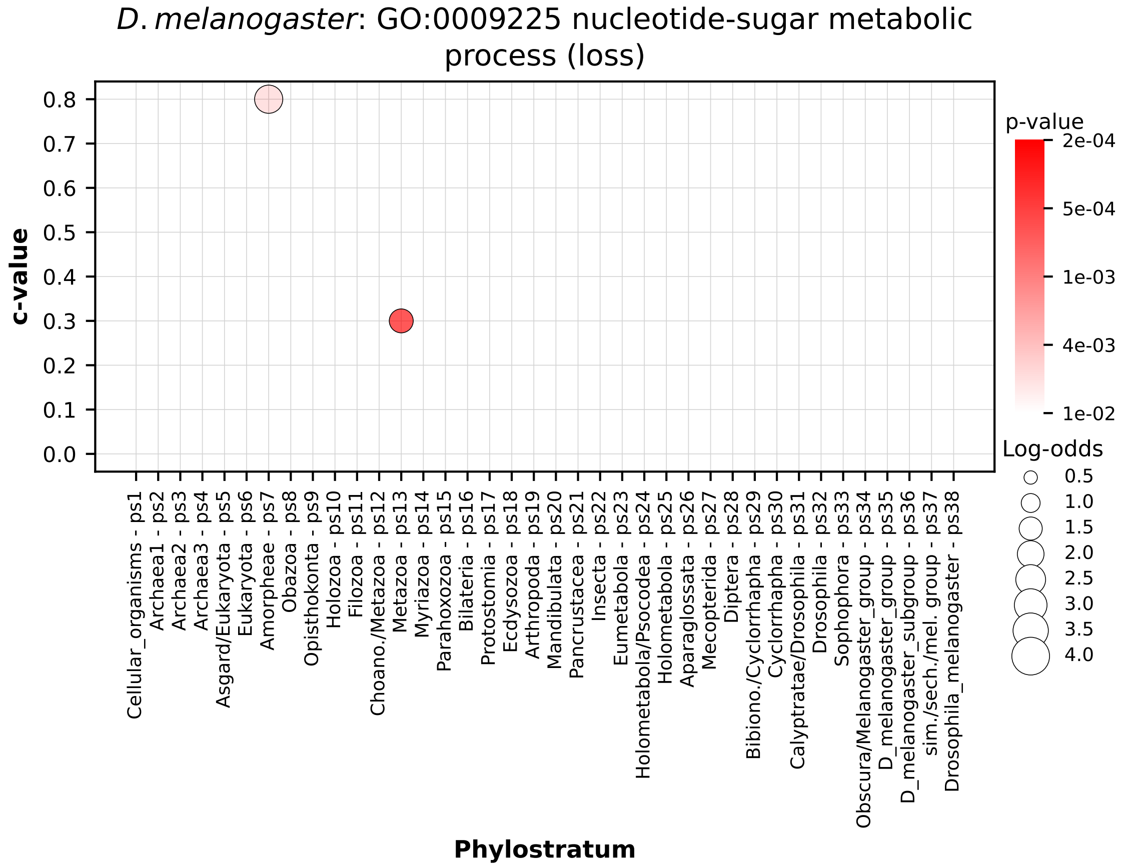

D. melanogaster: GO:0009225 nucleotide-sugar metabolic process (loss)
c-value
0.8
0.7
0.6
0.5
0.4
0.3
0.2
0.1
0.0
Phylostratum
Cellular_organisms - ps1
Archaea1 - ps2
Archaea2 - ps3
Archaea3 - ps4
Asgard/Eukaryota - ps5
Eukaryota - ps6
Amorpheae - ps7
Obazoa - ps8
Opisthokonta - ps9
Holozoa - ps10
Filozoa - ps11
Choano./Metazoa - ps12
Metazoa - ps13
Myriazoa - ps14
Parahoxozoa - ps15
Bilateria - ps16
Protostomia - ps17
Ecdysozoa - ps18
Arthropoda - ps19
Mandibulata - ps20
Pancrustacea - ps21
Insecta - ps22
Eumetabola - ps23
Holometabola/Psocodea - ps24
Holometabola - ps25
Aparaglossata - ps26
Mecopterida - ps27
Diptera - ps28
Bibiono./Cyclorrhapha - ps29
Cyclorrhapha - ps30
Calyptratae/Drosophila - ps31
Drosophila - ps32
Sophophora - ps33
Obscura/Melanogaster_group - ps34
D_melanogaster_group - ps35
D_melanogaster_subgroup - ps36
sim./sech./mel. group - ps37
Drosophila_melanogaster - ps38
p-value
2e-04
5e-04
1e-03
4e-03
1e-02
Log-odds
0.5
1.0
1.5
2.0
2.5
3.0
3.5
4.0

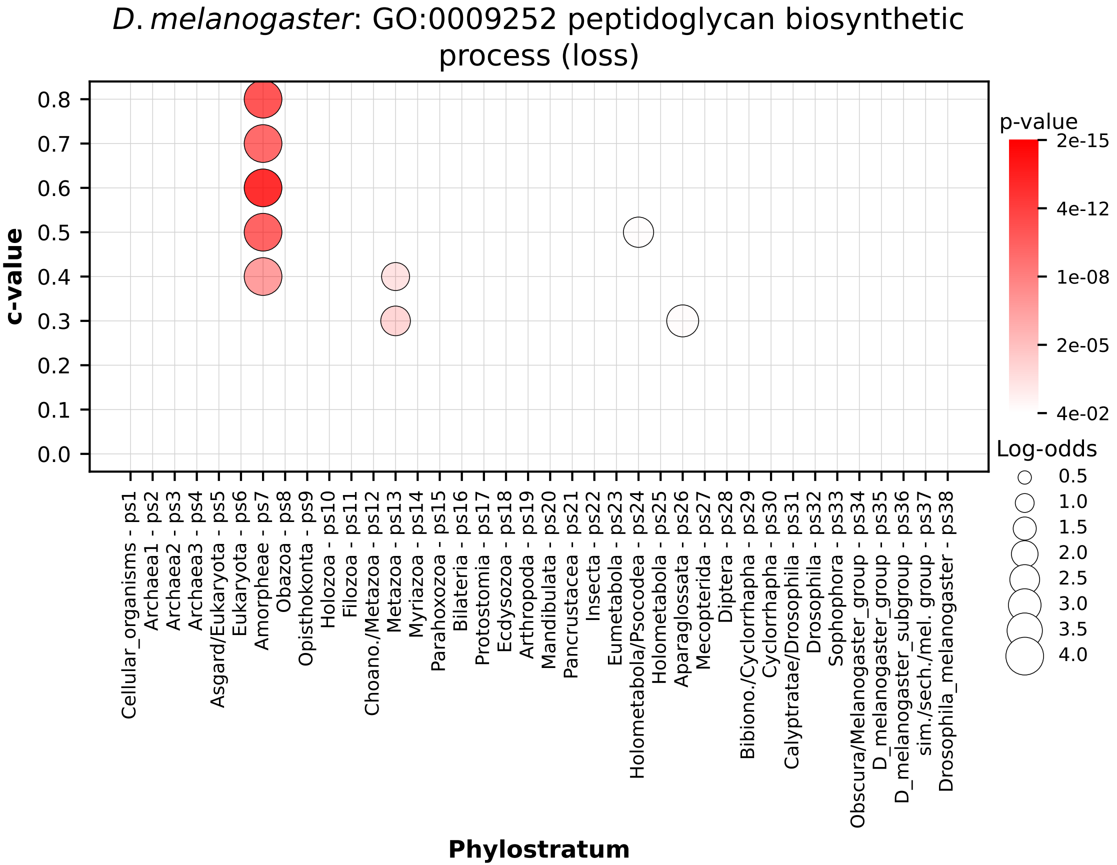

D. melanogaster: GO:0009252 peptidoglycan biosynthetic process (loss)
c-value
0.8
0.7
0.6
0.5
0.4
0.3
0.2
0.1
0.0
Phylostratum
Cellular_organisms - ps1
Archaea1 - ps2
Archaea2 - ps3
Archaea3 - ps4
Asgard/Eukaryota - ps5
Eukaryota - ps6
Amorpheae - ps7
Obazoa - ps8
Opisthokonta - ps9
Holozoa - ps10
Filozoa - ps11
Choano./Metazoa - ps12
Metazoa - ps13
Myriazoa - ps14
Parahoxozoa - ps15
Bilateria - ps16
Protostomia - ps17
Ecdysozoa - ps18
Arthropoda - ps19
Mandibulata - ps20
Pancrustacea - ps21
Insecta - ps22
Eumetabola - ps23
Holometabola/Psocodea - ps24
Holometabola - ps25
Aparaglossata - ps26
Mecopterida - ps27
Diptera - ps28
Bibiono./Cyclorrhapha - ps29
Cyclorrhapha - ps30
Calyptratae/Drosophila - ps31
Drosophila - ps32
Sophophora - ps33
Obscura/Melanogaster_group - ps34
D_melanogaster_group - ps35
D_melanogaster_subgroup - ps36
sim./sech./mel. group - ps37
Drosophila_melanogaster - ps38
p-value
2e-15
4e-12
1e-08
2e-05
4e-02
Log-odds
0.5
1.0
1.5
2.0
2.5
3.0
3.5
4.0

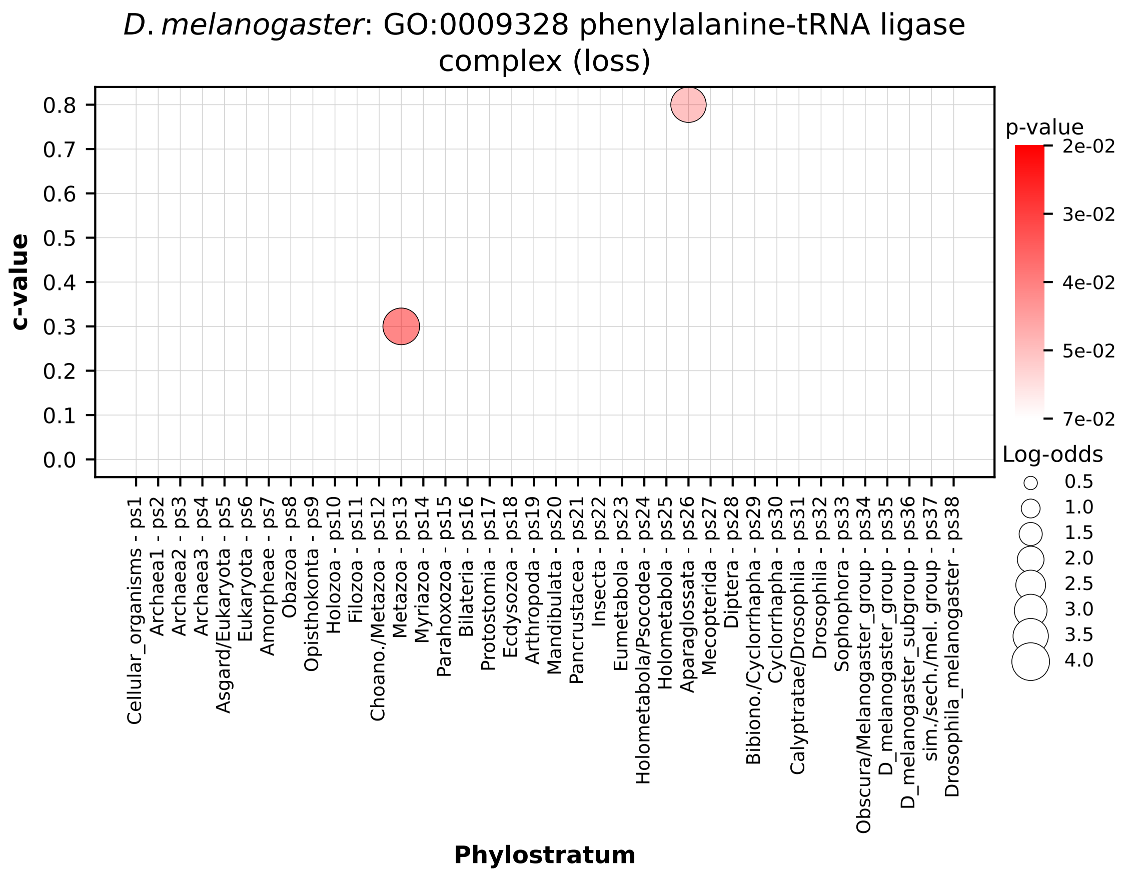
D. melanogaster: GO:0009328 phenylalanine-tRNA ligase complex (loss)
c-value
0.0
0.1
0.2
0.3
0.4
0.5
0.6
0.7
0.8
Phylostratum
Cellular_organisms - ps1
Archaea1 - ps2
Archaea2 - ps3
Archaea3 - ps4
Asgard/Eukaryota - ps5
Eukaryota - ps6
Amorpheae - ps7
Obazoa - ps8
Opisthokonta - ps9
Holozoa - ps10
Filozoa - ps11
Choano./Metazoa - ps12
Metazoa - ps13
Myriazoa - ps14
Parahoxozoa - ps15
Bilateria - ps16
Protostomia - ps17
Ecdysozoa - ps18
Arthropoda - ps19
Mandibulata - ps20
Pancrustacea - ps21
Insecta - ps22
Eumetabola - ps23
Holometabola/Psocodea - ps24
Holometabola - ps25
Aparaglossata - ps26
Mecopterida - ps27
Diptera - ps28
Bibiono./Cyclorrhapha - ps29
Cyclorrhapha - ps30
Calyptratae/Drosophila - ps31
Drosophila - ps32
Sophophora - ps33
Obscura/Melanogaster_group - ps34
D_melanogaster_group - ps35
D_melanogaster_subgroup - ps36
sim./sech./mel. group - ps37
Drosophila_melanogaster - ps38
p-value
2e-02
3e-02
4e-02
5e-02
7e-02
Log-odds
0.5
1.0
1.5
2.0
2.5
3.0
3.5
4.0

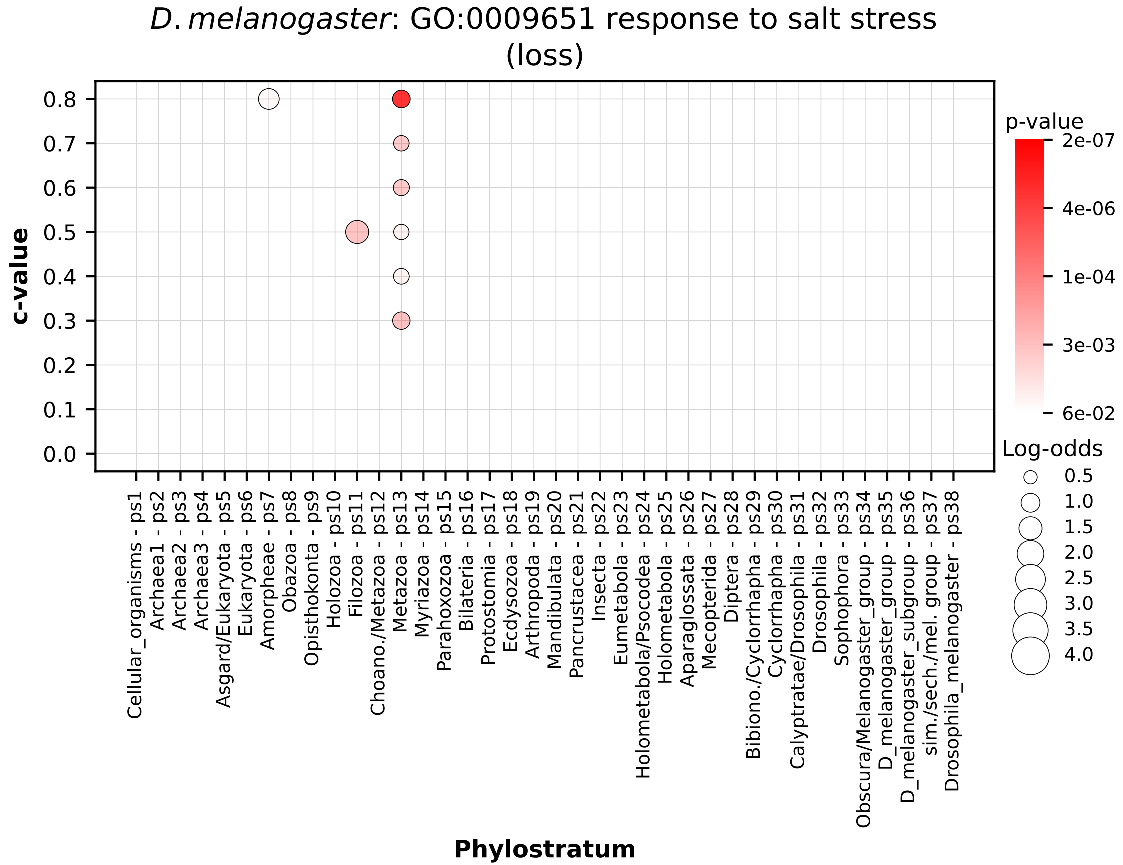
D. melanogaster: GO:0009651 response to salt stress
(loss)
c-value
0.8
0.7
0.6
0.5
0.4
0.3
0.2
0.1
0.0
Phylostratum
Cellular_organisms - ps1
Archaea1 - ps2
Archaea2 - ps3
Archaea3 - ps4
Asgard/Eukaryota - ps5
Eukaryota - ps6
Amorpheae - ps7
Obazoa - ps8
Opisthokonta - ps9
Holozoa - ps10
Filozoa - ps11
Choano./Metazoa - ps12
Metazoa - ps13
Myriazoa - ps14
Parahoxozoa - ps15
Bilateria - ps16
Protostomia - ps17
Ecdysozoa - ps18
Arthropoda - ps19
Mandibulata - ps20
Pancrustacea - ps21
Insecta - ps22
Eumetabola - ps23
Holometabola/Psocodea - ps24
Holometabola - ps25
Aparaglossata - ps26
Mecopterida - ps27
Diptera - ps28
Bibiono./Cyclorrhapha - ps29
Cyclorrhapha - ps30
Calyptratae/Drosophila - ps31
Drosophila - ps32
Sophophora - ps33
Obscura/Melanogaster_group - ps34
D_melanogaster_group - ps35
D_melanogaster_subgroup - ps36
sim./sech./mel. group - ps37
Drosophila_melanogaster - ps38
p-value
2e-07
4e-06
1e-04
3e-03
6e-02
Log-odds
0.5
1.0
1.5
2.0
2.5
3.0
3.5
4.0

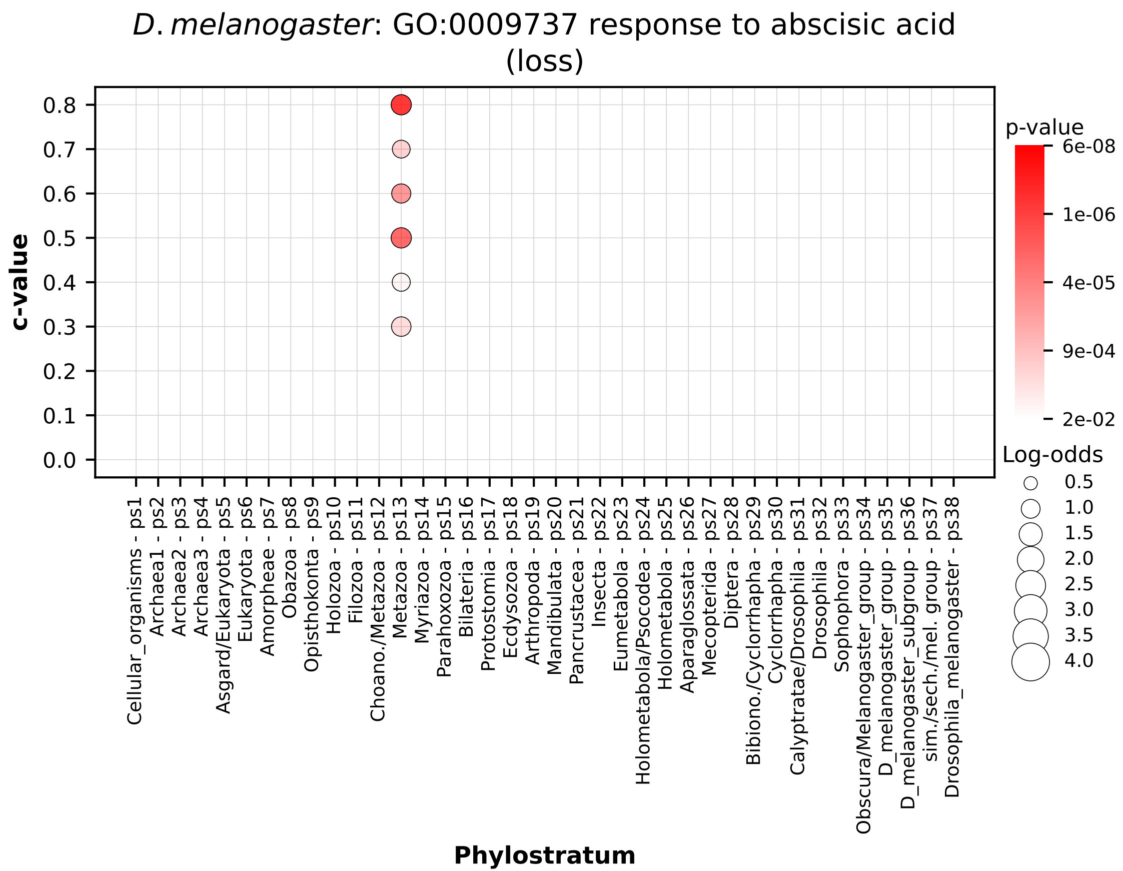
D. melanogaster: GO:0009737 response to abscisic acid (loss)
c-value
0.8
0.7
0.6
0.5
0.4
0.3
0.2
0.1
0.0
Phylostratum
Cellular_organisms - ps1
Archaea1 - ps2
Archaea2 - ps3
Archaea3 - ps4
Asgard/Eukaryota - ps5
Eukaryota - ps6
Amorpheae - ps7
Obazoa - ps8
Opisthokonta - ps9
Holozoa - ps10
Filozoa - ps11
Choano./Metazoa - ps12
Metazoa - ps13
Myriazoa - ps14
Parahoxozoa - ps15
Bilateria - ps16
Protostomia - ps17
Ecdysozoa - ps18
Arthropoda - ps19
Mandibulata - ps20
Pancrustacea - ps21
Insecta - ps22
Eumetabola - ps23
Holometabola/Psocodea - ps24
Holometabola - ps25
Aparaglossata - ps26
Mecopterida - ps27
Diptera - ps28
Bibiono./Cyclorrhapha - ps29
Cyclorrhapha - ps30
Calyptratae/Drosophila - ps31
Drosophila - ps32
Sophophora - ps33
Obscura/Melanogaster_group - ps34
D_melanogaster_group - ps35
D_melanogaster_subgroup - ps36
sim./sech./mel. group - ps37
Drosophila_melanogaster - ps38
p-value
6e-08
1e-06
4e-05
9e-04
2e-02
Log-odds
0.5
1.0
1.5
2.0
2.5
3.0
3.5
4.0

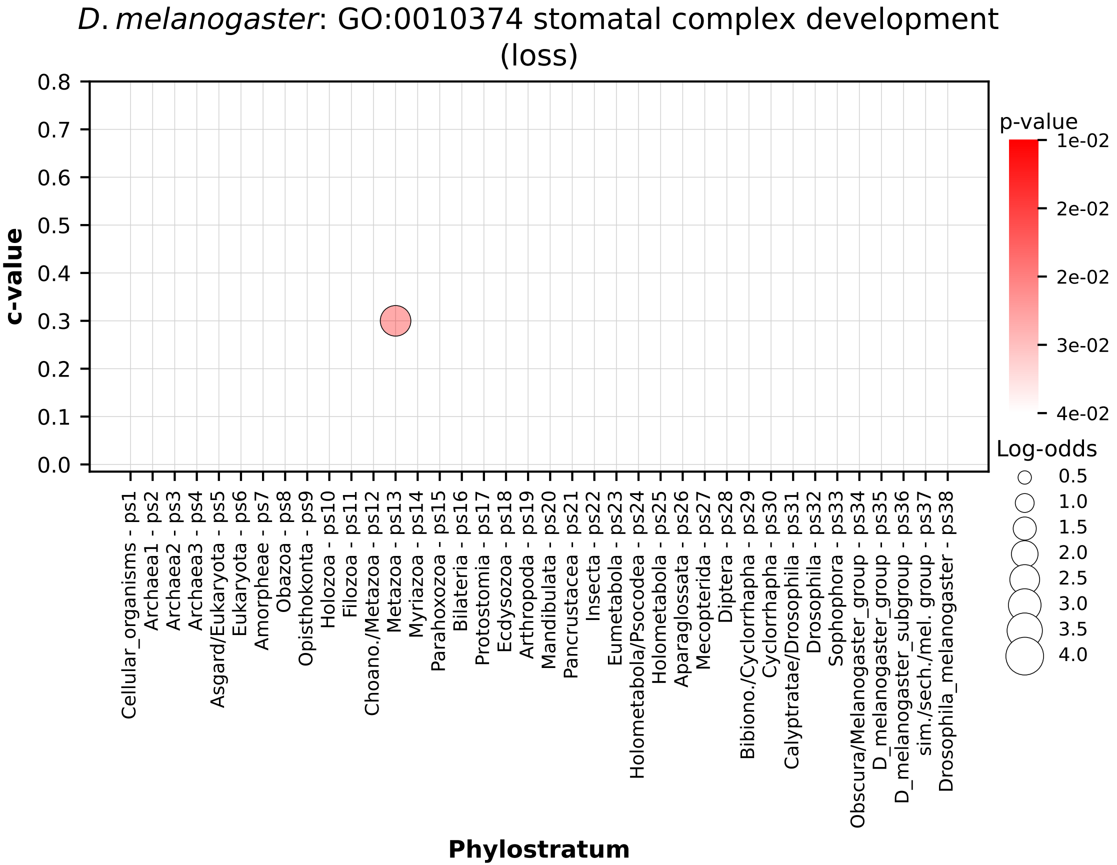

D. melanogaster: GO:0010374 stomatal complex development
(loss)
c-value
0.8
0.7
0.6
0.5
0.4
0.3
0.2
0.1
0.0
Phylostratum
Cellular_organisms - ps1
Archaea1 - ps2
Archaea2 - ps3
Archaea3 - ps4
Asgard/Eukaryota - ps5
Eukaryota - ps6
Amorpheae - ps7
Obazoa - ps8
Opisthokonta - ps9
Holozoa - ps10
Filozoa - ps11
Choano./Metazoa - ps12
Metazoa - ps13
Myriazoa - ps14
Parahoxozoa - ps15
Bilateria - ps16
Protostomia - ps17
Ecdysozoa - ps18
Arthropoda - ps19
Mandibulata - ps20
Pancrustacea - ps21
Insecta - ps22
Eumetabola - ps23
Holometabola/Psocodea - ps24
Holometabola - ps25
Aparaglossata - ps26
Mecopterida - ps27
Diptera - ps28
Bibiono./Cyclorrhapha - ps29
Cyclorrhapha - ps30
Calyptratae/Drosophila - ps31
Drosophila - ps32
Sophophora - ps33
Obscura/Melanogaster_group - ps34
D_melanogaster_group - ps35
D_melanogaster_subgroup - ps36
sim./sech./mel. group - ps37
Drosophila_melanogaster - ps38
p-value
1e-02
2e-02
2e-02
3e-02
4e-02
Log-odds
0.5
1.0
1.5
2.0
2.5
3.0
3.5
4.0

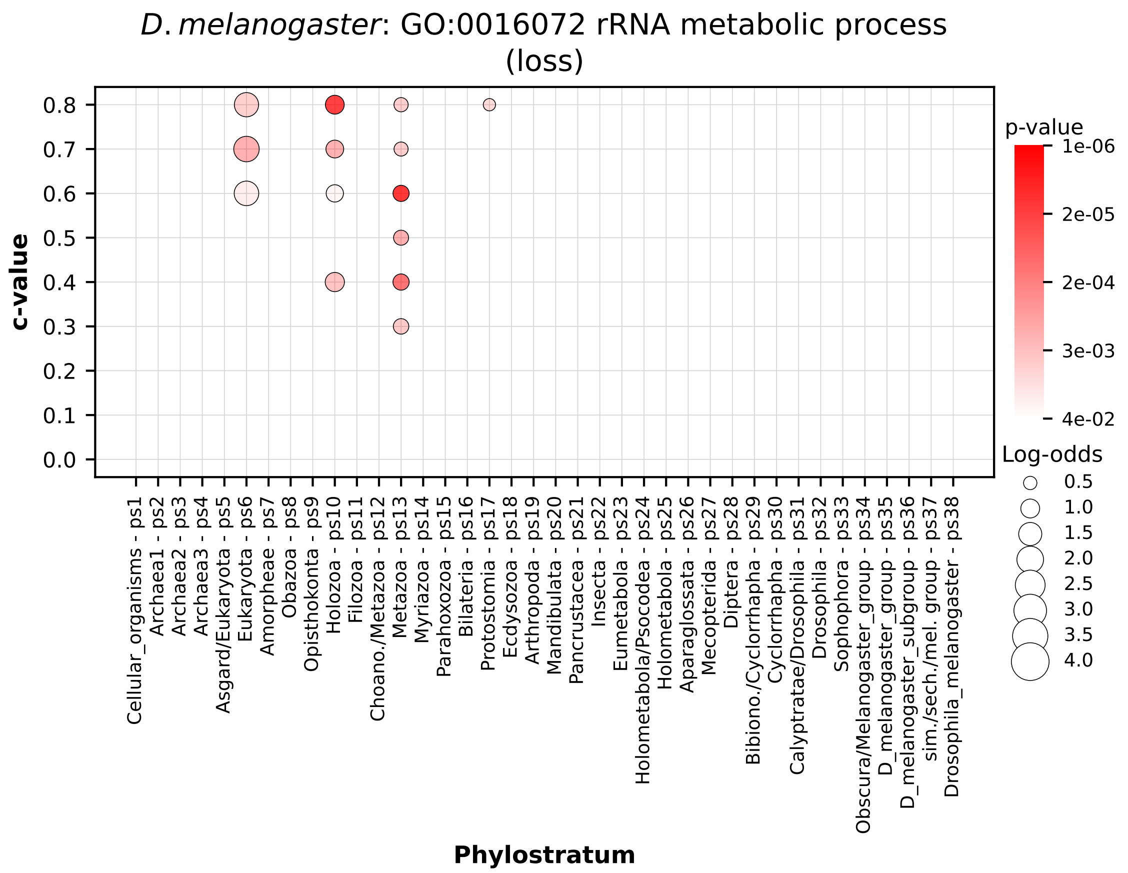

D. melanogaster: GO:0016072 rRNA metabolic process
(loss)
c-value
0.8
0.7
0.6
0.5
0.4
0.3
0.2
0.1
0.0
Phylostratum
Cellular_organisms - ps1
Archaea1 - ps2
Archaea2 - ps3
Archaea3 - ps4
Asgard/Eukaryota - ps5
Eukaryota - ps6
Amorpheae - ps7
Obazoa - ps8
Opisthokonta - ps9
Holozoa - ps10
Filozoa - ps11
Choano./Metazoa - ps12
Metazoa - ps13
Myriazoa - ps14
Parahoxozoa - ps15
Bilateria - ps16
Protostomia - ps17
Ecdysozoa - ps18
Arthropoda - ps19
Mandibulata - ps20
Pancrustacea - ps21
Insecta - ps22
Eumetabola - ps23
Holometabola/Psocodea - ps24
Holometabola - ps25
Aparaglossata - ps26
Mecopterida - ps27
Diptera - ps28
Bibiono./Cyclorrhapha - ps29
Cyclorrhapha - ps30
Calyptratae/Drosophila - ps31
Drosophila - ps32
Sophophora - ps33
Obscura/Melanogaster_group - ps34
D_melanogaster_group - ps35
D_melanogaster_subgroup - ps36
sim./sech./mel. group - ps37
Drosophila_melanogaster - ps38
p-value
1e-06
2e-05
2e-04
3e-03
4e-02
Log-odds
0.5
1.0
1.5
2.0
2.5
3.0
3.5
4.0

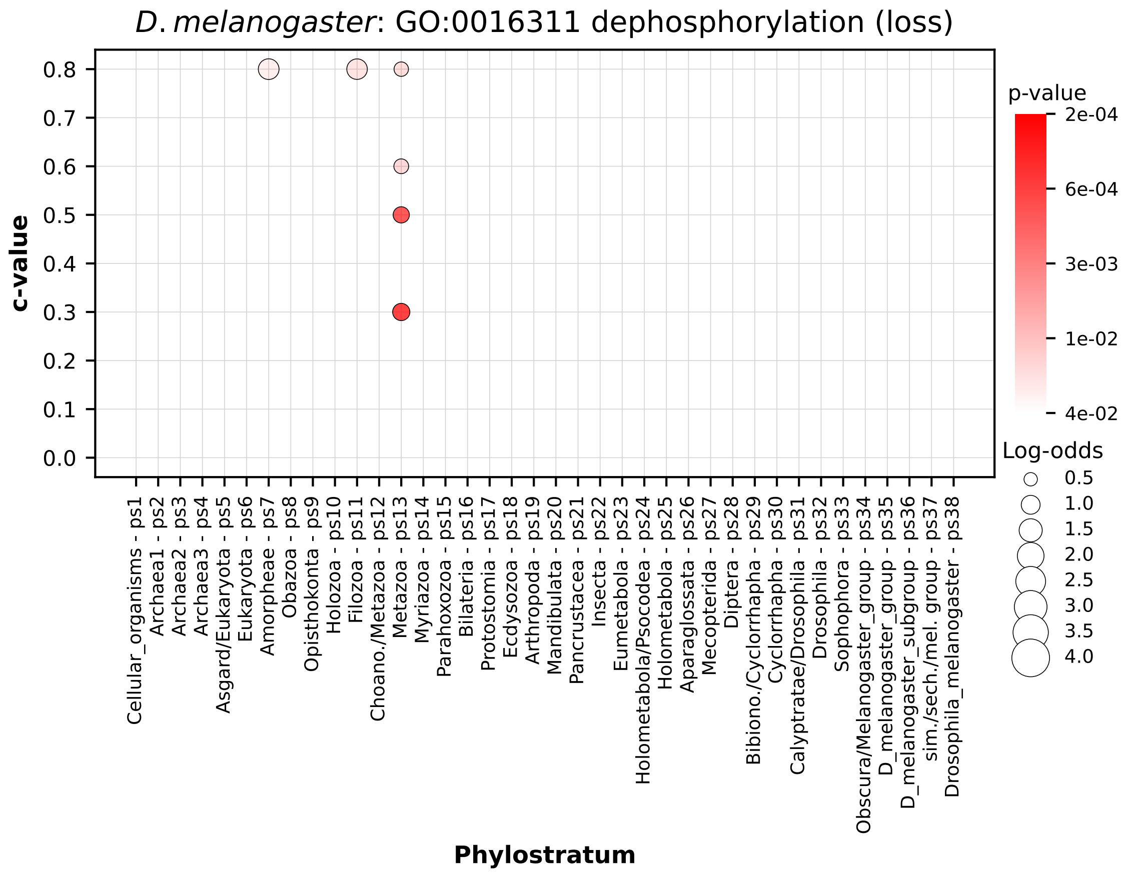

D. melanogaster: GO:0016311 dephosphorylation (loss)
c-value
0.0
0.1
0.2
0.3
0.4
0.5
0.6
0.7
0.8
p-value
2e-04
6e-04
3e-03
1e-02
4e-02
Log-odds
0.5
1.0
1.5
2.0
2.5
3.0
3.5
4.0
Cellular_organisms - ps1
Archaea1 - ps2
Archaea2 - ps3
Archaea3 - ps4
Asgard/Eukaryota - ps5
Eukaryota - ps6
Amorpheae - ps7
Obazoa - ps8
Opisthokonta - ps9
Holozoa - ps10
Filozoa - ps11
Choano./Metazoa - ps12
Metazoa - ps13
Myriazoa - ps14
Parahoxozoa - ps15
Bilateria - ps16
Protostomia - ps17
Ecdysozoa - ps18
Arthropoda - ps19
Mandibulata - ps20
Pancrustacea - ps21
Insecta - ps22
Eumetabola - ps23
Holometabola/Psocodea - ps24
Holometabola - ps25
Aparaglossata - ps26
Mecopterida - ps27
Diptera - ps28
Bibiono./Cyclorrhapha - ps29
Cyclorrhapha - ps30
Calyptratae/Drosophila - ps31
Drosophila - ps32
Sophophora - ps33
Obscura/Melanogaster_group - ps34
D_melanogaster_group - ps35
D_melanogaster_subgroup - ps36
sim./sech./mel. group - ps37
Drosophila_melanogaster - ps38
Phylostratum

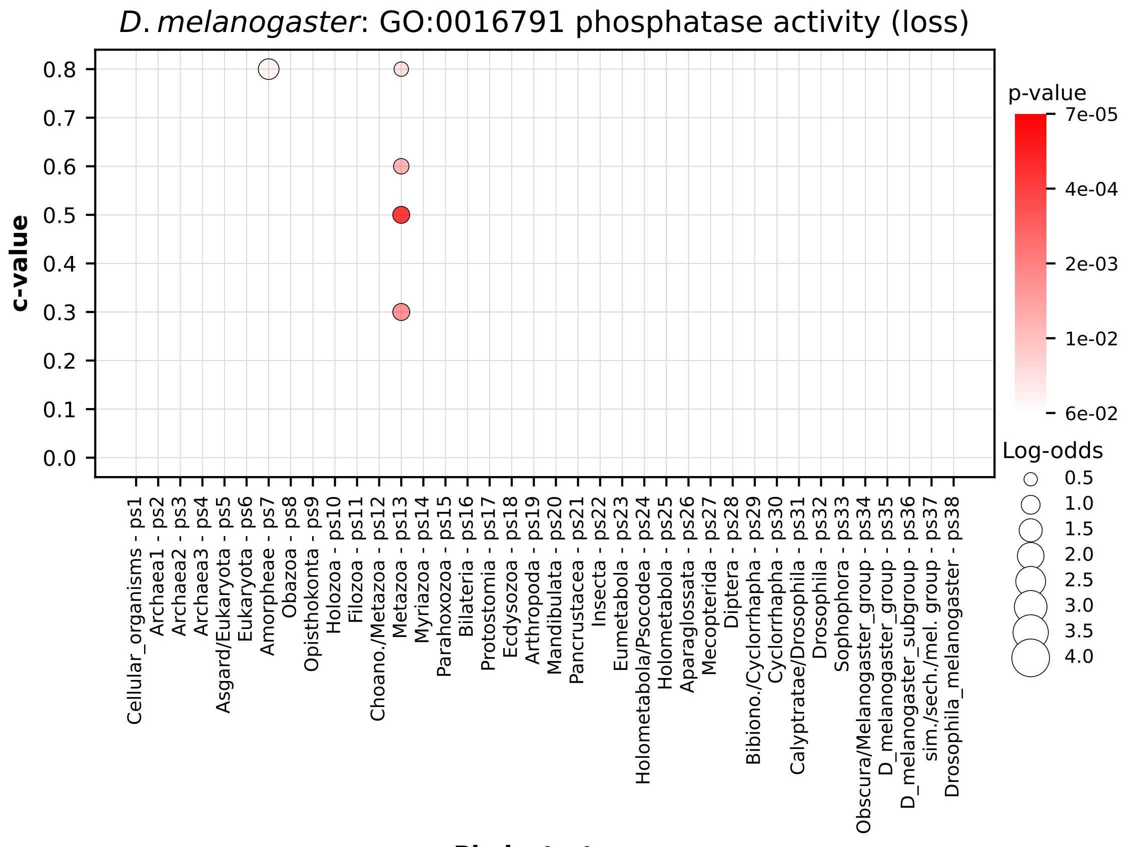

D. melanogaster: GO:0016791 phosphatase activity (loss)
c-value
0.8
0.7
0.6
0.5
0.4
0.3
0.2
0.1
0.0
Phylostratum
Cellular_organisms - ps1
Archaea1 - ps2
Archaea2 - ps3
Archaea3 - ps4
Asgard/Eukaryota - ps5
Eukaryota - ps6
Amorpheae - ps7
Obazoa - ps8
Opisthokonta - ps9
Holozoa - ps10
Filozoa - ps11
Choano./Metazoa - ps12
Metazoa - ps13
Myriazoa - ps14
Parahoxozoa - ps15
Bilateria - ps16
Protostomia - ps17
Ecdysozoa - ps18
Arthropoda - ps19
Mandibulata - ps20
Pancrustacea - ps21
Insecta - ps22
Eumetabola - ps23
Holometabola/Psocodea - ps24
Holometabola - ps25
Aparaglossata - ps26
Mecopterida - ps27
Diptera - ps28
Bibiono./Cyclorrhapha - ps29
Cyclorrhapha - ps30
Calyptratae/Drosophila - ps31
Drosophila - ps32
Sophophora - ps33
Obscura/Melanogaster_group - ps34
D_melanogaster_group - ps35
D_melanogaster_subgroup - ps36
sim./sech./mel. group - ps37
Drosophila_melanogaster - ps38
p-value
7e-05
4e-04
2e-03
1e-02
6e-02
Log-odds
0.5
1.0
1.5
2.0
2.5
3.0
3.5
4.0

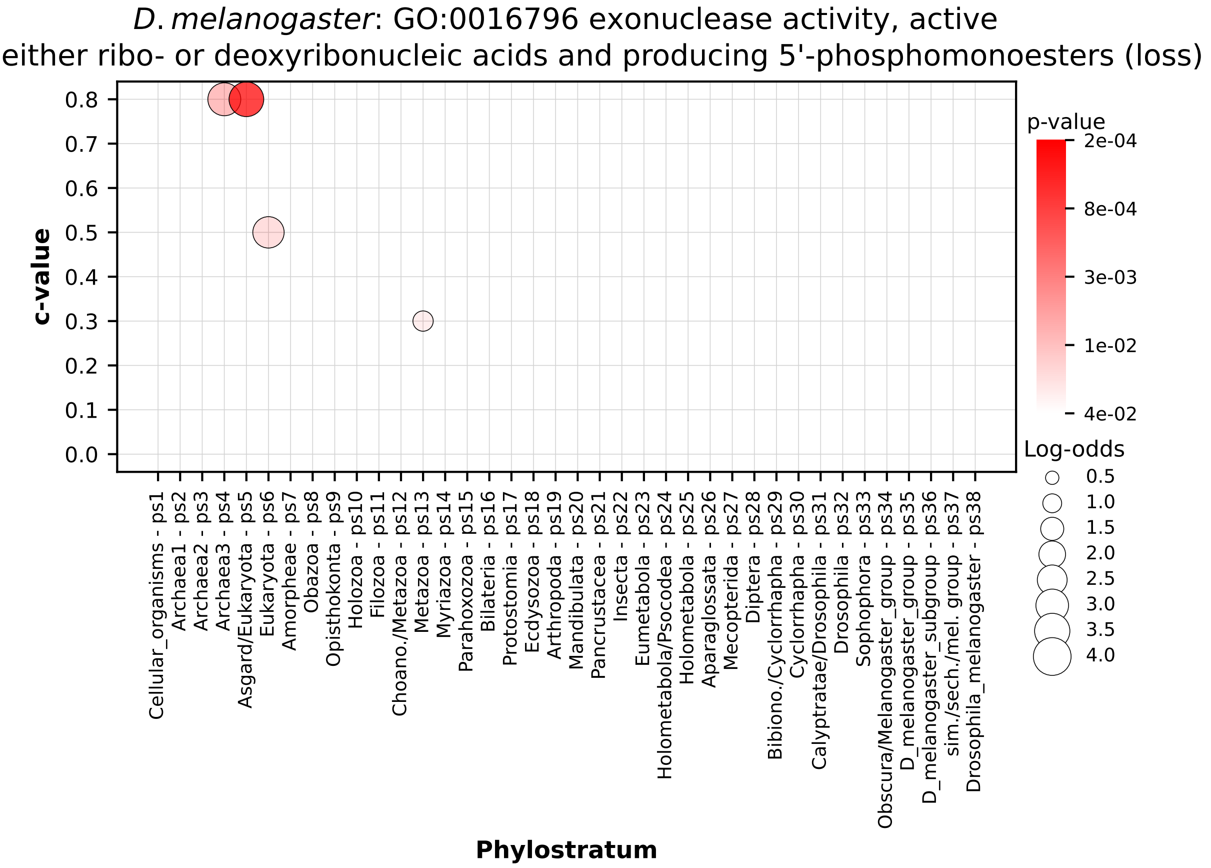
D. melanogaster: GO:0016796 exonuclease activity, active
either ribo- or deoxyribonucleic acids and producing 5'-phosphomonoesters (loss)
c-value
0.0
0.1
0.2
0.3
0.4
0.5
0.6
0.7
0.8
p-value
2e-04
8e-04
3e-03
1e-02
4e-02
Log-odds
0.5
1.0
1.5
2.0
2.5
3.0
3.5
4.0
Cellular_organisms - ps1
Archaea1 - ps2
Archaea2 - ps3
Archaea3 - ps4
Asgard/Eukaryota - ps5
Eukaryota - ps6
Amorpheae - ps7
Obazoa - ps8
Opisthokonta - ps9
Holozoa - ps10
Filozoa - ps11
Choano./Metazoa - ps12
Metazoa - ps13
Myriazoa - ps14
Parahoxozoa - ps15
Bilateria - ps16
Protostomia - ps17
Ecdysozoa - ps18
Arthropoda - ps19
Mandibulata - ps20
Pancrustacea - ps21
Insecta - ps22
Eumetabola - ps23
Holometabola/Psocodea - ps24
Holometabola - ps25
Aparaglossata - ps26
Mecopterida - ps27
Diptera - ps28
Bibiono./Cyclorrhapha - ps29
Cyclorrhapha - ps30
Calyptratae/Drosophila - ps31
Drosophila - ps32
Sophophora - ps33
Obscura/Melanogaster_group - ps34
D_melanogaster_group - ps35
D_melanogaster_subgroup - ps36
sim./sech./mel. group - ps37
Drosophila_melanogaster - ps38
Phylostratum

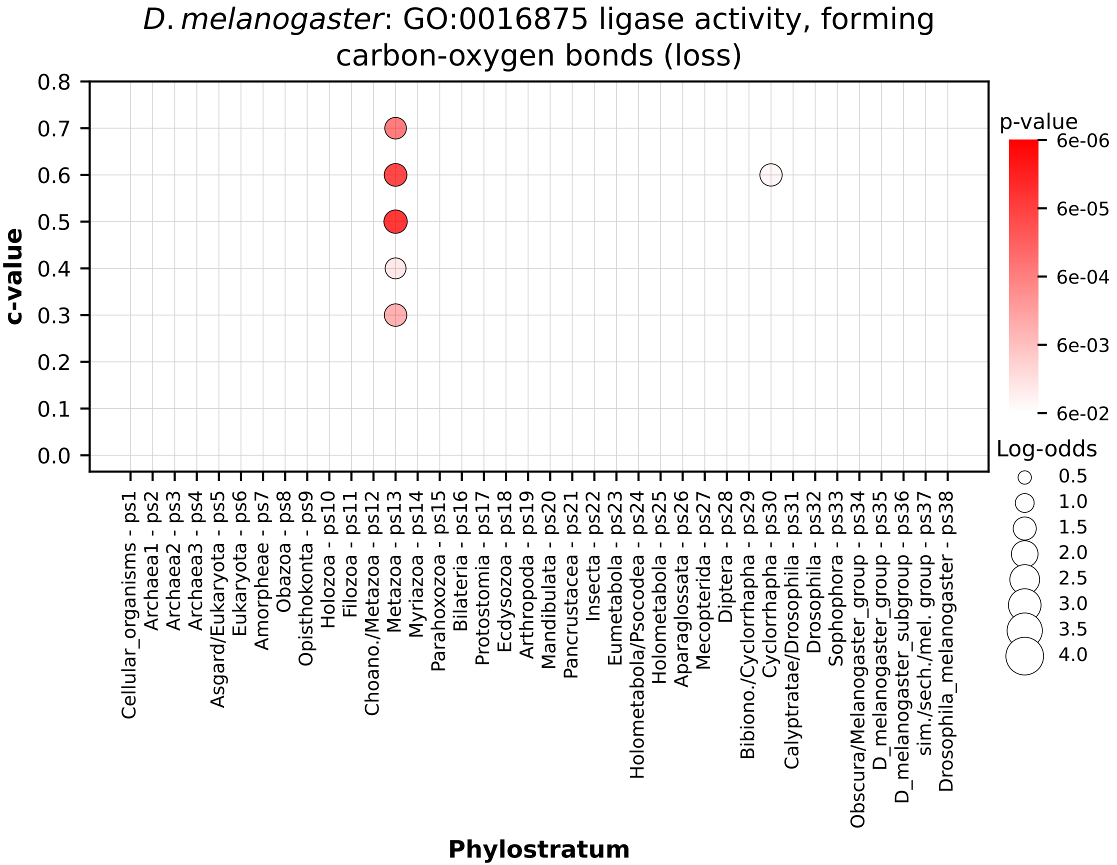

D. melanogaster: GO:0016875 ligase activity, forming carbon-oxygen bonds (loss)
c-value
0.8
0.7
0.6
0.5
0.4
0.3
0.2
0.1
0.0
p-value
6e-06
6e-05
6e-04
6e-03
6e-02
Log-odds
0.5
1.0
1.5
2.0
2.5
3.0
3.5
4.0
Cellular_organisms - ps1
Archaea1 - ps2
Archaea2 - ps3
Archaea3 - ps4
Asgard/Eukaryota - ps5
Eukaryota - ps6
Amorpheae - ps7
Obazoa - ps8
Opisthokonta - ps9
Holozoa - ps10
Filozoa - ps11
Choano./Metazoa - ps12
Metazoa - ps13
Myriazoa - ps14
Parahoxozoa - ps15
Bilateria - ps16
Protostomia - ps17
Ecdysozoa - ps18
Arthropoda - ps19
Mandibulata - ps20
Pancrustacea - ps21
Insecta - ps22
Eumetabola - ps23
Holometabola/Psocodea - ps24
Holometabola - ps25
Aparaglossata - ps26
Mecopterida - ps27
Diptera - ps28
Bibiono./Cyclorrhapha - ps29
Cyclorrhapha - ps30
Calyptratae/Drosophila - ps31
Drosophila - ps32
Sophophora - ps33
Obscura/Melanogaster_group - ps34
D_melanogaster_group - ps35
D_melanogaster_subgroup - ps36
sim./sech./mel. group - ps37
Drosophila_melanogaster - ps38
Phylostratum

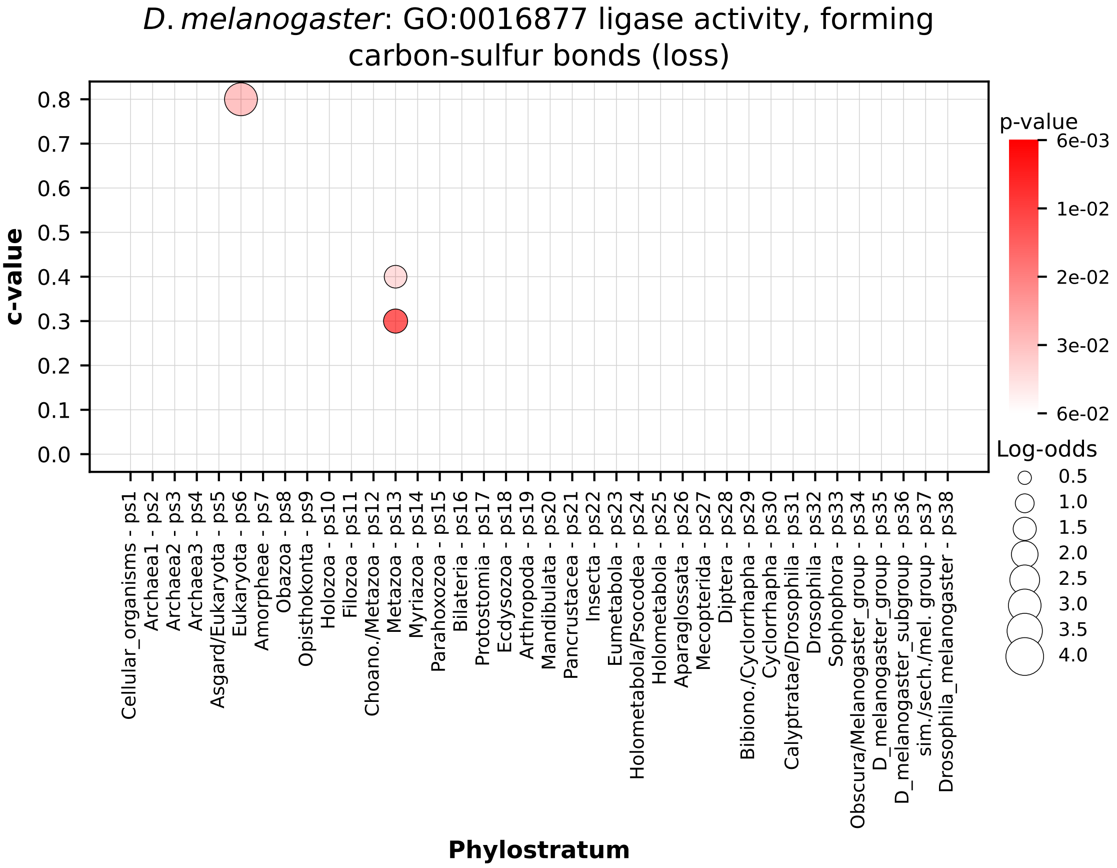

D. melanogaster: GO:0016877 ligase activity, forming carbon-sulfur bonds (loss)
c-value
0.0
0.1
0.2
0.3
0.4
0.5
0.6
0.7
0.8
Phylostratum
Cellular_organisms - ps1
Archaea1 - ps2
Archaea2 - ps3
Archaea3 - ps4
Asgard/Eukaryota - ps5
Eukaryota - ps6
Amorpheae - ps7
Obazoa - ps8
Opisthokonta - ps9
Holozoa - ps10
Filozoa - ps11
Choano./Metazoa - ps12
Metazoa - ps13
Myriazoa - ps14
Parahoxozoa - ps15
Bilateria - ps16
Protostomia - ps17
Ecdysozoa - ps18
Arthropoda - ps19
Mandibulata - ps20
Pancrustacea - ps21
Insecta - ps22
Eumetabola - ps23
Holometabola/Psocodea - ps24
Holometabola - ps25
Aparaglossata - ps26
Mecopterida - ps27
Diptera - ps28
Bibiono./Cyclorrhapha - ps29
Cyclorrhapha - ps30
Calyptratae/Drosophila - ps31
Drosophila - ps32
Sophophora - ps33
Obscura/Melanogaster_group - ps34
D_melanogaster_group - ps35
D_melanogaster_subgroup - ps36
sim./sech./mel. group - ps37
Drosophila_melanogaster - ps38
p-value
6e-03
1e-02
2e-02
3e-02
6e-02
Log-odds
0.5
1.0
1.5
2.0
2.5
3.0
3.5
4.0

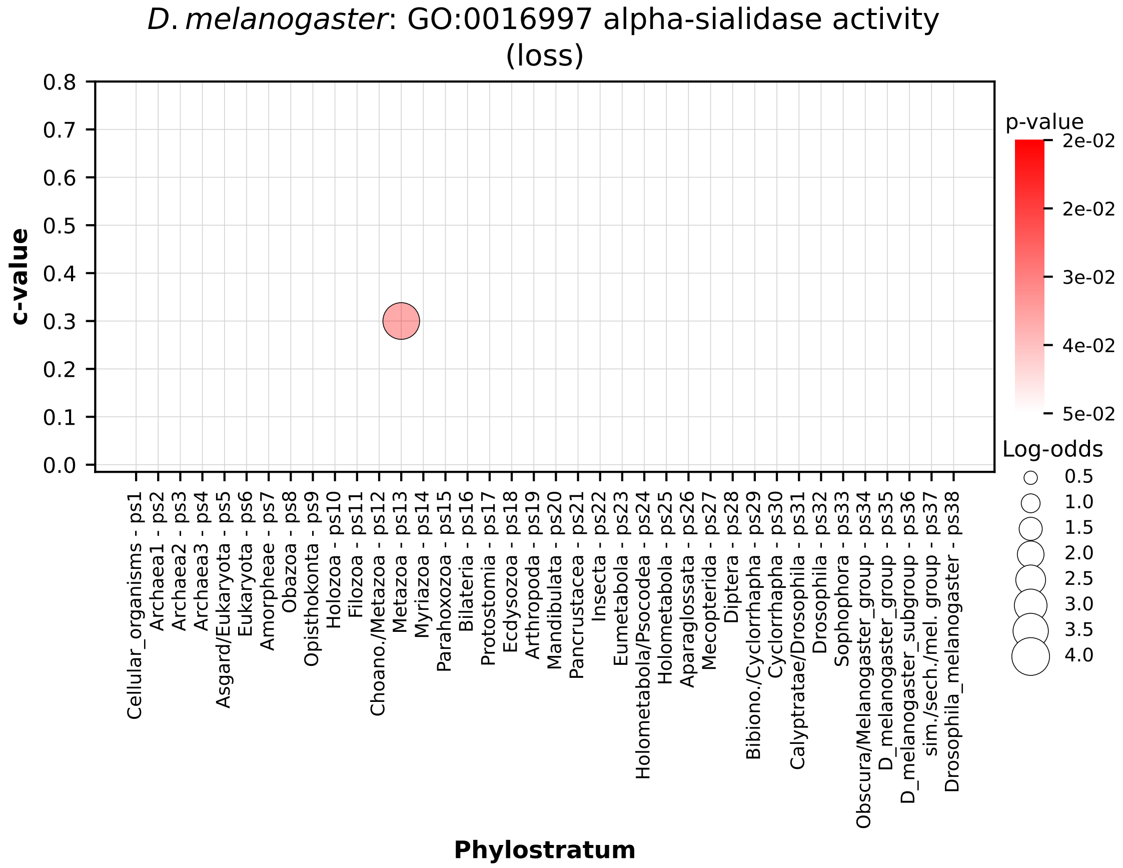
D. melanogaster: GO:0016997 alpha-sialidase activity
(loss)
c-value
0.8
0.7
0.6
0.5
0.4
0.3
0.2
0.1
0.0
p-value
2e-02
2e-02
3e-02
4e-02
5e-02
Log-odds
0.5
1.0
1.5
2.0
2.5
3.0
3.5
4.0
Cellular_organisms - ps1
Archaea1 - ps2
Archaea2 - ps3
Archaea3 - ps4
Asgard/Eukaryota - ps5
Eukaryota - ps6
Amorpheae - ps7
Obazoa - ps8
Opisthokonta - ps9
Holozoa - ps10
Filozoa - ps11
Choano./Metazoa - ps12
Metazoa - ps13
Myriazoa - ps14
Parahoxozoa - ps15
Bilateria - ps16
Protostomia - ps17
Ecdysozoa - ps18
Arthropoda - ps19
Mandibulata - ps20
Pancrustacea - ps21
Insecta - ps22
Eumetabola - ps23
Holometabola/Psocodea - ps24
Holometabola - ps25
Aparaglossata - ps26
Mecopterida - ps27
Diptera - ps28
Bibiono./Cyclorrhapha - ps29
Cyclorrhapha - ps30
Calyptratae/Drosophila - ps31
Drosophila - ps32
Sophophora - ps33
Obscura/Melanogaster_group - ps34
D_melanogaster_group - ps35
D_melanogaster_subgroup - ps36
sim./sech./mel. group - ps37
Drosophila_melanogaster - ps38
Phylostratum

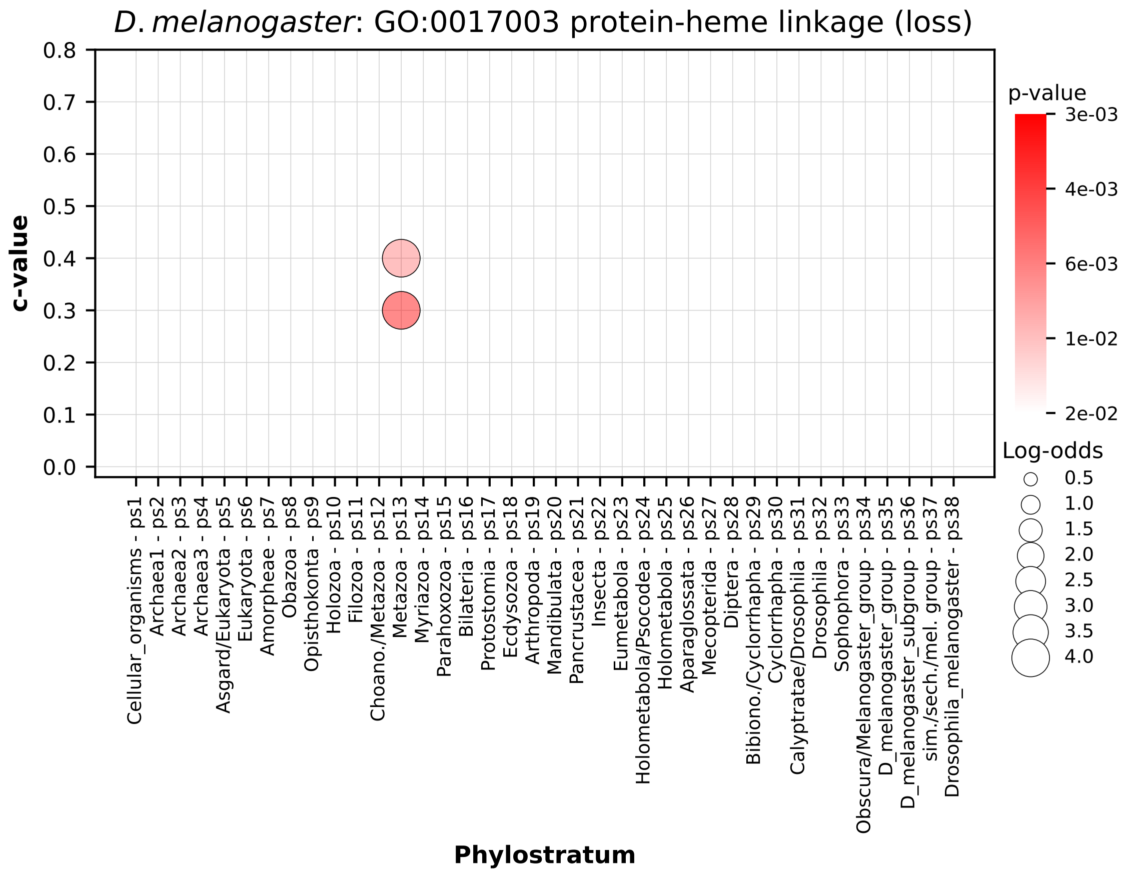
D. melanogaster: GO:0017003 protein-heme linkage (loss)
c-value
0.0
0.1
0.2
0.3
0.4
0.5
0.6
0.7
0.8
Phylostratum
Cellular_organisms - ps1
Archaea1 - ps2
Archaea2 - ps3
Archaea3 - ps4
Asgard/Eukaryota - ps5
Eukaryota - ps6
Amorpheae - ps7
Obazoa - ps8
Opisthokonta - ps9
Holozoa - ps10
Filozoa - ps11
Choano./Metazoa - ps12
Metazoa - ps13
Myriazoa - ps14
Parahoxozoa - ps15
Bilateria - ps16
Protostomia - ps17
Ecdysozoa - ps18
Arthropoda - ps19
Mandibulata - ps20
Pancrustacea - ps21
Insecta - ps22
Eumetabola - ps23
Holometabola/Psocodea - ps24
Holometabola - ps25
Aparaglossata - ps26
Mecopterida - ps27
Diptera - ps28
Bibiono./Cyclorrhapha - ps29
Cyclorrhapha - ps30
Calyptratae/Drosophila - ps31
Drosophila - ps32
Sophophora - ps33
Obscura/Melanogaster_group - ps34
D_melanogaster_group - ps35
D_melanogaster_subgroup - ps36
sim./sech./mel. group - ps37
Drosophila_melanogaster - ps38
p-value
3e-03
4e-03
6e-03
1e-02
2e-02
Log-odds
0.5
1.0
1.5
2.0
2.5
3.0
3.5
4.0

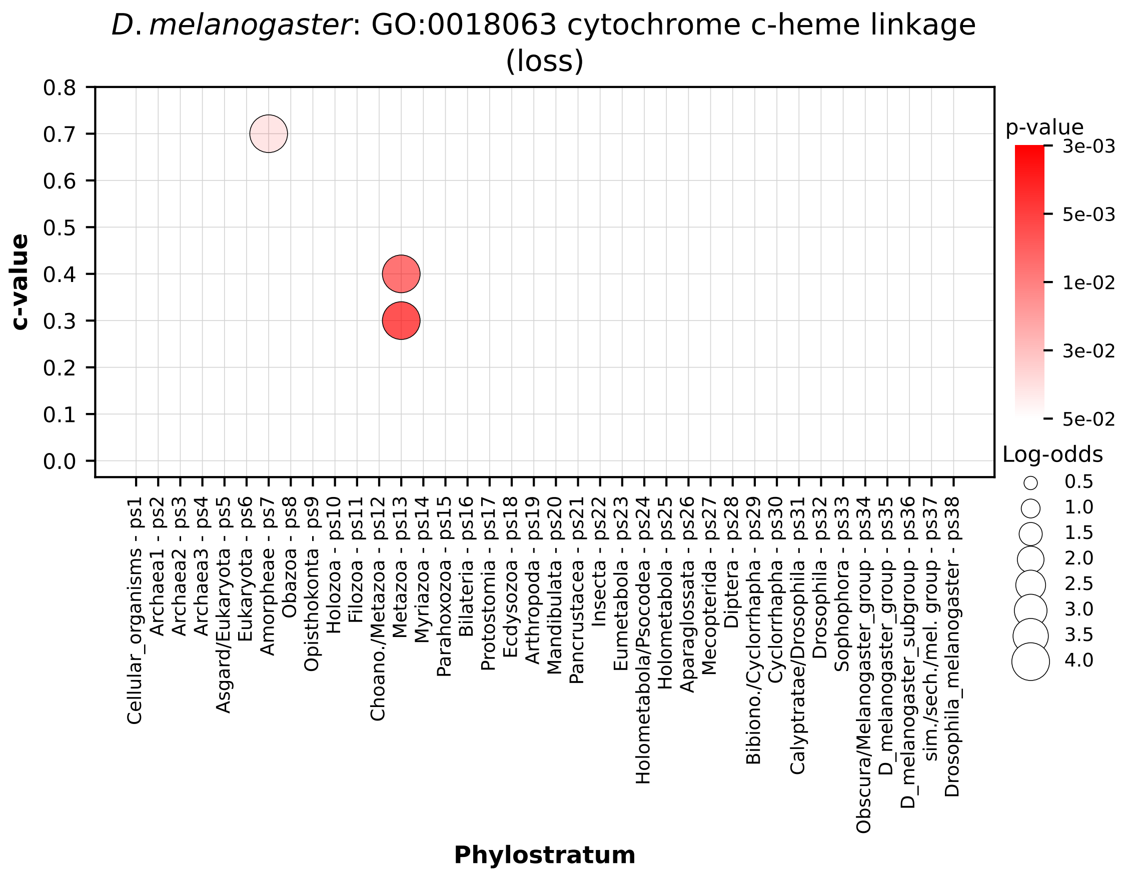
D. melanogaster: GO:0018063 cytochrome c-heme linkage
(loss)
c-value
0.8
0.7
0.6
0.5
0.4
0.3
0.2
0.1
0.0
p-value
3e-03
5e-03
1e-02
3e-02
5e-02
Log-odds
0.5
1.0
1.5
2.0
2.5
3.0
3.5
4.0
Cellular_organisms - ps1
Archaea1 - ps2
Archaea2 - ps3
Archaea3 - ps4
Asgard/Eukaryota - ps5
Eukaryota - ps6
Amorpheae - ps7
Obazoa - ps8
Opisthokonta - ps9
Holozoa - ps10
Filozoa - ps11
Choano./Metazoa - ps12
Metazoa - ps13
Myriazoa - ps14
Parahoxozoa - ps15
Bilateria - ps16
Protostomia - ps17
Ecdysozoa - ps18
Arthropoda - ps19
Mandibulata - ps20
Pancrustacea - ps21
Insecta - ps22
Eumetabola - ps23
Holometabola/Psocodea - ps24
Holometabola - ps25
Aparaglossata - ps26
Mecopterida - ps27
Diptera - ps28
Bibiono./Cyclorrhapha - ps29
Cyclorrhapha - ps30
Calyptratae/Drosophila - ps31
Drosophila - ps32
Sophophora - ps33
Obscura/Melanogaster_group - ps34
D_melanogaster_group - ps35
D_melanogaster_subgroup - ps36
sim./sech./mel. group - ps37
Drosophila_melanogaster - ps38
Phylostratum

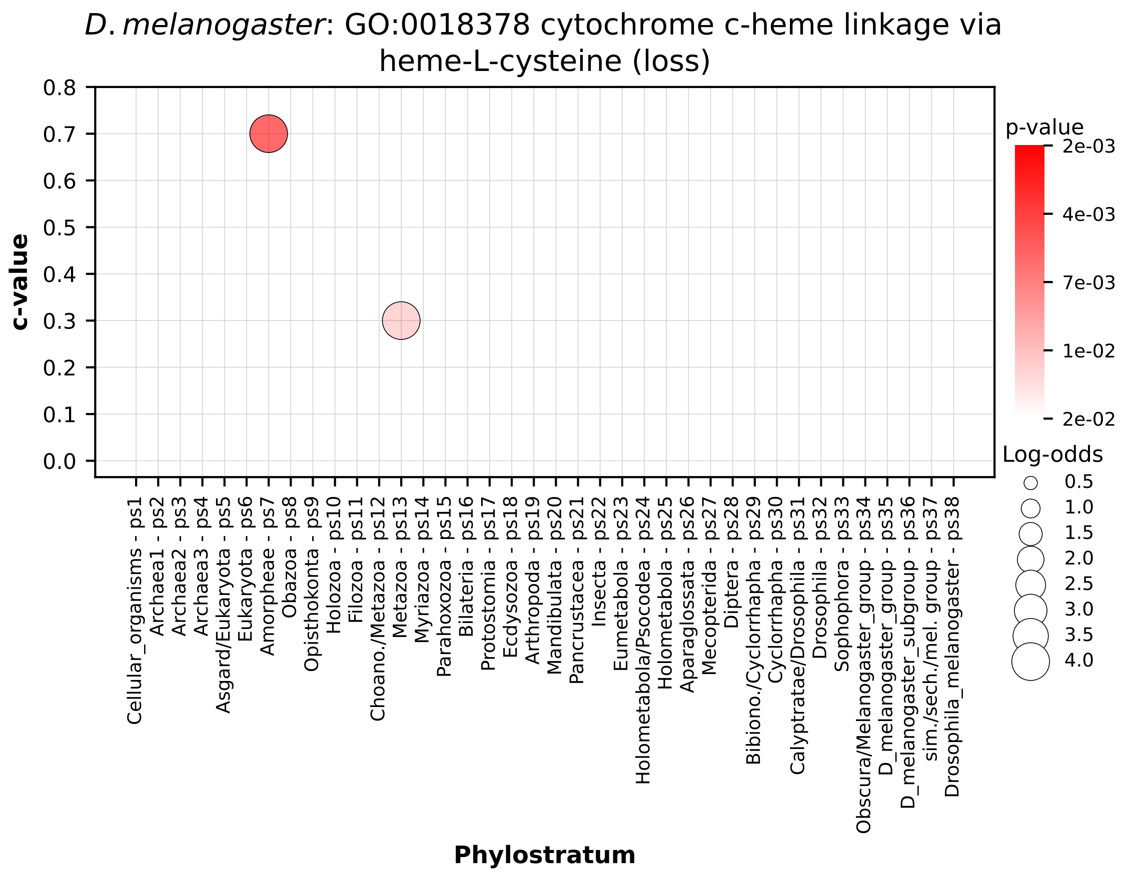

D. melanogaster: GO:0018378 cytochrome c-heme linkage via heme-L-cysteine (loss)
c-value
0.8
0.7
0.6
0.5
0.4
0.3
0.2
0.1
0.0
p-value
2e-03
4e-03
7e-03
1e-02
2e-02
Log-odds
0.5
1.0
1.5
2.0
2.5
3.0
3.5
4.0
Cellular_organisms - ps1
Archaea1 - ps2
Archaea2 - ps3
Archaea3 - ps4
Asgard/Eukaryota - ps5
Eukaryota - ps6
Amorpheae - ps7
Obazoa - ps8
Opisthokonta - ps9
Holozoa - ps10
Filozoa - ps11
Choano./Metazoa - ps12
Metazoa - ps13
Myriazoa - ps14
Parahoxozoa - ps15
Bilateria - ps16
Protostomia - ps17
Ecdysozoa - ps18
Arthropoda - ps19
Mandibulata - ps20
Pancrustacea - ps21
Insecta - ps22
Eumetabola - ps23
Holometabola/Psocodea - ps24
Holometabola - ps25
Aparaglossata - ps26
Mecopterida - ps27
Diptera - ps28
Bibiono./Cyclorrhapha - ps29
Cyclorrhapha - ps30
Calyptratae/Drosophila - ps31
Drosophila - ps32
Sophophora - ps33
Obscura/Melanogaster_group - ps34
D_melanogaster_group - ps35
D_melanogaster_subgroup - ps36
sim./sech./mel. group - ps37
Drosophila_melanogaster - ps38
Phylostratum

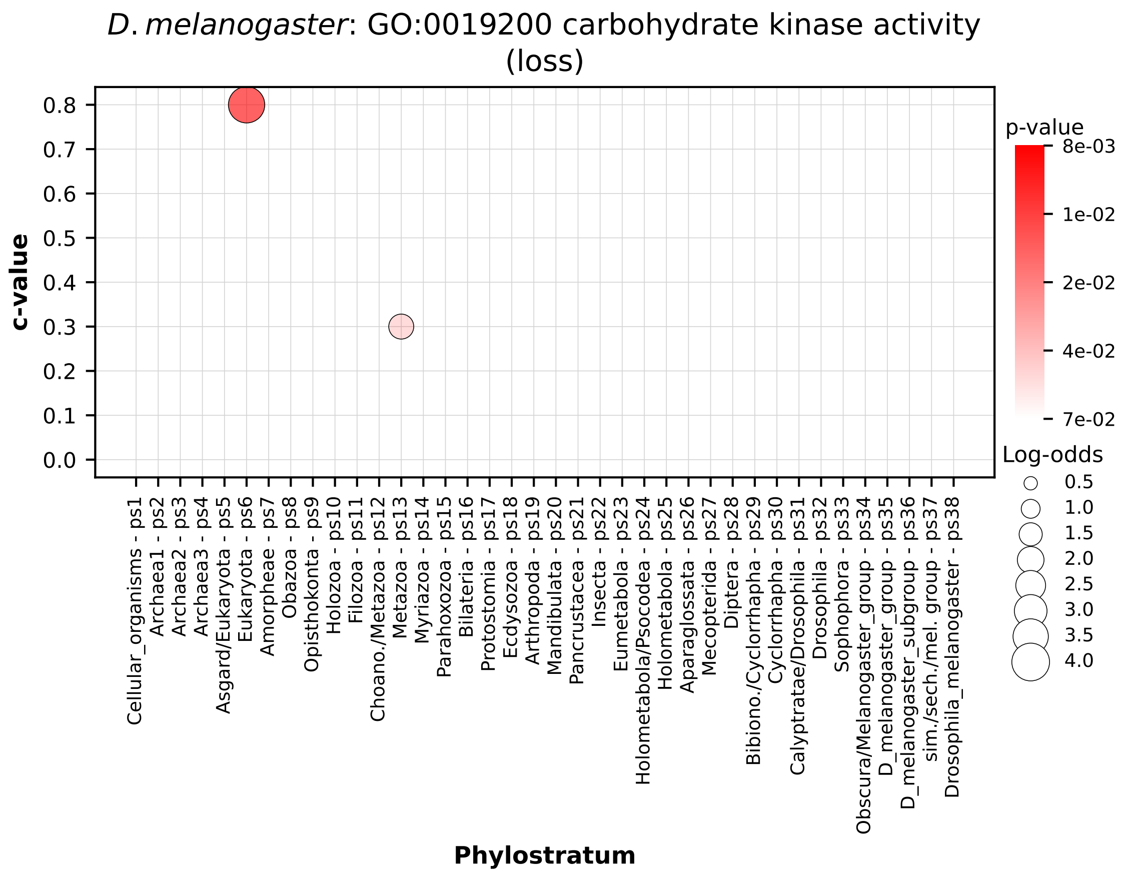

D. melanogaster: GO:0019200 carbohydrate kinase activity (loss)
c-value
0.0
0.1
0.2
0.3
0.4
0.5
0.6
0.7
0.8
p-value
8e-03
1e-02
2e-02
4e-02
7e-02
Log-odds
0.5
1.0
1.5
2.0
2.5
3.0
3.5
4.0
Cellular_organisms - ps1
Archaea1 - ps2
Archaea2 - ps3
Archaea3 - ps4
Asgard/Eukaryota - ps5
Eukaryota - ps6
Amorpheae - ps7
Obazoa - ps8
Opisthokonta - ps9
Holozoa - ps10
Filozoa - ps11
Choano./Metazoa - ps12
Metazoa - ps13
Myriazoa - ps14
Parahoxozoa - ps15
Bilateria - ps16
Protostomia - ps17
Ecdysozoa - ps18
Arthropoda - ps19
Mandibulata - ps20
Pancrustacea - ps21
Insecta - ps22
Eumetabola - ps23
Holometabola/Psocodea - ps24
Holometabola - ps25
Aparaglossata - ps26
Mecopterida - ps27
Diptera - ps28
Bibiono./Cyclorrhapha - ps29
Cyclorrhapha - ps30
Calyptratae/Drosophila - ps31
Drosophila - ps32
Sophophora - ps33
Obscura/Melanogaster_group - ps34
D_melanogaster_group - ps35
D_melanogaster_subgroup - ps36
sim./sech./mel. group - ps37
Drosophila_melanogaster - ps38
Phylostratum

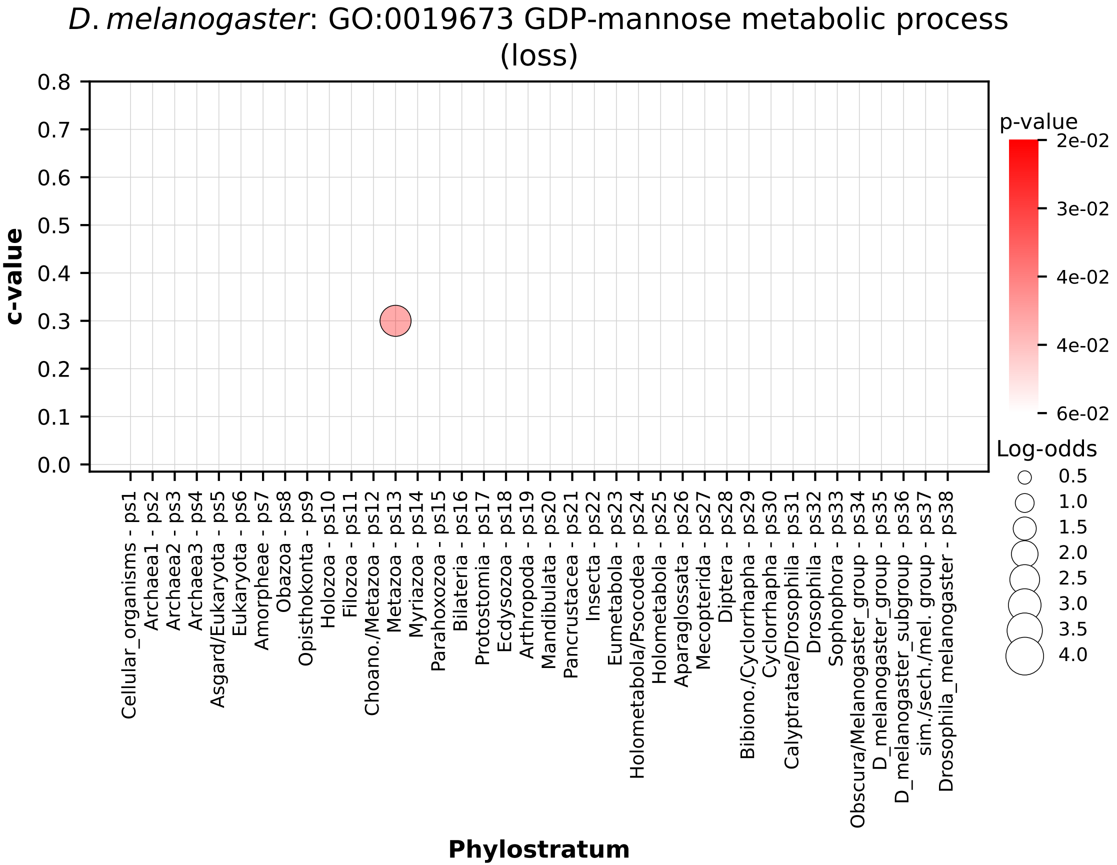

D. melanogaster: GO:0019673 GDP-mannose metabolic process (loss)
c-value
0.0
0.1
0.2
0.3
0.4
0.5
0.6
0.7
0.8
Phylostratum
Cellular_organisms - ps1
Archaea1 - ps2
Archaea2 - ps3
Archaea3 - ps4
Asgard/Eukaryota - ps5
Eukaryota - ps6
Amorpheae - ps7
Obazoa - ps8
Opisthokonta - ps9
Holozoa - ps10
Filozoa - ps11
Choano./Metazoa - ps12
Metazoa - ps13
Myriazoa - ps14
Parahoxozoa - ps15
Bilateria - ps16
Protostomia - ps17
Ecdysozoa - ps18
Arthropoda - ps19
Mandibulata - ps20
Pancrustacea - ps21
Insecta - ps22
Eumetabola - ps23
Holometabola/Psocodea - ps24
Holometabola - ps25
Aparaglossata - ps26
Mecopterida - ps27
Diptera - ps28
Bibiono./Cyclorrhapha - ps29
Cyclorrhapha - ps30
Calyptratae/Drosophila - ps31
Drosophila - ps32
Sophophora - ps33
Obscura/Melanogaster_group - ps34
D_melanogaster_group - ps35
D_melanogaster_subgroup - ps36
sim./sech./mel. group - ps37
Drosophila_melanogaster - ps38
p-value
2e-02
3e-02
4e-02
4e-02
6e-02
Log-odds
0.5
1.0
1.5
2.0
2.5
3.0
3.5
4.0

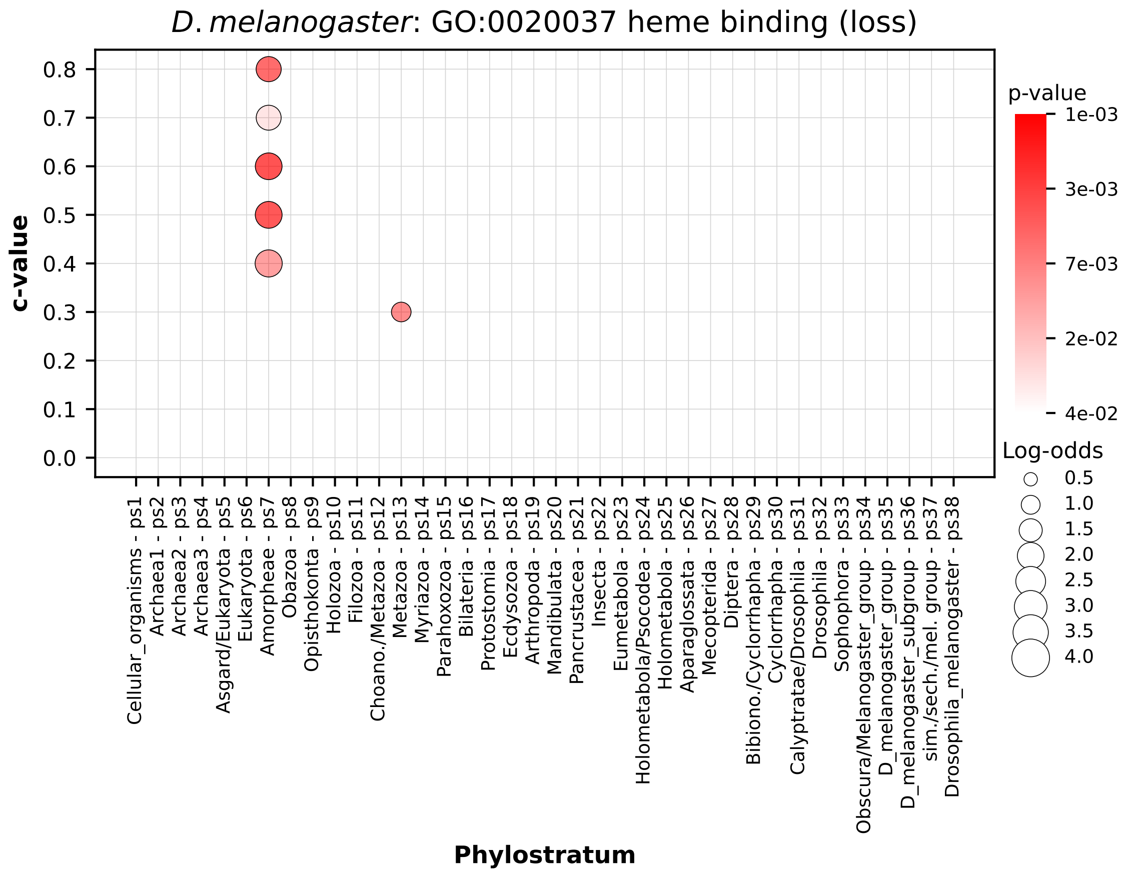

D. melanogaster: GO:0020037 heme binding (loss)
c-value
0.0
0.1
0.2
0.3
0.4
0.5
0.6
0.7
0.8
Phylostratum
Cellular_organisms - ps1
Archaea1 - ps2
Archaea2 - ps3
Archaea3 - ps4
Asgard/Eukaryota - ps5
Eukaryota - ps6
Amorpheae - ps7
Obazoa - ps8
Opisthokonta - ps9
Holozoa - ps10
Filozoa - ps11
Choano./Metazoa - ps12
Metazoa - ps13
Myriazoa - ps14
Parahoxozoa - ps15
Bilateria - ps16
Protostomia - ps17
Ecdysozoa - ps18
Arthropoda - ps19
Mandibulata - ps20
Pancrustacea - ps21
Insecta - ps22
Eumetabola - ps23
Holometabola/Psocodea - ps24
Holometabola - ps25
Aparaglossata - ps26
Mecopterida - ps27
Diptera - ps28
Bibiono./Cyclorrhapha - ps29
Cyclorrhapha - ps30
Calyptratae/Drosophila - ps31
Drosophila - ps32
Sophophora - ps33
Obscura/Melanogaster_group - ps34
D_melanogaster_group - ps35
D_melanogaster_subgroup - ps36
sim./sech./mel. group - ps37
Drosophila_melanogaster - ps38
p-value
1e-03
3e-03
7e-03
2e-02
4e-02
Log-odds
0.5
1.0
1.5
2.0
2.5
3.0
3.5
4.0

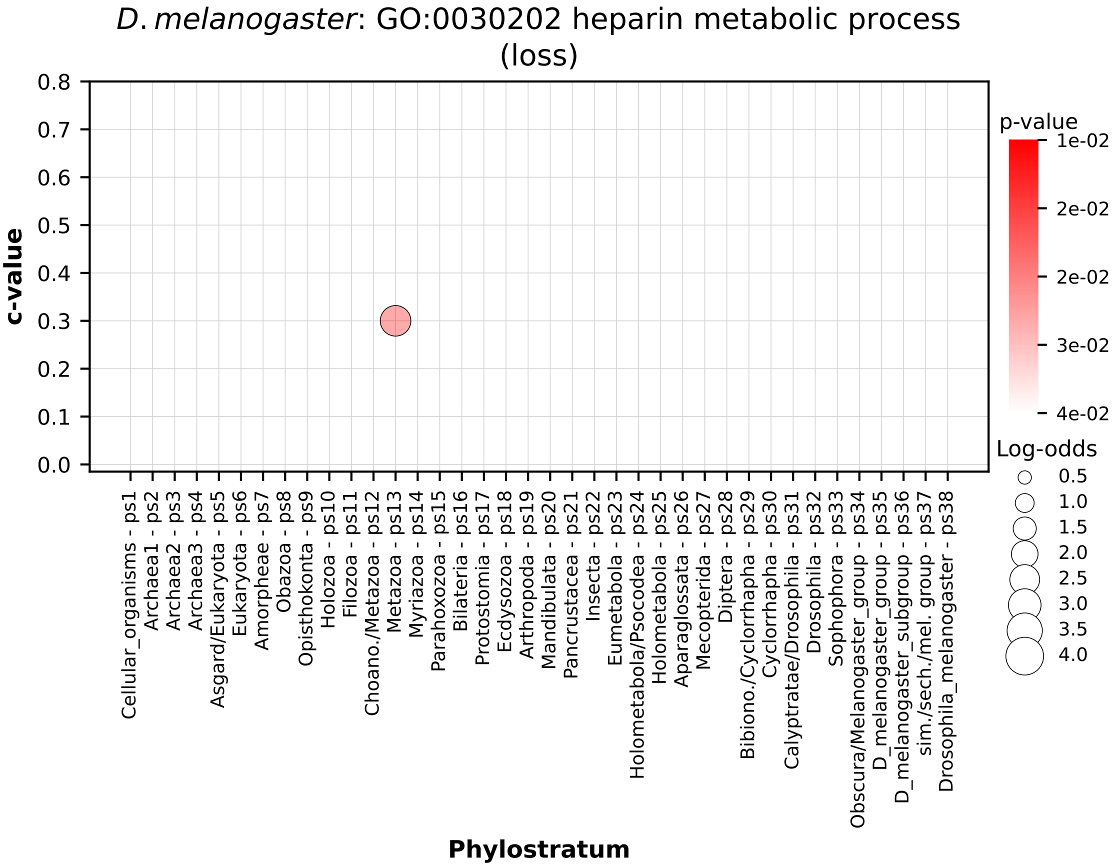

D. melanogaster: GO:0030202 heparin metabolic process (loss)
c-value
0.8
0.7
0.6
0.5
0.4
0.3
0.2
0.1
0.0
Phylostratum
Cellular_organisms - ps1
Archaea1 - ps2
Archaea2 - ps3
Archaea3 - ps4
Asgard/Eukaryota - ps5
Eukaryota - ps6
Amorpheae - ps7
Obazoa - ps8
Opisthokonta - ps9
Holozoa - ps10
Filozoa - ps11
Choano./Metazoa - ps12
Metazoa - ps13
Myriazoa - ps14
Parahoxozoa - ps15
Bilateria - ps16
Protostomia - ps17
Ecdysozoa - ps18
Arthropoda - ps19
Mandibulata - ps20
Pancrustacea - ps21
Insecta - ps22
Eumetabola - ps23
Holometabola/Psocodea - ps24
Holometabola - ps25
Aparaglossata - ps26
Mecopterida - ps27
Diptera - ps28
Bibiono./Cyclorrhapha - ps29
Cyclorrhapha - ps30
Calyptratae/Drosophila - ps31
Drosophila - ps32
Sophophora - ps33
Obscura/Melanogaster_group - ps34
D_melanogaster_group - ps35
D_melanogaster_subgroup - ps36
sim./sech./mel. group - ps37
Drosophila_melanogaster - ps38
p-value
1e-02
2e-02
2e-02
3e-02
4e-02
Log-odds
0.5
1.0
1.5
2.0
2.5
3.0
3.5
4.0

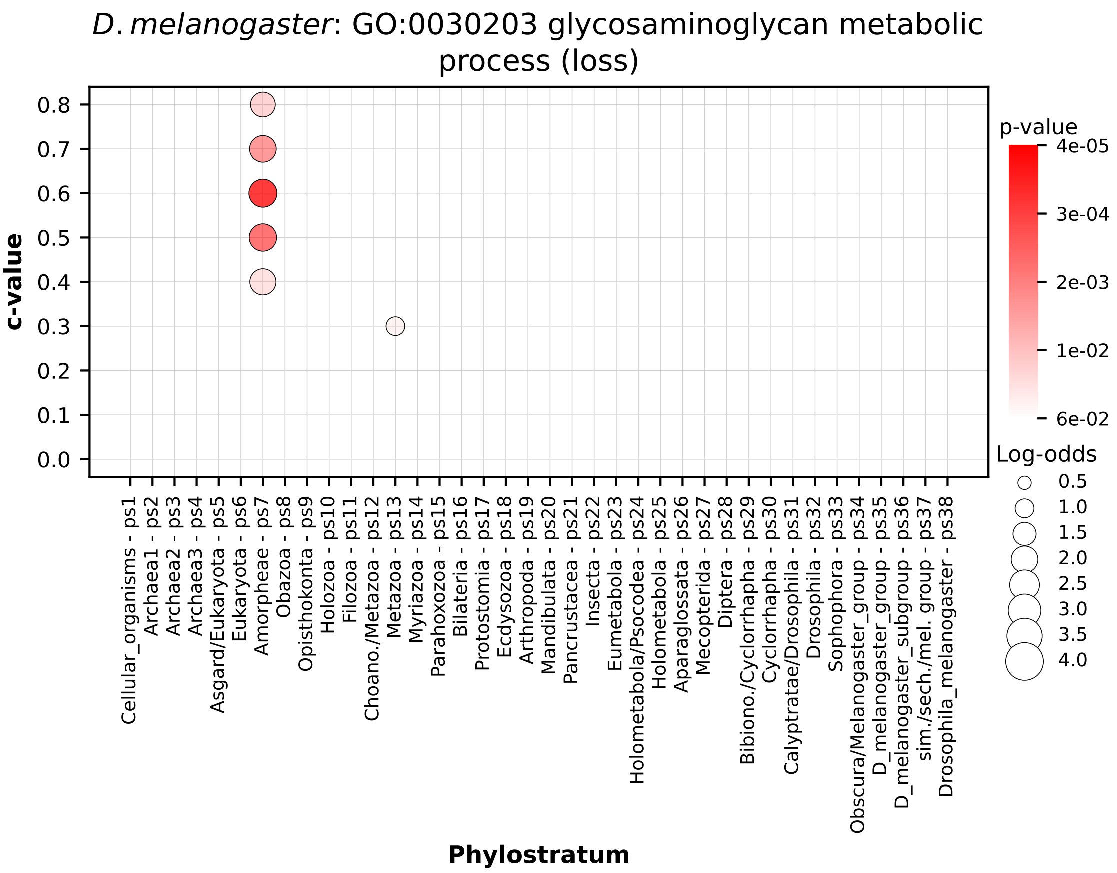

D. melanogaster: GO:0030203 glycosaminoglycan metabolic process (loss)
c-value
0.0
0.1
0.2
0.3
0.4
0.5
0.6
0.7
0.8
p-value
4e-05
3e-04
2e-03
1e-02
6e-02
Log-odds
0.5
1.0
1.5
2.0
2.5
3.0
3.5
4.0
Cellular_organisms - ps1
Archaea1 - ps2
Archaea2 - ps3
Archaea3 - ps4
Asgard/Eukaryota - ps5
Eukaryota - ps6
Amorpheae - ps7
Obazoa - ps8
Opisthokonta - ps9
Holozoa - ps10
Filozoa - ps11
Choano./Metazoa - ps12
Metazoa - ps13
Myriazoa - ps14
Parahoxozoa - ps15
Bilateria - ps16
Protostomia - ps17
Ecdysozoa - ps18
Arthropoda - ps19
Mandibulata - ps20
Pancrustacea - ps21
Insecta - ps22
Eumetabola - ps23
Holometabola/Psocodea - ps24
Holometabola - ps25
Aparaglossata - ps26
Mecopterida - ps27
Diptera - ps28
Bibiono./Cyclorrhapha - ps29
Cyclorrhapha - ps30
Calyptratae/Drosophila - ps31
Drosophila - ps32
Sophophora - ps33
Obscura/Melanogaster_group - ps34
D_melanogaster_group - ps35
D_melanogaster_subgroup - ps36
sim./sech./mel. group - ps37
Drosophila_melanogaster - ps38
Phylostratum

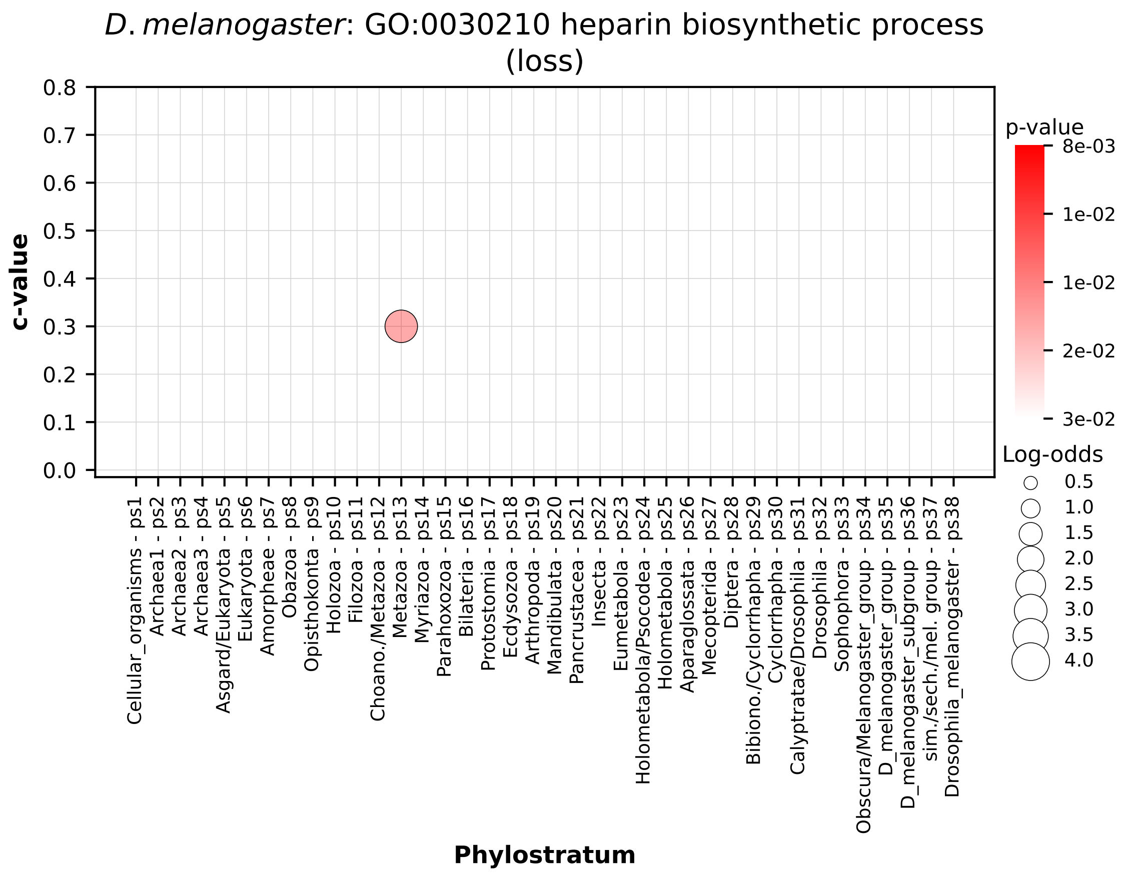
D. melanogaster: GO:0030210 heparin biosynthetic process (loss)
c-value
0.8
0.7
0.6
0.5
0.4
0.3
0.2
0.1
0.0
Phylostratum
Cellular_organisms - ps1
Archaea1 - ps2
Archaea2 - ps3
Archaea3 - ps4
Asgard/Eukaryota - ps5
Eukaryota - ps6
Amorpheae - ps7
Obazoa - ps8
Opisthokonta - ps9
Holozoa - ps10
Filozoa - ps11
Choano./Metazoa - ps12
Metazoa - ps13
Myriazoa - ps14
Parahoxozoa - ps15
Bilateria - ps16
Protostomia - ps17
Ecdysozoa - ps18
Arthropoda - ps19
Mandibulata - ps20
Pancrustacea - ps21
Insecta - ps22
Eumetabola - ps23
Holometabola/Psocodea - ps24
Holometabola - ps25
Aparaglossata - ps26
Mecopterida - ps27
Diptera - ps28
Bibiono./Cyclorrhapha - ps29
Cyclorrhapha - ps30
Calyptratae/Drosophila - ps31
Drosophila - ps32
Sophophora - ps33
Obscura/Melanogaster_group - ps34
D_melanogaster_group - ps35
D_melanogaster_subgroup - ps36
sim./sech./mel. group - ps37
Drosophila_melanogaster - ps38
p-value
8e-03
1e-02
1e-02
2e-02
3e-02
Log-odds
0.5
1.0
1.5
2.0
2.5
3.0
3.5
4.0

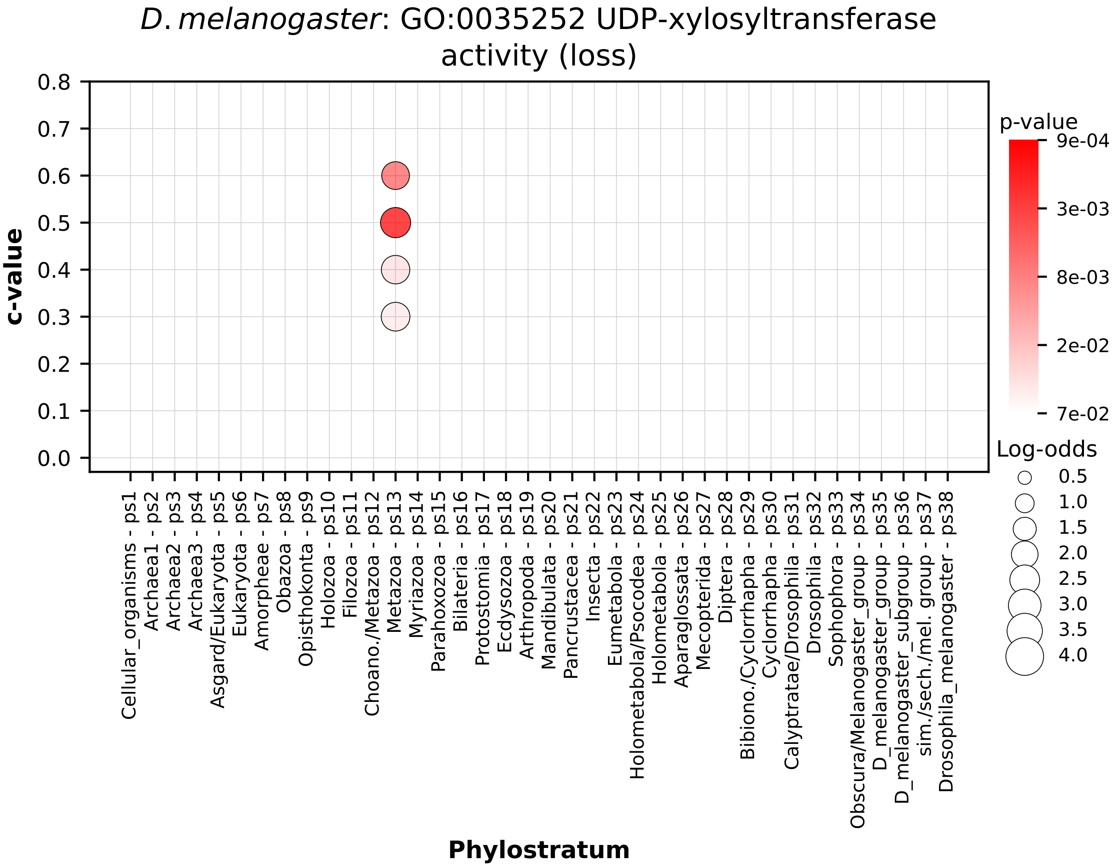

$D. melanogaster$: GO:0035252 UDP-xylosyltransferase activity (loss)
c-value
0.0
0.1
0.2
0.3
0.4
0.5
0.6
0.7
0.8
Phylostratum
Cellular_organisms - ps1
Archaea1 - ps2
Archaea2 - ps3
Archaea3 - ps4
Asgard/Eukaryota - ps5
Eukaryota - ps6
Amorpheae - ps7
Obazoa - ps8
Opisthokonta - ps9
Holozoa - ps10
Filozoa - ps11
Choano./Metazoa - ps12
Metazoa - ps13
Myriazoa - ps14
Parahoxozoa - ps15
Bilateria - ps16
Protostomia - ps17
Ecdysozoa - ps18
Arthropoda - ps19
Mandibulata - ps20
Pancrustacea - ps21
Insecta - ps22
Eumetabola - ps23
Holometabola/Psocodea - ps24
Holometabola - ps25
Aparaglossata - ps26
Mecopterida - ps27
Diptera - ps28
Bibiono./Cyclorrhapha - ps29
Cyclorrhapha - ps30
Calyptratae/Drosophila - ps31
Drosophila - ps32
Sophophora - ps33
Obscura/Melanogaster_group - ps34
D_melanogaster_group - ps35
D_melanogaster_subgroup - ps36
sim./sech./mel. group - ps37
Drosophila_melanogaster - ps38
p-value
9e-04
3e-03
8e-03
2e-02
7e-02
Log-odds
0.5
1.0
1.5
2.0
2.5
3.0
3.5
4.0

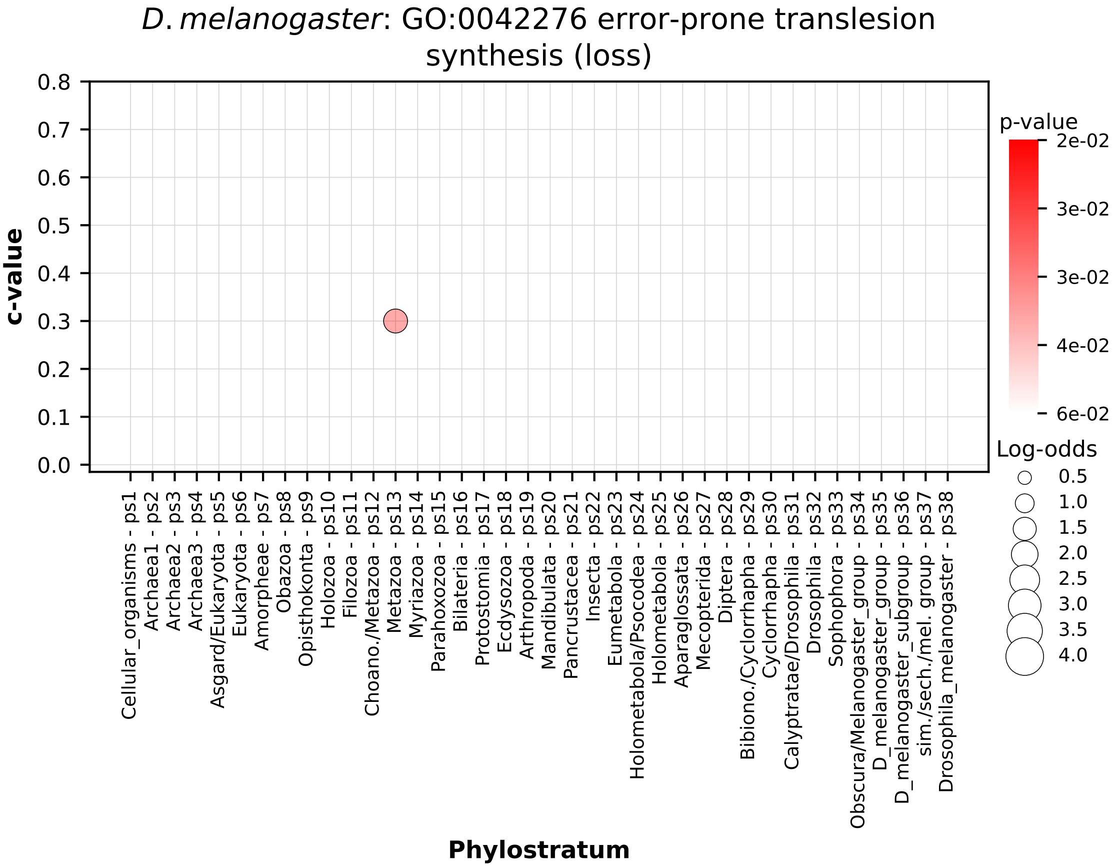

D. melanogaster: GO:0042276 error-prone translesion synthesis (loss)
c-value
0.8
0.7
0.6
0.5
0.4
0.3
0.2
0.1
0.0
p-value
2e-02
3e-02
3e-02
4e-02
6e-02
Log-odds
0.5
1.0
1.5
2.0
2.5
3.0
3.5
4.0
Cellular_organisms - ps1
Archaea1 - ps2
Archaea2 - ps3
Archaea3 - ps4
Asgard/Eukaryota - ps5
Eukaryota - ps6
Amorpheae - ps7
Obazoa - ps8
Opisthokonta - ps9
Holozoa - ps10
Filozoa - ps11
Choano./Metazoa - ps12
Metazoa - ps13
Myriazoa - ps14
Parahoxozoa - ps15
Bilateria - ps16
Protostomia - ps17
Ecdysozoa - ps18
Arthropoda - ps19
Mandibulata - ps20
Pancrustacea - ps21
Insecta - ps22
Eumetabola - ps23
Holometabola/Psocodea - ps24
Holometabola - ps25
Aparaglossata - ps26
Mecopterida - ps27
Diptera - ps28
Bibiono./Cyclorrhapha - ps29
Cyclorrhapha - ps30
Calyptratae/Drosophila - ps31
Drosophila - ps32
Sophophora - ps33
Obscura/Melanogaster_group - ps34
D_melanogaster_group - ps35
D_melanogaster_subgroup - ps36
sim./sech./mel. group - ps37
Drosophila_melanogaster - ps38
Phylostratum

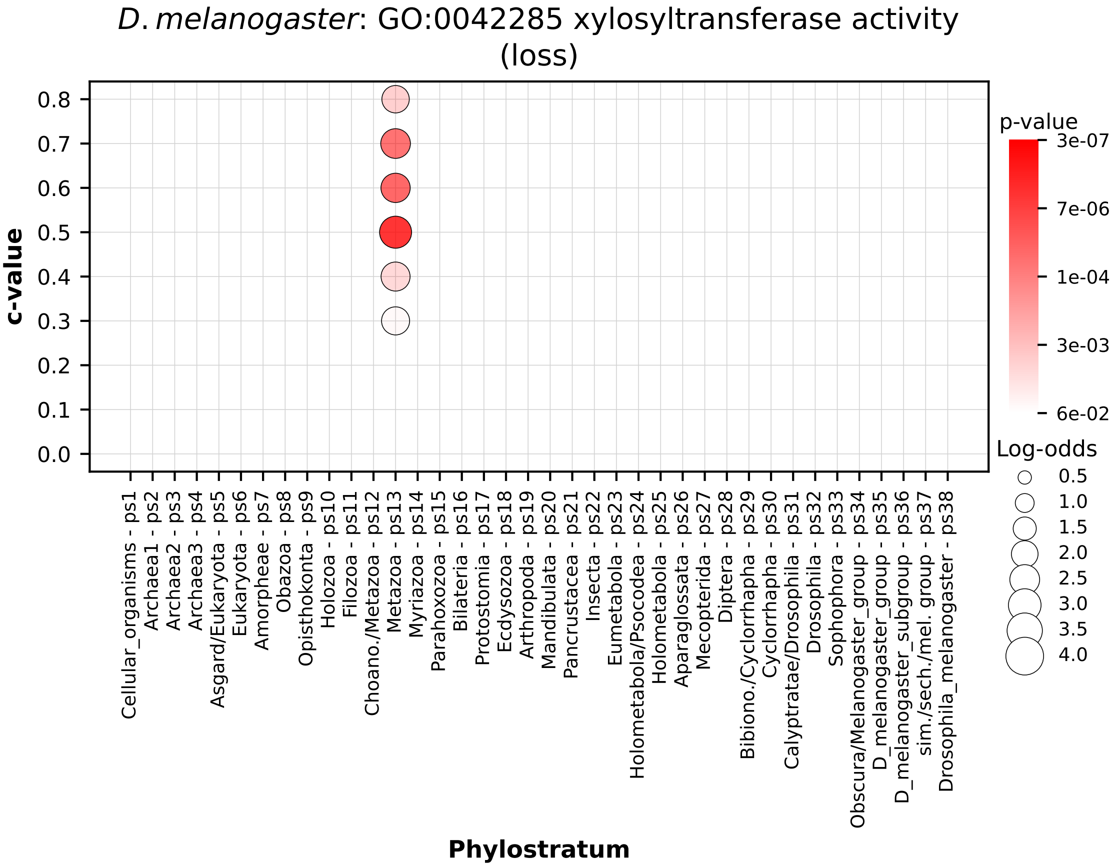

D. melanogaster: GO:0042285 xylosyltransferase activity (loss)
c-value
0.0
0.1
0.2
0.3
0.4
0.5
0.6
0.7
0.8
Phylostratum
Cellular_organisms - ps1
Archaea1 - ps2
Archaea2 - ps3
Archaea3 - ps4
Asgard/Eukaryota - ps5
Eukaryota - ps6
Amorpheae - ps7
Obazoa - ps8
Opisthokonta - ps9
Holozoa - ps10
Filozoa - ps11
Choano./Metazoa - ps12
Metazoa - ps13
Myriazoa - ps14
Parahoxozoa - ps15
Bilateria - ps16
Protostomia - ps17
Ecdysozoa - ps18
Arthropoda - ps19
Mandibulata - ps20
Pancrustacea - ps21
Insecta - ps22
Eumetabola - ps23
Holometabola/Psocodea - ps24
Holometabola - ps25
Aparaglossata - ps26
Mecopterida - ps27
Diptera - ps28
Bibiono./Cyclorrhapha - ps29
Cyclorrhapha - ps30
Calyptratae/Drosophila - ps31
Drosophila - ps32
Sophophora - ps33
Obscura/Melanogaster_group - ps34
D_melanogaster_group - ps35
D_melanogaster_subgroup - ps36
sim./sech./mel. group - ps37
Drosophila_melanogaster - ps38
p-value
3e-07
7e-06
1e-04
3e-03
6e-02
Log-odds
0.5
1.0
1.5
2.0
2.5
3.0
3.5
4.0

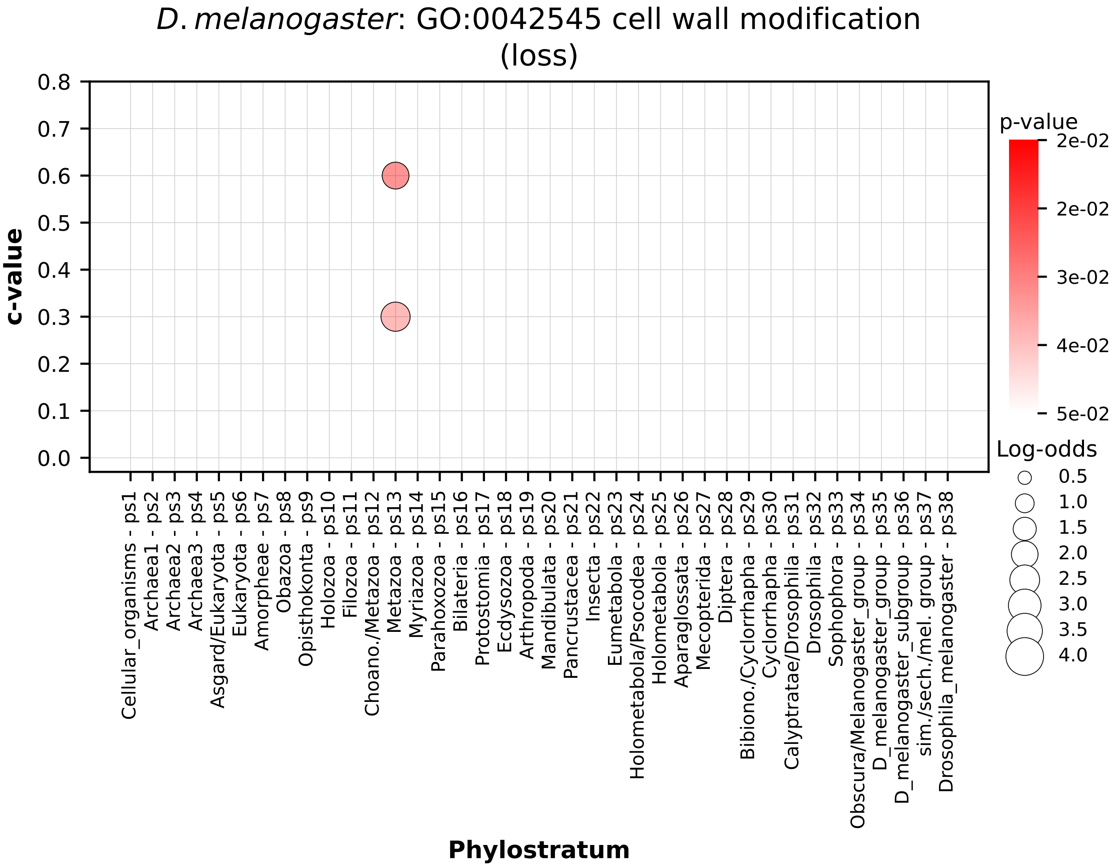
D. melanogaster: GO:0042545 cell wall modification
(loss)
c-value
0.8
0.7
0.6
0.5
0.4
0.3
0.2
0.1
0.0
Phylostratum
Cellular_organisms - ps1
Archaea1 - ps2
Archaea2 - ps3
Archaea3 - ps4
Asgard/Eukaryota - ps5
Eukaryota - ps6
Amorpheae - ps7
Obazoa - ps8
Opisthokonta - ps9
Holozoa - ps10
Filozoa - ps11
Choano./Metazoa - ps12
Metazoa - ps13
Myriazoa - ps14
Parahoxozoa - ps15
Bilateria - ps16
Protostomia - ps17
Ecdysozoa - ps18
Arthropoda - ps19
Mandibulata - ps20
Pancrustacea - ps21
Insecta - ps22
Eumetabola - ps23
Holometabola/Psocodea - ps24
Holometabola - ps25
Aparaglossata - ps26
Mecopterida - ps27
Diptera - ps28
Bibiono./Cyclorrhapha - ps29
Cyclorrhapha - ps30
Calyptratae/Drosophila - ps31
Drosophila - ps32
Sophophora - ps33
Obscura/Melanogaster_group - ps34
D_melanogaster_group - ps35
D_melanogaster_subgroup - ps36
sim./sech./mel. group - ps37
Drosophila_melanogaster - ps38
p-value
2e-02
2e-02
3e-02
4e-02
5e-02
Log-odds
0.5
1.0
1.5
2.0
2.5
3.0
3.5
4.0

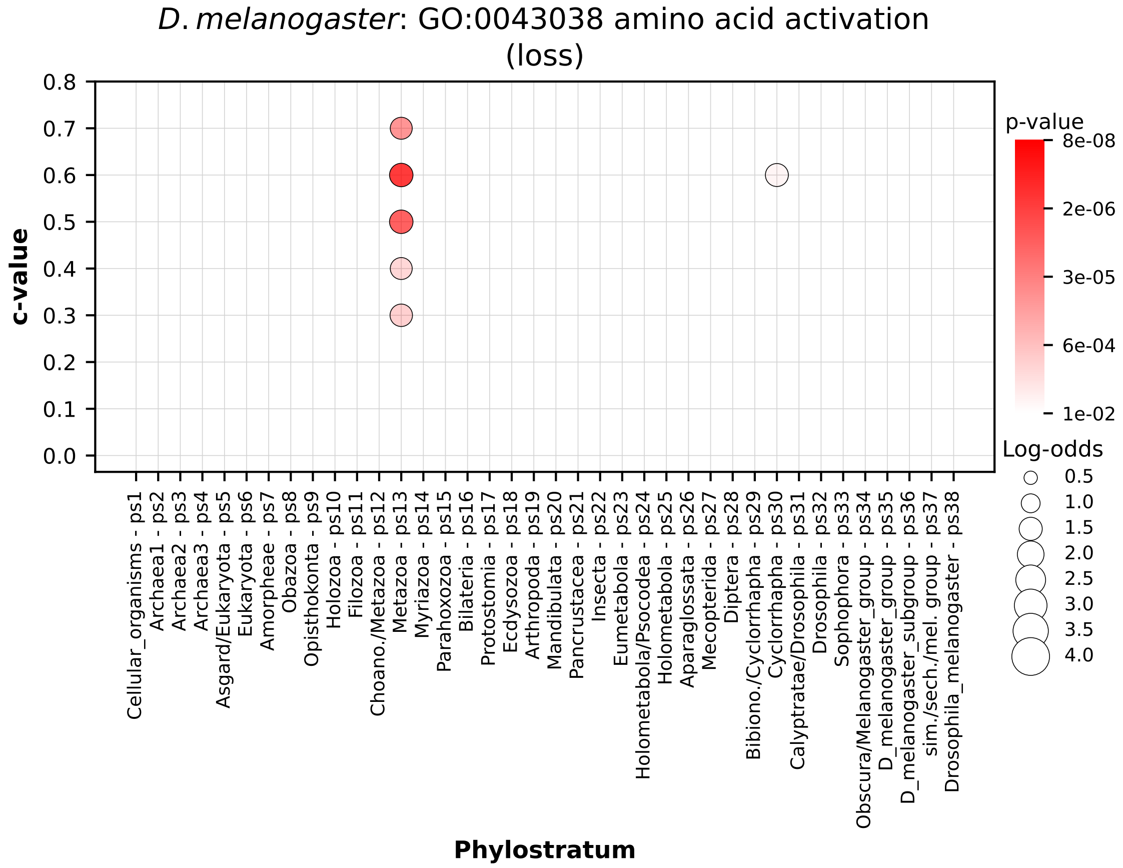
D. melanogaster: GO:0043038 amino acid activation
(loss)
c-value
0.0
0.1
0.2
0.3
0.4
0.5
0.6
0.7
0.8
p-value
8e-08
2e-06
3e-05
6e-04
1e-02
Log-odds
0.5
1.0
1.5
2.0
2.5
3.0
3.5
4.0
Cellular_organisms - ps1
Archaea1 - ps2
Archaea2 - ps3
Archaea3 - ps4
Asgard/Eukaryota - ps5
Eukaryota - ps6
Amorpheae - ps7
Obazoa - ps8
Opisthokonta - ps9
Holozoa - ps10
Filozoa - ps11
Choano./Metazoa - ps12
Metazoa - ps13
Myriazoa - ps14
Parahoxozoa - ps15
Bilateria - ps16
Protostomia - ps17
Ecdysozoa - ps18
Arthropoda - ps19
Mandibulata - ps20
Pancrustacea - ps21
Insecta - ps22
Eumetabola - ps23
Holometabola/Psocodea - ps24
Holometabola - ps25
Aparaglossata - ps26
Mecopterida - ps27
Diptera - ps28
Bibiono./Cyclorrhapha - ps29
Cyclorrhapha - ps30
Calyptratae/Drosophila - ps31
Drosophila - ps32
Sophophora - ps33
Obscura/Melanogaster_group - ps34
D_melanogaster_group - ps35
D_melanogaster_subgroup - ps36
sim./sech./mel. group - ps37
Drosophila_melanogaster - ps38
Phylostratum

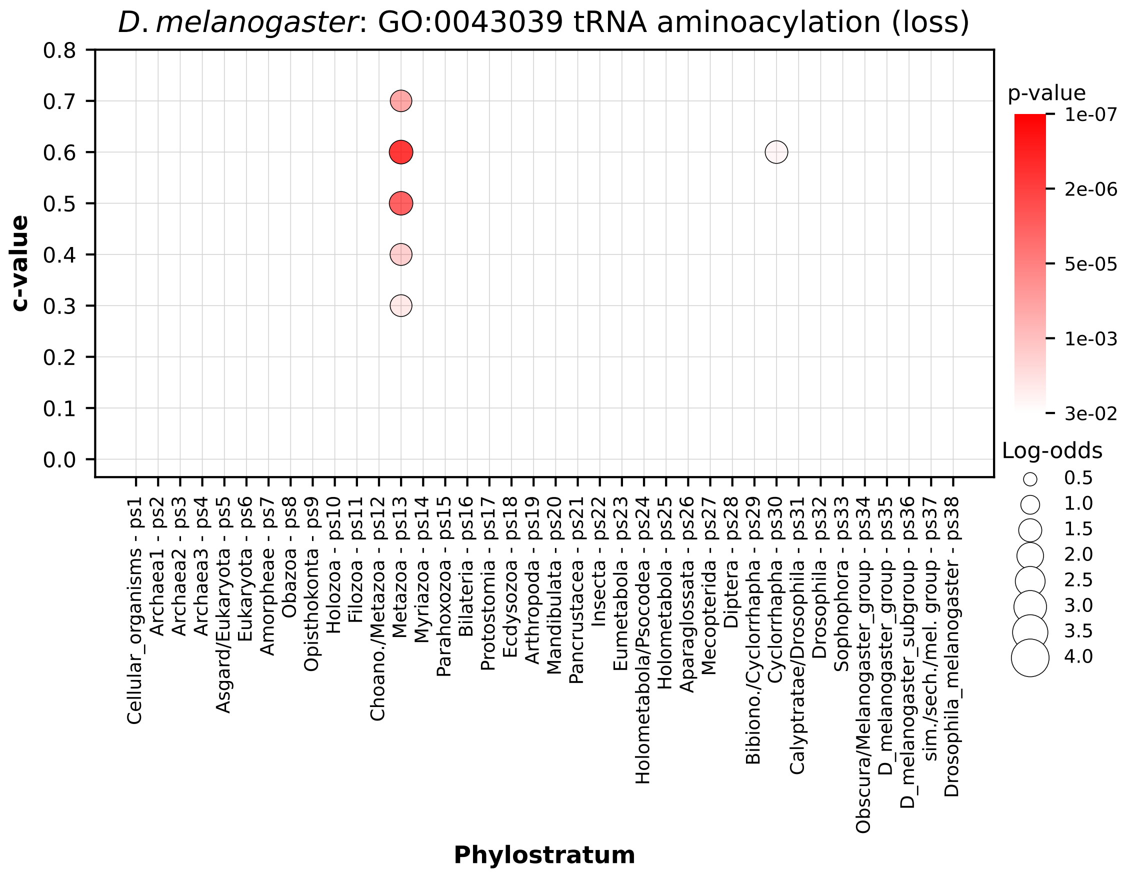
D. melanogaster: GO:0043039 tRNA aminoacylation (loss)
c-value
Phylostratum
p-value
1e-07
2e-06
5e-05
1e-03
3e-02
Log-odds
0.5
1.0
1.5
2.0
2.5
3.0
3.5
4.0
Cellular_organisms - ps1
Archaea1 - ps2
Archaea2 - ps3
Archaea3 - ps4
Asgard/Eukaryota - ps5
Eukaryota - ps6
Amorpheae - ps7
Obazoa - ps8
Opisthokonta - ps9
Holozoa - ps10
Filozoa - ps11
Choano./Metazoa - ps12
Metazoa - ps13
Myriazoa - ps14
Parahoxozoa - ps15
Bilateria - ps16
Protostomia - ps17
Ecdysozoa - ps18
Arthropoda - ps19
Mandibulata - ps20
Pancrustacea - ps21
Insecta - ps22
Eumetabola - ps23
Holometabola/Psocodea - ps24
Holometabola - ps25
Aparaglossata - ps26
Mecopterida - ps27
Diptera - ps28
Bibiono./Cyclorrhapha - ps29
Cyclorrhapha - ps30
Calyptratae/Drosophila - ps31
Drosophila - ps32
Sophophora - ps33
Obscura/Melanogaster_group - ps34
D_melanogaster_group - ps35
D_melanogaster_subgroup - ps36
sim./sech./mel. group - ps37
Drosophila_melanogaster - ps38

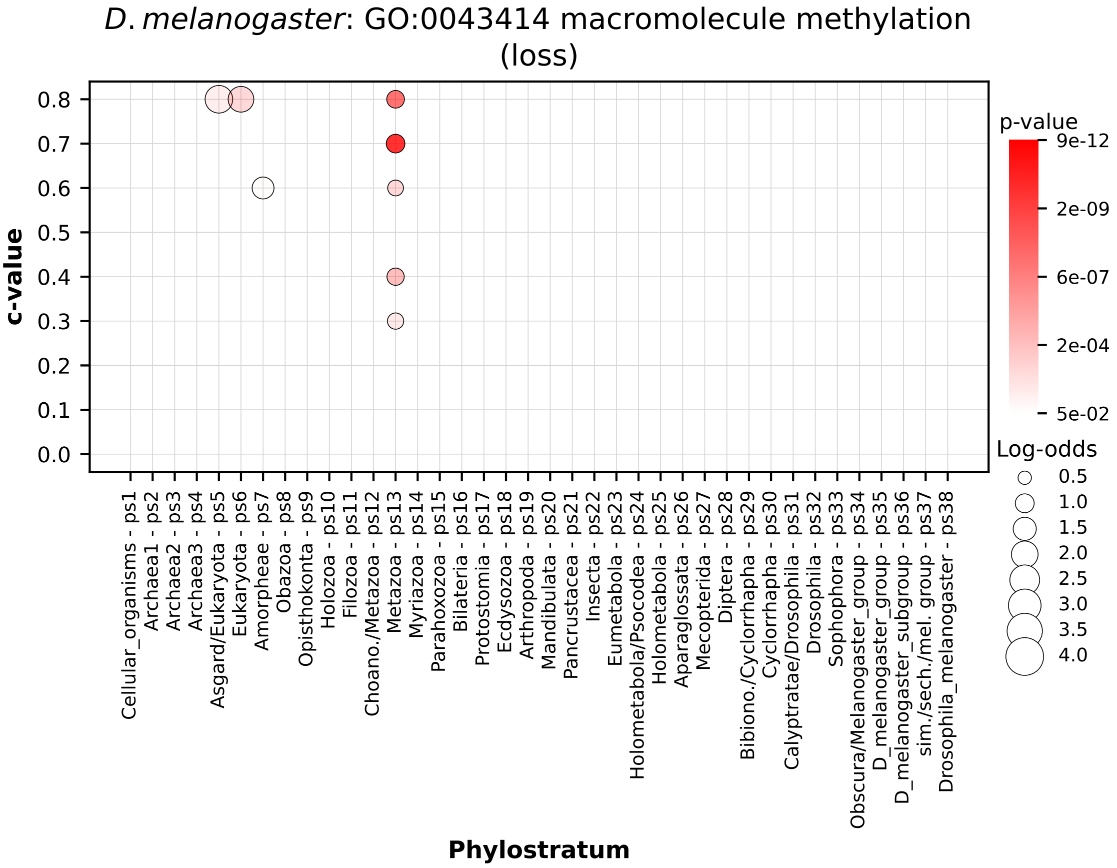

D. melanogaster: GO:0043414 macromolecule methylation (loss)
c-value
0.8
0.7
0.6
0.5
0.4
0.3
0.2
0.1
0.0
p-value
9e-12
2e-09
6e-07
2e-04
5e-02
Log-odds
0.5
1.0
1.5
2.0
2.5
3.0
3.5
4.0
Cellular_organisms - ps1
Archaea1 - ps2
Archaea2 - ps3
Archaea3 - ps4
Asgard/Eukaryota - ps5
Eukaryota - ps6
Amorpheae - ps7
Obazoa - ps8
Opisthokonta - ps9
Holozoa - ps10
Filozoa - ps11
Choano./Metazoa - ps12
Metazoa - ps13
Myriazoa - ps14
Parahoxozoa - ps15
Bilateria - ps16
Protostomia - ps17
Ecdysozoa - ps18
Arthropoda - ps19
Mandibulata - ps20
Pancrustacea - ps21
Insecta - ps22
Eumetabola - ps23
Holometabola/Psocodea - ps24
Holometabola - ps25
Aparaglossata - ps26
Mecopterida - ps27
Diptera - ps28
Bibiono./Cyclorrhapha - ps29
Cyclorrhapha - ps30
Calyptratae/Drosophila - ps31
Drosophila - ps32
Sophophora - ps33
Obscura/Melanogaster_group - ps34
D_melanogaster_group - ps35
D_melanogaster_subgroup - ps36
sim./sech./mel. group - ps37
Drosophila_melanogaster - ps38
Phylostratum

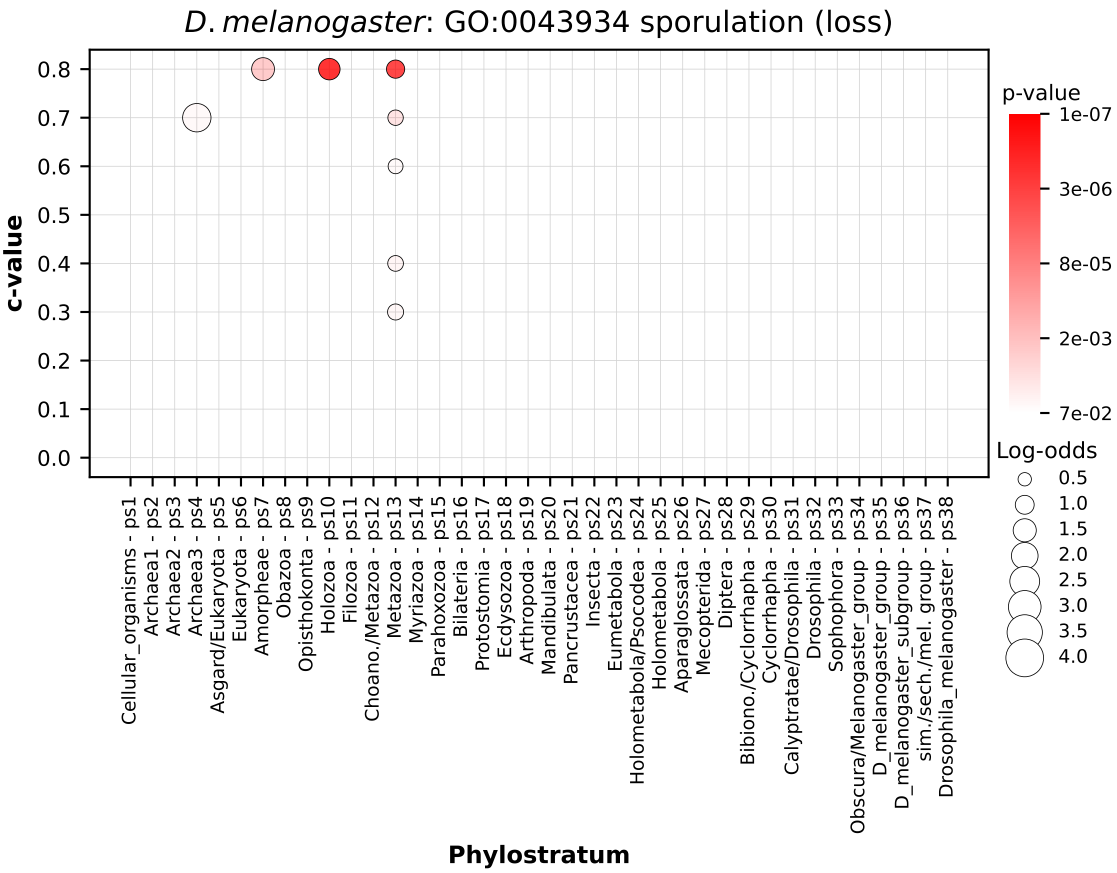
D. melanogaster: GO:0043934 sporulation (loss)
c-value
0.8
0.7
0.6
0.5
0.4
0.3
0.2
0.1
0.0
p-value
1e-07
3e-06
8e-05
2e-03
7e-02
Log-odds
0.5
1.0
1.5
2.0
2.5
3.0
3.5
4.0
Cellular_organisms - ps1
Archaea1 - ps2
Archaea2 - ps3
Archaea3 - ps4
Asgard/Eukaryota - ps5
Eukaryota - ps6
Amorpheae - ps7
Obazoa - ps8
Opisthokonta - ps9
Holozoa - ps10
Filozoa - ps11
Choano./Metazoa - ps12
Metazoa - ps13
Myriazoa - ps14
Parahoxozoa - ps15
Bilateria - ps16
Protostomia - ps17
Ecdysozoa - ps18
Arthropoda - ps19
Mandibulata - ps20
Pancrustacea - ps21
Insecta - ps22
Eumetabola - ps23
Holometabola/Psocodea - ps24
Holometabola - ps25
Aparaglossata - ps26
Mecopterida - ps27
Diptera - ps28
Bibiono./Cyclorrhapha - ps29
Cyclorrhapha - ps30
Calyptratae/Drosophila - ps31
Drosophila - ps32
Sophophora - ps33
Obscura/Melanogaster_group - ps34
D_melanogaster_group - ps35
D_melanogaster_subgroup - ps36
sim./sech./mel. group - ps37
Drosophila_melanogaster - ps38
Phylostratum

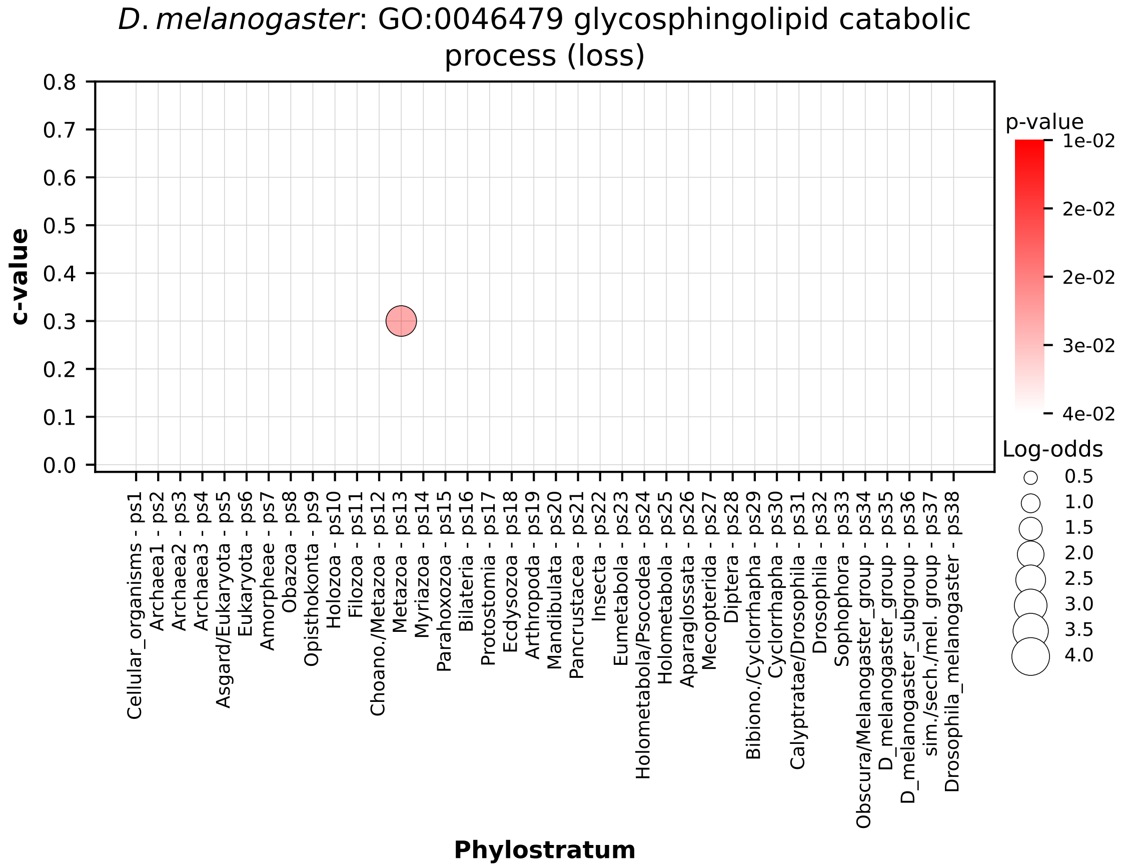
D. melanogaster: GO:0046479 glycosphingolipid catabolic process (loss)
c-value
0.8
0.7
0.6
0.5
0.4
0.3
0.2
0.1
0.0
p-value
1e-02
2e-02
2e-02
3e-02
4e-02
Log-odds
0.5
1.0
1.5
2.0
2.5
3.0
3.5
4.0
Cellular_organisms - ps1
Archaea1 - ps2
Archaea2 - ps3
Archaea3 - ps4
Asgard/Eukaryota - ps5
Eukaryota - ps6
Amorpheae - ps7
Obazoa - ps8
Opisthokonta - ps9
Holozoa - ps10
Filozoa - ps11
Choano./Metazoa - ps12
Metazoa - ps13
Myriazoa - ps14
Parahoxozoa - ps15
Bilateria - ps16
Protostomia - ps17
Ecdysozoa - ps18
Arthropoda - ps19
Mandibulata - ps20
Pancrustacea - ps21
Insecta - ps22
Eumetabola - ps23
Holometabola/Psocodea - ps24
Holometabola - ps25
Aparaglossata - ps26
Mecopterida - ps27
Diptera - ps28
Bibiono./Cyclorrhapha - ps29
Cyclorrhapha - ps30
Calyptratae/Drosophila - ps31
Drosophila - ps32
Sophophora - ps33
Obscura/Melanogaster_group - ps34
D_melanogaster_group - ps35
D_melanogaster_subgroup - ps36
sim./sech./mel. group - ps37
Drosophila_melanogaster - ps38
Phylostratum

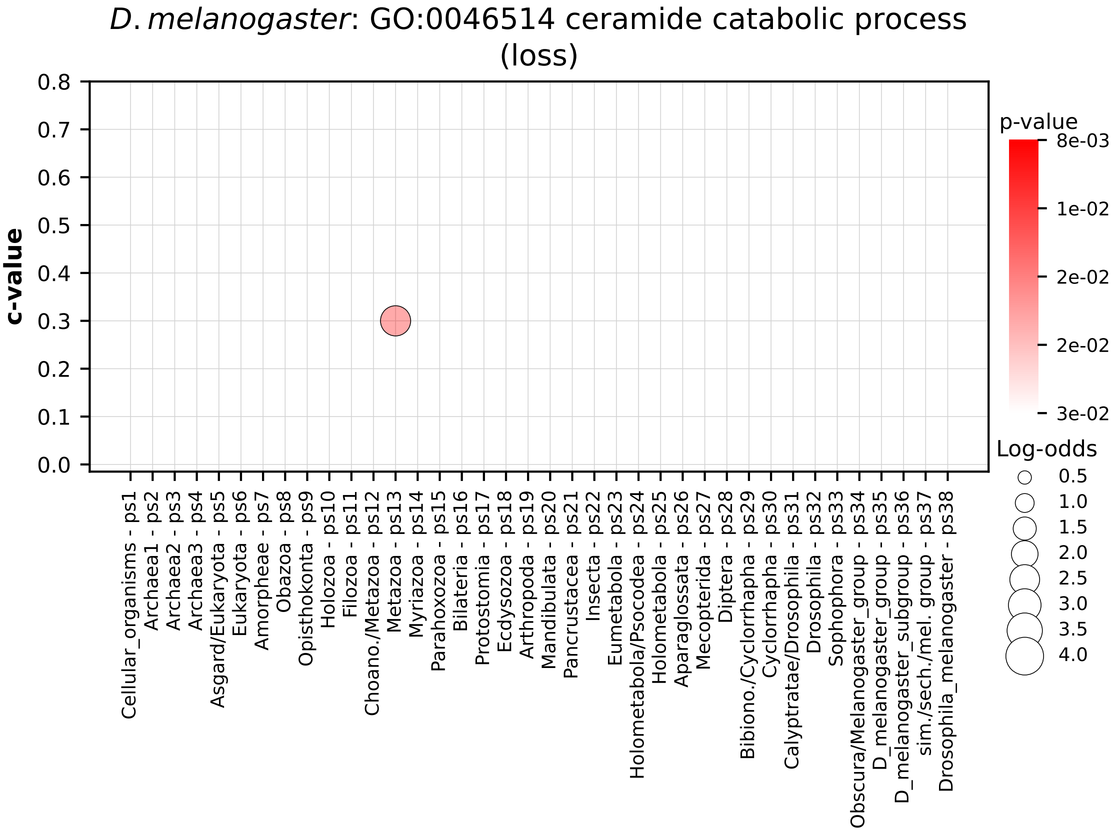
D. melanogaster: GO:0046514 ceramide catabolic process
(loss)
c-value
0.8
0.7
0.6
0.5
0.4
0.3
0.2
0.1
0.0
Phylostratum
Cellular_organisms - ps1
Archaea1 - ps2
Archaea2 - ps3
Archaea3 - ps4
Asgard/Eukaryota - ps5
Eukaryota - ps6
Amorpheae - ps7
Obazoa - ps8
Opisthokonta - ps9
Holozoa - ps10
Filozoa - ps11
Choano./Metazoa - ps12
Metazoa - ps13
Myriazoa - ps14
Parahoxozoa - ps15
Bilateria - ps16
Protostomia - ps17
Ecdysozoa - ps18
Arthropoda - ps19
Mandibulata - ps20
Pancrustacea - ps21
Insecta - ps22
Eumetabola - ps23
Holometabola/Psocodea - ps24
Holometabola - ps25
Aparaglossata - ps26
Mecopterida - ps27
Diptera - ps28
Bibiono./Cyclorrhapha - ps29
Cyclorrhapha - ps30
Calyptratae/Drosophila - ps31
Drosophila - ps32
Sophophora - ps33
Obscura/Melanogaster_group - ps34
D_melanogaster_group - ps35
D_melanogaster_subgroup - ps36
sim./sech./mel. group - ps37
Drosophila_melanogaster - ps38
p-value
8e-03
1e-02
2e-02
2e-02
3e-02
Log-odds
0.5
1.0
1.5
2.0
2.5
3.0
3.5
4.0

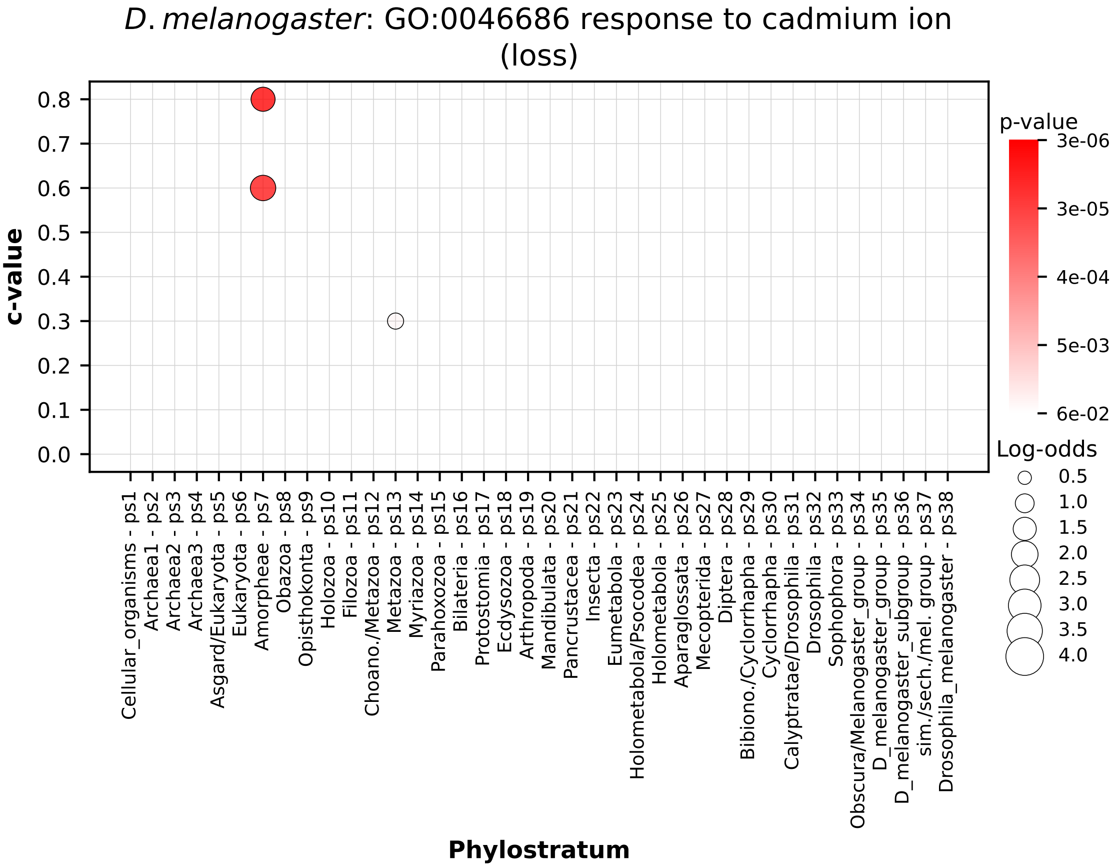

D. melanogaster: GO:0046686 response to cadmium ion (loss)
c-value
0.0
0.1
0.2
0.3
0.4
0.5
0.6
0.7
0.8
Phylostratum
Cellular_organisms - ps1
Archaea1 - ps2
Archaea2 - ps3
Archaea3 - ps4
Asgard/Eukaryota - ps5
Eukaryota - ps6
Amorpheae - ps7
Obazoa - ps8
Opisthokonta - ps9
Holozoa - ps10
Filozoa - ps11
Choano./Metazoa - ps12
Metazoa - ps13
Myriazoa - ps14
Parahoxozoa - ps15
Bilateria - ps16
Protostomia - ps17
Ecdysozoa - ps18
Arthropoda - ps19
Mandibulata - ps20
Pancrustacea - ps21
Insecta - ps22
Eumetabola - ps23
Holometabola/Psocodea - ps24
Holometabola - ps25
Aparaglossata - ps26
Mecopterida - ps27
Diptera - ps28
Bibiono./Cyclorrhapha - ps29
Cyclorrhapha - ps30
Calyptratae/Drosophila - ps31
Drosophila - ps32
Sophophora - ps33
Obscura/Melanogaster_group - ps34
D_melanogaster_group - ps35
D_melanogaster_subgroup - ps36
sim./sech./mel. group - ps37
Drosophila_melanogaster - ps38
p-value
3e-06
3e-05
4e-04
5e-03
6e-02
Log-odds
0.5
1.0
1.5
2.0
2.5
3.0
3.5
4.0

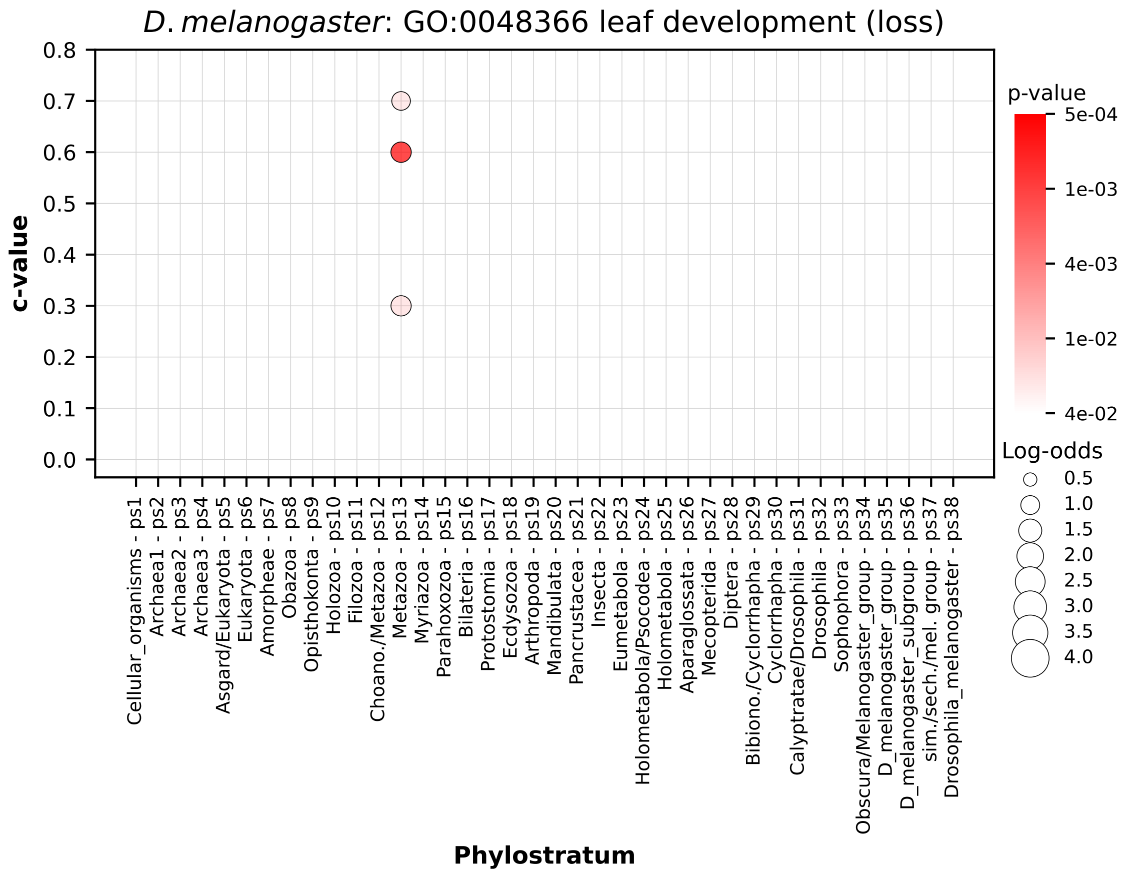

D. melanogaster: GO:0048366 leaf development (loss)
c-value
0.8
0.7
0.6
0.5
0.4
0.3
0.2
0.1
0.0
p-value
5e-04
1e-03
4e-03
1e-02
4e-02
Log-odds
0.5
1.0
1.5
2.0
2.5
3.0
3.5
4.0
Cellular_organisms - ps1
Archaea1 - ps2
Archaea2 - ps3
Archaea3 - ps4
Asgard/Eukaryota - ps5
Eukaryota - ps6
Amorpheae - ps7
Obazoa - ps8
Opisthokonta - ps9
Holozoa - ps10
Filozoa - ps11
Choano./Metazoa - ps12
Metazoa - ps13
Myriazoa - ps14
Parahoxozoa - ps15
Bilateria - ps16
Protostomia - ps17
Ecdysozoa - ps18
Arthropoda - ps19
Mandibulata - ps20
Pancrustacea - ps21
Insecta - ps22
Eumetabola - ps23
Holometabola/Psocodea - ps24
Holometabola - ps25
Aparaglossata - ps26
Mecopterida - ps27
Diptera - ps28
Bibiono./Cyclorrhapha - ps29
Cyclorrhapha - ps30
Calyptratae/Drosophila - ps31
Drosophila - ps32
Sophophora - ps33
Obscura/Melanogaster_group - ps34
D_melanogaster_group - ps35
D_melanogaster_subgroup - ps36
sim./sech./mel. group - ps37
Drosophila_melanogaster - ps38
Phylostratum

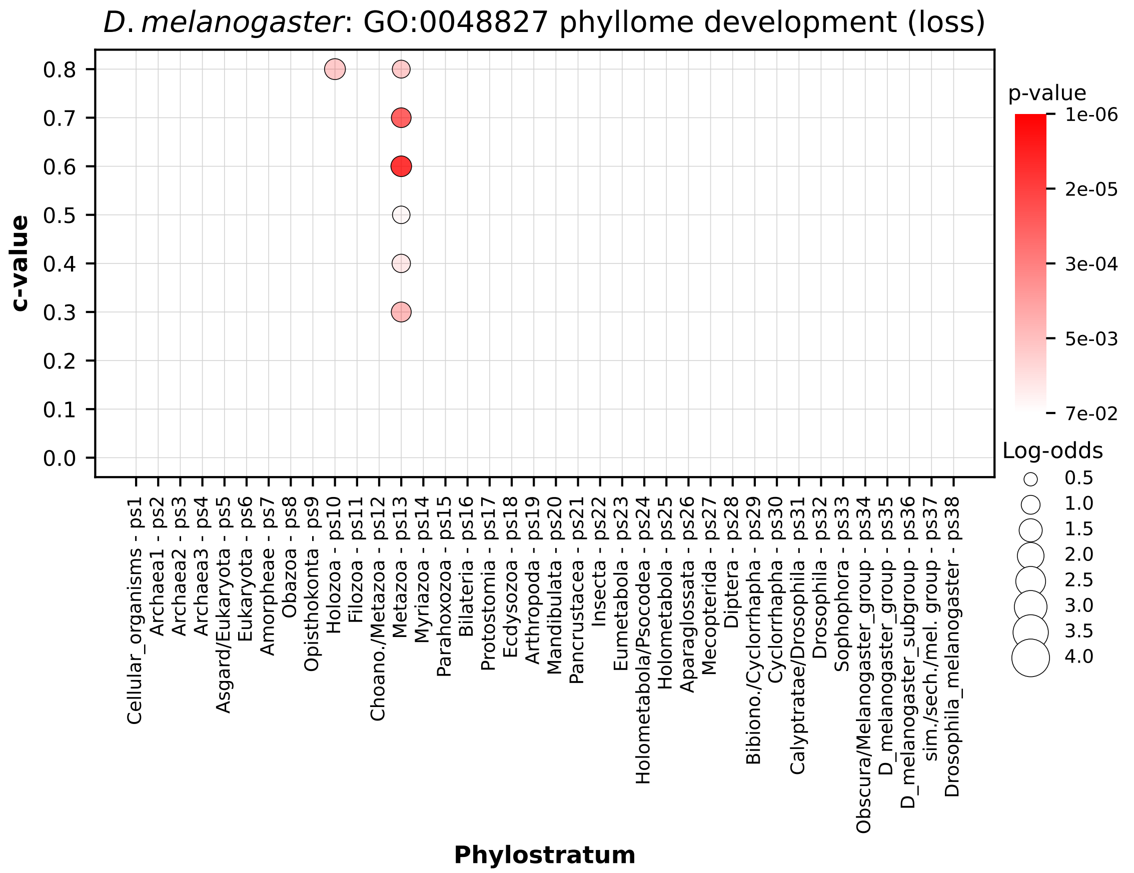

D. melanogaster: GO:0048827 phyllome development (loss)
c-value
0.8
0.7
0.6
0.5
0.4
0.3
0.2
0.1
0.0
p-value
1e-06
2e-05
3e-04
5e-03
7e-02
Log-odds
0.5
1.0
1.5
2.0
2.5
3.0
3.5
4.0
Cellular_organisms - ps1
Archaea1 - ps2
Archaea2 - ps3
Archaea3 - ps4
Asgard/Eukaryota - ps5
Eukaryota - ps6
Amorpheae - ps7
Obazoa - ps8
Opisthokonta - ps9
Holozoa - ps10
Filozoa - ps11
Choano./Metazoa - ps12
Metazoa - ps13
Myriazoa - ps14
Parahoxozoa - ps15
Bilateria - ps16
Protostomia - ps17
Ecdysozoa - ps18
Arthropoda - ps19
Mandibulata - ps20
Pancrustacea - ps21
Insecta - ps22
Eumetabola - ps23
Holometabola/Psocodea - ps24
Holometabola - ps25
Aparaglossata - ps26
Mecopterida - ps27
Diptera - ps28
Bibiono./Cyclorrhapha - ps29
Cyclorrhapha - ps30
Calyptratae/Drosophila - ps31
Drosophila - ps32
Sophophora - ps33
Obscura/Melanogaster_group - ps34
D_melanogaster_group - ps35
D_melanogaster_subgroup - ps36
sim./sech./mel. group - ps37
Drosophila_melanogaster - ps38
Phylostratum

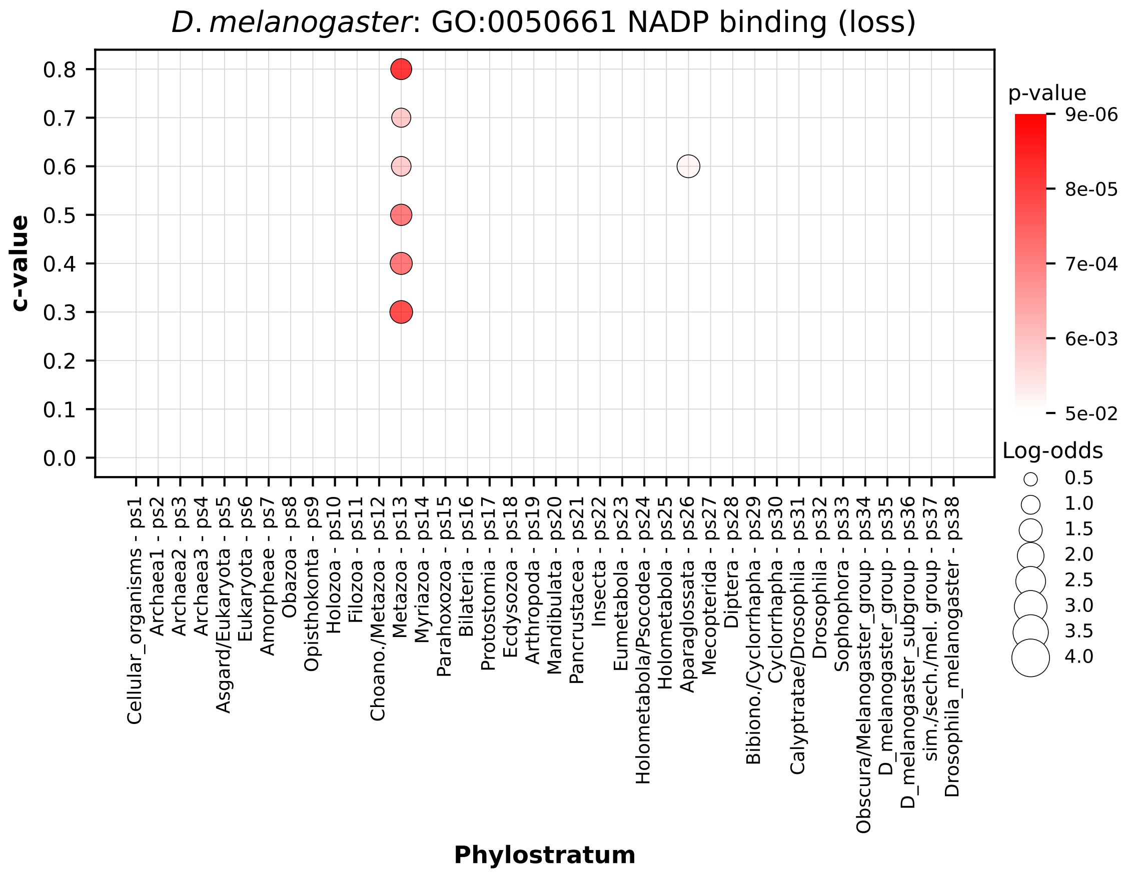

D. melanogaster: GO:0050661 NADP binding (loss)
c-value
0.0
0.1
0.2
0.3
0.4
0.5
0.6
0.7
0.8
Phylostratum
Cellular_organisms - ps1
Archaea1 - ps2
Archaea2 - ps3
Archaea3 - ps4
Asgard/Eukaryota - ps5
Eukaryota - ps6
Amorpheae - ps7
Obazoa - ps8
Opisthokonta - ps9
Holozoa - ps10
Filozoa - ps11
Choano./Metazoa - ps12
Metazoa - ps13
Myriazoa - ps14
Parahoxozoa - ps15
Bilateria - ps16
Protostomia - ps17
Ecdysozoa - ps18
Arthropoda - ps19
Mandibulata - ps20
Pancrustacea - ps21
Insecta - ps22
Eumetabola - ps23
Holometabola/Psocodea - ps24
Holometabola - ps25
Aparaglossata - ps26
Mecopterida - ps27
Diptera - ps28
Bibiono./Cyclorrhapha - ps29
Cyclorrhapha - ps30
Calyptratae/Drosophila - ps31
Drosophila - ps32
Sophophora - ps33
Obscura/Melanogaster_group - ps34
D_melanogaster_group - ps35
D_melanogaster_subgroup - ps36
sim./sech./mel. group - ps37
Drosophila_melanogaster - ps38
p-value
9e-06
8e-05
7e-04
6e-03
5e-02
Log-odds
0.5
1.0
1.5
2.0
2.5
3.0
3.5
4.0

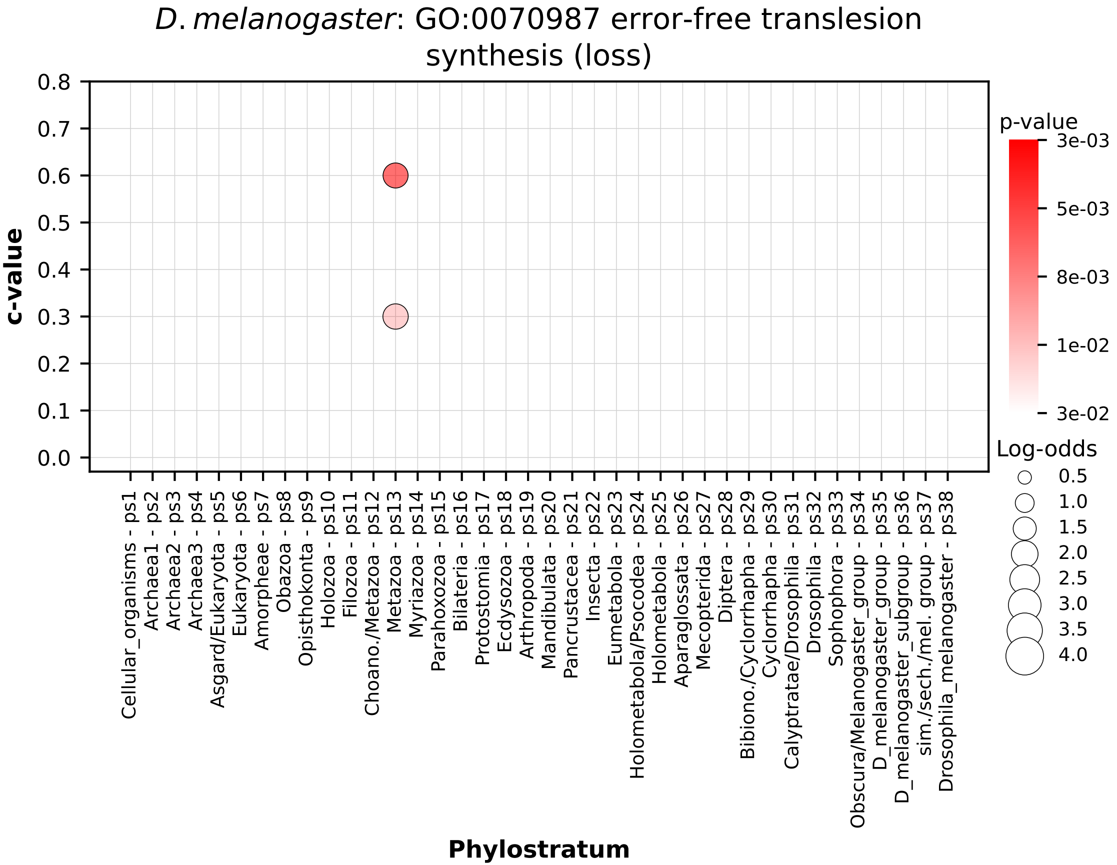

D. melanogaster: GO:0070987 error-free translesion synthesis (loss)
c-value
0.8
0.7
0.6
0.5
0.4
0.3
0.2
0.1
0.0
Phylostratum
Cellular_organisms - ps1
Archaea1 - ps2
Archaea2 - ps3
Archaea3 - ps4
Asgard/Eukaryota - ps5
Eukaryota - ps6
Amorpheae - ps7
Obazoa - ps8
Opisthokonta - ps9
Holozoa - ps10
Filozoa - ps11
Choano./Metazoa - ps12
Metazoa - ps13
Myriazoa - ps14
Parahoxozoa - ps15
Bilateria - ps16
Protostomia - ps17
Ecdysozoa - ps18
Arthropoda - ps19
Mandibulata - ps20
Pancrustacea - ps21
Insecta - ps22
Eumetabola - ps23
Holometabola/Psocodea - ps24
Holometabola - ps25
Aparaglossata - ps26
Mecopterida - ps27
Diptera - ps28
Bibiono./Cyclorrhapha - ps29
Cyclorrhapha - ps30
Calyptratae/Drosophila - ps31
Drosophila - ps32
Sophophora - ps33
Obscura/Melanogaster_group - ps34
D_melanogaster_group - ps35
D_melanogaster_subgroup - ps36
sim./sech./mel. group - ps37
Drosophila_melanogaster - ps38
p-value
3e-03
5e-03
8e-03
1e-02
3e-02
Log-odds
0.5
1.0
1.5
2.0
2.5
3.0
3.5
4.0

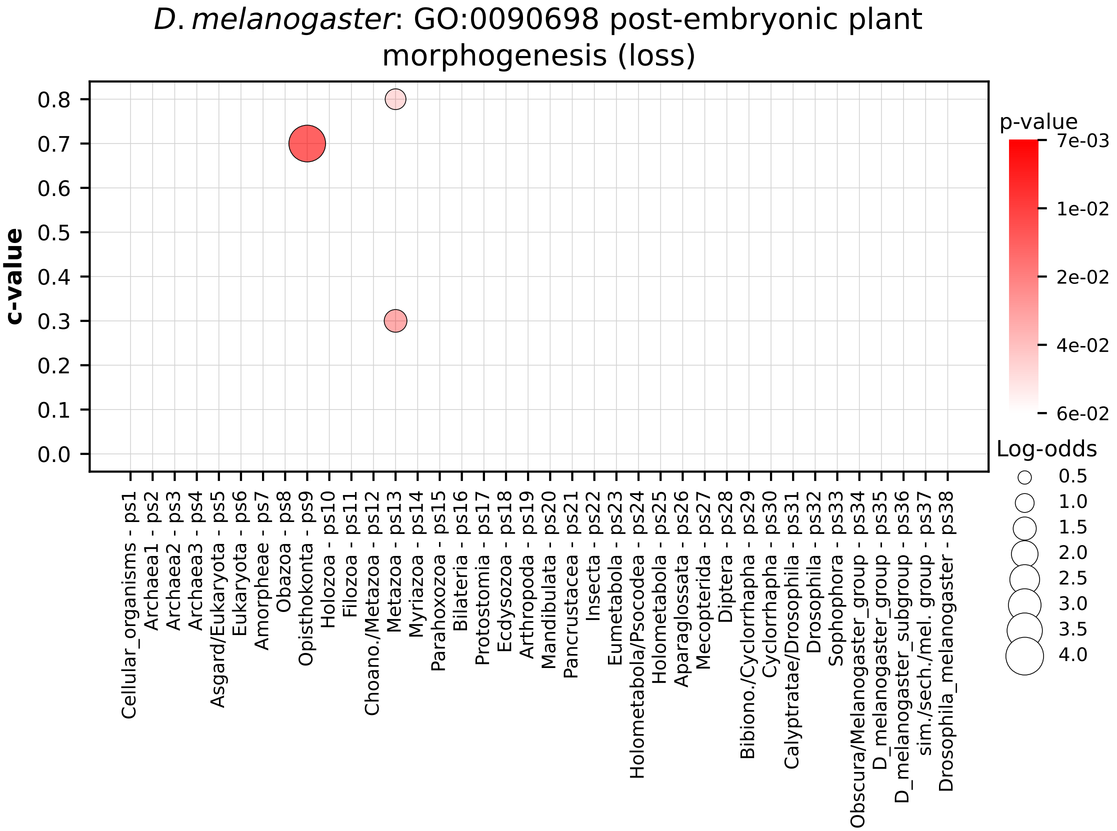

D. melanogaster: GO:0090698 post-embryonic plant morphogenesis (loss)
c-value
0.0
0.1
0.2
0.3
0.4
0.5
0.6
0.7
0.8
Phylostratum
Cellular_organisms - ps1
Archaea1 - ps2
Archaea2 - ps3
Archaea3 - ps4
Asgard/Eukaryota - ps5
Eukaryota - ps6
Amorpheae - ps7
Obazoa - ps8
Opisthokonta - ps9
Holozoa - ps10
Filozoa - ps11
Choano./Metazoa - ps12
Metazoa - ps13
Myriazoa - ps14
Parahoxozoa - ps15
Bilateria - ps16
Protostomia - ps17
Ecdysozoa - ps18
Arthropoda - ps19
Mandibulata - ps20
Pancrustacea - ps21
Insecta - ps22
Eumetabola - ps23
Holometabola/Psocodea - ps24
Holometabola - ps25
Aparaglossata - ps26
Mecopterida - ps27
Diptera - ps28
Bibiono./Cyclorrhapha - ps29
Cyclorrhapha - ps30
Calyptratae/Drosophila - ps31
Drosophila - ps32
Sophophora - ps33
Obscura/Melanogaster_group - ps34
D_melanogaster_group - ps35
D_melanogaster_subgroup - ps36
sim./sech./mel. group - ps37
Drosophila_melanogaster - ps38
p-value
7e-03
1e-02
2e-02
4e-02
6e-02
Log-odds
0.5
1.0
1.5
2.0
2.5
3.0
3.5
4.0

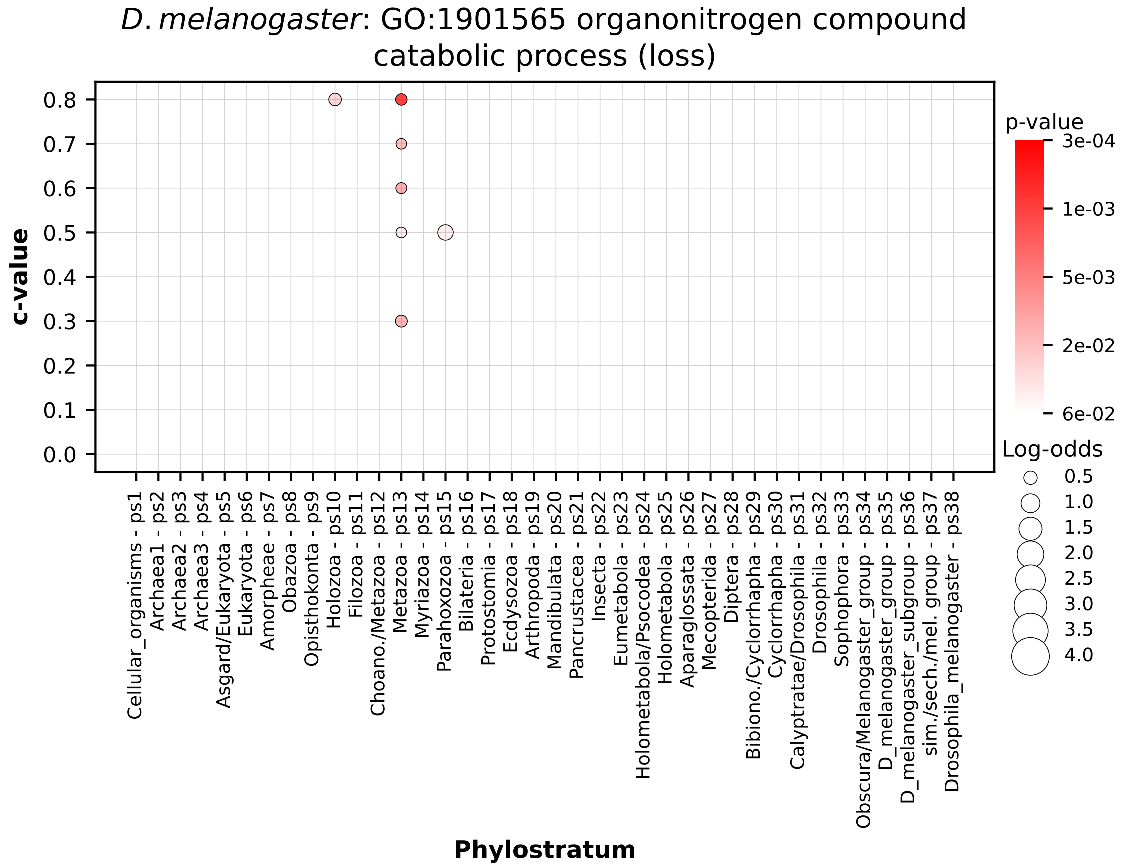

D. melanogaster: GO:1901565 organonitrogen compound catabolic process (loss)
c-value
0.8
0.7
0.6
0.5
0.4
0.3
0.2
0.1
0.0
p-value
3e-04
1e-03
5e-03
2e-02
6e-02
Log-odds
0.5
1.0
1.5
2.0
2.5
3.0
3.5
4.0
Cellular_organisms - ps1
Archaea1 - ps2
Archaea2 - ps3
Archaea3 - ps4
Asgard/Eukaryota - ps5
Eukaryota - ps6
Amorpheae - ps7
Obazoa - ps8
Opisthokonta - ps9
Holozoa - ps10
Filozoa - ps11
Choano./Metazoa - ps12
Metazoa - ps13
Myriazoa - ps14
Parahoxozoa - ps15
Bilateria - ps16
Protostomia - ps17
Ecdysozoa - ps18
Arthropoda - ps19
Mandibulata - ps20
Pancrustacea - ps21
Insecta - ps22
Eumetabola - ps23
Holometabola/Psocodea - ps24
Holometabola - ps25
Aparaglossata - ps26
Mecopterida - ps27
Diptera - ps28
Bibiono./Cyclorrhapha - ps29
Cyclorrhapha - ps30
Calyptratae/Drosophila - ps31
Drosophila - ps32
Sophophora - ps33
Obscura/Melanogaster_group - ps34
D_melanogaster_group - ps35
D_melanogaster_subgroup - ps36
sim./sech./mel. group - ps37
Drosophila_melanogaster - ps38
Phylostratum

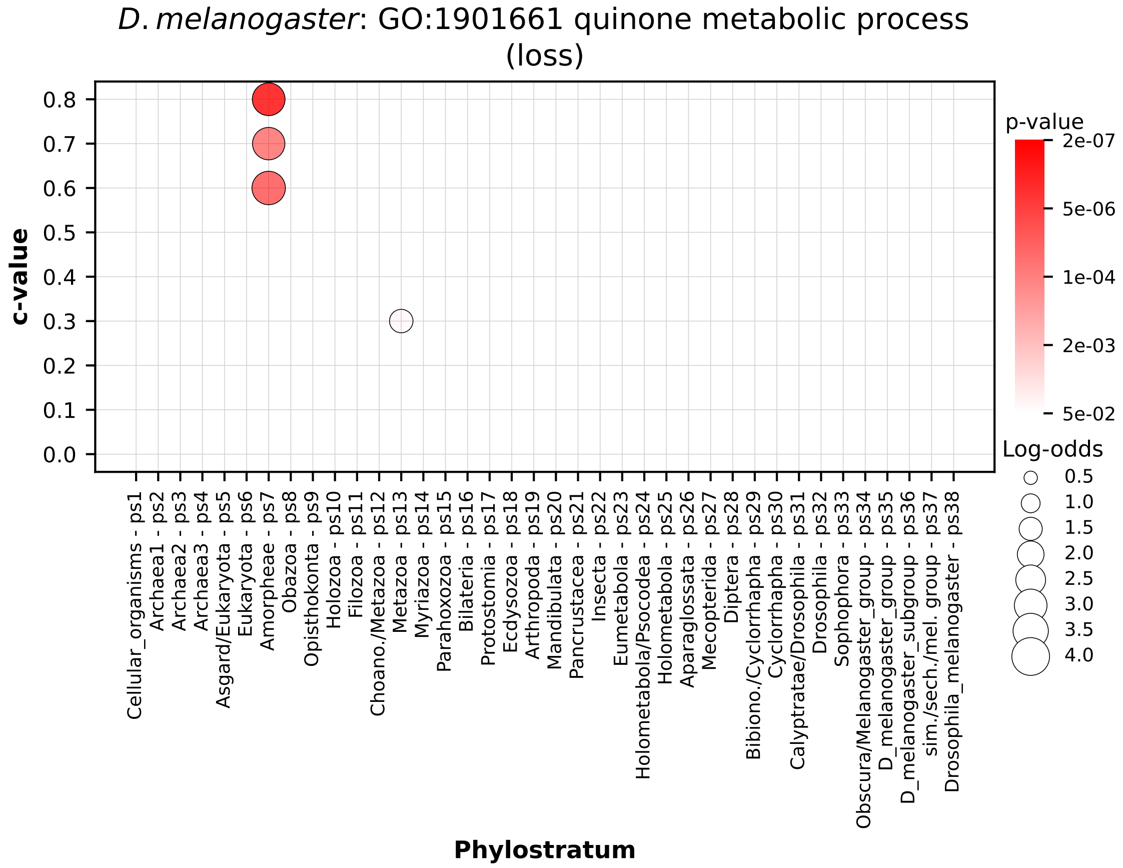

D. melanogaster: GO:1901661 quinone metabolic process (loss)
c-value
0.8
0.7
0.6
0.5
0.4
0.3
0.2
0.1
0.0
p-value
2e-07
5e-06
1e-04
2e-03
5e-02
Log-odds
0.5
1.0
1.5
2.0
2.5
3.0
3.5
4.0
Cellular_organisms - ps1
Archaea1 - ps2
Archaea2 - ps3
Archaea3 - ps4
Asgard/Eukaryota - ps5
Eukaryota - ps6
Amorpheae - ps7
Obazoa - ps8
Opisthokonta - ps9
Holozoa - ps10
Filozoa - ps11
Choano./Metazoa - ps12
Metazoa - ps13
Myriazoa - ps14
Parahoxozoa - ps15
Bilateria - ps16
Protostomia - ps17
Ecdysozoa - ps18
Arthropoda - ps19
Mandibulata - ps20
Pancrustacea - ps21
Insecta - ps22
Eumetabola - ps23
Holometabola/Psocodea - ps24
Holometabola - ps25
Aparaglossata - ps26
Mecopterida - ps27
Diptera - ps28
Bibiono./Cyclorrhapha - ps29
Cyclorrhapha - ps30
Calyptratae/Drosophila - ps31
Drosophila - ps32
Sophophora - ps33
Obscura/Melanogaster_group - ps34
D_melanogaster_group - ps35
D_melanogaster_subgroup - ps36
sim./sech./mel. group - ps37
Drosophila_melanogaster - ps38
Phylostratum

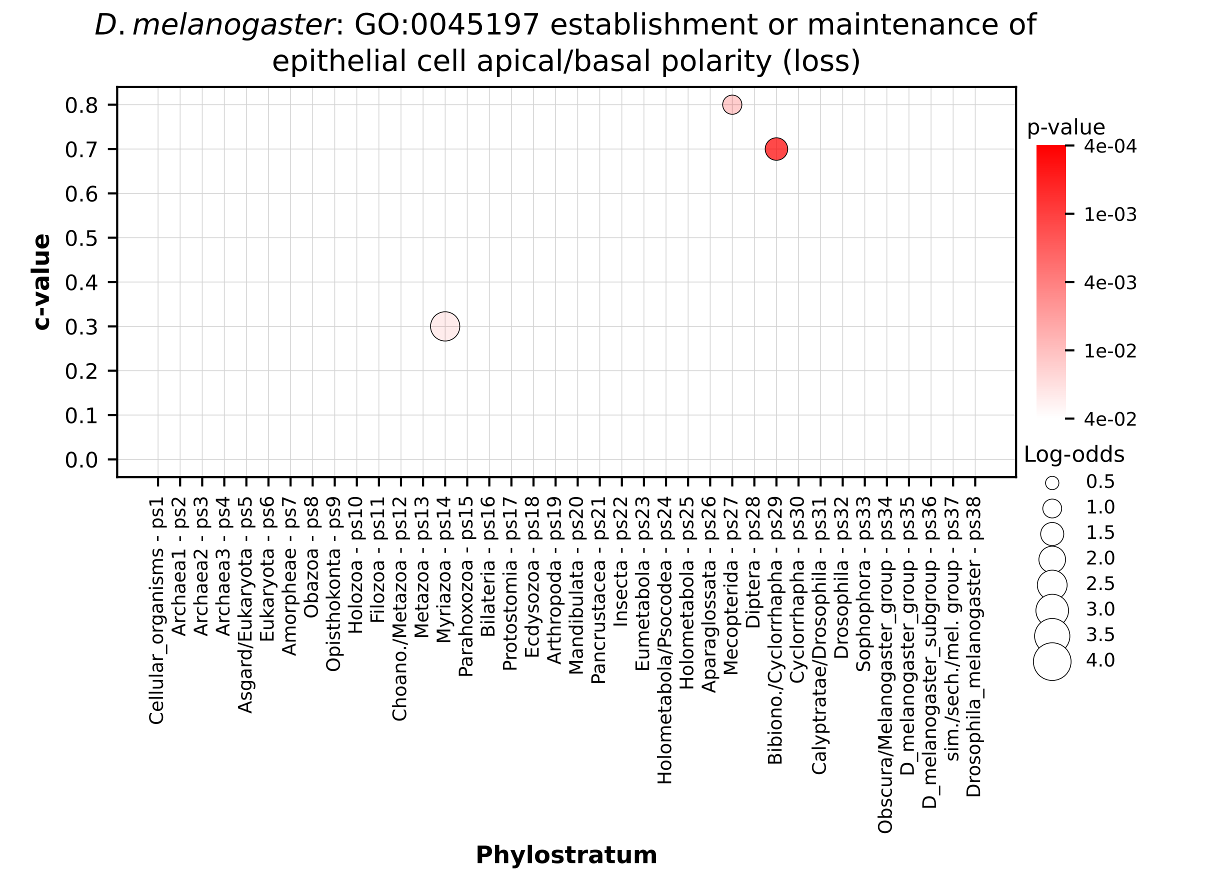

D. melanogaster: GO:0045197 establishment or maintenance of epithelial cell apical/basal polarity (loss)
c-value
0.8
0.7
0.6
0.5
0.4
0.3
0.2
0.1
0.0
p-value
4e-04
1e-03
4e-03
1e-02
4e-02
Log-odds
0.5
1.0
1.5
2.0
2.5
3.0
3.5
4.0
Cellular_organisms - ps1
Archaea1 - ps2
Archaea2 - ps3
Archaea3 - ps4
Asgard/Eukaryota - ps5
Eukaryota - ps6
Amorpheae - ps7
Obazoa - ps8
Opisthokonta - ps9
Holozoa - ps10
Filozoa - ps11
Choano./Metazoa - ps12
Metazoa - ps13
Myriazoa - ps14
Parahoxozoa - ps15
Bilateria - ps16
Protostomia - ps17
Ecdysozoa - ps18
Arthropoda - ps19
Mandibulata - ps20
Pancrustacea - ps21
Insecta - ps22
Eumetabola - ps23
Holometabola/Psocodea - ps24
Holometabola - ps25
Aparaglossata - ps26
Mecopterida - ps27
Diptera - ps28
Bibiono./Cyclorrhapha - ps29
Cyclorrhapha - ps30
Calyptratae/Drosophila - ps31
Drosophila - ps32
Sophophora - ps33
Obscura/Melanogaster_group - ps34
D_melanogaster_group - ps35
D_melanogaster_subgroup - ps36
sim./sech./mel. group - ps37
Drosophila_melanogaster - ps38
Phylostratum

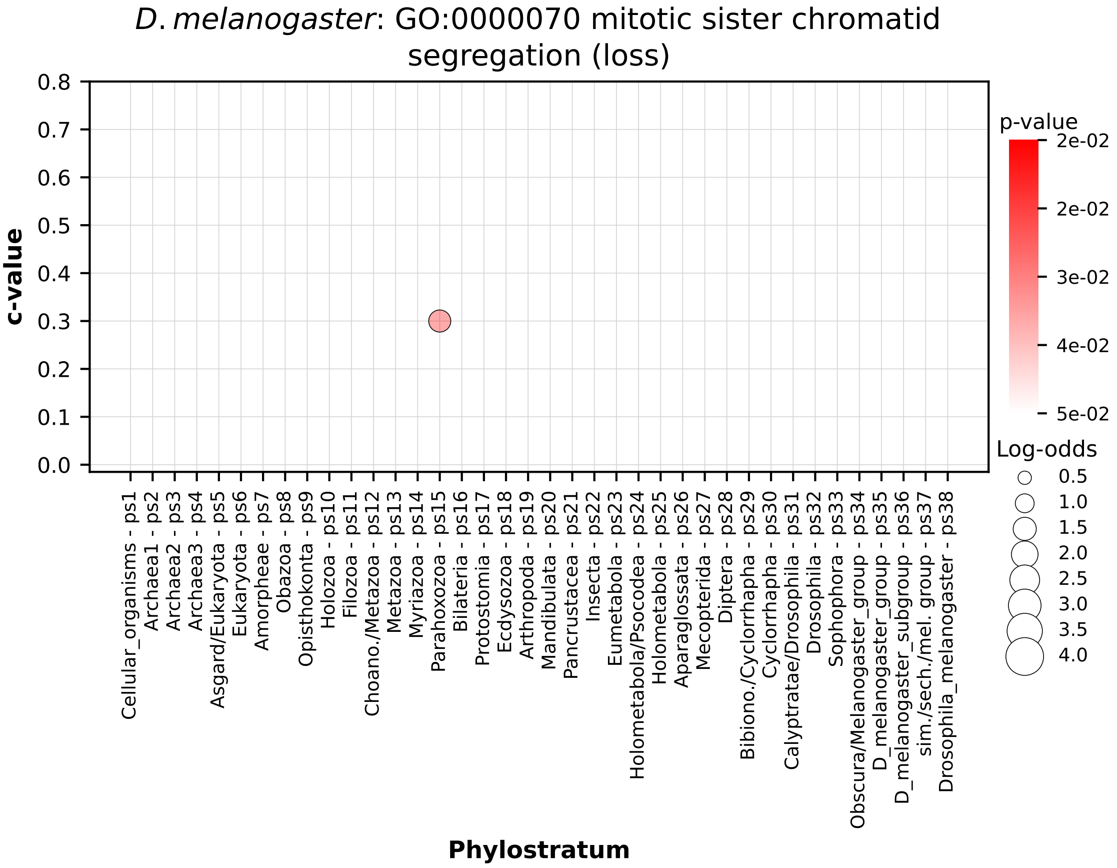

D. melanogaster: GO:0000070 mitotic sister chromatid segregation (loss)
c-value
0.8
0.7
0.6
0.5
0.4
0.3
0.2
0.1
0.0
p-value
2e-02
2e-02
3e-02
4e-02
5e-02
Log-odds
0.5
1.0
1.5
2.0
2.5
3.0
3.5
4.0
Cellular_organisms - ps1
Archaea1 - ps2
Archaea2 - ps3
Archaea3 - ps4
Asgard/Eukaryota - ps5
Eukaryota - ps6
Amorpheae - ps7
Obazoa - ps8
Opisthokonta - ps9
Holozoa - ps10
Filozoa - ps11
Choano./Metazoa - ps12
Metazoa - ps13
Myriazoa - ps14
Parahoxozoa - ps15
Bilateria - ps16
Protostomia - ps17
Ecdysozoa - ps18
Arthropoda - ps19
Mandibulata - ps20
Pancrustacea - ps21
Insecta - ps22
Eumetabola - ps23
Holometabola/Psocodea - ps24
Holometabola - ps25
Aparaglossata - ps26
Mecopterida - ps27
Diptera - ps28
Bibiono./Cyclorrhapha - ps29
Cyclorrhapha - ps30
Calyptratae/Drosophila - ps31
Drosophila - ps32
Sophophora - ps33
Obscura/Melanogaster_group - ps34
D_melanogaster_group - ps35
D_melanogaster_subgroup - ps36
sim./sech./mel. group - ps37
Drosophila_melanogaster - ps38
Phylostratum

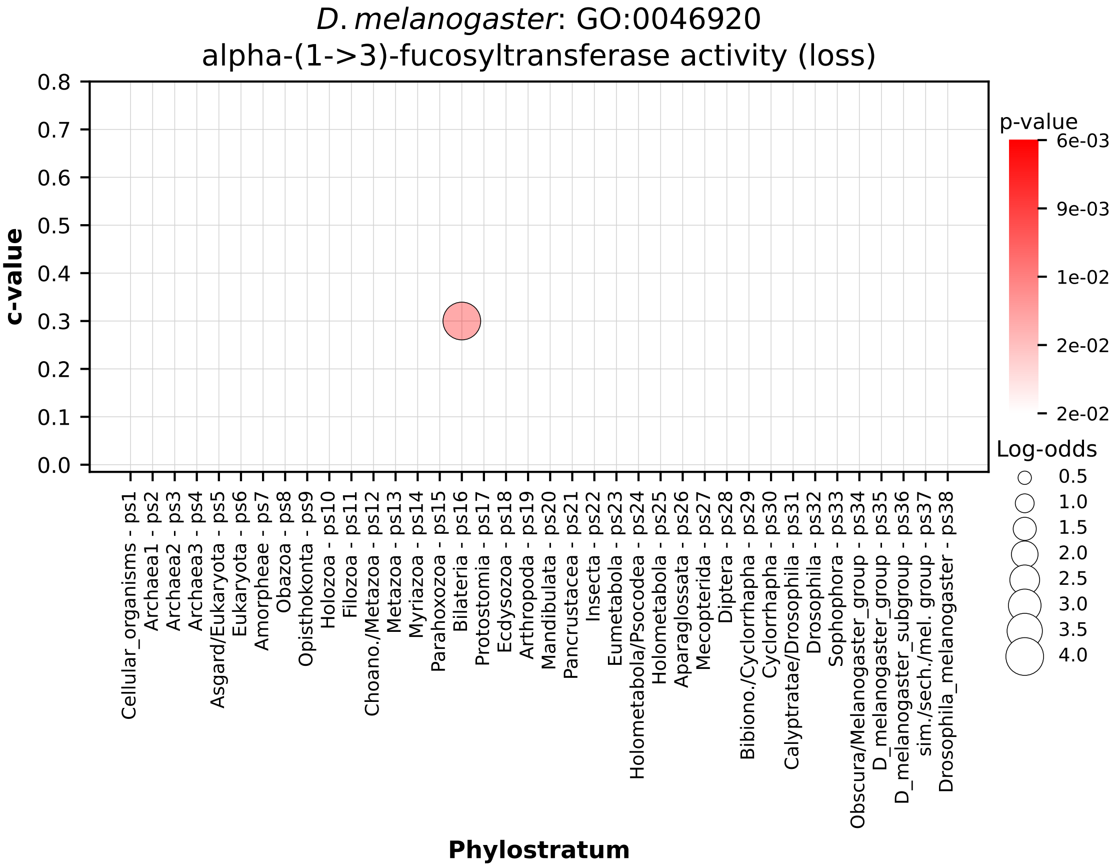
D. melanogaster: GO:0046920
alpha-(1->3)-fucosyltransferase activity (loss)
c-value
0.8
0.7
0.6
0.5
0.4
0.3
0.2
0.1
0.0
p-value
6e-03
9e-03
1e-02
2e-02
2e-02
Log-odds
0.5
1.0
1.5
2.0
2.5
3.0
3.5
4.0
Cellular_organisms - ps1
Archaea1 - ps2
Archaea2 - ps3
Archaea3 - ps4
Asgard/Eukaryota - ps5
Eukaryota - ps6
Amorpheae - ps7
Obazoa - ps8
Opisthokonta - ps9
Holozoa - ps10
Filozoa - ps11
Choano./Metazoa - ps12
Metazoa - ps13
Myriazoa - ps14
Parahoxozoa - ps15
Bilateria - ps16
Protostomia - ps17
Ecdysozoa - ps18
Arthropoda - ps19
Mandibulata - ps20
Pancrustacea - ps21
Insecta - ps22
Eumetabola - ps23
Holometabola/Psocodea - ps24
Holometabola - ps25
Aparaglossata - ps26
Mecopterida - ps27
Diptera - ps28
Bibiono./Cyclorrhapha - ps29
Cyclorrhapha - ps30
Calyptratae/Drosophila - ps31
Drosophila - ps32
Sophophora - ps33
Obscura/Melanogaster_group - ps34
D_melanogaster_group - ps35
D_melanogaster_subgroup - ps36
sim./sech./mel. group - ps37
Drosophila_melanogaster - ps38
Phylostratum

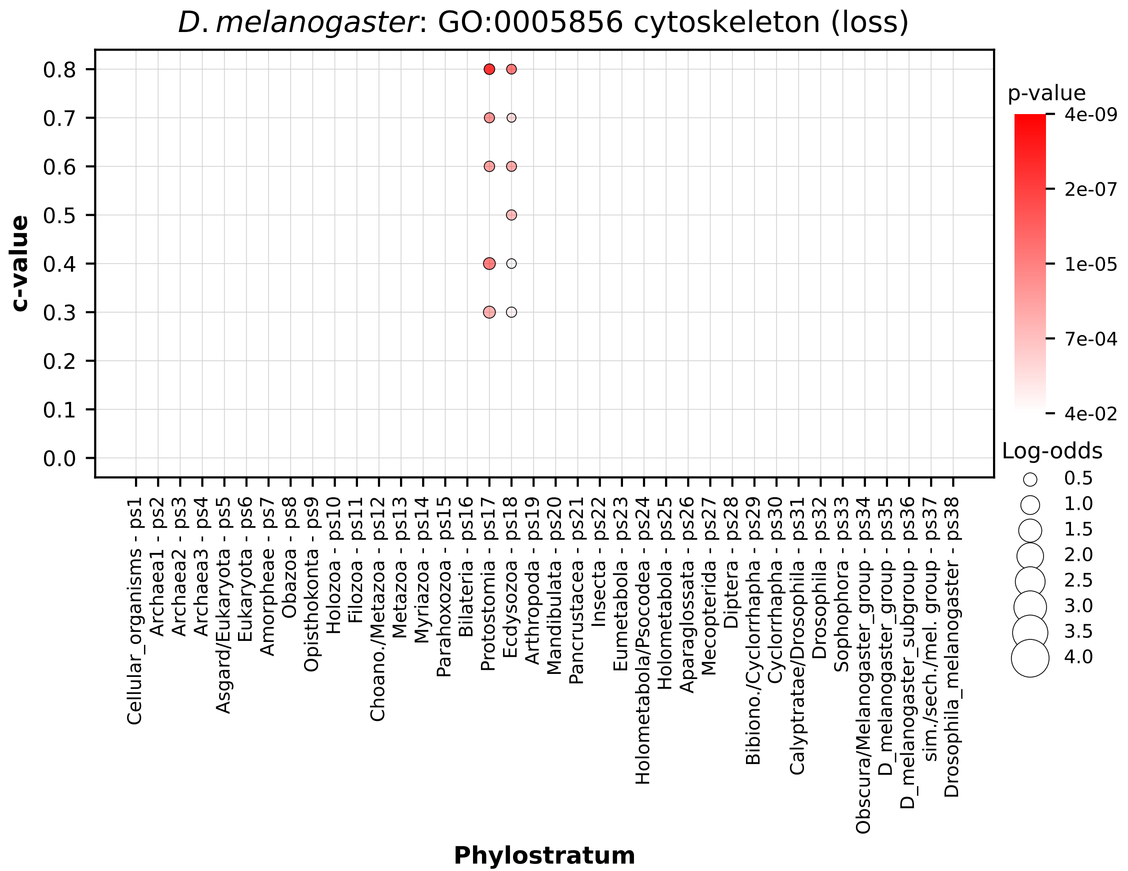

D. melanogaster: GO:0005856 cytoskeleton (loss)
c-value
0.8
0.7
0.6
0.5
0.4
0.3
0.2
0.1
0.0
p-value
4e-09
2e-07
1e-05
7e-04
4e-02
Log-odds
0.5
1.0
1.5
2.0
2.5
3.0
3.5
4.0
Cellular_organisms - ps1
Archaea1 - ps2
Archaea2 - ps3
Archaea3 - ps4
Asgard/Eukaryota - ps5
Eukaryota - ps6
Amorpheae - ps7
Obazoa - ps8
Opisthokonta - ps9
Holozoa - ps10
Filozoa - ps11
Choano./Metazoa - ps12
Metazoa - ps13
Myriazoa - ps14
Parahoxozoa - ps15
Bilateria - ps16
Protostomia - ps17
Ecdysozoa - ps18
Arthropoda - ps19
Mandibulata - ps20
Pancrustacea - ps21
Insecta - ps22
Eumetabola - ps23
Holometabola/Psocodea - ps24
Holometabola - ps25
Aparaglossata - ps26
Mecopterida - ps27
Diptera - ps28
Bibiono./Cyclorrhapha - ps29
Cyclorrhapha - ps30
Calyptratae/Drosophila - ps31
Drosophila - ps32
Sophophora - ps33
Obscura/Melanogaster_group - ps34
D_melanogaster_group - ps35
D_melanogaster_subgroup - ps36
sim./sech./mel. group - ps37
Drosophila_melanogaster - ps38
Phylostratum

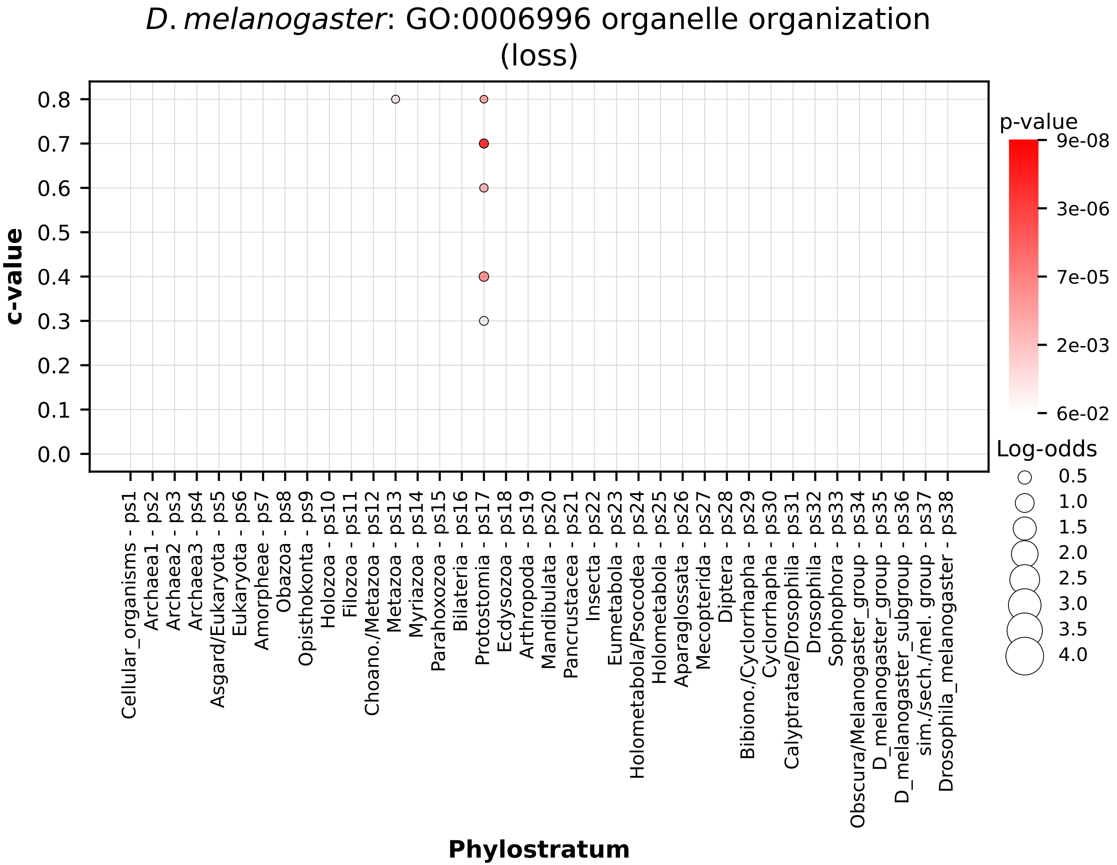

D. melanogaster: GO:0006996 organelle organization
(loss)
c-value
0.8
0.7
0.6
0.5
0.4
0.3
0.2
0.1
0.0
p-value
9e-08
3e-06
7e-05
2e-03
6e-02
Log-odds
0.5
1.0
1.5
2.0
2.5
3.0
3.5
4.0
Cellular_organisms - ps1
Archaea1 - ps2
Archaea2 - ps3
Archaea3 - ps4
Asgard/Eukaryota - ps5
Eukaryota - ps6
Amorpheae - ps7
Obazoa - ps8
Opisthokonta - ps9
Holozoa - ps10
Filozoa - ps11
Choano./Metazoa - ps12
Metazoa - ps13
Myriazoa - ps14
Parahoxozoa - ps15
Bilateria - ps16
Protostomia - ps17
Ecdysozoa - ps18
Arthropoda - ps19
Mandibulata - ps20
Pancrustacea - ps21
Insecta - ps22
Eumetabola - ps23
Holometabola/Psocodea - ps24
Holometabola - ps25
Aparaglossata - ps26
Mecopterida - ps27
Diptera - ps28
Bibiono./Cyclorrhapha - ps29
Cyclorrhapha - ps30
Calyptratae/Drosophila - ps31
Drosophila - ps32
Sophophora - ps33
Obscura/Melanogaster_group - ps34
D_melanogaster_group - ps35
D_melanogaster_subgroup - ps36
sim./sech./mel. group - ps37
Drosophila_melanogaster - ps38
Phylostratum

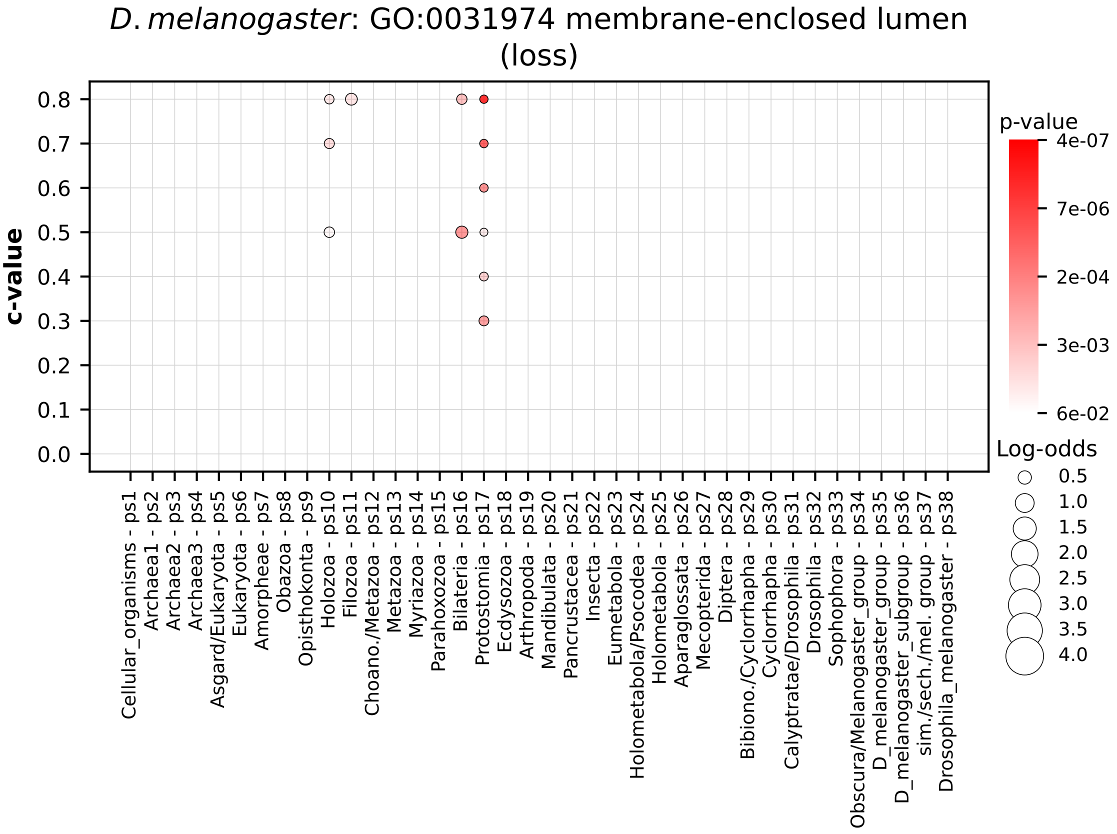

$D. melanogaster$: GO:0031974 membrane-enclosed lumen (loss)
c-value
0.0
0.1
0.2
0.3
0.4
0.5
0.6
0.7
0.8
Phylostratum
Cellular_organisms - ps1
Archaea1 - ps2
Archaea2 - ps3
Archaea3 - ps4
Asgard/Eukaryota - ps5
Eukaryota - ps6
Amorpheae - ps7
Obazoa - ps8
Opisthokonta - ps9
Holozoa - ps10
Filozoa - ps11
Choano./Metazoa - ps12
Metazoa - ps13
Myriazoa - ps14
Parahoxozoa - ps15
Bilateria - ps16
Protostomia - ps17
Ecdysozoa - ps18
Arthropoda - ps19
Mandibulata - ps20
Pancrustacea - ps21
Insecta - ps22
Eumetabola - ps23
Holometabola/Psocodea - ps24
Holometabola - ps25
Aparaglossata - ps26
Mecopterida - ps27
Diptera - ps28
Bibiono./Cyclorrhapha - ps29
Cyclorrhapha - ps30
Calyptratae/Drosophila - ps31
Drosophila - ps32
Sophophora - ps33
Obscura/Melanogaster_group - ps34
D_melanogaster_group - ps35
D_melanogaster_subgroup - ps36
sim./sech./mel. group - ps37
Drosophila_melanogaster - ps38
p-value
4e-07
7e-06
2e-04
3e-03
6e-02
Log-odds
0.5
1.0
1.5
2.0
2.5
3.0
3.5
4.0

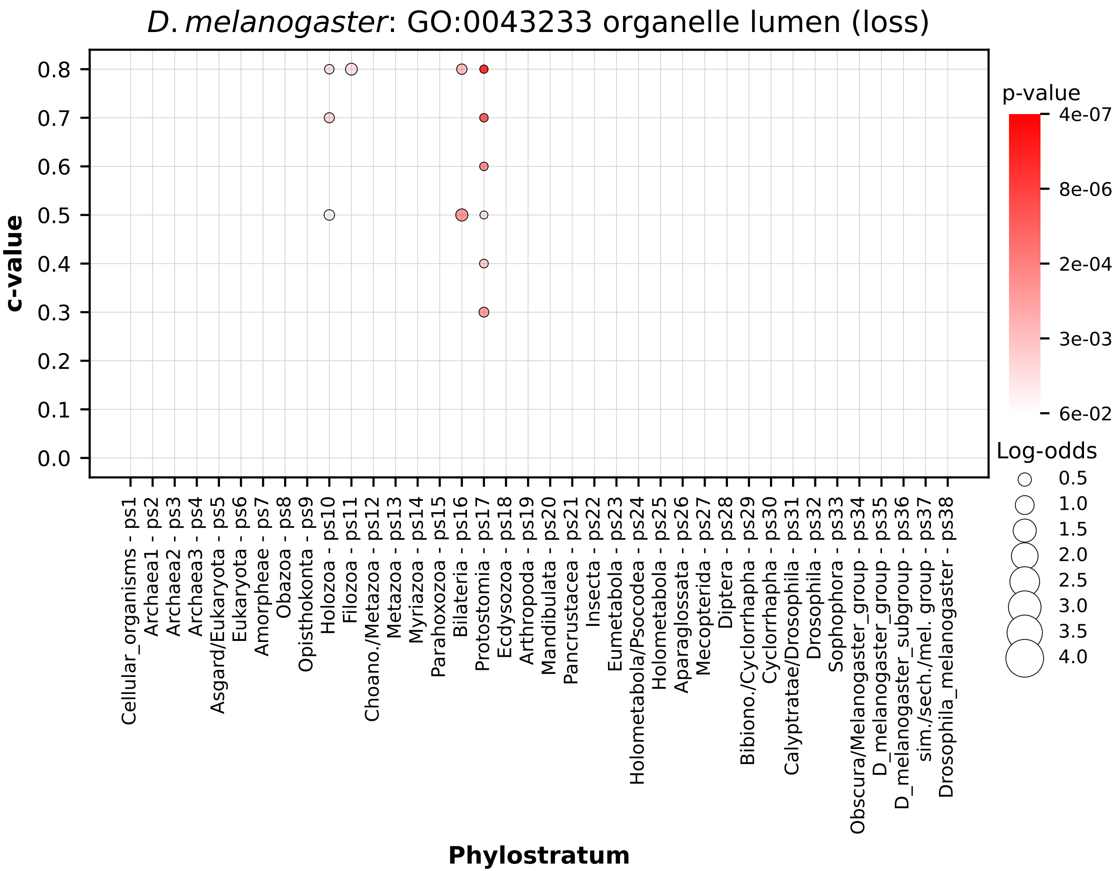
D. melanogaster: GO:0043233 organelle lumen (loss)
c-value
0.8
0.7
0.6
0.5
0.4
0.3
0.2
0.1
0.0
Phylostratum
Cellular_organisms - ps1
Archaea1 - ps2
Archaea2 - ps3
Archaea3 - ps4
Asgard/Eukaryota - ps5
Eukaryota - ps6
Amorpheae - ps7
Obazoa - ps8
Opisthokonta - ps9
Holozoa - ps10
Filozoa - ps11
Choano./Metazoa - ps12
Metazoa - ps13
Myriazoa - ps14
Parahoxozoa - ps15
Bilateria - ps16
Protostomia - ps17
Ecdysozoa - ps18
Arthropoda - ps19
Mandibulata - ps20
Pancrustacea - ps21
Insecta - ps22
Eumetabola - ps23
Holometabola/Psocodea - ps24
Holometabola - ps25
Aparaglossata - ps26
Mecopterida - ps27
Diptera - ps28
Bibiono./Cyclorrhapha - ps29
Cyclorrhapha - ps30
Calyptratae/Drosophila - ps31
Drosophila - ps32
Sophophora - ps33
Obscura/Melanogaster_group - ps34
D_melanogaster_group - ps35
D_melanogaster_subgroup - ps36
sim./sech./mel. group - ps37
Drosophila_melanogaster - ps38
p-value
4e-07
8e-06
2e-04
3e-03
6e-02
Log-odds
0.5
1.0
1.5
2.0
2.5
3.0
3.5
4.0

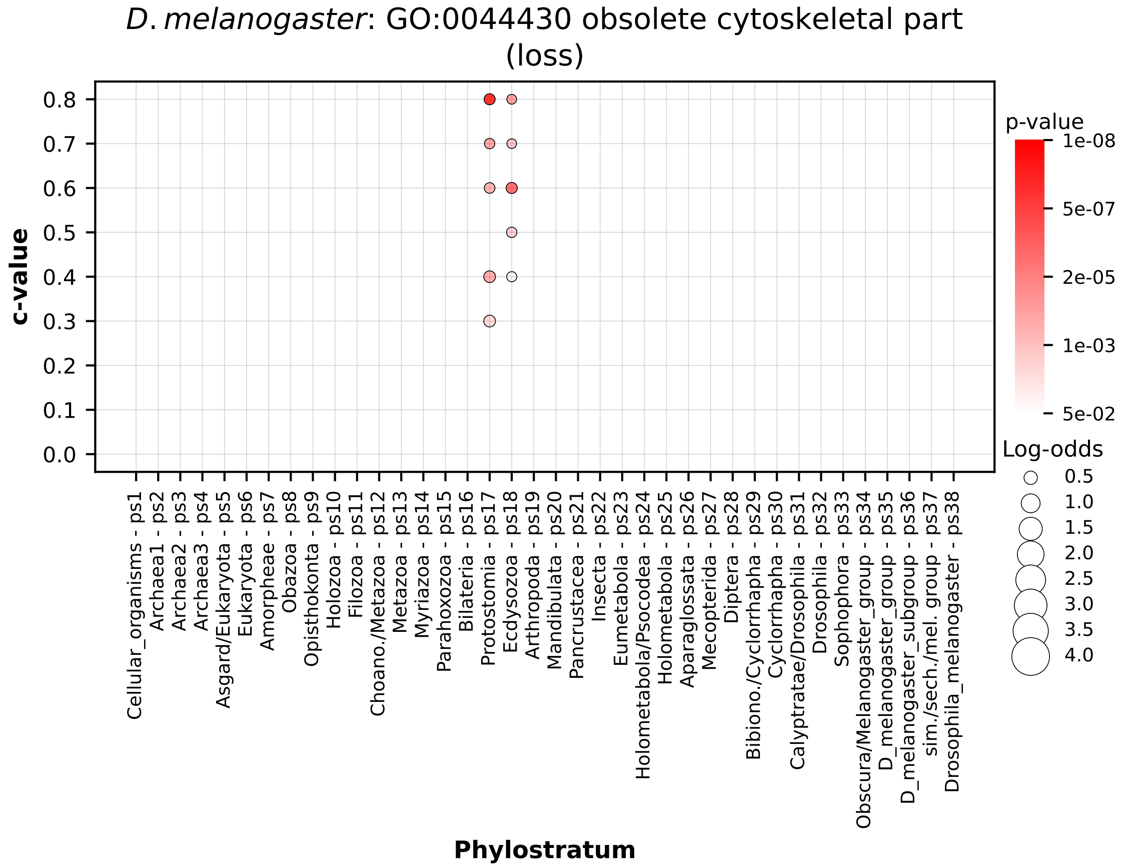

D. melanogaster: GO:0044430 obsolete cytoskeletal part (loss)
c-value
0.8
0.7
0.6
0.5
0.4
0.3
0.2
0.1
0.0
p-value
1e-08
5e-07
2e-05
1e-03
5e-02
Log-odds
0.5
1.0
1.5
2.0
2.5
3.0
3.5
4.0
Cellular_organisms - ps1
Archaea1 - ps2
Archaea2 - ps3
Archaea3 - ps4
Asgard/Eukaryota - ps5
Eukaryota - ps6
Amorpheae - ps7
Obazoa - ps8
Opisthokonta - ps9
Holozoa - ps10
Filozoa - ps11
Choano./Metazoa - ps12
Metazoa - ps13
Myriazoa - ps14
Parahoxozoa - ps15
Bilateria - ps16
Protostomia - ps17
Ecdysozoa - ps18
Arthropoda - ps19
Mandibulata - ps20
Pancrustacea - ps21
Insecta - ps22
Eumetabola - ps23
Holometabola/Psocodea - ps24
Holometabola - ps25
Aparaglossata - ps26
Mecopterida - ps27
Diptera - ps28
Bibiono./Cyclorrhapha - ps29
Cyclorrhapha - ps30
Calyptratae/Drosophila - ps31
Drosophila - ps32
Sophophora - ps33
Obscura/Melanogaster_group - ps34
D_melanogaster_group - ps35
D_melanogaster_subgroup - ps36
sim./sech./mel. group - ps37
Drosophila_melanogaster - ps38
Phylostratum

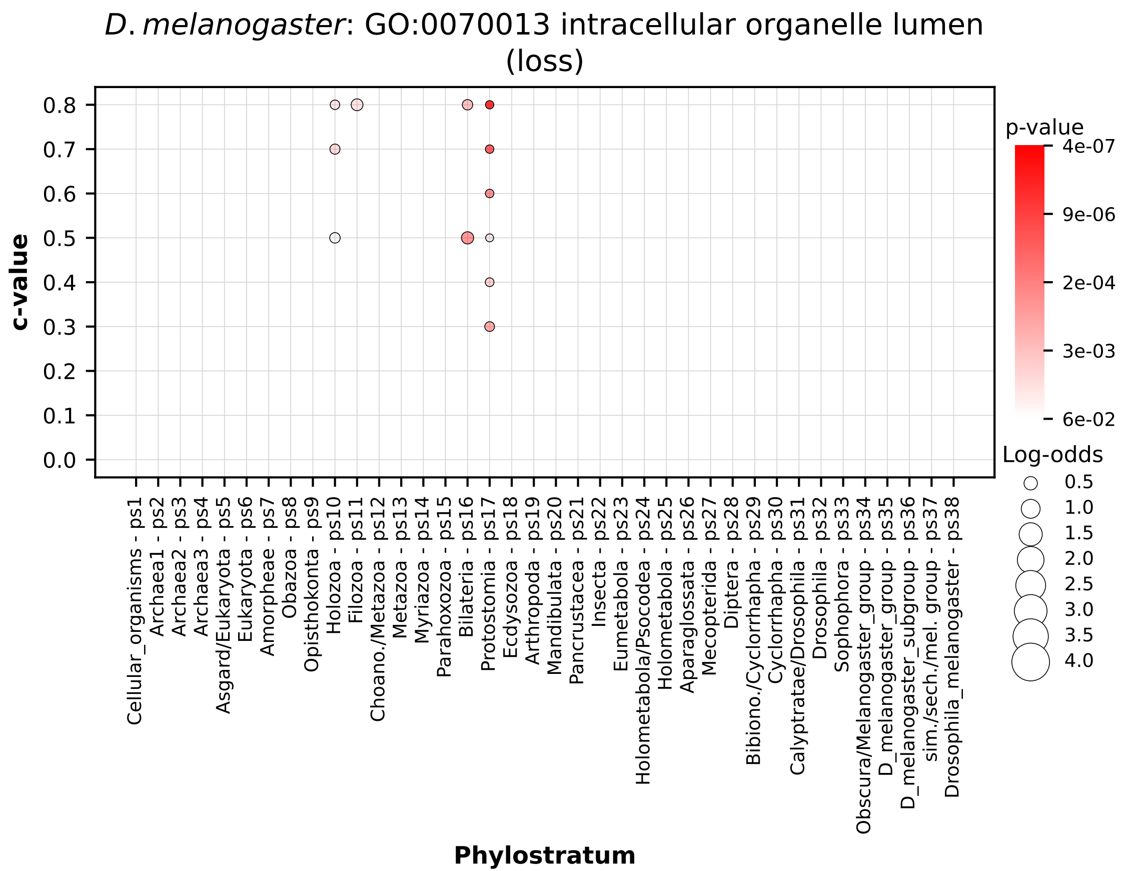

D. melanogaster: GO:0070013 intracellular organelle lumen (loss)
c-value
Phylostratum
p-value
4e-07
9e-06
2e-04
3e-03
6e-02
Log-odds
0.5
1.0
1.5
2.0
2.5
3.0
3.5
4.0
Cellular_organisms - ps1
Archaea1 - ps2
Archaea2 - ps3
Archaea3 - ps4
Asgard/Eukaryota - ps5
Eukaryota - ps6
Amorpheae - ps7
Obazoa - ps8
Opisthokonta - ps9
Holozoa - ps10
Filozoa - ps11
Choano./Metazoa - ps12
Metazoa - ps13
Myriazoa - ps14
Parahoxozoa - ps15
Bilateria - ps16
Protostomia - ps17
Ecdysozoa - ps18
Arthropoda - ps19
Mandibulata - ps20
Pancrustacea - ps21
Insecta - ps22
Eumetabola - ps23
Holometabola/Psocodea - ps24
Holometabola - ps25
Aparaglossata - ps26
Mecopterida - ps27
Diptera - ps28
Bibiono./Cyclorrhapha - ps29
Cyclorrhapha - ps30
Calyptratae/Drosophila - ps31
Drosophila - ps32
Sophophora - ps33
Obscura/Melanogaster_group - ps34
D_melanogaster_group - ps35
D_melanogaster_subgroup - ps36
sim./sech./mel. group - ps37
Drosophila_melanogaster - ps38

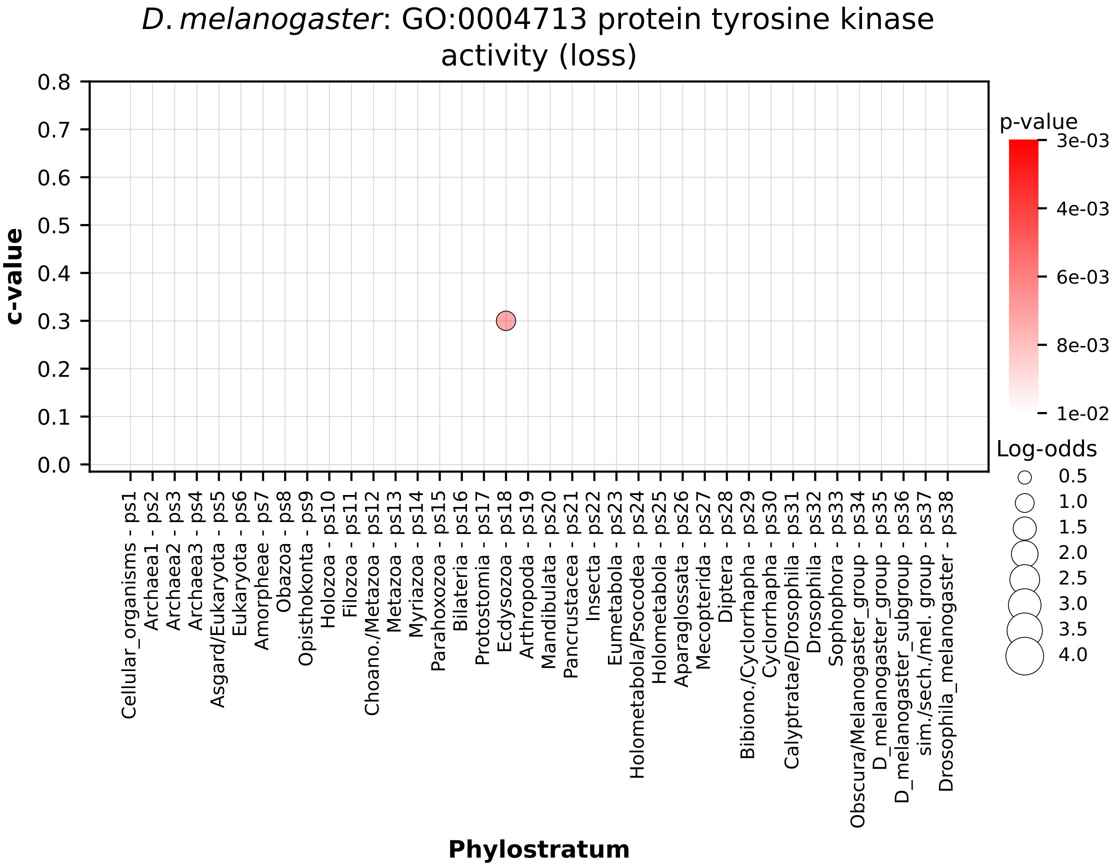

$D. melanogaster$: GO:0004713 protein tyrosine kinase activity (loss)
c-value
0.8
0.7
0.6
0.5
0.4
0.3
0.2
0.1
0.0
p-value
3e-03
4e-03
6e-03
8e-03
1e-02
Log-odds
0.5
1.0
1.5
2.0
2.5
3.0
3.5
4.0
Cellular_organisms - ps1
Archaea1 - ps2
Archaea2 - ps3
Archaea3 - ps4
Asgard/Eukaryota - ps5
Eukaryota - ps6
Amorpheae - ps7
Obazoa - ps8
Opisthokonta - ps9
Holozoa - ps10
Filozoa - ps11
Choano./Metazoa - ps12
Metazoa - ps13
Myriazoa - ps14
Parahoxozoa - ps15
Bilateria - ps16
Protostomia - ps17
Ecdysozoa - ps18
Arthropoda - ps19
Mandibulata - ps20
Pancrustacea - ps21
Insecta - ps22
Eumetabola - ps23
Holometabola/Psocodea - ps24
Holometabola - ps25
Aparaglossata - ps26
Mecopterida - ps27
Diptera - ps28
Bibiono./Cyclorrhapha - ps29
Cyclorrhapha - ps30
Calyptratae/Drosophila - ps31
Drosophila - ps32
Sophophora - ps33
Obscura/Melanogaster_group - ps34
D_melanogaster_group - ps35
D_melanogaster_subgroup - ps36
sim./sech./mel. group - ps37
Drosophila_melanogaster - ps38
Phylostratum

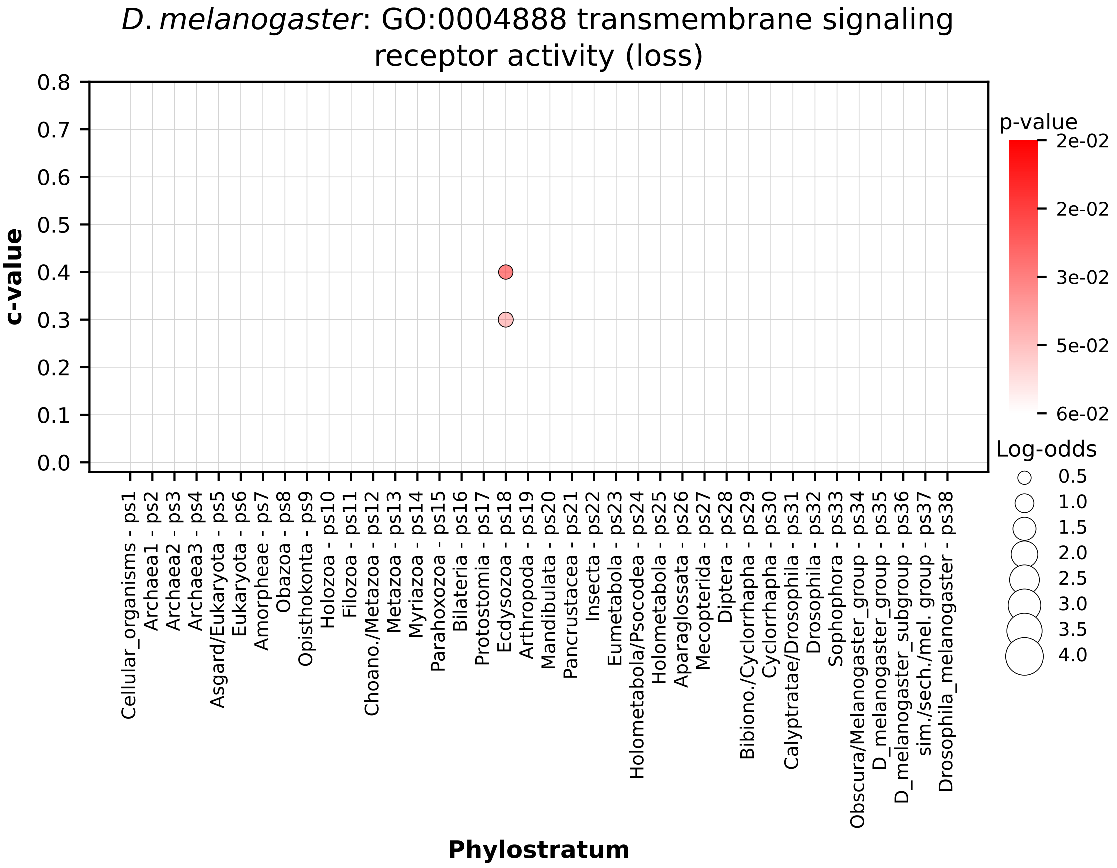

D. melanogaster: GO:0004888 transmembrane signaling receptor activity (loss)
c-value
0.8
0.7
0.6
0.5
0.4
0.3
0.2
0.1
0.0
Phylostratum
Cellular_organisms - ps1
Archaea1 - ps2
Archaea2 - ps3
Archaea3 - ps4
Asgard/Eukaryota - ps5
Eukaryota - ps6
Amorpheae - ps7
Obazoa - ps8
Opisthokonta - ps9
Holozoa - ps10
Filozoa - ps11
Choano./Metazoa - ps12
Metazoa - ps13
Myriazoa - ps14
Parahoxozoa - ps15
Bilateria - ps16
Protostomia - ps17
Ecdysozoa - ps18
Arthropoda - ps19
Mandibulata - ps20
Pancrustacea - ps21
Insecta - ps22
Eumetabola - ps23
Holometabola/Psocodea - ps24
Holometabola - ps25
Aparaglossata - ps26
Mecopterida - ps27
Diptera - ps28
Bibiono./Cyclorrhapha - ps29
Cyclorrhapha - ps30
Calyptratae/Drosophila - ps31
Drosophila - ps32
Sophophora - ps33
Obscura/Melanogaster_group - ps34
D_melanogaster_group - ps35
D_melanogaster_subgroup - ps36
sim./sech./mel. group - ps37
Drosophila_melanogaster - ps38
p-value
2e-02
2e-02
3e-02
5e-02
6e-02
Log-odds
0.5
1.0
1.5
2.0
2.5
3.0
3.5
4.0

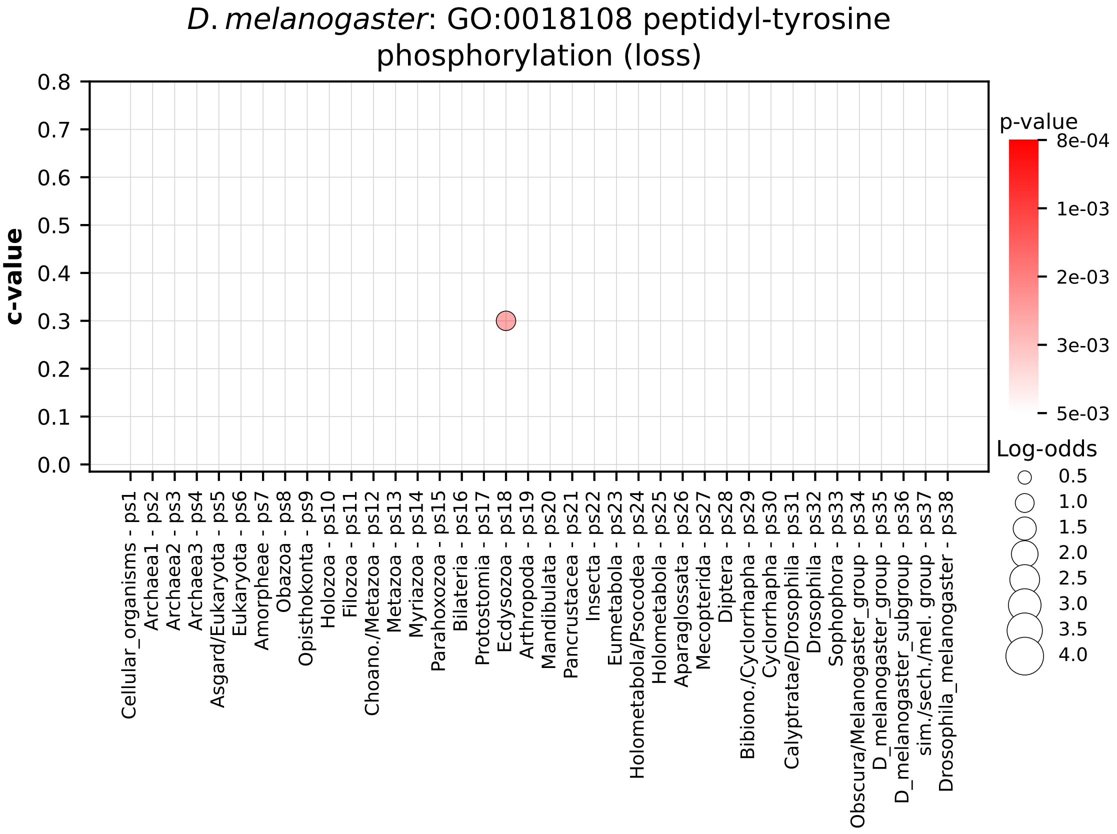
D. melanogaster: GO:0018108 peptidyl-tyrosine phosphorylation (loss)
c-value
0.8
0.7
0.6
0.5
0.4
0.3
0.2
0.1
0.0
p-value
8e-04
1e-03
2e-03
3e-03
5e-03
Log-odds
0.5
1.0
1.5
2.0
2.5
3.0
3.5
4.0
Cellular_organisms - ps1
Archaea1 - ps2
Archaea2 - ps3
Archaea3 - ps4
Asgard/Eukaryota - ps5
Eukaryota - ps6
Amorpheae - ps7
Obazoa - ps8
Opisthokonta - ps9
Holozoa - ps10
Filozoa - ps11
Choano./Metazoa - ps12
Metazoa - ps13
Myriazoa - ps14
Parahoxozoa - ps15
Bilateria - ps16
Protostomia - ps17
Ecdysozoa - ps18
Arthropoda - ps19
Mandibulata - ps20
Pancrustacea - ps21
Insecta - ps22
Eumetabola - ps23
Holometabola/Psocodea - ps24
Holometabola - ps25
Aparaglossata - ps26
Mecopterida - ps27
Diptera - ps28
Bibiono./Cyclorrhapha - ps29
Cyclorrhapha - ps30
Calyptratae/Drosophila - ps31
Drosophila - ps32
Sophophora - ps33
Obscura/Melanogaster_group - ps34
D_melanogaster_group - ps35
D_melanogaster_subgroup - ps36
sim./sech./mel. group - ps37
Drosophila_melanogaster - ps38
Phylostratum

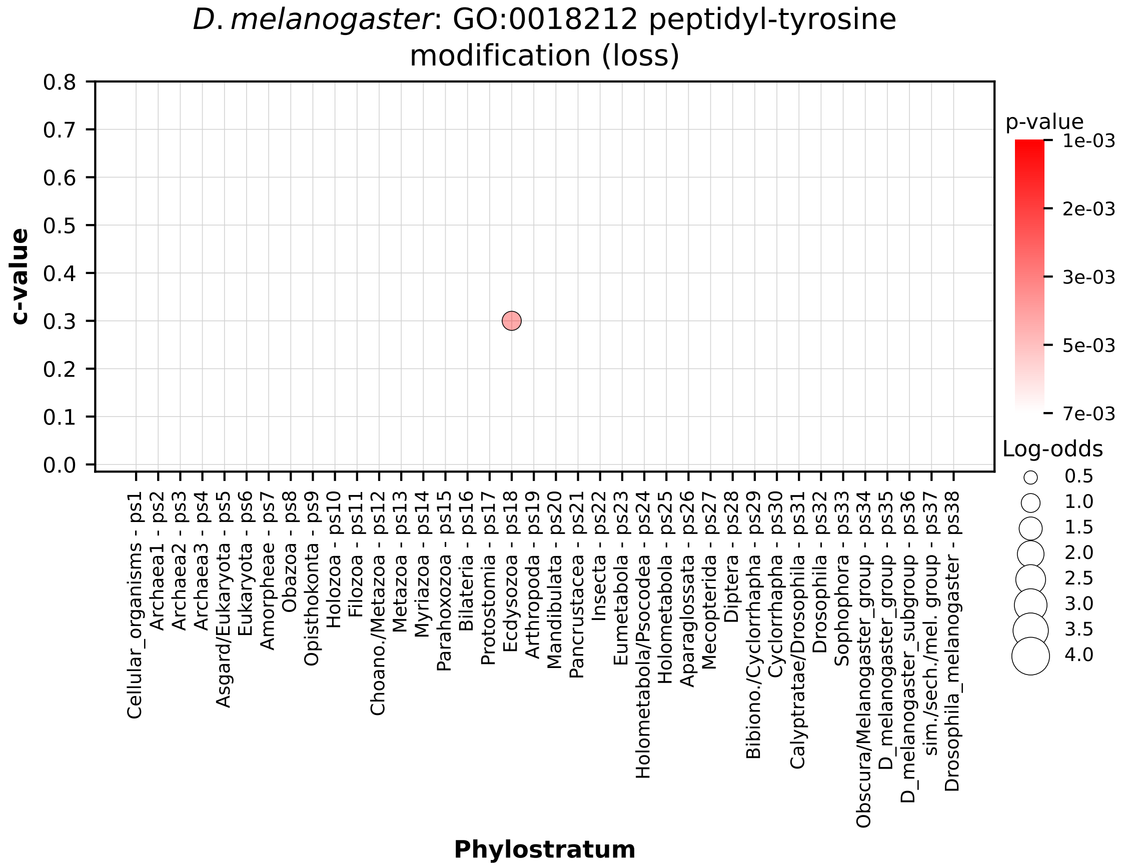
D. melanogaster: GO:0018212 peptidyl-tyrosine modification (loss)
c-value
0.8
0.7
0.6
0.5
0.4
0.3
0.2
0.1
0.0
Phylostratum
Cellular_organisms - ps1
Archaea1 - ps2
Archaea2 - ps3
Archaea3 - ps4
Asgard/Eukaryota - ps5
Eukaryota - ps6
Amorpheae - ps7
Obazoa - ps8
Opisthokonta - ps9
Holozoa - ps10
Filozoa - ps11
Choano./Metazoa - ps12
Metazoa - ps13
Myriazoa - ps14
Parahoxozoa - ps15
Bilateria - ps16
Protostomia - ps17
Ecdysozoa - ps18
Arthropoda - ps19
Mandibulata - ps20
Pancrustacea - ps21
Insecta - ps22
Eumetabola - ps23
Holometabola/Psocodea - ps24
Holometabola - ps25
Aparaglossata - ps26
Mecopterida - ps27
Diptera - ps28
Bibiono./Cyclorrhapha - ps29
Cyclorrhapha - ps30
Calyptratae/Drosophila - ps31
Drosophila - ps32
Sophophora - ps33
Obscura/Melanogaster_group - ps34
D_melanogaster_group - ps35
D_melanogaster_subgroup - ps36
sim./sech./mel. group - ps37
Drosophila_melanogaster - ps38
p-value
1e-03
2e-03
3e-03
5e-03
7e-03
Log-odds
0.5
1.0
1.5
2.0
2.5
3.0
3.5
4.0

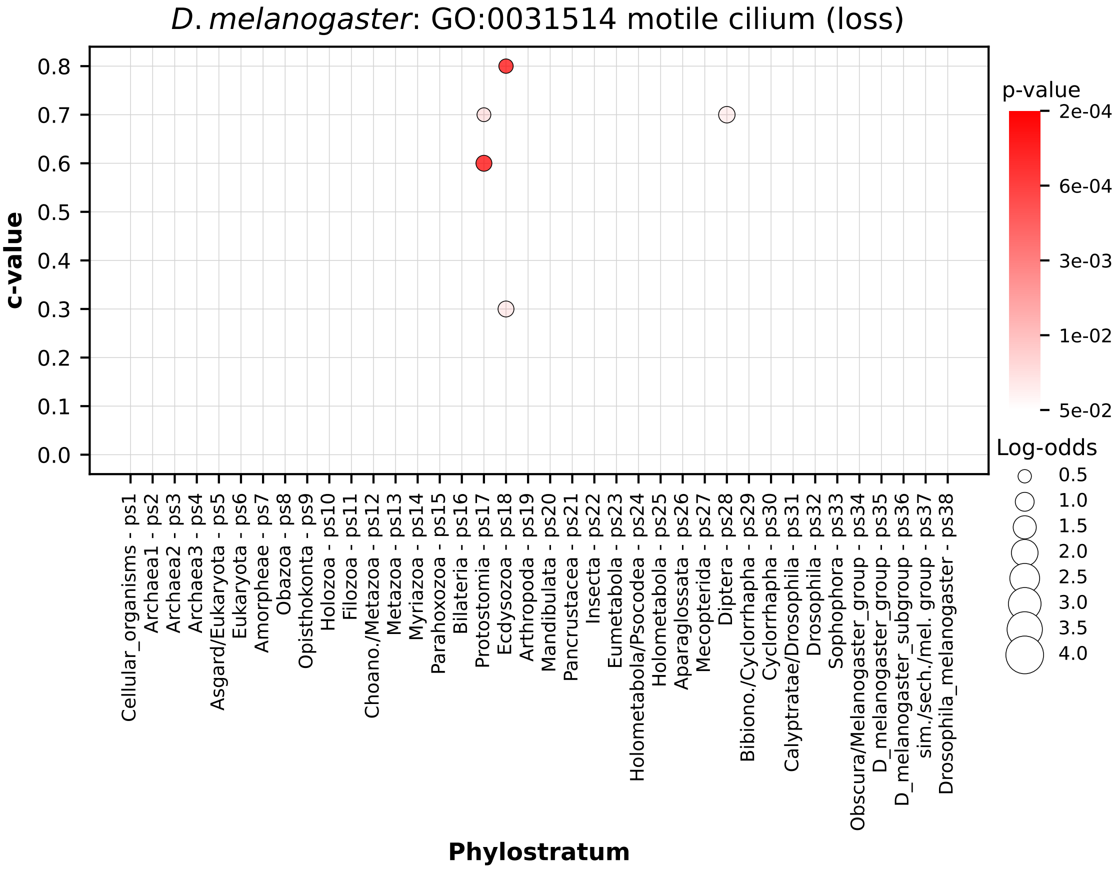
D. melanogaster: GO:0031514 motile cilium (loss)
c-value
0.8
0.7
0.6
0.5
0.4
0.3
0.2
0.1
0.0
Phylostratum
Cellular_organisms - ps1
Archaea1 - ps2
Archaea2 - ps3
Archaea3 - ps4
Asgard/Eukaryota - ps5
Eukaryota - ps6
Amorpheae - ps7
Obazoa - ps8
Opisthokonta - ps9
Holozoa - ps10
Filozoa - ps11
Choano./Metazoa - ps12
Metazoa - ps13
Myriazoa - ps14
Parahoxozoa - ps15
Bilateria - ps16
Protostomia - ps17
Ecdysozoa - ps18
Arthropoda - ps19
Mandibulata - ps20
Pancrustacea - ps21
Insecta - ps22
Eumetabola - ps23
Holometabola/Psocodea - ps24
Holometabola - ps25
Aparaglossata - ps26
Mecopterida - ps27
Diptera - ps28
Bibiono./Cyclorrhapha - ps29
Cyclorrhapha - ps30
Calyptratae/Drosophila - ps31
Drosophila - ps32
Sophophora - ps33
Obscura/Melanogaster_group - ps34
D_melanogaster_group - ps35
D_melanogaster_subgroup - ps36
sim./sech./mel. group - ps37
Drosophila_melanogaster - ps38
p-value
2e-04
6e-04
3e-03
1e-02
5e-02
Log-odds
0.5
1.0
1.5
2.0
2.5
3.0
3.5
4.0

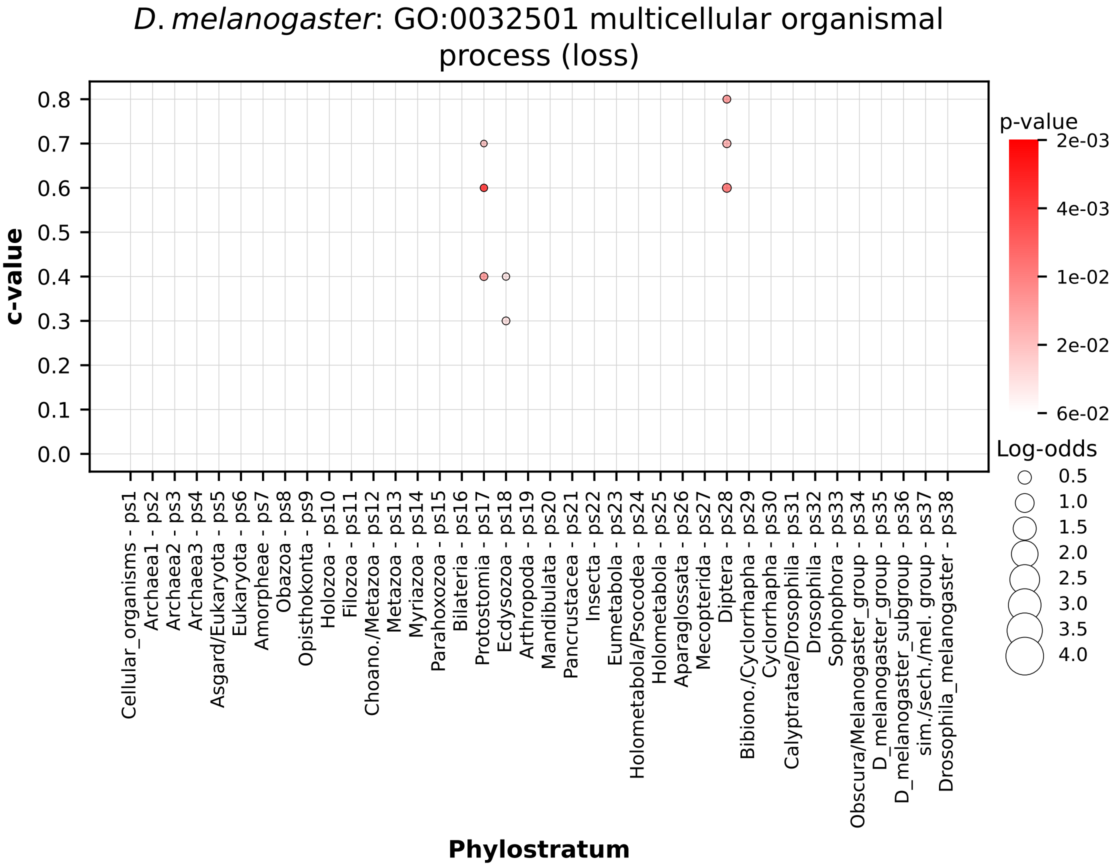

D. melanogaster: GO:0032501 multicellular organismal process (loss)
c-value
0.0
0.1
0.2
0.3
0.4
0.5
0.6
0.7
0.8
Phylostratum
Cellular_organisms - ps1
Archaea1 - ps2
Archaea2 - ps3
Archaea3 - ps4
Asgard/Eukaryota - ps5
Eukaryota - ps6
Amorpheae - ps7
Obazoa - ps8
Opisthokonta - ps9
Holozoa - ps10
Filozoa - ps11
Choano./Metazoa - ps12
Metazoa - ps13
Myriazoa - ps14
Parahoxozoa - ps15
Bilateria - ps16
Protostomia - ps17
Ecdysozoa - ps18
Arthropoda - ps19
Mandibulata - ps20
Pancrustacea - ps21
Insecta - ps22
Eumetabola - ps23
Holometabola/Psocodea - ps24
Holometabola - ps25
Aparaglossata - ps26
Mecopterida - ps27
Diptera - ps28
Bibiono./Cyclorrhapha - ps29
Cyclorrhapha - ps30
Calyptratae/Drosophila - ps31
Drosophila - ps32
Sophophora - ps33
Obscura/Melanogaster_group - ps34
D_melanogaster_group - ps35
D_melanogaster_subgroup - ps36
sim./sech./mel. group - ps37
Drosophila_melanogaster - ps38
p-value
2e-03
4e-03
1e-02
2e-02
6e-02
Log-odds
0.5
1.0
1.5
2.0
2.5
3.0
3.5
4.0

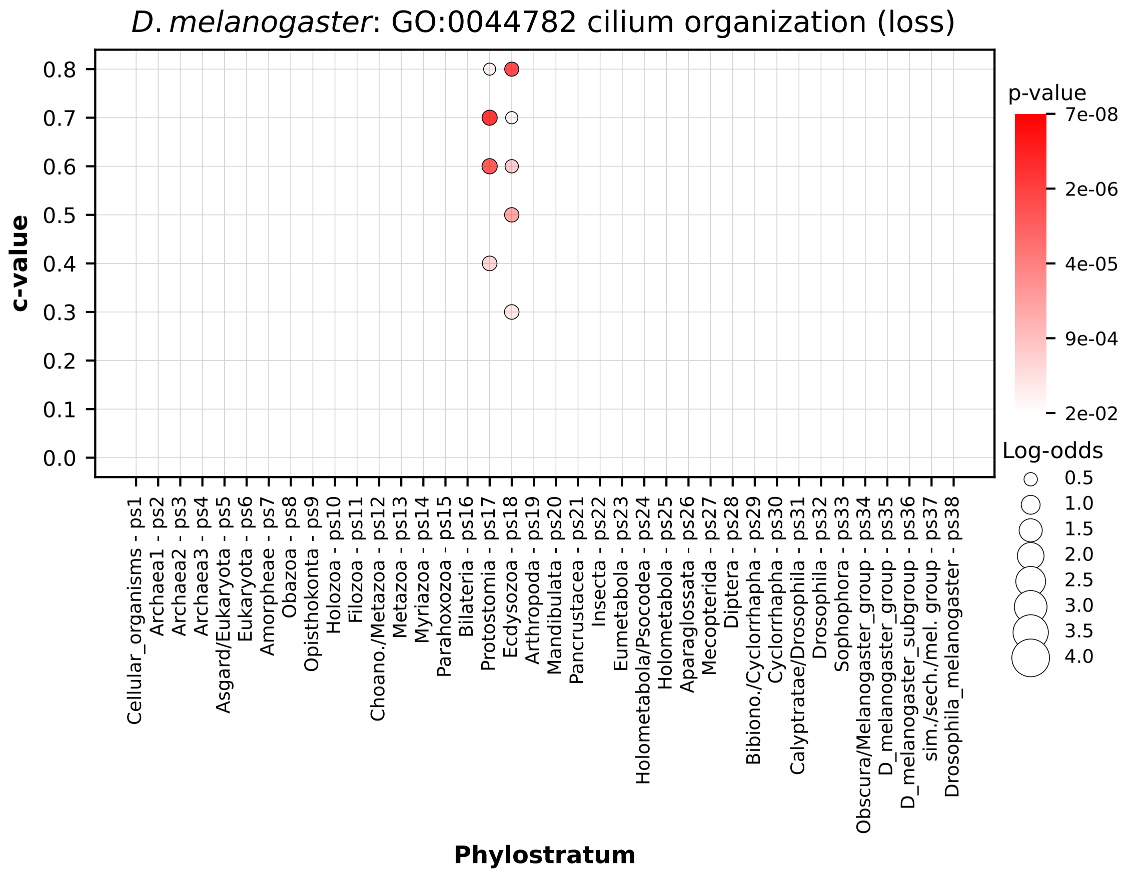

D. melanogaster: GO:0044782 cilium organization (loss)
c-value
0.8
0.7
0.6
0.5
0.4
0.3
0.2
0.1
0.0
p-value
7e-08
2e-06
4e-05
9e-04
2e-02
Log-odds
0.5
1.0
1.5
2.0
2.5
3.0
3.5
4.0
Cellular_organisms - ps1
Archaea1 - ps2
Archaea2 - ps3
Archaea3 - ps4
Asgard/Eukaryota - ps5
Eukaryota - ps6
Amorpheae - ps7
Obazoa - ps8
Opisthokonta - ps9
Holozoa - ps10
Filozoa - ps11
Choano./Metazoa - ps12
Metazoa - ps13
Myriazoa - ps14
Parahoxozoa - ps15
Bilateria - ps16
Protostomia - ps17
Ecdysozoa - ps18
Arthropoda - ps19
Mandibulata - ps20
Pancrustacea - ps21
Insecta - ps22
Eumetabola - ps23
Holometabola/Psocodea - ps24
Holometabola - ps25
Aparaglossata - ps26
Mecopterida - ps27
Diptera - ps28
Bibiono./Cyclorrhapha - ps29
Cyclorrhapha - ps30
Calyptratae/Drosophila - ps31
Drosophila - ps32
Sophophora - ps33
Obscura/Melanogaster_group - ps34
D_melanogaster_group - ps35
D_melanogaster_subgroup - ps36
sim./sech./mel. group - ps37
Drosophila_melanogaster - ps38
Phylostratum

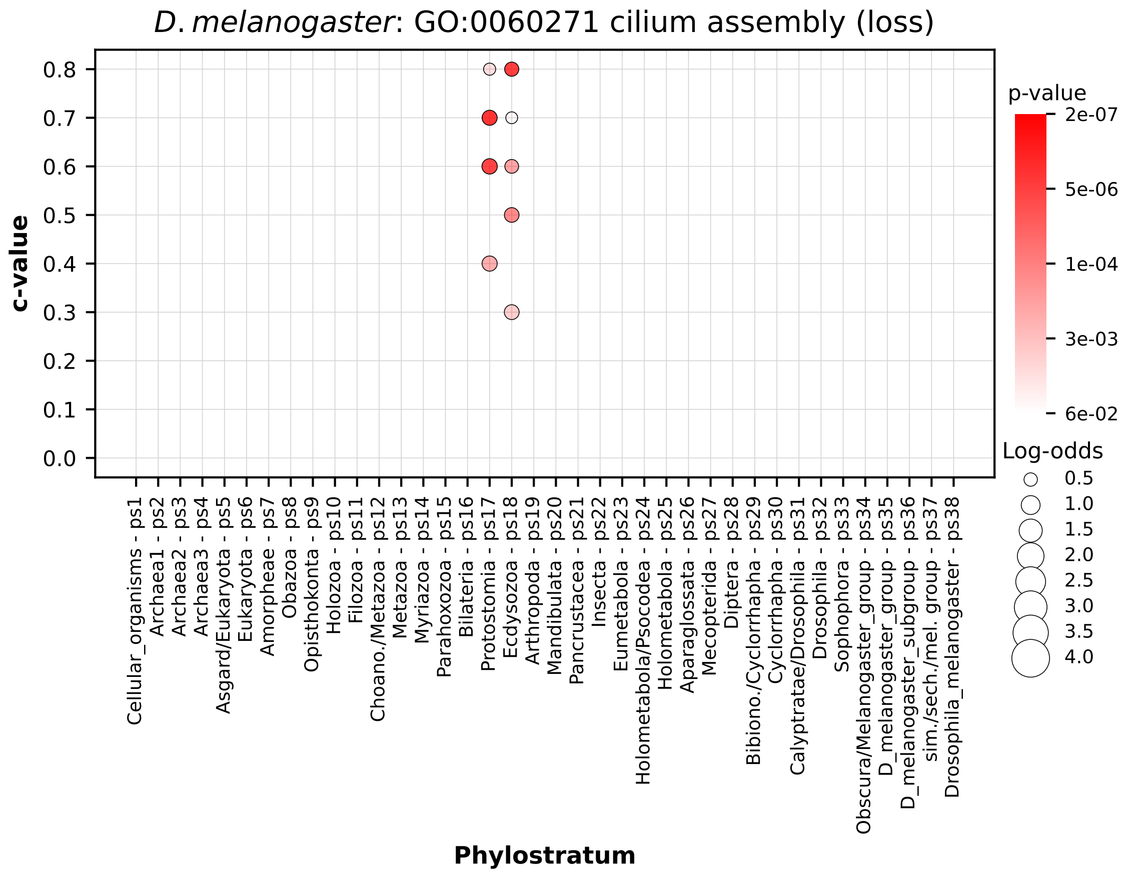
D. melanogaster: GO:0060271 cilium assembly (loss)
c-value
0.0
0.1
0.2
0.3
0.4
0.5
0.6
0.7
0.8
p-value
2e-07
5e-06
1e-04
3e-03
6e-02
Log-odds
0.5
1.0
1.5
2.0
2.5
3.0
3.5
4.0
Cellular_organisms - ps1
Archaea1 - ps2
Archaea2 - ps3
Archaea3 - ps4
Asgard/Eukaryota - ps5
Eukaryota - ps6
Amorpheae - ps7
Obazoa - ps8
Opisthokonta - ps9
Holozoa - ps10
Filozoa - ps11
Choano./Metazoa - ps12
Metazoa - ps13
Myriazoa - ps14
Parahoxozoa - ps15
Bilateria - ps16
Protostomia - ps17
Ecdysozoa - ps18
Arthropoda - ps19
Mandibulata - ps20
Pancrustacea - ps21
Insecta - ps22
Eumetabola - ps23
Holometabola/Psocodea - ps24
Holometabola - ps25
Aparaglossata - ps26
Mecopterida - ps27
Diptera - ps28
Bibiono./Cyclorrhapha - ps29
Cyclorrhapha - ps30
Calyptratae/Drosophila - ps31
Drosophila - ps32
Sophophora - ps33
Obscura/Melanogaster_group - ps34
D_melanogaster_group - ps35
D_melanogaster_subgroup - ps36
sim./sech./mel. group - ps37
Drosophila_melanogaster - ps38
Phylostratum

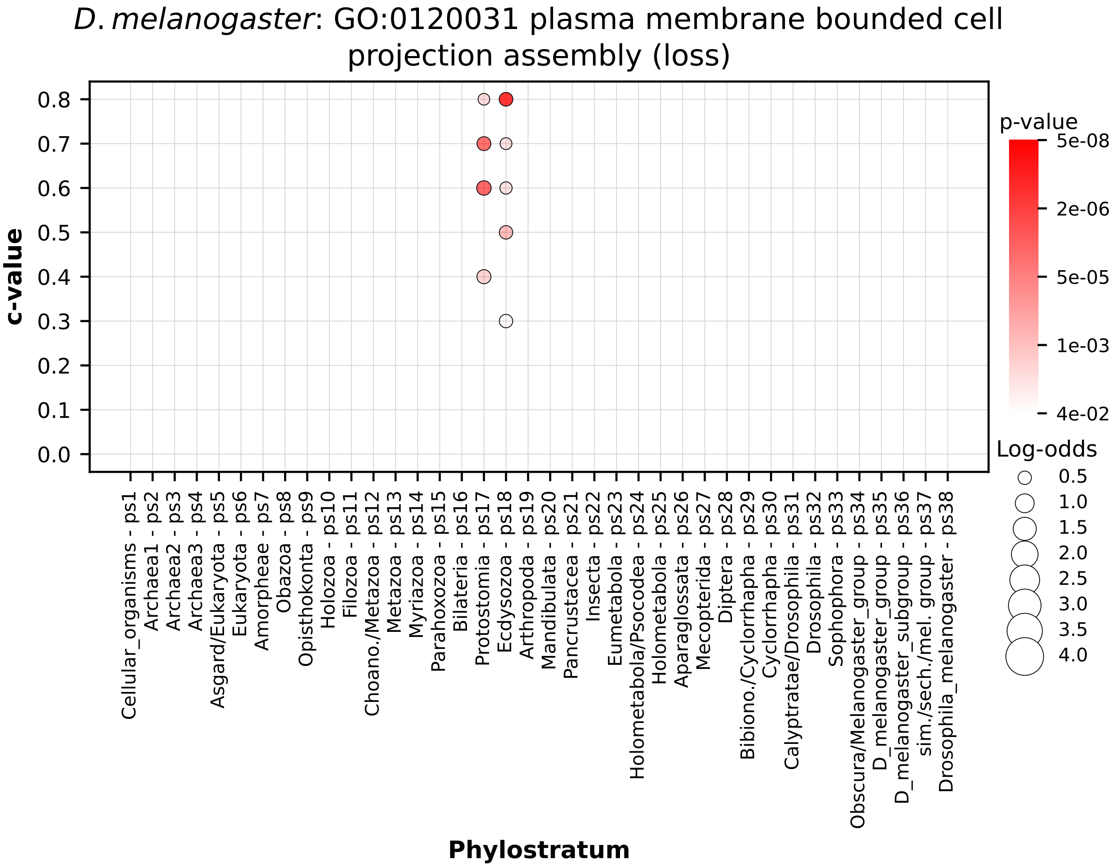
D. melanogaster: GO:0120031 plasma membrane bounded cell projection assembly (loss)
c-value
0.8
0.7
0.6
0.5
0.4
0.3
0.2
0.1
0.0
p-value
5e-08
2e-06
5e-05
1e-03
4e-02
Log-odds
0.5
1.0
1.5
2.0
2.5
3.0
3.5
4.0
Cellular_organisms - ps1
Archaea1 - ps2
Archaea2 - ps3
Archaea3 - ps4
Asgard/Eukaryota - ps5
Eukaryota - ps6
Amorpheae - ps7
Obazoa - ps8
Opisthokonta - ps9
Holozoa - ps10
Filozoa - ps11
Choano./Metazoa - ps12
Metazoa - ps13
Myriazoa - ps14
Parahoxozoa - ps15
Bilateria - ps16
Protostomia - ps17
Ecdysozoa - ps18
Arthropoda - ps19
Mandibulata - ps20
Pancrustacea - ps21
Insecta - ps22
Eumetabola - ps23
Holometabola/Psocodea - ps24
Holometabola - ps25
Aparaglossata - ps26
Mecopterida - ps27
Diptera - ps28
Bibiono./Cyclorrhapha - ps29
Cyclorrhapha - ps30
Calyptratae/Drosophila - ps31
Drosophila - ps32
Sophophora - ps33
Obscura/Melanogaster_group - ps34
D_melanogaster_group - ps35
D_melanogaster_subgroup - ps36
sim./sech./mel. group - ps37
Drosophila_melanogaster - ps38
Phylostratum

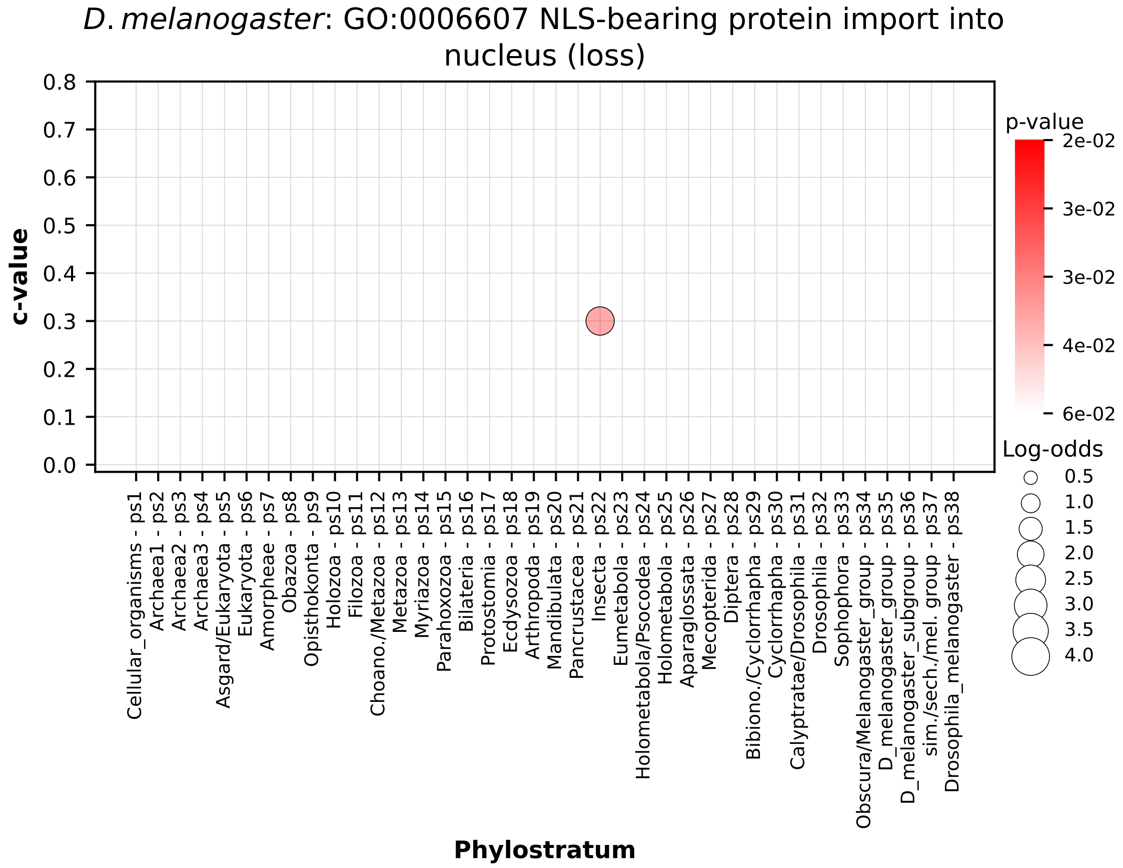

D. melanogaster: GO:0006607 NLS-bearing protein import into nucleus (loss)
c-value
0.8
0.7
0.6
0.5
0.4
0.3
0.2
0.1
0.0
p-value
2e-02
3e-02
3e-02
4e-02
6e-02
Log-odds
0.5
1.0
1.5
2.0
2.5
3.0
3.5
4.0
Cellular_organisms - ps1
Archaea1 - ps2
Archaea2 - ps3
Archaea3 - ps4
Asgard/Eukaryota - ps5
Eukaryota - ps6
Amorpheae - ps7
Obazoa - ps8
Opisthokonta - ps9
Holozoa - ps10
Filozoa - ps11
Choano./Metazoa - ps12
Metazoa - ps13
Myriazoa - ps14
Parahoxozoa - ps15
Bilateria - ps16
Protostomia - ps17
Ecdysozoa - ps18
Arthropoda - ps19
Mandibulata - ps20
Pancrustacea - ps21
Insecta - ps22
Eumetabola - ps23
Holometabola/Psocodea - ps24
Holometabola - ps25
Aparaglossata - ps26
Mecopterida - ps27
Diptera - ps28
Bibiono./Cyclorrhapha - ps29
Cyclorrhapha - ps30
Calyptratae/Drosophila - ps31
Drosophila - ps32
Sophophora - ps33
Obscura/Melanogaster_group - ps34
D_melanogaster_group - ps35
D_melanogaster_subgroup - ps36
sim./sech./mel. group - ps37
Drosophila_melanogaster - ps38
Phylostratum

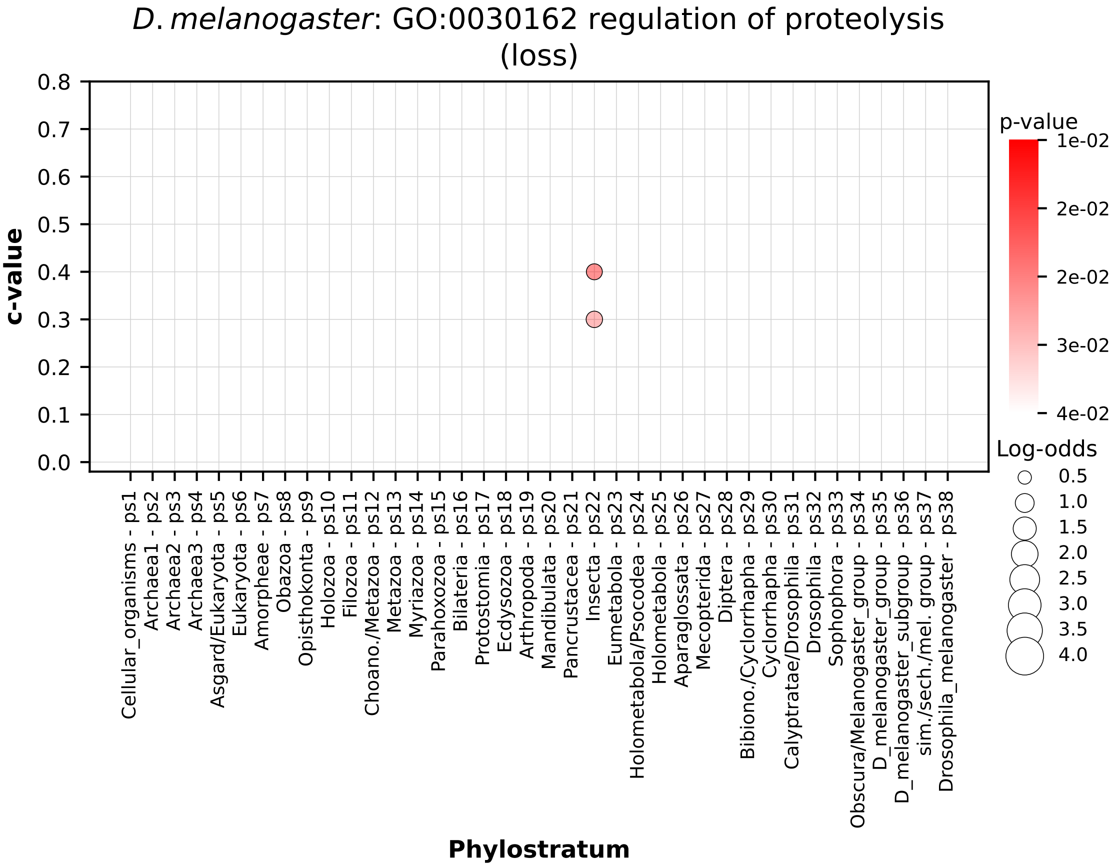
D. melanogaster: GO:0030162 regulation of proteolysis (loss)
c-value
0.8
0.7
0.6
0.5
0.4
0.3
0.2
0.1
0.0
p-value
1e-02
2e-02
2e-02
3e-02
4e-02
Log-odds
0.5
1.0
1.5
2.0
2.5
3.0
3.5
4.0
Cellular_organisms - ps1
Archaea1 - ps2
Archaea2 - ps3
Archaea3 - ps4
Asgard/Eukaryota - ps5
Eukaryota - ps6
Amorpheae - ps7
Obazoa - ps8
Opisthokonta - ps9
Holozoa - ps10
Filozoa - ps11
Choano./Metazoa - ps12
Metazoa - ps13
Myriazoa - ps14
Parahoxozoa - ps15
Bilateria - ps16
Protostomia - ps17
Ecdysozoa - ps18
Arthropoda - ps19
Mandibulata - ps20
Pancrustacea - ps21
Insecta - ps22
Eumetabola - ps23
Holometabola/Psocodea - ps24
Holometabola - ps25
Aparaglossata - ps26
Mecopterida - ps27
Diptera - ps28
Bibiono./Cyclorrhapha - ps29
Cyclorrhapha - ps30
Calyptratae/Drosophila - ps31
Drosophila - ps32
Sophophora - ps33
Obscura/Melanogaster_group - ps34
D_melanogaster_group - ps35
D_melanogaster_subgroup - ps36
sim./sech./mel. group - ps37
Drosophila_melanogaster - ps38
Phylostratum

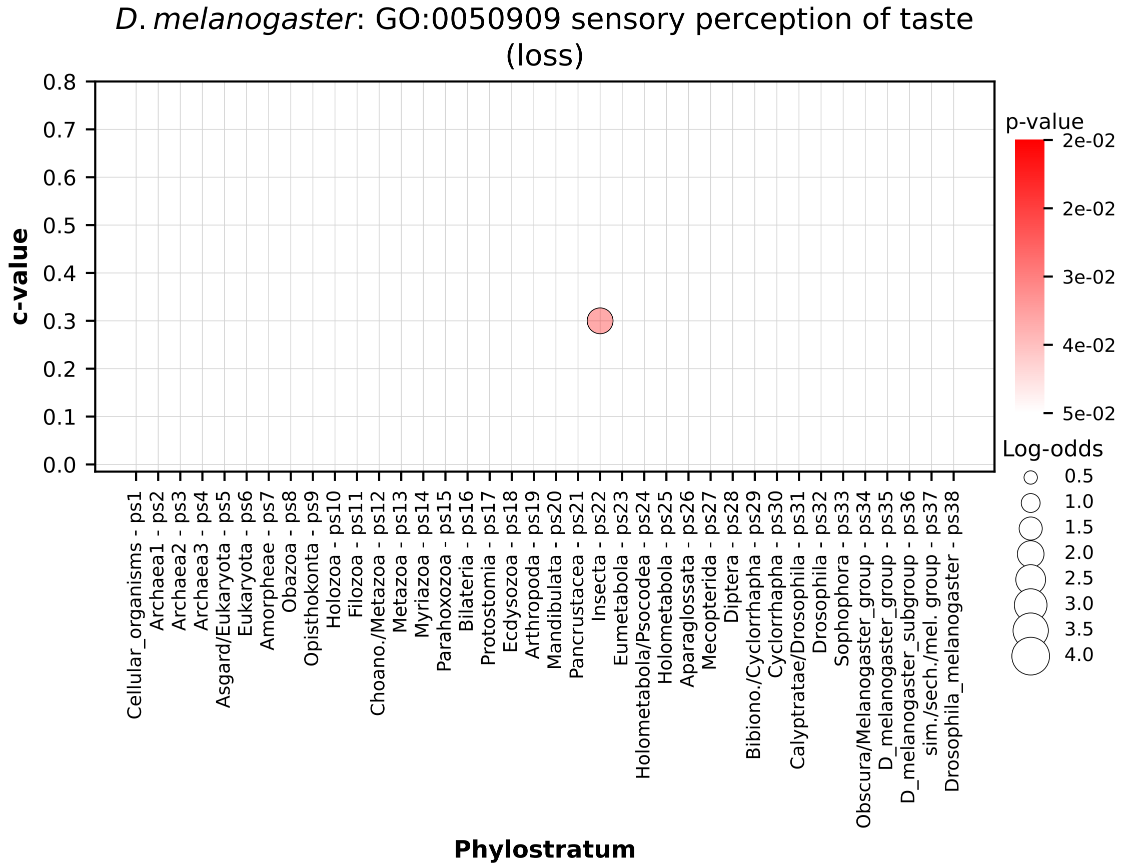
D. melanogaster: GO:0050909 sensory perception of taste
(loss)
c-value
0.0
0.1
0.2
0.3
0.4
0.5
0.6
0.7
0.8
Phylostratum
Cellular_organisms - ps1
Archaea1 - ps2
Archaea2 - ps3
Archaea3 - ps4
Asgard/Eukaryota - ps5
Eukaryota - ps6
Amorpheae - ps7
Obazoa - ps8
Opisthokonta - ps9
Holozoa - ps10
Filozoa - ps11
Choano./Metazoa - ps12
Metazoa - ps13
Myriazoa - ps14
Parahoxozoa - ps15
Bilateria - ps16
Protostomia - ps17
Ecdysozoa - ps18
Arthropoda - ps19
Mandibulata - ps20
Pancrustacea - ps21
Insecta - ps22
Eumetabola - ps23
Holometabola/Psocodea - ps24
Holometabola - ps25
Aparaglossata - ps26
Mecopterida - ps27
Diptera - ps28
Bibiono./Cyclorrhapha - ps29
Cyclorrhapha - ps30
Calyptratae/Drosophila - ps31
Drosophila - ps32
Sophophora - ps33
Obscura/Melanogaster_group - ps34
D_melanogaster_group - ps35
D_melanogaster_subgroup - ps36
sim./sech./mel. group - ps37
Drosophila_melanogaster - ps38
p-value
2e-02
2e-02
3e-02
4e-02
5e-02
Log-odds
0.5
1.0
1.5
2.0
2.5
3.0
3.5
4.0

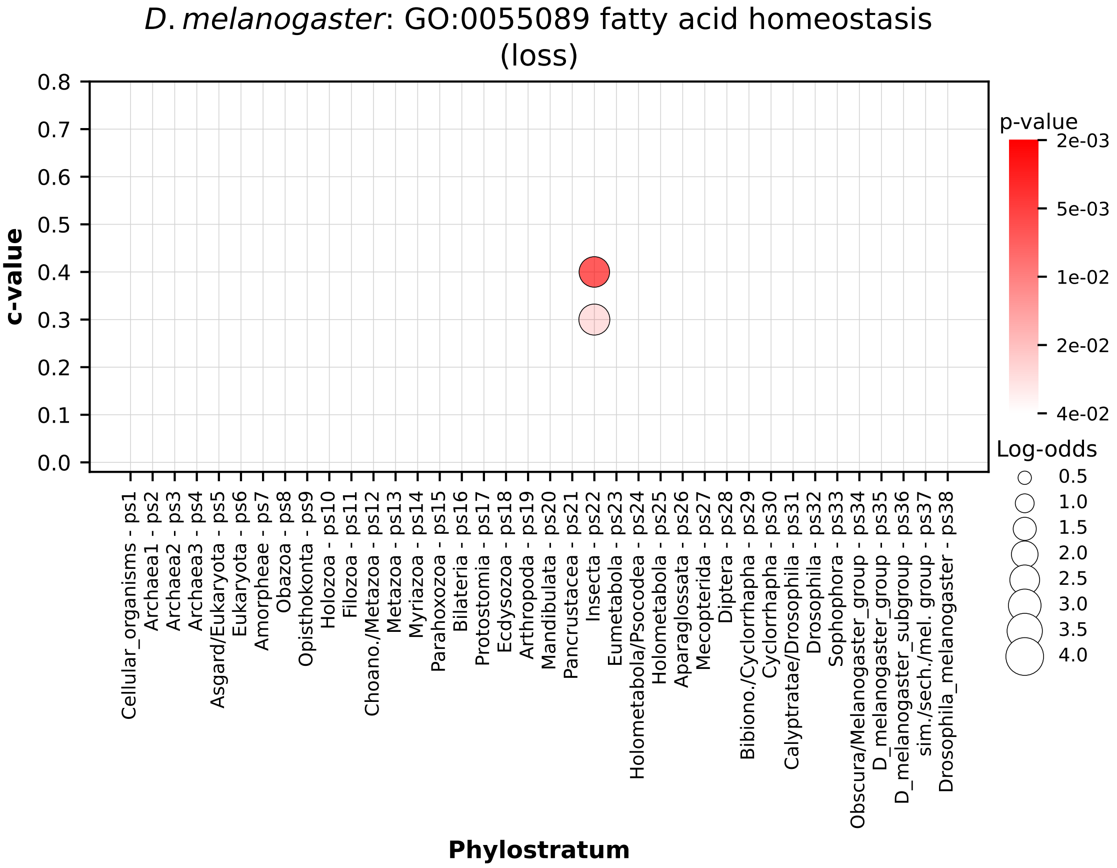

D. melanogaster: GO:0055089 fatty acid homeostasis
(loss)
c-value
0.8
0.7
0.6
0.5
0.4
0.3
0.2
0.1
0.0
p-value
2e-03
5e-03
1e-02
2e-02
4e-02
Log-odds
0.5
1.0
1.5
2.0
2.5
3.0
3.5
4.0
Cellular_organisms - ps1
Archaea1 - ps2
Archaea2 - ps3
Archaea3 - ps4
Asgard/Eukaryota - ps5
Eukaryota - ps6
Amorpheae - ps7
Obazoa - ps8
Opisthokonta - ps9
Holozoa - ps10
Filozoa - ps11
Choano./Metazoa - ps12
Metazoa - ps13
Myriazoa - ps14
Parahoxozoa - ps15
Bilateria - ps16
Protostomia - ps17
Ecdysozoa - ps18
Arthropoda - ps19
Mandibulata - ps20
Pancrustacea - ps21
Insecta - ps22
Eumetabola - ps23
Holometabola/Psocodea - ps24
Holometabola - ps25
Aparaglossata - ps26
Mecopterida - ps27
Diptera - ps28
Bibiono./Cyclorrhapha - ps29
Cyclorrhapha - ps30
Calyptratae/Drosophila - ps31
Drosophila - ps32
Sophophora - ps33
Obscura/Melanogaster_group - ps34
D_melanogaster_group - ps35
D_melanogaster_subgroup - ps36
sim./sech./mel. group - ps37
Drosophila_melanogaster - ps38
Phylostratum

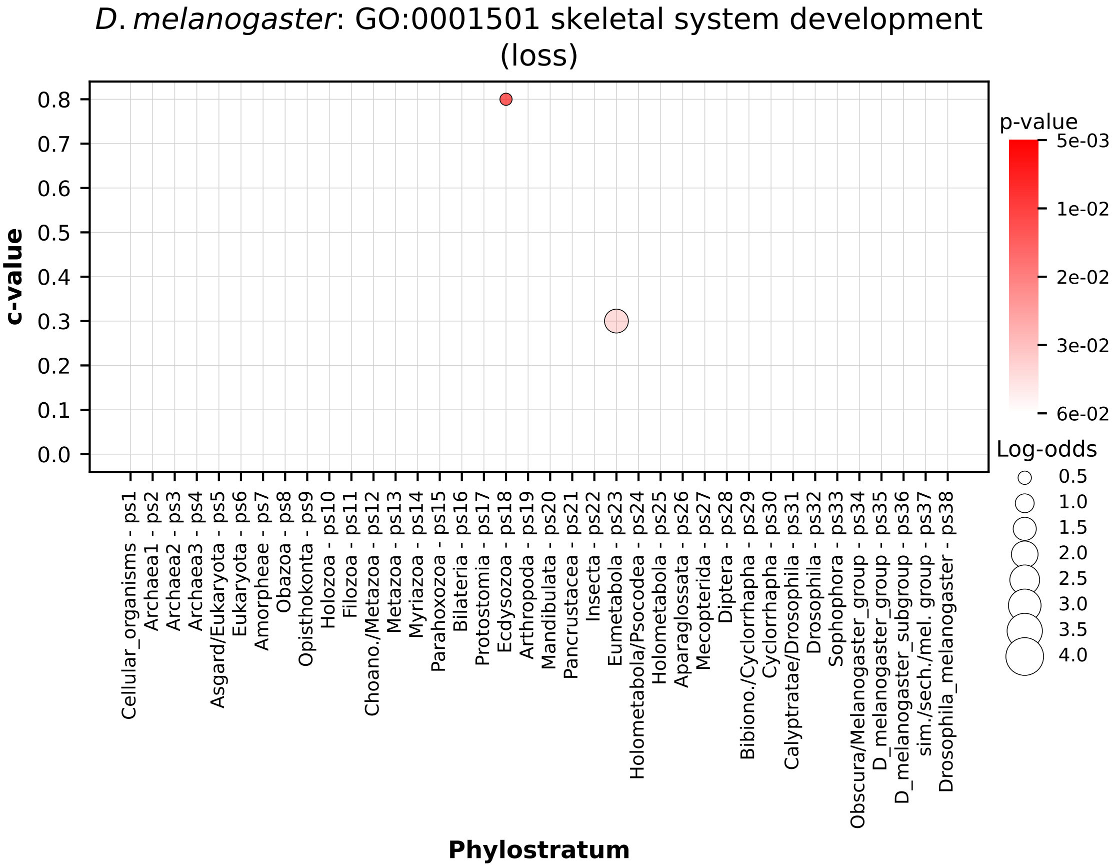

D. melanogaster: GO:0001501 skeletal system development (loss)
c-value
0.8
0.7
0.6
0.5
0.4
0.3
0.2
0.1
0.0
p-value
5e-03
1e-02
2e-02
3e-02
6e-02
Log-odds
0.5
1.0
1.5
2.0
2.5
3.0
3.5
4.0
Cellular_organisms - ps1
Archaea1 - ps2
Archaea2 - ps3
Archaea3 - ps4
Asgard/Eukaryota - ps5
Eukaryota - ps6
Amorpheae - ps7
Obazoa - ps8
Opisthokonta - ps9
Holozoa - ps10
Filozoa - ps11
Choano./Metazoa - ps12
Metazoa - ps13
Myriazoa - ps14
Parahoxozoa - ps15
Bilateria - ps16
Protostomia - ps17
Ecdysozoa - ps18
Arthropoda - ps19
Mandibulata - ps20
Pancrustacea - ps21
Insecta - ps22
Eumetabola - ps23
Holometabola/Psocodea - ps24
Holometabola - ps25
Aparaglossata - ps26
Mecopterida - ps27
Diptera - ps28
Bibiono./Cyclorrhapha - ps29
Cyclorrhapha - ps30
Calyptratae/Drosophila - ps31
Drosophila - ps32
Sophophora - ps33
Obscura/Melanogaster_group - ps34
D_melanogaster_group - ps35
D_melanogaster_subgroup - ps36
sim./sech./mel. group - ps37
Drosophila_melanogaster - ps38
Phylostratum

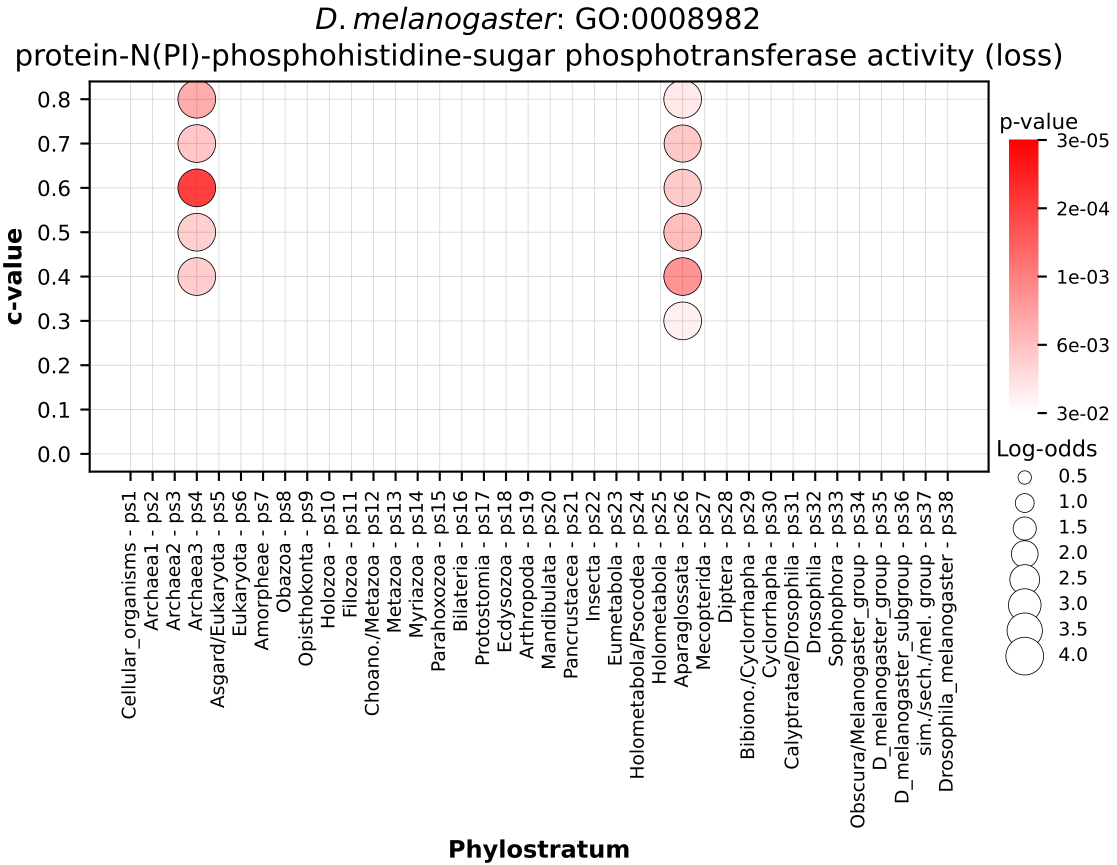

D. melanogaster: GO:0008982
protein-N(PI)-phosphohistidine-sugar phosphotransferase activity (loss)
c-value
0.8
0.7
0.6
0.5
0.4
0.3
0.2
0.1
0.0
p-value
3e-05
2e-04
1e-03
6e-03
3e-02
Log-odds
0.5
1.0
1.5
2.0
2.5
3.0
3.5
4.0
Cellular_organisms - ps1
Archaea1 - ps2
Archaea2 - ps3
Archaea3 - ps4
Asgard/Eukaryota - ps5
Eukaryota - ps6
Amorpheae - ps7
Obazoa - ps8
Opisthokonta - ps9
Holozoa - ps10
Filozoa - ps11
Choano./Metazoa - ps12
Metazoa - ps13
Myriazoa - ps14
Parahoxozoa - ps15
Bilateria - ps16
Protostomia - ps17
Ecdysozoa - ps18
Arthropoda - ps19
Mandibulata - ps20
Pancrustacea - ps21
Insecta - ps22
Eumetabola - ps23
Holometabola/Psocodea - ps24
Holometabola - ps25
Aparaglossata - ps26
Mecopterida - ps27
Diptera - ps28
Bibiono./Cyclorrhapha - ps29
Cyclorrhapha - ps30
Calyptratae/Drosophila - ps31
Drosophila - ps32
Sophophora - ps33
Obscura/Melanogaster_group - ps34
D_melanogaster_group - ps35
D_melanogaster_subgroup - ps36
sim./sech./mel. group - ps37
Drosophila_melanogaster - ps38
Phylostratum

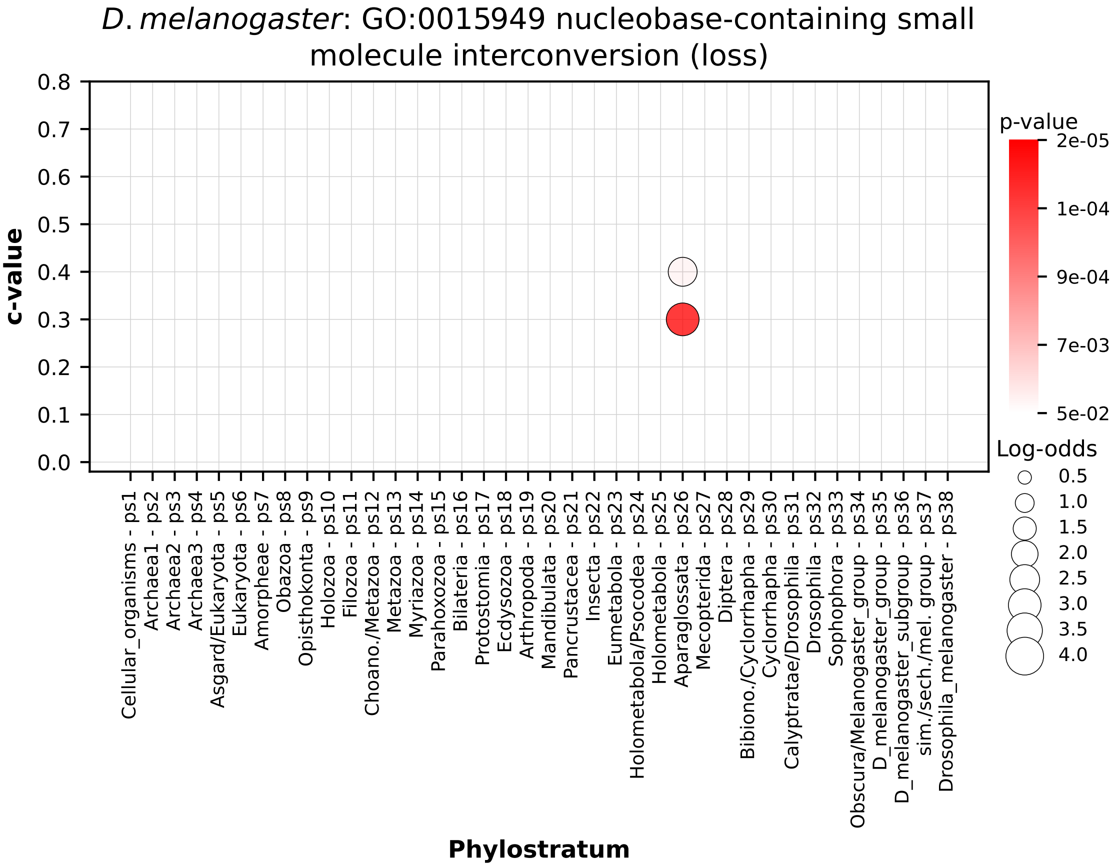

D. melanogaster: GO:0015949 nucleobase-containing small molecule interconversion (loss)
c-value
0.0
0.1
0.2
0.3
0.4
0.5
0.6
0.7
0.8
Phylostratum
Cellular_organisms - ps1
Archaea1 - ps2
Archaea2 - ps3
Archaea3 - ps4
Asgard/Eukaryota - ps5
Eukaryota - ps6
Amorpheae - ps7
Obazoa - ps8
Opisthokonta - ps9
Holozoa - ps10
Filozoa - ps11
Choano./Metazoa - ps12
Metazoa - ps13
Myriazoa - ps14
Parahoxozoa - ps15
Bilateria - ps16
Protostomia - ps17
Ecdysozoa - ps18
Arthropoda - ps19
Mandibulata - ps20
Pancrustacea - ps21
Insecta - ps22
Eumetabola - ps23
Holometabola/Psocodea - ps24
Holometabola - ps25
Aparaglossata - ps26
Mecopterida - ps27
Diptera - ps28
Bibiono./Cyclorrhapha - ps29
Cyclorrhapha - ps30
Calyptratae/Drosophila - ps31
Drosophila - ps32
Sophophora - ps33
Obscura/Melanogaster_group - ps34
D_melanogaster_group - ps35
D_melanogaster_subgroup - ps36
sim./sech./mel. group - ps37
Drosophila_melanogaster - ps38
p-value
2e-05
1e-04
9e-04
7e-03
5e-02
Log-odds
0.5
1.0
1.5
2.0
2.5
3.0
3.5
4.0

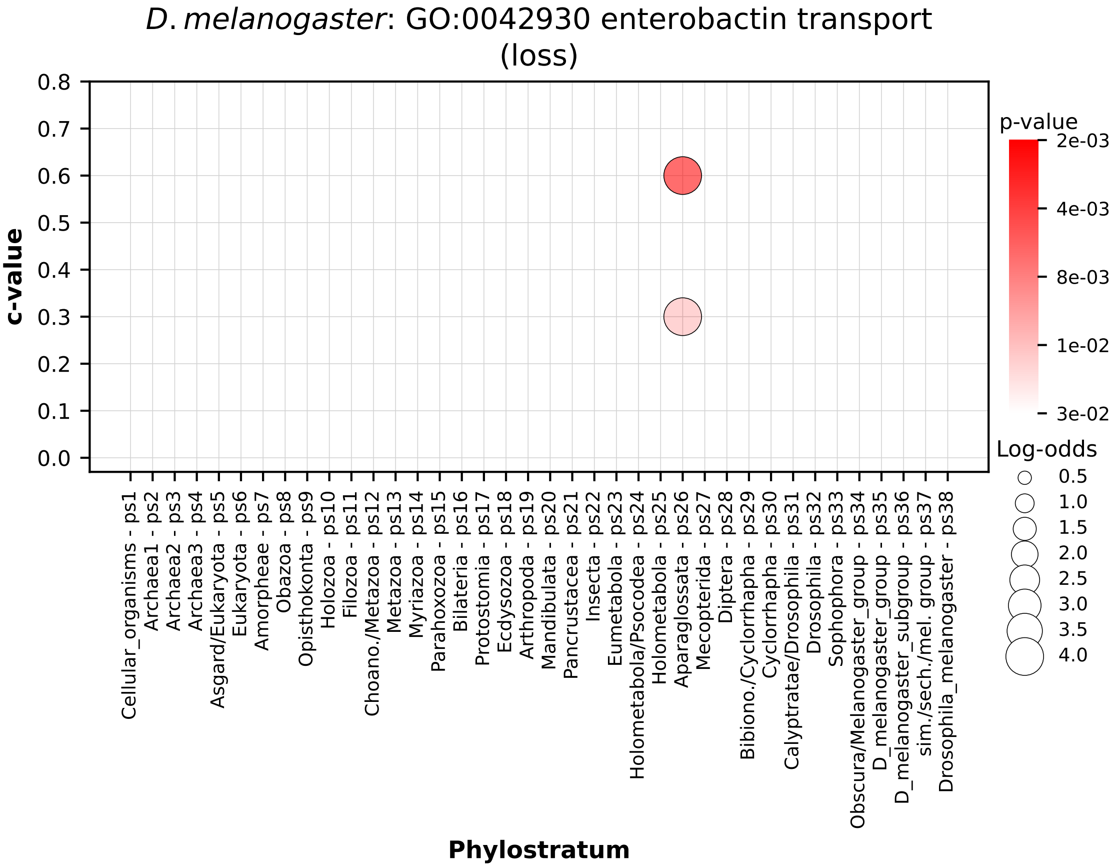

D. melanogaster: GO:0042930 enterobactin transport
(loss)
c-value
0.0
0.1
0.2
0.3
0.4
0.5
0.6
0.7
0.8
Phylostratum
Cellular_organisms - ps1
Archaea1 - ps2
Archaea2 - ps3
Archaea3 - ps4
Asgard/Eukaryota - ps5
Eukaryota - ps6
Amorpheae - ps7
Obazoa - ps8
Opisthokonta - ps9
Holozoa - ps10
Filozoa - ps11
Choano./Metazoa - ps12
Metazoa - ps13
Myriazoa - ps14
Parahoxozoa - ps15
Bilateria - ps16
Protostomia - ps17
Ecdysozoa - ps18
Arthropoda - ps19
Mandibulata - ps20
Pancrustacea - ps21
Insecta - ps22
Eumetabola - ps23
Holometabola/Psocodea - ps24
Holometabola - ps25
Aparaglossata - ps26
Mecopterida - ps27
Diptera - ps28
Bibiono./Cyclorrhapha - ps29
Cyclorrhapha - ps30
Calyptratae/Drosophila - ps31
Drosophila - ps32
Sophophora - ps33
Obscura/Melanogaster_group - ps34
D_melanogaster_group - ps35
D_melanogaster_subgroup - ps36
sim./sech./mel. group - ps37
Drosophila_melanogaster - ps38
p-value
2e-03
4e-03
8e-03
1e-02
3e-02
Log-odds
0.5
1.0
1.5
2.0
2.5
3.0
3.5
4.0

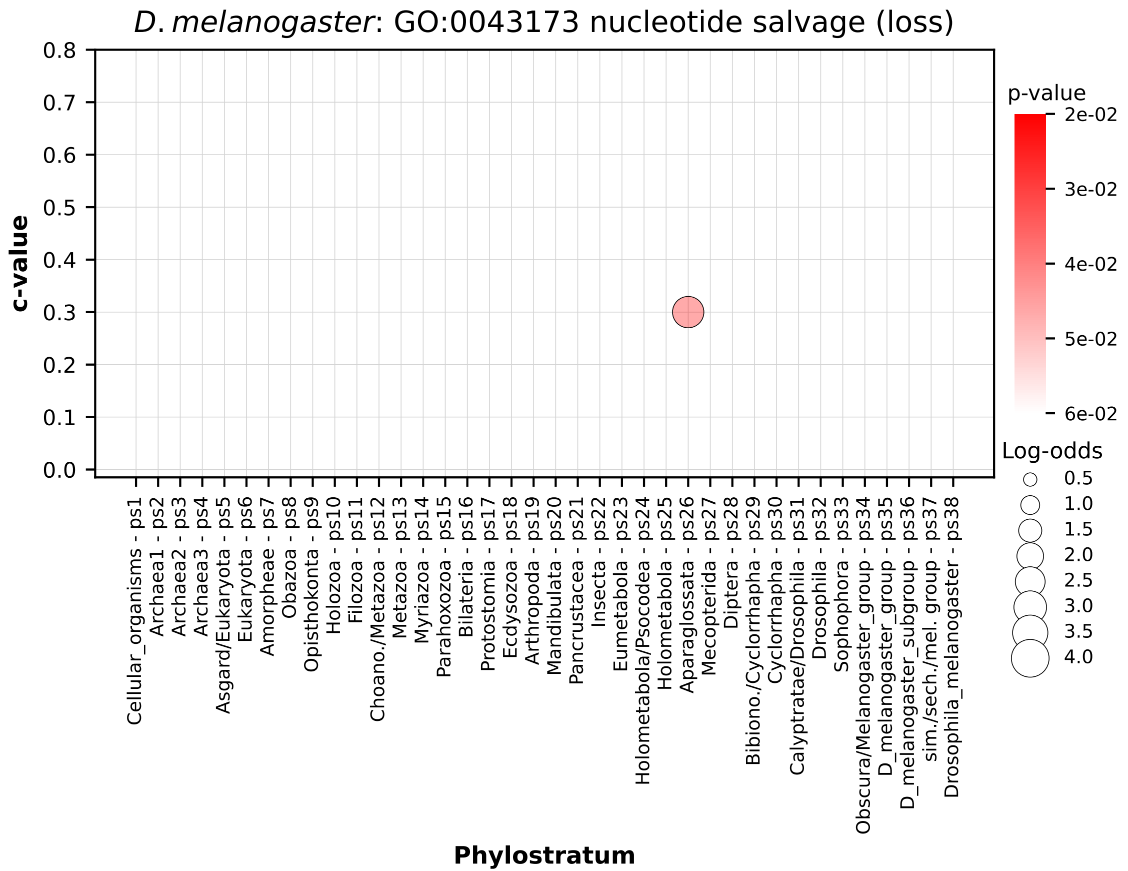
D. melanogaster: GO:0043173 nucleotide salvage (loss)
c-value
0.8
0.7
0.6
0.5
0.4
0.3
0.2
0.1
0.0
p-value
2e-02
3e-02
4e-02
5e-02
6e-02
Log-odds
0.5
1.0
1.5
2.0
2.5
3.0
3.5
4.0
Cellular_organisms - ps1
Archaea1 - ps2
Archaea2 - ps3
Archaea3 - ps4
Asgard/Eukaryota - ps5
Eukaryota - ps6
Amorpheae - ps7
Obazoa - ps8
Opisthokonta - ps9
Holozoa - ps10
Filozoa - ps11
Choano./Metazoa - ps12
Metazoa - ps13
Myriazoa - ps14
Parahoxozoa - ps15
Bilateria - ps16
Protostomia - ps17
Ecdysozoa - ps18
Arthropoda - ps19
Mandibulata - ps20
Pancrustacea - ps21
Insecta - ps22
Eumetabola - ps23
Holometabola/Psocodea - ps24
Holometabola - ps25
Aparaglossata - ps26
Mecopterida - ps27
Diptera - ps28
Bibiono./Cyclorrhapha - ps29
Cyclorrhapha - ps30
Calyptratae/Drosophila - ps31
Drosophila - ps32
Sophophora - ps33
Obscura/Melanogaster_group - ps34
D_melanogaster_group - ps35
D_melanogaster_subgroup - ps36
sim./sech./mel. group - ps37
Drosophila_melanogaster - ps38
Phylostratum

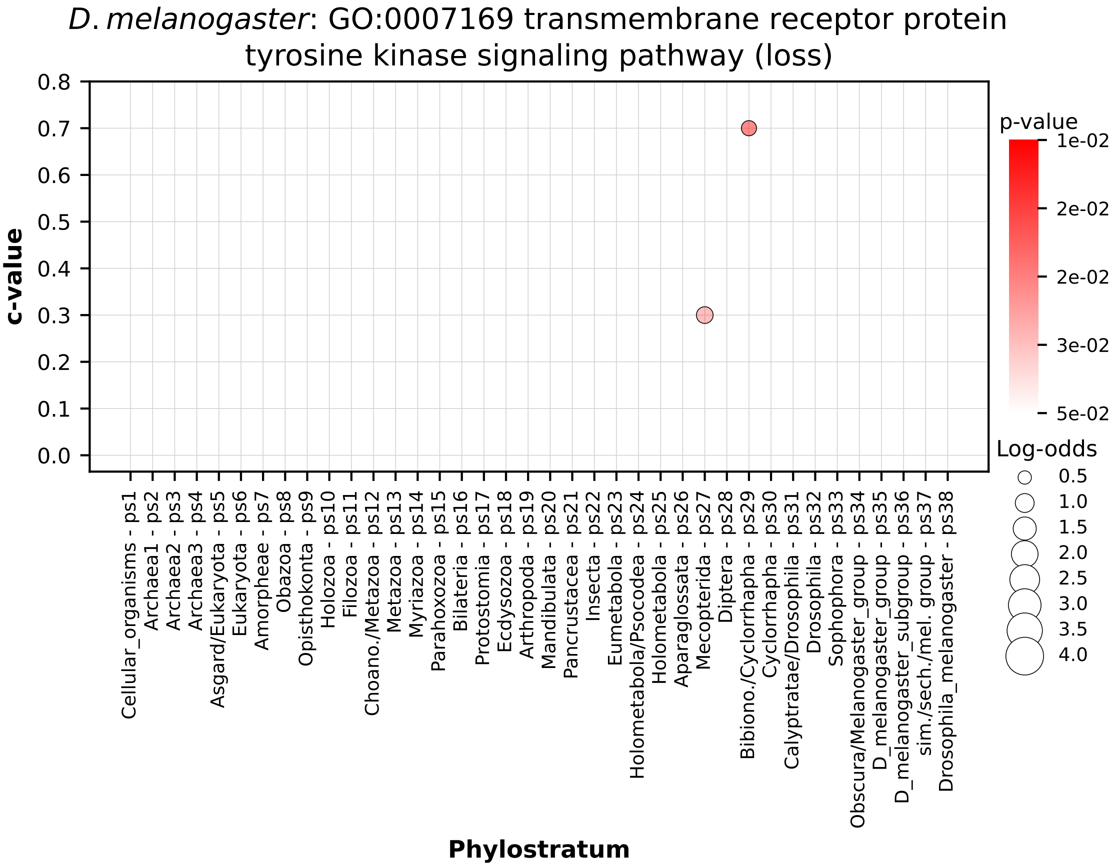

D. melanogaster: GO:0007169 transmembrane receptor protein tyrosine kinase signaling pathway (loss)
c-value
0.8
0.7
0.6
0.5
0.4
0.3
0.2
0.1
0.0
p-value
1e-02
2e-02
2e-02
3e-02
5e-02
Log-odds
0.5
1.0
1.5
2.0
2.5
3.0
3.5
4.0
Cellular_organisms - ps1
Archaea1 - ps2
Archaea2 - ps3
Archaea3 - ps4
Asgard/Eukaryota - ps5
Eukaryota - ps6
Amorpheae - ps7
Obazoa - ps8
Opisthokonta - ps9
Holozoa - ps10
Filozoa - ps11
Choano./Metazoa - ps12
Metazoa - ps13
Myriazoa - ps14
Parahoxozoa - ps15
Bilateria - ps16
Protostomia - ps17
Ecdysozoa - ps18
Arthropoda - ps19
Mandibulata - ps20
Pancrustacea - ps21
Insecta - ps22
Eumetabola - ps23
Holometabola/Psocodea - ps24
Holometabola - ps25
Aparaglossata - ps26
Mecopterida - ps27
Diptera - ps28
Bibiono./Cyclorrhapha - ps29
Cyclorrhapha - ps30
Calyptratae/Drosophila - ps31
Drosophila - ps32
Sophophora - ps33
Obscura/Melanogaster_group - ps34
D_melanogaster_group - ps35
D_melanogaster_subgroup - ps36
sim./sech./mel. group - ps37
Drosophila_melanogaster - ps38
Phylostratum

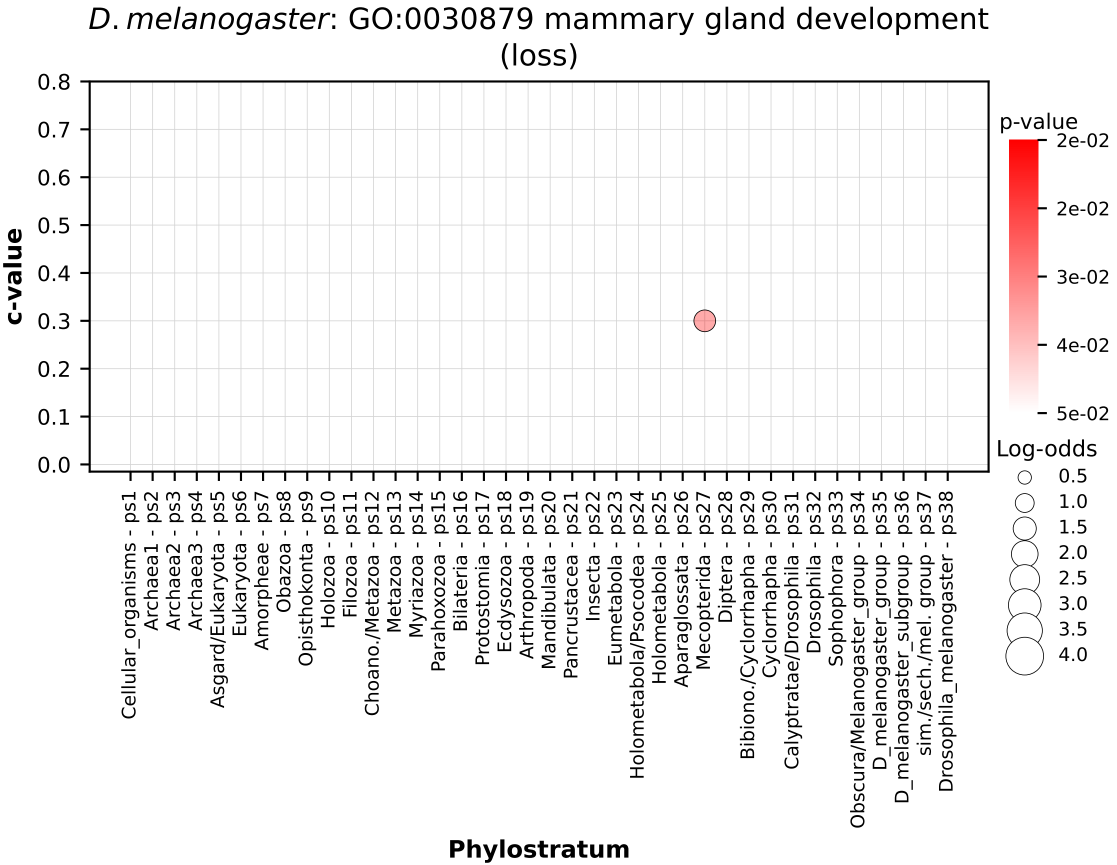
D. melanogaster: GO:0030879 mammary gland development
(loss)
c-value
0.8
0.7
0.6
0.5
0.4
0.3
0.2
0.1
0.0
p-value
2e-02
2e-02
3e-02
4e-02
5e-02
Log-odds
0.5
1.0
1.5
2.0
2.5
3.0
3.5
4.0
Cellular_organisms - ps1
Archaea1 - ps2
Archaea2 - ps3
Archaea3 - ps4
Asgard/Eukaryota - ps5
Eukaryota - ps6
Amorpheae - ps7
Obazoa - ps8
Opisthokonta - ps9
Holozoa - ps10
Filozoa - ps11
Choano./Metazoa - ps12
Metazoa - ps13
Myriazoa - ps14
Parahoxozoa - ps15
Bilateria - ps16
Protostomia - ps17
Ecdysozoa - ps18
Arthropoda - ps19
Mandibulata - ps20
Pancrustacea - ps21
Insecta - ps22
Eumetabola - ps23
Holometabola/Psocodea - ps24
Holometabola - ps25
Aparaglossata - ps26
Mecopterida - ps27
Diptera - ps28
Bibiono./Cyclorrhapha - ps29
Cyclorrhapha - ps30
Calyptratae/Drosophila - ps31
Drosophila - ps32
Sophophora - ps33
Obscura/Melanogaster_group - ps34
D_melanogaster_group - ps35
D_melanogaster_subgroup - ps36
sim./sech./mel. group - ps37
Drosophila_melanogaster - ps38
Phylostratum

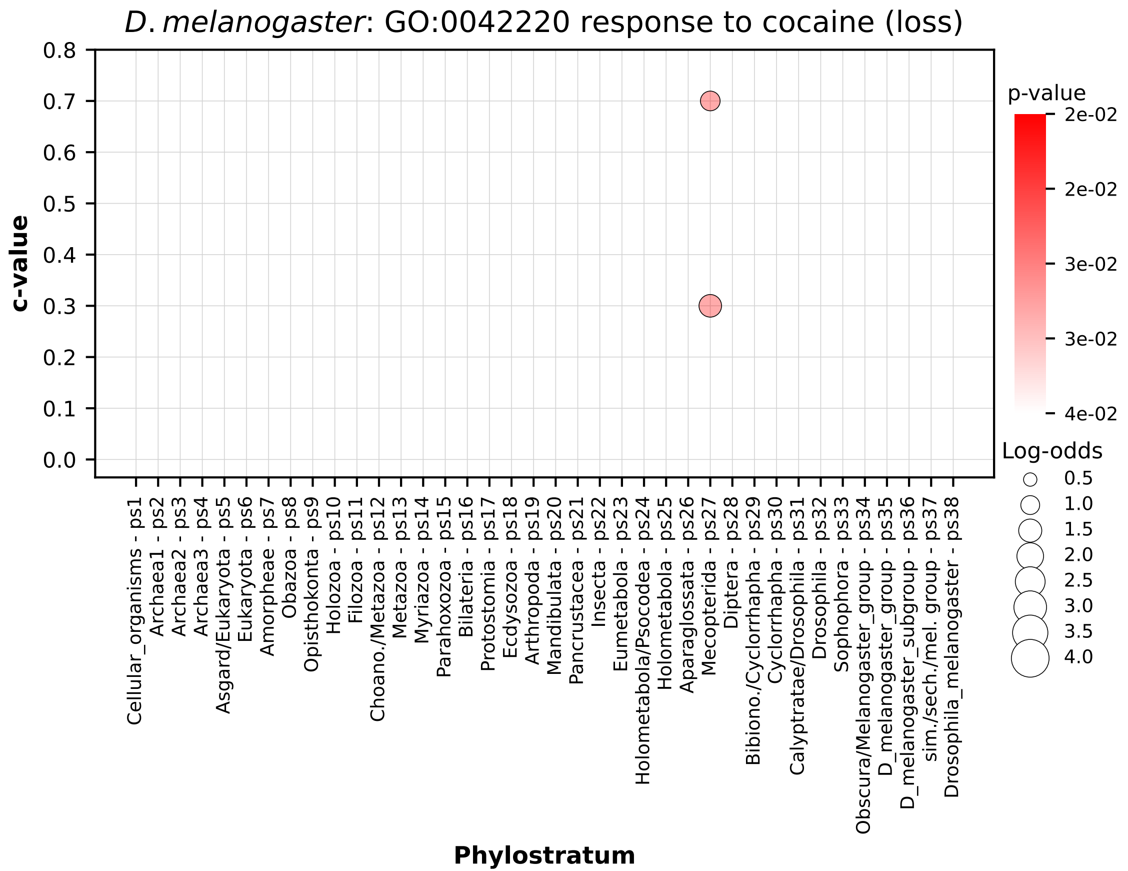
D. melanogaster: GO:0042220 response to cocaine (loss)
c-value
0.8
0.7
0.6
0.5
0.4
0.3
0.2
0.1
0.0
Phylostratum
Cellular_organisms - ps1
Archaea1 - ps2
Archaea2 - ps3
Archaea3 - ps4
Asgard/Eukaryota - ps5
Eukaryota - ps6
Amorpheae - ps7
Obazoa - ps8
Opisthokonta - ps9
Holozoa - ps10
Filozoa - ps11
Choano./Metazoa - ps12
Metazoa - ps13
Myriazoa - ps14
Parahoxozoa - ps15
Bilateria - ps16
Protostomia - ps17
Ecdysozoa - ps18
Arthropoda - ps19
Mandibulata - ps20
Pancrustacea - ps21
Insecta - ps22
Eumetabola - ps23
Holometabola/Psocodea - ps24
Holometabola - ps25
Aparaglossata - ps26
Mecopterida - ps27
Diptera - ps28
Bibiono./Cyclorrhapha - ps29
Cyclorrhapha - ps30
Calyptratae/Drosophila - ps31
Drosophila - ps32
Sophophora - ps33
Obscura/Melanogaster_group - ps34
D_melanogaster_group - ps35
D_melanogaster_subgroup - ps36
sim./sech./mel. group - ps37
Drosophila_melanogaster - ps38
p-value
2e-02
2e-02
3e-02
3e-02
4e-02
Log-odds
0.5
1.0
1.5
2.0
2.5
3.0
3.5
4.0

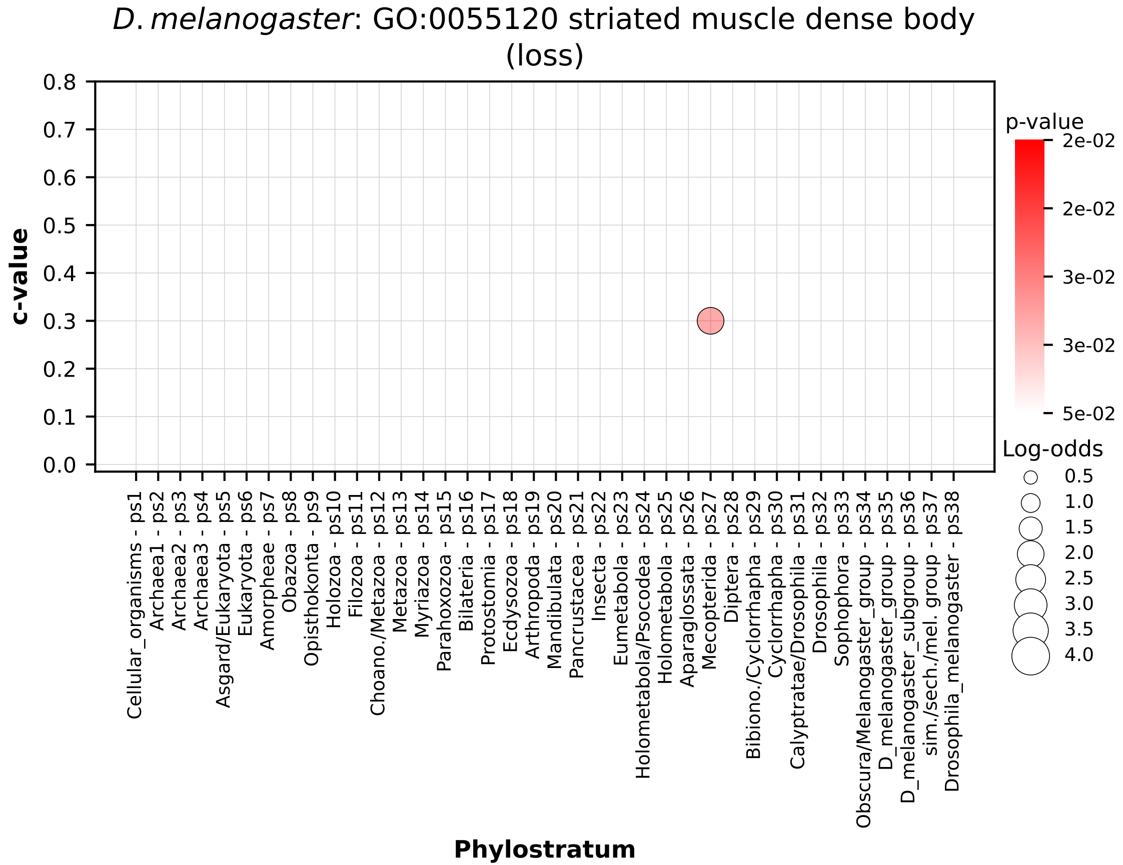

D. melanogaster: GO:0055120 striated muscle dense body (loss)
c-value
0.8
0.7
0.6
0.5
0.4
0.3
0.2
0.1
0.0
p-value
2e-02
2e-02
3e-02
3e-02
5e-02
Log-odds
0.5
1.0
1.5
2.0
2.5
3.0
3.5
4.0
Cellular_organisms - ps1
Archaea1 - ps2
Archaea2 - ps3
Archaea3 - ps4
Asgard/Eukaryota - ps5
Eukaryota - ps6
Amorpheae - ps7
Obazoa - ps8
Opisthokonta - ps9
Holozoa - ps10
Filozoa - ps11
Choano./Metazoa - ps12
Metazoa - ps13
Myriazoa - ps14
Parahoxozoa - ps15
Bilateria - ps16
Protostomia - ps17
Ecdysozoa - ps18
Arthropoda - ps19
Mandibulata - ps20
Pancrustacea - ps21
Insecta - ps22
Eumetabola - ps23
Holometabola/Psocodea - ps24
Holometabola - ps25
Aparaglossata - ps26
Mecopterida - ps27
Diptera - ps28
Bibiono./Cyclorrhapha - ps29
Cyclorrhapha - ps30
Calyptratae/Drosophila - ps31
Drosophila - ps32
Sophophora - ps33
Obscura/Melanogaster_group - ps34
D_melanogaster_group - ps35
D_melanogaster_subgroup - ps36
sim./sech./mel. group - ps37
Drosophila_melanogaster - ps38
Phylostratum

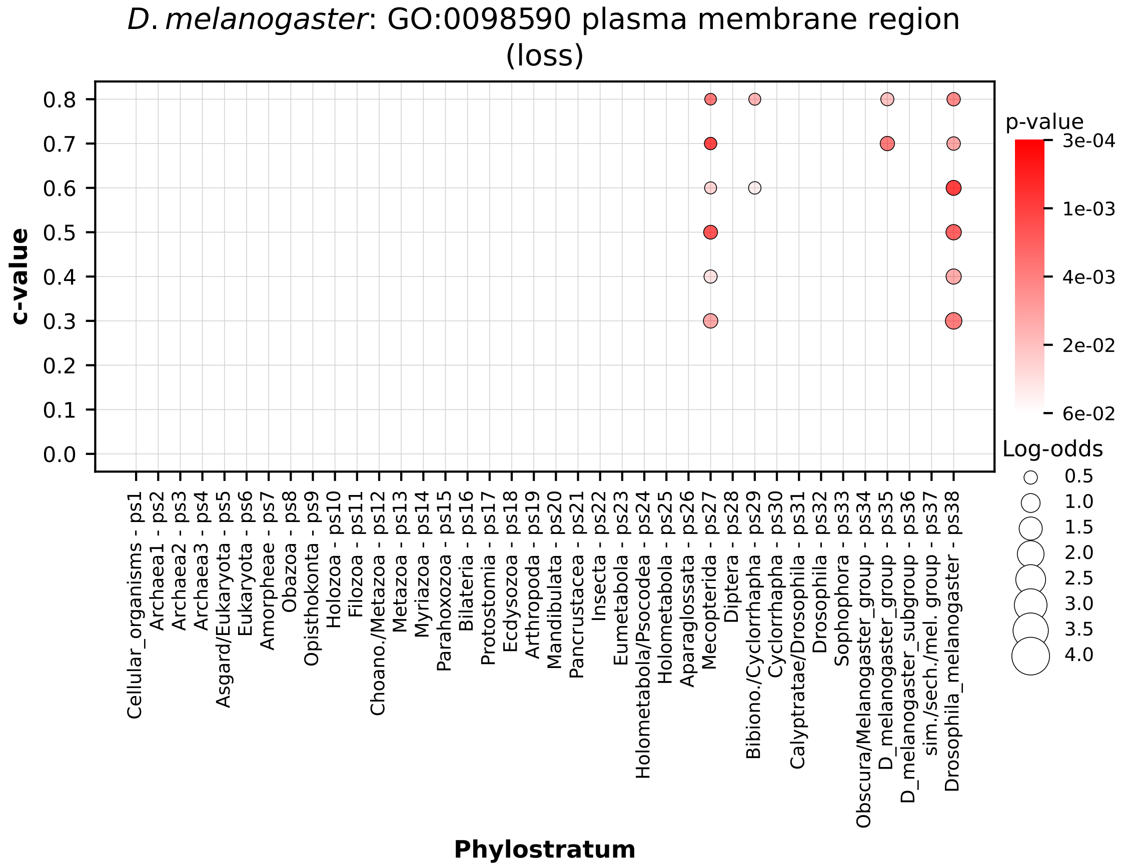

D. melanogaster: GO:0098590 plasma membrane region
(loss)
c-value
0.0
0.1
0.2
0.3
0.4
0.5
0.6
0.7
0.8
p-value
3e-04
1e-03
4e-03
2e-02
6e-02
Log-odds
0.5
1.0
1.5
2.0
2.5
3.0
3.5
4.0
Cellular_organisms - ps1
Archaea1 - ps2
Archaea2 - ps3
Archaea3 - ps4
Asgard/Eukaryota - ps5
Eukaryota - ps6
Amorpheae - ps7
Obazoa - ps8
Opisthokonta - ps9
Holozoa - ps10
Filozoa - ps11
Choano./Metazoa - ps12
Metazoa - ps13
Myriazoa - ps14
Parahoxozoa - ps15
Bilateria - ps16
Protostomia - ps17
Ecdysozoa - ps18
Arthropoda - ps19
Mandibulata - ps20
Pancrustacea - ps21
Insecta - ps22
Eumetabola - ps23
Holometabola/Psocodea - ps24
Holometabola - ps25
Aparaglossata - ps26
Mecopterida - ps27
Diptera - ps28
Bibiono./Cyclorrhapha - ps29
Cyclorrhapha - ps30
Calyptratae/Drosophila - ps31
Drosophila - ps32
Sophophora - ps33
Obscura/Melanogaster_group - ps34
D_melanogaster_group - ps35
D_melanogaster_subgroup - ps36
sim./sech./mel. group - ps37
Drosophila_melanogaster - ps38
Phylostratum

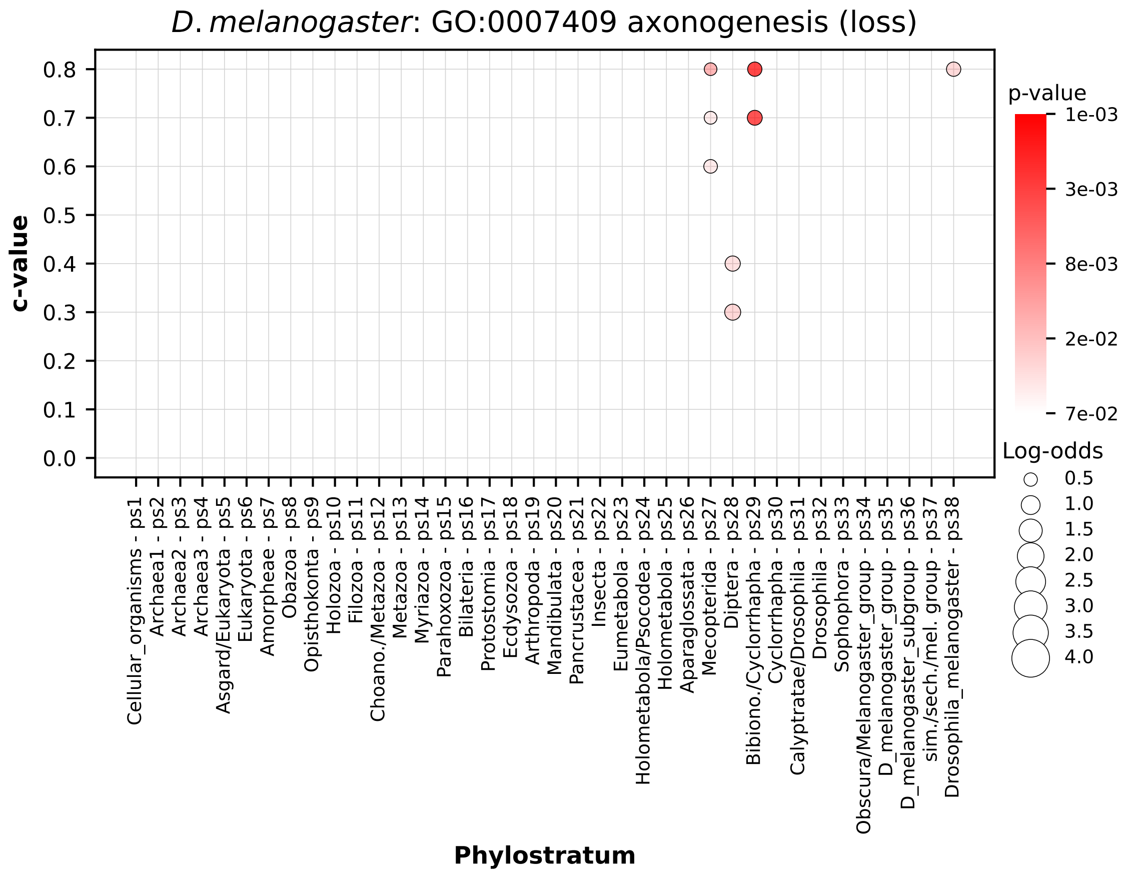

D. melanogaster: GO:0007409 axonogenesis (loss)
c-value
0.0
0.1
0.2
0.3
0.4
0.5
0.6
0.7
0.8
p-value
1e-03
3e-03
8e-03
2e-02
7e-02
Log-odds
0.5
1.0
1.5
2.0
2.5
3.0
3.5
4.0
Cellular_organisms - ps1
Archaea1 - ps2
Archaea2 - ps3
Archaea3 - ps4
Asgard/Eukaryota - ps5
Eukaryota - ps6
Amorpheae - ps7
Obazoa - ps8
Opisthokonta - ps9
Holozoa - ps10
Filozoa - ps11
Choano./Metazoa - ps12
Metazoa - ps13
Myriazoa - ps14
Parahoxozoa - ps15
Bilateria - ps16
Protostomia - ps17
Ecdysozoa - ps18
Arthropoda - ps19
Mandibulata - ps20
Pancrustacea - ps21
Insecta - ps22
Eumetabola - ps23
Holometabola/Psocodea - ps24
Holometabola - ps25
Aparaglossata - ps26
Mecopterida - ps27
Diptera - ps28
Bibiono./Cyclorrhapha - ps29
Cyclorrhapha - ps30
Calyptratae/Drosophila - ps31
Drosophila - ps32
Sophophora - ps33
Obscura/Melanogaster_group - ps34
D_melanogaster_group - ps35
D_melanogaster_subgroup - ps36
sim./sech./mel. group - ps37
Drosophila_melanogaster - ps38
Phylostratum

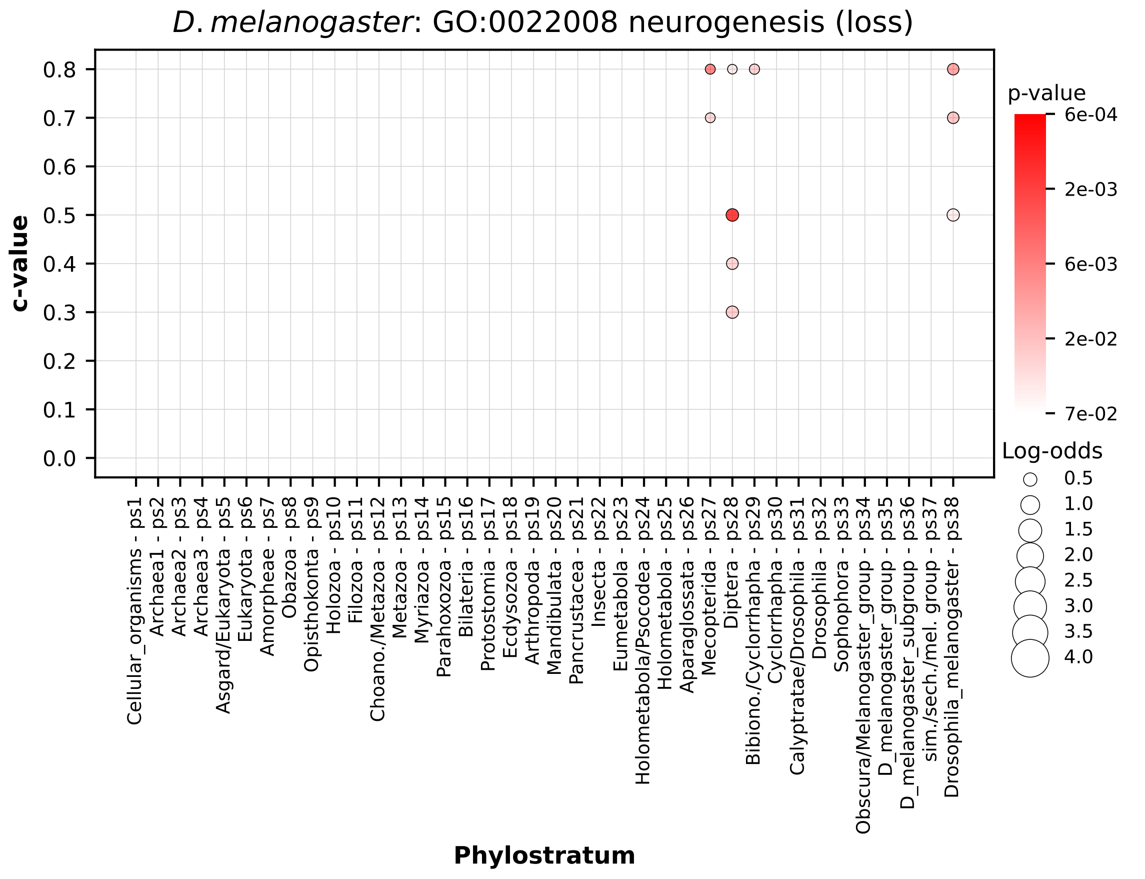

D. melanogaster: GO:0022008 neurogenesis (loss)
c-value
0.8
0.7
0.6
0.5
0.4
0.3
0.2
0.1
0.0
p-value
6e-04
2e-03
6e-03
2e-02
7e-02
Log-odds
0.5
1.0
1.5
2.0
2.5
3.0
3.5
4.0
Cellular_organisms - ps1
Archaea1 - ps2
Archaea2 - ps3
Archaea3 - ps4
Asgard/Eukaryota - ps5
Eukaryota - ps6
Amorpheae - ps7
Obazoa - ps8
Opisthokonta - ps9
Holozoa - ps10
Filozoa - ps11
Choano./Metazoa - ps12
Metazoa - ps13
Myriazoa - ps14
Parahoxozoa - ps15
Bilateria - ps16
Protostomia - ps17
Ecdysozoa - ps18
Arthropoda - ps19
Mandibulata - ps20
Pancrustacea - ps21
Insecta - ps22
Eumetabola - ps23
Holometabola/Psocodea - ps24
Holometabola - ps25
Aparaglossata - ps26
Mecopterida - ps27
Diptera - ps28
Bibiono./Cyclorrhapha - ps29
Cyclorrhapha - ps30
Calyptratae/Drosophila - ps31
Drosophila - ps32
Sophophora - ps33
Obscura/Melanogaster_group - ps34
D_melanogaster_group - ps35
D_melanogaster_subgroup - ps36
sim./sech./mel. group - ps37
Drosophila_melanogaster - ps38
Phylostratum

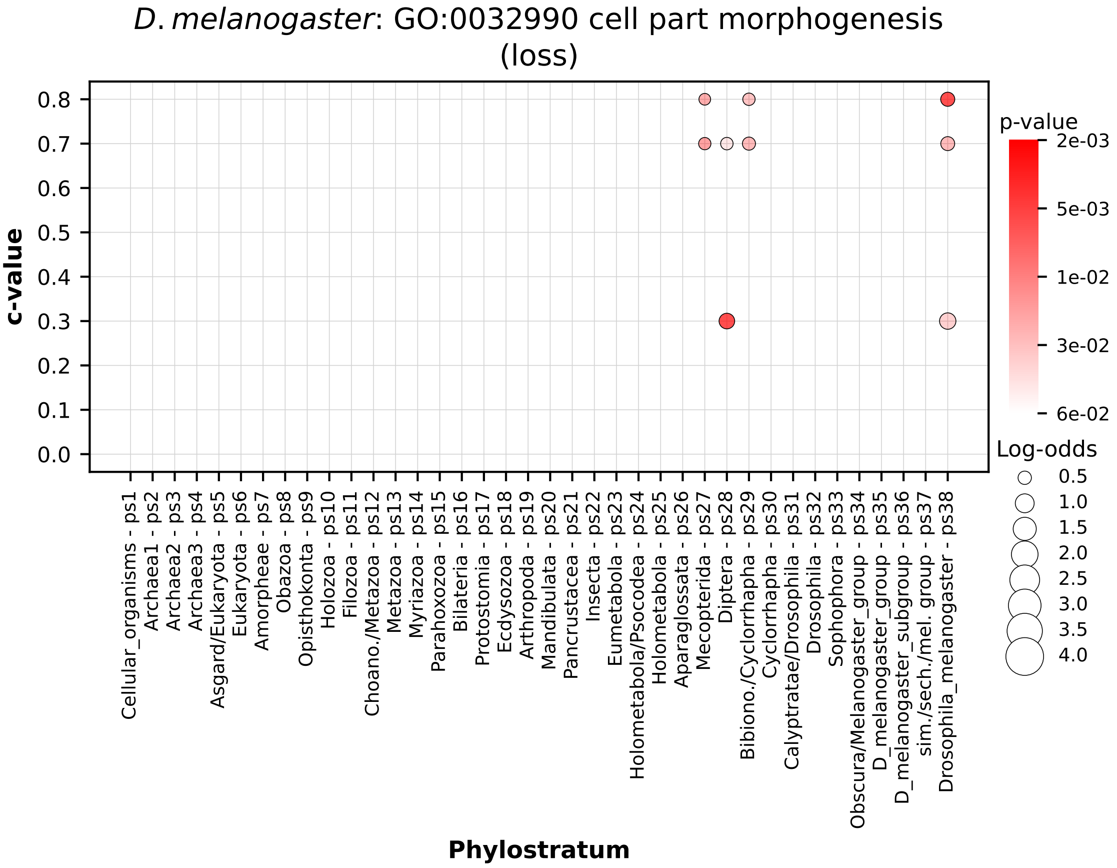

D. melanogaster: GO:0032990 cell part morphogenesis
(loss)
c-value
0.0
0.1
0.2
0.3
0.4
0.5
0.6
0.7
0.8
p-value
2e-03
5e-03
1e-02
3e-02
6e-02
Log-odds
0.5
1.0
1.5
2.0
2.5
3.0
3.5
4.0
Cellular_organisms - ps1
Archaea1 - ps2
Archaea2 - ps3
Archaea3 - ps4
Asgard/Eukaryota - ps5
Eukaryota - ps6
Amorpheae - ps7
Obazoa - ps8
Opisthokonta - ps9
Holozoa - ps10
Filozoa - ps11
Choano./Metazoa - ps12
Metazoa - ps13
Myriazoa - ps14
Parahoxozoa - ps15
Bilateria - ps16
Protostomia - ps17
Ecdysozoa - ps18
Arthropoda - ps19
Mandibulata - ps20
Pancrustacea - ps21
Insecta - ps22
Eumetabola - ps23
Holometabola/Psocodea - ps24
Holometabola - ps25
Aparaglossata - ps26
Mecopterida - ps27
Diptera - ps28
Bibiono./Cyclorrhapha - ps29
Cyclorrhapha - ps30
Calyptratae/Drosophila - ps31
Drosophila - ps32
Sophophora - ps33
Obscura/Melanogaster_group - ps34
D_melanogaster_group - ps35
D_melanogaster_subgroup - ps36
sim./sech./mel. group - ps37
Drosophila_melanogaster - ps38
Phylostratum

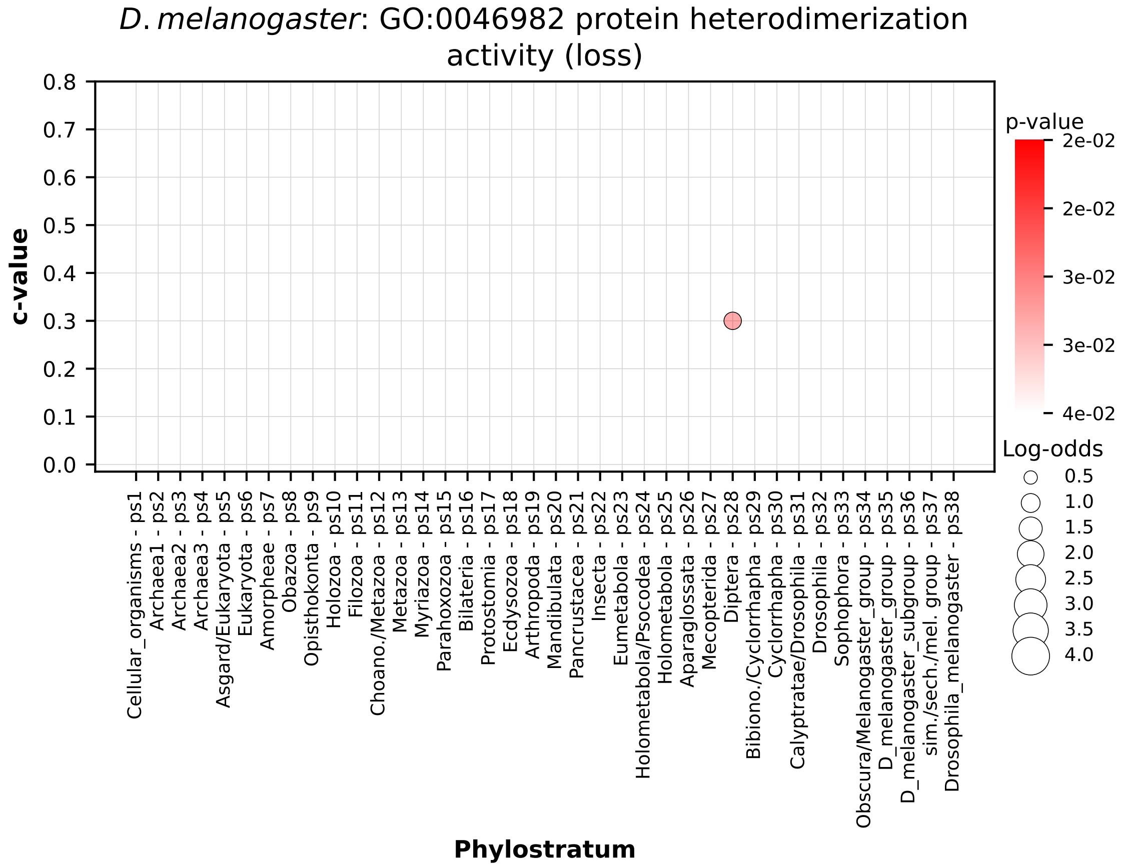
D. melanogaster: GO:0046982 protein heterodimerization activity (loss)
c-value
0.0
0.1
0.2
0.3
0.4
0.5
0.6
0.7
0.8
p-value
2e-02
2e-02
3e-02
3e-02
4e-02
Log-odds
0.5
1.0
1.5
2.0
2.5
3.0
3.5
4.0
Cellular_organisms - ps1
Archaea1 - ps2
Archaea2 - ps3
Archaea3 - ps4
Asgard/Eukaryota - ps5
Eukaryota - ps6
Amorpheae - ps7
Obazoa - ps8
Opisthokonta - ps9
Holozoa - ps10
Filozoa - ps11
Choano./Metazoa - ps12
Metazoa - ps13
Myriazoa - ps14
Parahoxozoa - ps15
Bilateria - ps16
Protostomia - ps17
Ecdysozoa - ps18
Arthropoda - ps19
Mandibulata - ps20
Pancrustacea - ps21
Insecta - ps22
Eumetabola - ps23
Holometabola/Psocodea - ps24
Holometabola - ps25
Aparaglossata - ps26
Mecopterida - ps27
Diptera - ps28
Bibiono./Cyclorrhapha - ps29
Cyclorrhapha - ps30
Calyptratae/Drosophila - ps31
Drosophila - ps32
Sophophora - ps33
Obscura/Melanogaster_group - ps34
D_melanogaster_group - ps35
D_melanogaster_subgroup - ps36
sim./sech./mel. group - ps37
Drosophila_melanogaster - ps38
Phylostratum

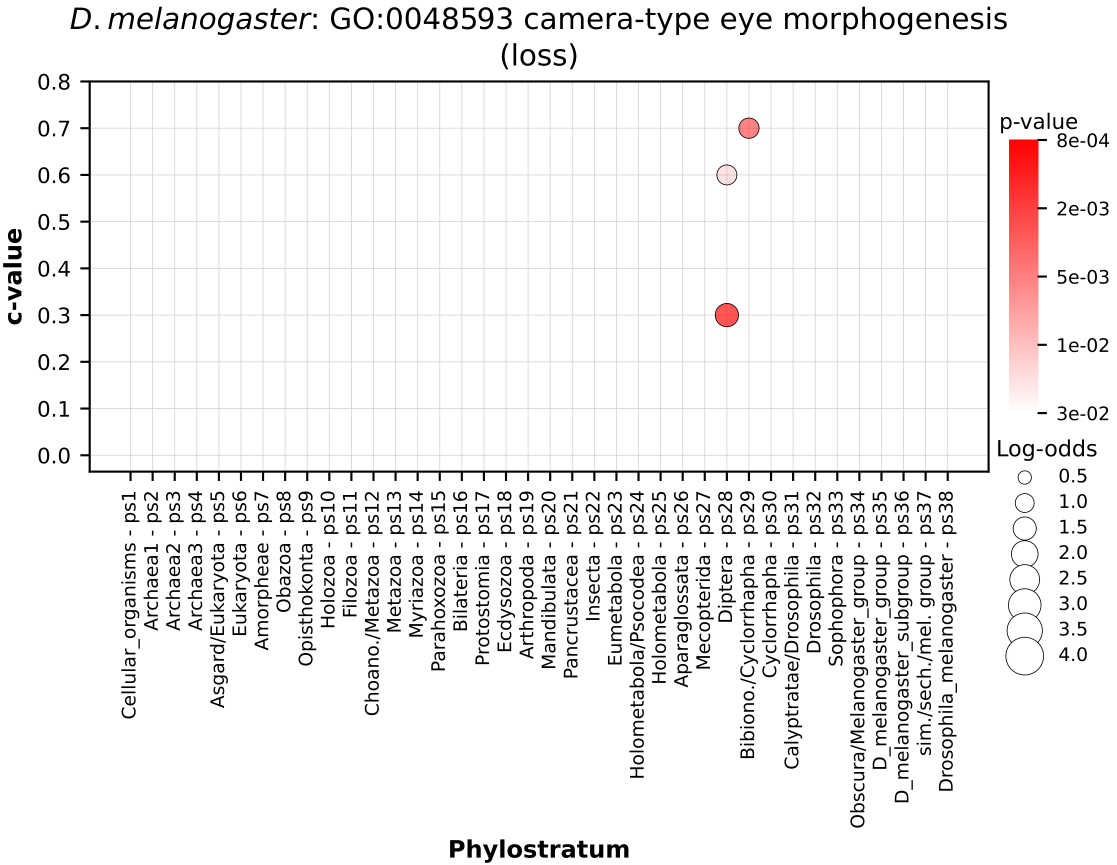

D. melanogaster: GO:0048593 camera-type eye morphogenesis
(loss)
c-value
0.8
0.7
0.6
0.5
0.4
0.3
0.2
0.1
0.0
Phylostratum
Cellular_organisms - ps1
Archaea1 - ps2
Archaea2 - ps3
Archaea3 - ps4
Asgard/Eukaryota - ps5
Eukaryota - ps6
Amorpheae - ps7
Obazoa - ps8
Opisthokonta - ps9
Holozoa - ps10
Filozoa - ps11
Choano./Metazoa - ps12
Metazoa - ps13
Myriazoa - ps14
Parahoxozoa - ps15
Bilateria - ps16
Protostomia - ps17
Ecdysozoa - ps18
Arthropoda - ps19
Mandibulata - ps20
Pancrustacea - ps21
Insecta - ps22
Eumetabola - ps23
Holometabola/Psocodea - ps24
Holometabola - ps25
Aparaglossata - ps26
Mecopterida - ps27
Diptera - ps28
Bibiono./Cyclorrhapha - ps29
Cyclorrhapha - ps30
Calyptratae/Drosophila - ps31
Drosophila - ps32
Sophophora - ps33
Obscura/Melanogaster_group - ps34
D_melanogaster_group - ps35
D_melanogaster_subgroup - ps36
sim./sech./mel. group - ps37
Drosophila_melanogaster - ps38
p-value
8e-04
2e-03
5e-03
1e-02
3e-02
Log-odds
0.5
1.0
1.5
2.0
2.5
3.0
3.5
4.0

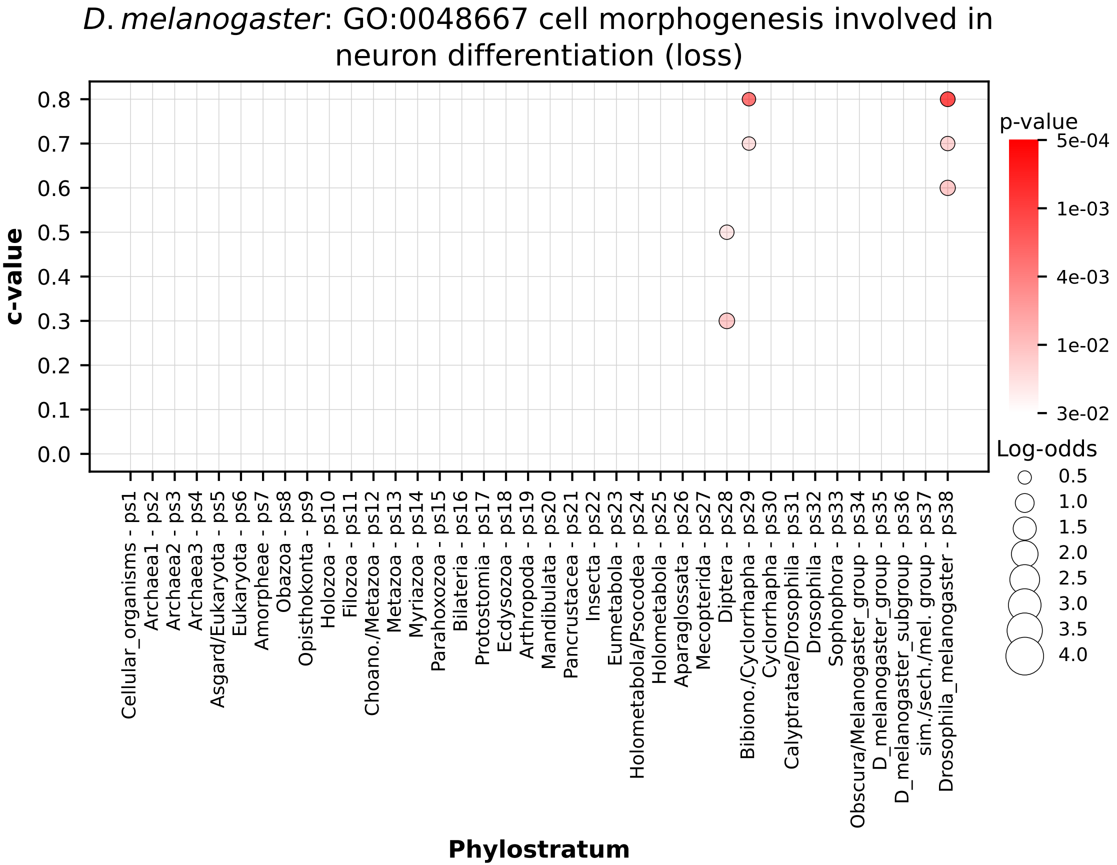

D. melanogaster: GO:0048667 cell morphogenesis involved in neuron differentiation (loss)
c-value
0.8
0.7
0.6
0.5
0.4
0.3
0.2
0.1
0.0
Phylostratum
Cellular_organisms - ps1
Archaea1 - ps2
Archaea2 - ps3
Archaea3 - ps4
Asgard/Eukaryota - ps5
Eukaryota - ps6
Amorpheae - ps7
Obazoa - ps8
Opisthokonta - ps9
Holozoa - ps10
Filozoa - ps11
Choano./Metazoa - ps12
Metazoa - ps13
Myriazoa - ps14
Parahoxozoa - ps15
Bilateria - ps16
Protostomia - ps17
Ecdysozoa - ps18
Arthropoda - ps19
Mandibulata - ps20
Pancrustacea - ps21
Insecta - ps22
Eumetabola - ps23
Holometabola/Psocodea - ps24
Holometabola - ps25
Aparaglossata - ps26
Mecopterida - ps27
Diptera - ps28
Bibiono./Cyclorrhapha - ps29
Cyclorrhapha - ps30
Calyptratae/Drosophila - ps31
Drosophila - ps32
Sophophora - ps33
Obscura/Melanogaster_group - ps34
D_melanogaster_group - ps35
D_melanogaster_subgroup - ps36
sim./sech./mel. group - ps37
Drosophila_melanogaster - ps38
p-value
5e-04
1e-03
4e-03
1e-02
3e-02
Log-odds
0.5
1.0
1.5
2.0
2.5
3.0
3.5
4.0

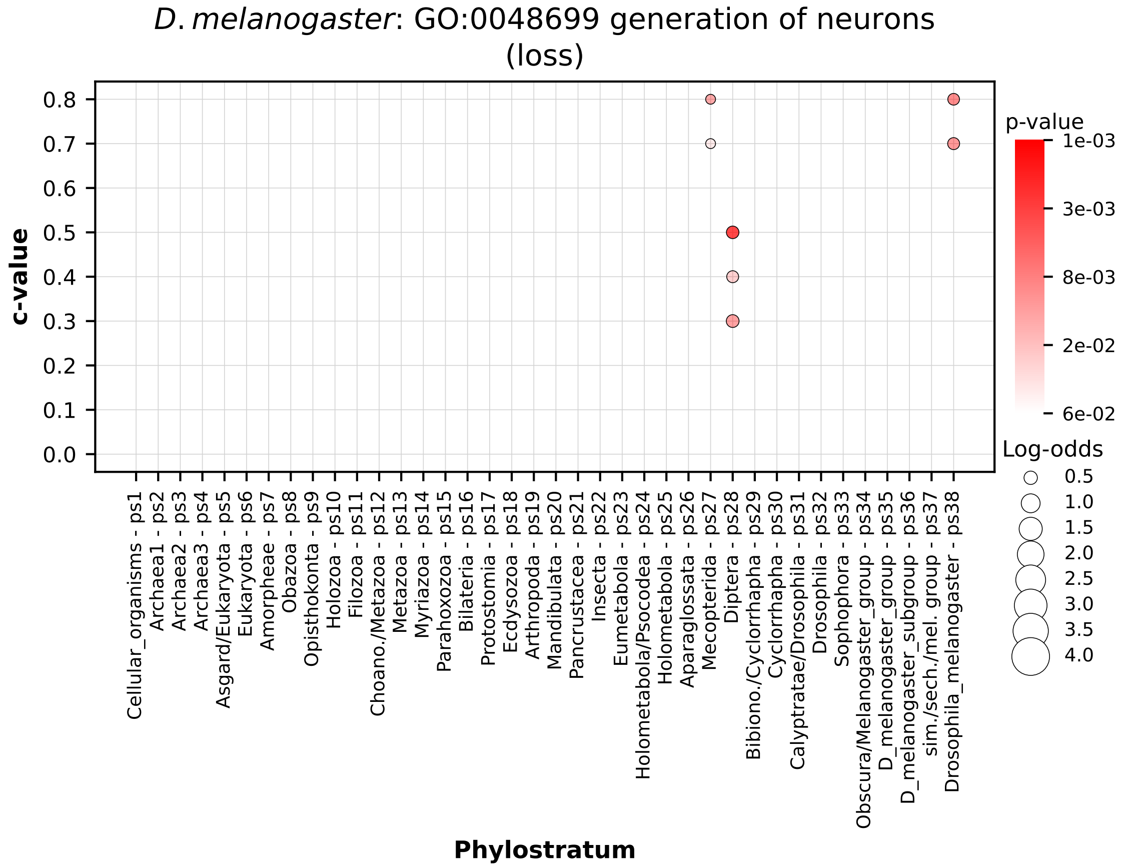

D. melanogaster: GO:0048699 generation of neurons (loss)
c-value
0.0
0.1
0.2
0.3
0.4
0.5
0.6
0.7
0.8
Phylostratum
Cellular_organisms - ps1
Archaea1 - ps2
Archaea2 - ps3
Archaea3 - ps4
Asgard/Eukaryota - ps5
Eukaryota - ps6
Amorpheae - ps7
Obazoa - ps8
Opisthokonta - ps9
Holozoa - ps10
Filozoa - ps11
Choano./Metazoa - ps12
Metazoa - ps13
Myriazoa - ps14
Parahoxozoa - ps15
Bilateria - ps16
Protostomia - ps17
Ecdysozoa - ps18
Arthropoda - ps19
Mandibulata - ps20
Pancrustacea - ps21
Insecta - ps22
Eumetabola - ps23
Holometabola/Psocodea - ps24
Holometabola - ps25
Aparaglossata - ps26
Mecopterida - ps27
Diptera - ps28
Bibiono./Cyclorrhapha - ps29
Cyclorrhapha - ps30
Calyptratae/Drosophila - ps31
Drosophila - ps32
Sophophora - ps33
Obscura/Melanogaster_group - ps34
D_melanogaster_group - ps35
D_melanogaster_subgroup - ps36
sim./sech./mel. group - ps37
Drosophila_melanogaster - ps38
p-value
1e-03
3e-03
8e-03
2e-02
6e-02
Log-odds
0.5
1.0
1.5
2.0
2.5
3.0
3.5
4.0

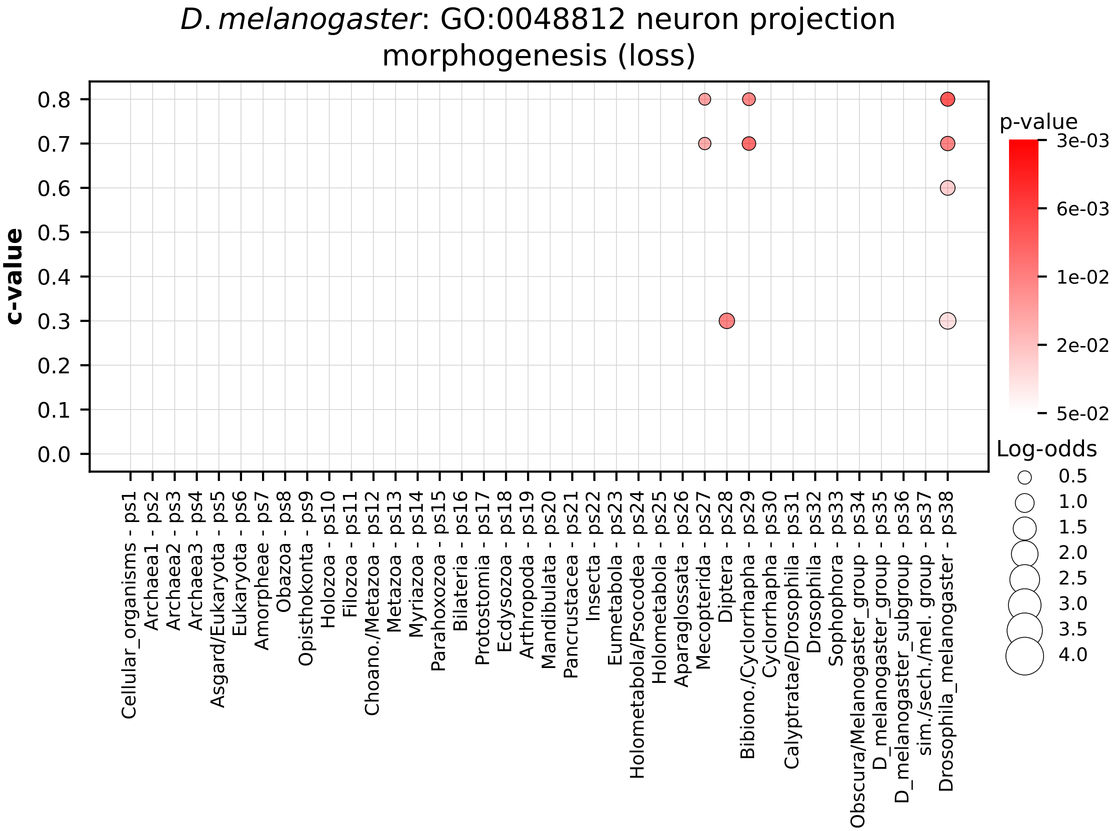

D. melanogaster: GO:0048812 neuron projection morphogenesis (loss)
c-value
0.8
0.7
0.6
0.5
0.4
0.3
0.2
0.1
0.0
p-value
3e-03
6e-03
1e-02
2e-02
5e-02
Log-odds
0.5
1.0
1.5
2.0
2.5
3.0
3.5
4.0
Cellular_organisms - ps1
Archaea1 - ps2
Archaea2 - ps3
Archaea3 - ps4
Asgard/Eukaryota - ps5
Eukaryota - ps6
Amorpheae - ps7
Obazoa - ps8
Opisthokonta - ps9
Holozoa - ps10
Filozoa - ps11
Choano./Metazoa - ps12
Metazoa - ps13
Myriazoa - ps14
Parahoxozoa - ps15
Bilateria - ps16
Protostomia - ps17
Ecdysozoa - ps18
Arthropoda - ps19
Mandibulata - ps20
Pancrustacea - ps21
Insecta - ps22
Eumetabola - ps23
Holometabola/Psocodea - ps24
Holometabola - ps25
Aparaglossata - ps26
Mecopterida - ps27
Diptera - ps28
Bibiono./Cyclorrhapha - ps29
Cyclorrhapha - ps30
Calyptratae/Drosophila - ps31
Drosophila - ps32
Sophophora - ps33
Obscura/Melanogaster_group - ps34
D_melanogaster_group - ps35
D_melanogaster_subgroup - ps36
sim./sech./mel. group - ps37
Drosophila_melanogaster - ps38
Phylostratum

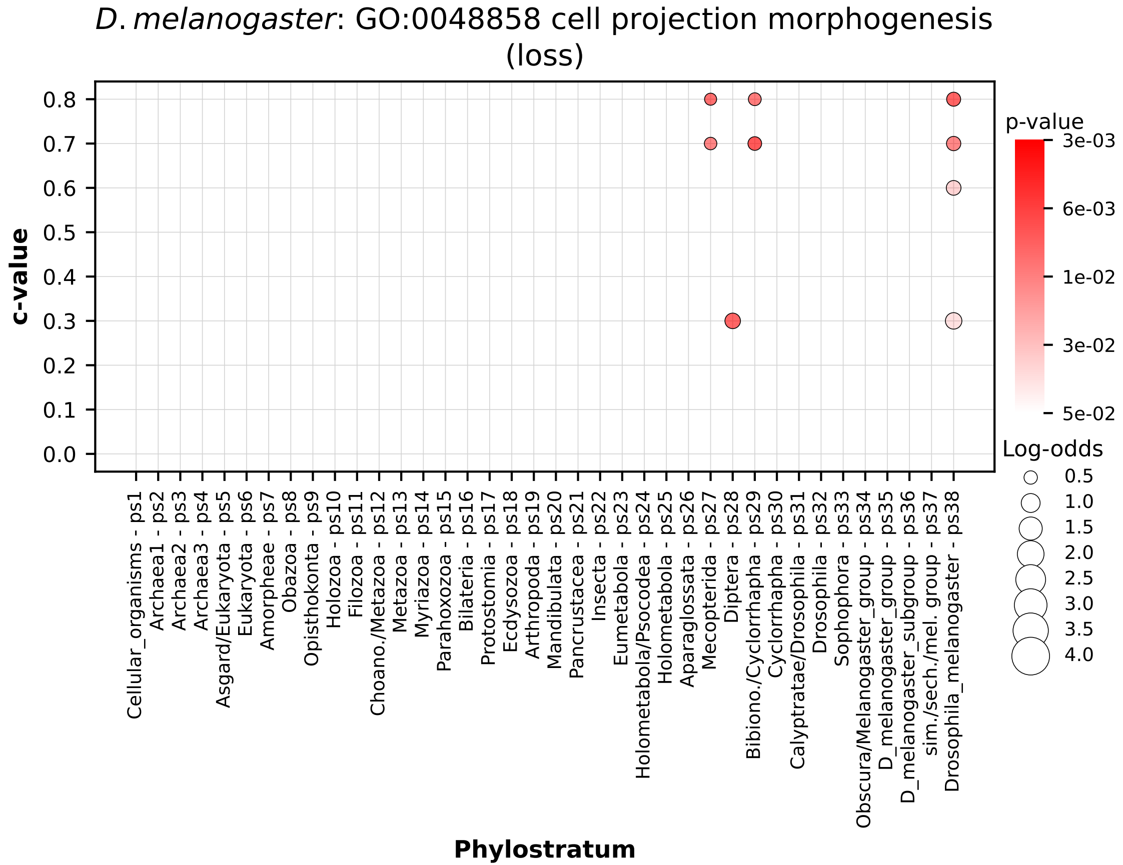

D. melanogaster: GO:0048858 cell projection morphogenesis (loss)
c-value
0.8
0.7
0.6
0.5
0.4
0.3
0.2
0.1
0.0
p-value
3e-03
6e-03
1e-02
3e-02
5e-02
Log-odds
0.5
1.0
1.5
2.0
2.5
3.0
3.5
4.0
Cellular_organisms - ps1
Archaea1 - ps2
Archaea2 - ps3
Archaea3 - ps4
Asgard/Eukaryota - ps5
Eukaryota - ps6
Amorpheae - ps7
Obazoa - ps8
Opisthokonta - ps9
Holozoa - ps10
Filozoa - ps11
Choano./Metazoa - ps12
Metazoa - ps13
Myriazoa - ps14
Parahoxozoa - ps15
Bilateria - ps16
Protostomia - ps17
Ecdysozoa - ps18
Arthropoda - ps19
Mandibulata - ps20
Pancrustacea - ps21
Insecta - ps22
Eumetabola - ps23
Holometabola/Psocodea - ps24
Holometabola - ps25
Aparaglossata - ps26
Mecopterida - ps27
Diptera - ps28
Bibiono./Cyclorrhapha - ps29
Cyclorrhapha - ps30
Calyptratae/Drosophila - ps31
Drosophila - ps32
Sophophora - ps33
Obscura/Melanogaster_group - ps34
D_melanogaster_group - ps35
D_melanogaster_subgroup - ps36
sim./sech./mel. group - ps37
Drosophila_melanogaster - ps38
Phylostratum

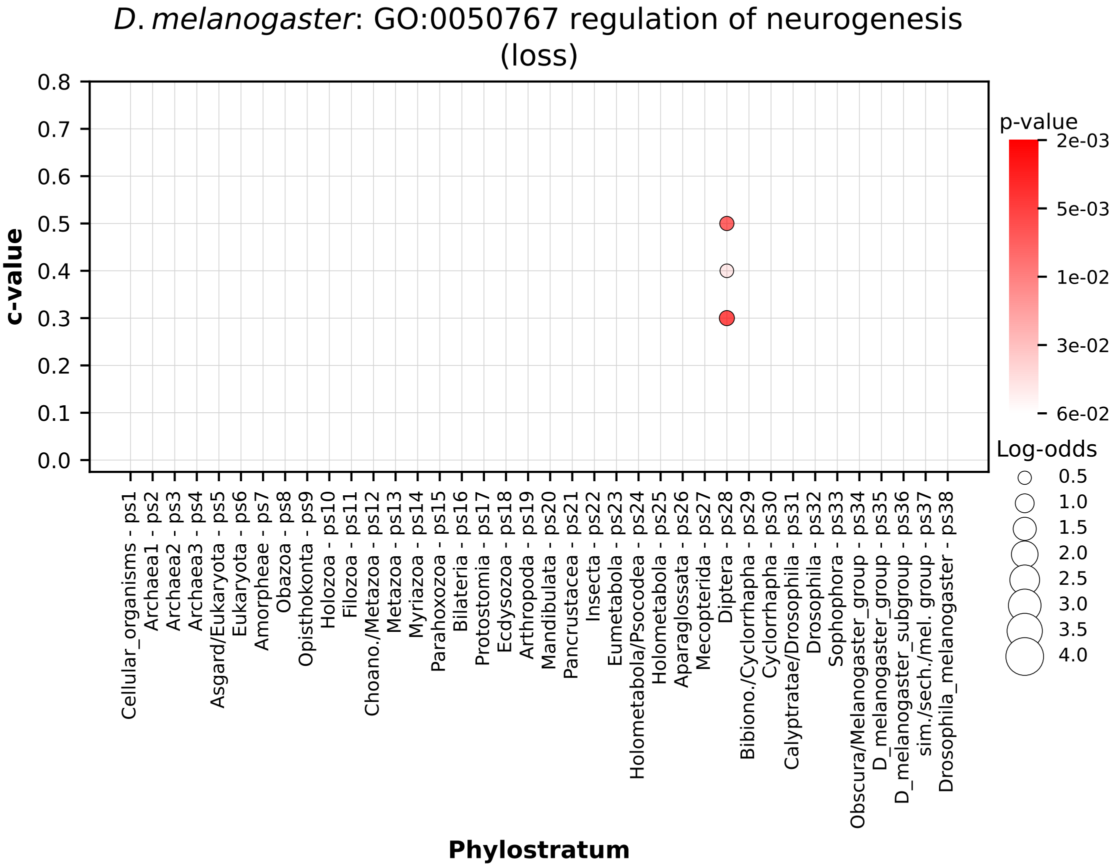

D. melanogaster: GO:0050767 regulation of neurogenesis (loss)
c-value
0.0
0.1
0.2
0.3
0.4
0.5
0.6
0.7
0.8
Phylostratum
Cellular_organisms - ps1
Archaea1 - ps2
Archaea2 - ps3
Archaea3 - ps4
Asgard/Eukaryota - ps5
Eukaryota - ps6
Amorpheae - ps7
Obazoa - ps8
Opisthokonta - ps9
Holozoa - ps10
Filozoa - ps11
Choano./Metazoa - ps12
Metazoa - ps13
Myriazoa - ps14
Parahoxozoa - ps15
Bilateria - ps16
Protostomia - ps17
Ecdysozoa - ps18
Arthropoda - ps19
Mandibulata - ps20
Pancrustacea - ps21
Insecta - ps22
Eumetabola - ps23
Holometabola/Psocodea - ps24
Holometabola - ps25
Aparaglossata - ps26
Mecopterida - ps27
Diptera - ps28
Bibiono./Cyclorrhapha - ps29
Cyclorrhapha - ps30
Calyptratae/Drosophila - ps31
Drosophila - ps32
Sophophora - ps33
Obscura/Melanogaster_group - ps34
D_melanogaster_group - ps35
D_melanogaster_subgroup - ps36
sim./sech./mel. group - ps37
Drosophila_melanogaster - ps38
p-value
2e-03
5e-03
1e-02
3e-02
6e-02
Log-odds
0.5
1.0
1.5
2.0
2.5
3.0
3.5
4.0

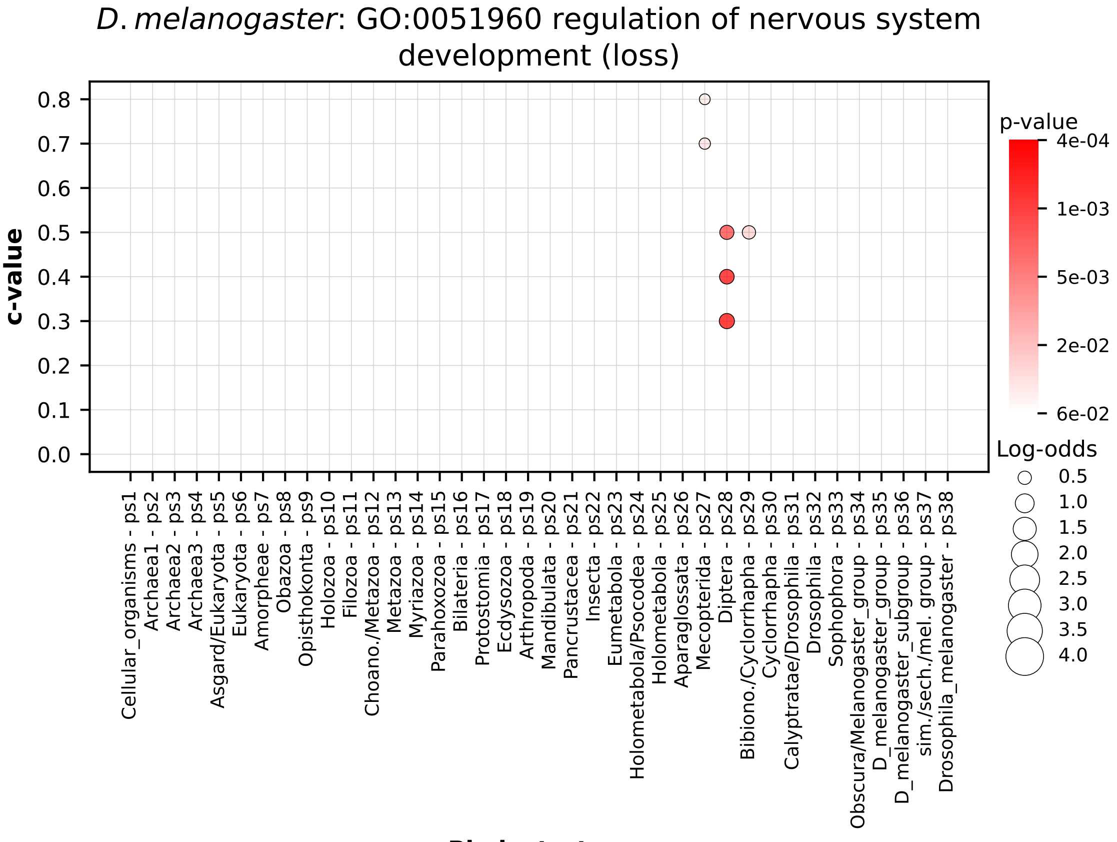

D. melanogaster: GO:0051960 regulation of nervous system development (loss)
c-value
0.8
0.7
0.6
0.5
0.4
0.3
0.2
0.1
0.0
Phylostratum
Cellular_organisms - ps1
Archaea1 - ps2
Archaea2 - ps3
Archaea3 - ps4
Asgard/Eukaryota - ps5
Eukaryota - ps6
Amorpheae - ps7
Obazoa - ps8
Opisthokonta - ps9
Holozoa - ps10
Filozoa - ps11
Choano./Metazoa - ps12
Metazoa - ps13
Myriazoa - ps14
Parahoxozoa - ps15
Bilateria - ps16
Protostomia - ps17
Ecdysozoa - ps18
Arthropoda - ps19
Mandibulata - ps20
Pancrustacea - ps21
Insecta - ps22
Eumetabola - ps23
Holometabola/Psocodea - ps24
Holometabola - ps25
Aparaglossata - ps26
Mecopterida - ps27
Diptera - ps28
Bibiono./Cyclorrhapha - ps29
Cyclorrhapha - ps30
Calyptratae/Drosophila - ps31
Drosophila - ps32
Sophophora - ps33
Obscura/Melanogaster_group - ps34
D_melanogaster_group - ps35
D_melanogaster_subgroup - ps36
sim./sech./mel. group - ps37
Drosophila_melanogaster - ps38
p-value
4e-04
1e-03
5e-03
2e-02
6e-02
Log-odds
0.5
1.0
1.5
2.0
2.5
3.0
3.5
4.0

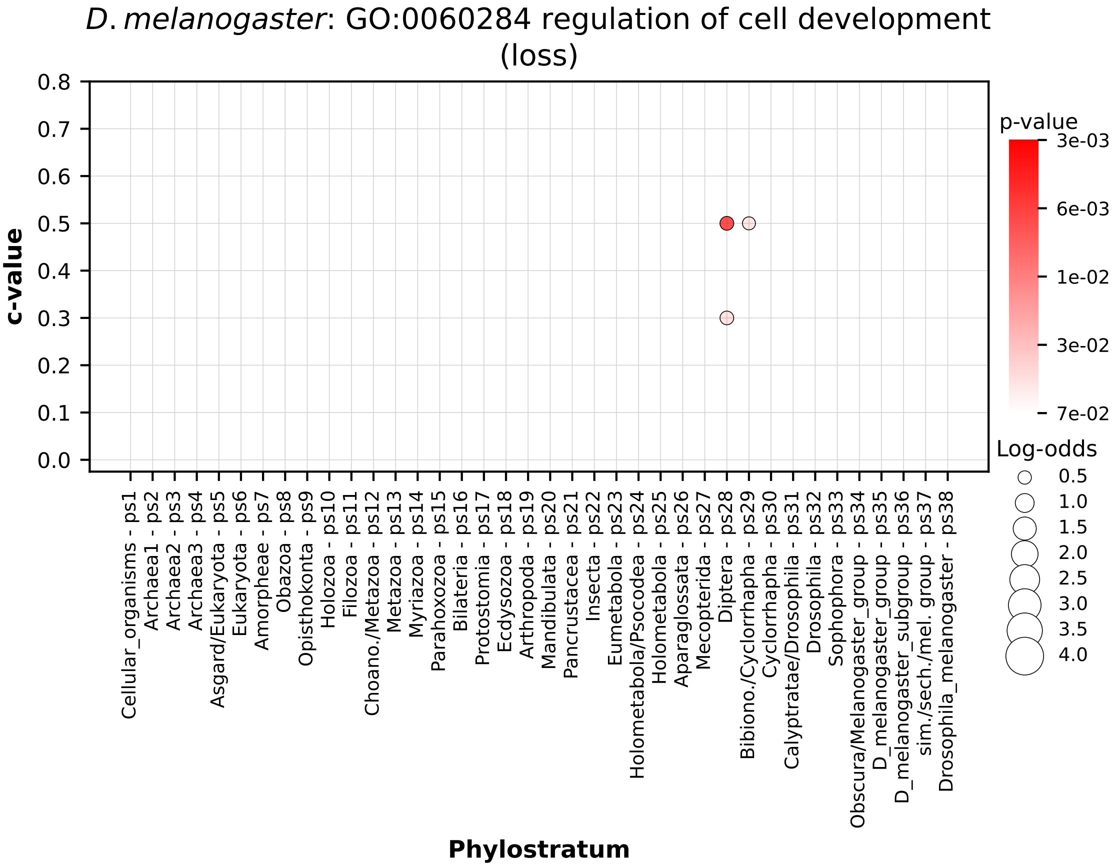

D. melanogaster: GO:0060284 regulation of cell development
(loss)
c-value
0.8
0.7
0.6
0.5
0.4
0.3
0.2
0.1
0.0
Phylostratum
Cellular_organisms - ps1
Archaea1 - ps2
Archaea2 - ps3
Archaea3 - ps4
Asgard/Eukaryota - ps5
Eukaryota - ps6
Amorpheae - ps7
Obazoa - ps8
Opisthokonta - ps9
Holozoa - ps10
Filozoa - ps11
Choano./Metazoa - ps12
Metazoa - ps13
Myriazoa - ps14
Parahoxozoa - ps15
Bilateria - ps16
Protostomia - ps17
Ecdysozoa - ps18
Arthropoda - ps19
Mandibulata - ps20
Pancrustacea - ps21
Insecta - ps22
Eumetabola - ps23
Holometabola/Psocodea - ps24
Holometabola - ps25
Aparaglossata - ps26
Mecopterida - ps27
Diptera - ps28
Bibiono./Cyclorrhapha - ps29
Cyclorrhapha - ps30
Calyptratae/Drosophila - ps31
Drosophila - ps32
Sophophora - ps33
Obscura/Melanogaster_group - ps34
D_melanogaster_group - ps35
D_melanogaster_subgroup - ps36
sim./sech./mel. group - ps37
Drosophila_melanogaster - ps38
p-value
3e-03
6e-03
1e-02
3e-02
7e-02
Log-odds
0.5
1.0
1.5
2.0
2.5
3.0
3.5
4.0

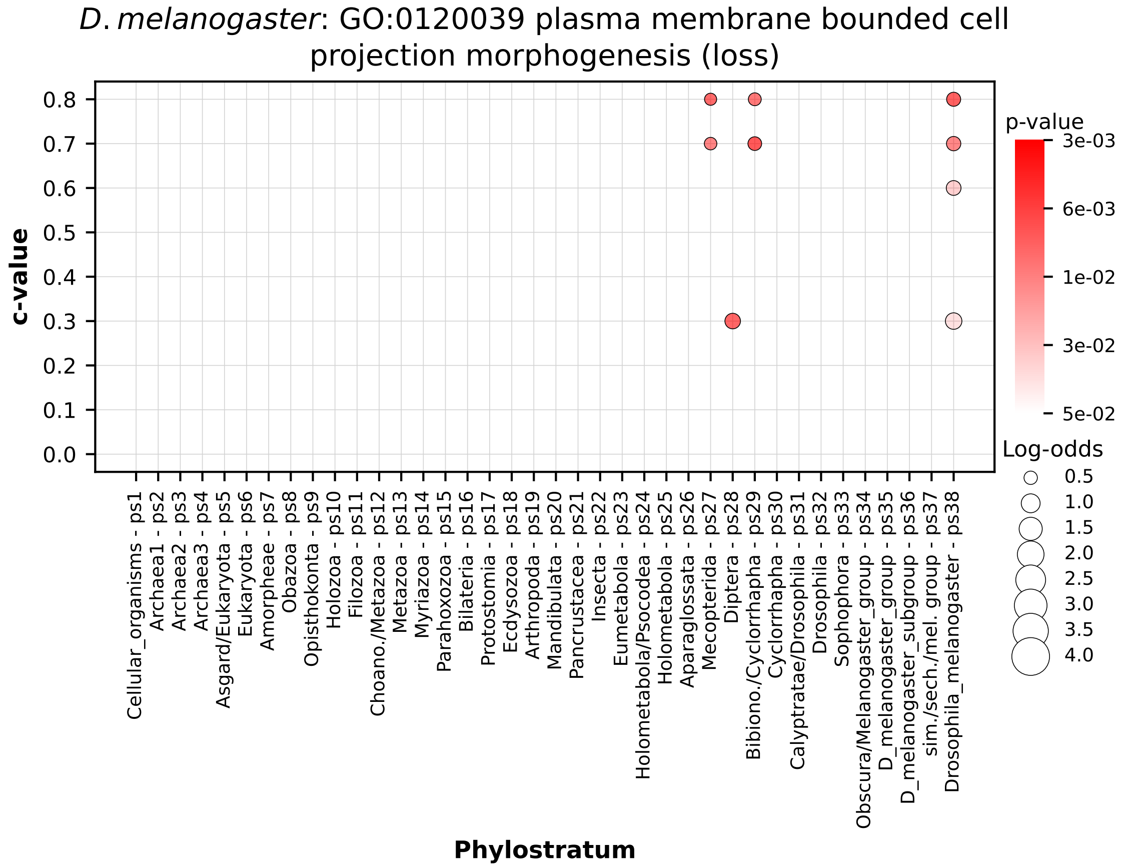

D. melanogaster: GO:0120039 plasma membrane bounded cell projection morphogenesis (loss)
c-value
0.8
0.7
0.6
0.5
0.4
0.3
0.2
0.1
0.0
p-value
3e-03
6e-03
1e-02
3e-02
5e-02
Log-odds
0.5
1.0
1.5
2.0
2.5
3.0
3.5
4.0
Cellular_organisms - ps1
Archaea1 - ps2
Archaea2 - ps3
Archaea3 - ps4
Asgard/Eukaryota - ps5
Eukaryota - ps6
Amorpheae - ps7
Obazoa - ps8
Opisthokonta - ps9
Holozoa - ps10
Filozoa - ps11
Choano./Metazoa - ps12
Metazoa - ps13
Myriazoa - ps14
Parahoxozoa - ps15
Bilateria - ps16
Protostomia - ps17
Ecdysozoa - ps18
Arthropoda - ps19
Mandibulata - ps20
Pancrustacea - ps21
Insecta - ps22
Eumetabola - ps23
Holometabola/Psocodea - ps24
Holometabola - ps25
Aparaglossata - ps26
Mecopterida - ps27
Diptera - ps28
Bibiono./Cyclorrhapha - ps29
Cyclorrhapha - ps30
Calyptratae/Drosophila - ps31
Drosophila - ps32
Sophophora - ps33
Obscura/Melanogaster_group - ps34
D_melanogaster_group - ps35
D_melanogaster_subgroup - ps36
sim./sech./mel. group - ps37
Drosophila_melanogaster - ps38
Phylostratum

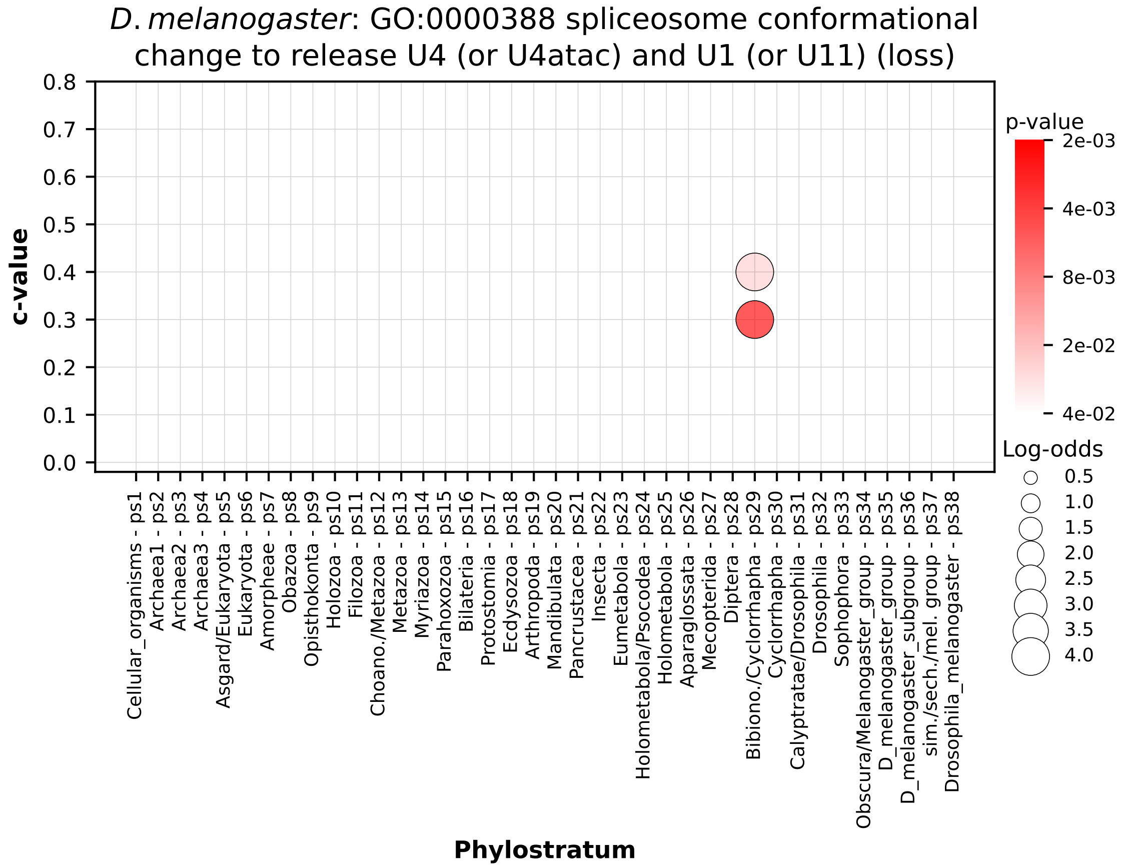
D. melanogaster: GO:0000388 spliceosome conformational change to release U4 (or U4atac) and U1 (or U11) (loss)
c-value
0.0
0.1
0.2
0.3
0.4
0.5
0.6
0.7
0.8
Phylostratum
Cellular_organisms - ps1
Archaea1 - ps2
Archaea2 - ps3
Archaea3 - ps4
Asgard/Eukaryota - ps5
Eukaryota - ps6
Amorpheae - ps7
Obazoa - ps8
Opisthokonta - ps9
Holozoa - ps10
Filozoa - ps11
Choano./Metazoa - ps12
Metazoa - ps13
Myriazoa - ps14
Parahoxozoa - ps15
Bilateria - ps16
Protostomia - ps17
Ecdysozoa - ps18
Arthropoda - ps19
Mandibulata - ps20
Pancrustacea - ps21
Insecta - ps22
Eumetabola - ps23
Holometabola/Psocodea - ps24
Holometabola - ps25
Aparaglossata - ps26
Mecopterida - ps27
Diptera - ps28
Bibiono./Cyclorrhapha - ps29
Cyclorrhapha - ps30
Calyptratae/Drosophila - ps31
Drosophila - ps32
Sophophora - ps33
Obscura/Melanogaster_group - ps34
D_melanogaster_group - ps35
D_melanogaster_subgroup - ps36
sim./sech./mel. group - ps37
Drosophila_melanogaster - ps38
p-value
2e-03
4e-03
8e-03
2e-02
4e-02
Log-odds
0.5
1.0
1.5
2.0
2.5
3.0
3.5
4.0

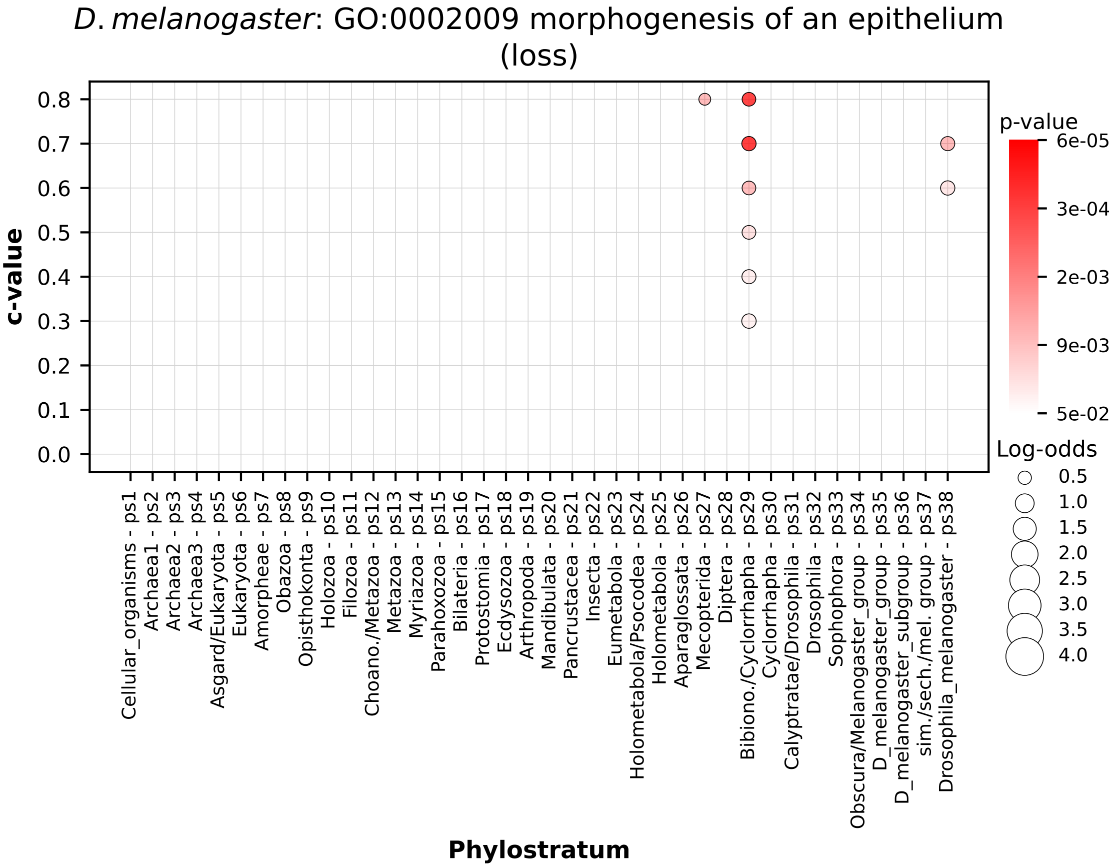

D. melanogaster: GO:0002009 morphogenesis of an epithelium (loss)
c-value
0.8
0.7
0.6
0.5
0.4
0.3
0.2
0.1
0.0
Phylostratum
Cellular_organisms - ps1
Archaea1 - ps2
Archaea2 - ps3
Archaea3 - ps4
Asgard/Eukaryota - ps5
Eukaryota - ps6
Amorpheae - ps7
Obazoa - ps8
Opisthokonta - ps9
Holozoa - ps10
Filozoa - ps11
Choano./Metazoa - ps12
Metazoa - ps13
Myriazoa - ps14
Parahoxozoa - ps15
Bilateria - ps16
Protostomia - ps17
Ecdysozoa - ps18
Arthropoda - ps19
Mandibulata - ps20
Pancrustacea - ps21
Insecta - ps22
Eumetabola - ps23
Holometabola/Psocodea - ps24
Holometabola - ps25
Aparaglossata - ps26
Mecopterida - ps27
Diptera - ps28
Bibiono./Cyclorrhapha - ps29
Cyclorrhapha - ps30
Calyptratae/Drosophila - ps31
Drosophila - ps32
Sophophora - ps33
Obscura/Melanogaster_group - ps34
D_melanogaster_group - ps35
D_melanogaster_subgroup - ps36
sim./sech./mel. group - ps37
Drosophila_melanogaster - ps38
p-value
6e-05
3e-04
2e-03
9e-03
5e-02
Log-odds
0.5
1.0
1.5
2.0
2.5
3.0
3.5
4.0

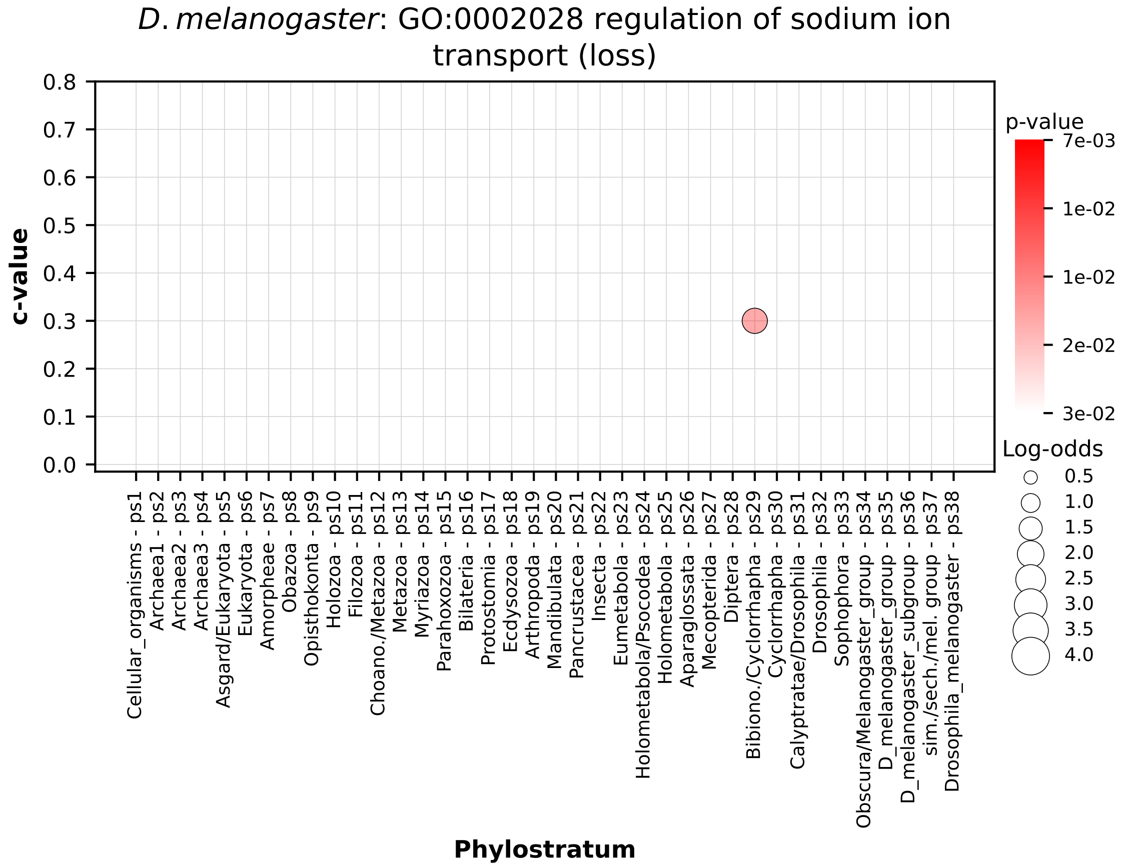

D. melanogaster: GO:0002028 regulation of sodium ion transport (loss)
c-value
0.8
0.7
0.6
0.5
0.4
0.3
0.2
0.1
0.0
Phylostratum
Cellular_organisms - ps1
Archaea1 - ps2
Archaea2 - ps3
Archaea3 - ps4
Asgard/Eukaryota - ps5
Eukaryota - ps6
Amorpheae - ps7
Obazoa - ps8
Opisthokonta - ps9
Holozoa - ps10
Filozoa - ps11
Choano./Metazoa - ps12
Metazoa - ps13
Myriazoa - ps14
Parahoxozoa - ps15
Bilateria - ps16
Protostomia - ps17
Ecdysozoa - ps18
Arthropoda - ps19
Mandibulata - ps20
Pancrustacea - ps21
Insecta - ps22
Eumetabola - ps23
Holometabola/Psocodea - ps24
Holometabola - ps25
Aparaglossata - ps26
Mecopterida - ps27
Diptera - ps28
Bibiono./Cyclorrhapha - ps29
Cyclorrhapha - ps30
Calyptratae/Drosophila - ps31
Drosophila - ps32
Sophophora - ps33
Obscura/Melanogaster_group - ps34
D_melanogaster_group - ps35
D_melanogaster_subgroup - ps36
sim./sech./mel. group - ps37
Drosophila_melanogaster - ps38
p-value
7e-03
1e-02
1e-02
2e-02
3e-02
Log-odds
0.5
1.0
1.5
2.0
2.5
3.0
3.5
4.0

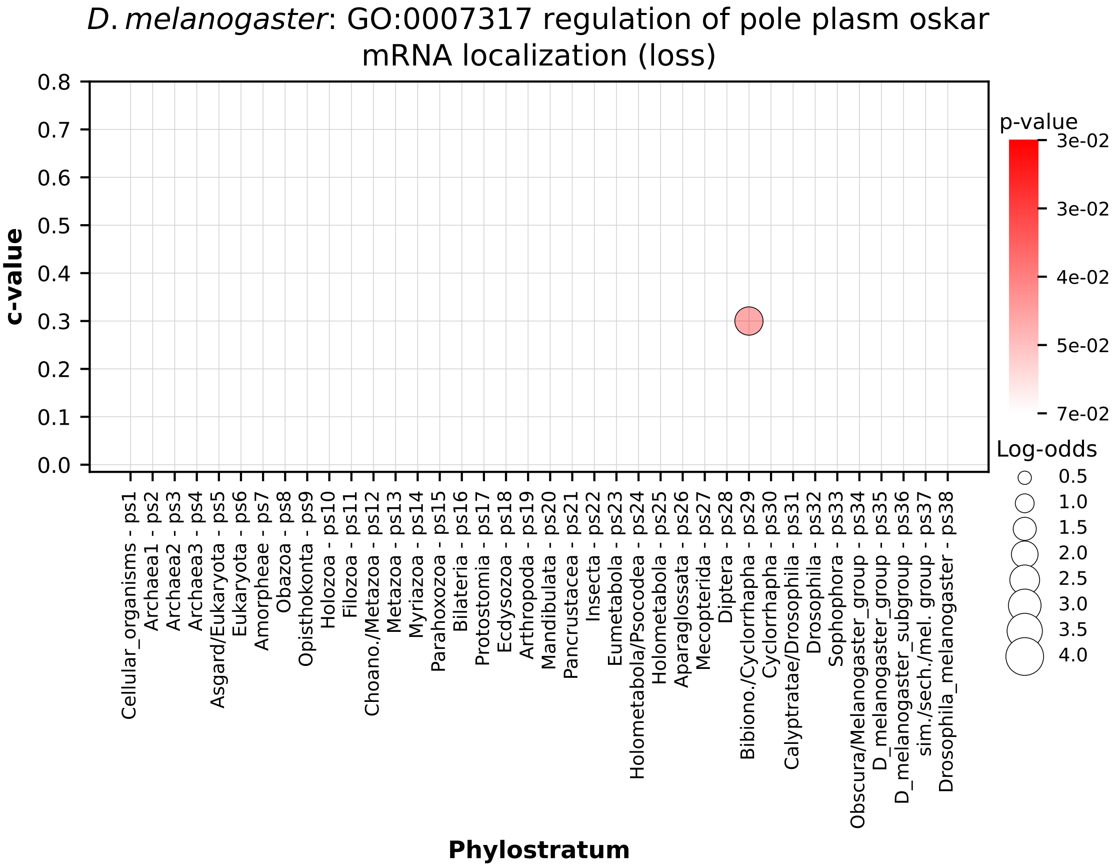

D. melanogaster: GO:0007317 regulation of pole plasm oskar mRNA localization (loss)
c-value
0.8
0.7
0.6
0.5
0.4
0.3
0.2
0.1
0.0
p-value
3e-02
3e-02
4e-02
5e-02
7e-02
Log-odds
0.5
1.0
1.5
2.0
2.5
3.0
3.5
4.0
Cellular_organisms - ps1
Archaea1 - ps2
Archaea2 - ps3
Archaea3 - ps4
Asgard/Eukaryota - ps5
Eukaryota - ps6
Amorpheae - ps7
Obazoa - ps8
Opisthokonta - ps9
Holozoa - ps10
Filozoa - ps11
Choano./Metazoa - ps12
Metazoa - ps13
Myriazoa - ps14
Parahoxozoa - ps15
Bilateria - ps16
Protostomia - ps17
Ecdysozoa - ps18
Arthropoda - ps19
Mandibulata - ps20
Pancrustacea - ps21
Insecta - ps22
Eumetabola - ps23
Holometabola/Psocodea - ps24
Holometabola - ps25
Aparaglossata - ps26
Mecopterida - ps27
Diptera - ps28
Bibiono./Cyclorrhapha - ps29
Cyclorrhapha - ps30
Calyptratae/Drosophila - ps31
Drosophila - ps32
Sophophora - ps33
Obscura/Melanogaster_group - ps34
D_melanogaster_group - ps35
D_melanogaster_subgroup - ps36
sim./sech./mel. group - ps37
Drosophila_melanogaster - ps38
Phylostratum

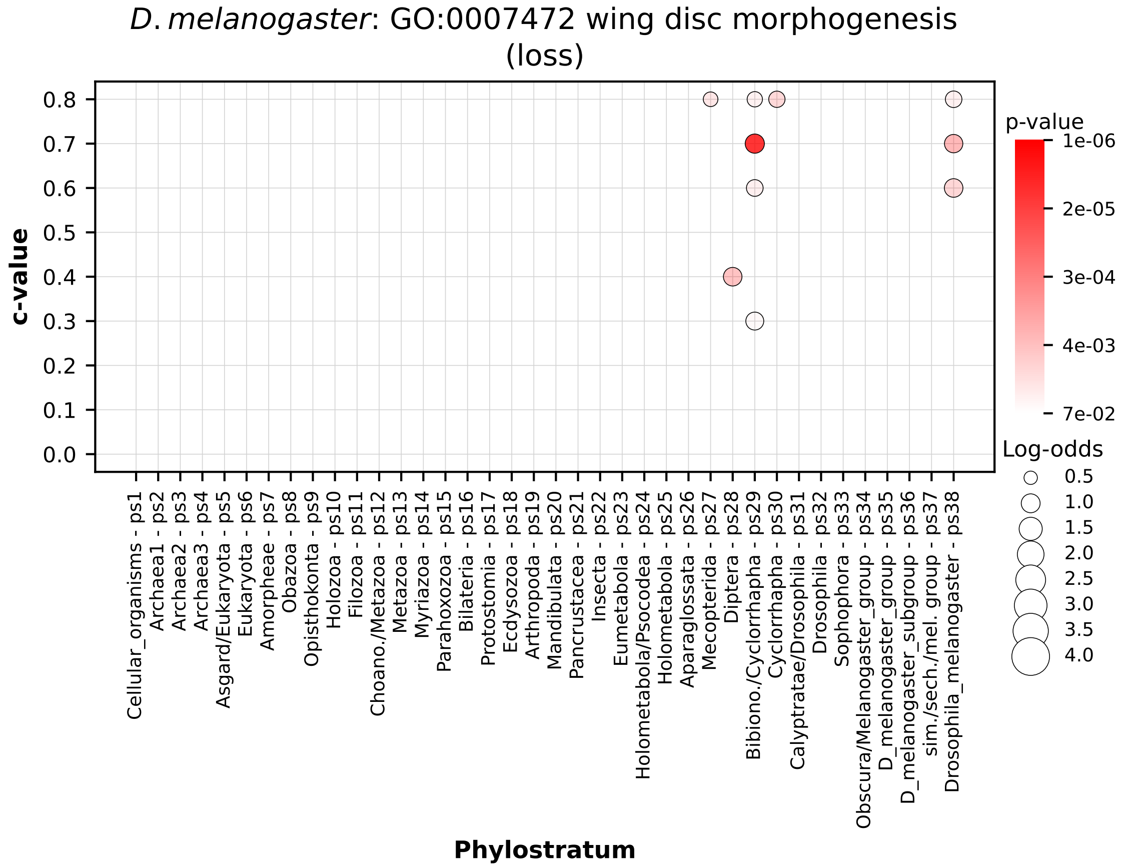
D. melanogaster: GO:0007472 wing disc morphogenesis
(loss)
c-value
0.0
0.1
0.2
0.3
0.4
0.5
0.6
0.7
0.8
p-value
1e-06
2e-05
3e-04
4e-03
7e-02
Log-odds
0.5
1.0
1.5
2.0
2.5
3.0
3.5
4.0
Cellular_organisms - ps1
Archaea1 - ps2
Archaea2 - ps3
Archaea3 - ps4
Asgard/Eukaryota - ps5
Eukaryota - ps6
Amorpheae - ps7
Obazoa - ps8
Opisthokonta - ps9
Holozoa - ps10
Filozoa - ps11
Choano./Metazoa - ps12
Metazoa - ps13
Myriazoa - ps14
Parahoxozoa - ps15
Bilateria - ps16
Protostomia - ps17
Ecdysozoa - ps18
Arthropoda - ps19
Mandibulata - ps20
Pancrustacea - ps21
Insecta - ps22
Eumetabola - ps23
Holometabola/Psocodea - ps24
Holometabola - ps25
Aparaglossata - ps26
Mecopterida - ps27
Diptera - ps28
Bibiono./Cyclorrhapha - ps29
Cyclorrhapha - ps30
Calyptratae/Drosophila - ps31
Drosophila - ps32
Sophophora - ps33
Obscura/Melanogaster_group - ps34
D_melanogaster_group - ps35
D_melanogaster_subgroup - ps36
sim./sech./mel. group - ps37
Drosophila_melanogaster - ps38
Phylostratum

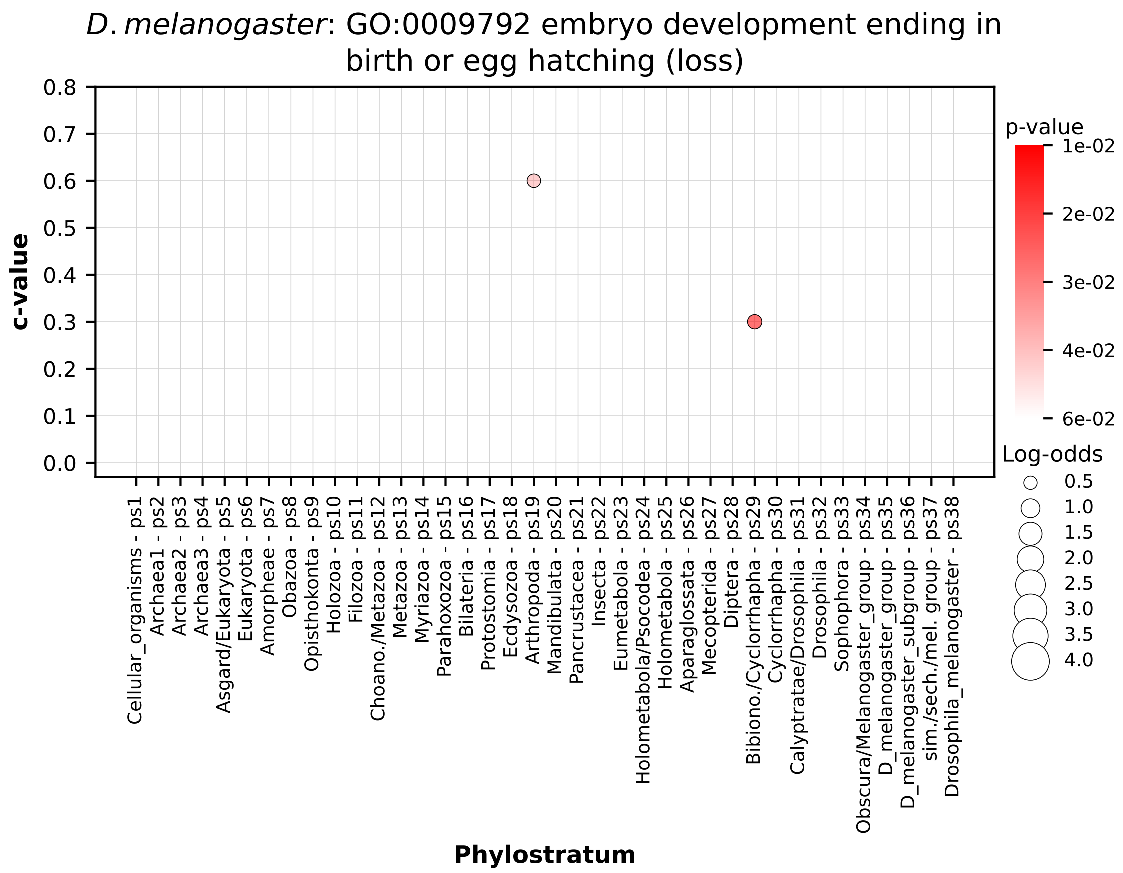

D. melanogaster: GO:0009792 embryo development ending in birth or egg hatching (loss)
c-value
0.8
0.7
0.6
0.5
0.4
0.3
0.2
0.1
0.0
Phylostratum
Cellular_organisms - ps1
Archaea1 - ps2
Archaea2 - ps3
Archaea3 - ps4
Asgard/Eukaryota - ps5
Eukaryota - ps6
Amorpheae - ps7
Obazoa - ps8
Opisthokonta - ps9
Holozoa - ps10
Filozoa - ps11
Choano./Metazoa - ps12
Metazoa - ps13
Myriazoa - ps14
Parahoxozoa - ps15
Bilateria - ps16
Protostomia - ps17
Ecdysozoa - ps18
Arthropoda - ps19
Mandibulata - ps20
Pancrustacea - ps21
Insecta - ps22
Eumetabola - ps23
Holometabola/Psocodea - ps24
Holometabola - ps25
Aparaglossata - ps26
Mecopterida - ps27
Diptera - ps28
Bibiono./Cyclorrhapha - ps29
Cyclorrhapha - ps30
Calyptratae/Drosophila - ps31
Drosophila - ps32
Sophophora - ps33
Obscura/Melanogaster_group - ps34
D_melanogaster_group - ps35
D_melanogaster_subgroup - ps36
sim./sech./mel. group - ps37
Drosophila_melanogaster - ps38
p-value
1e-02
2e-02
3e-02
4e-02
6e-02
Log-odds
0.5
1.0
1.5
2.0
2.5
3.0
3.5
4.0

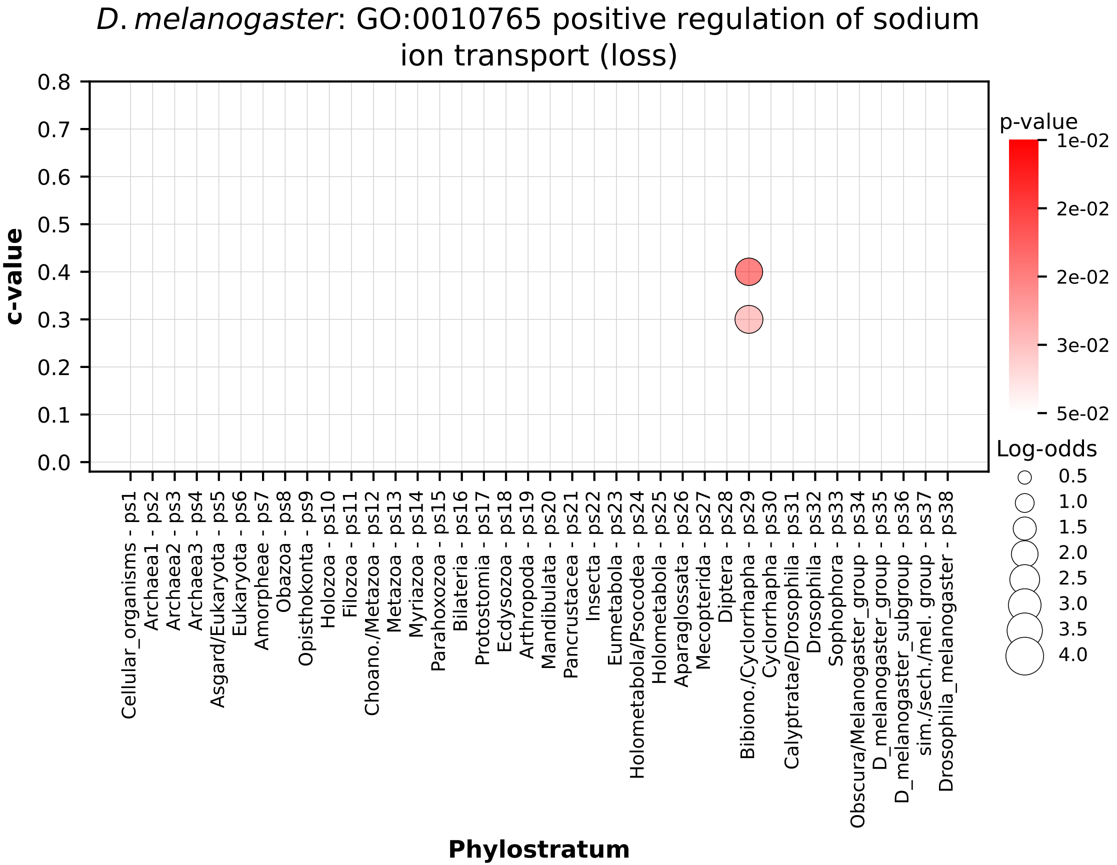

D. melanogaster: GO:0010765 positive regulation of sodium ion transport (loss)
c-value
0.8
0.7
0.6
0.5
0.4
0.3
0.2
0.1
0.0
Phylostratum
Cellular_organisms - ps1
Archaea1 - ps2
Archaea2 - ps3
Archaea3 - ps4
Asgard/Eukaryota - ps5
Eukaryota - ps6
Amorpheae - ps7
Obazoa - ps8
Opisthokonta - ps9
Holozoa - ps10
Filozoa - ps11
Choano./Metazoa - ps12
Metazoa - ps13
Myriazoa - ps14
Parahoxozoa - ps15
Bilateria - ps16
Protostomia - ps17
Ecdysozoa - ps18
Arthropoda - ps19
Mandibulata - ps20
Pancrustacea - ps21
Insecta - ps22
Eumetabola - ps23
Holometabola/Psocodea - ps24
Holometabola - ps25
Aparaglossata - ps26
Mecopterida - ps27
Diptera - ps28
Bibiono./Cyclorrhapha - ps29
Cyclorrhapha - ps30
Calyptratae/Drosophila - ps31
Drosophila - ps32
Sophophora - ps33
Obscura/Melanogaster_group - ps34
D_melanogaster_group - ps35
D_melanogaster_subgroup - ps36
sim./sech./mel. group - ps37
Drosophila_melanogaster - ps38
p-value
1e-02
2e-02
2e-02
3e-02
5e-02
Log-odds
0.5
1.0
1.5
2.0
2.5
3.0
3.5
4.0

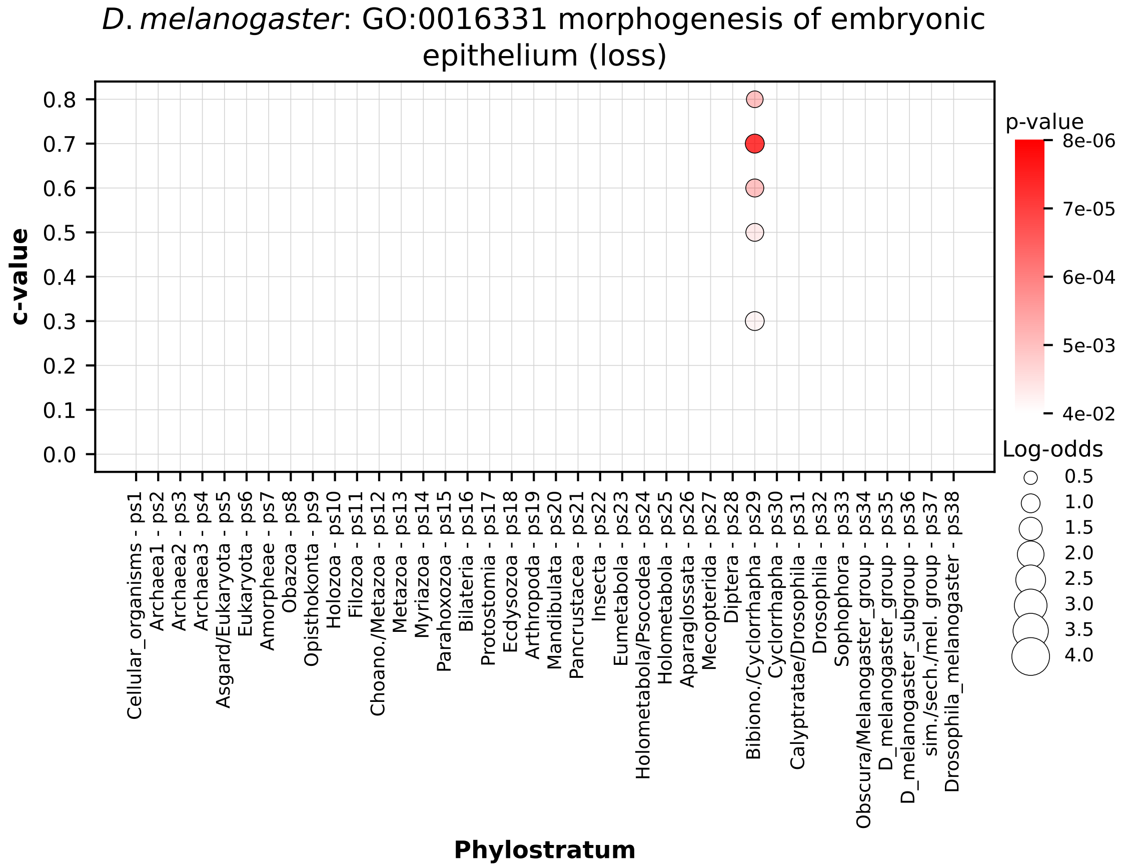

D. melanogaster: GO:0016331 morphogenesis of embryonic epithelium (loss)
c-value
0.8
0.7
0.6
0.5
0.4
0.3
0.2
0.1
0.0
p-value
8e-06
7e-05
6e-04
5e-03
4e-02
Log-odds
0.5
1.0
1.5
2.0
2.5
3.0
3.5
4.0
Cellular_organisms - ps1
Archaea1 - ps2
Archaea2 - ps3
Archaea3 - ps4
Asgard/Eukaryota - ps5
Eukaryota - ps6
Amorpheae - ps7
Obazoa - ps8
Opisthokonta - ps9
Holozoa - ps10
Filozoa - ps11
Choano./Metazoa - ps12
Metazoa - ps13
Myriazoa - ps14
Parahoxozoa - ps15
Bilateria - ps16
Protostomia - ps17
Ecdysozoa - ps18
Arthropoda - ps19
Mandibulata - ps20
Pancrustacea - ps21
Insecta - ps22
Eumetabola - ps23
Holometabola/Psocodea - ps24
Holometabola - ps25
Aparaglossata - ps26
Mecopterida - ps27
Diptera - ps28
Bibiono./Cyclorrhapha - ps29
Cyclorrhapha - ps30
Calyptratae/Drosophila - ps31
Drosophila - ps32
Sophophora - ps33
Obscura/Melanogaster_group - ps34
D_melanogaster_group - ps35
D_melanogaster_subgroup - ps36
sim./sech./mel. group - ps37
Drosophila_melanogaster - ps38
Phylostratum

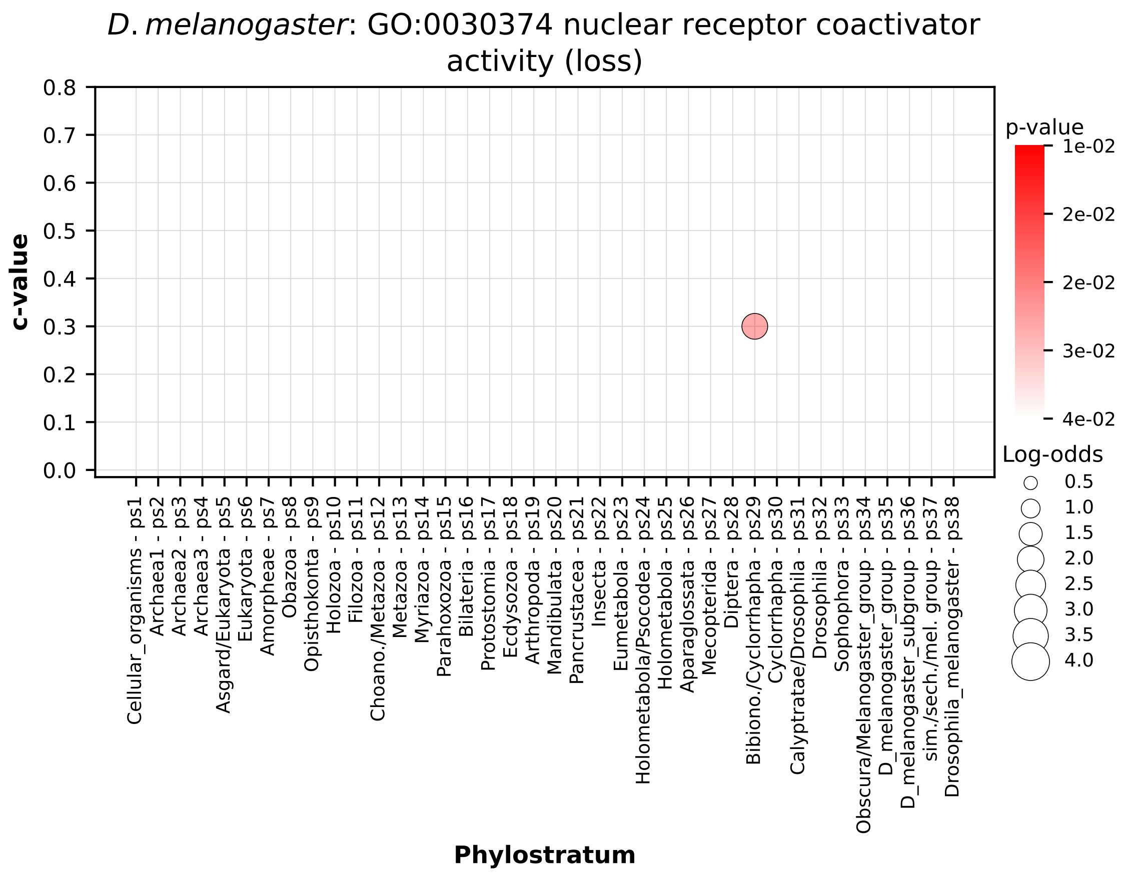
D. melanogaster: GO:0030374 nuclear receptor coactivator activity (loss)
c-value
0.8
0.7
0.6
0.5
0.4
0.3
0.2
0.1
0.0
p-value
1e-02
2e-02
2e-02
3e-02
4e-02
Log-odds
0.5
1.0
1.5
2.0
2.5
3.0
3.5
4.0
Cellular_organisms - ps1
Archaea1 - ps2
Archaea2 - ps3
Archaea3 - ps4
Asgard/Eukaryota - ps5
Eukaryota - ps6
Amorpheae - ps7
Obazoa - ps8
Opisthokonta - ps9
Holozoa - ps10
Filozoa - ps11
Choano./Metazoa - ps12
Metazoa - ps13
Myriazoa - ps14
Parahoxozoa - ps15
Bilateria - ps16
Protostomia - ps17
Ecdysozoa - ps18
Arthropoda - ps19
Mandibulata - ps20
Pancrustacea - ps21
Insecta - ps22
Eumetabola - ps23
Holometabola/Psocodea - ps24
Holometabola - ps25
Aparaglossata - ps26
Mecopterida - ps27
Diptera - ps28
Bibiono./Cyclorrhapha - ps29
Cyclorrhapha - ps30
Calyptratae/Drosophila - ps31
Drosophila - ps32
Sophophora - ps33
Obscura/Melanogaster_group - ps34
D_melanogaster_group - ps35
D_melanogaster_subgroup - ps36
sim./sech./mel. group - ps37
Drosophila_melanogaster - ps38
Phylostratum

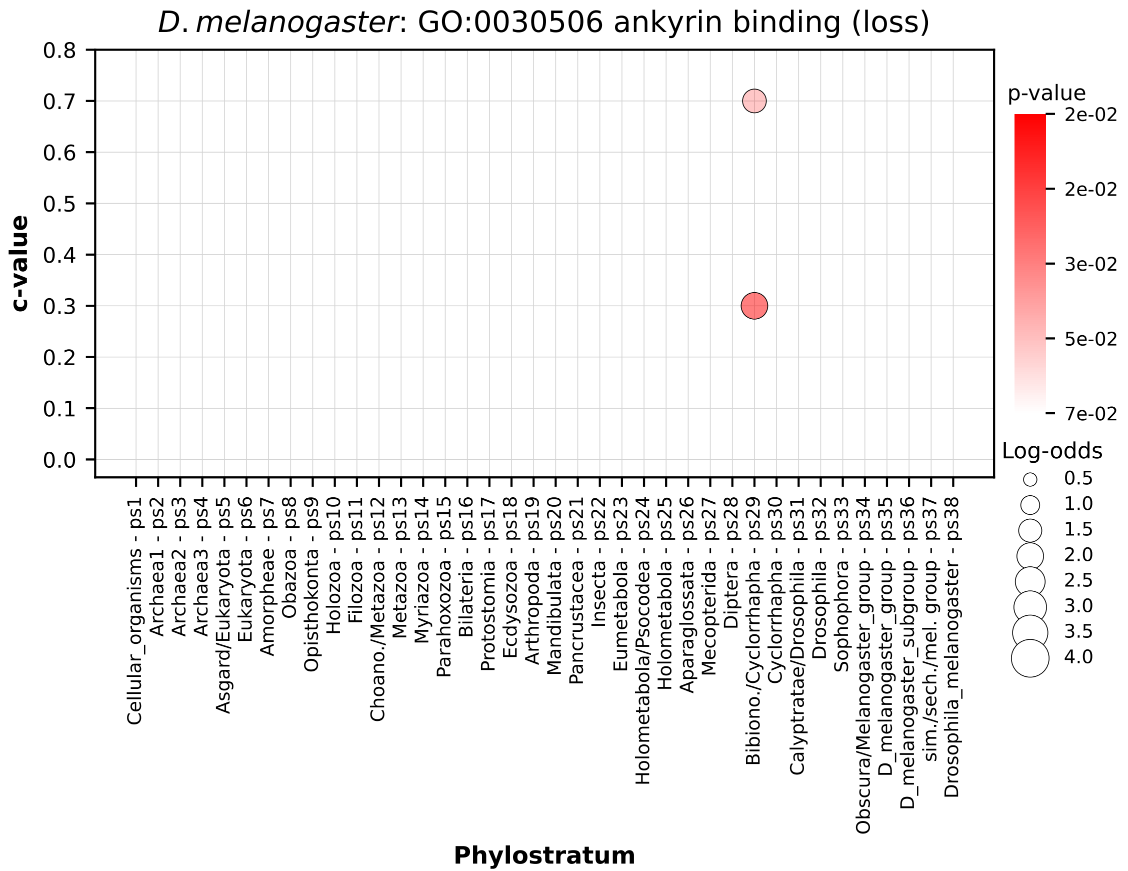

D. melanogaster: GO:0030506 ankyrin binding (loss)
c-value
0.8
0.7
0.6
0.5
0.4
0.3
0.2
0.1
0.0
Phylostratum
Cellular_organisms - ps1
Archaea1 - ps2
Archaea2 - ps3
Archaea3 - ps4
Asgard/Eukaryota - ps5
Eukaryota - ps6
Amorpheae - ps7
Obazoa - ps8
Opisthokonta - ps9
Holozoa - ps10
Filozoa - ps11
Choano./Metazoa - ps12
Metazoa - ps13
Myriazoa - ps14
Parahoxozoa - ps15
Bilateria - ps16
Protostomia - ps17
Ecdysozoa - ps18
Arthropoda - ps19
Mandibulata - ps20
Pancrustacea - ps21
Insecta - ps22
Eumetabola - ps23
Holometabola/Psocodea - ps24
Holometabola - ps25
Aparaglossata - ps26
Mecopterida - ps27
Diptera - ps28
Bibiono./Cyclorrhapha - ps29
Cyclorrhapha - ps30
Calyptratae/Drosophila - ps31
Drosophila - ps32
Sophophora - ps33
Obscura/Melanogaster_group - ps34
D_melanogaster_group - ps35
D_melanogaster_subgroup - ps36
sim./sech./mel. group - ps37
Drosophila_melanogaster - ps38
p-value
2e-02
2e-02
3e-02
5e-02
7e-02
Log-odds
0.5
1.0
1.5
2.0
2.5
3.0
3.5
4.0

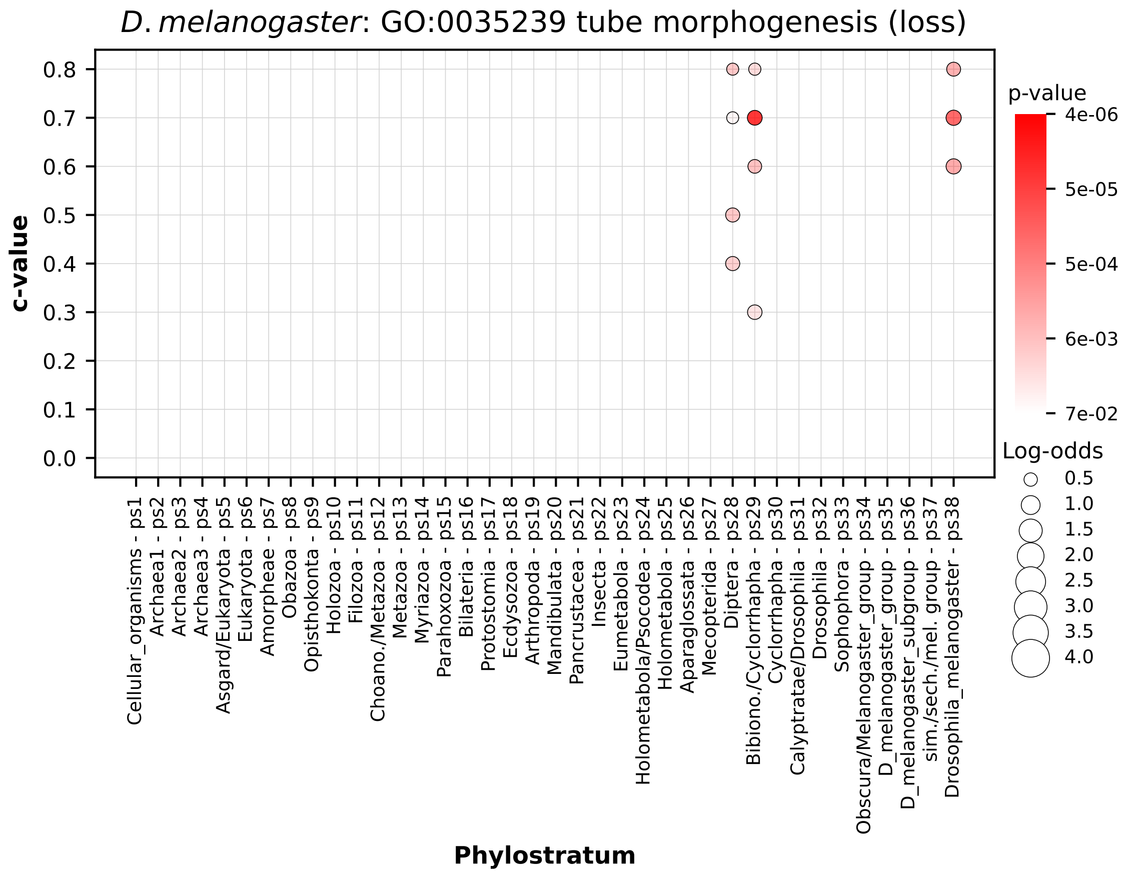

D. melanogaster: GO:0035239 tube morphogenesis (loss)
c-value
0.0
0.1
0.2
0.3
0.4
0.5
0.6
0.7
0.8
Phylostratum
Cellular_organisms - ps1
Archaea1 - ps2
Archaea2 - ps3
Archaea3 - ps4
Asgard/Eukaryota - ps5
Eukaryota - ps6
Amorpheae - ps7
Obazoa - ps8
Opisthokonta - ps9
Holozoa - ps10
Filozoa - ps11
Choano./Metazoa - ps12
Metazoa - ps13
Myriazoa - ps14
Parahoxozoa - ps15
Bilateria - ps16
Protostomia - ps17
Ecdysozoa - ps18
Arthropoda - ps19
Mandibulata - ps20
Pancrustacea - ps21
Insecta - ps22
Eumetabola - ps23
Holometabola/Psocodea - ps24
Holometabola - ps25
Aparaglossata - ps26
Mecopterida - ps27
Diptera - ps28
Bibiono./Cyclorrhapha - ps29
Cyclorrhapha - ps30
Calyptratae/Drosophila - ps31
Drosophila - ps32
Sophophora - ps33
Obscura/Melanogaster_group - ps34
D_melanogaster_group - ps35
D_melanogaster_subgroup - ps36
sim./sech./mel. group - ps37
Drosophila_melanogaster - ps38
p-value
4e-06
5e-05
5e-04
6e-03
7e-02
Log-odds
0.5
1.0
1.5
2.0
2.5
3.0
3.5
4.0

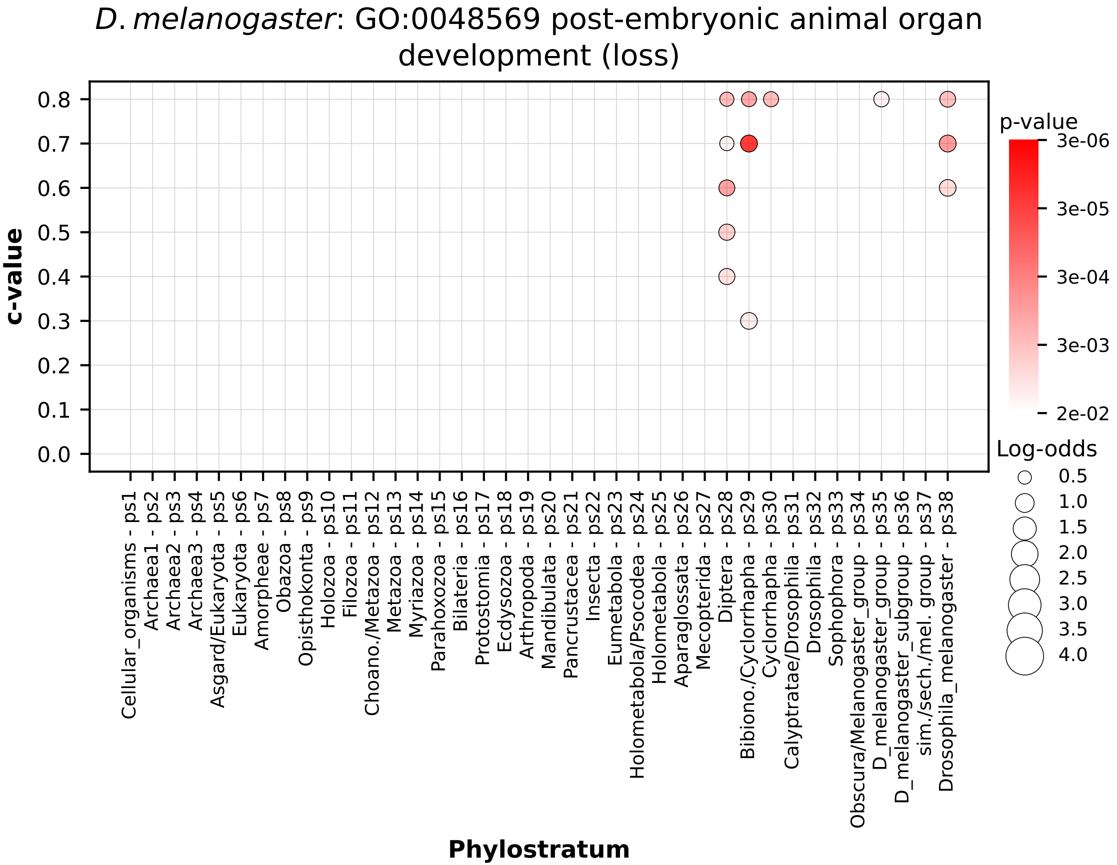

D. melanogaster: GO:0048569 post-embryonic animal organ development (loss)
c-value
0.8
0.7
0.6
0.5
0.4
0.3
0.2
0.1
0.0
Phylostratum
Cellular_organisms - ps1
Archaea1 - ps2
Archaea2 - ps3
Archaea3 - ps4
Asgard/Eukaryota - ps5
Eukaryota - ps6
Amorpheae - ps7
Obazoa - ps8
Opisthokonta - ps9
Holozoa - ps10
Filozoa - ps11
Choano./Metazoa - ps12
Metazoa - ps13
Myriazoa - ps14
Parahoxozoa - ps15
Bilateria - ps16
Protostomia - ps17
Ecdysozoa - ps18
Arthropoda - ps19
Mandibulata - ps20
Pancrustacea - ps21
Insecta - ps22
Eumetabola - ps23
Holometabola/Psocodea - ps24
Holometabola - ps25
Aparaglossata - ps26
Mecopterida - ps27
Diptera - ps28
Bibiono./Cyclorrhapha - ps29
Cyclorrhapha - ps30
Calyptratae/Drosophila - ps31
Drosophila - ps32
Sophophora - ps33
Obscura/Melanogaster_group - ps34
D_melanogaster_group - ps35
D_melanogaster_subgroup - ps36
sim./sech./mel. group - ps37
Drosophila_melanogaster - ps38
p-value
3e-06
3e-05
3e-04
3e-03
2e-02
Log-odds
0.5
1.0
1.5
2.0
2.5
3.0
3.5
4.0

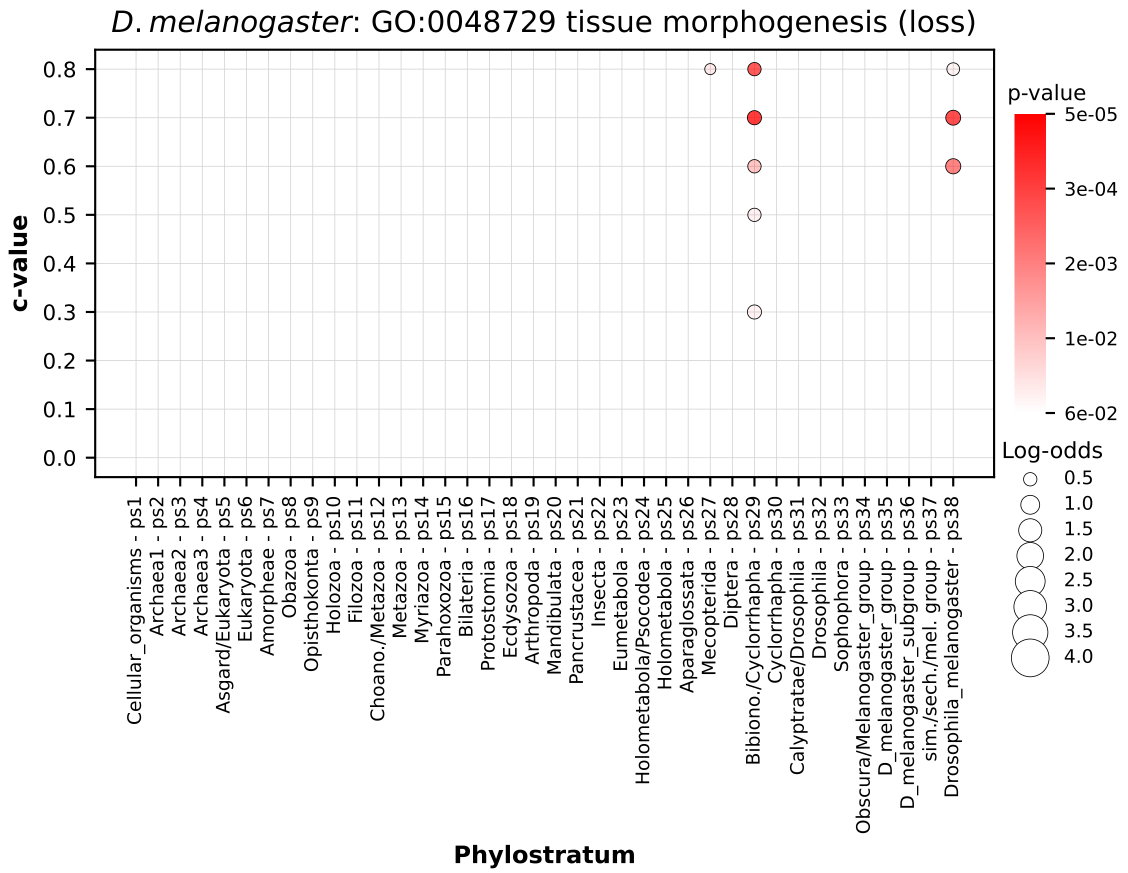

D. melanogaster: GO:0048729 tissue morphogenesis (loss)
c-value
0.0
0.1
0.2
0.3
0.4
0.5
0.6
0.7
0.8
p-value
5e-05
3e-04
2e-03
1e-02
6e-02
Log-odds
0.5
1.0
1.5
2.0
2.5
3.0
3.5
4.0
Cellular_organisms - ps1
Archaea1 - ps2
Archaea2 - ps3
Archaea3 - ps4
Asgard/Eukaryota - ps5
Eukaryota - ps6
Amorpheae - ps7
Obazoa - ps8
Opisthokonta - ps9
Holozoa - ps10
Filozoa - ps11
Choano./Metazoa - ps12
Metazoa - ps13
Myriazoa - ps14
Parahoxozoa - ps15
Bilateria - ps16
Protostomia - ps17
Ecdysozoa - ps18
Arthropoda - ps19
Mandibulata - ps20
Pancrustacea - ps21
Insecta - ps22
Eumetabola - ps23
Holometabola/Psocodea - ps24
Holometabola - ps25
Aparaglossata - ps26
Mecopterida - ps27
Diptera - ps28
Bibiono./Cyclorrhapha - ps29
Cyclorrhapha - ps30
Calyptratae/Drosophila - ps31
Drosophila - ps32
Sophophora - ps33
Obscura/Melanogaster_group - ps34
D_melanogaster_group - ps35
D_melanogaster_subgroup - ps36
sim./sech./mel. group - ps37
Drosophila_melanogaster - ps38
Phylostratum

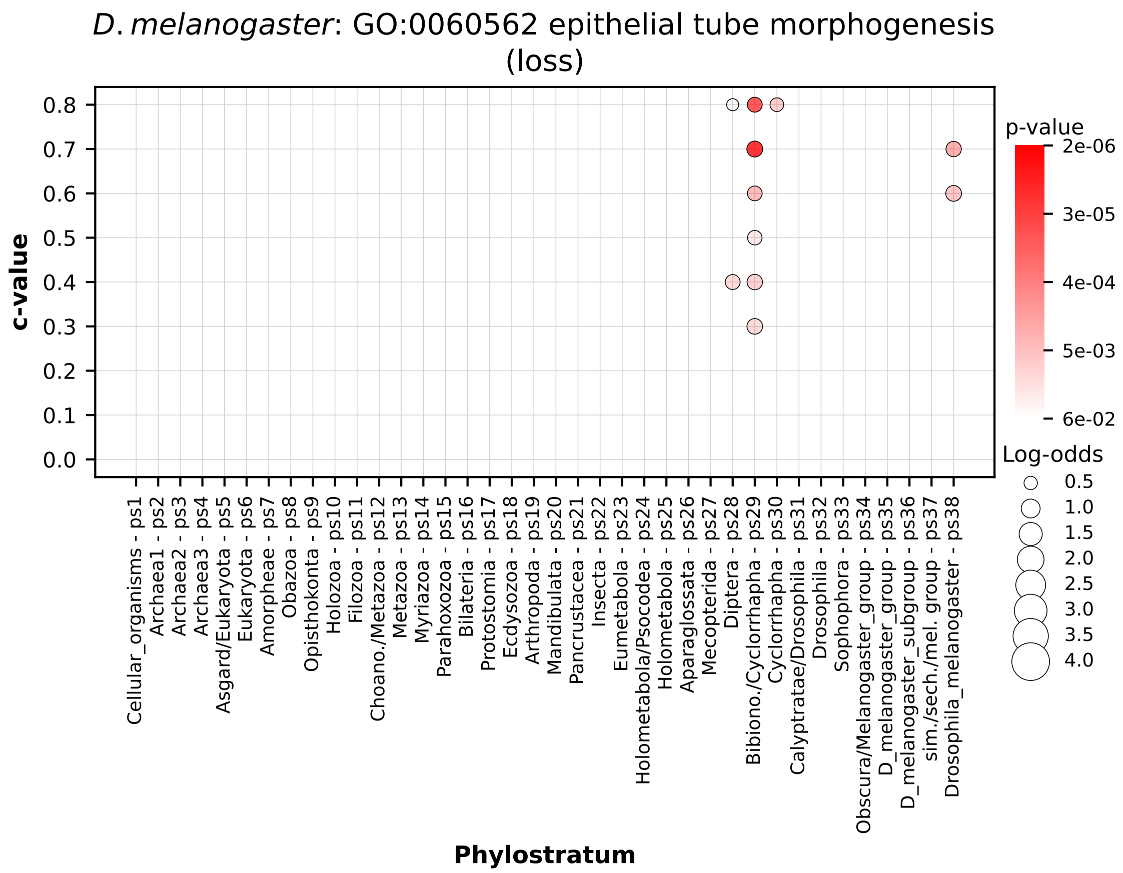

D. melanogaster: GO:0060562 epithelial tube morphogenesis (loss)
c-value
0.0
0.1
0.2
0.3
0.4
0.5
0.6
0.7
0.8
p-value
2e-06
3e-05
4e-04
5e-03
6e-02
Log-odds
0.5
1.0
1.5
2.0
2.5
3.0
3.5
4.0
Cellular_organisms - ps1
Archaea1 - ps2
Archaea2 - ps3
Archaea3 - ps4
Asgard/Eukaryota - ps5
Eukaryota - ps6
Amorpheae - ps7
Obazoa - ps8
Opisthokonta - ps9
Holozoa - ps10
Filozoa - ps11
Choano./Metazoa - ps12
Metazoa - ps13
Myriazoa - ps14
Parahoxozoa - ps15
Bilateria - ps16
Protostomia - ps17
Ecdysozoa - ps18
Arthropoda - ps19
Mandibulata - ps20
Pancrustacea - ps21
Insecta - ps22
Eumetabola - ps23
Holometabola/Psocodea - ps24
Holometabola - ps25
Aparaglossata - ps26
Mecopterida - ps27
Diptera - ps28
Bibiono./Cyclorrhapha - ps29
Cyclorrhapha - ps30
Calyptratae/Drosophila - ps31
Drosophila - ps32
Sophophora - ps33
Obscura/Melanogaster_group - ps34
D_melanogaster_group - ps35
D_melanogaster_subgroup - ps36
sim./sech./mel. group - ps37
Drosophila_melanogaster - ps38
Phylostratum

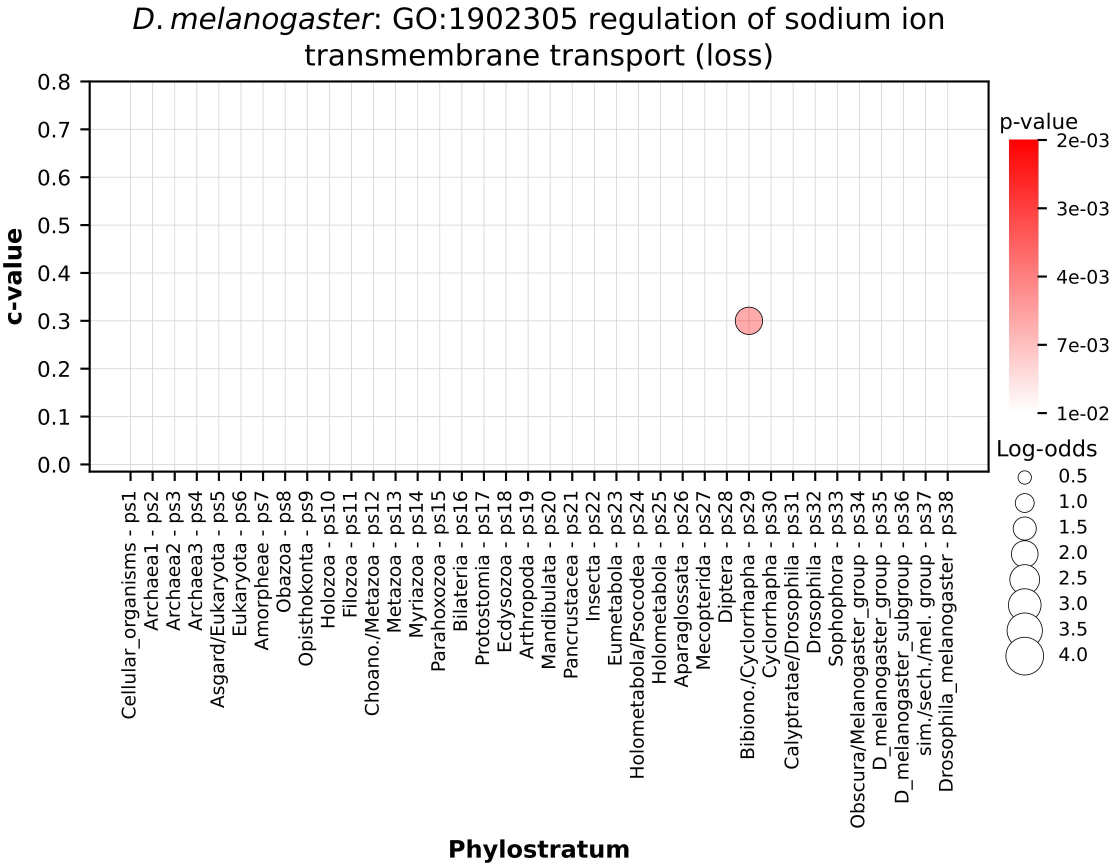

D. melanogaster: GO:1902305 regulation of sodium ion transmembrane transport (loss)
c-value
0.8
0.7
0.6
0.5
0.4
0.3
0.2
0.1
0.0
p-value
2e-03
3e-03
4e-03
7e-03
1e-02
Log-odds
0.5
1.0
1.5
2.0
2.5
3.0
3.5
4.0
Cellular_organisms - ps1
Archaea1 - ps2
Archaea2 - ps3
Archaea3 - ps4
Asgard/Eukaryota - ps5
Eukaryota - ps6
Amorpheae - ps7
Obazoa - ps8
Opisthokonta - ps9
Holozoa - ps10
Filozoa - ps11
Choano./Metazoa - ps12
Metazoa - ps13
Myriazoa - ps14
Parahoxozoa - ps15
Bilateria - ps16
Protostomia - ps17
Ecdysozoa - ps18
Arthropoda - ps19
Mandibulata - ps20
Pancrustacea - ps21
Insecta - ps22
Eumetabola - ps23
Holometabola/Psocodea - ps24
Holometabola - ps25
Aparaglossata - ps26
Mecopterida - ps27
Diptera - ps28
Bibiono./Cyclorrhapha - ps29
Cyclorrhapha - ps30
Calyptratae/Drosophila - ps31
Drosophila - ps32
Sophophora - ps33
Obscura/Melanogaster_group - ps34
D_melanogaster_group - ps35
D_melanogaster_subgroup - ps36
sim./sech./mel. group - ps37
Drosophila_melanogaster - ps38
Phylostratum

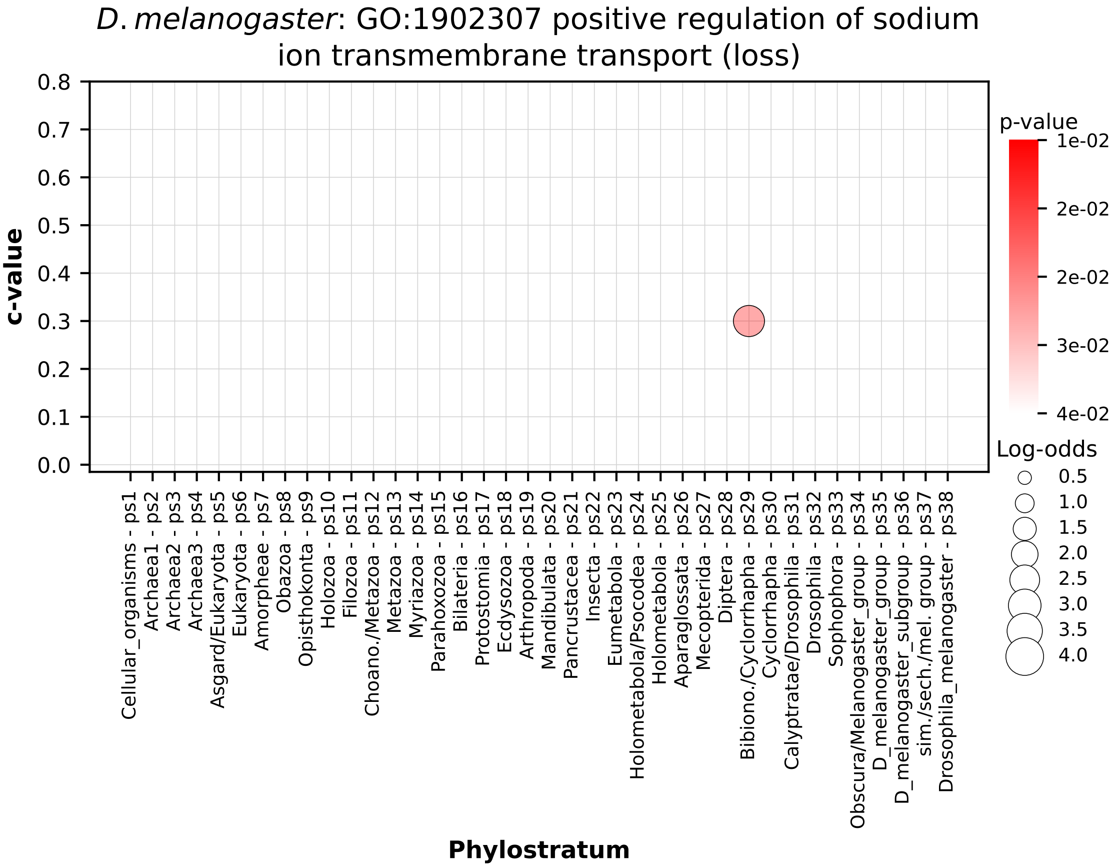

D. melanogaster: GO:1902307 positive regulation of sodium ion transmembrane transport (loss)
c-value
0.8
0.7
0.6
0.5
0.4
0.3
0.2
0.1
0.0
p-value
1e-02
2e-02
2e-02
3e-02
4e-02
Log-odds
0.5
1.0
1.5
2.0
2.5
3.0
3.5
4.0
Cellular_organisms - ps1
Archaea1 - ps2
Archaea2 - ps3
Archaea3 - ps4
Asgard/Eukaryota - ps5
Eukaryota - ps6
Amorpheae - ps7
Obazoa - ps8
Opisthokonta - ps9
Holozoa - ps10
Filozoa - ps11
Choano./Metazoa - ps12
Metazoa - ps13
Myriazoa - ps14
Parahoxozoa - ps15
Bilateria - ps16
Protostomia - ps17
Ecdysozoa - ps18
Arthropoda - ps19
Mandibulata - ps20
Pancrustacea - ps21
Insecta - ps22
Eumetabola - ps23
Holometabola/Psocodea - ps24
Holometabola - ps25
Aparaglossata - ps26
Mecopterida - ps27
Diptera - ps28
Bibiono./Cyclorrhapha - ps29
Cyclorrhapha - ps30
Calyptratae/Drosophila - ps31
Drosophila - ps32
Sophophora - ps33
Obscura/Melanogaster_group - ps34
D_melanogaster_group - ps35
D_melanogaster_subgroup - ps36
sim./sech./mel. group - ps37
Drosophila_melanogaster - ps38
Phylostratum

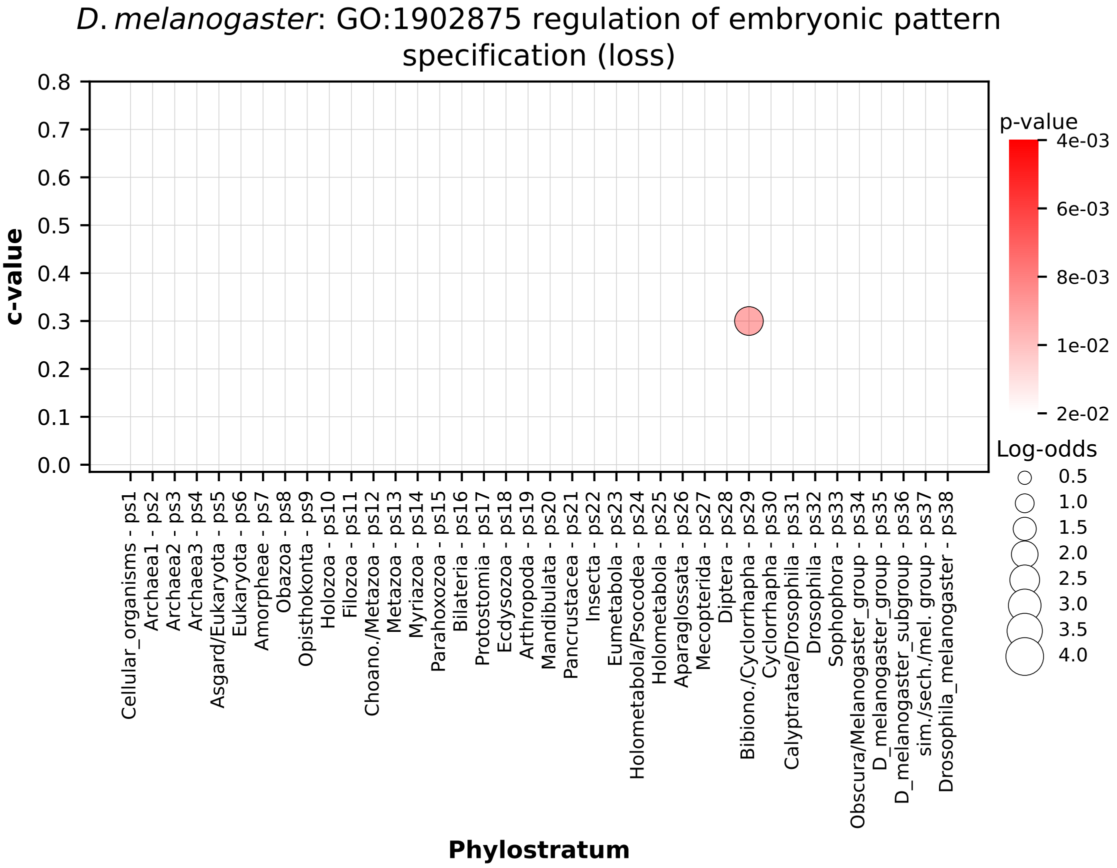

D. melanogaster: GO:1902875 regulation of embryonic pattern specification (loss)
c-value
0.8
0.7
0.6
0.5
0.4
0.3
0.2
0.1
0.0
Phylostratum
Cellular_organisms - ps1
Archaea1 - ps2
Archaea2 - ps3
Archaea3 - ps4
Asgard/Eukaryota - ps5
Eukaryota - ps6
Amorpheae - ps7
Obazoa - ps8
Opisthokonta - ps9
Holozoa - ps10
Filozoa - ps11
Choano./Metazoa - ps12
Metazoa - ps13
Myriazoa - ps14
Parahoxozoa - ps15
Bilateria - ps16
Protostomia - ps17
Ecdysozoa - ps18
Arthropoda - ps19
Mandibulata - ps20
Pancrustacea - ps21
Insecta - ps22
Eumetabola - ps23
Holometabola/Psocodea - ps24
Holometabola - ps25
Aparaglossata - ps26
Mecopterida - ps27
Diptera - ps28
Bibiono./Cyclorrhapha - ps29
Cyclorrhapha - ps30
Calyptratae/Drosophila - ps31
Drosophila - ps32
Sophophora - ps33
Obscura/Melanogaster_group - ps34
D_melanogaster_group - ps35
D_melanogaster_subgroup - ps36
sim./sech./mel. group - ps37
Drosophila_melanogaster - ps38
p-value
4e-03
6e-03
8e-03
1e-02
2e-02
Log-odds
0.5
1.0
1.5
2.0
2.5
3.0
3.5
4.0

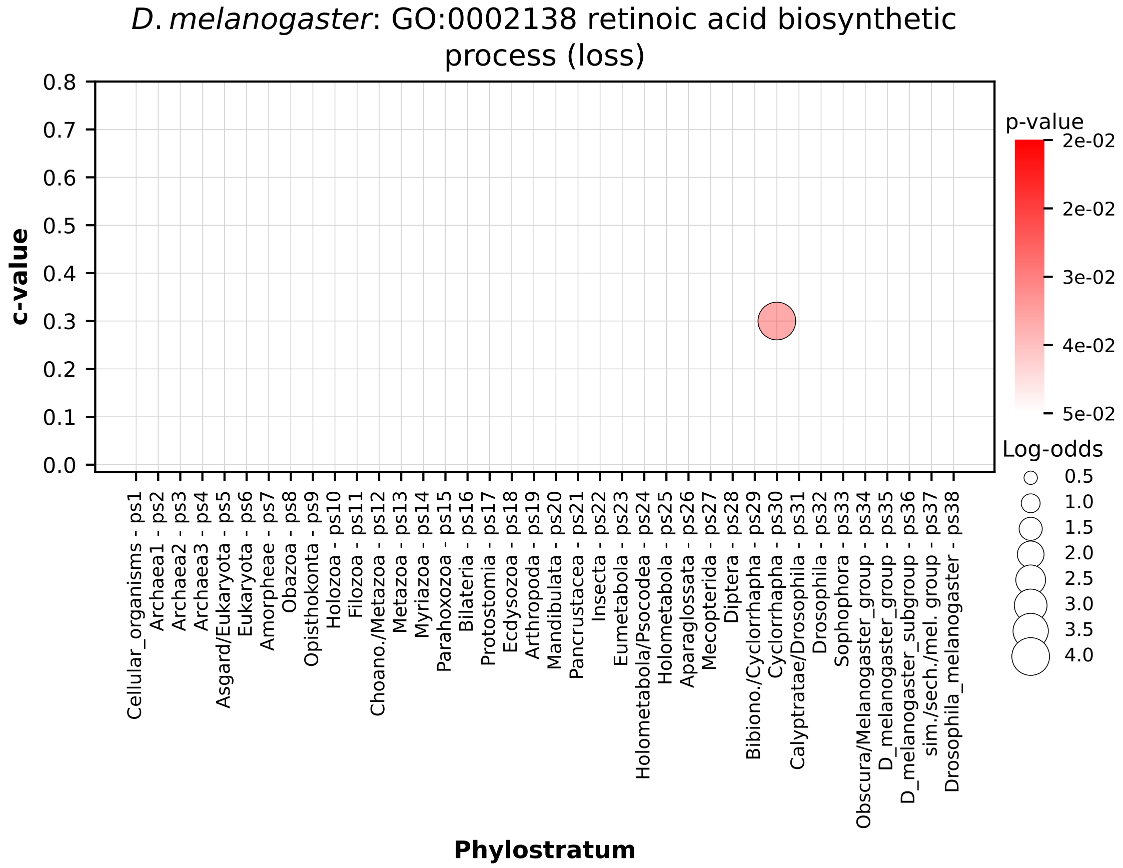

D. melanogaster: GO:0002138 retinoic acid biosynthetic process (loss)
c-value
0.8
0.7
0.6
0.5
0.4
0.3
0.2
0.1
0.0
p-value
2e-02
2e-02
3e-02
4e-02
5e-02
Log-odds
0.5
1.0
1.5
2.0
2.5
3.0
3.5
4.0
Cellular_organisms - ps1
Archaea1 - ps2
Archaea2 - ps3
Archaea3 - ps4
Asgard/Eukaryota - ps5
Eukaryota - ps6
Amorpheae - ps7
Obazoa - ps8
Opisthokonta - ps9
Holozoa - ps10
Filozoa - ps11
Choano./Metazoa - ps12
Metazoa - ps13
Myriazoa - ps14
Parahoxozoa - ps15
Bilateria - ps16
Protostomia - ps17
Ecdysozoa - ps18
Arthropoda - ps19
Mandibulata - ps20
Pancrustacea - ps21
Insecta - ps22
Eumetabola - ps23
Holometabola/Psocodea - ps24
Holometabola - ps25
Aparaglossata - ps26
Mecopterida - ps27
Diptera - ps28
Bibiono./Cyclorrhapha - ps29
Cyclorrhapha - ps30
Calyptratae/Drosophila - ps31
Drosophila - ps32
Sophophora - ps33
Obscura/Melanogaster_group - ps34
D_melanogaster_group - ps35
D_melanogaster_subgroup - ps36
sim./sech./mel. group - ps37
Drosophila_melanogaster - ps38
Phylostratum

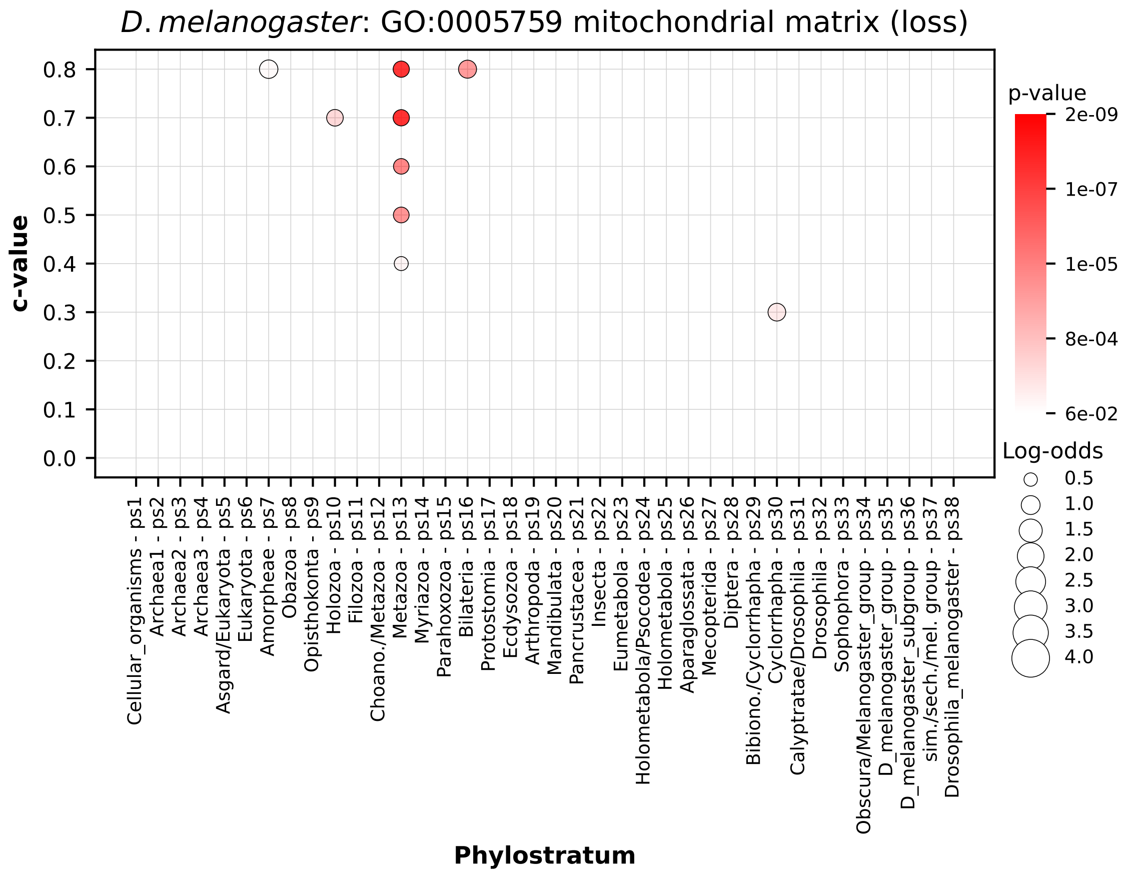

D. melanogaster: GO:0005759 mitochondrial matrix (loss)
c-value
0.0
0.1
0.2
0.3
0.4
0.5
0.6
0.7
0.8
Phylostratum
Cellular_organisms - ps1
Archaea1 - ps2
Archaea2 - ps3
Archaea3 - ps4
Asgard/Eukaryota - ps5
Eukaryota - ps6
Amorpheae - ps7
Obazoa - ps8
Opisthokonta - ps9
Holozoa - ps10
Filozoa - ps11
Choano./Metazoa - ps12
Metazoa - ps13
Myriazoa - ps14
Parahoxozoa - ps15
Bilateria - ps16
Protostomia - ps17
Ecdysozoa - ps18
Arthropoda - ps19
Mandibulata - ps20
Pancrustacea - ps21
Insecta - ps22
Eumetabola - ps23
Holometabola/Psocodea - ps24
Holometabola - ps25
Aparaglossata - ps26
Mecopterida - ps27
Diptera - ps28
Bibiono./Cyclorrhapha - ps29
Cyclorrhapha - ps30
Calyptratae/Drosophila - ps31
Drosophila - ps32
Sophophora - ps33
Obscura/Melanogaster_group - ps34
D_melanogaster_group - ps35
D_melanogaster_subgroup - ps36
sim./sech./mel. group - ps37
Drosophila_melanogaster - ps38
p-value
2e-09
1e-07
1e-05
8e-04
6e-02
Log-odds
0.5
1.0
1.5
2.0
2.5
3.0
3.5
4.0

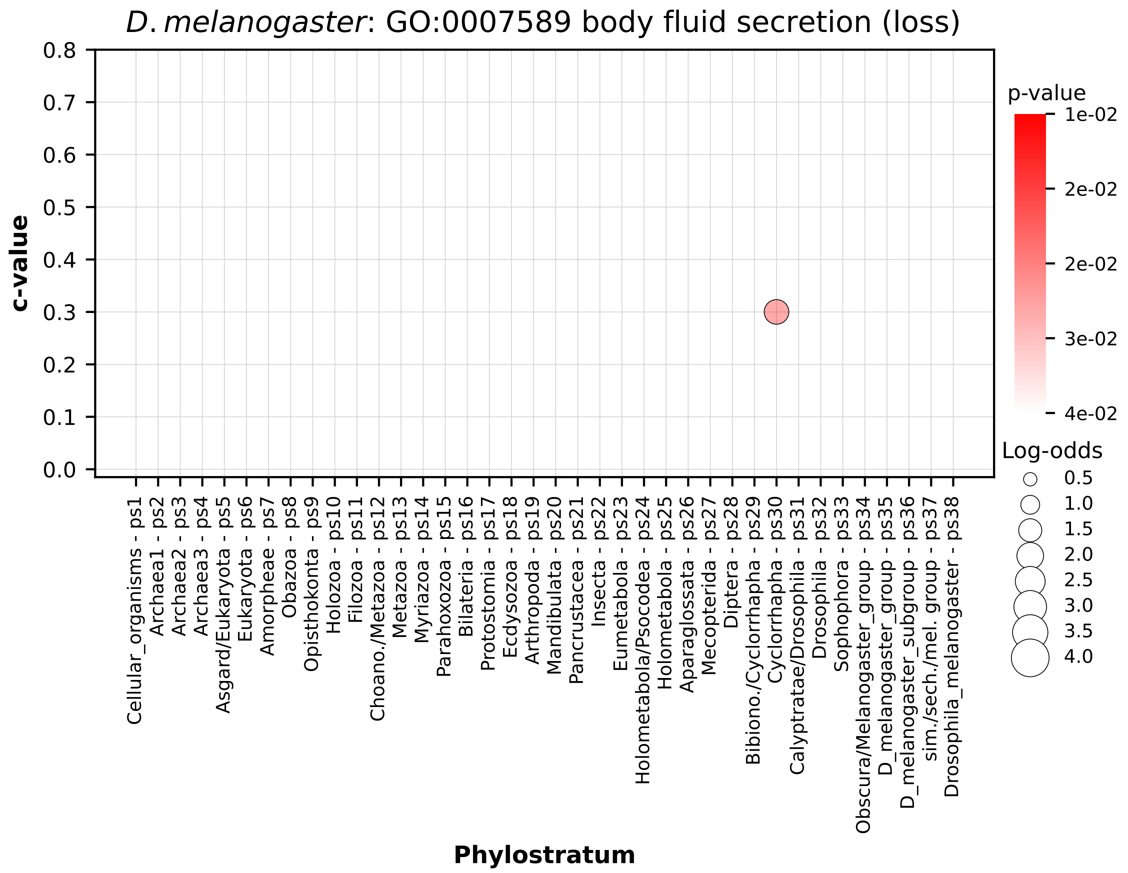
D. melanogaster: GO:0007589 body fluid secretion (loss)
c-value
0.8
0.7
0.6
0.5
0.4
0.3
0.2
0.1
0.0
p-value
1e-02
2e-02
2e-02
3e-02
4e-02
Log-odds
0.5
1.0
1.5
2.0
2.5
3.0
3.5
4.0
Cellular_organisms - ps1
Archaea1 - ps2
Archaea2 - ps3
Archaea3 - ps4
Asgard/Eukaryota - ps5
Eukaryota - ps6
Amorpheae - ps7
Obazoa - ps8
Opisthokonta - ps9
Holozoa - ps10
Filozoa - ps11
Choano./Metazoa - ps12
Metazoa - ps13
Myriazoa - ps14
Parahoxozoa - ps15
Bilateria - ps16
Protostomia - ps17
Ecdysozoa - ps18
Arthropoda - ps19
Mandibulata - ps20
Pancrustacea - ps21
Insecta - ps22
Eumetabola - ps23
Holometabola/Psocodea - ps24
Holometabola - ps25
Aparaglossata - ps26
Mecopterida - ps27
Diptera - ps28
Bibiono./Cyclorrhapha - ps29
Cyclorrhapha - ps30
Calyptratae/Drosophila - ps31
Drosophila - ps32
Sophophora - ps33
Obscura/Melanogaster_group - ps34
D_melanogaster_group - ps35
D_melanogaster_subgroup - ps36
sim./sech./mel. group - ps37
Drosophila_melanogaster - ps38
Phylostratum

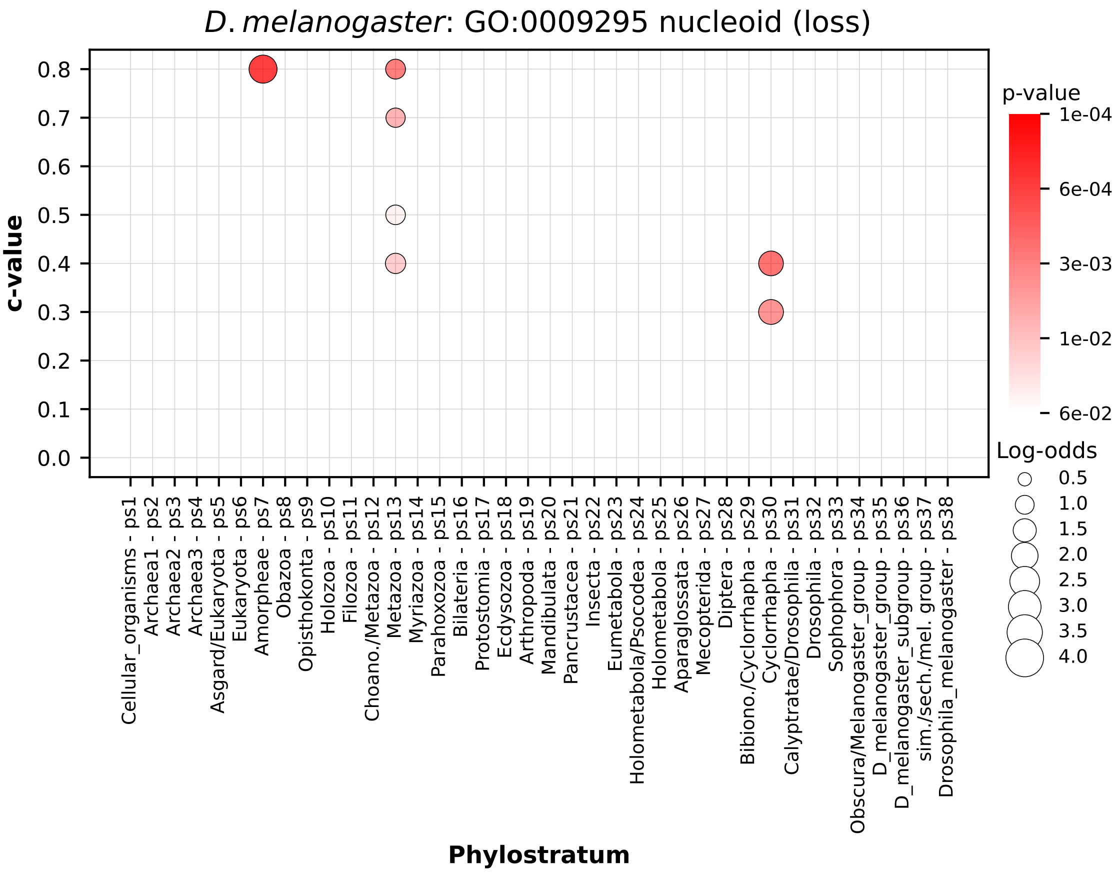

D. melanogaster: GO:0009295 nucleoid (loss)
c-value
0.0
0.1
0.2
0.3
0.4
0.5
0.6
0.7
0.8
Phylostratum
Cellular_organisms - ps1
Archaea1 - ps2
Archaea2 - ps3
Archaea3 - ps4
Asgard/Eukaryota - ps5
Eukaryota - ps6
Amorpheae - ps7
Obazoa - ps8
Opisthokonta - ps9
Holozoa - ps10
Filozoa - ps11
Choano./Metazoa - ps12
Metazoa - ps13
Myriazoa - ps14
Parahoxozoa - ps15
Bilateria - ps16
Protostomia - ps17
Ecdysozoa - ps18
Arthropoda - ps19
Mandibulata - ps20
Pancrustacea - ps21
Insecta - ps22
Eumetabola - ps23
Holometabola/Psocodea - ps24
Holometabola - ps25
Aparaglossata - ps26
Mecopterida - ps27
Diptera - ps28
Bibiono./Cyclorrhapha - ps29
Cyclorrhapha - ps30
Calyptratae/Drosophila - ps31
Drosophila - ps32
Sophophora - ps33
Obscura/Melanogaster_group - ps34
D_melanogaster_group - ps35
D_melanogaster_subgroup - ps36
sim./sech./mel. group - ps37
Drosophila_melanogaster - ps38
p-value
1e-04
6e-04
3e-03
1e-02
6e-02
Log-odds
0.5
1.0
1.5
2.0
2.5
3.0
3.5
4.0

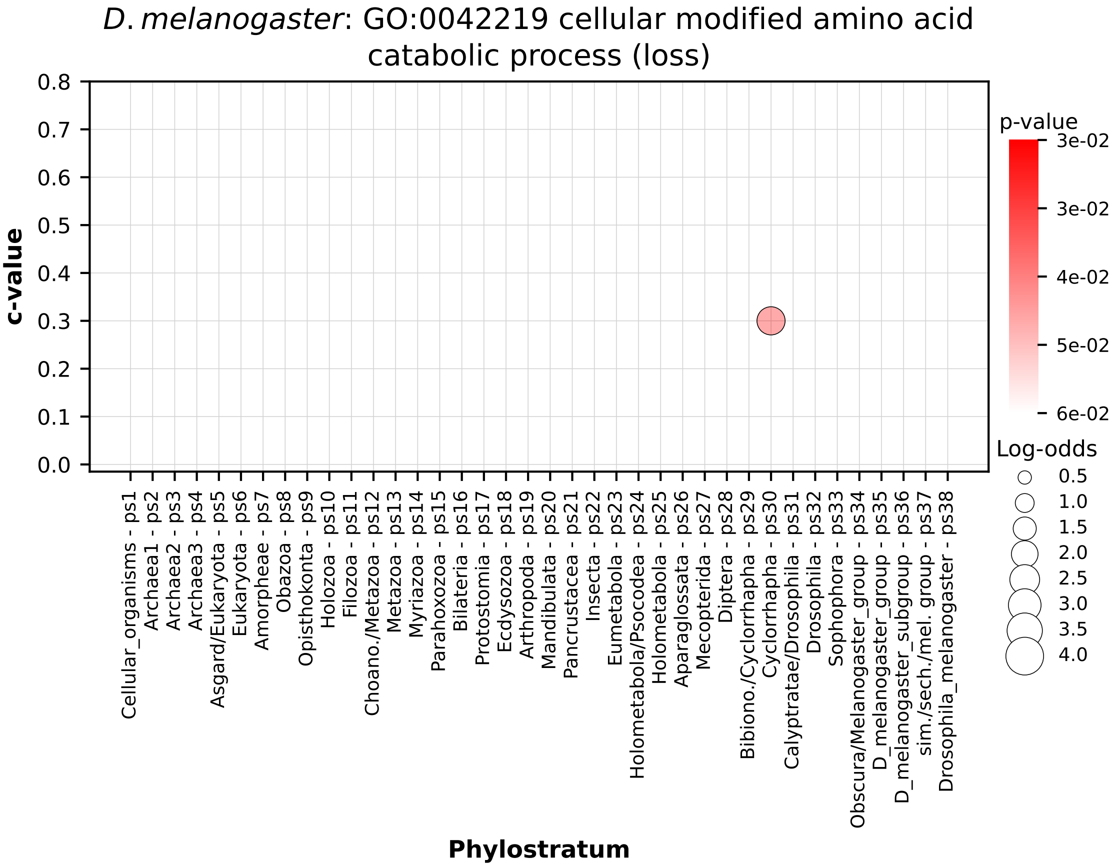
D. melanogaster: GO:0042219 cellular modified amino acid catabolic process (loss)
c-value
0.0
0.1
0.2
0.3
0.4
0.5
0.6
0.7
0.8
Phylostratum
Cellular_organisms - ps1
Archaea1 - ps2
Archaea2 - ps3
Archaea3 - ps4
Asgard/Eukaryota - ps5
Eukaryota - ps6
Amorpheae - ps7
Obazoa - ps8
Opisthokonta - ps9
Holozoa - ps10
Filozoa - ps11
Choano./Metazoa - ps12
Metazoa - ps13
Myriazoa - ps14
Parahoxozoa - ps15
Bilateria - ps16
Protostomia - ps17
Ecdysozoa - ps18
Arthropoda - ps19
Mandibulata - ps20
Pancrustacea - ps21
Insecta - ps22
Eumetabola - ps23
Holometabola/Psocodea - ps24
Holometabola - ps25
Aparaglossata - ps26
Mecopterida - ps27
Diptera - ps28
Bibiono./Cyclorrhapha - ps29
Cyclorrhapha - ps30
Calyptratae/Drosophila - ps31
Drosophila - ps32
Sophophora - ps33
Obscura/Melanogaster_group - ps34
D_melanogaster_group - ps35
D_melanogaster_subgroup - ps36
sim./sech./mel. group - ps37
Drosophila_melanogaster - ps38
p-value
3e-02
3e-02
4e-02
5e-02
6e-02
Log-odds
0.5
1.0
1.5
2.0
2.5
3.0
3.5
4.0

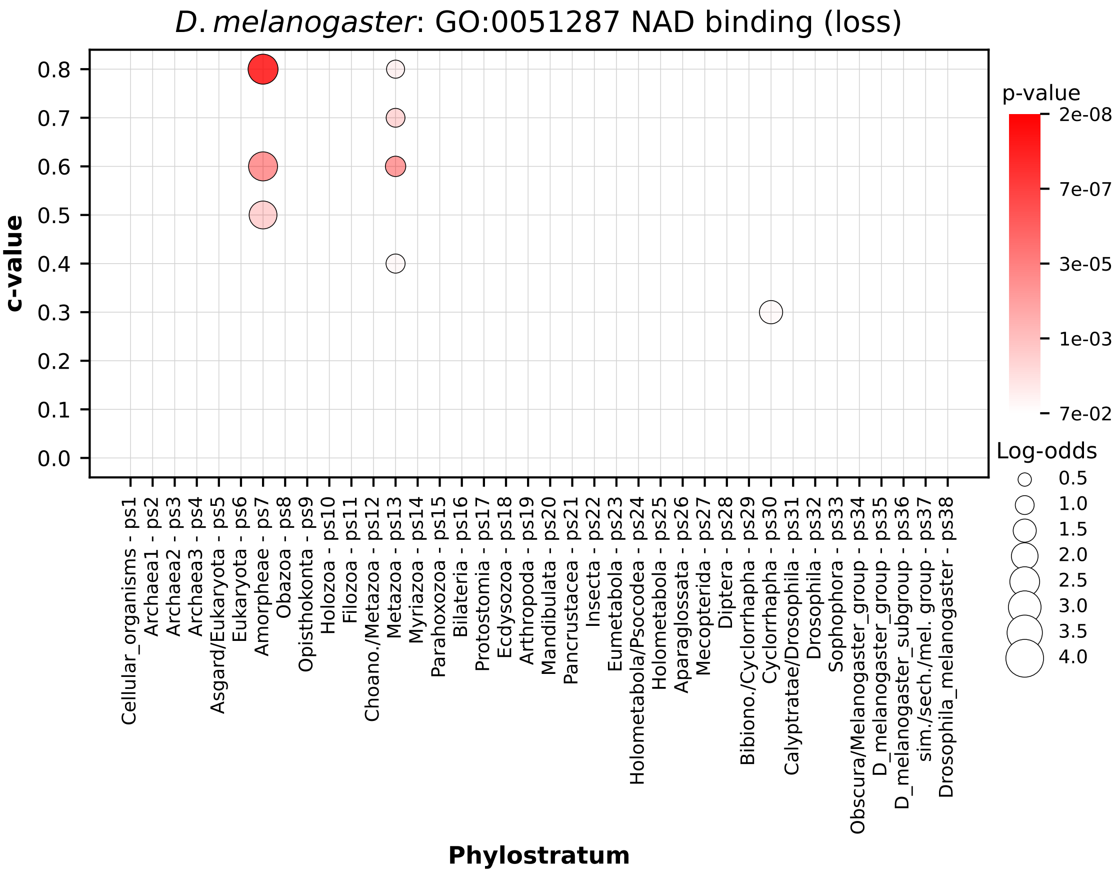

D. melanogaster: GO:0051287 NAD binding (loss)
c-value
0.0
0.1
0.2
0.3
0.4
0.5
0.6
0.7
0.8
Phylostratum
Cellular_organisms - ps1
Archaea1 - ps2
Archaea2 - ps3
Archaea3 - ps4
Asgard/Eukaryota - ps5
Eukaryota - ps6
Amorpheae - ps7
Obazoa - ps8
Opisthokonta - ps9
Holozoa - ps10
Filozoa - ps11
Choano./Metazoa - ps12
Metazoa - ps13
Myriazoa - ps14
Parahoxozoa - ps15
Bilateria - ps16
Protostomia - ps17
Ecdysozoa - ps18
Arthropoda - ps19
Mandibulata - ps20
Pancrustacea - ps21
Insecta - ps22
Eumetabola - ps23
Holometabola/Psocodea - ps24
Holometabola - ps25
Aparaglossata - ps26
Mecopterida - ps27
Diptera - ps28
Bibiono./Cyclorrhapha - ps29
Cyclorrhapha - ps30
Calyptratae/Drosophila - ps31
Drosophila - ps32
Sophophora - ps33
Obscura/Melanogaster_group - ps34
D_melanogaster_group - ps35
D_melanogaster_subgroup - ps36
sim./sech./mel. group - ps37
Drosophila_melanogaster - ps38
p-value
2e-08
7e-07
3e-05
1e-03
7e-02
Log-odds
0.5
1.0
1.5
2.0
2.5
3.0
3.5
4.0

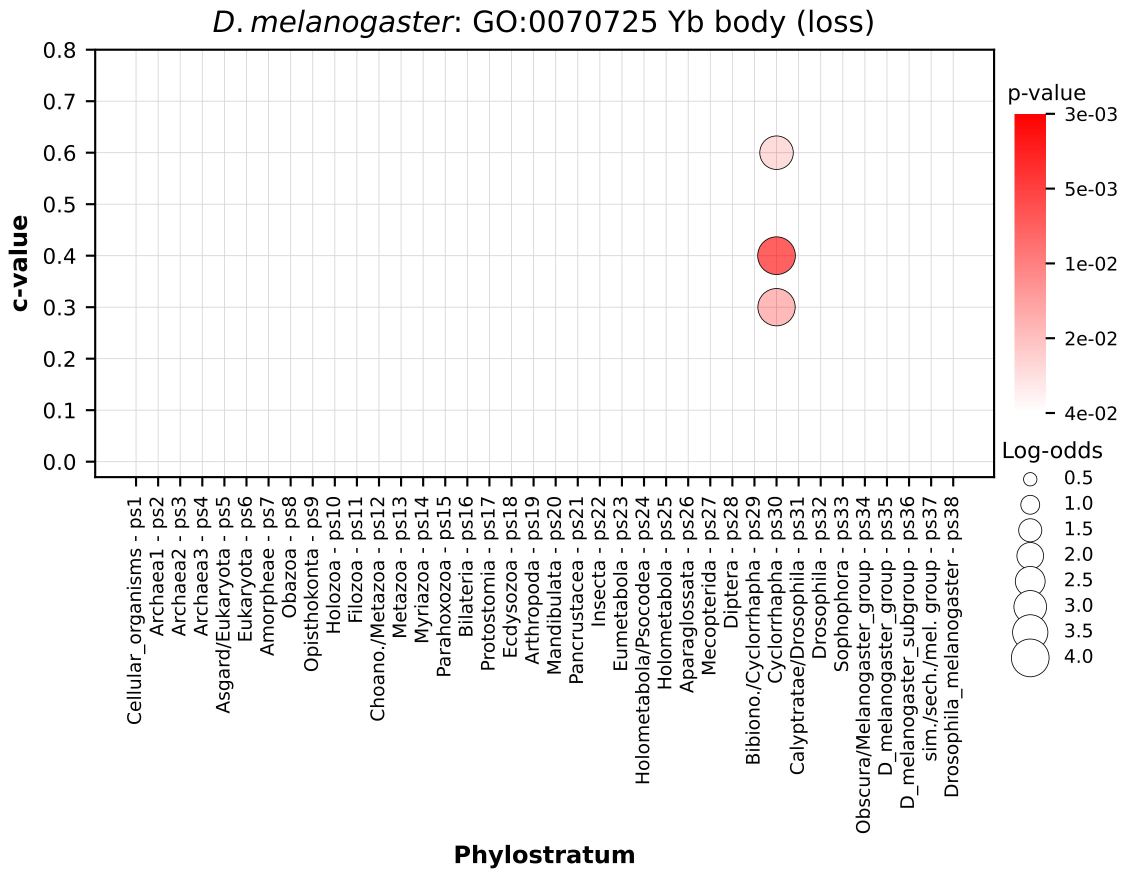

D. melanogaster: GO:0070725 Yb body (loss)
c-value
0.0
0.1
0.2
0.3
0.4
0.5
0.6
0.7
0.8
Phylostratum
Cellular_organisms - ps1
Archaea1 - ps2
Archaea2 - ps3
Archaea3 - ps4
Asgard/Eukaryota - ps5
Eukaryota - ps6
Amorpheae - ps7
Obazoa - ps8
Opisthokonta - ps9
Holozoa - ps10
Filozoa - ps11
Choano./Metazoa - ps12
Metazoa - ps13
Myriazoa - ps14
Parahoxozoa - ps15
Bilateria - ps16
Protostomia - ps17
Ecdysozoa - ps18
Arthropoda - ps19
Mandibulata - ps20
Pancrustacea - ps21
Insecta - ps22
Eumetabola - ps23
Holometabola/Psocodea - ps24
Holometabola - ps25
Aparaglossata - ps26
Mecopterida - ps27
Diptera - ps28
Bibiono./Cyclorrhapha - ps29
Cyclorrhapha - ps30
Calyptratae/Drosophila - ps31
Drosophila - ps32
Sophophora - ps33
Obscura/Melanogaster_group - ps34
D_melanogaster_group - ps35
D_melanogaster_subgroup - ps36
sim./sech./mel. group - ps37
Drosophila_melanogaster - ps38
p-value
3e-03
5e-03
1e-02
2e-02
4e-02
Log-odds
0.5
1.0
1.5
2.0
2.5
3.0
3.5
4.0

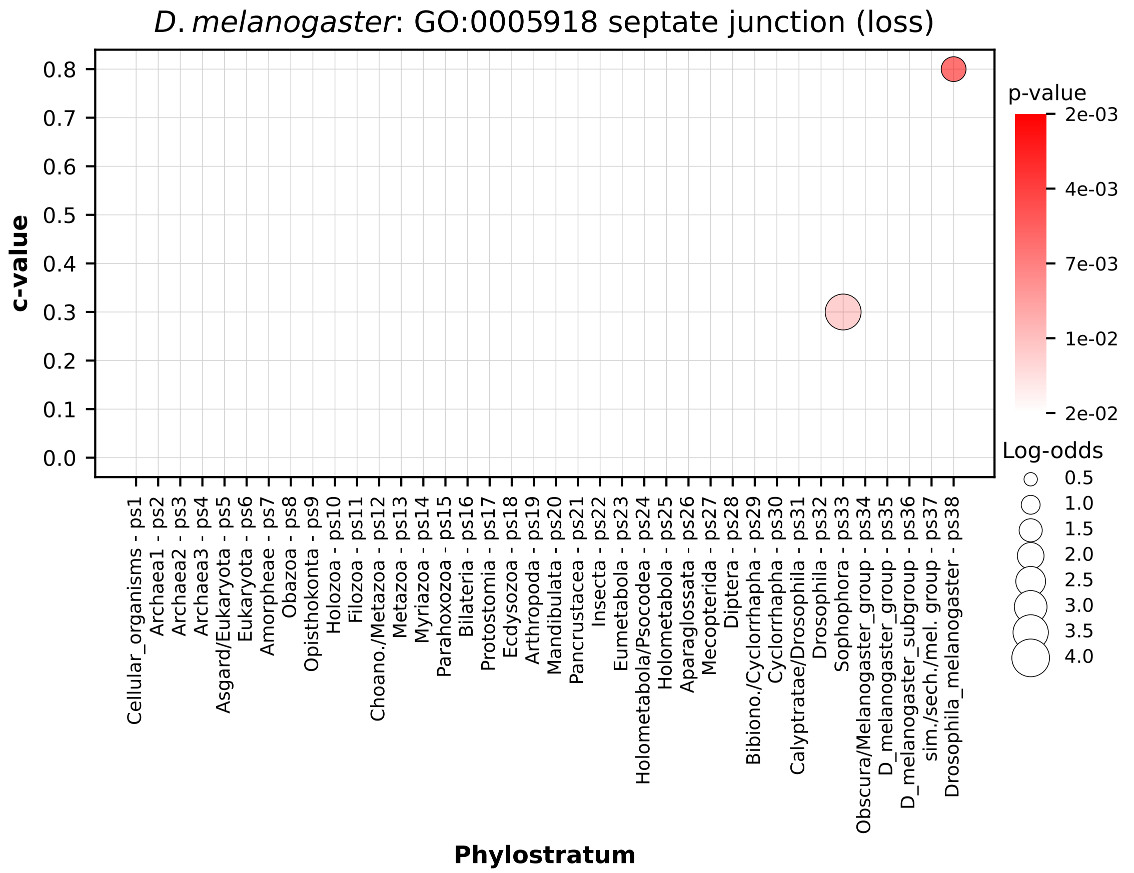

D. melanogaster: GO:0005918 septate junction (loss)
c-value
0.0
0.1
0.2
0.3
0.4
0.5
0.6
0.7
0.8
p-value
2e-03
4e-03
7e-03
1e-02
2e-02
Log-odds
0.5
1.0
1.5
2.0
2.5
3.0
3.5
4.0
Cellular_organisms - ps1
Archaea1 - ps2
Archaea2 - ps3
Archaea3 - ps4
Asgard/Eukaryota - ps5
Eukaryota - ps6
Amorpheae - ps7
Obazoa - ps8
Opisthokonta - ps9
Holozoa - ps10
Filozoa - ps11
Choano./Metazoa - ps12
Metazoa - ps13
Myriazoa - ps14
Parahoxozoa - ps15
Bilateria - ps16
Protostomia - ps17
Ecdysozoa - ps18
Arthropoda - ps19
Mandibulata - ps20
Pancrustacea - ps21
Insecta - ps22
Eumetabola - ps23
Holometabola/Psocodea - ps24
Holometabola - ps25
Aparaglossata - ps26
Mecopterida - ps27
Diptera - ps28
Bibiono./Cyclorrhapha - ps29
Cyclorrhapha - ps30
Calyptratae/Drosophila - ps31
Drosophila - ps32
Sophophora - ps33
Obscura/Melanogaster_group - ps34
D_melanogaster_group - ps35
D_melanogaster_subgroup - ps36
sim./sech./mel. group - ps37
Drosophila_melanogaster - ps38
Phylostratum

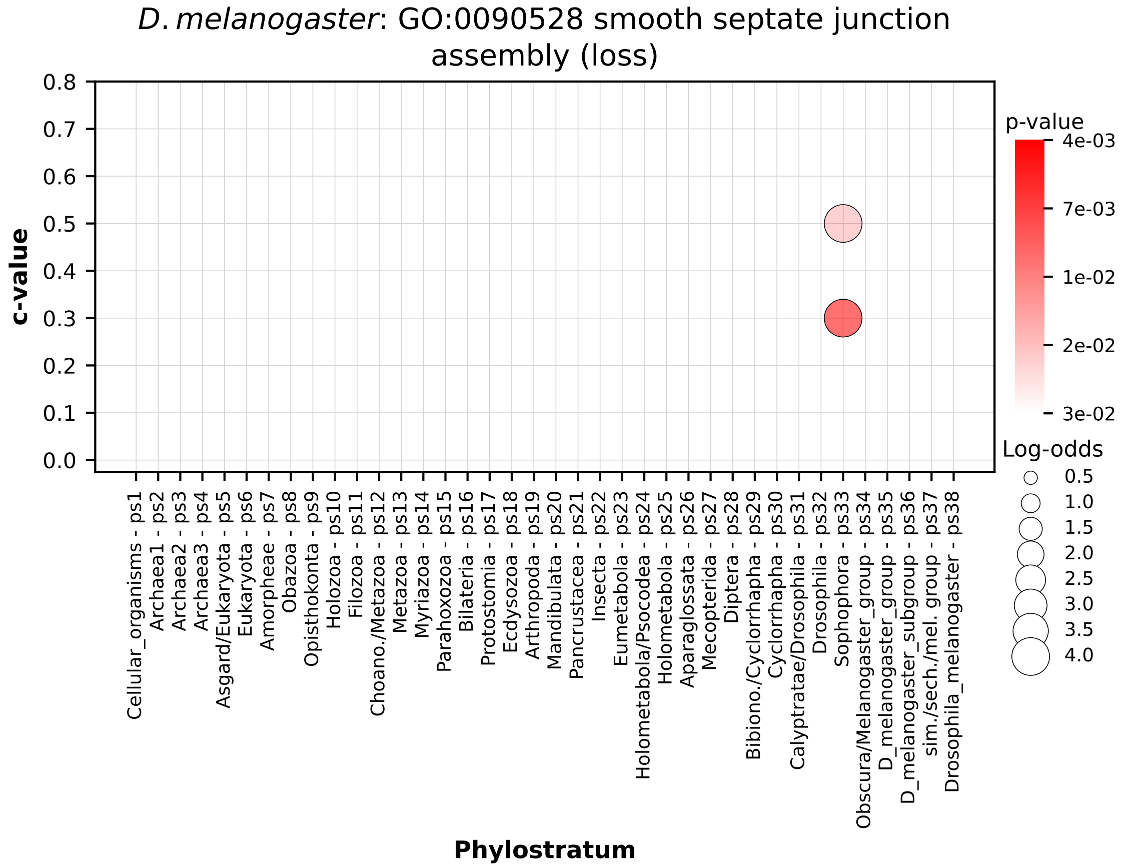

D. melanogaster: GO:0090528 smooth septate junction assembly (loss)
c-value
0.8
0.7
0.6
0.5
0.4
0.3
0.2
0.1
0.0
Phylostratum
Cellular_organisms - ps1
Archaea1 - ps2
Archaea2 - ps3
Archaea3 - ps4
Asgard/Eukaryota - ps5
Eukaryota - ps6
Amorpheae - ps7
Obazoa - ps8
Opisthokonta - ps9
Holozoa - ps10
Filozoa - ps11
Choano./Metazoa - ps12
Metazoa - ps13
Myriazoa - ps14
Parahoxozoa - ps15
Bilateria - ps16
Protostomia - ps17
Ecdysozoa - ps18
Arthropoda - ps19
Mandibulata - ps20
Pancrustacea - ps21
Insecta - ps22
Eumetabola - ps23
Holometabola/Psocodea - ps24
Holometabola - ps25
Aparaglossata - ps26
Mecopterida - ps27
Diptera - ps28
Bibiono./Cyclorrhapha - ps29
Cyclorrhapha - ps30
Calyptratae/Drosophila - ps31
Drosophila - ps32
Sophophora - ps33
Obscura/Melanogaster_group - ps34
D_melanogaster_group - ps35
D_melanogaster_subgroup - ps36
sim./sech./mel. group - ps37
Drosophila_melanogaster - ps38
p-value
4e-03
7e-03
1e-02
2e-02
3e-02
Log-odds
0.5
1.0
1.5
2.0
2.5
3.0
3.5
4.0

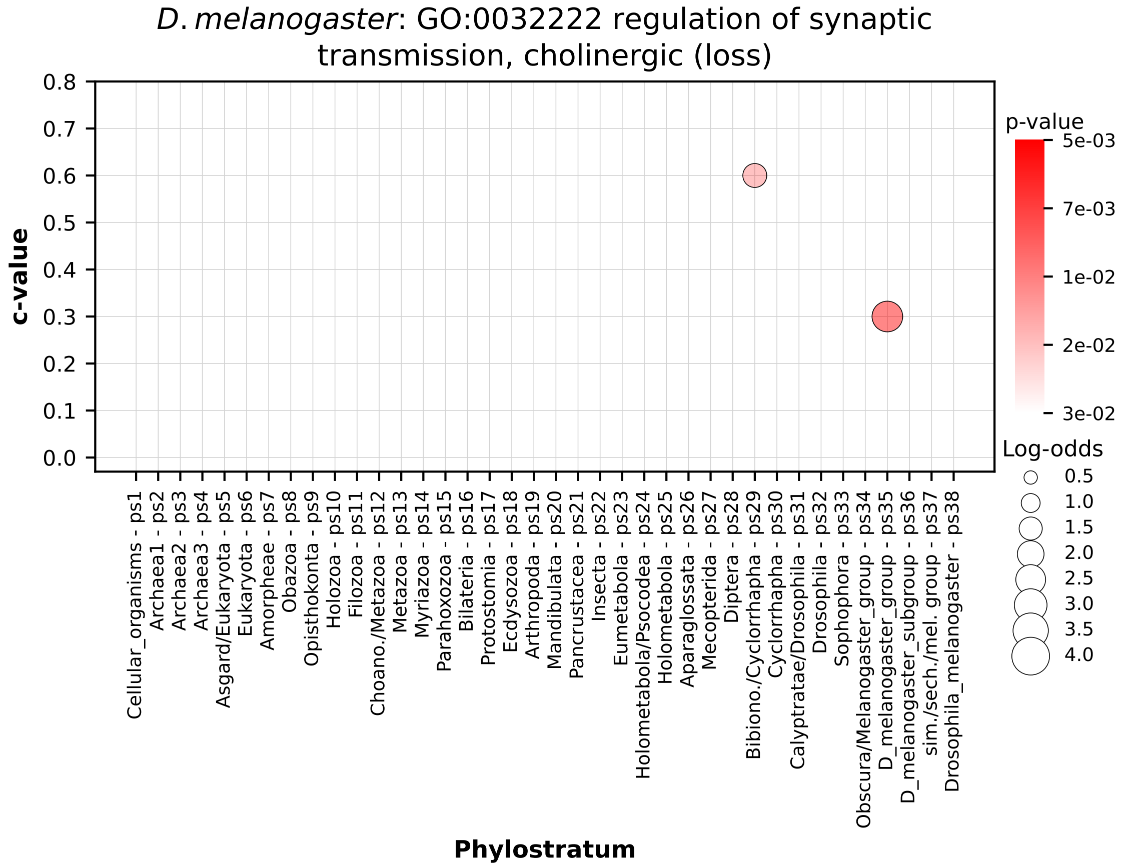

D. melanogaster: GO:0032222 regulation of synaptic transmission, cholinergic (loss)
c-value
0.8
0.7
0.6
0.5
0.4
0.3
0.2
0.1
0.0
p-value
5e-03
7e-03
1e-02
2e-02
3e-02
Log-odds
0.5
1.0
1.5
2.0
2.5
3.0
3.5
4.0
Cellular_organisms - ps1
Archaea1 - ps2
Archaea2 - ps3
Archaea3 - ps4
Asgard/Eukaryota - ps5
Eukaryota - ps6
Amorpheae - ps7
Obazoa - ps8
Opisthokonta - ps9
Holozoa - ps10
Filozoa - ps11
Choano./Metazoa - ps12
Metazoa - ps13
Myriazoa - ps14
Parahoxozoa - ps15
Bilateria - ps16
Protostomia - ps17
Ecdysozoa - ps18
Arthropoda - ps19
Mandibulata - ps20
Pancrustacea - ps21
Insecta - ps22
Eumetabola - ps23
Holometabola/Psocodea - ps24
Holometabola - ps25
Aparaglossata - ps26
Mecopterida - ps27
Diptera - ps28
Bibiono./Cyclorrhapha - ps29
Cyclorrhapha - ps30
Calyptratae/Drosophila - ps31
Drosophila - ps32
Sophophora - ps33
Obscura/Melanogaster_group - ps34
D_melanogaster_group - ps35
D_melanogaster_subgroup - ps36
sim./sech./mel. group - ps37
Drosophila_melanogaster - ps38
Phylostratum

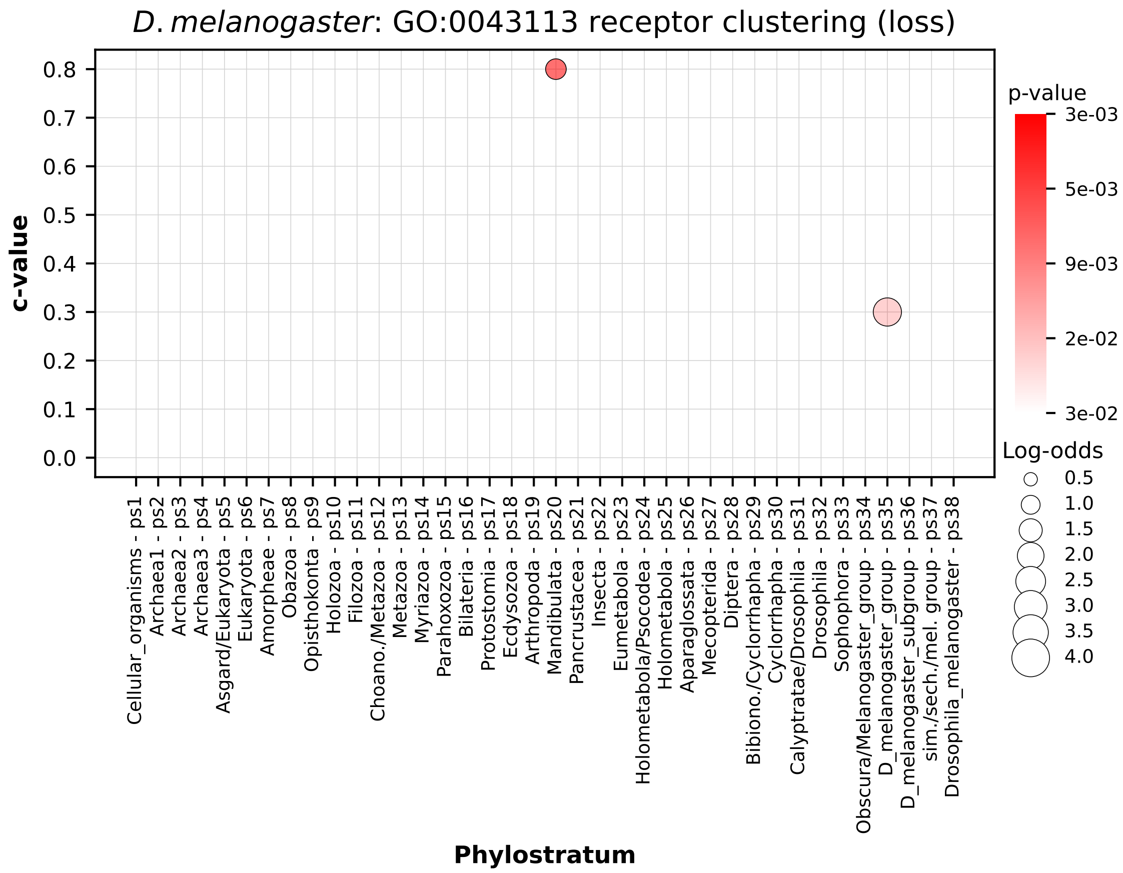

D. melanogaster: GO:0043113 receptor clustering (loss)
c-value
0.0
0.1
0.2
0.3
0.4
0.5
0.6
0.7
0.8
p-value
3e-03
5e-03
9e-03
2e-02
3e-02
Log-odds
0.5
1.0
1.5
2.0
2.5
3.0
3.5
4.0
Cellular_organisms - ps1
Archaea1 - ps2
Archaea2 - ps3
Archaea3 - ps4
Asgard/Eukaryota - ps5
Eukaryota - ps6
Amorpheae - ps7
Obazoa - ps8
Opisthokonta - ps9
Holozoa - ps10
Filozoa - ps11
Choano./Metazoa - ps12
Metazoa - ps13
Myriazoa - ps14
Parahoxozoa - ps15
Bilateria - ps16
Protostomia - ps17
Ecdysozoa - ps18
Arthropoda - ps19
Mandibulata - ps20
Pancrustacea - ps21
Insecta - ps22
Eumetabola - ps23
Holometabola/Psocodea - ps24
Holometabola - ps25
Aparaglossata - ps26
Mecopterida - ps27
Diptera - ps28
Bibiono./Cyclorrhapha - ps29
Cyclorrhapha - ps30
Calyptratae/Drosophila - ps31
Drosophila - ps32
Sophophora - ps33
Obscura/Melanogaster_group - ps34
D_melanogaster_group - ps35
D_melanogaster_subgroup - ps36
sim./sech./mel. group - ps37
Drosophila_melanogaster - ps38
Phylostratum

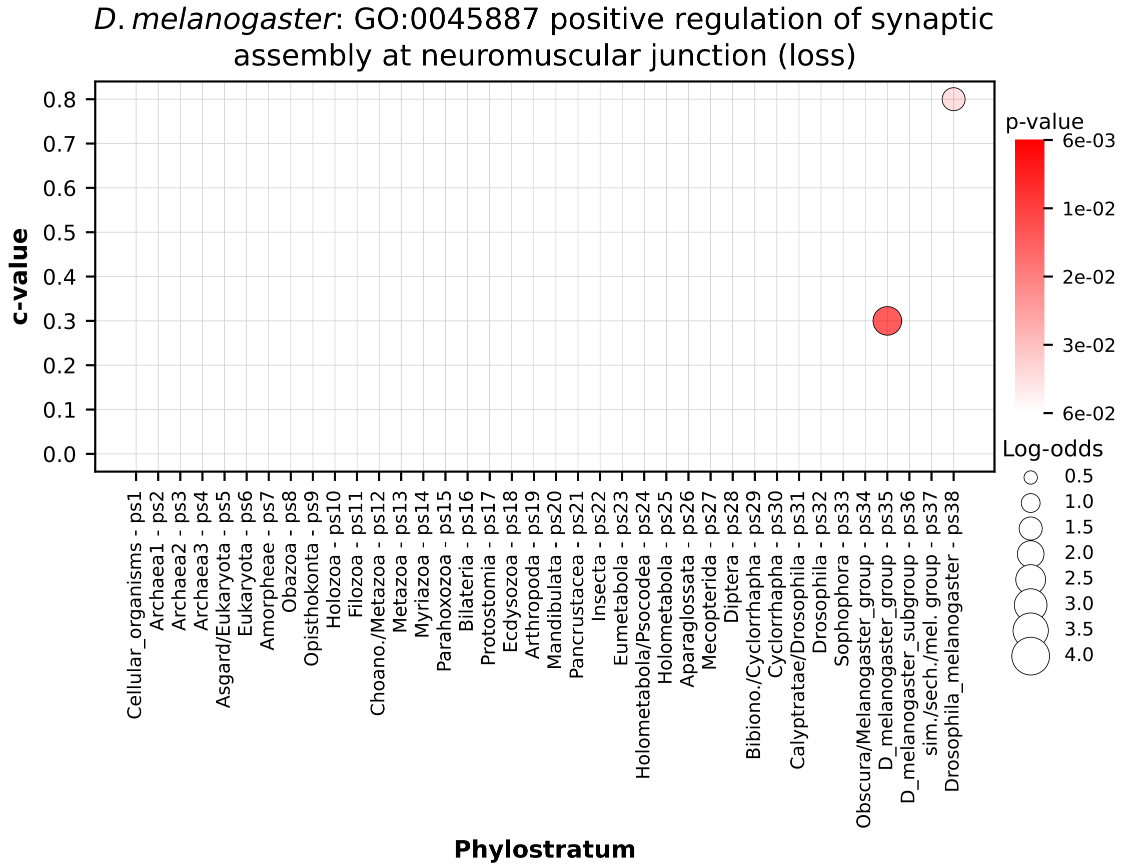

D. melanogaster: GO:0045887 positive regulation of synaptic assembly at neuromuscular junction (loss)
c-value
0.8
0.7
0.6
0.5
0.4
0.3
0.2
0.1
0.0
p-value
6e-03
1e-02
2e-02
3e-02
6e-02
Log-odds
0.5
1.0
1.5
2.0
2.5
3.0
3.5
4.0
Cellular_organisms - ps1
Archaea1 - ps2
Archaea2 - ps3
Archaea3 - ps4
Asgard/Eukaryota - ps5
Eukaryota - ps6
Amorpheae - ps7
Obazoa - ps8
Opisthokonta - ps9
Holozoa - ps10
Filozoa - ps11
Choano./Metazoa - ps12
Metazoa - ps13
Myriazoa - ps14
Parahoxozoa - ps15
Bilateria - ps16
Protostomia - ps17
Ecdysozoa - ps18
Arthropoda - ps19
Mandibulata - ps20
Pancrustacea - ps21
Insecta - ps22
Eumetabola - ps23
Holometabola/Psocodea - ps24
Holometabola - ps25
Aparaglossata - ps26
Mecopterida - ps27
Diptera - ps28
Bibiono./Cyclorrhapha - ps29
Cyclorrhapha - ps30
Calyptratae/Drosophila - ps31
Drosophila - ps32
Sophophora - ps33
Obscura/Melanogaster_group - ps34
D_melanogaster_group - ps35
D_melanogaster_subgroup - ps36
sim./sech./mel. group - ps37
Drosophila_melanogaster - ps38
Phylostratum

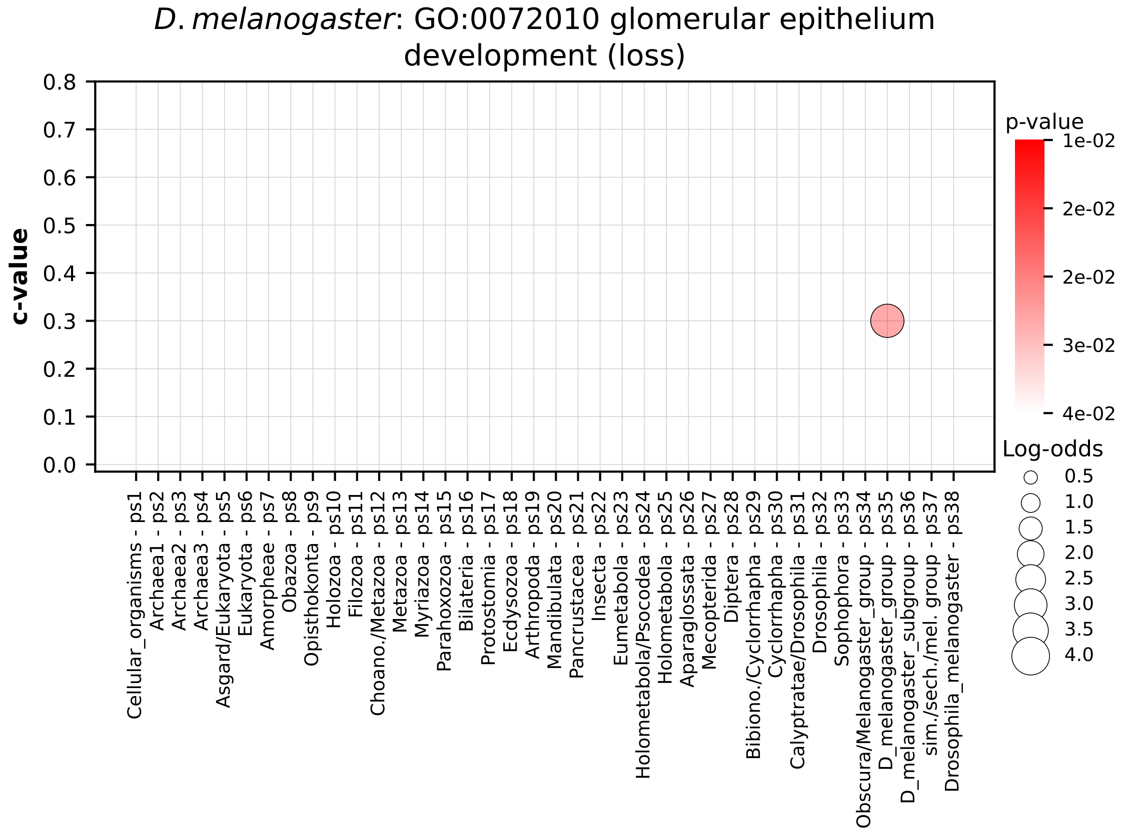
D. melanogaster: GO:0072010 glomerular epithelium development (loss)
c-value
0.0
0.1
0.2
0.3
0.4
0.5
0.6
0.7
0.8
p-value
1e-02
2e-02
2e-02
3e-02
4e-02
Log-odds
0.5
1.0
1.5
2.0
2.5
3.0
3.5
4.0
Cellular_organisms - ps1
Archaea1 - ps2
Archaea2 - ps3
Archaea3 - ps4
Asgard/Eukaryota - ps5
Eukaryota - ps6
Amorpheae - ps7
Obazoa - ps8
Opisthokonta - ps9
Holozoa - ps10
Filozoa - ps11
Choano./Metazoa - ps12
Metazoa - ps13
Myriazoa - ps14
Parahoxozoa - ps15
Bilateria - ps16
Protostomia - ps17
Ecdysozoa - ps18
Arthropoda - ps19
Mandibulata - ps20
Pancrustacea - ps21
Insecta - ps22
Eumetabola - ps23
Holometabola/Psocodea - ps24
Holometabola - ps25
Aparaglossata - ps26
Mecopterida - ps27
Diptera - ps28
Bibiono./Cyclorrhapha - ps29
Cyclorrhapha - ps30
Calyptratae/Drosophila - ps31
Drosophila - ps32
Sophophora - ps33
Obscura/Melanogaster_group - ps34
D_melanogaster_group - ps35
D_melanogaster_subgroup - ps36
sim./sech./mel. group - ps37
Drosophila_melanogaster - ps38
Phylostratum

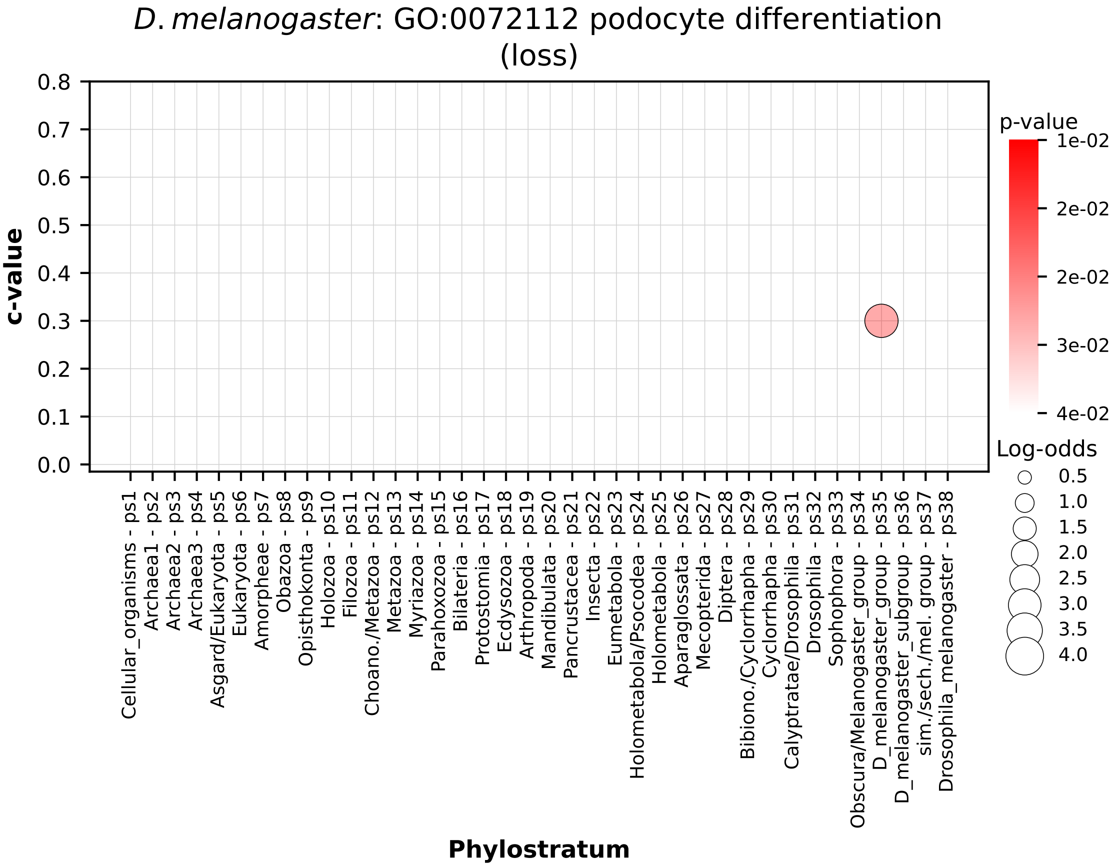
D. melanogaster: GO:0072112 podocyte differentiation
(loss)
c-value
0.8
0.7
0.6
0.5
0.4
0.3
0.2
0.1
0.0
p-value
1e-02
2e-02
2e-02
3e-02
4e-02
Log-odds
0.5
1.0
1.5
2.0
2.5
3.0
3.5
4.0
Cellular_organisms - ps1
Archaea1 - ps2
Archaea2 - ps3
Archaea3 - ps4
Asgard/Eukaryota - ps5
Eukaryota - ps6
Amorpheae - ps7
Obazoa - ps8
Opisthokonta - ps9
Holozoa - ps10
Filozoa - ps11
Choano./Metazoa - ps12
Metazoa - ps13
Myriazoa - ps14
Parahoxozoa - ps15
Bilateria - ps16
Protostomia - ps17
Ecdysozoa - ps18
Arthropoda - ps19
Mandibulata - ps20
Pancrustacea - ps21
Insecta - ps22
Eumetabola - ps23
Holometabola/Psocodea - ps24
Holometabola - ps25
Aparaglossata - ps26
Mecopterida - ps27
Diptera - ps28
Bibiono./Cyclorrhapha - ps29
Cyclorrhapha - ps30
Calyptratae/Drosophila - ps31
Drosophila - ps32
Sophophora - ps33
Obscura/Melanogaster_group - ps34
D_melanogaster_group - ps35
D_melanogaster_subgroup - ps36
sim./sech./mel. group - ps37
Drosophila_melanogaster - ps38
Phylostratum

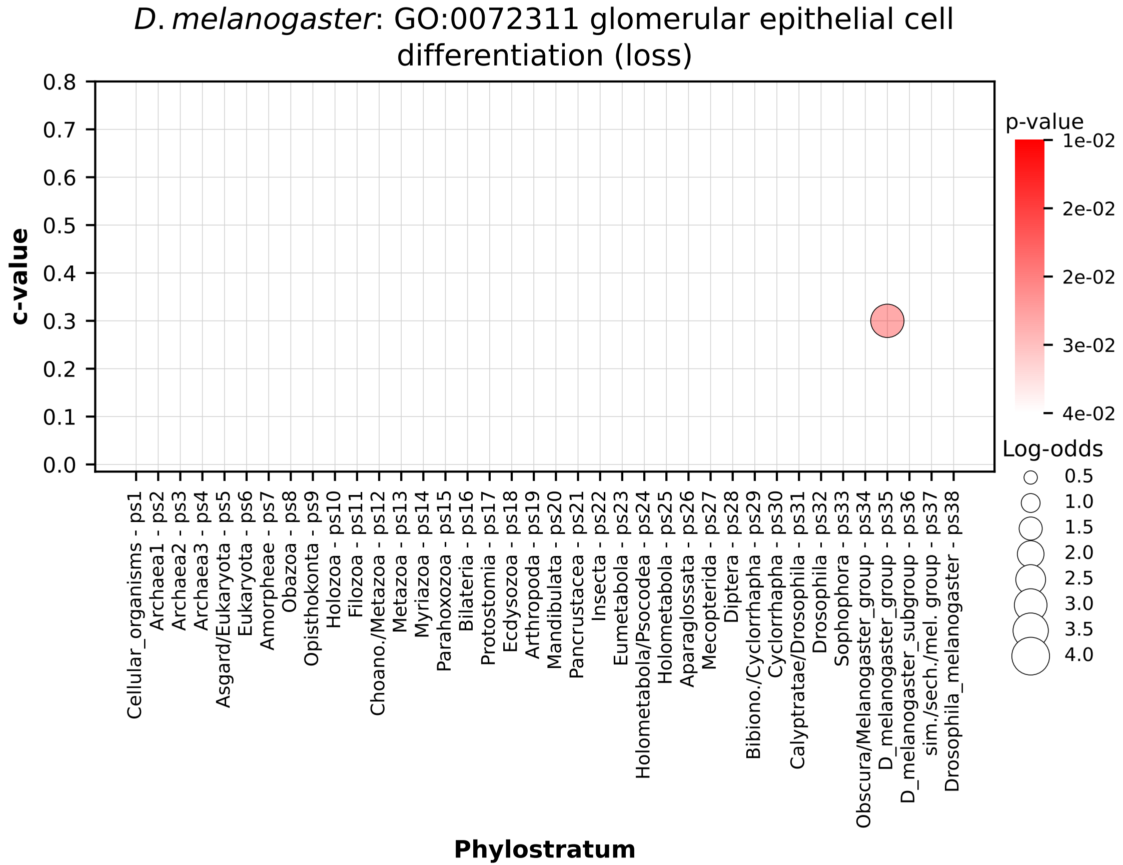

D. melanogaster: GO:0072311 glomerular epithelial cell differentiation (loss)
c-value
0.0
0.1
0.2
0.3
0.4
0.5
0.6
0.7
0.8
Phylostratum
Cellular_organisms - ps1
Archaea1 - ps2
Archaea2 - ps3
Archaea3 - ps4
Asgard/Eukaryota - ps5
Eukaryota - ps6
Amorpheae - ps7
Obazoa - ps8
Opisthokonta - ps9
Holozoa - ps10
Filozoa - ps11
Choano./Metazoa - ps12
Metazoa - ps13
Myriazoa - ps14
Parahoxozoa - ps15
Bilateria - ps16
Protostomia - ps17
Ecdysozoa - ps18
Arthropoda - ps19
Mandibulata - ps20
Pancrustacea - ps21
Insecta - ps22
Eumetabola - ps23
Holometabola/Psocodea - ps24
Holometabola - ps25
Aparaglossata - ps26
Mecopterida - ps27
Diptera - ps28
Bibiono./Cyclorrhapha - ps29
Cyclorrhapha - ps30
Calyptratae/Drosophila - ps31
Drosophila - ps32
Sophophora - ps33
Obscura/Melanogaster_group - ps34
D_melanogaster_group - ps35
D_melanogaster_subgroup - ps36
sim./sech./mel. group - ps37
Drosophila_melanogaster - ps38
p-value
1e-02
2e-02
2e-02
3e-02
4e-02
Log-odds
0.5
1.0
1.5
2.0
2.5
3.0
3.5
4.0

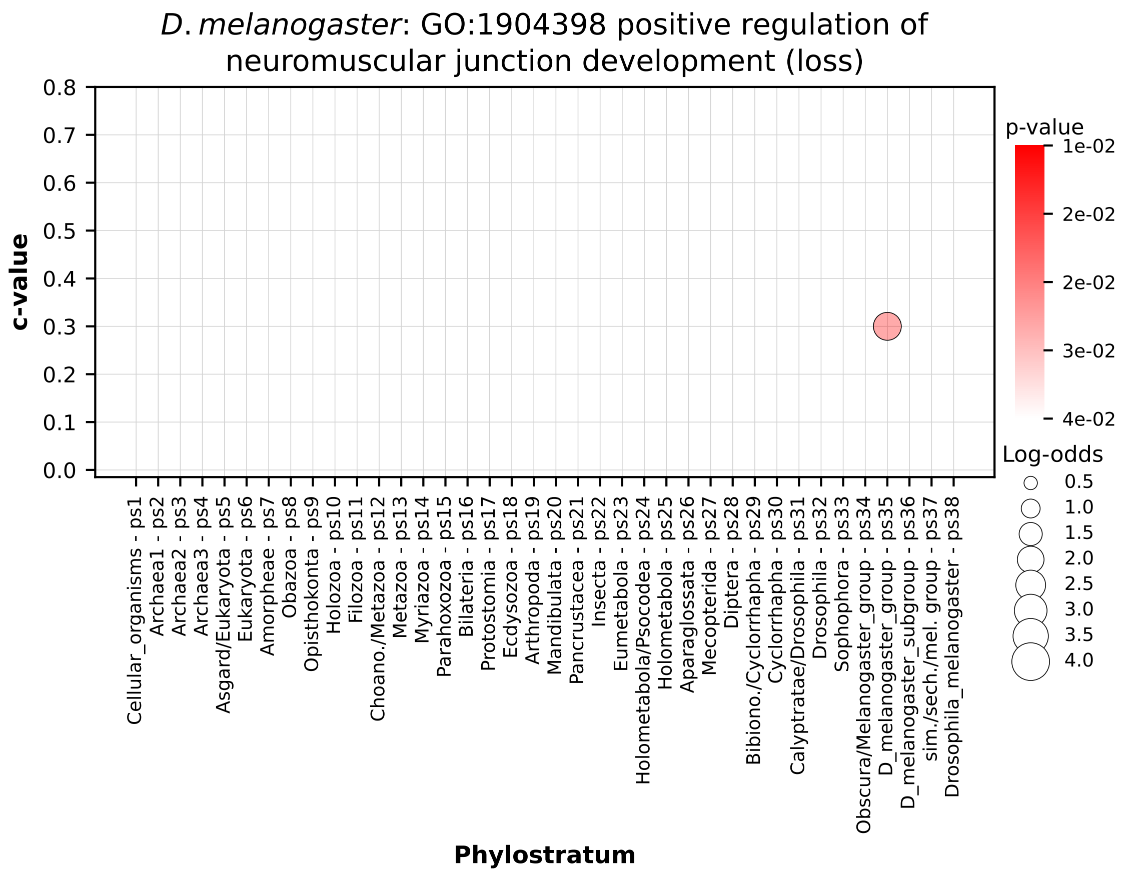

D. melanogaster: GO:1904398 positive regulation of neuromuscular junction development (loss)
c-value
0.8
0.7
0.6
0.5
0.4
0.3
0.2
0.1
0.0
Phylostratum
Cellular_organisms - ps1
Archaea1 - ps2
Archaea2 - ps3
Archaea3 - ps4
Asgard/Eukaryota - ps5
Eukaryota - ps6
Amorpheae - ps7
Obazoa - ps8
Opisthokonta - ps9
Holozoa - ps10
Filozoa - ps11
Choano./Metazoa - ps12
Metazoa - ps13
Myriazoa - ps14
Parahoxozoa - ps15
Bilateria - ps16
Protostomia - ps17
Ecdysozoa - ps18
Arthropoda - ps19
Mandibulata - ps20
Pancrustacea - ps21
Insecta - ps22
Eumetabola - ps23
Holometabola/Psocodea - ps24
Holometabola - ps25
Aparaglossata - ps26
Mecopterida - ps27
Diptera - ps28
Bibiono./Cyclorrhapha - ps29
Cyclorrhapha - ps30
Calyptratae/Drosophila - ps31
Drosophila - ps32
Sophophora - ps33
Obscura/Melanogaster_group - ps34
D_melanogaster_group - ps35
D_melanogaster_subgroup - ps36
sim./sech./mel. group - ps37
Drosophila_melanogaster - ps38
p-value
1e-02
2e-02
2e-02
3e-02
4e-02
Log-odds
0.5
1.0
1.5
2.0
2.5
3.0
3.5
4.0

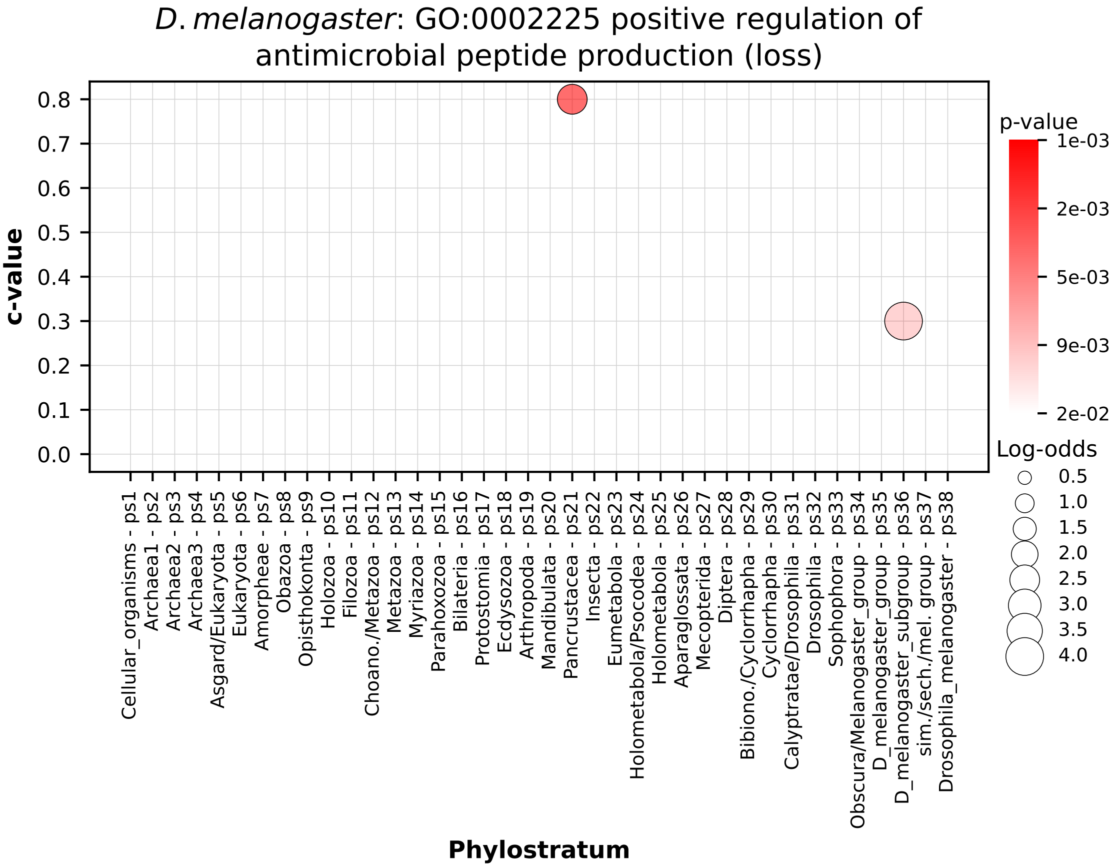

$D. melanogaster$: GO:0002225 positive regulation of antimicrobial peptide production (loss)
c-value
0.8
0.7
0.6
0.5
0.4
0.3
0.2
0.1
0.0
p-value
1e-03
2e-03
5e-03
9e-03
2e-02
Log-odds
0.5
1.0
1.5
2.0
2.5
3.0
3.5
4.0
Cellular_organisms - ps1
Archaea1 - ps2
Archaea2 - ps3
Archaea3 - ps4
Asgard/Eukaryota - ps5
Eukaryota - ps6
Amorpheae - ps7
Obazoa - ps8
Opisthokonta - ps9
Holozoa - ps10
Filozoa - ps11
Choano./Metazoa - ps12
Metazoa - ps13
Myriazoa - ps14
Parahoxozoa - ps15
Bilateria - ps16
Protostomia - ps17
Ecdysozoa - ps18
Arthropoda - ps19
Mandibulata - ps20
Pancrustacea - ps21
Insecta - ps22
Eumetabola - ps23
Holometabola/Psocodea - ps24
Holometabola - ps25
Aparaglossata - ps26
Mecopterida - ps27
Diptera - ps28
Bibiono./Cyclorrhapha - ps29
Cyclorrhapha - ps30
Calyptratae/Drosophila - ps31
Drosophila - ps32
Sophophora - ps33
Obscura/Melanogaster_group - ps34
D_melanogaster_group - ps35
D_melanogaster_subgroup - ps36
sim./sech./mel. group - ps37
Drosophila_melanogaster - ps38
Phylostratum

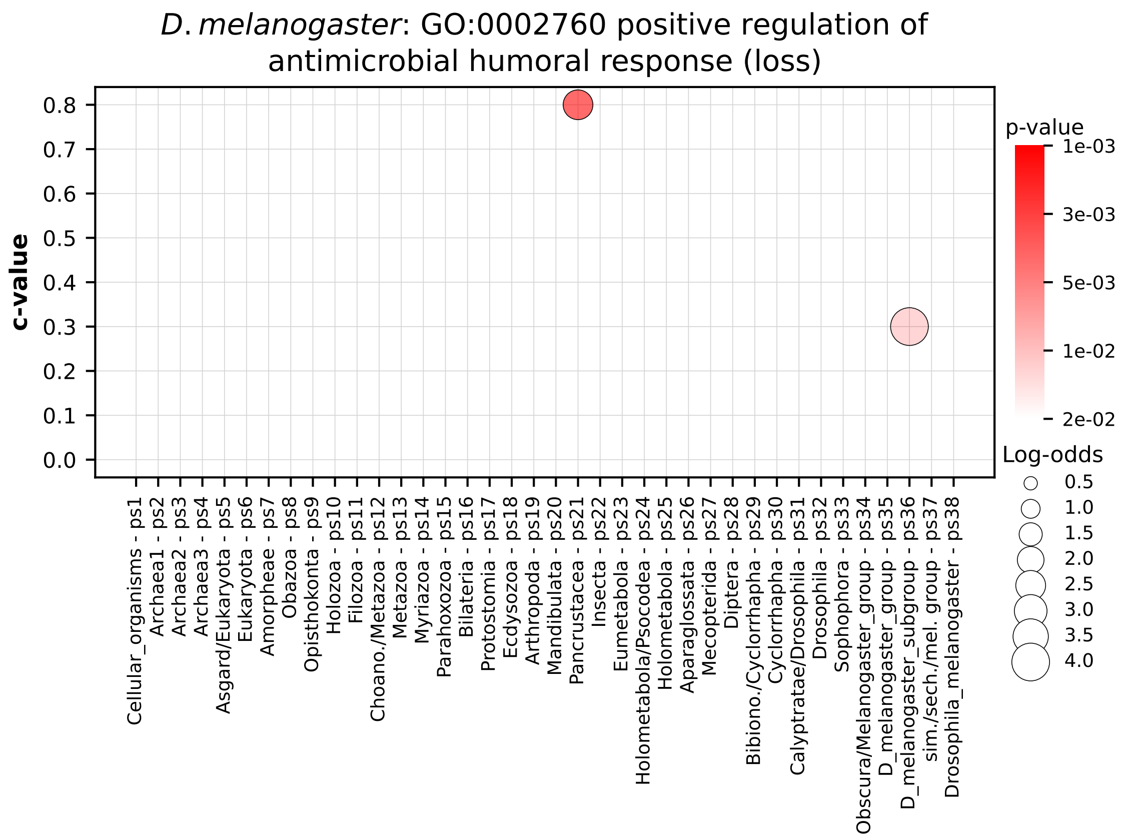

D. melanogaster: GO:0002760 positive regulation of antimicrobial humoral response (loss)
c-value
0.0
0.1
0.2
0.3
0.4
0.5
0.6
0.7
0.8
Phylostratum
Cellular_organisms - ps1
Archaea1 - ps2
Archaea2 - ps3
Archaea3 - ps4
Asgard/Eukaryota - ps5
Eukaryota - ps6
Amorpheae - ps7
Obazoa - ps8
Opisthokonta - ps9
Holozoa - ps10
Filozoa - ps11
Choano./Metazoa - ps12
Metazoa - ps13
Myriazoa - ps14
Parahoxozoa - ps15
Bilateria - ps16
Protostomia - ps17
Ecdysozoa - ps18
Arthropoda - ps19
Mandibulata - ps20
Pancrustacea - ps21
Insecta - ps22
Eumetabola - ps23
Holometabola/Psocodea - ps24
Holometabola - ps25
Aparaglossata - ps26
Mecopterida - ps27
Diptera - ps28
Bibiono./Cyclorrhapha - ps29
Cyclorrhapha - ps30
Calyptratae/Drosophila - ps31
Drosophila - ps32
Sophophora - ps33
Obscura/Melanogaster_group - ps34
D_melanogaster_group - ps35
D_melanogaster_subgroup - ps36
sim./sech./mel. group - ps37
Drosophila_melanogaster - ps38
p-value
1e-03
3e-03
5e-03
1e-02
2e-02
Log-odds
0.5
1.0
1.5
2.0
2.5
3.0
3.5
4.0

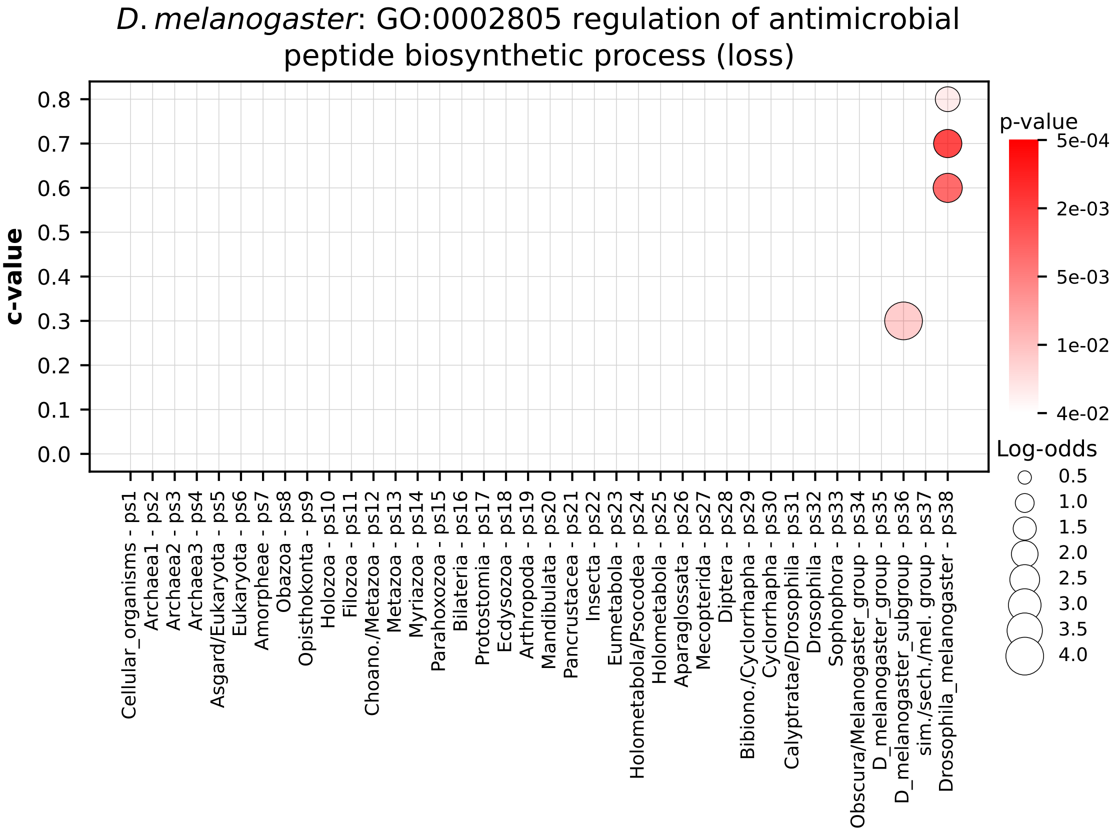

D. melanogaster: GO:0002805 regulation of antimicrobial peptide biosynthetic process (loss)
c-value
0.0
0.1
0.2
0.3
0.4
0.5
0.6
0.7
0.8
Phylostratum
Cellular_organisms - ps1
Archaea1 - ps2
Archaea2 - ps3
Archaea3 - ps4
Asgard/Eukaryota - ps5
Eukaryota - ps6
Amorpheae - ps7
Obazoa - ps8
Opisthokonta - ps9
Holozoa - ps10
Filozoa - ps11
Choano./Metazoa - ps12
Metazoa - ps13
Myriazoa - ps14
Parahoxozoa - ps15
Bilateria - ps16
Protostomia - ps17
Ecdysozoa - ps18
Arthropoda - ps19
Mandibulata - ps20
Pancrustacea - ps21
Insecta - ps22
Eumetabola - ps23
Holometabola/Psocodea - ps24
Holometabola - ps25
Aparaglossata - ps26
Mecopterida - ps27
Diptera - ps28
Bibiono./Cyclorrhapha - ps29
Cyclorrhapha - ps30
Calyptratae/Drosophila - ps31
Drosophila - ps32
Sophophora - ps33
Obscura/Melanogaster_group - ps34
D_melanogaster_group - ps35
D_melanogaster_subgroup - ps36
sim./sech./mel. group - ps37
Drosophila_melanogaster - ps38
p-value
5e-04
2e-03
5e-03
1e-02
4e-02
Log-odds
0.5
1.0
1.5
2.0
2.5
3.0
3.5
4.0

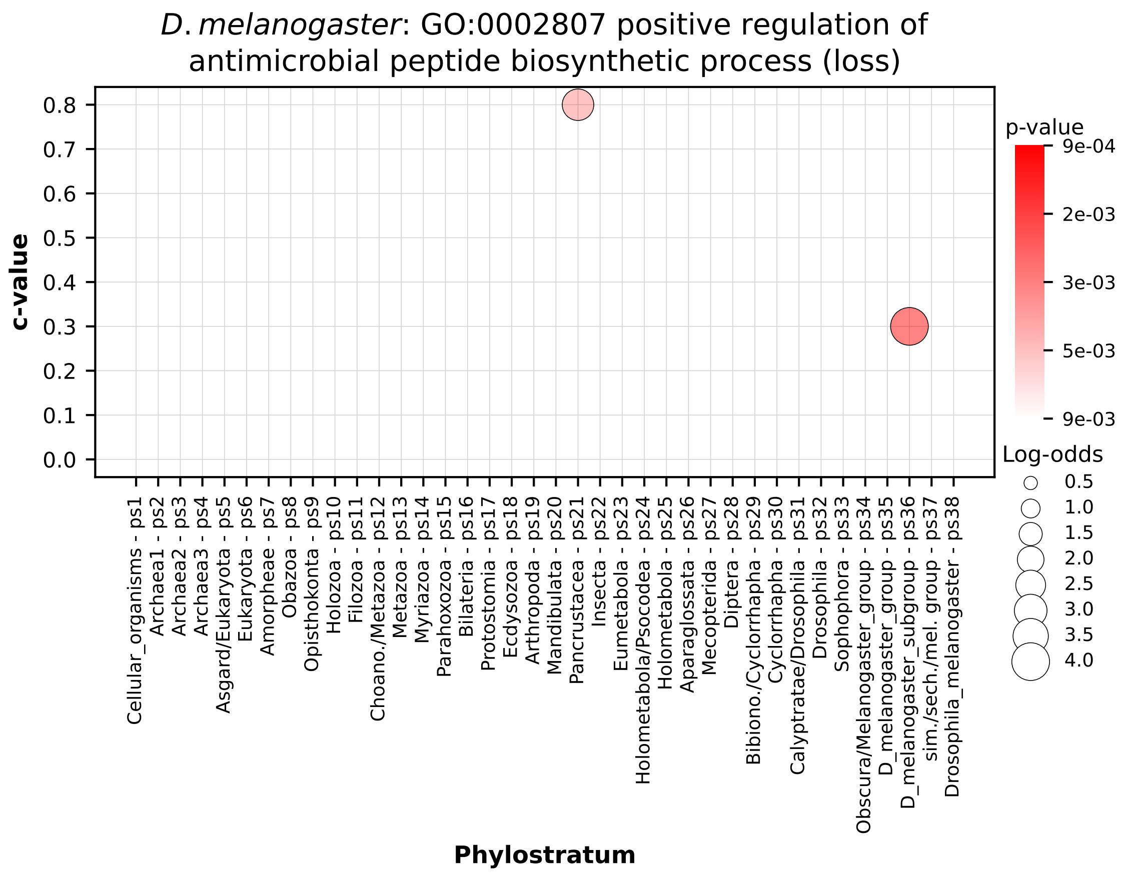

D. melanogaster: GO:0002807 positive regulation of antimicrobial peptide biosynthetic process (loss)
c-value
0.8
0.7
0.6
0.5
0.4
0.3
0.2
0.1
0.0
p-value
9e-04
2e-03
3e-03
5e-03
9e-03
Log-odds
0.5
1.0
1.5
2.0
2.5
3.0
3.5
4.0
Cellular_organisms - ps1
Archaea1 - ps2
Archaea2 - ps3
Archaea3 - ps4
Asgard/Eukaryota - ps5
Eukaryota - ps6
Amorpheae - ps7
Obazoa - ps8
Opisthokonta - ps9
Holozoa - ps10
Filozoa - ps11
Choano./Metazoa - ps12
Metazoa - ps13
Myriazoa - ps14
Parahoxozoa - ps15
Bilateria - ps16
Protostomia - ps17
Ecdysozoa - ps18
Arthropoda - ps19
Mandibulata - ps20
Pancrustacea - ps21
Insecta - ps22
Eumetabola - ps23
Holometabola/Psocodea - ps24
Holometabola - ps25
Aparaglossata - ps26
Mecopterida - ps27
Diptera - ps28
Bibiono./Cyclorrhapha - ps29
Cyclorrhapha - ps30
Calyptratae/Drosophila - ps31
Drosophila - ps32
Sophophora - ps33
Obscura/Melanogaster_group - ps34
D_melanogaster_group - ps35
D_melanogaster_subgroup - ps36
sim./sech./mel. group - ps37
Drosophila_melanogaster - ps38
Phylostratum

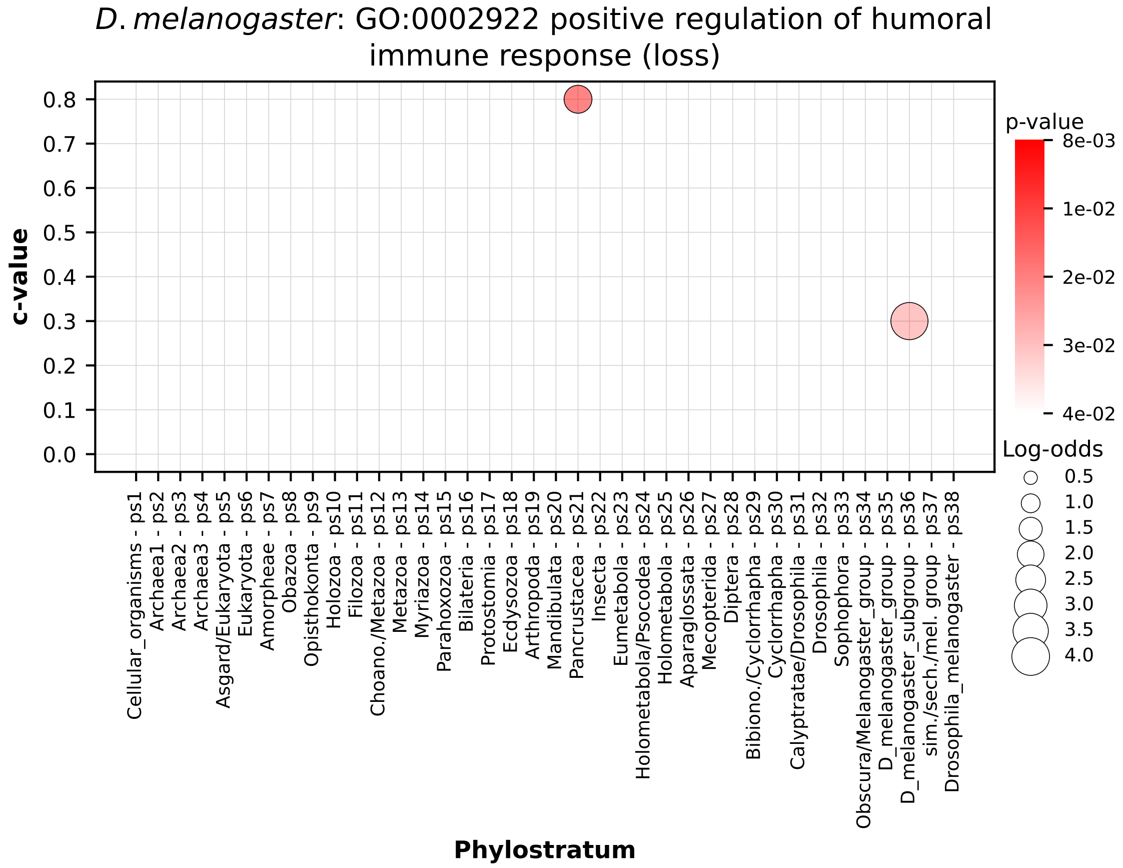

D. melanogaster: GO:0002922 positive regulation of humoral immune response (loss)
c-value
0.8
0.7
0.6
0.5
0.4
0.3
0.2
0.1
0.0
p-value
8e-03
1e-02
2e-02
3e-02
4e-02
Log-odds
0.5
1.0
1.5
2.0
2.5
3.0
3.5
4.0
Cellular_organisms - ps1
Archaea1 - ps2
Archaea2 - ps3
Archaea3 - ps4
Asgard/Eukaryota - ps5
Eukaryota - ps6
Amorpheae - ps7
Obazoa - ps8
Opisthokonta - ps9
Holozoa - ps10
Filozoa - ps11
Choano./Metazoa - ps12
Metazoa - ps13
Myriazoa - ps14
Parahoxozoa - ps15
Bilateria - ps16
Protostomia - ps17
Ecdysozoa - ps18
Arthropoda - ps19
Mandibulata - ps20
Pancrustacea - ps21
Insecta - ps22
Eumetabola - ps23
Holometabola/Psocodea - ps24
Holometabola - ps25
Aparaglossata - ps26
Mecopterida - ps27
Diptera - ps28
Bibiono./Cyclorrhapha - ps29
Cyclorrhapha - ps30
Calyptratae/Drosophila - ps31
Drosophila - ps32
Sophophora - ps33
Obscura/Melanogaster_group - ps34
D_melanogaster_group - ps35
D_melanogaster_subgroup - ps36
sim./sech./mel. group - ps37
Drosophila_melanogaster - ps38
Phylostratum

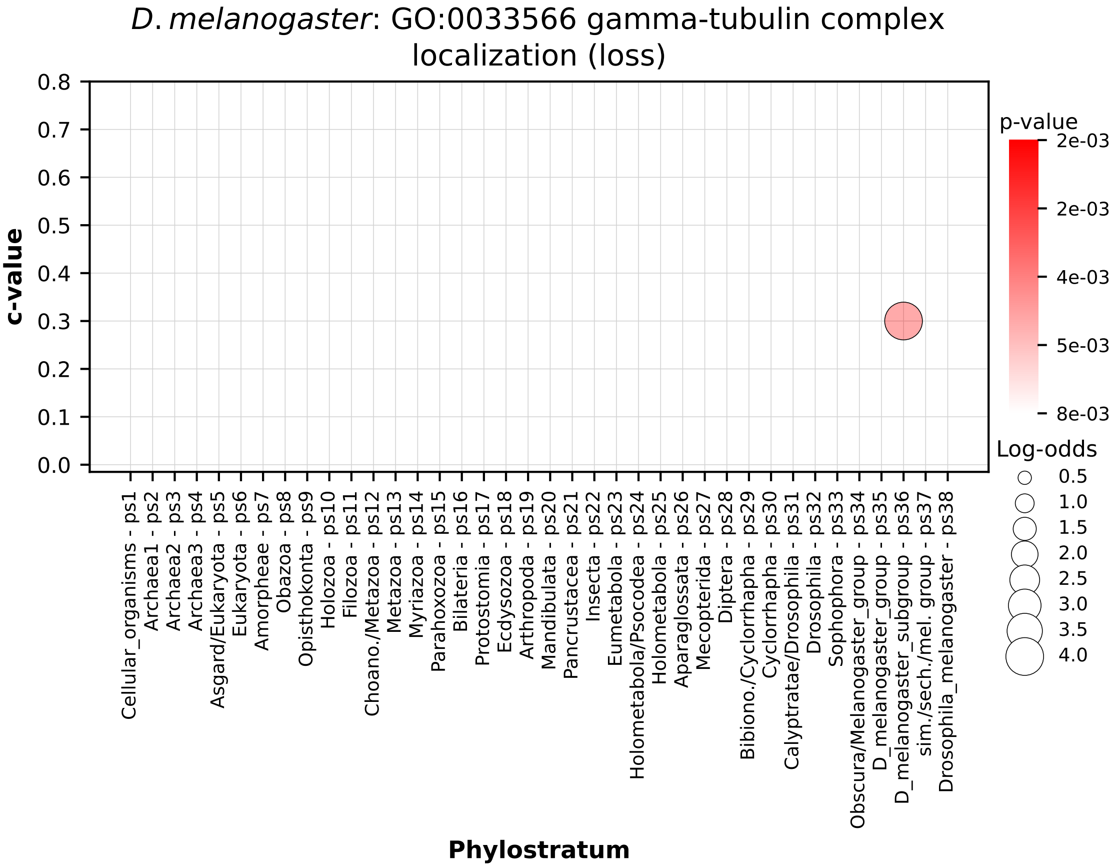
D. melanogaster: GO:0033566 gamma-tubulin complex localization (loss)
c-value
0.0
0.1
0.2
0.3
0.4
0.5
0.6
0.7
0.8
Phylostratum
Cellular_organisms - ps1
Archaea1 - ps2
Archaea2 - ps3
Archaea3 - ps4
Asgard/Eukaryota - ps5
Eukaryota - ps6
Amorpheae - ps7
Obazoa - ps8
Opisthokonta - ps9
Holozoa - ps10
Filozoa - ps11
Choano./Metazoa - ps12
Metazoa - ps13
Myriazoa - ps14
Parahoxozoa - ps15
Bilateria - ps16
Protostomia - ps17
Ecdysozoa - ps18
Arthropoda - ps19
Mandibulata - ps20
Pancrustacea - ps21
Insecta - ps22
Eumetabola - ps23
Holometabola/Psocodea - ps24
Holometabola - ps25
Aparaglossata - ps26
Mecopterida - ps27
Diptera - ps28
Bibiono./Cyclorrhapha - ps29
Cyclorrhapha - ps30
Calyptratae/Drosophila - ps31
Drosophila - ps32
Sophophora - ps33
Obscura/Melanogaster_group - ps34
D_melanogaster_group - ps35
D_melanogaster_subgroup - ps36
sim./sech./mel. group - ps37
Drosophila_melanogaster - ps38
p-value
2e-03
2e-03
4e-03
5e-03
8e-03
Log-odds
0.5
1.0
1.5
2.0
2.5
3.0
3.5
4.0

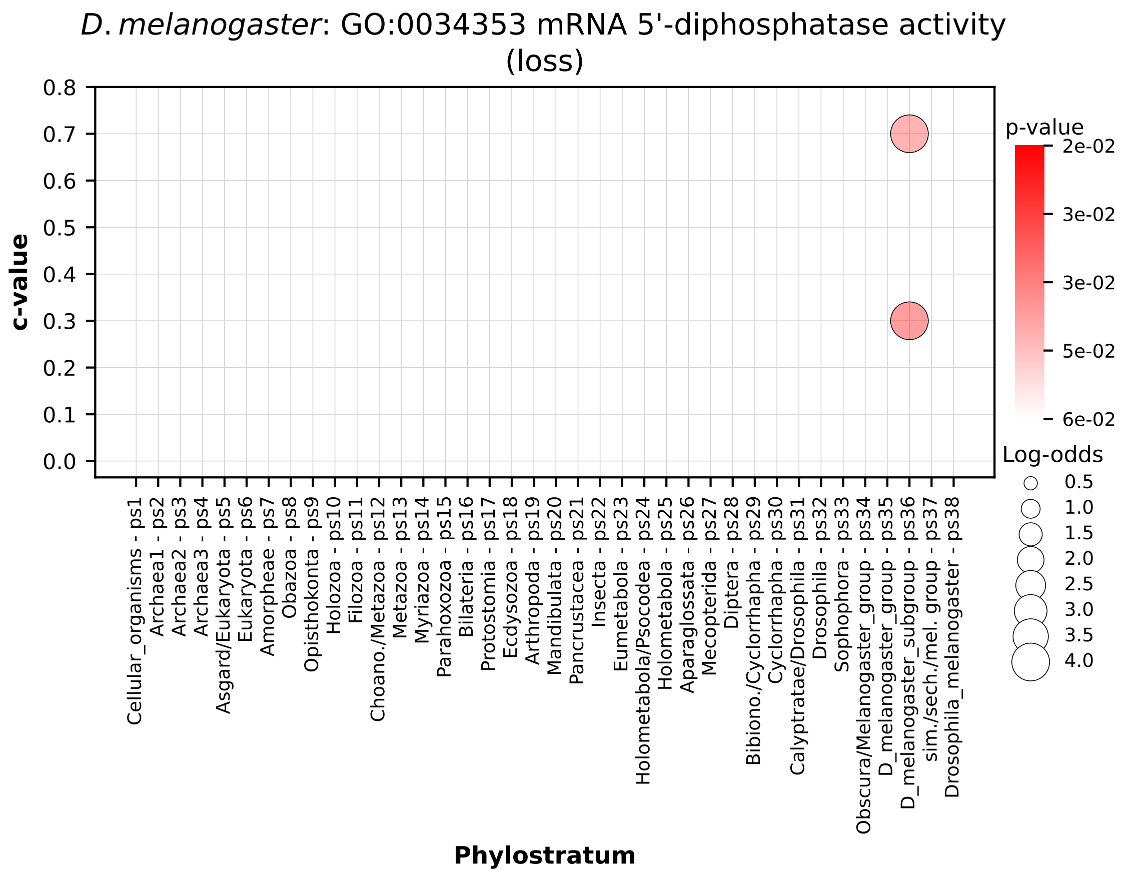

D. melanogaster: GO:0034353 mRNA 5'-diphosphatase activity
(loss)
c-value
0.8
0.7
0.6
0.5
0.4
0.3
0.2
0.1
0.0
p-value
2e-02
3e-02
3e-02
5e-02
6e-02
Log-odds
0.5
1.0
1.5
2.0
2.5
3.0
3.5
4.0
Cellular_organisms - ps1
Archaea1 - ps2
Archaea2 - ps3
Archaea3 - ps4
Asgard/Eukaryota - ps5
Eukaryota - ps6
Amorpheae - ps7
Obazoa - ps8
Opisthokonta - ps9
Holozoa - ps10
Filozoa - ps11
Choano./Metazoa - ps12
Metazoa - ps13
Myriazoa - ps14
Parahoxozoa - ps15
Bilateria - ps16
Protostomia - ps17
Ecdysozoa - ps18
Arthropoda - ps19
Mandibulata - ps20
Pancrustacea - ps21
Insecta - ps22
Eumetabola - ps23
Holometabola/Psocodea - ps24
Holometabola - ps25
Aparaglossata - ps26
Mecopterida - ps27
Diptera - ps28
Bibiono./Cyclorrhapha - ps29
Cyclorrhapha - ps30
Calyptratae/Drosophila - ps31
Drosophila - ps32
Sophophora - ps33
Obscura/Melanogaster_group - ps34
D_melanogaster_group - ps35
D_melanogaster_subgroup - ps36
sim./sech./mel. group - ps37
Drosophila_melanogaster - ps38
Phylostratum

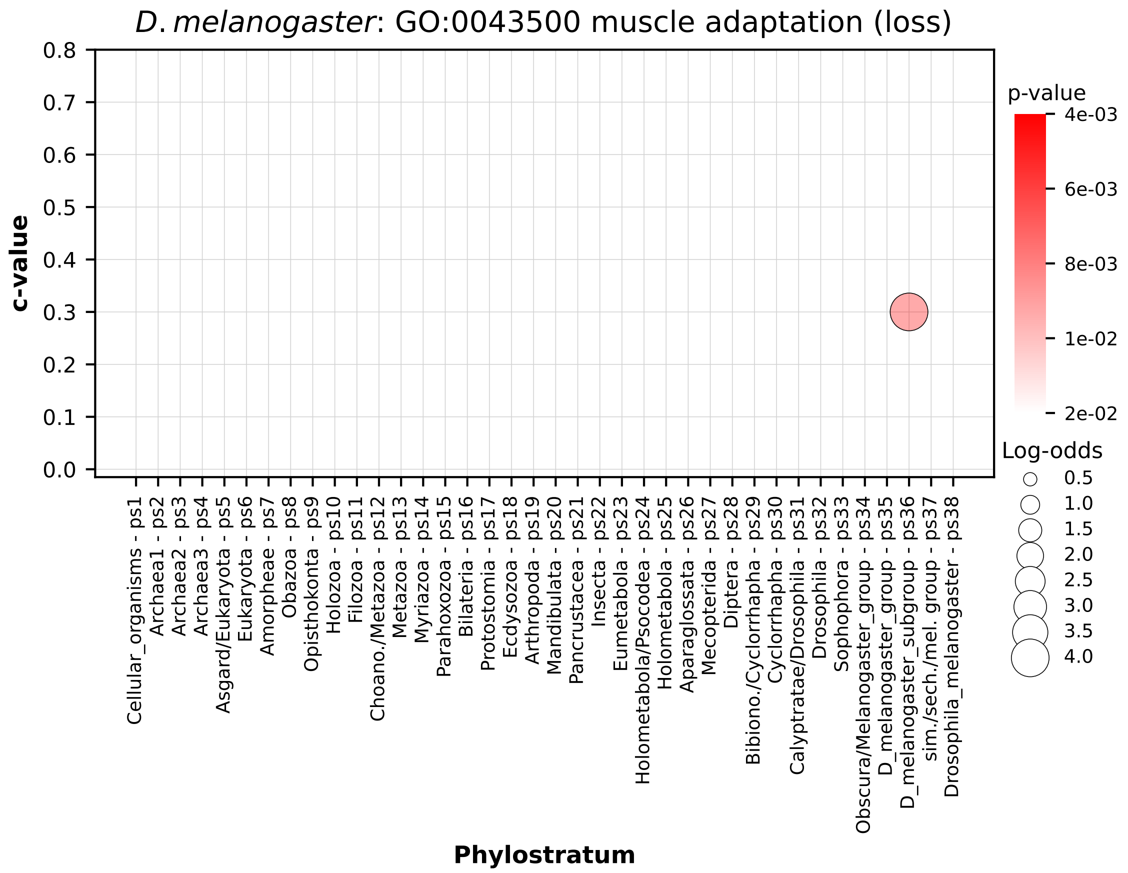

D. melanogaster: GO:0043500 muscle adaptation (loss)
c-value
0.8
0.7
0.6
0.5
0.4
0.3
0.2
0.1
0.0
Phylostratum
Cellular_organisms - ps1
Archaea1 - ps2
Archaea2 - ps3
Archaea3 - ps4
Asgard/Eukaryota - ps5
Eukaryota - ps6
Amorpheae - ps7
Obazoa - ps8
Opisthokonta - ps9
Holozoa - ps10
Filozoa - ps11
Choano./Metazoa - ps12
Metazoa - ps13
Myriazoa - ps14
Parahoxozoa - ps15
Bilateria - ps16
Protostomia - ps17
Ecdysozoa - ps18
Arthropoda - ps19
Mandibulata - ps20
Pancrustacea - ps21
Insecta - ps22
Eumetabola - ps23
Holometabola/Psocodea - ps24
Holometabola - ps25
Aparaglossata - ps26
Mecopterida - ps27
Diptera - ps28
Bibiono./Cyclorrhapha - ps29
Cyclorrhapha - ps30
Calyptratae/Drosophila - ps31
Drosophila - ps32
Sophophora - ps33
Obscura/Melanogaster_group - ps34
D_melanogaster_group - ps35
D_melanogaster_subgroup - ps36
sim./sech./mel. group - ps37
Drosophila_melanogaster - ps38
p-value
4e-03
6e-03
8e-03
1e-02
2e-02
Log-odds
0.5
1.0
1.5
2.0
2.5
3.0
3.5
4.0

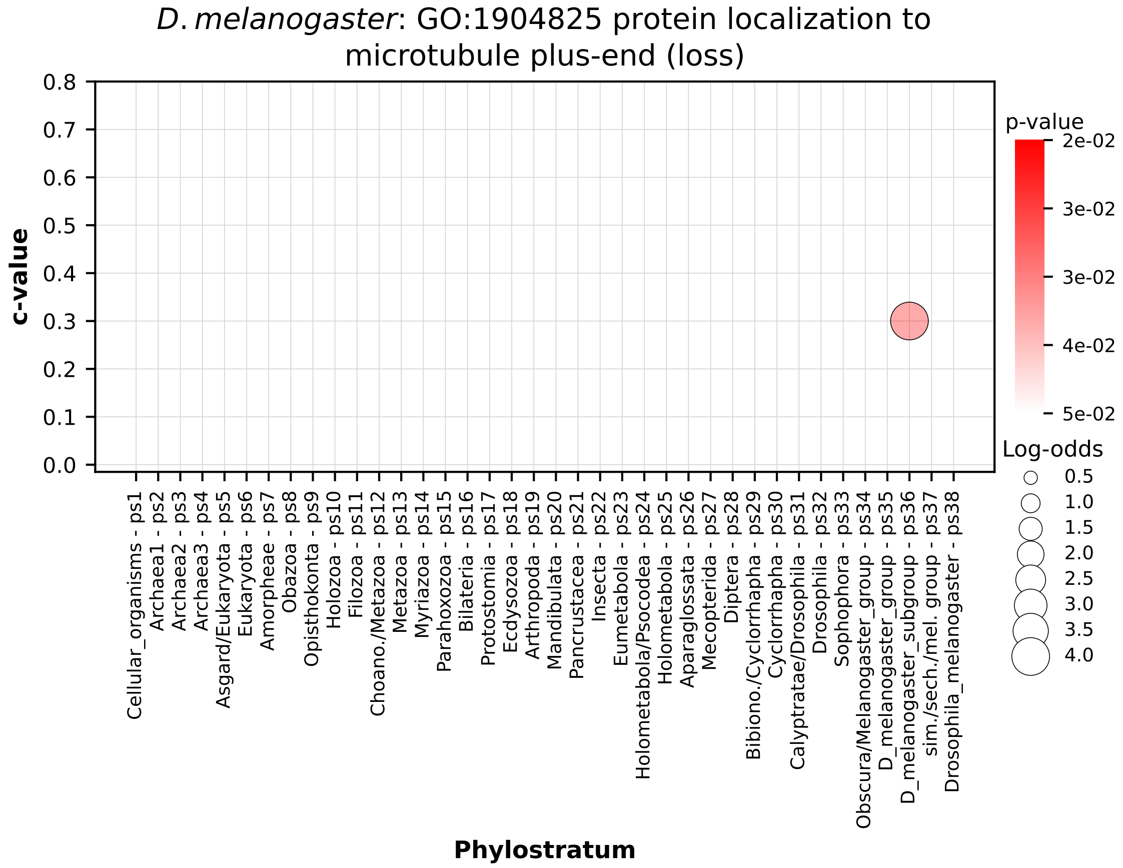

D. melanogaster: GO:1904825 protein localization to microtubule plus-end (loss)
c-value
0.8
0.7
0.6
0.5
0.4
0.3
0.2
0.1
0.0
p-value
2e-02
3e-02
3e-02
4e-02
5e-02
Log-odds
0.5
1.0
1.5
2.0
2.5
3.0
3.5
4.0
Cellular_organisms - ps1
Archaea1 - ps2
Archaea2 - ps3
Archaea3 - ps4
Asgard/Eukaryota - ps5
Eukaryota - ps6
Amorpheae - ps7
Obazoa - ps8
Opisthokonta - ps9
Holozoa - ps10
Filozoa - ps11
Choano./Metazoa - ps12
Metazoa - ps13
Myriazoa - ps14
Parahoxozoa - ps15
Bilateria - ps16
Protostomia - ps17
Ecdysozoa - ps18
Arthropoda - ps19
Mandibulata - ps20
Pancrustacea - ps21
Insecta - ps22
Eumetabola - ps23
Holometabola/Psocodea - ps24
Holometabola - ps25
Aparaglossata - ps26
Mecopterida - ps27
Diptera - ps28
Bibiono./Cyclorrhapha - ps29
Cyclorrhapha - ps30
Calyptratae/Drosophila - ps31
Drosophila - ps32
Sophophora - ps33
Obscura/Melanogaster_group - ps34
D_melanogaster_group - ps35
D_melanogaster_subgroup - ps36
sim./sech./mel. group - ps37
Drosophila_melanogaster - ps38
Phylostratum

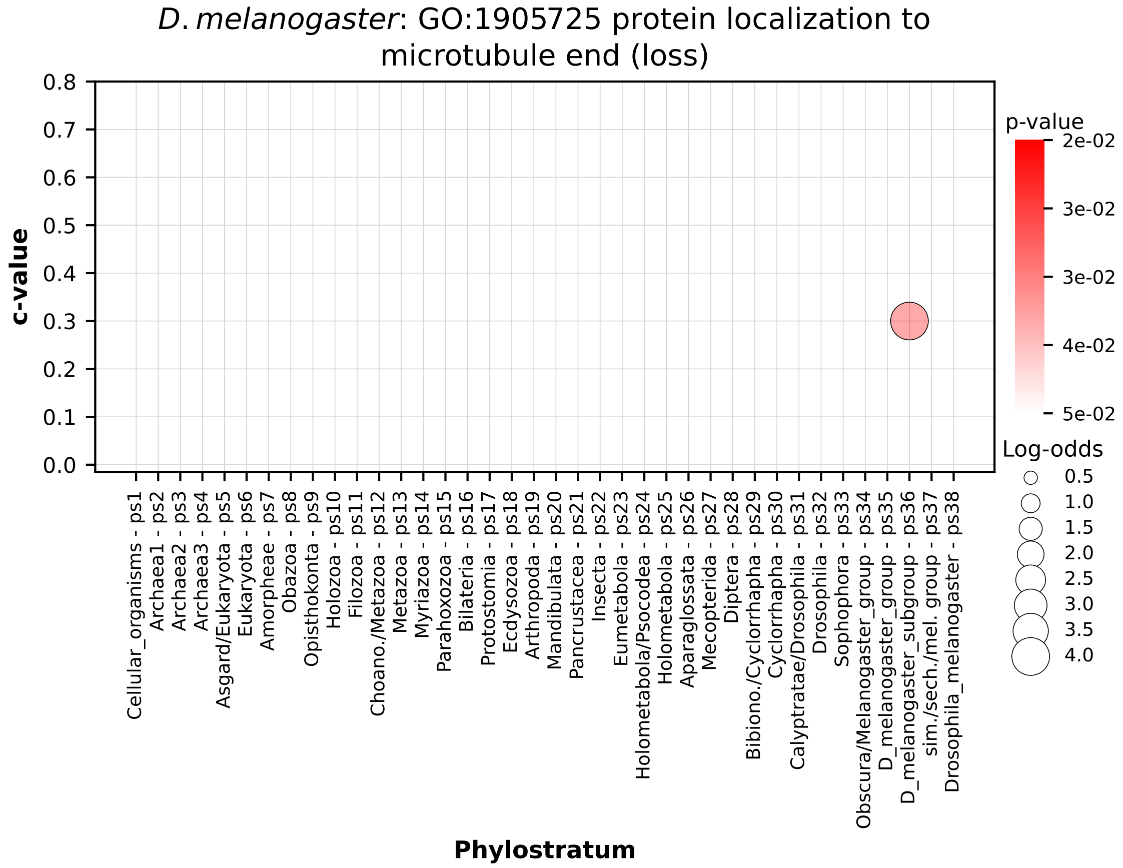
D. melanogaster: GO:1905725 protein localization to microtubule end (loss)
c-value
0.0
0.1
0.2
0.3
0.4
0.5
0.6
0.7
0.8
Phylostratum
Cellular_organisms - ps1
Archaea1 - ps2
Archaea2 - ps3
Archaea3 - ps4
Asgard/Eukaryota - ps5
Eukaryota - ps6
Amorpheae - ps7
Obazoa - ps8
Opisthokonta - ps9
Holozoa - ps10
Filozoa - ps11
Choano./Metazoa - ps12
Metazoa - ps13
Myriazoa - ps14
Parahoxozoa - ps15
Bilateria - ps16
Protostomia - ps17
Ecdysozoa - ps18
Arthropoda - ps19
Mandibulata - ps20
Pancrustacea - ps21
Insecta - ps22
Eumetabola - ps23
Holometabola/Psocodea - ps24
Holometabola - ps25
Aparaglossata - ps26
Mecopterida - ps27
Diptera - ps28
Bibiono./Cyclorrhapha - ps29
Cyclorrhapha - ps30
Calyptratae/Drosophila - ps31
Drosophila - ps32
Sophophora - ps33
Obscura/Melanogaster_group - ps34
D_melanogaster_group - ps35
D_melanogaster_subgroup - ps36
sim./sech./mel. group - ps37
Drosophila_melanogaster - ps38
p-value
2e-02
3e-02
3e-02
4e-02
5e-02
Log-odds
0.5
1.0
1.5
2.0
2.5
3.0
3.5
4.0

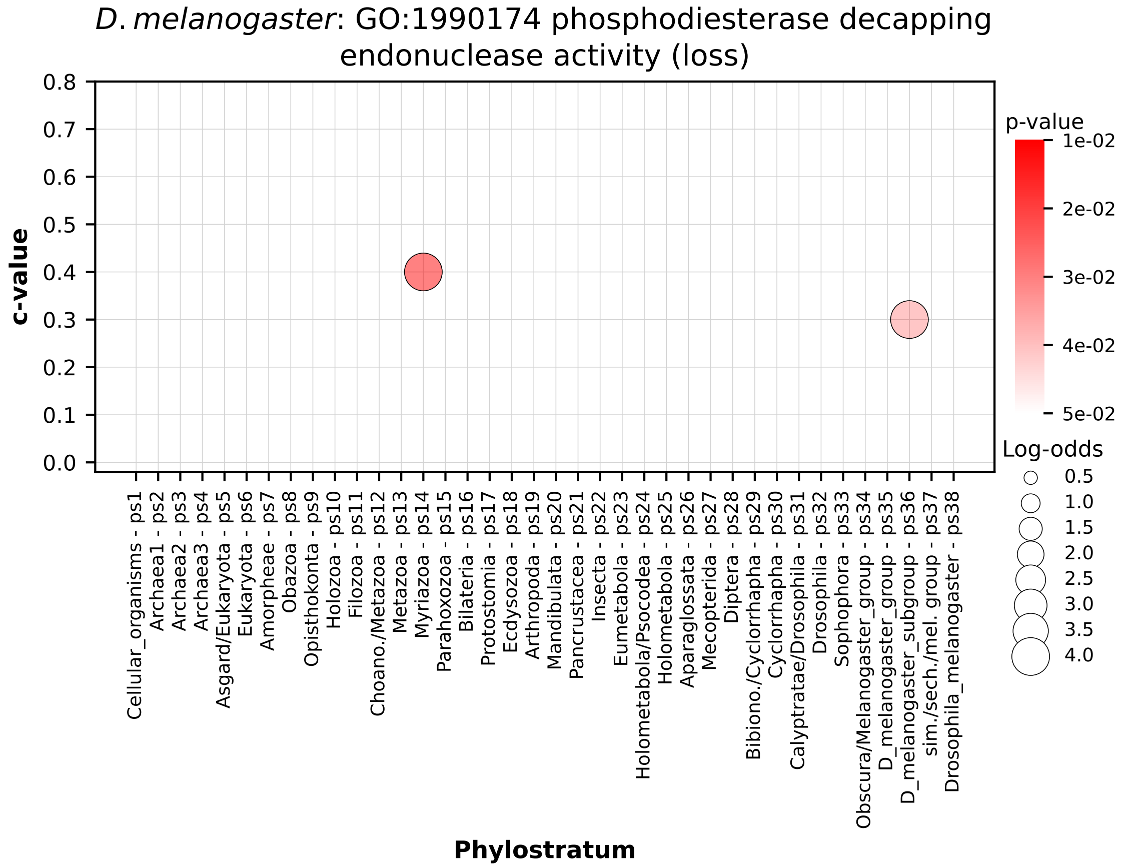

D. melanogaster: GO:1990174 phosphodiesterase decapping endonuclease activity (loss)
c-value
0.8
0.7
0.6
0.5
0.4
0.3
0.2
0.1
0.0
Phylostratum
Cellular_organisms - ps1
Archaea1 - ps2
Archaea2 - ps3
Archaea3 - ps4
Asgard/Eukaryota - ps5
Eukaryota - ps6
Amorpheae - ps7
Obazoa - ps8
Opisthokonta - ps9
Holozoa - ps10
Filozoa - ps11
Choano./Metazoa - ps12
Metazoa - ps13
Myriazoa - ps14
Parahoxozoa - ps15
Bilateria - ps16
Protostomia - ps17
Ecdysozoa - ps18
Arthropoda - ps19
Mandibulata - ps20
Pancrustacea - ps21
Insecta - ps22
Eumetabola - ps23
Holometabola/Psocodea - ps24
Holometabola - ps25
Aparaglossata - ps26
Mecopterida - ps27
Diptera - ps28
Bibiono./Cyclorrhapha - ps29
Cyclorrhapha - ps30
Calyptratae/Drosophila - ps31
Drosophila - ps32
Sophophora - ps33
Obscura/Melanogaster_group - ps34
D_melanogaster_group - ps35
D_melanogaster_subgroup - ps36
sim./sech./mel. group - ps37
Drosophila_melanogaster - ps38
p-value
1e-02
2e-02
3e-02
4e-02
5e-02
Log-odds
0.5
1.0
1.5
2.0
2.5
3.0
3.5
4.0

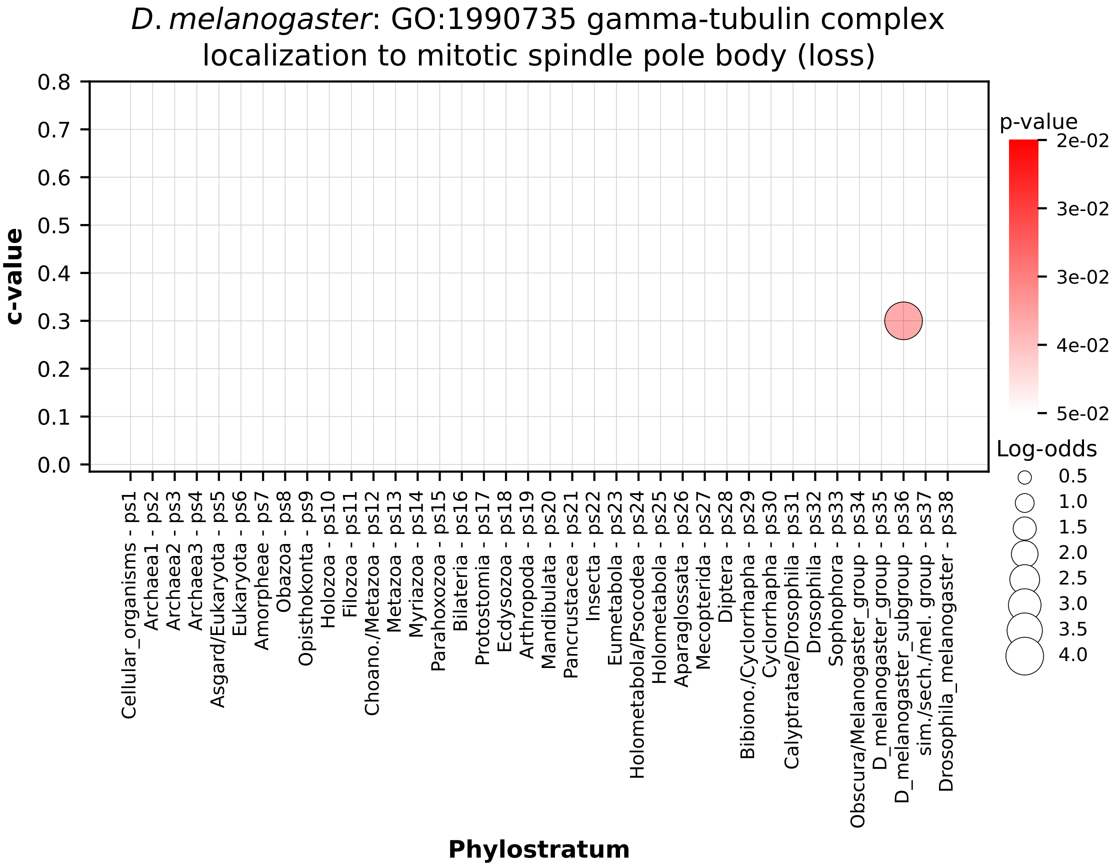
D. melanogaster: GO:1990735 gamma-tubulin complex localization to mitotic spindle pole body (loss)
c-value
0.8
0.7
0.6
0.5
0.4
0.3
0.2
0.1
0.0
Phylostratum
Cellular_organisms - ps1
Archaea1 - ps2
Archaea2 - ps3
Archaea3 - ps4
Asgard/Eukaryota - ps5
Eukaryota - ps6
Amorpheae - ps7
Obazoa - ps8
Opisthokonta - ps9
Holozoa - ps10
Filozoa - ps11
Choano./Metazoa - ps12
Metazoa - ps13
Myriazoa - ps14
Parahoxozoa - ps15
Bilateria - ps16
Protostomia - ps17
Ecdysozoa - ps18
Arthropoda - ps19
Mandibulata - ps20
Pancrustacea - ps21
Insecta - ps22
Eumetabola - ps23
Holometabola/Psocodea - ps24
Holometabola - ps25
Aparaglossata - ps26
Mecopterida - ps27
Diptera - ps28
Bibiono./Cyclorrhapha - ps29
Cyclorrhapha - ps30
Calyptratae/Drosophila - ps31
Drosophila - ps32
Sophophora - ps33
Obscura/Melanogaster_group - ps34
D_melanogaster_group - ps35
D_melanogaster_subgroup - ps36
sim./sech./mel. group - ps37
Drosophila_melanogaster - ps38
p-value
2e-02
3e-02
3e-02
4e-02
5e-02
Log-odds
0.5
1.0
1.5
2.0
2.5
3.0
3.5
4.0

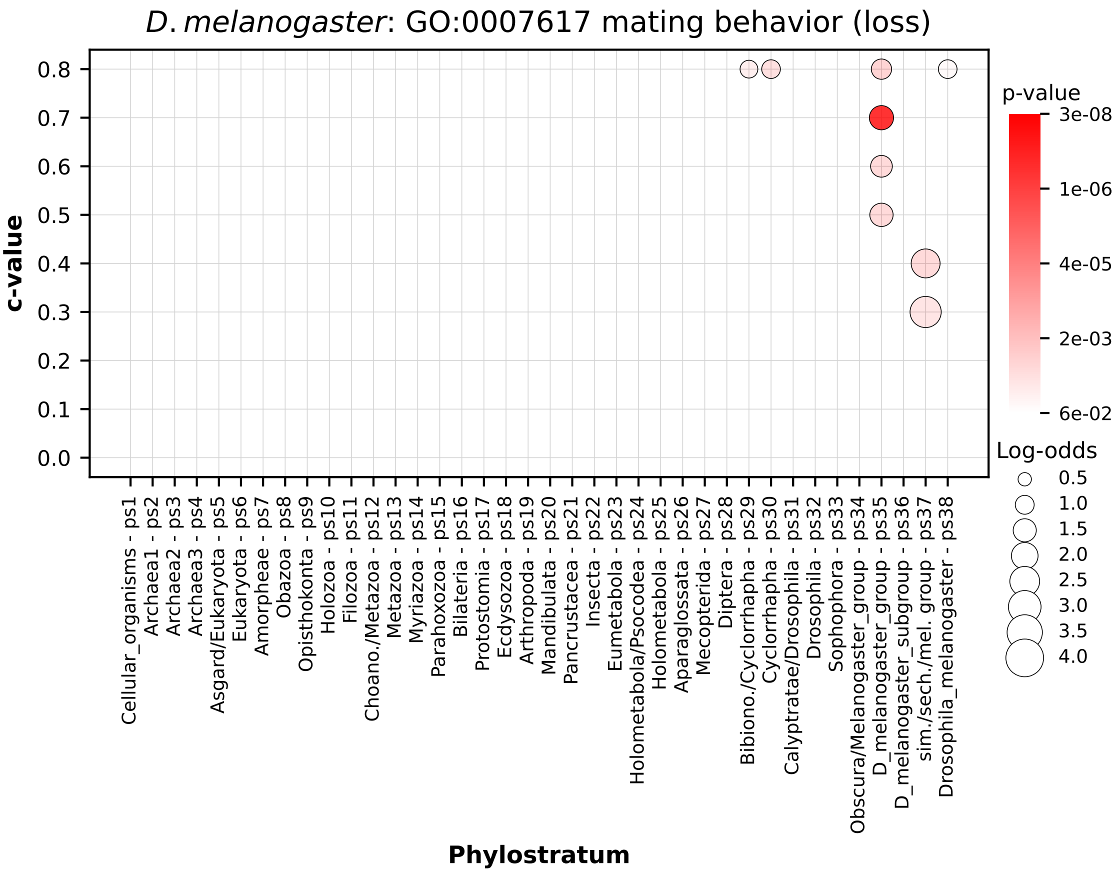

D. melanogaster: GO:0007617 mating behavior (loss)
c-value
0.0
0.1
0.2
0.3
0.4
0.5
0.6
0.7
0.8
p-value
3e-08
1e-06
4e-05
2e-03
6e-02
Log-odds
0.5
1.0
1.5
2.0
2.5
3.0
3.5
4.0
Cellular_organisms - ps1
Archaea1 - ps2
Archaea2 - ps3
Archaea3 - ps4
Asgard/Eukaryota - ps5
Eukaryota - ps6
Amorpheae - ps7
Obazoa - ps8
Opisthokonta - ps9
Holozoa - ps10
Filozoa - ps11
Choano./Metazoa - ps12
Metazoa - ps13
Myriazoa - ps14
Parahoxozoa - ps15
Bilateria - ps16
Protostomia - ps17
Ecdysozoa - ps18
Arthropoda - ps19
Mandibulata - ps20
Pancrustacea - ps21
Insecta - ps22
Eumetabola - ps23
Holometabola/Psocodea - ps24
Holometabola - ps25
Aparaglossata - ps26
Mecopterida - ps27
Diptera - ps28
Bibiono./Cyclorrhapha - ps29
Cyclorrhapha - ps30
Calyptratae/Drosophila - ps31
Drosophila - ps32
Sophophora - ps33
Obscura/Melanogaster_group - ps34
D_melanogaster_group - ps35
D_melanogaster_subgroup - ps36
sim./sech./mel. group - ps37
Drosophila_melanogaster - ps38
Phylostratum

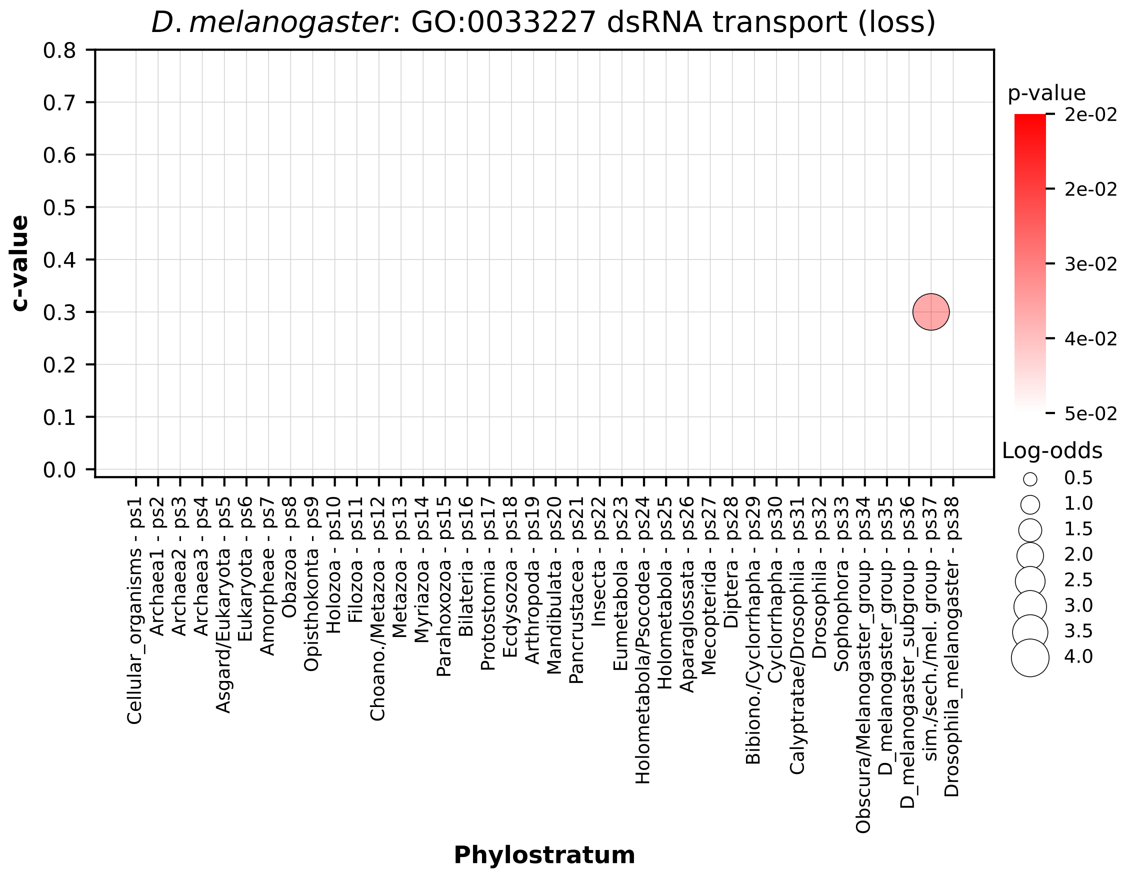

D. melanogaster: GO:0033227 dsRNA transport (loss)
c-value
0.8
0.7
0.6
0.5
0.4
0.3
0.2
0.1
0.0
p-value
2e-02
2e-02
3e-02
4e-02
5e-02
Log-odds
0.5
1.0
1.5
2.0
2.5
3.0
3.5
4.0
Cellular_organisms - ps1
Archaea1 - ps2
Archaea2 - ps3
Archaea3 - ps4
Asgard/Eukaryota - ps5
Eukaryota - ps6
Amorpheae - ps7
Obazoa - ps8
Opisthokonta - ps9
Holozoa - ps10
Filozoa - ps11
Choano./Metazoa - ps12
Metazoa - ps13
Myriazoa - ps14
Parahoxozoa - ps15
Bilateria - ps16
Protostomia - ps17
Ecdysozoa - ps18
Arthropoda - ps19
Mandibulata - ps20
Pancrustacea - ps21
Insecta - ps22
Eumetabola - ps23
Holometabola/Psocodea - ps24
Holometabola - ps25
Aparaglossata - ps26
Mecopterida - ps27
Diptera - ps28
Bibiono./Cyclorrhapha - ps29
Cyclorrhapha - ps30
Calyptratae/Drosophila - ps31
Drosophila - ps32
Sophophora - ps33
Obscura/Melanogaster_group - ps34
D_melanogaster_group - ps35
D_melanogaster_subgroup - ps36
sim./sech./mel. group - ps37
Drosophila_melanogaster - ps38
Phylostratum

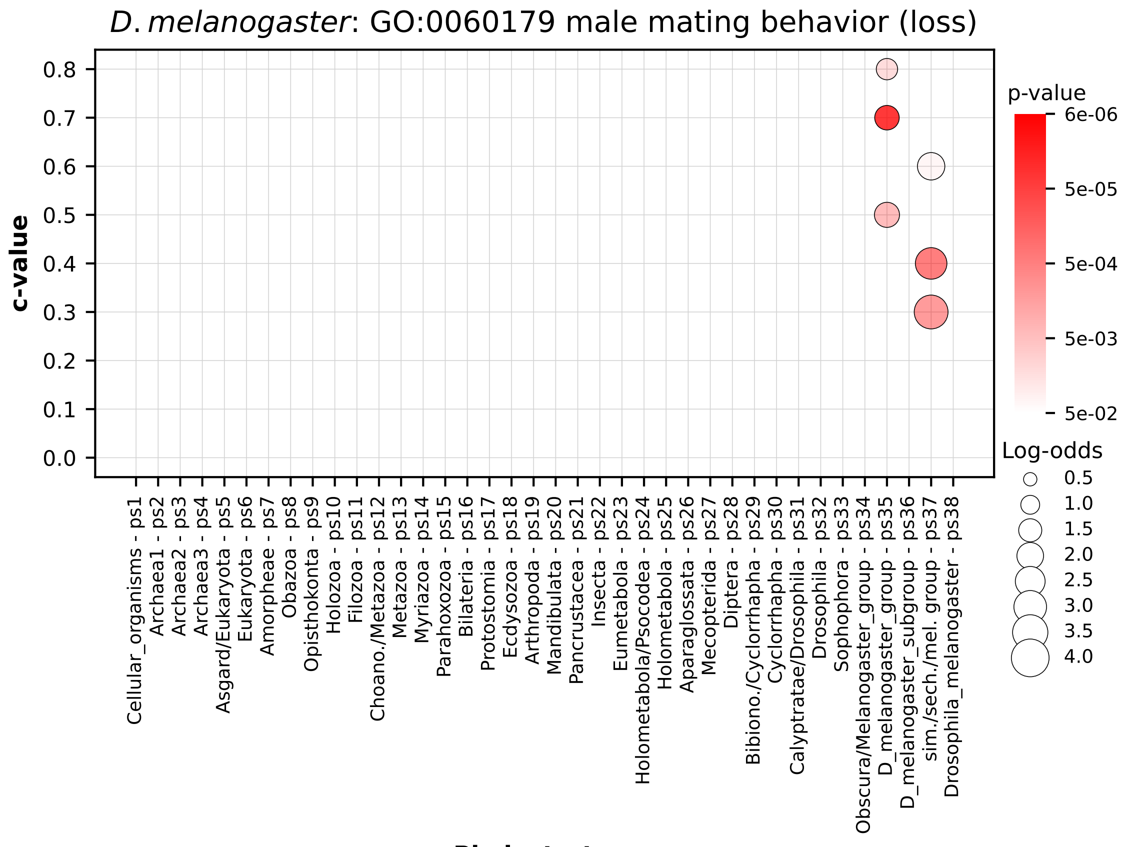
D. melanogaster: GO:0060179 male mating behavior (loss)
c-value
0.8
0.7
0.6
0.5
0.4
0.3
0.2
0.1
0.0
p-value
6e-06
5e-05
5e-04
5e-03
5e-02
Log-odds
0.5
1.0
1.5
2.0
2.5
3.0
3.5
4.0
Cellular_organisms - ps1
Archaea1 - ps2
Archaea2 - ps3
Archaea3 - ps4
Asgard/Eukaryota - ps5
Eukaryota - ps6
Amorpheae - ps7
Obazoa - ps8
Opisthokonta - ps9
Holozoa - ps10
Filozoa - ps11
Choano./Metazoa - ps12
Metazoa - ps13
Myriazoa - ps14
Parahoxozoa - ps15
Bilateria - ps16
Protostomia - ps17
Ecdysozoa - ps18
Arthropoda - ps19
Mandibulata - ps20
Pancrustacea - ps21
Insecta - ps22
Eumetabola - ps23
Holometabola/Psocodea - ps24
Holometabola - ps25
Aparaglossata - ps26
Mecopterida - ps27
Diptera - ps28
Bibiono./Cyclorrhapha - ps29
Cyclorrhapha - ps30
Calyptratae/Drosophila - ps31
Drosophila - ps32
Sophophora - ps33
Obscura/Melanogaster_group - ps34
D_melanogaster_group - ps35
D_melanogaster_subgroup - ps36
sim./sech./mel. group - ps37
Drosophila_melanogaster - ps38
Phylostratum

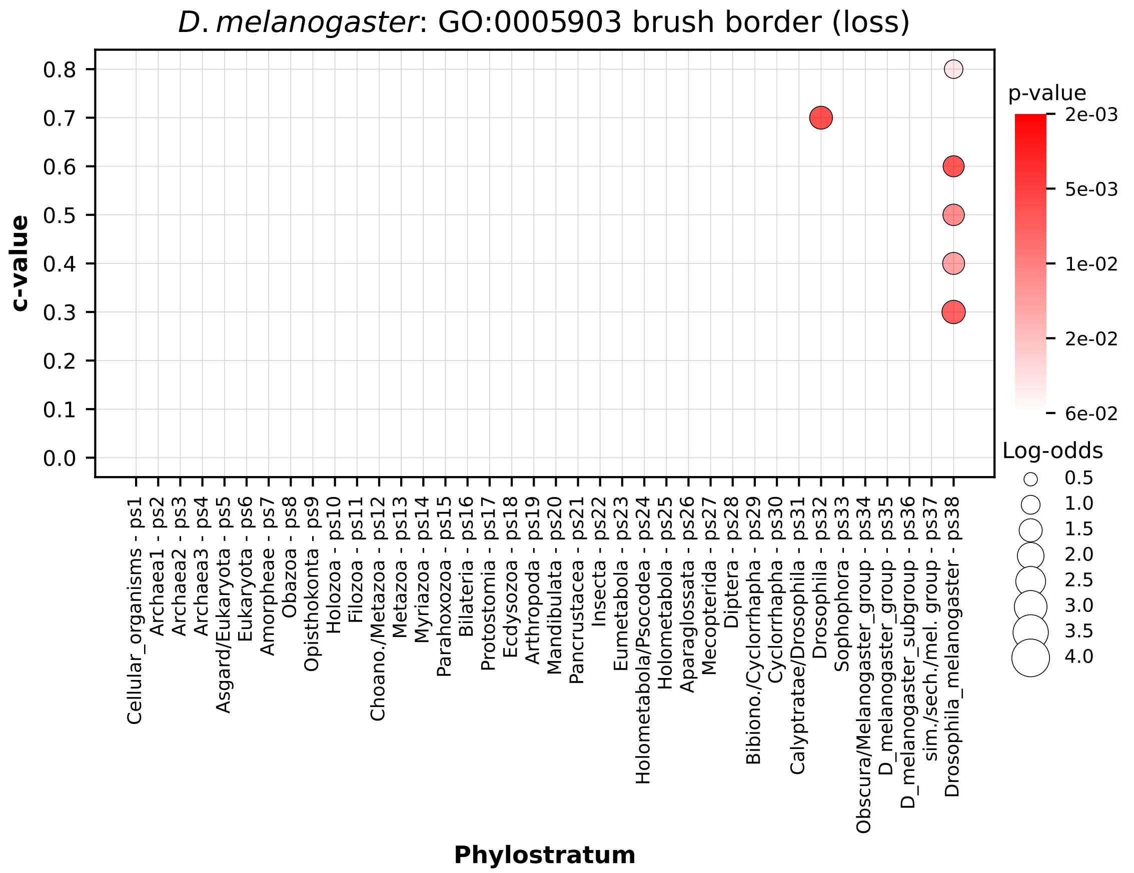

D. melanogaster: GO:0005903 brush border (loss)
c-value
0.0
0.1
0.2
0.3
0.4
0.5
0.6
0.7
0.8
Phylostratum
Cellular_organisms - ps1
Archaea1 - ps2
Archaea2 - ps3
Archaea3 - ps4
Asgard/Eukaryota - ps5
Eukaryota - ps6
Amorpheae - ps7
Obazoa - ps8
Opisthokonta - ps9
Holozoa - ps10
Filozoa - ps11
Choano./Metazoa - ps12
Metazoa - ps13
Myriazoa - ps14
Parahoxozoa - ps15
Bilateria - ps16
Protostomia - ps17
Ecdysozoa - ps18
Arthropoda - ps19
Mandibulata - ps20
Pancrustacea - ps21
Insecta - ps22
Eumetabola - ps23
Holometabola/Psocodea - ps24
Holometabola - ps25
Aparaglossata - ps26
Mecopterida - ps27
Diptera - ps28
Bibiono./Cyclorrhapha - ps29
Cyclorrhapha - ps30
Calyptratae/Drosophila - ps31
Drosophila - ps32
Sophophora - ps33
Obscura/Melanogaster_group - ps34
D_melanogaster_group - ps35
D_melanogaster_subgroup - ps36
sim./sech./mel. group - ps37
Drosophila_melanogaster - ps38
p-value
2e-03
5e-03
1e-02
2e-02
6e-02
Log-odds
0.5
1.0
1.5
2.0
2.5
3.0
3.5
4.0

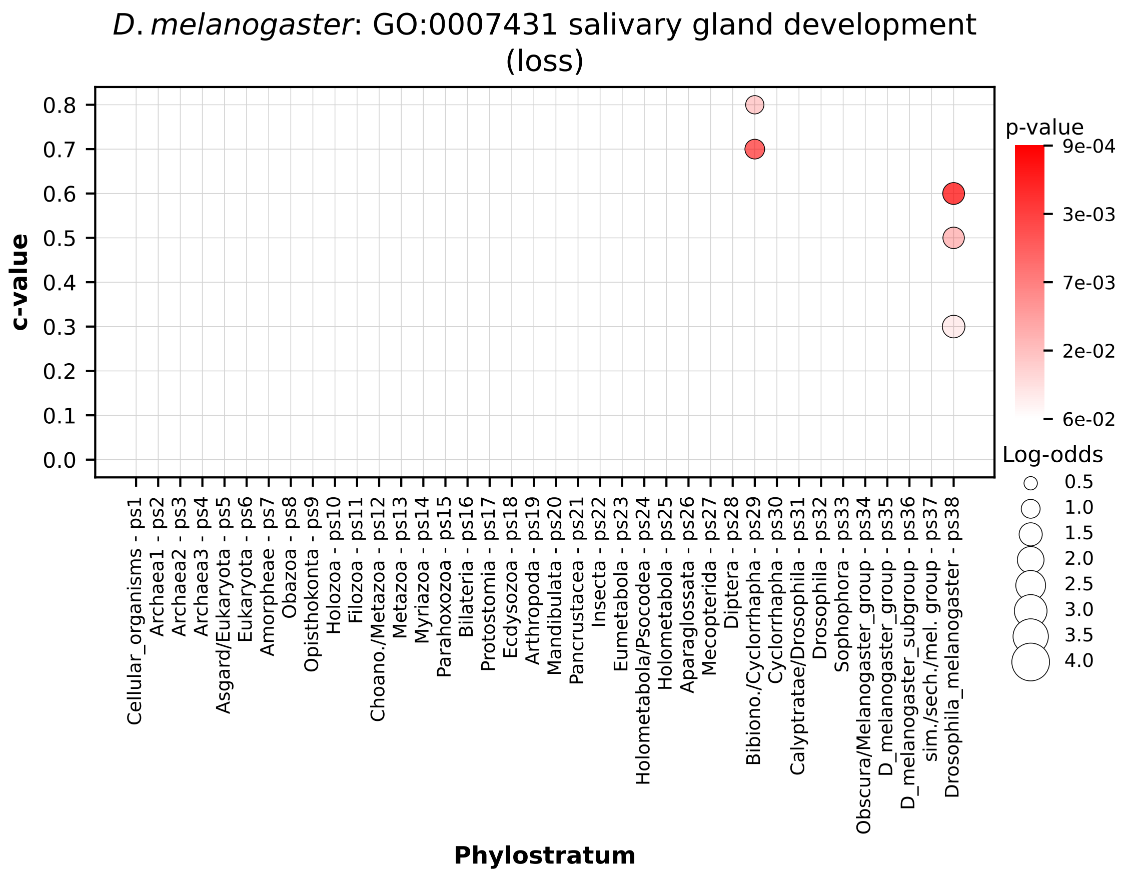
D. melanogaster: GO:0007431 salivary gland development
(loss)
c-value
0.8
0.7
0.6
0.5
0.4
0.3
0.2
0.1
0.0
p-value
9e-04
3e-03
7e-03
2e-02
6e-02
Log-odds
0.5
1.0
1.5
2.0
2.5
3.0
3.5
4.0
Cellular_organisms - ps1
Archaea1 - ps2
Archaea2 - ps3
Archaea3 - ps4
Asgard/Eukaryota - ps5
Eukaryota - ps6
Amorpheae - ps7
Obazoa - ps8
Opisthokonta - ps9
Holozoa - ps10
Filozoa - ps11
Choano./Metazoa - ps12
Metazoa - ps13
Myriazoa - ps14
Parahoxozoa - ps15
Bilateria - ps16
Protostomia - ps17
Ecdysozoa - ps18
Arthropoda - ps19
Mandibulata - ps20
Pancrustacea - ps21
Insecta - ps22
Eumetabola - ps23
Holometabola/Psocodea - ps24
Holometabola - ps25
Aparaglossata - ps26
Mecopterida - ps27
Diptera - ps28
Bibiono./Cyclorrhapha - ps29
Cyclorrhapha - ps30
Calyptratae/Drosophila - ps31
Drosophila - ps32
Sophophora - ps33
Obscura/Melanogaster_group - ps34
D_melanogaster_group - ps35
D_melanogaster_subgroup - ps36
sim./sech./mel. group - ps37
Drosophila_melanogaster - ps38
Phylostratum

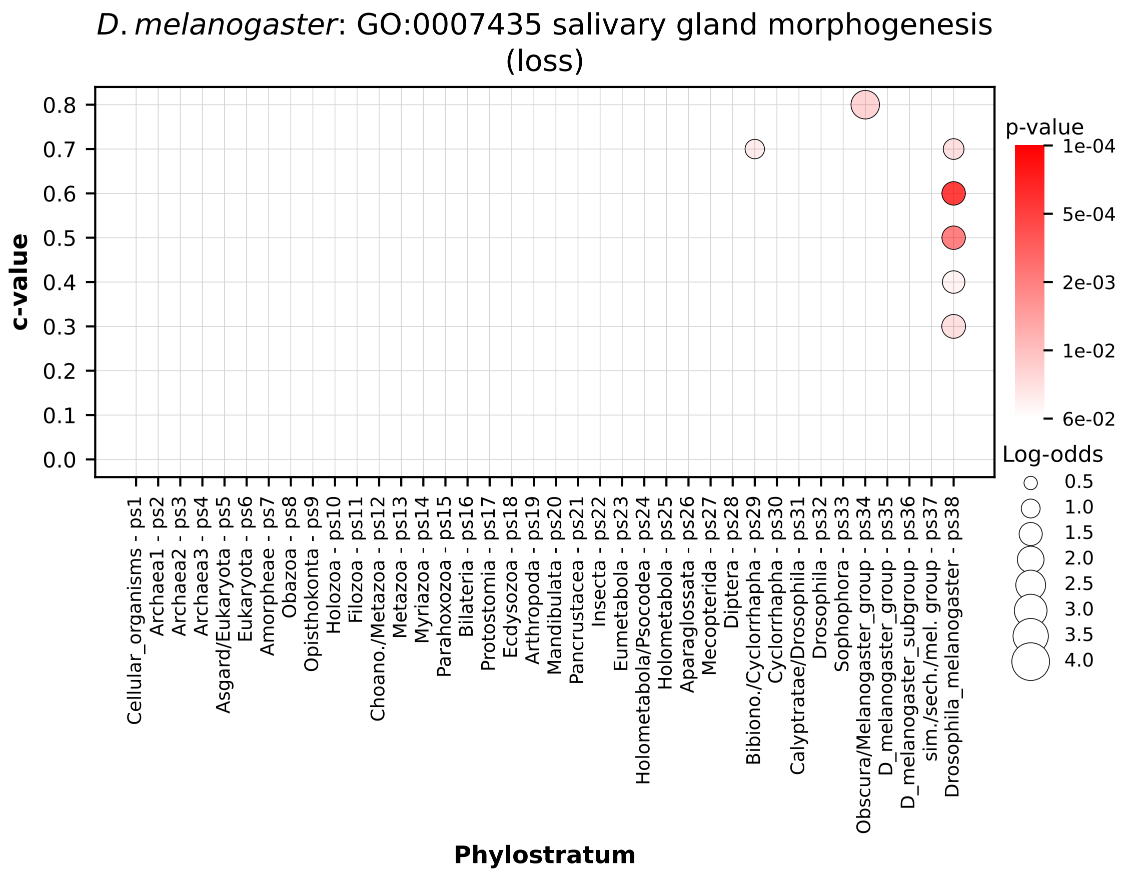

D. melanogaster: GO:0007435 salivary gland morphogenesis (loss)
c-value
0.8
0.7
0.6
0.5
0.4
0.3
0.2
0.1
0.0
Phylostratum
Cellular_organisms - ps1
Archaea1 - ps2
Archaea2 - ps3
Archaea3 - ps4
Asgard/Eukaryota - ps5
Eukaryota - ps6
Amorpheae - ps7
Obazoa - ps8
Opisthokonta - ps9
Holozoa - ps10
Filozoa - ps11
Choano./Metazoa - ps12
Metazoa - ps13
Myriazoa - ps14
Parahoxozoa - ps15
Bilateria - ps16
Protostomia - ps17
Ecdysozoa - ps18
Arthropoda - ps19
Mandibulata - ps20
Pancrustacea - ps21
Insecta - ps22
Eumetabola - ps23
Holometabola/Psocodea - ps24
Holometabola - ps25
Aparaglossata - ps26
Mecopterida - ps27
Diptera - ps28
Bibiono./Cyclorrhapha - ps29
Cyclorrhapha - ps30
Calyptratae/Drosophila - ps31
Drosophila - ps32
Sophophora - ps33
Obscura/Melanogaster_group - ps34
D_melanogaster_group - ps35
D_melanogaster_subgroup - ps36
sim./sech./mel. group - ps37
Drosophila_melanogaster - ps38
p-value
1e-04
5e-04
2e-03
1e-02
6e-02
Log-odds
0.5
1.0
1.5
2.0
2.5
3.0
3.5
4.0

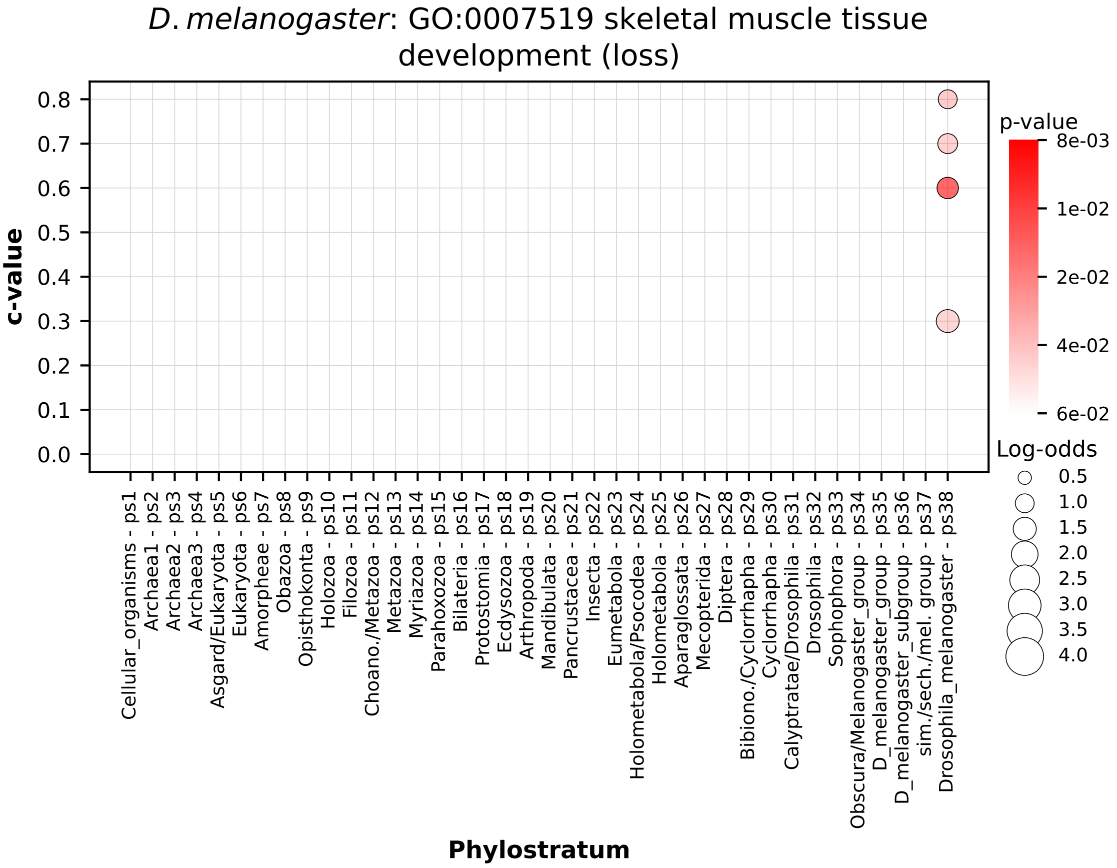
D. melanogaster: GO:0007519 skeletal muscle tissue development (loss)
c-value
0.0
0.1
0.2
0.3
0.4
0.5
0.6
0.7
0.8
Phylostratum
Cellular_organisms - ps1
Archaea1 - ps2
Archaea2 - ps3
Archaea3 - ps4
Asgard/Eukaryota - ps5
Eukaryota - ps6
Amorpheae - ps7
Obazoa - ps8
Opisthokonta - ps9
Holozoa - ps10
Filozoa - ps11
Choano./Metazoa - ps12
Metazoa - ps13
Myriazoa - ps14
Parahoxozoa - ps15
Bilateria - ps16
Protostomia - ps17
Ecdysozoa - ps18
Arthropoda - ps19
Mandibulata - ps20
Pancrustacea - ps21
Insecta - ps22
Eumetabola - ps23
Holometabola/Psocodea - ps24
Holometabola - ps25
Aparaglossata - ps26
Mecopterida - ps27
Diptera - ps28
Bibiono./Cyclorrhapha - ps29
Cyclorrhapha - ps30
Calyptratae/Drosophila - ps31
Drosophila - ps32
Sophophora - ps33
Obscura/Melanogaster_group - ps34
D_melanogaster_group - ps35
D_melanogaster_subgroup - ps36
sim./sech./mel. group - ps37
Drosophila_melanogaster - ps38
p-value
8e-03
1e-02
2e-02
4e-02
6e-02
Log-odds
0.5
1.0
1.5
2.0
2.5
3.0
3.5
4.0

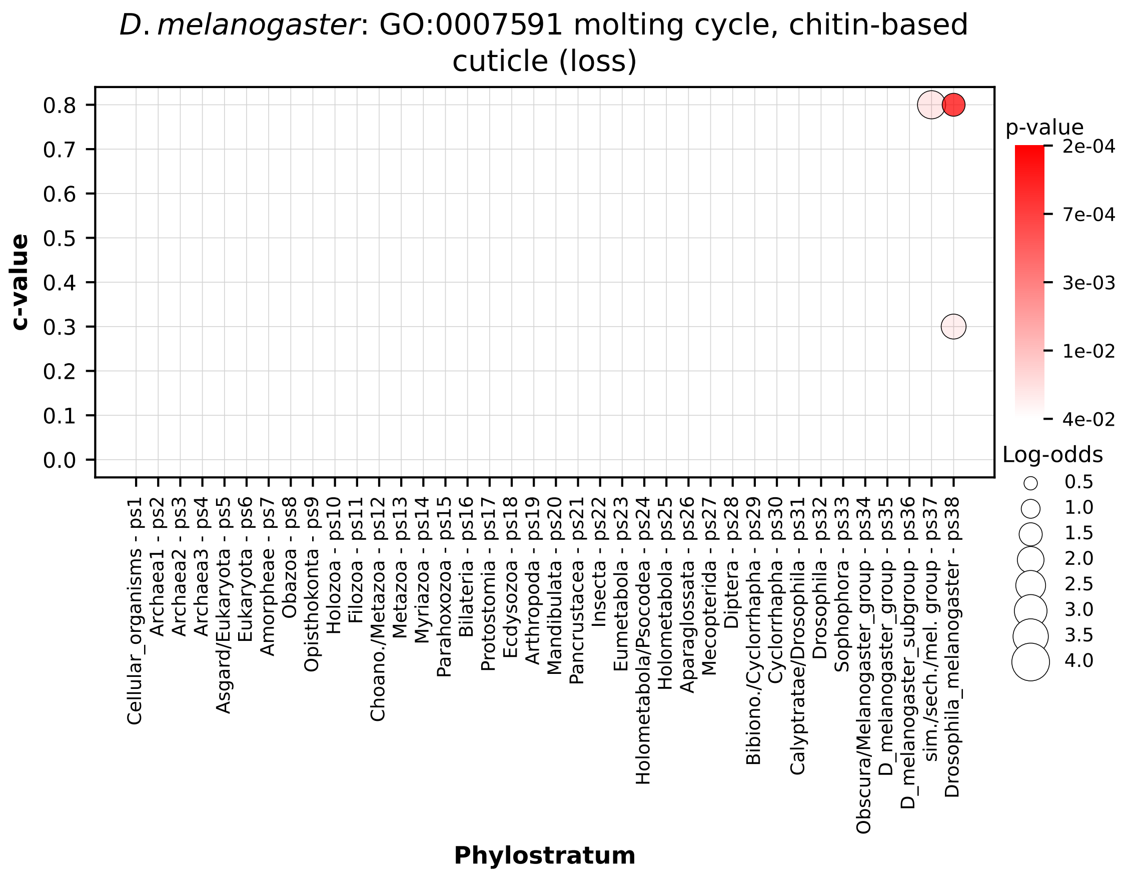

D. melanogaster: GO:0007591 molting cycle, chitin-based cuticle (loss)
c-value
0.8
0.7
0.6
0.5
0.4
0.3
0.2
0.1
0.0
Phylostratum
Cellular_organisms - ps1
Archaea1 - ps2
Archaea2 - ps3
Archaea3 - ps4
Asgard/Eukaryota - ps5
Eukaryota - ps6
Amorpheae - ps7
Obazoa - ps8
Opisthokonta - ps9
Holozoa - ps10
Filozoa - ps11
Choano./Metazoa - ps12
Metazoa - ps13
Myriazoa - ps14
Parahoxozoa - ps15
Bilateria - ps16
Protostomia - ps17
Ecdysozoa - ps18
Arthropoda - ps19
Mandibulata - ps20
Pancrustacea - ps21
Insecta - ps22
Eumetabola - ps23
Holometabola/Psocodea - ps24
Holometabola - ps25
Aparaglossata - ps26
Mecopterida - ps27
Diptera - ps28
Bibiono./Cyclorrhapha - ps29
Cyclorrhapha - ps30
Calyptratae/Drosophila - ps31
Drosophila - ps32
Sophophora - ps33
Obscura/Melanogaster_group - ps34
D_melanogaster_group - ps35
D_melanogaster_subgroup - ps36
sim./sech./mel. group - ps37
Drosophila_melanogaster - ps38
p-value
2e-04
7e-04
3e-03
1e-02
4e-02
Log-odds
0.5
1.0
1.5
2.0
2.5
3.0
3.5
4.0

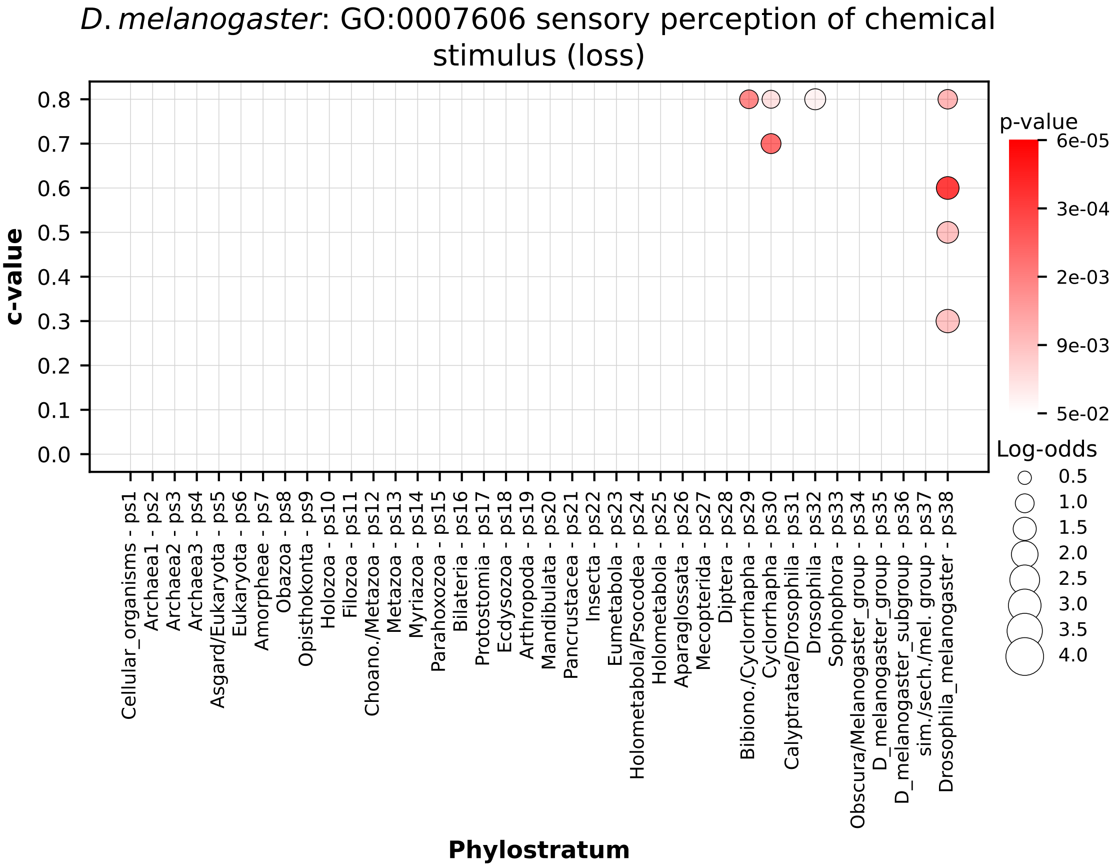

D. melanogaster: GO:0007606 sensory perception of chemical stimulus (loss)
c-value
0.8
0.7
0.6
0.5
0.4
0.3
0.2
0.1
0.0
Phylostratum
Cellular_organisms - ps1
Archaea1 - ps2
Archaea2 - ps3
Archaea3 - ps4
Asgard/Eukaryota - ps5
Eukaryota - ps6
Amorpheae - ps7
Obazoa - ps8
Opisthokonta - ps9
Holozoa - ps10
Filozoa - ps11
Choano./Metazoa - ps12
Metazoa - ps13
Myriazoa - ps14
Parahoxozoa - ps15
Bilateria - ps16
Protostomia - ps17
Ecdysozoa - ps18
Arthropoda - ps19
Mandibulata - ps20
Pancrustacea - ps21
Insecta - ps22
Eumetabola - ps23
Holometabola/Psocodea - ps24
Holometabola - ps25
Aparaglossata - ps26
Mecopterida - ps27
Diptera - ps28
Bibiono./Cyclorrhapha - ps29
Cyclorrhapha - ps30
Calyptratae/Drosophila - ps31
Drosophila - ps32
Sophophora - ps33
Obscura/Melanogaster_group - ps34
D_melanogaster_group - ps35
D_melanogaster_subgroup - ps36
sim./sech./mel. group - ps37
Drosophila_melanogaster - ps38
p-value
6e-05
3e-04
2e-03
9e-03
5e-02
Log-odds
0.5
1.0
1.5
2.0
2.5
3.0
3.5
4.0

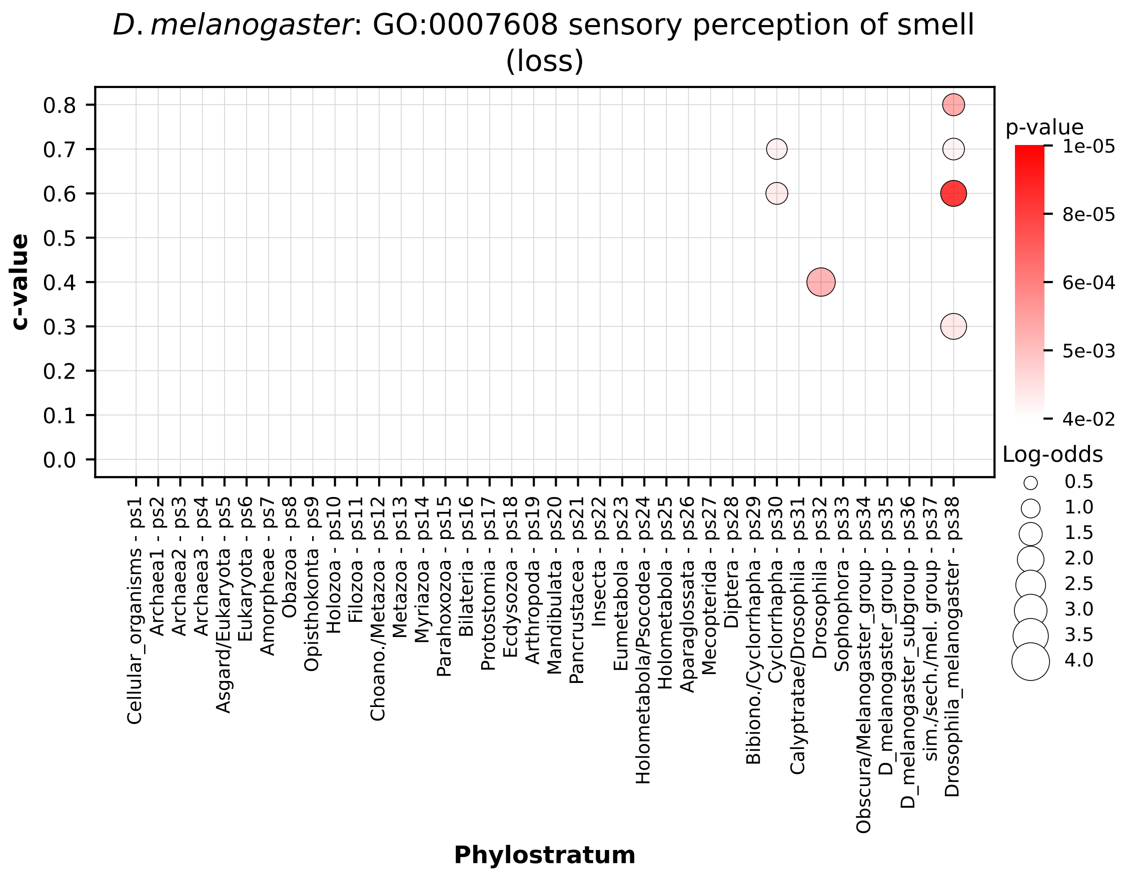
D. melanogaster: GO:0007608 sensory perception of smell
(loss)
c-value
0.0
0.1
0.2
0.3
0.4
0.5
0.6
0.7
0.8
p-value
1e-05
8e-05
6e-04
5e-03
4e-02
Log-odds
0.5
1.0
1.5
2.0
2.5
3.0
3.5
4.0
Cellular_organisms - ps1
Archaea1 - ps2
Archaea2 - ps3
Archaea3 - ps4
Asgard/Eukaryota - ps5
Eukaryota - ps6
Amorpheae - ps7
Obazoa - ps8
Opisthokonta - ps9
Holozoa - ps10
Filozoa - ps11
Choano./Metazoa - ps12
Metazoa - ps13
Myriazoa - ps14
Parahoxozoa - ps15
Bilateria - ps16
Protostomia - ps17
Ecdysozoa - ps18
Arthropoda - ps19
Mandibulata - ps20
Pancrustacea - ps21
Insecta - ps22
Eumetabola - ps23
Holometabola/Psocodea - ps24
Holometabola - ps25
Aparaglossata - ps26
Mecopterida - ps27
Diptera - ps28
Bibiono./Cyclorrhapha - ps29
Cyclorrhapha - ps30
Calyptratae/Drosophila - ps31
Drosophila - ps32
Sophophora - ps33
Obscura/Melanogaster_group - ps34
D_melanogaster_group - ps35
D_melanogaster_subgroup - ps36
sim./sech./mel. group - ps37
Drosophila_melanogaster - ps38
Phylostratum

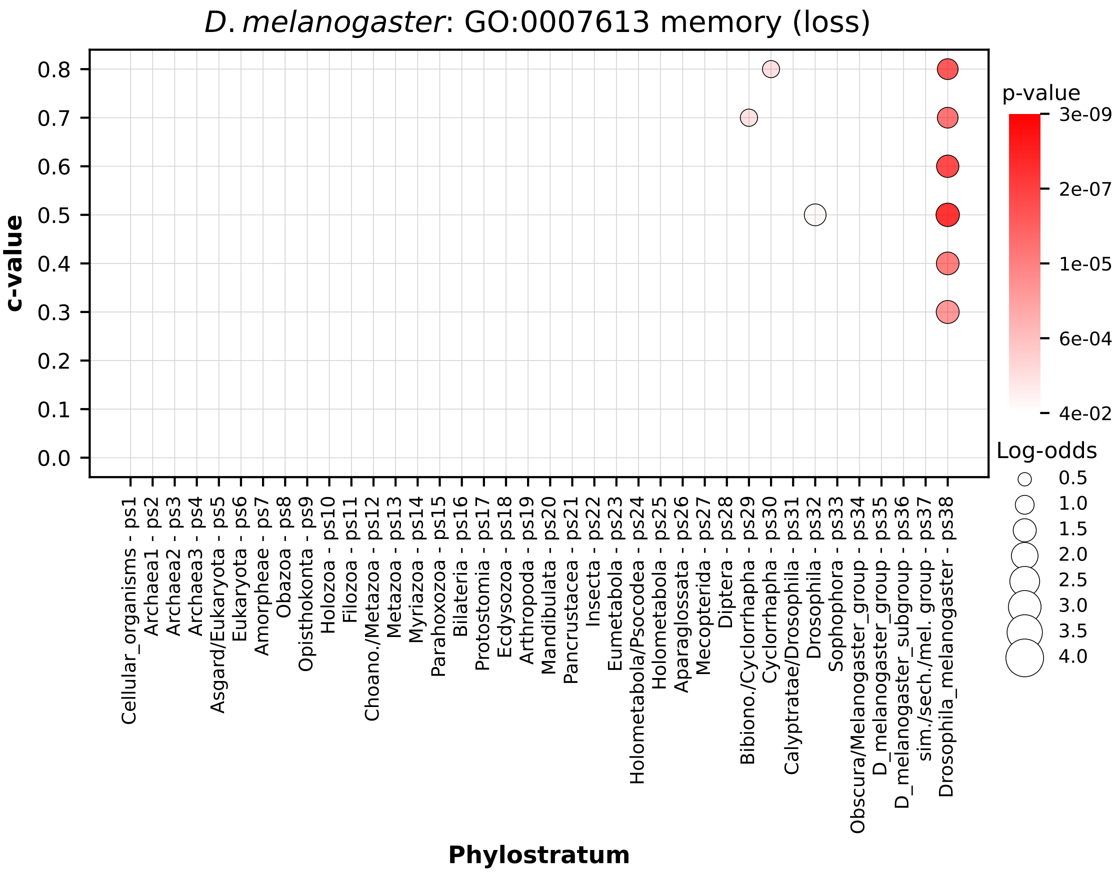

D. melanogaster: GO:0007613 memory (loss)
c-value
0.8
0.7
0.6
0.5
0.4
0.3
0.2
0.1
0.0
Phylostratum
Cellular_organisms - ps1
Archaea1 - ps2
Archaea2 - ps3
Archaea3 - ps4
Asgard/Eukaryota - ps5
Eukaryota - ps6
Amorpheae - ps7
Obazoa - ps8
Opisthokonta - ps9
Holozoa - ps10
Filozoa - ps11
Choano./Metazoa - ps12
Metazoa - ps13
Myriazoa - ps14
Parahoxozoa - ps15
Bilateria - ps16
Protostomia - ps17
Ecdysozoa - ps18
Arthropoda - ps19
Mandibulata - ps20
Pancrustacea - ps21
Insecta - ps22
Eumetabola - ps23
Holometabola/Psocodea - ps24
Holometabola - ps25
Aparaglossata - ps26
Mecopterida - ps27
Diptera - ps28
Bibiono./Cyclorrhapha - ps29
Cyclorrhapha - ps30
Calyptratae/Drosophila - ps31
Drosophila - ps32
Sophophora - ps33
Obscura/Melanogaster_group - ps34
D_melanogaster_group - ps35
D_melanogaster_subgroup - ps36
sim./sech./mel. group - ps37
Drosophila_melanogaster - ps38
p-value
3e-09
2e-07
1e-05
6e-04
4e-02
Log-odds
0.5
1.0
1.5
2.0
2.5
3.0
3.5
4.0

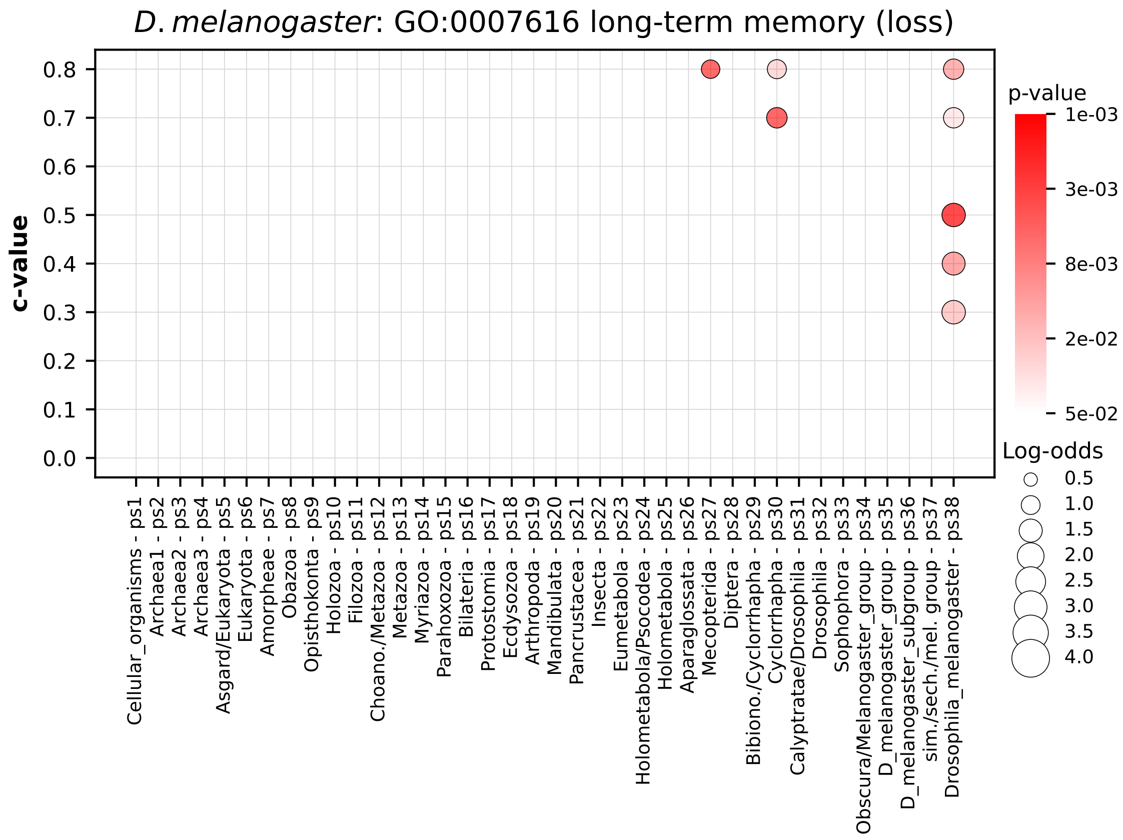

D. melanogaster: GO:0007616 long-term memory (loss)
c-value
0.0
0.1
0.2
0.3
0.4
0.5
0.6
0.7
0.8
Phylostratum
Cellular_organisms - ps1
Archaea1 - ps2
Archaea2 - ps3
Archaea3 - ps4
Asgard/Eukaryota - ps5
Eukaryota - ps6
Amorpheae - ps7
Obazoa - ps8
Opisthokonta - ps9
Holozoa - ps10
Filozoa - ps11
Choano./Metazoa - ps12
Metazoa - ps13
Myriazoa - ps14
Parahoxozoa - ps15
Bilateria - ps16
Protostomia - ps17
Ecdysozoa - ps18
Arthropoda - ps19
Mandibulata - ps20
Pancrustacea - ps21
Insecta - ps22
Eumetabola - ps23
Holometabola/Psocodea - ps24
Holometabola - ps25
Aparaglossata - ps26
Mecopterida - ps27
Diptera - ps28
Bibiono./Cyclorrhapha - ps29
Cyclorrhapha - ps30
Calyptratae/Drosophila - ps31
Drosophila - ps32
Sophophora - ps33
Obscura/Melanogaster_group - ps34
D_melanogaster_group - ps35
D_melanogaster_subgroup - ps36
sim./sech./mel. group - ps37
Drosophila_melanogaster - ps38
p-value
1e-03
3e-03
8e-03
2e-02
5e-02
Log-odds
0.5
1.0
1.5
2.0
2.5
3.0
3.5
4.0

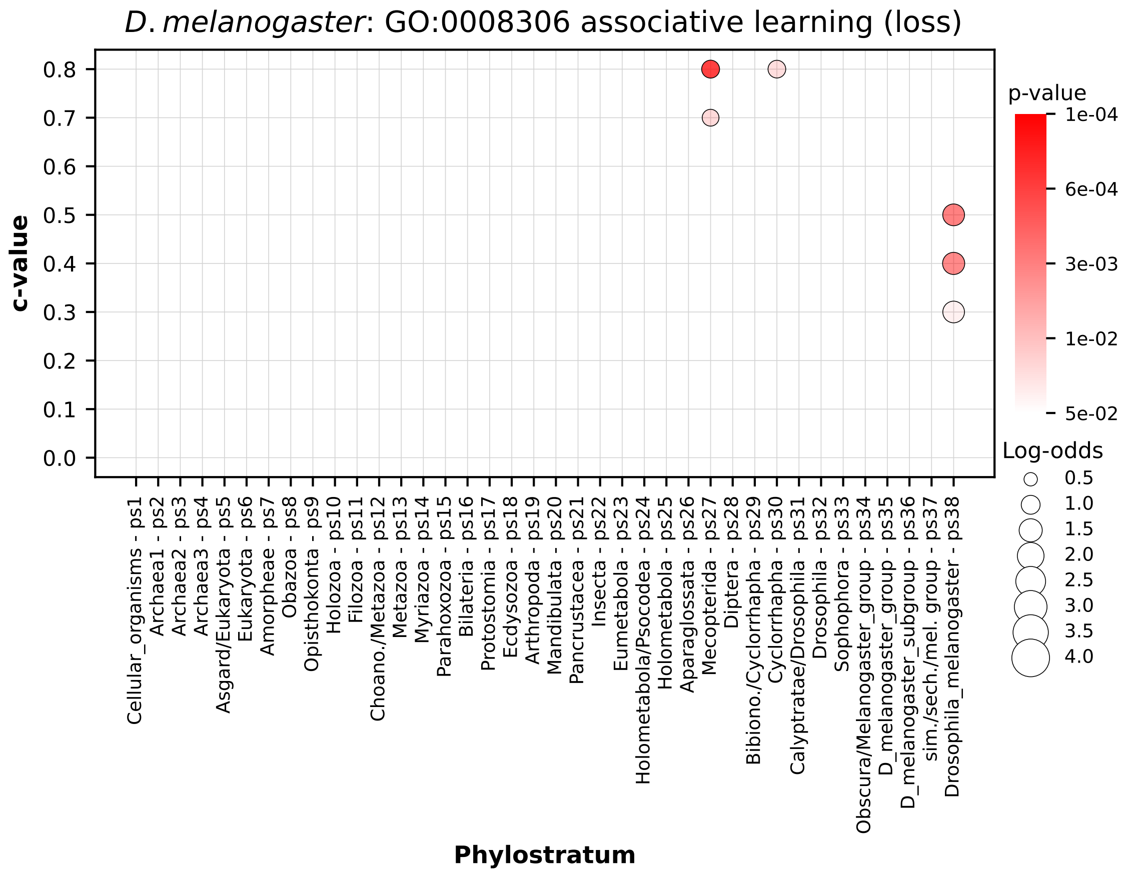

D. melanogaster: GO:0008306 associative learning (loss)
c-value
0.0
0.1
0.2
0.3
0.4
0.5
0.6
0.7
0.8
Phylostratum
Cellular_organisms - ps1
Archaea1 - ps2
Archaea2 - ps3
Archaea3 - ps4
Asgard/Eukaryota - ps5
Eukaryota - ps6
Amorpheae - ps7
Obazoa - ps8
Opisthokonta - ps9
Holozoa - ps10
Filozoa - ps11
Choano./Metazoa - ps12
Metazoa - ps13
Myriazoa - ps14
Parahoxozoa - ps15
Bilateria - ps16
Protostomia - ps17
Ecdysozoa - ps18
Arthropoda - ps19
Mandibulata - ps20
Pancrustacea - ps21
Insecta - ps22
Eumetabola - ps23
Holometabola/Psocodea - ps24
Holometabola - ps25
Aparaglossata - ps26
Mecopterida - ps27
Diptera - ps28
Bibiono./Cyclorrhapha - ps29
Cyclorrhapha - ps30
Calyptratae/Drosophila - ps31
Drosophila - ps32
Sophophora - ps33
Obscura/Melanogaster_group - ps34
D_melanogaster_group - ps35
D_melanogaster_subgroup - ps36
sim./sech./mel. group - ps37
Drosophila_melanogaster - ps38
p-value
1e-04
6e-04
3e-03
1e-02
5e-02
Log-odds
0.5
1.0
1.5
2.0
2.5
3.0
3.5
4.0

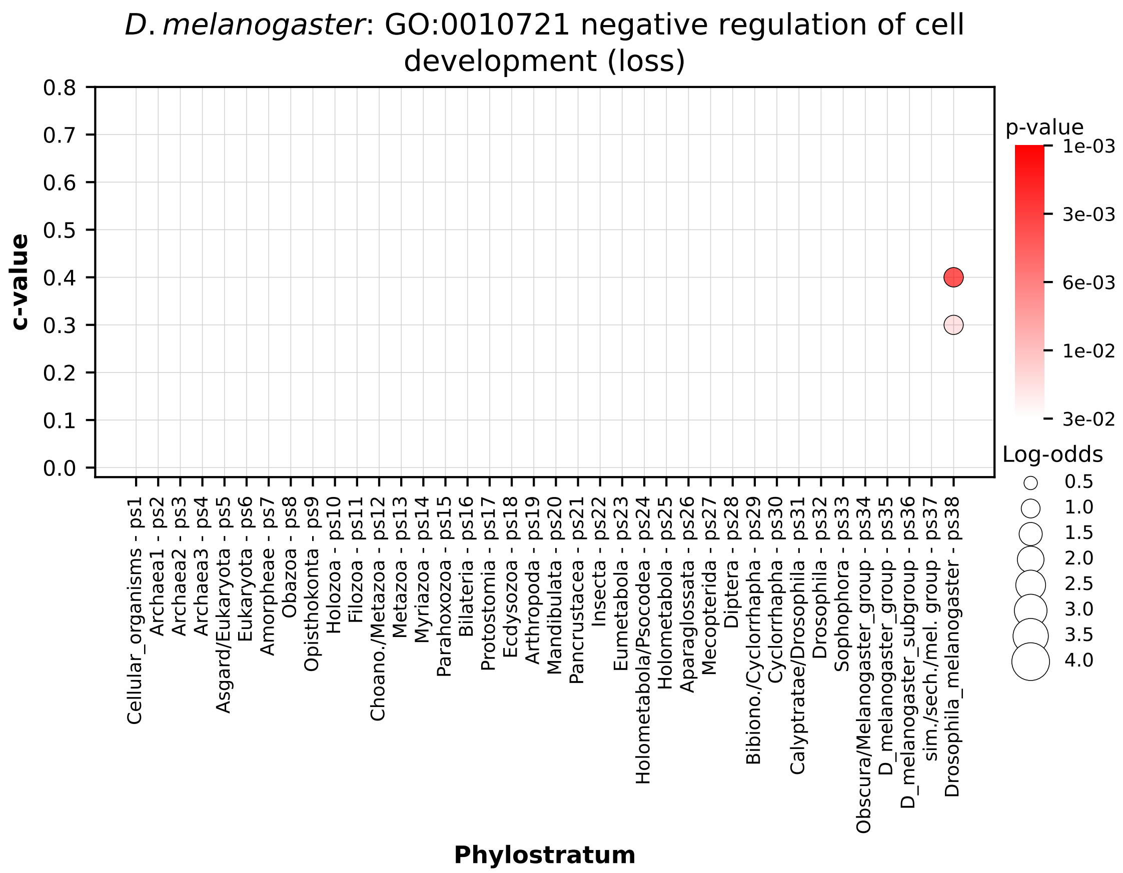
D. melanogaster: GO:0010721 negative regulation of cell development (loss)
c-value
0.8
0.7
0.6
0.5
0.4
0.3
0.2
0.1
0.0
Phylostratum
Cellular_organisms - ps1
Archaea1 - ps2
Archaea2 - ps3
Archaea3 - ps4
Asgard/Eukaryota - ps5
Eukaryota - ps6
Amorpheae - ps7
Obazoa - ps8
Opisthokonta - ps9
Holozoa - ps10
Filozoa - ps11
Choano./Metazoa - ps12
Metazoa - ps13
Myriazoa - ps14
Parahoxozoa - ps15
Bilateria - ps16
Protostomia - ps17
Ecdysozoa - ps18
Arthropoda - ps19
Mandibulata - ps20
Pancrustacea - ps21
Insecta - ps22
Eumetabola - ps23
Holometabola/Psocodea - ps24
Holometabola - ps25
Aparaglossata - ps26
Mecopterida - ps27
Diptera - ps28
Bibiono./Cyclorrhapha - ps29
Cyclorrhapha - ps30
Calyptratae/Drosophila - ps31
Drosophila - ps32
Sophophora - ps33
Obscura/Melanogaster_group - ps34
D_melanogaster_group - ps35
D_melanogaster_subgroup - ps36
sim./sech./mel. group - ps37
Drosophila_melanogaster - ps38
p-value
1e-03
3e-03
6e-03
1e-02
3e-02
Log-odds
0.5
1.0
1.5
2.0
2.5
3.0
3.5
4.0

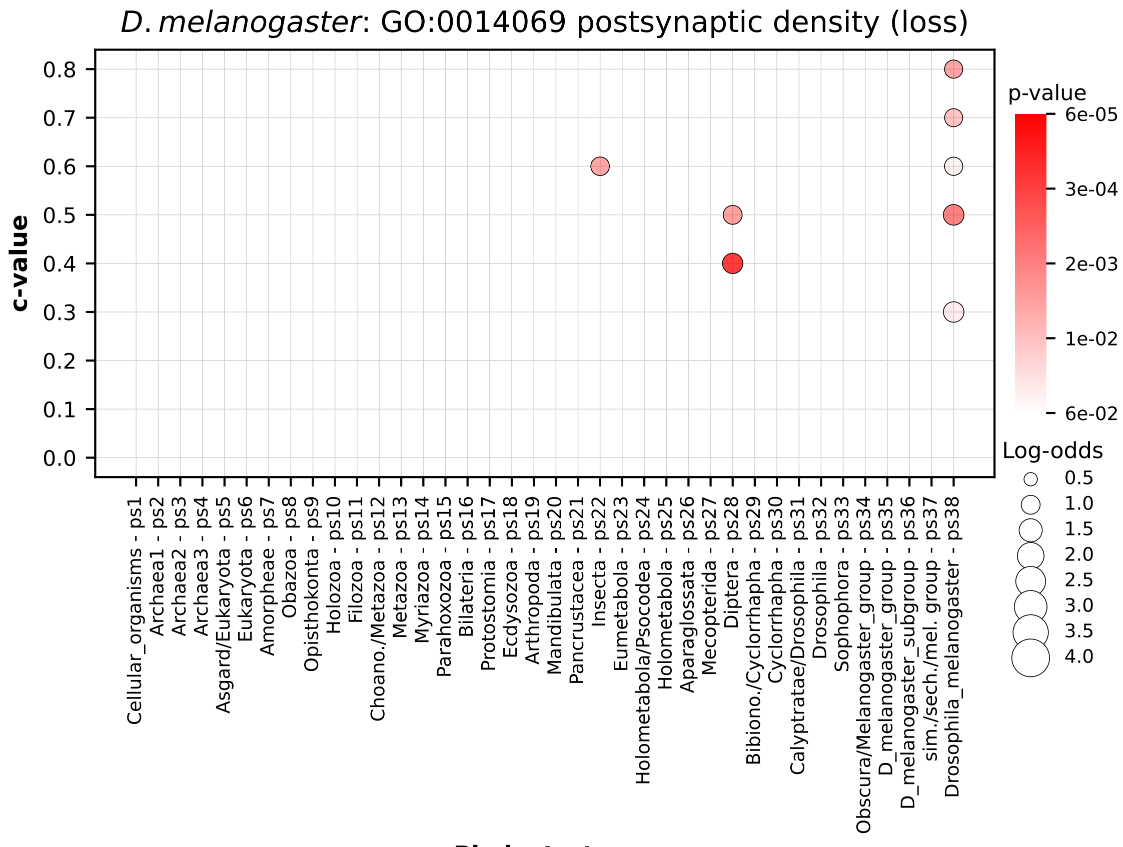

D. melanogaster: GO:0014069 postsynaptic density (loss)
c-value
0.0
0.1
0.2
0.3
0.4
0.5
0.6
0.7
0.8
Phylostratum
Cellular_organisms - ps1
Archaea1 - ps2
Archaea2 - ps3
Archaea3 - ps4
Asgard/Eukaryota - ps5
Eukaryota - ps6
Amorpheae - ps7
Obazoa - ps8
Opisthokonta - ps9
Holozoa - ps10
Filozoa - ps11
Choano./Metazoa - ps12
Metazoa - ps13
Myriazoa - ps14
Parahoxozoa - ps15
Bilateria - ps16
Protostomia - ps17
Ecdysozoa - ps18
Arthropoda - ps19
Mandibulata - ps20
Pancrustacea - ps21
Insecta - ps22
Eumetabola - ps23
Holometabola/Psocodea - ps24
Holometabola - ps25
Aparaglossata - ps26
Mecopterida - ps27
Diptera - ps28
Bibiono./Cyclorrhapha - ps29
Cyclorrhapha - ps30
Calyptratae/Drosophila - ps31
Drosophila - ps32
Sophophora - ps33
Obscura/Melanogaster_group - ps34
D_melanogaster_group - ps35
D_melanogaster_subgroup - ps36
sim./sech./mel. group - ps37
Drosophila_melanogaster - ps38
p-value
6e-05
3e-04
2e-03
1e-02
6e-02
Log-odds
0.5
1.0
1.5
2.0
2.5
3.0
3.5
4.0

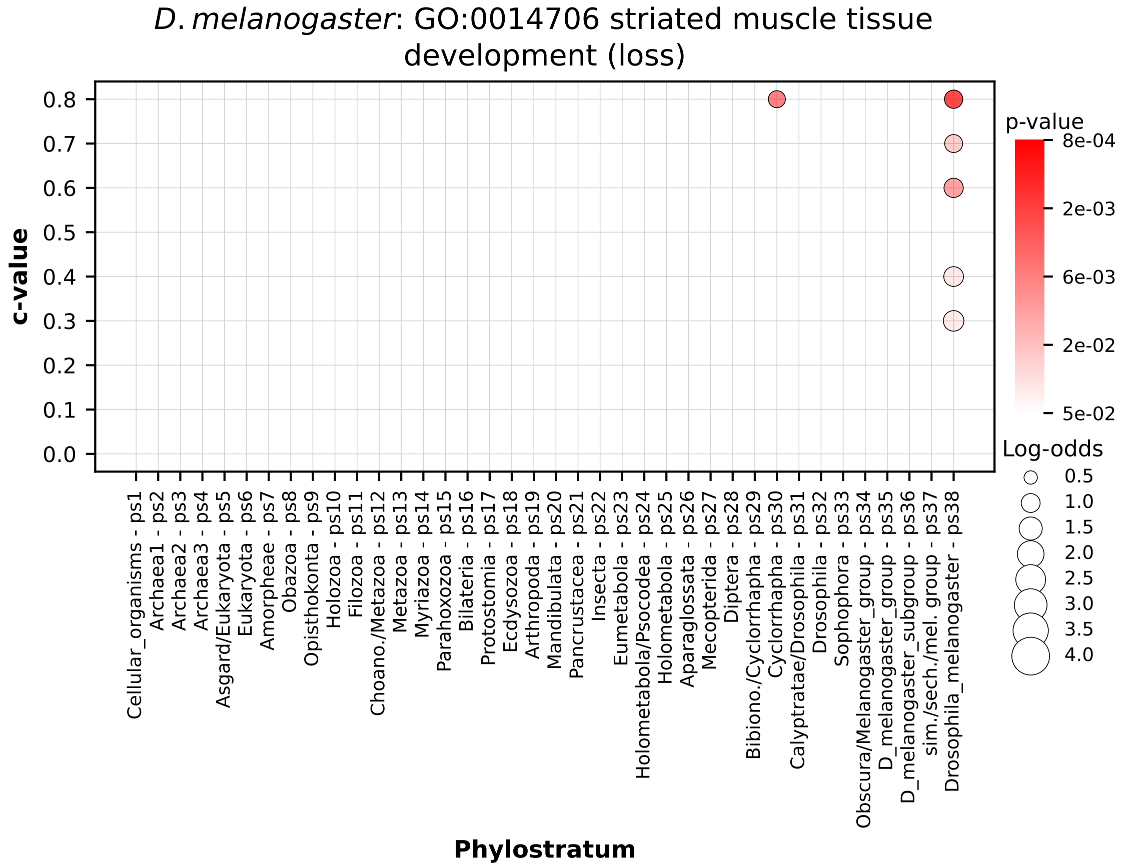

D. melanogaster: GO:0014706 striated muscle tissue development (loss)
c-value
0.8
0.7
0.6
0.5
0.4
0.3
0.2
0.1
0.0
p-value
8e-04
2e-03
6e-03
2e-02
5e-02
Log-odds
0.5
1.0
1.5
2.0
2.5
3.0
3.5
4.0
Cellular_organisms - ps1
Archaea1 - ps2
Archaea2 - ps3
Archaea3 - ps4
Asgard/Eukaryota - ps5
Eukaryota - ps6
Amorpheae - ps7
Obazoa - ps8
Opisthokonta - ps9
Holozoa - ps10
Filozoa - ps11
Choano./Metazoa - ps12
Metazoa - ps13
Myriazoa - ps14
Parahoxozoa - ps15
Bilateria - ps16
Protostomia - ps17
Ecdysozoa - ps18
Arthropoda - ps19
Mandibulata - ps20
Pancrustacea - ps21
Insecta - ps22
Eumetabola - ps23
Holometabola/Psocodea - ps24
Holometabola - ps25
Aparaglossata - ps26
Mecopterida - ps27
Diptera - ps28
Bibiono./Cyclorrhapha - ps29
Cyclorrhapha - ps30
Calyptratae/Drosophila - ps31
Drosophila - ps32
Sophophora - ps33
Obscura/Melanogaster_group - ps34
D_melanogaster_group - ps35
D_melanogaster_subgroup - ps36
sim./sech./mel. group - ps37
Drosophila_melanogaster - ps38
Phylostratum

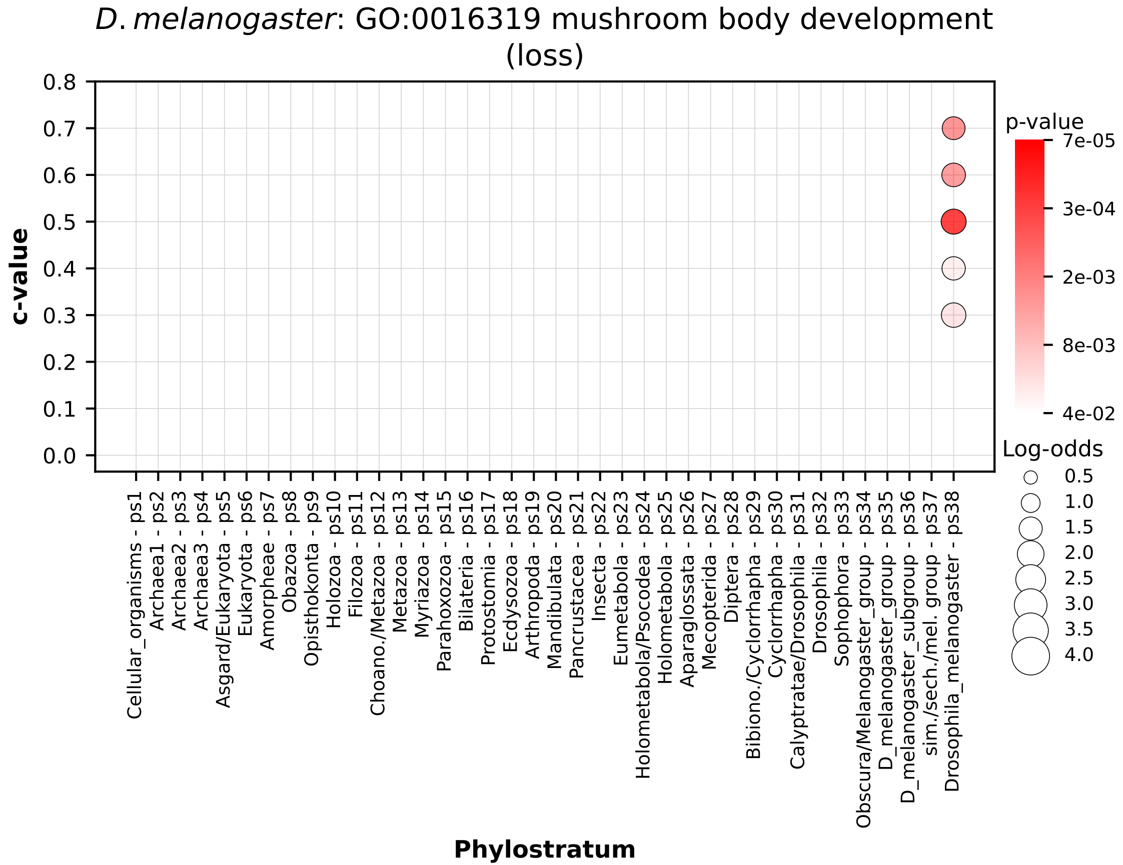

D. melanogaster: GO:0016319 mushroom body development (loss)
c-value
0.8
0.7
0.6
0.5
0.4
0.3
0.2
0.1
0.0
Phylostratum
Cellular_organisms - ps1
Archaea1 - ps2
Archaea2 - ps3
Archaea3 - ps4
Asgard/Eukaryota - ps5
Eukaryota - ps6
Amorpheae - ps7
Obazoa - ps8
Opisthokonta - ps9
Holozoa - ps10
Filozoa - ps11
Choano./Metazoa - ps12
Metazoa - ps13
Myriazoa - ps14
Parahoxozoa - ps15
Bilateria - ps16
Protostomia - ps17
Ecdysozoa - ps18
Arthropoda - ps19
Mandibulata - ps20
Pancrustacea - ps21
Insecta - ps22
Eumetabola - ps23
Holometabola/Psocodea - ps24
Holometabola - ps25
Aparaglossata - ps26
Mecopterida - ps27
Diptera - ps28
Bibiono./Cyclorrhapha - ps29
Cyclorrhapha - ps30
Calyptratae/Drosophila - ps31
Drosophila - ps32
Sophophora - ps33
Obscura/Melanogaster_group - ps34
D_melanogaster_group - ps35
D_melanogaster_subgroup - ps36
sim./sech./mel. group - ps37
Drosophila_melanogaster - ps38
p-value
7e-05
3e-04
2e-03
8e-03
4e-02
Log-odds
0.5
1.0
1.5
2.0
2.5
3.0
3.5
4.0

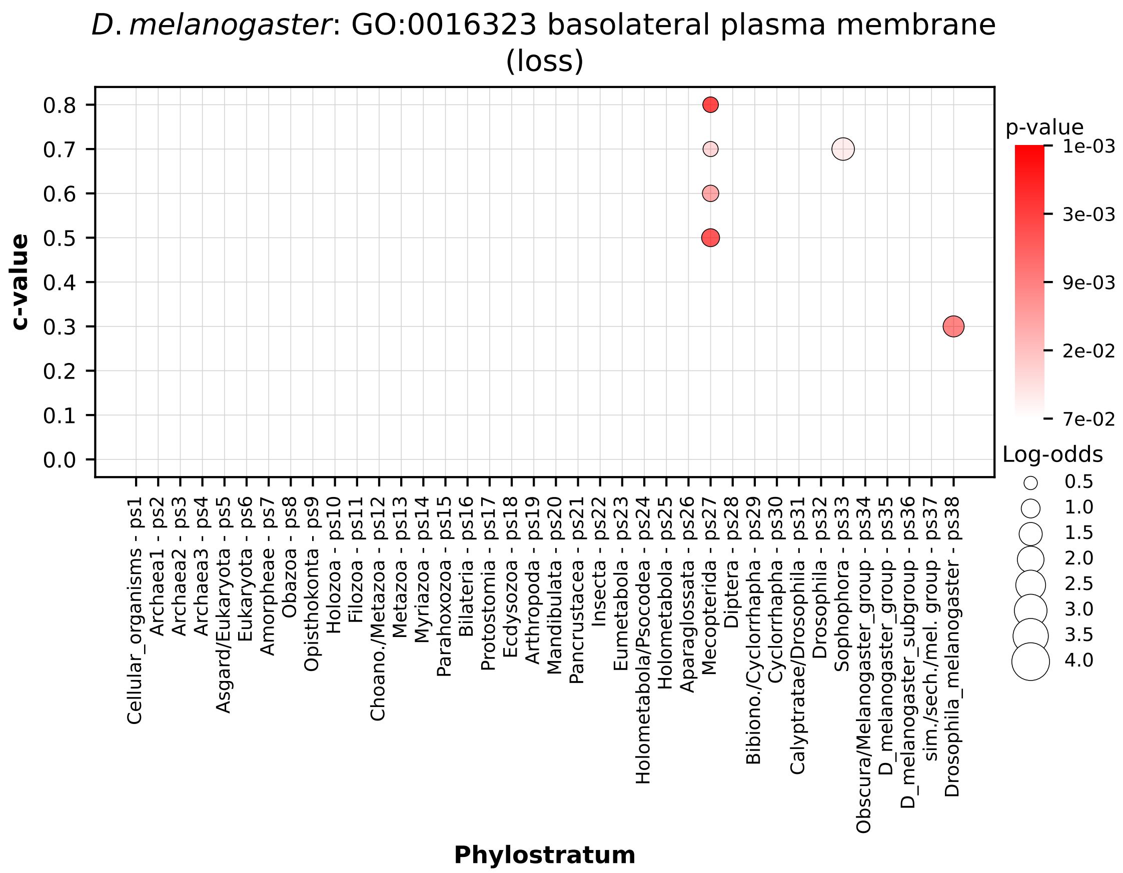

D. melanogaster: GO:0016323 basolateral plasma membrane (loss)
c-value
0.8
0.7
0.6
0.5
0.4
0.3
0.2
0.1
0.0
Phylostratum
Cellular_organisms - ps1
Archaea1 - ps2
Archaea2 - ps3
Archaea3 - ps4
Asgard/Eukaryota - ps5
Eukaryota - ps6
Amorpheae - ps7
Obazoa - ps8
Opisthokonta - ps9
Holozoa - ps10
Filozoa - ps11
Choano./Metazoa - ps12
Metazoa - ps13
Myriazoa - ps14
Parahoxozoa - ps15
Bilateria - ps16
Protostomia - ps17
Ecdysozoa - ps18
Arthropoda - ps19
Mandibulata - ps20
Pancrustacea - ps21
Insecta - ps22
Eumetabola - ps23
Holometabola/Psocodea - ps24
Holometabola - ps25
Aparaglossata - ps26
Mecopterida - ps27
Diptera - ps28
Bibiono./Cyclorrhapha - ps29
Cyclorrhapha - ps30
Calyptratae/Drosophila - ps31
Drosophila - ps32
Sophophora - ps33
Obscura/Melanogaster_group - ps34
D_melanogaster_group - ps35
D_melanogaster_subgroup - ps36
sim./sech./mel. group - ps37
Drosophila_melanogaster - ps38
p-value
1e-03
3e-03
9e-03
2e-02
7e-02
Log-odds
0.5
1.0
1.5
2.0
2.5
3.0
3.5
4.0

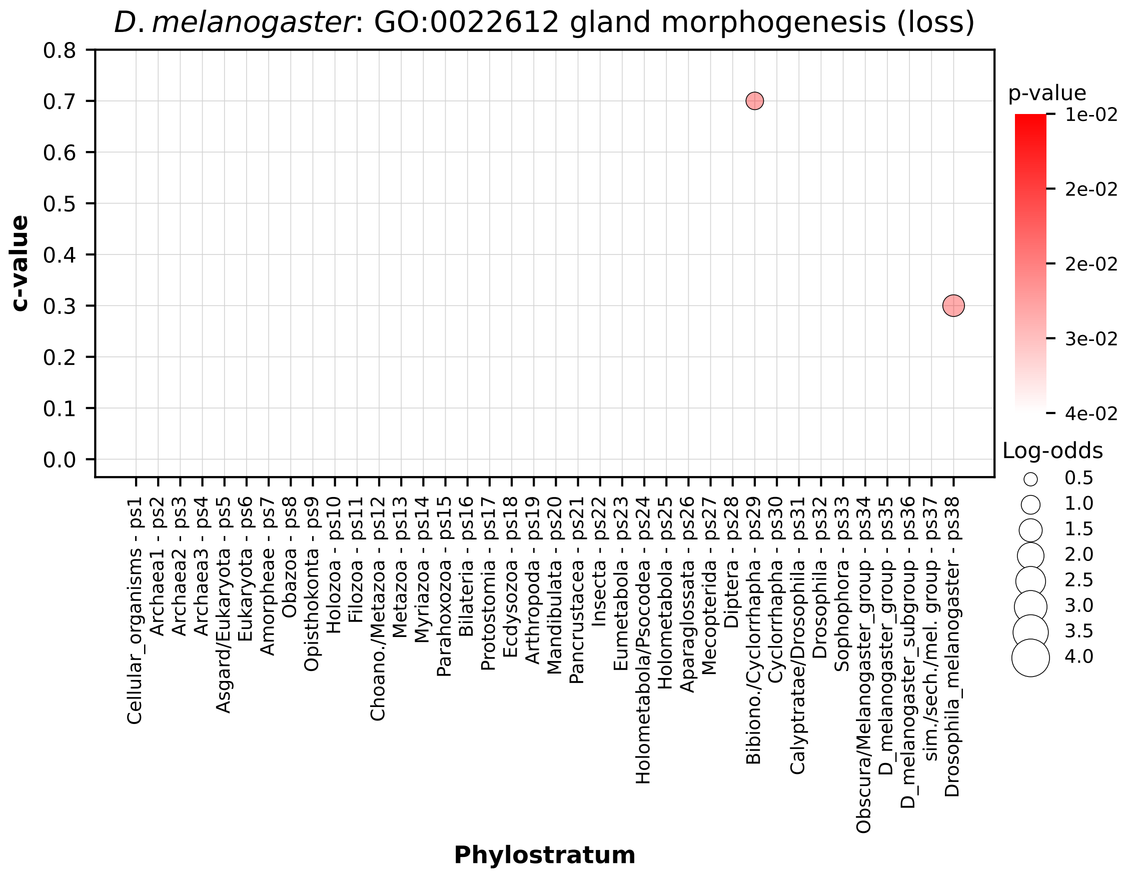

D. melanogaster: GO:0022612 gland morphogenesis (loss)
c-value
0.8
0.7
0.6
0.5
0.4
0.3
0.2
0.1
0.0
p-value
1e-02
2e-02
2e-02
3e-02
4e-02
Log-odds
0.5
1.0
1.5
2.0
2.5
3.0
3.5
4.0
Cellular_organisms - ps1
Archaea1 - ps2
Archaea2 - ps3
Archaea3 - ps4
Asgard/Eukaryota - ps5
Eukaryota - ps6
Amorpheae - ps7
Obazoa - ps8
Opisthokonta - ps9
Holozoa - ps10
Filozoa - ps11
Choano./Metazoa - ps12
Metazoa - ps13
Myriazoa - ps14
Parahoxozoa - ps15
Bilateria - ps16
Protostomia - ps17
Ecdysozoa - ps18
Arthropoda - ps19
Mandibulata - ps20
Pancrustacea - ps21
Insecta - ps22
Eumetabola - ps23
Holometabola/Psocodea - ps24
Holometabola - ps25
Aparaglossata - ps26
Mecopterida - ps27
Diptera - ps28
Bibiono./Cyclorrhapha - ps29
Cyclorrhapha - ps30
Calyptratae/Drosophila - ps31
Drosophila - ps32
Sophophora - ps33
Obscura/Melanogaster_group - ps34
D_melanogaster_group - ps35
D_melanogaster_subgroup - ps36
sim./sech./mel. group - ps37
Drosophila_melanogaster - ps38
Phylostratum

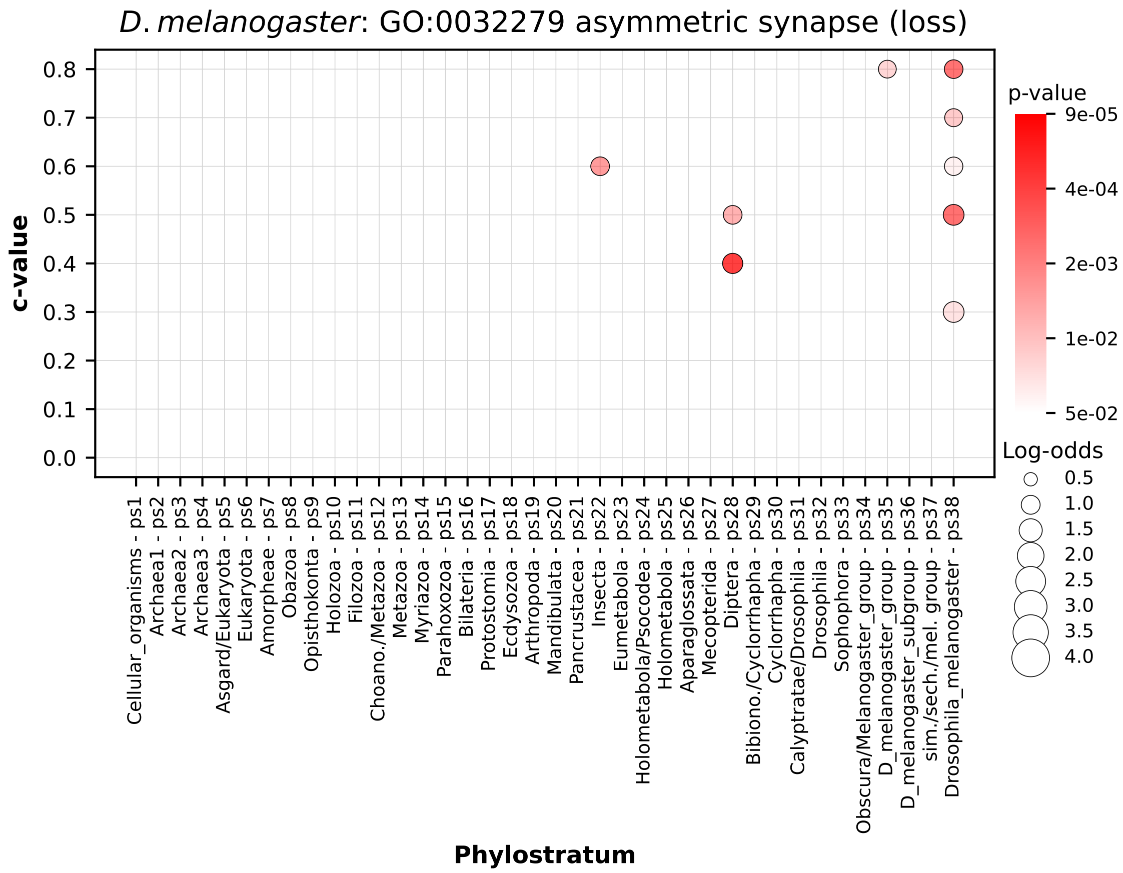
D. melanogaster: GO:0032279 asymmetric synapse (loss)
c-value
0.0
0.1
0.2
0.3
0.4
0.5
0.6
0.7
0.8
Phylostratum
Cellular_organisms - ps1
Archaea1 - ps2
Archaea2 - ps3
Archaea3 - ps4
Asgard/Eukaryota - ps5
Eukaryota - ps6
Amorpheae - ps7
Obazoa - ps8
Opisthokonta - ps9
Holozoa - ps10
Filozoa - ps11
Choano./Metazoa - ps12
Metazoa - ps13
Myriazoa - ps14
Parahoxozoa - ps15
Bilateria - ps16
Protostomia - ps17
Ecdysozoa - ps18
Arthropoda - ps19
Mandibulata - ps20
Pancrustacea - ps21
Insecta - ps22
Eumetabola - ps23
Holometabola/Psocodea - ps24
Holometabola - ps25
Aparaglossata - ps26
Mecopterida - ps27
Diptera - ps28
Bibiono./Cyclorrhapha - ps29
Cyclorrhapha - ps30
Calyptratae/Drosophila - ps31
Drosophila - ps32
Sophophora - ps33
Obscura/Melanogaster_group - ps34
D_melanogaster_group - ps35
D_melanogaster_subgroup - ps36
sim./sech./mel. group - ps37
Drosophila_melanogaster - ps38
p-value
9e-05
4e-04
2e-03
1e-02
5e-02
Log-odds
0.5
1.0
1.5
2.0
2.5
3.0
3.5
4.0

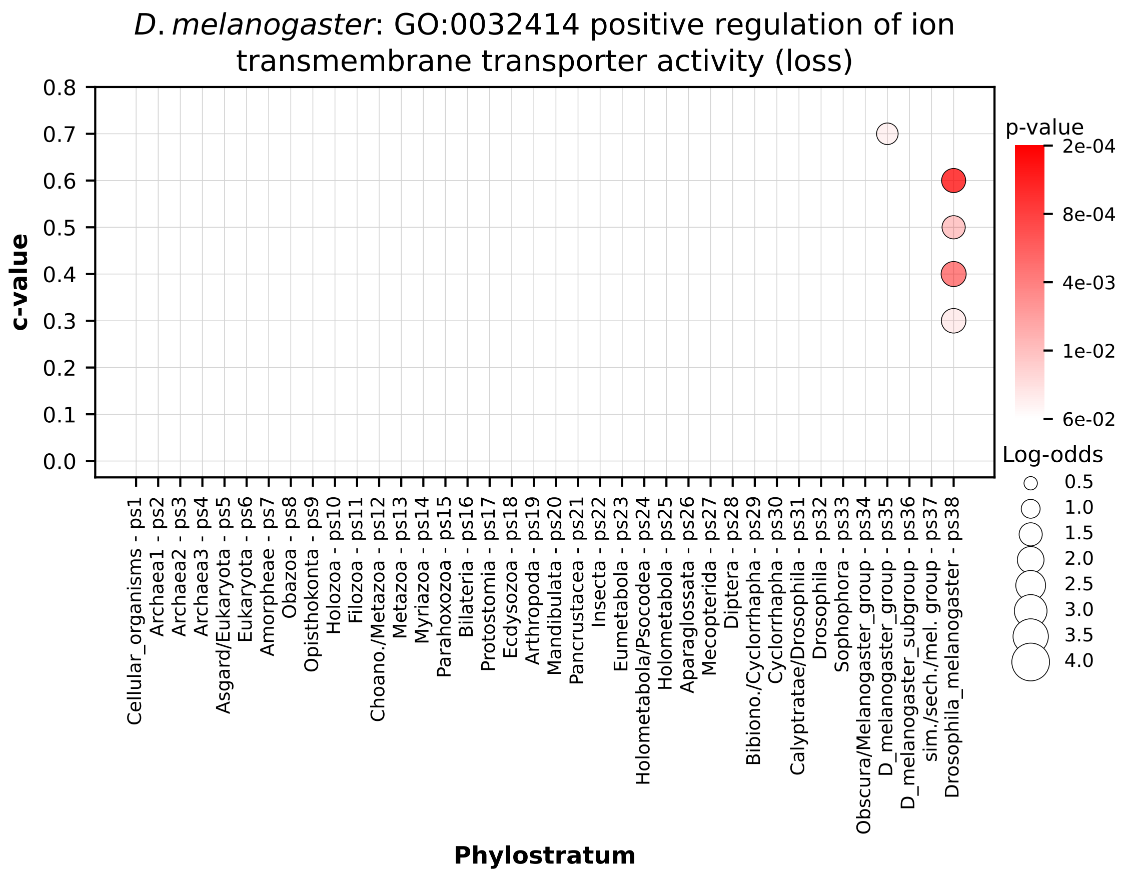

D. melanogaster: GO:0032414 positive regulation of ion transmembrane transporter activity (loss)
c-value
0.0
0.1
0.2
0.3
0.4
0.5
0.6
0.7
0.8
Phylostratum
Cellular_organisms - ps1
Archaea1 - ps2
Archaea2 - ps3
Archaea3 - ps4
Asgard/Eukaryota - ps5
Eukaryota - ps6
Amorpheae - ps7
Obazoa - ps8
Opisthokonta - ps9
Holozoa - ps10
Filozoa - ps11
Choano./Metazoa - ps12
Metazoa - ps13
Myriazoa - ps14
Parahoxozoa - ps15
Bilateria - ps16
Protostomia - ps17
Ecdysozoa - ps18
Arthropoda - ps19
Mandibulata - ps20
Pancrustacea - ps21
Insecta - ps22
Eumetabola - ps23
Holometabola/Psocodea - ps24
Holometabola - ps25
Aparaglossata - ps26
Mecopterida - ps27
Diptera - ps28
Bibiono./Cyclorrhapha - ps29
Cyclorrhapha - ps30
Calyptratae/Drosophila - ps31
Drosophila - ps32
Sophophora - ps33
Obscura/Melanogaster_group - ps34
D_melanogaster_group - ps35
D_melanogaster_subgroup - ps36
sim./sech./mel. group - ps37
Drosophila_melanogaster - ps38
p-value
2e-04
8e-04
4e-03
1e-02
6e-02
Log-odds
0.5
1.0
1.5
2.0
2.5
3.0
3.5
4.0

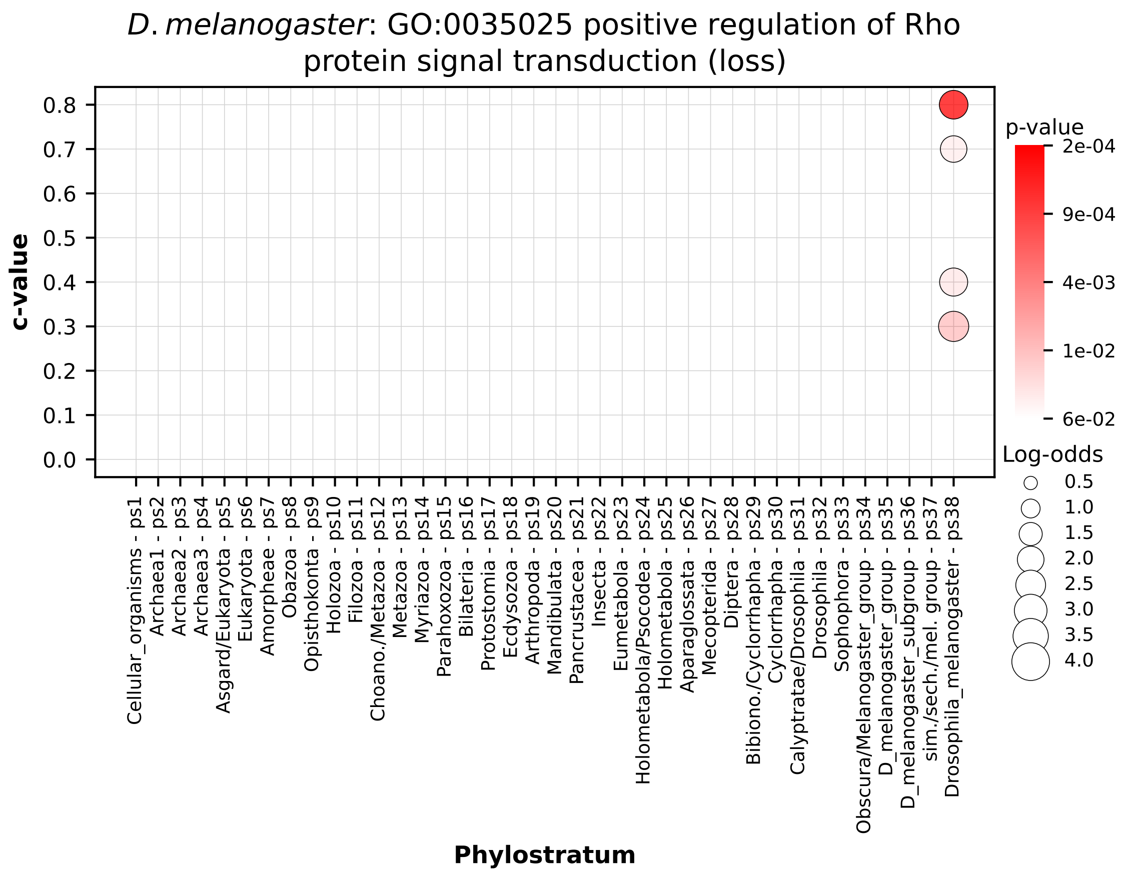
D. melanogaster: GO:0035025 positive regulation of Rho protein signal transduction (loss)
c-value
0.8
0.7
0.6
0.5
0.4
0.3
0.2
0.1
0.0
p-value
2e-04
9e-04
4e-03
1e-02
6e-02
Log-odds
0.5
1.0
1.5
2.0
2.5
3.0
3.5
4.0
Cellular_organisms - ps1
Archaea1 - ps2
Archaea2 - ps3
Archaea3 - ps4
Asgard/Eukaryota - ps5
Eukaryota - ps6
Amorpheae - ps7
Obazoa - ps8
Opisthokonta - ps9
Holozoa - ps10
Filozoa - ps11
Choano./Metazoa - ps12
Metazoa - ps13
Myriazoa - ps14
Parahoxozoa - ps15
Bilateria - ps16
Protostomia - ps17
Ecdysozoa - ps18
Arthropoda - ps19
Mandibulata - ps20
Pancrustacea - ps21
Insecta - ps22
Eumetabola - ps23
Holometabola/Psocodea - ps24
Holometabola - ps25
Aparaglossata - ps26
Mecopterida - ps27
Diptera - ps28
Bibiono./Cyclorrhapha - ps29
Cyclorrhapha - ps30
Calyptratae/Drosophila - ps31
Drosophila - ps32
Sophophora - ps33
Obscura/Melanogaster_group - ps34
D_melanogaster_group - ps35
D_melanogaster_subgroup - ps36
sim./sech./mel. group - ps37
Drosophila_melanogaster - ps38
Phylostratum

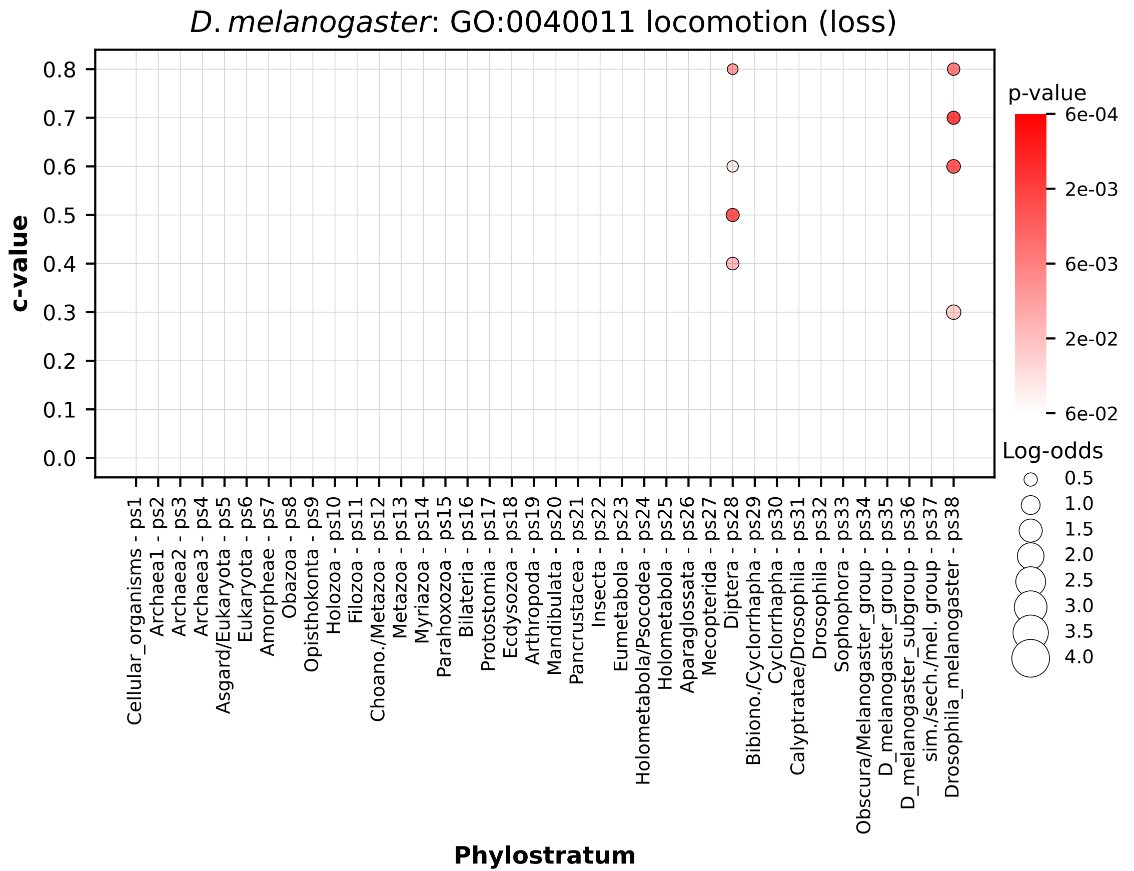

D. melanogaster: GO:0040011 locomotion (loss)
c-value
0.8
0.7
0.6
0.5
0.4
0.3
0.2
0.1
0.0
Phylostratum
Cellular_organisms - ps1
Archaea1 - ps2
Archaea2 - ps3
Archaea3 - ps4
Asgard/Eukaryota - ps5
Eukaryota - ps6
Amorpheae - ps7
Obazoa - ps8
Opisthokonta - ps9
Holozoa - ps10
Filozoa - ps11
Choano./Metazoa - ps12
Metazoa - ps13
Myriazoa - ps14
Parahoxozoa - ps15
Bilateria - ps16
Protostomia - ps17
Ecdysozoa - ps18
Arthropoda - ps19
Mandibulata - ps20
Pancrustacea - ps21
Insecta - ps22
Eumetabola - ps23
Holometabola/Psocodea - ps24
Holometabola - ps25
Aparaglossata - ps26
Mecopterida - ps27
Diptera - ps28
Bibiono./Cyclorrhapha - ps29
Cyclorrhapha - ps30
Calyptratae/Drosophila - ps31
Drosophila - ps32
Sophophora - ps33
Obscura/Melanogaster_group - ps34
D_melanogaster_group - ps35
D_melanogaster_subgroup - ps36
sim./sech./mel. group - ps37
Drosophila_melanogaster - ps38
p-value
6e-04
2e-03
6e-03
2e-02
6e-02
Log-odds
0.5
1.0
1.5
2.0
2.5
3.0
3.5
4.0

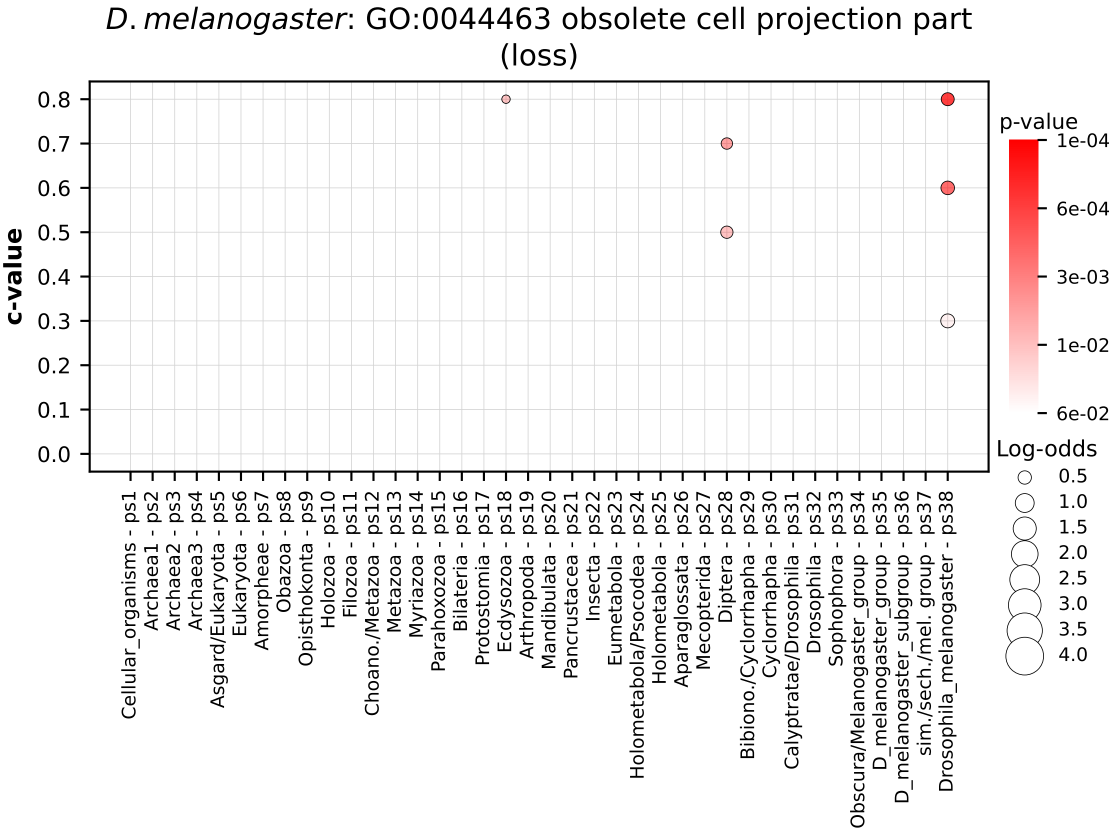

D. melanogaster: GO:0044463 obsolete cell projection part (loss)
c-value
0.8
0.7
0.6
0.5
0.4
0.3
0.2
0.1
0.0
Phylostratum
Cellular_organisms - ps1
Archaea1 - ps2
Archaea2 - ps3
Archaea3 - ps4
Asgard/Eukaryota - ps5
Eukaryota - ps6
Amorpheae - ps7
Obazoa - ps8
Opisthokonta - ps9
Holozoa - ps10
Filozoa - ps11
Choano./Metazoa - ps12
Metazoa - ps13
Myriazoa - ps14
Parahoxozoa - ps15
Bilateria - ps16
Protostomia - ps17
Ecdysozoa - ps18
Arthropoda - ps19
Mandibulata - ps20
Pancrustacea - ps21
Insecta - ps22
Eumetabola - ps23
Holometabola/Psocodea - ps24
Holometabola - ps25
Aparaglossata - ps26
Mecopterida - ps27
Diptera - ps28
Bibiono./Cyclorrhapha - ps29
Cyclorrhapha - ps30
Calyptratae/Drosophila - ps31
Drosophila - ps32
Sophophora - ps33
Obscura/Melanogaster_group - ps34
D_melanogaster_group - ps35
D_melanogaster_subgroup - ps36
sim./sech./mel. group - ps37
Drosophila_melanogaster - ps38
p-value
1e-04
6e-04
3e-03
1e-02
6e-02
Log-odds
0.5
1.0
1.5
2.0
2.5
3.0
3.5
4.0

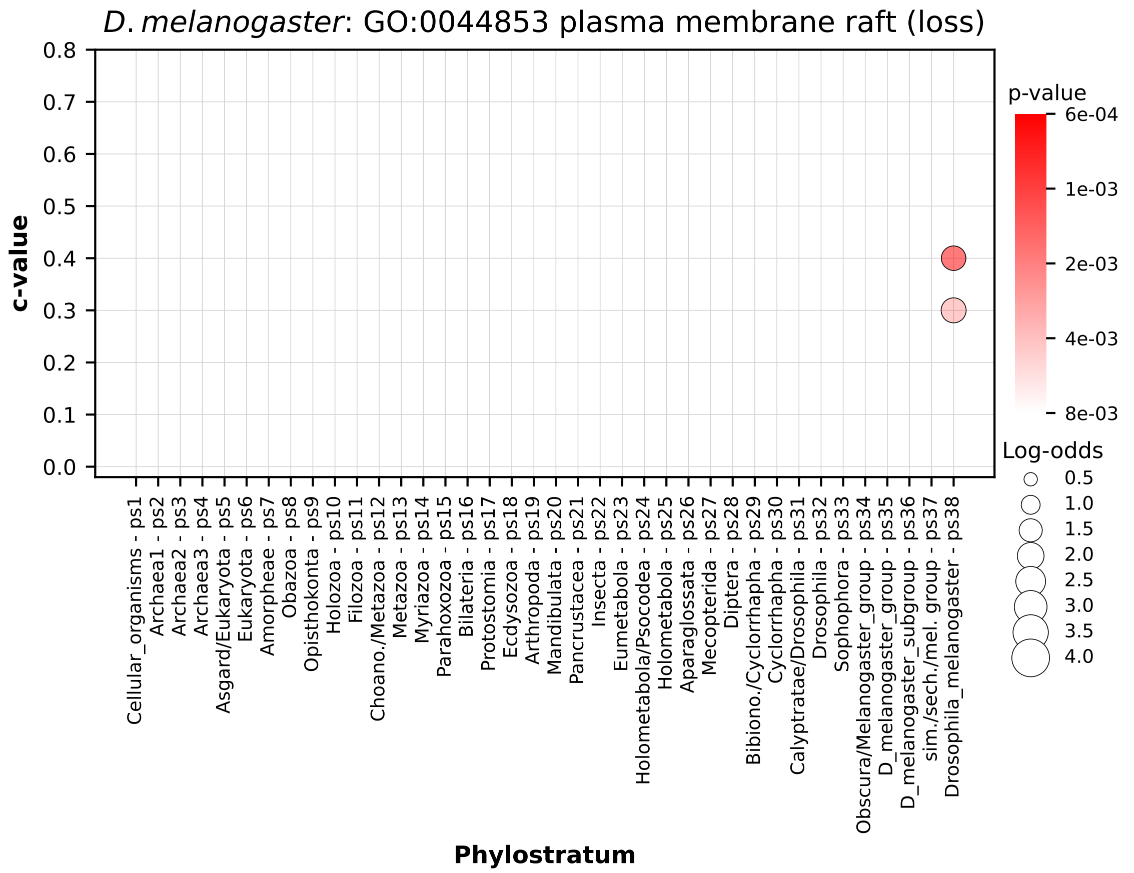

D. melanogaster: GO:0044853 plasma membrane raft (loss)
c-value
0.0
0.1
0.2
0.3
0.4
0.5
0.6
0.7
0.8
Phylostratum
Cellular_organisms - ps1
Archaea1 - ps2
Archaea2 - ps3
Archaea3 - ps4
Asgard/Eukaryota - ps5
Eukaryota - ps6
Amorpheae - ps7
Obazoa - ps8
Opisthokonta - ps9
Holozoa - ps10
Filozoa - ps11
Choano./Metazoa - ps12
Metazoa - ps13
Myriazoa - ps14
Parahoxozoa - ps15
Bilateria - ps16
Protostomia - ps17
Ecdysozoa - ps18
Arthropoda - ps19
Mandibulata - ps20
Pancrustacea - ps21
Insecta - ps22
Eumetabola - ps23
Holometabola/Psocodea - ps24
Holometabola - ps25
Aparaglossata - ps26
Mecopterida - ps27
Diptera - ps28
Bibiono./Cyclorrhapha - ps29
Cyclorrhapha - ps30
Calyptratae/Drosophila - ps31
Drosophila - ps32
Sophophora - ps33
Obscura/Melanogaster_group - ps34
D_melanogaster_group - ps35
D_melanogaster_subgroup - ps36
sim./sech./mel. group - ps37
Drosophila_melanogaster - ps38
p-value
6e-04
1e-03
2e-03
4e-03
8e-03
Log-odds
0.5
1.0
1.5
2.0
2.5
3.0
3.5
4.0

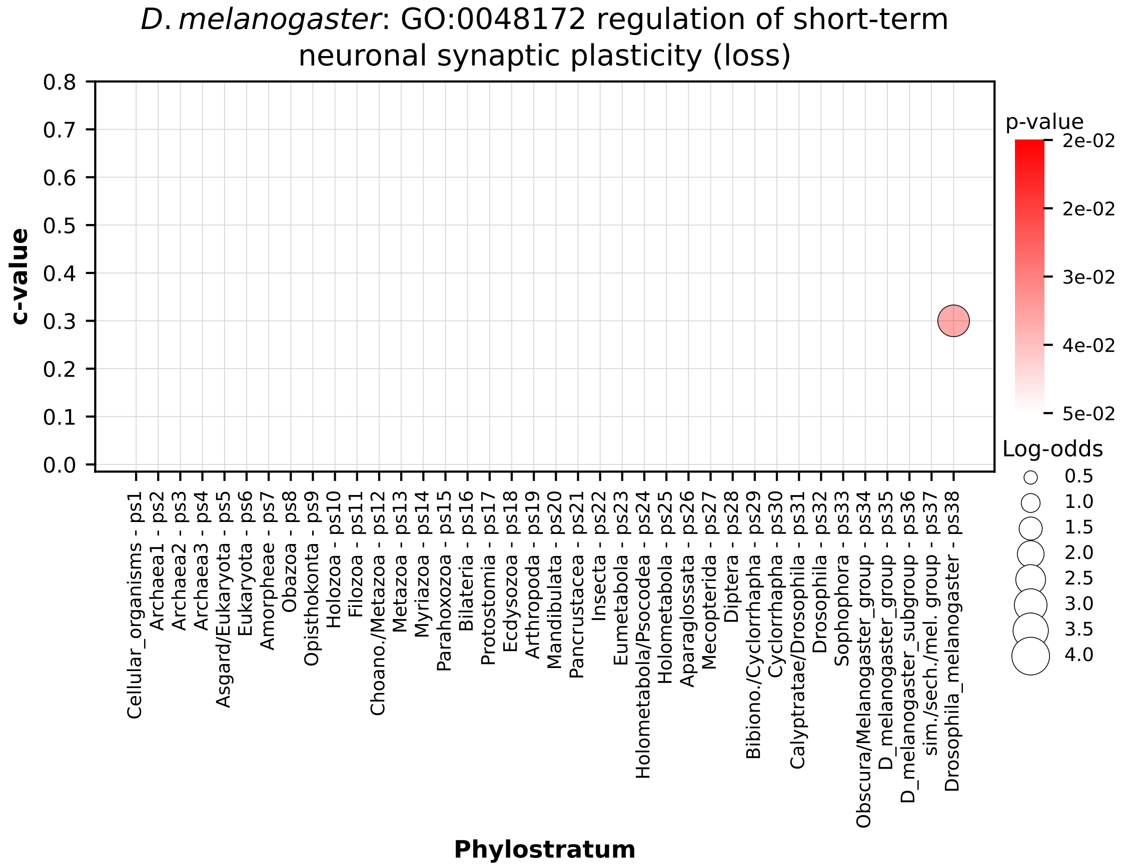
D. melanogaster: GO:0048172 regulation of short-term neuronal synaptic plasticity (loss)
c-value
0.8
0.7
0.6
0.5
0.4
0.3
0.2
0.1
0.0
p-value
2e-02
2e-02
3e-02
4e-02
5e-02
Log-odds
0.5
1.0
1.5
2.0
2.5
3.0
3.5
4.0
Cellular_organisms - ps1
Archaea1 - ps2
Archaea2 - ps3
Archaea3 - ps4
Asgard/Eukaryota - ps5
Eukaryota - ps6
Amorpheae - ps7
Obazoa - ps8
Opisthokonta - ps9
Holozoa - ps10
Filozoa - ps11
Choano./Metazoa - ps12
Metazoa - ps13
Myriazoa - ps14
Parahoxozoa - ps15
Bilateria - ps16
Protostomia - ps17
Ecdysozoa - ps18
Arthropoda - ps19
Mandibulata - ps20
Pancrustacea - ps21
Insecta - ps22
Eumetabola - ps23
Holometabola/Psocodea - ps24
Holometabola - ps25
Aparaglossata - ps26
Mecopterida - ps27
Diptera - ps28
Bibiono./Cyclorrhapha - ps29
Cyclorrhapha - ps30
Calyptratae/Drosophila - ps31
Drosophila - ps32
Sophophora - ps33
Obscura/Melanogaster_group - ps34
D_melanogaster_group - ps35
D_melanogaster_subgroup - ps36
sim./sech./mel. group - ps37
Drosophila_melanogaster - ps38
Phylostratum

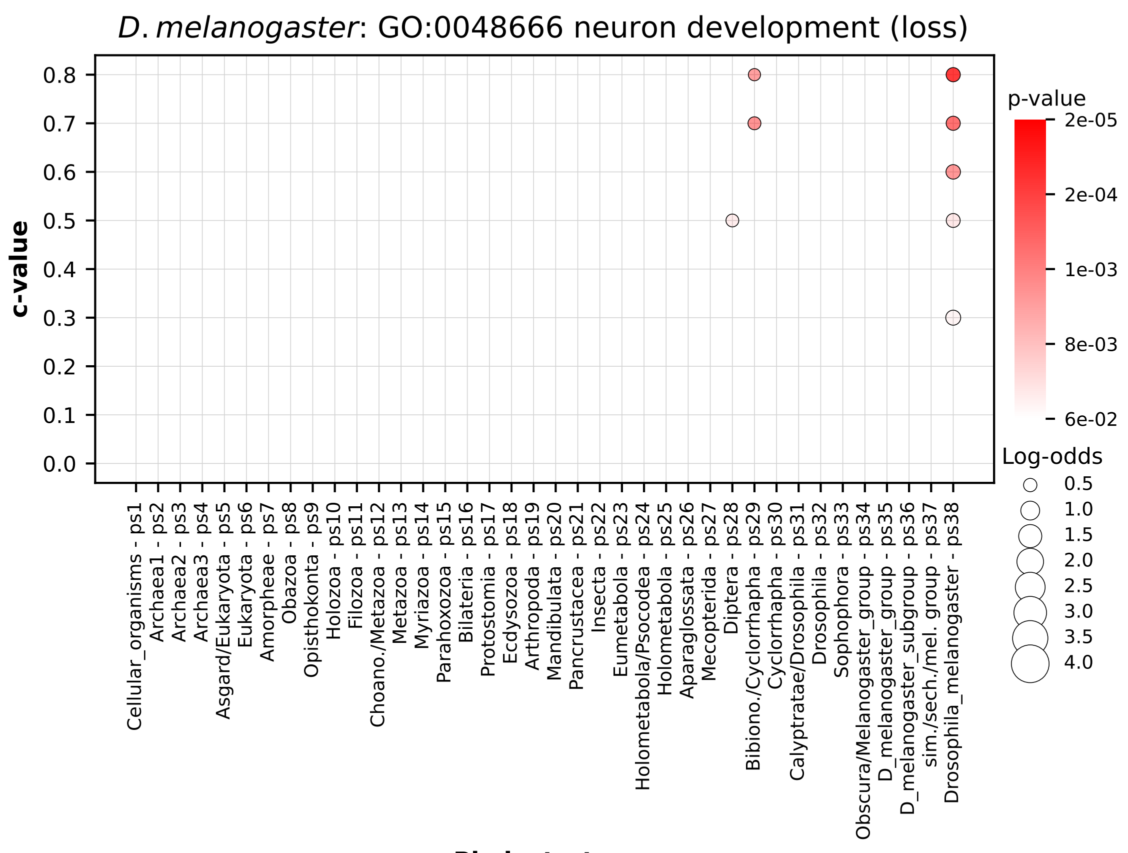

D. melanogaster: GO:0048666 neuron development (loss)
c-value
0.0
0.1
0.2
0.3
0.4
0.5
0.6
0.7
0.8
Phylostratum
Cellular_organisms - ps1
Archaea1 - ps2
Archaea2 - ps3
Archaea3 - ps4
Asgard/Eukaryota - ps5
Eukaryota - ps6
Amorpheae - ps7
Obazoa - ps8
Opisthokonta - ps9
Holozoa - ps10
Filozoa - ps11
Choano./Metazoa - ps12
Metazoa - ps13
Myriazoa - ps14
Parahoxozoa - ps15
Bilateria - ps16
Protostomia - ps17
Ecdysozoa - ps18
Arthropoda - ps19
Mandibulata - ps20
Pancrustacea - ps21
Insecta - ps22
Eumetabola - ps23
Holometabola/Psocodea - ps24
Holometabola - ps25
Aparaglossata - ps26
Mecopterida - ps27
Diptera - ps28
Bibiono./Cyclorrhapha - ps29
Cyclorrhapha - ps30
Calyptratae/Drosophila - ps31
Drosophila - ps32
Sophophora - ps33
Obscura/Melanogaster_group - ps34
D_melanogaster_group - ps35
D_melanogaster_subgroup - ps36
sim./sech./mel. group - ps37
Drosophila_melanogaster - ps38
p-value
2e-05
2e-04
1e-03
8e-03
6e-02
Log-odds
0.5
1.0
1.5
2.0
2.5
3.0
3.5
4.0

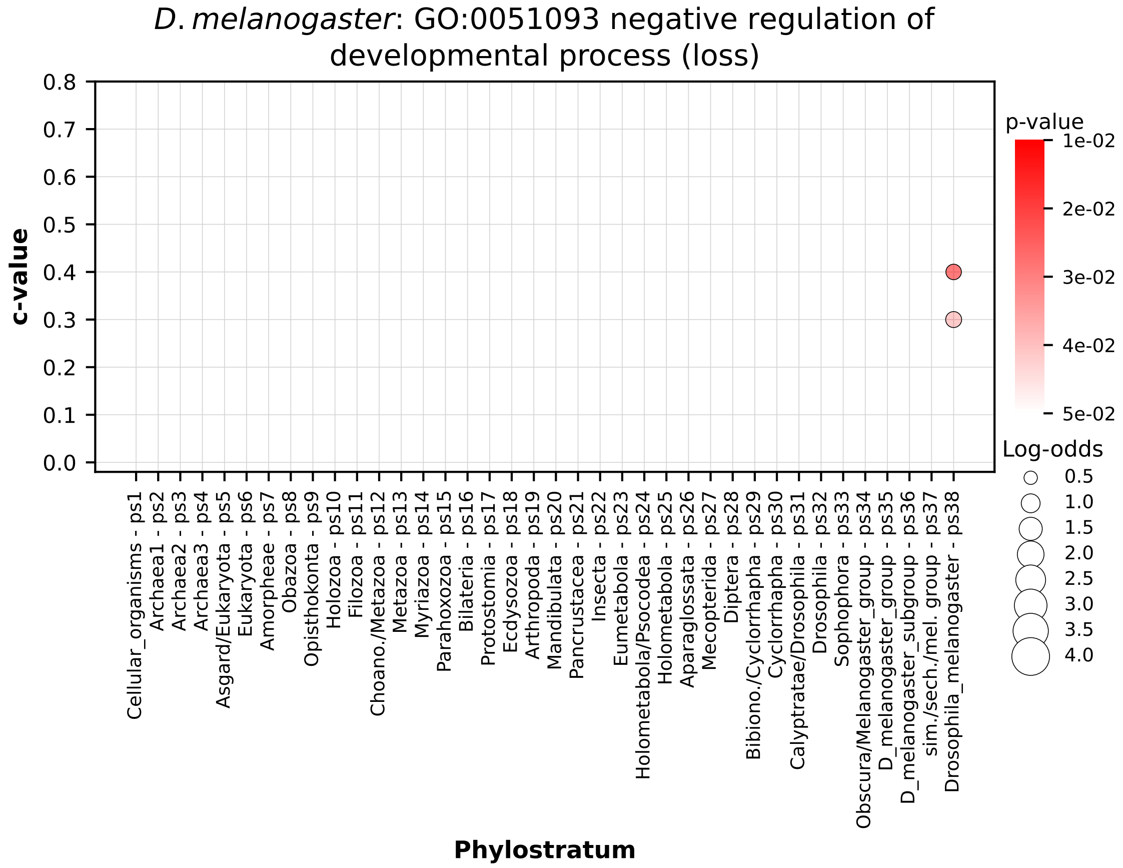
D. melanogaster: GO:0051093 negative regulation of developmental process (loss)
c-value
0.8
0.7
0.6
0.5
0.4
0.3
0.2
0.1
0.0
p-value
1e-02
2e-02
3e-02
4e-02
5e-02
Log-odds
0.5
1.0
1.5
2.0
2.5
3.0
3.5
4.0
Cellular_organisms - ps1
Archaea1 - ps2
Archaea2 - ps3
Archaea3 - ps4
Asgard/Eukaryota - ps5
Eukaryota - ps6
Amorpheae - ps7
Obazoa - ps8
Opisthokonta - ps9
Holozoa - ps10
Filozoa - ps11
Choano./Metazoa - ps12
Metazoa - ps13
Myriazoa - ps14
Parahoxozoa - ps15
Bilateria - ps16
Protostomia - ps17
Ecdysozoa - ps18
Arthropoda - ps19
Mandibulata - ps20
Pancrustacea - ps21
Insecta - ps22
Eumetabola - ps23
Holometabola/Psocodea - ps24
Holometabola - ps25
Aparaglossata - ps26
Mecopterida - ps27
Diptera - ps28
Bibiono./Cyclorrhapha - ps29
Cyclorrhapha - ps30
Calyptratae/Drosophila - ps31
Drosophila - ps32
Sophophora - ps33
Obscura/Melanogaster_group - ps34
D_melanogaster_group - ps35
D_melanogaster_subgroup - ps36
sim./sech./mel. group - ps37
Drosophila_melanogaster - ps38
Phylostratum

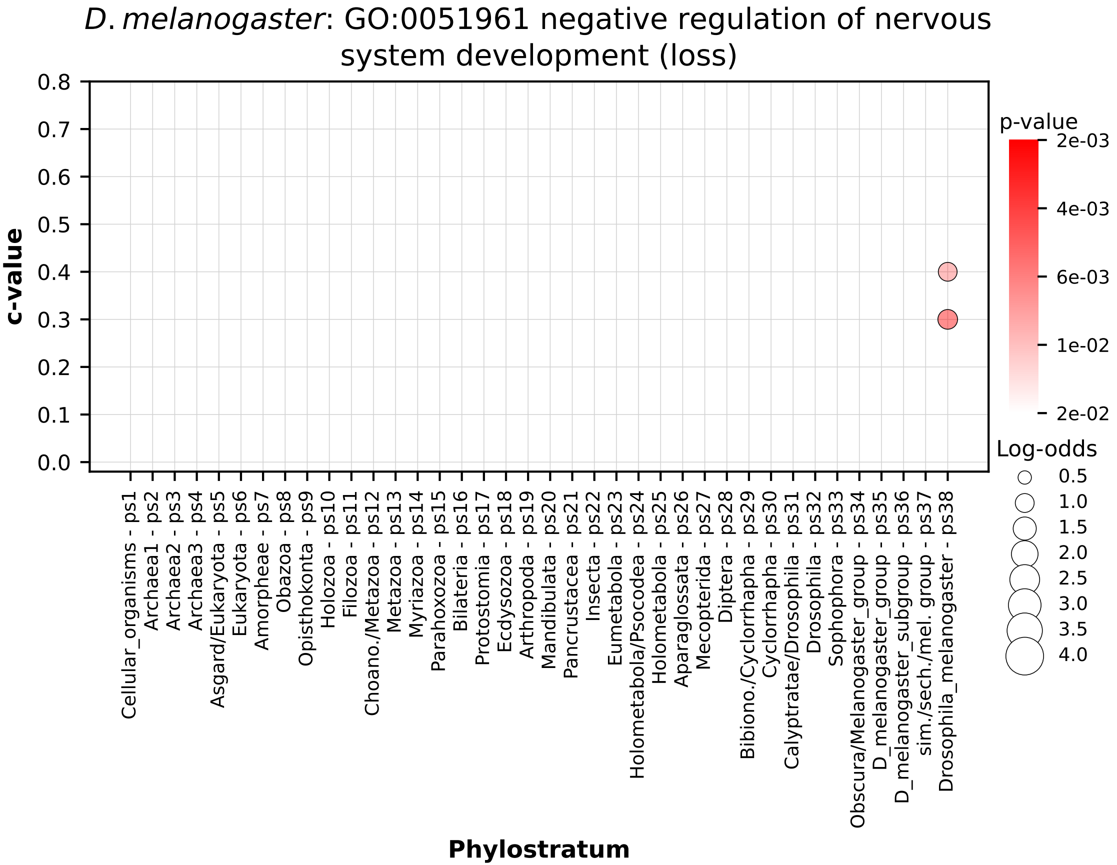
D. melanogaster: GO:0051961 negative regulation of nervous system development (loss)
c-value
0.8
0.7
0.6
0.5
0.4
0.3
0.2
0.1
0.0
Phylostratum
Cellular_organisms - ps1
Archaea1 - ps2
Archaea2 - ps3
Archaea3 - ps4
Asgard/Eukaryota - ps5
Eukaryota - ps6
Amorpheae - ps7
Obazoa - ps8
Opisthokonta - ps9
Holozoa - ps10
Filozoa - ps11
Choano./Metazoa - ps12
Metazoa - ps13
Myriazoa - ps14
Parahoxozoa - ps15
Bilateria - ps16
Protostomia - ps17
Ecdysozoa - ps18
Arthropoda - ps19
Mandibulata - ps20
Pancrustacea - ps21
Insecta - ps22
Eumetabola - ps23
Holometabola/Psocodea - ps24
Holometabola - ps25
Aparaglossata - ps26
Mecopterida - ps27
Diptera - ps28
Bibiono./Cyclorrhapha - ps29
Cyclorrhapha - ps30
Calyptratae/Drosophila - ps31
Drosophila - ps32
Sophophora - ps33
Obscura/Melanogaster_group - ps34
D_melanogaster_group - ps35
D_melanogaster_subgroup - ps36
sim./sech./mel. group - ps37
Drosophila_melanogaster - ps38
p-value
2e-03
4e-03
6e-03
1e-02
2e-02
Log-odds
0.5
1.0
1.5
2.0
2.5
3.0
3.5
4.0

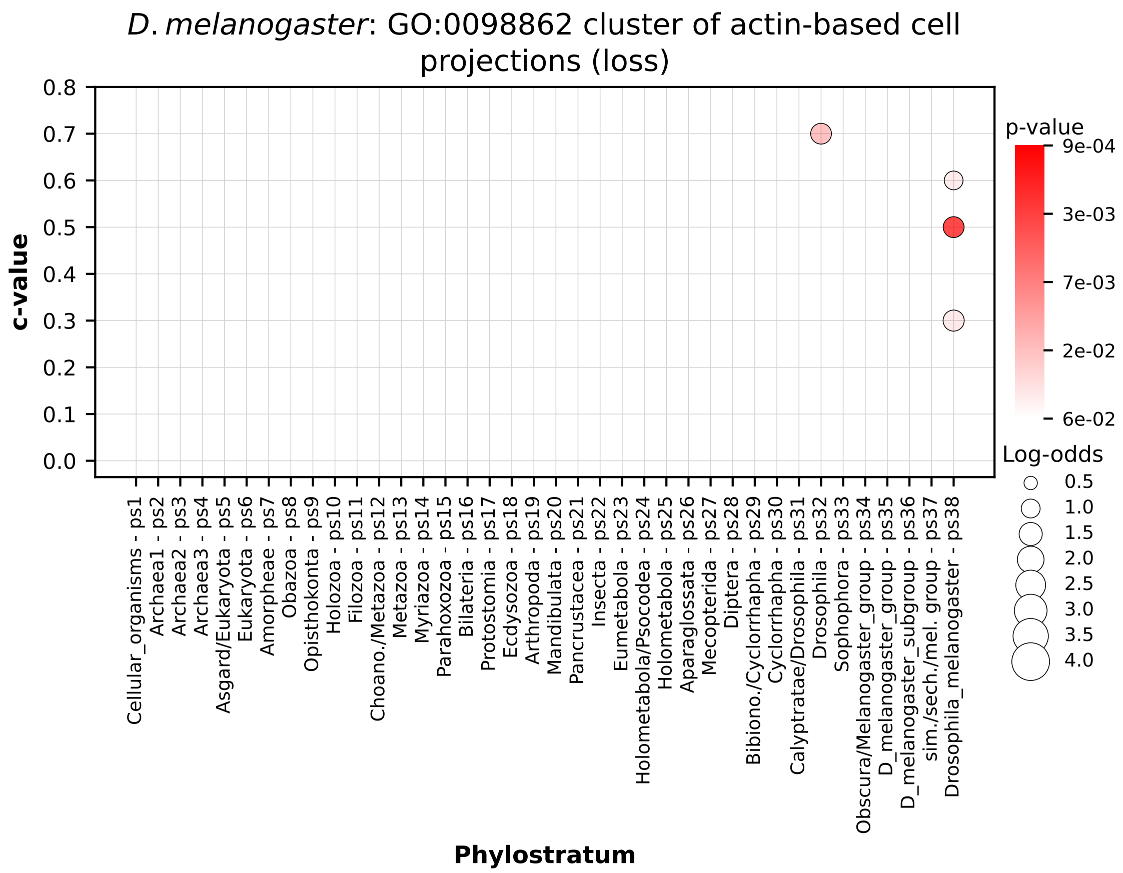

D. melanogaster: GO:0098862 cluster of actin-based cell projections (loss)
c-value
0.8
0.7
0.6
0.5
0.4
0.3
0.2
0.1
0.0
p-value
9e-04
3e-03
7e-03
2e-02
6e-02
Log-odds
0.5
1.0
1.5
2.0
2.5
3.0
3.5
4.0
Cellular_organisms - ps1
Archaea1 - ps2
Archaea2 - ps3
Archaea3 - ps4
Asgard/Eukaryota - ps5
Eukaryota - ps6
Amorpheae - ps7
Obazoa - ps8
Opisthokonta - ps9
Holozoa - ps10
Filozoa - ps11
Choano./Metazoa - ps12
Metazoa - ps13
Myriazoa - ps14
Parahoxozoa - ps15
Bilateria - ps16
Protostomia - ps17
Ecdysozoa - ps18
Arthropoda - ps19
Mandibulata - ps20
Pancrustacea - ps21
Insecta - ps22
Eumetabola - ps23
Holometabola/Psocodea - ps24
Holometabola - ps25
Aparaglossata - ps26
Mecopterida - ps27
Diptera - ps28
Bibiono./Cyclorrhapha - ps29
Cyclorrhapha - ps30
Calyptratae/Drosophila - ps31
Drosophila - ps32
Sophophora - ps33
Obscura/Melanogaster_group - ps34
D_melanogaster_group - ps35
D_melanogaster_subgroup - ps36
sim./sech./mel. group - ps37
Drosophila_melanogaster - ps38
Phylostratum

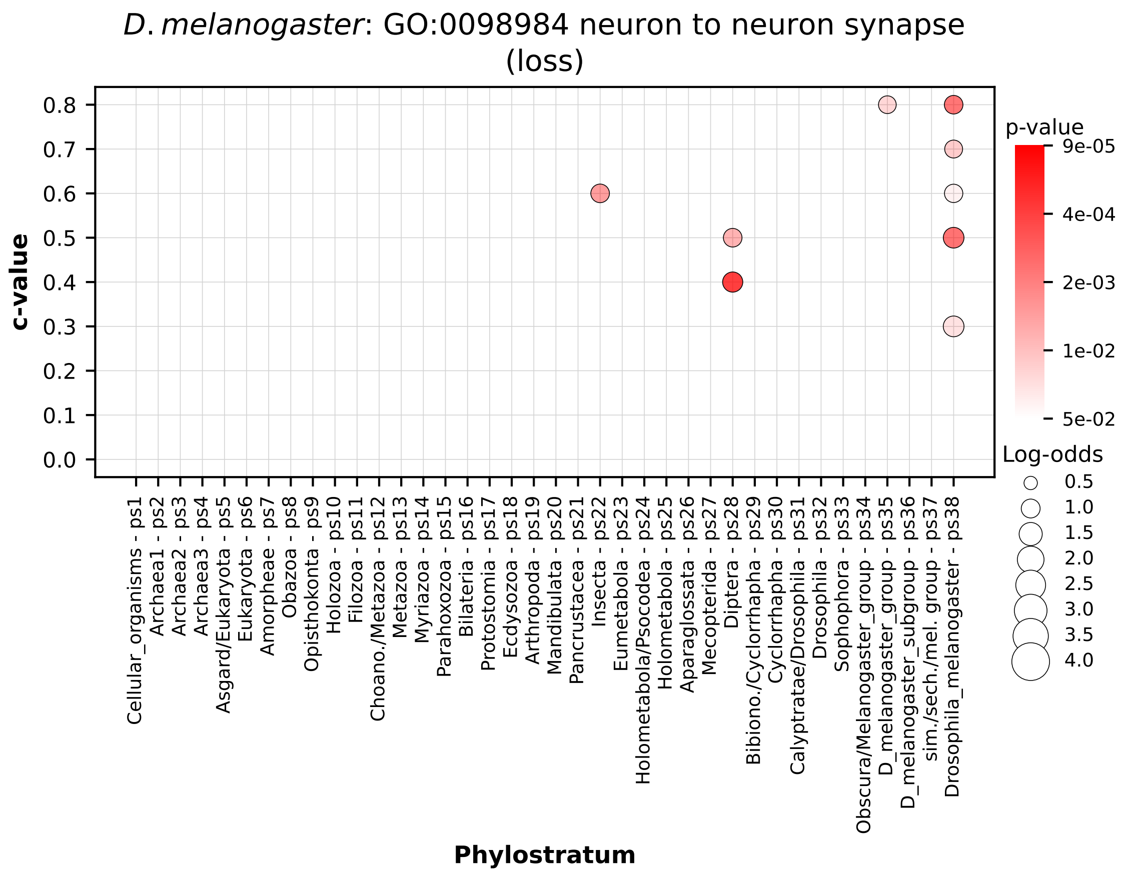

D. melanogaster: GO:0098984 neuron to neuron synapse
(loss)
c-value
0.0
0.1
0.2
0.3
0.4
0.5
0.6
0.7
0.8
p-value
9e-05
4e-04
2e-03
1e-02
5e-02
Log-odds
0.5
1.0
1.5
2.0
2.5
3.0
3.5
4.0
Cellular_organisms - ps1
Archaea1 - ps2
Archaea2 - ps3
Archaea3 - ps4
Asgard/Eukaryota - ps5
Eukaryota - ps6
Amorpheae - ps7
Obazoa - ps8
Opisthokonta - ps9
Holozoa - ps10
Filozoa - ps11
Choano./Metazoa - ps12
Metazoa - ps13
Myriazoa - ps14
Parahoxozoa - ps15
Bilateria - ps16
Protostomia - ps17
Ecdysozoa - ps18
Arthropoda - ps19
Mandibulata - ps20
Pancrustacea - ps21
Insecta - ps22
Eumetabola - ps23
Holometabola/Psocodea - ps24
Holometabola - ps25
Aparaglossata - ps26
Mecopterida - ps27
Diptera - ps28
Bibiono./Cyclorrhapha - ps29
Cyclorrhapha - ps30
Calyptratae/Drosophila - ps31
Drosophila - ps32
Sophophora - ps33
Obscura/Melanogaster_group - ps34
D_melanogaster_group - ps35
D_melanogaster_subgroup - ps36
sim./sech./mel. group - ps37
Drosophila_melanogaster - ps38
Phylostratum

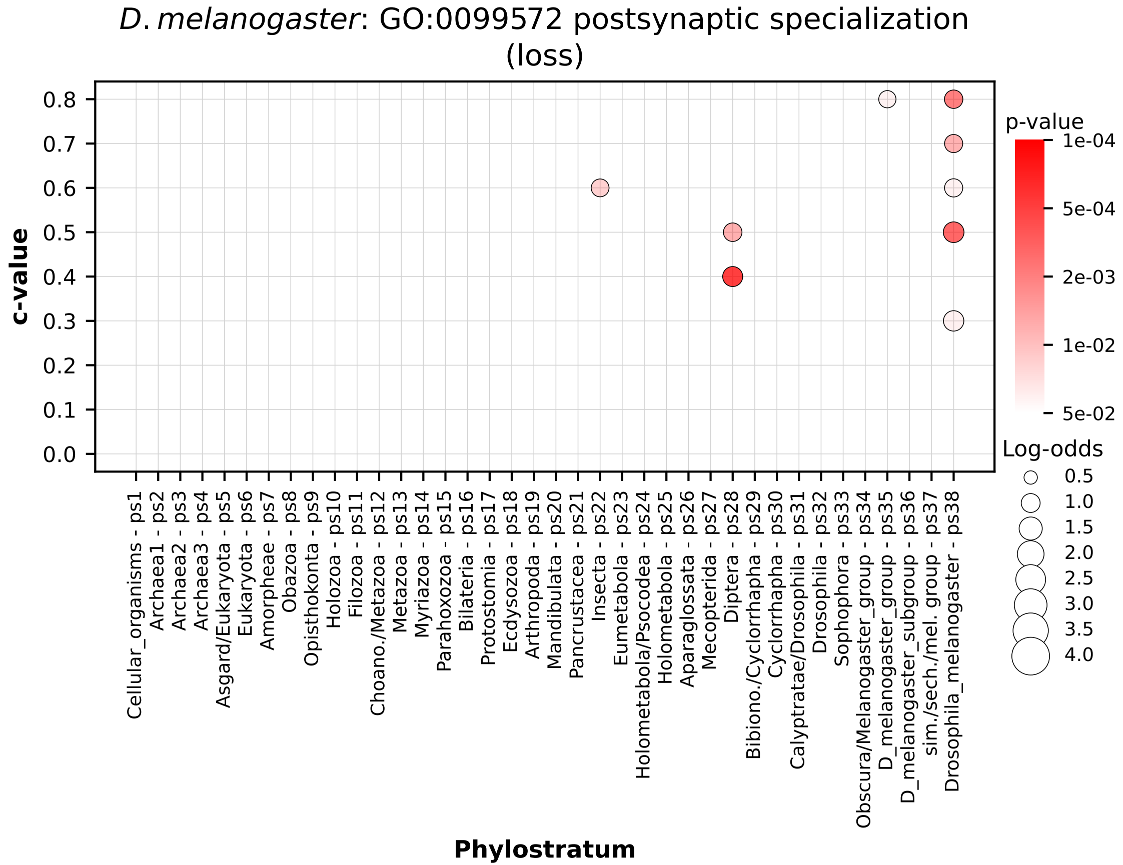
D. melanogaster: GO:0099572 postsynaptic specialization
(loss)
c-value
0.8
0.7
0.6
0.5
0.4
0.3
0.2
0.1
0.0
p-value
1e-04
5e-04
2e-03
1e-02
5e-02
Log-odds
0.5
1.0
1.5
2.0
2.5
3.0
3.5
4.0
Cellular_organisms - ps1
Archaea1 - ps2
Archaea2 - ps3
Archaea3 - ps4
Asgard/Eukaryota - ps5
Eukaryota - ps6
Amorpheae - ps7
Obazoa - ps8
Opisthokonta - ps9
Holozoa - ps10
Filozoa - ps11
Choano./Metazoa - ps12
Metazoa - ps13
Myriazoa - ps14
Parahoxozoa - ps15
Bilateria - ps16
Protostomia - ps17
Ecdysozoa - ps18
Arthropoda - ps19
Mandibulata - ps20
Pancrustacea - ps21
Insecta - ps22
Eumetabola - ps23
Holometabola/Psocodea - ps24
Holometabola - ps25
Aparaglossata - ps26
Mecopterida - ps27
Diptera - ps28
Bibiono./Cyclorrhapha - ps29
Cyclorrhapha - ps30
Calyptratae/Drosophila - ps31
Drosophila - ps32
Sophophora - ps33
Obscura/Melanogaster_group - ps34
D_melanogaster_group - ps35
D_melanogaster_subgroup - ps36
sim./sech./mel. group - ps37
Drosophila_melanogaster - ps38
Phylostratum

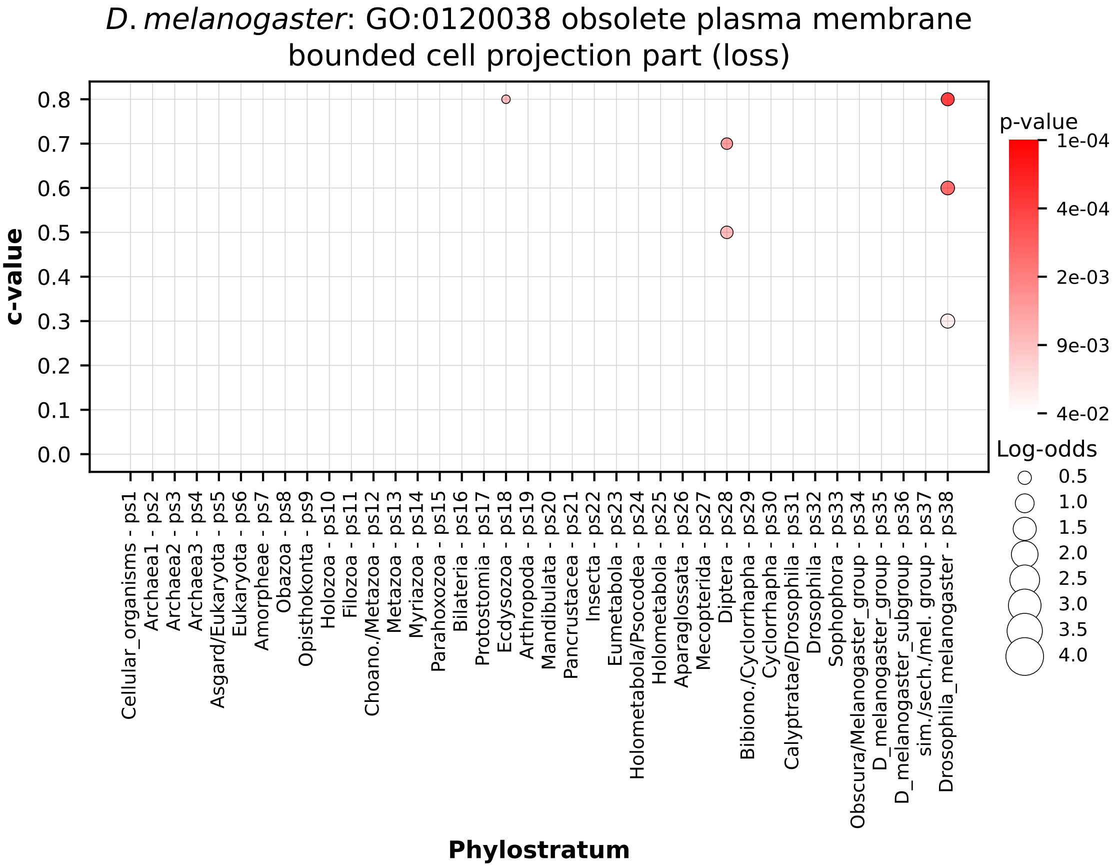

D. melanogaster: GO:0120038 obsolete plasma membrane bounded cell projection part (loss)
c-value
0.8
0.7
0.6
0.5
0.4
0.3
0.2
0.1
0.0
Phylostratum
Cellular_organisms - ps1
Archaea1 - ps2
Archaea2 - ps3
Archaea3 - ps4
Asgard/Eukaryota - ps5
Eukaryota - ps6
Amorpheae - ps7
Obazoa - ps8
Opisthokonta - ps9
Holozoa - ps10
Filozoa - ps11
Choano./Metazoa - ps12
Metazoa - ps13
Myriazoa - ps14
Parahoxozoa - ps15
Bilateria - ps16
Protostomia - ps17
Ecdysozoa - ps18
Arthropoda - ps19
Mandibulata - ps20
Pancrustacea - ps21
Insecta - ps22
Eumetabola - ps23
Holometabola/Psocodea - ps24
Holometabola - ps25
Aparaglossata - ps26
Mecopterida - ps27
Diptera - ps28
Bibiono./Cyclorrhapha - ps29
Cyclorrhapha - ps30
Calyptratae/Drosophila - ps31
Drosophila - ps32
Sophophora - ps33
Obscura/Melanogaster_group - ps34
D_melanogaster_group - ps35
D_melanogaster_subgroup - ps36
sim./sech./mel. group - ps37
Drosophila_melanogaster - ps38
p-value
1e-04
4e-04
2e-03
9e-03
4e-02
Log-odds
0.5
1.0
1.5
2.0
2.5
3.0
3.5
4.0

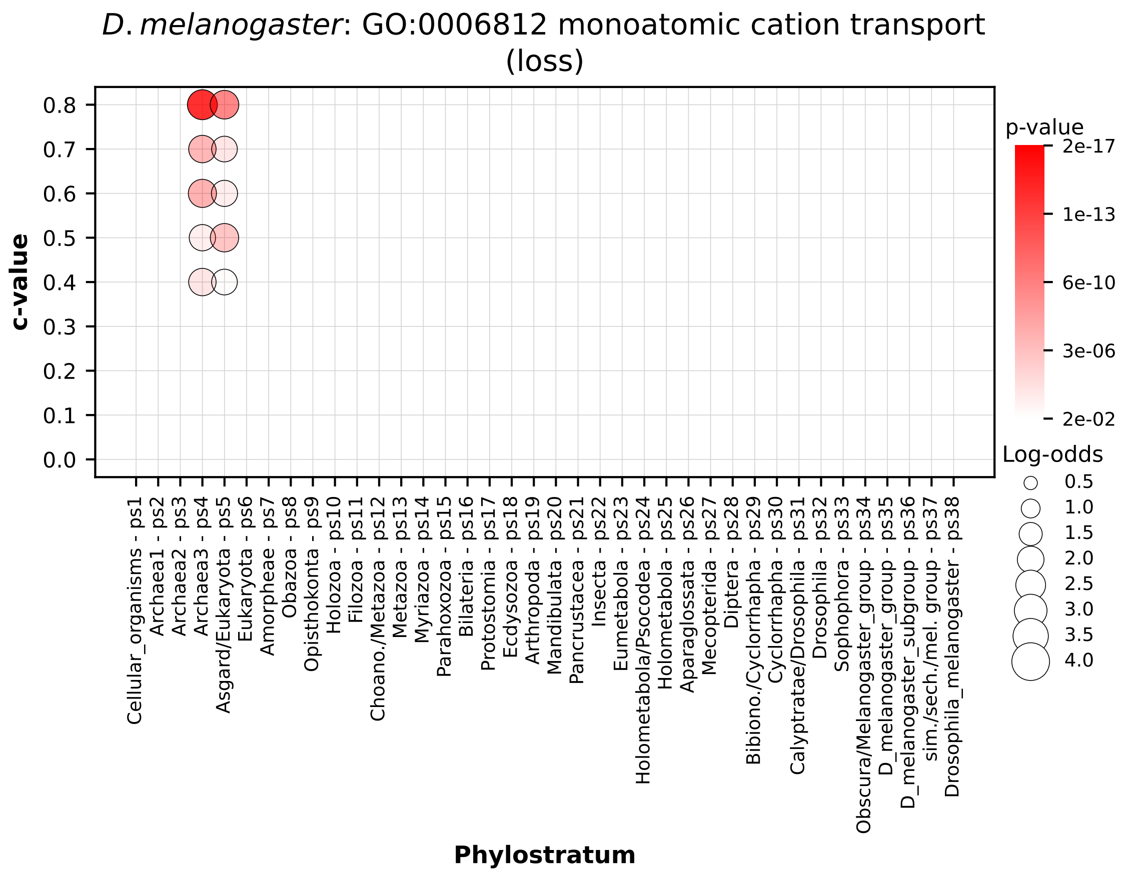

D. melanogaster: GO:0006812 monoatomic cation transport
(loss)
c-value
0.8
0.7
0.6
0.5
0.4
0.3
0.2
0.1
0.0
p-value
2e-17
1e-13
6e-10
3e-06
2e-02
Log-odds
0.5
1.0
1.5
2.0
2.5
3.0
3.5
4.0
Cellular_organisms - ps1
Archaea1 - ps2
Archaea2 - ps3
Archaea3 - ps4
Asgard/Eukaryota - ps5
Eukaryota - ps6
Amorpheae - ps7
Obazoa - ps8
Opisthokonta - ps9
Holozoa - ps10
Filozoa - ps11
Choano./Metazoa - ps12
Metazoa - ps13
Myriazoa - ps14
Parahoxozoa - ps15
Bilateria - ps16
Protostomia - ps17
Ecdysozoa - ps18
Arthropoda - ps19
Mandibulata - ps20
Pancrustacea - ps21
Insecta - ps22
Eumetabola - ps23
Holometabola/Psocodea - ps24
Holometabola - ps25
Aparaglossata - ps26
Mecopterida - ps27
Diptera - ps28
Bibiono./Cyclorrhapha - ps29
Cyclorrhapha - ps30
Calyptratae/Drosophila - ps31
Drosophila - ps32
Sophophora - ps33
Obscura/Melanogaster_group - ps34
D_melanogaster_group - ps35
D_melanogaster_subgroup - ps36
sim./sech./mel. group - ps37
Drosophila_melanogaster - ps38
Phylostratum

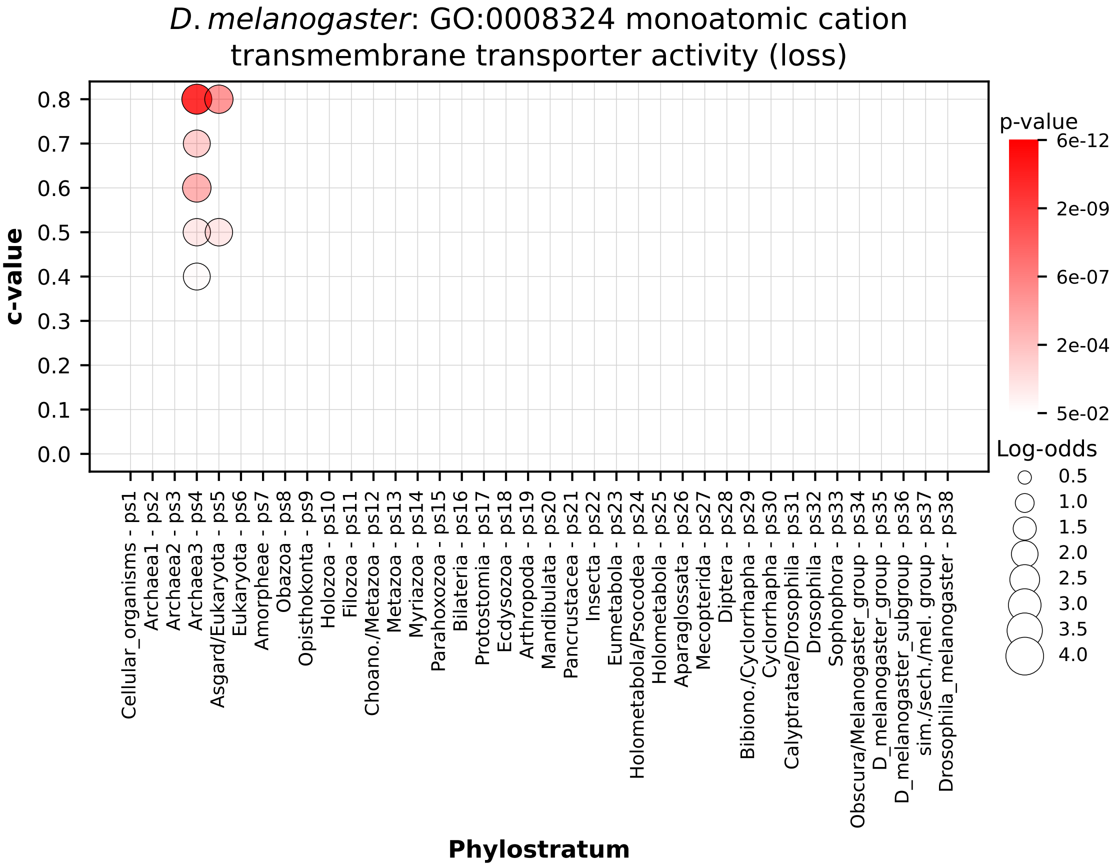

D. melanogaster: GO:0008324 monoatomic cation transmembrane transporter activity (loss)
c-value
0.8
0.7
0.6
0.5
0.4
0.3
0.2
0.1
0.0
Phylostratum
Cellular_organisms - ps1
Archaea1 - ps2
Archaea2 - ps3
Archaea3 - ps4
Asgard/Eukaryota - ps5
Eukaryota - ps6
Amorpheae - ps7
Obazoa - ps8
Opisthokonta - ps9
Holozoa - ps10
Filozoa - ps11
Choano./Metazoa - ps12
Metazoa - ps13
Myriazoa - ps14
Parahoxozoa - ps15
Bilateria - ps16
Protostomia - ps17
Ecdysozoa - ps18
Arthropoda - ps19
Mandibulata - ps20
Pancrustacea - ps21
Insecta - ps22
Eumetabola - ps23
Holometabola/Psocodea - ps24
Holometabola - ps25
Aparaglossata - ps26
Mecopterida - ps27
Diptera - ps28
Bibiono./Cyclorrhapha - ps29
Cyclorrhapha - ps30
Calyptratae/Drosophila - ps31
Drosophila - ps32
Sophophora - ps33
Obscura/Melanogaster_group - ps34
D_melanogaster_group - ps35
D_melanogaster_subgroup - ps36
sim./sech./mel. group - ps37
Drosophila_melanogaster - ps38
p-value
6e-12
2e-09
6e-07
2e-04
5e-02
Log-odds
0.5
1.0
1.5
2.0
2.5
3.0
3.5
4.0

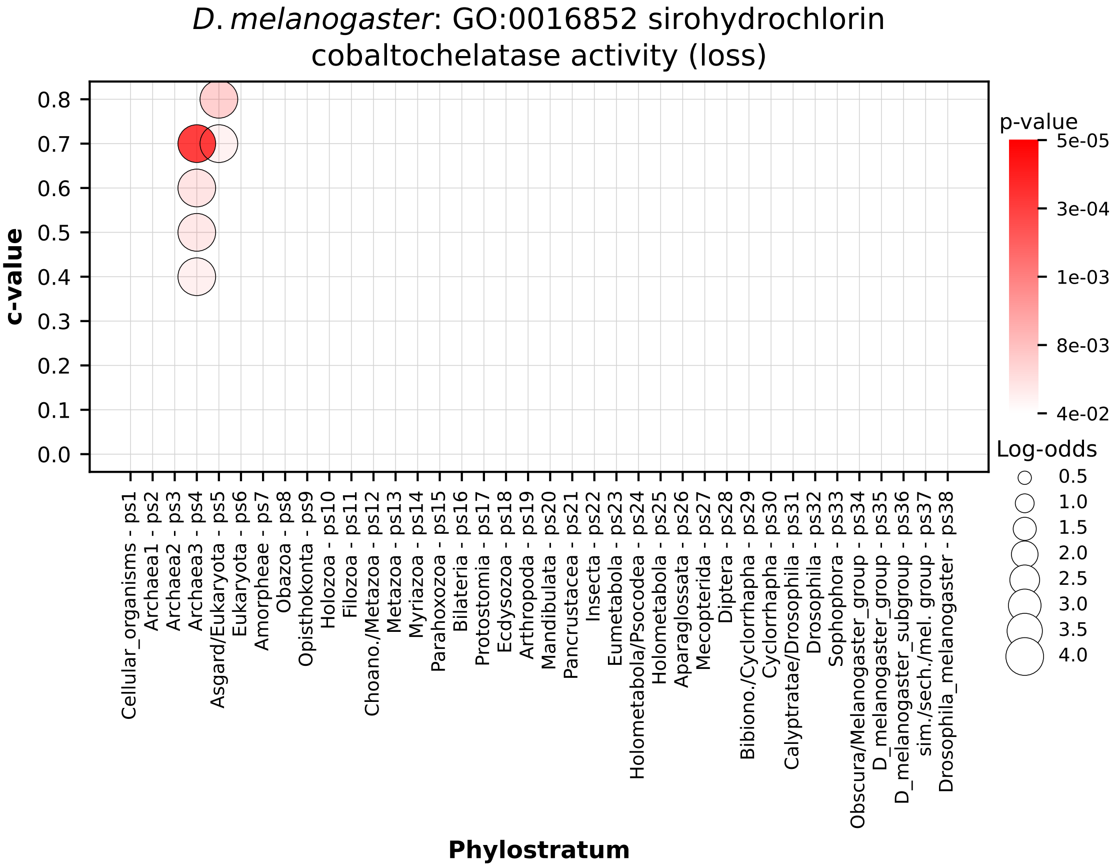

D. melanogaster: GO:0016852 sirohydrochlorin cobaltochelatase activity (loss)
c-value
0.8
0.7
0.6
0.5
0.4
0.3
0.2
0.1
0.0
p-value
5e-05
3e-04
1e-03
8e-03
4e-02
Log-odds
0.5
1.0
1.5
2.0
2.5
3.0
3.5
4.0
Cellular_organisms - ps1
Archaea1 - ps2
Archaea2 - ps3
Archaea3 - ps4
Asgard/Eukaryota - ps5
Eukaryota - ps6
Amorpheae - ps7
Obazoa - ps8
Opisthokonta - ps9
Holozoa - ps10
Filozoa - ps11
Choano./Metazoa - ps12
Metazoa - ps13
Myriazoa - ps14
Parahoxozoa - ps15
Bilateria - ps16
Protostomia - ps17
Ecdysozoa - ps18
Arthropoda - ps19
Mandibulata - ps20
Pancrustacea - ps21
Insecta - ps22
Eumetabola - ps23
Holometabola/Psocodea - ps24
Holometabola - ps25
Aparaglossata - ps26
Mecopterida - ps27
Diptera - ps28
Bibiono./Cyclorrhapha - ps29
Cyclorrhapha - ps30
Calyptratae/Drosophila - ps31
Drosophila - ps32
Sophophora - ps33
Obscura/Melanogaster_group - ps34
D_melanogaster_group - ps35
D_melanogaster_subgroup - ps36
sim./sech./mel. group - ps37
Drosophila_melanogaster - ps38
Phylostratum

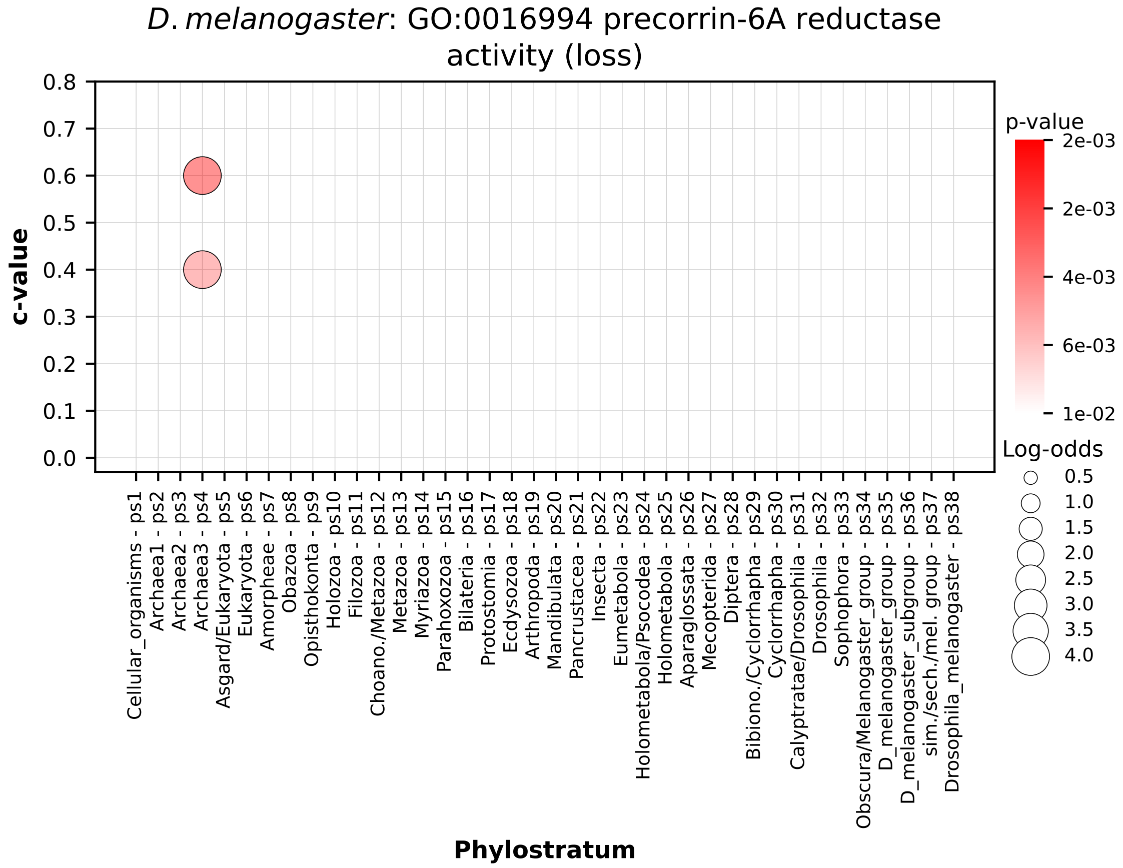

D. melanogaster: GO:0016994 precorrin-6A reductase activity (loss)
c-value
0.8
0.7
0.6
0.5
0.4
0.3
0.2
0.1
0.0
Phylostratum
Cellular_organisms - ps1
Archaea1 - ps2
Archaea2 - ps3
Archaea3 - ps4
Asgard/Eukaryota - ps5
Eukaryota - ps6
Amorpheae - ps7
Obazoa - ps8
Opisthokonta - ps9
Holozoa - ps10
Filozoa - ps11
Choano./Metazoa - ps12
Metazoa - ps13
Myriazoa - ps14
Parahoxozoa - ps15
Bilateria - ps16
Protostomia - ps17
Ecdysozoa - ps18
Arthropoda - ps19
Mandibulata - ps20
Pancrustacea - ps21
Insecta - ps22
Eumetabola - ps23
Holometabola/Psocodea - ps24
Holometabola - ps25
Aparaglossata - ps26
Mecopterida - ps27
Diptera - ps28
Bibiono./Cyclorrhapha - ps29
Cyclorrhapha - ps30
Calyptratae/Drosophila - ps31
Drosophila - ps32
Sophophora - ps33
Obscura/Melanogaster_group - ps34
D_melanogaster_group - ps35
D_melanogaster_subgroup - ps36
sim./sech./mel. group - ps37
Drosophila_melanogaster - ps38
p-value
2e-03
2e-03
4e-03
6e-03
1e-02
Log-odds
0.5
1.0
1.5
2.0
2.5
3.0
3.5
4.0

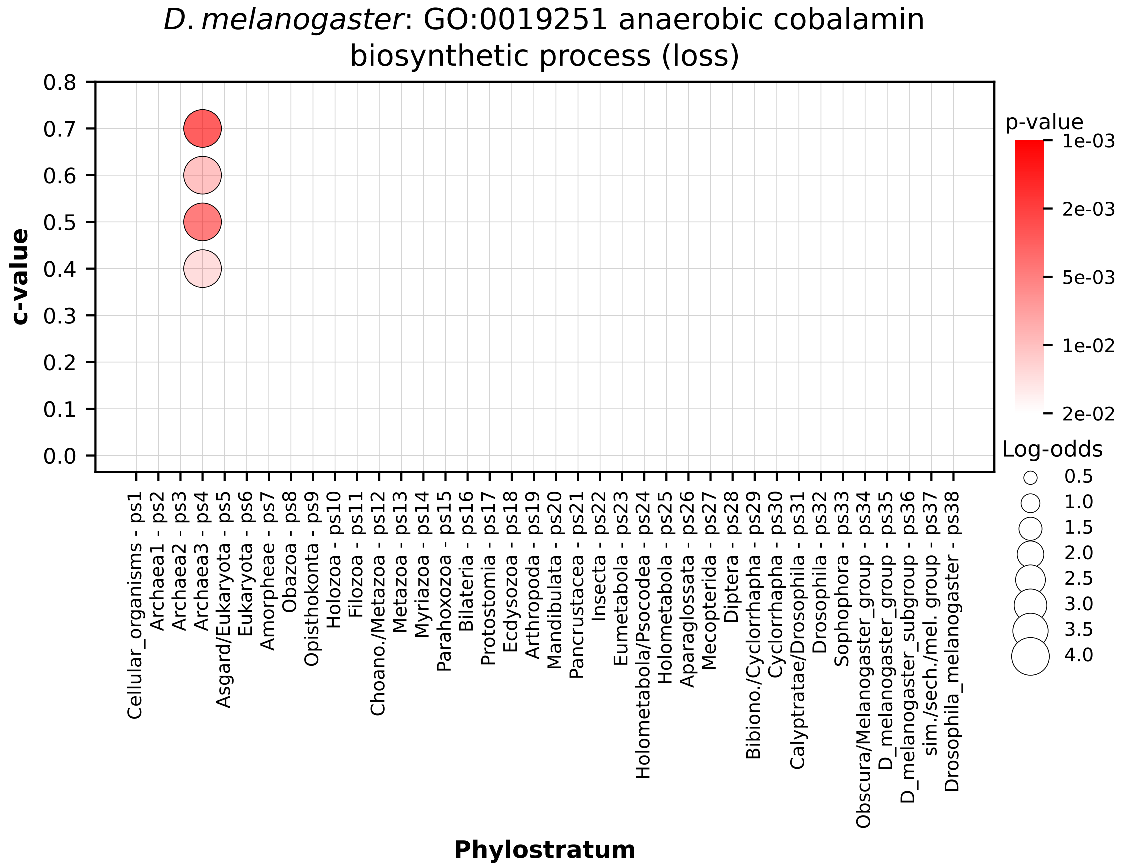

D. melanogaster: GO:0019251 anaerobic cobalamin biosynthetic process (loss)
c-value
0.8
0.7
0.6
0.5
0.4
0.3
0.2
0.1
0.0
p-value
1e-03
2e-03
5e-03
1e-02
2e-02
Log-odds
0.5
1.0
1.5
2.0
2.5
3.0
3.5
4.0
Cellular_organisms - ps1
Archaea1 - ps2
Archaea2 - ps3
Archaea3 - ps4
Asgard/Eukaryota - ps5
Eukaryota - ps6
Amorpheae - ps7
Obazoa - ps8
Opisthokonta - ps9
Holozoa - ps10
Filozoa - ps11
Choano./Metazoa - ps12
Metazoa - ps13
Myriazoa - ps14
Parahoxozoa - ps15
Bilateria - ps16
Protostomia - ps17
Ecdysozoa - ps18
Arthropoda - ps19
Mandibulata - ps20
Pancrustacea - ps21
Insecta - ps22
Eumetabola - ps23
Holometabola/Psocodea - ps24
Holometabola - ps25
Aparaglossata - ps26
Mecopterida - ps27
Diptera - ps28
Bibiono./Cyclorrhapha - ps29
Cyclorrhapha - ps30
Calyptratae/Drosophila - ps31
Drosophila - ps32
Sophophora - ps33
Obscura/Melanogaster_group - ps34
D_melanogaster_group - ps35
D_melanogaster_subgroup - ps36
sim./sech./mel. group - ps37
Drosophila_melanogaster - ps38
Phylostratum

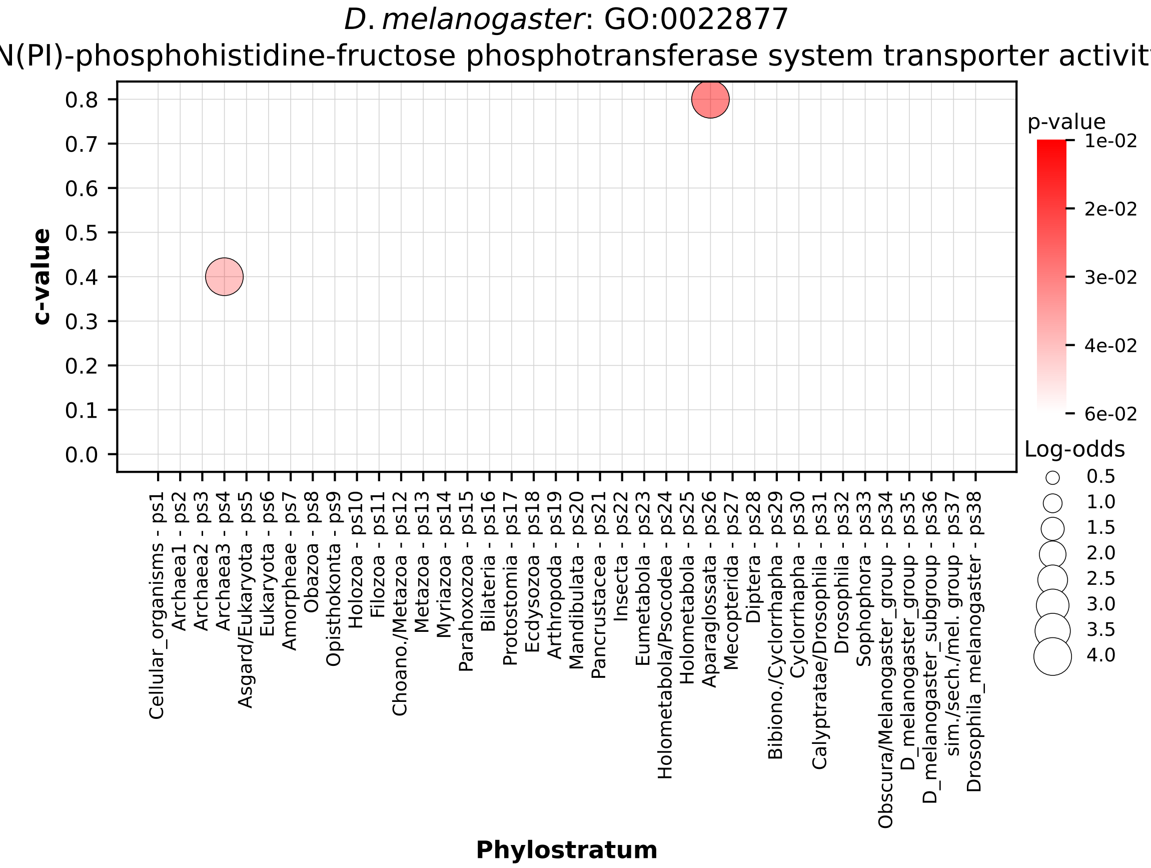

D. melanogaster: GO:0022877
N(PI)-phosphohistidine-fructose phosphotransferase system transporter activity (los
c-value
0.0
0.1
0.2
0.3
0.4
0.5
0.6
0.7
0.8
Phylostratum
Cellular_organisms - ps1
Archaea1 - ps2
Archaea2 - ps3
Archaea3 - ps4
Asgard/Eukaryota - ps5
Eukaryota - ps6
Amorpheae - ps7
Obazoa - ps8
Opisthokonta - ps9
Holozoa - ps10
Filozoa - ps11
Choano./Metazoa - ps12
Metazoa - ps13
Myriazoa - ps14
Parahoxozoa - ps15
Bilateria - ps16
Protostomia - ps17
Ecdysozoa - ps18
Arthropoda - ps19
Mandibulata - ps20
Pancrustacea - ps21
Insecta - ps22
Eumetabola - ps23
Holometabola/Psocodea - ps24
Holometabola - ps25
Aparaglossata - ps26
Mecopterida - ps27
Diptera - ps28
Bibiono./Cyclorrhapha - ps29
Cyclorrhapha - ps30
Calyptratae/Drosophila - ps31
Drosophila - ps32
Sophophora - ps33
Obscura/Melanogaster_group - ps34
D_melanogaster_group - ps35
D_melanogaster_subgroup - ps36
sim./sech./mel. group - ps37
Drosophila_melanogaster - ps38
p-value
1e-02
2e-02
3e-02
4e-02
6e-02
Log-odds
0.5
1.0
1.5
2.0
2.5
3.0
3.5
4.0

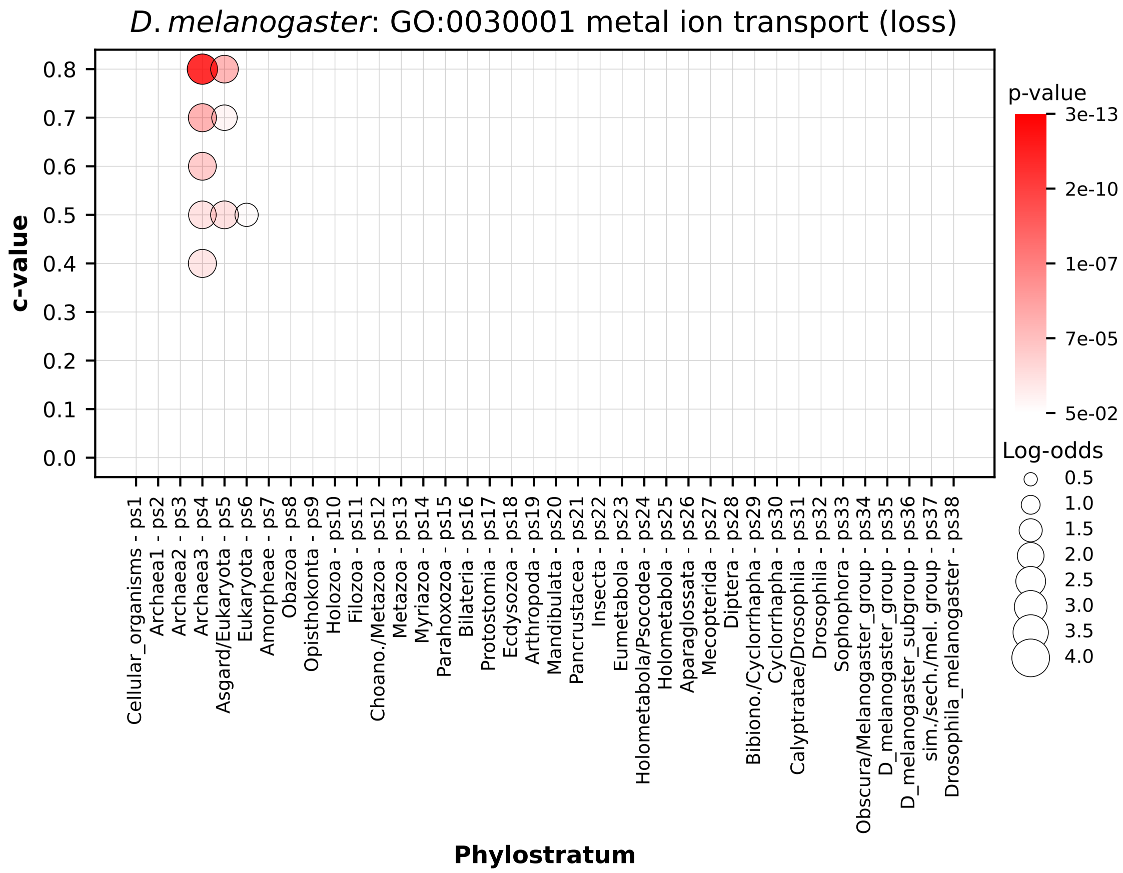

D. melanogaster: GO:0030001 metal ion transport (loss)
c-value
0.8
0.7
0.6
0.5
0.4
0.3
0.2
0.1
0.0
p-value
3e-13
2e-10
1e-07
7e-05
5e-02
Log-odds
0.5
1.0
1.5
2.0
2.5
3.0
3.5
4.0
Cellular_organisms - ps1
Archaea1 - ps2
Archaea2 - ps3
Archaea3 - ps4
Asgard/Eukaryota - ps5
Eukaryota - ps6
Amorpheae - ps7
Obazoa - ps8
Opisthokonta - ps9
Holozoa - ps10
Filozoa - ps11
Choano./Metazoa - ps12
Metazoa - ps13
Myriazoa - ps14
Parahoxozoa - ps15
Bilateria - ps16
Protostomia - ps17
Ecdysozoa - ps18
Arthropoda - ps19
Mandibulata - ps20
Pancrustacea - ps21
Insecta - ps22
Eumetabola - ps23
Holometabola/Psocodea - ps24
Holometabola - ps25
Aparaglossata - ps26
Mecopterida - ps27
Diptera - ps28
Bibiono./Cyclorrhapha - ps29
Cyclorrhapha - ps30
Calyptratae/Drosophila - ps31
Drosophila - ps32
Sophophora - ps33
Obscura/Melanogaster_group - ps34
D_melanogaster_group - ps35
D_melanogaster_subgroup - ps36
sim./sech./mel. group - ps37
Drosophila_melanogaster - ps38
Phylostratum

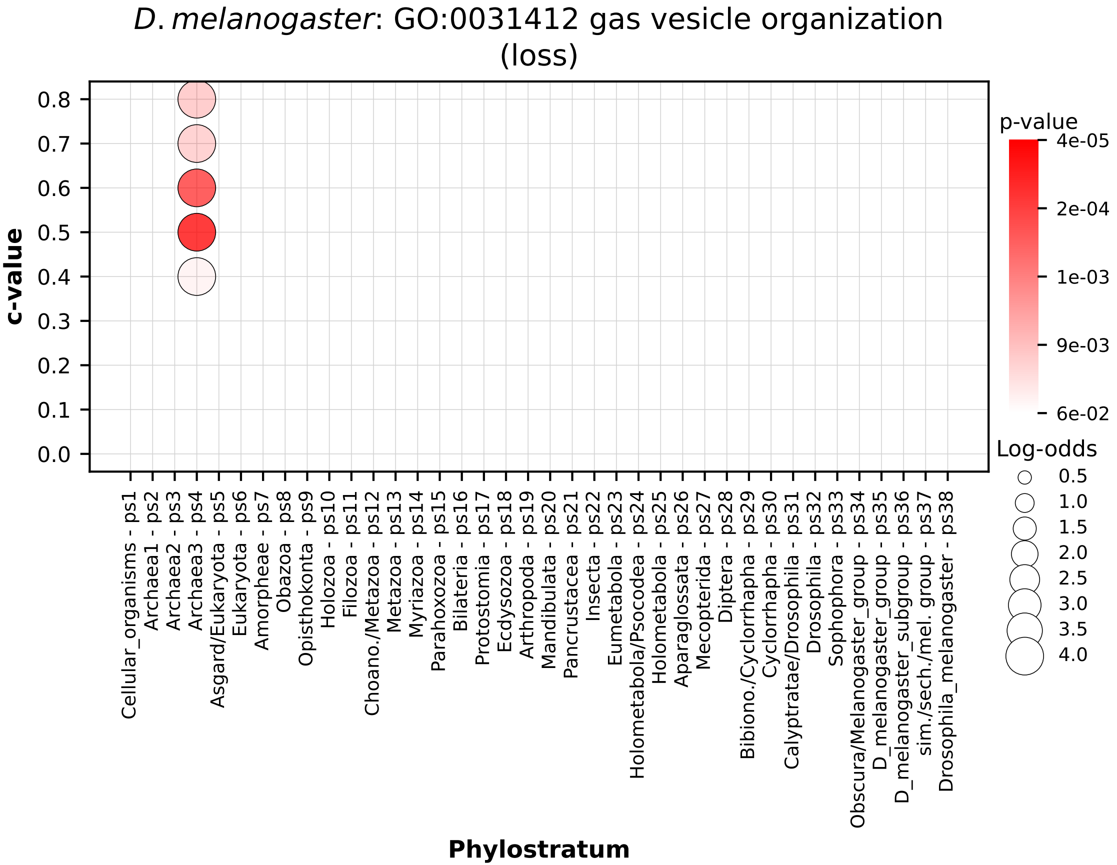
D. melanogaster: GO:0031412 gas vesicle organization
(loss)
c-value
0.8
0.7
0.6
0.5
0.4
0.3
0.2
0.1
0.0
p-value
4e-05
2e-04
1e-03
9e-03
6e-02
Log-odds
0.5
1.0
1.5
2.0
2.5
3.0
3.5
4.0
Cellular_organisms - ps1
Archaea1 - ps2
Archaea2 - ps3
Archaea3 - ps4
Asgard/Eukaryota - ps5
Eukaryota - ps6
Amorpheae - ps7
Obazoa - ps8
Opisthokonta - ps9
Holozoa - ps10
Filozoa - ps11
Choano./Metazoa - ps12
Metazoa - ps13
Myriazoa - ps14
Parahoxozoa - ps15
Bilateria - ps16
Protostomia - ps17
Ecdysozoa - ps18
Arthropoda - ps19
Mandibulata - ps20
Pancrustacea - ps21
Insecta - ps22
Eumetabola - ps23
Holometabola/Psocodea - ps24
Holometabola - ps25
Aparaglossata - ps26
Mecopterida - ps27
Diptera - ps28
Bibiono./Cyclorrhapha - ps29
Cyclorrhapha - ps30
Calyptratae/Drosophila - ps31
Drosophila - ps32
Sophophora - ps33
Obscura/Melanogaster_group - ps34
D_melanogaster_group - ps35
D_melanogaster_subgroup - ps36
sim./sech./mel. group - ps37
Drosophila_melanogaster - ps38
Phylostratum

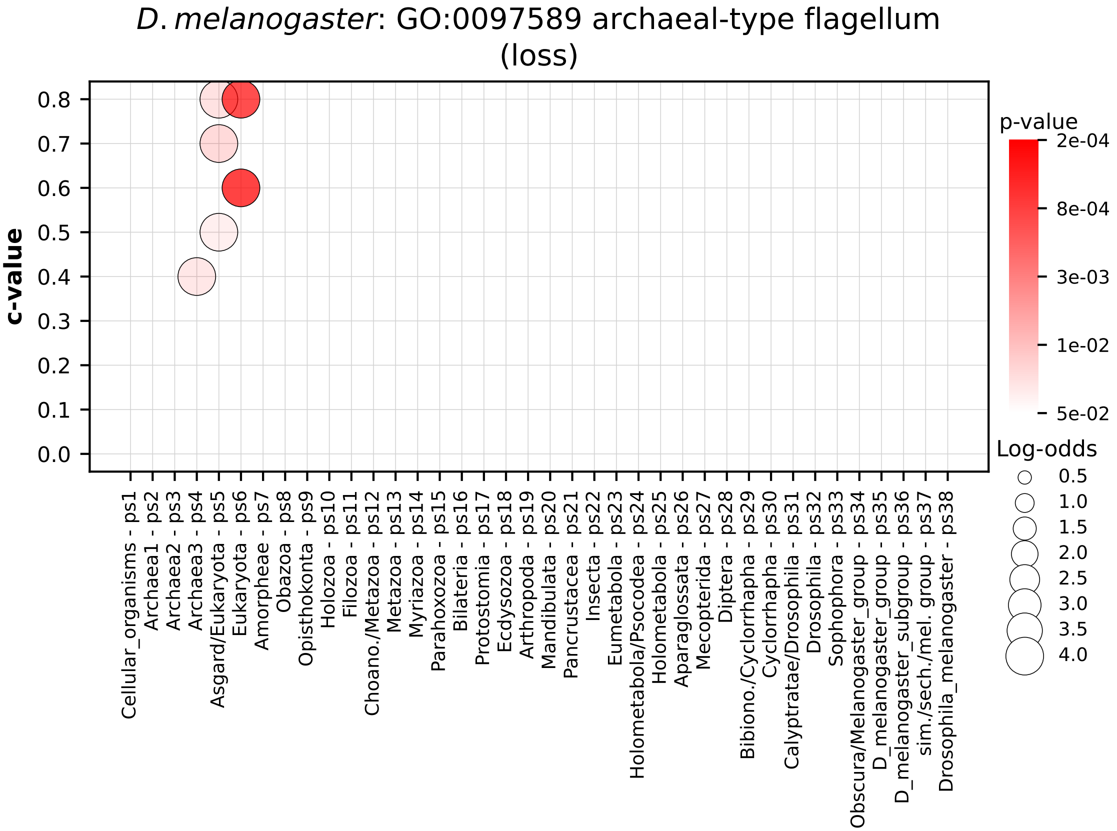

D. melanogaster: GO:0097589 archaeal-type flagellum
(loss)
c-value
0.8
0.7
0.6
0.5
0.4
0.3
0.2
0.1
0.0
p-value
2e-04
8e-04
3e-03
1e-02
5e-02
Log-odds
0.5
1.0
1.5
2.0
2.5
3.0
3.5
4.0
Cellular_organisms - ps1
Archaea1 - ps2
Archaea2 - ps3
Archaea3 - ps4
Asgard/Eukaryota - ps5
Eukaryota - ps6
Amorpheae - ps7
Obazoa - ps8
Opisthokonta - ps9
Holozoa - ps10
Filozoa - ps11
Choano./Metazoa - ps12
Metazoa - ps13
Myriazoa - ps14
Parahoxozoa - ps15
Bilateria - ps16
Protostomia - ps17
Ecdysozoa - ps18
Arthropoda - ps19
Mandibulata - ps20
Pancrustacea - ps21
Insecta - ps22
Eumetabola - ps23
Holometabola/Psocodea - ps24
Holometabola - ps25
Aparaglossata - ps26
Mecopterida - ps27
Diptera - ps28
Bibiono./Cyclorrhapha - ps29
Cyclorrhapha - ps30
Calyptratae/Drosophila - ps31
Drosophila - ps32
Sophophora - ps33
Obscura/Melanogaster_group - ps34
D_melanogaster_group - ps35
D_melanogaster_subgroup - ps36
sim./sech./mel. group - ps37
Drosophila_melanogaster - ps38
Phylostratum

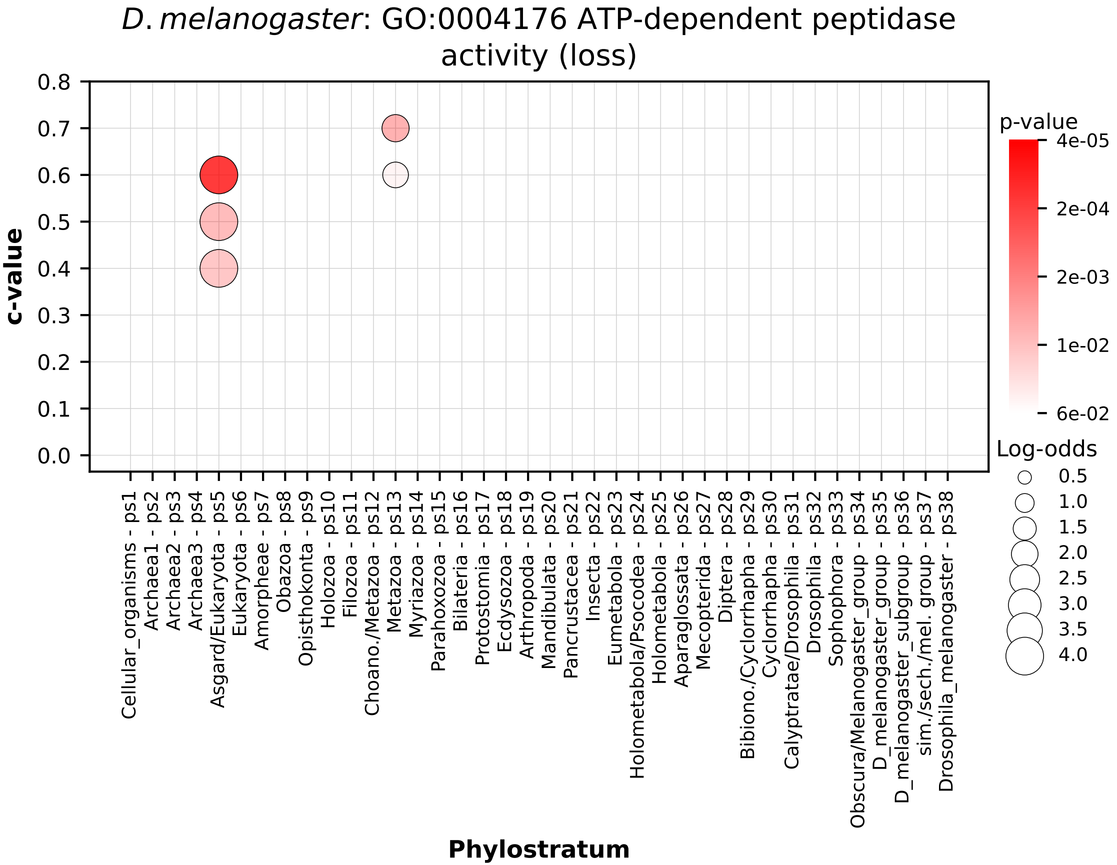

D. melanogaster: GO:0004176 ATP-dependent peptidase activity (loss)
c-value
0.8
0.7
0.6
0.5
0.4
0.3
0.2
0.1
0.0
Phylostratum
Cellular_organisms - ps1
Archaea1 - ps2
Archaea2 - ps3
Archaea3 - ps4
Asgard/Eukaryota - ps5
Eukaryota - ps6
Amorpheae - ps7
Obazoa - ps8
Opisthokonta - ps9
Holozoa - ps10
Filozoa - ps11
Choano./Metazoa - ps12
Metazoa - ps13
Myriazoa - ps14
Parahoxozoa - ps15
Bilateria - ps16
Protostomia - ps17
Ecdysozoa - ps18
Arthropoda - ps19
Mandibulata - ps20
Pancrustacea - ps21
Insecta - ps22
Eumetabola - ps23
Holometabola/Psocodea - ps24
Holometabola - ps25
Aparaglossata - ps26
Mecopterida - ps27
Diptera - ps28
Bibiono./Cyclorrhapha - ps29
Cyclorrhapha - ps30
Calyptratae/Drosophila - ps31
Drosophila - ps32
Sophophora - ps33
Obscura/Melanogaster_group - ps34
D_melanogaster_group - ps35
D_melanogaster_subgroup - ps36
sim./sech./mel. group - ps37
Drosophila_melanogaster - ps38
p-value
4e-05
2e-04
2e-03
1e-02
6e-02
Log-odds
0.5
1.0
1.5
2.0
2.5
3.0
3.5
4.0

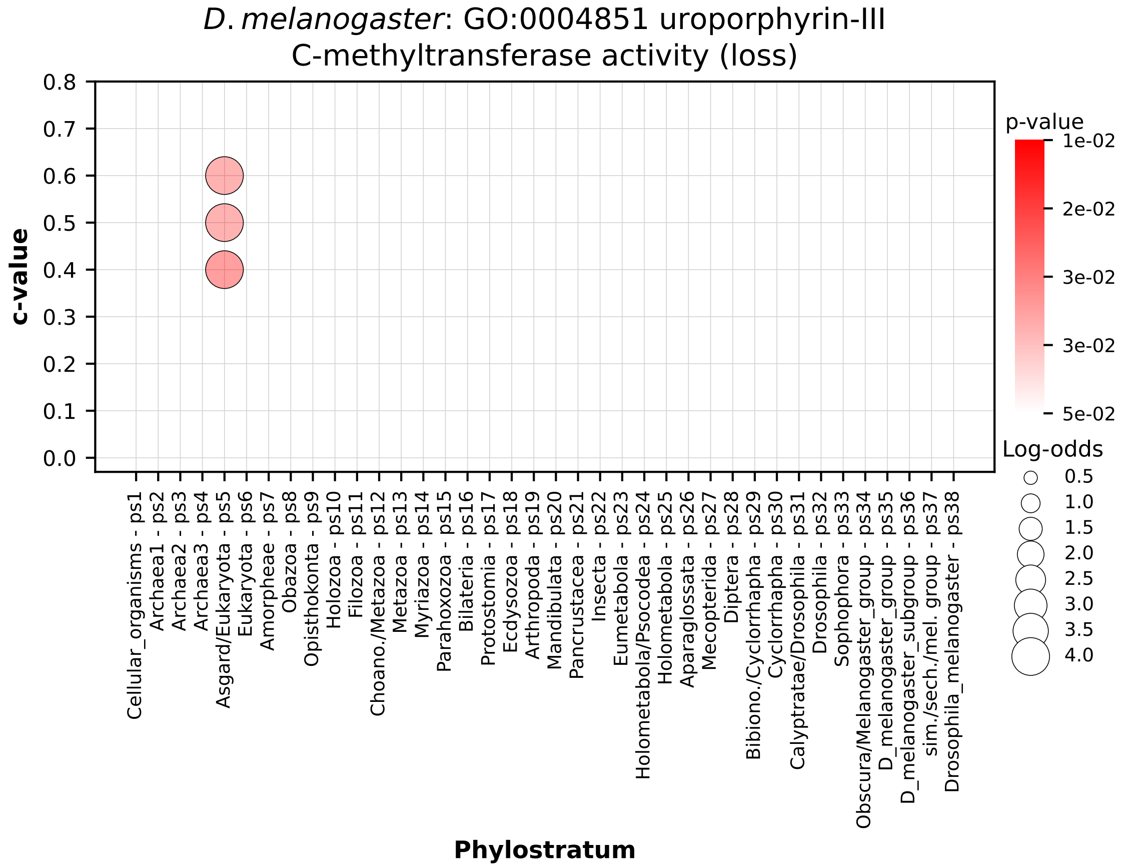
D. melanogaster: GO:0004851 uroporphyrin-III C-methyltransferase activity (loss)
c-value
0.0
0.1
0.2
0.3
0.4
0.5
0.6
0.7
0.8
p-value
1e-02
2e-02
3e-02
3e-02
5e-02
Log-odds
0.5
1.0
1.5
2.0
2.5
3.0
3.5
4.0
Cellular_organisms - ps1
Archaea1 - ps2
Archaea2 - ps3
Archaea3 - ps4
Asgard/Eukaryota - ps5
Eukaryota - ps6
Amorpheae - ps7
Obazoa - ps8
Opisthokonta - ps9
Holozoa - ps10
Filozoa - ps11
Choano./Metazoa - ps12
Metazoa - ps13
Myriazoa - ps14
Parahoxozoa - ps15
Bilateria - ps16
Protostomia - ps17
Ecdysozoa - ps18
Arthropoda - ps19
Mandibulata - ps20
Pancrustacea - ps21
Insecta - ps22
Eumetabola - ps23
Holometabola/Psocodea - ps24
Holometabola - ps25
Aparaglossata - ps26
Mecopterida - ps27
Diptera - ps28
Bibiono./Cyclorrhapha - ps29
Cyclorrhapha - ps30
Calyptratae/Drosophila - ps31
Drosophila - ps32
Sophophora - ps33
Obscura/Melanogaster_group - ps34
D_melanogaster_group - ps35
D_melanogaster_subgroup - ps36
sim./sech./mel. group - ps37
Drosophila_melanogaster - ps38
Phylostratum

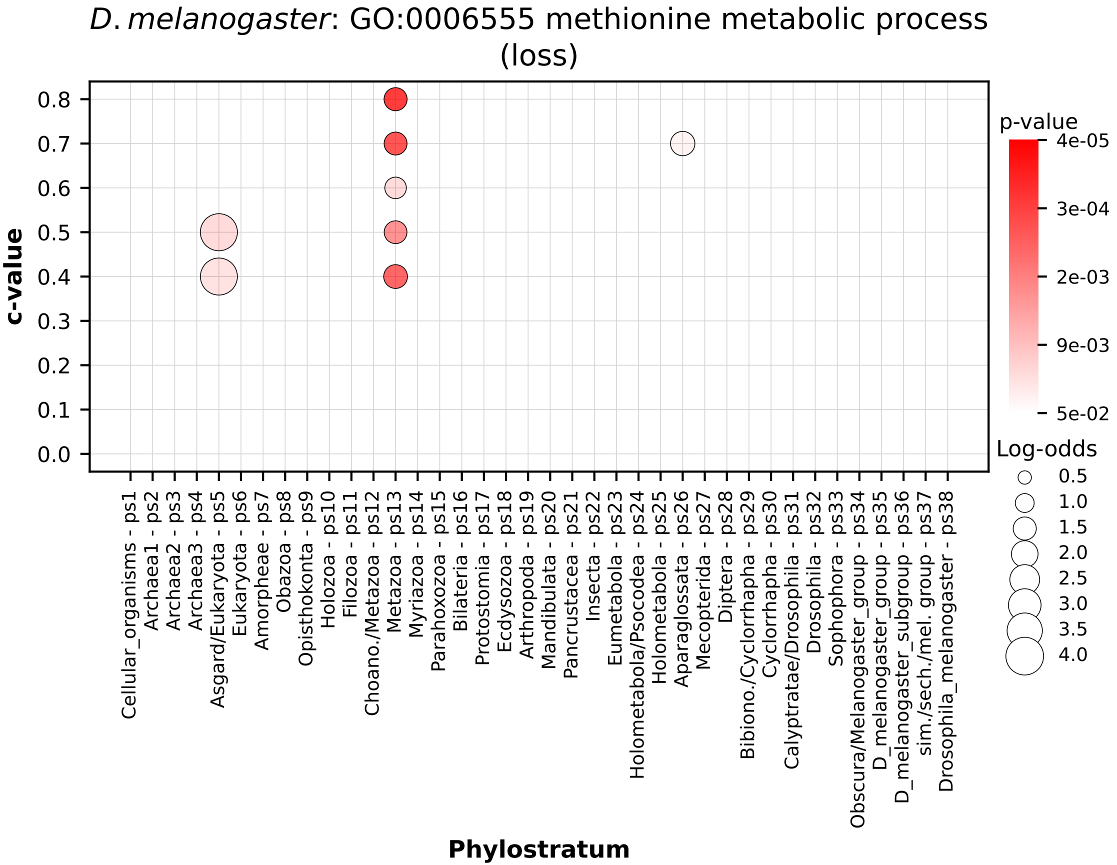

D. melanogaster: GO:0006555 methionine metabolic process (loss)
c-value
0.8
0.7
0.6
0.5
0.4
0.3
0.2
0.1
0.0
p-value
4e-05
3e-04
2e-03
9e-03
5e-02
Log-odds
0.5
1.0
1.5
2.0
2.5
3.0
3.5
4.0
Cellular_organisms - ps1
Archaea1 - ps2
Archaea2 - ps3
Archaea3 - ps4
Asgard/Eukaryota - ps5
Eukaryota - ps6
Amorpheae - ps7
Obazoa - ps8
Opisthokonta - ps9
Holozoa - ps10
Filozoa - ps11
Choano./Metazoa - ps12
Metazoa - ps13
Myriazoa - ps14
Parahoxozoa - ps15
Bilateria - ps16
Protostomia - ps17
Ecdysozoa - ps18
Arthropoda - ps19
Mandibulata - ps20
Pancrustacea - ps21
Insecta - ps22
Eumetabola - ps23
Holometabola/Psocodea - ps24
Holometabola - ps25
Aparaglossata - ps26
Mecopterida - ps27
Diptera - ps28
Bibiono./Cyclorrhapha - ps29
Cyclorrhapha - ps30
Calyptratae/Drosophila - ps31
Drosophila - ps32
Sophophora - ps33
Obscura/Melanogaster_group - ps34
D_melanogaster_group - ps35
D_melanogaster_subgroup - ps36
sim./sech./mel. group - ps37
Drosophila_melanogaster - ps38
Phylostratum

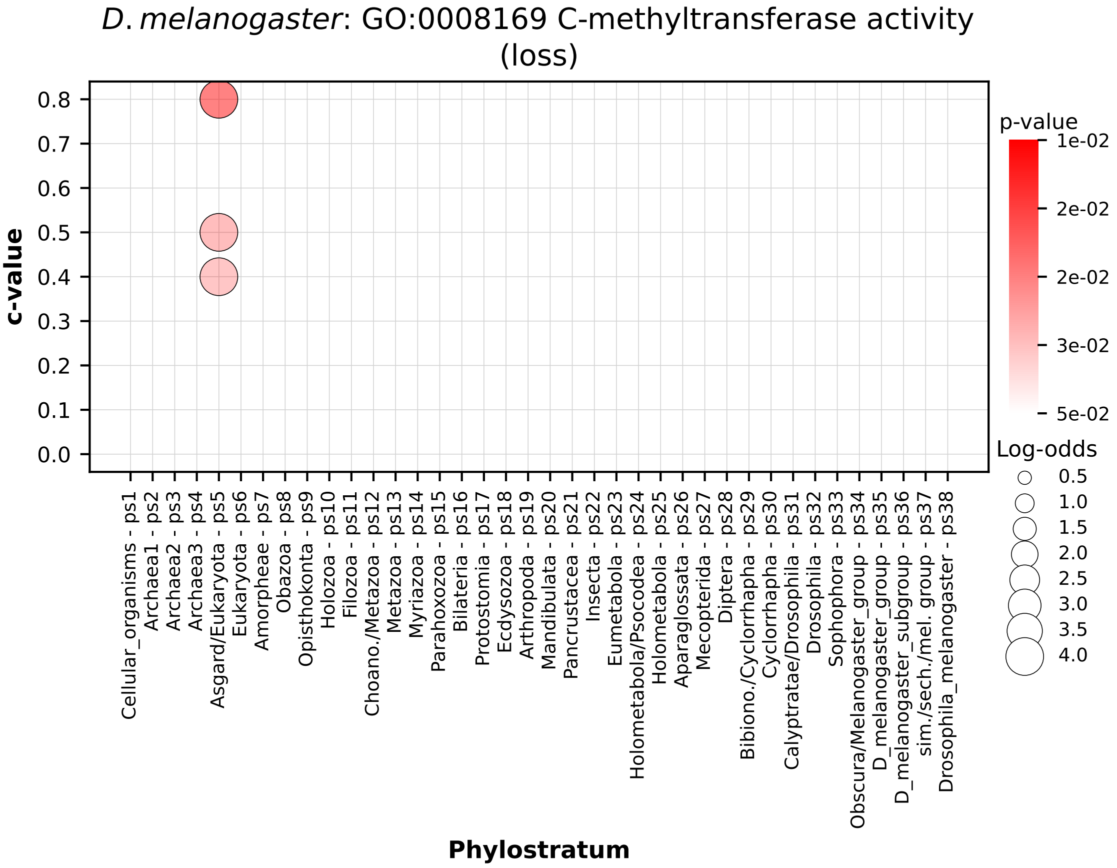

D. melanogaster: GO:0008169 C-methyltransferase activity
(loss)
c-value
0.8
0.7
0.6
0.5
0.4
0.3
0.2
0.1
0.0
p-value
1e-02
2e-02
2e-02
3e-02
5e-02
Log-odds
0.5
1.0
1.5
2.0
2.5
3.0
3.5
4.0
Cellular_organisms - ps1
Archaea1 - ps2
Archaea2 - ps3
Archaea3 - ps4
Asgard/Eukaryota - ps5
Eukaryota - ps6
Amorpheae - ps7
Obazoa - ps8
Opisthokonta - ps9
Holozoa - ps10
Filozoa - ps11
Choano./Metazoa - ps12
Metazoa - ps13
Myriazoa - ps14
Parahoxozoa - ps15
Bilateria - ps16
Protostomia - ps17
Ecdysozoa - ps18
Arthropoda - ps19
Mandibulata - ps20
Pancrustacea - ps21
Insecta - ps22
Eumetabola - ps23
Holometabola/Psocodea - ps24
Holometabola - ps25
Aparaglossata - ps26
Mecopterida - ps27
Diptera - ps28
Bibiono./Cyclorrhapha - ps29
Cyclorrhapha - ps30
Calyptratae/Drosophila - ps31
Drosophila - ps32
Sophophora - ps33
Obscura/Melanogaster_group - ps34
D_melanogaster_group - ps35
D_melanogaster_subgroup - ps36
sim./sech./mel. group - ps37
Drosophila_melanogaster - ps38
Phylostratum

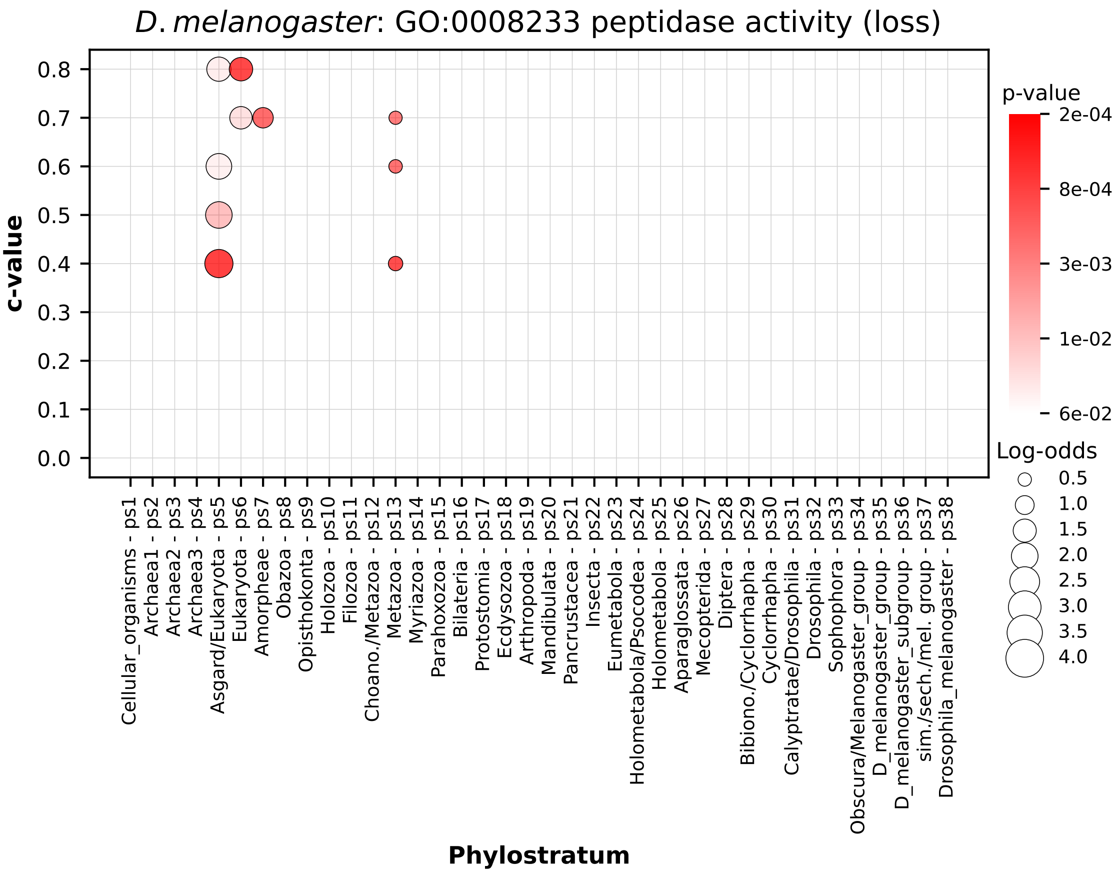

D. melanogaster: GO:0008233 peptidase activity (loss)
c-value
0.8
0.7
0.6
0.5
0.4
0.3
0.2
0.1
0.0
Phylostratum
Cellular_organisms - ps1
Archaea1 - ps2
Archaea2 - ps3
Archaea3 - ps4
Asgard/Eukaryota - ps5
Eukaryota - ps6
Amorpheae - ps7
Obazoa - ps8
Opisthokonta - ps9
Holozoa - ps10
Filozoa - ps11
Choano./Metazoa - ps12
Metazoa - ps13
Myriazoa - ps14
Parahoxozoa - ps15
Bilateria - ps16
Protostomia - ps17
Ecdysozoa - ps18
Arthropoda - ps19
Mandibulata - ps20
Pancrustacea - ps21
Insecta - ps22
Eumetabola - ps23
Holometabola/Psocodea - ps24
Holometabola - ps25
Aparaglossata - ps26
Mecopterida - ps27
Diptera - ps28
Bibiono./Cyclorrhapha - ps29
Cyclorrhapha - ps30
Calyptratae/Drosophila - ps31
Drosophila - ps32
Sophophora - ps33
Obscura/Melanogaster_group - ps34
D_melanogaster_group - ps35
D_melanogaster_subgroup - ps36
sim./sech./mel. group - ps37
Drosophila_melanogaster - ps38
p-value
2e-04
8e-04
3e-03
1e-02
6e-02
Log-odds
0.5
1.0
1.5
2.0
2.5
3.0
3.5
4.0

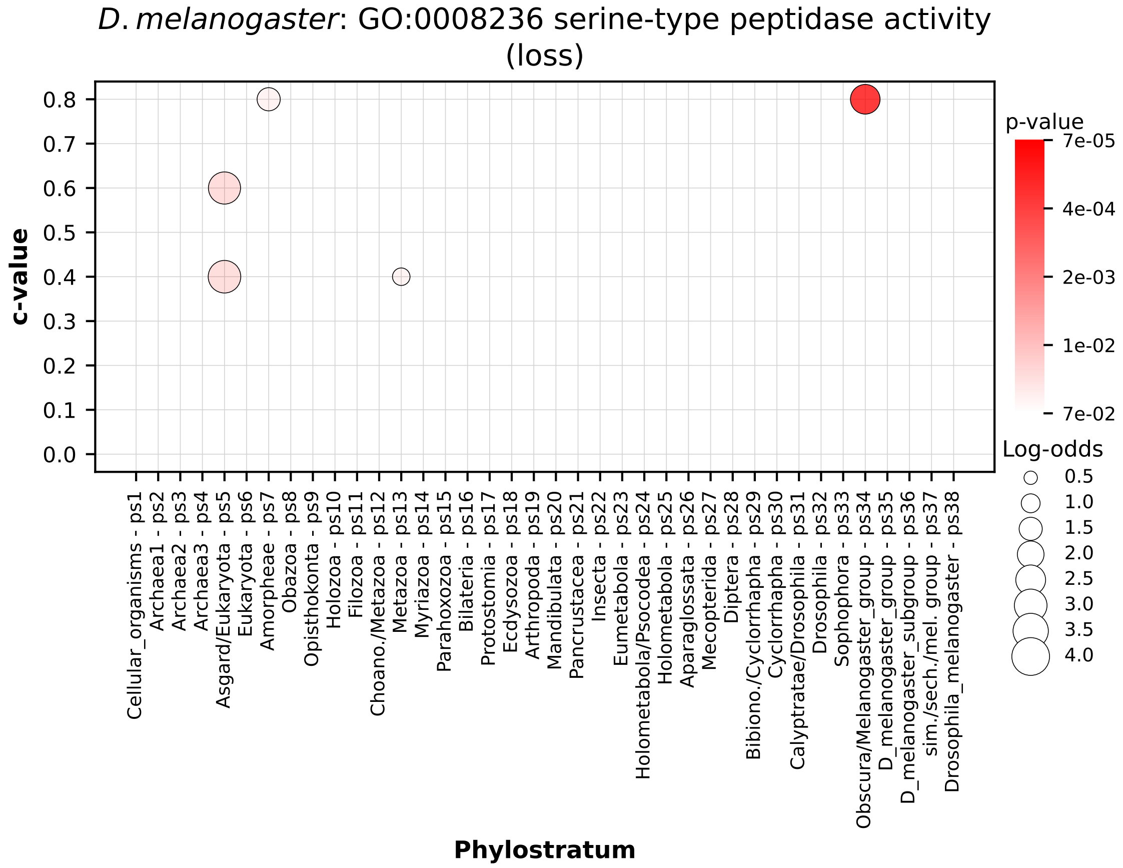

D. melanogaster: GO:0008236 serine-type peptidase activity (loss)
c-value
0.0
0.1
0.2
0.3
0.4
0.5
0.6
0.7
0.8
p-value
7e-05
4e-04
2e-03
1e-02
7e-02
Log-odds
0.5
1.0
1.5
2.0
2.5
3.0
3.5
4.0
Cellular_organisms - ps1
Archaea1 - ps2
Archaea2 - ps3
Archaea3 - ps4
Asgard/Eukaryota - ps5
Eukaryota - ps6
Amorpheae - ps7
Obazoa - ps8
Opisthokonta - ps9
Holozoa - ps10
Filozoa - ps11
Choano./Metazoa - ps12
Metazoa - ps13
Myriazoa - ps14
Parahoxozoa - ps15
Bilateria - ps16
Protostomia - ps17
Ecdysozoa - ps18
Arthropoda - ps19
Mandibulata - ps20
Pancrustacea - ps21
Insecta - ps22
Eumetabola - ps23
Holometabola/Psocodea - ps24
Holometabola - ps25
Aparaglossata - ps26
Mecopterida - ps27
Diptera - ps28
Bibiono./Cyclorrhapha - ps29
Cyclorrhapha - ps30
Calyptratae/Drosophila - ps31
Drosophila - ps32
Sophophora - ps33
Obscura/Melanogaster_group - ps34
D_melanogaster_group - ps35
D_melanogaster_subgroup - ps36
sim./sech./mel. group - ps37
Drosophila_melanogaster - ps38
Phylostratum

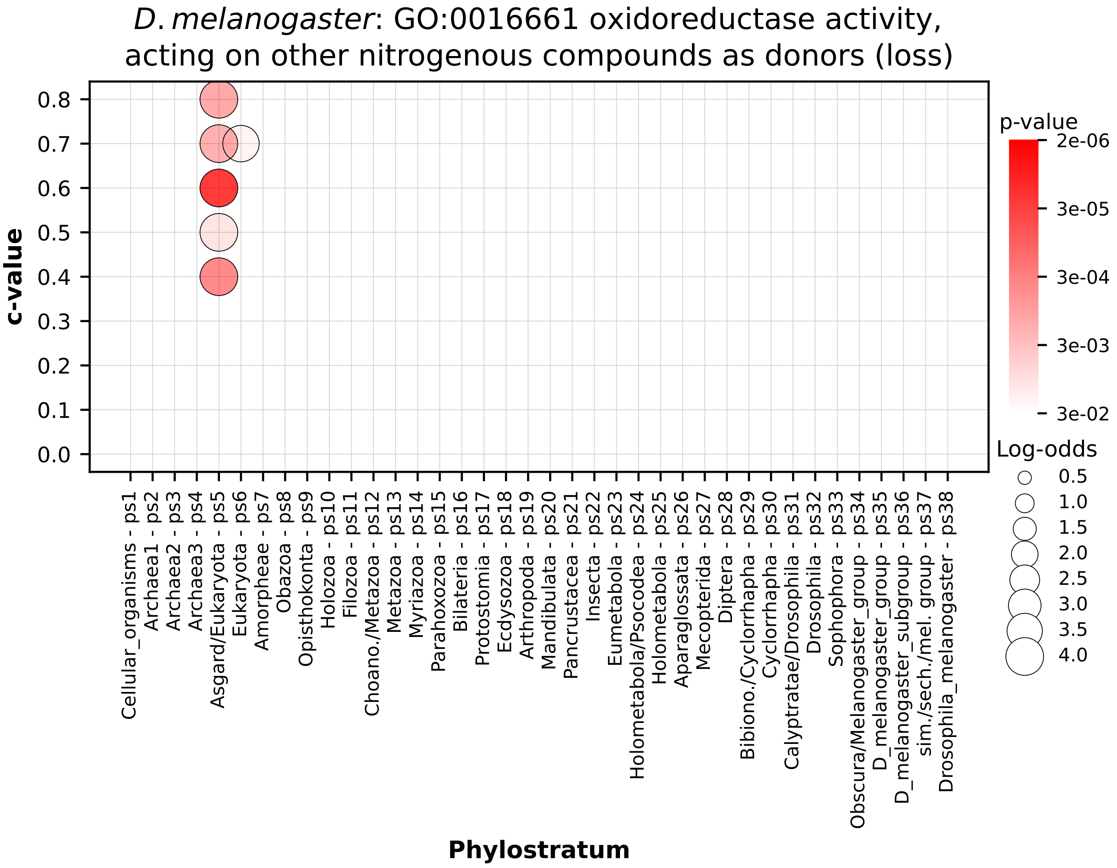

$D. melanogaster$: GO:0016661 oxidoreductase activity, acting on other nitrogenous compounds as donors (loss)
c-value
0.8
0.7
0.6
0.5
0.4
0.3
0.2
0.1
0.0
Phylostratum
Cellular_organisms - ps1
Archaea1 - ps2
Archaea2 - ps3
Archaea3 - ps4
Asgard/Eukaryota - ps5
Eukaryota - ps6
Amorpheae - ps7
Obazoa - ps8
Opisthokonta - ps9
Holozoa - ps10
Filozoa - ps11
Choano./Metazoa - ps12
Metazoa - ps13
Myriazoa - ps14
Parahoxozoa - ps15
Bilateria - ps16
Protostomia - ps17
Ecdysozoa - ps18
Arthropoda - ps19
Mandibulata - ps20
Pancrustacea - ps21
Insecta - ps22
Eumetabola - ps23
Holometabola/Psocodea - ps24
Holometabola - ps25
Aparaglossata - ps26
Mecopterida - ps27
Diptera - ps28
Bibiono./Cyclorrhapha - ps29
Cyclorrhapha - ps30
Calyptratae/Drosophila - ps31
Drosophila - ps32
Sophophora - ps33
Obscura/Melanogaster_group - ps34
D_melanogaster_group - ps35
D_melanogaster_subgroup - ps36
sim./sech./mel. group - ps37
Drosophila_melanogaster - ps38
p-value
2e-06
3e-05
3e-04
3e-03
3e-02
Log-odds
0.5
1.0
1.5
2.0
2.5
3.0
3.5
4.0

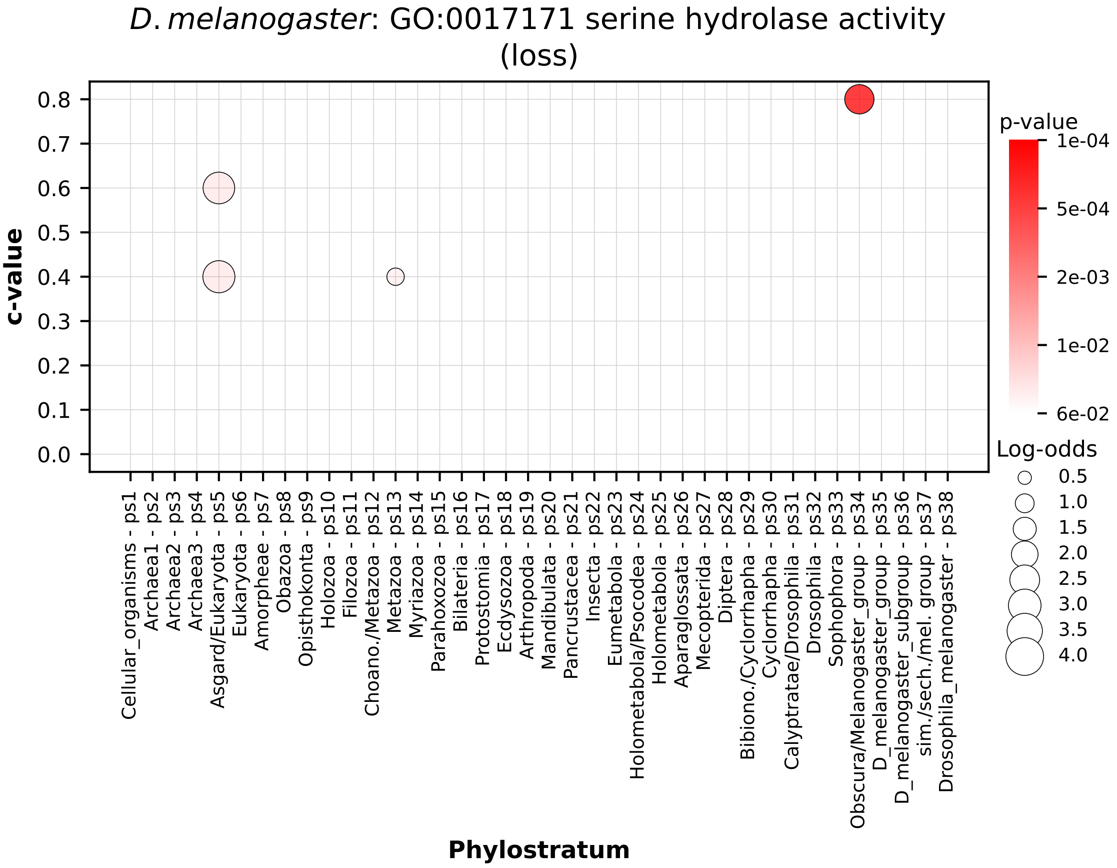

D. melanogaster: GO:0017171 serine hydrolase activity
(loss)
c-value
0.8
0.7
0.6
0.5
0.4
0.3
0.2
0.1
0.0
Phylostratum
Cellular_organisms - ps1
Archaea1 - ps2
Archaea2 - ps3
Archaea3 - ps4
Asgard/Eukaryota - ps5
Eukaryota - ps6
Amorpheae - ps7
Obazoa - ps8
Opisthokonta - ps9
Holozoa - ps10
Filozoa - ps11
Choano./Metazoa - ps12
Metazoa - ps13
Myriazoa - ps14
Parahoxozoa - ps15
Bilateria - ps16
Protostomia - ps17
Ecdysozoa - ps18
Arthropoda - ps19
Mandibulata - ps20
Pancrustacea - ps21
Insecta - ps22
Eumetabola - ps23
Holometabola/Psocodea - ps24
Holometabola - ps25
Aparaglossata - ps26
Mecopterida - ps27
Diptera - ps28
Bibiono./Cyclorrhapha - ps29
Cyclorrhapha - ps30
Calyptratae/Drosophila - ps31
Drosophila - ps32
Sophophora - ps33
Obscura/Melanogaster_group - ps34
D_melanogaster_group - ps35
D_melanogaster_subgroup - ps36
sim./sech./mel. group - ps37
Drosophila_melanogaster - ps38
p-value
1e-04
5e-04
2e-03
1e-02
6e-02
Log-odds
0.5
1.0
1.5
2.0
2.5
3.0
3.5
4.0

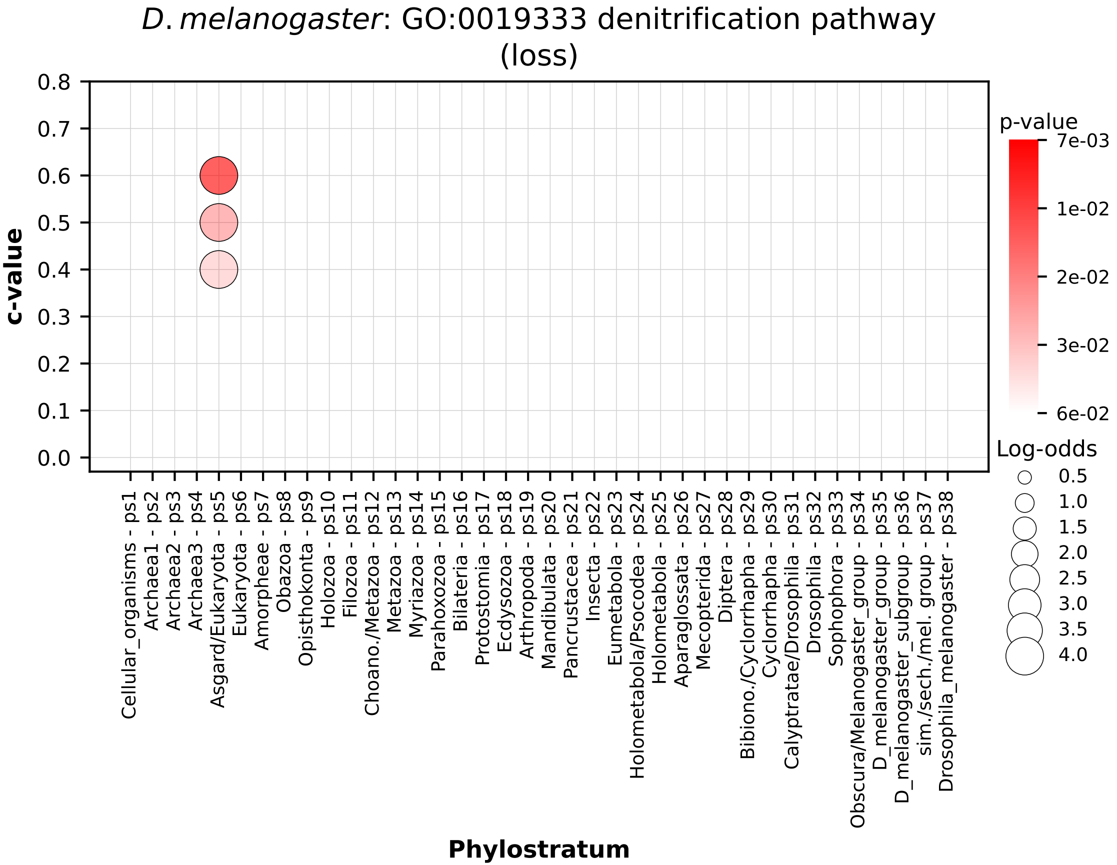

D. melanogaster: GO:0019333 denitrification pathway (loss)
c-value
0.8
0.7
0.6
0.5
0.4
0.3
0.2
0.1
0.0
Phylostratum
Cellular_organisms - ps1
Archaea1 - ps2
Archaea2 - ps3
Archaea3 - ps4
Asgard/Eukaryota - ps5
Eukaryota - ps6
Amorpheae - ps7
Obazoa - ps8
Opisthokonta - ps9
Holozoa - ps10
Filozoa - ps11
Choano./Metazoa - ps12
Metazoa - ps13
Myriazoa - ps14
Parahoxozoa - ps15
Bilateria - ps16
Protostomia - ps17
Ecdysozoa - ps18
Arthropoda - ps19
Mandibulata - ps20
Pancrustacea - ps21
Insecta - ps22
Eumetabola - ps23
Holometabola/Psocodea - ps24
Holometabola - ps25
Aparaglossata - ps26
Mecopterida - ps27
Diptera - ps28
Bibiono./Cyclorrhapha - ps29
Cyclorrhapha - ps30
Calyptratae/Drosophila - ps31
Drosophila - ps32
Sophophora - ps33
Obscura/Melanogaster_group - ps34
D_melanogaster_group - ps35
D_melanogaster_subgroup - ps36
sim./sech./mel. group - ps37
Drosophila_melanogaster - ps38
p-value
7e-03
1e-02
2e-02
3e-02
6e-02
Log-odds
0.5
1.0
1.5
2.0
2.5
3.0
3.5
4.0

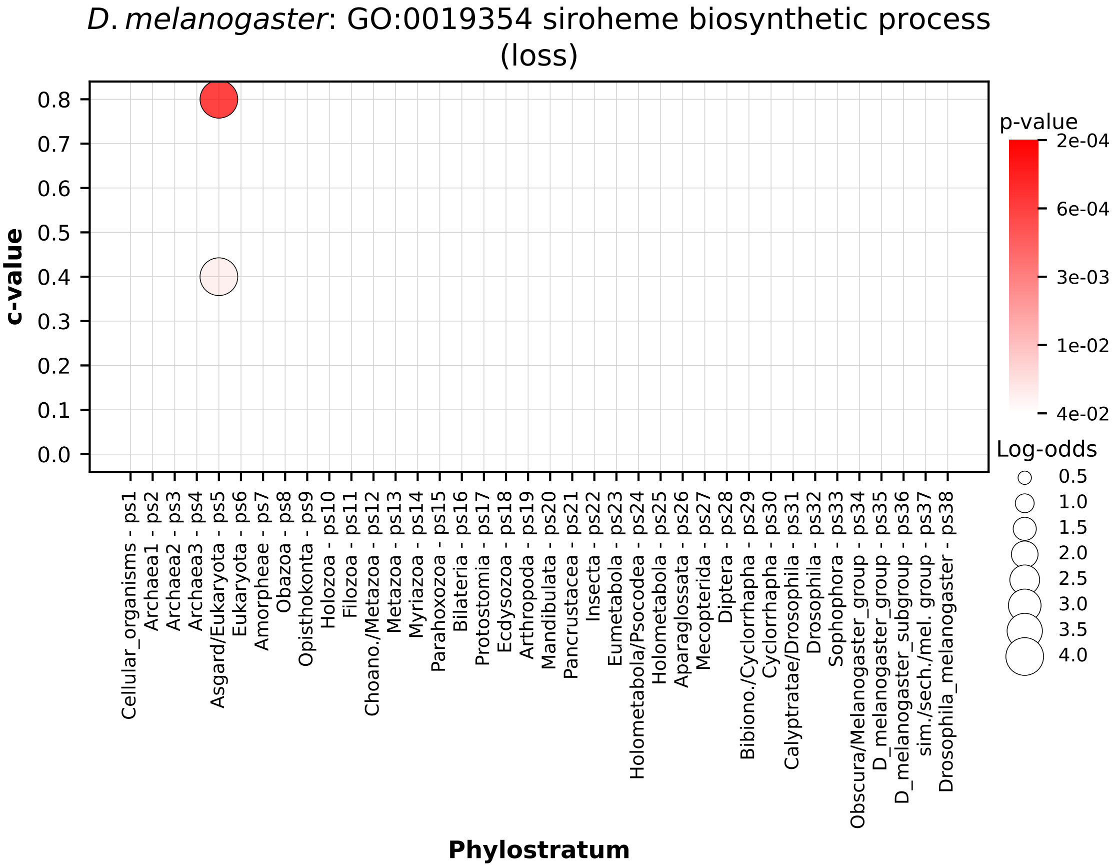
D. melanogaster: GO:0019354 siroheme biosynthetic process (loss)
c-value
0.8
0.7
0.6
0.5
0.4
0.3
0.2
0.1
0.0
p-value
2e-04
6e-04
3e-03
1e-02
4e-02
Log-odds
0.5
1.0
1.5
2.0
2.5
3.0
3.5
4.0
Cellular_organisms - ps1
Archaea1 - ps2
Archaea2 - ps3
Archaea3 - ps4
Asgard/Eukaryota - ps5
Eukaryota - ps6
Amorpheae - ps7
Obazoa - ps8
Opisthokonta - ps9
Holozoa - ps10
Filozoa - ps11
Choano./Metazoa - ps12
Metazoa - ps13
Myriazoa - ps14
Parahoxozoa - ps15
Bilateria - ps16
Protostomia - ps17
Ecdysozoa - ps18
Arthropoda - ps19
Mandibulata - ps20
Pancrustacea - ps21
Insecta - ps22
Eumetabola - ps23
Holometabola/Psocodea - ps24
Holometabola - ps25
Aparaglossata - ps26
Mecopterida - ps27
Diptera - ps28
Bibiono./Cyclorrhapha - ps29
Cyclorrhapha - ps30
Calyptratae/Drosophila - ps31
Drosophila - ps32
Sophophora - ps33
Obscura/Melanogaster_group - ps34
D_melanogaster_group - ps35
D_melanogaster_subgroup - ps36
sim./sech./mel. group - ps37
Drosophila_melanogaster - ps38
Phylostratum

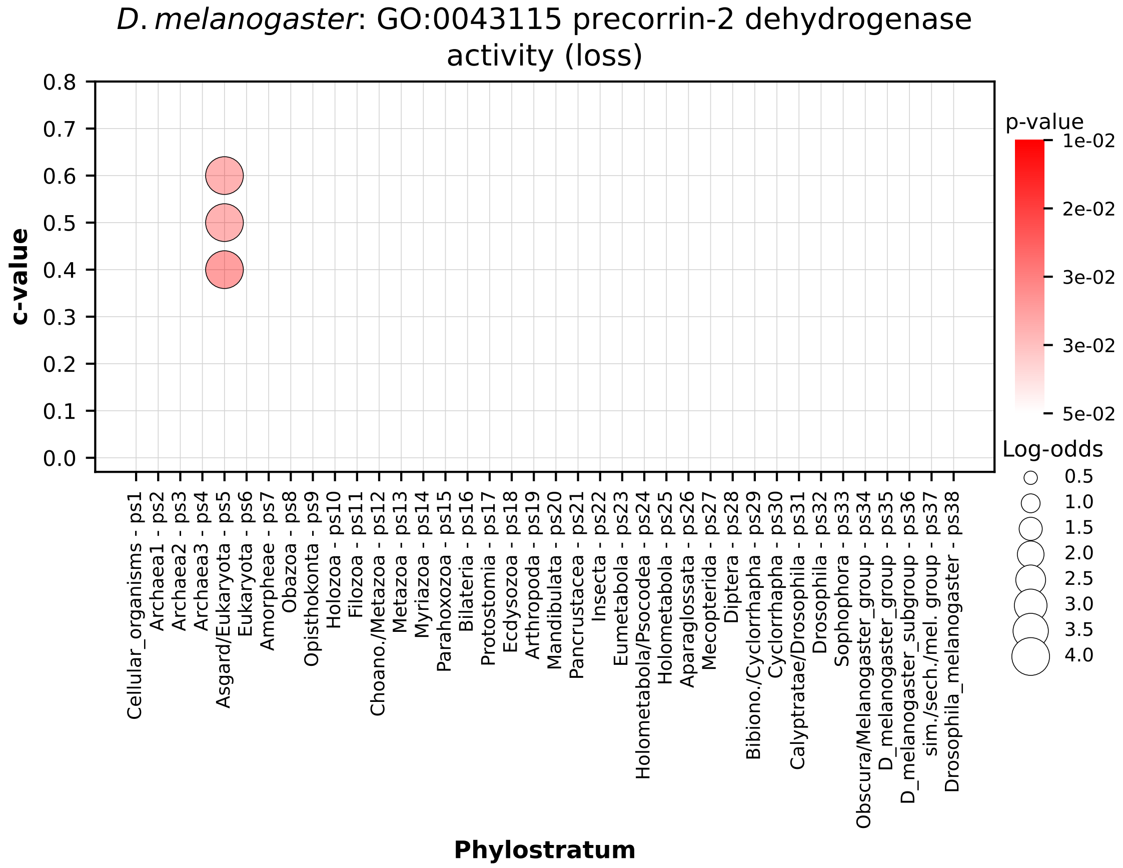

D. melanogaster: GO:0043115 precorrin-2 dehydrogenase activity (loss)
c-value
0.8
0.7
0.6
0.5
0.4
0.3
0.2
0.1
0.0
Phylostratum
Cellular_organisms - ps1
Archaea1 - ps2
Archaea2 - ps3
Archaea3 - ps4
Asgard/Eukaryota - ps5
Eukaryota - ps6
Amorpheae - ps7
Obazoa - ps8
Opisthokonta - ps9
Holozoa - ps10
Filozoa - ps11
Choano./Metazoa - ps12
Metazoa - ps13
Myriazoa - ps14
Parahoxozoa - ps15
Bilateria - ps16
Protostomia - ps17
Ecdysozoa - ps18
Arthropoda - ps19
Mandibulata - ps20
Pancrustacea - ps21
Insecta - ps22
Eumetabola - ps23
Holometabola/Psocodea - ps24
Holometabola - ps25
Aparaglossata - ps26
Mecopterida - ps27
Diptera - ps28
Bibiono./Cyclorrhapha - ps29
Cyclorrhapha - ps30
Calyptratae/Drosophila - ps31
Drosophila - ps32
Sophophora - ps33
Obscura/Melanogaster_group - ps34
D_melanogaster_group - ps35
D_melanogaster_subgroup - ps36
sim./sech./mel. group - ps37
Drosophila_melanogaster - ps38
p-value
1e-02
2e-02
3e-02
3e-02
5e-02
Log-odds
0.5
1.0
1.5
2.0
2.5
3.0
3.5
4.0

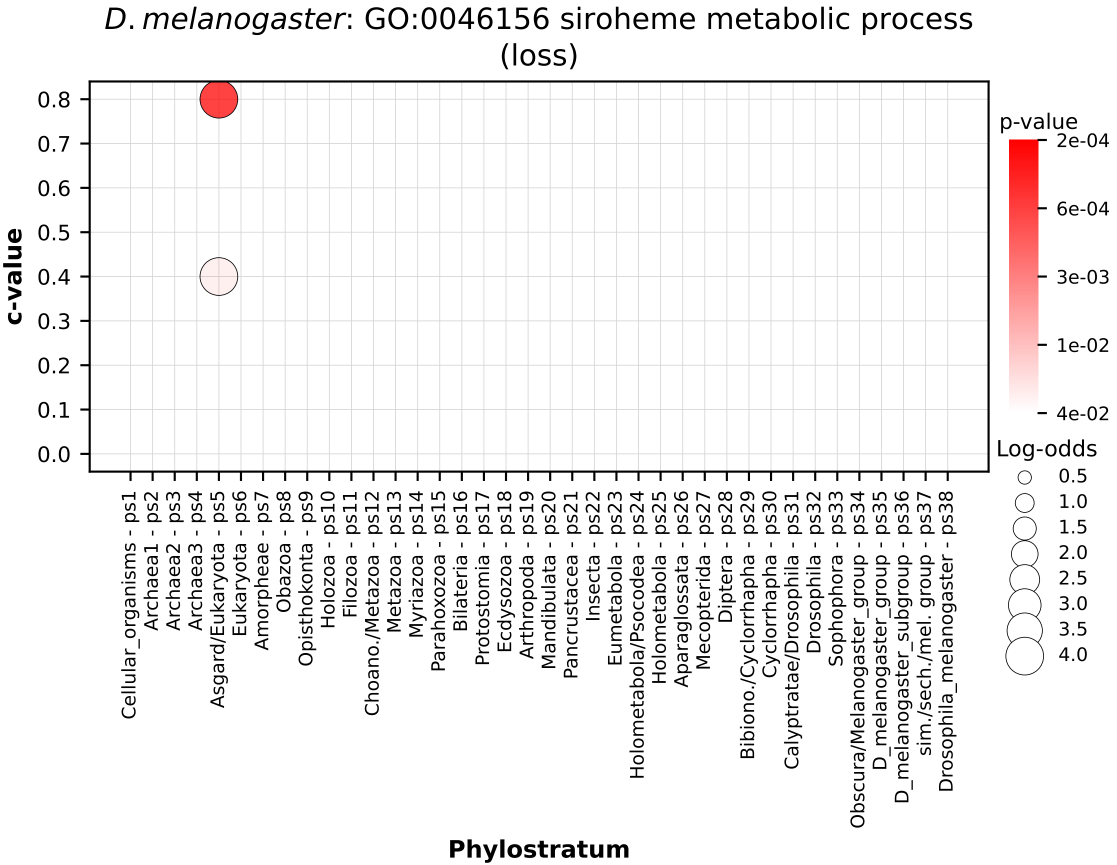

D. melanogaster: GO:0046156 siroheme metabolic process (loss)
c-value
0.8
0.7
0.6
0.5
0.4
0.3
0.2
0.1
0.0
p-value
2e-04
6e-04
3e-03
1e-02
4e-02
Log-odds
0.5
1.0
1.5
2.0
2.5
3.0
3.5
4.0
Cellular_organisms - ps1
Archaea1 - ps2
Archaea2 - ps3
Archaea3 - ps4
Asgard/Eukaryota - ps5
Eukaryota - ps6
Amorpheae - ps7
Obazoa - ps8
Opisthokonta - ps9
Holozoa - ps10
Filozoa - ps11
Choano./Metazoa - ps12
Metazoa - ps13
Myriazoa - ps14
Parahoxozoa - ps15
Bilateria - ps16
Protostomia - ps17
Ecdysozoa - ps18
Arthropoda - ps19
Mandibulata - ps20
Pancrustacea - ps21
Insecta - ps22
Eumetabola - ps23
Holometabola/Psocodea - ps24
Holometabola - ps25
Aparaglossata - ps26
Mecopterida - ps27
Diptera - ps28
Bibiono./Cyclorrhapha - ps29
Cyclorrhapha - ps30
Calyptratae/Drosophila - ps31
Drosophila - ps32
Sophophora - ps33
Obscura/Melanogaster_group - ps34
D_melanogaster_group - ps35
D_melanogaster_subgroup - ps36
sim./sech./mel. group - ps37
Drosophila_melanogaster - ps38
Phylostratum

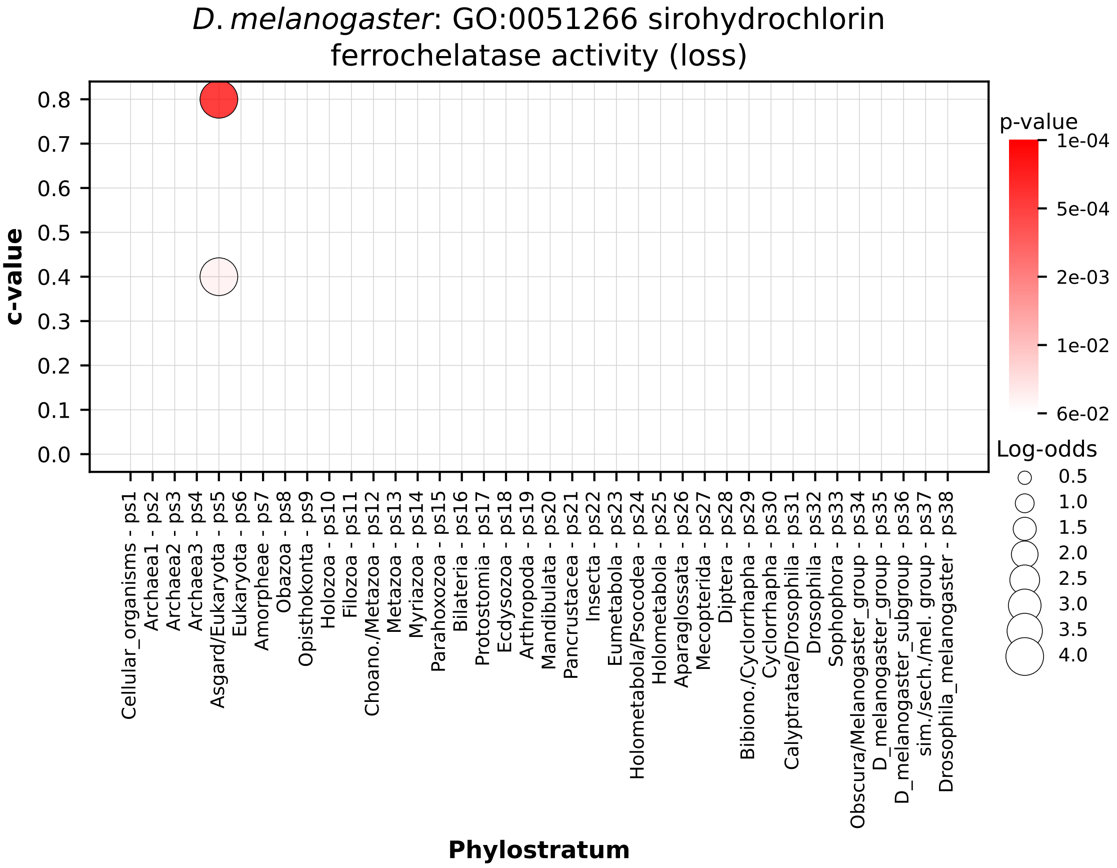

D. melanogaster: GO:0051266 sirohydrochlorin ferrochelatase activity (loss)
c-value
0.0
0.1
0.2
0.3
0.4
0.5
0.6
0.7
0.8
Phylostratum
Cellular_organisms - ps1
Archaea1 - ps2
Archaea2 - ps3
Archaea3 - ps4
Asgard/Eukaryota - ps5
Eukaryota - ps6
Amorpheae - ps7
Obazoa - ps8
Opisthokonta - ps9
Holozoa - ps10
Filozoa - ps11
Choano./Metazoa - ps12
Metazoa - ps13
Myriazoa - ps14
Parahoxozoa - ps15
Bilateria - ps16
Protostomia - ps17
Ecdysozoa - ps18
Arthropoda - ps19
Mandibulata - ps20
Pancrustacea - ps21
Insecta - ps22
Eumetabola - ps23
Holometabola/Psocodea - ps24
Holometabola - ps25
Aparaglossata - ps26
Mecopterida - ps27
Diptera - ps28
Bibiono./Cyclorrhapha - ps29
Cyclorrhapha - ps30
Calyptratae/Drosophila - ps31
Drosophila - ps32
Sophophora - ps33
Obscura/Melanogaster_group - ps34
D_melanogaster_group - ps35
D_melanogaster_subgroup - ps36
sim./sech./mel. group - ps37
Drosophila_melanogaster - ps38
p-value
1e-04
5e-04
2e-03
1e-02
6e-02
Log-odds
0.5
1.0
1.5
2.0
2.5
3.0
3.5
4.0

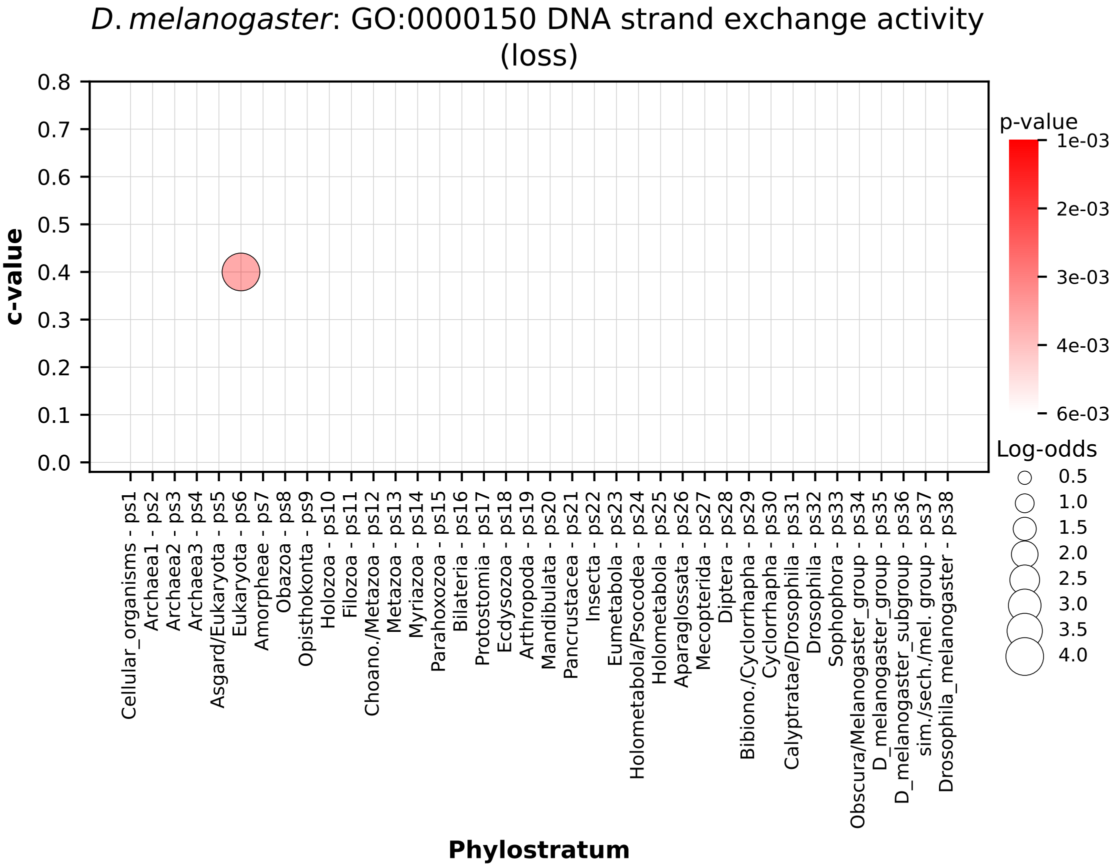
D. melanogaster: GO:0000150 DNA strand exchange activity
(loss)
c-value
0.8
0.7
0.6
0.5
0.4
0.3
0.2
0.1
0.0
Phylostratum
Cellular_organisms - ps1
Archaea1 - ps2
Archaea2 - ps3
Archaea3 - ps4
Asgard/Eukaryota - ps5
Eukaryota - ps6
Amorpheae - ps7
Obazoa - ps8
Opisthokonta - ps9
Holozoa - ps10
Filozoa - ps11
Choano./Metazoa - ps12
Metazoa - ps13
Myriazoa - ps14
Parahoxozoa - ps15
Bilateria - ps16
Protostomia - ps17
Ecdysozoa - ps18
Arthropoda - ps19
Mandibulata - ps20
Pancrustacea - ps21
Insecta - ps22
Eumetabola - ps23
Holometabola/Psocodea - ps24
Holometabola - ps25
Aparaglossata - ps26
Mecopterida - ps27
Diptera - ps28
Bibiono./Cyclorrhapha - ps29
Cyclorrhapha - ps30
Calyptratae/Drosophila - ps31
Drosophila - ps32
Sophophora - ps33
Obscura/Melanogaster_group - ps34
D_melanogaster_group - ps35
D_melanogaster_subgroup - ps36
sim./sech./mel. group - ps37
Drosophila_melanogaster - ps38
p-value
1e-03
2e-03
3e-03
4e-03
6e-03
Log-odds
0.5
1.0
1.5
2.0
2.5
3.0
3.5
4.0

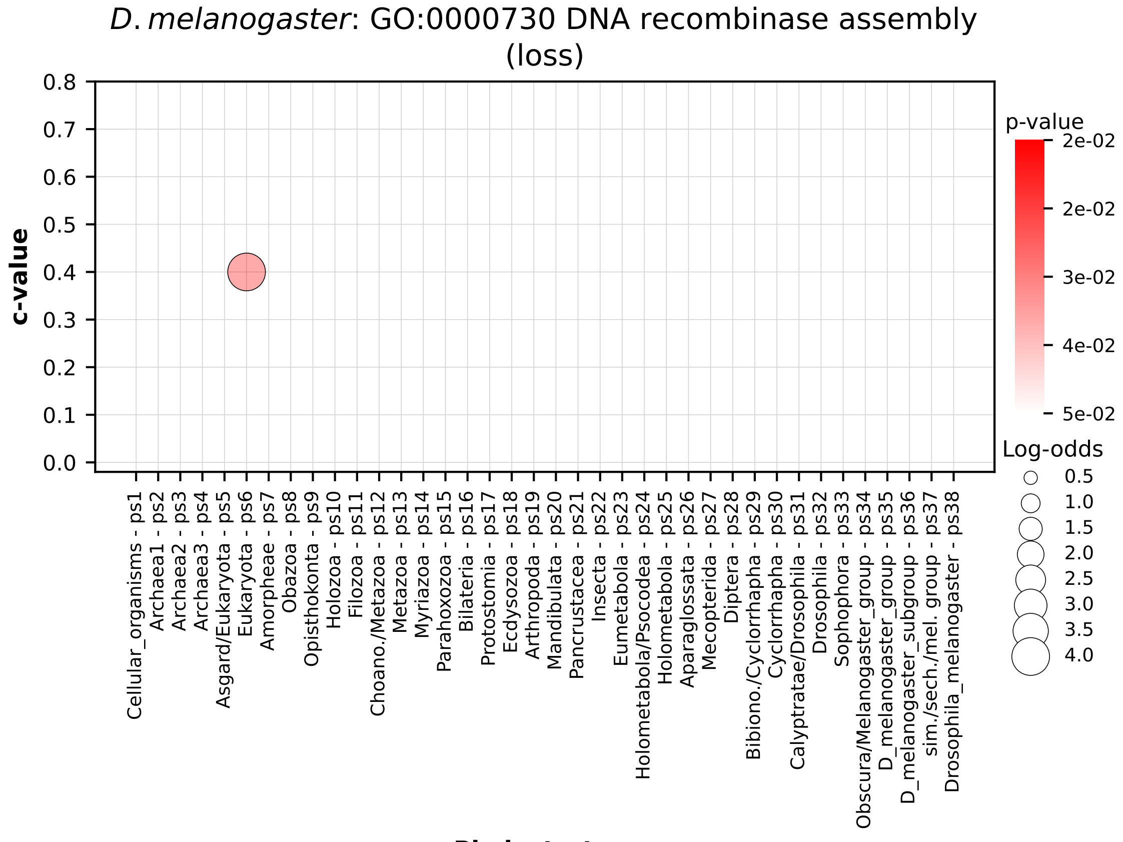

D. melanogaster: GO:0000730 DNA recombinase assembly (loss)
c-value
0.8
0.7
0.6
0.5
0.4
0.3
0.2
0.1
0.0
p-value
2e-02
2e-02
3e-02
4e-02
5e-02
Log-odds
0.5
1.0
1.5
2.0
2.5
3.0
3.5
4.0
Cellular_organisms - ps1
Archaea1 - ps2
Archaea2 - ps3
Archaea3 - ps4
Asgard/Eukaryota - ps5
Eukaryota - ps6
Amorpheae - ps7
Obazoa - ps8
Opisthokonta - ps9
Holozoa - ps10
Filozoa - ps11
Choano./Metazoa - ps12
Metazoa - ps13
Myriazoa - ps14
Parahoxozoa - ps15
Bilateria - ps16
Protostomia - ps17
Ecdysozoa - ps18
Arthropoda - ps19
Mandibulata - ps20
Pancrustacea - ps21
Insecta - ps22
Eumetabola - ps23
Holometabola/Psocodea - ps24
Holometabola - ps25
Aparaglossata - ps26
Mecopterida - ps27
Diptera - ps28
Bibiono./Cyclorrhapha - ps29
Cyclorrhapha - ps30
Calyptratae/Drosophila - ps31
Drosophila - ps32
Sophophora - ps33
Obscura/Melanogaster_group - ps34
D_melanogaster_group - ps35
D_melanogaster_subgroup - ps36
sim./sech./mel. group - ps37
Drosophila_melanogaster - ps38
Phylostratum

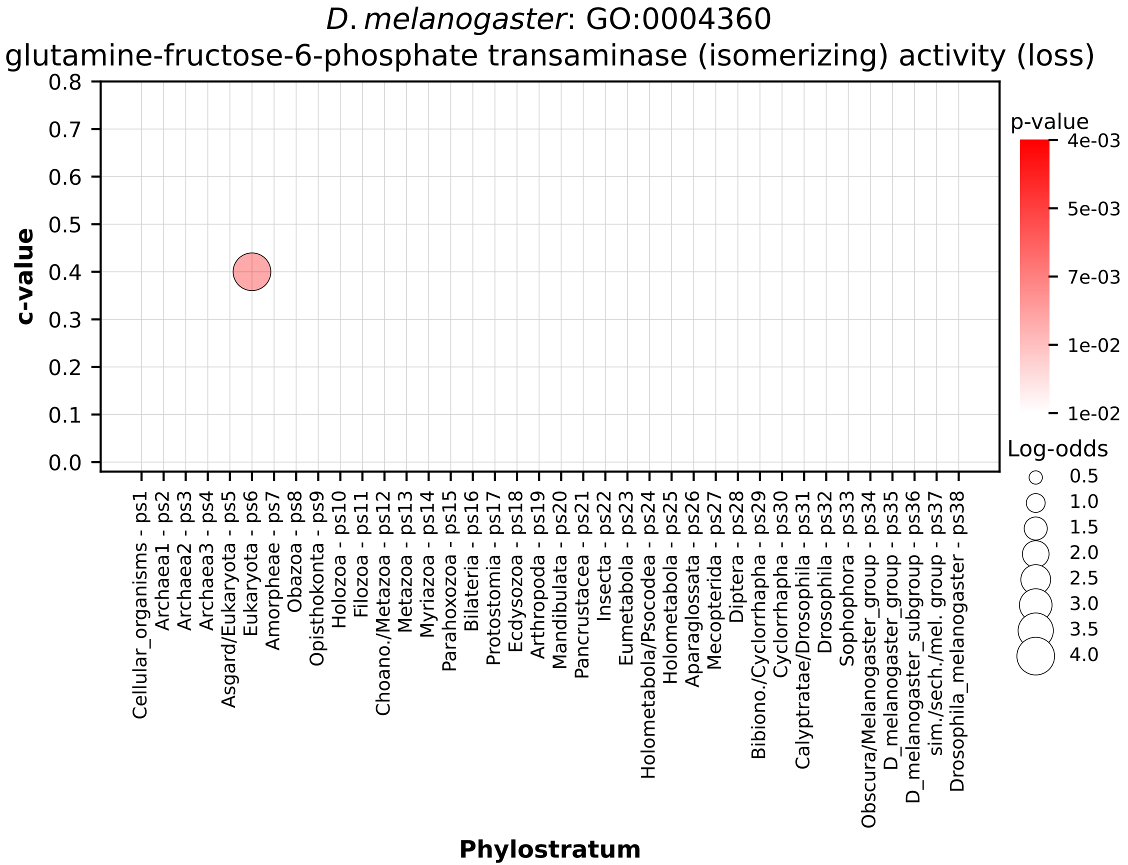

D. melanogaster: GO:0004360
glutamine-fructose-6-phosphate transaminase (isomerizing) activity (loss)
c-value
0.8
0.7
0.6
0.5
0.4
0.3
0.2
0.1
0.0
p-value
4e-03
5e-03
7e-03
1e-02
1e-02
Log-odds
0.5
1.0
1.5
2.0
2.5
3.0
3.5
4.0
Cellular_organisms - ps1
Archaea1 - ps2
Archaea2 - ps3
Archaea3 - ps4
Asgard/Eukaryota - ps5
Eukaryota - ps6
Amorpheae - ps7
Obazoa - ps8
Opisthokonta - ps9
Holozoa - ps10
Filozoa - ps11
Choano./Metazoa - ps12
Metazoa - ps13
Myriazoa - ps14
Parahoxozoa - ps15
Bilateria - ps16
Protostomia - ps17
Ecdysozoa - ps18
Arthropoda - ps19
Mandibulata - ps20
Pancrustacea - ps21
Insecta - ps22
Eumetabola - ps23
Holometabola/Psocodea - ps24
Holometabola - ps25
Aparaglossata - ps26
Mecopterida - ps27
Diptera - ps28
Bibiono./Cyclorrhapha - ps29
Cyclorrhapha - ps30
Calyptratae/Drosophila - ps31
Drosophila - ps32
Sophophora - ps33
Obscura/Melanogaster_group - ps34
D_melanogaster_group - ps35
D_melanogaster_subgroup - ps36
sim./sech./mel. group - ps37
Drosophila_melanogaster - ps38
Phylostratum

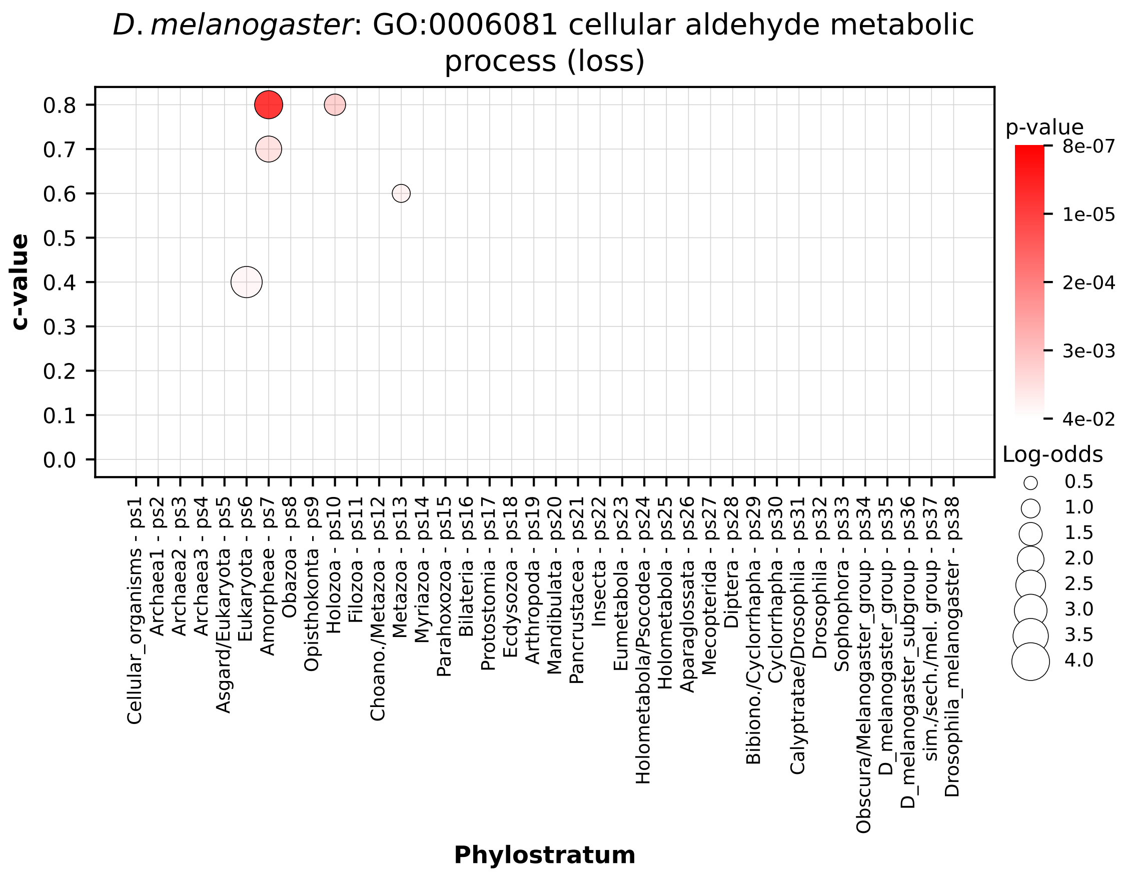
D. melanogaster: GO:0006081 cellular aldehyde metabolic process (loss)
c-value
0.0
0.1
0.2
0.3
0.4
0.5
0.6
0.7
0.8
Phylostratum
Cellular_organisms - ps1
Archaea1 - ps2
Archaea2 - ps3
Archaea3 - ps4
Asgard/Eukaryota - ps5
Eukaryota - ps6
Amorpheae - ps7
Obazoa - ps8
Opisthokonta - ps9
Holozoa - ps10
Filozoa - ps11
Choano./Metazoa - ps12
Metazoa - ps13
Myriazoa - ps14
Parahoxozoa - ps15
Bilateria - ps16
Protostomia - ps17
Ecdysozoa - ps18
Arthropoda - ps19
Mandibulata - ps20
Pancrustacea - ps21
Insecta - ps22
Eumetabola - ps23
Holometabola/Psocodea - ps24
Holometabola - ps25
Aparaglossata - ps26
Mecopterida - ps27
Diptera - ps28
Bibiono./Cyclorrhapha - ps29
Cyclorrhapha - ps30
Calyptratae/Drosophila - ps31
Drosophila - ps32
Sophophora - ps33
Obscura/Melanogaster_group - ps34
D_melanogaster_group - ps35
D_melanogaster_subgroup - ps36
sim./sech./mel. group - ps37
Drosophila_melanogaster - ps38
p-value
8e-07
1e-05
2e-04
3e-03
4e-02
Log-odds
0.5
1.0
1.5
2.0
2.5
3.0
3.5
4.0

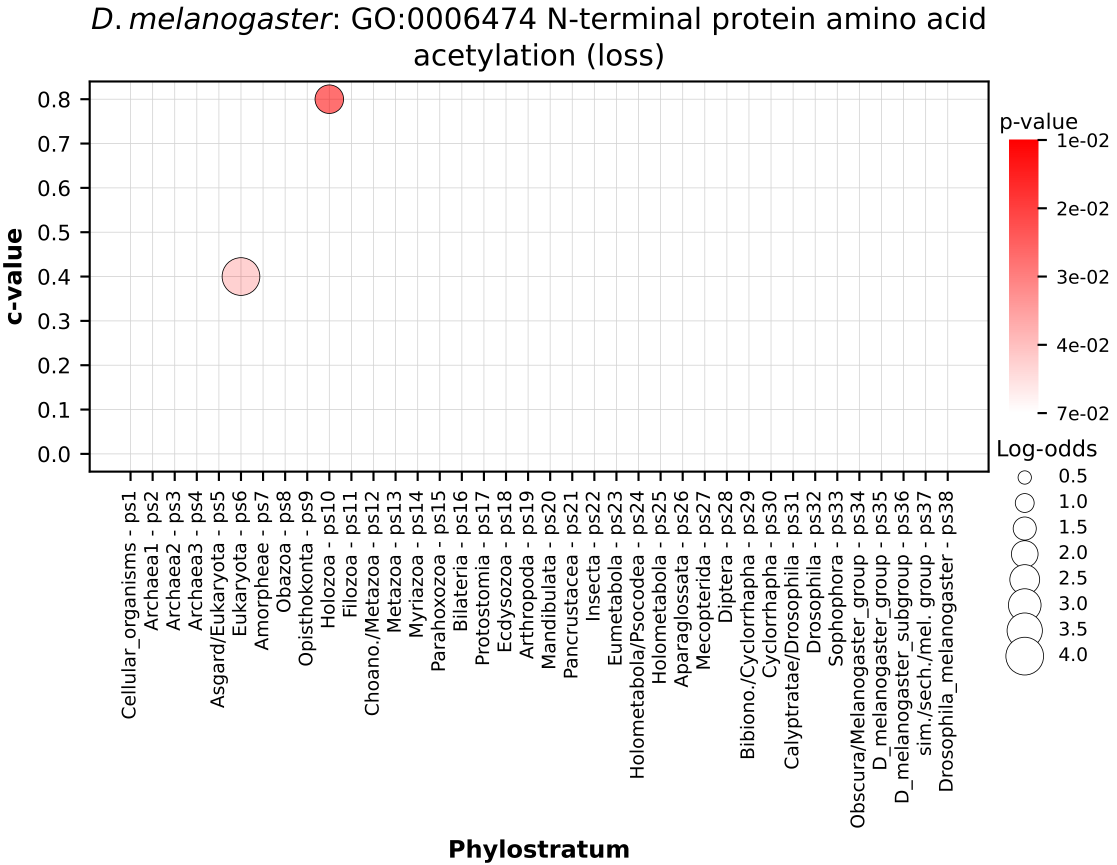

D. melanogaster: GO:0006474 N-terminal protein amino acid acetylation (loss)
c-value
0.8
0.7
0.6
0.5
0.4
0.3
0.2
0.1
0.0
p-value
1e-02
2e-02
3e-02
4e-02
7e-02
Log-odds
0.5
1.0
1.5
2.0
2.5
3.0
3.5
4.0
Cellular_organisms - ps1
Archaea1 - ps2
Archaea2 - ps3
Archaea3 - ps4
Asgard/Eukaryota - ps5
Eukaryota - ps6
Amorpheae - ps7
Obazoa - ps8
Opisthokonta - ps9
Holozoa - ps10
Filozoa - ps11
Choano./Metazoa - ps12
Metazoa - ps13
Myriazoa - ps14
Parahoxozoa - ps15
Bilateria - ps16
Protostomia - ps17
Ecdysozoa - ps18
Arthropoda - ps19
Mandibulata - ps20
Pancrustacea - ps21
Insecta - ps22
Eumetabola - ps23
Holometabola/Psocodea - ps24
Holometabola - ps25
Aparaglossata - ps26
Mecopterida - ps27
Diptera - ps28
Bibiono./Cyclorrhapha - ps29
Cyclorrhapha - ps30
Calyptratae/Drosophila - ps31
Drosophila - ps32
Sophophora - ps33
Obscura/Melanogaster_group - ps34
D_melanogaster_group - ps35
D_melanogaster_subgroup - ps36
sim./sech./mel. group - ps37
Drosophila_melanogaster - ps38
Phylostratum

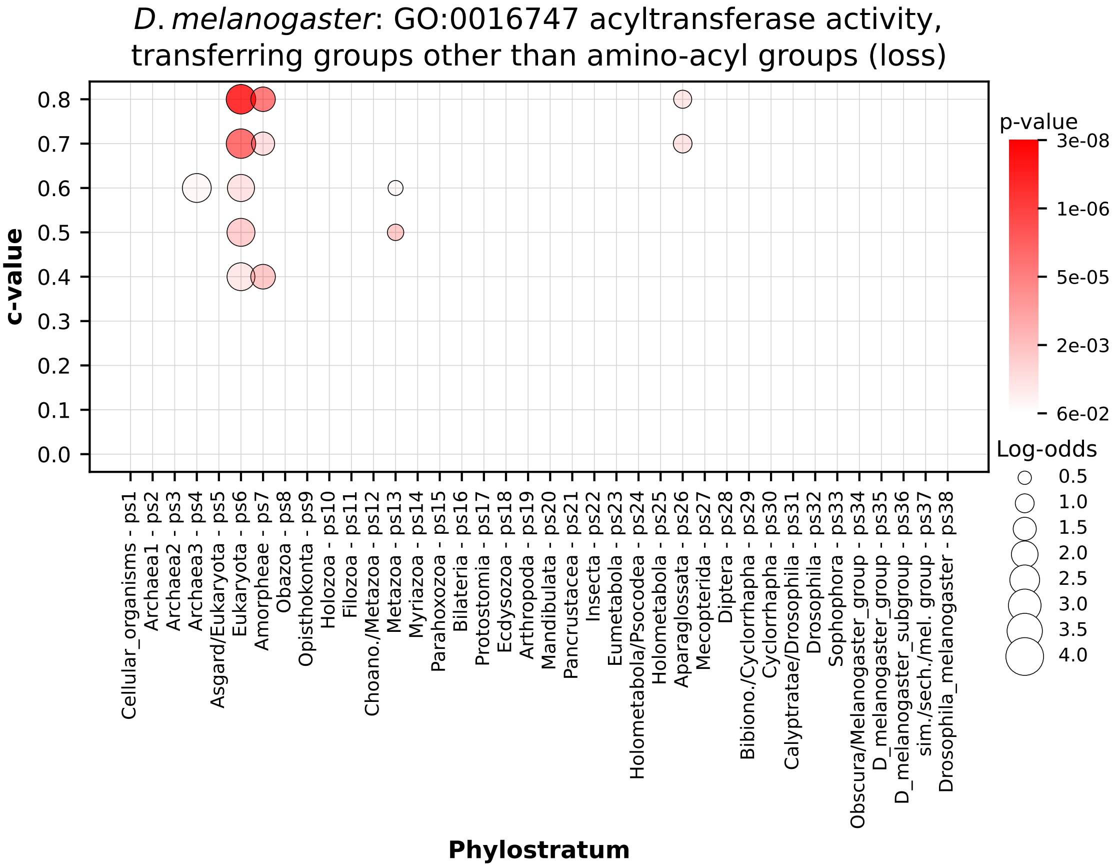
D. melanogaster: GO:0016747 acyltransferase activity, transferring groups other than amino-acyl groups (loss)
c-value
0.8
0.7
0.6
0.5
0.4
0.3
0.2
0.1
0.0
Phylostratum
Cellular_organisms - ps1
Archaea1 - ps2
Archaea2 - ps3
Archaea3 - ps4
Asgard/Eukaryota - ps5
Eukaryota - ps6
Amorpheae - ps7
Obazoa - ps8
Opisthokonta - ps9
Holozoa - ps10
Filozoa - ps11
Choano./Metazoa - ps12
Metazoa - ps13
Myriazoa - ps14
Parahoxozoa - ps15
Bilateria - ps16
Protostomia - ps17
Ecdysozoa - ps18
Arthropoda - ps19
Mandibulata - ps20
Pancrustacea - ps21
Insecta - ps22
Eumetabola - ps23
Holometabola/Psocodea - ps24
Holometabola - ps25
Aparaglossata - ps26
Mecopterida - ps27
Diptera - ps28
Bibiono./Cyclorrhapha - ps29
Cyclorrhapha - ps30
Calyptratae/Drosophila - ps31
Drosophila - ps32
Sophophora - ps33
Obscura/Melanogaster_group - ps34
D_melanogaster_group - ps35
D_melanogaster_subgroup - ps36
sim./sech./mel. group - ps37
Drosophila_melanogaster - ps38
p-value
3e-08
1e-06
5e-05
2e-03
6e-02
Log-odds
0.5
1.0
1.5
2.0
2.5
3.0
3.5
4.0

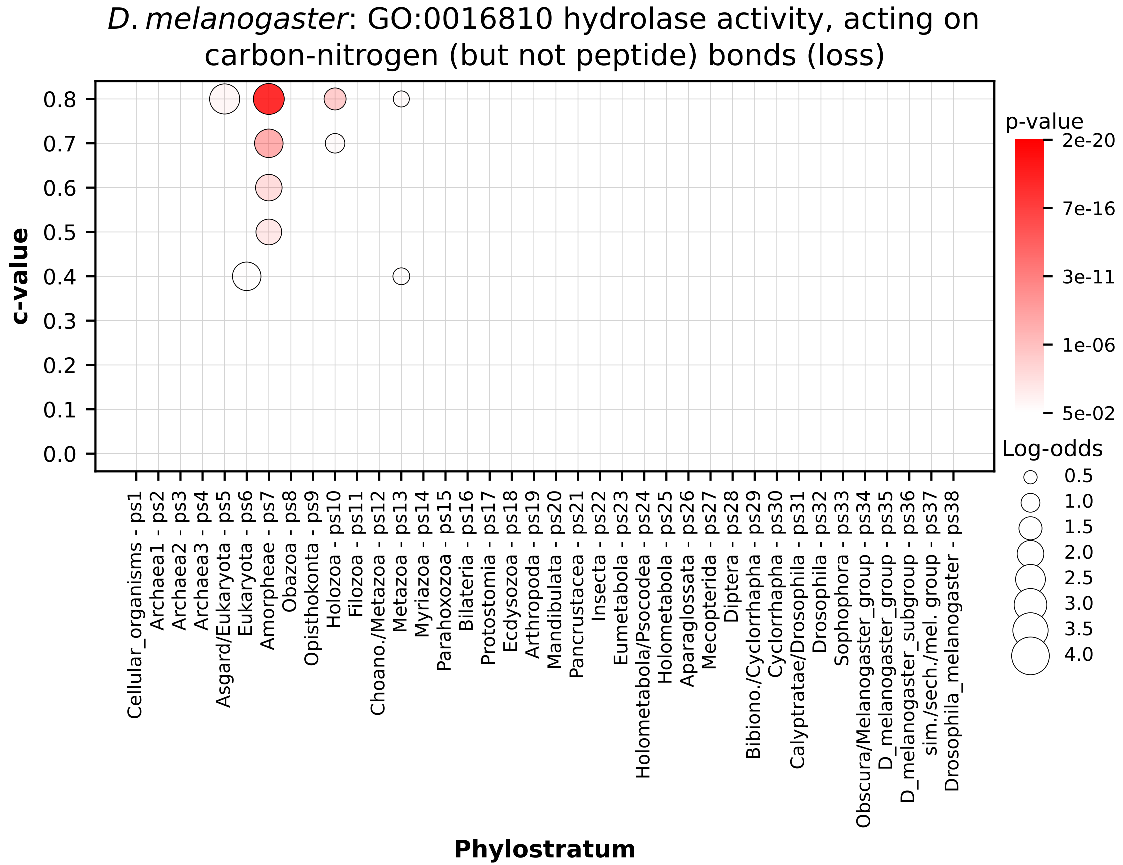

D. melanogaster: GO:0016810 hydrolase activity, acting on carbon-nitrogen (but not peptide) bonds (loss)
c-value
0.8
0.7
0.6
0.5
0.4
0.3
0.2
0.1
0.0
p-value
2e-20
7e-16
3e-11
1e-06
5e-02
Log-odds
0.5
1.0
1.5
2.0
2.5
3.0
3.5
4.0
Cellular_organisms - ps1
Archaea1 - ps2
Archaea2 - ps3
Archaea3 - ps4
Asgard/Eukaryota - ps5
Eukaryota - ps6
Amorpheae - ps7
Obazoa - ps8
Opisthokonta - ps9
Holozoa - ps10
Filozoa - ps11
Choano./Metazoa - ps12
Metazoa - ps13
Myriazoa - ps14
Parahoxozoa - ps15
Bilateria - ps16
Protostomia - ps17
Ecdysozoa - ps18
Arthropoda - ps19
Mandibulata - ps20
Pancrustacea - ps21
Insecta - ps22
Eumetabola - ps23
Holometabola/Psocodea - ps24
Holometabola - ps25
Aparaglossata - ps26
Mecopterida - ps27
Diptera - ps28
Bibiono./Cyclorrhapha - ps29
Cyclorrhapha - ps30
Calyptratae/Drosophila - ps31
Drosophila - ps32
Sophophora - ps33
Obscura/Melanogaster_group - ps34
D_melanogaster_group - ps35
D_melanogaster_subgroup - ps36
sim./sech./mel. group - ps37
Drosophila_melanogaster - ps38
Phylostratum

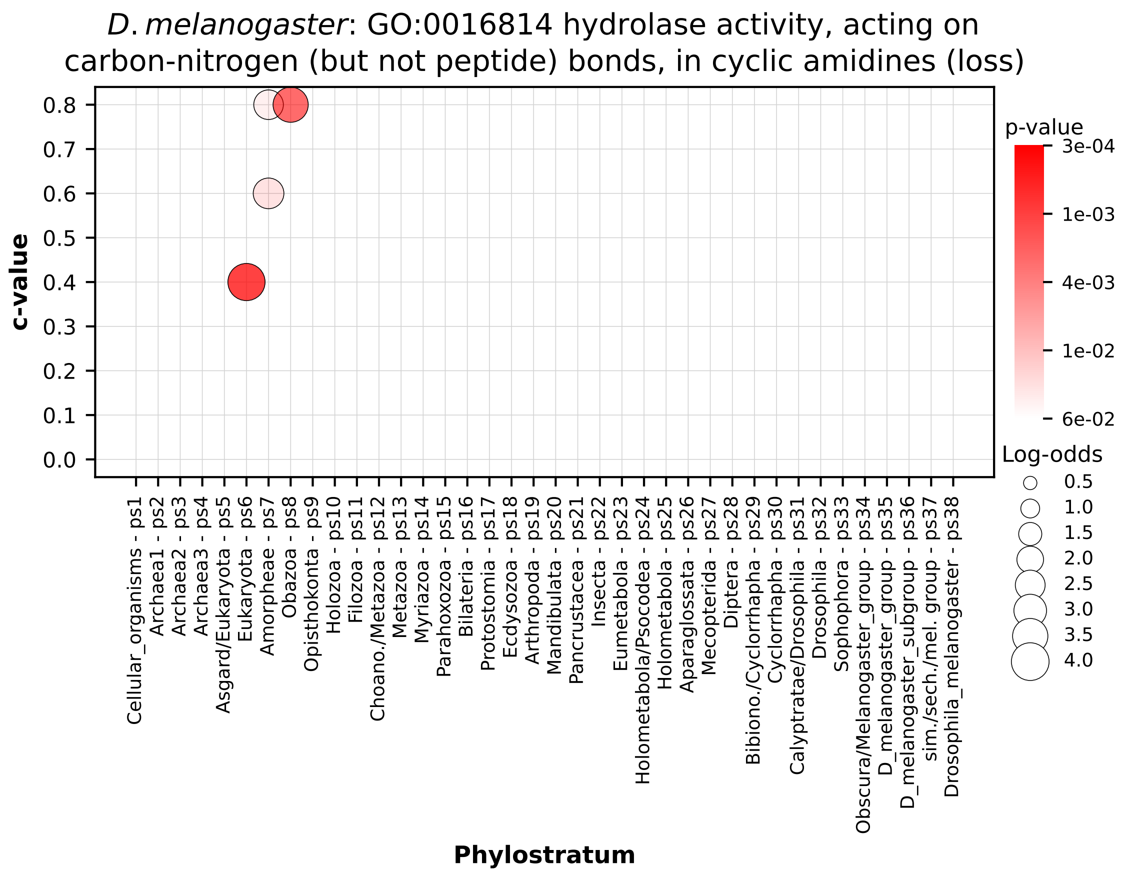

D. melanogaster: GO:0016814 hydrolase activity, acting on carbon-nitrogen (but not peptide) bonds, in cyclic amidines (loss)
c-value
0.0
0.1
0.2
0.3
0.4
0.5
0.6
0.7
0.8
p-value
3e-04
1e-03
4e-03
1e-02
6e-02
Log-odds
0.5
1.0
1.5
2.0
2.5
3.0
3.5
4.0
Cellular_organisms - ps1
Archaea1 - ps2
Archaea2 - ps3
Archaea3 - ps4
Asgard/Eukaryota - ps5
Eukaryota - ps6
Amorpheae - ps7
Obazoa - ps8
Opisthokonta - ps9
Holozoa - ps10
Filozoa - ps11
Choano./Metazoa - ps12
Metazoa - ps13
Myriazoa - ps14
Parahoxozoa - ps15
Bilateria - ps16
Protostomia - ps17
Ecdysozoa - ps18
Arthropoda - ps19
Mandibulata - ps20
Pancrustacea - ps21
Insecta - ps22
Eumetabola - ps23
Holometabola/Psocodea - ps24
Holometabola - ps25
Aparaglossata - ps26
Mecopterida - ps27
Diptera - ps28
Bibiono./Cyclorrhapha - ps29
Cyclorrhapha - ps30
Calyptratae/Drosophila - ps31
Drosophila - ps32
Sophophora - ps33
Obscura/Melanogaster_group - ps34
D_melanogaster_group - ps35
D_melanogaster_subgroup - ps36
sim./sech./mel. group - ps37
Drosophila_melanogaster - ps38
Phylostratum

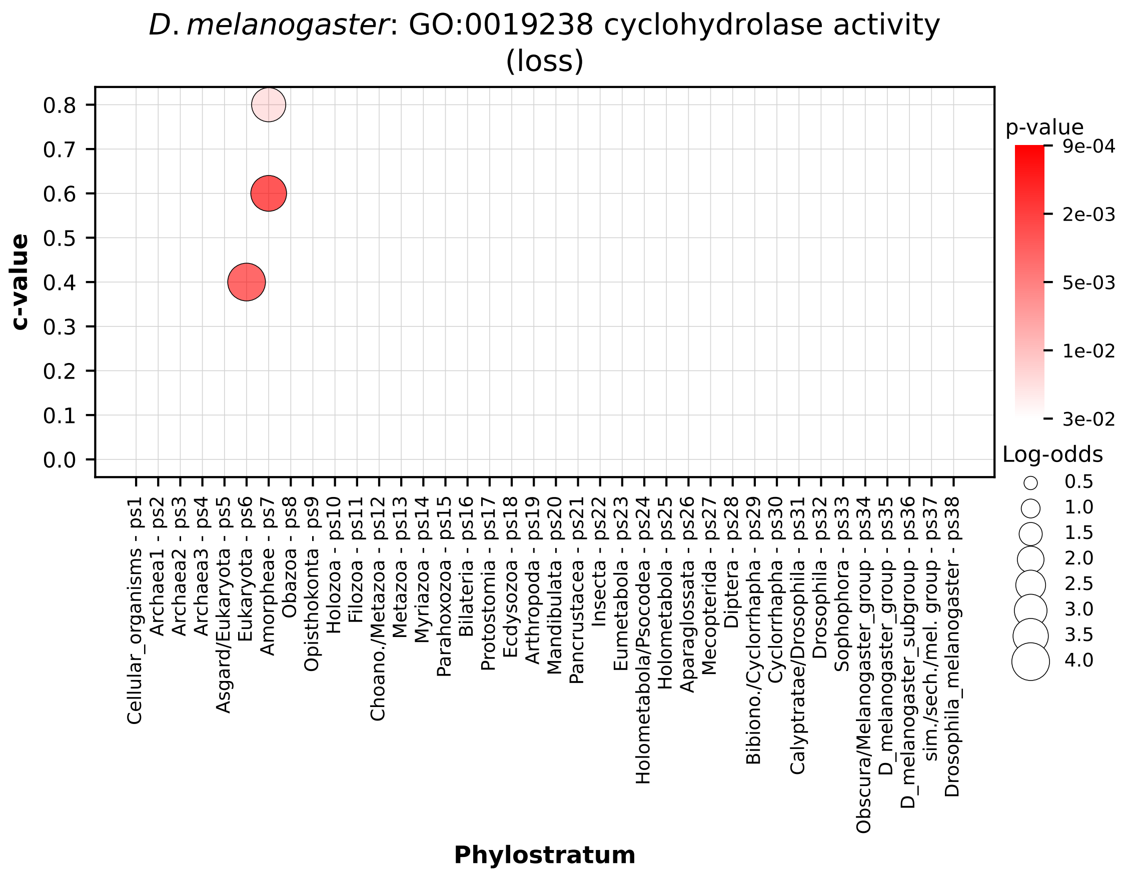

D. melanogaster: GO:0019238 cyclohydrolase activity (loss)
c-value
0.0
0.1
0.2
0.3
0.4
0.5
0.6
0.7
0.8
p-value
9e-04
2e-03
5e-03
1e-02
3e-02
Log-odds
0.5
1.0
1.5
2.0
2.5
3.0
3.5
4.0
Cellular_organisms - ps1
Archaea1 - ps2
Archaea2 - ps3
Archaea3 - ps4
Asgard/Eukaryota - ps5
Eukaryota - ps6
Amorpheae - ps7
Obazoa - ps8
Opisthokonta - ps9
Holozoa - ps10
Filozoa - ps11
Choano./Metazoa - ps12
Metazoa - ps13
Myriazoa - ps14
Parahoxozoa - ps15
Bilateria - ps16
Protostomia - ps17
Ecdysozoa - ps18
Arthropoda - ps19
Mandibulata - ps20
Pancrustacea - ps21
Insecta - ps22
Eumetabola - ps23
Holometabola/Psocodea - ps24
Holometabola - ps25
Aparaglossata - ps26
Mecopterida - ps27
Diptera - ps28
Bibiono./Cyclorrhapha - ps29
Cyclorrhapha - ps30
Calyptratae/Drosophila - ps31
Drosophila - ps32
Sophophora - ps33
Obscura/Melanogaster_group - ps34
D_melanogaster_group - ps35
D_melanogaster_subgroup - ps36
sim./sech./mel. group - ps37
Drosophila_melanogaster - ps38
Phylostratum

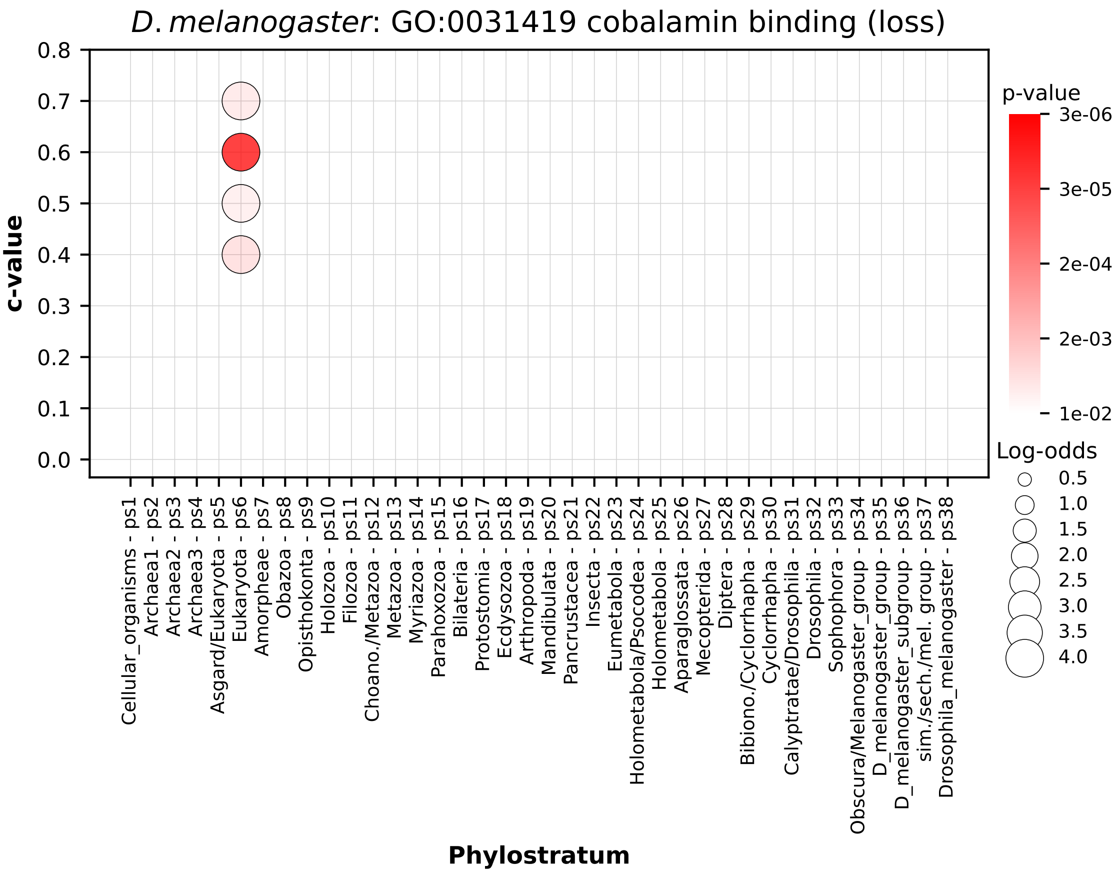

D. melanogaster: GO:0031419 cobalamin binding (loss)
c-value
0.8
0.7
0.6
0.5
0.4
0.3
0.2
0.1
0.0
p-value
3e-06
3e-05
2e-04
2e-03
1e-02
Log-odds
0.5
1.0
1.5
2.0
2.5
3.0
3.5
4.0
Cellular_organisms - ps1
Archaea1 - ps2
Archaea2 - ps3
Archaea3 - ps4
Asgard/Eukaryota - ps5
Eukaryota - ps6
Amorpheae - ps7
Obazoa - ps8
Opisthokonta - ps9
Holozoa - ps10
Filozoa - ps11
Choano./Metazoa - ps12
Metazoa - ps13
Myriazoa - ps14
Parahoxozoa - ps15
Bilateria - ps16
Protostomia - ps17
Ecdysozoa - ps18
Arthropoda - ps19
Mandibulata - ps20
Pancrustacea - ps21
Insecta - ps22
Eumetabola - ps23
Holometabola/Psocodea - ps24
Holometabola - ps25
Aparaglossata - ps26
Mecopterida - ps27
Diptera - ps28
Bibiono./Cyclorrhapha - ps29
Cyclorrhapha - ps30
Calyptratae/Drosophila - ps31
Drosophila - ps32
Sophophora - ps33
Obscura/Melanogaster_group - ps34
D_melanogaster_group - ps35
D_melanogaster_subgroup - ps36
sim./sech./mel. group - ps37
Drosophila_melanogaster - ps38
Phylostratum

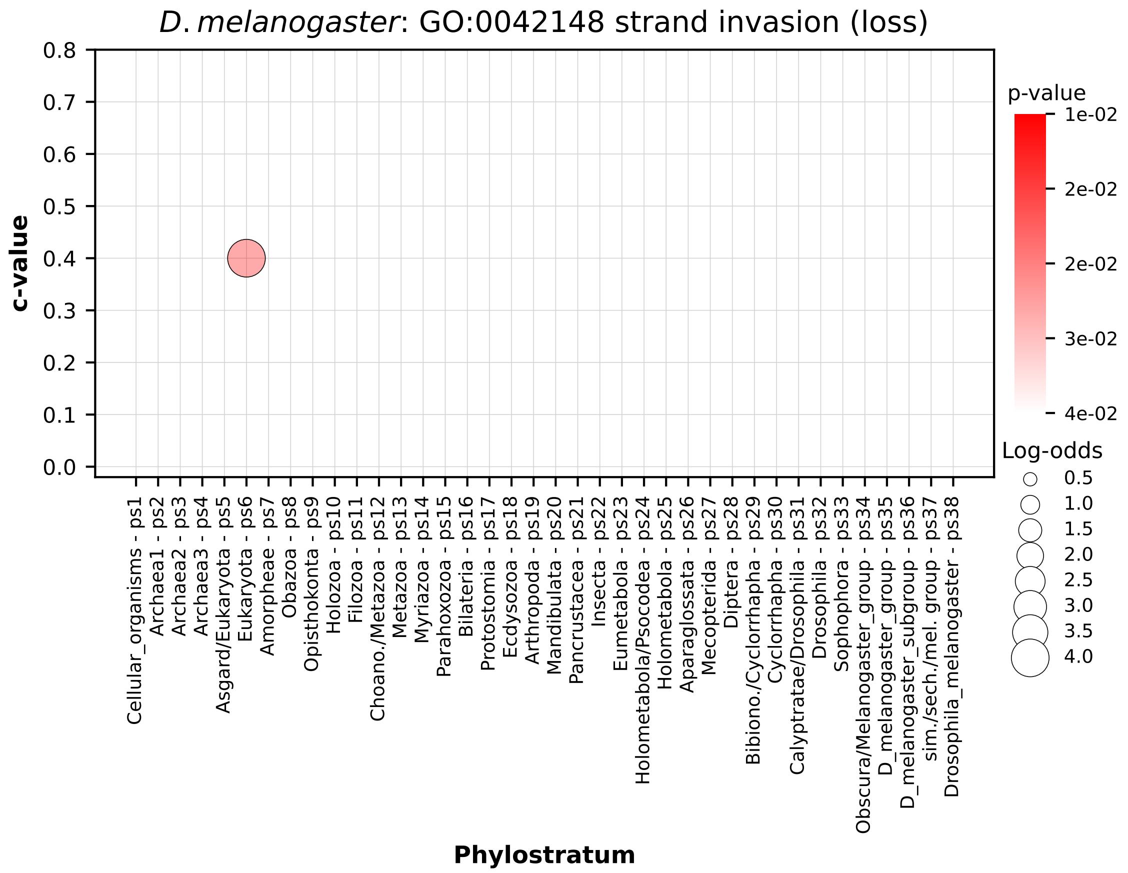

D. melanogaster: GO:0042148 strand invasion (loss)
c-value
0.0
0.1
0.2
0.3
0.4
0.5
0.6
0.7
0.8
Phylostratum
Cellular_organisms - ps1
Archaea1 - ps2
Archaea2 - ps3
Archaea3 - ps4
Asgard/Eukaryota - ps5
Eukaryota - ps6
Amorpheae - ps7
Obazoa - ps8
Opisthokonta - ps9
Holozoa - ps10
Filozoa - ps11
Choano./Metazoa - ps12
Metazoa - ps13
Myriazoa - ps14
Parahoxozoa - ps15
Bilateria - ps16
Protostomia - ps17
Ecdysozoa - ps18
Arthropoda - ps19
Mandibulata - ps20
Pancrustacea - ps21
Insecta - ps22
Eumetabola - ps23
Holometabola/Psocodea - ps24
Holometabola - ps25
Aparaglossata - ps26
Mecopterida - ps27
Diptera - ps28
Bibiono./Cyclorrhapha - ps29
Cyclorrhapha - ps30
Calyptratae/Drosophila - ps31
Drosophila - ps32
Sophophora - ps33
Obscura/Melanogaster_group - ps34
D_melanogaster_group - ps35
D_melanogaster_subgroup - ps36
sim./sech./mel. group - ps37
Drosophila_melanogaster - ps38
p-value
1e-02
2e-02
2e-02
3e-02
4e-02
Log-odds
0.5
1.0
1.5
2.0
2.5
3.0
3.5
4.0

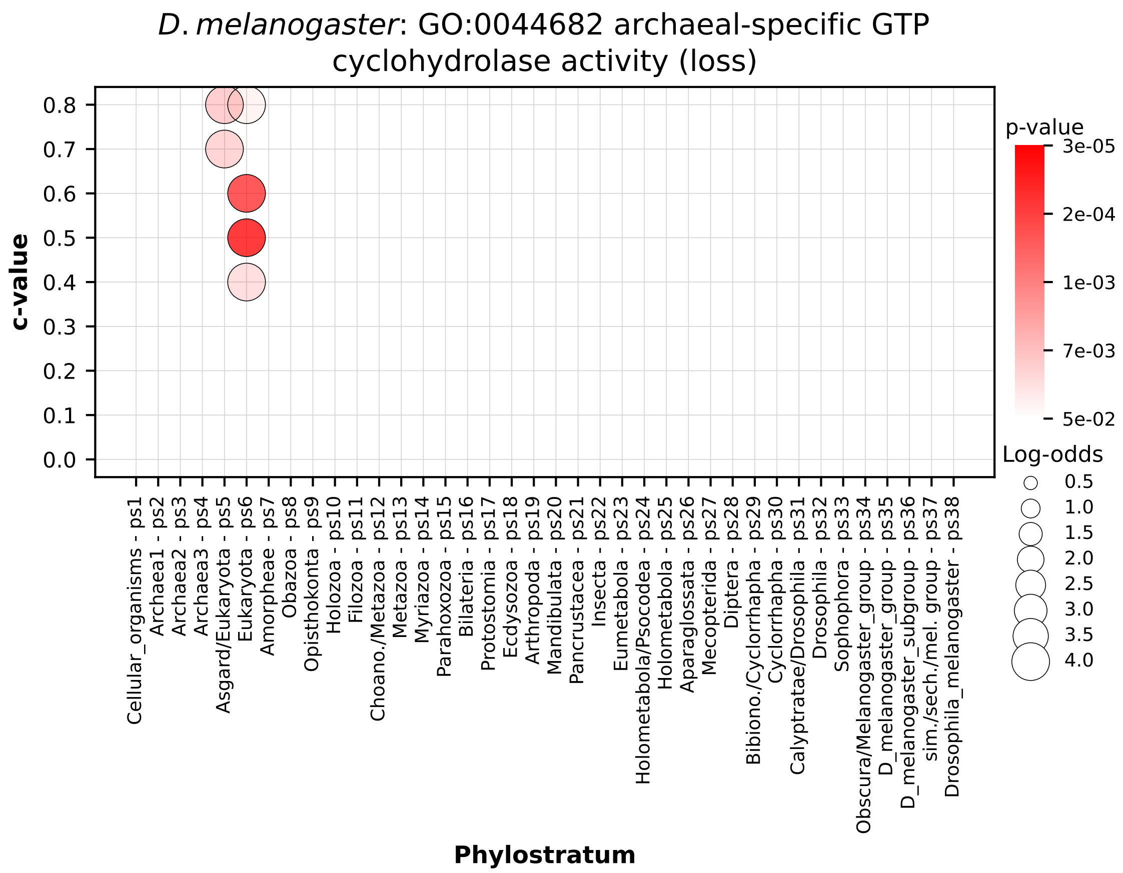

D. melanogaster: GO:0044682 archaeal-specific GTP cyclohydrolase activity (loss)
c-value
0.0
0.1
0.2
0.3
0.4
0.5
0.6
0.7
0.8
Phylostratum
Cellular_organisms - ps1
Archaea1 - ps2
Archaea2 - ps3
Archaea3 - ps4
Asgard/Eukaryota - ps5
Eukaryota - ps6
Amorpheae - ps7
Obazoa - ps8
Opisthokonta - ps9
Holozoa - ps10
Filozoa - ps11
Choano./Metazoa - ps12
Metazoa - ps13
Myriazoa - ps14
Parahoxozoa - ps15
Bilateria - ps16
Protostomia - ps17
Ecdysozoa - ps18
Arthropoda - ps19
Mandibulata - ps20
Pancrustacea - ps21
Insecta - ps22
Eumetabola - ps23
Holometabola/Psocodea - ps24
Holometabola - ps25
Aparaglossata - ps26
Mecopterida - ps27
Diptera - ps28
Bibiono./Cyclorrhapha - ps29
Cyclorrhapha - ps30
Calyptratae/Drosophila - ps31
Drosophila - ps32
Sophophora - ps33
Obscura/Melanogaster_group - ps34
D_melanogaster_group - ps35
D_melanogaster_subgroup - ps36
sim./sech./mel. group - ps37
Drosophila_melanogaster - ps38
p-value
3e-05
2e-04
1e-03
7e-03
5e-02
Log-odds
0.5
1.0
1.5
2.0
2.5
3.0
3.5
4.0

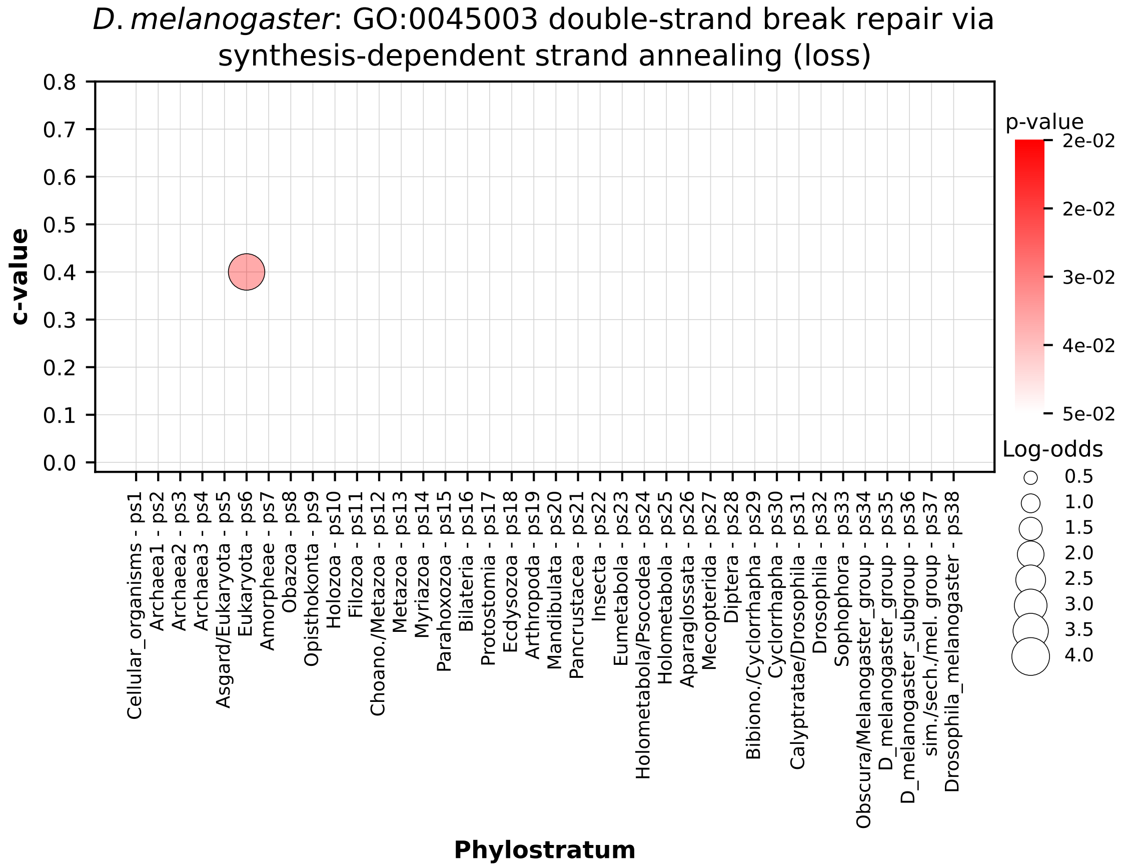
D. melanogaster: GO:0045003 double-strand break repair via synthesis-dependent strand annealing (loss)
c-value
0.8
0.7
0.6
0.5
0.4
0.3
0.2
0.1
0.0
Phylostratum
Cellular_organisms - ps1
Archaea1 - ps2
Archaea2 - ps3
Archaea3 - ps4
Asgard/Eukaryota - ps5
Eukaryota - ps6
Amorpheae - ps7
Obazoa - ps8
Opisthokonta - ps9
Holozoa - ps10
Filozoa - ps11
Choano./Metazoa - ps12
Metazoa - ps13
Myriazoa - ps14
Parahoxozoa - ps15
Bilateria - ps16
Protostomia - ps17
Ecdysozoa - ps18
Arthropoda - ps19
Mandibulata - ps20
Pancrustacea - ps21
Insecta - ps22
Eumetabola - ps23
Holometabola/Psocodea - ps24
Holometabola - ps25
Aparaglossata - ps26
Mecopterida - ps27
Diptera - ps28
Bibiono./Cyclorrhapha - ps29
Cyclorrhapha - ps30
Calyptratae/Drosophila - ps31
Drosophila - ps32
Sophophora - ps33
Obscura/Melanogaster_group - ps34
D_melanogaster_group - ps35
D_melanogaster_subgroup - ps36
sim./sech./mel. group - ps37
Drosophila_melanogaster - ps38
p-value
2e-02
2e-02
3e-02
4e-02
5e-02
Log-odds
0.5
1.0
1.5
2.0
2.5
3.0
3.5
4.0

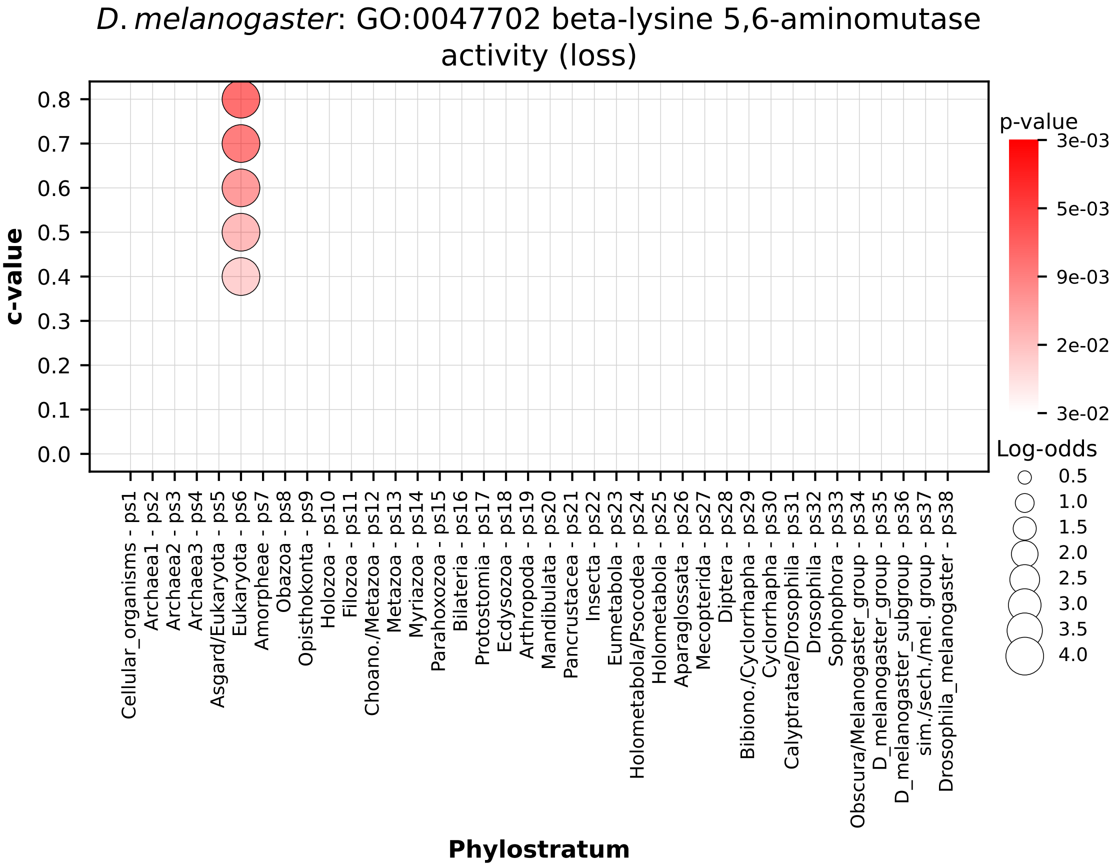

D. melanogaster: GO:0047702 beta-lysine 5,6-aminomutase activity (loss)
c-value
0.8
0.7
0.6
0.5
0.4
0.3
0.2
0.1
0.0
p-value
3e-03
5e-03
9e-03
2e-02
3e-02
Log-odds
0.5
1.0
1.5
2.0
2.5
3.0
3.5
4.0
Cellular_organisms - ps1
Archaea1 - ps2
Archaea2 - ps3
Archaea3 - ps4
Asgard/Eukaryota - ps5
Eukaryota - ps6
Amorpheae - ps7
Obazoa - ps8
Opisthokonta - ps9
Holozoa - ps10
Filozoa - ps11
Choano./Metazoa - ps12
Metazoa - ps13
Myriazoa - ps14
Parahoxozoa - ps15
Bilateria - ps16
Protostomia - ps17
Ecdysozoa - ps18
Arthropoda - ps19
Mandibulata - ps20
Pancrustacea - ps21
Insecta - ps22
Eumetabola - ps23
Holometabola/Psocodea - ps24
Holometabola - ps25
Aparaglossata - ps26
Mecopterida - ps27
Diptera - ps28
Bibiono./Cyclorrhapha - ps29
Cyclorrhapha - ps30
Calyptratae/Drosophila - ps31
Drosophila - ps32
Sophophora - ps33
Obscura/Melanogaster_group - ps34
D_melanogaster_group - ps35
D_melanogaster_subgroup - ps36
sim./sech./mel. group - ps37
Drosophila_melanogaster - ps38
Phylostratum

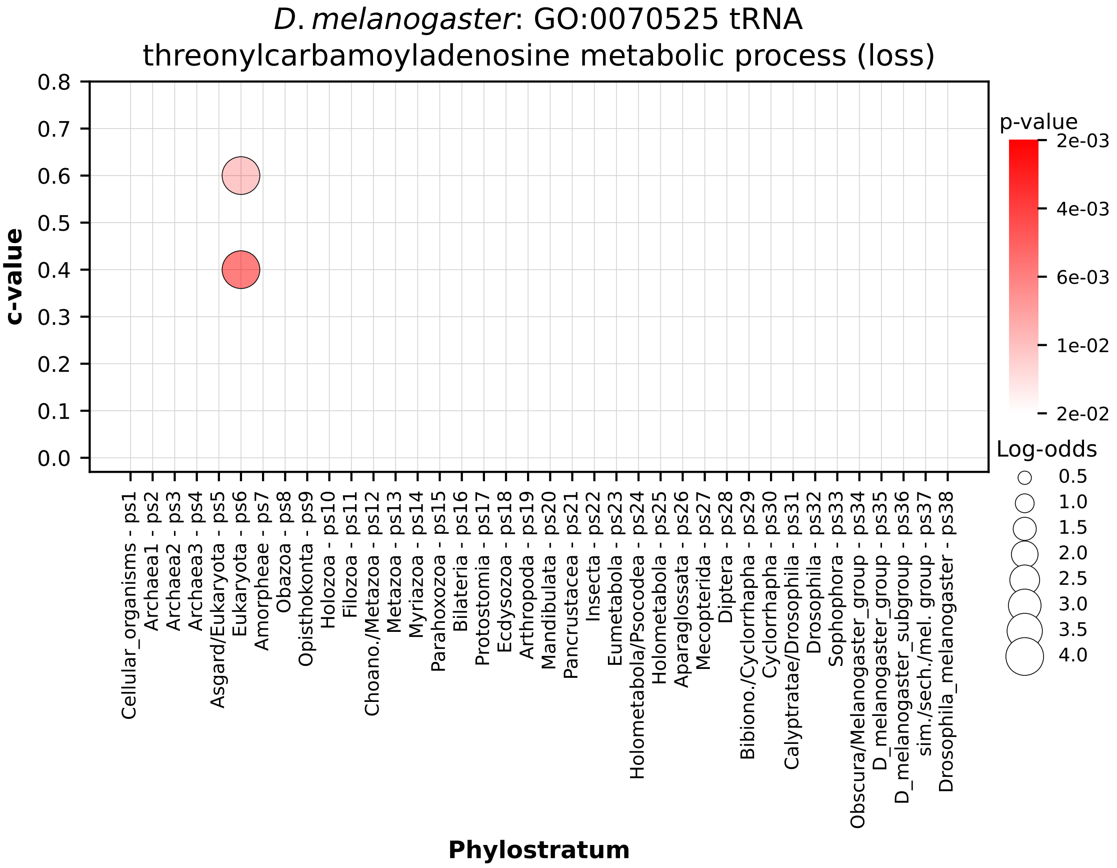

D. melanogaster: GO:0070525 tRNA threonylcarbamoyladenosine metabolic process (loss)
c-value
0.8
0.7
0.6
0.5
0.4
0.3
0.2
0.1
0.0
Phylostratum
Cellular_organisms - ps1
Archaea1 - ps2
Archaea2 - ps3
Archaea3 - ps4
Asgard/Eukaryota - ps5
Eukaryota - ps6
Amorpheae - ps7
Obazoa - ps8
Opisthokonta - ps9
Holozoa - ps10
Filozoa - ps11
Choano./Metazoa - ps12
Metazoa - ps13
Myriazoa - ps14
Parahoxozoa - ps15
Bilateria - ps16
Protostomia - ps17
Ecdysozoa - ps18
Arthropoda - ps19
Mandibulata - ps20
Pancrustacea - ps21
Insecta - ps22
Eumetabola - ps23
Holometabola/Psocodea - ps24
Holometabola - ps25
Aparaglossata - ps26
Mecopterida - ps27
Diptera - ps28
Bibiono./Cyclorrhapha - ps29
Cyclorrhapha - ps30
Calyptratae/Drosophila - ps31
Drosophila - ps32
Sophophora - ps33
Obscura/Melanogaster_group - ps34
D_melanogaster_group - ps35
D_melanogaster_subgroup - ps36
sim./sech./mel. group - ps37
Drosophila_melanogaster - ps38
p-value
2e-03
4e-03
6e-03
1e-02
2e-02
Log-odds
0.5
1.0
1.5
2.0
2.5
3.0
3.5
4.0

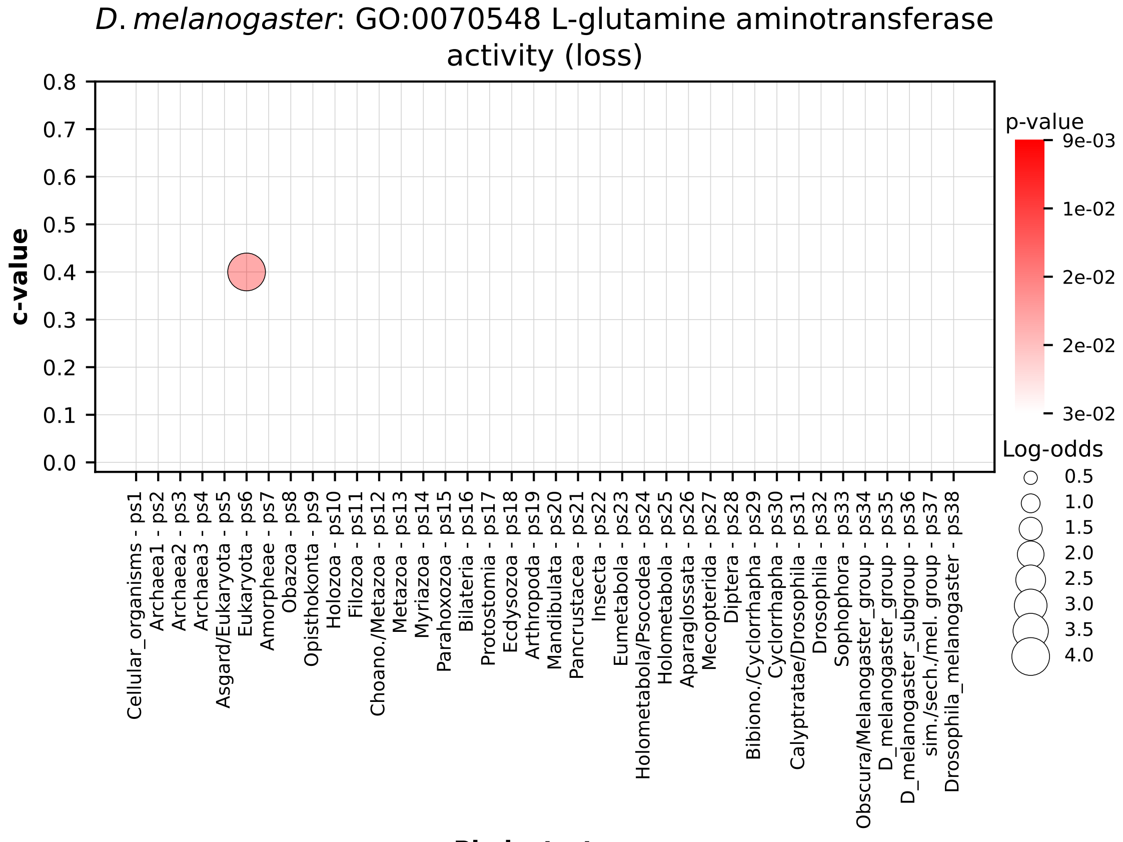

D. melanogaster: GO:0070548 L-glutamine aminotransferase activity (loss)
c-value
0.8
0.7
0.6
0.5
0.4
0.3
0.2
0.1
0.0
Phylostratum
Cellular_organisms - ps1
Archaea1 - ps2
Archaea2 - ps3
Archaea3 - ps4
Asgard/Eukaryota - ps5
Eukaryota - ps6
Amorpheae - ps7
Obazoa - ps8
Opisthokonta - ps9
Holozoa - ps10
Filozoa - ps11
Choano./Metazoa - ps12
Metazoa - ps13
Myriazoa - ps14
Parahoxozoa - ps15
Bilateria - ps16
Protostomia - ps17
Ecdysozoa - ps18
Arthropoda - ps19
Mandibulata - ps20
Pancrustacea - ps21
Insecta - ps22
Eumetabola - ps23
Holometabola/Psocodea - ps24
Holometabola - ps25
Aparaglossata - ps26
Mecopterida - ps27
Diptera - ps28
Bibiono./Cyclorrhapha - ps29
Cyclorrhapha - ps30
Calyptratae/Drosophila - ps31
Drosophila - ps32
Sophophora - ps33
Obscura/Melanogaster_group - ps34
D_melanogaster_group - ps35
D_melanogaster_subgroup - ps36
sim./sech./mel. group - ps37
Drosophila_melanogaster - ps38
p-value
9e-03
1e-02
2e-02
2e-02
3e-02
Log-odds
0.5
1.0
1.5
2.0
2.5
3.0
3.5
4.0

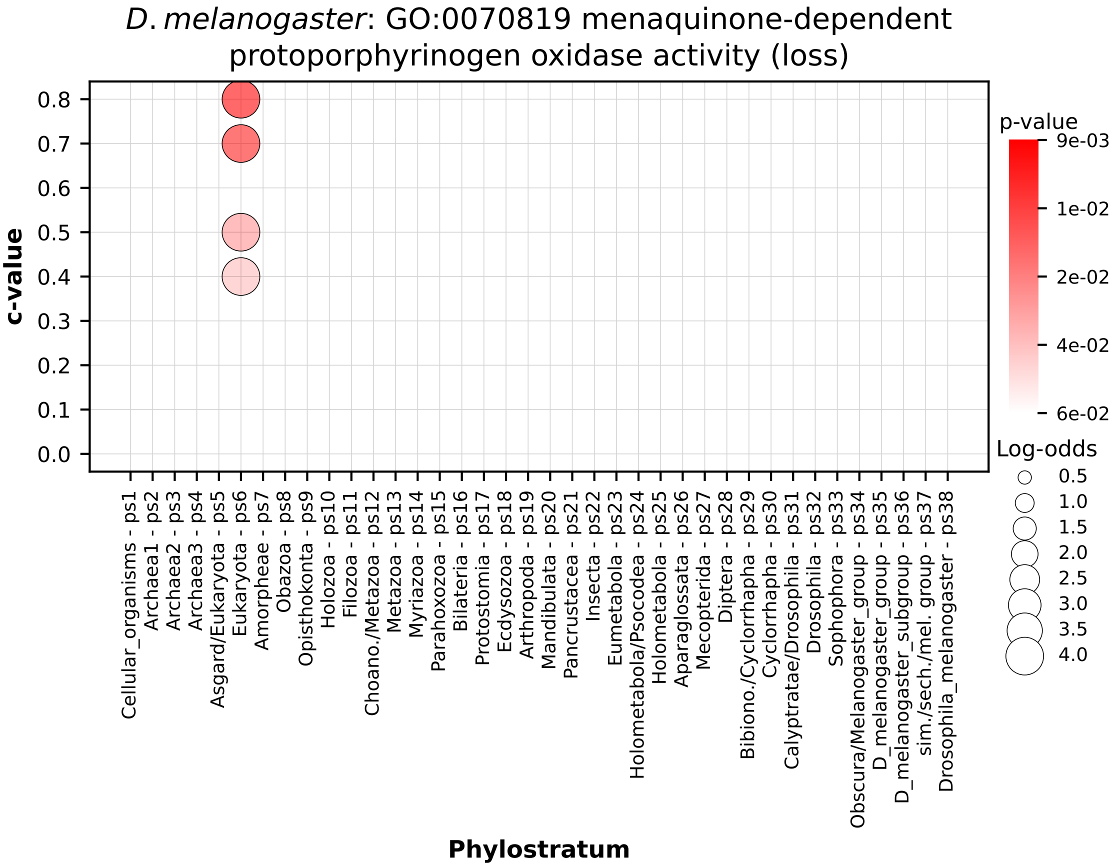

D. melanogaster: GO:0070819 menaquinone-dependent protoporphyrinogen oxidase activity (loss)
c-value
0.8
0.7
0.6
0.5
0.4
0.3
0.2
0.1
0.0
p-value
9e-03
1e-02
2e-02
4e-02
6e-02
Log-odds
0.5
1.0
1.5
2.0
2.5
3.0
3.5
4.0
Cellular_organisms - ps1
Archaea1 - ps2
Archaea2 - ps3
Archaea3 - ps4
Asgard/Eukaryota - ps5
Eukaryota - ps6
Amorpheae - ps7
Obazoa - ps8
Opisthokonta - ps9
Holozoa - ps10
Filozoa - ps11
Choano./Metazoa - ps12
Metazoa - ps13
Myriazoa - ps14
Parahoxozoa - ps15
Bilateria - ps16
Protostomia - ps17
Ecdysozoa - ps18
Arthropoda - ps19
Mandibulata - ps20
Pancrustacea - ps21
Insecta - ps22
Eumetabola - ps23
Holometabola/Psocodea - ps24
Holometabola - ps25
Aparaglossata - ps26
Mecopterida - ps27
Diptera - ps28
Bibiono./Cyclorrhapha - ps29
Cyclorrhapha - ps30
Calyptratae/Drosophila - ps31
Drosophila - ps32
Sophophora - ps33
Obscura/Melanogaster_group - ps34
D_melanogaster_group - ps35
D_melanogaster_subgroup - ps36
sim./sech./mel. group - ps37
Drosophila_melanogaster - ps38
Phylostratum

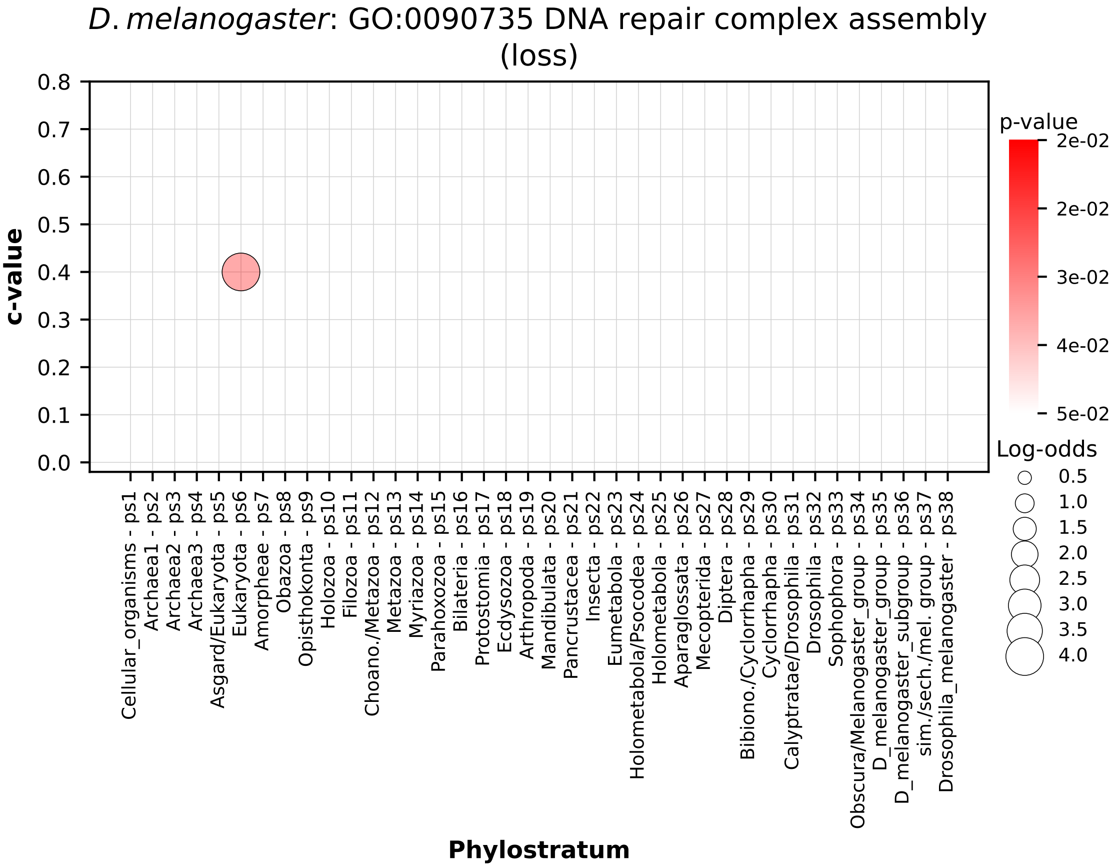

D. melanogaster: GO:0090735 DNA repair complex assembly (loss)
c-value
0.0
0.1
0.2
0.3
0.4
0.5
0.6
0.7
0.8
p-value
2e-02
2e-02
3e-02
4e-02
5e-02
Log-odds
0.5
1.0
1.5
2.0
2.5
3.0
3.5
4.0
Cellular_organisms - ps1
Archaea1 - ps2
Archaea2 - ps3
Archaea3 - ps4
Asgard/Eukaryota - ps5
Eukaryota - ps6
Amorpheae - ps7
Obazoa - ps8
Opisthokonta - ps9
Holozoa - ps10
Filozoa - ps11
Choano./Metazoa - ps12
Metazoa - ps13
Myriazoa - ps14
Parahoxozoa - ps15
Bilateria - ps16
Protostomia - ps17
Ecdysozoa - ps18
Arthropoda - ps19
Mandibulata - ps20
Pancrustacea - ps21
Insecta - ps22
Eumetabola - ps23
Holometabola/Psocodea - ps24
Holometabola - ps25
Aparaglossata - ps26
Mecopterida - ps27
Diptera - ps28
Bibiono./Cyclorrhapha - ps29
Cyclorrhapha - ps30
Calyptratae/Drosophila - ps31
Drosophila - ps32
Sophophora - ps33
Obscura/Melanogaster_group - ps34
D_melanogaster_group - ps35
D_melanogaster_subgroup - ps36
sim./sech./mel. group - ps37
Drosophila_melanogaster - ps38
Phylostratum

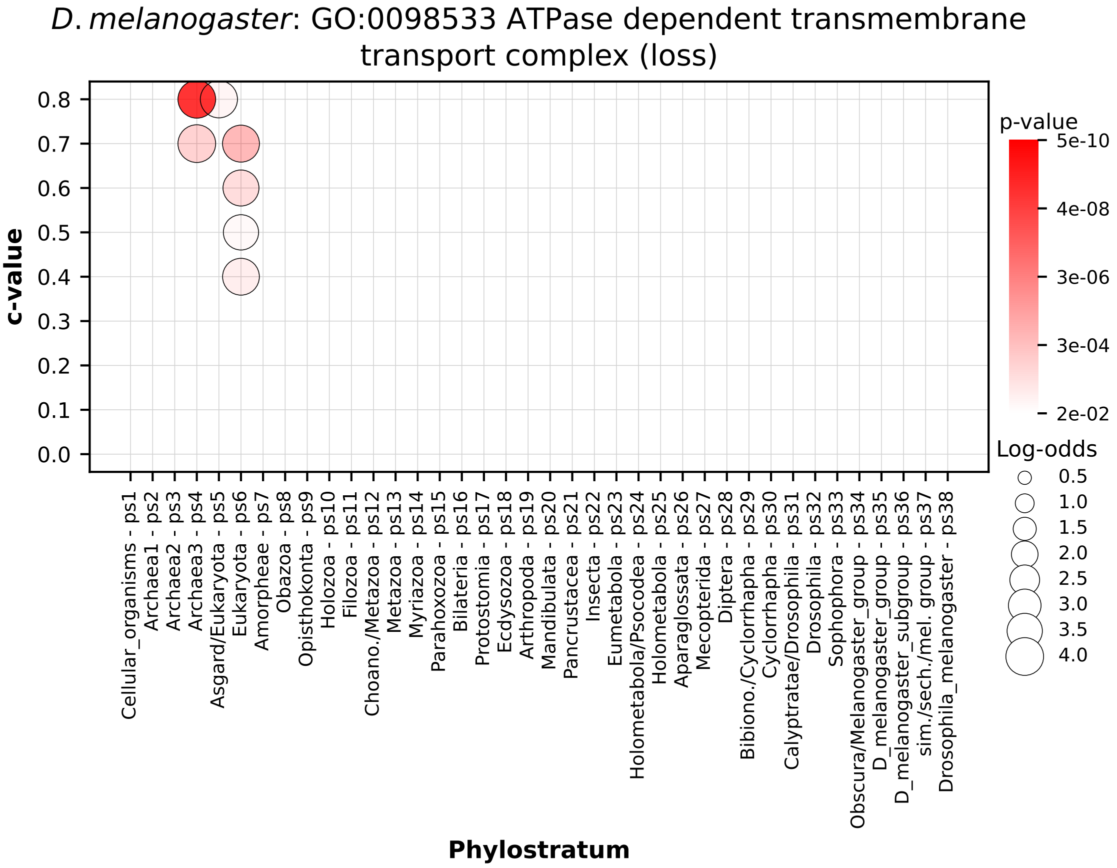

D. melanogaster: GO:0098533 ATPase dependent transmembrane transport complex (loss)
c-value
0.8
0.7
0.6
0.5
0.4
0.3
0.2
0.1
0.0
Phylostratum
Cellular_organisms - ps1
Archaea1 - ps2
Archaea2 - ps3
Archaea3 - ps4
Asgard/Eukaryota - ps5
Eukaryota - ps6
Amorpheae - ps7
Obazoa - ps8
Opisthokonta - ps9
Holozoa - ps10
Filozoa - ps11
Choano./Metazoa - ps12
Metazoa - ps13
Myriazoa - ps14
Parahoxozoa - ps15
Bilateria - ps16
Protostomia - ps17
Ecdysozoa - ps18
Arthropoda - ps19
Mandibulata - ps20
Pancrustacea - ps21
Insecta - ps22
Eumetabola - ps23
Holometabola/Psocodea - ps24
Holometabola - ps25
Aparaglossata - ps26
Mecopterida - ps27
Diptera - ps28
Bibiono./Cyclorrhapha - ps29
Cyclorrhapha - ps30
Calyptratae/Drosophila - ps31
Drosophila - ps32
Sophophora - ps33
Obscura/Melanogaster_group - ps34
D_melanogaster_group - ps35
D_melanogaster_subgroup - ps36
sim./sech./mel. group - ps37
Drosophila_melanogaster - ps38
p-value
5e-10
4e-08
3e-06
3e-04
2e-02
Log-odds
0.5
1.0
1.5
2.0
2.5
3.0
3.5
4.0

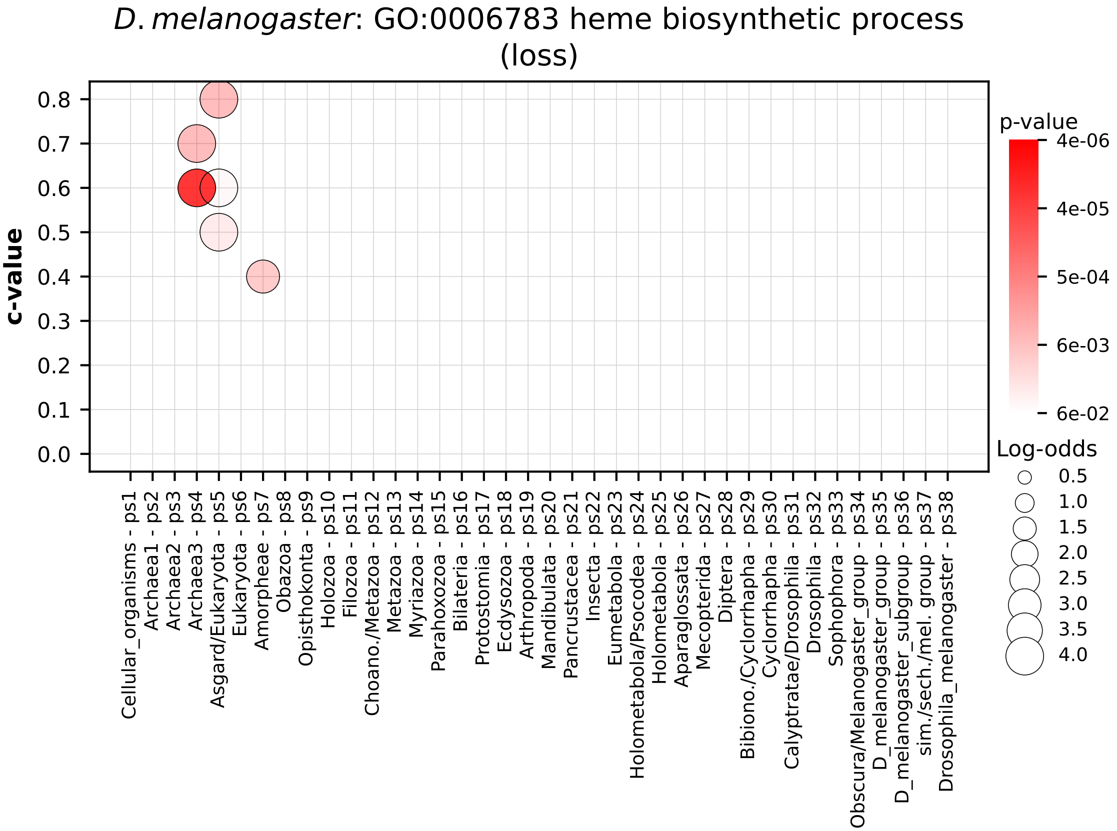

D. melanogaster: GO:0006783 heme biosynthetic process
(loss)
c-value
0.8
0.7
0.6
0.5
0.4
0.3
0.2
0.1
0.0
p-value
4e-06
4e-05
5e-04
6e-03
6e-02
Log-odds
0.5
1.0
1.5
2.0
2.5
3.0
3.5
4.0
Cellular_organisms - ps1
Archaea1 - ps2
Archaea2 - ps3
Archaea3 - ps4
Asgard/Eukaryota - ps5
Eukaryota - ps6
Amorpheae - ps7
Obazoa - ps8
Opisthokonta - ps9
Holozoa - ps10
Filozoa - ps11
Choano./Metazoa - ps12
Metazoa - ps13
Myriazoa - ps14
Parahoxozoa - ps15
Bilateria - ps16
Protostomia - ps17
Ecdysozoa - ps18
Arthropoda - ps19
Mandibulata - ps20
Pancrustacea - ps21
Insecta - ps22
Eumetabola - ps23
Holometabola/Psocodea - ps24
Holometabola - ps25
Aparaglossata - ps26
Mecopterida - ps27
Diptera - ps28
Bibiono./Cyclorrhapha - ps29
Cyclorrhapha - ps30
Calyptratae/Drosophila - ps31
Drosophila - ps32
Sophophora - ps33
Obscura/Melanogaster_group - ps34
D_melanogaster_group - ps35
D_melanogaster_subgroup - ps36
sim./sech./mel. group - ps37
Drosophila_melanogaster - ps38
Phylostratum

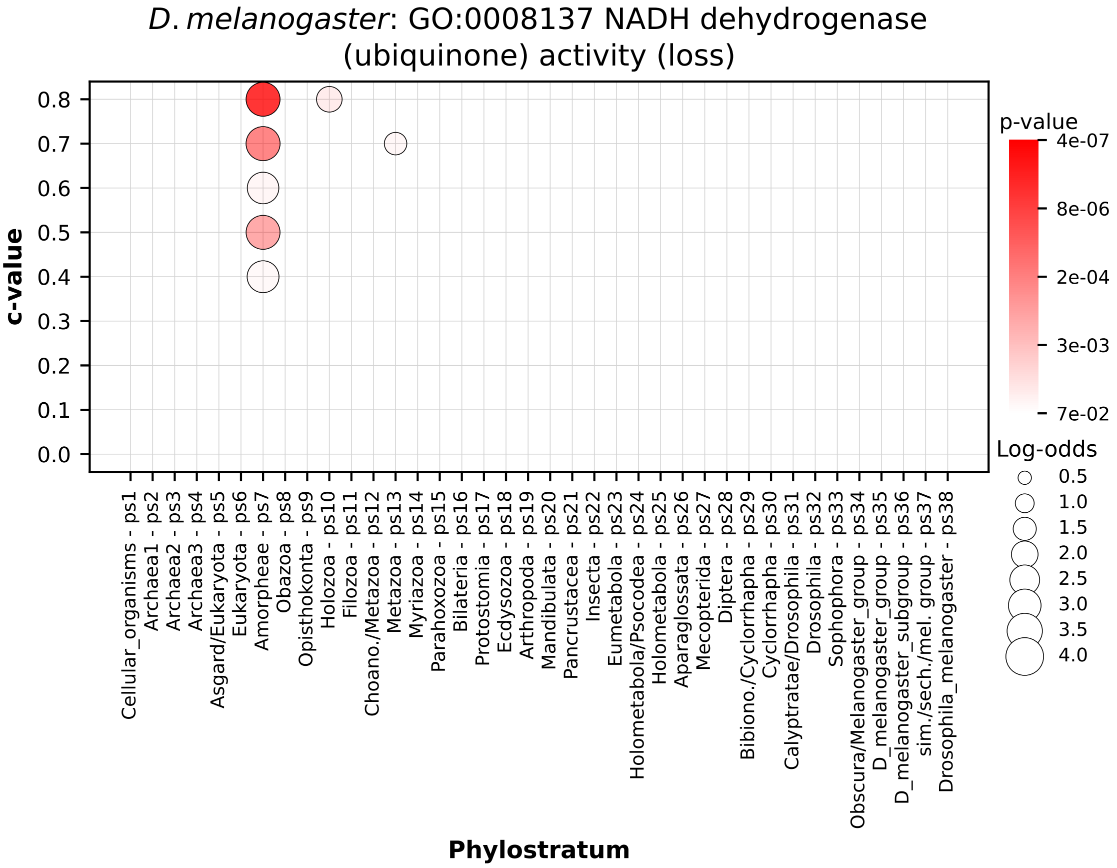

D. melanogaster: GO:0008137 NADH dehydrogenase (ubiquinone) activity (loss)
c-value
0.0
0.1
0.2
0.3
0.4
0.5
0.6
0.7
0.8
Phylostratum
Cellular_organisms - ps1
Archaea1 - ps2
Archaea2 - ps3
Archaea3 - ps4
Asgard/Eukaryota - ps5
Eukaryota - ps6
Amorpheae - ps7
Obazoa - ps8
Opisthokonta - ps9
Holozoa - ps10
Filozoa - ps11
Choano./Metazoa - ps12
Metazoa - ps13
Myriazoa - ps14
Parahoxozoa - ps15
Bilateria - ps16
Protostomia - ps17
Ecdysozoa - ps18
Arthropoda - ps19
Mandibulata - ps20
Pancrustacea - ps21
Insecta - ps22
Eumetabola - ps23
Holometabola/Psocodea - ps24
Holometabola - ps25
Aparaglossata - ps26
Mecopterida - ps27
Diptera - ps28
Bibiono./Cyclorrhapha - ps29
Cyclorrhapha - ps30
Calyptratae/Drosophila - ps31
Drosophila - ps32
Sophophora - ps33
Obscura/Melanogaster_group - ps34
D_melanogaster_group - ps35
D_melanogaster_subgroup - ps36
sim./sech./mel. group - ps37
Drosophila_melanogaster - ps38
p-value
4e-07
8e-06
2e-04
3e-03
7e-02
Log-odds
0.5
1.0
1.5
2.0
2.5
3.0
3.5
4.0

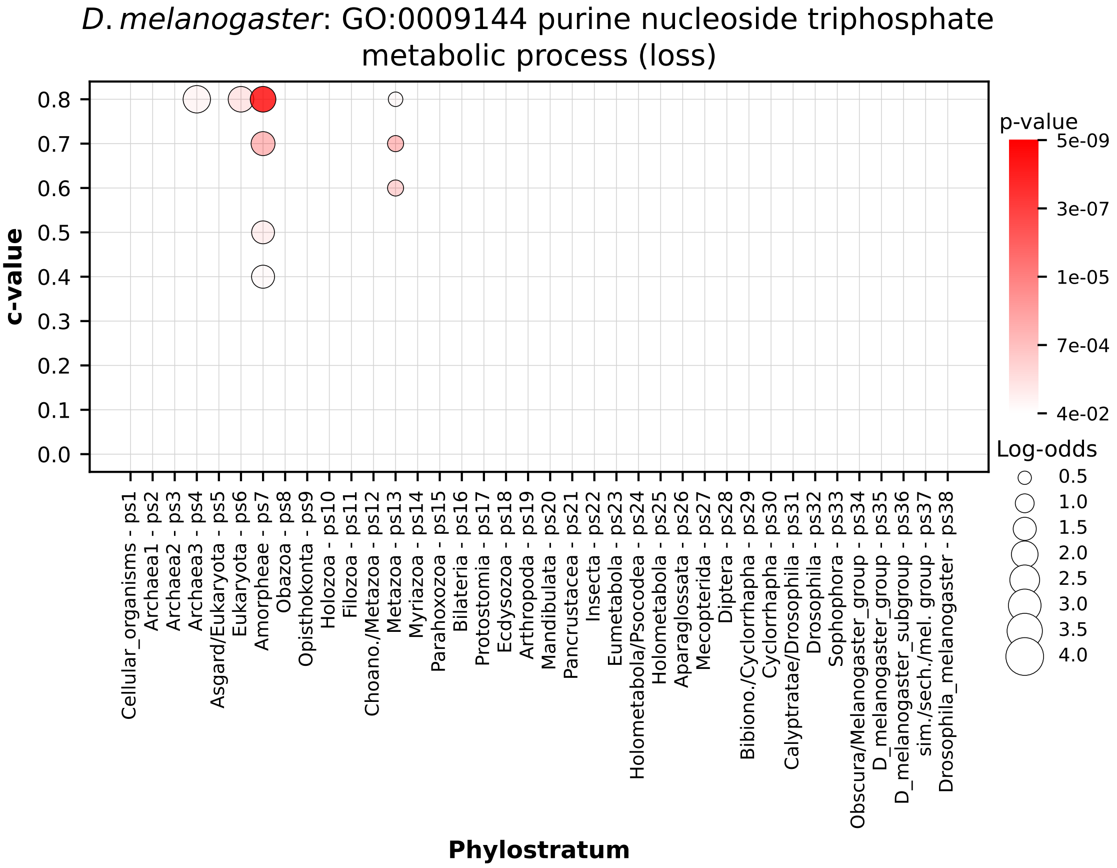

D. melanogaster: GO:0009144 purine nucleoside triphosphate metabolic process (loss)
c-value
0.8
0.7
0.6
0.5
0.4
0.3
0.2
0.1
0.0
p-value
5e-09
3e-07
1e-05
7e-04
4e-02
Log-odds
0.5
1.0
1.5
2.0
2.5
3.0
3.5
4.0
Cellular_organisms - ps1
Archaea1 - ps2
Archaea2 - ps3
Archaea3 - ps4
Asgard/Eukaryota - ps5
Eukaryota - ps6
Amorpheae - ps7
Obazoa - ps8
Opisthokonta - ps9
Holozoa - ps10
Filozoa - ps11
Choano./Metazoa - ps12
Metazoa - ps13
Myriazoa - ps14
Parahoxozoa - ps15
Bilateria - ps16
Protostomia - ps17
Ecdysozoa - ps18
Arthropoda - ps19
Mandibulata - ps20
Pancrustacea - ps21
Insecta - ps22
Eumetabola - ps23
Holometabola/Psocodea - ps24
Holometabola - ps25
Aparaglossata - ps26
Mecopterida - ps27
Diptera - ps28
Bibiono./Cyclorrhapha - ps29
Cyclorrhapha - ps30
Calyptratae/Drosophila - ps31
Drosophila - ps32
Sophophora - ps33
Obscura/Melanogaster_group - ps34
D_melanogaster_group - ps35
D_melanogaster_subgroup - ps36
sim./sech./mel. group - ps37
Drosophila_melanogaster - ps38
Phylostratum

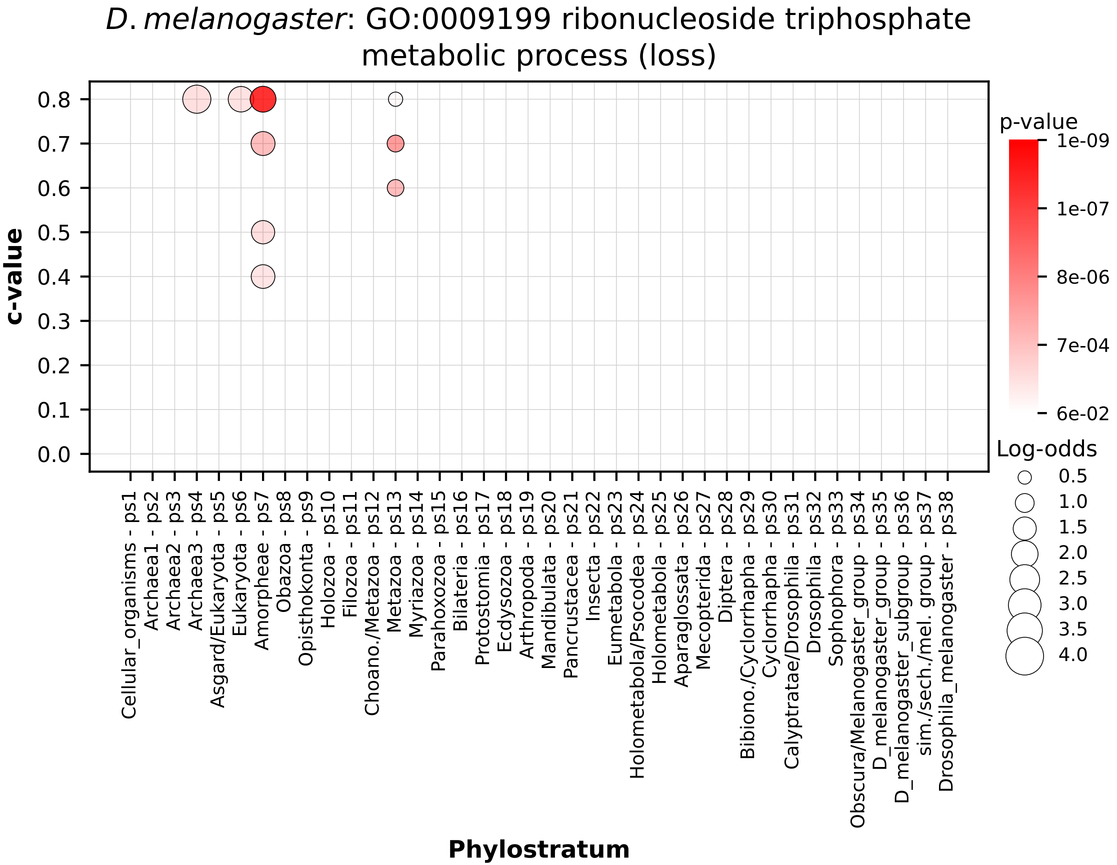

$D. melanogaster$: GO:0009199 ribonucleoside triphosphate metabolic process (loss)
c-value
0.0
0.1
0.2
0.3
0.4
0.5
0.6
0.7
0.8
Phylostratum
Cellular_organisms - ps1
Archaea1 - ps2
Archaea2 - ps3
Archaea3 - ps4
Asgard/Eukaryota - ps5
Eukaryota - ps6
Amorpheae - ps7
Obazoa - ps8
Opisthokonta - ps9
Holozoa - ps10
Filozoa - ps11
Choano./Metazoa - ps12
Metazoa - ps13
Myriazoa - ps14
Parahoxozoa - ps15
Bilateria - ps16
Protostomia - ps17
Ecdysozoa - ps18
Arthropoda - ps19
Mandibulata - ps20
Pancrustacea - ps21
Insecta - ps22
Eumetabola - ps23
Holometabola/Psocodea - ps24
Holometabola - ps25
Aparaglossata - ps26
Mecopterida - ps27
Diptera - ps28
Bibiono./Cyclorrhapha - ps29
Cyclorrhapha - ps30
Calyptratae/Drosophila - ps31
Drosophila - ps32
Sophophora - ps33
Obscura/Melanogaster_group - ps34
D_melanogaster_group - ps35
D_melanogaster_subgroup - ps36
sim./sech./mel. group - ps37
Drosophila_melanogaster - ps38
p-value
1e-09
1e-07
8e-06
7e-04
6e-02
Log-odds
0.5
1.0
1.5
2.0
2.5
3.0
3.5
4.0

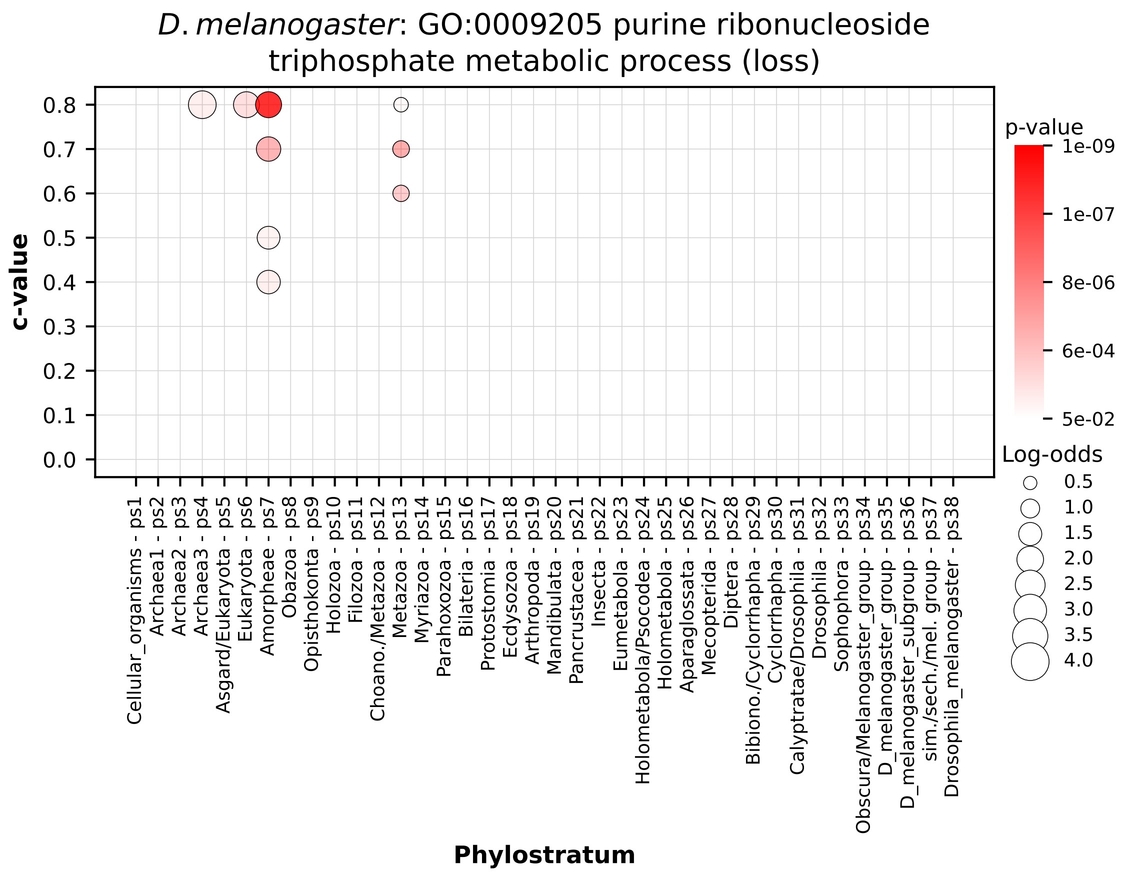

D. melanogaster: GO:0009205 purine ribonucleoside triphosphate metabolic process (loss)
c-value
0.8
0.7
0.6
0.5
0.4
0.3
0.2
0.1
0.0
p-value
1e-09
1e-07
8e-06
6e-04
5e-02
Log-odds
0.5
1.0
1.5
2.0
2.5
3.0
3.5
4.0
Cellular_organisms - ps1
Archaea1 - ps2
Archaea2 - ps3
Archaea3 - ps4
Asgard/Eukaryota - ps5
Eukaryota - ps6
Amorpheae - ps7
Obazoa - ps8
Opisthokonta - ps9
Holozoa - ps10
Filozoa - ps11
Choano./Metazoa - ps12
Metazoa - ps13
Myriazoa - ps14
Parahoxozoa - ps15
Bilateria - ps16
Protostomia - ps17
Ecdysozoa - ps18
Arthropoda - ps19
Mandibulata - ps20
Pancrustacea - ps21
Insecta - ps22
Eumetabola - ps23
Holometabola/Psocodea - ps24
Holometabola - ps25
Aparaglossata - ps26
Mecopterida - ps27
Diptera - ps28
Bibiono./Cyclorrhapha - ps29
Cyclorrhapha - ps30
Calyptratae/Drosophila - ps31
Drosophila - ps32
Sophophora - ps33
Obscura/Melanogaster_group - ps34
D_melanogaster_group - ps35
D_melanogaster_subgroup - ps36
sim./sech./mel. group - ps37
Drosophila_melanogaster - ps38
Phylostratum

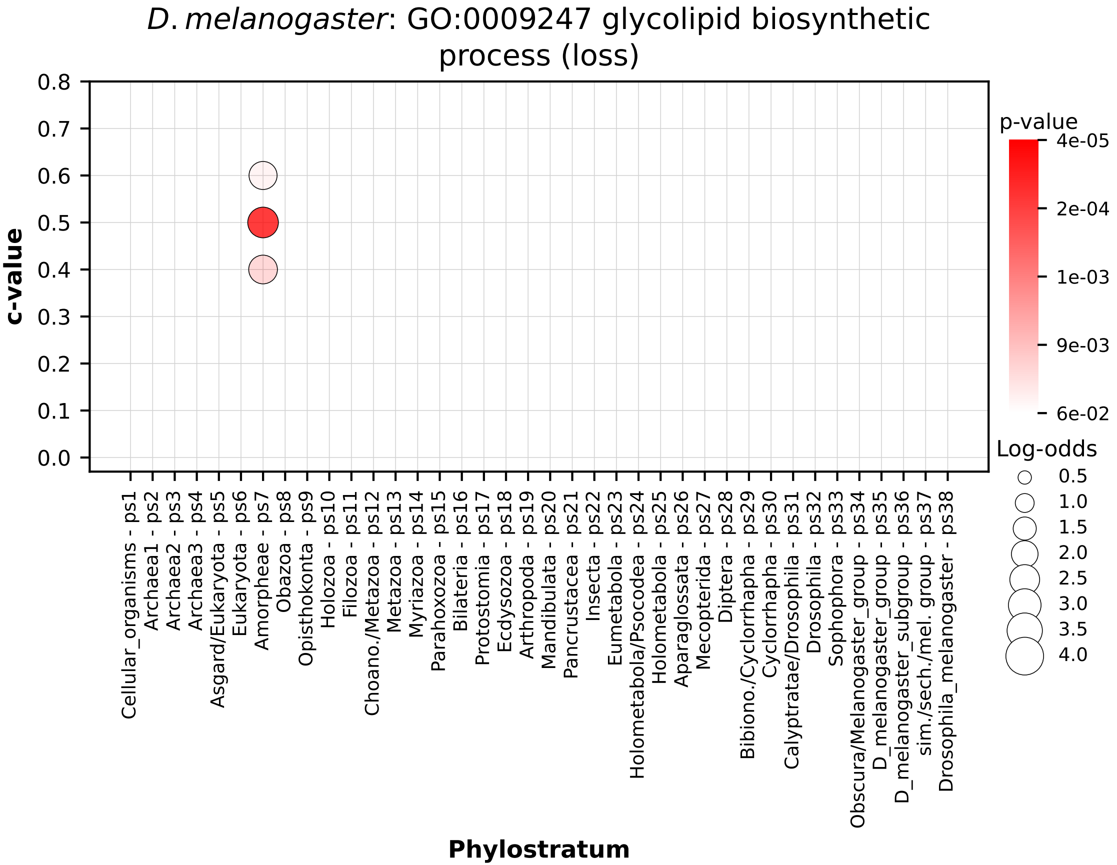

$D. melanogaster$: GO:0009247 glycolipid biosynthetic process (loss)
c-value
0.8
0.7
0.6
0.5
0.4
0.3
0.2
0.1
0.0
Phylostratum
Cellular_organisms - ps1
Archaea1 - ps2
Archaea2 - ps3
Archaea3 - ps4
Asgard/Eukaryota - ps5
Eukaryota - ps6
Amorpheae - ps7
Obazoa - ps8
Opisthokonta - ps9
Holozoa - ps10
Filozoa - ps11
Choano./Metazoa - ps12
Metazoa - ps13
Myriazoa - ps14
Parahoxozoa - ps15
Bilateria - ps16
Protostomia - ps17
Ecdysozoa - ps18
Arthropoda - ps19
Mandibulata - ps20
Pancrustacea - ps21
Insecta - ps22
Eumetabola - ps23
Holometabola/Psocodea - ps24
Holometabola - ps25
Aparaglossata - ps26
Mecopterida - ps27
Diptera - ps28
Bibiono./Cyclorrhapha - ps29
Cyclorrhapha - ps30
Calyptratae/Drosophila - ps31
Drosophila - ps32
Sophophora - ps33
Obscura/Melanogaster_group - ps34
D_melanogaster_group - ps35
D_melanogaster_subgroup - ps36
sim./sech./mel. group - ps37
Drosophila_melanogaster - ps38
p-value
4e-05
2e-04
1e-03
9e-03
6e-02
Log-odds
0.5
1.0
1.5
2.0
2.5
3.0
3.5
4.0

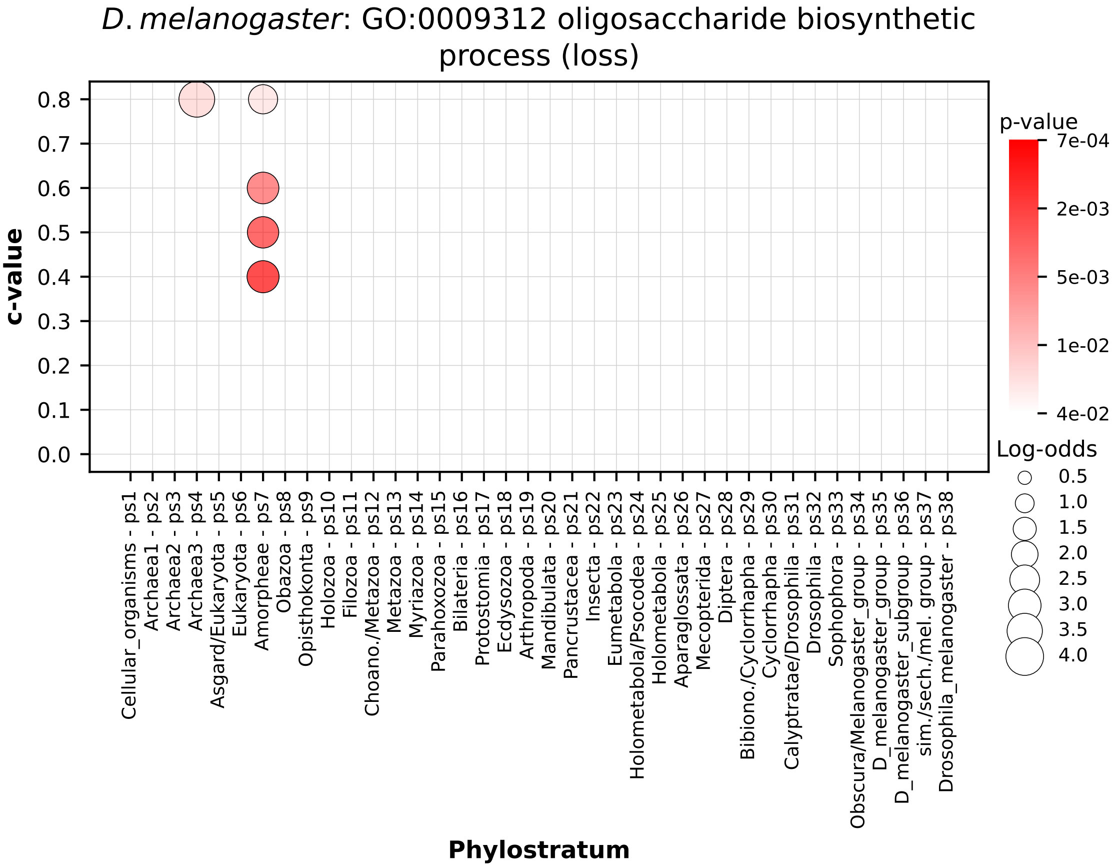

D. melanogaster: GO:0009312 oligosaccharide biosynthetic process (loss)
c-value
0.0
0.1
0.2
0.3
0.4
0.5
0.6
0.7
0.8
Phylostratum
Cellular_organisms - ps1
Archaea1 - ps2
Archaea2 - ps3
Archaea3 - ps4
Asgard/Eukaryota - ps5
Eukaryota - ps6
Amorpheae - ps7
Obazoa - ps8
Opisthokonta - ps9
Holozoa - ps10
Filozoa - ps11
Choano./Metazoa - ps12
Metazoa - ps13
Myriazoa - ps14
Parahoxozoa - ps15
Bilateria - ps16
Protostomia - ps17
Ecdysozoa - ps18
Arthropoda - ps19
Mandibulata - ps20
Pancrustacea - ps21
Insecta - ps22
Eumetabola - ps23
Holometabola/Psocodea - ps24
Holometabola - ps25
Aparaglossata - ps26
Mecopterida - ps27
Diptera - ps28
Bibiono./Cyclorrhapha - ps29
Cyclorrhapha - ps30
Calyptratae/Drosophila - ps31
Drosophila - ps32
Sophophora - ps33
Obscura/Melanogaster_group - ps34
D_melanogaster_group - ps35
D_melanogaster_subgroup - ps36
sim./sech./mel. group - ps37
Drosophila_melanogaster - ps38
p-value
7e-04
2e-03
5e-03
1e-02
4e-02
Log-odds
0.5
1.0
1.5
2.0
2.5
3.0
3.5
4.0

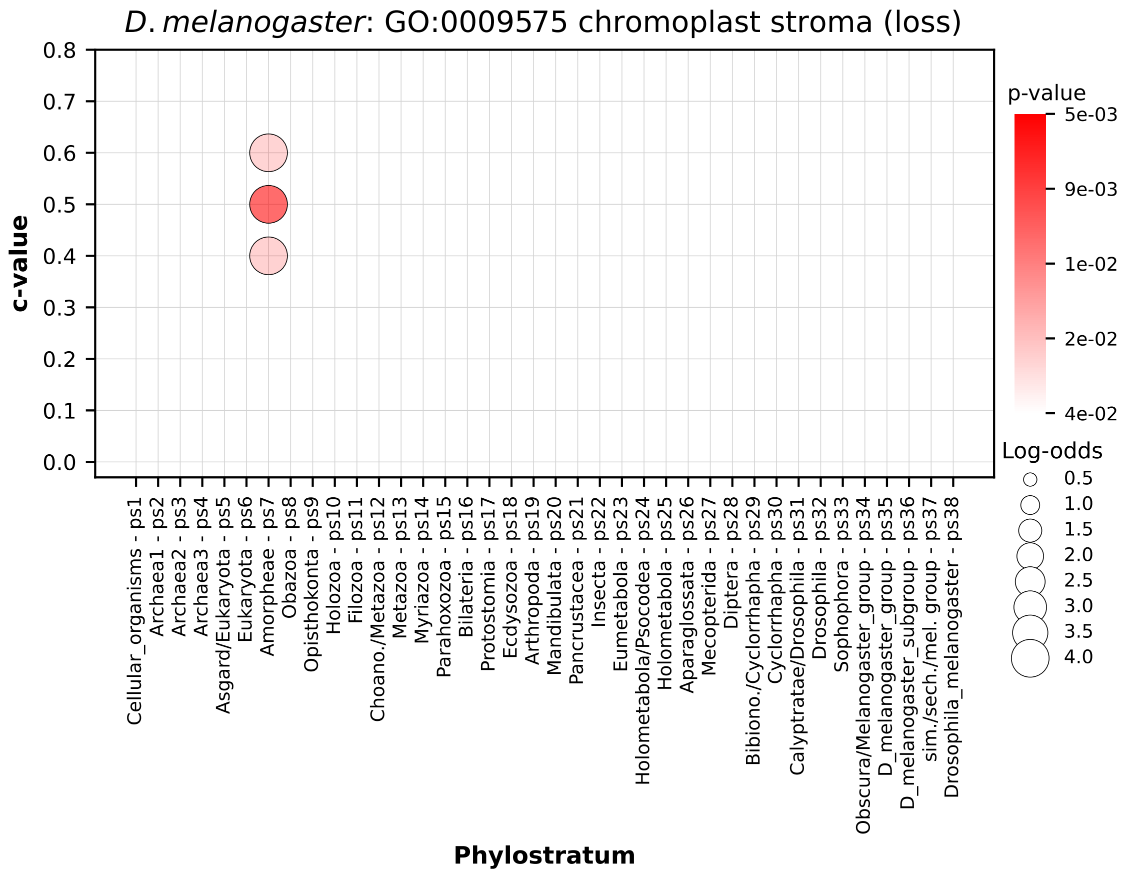
D. melanogaster: GO:0009575 chromoplast stroma (loss)
c-value
0.8
0.7
0.6
0.5
0.4
0.3
0.2
0.1
0.0
p-value
5e-03
9e-03
1e-02
2e-02
4e-02
Log-odds
0.5
1.0
1.5
2.0
2.5
3.0
3.5
4.0
Cellular_organisms - ps1
Archaea1 - ps2
Archaea2 - ps3
Archaea3 - ps4
Asgard/Eukaryota - ps5
Eukaryota - ps6
Amorpheae - ps7
Obazoa - ps8
Opisthokonta - ps9
Holozoa - ps10
Filozoa - ps11
Choano./Metazoa - ps12
Metazoa - ps13
Myriazoa - ps14
Parahoxozoa - ps15
Bilateria - ps16
Protostomia - ps17
Ecdysozoa - ps18
Arthropoda - ps19
Mandibulata - ps20
Pancrustacea - ps21
Insecta - ps22
Eumetabola - ps23
Holometabola/Psocodea - ps24
Holometabola - ps25
Aparaglossata - ps26
Mecopterida - ps27
Diptera - ps28
Bibiono./Cyclorrhapha - ps29
Cyclorrhapha - ps30
Calyptratae/Drosophila - ps31
Drosophila - ps32
Sophophora - ps33
Obscura/Melanogaster_group - ps34
D_melanogaster_group - ps35
D_melanogaster_subgroup - ps36
sim./sech./mel. group - ps37
Drosophila_melanogaster - ps38
Phylostratum

D. melanogaster: GO:0009635 response to herbicide (loss)

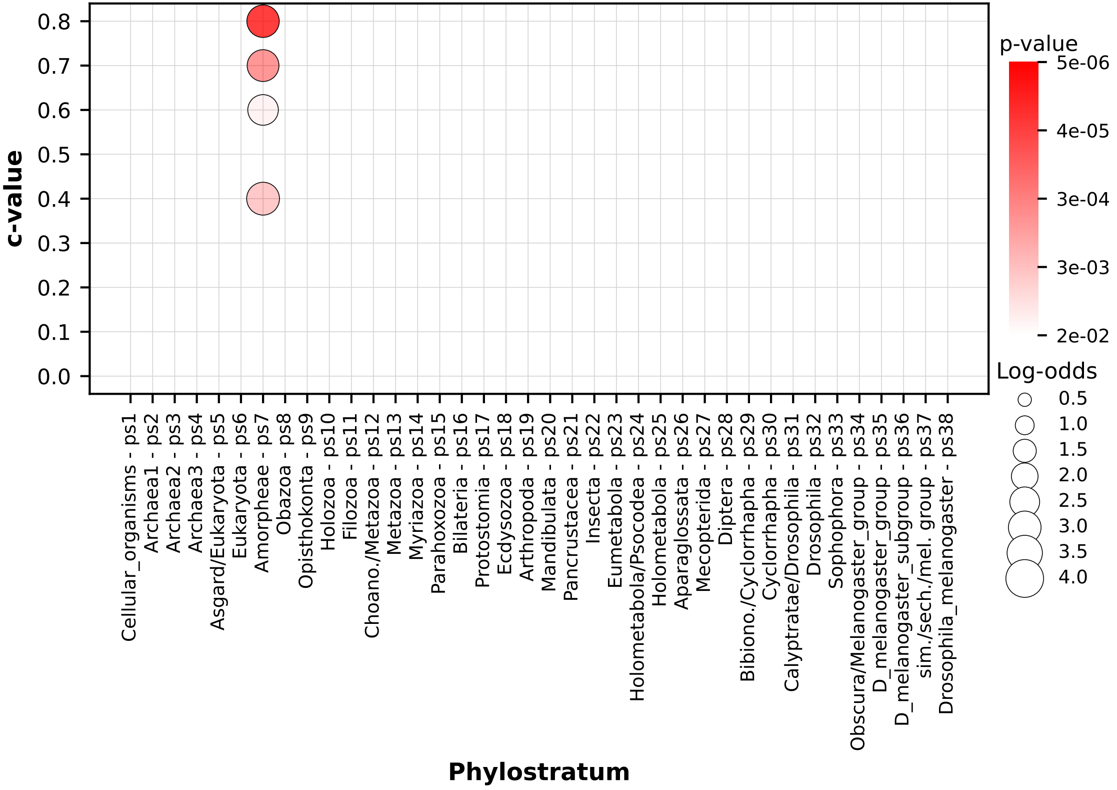

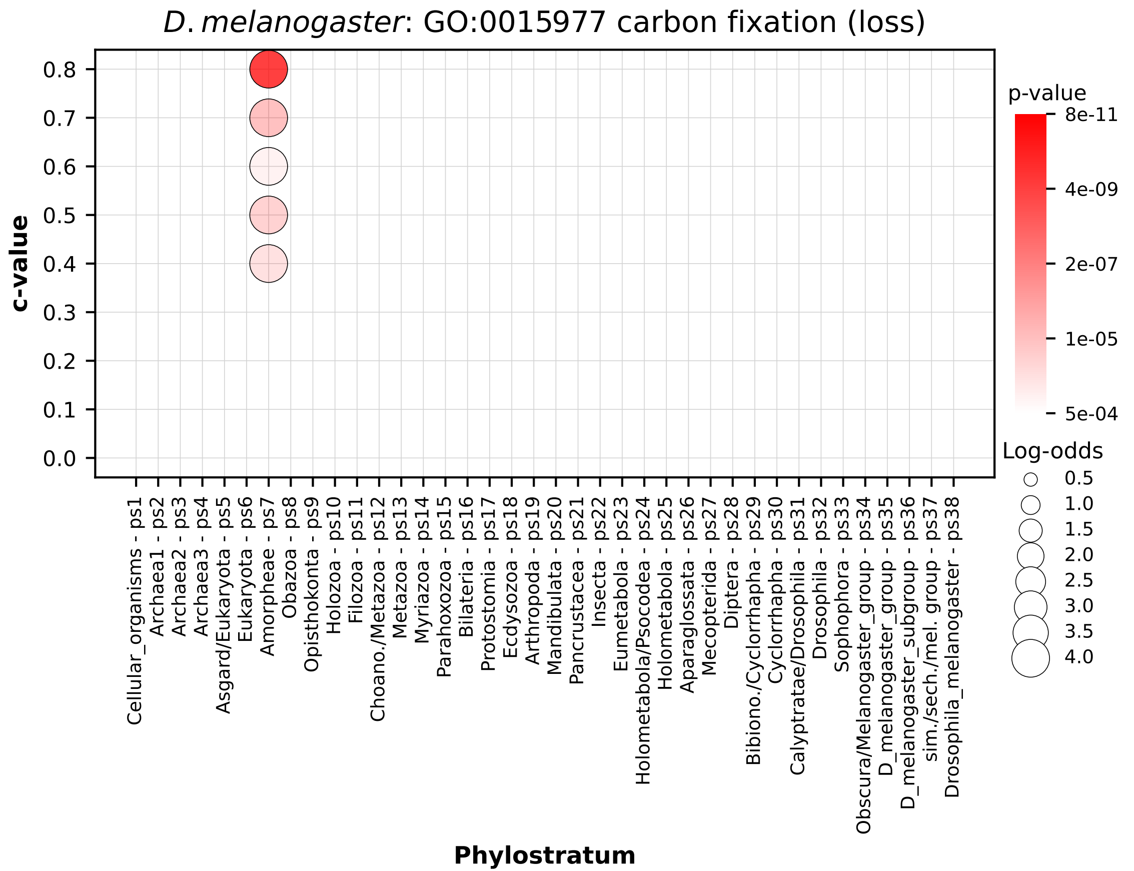

D. melanogaster: GO:0015977 carbon fixation (loss)
c-value
0.0
0.1
0.2
0.3
0.4
0.5
0.6
0.7
0.8
Phylostratum
Cellular_organisms - ps1
Archaea1 - ps2
Archaea2 - ps3
Archaea3 - ps4
Asgard/Eukaryota - ps5
Eukaryota - ps6
Amorpheae - ps7
Obazoa - ps8
Opisthokonta - ps9
Holozoa - ps10
Filozoa - ps11
Choano./Metazoa - ps12
Metazoa - ps13
Myriazoa - ps14
Parahoxozoa - ps15
Bilateria - ps16
Protostomia - ps17
Ecdysozoa - ps18
Arthropoda - ps19
Mandibulata - ps20
Pancrustacea - ps21
Insecta - ps22
Eumetabola - ps23
Holometabola/Psocodea - ps24
Holometabola - ps25
Aparaglossata - ps26
Mecopterida - ps27
Diptera - ps28
Bibiono./Cyclorrhapha - ps29
Cyclorrhapha - ps30
Calyptratae/Drosophila - ps31
Drosophila - ps32
Sophophora - ps33
Obscura/Melanogaster_group - ps34
D_melanogaster_group - ps35
D_melanogaster_subgroup - ps36
sim./sech./mel. group - ps37
Drosophila_melanogaster - ps38
p-value
8e-11
4e-09
2e-07
1e-05
5e-04
Log-odds
0.5
1.0
1.5
2.0
2.5
3.0
3.5
4.0

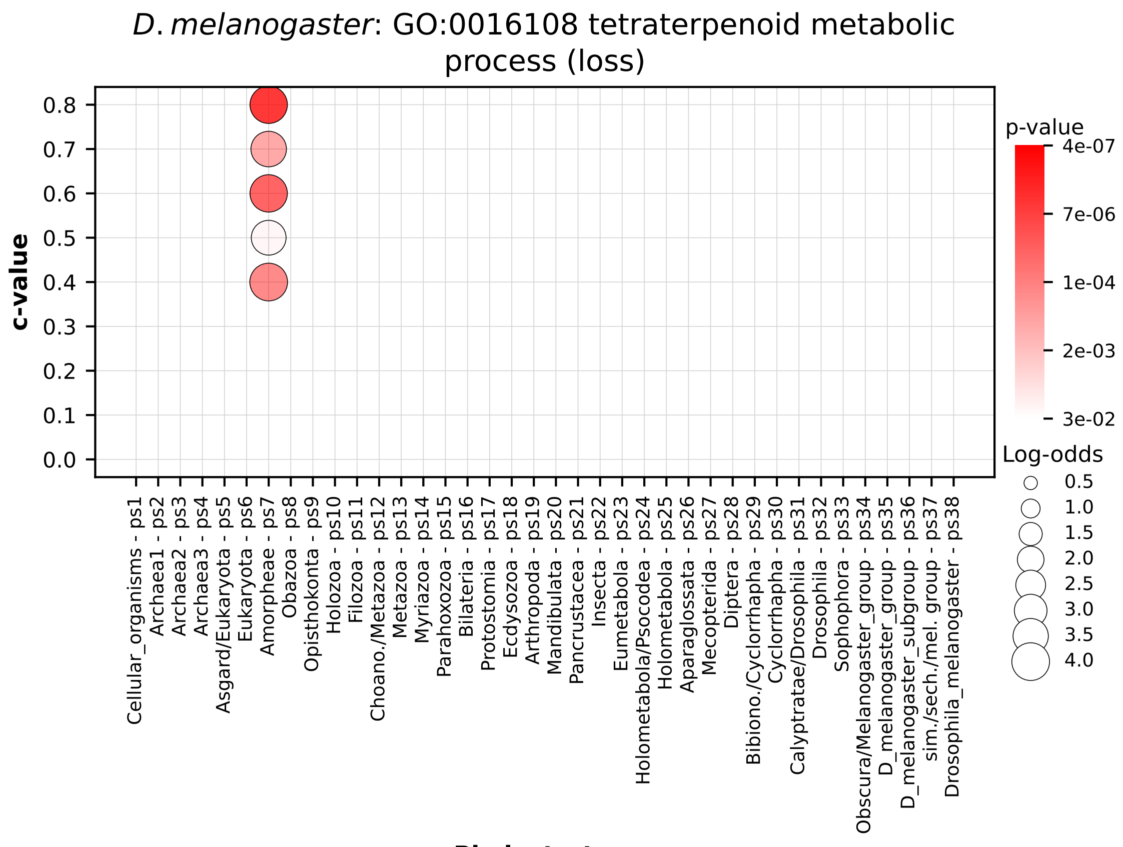

D. melanogaster: GO:0016108 tetraterpenoid metabolic process (loss)
c-value
0.8
0.7
0.6
0.5
0.4
0.3
0.2
0.1
0.0
Phylostratum
Cellular_organisms - ps1
Archaea1 - ps2
Archaea2 - ps3
Archaea3 - ps4
Asgard/Eukaryota - ps5
Eukaryota - ps6
Amorpheae - ps7
Obazoa - ps8
Opisthokonta - ps9
Holozoa - ps10
Filozoa - ps11
Choano./Metazoa - ps12
Metazoa - ps13
Myriazoa - ps14
Parahoxozoa - ps15
Bilateria - ps16
Protostomia - ps17
Ecdysozoa - ps18
Arthropoda - ps19
Mandibulata - ps20
Pancrustacea - ps21
Insecta - ps22
Eumetabola - ps23
Holometabola/Psocodea - ps24
Holometabola - ps25
Aparaglossata - ps26
Mecopterida - ps27
Diptera - ps28
Bibiono./Cyclorrhapha - ps29
Cyclorrhapha - ps30
Calyptratae/Drosophila - ps31
Drosophila - ps32
Sophophora - ps33
Obscura/Melanogaster_group - ps34
D_melanogaster_group - ps35
D_melanogaster_subgroup - ps36
sim./sech./mel. group - ps37
Drosophila_melanogaster - ps38
p-value
4e-07
7e-06
1e-04
2e-03
3e-02
Log-odds
0.5
1.0
1.5
2.0
2.5
3.0
3.5
4.0

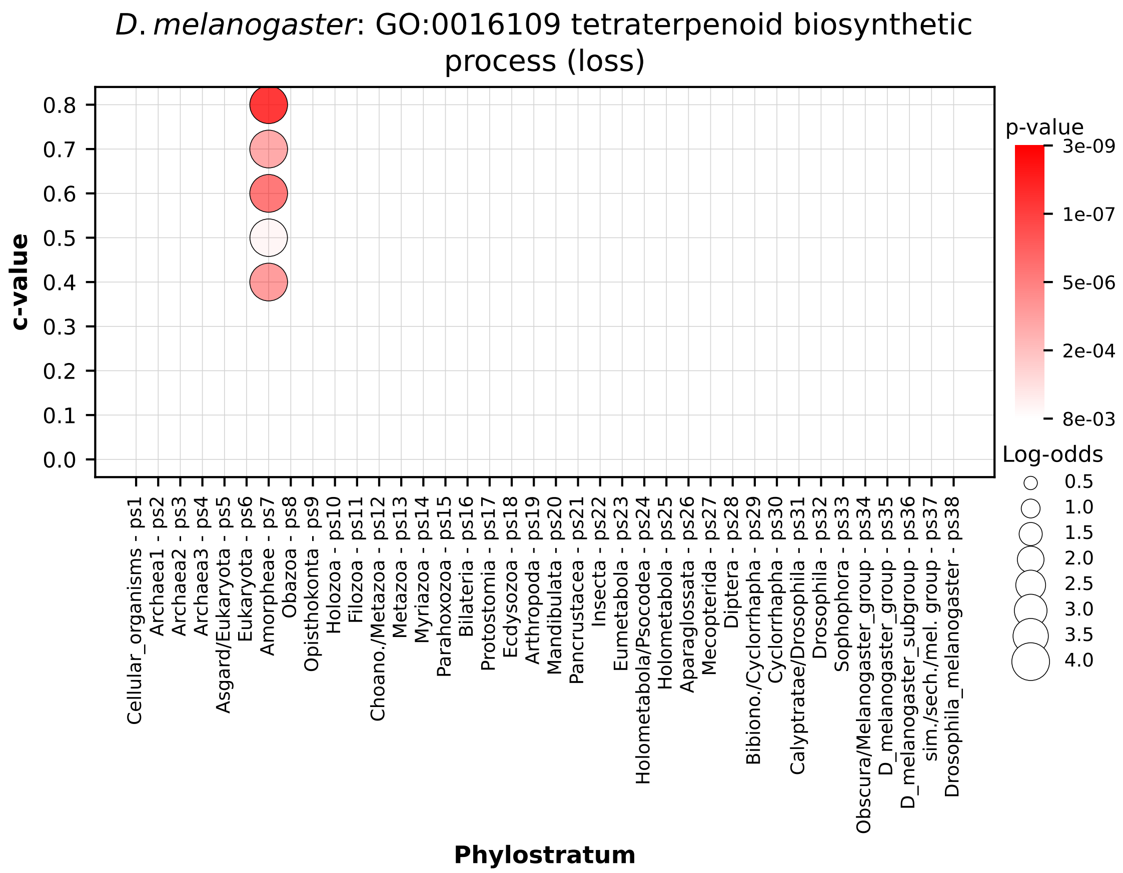

$D. melanogaster$: GO:0016109 tetraterpenoid biosynthetic process (loss)
c-value
0.8
0.7
0.6
0.5
0.4
0.3
0.2
0.1
0.0
Phylostratum
Cellular_organisms - ps1
Archaea1 - ps2
Archaea2 - ps3
Archaea3 - ps4
Asgard/Eukaryota - ps5
Eukaryota - ps6
Amorpheae - ps7
Obazoa - ps8
Opisthokonta - ps9
Holozoa - ps10
Filozoa - ps11
Choano./Metazoa - ps12
Metazoa - ps13
Myriazoa - ps14
Parahoxozoa - ps15
Bilateria - ps16
Protostomia - ps17
Ecdysozoa - ps18
Arthropoda - ps19
Mandibulata - ps20
Pancrustacea - ps21
Insecta - ps22
Eumetabola - ps23
Holometabola/Psocodea - ps24
Holometabola - ps25
Aparaglossata - ps26
Mecopterida - ps27
Diptera - ps28
Bibiono./Cyclorrhapha - ps29
Cyclorrhapha - ps30
Calyptratae/Drosophila - ps31
Drosophila - ps32
Sophophora - ps33
Obscura/Melanogaster_group - ps34
D_melanogaster_group - ps35
D_melanogaster_subgroup - ps36
sim./sech./mel. group - ps37
Drosophila_melanogaster - ps38
p-value
3e-09
1e-07
5e-06
2e-04
8e-03
Log-odds
0.5
1.0
1.5
2.0
2.5
3.0
3.5
4.0

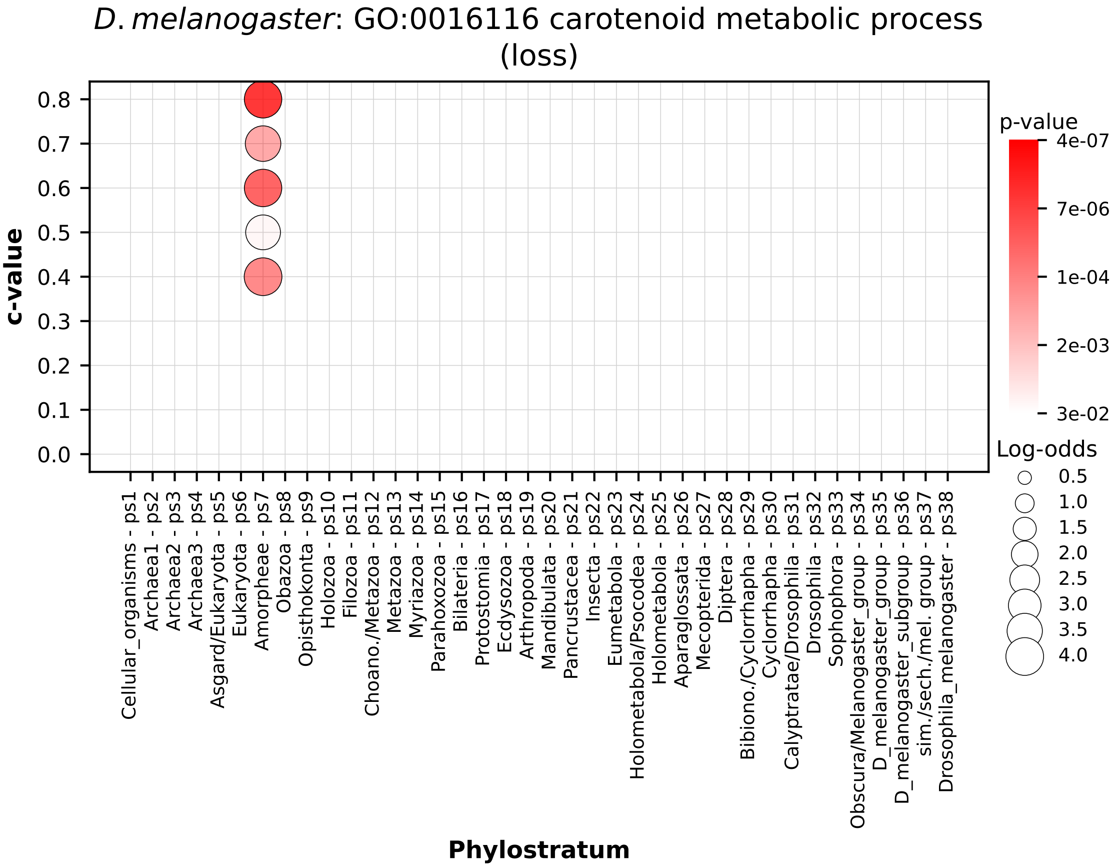
D. melanogaster: GO:0016116 carotenoid metabolic process
(loss)
c-value
0.0
0.1
0.2
0.3
0.4
0.5
0.6
0.7
0.8
p-value
4e-07
7e-06
1e-04
2e-03
3e-02
Log-odds
0.5
1.0
1.5
2.0
2.5
3.0
3.5
4.0
Cellular_organisms - ps1
Archaea1 - ps2
Archaea2 - ps3
Archaea3 - ps4
Asgard/Eukaryota - ps5
Eukaryota - ps6
Amorpheae - ps7
Obazoa - ps8
Opisthokonta - ps9
Holozoa - ps10
Filozoa - ps11
Choano./Metazoa - ps12
Metazoa - ps13
Myriazoa - ps14
Parahoxozoa - ps15
Bilateria - ps16
Protostomia - ps17
Ecdysozoa - ps18
Arthropoda - ps19
Mandibulata - ps20
Pancrustacea - ps21
Insecta - ps22
Eumetabola - ps23
Holometabola/Psocodea - ps24
Holometabola - ps25
Aparaglossata - ps26
Mecopterida - ps27
Diptera - ps28
Bibiono./Cyclorrhapha - ps29
Cyclorrhapha - ps30
Calyptratae/Drosophila - ps31
Drosophila - ps32
Sophophora - ps33
Obscura/Melanogaster_group - ps34
D_melanogaster_group - ps35
D_melanogaster_subgroup - ps36
sim./sech./mel. group - ps37
Drosophila_melanogaster - ps38
Phylostratum

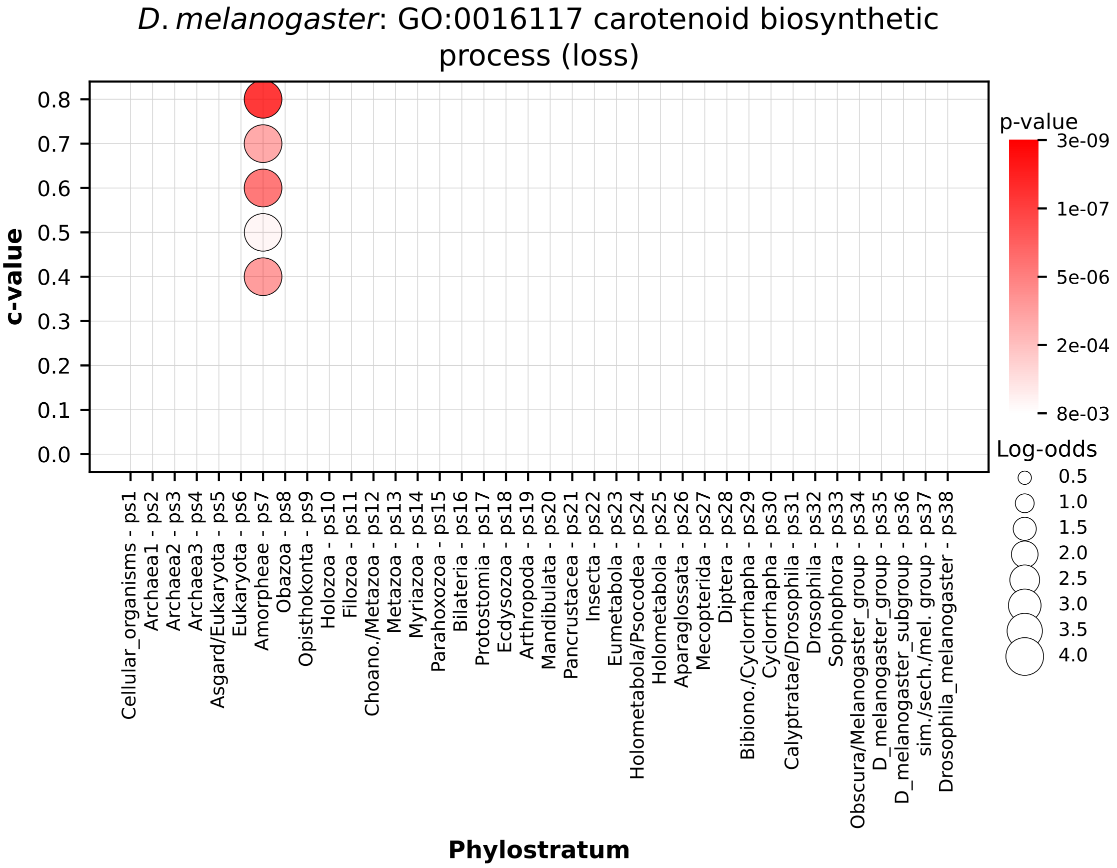

D. melanogaster: GO:0016117 carotenoid biosynthetic process (loss)
c-value
0.8
0.7
0.6
0.5
0.4
0.3
0.2
0.1
0.0
Phylostratum
Cellular_organisms - ps1
Archaea1 - ps2
Archaea2 - ps3
Archaea3 - ps4
Asgard/Eukaryota - ps5
Eukaryota - ps6
Amorpheae - ps7
Obazoa - ps8
Opisthokonta - ps9
Holozoa - ps10
Filozoa - ps11
Choano./Metazoa - ps12
Metazoa - ps13
Myriazoa - ps14
Parahoxozoa - ps15
Bilateria - ps16
Protostomia - ps17
Ecdysozoa - ps18
Arthropoda - ps19
Mandibulata - ps20
Pancrustacea - ps21
Insecta - ps22
Eumetabola - ps23
Holometabola/Psocodea - ps24
Holometabola - ps25
Aparaglossata - ps26
Mecopterida - ps27
Diptera - ps28
Bibiono./Cyclorrhapha - ps29
Cyclorrhapha - ps30
Calyptratae/Drosophila - ps31
Drosophila - ps32
Sophophora - ps33
Obscura/Melanogaster_group - ps34
D_melanogaster_group - ps35
D_melanogaster_subgroup - ps36
sim./sech./mel. group - ps37
Drosophila_melanogaster - ps38
p-value
3e-09
1e-07
5e-06
2e-04
8e-03
Log-odds
0.5
1.0
1.5
2.0
2.5
3.0
3.5
4.0

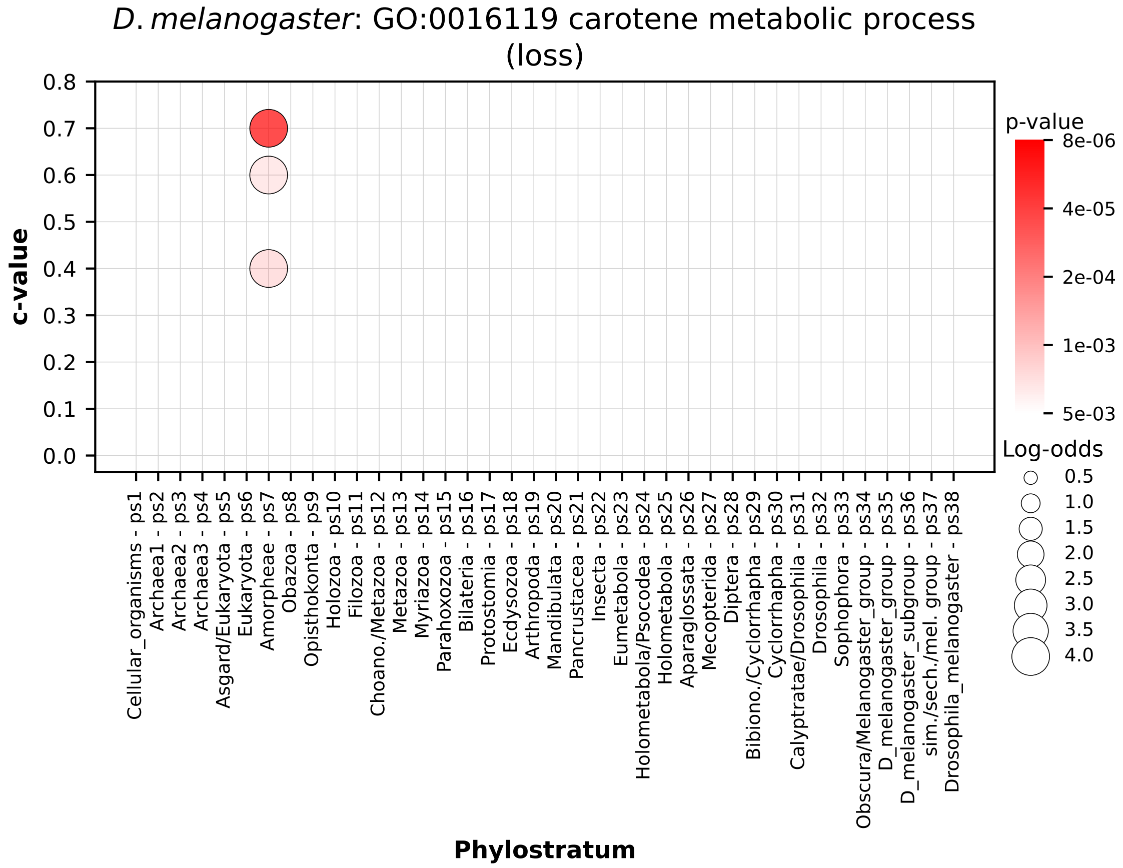

D. melanogaster: GO:0016119 carotene metabolic process (loss)
c-value
0.8
0.7
0.6
0.5
0.4
0.3
0.2
0.1
0.0
p-value
8e-06
4e-05
2e-04
1e-03
5e-03
Log-odds
0.5
1.0
1.5
2.0
2.5
3.0
3.5
4.0
Cellular_organisms - ps1
Archaea1 - ps2
Archaea2 - ps3
Archaea3 - ps4
Asgard/Eukaryota - ps5
Eukaryota - ps6
Amorpheae - ps7
Obazoa - ps8
Opisthokonta - ps9
Holozoa - ps10
Filozoa - ps11
Choano./Metazoa - ps12
Metazoa - ps13
Myriazoa - ps14
Parahoxozoa - ps15
Bilateria - ps16
Protostomia - ps17
Ecdysozoa - ps18
Arthropoda - ps19
Mandibulata - ps20
Pancrustacea - ps21
Insecta - ps22
Eumetabola - ps23
Holometabola/Psocodea - ps24
Holometabola - ps25
Aparaglossata - ps26
Mecopterida - ps27
Diptera - ps28
Bibiono./Cyclorrhapha - ps29
Cyclorrhapha - ps30
Calyptratae/Drosophila - ps31
Drosophila - ps32
Sophophora - ps33
Obscura/Melanogaster_group - ps34
D_melanogaster_group - ps35
D_melanogaster_subgroup - ps36
sim./sech./mel. group - ps37
Drosophila_melanogaster - ps38
Phylostratum

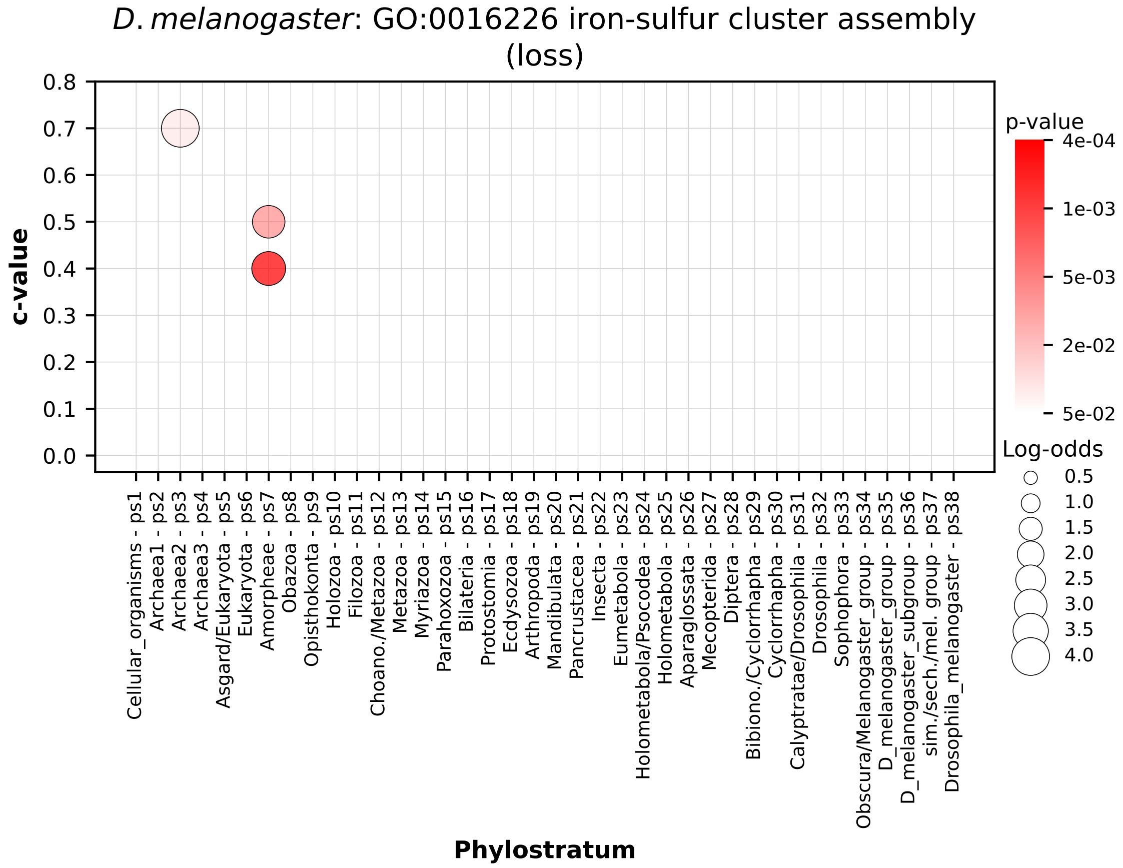

D. melanogaster: GO:0016226 iron-sulfur cluster assembly (loss)
c-value
0.8
0.7
0.6
0.5
0.4
0.3
0.2
0.1
0.0
Phylostratum
Cellular_organisms - ps1
Archaea1 - ps2
Archaea2 - ps3
Archaea3 - ps4
Asgard/Eukaryota - ps5
Eukaryota - ps6
Amorpheae - ps7
Obazoa - ps8
Opisthokonta - ps9
Holozoa - ps10
Filozoa - ps11
Choano./Metazoa - ps12
Metazoa - ps13
Myriazoa - ps14
Parahoxozoa - ps15
Bilateria - ps16
Protostomia - ps17
Ecdysozoa - ps18
Arthropoda - ps19
Mandibulata - ps20
Pancrustacea - ps21
Insecta - ps22
Eumetabola - ps23
Holometabola/Psocodea - ps24
Holometabola - ps25
Aparaglossata - ps26
Mecopterida - ps27
Diptera - ps28
Bibiono./Cyclorrhapha - ps29
Cyclorrhapha - ps30
Calyptratae/Drosophila - ps31
Drosophila - ps32
Sophophora - ps33
Obscura/Melanogaster_group - ps34
D_melanogaster_group - ps35
D_melanogaster_subgroup - ps36
sim./sech./mel. group - ps37
Drosophila_melanogaster - ps38
p-value
4e-04
1e-03
5e-03
2e-02
5e-02
Log-odds
0.5
1.0
1.5
2.0
2.5
3.0
3.5
4.0

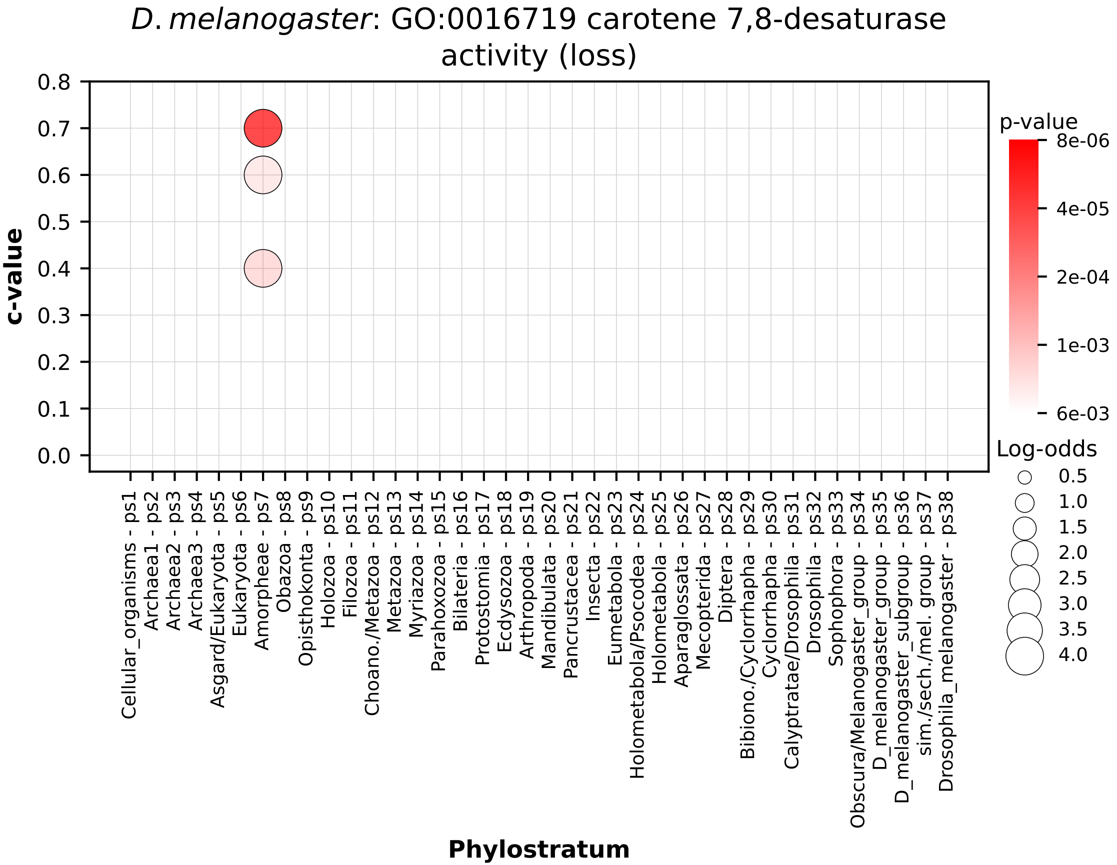

D. melanogaster: GO:0016719 carotene 7,8-desaturase activity (loss)
c-value
0.8
0.7
0.6
0.5
0.4
0.3
0.2
0.1
0.0
Phylostratum
Cellular_organisms - ps1
Archaea1 - ps2
Archaea2 - ps3
Archaea3 - ps4
Asgard/Eukaryota - ps5
Eukaryota - ps6
Amorpheae - ps7
Obazoa - ps8
Opisthokonta - ps9
Holozoa - ps10
Filozoa - ps11
Choano./Metazoa - ps12
Metazoa - ps13
Myriazoa - ps14
Parahoxozoa - ps15
Bilateria - ps16
Protostomia - ps17
Ecdysozoa - ps18
Arthropoda - ps19
Mandibulata - ps20
Pancrustacea - ps21
Insecta - ps22
Eumetabola - ps23
Holometabola/Psocodea - ps24
Holometabola - ps25
Aparaglossata - ps26
Mecopterida - ps27
Diptera - ps28
Bibiono./Cyclorrhapha - ps29
Cyclorrhapha - ps30
Calyptratae/Drosophila - ps31
Drosophila - ps32
Sophophora - ps33
Obscura/Melanogaster_group - ps34
D_melanogaster_group - ps35
D_melanogaster_subgroup - ps36
sim./sech./mel. group - ps37
Drosophila_melanogaster - ps38
p-value
8e-06
4e-05
2e-04
1e-03
6e-03
Log-odds
0.5
1.0
1.5
2.0
2.5
3.0
3.5
4.0

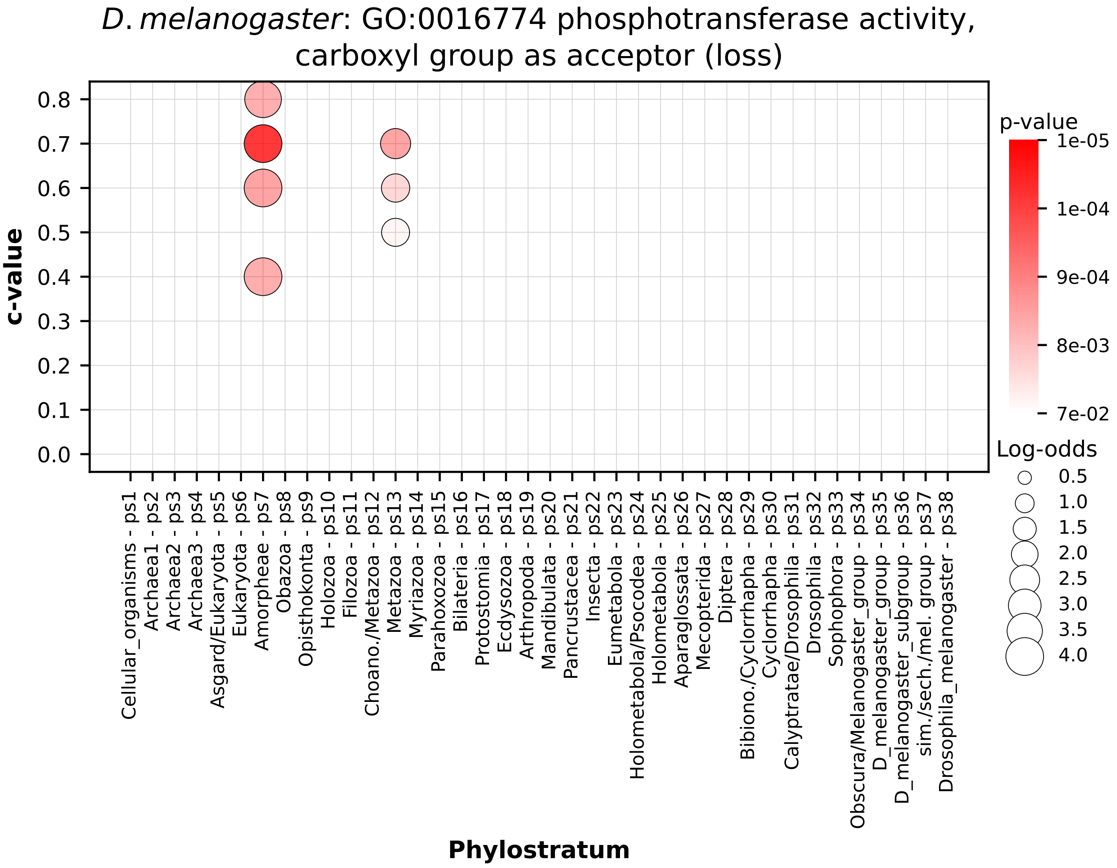
D. melanogaster: GO:0016774 phosphotransferase activity, carboxyl group as acceptor (loss)
c-value
0.8
0.7
0.6
0.5
0.4
0.3
0.2
0.1
0.0
Phylostratum
Cellular_organisms - ps1
Archaea1 - ps2
Archaea2 - ps3
Archaea3 - ps4
Asgard/Eukaryota - ps5
Eukaryota - ps6
Amorpheae - ps7
Obazoa - ps8
Opisthokonta - ps9
Holozoa - ps10
Filozoa - ps11
Choano./Metazoa - ps12
Metazoa - ps13
Myriazoa - ps14
Parahoxozoa - ps15
Bilateria - ps16
Protostomia - ps17
Ecdysozoa - ps18
Arthropoda - ps19
Mandibulata - ps20
Pancrustacea - ps21
Insecta - ps22
Eumetabola - ps23
Holometabola/Psocodea - ps24
Holometabola - ps25
Aparaglossata - ps26
Mecopterida - ps27
Diptera - ps28
Bibiono./Cyclorrhapha - ps29
Cyclorrhapha - ps30
Calyptratae/Drosophila - ps31
Drosophila - ps32
Sophophora - ps33
Obscura/Melanogaster_group - ps34
D_melanogaster_group - ps35
D_melanogaster_subgroup - ps36
sim./sech./mel. group - ps37
Drosophila_melanogaster - ps38
p-value
1e-05
1e-04
9e-04
8e-03
7e-02
Log-odds
0.5
1.0
1.5
2.0
2.5
3.0
3.5
4.0

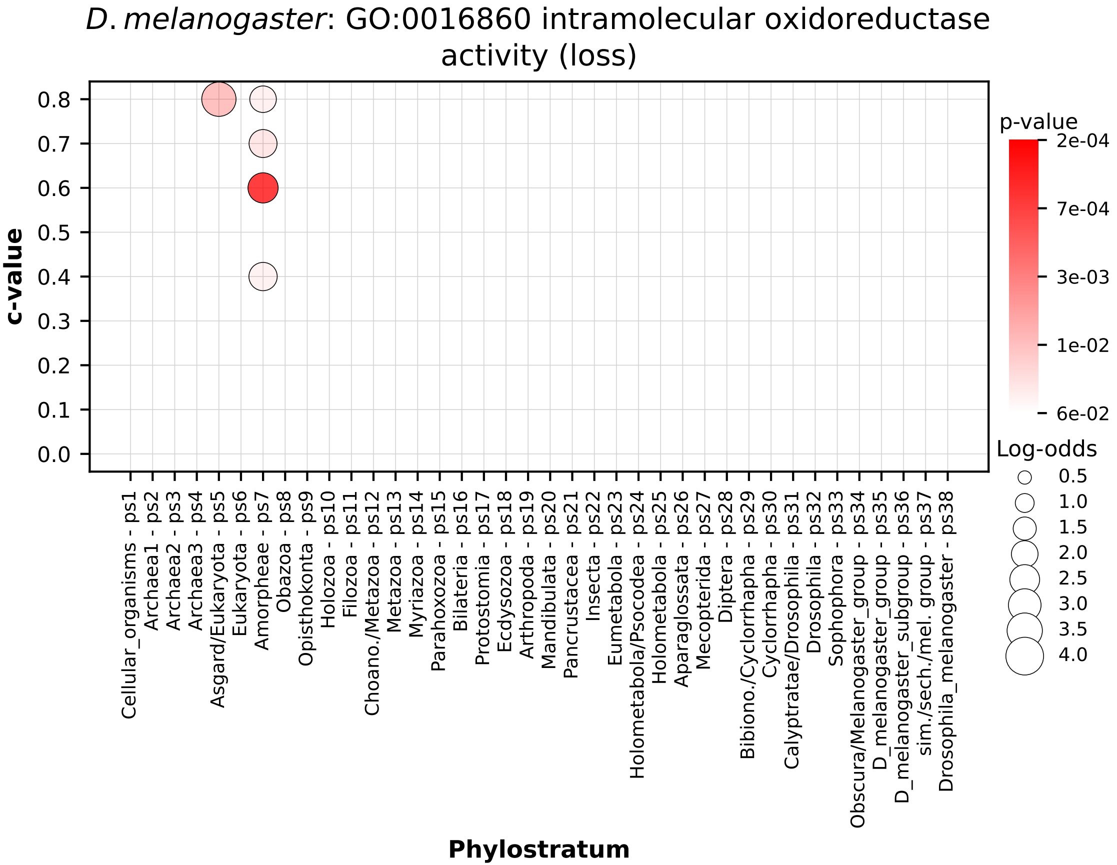

D. melanogaster: GO:0016860 intramolecular oxidoreductase activity (loss)
c-value
0.0
0.1
0.2
0.3
0.4
0.5
0.6
0.7
0.8
Phylostratum
Cellular_organisms - ps1
Archaea1 - ps2
Archaea2 - ps3
Archaea3 - ps4
Asgard/Eukaryota - ps5
Eukaryota - ps6
Amorpheae - ps7
Obazoa - ps8
Opisthokonta - ps9
Holozoa - ps10
Filozoa - ps11
Choano./Metazoa - ps12
Metazoa - ps13
Myriazoa - ps14
Parahoxozoa - ps15
Bilateria - ps16
Protostomia - ps17
Ecdysozoa - ps18
Arthropoda - ps19
Mandibulata - ps20
Pancrustacea - ps21
Insecta - ps22
Eumetabola - ps23
Holometabola/Psocodea - ps24
Holometabola - ps25
Aparaglossata - ps26
Mecopterida - ps27
Diptera - ps28
Bibiono./Cyclorrhapha - ps29
Cyclorrhapha - ps30
Calyptratae/Drosophila - ps31
Drosophila - ps32
Sophophora - ps33
Obscura/Melanogaster_group - ps34
D_melanogaster_group - ps35
D_melanogaster_subgroup - ps36
sim./sech./mel. group - ps37
Drosophila_melanogaster - ps38
p-value
2e-04
7e-04
3e-03
1e-02
6e-02
Log-odds
0.5
1.0
1.5
2.0
2.5
3.0
3.5
4.0

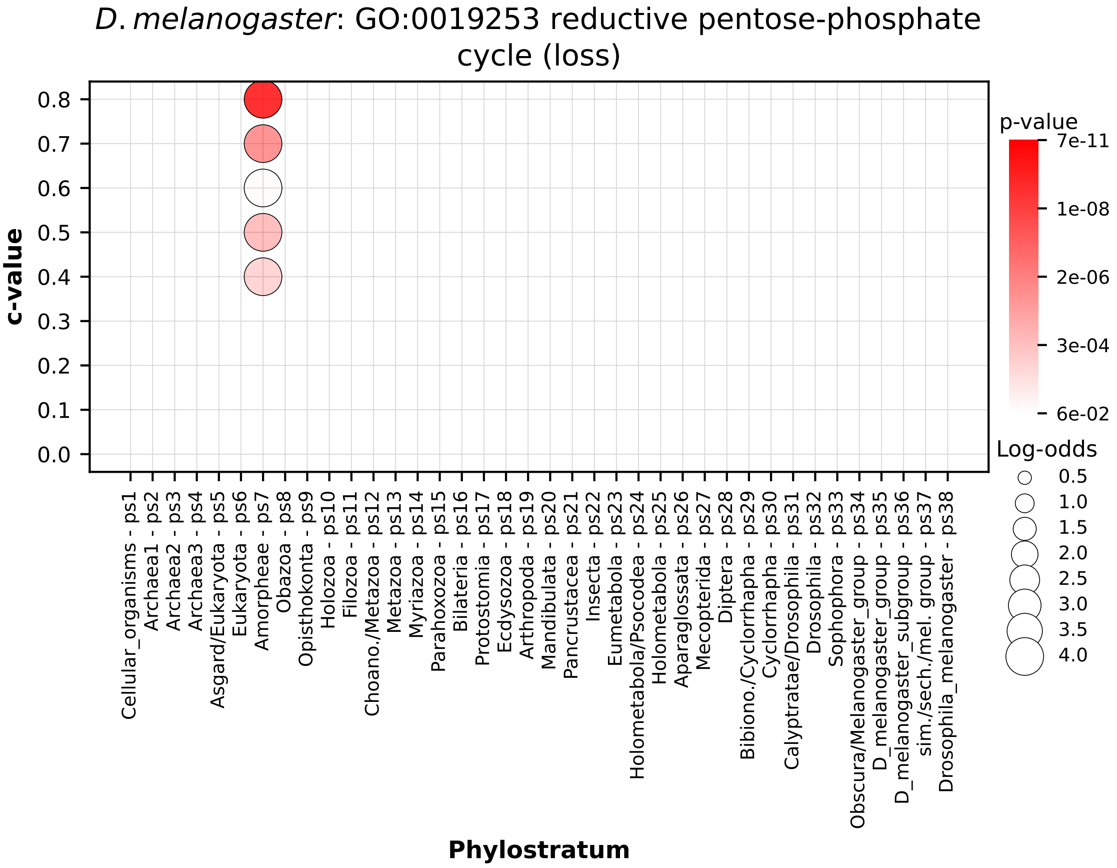

D. melanogaster: GO:0019253 reductive pentose-phosphate cycle (loss)
c-value
0.8
0.7
0.6
0.5
0.4
0.3
0.2
0.1
0.0
Phylostratum
Cellular_organisms - ps1
Archaea1 - ps2
Archaea2 - ps3
Archaea3 - ps4
Asgard/Eukaryota - ps5
Eukaryota - ps6
Amorpheae - ps7
Obazoa - ps8
Opisthokonta - ps9
Holozoa - ps10
Filozoa - ps11
Choano./Metazoa - ps12
Metazoa - ps13
Myriazoa - ps14
Parahoxozoa - ps15
Bilateria - ps16
Protostomia - ps17
Ecdysozoa - ps18
Arthropoda - ps19
Mandibulata - ps20
Pancrustacea - ps21
Insecta - ps22
Eumetabola - ps23
Holometabola/Psocodea - ps24
Holometabola - ps25
Aparaglossata - ps26
Mecopterida - ps27
Diptera - ps28
Bibiono./Cyclorrhapha - ps29
Cyclorrhapha - ps30
Calyptratae/Drosophila - ps31
Drosophila - ps32
Sophophora - ps33
Obscura/Melanogaster_group - ps34
D_melanogaster_group - ps35
D_melanogaster_subgroup - ps36
sim./sech./mel. group - ps37
Drosophila_melanogaster - ps38
p-value
7e-11
1e-08
2e-06
3e-04
6e-02
Log-odds
0.5
1.0
1.5
2.0
2.5
3.0
3.5
4.0

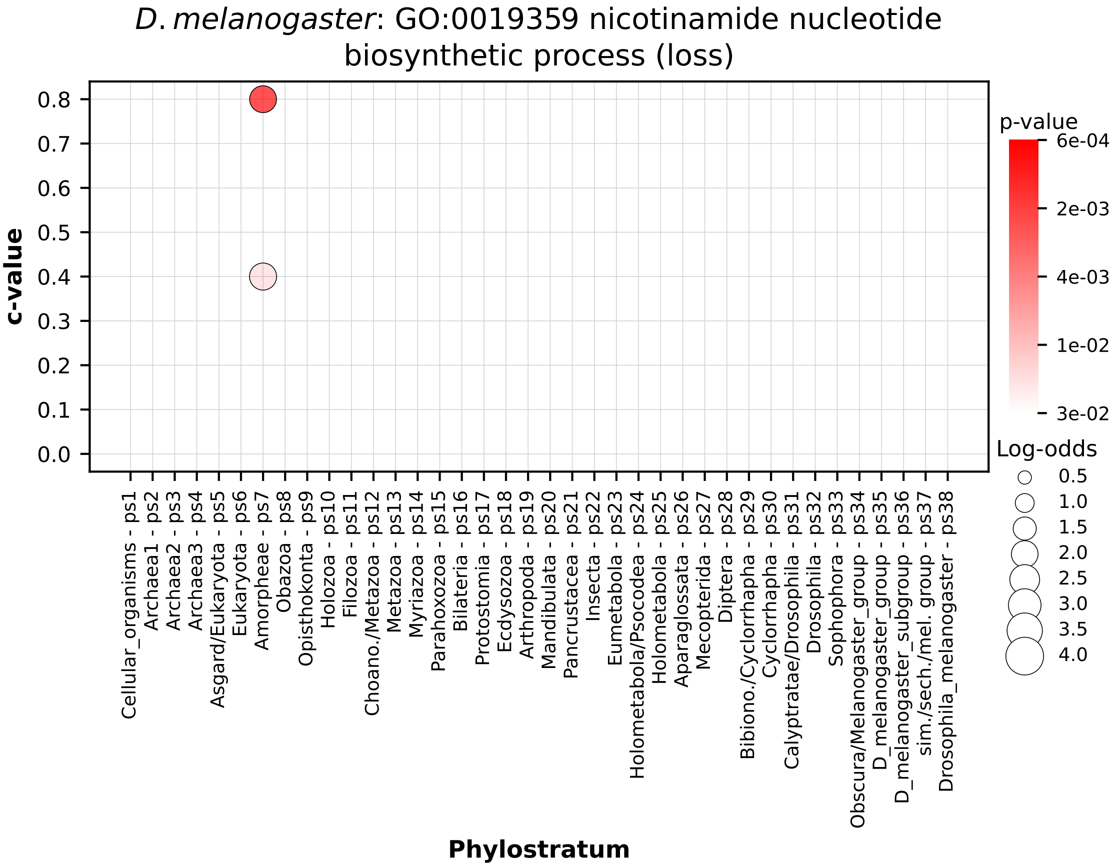

D. melanogaster: GO:0019359 nicotinamide nucleotide biosynthetic process (loss)
c-value
0.0
0.1
0.2
0.3
0.4
0.5
0.6
0.7
0.8
Phylostratum
Cellular_organisms - ps1
Archaea1 - ps2
Archaea2 - ps3
Archaea3 - ps4
Asgard/Eukaryota - ps5
Eukaryota - ps6
Amorpheae - ps7
Obazoa - ps8
Opisthokonta - ps9
Holozoa - ps10
Filozoa - ps11
Choano./Metazoa - ps12
Metazoa - ps13
Myriazoa - ps14
Parahoxozoa - ps15
Bilateria - ps16
Protostomia - ps17
Ecdysozoa - ps18
Arthropoda - ps19
Mandibulata - ps20
Pancrustacea - ps21
Insecta - ps22
Eumetabola - ps23
Holometabola/Psocodea - ps24
Holometabola - ps25
Aparaglossata - ps26
Mecopterida - ps27
Diptera - ps28
Bibiono./Cyclorrhapha - ps29
Cyclorrhapha - ps30
Calyptratae/Drosophila - ps31
Drosophila - ps32
Sophophora - ps33
Obscura/Melanogaster_group - ps34
D_melanogaster_group - ps35
D_melanogaster_subgroup - ps36
sim./sech./mel. group - ps37
Drosophila_melanogaster - ps38
p-value
6e-04
2e-03
4e-03
1e-02
3e-02
Log-odds
0.5
1.0
1.5
2.0
2.5
3.0
3.5
4.0

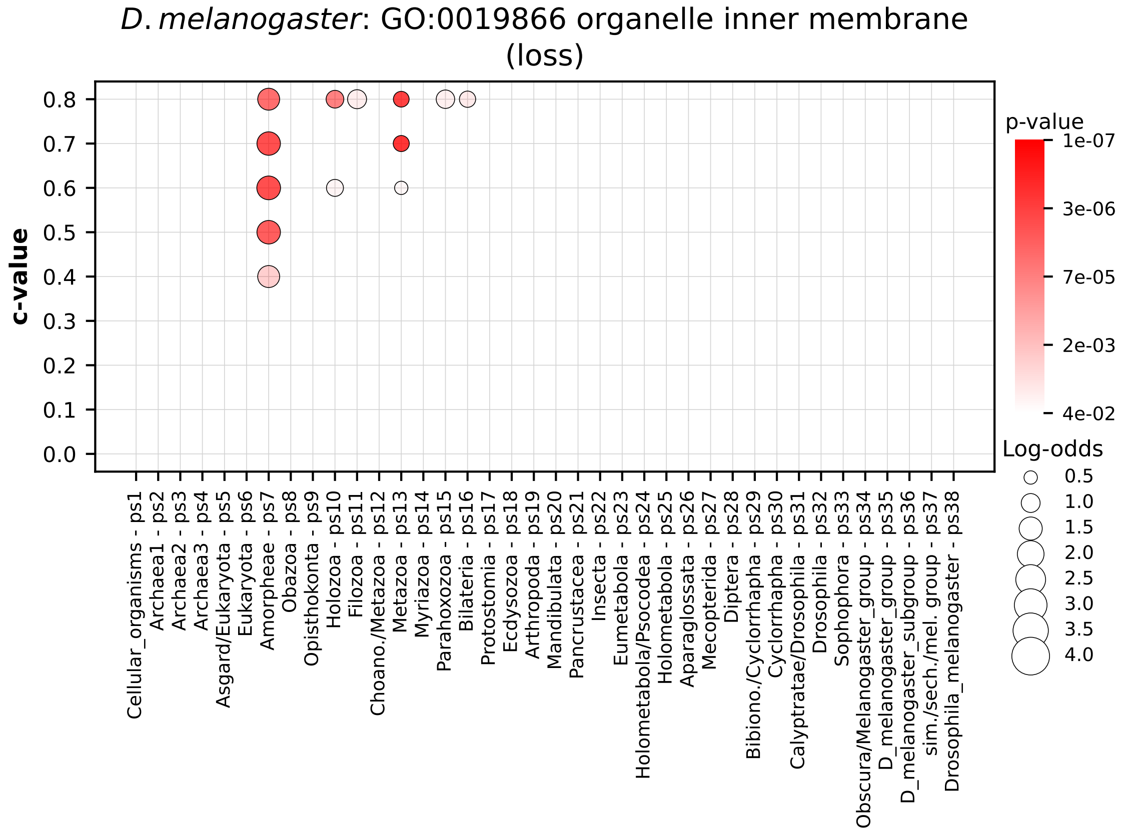

D. melanogaster: GO:0019866 organelle inner membrane (loss)
c-value
0.8
0.7
0.6
0.5
0.4
0.3
0.2
0.1
0.0
p-value
1e-07
3e-06
7e-05
2e-03
4e-02
Log-odds
0.5
1.0
1.5
2.0
2.5
3.0
3.5
4.0
Cellular_organisms - ps1
Archaea1 - ps2
Archaea2 - ps3
Archaea3 - ps4
Asgard/Eukaryota - ps5
Eukaryota - ps6
Amorpheae - ps7
Obazoa - ps8
Opisthokonta - ps9
Holozoa - ps10
Filozoa - ps11
Choano./Metazoa - ps12
Metazoa - ps13
Myriazoa - ps14
Parahoxozoa - ps15
Bilateria - ps16
Protostomia - ps17
Ecdysozoa - ps18
Arthropoda - ps19
Mandibulata - ps20
Pancrustacea - ps21
Insecta - ps22
Eumetabola - ps23
Holometabola/Psocodea - ps24
Holometabola - ps25
Aparaglossata - ps26
Mecopterida - ps27
Diptera - ps28
Bibiono./Cyclorrhapha - ps29
Cyclorrhapha - ps30
Calyptratae/Drosophila - ps31
Drosophila - ps32
Sophophora - ps33
Obscura/Melanogaster_group - ps34
D_melanogaster_group - ps35
D_melanogaster_subgroup - ps36
sim./sech./mel. group - ps37
Drosophila_melanogaster - ps38
Phylostratum

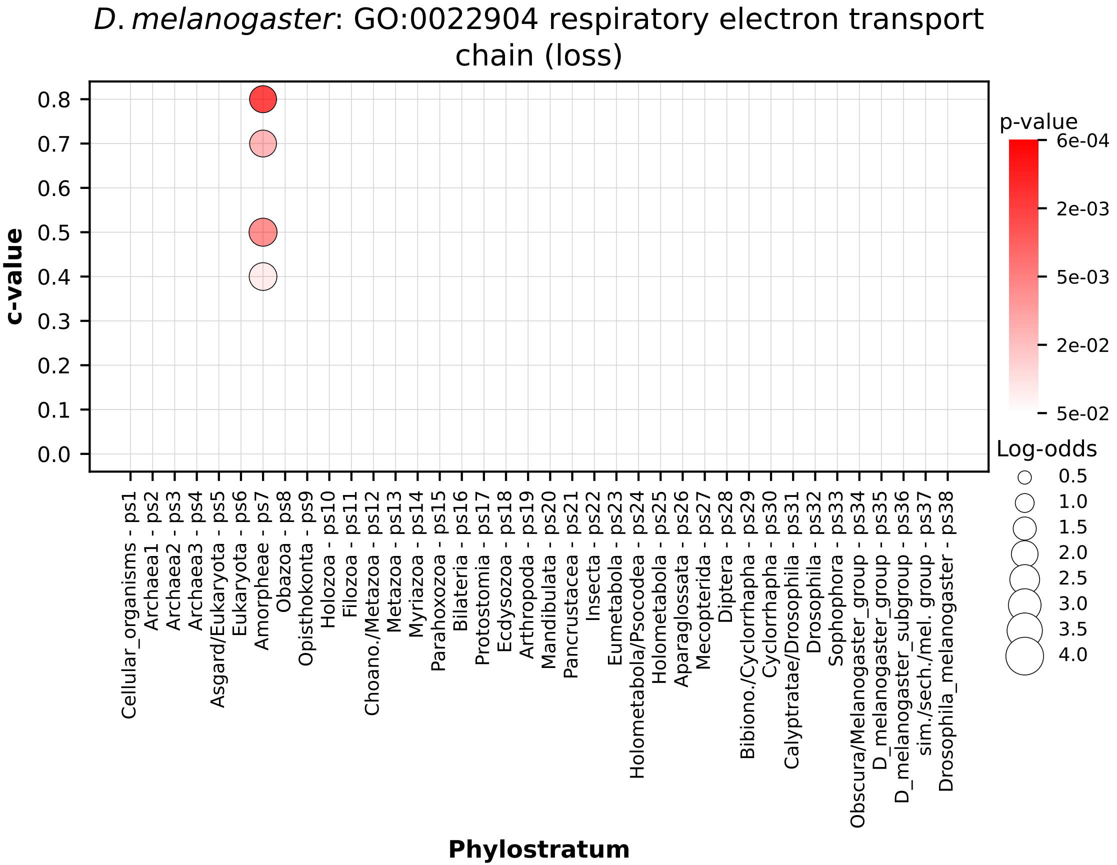

D. melanogaster: GO:0022904 respiratory electron transport chain (loss)
c-value
0.8
0.7
0.6
0.5
0.4
0.3
0.2
0.1
0.0
Phylostratum
Cellular_organisms - ps1
Archaea1 - ps2
Archaea2 - ps3
Archaea3 - ps4
Asgard/Eukaryota - ps5
Eukaryota - ps6
Amorpheae - ps7
Obazoa - ps8
Opisthokonta - ps9
Holozoa - ps10
Filozoa - ps11
Choano./Metazoa - ps12
Metazoa - ps13
Myriazoa - ps14
Parahoxozoa - ps15
Bilateria - ps16
Protostomia - ps17
Ecdysozoa - ps18
Arthropoda - ps19
Mandibulata - ps20
Pancrustacea - ps21
Insecta - ps22
Eumetabola - ps23
Holometabola/Psocodea - ps24
Holometabola - ps25
Aparaglossata - ps26
Mecopterida - ps27
Diptera - ps28
Bibiono./Cyclorrhapha - ps29
Cyclorrhapha - ps30
Calyptratae/Drosophila - ps31
Drosophila - ps32
Sophophora - ps33
Obscura/Melanogaster_group - ps34
D_melanogaster_group - ps35
D_melanogaster_subgroup - ps36
sim./sech./mel. group - ps37
Drosophila_melanogaster - ps38
p-value
6e-04
2e-03
5e-03
2e-02
5e-02
Log-odds
0.5
1.0
1.5
2.0
2.5
3.0
3.5
4.0

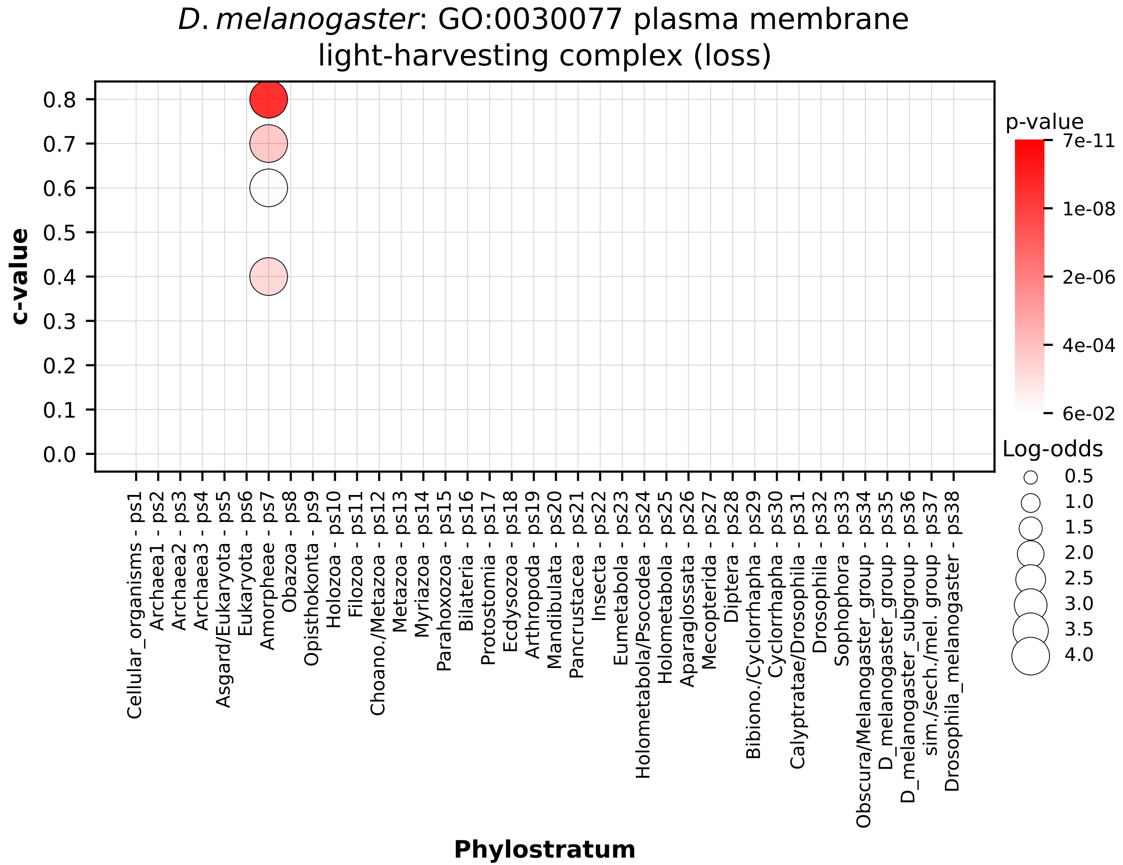

D. melanogaster: GO:0030077 plasma membrane light-harvesting complex (loss)
c-value
0.8
0.7
0.6
0.5
0.4
0.3
0.2
0.1
0.0
p-value
7e-11
1e-08
2e-06
4e-04
6e-02
Log-odds
0.5
1.0
1.5
2.0
2.5
3.0
3.5
4.0
Cellular_organisms - ps1
Archaea1 - ps2
Archaea2 - ps3
Archaea3 - ps4
Asgard/Eukaryota - ps5
Eukaryota - ps6
Amorpheae - ps7
Obazoa - ps8
Opisthokonta - ps9
Holozoa - ps10
Filozoa - ps11
Choano./Metazoa - ps12
Metazoa - ps13
Myriazoa - ps14
Parahoxozoa - ps15
Bilateria - ps16
Protostomia - ps17
Ecdysozoa - ps18
Arthropoda - ps19
Mandibulata - ps20
Pancrustacea - ps21
Insecta - ps22
Eumetabola - ps23
Holometabola/Psocodea - ps24
Holometabola - ps25
Aparaglossata - ps26
Mecopterida - ps27
Diptera - ps28
Bibiono./Cyclorrhapha - ps29
Cyclorrhapha - ps30
Calyptratae/Drosophila - ps31
Drosophila - ps32
Sophophora - ps33
Obscura/Melanogaster_group - ps34
D_melanogaster_group - ps35
D_melanogaster_subgroup - ps36
sim./sech./mel. group - ps37
Drosophila_melanogaster - ps38
Phylostratum

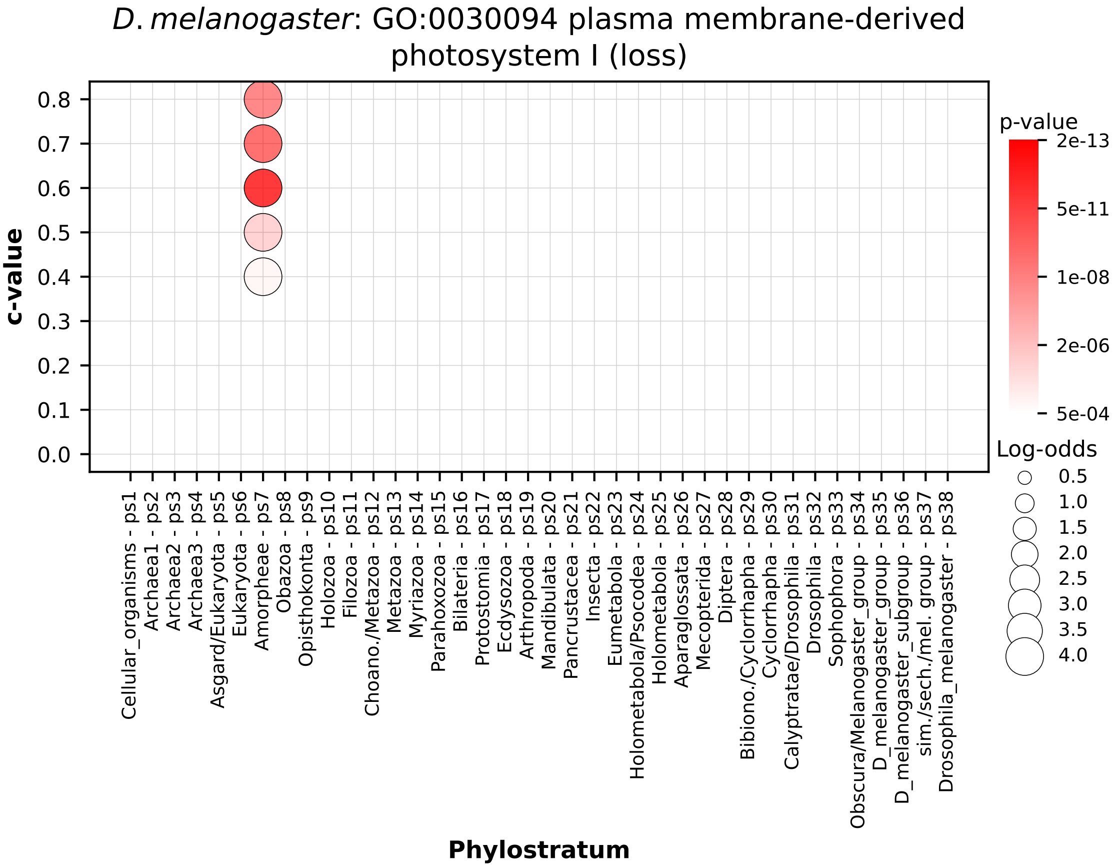

D. melanogaster: GO:0030094 plasma membrane-derived photosystem I (loss)
c-value
0.0
0.1
0.2
0.3
0.4
0.5
0.6
0.7
0.8
Phylostratum
Cellular_organisms - ps1
Archaea1 - ps2
Archaea2 - ps3
Archaea3 - ps4
Asgard/Eukaryota - ps5
Eukaryota - ps6
Amorpheae - ps7
Obazoa - ps8
Opisthokonta - ps9
Holozoa - ps10
Filozoa - ps11
Choano./Metazoa - ps12
Metazoa - ps13
Myriazoa - ps14
Parahoxozoa - ps15
Bilateria - ps16
Protostomia - ps17
Ecdysozoa - ps18
Arthropoda - ps19
Mandibulata - ps20
Pancrustacea - ps21
Insecta - ps22
Eumetabola - ps23
Holometabola/Psocodea - ps24
Holometabola - ps25
Aparaglossata - ps26
Mecopterida - ps27
Diptera - ps28
Bibiono./Cyclorrhapha - ps29
Cyclorrhapha - ps30
Calyptratae/Drosophila - ps31
Drosophila - ps32
Sophophora - ps33
Obscura/Melanogaster_group - ps34
D_melanogaster_group - ps35
D_melanogaster_subgroup - ps36
sim./sech./mel. group - ps37
Drosophila_melanogaster - ps38
p-value
2e-13
5e-11
1e-08
2e-06
5e-04
Log-odds
0.5
1.0
1.5
2.0
2.5
3.0
3.5
4.0

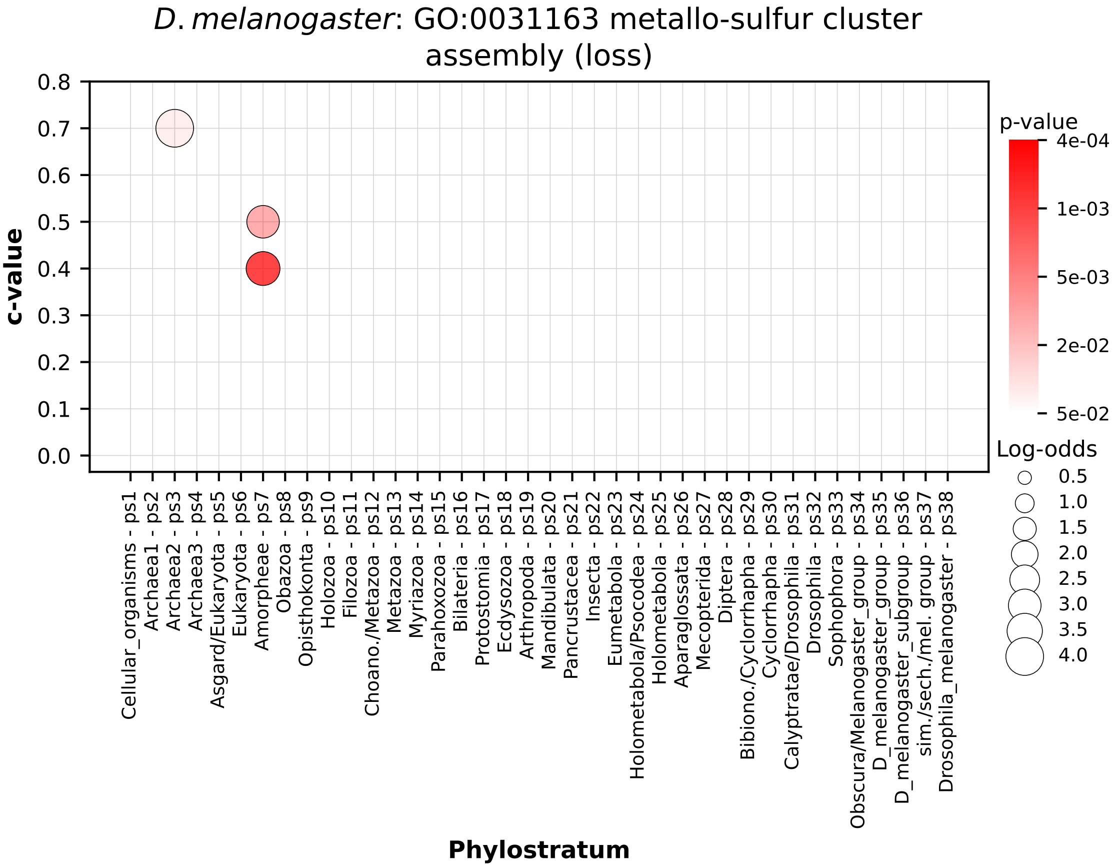

D. melanogaster: GO:0031163 metallo-sulfur cluster assembly (loss)
c-value
0.0
0.1
0.2
0.3
0.4
0.5
0.6
0.7
0.8
Phylostratum
Cellular_organisms - ps1
Archaea1 - ps2
Archaea2 - ps3
Archaea3 - ps4
Asgard/Eukaryota - ps5
Eukaryota - ps6
Amorpheae - ps7
Obazoa - ps8
Opisthokonta - ps9
Holozoa - ps10
Filozoa - ps11
Choano./Metazoa - ps12
Metazoa - ps13
Myriazoa - ps14
Parahoxozoa - ps15
Bilateria - ps16
Protostomia - ps17
Ecdysozoa - ps18
Arthropoda - ps19
Mandibulata - ps20
Pancrustacea - ps21
Insecta - ps22
Eumetabola - ps23
Holometabola/Psocodea - ps24
Holometabola - ps25
Aparaglossata - ps26
Mecopterida - ps27
Diptera - ps28
Bibiono./Cyclorrhapha - ps29
Cyclorrhapha - ps30
Calyptratae/Drosophila - ps31
Drosophila - ps32
Sophophora - ps33
Obscura/Melanogaster_group - ps34
D_melanogaster_group - ps35
D_melanogaster_subgroup - ps36
sim./sech./mel. group - ps37
Drosophila_melanogaster - ps38
p-value
4e-04
1e-03
5e-03
2e-02
5e-02
Log-odds
0.5
1.0
1.5
2.0
2.5
3.0
3.5
4.0

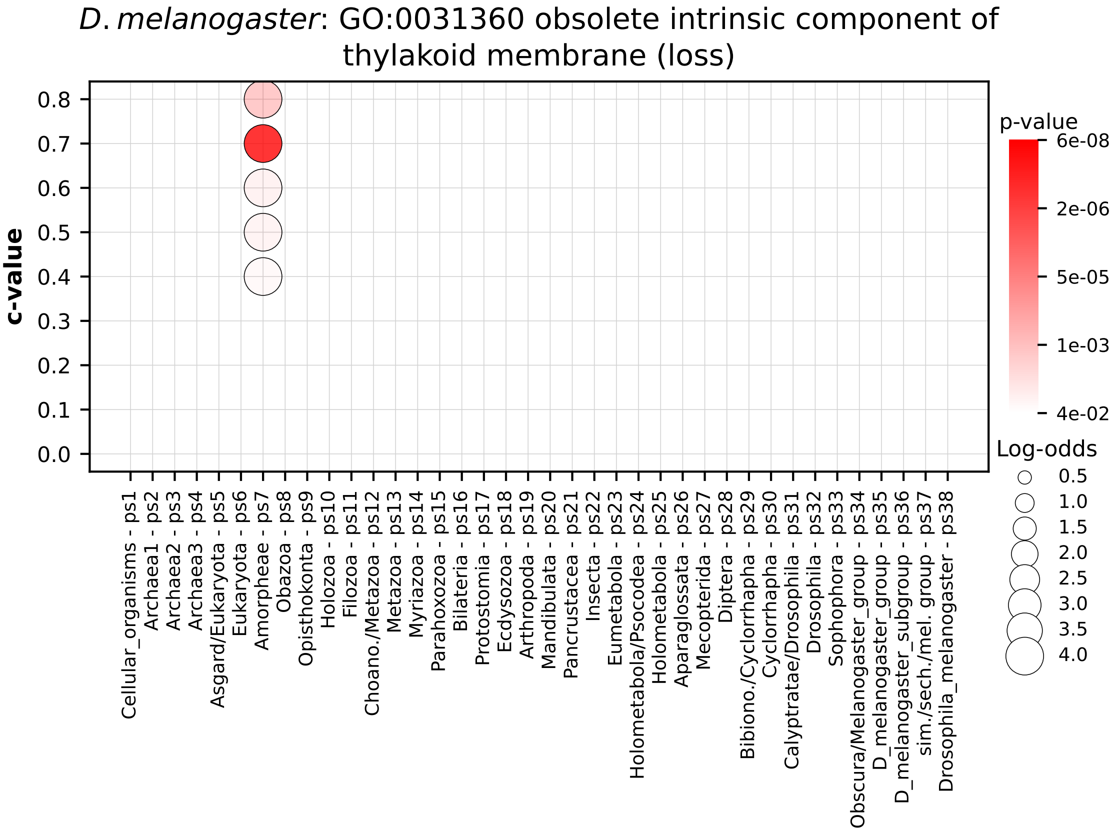

D. melanogaster: GO:0031360 obsolete intrinsic component of thylakoid membrane (loss)
c-value
0.0
0.1
0.2
0.3
0.4
0.5
0.6
0.7
0.8
Phylostratum
Cellular_organisms - ps1
Archaea1 - ps2
Archaea2 - ps3
Archaea3 - ps4
Asgard/Eukaryota - ps5
Eukaryota - ps6
Amorpheae - ps7
Obazoa - ps8
Opisthokonta - ps9
Holozoa - ps10
Filozoa - ps11
Choano./Metazoa - ps12
Metazoa - ps13
Myriazoa - ps14
Parahoxozoa - ps15
Bilateria - ps16
Protostomia - ps17
Ecdysozoa - ps18
Arthropoda - ps19
Mandibulata - ps20
Pancrustacea - ps21
Insecta - ps22
Eumetabola - ps23
Holometabola/Psocodea - ps24
Holometabola - ps25
Aparaglossata - ps26
Mecopterida - ps27
Diptera - ps28
Bibiono./Cyclorrhapha - ps29
Cyclorrhapha - ps30
Calyptratae/Drosophila - ps31
Drosophila - ps32
Sophophora - ps33
Obscura/Melanogaster_group - ps34
D_melanogaster_group - ps35
D_melanogaster_subgroup - ps36
sim./sech./mel. group - ps37
Drosophila_melanogaster - ps38
p-value
6e-08
2e-06
5e-05
1e-03
4e-02
Log-odds
0.5
1.0
1.5
2.0
2.5
3.0
3.5
4.0

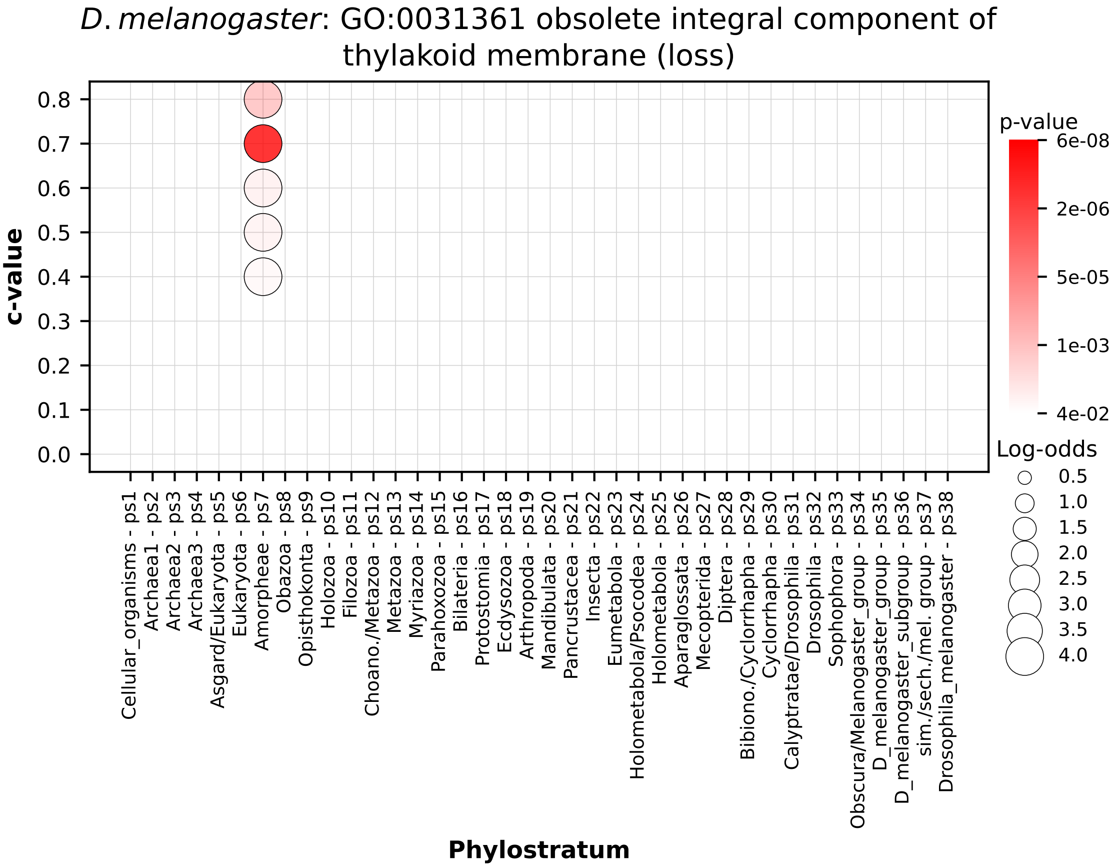

D. melanogaster: GO:0031361 obsolete integral component of thylakoid membrane (loss)
c-value
0.8
0.7
0.6
0.5
0.4
0.3
0.2
0.1
0.0
p-value
6e-08
2e-06
5e-05
1e-03
4e-02
Log-odds
0.5
1.0
1.5
2.0
2.5
3.0
3.5
4.0
Cellular_organisms - ps1
Archaea1 - ps2
Archaea2 - ps3
Archaea3 - ps4
Asgard/Eukaryota - ps5
Eukaryota - ps6
Amorpheae - ps7
Obazoa - ps8
Opisthokonta - ps9
Holozoa - ps10
Filozoa - ps11
Choano./Metazoa - ps12
Metazoa - ps13
Myriazoa - ps14
Parahoxozoa - ps15
Bilateria - ps16
Protostomia - ps17
Ecdysozoa - ps18
Arthropoda - ps19
Mandibulata - ps20
Pancrustacea - ps21
Insecta - ps22
Eumetabola - ps23
Holometabola/Psocodea - ps24
Holometabola - ps25
Aparaglossata - ps26
Mecopterida - ps27
Diptera - ps28
Bibiono./Cyclorrhapha - ps29
Cyclorrhapha - ps30
Calyptratae/Drosophila - ps31
Drosophila - ps32
Sophophora - ps33
Obscura/Melanogaster_group - ps34
D_melanogaster_group - ps35
D_melanogaster_subgroup - ps36
sim./sech./mel. group - ps37
Drosophila_melanogaster - ps38
Phylostratum

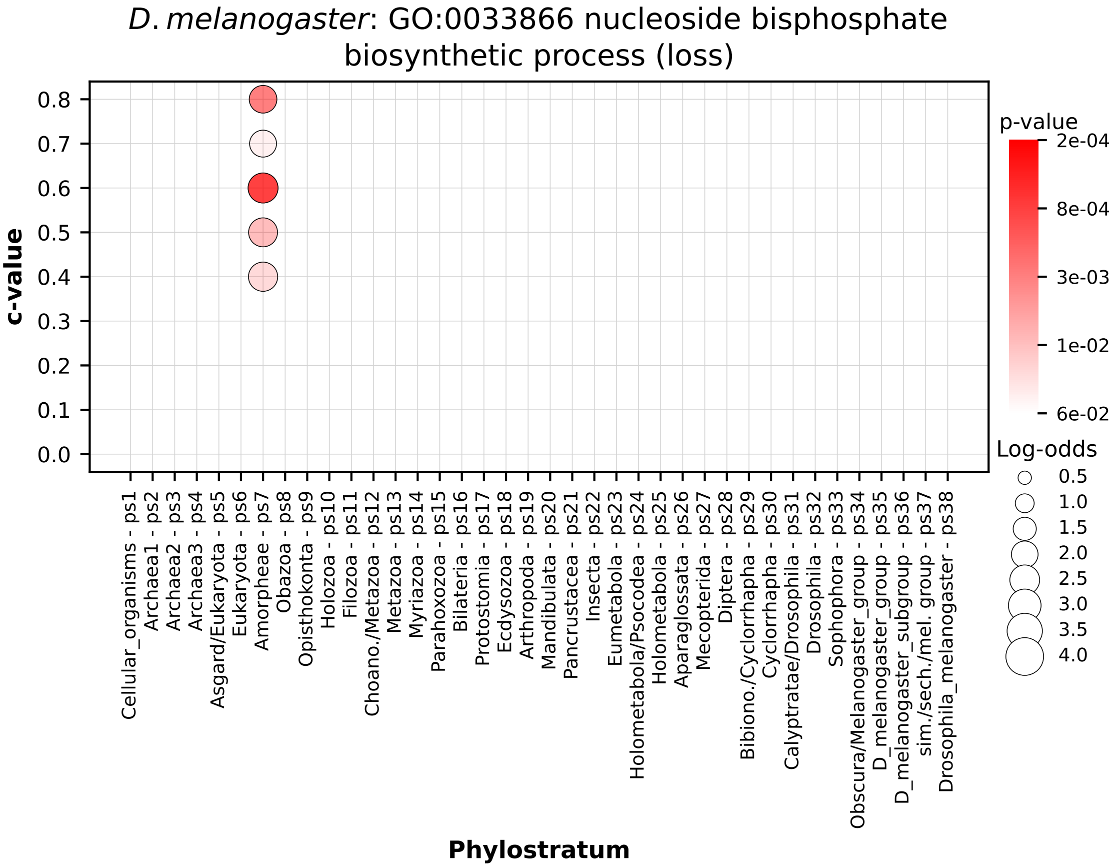
D. melanogaster: GO:0033866 nucleoside bisphosphate biosynthetic process (loss)
c-value
0.0
0.1
0.2
0.3
0.4
0.5
0.6
0.7
0.8
Phylostratum
Cellular_organisms - ps1
Archaea1 - ps2
Archaea2 - ps3
Archaea3 - ps4
Asgard/Eukaryota - ps5
Eukaryota - ps6
Amorpheae - ps7
Obazoa - ps8
Opisthokonta - ps9
Holozoa - ps10
Filozoa - ps11
Choano./Metazoa - ps12
Metazoa - ps13
Myriazoa - ps14
Parahoxozoa - ps15
Bilateria - ps16
Protostomia - ps17
Ecdysozoa - ps18
Arthropoda - ps19
Mandibulata - ps20
Pancrustacea - ps21
Insecta - ps22
Eumetabola - ps23
Holometabola/Psocodea - ps24
Holometabola - ps25
Aparaglossata - ps26
Mecopterida - ps27
Diptera - ps28
Bibiono./Cyclorrhapha - ps29
Cyclorrhapha - ps30
Calyptratae/Drosophila - ps31
Drosophila - ps32
Sophophora - ps33
Obscura/Melanogaster_group - ps34
D_melanogaster_group - ps35
D_melanogaster_subgroup - ps36
sim./sech./mel. group - ps37
Drosophila_melanogaster - ps38
p-value
2e-04
8e-04
3e-03
1e-02
6e-02
Log-odds
0.5
1.0
1.5
2.0
2.5
3.0
3.5
4.0

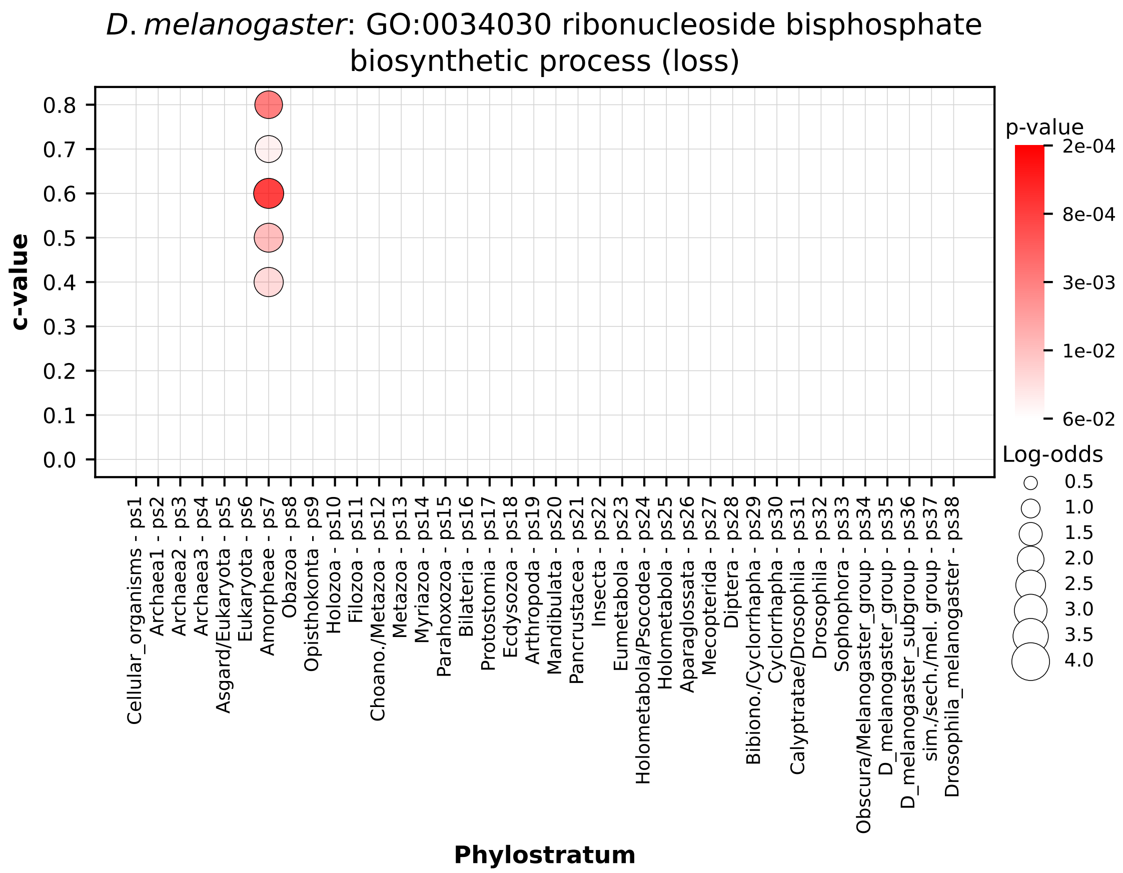

D. melanogaster: GO:0034030 ribonucleoside bisphosphate biosynthetic process (loss)
c-value
0.8
0.7
0.6
0.5
0.4
0.3
0.2
0.1
0.0
p-value
2e-04
8e-04
3e-03
1e-02
6e-02
Log-odds
0.5
1.0
1.5
2.0
2.5
3.0
3.5
4.0
Cellular_organisms - ps1
Archaea1 - ps2
Archaea2 - ps3
Archaea3 - ps4
Asgard/Eukaryota - ps5
Eukaryota - ps6
Amorpheae - ps7
Obazoa - ps8
Opisthokonta - ps9
Holozoa - ps10
Filozoa - ps11
Choano./Metazoa - ps12
Metazoa - ps13
Myriazoa - ps14
Parahoxozoa - ps15
Bilateria - ps16
Protostomia - ps17
Ecdysozoa - ps18
Arthropoda - ps19
Mandibulata - ps20
Pancrustacea - ps21
Insecta - ps22
Eumetabola - ps23
Holometabola/Psocodea - ps24
Holometabola - ps25
Aparaglossata - ps26
Mecopterida - ps27
Diptera - ps28
Bibiono./Cyclorrhapha - ps29
Cyclorrhapha - ps30
Calyptratae/Drosophila - ps31
Drosophila - ps32
Sophophora - ps33
Obscura/Melanogaster_group - ps34
D_melanogaster_group - ps35
D_melanogaster_subgroup - ps36
sim./sech./mel. group - ps37
Drosophila_melanogaster - ps38
Phylostratum

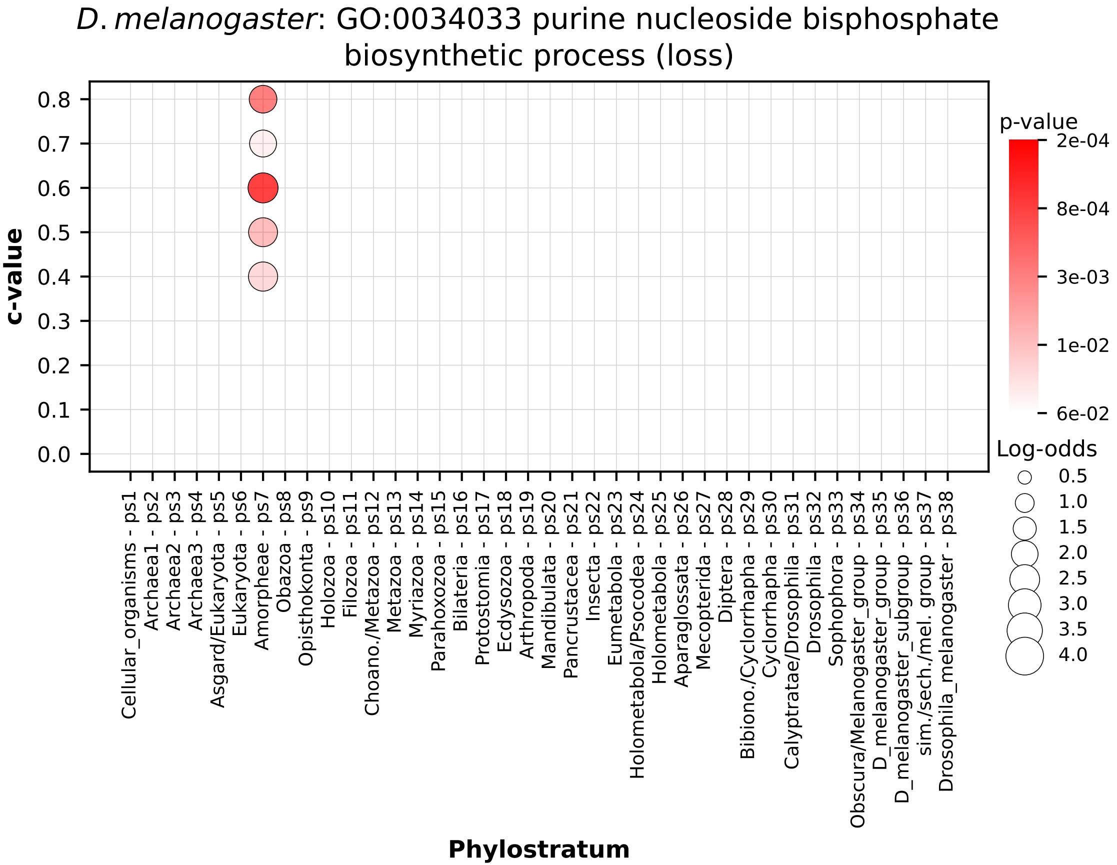

D. melanogaster: GO:0034033 purine nucleoside bisphosphate biosynthetic process (loss)
c-value
0.8
0.7
0.6
0.5
0.4
0.3
0.2
0.1
0.0
Phylostratum
Cellular_organisms - ps1
Archaea1 - ps2
Archaea2 - ps3
Archaea3 - ps4
Asgard/Eukaryota - ps5
Eukaryota - ps6
Amorpheae - ps7
Obazoa - ps8
Opisthokonta - ps9
Holozoa - ps10
Filozoa - ps11
Choano./Metazoa - ps12
Metazoa - ps13
Myriazoa - ps14
Parahoxozoa - ps15
Bilateria - ps16
Protostomia - ps17
Ecdysozoa - ps18
Arthropoda - ps19
Mandibulata - ps20
Pancrustacea - ps21
Insecta - ps22
Eumetabola - ps23
Holometabola/Psocodea - ps24
Holometabola - ps25
Aparaglossata - ps26
Mecopterida - ps27
Diptera - ps28
Bibiono./Cyclorrhapha - ps29
Cyclorrhapha - ps30
Calyptratae/Drosophila - ps31
Drosophila - ps32
Sophophora - ps33
Obscura/Melanogaster_group - ps34
D_melanogaster_group - ps35
D_melanogaster_subgroup - ps36
sim./sech./mel. group - ps37
Drosophila_melanogaster - ps38
p-value
2e-04
8e-04
3e-03
1e-02
6e-02
Log-odds
0.5
1.0
1.5
2.0
2.5
3.0
3.5
4.0

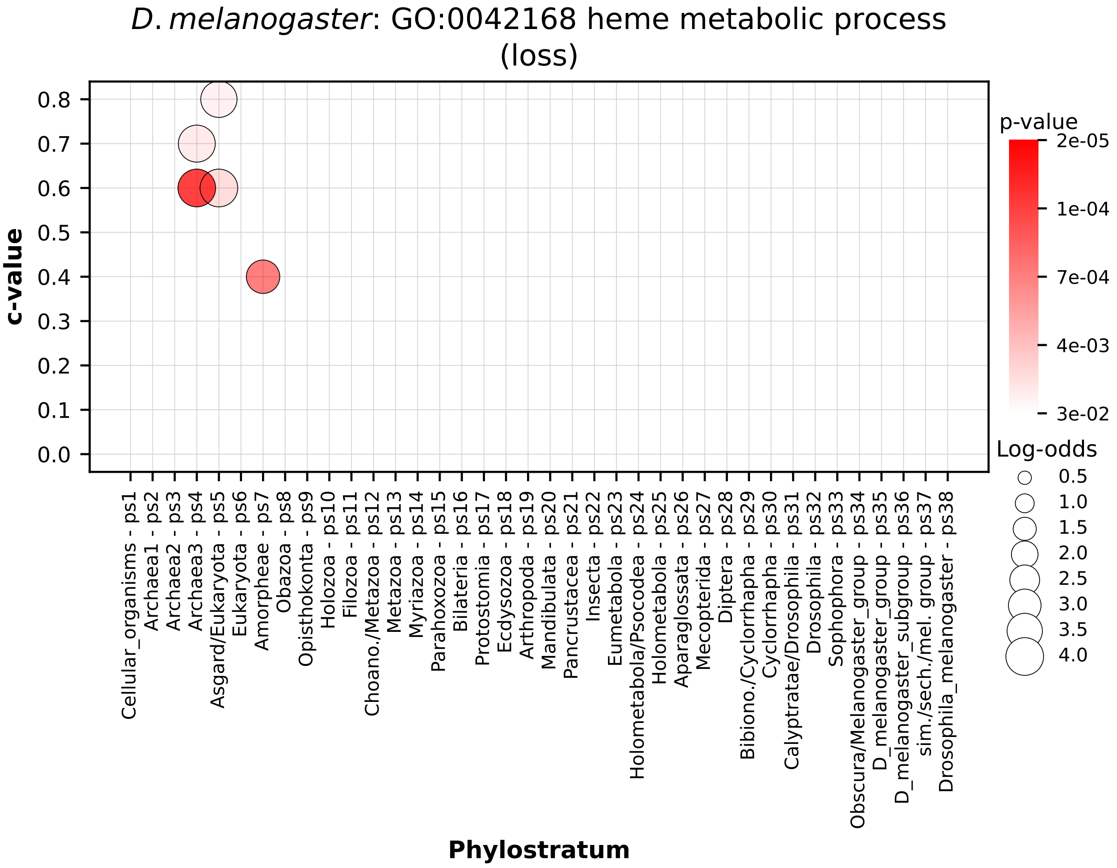

D. melanogaster: GO:0042168 heme metabolic process (loss)
c-value
0.8
0.7
0.6
0.5
0.4
0.3
0.2
0.1
0.0
Phylostratum
Cellular_organisms - ps1
Archaea1 - ps2
Archaea2 - ps3
Archaea3 - ps4
Asgard/Eukaryota - ps5
Eukaryota - ps6
Amorpheae - ps7
Obazoa - ps8
Opisthokonta - ps9
Holozoa - ps10
Filozoa - ps11
Choano./Metazoa - ps12
Metazoa - ps13
Myriazoa - ps14
Parahoxozoa - ps15
Bilateria - ps16
Protostomia - ps17
Ecdysozoa - ps18
Arthropoda - ps19
Mandibulata - ps20
Pancrustacea - ps21
Insecta - ps22
Eumetabola - ps23
Holometabola/Psocodea - ps24
Holometabola - ps25
Aparaglossata - ps26
Mecopterida - ps27
Diptera - ps28
Bibiono./Cyclorrhapha - ps29
Cyclorrhapha - ps30
Calyptratae/Drosophila - ps31
Drosophila - ps32
Sophophora - ps33
Obscura/Melanogaster_group - ps34
D_melanogaster_group - ps35
D_melanogaster_subgroup - ps36
sim./sech./mel. group - ps37
Drosophila_melanogaster - ps38
p-value
2e-05
1e-04
7e-04
4e-03
3e-02
Log-odds
0.5
1.0
1.5
2.0
2.5
3.0
3.5
4.0

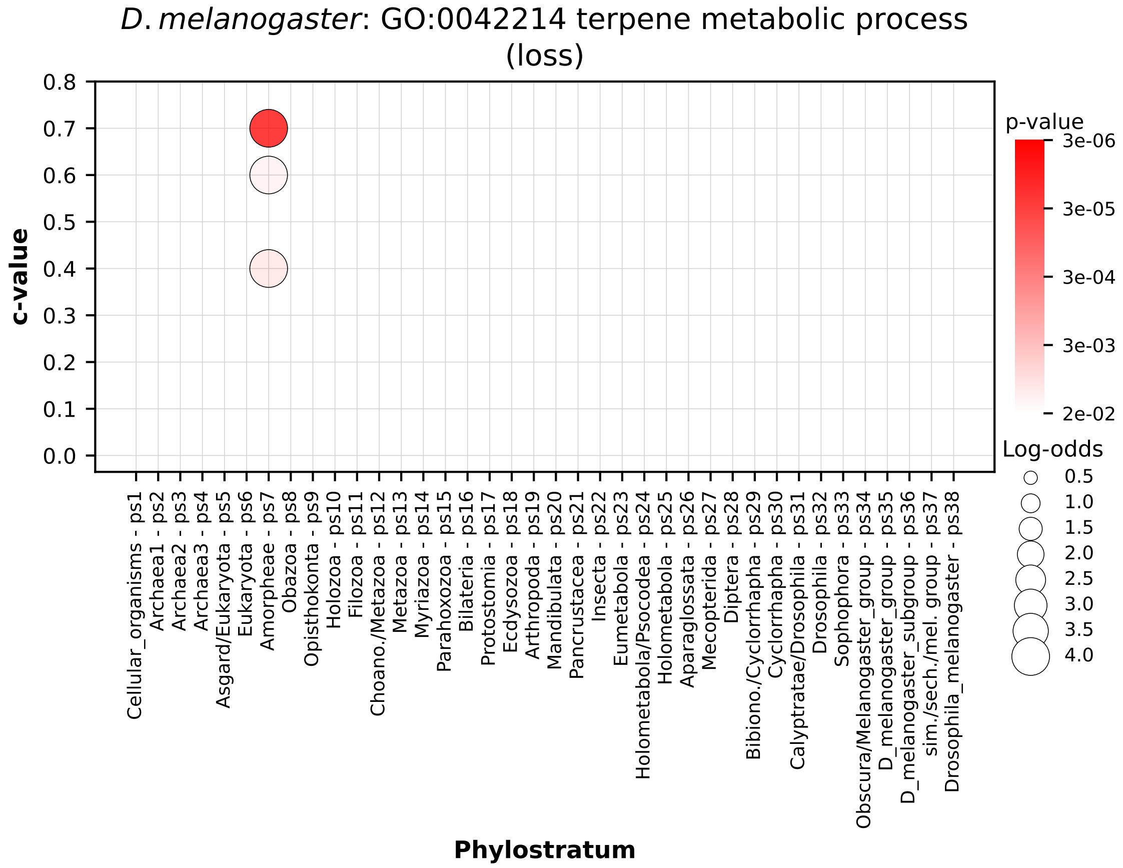
D. melanogaster: GO:0042214 terpene metabolic process
(loss)
c-value
0.0
0.1
0.2
0.3
0.4
0.5
0.6
0.7
0.8
Phylostratum
Cellular_organisms - ps1
Archaea1 - ps2
Archaea2 - ps3
Archaea3 - ps4
Asgard/Eukaryota - ps5
Eukaryota - ps6
Amorpheae - ps7
Obazoa - ps8
Opisthokonta - ps9
Holozoa - ps10
Filozoa - ps11
Choano./Metazoa - ps12
Metazoa - ps13
Myriazoa - ps14
Parahoxozoa - ps15
Bilateria - ps16
Protostomia - ps17
Ecdysozoa - ps18
Arthropoda - ps19
Mandibulata - ps20
Pancrustacea - ps21
Insecta - ps22
Eumetabola - ps23
Holometabola/Psocodea - ps24
Holometabola - ps25
Aparaglossata - ps26
Mecopterida - ps27
Diptera - ps28
Bibiono./Cyclorrhapha - ps29
Cyclorrhapha - ps30
Calyptratae/Drosophila - ps31
Drosophila - ps32
Sophophora - ps33
Obscura/Melanogaster_group - ps34
D_melanogaster_group - ps35
D_melanogaster_subgroup - ps36
sim./sech./mel. group - ps37
Drosophila_melanogaster - ps38
p-value
3e-06
3e-05
3e-04
3e-03
2e-02
Log-odds
0.5
1.0
1.5
2.0
2.5
3.0
3.5
4.0

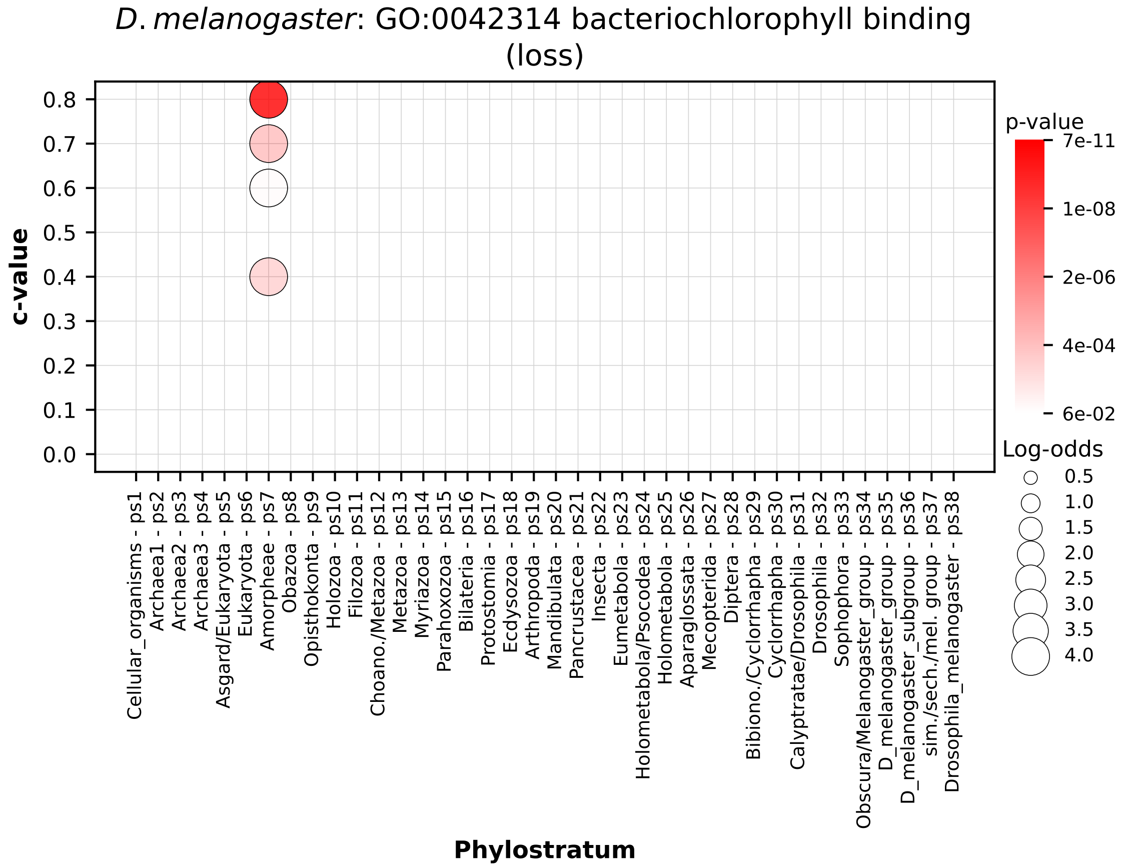

D. melanogaster: GO:0042314 bacteriochlorophyll binding (loss)
c-value
0.0
0.1
0.2
0.3
0.4
0.5
0.6
0.7
0.8
Phylostratum
Cellular_organisms - ps1
Archaea1 - ps2
Archaea2 - ps3
Archaea3 - ps4
Asgard/Eukaryota - ps5
Eukaryota - ps6
Amorpheae - ps7
Obazoa - ps8
Opisthokonta - ps9
Holozoa - ps10
Filozoa - ps11
Choano./Metazoa - ps12
Metazoa - ps13
Myriazoa - ps14
Parahoxozoa - ps15
Bilateria - ps16
Protostomia - ps17
Ecdysozoa - ps18
Arthropoda - ps19
Mandibulata - ps20
Pancrustacea - ps21
Insecta - ps22
Eumetabola - ps23
Holometabola/Psocodea - ps24
Holometabola - ps25
Aparaglossata - ps26
Mecopterida - ps27
Diptera - ps28
Bibiono./Cyclorrhapha - ps29
Cyclorrhapha - ps30
Calyptratae/Drosophila - ps31
Drosophila - ps32
Sophophora - ps33
Obscura/Melanogaster_group - ps34
D_melanogaster_group - ps35
D_melanogaster_subgroup - ps36
sim./sech./mel. group - ps37
Drosophila_melanogaster - ps38
p-value
7e-11
1e-08
2e-06
4e-04
6e-02
Log-odds
0.5
1.0
1.5
2.0
2.5
3.0
3.5
4.0

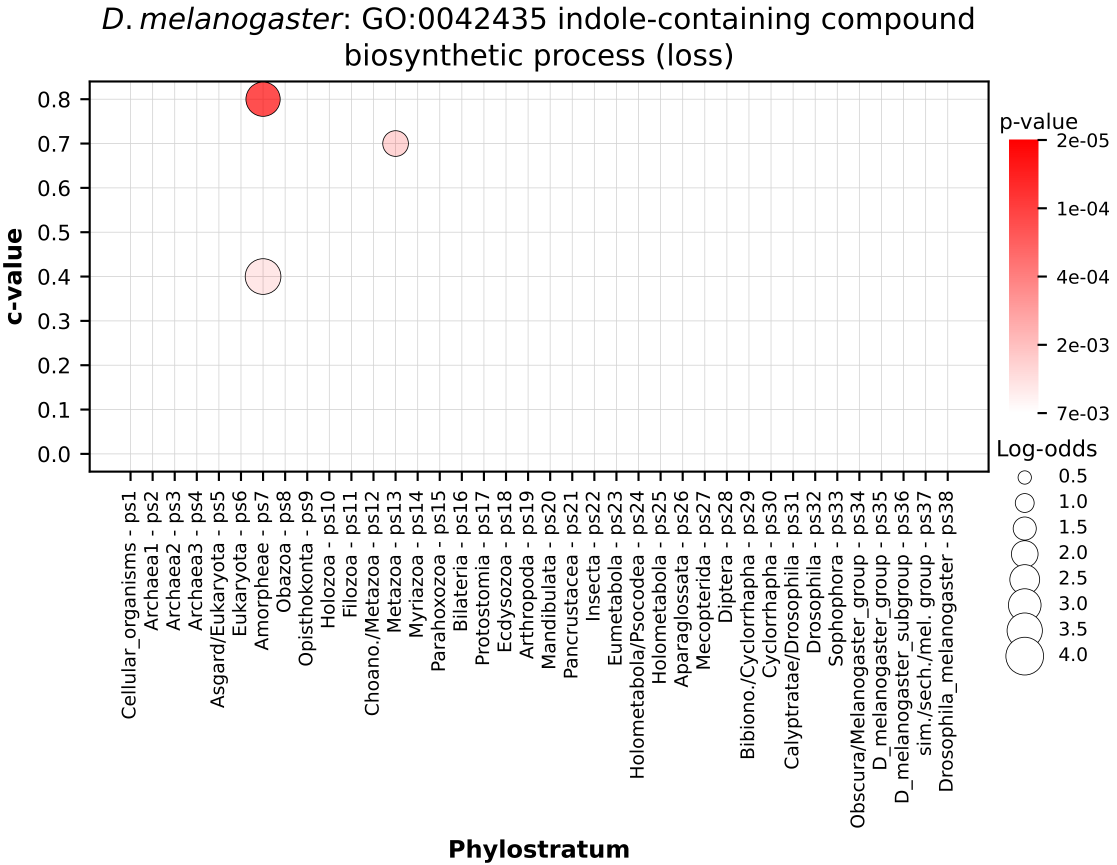

D. melanogaster: GO:0042435 indole-containing compound biosynthetic process (loss)
c-value
0.8
0.7
0.6
0.5
0.4
0.3
0.2
0.1
0.0
p-value
2e-05
1e-04
4e-04
2e-03
7e-03
Log-odds
0.5
1.0
1.5
2.0
2.5
3.0
3.5
4.0
Cellular_organisms - ps1
Archaea1 - ps2
Archaea2 - ps3
Archaea3 - ps4
Asgard/Eukaryota - ps5
Eukaryota - ps6
Amorpheae - ps7
Obazoa - ps8
Opisthokonta - ps9
Holozoa - ps10
Filozoa - ps11
Choano./Metazoa - ps12
Metazoa - ps13
Myriazoa - ps14
Parahoxozoa - ps15
Bilateria - ps16
Protostomia - ps17
Ecdysozoa - ps18
Arthropoda - ps19
Mandibulata - ps20
Pancrustacea - ps21
Insecta - ps22
Eumetabola - ps23
Holometabola/Psocodea - ps24
Holometabola - ps25
Aparaglossata - ps26
Mecopterida - ps27
Diptera - ps28
Bibiono./Cyclorrhapha - ps29
Cyclorrhapha - ps30
Calyptratae/Drosophila - ps31
Drosophila - ps32
Sophophora - ps33
Obscura/Melanogaster_group - ps34
D_melanogaster_group - ps35
D_melanogaster_subgroup - ps36
sim./sech./mel. group - ps37
Drosophila_melanogaster - ps38
Phylostratum

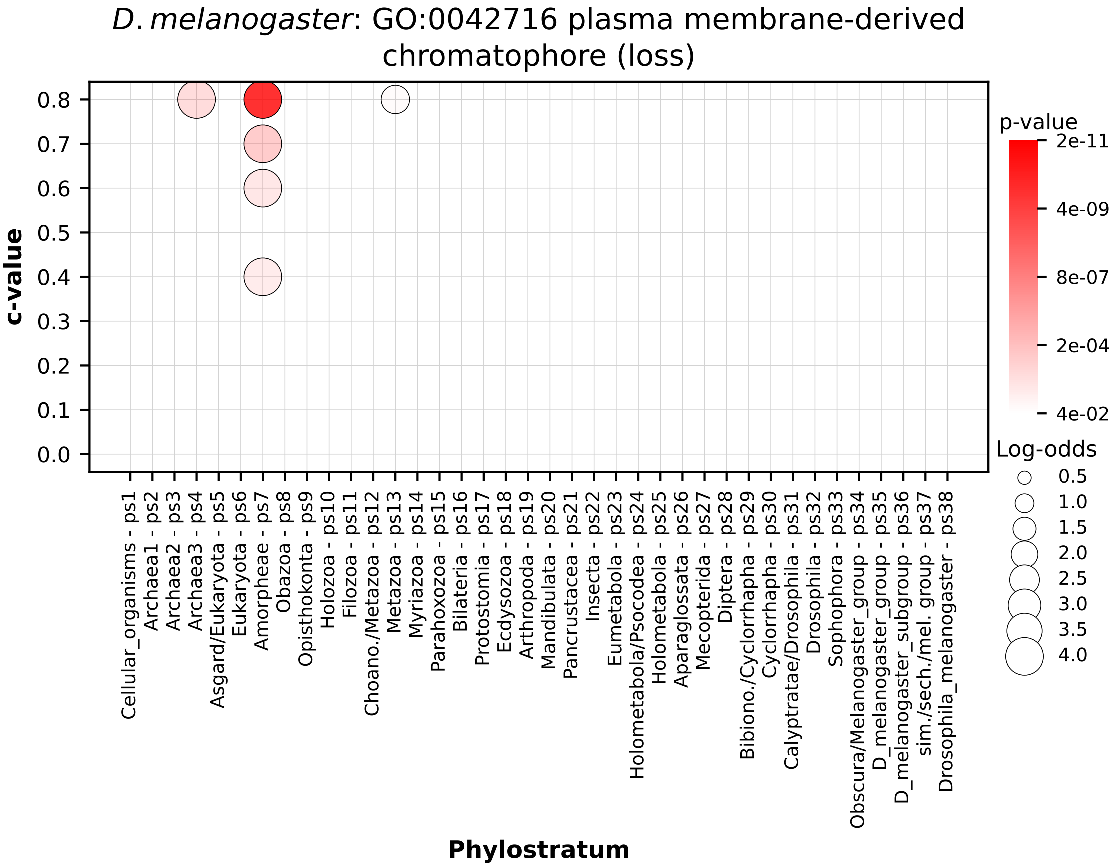

D. melanogaster: GO:0042716 plasma membrane-derived chromatophore (loss)
c-value
0.8
0.7
0.6
0.5
0.4
0.3
0.2
0.1
0.0
p-value
2e-11
4e-09
8e-07
2e-04
4e-02
Log-odds
0.5
1.0
1.5
2.0
2.5
3.0
3.5
4.0
Cellular_organisms - ps1
Archaea1 - ps2
Archaea2 - ps3
Archaea3 - ps4
Asgard/Eukaryota - ps5
Eukaryota - ps6
Amorpheae - ps7
Obazoa - ps8
Opisthokonta - ps9
Holozoa - ps10
Filozoa - ps11
Choano./Metazoa - ps12
Metazoa - ps13
Myriazoa - ps14
Parahoxozoa - ps15
Bilateria - ps16
Protostomia - ps17
Ecdysozoa - ps18
Arthropoda - ps19
Mandibulata - ps20
Pancrustacea - ps21
Insecta - ps22
Eumetabola - ps23
Holometabola/Psocodea - ps24
Holometabola - ps25
Aparaglossata - ps26
Mecopterida - ps27
Diptera - ps28
Bibiono./Cyclorrhapha - ps29
Cyclorrhapha - ps30
Calyptratae/Drosophila - ps31
Drosophila - ps32
Sophophora - ps33
Obscura/Melanogaster_group - ps34
D_melanogaster_group - ps35
D_melanogaster_subgroup - ps36
sim./sech./mel. group - ps37
Drosophila_melanogaster - ps38
Phylostratum

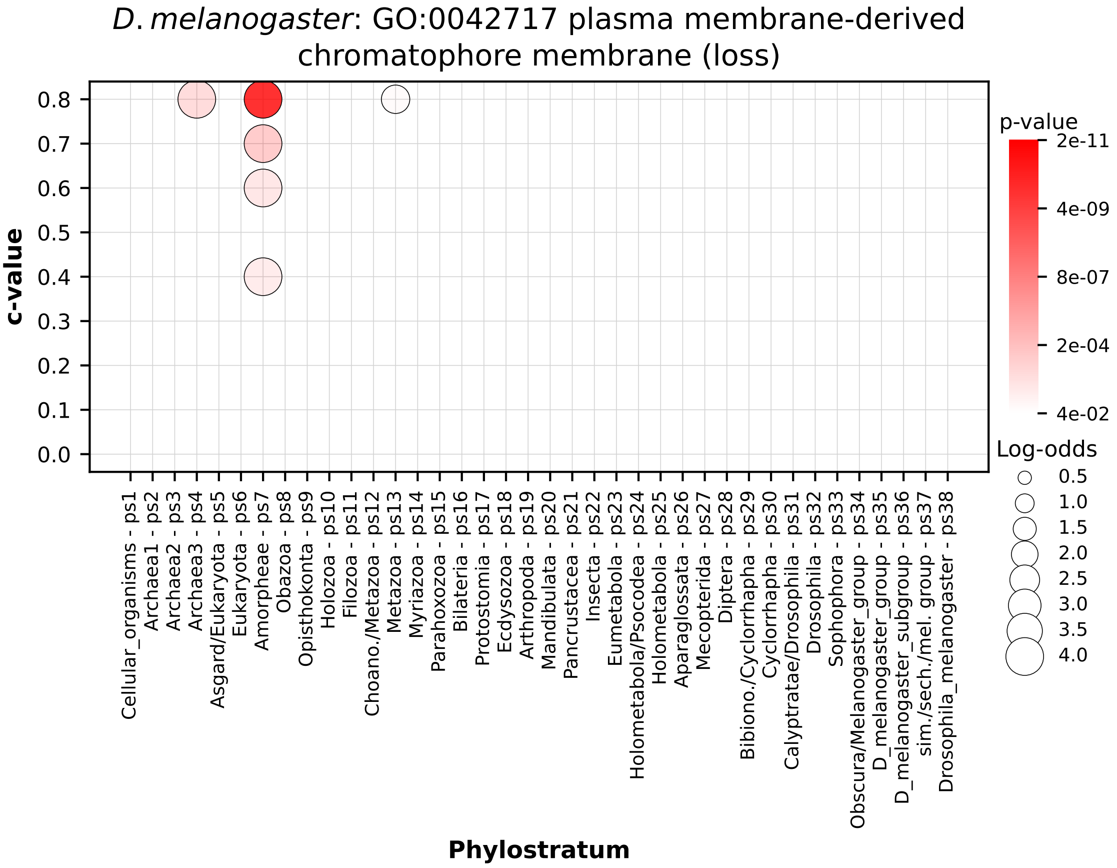

D. melanogaster: GO:0042717 plasma membrane-derived chromatophore membrane (loss)
c-value
0.0
0.1
0.2
0.3
0.4
0.5
0.6
0.7
0.8
p-value
2e-11
4e-09
8e-07
2e-04
4e-02
Log-odds
0.5
1.0
1.5
2.0
2.5
3.0
3.5
4.0
Cellular_organisms - ps1
Archaea1 - ps2
Archaea2 - ps3
Archaea3 - ps4
Asgard/Eukaryota - ps5
Eukaryota - ps6
Amorpheae - ps7
Obazoa - ps8
Opisthokonta - ps9
Holozoa - ps10
Filozoa - ps11
Choano./Metazoa - ps12
Metazoa - ps13
Myriazoa - ps14
Parahoxozoa - ps15
Bilateria - ps16
Protostomia - ps17
Ecdysozoa - ps18
Arthropoda - ps19
Mandibulata - ps20
Pancrustacea - ps21
Insecta - ps22
Eumetabola - ps23
Holometabola/Psocodea - ps24
Holometabola - ps25
Aparaglossata - ps26
Mecopterida - ps27
Diptera - ps28
Bibiono./Cyclorrhapha - ps29
Cyclorrhapha - ps30
Calyptratae/Drosophila - ps31
Drosophila - ps32
Sophophora - ps33
Obscura/Melanogaster_group - ps34
D_melanogaster_group - ps35
D_melanogaster_subgroup - ps36
sim./sech./mel. group - ps37
Drosophila_melanogaster - ps38
Phylostratum

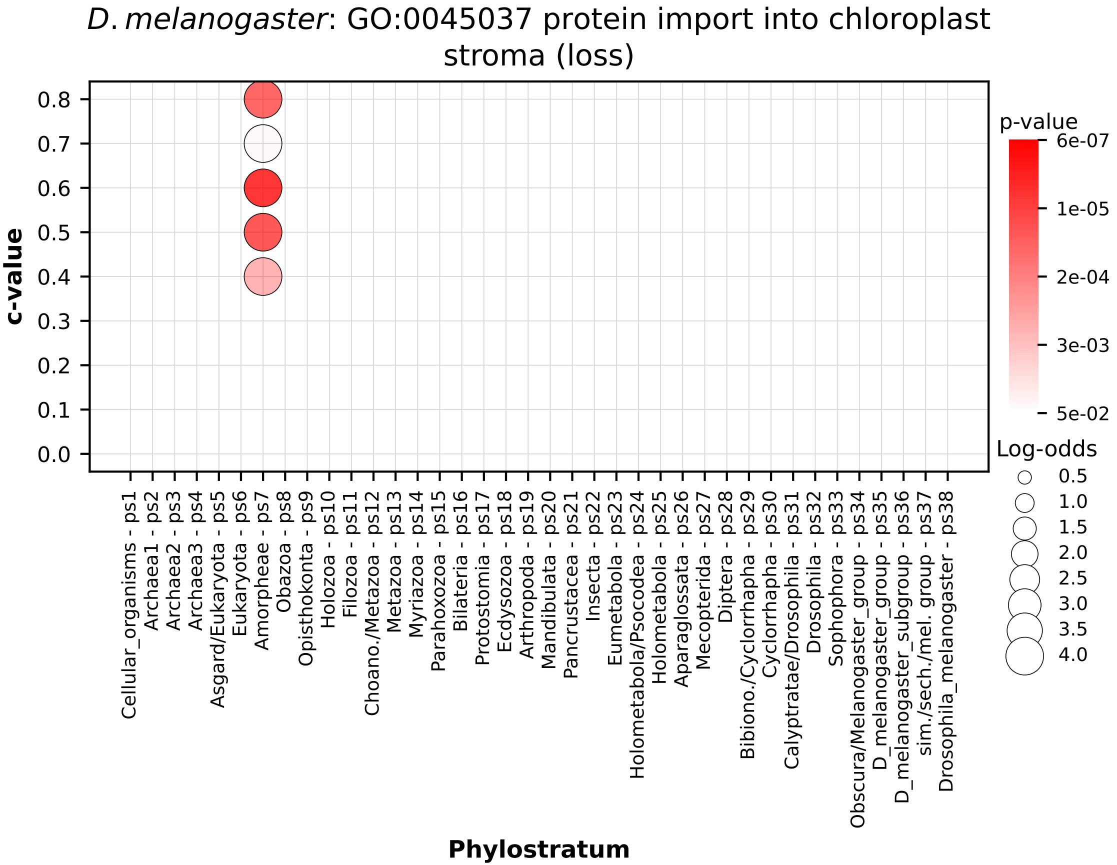

D. melanogaster: GO:0045037 protein import into chloroplast stroma (loss)
c-value
0.8
0.7
0.6
0.5
0.4
0.3
0.2
0.1
0.0
p-value
6e-07
1e-05
2e-04
3e-03
5e-02
Log-odds
0.5
1.0
1.5
2.0
2.5
3.0
3.5
4.0
Cellular_organisms - ps1
Archaea1 - ps2
Archaea2 - ps3
Archaea3 - ps4
Asgard/Eukaryota - ps5
Eukaryota - ps6
Amorpheae - ps7
Obazoa - ps8
Opisthokonta - ps9
Holozoa - ps10
Filozoa - ps11
Choano./Metazoa - ps12
Metazoa - ps13
Myriazoa - ps14
Parahoxozoa - ps15
Bilateria - ps16
Protostomia - ps17
Ecdysozoa - ps18
Arthropoda - ps19
Mandibulata - ps20
Pancrustacea - ps21
Insecta - ps22
Eumetabola - ps23
Holometabola/Psocodea - ps24
Holometabola - ps25
Aparaglossata - ps26
Mecopterida - ps27
Diptera - ps28
Bibiono./Cyclorrhapha - ps29
Cyclorrhapha - ps30
Calyptratae/Drosophila - ps31
Drosophila - ps32
Sophophora - ps33
Obscura/Melanogaster_group - ps34
D_melanogaster_group - ps35
D_melanogaster_subgroup - ps36
sim./sech./mel. group - ps37
Drosophila_melanogaster - ps38
Phylostratum

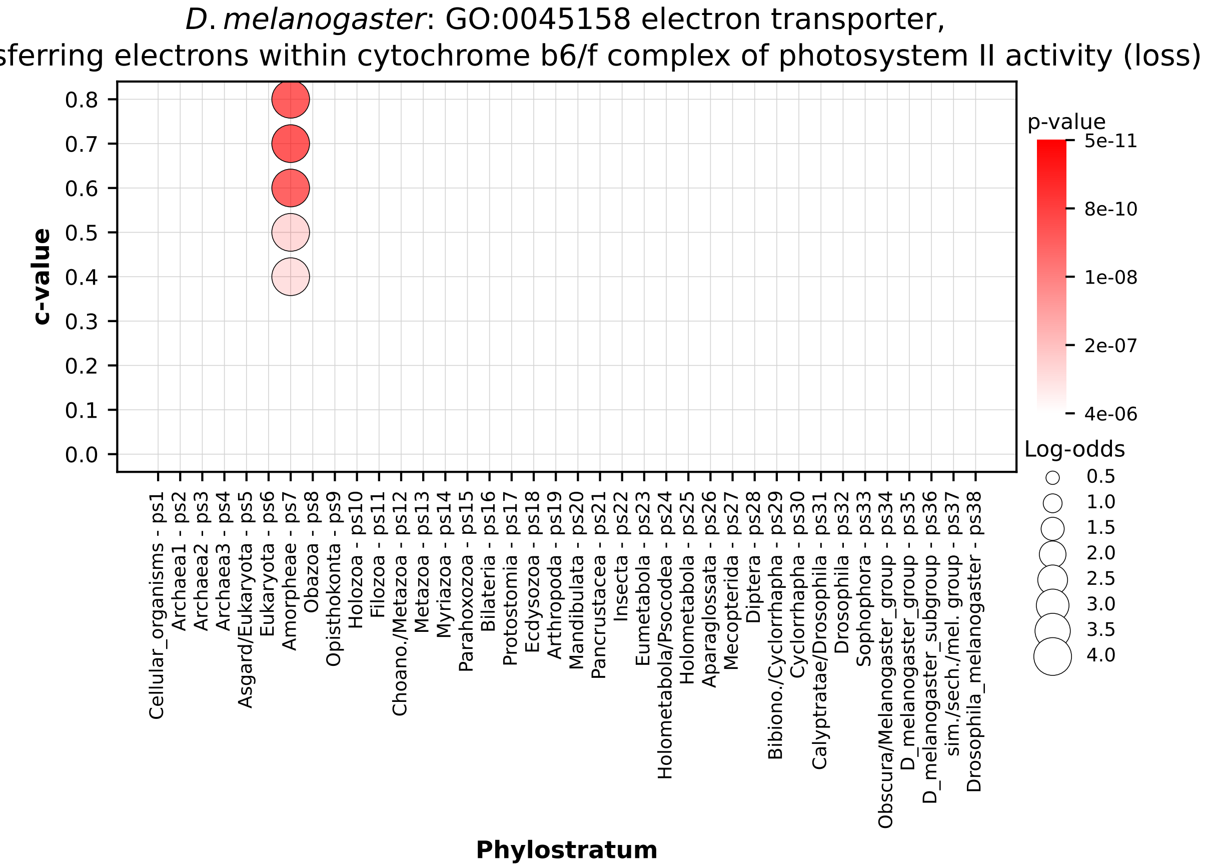
D. melanogaster: GO:0045158 electron transporter,
sferring electrons within cytochrome b6/f complex of photosystem II activity (loss)
c-value
0.8
0.7
0.6
0.5
0.4
0.3
0.2
0.1
0.0
p-value
5e-11
8e-10
1e-08
2e-07
4e-06
Log-odds
0.5
1.0
1.5
2.0
2.5
3.0
3.5
4.0
Cellular_organisms - ps1
Archaea1 - ps2
Archaea2 - ps3
Archaea3 - ps4
Asgard/Eukaryota - ps5
Eukaryota - ps6
Amorpheae - ps7
Obazoa - ps8
Opisthokonta - ps9
Holozoa - ps10
Filozoa - ps11
Choano./Metazoa - ps12
Metazoa - ps13
Myriazoa - ps14
Parahoxozoa - ps15
Bilateria - ps16
Protostomia - ps17
Ecdysozoa - ps18
Arthropoda - ps19
Mandibulata - ps20
Pancrustacea - ps21
Insecta - ps22
Eumetabola - ps23
Holometabola/Psocodea - ps24
Holometabola - ps25
Aparaglossata - ps26
Mecopterida - ps27
Diptera - ps28
Bibiono./Cyclorrhapha - ps29
Cyclorrhapha - ps30
Calyptratae/Drosophila - ps31
Drosophila - ps32
Sophophora - ps33
Obscura/Melanogaster_group - ps34
D_melanogaster_group - ps35
D_melanogaster_subgroup - ps36
sim./sech./mel. group - ps37
Drosophila_melanogaster - ps38
Phylostratum

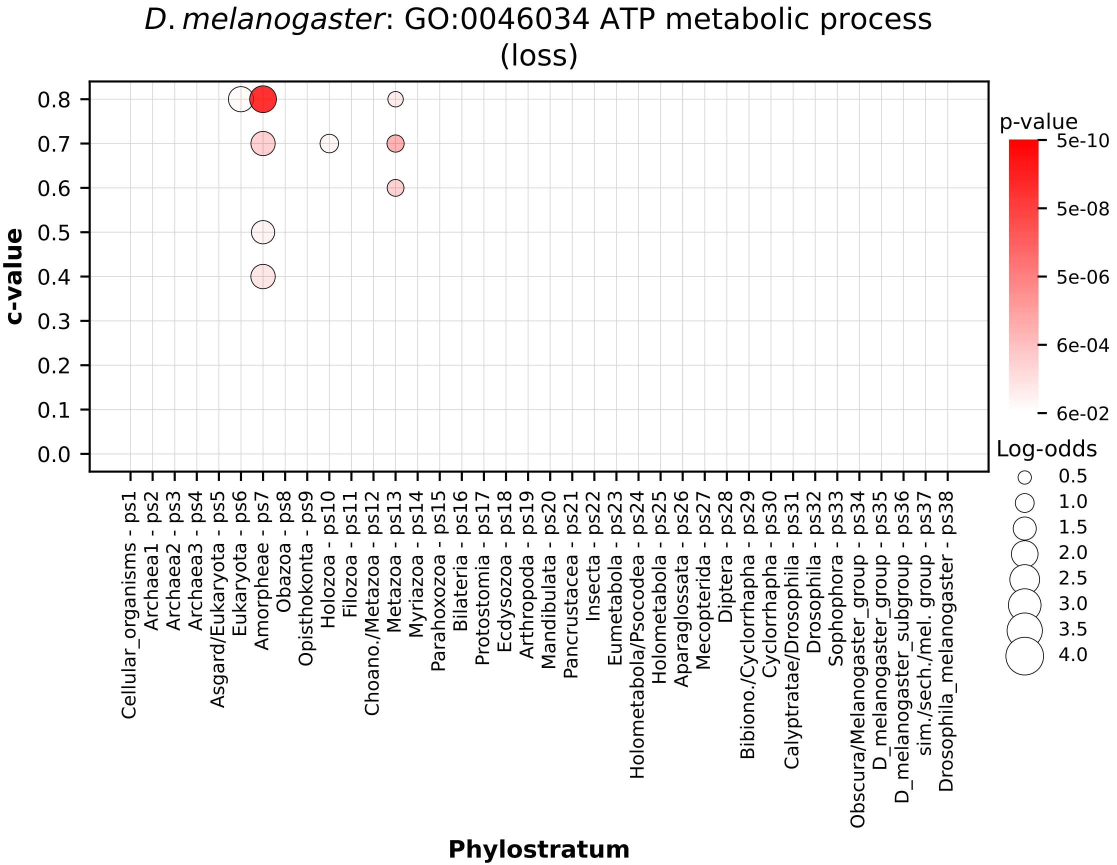

D. melanogaster: GO:0046034 ATP metabolic process
(loss)
c-value
0.0
0.1
0.2
0.3
0.4
0.5
0.6
0.7
0.8
Phylostratum
Cellular_organisms - ps1
Archaea1 - ps2
Archaea2 - ps3
Archaea3 - ps4
Asgard/Eukaryota - ps5
Eukaryota - ps6
Amorpheae - ps7
Obazoa - ps8
Opisthokonta - ps9
Holozoa - ps10
Filozoa - ps11
Choano./Metazoa - ps12
Metazoa - ps13
Myriazoa - ps14
Parahoxozoa - ps15
Bilateria - ps16
Protostomia - ps17
Ecdysozoa - ps18
Arthropoda - ps19
Mandibulata - ps20
Pancrustacea - ps21
Insecta - ps22
Eumetabola - ps23
Holometabola/Psocodea - ps24
Holometabola - ps25
Aparaglossata - ps26
Mecopterida - ps27
Diptera - ps28
Bibiono./Cyclorrhapha - ps29
Cyclorrhapha - ps30
Calyptratae/Drosophila - ps31
Drosophila - ps32
Sophophora - ps33
Obscura/Melanogaster_group - ps34
D_melanogaster_group - ps35
D_melanogaster_subgroup - ps36
sim./sech./mel. group - ps37
Drosophila_melanogaster - ps38
p-value
5e-10
5e-08
5e-06
6e-04
6e-02
Log-odds
0.5
1.0
1.5
2.0
2.5
3.0
3.5
4.0

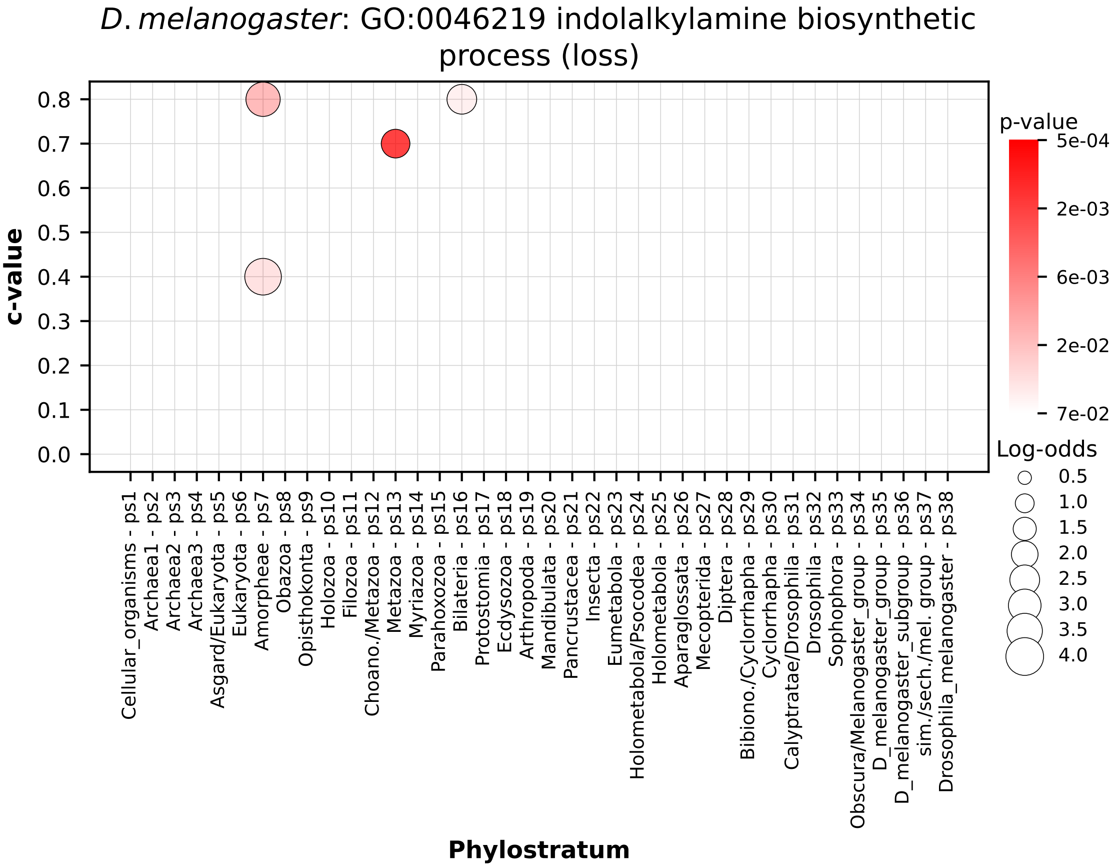
D. melanogaster: GO:0046219 indolalkylamine biosynthetic process (loss)
c-value
0.0
0.1
0.2
0.3
0.4
0.5
0.6
0.7
0.8
Phylostratum
Cellular_organisms - ps1
Archaea1 - ps2
Archaea2 - ps3
Archaea3 - ps4
Asgard/Eukaryota - ps5
Eukaryota - ps6
Amorpheae - ps7
Obazoa - ps8
Opisthokonta - ps9
Holozoa - ps10
Filozoa - ps11
Choano./Metazoa - ps12
Metazoa - ps13
Myriazoa - ps14
Parahoxozoa - ps15
Bilateria - ps16
Protostomia - ps17
Ecdysozoa - ps18
Arthropoda - ps19
Mandibulata - ps20
Pancrustacea - ps21
Insecta - ps22
Eumetabola - ps23
Holometabola/Psocodea - ps24
Holometabola - ps25
Aparaglossata - ps26
Mecopterida - ps27
Diptera - ps28
Bibiono./Cyclorrhapha - ps29
Cyclorrhapha - ps30
Calyptratae/Drosophila - ps31
Drosophila - ps32
Sophophora - ps33
Obscura/Melanogaster_group - ps34
D_melanogaster_group - ps35
D_melanogaster_subgroup - ps36
sim./sech./mel. group - ps37
Drosophila_melanogaster - ps38
p-value
5e-04
2e-03
6e-03
2e-02
7e-02
Log-odds
0.5
1.0
1.5
2.0
2.5
3.0
3.5
4.0

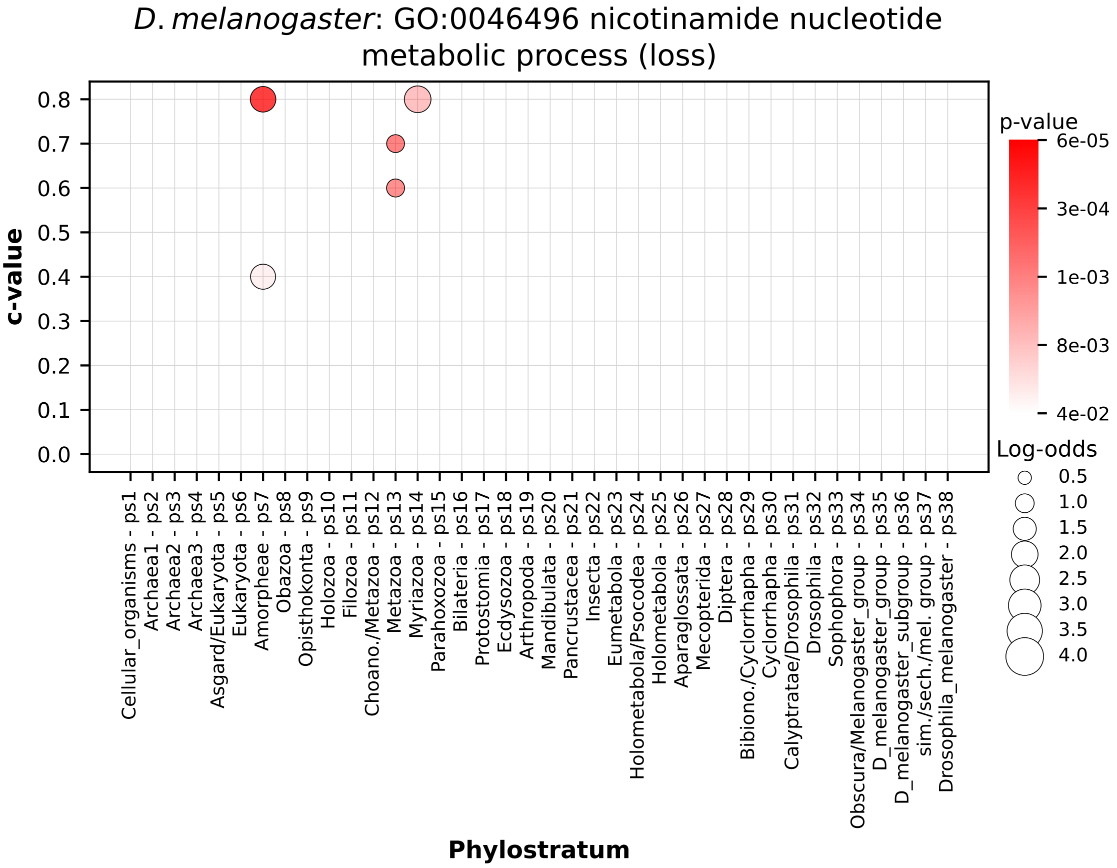

D. melanogaster: GO:0046496 nicotinamide nucleotide metabolic process (loss)
c-value
0.0
0.1
0.2
0.3
0.4
0.5
0.6
0.7
0.8
p-value
6e-05
3e-04
1e-03
8e-03
4e-02
Log-odds
0.5
1.0
1.5
2.0
2.5
3.0
3.5
4.0
Cellular_organisms - ps1
Archaea1 - ps2
Archaea2 - ps3
Archaea3 - ps4
Asgard/Eukaryota - ps5
Eukaryota - ps6
Amorpheae - ps7
Obazoa - ps8
Opisthokonta - ps9
Holozoa - ps10
Filozoa - ps11
Choano./Metazoa - ps12
Metazoa - ps13
Myriazoa - ps14
Parahoxozoa - ps15
Bilateria - ps16
Protostomia - ps17
Ecdysozoa - ps18
Arthropoda - ps19
Mandibulata - ps20
Pancrustacea - ps21
Insecta - ps22
Eumetabola - ps23
Holometabola/Psocodea - ps24
Holometabola - ps25
Aparaglossata - ps26
Mecopterida - ps27
Diptera - ps28
Bibiono./Cyclorrhapha - ps29
Cyclorrhapha - ps30
Calyptratae/Drosophila - ps31
Drosophila - ps32
Sophophora - ps33
Obscura/Melanogaster_group - ps34
D_melanogaster_group - ps35
D_melanogaster_subgroup - ps36
sim./sech./mel. group - ps37
Drosophila_melanogaster - ps38
Phylostratum

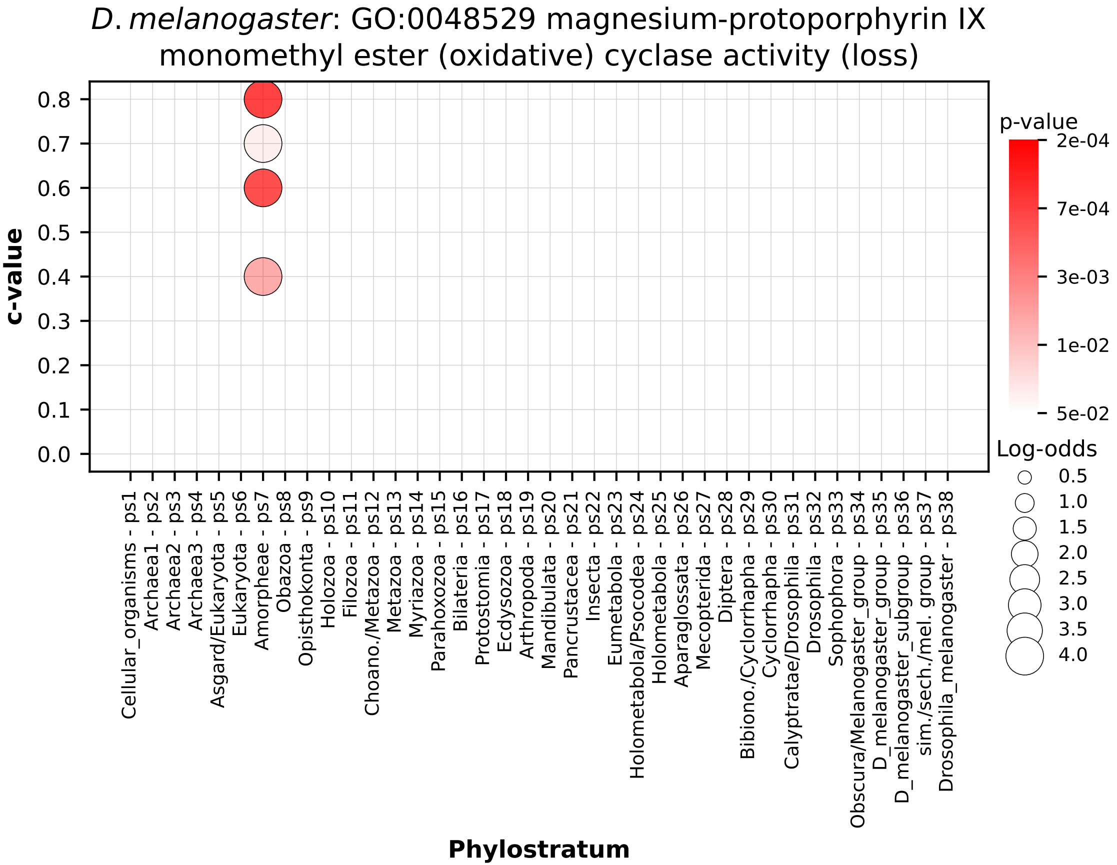

D. melanogaster: GO:0048529 magnesium-protoporphyrin IX monomethyl ester (oxidative) cyclase activity (loss)
c-value
0.0
0.1
0.2
0.3
0.4
0.5
0.6
0.7
0.8
Phylostratum
Cellular_organisms - ps1
Archaea1 - ps2
Archaea2 - ps3
Archaea3 - ps4
Asgard/Eukaryota - ps5
Eukaryota - ps6
Amorpheae - ps7
Obazoa - ps8
Opisthokonta - ps9
Holozoa - ps10
Filozoa - ps11
Choano./Metazoa - ps12
Metazoa - ps13
Myriazoa - ps14
Parahoxozoa - ps15
Bilateria - ps16
Protostomia - ps17
Ecdysozoa - ps18
Arthropoda - ps19
Mandibulata - ps20
Pancrustacea - ps21
Insecta - ps22
Eumetabola - ps23
Holometabola/Psocodea - ps24
Holometabola - ps25
Aparaglossata - ps26
Mecopterida - ps27
Diptera - ps28
Bibiono./Cyclorrhapha - ps29
Cyclorrhapha - ps30
Calyptratae/Drosophila - ps31
Drosophila - ps32
Sophophora - ps33
Obscura/Melanogaster_group - ps34
D_melanogaster_group - ps35
D_melanogaster_subgroup - ps36
sim./sech./mel. group - ps37
Drosophila_melanogaster - ps38
p-value
2e-04
7e-04
3e-03
1e-02
5e-02
Log-odds
0.5
1.0
1.5
2.0
2.5
3.0
3.5
4.0

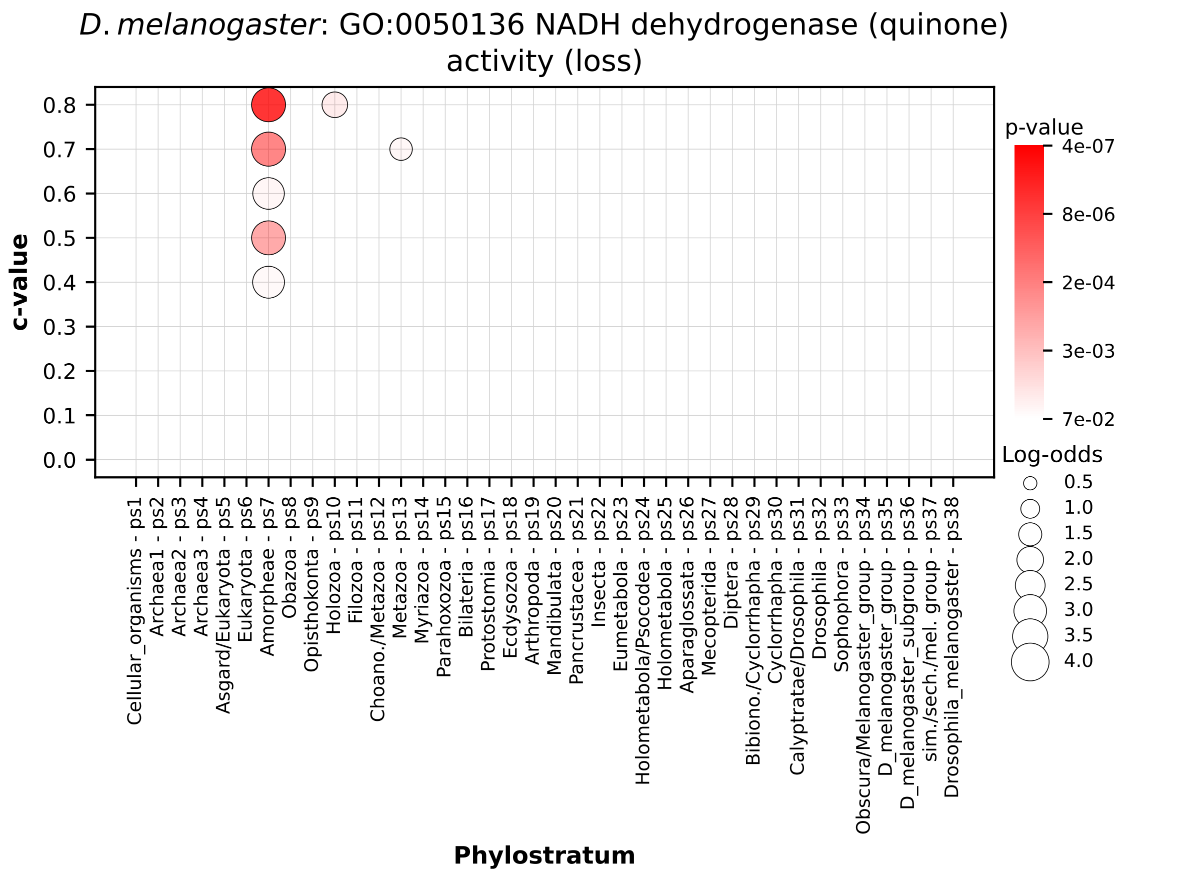

D. melanogaster: GO:0050136 NADH dehydrogenase (quinone) activity (loss)
c-value
0.8
0.7
0.6
0.5
0.4
0.3
0.2
0.1
0.0
Phylostratum
Cellular_organisms - ps1
Archaea1 - ps2
Archaea2 - ps3
Archaea3 - ps4
Asgard/Eukaryota - ps5
Eukaryota - ps6
Amorpheae - ps7
Obazoa - ps8
Opisthokonta - ps9
Holozoa - ps10
Filozoa - ps11
Choano./Metazoa - ps12
Metazoa - ps13
Myriazoa - ps14
Parahoxozoa - ps15
Bilateria - ps16
Protostomia - ps17
Ecdysozoa - ps18
Arthropoda - ps19
Mandibulata - ps20
Pancrustacea - ps21
Insecta - ps22
Eumetabola - ps23
Holometabola/Psocodea - ps24
Holometabola - ps25
Aparaglossata - ps26
Mecopterida - ps27
Diptera - ps28
Bibiono./Cyclorrhapha - ps29
Cyclorrhapha - ps30
Calyptratae/Drosophila - ps31
Drosophila - ps32
Sophophora - ps33
Obscura/Melanogaster_group - ps34
D_melanogaster_group - ps35
D_melanogaster_subgroup - ps36
sim./sech./mel. group - ps37
Drosophila_melanogaster - ps38
p-value
4e-07
8e-06
2e-04
3e-03
7e-02
Log-odds
0.5
1.0
1.5
2.0
2.5
3.0
3.5
4.0

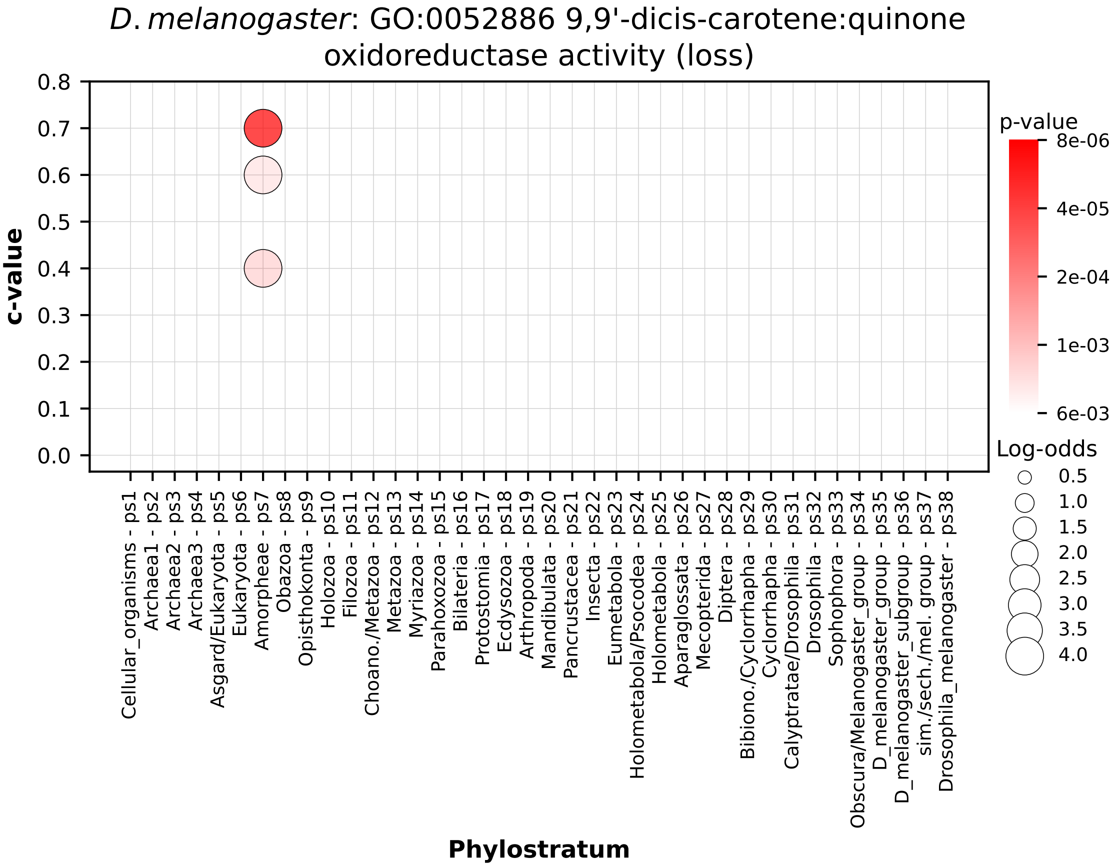

D. melanogaster: GO:0052886 9,9'-dicis-carotene:quinone oxidoreductase activity (loss)
c-value
0.8
0.7
0.6
0.5
0.4
0.3
0.2
0.1
0.0
p-value
8e-06
4e-05
2e-04
1e-03
6e-03
Log-odds
0.5
1.0
1.5
2.0
2.5
3.0
3.5
4.0
Cellular_organisms - ps1
Archaea1 - ps2
Archaea2 - ps3
Archaea3 - ps4
Asgard/Eukaryota - ps5
Eukaryota - ps6
Amorpheae - ps7
Obazoa - ps8
Opisthokonta - ps9
Holozoa - ps10
Filozoa - ps11
Choano./Metazoa - ps12
Metazoa - ps13
Myriazoa - ps14
Parahoxozoa - ps15
Bilateria - ps16
Protostomia - ps17
Ecdysozoa - ps18
Arthropoda - ps19
Mandibulata - ps20
Pancrustacea - ps21
Insecta - ps22
Eumetabola - ps23
Holometabola/Psocodea - ps24
Holometabola - ps25
Aparaglossata - ps26
Mecopterida - ps27
Diptera - ps28
Bibiono./Cyclorrhapha - ps29
Cyclorrhapha - ps30
Calyptratae/Drosophila - ps31
Drosophila - ps32
Sophophora - ps33
Obscura/Melanogaster_group - ps34
D_melanogaster_group - ps35
D_melanogaster_subgroup - ps36
sim./sech./mel. group - ps37
Drosophila_melanogaster - ps38
Phylostratum

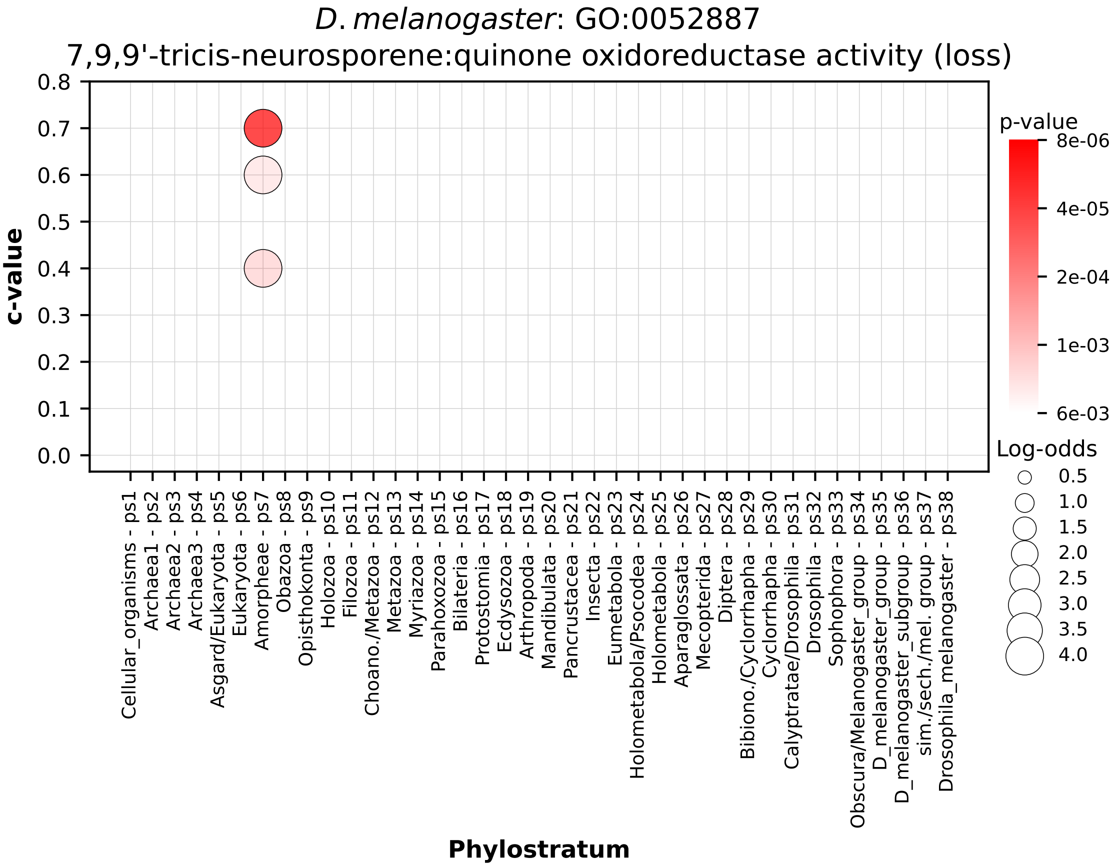

D. melanogaster: GO:0052887
7,9,9'-tricis-neurosporene:quinone oxidoreductase activity (loss)
c-value
0.8
0.7
0.6
0.5
0.4
0.3
0.2
0.1
0.0
p-value
8e-06
4e-05
2e-04
1e-03
6e-03
Log-odds
0.5
1.0
1.5
2.0
2.5
3.0
3.5
4.0
Cellular_organisms - ps1
Archaea1 - ps2
Archaea2 - ps3
Archaea3 - ps4
Asgard/Eukaryota - ps5
Eukaryota - ps6
Amorpheae - ps7
Obazoa - ps8
Opisthokonta - ps9
Holozoa - ps10
Filozoa - ps11
Choano./Metazoa - ps12
Metazoa - ps13
Myriazoa - ps14
Parahoxozoa - ps15
Bilateria - ps16
Protostomia - ps17
Ecdysozoa - ps18
Arthropoda - ps19
Mandibulata - ps20
Pancrustacea - ps21
Insecta - ps22
Eumetabola - ps23
Holometabola/Psocodea - ps24
Holometabola - ps25
Aparaglossata - ps26
Mecopterida - ps27
Diptera - ps28
Bibiono./Cyclorrhapha - ps29
Cyclorrhapha - ps30
Calyptratae/Drosophila - ps31
Drosophila - ps32
Sophophora - ps33
Obscura/Melanogaster_group - ps34
D_melanogaster_group - ps35
D_melanogaster_subgroup - ps36
sim./sech./mel. group - ps37
Drosophila_melanogaster - ps38
Phylostratum

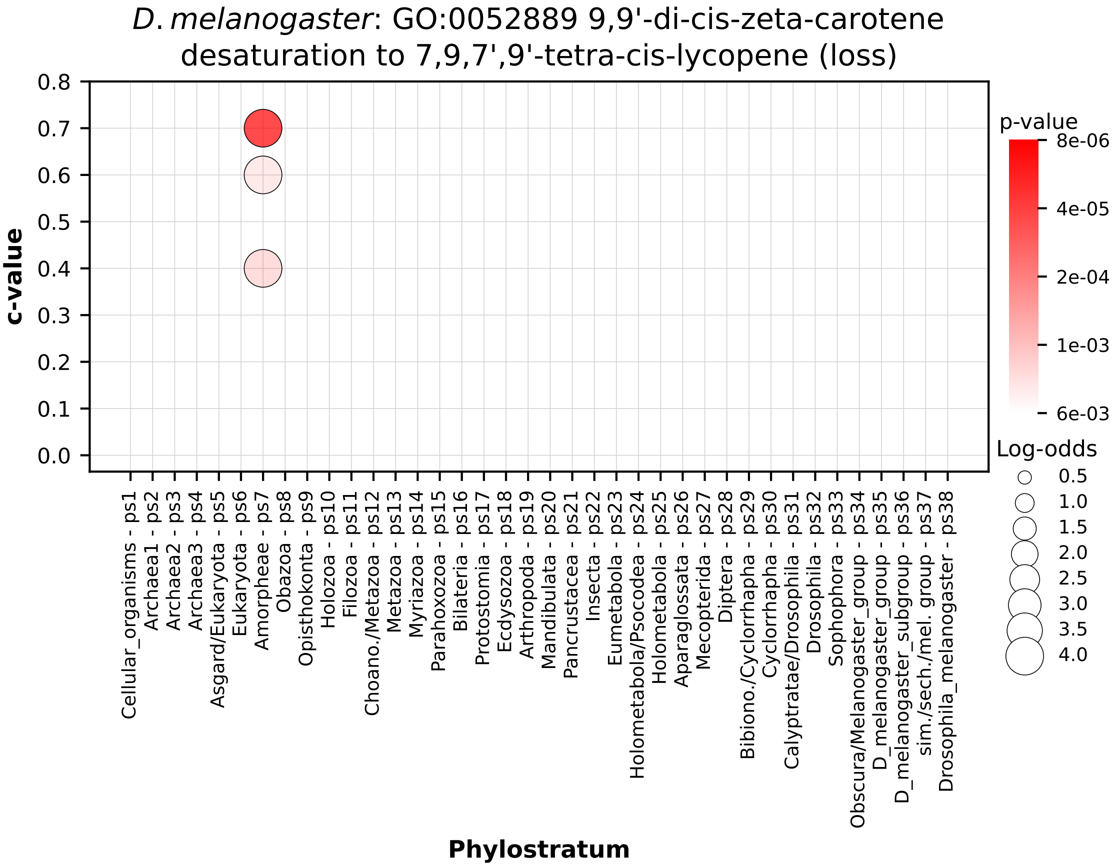

D. melanogaster: GO:0052889 9,9'-di-cis-zeta-carotene desaturation to 7,9,7',9'-tetra-cis-lycopene (loss)
c-value
0.0
0.1
0.2
0.3
0.4
0.5
0.6
0.7
0.8
Phylostratum
Cellular_organisms - ps1
Archaea1 - ps2
Archaea2 - ps3
Archaea3 - ps4
Asgard/Eukaryota - ps5
Eukaryota - ps6
Amorpheae - ps7
Obazoa - ps8
Opisthokonta - ps9
Holozoa - ps10
Filozoa - ps11
Choano./Metazoa - ps12
Metazoa - ps13
Myriazoa - ps14
Parahoxozoa - ps15
Bilateria - ps16
Protostomia - ps17
Ecdysozoa - ps18
Arthropoda - ps19
Mandibulata - ps20
Pancrustacea - ps21
Insecta - ps22
Eumetabola - ps23
Holometabola/Psocodea - ps24
Holometabola - ps25
Aparaglossata - ps26
Mecopterida - ps27
Diptera - ps28
Bibiono./Cyclorrhapha - ps29
Cyclorrhapha - ps30
Calyptratae/Drosophila - ps31
Drosophila - ps32
Sophophora - ps33
Obscura/Melanogaster_group - ps34
D_melanogaster_group - ps35
D_melanogaster_subgroup - ps36
sim./sech./mel. group - ps37
Drosophila_melanogaster - ps38
p-value
8e-06
4e-05
2e-04
1e-03
6e-03
Log-odds
0.5
1.0
1.5
2.0
2.5
3.0
3.5
4.0

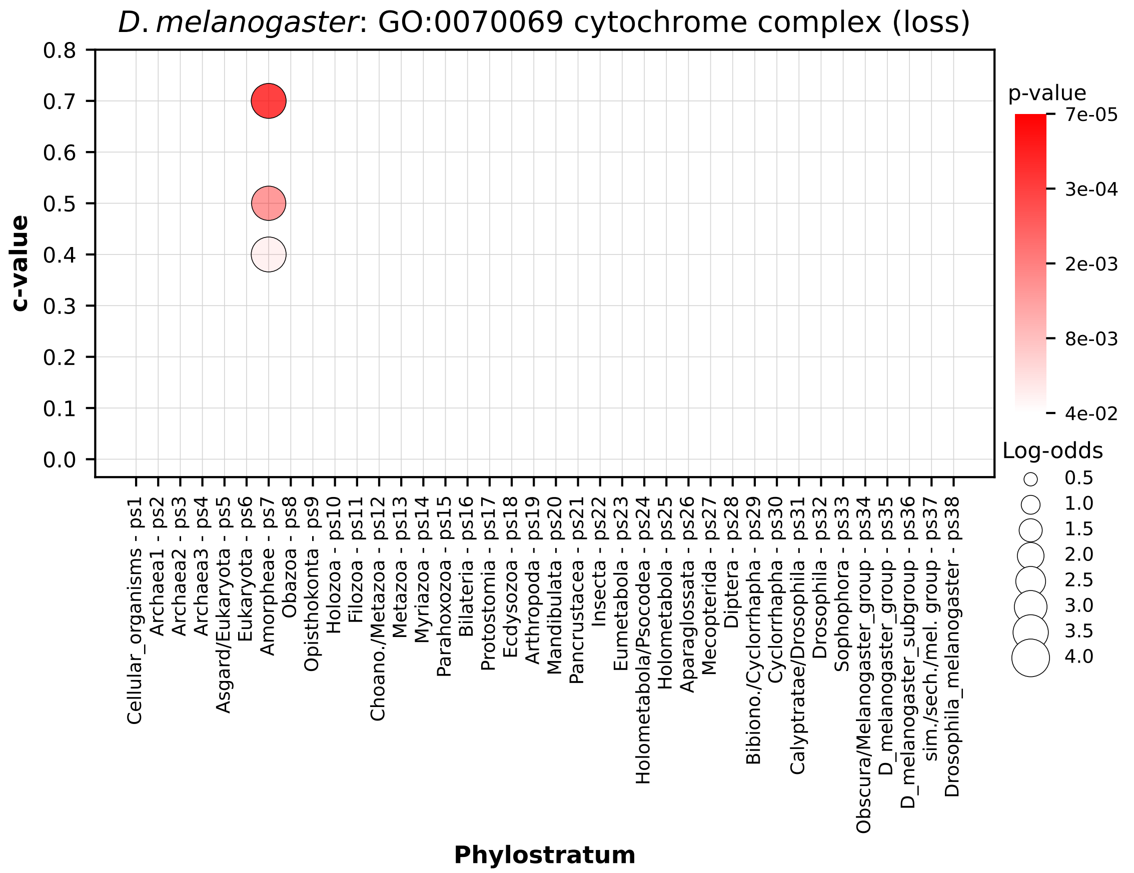

D. melanogaster: GO:0070069 cytochrome complex (loss)
c-value
0.0
0.1
0.2
0.3
0.4
0.5
0.6
0.7
0.8
Phylostratum
Cellular_organisms - ps1
Archaea1 - ps2
Archaea2 - ps3
Archaea3 - ps4
Asgard/Eukaryota - ps5
Eukaryota - ps6
Amorpheae - ps7
Obazoa - ps8
Opisthokonta - ps9
Holozoa - ps10
Filozoa - ps11
Choano./Metazoa - ps12
Metazoa - ps13
Myriazoa - ps14
Parahoxozoa - ps15
Bilateria - ps16
Protostomia - ps17
Ecdysozoa - ps18
Arthropoda - ps19
Mandibulata - ps20
Pancrustacea - ps21
Insecta - ps22
Eumetabola - ps23
Holometabola/Psocodea - ps24
Holometabola - ps25
Aparaglossata - ps26
Mecopterida - ps27
Diptera - ps28
Bibiono./Cyclorrhapha - ps29
Cyclorrhapha - ps30
Calyptratae/Drosophila - ps31
Drosophila - ps32
Sophophora - ps33
Obscura/Melanogaster_group - ps34
D_melanogaster_group - ps35
D_melanogaster_subgroup - ps36
sim./sech./mel. group - ps37
Drosophila_melanogaster - ps38
p-value
7e-05
3e-04
2e-03
8e-03
4e-02
Log-odds
0.5
1.0
1.5
2.0
2.5
3.0
3.5
4.0

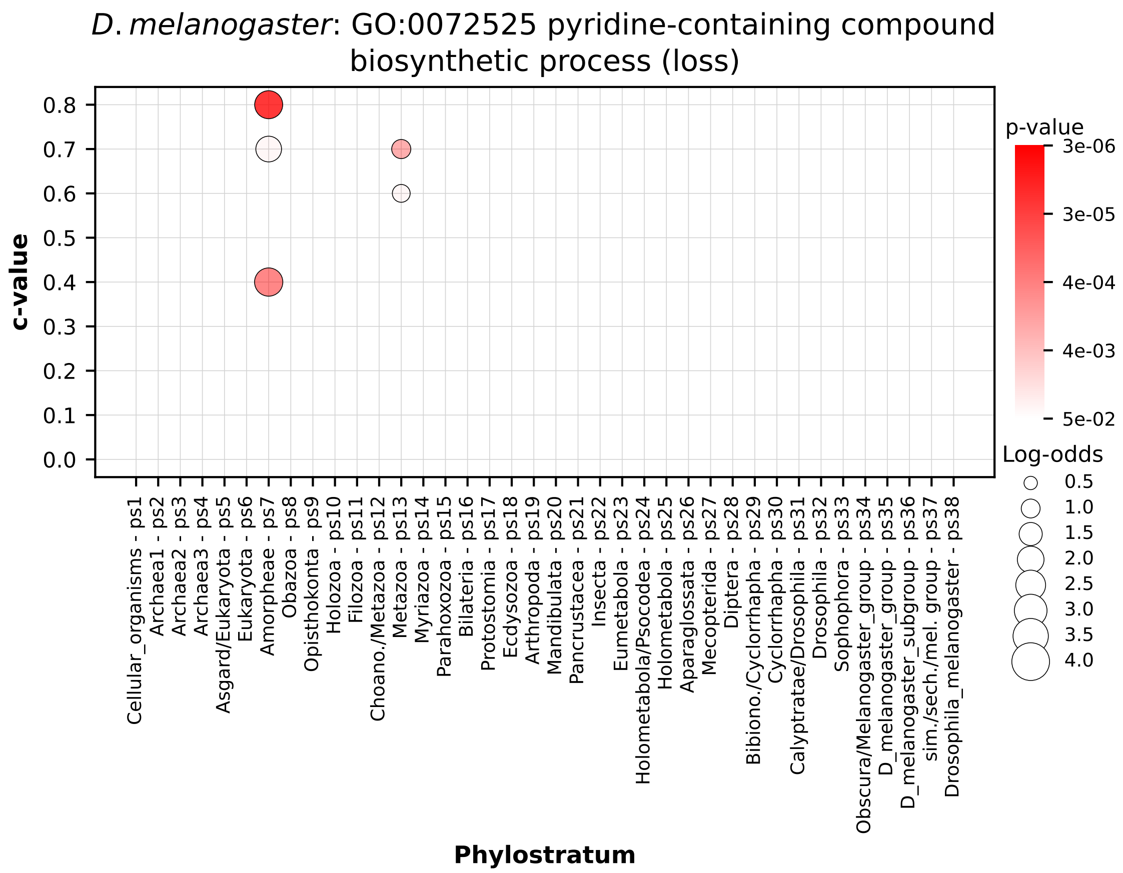

D. melanogaster: GO:0072525 pyridine-containing compound biosynthetic process (loss)
c-value
0.8
0.7
0.6
0.5
0.4
0.3
0.2
0.1
0.0
p-value
3e-06
3e-05
4e-04
4e-03
5e-02
Log-odds
0.5
1.0
1.5
2.0
2.5
3.0
3.5
4.0
Cellular_organisms - ps1
Archaea1 - ps2
Archaea2 - ps3
Archaea3 - ps4
Asgard/Eukaryota - ps5
Eukaryota - ps6
Amorpheae - ps7
Obazoa - ps8
Opisthokonta - ps9
Holozoa - ps10
Filozoa - ps11
Choano./Metazoa - ps12
Metazoa - ps13
Myriazoa - ps14
Parahoxozoa - ps15
Bilateria - ps16
Protostomia - ps17
Ecdysozoa - ps18
Arthropoda - ps19
Mandibulata - ps20
Pancrustacea - ps21
Insecta - ps22
Eumetabola - ps23
Holometabola/Psocodea - ps24
Holometabola - ps25
Aparaglossata - ps26
Mecopterida - ps27
Diptera - ps28
Bibiono./Cyclorrhapha - ps29
Cyclorrhapha - ps30
Calyptratae/Drosophila - ps31
Drosophila - ps32
Sophophora - ps33
Obscura/Melanogaster_group - ps34
D_melanogaster_group - ps35
D_melanogaster_subgroup - ps36
sim./sech./mel. group - ps37
Drosophila_melanogaster - ps38
Phylostratum

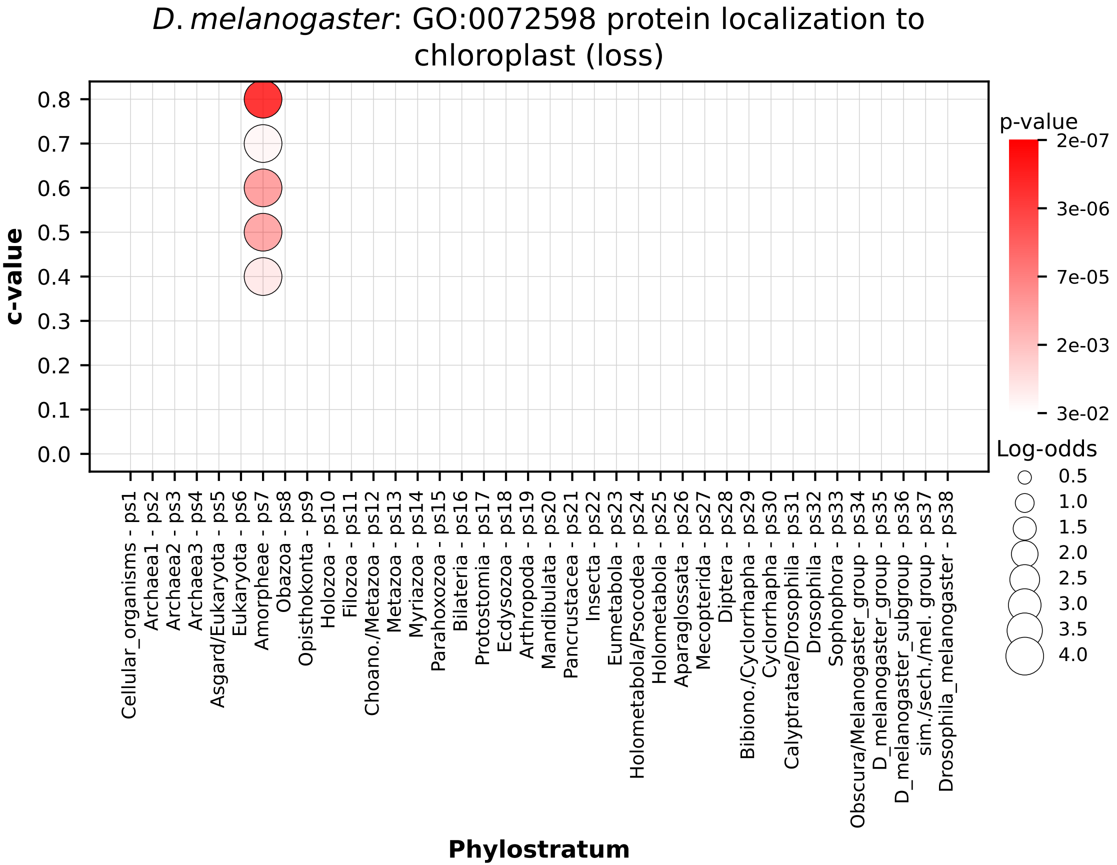
D. melanogaster: GO:0072598 protein localization to chloroplast (loss)
c-value
0.0
0.1
0.2
0.3
0.4
0.5
0.6
0.7
0.8
Phylostratum
Cellular_organisms - ps1
Archaea1 - ps2
Archaea2 - ps3
Archaea3 - ps4
Asgard/Eukaryota - ps5
Eukaryota - ps6
Amorpheae - ps7
Obazoa - ps8
Opisthokonta - ps9
Holozoa - ps10
Filozoa - ps11
Choano./Metazoa - ps12
Metazoa - ps13
Myriazoa - ps14
Parahoxozoa - ps15
Bilateria - ps16
Protostomia - ps17
Ecdysozoa - ps18
Arthropoda - ps19
Mandibulata - ps20
Pancrustacea - ps21
Insecta - ps22
Eumetabola - ps23
Holometabola/Psocodea - ps24
Holometabola - ps25
Aparaglossata - ps26
Mecopterida - ps27
Diptera - ps28
Bibiono./Cyclorrhapha - ps29
Cyclorrhapha - ps30
Calyptratae/Drosophila - ps31
Drosophila - ps32
Sophophora - ps33
Obscura/Melanogaster_group - ps34
D_melanogaster_group - ps35
D_melanogaster_subgroup - ps36
sim./sech./mel. group - ps37
Drosophila_melanogaster - ps38
p-value
2e-07
3e-06
7e-05
2e-03
3e-02
Log-odds
0.5
1.0
1.5
2.0
2.5
3.0
3.5
4.0

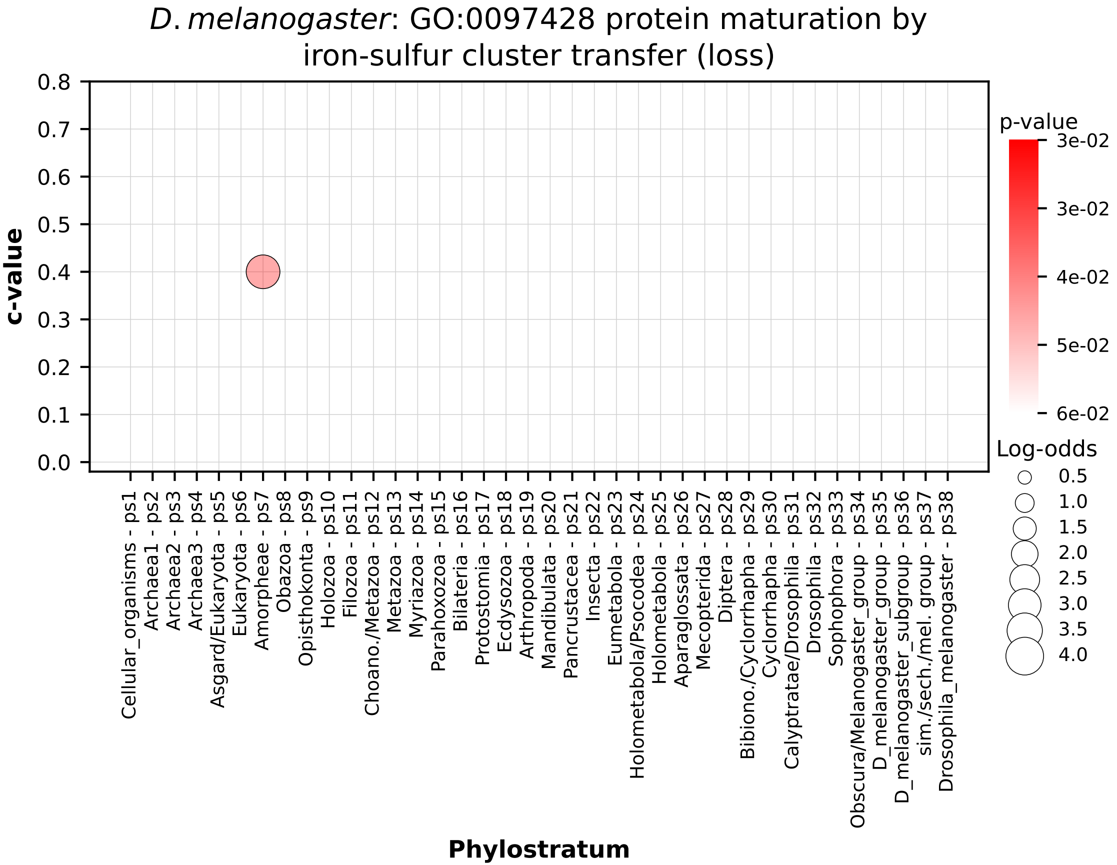

D. melanogaster: GO:0097428 protein maturation by iron-sulfur cluster transfer (loss)
c-value
0.8
0.7
0.6
0.5
0.4
0.3
0.2
0.1
0.0
p-value
3e-02
3e-02
4e-02
5e-02
6e-02
Log-odds
0.5
1.0
1.5
2.0
2.5
3.0
3.5
4.0
Cellular_organisms - ps1
Archaea1 - ps2
Archaea2 - ps3
Archaea3 - ps4
Asgard/Eukaryota - ps5
Eukaryota - ps6
Amorpheae - ps7
Obazoa - ps8
Opisthokonta - ps9
Holozoa - ps10
Filozoa - ps11
Choano./Metazoa - ps12
Metazoa - ps13
Myriazoa - ps14
Parahoxozoa - ps15
Bilateria - ps16
Protostomia - ps17
Ecdysozoa - ps18
Arthropoda - ps19
Mandibulata - ps20
Pancrustacea - ps21
Insecta - ps22
Eumetabola - ps23
Holometabola/Psocodea - ps24
Holometabola - ps25
Aparaglossata - ps26
Mecopterida - ps27
Diptera - ps28
Bibiono./Cyclorrhapha - ps29
Cyclorrhapha - ps30
Calyptratae/Drosophila - ps31
Drosophila - ps32
Sophophora - ps33
Obscura/Melanogaster_group - ps34
D_melanogaster_group - ps35
D_melanogaster_subgroup - ps36
sim./sech./mel. group - ps37
Drosophila_melanogaster - ps38
Phylostratum

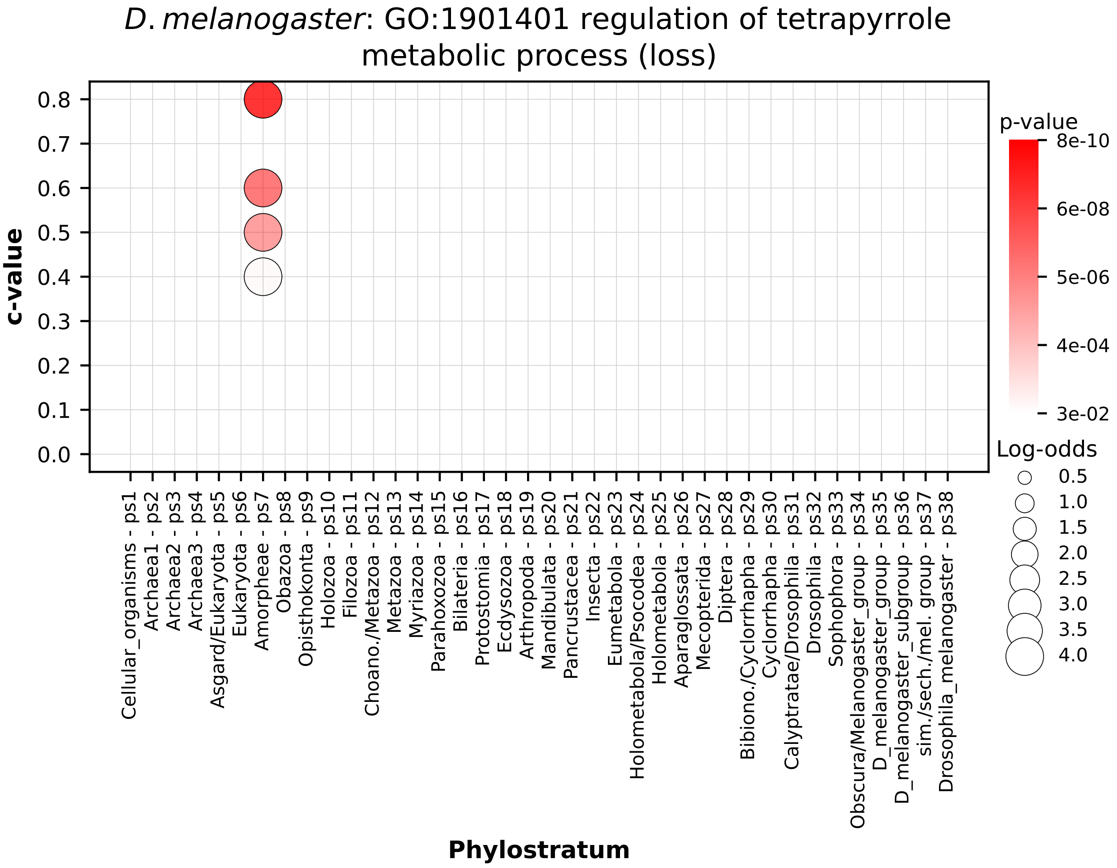

D. melanogaster: GO:1901401 regulation of tetrapyrrole metabolic process (loss)
c-value
0.0
0.1
0.2
0.3
0.4
0.5
0.6
0.7
0.8
Phylostratum
Cellular_organisms - ps1
Archaea1 - ps2
Archaea2 - ps3
Archaea3 - ps4
Asgard/Eukaryota - ps5
Eukaryota - ps6
Amorpheae - ps7
Obazoa - ps8
Opisthokonta - ps9
Holozoa - ps10
Filozoa - ps11
Choano./Metazoa - ps12
Metazoa - ps13
Myriazoa - ps14
Parahoxozoa - ps15
Bilateria - ps16
Protostomia - ps17
Ecdysozoa - ps18
Arthropoda - ps19
Mandibulata - ps20
Pancrustacea - ps21
Insecta - ps22
Eumetabola - ps23
Holometabola/Psocodea - ps24
Holometabola - ps25
Aparaglossata - ps26
Mecopterida - ps27
Diptera - ps28
Bibiono./Cyclorrhapha - ps29
Cyclorrhapha - ps30
Calyptratae/Drosophila - ps31
Drosophila - ps32
Sophophora - ps33
Obscura/Melanogaster_group - ps34
D_melanogaster_group - ps35
D_melanogaster_subgroup - ps36
sim./sech./mel. group - ps37
Drosophila_melanogaster - ps38
p-value
8e-10
6e-08
5e-06
4e-04
3e-02
Log-odds
0.5
1.0
1.5
2.0
2.5
3.0
3.5
4.0

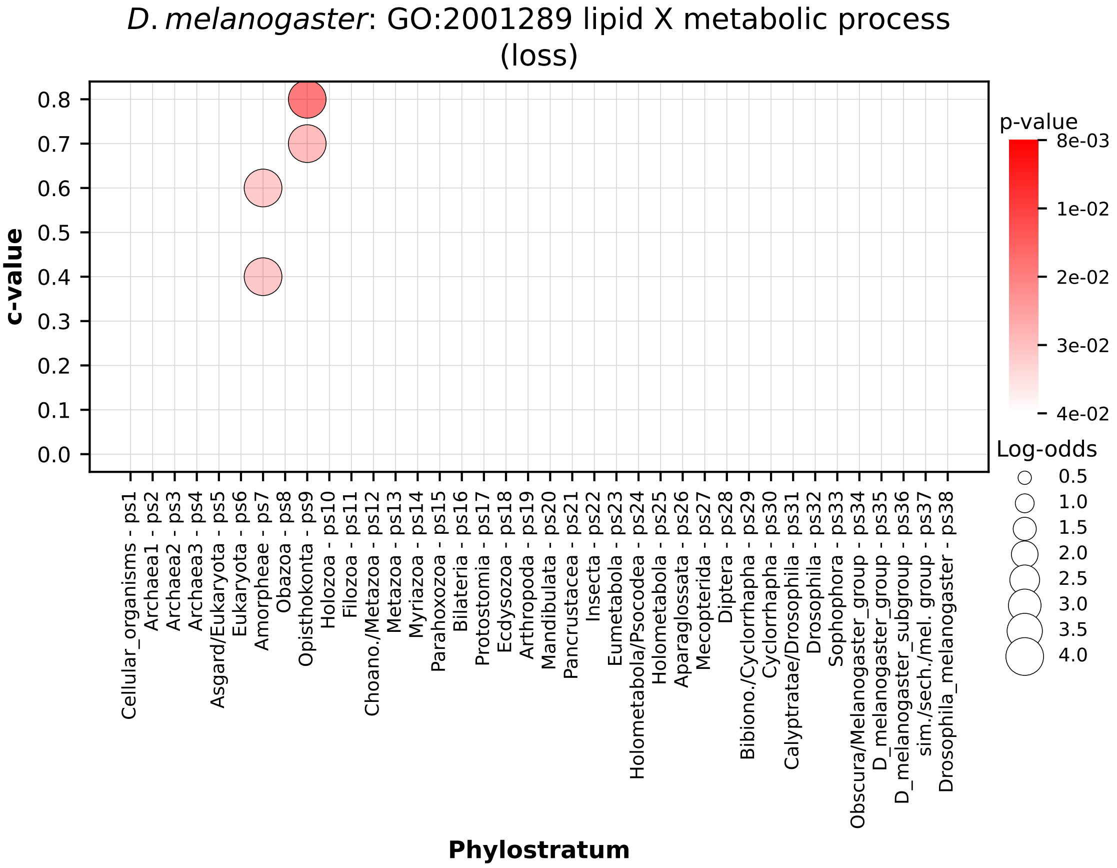

D. melanogaster: GO:2001289 lipid X metabolic process
(loss)
c-value
0.0
0.1
0.2
0.3
0.4
0.5
0.6
0.7
0.8
p-value
8e-03
1e-02
2e-02
3e-02
4e-02
Log-odds
0.5
1.0
1.5
2.0
2.5
3.0
3.5
4.0
Cellular_organisms - ps1
Archaea1 - ps2
Archaea2 - ps3
Archaea3 - ps4
Asgard/Eukaryota - ps5
Eukaryota - ps6
Amorpheae - ps7
Obazoa - ps8
Opisthokonta - ps9
Holozoa - ps10
Filozoa - ps11
Choano./Metazoa - ps12
Metazoa - ps13
Myriazoa - ps14
Parahoxozoa - ps15
Bilateria - ps16
Protostomia - ps17
Ecdysozoa - ps18
Arthropoda - ps19
Mandibulata - ps20
Pancrustacea - ps21
Insecta - ps22
Eumetabola - ps23
Holometabola/Psocodea - ps24
Holometabola - ps25
Aparaglossata - ps26
Mecopterida - ps27
Diptera - ps28
Bibiono./Cyclorrhapha - ps29
Cyclorrhapha - ps30
Calyptratae/Drosophila - ps31
Drosophila - ps32
Sophophora - ps33
Obscura/Melanogaster_group - ps34
D_melanogaster_group - ps35
D_melanogaster_subgroup - ps36
sim./sech./mel. group - ps37
Drosophila_melanogaster - ps38
Phylostratum

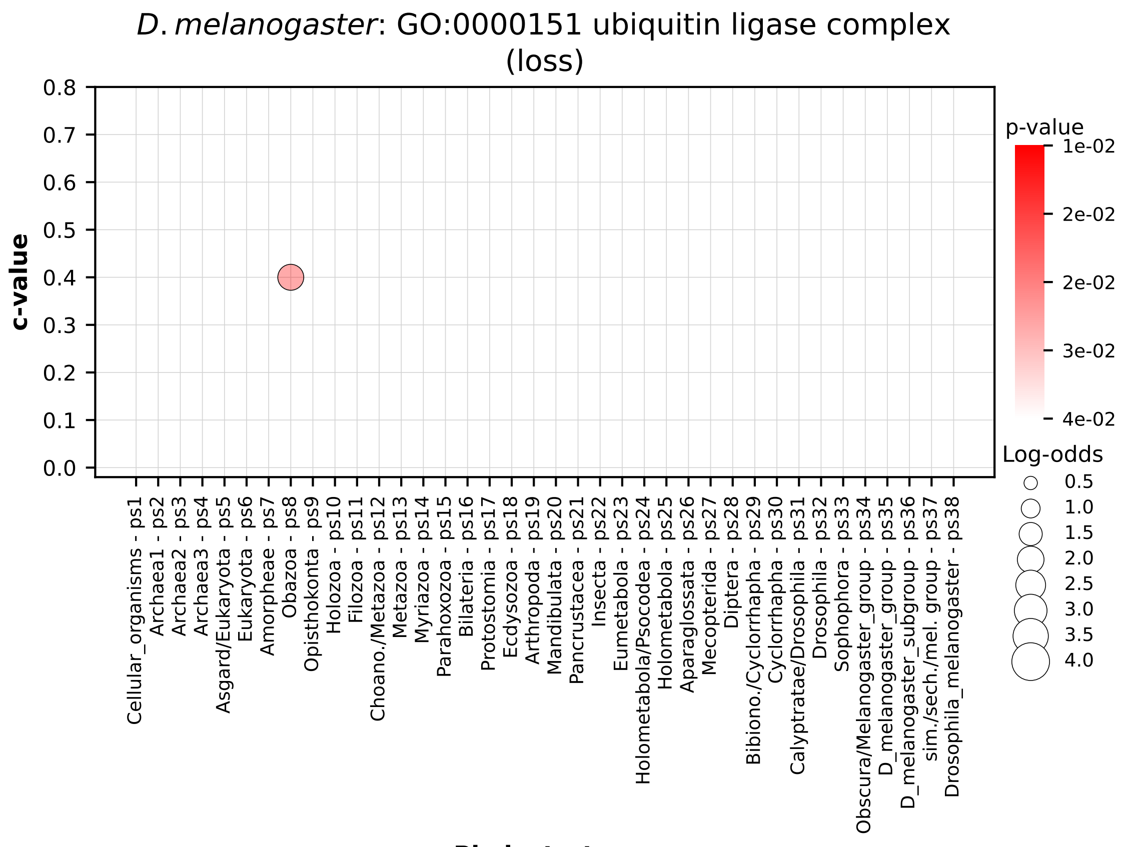

D. melanogaster: GO:0000151 ubiquitin ligase complex
(loss)
c-value
0.8
0.7
0.6
0.5
0.4
0.3
0.2
0.1
0.0
Phylostratum
Cellular_organisms - ps1
Archaea1 - ps2
Archaea2 - ps3
Archaea3 - ps4
Asgard/Eukaryota - ps5
Eukaryota - ps6
Amorpheae - ps7
Obazoa - ps8
Opisthokonta - ps9
Holozoa - ps10
Filozoa - ps11
Choano./Metazoa - ps12
Metazoa - ps13
Myriazoa - ps14
Parahoxozoa - ps15
Bilateria - ps16
Protostomia - ps17
Ecdysozoa - ps18
Arthropoda - ps19
Mandibulata - ps20
Pancrustacea - ps21
Insecta - ps22
Eumetabola - ps23
Holometabola/Psocodea - ps24
Holometabola - ps25
Aparaglossata - ps26
Mecopterida - ps27
Diptera - ps28
Bibiono./Cyclorrhapha - ps29
Cyclorrhapha - ps30
Calyptratae/Drosophila - ps31
Drosophila - ps32
Sophophora - ps33
Obscura/Melanogaster_group - ps34
D_melanogaster_group - ps35
D_melanogaster_subgroup - ps36
sim./sech./mel. group - ps37
Drosophila_melanogaster - ps38
p-value
1e-02
2e-02
2e-02
3e-02
4e-02
Log-odds
0.5
1.0
1.5
2.0
2.5
3.0
3.5
4.0

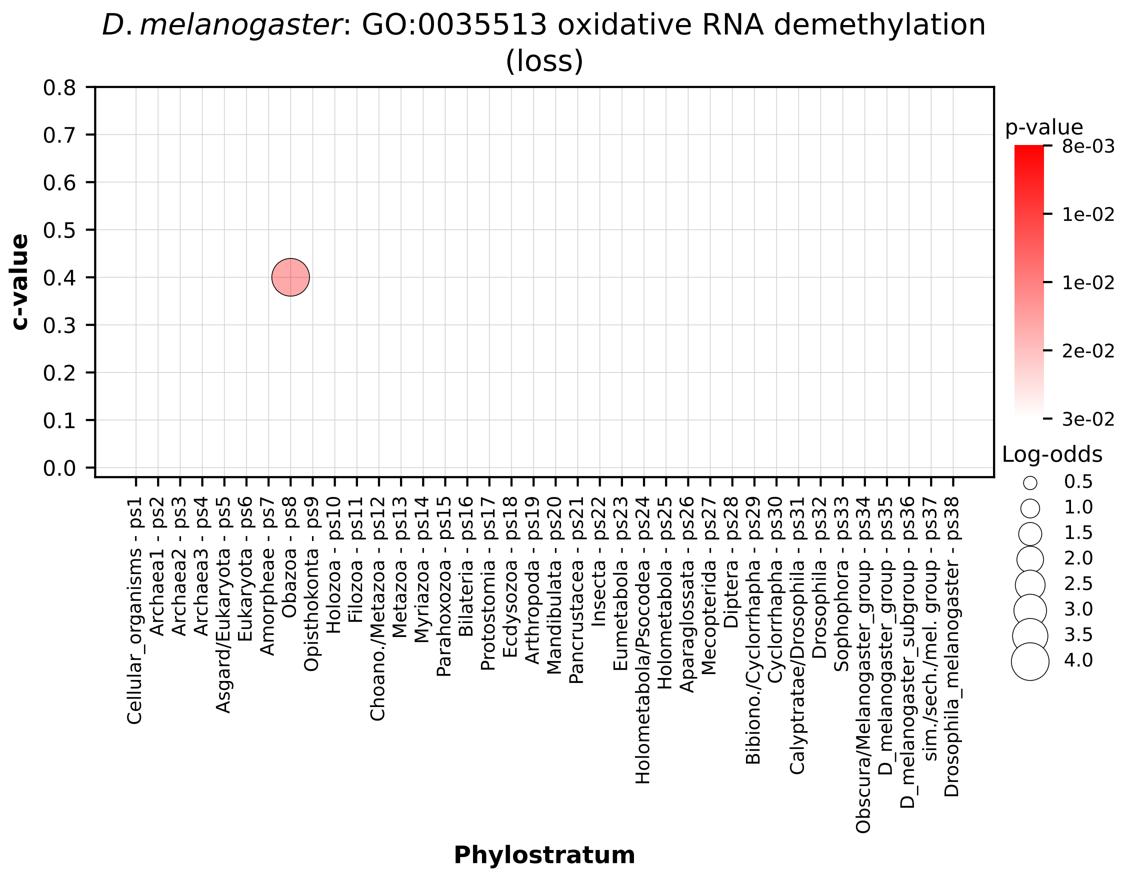

D. melanogaster: GO:0035513 oxidative RNA demethylation (loss)
c-value
0.8
0.7
0.6
0.5
0.4
0.3
0.2
0.1
0.0
p-value
8e-03
1e-02
1e-02
2e-02
3e-02
Log-odds
0.5
1.0
1.5
2.0
2.5
3.0
3.5
4.0
Cellular_organisms - ps1
Archaea1 - ps2
Archaea2 - ps3
Archaea3 - ps4
Asgard/Eukaryota - ps5
Eukaryota - ps6
Amorpheae - ps7
Obazoa - ps8
Opisthokonta - ps9
Holozoa - ps10
Filozoa - ps11
Choano./Metazoa - ps12
Metazoa - ps13
Myriazoa - ps14
Parahoxozoa - ps15
Bilateria - ps16
Protostomia - ps17
Ecdysozoa - ps18
Arthropoda - ps19
Mandibulata - ps20
Pancrustacea - ps21
Insecta - ps22
Eumetabola - ps23
Holometabola/Psocodea - ps24
Holometabola - ps25
Aparaglossata - ps26
Mecopterida - ps27
Diptera - ps28
Bibiono./Cyclorrhapha - ps29
Cyclorrhapha - ps30
Calyptratae/Drosophila - ps31
Drosophila - ps32
Sophophora - ps33
Obscura/Melanogaster_group - ps34
D_melanogaster_group - ps35
D_melanogaster_subgroup - ps36
sim./sech./mel. group - ps37
Drosophila_melanogaster - ps38
Phylostratum

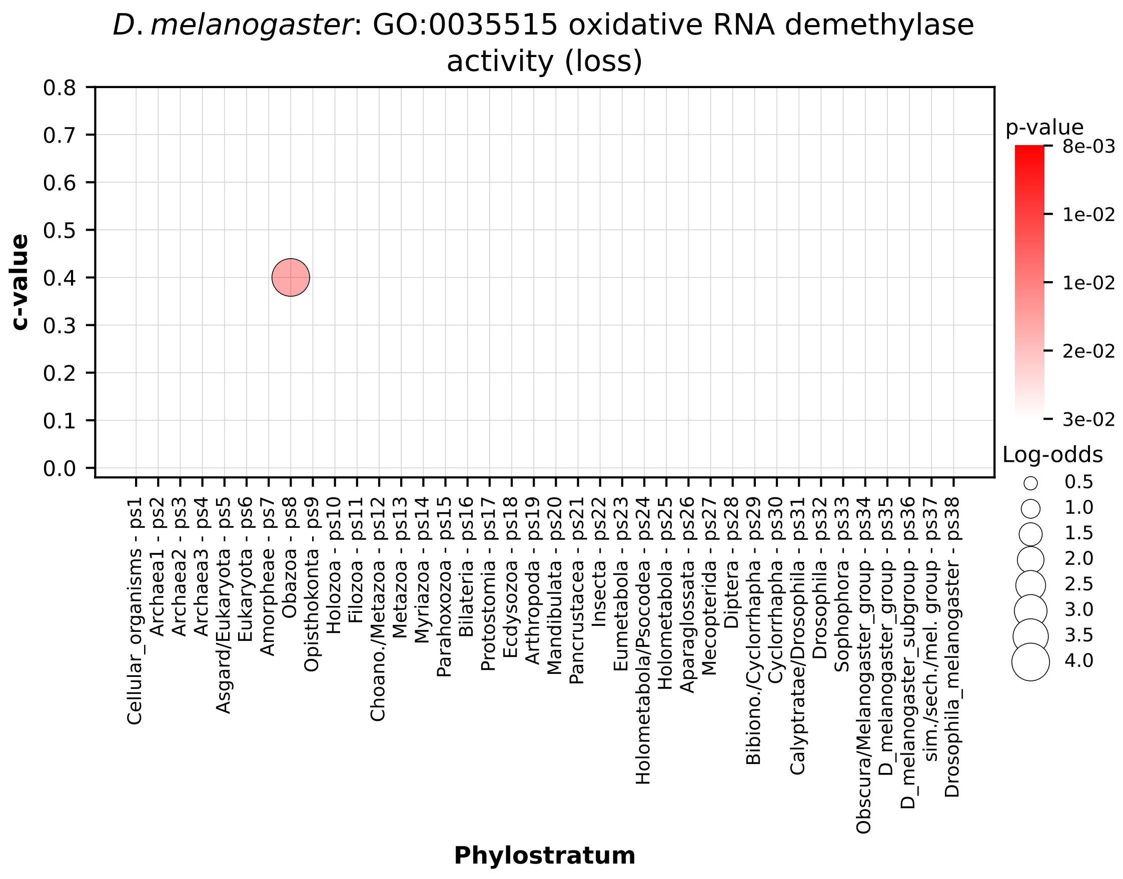

D. melanogaster: GO:0035515 oxidative RNA demethylase activity (loss)
c-value
0.8
0.7
0.6
0.5
0.4
0.3
0.2
0.1
0.0
Phylostratum
Cellular_organisms - ps1
Archaea1 - ps2
Archaea2 - ps3
Archaea3 - ps4
Asgard/Eukaryota - ps5
Eukaryota - ps6
Amorpheae - ps7
Obazoa - ps8
Opisthokonta - ps9
Holozoa - ps10
Filozoa - ps11
Choano./Metazoa - ps12
Metazoa - ps13
Myriazoa - ps14
Parahoxozoa - ps15
Bilateria - ps16
Protostomia - ps17
Ecdysozoa - ps18
Arthropoda - ps19
Mandibulata - ps20
Pancrustacea - ps21
Insecta - ps22
Eumetabola - ps23
Holometabola/Psocodea - ps24
Holometabola - ps25
Aparaglossata - ps26
Mecopterida - ps27
Diptera - ps28
Bibiono./Cyclorrhapha - ps29
Cyclorrhapha - ps30
Calyptratae/Drosophila - ps31
Drosophila - ps32
Sophophora - ps33
Obscura/Melanogaster_group - ps34
D_melanogaster_group - ps35
D_melanogaster_subgroup - ps36
sim./sech./mel. group - ps37
Drosophila_melanogaster - ps38
p-value
8e-03
1e-02
1e-02
2e-02
3e-02
Log-odds
0.5
1.0
1.5
2.0
2.5
3.0
3.5
4.0

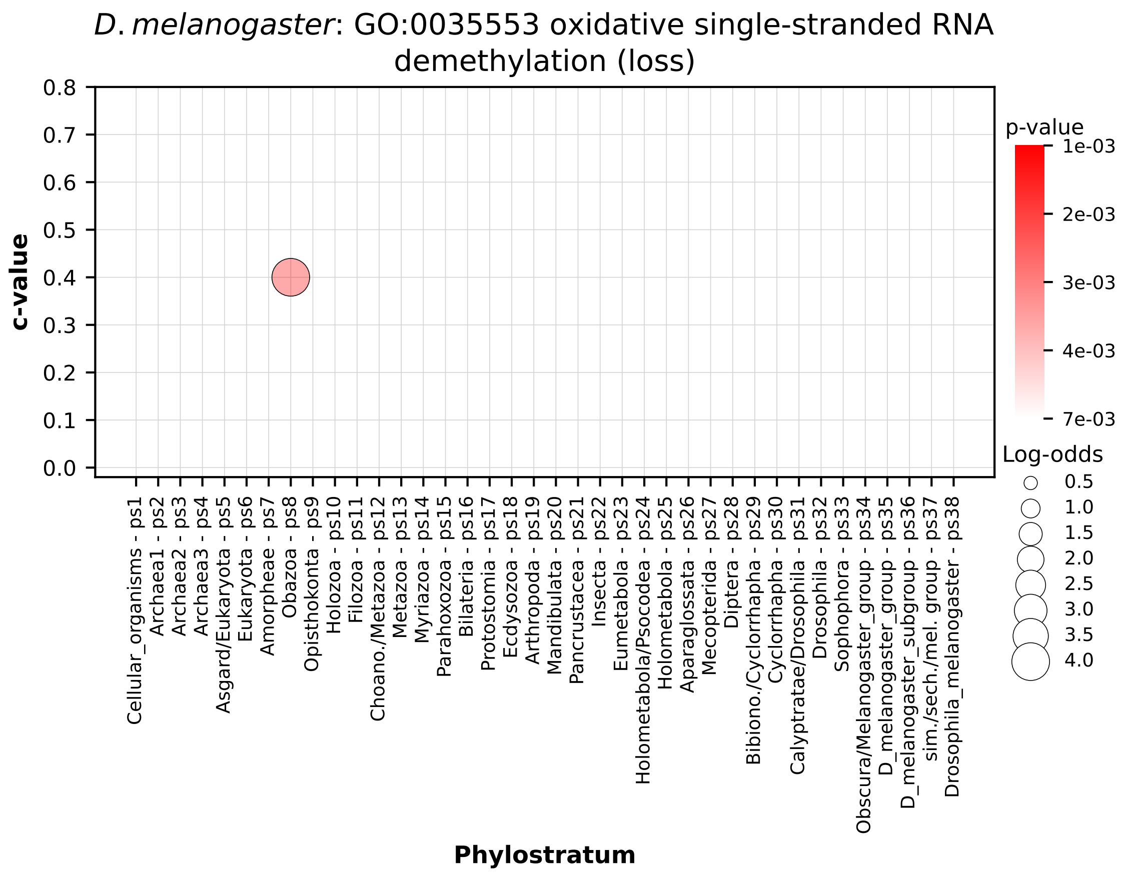

D. melanogaster: GO:0035553 oxidative single-stranded RNA demethylation (loss)
c-value
0.8
0.7
0.6
0.5
0.4
0.3
0.2
0.1
0.0
Phylostratum
Cellular_organisms - ps1
Archaea1 - ps2
Archaea2 - ps3
Archaea3 - ps4
Asgard/Eukaryota - ps5
Eukaryota - ps6
Amorpheae - ps7
Obazoa - ps8
Opisthokonta - ps9
Holozoa - ps10
Filozoa - ps11
Choano./Metazoa - ps12
Metazoa - ps13
Myriazoa - ps14
Parahoxozoa - ps15
Bilateria - ps16
Protostomia - ps17
Ecdysozoa - ps18
Arthropoda - ps19
Mandibulata - ps20
Pancrustacea - ps21
Insecta - ps22
Eumetabola - ps23
Holometabola/Psocodea - ps24
Holometabola - ps25
Aparaglossata - ps26
Mecopterida - ps27
Diptera - ps28
Bibiono./Cyclorrhapha - ps29
Cyclorrhapha - ps30
Calyptratae/Drosophila - ps31
Drosophila - ps32
Sophophora - ps33
Obscura/Melanogaster_group - ps34
D_melanogaster_group - ps35
D_melanogaster_subgroup - ps36
sim./sech./mel. group - ps37
Drosophila_melanogaster - ps38
p-value
1e-03
2e-03
3e-03
4e-03
7e-03
Log-odds
0.5
1.0
1.5
2.0
2.5
3.0
3.5
4.0

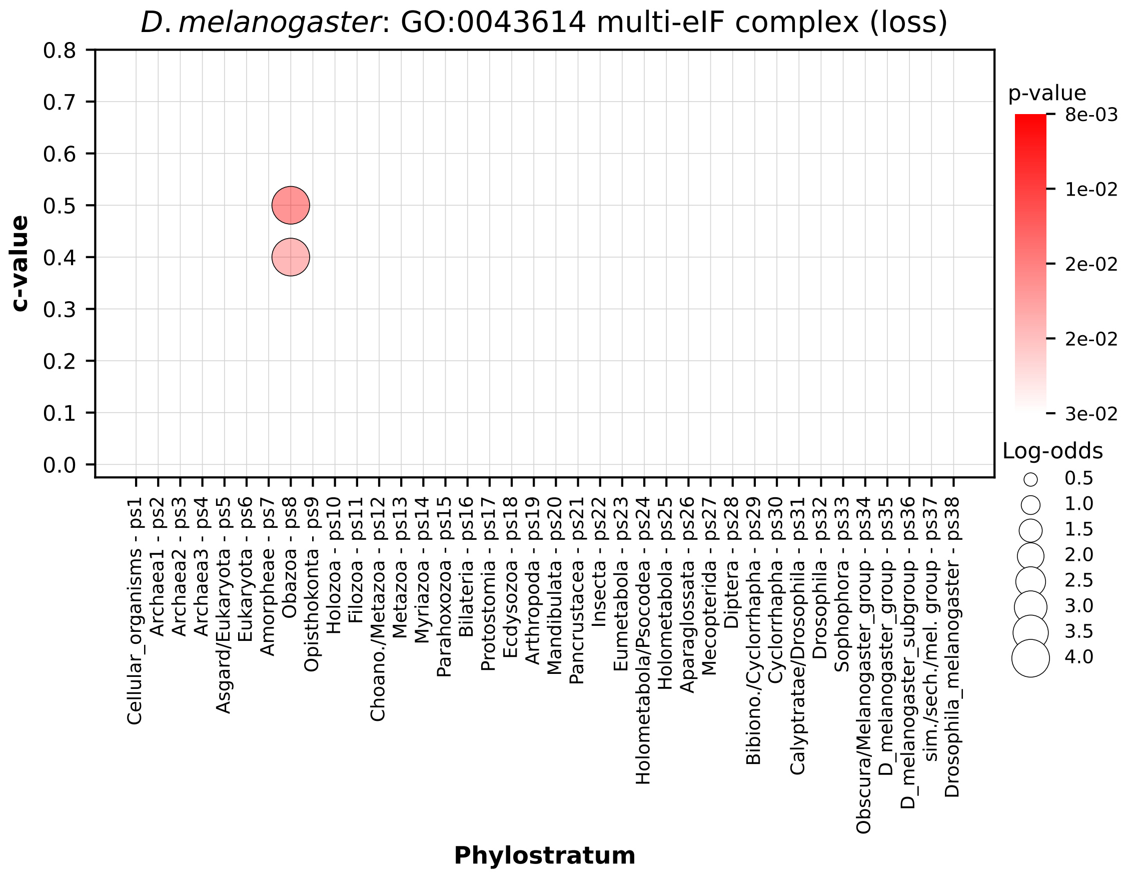
D. melanogaster: GO:0043614 multi-eIF complex (loss)
c-value
0.0
0.1
0.2
0.3
0.4
0.5
0.6
0.7
0.8
p-value
8e-03
1e-02
2e-02
2e-02
3e-02
Log-odds
0.5
1.0
1.5
2.0
2.5
3.0
3.5
4.0
Cellular_organisms - ps1
Archaea1 - ps2
Archaea2 - ps3
Archaea3 - ps4
Asgard/Eukaryota - ps5
Eukaryota - ps6
Amorpheae - ps7
Obazoa - ps8
Opisthokonta - ps9
Holozoa - ps10
Filozoa - ps11
Choano./Metazoa - ps12
Metazoa - ps13
Myriazoa - ps14
Parahoxozoa - ps15
Bilateria - ps16
Protostomia - ps17
Ecdysozoa - ps18
Arthropoda - ps19
Mandibulata - ps20
Pancrustacea - ps21
Insecta - ps22
Eumetabola - ps23
Holometabola/Psocodea - ps24
Holometabola - ps25
Aparaglossata - ps26
Mecopterida - ps27
Diptera - ps28
Bibiono./Cyclorrhapha - ps29
Cyclorrhapha - ps30
Calyptratae/Drosophila - ps31
Drosophila - ps32
Sophophora - ps33
Obscura/Melanogaster_group - ps34
D_melanogaster_group - ps35
D_melanogaster_subgroup - ps36
sim./sech./mel. group - ps37
Drosophila_melanogaster - ps38
Phylostratum

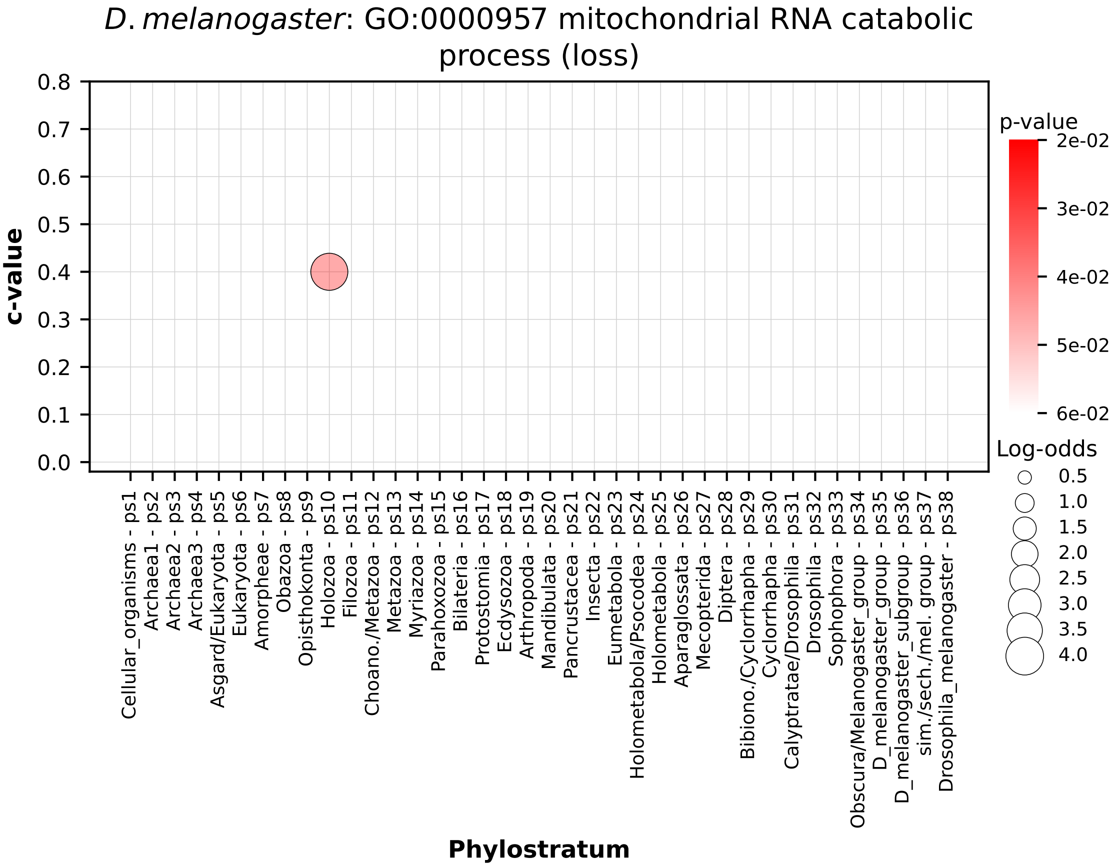

D. melanogaster: GO:0000957 mitochondrial RNA catabolic process (loss)
c-value
0.8
0.7
0.6
0.5
0.4
0.3
0.2
0.1
0.0
p-value
2e-02
3e-02
4e-02
5e-02
6e-02
Log-odds
0.5
1.0
1.5
2.0
2.5
3.0
3.5
4.0
Cellular_organisms - ps1
Archaea1 - ps2
Archaea2 - ps3
Archaea3 - ps4
Asgard/Eukaryota - ps5
Eukaryota - ps6
Amorpheae - ps7
Obazoa - ps8
Opisthokonta - ps9
Holozoa - ps10
Filozoa - ps11
Choano./Metazoa - ps12
Metazoa - ps13
Myriazoa - ps14
Parahoxozoa - ps15
Bilateria - ps16
Protostomia - ps17
Ecdysozoa - ps18
Arthropoda - ps19
Mandibulata - ps20
Pancrustacea - ps21
Insecta - ps22
Eumetabola - ps23
Holometabola/Psocodea - ps24
Holometabola - ps25
Aparaglossata - ps26
Mecopterida - ps27
Diptera - ps28
Bibiono./Cyclorrhapha - ps29
Cyclorrhapha - ps30
Calyptratae/Drosophila - ps31
Drosophila - ps32
Sophophora - ps33
Obscura/Melanogaster_group - ps34
D_melanogaster_group - ps35
D_melanogaster_subgroup - ps36
sim./sech./mel. group - ps37
Drosophila_melanogaster - ps38
Phylostratum

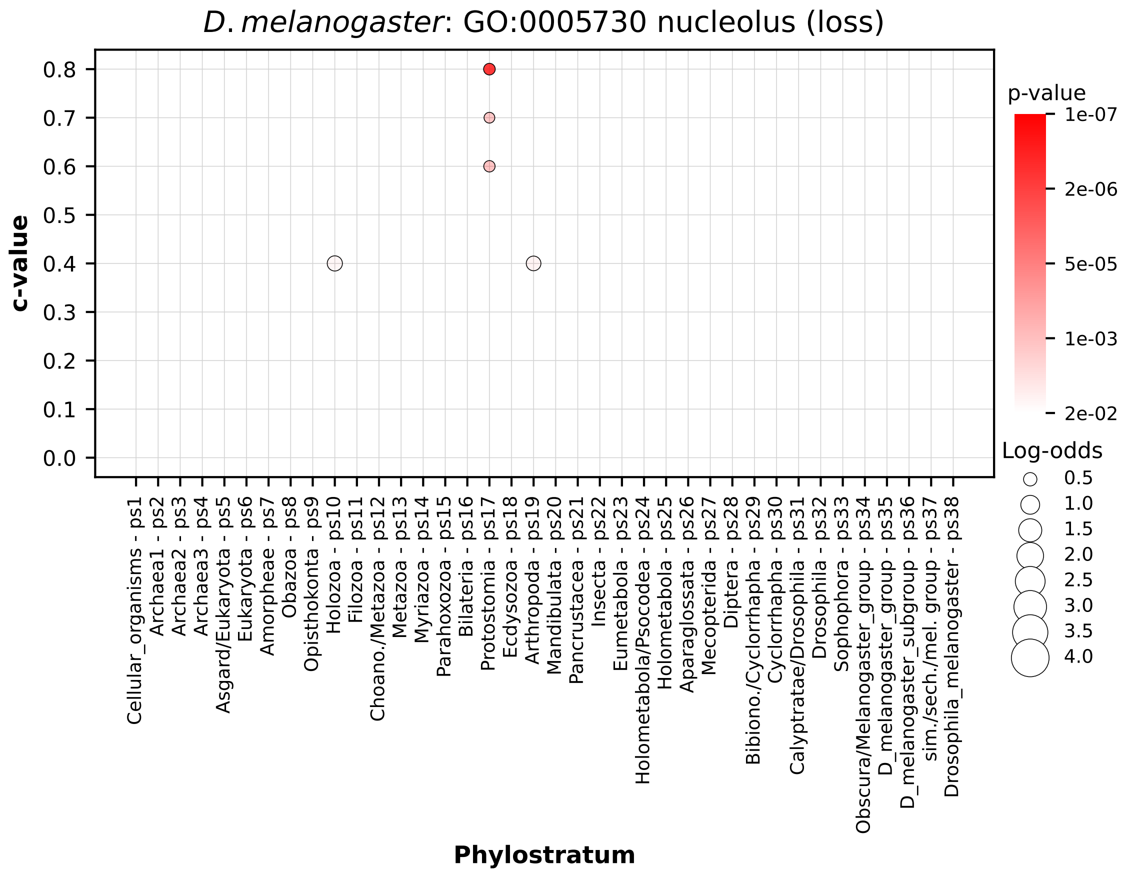

D. melanogaster: GO:0005730 nucleolus (loss)
c-value
Phylostratum
p-value
1e-07
2e-06
5e-05
1e-03
2e-02
Log-odds
0.5
1.0
1.5
2.0
2.5
3.0
3.5
4.0
Cellular_organisms - ps1
Archaea1 - ps2
Archaea2 - ps3
Archaea3 - ps4
Asgard/Eukaryota - ps5
Eukaryota - ps6
Amorpheae - ps7
Obazoa - ps8
Opisthokonta - ps9
Holozoa - ps10
Filozoa - ps11
Choano./Metazoa - ps12
Metazoa - ps13
Myriazoa - ps14
Parahoxozoa - ps15
Bilateria - ps16
Protostomia - ps17
Ecdysozoa - ps18
Arthropoda - ps19
Mandibulata - ps20
Pancrustacea - ps21
Insecta - ps22
Eumetabola - ps23
Holometabola/Psocodea - ps24
Holometabola - ps25
Aparaglossata - ps26
Mecopterida - ps27
Diptera - ps28
Bibiono./Cyclorrhapha - ps29
Cyclorrhapha - ps30
Calyptratae/Drosophila - ps31
Drosophila - ps32
Sophophora - ps33
Obscura/Melanogaster_group - ps34
D_melanogaster_group - ps35
D_melanogaster_subgroup - ps36
sim./sech./mel. group - ps37
Drosophila_melanogaster - ps38

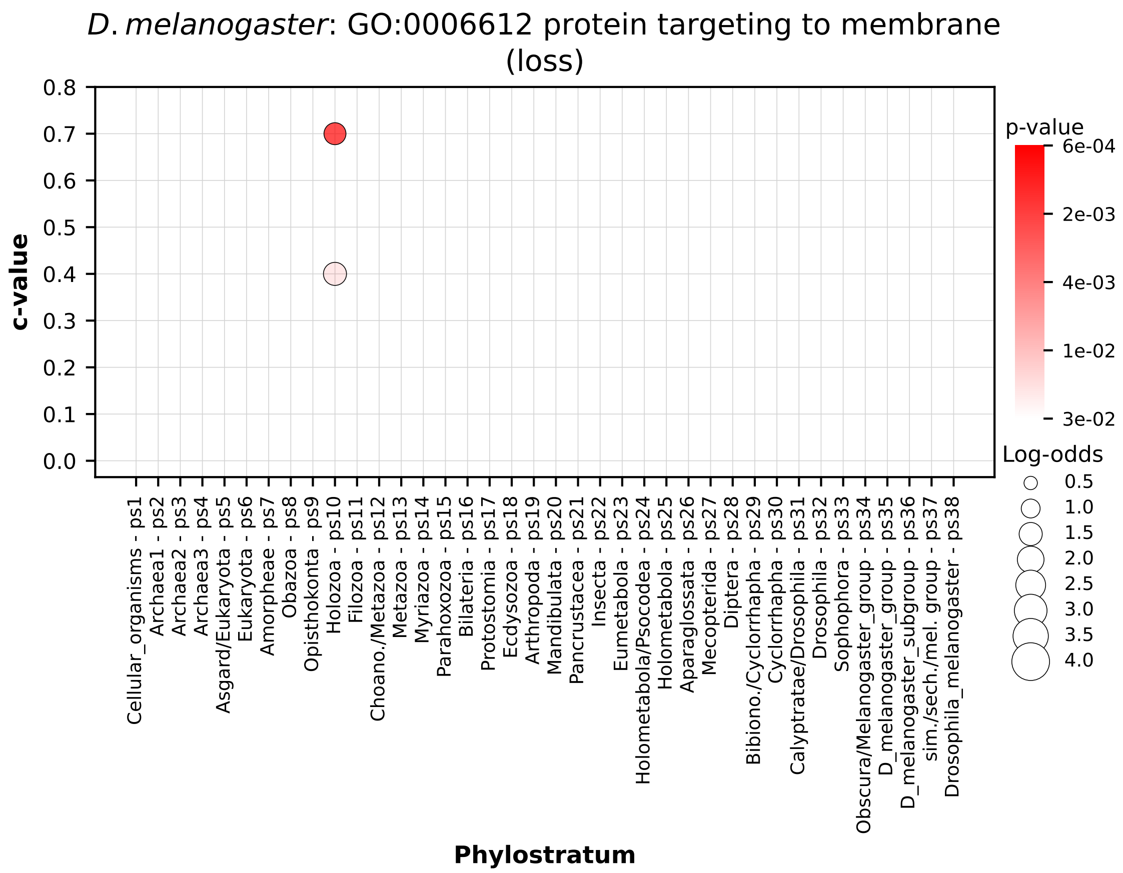
D. melanogaster: GO:0006612 protein targeting to membrane
(loss)
c-value
0.8
0.7
0.6
0.5
0.4
0.3
0.2
0.1
0.0
Phylostratum
Cellular_organisms - ps1
Archaea1 - ps2
Archaea2 - ps3
Archaea3 - ps4
Asgard/Eukaryota - ps5
Eukaryota - ps6
Amorpheae - ps7
Obazoa - ps8
Opisthokonta - ps9
Holozoa - ps10
Filozoa - ps11
Choano./Metazoa - ps12
Metazoa - ps13
Myriazoa - ps14
Parahoxozoa - ps15
Bilateria - ps16
Protostomia - ps17
Ecdysozoa - ps18
Arthropoda - ps19
Mandibulata - ps20
Pancrustacea - ps21
Insecta - ps22
Eumetabola - ps23
Holometabola/Psocodea - ps24
Holometabola - ps25
Aparaglossata - ps26
Mecopterida - ps27
Diptera - ps28
Bibiono./Cyclorrhapha - ps29
Cyclorrhapha - ps30
Calyptratae/Drosophila - ps31
Drosophila - ps32
Sophophora - ps33
Obscura/Melanogaster_group - ps34
D_melanogaster_group - ps35
D_melanogaster_subgroup - ps36
sim./sech./mel. group - ps37
Drosophila_melanogaster - ps38
p-value
6e-04
2e-03
4e-03
1e-02
3e-02
Log-odds
0.5
1.0
1.5
2.0
2.5
3.0
3.5
4.0

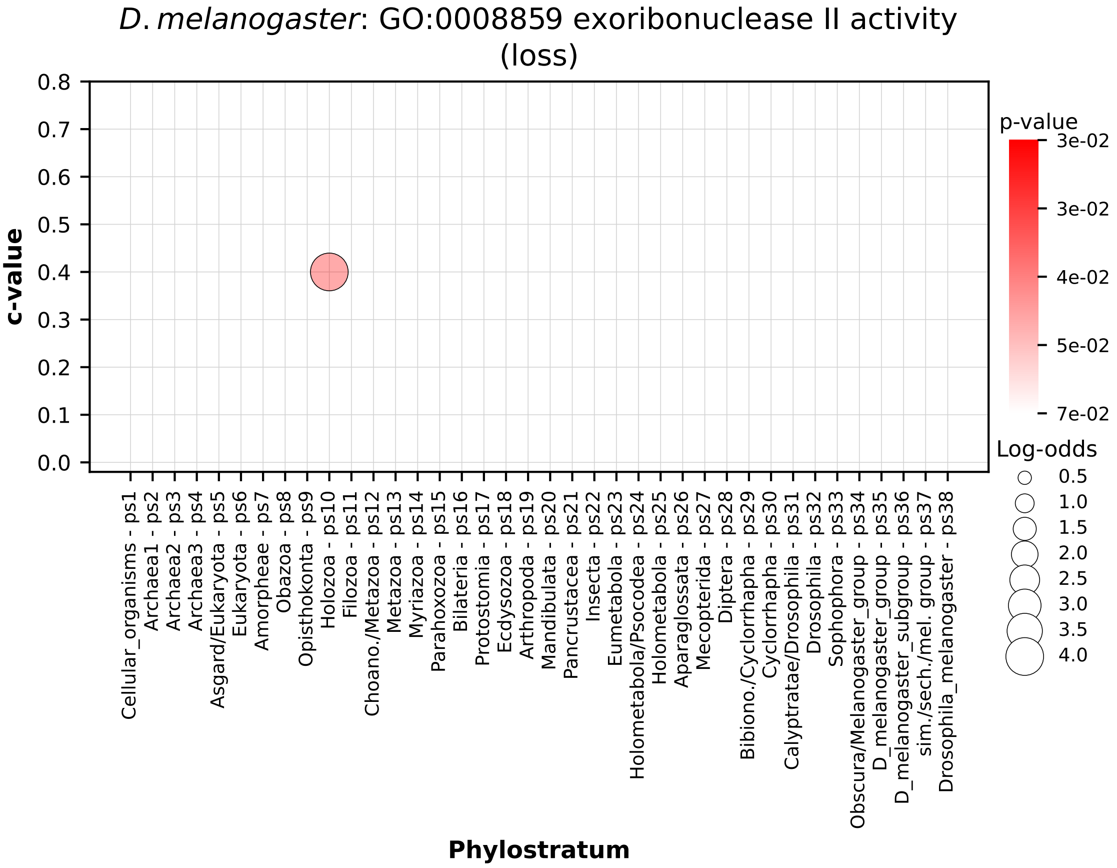

D. melanogaster: GO:0008859 exoribonuclease II activity
(loss)
c-value
0.0
0.1
0.2
0.3
0.4
0.5
0.6
0.7
0.8
Phylostratum
Cellular_organisms - ps1
Archaea1 - ps2
Archaea2 - ps3
Archaea3 - ps4
Asgard/Eukaryota - ps5
Eukaryota - ps6
Amorpheae - ps7
Obazoa - ps8
Opisthokonta - ps9
Holozoa - ps10
Filozoa - ps11
Choano./Metazoa - ps12
Metazoa - ps13
Myriazoa - ps14
Parahoxozoa - ps15
Bilateria - ps16
Protostomia - ps17
Ecdysozoa - ps18
Arthropoda - ps19
Mandibulata - ps20
Pancrustacea - ps21
Insecta - ps22
Eumetabola - ps23
Holometabola/Psocodea - ps24
Holometabola - ps25
Aparaglossata - ps26
Mecopterida - ps27
Diptera - ps28
Bibiono./Cyclorrhapha - ps29
Cyclorrhapha - ps30
Calyptratae/Drosophila - ps31
Drosophila - ps32
Sophophora - ps33
Obscura/Melanogaster_group - ps34
D_melanogaster_group - ps35
D_melanogaster_subgroup - ps36
sim./sech./mel. group - ps37
Drosophila_melanogaster - ps38
p-value
3e-02
3e-02
4e-02
5e-02
7e-02
Log-odds
0.5
1.0
1.5
2.0
2.5
3.0
3.5
4.0

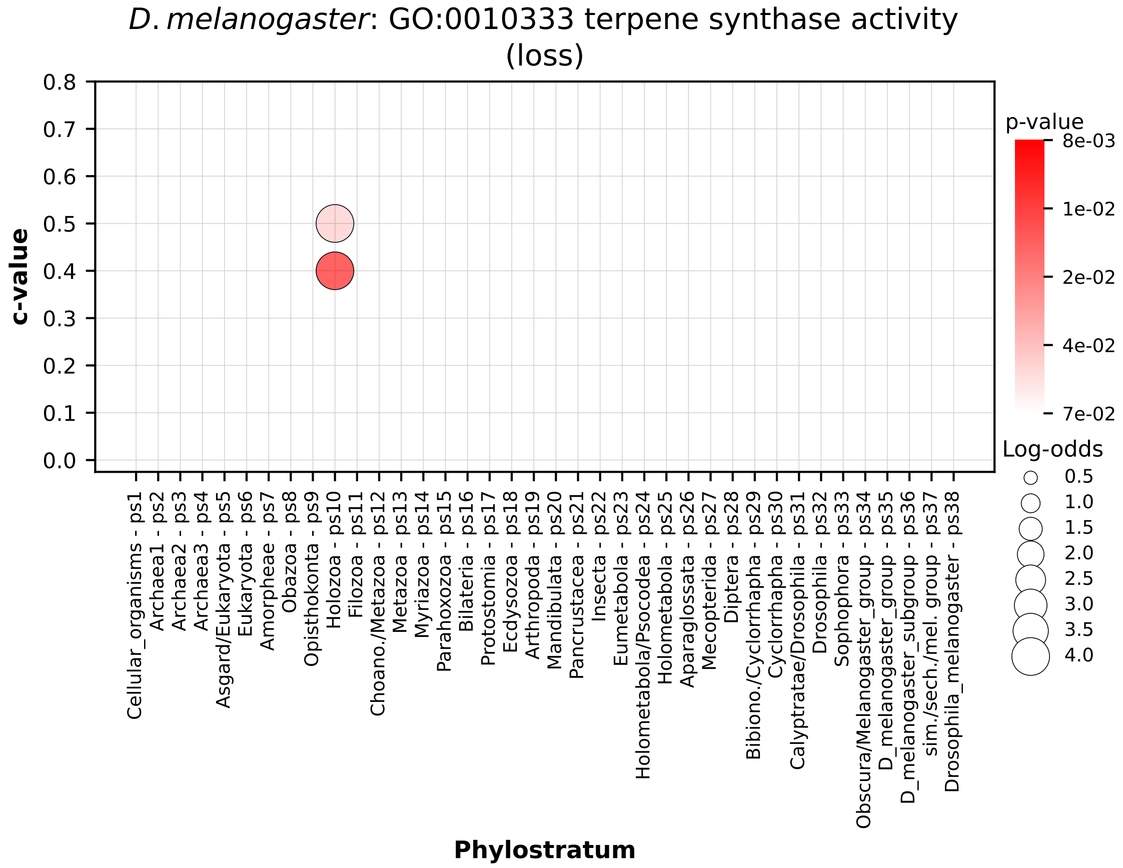

D. melanogaster: GO:0010333 terpene synthase activity (loss)
c-value
0.8
0.7
0.6
0.5
0.4
0.3
0.2
0.1
0.0
Phylostratum
Cellular_organisms - ps1
Archaea1 - ps2
Archaea2 - ps3
Archaea3 - ps4
Asgard/Eukaryota - ps5
Eukaryota - ps6
Amorpheae - ps7
Obazoa - ps8
Opisthokonta - ps9
Holozoa - ps10
Filozoa - ps11
Choano./Metazoa - ps12
Metazoa - ps13
Myriazoa - ps14
Parahoxozoa - ps15
Bilateria - ps16
Protostomia - ps17
Ecdysozoa - ps18
Arthropoda - ps19
Mandibulata - ps20
Pancrustacea - ps21
Insecta - ps22
Eumetabola - ps23
Holometabola/Psocodea - ps24
Holometabola - ps25
Aparaglossata - ps26
Mecopterida - ps27
Diptera - ps28
Bibiono./Cyclorrhapha - ps29
Cyclorrhapha - ps30
Calyptratae/Drosophila - ps31
Drosophila - ps32
Sophophora - ps33
Obscura/Melanogaster_group - ps34
D_melanogaster_group - ps35
D_melanogaster_subgroup - ps36
sim./sech./mel. group - ps37
Drosophila_melanogaster - ps38
p-value
8e-03
1e-02
2e-02
4e-02
7e-02
Log-odds
0.5
1.0
1.5
2.0
2.5
3.0
3.5
4.0

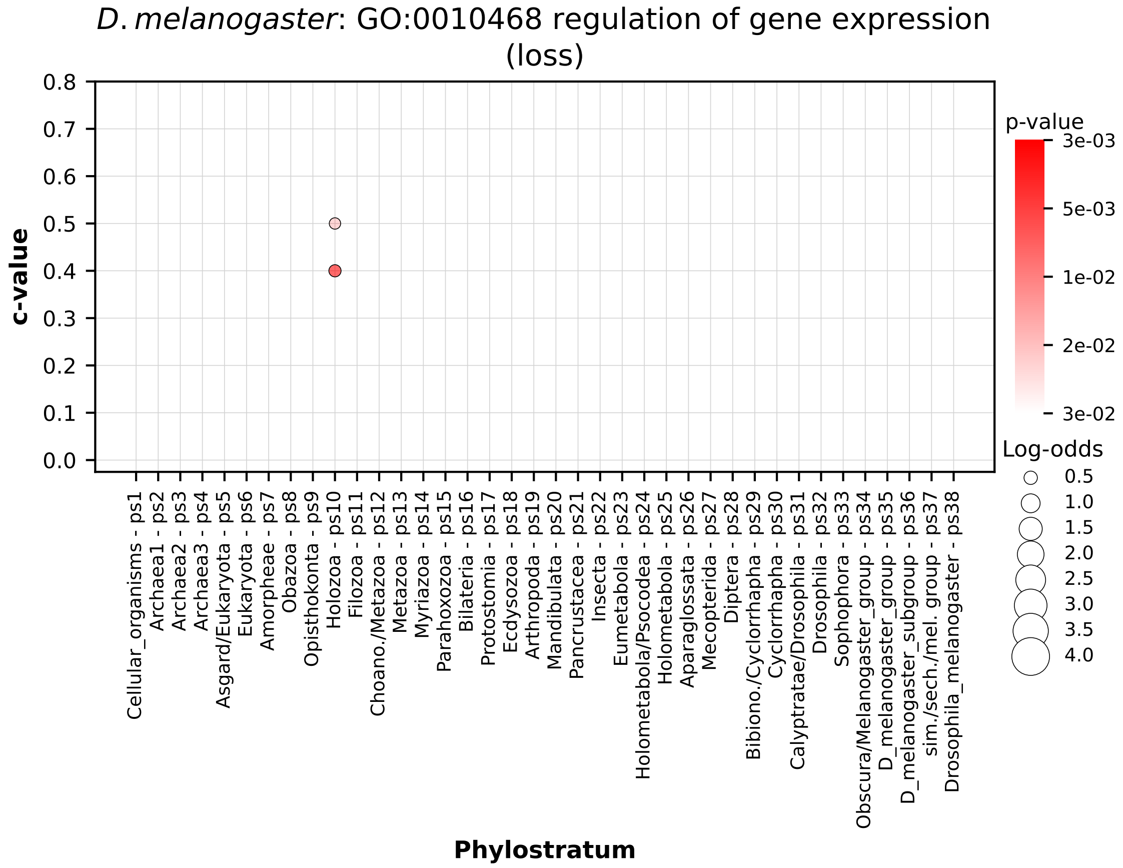

D. melanogaster: GO:0010468 regulation of gene expression (loss)
c-value
0.8
0.7
0.6
0.5
0.4
0.3
0.2
0.1
0.0
p-value
3e-03
5e-03
1e-02
2e-02
3e-02
Log-odds
0.5
1.0
1.5
2.0
2.5
3.0
3.5
4.0
Cellular_organisms - ps1
Archaea1 - ps2
Archaea2 - ps3
Archaea3 - ps4
Asgard/Eukaryota - ps5
Eukaryota - ps6
Amorpheae - ps7
Obazoa - ps8
Opisthokonta - ps9
Holozoa - ps10
Filozoa - ps11
Choano./Metazoa - ps12
Metazoa - ps13
Myriazoa - ps14
Parahoxozoa - ps15
Bilateria - ps16
Protostomia - ps17
Ecdysozoa - ps18
Arthropoda - ps19
Mandibulata - ps20
Pancrustacea - ps21
Insecta - ps22
Eumetabola - ps23
Holometabola/Psocodea - ps24
Holometabola - ps25
Aparaglossata - ps26
Mecopterida - ps27
Diptera - ps28
Bibiono./Cyclorrhapha - ps29
Cyclorrhapha - ps30
Calyptratae/Drosophila - ps31
Drosophila - ps32
Sophophora - ps33
Obscura/Melanogaster_group - ps34
D_melanogaster_group - ps35
D_melanogaster_subgroup - ps36
sim./sech./mel. group - ps37
Drosophila_melanogaster - ps38
Phylostratum

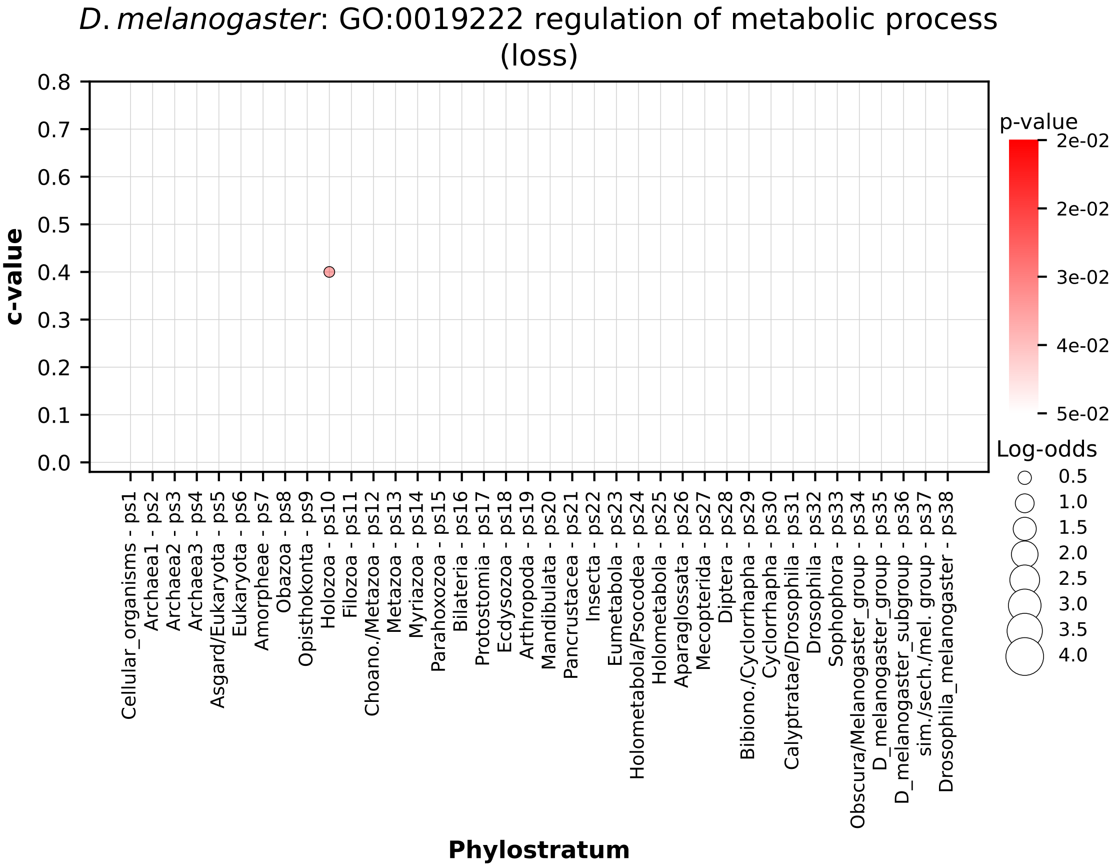

D. melanogaster: GO:0019222 regulation of metabolic process (loss)
c-value
0.8
0.7
0.6
0.5
0.4
0.3
0.2
0.1
0.0
p-value
2e-02
2e-02
3e-02
4e-02
5e-02
Log-odds
0.5
1.0
1.5
2.0
2.5
3.0
3.5
4.0
Cellular_organisms - ps1
Archaea1 - ps2
Archaea2 - ps3
Archaea3 - ps4
Asgard/Eukaryota - ps5
Eukaryota - ps6
Amorpheae - ps7
Obazoa - ps8
Opisthokonta - ps9
Holozoa - ps10
Filozoa - ps11
Choano./Metazoa - ps12
Metazoa - ps13
Myriazoa - ps14
Parahoxozoa - ps15
Bilateria - ps16
Protostomia - ps17
Ecdysozoa - ps18
Arthropoda - ps19
Mandibulata - ps20
Pancrustacea - ps21
Insecta - ps22
Eumetabola - ps23
Holometabola/Psocodea - ps24
Holometabola - ps25
Aparaglossata - ps26
Mecopterida - ps27
Diptera - ps28
Bibiono./Cyclorrhapha - ps29
Cyclorrhapha - ps30
Calyptratae/Drosophila - ps31
Drosophila - ps32
Sophophora - ps33
Obscura/Melanogaster_group - ps34
D_melanogaster_group - ps35
D_melanogaster_subgroup - ps36
sim./sech./mel. group - ps37
Drosophila_melanogaster - ps38
Phylostratum

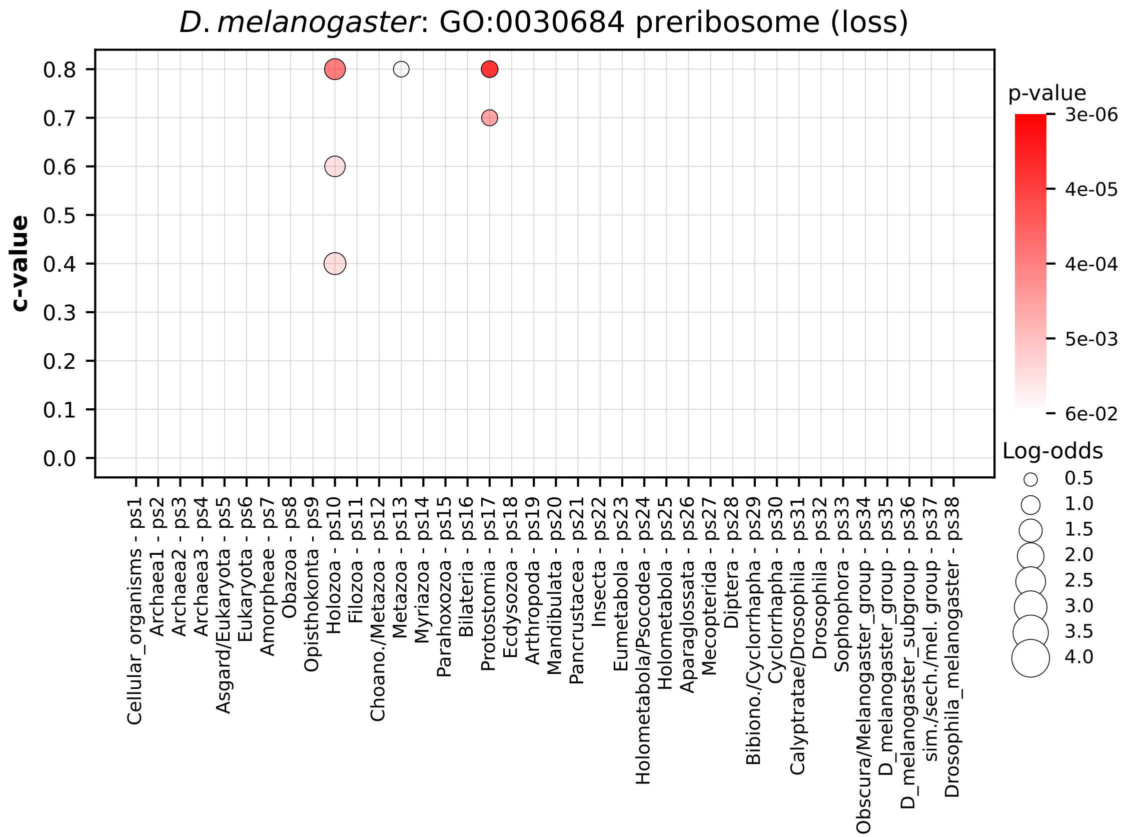
D. melanogaster: GO:0030684 preribosome (loss)
c-value
0.0
0.1
0.2
0.3
0.4
0.5
0.6
0.7
0.8
Phylostratum
Cellular_organisms - ps1
Archaea1 - ps2
Archaea2 - ps3
Archaea3 - ps4
Asgard/Eukaryota - ps5
Eukaryota - ps6
Amorpheae - ps7
Obazoa - ps8
Opisthokonta - ps9
Holozoa - ps10
Filozoa - ps11
Choano./Metazoa - ps12
Metazoa - ps13
Myriazoa - ps14
Parahoxozoa - ps15
Bilateria - ps16
Protostomia - ps17
Ecdysozoa - ps18
Arthropoda - ps19
Mandibulata - ps20
Pancrustacea - ps21
Insecta - ps22
Eumetabola - ps23
Holometabola/Psocodea - ps24
Holometabola - ps25
Aparaglossata - ps26
Mecopterida - ps27
Diptera - ps28
Bibiono./Cyclorrhapha - ps29
Cyclorrhapha - ps30
Calyptratae/Drosophila - ps31
Drosophila - ps32
Sophophora - ps33
Obscura/Melanogaster_group - ps34
D_melanogaster_group - ps35
D_melanogaster_subgroup - ps36
sim./sech./mel. group - ps37
Drosophila_melanogaster - ps38
p-value
3e-06
4e-05
4e-04
5e-03
6e-02
Log-odds
0.5
1.0
1.5
2.0
2.5
3.0
3.5
4.0

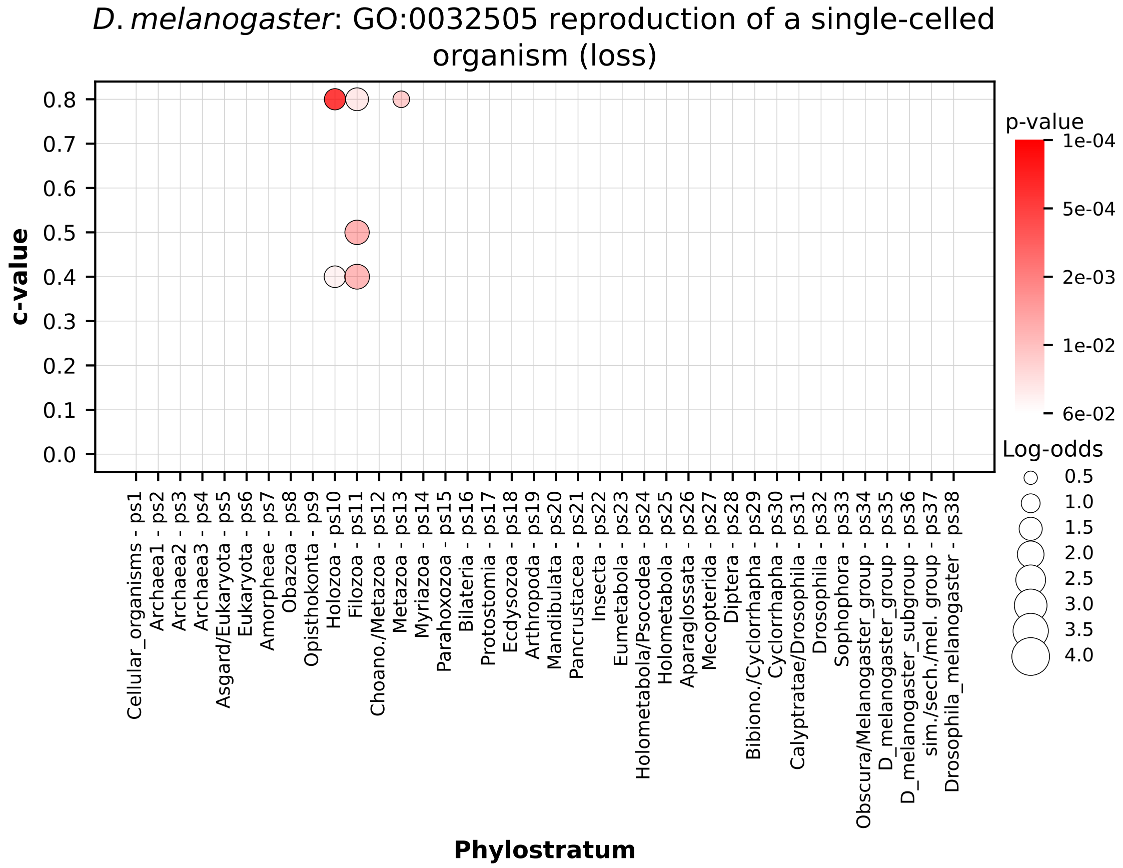

D. melanogaster: GO:0032505 reproduction of a single-celled organism (loss)
c-value
0.8
0.7
0.6
0.5
0.4
0.3
0.2
0.1
0.0
p-value
1e-04
5e-04
2e-03
1e-02
6e-02
Log-odds
0.5
1.0
1.5
2.0
2.5
3.0
3.5
4.0
Cellular_organisms - ps1
Archaea1 - ps2
Archaea2 - ps3
Archaea3 - ps4
Asgard/Eukaryota - ps5
Eukaryota - ps6
Amorpheae - ps7
Obazoa - ps8
Opisthokonta - ps9
Holozoa - ps10
Filozoa - ps11
Choano./Metazoa - ps12
Metazoa - ps13
Myriazoa - ps14
Parahoxozoa - ps15
Bilateria - ps16
Protostomia - ps17
Ecdysozoa - ps18
Arthropoda - ps19
Mandibulata - ps20
Pancrustacea - ps21
Insecta - ps22
Eumetabola - ps23
Holometabola/Psocodea - ps24
Holometabola - ps25
Aparaglossata - ps26
Mecopterida - ps27
Diptera - ps28
Bibiono./Cyclorrhapha - ps29
Cyclorrhapha - ps30
Calyptratae/Drosophila - ps31
Drosophila - ps32
Sophophora - ps33
Obscura/Melanogaster_group - ps34
D_melanogaster_group - ps35
D_melanogaster_subgroup - ps36
sim./sech./mel. group - ps37
Drosophila_melanogaster - ps38
Phylostratum

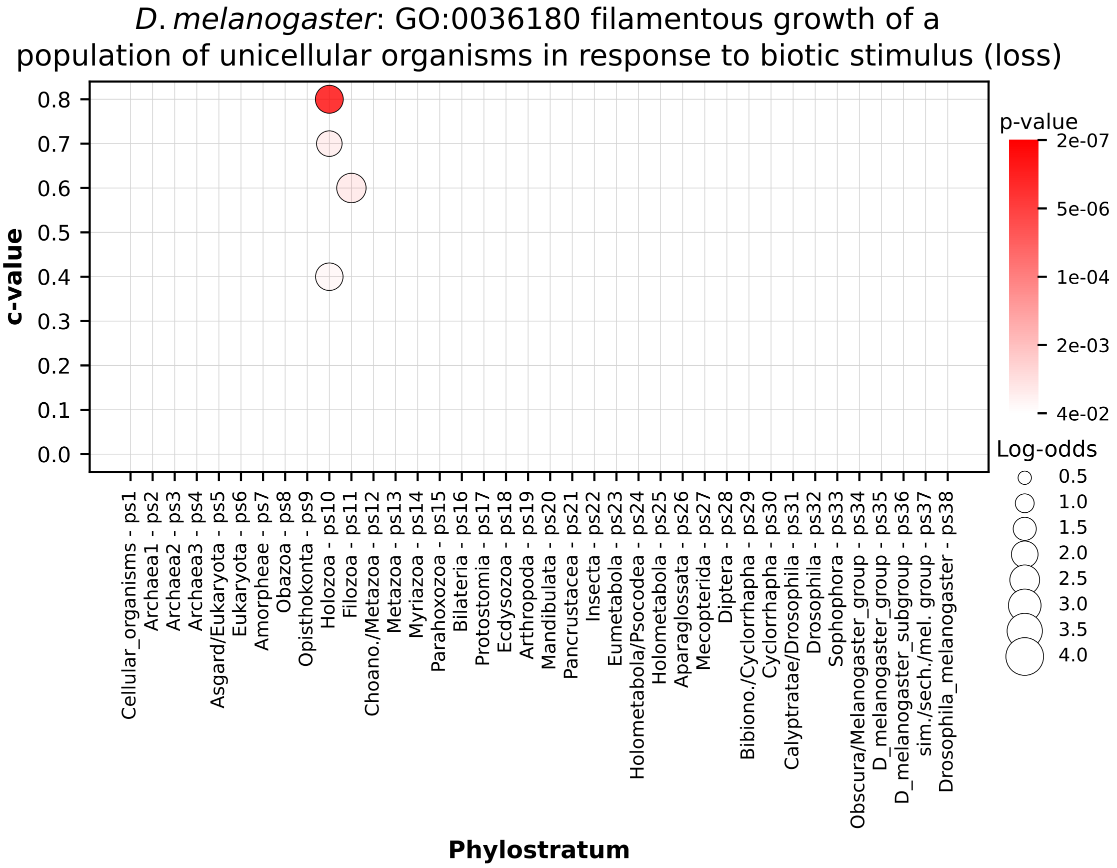

D. melanogaster: GO:0036180 filamentous growth of a population of unicellular organisms in response to biotic stimulus (loss)
c-value
0.0
0.1
0.2
0.3
0.4
0.5
0.6
0.7
0.8
p-value
2e-07
5e-06
1e-04
2e-03
4e-02
Log-odds
0.5
1.0
1.5
2.0
2.5
3.0
3.5
4.0
Cellular_organisms - ps1
Archaea1 - ps2
Archaea2 - ps3
Archaea3 - ps4
Asgard/Eukaryota - ps5
Eukaryota - ps6
Amorpheae - ps7
Obazoa - ps8
Opisthokonta - ps9
Holozoa - ps10
Filozoa - ps11
Choano./Metazoa - ps12
Metazoa - ps13
Myriazoa - ps14
Parahoxozoa - ps15
Bilateria - ps16
Protostomia - ps17
Ecdysozoa - ps18
Arthropoda - ps19
Mandibulata - ps20
Pancrustacea - ps21
Insecta - ps22
Eumetabola - ps23
Holometabola/Psocodea - ps24
Holometabola - ps25
Aparaglossata - ps26
Mecopterida - ps27
Diptera - ps28
Bibiono./Cyclorrhapha - ps29
Cyclorrhapha - ps30
Calyptratae/Drosophila - ps31
Drosophila - ps32
Sophophora - ps33
Obscura/Melanogaster_group - ps34
D_melanogaster_group - ps35
D_melanogaster_subgroup - ps36
sim./sech./mel. group - ps37
Drosophila_melanogaster - ps38
Phylostratum

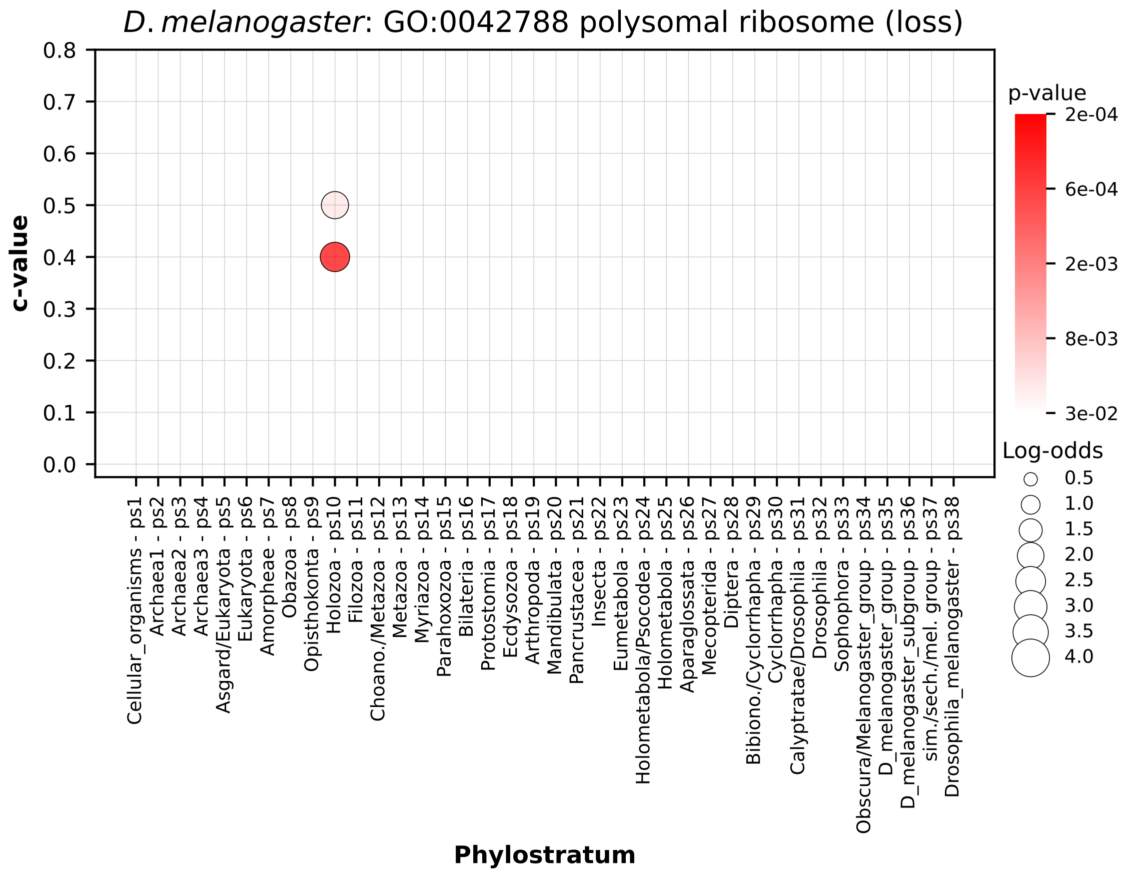

D. melanogaster: GO:0042788 polysomal ribosome (loss)
c-value
0.8
0.7
0.6
0.5
0.4
0.3
0.2
0.1
0.0
Phylostratum
Cellular_organisms - ps1
Archaea1 - ps2
Archaea2 - ps3
Archaea3 - ps4
Asgard/Eukaryota - ps5
Eukaryota - ps6
Amorpheae - ps7
Obazoa - ps8
Opisthokonta - ps9
Holozoa - ps10
Filozoa - ps11
Choano./Metazoa - ps12
Metazoa - ps13
Myriazoa - ps14
Parahoxozoa - ps15
Bilateria - ps16
Protostomia - ps17
Ecdysozoa - ps18
Arthropoda - ps19
Mandibulata - ps20
Pancrustacea - ps21
Insecta - ps22
Eumetabola - ps23
Holometabola/Psocodea - ps24
Holometabola - ps25
Aparaglossata - ps26
Mecopterida - ps27
Diptera - ps28
Bibiono./Cyclorrhapha - ps29
Cyclorrhapha - ps30
Calyptratae/Drosophila - ps31
Drosophila - ps32
Sophophora - ps33
Obscura/Melanogaster_group - ps34
D_melanogaster_group - ps35
D_melanogaster_subgroup - ps36
sim./sech./mel. group - ps37
Drosophila_melanogaster - ps38
p-value
2e-04
6e-04
2e-03
8e-03
3e-02
Log-odds
0.5
1.0
1.5
2.0
2.5
3.0
3.5
4.0

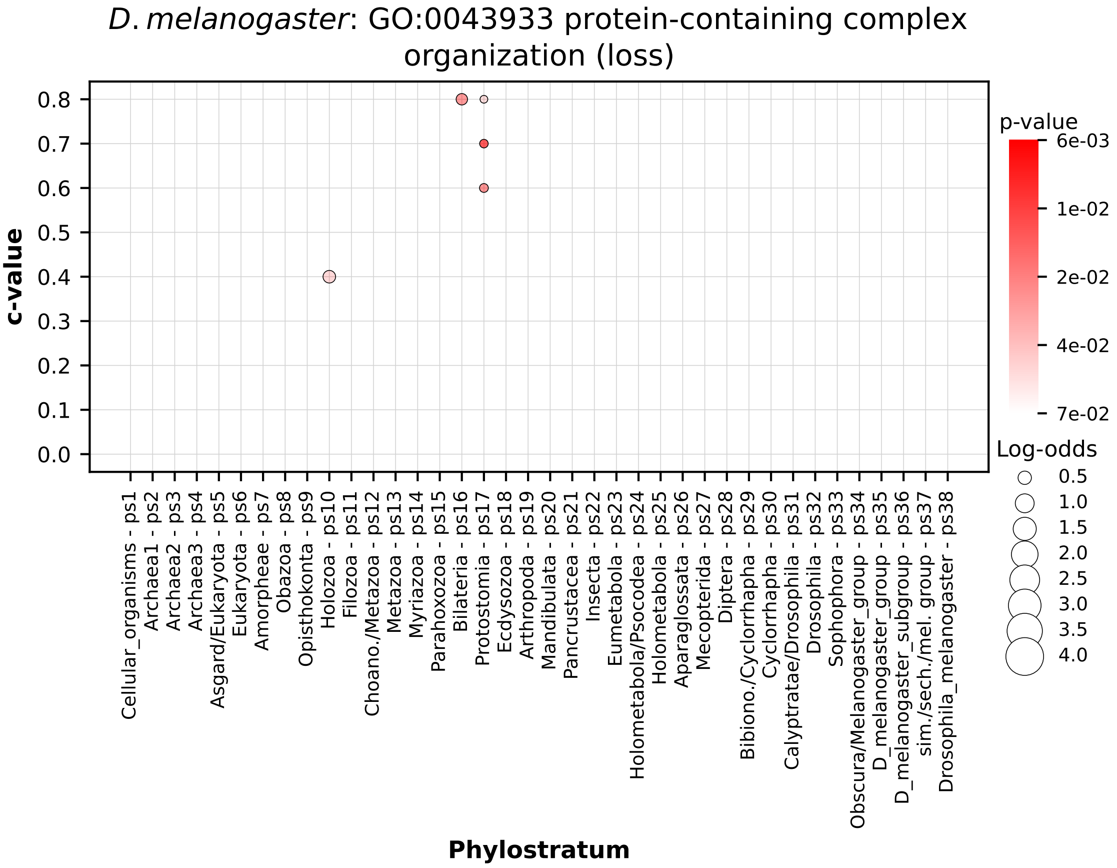
D. melanogaster: GO:0043933 protein-containing complex organization (loss)
c-value
0.0
0.1
0.2
0.3
0.4
0.5
0.6
0.7
0.8
Phylostratum
Cellular_organisms - ps1
Archaea1 - ps2
Archaea2 - ps3
Archaea3 - ps4
Asgard/Eukaryota - ps5
Eukaryota - ps6
Amorpheae - ps7
Obazoa - ps8
Opisthokonta - ps9
Holozoa - ps10
Filozoa - ps11
Choano./Metazoa - ps12
Metazoa - ps13
Myriazoa - ps14
Parahoxozoa - ps15
Bilateria - ps16
Protostomia - ps17
Ecdysozoa - ps18
Arthropoda - ps19
Mandibulata - ps20
Pancrustacea - ps21
Insecta - ps22
Eumetabola - ps23
Holometabola/Psocodea - ps24
Holometabola - ps25
Aparaglossata - ps26
Mecopterida - ps27
Diptera - ps28
Bibiono./Cyclorrhapha - ps29
Cyclorrhapha - ps30
Calyptratae/Drosophila - ps31
Drosophila - ps32
Sophophora - ps33
Obscura/Melanogaster_group - ps34
D_melanogaster_group - ps35
D_melanogaster_subgroup - ps36
sim./sech./mel. group - ps37
Drosophila_melanogaster - ps38
p-value
6e-03
1e-02
2e-02
4e-02
7e-02
Log-odds
0.5
1.0
1.5
2.0
2.5
3.0
3.5
4.0

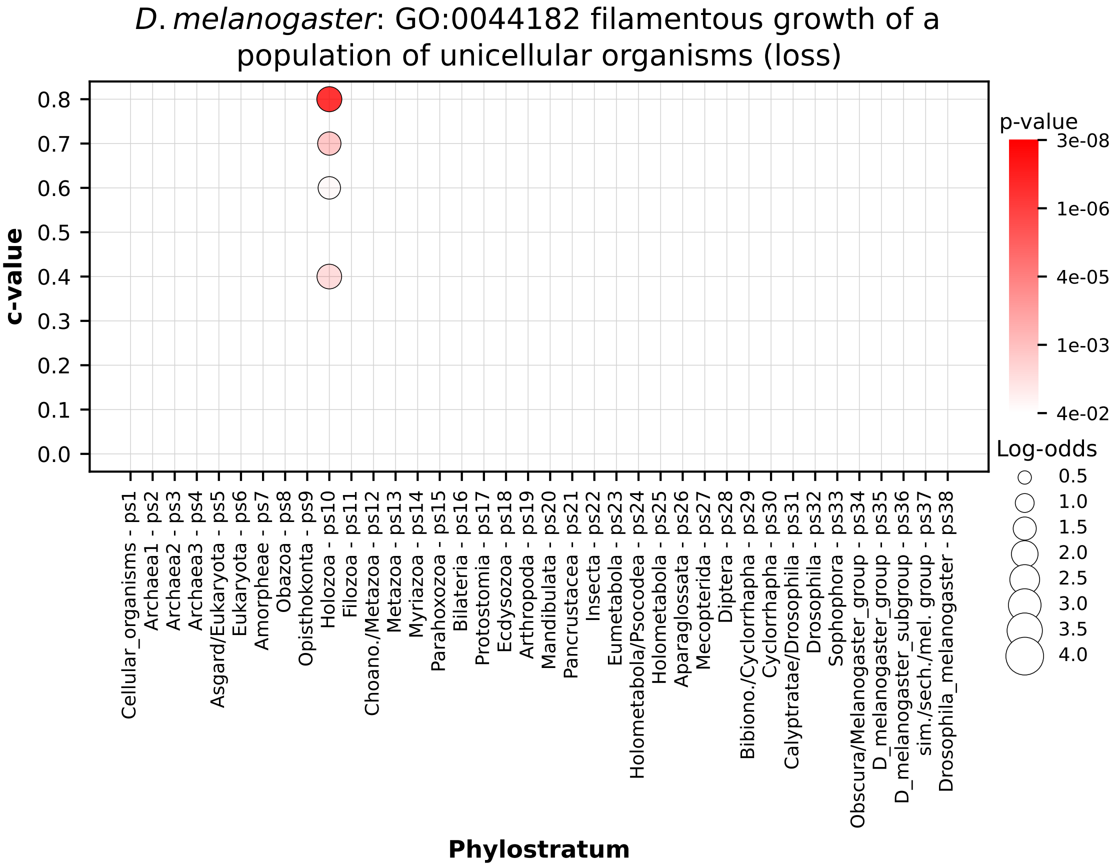

D. melanogaster: GO:0044182 filamentous growth of a population of unicellular organisms (loss)
c-value
0.8
0.7
0.6
0.5
0.4
0.3
0.2
0.1
0.0
p-value
3e-08
1e-06
4e-05
1e-03
4e-02
Log-odds
0.5
1.0
1.5
2.0
2.5
3.0
3.5
4.0
Cellular_organisms - ps1
Archaea1 - ps2
Archaea2 - ps3
Archaea3 - ps4
Asgard/Eukaryota - ps5
Eukaryota - ps6
Amorpheae - ps7
Obazoa - ps8
Opisthokonta - ps9
Holozoa - ps10
Filozoa - ps11
Choano./Metazoa - ps12
Metazoa - ps13
Myriazoa - ps14
Parahoxozoa - ps15
Bilateria - ps16
Protostomia - ps17
Ecdysozoa - ps18
Arthropoda - ps19
Mandibulata - ps20
Pancrustacea - ps21
Insecta - ps22
Eumetabola - ps23
Holometabola/Psocodea - ps24
Holometabola - ps25
Aparaglossata - ps26
Mecopterida - ps27
Diptera - ps28
Bibiono./Cyclorrhapha - ps29
Cyclorrhapha - ps30
Calyptratae/Drosophila - ps31
Drosophila - ps32
Sophophora - ps33
Obscura/Melanogaster_group - ps34
D_melanogaster_group - ps35
D_melanogaster_subgroup - ps36
sim./sech./mel. group - ps37
Drosophila_melanogaster - ps38
Phylostratum

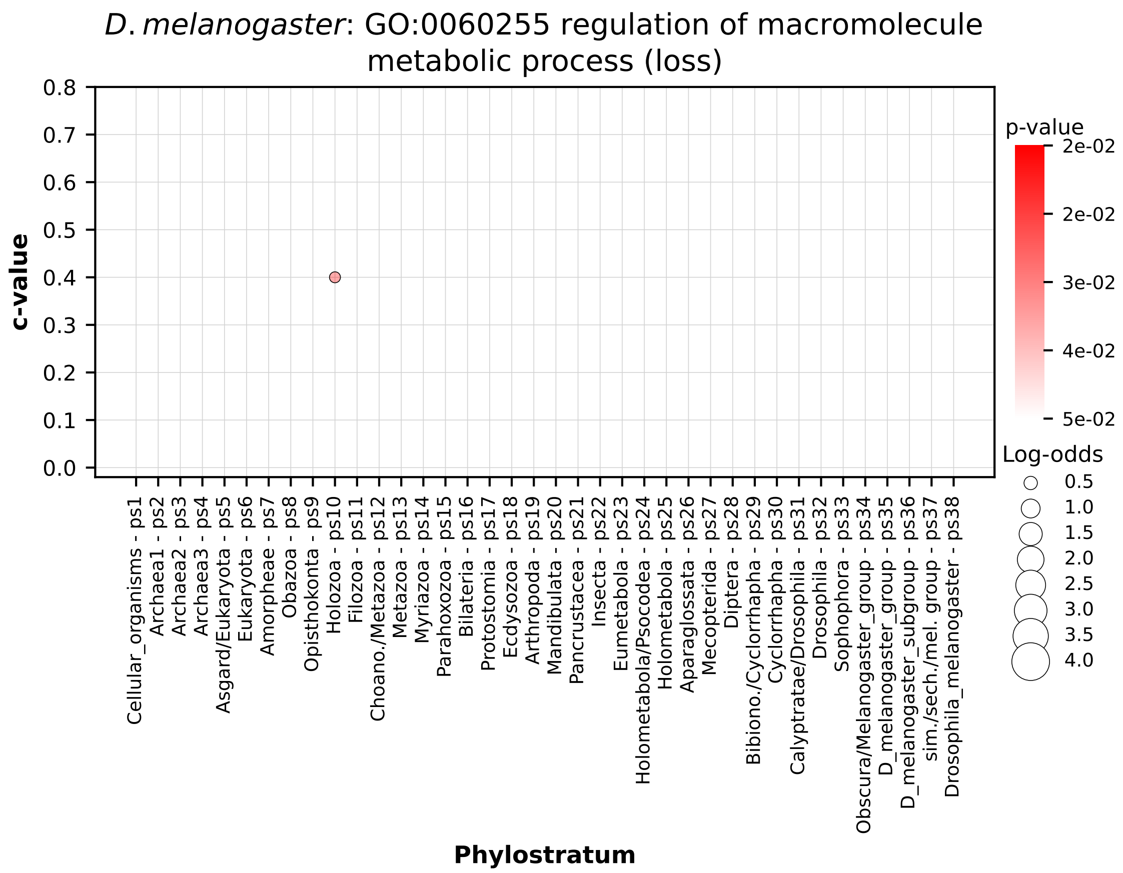

D. melanogaster: GO:0060255 regulation of macromolecule metabolic process (loss)
c-value
0.8
0.7
0.6
0.5
0.4
0.3
0.2
0.1
0.0
Phylostratum
Cellular_organisms - ps1
Archaea1 - ps2
Archaea2 - ps3
Archaea3 - ps4
Asgard/Eukaryota - ps5
Eukaryota - ps6
Amorpheae - ps7
Obazoa - ps8
Opisthokonta - ps9
Holozoa - ps10
Filozoa - ps11
Choano./Metazoa - ps12
Metazoa - ps13
Myriazoa - ps14
Parahoxozoa - ps15
Bilateria - ps16
Protostomia - ps17
Ecdysozoa - ps18
Arthropoda - ps19
Mandibulata - ps20
Pancrustacea - ps21
Insecta - ps22
Eumetabola - ps23
Holometabola/Psocodea - ps24
Holometabola - ps25
Aparaglossata - ps26
Mecopterida - ps27
Diptera - ps28
Bibiono./Cyclorrhapha - ps29
Cyclorrhapha - ps30
Calyptratae/Drosophila - ps31
Drosophila - ps32
Sophophora - ps33
Obscura/Melanogaster_group - ps34
D_melanogaster_group - ps35
D_melanogaster_subgroup - ps36
sim./sech./mel. group - ps37
Drosophila_melanogaster - ps38
p-value
2e-02
2e-02
3e-02
4e-02
5e-02
Log-odds
0.5
1.0
1.5
2.0
2.5
3.0
3.5
4.0

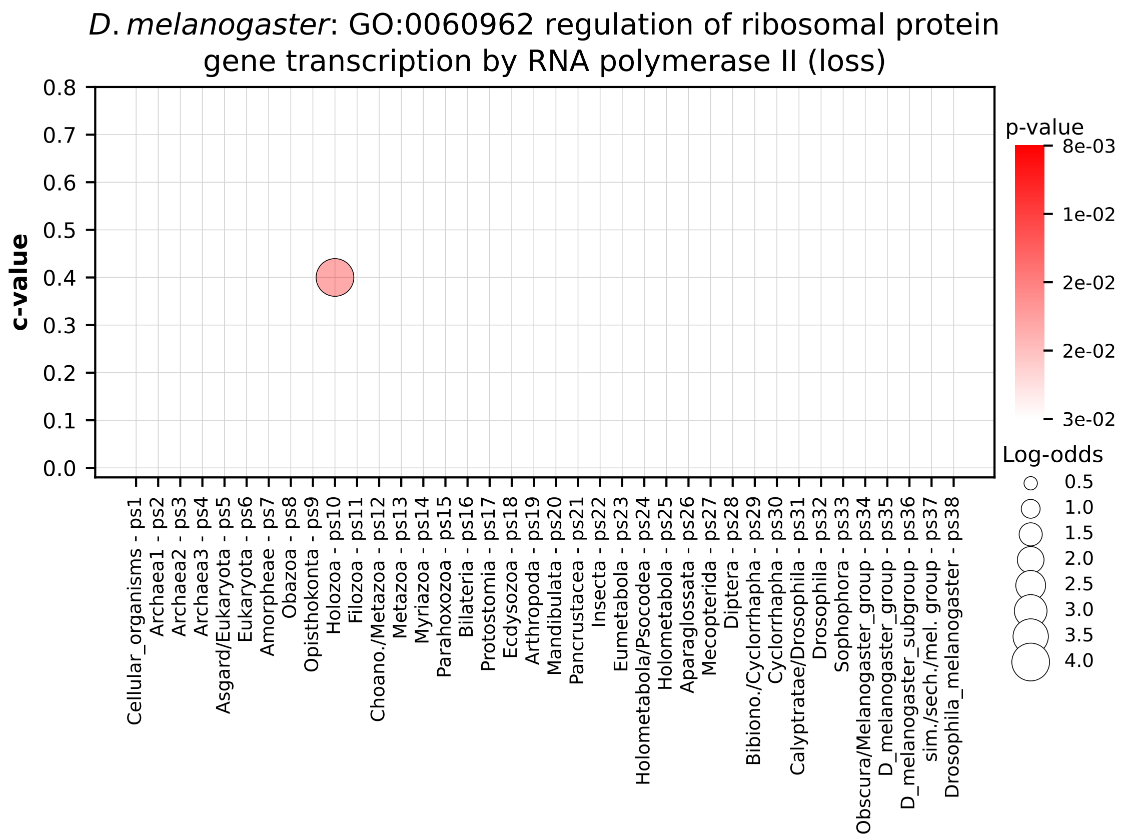
D. melanogaster: GO:0060962 regulation of ribosomal protein gene transcription by RNA polymerase II (loss)
c-value
0.0
0.1
0.2
0.3
0.4
0.5
0.6
0.7
0.8
Phylostratum
Cellular_organisms - ps1
Archaea1 - ps2
Archaea2 - ps3
Archaea3 - ps4
Asgard/Eukaryota - ps5
Eukaryota - ps6
Amorpheae - ps7
Obazoa - ps8
Opisthokonta - ps9
Holozoa - ps10
Filozoa - ps11
Choano./Metazoa - ps12
Metazoa - ps13
Myriazoa - ps14
Parahoxozoa - ps15
Bilateria - ps16
Protostomia - ps17
Ecdysozoa - ps18
Arthropoda - ps19
Mandibulata - ps20
Pancrustacea - ps21
Insecta - ps22
Eumetabola - ps23
Holometabola/Psocodea - ps24
Holometabola - ps25
Aparaglossata - ps26
Mecopterida - ps27
Diptera - ps28
Bibiono./Cyclorrhapha - ps29
Cyclorrhapha - ps30
Calyptratae/Drosophila - ps31
Drosophila - ps32
Sophophora - ps33
Obscura/Melanogaster_group - ps34
D_melanogaster_group - ps35
D_melanogaster_subgroup - ps36
sim./sech./mel. group - ps37
Drosophila_melanogaster - ps38
p-value
8e-03
1e-02
2e-02
2e-02
3e-02
Log-odds
0.5
1.0
1.5
2.0
2.5
3.0
3.5
4.0

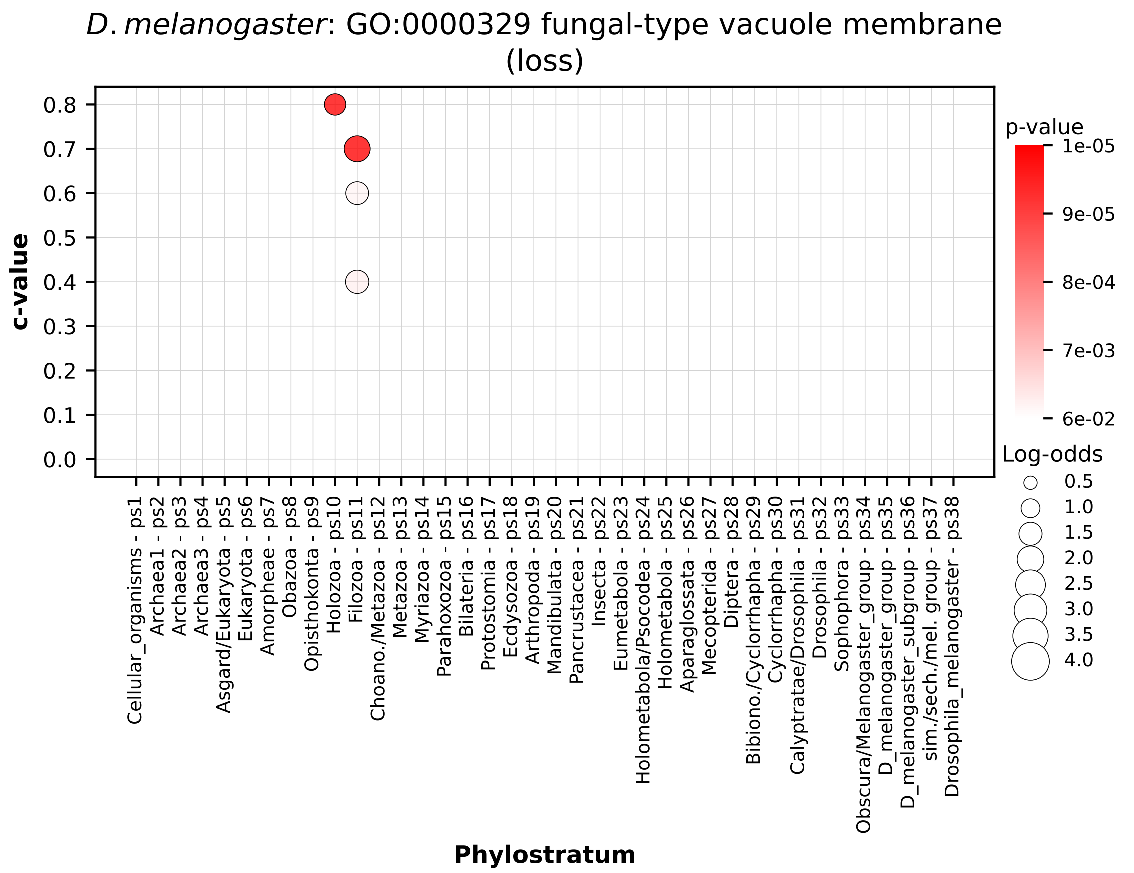
D. melanogaster: GO:0000329 fungal-type vacuole membrane
(loss)
c-value
Phylostratum
p-value
1e-05
9e-05
8e-04
7e-03
6e-02
Log-odds
0.5
1.0
1.5
2.0
2.5
3.0
3.5
4.0
Cellular_organisms - ps1
Archaea1 - ps2
Archaea2 - ps3
Archaea3 - ps4
Asgard/Eukaryota - ps5
Eukaryota - ps6
Amorpheae - ps7
Obazoa - ps8
Opisthokonta - ps9
Holozoa - ps10
Filozoa - ps11
Choano./Metazoa - ps12
Metazoa - ps13
Myriazoa - ps14
Parahoxozoa - ps15
Bilateria - ps16
Protostomia - ps17
Ecdysozoa - ps18
Arthropoda - ps19
Mandibulata - ps20
Pancrustacea - ps21
Insecta - ps22
Eumetabola - ps23
Holometabola/Psocodea - ps24
Holometabola - ps25
Aparaglossata - ps26
Mecopterida - ps27
Diptera - ps28
Bibiono./Cyclorrhapha - ps29
Cyclorrhapha - ps30
Calyptratae/Drosophila - ps31
Drosophila - ps32
Sophophora - ps33
Obscura/Melanogaster_group - ps34
D_melanogaster_group - ps35
D_melanogaster_subgroup - ps36
sim./sech./mel. group - ps37
Drosophila_melanogaster - ps38

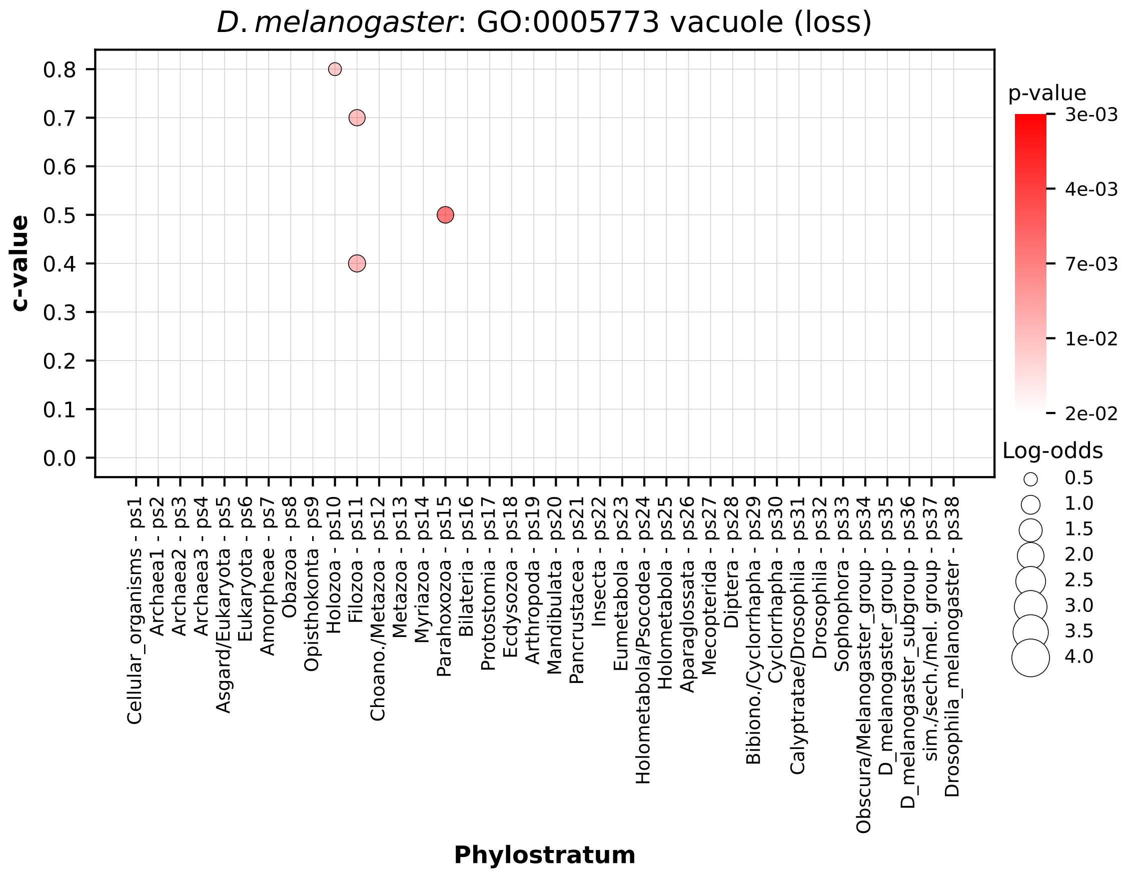

D. melanogaster: GO:0005773 vacuole (loss)
c-value
0.8
0.7
0.6
0.5
0.4
0.3
0.2
0.1
0.0
Phylostratum
Cellular_organisms - ps1
Archaea1 - ps2
Archaea2 - ps3
Archaea3 - ps4
Asgard/Eukaryota - ps5
Eukaryota - ps6
Amorpheae - ps7
Obazoa - ps8
Opisthokonta - ps9
Holozoa - ps10
Filozoa - ps11
Choano./Metazoa - ps12
Metazoa - ps13
Myriazoa - ps14
Parahoxozoa - ps15
Bilateria - ps16
Protostomia - ps17
Ecdysozoa - ps18
Arthropoda - ps19
Mandibulata - ps20
Pancrustacea - ps21
Insecta - ps22
Eumetabola - ps23
Holometabola/Psocodea - ps24
Holometabola - ps25
Aparaglossata - ps26
Mecopterida - ps27
Diptera - ps28
Bibiono./Cyclorrhapha - ps29
Cyclorrhapha - ps30
Calyptratae/Drosophila - ps31
Drosophila - ps32
Sophophora - ps33
Obscura/Melanogaster_group - ps34
D_melanogaster_group - ps35
D_melanogaster_subgroup - ps36
sim./sech./mel. group - ps37
Drosophila_melanogaster - ps38
p-value
3e-03
4e-03
7e-03
1e-02
2e-02
Log-odds
0.5
1.0
1.5
2.0
2.5
3.0
3.5
4.0

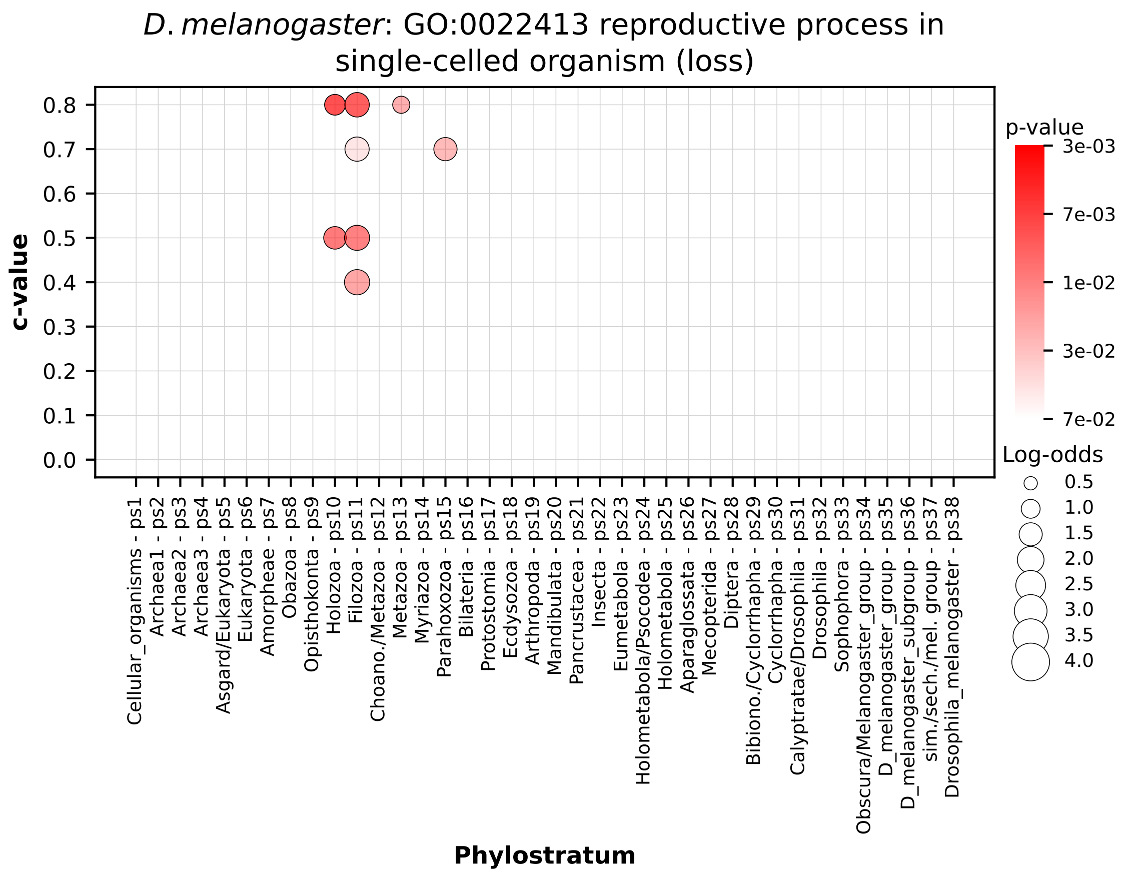

D. melanogaster: GO:0022413 reproductive process in single-celled organism (loss)
c-value
0.8
0.7
0.6
0.5
0.4
0.3
0.2
0.1
0.0
Phylostratum
Cellular_organisms - ps1
Archaea1 - ps2
Archaea2 - ps3
Archaea3 - ps4
Asgard/Eukaryota - ps5
Eukaryota - ps6
Amorpheae - ps7
Obazoa - ps8
Opisthokonta - ps9
Holozoa - ps10
Filozoa - ps11
Choano./Metazoa - ps12
Metazoa - ps13
Myriazoa - ps14
Parahoxozoa - ps15
Bilateria - ps16
Protostomia - ps17
Ecdysozoa - ps18
Arthropoda - ps19
Mandibulata - ps20
Pancrustacea - ps21
Insecta - ps22
Eumetabola - ps23
Holometabola/Psocodea - ps24
Holometabola - ps25
Aparaglossata - ps26
Mecopterida - ps27
Diptera - ps28
Bibiono./Cyclorrhapha - ps29
Cyclorrhapha - ps30
Calyptratae/Drosophila - ps31
Drosophila - ps32
Sophophora - ps33
Obscura/Melanogaster_group - ps34
D_melanogaster_group - ps35
D_melanogaster_subgroup - ps36
sim./sech./mel. group - ps37
Drosophila_melanogaster - ps38
p-value
3e-03
7e-03
1e-02
3e-02
7e-02
Log-odds
0.5
1.0
1.5
2.0
2.5
3.0
3.5
4.0

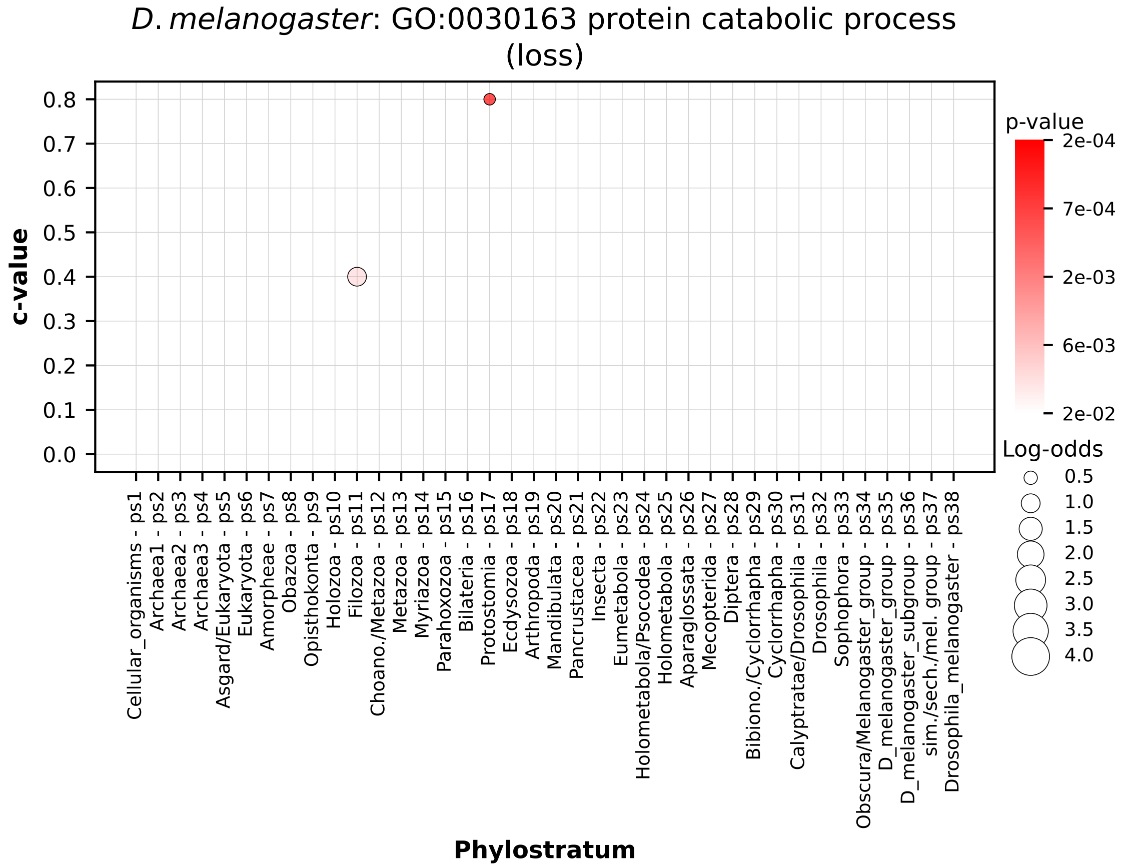
D. melanogaster: GO:0030163 protein catabolic process
(loss)
c-value
0.8
0.7
0.6
0.5
0.4
0.3
0.2
0.1
0.0
p-value
2e-04
7e-04
2e-03
6e-03
2e-02
Log-odds
0.5
1.0
1.5
2.0
2.5
3.0
3.5
4.0
Cellular_organisms - ps1
Archaea1 - ps2
Archaea2 - ps3
Archaea3 - ps4
Asgard/Eukaryota - ps5
Eukaryota - ps6
Amorpheae - ps7
Obazoa - ps8
Opisthokonta - ps9
Holozoa - ps10
Filozoa - ps11
Choano./Metazoa - ps12
Metazoa - ps13
Myriazoa - ps14
Parahoxozoa - ps15
Bilateria - ps16
Protostomia - ps17
Ecdysozoa - ps18
Arthropoda - ps19
Mandibulata - ps20
Pancrustacea - ps21
Insecta - ps22
Eumetabola - ps23
Holometabola/Psocodea - ps24
Holometabola - ps25
Aparaglossata - ps26
Mecopterida - ps27
Diptera - ps28
Bibiono./Cyclorrhapha - ps29
Cyclorrhapha - ps30
Calyptratae/Drosophila - ps31
Drosophila - ps32
Sophophora - ps33
Obscura/Melanogaster_group - ps34
D_melanogaster_group - ps35
D_melanogaster_subgroup - ps36
sim./sech./mel. group - ps37
Drosophila_melanogaster - ps38
Phylostratum

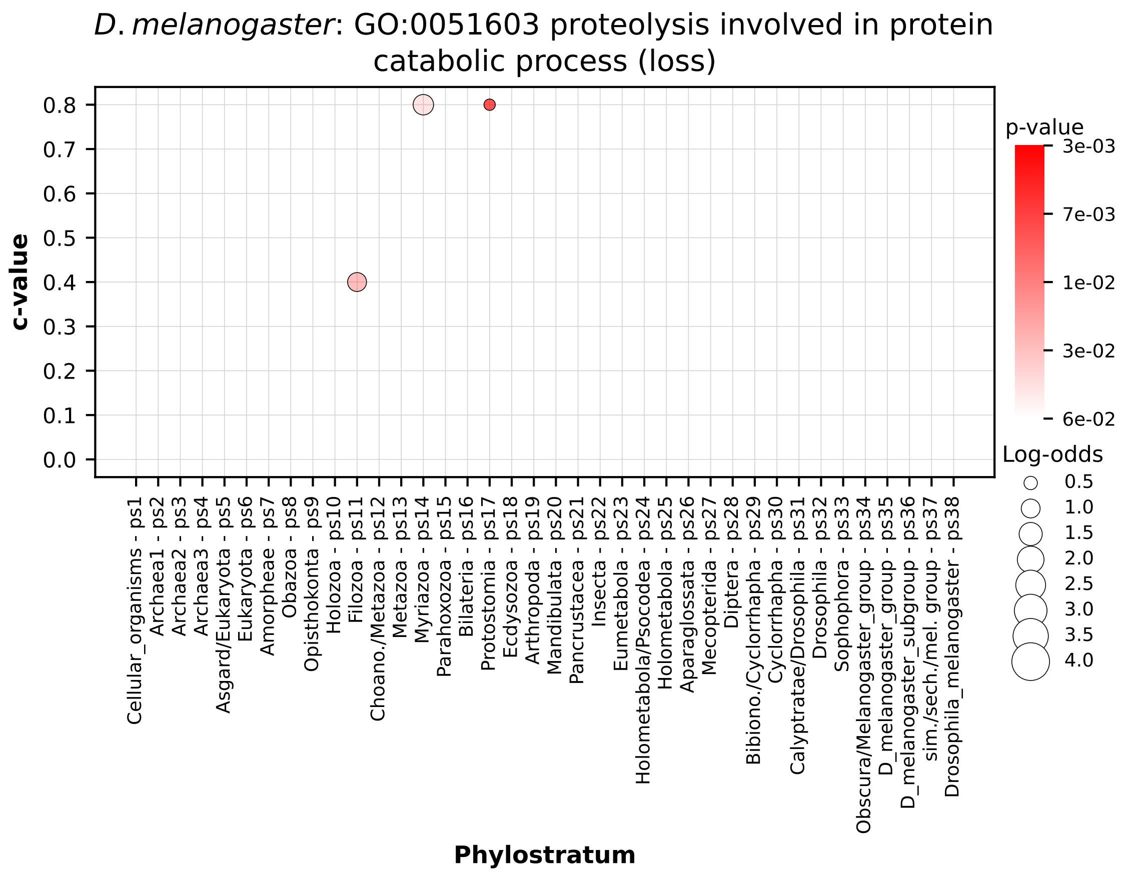

D. melanogaster: GO:0051603 proteolysis involved in protein catabolic process (loss)
c-value
0.8
0.7
0.6
0.5
0.4
0.3
0.2
0.1
0.0
p-value
3e-03
7e-03
1e-02
3e-02
6e-02
Log-odds
0.5
1.0
1.5
2.0
2.5
3.0
3.5
4.0
Cellular_organisms - ps1
Archaea1 - ps2
Archaea2 - ps3
Archaea3 - ps4
Asgard/Eukaryota - ps5
Eukaryota - ps6
Amorpheae - ps7
Obazoa - ps8
Opisthokonta - ps9
Holozoa - ps10
Filozoa - ps11
Choano./Metazoa - ps12
Metazoa - ps13
Myriazoa - ps14
Parahoxozoa - ps15
Bilateria - ps16
Protostomia - ps17
Ecdysozoa - ps18
Arthropoda - ps19
Mandibulata - ps20
Pancrustacea - ps21
Insecta - ps22
Eumetabola - ps23
Holometabola/Psocodea - ps24
Holometabola - ps25
Aparaglossata - ps26
Mecopterida - ps27
Diptera - ps28
Bibiono./Cyclorrhapha - ps29
Cyclorrhapha - ps30
Calyptratae/Drosophila - ps31
Drosophila - ps32
Sophophora - ps33
Obscura/Melanogaster_group - ps34
D_melanogaster_group - ps35
D_melanogaster_subgroup - ps36
sim./sech./mel. group - ps37
Drosophila_melanogaster - ps38
Phylostratum

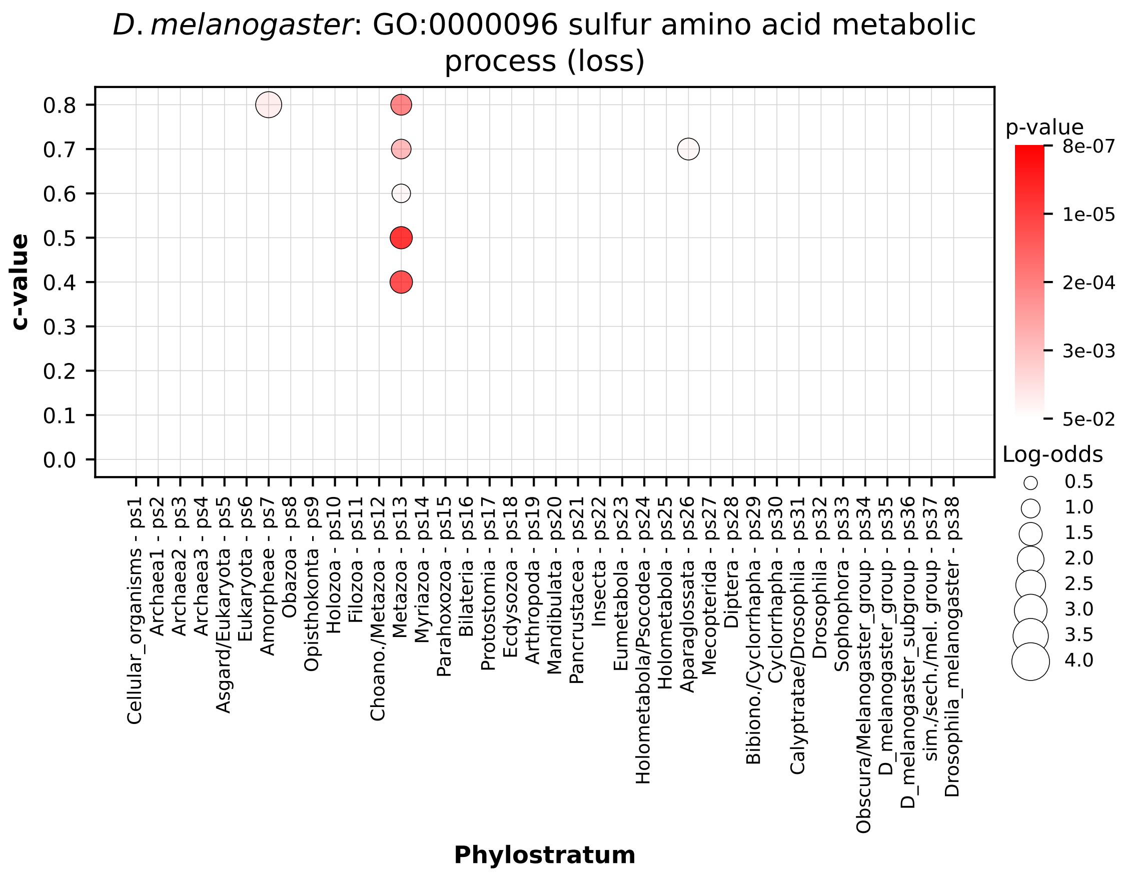

D. melanogaster: GO:0000096 sulfur amino acid metabolic process (loss)
c-value
0.8
0.7
0.6
0.5
0.4
0.3
0.2
0.1
0.0
p-value
8e-07
1e-05
2e-04
3e-03
5e-02
Log-odds
0.5
1.0
1.5
2.0
2.5
3.0
3.5
4.0
Cellular_organisms - ps1
Archaea1 - ps2
Archaea2 - ps3
Archaea3 - ps4
Asgard/Eukaryota - ps5
Eukaryota - ps6
Amorpheae - ps7
Obazoa - ps8
Opisthokonta - ps9
Holozoa - ps10
Filozoa - ps11
Choano./Metazoa - ps12
Metazoa - ps13
Myriazoa - ps14
Parahoxozoa - ps15
Bilateria - ps16
Protostomia - ps17
Ecdysozoa - ps18
Arthropoda - ps19
Mandibulata - ps20
Pancrustacea - ps21
Insecta - ps22
Eumetabola - ps23
Holometabola/Psocodea - ps24
Holometabola - ps25
Aparaglossata - ps26
Mecopterida - ps27
Diptera - ps28
Bibiono./Cyclorrhapha - ps29
Cyclorrhapha - ps30
Calyptratae/Drosophila - ps31
Drosophila - ps32
Sophophora - ps33
Obscura/Melanogaster_group - ps34
D_melanogaster_group - ps35
D_melanogaster_subgroup - ps36
sim./sech./mel. group - ps37
Drosophila_melanogaster - ps38
Phylostratum

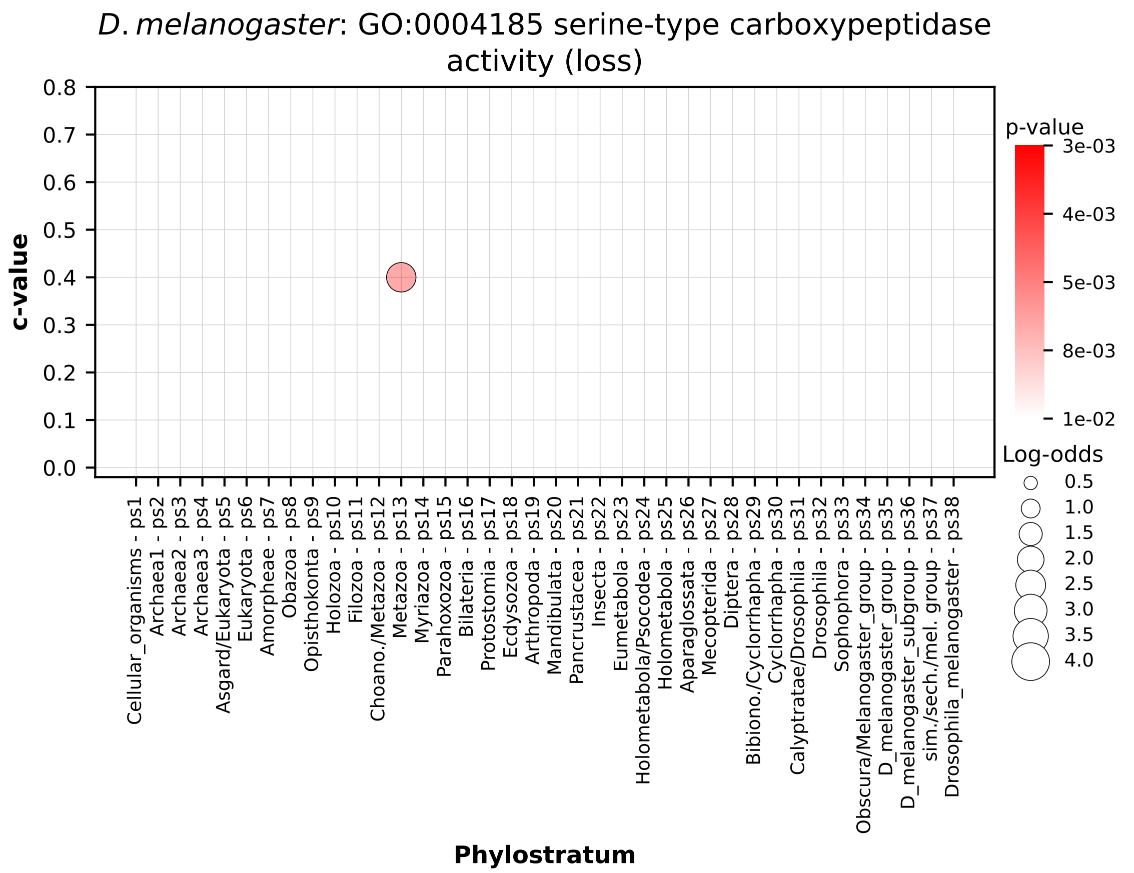
D. melanogaster: GO:0004185 serine-type carboxypeptidase activity (loss)
c-value
Phylostratum
p-value
3e-03
4e-03
5e-03
8e-03
1e-02
Log-odds
0.5
1.0
1.5
2.0
2.5
3.0
3.5
4.0
Cellular_organisms - ps1
Archaea1 - ps2
Archaea2 - ps3
Archaea3 - ps4
Asgard/Eukaryota - ps5
Eukaryota - ps6
Amorpheae - ps7
Obazoa - ps8
Opisthokonta - ps9
Holozoa - ps10
Filozoa - ps11
Choano./Metazoa - ps12
Metazoa - ps13
Myriazoa - ps14
Parahoxozoa - ps15
Bilateria - ps16
Protostomia - ps17
Ecdysozoa - ps18
Arthropoda - ps19
Mandibulata - ps20
Pancrustacea - ps21
Insecta - ps22
Eumetabola - ps23
Holometabola/Psocodea - ps24
Holometabola - ps25
Aparaglossata - ps26
Mecopterida - ps27
Diptera - ps28
Bibiono./Cyclorrhapha - ps29
Cyclorrhapha - ps30
Calyptratae/Drosophila - ps31
Drosophila - ps32
Sophophora - ps33
Obscura/Melanogaster_group - ps34
D_melanogaster_group - ps35
D_melanogaster_subgroup - ps36
sim./sech./mel. group - ps37
Drosophila_melanogaster - ps38

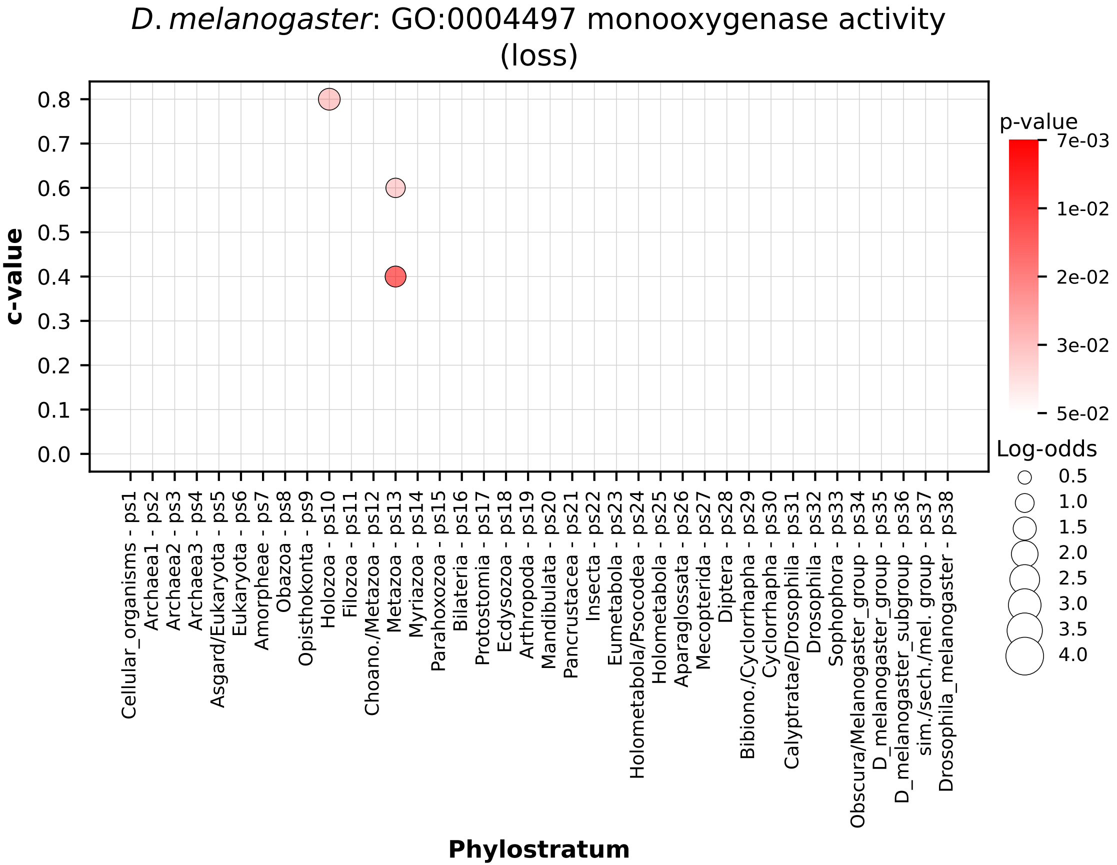
D. melanogaster: GO:0004497 monooxygenase activity
(loss)
c-value
0.0
0.1
0.2
0.3
0.4
0.5
0.6
0.7
0.8
Phylostratum
Cellular_organisms - ps1
Archaea1 - ps2
Archaea2 - ps3
Archaea3 - ps4
Asgard/Eukaryota - ps5
Eukaryota - ps6
Amorpheae - ps7
Obazoa - ps8
Opisthokonta - ps9
Holozoa - ps10
Filozoa - ps11
Choano./Metazoa - ps12
Metazoa - ps13
Myriazoa - ps14
Parahoxozoa - ps15
Bilateria - ps16
Protostomia - ps17
Ecdysozoa - ps18
Arthropoda - ps19
Mandibulata - ps20
Pancrustacea - ps21
Insecta - ps22
Eumetabola - ps23
Holometabola/Psocodea - ps24
Holometabola - ps25
Aparaglossata - ps26
Mecopterida - ps27
Diptera - ps28
Bibiono./Cyclorrhapha - ps29
Cyclorrhapha - ps30
Calyptratae/Drosophila - ps31
Drosophila - ps32
Sophophora - ps33
Obscura/Melanogaster_group - ps34
D_melanogaster_group - ps35
D_melanogaster_subgroup - ps36
sim./sech./mel. group - ps37
Drosophila_melanogaster - ps38
p-value
7e-03
1e-02
2e-02
3e-02
5e-02
Log-odds
0.5
1.0
1.5
2.0
2.5
3.0
3.5
4.0

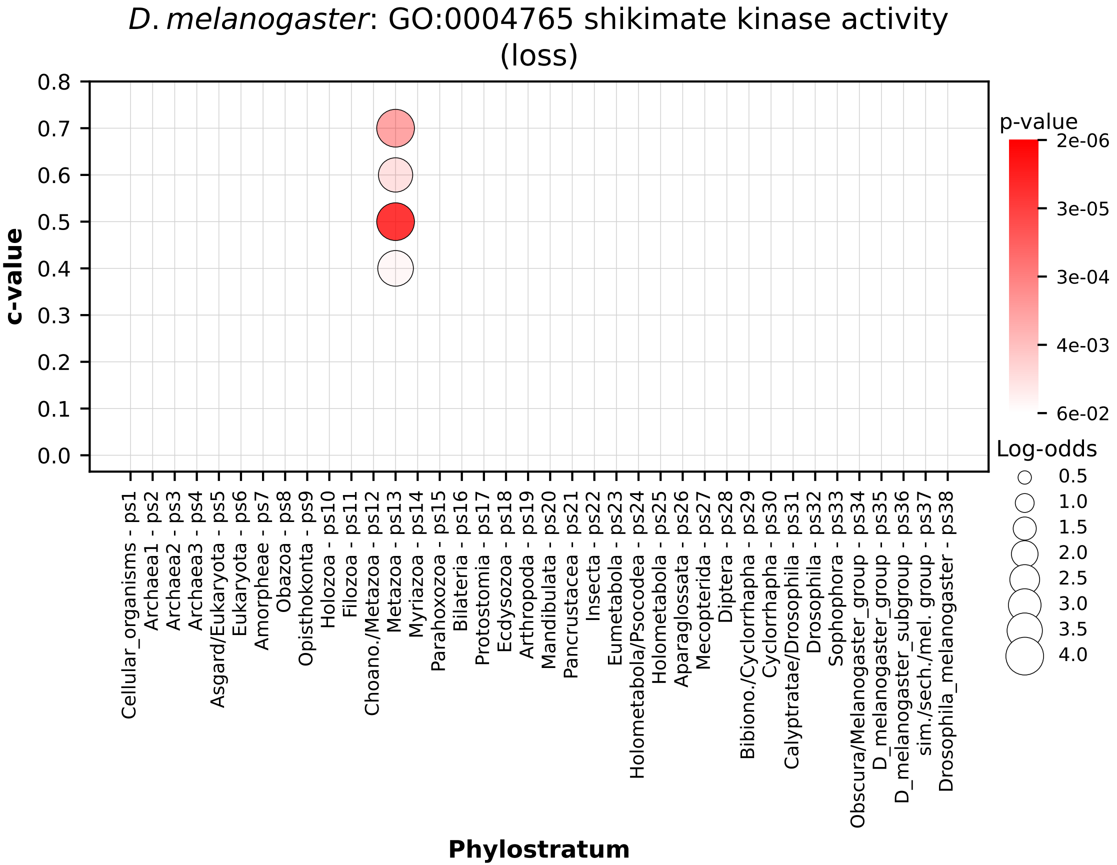

D. melanogaster: GO:0004765 shikimate kinase activity (loss)
c-value
0.0
0.1
0.2
0.3
0.4
0.5
0.6
0.7
0.8
Phylostratum
Cellular_organisms - ps1
Archaea1 - ps2
Archaea2 - ps3
Archaea3 - ps4
Asgard/Eukaryota - ps5
Eukaryota - ps6
Amorpheae - ps7
Obazoa - ps8
Opisthokonta - ps9
Holozoa - ps10
Filozoa - ps11
Choano./Metazoa - ps12
Metazoa - ps13
Myriazoa - ps14
Parahoxozoa - ps15
Bilateria - ps16
Protostomia - ps17
Ecdysozoa - ps18
Arthropoda - ps19
Mandibulata - ps20
Pancrustacea - ps21
Insecta - ps22
Eumetabola - ps23
Holometabola/Psocodea - ps24
Holometabola - ps25
Aparaglossata - ps26
Mecopterida - ps27
Diptera - ps28
Bibiono./Cyclorrhapha - ps29
Cyclorrhapha - ps30
Calyptratae/Drosophila - ps31
Drosophila - ps32
Sophophora - ps33
Obscura/Melanogaster_group - ps34
D_melanogaster_group - ps35
D_melanogaster_subgroup - ps36
sim./sech./mel. group - ps37
Drosophila_melanogaster - ps38
p-value
2e-06
3e-05
3e-04
4e-03
6e-02
Log-odds
0.5
1.0
1.5
2.0
2.5
3.0
3.5
4.0

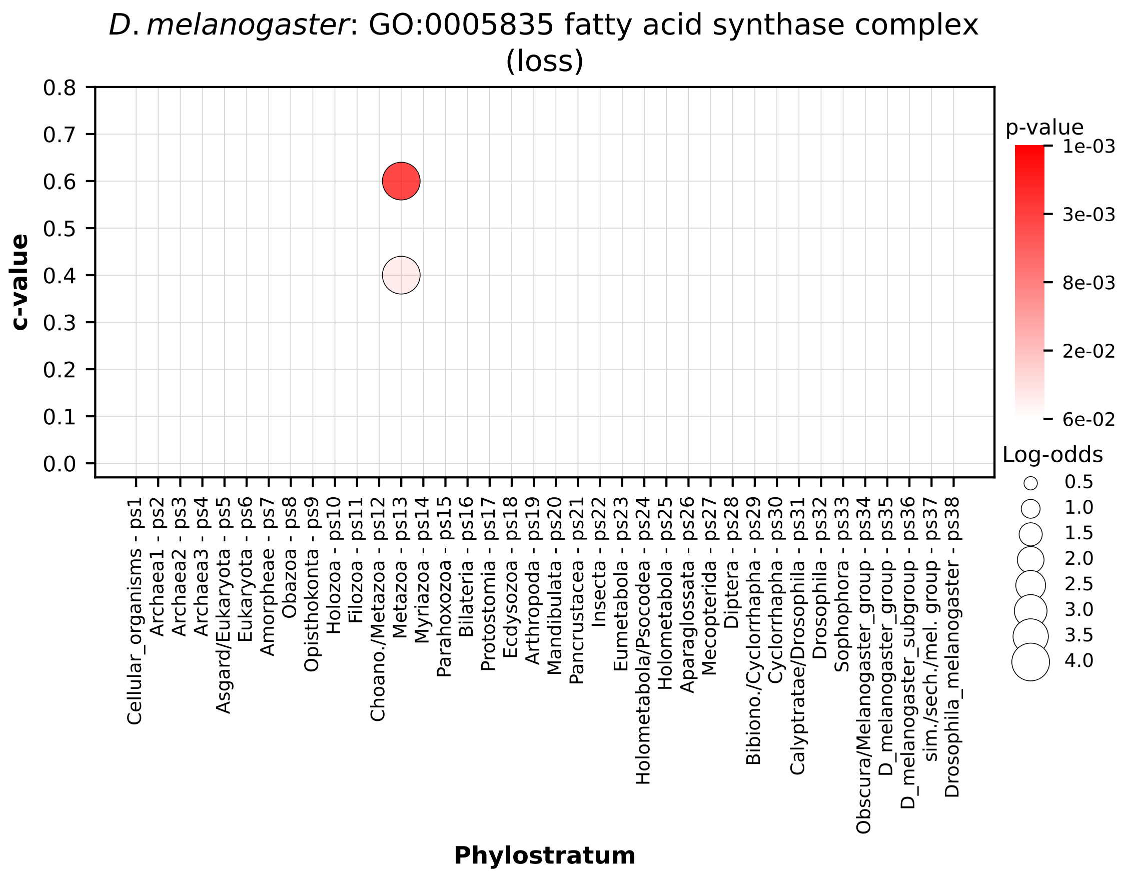
D. melanogaster: GO:0005835 fatty acid synthase complex
(loss)
c-value
0.8
0.7
0.6
0.5
0.4
0.3
0.2
0.1
0.0
Phylostratum
Cellular_organisms - ps1
Archaea1 - ps2
Archaea2 - ps3
Archaea3 - ps4
Asgard/Eukaryota - ps5
Eukaryota - ps6
Amorpheae - ps7
Obazoa - ps8
Opisthokonta - ps9
Holozoa - ps10
Filozoa - ps11
Choano./Metazoa - ps12
Metazoa - ps13
Myriazoa - ps14
Parahoxozoa - ps15
Bilateria - ps16
Protostomia - ps17
Ecdysozoa - ps18
Arthropoda - ps19
Mandibulata - ps20
Pancrustacea - ps21
Insecta - ps22
Eumetabola - ps23
Holometabola/Psocodea - ps24
Holometabola - ps25
Aparaglossata - ps26
Mecopterida - ps27
Diptera - ps28
Bibiono./Cyclorrhapha - ps29
Cyclorrhapha - ps30
Calyptratae/Drosophila - ps31
Drosophila - ps32
Sophophora - ps33
Obscura/Melanogaster_group - ps34
D_melanogaster_group - ps35
D_melanogaster_subgroup - ps36
sim./sech./mel. group - ps37
Drosophila_melanogaster - ps38
p-value
1e-03
3e-03
8e-03
2e-02
6e-02
Log-odds
0.5
1.0
1.5
2.0
2.5
3.0
3.5
4.0

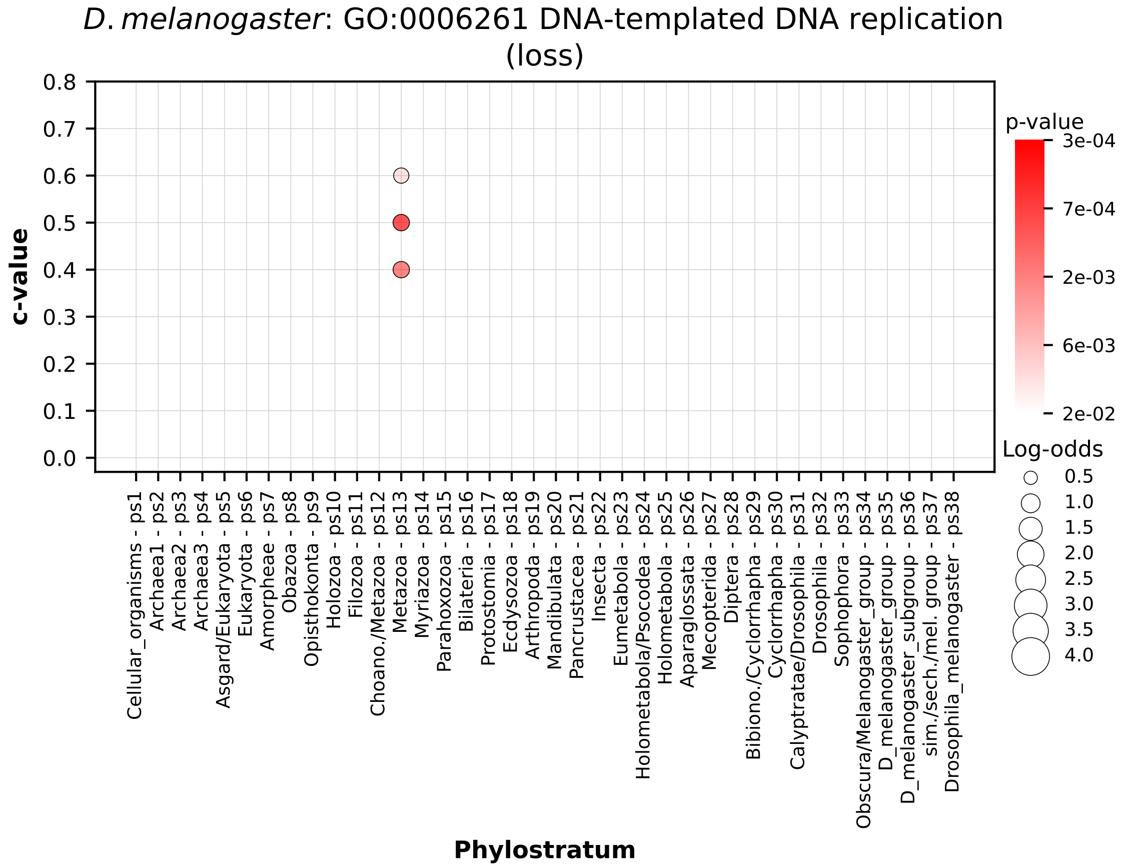

D. melanogaster: GO:0006261 DNA-templated DNA replication (loss)
c-value
0.8
0.7
0.6
0.5
0.4
0.3
0.2
0.1
0.0
p-value
3e-04
7e-04
2e-03
6e-03
2e-02
Log-odds
0.5
1.0
1.5
2.0
2.5
3.0
3.5
4.0
Cellular_organisms - ps1
Archaea1 - ps2
Archaea2 - ps3
Archaea3 - ps4
Asgard/Eukaryota - ps5
Eukaryota - ps6
Amorpheae - ps7
Obazoa - ps8
Opisthokonta - ps9
Holozoa - ps10
Filozoa - ps11
Choano./Metazoa - ps12
Metazoa - ps13
Myriazoa - ps14
Parahoxozoa - ps15
Bilateria - ps16
Protostomia - ps17
Ecdysozoa - ps18
Arthropoda - ps19
Mandibulata - ps20
Pancrustacea - ps21
Insecta - ps22
Eumetabola - ps23
Holometabola/Psocodea - ps24
Holometabola - ps25
Aparaglossata - ps26
Mecopterida - ps27
Diptera - ps28
Bibiono./Cyclorrhapha - ps29
Cyclorrhapha - ps30
Calyptratae/Drosophila - ps31
Drosophila - ps32
Sophophora - ps33
Obscura/Melanogaster_group - ps34
D_melanogaster_group - ps35
D_melanogaster_subgroup - ps36
sim./sech./mel. group - ps37
Drosophila_melanogaster - ps38
Phylostratum

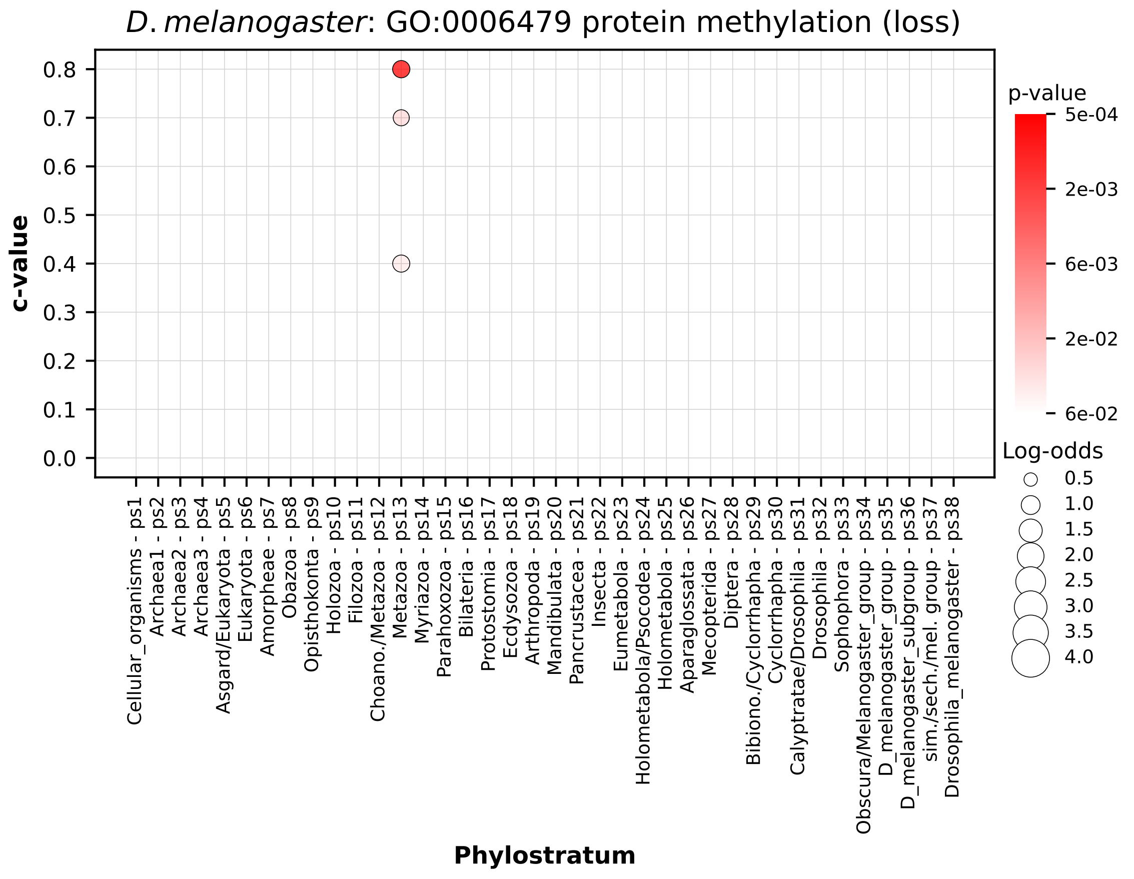

D. melanogaster: GO:0006479 protein methylation (loss)
c-value
0.0
0.1
0.2
0.3
0.4
0.5
0.6
0.7
0.8
Phylostratum
Cellular_organisms - ps1
Archaea1 - ps2
Archaea2 - ps3
Archaea3 - ps4
Asgard/Eukaryota - ps5
Eukaryota - ps6
Amorpheae - ps7
Obazoa - ps8
Opisthokonta - ps9
Holozoa - ps10
Filozoa - ps11
Choano./Metazoa - ps12
Metazoa - ps13
Myriazoa - ps14
Parahoxozoa - ps15
Bilateria - ps16
Protostomia - ps17
Ecdysozoa - ps18
Arthropoda - ps19
Mandibulata - ps20
Pancrustacea - ps21
Insecta - ps22
Eumetabola - ps23
Holometabola/Psocodea - ps24
Holometabola - ps25
Aparaglossata - ps26
Mecopterida - ps27
Diptera - ps28
Bibiono./Cyclorrhapha - ps29
Cyclorrhapha - ps30
Calyptratae/Drosophila - ps31
Drosophila - ps32
Sophophora - ps33
Obscura/Melanogaster_group - ps34
D_melanogaster_group - ps35
D_melanogaster_subgroup - ps36
sim./sech./mel. group - ps37
Drosophila_melanogaster - ps38
p-value
5e-04
2e-03
6e-03
2e-02
6e-02
Log-odds
0.5
1.0
1.5
2.0
2.5
3.0
3.5
4.0

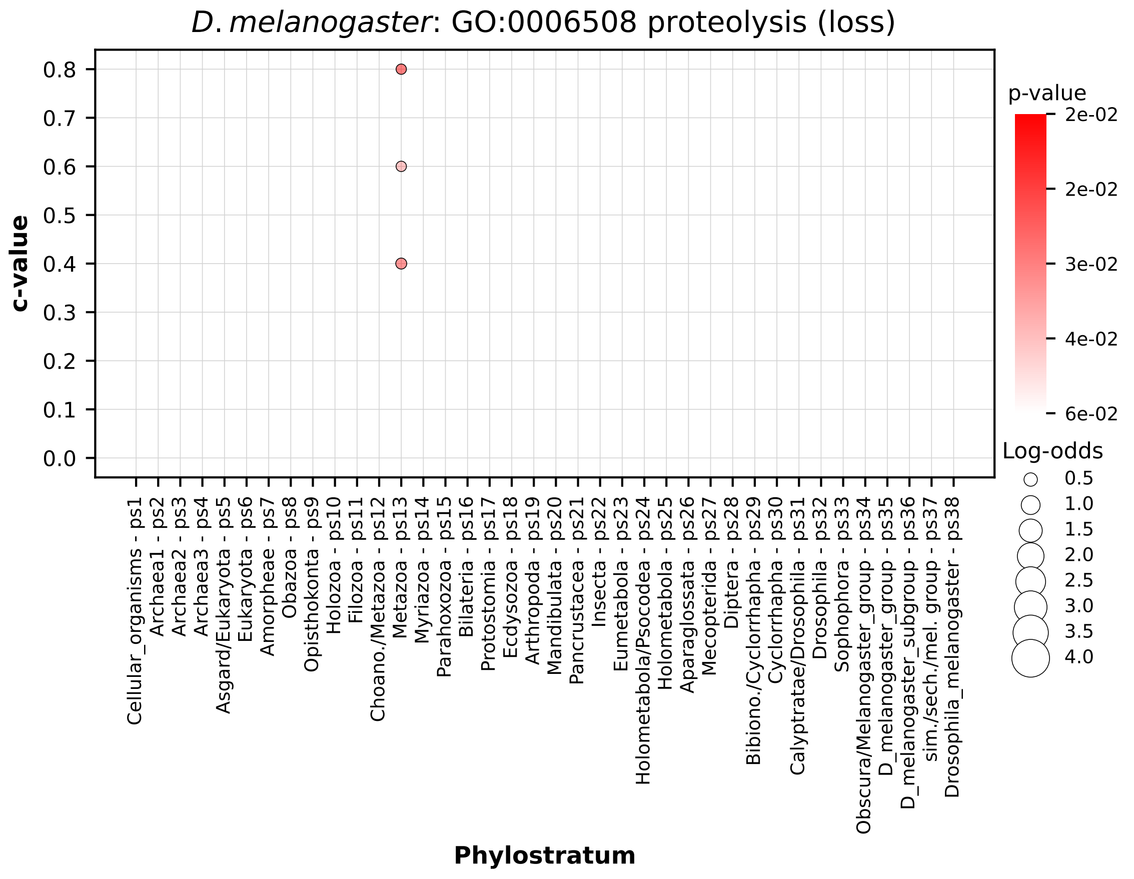

D. melanogaster: GO:0006508 proteolysis (loss)
c-value
0.0
0.1
0.2
0.3
0.4
0.5
0.6
0.7
0.8
p-value
2e-02
2e-02
3e-02
4e-02
6e-02
Log-odds
0.5
1.0
1.5
2.0
2.5
3.0
3.5
4.0
Cellular_organisms - ps1
Archaea1 - ps2
Archaea2 - ps3
Archaea3 - ps4
Asgard/Eukaryota - ps5
Eukaryota - ps6
Amorpheae - ps7
Obazoa - ps8
Opisthokonta - ps9
Holozoa - ps10
Filozoa - ps11
Choano./Metazoa - ps12
Metazoa - ps13
Myriazoa - ps14
Parahoxozoa - ps15
Bilateria - ps16
Protostomia - ps17
Ecdysozoa - ps18
Arthropoda - ps19
Mandibulata - ps20
Pancrustacea - ps21
Insecta - ps22
Eumetabola - ps23
Holometabola/Psocodea - ps24
Holometabola - ps25
Aparaglossata - ps26
Mecopterida - ps27
Diptera - ps28
Bibiono./Cyclorrhapha - ps29
Cyclorrhapha - ps30
Calyptratae/Drosophila - ps31
Drosophila - ps32
Sophophora - ps33
Obscura/Melanogaster_group - ps34
D_melanogaster_group - ps35
D_melanogaster_subgroup - ps36
sim./sech./mel. group - ps37
Drosophila_melanogaster - ps38
Phylostratum

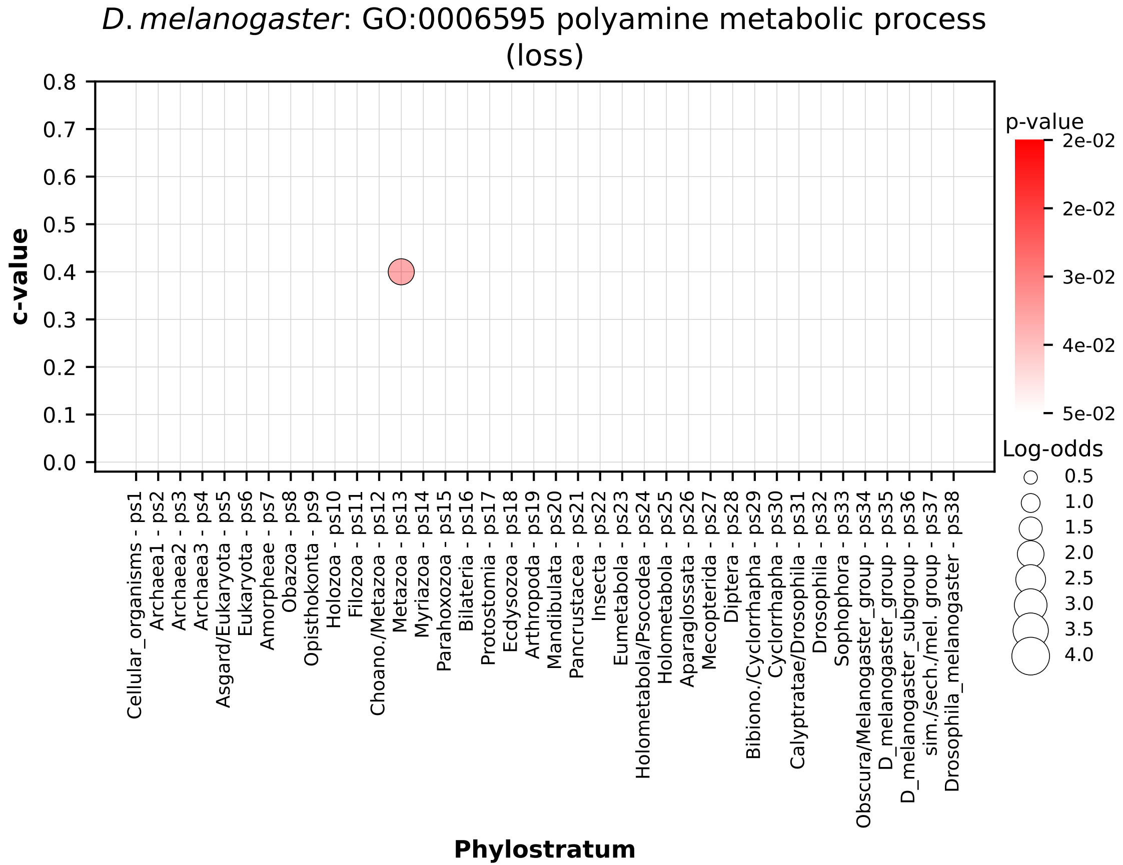

D. melanogaster: GO:0006595 polyamine metabolic process (loss)
c-value
0.8
0.7
0.6
0.5
0.4
0.3
0.2
0.1
0.0
Phylostratum
Cellular_organisms - ps1
Archaea1 - ps2
Archaea2 - ps3
Archaea3 - ps4
Asgard/Eukaryota - ps5
Eukaryota - ps6
Amorpheae - ps7
Obazoa - ps8
Opisthokonta - ps9
Holozoa - ps10
Filozoa - ps11
Choano./Metazoa - ps12
Metazoa - ps13
Myriazoa - ps14
Parahoxozoa - ps15
Bilateria - ps16
Protostomia - ps17
Ecdysozoa - ps18
Arthropoda - ps19
Mandibulata - ps20
Pancrustacea - ps21
Insecta - ps22
Eumetabola - ps23
Holometabola/Psocodea - ps24
Holometabola - ps25
Aparaglossata - ps26
Mecopterida - ps27
Diptera - ps28
Bibiono./Cyclorrhapha - ps29
Cyclorrhapha - ps30
Calyptratae/Drosophila - ps31
Drosophila - ps32
Sophophora - ps33
Obscura/Melanogaster_group - ps34
D_melanogaster_group - ps35
D_melanogaster_subgroup - ps36
sim./sech./mel. group - ps37
Drosophila_melanogaster - ps38
p-value
2e-02
2e-02
3e-02
4e-02
5e-02
Log-odds
0.5
1.0
1.5
2.0
2.5
3.0
3.5
4.0

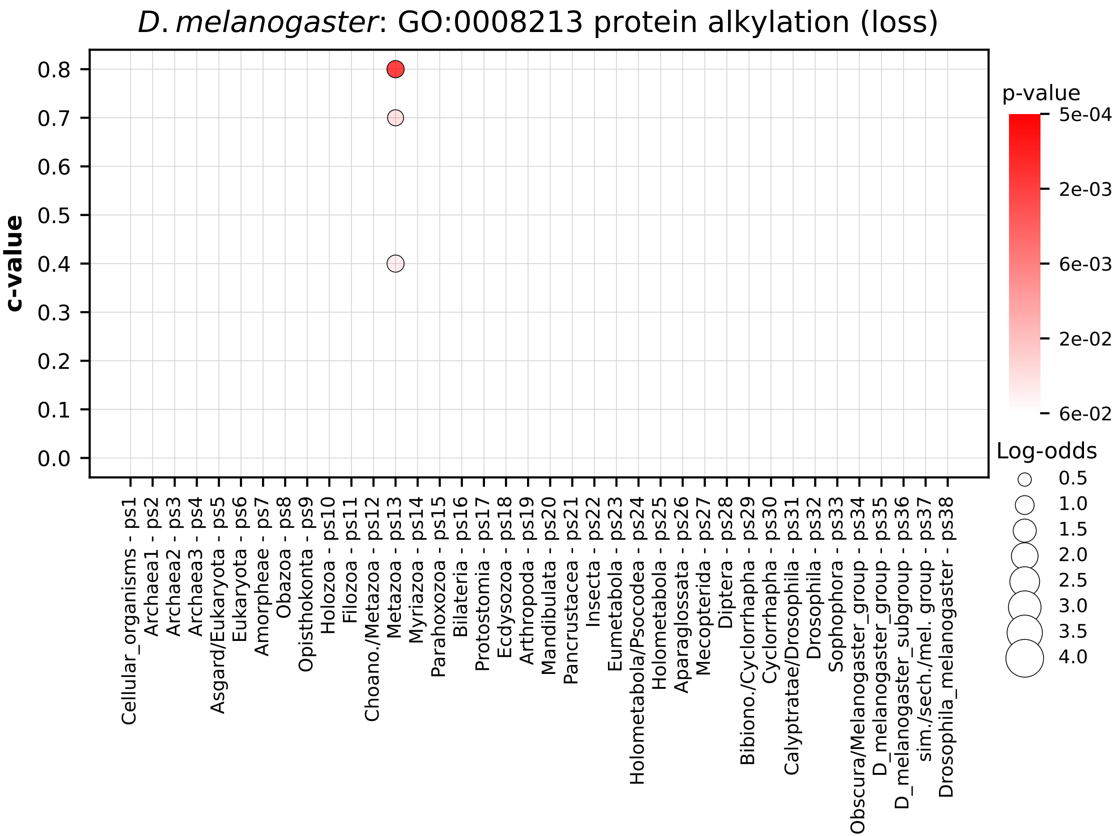

D. melanogaster: GO:0008213 protein alkylation (loss)
c-value
0.8
0.7
0.6
0.5
0.4
0.3
0.2
0.1
0.0
p-value
5e-04
2e-03
6e-03
2e-02
6e-02
Log-odds
0.5
1.0
1.5
2.0
2.5
3.0
3.5
4.0
Cellular_organisms - ps1
Archaea1 - ps2
Archaea2 - ps3
Archaea3 - ps4
Asgard/Eukaryota - ps5
Eukaryota - ps6
Amorpheae - ps7
Obazoa - ps8
Opisthokonta - ps9
Holozoa - ps10
Filozoa - ps11
Choano./Metazoa - ps12
Metazoa - ps13
Myriazoa - ps14
Parahoxozoa - ps15
Bilateria - ps16
Protostomia - ps17
Ecdysozoa - ps18
Arthropoda - ps19
Mandibulata - ps20
Pancrustacea - ps21
Insecta - ps22
Eumetabola - ps23
Holometabola/Psocodea - ps24
Holometabola - ps25
Aparaglossata - ps26
Mecopterida - ps27
Diptera - ps28
Bibiono./Cyclorrhapha - ps29
Cyclorrhapha - ps30
Calyptratae/Drosophila - ps31
Drosophila - ps32
Sophophora - ps33
Obscura/Melanogaster_group - ps34
D_melanogaster_group - ps35
D_melanogaster_subgroup - ps36
sim./sech./mel. group - ps37
Drosophila_melanogaster - ps38
Phylostratum

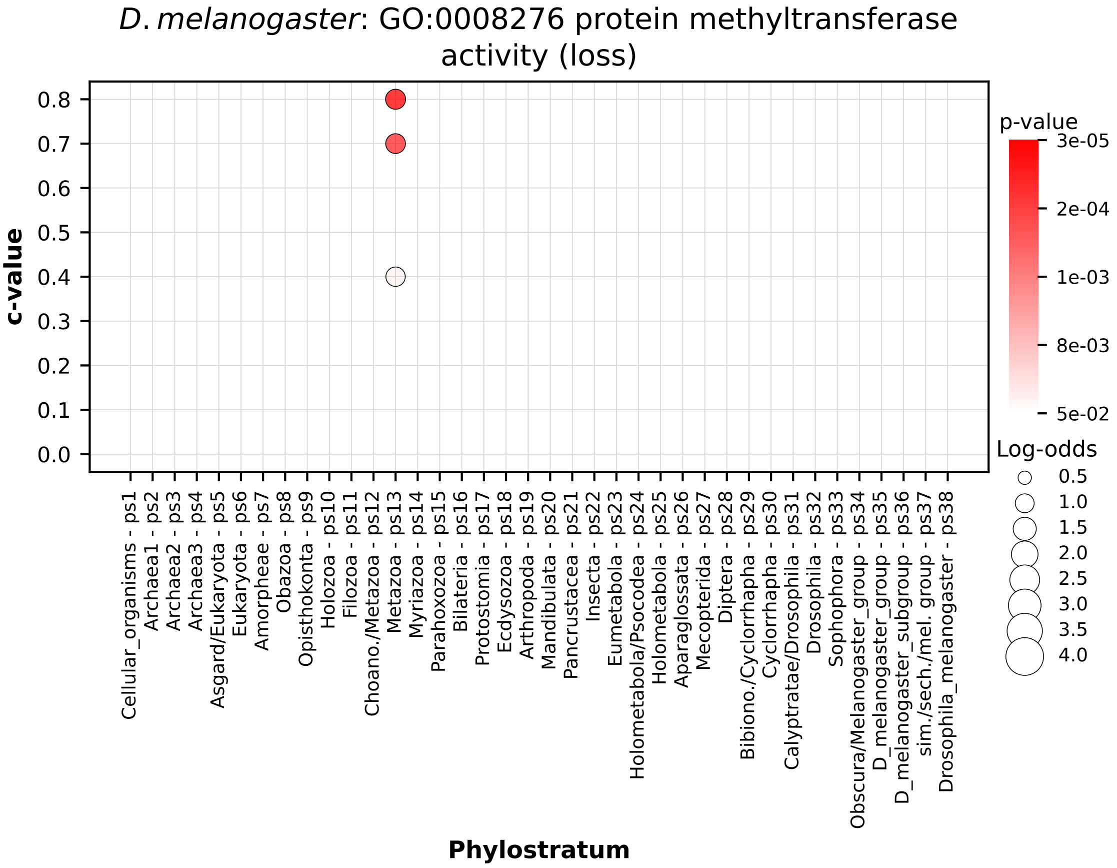
D. melanogaster: GO:0008276 protein methyltransferase activity (loss)
c-value
0.8
0.7
0.6
0.5
0.4
0.3
0.2
0.1
0.0
Phylostratum
Cellular_organisms - ps1
Archaea1 - ps2
Archaea2 - ps3
Archaea3 - ps4
Asgard/Eukaryota - ps5
Eukaryota - ps6
Amorpheae - ps7
Obazoa - ps8
Opisthokonta - ps9
Holozoa - ps10
Filozoa - ps11
Choano./Metazoa - ps12
Metazoa - ps13
Myriazoa - ps14
Parahoxozoa - ps15
Bilateria - ps16
Protostomia - ps17
Ecdysozoa - ps18
Arthropoda - ps19
Mandibulata - ps20
Pancrustacea - ps21
Insecta - ps22
Eumetabola - ps23
Holometabola/Psocodea - ps24
Holometabola - ps25
Aparaglossata - ps26
Mecopterida - ps27
Diptera - ps28
Bibiono./Cyclorrhapha - ps29
Cyclorrhapha - ps30
Calyptratae/Drosophila - ps31
Drosophila - ps32
Sophophora - ps33
Obscura/Melanogaster_group - ps34
D_melanogaster_group - ps35
D_melanogaster_subgroup - ps36
sim./sech./mel. group - ps37
Drosophila_melanogaster - ps38
p-value
3e-05
2e-04
1e-03
8e-03
5e-02
Log-odds
0.5
1.0
1.5
2.0
2.5
3.0
3.5
4.0

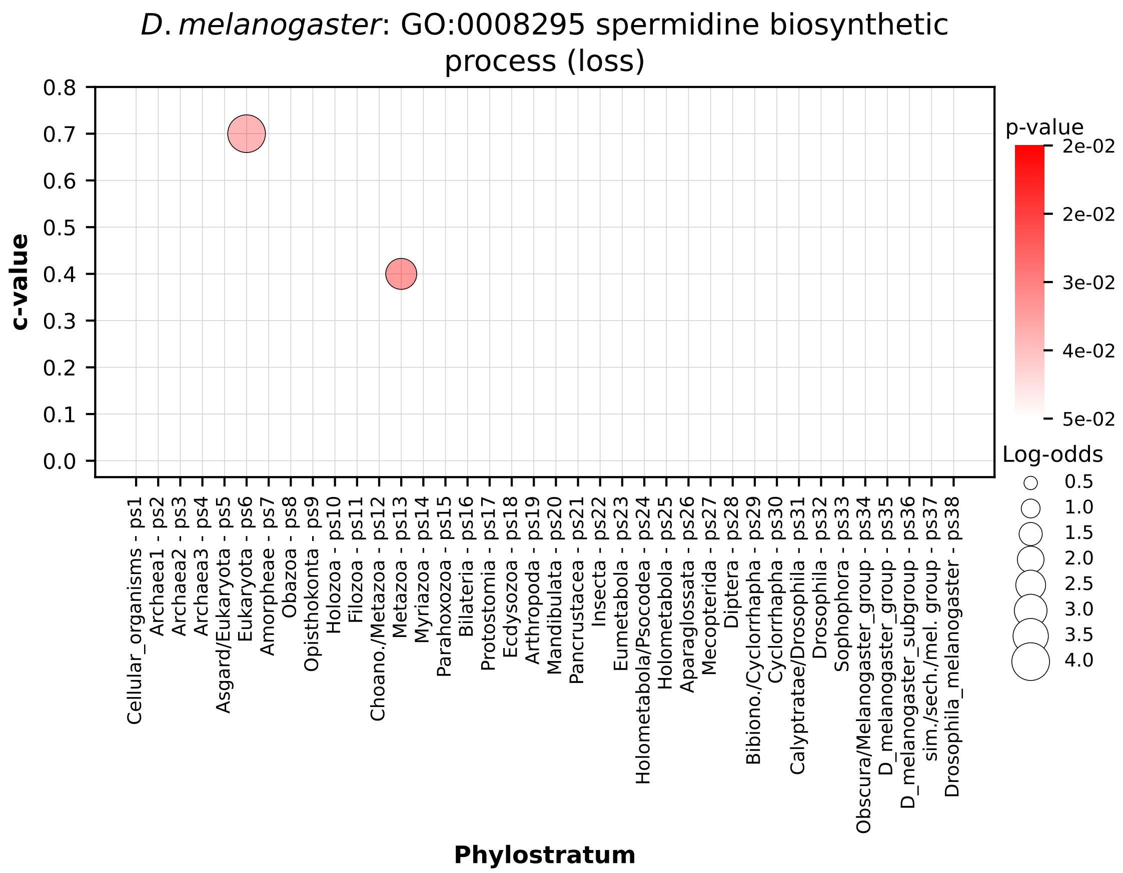

D. melanogaster: GO:0008295 spermidine biosynthetic process (loss)
c-value
0.8
0.7
0.6
0.5
0.4
0.3
0.2
0.1
0.0
Phylostratum
Cellular_organisms - ps1
Archaea1 - ps2
Archaea2 - ps3
Archaea3 - ps4
Asgard/Eukaryota - ps5
Eukaryota - ps6
Amorpheae - ps7
Obazoa - ps8
Opisthokonta - ps9
Holozoa - ps10
Filozoa - ps11
Choano./Metazoa - ps12
Metazoa - ps13
Myriazoa - ps14
Parahoxozoa - ps15
Bilateria - ps16
Protostomia - ps17
Ecdysozoa - ps18
Arthropoda - ps19
Mandibulata - ps20
Pancrustacea - ps21
Insecta - ps22
Eumetabola - ps23
Holometabola/Psocodea - ps24
Holometabola - ps25
Aparaglossata - ps26
Mecopterida - ps27
Diptera - ps28
Bibiono./Cyclorrhapha - ps29
Cyclorrhapha - ps30
Calyptratae/Drosophila - ps31
Drosophila - ps32
Sophophora - ps33
Obscura/Melanogaster_group - ps34
D_melanogaster_group - ps35
D_melanogaster_subgroup - ps36
sim./sech./mel. group - ps37
Drosophila_melanogaster - ps38
p-value
2e-02
2e-02
3e-02
4e-02
5e-02
Log-odds
0.5
1.0
1.5
2.0
2.5
3.0
3.5
4.0

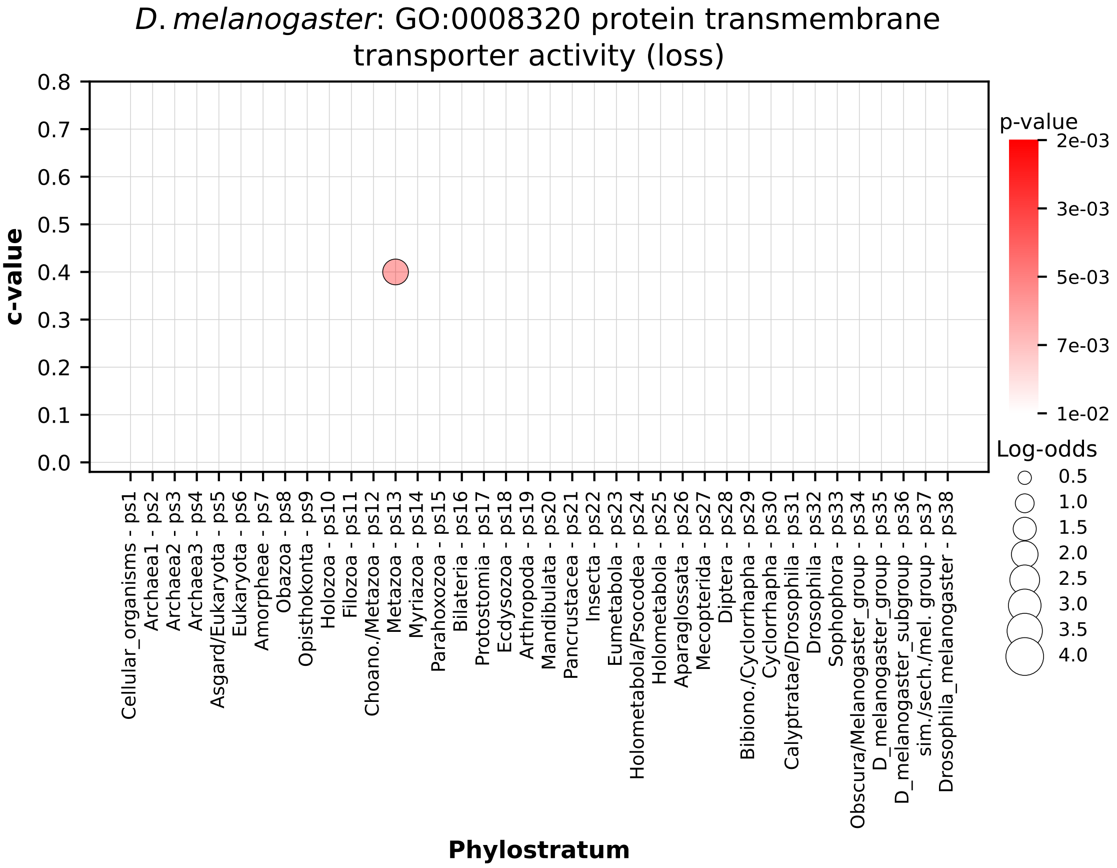
D. melanogaster: GO:0008320 protein transmembrane transporter activity (loss)
c-value
0.8
0.7
0.6
0.5
0.4
0.3
0.2
0.1
0.0
p-value
2e-03
3e-03
5e-03
7e-03
1e-02
Log-odds
0.5
1.0
1.5
2.0
2.5
3.0
3.5
4.0
Cellular_organisms - ps1
Archaea1 - ps2
Archaea2 - ps3
Archaea3 - ps4
Asgard/Eukaryota - ps5
Eukaryota - ps6
Amorpheae - ps7
Obazoa - ps8
Opisthokonta - ps9
Holozoa - ps10
Filozoa - ps11
Choano./Metazoa - ps12
Metazoa - ps13
Myriazoa - ps14
Parahoxozoa - ps15
Bilateria - ps16
Protostomia - ps17
Ecdysozoa - ps18
Arthropoda - ps19
Mandibulata - ps20
Pancrustacea - ps21
Insecta - ps22
Eumetabola - ps23
Holometabola/Psocodea - ps24
Holometabola - ps25
Aparaglossata - ps26
Mecopterida - ps27
Diptera - ps28
Bibiono./Cyclorrhapha - ps29
Cyclorrhapha - ps30
Calyptratae/Drosophila - ps31
Drosophila - ps32
Sophophora - ps33
Obscura/Melanogaster_group - ps34
D_melanogaster_group - ps35
D_melanogaster_subgroup - ps36
sim./sech./mel. group - ps37
Drosophila_melanogaster - ps38
Phylostratum

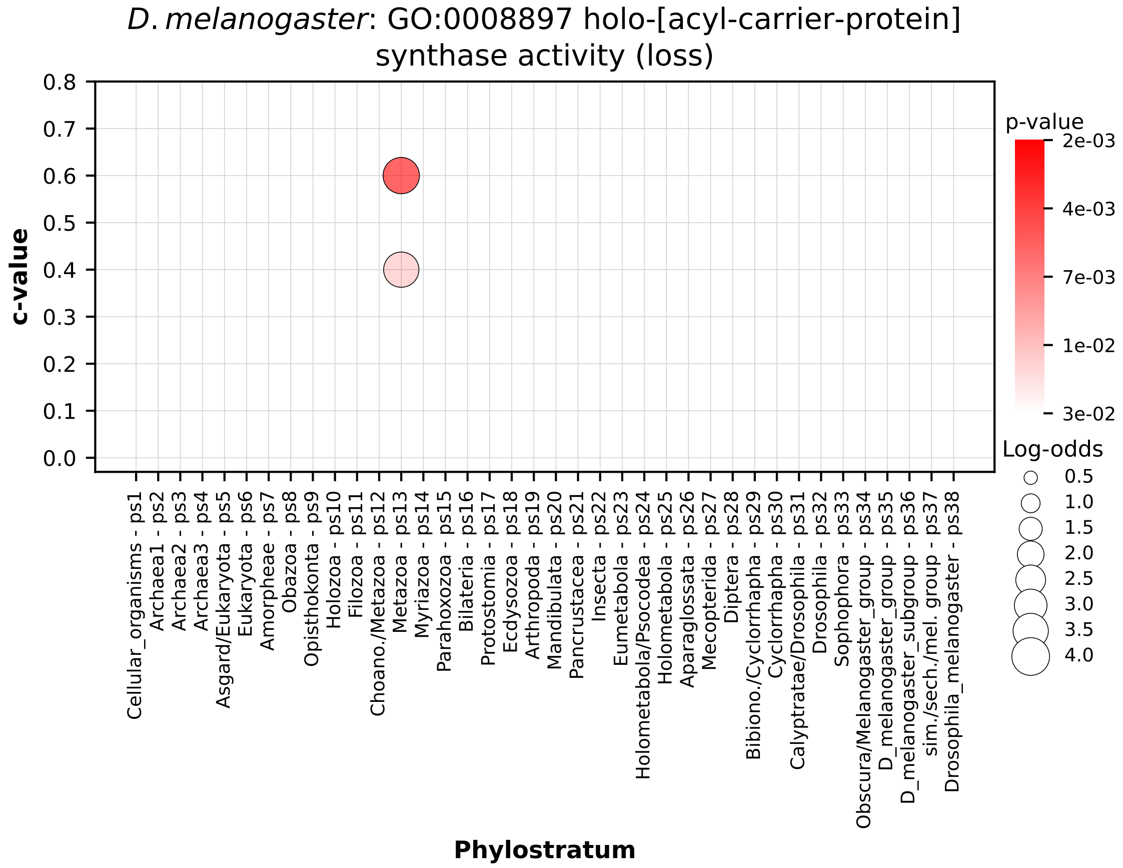

D. melanogaster: GO:0008897 holo-[acyl-carrier-protein] synthase activity (loss)
c-value
0.8
0.7
0.6
0.5
0.4
0.3
0.2
0.1
0.0
p-value
2e-03
4e-03
7e-03
1e-02
3e-02
Log-odds
0.5
1.0
1.5
2.0
2.5
3.0
3.5
4.0
Cellular_organisms - ps1
Archaea1 - ps2
Archaea2 - ps3
Archaea3 - ps4
Asgard/Eukaryota - ps5
Eukaryota - ps6
Amorpheae - ps7
Obazoa - ps8
Opisthokonta - ps9
Holozoa - ps10
Filozoa - ps11
Choano./Metazoa - ps12
Metazoa - ps13
Myriazoa - ps14
Parahoxozoa - ps15
Bilateria - ps16
Protostomia - ps17
Ecdysozoa - ps18
Arthropoda - ps19
Mandibulata - ps20
Pancrustacea - ps21
Insecta - ps22
Eumetabola - ps23
Holometabola/Psocodea - ps24
Holometabola - ps25
Aparaglossata - ps26
Mecopterida - ps27
Diptera - ps28
Bibiono./Cyclorrhapha - ps29
Cyclorrhapha - ps30
Calyptratae/Drosophila - ps31
Drosophila - ps32
Sophophora - ps33
Obscura/Melanogaster_group - ps34
D_melanogaster_group - ps35
D_melanogaster_subgroup - ps36
sim./sech./mel. group - ps37
Drosophila_melanogaster - ps38
Phylostratum

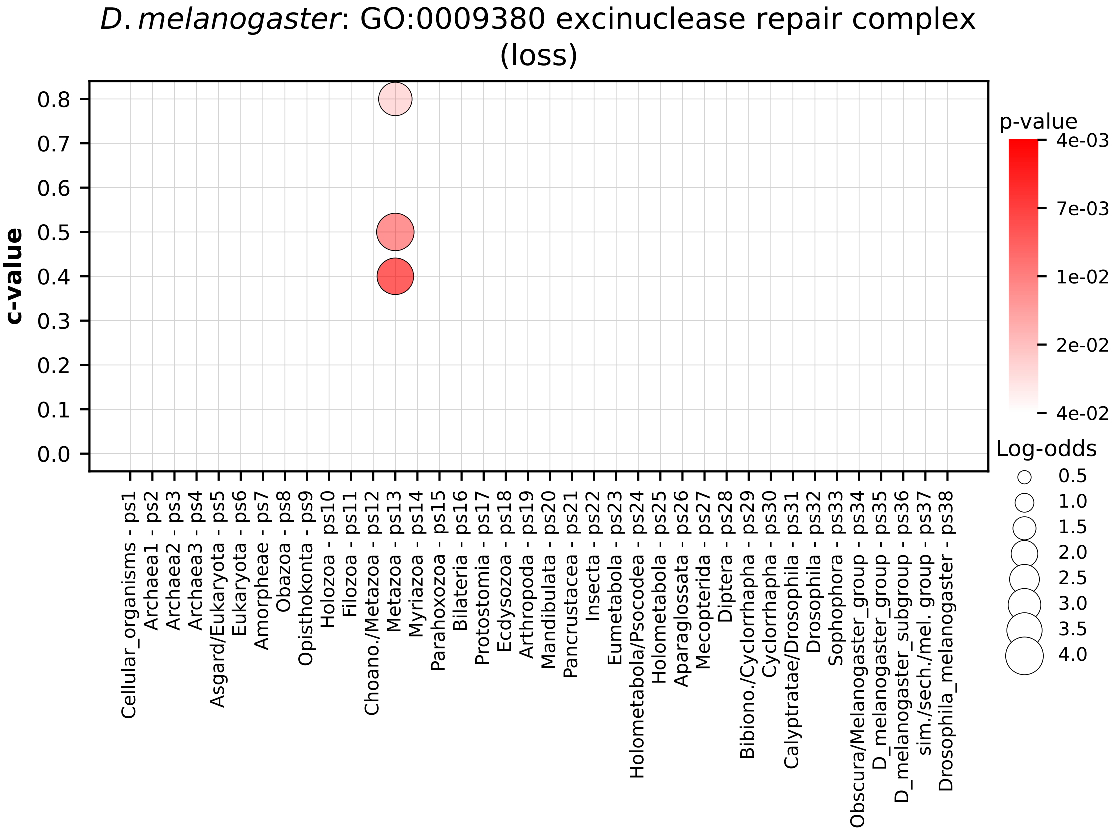

D. melanogaster: GO:0009380 excinuclease repair complex
(loss)
c-value
0.8
0.7
0.6
0.5
0.4
0.3
0.2
0.1
0.0
Phylostratum
Cellular_organisms - ps1
Archaea1 - ps2
Archaea2 - ps3
Archaea3 - ps4
Asgard/Eukaryota - ps5
Eukaryota - ps6
Amorpheae - ps7
Obazoa - ps8
Opisthokonta - ps9
Holozoa - ps10
Filozoa - ps11
Choano./Metazoa - ps12
Metazoa - ps13
Myriazoa - ps14
Parahoxozoa - ps15
Bilateria - ps16
Protostomia - ps17
Ecdysozoa - ps18
Arthropoda - ps19
Mandibulata - ps20
Pancrustacea - ps21
Insecta - ps22
Eumetabola - ps23
Holometabola/Psocodea - ps24
Holometabola - ps25
Aparaglossata - ps26
Mecopterida - ps27
Diptera - ps28
Bibiono./Cyclorrhapha - ps29
Cyclorrhapha - ps30
Calyptratae/Drosophila - ps31
Drosophila - ps32
Sophophora - ps33
Obscura/Melanogaster_group - ps34
D_melanogaster_group - ps35
D_melanogaster_subgroup - ps36
sim./sech./mel. group - ps37
Drosophila_melanogaster - ps38
p-value
4e-03
7e-03
1e-02
2e-02
4e-02
Log-odds
0.5
1.0
1.5
2.0
2.5
3.0
3.5
4.0

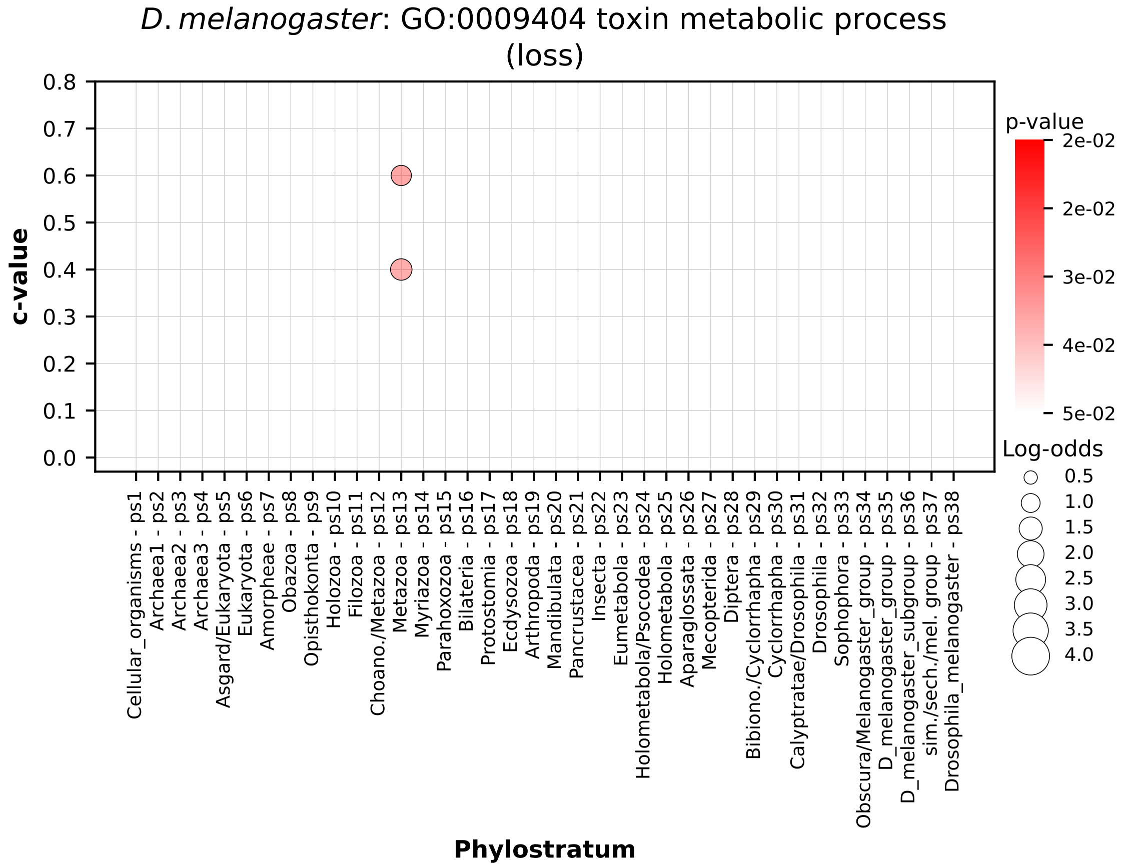
D. melanogaster: GO:0009404 toxin metabolic process
(loss)
c-value
0.8
0.7
0.6
0.5
0.4
0.3
0.2
0.1
0.0
p-value
2e-02
2e-02
3e-02
4e-02
5e-02
Log-odds
0.5
1.0
1.5
2.0
2.5
3.0
3.5
4.0
Cellular_organisms - ps1
Archaea1 - ps2
Archaea2 - ps3
Archaea3 - ps4
Asgard/Eukaryota - ps5
Eukaryota - ps6
Amorpheae - ps7
Obazoa - ps8
Opisthokonta - ps9
Holozoa - ps10
Filozoa - ps11
Choano./Metazoa - ps12
Metazoa - ps13
Myriazoa - ps14
Parahoxozoa - ps15
Bilateria - ps16
Protostomia - ps17
Ecdysozoa - ps18
Arthropoda - ps19
Mandibulata - ps20
Pancrustacea - ps21
Insecta - ps22
Eumetabola - ps23
Holometabola/Psocodea - ps24
Holometabola - ps25
Aparaglossata - ps26
Mecopterida - ps27
Diptera - ps28
Bibiono./Cyclorrhapha - ps29
Cyclorrhapha - ps30
Calyptratae/Drosophila - ps31
Drosophila - ps32
Sophophora - ps33
Obscura/Melanogaster_group - ps34
D_melanogaster_group - ps35
D_melanogaster_subgroup - ps36
sim./sech./mel. group - ps37
Drosophila_melanogaster - ps38
Phylostratum

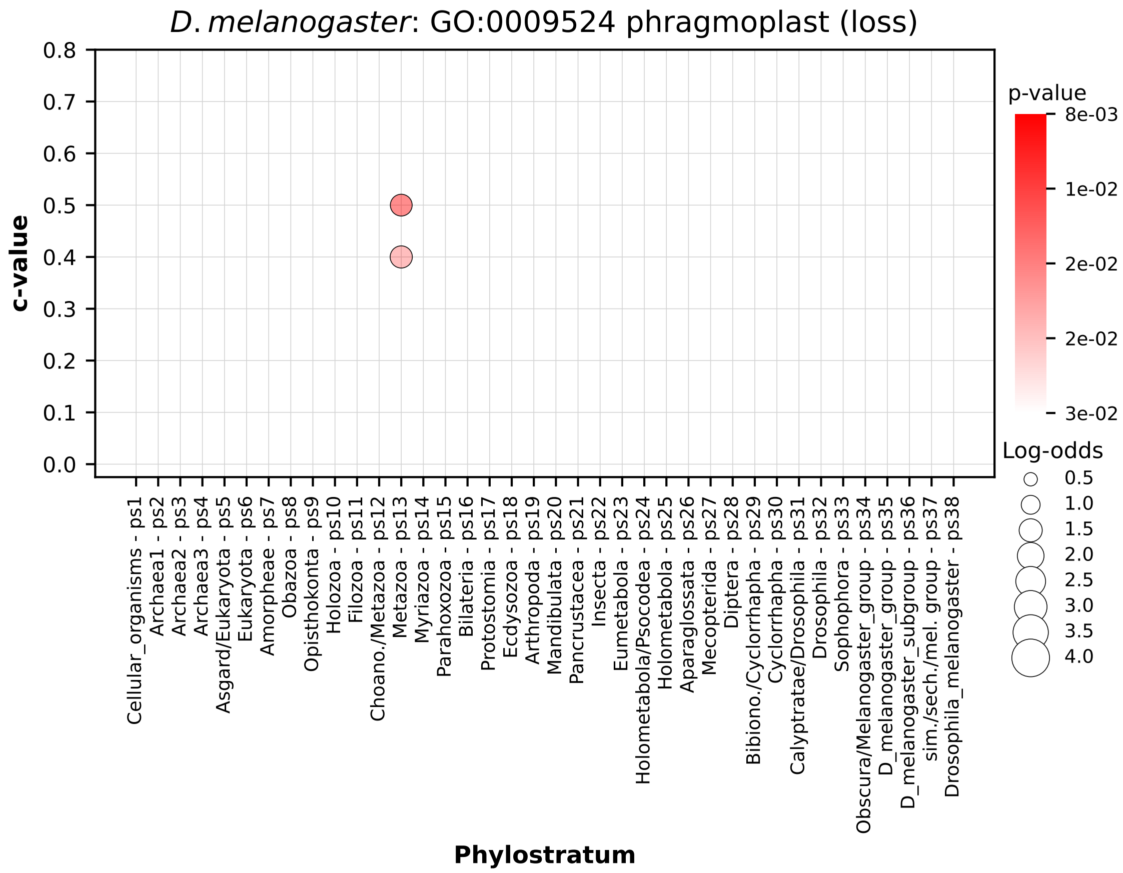

D. melanogaster: GO:0009524 phragmoplast (loss)
c-value
0.8
0.7
0.6
0.5
0.4
0.3
0.2
0.1
0.0
Phylostratum
Cellular_organisms - ps1
Archaea1 - ps2
Archaea2 - ps3
Archaea3 - ps4
Asgard/Eukaryota - ps5
Eukaryota - ps6
Amorpheae - ps7
Obazoa - ps8
Opisthokonta - ps9
Holozoa - ps10
Filozoa - ps11
Choano./Metazoa - ps12
Metazoa - ps13
Myriazoa - ps14
Parahoxozoa - ps15
Bilateria - ps16
Protostomia - ps17
Ecdysozoa - ps18
Arthropoda - ps19
Mandibulata - ps20
Pancrustacea - ps21
Insecta - ps22
Eumetabola - ps23
Holometabola/Psocodea - ps24
Holometabola - ps25
Aparaglossata - ps26
Mecopterida - ps27
Diptera - ps28
Bibiono./Cyclorrhapha - ps29
Cyclorrhapha - ps30
Calyptratae/Drosophila - ps31
Drosophila - ps32
Sophophora - ps33
Obscura/Melanogaster_group - ps34
D_melanogaster_group - ps35
D_melanogaster_subgroup - ps36
sim./sech./mel. group - ps37
Drosophila_melanogaster - ps38
p-value
8e-03
1e-02
2e-02
2e-02
3e-02
Log-odds
0.5
1.0
1.5
2.0
2.5
3.0
3.5
4.0

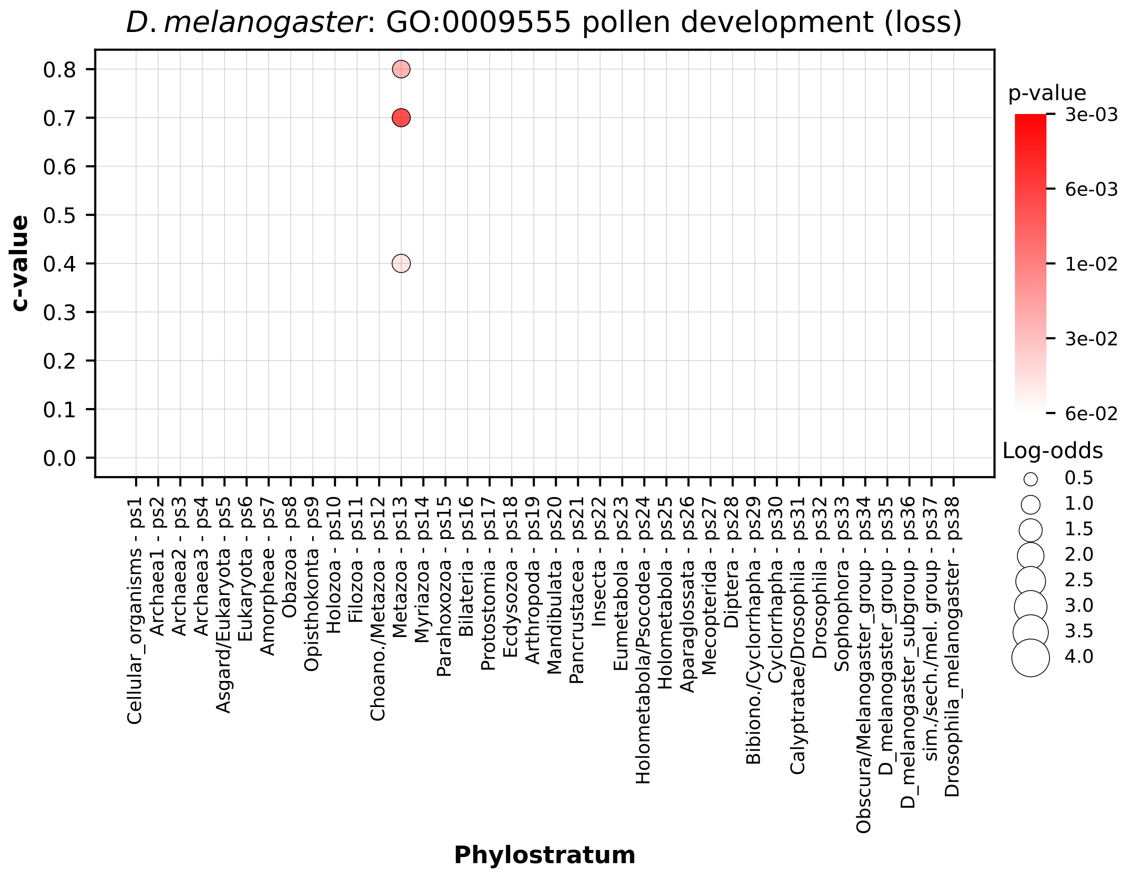
D. melanogaster: GO:0009555 pollen development (loss)
c-value
0.8
0.7
0.6
0.5
0.4
0.3
0.2
0.1
0.0
Phylostratum
Cellular_organisms - ps1
Archaea1 - ps2
Archaea2 - ps3
Archaea3 - ps4
Asgard/Eukaryota - ps5
Eukaryota - ps6
Amorpheae - ps7
Obazoa - ps8
Opisthokonta - ps9
Holozoa - ps10
Filozoa - ps11
Choano./Metazoa - ps12
Metazoa - ps13
Myriazoa - ps14
Parahoxozoa - ps15
Bilateria - ps16
Protostomia - ps17
Ecdysozoa - ps18
Arthropoda - ps19
Mandibulata - ps20
Pancrustacea - ps21
Insecta - ps22
Eumetabola - ps23
Holometabola/Psocodea - ps24
Holometabola - ps25
Aparaglossata - ps26
Mecopterida - ps27
Diptera - ps28
Bibiono./Cyclorrhapha - ps29
Cyclorrhapha - ps30
Calyptratae/Drosophila - ps31
Drosophila - ps32
Sophophora - ps33
Obscura/Melanogaster_group - ps34
D_melanogaster_group - ps35
D_melanogaster_subgroup - ps36
sim./sech./mel. group - ps37
Drosophila_melanogaster - ps38
p-value
3e-03
6e-03
1e-02
3e-02
6e-02
Log-odds
0.5
1.0
1.5
2.0
2.5
3.0
3.5
4.0

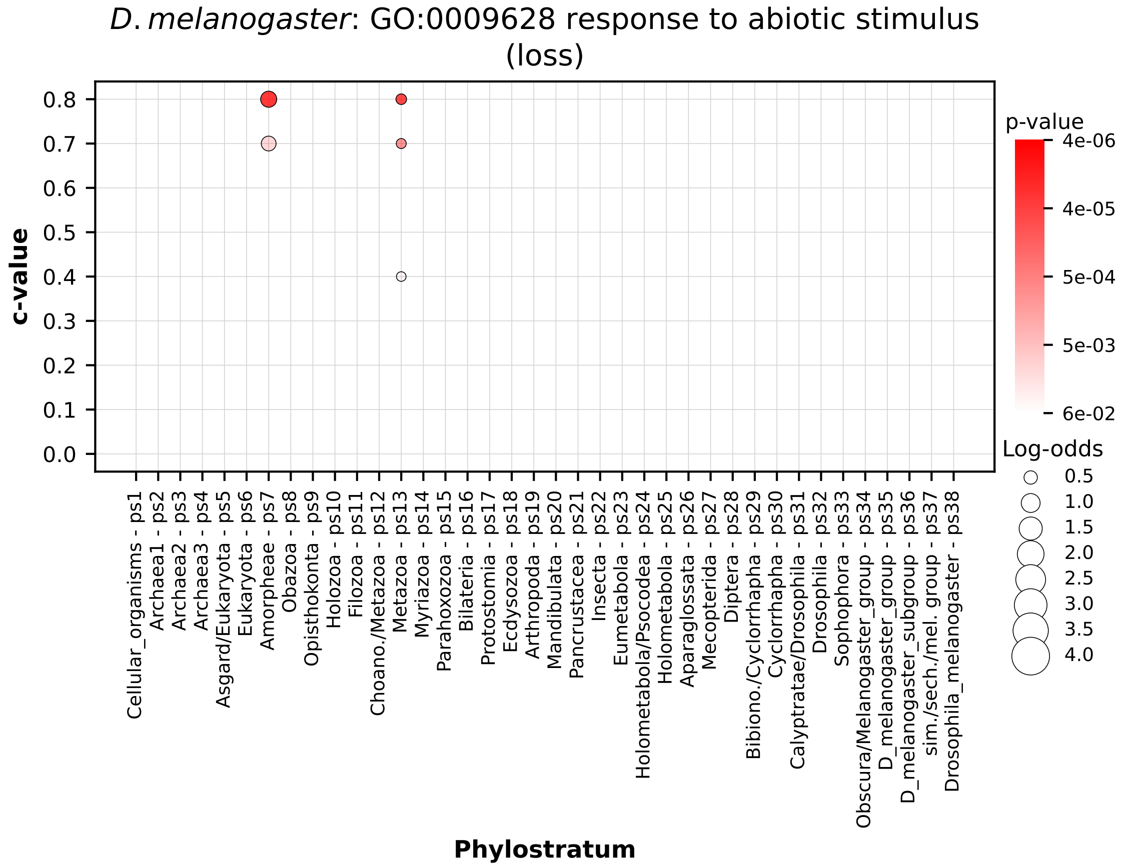

D. melanogaster: GO:0009628 response to abiotic stimulus
(loss)
c-value
0.8
0.7
0.6
0.5
0.4
0.3
0.2
0.1
0.0
Phylostratum
Cellular_organisms - ps1
Archaea1 - ps2
Archaea2 - ps3
Archaea3 - ps4
Asgard/Eukaryota - ps5
Eukaryota - ps6
Amorpheae - ps7
Obazoa - ps8
Opisthokonta - ps9
Holozoa - ps10
Filozoa - ps11
Choano./Metazoa - ps12
Metazoa - ps13
Myriazoa - ps14
Parahoxozoa - ps15
Bilateria - ps16
Protostomia - ps17
Ecdysozoa - ps18
Arthropoda - ps19
Mandibulata - ps20
Pancrustacea - ps21
Insecta - ps22
Eumetabola - ps23
Holometabola/Psocodea - ps24
Holometabola - ps25
Aparaglossata - ps26
Mecopterida - ps27
Diptera - ps28
Bibiono./Cyclorrhapha - ps29
Cyclorrhapha - ps30
Calyptratae/Drosophila - ps31
Drosophila - ps32
Sophophora - ps33
Obscura/Melanogaster_group - ps34
D_melanogaster_group - ps35
D_melanogaster_subgroup - ps36
sim./sech./mel. group - ps37
Drosophila_melanogaster - ps38
p-value
4e-06
4e-05
5e-04
5e-03
6e-02
Log-odds
0.5
1.0
1.5
2.0
2.5
3.0
3.5
4.0

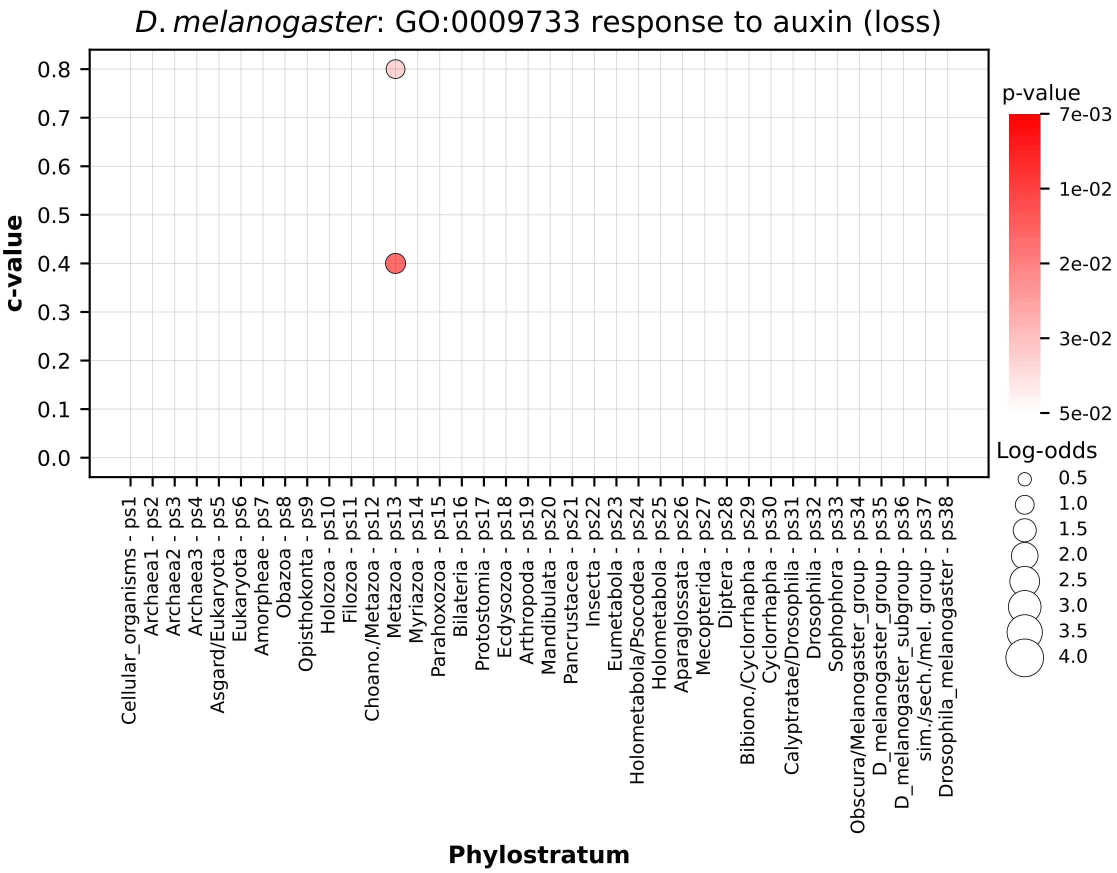

D. melanogaster: GO:0009733 response to auxin (loss)
c-value
0.0
0.1
0.2
0.3
0.4
0.5
0.6
0.7
0.8
p-value
7e-03
1e-02
2e-02
3e-02
5e-02
Log-odds
0.5
1.0
1.5
2.0
2.5
3.0
3.5
4.0
Cellular_organisms - ps1
Archaea1 - ps2
Archaea2 - ps3
Archaea3 - ps4
Asgard/Eukaryota - ps5
Eukaryota - ps6
Amorpheae - ps7
Obazoa - ps8
Opisthokonta - ps9
Holozoa - ps10
Filozoa - ps11
Choano./Metazoa - ps12
Metazoa - ps13
Myriazoa - ps14
Parahoxozoa - ps15
Bilateria - ps16
Protostomia - ps17
Ecdysozoa - ps18
Arthropoda - ps19
Mandibulata - ps20
Pancrustacea - ps21
Insecta - ps22
Eumetabola - ps23
Holometabola/Psocodea - ps24
Holometabola - ps25
Aparaglossata - ps26
Mecopterida - ps27
Diptera - ps28
Bibiono./Cyclorrhapha - ps29
Cyclorrhapha - ps30
Calyptratae/Drosophila - ps31
Drosophila - ps32
Sophophora - ps33
Obscura/Melanogaster_group - ps34
D_melanogaster_group - ps35
D_melanogaster_subgroup - ps36
sim./sech./mel. group - ps37
Drosophila_melanogaster - ps38
Phylostratum

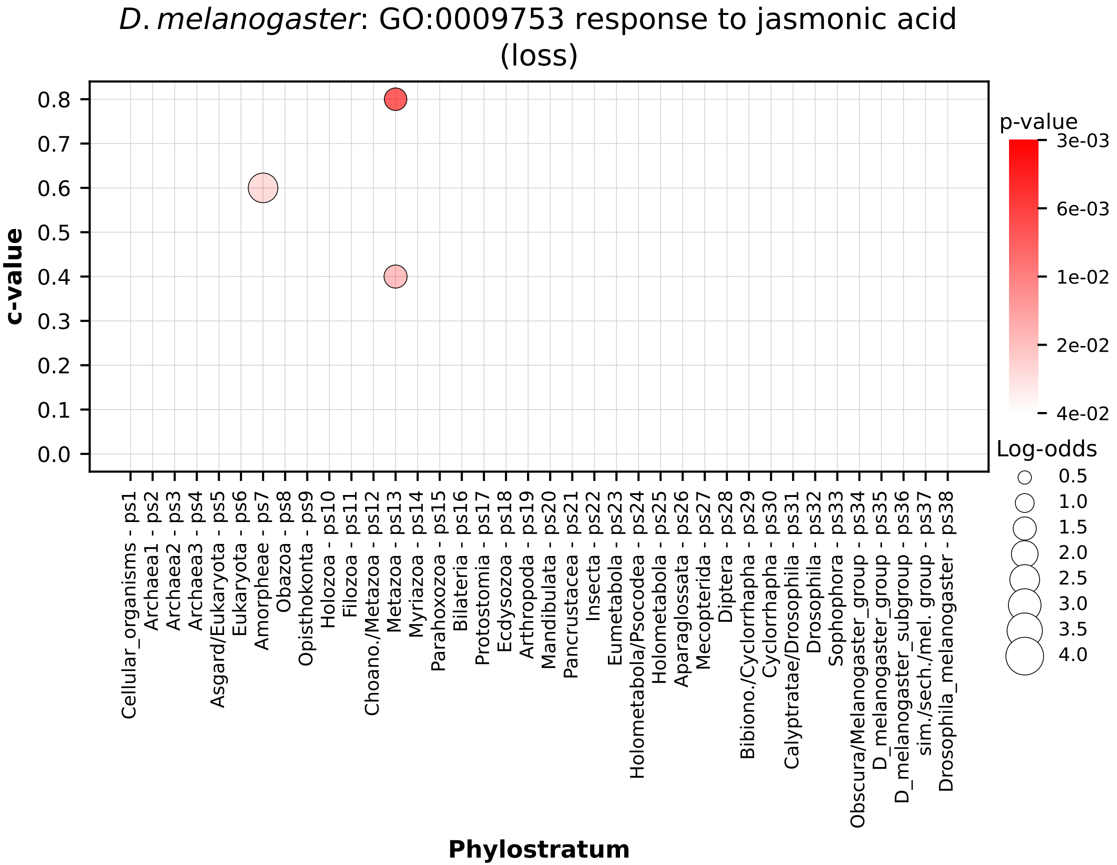
D. melanogaster: GO:0009753 response to jasmonic acid
(loss)
c-value
0.0
0.1
0.2
0.3
0.4
0.5
0.6
0.7
0.8
p-value
3e-03
6e-03
1e-02
2e-02
4e-02
Log-odds
0.5
1.0
1.5
2.0
2.5
3.0
3.5
4.0
Cellular_organisms - ps1
Archaea1 - ps2
Archaea2 - ps3
Archaea3 - ps4
Asgard/Eukaryota - ps5
Eukaryota - ps6
Amorpheae - ps7
Obazoa - ps8
Opisthokonta - ps9
Holozoa - ps10
Filozoa - ps11
Choano./Metazoa - ps12
Metazoa - ps13
Myriazoa - ps14
Parahoxozoa - ps15
Bilateria - ps16
Protostomia - ps17
Ecdysozoa - ps18
Arthropoda - ps19
Mandibulata - ps20
Pancrustacea - ps21
Insecta - ps22
Eumetabola - ps23
Holometabola/Psocodea - ps24
Holometabola - ps25
Aparaglossata - ps26
Mecopterida - ps27
Diptera - ps28
Bibiono./Cyclorrhapha - ps29
Cyclorrhapha - ps30
Calyptratae/Drosophila - ps31
Drosophila - ps32
Sophophora - ps33
Obscura/Melanogaster_group - ps34
D_melanogaster_group - ps35
D_melanogaster_subgroup - ps36
sim./sech./mel. group - ps37
Drosophila_melanogaster - ps38
Phylostratum

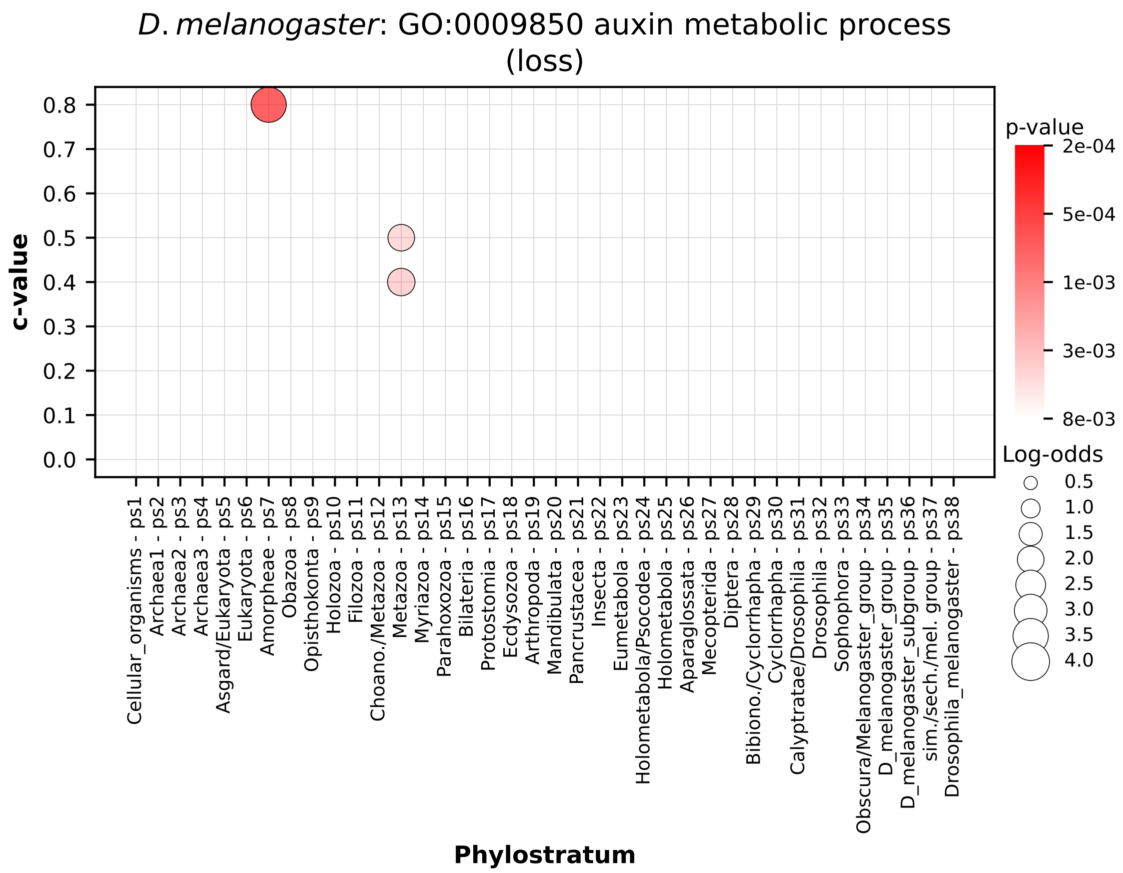
D. melanogaster: GO:0009850 auxin metabolic process
(loss)
c-value
0.8
0.7
0.6
0.5
0.4
0.3
0.2
0.1
0.0
p-value
2e-04
5e-04
1e-03
3e-03
8e-03
Log-odds
0.5
1.0
1.5
2.0
2.5
3.0
3.5
4.0
Cellular_organisms - ps1
Archaea1 - ps2
Archaea2 - ps3
Archaea3 - ps4
Asgard/Eukaryota - ps5
Eukaryota - ps6
Amorpheae - ps7
Obazoa - ps8
Opisthokonta - ps9
Holozoa - ps10
Filozoa - ps11
Choano./Metazoa - ps12
Metazoa - ps13
Myriazoa - ps14
Parahoxozoa - ps15
Bilateria - ps16
Protostomia - ps17
Ecdysozoa - ps18
Arthropoda - ps19
Mandibulata - ps20
Pancrustacea - ps21
Insecta - ps22
Eumetabola - ps23
Holometabola/Psocodea - ps24
Holometabola - ps25
Aparaglossata - ps26
Mecopterida - ps27
Diptera - ps28
Bibiono./Cyclorrhapha - ps29
Cyclorrhapha - ps30
Calyptratae/Drosophila - ps31
Drosophila - ps32
Sophophora - ps33
Obscura/Melanogaster_group - ps34
D_melanogaster_group - ps35
D_melanogaster_subgroup - ps36
sim./sech./mel. group - ps37
Drosophila_melanogaster - ps38
Phylostratum

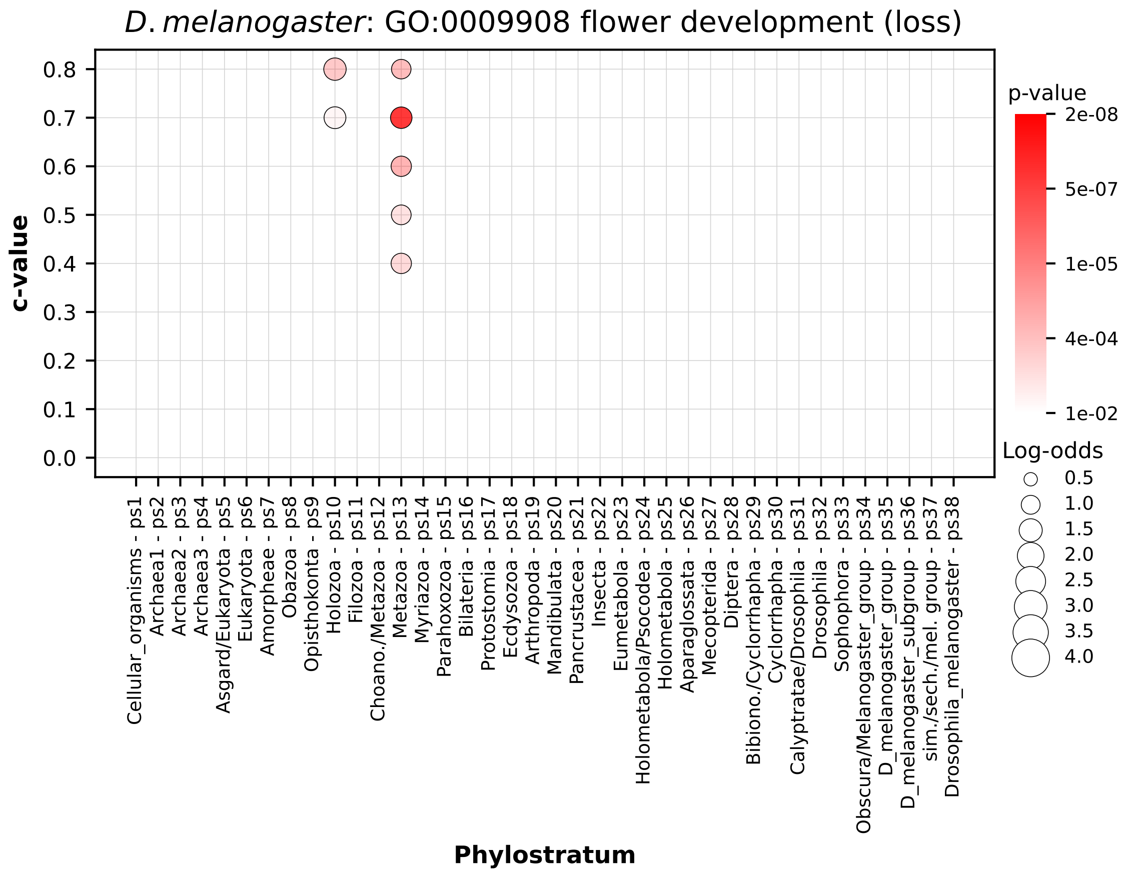
D. melanogaster: GO:0009908 flower development (loss)
c-value
0.8
0.7
0.6
0.5
0.4
0.3
0.2
0.1
0.0
p-value
2e-08
5e-07
1e-05
4e-04
1e-02
Log-odds
0.5
1.0
1.5
2.0
2.5
3.0
3.5
4.0
Cellular_organisms - ps1
Archaea1 - ps2
Archaea2 - ps3
Archaea3 - ps4
Asgard/Eukaryota - ps5
Eukaryota - ps6
Amorpheae - ps7
Obazoa - ps8
Opisthokonta - ps9
Holozoa - ps10
Filozoa - ps11
Choano./Metazoa - ps12
Metazoa - ps13
Myriazoa - ps14
Parahoxozoa - ps15
Bilateria - ps16
Protostomia - ps17
Ecdysozoa - ps18
Arthropoda - ps19
Mandibulata - ps20
Pancrustacea - ps21
Insecta - ps22
Eumetabola - ps23
Holometabola/Psocodea - ps24
Holometabola - ps25
Aparaglossata - ps26
Mecopterida - ps27
Diptera - ps28
Bibiono./Cyclorrhapha - ps29
Cyclorrhapha - ps30
Calyptratae/Drosophila - ps31
Drosophila - ps32
Sophophora - ps33
Obscura/Melanogaster_group - ps34
D_melanogaster_group - ps35
D_melanogaster_subgroup - ps36
sim./sech./mel. group - ps37
Drosophila_melanogaster - ps38
Phylostratum

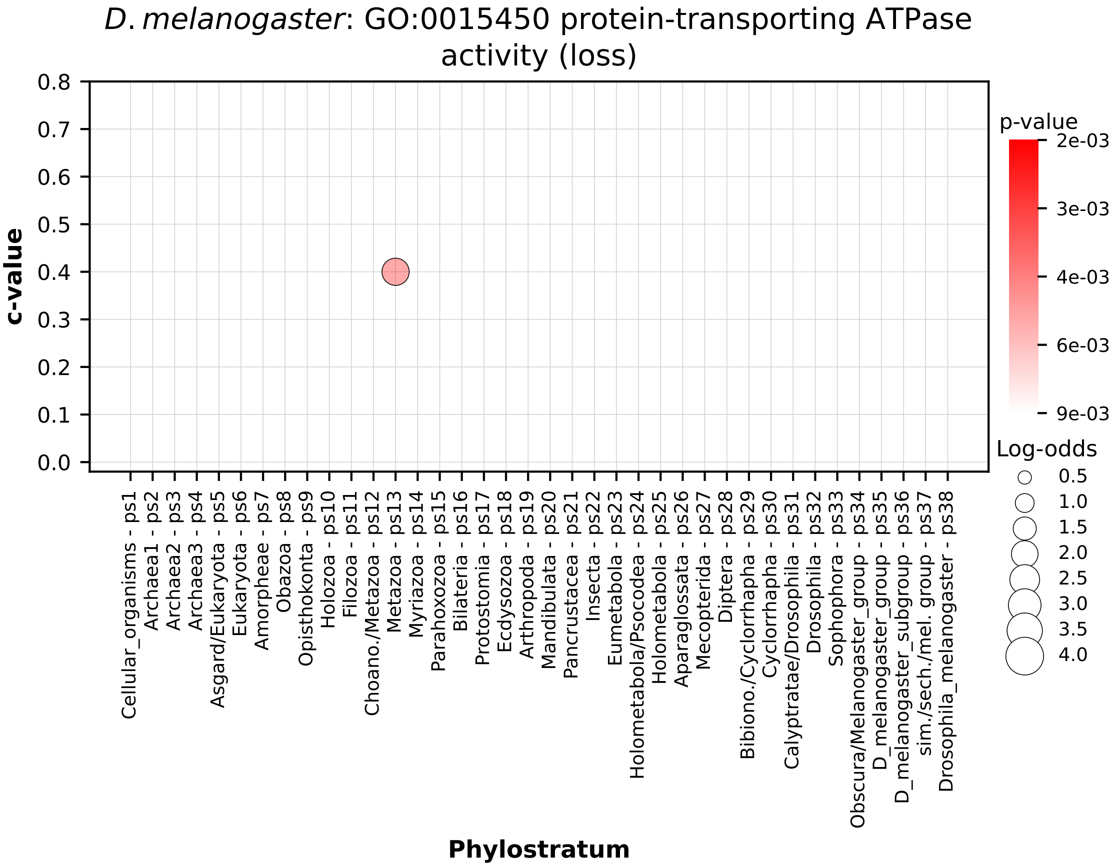
D. melanogaster: GO:0015450 protein-transporting ATPase activity (loss)
c-value
0.0
0.1
0.2
0.3
0.4
0.5
0.6
0.7
0.8
Phylostratum
Cellular_organisms - ps1
Archaea1 - ps2
Archaea2 - ps3
Archaea3 - ps4
Asgard/Eukaryota - ps5
Eukaryota - ps6
Amorpheae - ps7
Obazoa - ps8
Opisthokonta - ps9
Holozoa - ps10
Filozoa - ps11
Choano./Metazoa - ps12
Metazoa - ps13
Myriazoa - ps14
Parahoxozoa - ps15
Bilateria - ps16
Protostomia - ps17
Ecdysozoa - ps18
Arthropoda - ps19
Mandibulata - ps20
Pancrustacea - ps21
Insecta - ps22
Eumetabola - ps23
Holometabola/Psocodea - ps24
Holometabola - ps25
Aparaglossata - ps26
Mecopterida - ps27
Diptera - ps28
Bibiono./Cyclorrhapha - ps29
Cyclorrhapha - ps30
Calyptratae/Drosophila - ps31
Drosophila - ps32
Sophophora - ps33
Obscura/Melanogaster_group - ps34
D_melanogaster_group - ps35
D_melanogaster_subgroup - ps36
sim./sech./mel. group - ps37
Drosophila_melanogaster - ps38
p-value
2e-03
3e-03
4e-03
6e-03
9e-03
Log-odds
0.5
1.0
1.5
2.0
2.5
3.0
3.5
4.0

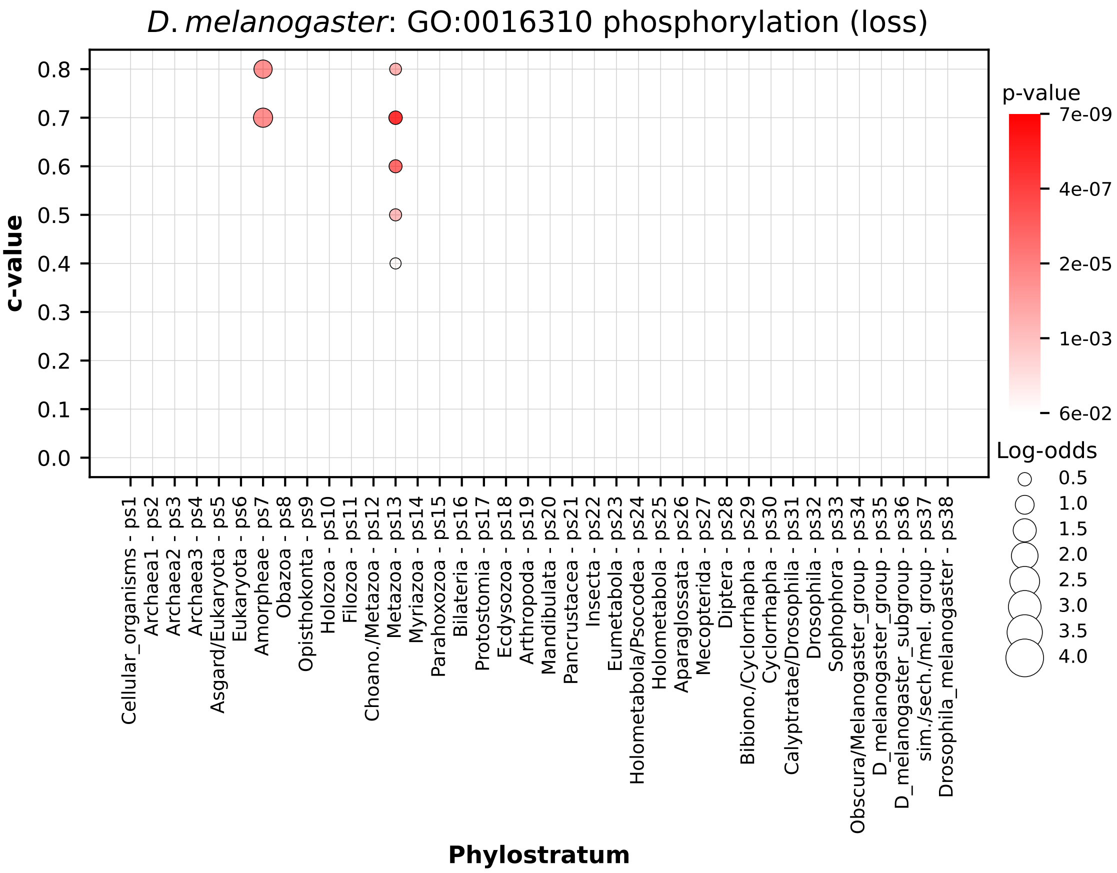
D. melanogaster: GO:0016310 phosphorylation (loss)
c-value
0.0
0.1
0.2
0.3
0.4
0.5
0.6
0.7
0.8
Phylostratum
Cellular_organisms - ps1
Archaea1 - ps2
Archaea2 - ps3
Archaea3 - ps4
Asgard/Eukaryota - ps5
Eukaryota - ps6
Amorpheae - ps7
Obazoa - ps8
Opisthokonta - ps9
Holozoa - ps10
Filozoa - ps11
Choano./Metazoa - ps12
Metazoa - ps13
Myriazoa - ps14
Parahoxozoa - ps15
Bilateria - ps16
Protostomia - ps17
Ecdysozoa - ps18
Arthropoda - ps19
Mandibulata - ps20
Pancrustacea - ps21
Insecta - ps22
Eumetabola - ps23
Holometabola/Psocodea - ps24
Holometabola - ps25
Aparaglossata - ps26
Mecopterida - ps27
Diptera - ps28
Bibiono./Cyclorrhapha - ps29
Cyclorrhapha - ps30
Calyptratae/Drosophila - ps31
Drosophila - ps32
Sophophora - ps33
Obscura/Melanogaster_group - ps34
D_melanogaster_group - ps35
D_melanogaster_subgroup - ps36
sim./sech./mel. group - ps37
Drosophila_melanogaster - ps38
p-value
7e-09
4e-07
2e-05
1e-03
6e-02
Log-odds
0.5
1.0
1.5
2.0
2.5
3.0
3.5
4.0

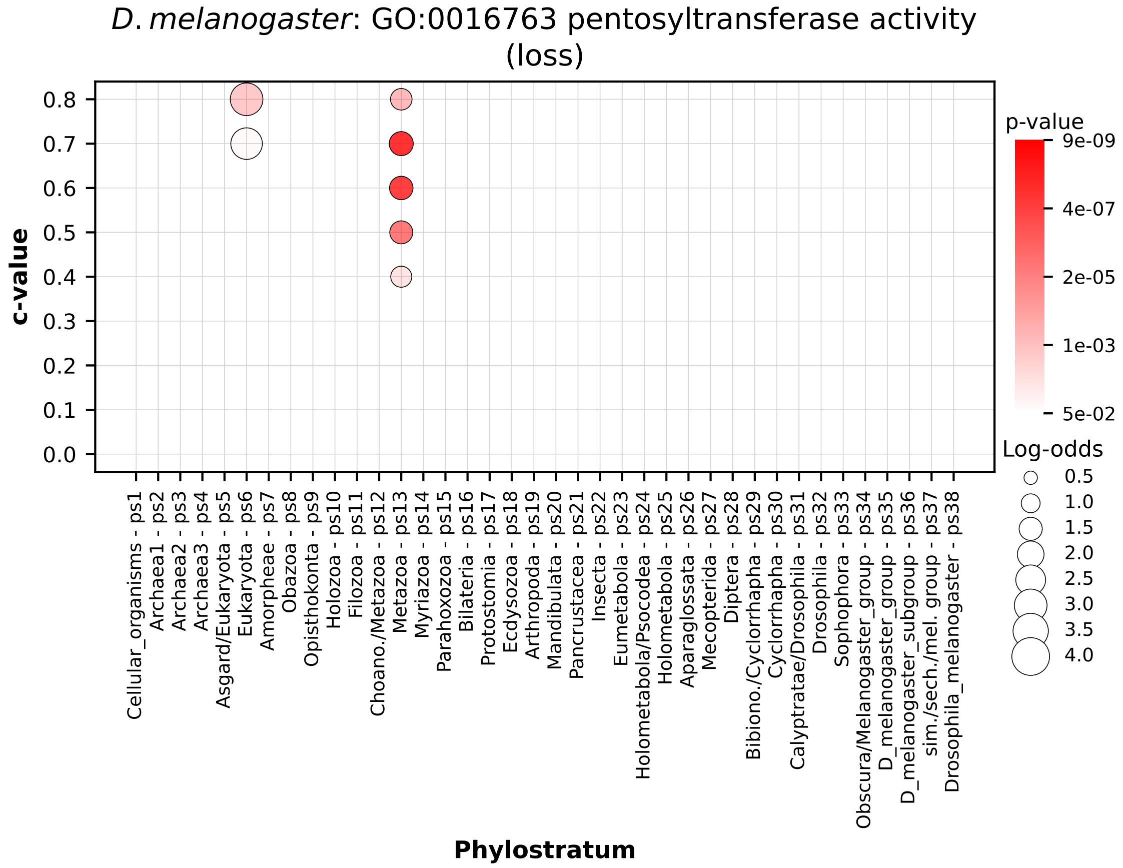

D. melanogaster: GO:0016763 pentosyltransferase activity
(loss)
c-value
0.8
0.7
0.6
0.5
0.4
0.3
0.2
0.1
0.0
p-value
9e-09
4e-07
2e-05
1e-03
5e-02
Log-odds
0.5
1.0
1.5
2.0
2.5
3.0
3.5
4.0
Cellular_organisms - ps1
Archaea1 - ps2
Archaea2 - ps3
Archaea3 - ps4
Asgard/Eukaryota - ps5
Eukaryota - ps6
Amorpheae - ps7
Obazoa - ps8
Opisthokonta - ps9
Holozoa - ps10
Filozoa - ps11
Choano./Metazoa - ps12
Metazoa - ps13
Myriazoa - ps14
Parahoxozoa - ps15
Bilateria - ps16
Protostomia - ps17
Ecdysozoa - ps18
Arthropoda - ps19
Mandibulata - ps20
Pancrustacea - ps21
Insecta - ps22
Eumetabola - ps23
Holometabola/Psocodea - ps24
Holometabola - ps25
Aparaglossata - ps26
Mecopterida - ps27
Diptera - ps28
Bibiono./Cyclorrhapha - ps29
Cyclorrhapha - ps30
Calyptratae/Drosophila - ps31
Drosophila - ps32
Sophophora - ps33
Obscura/Melanogaster_group - ps34
D_melanogaster_group - ps35
D_melanogaster_subgroup - ps36
sim./sech./mel. group - ps37
Drosophila_melanogaster - ps38
Phylostratum

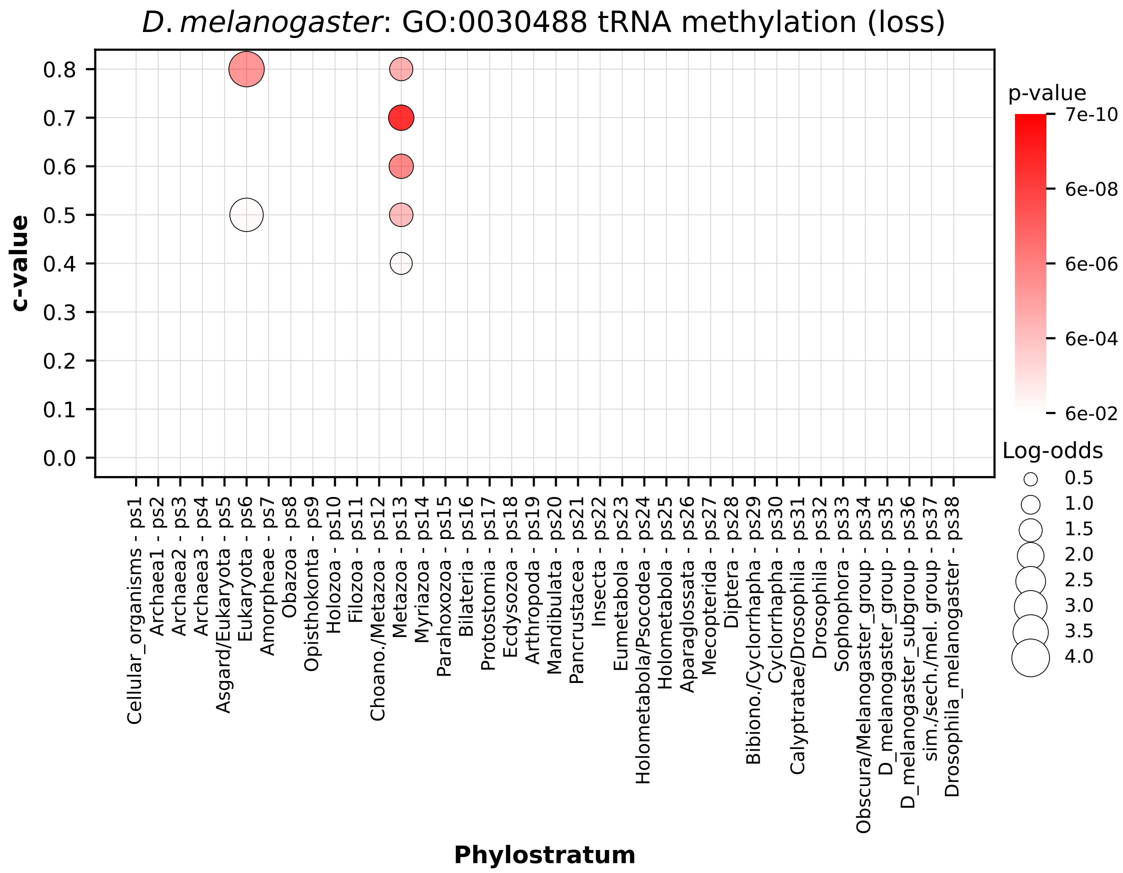

D. melanogaster: GO:0030488 tRNA methylation (loss)
c-value
0.0
0.1
0.2
0.3
0.4
0.5
0.6
0.7
0.8
Phylostratum
Cellular_organisms - ps1
Archaea1 - ps2
Archaea2 - ps3
Archaea3 - ps4
Asgard/Eukaryota - ps5
Eukaryota - ps6
Amorpheae - ps7
Obazoa - ps8
Opisthokonta - ps9
Holozoa - ps10
Filozoa - ps11
Choano./Metazoa - ps12
Metazoa - ps13
Myriazoa - ps14
Parahoxozoa - ps15
Bilateria - ps16
Protostomia - ps17
Ecdysozoa - ps18
Arthropoda - ps19
Mandibulata - ps20
Pancrustacea - ps21
Insecta - ps22
Eumetabola - ps23
Holometabola/Psocodea - ps24
Holometabola - ps25
Aparaglossata - ps26
Mecopterida - ps27
Diptera - ps28
Bibiono./Cyclorrhapha - ps29
Cyclorrhapha - ps30
Calyptratae/Drosophila - ps31
Drosophila - ps32
Sophophora - ps33
Obscura/Melanogaster_group - ps34
D_melanogaster_group - ps35
D_melanogaster_subgroup - ps36
sim./sech./mel. group - ps37
Drosophila_melanogaster - ps38
p-value
7e-10
6e-08
6e-06
6e-04
6e-02
Log-odds
0.5
1.0
1.5
2.0
2.5
3.0
3.5
4.0

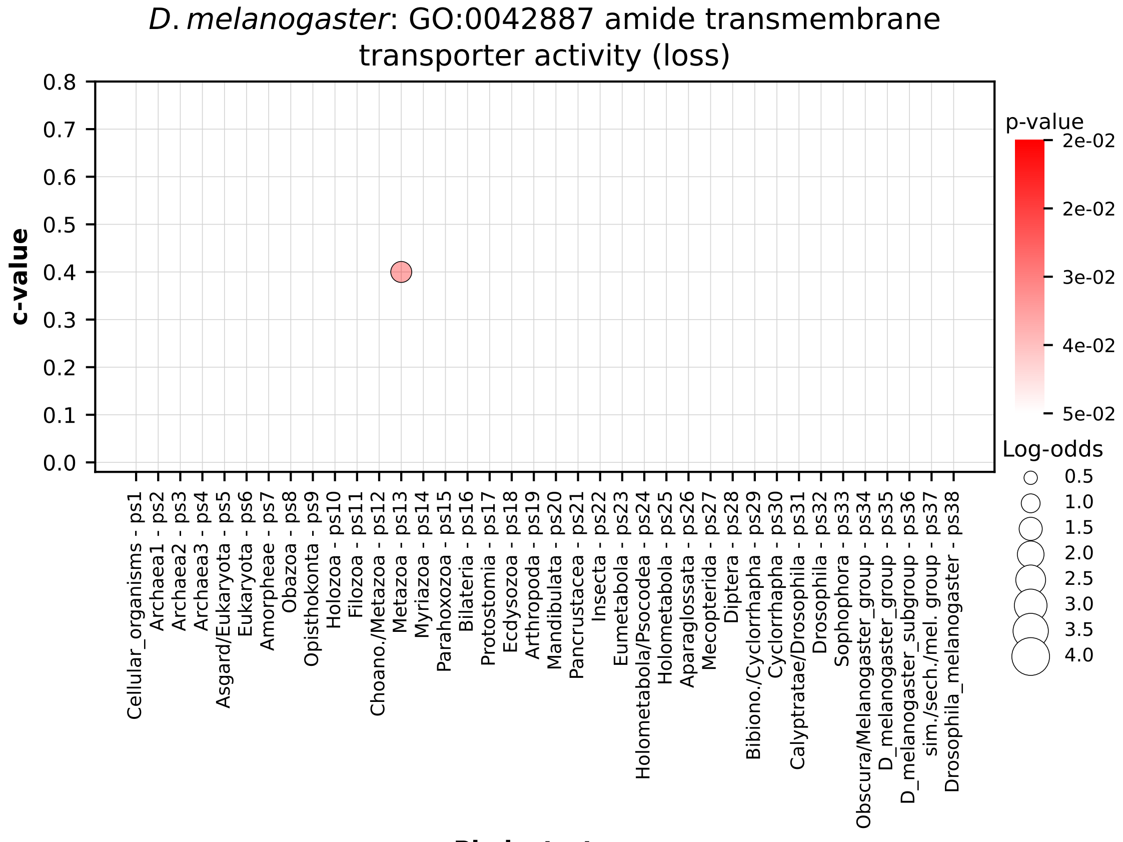

D. melanogaster: GO:0042887 amide transmembrane transporter activity (loss)
c-value
0.8
0.7
0.6
0.5
0.4
0.3
0.2
0.1
0.0
p-value
2e-02
2e-02
3e-02
4e-02
5e-02
Log-odds
0.5
1.0
1.5
2.0
2.5
3.0
3.5
4.0
Cellular_organisms - ps1
Archaea1 - ps2
Archaea2 - ps3
Archaea3 - ps4
Asgard/Eukaryota - ps5
Eukaryota - ps6
Amorpheae - ps7
Obazoa - ps8
Opisthokonta - ps9
Holozoa - ps10
Filozoa - ps11
Choano./Metazoa - ps12
Metazoa - ps13
Myriazoa - ps14
Parahoxozoa - ps15
Bilateria - ps16
Protostomia - ps17
Ecdysozoa - ps18
Arthropoda - ps19
Mandibulata - ps20
Pancrustacea - ps21
Insecta - ps22
Eumetabola - ps23
Holometabola/Psocodea - ps24
Holometabola - ps25
Aparaglossata - ps26
Mecopterida - ps27
Diptera - ps28
Bibiono./Cyclorrhapha - ps29
Cyclorrhapha - ps30
Calyptratae/Drosophila - ps31
Drosophila - ps32
Sophophora - ps33
Obscura/Melanogaster_group - ps34
D_melanogaster_group - ps35
D_melanogaster_subgroup - ps36
sim./sech./mel. group - ps37
Drosophila_melanogaster - ps38
Phylostratum

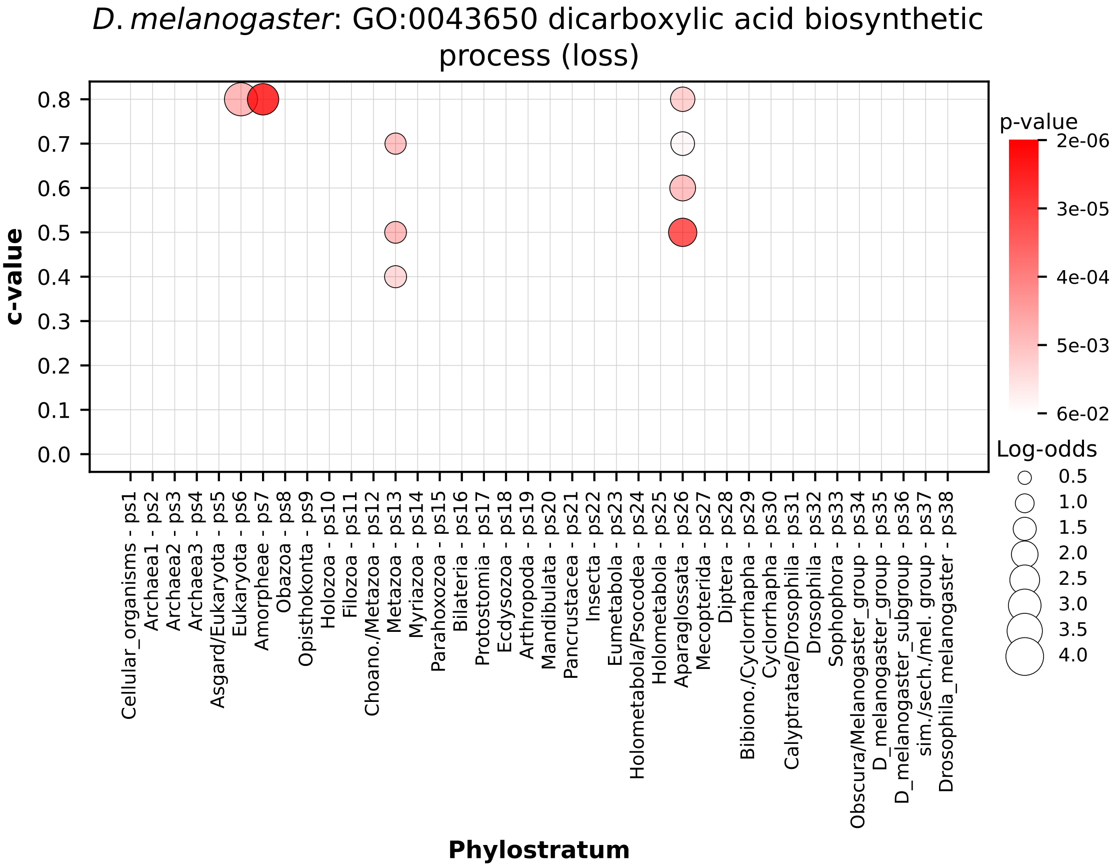

D. melanogaster: GO:0043650 dicarboxylic acid biosynthetic process (loss)
c-value
0.0
0.1
0.2
0.3
0.4
0.5
0.6
0.7
0.8
Phylostratum
Cellular_organisms - ps1
Archaea1 - ps2
Archaea2 - ps3
Archaea3 - ps4
Asgard/Eukaryota - ps5
Eukaryota - ps6
Amorpheae - ps7
Obazoa - ps8
Opisthokonta - ps9
Holozoa - ps10
Filozoa - ps11
Choano./Metazoa - ps12
Metazoa - ps13
Myriazoa - ps14
Parahoxozoa - ps15
Bilateria - ps16
Protostomia - ps17
Ecdysozoa - ps18
Arthropoda - ps19
Mandibulata - ps20
Pancrustacea - ps21
Insecta - ps22
Eumetabola - ps23
Holometabola/Psocodea - ps24
Holometabola - ps25
Aparaglossata - ps26
Mecopterida - ps27
Diptera - ps28
Bibiono./Cyclorrhapha - ps29
Cyclorrhapha - ps30
Calyptratae/Drosophila - ps31
Drosophila - ps32
Sophophora - ps33
Obscura/Melanogaster_group - ps34
D_melanogaster_group - ps35
D_melanogaster_subgroup - ps36
sim./sech./mel. group - ps37
Drosophila_melanogaster - ps38
p-value
2e-06
3e-05
4e-04
5e-03
6e-02
Log-odds
0.5
1.0
1.5
2.0
2.5
3.0
3.5
4.0

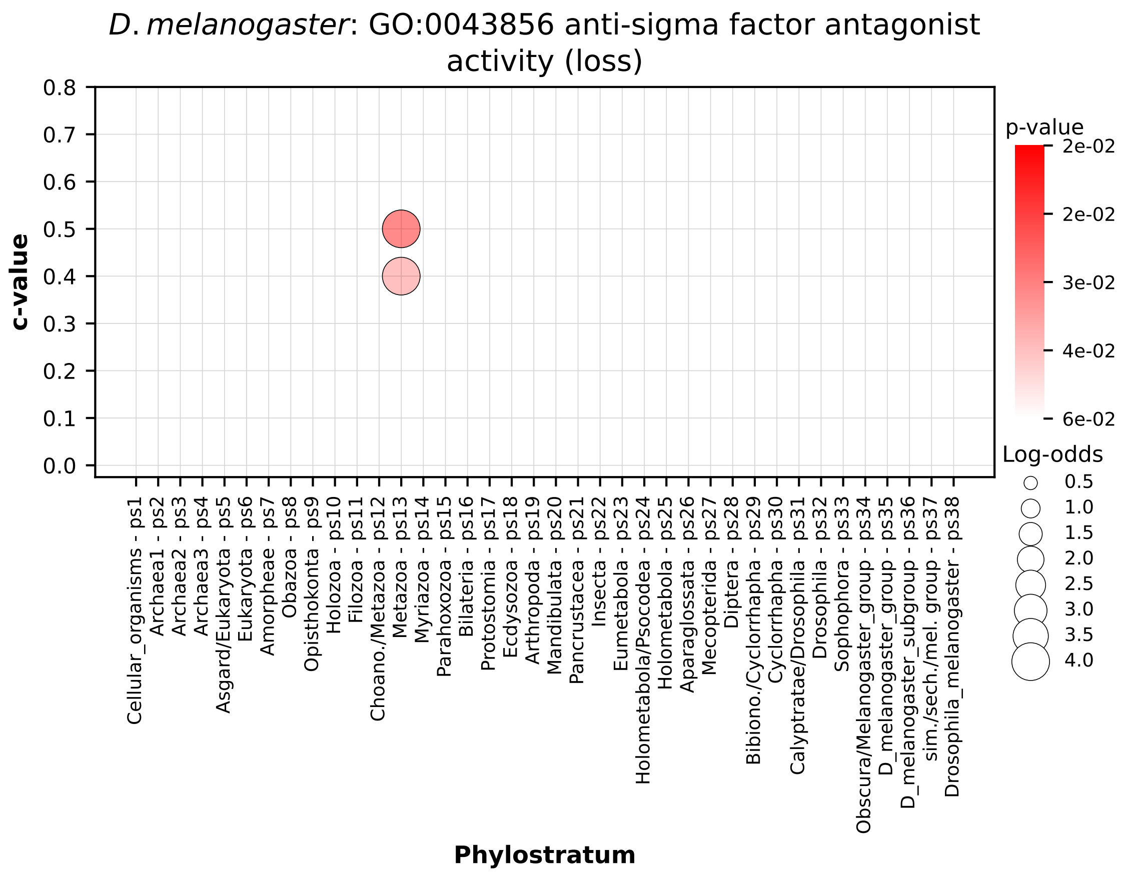

D. melanogaster: GO:0043856 anti-sigma factor antagonist activity (loss)
c-value
0.8
0.7
0.6
0.5
0.4
0.3
0.2
0.1
0.0
Phylostratum
Cellular_organisms - ps1
Archaea1 - ps2
Archaea2 - ps3
Archaea3 - ps4
Asgard/Eukaryota - ps5
Eukaryota - ps6
Amorpheae - ps7
Obazoa - ps8
Opisthokonta - ps9
Holozoa - ps10
Filozoa - ps11
Choano./Metazoa - ps12
Metazoa - ps13
Myriazoa - ps14
Parahoxozoa - ps15
Bilateria - ps16
Protostomia - ps17
Ecdysozoa - ps18
Arthropoda - ps19
Mandibulata - ps20
Pancrustacea - ps21
Insecta - ps22
Eumetabola - ps23
Holometabola/Psocodea - ps24
Holometabola - ps25
Aparaglossata - ps26
Mecopterida - ps27
Diptera - ps28
Bibiono./Cyclorrhapha - ps29
Cyclorrhapha - ps30
Calyptratae/Drosophila - ps31
Drosophila - ps32
Sophophora - ps33
Obscura/Melanogaster_group - ps34
D_melanogaster_group - ps35
D_melanogaster_subgroup - ps36
sim./sech./mel. group - ps37
Drosophila_melanogaster - ps38
p-value
2e-02
2e-02
3e-02
4e-02
6e-02
Log-odds
0.5
1.0
1.5
2.0
2.5
3.0
3.5
4.0

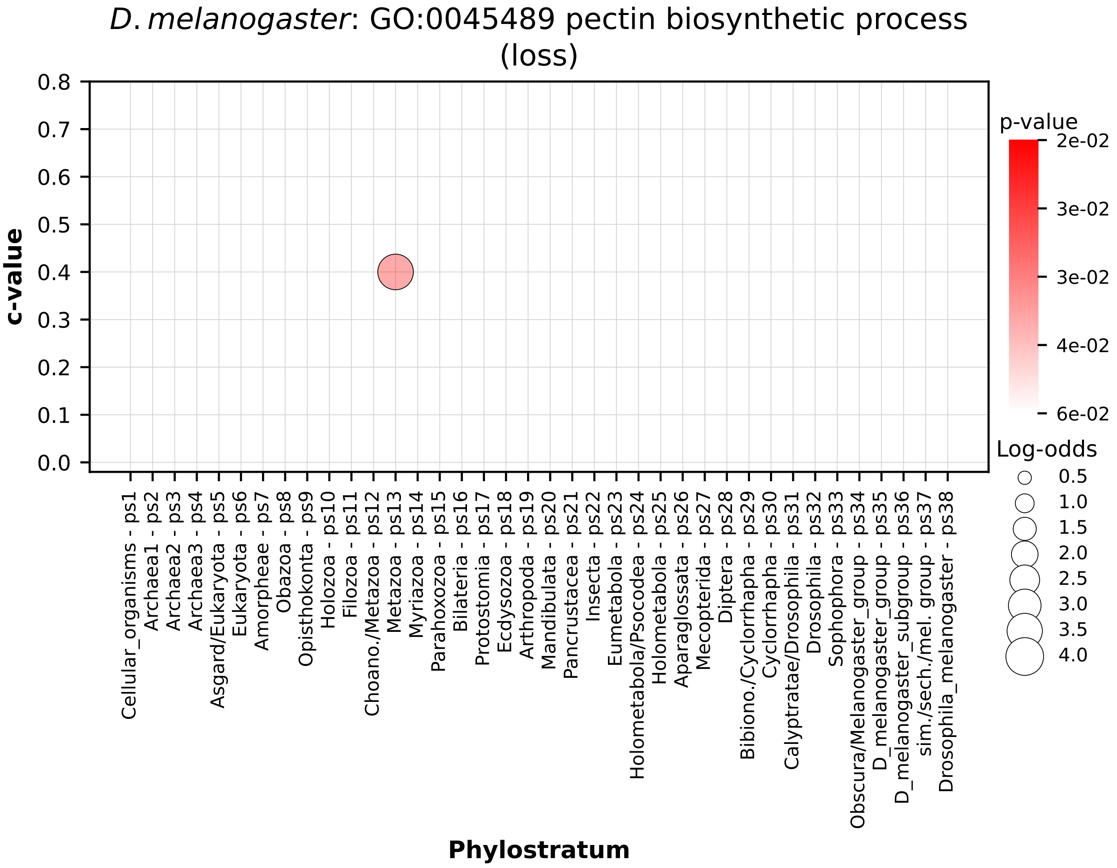

D. melanogaster: GO:0045489 pectin biosynthetic process
(loss)
c-value
0.8
0.7
0.6
0.5
0.4
0.3
0.2
0.1
0.0
Phylostratum
Cellular_organisms - ps1
Archaea1 - ps2
Archaea2 - ps3
Archaea3 - ps4
Asgard/Eukaryota - ps5
Eukaryota - ps6
Amorpheae - ps7
Obazoa - ps8
Opisthokonta - ps9
Holozoa - ps10
Filozoa - ps11
Choano./Metazoa - ps12
Metazoa - ps13
Myriazoa - ps14
Parahoxozoa - ps15
Bilateria - ps16
Protostomia - ps17
Ecdysozoa - ps18
Arthropoda - ps19
Mandibulata - ps20
Pancrustacea - ps21
Insecta - ps22
Eumetabola - ps23
Holometabola/Psocodea - ps24
Holometabola - ps25
Aparaglossata - ps26
Mecopterida - ps27
Diptera - ps28
Bibiono./Cyclorrhapha - ps29
Cyclorrhapha - ps30
Calyptratae/Drosophila - ps31
Drosophila - ps32
Sophophora - ps33
Obscura/Melanogaster_group - ps34
D_melanogaster_group - ps35
D_melanogaster_subgroup - ps36
sim./sech./mel. group - ps37
Drosophila_melanogaster - ps38
p-value
2e-02
3e-02
3e-02
4e-02
6e-02
Log-odds
0.5
1.0
1.5
2.0
2.5
3.0
3.5
4.0

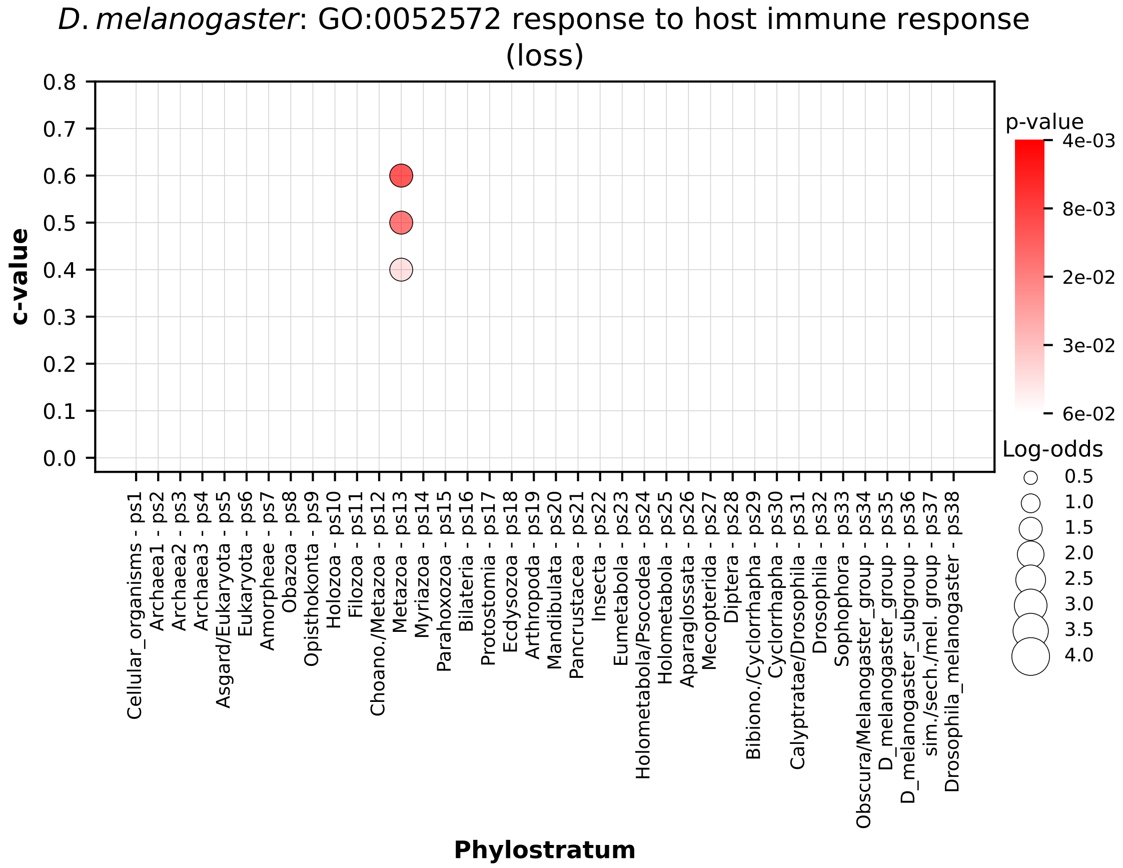

$D. melanogaster$: GO:0052572 response to host immune response (loss)
c-value
0.8
0.7
0.6
0.5
0.4
0.3
0.2
0.1
0.0
Phylostratum
Cellular_organisms - ps1
Archaea1 - ps2
Archaea2 - ps3
Archaea3 - ps4
Asgard/Eukaryota - ps5
Eukaryota - ps6
Amorpheae - ps7
Obazoa - ps8
Opisthokonta - ps9
Holozoa - ps10
Filozoa - ps11
Choano./Metazoa - ps12
Metazoa - ps13
Myriazoa - ps14
Parahoxozoa - ps15
Bilateria - ps16
Protostomia - ps17
Ecdysozoa - ps18
Arthropoda - ps19
Mandibulata - ps20
Pancrustacea - ps21
Insecta - ps22
Eumetabola - ps23
Holometabola/Psocodea - ps24
Holometabola - ps25
Aparaglossata - ps26
Mecopterida - ps27
Diptera - ps28
Bibiono./Cyclorrhapha - ps29
Cyclorrhapha - ps30
Calyptratae/Drosophila - ps31
Drosophila - ps32
Sophophora - ps33
Obscura/Melanogaster_group - ps34
D_melanogaster_group - ps35
D_melanogaster_subgroup - ps36
sim./sech./mel. group - ps37
Drosophila_melanogaster - ps38
p-value
4e-03
8e-03
2e-02
3e-02
6e-02
Log-odds
0.5
1.0
1.5
2.0
2.5
3.0
3.5
4.0

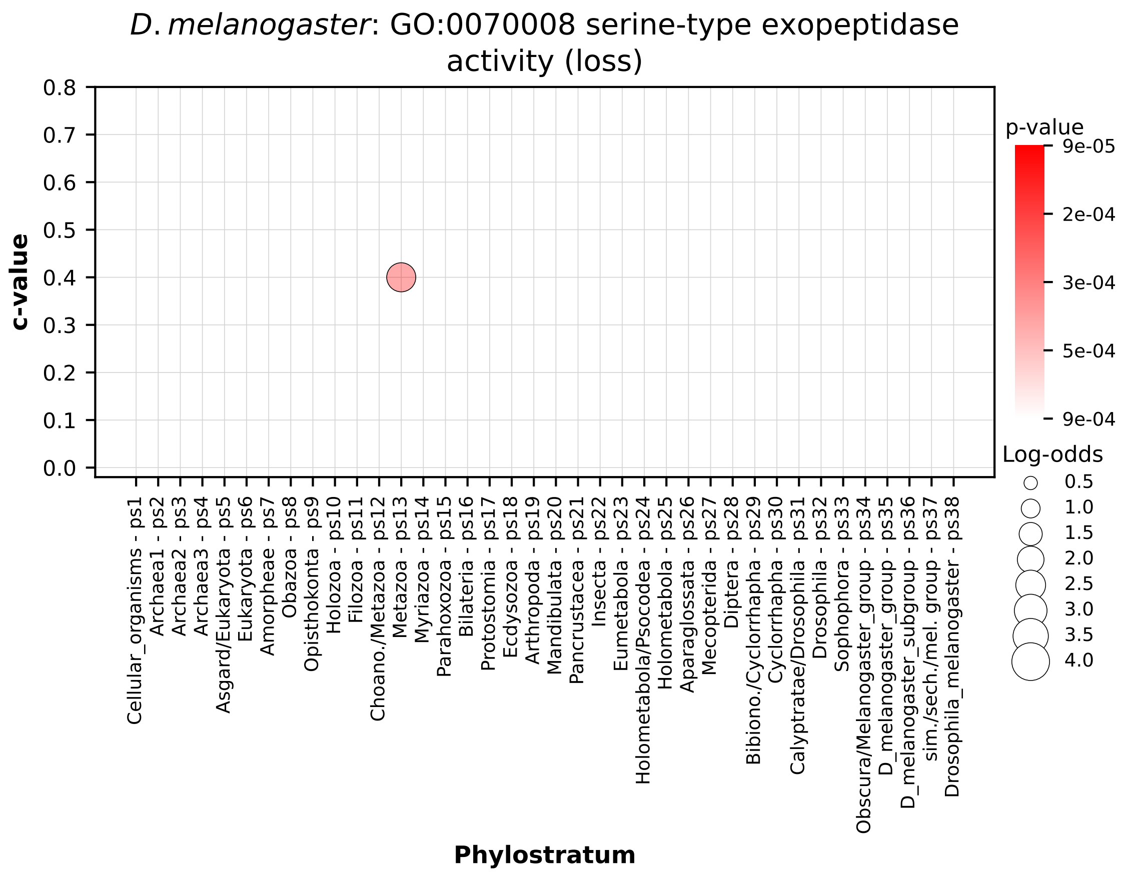

D. melanogaster: GO:0070008 serine-type exopeptidase activity (loss)
c-value
0.8
0.7
0.6
0.5
0.4
0.3
0.2
0.1
0.0
Phylostratum
Cellular_organisms - ps1
Archaea1 - ps2
Archaea2 - ps3
Archaea3 - ps4
Asgard/Eukaryota - ps5
Eukaryota - ps6
Amorpheae - ps7
Obazoa - ps8
Opisthokonta - ps9
Holozoa - ps10
Filozoa - ps11
Choano./Metazoa - ps12
Metazoa - ps13
Myriazoa - ps14
Parahoxozoa - ps15
Bilateria - ps16
Protostomia - ps17
Ecdysozoa - ps18
Arthropoda - ps19
Mandibulata - ps20
Pancrustacea - ps21
Insecta - ps22
Eumetabola - ps23
Holometabola/Psocodea - ps24
Holometabola - ps25
Aparaglossata - ps26
Mecopterida - ps27
Diptera - ps28
Bibiono./Cyclorrhapha - ps29
Cyclorrhapha - ps30
Calyptratae/Drosophila - ps31
Drosophila - ps32
Sophophora - ps33
Obscura/Melanogaster_group - ps34
D_melanogaster_group - ps35
D_melanogaster_subgroup - ps36
sim./sech./mel. group - ps37
Drosophila_melanogaster - ps38
p-value
9e-05
2e-04
3e-04
5e-04
9e-04
Log-odds
0.5
1.0
1.5
2.0
2.5
3.0
3.5
4.0

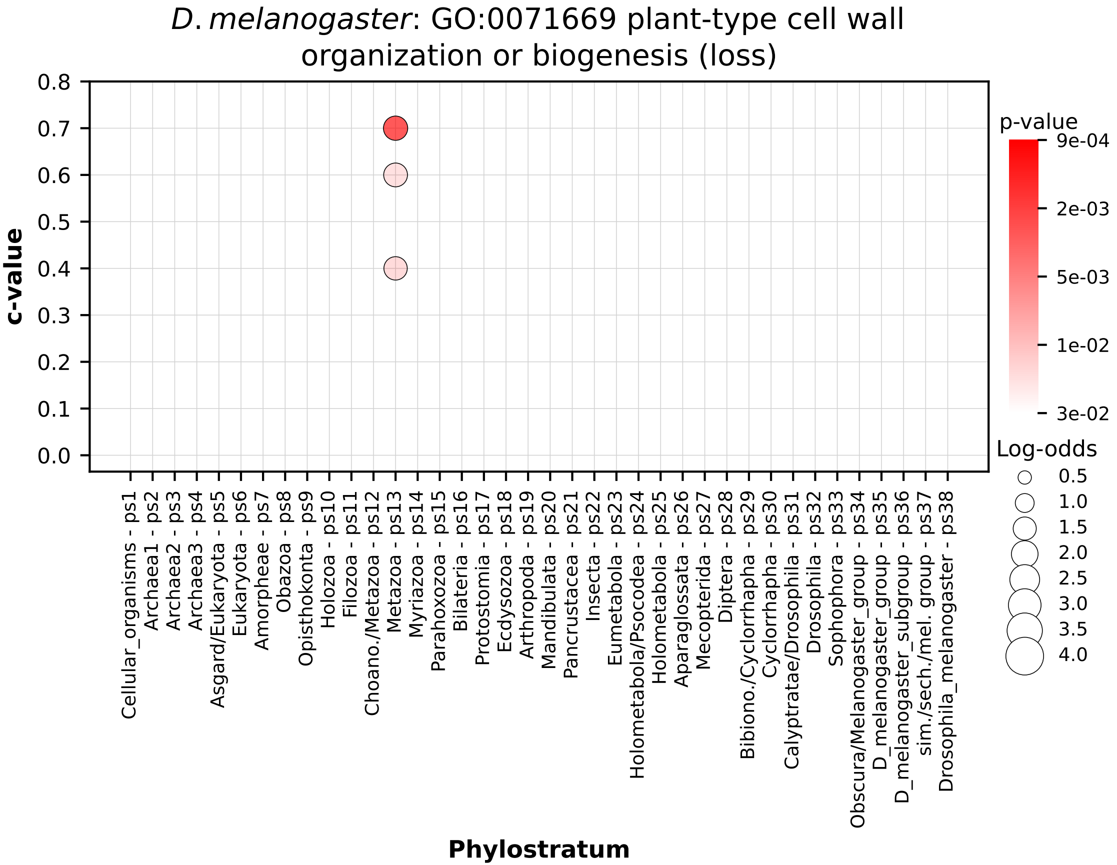

D. melanogaster: GO:0071669 plant-type cell wall organization or biogenesis (loss)
c-value
0.8
0.7
0.6
0.5
0.4
0.3
0.2
0.1
0.0
Phylostratum
Cellular_organisms - ps1
Archaea1 - ps2
Archaea2 - ps3
Archaea3 - ps4
Asgard/Eukaryota - ps5
Eukaryota - ps6
Amorpheae - ps7
Obazoa - ps8
Opisthokonta - ps9
Holozoa - ps10
Filozoa - ps11
Choano./Metazoa - ps12
Metazoa - ps13
Myriazoa - ps14
Parahoxozoa - ps15
Bilateria - ps16
Protostomia - ps17
Ecdysozoa - ps18
Arthropoda - ps19
Mandibulata - ps20
Pancrustacea - ps21
Insecta - ps22
Eumetabola - ps23
Holometabola/Psocodea - ps24
Holometabola - ps25
Aparaglossata - ps26
Mecopterida - ps27
Diptera - ps28
Bibiono./Cyclorrhapha - ps29
Cyclorrhapha - ps30
Calyptratae/Drosophila - ps31
Drosophila - ps32
Sophophora - ps33
Obscura/Melanogaster_group - ps34
D_melanogaster_group - ps35
D_melanogaster_subgroup - ps36
sim./sech./mel. group - ps37
Drosophila_melanogaster - ps38
p-value
9e-04
2e-03
5e-03
1e-02
3e-02
Log-odds
0.5
1.0
1.5
2.0
2.5
3.0
3.5
4.0

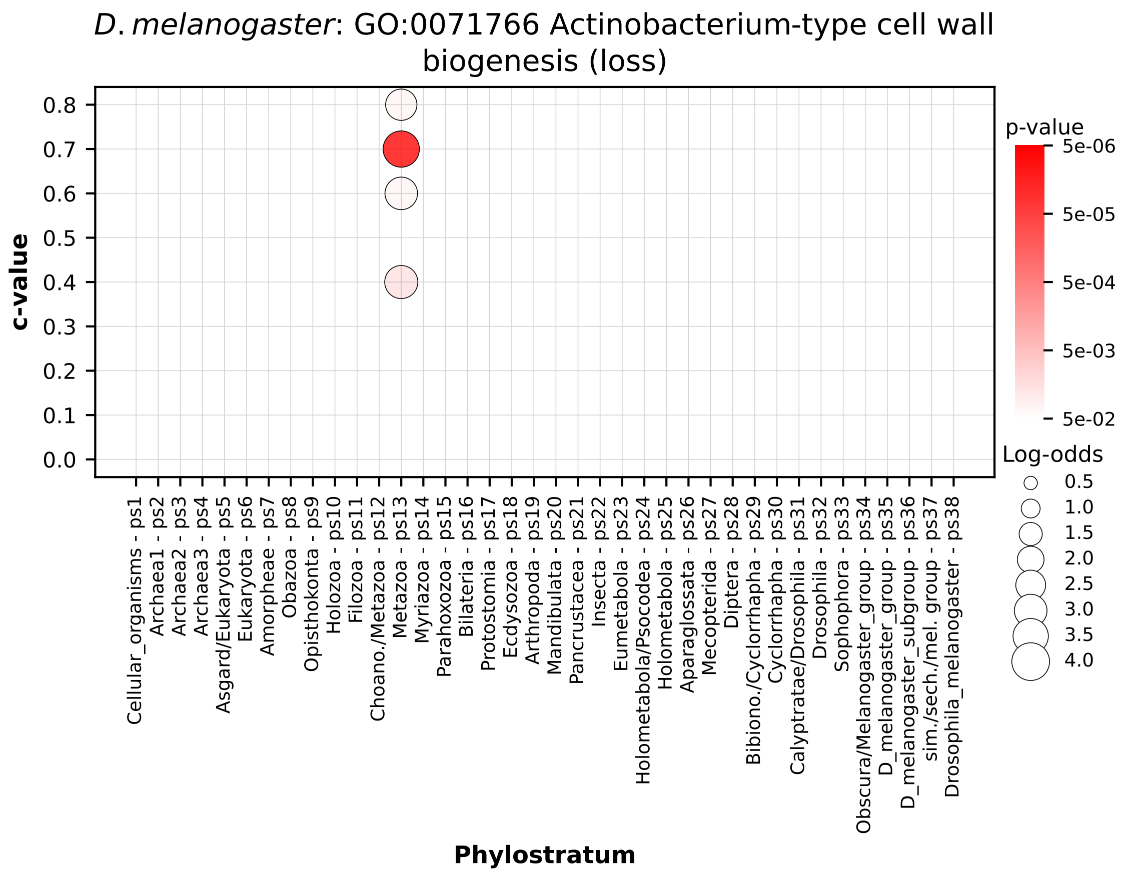

D. melanogaster: GO:0071766 Actinobacterium-type cell wall biogenesis (loss)
c-value
0.8
0.7
0.6
0.5
0.4
0.3
0.2
0.1
0.0
p-value
5e-06
5e-05
5e-04
5e-03
5e-02
Log-odds
0.5
1.0
1.5
2.0
2.5
3.0
3.5
4.0
Cellular_organisms - ps1
Archaea1 - ps2
Archaea2 - ps3
Archaea3 - ps4
Asgard/Eukaryota - ps5
Eukaryota - ps6
Amorpheae - ps7
Obazoa - ps8
Opisthokonta - ps9
Holozoa - ps10
Filozoa - ps11
Choano./Metazoa - ps12
Metazoa - ps13
Myriazoa - ps14
Parahoxozoa - ps15
Bilateria - ps16
Protostomia - ps17
Ecdysozoa - ps18
Arthropoda - ps19
Mandibulata - ps20
Pancrustacea - ps21
Insecta - ps22
Eumetabola - ps23
Holometabola/Psocodea - ps24
Holometabola - ps25
Aparaglossata - ps26
Mecopterida - ps27
Diptera - ps28
Bibiono./Cyclorrhapha - ps29
Cyclorrhapha - ps30
Calyptratae/Drosophila - ps31
Drosophila - ps32
Sophophora - ps33
Obscura/Melanogaster_group - ps34
D_melanogaster_group - ps35
D_melanogaster_subgroup - ps36
sim./sech./mel. group - ps37
Drosophila_melanogaster - ps38
Phylostratum

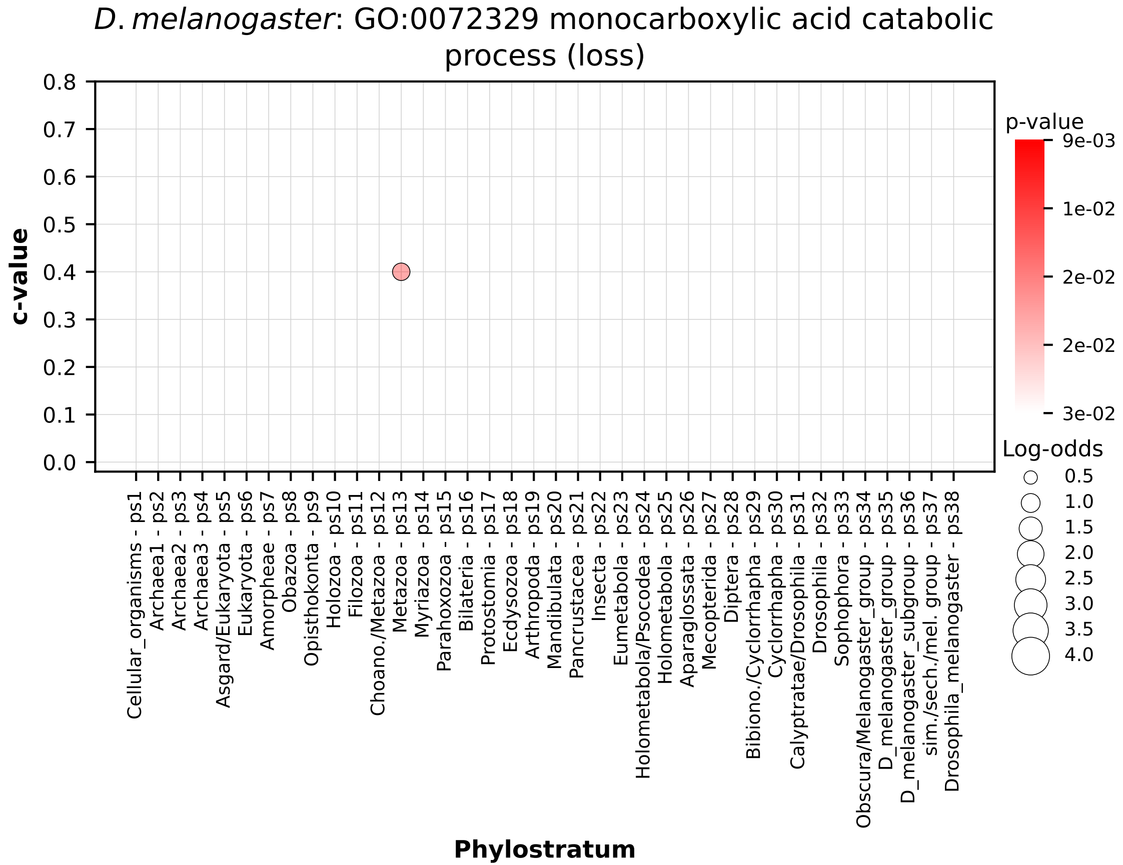
D. melanogaster: GO:0072329 monocarboxylic acid catabolic process (loss)
c-value
Phylostratum
p-value
9e-03
1e-02
2e-02
2e-02
3e-02
Log-odds
0.5
1.0
1.5
2.0
2.5
3.0
3.5
4.0
Cellular_organisms - ps1
Archaea1 - ps2
Archaea2 - ps3
Archaea3 - ps4
Asgard/Eukaryota - ps5
Eukaryota - ps6
Amorpheae - ps7
Obazoa - ps8
Opisthokonta - ps9
Holozoa - ps10
Filozoa - ps11
Choano./Metazoa - ps12
Metazoa - ps13
Myriazoa - ps14
Parahoxozoa - ps15
Bilateria - ps16
Protostomia - ps17
Ecdysozoa - ps18
Arthropoda - ps19
Mandibulata - ps20
Pancrustacea - ps21
Insecta - ps22
Eumetabola - ps23
Holometabola/Psocodea - ps24
Holometabola - ps25
Aparaglossata - ps26
Mecopterida - ps27
Diptera - ps28
Bibiono./Cyclorrhapha - ps29
Cyclorrhapha - ps30
Calyptratae/Drosophila - ps31
Drosophila - ps32
Sophophora - ps33
Obscura/Melanogaster_group - ps34
D_melanogaster_group - ps35
D_melanogaster_subgroup - ps36
sim./sech./mel. group - ps37
Drosophila_melanogaster - ps38

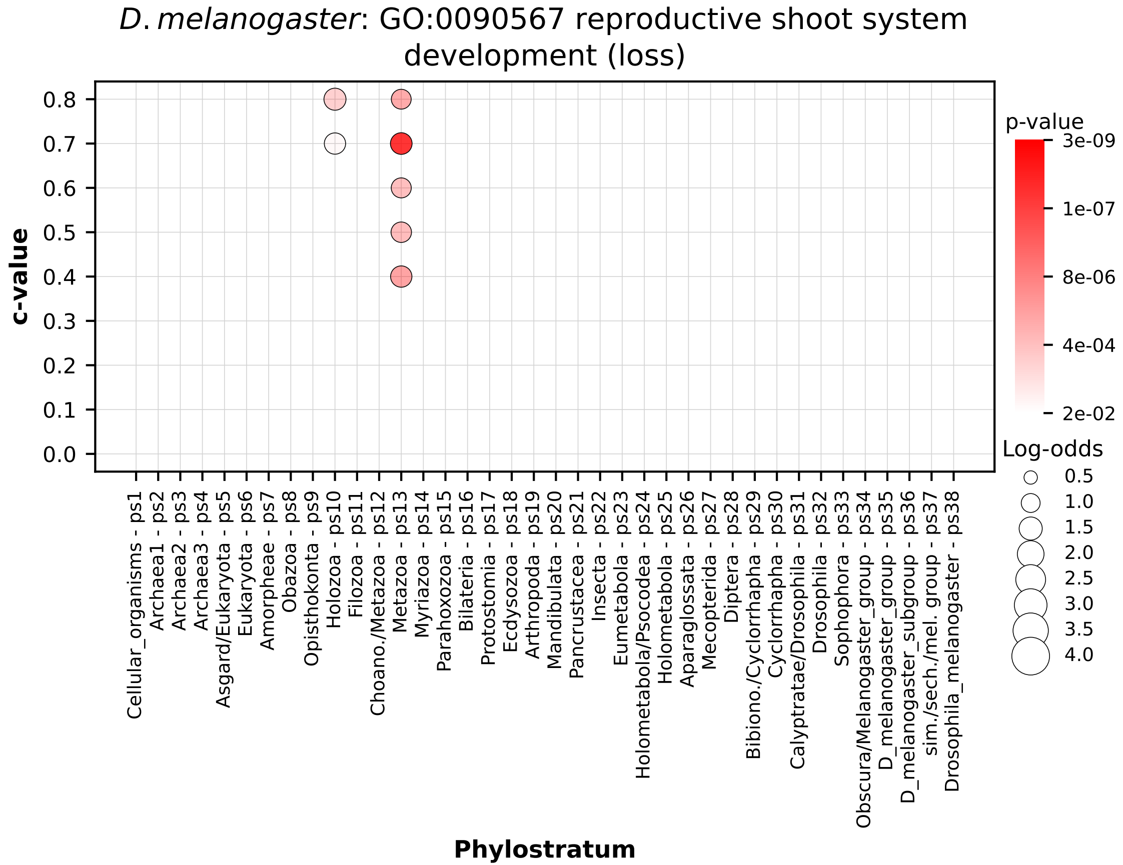
D. melanogaster: GO:0090567 reproductive shoot system development (loss)
c-value
0.0
0.1
0.2
0.3
0.4
0.5
0.6
0.7
0.8
Phylostratum
Cellular_organisms - ps1
Archaea1 - ps2
Archaea2 - ps3
Archaea3 - ps4
Asgard/Eukaryota - ps5
Eukaryota - ps6
Amorpheae - ps7
Obazoa - ps8
Opisthokonta - ps9
Holozoa - ps10
Filozoa - ps11
Choano./Metazoa - ps12
Metazoa - ps13
Myriazoa - ps14
Parahoxozoa - ps15
Bilateria - ps16
Protostomia - ps17
Ecdysozoa - ps18
Arthropoda - ps19
Mandibulata - ps20
Pancrustacea - ps21
Insecta - ps22
Eumetabola - ps23
Holometabola/Psocodea - ps24
Holometabola - ps25
Aparaglossata - ps26
Mecopterida - ps27
Diptera - ps28
Bibiono./Cyclorrhapha - ps29
Cyclorrhapha - ps30
Calyptratae/Drosophila - ps31
Drosophila - ps32
Sophophora - ps33
Obscura/Melanogaster_group - ps34
D_melanogaster_group - ps35
D_melanogaster_subgroup - ps36
sim./sech./mel. group - ps37
Drosophila_melanogaster - ps38
p-value
3e-09
1e-07
8e-06
4e-04
2e-02
Log-odds
0.5
1.0
1.5
2.0
2.5
3.0
3.5
4.0

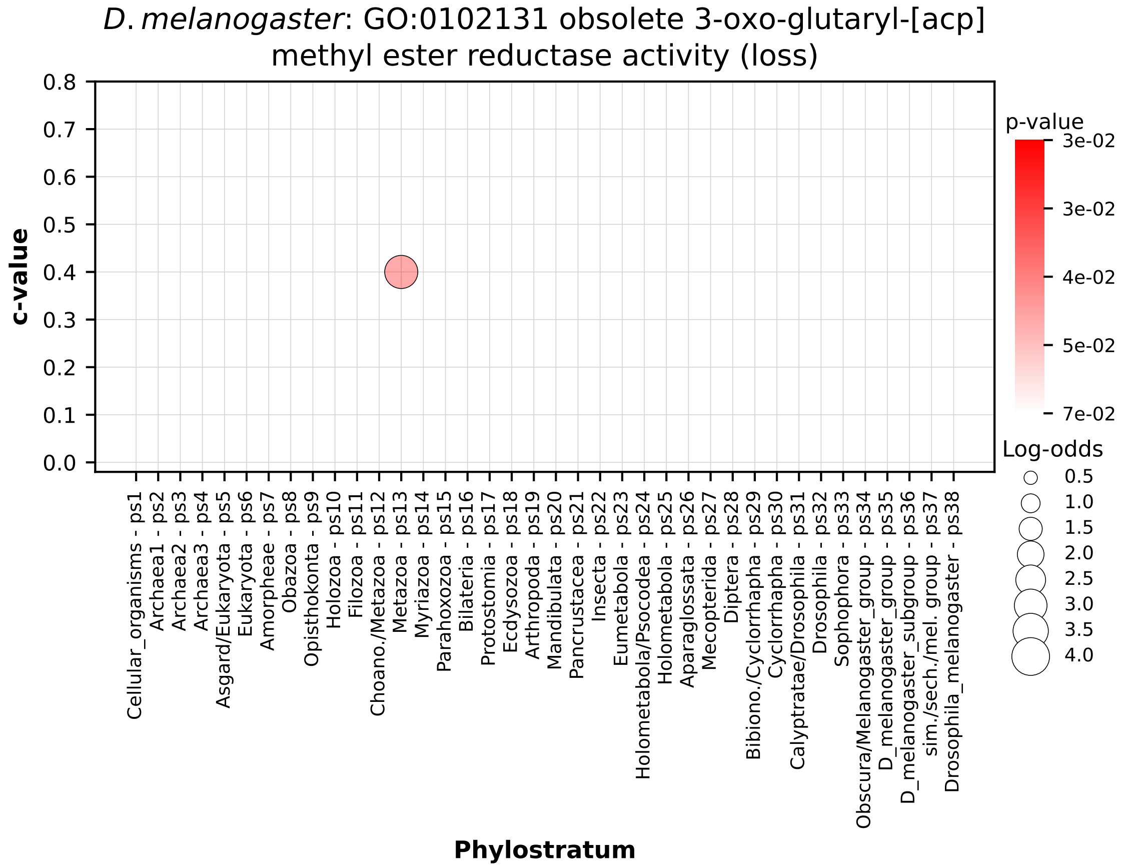

D. melanogaster: GO:0102131 obsolete 3-oxo-glutaryl-[acp] methyl ester reductase activity (loss)
c-value
0.8
0.7
0.6
0.5
0.4
0.3
0.2
0.1
0.0
Phylostratum
Cellular_organisms - ps1
Archaea1 - ps2
Archaea2 - ps3
Archaea3 - ps4
Asgard/Eukaryota - ps5
Eukaryota - ps6
Amorpheae - ps7
Obazoa - ps8
Opisthokonta - ps9
Holozoa - ps10
Filozoa - ps11
Choano./Metazoa - ps12
Metazoa - ps13
Myriazoa - ps14
Parahoxozoa - ps15
Bilateria - ps16
Protostomia - ps17
Ecdysozoa - ps18
Arthropoda - ps19
Mandibulata - ps20
Pancrustacea - ps21
Insecta - ps22
Eumetabola - ps23
Holometabola/Psocodea - ps24
Holometabola - ps25
Aparaglossata - ps26
Mecopterida - ps27
Diptera - ps28
Bibiono./Cyclorrhapha - ps29
Cyclorrhapha - ps30
Calyptratae/Drosophila - ps31
Drosophila - ps32
Sophophora - ps33
Obscura/Melanogaster_group - ps34
D_melanogaster_group - ps35
D_melanogaster_subgroup - ps36
sim./sech./mel. group - ps37
Drosophila_melanogaster - ps38
p-value
3e-02
3e-02
4e-02
5e-02
7e-02
Log-odds
0.5
1.0
1.5
2.0
2.5
3.0
3.5
4.0

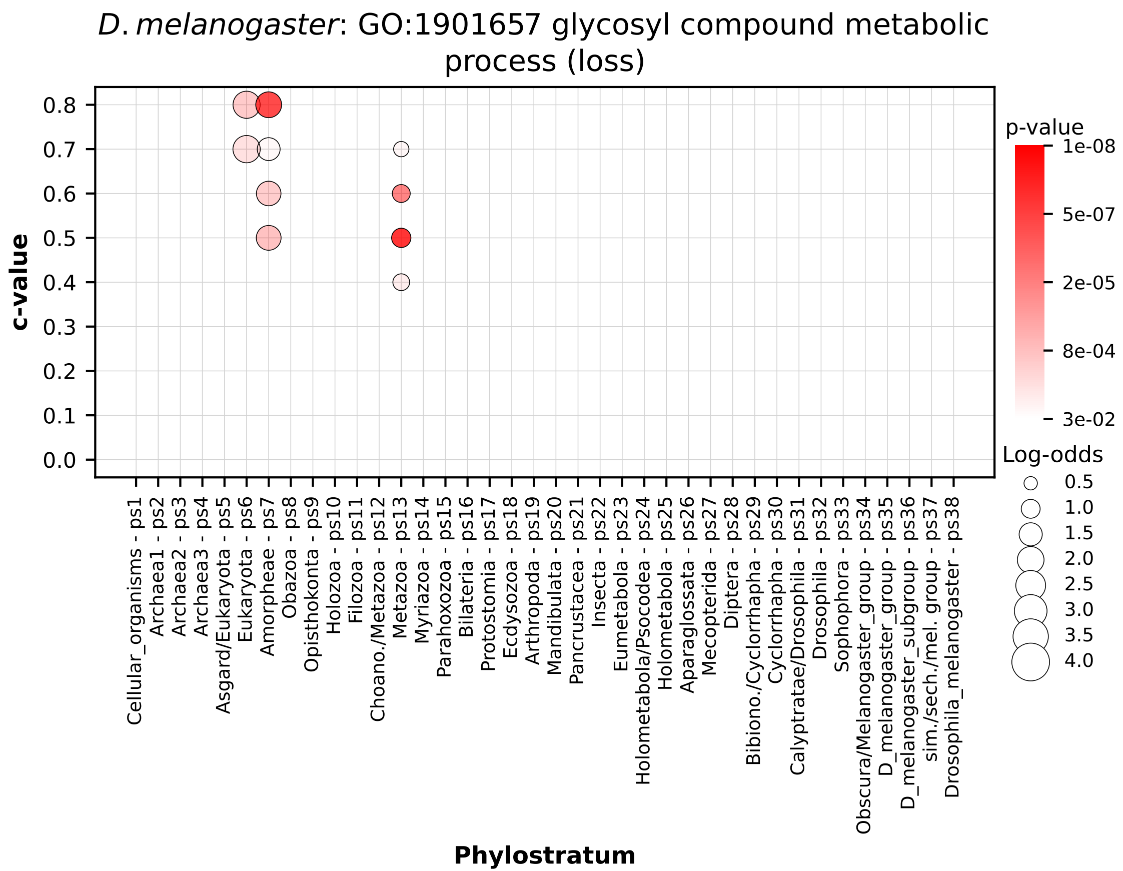

D. melanogaster: GO:1901657 glycosyl compound metabolic process (loss)
c-value
0.8
0.7
0.6
0.5
0.4
0.3
0.2
0.1
0.0
Phylostratum
Cellular_organisms - ps1
Archaea1 - ps2
Archaea2 - ps3
Archaea3 - ps4
Asgard/Eukaryota - ps5
Eukaryota - ps6
Amorpheae - ps7
Obazoa - ps8
Opisthokonta - ps9
Holozoa - ps10
Filozoa - ps11
Choano./Metazoa - ps12
Metazoa - ps13
Myriazoa - ps14
Parahoxozoa - ps15
Bilateria - ps16
Protostomia - ps17
Ecdysozoa - ps18
Arthropoda - ps19
Mandibulata - ps20
Pancrustacea - ps21
Insecta - ps22
Eumetabola - ps23
Holometabola/Psocodea - ps24
Holometabola - ps25
Aparaglossata - ps26
Mecopterida - ps27
Diptera - ps28
Bibiono./Cyclorrhapha - ps29
Cyclorrhapha - ps30
Calyptratae/Drosophila - ps31
Drosophila - ps32
Sophophora - ps33
Obscura/Melanogaster_group - ps34
D_melanogaster_group - ps35
D_melanogaster_subgroup - ps36
sim./sech./mel. group - ps37
Drosophila_melanogaster - ps38
p-value
1e-08
5e-07
2e-05
8e-04
3e-02
Log-odds
0.5
1.0
1.5
2.0
2.5
3.0
3.5
4.0

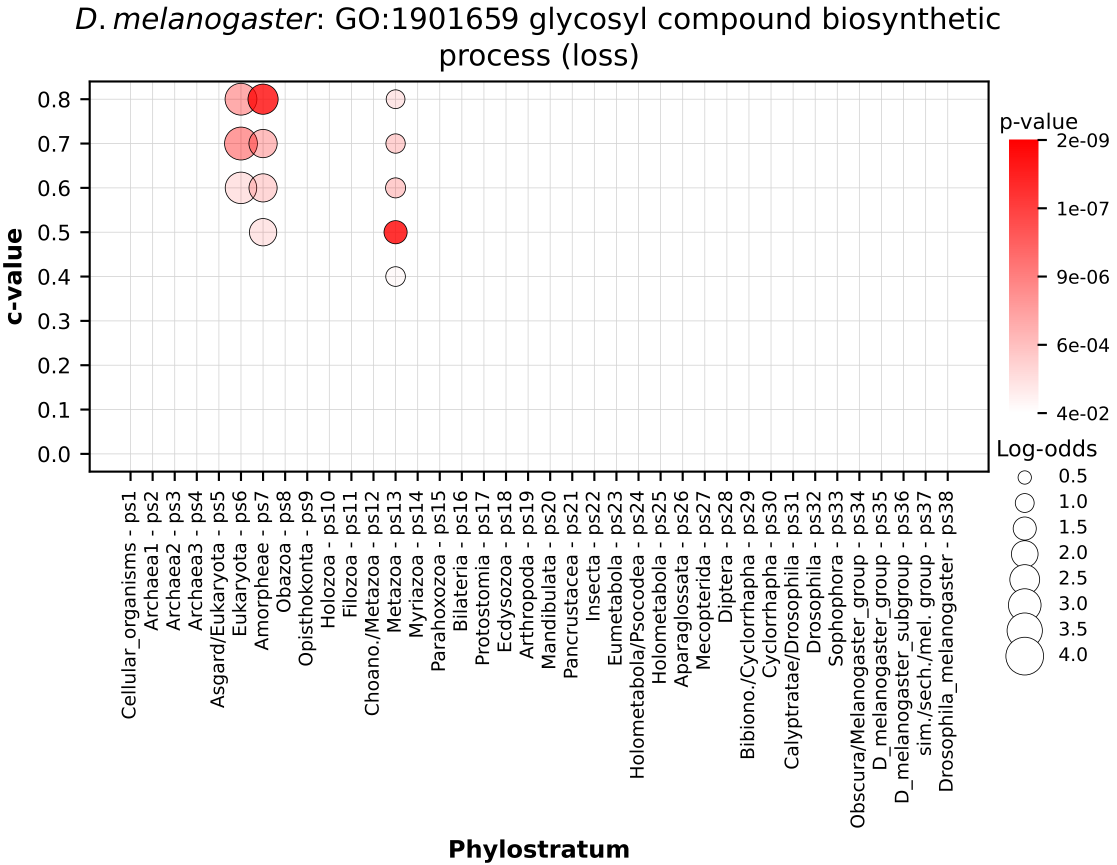

D. melanogaster: GO:1901659 glycosyl compound biosynthetic process (loss)
c-value
0.8
0.7
0.6
0.5
0.4
0.3
0.2
0.1
0.0
p-value
2e-09
1e-07
9e-06
6e-04
4e-02
Log-odds
0.5
1.0
1.5
2.0
2.5
3.0
3.5
4.0
Cellular_organisms - ps1
Archaea1 - ps2
Archaea2 - ps3
Archaea3 - ps4
Asgard/Eukaryota - ps5
Eukaryota - ps6
Amorpheae - ps7
Obazoa - ps8
Opisthokonta - ps9
Holozoa - ps10
Filozoa - ps11
Choano./Metazoa - ps12
Metazoa - ps13
Myriazoa - ps14
Parahoxozoa - ps15
Bilateria - ps16
Protostomia - ps17
Ecdysozoa - ps18
Arthropoda - ps19
Mandibulata - ps20
Pancrustacea - ps21
Insecta - ps22
Eumetabola - ps23
Holometabola/Psocodea - ps24
Holometabola - ps25
Aparaglossata - ps26
Mecopterida - ps27
Diptera - ps28
Bibiono./Cyclorrhapha - ps29
Cyclorrhapha - ps30
Calyptratae/Drosophila - ps31
Drosophila - ps32
Sophophora - ps33
Obscura/Melanogaster_group - ps34
D_melanogaster_group - ps35
D_melanogaster_subgroup - ps36
sim./sech./mel. group - ps37
Drosophila_melanogaster - ps38
Phylostratum

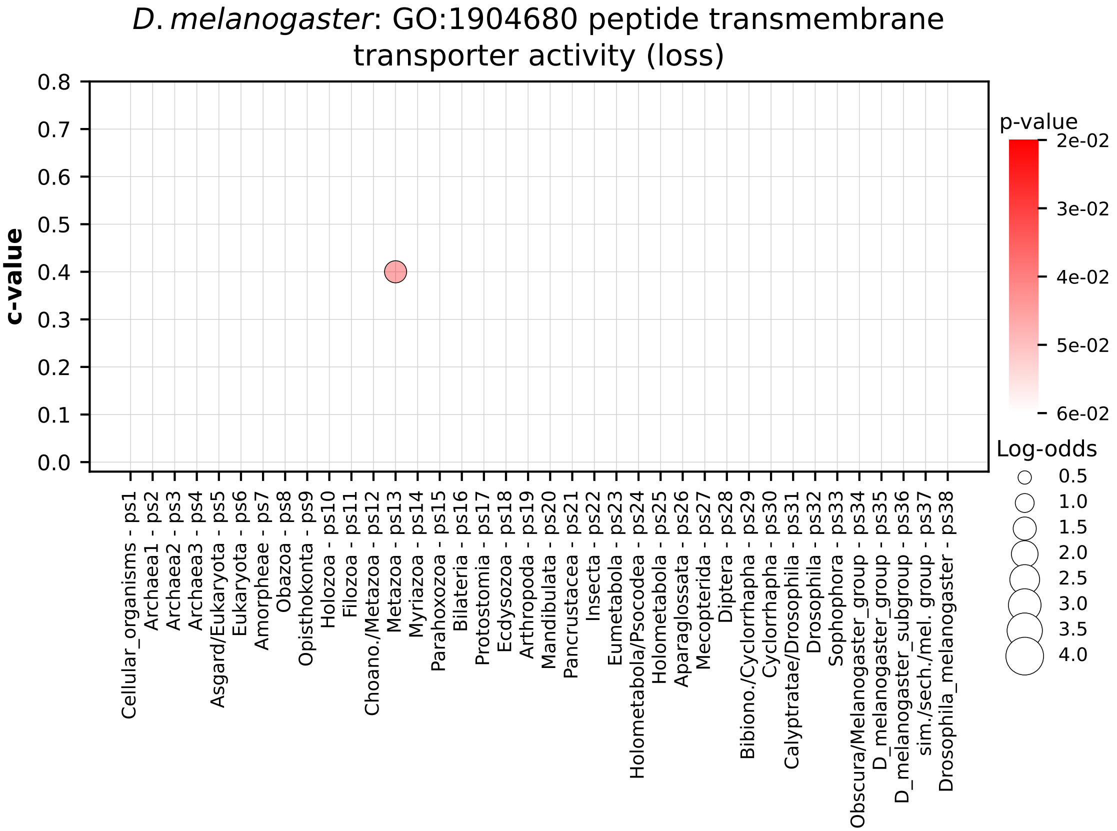

D. melanogaster: GO:1904680 peptide transmembrane transporter activity (loss)
c-value
0.8
0.7
0.6
0.5
0.4
0.3
0.2
0.1
0.0
Phylostratum
Cellular_organisms - ps1
Archaea1 - ps2
Archaea2 - ps3
Archaea3 - ps4
Asgard/Eukaryota - ps5
Eukaryota - ps6
Amorpheae - ps7
Obazoa - ps8
Opisthokonta - ps9
Holozoa - ps10
Filozoa - ps11
Choano./Metazoa - ps12
Metazoa - ps13
Myriazoa - ps14
Parahoxozoa - ps15
Bilateria - ps16
Protostomia - ps17
Ecdysozoa - ps18
Arthropoda - ps19
Mandibulata - ps20
Pancrustacea - ps21
Insecta - ps22
Eumetabola - ps23
Holometabola/Psocodea - ps24
Holometabola - ps25
Aparaglossata - ps26
Mecopterida - ps27
Diptera - ps28
Bibiono./Cyclorrhapha - ps29
Cyclorrhapha - ps30
Calyptratae/Drosophila - ps31
Drosophila - ps32
Sophophora - ps33
Obscura/Melanogaster_group - ps34
D_melanogaster_group - ps35
D_melanogaster_subgroup - ps36
sim./sech./mel. group - ps37
Drosophila_melanogaster - ps38
p-value
2e-02
3e-02
4e-02
5e-02
6e-02
Log-odds
0.5
1.0
1.5
2.0
2.5
3.0
3.5
4.0

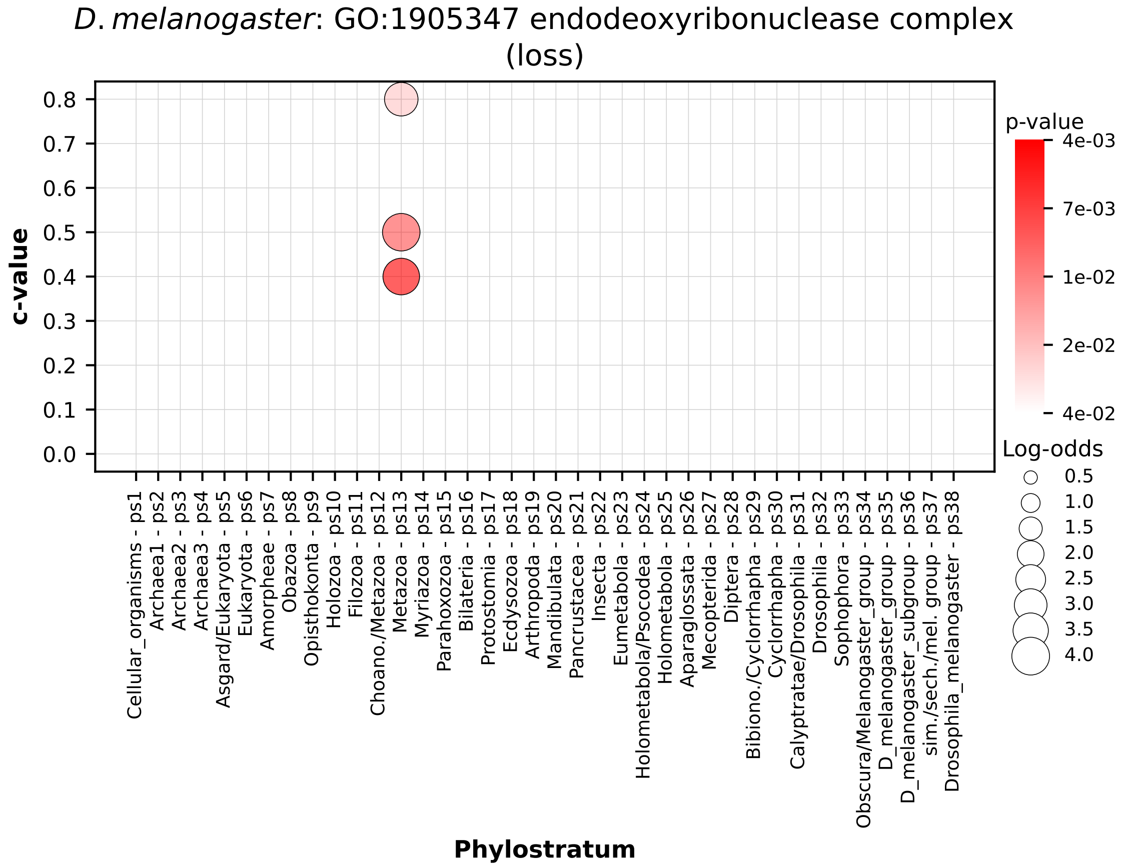

D. melanogaster: GO:1905347 endodeoxyribonuclease complex (loss)
c-value
0.8
0.7
0.6
0.5
0.4
0.3
0.2
0.1
0.0
p-value
4e-03
7e-03
1e-02
2e-02
4e-02
Log-odds
0.5
1.0
1.5
2.0
2.5
3.0
3.5
4.0
Cellular_organisms - ps1
Archaea1 - ps2
Archaea2 - ps3
Archaea3 - ps4
Asgard/Eukaryota - ps5
Eukaryota - ps6
Amorpheae - ps7
Obazoa - ps8
Opisthokonta - ps9
Holozoa - ps10
Filozoa - ps11
Choano./Metazoa - ps12
Metazoa - ps13
Myriazoa - ps14
Parahoxozoa - ps15
Bilateria - ps16
Protostomia - ps17
Ecdysozoa - ps18
Arthropoda - ps19
Mandibulata - ps20
Pancrustacea - ps21
Insecta - ps22
Eumetabola - ps23
Holometabola/Psocodea - ps24
Holometabola - ps25
Aparaglossata - ps26
Mecopterida - ps27
Diptera - ps28
Bibiono./Cyclorrhapha - ps29
Cyclorrhapha - ps30
Calyptratae/Drosophila - ps31
Drosophila - ps32
Sophophora - ps33
Obscura/Melanogaster_group - ps34
D_melanogaster_group - ps35
D_melanogaster_subgroup - ps36
sim./sech./mel. group - ps37
Drosophila_melanogaster - ps38
Phylostratum

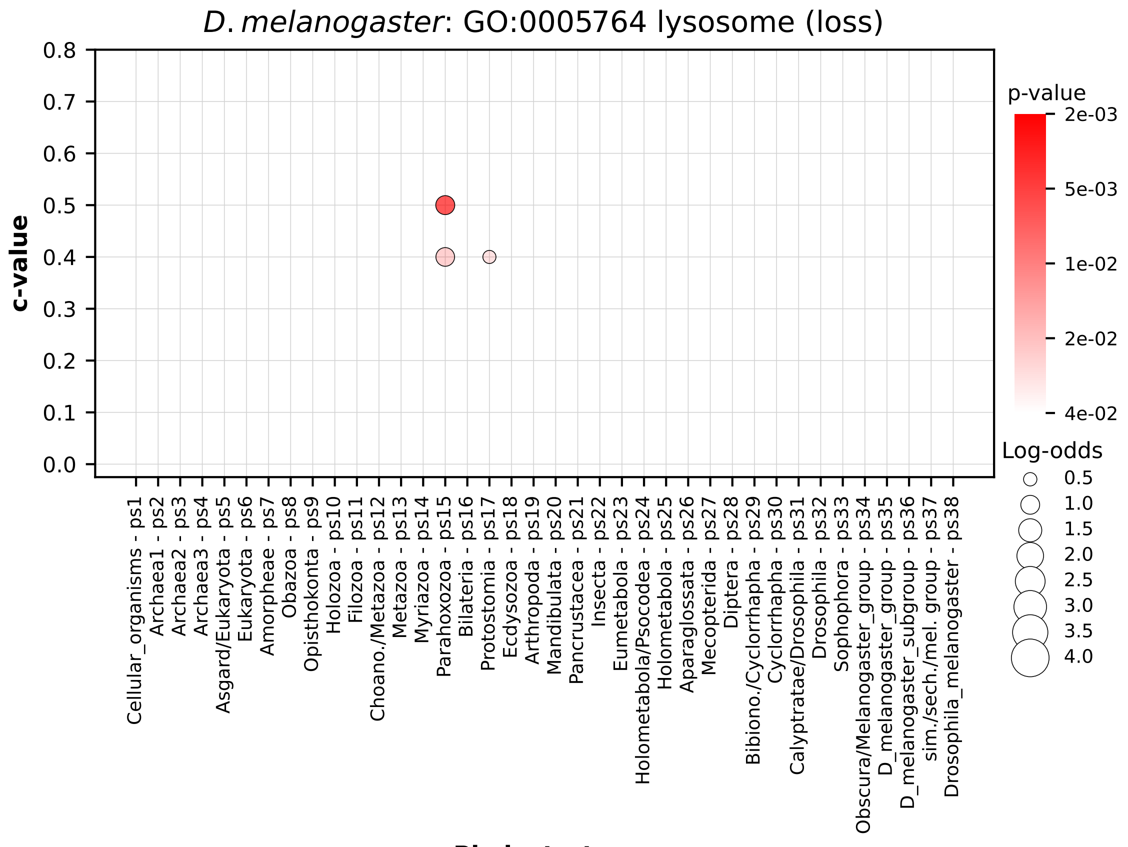

D. melanogaster: GO:0005764 lysosome (loss)
c-value
0.8
0.7
0.6
0.5
0.4
0.3
0.2
0.1
0.0
p-value
2e-03
5e-03
1e-02
2e-02
4e-02
Log-odds
0.5
1.0
1.5
2.0
2.5
3.0
3.5
4.0
Cellular_organisms - ps1
Archaea1 - ps2
Archaea2 - ps3
Archaea3 - ps4
Asgard/Eukaryota - ps5
Eukaryota - ps6
Amorpheae - ps7
Obazoa - ps8
Opisthokonta - ps9
Holozoa - ps10
Filozoa - ps11
Choano./Metazoa - ps12
Metazoa - ps13
Myriazoa - ps14
Parahoxozoa - ps15
Bilateria - ps16
Protostomia - ps17
Ecdysozoa - ps18
Arthropoda - ps19
Mandibulata - ps20
Pancrustacea - ps21
Insecta - ps22
Eumetabola - ps23
Holometabola/Psocodea - ps24
Holometabola - ps25
Aparaglossata - ps26
Mecopterida - ps27
Diptera - ps28
Bibiono./Cyclorrhapha - ps29
Cyclorrhapha - ps30
Calyptratae/Drosophila - ps31
Drosophila - ps32
Sophophora - ps33
Obscura/Melanogaster_group - ps34
D_melanogaster_group - ps35
D_melanogaster_subgroup - ps36
sim./sech./mel. group - ps37
Drosophila_melanogaster - ps38
Phylostratum

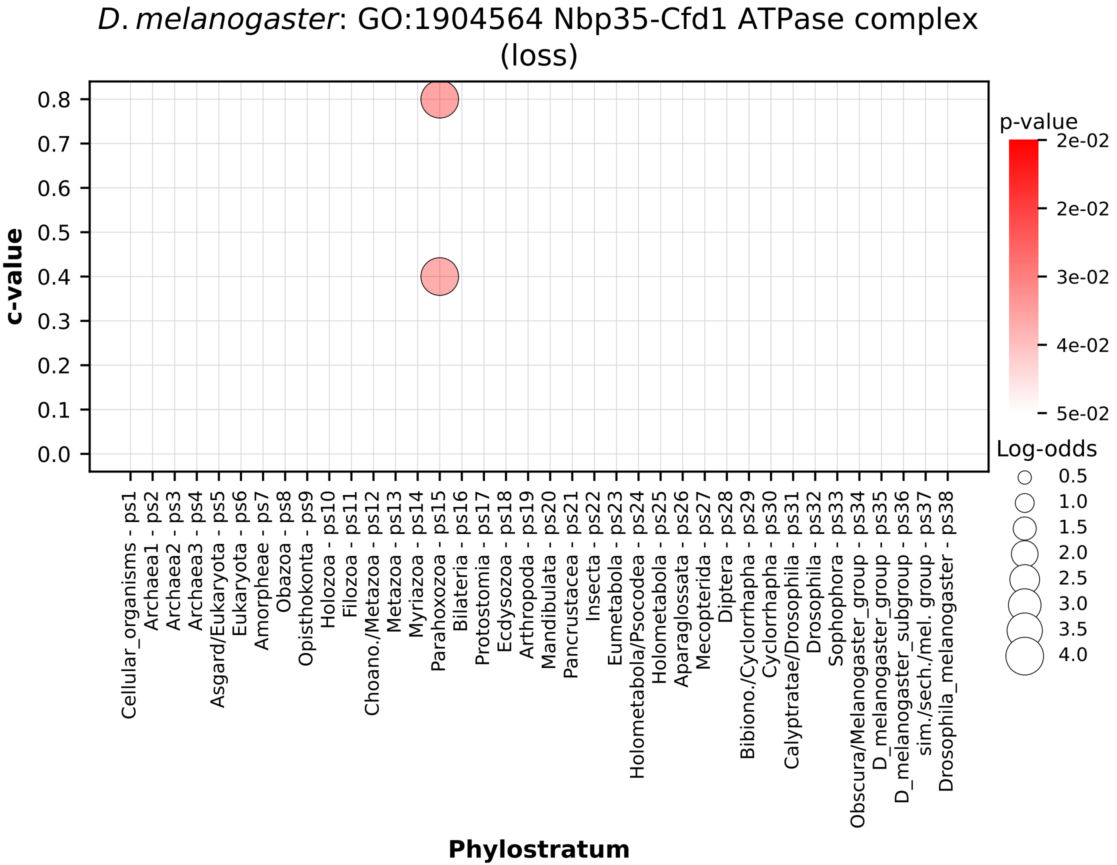

D. melanogaster: GO:1904564 Nbp35-Cfd1 ATPase complex
(loss)
c-value
0.0
0.1
0.2
0.3
0.4
0.5
0.6
0.7
0.8
p-value
2e-02
2e-02
3e-02
4e-02
5e-02
Log-odds
0.5
1.0
1.5
2.0
2.5
3.0
3.5
4.0
Cellular_organisms - ps1
Archaea1 - ps2
Archaea2 - ps3
Archaea3 - ps4
Asgard/Eukaryota - ps5
Eukaryota - ps6
Amorpheae - ps7
Obazoa - ps8
Opisthokonta - ps9
Holozoa - ps10
Filozoa - ps11
Choano./Metazoa - ps12
Metazoa - ps13
Myriazoa - ps14
Parahoxozoa - ps15
Bilateria - ps16
Protostomia - ps17
Ecdysozoa - ps18
Arthropoda - ps19
Mandibulata - ps20
Pancrustacea - ps21
Insecta - ps22
Eumetabola - ps23
Holometabola/Psocodea - ps24
Holometabola - ps25
Aparaglossata - ps26
Mecopterida - ps27
Diptera - ps28
Bibiono./Cyclorrhapha - ps29
Cyclorrhapha - ps30
Calyptratae/Drosophila - ps31
Drosophila - ps32
Sophophora - ps33
Obscura/Melanogaster_group - ps34
D_melanogaster_group - ps35
D_melanogaster_subgroup - ps36
sim./sech./mel. group - ps37
Drosophila_melanogaster - ps38
Phylostratum

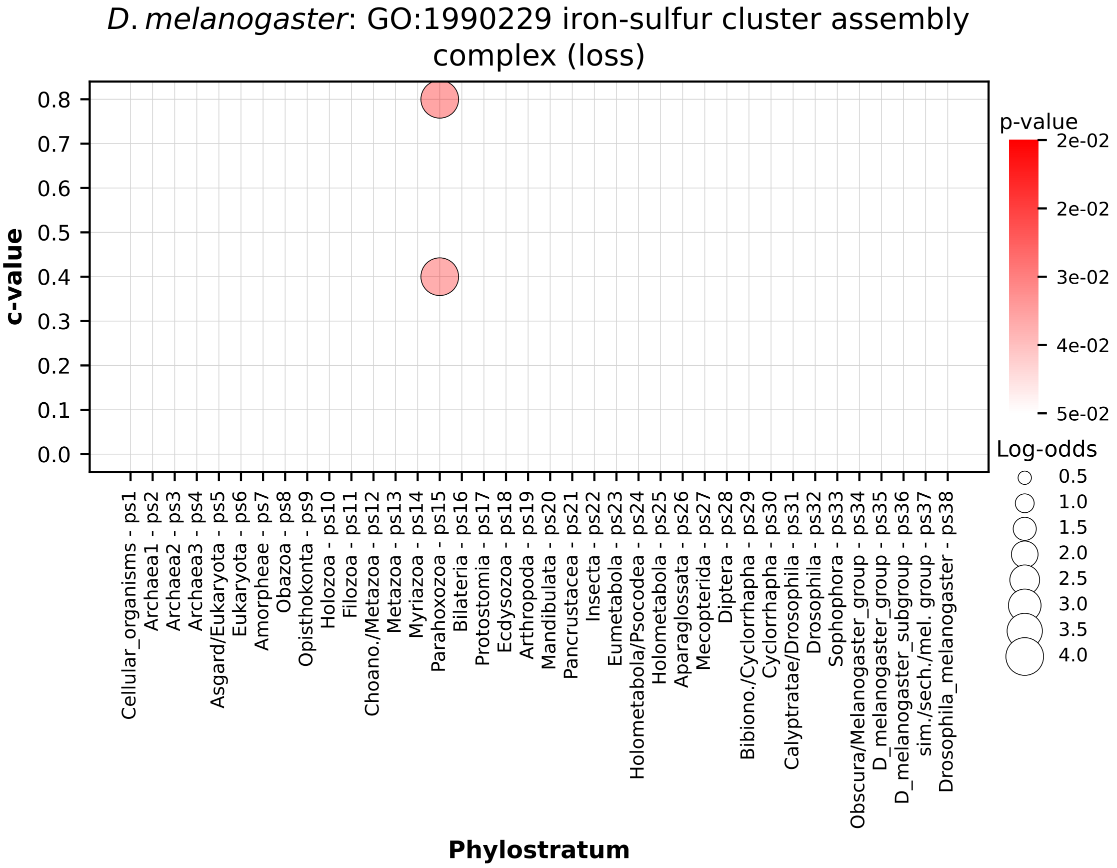

D. melanogaster: GO:1990229 iron-sulfur cluster assembly complex (loss)
c-value
0.0
0.1
0.2
0.3
0.4
0.5
0.6
0.7
0.8
p-value
2e-02
2e-02
3e-02
4e-02
5e-02
Log-odds
0.5
1.0
1.5
2.0
2.5
3.0
3.5
4.0
Cellular_organisms - ps1
Archaea1 - ps2
Archaea2 - ps3
Archaea3 - ps4
Asgard/Eukaryota - ps5
Eukaryota - ps6
Amorpheae - ps7
Obazoa - ps8
Opisthokonta - ps9
Holozoa - ps10
Filozoa - ps11
Choano./Metazoa - ps12
Metazoa - ps13
Myriazoa - ps14
Parahoxozoa - ps15
Bilateria - ps16
Protostomia - ps17
Ecdysozoa - ps18
Arthropoda - ps19
Mandibulata - ps20
Pancrustacea - ps21
Insecta - ps22
Eumetabola - ps23
Holometabola/Psocodea - ps24
Holometabola - ps25
Aparaglossata - ps26
Mecopterida - ps27
Diptera - ps28
Bibiono./Cyclorrhapha - ps29
Cyclorrhapha - ps30
Calyptratae/Drosophila - ps31
Drosophila - ps32
Sophophora - ps33
Obscura/Melanogaster_group - ps34
D_melanogaster_group - ps35
D_melanogaster_subgroup - ps36
sim./sech./mel. group - ps37
Drosophila_melanogaster - ps38
Phylostratum

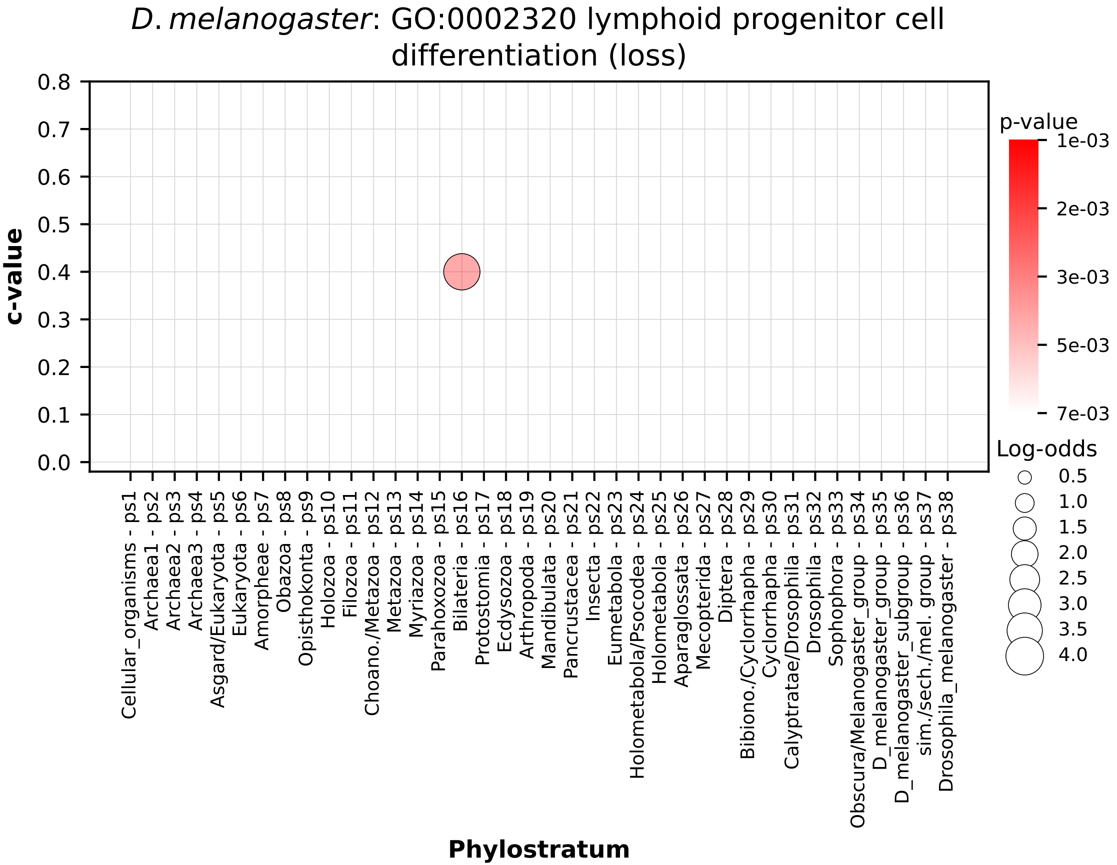

D. melanogaster: GO:0002320 lymphoid progenitor cell differentiation (loss)
c-value
0.8
0.7
0.6
0.5
0.4
0.3
0.2
0.1
0.0
Phylostratum
Cellular_organisms - ps1
Archaea1 - ps2
Archaea2 - ps3
Archaea3 - ps4
Asgard/Eukaryota - ps5
Eukaryota - ps6
Amorpheae - ps7
Obazoa - ps8
Opisthokonta - ps9
Holozoa - ps10
Filozoa - ps11
Choano./Metazoa - ps12
Metazoa - ps13
Myriazoa - ps14
Parahoxozoa - ps15
Bilateria - ps16
Protostomia - ps17
Ecdysozoa - ps18
Arthropoda - ps19
Mandibulata - ps20
Pancrustacea - ps21
Insecta - ps22
Eumetabola - ps23
Holometabola/Psocodea - ps24
Holometabola - ps25
Aparaglossata - ps26
Mecopterida - ps27
Diptera - ps28
Bibiono./Cyclorrhapha - ps29
Cyclorrhapha - ps30
Calyptratae/Drosophila - ps31
Drosophila - ps32
Sophophora - ps33
Obscura/Melanogaster_group - ps34
D_melanogaster_group - ps35
D_melanogaster_subgroup - ps36
sim./sech./mel. group - ps37
Drosophila_melanogaster - ps38
p-value
1e-03
2e-03
3e-03
5e-03
7e-03
Log-odds
0.5
1.0
1.5
2.0
2.5
3.0
3.5
4.0

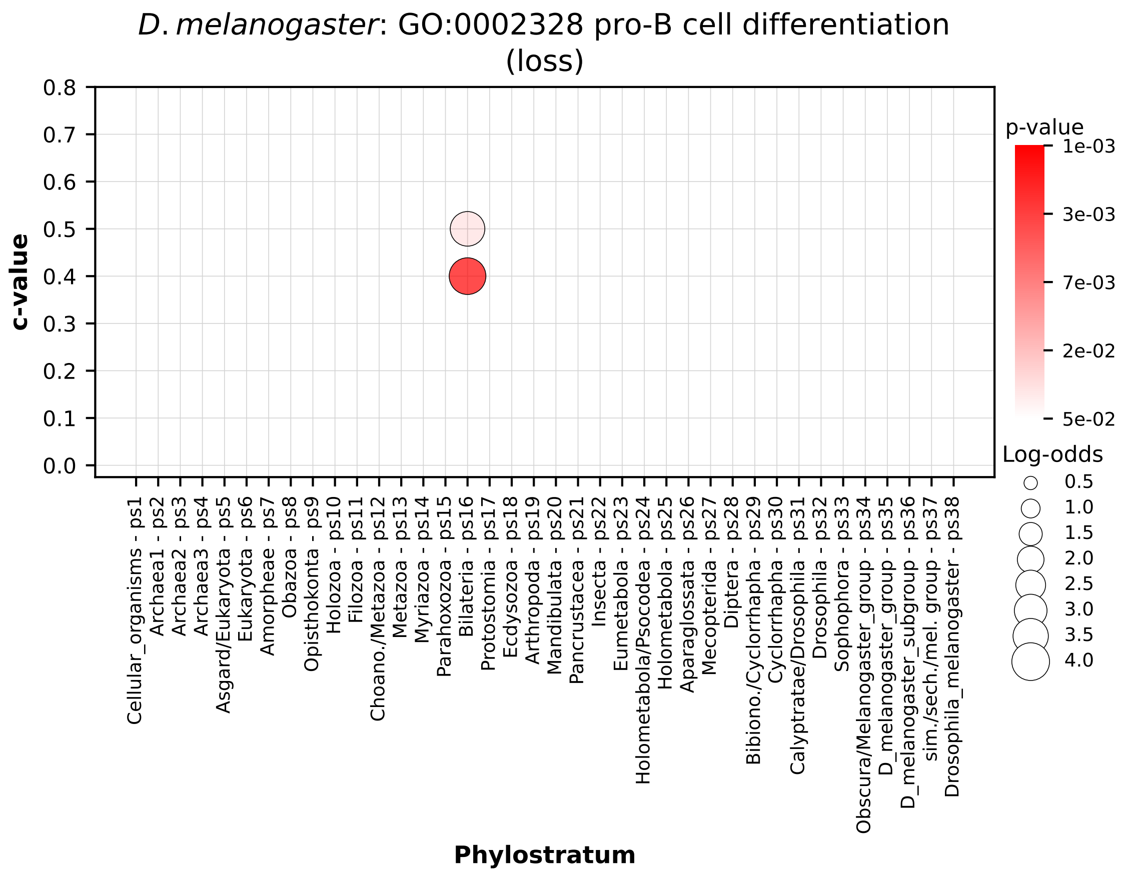
D. melanogaster: GO:0002328 pro-B cell differentiation (loss)
c-value
0.8
0.7
0.6
0.5
0.4
0.3
0.2
0.1
0.0
p-value
1e-03
3e-03
7e-03
2e-02
5e-02
Log-odds
0.5
1.0
1.5
2.0
2.5
3.0
3.5
4.0
Cellular_organisms - ps1
Archaea1 - ps2
Archaea2 - ps3
Archaea3 - ps4
Asgard/Eukaryota - ps5
Eukaryota - ps6
Amorpheae - ps7
Obazoa - ps8
Opisthokonta - ps9
Holozoa - ps10
Filozoa - ps11
Choano./Metazoa - ps12
Metazoa - ps13
Myriazoa - ps14
Parahoxozoa - ps15
Bilateria - ps16
Protostomia - ps17
Ecdysozoa - ps18
Arthropoda - ps19
Mandibulata - ps20
Pancrustacea - ps21
Insecta - ps22
Eumetabola - ps23
Holometabola/Psocodea - ps24
Holometabola - ps25
Aparaglossata - ps26
Mecopterida - ps27
Diptera - ps28
Bibiono./Cyclorrhapha - ps29
Cyclorrhapha - ps30
Calyptratae/Drosophila - ps31
Drosophila - ps32
Sophophora - ps33
Obscura/Melanogaster_group - ps34
D_melanogaster_group - ps35
D_melanogaster_subgroup - ps36
sim./sech./mel. group - ps37
Drosophila_melanogaster - ps38
Phylostratum

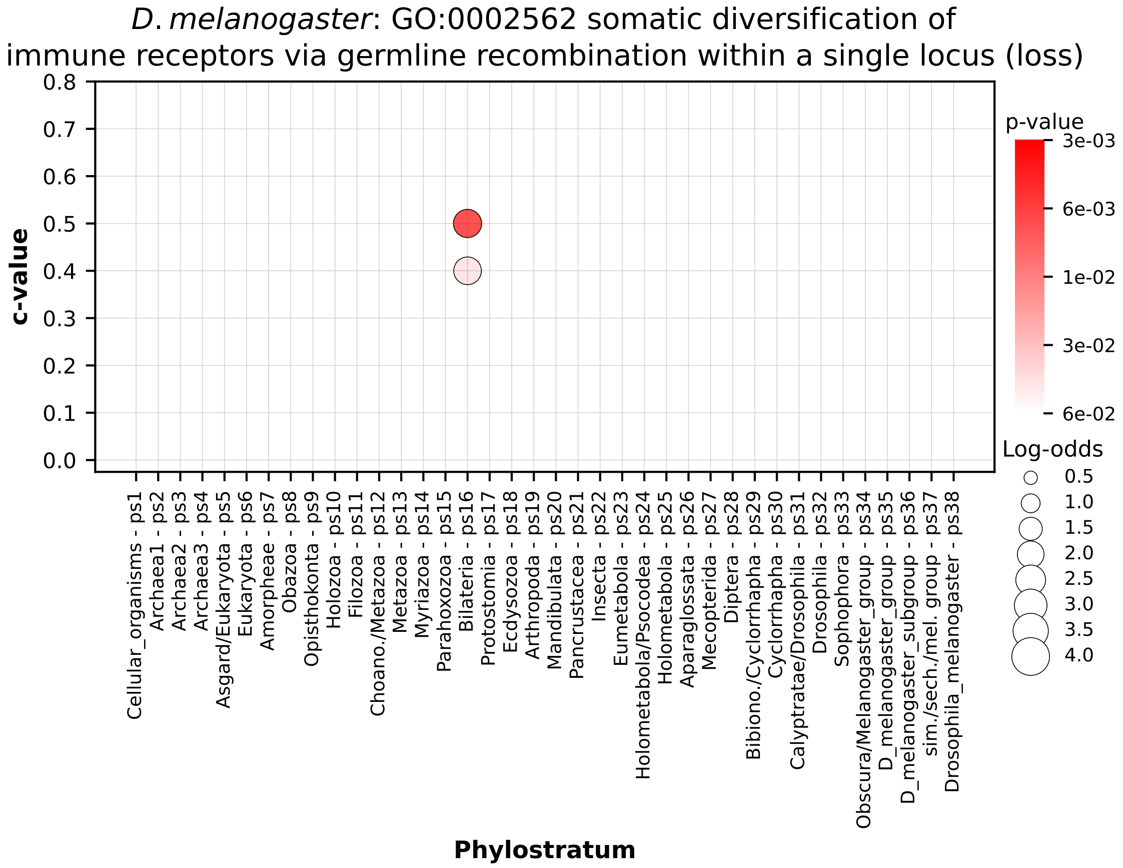
D. melanogaster: GO:0002562 somatic diversification of
immune receptors via germline recombination within a single locus (loss)
c-value
0.8
0.7
0.6
0.5
0.4
0.3
0.2
0.1
0.0
Phylostratum
Cellular_organisms - ps1
Archaea1 - ps2
Archaea2 - ps3
Archaea3 - ps4
Asgard/Eukaryota - ps5
Eukaryota - ps6
Amorpheae - ps7
Obazoa - ps8
Opisthokonta - ps9
Holozoa - ps10
Filozoa - ps11
Choano./Metazoa - ps12
Metazoa - ps13
Myriazoa - ps14
Parahoxozoa - ps15
Bilateria - ps16
Protostomia - ps17
Ecdysozoa - ps18
Arthropoda - ps19
Mandibulata - ps20
Pancrustacea - ps21
Insecta - ps22
Eumetabola - ps23
Holometabola/Psocodea - ps24
Holometabola - ps25
Aparaglossata - ps26
Mecopterida - ps27
Diptera - ps28
Bibiono./Cyclorrhapha - ps29
Cyclorrhapha - ps30
Calyptratae/Drosophila - ps31
Drosophila - ps32
Sophophora - ps33
Obscura/Melanogaster_group - ps34
D_melanogaster_group - ps35
D_melanogaster_subgroup - ps36
sim./sech./mel. group - ps37
Drosophila_melanogaster - ps38
p-value
3e-03
6e-03
1e-02
3e-02
6e-02
Log-odds
0.5
1.0
1.5
2.0
2.5
3.0
3.5
4.0

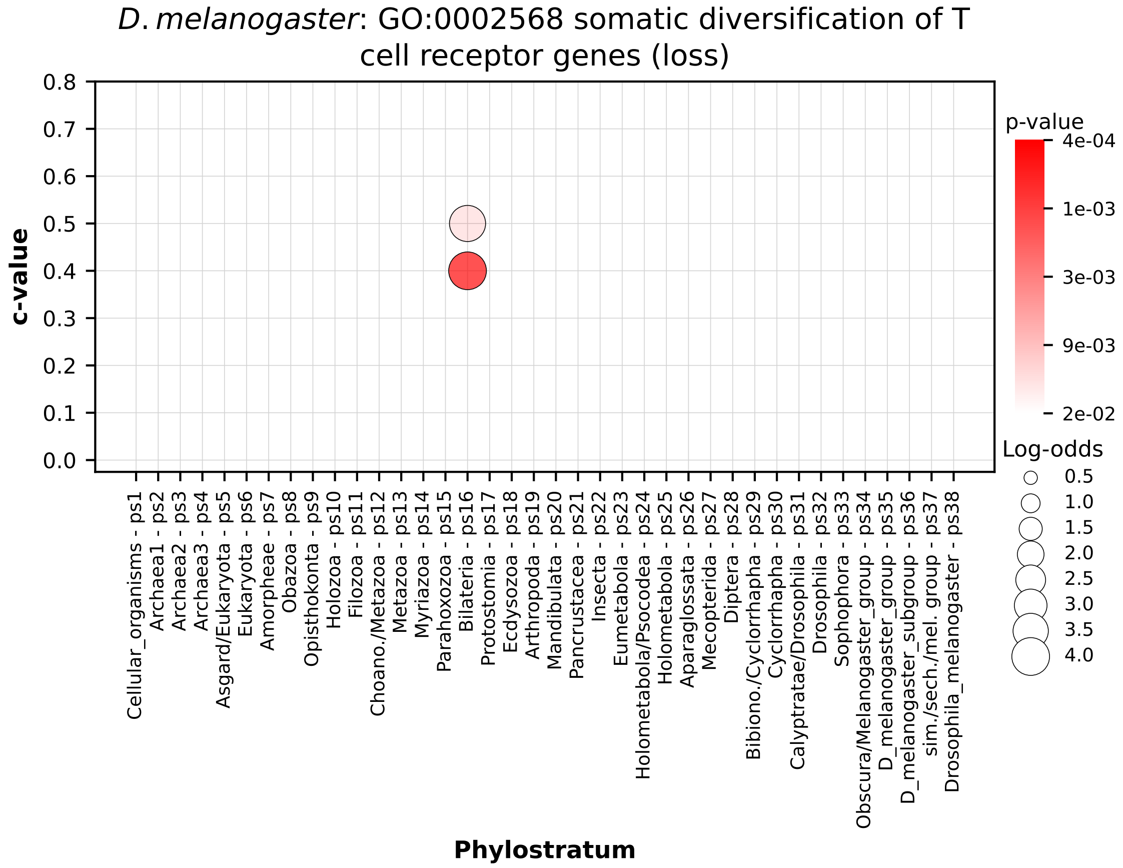

D. melanogaster: GO:0002568 somatic diversification of T cell receptor genes (loss)
c-value
0.8
0.7
0.6
0.5
0.4
0.3
0.2
0.1
0.0
p-value
4e-04
1e-03
3e-03
9e-03
2e-02
Log-odds
0.5
1.0
1.5
2.0
2.5
3.0
3.5
4.0
Cellular_organisms - ps1
Archaea1 - ps2
Archaea2 - ps3
Archaea3 - ps4
Asgard/Eukaryota - ps5
Eukaryota - ps6
Amorpheae - ps7
Obazoa - ps8
Opisthokonta - ps9
Holozoa - ps10
Filozoa - ps11
Choano./Metazoa - ps12
Metazoa - ps13
Myriazoa - ps14
Parahoxozoa - ps15
Bilateria - ps16
Protostomia - ps17
Ecdysozoa - ps18
Arthropoda - ps19
Mandibulata - ps20
Pancrustacea - ps21
Insecta - ps22
Eumetabola - ps23
Holometabola/Psocodea - ps24
Holometabola - ps25
Aparaglossata - ps26
Mecopterida - ps27
Diptera - ps28
Bibiono./Cyclorrhapha - ps29
Cyclorrhapha - ps30
Calyptratae/Drosophila - ps31
Drosophila - ps32
Sophophora - ps33
Obscura/Melanogaster_group - ps34
D_melanogaster_group - ps35
D_melanogaster_subgroup - ps36
sim./sech./mel. group - ps37
Drosophila_melanogaster - ps38
Phylostratum

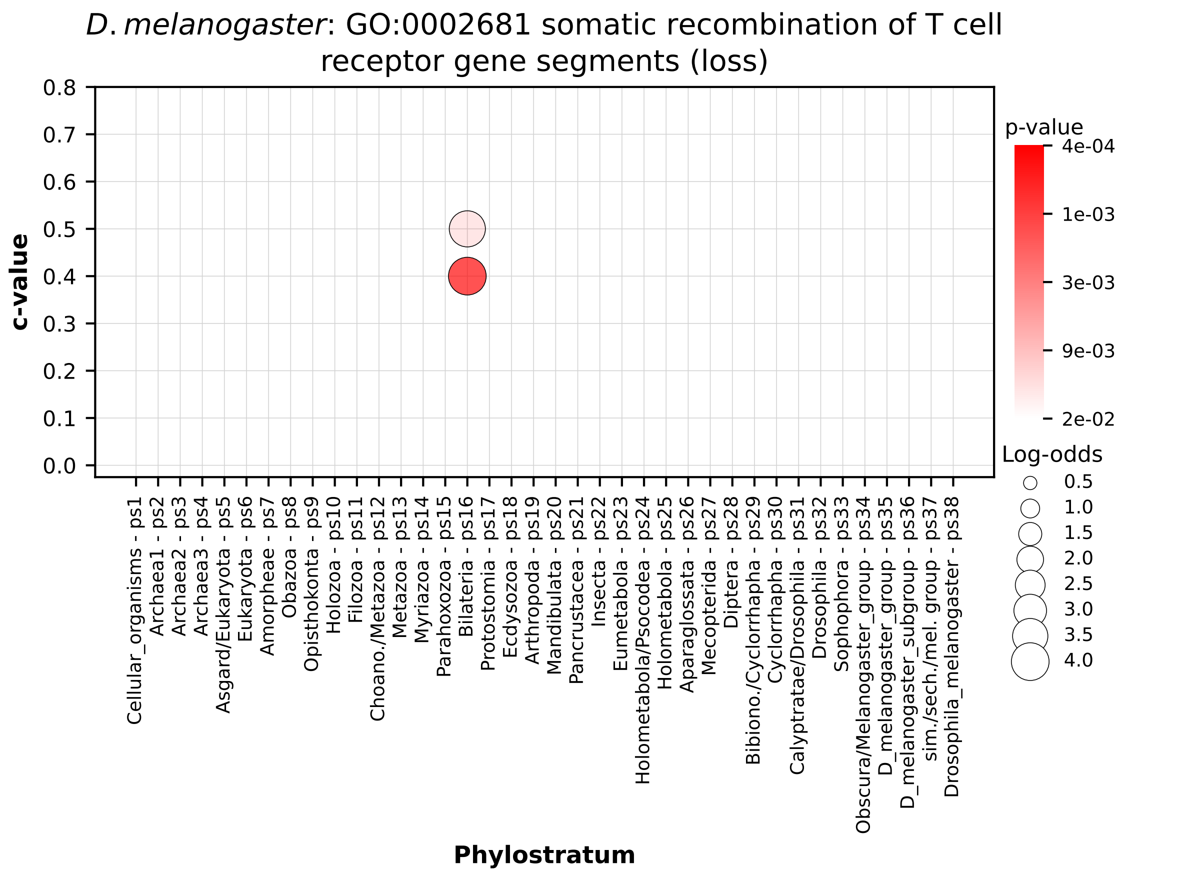
D. melanogaster: GO:0002681 somatic recombination of T cell receptor gene segments (loss)
c-value
0.8
0.7
0.6
0.5
0.4
0.3
0.2
0.1
0.0
p-value
4e-04
1e-03
3e-03
9e-03
2e-02
Log-odds
0.5
1.0
1.5
2.0
2.5
3.0
3.5
4.0
Cellular_organisms - ps1
Archaea1 - ps2
Archaea2 - ps3
Archaea3 - ps4
Asgard/Eukaryota - ps5
Eukaryota - ps6
Amorpheae - ps7
Obazoa - ps8
Opisthokonta - ps9
Holozoa - ps10
Filozoa - ps11
Choano./Metazoa - ps12
Metazoa - ps13
Myriazoa - ps14
Parahoxozoa - ps15
Bilateria - ps16
Protostomia - ps17
Ecdysozoa - ps18
Arthropoda - ps19
Mandibulata - ps20
Pancrustacea - ps21
Insecta - ps22
Eumetabola - ps23
Holometabola/Psocodea - ps24
Holometabola - ps25
Aparaglossata - ps26
Mecopterida - ps27
Diptera - ps28
Bibiono./Cyclorrhapha - ps29
Cyclorrhapha - ps30
Calyptratae/Drosophila - ps31
Drosophila - ps32
Sophophora - ps33
Obscura/Melanogaster_group - ps34
D_melanogaster_group - ps35
D_melanogaster_subgroup - ps36
sim./sech./mel. group - ps37
Drosophila_melanogaster - ps38
Phylostratum

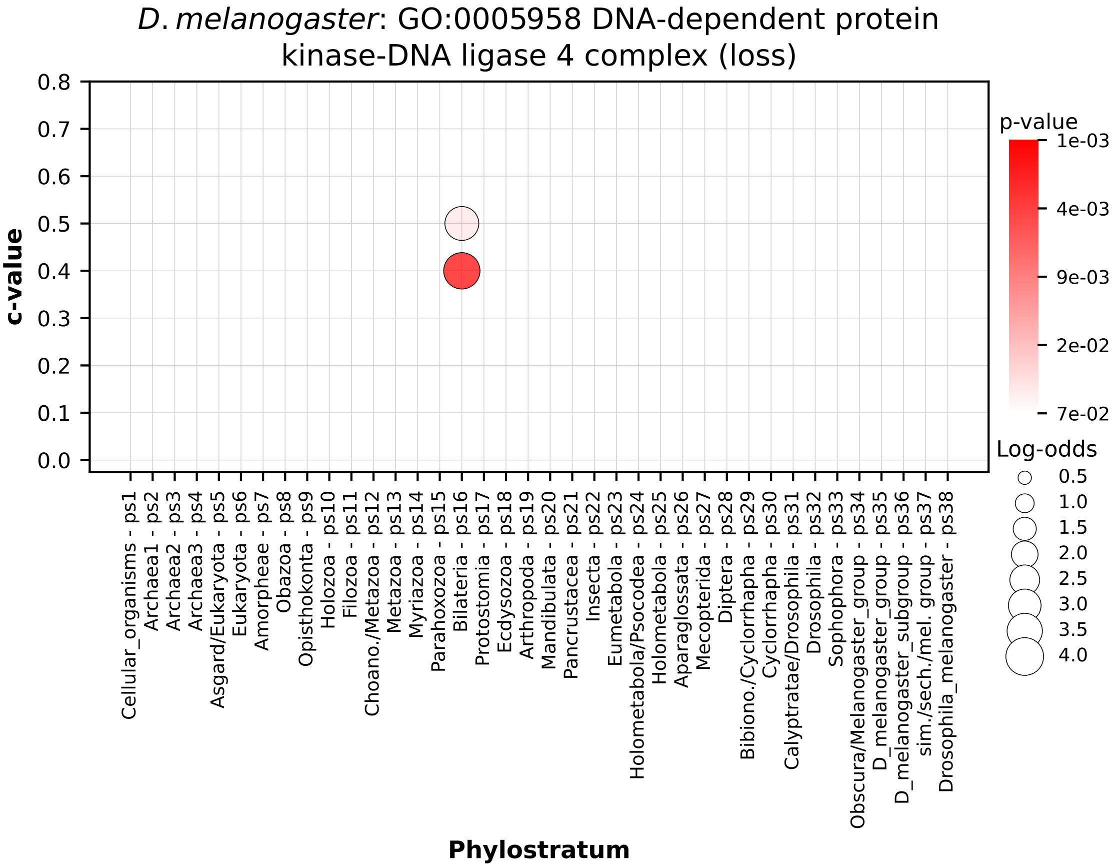

D. melanogaster: GO:0005958 DNA-dependent protein kinase-DNA ligase 4 complex (loss)
c-value
0.8
0.7
0.6
0.5
0.4
0.3
0.2
0.1
0.0
Phylostratum
Cellular_organisms - ps1
Archaea1 - ps2
Archaea2 - ps3
Archaea3 - ps4
Asgard/Eukaryota - ps5
Eukaryota - ps6
Amorpheae - ps7
Obazoa - ps8
Opisthokonta - ps9
Holozoa - ps10
Filozoa - ps11
Choano./Metazoa - ps12
Metazoa - ps13
Myriazoa - ps14
Parahoxozoa - ps15
Bilateria - ps16
Protostomia - ps17
Ecdysozoa - ps18
Arthropoda - ps19
Mandibulata - ps20
Pancrustacea - ps21
Insecta - ps22
Eumetabola - ps23
Holometabola/Psocodea - ps24
Holometabola - ps25
Aparaglossata - ps26
Mecopterida - ps27
Diptera - ps28
Bibiono./Cyclorrhapha - ps29
Cyclorrhapha - ps30
Calyptratae/Drosophila - ps31
Drosophila - ps32
Sophophora - ps33
Obscura/Melanogaster_group - ps34
D_melanogaster_group - ps35
D_melanogaster_subgroup - ps36
sim./sech./mel. group - ps37
Drosophila_melanogaster - ps38
p-value
1e-03
4e-03
9e-03
2e-02
7e-02
Log-odds
0.5
1.0
1.5
2.0
2.5
3.0
3.5
4.0

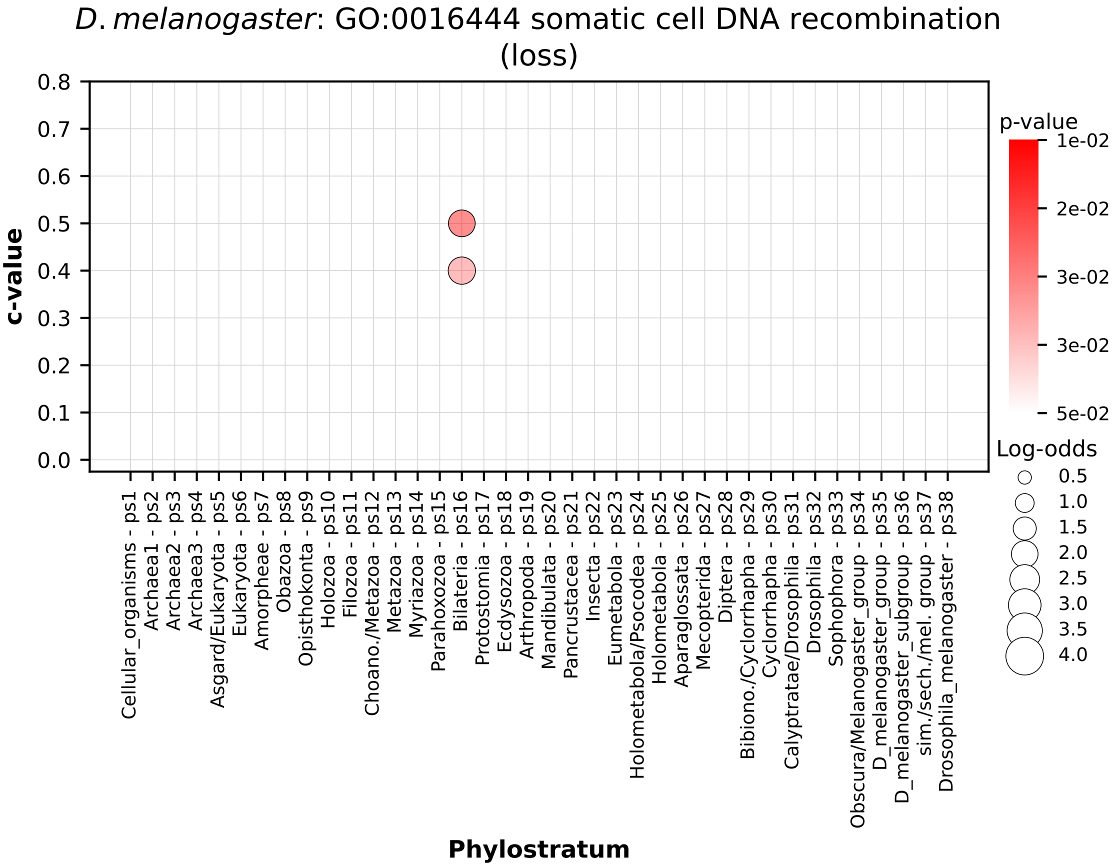

D. melanogaster: GO:0016444 somatic cell DNA recombination (loss)
c-value
0.8
0.7
0.6
0.5
0.4
0.3
0.2
0.1
0.0
p-value
1e-02
2e-02
3e-02
3e-02
5e-02
Log-odds
0.5
1.0
1.5
2.0
2.5
3.0
3.5
4.0
Cellular_organisms - ps1
Archaea1 - ps2
Archaea2 - ps3
Archaea3 - ps4
Asgard/Eukaryota - ps5
Eukaryota - ps6
Amorpheae - ps7
Obazoa - ps8
Opisthokonta - ps9
Holozoa - ps10
Filozoa - ps11
Choano./Metazoa - ps12
Metazoa - ps13
Myriazoa - ps14
Parahoxozoa - ps15
Bilateria - ps16
Protostomia - ps17
Ecdysozoa - ps18
Arthropoda - ps19
Mandibulata - ps20
Pancrustacea - ps21
Insecta - ps22
Eumetabola - ps23
Holometabola/Psocodea - ps24
Holometabola - ps25
Aparaglossata - ps26
Mecopterida - ps27
Diptera - ps28
Bibiono./Cyclorrhapha - ps29
Cyclorrhapha - ps30
Calyptratae/Drosophila - ps31
Drosophila - ps32
Sophophora - ps33
Obscura/Melanogaster_group - ps34
D_melanogaster_group - ps35
D_melanogaster_subgroup - ps36
sim./sech./mel. group - ps37
Drosophila_melanogaster - ps38
Phylostratum

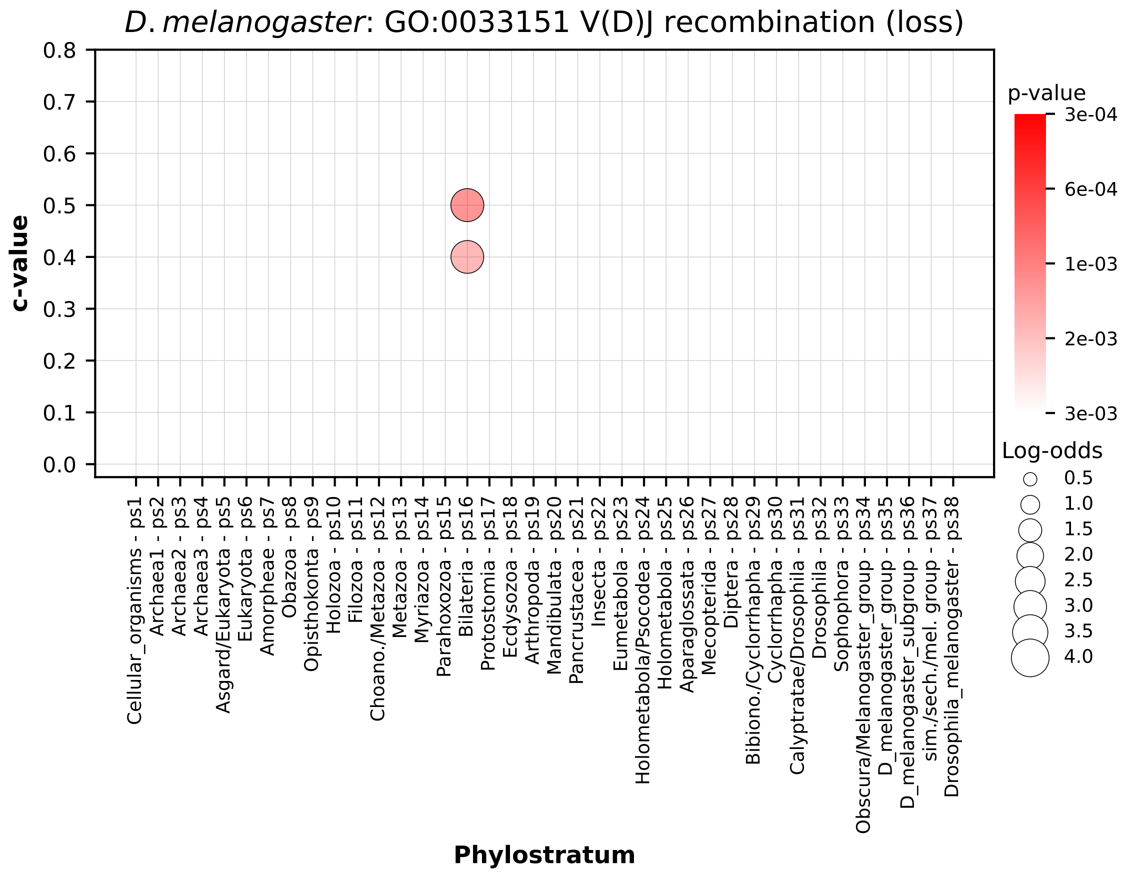

D. melanogaster: GO:0033151 V(D)J recombination (loss)
c-value
0.8
0.7
0.6
0.5
0.4
0.3
0.2
0.1
0.0
Phylostratum
Cellular_organisms - ps1
Archaea1 - ps2
Archaea2 - ps3
Archaea3 - ps4
Asgard/Eukaryota - ps5
Eukaryota - ps6
Amorpheae - ps7
Obazoa - ps8
Opisthokonta - ps9
Holozoa - ps10
Filozoa - ps11
Choano./Metazoa - ps12
Metazoa - ps13
Myriazoa - ps14
Parahoxozoa - ps15
Bilateria - ps16
Protostomia - ps17
Ecdysozoa - ps18
Arthropoda - ps19
Mandibulata - ps20
Pancrustacea - ps21
Insecta - ps22
Eumetabola - ps23
Holometabola/Psocodea - ps24
Holometabola - ps25
Aparaglossata - ps26
Mecopterida - ps27
Diptera - ps28
Bibiono./Cyclorrhapha - ps29
Cyclorrhapha - ps30
Calyptratae/Drosophila - ps31
Drosophila - ps32
Sophophora - ps33
Obscura/Melanogaster_group - ps34
D_melanogaster_group - ps35
D_melanogaster_subgroup - ps36
sim./sech./mel. group - ps37
Drosophila_melanogaster - ps38
p-value
3e-04
6e-04
1e-03
2e-03
3e-03
Log-odds
0.5
1.0
1.5
2.0
2.5
3.0
3.5
4.0

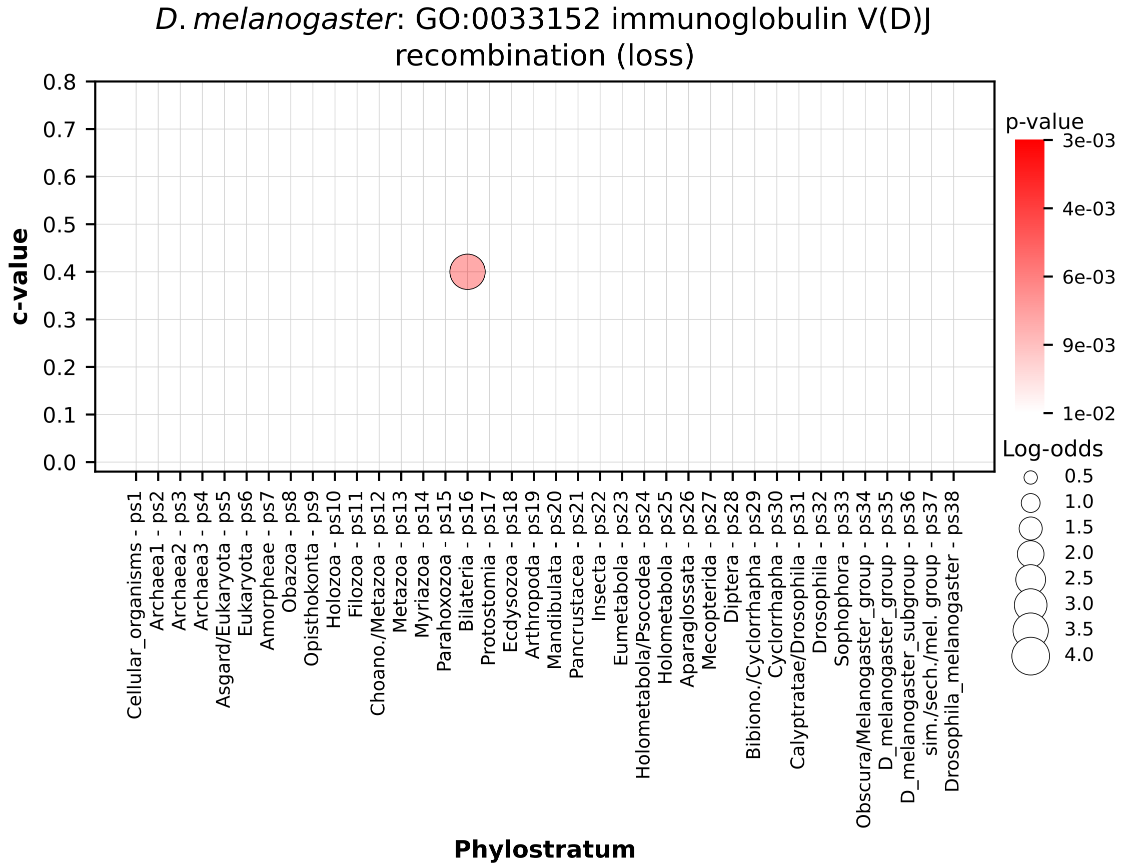
D. melanogaster: GO:0033152 immunoglobulin V(D)J recombination (loss)
c-value
0.8
0.7
0.6
0.5
0.4
0.3
0.2
0.1
0.0
Phylostratum
Cellular_organisms - ps1
Archaea1 - ps2
Archaea2 - ps3
Archaea3 - ps4
Asgard/Eukaryota - ps5
Eukaryota - ps6
Amorpheae - ps7
Obazoa - ps8
Opisthokonta - ps9
Holozoa - ps10
Filozoa - ps11
Choano./Metazoa - ps12
Metazoa - ps13
Myriazoa - ps14
Parahoxozoa - ps15
Bilateria - ps16
Protostomia - ps17
Ecdysozoa - ps18
Arthropoda - ps19
Mandibulata - ps20
Pancrustacea - ps21
Insecta - ps22
Eumetabola - ps23
Holometabola/Psocodea - ps24
Holometabola - ps25
Aparaglossata - ps26
Mecopterida - ps27
Diptera - ps28
Bibiono./Cyclorrhapha - ps29
Cyclorrhapha - ps30
Calyptratae/Drosophila - ps31
Drosophila - ps32
Sophophora - ps33
Obscura/Melanogaster_group - ps34
D_melanogaster_group - ps35
D_melanogaster_subgroup - ps36
sim./sech./mel. group - ps37
Drosophila_melanogaster - ps38
p-value
3e-03
4e-03
6e-03
9e-03
1e-02
Log-odds
0.5
1.0
1.5
2.0
2.5
3.0
3.5
4.0

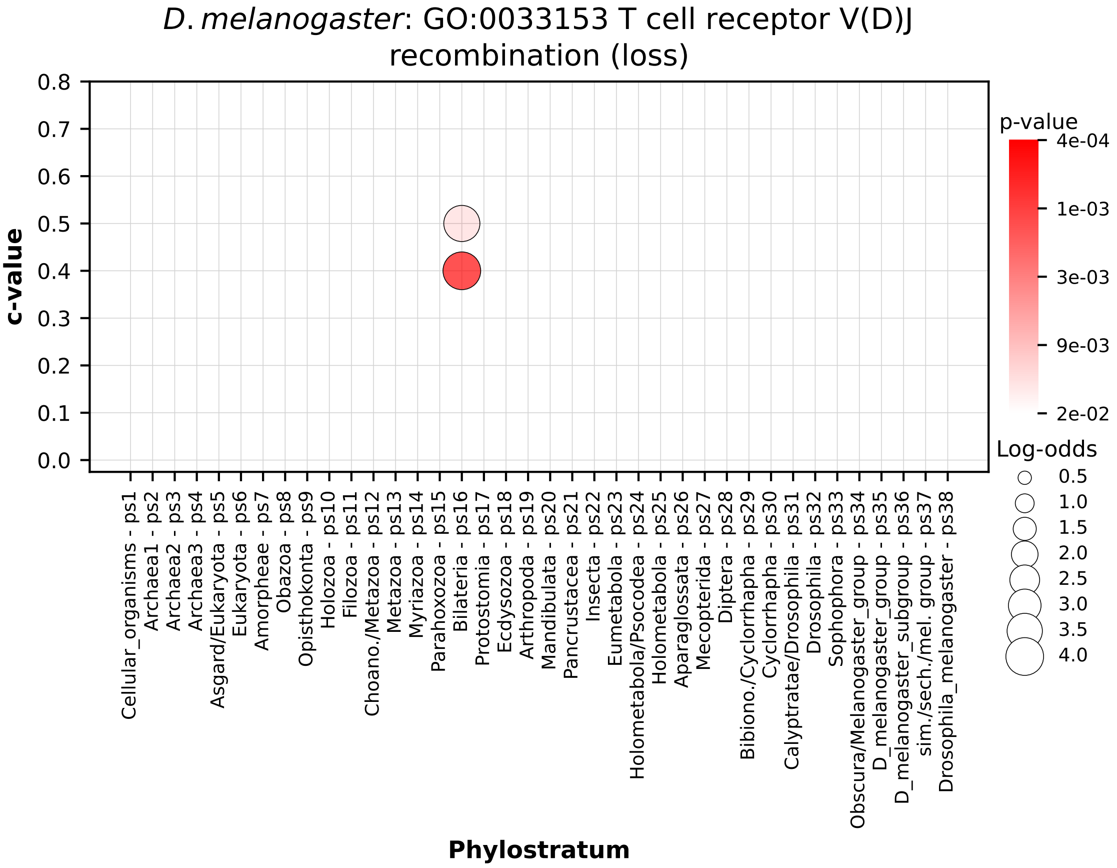

D. melanogaster: GO:0033153 T cell receptor V(D)J recombination (loss)
c-value
0.0
0.1
0.2
0.3
0.4
0.5
0.6
0.7
0.8
p-value
4e-04
1e-03
3e-03
9e-03
2e-02
Log-odds
0.5
1.0
1.5
2.0
2.5
3.0
3.5
4.0
Cellular_organisms - ps1
Archaea1 - ps2
Archaea2 - ps3
Archaea3 - ps4
Asgard/Eukaryota - ps5
Eukaryota - ps6
Amorpheae - ps7
Obazoa - ps8
Opisthokonta - ps9
Holozoa - ps10
Filozoa - ps11
Choano./Metazoa - ps12
Metazoa - ps13
Myriazoa - ps14
Parahoxozoa - ps15
Bilateria - ps16
Protostomia - ps17
Ecdysozoa - ps18
Arthropoda - ps19
Mandibulata - ps20
Pancrustacea - ps21
Insecta - ps22
Eumetabola - ps23
Holometabola/Psocodea - ps24
Holometabola - ps25
Aparaglossata - ps26
Mecopterida - ps27
Diptera - ps28
Bibiono./Cyclorrhapha - ps29
Cyclorrhapha - ps30
Calyptratae/Drosophila - ps31
Drosophila - ps32
Sophophora - ps33
Obscura/Melanogaster_group - ps34
D_melanogaster_group - ps35
D_melanogaster_subgroup - ps36
sim./sech./mel. group - ps37
Drosophila_melanogaster - ps38
Phylostratum

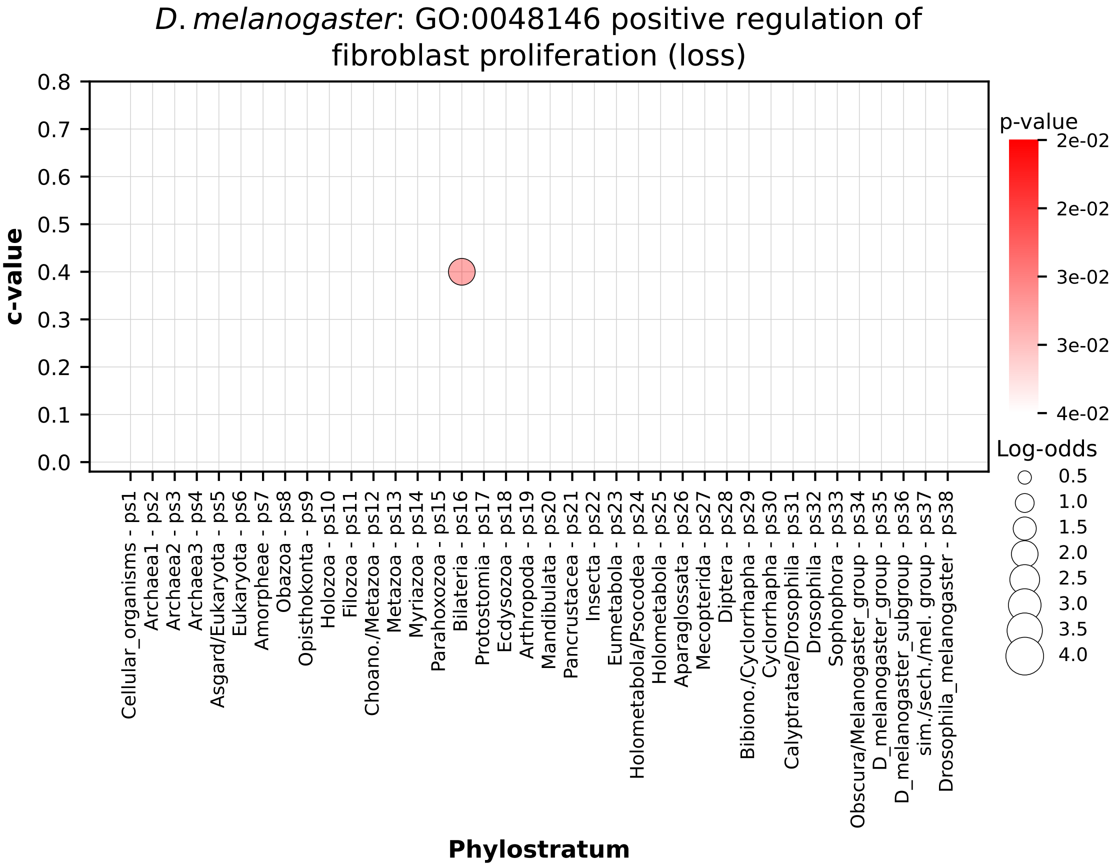

D. melanogaster: GO:0048146 positive regulation of fibroblast proliferation (loss)
c-value
0.8
0.7
0.6
0.5
0.4
0.3
0.2
0.1
0.0
p-value
2e-02
2e-02
3e-02
3e-02
4e-02
Log-odds
0.5
1.0
1.5
2.0
2.5
3.0
3.5
4.0
Cellular_organisms - ps1
Archaea1 - ps2
Archaea2 - ps3
Archaea3 - ps4
Asgard/Eukaryota - ps5
Eukaryota - ps6
Amorpheae - ps7
Obazoa - ps8
Opisthokonta - ps9
Holozoa - ps10
Filozoa - ps11
Choano./Metazoa - ps12
Metazoa - ps13
Myriazoa - ps14
Parahoxozoa - ps15
Bilateria - ps16
Protostomia - ps17
Ecdysozoa - ps18
Arthropoda - ps19
Mandibulata - ps20
Pancrustacea - ps21
Insecta - ps22
Eumetabola - ps23
Holometabola/Psocodea - ps24
Holometabola - ps25
Aparaglossata - ps26
Mecopterida - ps27
Diptera - ps28
Bibiono./Cyclorrhapha - ps29
Cyclorrhapha - ps30
Calyptratae/Drosophila - ps31
Drosophila - ps32
Sophophora - ps33
Obscura/Melanogaster_group - ps34
D_melanogaster_group - ps35
D_melanogaster_subgroup - ps36
sim./sech./mel. group - ps37
Drosophila_melanogaster - ps38
Phylostratum

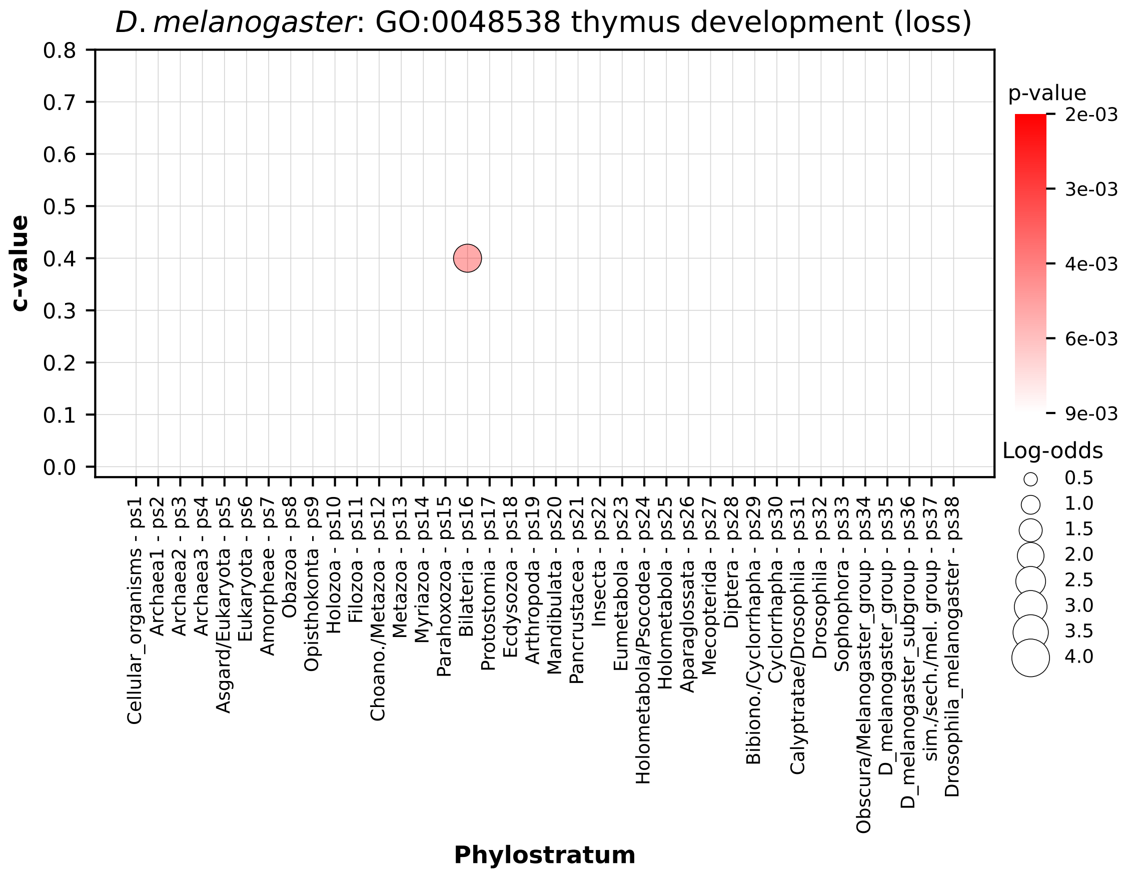
D. melanogaster: GO:0048538 thymus development (loss)
c-value
0.0
0.1
0.2
0.3
0.4
0.5
0.6
0.7
0.8
Phylostratum
Cellular_organisms - ps1
Archaea1 - ps2
Archaea2 - ps3
Archaea3 - ps4
Asgard/Eukaryota - ps5
Eukaryota - ps6
Amorpheae - ps7
Obazoa - ps8
Opisthokonta - ps9
Holozoa - ps10
Filozoa - ps11
Choano./Metazoa - ps12
Metazoa - ps13
Myriazoa - ps14
Parahoxozoa - ps15
Bilateria - ps16
Protostomia - ps17
Ecdysozoa - ps18
Arthropoda - ps19
Mandibulata - ps20
Pancrustacea - ps21
Insecta - ps22
Eumetabola - ps23
Holometabola/Psocodea - ps24
Holometabola - ps25
Aparaglossata - ps26
Mecopterida - ps27
Diptera - ps28
Bibiono./Cyclorrhapha - ps29
Cyclorrhapha - ps30
Calyptratae/Drosophila - ps31
Drosophila - ps32
Sophophora - ps33
Obscura/Melanogaster_group - ps34
D_melanogaster_group - ps35
D_melanogaster_subgroup - ps36
sim./sech./mel. group - ps37
Drosophila_melanogaster - ps38
p-value
2e-03
3e-03
4e-03
6e-03
9e-03
Log-odds
0.5
1.0
1.5
2.0
2.5
3.0
3.5
4.0

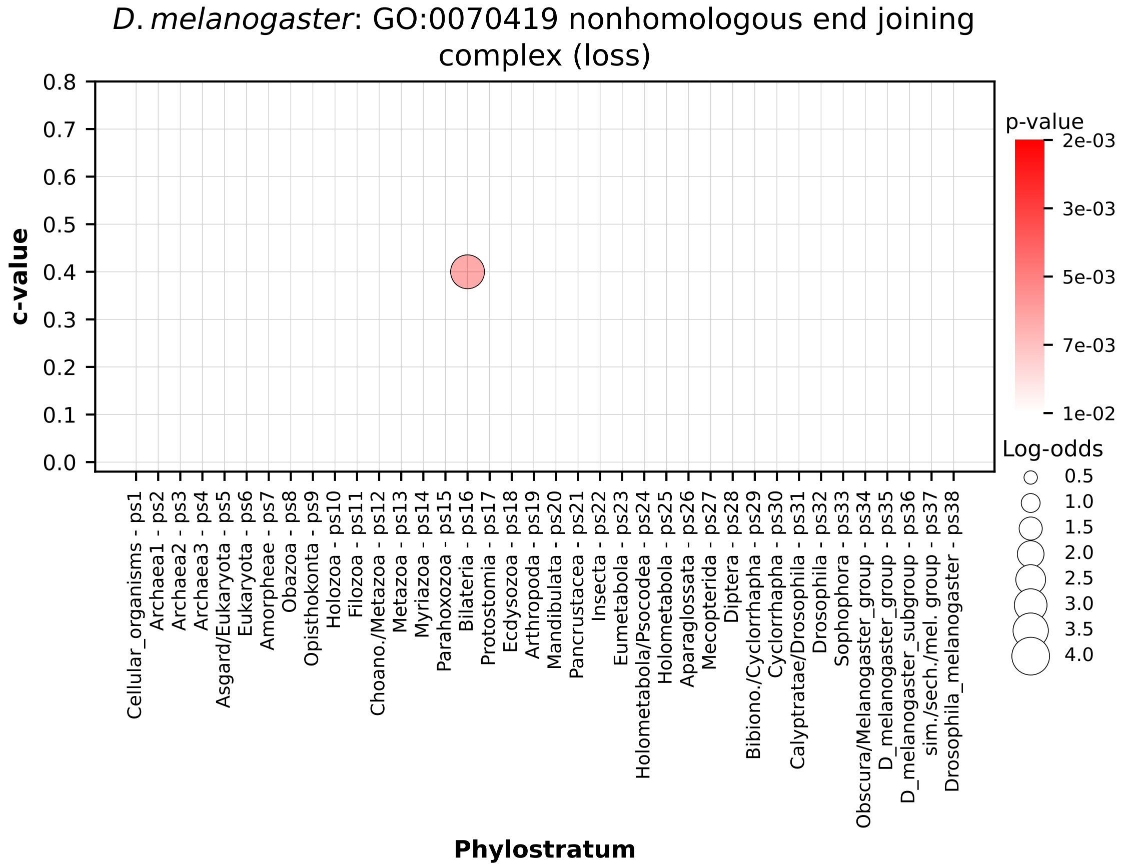

D. melanogaster: GO:0070419 nonhomologous end joining complex (loss)
c-value
0.8
0.7
0.6
0.5
0.4
0.3
0.2
0.1
0.0
Phylostratum
Cellular_organisms - ps1
Archaea1 - ps2
Archaea2 - ps3
Archaea3 - ps4
Asgard/Eukaryota - ps5
Eukaryota - ps6
Amorpheae - ps7
Obazoa - ps8
Opisthokonta - ps9
Holozoa - ps10
Filozoa - ps11
Choano./Metazoa - ps12
Metazoa - ps13
Myriazoa - ps14
Parahoxozoa - ps15
Bilateria - ps16
Protostomia - ps17
Ecdysozoa - ps18
Arthropoda - ps19
Mandibulata - ps20
Pancrustacea - ps21
Insecta - ps22
Eumetabola - ps23
Holometabola/Psocodea - ps24
Holometabola - ps25
Aparaglossata - ps26
Mecopterida - ps27
Diptera - ps28
Bibiono./Cyclorrhapha - ps29
Cyclorrhapha - ps30
Calyptratae/Drosophila - ps31
Drosophila - ps32
Sophophora - ps33
Obscura/Melanogaster_group - ps34
D_melanogaster_group - ps35
D_melanogaster_subgroup - ps36
sim./sech./mel. group - ps37
Drosophila_melanogaster - ps38
p-value
2e-03
3e-03
5e-03
7e-03
1e-02
Log-odds
0.5
1.0
1.5
2.0
2.5
3.0
3.5
4.0

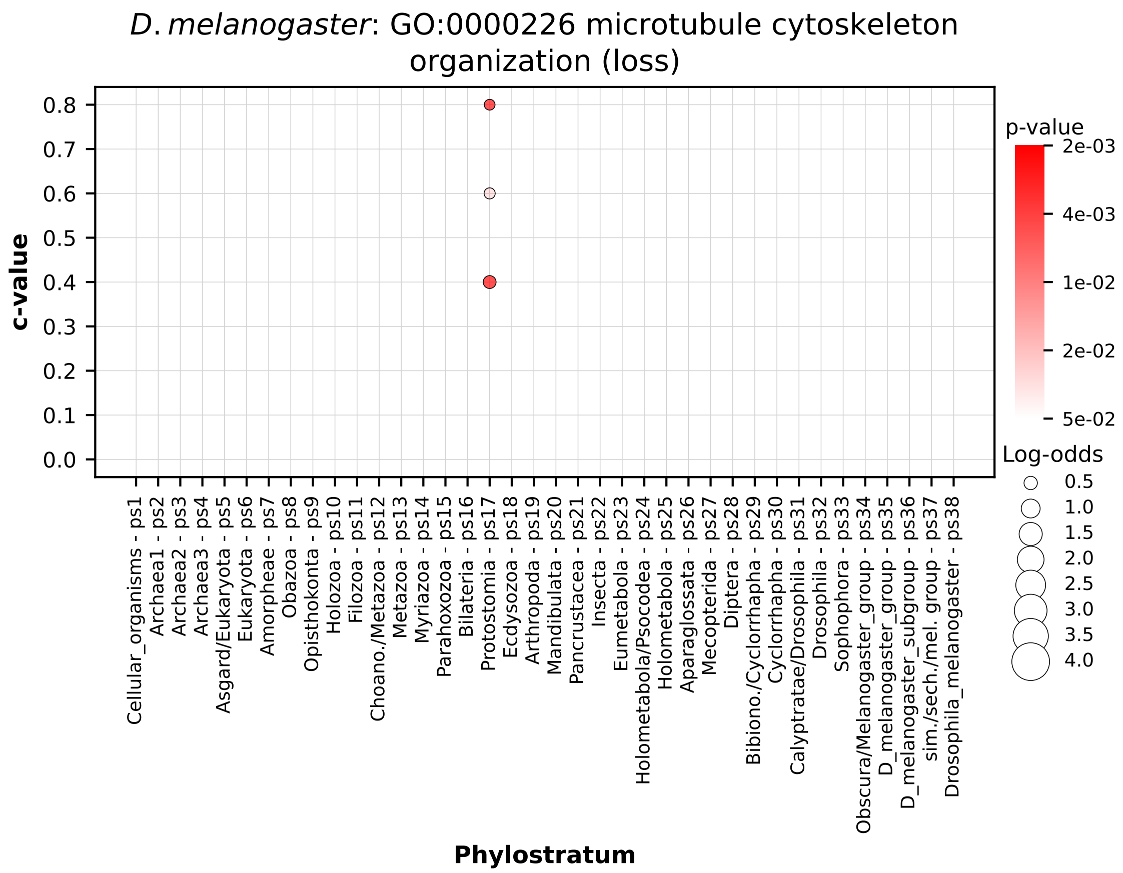

$D. melanogaster$: GO:0000226 microtubule cytoskeleton organization (loss)
c-value
0.0
0.1
0.2
0.3
0.4
0.5
0.6
0.7
0.8
Phylostratum
Cellular_organisms - ps1
Archaea1 - ps2
Archaea2 - ps3
Archaea3 - ps4
Asgard/Eukaryota - ps5
Eukaryota - ps6
Amorpheae - ps7
Obazoa - ps8
Opisthokonta - ps9
Holozoa - ps10
Filozoa - ps11
Choano./Metazoa - ps12
Metazoa - ps13
Myriazoa - ps14
Parahoxozoa - ps15
Bilateria - ps16
Protostomia - ps17
Ecdysozoa - ps18
Arthropoda - ps19
Mandibulata - ps20
Pancrustacea - ps21
Insecta - ps22
Eumetabola - ps23
Holometabola/Psocodea - ps24
Holometabola - ps25
Aparaglossata - ps26
Mecopterida - ps27
Diptera - ps28
Bibiono./Cyclorrhapha - ps29
Cyclorrhapha - ps30
Calyptratae/Drosophila - ps31
Drosophila - ps32
Sophophora - ps33
Obscura/Melanogaster_group - ps34
D_melanogaster_group - ps35
D_melanogaster_subgroup - ps36
sim./sech./mel. group - ps37
Drosophila_melanogaster - ps38
p-value
2e-03
4e-03
1e-02
2e-02
5e-02
Log-odds
0.5
1.0
1.5
2.0
2.5
3.0
3.5
4.0

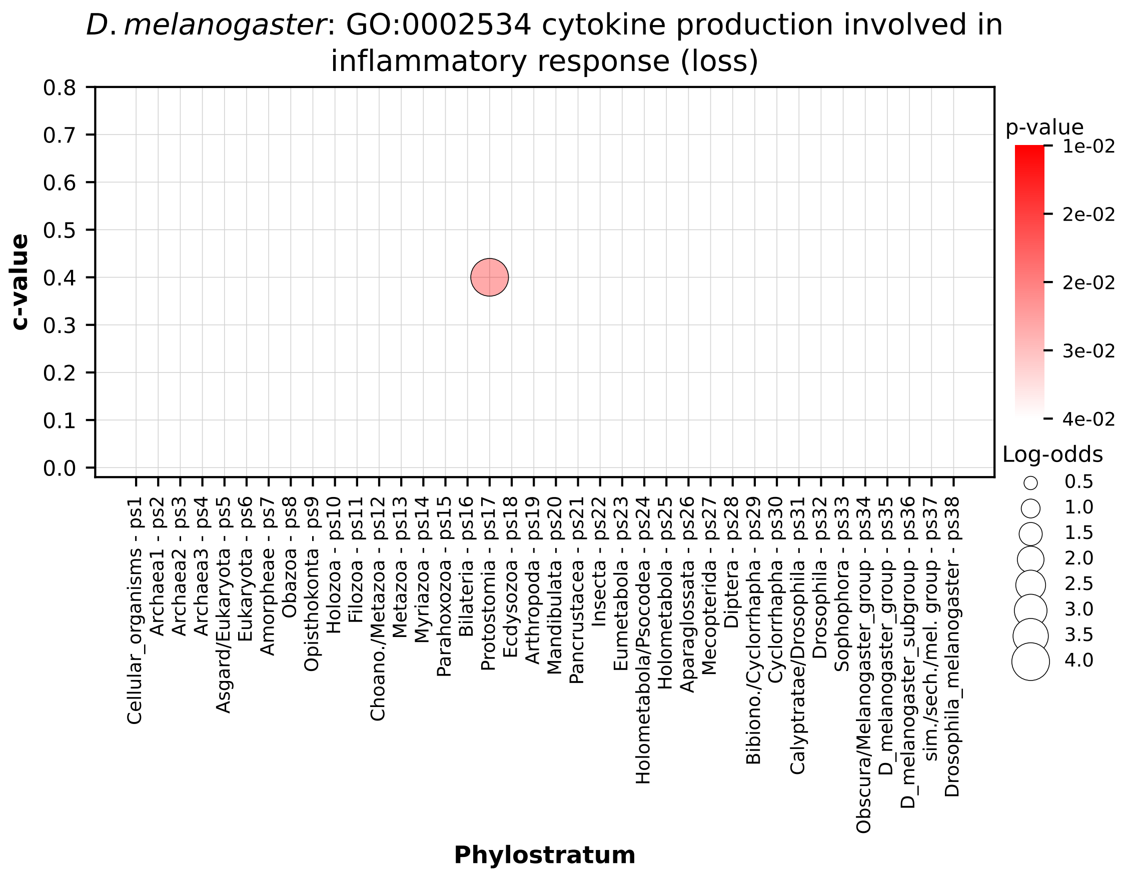
D. melanogaster: GO:0002534 cytokine production involved in inflammatory response (loss)
c-value
0.8
0.7
0.6
0.5
0.4
0.3
0.2
0.1
0.0
Phylostratum
Cellular_organisms - ps1
Archaea1 - ps2
Archaea2 - ps3
Archaea3 - ps4
Asgard/Eukaryota - ps5
Eukaryota - ps6
Amorpheae - ps7
Obazoa - ps8
Opisthokonta - ps9
Holozoa - ps10
Filozoa - ps11
Choano./Metazoa - ps12
Metazoa - ps13
Myriazoa - ps14
Parahoxozoa - ps15
Bilateria - ps16
Protostomia - ps17
Ecdysozoa - ps18
Arthropoda - ps19
Mandibulata - ps20
Pancrustacea - ps21
Insecta - ps22
Eumetabola - ps23
Holometabola/Psocodea - ps24
Holometabola - ps25
Aparaglossata - ps26
Mecopterida - ps27
Diptera - ps28
Bibiono./Cyclorrhapha - ps29
Cyclorrhapha - ps30
Calyptratae/Drosophila - ps31
Drosophila - ps32
Sophophora - ps33
Obscura/Melanogaster_group - ps34
D_melanogaster_group - ps35
D_melanogaster_subgroup - ps36
sim./sech./mel. group - ps37
Drosophila_melanogaster - ps38
p-value
1e-02
2e-02
2e-02
3e-02
4e-02
Log-odds
0.5
1.0
1.5
2.0
2.5
3.0
3.5
4.0

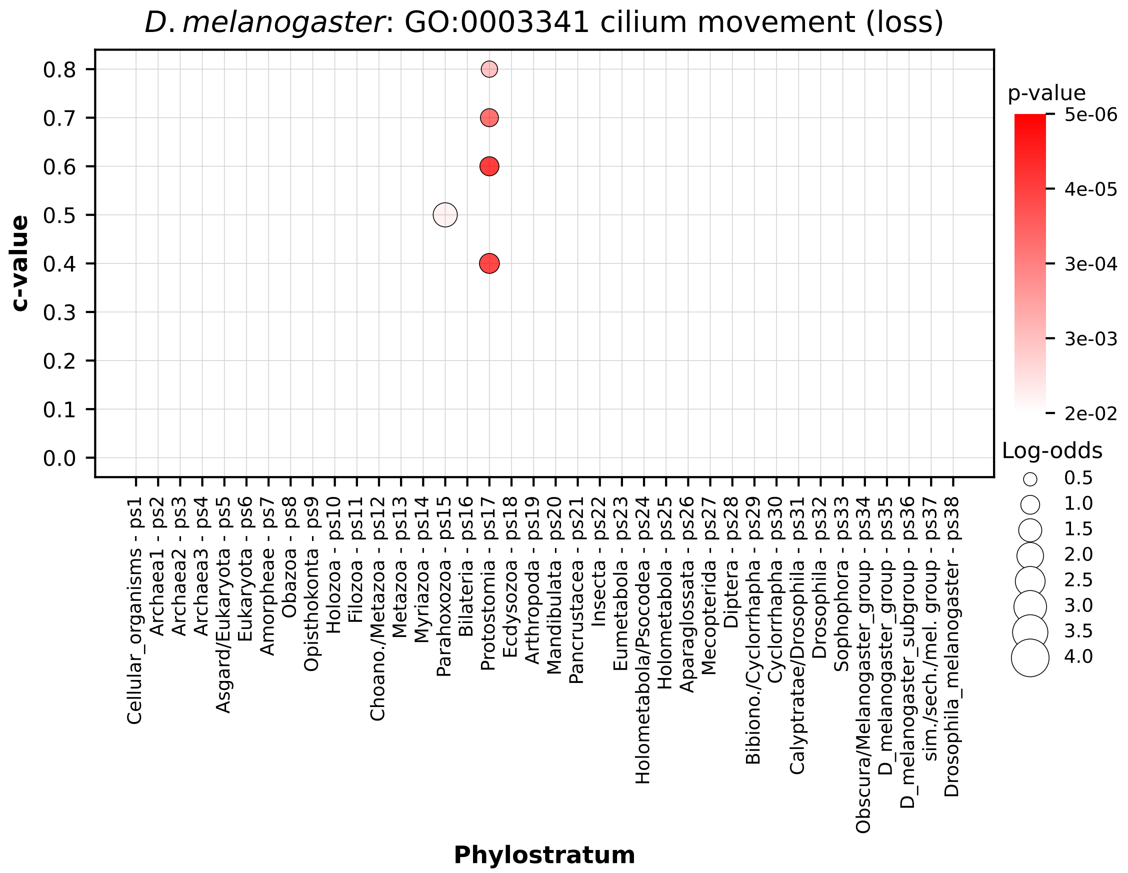

D. melanogaster: GO:0003341 cilium movement (loss)
c-value
0.8
0.7
0.6
0.5
0.4
0.3
0.2
0.1
0.0
Phylostratum
Cellular_organisms - ps1
Archaea1 - ps2
Archaea2 - ps3
Archaea3 - ps4
Asgard/Eukaryota - ps5
Eukaryota - ps6
Amorpheae - ps7
Obazoa - ps8
Opisthokonta - ps9
Holozoa - ps10
Filozoa - ps11
Choano./Metazoa - ps12
Metazoa - ps13
Myriazoa - ps14
Parahoxozoa - ps15
Bilateria - ps16
Protostomia - ps17
Ecdysozoa - ps18
Arthropoda - ps19
Mandibulata - ps20
Pancrustacea - ps21
Insecta - ps22
Eumetabola - ps23
Holometabola/Psocodea - ps24
Holometabola - ps25
Aparaglossata - ps26
Mecopterida - ps27
Diptera - ps28
Bibiono./Cyclorrhapha - ps29
Cyclorrhapha - ps30
Calyptratae/Drosophila - ps31
Drosophila - ps32
Sophophora - ps33
Obscura/Melanogaster_group - ps34
D_melanogaster_group - ps35
D_melanogaster_subgroup - ps36
sim./sech./mel. group - ps37
Drosophila_melanogaster - ps38
p-value
5e-06
4e-05
3e-04
3e-03
2e-02
Log-odds
0.5
1.0
1.5
2.0
2.5
3.0
3.5
4.0

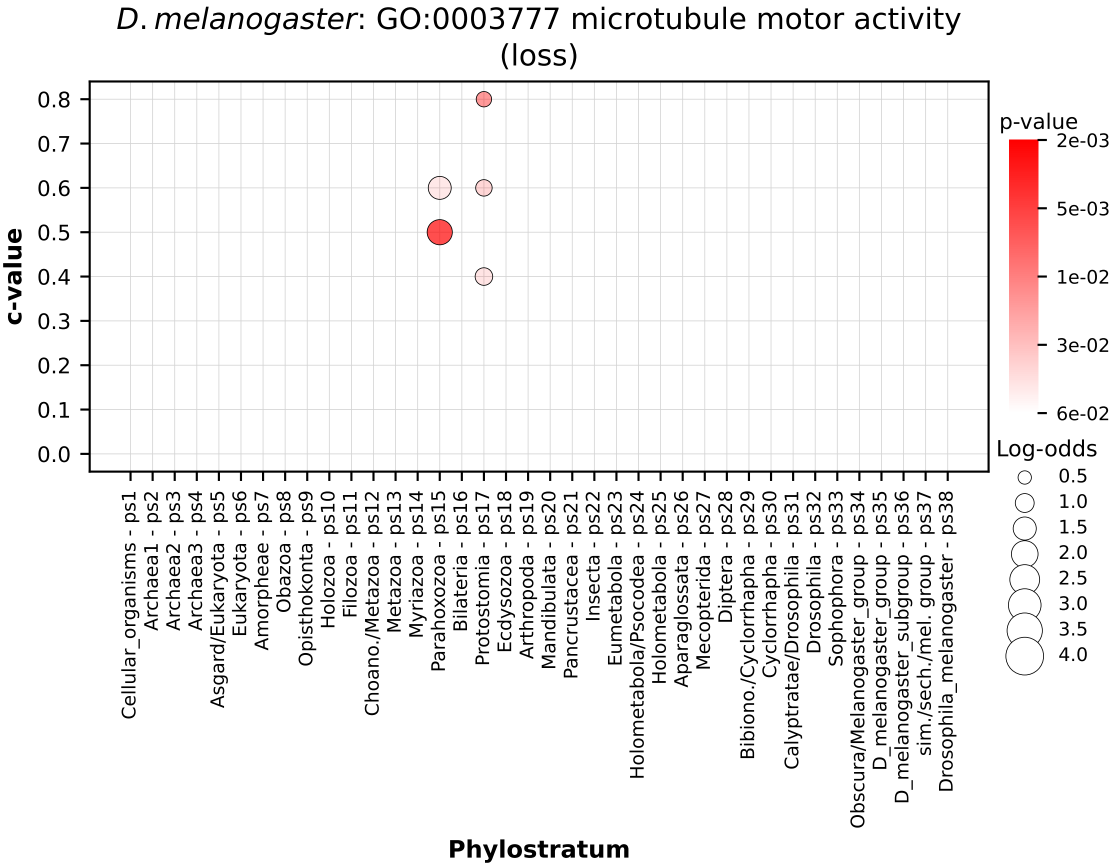

D. melanogaster: GO:0003777 microtubule motor activity (loss)
c-value
0.8
0.7
0.6
0.5
0.4
0.3
0.2
0.1
0.0
Phylostratum
Cellular_organisms - ps1
Archaea1 - ps2
Archaea2 - ps3
Archaea3 - ps4
Asgard/Eukaryota - ps5
Eukaryota - ps6
Amorpheae - ps7
Obazoa - ps8
Opisthokonta - ps9
Holozoa - ps10
Filozoa - ps11
Choano./Metazoa - ps12
Metazoa - ps13
Myriazoa - ps14
Parahoxozoa - ps15
Bilateria - ps16
Protostomia - ps17
Ecdysozoa - ps18
Arthropoda - ps19
Mandibulata - ps20
Pancrustacea - ps21
Insecta - ps22
Eumetabola - ps23
Holometabola/Psocodea - ps24
Holometabola - ps25
Aparaglossata - ps26
Mecopterida - ps27
Diptera - ps28
Bibiono./Cyclorrhapha - ps29
Cyclorrhapha - ps30
Calyptratae/Drosophila - ps31
Drosophila - ps32
Sophophora - ps33
Obscura/Melanogaster_group - ps34
D_melanogaster_group - ps35
D_melanogaster_subgroup - ps36
sim./sech./mel. group - ps37
Drosophila_melanogaster - ps38
p-value
2e-03
5e-03
1e-02
3e-02
6e-02
Log-odds
0.5
1.0
1.5
2.0
2.5
3.0
3.5
4.0

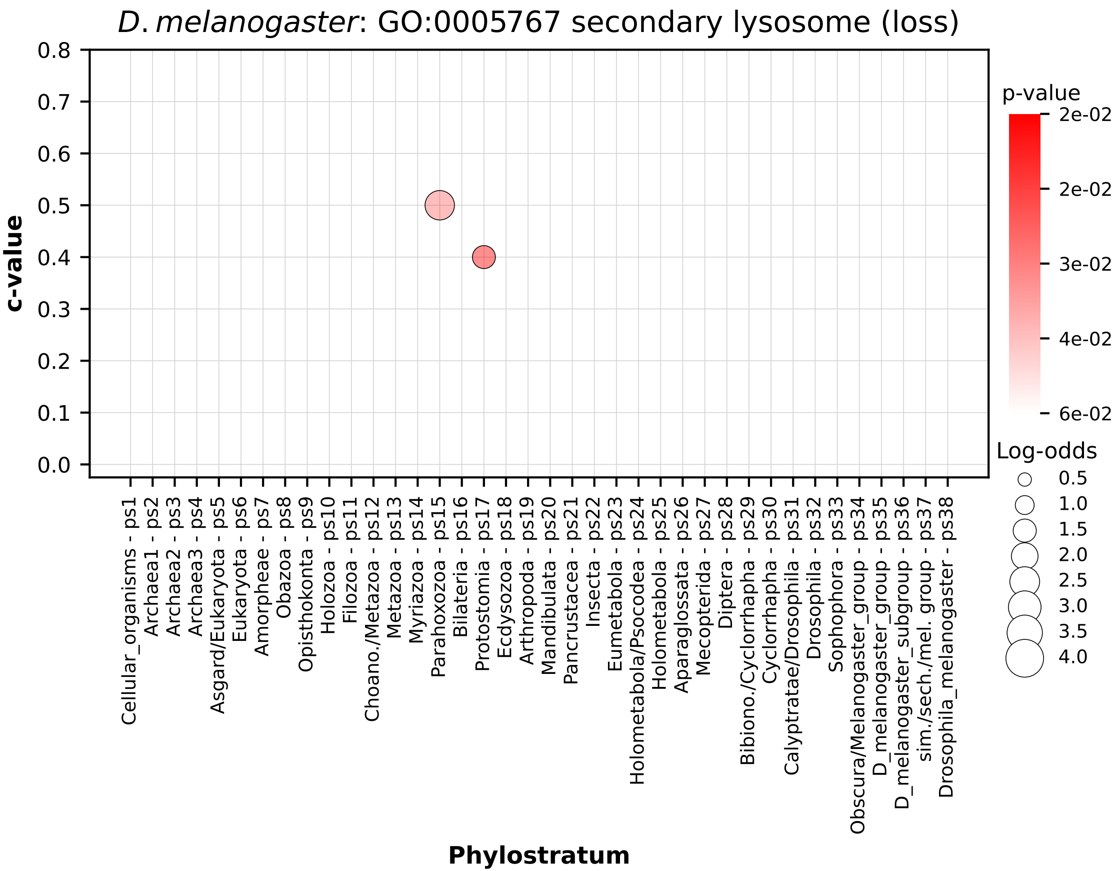

D. melanogaster: GO:0005767 secondary lysosome (loss)
c-value
0.0
0.1
0.2
0.3
0.4
0.5
0.6
0.7
0.8
Phylostratum
Cellular_organisms - ps1
Archaea1 - ps2
Archaea2 - ps3
Archaea3 - ps4
Asgard/Eukaryota - ps5
Eukaryota - ps6
Amorpheae - ps7
Obazoa - ps8
Opisthokonta - ps9
Holozoa - ps10
Filozoa - ps11
Choano./Metazoa - ps12
Metazoa - ps13
Myriazoa - ps14
Parahoxozoa - ps15
Bilateria - ps16
Protostomia - ps17
Ecdysozoa - ps18
Arthropoda - ps19
Mandibulata - ps20
Pancrustacea - ps21
Insecta - ps22
Eumetabola - ps23
Holometabola/Psocodea - ps24
Holometabola - ps25
Aparaglossata - ps26
Mecopterida - ps27
Diptera - ps28
Bibiono./Cyclorrhapha - ps29
Cyclorrhapha - ps30
Calyptratae/Drosophila - ps31
Drosophila - ps32
Sophophora - ps33
Obscura/Melanogaster_group - ps34
D_melanogaster_group - ps35
D_melanogaster_subgroup - ps36
sim./sech./mel. group - ps37
Drosophila_melanogaster - ps38
p-value
2e-02
2e-02
3e-02
4e-02
6e-02
Log-odds
0.5
1.0
1.5
2.0
2.5
3.0
3.5
4.0

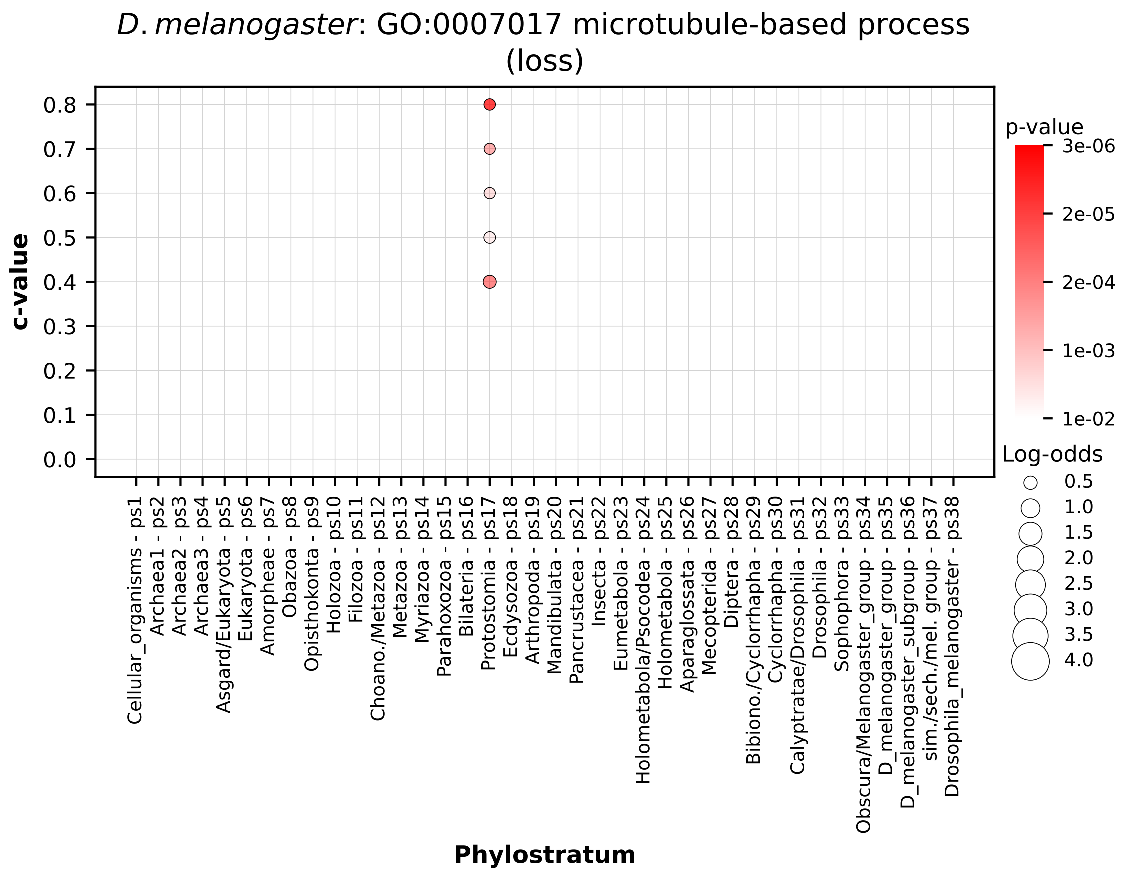

D. melanogaster: GO:0007017 microtubule-based process (loss)
c-value
0.0
0.1
0.2
0.3
0.4
0.5
0.6
0.7
0.8
p-value
3e-06
2e-05
2e-04
1e-03
1e-02
Log-odds
0.5
1.0
1.5
2.0
2.5
3.0
3.5
4.0
Cellular_organisms - ps1
Archaea1 - ps2
Archaea2 - ps3
Archaea3 - ps4
Asgard/Eukaryota - ps5
Eukaryota - ps6
Amorpheae - ps7
Obazoa - ps8
Opisthokonta - ps9
Holozoa - ps10
Filozoa - ps11
Choano./Metazoa - ps12
Metazoa - ps13
Myriazoa - ps14
Parahoxozoa - ps15
Bilateria - ps16
Protostomia - ps17
Ecdysozoa - ps18
Arthropoda - ps19
Mandibulata - ps20
Pancrustacea - ps21
Insecta - ps22
Eumetabola - ps23
Holometabola/Psocodea - ps24
Holometabola - ps25
Aparaglossata - ps26
Mecopterida - ps27
Diptera - ps28
Bibiono./Cyclorrhapha - ps29
Cyclorrhapha - ps30
Calyptratae/Drosophila - ps31
Drosophila - ps32
Sophophora - ps33
Obscura/Melanogaster_group - ps34
D_melanogaster_group - ps35
D_melanogaster_subgroup - ps36
sim./sech./mel. group - ps37
Drosophila_melanogaster - ps38
Phylostratum

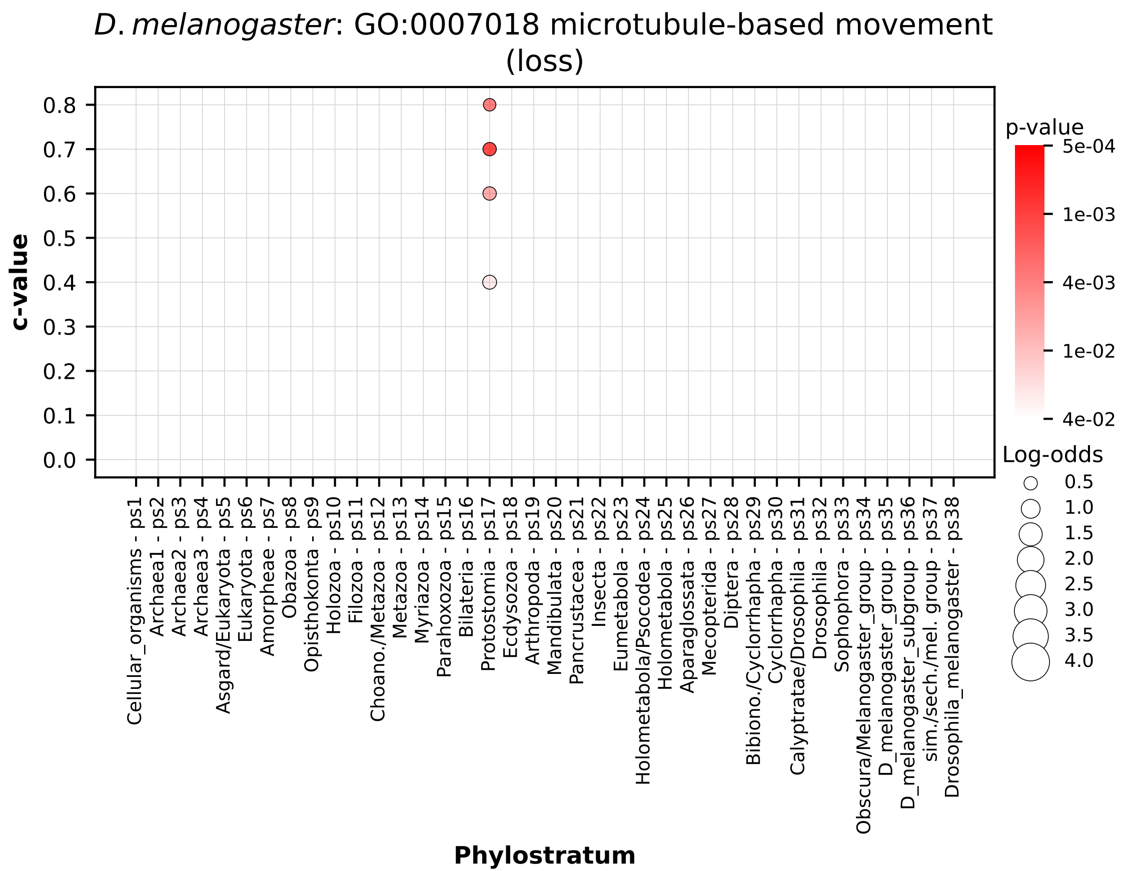
D. melanogaster: GO:0007018 microtubule-based movement (loss)
c-value
0.8
0.7
0.6
0.5
0.4
0.3
0.2
0.1
0.0
Phylostratum
Cellular_organisms - ps1
Archaea1 - ps2
Archaea2 - ps3
Archaea3 - ps4
Asgard/Eukaryota - ps5
Eukaryota - ps6
Amorpheae - ps7
Obazoa - ps8
Opisthokonta - ps9
Holozoa - ps10
Filozoa - ps11
Choano./Metazoa - ps12
Metazoa - ps13
Myriazoa - ps14
Parahoxozoa - ps15
Bilateria - ps16
Protostomia - ps17
Ecdysozoa - ps18
Arthropoda - ps19
Mandibulata - ps20
Pancrustacea - ps21
Insecta - ps22
Eumetabola - ps23
Holometabola/Psocodea - ps24
Holometabola - ps25
Aparaglossata - ps26
Mecopterida - ps27
Diptera - ps28
Bibiono./Cyclorrhapha - ps29
Cyclorrhapha - ps30
Calyptratae/Drosophila - ps31
Drosophila - ps32
Sophophora - ps33
Obscura/Melanogaster_group - ps34
D_melanogaster_group - ps35
D_melanogaster_subgroup - ps36
sim./sech./mel. group - ps37
Drosophila_melanogaster - ps38
p-value
5e-04
1e-03
4e-03
1e-02
4e-02
Log-odds
0.5
1.0
1.5
2.0
2.5
3.0
3.5
4.0

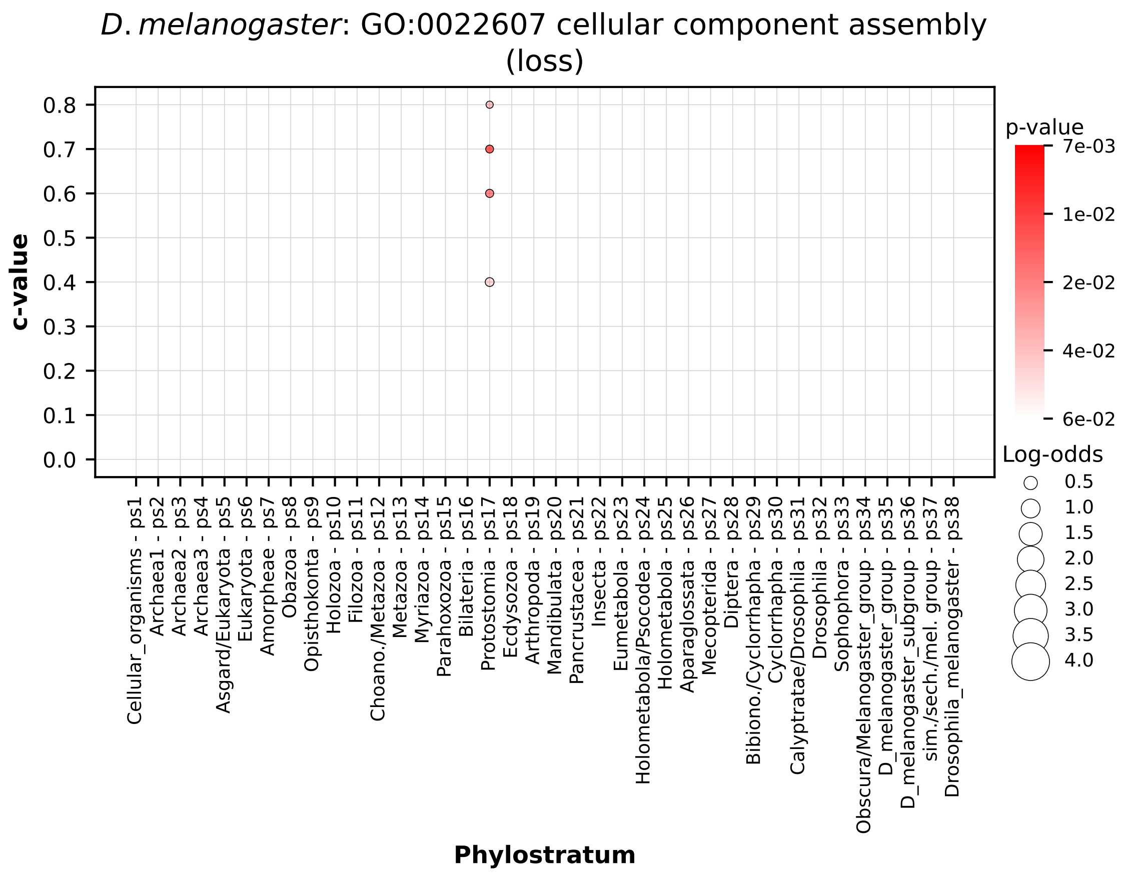

D. melanogaster: GO:0022607 cellular component assembly (loss)
c-value
0.0
0.1
0.2
0.3
0.4
0.5
0.6
0.7
0.8
Phylostratum
Cellular_organisms - ps1
Archaea1 - ps2
Archaea2 - ps3
Archaea3 - ps4
Asgard/Eukaryota - ps5
Eukaryota - ps6
Amorpheae - ps7
Obazoa - ps8
Opisthokonta - ps9
Holozoa - ps10
Filozoa - ps11
Choano./Metazoa - ps12
Metazoa - ps13
Myriazoa - ps14
Parahoxozoa - ps15
Bilateria - ps16
Protostomia - ps17
Ecdysozoa - ps18
Arthropoda - ps19
Mandibulata - ps20
Pancrustacea - ps21
Insecta - ps22
Eumetabola - ps23
Holometabola/Psocodea - ps24
Holometabola - ps25
Aparaglossata - ps26
Mecopterida - ps27
Diptera - ps28
Bibiono./Cyclorrhapha - ps29
Cyclorrhapha - ps30
Calyptratae/Drosophila - ps31
Drosophila - ps32
Sophophora - ps33
Obscura/Melanogaster_group - ps34
D_melanogaster_group - ps35
D_melanogaster_subgroup - ps36
sim./sech./mel. group - ps37
Drosophila_melanogaster - ps38
p-value
7e-03
1e-02
2e-02
4e-02
6e-02
Log-odds
0.5
1.0
1.5
2.0
2.5
3.0
3.5
4.0

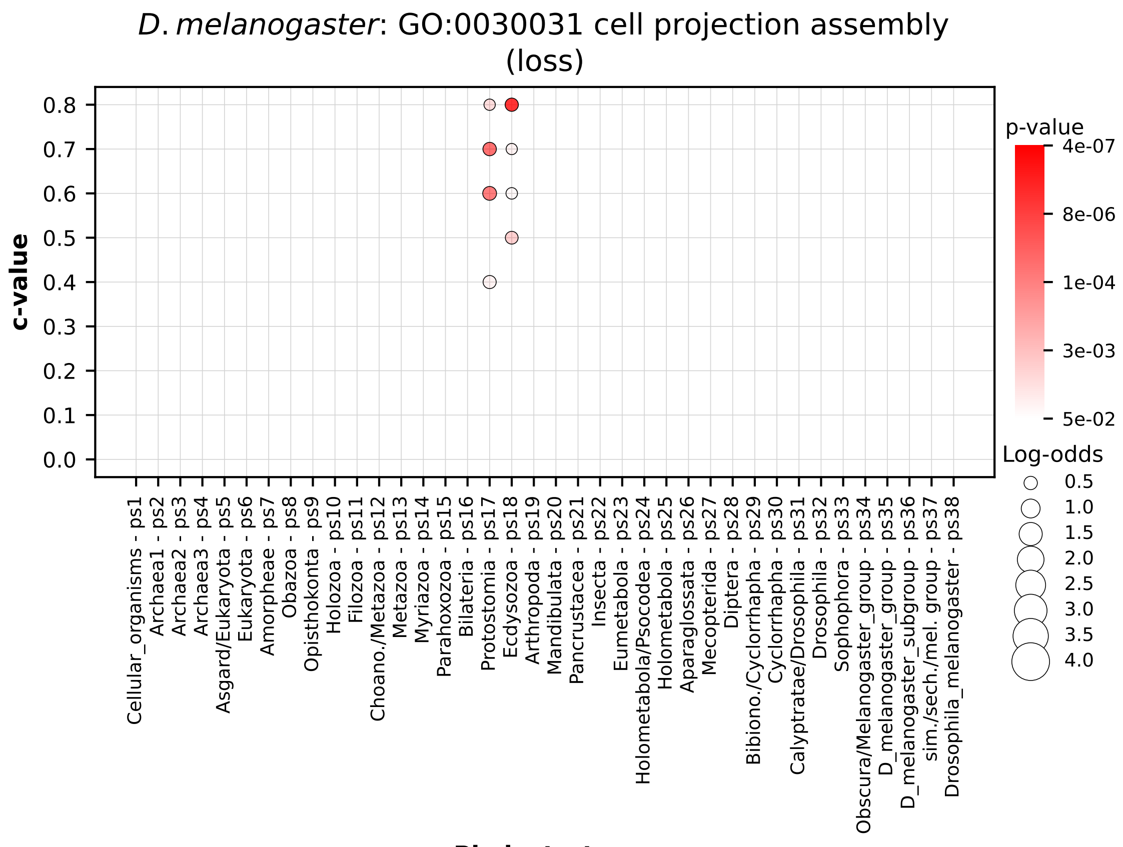

D. melanogaster: GO:0030031 cell projection assembly (loss)
c-value
0.0
0.1
0.2
0.3
0.4
0.5
0.6
0.7
0.8
Phylostratum
Cellular_organisms - ps1
Archaea1 - ps2
Archaea2 - ps3
Archaea3 - ps4
Asgard/Eukaryota - ps5
Eukaryota - ps6
Amorpheae - ps7
Obazoa - ps8
Opisthokonta - ps9
Holozoa - ps10
Filozoa - ps11
Choano./Metazoa - ps12
Metazoa - ps13
Myriazoa - ps14
Parahoxozoa - ps15
Bilateria - ps16
Protostomia - ps17
Ecdysozoa - ps18
Arthropoda - ps19
Mandibulata - ps20
Pancrustacea - ps21
Insecta - ps22
Eumetabola - ps23
Holometabola/Psocodea - ps24
Holometabola - ps25
Aparaglossata - ps26
Mecopterida - ps27
Diptera - ps28
Bibiono./Cyclorrhapha - ps29
Cyclorrhapha - ps30
Calyptratae/Drosophila - ps31
Drosophila - ps32
Sophophora - ps33
Obscura/Melanogaster_group - ps34
D_melanogaster_group - ps35
D_melanogaster_subgroup - ps36
sim./sech./mel. group - ps37
Drosophila_melanogaster - ps38
p-value
4e-07
8e-06
1e-04
3e-03
5e-02
Log-odds
0.5
1.0
1.5
2.0
2.5
3.0
3.5
4.0

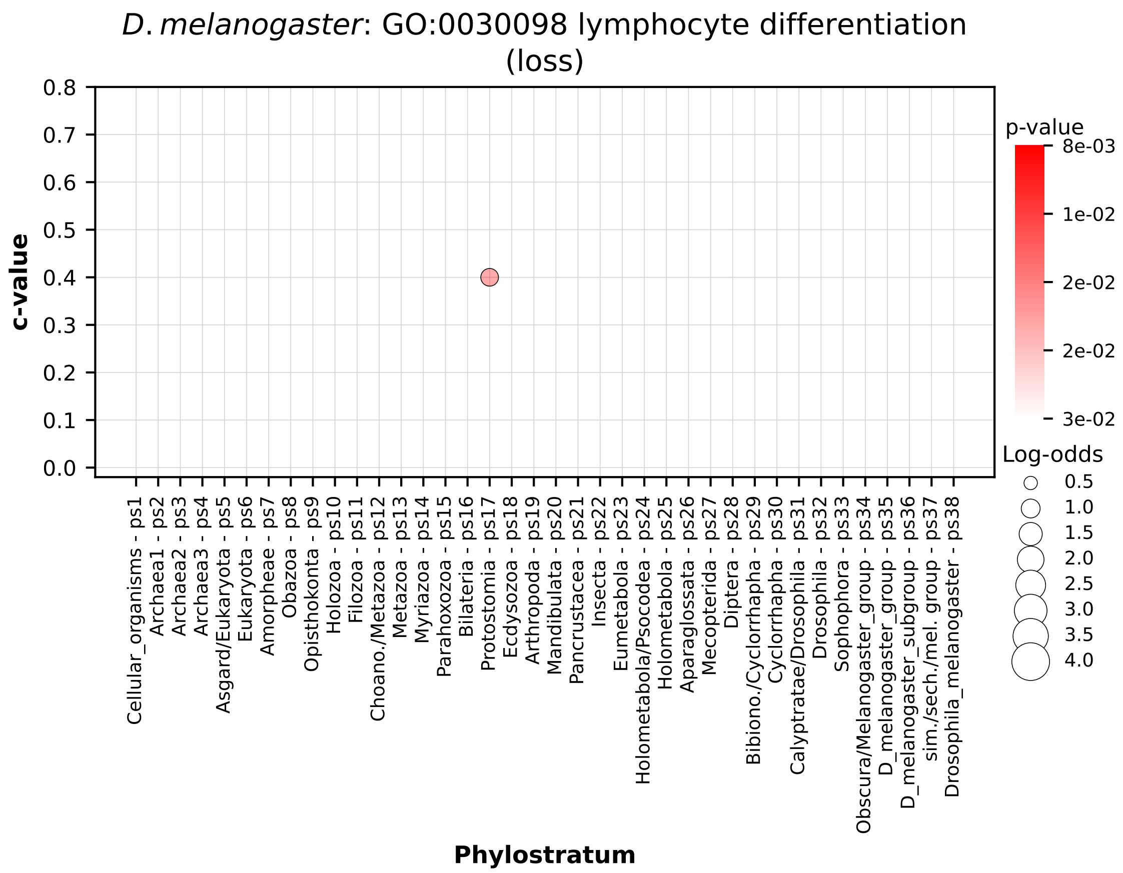

D. melanogaster: GO:0030098 lymphocyte differentiation (loss)
c-value
0.0
0.1
0.2
0.3
0.4
0.5
0.6
0.7
0.8
p-value
8e-03
1e-02
2e-02
2e-02
3e-02
Log-odds
0.5
1.0
1.5
2.0
2.5
3.0
3.5
4.0
Cellular_organisms - ps1
Archaea1 - ps2
Archaea2 - ps3
Archaea3 - ps4
Asgard/Eukaryota - ps5
Eukaryota - ps6
Amorpheae - ps7
Obazoa - ps8
Opisthokonta - ps9
Holozoa - ps10
Filozoa - ps11
Choano./Metazoa - ps12
Metazoa - ps13
Myriazoa - ps14
Parahoxozoa - ps15
Bilateria - ps16
Protostomia - ps17
Ecdysozoa - ps18
Arthropoda - ps19
Mandibulata - ps20
Pancrustacea - ps21
Insecta - ps22
Eumetabola - ps23
Holometabola/Psocodea - ps24
Holometabola - ps25
Aparaglossata - ps26
Mecopterida - ps27
Diptera - ps28
Bibiono./Cyclorrhapha - ps29
Cyclorrhapha - ps30
Calyptratae/Drosophila - ps31
Drosophila - ps32
Sophophora - ps33
Obscura/Melanogaster_group - ps34
D_melanogaster_group - ps35
D_melanogaster_subgroup - ps36
sim./sech./mel. group - ps37
Drosophila_melanogaster - ps38
Phylostratum

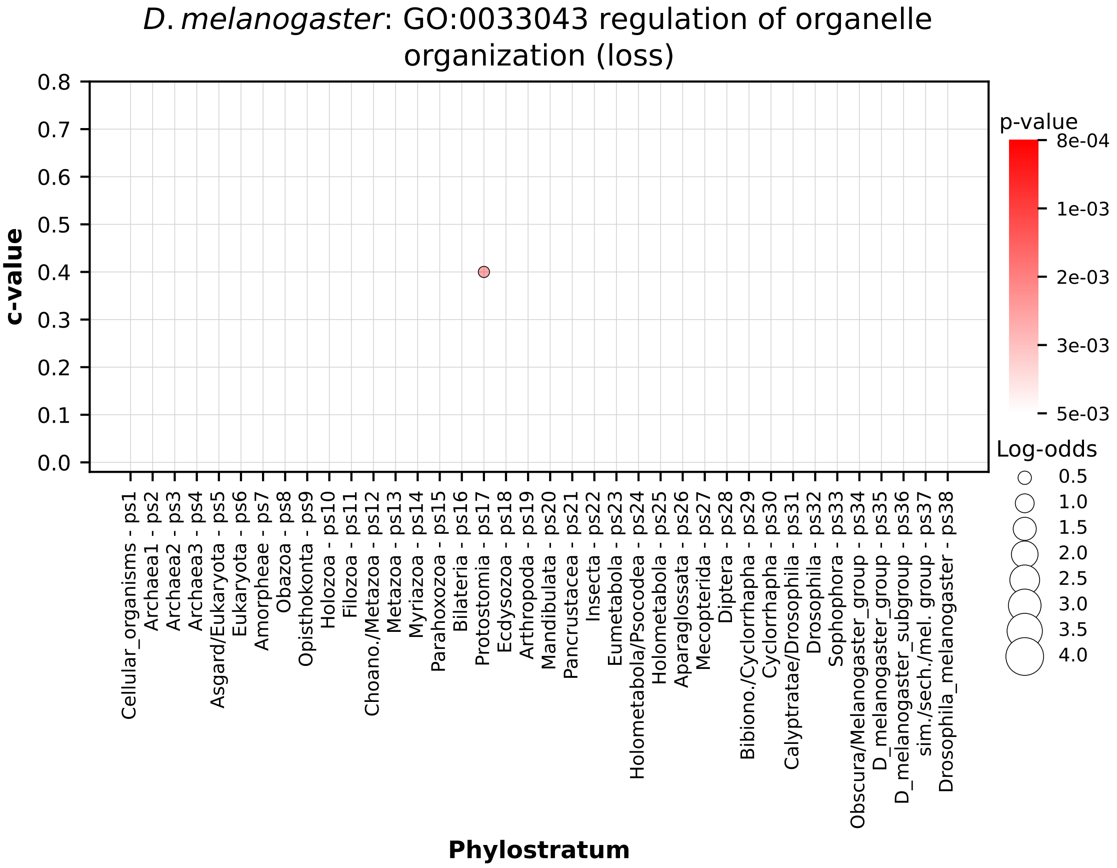

D. melanogaster: GO:0033043 regulation of organelle organization (loss)
c-value
0.8
0.7
0.6
0.5
0.4
0.3
0.2
0.1
0.0
Phylostratum
Cellular_organisms - ps1
Archaea1 - ps2
Archaea2 - ps3
Archaea3 - ps4
Asgard/Eukaryota - ps5
Eukaryota - ps6
Amorpheae - ps7
Obazoa - ps8
Opisthokonta - ps9
Holozoa - ps10
Filozoa - ps11
Choano./Metazoa - ps12
Metazoa - ps13
Myriazoa - ps14
Parahoxozoa - ps15
Bilateria - ps16
Protostomia - ps17
Ecdysozoa - ps18
Arthropoda - ps19
Mandibulata - ps20
Pancrustacea - ps21
Insecta - ps22
Eumetabola - ps23
Holometabola/Psocodea - ps24
Holometabola - ps25
Aparaglossata - ps26
Mecopterida - ps27
Diptera - ps28
Bibiono./Cyclorrhapha - ps29
Cyclorrhapha - ps30
Calyptratae/Drosophila - ps31
Drosophila - ps32
Sophophora - ps33
Obscura/Melanogaster_group - ps34
D_melanogaster_group - ps35
D_melanogaster_subgroup - ps36
sim./sech./mel. group - ps37
Drosophila_melanogaster - ps38
p-value
8e-04
1e-03
2e-03
3e-03
5e-03
Log-odds
0.5
1.0
1.5
2.0
2.5
3.0
3.5
4.0

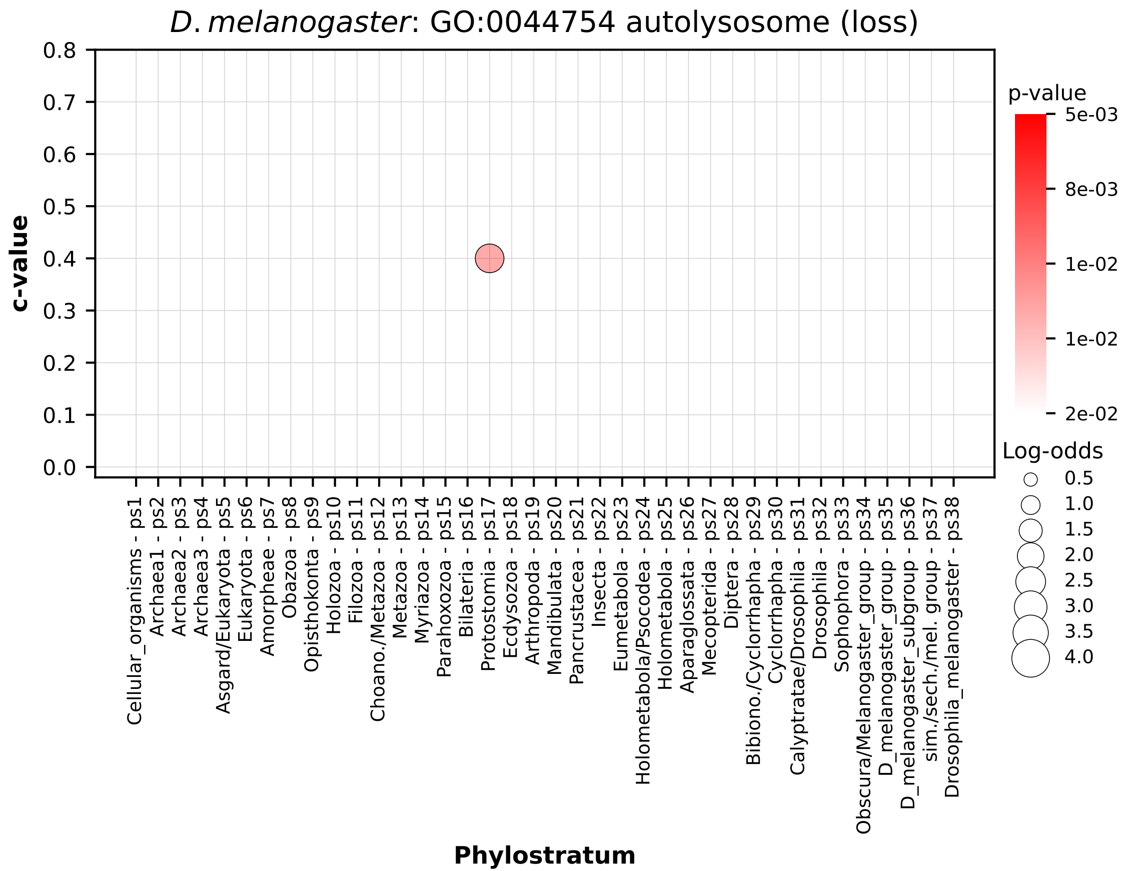

D. melanogaster: GO:0044754 autolysosome (loss)
c-value
0.8
0.7
0.6
0.5
0.4
0.3
0.2
0.1
0.0
Phylostratum
Cellular_organisms - ps1
Archaea1 - ps2
Archaea2 - ps3
Archaea3 - ps4
Asgard/Eukaryota - ps5
Eukaryota - ps6
Amorpheae - ps7
Obazoa - ps8
Opisthokonta - ps9
Holozoa - ps10
Filozoa - ps11
Choano./Metazoa - ps12
Metazoa - ps13
Myriazoa - ps14
Parahoxozoa - ps15
Bilateria - ps16
Protostomia - ps17
Ecdysozoa - ps18
Arthropoda - ps19
Mandibulata - ps20
Pancrustacea - ps21
Insecta - ps22
Eumetabola - ps23
Holometabola/Psocodea - ps24
Holometabola - ps25
Aparaglossata - ps26
Mecopterida - ps27
Diptera - ps28
Bibiono./Cyclorrhapha - ps29
Cyclorrhapha - ps30
Calyptratae/Drosophila - ps31
Drosophila - ps32
Sophophora - ps33
Obscura/Melanogaster_group - ps34
D_melanogaster_group - ps35
D_melanogaster_subgroup - ps36
sim./sech./mel. group - ps37
Drosophila_melanogaster - ps38
p-value
5e-03
8e-03
1e-02
1e-02
2e-02
Log-odds
0.5
1.0
1.5
2.0
2.5
3.0
3.5
4.0

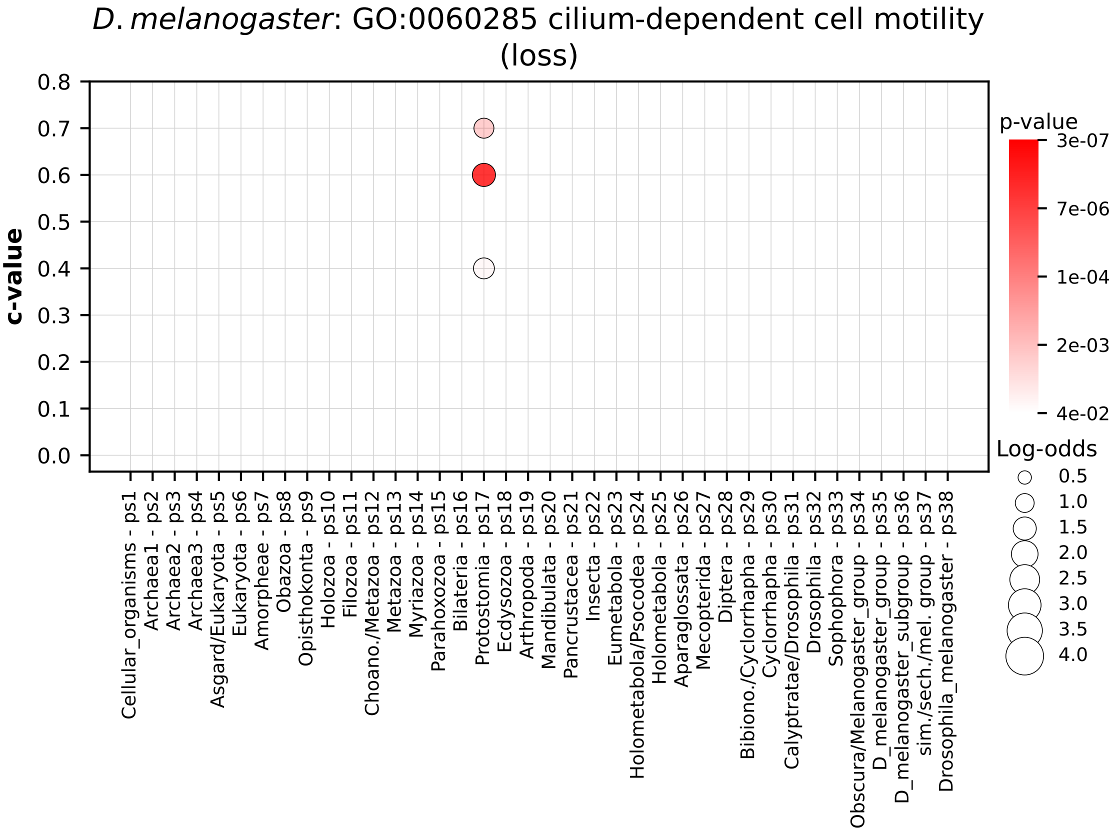

D. melanogaster: GO:0060285 cilium-dependent cell motility
(loss)
c-value
0.8
0.7
0.6
0.5
0.4
0.3
0.2
0.1
0.0
Phylostratum
Cellular_organisms - ps1
Archaea1 - ps2
Archaea2 - ps3
Archaea3 - ps4
Asgard/Eukaryota - ps5
Eukaryota - ps6
Amorpheae - ps7
Obazoa - ps8
Opisthokonta - ps9
Holozoa - ps10
Filozoa - ps11
Choano./Metazoa - ps12
Metazoa - ps13
Myriazoa - ps14
Parahoxozoa - ps15
Bilateria - ps16
Protostomia - ps17
Ecdysozoa - ps18
Arthropoda - ps19
Mandibulata - ps20
Pancrustacea - ps21
Insecta - ps22
Eumetabola - ps23
Holometabola/Psocodea - ps24
Holometabola - ps25
Aparaglossata - ps26
Mecopterida - ps27
Diptera - ps28
Bibiono./Cyclorrhapha - ps29
Cyclorrhapha - ps30
Calyptratae/Drosophila - ps31
Drosophila - ps32
Sophophora - ps33
Obscura/Melanogaster_group - ps34
D_melanogaster_group - ps35
D_melanogaster_subgroup - ps36
sim./sech./mel. group - ps37
Drosophila_melanogaster - ps38
p-value
3e-07
7e-06
1e-04
2e-03
4e-02
Log-odds
0.5
1.0
1.5
2.0
2.5
3.0
3.5
4.0

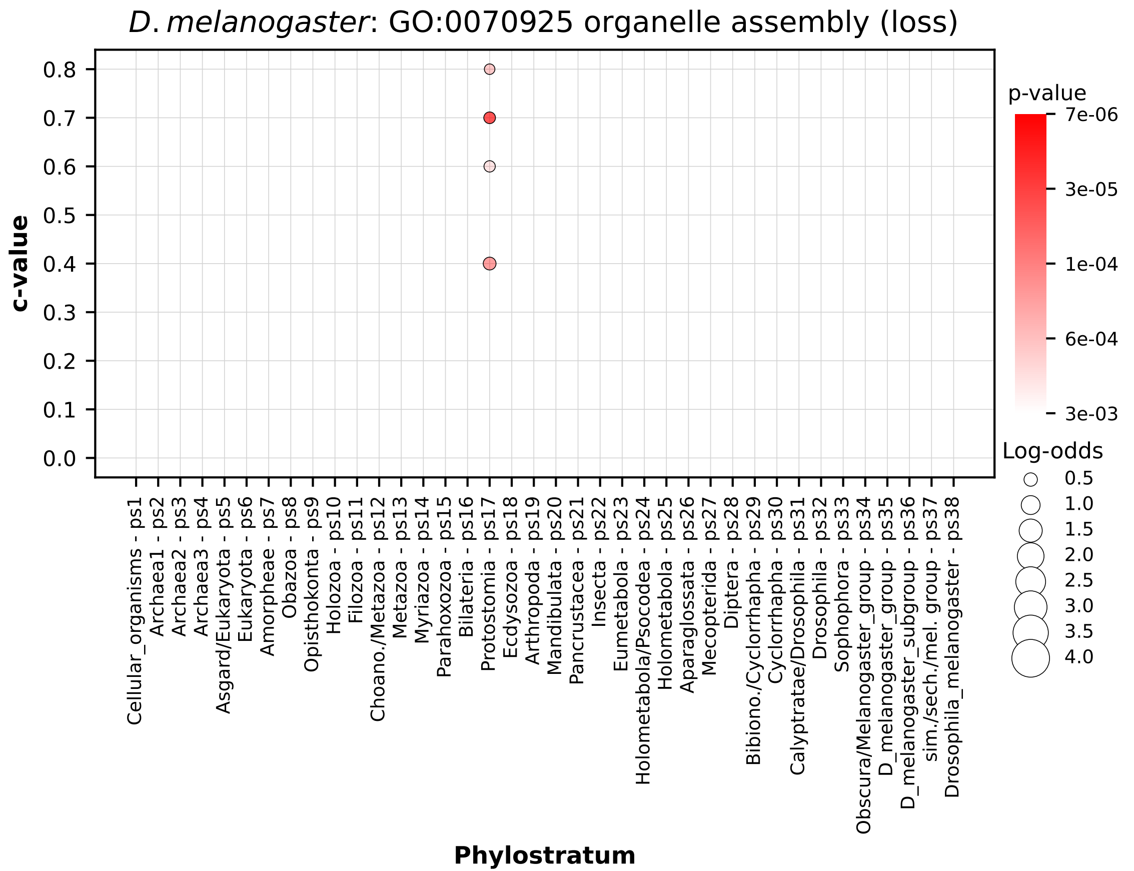

D. melanogaster: GO:0070925 organelle assembly (loss)
c-value
0.0
0.1
0.2
0.3
0.4
0.5
0.6
0.7
0.8
Phylostratum
Cellular_organisms - ps1
Archaea1 - ps2
Archaea2 - ps3
Archaea3 - ps4
Asgard/Eukaryota - ps5
Eukaryota - ps6
Amorpheae - ps7
Obazoa - ps8
Opisthokonta - ps9
Holozoa - ps10
Filozoa - ps11
Choano./Metazoa - ps12
Metazoa - ps13
Myriazoa - ps14
Parahoxozoa - ps15
Bilateria - ps16
Protostomia - ps17
Ecdysozoa - ps18
Arthropoda - ps19
Mandibulata - ps20
Pancrustacea - ps21
Insecta - ps22
Eumetabola - ps23
Holometabola/Psocodea - ps24
Holometabola - ps25
Aparaglossata - ps26
Mecopterida - ps27
Diptera - ps28
Bibiono./Cyclorrhapha - ps29
Cyclorrhapha - ps30
Calyptratae/Drosophila - ps31
Drosophila - ps32
Sophophora - ps33
Obscura/Melanogaster_group - ps34
D_melanogaster_group - ps35
D_melanogaster_subgroup - ps36
sim./sech./mel. group - ps37
Drosophila_melanogaster - ps38
p-value
7e-06
3e-05
1e-04
6e-04
3e-03
Log-odds
0.5
1.0
1.5
2.0
2.5
3.0
3.5
4.0

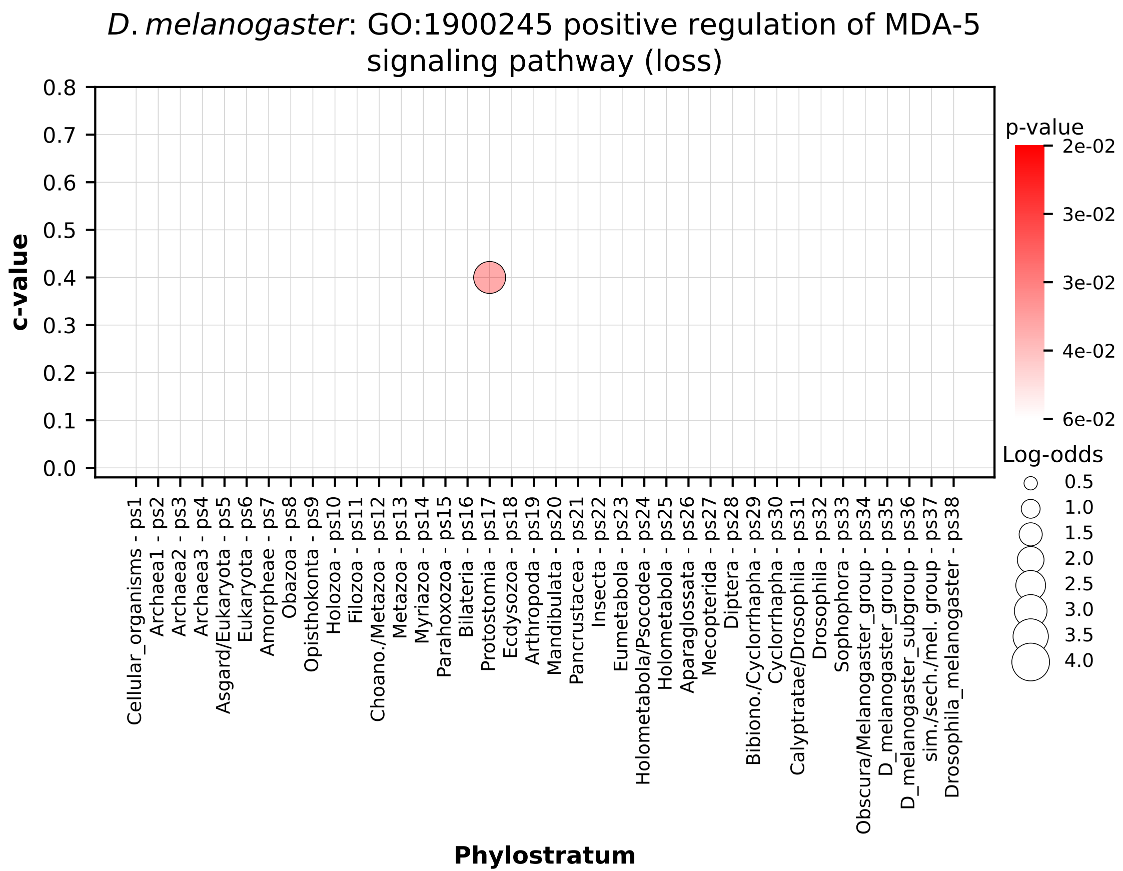

$D. melanogaster$: GO:1900245 positive regulation of MDA-5 signaling pathway (loss)
c-value
0.8
0.7
0.6
0.5
0.4
0.3
0.2
0.1
0.0
p-value
2e-02
3e-02
3e-02
4e-02
6e-02
Log-odds
0.5
1.0
1.5
2.0
2.5
3.0
3.5
4.0
Cellular_organisms - ps1
Archaea1 - ps2
Archaea2 - ps3
Archaea3 - ps4
Asgard/Eukaryota - ps5
Eukaryota - ps6
Amorpheae - ps7
Obazoa - ps8
Opisthokonta - ps9
Holozoa - ps10
Filozoa - ps11
Choano./Metazoa - ps12
Metazoa - ps13
Myriazoa - ps14
Parahoxozoa - ps15
Bilateria - ps16
Protostomia - ps17
Ecdysozoa - ps18
Arthropoda - ps19
Mandibulata - ps20
Pancrustacea - ps21
Insecta - ps22
Eumetabola - ps23
Holometabola/Psocodea - ps24
Holometabola - ps25
Aparaglossata - ps26
Mecopterida - ps27
Diptera - ps28
Bibiono./Cyclorrhapha - ps29
Cyclorrhapha - ps30
Calyptratae/Drosophila - ps31
Drosophila - ps32
Sophophora - ps33
Obscura/Melanogaster_group - ps34
D_melanogaster_group - ps35
D_melanogaster_subgroup - ps36
sim./sech./mel. group - ps37
Drosophila_melanogaster - ps38
Phylostratum

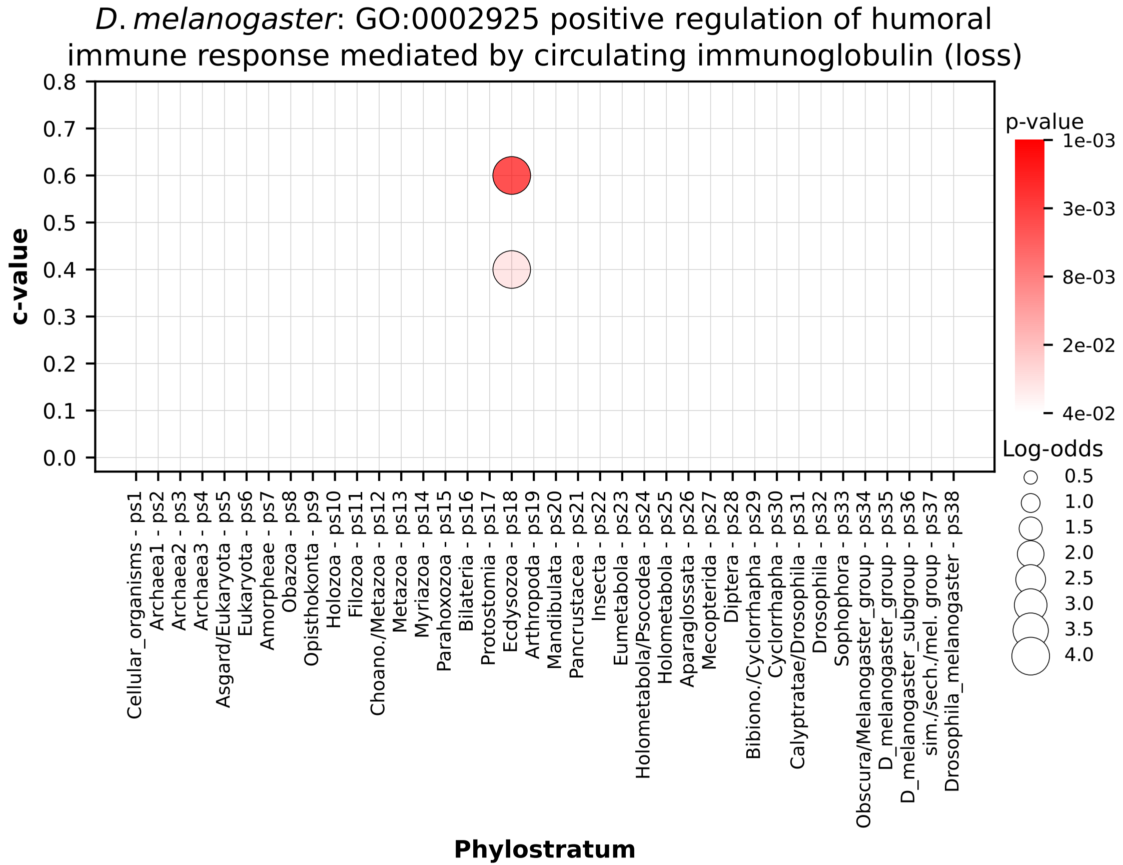

$D. melanogaster$: GO:0002925 positive regulation of humoral immune response mediated by circulating immunoglobulin (loss)
c-value
0.8
0.7
0.6
0.5
0.4
0.3
0.2
0.1
0.0
p-value
1e-03
3e-03
8e-03
2e-02
4e-02
Log-odds
0.5
1.0
1.5
2.0
2.5
3.0
3.5
4.0
Cellular_organisms - ps1
Archaea1 - ps2
Archaea2 - ps3
Archaea3 - ps4
Asgard/Eukaryota - ps5
Eukaryota - ps6
Amorpheae - ps7
Obazoa - ps8
Opisthokonta - ps9
Holozoa - ps10
Filozoa - ps11
Choano./Metazoa - ps12
Metazoa - ps13
Myriazoa - ps14
Parahoxozoa - ps15
Bilateria - ps16
Protostomia - ps17
Ecdysozoa - ps18
Arthropoda - ps19
Mandibulata - ps20
Pancrustacea - ps21
Insecta - ps22
Eumetabola - ps23
Holometabola/Psocodea - ps24
Holometabola - ps25
Aparaglossata - ps26
Mecopterida - ps27
Diptera - ps28
Bibiono./Cyclorrhapha - ps29
Cyclorrhapha - ps30
Calyptratae/Drosophila - ps31
Drosophila - ps32
Sophophora - ps33
Obscura/Melanogaster_group - ps34
D_melanogaster_group - ps35
D_melanogaster_subgroup - ps36
sim./sech./mel. group - ps37
Drosophila_melanogaster - ps38
Phylostratum

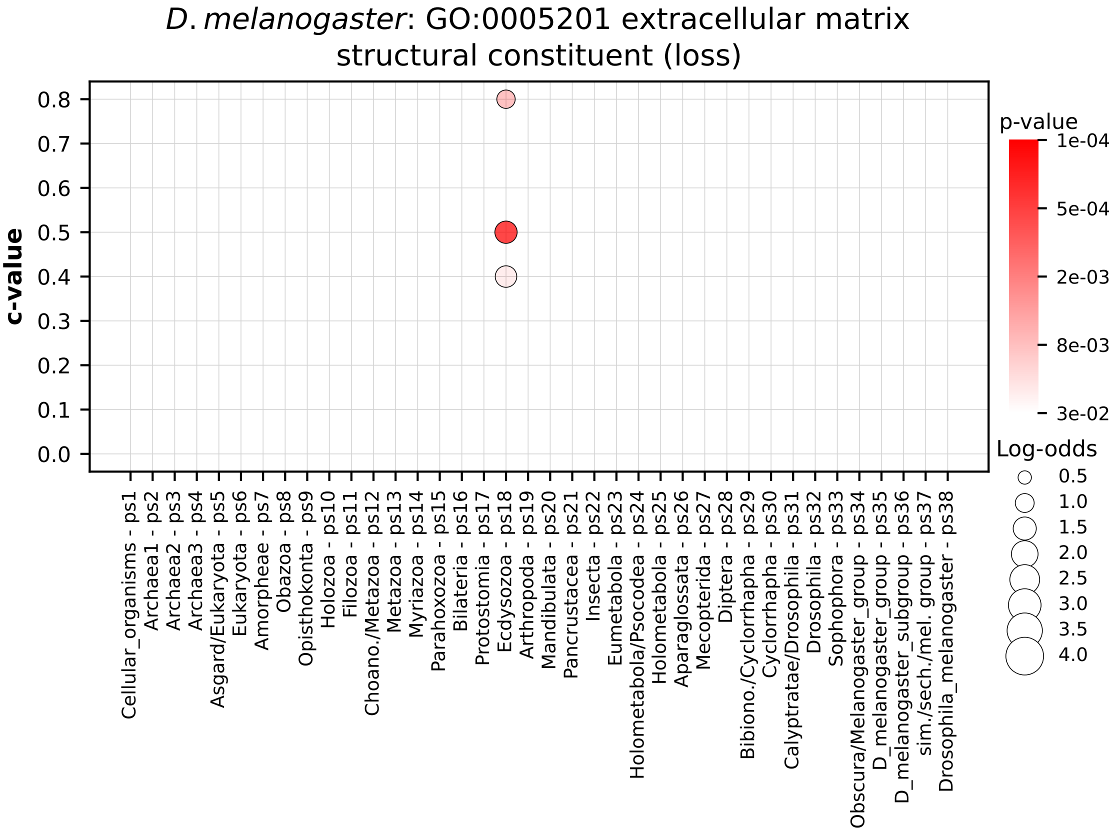
D. melanogaster: GO:0005201 extracellular matrix structural constituent (loss)
c-value
0.0
0.1
0.2
0.3
0.4
0.5
0.6
0.7
0.8
p-value
1e-04
5e-04
2e-03
8e-03
3e-02
Log-odds
0.5
1.0
1.5
2.0
2.5
3.0
3.5
4.0
Cellular_organisms - ps1
Archaea1 - ps2
Archaea2 - ps3
Archaea3 - ps4
Asgard/Eukaryota - ps5
Eukaryota - ps6
Amorpheae - ps7
Obazoa - ps8
Opisthokonta - ps9
Holozoa - ps10
Filozoa - ps11
Choano./Metazoa - ps12
Metazoa - ps13
Myriazoa - ps14
Parahoxozoa - ps15
Bilateria - ps16
Protostomia - ps17
Ecdysozoa - ps18
Arthropoda - ps19
Mandibulata - ps20
Pancrustacea - ps21
Insecta - ps22
Eumetabola - ps23
Holometabola/Psocodea - ps24
Holometabola - ps25
Aparaglossata - ps26
Mecopterida - ps27
Diptera - ps28
Bibiono./Cyclorrhapha - ps29
Cyclorrhapha - ps30
Calyptratae/Drosophila - ps31
Drosophila - ps32
Sophophora - ps33
Obscura/Melanogaster_group - ps34
D_melanogaster_group - ps35
D_melanogaster_subgroup - ps36
sim./sech./mel. group - ps37
Drosophila_melanogaster - ps38
Phylostratum

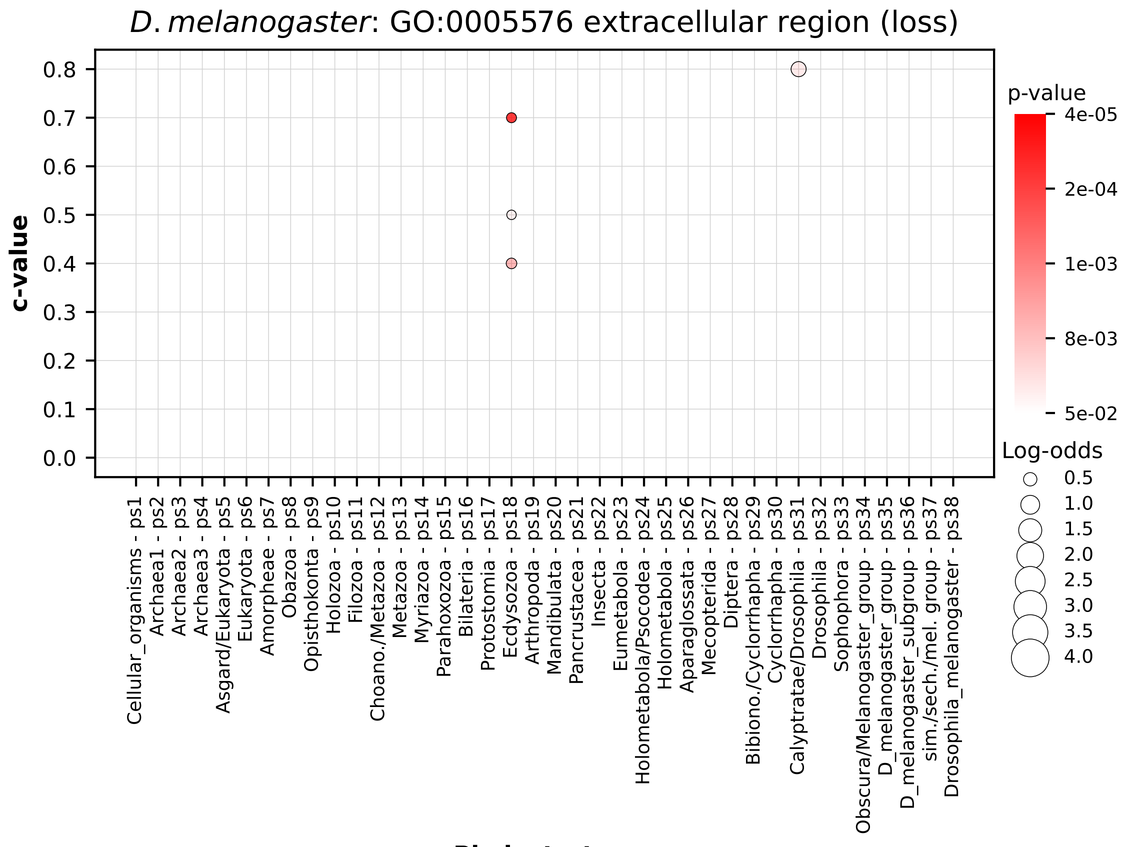

D. melanogaster: GO:0005576 extracellular region (loss)
c-value
0.0
0.1
0.2
0.3
0.4
0.5
0.6
0.7
0.8
p-value
4e-05
2e-04
1e-03
8e-03
5e-02
Log-odds
0.5
1.0
1.5
2.0
2.5
3.0
3.5
4.0
Cellular_organisms - ps1
Archaea1 - ps2
Archaea2 - ps3
Archaea3 - ps4
Asgard/Eukaryota - ps5
Eukaryota - ps6
Amorpheae - ps7
Obazoa - ps8
Opisthokonta - ps9
Holozoa - ps10
Filozoa - ps11
Choano./Metazoa - ps12
Metazoa - ps13
Myriazoa - ps14
Parahoxozoa - ps15
Bilateria - ps16
Protostomia - ps17
Ecdysozoa - ps18
Arthropoda - ps19
Mandibulata - ps20
Pancrustacea - ps21
Insecta - ps22
Eumetabola - ps23
Holometabola/Psocodea - ps24
Holometabola - ps25
Aparaglossata - ps26
Mecopterida - ps27
Diptera - ps28
Bibiono./Cyclorrhapha - ps29
Cyclorrhapha - ps30
Calyptratae/Drosophila - ps31
Drosophila - ps32
Sophophora - ps33
Obscura/Melanogaster_group - ps34
D_melanogaster_group - ps35
D_melanogaster_subgroup - ps36
sim./sech./mel. group - ps37
Drosophila_melanogaster - ps38
Phylostratum

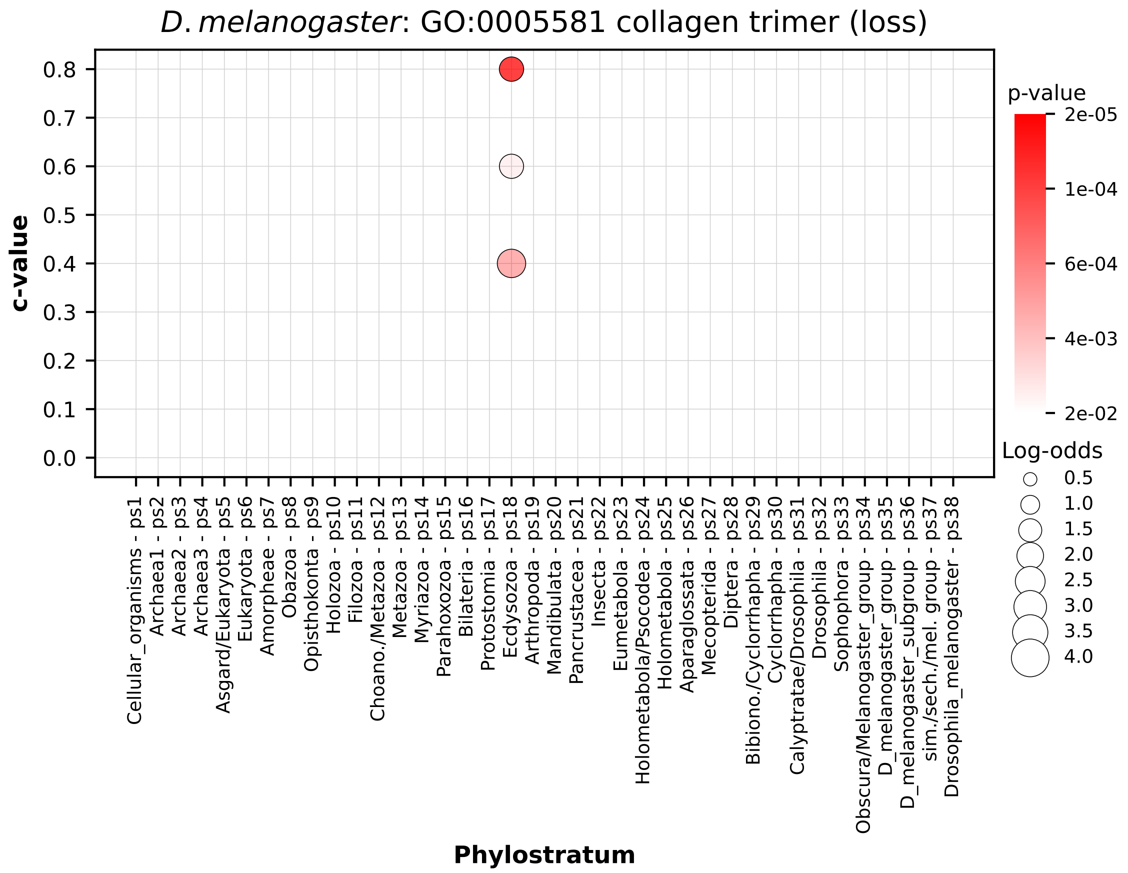

D. melanogaster: GO:0005581 collagen trimer (loss)
c-value
0.0
0.1
0.2
0.3
0.4
0.5
0.6
0.7
0.8
Phylostratum
Cellular_organisms - ps1
Archaea1 - ps2
Archaea2 - ps3
Archaea3 - ps4
Asgard/Eukaryota - ps5
Eukaryota - ps6
Amorpheae - ps7
Obazoa - ps8
Opisthokonta - ps9
Holozoa - ps10
Filozoa - ps11
Choano./Metazoa - ps12
Metazoa - ps13
Myriazoa - ps14
Parahoxozoa - ps15
Bilateria - ps16
Protostomia - ps17
Ecdysozoa - ps18
Arthropoda - ps19
Mandibulata - ps20
Pancrustacea - ps21
Insecta - ps22
Eumetabola - ps23
Holometabola/Psocodea - ps24
Holometabola - ps25
Aparaglossata - ps26
Mecopterida - ps27
Diptera - ps28
Bibiono./Cyclorrhapha - ps29
Cyclorrhapha - ps30
Calyptratae/Drosophila - ps31
Drosophila - ps32
Sophophora - ps33
Obscura/Melanogaster_group - ps34
D_melanogaster_group - ps35
D_melanogaster_subgroup - ps36
sim./sech./mel. group - ps37
Drosophila_melanogaster - ps38
p-value
2e-05
1e-04
6e-04
4e-03
2e-02
Log-odds
0.5
1.0
1.5
2.0
2.5
3.0
3.5
4.0

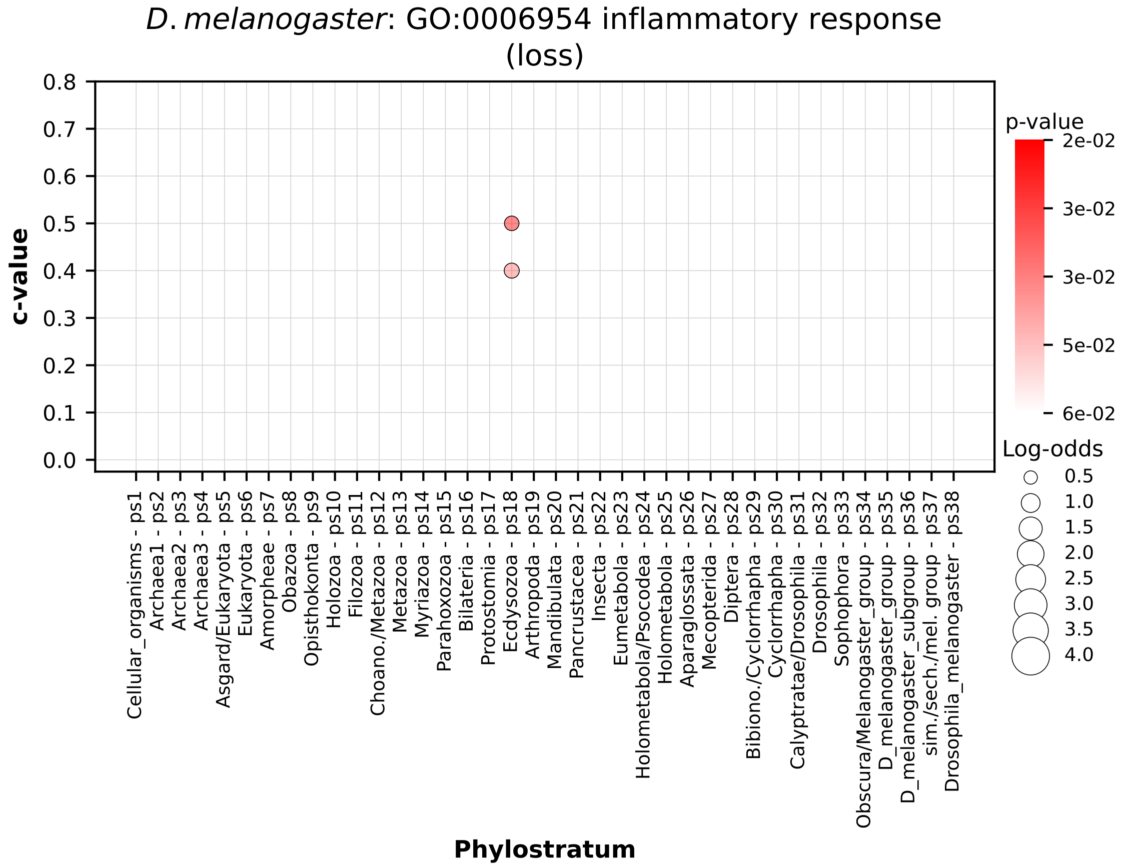

D. melanogaster: GO:0006954 inflammatory response
(loss)
c-value
0.8
0.7
0.6
0.5
0.4
0.3
0.2
0.1
0.0
Phylostratum
Cellular_organisms - ps1
Archaea1 - ps2
Archaea2 - ps3
Archaea3 - ps4
Asgard/Eukaryota - ps5
Eukaryota - ps6
Amorpheae - ps7
Obazoa - ps8
Opisthokonta - ps9
Holozoa - ps10
Filozoa - ps11
Choano./Metazoa - ps12
Metazoa - ps13
Myriazoa - ps14
Parahoxozoa - ps15
Bilateria - ps16
Protostomia - ps17
Ecdysozoa - ps18
Arthropoda - ps19
Mandibulata - ps20
Pancrustacea - ps21
Insecta - ps22
Eumetabola - ps23
Holometabola/Psocodea - ps24
Holometabola - ps25
Aparaglossata - ps26
Mecopterida - ps27
Diptera - ps28
Bibiono./Cyclorrhapha - ps29
Cyclorrhapha - ps30
Calyptratae/Drosophila - ps31
Drosophila - ps32
Sophophora - ps33
Obscura/Melanogaster_group - ps34
D_melanogaster_group - ps35
D_melanogaster_subgroup - ps36
sim./sech./mel. group - ps37
Drosophila_melanogaster - ps38
p-value
2e-02
3e-02
3e-02
5e-02
6e-02
Log-odds
0.5
1.0
1.5
2.0
2.5
3.0
3.5
4.0

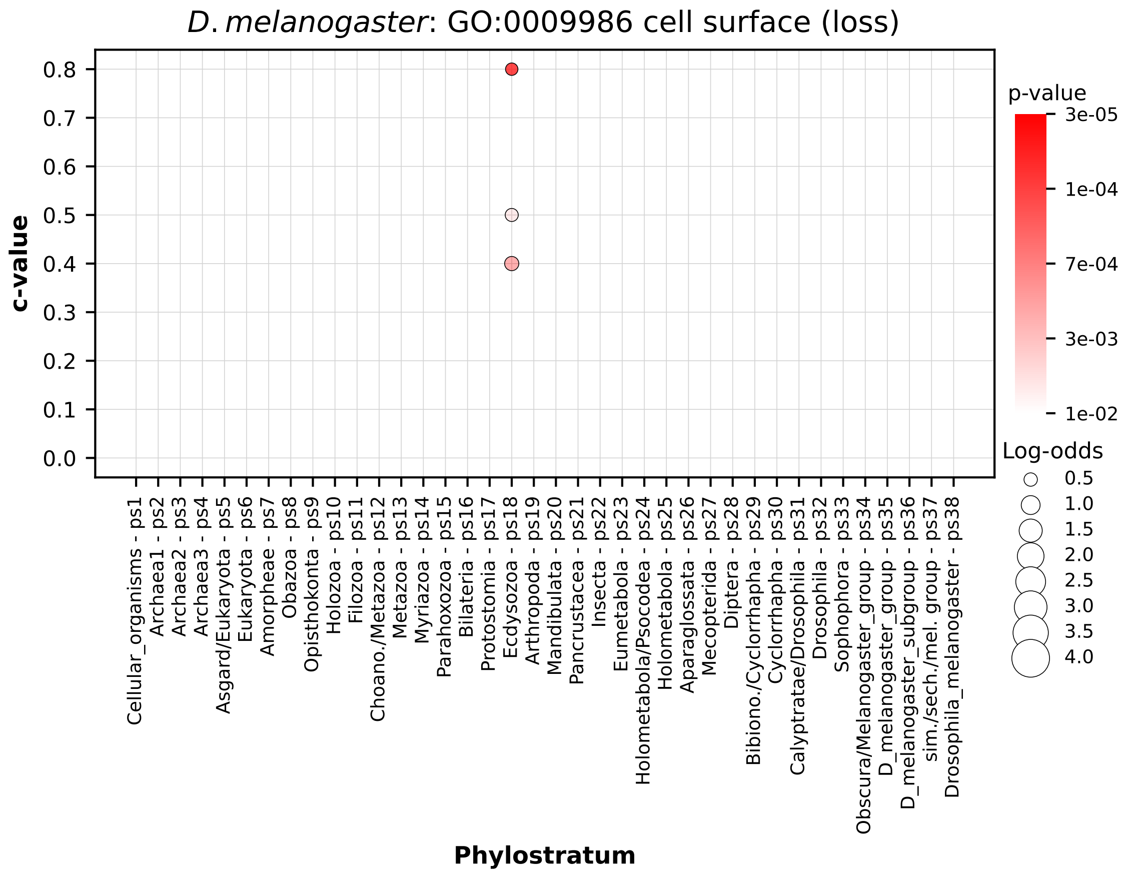

D. melanogaster: GO:0009986 cell surface (loss)
c-value
0.0
0.1
0.2
0.3
0.4
0.5
0.6
0.7
0.8
Phylostratum
Cellular_organisms - ps1
Archaea1 - ps2
Archaea2 - ps3
Archaea3 - ps4
Asgard/Eukaryota - ps5
Eukaryota - ps6
Amorpheae - ps7
Obazoa - ps8
Opisthokonta - ps9
Holozoa - ps10
Filozoa - ps11
Choano./Metazoa - ps12
Metazoa - ps13
Myriazoa - ps14
Parahoxozoa - ps15
Bilateria - ps16
Protostomia - ps17
Ecdysozoa - ps18
Arthropoda - ps19
Mandibulata - ps20
Pancrustacea - ps21
Insecta - ps22
Eumetabola - ps23
Holometabola/Psocodea - ps24
Holometabola - ps25
Aparaglossata - ps26
Mecopterida - ps27
Diptera - ps28
Bibiono./Cyclorrhapha - ps29
Cyclorrhapha - ps30
Calyptratae/Drosophila - ps31
Drosophila - ps32
Sophophora - ps33
Obscura/Melanogaster_group - ps34
D_melanogaster_group - ps35
D_melanogaster_subgroup - ps36
sim./sech./mel. group - ps37
Drosophila_melanogaster - ps38
p-value
3e-05
1e-04
7e-04
3e-03
1e-02
Log-odds
0.5
1.0
1.5
2.0
2.5
3.0
3.5
4.0

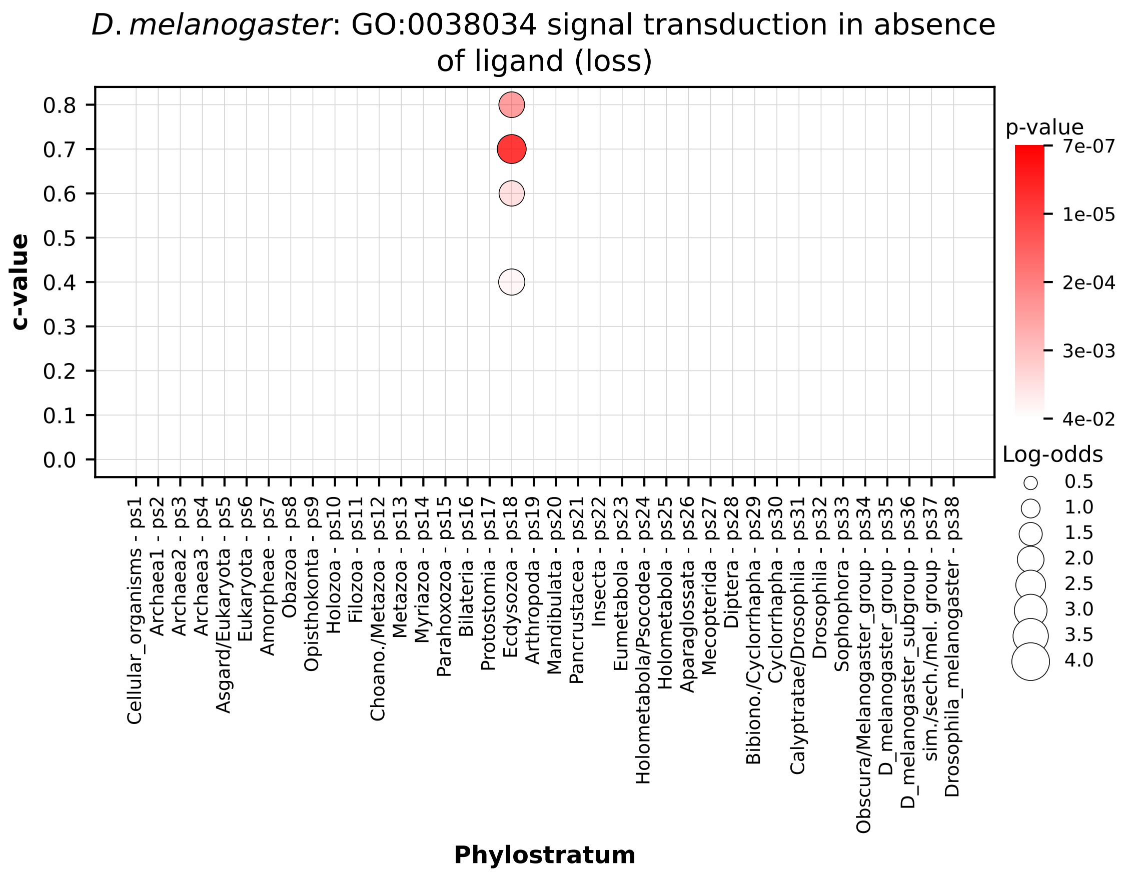

D. melanogaster: GO:0038034 signal transduction in absence of ligand (loss)
c-value
0.8
0.7
0.6
0.5
0.4
0.3
0.2
0.1
0.0
p-value
7e-07
1e-05
2e-04
3e-03
4e-02
Log-odds
0.5
1.0
1.5
2.0
2.5
3.0
3.5
4.0
Cellular_organisms - ps1
Archaea1 - ps2
Archaea2 - ps3
Archaea3 - ps4
Asgard/Eukaryota - ps5
Eukaryota - ps6
Amorpheae - ps7
Obazoa - ps8
Opisthokonta - ps9
Holozoa - ps10
Filozoa - ps11
Choano./Metazoa - ps12
Metazoa - ps13
Myriazoa - ps14
Parahoxozoa - ps15
Bilateria - ps16
Protostomia - ps17
Ecdysozoa - ps18
Arthropoda - ps19
Mandibulata - ps20
Pancrustacea - ps21
Insecta - ps22
Eumetabola - ps23
Holometabola/Psocodea - ps24
Holometabola - ps25
Aparaglossata - ps26
Mecopterida - ps27
Diptera - ps28
Bibiono./Cyclorrhapha - ps29
Cyclorrhapha - ps30
Calyptratae/Drosophila - ps31
Drosophila - ps32
Sophophora - ps33
Obscura/Melanogaster_group - ps34
D_melanogaster_group - ps35
D_melanogaster_subgroup - ps36
sim./sech./mel. group - ps37
Drosophila_melanogaster - ps38
Phylostratum

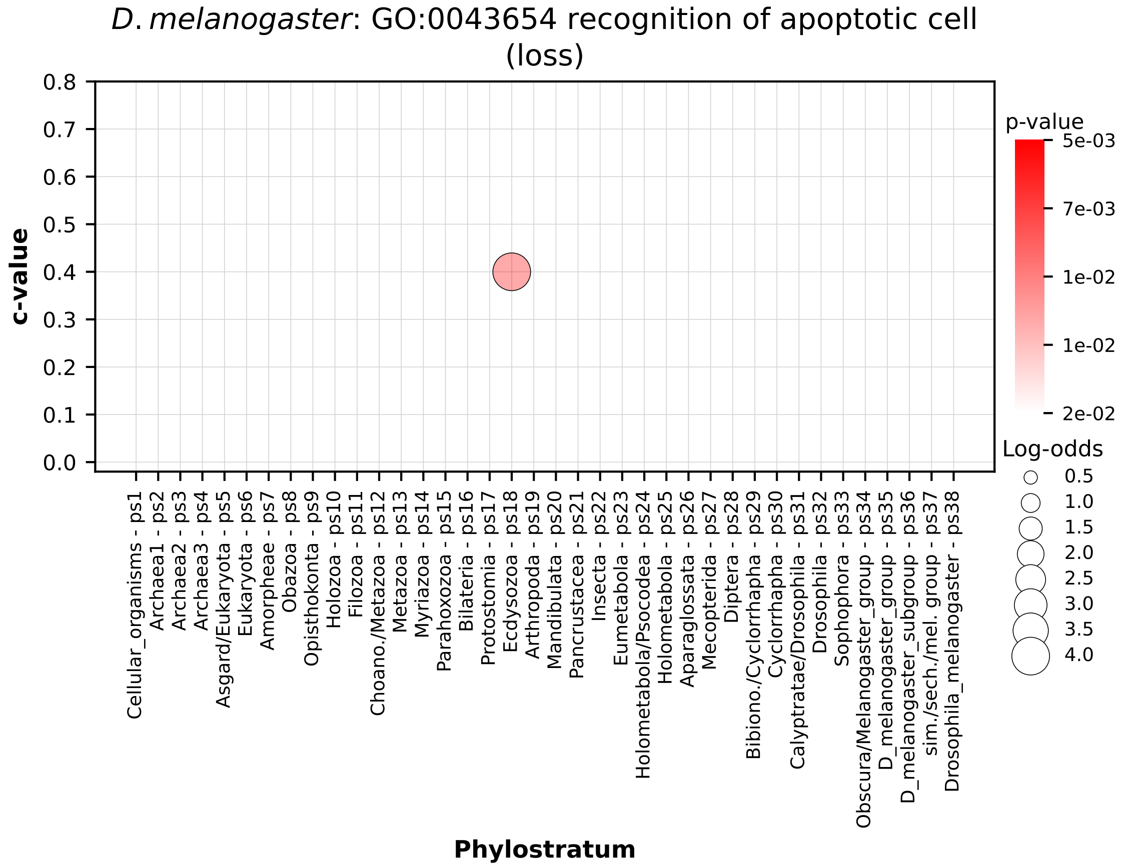
D. melanogaster: GO:0043654 recognition of apoptotic cell (loss)
c-value
0.8
0.7
0.6
0.5
0.4
0.3
0.2
0.1
0.0
Phylostratum
Cellular_organisms - ps1
Archaea1 - ps2
Archaea2 - ps3
Archaea3 - ps4
Asgard/Eukaryota - ps5
Eukaryota - ps6
Amorpheae - ps7
Obazoa - ps8
Opisthokonta - ps9
Holozoa - ps10
Filozoa - ps11
Choano./Metazoa - ps12
Metazoa - ps13
Myriazoa - ps14
Parahoxozoa - ps15
Bilateria - ps16
Protostomia - ps17
Ecdysozoa - ps18
Arthropoda - ps19
Mandibulata - ps20
Pancrustacea - ps21
Insecta - ps22
Eumetabola - ps23
Holometabola/Psocodea - ps24
Holometabola - ps25
Aparaglossata - ps26
Mecopterida - ps27
Diptera - ps28
Bibiono./Cyclorrhapha - ps29
Cyclorrhapha - ps30
Calyptratae/Drosophila - ps31
Drosophila - ps32
Sophophora - ps33
Obscura/Melanogaster_group - ps34
D_melanogaster_group - ps35
D_melanogaster_subgroup - ps36
sim./sech./mel. group - ps37
Drosophila_melanogaster - ps38
p-value
5e-03
7e-03
1e-02
1e-02
2e-02
Log-odds
0.5
1.0
1.5
2.0
2.5
3.0
3.5
4.0

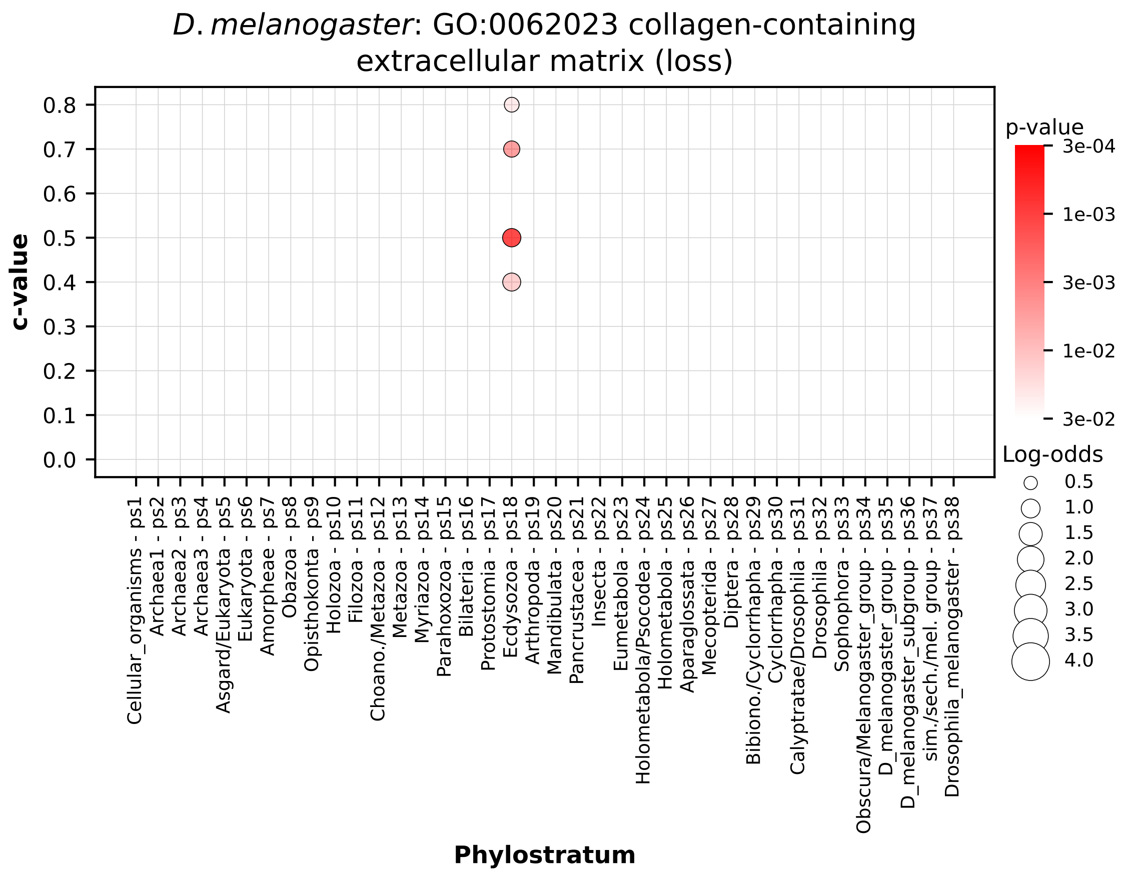

D. melanogaster: GO:0062023 collagen-containing extracellular matrix (loss)
c-value
0.0
0.1
0.2
0.3
0.4
0.5
0.6
0.7
0.8
p-value
3e-04
1e-03
3e-03
1e-02
3e-02
Log-odds
0.5
1.0
1.5
2.0
2.5
3.0
3.5
4.0
Cellular_organisms - ps1
Archaea1 - ps2
Archaea2 - ps3
Archaea3 - ps4
Asgard/Eukaryota - ps5
Eukaryota - ps6
Amorpheae - ps7
Obazoa - ps8
Opisthokonta - ps9
Holozoa - ps10
Filozoa - ps11
Choano./Metazoa - ps12
Metazoa - ps13
Myriazoa - ps14
Parahoxozoa - ps15
Bilateria - ps16
Protostomia - ps17
Ecdysozoa - ps18
Arthropoda - ps19
Mandibulata - ps20
Pancrustacea - ps21
Insecta - ps22
Eumetabola - ps23
Holometabola/Psocodea - ps24
Holometabola - ps25
Aparaglossata - ps26
Mecopterida - ps27
Diptera - ps28
Bibiono./Cyclorrhapha - ps29
Cyclorrhapha - ps30
Calyptratae/Drosophila - ps31
Drosophila - ps32
Sophophora - ps33
Obscura/Melanogaster_group - ps34
D_melanogaster_group - ps35
D_melanogaster_subgroup - ps36
sim./sech./mel. group - ps37
Drosophila_melanogaster - ps38
Phylostratum

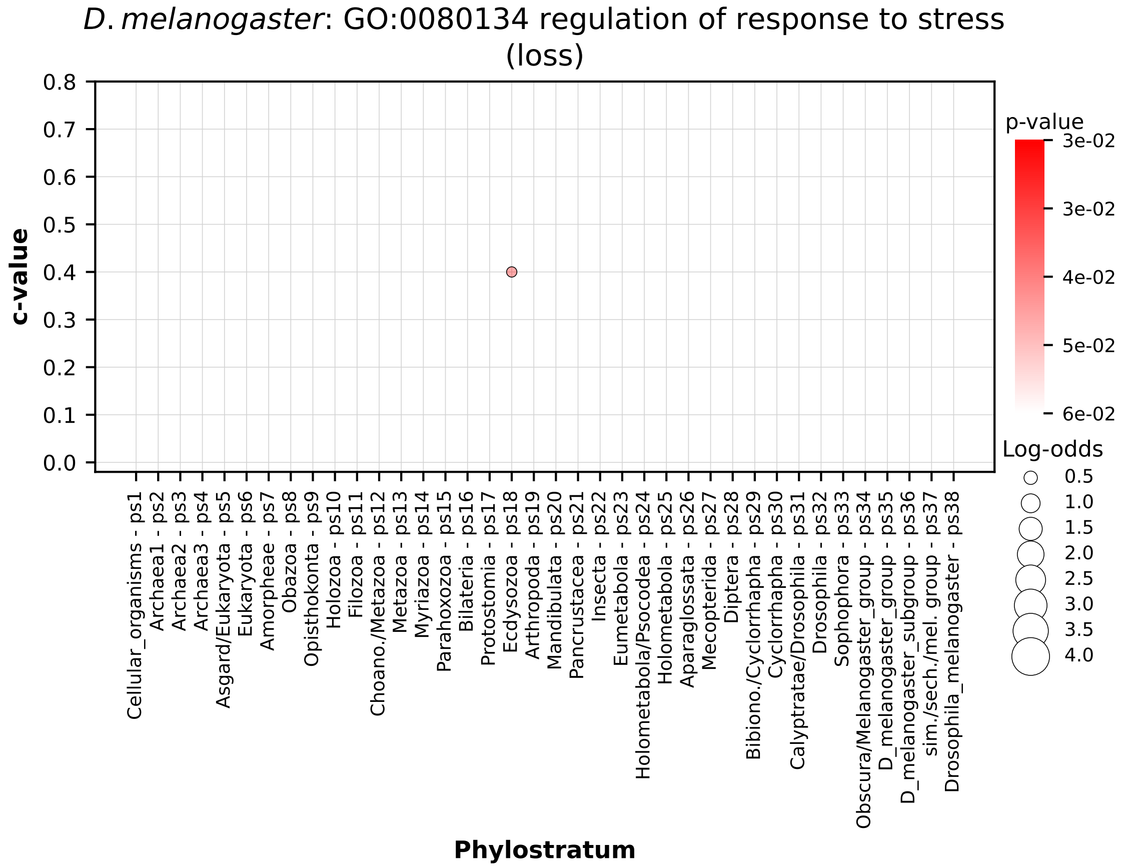

D. melanogaster: GO:0080134 regulation of response to stress (loss)
c-value
0.8
0.7
0.6
0.5
0.4
0.3
0.2
0.1
0.0
p-value
3e-02
3e-02
4e-02
5e-02
6e-02
Log-odds
0.5
1.0
1.5
2.0
2.5
3.0
3.5
4.0
Cellular_organisms - ps1
Archaea1 - ps2
Archaea2 - ps3
Archaea3 - ps4
Asgard/Eukaryota - ps5
Eukaryota - ps6
Amorpheae - ps7
Obazoa - ps8
Opisthokonta - ps9
Holozoa - ps10
Filozoa - ps11
Choano./Metazoa - ps12
Metazoa - ps13
Myriazoa - ps14
Parahoxozoa - ps15
Bilateria - ps16
Protostomia - ps17
Ecdysozoa - ps18
Arthropoda - ps19
Mandibulata - ps20
Pancrustacea - ps21
Insecta - ps22
Eumetabola - ps23
Holometabola/Psocodea - ps24
Holometabola - ps25
Aparaglossata - ps26
Mecopterida - ps27
Diptera - ps28
Bibiono./Cyclorrhapha - ps29
Cyclorrhapha - ps30
Calyptratae/Drosophila - ps31
Drosophila - ps32
Sophophora - ps33
Obscura/Melanogaster_group - ps34
D_melanogaster_group - ps35
D_melanogaster_subgroup - ps36
sim./sech./mel. group - ps37
Drosophila_melanogaster - ps38
Phylostratum

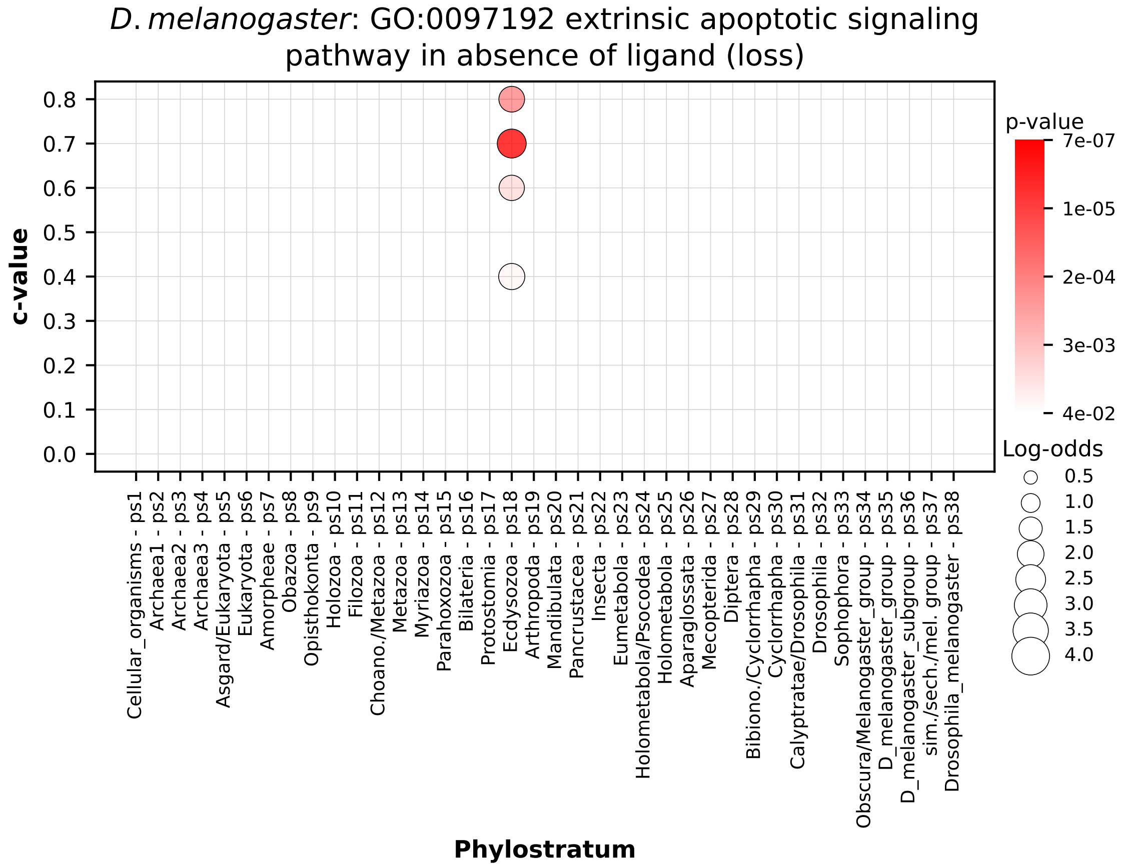

D. melanogaster: GO:0097192 extrinsic apoptotic signaling pathway in absence of ligand (loss)
c-value
0.0
0.1
0.2
0.3
0.4
0.5
0.6
0.7
0.8
Phylostratum
Cellular_organisms - ps1
Archaea1 - ps2
Archaea2 - ps3
Archaea3 - ps4
Asgard/Eukaryota - ps5
Eukaryota - ps6
Amorpheae - ps7
Obazoa - ps8
Opisthokonta - ps9
Holozoa - ps10
Filozoa - ps11
Choano./Metazoa - ps12
Metazoa - ps13
Myriazoa - ps14
Parahoxozoa - ps15
Bilateria - ps16
Protostomia - ps17
Ecdysozoa - ps18
Arthropoda - ps19
Mandibulata - ps20
Pancrustacea - ps21
Insecta - ps22
Eumetabola - ps23
Holometabola/Psocodea - ps24
Holometabola - ps25
Aparaglossata - ps26
Mecopterida - ps27
Diptera - ps28
Bibiono./Cyclorrhapha - ps29
Cyclorrhapha - ps30
Calyptratae/Drosophila - ps31
Drosophila - ps32
Sophophora - ps33
Obscura/Melanogaster_group - ps34
D_melanogaster_group - ps35
D_melanogaster_subgroup - ps36
sim./sech./mel. group - ps37
Drosophila_melanogaster - ps38
p-value
7e-07
1e-05
2e-04
3e-03
4e-02
Log-odds
0.5
1.0
1.5
2.0
2.5
3.0
3.5
4.0

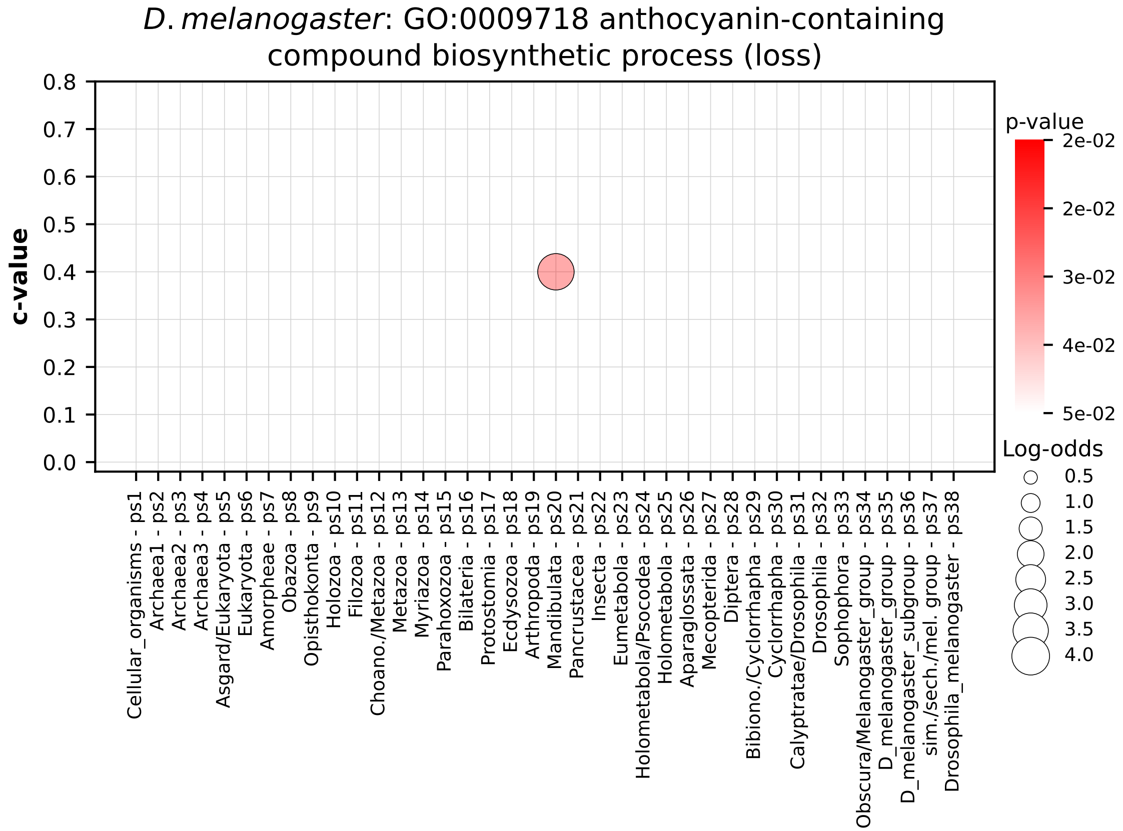
D. melanogaster: GO:0009718 anthocyanin-containing compound biosynthetic process (loss)
c-value
0.8
0.7
0.6
0.5
0.4
0.3
0.2
0.1
0.0
Phylostratum
Cellular_organisms - ps1
Archaea1 - ps2
Archaea2 - ps3
Archaea3 - ps4
Asgard/Eukaryota - ps5
Eukaryota - ps6
Amorpheae - ps7
Obazoa - ps8
Opisthokonta - ps9
Holozoa - ps10
Filozoa - ps11
Choano./Metazoa - ps12
Metazoa - ps13
Myriazoa - ps14
Parahoxozoa - ps15
Bilateria - ps16
Protostomia - ps17
Ecdysozoa - ps18
Arthropoda - ps19
Mandibulata - ps20
Pancrustacea - ps21
Insecta - ps22
Eumetabola - ps23
Holometabola/Psocodea - ps24
Holometabola - ps25
Aparaglossata - ps26
Mecopterida - ps27
Diptera - ps28
Bibiono./Cyclorrhapha - ps29
Cyclorrhapha - ps30
Calyptratae/Drosophila - ps31
Drosophila - ps32
Sophophora - ps33
Obscura/Melanogaster_group - ps34
D_melanogaster_group - ps35
D_melanogaster_subgroup - ps36
sim./sech./mel. group - ps37
Drosophila_melanogaster - ps38
p-value
2e-02
2e-02
3e-02
4e-02
5e-02
Log-odds
0.5
1.0
1.5
2.0
2.5
3.0
3.5
4.0

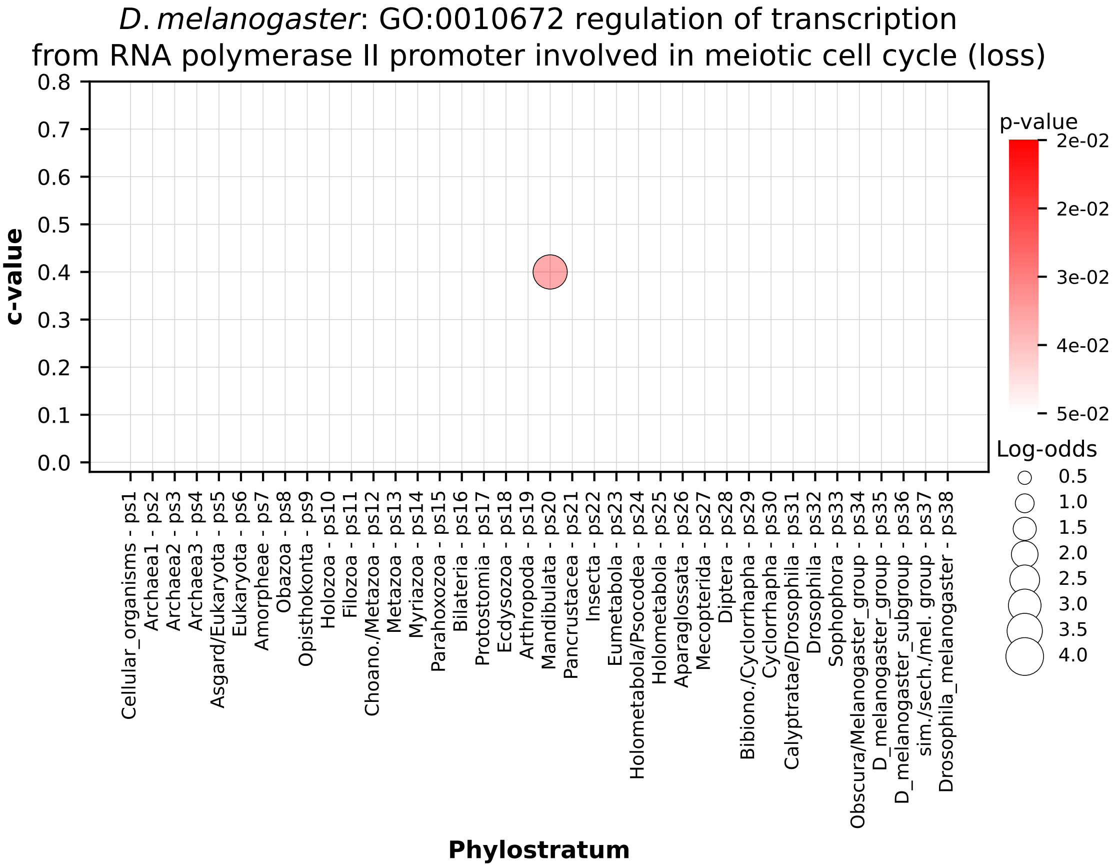

D. melanogaster: GO:0010672 regulation of transcription from RNA polymerase II promoter involved in meiotic cell cycle (loss)
c-value
0.8
0.7
0.6
0.5
0.4
0.3
0.2
0.1
0.0
p-value
2e-02
2e-02
3e-02
4e-02
5e-02
Log-odds
0.5
1.0
1.5
2.0
2.5
3.0
3.5
4.0
Cellular_organisms - ps1
Archaea1 - ps2
Archaea2 - ps3
Archaea3 - ps4
Asgard/Eukaryota - ps5
Eukaryota - ps6
Amorpheae - ps7
Obazoa - ps8
Opisthokonta - ps9
Holozoa - ps10
Filozoa - ps11
Choano./Metazoa - ps12
Metazoa - ps13
Myriazoa - ps14
Parahoxozoa - ps15
Bilateria - ps16
Protostomia - ps17
Ecdysozoa - ps18
Arthropoda - ps19
Mandibulata - ps20
Pancrustacea - ps21
Insecta - ps22
Eumetabola - ps23
Holometabola/Psocodea - ps24
Holometabola - ps25
Aparaglossata - ps26
Mecopterida - ps27
Diptera - ps28
Bibiono./Cyclorrhapha - ps29
Cyclorrhapha - ps30
Calyptratae/Drosophila - ps31
Drosophila - ps32
Sophophora - ps33
Obscura/Melanogaster_group - ps34
D_melanogaster_group - ps35
D_melanogaster_subgroup - ps36
sim./sech./mel. group - ps37
Drosophila_melanogaster - ps38
Phylostratum

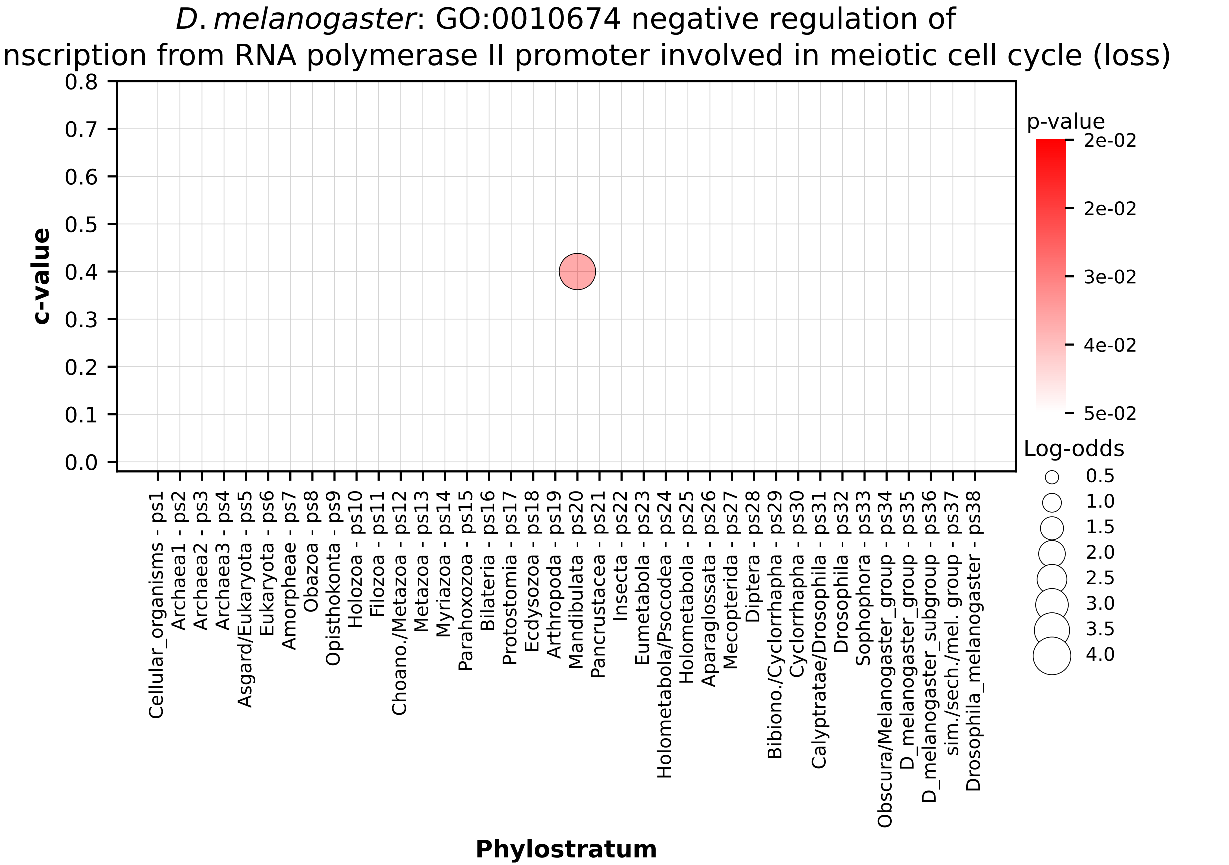

D. melanogaster: GO:0010674 negative regulation of
nscription from RNA polymerase II promoter involved in meiotic cell cycle (loss)
c-value
0.8
0.7
0.6
0.5
0.4
0.3
0.2
0.1
0.0
p-value
2e-02
2e-02
3e-02
4e-02
5e-02
Log-odds
0.5
1.0
1.5
2.0
2.5
3.0
3.5
4.0
Cellular_organisms - ps1
Archaea1 - ps2
Archaea2 - ps3
Archaea3 - ps4
Asgard/Eukaryota - ps5
Eukaryota - ps6
Amorpheae - ps7
Obazoa - ps8
Opisthokonta - ps9
Holozoa - ps10
Filozoa - ps11
Choano./Metazoa - ps12
Metazoa - ps13
Myriazoa - ps14
Parahoxozoa - ps15
Bilateria - ps16
Protostomia - ps17
Ecdysozoa - ps18
Arthropoda - ps19
Mandibulata - ps20
Pancrustacea - ps21
Insecta - ps22
Eumetabola - ps23
Holometabola/Psocodea - ps24
Holometabola - ps25
Aparaglossata - ps26
Mecopterida - ps27
Diptera - ps28
Bibiono./Cyclorrhapha - ps29
Cyclorrhapha - ps30
Calyptratae/Drosophila - ps31
Drosophila - ps32
Sophophora - ps33
Obscura/Melanogaster_group - ps34
D_melanogaster_group - ps35
D_melanogaster_subgroup - ps36
sim./sech./mel. group - ps37
Drosophila_melanogaster - ps38
Phylostratum

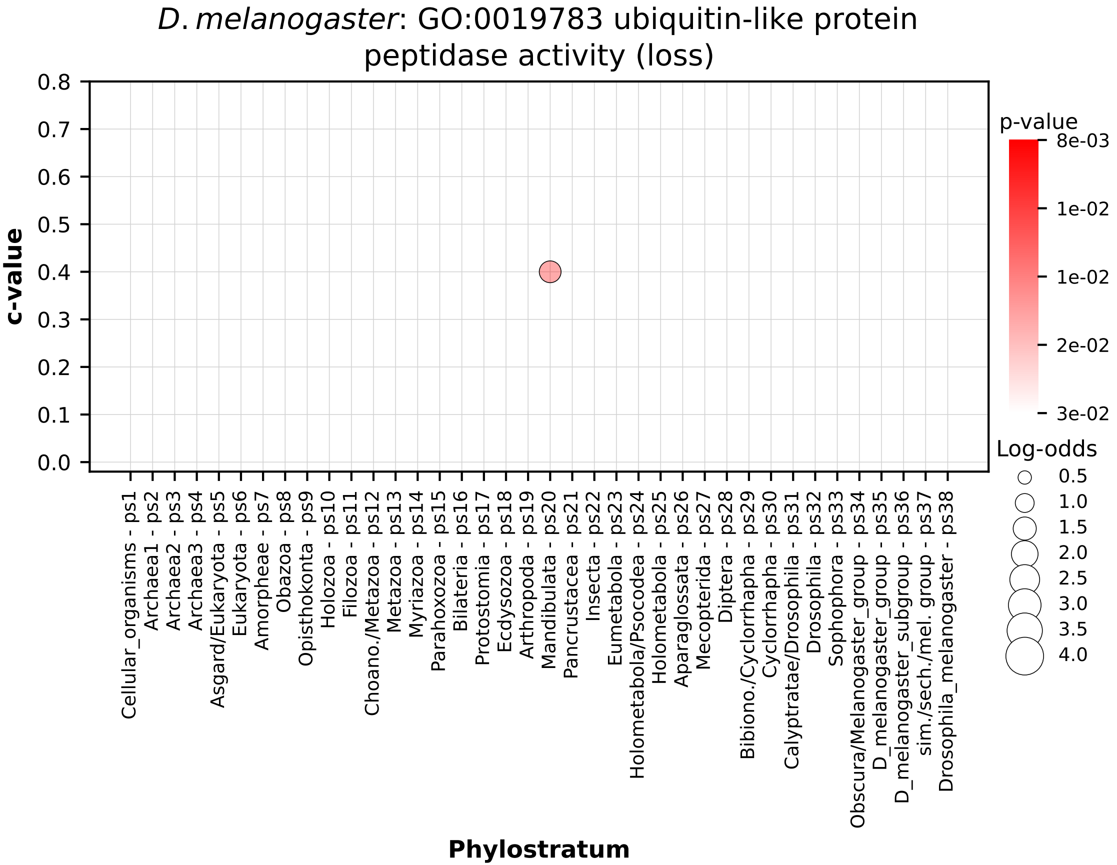

D. melanogaster: GO:0019783 ubiquitin-like protein peptidase activity (loss)
c-value
0.8
0.7
0.6
0.5
0.4
0.3
0.2
0.1
0.0
p-value
8e-03
1e-02
1e-02
2e-02
3e-02
Log-odds
0.5
1.0
1.5
2.0
2.5
3.0
3.5
4.0
Cellular_organisms - ps1
Archaea1 - ps2
Archaea2 - ps3
Archaea3 - ps4
Asgard/Eukaryota - ps5
Eukaryota - ps6
Amorpheae - ps7
Obazoa - ps8
Opisthokonta - ps9
Holozoa - ps10
Filozoa - ps11
Choano./Metazoa - ps12
Metazoa - ps13
Myriazoa - ps14
Parahoxozoa - ps15
Bilateria - ps16
Protostomia - ps17
Ecdysozoa - ps18
Arthropoda - ps19
Mandibulata - ps20
Pancrustacea - ps21
Insecta - ps22
Eumetabola - ps23
Holometabola/Psocodea - ps24
Holometabola - ps25
Aparaglossata - ps26
Mecopterida - ps27
Diptera - ps28
Bibiono./Cyclorrhapha - ps29
Cyclorrhapha - ps30
Calyptratae/Drosophila - ps31
Drosophila - ps32
Sophophora - ps33
Obscura/Melanogaster_group - ps34
D_melanogaster_group - ps35
D_melanogaster_subgroup - ps36
sim./sech./mel. group - ps37
Drosophila_melanogaster - ps38
Phylostratum

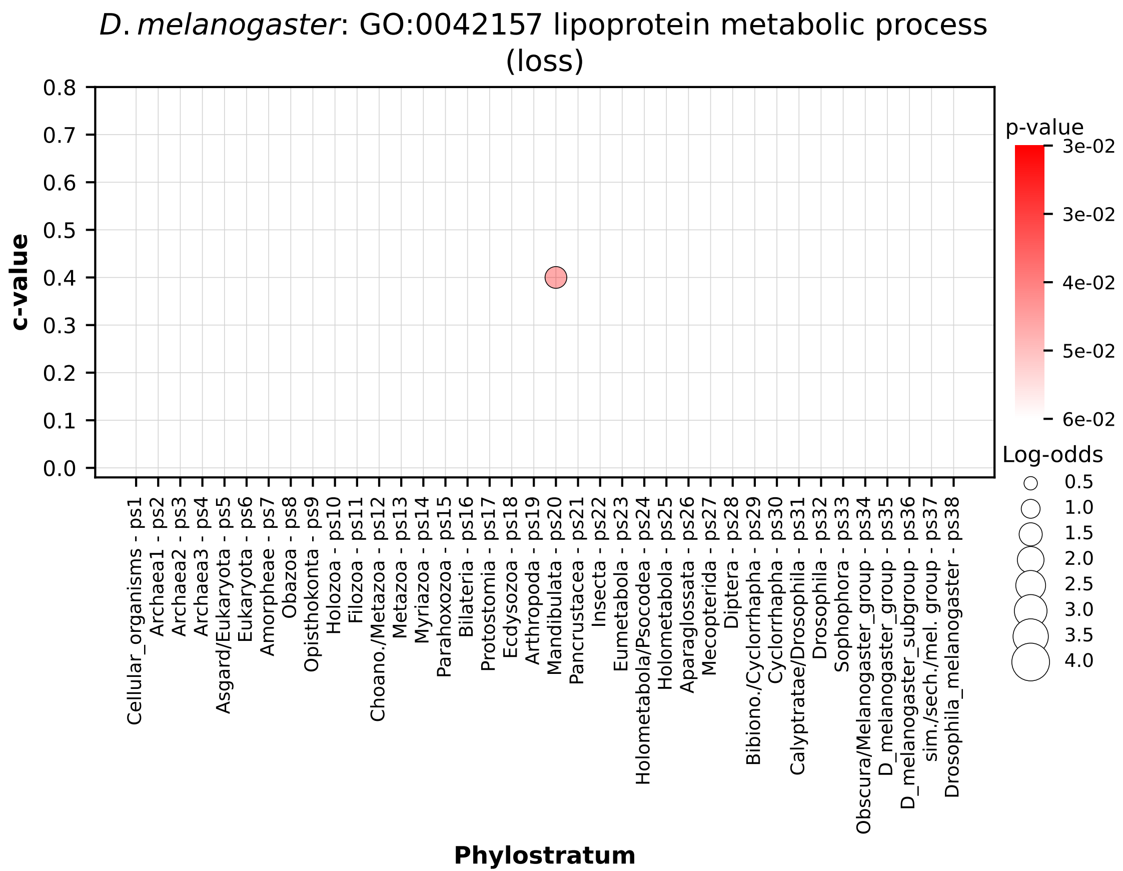

D. melanogaster: GO:0042157 lipoprotein metabolic process (loss)
c-value
0.8
0.7
0.6
0.5
0.4
0.3
0.2
0.1
0.0
p-value
3e-02
3e-02
4e-02
5e-02
6e-02
Log-odds
0.5
1.0
1.5
2.0
2.5
3.0
3.5
4.0
Cellular_organisms - ps1
Archaea1 - ps2
Archaea2 - ps3
Archaea3 - ps4
Asgard/Eukaryota - ps5
Eukaryota - ps6
Amorpheae - ps7
Obazoa - ps8
Opisthokonta - ps9
Holozoa - ps10
Filozoa - ps11
Choano./Metazoa - ps12
Metazoa - ps13
Myriazoa - ps14
Parahoxozoa - ps15
Bilateria - ps16
Protostomia - ps17
Ecdysozoa - ps18
Arthropoda - ps19
Mandibulata - ps20
Pancrustacea - ps21
Insecta - ps22
Eumetabola - ps23
Holometabola/Psocodea - ps24
Holometabola - ps25
Aparaglossata - ps26
Mecopterida - ps27
Diptera - ps28
Bibiono./Cyclorrhapha - ps29
Cyclorrhapha - ps30
Calyptratae/Drosophila - ps31
Drosophila - ps32
Sophophora - ps33
Obscura/Melanogaster_group - ps34
D_melanogaster_group - ps35
D_melanogaster_subgroup - ps36
sim./sech./mel. group - ps37
Drosophila_melanogaster - ps38
Phylostratum

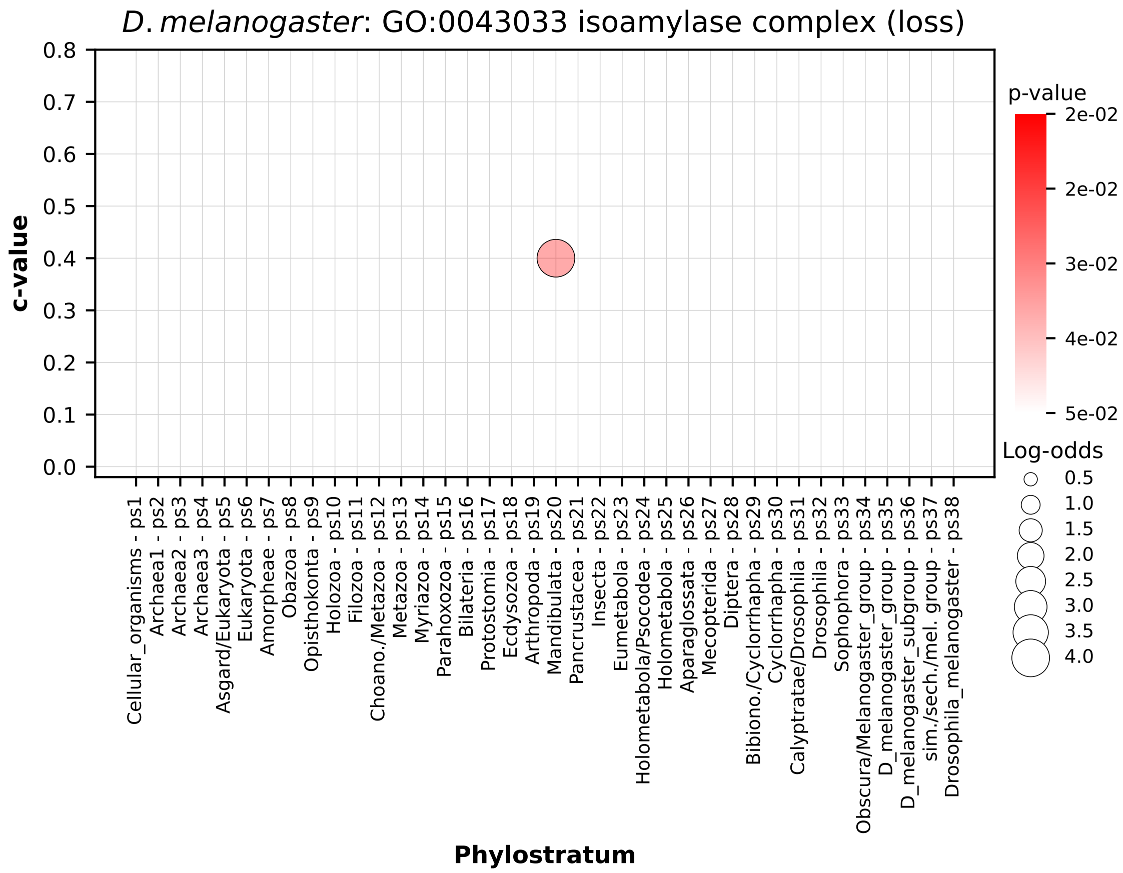

D. melanogaster: GO:0043033 isoamylase complex (loss)
c-value
0.0
0.1
0.2
0.3
0.4
0.5
0.6
0.7
0.8
Phylostratum
Cellular_organisms - ps1
Archaea1 - ps2
Archaea2 - ps3
Archaea3 - ps4
Asgard/Eukaryota - ps5
Eukaryota - ps6
Amorpheae - ps7
Obazoa - ps8
Opisthokonta - ps9
Holozoa - ps10
Filozoa - ps11
Choano./Metazoa - ps12
Metazoa - ps13
Myriazoa - ps14
Parahoxozoa - ps15
Bilateria - ps16
Protostomia - ps17
Ecdysozoa - ps18
Arthropoda - ps19
Mandibulata - ps20
Pancrustacea - ps21
Insecta - ps22
Eumetabola - ps23
Holometabola/Psocodea - ps24
Holometabola - ps25
Aparaglossata - ps26
Mecopterida - ps27
Diptera - ps28
Bibiono./Cyclorrhapha - ps29
Cyclorrhapha - ps30
Calyptratae/Drosophila - ps31
Drosophila - ps32
Sophophora - ps33
Obscura/Melanogaster_group - ps34
D_melanogaster_group - ps35
D_melanogaster_subgroup - ps36
sim./sech./mel. group - ps37
Drosophila_melanogaster - ps38
p-value
2e-02
2e-02
3e-02
4e-02
5e-02
Log-odds
0.5
1.0
1.5
2.0
2.5
3.0
3.5
4.0

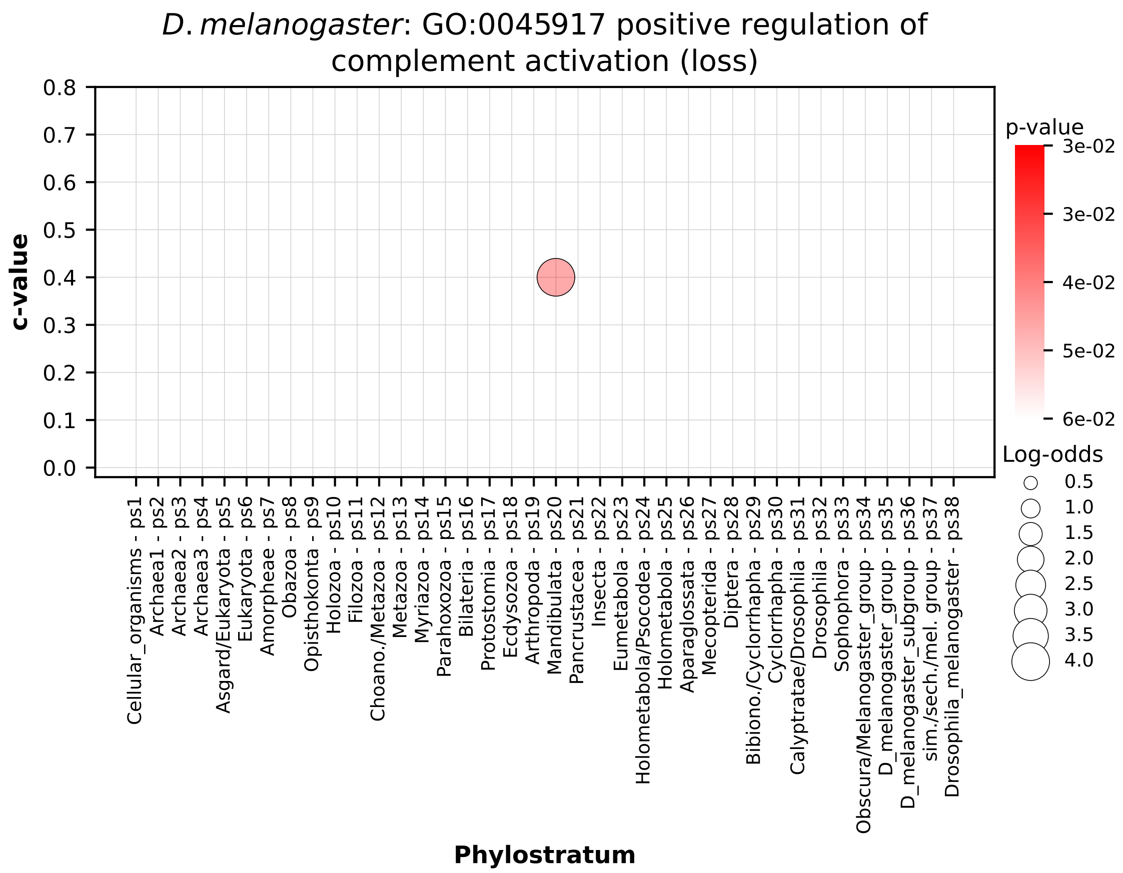

D. melanogaster: GO:0045917 positive regulation of complement activation (loss)
c-value
0.8
0.7
0.6
0.5
0.4
0.3
0.2
0.1
0.0
Phylostratum
Cellular_organisms - ps1
Archaea1 - ps2
Archaea2 - ps3
Archaea3 - ps4
Asgard/Eukaryota - ps5
Eukaryota - ps6
Amorpheae - ps7
Obazoa - ps8
Opisthokonta - ps9
Holozoa - ps10
Filozoa - ps11
Choano./Metazoa - ps12
Metazoa - ps13
Myriazoa - ps14
Parahoxozoa - ps15
Bilateria - ps16
Protostomia - ps17
Ecdysozoa - ps18
Arthropoda - ps19
Mandibulata - ps20
Pancrustacea - ps21
Insecta - ps22
Eumetabola - ps23
Holometabola/Psocodea - ps24
Holometabola - ps25
Aparaglossata - ps26
Mecopterida - ps27
Diptera - ps28
Bibiono./Cyclorrhapha - ps29
Cyclorrhapha - ps30
Calyptratae/Drosophila - ps31
Drosophila - ps32
Sophophora - ps33
Obscura/Melanogaster_group - ps34
D_melanogaster_group - ps35
D_melanogaster_subgroup - ps36
sim./sech./mel. group - ps37
Drosophila_melanogaster - ps38
p-value
3e-02
3e-02
4e-02
5e-02
6e-02
Log-odds
0.5
1.0
1.5
2.0
2.5
3.0
3.5
4.0

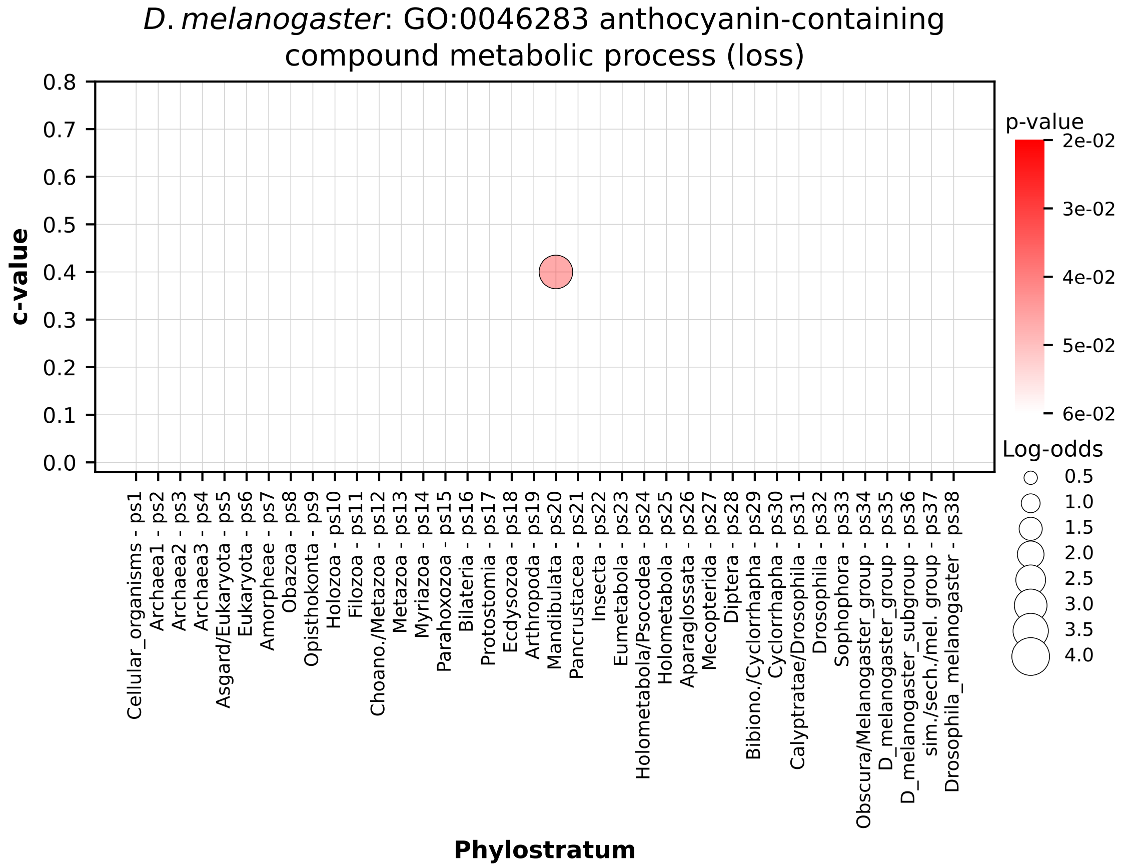
D. melanogaster: GO:0046283 anthocyanin-containing compound metabolic process (loss)
c-value
0.8
0.7
0.6
0.5
0.4
0.3
0.2
0.1
0.0
p-value
2e-02
3e-02
4e-02
5e-02
6e-02
Log-odds
0.5
1.0
1.5
2.0
2.5
3.0
3.5
4.0
Cellular_organisms - ps1
Archaea1 - ps2
Archaea2 - ps3
Archaea3 - ps4
Asgard/Eukaryota - ps5
Eukaryota - ps6
Amorpheae - ps7
Obazoa - ps8
Opisthokonta - ps9
Holozoa - ps10
Filozoa - ps11
Choano./Metazoa - ps12
Metazoa - ps13
Myriazoa - ps14
Parahoxozoa - ps15
Bilateria - ps16
Protostomia - ps17
Ecdysozoa - ps18
Arthropoda - ps19
Mandibulata - ps20
Pancrustacea - ps21
Insecta - ps22
Eumetabola - ps23
Holometabola/Psocodea - ps24
Holometabola - ps25
Aparaglossata - ps26
Mecopterida - ps27
Diptera - ps28
Bibiono./Cyclorrhapha - ps29
Cyclorrhapha - ps30
Calyptratae/Drosophila - ps31
Drosophila - ps32
Sophophora - ps33
Obscura/Melanogaster_group - ps34
D_melanogaster_group - ps35
D_melanogaster_subgroup - ps36
sim./sech./mel. group - ps37
Drosophila_melanogaster - ps38
Phylostratum

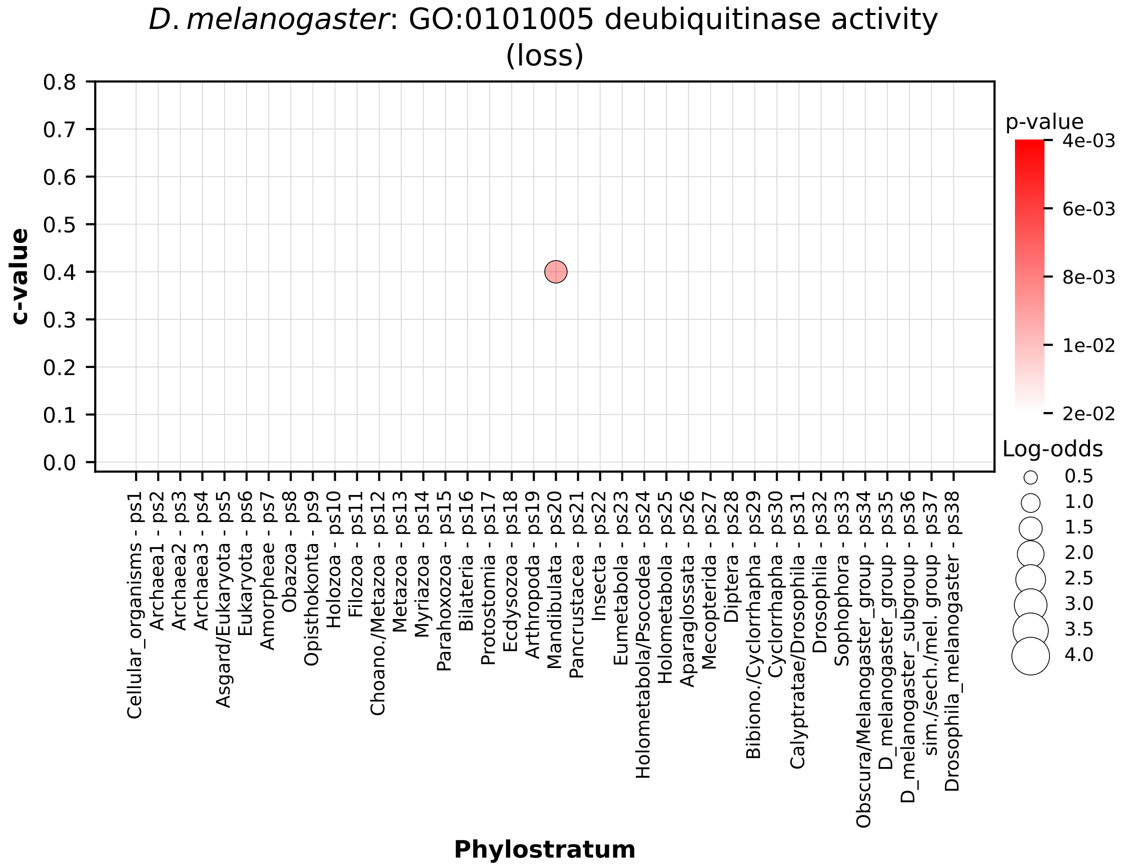
D. melanogaster: GO:0101005 deubiquitinase activity
(loss)
c-value
0.8
0.7
0.6
0.5
0.4
0.3
0.2
0.1
0.0
p-value
4e-03
6e-03
8e-03
1e-02
2e-02
Log-odds
0.5
1.0
1.5
2.0
2.5
3.0
3.5
4.0
Cellular_organisms - ps1
Archaea1 - ps2
Archaea2 - ps3
Archaea3 - ps4
Asgard/Eukaryota - ps5
Eukaryota - ps6
Amorpheae - ps7
Obazoa - ps8
Opisthokonta - ps9
Holozoa - ps10
Filozoa - ps11
Choano./Metazoa - ps12
Metazoa - ps13
Myriazoa - ps14
Parahoxozoa - ps15
Bilateria - ps16
Protostomia - ps17
Ecdysozoa - ps18
Arthropoda - ps19
Mandibulata - ps20
Pancrustacea - ps21
Insecta - ps22
Eumetabola - ps23
Holometabola/Psocodea - ps24
Holometabola - ps25
Aparaglossata - ps26
Mecopterida - ps27
Diptera - ps28
Bibiono./Cyclorrhapha - ps29
Cyclorrhapha - ps30
Calyptratae/Drosophila - ps31
Drosophila - ps32
Sophophora - ps33
Obscura/Melanogaster_group - ps34
D_melanogaster_group - ps35
D_melanogaster_subgroup - ps36
sim./sech./mel. group - ps37
Drosophila_melanogaster - ps38
Phylostratum

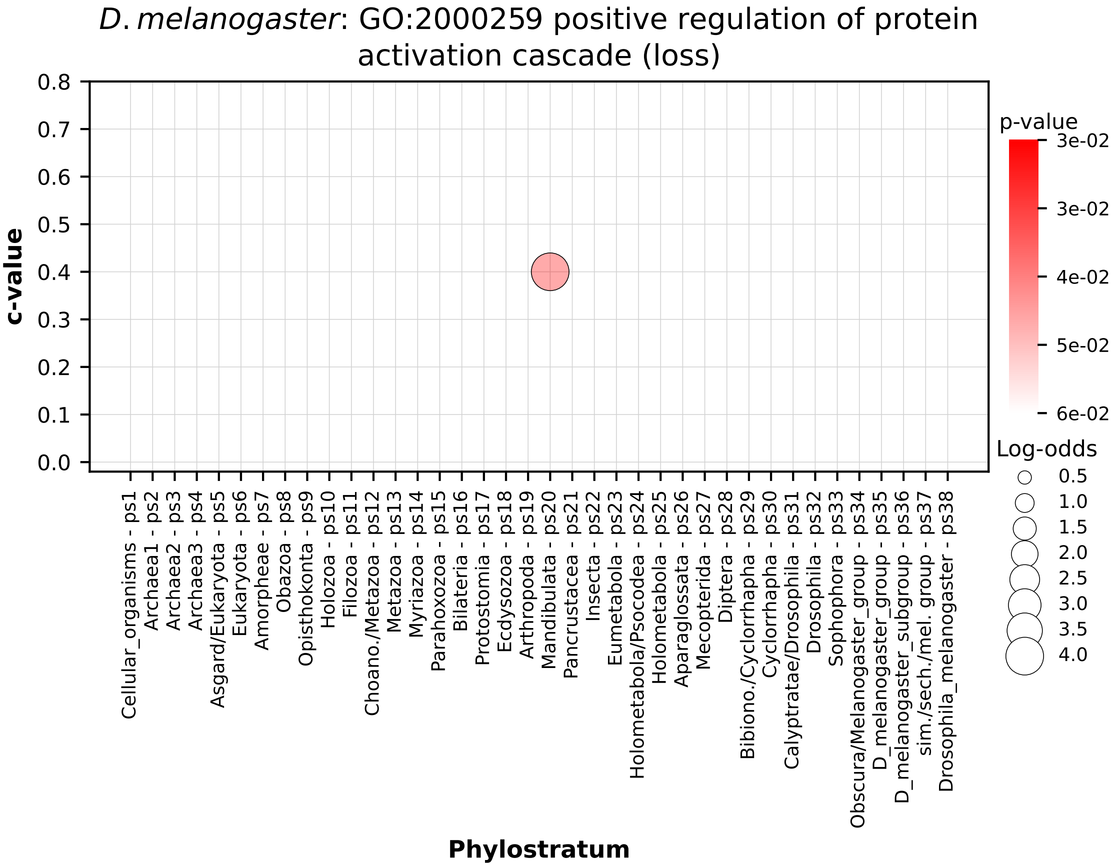
D. melanogaster: GO:2000259 positive regulation of protein activation cascade (loss)
c-value
0.0
0.1
0.2
0.3
0.4
0.5
0.6
0.7
0.8
p-value
3e-02
3e-02
4e-02
5e-02
6e-02
Log-odds
0.5
1.0
1.5
2.0
2.5
3.0
3.5
4.0
Cellular_organisms - ps1
Archaea1 - ps2
Archaea2 - ps3
Archaea3 - ps4
Asgard/Eukaryota - ps5
Eukaryota - ps6
Amorpheae - ps7
Obazoa - ps8
Opisthokonta - ps9
Holozoa - ps10
Filozoa - ps11
Choano./Metazoa - ps12
Metazoa - ps13
Myriazoa - ps14
Parahoxozoa - ps15
Bilateria - ps16
Protostomia - ps17
Ecdysozoa - ps18
Arthropoda - ps19
Mandibulata - ps20
Pancrustacea - ps21
Insecta - ps22
Eumetabola - ps23
Holometabola/Psocodea - ps24
Holometabola - ps25
Aparaglossata - ps26
Mecopterida - ps27
Diptera - ps28
Bibiono./Cyclorrhapha - ps29
Cyclorrhapha - ps30
Calyptratae/Drosophila - ps31
Drosophila - ps32
Sophophora - ps33
Obscura/Melanogaster_group - ps34
D_melanogaster_group - ps35
D_melanogaster_subgroup - ps36
sim./sech./mel. group - ps37
Drosophila_melanogaster - ps38
Phylostratum

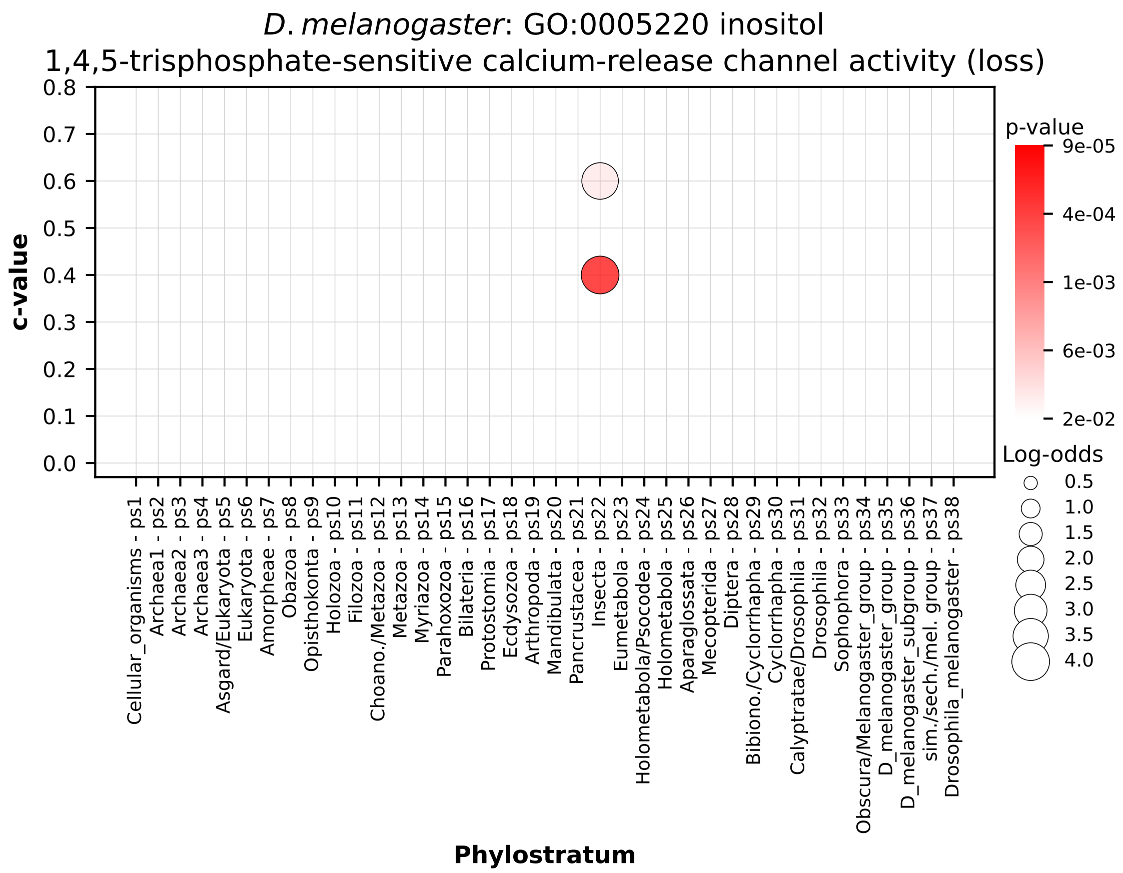

D. melanogaster: GO:0005220 inositol 1,4,5-trisphosphate-sensitive calcium-release channel activity (loss)
c-value
0.8
0.7
0.6
0.5
0.4
0.3
0.2
0.1
0.0
p-value
9e-05
4e-04
1e-03
6e-03
2e-02
Log-odds
0.5
1.0
1.5
2.0
2.5
3.0
3.5
4.0
Cellular_organisms - ps1
Archaea1 - ps2
Archaea2 - ps3
Archaea3 - ps4
Asgard/Eukaryota - ps5
Eukaryota - ps6
Amorpheae - ps7
Obazoa - ps8
Opisthokonta - ps9
Holozoa - ps10
Filozoa - ps11
Choano./Metazoa - ps12
Metazoa - ps13
Myriazoa - ps14
Parahoxozoa - ps15
Bilateria - ps16
Protostomia - ps17
Ecdysozoa - ps18
Arthropoda - ps19
Mandibulata - ps20
Pancrustacea - ps21
Insecta - ps22
Eumetabola - ps23
Holometabola/Psocodea - ps24
Holometabola - ps25
Aparaglossata - ps26
Mecopterida - ps27
Diptera - ps28
Bibiono./Cyclorrhapha - ps29
Cyclorrhapha - ps30
Calyptratae/Drosophila - ps31
Drosophila - ps32
Sophophora - ps33
Obscura/Melanogaster_group - ps34
D_melanogaster_group - ps35
D_melanogaster_subgroup - ps36
sim./sech./mel. group - ps37
Drosophila_melanogaster - ps38
Phylostratum

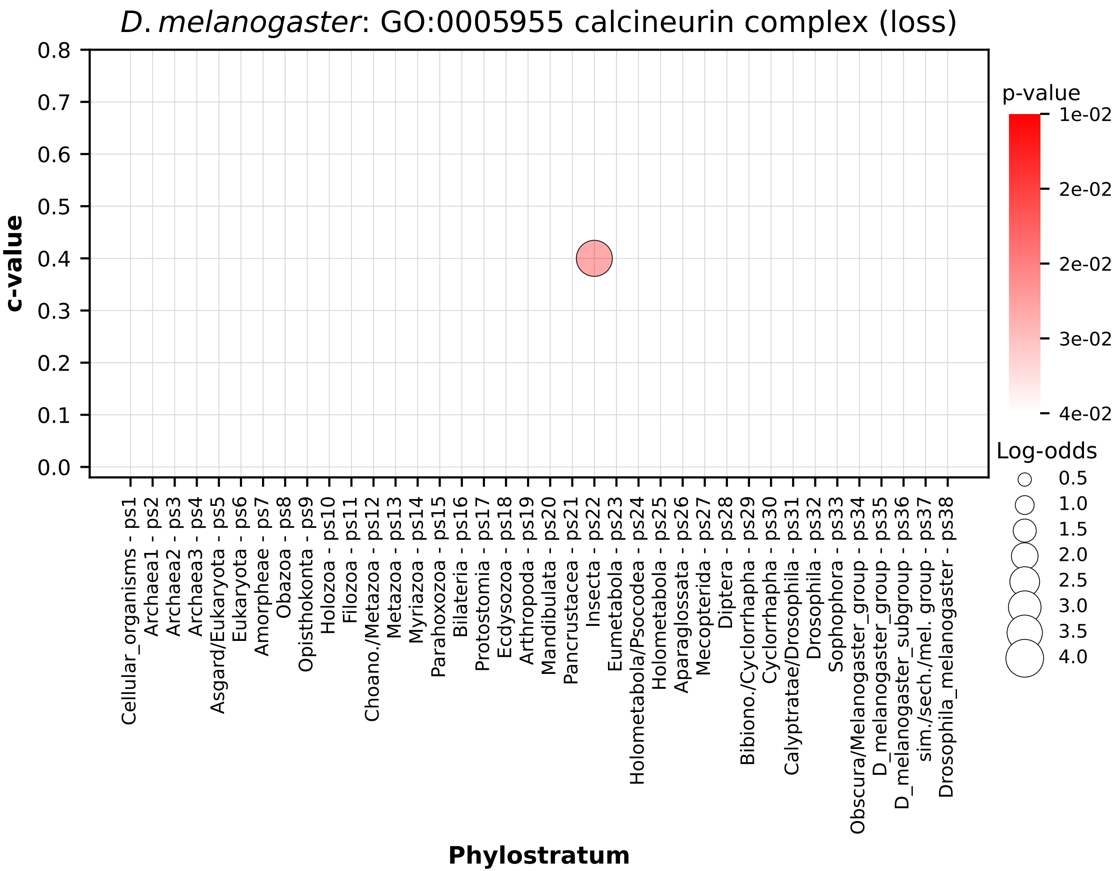
D. melanogaster: GO:0005955 calcineurin complex (loss)
c-value
0.8
0.7
0.6
0.5
0.4
0.3
0.2
0.1
0.0
p-value
1e-02
2e-02
2e-02
3e-02
4e-02
Log-odds
0.5
1.0
1.5
2.0
2.5
3.0
3.5
4.0
Cellular_organisms - ps1
Archaea1 - ps2
Archaea2 - ps3
Archaea3 - ps4
Asgard/Eukaryota - ps5
Eukaryota - ps6
Amorpheae - ps7
Obazoa - ps8
Opisthokonta - ps9
Holozoa - ps10
Filozoa - ps11
Choano./Metazoa - ps12
Metazoa - ps13
Myriazoa - ps14
Parahoxozoa - ps15
Bilateria - ps16
Protostomia - ps17
Ecdysozoa - ps18
Arthropoda - ps19
Mandibulata - ps20
Pancrustacea - ps21
Insecta - ps22
Eumetabola - ps23
Holometabola/Psocodea - ps24
Holometabola - ps25
Aparaglossata - ps26
Mecopterida - ps27
Diptera - ps28
Bibiono./Cyclorrhapha - ps29
Cyclorrhapha - ps30
Calyptratae/Drosophila - ps31
Drosophila - ps32
Sophophora - ps33
Obscura/Melanogaster_group - ps34
D_melanogaster_group - ps35
D_melanogaster_subgroup - ps36
sim./sech./mel. group - ps37
Drosophila_melanogaster - ps38
Phylostratum

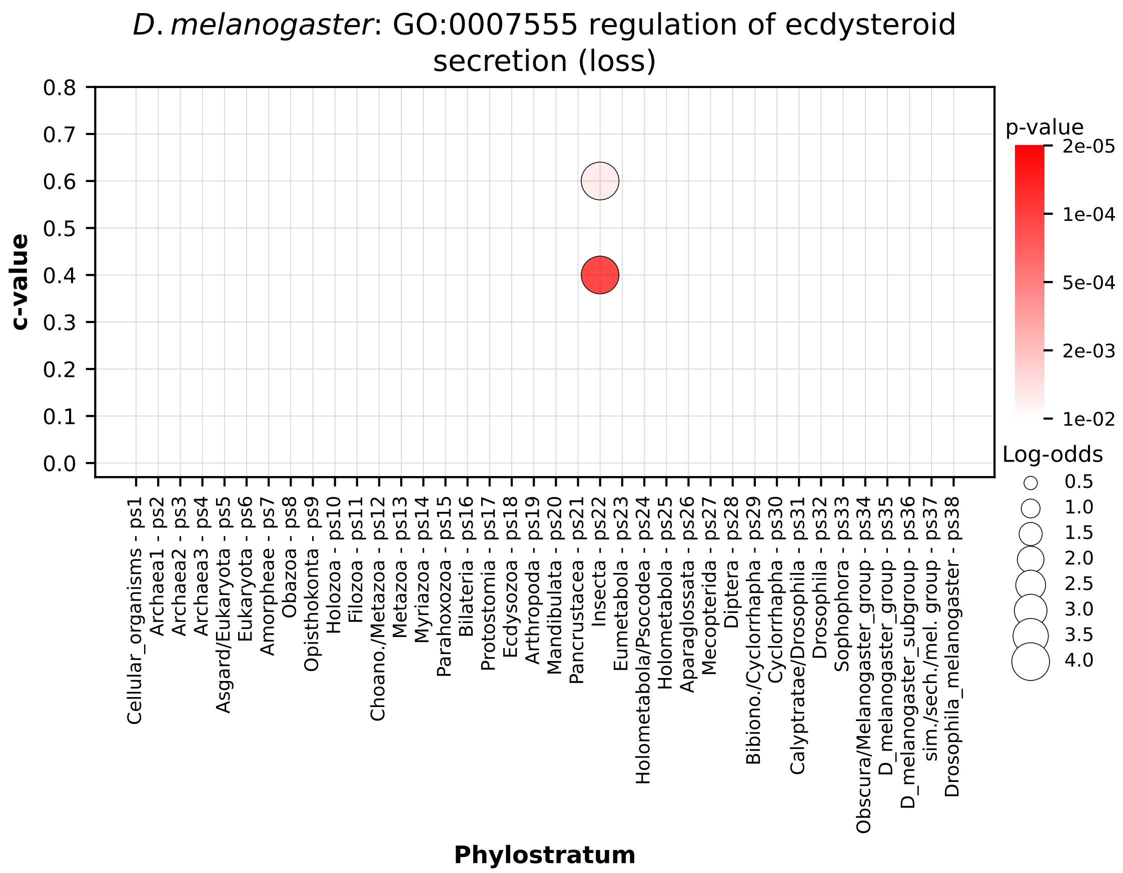

D. melanogaster: GO:0007555 regulation of ecdysteroid secretion (loss)
c-value
0.8
0.7
0.6
0.5
0.4
0.3
0.2
0.1
0.0
Phylostratum
Cellular_organisms - ps1
Archaea1 - ps2
Archaea2 - ps3
Archaea3 - ps4
Asgard/Eukaryota - ps5
Eukaryota - ps6
Amorpheae - ps7
Obazoa - ps8
Opisthokonta - ps9
Holozoa - ps10
Filozoa - ps11
Choano./Metazoa - ps12
Metazoa - ps13
Myriazoa - ps14
Parahoxozoa - ps15
Bilateria - ps16
Protostomia - ps17
Ecdysozoa - ps18
Arthropoda - ps19
Mandibulata - ps20
Pancrustacea - ps21
Insecta - ps22
Eumetabola - ps23
Holometabola/Psocodea - ps24
Holometabola - ps25
Aparaglossata - ps26
Mecopterida - ps27
Diptera - ps28
Bibiono./Cyclorrhapha - ps29
Cyclorrhapha - ps30
Calyptratae/Drosophila - ps31
Drosophila - ps32
Sophophora - ps33
Obscura/Melanogaster_group - ps34
D_melanogaster_group - ps35
D_melanogaster_subgroup - ps36
sim./sech./mel. group - ps37
Drosophila_melanogaster - ps38
p-value
2e-05
1e-04
5e-04
2e-03
1e-02
Log-odds
0.5
1.0
1.5
2.0
2.5
3.0
3.5
4.0

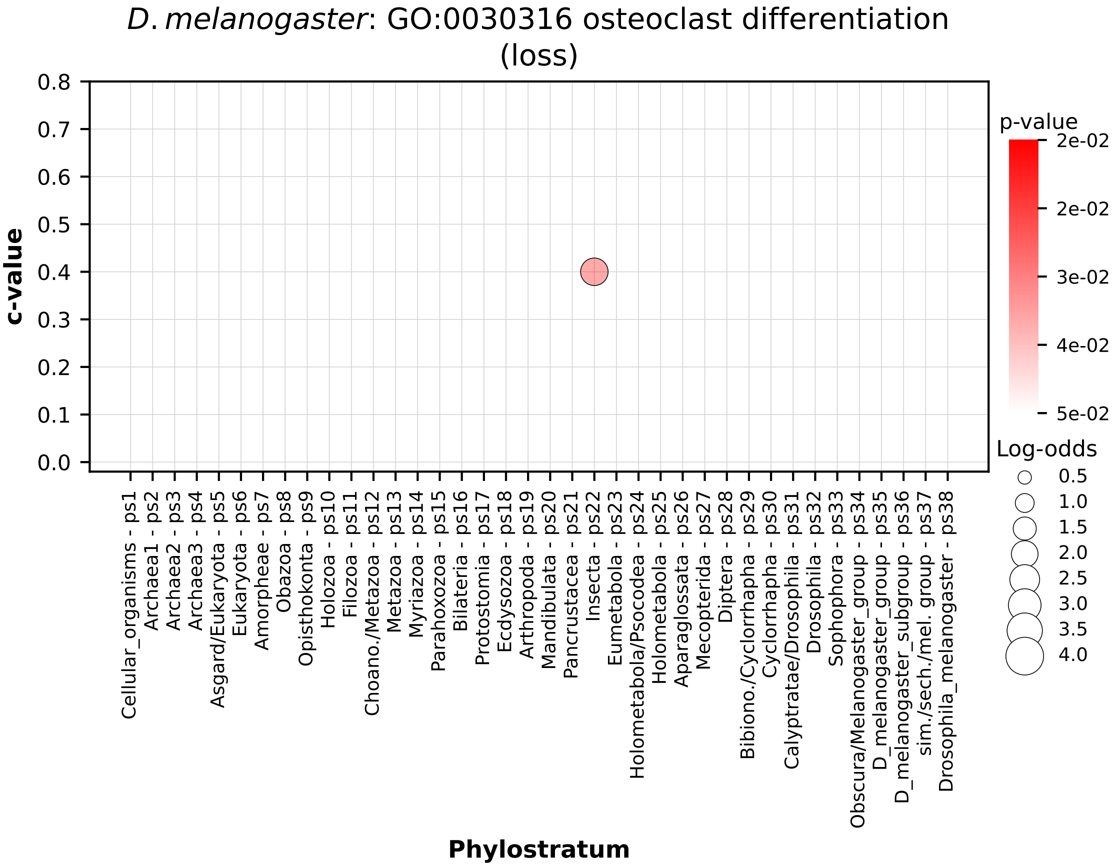

D. melanogaster: GO:0030316 osteoclast differentiation
(loss)
c-value
0.8
0.7
0.6
0.5
0.4
0.3
0.2
0.1
0.0
Phylostratum
Cellular_organisms - ps1
Archaea1 - ps2
Archaea2 - ps3
Archaea3 - ps4
Asgard/Eukaryota - ps5
Eukaryota - ps6
Amorpheae - ps7
Obazoa - ps8
Opisthokonta - ps9
Holozoa - ps10
Filozoa - ps11
Choano./Metazoa - ps12
Metazoa - ps13
Myriazoa - ps14
Parahoxozoa - ps15
Bilateria - ps16
Protostomia - ps17
Ecdysozoa - ps18
Arthropoda - ps19
Mandibulata - ps20
Pancrustacea - ps21
Insecta - ps22
Eumetabola - ps23
Holometabola/Psocodea - ps24
Holometabola - ps25
Aparaglossata - ps26
Mecopterida - ps27
Diptera - ps28
Bibiono./Cyclorrhapha - ps29
Cyclorrhapha - ps30
Calyptratae/Drosophila - ps31
Drosophila - ps32
Sophophora - ps33
Obscura/Melanogaster_group - ps34
D_melanogaster_group - ps35
D_melanogaster_subgroup - ps36
sim./sech./mel. group - ps37
Drosophila_melanogaster - ps38
p-value
2e-02
2e-02
3e-02
4e-02
5e-02
Log-odds
0.5
1.0
1.5
2.0
2.5
3.0
3.5
4.0

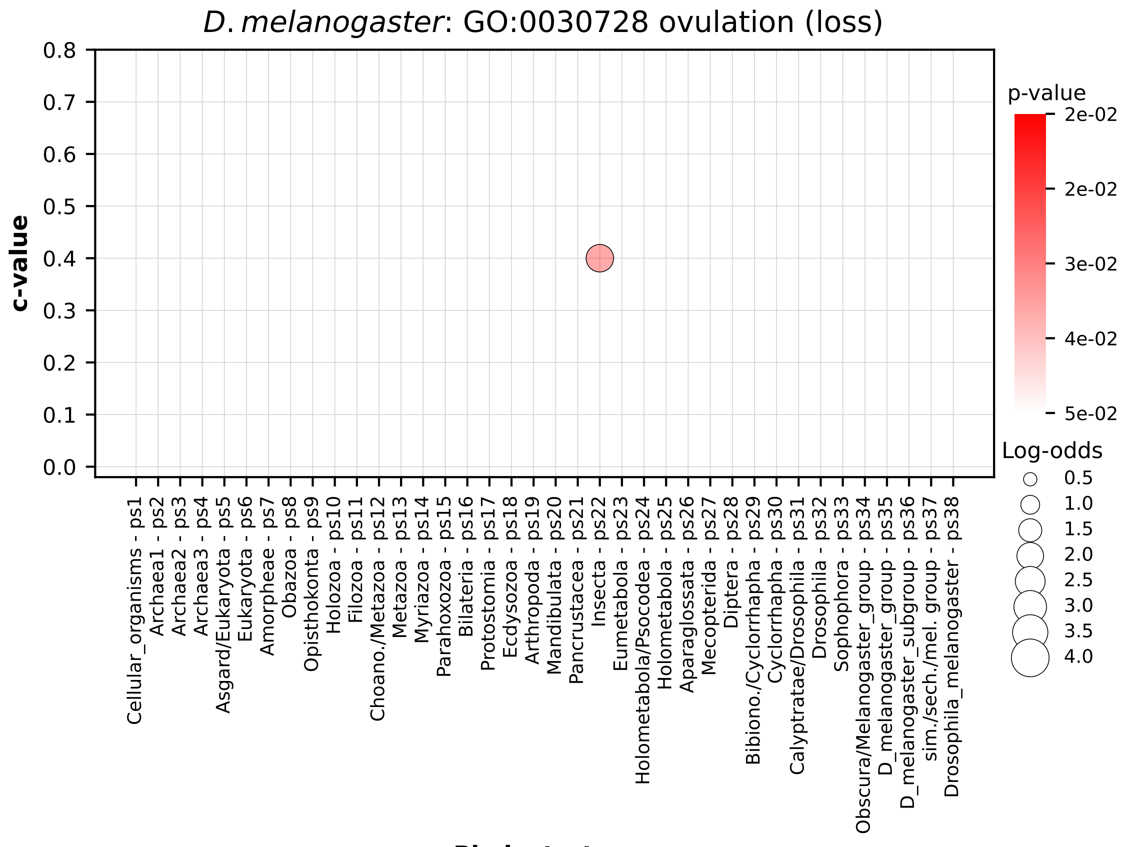

D. melanogaster: GO:0030728 ovulation (loss)
c-value
0.8
0.7
0.6
0.5
0.4
0.3
0.2
0.1
0.0
Phylostratum
Cellular_organisms - ps1
Archaea1 - ps2
Archaea2 - ps3
Archaea3 - ps4
Asgard/Eukaryota - ps5
Eukaryota - ps6
Amorpheae - ps7
Obazoa - ps8
Opisthokonta - ps9
Holozoa - ps10
Filozoa - ps11
Choano./Metazoa - ps12
Metazoa - ps13
Myriazoa - ps14
Parahoxozoa - ps15
Bilateria - ps16
Protostomia - ps17
Ecdysozoa - ps18
Arthropoda - ps19
Mandibulata - ps20
Pancrustacea - ps21
Insecta - ps22
Eumetabola - ps23
Holometabola/Psocodea - ps24
Holometabola - ps25
Aparaglossata - ps26
Mecopterida - ps27
Diptera - ps28
Bibiono./Cyclorrhapha - ps29
Cyclorrhapha - ps30
Calyptratae/Drosophila - ps31
Drosophila - ps32
Sophophora - ps33
Obscura/Melanogaster_group - ps34
D_melanogaster_group - ps35
D_melanogaster_subgroup - ps36
sim./sech./mel. group - ps37
Drosophila_melanogaster - ps38
p-value
2e-02
2e-02
3e-02
4e-02
5e-02
Log-odds
0.5
1.0
1.5
2.0
2.5
3.0
3.5
4.0

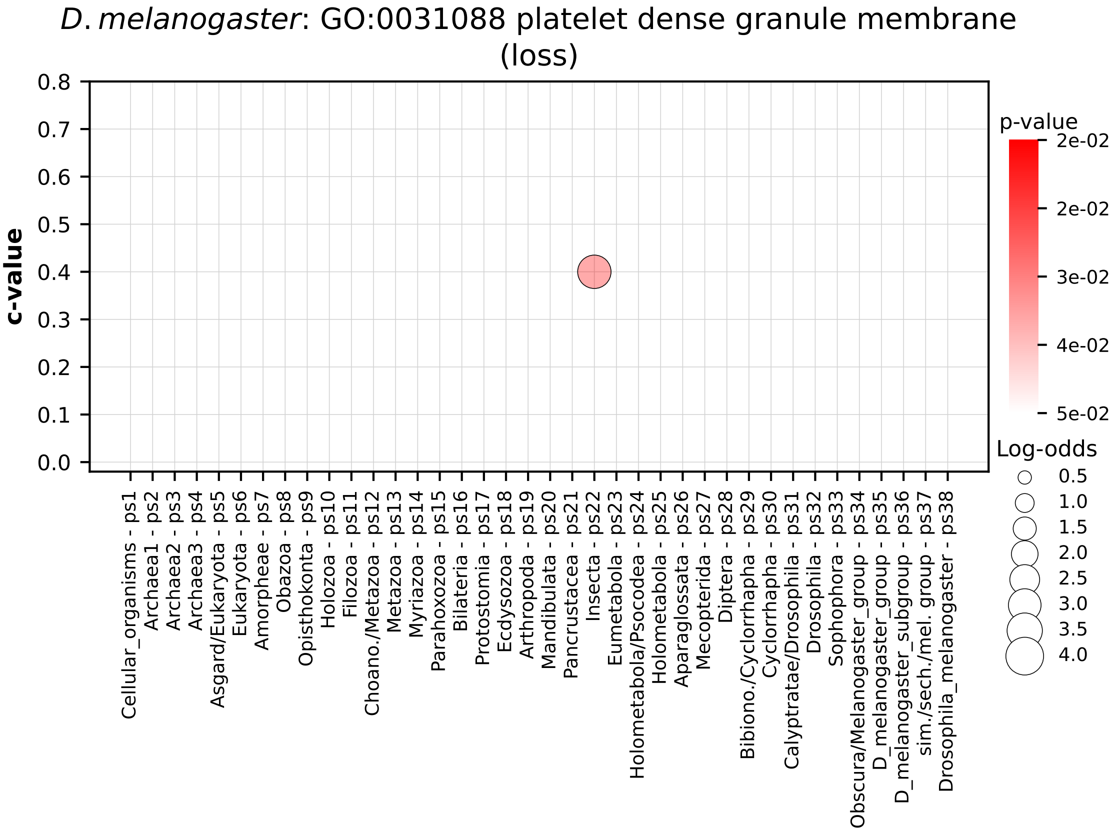

D. melanogaster: GO:0031088 platelet dense granule membrane (loss)
c-value
0.0
0.1
0.2
0.3
0.4
0.5
0.6
0.7
0.8
Phylostratum
Cellular_organisms - ps1
Archaea1 - ps2
Archaea2 - ps3
Archaea3 - ps4
Asgard/Eukaryota - ps5
Eukaryota - ps6
Amorpheae - ps7
Obazoa - ps8
Opisthokonta - ps9
Holozoa - ps10
Filozoa - ps11
Choano./Metazoa - ps12
Metazoa - ps13
Myriazoa - ps14
Parahoxozoa - ps15
Bilateria - ps16
Protostomia - ps17
Ecdysozoa - ps18
Arthropoda - ps19
Mandibulata - ps20
Pancrustacea - ps21
Insecta - ps22
Eumetabola - ps23
Holometabola/Psocodea - ps24
Holometabola - ps25
Aparaglossata - ps26
Mecopterida - ps27
Diptera - ps28
Bibiono./Cyclorrhapha - ps29
Cyclorrhapha - ps30
Calyptratae/Drosophila - ps31
Drosophila - ps32
Sophophora - ps33
Obscura/Melanogaster_group - ps34
D_melanogaster_group - ps35
D_melanogaster_subgroup - ps36
sim./sech./mel. group - ps37
Drosophila_melanogaster - ps38
p-value
2e-02
2e-02
3e-02
4e-02
5e-02
Log-odds
0.5
1.0
1.5
2.0
2.5
3.0
3.5
4.0

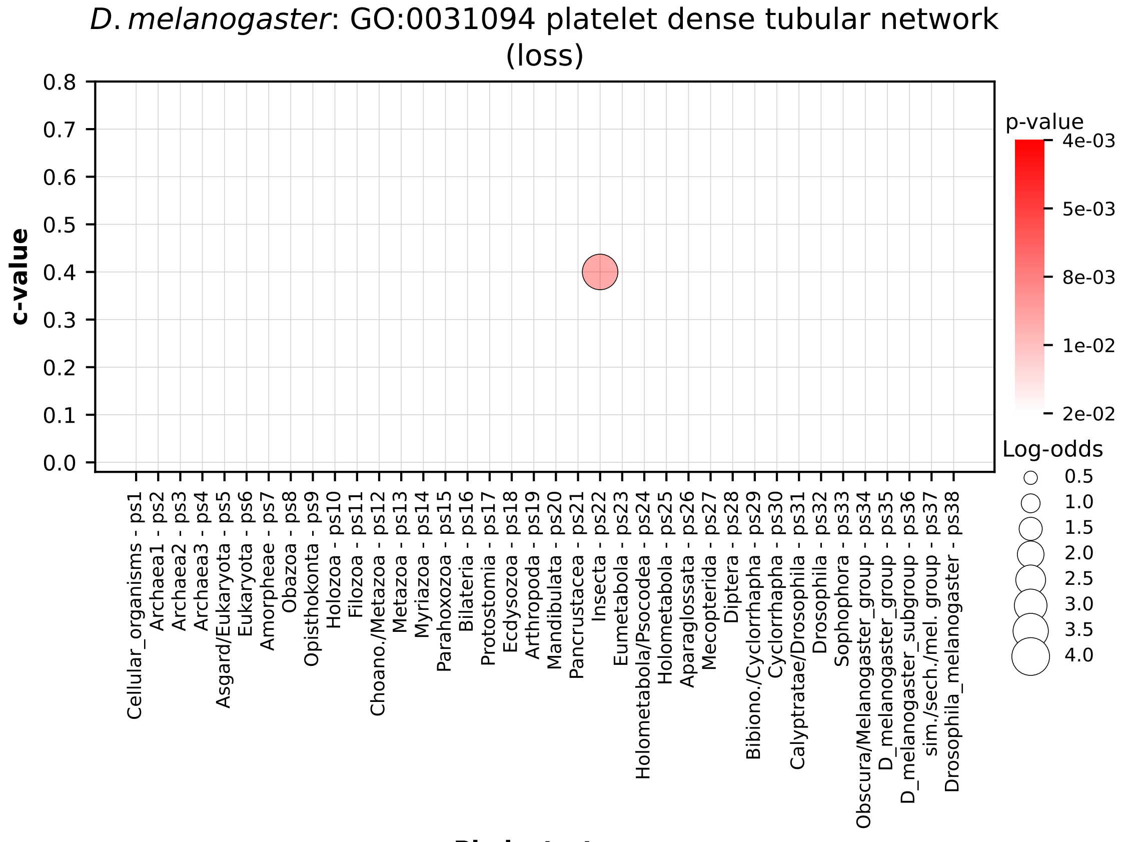

D. melanogaster: GO:0031094 platelet dense tubular network
(loss)
c-value
0.8
0.7
0.6
0.5
0.4
0.3
0.2
0.1
0.0
p-value
4e-03
5e-03
8e-03
1e-02
2e-02
Log-odds
0.5
1.0
1.5
2.0
2.5
3.0
3.5
4.0
Cellular_organisms - ps1
Archaea1 - ps2
Archaea2 - ps3
Archaea3 - ps4
Asgard/Eukaryota - ps5
Eukaryota - ps6
Amorpheae - ps7
Obazoa - ps8
Opisthokonta - ps9
Holozoa - ps10
Filozoa - ps11
Choano./Metazoa - ps12
Metazoa - ps13
Myriazoa - ps14
Parahoxozoa - ps15
Bilateria - ps16
Protostomia - ps17
Ecdysozoa - ps18
Arthropoda - ps19
Mandibulata - ps20
Pancrustacea - ps21
Insecta - ps22
Eumetabola - ps23
Holometabola/Psocodea - ps24
Holometabola - ps25
Aparaglossata - ps26
Mecopterida - ps27
Diptera - ps28
Bibiono./Cyclorrhapha - ps29
Cyclorrhapha - ps30
Calyptratae/Drosophila - ps31
Drosophila - ps32
Sophophora - ps33
Obscura/Melanogaster_group - ps34
D_melanogaster_group - ps35
D_melanogaster_subgroup - ps36
sim./sech./mel. group - ps37
Drosophila_melanogaster - ps38
Phylostratum

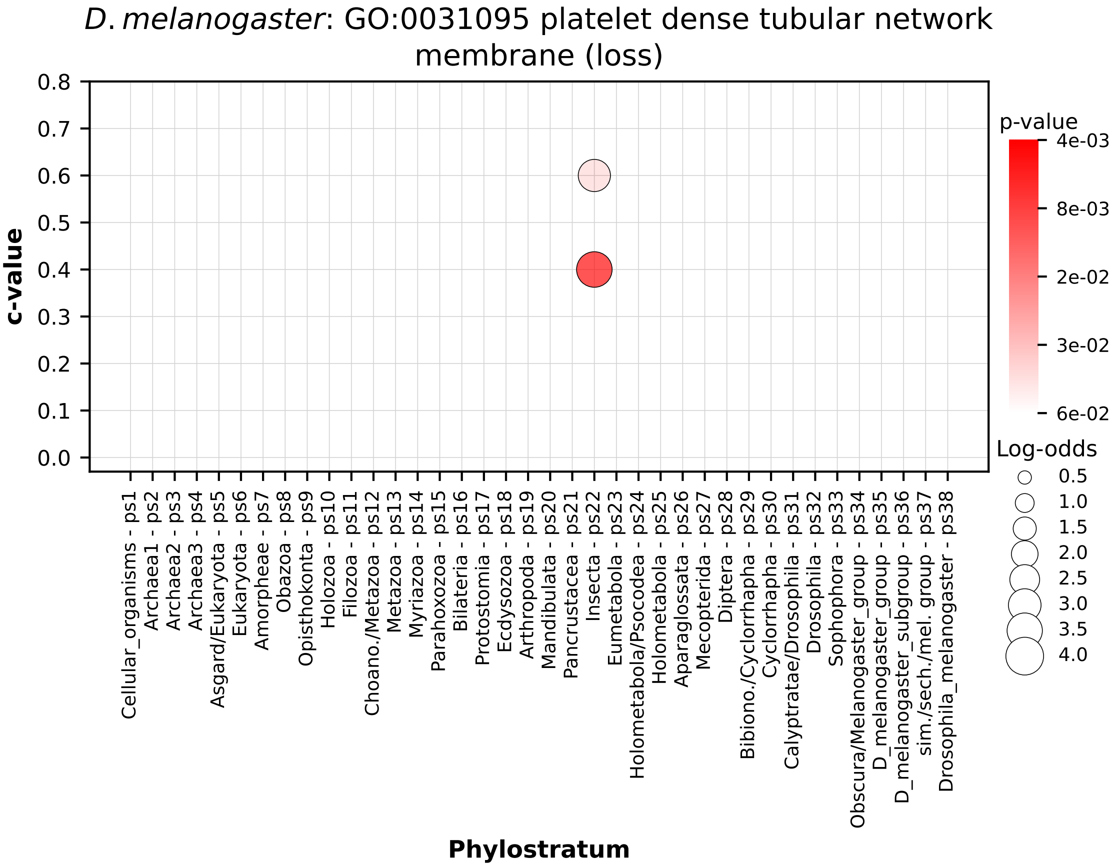

$D. melanogaster$: GO:0031095 platelet dense tubular network membrane (loss)
c-value
0.8
0.7
0.6
0.5
0.4
0.3
0.2
0.1
0.0
p-value
4e-03
8e-03
2e-02
3e-02
6e-02
Log-odds
0.5
1.0
1.5
2.0
2.5
3.0
3.5
4.0
Cellular_organisms - ps1
Archaea1 - ps2
Archaea2 - ps3
Archaea3 - ps4
Asgard/Eukaryota - ps5
Eukaryota - ps6
Amorpheae - ps7
Obazoa - ps8
Opisthokonta - ps9
Holozoa - ps10
Filozoa - ps11
Choano./Metazoa - ps12
Metazoa - ps13
Myriazoa - ps14
Parahoxozoa - ps15
Bilateria - ps16
Protostomia - ps17
Ecdysozoa - ps18
Arthropoda - ps19
Mandibulata - ps20
Pancrustacea - ps21
Insecta - ps22
Eumetabola - ps23
Holometabola/Psocodea - ps24
Holometabola - ps25
Aparaglossata - ps26
Mecopterida - ps27
Diptera - ps28
Bibiono./Cyclorrhapha - ps29
Cyclorrhapha - ps30
Calyptratae/Drosophila - ps31
Drosophila - ps32
Sophophora - ps33
Obscura/Melanogaster_group - ps34
D_melanogaster_group - ps35
D_melanogaster_subgroup - ps36
sim./sech./mel. group - ps37
Drosophila_melanogaster - ps38
Phylostratum

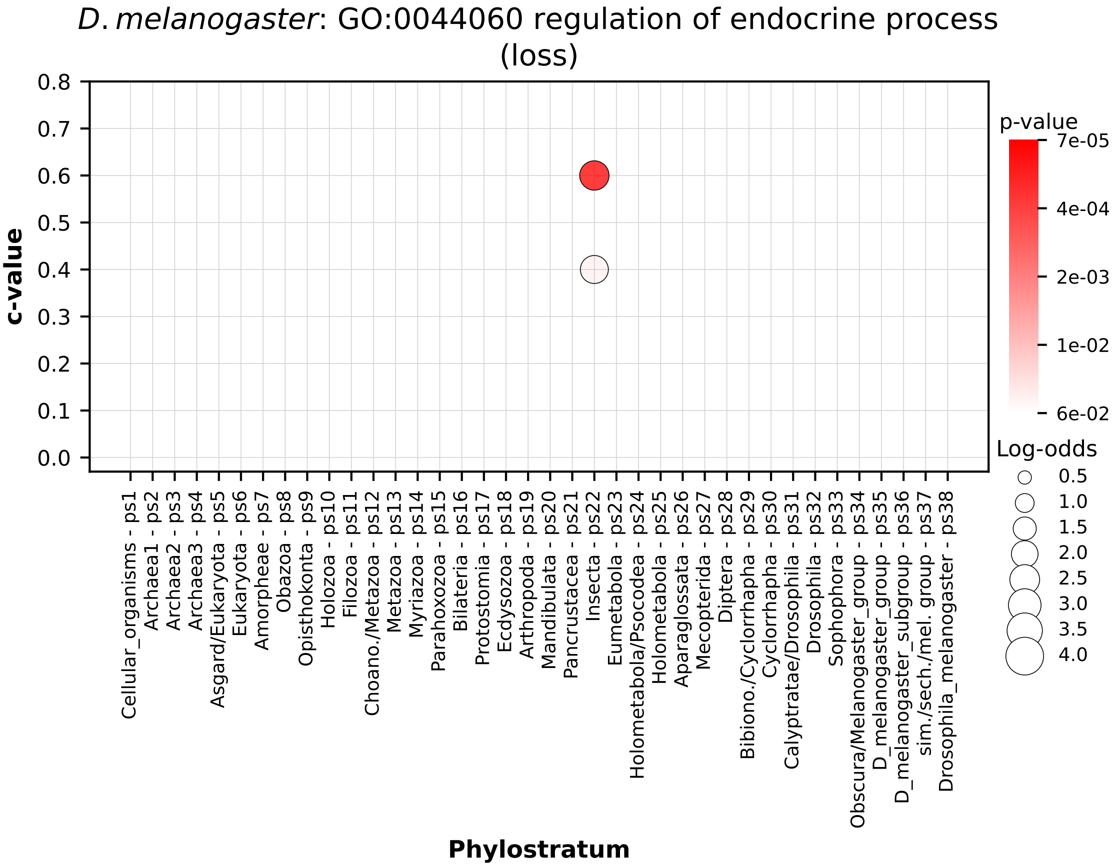
D. melanogaster: GO:0044060 regulation of endocrine process
(loss)
c-value
0.8
0.7
0.6
0.5
0.4
0.3
0.2
0.1
0.0
p-value
7e-05
4e-04
2e-03
1e-02
6e-02
Log-odds
0.5
1.0
1.5
2.0
2.5
3.0
3.5
4.0
Cellular_organisms - ps1
Archaea1 - ps2
Archaea2 - ps3
Archaea3 - ps4
Asgard/Eukaryota - ps5
Eukaryota - ps6
Amorpheae - ps7
Obazoa - ps8
Opisthokonta - ps9
Holozoa - ps10
Filozoa - ps11
Choano./Metazoa - ps12
Metazoa - ps13
Myriazoa - ps14
Parahoxozoa - ps15
Bilateria - ps16
Protostomia - ps17
Ecdysozoa - ps18
Arthropoda - ps19
Mandibulata - ps20
Pancrustacea - ps21
Insecta - ps22
Eumetabola - ps23
Holometabola/Psocodea - ps24
Holometabola - ps25
Aparaglossata - ps26
Mecopterida - ps27
Diptera - ps28
Bibiono./Cyclorrhapha - ps29
Cyclorrhapha - ps30
Calyptratae/Drosophila - ps31
Drosophila - ps32
Sophophora - ps33
Obscura/Melanogaster_group - ps34
D_melanogaster_group - ps35
D_melanogaster_subgroup - ps36
sim./sech./mel. group - ps37
Drosophila_melanogaster - ps38
Phylostratum

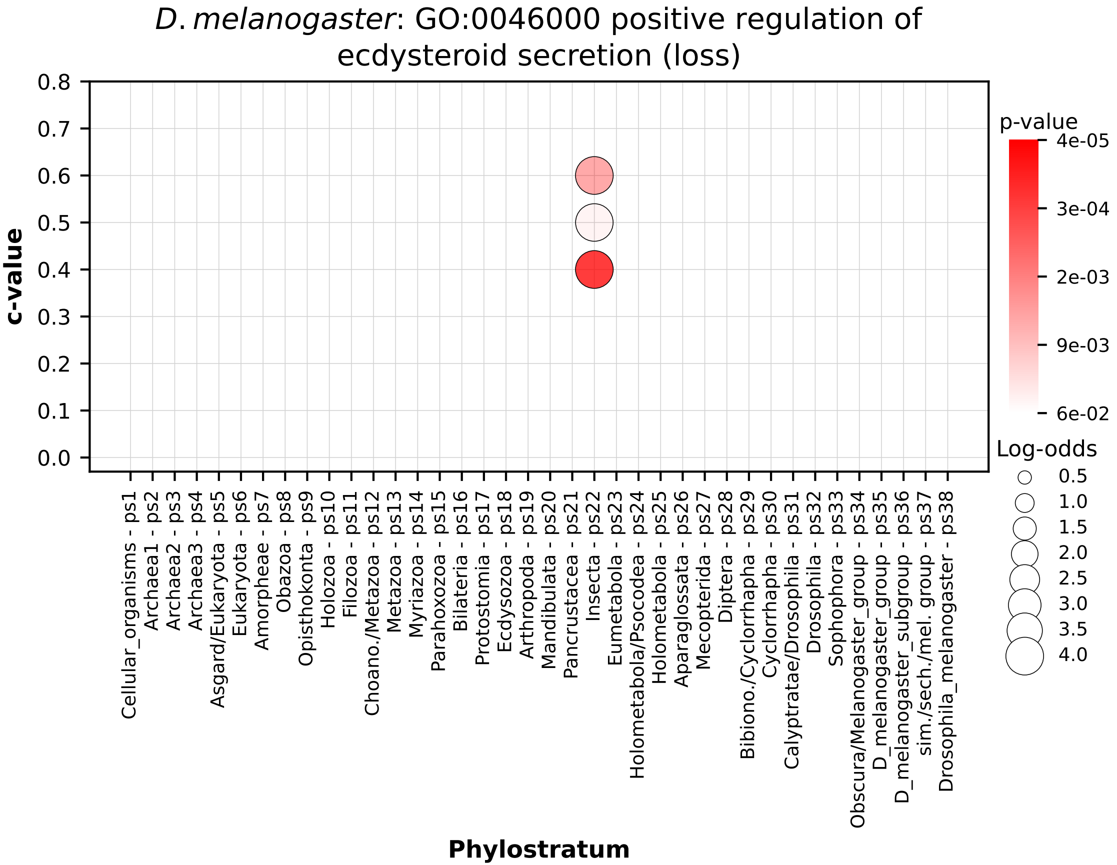

D. melanogaster: GO:0046000 positive regulation of ecdysteroid secretion (loss)
c-value
0.8
0.7
0.6
0.5
0.4
0.3
0.2
0.1
0.0
Phylostratum
Cellular_organisms - ps1
Archaea1 - ps2
Archaea2 - ps3
Archaea3 - ps4
Asgard/Eukaryota - ps5
Eukaryota - ps6
Amorpheae - ps7
Obazoa - ps8
Opisthokonta - ps9
Holozoa - ps10
Filozoa - ps11
Choano./Metazoa - ps12
Metazoa - ps13
Myriazoa - ps14
Parahoxozoa - ps15
Bilateria - ps16
Protostomia - ps17
Ecdysozoa - ps18
Arthropoda - ps19
Mandibulata - ps20
Pancrustacea - ps21
Insecta - ps22
Eumetabola - ps23
Holometabola/Psocodea - ps24
Holometabola - ps25
Aparaglossata - ps26
Mecopterida - ps27
Diptera - ps28
Bibiono./Cyclorrhapha - ps29
Cyclorrhapha - ps30
Calyptratae/Drosophila - ps31
Drosophila - ps32
Sophophora - ps33
Obscura/Melanogaster_group - ps34
D_melanogaster_group - ps35
D_melanogaster_subgroup - ps36
sim./sech./mel. group - ps37
Drosophila_melanogaster - ps38
p-value
4e-05
3e-04
2e-03
9e-03
6e-02
Log-odds
0.5
1.0
1.5
2.0
2.5
3.0
3.5
4.0

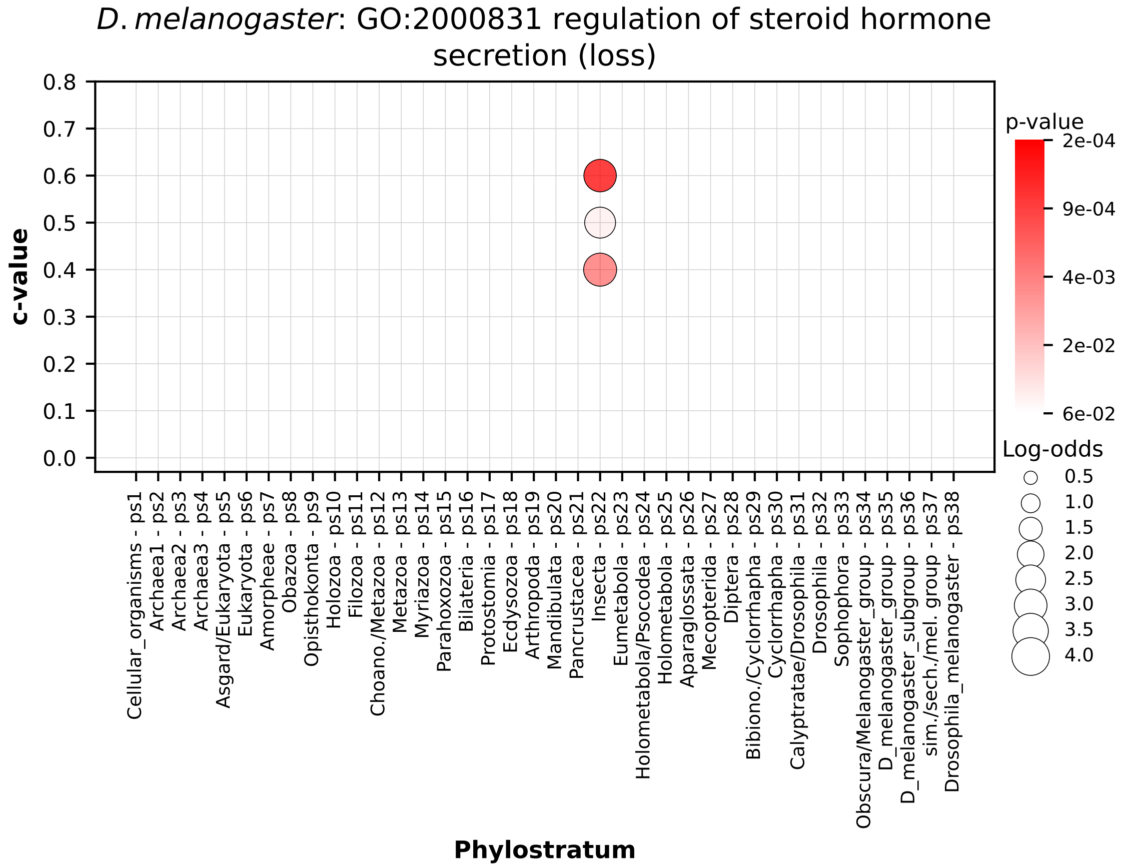

D. melanogaster: GO:2000831 regulation of steroid hormone secretion (loss)
c-value
0.8
0.7
0.6
0.5
0.4
0.3
0.2
0.1
0.0
Phylostratum
Cellular_organisms - ps1
Archaea1 - ps2
Archaea2 - ps3
Archaea3 - ps4
Asgard/Eukaryota - ps5
Eukaryota - ps6
Amorpheae - ps7
Obazoa - ps8
Opisthokonta - ps9
Holozoa - ps10
Filozoa - ps11
Choano./Metazoa - ps12
Metazoa - ps13
Myriazoa - ps14
Parahoxozoa - ps15
Bilateria - ps16
Protostomia - ps17
Ecdysozoa - ps18
Arthropoda - ps19
Mandibulata - ps20
Pancrustacea - ps21
Insecta - ps22
Eumetabola - ps23
Holometabola/Psocodea - ps24
Holometabola - ps25
Aparaglossata - ps26
Mecopterida - ps27
Diptera - ps28
Bibiono./Cyclorrhapha - ps29
Cyclorrhapha - ps30
Calyptratae/Drosophila - ps31
Drosophila - ps32
Sophophora - ps33
Obscura/Melanogaster_group - ps34
D_melanogaster_group - ps35
D_melanogaster_subgroup - ps36
sim./sech./mel. group - ps37
Drosophila_melanogaster - ps38
p-value
2e-04
9e-04
4e-03
2e-02
6e-02
Log-odds
0.5
1.0
1.5
2.0
2.5
3.0
3.5
4.0

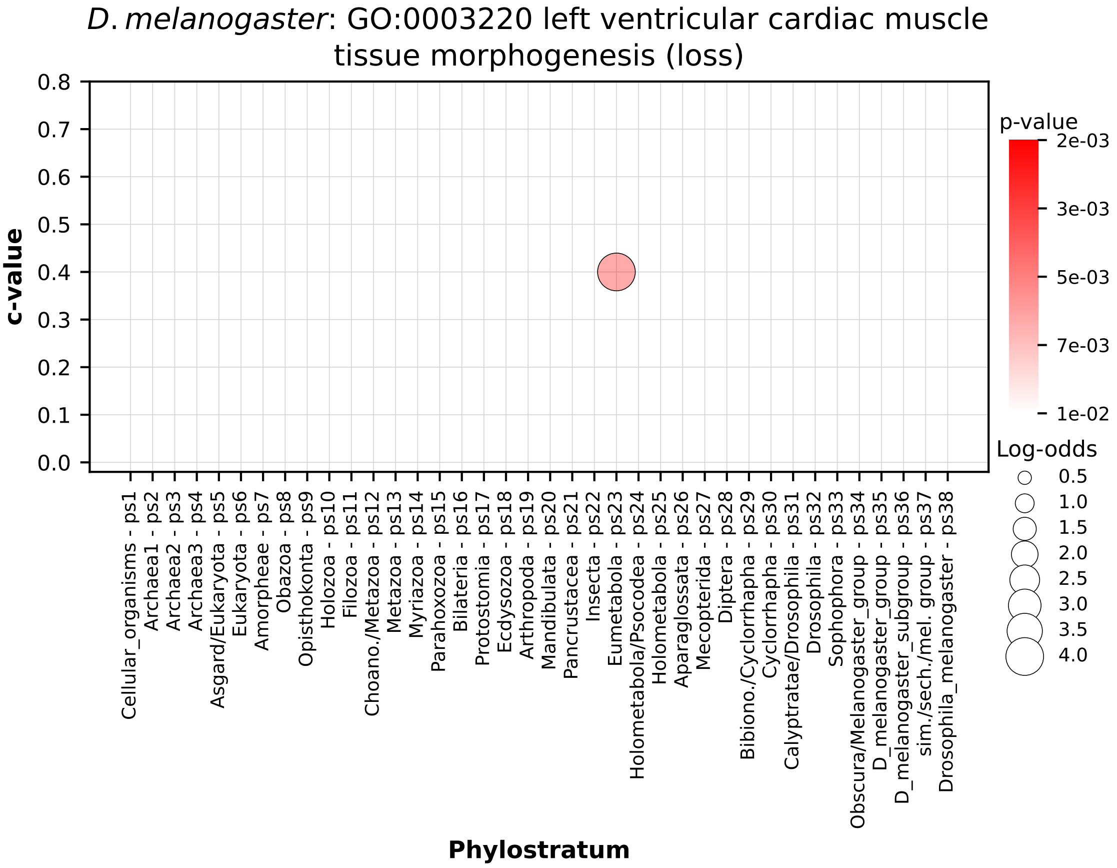
D. melanogaster: GO:0003220 left ventricular cardiac muscle tissue morphogenesis (loss)
c-value
0.8
0.7
0.6
0.5
0.4
0.3
0.2
0.1
0.0
p-value
2e-03
3e-03
5e-03
7e-03
1e-02
Log-odds
0.5
1.0
1.5
2.0
2.5
3.0
3.5
4.0
Cellular_organisms - ps1
Archaea1 - ps2
Archaea2 - ps3
Archaea3 - ps4
Asgard/Eukaryota - ps5
Eukaryota - ps6
Amorpheae - ps7
Obazoa - ps8
Opisthokonta - ps9
Holozoa - ps10
Filozoa - ps11
Choano./Metazoa - ps12
Metazoa - ps13
Myriazoa - ps14
Parahoxozoa - ps15
Bilateria - ps16
Protostomia - ps17
Ecdysozoa - ps18
Arthropoda - ps19
Mandibulata - ps20
Pancrustacea - ps21
Insecta - ps22
Eumetabola - ps23
Holometabola/Psocodea - ps24
Holometabola - ps25
Aparaglossata - ps26
Mecopterida - ps27
Diptera - ps28
Bibiono./Cyclorrhapha - ps29
Cyclorrhapha - ps30
Calyptratae/Drosophila - ps31
Drosophila - ps32
Sophophora - ps33
Obscura/Melanogaster_group - ps34
D_melanogaster_group - ps35
D_melanogaster_subgroup - ps36
sim./sech./mel. group - ps37
Drosophila_melanogaster - ps38
Phylostratum

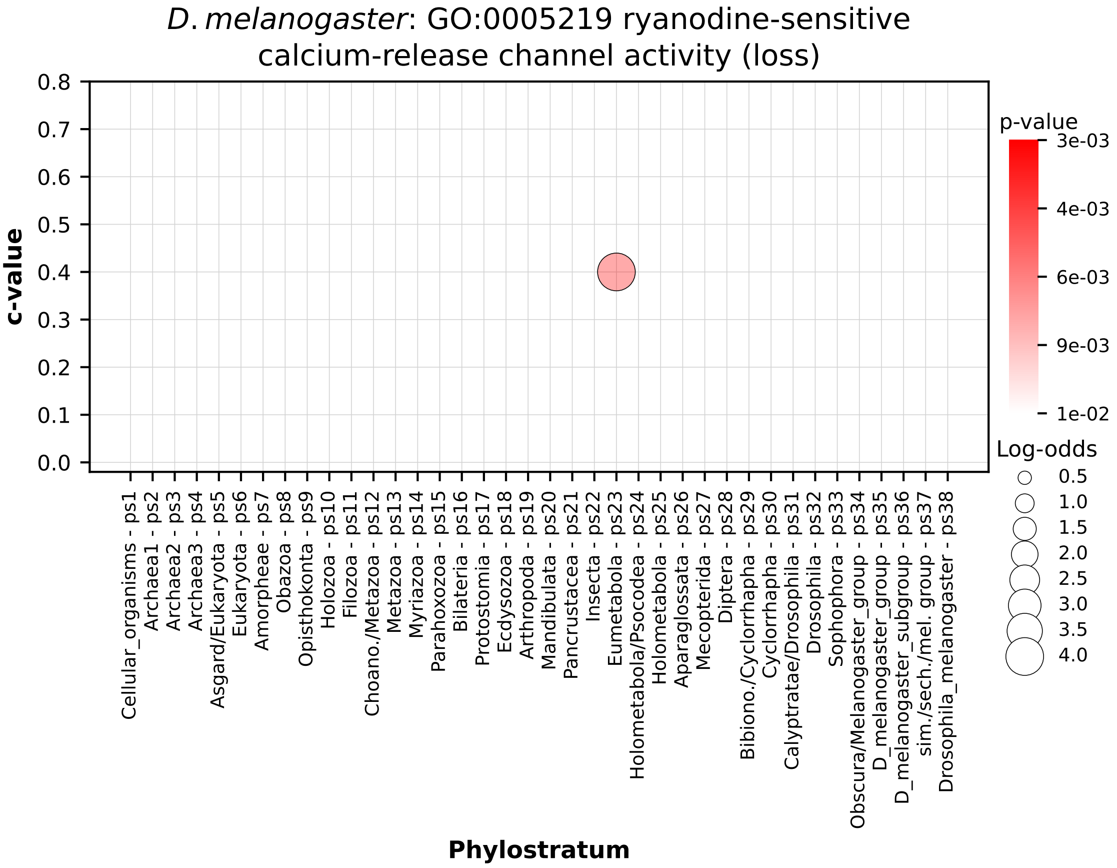
D. melanogaster: GO:0005219 ryanodine-sensitive calcium-release channel activity (loss)
c-value
0.0
0.1
0.2
0.3
0.4
0.5
0.6
0.7
0.8
Phylostratum
Cellular_organisms - ps1
Archaea1 - ps2
Archaea2 - ps3
Archaea3 - ps4
Asgard/Eukaryota - ps5
Eukaryota - ps6
Amorpheae - ps7
Obazoa - ps8
Opisthokonta - ps9
Holozoa - ps10
Filozoa - ps11
Choano./Metazoa - ps12
Metazoa - ps13
Myriazoa - ps14
Parahoxozoa - ps15
Bilateria - ps16
Protostomia - ps17
Ecdysozoa - ps18
Arthropoda - ps19
Mandibulata - ps20
Pancrustacea - ps21
Insecta - ps22
Eumetabola - ps23
Holometabola/Psocodea - ps24
Holometabola - ps25
Aparaglossata - ps26
Mecopterida - ps27
Diptera - ps28
Bibiono./Cyclorrhapha - ps29
Cyclorrhapha - ps30
Calyptratae/Drosophila - ps31
Drosophila - ps32
Sophophora - ps33
Obscura/Melanogaster_group - ps34
D_melanogaster_group - ps35
D_melanogaster_subgroup - ps36
sim./sech./mel. group - ps37
Drosophila_melanogaster - ps38
p-value
3e-03
4e-03
6e-03
9e-03
1e-02
Log-odds
0.5
1.0
1.5
2.0
2.5
3.0
3.5
4.0

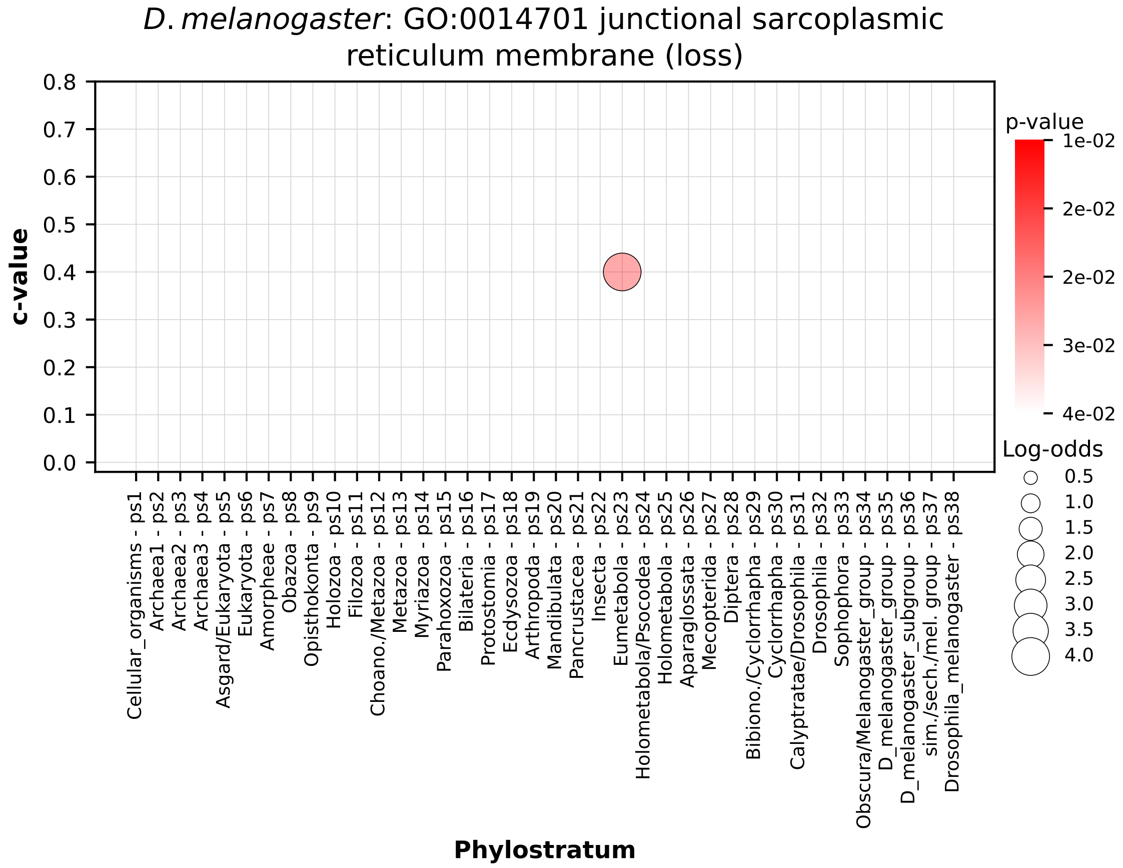
D. melanogaster: GO:0014701 junctional sarcoplasmic reticulum membrane (loss)
c-value
0.8
0.7
0.6
0.5
0.4
0.3
0.2
0.1
0.0
p-value
1e-02
2e-02
2e-02
3e-02
4e-02
Log-odds
0.5
1.0
1.5
2.0
2.5
3.0
3.5
4.0
Cellular_organisms - ps1
Archaea1 - ps2
Archaea2 - ps3
Archaea3 - ps4
Asgard/Eukaryota - ps5
Eukaryota - ps6
Amorpheae - ps7
Obazoa - ps8
Opisthokonta - ps9
Holozoa - ps10
Filozoa - ps11
Choano./Metazoa - ps12
Metazoa - ps13
Myriazoa - ps14
Parahoxozoa - ps15
Bilateria - ps16
Protostomia - ps17
Ecdysozoa - ps18
Arthropoda - ps19
Mandibulata - ps20
Pancrustacea - ps21
Insecta - ps22
Eumetabola - ps23
Holometabola/Psocodea - ps24
Holometabola - ps25
Aparaglossata - ps26
Mecopterida - ps27
Diptera - ps28
Bibiono./Cyclorrhapha - ps29
Cyclorrhapha - ps30
Calyptratae/Drosophila - ps31
Drosophila - ps32
Sophophora - ps33
Obscura/Melanogaster_group - ps34
D_melanogaster_group - ps35
D_melanogaster_subgroup - ps36
sim./sech./mel. group - ps37
Drosophila_melanogaster - ps38
Phylostratum

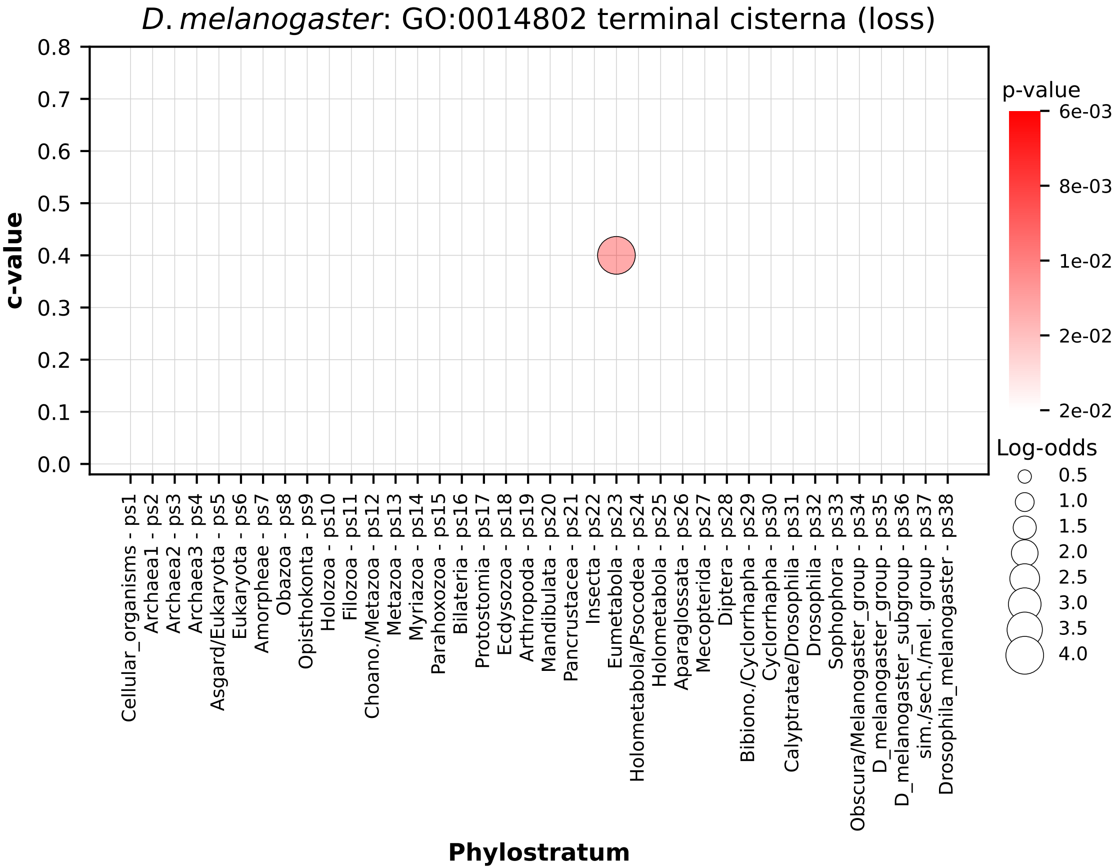

D. melanogaster: GO:0014802 terminal cisterna (loss)
c-value
0.8
0.7
0.6
0.5
0.4
0.3
0.2
0.1
0.0
Phylostratum
Cellular_organisms - ps1
Archaea1 - ps2
Archaea2 - ps3
Archaea3 - ps4
Asgard/Eukaryota - ps5
Eukaryota - ps6
Amorpheae - ps7
Obazoa - ps8
Opisthokonta - ps9
Holozoa - ps10
Filozoa - ps11
Choano./Metazoa - ps12
Metazoa - ps13
Myriazoa - ps14
Parahoxozoa - ps15
Bilateria - ps16
Protostomia - ps17
Ecdysozoa - ps18
Arthropoda - ps19
Mandibulata - ps20
Pancrustacea - ps21
Insecta - ps22
Eumetabola - ps23
Holometabola/Psocodea - ps24
Holometabola - ps25
Aparaglossata - ps26
Mecopterida - ps27
Diptera - ps28
Bibiono./Cyclorrhapha - ps29
Cyclorrhapha - ps30
Calyptratae/Drosophila - ps31
Drosophila - ps32
Sophophora - ps33
Obscura/Melanogaster_group - ps34
D_melanogaster_group - ps35
D_melanogaster_subgroup - ps36
sim./sech./mel. group - ps37
Drosophila_melanogaster - ps38
p-value
6e-03
8e-03
1e-02
2e-02
2e-02
Log-odds
0.5
1.0
1.5
2.0
2.5
3.0
3.5
4.0

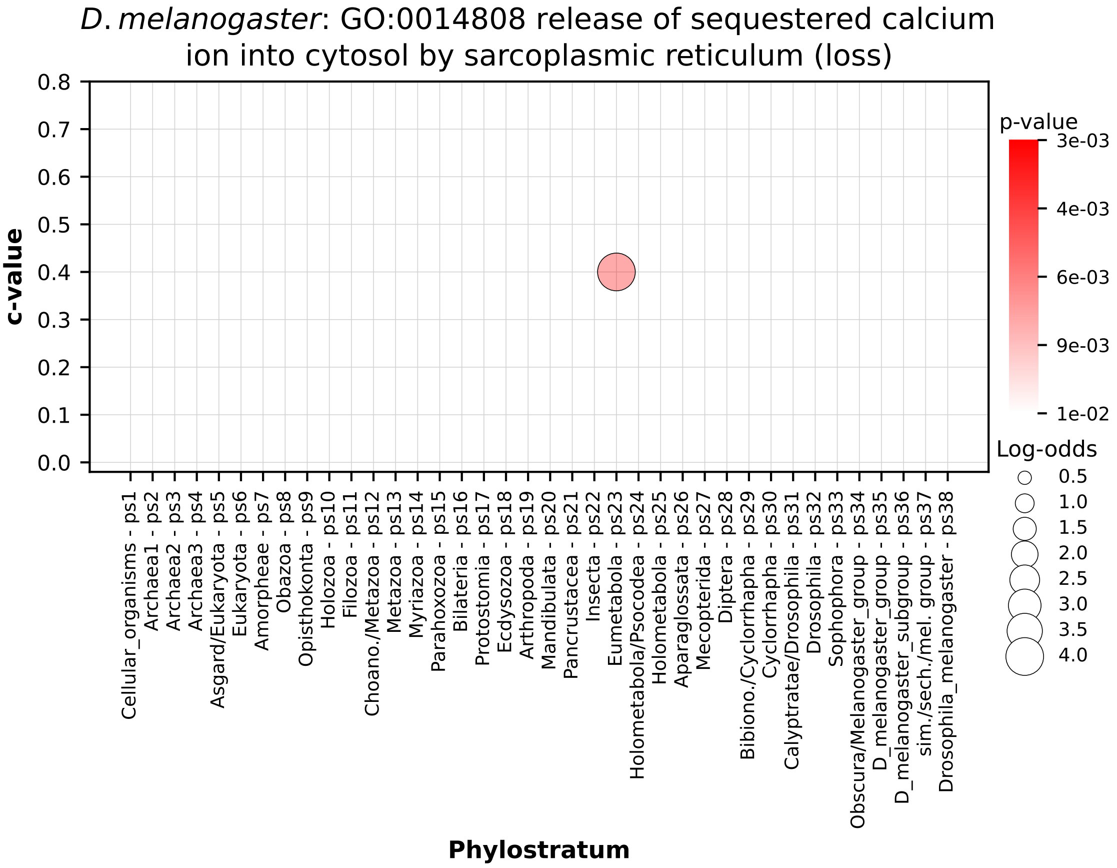

D. melanogaster: GO:0014808 release of sequestered calcium ion into cytosol by sarcoplasmic reticulum (loss)
c-value
0.8
0.7
0.6
0.5
0.4
0.3
0.2
0.1
0.0
Phylostratum
Cellular_organisms - ps1
Archaea1 - ps2
Archaea2 - ps3
Archaea3 - ps4
Asgard/Eukaryota - ps5
Eukaryota - ps6
Amorpheae - ps7
Obazoa - ps8
Opisthokonta - ps9
Holozoa - ps10
Filozoa - ps11
Choano./Metazoa - ps12
Metazoa - ps13
Myriazoa - ps14
Parahoxozoa - ps15
Bilateria - ps16
Protostomia - ps17
Ecdysozoa - ps18
Arthropoda - ps19
Mandibulata - ps20
Pancrustacea - ps21
Insecta - ps22
Eumetabola - ps23
Holometabola/Psocodea - ps24
Holometabola - ps25
Aparaglossata - ps26
Mecopterida - ps27
Diptera - ps28
Bibiono./Cyclorrhapha - ps29
Cyclorrhapha - ps30
Calyptratae/Drosophila - ps31
Drosophila - ps32
Sophophora - ps33
Obscura/Melanogaster_group - ps34
D_melanogaster_group - ps35
D_melanogaster_subgroup - ps36
sim./sech./mel. group - ps37
Drosophila_melanogaster - ps38
p-value
3e-03
4e-03
6e-03
9e-03
1e-02
Log-odds
0.5
1.0
1.5
2.0
2.5
3.0
3.5
4.0

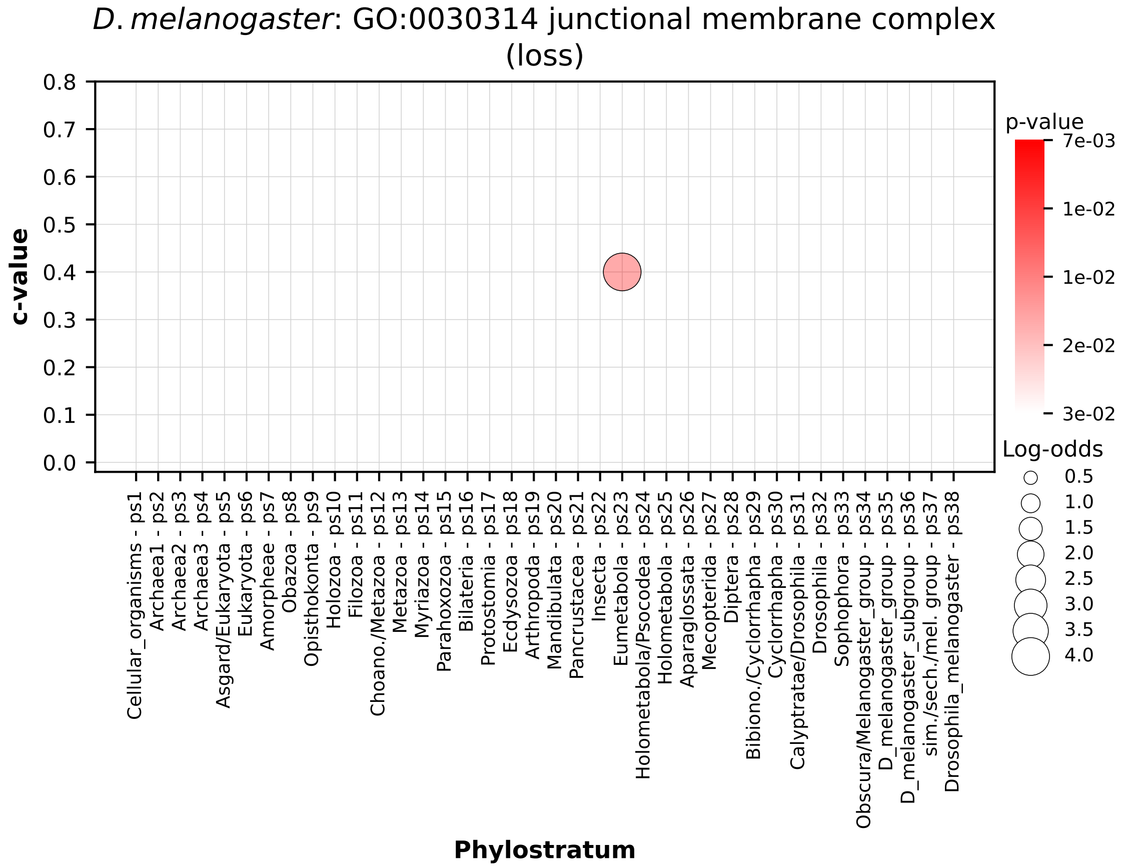

D. melanogaster: GO:0030314 junctional membrane complex (loss)
c-value
0.8
0.7
0.6
0.5
0.4
0.3
0.2
0.1
0.0
Phylostratum
Cellular_organisms - ps1
Archaea1 - ps2
Archaea2 - ps3
Archaea3 - ps4
Asgard/Eukaryota - ps5
Eukaryota - ps6
Amorpheae - ps7
Obazoa - ps8
Opisthokonta - ps9
Holozoa - ps10
Filozoa - ps11
Choano./Metazoa - ps12
Metazoa - ps13
Myriazoa - ps14
Parahoxozoa - ps15
Bilateria - ps16
Protostomia - ps17
Ecdysozoa - ps18
Arthropoda - ps19
Mandibulata - ps20
Pancrustacea - ps21
Insecta - ps22
Eumetabola - ps23
Holometabola/Psocodea - ps24
Holometabola - ps25
Aparaglossata - ps26
Mecopterida - ps27
Diptera - ps28
Bibiono./Cyclorrhapha - ps29
Cyclorrhapha - ps30
Calyptratae/Drosophila - ps31
Drosophila - ps32
Sophophora - ps33
Obscura/Melanogaster_group - ps34
D_melanogaster_group - ps35
D_melanogaster_subgroup - ps36
sim./sech./mel. group - ps37
Drosophila_melanogaster - ps38
p-value
7e-03
1e-02
1e-02
2e-02
3e-02
Log-odds
0.5
1.0
1.5
2.0
2.5
3.0
3.5
4.0

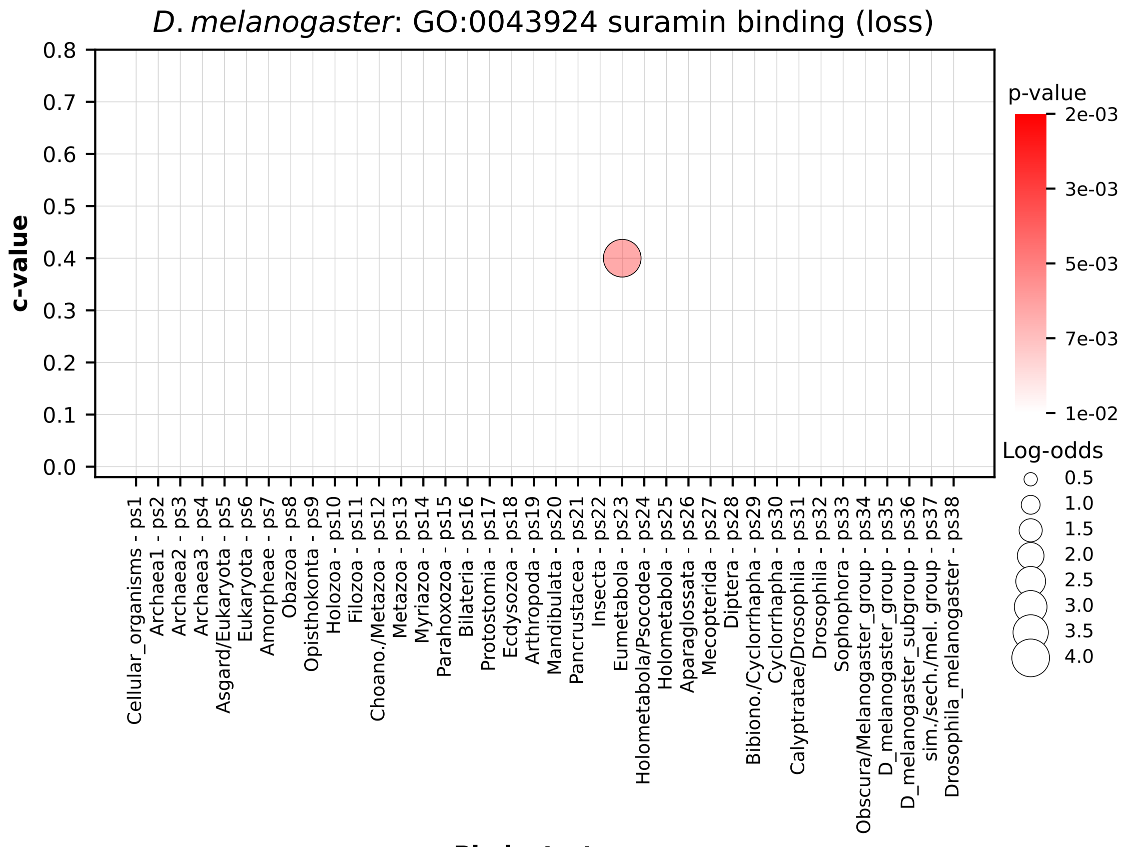
D. melanogaster: GO:0043924 suramin binding (loss)
c-value
0.8
0.7
0.6
0.5
0.4
0.3
0.2
0.1
0.0
Phylostratum
Cellular_organisms - ps1
Archaea1 - ps2
Archaea2 - ps3
Archaea3 - ps4
Asgard/Eukaryota - ps5
Eukaryota - ps6
Amorpheae - ps7
Obazoa - ps8
Opisthokonta - ps9
Holozoa - ps10
Filozoa - ps11
Choano./Metazoa - ps12
Metazoa - ps13
Myriazoa - ps14
Parahoxozoa - ps15
Bilateria - ps16
Protostomia - ps17
Ecdysozoa - ps18
Arthropoda - ps19
Mandibulata - ps20
Pancrustacea - ps21
Insecta - ps22
Eumetabola - ps23
Holometabola/Psocodea - ps24
Holometabola - ps25
Aparaglossata - ps26
Mecopterida - ps27
Diptera - ps28
Bibiono./Cyclorrhapha - ps29
Cyclorrhapha - ps30
Calyptratae/Drosophila - ps31
Drosophila - ps32
Sophophora - ps33
Obscura/Melanogaster_group - ps34
D_melanogaster_group - ps35
D_melanogaster_subgroup - ps36
sim./sech./mel. group - ps37
Drosophila_melanogaster - ps38
p-value
2e-03
3e-03
5e-03
7e-03
1e-02
Log-odds
0.5
1.0
1.5
2.0
2.5
3.0
3.5
4.0

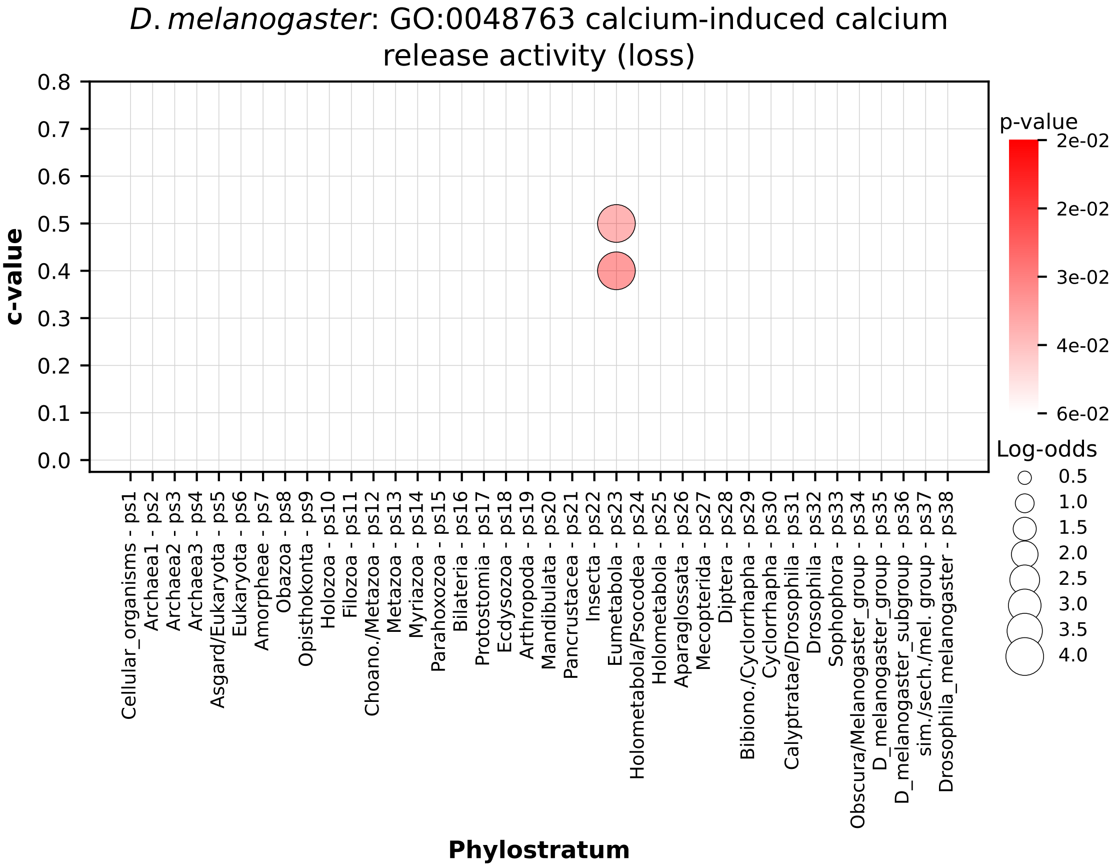

D. melanogaster: GO:0048763 calcium-induced calcium release activity (loss)
c-value
0.8
0.7
0.6
0.5
0.4
0.3
0.2
0.1
0.0
p-value
2e-02
2e-02
3e-02
4e-02
6e-02
Log-odds
0.5
1.0
1.5
2.0
2.5
3.0
3.5
4.0
Cellular_organisms - ps1
Archaea1 - ps2
Archaea2 - ps3
Archaea3 - ps4
Asgard/Eukaryota - ps5
Eukaryota - ps6
Amorpheae - ps7
Obazoa - ps8
Opisthokonta - ps9
Holozoa - ps10
Filozoa - ps11
Choano./Metazoa - ps12
Metazoa - ps13
Myriazoa - ps14
Parahoxozoa - ps15
Bilateria - ps16
Protostomia - ps17
Ecdysozoa - ps18
Arthropoda - ps19
Mandibulata - ps20
Pancrustacea - ps21
Insecta - ps22
Eumetabola - ps23
Holometabola/Psocodea - ps24
Holometabola - ps25
Aparaglossata - ps26
Mecopterida - ps27
Diptera - ps28
Bibiono./Cyclorrhapha - ps29
Cyclorrhapha - ps30
Calyptratae/Drosophila - ps31
Drosophila - ps32
Sophophora - ps33
Obscura/Melanogaster_group - ps34
D_melanogaster_group - ps35
D_melanogaster_subgroup - ps36
sim./sech./mel. group - ps37
Drosophila_melanogaster - ps38
Phylostratum

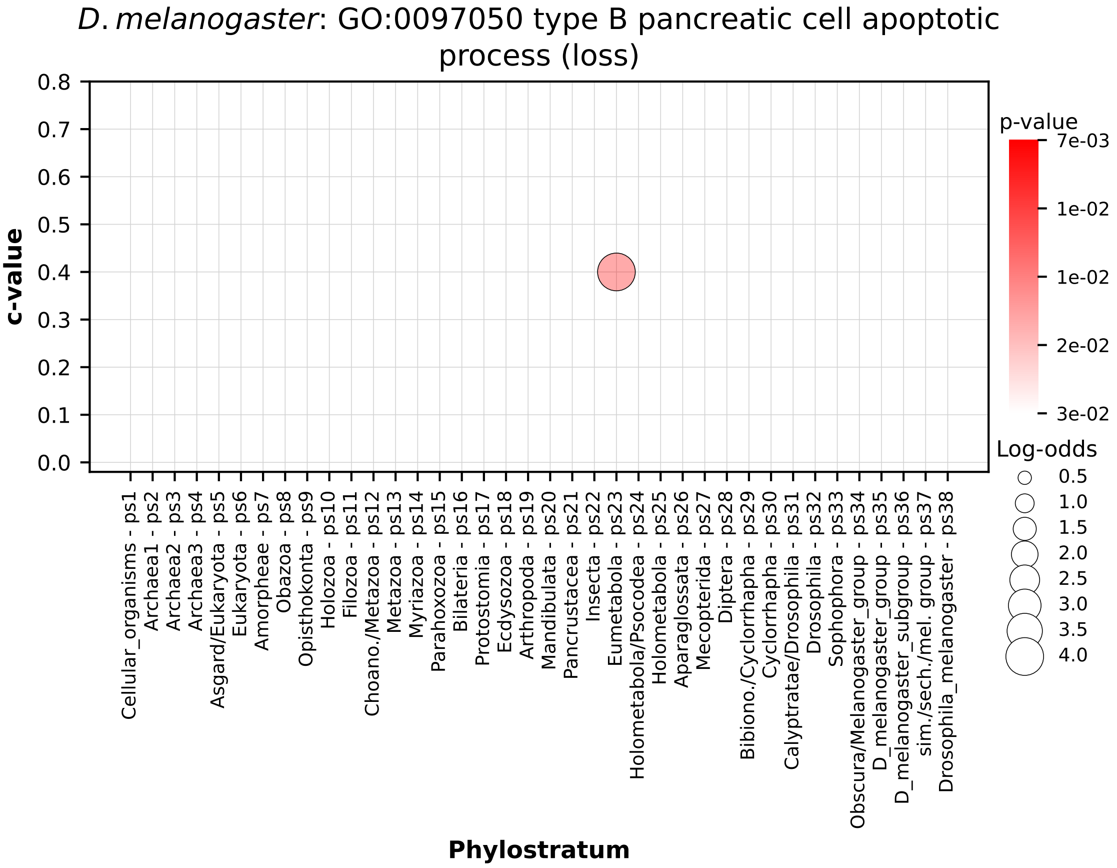

D. melanogaster: GO:0097050 type B pancreatic cell apoptotic process (loss)
c-value
0.8
0.7
0.6
0.5
0.4
0.3
0.2
0.1
0.0
Phylostratum
Cellular_organisms - ps1
Archaea1 - ps2
Archaea2 - ps3
Archaea3 - ps4
Asgard/Eukaryota - ps5
Eukaryota - ps6
Amorpheae - ps7
Obazoa - ps8
Opisthokonta - ps9
Holozoa - ps10
Filozoa - ps11
Choano./Metazoa - ps12
Metazoa - ps13
Myriazoa - ps14
Parahoxozoa - ps15
Bilateria - ps16
Protostomia - ps17
Ecdysozoa - ps18
Arthropoda - ps19
Mandibulata - ps20
Pancrustacea - ps21
Insecta - ps22
Eumetabola - ps23
Holometabola/Psocodea - ps24
Holometabola - ps25
Aparaglossata - ps26
Mecopterida - ps27
Diptera - ps28
Bibiono./Cyclorrhapha - ps29
Cyclorrhapha - ps30
Calyptratae/Drosophila - ps31
Drosophila - ps32
Sophophora - ps33
Obscura/Melanogaster_group - ps34
D_melanogaster_group - ps35
D_melanogaster_subgroup - ps36
sim./sech./mel. group - ps37
Drosophila_melanogaster - ps38
p-value
7e-03
1e-02
1e-02
2e-02
3e-02
Log-odds
0.5
1.0
1.5
2.0
2.5
3.0
3.5
4.0

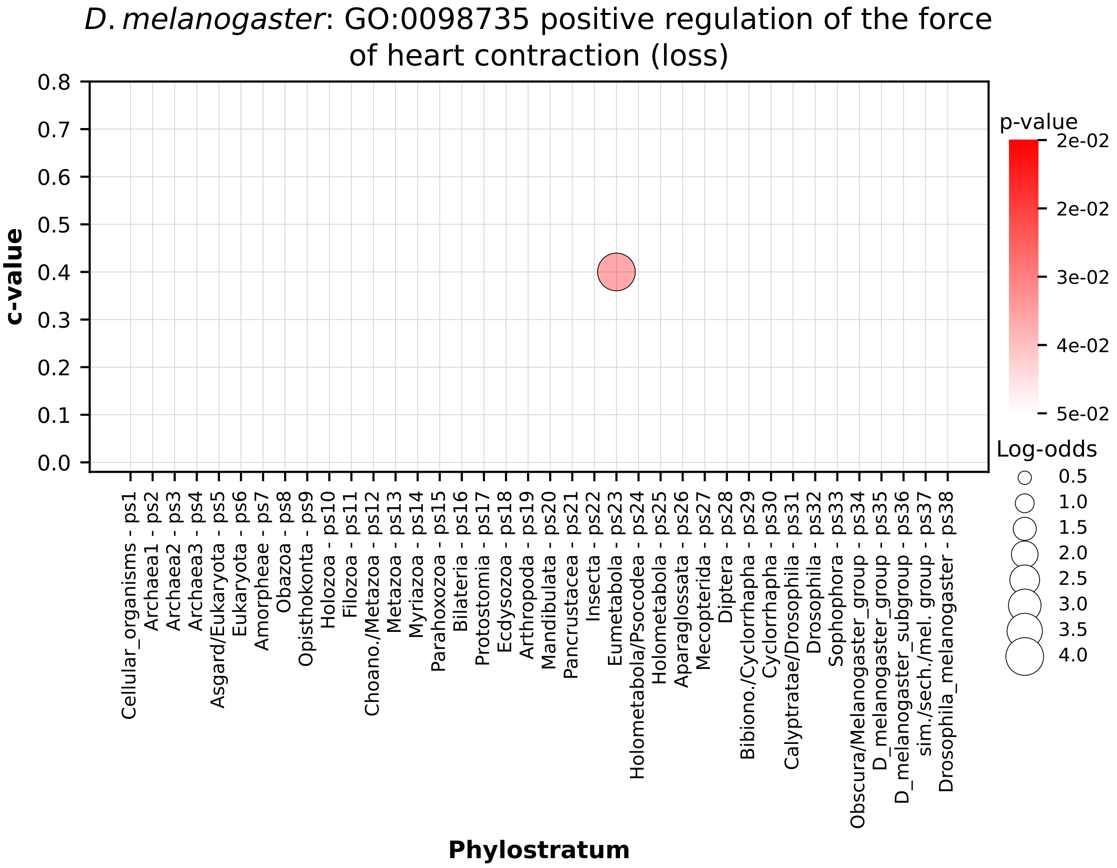

D. melanogaster: GO:0098735 positive regulation of the force of heart contraction (loss)
c-value
0.8
0.7
0.6
0.5
0.4
0.3
0.2
0.1
0.0
p-value
2e-02
2e-02
3e-02
4e-02
5e-02
Log-odds
0.5
1.0
1.5
2.0
2.5
3.0
3.5
4.0
Cellular_organisms - ps1
Archaea1 - ps2
Archaea2 - ps3
Archaea3 - ps4
Asgard/Eukaryota - ps5
Eukaryota - ps6
Amorpheae - ps7
Obazoa - ps8
Opisthokonta - ps9
Holozoa - ps10
Filozoa - ps11
Choano./Metazoa - ps12
Metazoa - ps13
Myriazoa - ps14
Parahoxozoa - ps15
Bilateria - ps16
Protostomia - ps17
Ecdysozoa - ps18
Arthropoda - ps19
Mandibulata - ps20
Pancrustacea - ps21
Insecta - ps22
Eumetabola - ps23
Holometabola/Psocodea - ps24
Holometabola - ps25
Aparaglossata - ps26
Mecopterida - ps27
Diptera - ps28
Bibiono./Cyclorrhapha - ps29
Cyclorrhapha - ps30
Calyptratae/Drosophila - ps31
Drosophila - ps32
Sophophora - ps33
Obscura/Melanogaster_group - ps34
D_melanogaster_group - ps35
D_melanogaster_subgroup - ps36
sim./sech./mel. group - ps37
Drosophila_melanogaster - ps38
Phylostratum

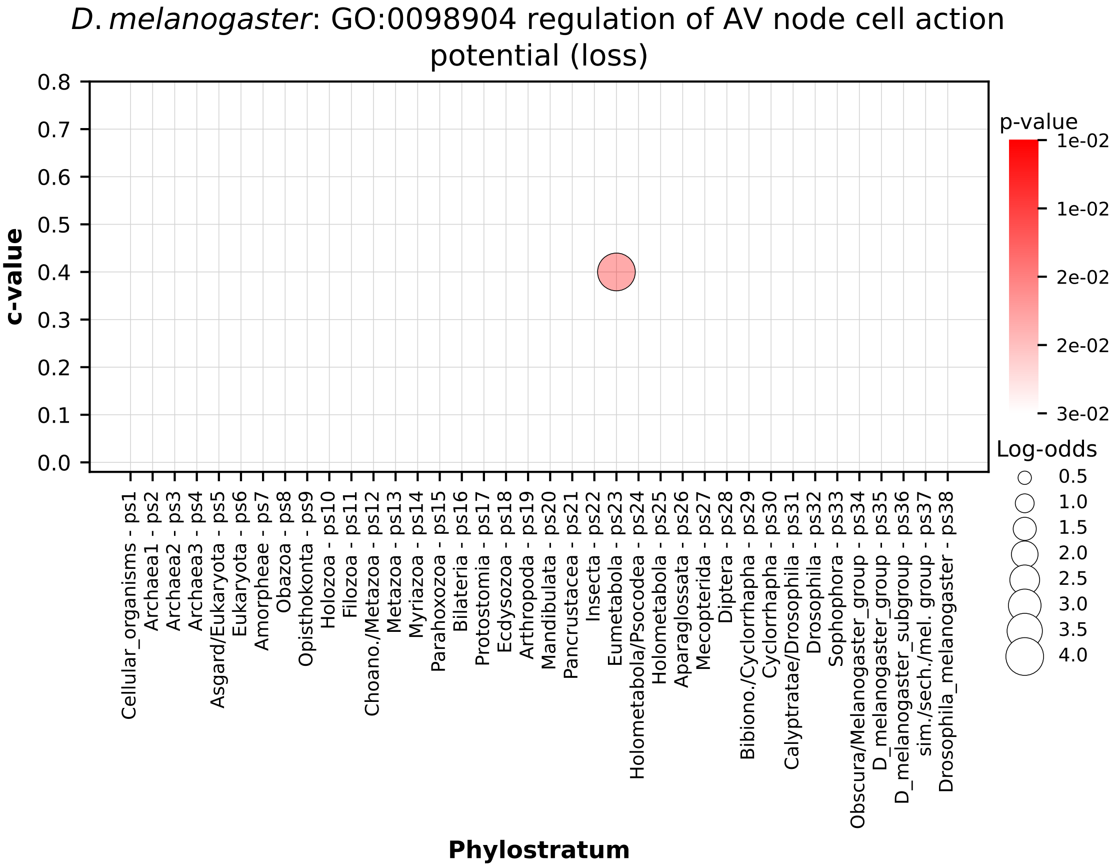

D. melanogaster: GO:0098904 regulation of AV node cell action potential (loss)
c-value
0.0
0.1
0.2
0.3
0.4
0.5
0.6
0.7
0.8
p-value
1e-02
1e-02
2e-02
2e-02
3e-02
Log-odds
0.5
1.0
1.5
2.0
2.5
3.0
3.5
4.0
Cellular_organisms - ps1
Archaea1 - ps2
Archaea2 - ps3
Archaea3 - ps4
Asgard/Eukaryota - ps5
Eukaryota - ps6
Amorpheae - ps7
Obazoa - ps8
Opisthokonta - ps9
Holozoa - ps10
Filozoa - ps11
Choano./Metazoa - ps12
Metazoa - ps13
Myriazoa - ps14
Parahoxozoa - ps15
Bilateria - ps16
Protostomia - ps17
Ecdysozoa - ps18
Arthropoda - ps19
Mandibulata - ps20
Pancrustacea - ps21
Insecta - ps22
Eumetabola - ps23
Holometabola/Psocodea - ps24
Holometabola - ps25
Aparaglossata - ps26
Mecopterida - ps27
Diptera - ps28
Bibiono./Cyclorrhapha - ps29
Cyclorrhapha - ps30
Calyptratae/Drosophila - ps31
Drosophila - ps32
Sophophora - ps33
Obscura/Melanogaster_group - ps34
D_melanogaster_group - ps35
D_melanogaster_subgroup - ps36
sim./sech./mel. group - ps37
Drosophila_melanogaster - ps38
Phylostratum

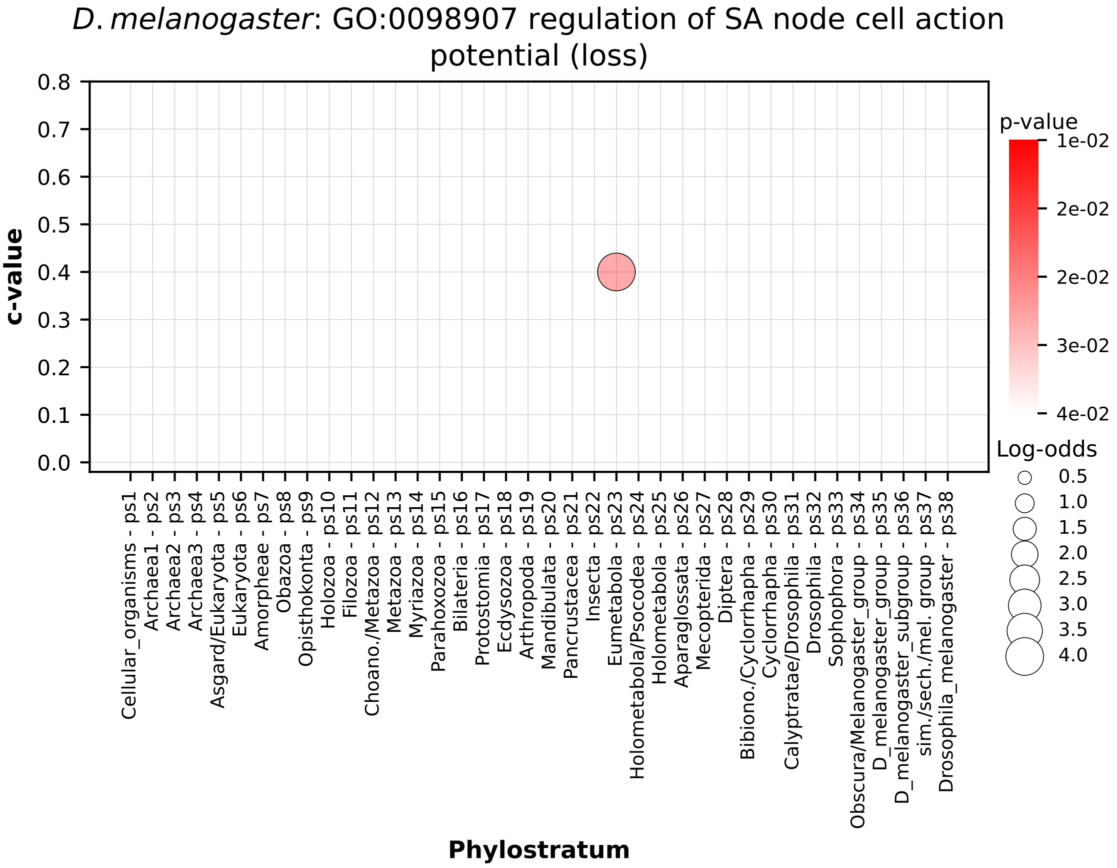

D. melanogaster: GO:0098907 regulation of SA node cell action potential (loss)
c-value
0.8
0.7
0.6
0.5
0.4
0.3
0.2
0.1
0.0
p-value
1e-02
2e-02
2e-02
3e-02
4e-02
Log-odds
0.5
1.0
1.5
2.0
2.5
3.0
3.5
4.0
Cellular_organisms - ps1
Archaea1 - ps2
Archaea2 - ps3
Archaea3 - ps4
Asgard/Eukaryota - ps5
Eukaryota - ps6
Amorpheae - ps7
Obazoa - ps8
Opisthokonta - ps9
Holozoa - ps10
Filozoa - ps11
Choano./Metazoa - ps12
Metazoa - ps13
Myriazoa - ps14
Parahoxozoa - ps15
Bilateria - ps16
Protostomia - ps17
Ecdysozoa - ps18
Arthropoda - ps19
Mandibulata - ps20
Pancrustacea - ps21
Insecta - ps22
Eumetabola - ps23
Holometabola/Psocodea - ps24
Holometabola - ps25
Aparaglossata - ps26
Mecopterida - ps27
Diptera - ps28
Bibiono./Cyclorrhapha - ps29
Cyclorrhapha - ps30
Calyptratae/Drosophila - ps31
Drosophila - ps32
Sophophora - ps33
Obscura/Melanogaster_group - ps34
D_melanogaster_group - ps35
D_melanogaster_subgroup - ps36
sim./sech./mel. group - ps37
Drosophila_melanogaster - ps38
Phylostratum

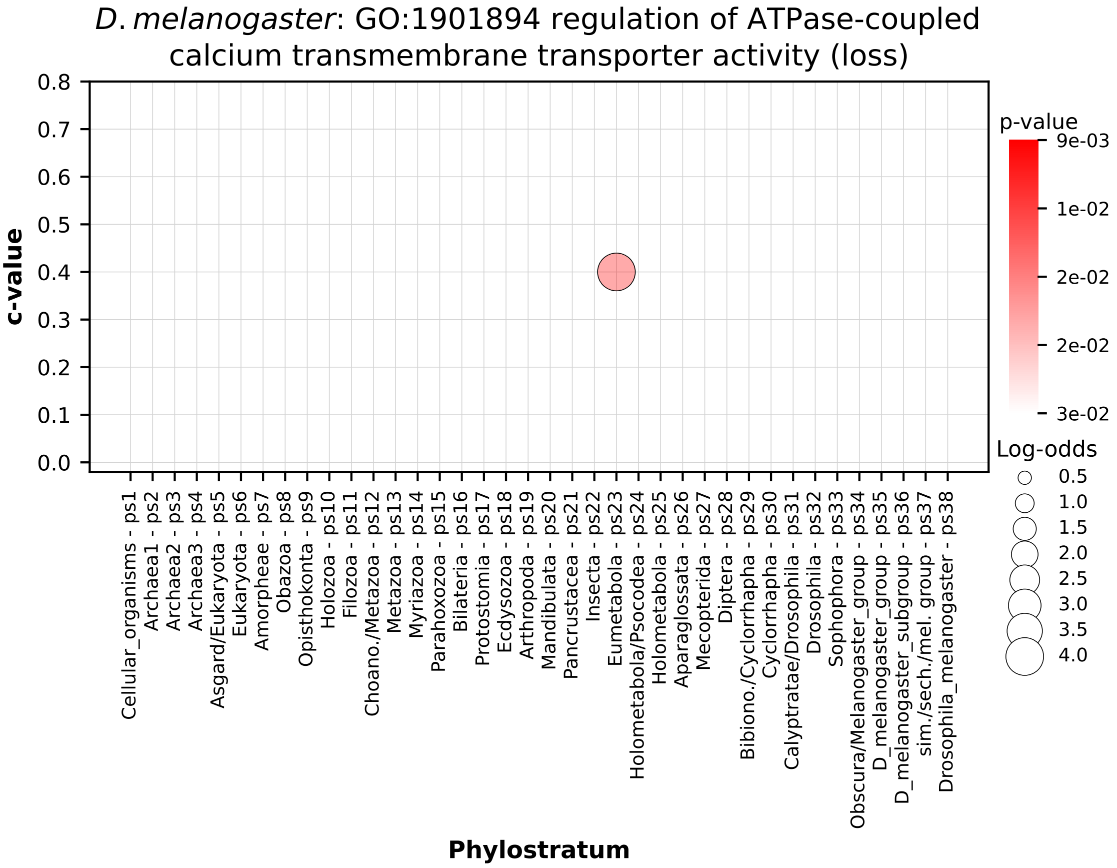

D. melanogaster: GO:1901894 regulation of ATPase-coupled calcium transmembrane transporter activity (loss)
c-value
0.8
0.7
0.6
0.5
0.4
0.3
0.2
0.1
0.0
p-value
9e-03
1e-02
2e-02
2e-02
3e-02
Log-odds
0.5
1.0
1.5
2.0
2.5
3.0
3.5
4.0
Cellular_organisms - ps1
Archaea1 - ps2
Archaea2 - ps3
Archaea3 - ps4
Asgard/Eukaryota - ps5
Eukaryota - ps6
Amorpheae - ps7
Obazoa - ps8
Opisthokonta - ps9
Holozoa - ps10
Filozoa - ps11
Choano./Metazoa - ps12
Metazoa - ps13
Myriazoa - ps14
Parahoxozoa - ps15
Bilateria - ps16
Protostomia - ps17
Ecdysozoa - ps18
Arthropoda - ps19
Mandibulata - ps20
Pancrustacea - ps21
Insecta - ps22
Eumetabola - ps23
Holometabola/Psocodea - ps24
Holometabola - ps25
Aparaglossata - ps26
Mecopterida - ps27
Diptera - ps28
Bibiono./Cyclorrhapha - ps29
Cyclorrhapha - ps30
Calyptratae/Drosophila - ps31
Drosophila - ps32
Sophophora - ps33
Obscura/Melanogaster_group - ps34
D_melanogaster_group - ps35
D_melanogaster_subgroup - ps36
sim./sech./mel. group - ps37
Drosophila_melanogaster - ps38
Phylostratum

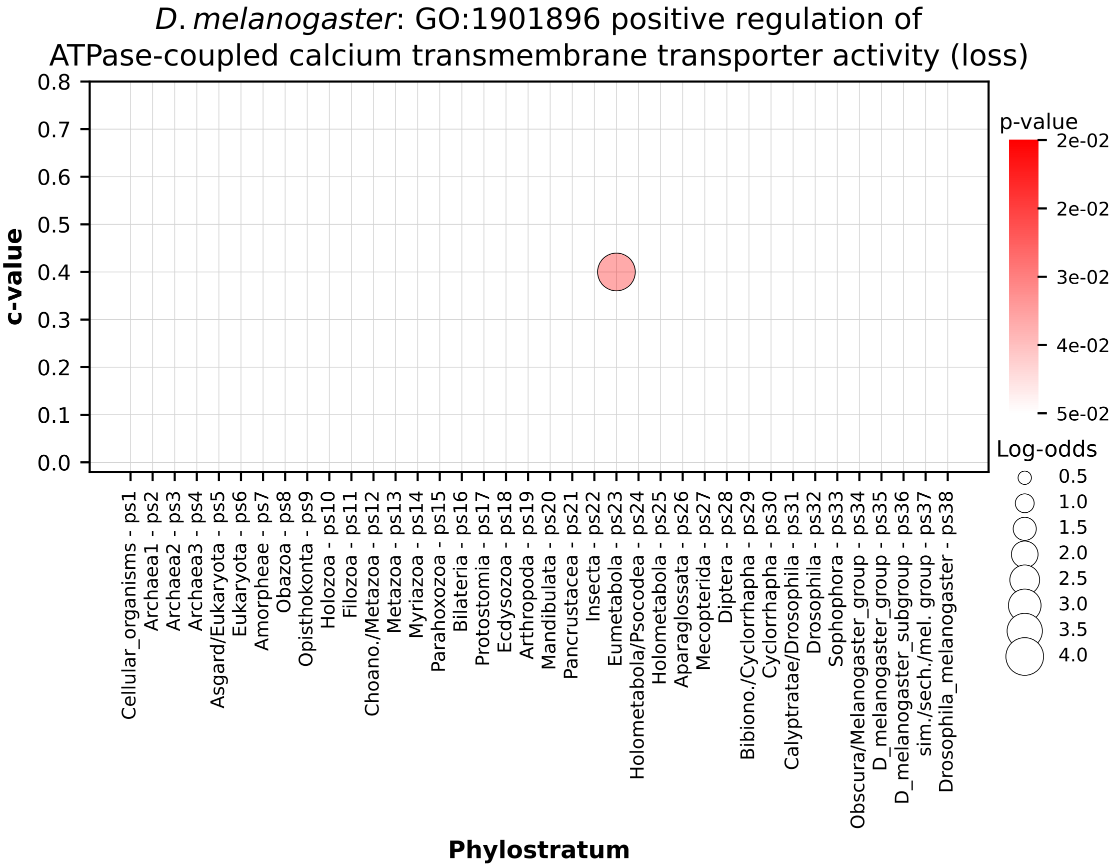

D. melanogaster: GO:1901896 positive regulation of ATPase-coupled calcium transmembrane transporter activity (loss)
c-value
0.8
0.7
0.6
0.5
0.4
0.3
0.2
0.1
0.0
Phylostratum
Cellular_organisms - ps1
Archaea1 - ps2
Archaea2 - ps3
Archaea3 - ps4
Asgard/Eukaryota - ps5
Eukaryota - ps6
Amorpheae - ps7
Obazoa - ps8
Opisthokonta - ps9
Holozoa - ps10
Filozoa - ps11
Choano./Metazoa - ps12
Metazoa - ps13
Myriazoa - ps14
Parahoxozoa - ps15
Bilateria - ps16
Protostomia - ps17
Ecdysozoa - ps18
Arthropoda - ps19
Mandibulata - ps20
Pancrustacea - ps21
Insecta - ps22
Eumetabola - ps23
Holometabola/Psocodea - ps24
Holometabola - ps25
Aparaglossata - ps26
Mecopterida - ps27
Diptera - ps28
Bibiono./Cyclorrhapha - ps29
Cyclorrhapha - ps30
Calyptratae/Drosophila - ps31
Drosophila - ps32
Sophophora - ps33
Obscura/Melanogaster_group - ps34
D_melanogaster_group - ps35
D_melanogaster_subgroup - ps36
sim./sech./mel. group - ps37
Drosophila_melanogaster - ps38
p-value
2e-02
2e-02
3e-02
4e-02
5e-02
Log-odds
0.5
1.0
1.5
2.0
2.5
3.0
3.5
4.0

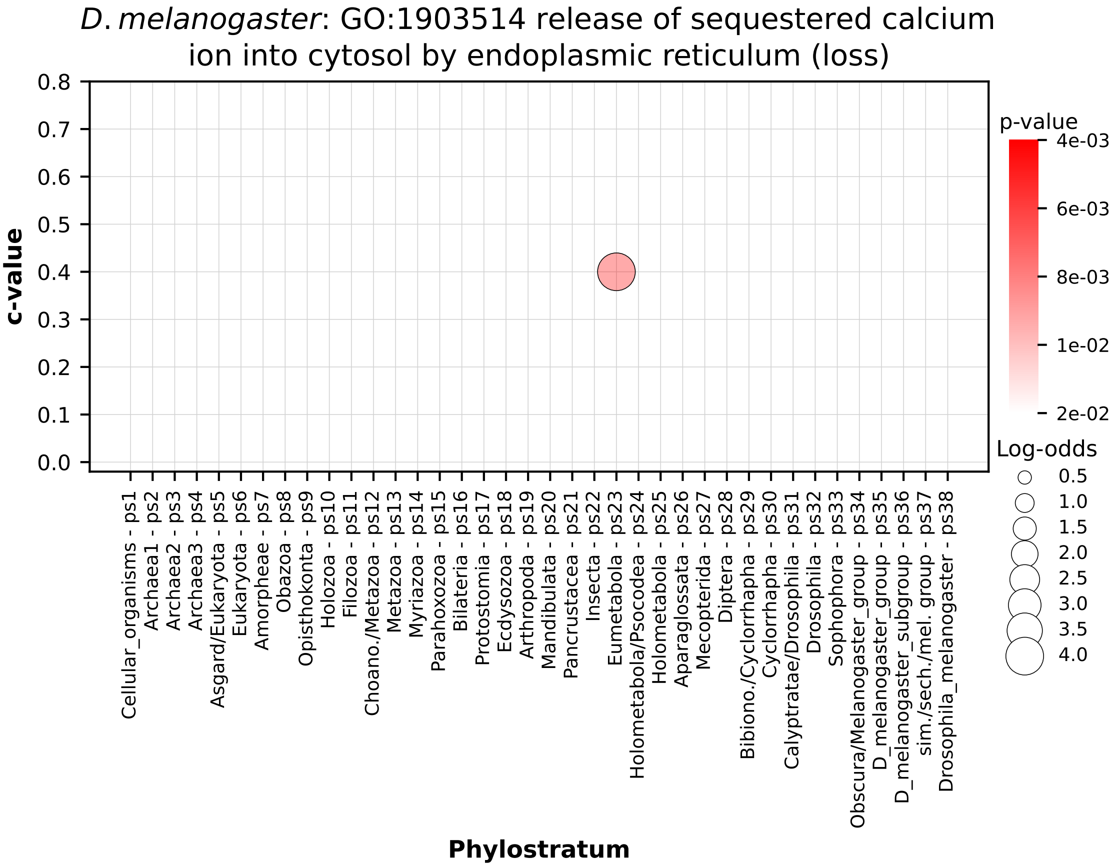

D. melanogaster: GO:1903514 release of sequestered calcium ion into cytosol by endoplasmic reticulum (loss)
c-value
0.8
0.7
0.6
0.5
0.4
0.3
0.2
0.1
0.0
p-value
4e-03
6e-03
8e-03
1e-02
2e-02
Log-odds
0.5
1.0
1.5
2.0
2.5
3.0
3.5
4.0
Cellular_organisms - ps1
Archaea1 - ps2
Archaea2 - ps3
Archaea3 - ps4
Asgard/Eukaryota - ps5
Eukaryota - ps6
Amorpheae - ps7
Obazoa - ps8
Opisthokonta - ps9
Holozoa - ps10
Filozoa - ps11
Choano./Metazoa - ps12
Metazoa - ps13
Myriazoa - ps14
Parahoxozoa - ps15
Bilateria - ps16
Protostomia - ps17
Ecdysozoa - ps18
Arthropoda - ps19
Mandibulata - ps20
Pancrustacea - ps21
Insecta - ps22
Eumetabola - ps23
Holometabola/Psocodea - ps24
Holometabola - ps25
Aparaglossata - ps26
Mecopterida - ps27
Diptera - ps28
Bibiono./Cyclorrhapha - ps29
Cyclorrhapha - ps30
Calyptratae/Drosophila - ps31
Drosophila - ps32
Sophophora - ps33
Obscura/Melanogaster_group - ps34
D_melanogaster_group - ps35
D_melanogaster_subgroup - ps36
sim./sech./mel. group - ps37
Drosophila_melanogaster - ps38
Phylostratum

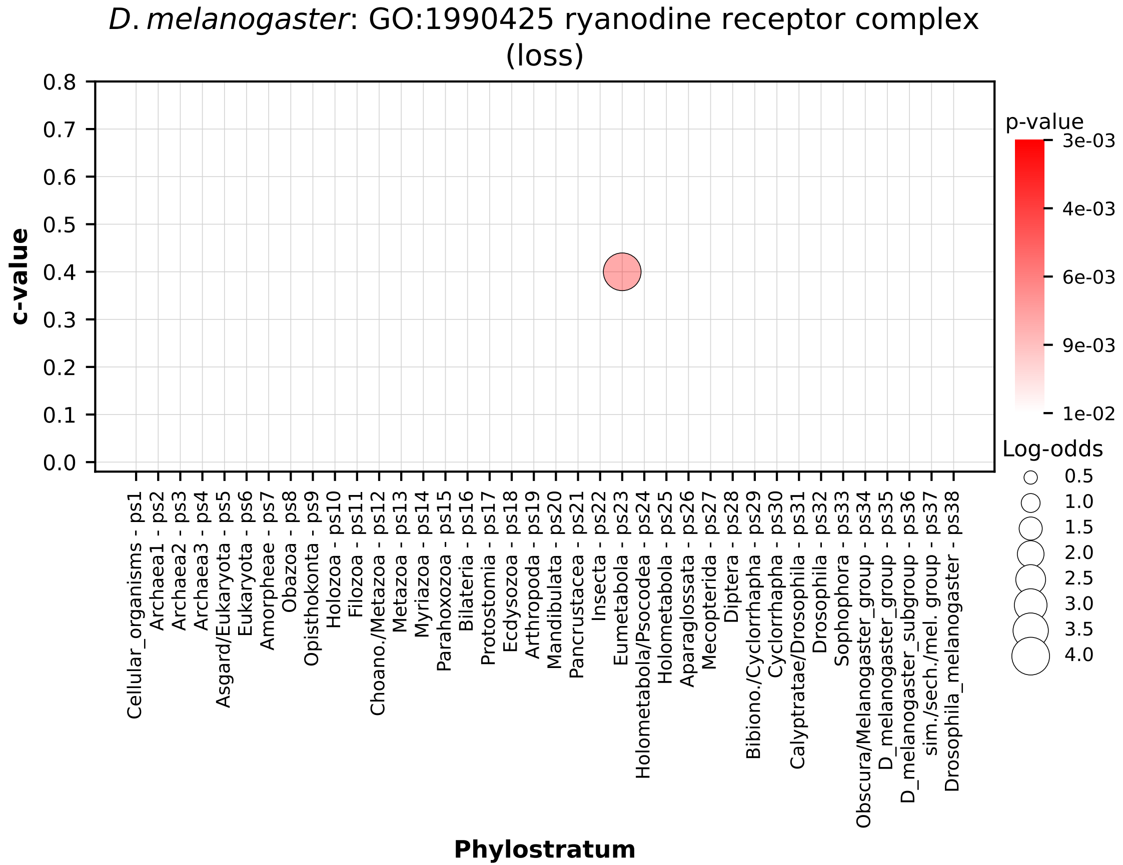
D. melanogaster: GO:1990425 ryanodine receptor complex (loss)
c-value
0.0
0.1
0.2
0.3
0.4
0.5
0.6
0.7
0.8
p-value
3e-03
4e-03
6e-03
9e-03
1e-02
Log-odds
0.5
1.0
1.5
2.0
2.5
3.0
3.5
4.0
Cellular_organisms - ps1
Archaea1 - ps2
Archaea2 - ps3
Archaea3 - ps4
Asgard/Eukaryota - ps5
Eukaryota - ps6
Amorpheae - ps7
Obazoa - ps8
Opisthokonta - ps9
Holozoa - ps10
Filozoa - ps11
Choano./Metazoa - ps12
Metazoa - ps13
Myriazoa - ps14
Parahoxozoa - ps15
Bilateria - ps16
Protostomia - ps17
Ecdysozoa - ps18
Arthropoda - ps19
Mandibulata - ps20
Pancrustacea - ps21
Insecta - ps22
Eumetabola - ps23
Holometabola/Psocodea - ps24
Holometabola - ps25
Aparaglossata - ps26
Mecopterida - ps27
Diptera - ps28
Bibiono./Cyclorrhapha - ps29
Cyclorrhapha - ps30
Calyptratae/Drosophila - ps31
Drosophila - ps32
Sophophora - ps33
Obscura/Melanogaster_group - ps34
D_melanogaster_group - ps35
D_melanogaster_subgroup - ps36
sim./sech./mel. group - ps37
Drosophila_melanogaster - ps38
Phylostratum

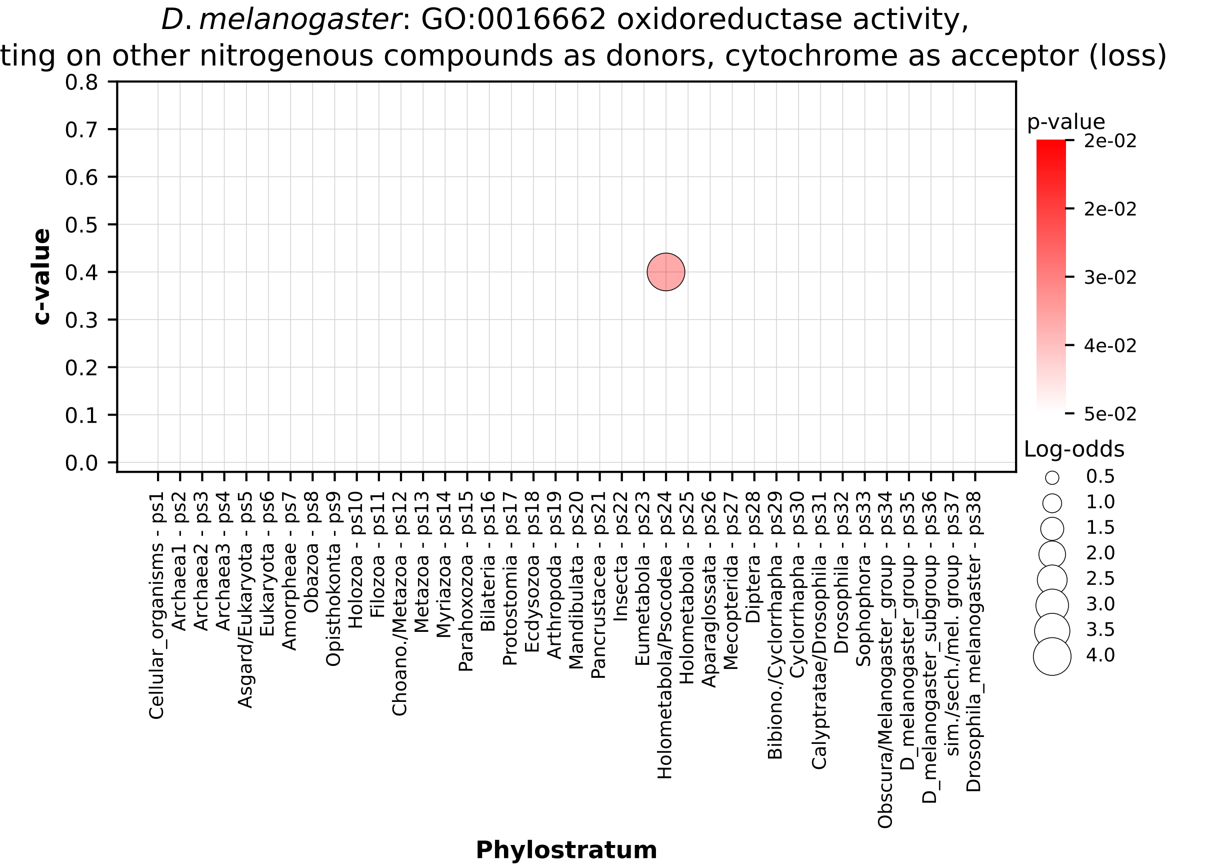
D. melanogaster: GO:0016662 oxidoreductase activity,
ting on other nitrogenous compounds as donors, cytochrome as acceptor (loss)
c-value
0.8
0.7
0.6
0.5
0.4
0.3
0.2
0.1
0.0
p-value
2e-02
2e-02
3e-02
4e-02
5e-02
Log-odds
0.5
1.0
1.5
2.0
2.5
3.0
3.5
4.0
Cellular_organisms - ps1
Archaea1 - ps2
Archaea2 - ps3
Archaea3 - ps4
Asgard/Eukaryota - ps5
Eukaryota - ps6
Amorpheae - ps7
Obazoa - ps8
Opisthokonta - ps9
Holozoa - ps10
Filozoa - ps11
Choano./Metazoa - ps12
Metazoa - ps13
Myriazoa - ps14
Parahoxozoa - ps15
Bilateria - ps16
Protostomia - ps17
Ecdysozoa - ps18
Arthropoda - ps19
Mandibulata - ps20
Pancrustacea - ps21
Insecta - ps22
Eumetabola - ps23
Holometabola/Psocodea - ps24
Holometabola - ps25
Aparaglossata - ps26
Mecopterida - ps27
Diptera - ps28
Bibiono./Cyclorrhapha - ps29
Cyclorrhapha - ps30
Calyptratae/Drosophila - ps31
Drosophila - ps32
Sophophora - ps33
Obscura/Melanogaster_group - ps34
D_melanogaster_group - ps35
D_melanogaster_subgroup - ps36
sim./sech./mel. group - ps37
Drosophila_melanogaster - ps38
Phylostratum

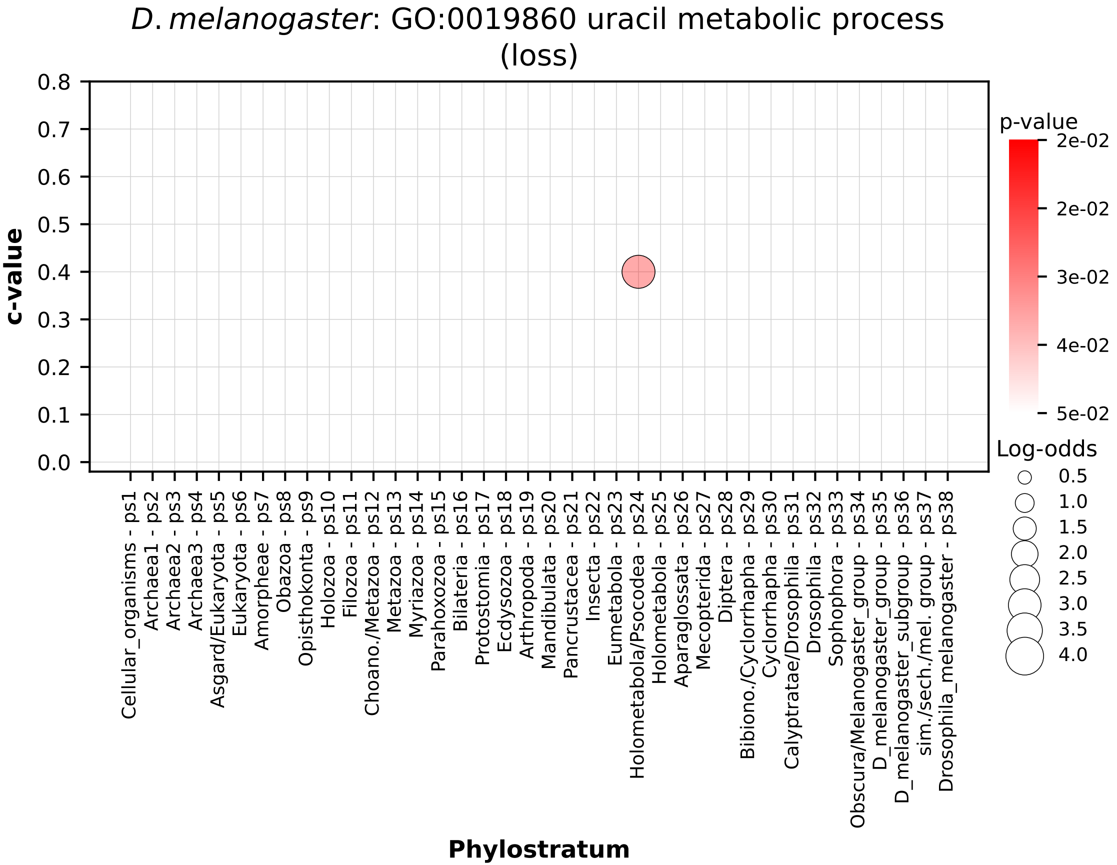

D. melanogaster: GO:0019860 uracil metabolic process
(loss)
c-value
0.8
0.7
0.6
0.5
0.4
0.3
0.2
0.1
0.0
p-value
2e-02
2e-02
3e-02
4e-02
5e-02
Log-odds
0.5
1.0
1.5
2.0
2.5
3.0
3.5
4.0
Cellular_organisms - ps1
Archaea1 - ps2
Archaea2 - ps3
Archaea3 - ps4
Asgard/Eukaryota - ps5
Eukaryota - ps6
Amorpheae - ps7
Obazoa - ps8
Opisthokonta - ps9
Holozoa - ps10
Filozoa - ps11
Choano./Metazoa - ps12
Metazoa - ps13
Myriazoa - ps14
Parahoxozoa - ps15
Bilateria - ps16
Protostomia - ps17
Ecdysozoa - ps18
Arthropoda - ps19
Mandibulata - ps20
Pancrustacea - ps21
Insecta - ps22
Eumetabola - ps23
Holometabola/Psocodea - ps24
Holometabola - ps25
Aparaglossata - ps26
Mecopterida - ps27
Diptera - ps28
Bibiono./Cyclorrhapha - ps29
Cyclorrhapha - ps30
Calyptratae/Drosophila - ps31
Drosophila - ps32
Sophophora - ps33
Obscura/Melanogaster_group - ps34
D_melanogaster_group - ps35
D_melanogaster_subgroup - ps36
sim./sech./mel. group - ps37
Drosophila_melanogaster - ps38
Phylostratum

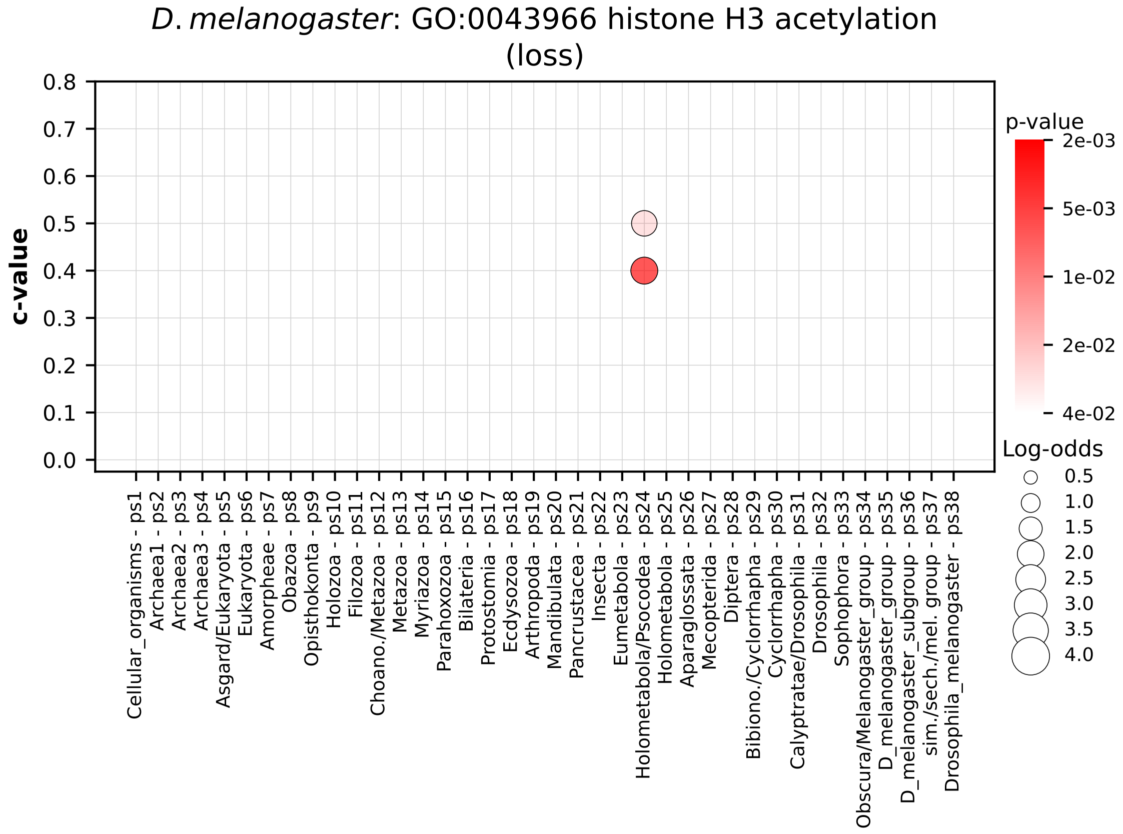

D. melanogaster: GO:0043966 histone H3 acetylation
(loss)
c-value
0.8
0.7
0.6
0.5
0.4
0.3
0.2
0.1
0.0
p-value
2e-03
5e-03
1e-02
2e-02
4e-02
Log-odds
0.5
1.0
1.5
2.0
2.5
3.0
3.5
4.0
Cellular_organisms - ps1
Archaea1 - ps2
Archaea2 - ps3
Archaea3 - ps4
Asgard/Eukaryota - ps5
Eukaryota - ps6
Amorpheae - ps7
Obazoa - ps8
Opisthokonta - ps9
Holozoa - ps10
Filozoa - ps11
Choano./Metazoa - ps12
Metazoa - ps13
Myriazoa - ps14
Parahoxozoa - ps15
Bilateria - ps16
Protostomia - ps17
Ecdysozoa - ps18
Arthropoda - ps19
Mandibulata - ps20
Pancrustacea - ps21
Insecta - ps22
Eumetabola - ps23
Holometabola/Psocodea - ps24
Holometabola - ps25
Aparaglossata - ps26
Mecopterida - ps27
Diptera - ps28
Bibiono./Cyclorrhapha - ps29
Cyclorrhapha - ps30
Calyptratae/Drosophila - ps31
Drosophila - ps32
Sophophora - ps33
Obscura/Melanogaster_group - ps34
D_melanogaster_group - ps35
D_melanogaster_subgroup - ps36
sim./sech./mel. group - ps37
Drosophila_melanogaster - ps38
Phylostratum

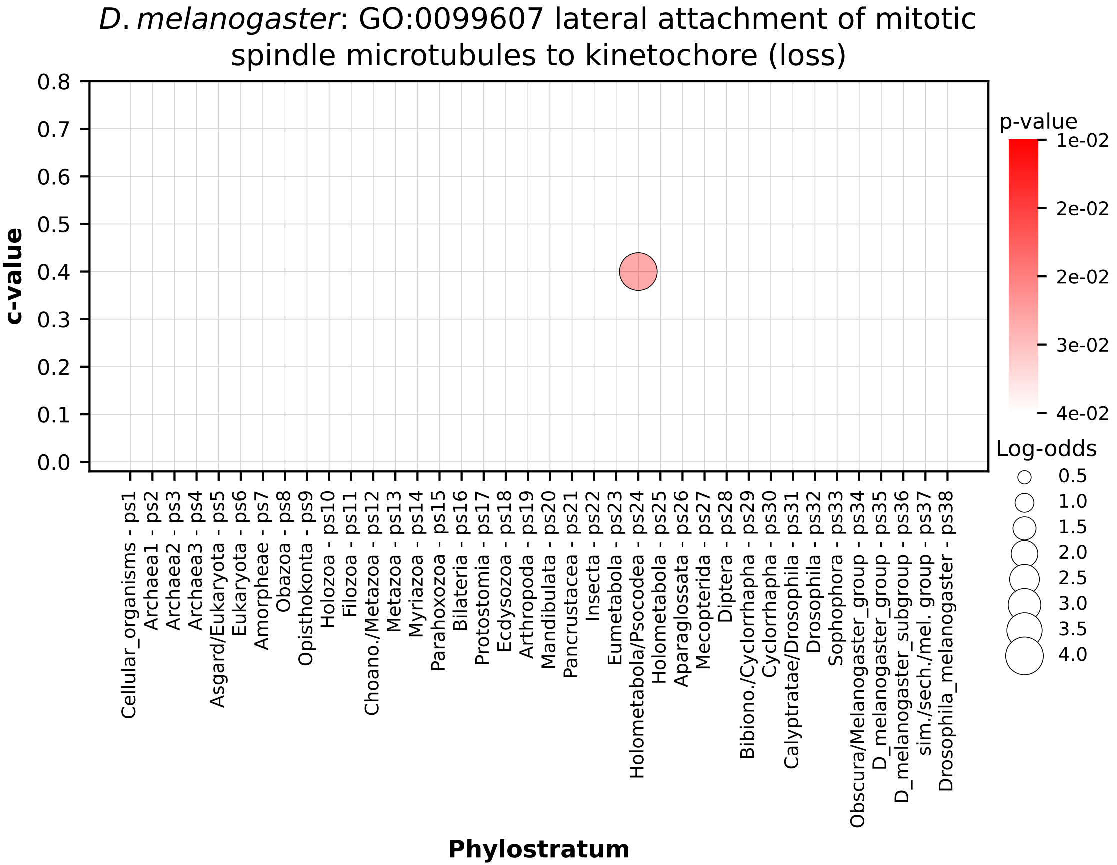

D. melanogaster: GO:0099607 lateral attachment of mitotic spindle microtubules to kinetochore (loss)
c-value
0.8
0.7
0.6
0.5
0.4
0.3
0.2
0.1
0.0
p-value
1e-02
2e-02
2e-02
3e-02
4e-02
Log-odds
0.5
1.0
1.5
2.0
2.5
3.0
3.5
4.0
Cellular_organisms - ps1
Archaea1 - ps2
Archaea2 - ps3
Archaea3 - ps4
Asgard/Eukaryota - ps5
Eukaryota - ps6
Amorpheae - ps7
Obazoa - ps8
Opisthokonta - ps9
Holozoa - ps10
Filozoa - ps11
Choano./Metazoa - ps12
Metazoa - ps13
Myriazoa - ps14
Parahoxozoa - ps15
Bilateria - ps16
Protostomia - ps17
Ecdysozoa - ps18
Arthropoda - ps19
Mandibulata - ps20
Pancrustacea - ps21
Insecta - ps22
Eumetabola - ps23
Holometabola/Psocodea - ps24
Holometabola - ps25
Aparaglossata - ps26
Mecopterida - ps27
Diptera - ps28
Bibiono./Cyclorrhapha - ps29
Cyclorrhapha - ps30
Calyptratae/Drosophila - ps31
Drosophila - ps32
Sophophora - ps33
Obscura/Melanogaster_group - ps34
D_melanogaster_group - ps35
D_melanogaster_subgroup - ps36
sim./sech./mel. group - ps37
Drosophila_melanogaster - ps38
Phylostratum

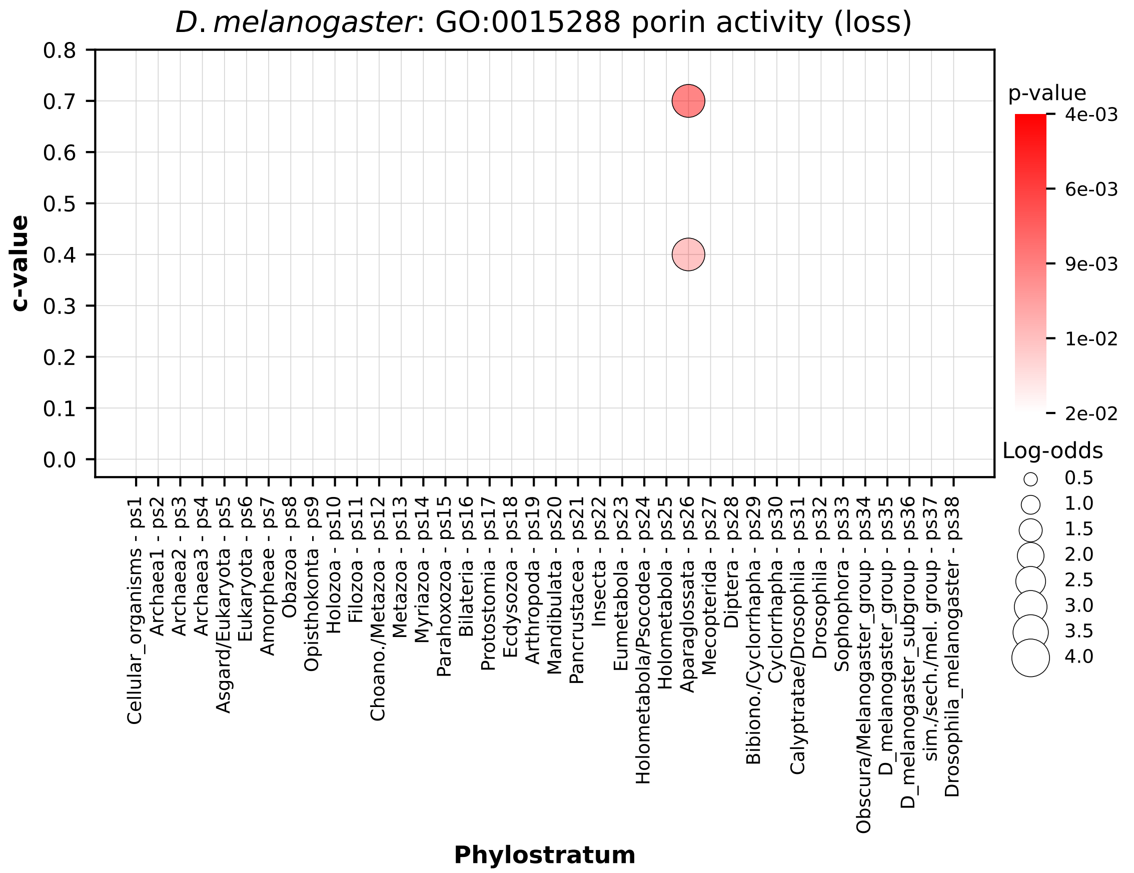

D. melanogaster: GO:0015288 porin activity (loss)
c-value
0.0
0.1
0.2
0.3
0.4
0.5
0.6
0.7
0.8
p-value
4e-03
6e-03
9e-03
1e-02
2e-02
Log-odds
0.5
1.0
1.5
2.0
2.5
3.0
3.5
4.0
Cellular_organisms - ps1
Archaea1 - ps2
Archaea2 - ps3
Archaea3 - ps4
Asgard/Eukaryota - ps5
Eukaryota - ps6
Amorpheae - ps7
Obazoa - ps8
Opisthokonta - ps9
Holozoa - ps10
Filozoa - ps11
Choano./Metazoa - ps12
Metazoa - ps13
Myriazoa - ps14
Parahoxozoa - ps15
Bilateria - ps16
Protostomia - ps17
Ecdysozoa - ps18
Arthropoda - ps19
Mandibulata - ps20
Pancrustacea - ps21
Insecta - ps22
Eumetabola - ps23
Holometabola/Psocodea - ps24
Holometabola - ps25
Aparaglossata - ps26
Mecopterida - ps27
Diptera - ps28
Bibiono./Cyclorrhapha - ps29
Cyclorrhapha - ps30
Calyptratae/Drosophila - ps31
Drosophila - ps32
Sophophora - ps33
Obscura/Melanogaster_group - ps34
D_melanogaster_group - ps35
D_melanogaster_subgroup - ps36
sim./sech./mel. group - ps37
Drosophila_melanogaster - ps38
Phylostratum

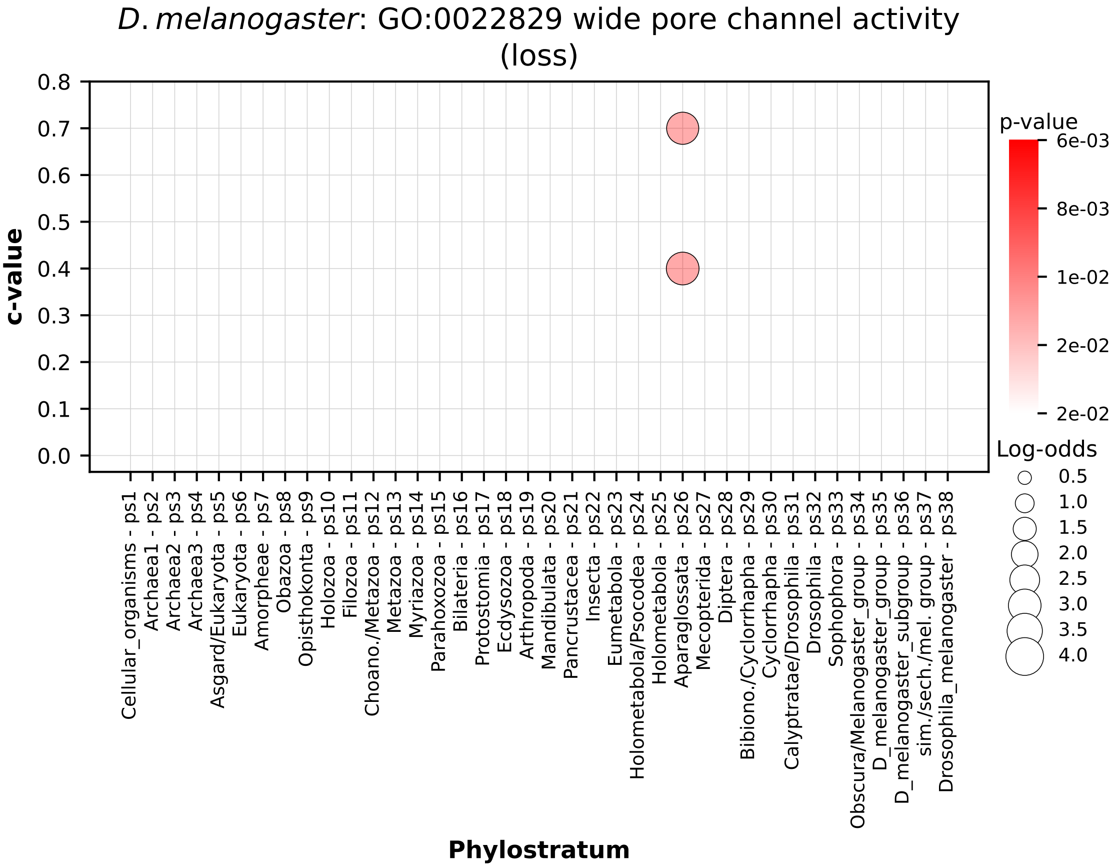

D. melanogaster: GO:0022829 wide pore channel activity (loss)
c-value
0.8
0.7
0.6
0.5
0.4
0.3
0.2
0.1
0.0
Phylostratum
Cellular_organisms - ps1
Archaea1 - ps2
Archaea2 - ps3
Archaea3 - ps4
Asgard/Eukaryota - ps5
Eukaryota - ps6
Amorpheae - ps7
Obazoa - ps8
Opisthokonta - ps9
Holozoa - ps10
Filozoa - ps11
Choano./Metazoa - ps12
Metazoa - ps13
Myriazoa - ps14
Parahoxozoa - ps15
Bilateria - ps16
Protostomia - ps17
Ecdysozoa - ps18
Arthropoda - ps19
Mandibulata - ps20
Pancrustacea - ps21
Insecta - ps22
Eumetabola - ps23
Holometabola/Psocodea - ps24
Holometabola - ps25
Aparaglossata - ps26
Mecopterida - ps27
Diptera - ps28
Bibiono./Cyclorrhapha - ps29
Cyclorrhapha - ps30
Calyptratae/Drosophila - ps31
Drosophila - ps32
Sophophora - ps33
Obscura/Melanogaster_group - ps34
D_melanogaster_group - ps35
D_melanogaster_subgroup - ps36
sim./sech./mel. group - ps37
Drosophila_melanogaster - ps38
p-value
6e-03
8e-03
1e-02
2e-02
2e-02
Log-odds
0.5
1.0
1.5
2.0
2.5
3.0
3.5
4.0

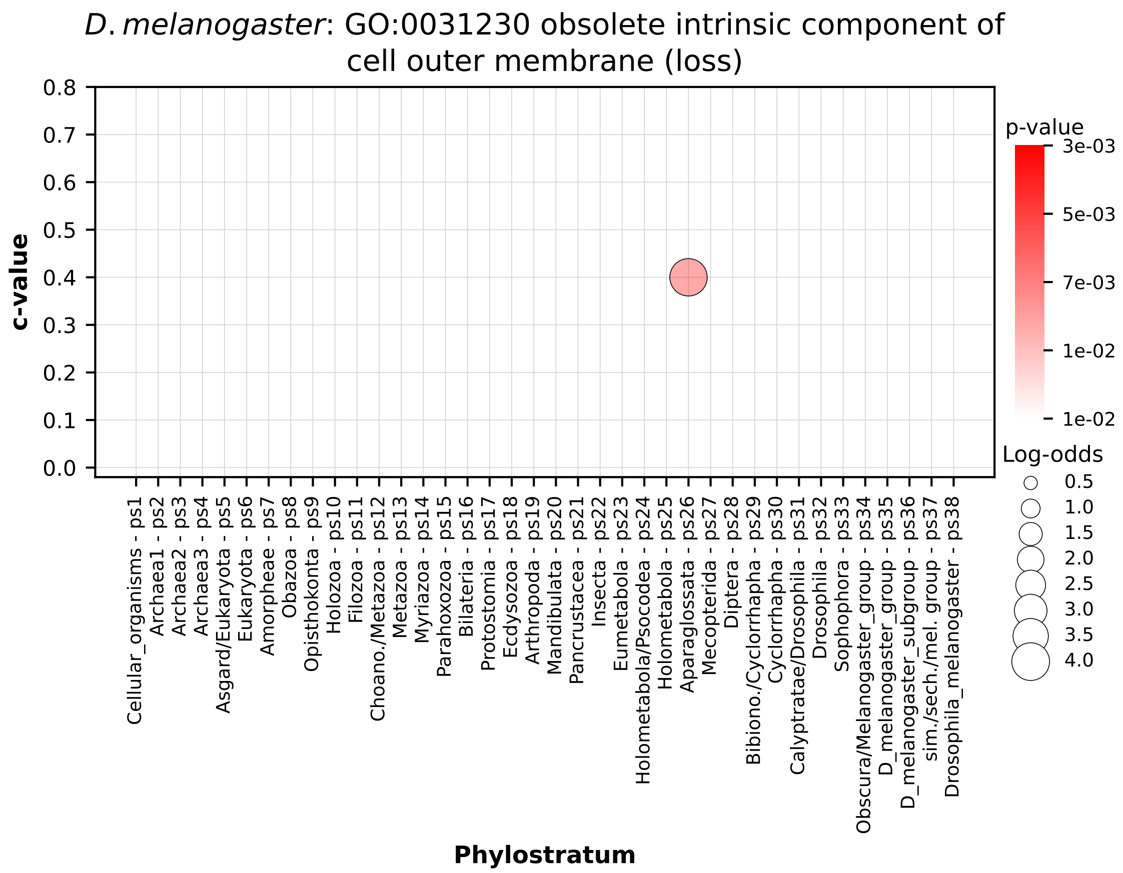

D. melanogaster: GO:0031230 obsolete intrinsic component of cell outer membrane (loss)
c-value
0.8
0.7
0.6
0.5
0.4
0.3
0.2
0.1
0.0
p-value
3e-03
5e-03
7e-03
1e-02
1e-02
Log-odds
0.5
1.0
1.5
2.0
2.5
3.0
3.5
4.0
Cellular_organisms - ps1
Archaea1 - ps2
Archaea2 - ps3
Archaea3 - ps4
Asgard/Eukaryota - ps5
Eukaryota - ps6
Amorpheae - ps7
Obazoa - ps8
Opisthokonta - ps9
Holozoa - ps10
Filozoa - ps11
Choano./Metazoa - ps12
Metazoa - ps13
Myriazoa - ps14
Parahoxozoa - ps15
Bilateria - ps16
Protostomia - ps17
Ecdysozoa - ps18
Arthropoda - ps19
Mandibulata - ps20
Pancrustacea - ps21
Insecta - ps22
Eumetabola - ps23
Holometabola/Psocodea - ps24
Holometabola - ps25
Aparaglossata - ps26
Mecopterida - ps27
Diptera - ps28
Bibiono./Cyclorrhapha - ps29
Cyclorrhapha - ps30
Calyptratae/Drosophila - ps31
Drosophila - ps32
Sophophora - ps33
Obscura/Melanogaster_group - ps34
D_melanogaster_group - ps35
D_melanogaster_subgroup - ps36
sim./sech./mel. group - ps37
Drosophila_melanogaster - ps38
Phylostratum

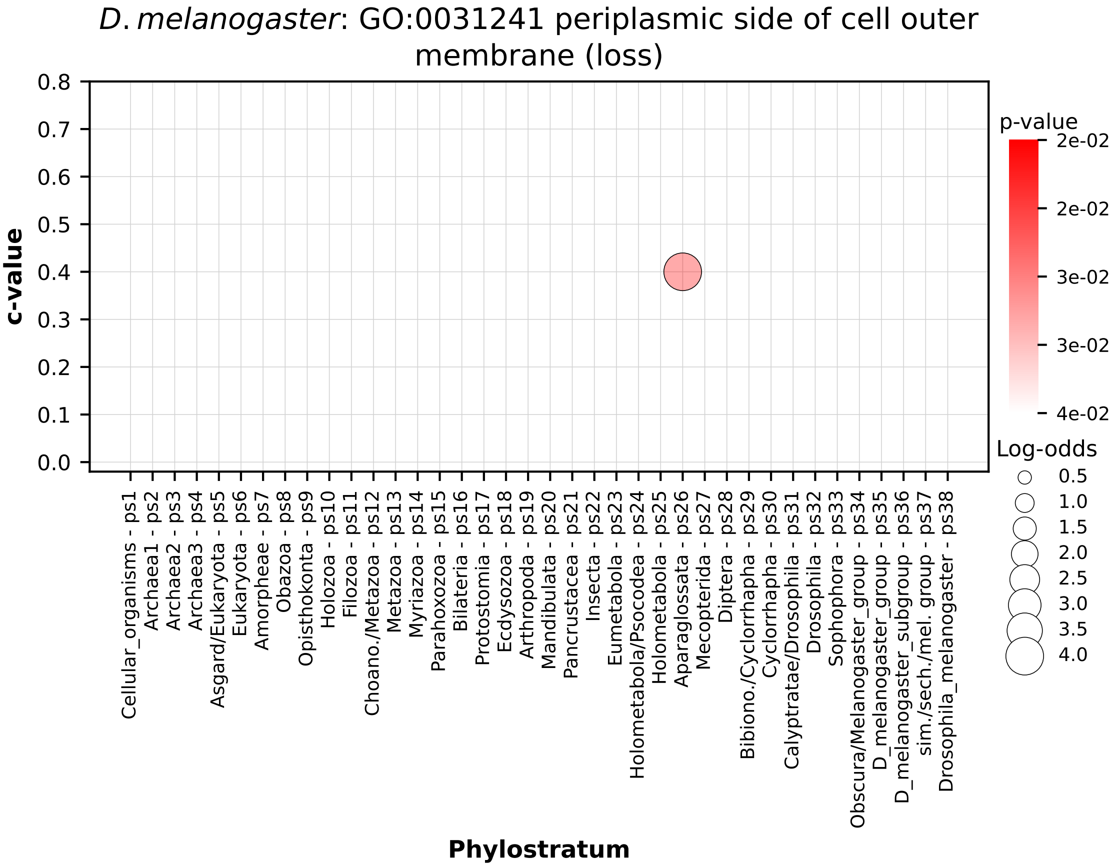

D. melanogaster: GO:0031241 periplasmic side of cell outer membrane (loss)
c-value
0.8
0.7
0.6
0.5
0.4
0.3
0.2
0.1
0.0
Phylostratum
Cellular_organisms - ps1
Archaea1 - ps2
Archaea2 - ps3
Archaea3 - ps4
Asgard/Eukaryota - ps5
Eukaryota - ps6
Amorpheae - ps7
Obazoa - ps8
Opisthokonta - ps9
Holozoa - ps10
Filozoa - ps11
Choano./Metazoa - ps12
Metazoa - ps13
Myriazoa - ps14
Parahoxozoa - ps15
Bilateria - ps16
Protostomia - ps17
Ecdysozoa - ps18
Arthropoda - ps19
Mandibulata - ps20
Pancrustacea - ps21
Insecta - ps22
Eumetabola - ps23
Holometabola/Psocodea - ps24
Holometabola - ps25
Aparaglossata - ps26
Mecopterida - ps27
Diptera - ps28
Bibiono./Cyclorrhapha - ps29
Cyclorrhapha - ps30
Calyptratae/Drosophila - ps31
Drosophila - ps32
Sophophora - ps33
Obscura/Melanogaster_group - ps34
D_melanogaster_group - ps35
D_melanogaster_subgroup - ps36
sim./sech./mel. group - ps37
Drosophila_melanogaster - ps38
p-value
2e-02
2e-02
3e-02
3e-02
4e-02
Log-odds
0.5
1.0
1.5
2.0
2.5
3.0
3.5
4.0

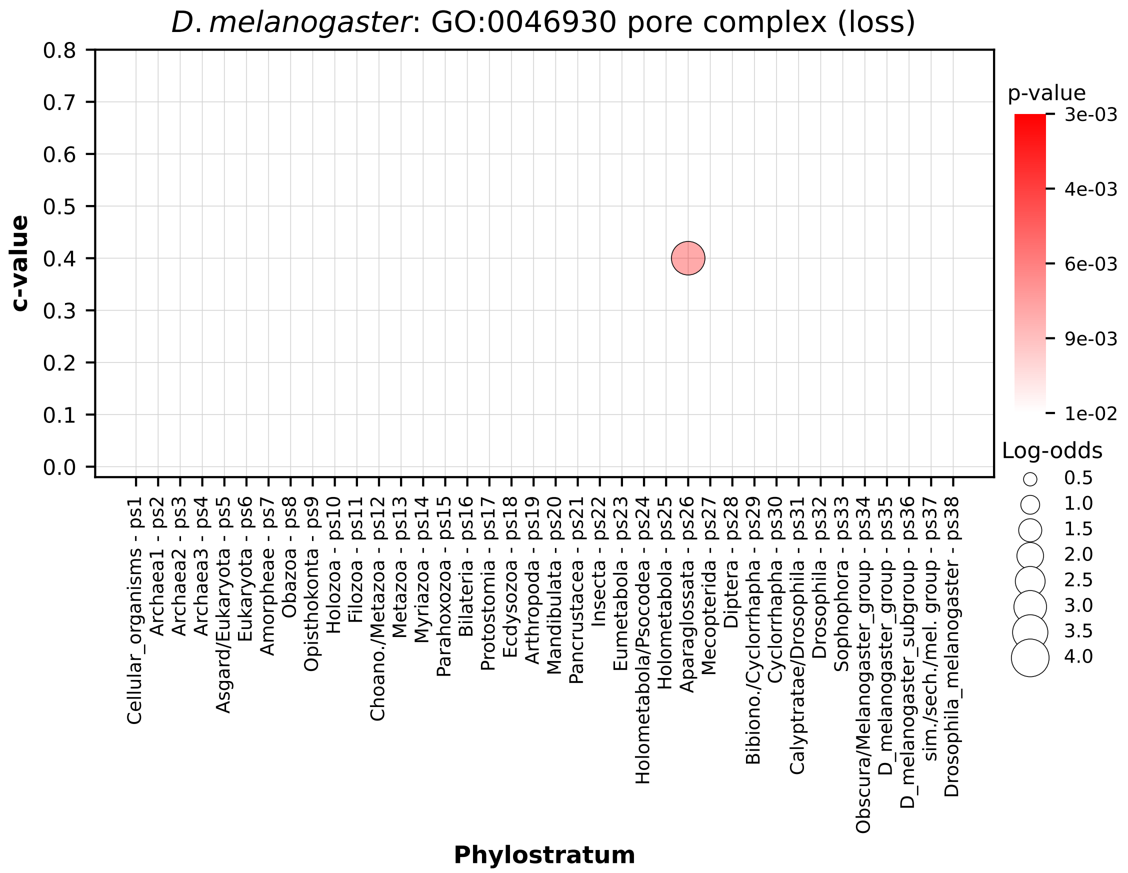

D. melanogaster: GO:0046930 pore complex (loss)
c-value
0.0
0.1
0.2
0.3
0.4
0.5
0.6
0.7
0.8
Phylostratum
Cellular_organisms - ps1
Archaea1 - ps2
Archaea2 - ps3
Archaea3 - ps4
Asgard/Eukaryota - ps5
Eukaryota - ps6
Amorpheae - ps7
Obazoa - ps8
Opisthokonta - ps9
Holozoa - ps10
Filozoa - ps11
Choano./Metazoa - ps12
Metazoa - ps13
Myriazoa - ps14
Parahoxozoa - ps15
Bilateria - ps16
Protostomia - ps17
Ecdysozoa - ps18
Arthropoda - ps19
Mandibulata - ps20
Pancrustacea - ps21
Insecta - ps22
Eumetabola - ps23
Holometabola/Psocodea - ps24
Holometabola - ps25
Aparaglossata - ps26
Mecopterida - ps27
Diptera - ps28
Bibiono./Cyclorrhapha - ps29
Cyclorrhapha - ps30
Calyptratae/Drosophila - ps31
Drosophila - ps32
Sophophora - ps33
Obscura/Melanogaster_group - ps34
D_melanogaster_group - ps35
D_melanogaster_subgroup - ps36
sim./sech./mel. group - ps37
Drosophila_melanogaster - ps38
p-value
3e-03
4e-03
6e-03
9e-03
1e-02
Log-odds
0.5
1.0
1.5
2.0
2.5
3.0
3.5
4.0

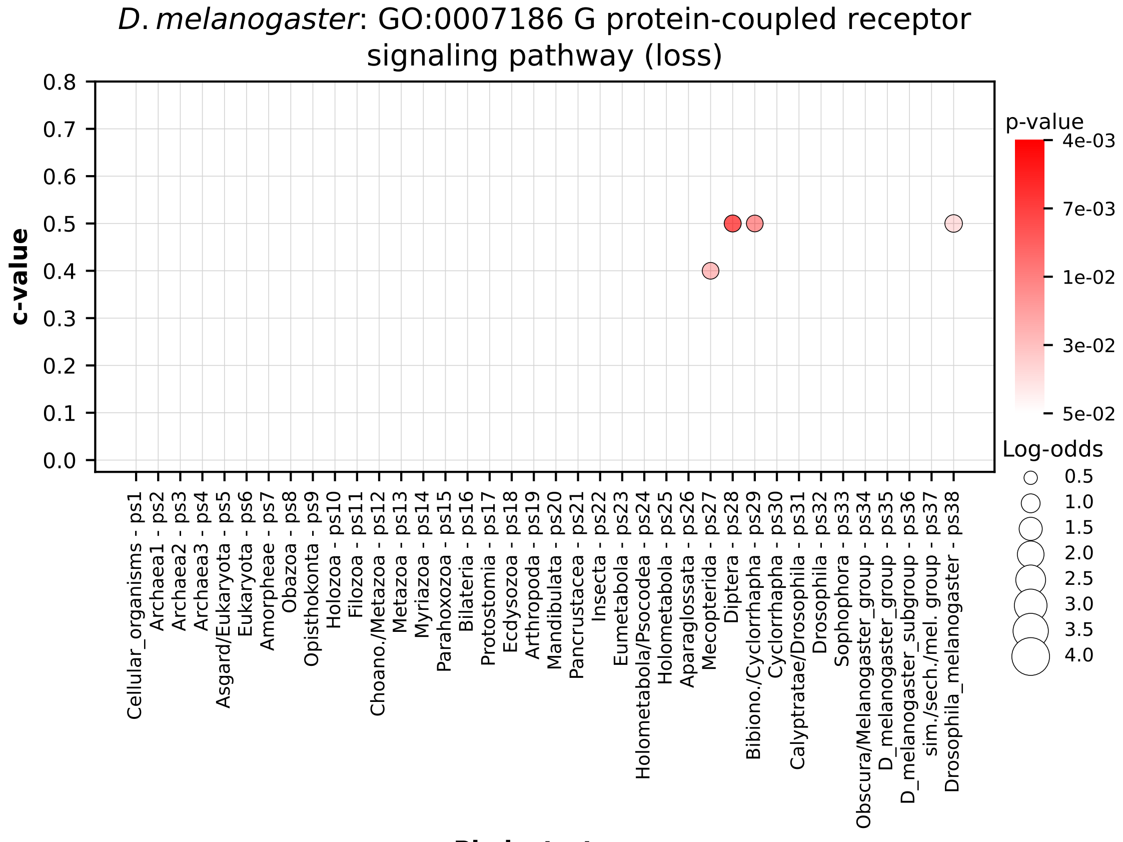

D. melanogaster: GO:0007186 G protein-coupled receptor signaling pathway (loss)
c-value
0.0
0.1
0.2
0.3
0.4
0.5
0.6
0.7
0.8
Phylostratum
Cellular_organisms - ps1
Archaea1 - ps2
Archaea2 - ps3
Archaea3 - ps4
Asgard/Eukaryota - ps5
Eukaryota - ps6
Amorpheae - ps7
Obazoa - ps8
Opisthokonta - ps9
Holozoa - ps10
Filozoa - ps11
Choano./Metazoa - ps12
Metazoa - ps13
Myriazoa - ps14
Parahoxozoa - ps15
Bilateria - ps16
Protostomia - ps17
Ecdysozoa - ps18
Arthropoda - ps19
Mandibulata - ps20
Pancrustacea - ps21
Insecta - ps22
Eumetabola - ps23
Holometabola/Psocodea - ps24
Holometabola - ps25
Aparaglossata - ps26
Mecopterida - ps27
Diptera - ps28
Bibiono./Cyclorrhapha - ps29
Cyclorrhapha - ps30
Calyptratae/Drosophila - ps31
Drosophila - ps32
Sophophora - ps33
Obscura/Melanogaster_group - ps34
D_melanogaster_group - ps35
D_melanogaster_subgroup - ps36
sim./sech./mel. group - ps37
Drosophila_melanogaster - ps38
p-value
4e-03
7e-03
1e-02
3e-02
5e-02
Log-odds
0.5
1.0
1.5
2.0
2.5
3.0
3.5
4.0

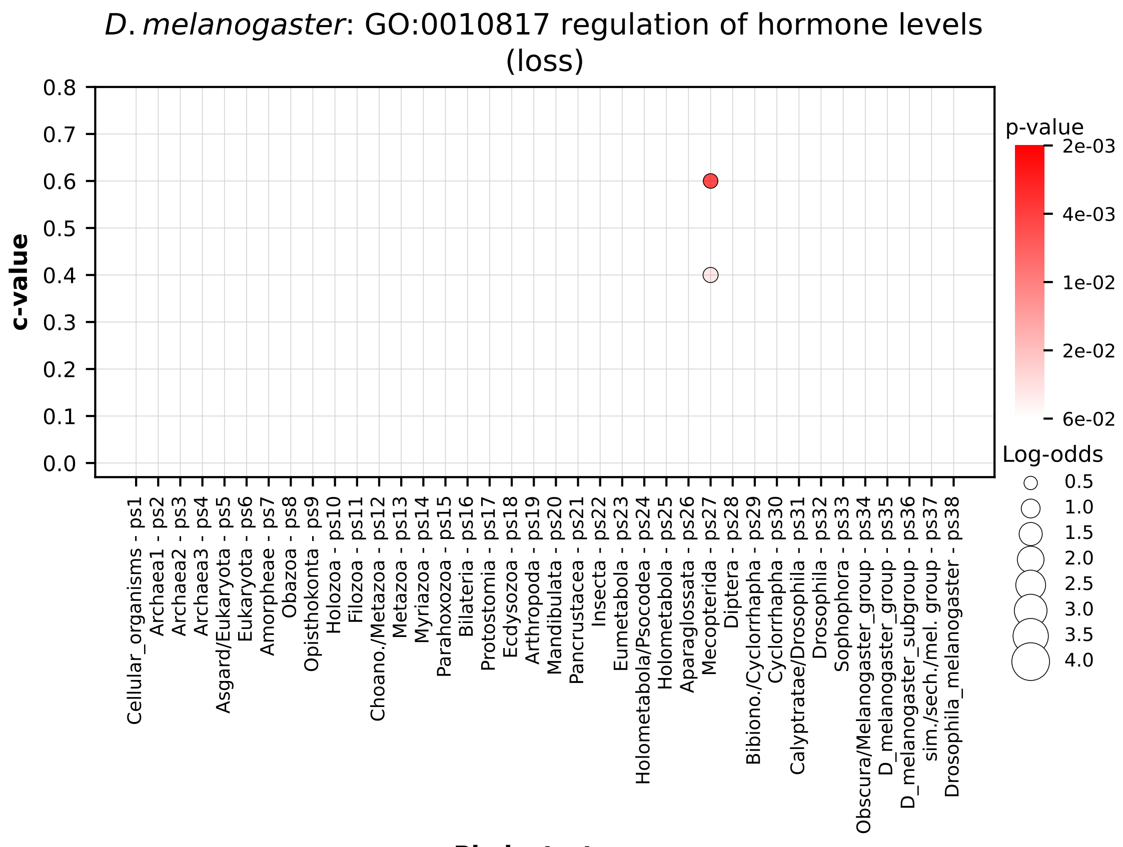
D. melanogaster: GO:0010817 regulation of hormone levels (loss)
c-value
0.0
0.1
0.2
0.3
0.4
0.5
0.6
0.7
0.8
Phylostratum
Cellular_organisms - ps1
Archaea1 - ps2
Archaea2 - ps3
Archaea3 - ps4
Asgard/Eukaryota - ps5
Eukaryota - ps6
Amorpheae - ps7
Obazoa - ps8
Opisthokonta - ps9
Holozoa - ps10
Filozoa - ps11
Choano./Metazoa - ps12
Metazoa - ps13
Myriazoa - ps14
Parahoxozoa - ps15
Bilateria - ps16
Protostomia - ps17
Ecdysozoa - ps18
Arthropoda - ps19
Mandibulata - ps20
Pancrustacea - ps21
Insecta - ps22
Eumetabola - ps23
Holometabola/Psocodea - ps24
Holometabola - ps25
Aparaglossata - ps26
Mecopterida - ps27
Diptera - ps28
Bibiono./Cyclorrhapha - ps29
Cyclorrhapha - ps30
Calyptratae/Drosophila - ps31
Drosophila - ps32
Sophophora - ps33
Obscura/Melanogaster_group - ps34
D_melanogaster_group - ps35
D_melanogaster_subgroup - ps36
sim./sech./mel. group - ps37
Drosophila_melanogaster - ps38
p-value
2e-03
4e-03
1e-02
2e-02
6e-02
Log-odds
0.5
1.0
1.5
2.0
2.5
3.0
3.5
4.0

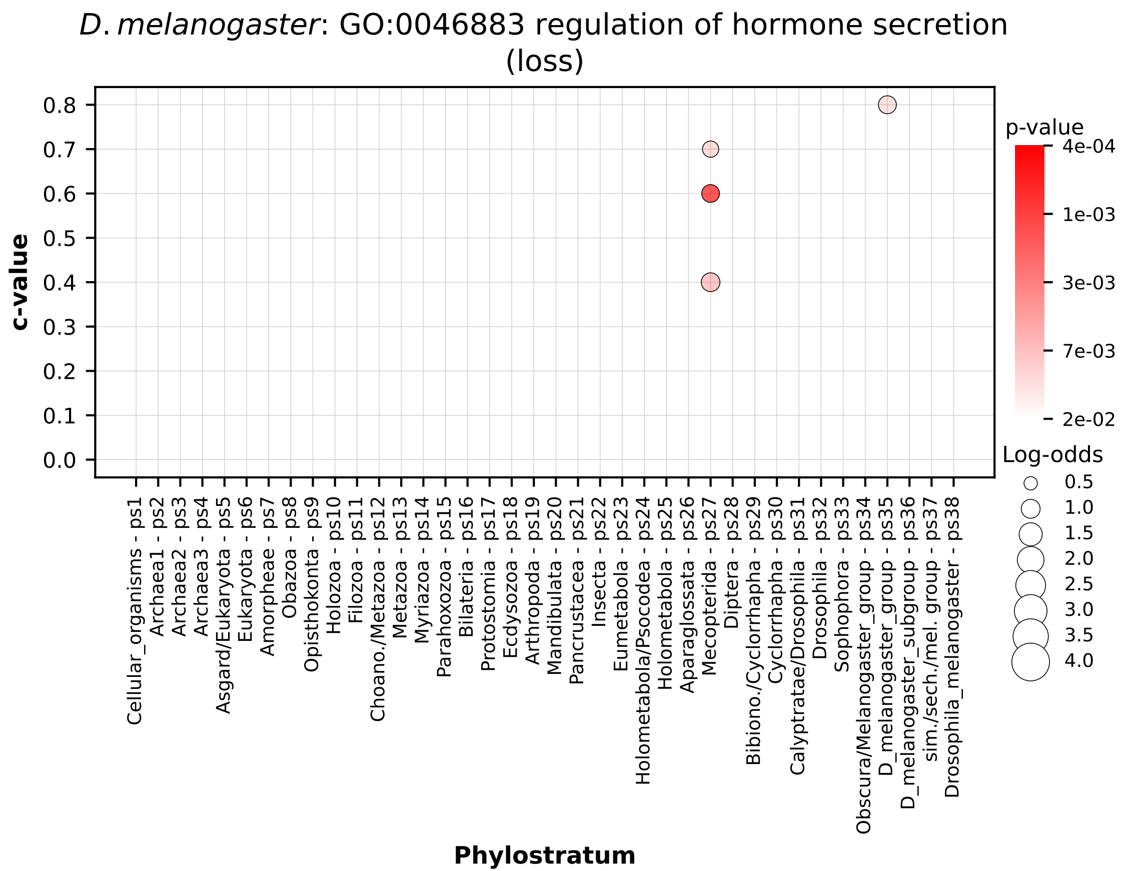

D. melanogaster: GO:0046883 regulation of hormone secretion (loss)
c-value
0.8
0.7
0.6
0.5
0.4
0.3
0.2
0.1
0.0
p-value
4e-04
1e-03
3e-03
7e-03
2e-02
Log-odds
0.5
1.0
1.5
2.0
2.5
3.0
3.5
4.0
Cellular_organisms - ps1
Archaea1 - ps2
Archaea2 - ps3
Archaea3 - ps4
Asgard/Eukaryota - ps5
Eukaryota - ps6
Amorpheae - ps7
Obazoa - ps8
Opisthokonta - ps9
Holozoa - ps10
Filozoa - ps11
Choano./Metazoa - ps12
Metazoa - ps13
Myriazoa - ps14
Parahoxozoa - ps15
Bilateria - ps16
Protostomia - ps17
Ecdysozoa - ps18
Arthropoda - ps19
Mandibulata - ps20
Pancrustacea - ps21
Insecta - ps22
Eumetabola - ps23
Holometabola/Psocodea - ps24
Holometabola - ps25
Aparaglossata - ps26
Mecopterida - ps27
Diptera - ps28
Bibiono./Cyclorrhapha - ps29
Cyclorrhapha - ps30
Calyptratae/Drosophila - ps31
Drosophila - ps32
Sophophora - ps33
Obscura/Melanogaster_group - ps34
D_melanogaster_group - ps35
D_melanogaster_subgroup - ps36
sim./sech./mel. group - ps37
Drosophila_melanogaster - ps38
Phylostratum

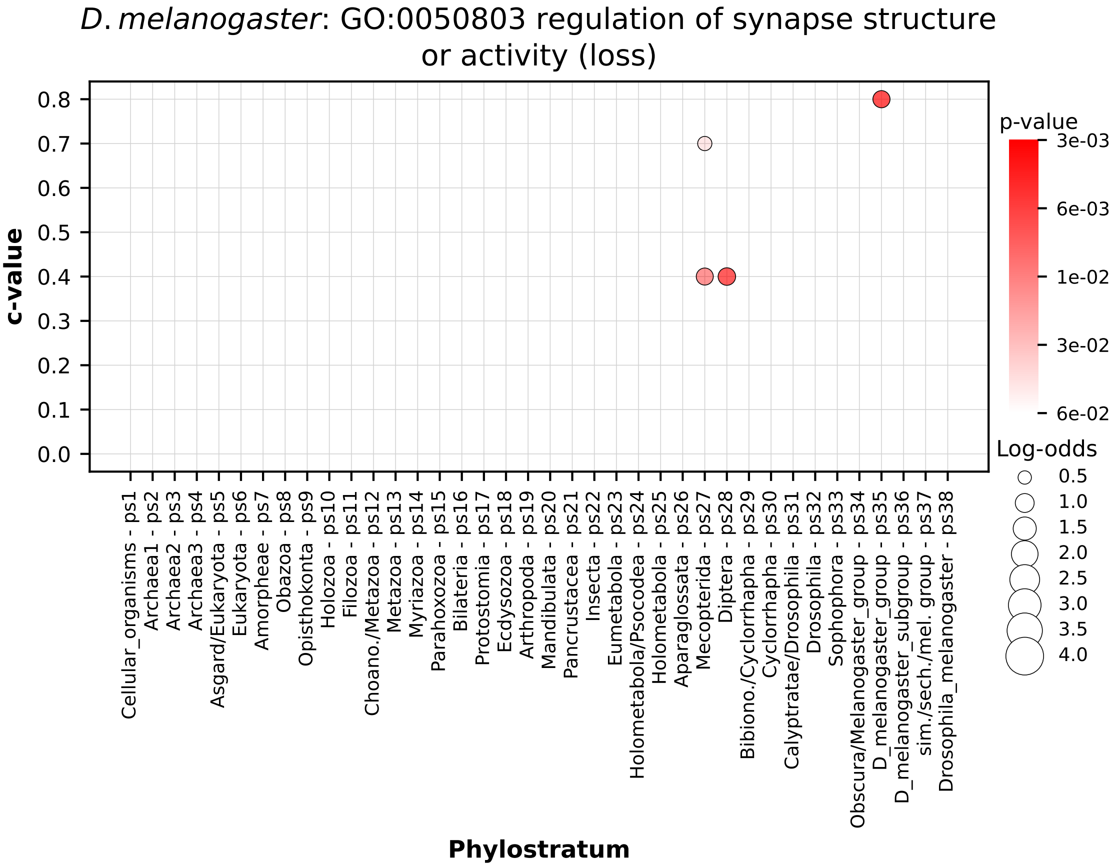

D. melanogaster: GO:0050803 regulation of synapse structure or activity (loss)
c-value
0.8
0.7
0.6
0.5
0.4
0.3
0.2
0.1
0.0
p-value
3e-03
6e-03
1e-02
3e-02
6e-02
Log-odds
0.5
1.0
1.5
2.0
2.5
3.0
3.5
4.0
Cellular_organisms - ps1
Archaea1 - ps2
Archaea2 - ps3
Archaea3 - ps4
Asgard/Eukaryota - ps5
Eukaryota - ps6
Amorpheae - ps7
Obazoa - ps8
Opisthokonta - ps9
Holozoa - ps10
Filozoa - ps11
Choano./Metazoa - ps12
Metazoa - ps13
Myriazoa - ps14
Parahoxozoa - ps15
Bilateria - ps16
Protostomia - ps17
Ecdysozoa - ps18
Arthropoda - ps19
Mandibulata - ps20
Pancrustacea - ps21
Insecta - ps22
Eumetabola - ps23
Holometabola/Psocodea - ps24
Holometabola - ps25
Aparaglossata - ps26
Mecopterida - ps27
Diptera - ps28
Bibiono./Cyclorrhapha - ps29
Cyclorrhapha - ps30
Calyptratae/Drosophila - ps31
Drosophila - ps32
Sophophora - ps33
Obscura/Melanogaster_group - ps34
D_melanogaster_group - ps35
D_melanogaster_subgroup - ps36
sim./sech./mel. group - ps37
Drosophila_melanogaster - ps38
Phylostratum

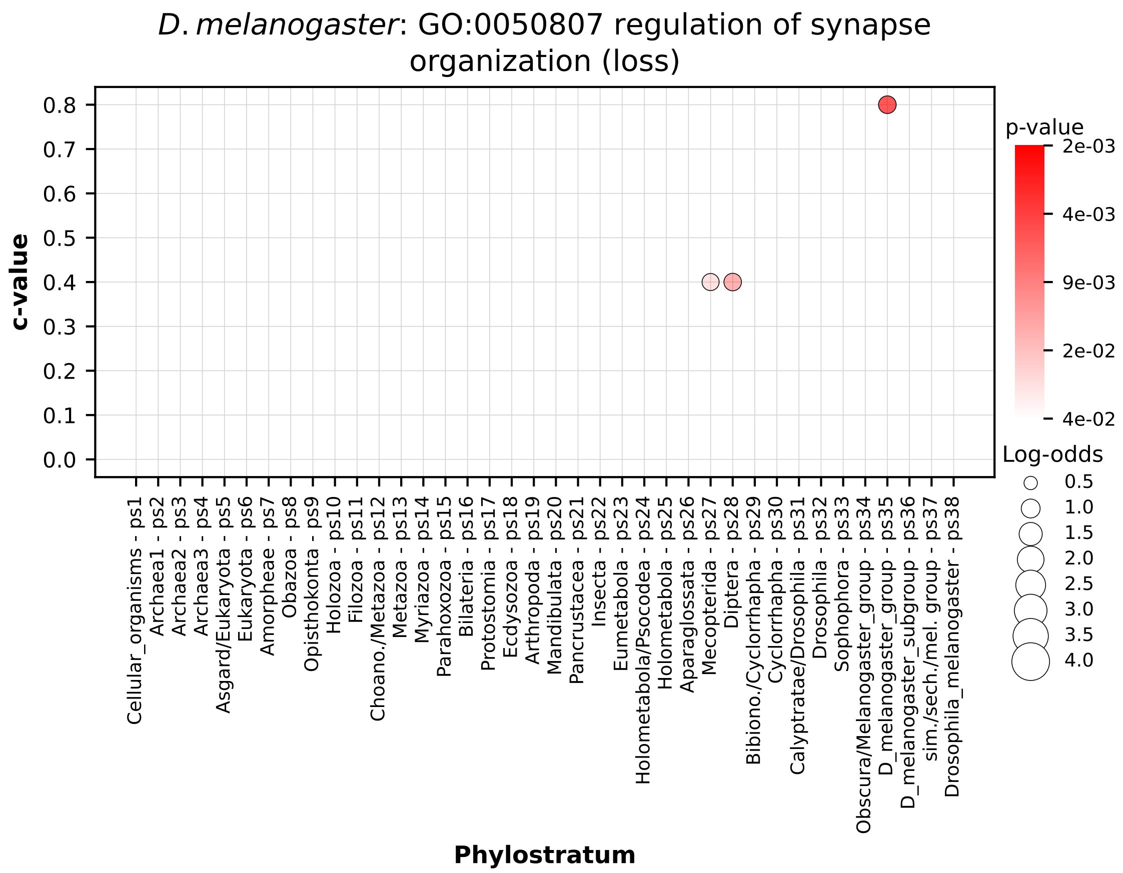
D. melanogaster: GO:0050807 regulation of synapse organization (loss)
c-value
0.8
0.7
0.6
0.5
0.4
0.3
0.2
0.1
0.0
p-value
2e-03
4e-03
9e-03
2e-02
4e-02
Log-odds
0.5
1.0
1.5
2.0
2.5
3.0
3.5
4.0
Cellular_organisms - ps1
Archaea1 - ps2
Archaea2 - ps3
Archaea3 - ps4
Asgard/Eukaryota - ps5
Eukaryota - ps6
Amorpheae - ps7
Obazoa - ps8
Opisthokonta - ps9
Holozoa - ps10
Filozoa - ps11
Choano./Metazoa - ps12
Metazoa - ps13
Myriazoa - ps14
Parahoxozoa - ps15
Bilateria - ps16
Protostomia - ps17
Ecdysozoa - ps18
Arthropoda - ps19
Mandibulata - ps20
Pancrustacea - ps21
Insecta - ps22
Eumetabola - ps23
Holometabola/Psocodea - ps24
Holometabola - ps25
Aparaglossata - ps26
Mecopterida - ps27
Diptera - ps28
Bibiono./Cyclorrhapha - ps29
Cyclorrhapha - ps30
Calyptratae/Drosophila - ps31
Drosophila - ps32
Sophophora - ps33
Obscura/Melanogaster_group - ps34
D_melanogaster_group - ps35
D_melanogaster_subgroup - ps36
sim./sech./mel. group - ps37
Drosophila_melanogaster - ps38
Phylostratum

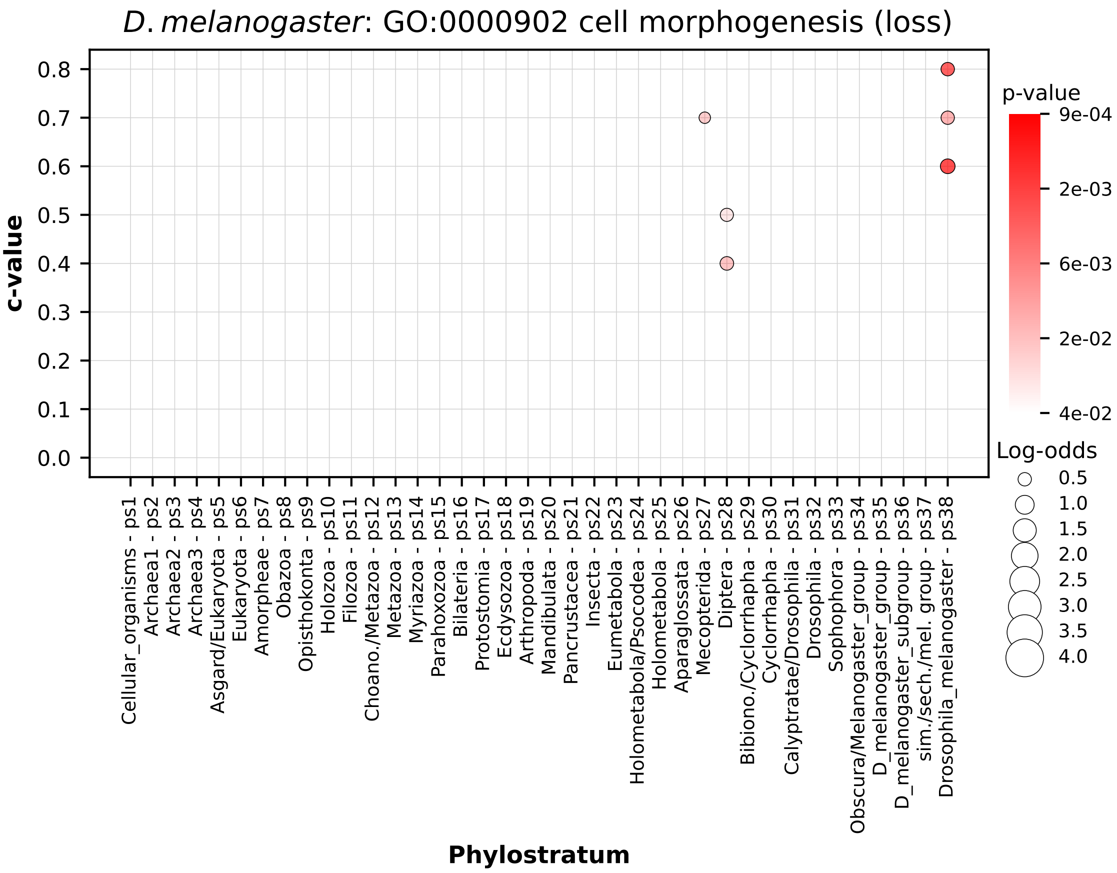

D. melanogaster: GO:0000902 cell morphogenesis (loss)
c-value
0.8
0.7
0.6
0.5
0.4
0.3
0.2
0.1
0.0
p-value
9e-04
2e-03
6e-03
2e-02
4e-02
Log-odds
0.5
1.0
1.5
2.0
2.5
3.0
3.5
4.0
Cellular_organisms - ps1
Archaea1 - ps2
Archaea2 - ps3
Archaea3 - ps4
Asgard/Eukaryota - ps5
Eukaryota - ps6
Amorpheae - ps7
Obazoa - ps8
Opisthokonta - ps9
Holozoa - ps10
Filozoa - ps11
Choano./Metazoa - ps12
Metazoa - ps13
Myriazoa - ps14
Parahoxozoa - ps15
Bilateria - ps16
Protostomia - ps17
Ecdysozoa - ps18
Arthropoda - ps19
Mandibulata - ps20
Pancrustacea - ps21
Insecta - ps22
Eumetabola - ps23
Holometabola/Psocodea - ps24
Holometabola - ps25
Aparaglossata - ps26
Mecopterida - ps27
Diptera - ps28
Bibiono./Cyclorrhapha - ps29
Cyclorrhapha - ps30
Calyptratae/Drosophila - ps31
Drosophila - ps32
Sophophora - ps33
Obscura/Melanogaster_group - ps34
D_melanogaster_group - ps35
D_melanogaster_subgroup - ps36
sim./sech./mel. group - ps37
Drosophila_melanogaster - ps38
Phylostratum

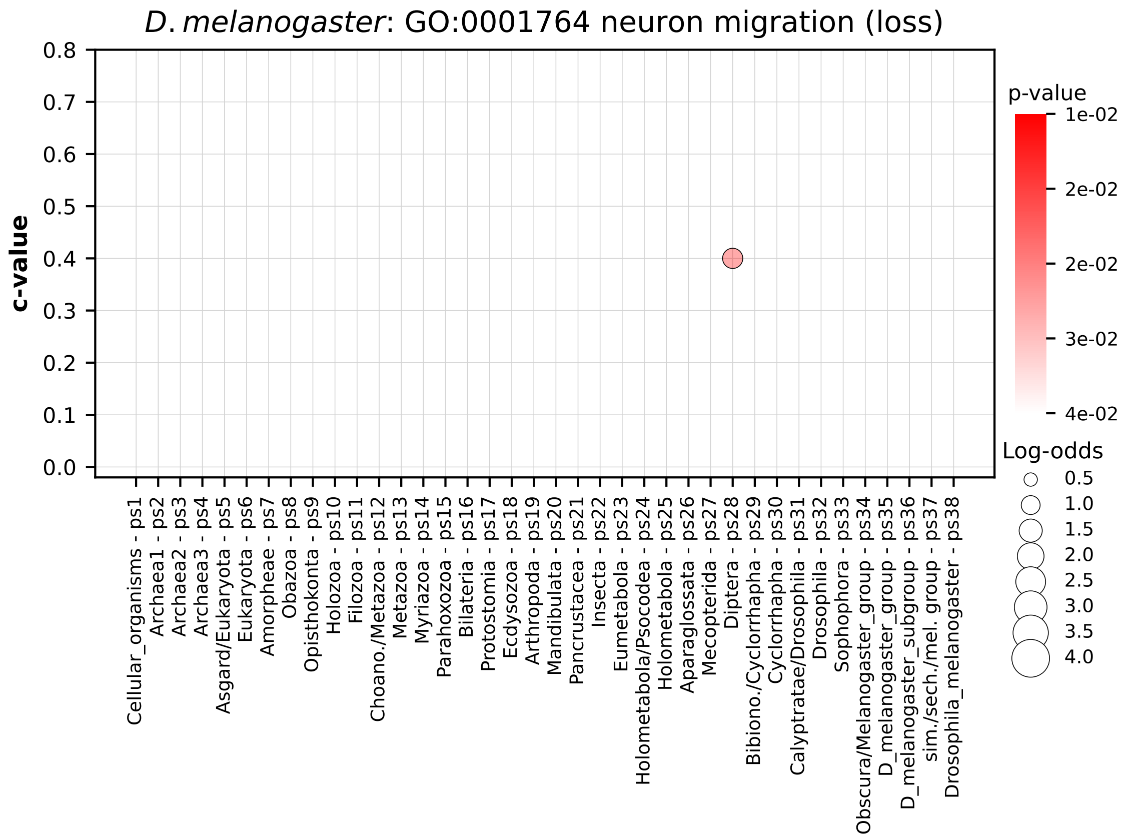

D. melanogaster: GO:0001764 neuron migration (loss)
c-value
0.8
0.7
0.6
0.5
0.4
0.3
0.2
0.1
0.0
p-value
1e-02
2e-02
2e-02
3e-02
4e-02
Log-odds
0.5
1.0
1.5
2.0
2.5
3.0
3.5
4.0
Cellular_organisms - ps1
Archaea1 - ps2
Archaea2 - ps3
Archaea3 - ps4
Asgard/Eukaryota - ps5
Eukaryota - ps6
Amorpheae - ps7
Obazoa - ps8
Opisthokonta - ps9
Holozoa - ps10
Filozoa - ps11
Choano./Metazoa - ps12
Metazoa - ps13
Myriazoa - ps14
Parahoxozoa - ps15
Bilateria - ps16
Protostomia - ps17
Ecdysozoa - ps18
Arthropoda - ps19
Mandibulata - ps20
Pancrustacea - ps21
Insecta - ps22
Eumetabola - ps23
Holometabola/Psocodea - ps24
Holometabola - ps25
Aparaglossata - ps26
Mecopterida - ps27
Diptera - ps28
Bibiono./Cyclorrhapha - ps29
Cyclorrhapha - ps30
Calyptratae/Drosophila - ps31
Drosophila - ps32
Sophophora - ps33
Obscura/Melanogaster_group - ps34
D_melanogaster_group - ps35
D_melanogaster_subgroup - ps36
sim./sech./mel. group - ps37
Drosophila_melanogaster - ps38
Phylostratum

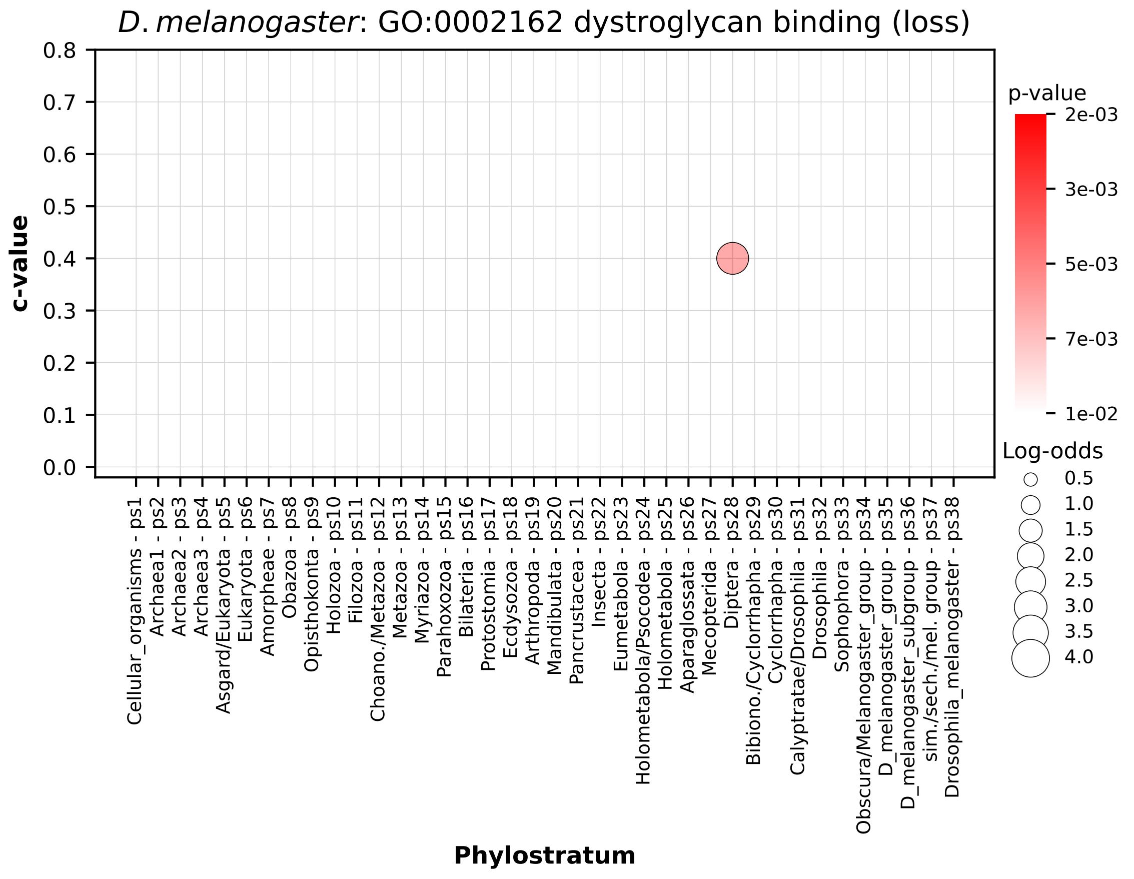

D. melanogaster: GO:0002162 dystroglycan binding (loss)
c-value
0.8
0.7
0.6
0.5
0.4
0.3
0.2
0.1
0.0
Phylostratum
Cellular_organisms - ps1
Archaea1 - ps2
Archaea2 - ps3
Archaea3 - ps4
Asgard/Eukaryota - ps5
Eukaryota - ps6
Amorpheae - ps7
Obazoa - ps8
Opisthokonta - ps9
Holozoa - ps10
Filozoa - ps11
Choano./Metazoa - ps12
Metazoa - ps13
Myriazoa - ps14
Parahoxozoa - ps15
Bilateria - ps16
Protostomia - ps17
Ecdysozoa - ps18
Arthropoda - ps19
Mandibulata - ps20
Pancrustacea - ps21
Insecta - ps22
Eumetabola - ps23
Holometabola/Psocodea - ps24
Holometabola - ps25
Aparaglossata - ps26
Mecopterida - ps27
Diptera - ps28
Bibiono./Cyclorrhapha - ps29
Cyclorrhapha - ps30
Calyptratae/Drosophila - ps31
Drosophila - ps32
Sophophora - ps33
Obscura/Melanogaster_group - ps34
D_melanogaster_group - ps35
D_melanogaster_subgroup - ps36
sim./sech./mel. group - ps37
Drosophila_melanogaster - ps38
p-value
2e-03
3e-03
5e-03
7e-03
1e-02
Log-odds
0.5
1.0
1.5
2.0
2.5
3.0
3.5
4.0

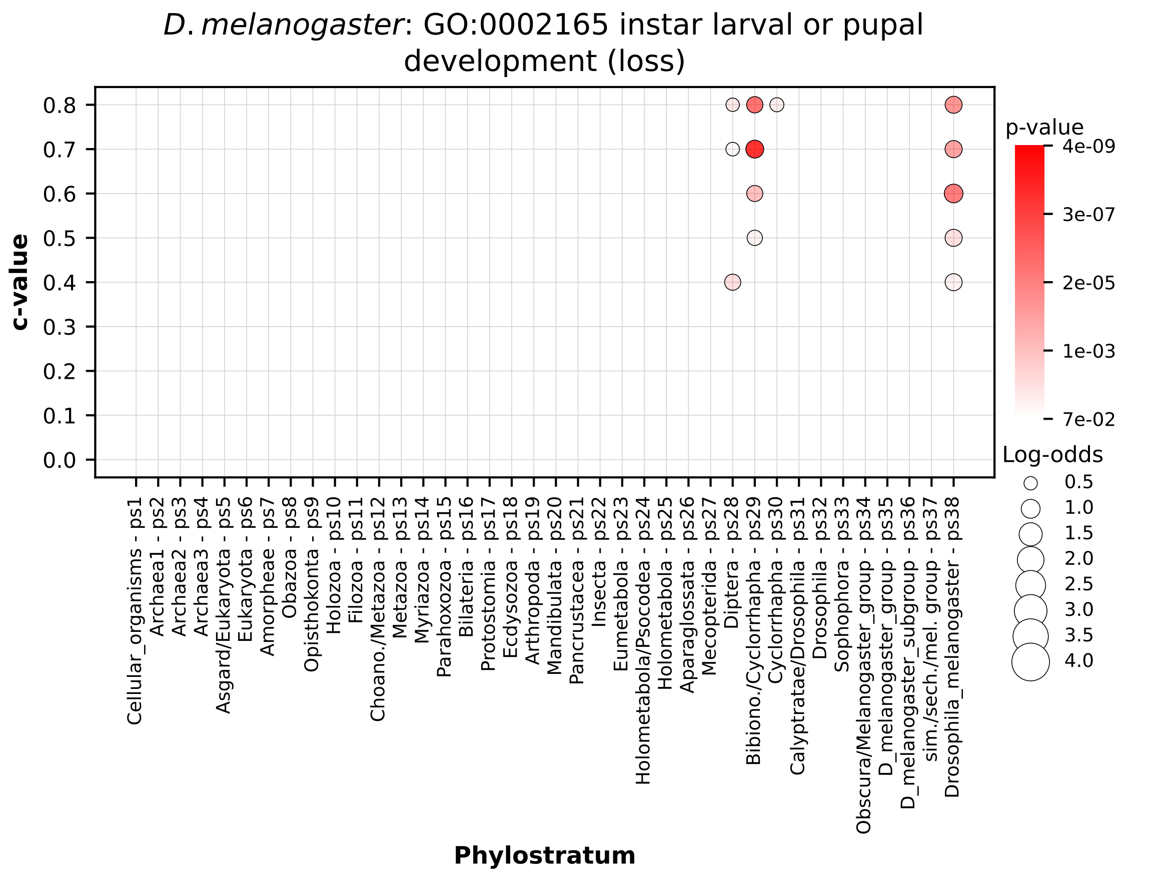

D. melanogaster: GO:0002165 instar larval or pupal development (loss)
c-value
0.0
0.1
0.2
0.3
0.4
0.5
0.6
0.7
0.8
Phylostratum
Cellular_organisms - ps1
Archaea1 - ps2
Archaea2 - ps3
Archaea3 - ps4
Asgard/Eukaryota - ps5
Eukaryota - ps6
Amorpheae - ps7
Obazoa - ps8
Opisthokonta - ps9
Holozoa - ps10
Filozoa - ps11
Choano./Metazoa - ps12
Metazoa - ps13
Myriazoa - ps14
Parahoxozoa - ps15
Bilateria - ps16
Protostomia - ps17
Ecdysozoa - ps18
Arthropoda - ps19
Mandibulata - ps20
Pancrustacea - ps21
Insecta - ps22
Eumetabola - ps23
Holometabola/Psocodea - ps24
Holometabola - ps25
Aparaglossata - ps26
Mecopterida - ps27
Diptera - ps28
Bibiono./Cyclorrhapha - ps29
Cyclorrhapha - ps30
Calyptratae/Drosophila - ps31
Drosophila - ps32
Sophophora - ps33
Obscura/Melanogaster_group - ps34
D_melanogaster_group - ps35
D_melanogaster_subgroup - ps36
sim./sech./mel. group - ps37
Drosophila_melanogaster - ps38
p-value
4e-09
3e-07
2e-05
1e-03
7e-02
Log-odds
0.5
1.0
1.5
2.0
2.5
3.0
3.5
4.0

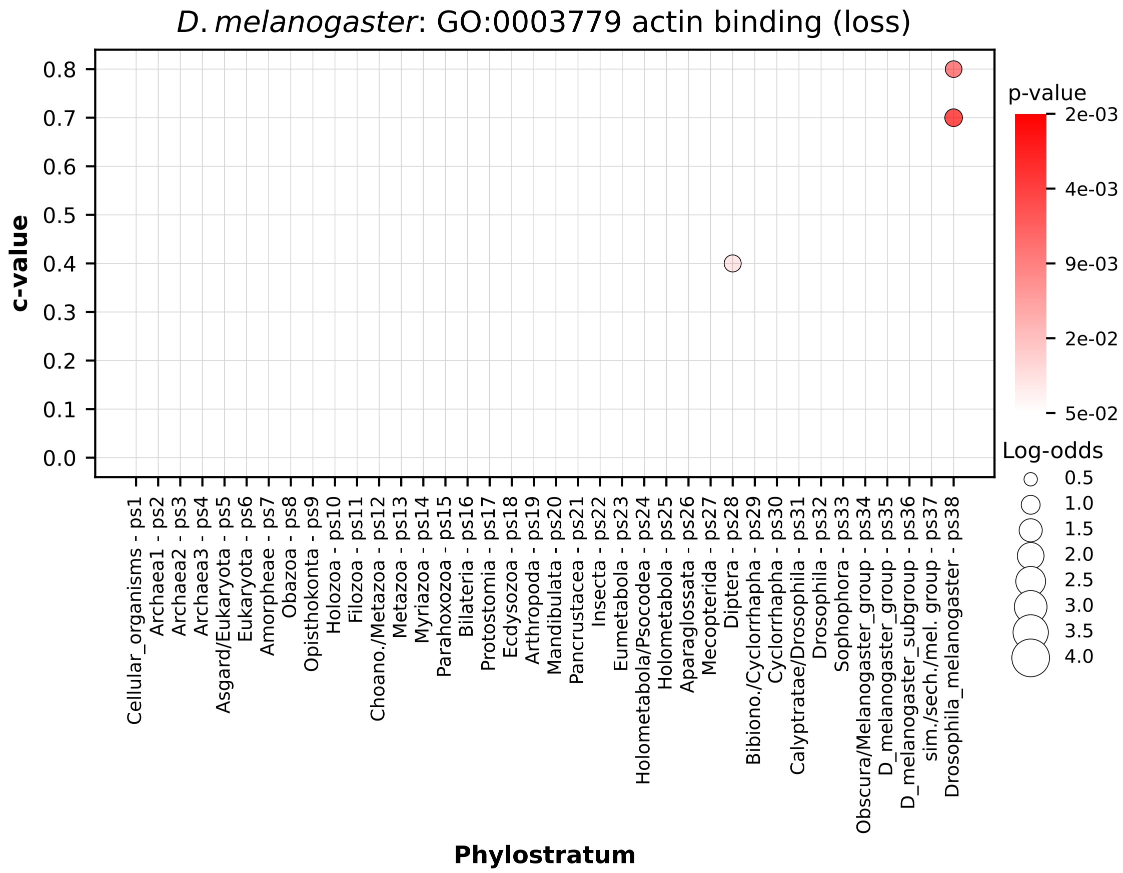
D. melanogaster: GO:0003779 actin binding (loss)
c-value
0.0
0.1
0.2
0.3
0.4
0.5
0.6
0.7
0.8
p-value
2e-03
4e-03
9e-03
2e-02
5e-02
Log-odds
0.5
1.0
1.5
2.0
2.5
3.0
3.5
4.0
Cellular_organisms - ps1
Archaea1 - ps2
Archaea2 - ps3
Archaea3 - ps4
Asgard/Eukaryota - ps5
Eukaryota - ps6
Amorpheae - ps7
Obazoa - ps8
Opisthokonta - ps9
Holozoa - ps10
Filozoa - ps11
Choano./Metazoa - ps12
Metazoa - ps13
Myriazoa - ps14
Parahoxozoa - ps15
Bilateria - ps16
Protostomia - ps17
Ecdysozoa - ps18
Arthropoda - ps19
Mandibulata - ps20
Pancrustacea - ps21
Insecta - ps22
Eumetabola - ps23
Holometabola/Psocodea - ps24
Holometabola - ps25
Aparaglossata - ps26
Mecopterida - ps27
Diptera - ps28
Bibiono./Cyclorrhapha - ps29
Cyclorrhapha - ps30
Calyptratae/Drosophila - ps31
Drosophila - ps32
Sophophora - ps33
Obscura/Melanogaster_group - ps34
D_melanogaster_group - ps35
D_melanogaster_subgroup - ps36
sim./sech./mel. group - ps37
Drosophila_melanogaster - ps38
Phylostratum

D. melanogaster: GO:0005924 obsolete cell-substrate adherens junction (loss)

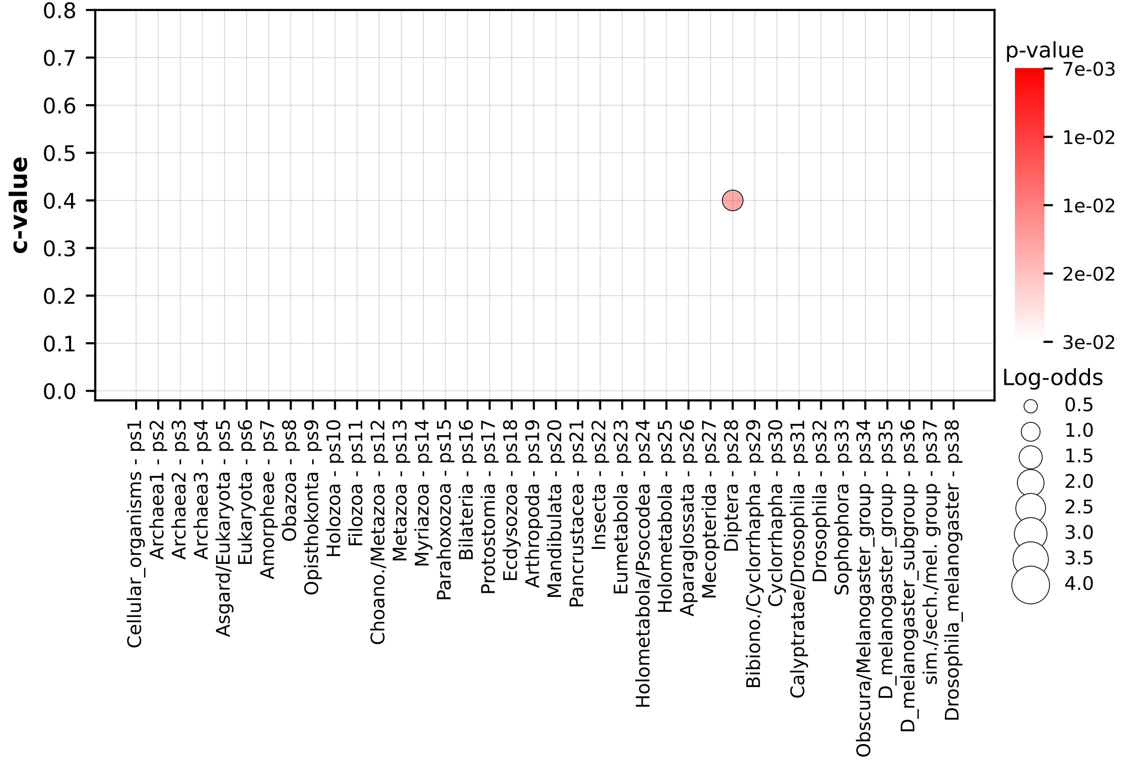

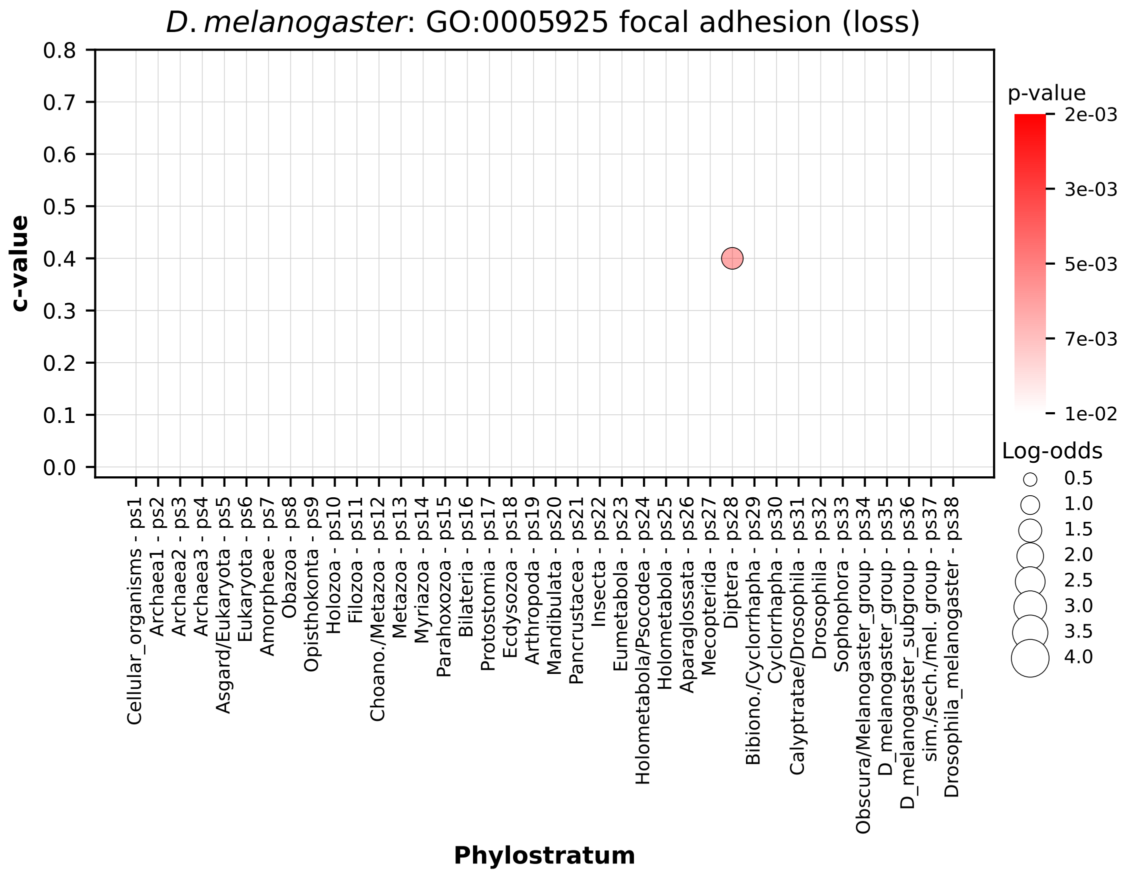

D. melanogaster: GO:0005925 focal adhesion (loss)
c-value
0.8
0.7
0.6
0.5
0.4
0.3
0.2
0.1
0.0
p-value
2e-03
3e-03
5e-03
7e-03
1e-02
Log-odds
0.5
1.0
1.5
2.0
2.5
3.0
3.5
4.0
Cellular_organisms - ps1
Archaea1 - ps2
Archaea2 - ps3
Archaea3 - ps4
Asgard/Eukaryota - ps5
Eukaryota - ps6
Amorpheae - ps7
Obazoa - ps8
Opisthokonta - ps9
Holozoa - ps10
Filozoa - ps11
Choano./Metazoa - ps12
Metazoa - ps13
Myriazoa - ps14
Parahoxozoa - ps15
Bilateria - ps16
Protostomia - ps17
Ecdysozoa - ps18
Arthropoda - ps19
Mandibulata - ps20
Pancrustacea - ps21
Insecta - ps22
Eumetabola - ps23
Holometabola/Psocodea - ps24
Holometabola - ps25
Aparaglossata - ps26
Mecopterida - ps27
Diptera - ps28
Bibiono./Cyclorrhapha - ps29
Cyclorrhapha - ps30
Calyptratae/Drosophila - ps31
Drosophila - ps32
Sophophora - ps33
Obscura/Melanogaster_group - ps34
D_melanogaster_group - ps35
D_melanogaster_subgroup - ps36
sim./sech./mel. group - ps37
Drosophila_melanogaster - ps38
Phylostratum

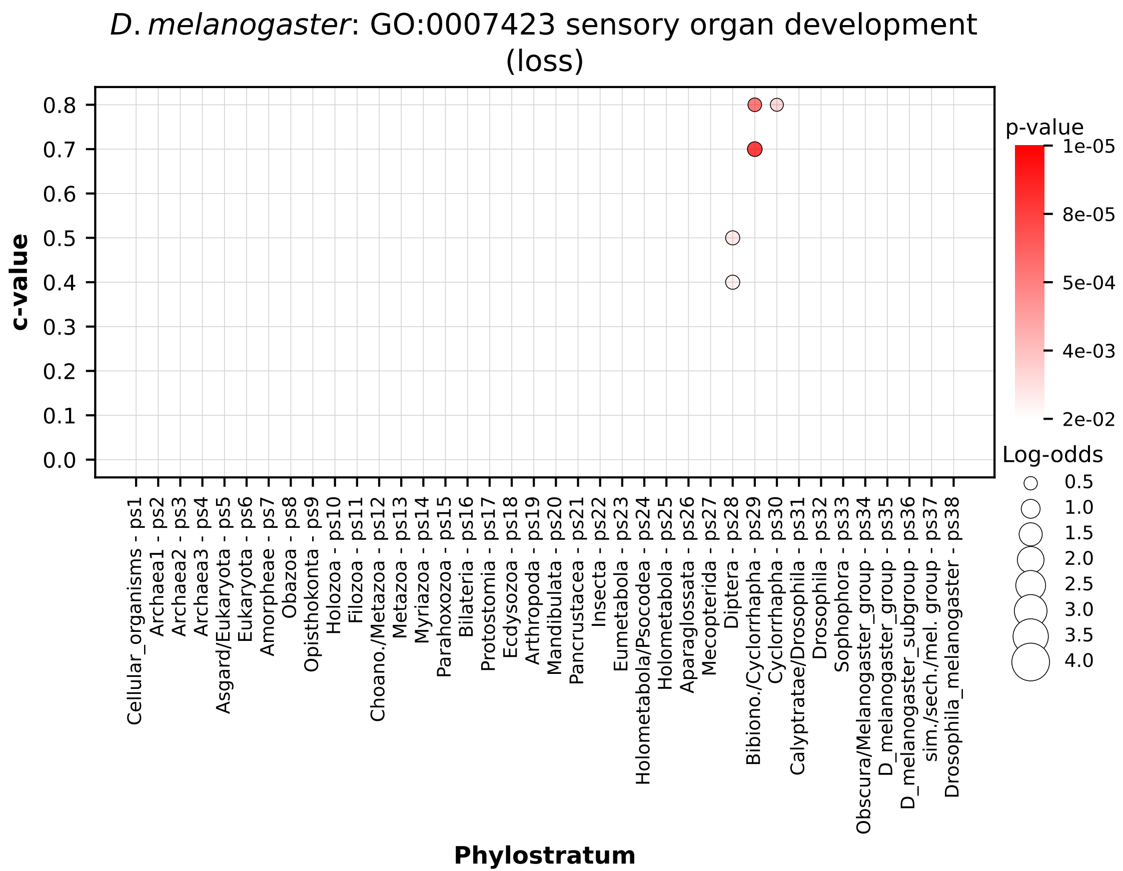

D. melanogaster: GO:0007423 sensory organ development
(loss)
c-value
0.0
0.1
0.2
0.3
0.4
0.5
0.6
0.7
0.8
Phylostratum
Cellular_organisms - ps1
Archaea1 - ps2
Archaea2 - ps3
Archaea3 - ps4
Asgard/Eukaryota - ps5
Eukaryota - ps6
Amorpheae - ps7
Obazoa - ps8
Opisthokonta - ps9
Holozoa - ps10
Filozoa - ps11
Choano./Metazoa - ps12
Metazoa - ps13
Myriazoa - ps14
Parahoxozoa - ps15
Bilateria - ps16
Protostomia - ps17
Ecdysozoa - ps18
Arthropoda - ps19
Mandibulata - ps20
Pancrustacea - ps21
Insecta - ps22
Eumetabola - ps23
Holometabola/Psocodea - ps24
Holometabola - ps25
Aparaglossata - ps26
Mecopterida - ps27
Diptera - ps28
Bibiono./Cyclorrhapha - ps29
Cyclorrhapha - ps30
Calyptratae/Drosophila - ps31
Drosophila - ps32
Sophophora - ps33
Obscura/Melanogaster_group - ps34
D_melanogaster_group - ps35
D_melanogaster_subgroup - ps36
sim./sech./mel. group - ps37
Drosophila_melanogaster - ps38
p-value
1e-05
8e-05
5e-04
4e-03
2e-02
Log-odds
0.5
1.0
1.5
2.0
2.5
3.0
3.5
4.0

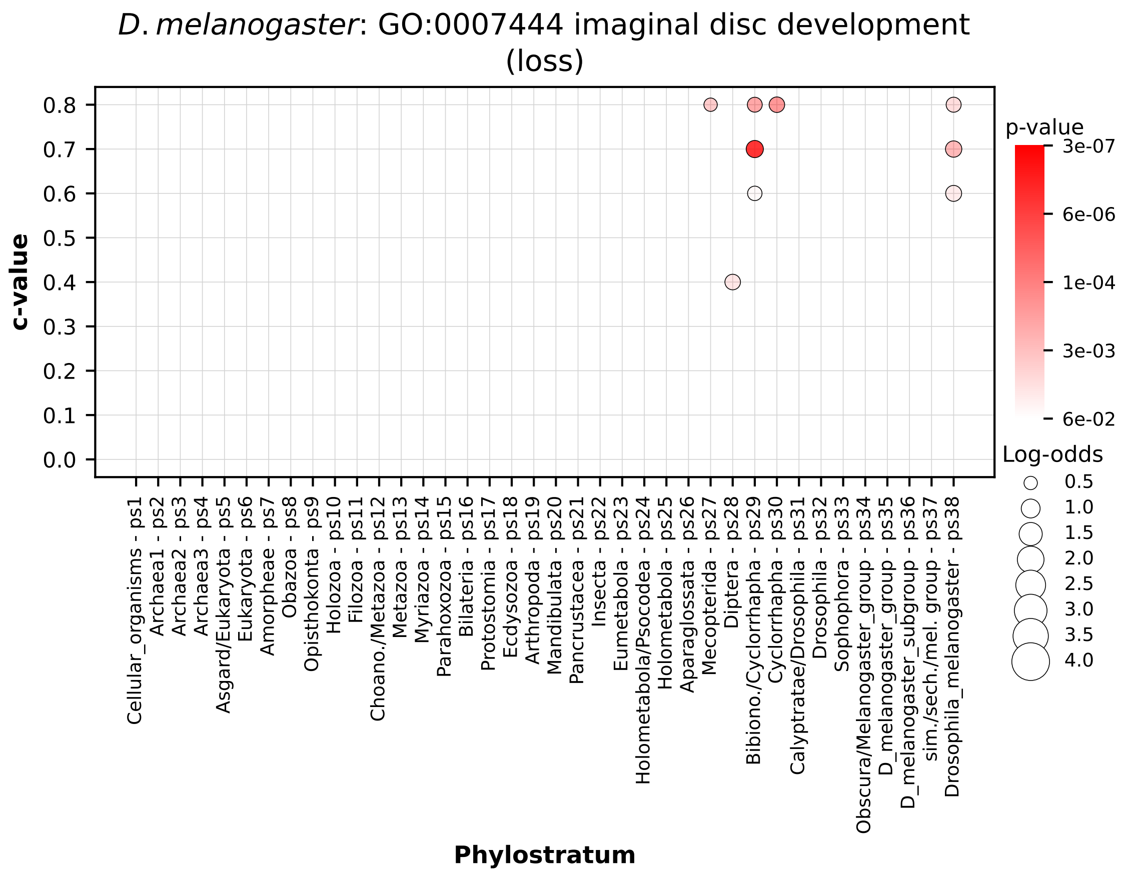

D. melanogaster: GO:0007444 imaginal disc development
(loss)
c-value
0.8
0.7
0.6
0.5
0.4
0.3
0.2
0.1
0.0
p-value
3e-07
6e-06
1e-04
3e-03
6e-02
Log-odds
0.5
1.0
1.5
2.0
2.5
3.0
3.5
4.0
Cellular_organisms - ps1
Archaea1 - ps2
Archaea2 - ps3
Archaea3 - ps4
Asgard/Eukaryota - ps5
Eukaryota - ps6
Amorpheae - ps7
Obazoa - ps8
Opisthokonta - ps9
Holozoa - ps10
Filozoa - ps11
Choano./Metazoa - ps12
Metazoa - ps13
Myriazoa - ps14
Parahoxozoa - ps15
Bilateria - ps16
Protostomia - ps17
Ecdysozoa - ps18
Arthropoda - ps19
Mandibulata - ps20
Pancrustacea - ps21
Insecta - ps22
Eumetabola - ps23
Holometabola/Psocodea - ps24
Holometabola - ps25
Aparaglossata - ps26
Mecopterida - ps27
Diptera - ps28
Bibiono./Cyclorrhapha - ps29
Cyclorrhapha - ps30
Calyptratae/Drosophila - ps31
Drosophila - ps32
Sophophora - ps33
Obscura/Melanogaster_group - ps34
D_melanogaster_group - ps35
D_melanogaster_subgroup - ps36
sim./sech./mel. group - ps37
Drosophila_melanogaster - ps38
Phylostratum

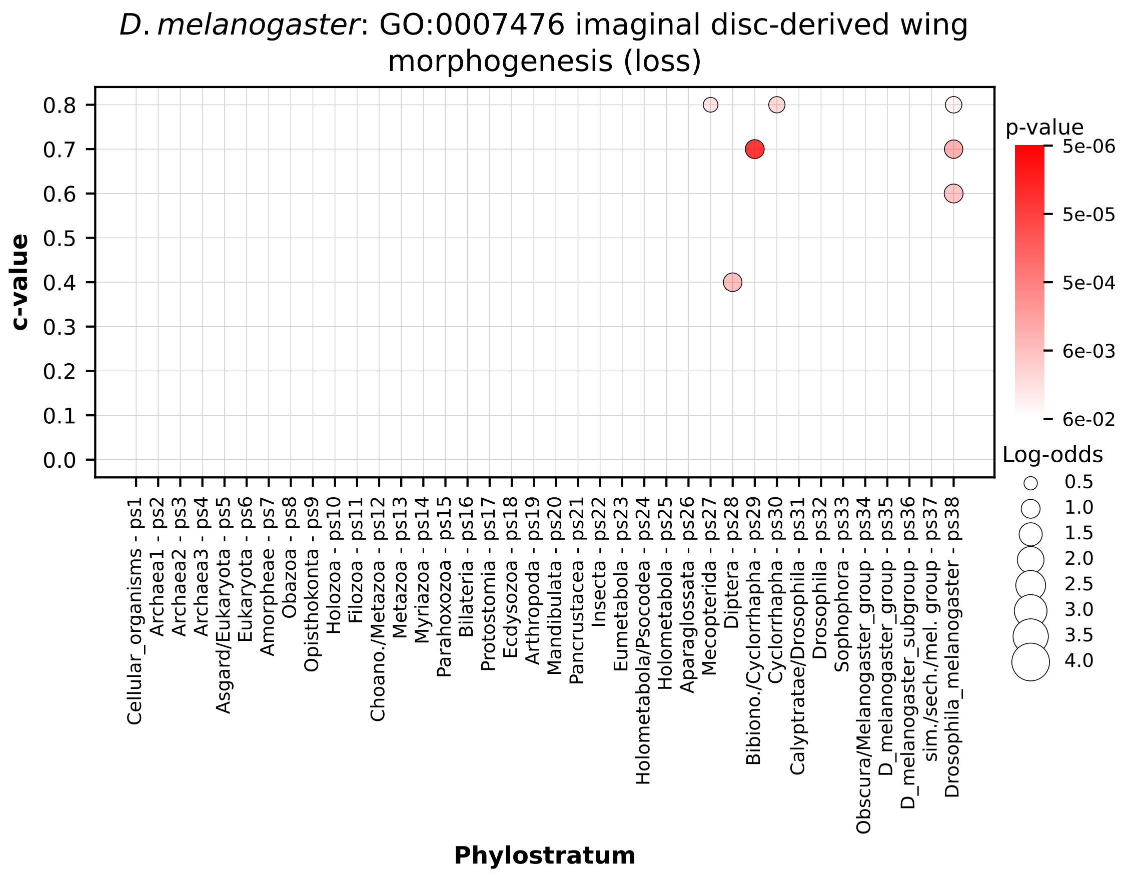
D. melanogaster: GO:0007476 imaginal disc-derived wing morphogenesis (loss)
c-value
0.8
0.7
0.6
0.5
0.4
0.3
0.2
0.1
0.0
p-value
5e-06
5e-05
5e-04
6e-03
6e-02
Log-odds
0.5
1.0
1.5
2.0
2.5
3.0
3.5
4.0
Cellular_organisms - ps1
Archaea1 - ps2
Archaea2 - ps3
Archaea3 - ps4
Asgard/Eukaryota - ps5
Eukaryota - ps6
Amorpheae - ps7
Obazoa - ps8
Opisthokonta - ps9
Holozoa - ps10
Filozoa - ps11
Choano./Metazoa - ps12
Metazoa - ps13
Myriazoa - ps14
Parahoxozoa - ps15
Bilateria - ps16
Protostomia - ps17
Ecdysozoa - ps18
Arthropoda - ps19
Mandibulata - ps20
Pancrustacea - ps21
Insecta - ps22
Eumetabola - ps23
Holometabola/Psocodea - ps24
Holometabola - ps25
Aparaglossata - ps26
Mecopterida - ps27
Diptera - ps28
Bibiono./Cyclorrhapha - ps29
Cyclorrhapha - ps30
Calyptratae/Drosophila - ps31
Drosophila - ps32
Sophophora - ps33
Obscura/Melanogaster_group - ps34
D_melanogaster_group - ps35
D_melanogaster_subgroup - ps36
sim./sech./mel. group - ps37
Drosophila_melanogaster - ps38
Phylostratum

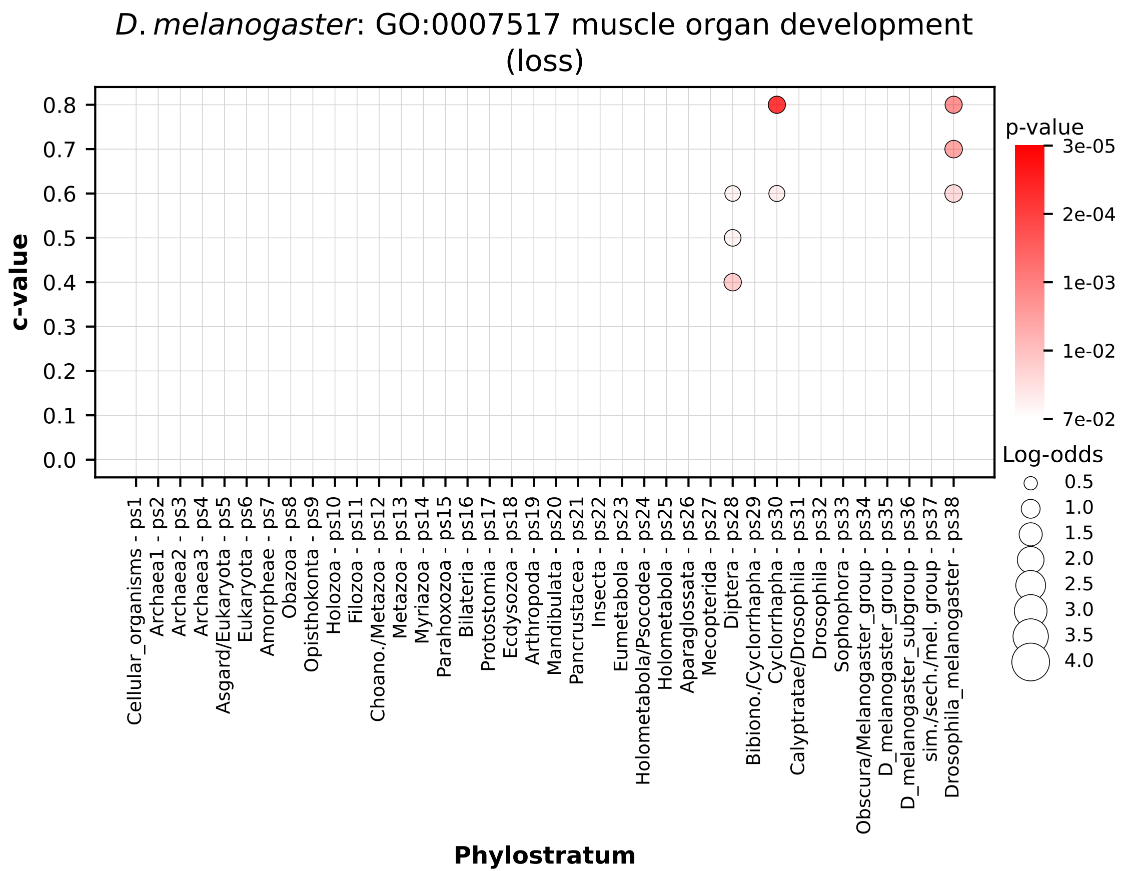

D. melanogaster: GO:0007517 muscle organ development
(loss)
c-value
0.8
0.7
0.6
0.5
0.4
0.3
0.2
0.1
0.0
p-value
3e-05
2e-04
1e-03
1e-02
7e-02
Log-odds
0.5
1.0
1.5
2.0
2.5
3.0
3.5
4.0
Cellular_organisms - ps1
Archaea1 - ps2
Archaea2 - ps3
Archaea3 - ps4
Asgard/Eukaryota - ps5
Eukaryota - ps6
Amorpheae - ps7
Obazoa - ps8
Opisthokonta - ps9
Holozoa - ps10
Filozoa - ps11
Choano./Metazoa - ps12
Metazoa - ps13
Myriazoa - ps14
Parahoxozoa - ps15
Bilateria - ps16
Protostomia - ps17
Ecdysozoa - ps18
Arthropoda - ps19
Mandibulata - ps20
Pancrustacea - ps21
Insecta - ps22
Eumetabola - ps23
Holometabola/Psocodea - ps24
Holometabola - ps25
Aparaglossata - ps26
Mecopterida - ps27
Diptera - ps28
Bibiono./Cyclorrhapha - ps29
Cyclorrhapha - ps30
Calyptratae/Drosophila - ps31
Drosophila - ps32
Sophophora - ps33
Obscura/Melanogaster_group - ps34
D_melanogaster_group - ps35
D_melanogaster_subgroup - ps36
sim./sech./mel. group - ps37
Drosophila_melanogaster - ps38
Phylostratum

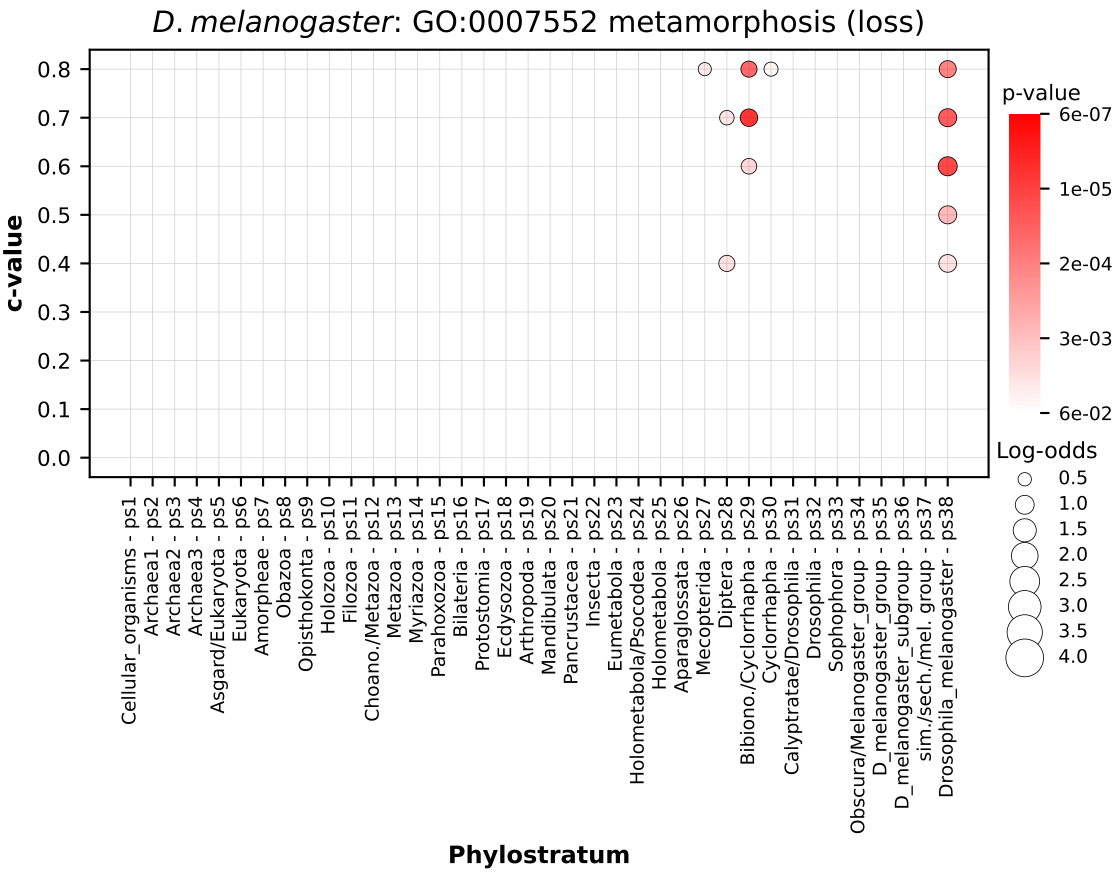

D. melanogaster: GO:0007552 metamorphosis (loss)
c-value
0.8
0.7
0.6
0.5
0.4
0.3
0.2
0.1
0.0
Phylostratum
Cellular_organisms - ps1
Archaea1 - ps2
Archaea2 - ps3
Archaea3 - ps4
Asgard/Eukaryota - ps5
Eukaryota - ps6
Amorpheae - ps7
Obazoa - ps8
Opisthokonta - ps9
Holozoa - ps10
Filozoa - ps11
Choano./Metazoa - ps12
Metazoa - ps13
Myriazoa - ps14
Parahoxozoa - ps15
Bilateria - ps16
Protostomia - ps17
Ecdysozoa - ps18
Arthropoda - ps19
Mandibulata - ps20
Pancrustacea - ps21
Insecta - ps22
Eumetabola - ps23
Holometabola/Psocodea - ps24
Holometabola - ps25
Aparaglossata - ps26
Mecopterida - ps27
Diptera - ps28
Bibiono./Cyclorrhapha - ps29
Cyclorrhapha - ps30
Calyptratae/Drosophila - ps31
Drosophila - ps32
Sophophora - ps33
Obscura/Melanogaster_group - ps34
D_melanogaster_group - ps35
D_melanogaster_subgroup - ps36
sim./sech./mel. group - ps37
Drosophila_melanogaster - ps38
p-value
6e-07
1e-05
2e-04
3e-03
6e-02
Log-odds
0.5
1.0
1.5
2.0
2.5
3.0
3.5
4.0

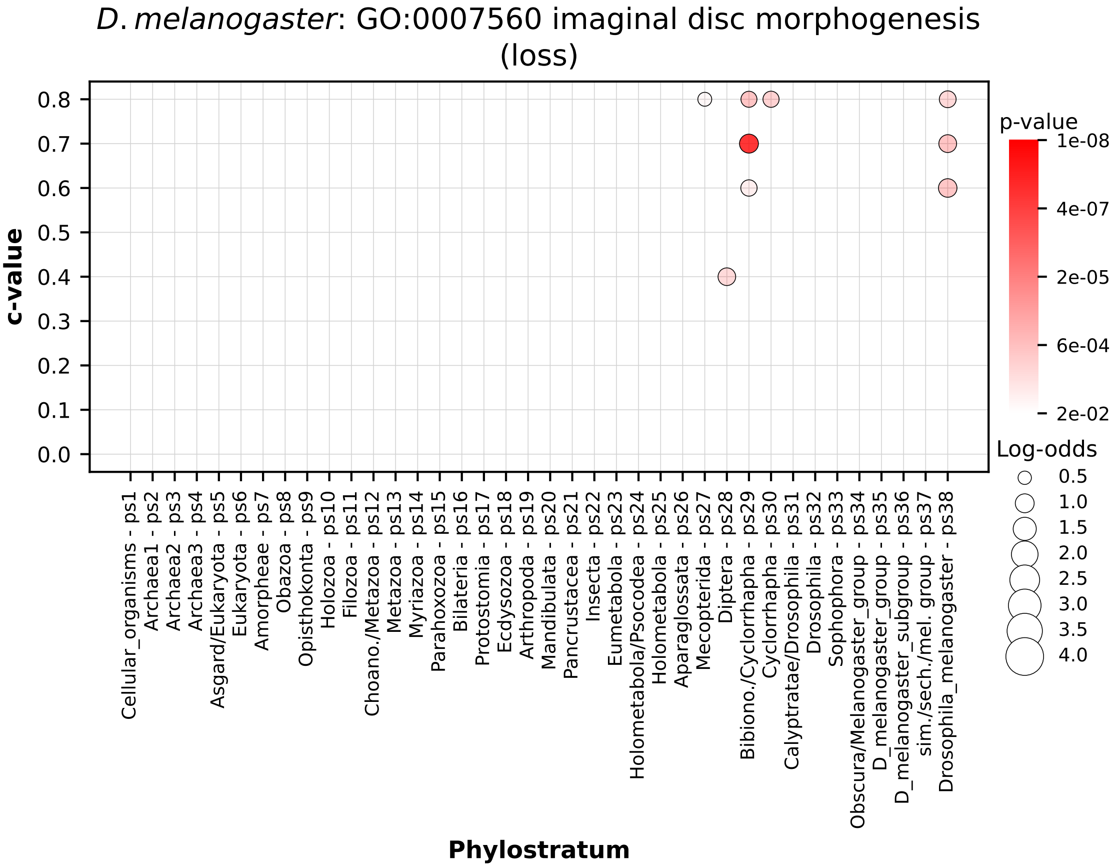

D. melanogaster: GO:0007560 imaginal disc morphogenesis
(loss)
c-value
0.8
0.7
0.6
0.5
0.4
0.3
0.2
0.1
0.0
p-value
1e-08
4e-07
2e-05
6e-04
2e-02
Log-odds
0.5
1.0
1.5
2.0
2.5
3.0
3.5
4.0
Cellular_organisms - ps1
Archaea1 - ps2
Archaea2 - ps3
Archaea3 - ps4
Asgard/Eukaryota - ps5
Eukaryota - ps6
Amorpheae - ps7
Obazoa - ps8
Opisthokonta - ps9
Holozoa - ps10
Filozoa - ps11
Choano./Metazoa - ps12
Metazoa - ps13
Myriazoa - ps14
Parahoxozoa - ps15
Bilateria - ps16
Protostomia - ps17
Ecdysozoa - ps18
Arthropoda - ps19
Mandibulata - ps20
Pancrustacea - ps21
Insecta - ps22
Eumetabola - ps23
Holometabola/Psocodea - ps24
Holometabola - ps25
Aparaglossata - ps26
Mecopterida - ps27
Diptera - ps28
Bibiono./Cyclorrhapha - ps29
Cyclorrhapha - ps30
Calyptratae/Drosophila - ps31
Drosophila - ps32
Sophophora - ps33
Obscura/Melanogaster_group - ps34
D_melanogaster_group - ps35
D_melanogaster_subgroup - ps36
sim./sech./mel. group - ps37
Drosophila_melanogaster - ps38
Phylostratum

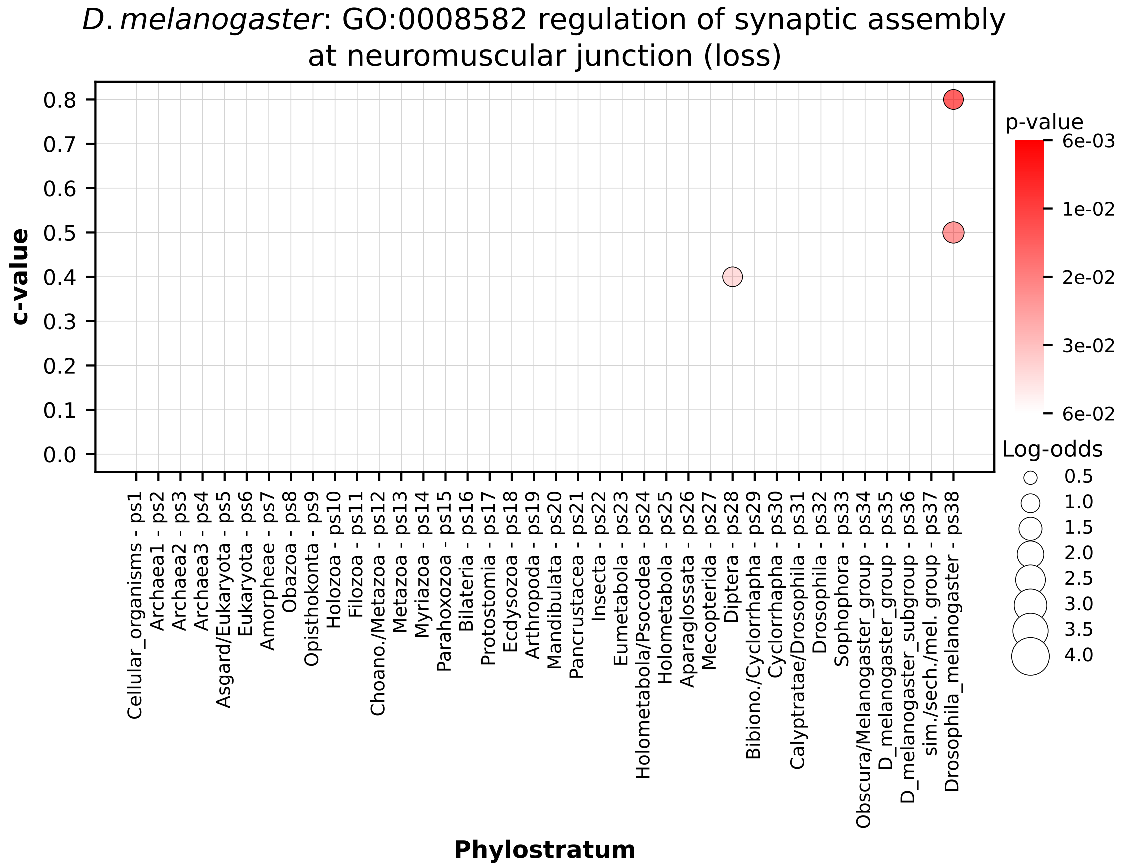

D. melanogaster: GO:0008582 regulation of synaptic assembly at neuromuscular junction (loss)
c-value
0.8
0.7
0.6
0.5
0.4
0.3
0.2
0.1
0.0
p-value
6e-03
1e-02
2e-02
3e-02
6e-02
Log-odds
0.5
1.0
1.5
2.0
2.5
3.0
3.5
4.0
Cellular_organisms - ps1
Archaea1 - ps2
Archaea2 - ps3
Archaea3 - ps4
Asgard/Eukaryota - ps5
Eukaryota - ps6
Amorpheae - ps7
Obazoa - ps8
Opisthokonta - ps9
Holozoa - ps10
Filozoa - ps11
Choano./Metazoa - ps12
Metazoa - ps13
Myriazoa - ps14
Parahoxozoa - ps15
Bilateria - ps16
Protostomia - ps17
Ecdysozoa - ps18
Arthropoda - ps19
Mandibulata - ps20
Pancrustacea - ps21
Insecta - ps22
Eumetabola - ps23
Holometabola/Psocodea - ps24
Holometabola - ps25
Aparaglossata - ps26
Mecopterida - ps27
Diptera - ps28
Bibiono./Cyclorrhapha - ps29
Cyclorrhapha - ps30
Calyptratae/Drosophila - ps31
Drosophila - ps32
Sophophora - ps33
Obscura/Melanogaster_group - ps34
D_melanogaster_group - ps35
D_melanogaster_subgroup - ps36
sim./sech./mel. group - ps37
Drosophila_melanogaster - ps38
Phylostratum

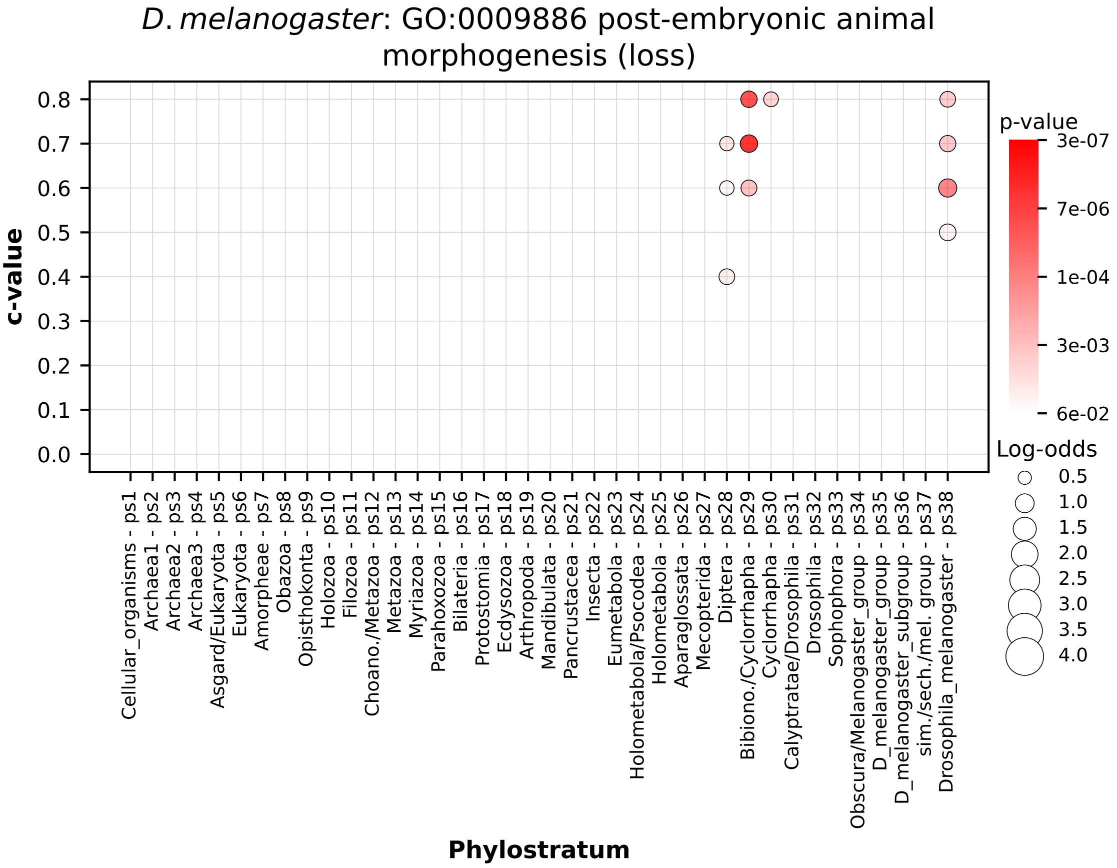

D. melanogaster: GO:0009886 post-embryonic animal morphogenesis (loss)
c-value
0.8
0.7
0.6
0.5
0.4
0.3
0.2
0.1
0.0
p-value
3e-07
7e-06
1e-04
3e-03
6e-02
Log-odds
0.5
1.0
1.5
2.0
2.5
3.0
3.5
4.0
Cellular_organisms - ps1
Archaea1 - ps2
Archaea2 - ps3
Archaea3 - ps4
Asgard/Eukaryota - ps5
Eukaryota - ps6
Amorpheae - ps7
Obazoa - ps8
Opisthokonta - ps9
Holozoa - ps10
Filozoa - ps11
Choano./Metazoa - ps12
Metazoa - ps13
Myriazoa - ps14
Parahoxozoa - ps15
Bilateria - ps16
Protostomia - ps17
Ecdysozoa - ps18
Arthropoda - ps19
Mandibulata - ps20
Pancrustacea - ps21
Insecta - ps22
Eumetabola - ps23
Holometabola/Psocodea - ps24
Holometabola - ps25
Aparaglossata - ps26
Mecopterida - ps27
Diptera - ps28
Bibiono./Cyclorrhapha - ps29
Cyclorrhapha - ps30
Calyptratae/Drosophila - ps31
Drosophila - ps32
Sophophora - ps33
Obscura/Melanogaster_group - ps34
D_melanogaster_group - ps35
D_melanogaster_subgroup - ps36
sim./sech./mel. group - ps37
Drosophila_melanogaster - ps38
Phylostratum

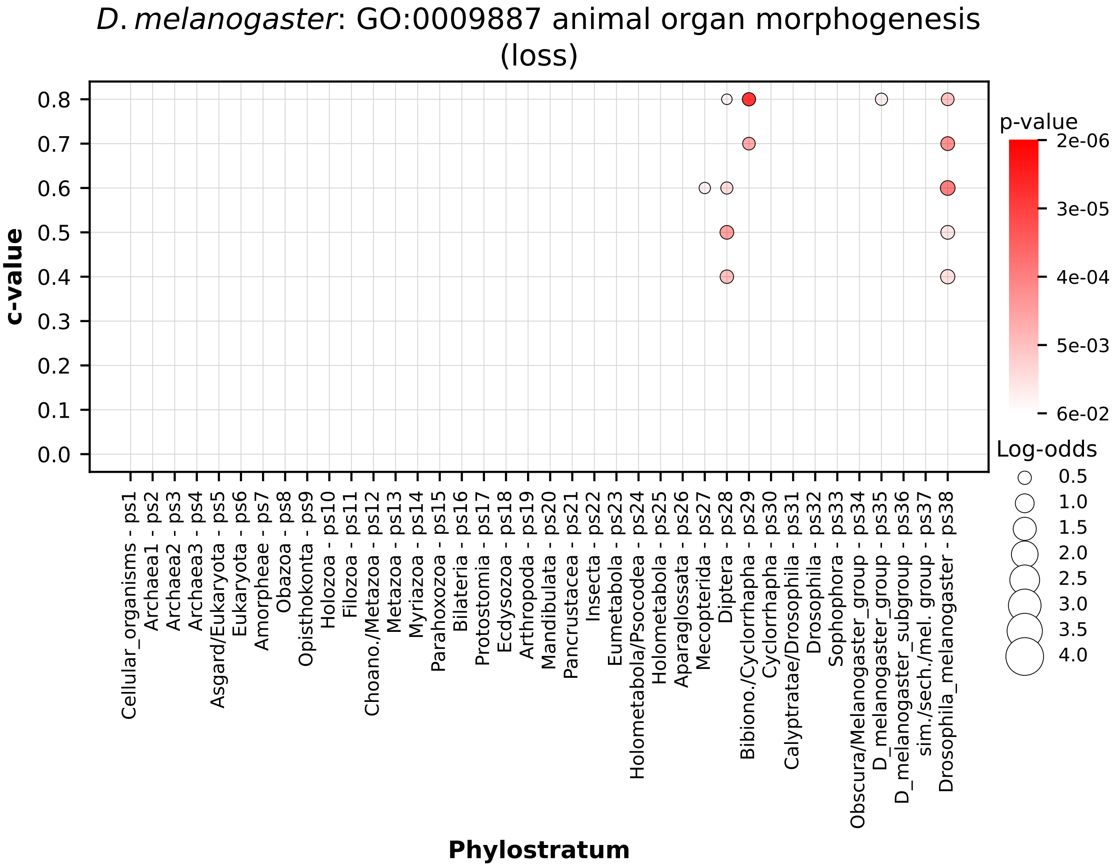

D. melanogaster: GO:0009887 animal organ morphogenesis (loss)
c-value
0.0
0.1
0.2
0.3
0.4
0.5
0.6
0.7
0.8
p-value
2e-06
3e-05
4e-04
5e-03
6e-02
Log-odds
0.5
1.0
1.5
2.0
2.5
3.0
3.5
4.0
Cellular_organisms - ps1
Archaea1 - ps2
Archaea2 - ps3
Archaea3 - ps4
Asgard/Eukaryota - ps5
Eukaryota - ps6
Amorpheae - ps7
Obazoa - ps8
Opisthokonta - ps9
Holozoa - ps10
Filozoa - ps11
Choano./Metazoa - ps12
Metazoa - ps13
Myriazoa - ps14
Parahoxozoa - ps15
Bilateria - ps16
Protostomia - ps17
Ecdysozoa - ps18
Arthropoda - ps19
Mandibulata - ps20
Pancrustacea - ps21
Insecta - ps22
Eumetabola - ps23
Holometabola/Psocodea - ps24
Holometabola - ps25
Aparaglossata - ps26
Mecopterida - ps27
Diptera - ps28
Bibiono./Cyclorrhapha - ps29
Cyclorrhapha - ps30
Calyptratae/Drosophila - ps31
Drosophila - ps32
Sophophora - ps33
Obscura/Melanogaster_group - ps34
D_melanogaster_group - ps35
D_melanogaster_subgroup - ps36
sim./sech./mel. group - ps37
Drosophila_melanogaster - ps38
Phylostratum

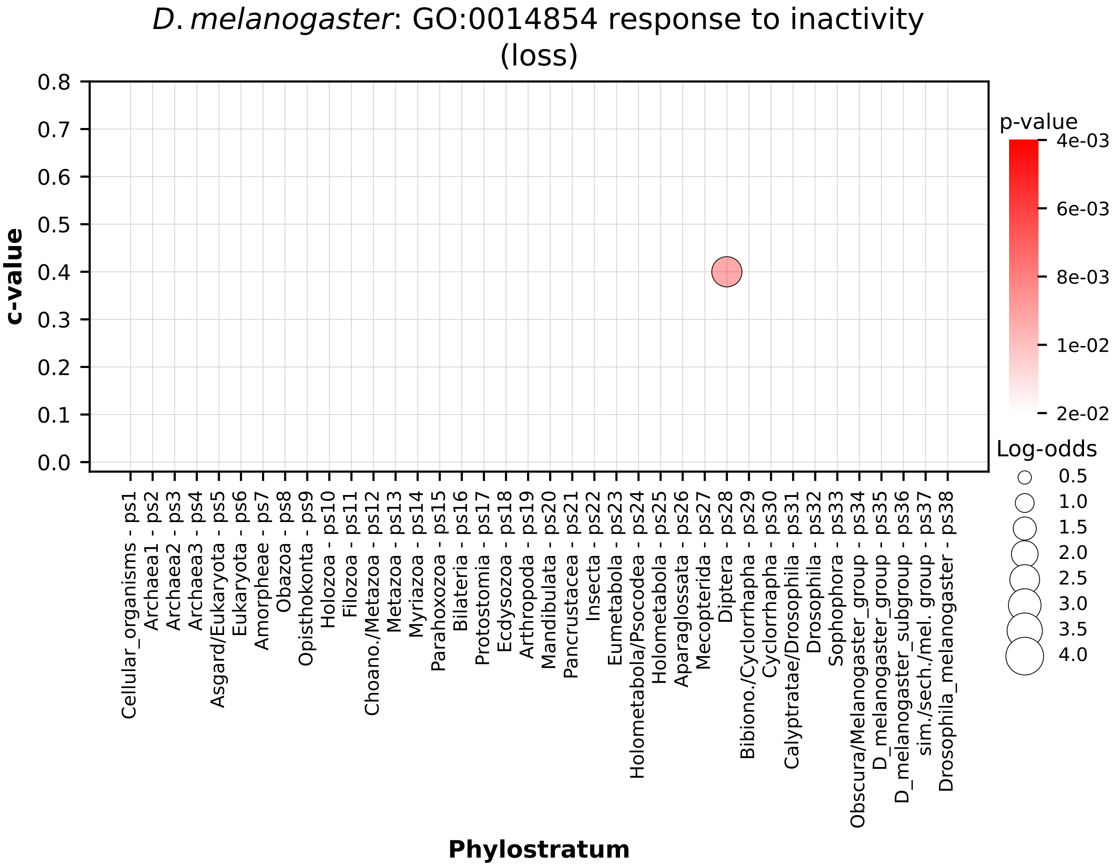

D. melanogaster: GO:0014854 response to inactivity
(loss)
c-value
0.8
0.7
0.6
0.5
0.4
0.3
0.2
0.1
0.0
Phylostratum
Cellular_organisms - ps1
Archaea1 - ps2
Archaea2 - ps3
Archaea3 - ps4
Asgard/Eukaryota - ps5
Eukaryota - ps6
Amorpheae - ps7
Obazoa - ps8
Opisthokonta - ps9
Holozoa - ps10
Filozoa - ps11
Choano./Metazoa - ps12
Metazoa - ps13
Myriazoa - ps14
Parahoxozoa - ps15
Bilateria - ps16
Protostomia - ps17
Ecdysozoa - ps18
Arthropoda - ps19
Mandibulata - ps20
Pancrustacea - ps21
Insecta - ps22
Eumetabola - ps23
Holometabola/Psocodea - ps24
Holometabola - ps25
Aparaglossata - ps26
Mecopterida - ps27
Diptera - ps28
Bibiono./Cyclorrhapha - ps29
Cyclorrhapha - ps30
Calyptratae/Drosophila - ps31
Drosophila - ps32
Sophophora - ps33
Obscura/Melanogaster_group - ps34
D_melanogaster_group - ps35
D_melanogaster_subgroup - ps36
sim./sech./mel. group - ps37
Drosophila_melanogaster - ps38
p-value
4e-03
6e-03
8e-03
1e-02
2e-02
Log-odds
0.5
1.0
1.5
2.0
2.5
3.0
3.5
4.0

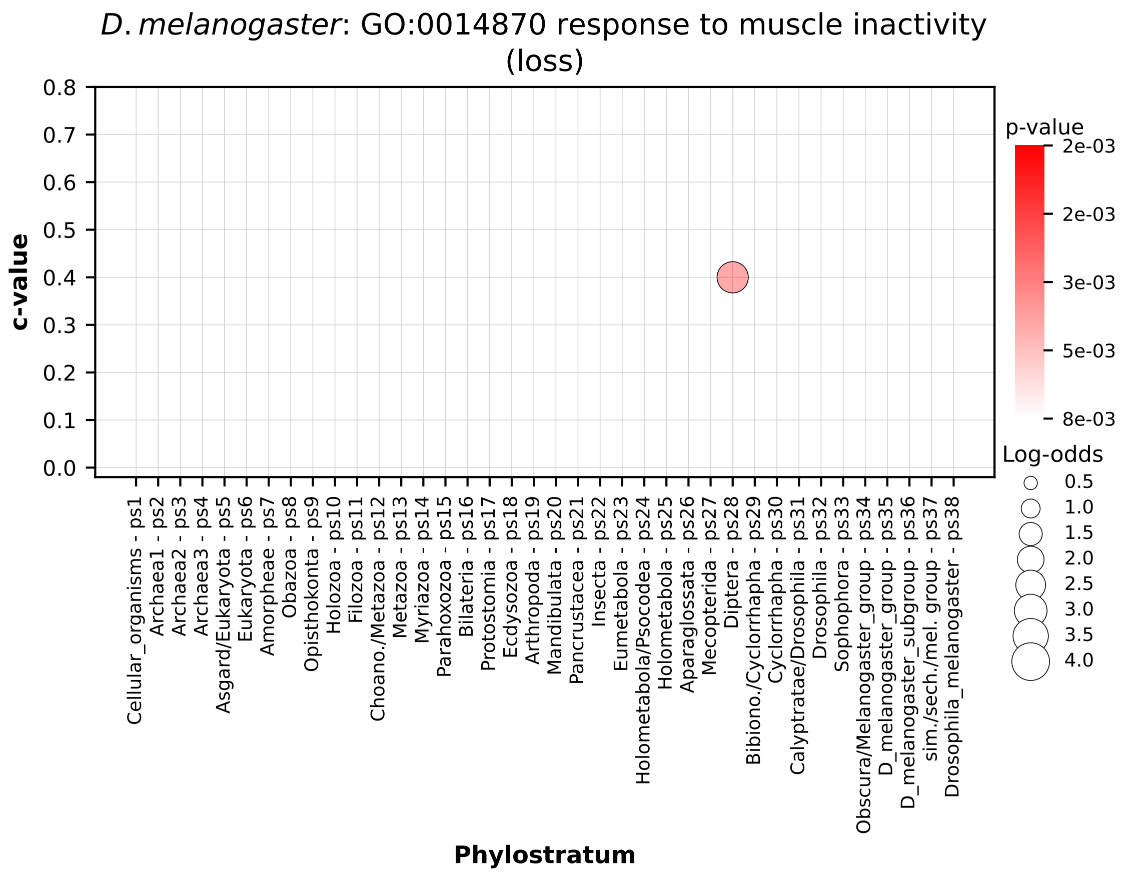
D. melanogaster: GO:0014870 response to muscle inactivity
(loss)
c-value
0.8
0.7
0.6
0.5
0.4
0.3
0.2
0.1
0.0
p-value
2e-03
2e-03
3e-03
5e-03
8e-03
Log-odds
0.5
1.0
1.5
2.0
2.5
3.0
3.5
4.0
Cellular_organisms - ps1
Archaea1 - ps2
Archaea2 - ps3
Archaea3 - ps4
Asgard/Eukaryota - ps5
Eukaryota - ps6
Amorpheae - ps7
Obazoa - ps8
Opisthokonta - ps9
Holozoa - ps10
Filozoa - ps11
Choano./Metazoa - ps12
Metazoa - ps13
Myriazoa - ps14
Parahoxozoa - ps15
Bilateria - ps16
Protostomia - ps17
Ecdysozoa - ps18
Arthropoda - ps19
Mandibulata - ps20
Pancrustacea - ps21
Insecta - ps22
Eumetabola - ps23
Holometabola/Psocodea - ps24
Holometabola - ps25
Aparaglossata - ps26
Mecopterida - ps27
Diptera - ps28
Bibiono./Cyclorrhapha - ps29
Cyclorrhapha - ps30
Calyptratae/Drosophila - ps31
Drosophila - ps32
Sophophora - ps33
Obscura/Melanogaster_group - ps34
D_melanogaster_group - ps35
D_melanogaster_subgroup - ps36
sim./sech./mel. group - ps37
Drosophila_melanogaster - ps38
Phylostratum

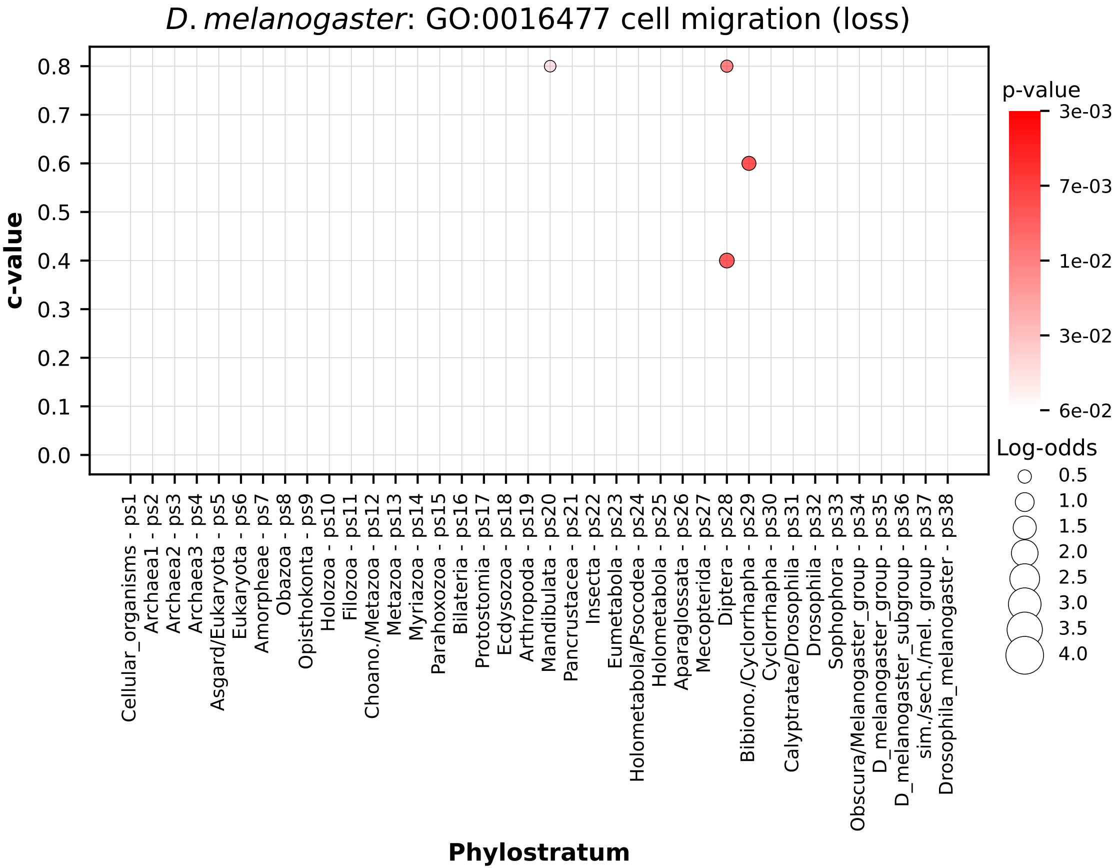

D. melanogaster: GO:0016477 cell migration (loss)
c-value
0.0
0.1
0.2
0.3
0.4
0.5
0.6
0.7
0.8
Phylostratum
Cellular_organisms - ps1
Archaea1 - ps2
Archaea2 - ps3
Archaea3 - ps4
Asgard/Eukaryota - ps5
Eukaryota - ps6
Amorpheae - ps7
Obazoa - ps8
Opisthokonta - ps9
Holozoa - ps10
Filozoa - ps11
Choano./Metazoa - ps12
Metazoa - ps13
Myriazoa - ps14
Parahoxozoa - ps15
Bilateria - ps16
Protostomia - ps17
Ecdysozoa - ps18
Arthropoda - ps19
Mandibulata - ps20
Pancrustacea - ps21
Insecta - ps22
Eumetabola - ps23
Holometabola/Psocodea - ps24
Holometabola - ps25
Aparaglossata - ps26
Mecopterida - ps27
Diptera - ps28
Bibiono./Cyclorrhapha - ps29
Cyclorrhapha - ps30
Calyptratae/Drosophila - ps31
Drosophila - ps32
Sophophora - ps33
Obscura/Melanogaster_group - ps34
D_melanogaster_group - ps35
D_melanogaster_subgroup - ps36
sim./sech./mel. group - ps37
Drosophila_melanogaster - ps38
p-value
3e-03
7e-03
1e-02
3e-02
6e-02
Log-odds
0.5
1.0
1.5
2.0
2.5
3.0
3.5
4.0

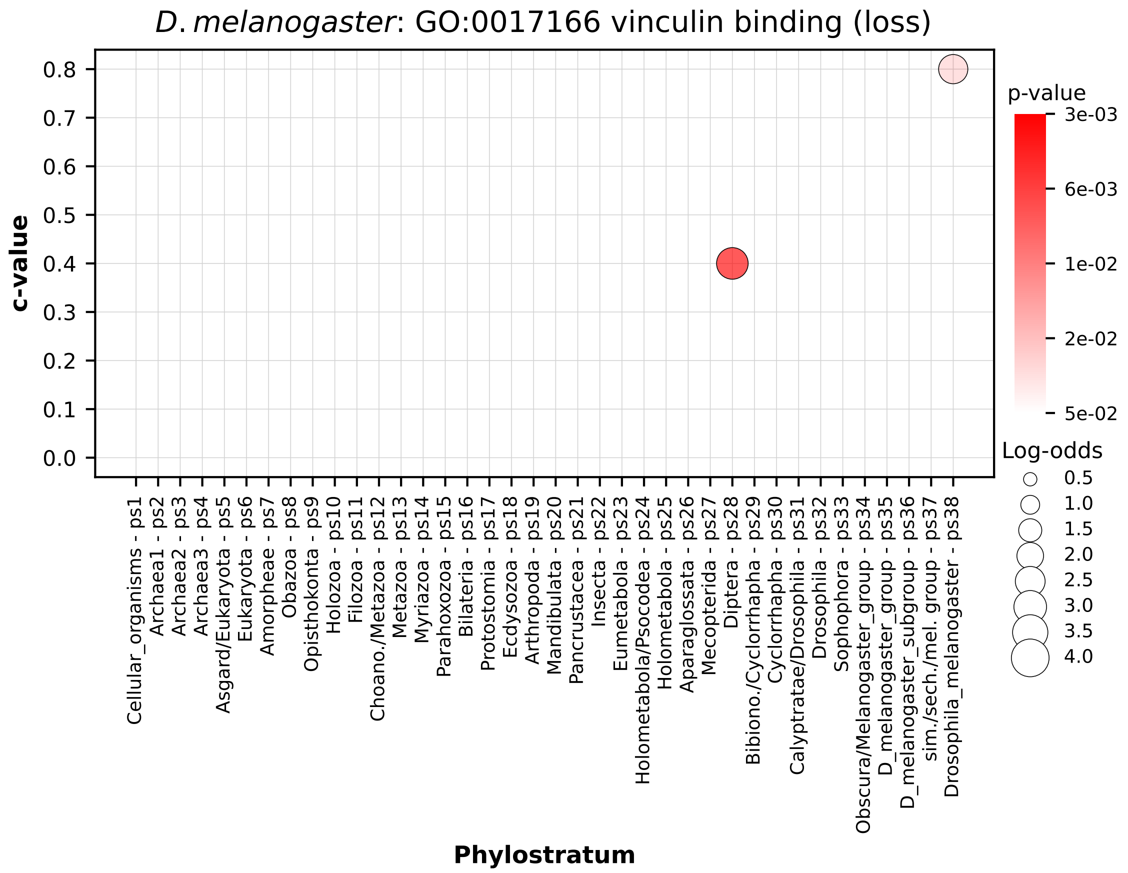

D. melanogaster: GO:0017166 vinculin binding (loss)
c-value
0.0
0.1
0.2
0.3
0.4
0.5
0.6
0.7
0.8
Phylostratum
Cellular_organisms - ps1
Archaea1 - ps2
Archaea2 - ps3
Archaea3 - ps4
Asgard/Eukaryota - ps5
Eukaryota - ps6
Amorpheae - ps7
Obazoa - ps8
Opisthokonta - ps9
Holozoa - ps10
Filozoa - ps11
Choano./Metazoa - ps12
Metazoa - ps13
Myriazoa - ps14
Parahoxozoa - ps15
Bilateria - ps16
Protostomia - ps17
Ecdysozoa - ps18
Arthropoda - ps19
Mandibulata - ps20
Pancrustacea - ps21
Insecta - ps22
Eumetabola - ps23
Holometabola/Psocodea - ps24
Holometabola - ps25
Aparaglossata - ps26
Mecopterida - ps27
Diptera - ps28
Bibiono./Cyclorrhapha - ps29
Cyclorrhapha - ps30
Calyptratae/Drosophila - ps31
Drosophila - ps32
Sophophora - ps33
Obscura/Melanogaster_group - ps34
D_melanogaster_group - ps35
D_melanogaster_subgroup - ps36
sim./sech./mel. group - ps37
Drosophila_melanogaster - ps38
p-value
3e-03
6e-03
1e-02
2e-02
5e-02
Log-odds
0.5
1.0
1.5
2.0
2.5
3.0
3.5
4.0

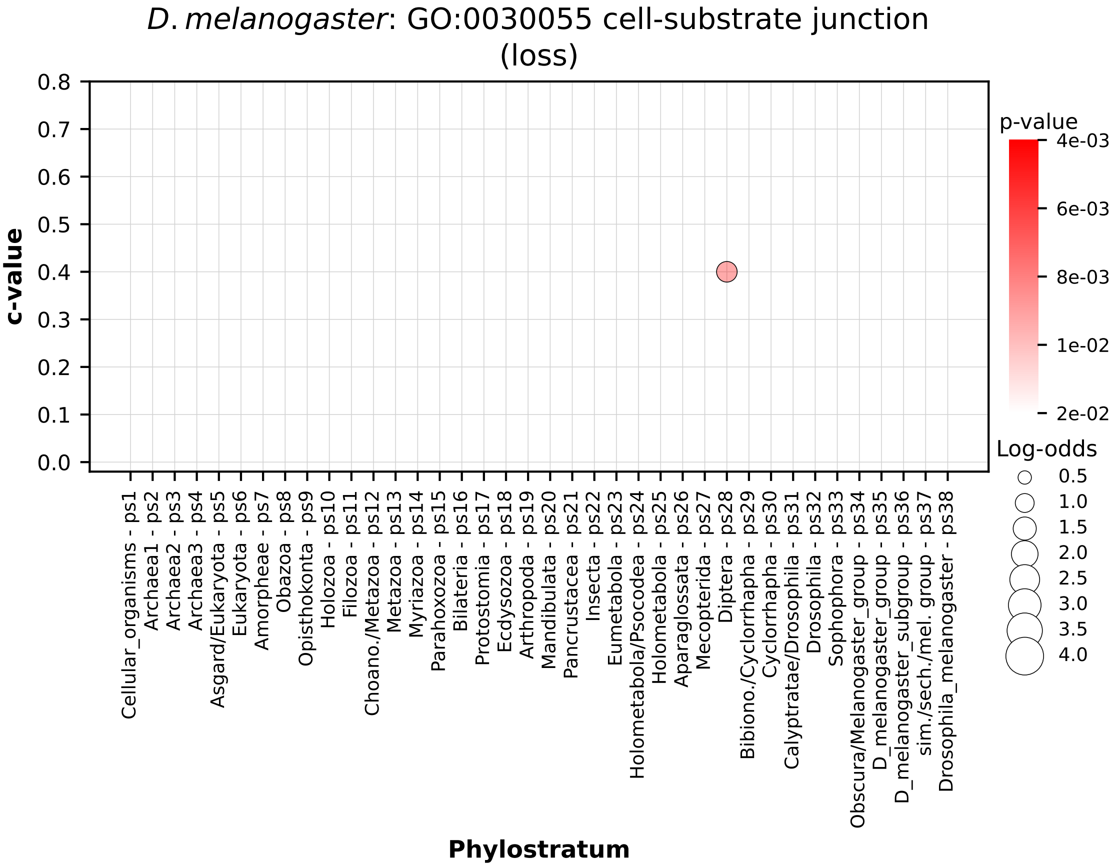

D. melanogaster: GO:0030055 cell-substrate junction (loss)
c-value
0.8
0.7
0.6
0.5
0.4
0.3
0.2
0.1
0.0
Phylostratum
Cellular_organisms - ps1
Archaea1 - ps2
Archaea2 - ps3
Archaea3 - ps4
Asgard/Eukaryota - ps5
Eukaryota - ps6
Amorpheae - ps7
Obazoa - ps8
Opisthokonta - ps9
Holozoa - ps10
Filozoa - ps11
Choano./Metazoa - ps12
Metazoa - ps13
Myriazoa - ps14
Parahoxozoa - ps15
Bilateria - ps16
Protostomia - ps17
Ecdysozoa - ps18
Arthropoda - ps19
Mandibulata - ps20
Pancrustacea - ps21
Insecta - ps22
Eumetabola - ps23
Holometabola/Psocodea - ps24
Holometabola - ps25
Aparaglossata - ps26
Mecopterida - ps27
Diptera - ps28
Bibiono./Cyclorrhapha - ps29
Cyclorrhapha - ps30
Calyptratae/Drosophila - ps31
Drosophila - ps32
Sophophora - ps33
Obscura/Melanogaster_group - ps34
D_melanogaster_group - ps35
D_melanogaster_subgroup - ps36
sim./sech./mel. group - ps37
Drosophila_melanogaster - ps38
p-value
4e-03
6e-03
8e-03
1e-02
2e-02
Log-odds
0.5
1.0
1.5
2.0
2.5
3.0
3.5
4.0

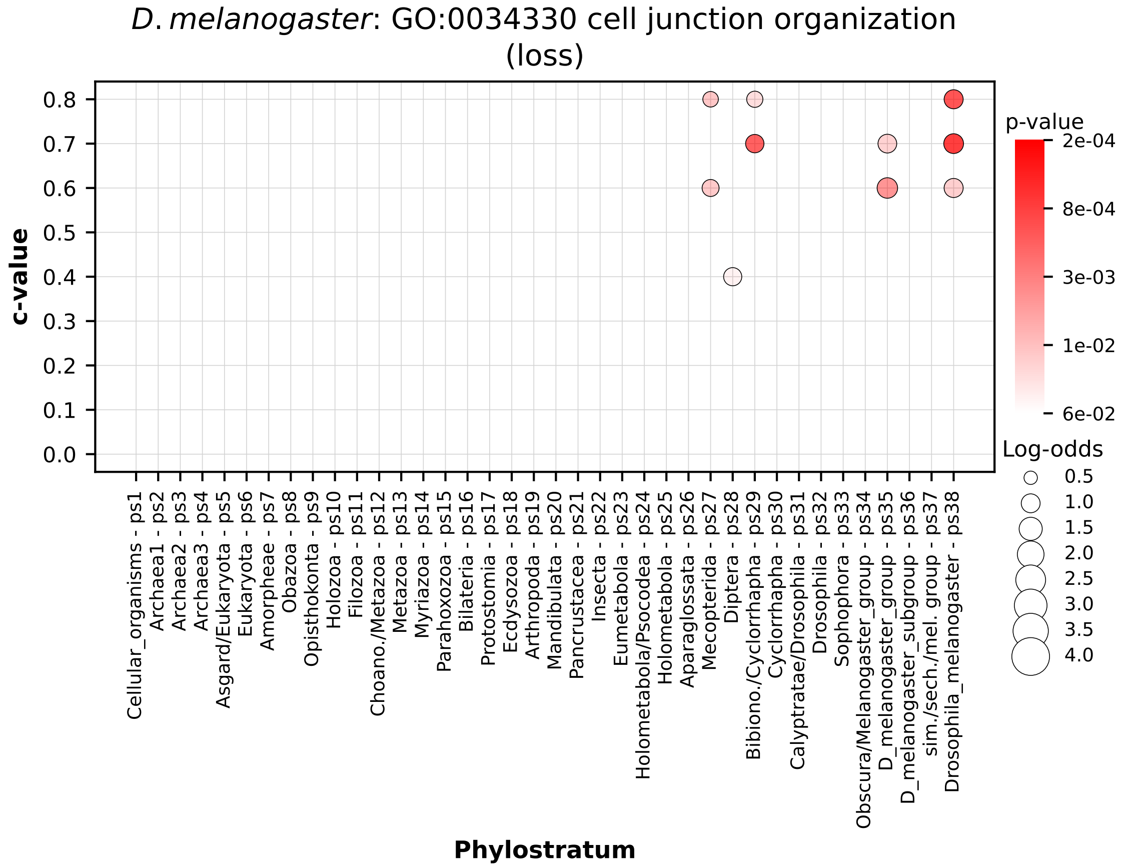

D. melanogaster: GO:0034330 cell junction organization
(loss)
c-value
0.8
0.7
0.6
0.5
0.4
0.3
0.2
0.1
0.0
p-value
2e-04
8e-04
3e-03
1e-02
6e-02
Log-odds
0.5
1.0
1.5
2.0
2.5
3.0
3.5
4.0
Cellular_organisms - ps1
Archaea1 - ps2
Archaea2 - ps3
Archaea3 - ps4
Asgard/Eukaryota - ps5
Eukaryota - ps6
Amorpheae - ps7
Obazoa - ps8
Opisthokonta - ps9
Holozoa - ps10
Filozoa - ps11
Choano./Metazoa - ps12
Metazoa - ps13
Myriazoa - ps14
Parahoxozoa - ps15
Bilateria - ps16
Protostomia - ps17
Ecdysozoa - ps18
Arthropoda - ps19
Mandibulata - ps20
Pancrustacea - ps21
Insecta - ps22
Eumetabola - ps23
Holometabola/Psocodea - ps24
Holometabola - ps25
Aparaglossata - ps26
Mecopterida - ps27
Diptera - ps28
Bibiono./Cyclorrhapha - ps29
Cyclorrhapha - ps30
Calyptratae/Drosophila - ps31
Drosophila - ps32
Sophophora - ps33
Obscura/Melanogaster_group - ps34
D_melanogaster_group - ps35
D_melanogaster_subgroup - ps36
sim./sech./mel. group - ps37
Drosophila_melanogaster - ps38
Phylostratum

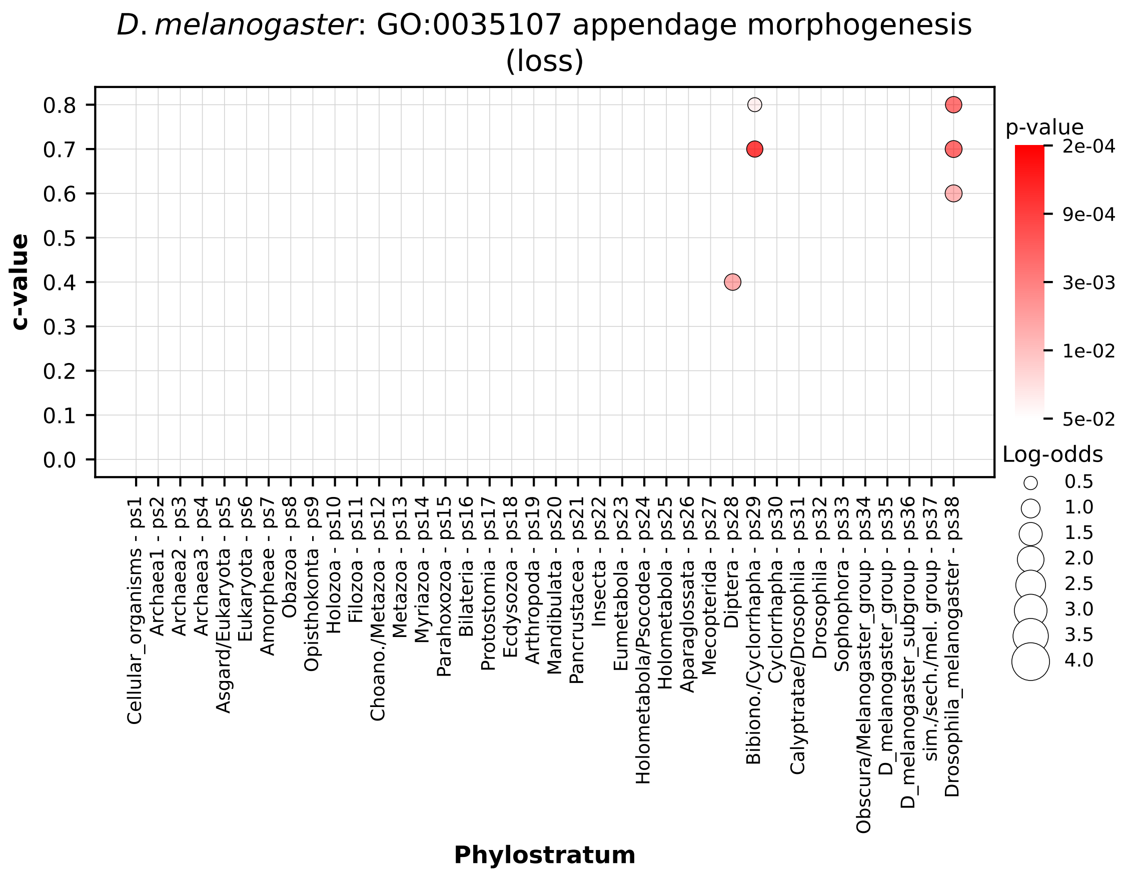

D. melanogaster: GO:0035107 appendage morphogenesis (loss)
c-value
0.8
0.7
0.6
0.5
0.4
0.3
0.2
0.1
0.0
p-value
2e-04
9e-04
3e-03
1e-02
5e-02
Log-odds
0.5
1.0
1.5
2.0
2.5
3.0
3.5
4.0
Cellular_organisms - ps1
Archaea1 - ps2
Archaea2 - ps3
Archaea3 - ps4
Asgard/Eukaryota - ps5
Eukaryota - ps6
Amorpheae - ps7
Obazoa - ps8
Opisthokonta - ps9
Holozoa - ps10
Filozoa - ps11
Choano./Metazoa - ps12
Metazoa - ps13
Myriazoa - ps14
Parahoxozoa - ps15
Bilateria - ps16
Protostomia - ps17
Ecdysozoa - ps18
Arthropoda - ps19
Mandibulata - ps20
Pancrustacea - ps21
Insecta - ps22
Eumetabola - ps23
Holometabola/Psocodea - ps24
Holometabola - ps25
Aparaglossata - ps26
Mecopterida - ps27
Diptera - ps28
Bibiono./Cyclorrhapha - ps29
Cyclorrhapha - ps30
Calyptratae/Drosophila - ps31
Drosophila - ps32
Sophophora - ps33
Obscura/Melanogaster_group - ps34
D_melanogaster_group - ps35
D_melanogaster_subgroup - ps36
sim./sech./mel. group - ps37
Drosophila_melanogaster - ps38
Phylostratum

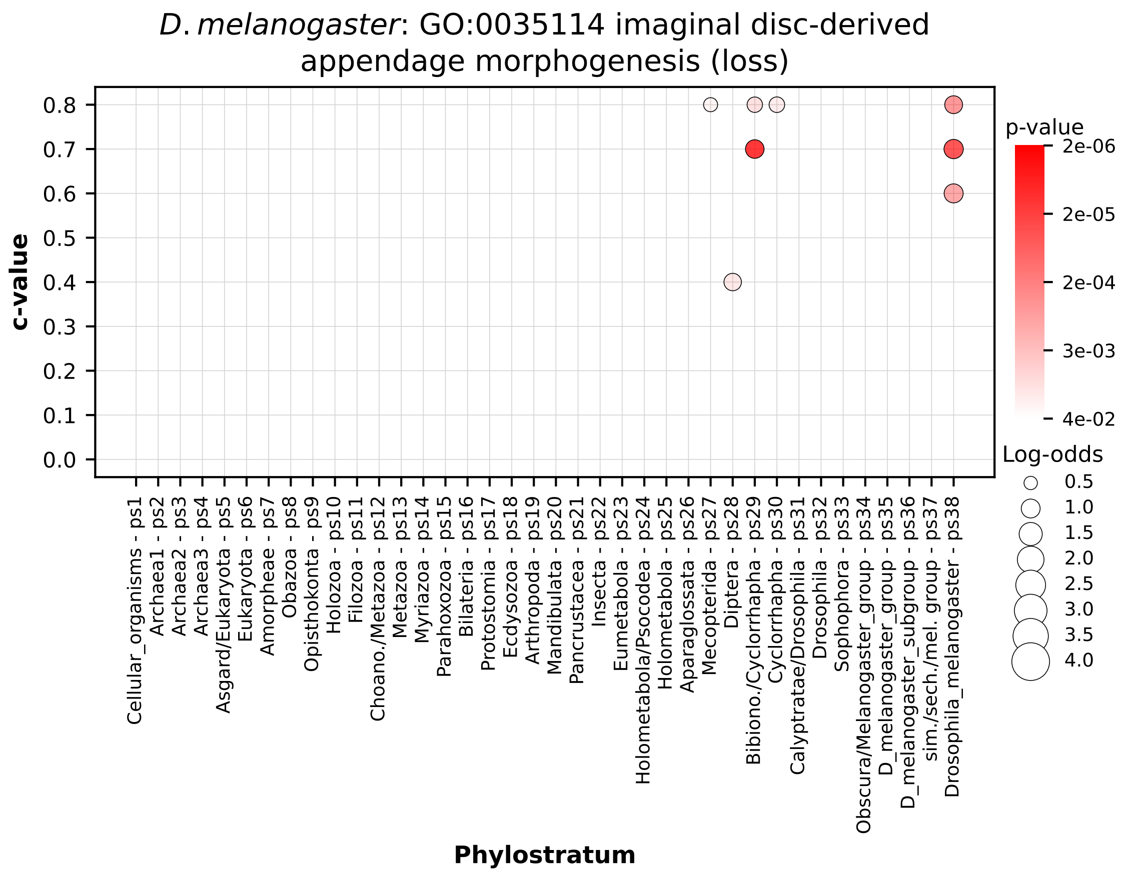

D. melanogaster: GO:0035114 imaginal disc-derived appendage morphogenesis (loss)
c-value
0.0
0.1
0.2
0.3
0.4
0.5
0.6
0.7
0.8
p-value
2e-06
2e-05
2e-04
3e-03
4e-02
Log-odds
0.5
1.0
1.5
2.0
2.5
3.0
3.5
4.0
Cellular_organisms - ps1
Archaea1 - ps2
Archaea2 - ps3
Archaea3 - ps4
Asgard/Eukaryota - ps5
Eukaryota - ps6
Amorpheae - ps7
Obazoa - ps8
Opisthokonta - ps9
Holozoa - ps10
Filozoa - ps11
Choano./Metazoa - ps12
Metazoa - ps13
Myriazoa - ps14
Parahoxozoa - ps15
Bilateria - ps16
Protostomia - ps17
Ecdysozoa - ps18
Arthropoda - ps19
Mandibulata - ps20
Pancrustacea - ps21
Insecta - ps22
Eumetabola - ps23
Holometabola/Psocodea - ps24
Holometabola - ps25
Aparaglossata - ps26
Mecopterida - ps27
Diptera - ps28
Bibiono./Cyclorrhapha - ps29
Cyclorrhapha - ps30
Calyptratae/Drosophila - ps31
Drosophila - ps32
Sophophora - ps33
Obscura/Melanogaster_group - ps34
D_melanogaster_group - ps35
D_melanogaster_subgroup - ps36
sim./sech./mel. group - ps37
Drosophila_melanogaster - ps38
Phylostratum

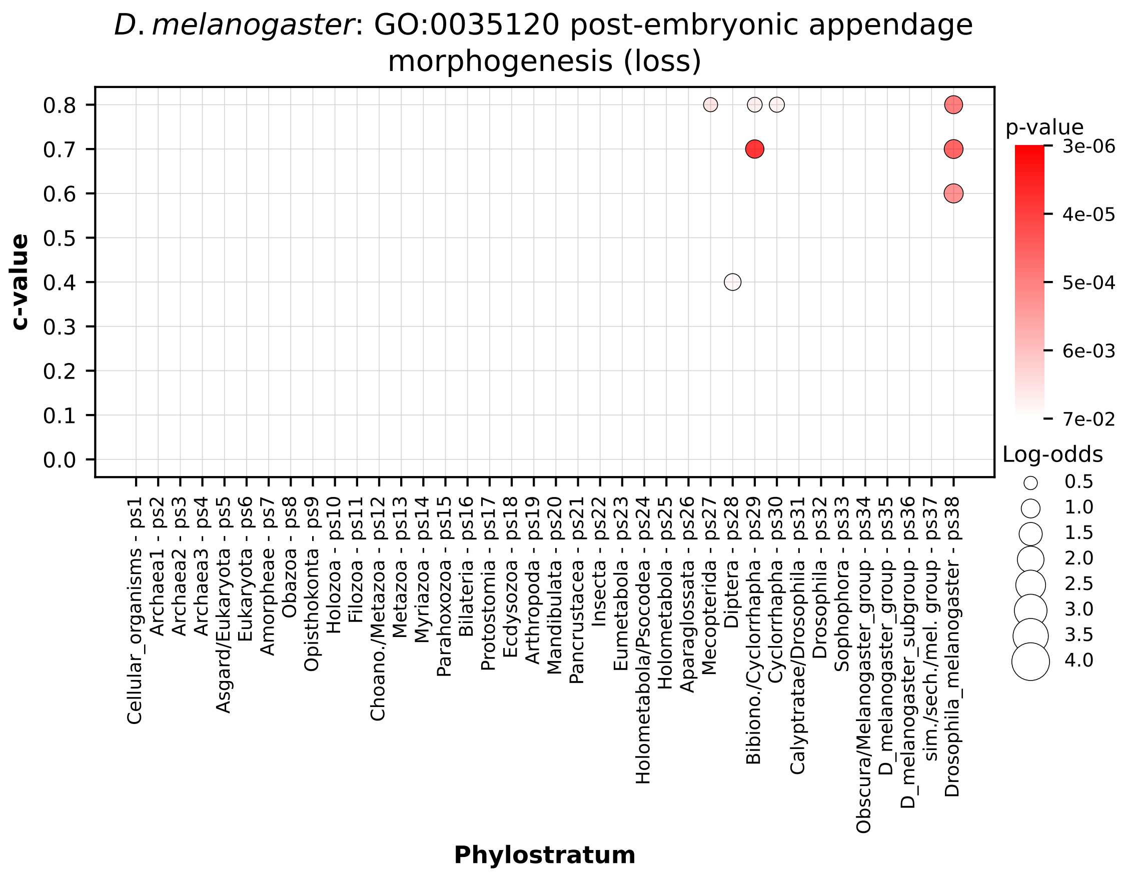

D. melanogaster: GO:0035120 post-embryonic appendage morphogenesis (loss)
c-value
0.8
0.7
0.6
0.5
0.4
0.3
0.2
0.1
0.0
p-value
3e-06
4e-05
5e-04
6e-03
7e-02
Log-odds
0.5
1.0
1.5
2.0
2.5
3.0
3.5
4.0
Cellular_organisms - ps1
Archaea1 - ps2
Archaea2 - ps3
Archaea3 - ps4
Asgard/Eukaryota - ps5
Eukaryota - ps6
Amorpheae - ps7
Obazoa - ps8
Opisthokonta - ps9
Holozoa - ps10
Filozoa - ps11
Choano./Metazoa - ps12
Metazoa - ps13
Myriazoa - ps14
Parahoxozoa - ps15
Bilateria - ps16
Protostomia - ps17
Ecdysozoa - ps18
Arthropoda - ps19
Mandibulata - ps20
Pancrustacea - ps21
Insecta - ps22
Eumetabola - ps23
Holometabola/Psocodea - ps24
Holometabola - ps25
Aparaglossata - ps26
Mecopterida - ps27
Diptera - ps28
Bibiono./Cyclorrhapha - ps29
Cyclorrhapha - ps30
Calyptratae/Drosophila - ps31
Drosophila - ps32
Sophophora - ps33
Obscura/Melanogaster_group - ps34
D_melanogaster_group - ps35
D_melanogaster_subgroup - ps36
sim./sech./mel. group - ps37
Drosophila_melanogaster - ps38
Phylostratum

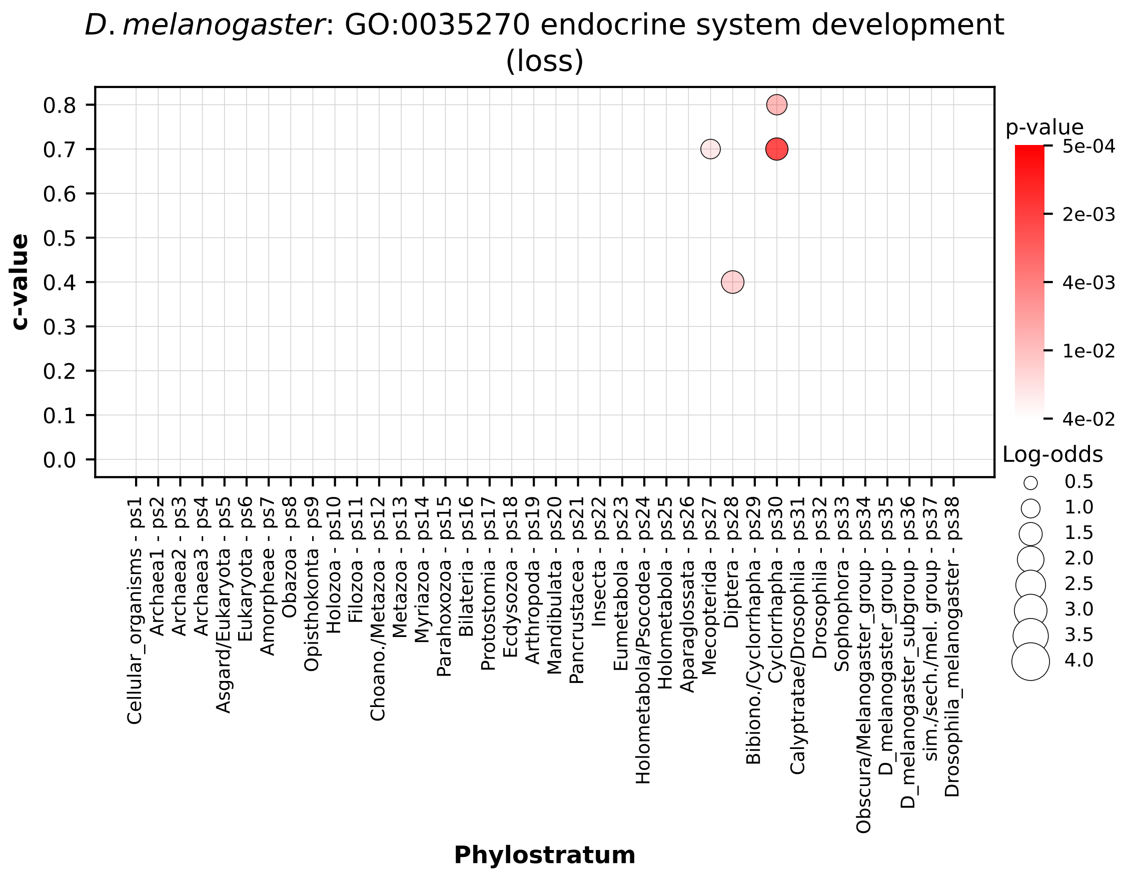

D. melanogaster: GO:0035270 endocrine system development
(loss)
c-value
0.0
0.1
0.2
0.3
0.4
0.5
0.6
0.7
0.8
p-value
5e-04
2e-03
4e-03
1e-02
4e-02
Log-odds
0.5
1.0
1.5
2.0
2.5
3.0
3.5
4.0
Cellular_organisms - ps1
Archaea1 - ps2
Archaea2 - ps3
Archaea3 - ps4
Asgard/Eukaryota - ps5
Eukaryota - ps6
Amorpheae - ps7
Obazoa - ps8
Opisthokonta - ps9
Holozoa - ps10
Filozoa - ps11
Choano./Metazoa - ps12
Metazoa - ps13
Myriazoa - ps14
Parahoxozoa - ps15
Bilateria - ps16
Protostomia - ps17
Ecdysozoa - ps18
Arthropoda - ps19
Mandibulata - ps20
Pancrustacea - ps21
Insecta - ps22
Eumetabola - ps23
Holometabola/Psocodea - ps24
Holometabola - ps25
Aparaglossata - ps26
Mecopterida - ps27
Diptera - ps28
Bibiono./Cyclorrhapha - ps29
Cyclorrhapha - ps30
Calyptratae/Drosophila - ps31
Drosophila - ps32
Sophophora - ps33
Obscura/Melanogaster_group - ps34
D_melanogaster_group - ps35
D_melanogaster_subgroup - ps36
sim./sech./mel. group - ps37
Drosophila_melanogaster - ps38
Phylostratum

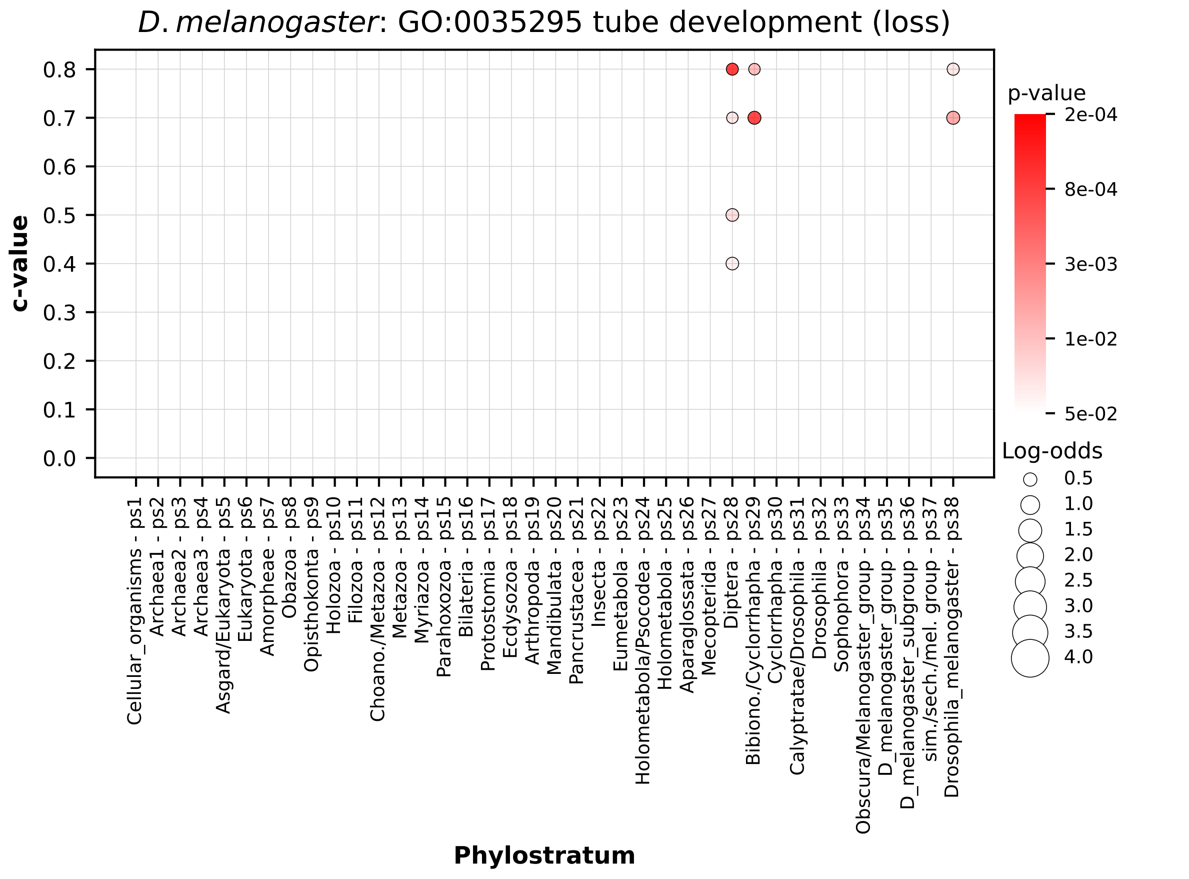

D. melanogaster: GO:0035295 tube development (loss)
c-value
0.0
0.1
0.2
0.3
0.4
0.5
0.6
0.7
0.8
p-value
2e-04
8e-04
3e-03
1e-02
5e-02
Log-odds
0.5
1.0
1.5
2.0
2.5
3.0
3.5
4.0
Cellular_organisms - ps1
Archaea1 - ps2
Archaea2 - ps3
Archaea3 - ps4
Asgard/Eukaryota - ps5
Eukaryota - ps6
Amorpheae - ps7
Obazoa - ps8
Opisthokonta - ps9
Holozoa - ps10
Filozoa - ps11
Choano./Metazoa - ps12
Metazoa - ps13
Myriazoa - ps14
Parahoxozoa - ps15
Bilateria - ps16
Protostomia - ps17
Ecdysozoa - ps18
Arthropoda - ps19
Mandibulata - ps20
Pancrustacea - ps21
Insecta - ps22
Eumetabola - ps23
Holometabola/Psocodea - ps24
Holometabola - ps25
Aparaglossata - ps26
Mecopterida - ps27
Diptera - ps28
Bibiono./Cyclorrhapha - ps29
Cyclorrhapha - ps30
Calyptratae/Drosophila - ps31
Drosophila - ps32
Sophophora - ps33
Obscura/Melanogaster_group - ps34
D_melanogaster_group - ps35
D_melanogaster_subgroup - ps36
sim./sech./mel. group - ps37
Drosophila_melanogaster - ps38
Phylostratum

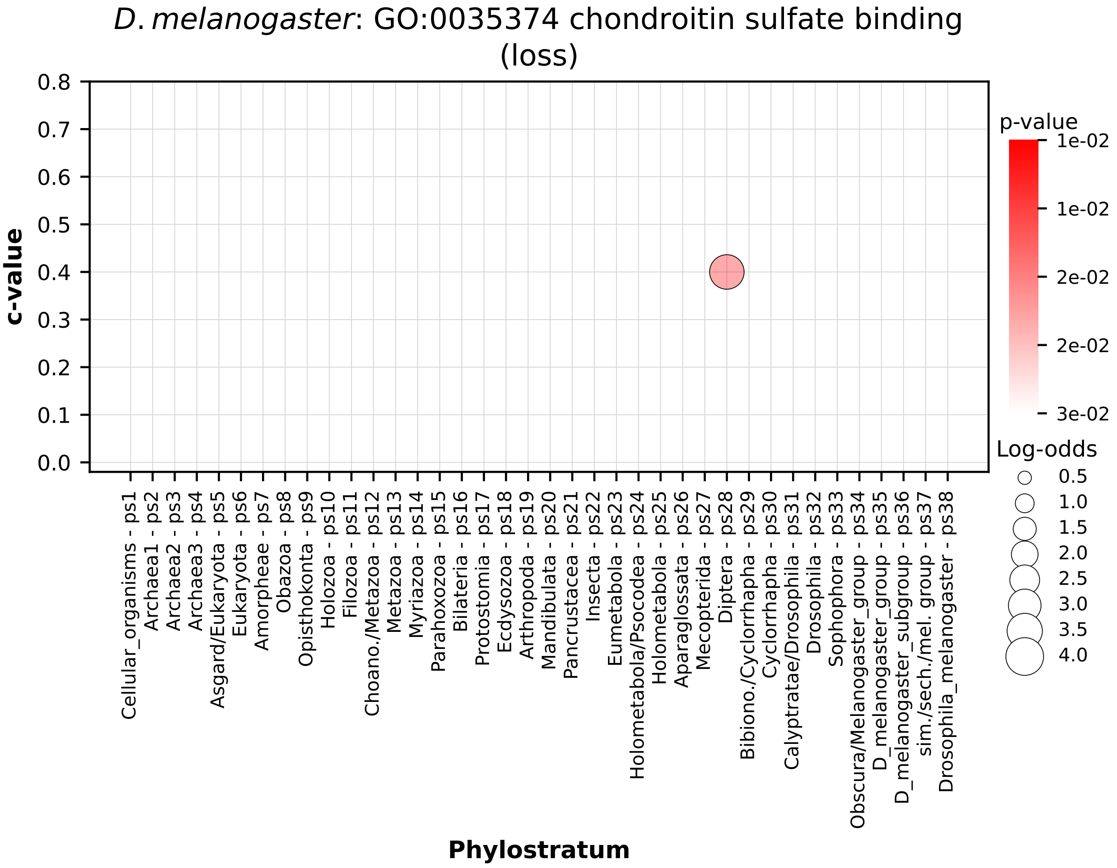

D. melanogaster: GO:0035374 chondroitin sulfate binding
(loss)
c-value
0.0
0.1
0.2
0.3
0.4
0.5
0.6
0.7
0.8
Phylostratum
Cellular_organisms - ps1
Archaea1 - ps2
Archaea2 - ps3
Archaea3 - ps4
Asgard/Eukaryota - ps5
Eukaryota - ps6
Amorpheae - ps7
Obazoa - ps8
Opisthokonta - ps9
Holozoa - ps10
Filozoa - ps11
Choano./Metazoa - ps12
Metazoa - ps13
Myriazoa - ps14
Parahoxozoa - ps15
Bilateria - ps16
Protostomia - ps17
Ecdysozoa - ps18
Arthropoda - ps19
Mandibulata - ps20
Pancrustacea - ps21
Insecta - ps22
Eumetabola - ps23
Holometabola/Psocodea - ps24
Holometabola - ps25
Aparaglossata - ps26
Mecopterida - ps27
Diptera - ps28
Bibiono./Cyclorrhapha - ps29
Cyclorrhapha - ps30
Calyptratae/Drosophila - ps31
Drosophila - ps32
Sophophora - ps33
Obscura/Melanogaster_group - ps34
D_melanogaster_group - ps35
D_melanogaster_subgroup - ps36
sim./sech./mel. group - ps37
Drosophila_melanogaster - ps38
p-value
1e-02
1e-02
2e-02
2e-02
3e-02
Log-odds
0.5
1.0
1.5
2.0
2.5
3.0
3.5
4.0

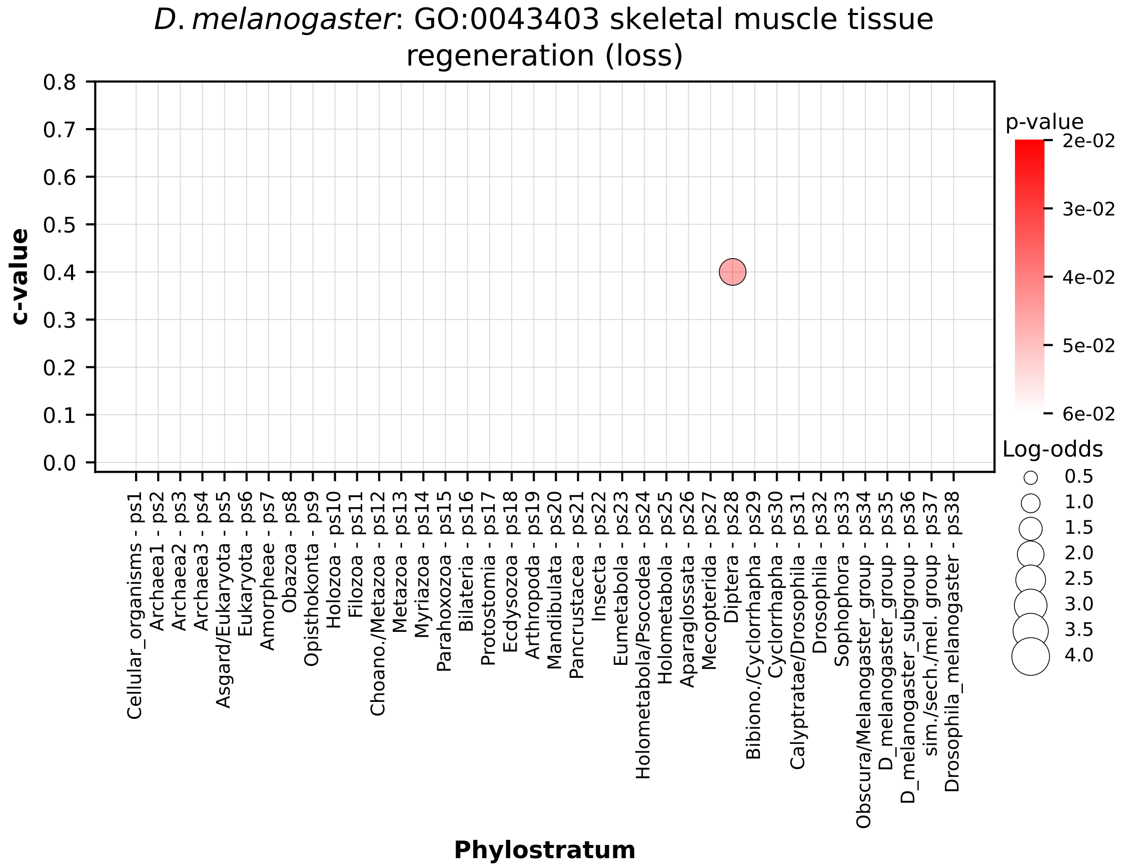
D. melanogaster: GO:0043403 skeletal muscle tissue regeneration (loss)
c-value
0.8
0.7
0.6
0.5
0.4
0.3
0.2
0.1
0.0
p-value
2e-02
3e-02
4e-02
5e-02
6e-02
Log-odds
0.5
1.0
1.5
2.0
2.5
3.0
3.5
4.0
Cellular_organisms - ps1
Archaea1 - ps2
Archaea2 - ps3
Archaea3 - ps4
Asgard/Eukaryota - ps5
Eukaryota - ps6
Amorpheae - ps7
Obazoa - ps8
Opisthokonta - ps9
Holozoa - ps10
Filozoa - ps11
Choano./Metazoa - ps12
Metazoa - ps13
Myriazoa - ps14
Parahoxozoa - ps15
Bilateria - ps16
Protostomia - ps17
Ecdysozoa - ps18
Arthropoda - ps19
Mandibulata - ps20
Pancrustacea - ps21
Insecta - ps22
Eumetabola - ps23
Holometabola/Psocodea - ps24
Holometabola - ps25
Aparaglossata - ps26
Mecopterida - ps27
Diptera - ps28
Bibiono./Cyclorrhapha - ps29
Cyclorrhapha - ps30
Calyptratae/Drosophila - ps31
Drosophila - ps32
Sophophora - ps33
Obscura/Melanogaster_group - ps34
D_melanogaster_group - ps35
D_melanogaster_subgroup - ps36
sim./sech./mel. group - ps37
Drosophila_melanogaster - ps38
Phylostratum

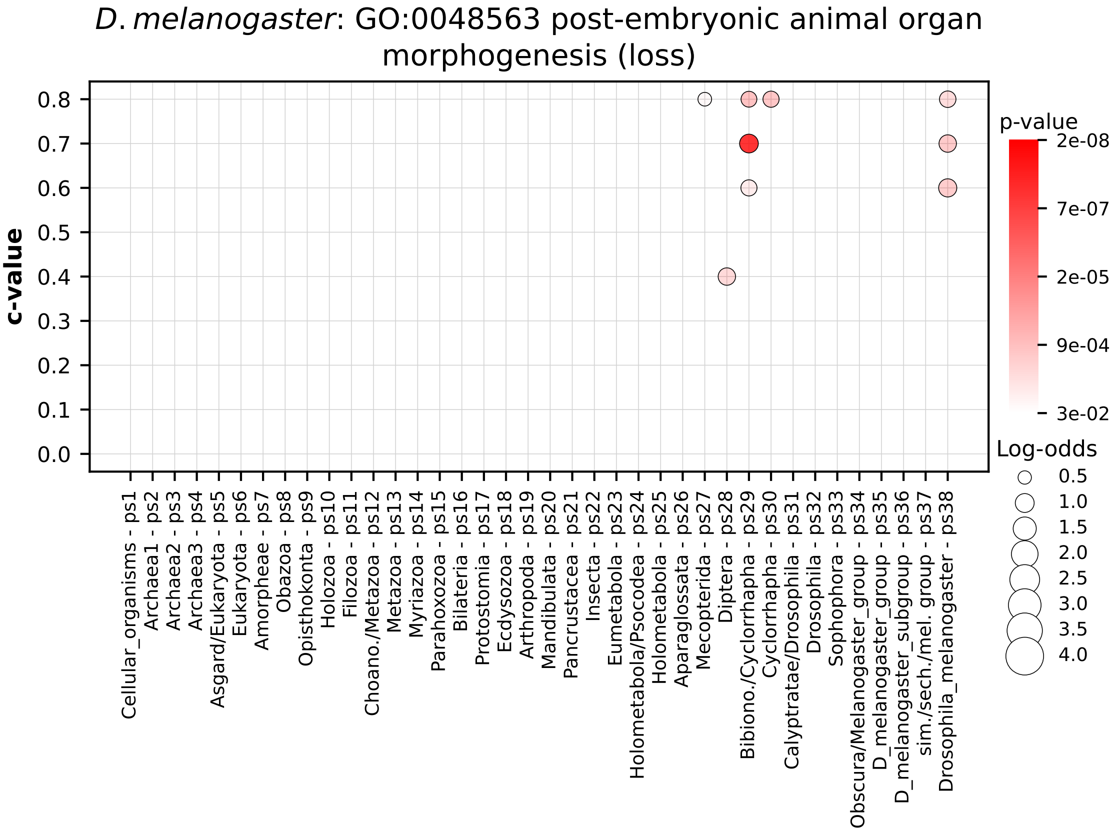
D. melanogaster: GO:0048563 post-embryonic animal organ morphogenesis (loss)
c-value
0.8
0.7
0.6
0.5
0.4
0.3
0.2
0.1
0.0
Phylostratum
Cellular_organisms - ps1
Archaea1 - ps2
Archaea2 - ps3
Archaea3 - ps4
Asgard/Eukaryota - ps5
Eukaryota - ps6
Amorpheae - ps7
Obazoa - ps8
Opisthokonta - ps9
Holozoa - ps10
Filozoa - ps11
Choano./Metazoa - ps12
Metazoa - ps13
Myriazoa - ps14
Parahoxozoa - ps15
Bilateria - ps16
Protostomia - ps17
Ecdysozoa - ps18
Arthropoda - ps19
Mandibulata - ps20
Pancrustacea - ps21
Insecta - ps22
Eumetabola - ps23
Holometabola/Psocodea - ps24
Holometabola - ps25
Aparaglossata - ps26
Mecopterida - ps27
Diptera - ps28
Bibiono./Cyclorrhapha - ps29
Cyclorrhapha - ps30
Calyptratae/Drosophila - ps31
Drosophila - ps32
Sophophora - ps33
Obscura/Melanogaster_group - ps34
D_melanogaster_group - ps35
D_melanogaster_subgroup - ps36
sim./sech./mel. group - ps37
Drosophila_melanogaster - ps38
p-value
2e-08
7e-07
2e-05
9e-04
3e-02
Log-odds
0.5
1.0
1.5
2.0
2.5
3.0
3.5
4.0

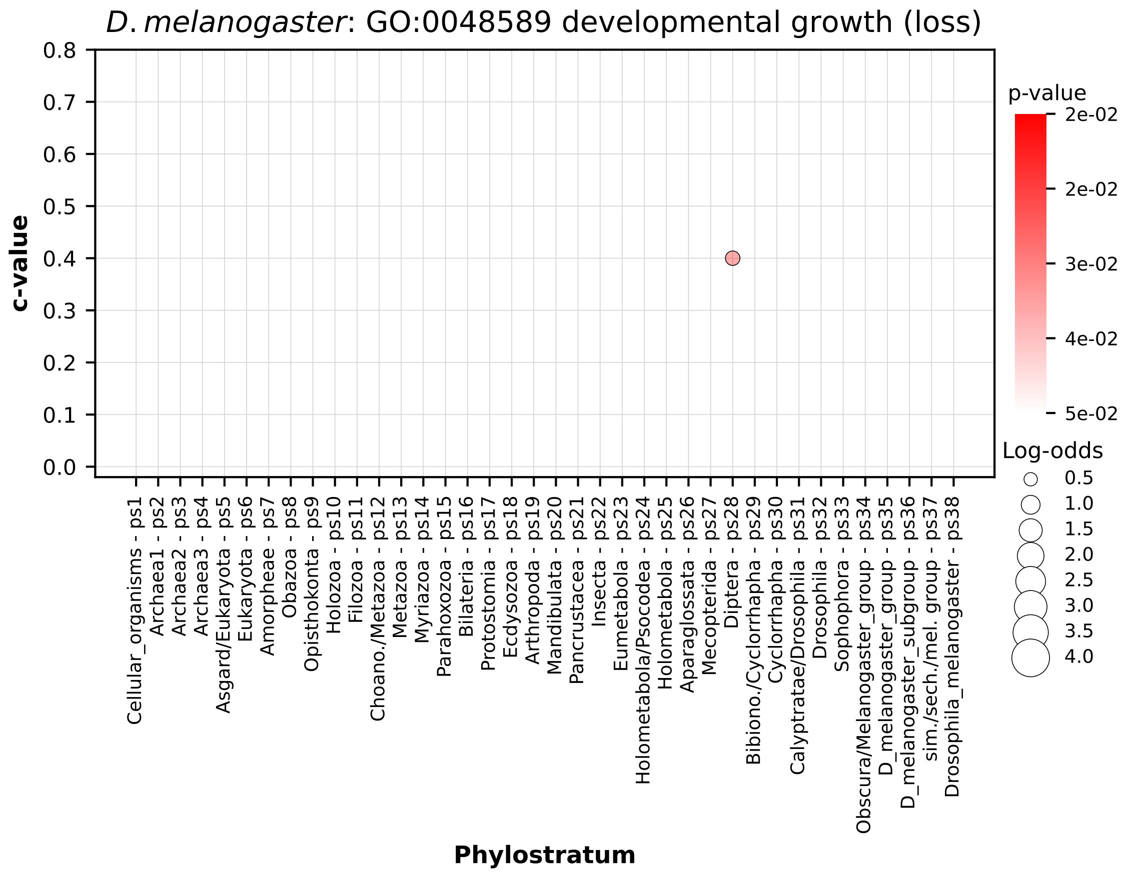

D. melanogaster: GO:0048589 developmental growth (loss)
c-value
0.0
0.1
0.2
0.3
0.4
0.5
0.6
0.7
0.8
Phylostratum
Cellular_organisms - ps1
Archaea1 - ps2
Archaea2 - ps3
Archaea3 - ps4
Asgard/Eukaryota - ps5
Eukaryota - ps6
Amorpheae - ps7
Obazoa - ps8
Opisthokonta - ps9
Holozoa - ps10
Filozoa - ps11
Choano./Metazoa - ps12
Metazoa - ps13
Myriazoa - ps14
Parahoxozoa - ps15
Bilateria - ps16
Protostomia - ps17
Ecdysozoa - ps18
Arthropoda - ps19
Mandibulata - ps20
Pancrustacea - ps21
Insecta - ps22
Eumetabola - ps23
Holometabola/Psocodea - ps24
Holometabola - ps25
Aparaglossata - ps26
Mecopterida - ps27
Diptera - ps28
Bibiono./Cyclorrhapha - ps29
Cyclorrhapha - ps30
Calyptratae/Drosophila - ps31
Drosophila - ps32
Sophophora - ps33
Obscura/Melanogaster_group - ps34
D_melanogaster_group - ps35
D_melanogaster_subgroup - ps36
sim./sech./mel. group - ps37
Drosophila_melanogaster - ps38
p-value
2e-02
2e-02
3e-02
4e-02
5e-02
Log-odds
0.5
1.0
1.5
2.0
2.5
3.0
3.5
4.0

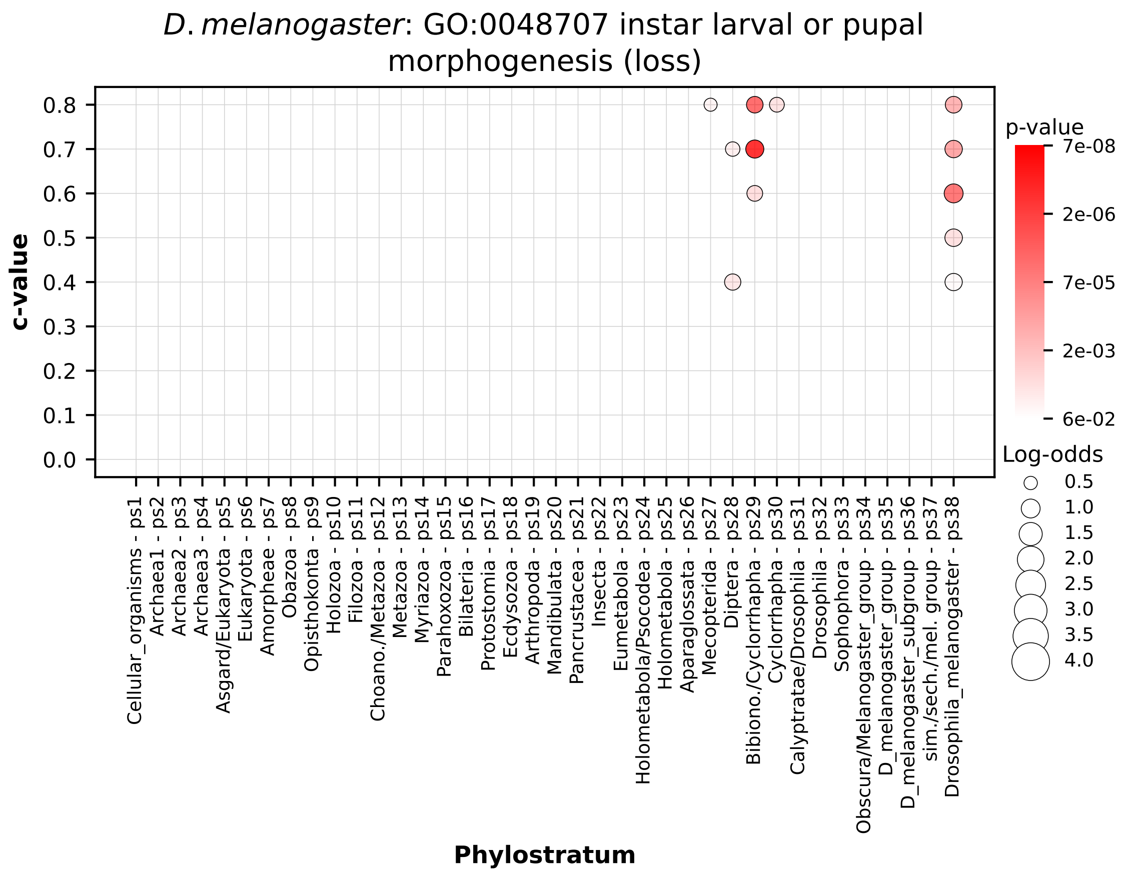

D. melanogaster: GO:0048707 instar larval or pupal morphogenesis (loss)
c-value
0.8
0.7
0.6
0.5
0.4
0.3
0.2
0.1
0.0
p-value
7e-08
2e-06
7e-05
2e-03
6e-02
Log-odds
0.5
1.0
1.5
2.0
2.5
3.0
3.5
4.0
Cellular_organisms - ps1
Archaea1 - ps2
Archaea2 - ps3
Archaea3 - ps4
Asgard/Eukaryota - ps5
Eukaryota - ps6
Amorpheae - ps7
Obazoa - ps8
Opisthokonta - ps9
Holozoa - ps10
Filozoa - ps11
Choano./Metazoa - ps12
Metazoa - ps13
Myriazoa - ps14
Parahoxozoa - ps15
Bilateria - ps16
Protostomia - ps17
Ecdysozoa - ps18
Arthropoda - ps19
Mandibulata - ps20
Pancrustacea - ps21
Insecta - ps22
Eumetabola - ps23
Holometabola/Psocodea - ps24
Holometabola - ps25
Aparaglossata - ps26
Mecopterida - ps27
Diptera - ps28
Bibiono./Cyclorrhapha - ps29
Cyclorrhapha - ps30
Calyptratae/Drosophila - ps31
Drosophila - ps32
Sophophora - ps33
Obscura/Melanogaster_group - ps34
D_melanogaster_group - ps35
D_melanogaster_subgroup - ps36
sim./sech./mel. group - ps37
Drosophila_melanogaster - ps38
Phylostratum

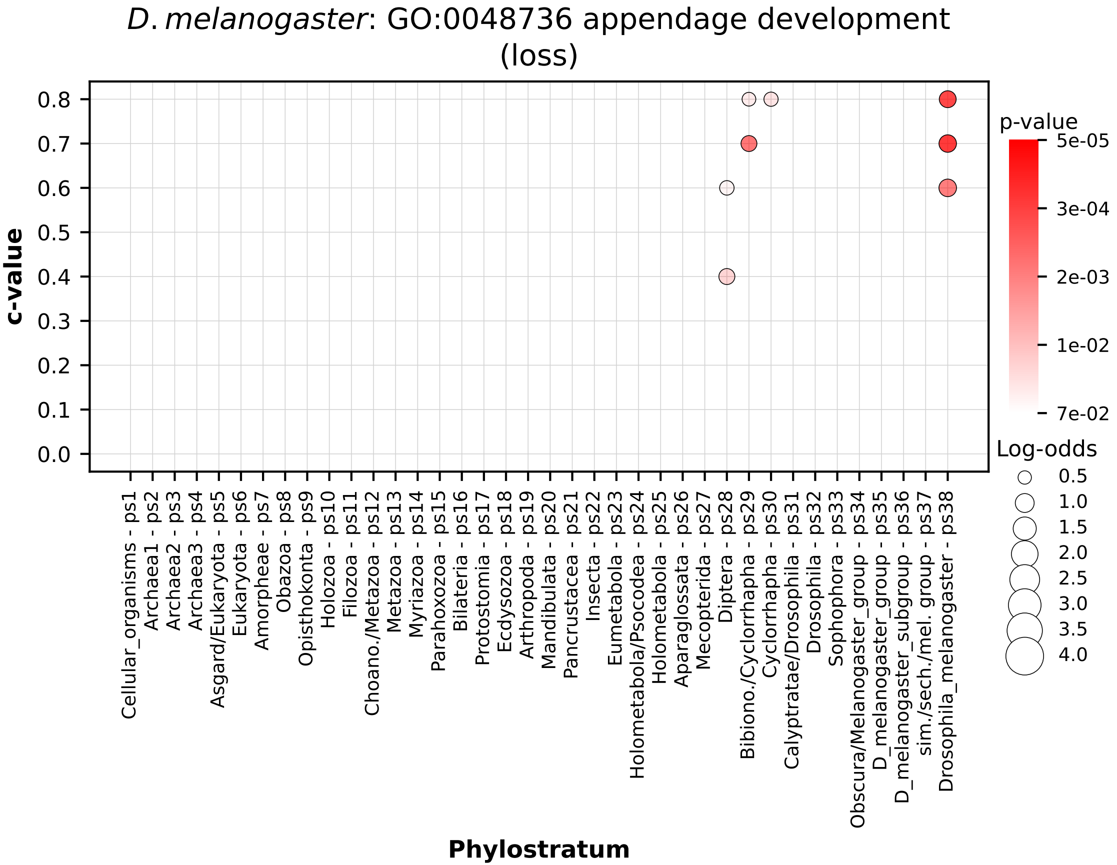

D. melanogaster: GO:0048736 appendage development
(loss)
c-value
0.8
0.7
0.6
0.5
0.4
0.3
0.2
0.1
0.0
p-value
5e-05
3e-04
2e-03
1e-02
7e-02
Log-odds
0.5
1.0
1.5
2.0
2.5
3.0
3.5
4.0
Cellular_organisms - ps1
Archaea1 - ps2
Archaea2 - ps3
Archaea3 - ps4
Asgard/Eukaryota - ps5
Eukaryota - ps6
Amorpheae - ps7
Obazoa - ps8
Opisthokonta - ps9
Holozoa - ps10
Filozoa - ps11
Choano./Metazoa - ps12
Metazoa - ps13
Myriazoa - ps14
Parahoxozoa - ps15
Bilateria - ps16
Protostomia - ps17
Ecdysozoa - ps18
Arthropoda - ps19
Mandibulata - ps20
Pancrustacea - ps21
Insecta - ps22
Eumetabola - ps23
Holometabola/Psocodea - ps24
Holometabola - ps25
Aparaglossata - ps26
Mecopterida - ps27
Diptera - ps28
Bibiono./Cyclorrhapha - ps29
Cyclorrhapha - ps30
Calyptratae/Drosophila - ps31
Drosophila - ps32
Sophophora - ps33
Obscura/Melanogaster_group - ps34
D_melanogaster_group - ps35
D_melanogaster_subgroup - ps36
sim./sech./mel. group - ps37
Drosophila_melanogaster - ps38
Phylostratum

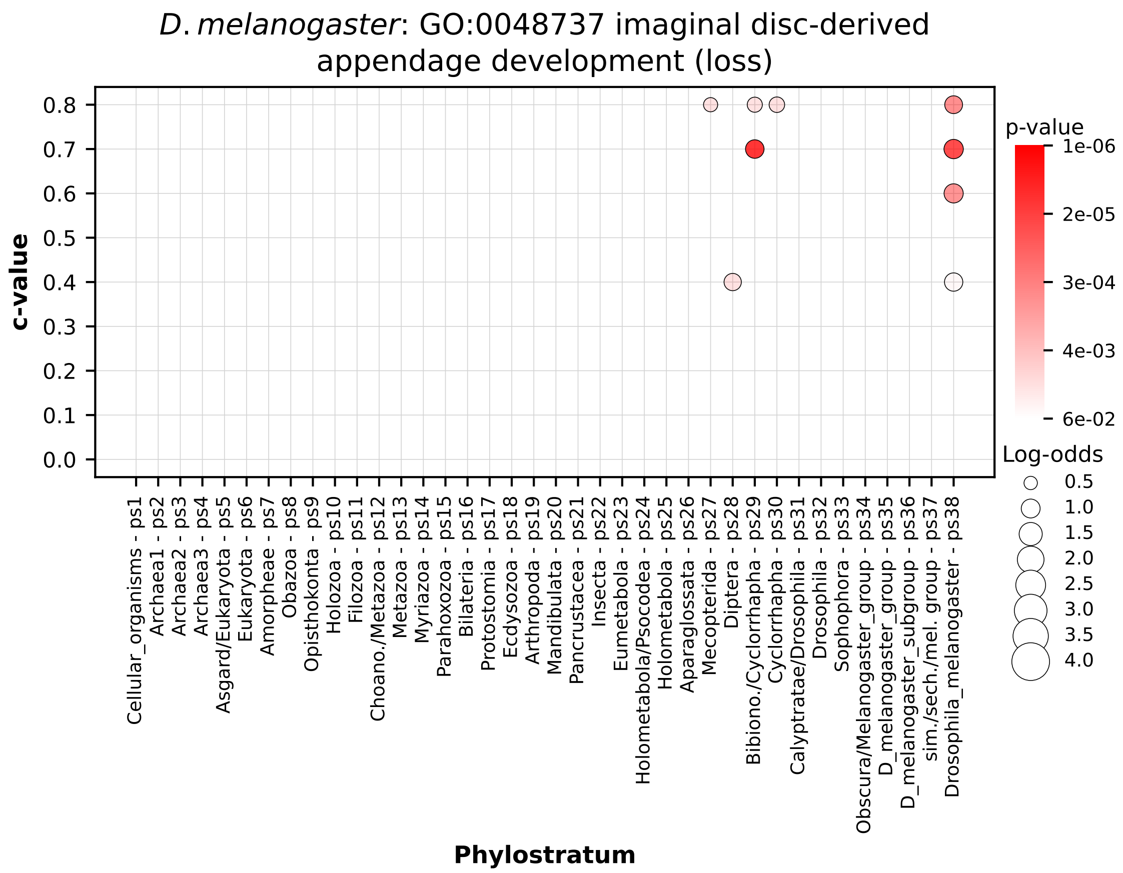

D. melanogaster: GO:0048737 imaginal disc-derived appendage development (loss)
c-value
0.8
0.7
0.6
0.5
0.4
0.3
0.2
0.1
0.0
p-value
1e-06
2e-05
3e-04
4e-03
6e-02
Log-odds
0.5
1.0
1.5
2.0
2.5
3.0
3.5
4.0
Cellular_organisms - ps1
Archaea1 - ps2
Archaea2 - ps3
Archaea3 - ps4
Asgard/Eukaryota - ps5
Eukaryota - ps6
Amorpheae - ps7
Obazoa - ps8
Opisthokonta - ps9
Holozoa - ps10
Filozoa - ps11
Choano./Metazoa - ps12
Metazoa - ps13
Myriazoa - ps14
Parahoxozoa - ps15
Bilateria - ps16
Protostomia - ps17
Ecdysozoa - ps18
Arthropoda - ps19
Mandibulata - ps20
Pancrustacea - ps21
Insecta - ps22
Eumetabola - ps23
Holometabola/Psocodea - ps24
Holometabola - ps25
Aparaglossata - ps26
Mecopterida - ps27
Diptera - ps28
Bibiono./Cyclorrhapha - ps29
Cyclorrhapha - ps30
Calyptratae/Drosophila - ps31
Drosophila - ps32
Sophophora - ps33
Obscura/Melanogaster_group - ps34
D_melanogaster_group - ps35
D_melanogaster_subgroup - ps36
sim./sech./mel. group - ps37
Drosophila_melanogaster - ps38
Phylostratum

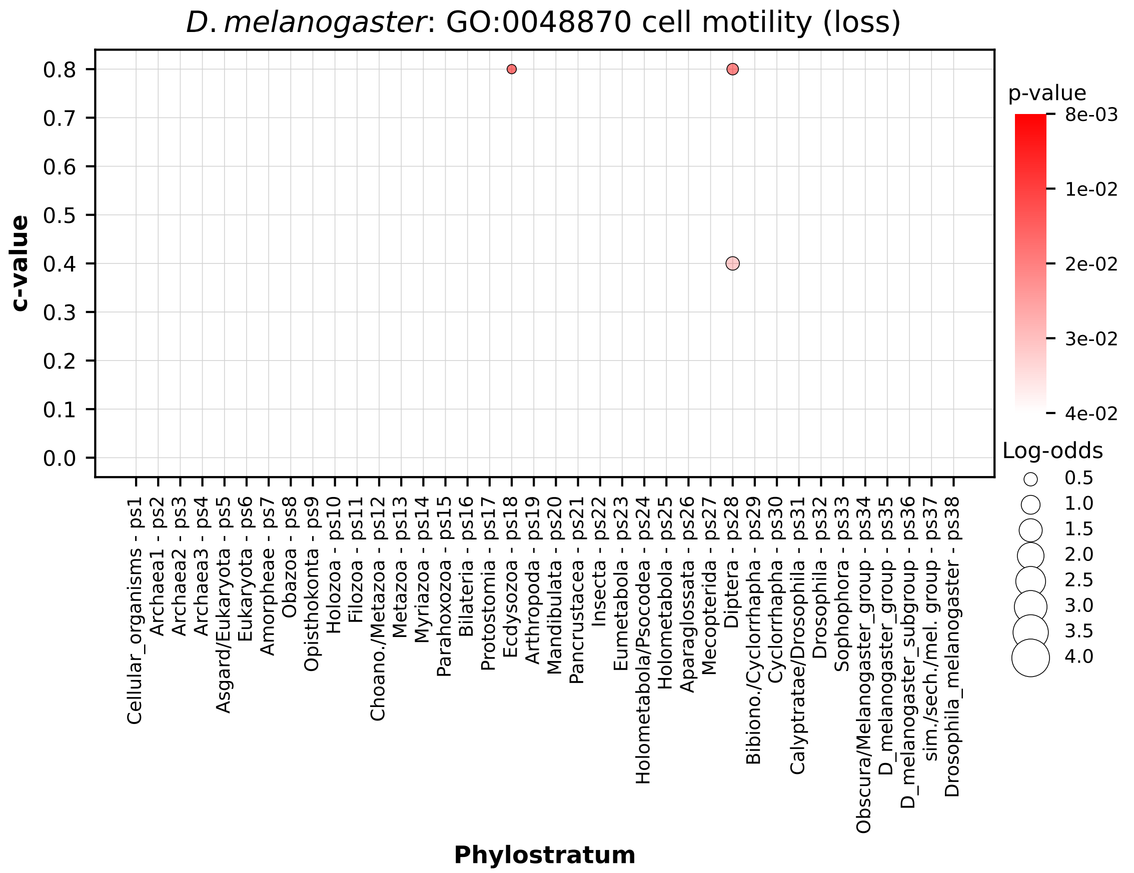

D. melanogaster: GO:0048870 cell motility (loss)
c-value
0.0
0.1
0.2
0.3
0.4
0.5
0.6
0.7
0.8
Phylostratum
Cellular_organisms - ps1
Archaea1 - ps2
Archaea2 - ps3
Archaea3 - ps4
Asgard/Eukaryota - ps5
Eukaryota - ps6
Amorpheae - ps7
Obazoa - ps8
Opisthokonta - ps9
Holozoa - ps10
Filozoa - ps11
Choano./Metazoa - ps12
Metazoa - ps13
Myriazoa - ps14
Parahoxozoa - ps15
Bilateria - ps16
Protostomia - ps17
Ecdysozoa - ps18
Arthropoda - ps19
Mandibulata - ps20
Pancrustacea - ps21
Insecta - ps22
Eumetabola - ps23
Holometabola/Psocodea - ps24
Holometabola - ps25
Aparaglossata - ps26
Mecopterida - ps27
Diptera - ps28
Bibiono./Cyclorrhapha - ps29
Cyclorrhapha - ps30
Calyptratae/Drosophila - ps31
Drosophila - ps32
Sophophora - ps33
Obscura/Melanogaster_group - ps34
D_melanogaster_group - ps35
D_melanogaster_subgroup - ps36
sim./sech./mel. group - ps37
Drosophila_melanogaster - ps38
p-value
8e-03
1e-02
2e-02
3e-02
4e-02
Log-odds
0.5
1.0
1.5
2.0
2.5
3.0
3.5
4.0

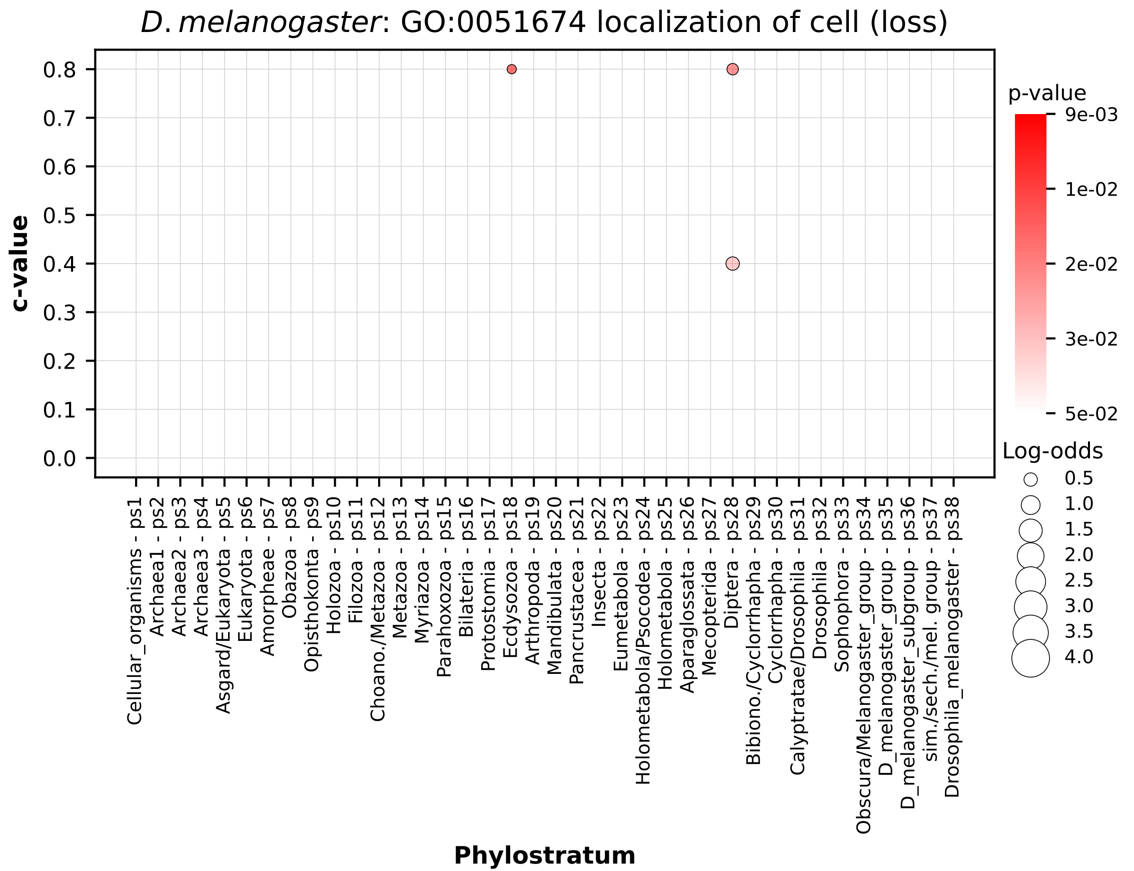

D. melanogaster: GO:0051674 localization of cell (loss)
c-value
0.0
0.1
0.2
0.3
0.4
0.5
0.6
0.7
0.8
Phylostratum
Cellular_organisms - ps1
Archaea1 - ps2
Archaea2 - ps3
Archaea3 - ps4
Asgard/Eukaryota - ps5
Eukaryota - ps6
Amorpheae - ps7
Obazoa - ps8
Opisthokonta - ps9
Holozoa - ps10
Filozoa - ps11
Choano./Metazoa - ps12
Metazoa - ps13
Myriazoa - ps14
Parahoxozoa - ps15
Bilateria - ps16
Protostomia - ps17
Ecdysozoa - ps18
Arthropoda - ps19
Mandibulata - ps20
Pancrustacea - ps21
Insecta - ps22
Eumetabola - ps23
Holometabola/Psocodea - ps24
Holometabola - ps25
Aparaglossata - ps26
Mecopterida - ps27
Diptera - ps28
Bibiono./Cyclorrhapha - ps29
Cyclorrhapha - ps30
Calyptratae/Drosophila - ps31
Drosophila - ps32
Sophophora - ps33
Obscura/Melanogaster_group - ps34
D_melanogaster_group - ps35
D_melanogaster_subgroup - ps36
sim./sech./mel. group - ps37
Drosophila_melanogaster - ps38
p-value
9e-03
1e-02
2e-02
3e-02
5e-02
Log-odds
0.5
1.0
1.5
2.0
2.5
3.0
3.5
4.0

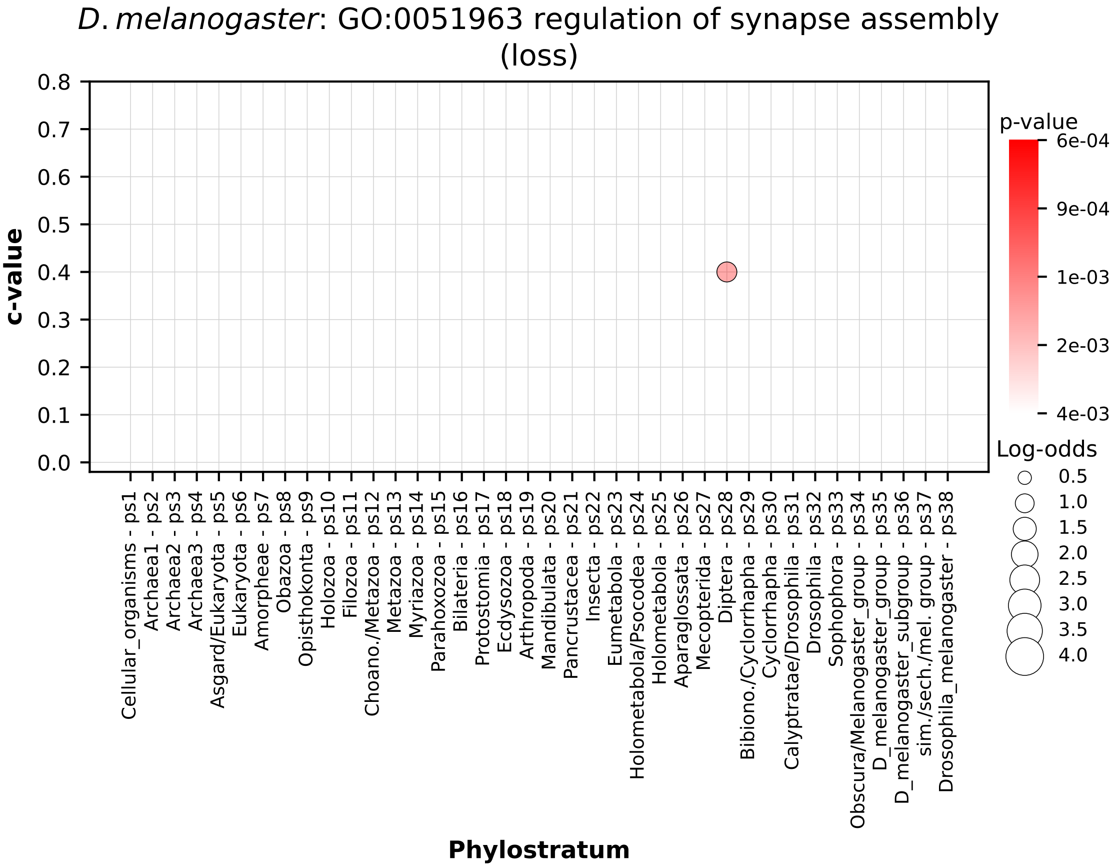

D. melanogaster: GO:0051963 regulation of synapse assembly (loss)
c-value
0.8
0.7
0.6
0.5
0.4
0.3
0.2
0.1
0.0
Phylostratum
Cellular_organisms - ps1
Archaea1 - ps2
Archaea2 - ps3
Archaea3 - ps4
Asgard/Eukaryota - ps5
Eukaryota - ps6
Amorpheae - ps7
Obazoa - ps8
Opisthokonta - ps9
Holozoa - ps10
Filozoa - ps11
Choano./Metazoa - ps12
Metazoa - ps13
Myriazoa - ps14
Parahoxozoa - ps15
Bilateria - ps16
Protostomia - ps17
Ecdysozoa - ps18
Arthropoda - ps19
Mandibulata - ps20
Pancrustacea - ps21
Insecta - ps22
Eumetabola - ps23
Holometabola/Psocodea - ps24
Holometabola - ps25
Aparaglossata - ps26
Mecopterida - ps27
Diptera - ps28
Bibiono./Cyclorrhapha - ps29
Cyclorrhapha - ps30
Calyptratae/Drosophila - ps31
Drosophila - ps32
Sophophora - ps33
Obscura/Melanogaster_group - ps34
D_melanogaster_group - ps35
D_melanogaster_subgroup - ps36
sim./sech./mel. group - ps37
Drosophila_melanogaster - ps38
p-value
6e-04
9e-04
1e-03
2e-03
4e-03
Log-odds
0.5
1.0
1.5
2.0
2.5
3.0
3.5
4.0

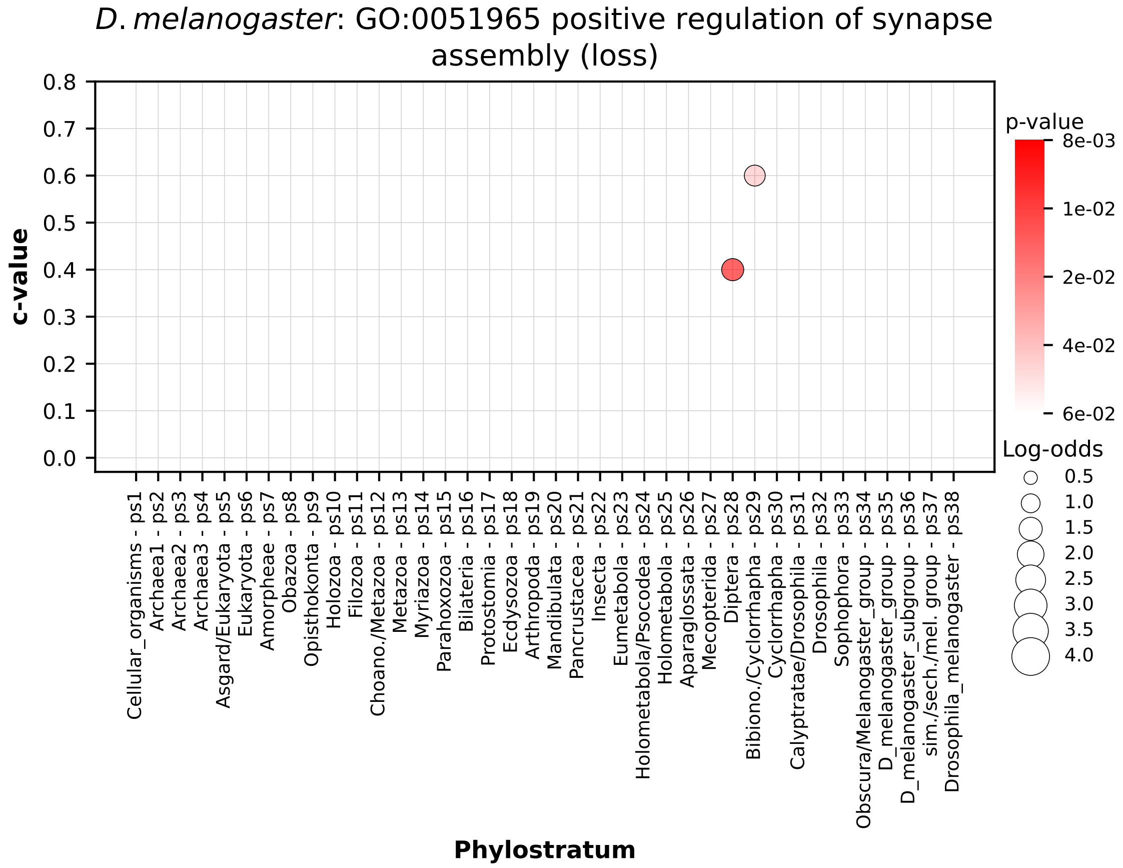

D. melanogaster: GO:0051965 positive regulation of synapse assembly (loss)
c-value
0.8
0.7
0.6
0.5
0.4
0.3
0.2
0.1
0.0
p-value
8e-03
1e-02
2e-02
4e-02
6e-02
Log-odds
0.5
1.0
1.5
2.0
2.5
3.0
3.5
4.0
Cellular_organisms - ps1
Archaea1 - ps2
Archaea2 - ps3
Archaea3 - ps4
Asgard/Eukaryota - ps5
Eukaryota - ps6
Amorpheae - ps7
Obazoa - ps8
Opisthokonta - ps9
Holozoa - ps10
Filozoa - ps11
Choano./Metazoa - ps12
Metazoa - ps13
Myriazoa - ps14
Parahoxozoa - ps15
Bilateria - ps16
Protostomia - ps17
Ecdysozoa - ps18
Arthropoda - ps19
Mandibulata - ps20
Pancrustacea - ps21
Insecta - ps22
Eumetabola - ps23
Holometabola/Psocodea - ps24
Holometabola - ps25
Aparaglossata - ps26
Mecopterida - ps27
Diptera - ps28
Bibiono./Cyclorrhapha - ps29
Cyclorrhapha - ps30
Calyptratae/Drosophila - ps31
Drosophila - ps32
Sophophora - ps33
Obscura/Melanogaster_group - ps34
D_melanogaster_group - ps35
D_melanogaster_subgroup - ps36
sim./sech./mel. group - ps37
Drosophila_melanogaster - ps38
Phylostratum

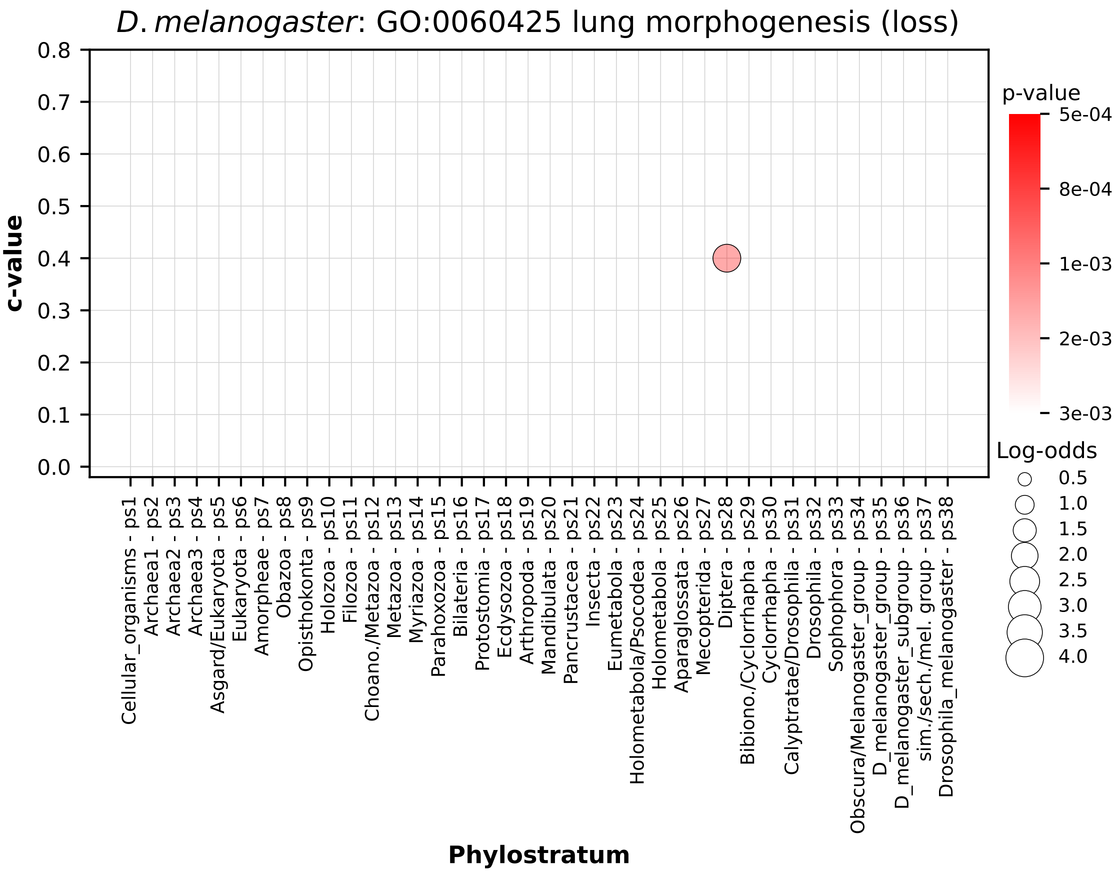
D. melanogaster: GO:0060425 lung morphogenesis (loss)
c-value
0.8
0.7
0.6
0.5
0.4
0.3
0.2
0.1
0.0
p-value
5e-04
8e-04
1e-03
2e-03
3e-03
Log-odds
0.5
1.0
1.5
2.0
2.5
3.0
3.5
4.0
Cellular_organisms - ps1
Archaea1 - ps2
Archaea2 - ps3
Archaea3 - ps4
Asgard/Eukaryota - ps5
Eukaryota - ps6
Amorpheae - ps7
Obazoa - ps8
Opisthokonta - ps9
Holozoa - ps10
Filozoa - ps11
Choano./Metazoa - ps12
Metazoa - ps13
Myriazoa - ps14
Parahoxozoa - ps15
Bilateria - ps16
Protostomia - ps17
Ecdysozoa - ps18
Arthropoda - ps19
Mandibulata - ps20
Pancrustacea - ps21
Insecta - ps22
Eumetabola - ps23
Holometabola/Psocodea - ps24
Holometabola - ps25
Aparaglossata - ps26
Mecopterida - ps27
Diptera - ps28
Bibiono./Cyclorrhapha - ps29
Cyclorrhapha - ps30
Calyptratae/Drosophila - ps31
Drosophila - ps32
Sophophora - ps33
Obscura/Melanogaster_group - ps34
D_melanogaster_group - ps35
D_melanogaster_subgroup - ps36
sim./sech./mel. group - ps37
Drosophila_melanogaster - ps38
Phylostratum

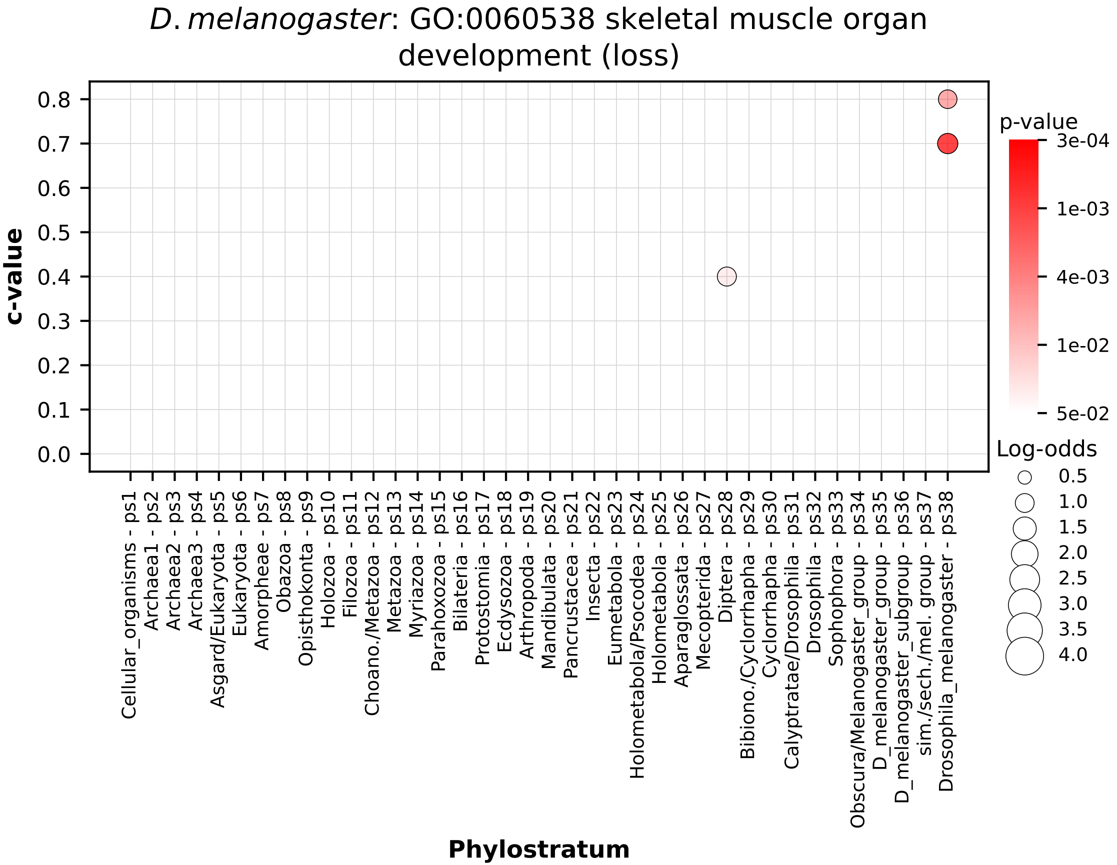

D. melanogaster: GO:0060538 skeletal muscle organ development (loss)
c-value
0.8
0.7
0.6
0.5
0.4
0.3
0.2
0.1
0.0
p-value
3e-04
1e-03
4e-03
1e-02
5e-02
Log-odds
0.5
1.0
1.5
2.0
2.5
3.0
3.5
4.0
Cellular_organisms - ps1
Archaea1 - ps2
Archaea2 - ps3
Archaea3 - ps4
Asgard/Eukaryota - ps5
Eukaryota - ps6
Amorpheae - ps7
Obazoa - ps8
Opisthokonta - ps9
Holozoa - ps10
Filozoa - ps11
Choano./Metazoa - ps12
Metazoa - ps13
Myriazoa - ps14
Parahoxozoa - ps15
Bilateria - ps16
Protostomia - ps17
Ecdysozoa - ps18
Arthropoda - ps19
Mandibulata - ps20
Pancrustacea - ps21
Insecta - ps22
Eumetabola - ps23
Holometabola/Psocodea - ps24
Holometabola - ps25
Aparaglossata - ps26
Mecopterida - ps27
Diptera - ps28
Bibiono./Cyclorrhapha - ps29
Cyclorrhapha - ps30
Calyptratae/Drosophila - ps31
Drosophila - ps32
Sophophora - ps33
Obscura/Melanogaster_group - ps34
D_melanogaster_group - ps35
D_melanogaster_subgroup - ps36
sim./sech./mel. group - ps37
Drosophila_melanogaster - ps38
Phylostratum

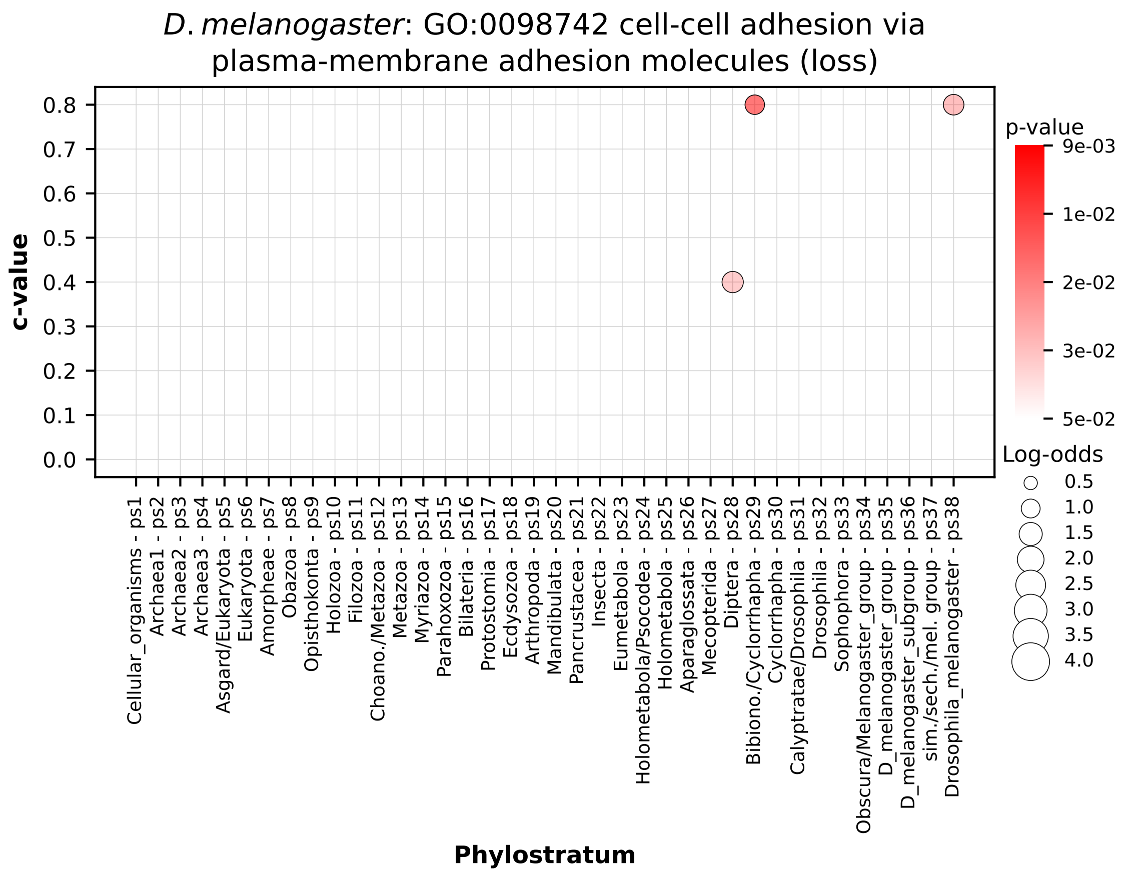

D. melanogaster: GO:0098742 cell-cell adhesion via plasma-membrane adhesion molecules (loss)
c-value
0.0
0.1
0.2
0.3
0.4
0.5
0.6
0.7
0.8
p-value
9e-03
1e-02
2e-02
3e-02
5e-02
Log-odds
0.5
1.0
1.5
2.0
2.5
3.0
3.5
4.0
Cellular_organisms - ps1
Archaea1 - ps2
Archaea2 - ps3
Archaea3 - ps4
Asgard/Eukaryota - ps5
Eukaryota - ps6
Amorpheae - ps7
Obazoa - ps8
Opisthokonta - ps9
Holozoa - ps10
Filozoa - ps11
Choano./Metazoa - ps12
Metazoa - ps13
Myriazoa - ps14
Parahoxozoa - ps15
Bilateria - ps16
Protostomia - ps17
Ecdysozoa - ps18
Arthropoda - ps19
Mandibulata - ps20
Pancrustacea - ps21
Insecta - ps22
Eumetabola - ps23
Holometabola/Psocodea - ps24
Holometabola - ps25
Aparaglossata - ps26
Mecopterida - ps27
Diptera - ps28
Bibiono./Cyclorrhapha - ps29
Cyclorrhapha - ps30
Calyptratae/Drosophila - ps31
Drosophila - ps32
Sophophora - ps33
Obscura/Melanogaster_group - ps34
D_melanogaster_group - ps35
D_melanogaster_subgroup - ps36
sim./sech./mel. group - ps37
Drosophila_melanogaster - ps38
Phylostratum

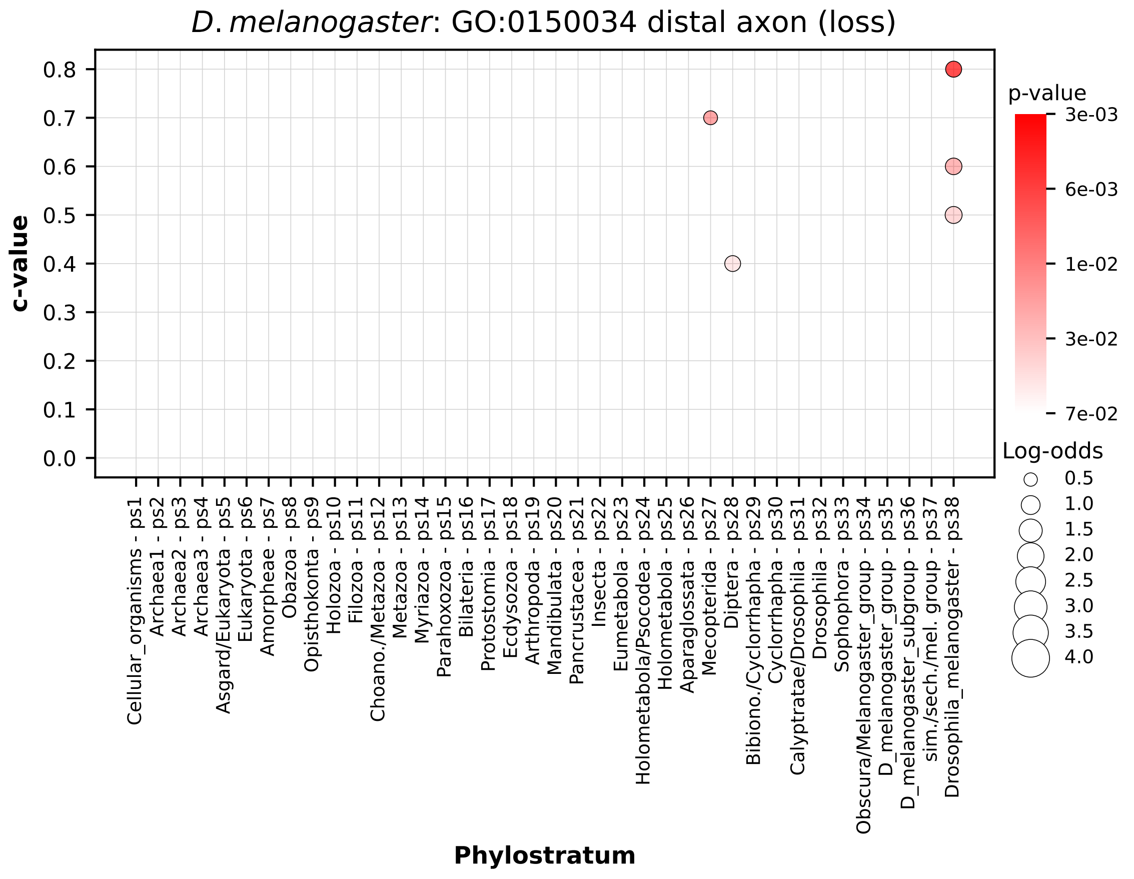

D. melanogaster: GO:0150034 distal axon (loss)
c-value
0.0
0.1
0.2
0.3
0.4
0.5
0.6
0.7
0.8
Phylostratum
Cellular_organisms - ps1
Archaea1 - ps2
Archaea2 - ps3
Archaea3 - ps4
Asgard/Eukaryota - ps5
Eukaryota - ps6
Amorpheae - ps7
Obazoa - ps8
Opisthokonta - ps9
Holozoa - ps10
Filozoa - ps11
Choano./Metazoa - ps12
Metazoa - ps13
Myriazoa - ps14
Parahoxozoa - ps15
Bilateria - ps16
Protostomia - ps17
Ecdysozoa - ps18
Arthropoda - ps19
Mandibulata - ps20
Pancrustacea - ps21
Insecta - ps22
Eumetabola - ps23
Holometabola/Psocodea - ps24
Holometabola - ps25
Aparaglossata - ps26
Mecopterida - ps27
Diptera - ps28
Bibiono./Cyclorrhapha - ps29
Cyclorrhapha - ps30
Calyptratae/Drosophila - ps31
Drosophila - ps32
Sophophora - ps33
Obscura/Melanogaster_group - ps34
D_melanogaster_group - ps35
D_melanogaster_subgroup - ps36
sim./sech./mel. group - ps37
Drosophila_melanogaster - ps38
p-value
3e-03
6e-03
1e-02
3e-02
7e-02
Log-odds
0.5
1.0
1.5
2.0
2.5
3.0
3.5
4.0

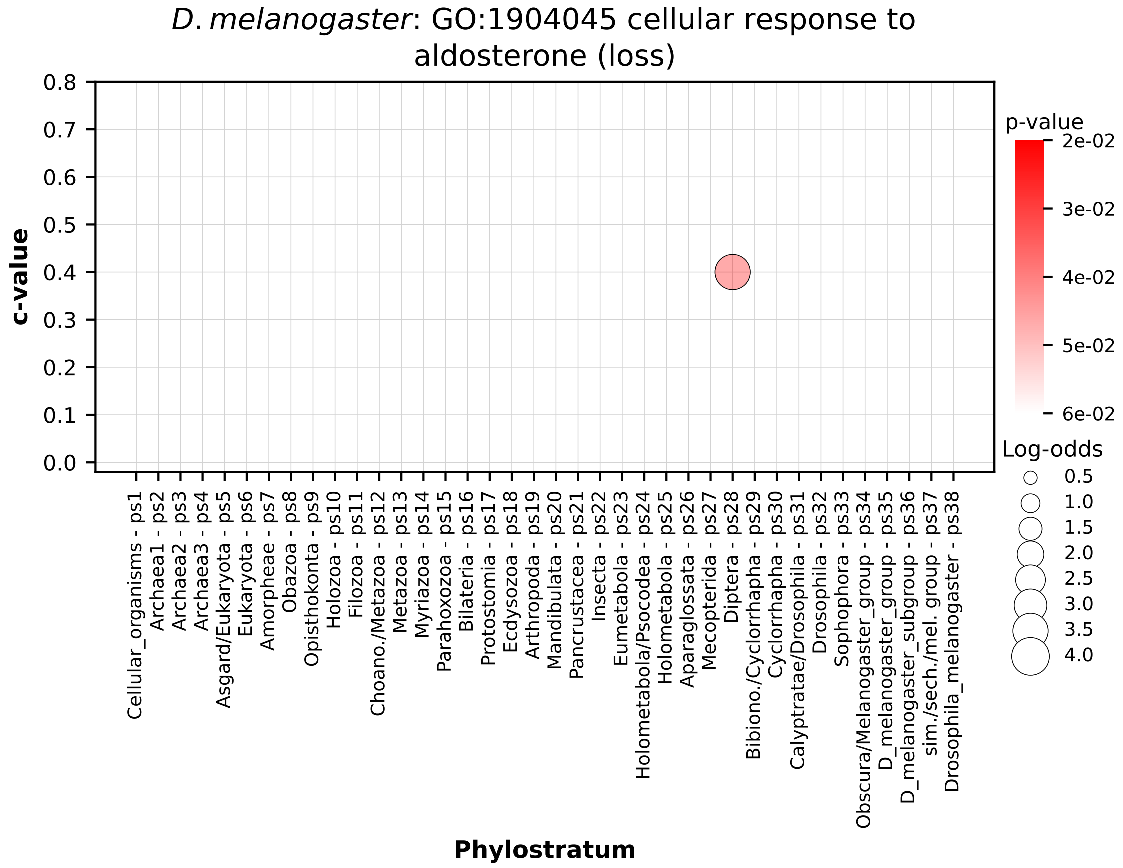

D. melanogaster: GO:1904045 cellular response to aldosterone (loss)
c-value
0.0
0.1
0.2
0.3
0.4
0.5
0.6
0.7
0.8
p-value
2e-02
3e-02
4e-02
5e-02
6e-02
Log-odds
0.5
1.0
1.5
2.0
2.5
3.0
3.5
4.0
Cellular_organisms - ps1
Archaea1 - ps2
Archaea2 - ps3
Archaea3 - ps4
Asgard/Eukaryota - ps5
Eukaryota - ps6
Amorpheae - ps7
Obazoa - ps8
Opisthokonta - ps9
Holozoa - ps10
Filozoa - ps11
Choano./Metazoa - ps12
Metazoa - ps13
Myriazoa - ps14
Parahoxozoa - ps15
Bilateria - ps16
Protostomia - ps17
Ecdysozoa - ps18
Arthropoda - ps19
Mandibulata - ps20
Pancrustacea - ps21
Insecta - ps22
Eumetabola - ps23
Holometabola/Psocodea - ps24
Holometabola - ps25
Aparaglossata - ps26
Mecopterida - ps27
Diptera - ps28
Bibiono./Cyclorrhapha - ps29
Cyclorrhapha - ps30
Calyptratae/Drosophila - ps31
Drosophila - ps32
Sophophora - ps33
Obscura/Melanogaster_group - ps34
D_melanogaster_group - ps35
D_melanogaster_subgroup - ps36
sim./sech./mel. group - ps37
Drosophila_melanogaster - ps38
Phylostratum

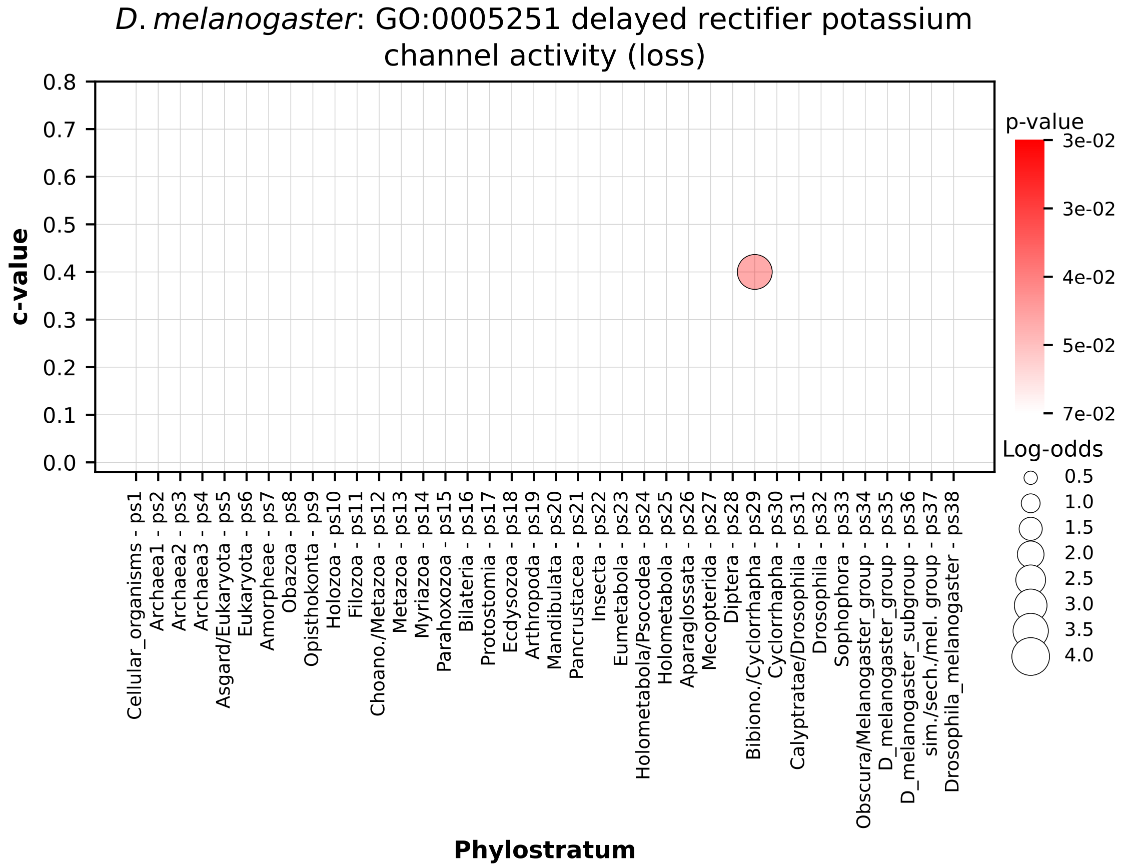

D. melanogaster: GO:0005251 delayed rectifier potassium channel activity (loss)
c-value
0.8
0.7
0.6
0.5
0.4
0.3
0.2
0.1
0.0
p-value
3e-02
3e-02
4e-02
5e-02
7e-02
Log-odds
0.5
1.0
1.5
2.0
2.5
3.0
3.5
4.0
Cellular_organisms - ps1
Archaea1 - ps2
Archaea2 - ps3
Archaea3 - ps4
Asgard/Eukaryota - ps5
Eukaryota - ps6
Amorpheae - ps7
Obazoa - ps8
Opisthokonta - ps9
Holozoa - ps10
Filozoa - ps11
Choano./Metazoa - ps12
Metazoa - ps13
Myriazoa - ps14
Parahoxozoa - ps15
Bilateria - ps16
Protostomia - ps17
Ecdysozoa - ps18
Arthropoda - ps19
Mandibulata - ps20
Pancrustacea - ps21
Insecta - ps22
Eumetabola - ps23
Holometabola/Psocodea - ps24
Holometabola - ps25
Aparaglossata - ps26
Mecopterida - ps27
Diptera - ps28
Bibiono./Cyclorrhapha - ps29
Cyclorrhapha - ps30
Calyptratae/Drosophila - ps31
Drosophila - ps32
Sophophora - ps33
Obscura/Melanogaster_group - ps34
D_melanogaster_group - ps35
D_melanogaster_subgroup - ps36
sim./sech./mel. group - ps37
Drosophila_melanogaster - ps38
Phylostratum

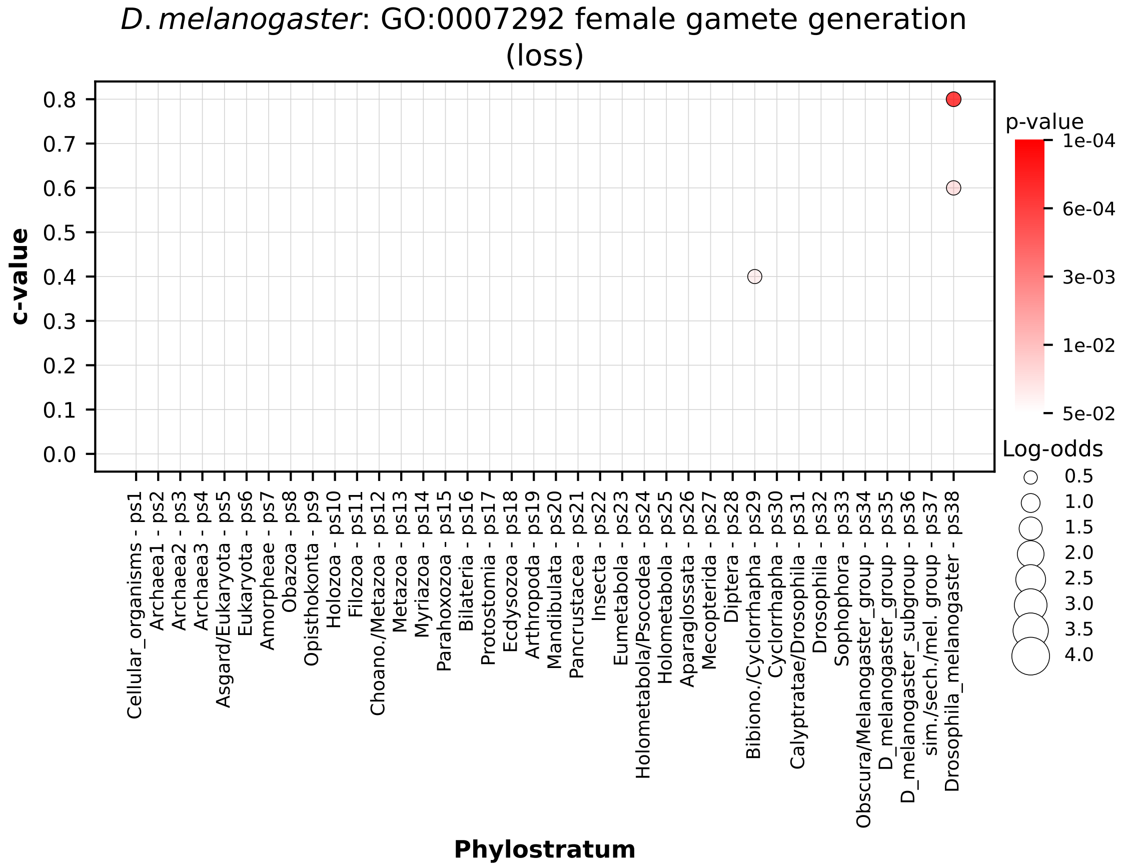

D. melanogaster: GO:0007292 female gamete generation
(loss)
c-value
0.0
0.1
0.2
0.3
0.4
0.5
0.6
0.7
0.8
Phylostratum
Cellular_organisms - ps1
Archaea1 - ps2
Archaea2 - ps3
Archaea3 - ps4
Asgard/Eukaryota - ps5
Eukaryota - ps6
Amorpheae - ps7
Obazoa - ps8
Opisthokonta - ps9
Holozoa - ps10
Filozoa - ps11
Choano./Metazoa - ps12
Metazoa - ps13
Myriazoa - ps14
Parahoxozoa - ps15
Bilateria - ps16
Protostomia - ps17
Ecdysozoa - ps18
Arthropoda - ps19
Mandibulata - ps20
Pancrustacea - ps21
Insecta - ps22
Eumetabola - ps23
Holometabola/Psocodea - ps24
Holometabola - ps25
Aparaglossata - ps26
Mecopterida - ps27
Diptera - ps28
Bibiono./Cyclorrhapha - ps29
Cyclorrhapha - ps30
Calyptratae/Drosophila - ps31
Drosophila - ps32
Sophophora - ps33
Obscura/Melanogaster_group - ps34
D_melanogaster_group - ps35
D_melanogaster_subgroup - ps36
sim./sech./mel. group - ps37
Drosophila_melanogaster - ps38
p-value
1e-04
6e-04
3e-03
1e-02
5e-02
Log-odds
0.5
1.0
1.5
2.0
2.5
3.0
3.5
4.0

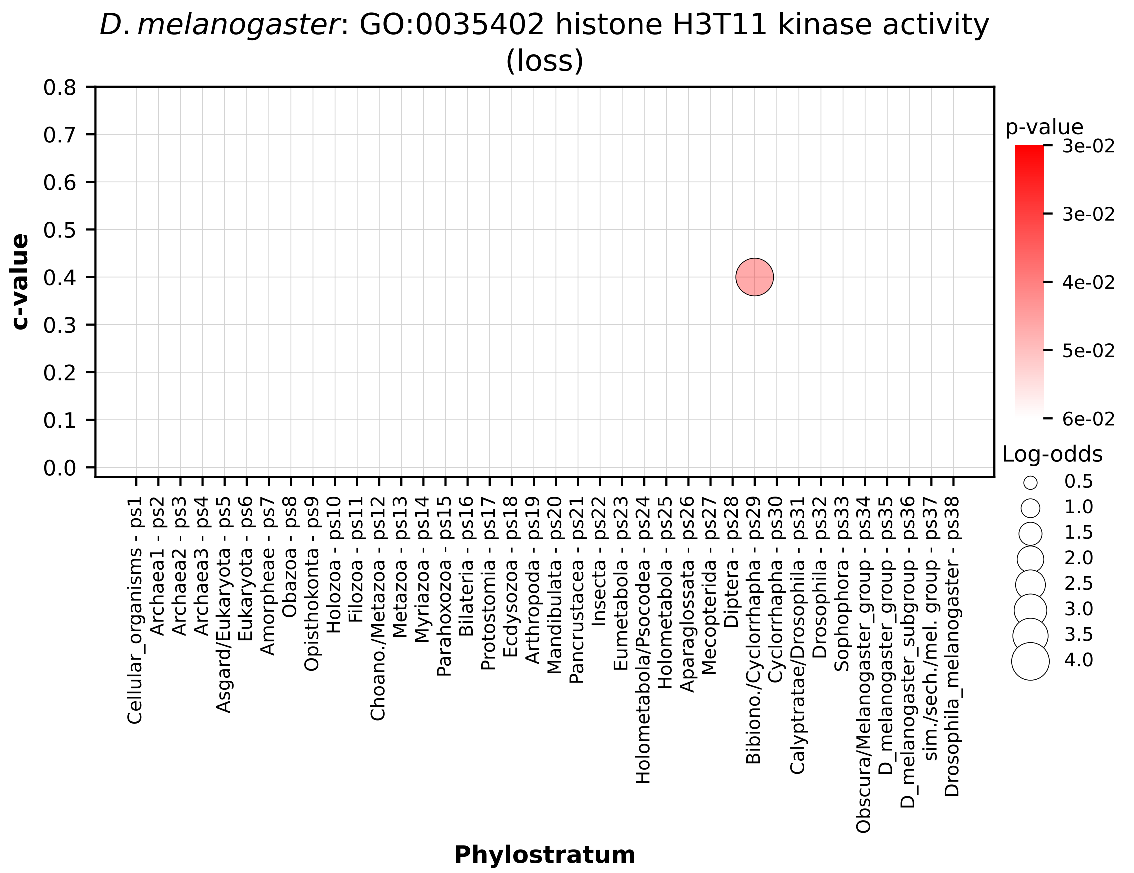

D. melanogaster: GO:0035402 histone H3T11 kinase activity (loss)
c-value
0.8
0.7
0.6
0.5
0.4
0.3
0.2
0.1
0.0
p-value
3e-02
3e-02
4e-02
5e-02
6e-02
Log-odds
0.5
1.0
1.5
2.0
2.5
3.0
3.5
4.0
Cellular_organisms - ps1
Archaea1 - ps2
Archaea2 - ps3
Archaea3 - ps4
Asgard/Eukaryota - ps5
Eukaryota - ps6
Amorpheae - ps7
Obazoa - ps8
Opisthokonta - ps9
Holozoa - ps10
Filozoa - ps11
Choano./Metazoa - ps12
Metazoa - ps13
Myriazoa - ps14
Parahoxozoa - ps15
Bilateria - ps16
Protostomia - ps17
Ecdysozoa - ps18
Arthropoda - ps19
Mandibulata - ps20
Pancrustacea - ps21
Insecta - ps22
Eumetabola - ps23
Holometabola/Psocodea - ps24
Holometabola - ps25
Aparaglossata - ps26
Mecopterida - ps27
Diptera - ps28
Bibiono./Cyclorrhapha - ps29
Cyclorrhapha - ps30
Calyptratae/Drosophila - ps31
Drosophila - ps32
Sophophora - ps33
Obscura/Melanogaster_group - ps34
D_melanogaster_group - ps35
D_melanogaster_subgroup - ps36
sim./sech./mel. group - ps37
Drosophila_melanogaster - ps38
Phylostratum

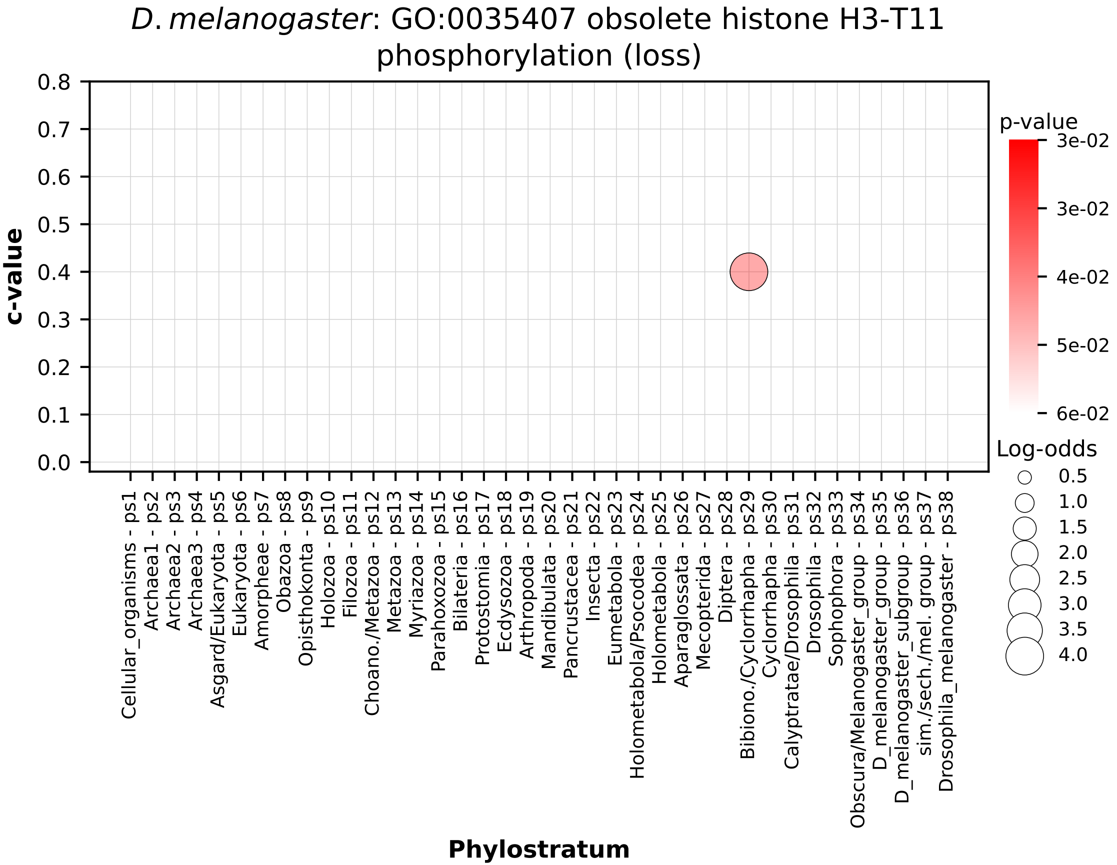

D. melanogaster: GO:0035407 obsolete histone H3-T11 phosphorylation (loss)
c-value
0.8
0.7
0.6
0.5
0.4
0.3
0.2
0.1
0.0
Phylostratum
Cellular_organisms - ps1
Archaea1 - ps2
Archaea2 - ps3
Archaea3 - ps4
Asgard/Eukaryota - ps5
Eukaryota - ps6
Amorpheae - ps7
Obazoa - ps8
Opisthokonta - ps9
Holozoa - ps10
Filozoa - ps11
Choano./Metazoa - ps12
Metazoa - ps13
Myriazoa - ps14
Parahoxozoa - ps15
Bilateria - ps16
Protostomia - ps17
Ecdysozoa - ps18
Arthropoda - ps19
Mandibulata - ps20
Pancrustacea - ps21
Insecta - ps22
Eumetabola - ps23
Holometabola/Psocodea - ps24
Holometabola - ps25
Aparaglossata - ps26
Mecopterida - ps27
Diptera - ps28
Bibiono./Cyclorrhapha - ps29
Cyclorrhapha - ps30
Calyptratae/Drosophila - ps31
Drosophila - ps32
Sophophora - ps33
Obscura/Melanogaster_group - ps34
D_melanogaster_group - ps35
D_melanogaster_subgroup - ps36
sim./sech./mel. group - ps37
Drosophila_melanogaster - ps38
p-value
3e-02
3e-02
4e-02
5e-02
6e-02
Log-odds
0.5
1.0
1.5
2.0
2.5
3.0
3.5
4.0

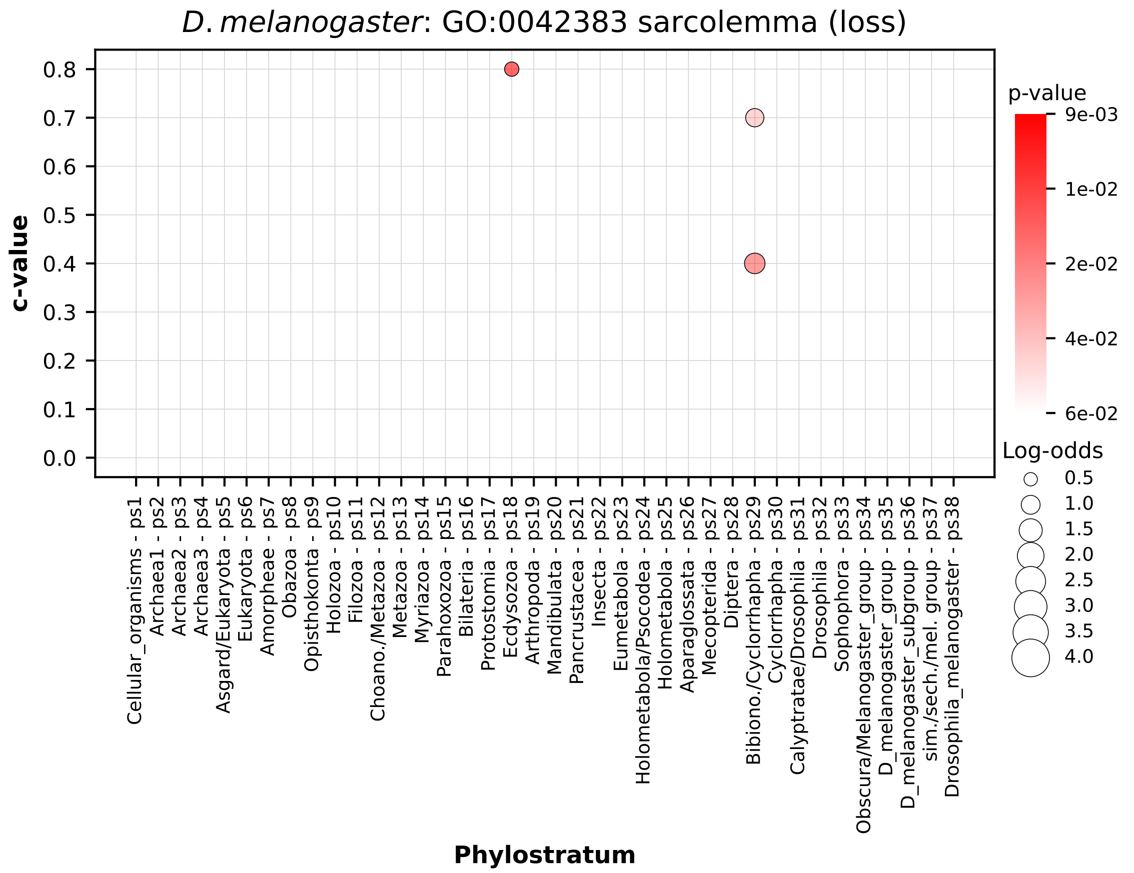
D. melanogaster: GO:0042383 sarcolemma (loss)
c-value
0.0
0.1
0.2
0.3
0.4
0.5
0.6
0.7
0.8
Phylostratum
Cellular_organisms - ps1
Archaea1 - ps2
Archaea2 - ps3
Archaea3 - ps4
Asgard/Eukaryota - ps5
Eukaryota - ps6
Amorpheae - ps7
Obazoa - ps8
Opisthokonta - ps9
Holozoa - ps10
Filozoa - ps11
Choano./Metazoa - ps12
Metazoa - ps13
Myriazoa - ps14
Parahoxozoa - ps15
Bilateria - ps16
Protostomia - ps17
Ecdysozoa - ps18
Arthropoda - ps19
Mandibulata - ps20
Pancrustacea - ps21
Insecta - ps22
Eumetabola - ps23
Holometabola/Psocodea - ps24
Holometabola - ps25
Aparaglossata - ps26
Mecopterida - ps27
Diptera - ps28
Bibiono./Cyclorrhapha - ps29
Cyclorrhapha - ps30
Calyptratae/Drosophila - ps31
Drosophila - ps32
Sophophora - ps33
Obscura/Melanogaster_group - ps34
D_melanogaster_group - ps35
D_melanogaster_subgroup - ps36
sim./sech./mel. group - ps37
Drosophila_melanogaster - ps38
p-value
9e-03
1e-02
2e-02
4e-02
6e-02
Log-odds
0.5
1.0
1.5
2.0
2.5
3.0
3.5
4.0

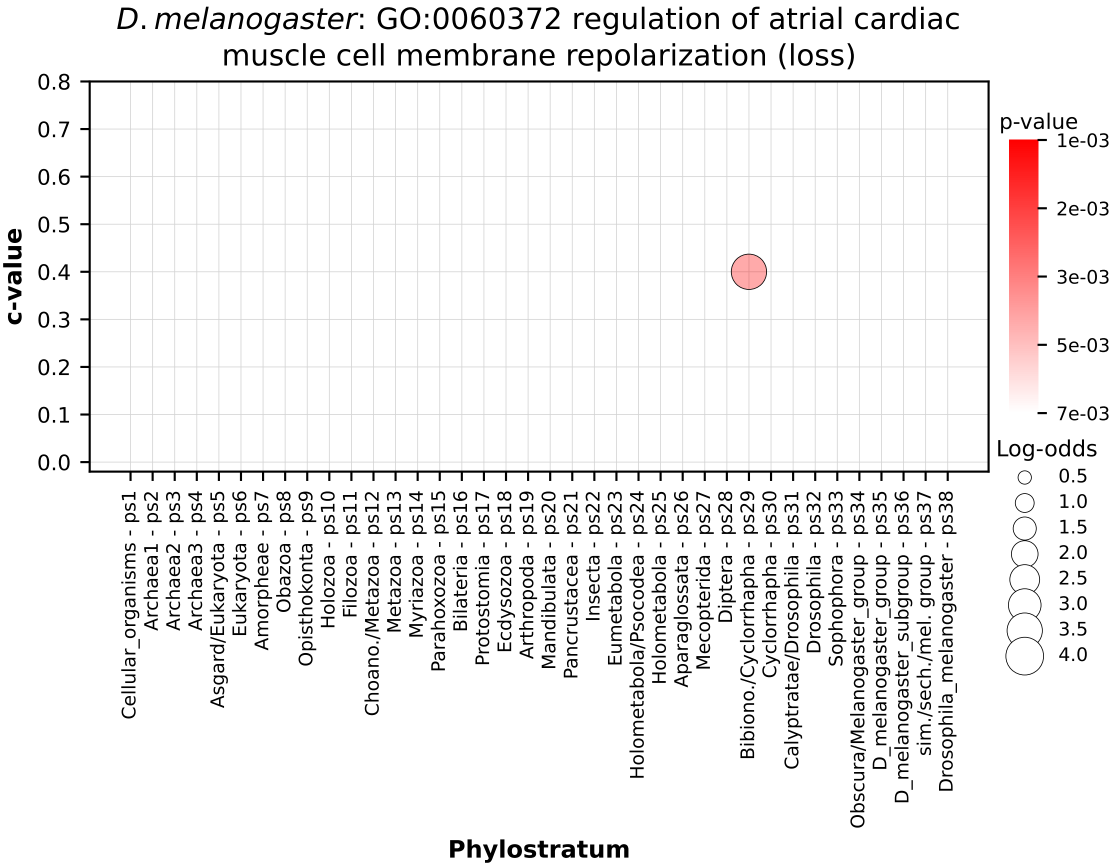
D. melanogaster: GO:0060372 regulation of atrial cardiac muscle cell membrane repolarization (loss)
c-value
0.8
0.7
0.6
0.5
0.4
0.3
0.2
0.1
0.0
p-value
1e-03
2e-03
3e-03
5e-03
7e-03
Log-odds
0.5
1.0
1.5
2.0
2.5
3.0
3.5
4.0
Cellular_organisms - ps1
Archaea1 - ps2
Archaea2 - ps3
Archaea3 - ps4
Asgard/Eukaryota - ps5
Eukaryota - ps6
Amorpheae - ps7
Obazoa - ps8
Opisthokonta - ps9
Holozoa - ps10
Filozoa - ps11
Choano./Metazoa - ps12
Metazoa - ps13
Myriazoa - ps14
Parahoxozoa - ps15
Bilateria - ps16
Protostomia - ps17
Ecdysozoa - ps18
Arthropoda - ps19
Mandibulata - ps20
Pancrustacea - ps21
Insecta - ps22
Eumetabola - ps23
Holometabola/Psocodea - ps24
Holometabola - ps25
Aparaglossata - ps26
Mecopterida - ps27
Diptera - ps28
Bibiono./Cyclorrhapha - ps29
Cyclorrhapha - ps30
Calyptratae/Drosophila - ps31
Drosophila - ps32
Sophophora - ps33
Obscura/Melanogaster_group - ps34
D_melanogaster_group - ps35
D_melanogaster_subgroup - ps36
sim./sech./mel. group - ps37
Drosophila_melanogaster - ps38
Phylostratum

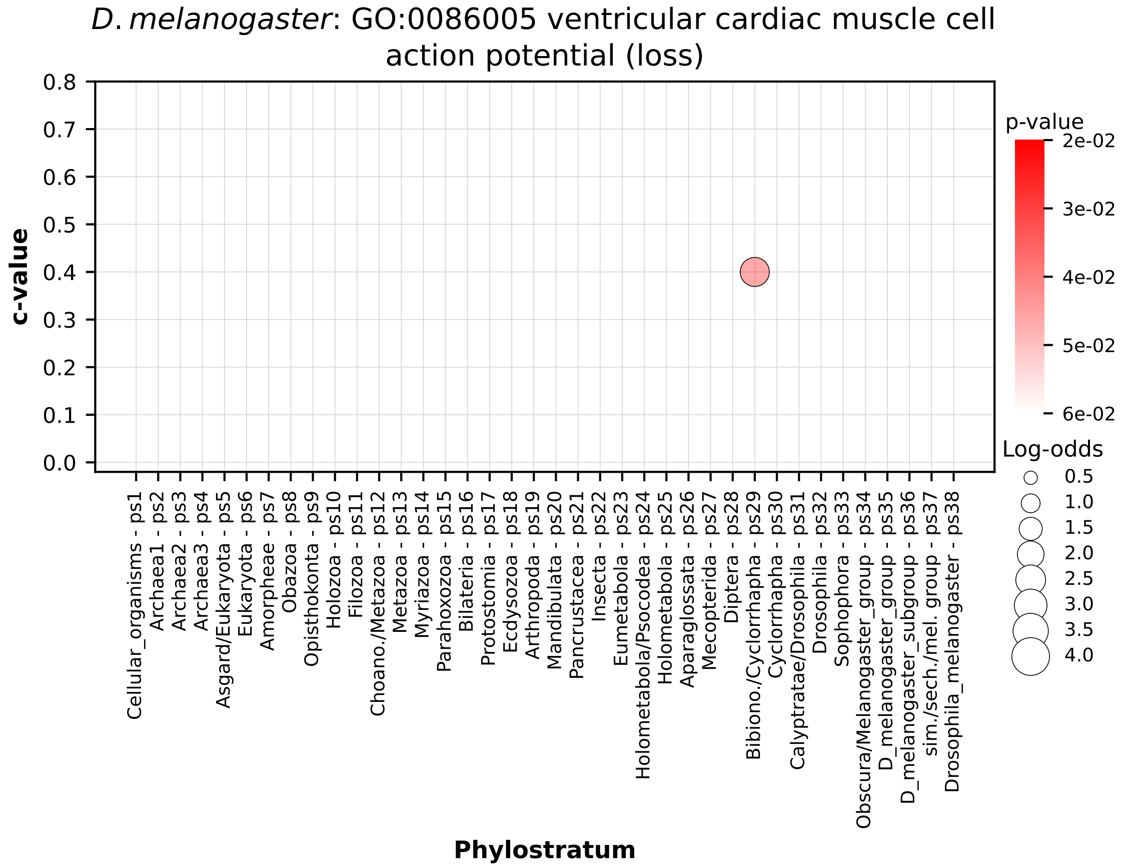

D. melanogaster: GO:0086005 ventricular cardiac muscle cell action potential (loss)
c-value
0.8
0.7
0.6
0.5
0.4
0.3
0.2
0.1
0.0
p-value
2e-02
3e-02
4e-02
5e-02
6e-02
Log-odds
0.5
1.0
1.5
2.0
2.5
3.0
3.5
4.0
Cellular_organisms - ps1
Archaea1 - ps2
Archaea2 - ps3
Archaea3 - ps4
Asgard/Eukaryota - ps5
Eukaryota - ps6
Amorpheae - ps7
Obazoa - ps8
Opisthokonta - ps9
Holozoa - ps10
Filozoa - ps11
Choano./Metazoa - ps12
Metazoa - ps13
Myriazoa - ps14
Parahoxozoa - ps15
Bilateria - ps16
Protostomia - ps17
Ecdysozoa - ps18
Arthropoda - ps19
Mandibulata - ps20
Pancrustacea - ps21
Insecta - ps22
Eumetabola - ps23
Holometabola/Psocodea - ps24
Holometabola - ps25
Aparaglossata - ps26
Mecopterida - ps27
Diptera - ps28
Bibiono./Cyclorrhapha - ps29
Cyclorrhapha - ps30
Calyptratae/Drosophila - ps31
Drosophila - ps32
Sophophora - ps33
Obscura/Melanogaster_group - ps34
D_melanogaster_group - ps35
D_melanogaster_subgroup - ps36
sim./sech./mel. group - ps37
Drosophila_melanogaster - ps38
Phylostratum

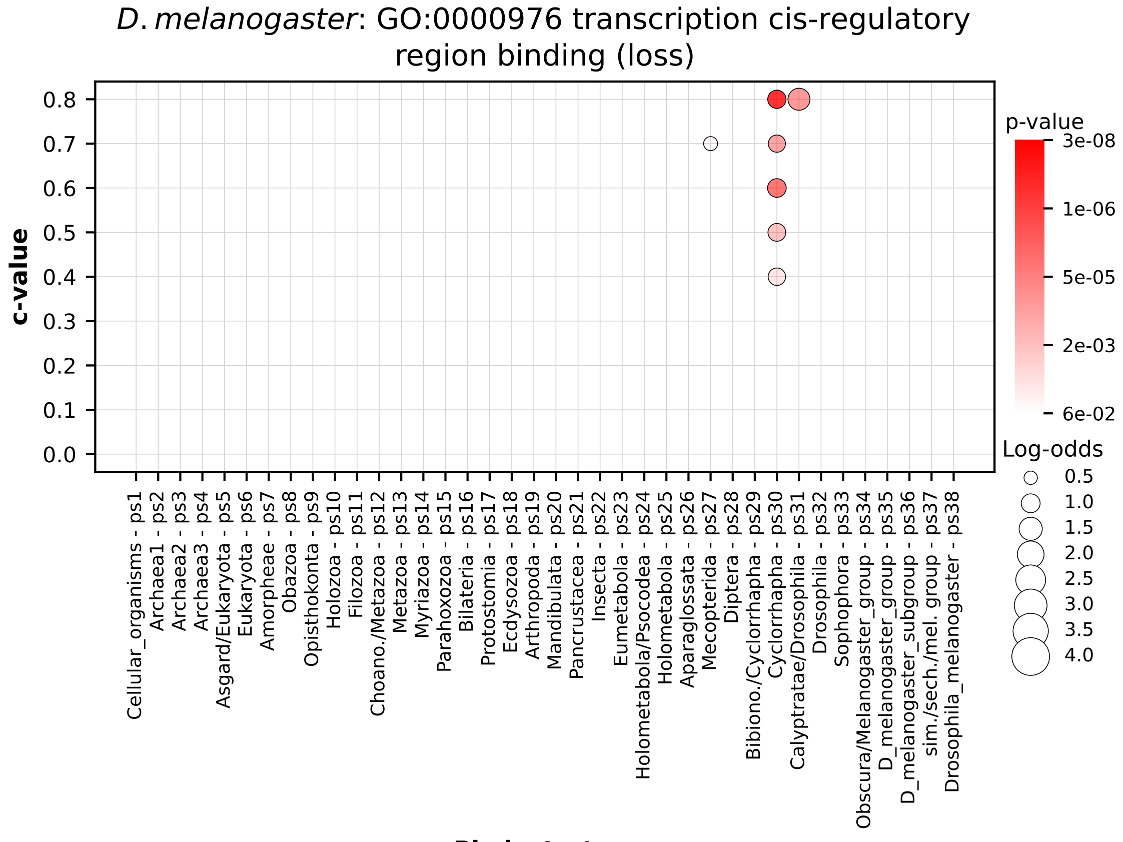

D. melanogaster: GO:0000976 transcription cis-regulatory region binding (loss)
c-value
0.8
0.7
0.6
0.5
0.4
0.3
0.2
0.1
0.0
Phylostratum
Cellular_organisms - ps1
Archaea1 - ps2
Archaea2 - ps3
Archaea3 - ps4
Asgard/Eukaryota - ps5
Eukaryota - ps6
Amorpheae - ps7
Obazoa - ps8
Opisthokonta - ps9
Holozoa - ps10
Filozoa - ps11
Choano./Metazoa - ps12
Metazoa - ps13
Myriazoa - ps14
Parahoxozoa - ps15
Bilateria - ps16
Protostomia - ps17
Ecdysozoa - ps18
Arthropoda - ps19
Mandibulata - ps20
Pancrustacea - ps21
Insecta - ps22
Eumetabola - ps23
Holometabola/Psocodea - ps24
Holometabola - ps25
Aparaglossata - ps26
Mecopterida - ps27
Diptera - ps28
Bibiono./Cyclorrhapha - ps29
Cyclorrhapha - ps30
Calyptratae/Drosophila - ps31
Drosophila - ps32
Sophophora - ps33
Obscura/Melanogaster_group - ps34
D_melanogaster_group - ps35
D_melanogaster_subgroup - ps36
sim./sech./mel. group - ps37
Drosophila_melanogaster - ps38
p-value
3e-08
1e-06
5e-05
2e-03
6e-02
Log-odds
0.5
1.0
1.5
2.0
2.5
3.0
3.5
4.0

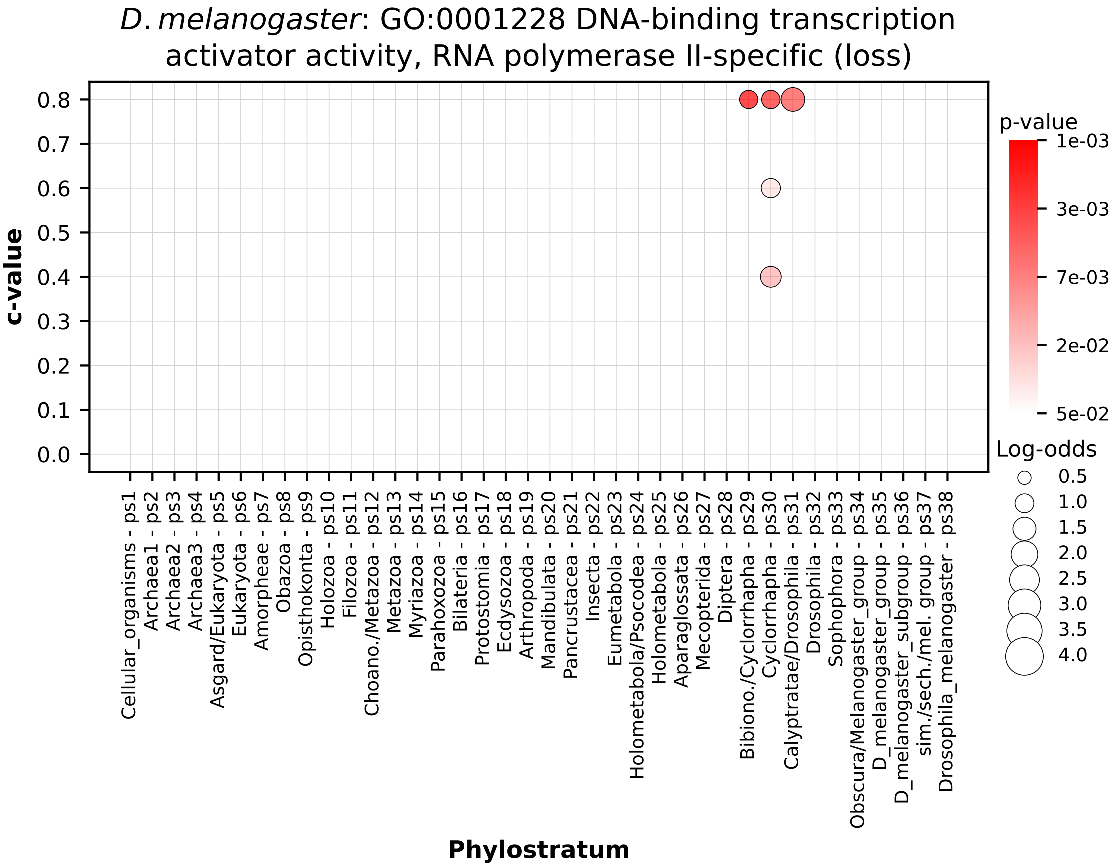

D. melanogaster: GO:0001228 DNA-binding transcription activator activity, RNA polymerase II-specific (loss)
c-value
0.8
0.7
0.6
0.5
0.4
0.3
0.2
0.1
0.0
Phylostratum
Cellular_organisms - ps1
Archaea1 - ps2
Archaea2 - ps3
Archaea3 - ps4
Asgard/Eukaryota - ps5
Eukaryota - ps6
Amorpheae - ps7
Obazoa - ps8
Opisthokonta - ps9
Holozoa - ps10
Filozoa - ps11
Choano./Metazoa - ps12
Metazoa - ps13
Myriazoa - ps14
Parahoxozoa - ps15
Bilateria - ps16
Protostomia - ps17
Ecdysozoa - ps18
Arthropoda - ps19
Mandibulata - ps20
Pancrustacea - ps21
Insecta - ps22
Eumetabola - ps23
Holometabola/Psocodea - ps24
Holometabola - ps25
Aparaglossata - ps26
Mecopterida - ps27
Diptera - ps28
Bibiono./Cyclorrhapha - ps29
Cyclorrhapha - ps30
Calyptratae/Drosophila - ps31
Drosophila - ps32
Sophophora - ps33
Obscura/Melanogaster_group - ps34
D_melanogaster_group - ps35
D_melanogaster_subgroup - ps36
sim./sech./mel. group - ps37
Drosophila_melanogaster - ps38
p-value
1e-03
3e-03
7e-03
2e-02
5e-02
Log-odds
0.5
1.0
1.5
2.0
2.5
3.0
3.5
4.0

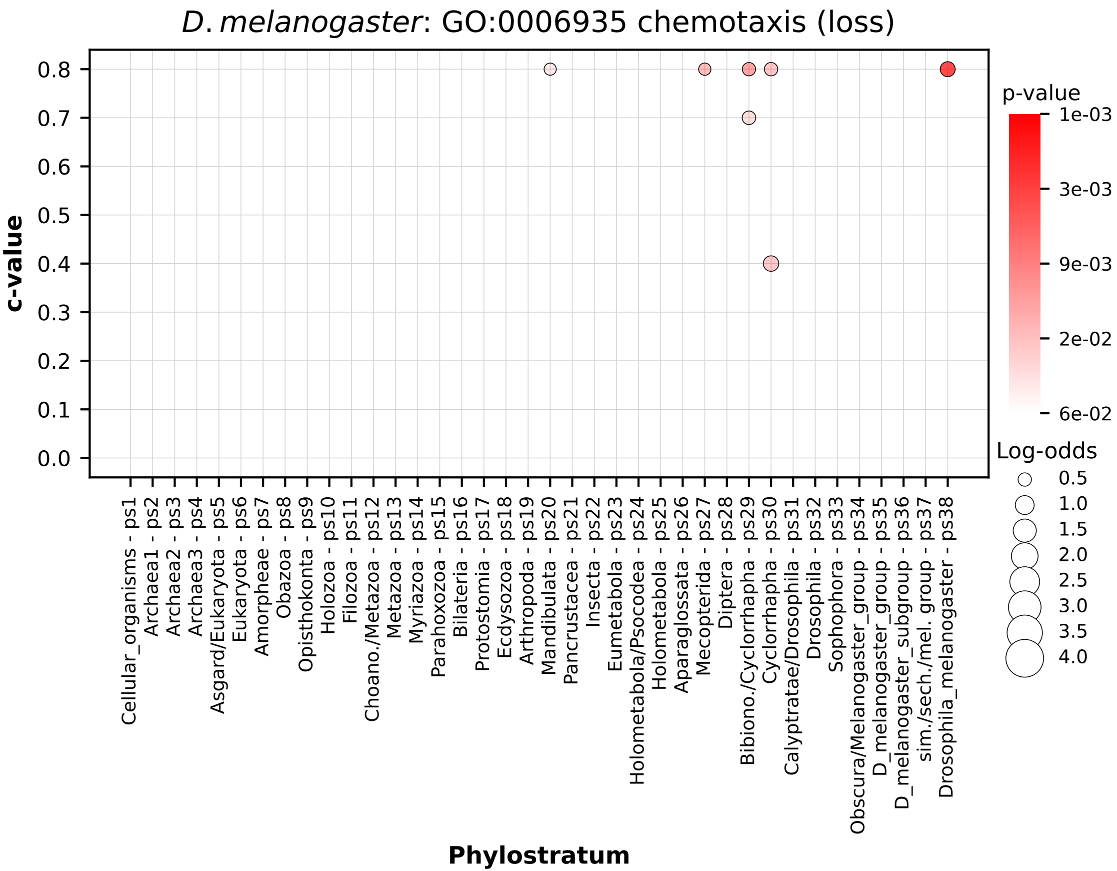
D. melanogaster: GO:0006935 chemotaxis (loss)
c-value
0.8
0.7
0.6
0.5
0.4
0.3
0.2
0.1
0.0
p-value
1e-03
3e-03
9e-03
2e-02
6e-02
Log-odds
0.5
1.0
1.5
2.0
2.5
3.0
3.5
4.0
Cellular_organisms - ps1
Archaea1 - ps2
Archaea2 - ps3
Archaea3 - ps4
Asgard/Eukaryota - ps5
Eukaryota - ps6
Amorpheae - ps7
Obazoa - ps8
Opisthokonta - ps9
Holozoa - ps10
Filozoa - ps11
Choano./Metazoa - ps12
Metazoa - ps13
Myriazoa - ps14
Parahoxozoa - ps15
Bilateria - ps16
Protostomia - ps17
Ecdysozoa - ps18
Arthropoda - ps19
Mandibulata - ps20
Pancrustacea - ps21
Insecta - ps22
Eumetabola - ps23
Holometabola/Psocodea - ps24
Holometabola - ps25
Aparaglossata - ps26
Mecopterida - ps27
Diptera - ps28
Bibiono./Cyclorrhapha - ps29
Cyclorrhapha - ps30
Calyptratae/Drosophila - ps31
Drosophila - ps32
Sophophora - ps33
Obscura/Melanogaster_group - ps34
D_melanogaster_group - ps35
D_melanogaster_subgroup - ps36
sim./sech./mel. group - ps37
Drosophila_melanogaster - ps38
Phylostratum

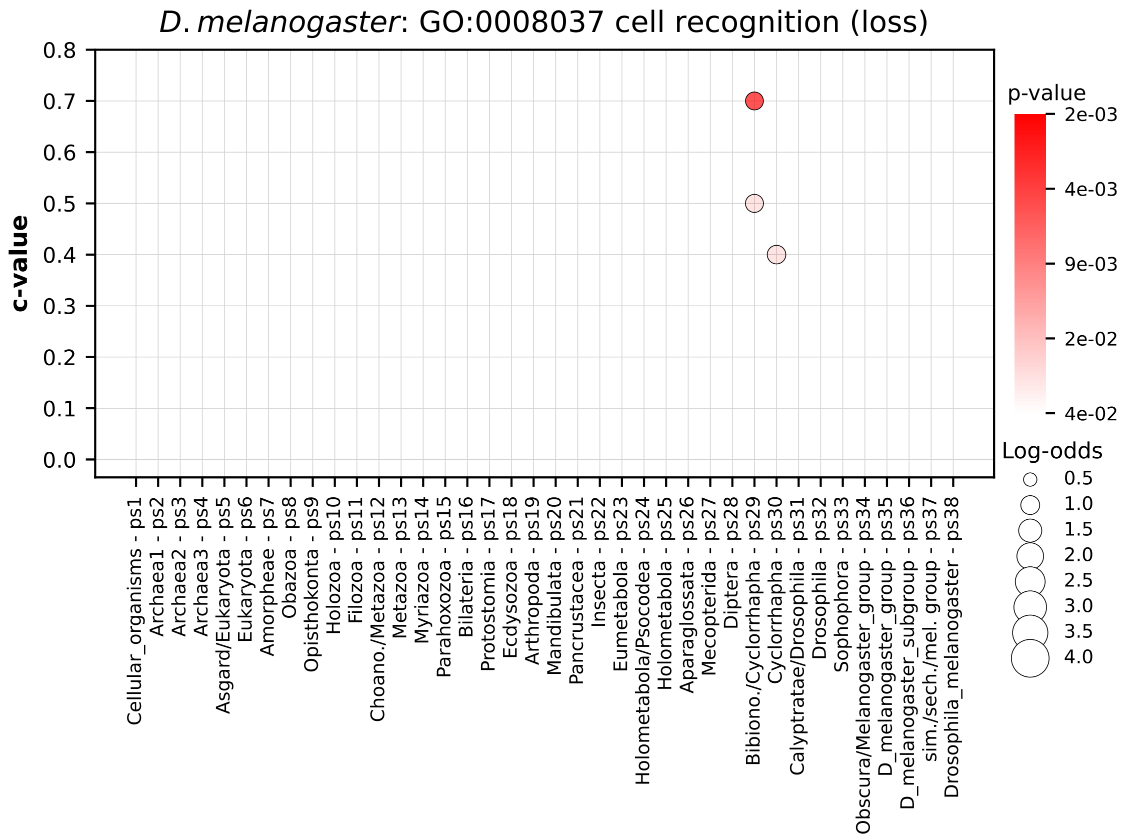
D. melanogaster: GO:0008037 cell recognition (loss)
c-value
0.8
0.7
0.6
0.5
0.4
0.3
0.2
0.1
0.0
Phylostratum
Cellular_organisms - ps1
Archaea1 - ps2
Archaea2 - ps3
Archaea3 - ps4
Asgard/Eukaryota - ps5
Eukaryota - ps6
Amorpheae - ps7
Obazoa - ps8
Opisthokonta - ps9
Holozoa - ps10
Filozoa - ps11
Choano./Metazoa - ps12
Metazoa - ps13
Myriazoa - ps14
Parahoxozoa - ps15
Bilateria - ps16
Protostomia - ps17
Ecdysozoa - ps18
Arthropoda - ps19
Mandibulata - ps20
Pancrustacea - ps21
Insecta - ps22
Eumetabola - ps23
Holometabola/Psocodea - ps24
Holometabola - ps25
Aparaglossata - ps26
Mecopterida - ps27
Diptera - ps28
Bibiono./Cyclorrhapha - ps29
Cyclorrhapha - ps30
Calyptratae/Drosophila - ps31
Drosophila - ps32
Sophophora - ps33
Obscura/Melanogaster_group - ps34
D_melanogaster_group - ps35
D_melanogaster_subgroup - ps36
sim./sech./mel. group - ps37
Drosophila_melanogaster - ps38
p-value
2e-03
4e-03
9e-03
2e-02
4e-02
Log-odds
0.5
1.0
1.5
2.0
2.5
3.0
3.5
4.0

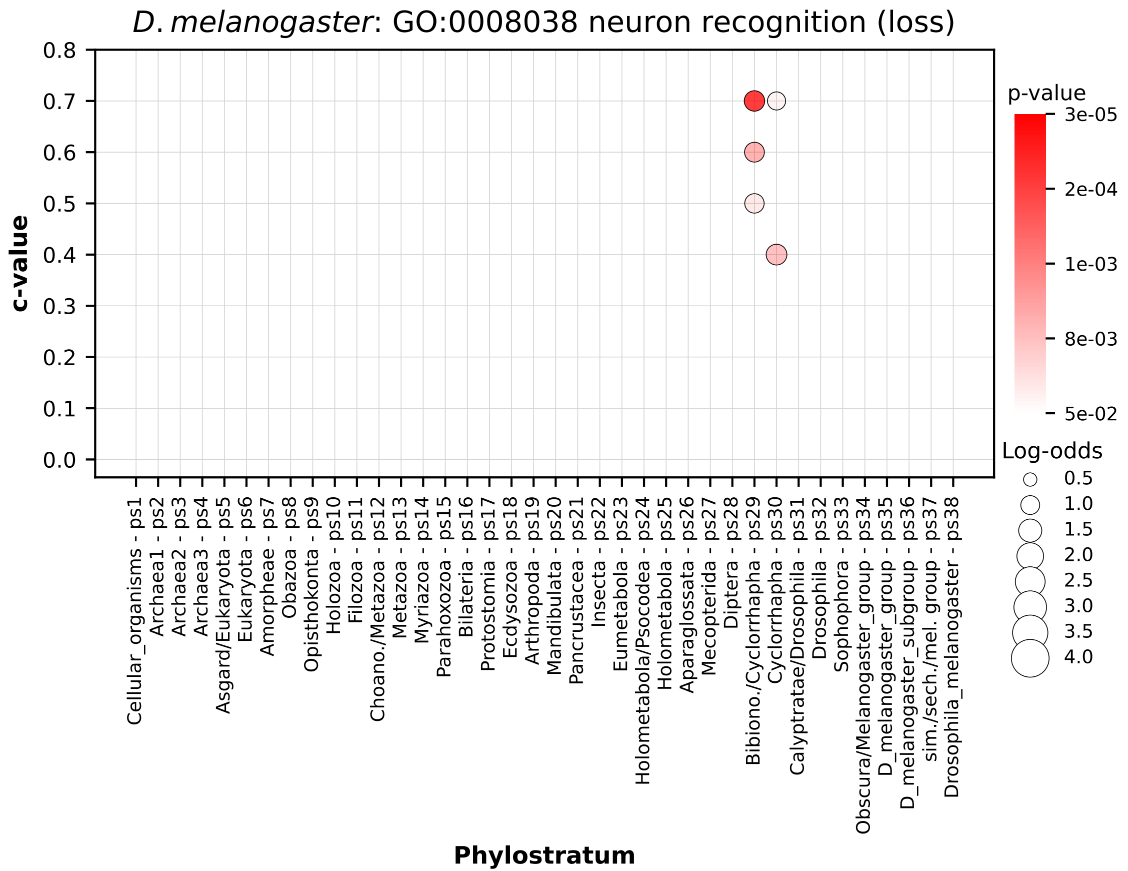

D. melanogaster: GO:0008038 neuron recognition (loss)
c-value
0.8
0.7
0.6
0.5
0.4
0.3
0.2
0.1
0.0
Phylostratum
Cellular_organisms - ps1
Archaea1 - ps2
Archaea2 - ps3
Archaea3 - ps4
Asgard/Eukaryota - ps5
Eukaryota - ps6
Amorpheae - ps7
Obazoa - ps8
Opisthokonta - ps9
Holozoa - ps10
Filozoa - ps11
Choano./Metazoa - ps12
Metazoa - ps13
Myriazoa - ps14
Parahoxozoa - ps15
Bilateria - ps16
Protostomia - ps17
Ecdysozoa - ps18
Arthropoda - ps19
Mandibulata - ps20
Pancrustacea - ps21
Insecta - ps22
Eumetabola - ps23
Holometabola/Psocodea - ps24
Holometabola - ps25
Aparaglossata - ps26
Mecopterida - ps27
Diptera - ps28
Bibiono./Cyclorrhapha - ps29
Cyclorrhapha - ps30
Calyptratae/Drosophila - ps31
Drosophila - ps32
Sophophora - ps33
Obscura/Melanogaster_group - ps34
D_melanogaster_group - ps35
D_melanogaster_subgroup - ps36
sim./sech./mel. group - ps37
Drosophila_melanogaster - ps38
p-value
3e-05
2e-04
1e-03
8e-03
5e-02
Log-odds
0.5
1.0
1.5
2.0
2.5
3.0
3.5
4.0

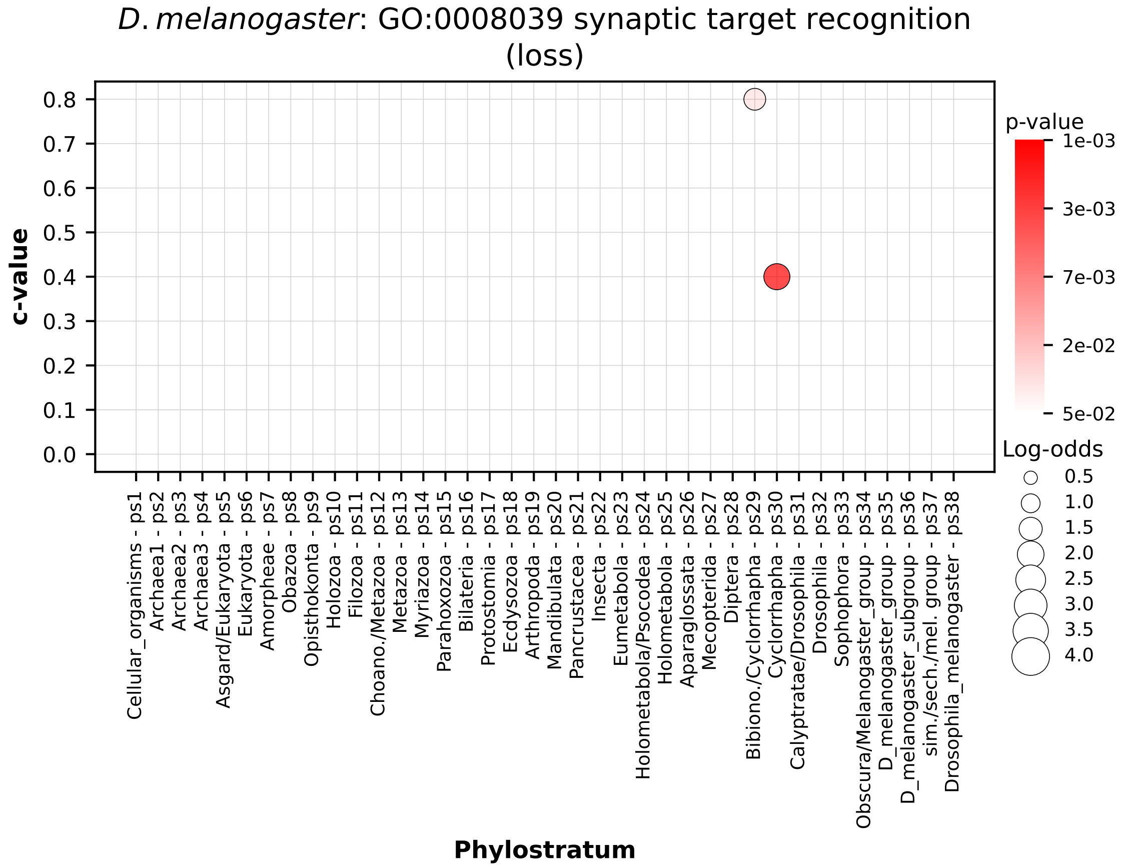

D. melanogaster: GO:0008039 synaptic target recognition
(loss)
c-value
0.8
0.7
0.6
0.5
0.4
0.3
0.2
0.1
0.0
p-value
1e-03
3e-03
7e-03
2e-02
5e-02
Log-odds
0.5
1.0
1.5
2.0
2.5
3.0
3.5
4.0
Cellular_organisms - ps1
Archaea1 - ps2
Archaea2 - ps3
Archaea3 - ps4
Asgard/Eukaryota - ps5
Eukaryota - ps6
Amorpheae - ps7
Obazoa - ps8
Opisthokonta - ps9
Holozoa - ps10
Filozoa - ps11
Choano./Metazoa - ps12
Metazoa - ps13
Myriazoa - ps14
Parahoxozoa - ps15
Bilateria - ps16
Protostomia - ps17
Ecdysozoa - ps18
Arthropoda - ps19
Mandibulata - ps20
Pancrustacea - ps21
Insecta - ps22
Eumetabola - ps23
Holometabola/Psocodea - ps24
Holometabola - ps25
Aparaglossata - ps26
Mecopterida - ps27
Diptera - ps28
Bibiono./Cyclorrhapha - ps29
Cyclorrhapha - ps30
Calyptratae/Drosophila - ps31
Drosophila - ps32
Sophophora - ps33
Obscura/Melanogaster_group - ps34
D_melanogaster_group - ps35
D_melanogaster_subgroup - ps36
sim./sech./mel. group - ps37
Drosophila_melanogaster - ps38
Phylostratum

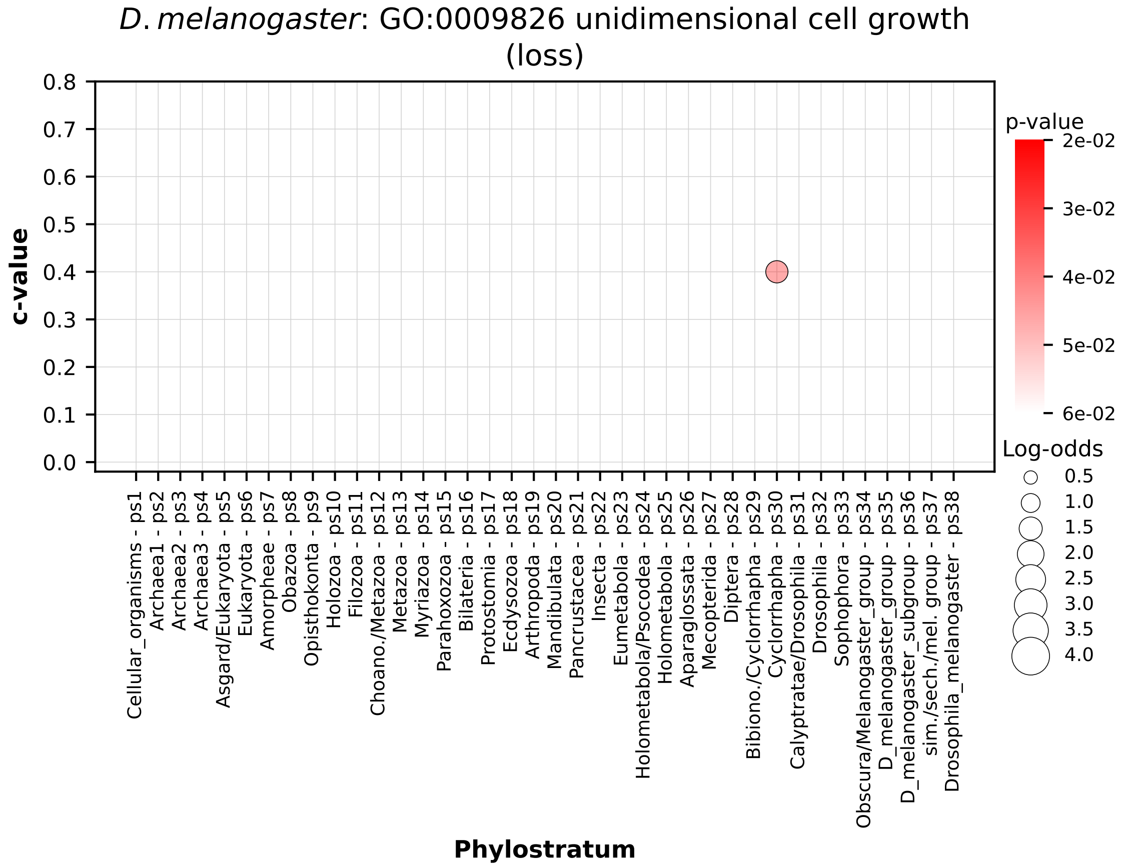

D. melanogaster: GO:0009826 unidimensional cell growth (loss)
c-value
0.8
0.7
0.6
0.5
0.4
0.3
0.2
0.1
0.0
Phylostratum
Cellular_organisms - ps1
Archaea1 - ps2
Archaea2 - ps3
Archaea3 - ps4
Asgard/Eukaryota - ps5
Eukaryota - ps6
Amorpheae - ps7
Obazoa - ps8
Opisthokonta - ps9
Holozoa - ps10
Filozoa - ps11
Choano./Metazoa - ps12
Metazoa - ps13
Myriazoa - ps14
Parahoxozoa - ps15
Bilateria - ps16
Protostomia - ps17
Ecdysozoa - ps18
Arthropoda - ps19
Mandibulata - ps20
Pancrustacea - ps21
Insecta - ps22
Eumetabola - ps23
Holometabola/Psocodea - ps24
Holometabola - ps25
Aparaglossata - ps26
Mecopterida - ps27
Diptera - ps28
Bibiono./Cyclorrhapha - ps29
Cyclorrhapha - ps30
Calyptratae/Drosophila - ps31
Drosophila - ps32
Sophophora - ps33
Obscura/Melanogaster_group - ps34
D_melanogaster_group - ps35
D_melanogaster_subgroup - ps36
sim./sech./mel. group - ps37
Drosophila_melanogaster - ps38
p-value
2e-02
3e-02
4e-02
5e-02
6e-02
Log-odds
0.5
1.0
1.5
2.0
2.5
3.0
3.5
4.0

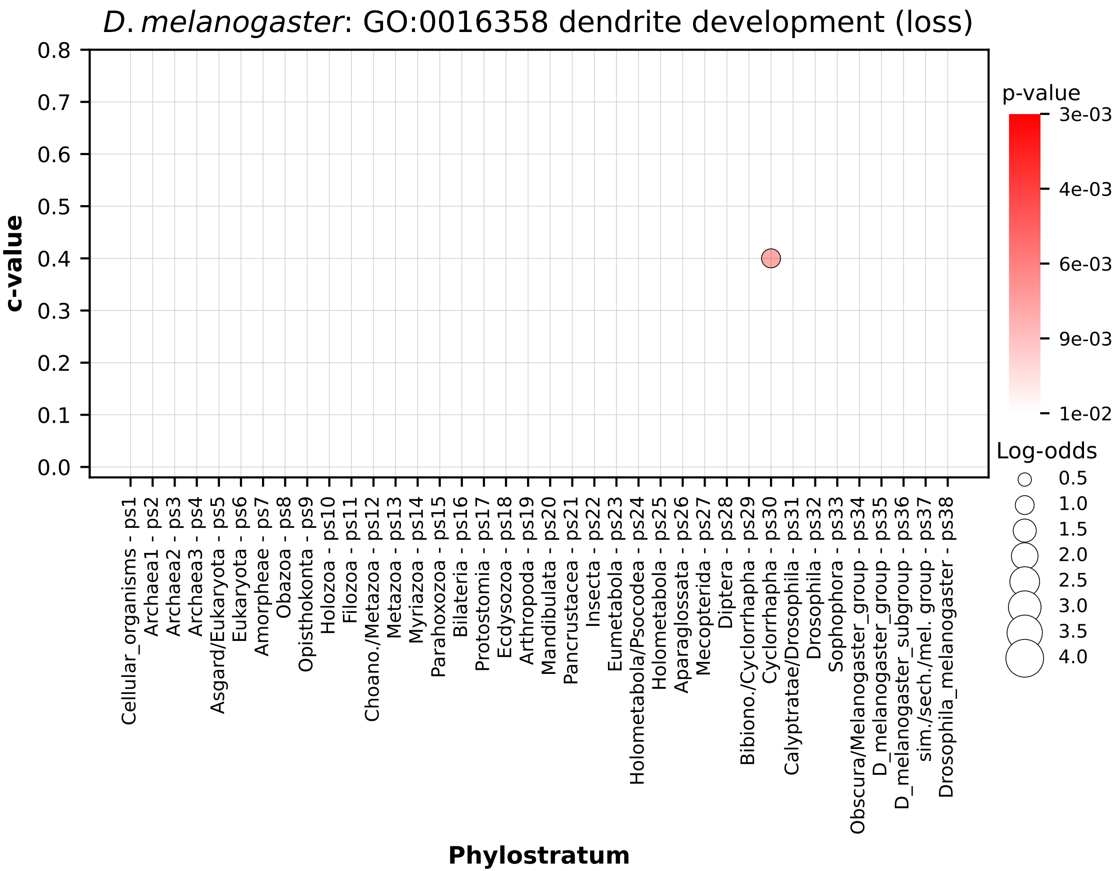
D. melanogaster: GO:0016358 dendrite development (loss)
c-value
0.0
0.1
0.2
0.3
0.4
0.5
0.6
0.7
0.8
Phylostratum
Cellular_organisms - ps1
Archaea1 - ps2
Archaea2 - ps3
Archaea3 - ps4
Asgard/Eukaryota - ps5
Eukaryota - ps6
Amorpheae - ps7
Obazoa - ps8
Opisthokonta - ps9
Holozoa - ps10
Filozoa - ps11
Choano./Metazoa - ps12
Metazoa - ps13
Myriazoa - ps14
Parahoxozoa - ps15
Bilateria - ps16
Protostomia - ps17
Ecdysozoa - ps18
Arthropoda - ps19
Mandibulata - ps20
Pancrustacea - ps21
Insecta - ps22
Eumetabola - ps23
Holometabola/Psocodea - ps24
Holometabola - ps25
Aparaglossata - ps26
Mecopterida - ps27
Diptera - ps28
Bibiono./Cyclorrhapha - ps29
Cyclorrhapha - ps30
Calyptratae/Drosophila - ps31
Drosophila - ps32
Sophophora - ps33
Obscura/Melanogaster_group - ps34
D_melanogaster_group - ps35
D_melanogaster_subgroup - ps36
sim./sech./mel. group - ps37
Drosophila_melanogaster - ps38
p-value
3e-03
4e-03
6e-03
9e-03
1e-02
Log-odds
0.5
1.0
1.5
2.0
2.5
3.0
3.5
4.0

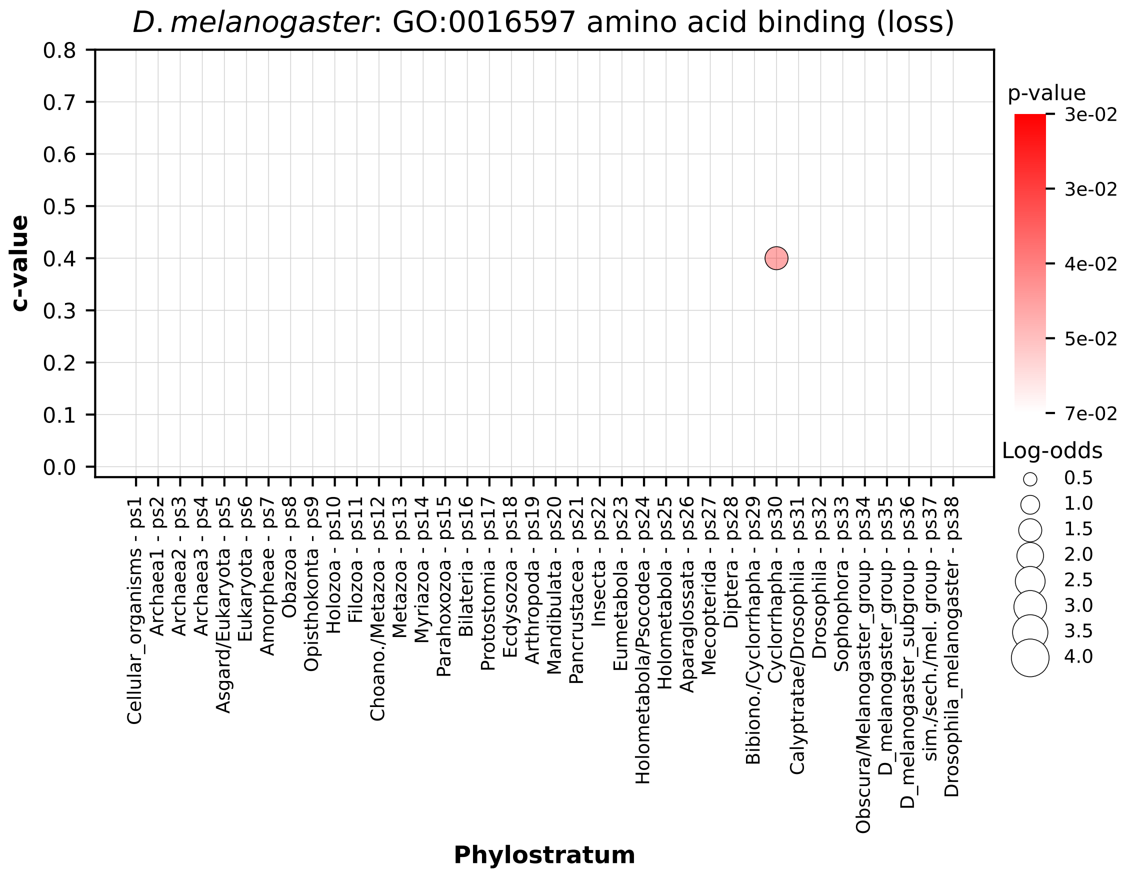

D. melanogaster: GO:0016597 amino acid binding (loss)
c-value
0.8
0.7
0.6
0.5
0.4
0.3
0.2
0.1
0.0
p-value
3e-02
3e-02
4e-02
5e-02
7e-02
Log-odds
0.5
1.0
1.5
2.0
2.5
3.0
3.5
4.0
Cellular_organisms - ps1
Archaea1 - ps2
Archaea2 - ps3
Archaea3 - ps4
Asgard/Eukaryota - ps5
Eukaryota - ps6
Amorpheae - ps7
Obazoa - ps8
Opisthokonta - ps9
Holozoa - ps10
Filozoa - ps11
Choano./Metazoa - ps12
Metazoa - ps13
Myriazoa - ps14
Parahoxozoa - ps15
Bilateria - ps16
Protostomia - ps17
Ecdysozoa - ps18
Arthropoda - ps19
Mandibulata - ps20
Pancrustacea - ps21
Insecta - ps22
Eumetabola - ps23
Holometabola/Psocodea - ps24
Holometabola - ps25
Aparaglossata - ps26
Mecopterida - ps27
Diptera - ps28
Bibiono./Cyclorrhapha - ps29
Cyclorrhapha - ps30
Calyptratae/Drosophila - ps31
Drosophila - ps32
Sophophora - ps33
Obscura/Melanogaster_group - ps34
D_melanogaster_group - ps35
D_melanogaster_subgroup - ps36
sim./sech./mel. group - ps37
Drosophila_melanogaster - ps38
Phylostratum

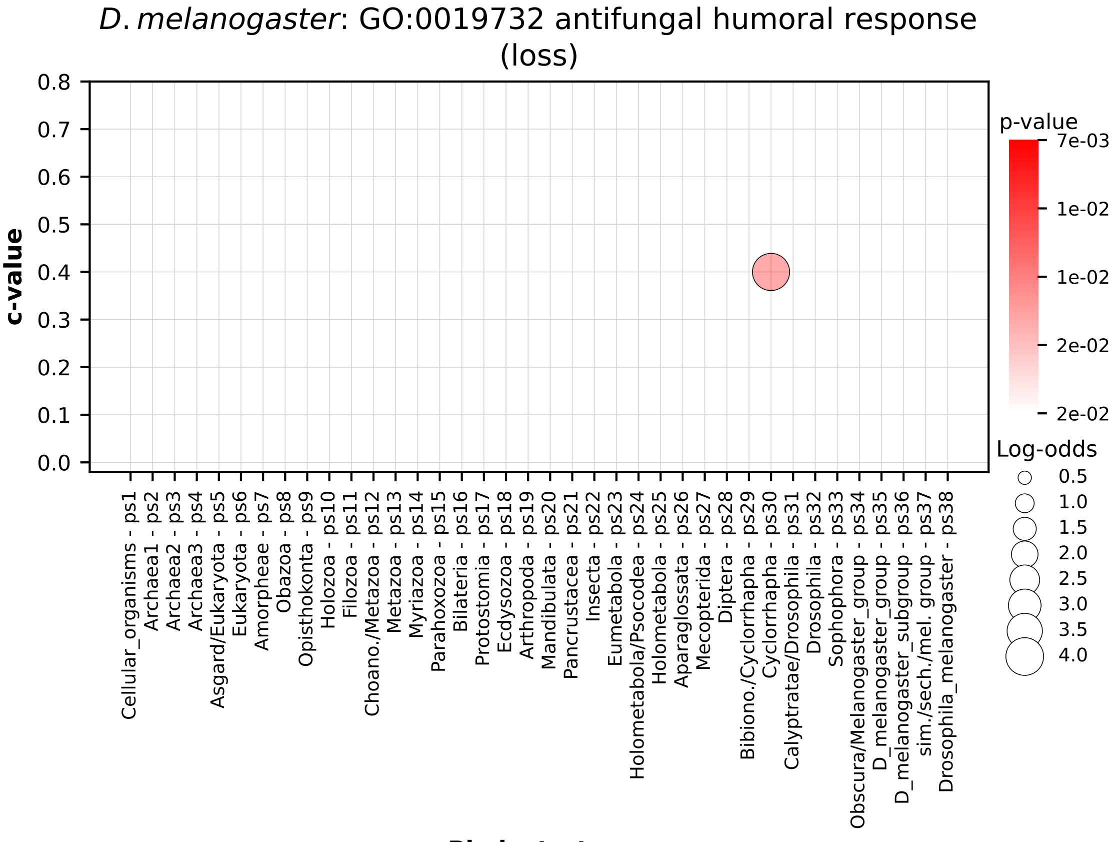
D. melanogaster: GO:0019732 antifungal humoral response
(loss)
c-value
0.8
0.7
0.6
0.5
0.4
0.3
0.2
0.1
0.0
p-value
7e-03
1e-02
1e-02
2e-02
2e-02
Log-odds
0.5
1.0
1.5
2.0
2.5
3.0
3.5
4.0
Cellular_organisms - ps1
Archaea1 - ps2
Archaea2 - ps3
Archaea3 - ps4
Asgard/Eukaryota - ps5
Eukaryota - ps6
Amorpheae - ps7
Obazoa - ps8
Opisthokonta - ps9
Holozoa - ps10
Filozoa - ps11
Choano./Metazoa - ps12
Metazoa - ps13
Myriazoa - ps14
Parahoxozoa - ps15
Bilateria - ps16
Protostomia - ps17
Ecdysozoa - ps18
Arthropoda - ps19
Mandibulata - ps20
Pancrustacea - ps21
Insecta - ps22
Eumetabola - ps23
Holometabola/Psocodea - ps24
Holometabola - ps25
Aparaglossata - ps26
Mecopterida - ps27
Diptera - ps28
Bibiono./Cyclorrhapha - ps29
Cyclorrhapha - ps30
Calyptratae/Drosophila - ps31
Drosophila - ps32
Sophophora - ps33
Obscura/Melanogaster_group - ps34
D_melanogaster_group - ps35
D_melanogaster_subgroup - ps36
sim./sech./mel. group - ps37
Drosophila_melanogaster - ps38
Phylostratum

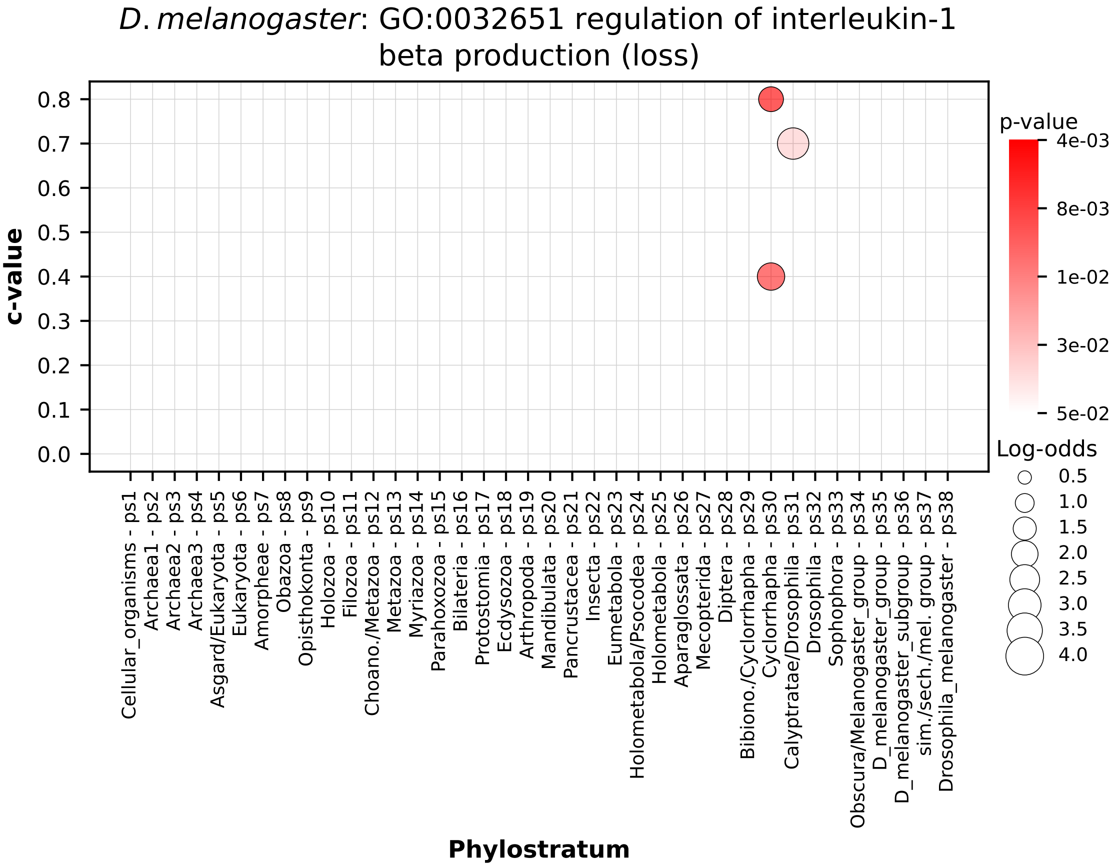

D. melanogaster: GO:0032651 regulation of interleukin-1 beta production (loss)
c-value
0.0
0.1
0.2
0.3
0.4
0.5
0.6
0.7
0.8
Phylostratum
Cellular_organisms - ps1
Archaea1 - ps2
Archaea2 - ps3
Archaea3 - ps4
Asgard/Eukaryota - ps5
Eukaryota - ps6
Amorpheae - ps7
Obazoa - ps8
Opisthokonta - ps9
Holozoa - ps10
Filozoa - ps11
Choano./Metazoa - ps12
Metazoa - ps13
Myriazoa - ps14
Parahoxozoa - ps15
Bilateria - ps16
Protostomia - ps17
Ecdysozoa - ps18
Arthropoda - ps19
Mandibulata - ps20
Pancrustacea - ps21
Insecta - ps22
Eumetabola - ps23
Holometabola/Psocodea - ps24
Holometabola - ps25
Aparaglossata - ps26
Mecopterida - ps27
Diptera - ps28
Bibiono./Cyclorrhapha - ps29
Cyclorrhapha - ps30
Calyptratae/Drosophila - ps31
Drosophila - ps32
Sophophora - ps33
Obscura/Melanogaster_group - ps34
D_melanogaster_group - ps35
D_melanogaster_subgroup - ps36
sim./sech./mel. group - ps37
Drosophila_melanogaster - ps38
p-value
4e-03
8e-03
1e-02
3e-02
5e-02
Log-odds
0.5
1.0
1.5
2.0
2.5
3.0
3.5
4.0

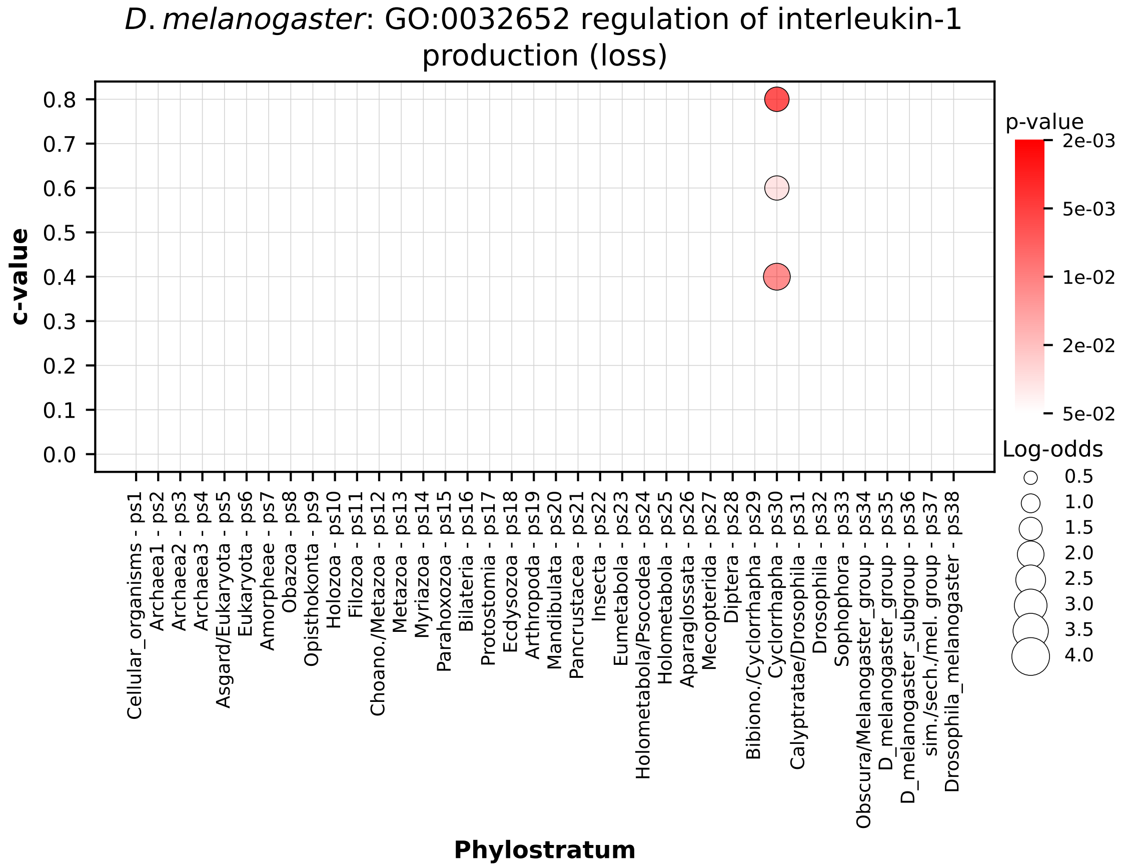

D. melanogaster: GO:0032652 regulation of interleukin-1 production (loss)
c-value
0.0
0.1
0.2
0.3
0.4
0.5
0.6
0.7
0.8
p-value
2e-03
5e-03
1e-02
2e-02
5e-02
Log-odds
0.5
1.0
1.5
2.0
2.5
3.0
3.5
4.0
Cellular_organisms - ps1
Archaea1 - ps2
Archaea2 - ps3
Archaea3 - ps4
Asgard/Eukaryota - ps5
Eukaryota - ps6
Amorpheae - ps7
Obazoa - ps8
Opisthokonta - ps9
Holozoa - ps10
Filozoa - ps11
Choano./Metazoa - ps12
Metazoa - ps13
Myriazoa - ps14
Parahoxozoa - ps15
Bilateria - ps16
Protostomia - ps17
Ecdysozoa - ps18
Arthropoda - ps19
Mandibulata - ps20
Pancrustacea - ps21
Insecta - ps22
Eumetabola - ps23
Holometabola/Psocodea - ps24
Holometabola - ps25
Aparaglossata - ps26
Mecopterida - ps27
Diptera - ps28
Bibiono./Cyclorrhapha - ps29
Cyclorrhapha - ps30
Calyptratae/Drosophila - ps31
Drosophila - ps32
Sophophora - ps33
Obscura/Melanogaster_group - ps34
D_melanogaster_group - ps35
D_melanogaster_subgroup - ps36
sim./sech./mel. group - ps37
Drosophila_melanogaster - ps38
Phylostratum

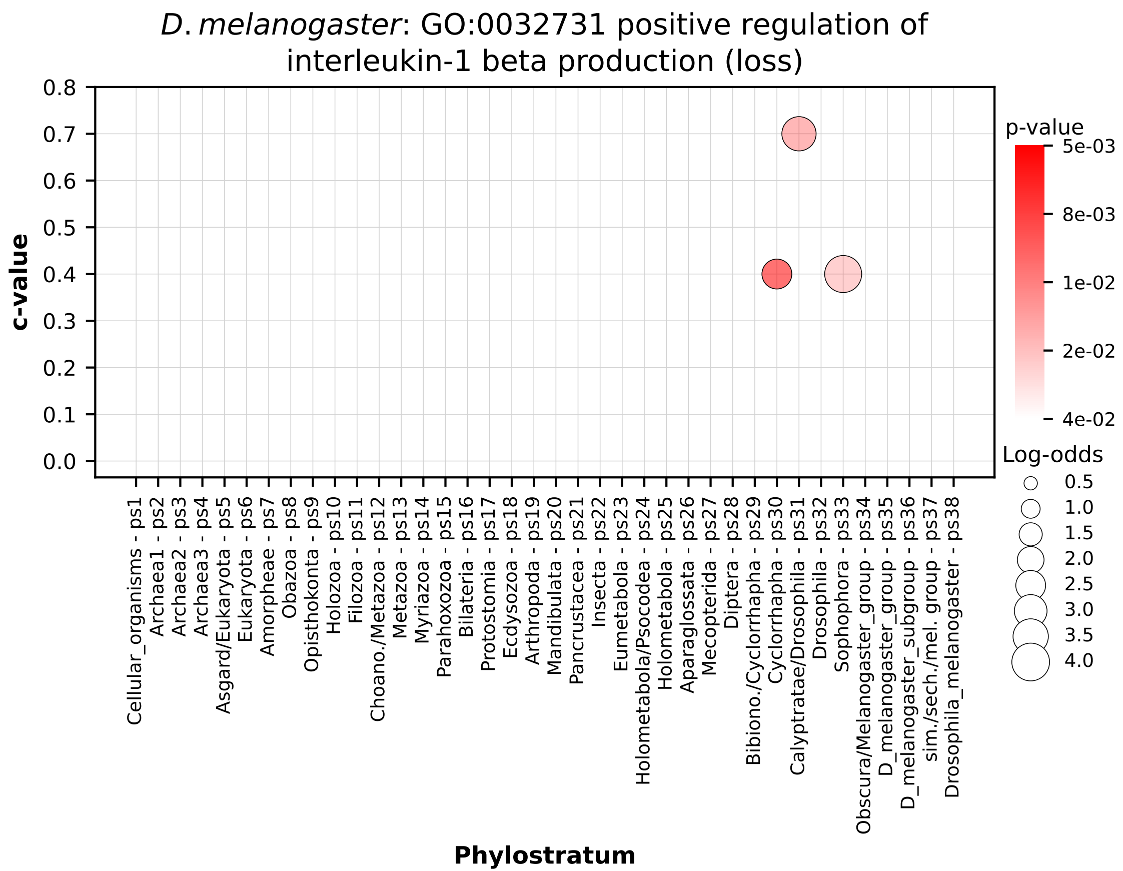

D. melanogaster: GO:0032731 positive regulation of interleukin-1 beta production (loss)
c-value
0.8
0.7
0.6
0.5
0.4
0.3
0.2
0.1
0.0
p-value
5e-03
8e-03
1e-02
2e-02
4e-02
Log-odds
0.5
1.0
1.5
2.0
2.5
3.0
3.5
4.0
Cellular_organisms - ps1
Archaea1 - ps2
Archaea2 - ps3
Archaea3 - ps4
Asgard/Eukaryota - ps5
Eukaryota - ps6
Amorpheae - ps7
Obazoa - ps8
Opisthokonta - ps9
Holozoa - ps10
Filozoa - ps11
Choano./Metazoa - ps12
Metazoa - ps13
Myriazoa - ps14
Parahoxozoa - ps15
Bilateria - ps16
Protostomia - ps17
Ecdysozoa - ps18
Arthropoda - ps19
Mandibulata - ps20
Pancrustacea - ps21
Insecta - ps22
Eumetabola - ps23
Holometabola/Psocodea - ps24
Holometabola - ps25
Aparaglossata - ps26
Mecopterida - ps27
Diptera - ps28
Bibiono./Cyclorrhapha - ps29
Cyclorrhapha - ps30
Calyptratae/Drosophila - ps31
Drosophila - ps32
Sophophora - ps33
Obscura/Melanogaster_group - ps34
D_melanogaster_group - ps35
D_melanogaster_subgroup - ps36
sim./sech./mel. group - ps37
Drosophila_melanogaster - ps38
Phylostratum

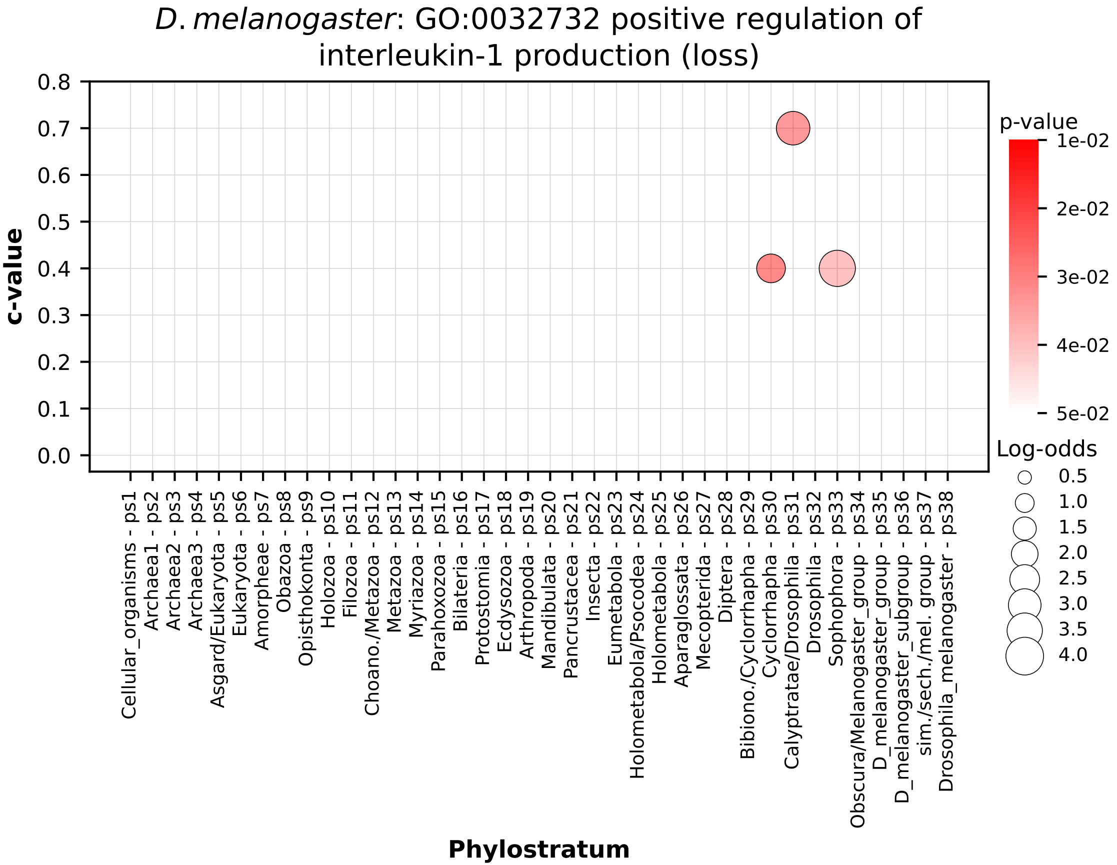

D. melanogaster: GO:0032732 positive regulation of interleukin-1 production (loss)
c-value
0.8
0.7
0.6
0.5
0.4
0.3
0.2
0.1
0.0
p-value
1e-02
2e-02
3e-02
4e-02
5e-02
Log-odds
0.5
1.0
1.5
2.0
2.5
3.0
3.5
4.0
Cellular_organisms - ps1
Archaea1 - ps2
Archaea2 - ps3
Archaea3 - ps4
Asgard/Eukaryota - ps5
Eukaryota - ps6
Amorpheae - ps7
Obazoa - ps8
Opisthokonta - ps9
Holozoa - ps10
Filozoa - ps11
Choano./Metazoa - ps12
Metazoa - ps13
Myriazoa - ps14
Parahoxozoa - ps15
Bilateria - ps16
Protostomia - ps17
Ecdysozoa - ps18
Arthropoda - ps19
Mandibulata - ps20
Pancrustacea - ps21
Insecta - ps22
Eumetabola - ps23
Holometabola/Psocodea - ps24
Holometabola - ps25
Aparaglossata - ps26
Mecopterida - ps27
Diptera - ps28
Bibiono./Cyclorrhapha - ps29
Cyclorrhapha - ps30
Calyptratae/Drosophila - ps31
Drosophila - ps32
Sophophora - ps33
Obscura/Melanogaster_group - ps34
D_melanogaster_group - ps35
D_melanogaster_subgroup - ps36
sim./sech./mel. group - ps37
Drosophila_melanogaster - ps38
Phylostratum

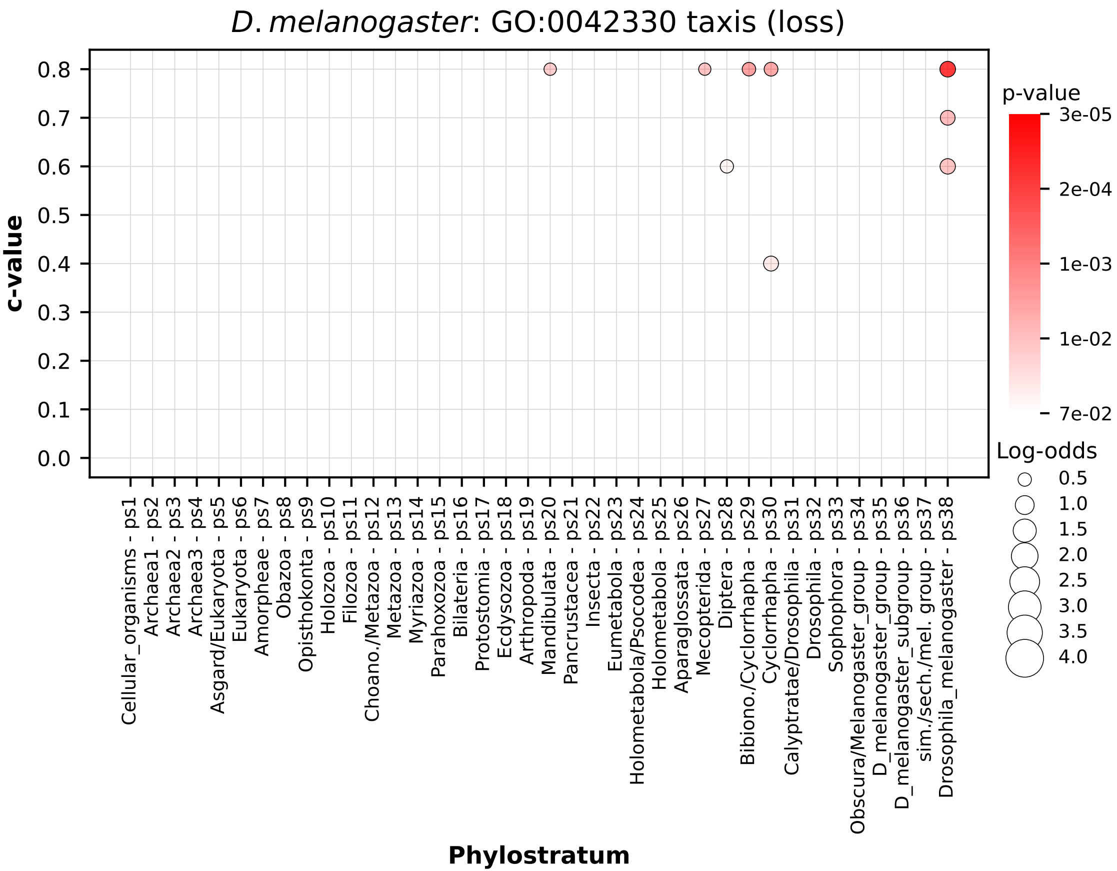

D. melanogaster: GO:0042330 taxis (loss)
c-value
0.0
0.1
0.2
0.3
0.4
0.5
0.6
0.7
0.8
Phylostratum
Cellular_organisms - ps1
Archaea1 - ps2
Archaea2 - ps3
Archaea3 - ps4
Asgard/Eukaryota - ps5
Eukaryota - ps6
Amorpheae - ps7
Obazoa - ps8
Opisthokonta - ps9
Holozoa - ps10
Filozoa - ps11
Choano./Metazoa - ps12
Metazoa - ps13
Myriazoa - ps14
Parahoxozoa - ps15
Bilateria - ps16
Protostomia - ps17
Ecdysozoa - ps18
Arthropoda - ps19
Mandibulata - ps20
Pancrustacea - ps21
Insecta - ps22
Eumetabola - ps23
Holometabola/Psocodea - ps24
Holometabola - ps25
Aparaglossata - ps26
Mecopterida - ps27
Diptera - ps28
Bibiono./Cyclorrhapha - ps29
Cyclorrhapha - ps30
Calyptratae/Drosophila - ps31
Drosophila - ps32
Sophophora - ps33
Obscura/Melanogaster_group - ps34
D_melanogaster_group - ps35
D_melanogaster_subgroup - ps36
sim./sech./mel. group - ps37
Drosophila_melanogaster - ps38
p-value
3e-05
2e-04
1e-03
1e-02
7e-02
Log-odds
0.5
1.0
1.5
2.0
2.5
3.0
3.5
4.0

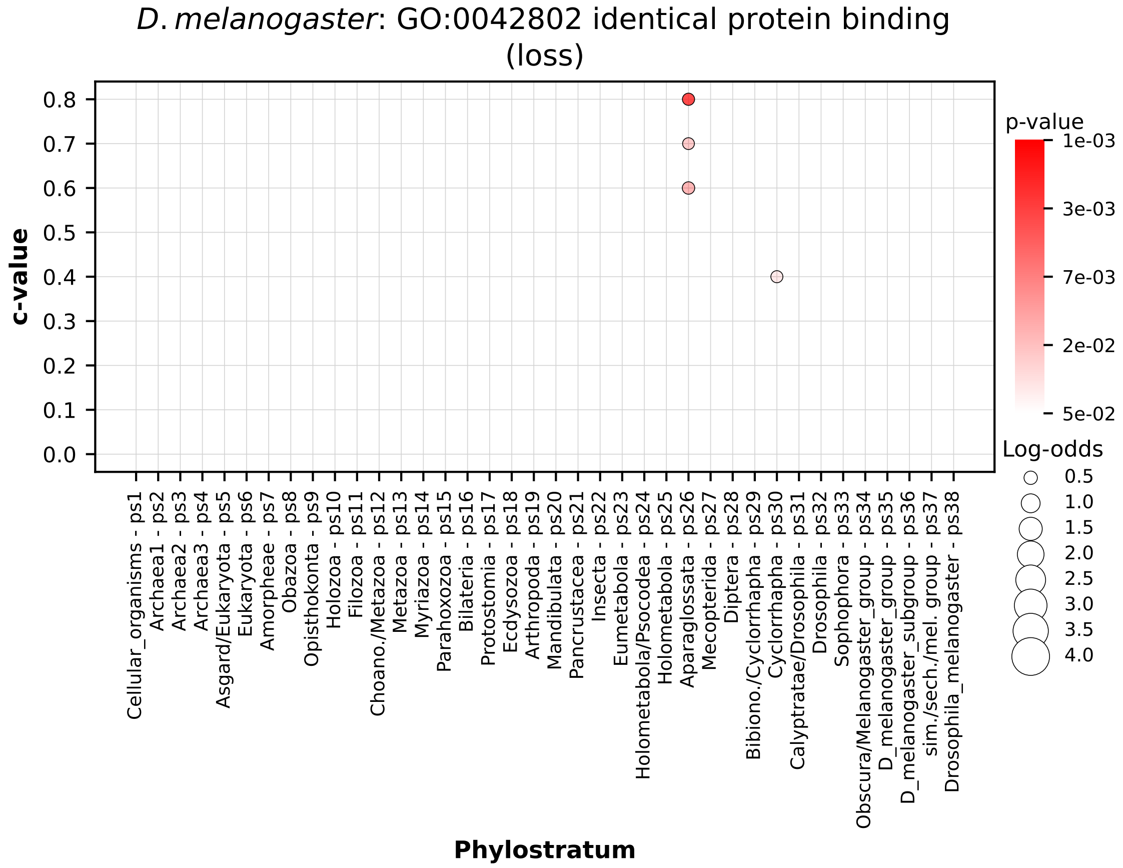

D. melanogaster: GO:0042802 identical protein binding (loss)
c-value
0.8
0.7
0.6
0.5
0.4
0.3
0.2
0.1
0.0
p-value
1e-03
3e-03
7e-03
2e-02
5e-02
Log-odds
0.5
1.0
1.5
2.0
2.5
3.0
3.5
4.0
Cellular_organisms - ps1
Archaea1 - ps2
Archaea2 - ps3
Archaea3 - ps4
Asgard/Eukaryota - ps5
Eukaryota - ps6
Amorpheae - ps7
Obazoa - ps8
Opisthokonta - ps9
Holozoa - ps10
Filozoa - ps11
Choano./Metazoa - ps12
Metazoa - ps13
Myriazoa - ps14
Parahoxozoa - ps15
Bilateria - ps16
Protostomia - ps17
Ecdysozoa - ps18
Arthropoda - ps19
Mandibulata - ps20
Pancrustacea - ps21
Insecta - ps22
Eumetabola - ps23
Holometabola/Psocodea - ps24
Holometabola - ps25
Aparaglossata - ps26
Mecopterida - ps27
Diptera - ps28
Bibiono./Cyclorrhapha - ps29
Cyclorrhapha - ps30
Calyptratae/Drosophila - ps31
Drosophila - ps32
Sophophora - ps33
Obscura/Melanogaster_group - ps34
D_melanogaster_group - ps35
D_melanogaster_subgroup - ps36
sim./sech./mel. group - ps37
Drosophila_melanogaster - ps38
Phylostratum

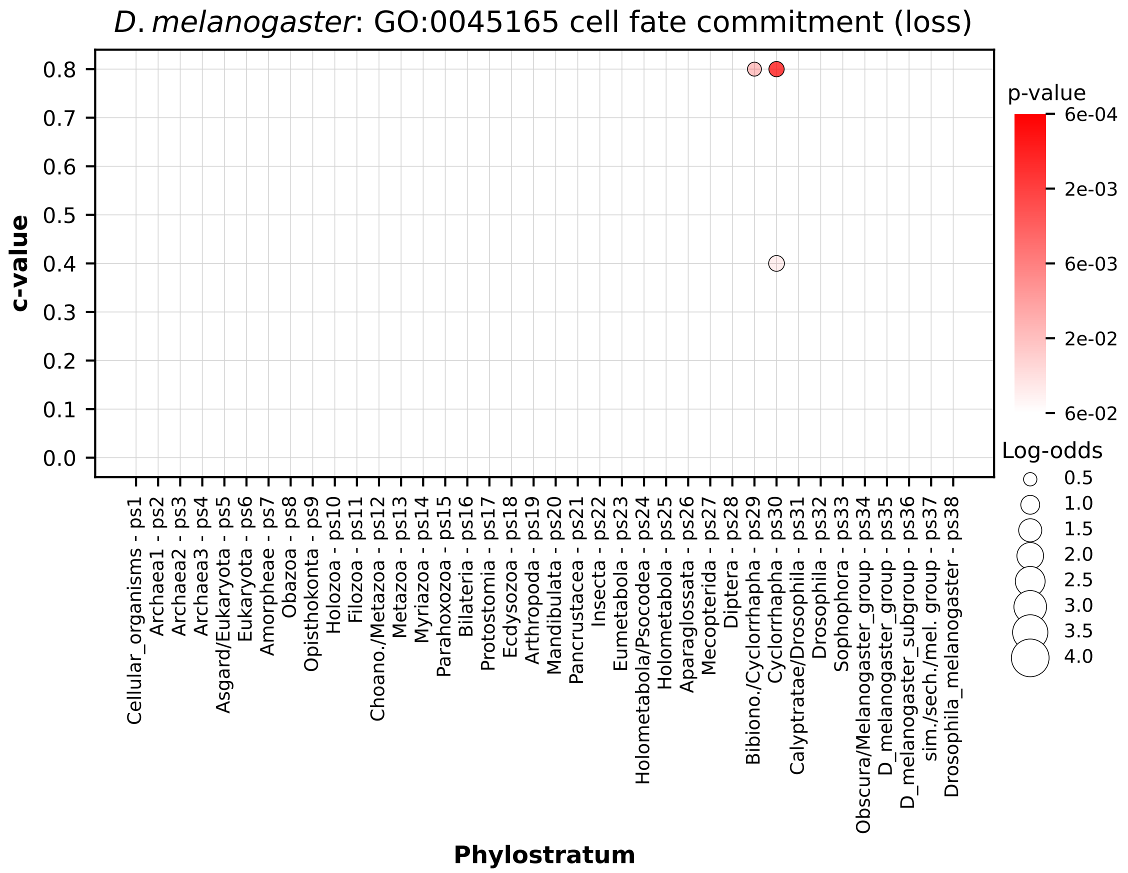
D. melanogaster: GO:0045165 cell fate commitment (loss)
c-value
0.0
0.1
0.2
0.3
0.4
0.5
0.6
0.7
0.8
Phylostratum
Cellular_organisms - ps1
Archaea1 - ps2
Archaea2 - ps3
Archaea3 - ps4
Asgard/Eukaryota - ps5
Eukaryota - ps6
Amorpheae - ps7
Obazoa - ps8
Opisthokonta - ps9
Holozoa - ps10
Filozoa - ps11
Choano./Metazoa - ps12
Metazoa - ps13
Myriazoa - ps14
Parahoxozoa - ps15
Bilateria - ps16
Protostomia - ps17
Ecdysozoa - ps18
Arthropoda - ps19
Mandibulata - ps20
Pancrustacea - ps21
Insecta - ps22
Eumetabola - ps23
Holometabola/Psocodea - ps24
Holometabola - ps25
Aparaglossata - ps26
Mecopterida - ps27
Diptera - ps28
Bibiono./Cyclorrhapha - ps29
Cyclorrhapha - ps30
Calyptratae/Drosophila - ps31
Drosophila - ps32
Sophophora - ps33
Obscura/Melanogaster_group - ps34
D_melanogaster_group - ps35
D_melanogaster_subgroup - ps36
sim./sech./mel. group - ps37
Drosophila_melanogaster - ps38
p-value
6e-04
2e-03
6e-03
2e-02
6e-02
Log-odds
0.5
1.0
1.5
2.0
2.5
3.0
3.5
4.0

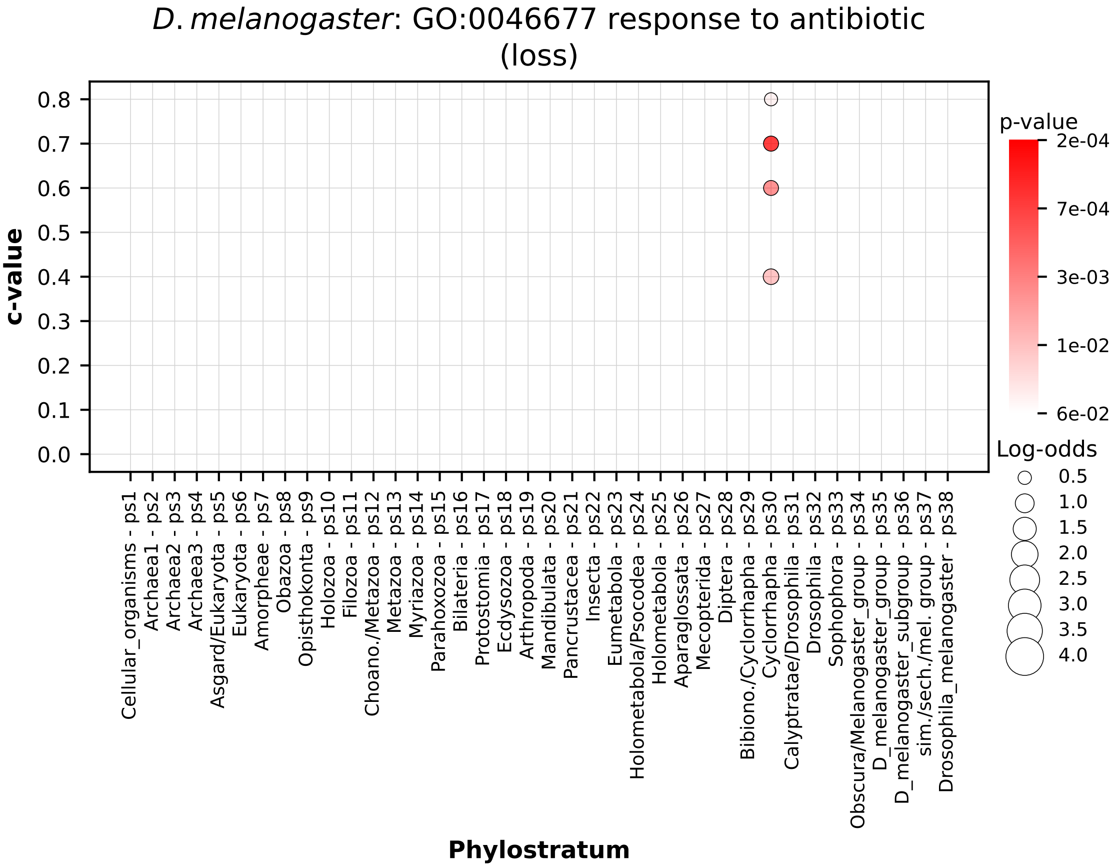

$D. melanogaster$: GO:0046677 response to antibiotic (loss)
c-value
0.8
0.7
0.6
0.5
0.4
0.3
0.2
0.1
0.0
p-value
2e-04
7e-04
3e-03
1e-02
6e-02
Log-odds
0.5
1.0
1.5
2.0
2.5
3.0
3.5
4.0
Cellular_organisms - ps1
Archaea1 - ps2
Archaea2 - ps3
Archaea3 - ps4
Asgard/Eukaryota - ps5
Eukaryota - ps6
Amorpheae - ps7
Obazoa - ps8
Opisthokonta - ps9
Holozoa - ps10
Filozoa - ps11
Choano./Metazoa - ps12
Metazoa - ps13
Myriazoa - ps14
Parahoxozoa - ps15
Bilateria - ps16
Protostomia - ps17
Ecdysozoa - ps18
Arthropoda - ps19
Mandibulata - ps20
Pancrustacea - ps21
Insecta - ps22
Eumetabola - ps23
Holometabola/Psocodea - ps24
Holometabola - ps25
Aparaglossata - ps26
Mecopterida - ps27
Diptera - ps28
Bibiono./Cyclorrhapha - ps29
Cyclorrhapha - ps30
Calyptratae/Drosophila - ps31
Drosophila - ps32
Sophophora - ps33
Obscura/Melanogaster_group - ps34
D_melanogaster_group - ps35
D_melanogaster_subgroup - ps36
sim./sech./mel. group - ps37
Drosophila_melanogaster - ps38
Phylostratum

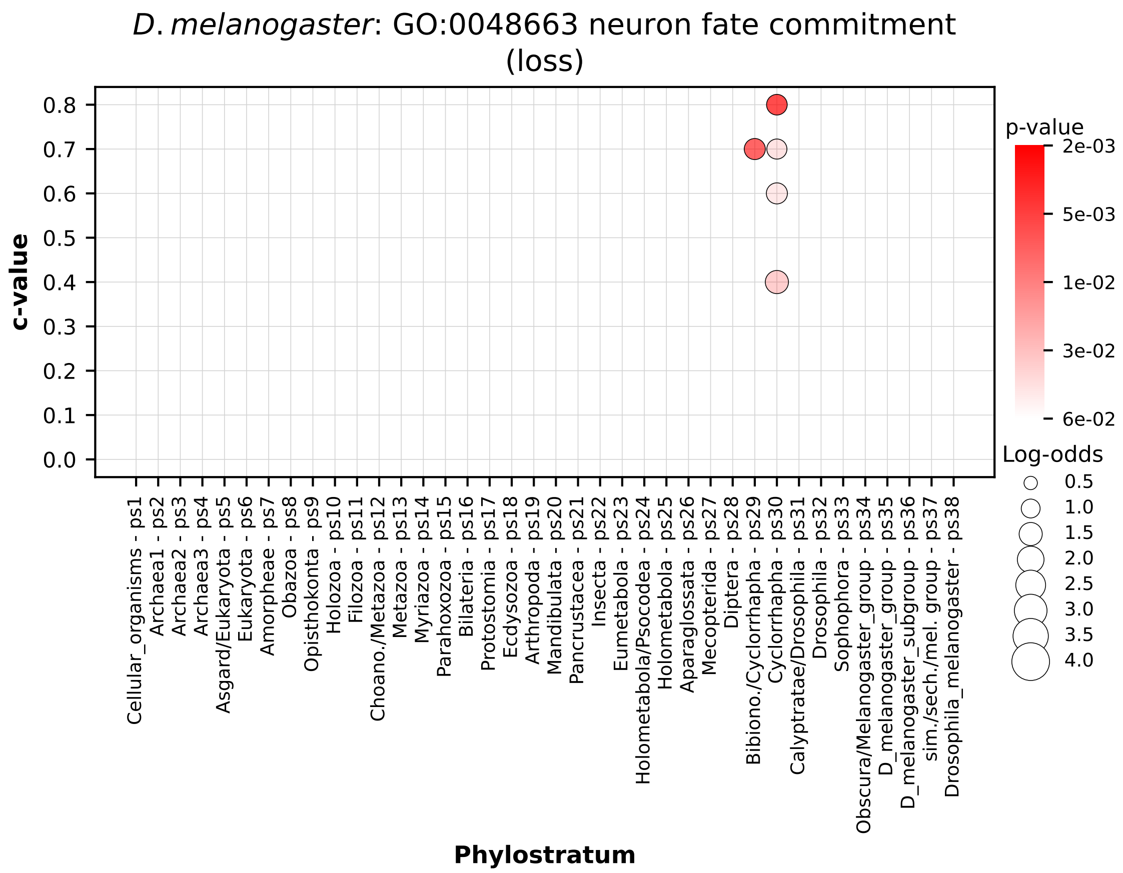

D. melanogaster: GO:0048663 neuron fate commitment
(loss)
c-value
0.0
0.1
0.2
0.3
0.4
0.5
0.6
0.7
0.8
Phylostratum
Cellular_organisms - ps1
Archaea1 - ps2
Archaea2 - ps3
Archaea3 - ps4
Asgard/Eukaryota - ps5
Eukaryota - ps6
Amorpheae - ps7
Obazoa - ps8
Opisthokonta - ps9
Holozoa - ps10
Filozoa - ps11
Choano./Metazoa - ps12
Metazoa - ps13
Myriazoa - ps14
Parahoxozoa - ps15
Bilateria - ps16
Protostomia - ps17
Ecdysozoa - ps18
Arthropoda - ps19
Mandibulata - ps20
Pancrustacea - ps21
Insecta - ps22
Eumetabola - ps23
Holometabola/Psocodea - ps24
Holometabola - ps25
Aparaglossata - ps26
Mecopterida - ps27
Diptera - ps28
Bibiono./Cyclorrhapha - ps29
Cyclorrhapha - ps30
Calyptratae/Drosophila - ps31
Drosophila - ps32
Sophophora - ps33
Obscura/Melanogaster_group - ps34
D_melanogaster_group - ps35
D_melanogaster_subgroup - ps36
sim./sech./mel. group - ps37
Drosophila_melanogaster - ps38
p-value
2e-03
5e-03
1e-02
3e-02
6e-02
Log-odds
0.5
1.0
1.5
2.0
2.5
3.0
3.5
4.0

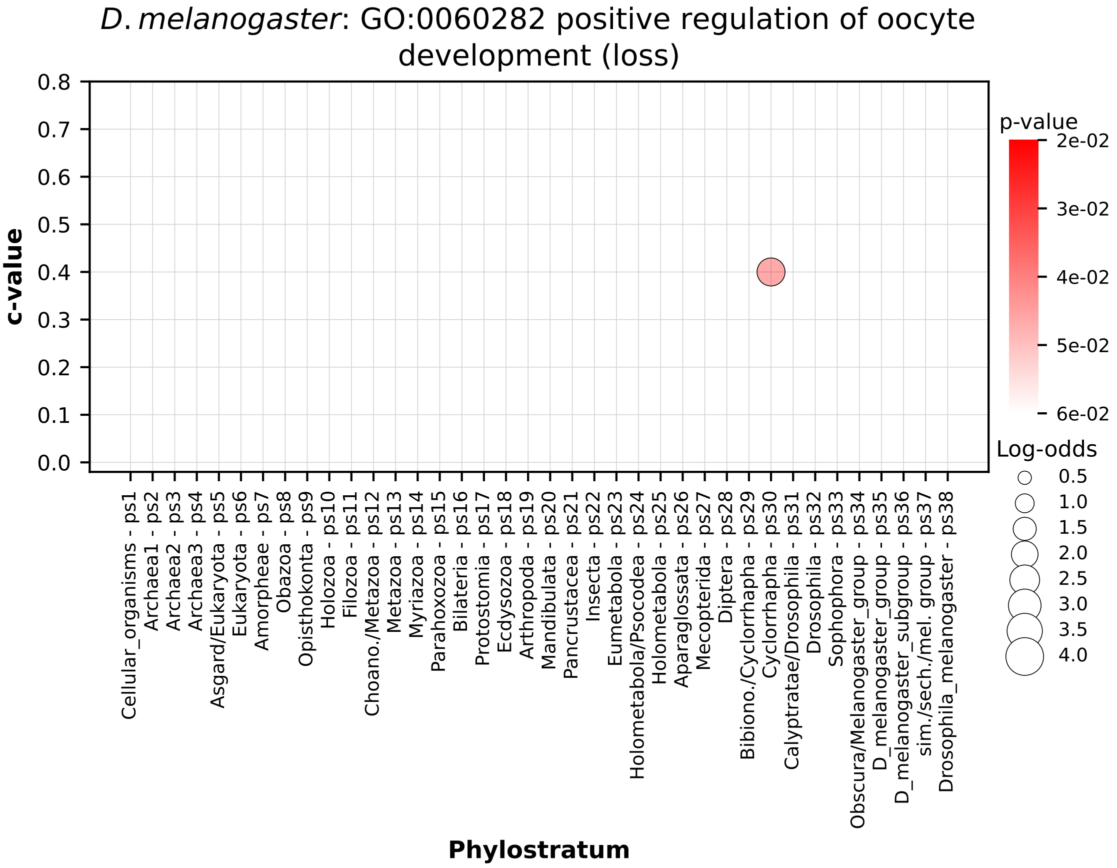

D. melanogaster: GO:0060282 positive regulation of oocyte development (loss)
c-value
0.8
0.7
0.6
0.5
0.4
0.3
0.2
0.1
0.0
p-value
2e-02
3e-02
4e-02
5e-02
6e-02
Log-odds
0.5
1.0
1.5
2.0
2.5
3.0
3.5
4.0
Cellular_organisms - ps1
Archaea1 - ps2
Archaea2 - ps3
Archaea3 - ps4
Asgard/Eukaryota - ps5
Eukaryota - ps6
Amorpheae - ps7
Obazoa - ps8
Opisthokonta - ps9
Holozoa - ps10
Filozoa - ps11
Choano./Metazoa - ps12
Metazoa - ps13
Myriazoa - ps14
Parahoxozoa - ps15
Bilateria - ps16
Protostomia - ps17
Ecdysozoa - ps18
Arthropoda - ps19
Mandibulata - ps20
Pancrustacea - ps21
Insecta - ps22
Eumetabola - ps23
Holometabola/Psocodea - ps24
Holometabola - ps25
Aparaglossata - ps26
Mecopterida - ps27
Diptera - ps28
Bibiono./Cyclorrhapha - ps29
Cyclorrhapha - ps30
Calyptratae/Drosophila - ps31
Drosophila - ps32
Sophophora - ps33
Obscura/Melanogaster_group - ps34
D_melanogaster_group - ps35
D_melanogaster_subgroup - ps36
sim./sech./mel. group - ps37
Drosophila_melanogaster - ps38
Phylostratum

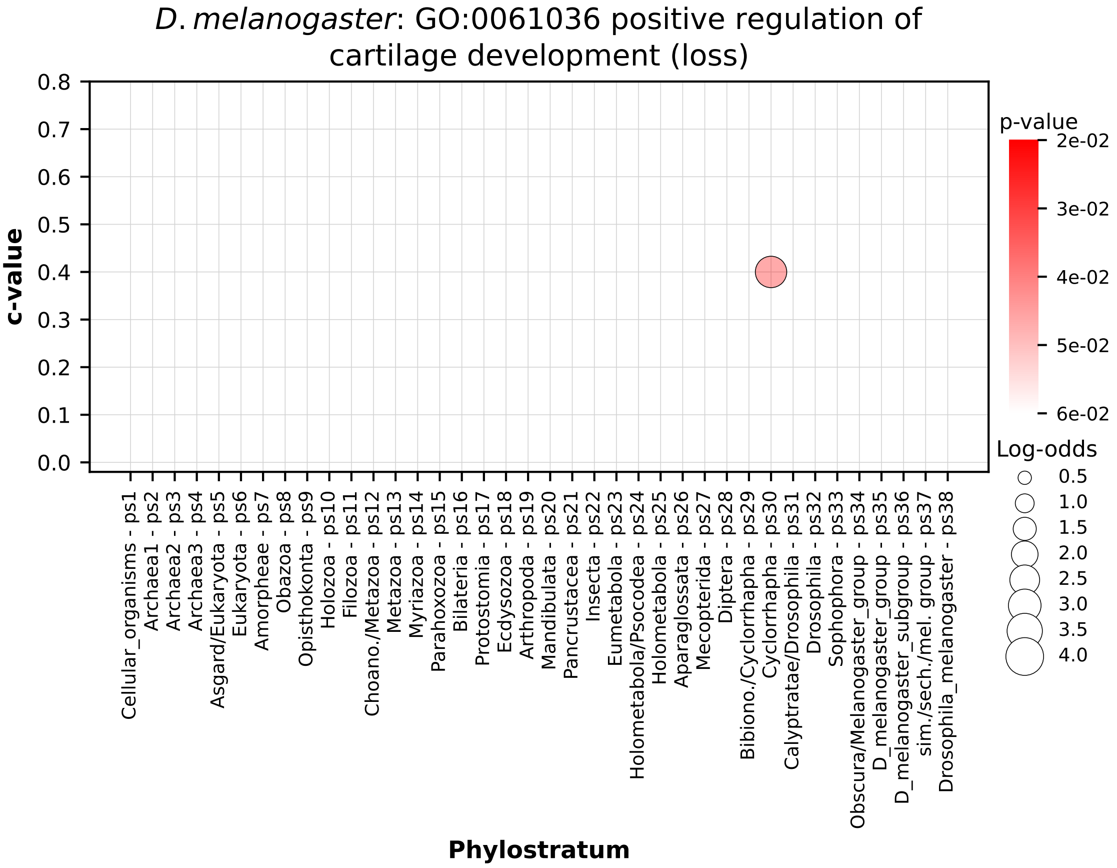
D. melanogaster: GO:0061036 positive regulation of cartilage development (loss)
c-value
0.0
0.1
0.2
0.3
0.4
0.5
0.6
0.7
0.8
Phylostratum
Cellular_organisms - ps1
Archaea1 - ps2
Archaea2 - ps3
Archaea3 - ps4
Asgard/Eukaryota - ps5
Eukaryota - ps6
Amorpheae - ps7
Obazoa - ps8
Opisthokonta - ps9
Holozoa - ps10
Filozoa - ps11
Choano./Metazoa - ps12
Metazoa - ps13
Myriazoa - ps14
Parahoxozoa - ps15
Bilateria - ps16
Protostomia - ps17
Ecdysozoa - ps18
Arthropoda - ps19
Mandibulata - ps20
Pancrustacea - ps21
Insecta - ps22
Eumetabola - ps23
Holometabola/Psocodea - ps24
Holometabola - ps25
Aparaglossata - ps26
Mecopterida - ps27
Diptera - ps28
Bibiono./Cyclorrhapha - ps29
Cyclorrhapha - ps30
Calyptratae/Drosophila - ps31
Drosophila - ps32
Sophophora - ps33
Obscura/Melanogaster_group - ps34
D_melanogaster_group - ps35
D_melanogaster_subgroup - ps36
sim./sech./mel. group - ps37
Drosophila_melanogaster - ps38
p-value
2e-02
3e-02
4e-02
5e-02
6e-02
Log-odds
0.5
1.0
1.5
2.0
2.5
3.0
3.5
4.0

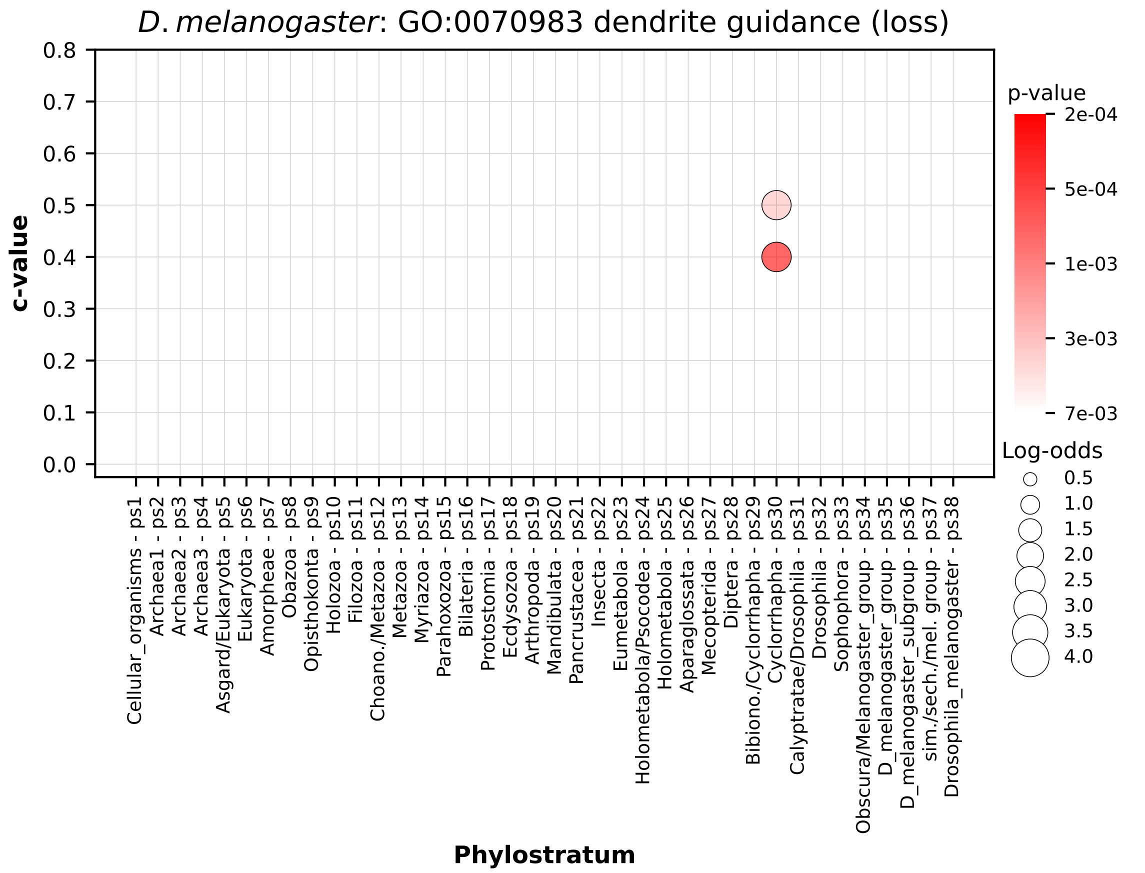

D. melanogaster: GO:0070983 dendrite guidance (loss)
c-value
0.8
0.7
0.6
0.5
0.4
0.3
0.2
0.1
0.0
Phylostratum
Cellular_organisms - ps1
Archaea1 - ps2
Archaea2 - ps3
Archaea3 - ps4
Asgard/Eukaryota - ps5
Eukaryota - ps6
Amorpheae - ps7
Obazoa - ps8
Opisthokonta - ps9
Holozoa - ps10
Filozoa - ps11
Choano./Metazoa - ps12
Metazoa - ps13
Myriazoa - ps14
Parahoxozoa - ps15
Bilateria - ps16
Protostomia - ps17
Ecdysozoa - ps18
Arthropoda - ps19
Mandibulata - ps20
Pancrustacea - ps21
Insecta - ps22
Eumetabola - ps23
Holometabola/Psocodea - ps24
Holometabola - ps25
Aparaglossata - ps26
Mecopterida - ps27
Diptera - ps28
Bibiono./Cyclorrhapha - ps29
Cyclorrhapha - ps30
Calyptratae/Drosophila - ps31
Drosophila - ps32
Sophophora - ps33
Obscura/Melanogaster_group - ps34
D_melanogaster_group - ps35
D_melanogaster_subgroup - ps36
sim./sech./mel. group - ps37
Drosophila_melanogaster - ps38
p-value
2e-04
5e-04
1e-03
3e-03
7e-03
Log-odds
0.5
1.0
1.5
2.0
2.5
3.0
3.5
4.0

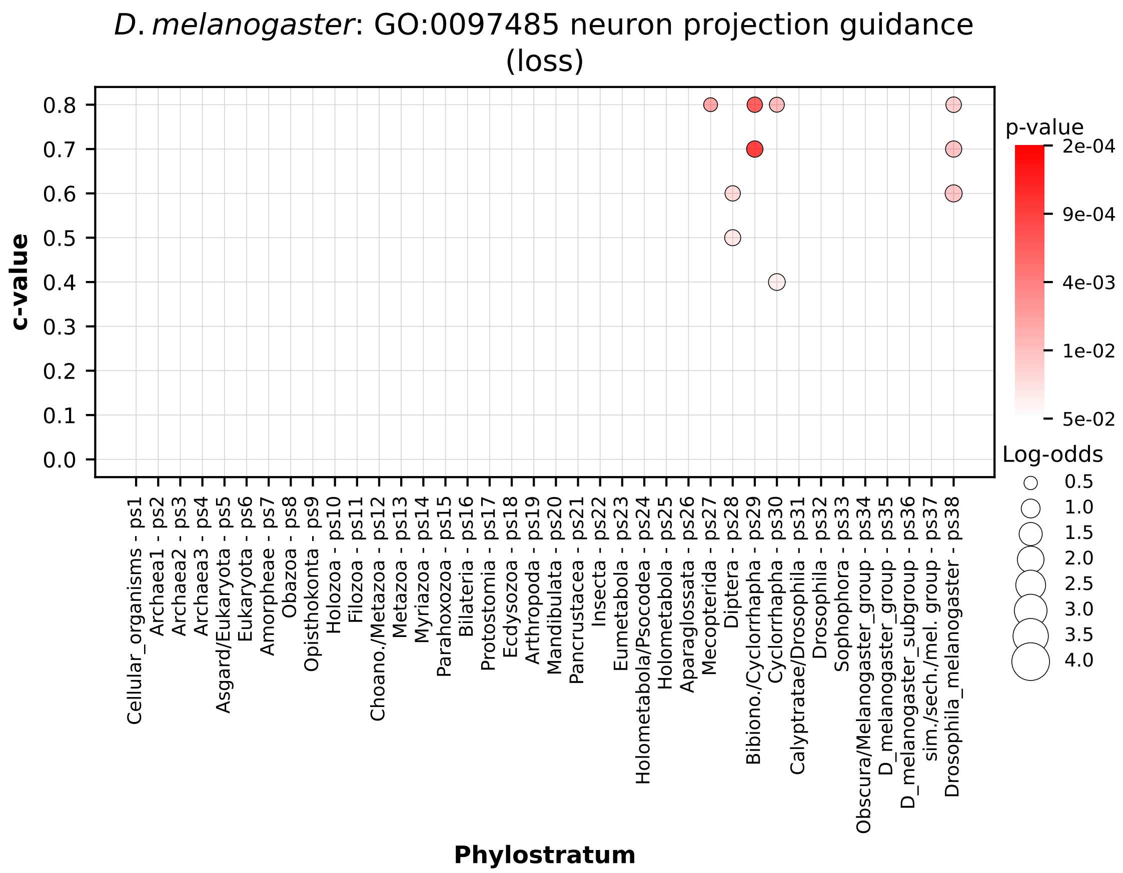

D. melanogaster: GO:0097485 neuron projection guidance (loss)
c-value
0.0
0.1
0.2
0.3
0.4
0.5
0.6
0.7
0.8
Phylostratum
Cellular_organisms - ps1
Archaea1 - ps2
Archaea2 - ps3
Archaea3 - ps4
Asgard/Eukaryota - ps5
Eukaryota - ps6
Amorpheae - ps7
Obazoa - ps8
Opisthokonta - ps9
Holozoa - ps10
Filozoa - ps11
Choano./Metazoa - ps12
Metazoa - ps13
Myriazoa - ps14
Parahoxozoa - ps15
Bilateria - ps16
Protostomia - ps17
Ecdysozoa - ps18
Arthropoda - ps19
Mandibulata - ps20
Pancrustacea - ps21
Insecta - ps22
Eumetabola - ps23
Holometabola/Psocodea - ps24
Holometabola - ps25
Aparaglossata - ps26
Mecopterida - ps27
Diptera - ps28
Bibiono./Cyclorrhapha - ps29
Cyclorrhapha - ps30
Calyptratae/Drosophila - ps31
Drosophila - ps32
Sophophora - ps33
Obscura/Melanogaster_group - ps34
D_melanogaster_group - ps35
D_melanogaster_subgroup - ps36
sim./sech./mel. group - ps37
Drosophila_melanogaster - ps38
p-value
2e-04
9e-04
4e-03
1e-02
5e-02
Log-odds
0.5
1.0
1.5
2.0
2.5
3.0
3.5
4.0

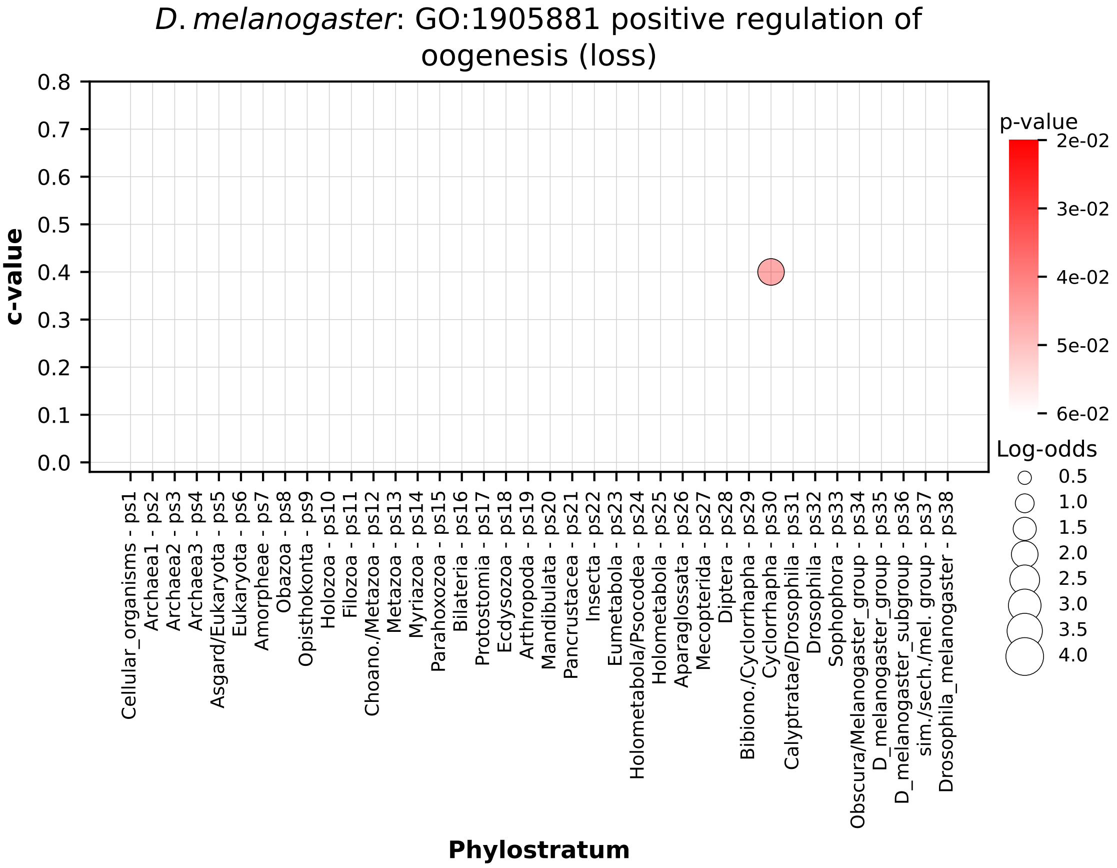

$D. melanogaster$: GO:1905881 positive regulation of oogenesis (loss)
c-value
Phylostratum
p-value
2e-02
3e-02
4e-02
5e-02
6e-02
Log-odds
0.5
1.0
1.5
2.0
2.5
3.0
3.5
4.0
0.0
0.1
0.2
0.3
0.4
0.5
0.6
0.7
0.8
Cellular_organisms - ps1
Archaea1 - ps2
Archaea2 - ps3
Archaea3 - ps4
Asgard/Eukaryota - ps5
Eukaryota - ps6
Amorpheae - ps7
Obazoa - ps8
Opisthokonta - ps9
Holozoa - ps10
Filozoa - ps11
Choano./Metazoa - ps12
Metazoa - ps13
Myriazoa - ps14
Parahoxozoa - ps15
Bilateria - ps16
Protostomia - ps17
Ecdysozoa - ps18
Arthropoda - ps19
Mandibulata - ps20
Pancrustacea - ps21
Insecta - ps22
Eumetabola - ps23
Holometabola/Psocodea - ps24
Holometabola - ps25
Aparaglossata - ps26
Mecopterida - ps27
Diptera - ps28
Bibiono./Cyclorrhapha - ps29
Cyclorrhapha - ps30
Calyptratae/Drosophila - ps31
Drosophila - ps32
Sophophora - ps33
Obscura/Melanogaster_group - ps34
D_melanogaster_group - ps35
D_melanogaster_subgroup - ps36
sim./sech./mel. group - ps37
Drosophila_melanogaster - ps38

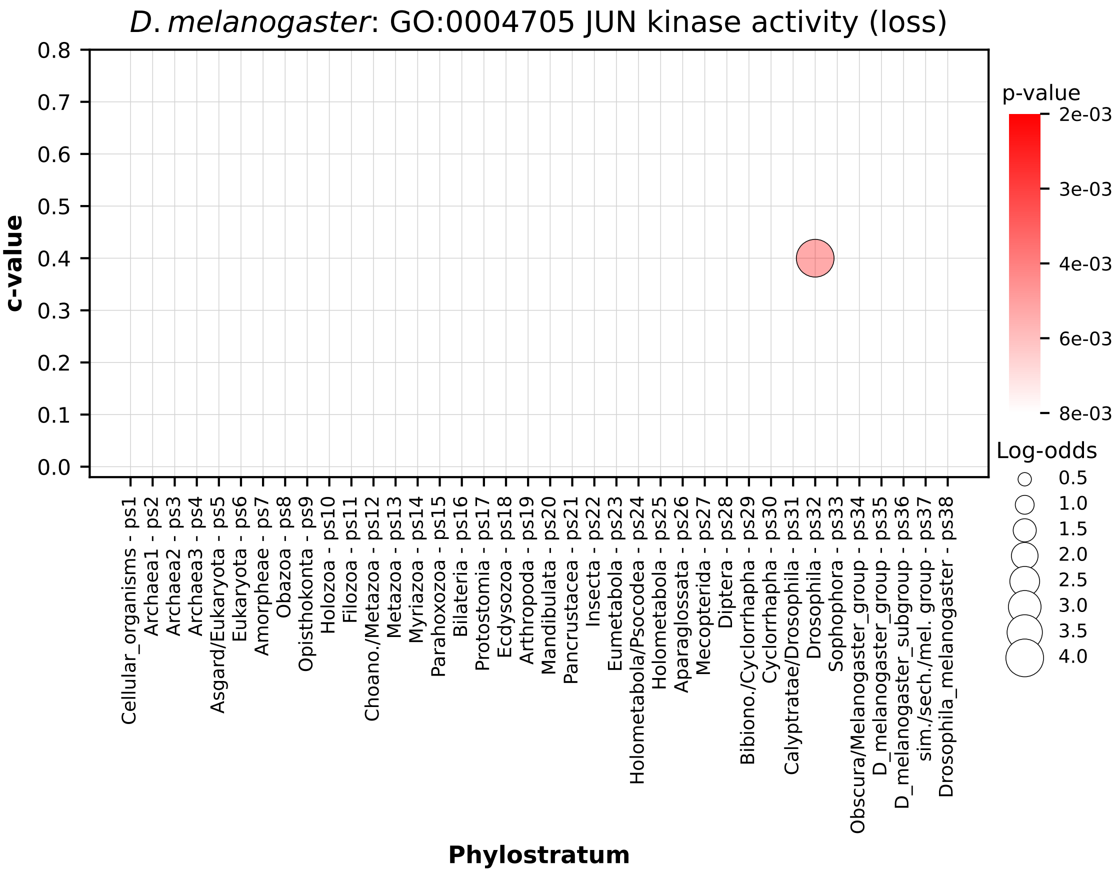

D. melanogaster: GO:0004705 JUN kinase activity (loss)
c-value
0.0
0.1
0.2
0.3
0.4
0.5
0.6
0.7
0.8
p-value
2e-03
3e-03
4e-03
6e-03
8e-03
Log-odds
0.5
1.0
1.5
2.0
2.5
3.0
3.5
4.0
Cellular_organisms - ps1
Archaea1 - ps2
Archaea2 - ps3
Archaea3 - ps4
Asgard/Eukaryota - ps5
Eukaryota - ps6
Amorpheae - ps7
Obazoa - ps8
Opisthokonta - ps9
Holozoa - ps10
Filozoa - ps11
Choano./Metazoa - ps12
Metazoa - ps13
Myriazoa - ps14
Parahoxozoa - ps15
Bilateria - ps16
Protostomia - ps17
Ecdysozoa - ps18
Arthropoda - ps19
Mandibulata - ps20
Pancrustacea - ps21
Insecta - ps22
Eumetabola - ps23
Holometabola/Psocodea - ps24
Holometabola - ps25
Aparaglossata - ps26
Mecopterida - ps27
Diptera - ps28
Bibiono./Cyclorrhapha - ps29
Cyclorrhapha - ps30
Calyptratae/Drosophila - ps31
Drosophila - ps32
Sophophora - ps33
Obscura/Melanogaster_group - ps34
D_melanogaster_group - ps35
D_melanogaster_subgroup - ps36
sim./sech./mel. group - ps37
Drosophila_melanogaster - ps38
Phylostratum

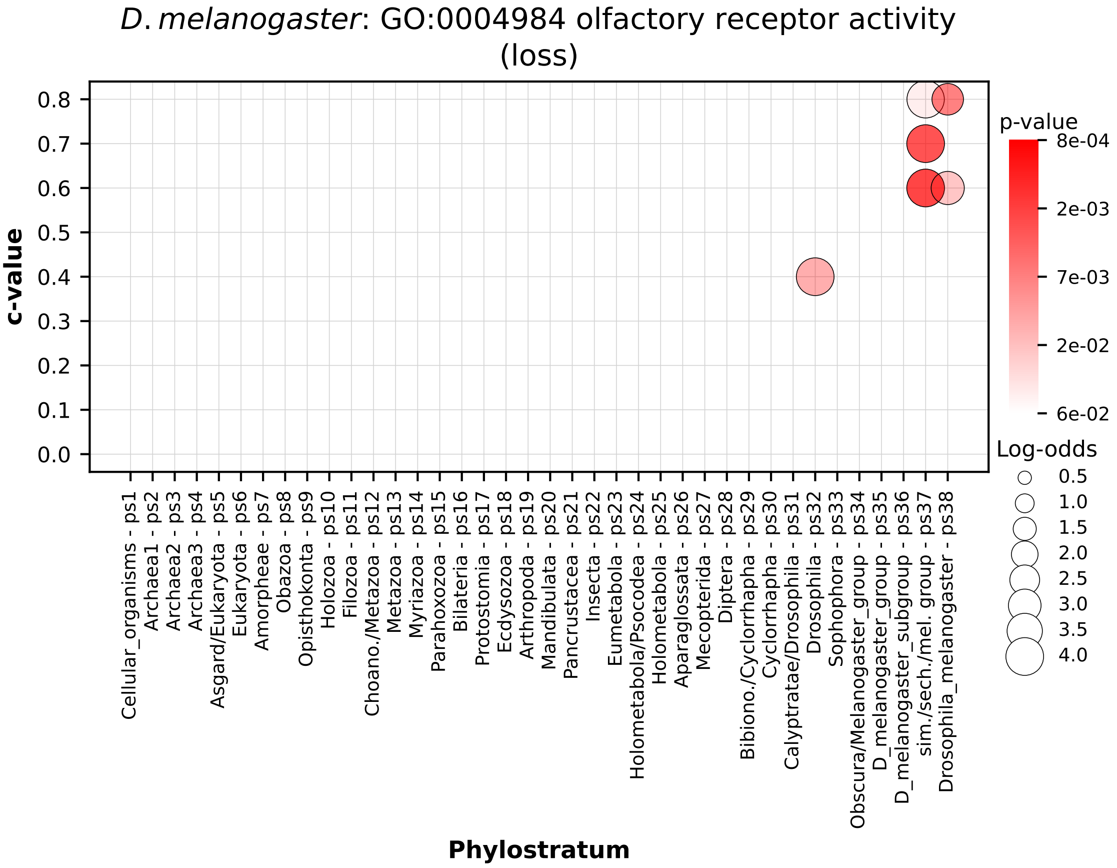
D. melanogaster: GO:0004984 olfactory receptor activity
(loss)
c-value
0.8
0.7
0.6
0.5
0.4
0.3
0.2
0.1
0.0
Phylostratum
Cellular_organisms - ps1
Archaea1 - ps2
Archaea2 - ps3
Archaea3 - ps4
Asgard/Eukaryota - ps5
Eukaryota - ps6
Amorpheae - ps7
Obazoa - ps8
Opisthokonta - ps9
Holozoa - ps10
Filozoa - ps11
Choano./Metazoa - ps12
Metazoa - ps13
Myriazoa - ps14
Parahoxozoa - ps15
Bilateria - ps16
Protostomia - ps17
Ecdysozoa - ps18
Arthropoda - ps19
Mandibulata - ps20
Pancrustacea - ps21
Insecta - ps22
Eumetabola - ps23
Holometabola/Psocodea - ps24
Holometabola - ps25
Aparaglossata - ps26
Mecopterida - ps27
Diptera - ps28
Bibiono./Cyclorrhapha - ps29
Cyclorrhapha - ps30
Calyptratae/Drosophila - ps31
Drosophila - ps32
Sophophora - ps33
Obscura/Melanogaster_group - ps34
D_melanogaster_group - ps35
D_melanogaster_subgroup - ps36
sim./sech./mel. group - ps37
Drosophila_melanogaster - ps38
p-value
8e-04
2e-03
7e-03
2e-02
6e-02
Log-odds
0.5
1.0
1.5
2.0
2.5
3.0
3.5
4.0

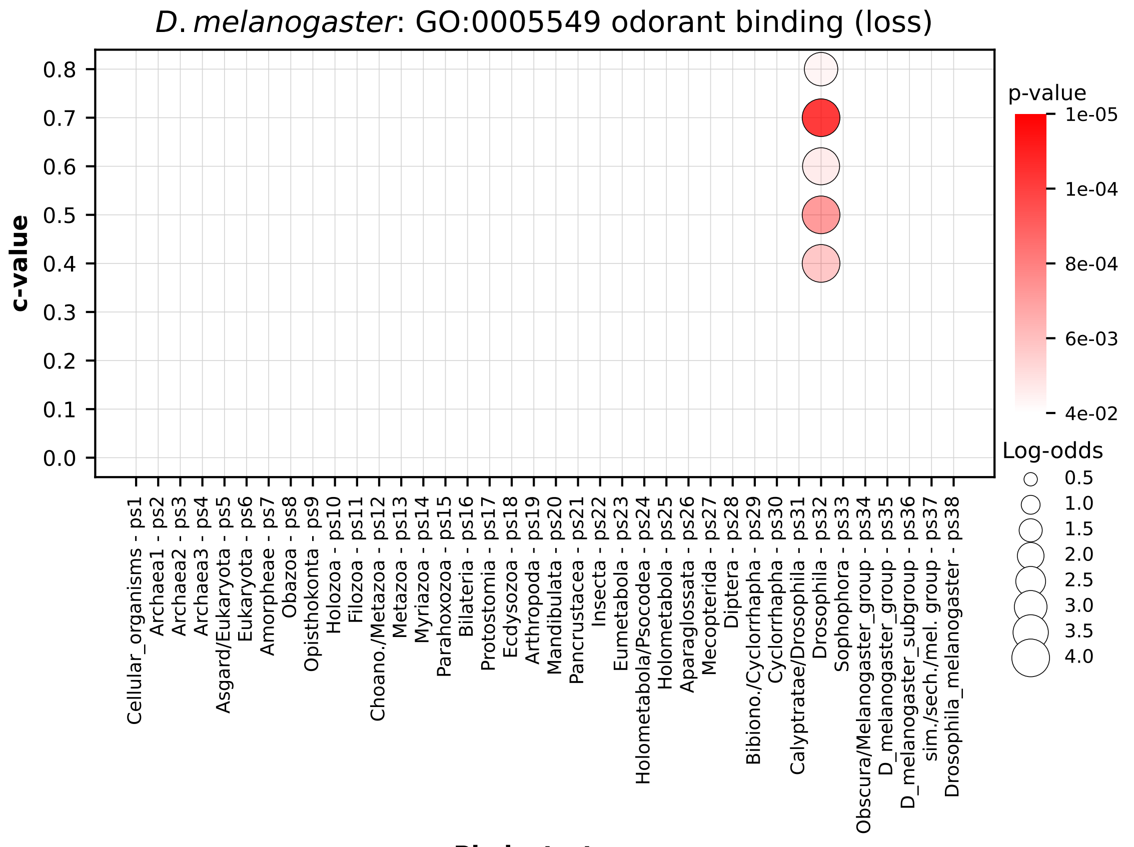
D. melanogaster: GO:0005549 odorant binding (loss)
c-value
0.8
0.7
0.6
0.5
0.4
0.3
0.2
0.1
0.0
p-value
1e-05
1e-04
8e-04
6e-03
4e-02
Log-odds
0.5
1.0
1.5
2.0
2.5
3.0
3.5
4.0
Cellular_organisms - ps1
Archaea1 - ps2
Archaea2 - ps3
Archaea3 - ps4
Asgard/Eukaryota - ps5
Eukaryota - ps6
Amorpheae - ps7
Obazoa - ps8
Opisthokonta - ps9
Holozoa - ps10
Filozoa - ps11
Choano./Metazoa - ps12
Metazoa - ps13
Myriazoa - ps14
Parahoxozoa - ps15
Bilateria - ps16
Protostomia - ps17
Ecdysozoa - ps18
Arthropoda - ps19
Mandibulata - ps20
Pancrustacea - ps21
Insecta - ps22
Eumetabola - ps23
Holometabola/Psocodea - ps24
Holometabola - ps25
Aparaglossata - ps26
Mecopterida - ps27
Diptera - ps28
Bibiono./Cyclorrhapha - ps29
Cyclorrhapha - ps30
Calyptratae/Drosophila - ps31
Drosophila - ps32
Sophophora - ps33
Obscura/Melanogaster_group - ps34
D_melanogaster_group - ps35
D_melanogaster_subgroup - ps36
sim./sech./mel. group - ps37
Drosophila_melanogaster - ps38
Phylostratum

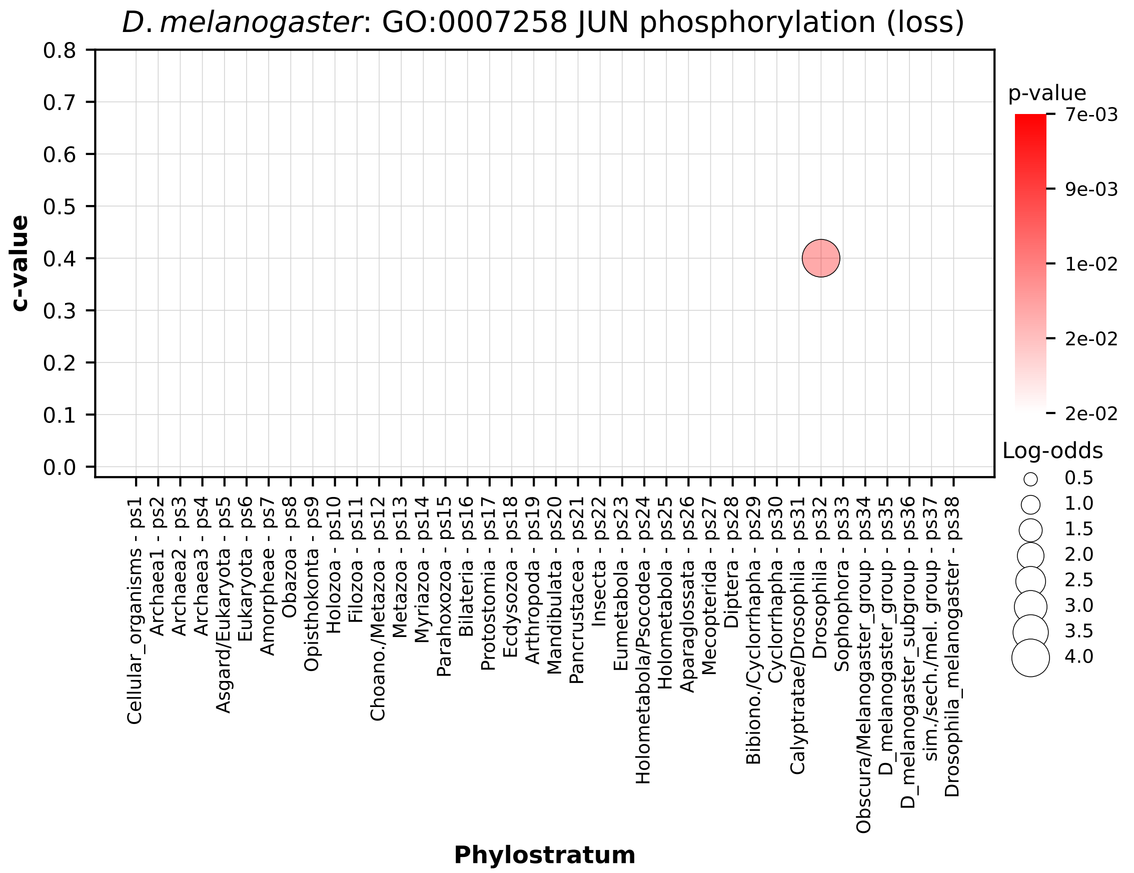
D. melanogaster: GO:0007258 JUN phosphorylation (loss)
c-value
0.0
0.1
0.2
0.3
0.4
0.5
0.6
0.7
0.8
Phylostratum
Cellular_organisms - ps1
Archaea1 - ps2
Archaea2 - ps3
Archaea3 - ps4
Asgard/Eukaryota - ps5
Eukaryota - ps6
Amorpheae - ps7
Obazoa - ps8
Opisthokonta - ps9
Holozoa - ps10
Filozoa - ps11
Choano./Metazoa - ps12
Metazoa - ps13
Myriazoa - ps14
Parahoxozoa - ps15
Bilateria - ps16
Protostomia - ps17
Ecdysozoa - ps18
Arthropoda - ps19
Mandibulata - ps20
Pancrustacea - ps21
Insecta - ps22
Eumetabola - ps23
Holometabola/Psocodea - ps24
Holometabola - ps25
Aparaglossata - ps26
Mecopterida - ps27
Diptera - ps28
Bibiono./Cyclorrhapha - ps29
Cyclorrhapha - ps30
Calyptratae/Drosophila - ps31
Drosophila - ps32
Sophophora - ps33
Obscura/Melanogaster_group - ps34
D_melanogaster_group - ps35
D_melanogaster_subgroup - ps36
sim./sech./mel. group - ps37
Drosophila_melanogaster - ps38
p-value
7e-03
9e-03
1e-02
2e-02
2e-02
Log-odds
0.5
1.0
1.5
2.0
2.5
3.0
3.5
4.0

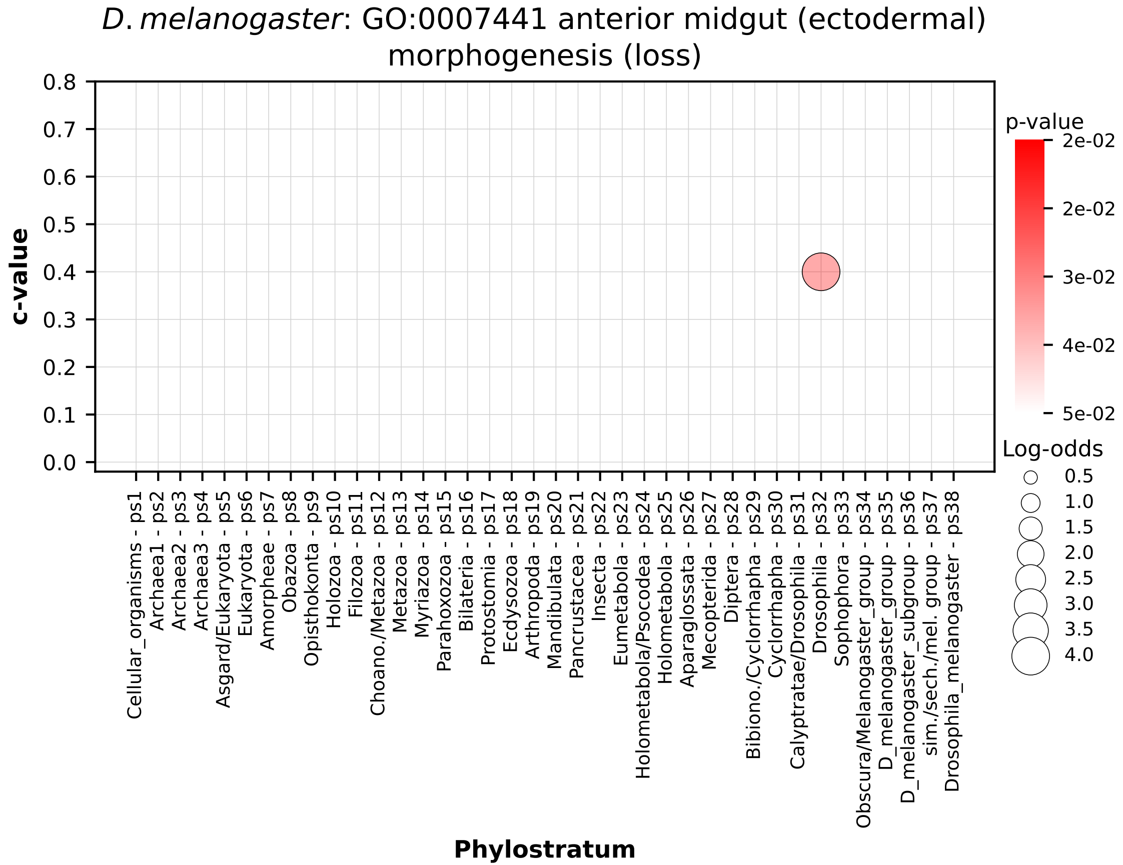

D. melanogaster: GO:0007441 anterior midgut (ectodermal) morphogenesis (loss)
c-value
0.8
0.7
0.6
0.5
0.4
0.3
0.2
0.1
0.0
p-value
2e-02
2e-02
3e-02
4e-02
5e-02
Log-odds
0.5
1.0
1.5
2.0
2.5
3.0
3.5
4.0
Cellular_organisms - ps1
Archaea1 - ps2
Archaea2 - ps3
Archaea3 - ps4
Asgard/Eukaryota - ps5
Eukaryota - ps6
Amorpheae - ps7
Obazoa - ps8
Opisthokonta - ps9
Holozoa - ps10
Filozoa - ps11
Choano./Metazoa - ps12
Metazoa - ps13
Myriazoa - ps14
Parahoxozoa - ps15
Bilateria - ps16
Protostomia - ps17
Ecdysozoa - ps18
Arthropoda - ps19
Mandibulata - ps20
Pancrustacea - ps21
Insecta - ps22
Eumetabola - ps23
Holometabola/Psocodea - ps24
Holometabola - ps25
Aparaglossata - ps26
Mecopterida - ps27
Diptera - ps28
Bibiono./Cyclorrhapha - ps29
Cyclorrhapha - ps30
Calyptratae/Drosophila - ps31
Drosophila - ps32
Sophophora - ps33
Obscura/Melanogaster_group - ps34
D_melanogaster_group - ps35
D_melanogaster_subgroup - ps36
sim./sech./mel. group - ps37
Drosophila_melanogaster - ps38
Phylostratum

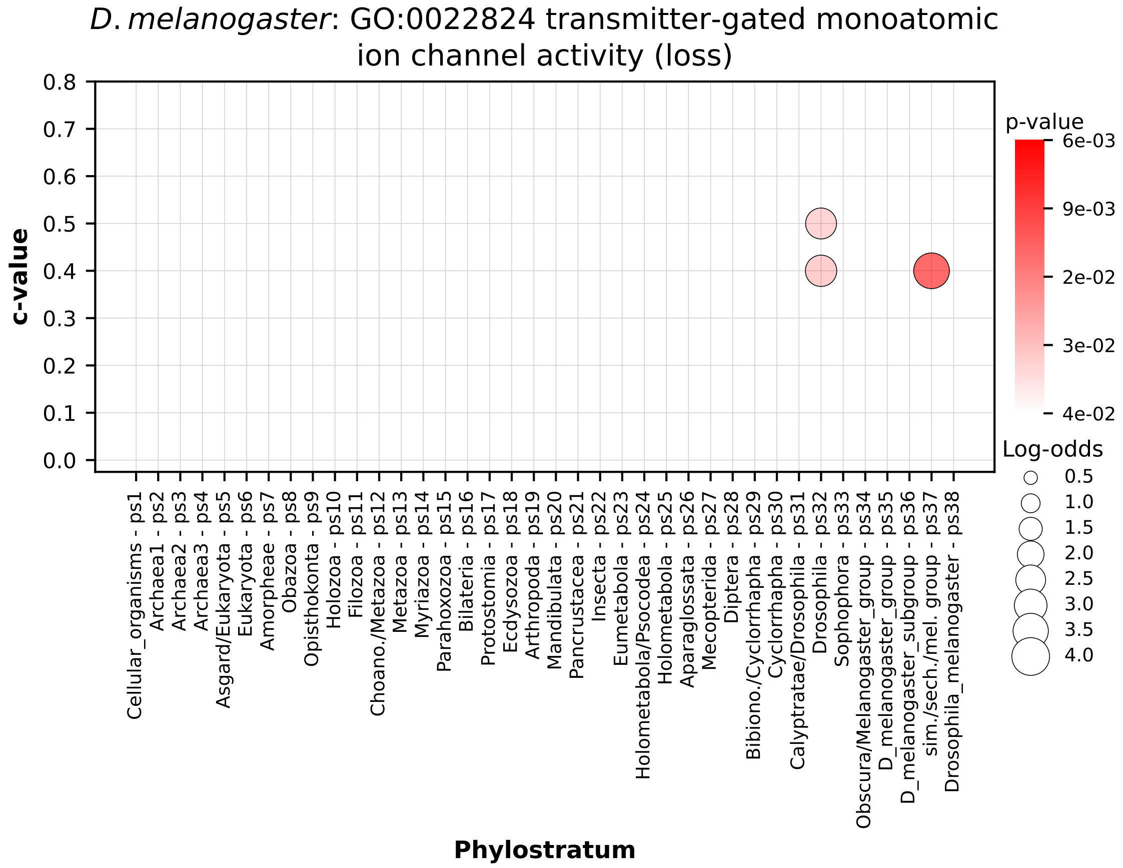
D. melanogaster: GO:0022824 transmitter-gated monoatomic ion channel activity (loss)
c-value
0.8
0.7
0.6
0.5
0.4
0.3
0.2
0.1
0.0
Phylostratum
Cellular_organisms - ps1
Archaea1 - ps2
Archaea2 - ps3
Archaea3 - ps4
Asgard/Eukaryota - ps5
Eukaryota - ps6
Amorpheae - ps7
Obazoa - ps8
Opisthokonta - ps9
Holozoa - ps10
Filozoa - ps11
Choano./Metazoa - ps12
Metazoa - ps13
Myriazoa - ps14
Parahoxozoa - ps15
Bilateria - ps16
Protostomia - ps17
Ecdysozoa - ps18
Arthropoda - ps19
Mandibulata - ps20
Pancrustacea - ps21
Insecta - ps22
Eumetabola - ps23
Holometabola/Psocodea - ps24
Holometabola - ps25
Aparaglossata - ps26
Mecopterida - ps27
Diptera - ps28
Bibiono./Cyclorrhapha - ps29
Cyclorrhapha - ps30
Calyptratae/Drosophila - ps31
Drosophila - ps32
Sophophora - ps33
Obscura/Melanogaster_group - ps34
D_melanogaster_group - ps35
D_melanogaster_subgroup - ps36
sim./sech./mel. group - ps37
Drosophila_melanogaster - ps38
p-value
6e-03
9e-03
2e-02
3e-02
4e-02
Log-odds
0.5
1.0
1.5
2.0
2.5
3.0
3.5
4.0

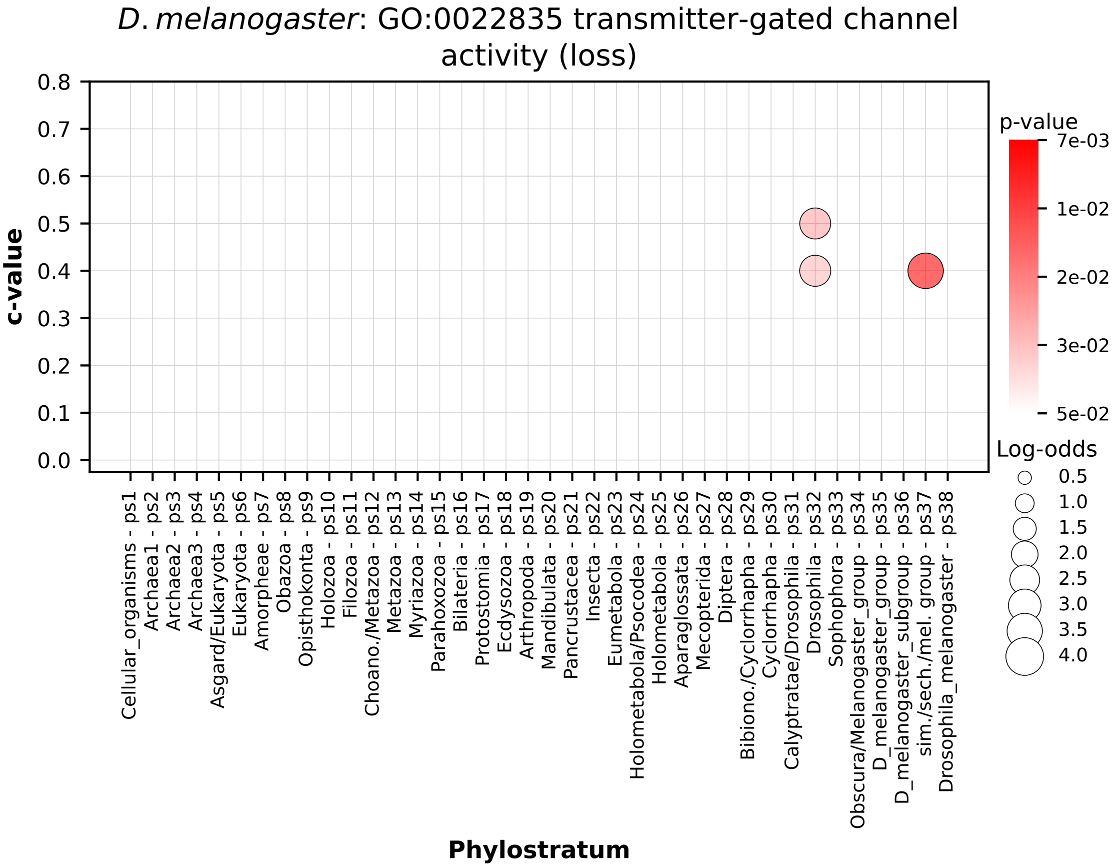
D. melanogaster: GO:0022835 transmitter-gated channel activity (loss)
c-value
0.8
0.7
0.6
0.5
0.4
0.3
0.2
0.1
0.0
p-value
7e-03
1e-02
2e-02
3e-02
5e-02
Log-odds
0.5
1.0
1.5
2.0
2.5
3.0
3.5
4.0
Cellular_organisms - ps1
Archaea1 - ps2
Archaea2 - ps3
Archaea3 - ps4
Asgard/Eukaryota - ps5
Eukaryota - ps6
Amorpheae - ps7
Obazoa - ps8
Opisthokonta - ps9
Holozoa - ps10
Filozoa - ps11
Choano./Metazoa - ps12
Metazoa - ps13
Myriazoa - ps14
Parahoxozoa - ps15
Bilateria - ps16
Protostomia - ps17
Ecdysozoa - ps18
Arthropoda - ps19
Mandibulata - ps20
Pancrustacea - ps21
Insecta - ps22
Eumetabola - ps23
Holometabola/Psocodea - ps24
Holometabola - ps25
Aparaglossata - ps26
Mecopterida - ps27
Diptera - ps28
Bibiono./Cyclorrhapha - ps29
Cyclorrhapha - ps30
Calyptratae/Drosophila - ps31
Drosophila - ps32
Sophophora - ps33
Obscura/Melanogaster_group - ps34
D_melanogaster_group - ps35
D_melanogaster_subgroup - ps36
sim./sech./mel. group - ps37
Drosophila_melanogaster - ps38
Phylostratum

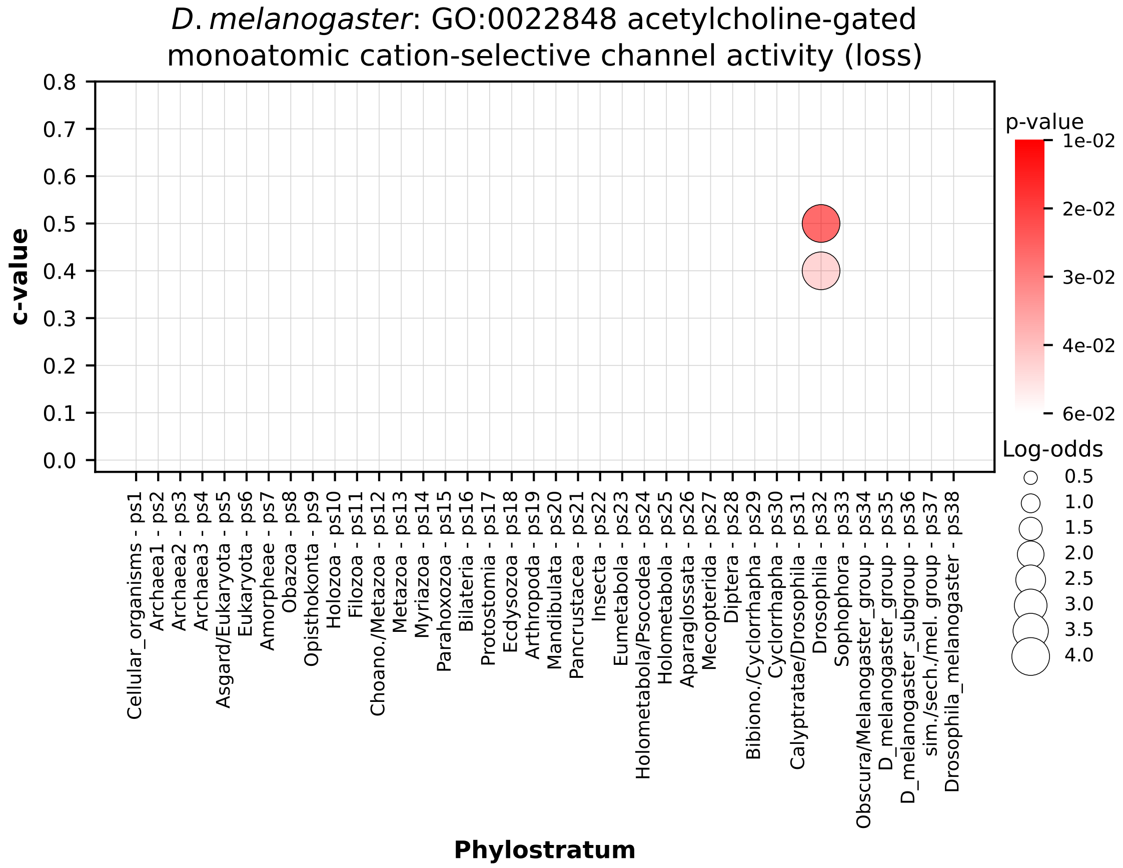
D. melanogaster: GO:0022848 acetylcholine-gated monoatomic cation-selective channel activity (loss)
c-value
0.0
0.1
0.2
0.3
0.4
0.5
0.6
0.7
0.8
p-value
1e-02
2e-02
3e-02
4e-02
6e-02
Log-odds
0.5
1.0
1.5
2.0
2.5
3.0
3.5
4.0
Cellular_organisms - ps1
Archaea1 - ps2
Archaea2 - ps3
Archaea3 - ps4
Asgard/Eukaryota - ps5
Eukaryota - ps6
Amorpheae - ps7
Obazoa - ps8
Opisthokonta - ps9
Holozoa - ps10
Filozoa - ps11
Choano./Metazoa - ps12
Metazoa - ps13
Myriazoa - ps14
Parahoxozoa - ps15
Bilateria - ps16
Protostomia - ps17
Ecdysozoa - ps18
Arthropoda - ps19
Mandibulata - ps20
Pancrustacea - ps21
Insecta - ps22
Eumetabola - ps23
Holometabola/Psocodea - ps24
Holometabola - ps25
Aparaglossata - ps26
Mecopterida - ps27
Diptera - ps28
Bibiono./Cyclorrhapha - ps29
Cyclorrhapha - ps30
Calyptratae/Drosophila - ps31
Drosophila - ps32
Sophophora - ps33
Obscura/Melanogaster_group - ps34
D_melanogaster_group - ps35
D_melanogaster_subgroup - ps36
sim./sech./mel. group - ps37
Drosophila_melanogaster - ps38
Phylostratum

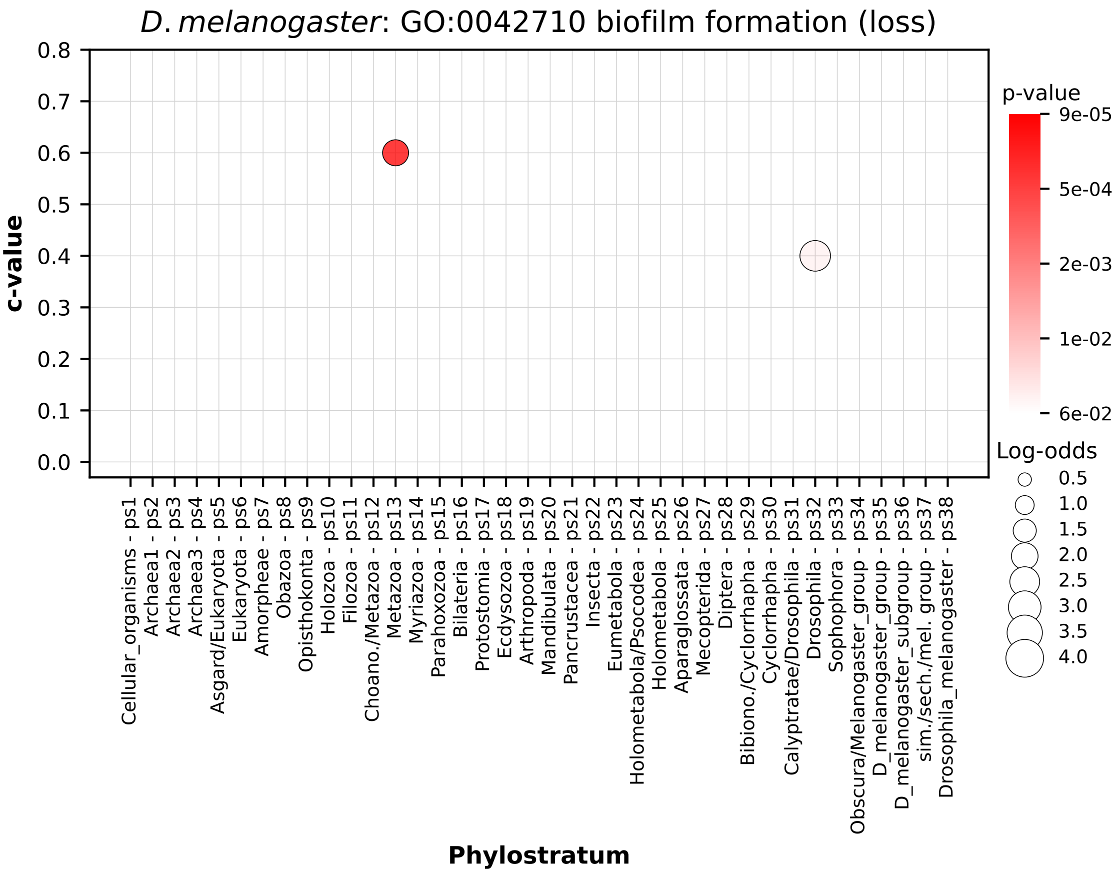

D. melanogaster: GO:0042710 biofilm formation (loss)
c-value
0.0
0.1
0.2
0.3
0.4
0.5
0.6
0.7
0.8
p-value
9e-05
5e-04
2e-03
1e-02
6e-02
Log-odds
0.5
1.0
1.5
2.0
2.5
3.0
3.5
4.0
Phylostratum
Cellular_organisms - ps1
Archaea1 - ps2
Archaea2 - ps3
Archaea3 - ps4
Asgard/Eukaryota - ps5
Eukaryota - ps6
Amorpheae - ps7
Obazoa - ps8
Opisthokonta - ps9
Holozoa - ps10
Filozoa - ps11
Choano./Metazoa - ps12
Metazoa - ps13
Myriazoa - ps14
Parahoxozoa - ps15
Bilateria - ps16
Protostomia - ps17
Ecdysozoa - ps18
Arthropoda - ps19
Mandibulata - ps20
Pancrustacea - ps21
Insecta - ps22
Eumetabola - ps23
Holometabola/Psocodea - ps24
Holometabola - ps25
Aparaglossata - ps26
Mecopterida - ps27
Diptera - ps28
Bibiono./Cyclorrhapha - ps29
Cyclorrhapha - ps30
Calyptratae/Drosophila - ps31
Drosophila - ps32
Sophophora - ps33
Obscura/Melanogaster_group - ps34
D_melanogaster_group - ps35
D_melanogaster_subgroup - ps36
sim./sech./mel. group - ps37
Drosophila_melanogaster - ps38

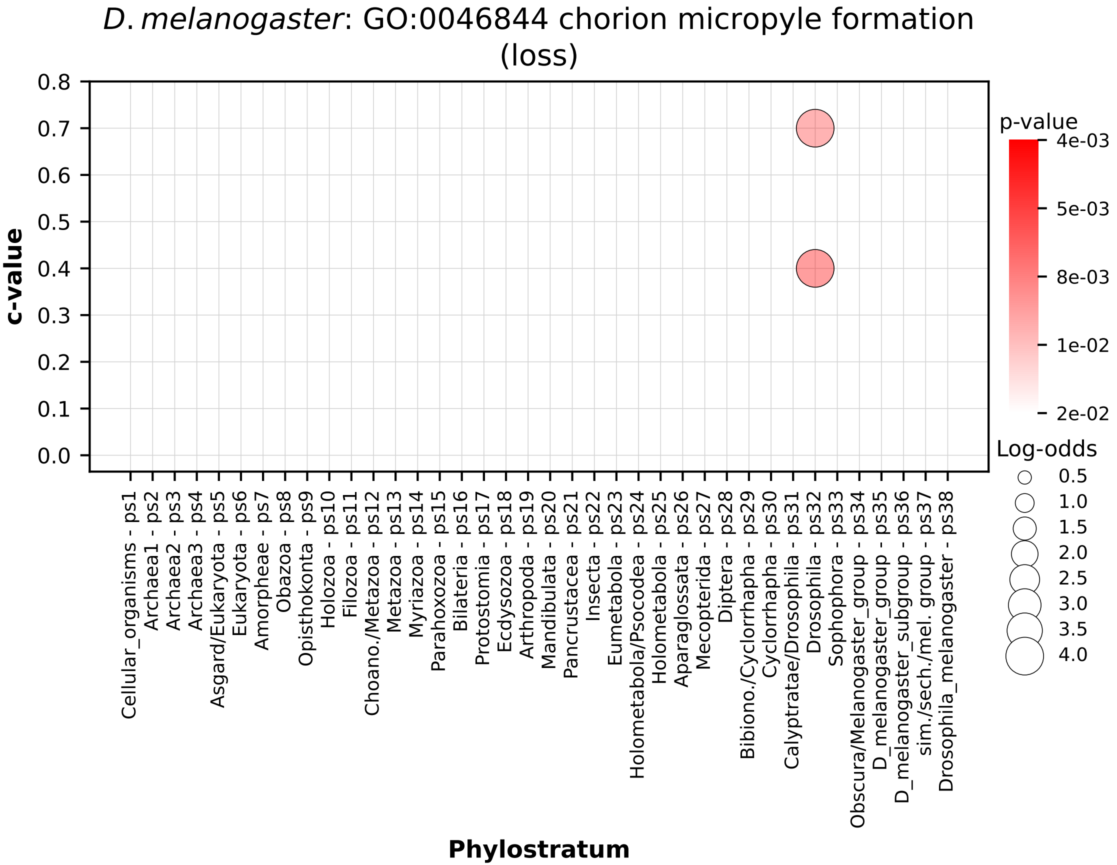

D. melanogaster: GO:0046844 chorion micropyle formation (loss)
c-value
0.8
0.7
0.6
0.5
0.4
0.3
0.2
0.1
0.0
p-value
4e-03
5e-03
8e-03
1e-02
2e-02
Log-odds
0.5
1.0
1.5
2.0
2.5
3.0
3.5
4.0
Cellular_organisms - ps1
Archaea1 - ps2
Archaea2 - ps3
Archaea3 - ps4
Asgard/Eukaryota - ps5
Eukaryota - ps6
Amorpheae - ps7
Obazoa - ps8
Opisthokonta - ps9
Holozoa - ps10
Filozoa - ps11
Choano./Metazoa - ps12
Metazoa - ps13
Myriazoa - ps14
Parahoxozoa - ps15
Bilateria - ps16
Protostomia - ps17
Ecdysozoa - ps18
Arthropoda - ps19
Mandibulata - ps20
Pancrustacea - ps21
Insecta - ps22
Eumetabola - ps23
Holometabola/Psocodea - ps24
Holometabola - ps25
Aparaglossata - ps26
Mecopterida - ps27
Diptera - ps28
Bibiono./Cyclorrhapha - ps29
Cyclorrhapha - ps30
Calyptratae/Drosophila - ps31
Drosophila - ps32
Sophophora - ps33
Obscura/Melanogaster_group - ps34
D_melanogaster_group - ps35
D_melanogaster_subgroup - ps36
sim./sech./mel. group - ps37
Drosophila_melanogaster - ps38
Phylostratum

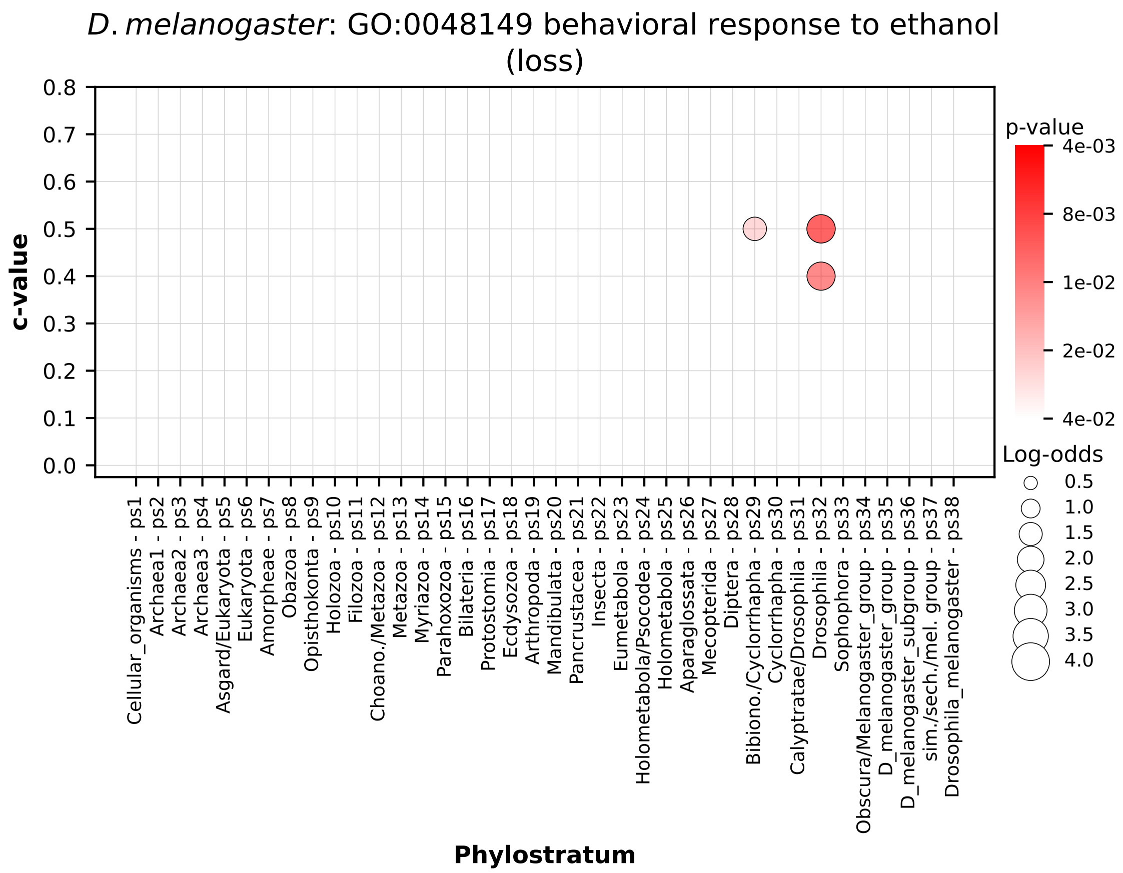

D. melanogaster: GO:0048149 behavioral response to ethanol (loss)
c-value
0.8
0.7
0.6
0.5
0.4
0.3
0.2
0.1
0.0
p-value
4e-03
8e-03
1e-02
2e-02
4e-02
Log-odds
0.5
1.0
1.5
2.0
2.5
3.0
3.5
4.0
Cellular_organisms - ps1
Archaea1 - ps2
Archaea2 - ps3
Archaea3 - ps4
Asgard/Eukaryota - ps5
Eukaryota - ps6
Amorpheae - ps7
Obazoa - ps8
Opisthokonta - ps9
Holozoa - ps10
Filozoa - ps11
Choano./Metazoa - ps12
Metazoa - ps13
Myriazoa - ps14
Parahoxozoa - ps15
Bilateria - ps16
Protostomia - ps17
Ecdysozoa - ps18
Arthropoda - ps19
Mandibulata - ps20
Pancrustacea - ps21
Insecta - ps22
Eumetabola - ps23
Holometabola/Psocodea - ps24
Holometabola - ps25
Aparaglossata - ps26
Mecopterida - ps27
Diptera - ps28
Bibiono./Cyclorrhapha - ps29
Cyclorrhapha - ps30
Calyptratae/Drosophila - ps31
Drosophila - ps32
Sophophora - ps33
Obscura/Melanogaster_group - ps34
D_melanogaster_group - ps35
D_melanogaster_subgroup - ps36
sim./sech./mel. group - ps37
Drosophila_melanogaster - ps38
Phylostratum

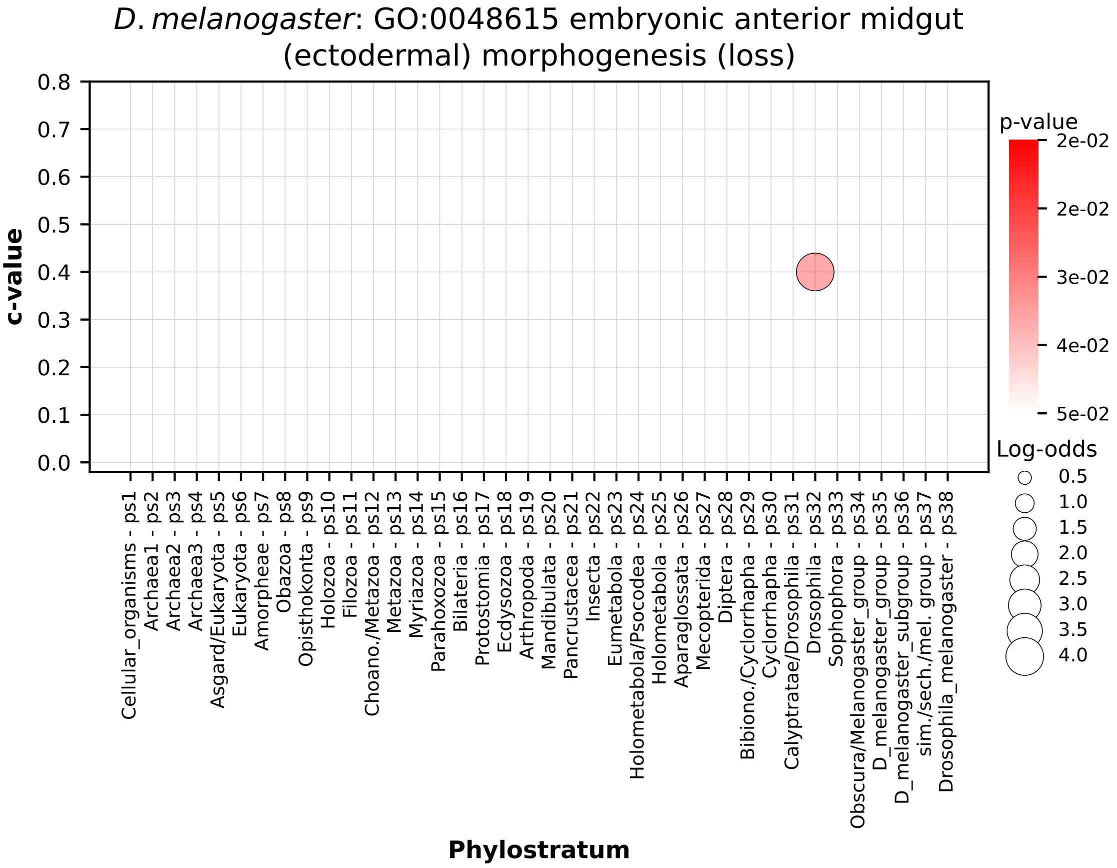

D. melanogaster: GO:0048615 embryonic anterior midgut (ectodermal) morphogenesis (loss)
c-value
0.8
0.7
0.6
0.5
0.4
0.3
0.2
0.1
0.0
Phylostratum
Cellular_organisms - ps1
Archaea1 - ps2
Archaea2 - ps3
Archaea3 - ps4
Asgard/Eukaryota - ps5
Eukaryota - ps6
Amorpheae - ps7
Obazoa - ps8
Opisthokonta - ps9
Holozoa - ps10
Filozoa - ps11
Choano./Metazoa - ps12
Metazoa - ps13
Myriazoa - ps14
Parahoxozoa - ps15
Bilateria - ps16
Protostomia - ps17
Ecdysozoa - ps18
Arthropoda - ps19
Mandibulata - ps20
Pancrustacea - ps21
Insecta - ps22
Eumetabola - ps23
Holometabola/Psocodea - ps24
Holometabola - ps25
Aparaglossata - ps26
Mecopterida - ps27
Diptera - ps28
Bibiono./Cyclorrhapha - ps29
Cyclorrhapha - ps30
Calyptratae/Drosophila - ps31
Drosophila - ps32
Sophophora - ps33
Obscura/Melanogaster_group - ps34
D_melanogaster_group - ps35
D_melanogaster_subgroup - ps36
sim./sech./mel. group - ps37
Drosophila_melanogaster - ps38
p-value
2e-02
2e-02
3e-02
4e-02
5e-02
Log-odds
0.5
1.0
1.5
2.0
2.5
3.0
3.5
4.0

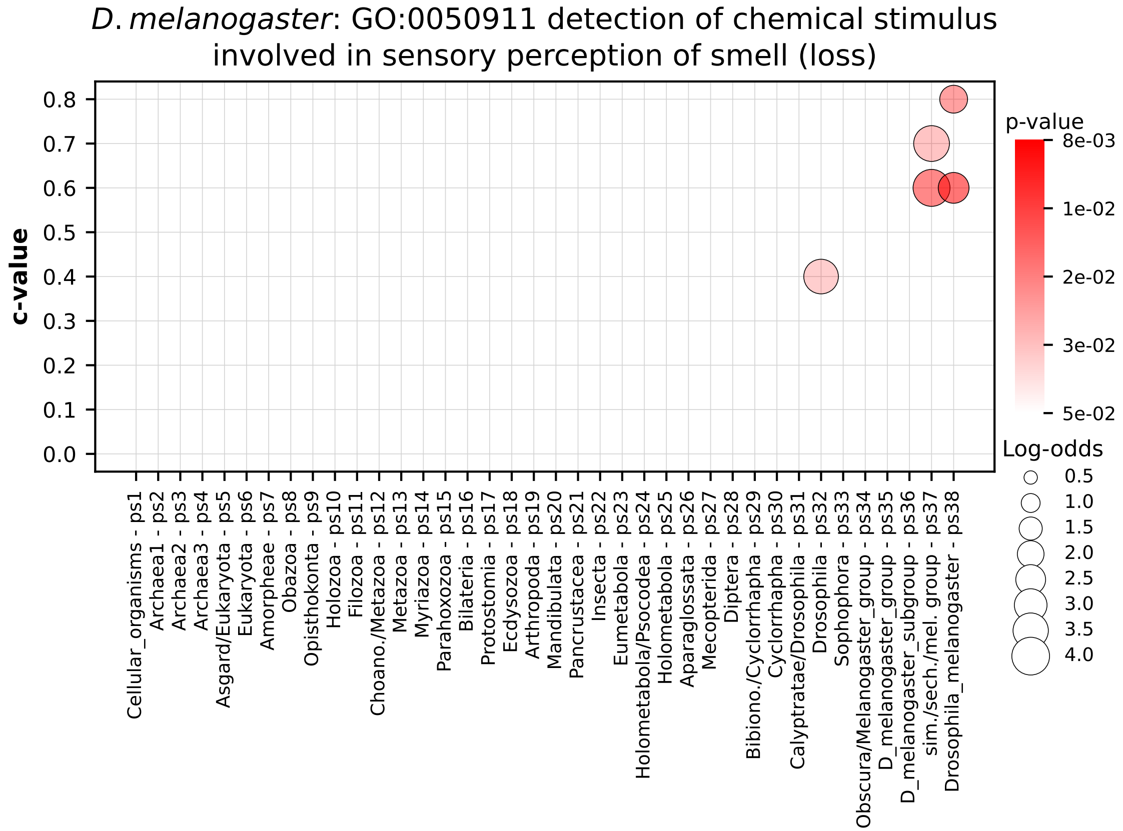

D. melanogaster: GO:0050911 detection of chemical stimulus involved in sensory perception of smell (loss)
c-value
0.0
0.1
0.2
0.3
0.4
0.5
0.6
0.7
0.8
p-value
8e-03
1e-02
2e-02
3e-02
5e-02
Log-odds
0.5
1.0
1.5
2.0
2.5
3.0
3.5
4.0
Cellular_organisms - ps1
Archaea1 - ps2
Archaea2 - ps3
Archaea3 - ps4
Asgard/Eukaryota - ps5
Eukaryota - ps6
Amorpheae - ps7
Obazoa - ps8
Opisthokonta - ps9
Holozoa - ps10
Filozoa - ps11
Choano./Metazoa - ps12
Metazoa - ps13
Myriazoa - ps14
Parahoxozoa - ps15
Bilateria - ps16
Protostomia - ps17
Ecdysozoa - ps18
Arthropoda - ps19
Mandibulata - ps20
Pancrustacea - ps21
Insecta - ps22
Eumetabola - ps23
Holometabola/Psocodea - ps24
Holometabola - ps25
Aparaglossata - ps26
Mecopterida - ps27
Diptera - ps28
Bibiono./Cyclorrhapha - ps29
Cyclorrhapha - ps30
Calyptratae/Drosophila - ps31
Drosophila - ps32
Sophophora - ps33
Obscura/Melanogaster_group - ps34
D_melanogaster_group - ps35
D_melanogaster_subgroup - ps36
sim./sech./mel. group - ps37
Drosophila_melanogaster - ps38
Phylostratum

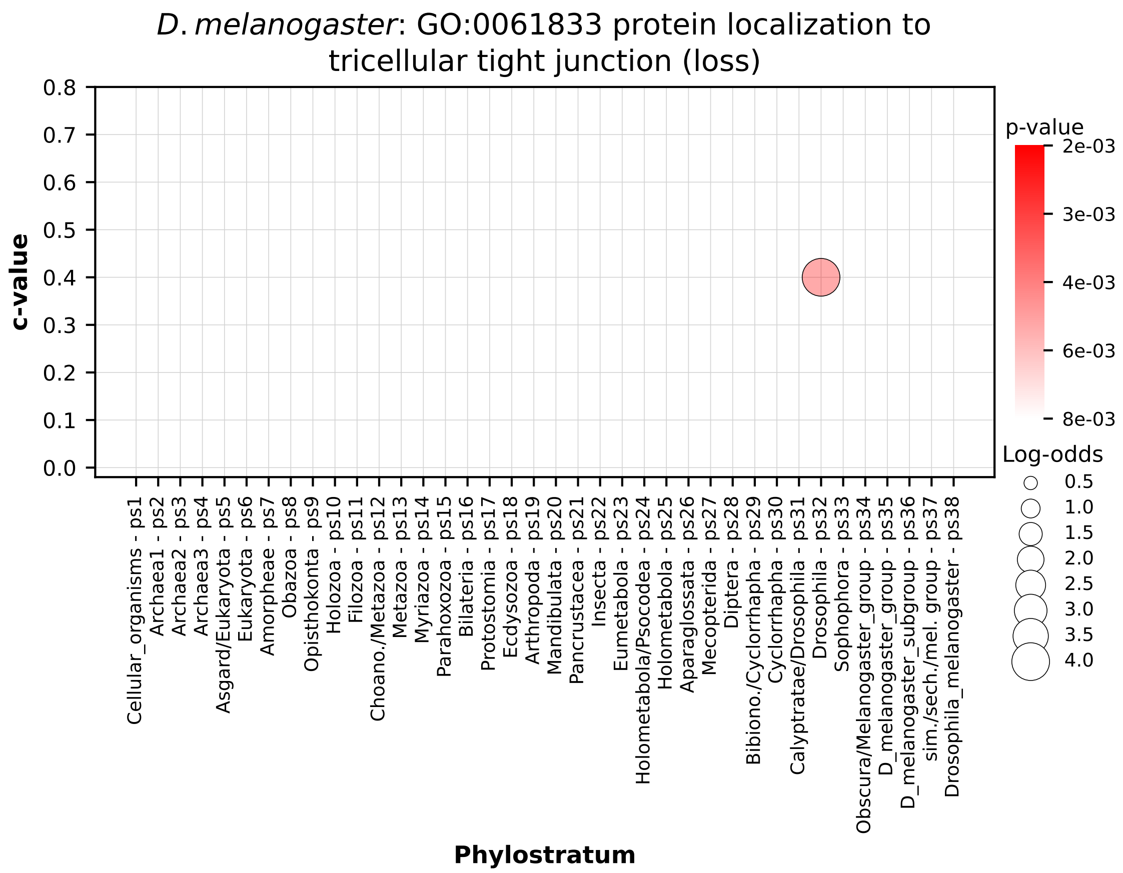

D. melanogaster: GO:0061833 protein localization to tricellular tight junction (loss)
c-value
0.8
0.7
0.6
0.5
0.4
0.3
0.2
0.1
0.0
p-value
2e-03
3e-03
4e-03
6e-03
8e-03
Log-odds
0.5
1.0
1.5
2.0
2.5
3.0
3.5
4.0
Cellular_organisms - ps1
Archaea1 - ps2
Archaea2 - ps3
Archaea3 - ps4
Asgard/Eukaryota - ps5
Eukaryota - ps6
Amorpheae - ps7
Obazoa - ps8
Opisthokonta - ps9
Holozoa - ps10
Filozoa - ps11
Choano./Metazoa - ps12
Metazoa - ps13
Myriazoa - ps14
Parahoxozoa - ps15
Bilateria - ps16
Protostomia - ps17
Ecdysozoa - ps18
Arthropoda - ps19
Mandibulata - ps20
Pancrustacea - ps21
Insecta - ps22
Eumetabola - ps23
Holometabola/Psocodea - ps24
Holometabola - ps25
Aparaglossata - ps26
Mecopterida - ps27
Diptera - ps28
Bibiono./Cyclorrhapha - ps29
Cyclorrhapha - ps30
Calyptratae/Drosophila - ps31
Drosophila - ps32
Sophophora - ps33
Obscura/Melanogaster_group - ps34
D_melanogaster_group - ps35
D_melanogaster_subgroup - ps36
sim./sech./mel. group - ps37
Drosophila_melanogaster - ps38
Phylostratum

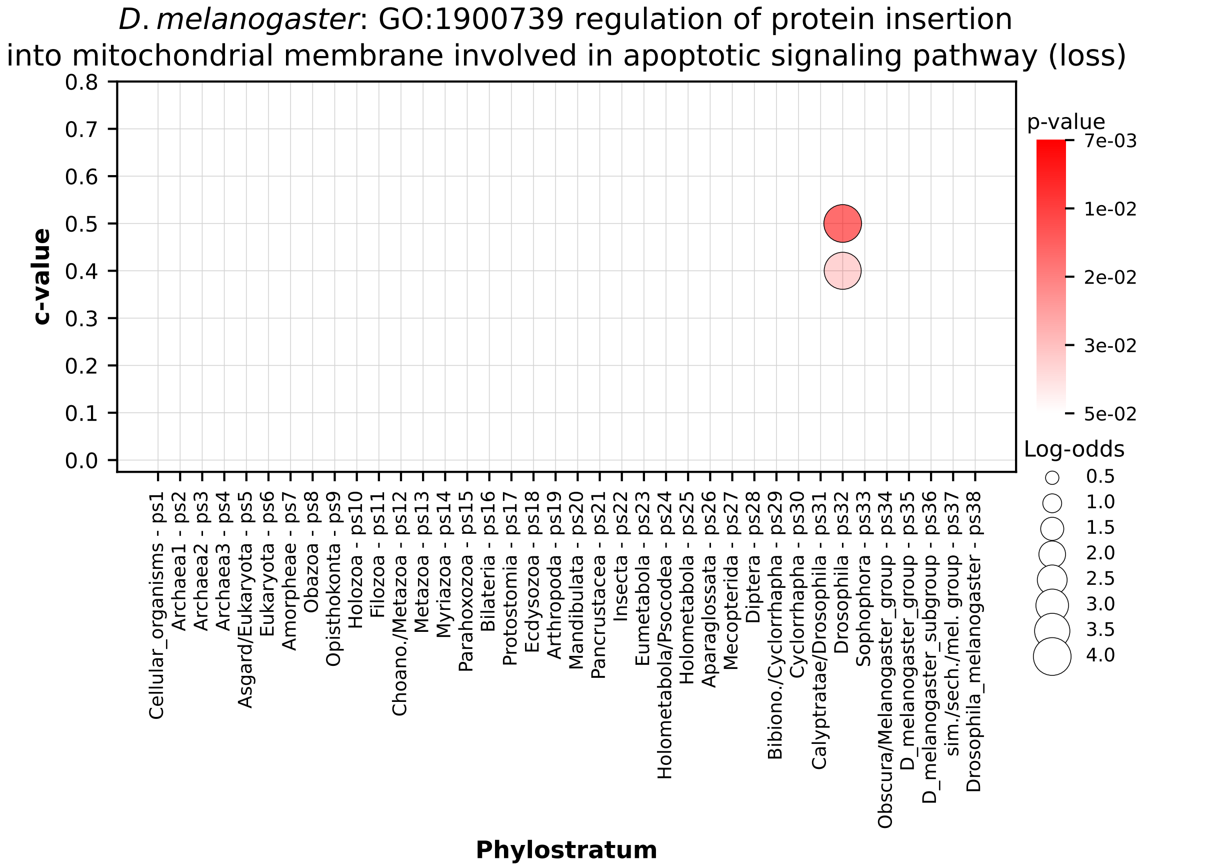

D. melanogaster: GO:1900739 regulation of protein insertion into mitochondrial membrane involved in apoptotic signaling pathway (loss)
c-value
0.8
0.7
0.6
0.5
0.4
0.3
0.2
0.1
0.0
Phylostratum
Cellular_organisms - ps1
Archaea1 - ps2
Archaea2 - ps3
Archaea3 - ps4
Asgard/Eukaryota - ps5
Eukaryota - ps6
Amorpheae - ps7
Obazoa - ps8
Opisthokonta - ps9
Holozoa - ps10
Filozoa - ps11
Choano./Metazoa - ps12
Metazoa - ps13
Myriazoa - ps14
Parahoxozoa - ps15
Bilateria - ps16
Protostomia - ps17
Ecdysozoa - ps18
Arthropoda - ps19
Mandibulata - ps20
Pancrustacea - ps21
Insecta - ps22
Eumetabola - ps23
Holometabola/Psocodea - ps24
Holometabola - ps25
Aparaglossata - ps26
Mecopterida - ps27
Diptera - ps28
Bibiono./Cyclorrhapha - ps29
Cyclorrhapha - ps30
Calyptratae/Drosophila - ps31
Drosophila - ps32
Sophophora - ps33
Obscura/Melanogaster_group - ps34
D_melanogaster_group - ps35
D_melanogaster_subgroup - ps36
sim./sech./mel. group - ps37
Drosophila_melanogaster - ps38
p-value
7e-03
1e-02
2e-02
3e-02
5e-02
Log-odds
0.5
1.0
1.5
2.0
2.5
3.0
3.5
4.0

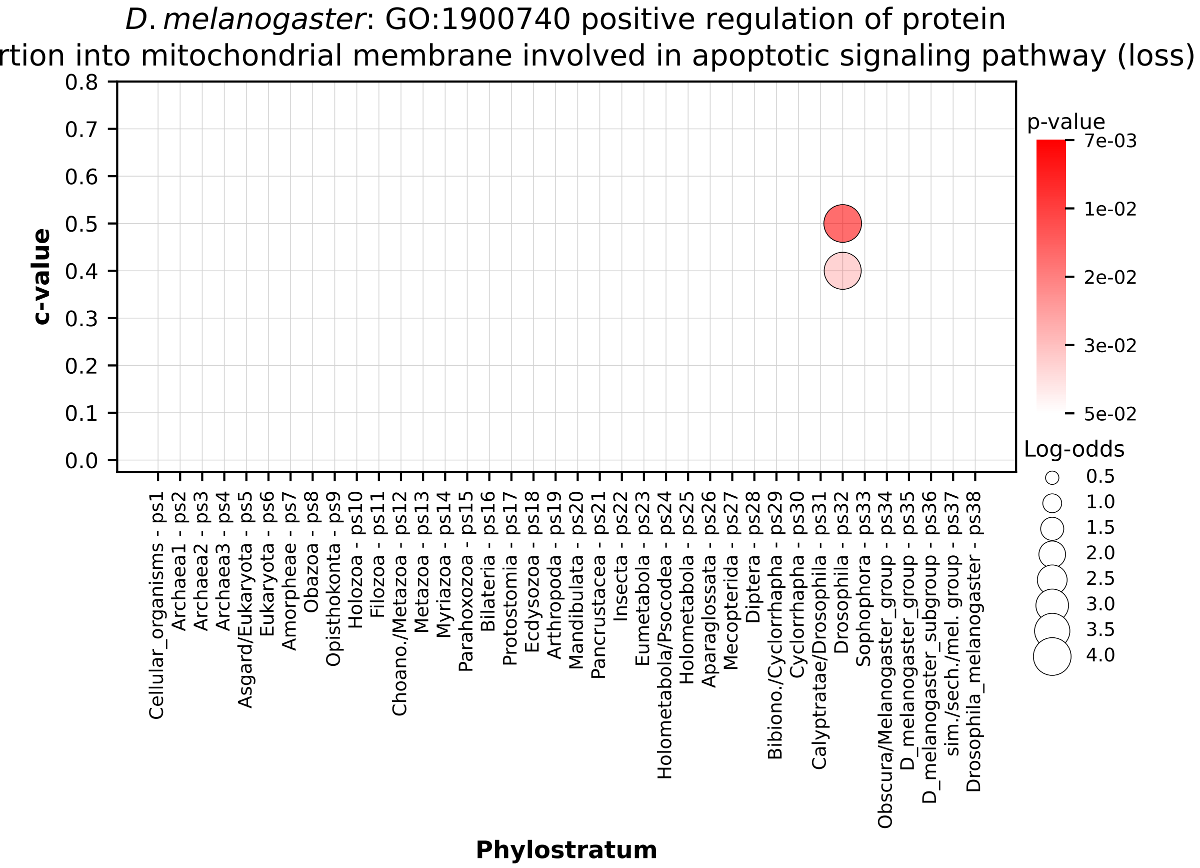
D. melanogaster: GO:1900740 positive regulation of protein
rtion into mitochondrial membrane involved in apoptotic signaling pathway (loss)
c-value
0.8
0.7
0.6
0.5
0.4
0.3
0.2
0.1
0.0
p-value
7e-03
1e-02
2e-02
3e-02
5e-02
Log-odds
0.5
1.0
1.5
2.0
2.5
3.0
3.5
4.0
Cellular_organisms - ps1
Archaea1 - ps2
Archaea2 - ps3
Archaea3 - ps4
Asgard/Eukaryota - ps5
Eukaryota - ps6
Amorpheae - ps7
Obazoa - ps8
Opisthokonta - ps9
Holozoa - ps10
Filozoa - ps11
Choano./Metazoa - ps12
Metazoa - ps13
Myriazoa - ps14
Parahoxozoa - ps15
Bilateria - ps16
Protostomia - ps17
Ecdysozoa - ps18
Arthropoda - ps19
Mandibulata - ps20
Pancrustacea - ps21
Insecta - ps22
Eumetabola - ps23
Holometabola/Psocodea - ps24
Holometabola - ps25
Aparaglossata - ps26
Mecopterida - ps27
Diptera - ps28
Bibiono./Cyclorrhapha - ps29
Cyclorrhapha - ps30
Calyptratae/Drosophila - ps31
Drosophila - ps32
Sophophora - ps33
Obscura/Melanogaster_group - ps34
D_melanogaster_group - ps35
D_melanogaster_subgroup - ps36
sim./sech./mel. group - ps37
Drosophila_melanogaster - ps38
Phylostratum

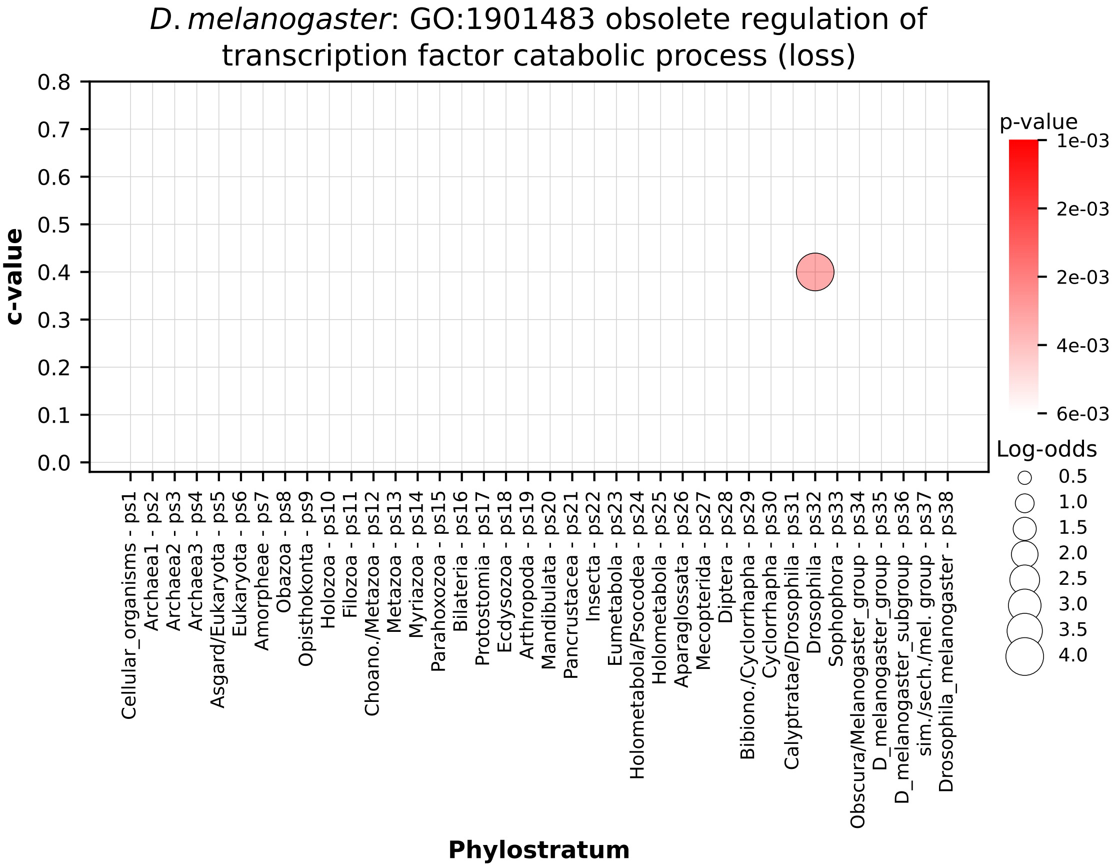
D. melanogaster: GO:1901483 obsolete regulation of transcription factor catabolic process (loss)
c-value
0.8
0.7
0.6
0.5
0.4
0.3
0.2
0.1
0.0
Phylostratum
Cellular_organisms - ps1
Archaea1 - ps2
Archaea2 - ps3
Archaea3 - ps4
Asgard/Eukaryota - ps5
Eukaryota - ps6
Amorpheae - ps7
Obazoa - ps8
Opisthokonta - ps9
Holozoa - ps10
Filozoa - ps11
Choano./Metazoa - ps12
Metazoa - ps13
Myriazoa - ps14
Parahoxozoa - ps15
Bilateria - ps16
Protostomia - ps17
Ecdysozoa - ps18
Arthropoda - ps19
Mandibulata - ps20
Pancrustacea - ps21
Insecta - ps22
Eumetabola - ps23
Holometabola/Psocodea - ps24
Holometabola - ps25
Aparaglossata - ps26
Mecopterida - ps27
Diptera - ps28
Bibiono./Cyclorrhapha - ps29
Cyclorrhapha - ps30
Calyptratae/Drosophila - ps31
Drosophila - ps32
Sophophora - ps33
Obscura/Melanogaster_group - ps34
D_melanogaster_group - ps35
D_melanogaster_subgroup - ps36
sim./sech./mel. group - ps37
Drosophila_melanogaster - ps38
p-value
1e-03
2e-03
2e-03
4e-03
6e-03
Log-odds
0.5
1.0
1.5
2.0
2.5
3.0
3.5
4.0

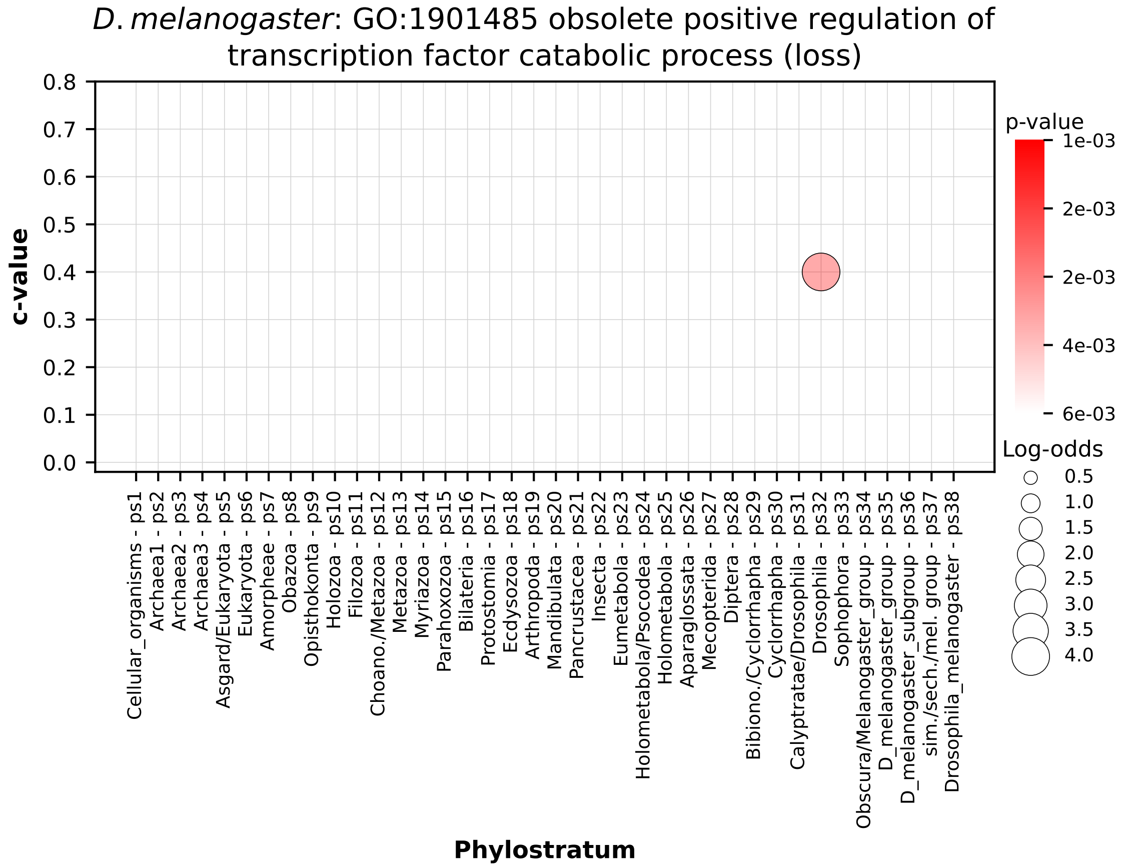
D. melanogaster: GO:1901485 obsolete positive regulation of transcription factor catabolic process (loss)
c-value
0.0
0.1
0.2
0.3
0.4
0.5
0.6
0.7
0.8
p-value
1e-03
2e-03
2e-03
4e-03
6e-03
Log-odds
0.5
1.0
1.5
2.0
2.5
3.0
3.5
4.0
Cellular_organisms - ps1
Archaea1 - ps2
Archaea2 - ps3
Archaea3 - ps4
Asgard/Eukaryota - ps5
Eukaryota - ps6
Amorpheae - ps7
Obazoa - ps8
Opisthokonta - ps9
Holozoa - ps10
Filozoa - ps11
Choano./Metazoa - ps12
Metazoa - ps13
Myriazoa - ps14
Parahoxozoa - ps15
Bilateria - ps16
Protostomia - ps17
Ecdysozoa - ps18
Arthropoda - ps19
Mandibulata - ps20
Pancrustacea - ps21
Insecta - ps22
Eumetabola - ps23
Holometabola/Psocodea - ps24
Holometabola - ps25
Aparaglossata - ps26
Mecopterida - ps27
Diptera - ps28
Bibiono./Cyclorrhapha - ps29
Cyclorrhapha - ps30
Calyptratae/Drosophila - ps31
Drosophila - ps32
Sophophora - ps33
Obscura/Melanogaster_group - ps34
D_melanogaster_group - ps35
D_melanogaster_subgroup - ps36
sim./sech./mel. group - ps37
Drosophila_melanogaster - ps38
Phylostratum

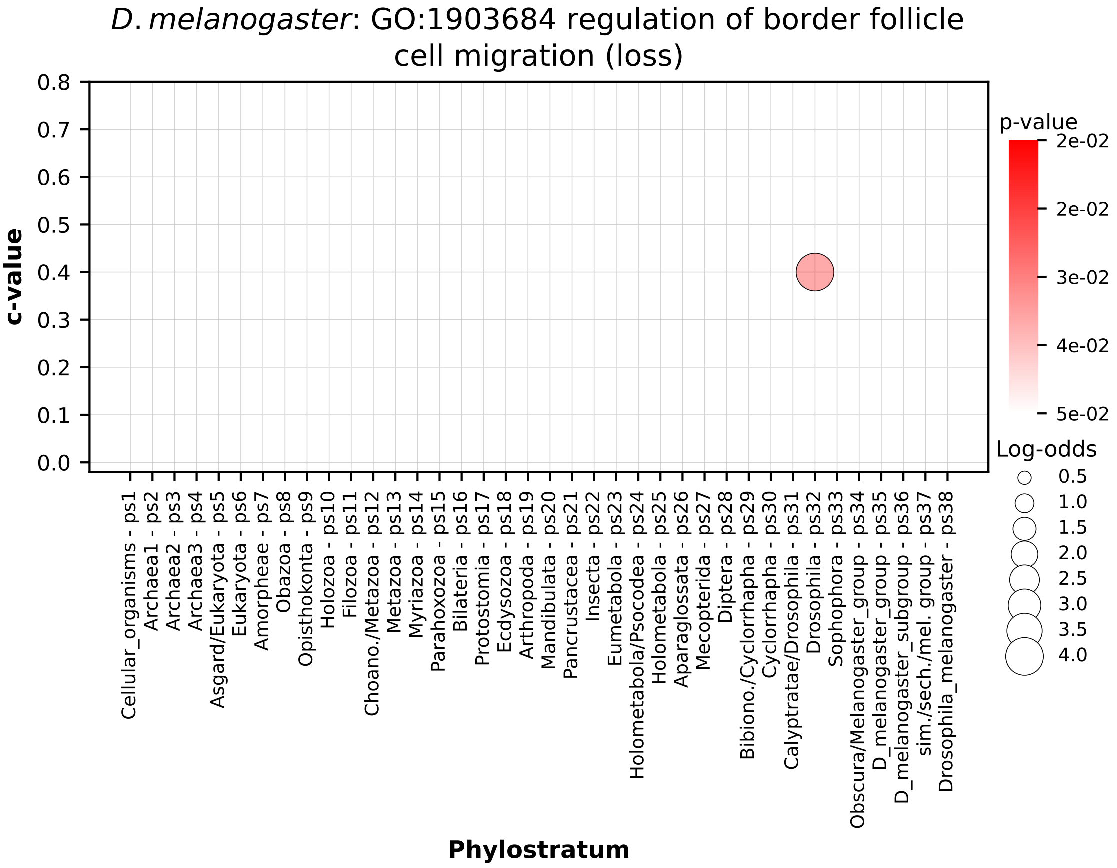

D. melanogaster: GO:1903684 regulation of border follicle cell migration (loss)
c-value
0.8
0.7
0.6
0.5
0.4
0.3
0.2
0.1
0.0
Phylostratum
Cellular_organisms - ps1
Archaea1 - ps2
Archaea2 - ps3
Archaea3 - ps4
Asgard/Eukaryota - ps5
Eukaryota - ps6
Amorpheae - ps7
Obazoa - ps8
Opisthokonta - ps9
Holozoa - ps10
Filozoa - ps11
Choano./Metazoa - ps12
Metazoa - ps13
Myriazoa - ps14
Parahoxozoa - ps15
Bilateria - ps16
Protostomia - ps17
Ecdysozoa - ps18
Arthropoda - ps19
Mandibulata - ps20
Pancrustacea - ps21
Insecta - ps22
Eumetabola - ps23
Holometabola/Psocodea - ps24
Holometabola - ps25
Aparaglossata - ps26
Mecopterida - ps27
Diptera - ps28
Bibiono./Cyclorrhapha - ps29
Cyclorrhapha - ps30
Calyptratae/Drosophila - ps31
Drosophila - ps32
Sophophora - ps33
Obscura/Melanogaster_group - ps34
D_melanogaster_group - ps35
D_melanogaster_subgroup - ps36
sim./sech./mel. group - ps37
Drosophila_melanogaster - ps38
p-value
2e-02
2e-02
3e-02
4e-02
5e-02
Log-odds
0.5
1.0
1.5
2.0
2.5
3.0
3.5
4.0

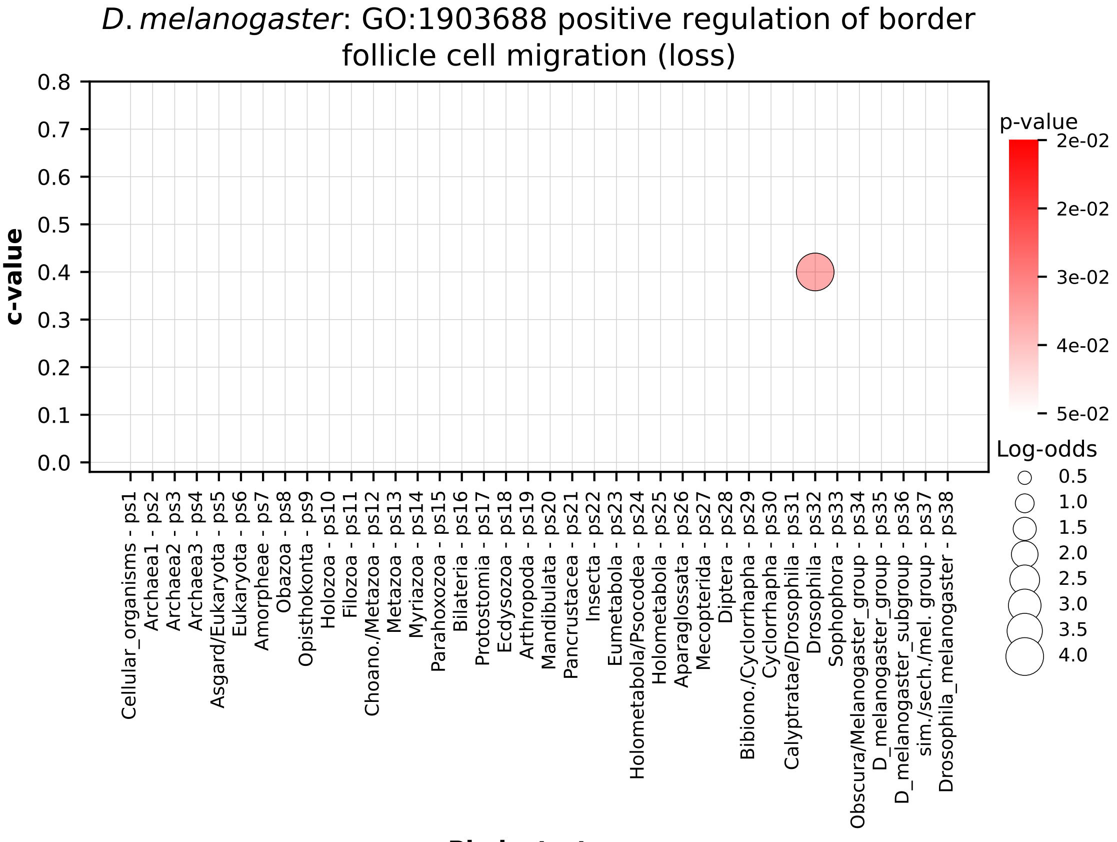

D. melanogaster: GO:1903688 positive regulation of border follicle cell migration (loss)
c-value
0.8
0.7
0.6
0.5
0.4
0.3
0.2
0.1
0.0
Phylostratum
Cellular_organisms - ps1
Archaea1 - ps2
Archaea2 - ps3
Archaea3 - ps4
Asgard/Eukaryota - ps5
Eukaryota - ps6
Amorpheae - ps7
Obazoa - ps8
Opisthokonta - ps9
Holozoa - ps10
Filozoa - ps11
Choano./Metazoa - ps12
Metazoa - ps13
Myriazoa - ps14
Parahoxozoa - ps15
Bilateria - ps16
Protostomia - ps17
Ecdysozoa - ps18
Arthropoda - ps19
Mandibulata - ps20
Pancrustacea - ps21
Insecta - ps22
Eumetabola - ps23
Holometabola/Psocodea - ps24
Holometabola - ps25
Aparaglossata - ps26
Mecopterida - ps27
Diptera - ps28
Bibiono./Cyclorrhapha - ps29
Cyclorrhapha - ps30
Calyptratae/Drosophila - ps31
Drosophila - ps32
Sophophora - ps33
Obscura/Melanogaster_group - ps34
D_melanogaster_group - ps35
D_melanogaster_subgroup - ps36
sim./sech./mel. group - ps37
Drosophila_melanogaster - ps38
p-value
2e-02
2e-02
3e-02
4e-02
5e-02
Log-odds
0.5
1.0
1.5
2.0
2.5
3.0
3.5
4.0

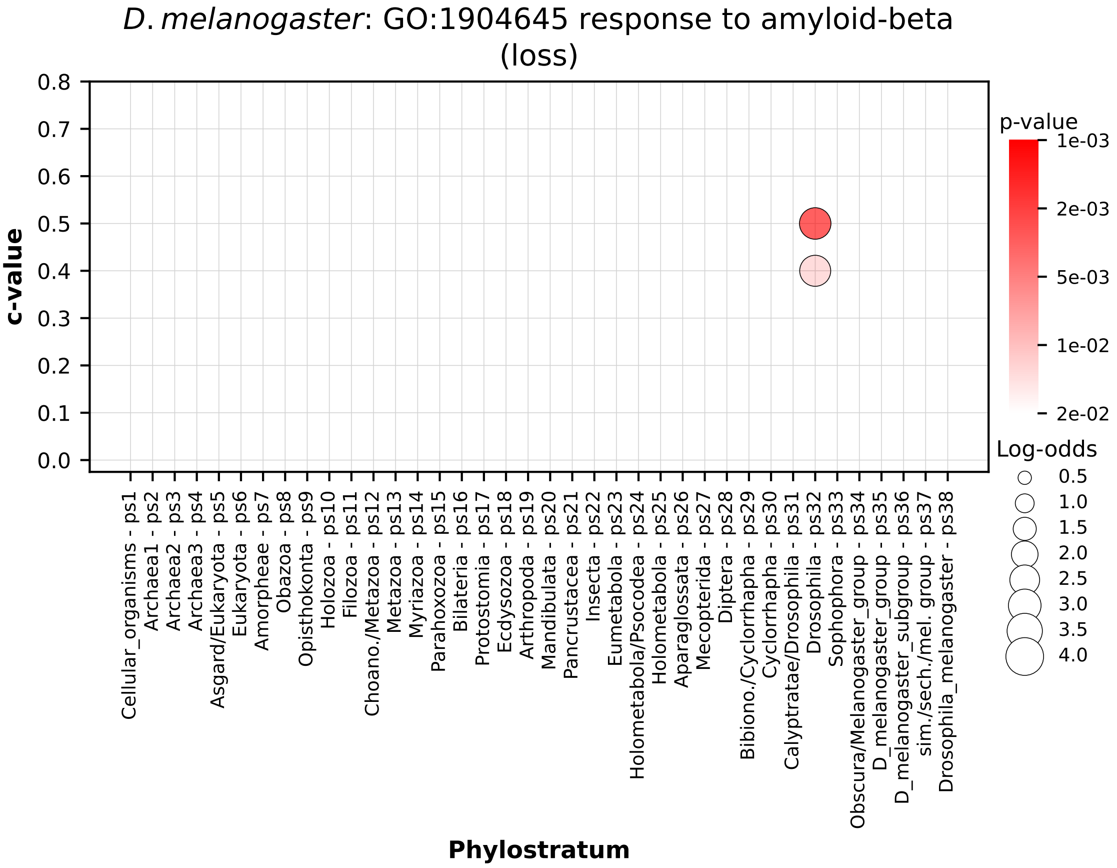

D. melanogaster: GO:1904645 response to amyloid-beta (loss)
c-value
0.8
0.7
0.6
0.5
0.4
0.3
0.2
0.1
0.0
p-value
1e-03
2e-03
5e-03
1e-02
2e-02
Log-odds
0.5
1.0
1.5
2.0
2.5
3.0
3.5
4.0
Cellular_organisms - ps1
Archaea1 - ps2
Archaea2 - ps3
Archaea3 - ps4
Asgard/Eukaryota - ps5
Eukaryota - ps6
Amorpheae - ps7
Obazoa - ps8
Opisthokonta - ps9
Holozoa - ps10
Filozoa - ps11
Choano./Metazoa - ps12
Metazoa - ps13
Myriazoa - ps14
Parahoxozoa - ps15
Bilateria - ps16
Protostomia - ps17
Ecdysozoa - ps18
Arthropoda - ps19
Mandibulata - ps20
Pancrustacea - ps21
Insecta - ps22
Eumetabola - ps23
Holometabola/Psocodea - ps24
Holometabola - ps25
Aparaglossata - ps26
Mecopterida - ps27
Diptera - ps28
Bibiono./Cyclorrhapha - ps29
Cyclorrhapha - ps30
Calyptratae/Drosophila - ps31
Drosophila - ps32
Sophophora - ps33
Obscura/Melanogaster_group - ps34
D_melanogaster_group - ps35
D_melanogaster_subgroup - ps36
sim./sech./mel. group - ps37
Drosophila_melanogaster - ps38
Phylostratum

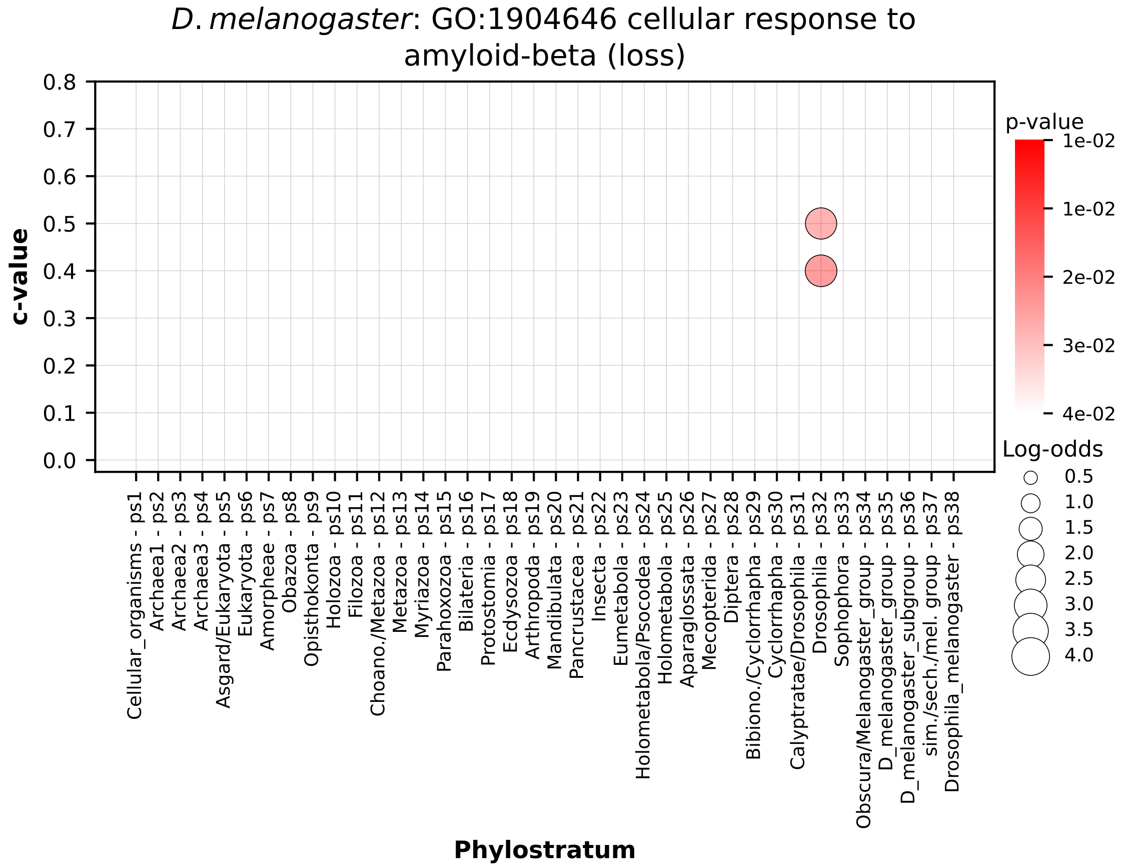
D. melanogaster: GO:1904646 cellular response to amyloid-beta (loss)
c-value
0.8
0.7
0.6
0.5
0.4
0.3
0.2
0.1
0.0
p-value
1e-02
1e-02
2e-02
3e-02
4e-02
Log-odds
0.5
1.0
1.5
2.0
2.5
3.0
3.5
4.0
Cellular_organisms - ps1
Archaea1 - ps2
Archaea2 - ps3
Archaea3 - ps4
Asgard/Eukaryota - ps5
Eukaryota - ps6
Amorpheae - ps7
Obazoa - ps8
Opisthokonta - ps9
Holozoa - ps10
Filozoa - ps11
Choano./Metazoa - ps12
Metazoa - ps13
Myriazoa - ps14
Parahoxozoa - ps15
Bilateria - ps16
Protostomia - ps17
Ecdysozoa - ps18
Arthropoda - ps19
Mandibulata - ps20
Pancrustacea - ps21
Insecta - ps22
Eumetabola - ps23
Holometabola/Psocodea - ps24
Holometabola - ps25
Aparaglossata - ps26
Mecopterida - ps27
Diptera - ps28
Bibiono./Cyclorrhapha - ps29
Cyclorrhapha - ps30
Calyptratae/Drosophila - ps31
Drosophila - ps32
Sophophora - ps33
Obscura/Melanogaster_group - ps34
D_melanogaster_group - ps35
D_melanogaster_subgroup - ps36
sim./sech./mel. group - ps37
Drosophila_melanogaster - ps38
Phylostratum

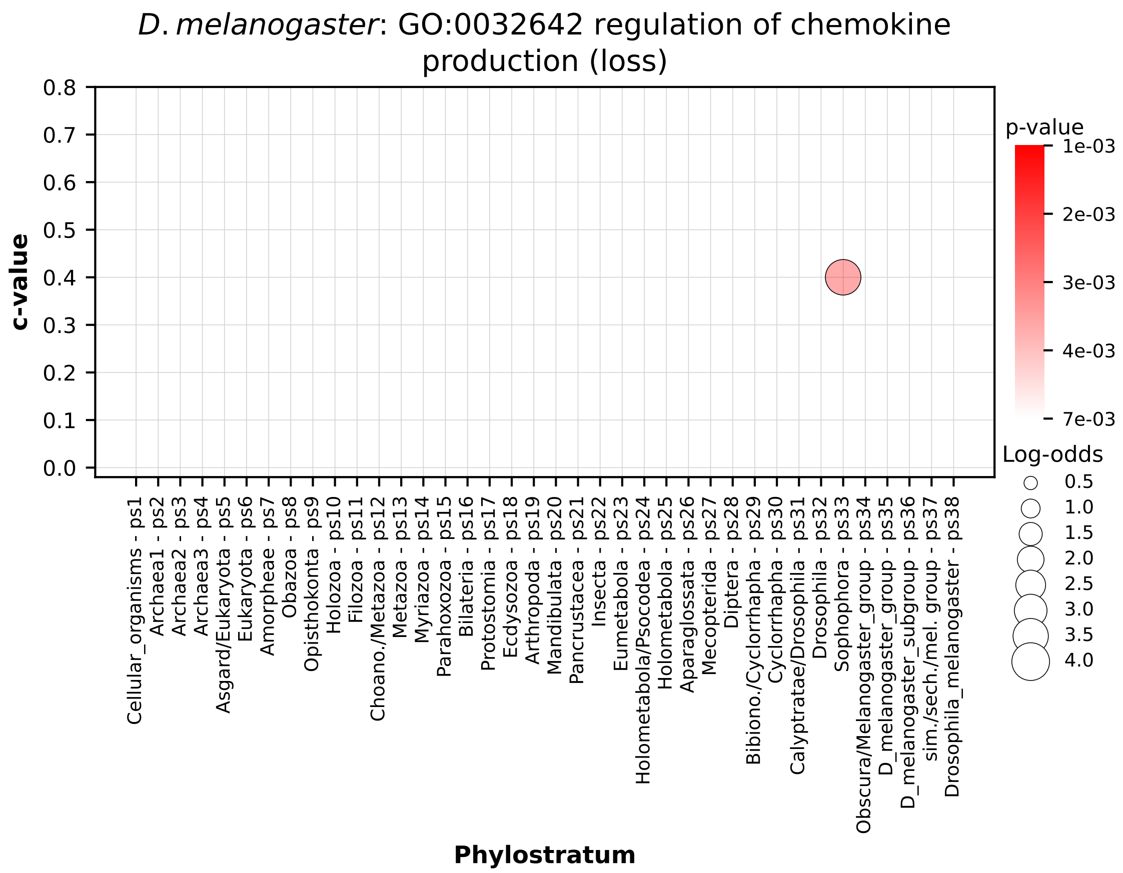

D. melanogaster: GO:0032642 regulation of chemokine production (loss)
c-value
0.8
0.7
0.6
0.5
0.4
0.3
0.2
0.1
0.0
Phylostratum
Cellular_organisms - ps1
Archaea1 - ps2
Archaea2 - ps3
Archaea3 - ps4
Asgard/Eukaryota - ps5
Eukaryota - ps6
Amorpheae - ps7
Obazoa - ps8
Opisthokonta - ps9
Holozoa - ps10
Filozoa - ps11
Choano./Metazoa - ps12
Metazoa - ps13
Myriazoa - ps14
Parahoxozoa - ps15
Bilateria - ps16
Protostomia - ps17
Ecdysozoa - ps18
Arthropoda - ps19
Mandibulata - ps20
Pancrustacea - ps21
Insecta - ps22
Eumetabola - ps23
Holometabola/Psocodea - ps24
Holometabola - ps25
Aparaglossata - ps26
Mecopterida - ps27
Diptera - ps28
Bibiono./Cyclorrhapha - ps29
Cyclorrhapha - ps30
Calyptratae/Drosophila - ps31
Drosophila - ps32
Sophophora - ps33
Obscura/Melanogaster_group - ps34
D_melanogaster_group - ps35
D_melanogaster_subgroup - ps36
sim./sech./mel. group - ps37
Drosophila_melanogaster - ps38
p-value
1e-03
2e-03
3e-03
4e-03
7e-03
Log-odds
0.5
1.0
1.5
2.0
2.5
3.0
3.5
4.0

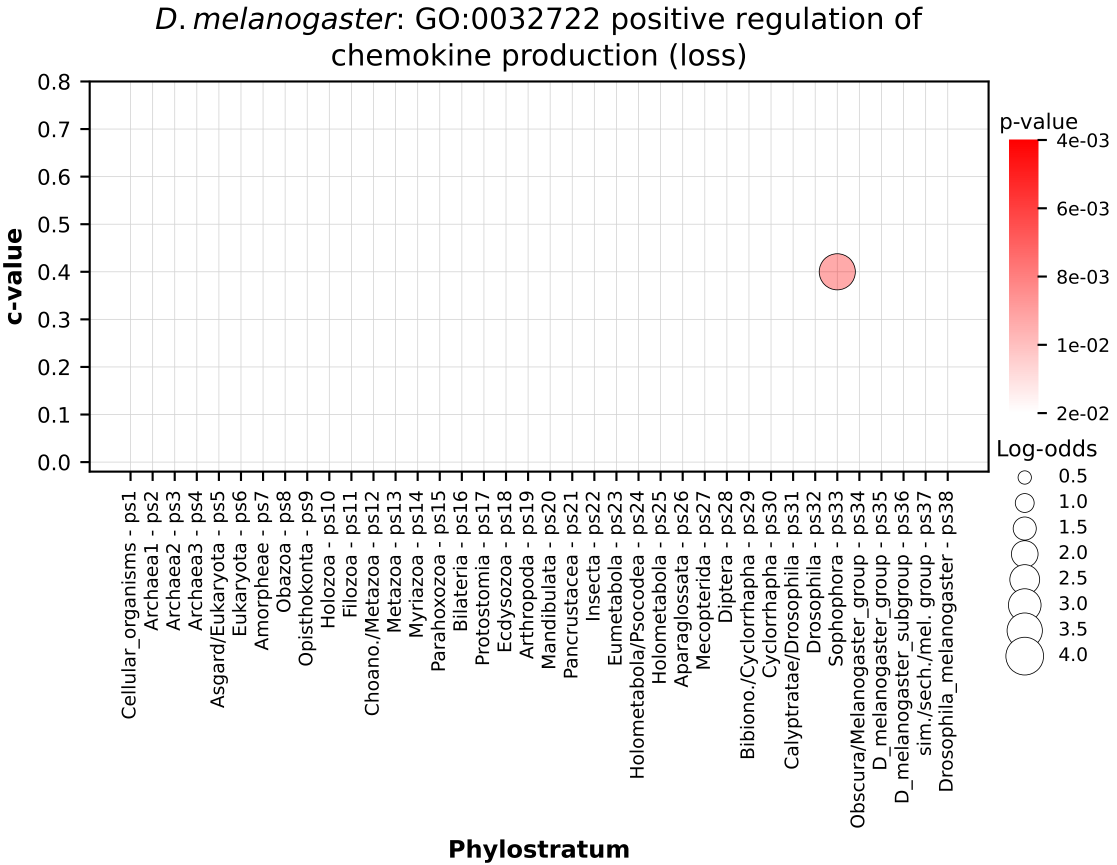

D. melanogaster: GO:0032722 positive regulation of chemokine production (loss)
c-value
0.0
0.1
0.2
0.3
0.4
0.5
0.6
0.7
0.8
p-value
4e-03
6e-03
8e-03
1e-02
2e-02
Log-odds
0.5
1.0
1.5
2.0
2.5
3.0
3.5
4.0
Cellular_organisms - ps1
Archaea1 - ps2
Archaea2 - ps3
Archaea3 - ps4
Asgard/Eukaryota - ps5
Eukaryota - ps6
Amorpheae - ps7
Obazoa - ps8
Opisthokonta - ps9
Holozoa - ps10
Filozoa - ps11
Choano./Metazoa - ps12
Metazoa - ps13
Myriazoa - ps14
Parahoxozoa - ps15
Bilateria - ps16
Protostomia - ps17
Ecdysozoa - ps18
Arthropoda - ps19
Mandibulata - ps20
Pancrustacea - ps21
Insecta - ps22
Eumetabola - ps23
Holometabola/Psocodea - ps24
Holometabola - ps25
Aparaglossata - ps26
Mecopterida - ps27
Diptera - ps28
Bibiono./Cyclorrhapha - ps29
Cyclorrhapha - ps30
Calyptratae/Drosophila - ps31
Drosophila - ps32
Sophophora - ps33
Obscura/Melanogaster_group - ps34
D_melanogaster_group - ps35
D_melanogaster_subgroup - ps36
sim./sech./mel. group - ps37
Drosophila_melanogaster - ps38
Phylostratum

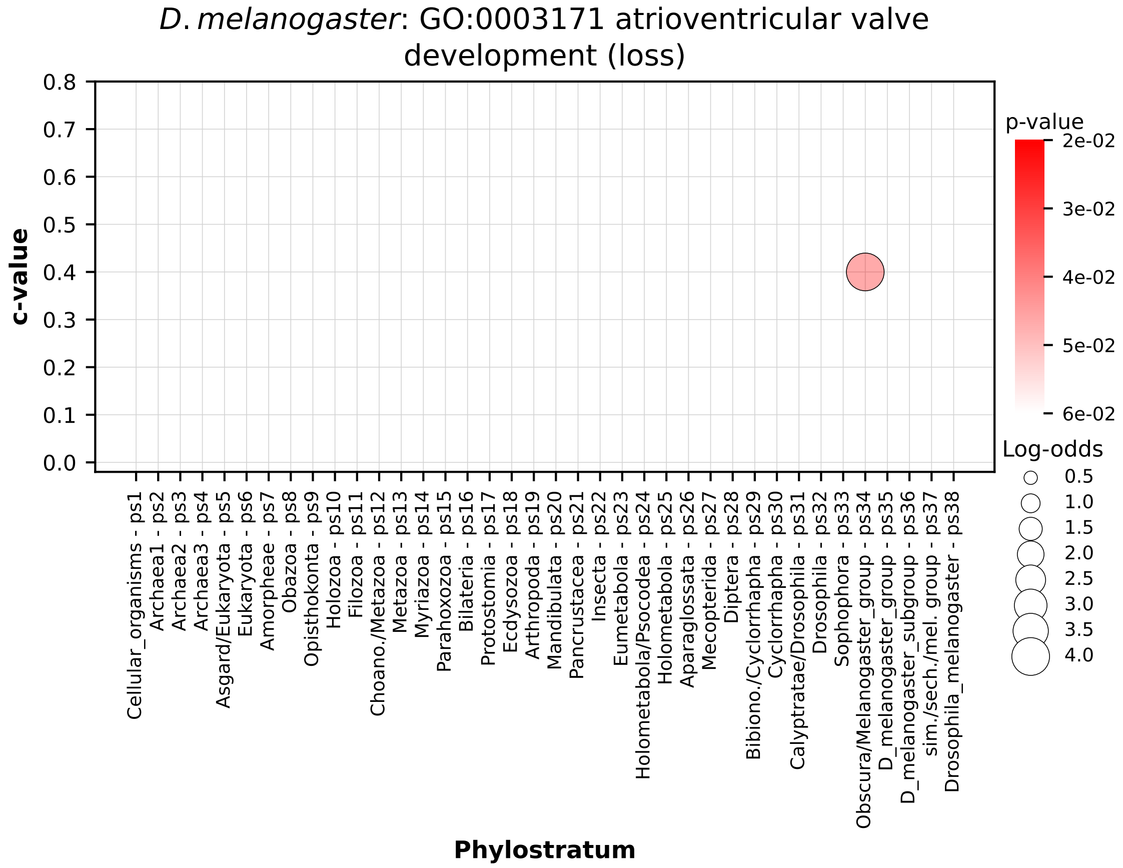
D. melanogaster: GO:0003171 atrioventricular valve development (loss)
c-value
0.0
0.1
0.2
0.3
0.4
0.5
0.6
0.7
0.8
p-value
2e-02
3e-02
4e-02
5e-02
6e-02
Log-odds
0.5
1.0
1.5
2.0
2.5
3.0
3.5
4.0
Cellular_organisms - ps1
Archaea1 - ps2
Archaea2 - ps3
Archaea3 - ps4
Asgard/Eukaryota - ps5
Eukaryota - ps6
Amorpheae - ps7
Obazoa - ps8
Opisthokonta - ps9
Holozoa - ps10
Filozoa - ps11
Choano./Metazoa - ps12
Metazoa - ps13
Myriazoa - ps14
Parahoxozoa - ps15
Bilateria - ps16
Protostomia - ps17
Ecdysozoa - ps18
Arthropoda - ps19
Mandibulata - ps20
Pancrustacea - ps21
Insecta - ps22
Eumetabola - ps23
Holometabola/Psocodea - ps24
Holometabola - ps25
Aparaglossata - ps26
Mecopterida - ps27
Diptera - ps28
Bibiono./Cyclorrhapha - ps29
Cyclorrhapha - ps30
Calyptratae/Drosophila - ps31
Drosophila - ps32
Sophophora - ps33
Obscura/Melanogaster_group - ps34
D_melanogaster_group - ps35
D_melanogaster_subgroup - ps36
sim./sech./mel. group - ps37
Drosophila_melanogaster - ps38
Phylostratum

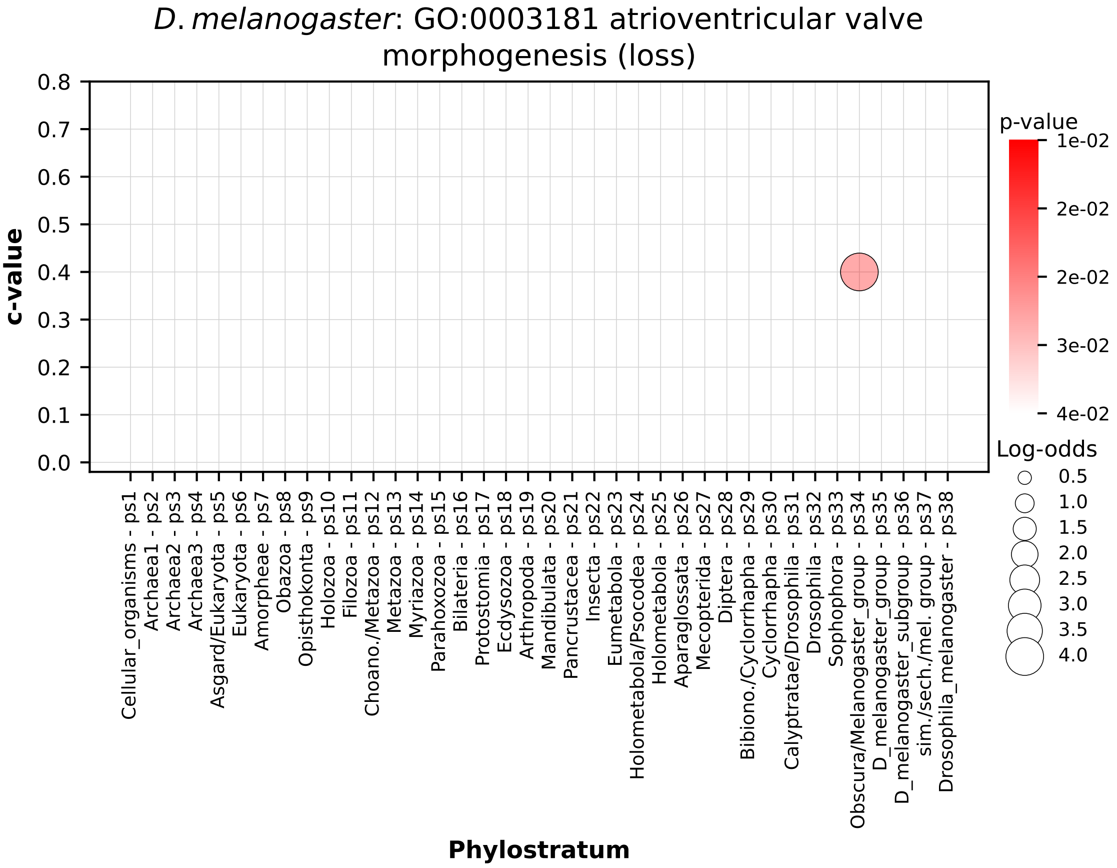

D. melanogaster: GO:0003181 atrioventricular valve morphogenesis (loss)
c-value
0.8
0.7
0.6
0.5
0.4
0.3
0.2
0.1
0.0
Phylostratum
Cellular_organisms - ps1
Archaea1 - ps2
Archaea2 - ps3
Archaea3 - ps4
Asgard/Eukaryota - ps5
Eukaryota - ps6
Amorpheae - ps7
Obazoa - ps8
Opisthokonta - ps9
Holozoa - ps10
Filozoa - ps11
Choano./Metazoa - ps12
Metazoa - ps13
Myriazoa - ps14
Parahoxozoa - ps15
Bilateria - ps16
Protostomia - ps17
Ecdysozoa - ps18
Arthropoda - ps19
Mandibulata - ps20
Pancrustacea - ps21
Insecta - ps22
Eumetabola - ps23
Holometabola/Psocodea - ps24
Holometabola - ps25
Aparaglossata - ps26
Mecopterida - ps27
Diptera - ps28
Bibiono./Cyclorrhapha - ps29
Cyclorrhapha - ps30
Calyptratae/Drosophila - ps31
Drosophila - ps32
Sophophora - ps33
Obscura/Melanogaster_group - ps34
D_melanogaster_group - ps35
D_melanogaster_subgroup - ps36
sim./sech./mel. group - ps37
Drosophila_melanogaster - ps38
p-value
1e-02
2e-02
2e-02
3e-02
4e-02
Log-odds
0.5
1.0
1.5
2.0
2.5
3.0
3.5
4.0

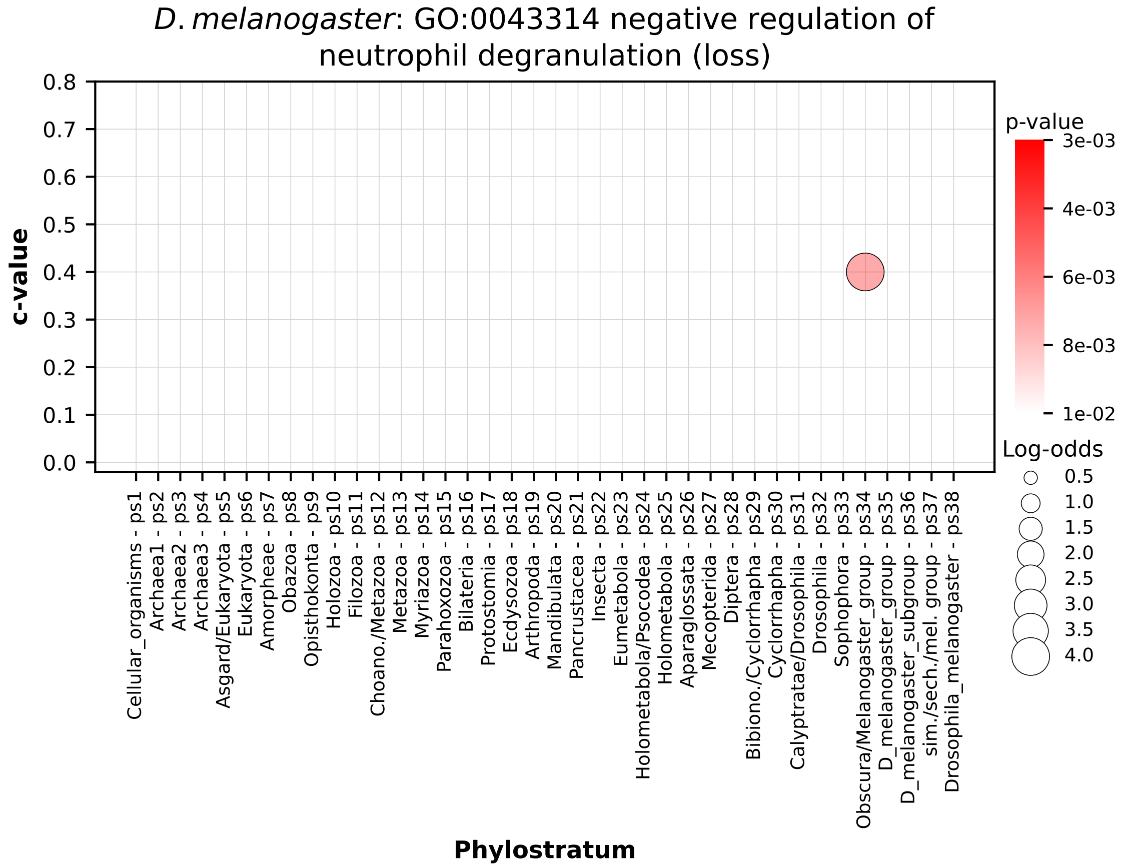
D. melanogaster: GO:0043314 negative regulation of neutrophil degranulation (loss)
c-value
0.8
0.7
0.6
0.5
0.4
0.3
0.2
0.1
0.0
Phylostratum
Cellular_organisms - ps1
Archaea1 - ps2
Archaea2 - ps3
Archaea3 - ps4
Asgard/Eukaryota - ps5
Eukaryota - ps6
Amorpheae - ps7
Obazoa - ps8
Opisthokonta - ps9
Holozoa - ps10
Filozoa - ps11
Choano./Metazoa - ps12
Metazoa - ps13
Myriazoa - ps14
Parahoxozoa - ps15
Bilateria - ps16
Protostomia - ps17
Ecdysozoa - ps18
Arthropoda - ps19
Mandibulata - ps20
Pancrustacea - ps21
Insecta - ps22
Eumetabola - ps23
Holometabola/Psocodea - ps24
Holometabola - ps25
Aparaglossata - ps26
Mecopterida - ps27
Diptera - ps28
Bibiono./Cyclorrhapha - ps29
Cyclorrhapha - ps30
Calyptratae/Drosophila - ps31
Drosophila - ps32
Sophophora - ps33
Obscura/Melanogaster_group - ps34
D_melanogaster_group - ps35
D_melanogaster_subgroup - ps36
sim./sech./mel. group - ps37
Drosophila_melanogaster - ps38
p-value
3e-03
4e-03
6e-03
8e-03
1e-02
Log-odds
0.5
1.0
1.5
2.0
2.5
3.0
3.5
4.0

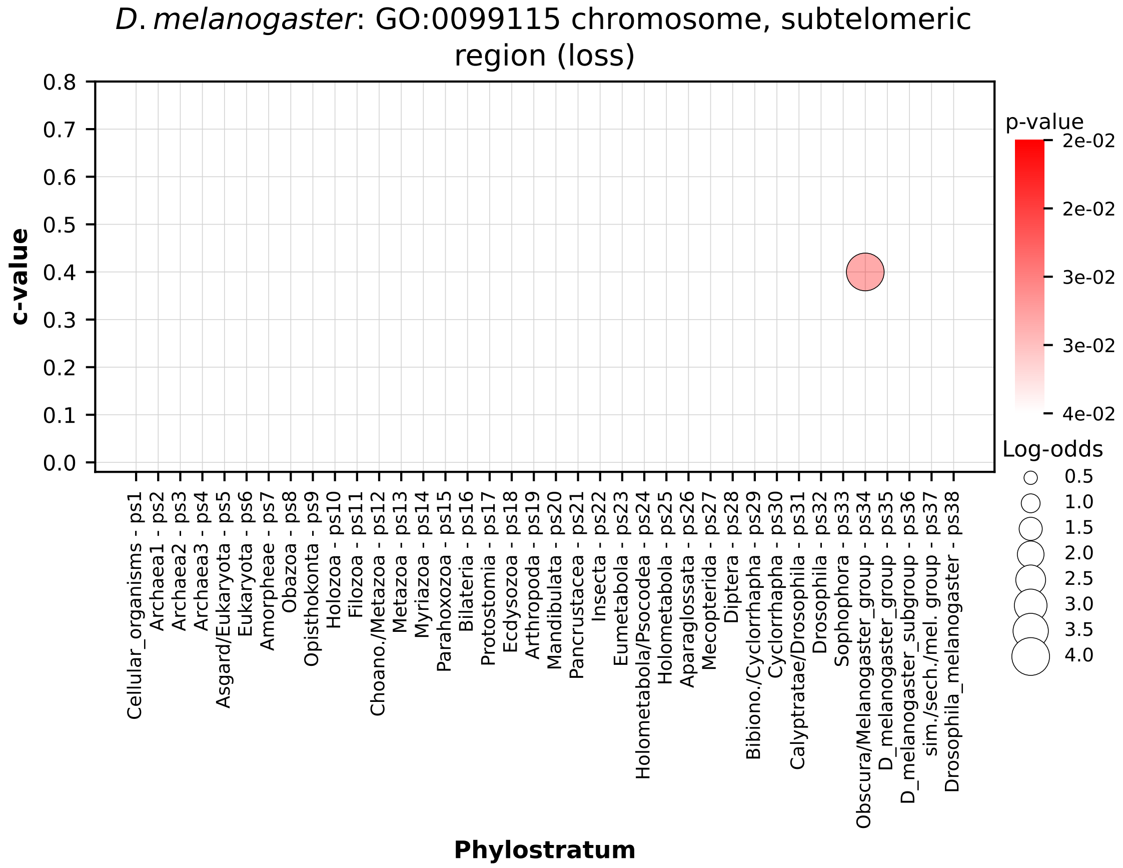

D. melanogaster: GO:0099115 chromosome, subtelomeric region (loss)
c-value
0.8
0.7
0.6
0.5
0.4
0.3
0.2
0.1
0.0
p-value
2e-02
2e-02
3e-02
3e-02
4e-02
Log-odds
0.5
1.0
1.5
2.0
2.5
3.0
3.5
4.0
Cellular_organisms - ps1
Archaea1 - ps2
Archaea2 - ps3
Archaea3 - ps4
Asgard/Eukaryota - ps5
Eukaryota - ps6
Amorpheae - ps7
Obazoa - ps8
Opisthokonta - ps9
Holozoa - ps10
Filozoa - ps11
Choano./Metazoa - ps12
Metazoa - ps13
Myriazoa - ps14
Parahoxozoa - ps15
Bilateria - ps16
Protostomia - ps17
Ecdysozoa - ps18
Arthropoda - ps19
Mandibulata - ps20
Pancrustacea - ps21
Insecta - ps22
Eumetabola - ps23
Holometabola/Psocodea - ps24
Holometabola - ps25
Aparaglossata - ps26
Mecopterida - ps27
Diptera - ps28
Bibiono./Cyclorrhapha - ps29
Cyclorrhapha - ps30
Calyptratae/Drosophila - ps31
Drosophila - ps32
Sophophora - ps33
Obscura/Melanogaster_group - ps34
D_melanogaster_group - ps35
D_melanogaster_subgroup - ps36
sim./sech./mel. group - ps37
Drosophila_melanogaster - ps38
Phylostratum

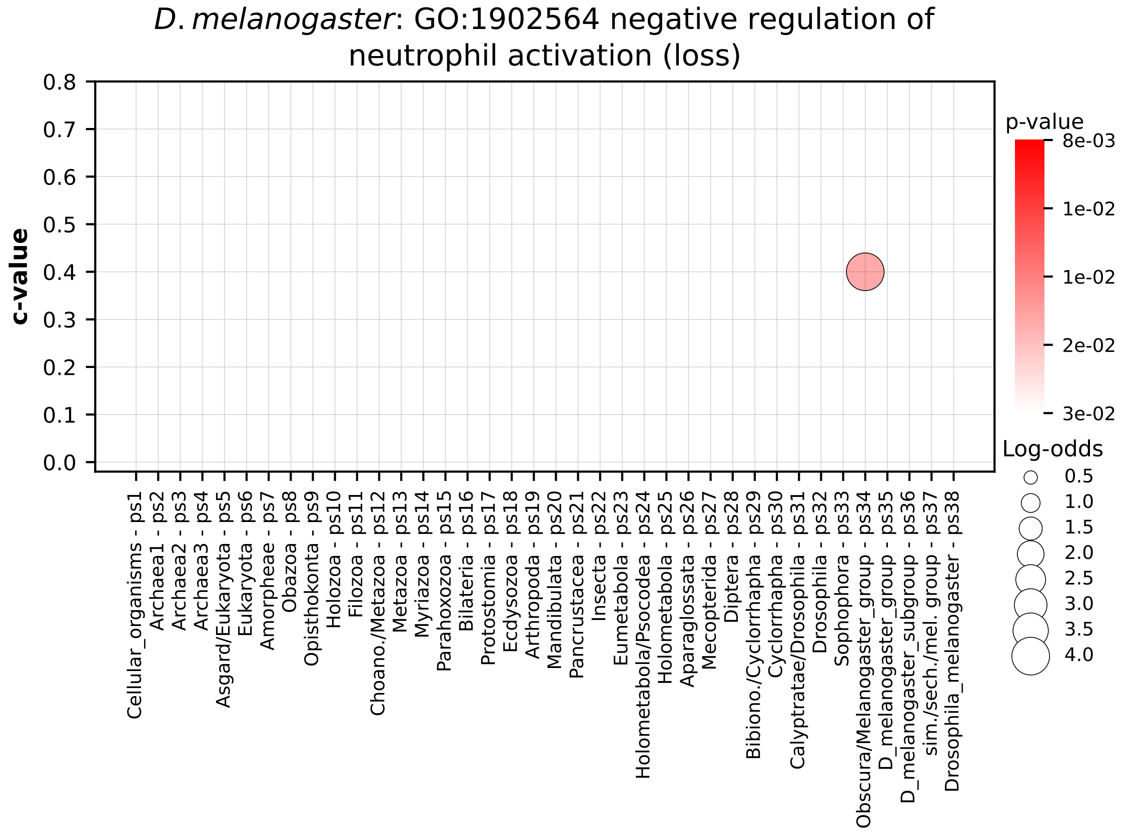

D. melanogaster: GO:1902564 negative regulation of neutrophil activation (loss)
c-value
0.0
0.1
0.2
0.3
0.4
0.5
0.6
0.7
0.8
Phylostratum
Cellular_organisms - ps1
Archaea1 - ps2
Archaea2 - ps3
Archaea3 - ps4
Asgard/Eukaryota - ps5
Eukaryota - ps6
Amorpheae - ps7
Obazoa - ps8
Opisthokonta - ps9
Holozoa - ps10
Filozoa - ps11
Choano./Metazoa - ps12
Metazoa - ps13
Myriazoa - ps14
Parahoxozoa - ps15
Bilateria - ps16
Protostomia - ps17
Ecdysozoa - ps18
Arthropoda - ps19
Mandibulata - ps20
Pancrustacea - ps21
Insecta - ps22
Eumetabola - ps23
Holometabola/Psocodea - ps24
Holometabola - ps25
Aparaglossata - ps26
Mecopterida - ps27
Diptera - ps28
Bibiono./Cyclorrhapha - ps29
Cyclorrhapha - ps30
Calyptratae/Drosophila - ps31
Drosophila - ps32
Sophophora - ps33
Obscura/Melanogaster_group - ps34
D_melanogaster_group - ps35
D_melanogaster_subgroup - ps36
sim./sech./mel. group - ps37
Drosophila_melanogaster - ps38
p-value
8e-03
1e-02
1e-02
2e-02
3e-02
Log-odds
0.5
1.0
1.5
2.0
2.5
3.0
3.5
4.0

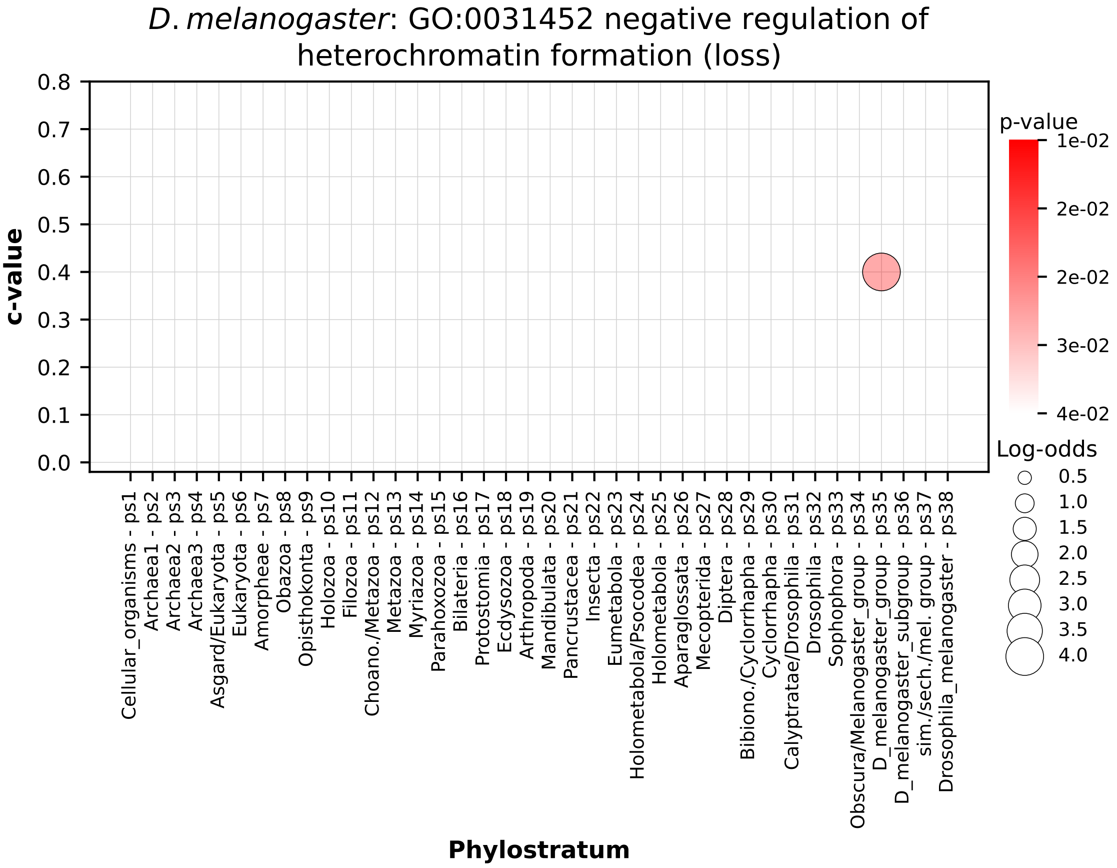

D. melanogaster: GO:0031452 negative regulation of heterochromatin formation (loss)
c-value
0.8
0.7
0.6
0.5
0.4
0.3
0.2
0.1
0.0
Phylostratum
Cellular_organisms - ps1
Archaea1 - ps2
Archaea2 - ps3
Archaea3 - ps4
Asgard/Eukaryota - ps5
Eukaryota - ps6
Amorpheae - ps7
Obazoa - ps8
Opisthokonta - ps9
Holozoa - ps10
Filozoa - ps11
Choano./Metazoa - ps12
Metazoa - ps13
Myriazoa - ps14
Parahoxozoa - ps15
Bilateria - ps16
Protostomia - ps17
Ecdysozoa - ps18
Arthropoda - ps19
Mandibulata - ps20
Pancrustacea - ps21
Insecta - ps22
Eumetabola - ps23
Holometabola/Psocodea - ps24
Holometabola - ps25
Aparaglossata - ps26
Mecopterida - ps27
Diptera - ps28
Bibiono./Cyclorrhapha - ps29
Cyclorrhapha - ps30
Calyptratae/Drosophila - ps31
Drosophila - ps32
Sophophora - ps33
Obscura/Melanogaster_group - ps34
D_melanogaster_group - ps35
D_melanogaster_subgroup - ps36
sim./sech./mel. group - ps37
Drosophila_melanogaster - ps38
p-value
1e-02
2e-02
2e-02
3e-02
4e-02
Log-odds
0.5
1.0
1.5
2.0
2.5
3.0
3.5
4.0

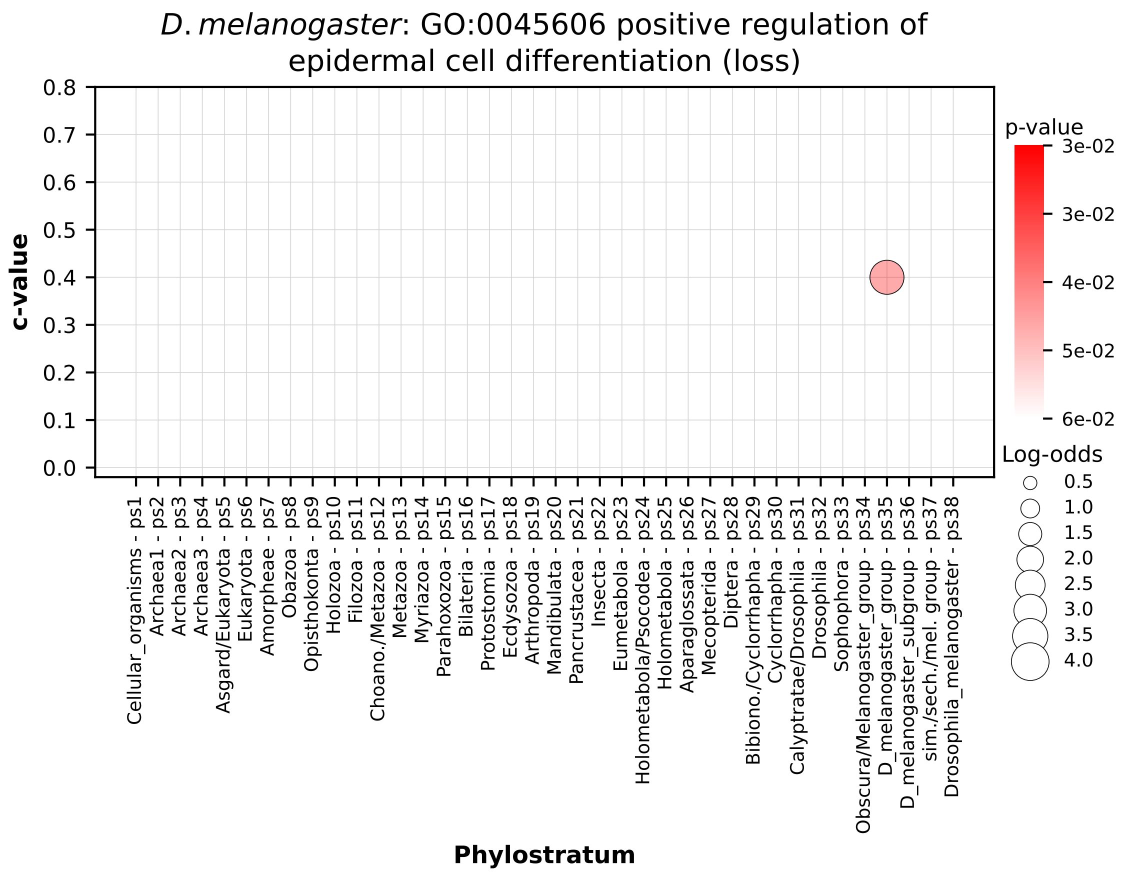

D. melanogaster: GO:0045606 positive regulation of epidermal cell differentiation (loss)
c-value
0.8
0.7
0.6
0.5
0.4
0.3
0.2
0.1
0.0
Phylostratum
Cellular_organisms - ps1
Archaea1 - ps2
Archaea2 - ps3
Archaea3 - ps4
Asgard/Eukaryota - ps5
Eukaryota - ps6
Amorpheae - ps7
Obazoa - ps8
Opisthokonta - ps9
Holozoa - ps10
Filozoa - ps11
Choano./Metazoa - ps12
Metazoa - ps13
Myriazoa - ps14
Parahoxozoa - ps15
Bilateria - ps16
Protostomia - ps17
Ecdysozoa - ps18
Arthropoda - ps19
Mandibulata - ps20
Pancrustacea - ps21
Insecta - ps22
Eumetabola - ps23
Holometabola/Psocodea - ps24
Holometabola - ps25
Aparaglossata - ps26
Mecopterida - ps27
Diptera - ps28
Bibiono./Cyclorrhapha - ps29
Cyclorrhapha - ps30
Calyptratae/Drosophila - ps31
Drosophila - ps32
Sophophora - ps33
Obscura/Melanogaster_group - ps34
D_melanogaster_group - ps35
D_melanogaster_subgroup - ps36
sim./sech./mel. group - ps37
Drosophila_melanogaster - ps38
p-value
3e-02
3e-02
4e-02
5e-02
6e-02
Log-odds
0.5
1.0
1.5
2.0
2.5
3.0
3.5
4.0

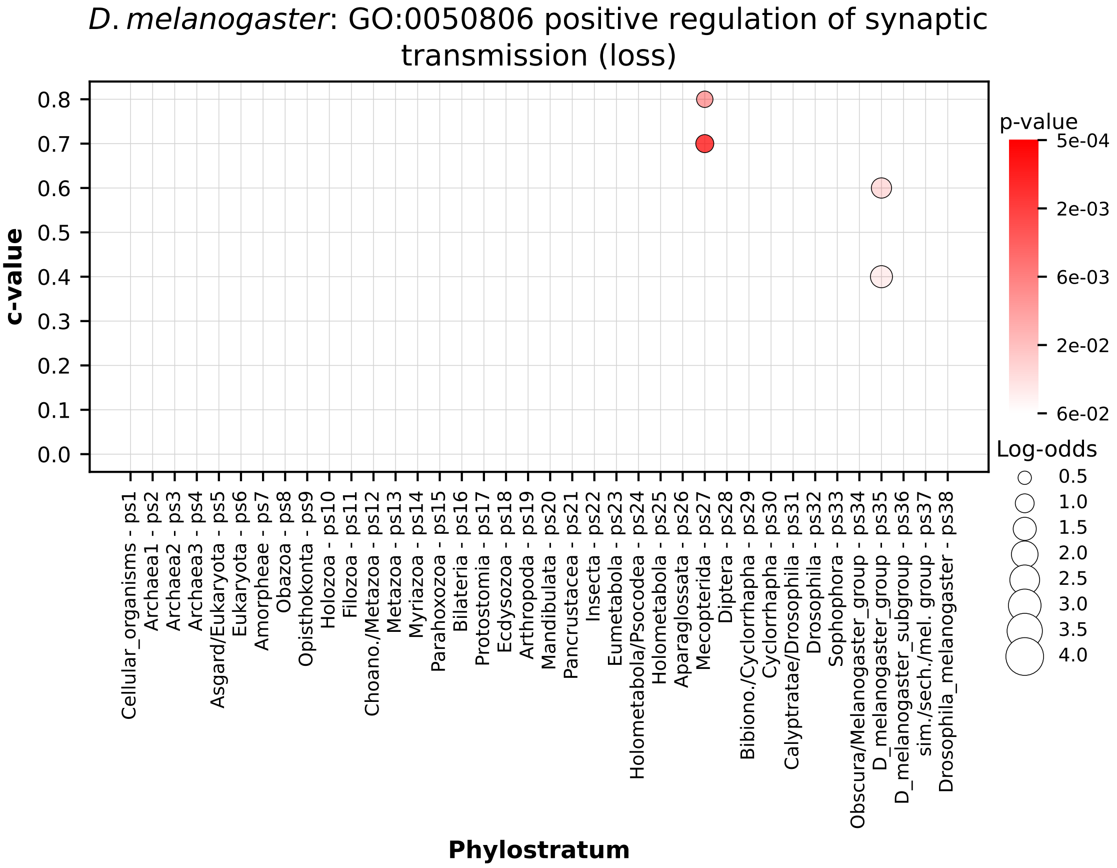
D. melanogaster: GO:0050806 positive regulation of synaptic transmission (loss)
c-value
0.8
0.7
0.6
0.5
0.4
0.3
0.2
0.1
0.0
Phylostratum
Cellular_organisms - ps1
Archaea1 - ps2
Archaea2 - ps3
Archaea3 - ps4
Asgard/Eukaryota - ps5
Eukaryota - ps6
Amorpheae - ps7
Obazoa - ps8
Opisthokonta - ps9
Holozoa - ps10
Filozoa - ps11
Choano./Metazoa - ps12
Metazoa - ps13
Myriazoa - ps14
Parahoxozoa - ps15
Bilateria - ps16
Protostomia - ps17
Ecdysozoa - ps18
Arthropoda - ps19
Mandibulata - ps20
Pancrustacea - ps21
Insecta - ps22
Eumetabola - ps23
Holometabola/Psocodea - ps24
Holometabola - ps25
Aparaglossata - ps26
Mecopterida - ps27
Diptera - ps28
Bibiono./Cyclorrhapha - ps29
Cyclorrhapha - ps30
Calyptratae/Drosophila - ps31
Drosophila - ps32
Sophophora - ps33
Obscura/Melanogaster_group - ps34
D_melanogaster_group - ps35
D_melanogaster_subgroup - ps36
sim./sech./mel. group - ps37
Drosophila_melanogaster - ps38
p-value
5e-04
2e-03
6e-03
2e-02
6e-02
Log-odds
0.5
1.0
1.5
2.0
2.5
3.0
3.5
4.0

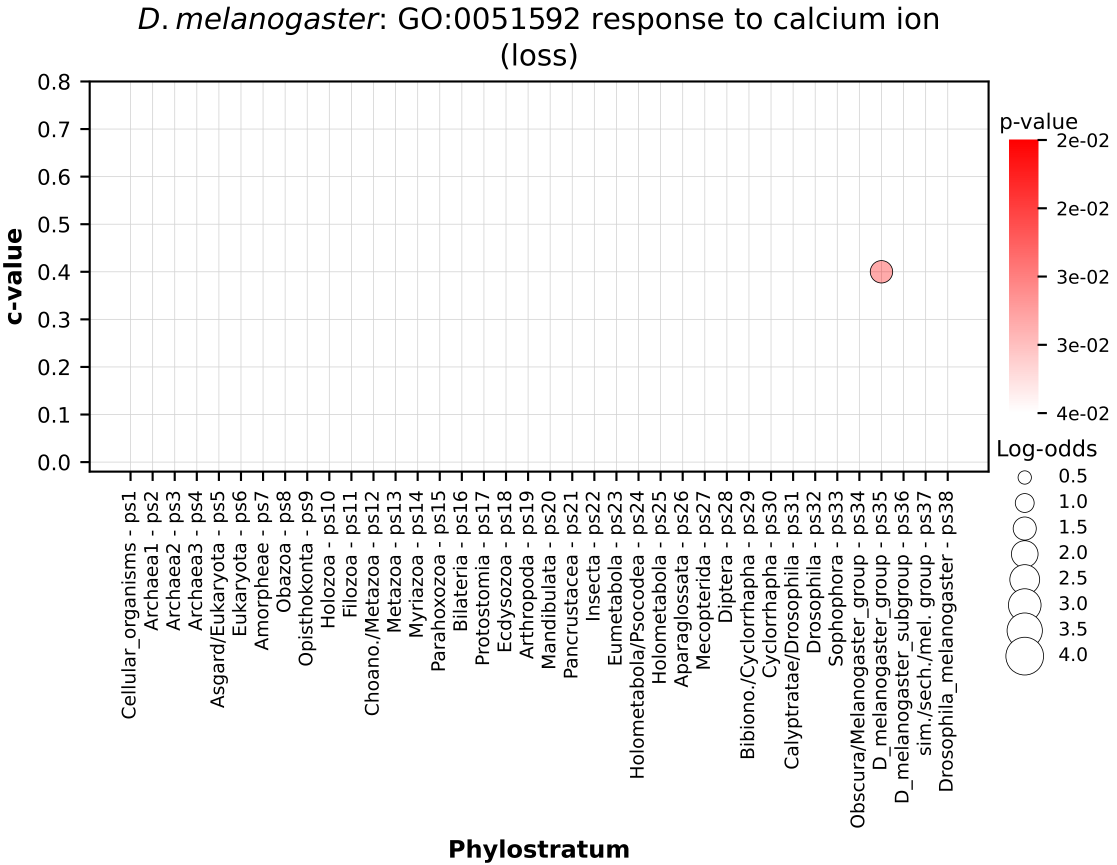

D. melanogaster: GO:0051592 response to calcium ion
(loss)
c-value
0.8
0.7
0.6
0.5
0.4
0.3
0.2
0.1
0.0
p-value
2e-02
2e-02
3e-02
3e-02
4e-02
Log-odds
0.5
1.0
1.5
2.0
2.5
3.0
3.5
4.0
Cellular_organisms - ps1
Archaea1 - ps2
Archaea2 - ps3
Archaea3 - ps4
Asgard/Eukaryota - ps5
Eukaryota - ps6
Amorpheae - ps7
Obazoa - ps8
Opisthokonta - ps9
Holozoa - ps10
Filozoa - ps11
Choano./Metazoa - ps12
Metazoa - ps13
Myriazoa - ps14
Parahoxozoa - ps15
Bilateria - ps16
Protostomia - ps17
Ecdysozoa - ps18
Arthropoda - ps19
Mandibulata - ps20
Pancrustacea - ps21
Insecta - ps22
Eumetabola - ps23
Holometabola/Psocodea - ps24
Holometabola - ps25
Aparaglossata - ps26
Mecopterida - ps27
Diptera - ps28
Bibiono./Cyclorrhapha - ps29
Cyclorrhapha - ps30
Calyptratae/Drosophila - ps31
Drosophila - ps32
Sophophora - ps33
Obscura/Melanogaster_group - ps34
D_melanogaster_group - ps35
D_melanogaster_subgroup - ps36
sim./sech./mel. group - ps37
Drosophila_melanogaster - ps38
Phylostratum

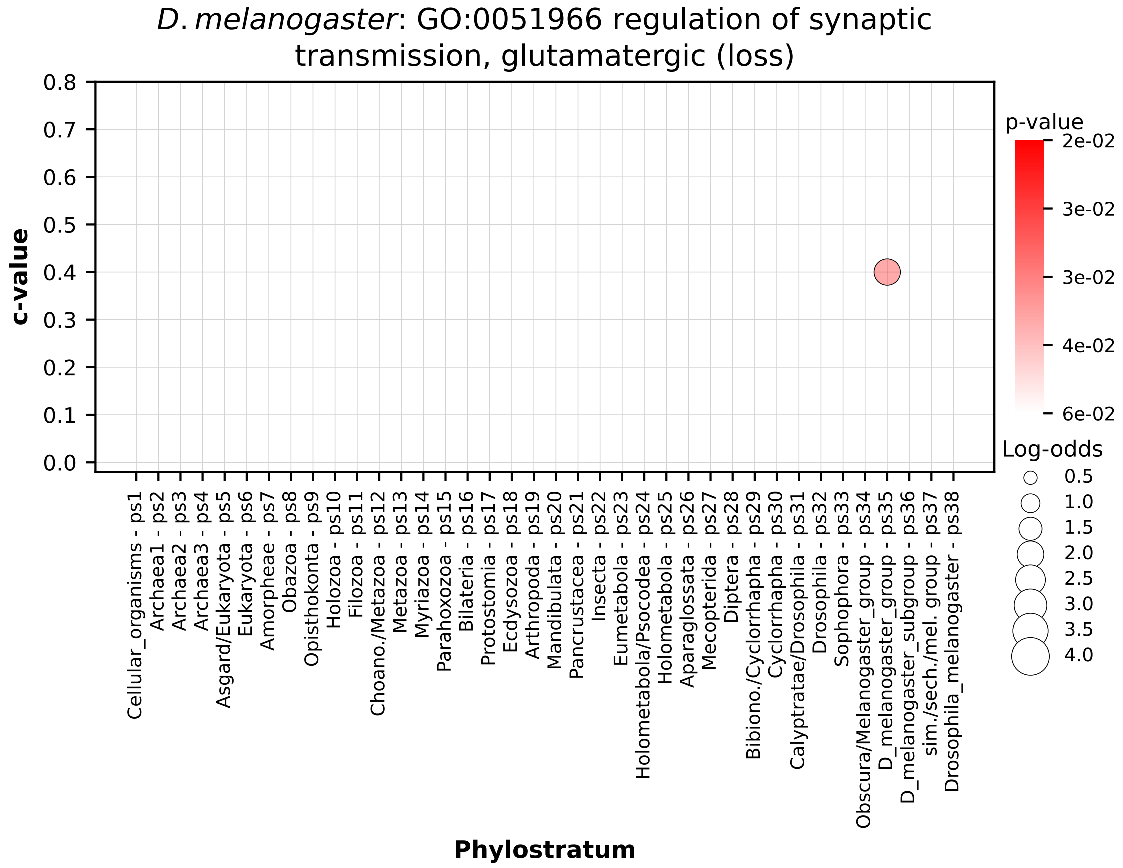

D. melanogaster: GO:0051966 regulation of synaptic transmission, glutamatergic (loss)
c-value
0.8
0.7
0.6
0.5
0.4
0.3
0.2
0.1
0.0
Phylostratum
Cellular_organisms - ps1
Archaea1 - ps2
Archaea2 - ps3
Archaea3 - ps4
Asgard/Eukaryota - ps5
Eukaryota - ps6
Amorpheae - ps7
Obazoa - ps8
Opisthokonta - ps9
Holozoa - ps10
Filozoa - ps11
Choano./Metazoa - ps12
Metazoa - ps13
Myriazoa - ps14
Parahoxozoa - ps15
Bilateria - ps16
Protostomia - ps17
Ecdysozoa - ps18
Arthropoda - ps19
Mandibulata - ps20
Pancrustacea - ps21
Insecta - ps22
Eumetabola - ps23
Holometabola/Psocodea - ps24
Holometabola - ps25
Aparaglossata - ps26
Mecopterida - ps27
Diptera - ps28
Bibiono./Cyclorrhapha - ps29
Cyclorrhapha - ps30
Calyptratae/Drosophila - ps31
Drosophila - ps32
Sophophora - ps33
Obscura/Melanogaster_group - ps34
D_melanogaster_group - ps35
D_melanogaster_subgroup - ps36
sim./sech./mel. group - ps37
Drosophila_melanogaster - ps38
p-value
2e-02
3e-02
3e-02
4e-02
6e-02
Log-odds
0.5
1.0
1.5
2.0
2.5
3.0
3.5
4.0

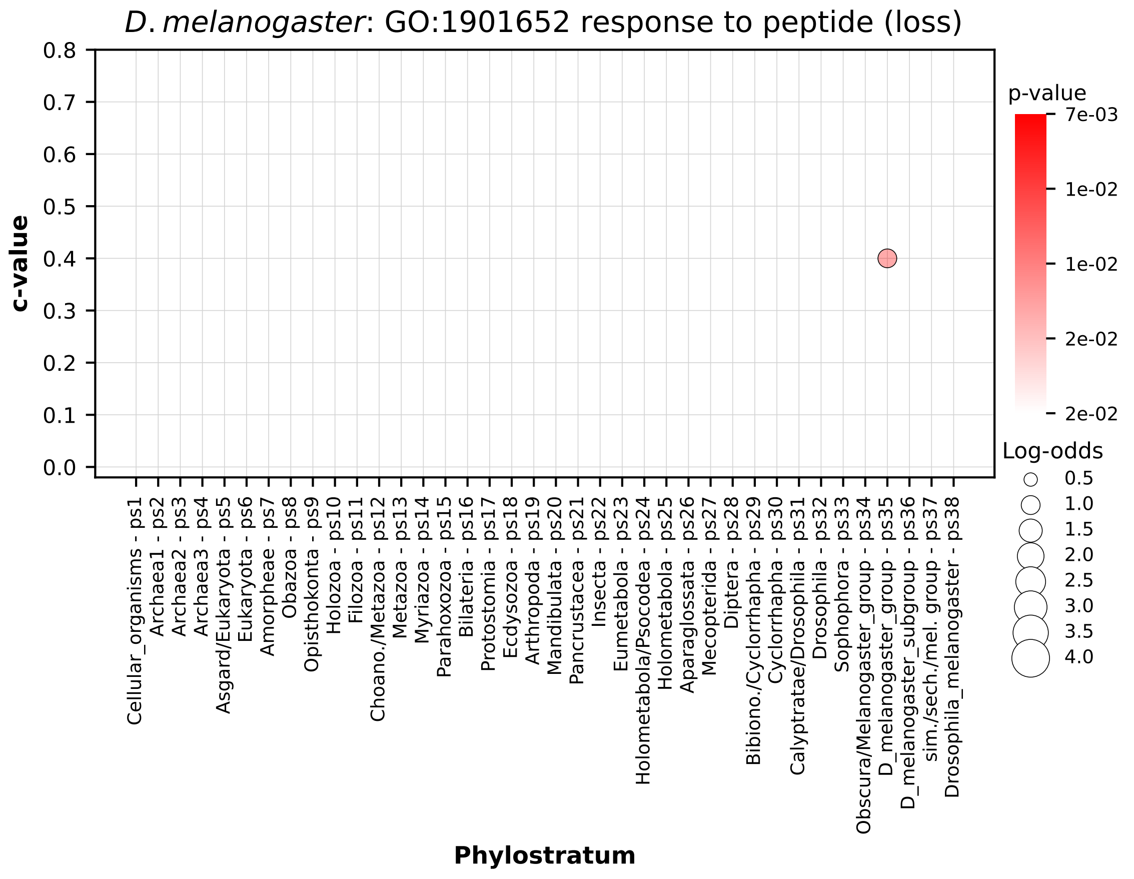
D. melanogaster: GO:1901652 response to peptide (loss)
c-value
0.0
0.1
0.2
0.3
0.4
0.5
0.6
0.7
0.8
Phylostratum
Cellular_organisms - ps1
Archaea1 - ps2
Archaea2 - ps3
Archaea3 - ps4
Asgard/Eukaryota - ps5
Eukaryota - ps6
Amorpheae - ps7
Obazoa - ps8
Opisthokonta - ps9
Holozoa - ps10
Filozoa - ps11
Choano./Metazoa - ps12
Metazoa - ps13
Myriazoa - ps14
Parahoxozoa - ps15
Bilateria - ps16
Protostomia - ps17
Ecdysozoa - ps18
Arthropoda - ps19
Mandibulata - ps20
Pancrustacea - ps21
Insecta - ps22
Eumetabola - ps23
Holometabola/Psocodea - ps24
Holometabola - ps25
Aparaglossata - ps26
Mecopterida - ps27
Diptera - ps28
Bibiono./Cyclorrhapha - ps29
Cyclorrhapha - ps30
Calyptratae/Drosophila - ps31
Drosophila - ps32
Sophophora - ps33
Obscura/Melanogaster_group - ps34
D_melanogaster_group - ps35
D_melanogaster_subgroup - ps36
sim./sech./mel. group - ps37
Drosophila_melanogaster - ps38
p-value
7e-03
1e-02
1e-02
2e-02
2e-02
Log-odds
0.5
1.0
1.5
2.0
2.5
3.0
3.5
4.0

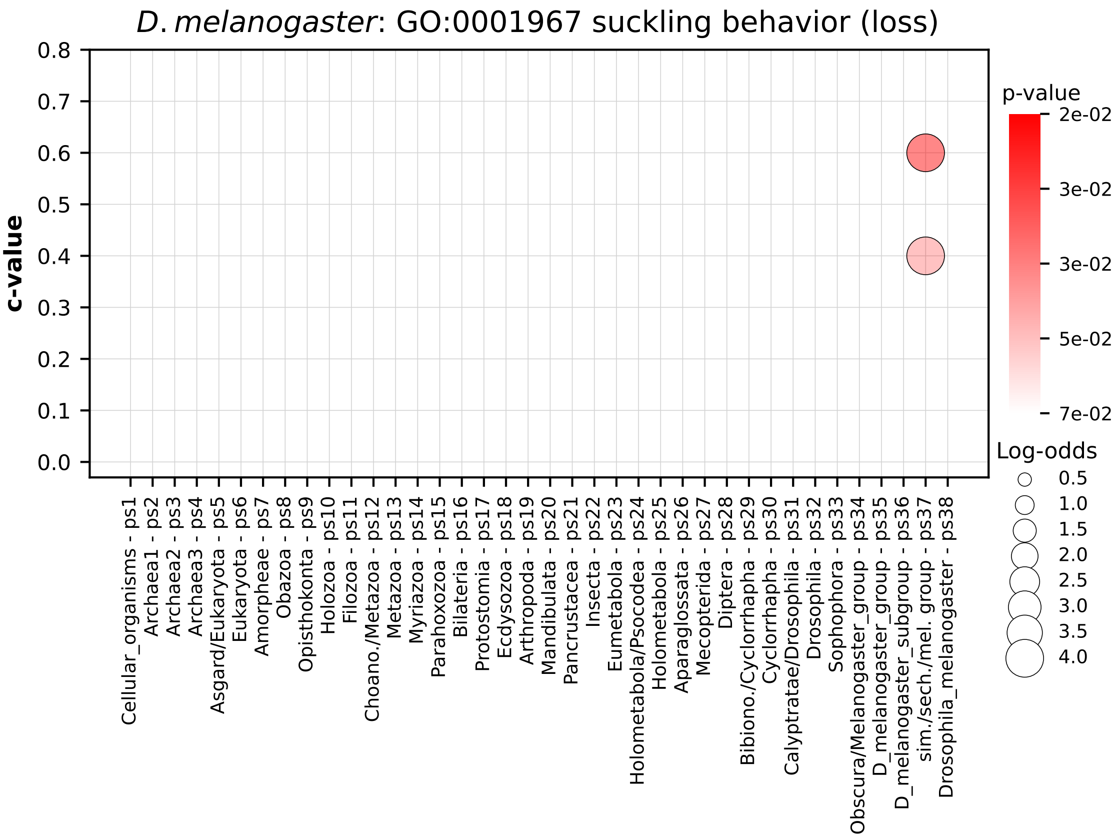
D. melanogaster: GO:0001967 suckling behavior (loss)
c-value
0.8
0.7
0.6
0.5
0.4
0.3
0.2
0.1
0.0
Phylostratum
Cellular_organisms - ps1
Archaea1 - ps2
Archaea2 - ps3
Archaea3 - ps4
Asgard/Eukaryota - ps5
Eukaryota - ps6
Amorpheae - ps7
Obazoa - ps8
Opisthokonta - ps9
Holozoa - ps10
Filozoa - ps11
Choano./Metazoa - ps12
Metazoa - ps13
Myriazoa - ps14
Parahoxozoa - ps15
Bilateria - ps16
Protostomia - ps17
Ecdysozoa - ps18
Arthropoda - ps19
Mandibulata - ps20
Pancrustacea - ps21
Insecta - ps22
Eumetabola - ps23
Holometabola/Psocodea - ps24
Holometabola - ps25
Aparaglossata - ps26
Mecopterida - ps27
Diptera - ps28
Bibiono./Cyclorrhapha - ps29
Cyclorrhapha - ps30
Calyptratae/Drosophila - ps31
Drosophila - ps32
Sophophora - ps33
Obscura/Melanogaster_group - ps34
D_melanogaster_group - ps35
D_melanogaster_subgroup - ps36
sim./sech./mel. group - ps37
Drosophila_melanogaster - ps38
p-value
2e-02
3e-02
3e-02
5e-02
7e-02
Log-odds
0.5
1.0
1.5
2.0
2.5
3.0
3.5
4.0

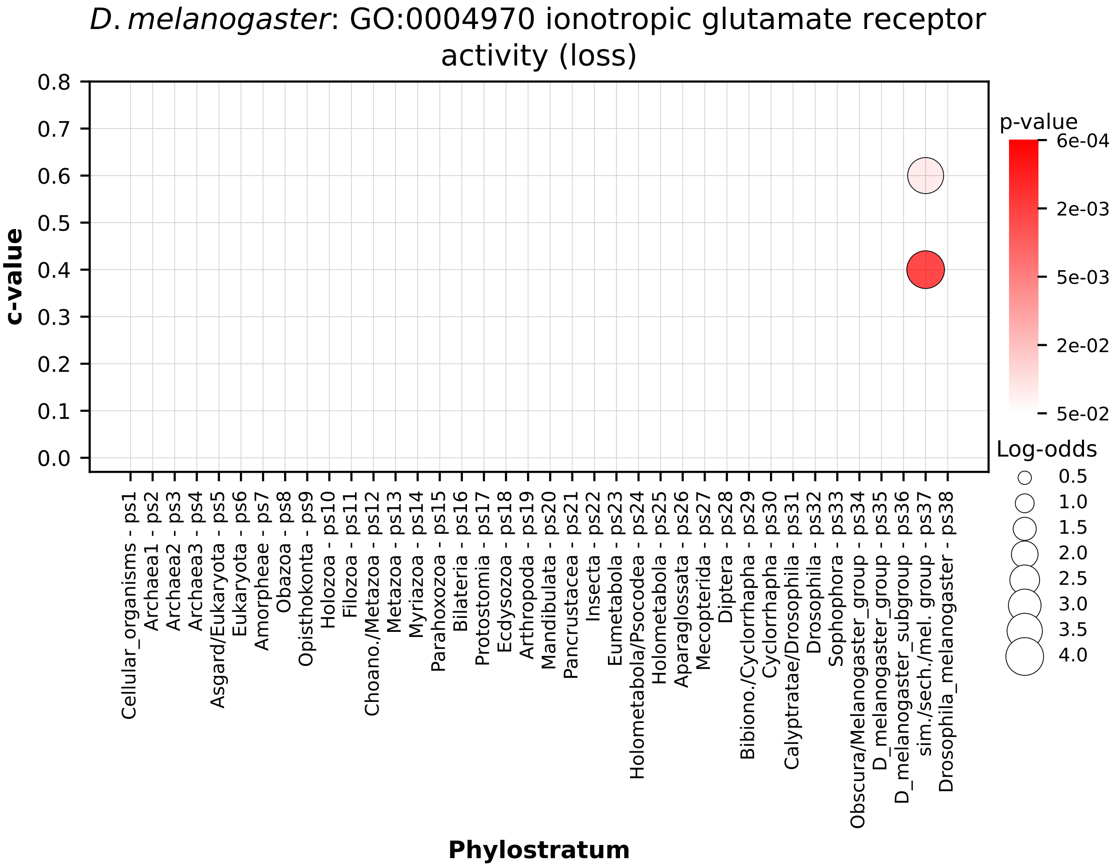

D. melanogaster: GO:0004970 ionotropic glutamate receptor activity (loss)
c-value
0.8
0.7
0.6
0.5
0.4
0.3
0.2
0.1
0.0
Phylostratum
Cellular_organisms - ps1
Archaea1 - ps2
Archaea2 - ps3
Archaea3 - ps4
Asgard/Eukaryota - ps5
Eukaryota - ps6
Amorpheae - ps7
Obazoa - ps8
Opisthokonta - ps9
Holozoa - ps10
Filozoa - ps11
Choano./Metazoa - ps12
Metazoa - ps13
Myriazoa - ps14
Parahoxozoa - ps15
Bilateria - ps16
Protostomia - ps17
Ecdysozoa - ps18
Arthropoda - ps19
Mandibulata - ps20
Pancrustacea - ps21
Insecta - ps22
Eumetabola - ps23
Holometabola/Psocodea - ps24
Holometabola - ps25
Aparaglossata - ps26
Mecopterida - ps27
Diptera - ps28
Bibiono./Cyclorrhapha - ps29
Cyclorrhapha - ps30
Calyptratae/Drosophila - ps31
Drosophila - ps32
Sophophora - ps33
Obscura/Melanogaster_group - ps34
D_melanogaster_group - ps35
D_melanogaster_subgroup - ps36
sim./sech./mel. group - ps37
Drosophila_melanogaster - ps38
p-value
6e-04
2e-03
5e-03
2e-02
5e-02
Log-odds
0.5
1.0
1.5
2.0
2.5
3.0
3.5
4.0

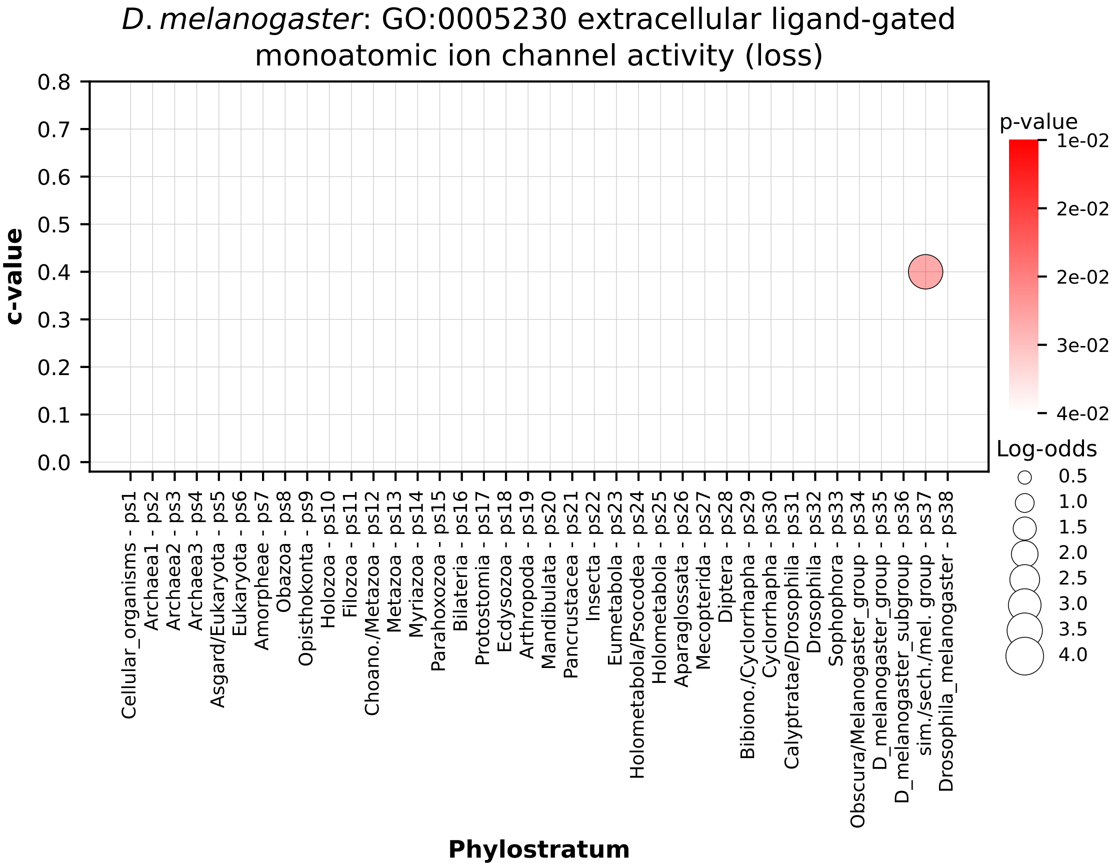

D. melanogaster: GO:0005230 extracellular ligand-gated monoatomic ion channel activity (loss)
c-value
0.8
0.7
0.6
0.5
0.4
0.3
0.2
0.1
0.0
Phylostratum
Cellular_organisms - ps1
Archaea1 - ps2
Archaea2 - ps3
Archaea3 - ps4
Asgard/Eukaryota - ps5
Eukaryota - ps6
Amorpheae - ps7
Obazoa - ps8
Opisthokonta - ps9
Holozoa - ps10
Filozoa - ps11
Choano./Metazoa - ps12
Metazoa - ps13
Myriazoa - ps14
Parahoxozoa - ps15
Bilateria - ps16
Protostomia - ps17
Ecdysozoa - ps18
Arthropoda - ps19
Mandibulata - ps20
Pancrustacea - ps21
Insecta - ps22
Eumetabola - ps23
Holometabola/Psocodea - ps24
Holometabola - ps25
Aparaglossata - ps26
Mecopterida - ps27
Diptera - ps28
Bibiono./Cyclorrhapha - ps29
Cyclorrhapha - ps30
Calyptratae/Drosophila - ps31
Drosophila - ps32
Sophophora - ps33
Obscura/Melanogaster_group - ps34
D_melanogaster_group - ps35
D_melanogaster_subgroup - ps36
sim./sech./mel. group - ps37
Drosophila_melanogaster - ps38
p-value
1e-02
2e-02
2e-02
3e-02
4e-02
Log-odds
0.5
1.0
1.5
2.0
2.5
3.0
3.5
4.0

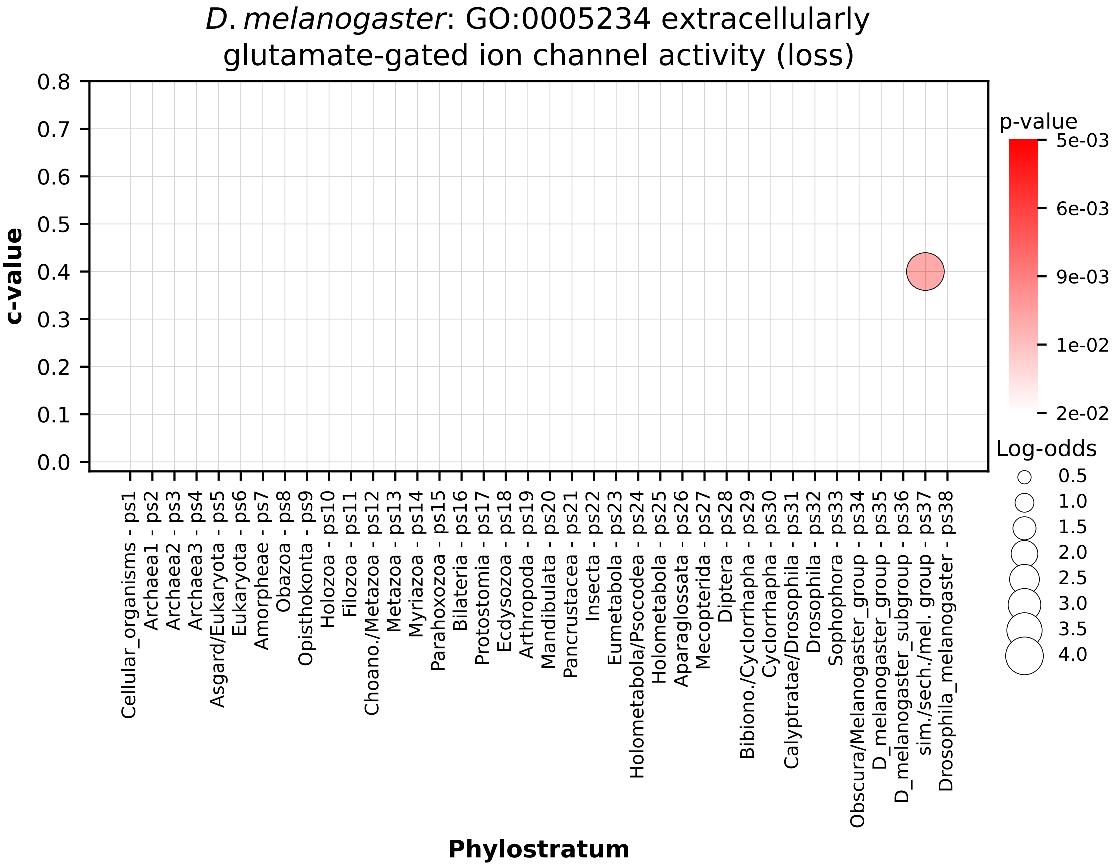
D. melanogaster: GO:0005234 extracellularly glutamate-gated ion channel activity (loss)
c-value
Phylostratum
p-value
5e-03
6e-03
9e-03
1e-02
2e-02
Log-odds
0.5
1.0
1.5
2.0
2.5
3.0
3.5
4.0
Cellular_organisms - ps1
Archaea1 - ps2
Archaea2 - ps3
Archaea3 - ps4
Asgard/Eukaryota - ps5
Eukaryota - ps6
Amorpheae - ps7
Obazoa - ps8
Opisthokonta - ps9
Holozoa - ps10
Filozoa - ps11
Choano./Metazoa - ps12
Metazoa - ps13
Myriazoa - ps14
Parahoxozoa - ps15
Bilateria - ps16
Protostomia - ps17
Ecdysozoa - ps18
Arthropoda - ps19
Mandibulata - ps20
Pancrustacea - ps21
Insecta - ps22
Eumetabola - ps23
Holometabola/Psocodea - ps24
Holometabola - ps25
Aparaglossata - ps26
Mecopterida - ps27
Diptera - ps28
Bibiono./Cyclorrhapha - ps29
Cyclorrhapha - ps30
Calyptratae/Drosophila - ps31
Drosophila - ps32
Sophophora - ps33
Obscura/Melanogaster_group - ps34
D_melanogaster_group - ps35
D_melanogaster_subgroup - ps36
sim./sech./mel. group - ps37
Drosophila_melanogaster - ps38

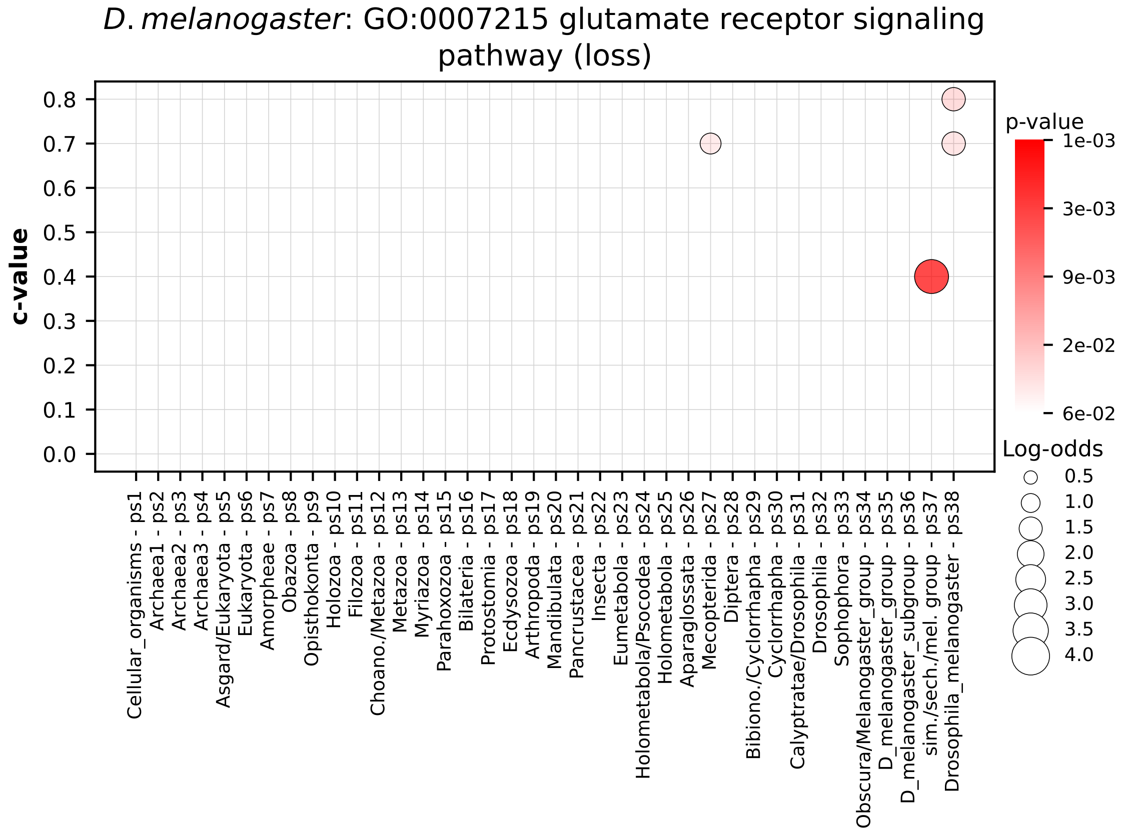

D. melanogaster: GO:0007215 glutamate receptor signaling pathway (loss)
c-value
0.8
0.7
0.6
0.5
0.4
0.3
0.2
0.1
0.0
Phylostratum
Cellular_organisms - ps1
Archaea1 - ps2
Archaea2 - ps3
Archaea3 - ps4
Asgard/Eukaryota - ps5
Eukaryota - ps6
Amorpheae - ps7
Obazoa - ps8
Opisthokonta - ps9
Holozoa - ps10
Filozoa - ps11
Choano./Metazoa - ps12
Metazoa - ps13
Myriazoa - ps14
Parahoxozoa - ps15
Bilateria - ps16
Protostomia - ps17
Ecdysozoa - ps18
Arthropoda - ps19
Mandibulata - ps20
Pancrustacea - ps21
Insecta - ps22
Eumetabola - ps23
Holometabola/Psocodea - ps24
Holometabola - ps25
Aparaglossata - ps26
Mecopterida - ps27
Diptera - ps28
Bibiono./Cyclorrhapha - ps29
Cyclorrhapha - ps30
Calyptratae/Drosophila - ps31
Drosophila - ps32
Sophophora - ps33
Obscura/Melanogaster_group - ps34
D_melanogaster_group - ps35
D_melanogaster_subgroup - ps36
sim./sech./mel. group - ps37
Drosophila_melanogaster - ps38
p-value
1e-03
3e-03
9e-03
2e-02
6e-02
Log-odds
0.5
1.0
1.5
2.0
2.5
3.0
3.5
4.0

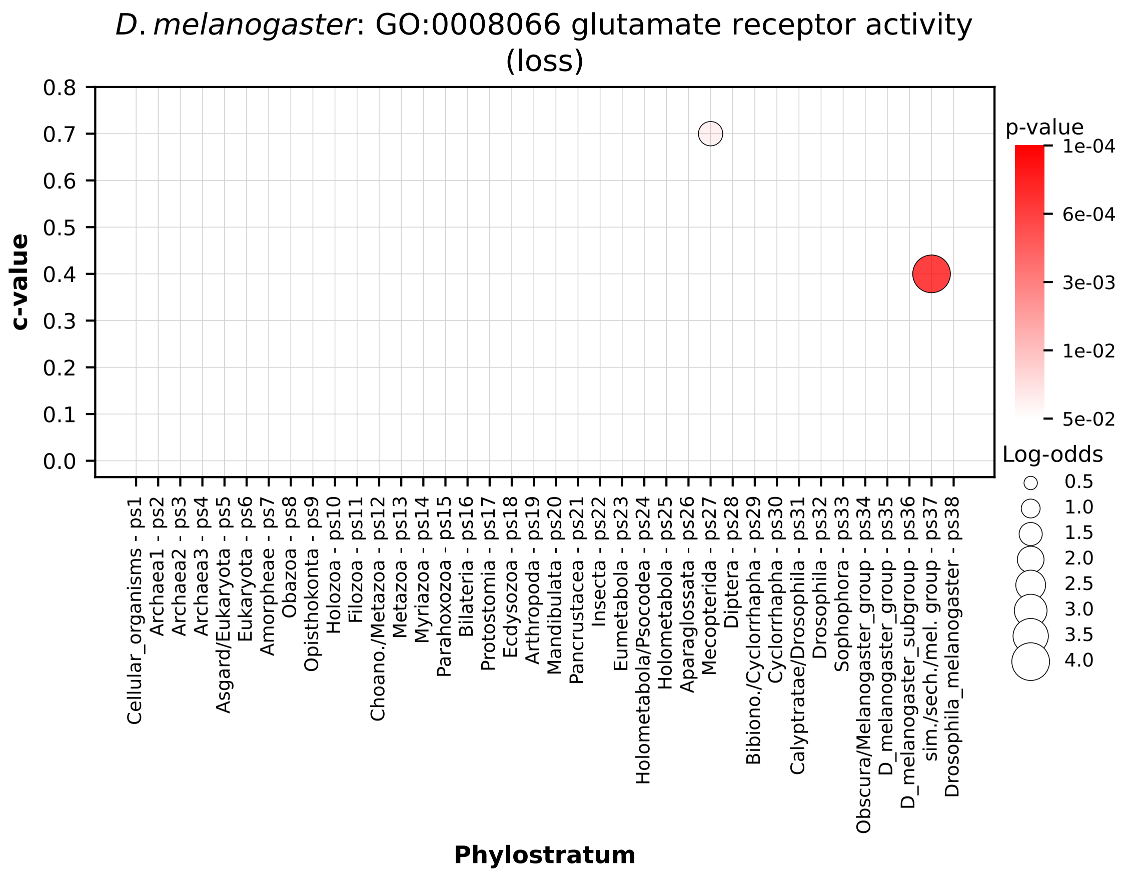

D. melanogaster: GO:0008066 glutamate receptor activity
(loss)
c-value
0.8
0.7
0.6
0.5
0.4
0.3
0.2
0.1
0.0
p-value
1e-04
6e-04
3e-03
1e-02
5e-02
Log-odds
0.5
1.0
1.5
2.0
2.5
3.0
3.5
4.0
Cellular_organisms - ps1
Archaea1 - ps2
Archaea2 - ps3
Archaea3 - ps4
Asgard/Eukaryota - ps5
Eukaryota - ps6
Amorpheae - ps7
Obazoa - ps8
Opisthokonta - ps9
Holozoa - ps10
Filozoa - ps11
Choano./Metazoa - ps12
Metazoa - ps13
Myriazoa - ps14
Parahoxozoa - ps15
Bilateria - ps16
Protostomia - ps17
Ecdysozoa - ps18
Arthropoda - ps19
Mandibulata - ps20
Pancrustacea - ps21
Insecta - ps22
Eumetabola - ps23
Holometabola/Psocodea - ps24
Holometabola - ps25
Aparaglossata - ps26
Mecopterida - ps27
Diptera - ps28
Bibiono./Cyclorrhapha - ps29
Cyclorrhapha - ps30
Calyptratae/Drosophila - ps31
Drosophila - ps32
Sophophora - ps33
Obscura/Melanogaster_group - ps34
D_melanogaster_group - ps35
D_melanogaster_subgroup - ps36
sim./sech./mel. group - ps37
Drosophila_melanogaster - ps38
Phylostratum

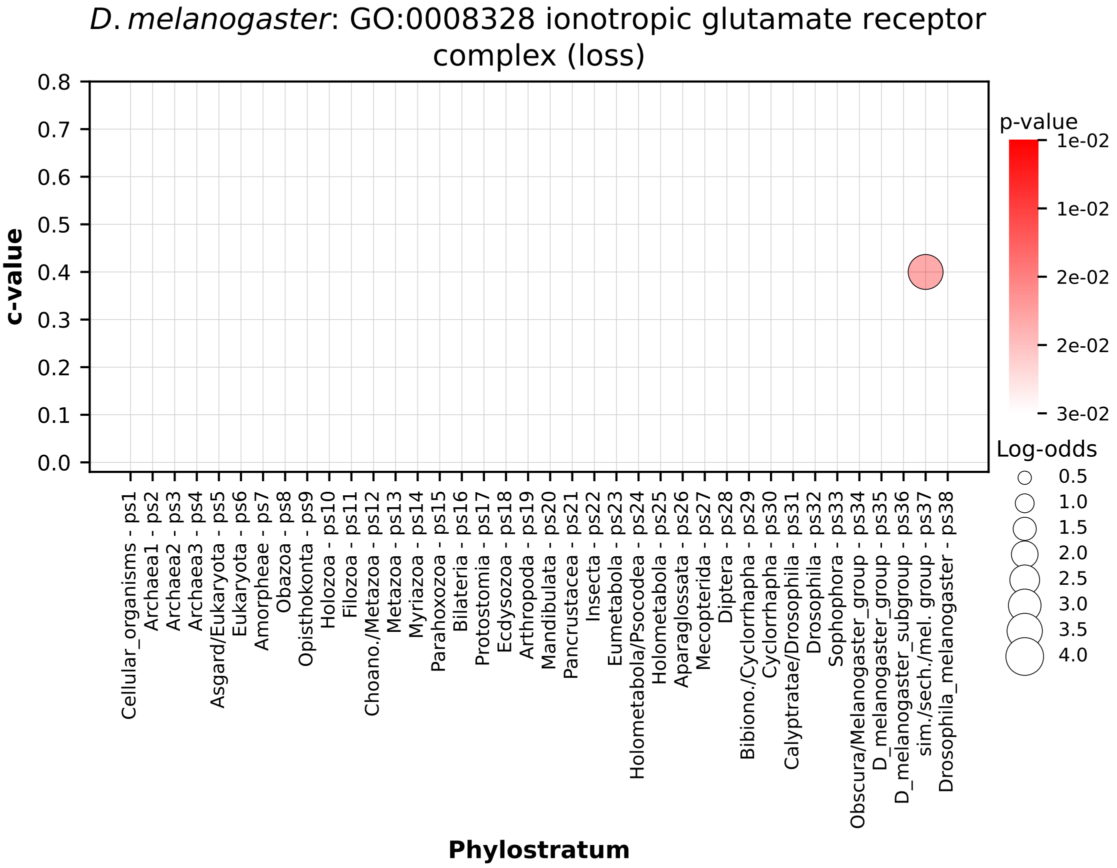

D. melanogaster: GO:0008328 ionotropic glutamate receptor complex (loss)
c-value
0.8
0.7
0.6
0.5
0.4
0.3
0.2
0.1
0.0
p-value
1e-02
1e-02
2e-02
2e-02
3e-02
Log-odds
0.5
1.0
1.5
2.0
2.5
3.0
3.5
4.0
Cellular_organisms - ps1
Archaea1 - ps2
Archaea2 - ps3
Archaea3 - ps4
Asgard/Eukaryota - ps5
Eukaryota - ps6
Amorpheae - ps7
Obazoa - ps8
Opisthokonta - ps9
Holozoa - ps10
Filozoa - ps11
Choano./Metazoa - ps12
Metazoa - ps13
Myriazoa - ps14
Parahoxozoa - ps15
Bilateria - ps16
Protostomia - ps17
Ecdysozoa - ps18
Arthropoda - ps19
Mandibulata - ps20
Pancrustacea - ps21
Insecta - ps22
Eumetabola - ps23
Holometabola/Psocodea - ps24
Holometabola - ps25
Aparaglossata - ps26
Mecopterida - ps27
Diptera - ps28
Bibiono./Cyclorrhapha - ps29
Cyclorrhapha - ps30
Calyptratae/Drosophila - ps31
Drosophila - ps32
Sophophora - ps33
Obscura/Melanogaster_group - ps34
D_melanogaster_group - ps35
D_melanogaster_subgroup - ps36
sim./sech./mel. group - ps37
Drosophila_melanogaster - ps38
Phylostratum

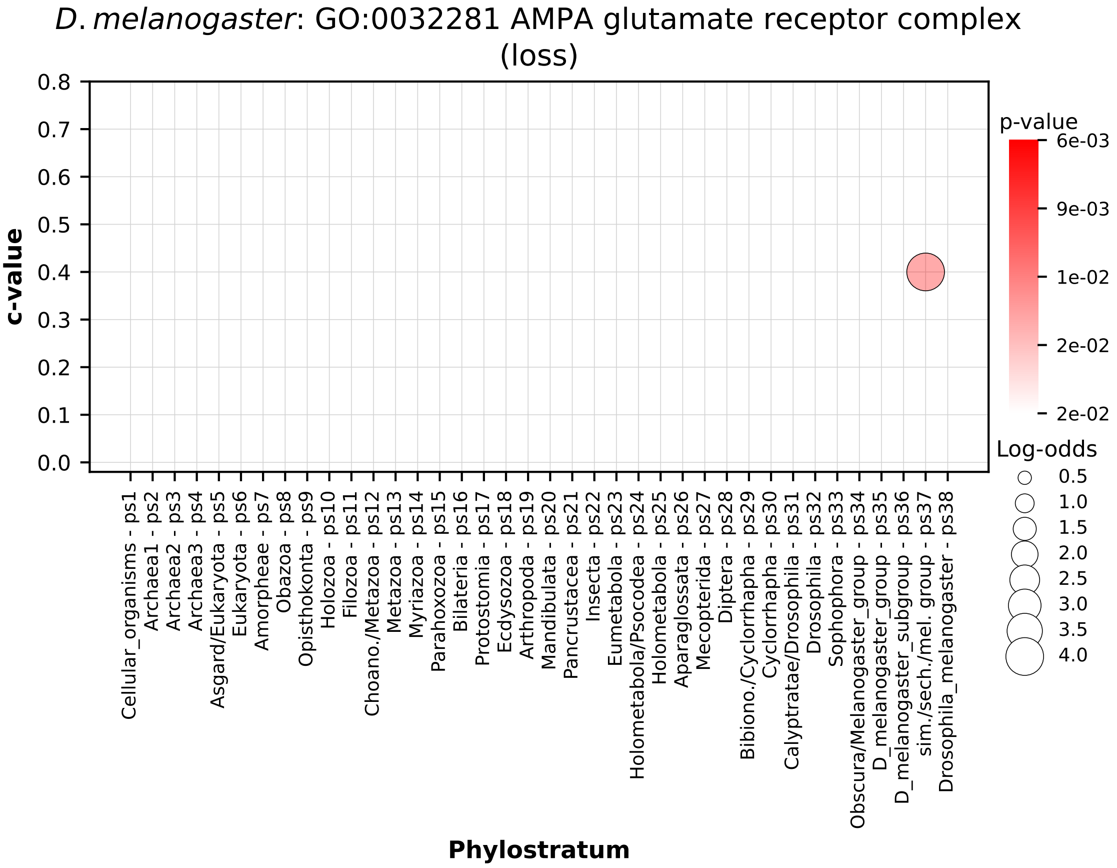

D. melanogaster: GO:0032281 AMPA glutamate receptor complex (loss)
c-value
0.8
0.7
0.6
0.5
0.4
0.3
0.2
0.1
0.0
p-value
6e-03
9e-03
1e-02
2e-02
2e-02
Log-odds
0.5
1.0
1.5
2.0
2.5
3.0
3.5
4.0
Cellular_organisms - ps1
Archaea1 - ps2
Archaea2 - ps3
Archaea3 - ps4
Asgard/Eukaryota - ps5
Eukaryota - ps6
Amorpheae - ps7
Obazoa - ps8
Opisthokonta - ps9
Holozoa - ps10
Filozoa - ps11
Choano./Metazoa - ps12
Metazoa - ps13
Myriazoa - ps14
Parahoxozoa - ps15
Bilateria - ps16
Protostomia - ps17
Ecdysozoa - ps18
Arthropoda - ps19
Mandibulata - ps20
Pancrustacea - ps21
Insecta - ps22
Eumetabola - ps23
Holometabola/Psocodea - ps24
Holometabola - ps25
Aparaglossata - ps26
Mecopterida - ps27
Diptera - ps28
Bibiono./Cyclorrhapha - ps29
Cyclorrhapha - ps30
Calyptratae/Drosophila - ps31
Drosophila - ps32
Sophophora - ps33
Obscura/Melanogaster_group - ps34
D_melanogaster_group - ps35
D_melanogaster_subgroup - ps36
sim./sech./mel. group - ps37
Drosophila_melanogaster - ps38
Phylostratum

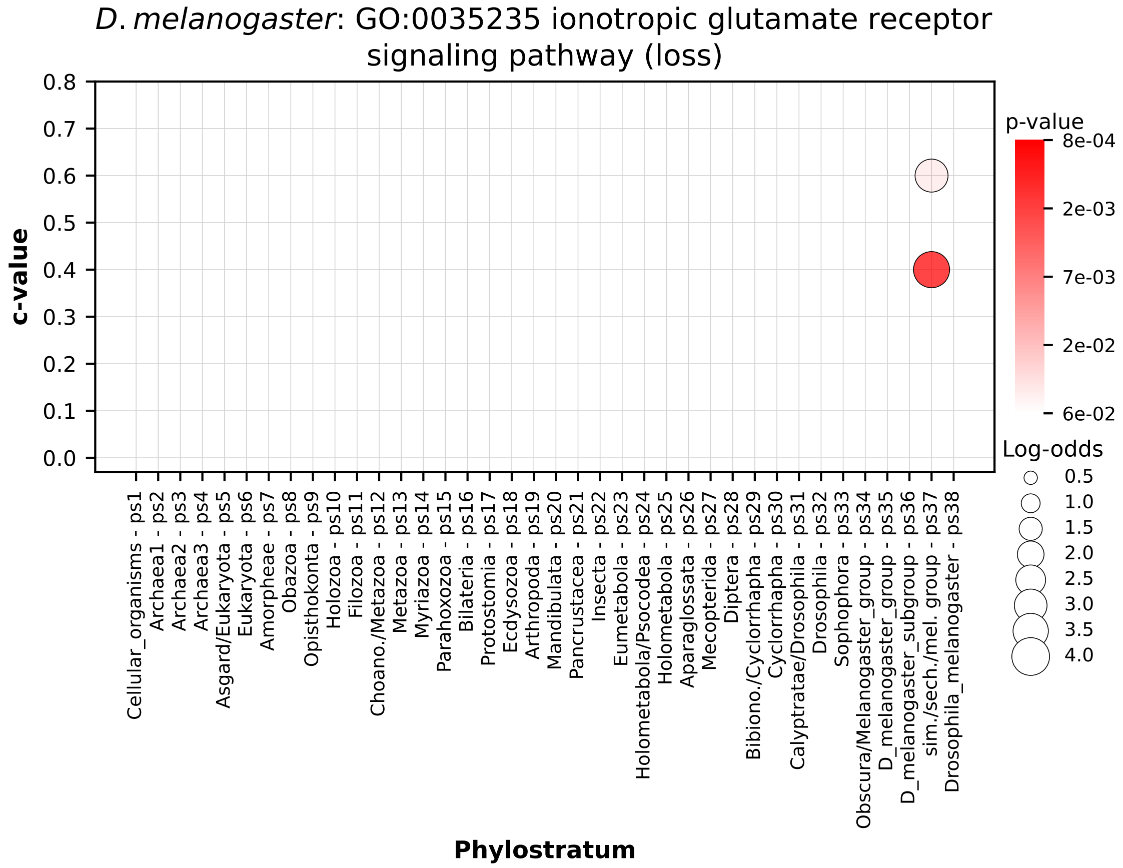

D. melanogaster: GO:0035235 ionotropic glutamate receptor signaling pathway (loss)
c-value
0.0
0.1
0.2
0.3
0.4
0.5
0.6
0.7
0.8
Phylostratum
Cellular_organisms - ps1
Archaea1 - ps2
Archaea2 - ps3
Archaea3 - ps4
Asgard/Eukaryota - ps5
Eukaryota - ps6
Amorpheae - ps7
Obazoa - ps8
Opisthokonta - ps9
Holozoa - ps10
Filozoa - ps11
Choano./Metazoa - ps12
Metazoa - ps13
Myriazoa - ps14
Parahoxozoa - ps15
Bilateria - ps16
Protostomia - ps17
Ecdysozoa - ps18
Arthropoda - ps19
Mandibulata - ps20
Pancrustacea - ps21
Insecta - ps22
Eumetabola - ps23
Holometabola/Psocodea - ps24
Holometabola - ps25
Aparaglossata - ps26
Mecopterida - ps27
Diptera - ps28
Bibiono./Cyclorrhapha - ps29
Cyclorrhapha - ps30
Calyptratae/Drosophila - ps31
Drosophila - ps32
Sophophora - ps33
Obscura/Melanogaster_group - ps34
D_melanogaster_group - ps35
D_melanogaster_subgroup - ps36
sim./sech./mel. group - ps37
Drosophila_melanogaster - ps38
p-value
8e-04
2e-03
7e-03
2e-02
6e-02
Log-odds
0.5
1.0
1.5
2.0
2.5
3.0
3.5
4.0

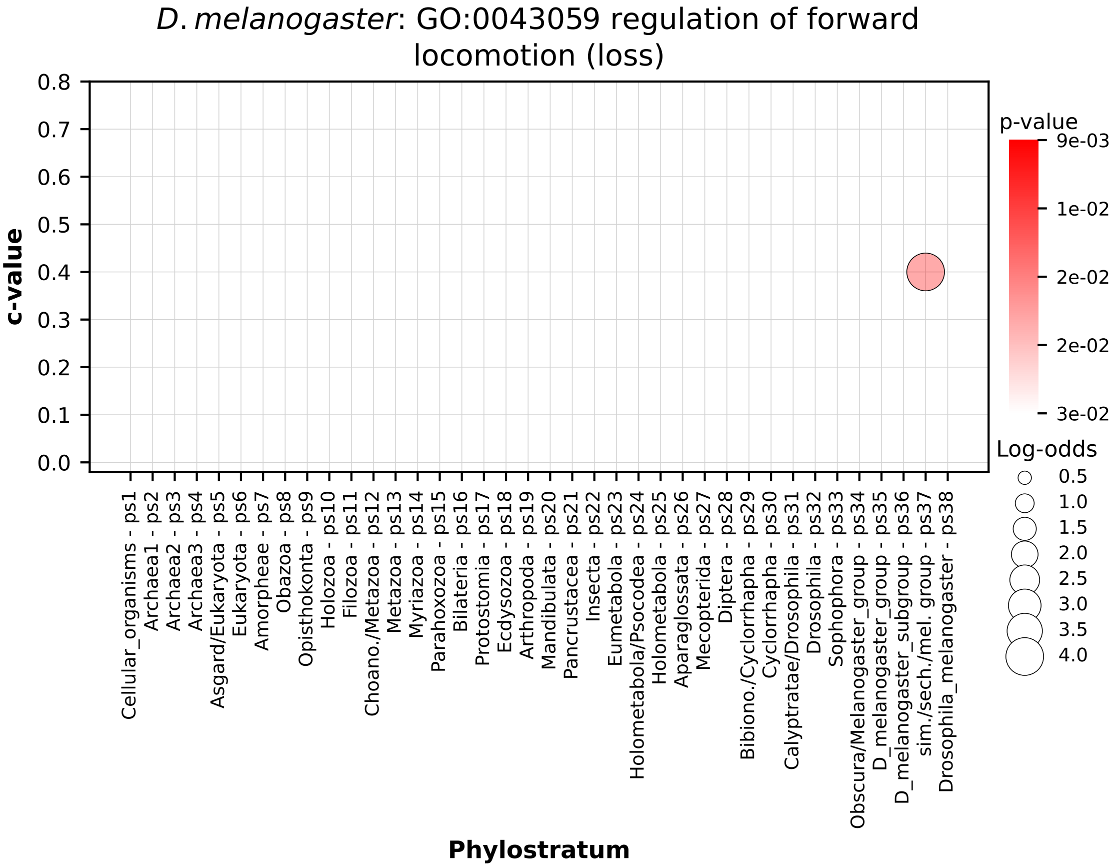
D. melanogaster: GO:0043059 regulation of forward locomotion (loss)
c-value
0.0
0.1
0.2
0.3
0.4
0.5
0.6
0.7
0.8
Phylostratum
Cellular_organisms - ps1
Archaea1 - ps2
Archaea2 - ps3
Archaea3 - ps4
Asgard/Eukaryota - ps5
Eukaryota - ps6
Amorpheae - ps7
Obazoa - ps8
Opisthokonta - ps9
Holozoa - ps10
Filozoa - ps11
Choano./Metazoa - ps12
Metazoa - ps13
Myriazoa - ps14
Parahoxozoa - ps15
Bilateria - ps16
Protostomia - ps17
Ecdysozoa - ps18
Arthropoda - ps19
Mandibulata - ps20
Pancrustacea - ps21
Insecta - ps22
Eumetabola - ps23
Holometabola/Psocodea - ps24
Holometabola - ps25
Aparaglossata - ps26
Mecopterida - ps27
Diptera - ps28
Bibiono./Cyclorrhapha - ps29
Cyclorrhapha - ps30
Calyptratae/Drosophila - ps31
Drosophila - ps32
Sophophora - ps33
Obscura/Melanogaster_group - ps34
D_melanogaster_group - ps35
D_melanogaster_subgroup - ps36
sim./sech./mel. group - ps37
Drosophila_melanogaster - ps38
p-value
9e-03
1e-02
2e-02
2e-02
3e-02
Log-odds
0.5
1.0
1.5
2.0
2.5
3.0
3.5
4.0

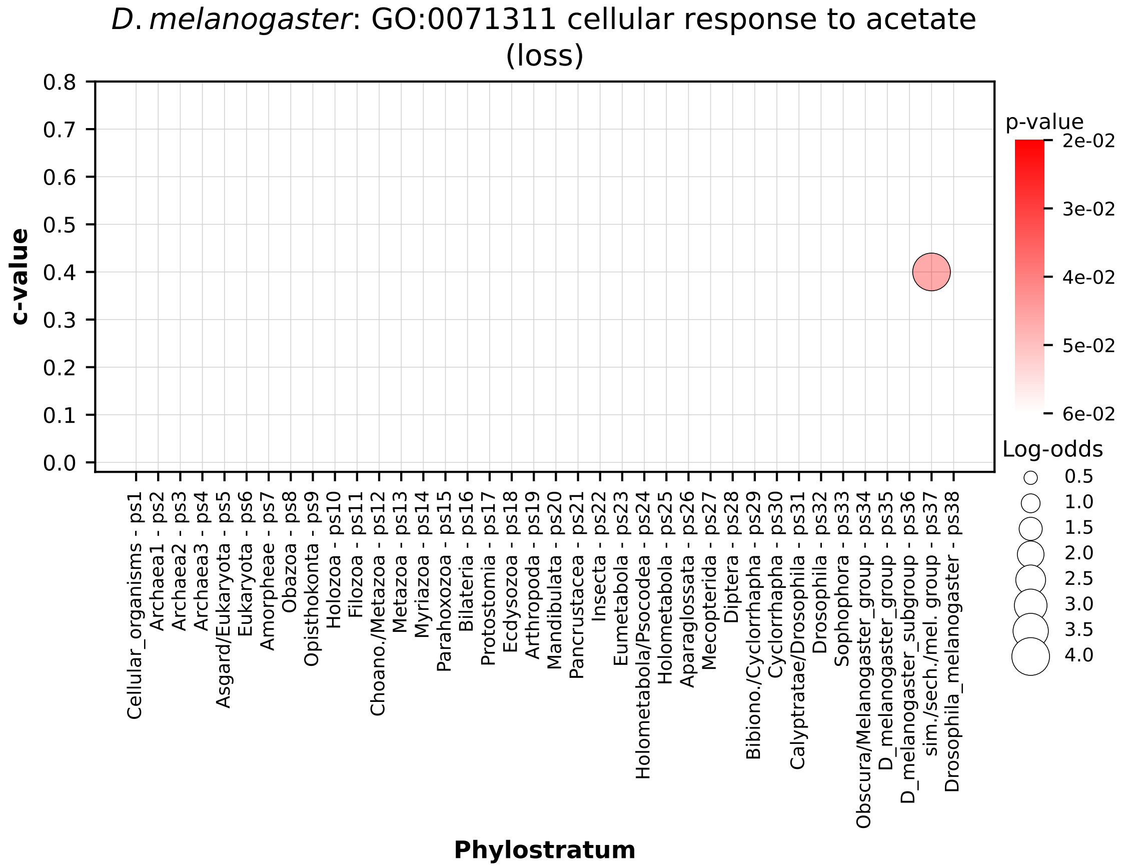
D. melanogaster: GO:0071311 cellular response to acetate (loss)
c-value
0.8
0.7
0.6
0.5
0.4
0.3
0.2
0.1
0.0
p-value
2e-02
3e-02
4e-02
5e-02
6e-02
Log-odds
0.5
1.0
1.5
2.0
2.5
3.0
3.5
4.0
Cellular_organisms - ps1
Archaea1 - ps2
Archaea2 - ps3
Archaea3 - ps4
Asgard/Eukaryota - ps5
Eukaryota - ps6
Amorpheae - ps7
Obazoa - ps8
Opisthokonta - ps9
Holozoa - ps10
Filozoa - ps11
Choano./Metazoa - ps12
Metazoa - ps13
Myriazoa - ps14
Parahoxozoa - ps15
Bilateria - ps16
Protostomia - ps17
Ecdysozoa - ps18
Arthropoda - ps19
Mandibulata - ps20
Pancrustacea - ps21
Insecta - ps22
Eumetabola - ps23
Holometabola/Psocodea - ps24
Holometabola - ps25
Aparaglossata - ps26
Mecopterida - ps27
Diptera - ps28
Bibiono./Cyclorrhapha - ps29
Cyclorrhapha - ps30
Calyptratae/Drosophila - ps31
Drosophila - ps32
Sophophora - ps33
Obscura/Melanogaster_group - ps34
D_melanogaster_group - ps35
D_melanogaster_subgroup - ps36
sim./sech./mel. group - ps37
Drosophila_melanogaster - ps38
Phylostratum

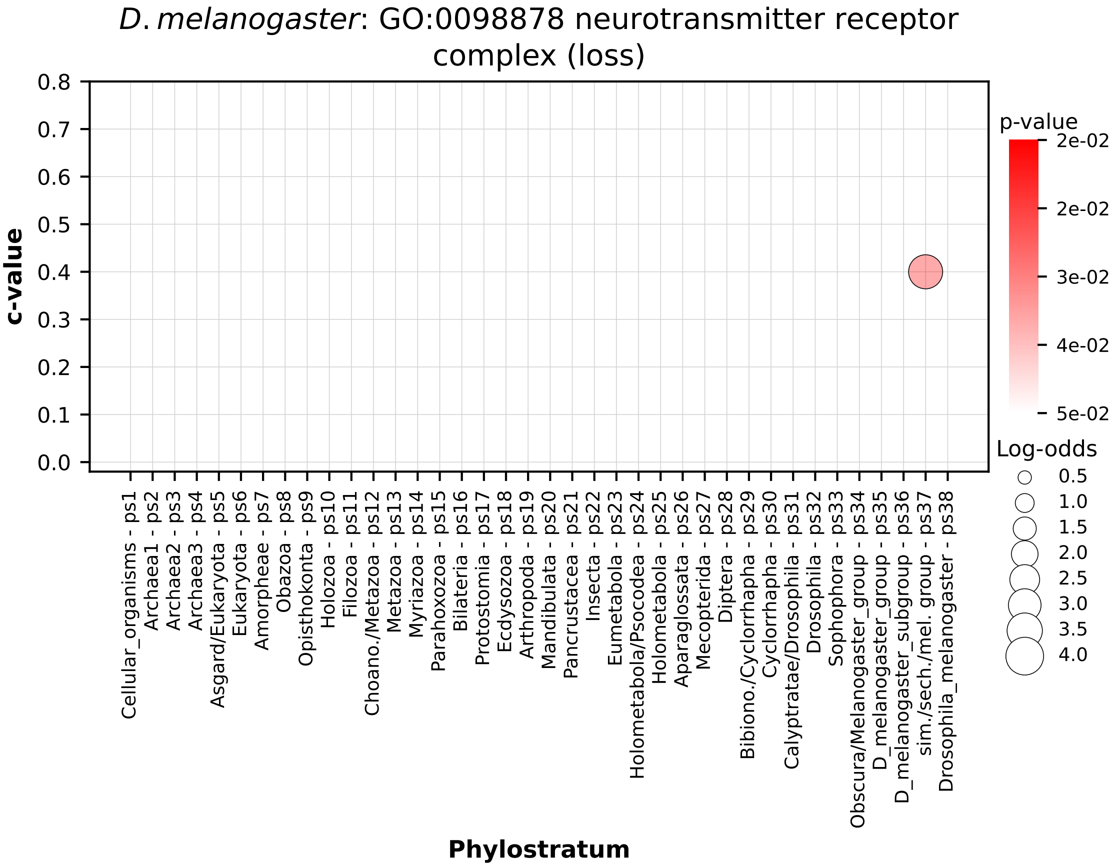

D. melanogaster: GO:0098878 neurotransmitter receptor complex (loss)
c-value
0.8
0.7
0.6
0.5
0.4
0.3
0.2
0.1
0.0
Phylostratum
Cellular_organisms - ps1
Archaea1 - ps2
Archaea2 - ps3
Archaea3 - ps4
Asgard/Eukaryota - ps5
Eukaryota - ps6
Amorpheae - ps7
Obazoa - ps8
Opisthokonta - ps9
Holozoa - ps10
Filozoa - ps11
Choano./Metazoa - ps12
Metazoa - ps13
Myriazoa - ps14
Parahoxozoa - ps15
Bilateria - ps16
Protostomia - ps17
Ecdysozoa - ps18
Arthropoda - ps19
Mandibulata - ps20
Pancrustacea - ps21
Insecta - ps22
Eumetabola - ps23
Holometabola/Psocodea - ps24
Holometabola - ps25
Aparaglossata - ps26
Mecopterida - ps27
Diptera - ps28
Bibiono./Cyclorrhapha - ps29
Cyclorrhapha - ps30
Calyptratae/Drosophila - ps31
Drosophila - ps32
Sophophora - ps33
Obscura/Melanogaster_group - ps34
D_melanogaster_group - ps35
D_melanogaster_subgroup - ps36
sim./sech./mel. group - ps37
Drosophila_melanogaster - ps38
p-value
2e-02
2e-02
3e-02
4e-02
5e-02
Log-odds
0.5
1.0
1.5
2.0
2.5
3.0
3.5
4.0

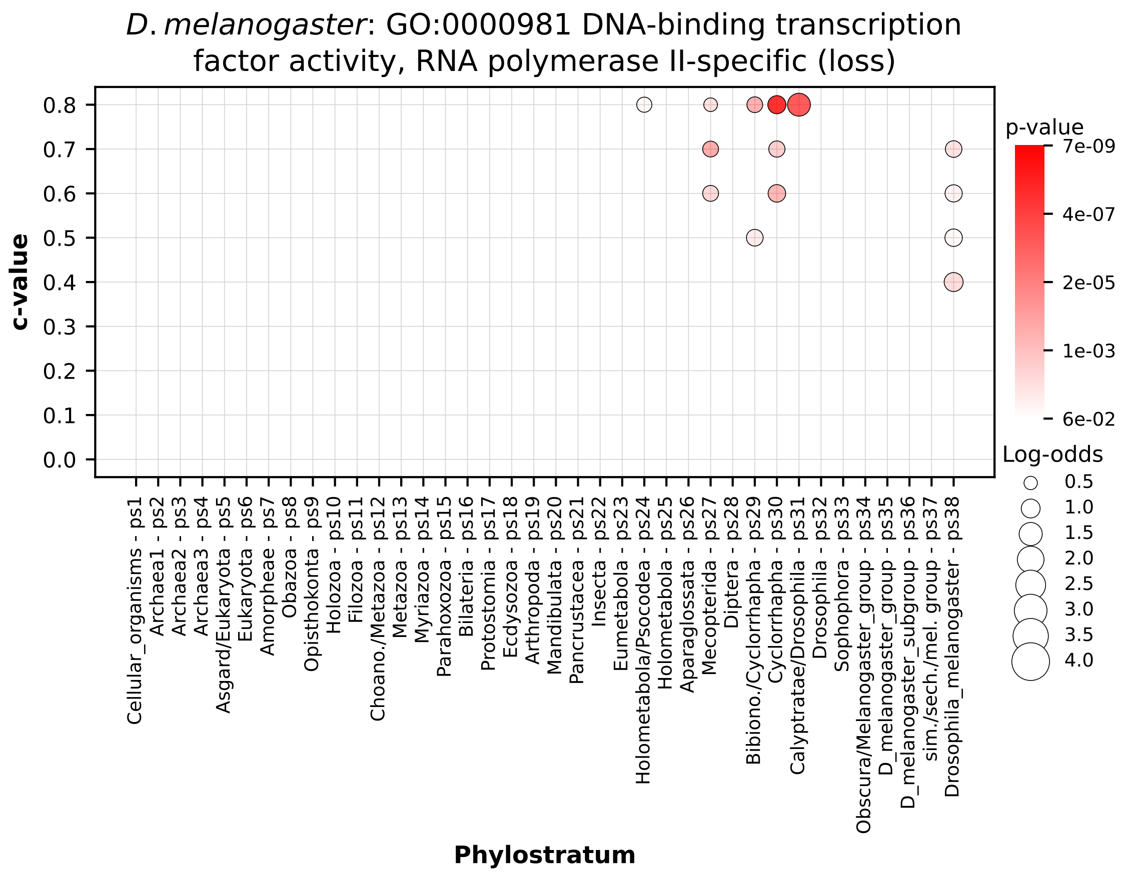

D. melanogaster: GO:0000981 DNA-binding transcription factor activity, RNA polymerase II-specific (loss)
c-value
0.8
0.7
0.6
0.5
0.4
0.3
0.2
0.1
0.0
p-value
7e-09
4e-07
2e-05
1e-03
6e-02
Log-odds
0.5
1.0
1.5
2.0
2.5
3.0
3.5
4.0
Cellular_organisms - ps1
Archaea1 - ps2
Archaea2 - ps3
Archaea3 - ps4
Asgard/Eukaryota - ps5
Eukaryota - ps6
Amorpheae - ps7
Obazoa - ps8
Opisthokonta - ps9
Holozoa - ps10
Filozoa - ps11
Choano./Metazoa - ps12
Metazoa - ps13
Myriazoa - ps14
Parahoxozoa - ps15
Bilateria - ps16
Protostomia - ps17
Ecdysozoa - ps18
Arthropoda - ps19
Mandibulata - ps20
Pancrustacea - ps21
Insecta - ps22
Eumetabola - ps23
Holometabola/Psocodea - ps24
Holometabola - ps25
Aparaglossata - ps26
Mecopterida - ps27
Diptera - ps28
Bibiono./Cyclorrhapha - ps29
Cyclorrhapha - ps30
Calyptratae/Drosophila - ps31
Drosophila - ps32
Sophophora - ps33
Obscura/Melanogaster_group - ps34
D_melanogaster_group - ps35
D_melanogaster_subgroup - ps36
sim./sech./mel. group - ps37
Drosophila_melanogaster - ps38
Phylostratum

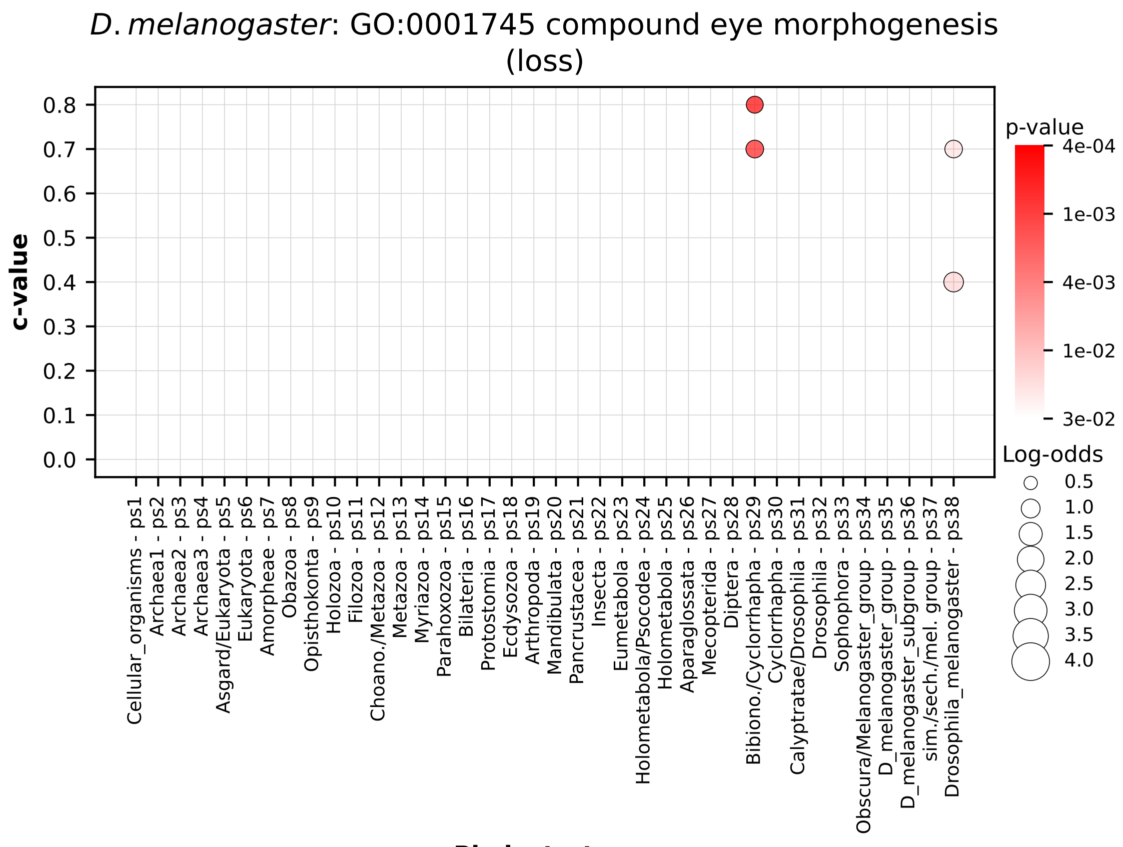
D. melanogaster: GO:0001745 compound eye morphogenesis (loss)
c-value
0.8
0.7
0.6
0.5
0.4
0.3
0.2
0.1
0.0
Phylostratum
Cellular_organisms - ps1
Archaea1 - ps2
Archaea2 - ps3
Archaea3 - ps4
Asgard/Eukaryota - ps5
Eukaryota - ps6
Amorpheae - ps7
Obazoa - ps8
Opisthokonta - ps9
Holozoa - ps10
Filozoa - ps11
Choano./Metazoa - ps12
Metazoa - ps13
Myriazoa - ps14
Parahoxozoa - ps15
Bilateria - ps16
Protostomia - ps17
Ecdysozoa - ps18
Arthropoda - ps19
Mandibulata - ps20
Pancrustacea - ps21
Insecta - ps22
Eumetabola - ps23
Holometabola/Psocodea - ps24
Holometabola - ps25
Aparaglossata - ps26
Mecopterida - ps27
Diptera - ps28
Bibiono./Cyclorrhapha - ps29
Cyclorrhapha - ps30
Calyptratae/Drosophila - ps31
Drosophila - ps32
Sophophora - ps33
Obscura/Melanogaster_group - ps34
D_melanogaster_group - ps35
D_melanogaster_subgroup - ps36
sim./sech./mel. group - ps37
Drosophila_melanogaster - ps38
p-value
4e-04
1e-03
4e-03
1e-02
3e-02
Log-odds
0.5
1.0
1.5
2.0
2.5
3.0
3.5
4.0

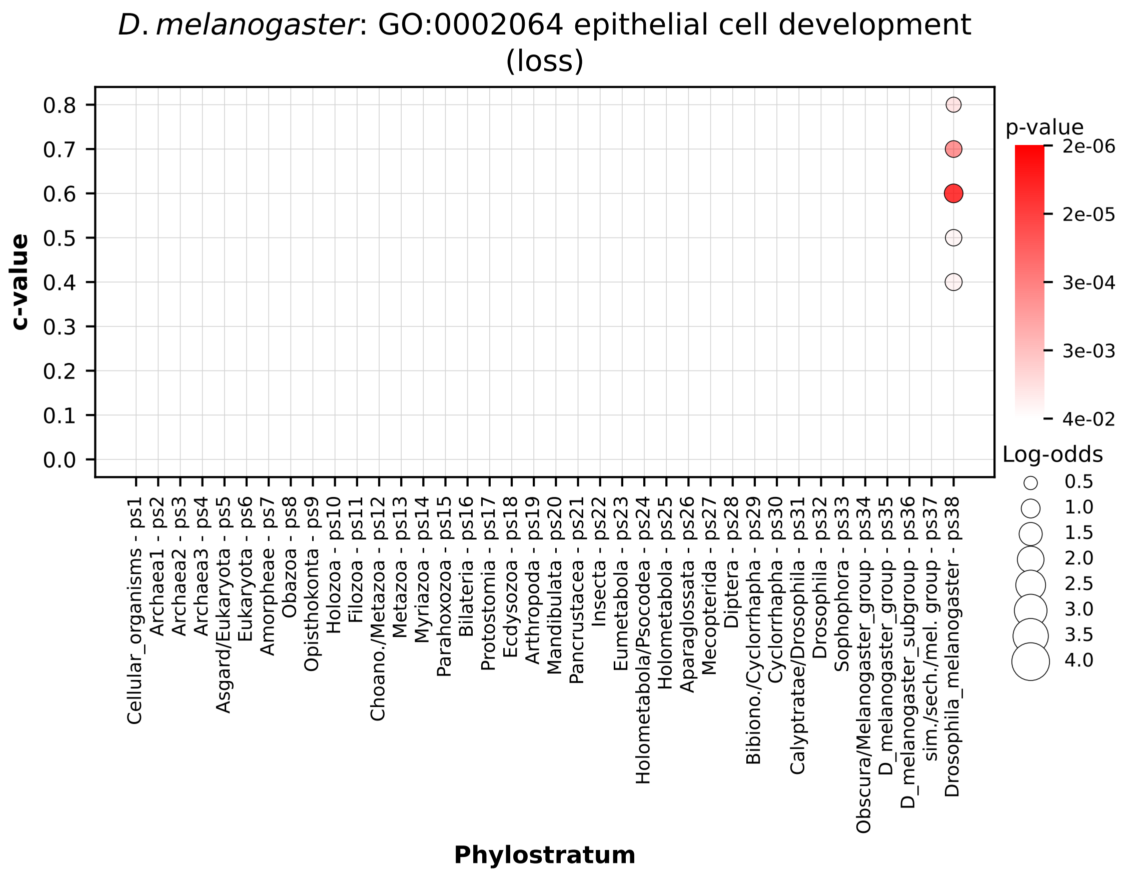
D. melanogaster: GO:0002064 epithelial cell development
(loss)
c-value
0.0
0.1
0.2
0.3
0.4
0.5
0.6
0.7
0.8
p-value
2e-06
2e-05
3e-04
3e-03
4e-02
Log-odds
0.5
1.0
1.5
2.0
2.5
3.0
3.5
4.0
Cellular_organisms - ps1
Archaea1 - ps2
Archaea2 - ps3
Archaea3 - ps4
Asgard/Eukaryota - ps5
Eukaryota - ps6
Amorpheae - ps7
Obazoa - ps8
Opisthokonta - ps9
Holozoa - ps10
Filozoa - ps11
Choano./Metazoa - ps12
Metazoa - ps13
Myriazoa - ps14
Parahoxozoa - ps15
Bilateria - ps16
Protostomia - ps17
Ecdysozoa - ps18
Arthropoda - ps19
Mandibulata - ps20
Pancrustacea - ps21
Insecta - ps22
Eumetabola - ps23
Holometabola/Psocodea - ps24
Holometabola - ps25
Aparaglossata - ps26
Mecopterida - ps27
Diptera - ps28
Bibiono./Cyclorrhapha - ps29
Cyclorrhapha - ps30
Calyptratae/Drosophila - ps31
Drosophila - ps32
Sophophora - ps33
Obscura/Melanogaster_group - ps34
D_melanogaster_group - ps35
D_melanogaster_subgroup - ps36
sim./sech./mel. group - ps37
Drosophila_melanogaster - ps38
Phylostratum

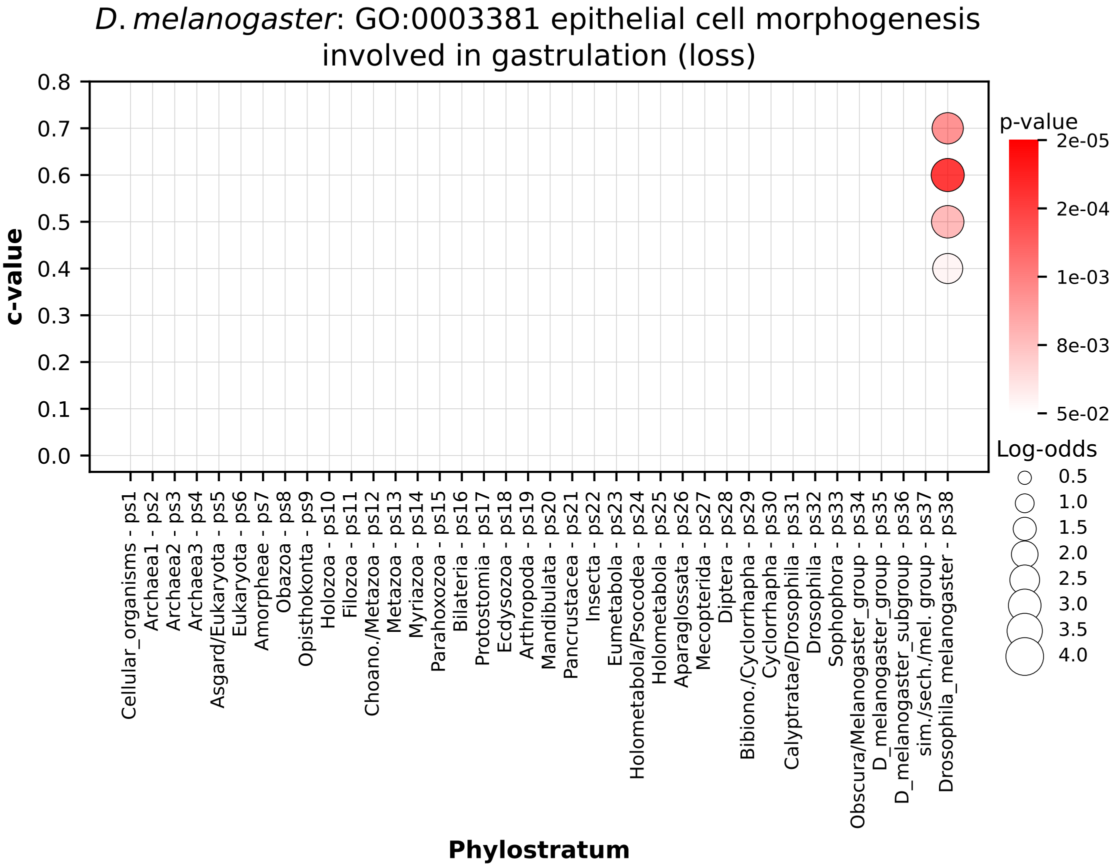

D. melanogaster: GO:0003381 epithelial cell morphogenesis involved in gastrulation (loss)
c-value
0.8
0.7
0.6
0.5
0.4
0.3
0.2
0.1
0.0
p-value
2e-05
2e-04
1e-03
8e-03
5e-02
Log-odds
0.5
1.0
1.5
2.0
2.5
3.0
3.5
4.0
Cellular_organisms - ps1
Archaea1 - ps2
Archaea2 - ps3
Archaea3 - ps4
Asgard/Eukaryota - ps5
Eukaryota - ps6
Amorpheae - ps7
Obazoa - ps8
Opisthokonta - ps9
Holozoa - ps10
Filozoa - ps11
Choano./Metazoa - ps12
Metazoa - ps13
Myriazoa - ps14
Parahoxozoa - ps15
Bilateria - ps16
Protostomia - ps17
Ecdysozoa - ps18
Arthropoda - ps19
Mandibulata - ps20
Pancrustacea - ps21
Insecta - ps22
Eumetabola - ps23
Holometabola/Psocodea - ps24
Holometabola - ps25
Aparaglossata - ps26
Mecopterida - ps27
Diptera - ps28
Bibiono./Cyclorrhapha - ps29
Cyclorrhapha - ps30
Calyptratae/Drosophila - ps31
Drosophila - ps32
Sophophora - ps33
Obscura/Melanogaster_group - ps34
D_melanogaster_group - ps35
D_melanogaster_subgroup - ps36
sim./sech./mel. group - ps37
Drosophila_melanogaster - ps38
Phylostratum

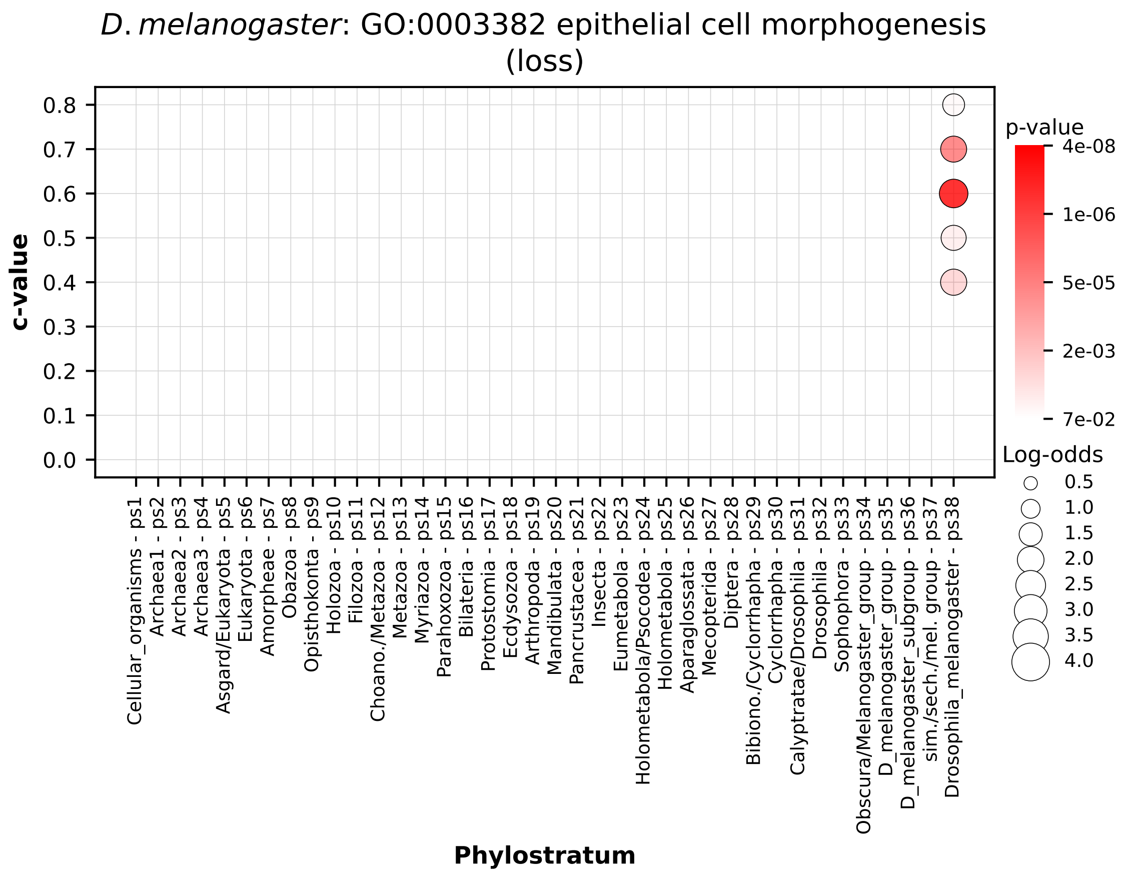
D. melanogaster: GO:0003382 epithelial cell morphogenesis (loss)
c-value
0.0
0.1
0.2
0.3
0.4
0.5
0.6
0.7
0.8
p-value
4e-08
1e-06
5e-05
2e-03
7e-02
Log-odds
0.5
1.0
1.5
2.0
2.5
3.0
3.5
4.0
Cellular_organisms - ps1
Archaea1 - ps2
Archaea2 - ps3
Archaea3 - ps4
Asgard/Eukaryota - ps5
Eukaryota - ps6
Amorpheae - ps7
Obazoa - ps8
Opisthokonta - ps9
Holozoa - ps10
Filozoa - ps11
Choano./Metazoa - ps12
Metazoa - ps13
Myriazoa - ps14
Parahoxozoa - ps15
Bilateria - ps16
Protostomia - ps17
Ecdysozoa - ps18
Arthropoda - ps19
Mandibulata - ps20
Pancrustacea - ps21
Insecta - ps22
Eumetabola - ps23
Holometabola/Psocodea - ps24
Holometabola - ps25
Aparaglossata - ps26
Mecopterida - ps27
Diptera - ps28
Bibiono./Cyclorrhapha - ps29
Cyclorrhapha - ps30
Calyptratae/Drosophila - ps31
Drosophila - ps32
Sophophora - ps33
Obscura/Melanogaster_group - ps34
D_melanogaster_group - ps35
D_melanogaster_subgroup - ps36
sim./sech./mel. group - ps37
Drosophila_melanogaster - ps38
Phylostratum

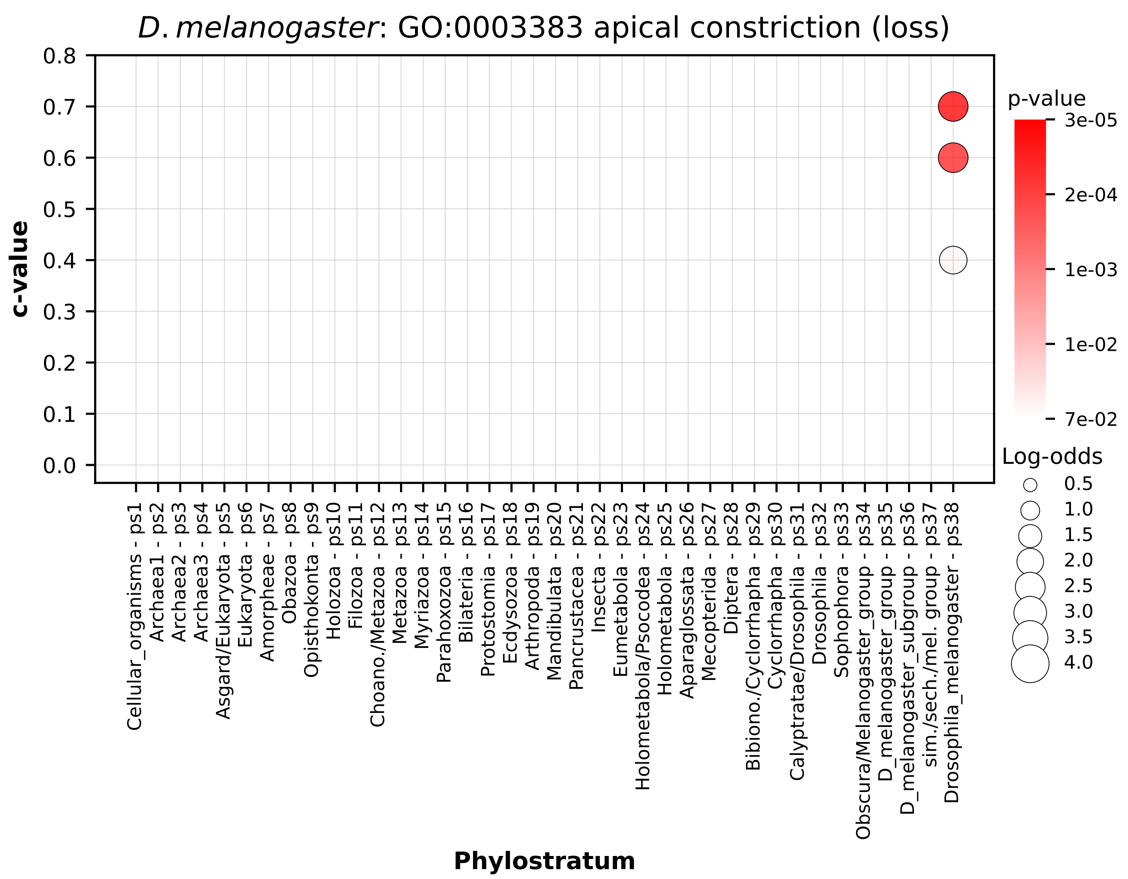

D. melanogaster: GO:0003383 apical constriction (loss)
c-value
0.8
0.7
0.6
0.5
0.4
0.3
0.2
0.1
0.0
p-value
3e-05
2e-04
1e-03
1e-02
7e-02
Log-odds
0.5
1.0
1.5
2.0
2.5
3.0
3.5
4.0
Cellular_organisms - ps1
Archaea1 - ps2
Archaea2 - ps3
Archaea3 - ps4
Asgard/Eukaryota - ps5
Eukaryota - ps6
Amorpheae - ps7
Obazoa - ps8
Opisthokonta - ps9
Holozoa - ps10
Filozoa - ps11
Choano./Metazoa - ps12
Metazoa - ps13
Myriazoa - ps14
Parahoxozoa - ps15
Bilateria - ps16
Protostomia - ps17
Ecdysozoa - ps18
Arthropoda - ps19
Mandibulata - ps20
Pancrustacea - ps21
Insecta - ps22
Eumetabola - ps23
Holometabola/Psocodea - ps24
Holometabola - ps25
Aparaglossata - ps26
Mecopterida - ps27
Diptera - ps28
Bibiono./Cyclorrhapha - ps29
Cyclorrhapha - ps30
Calyptratae/Drosophila - ps31
Drosophila - ps32
Sophophora - ps33
Obscura/Melanogaster_group - ps34
D_melanogaster_group - ps35
D_melanogaster_subgroup - ps36
sim./sech./mel. group - ps37
Drosophila_melanogaster - ps38
Phylostratum

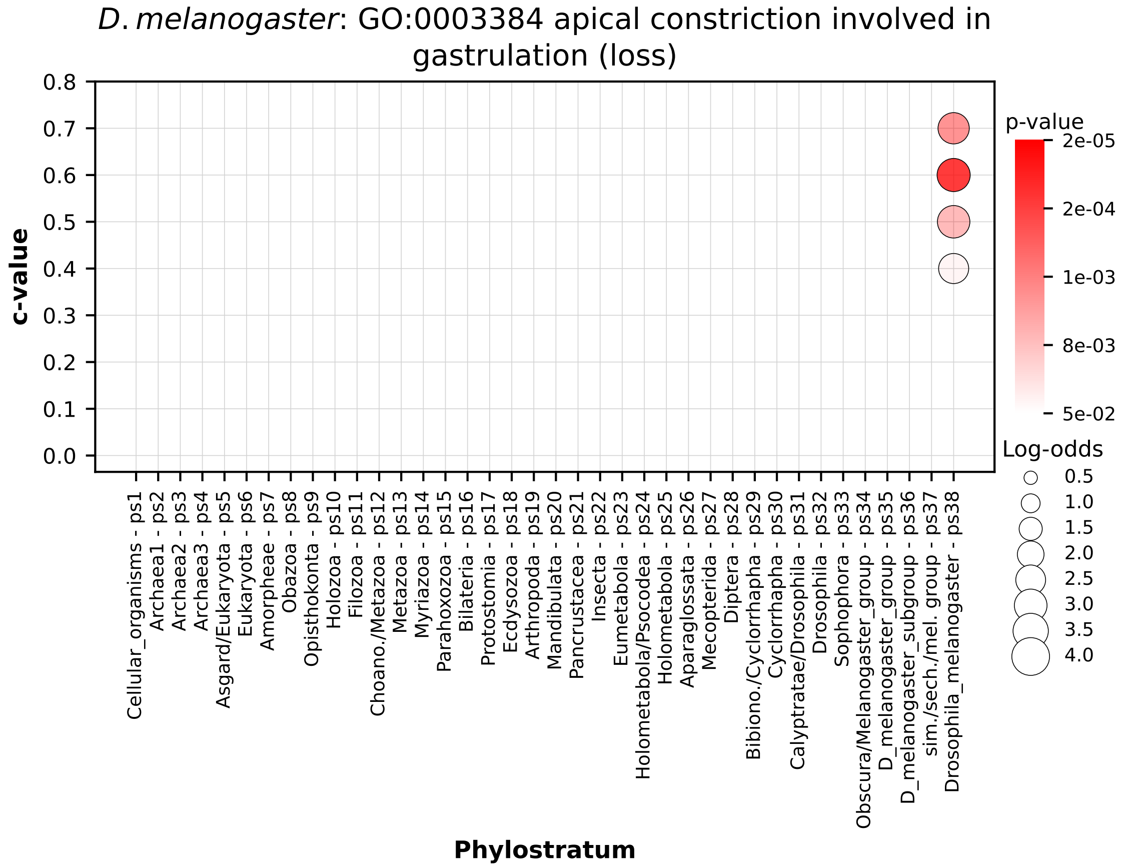

D. melanogaster: GO:0003384 apical constriction involved in gastrulation (loss)
c-value
0.8
0.7
0.6
0.5
0.4
0.3
0.2
0.1
0.0
p-value
2e-05
2e-04
1e-03
8e-03
5e-02
Log-odds
0.5
1.0
1.5
2.0
2.5
3.0
3.5
4.0
Cellular_organisms - ps1
Archaea1 - ps2
Archaea2 - ps3
Archaea3 - ps4
Asgard/Eukaryota - ps5
Eukaryota - ps6
Amorpheae - ps7
Obazoa - ps8
Opisthokonta - ps9
Holozoa - ps10
Filozoa - ps11
Choano./Metazoa - ps12
Metazoa - ps13
Myriazoa - ps14
Parahoxozoa - ps15
Bilateria - ps16
Protostomia - ps17
Ecdysozoa - ps18
Arthropoda - ps19
Mandibulata - ps20
Pancrustacea - ps21
Insecta - ps22
Eumetabola - ps23
Holometabola/Psocodea - ps24
Holometabola - ps25
Aparaglossata - ps26
Mecopterida - ps27
Diptera - ps28
Bibiono./Cyclorrhapha - ps29
Cyclorrhapha - ps30
Calyptratae/Drosophila - ps31
Drosophila - ps32
Sophophora - ps33
Obscura/Melanogaster_group - ps34
D_melanogaster_group - ps35
D_melanogaster_subgroup - ps36
sim./sech./mel. group - ps37
Drosophila_melanogaster - ps38
Phylostratum

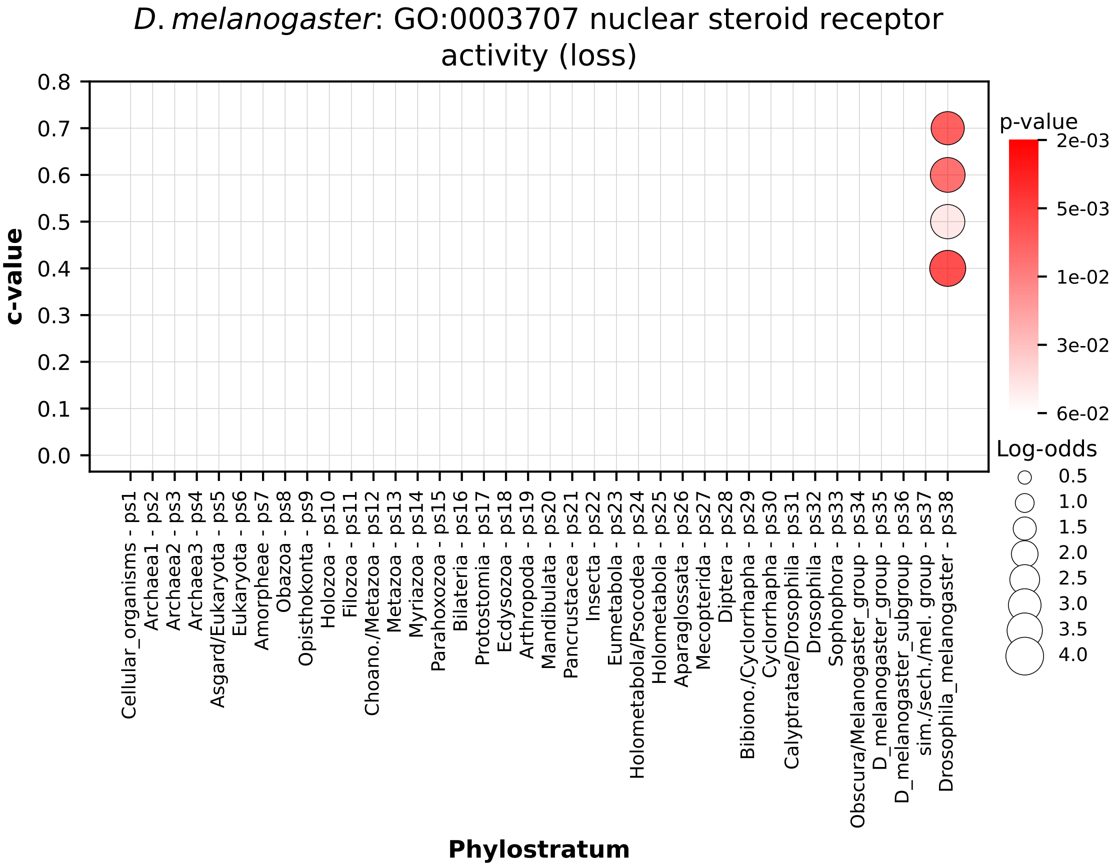

D. melanogaster: GO:0003707 nuclear steroid receptor activity (loss)
c-value
0.8
0.7
0.6
0.5
0.4
0.3
0.2
0.1
0.0
p-value
2e-03
5e-03
1e-02
3e-02
6e-02
Log-odds
0.5
1.0
1.5
2.0
2.5
3.0
3.5
4.0
Cellular_organisms - ps1
Archaea1 - ps2
Archaea2 - ps3
Archaea3 - ps4
Asgard/Eukaryota - ps5
Eukaryota - ps6
Amorpheae - ps7
Obazoa - ps8
Opisthokonta - ps9
Holozoa - ps10
Filozoa - ps11
Choano./Metazoa - ps12
Metazoa - ps13
Myriazoa - ps14
Parahoxozoa - ps15
Bilateria - ps16
Protostomia - ps17
Ecdysozoa - ps18
Arthropoda - ps19
Mandibulata - ps20
Pancrustacea - ps21
Insecta - ps22
Eumetabola - ps23
Holometabola/Psocodea - ps24
Holometabola - ps25
Aparaglossata - ps26
Mecopterida - ps27
Diptera - ps28
Bibiono./Cyclorrhapha - ps29
Cyclorrhapha - ps30
Calyptratae/Drosophila - ps31
Drosophila - ps32
Sophophora - ps33
Obscura/Melanogaster_group - ps34
D_melanogaster_group - ps35
D_melanogaster_subgroup - ps36
sim./sech./mel. group - ps37
Drosophila_melanogaster - ps38
Phylostratum

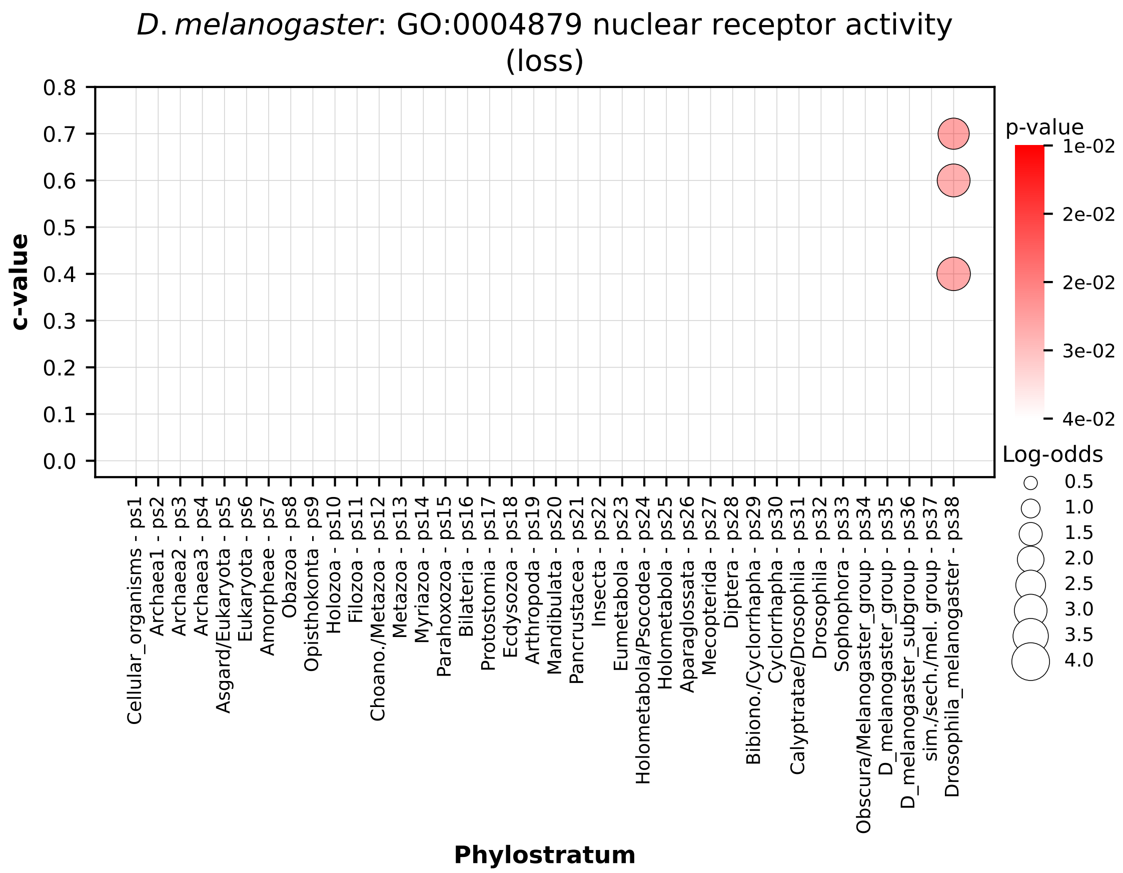

D. melanogaster: GO:0004879 nuclear receptor activity
(loss)
c-value
0.8
0.7
0.6
0.5
0.4
0.3
0.2
0.1
0.0
p-value
1e-02
2e-02
2e-02
3e-02
4e-02
Log-odds
0.5
1.0
1.5
2.0
2.5
3.0
3.5
4.0
Cellular_organisms - ps1
Archaea1 - ps2
Archaea2 - ps3
Archaea3 - ps4
Asgard/Eukaryota - ps5
Eukaryota - ps6
Amorpheae - ps7
Obazoa - ps8
Opisthokonta - ps9
Holozoa - ps10
Filozoa - ps11
Choano./Metazoa - ps12
Metazoa - ps13
Myriazoa - ps14
Parahoxozoa - ps15
Bilateria - ps16
Protostomia - ps17
Ecdysozoa - ps18
Arthropoda - ps19
Mandibulata - ps20
Pancrustacea - ps21
Insecta - ps22
Eumetabola - ps23
Holometabola/Psocodea - ps24
Holometabola - ps25
Aparaglossata - ps26
Mecopterida - ps27
Diptera - ps28
Bibiono./Cyclorrhapha - ps29
Cyclorrhapha - ps30
Calyptratae/Drosophila - ps31
Drosophila - ps32
Sophophora - ps33
Obscura/Melanogaster_group - ps34
D_melanogaster_group - ps35
D_melanogaster_subgroup - ps36
sim./sech./mel. group - ps37
Drosophila_melanogaster - ps38
Phylostratum

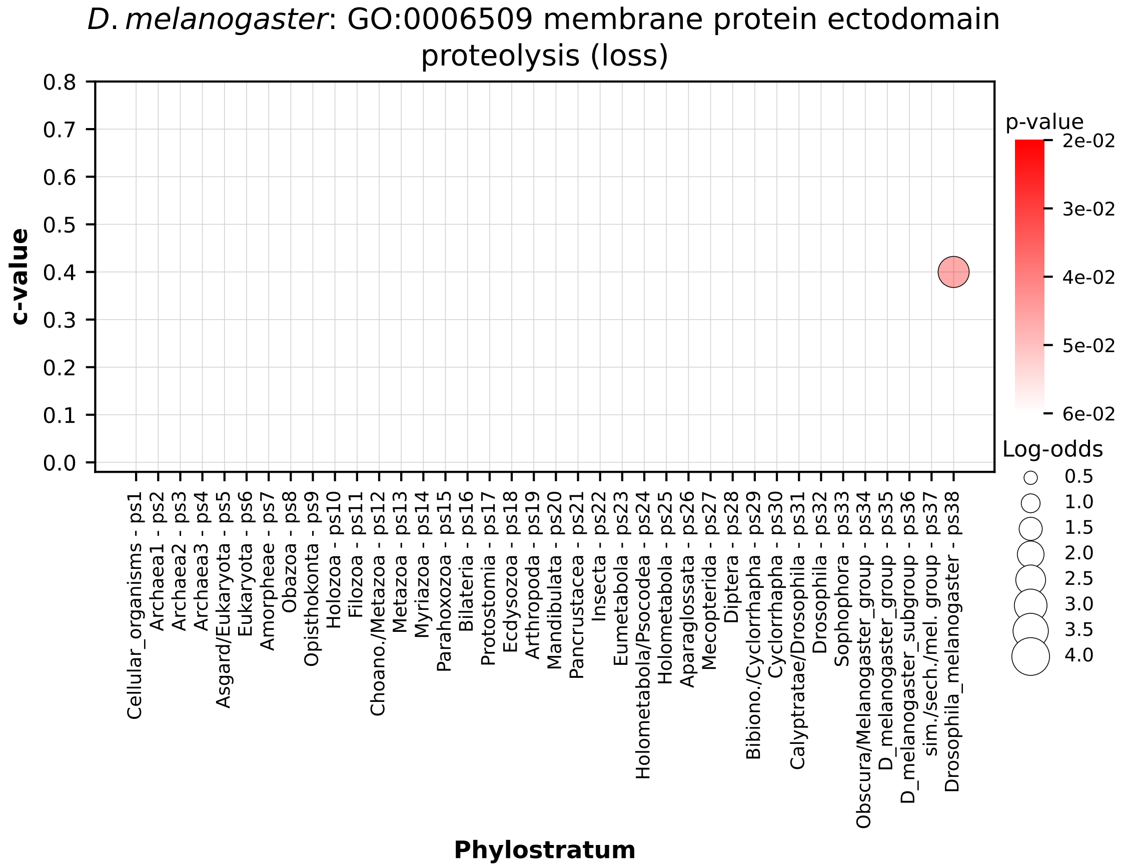
D. melanogaster: GO:0006509 membrane protein ectodomain proteolysis (loss)
c-value
Phylostratum
p-value
2e-02
3e-02
4e-02
5e-02
6e-02
Log-odds
0.5
1.0
1.5
2.0
2.5
3.0
3.5
4.0
0.0
0.1
0.2
0.3
0.4
0.5
0.6
0.7
0.8
Cellular_organisms - ps1
Archaea1 - ps2
Archaea2 - ps3
Archaea3 - ps4
Asgard/Eukaryota - ps5
Eukaryota - ps6
Amorpheae - ps7
Obazoa - ps8
Opisthokonta - ps9
Holozoa - ps10
Filozoa - ps11
Choano./Metazoa - ps12
Metazoa - ps13
Myriazoa - ps14
Parahoxozoa - ps15
Bilateria - ps16
Protostomia - ps17
Ecdysozoa - ps18
Arthropoda - ps19
Mandibulata - ps20
Pancrustacea - ps21
Insecta - ps22
Eumetabola - ps23
Holometabola/Psocodea - ps24
Holometabola - ps25
Aparaglossata - ps26
Mecopterida - ps27
Diptera - ps28
Bibiono./Cyclorrhapha - ps29
Cyclorrhapha - ps30
Calyptratae/Drosophila - ps31
Drosophila - ps32
Sophophora - ps33
Obscura/Melanogaster_group - ps34
D_melanogaster_group - ps35
D_melanogaster_subgroup - ps36
sim./sech./mel. group - ps37
Drosophila_melanogaster - ps38

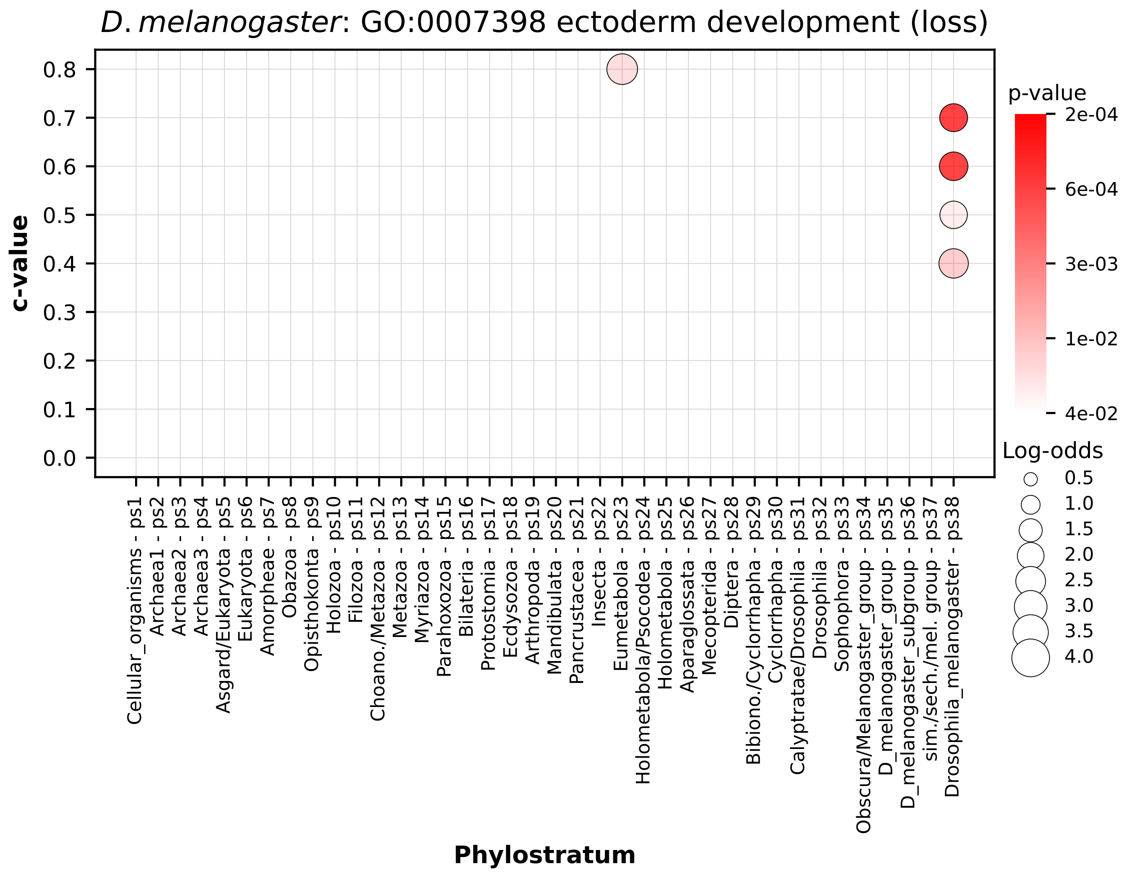
D. melanogaster: GO:0007398 ectoderm development (loss)
c-value
0.8
0.7
0.6
0.5
0.4
0.3
0.2
0.1
0.0
p-value
2e-04
6e-04
3e-03
1e-02
4e-02
Log-odds
0.5
1.0
1.5
2.0
2.5
3.0
3.5
4.0
Cellular_organisms - ps1
Archaea1 - ps2
Archaea2 - ps3
Archaea3 - ps4
Asgard/Eukaryota - ps5
Eukaryota - ps6
Amorpheae - ps7
Obazoa - ps8
Opisthokonta - ps9
Holozoa - ps10
Filozoa - ps11
Choano./Metazoa - ps12
Metazoa - ps13
Myriazoa - ps14
Parahoxozoa - ps15
Bilateria - ps16
Protostomia - ps17
Ecdysozoa - ps18
Arthropoda - ps19
Mandibulata - ps20
Pancrustacea - ps21
Insecta - ps22
Eumetabola - ps23
Holometabola/Psocodea - ps24
Holometabola - ps25
Aparaglossata - ps26
Mecopterida - ps27
Diptera - ps28
Bibiono./Cyclorrhapha - ps29
Cyclorrhapha - ps30
Calyptratae/Drosophila - ps31
Drosophila - ps32
Sophophora - ps33
Obscura/Melanogaster_group - ps34
D_melanogaster_group - ps35
D_melanogaster_subgroup - ps36
sim./sech./mel. group - ps37
Drosophila_melanogaster - ps38
Phylostratum

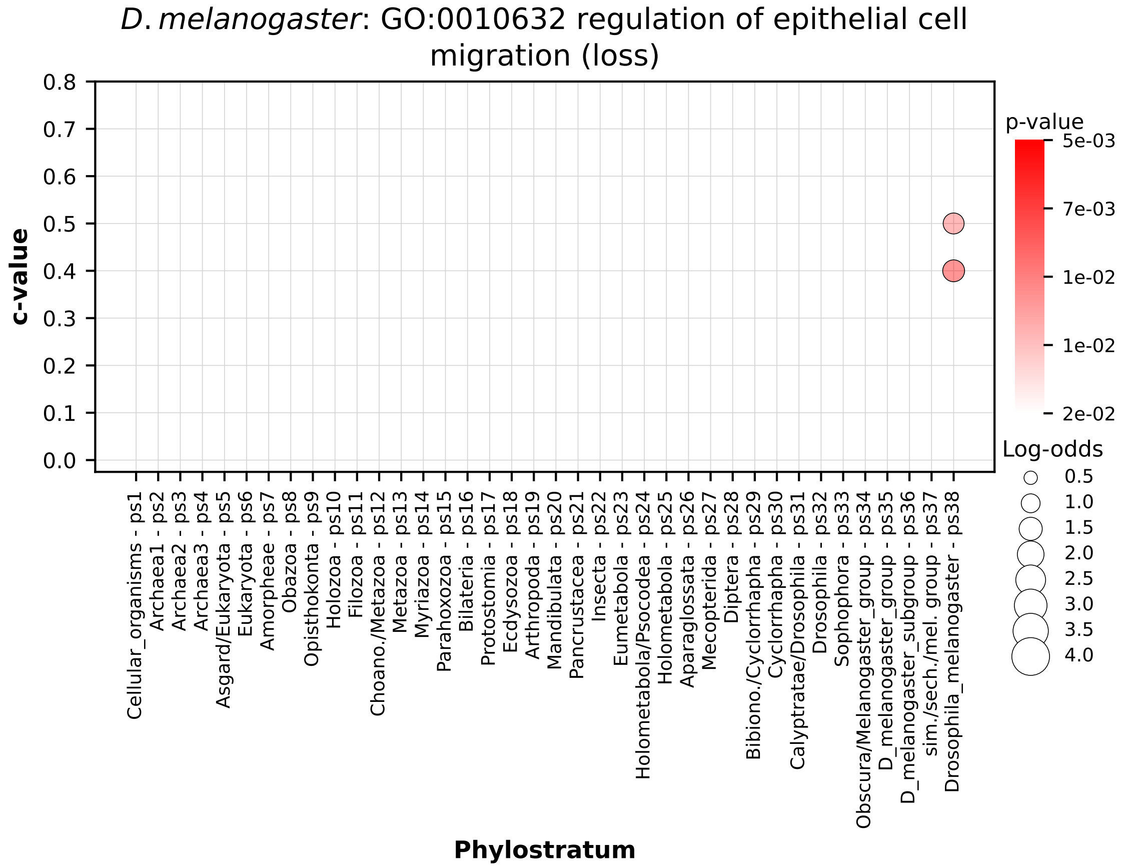

$D. melanogaster$: GO:0010632 regulation of epithelial cell migration (loss)
c-value
0.0
0.1
0.2
0.3
0.4
0.5
0.6
0.7
0.8
Phylostratum
Cellular_organisms - ps1
Archaea1 - ps2
Archaea2 - ps3
Archaea3 - ps4
Asgard/Eukaryota - ps5
Eukaryota - ps6
Amorpheae - ps7
Obazoa - ps8
Opisthokonta - ps9
Holozoa - ps10
Filozoa - ps11
Choano./Metazoa - ps12
Metazoa - ps13
Myriazoa - ps14
Parahoxozoa - ps15
Bilateria - ps16
Protostomia - ps17
Ecdysozoa - ps18
Arthropoda - ps19
Mandibulata - ps20
Pancrustacea - ps21
Insecta - ps22
Eumetabola - ps23
Holometabola/Psocodea - ps24
Holometabola - ps25
Aparaglossata - ps26
Mecopterida - ps27
Diptera - ps28
Bibiono./Cyclorrhapha - ps29
Cyclorrhapha - ps30
Calyptratae/Drosophila - ps31
Drosophila - ps32
Sophophora - ps33
Obscura/Melanogaster_group - ps34
D_melanogaster_group - ps35
D_melanogaster_subgroup - ps36
sim./sech./mel. group - ps37
Drosophila_melanogaster - ps38
p-value
5e-03
7e-03
1e-02
1e-02
2e-02
Log-odds
0.5
1.0
1.5
2.0
2.5
3.0
3.5
4.0

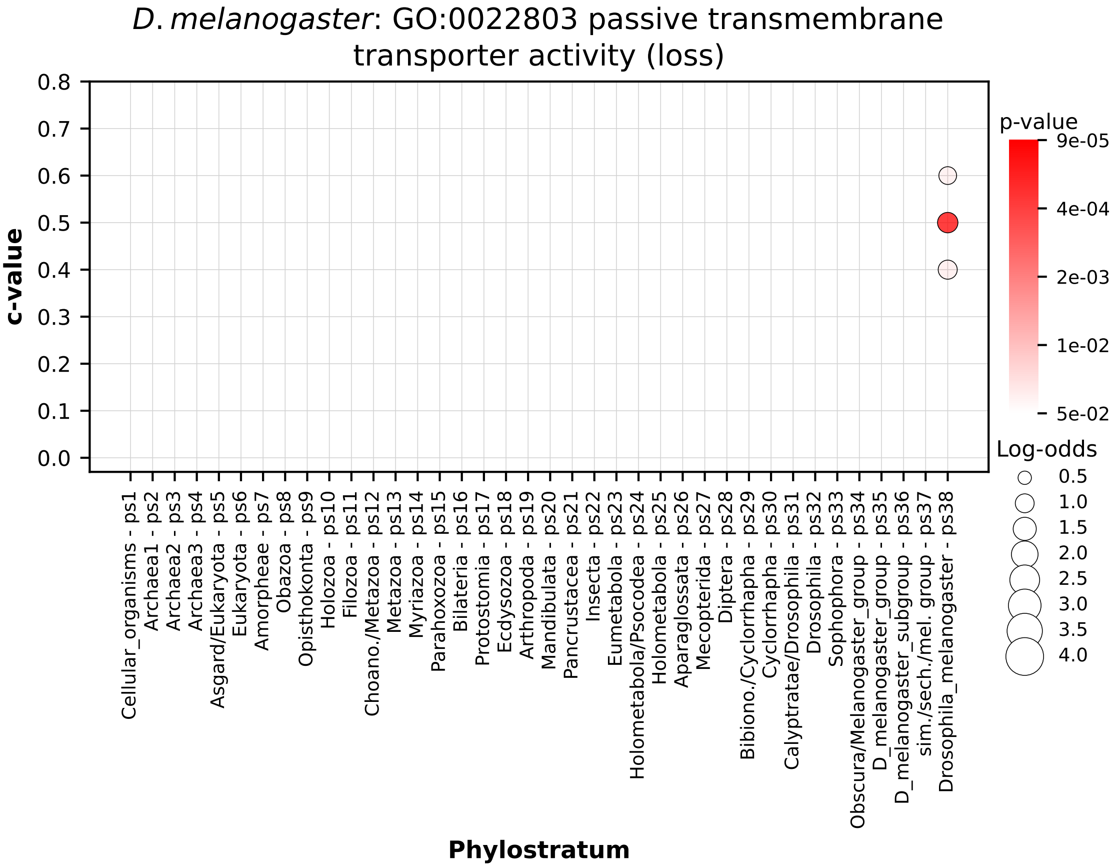

D. melanogaster: GO:0022803 passive transmembrane transporter activity (loss)
c-value
0.0
0.1
0.2
0.3
0.4
0.5
0.6
0.7
0.8
p-value
9e-05
4e-04
2e-03
1e-02
5e-02
Log-odds
0.5
1.0
1.5
2.0
2.5
3.0
3.5
4.0
Cellular_organisms - ps1
Archaea1 - ps2
Archaea2 - ps3
Archaea3 - ps4
Asgard/Eukaryota - ps5
Eukaryota - ps6
Amorpheae - ps7
Obazoa - ps8
Opisthokonta - ps9
Holozoa - ps10
Filozoa - ps11
Choano./Metazoa - ps12
Metazoa - ps13
Myriazoa - ps14
Parahoxozoa - ps15
Bilateria - ps16
Protostomia - ps17
Ecdysozoa - ps18
Arthropoda - ps19
Mandibulata - ps20
Pancrustacea - ps21
Insecta - ps22
Eumetabola - ps23
Holometabola/Psocodea - ps24
Holometabola - ps25
Aparaglossata - ps26
Mecopterida - ps27
Diptera - ps28
Bibiono./Cyclorrhapha - ps29
Cyclorrhapha - ps30
Calyptratae/Drosophila - ps31
Drosophila - ps32
Sophophora - ps33
Obscura/Melanogaster_group - ps34
D_melanogaster_group - ps35
D_melanogaster_subgroup - ps36
sim./sech./mel. group - ps37
Drosophila_melanogaster - ps38
Phylostratum

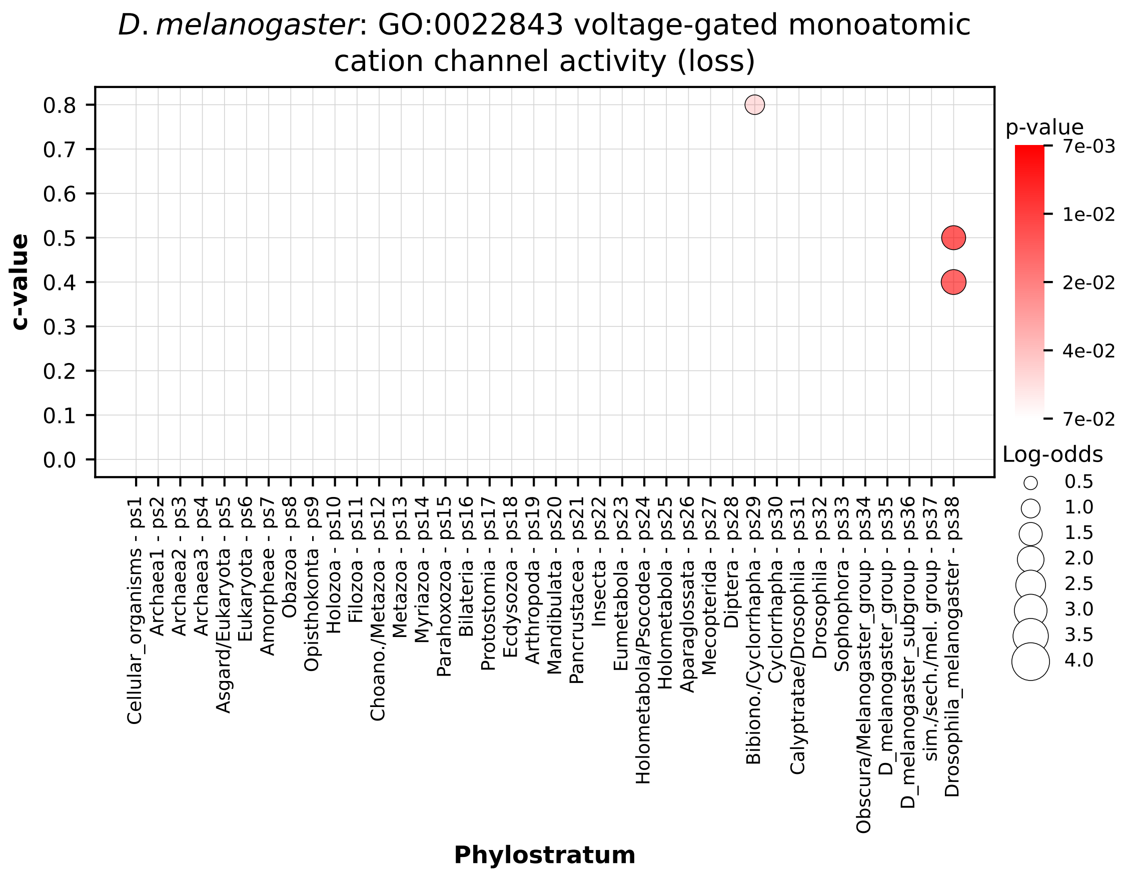

D. melanogaster: GO:0022843 voltage-gated monoatomic cation channel activity (loss)
c-value
0.0
0.1
0.2
0.3
0.4
0.5
0.6
0.7
0.8
Phylostratum
Cellular_organisms - ps1
Archaea1 - ps2
Archaea2 - ps3
Archaea3 - ps4
Asgard/Eukaryota - ps5
Eukaryota - ps6
Amorpheae - ps7
Obazoa - ps8
Opisthokonta - ps9
Holozoa - ps10
Filozoa - ps11
Choano./Metazoa - ps12
Metazoa - ps13
Myriazoa - ps14
Parahoxozoa - ps15
Bilateria - ps16
Protostomia - ps17
Ecdysozoa - ps18
Arthropoda - ps19
Mandibulata - ps20
Pancrustacea - ps21
Insecta - ps22
Eumetabola - ps23
Holometabola/Psocodea - ps24
Holometabola - ps25
Aparaglossata - ps26
Mecopterida - ps27
Diptera - ps28
Bibiono./Cyclorrhapha - ps29
Cyclorrhapha - ps30
Calyptratae/Drosophila - ps31
Drosophila - ps32
Sophophora - ps33
Obscura/Melanogaster_group - ps34
D_melanogaster_group - ps35
D_melanogaster_subgroup - ps36
sim./sech./mel. group - ps37
Drosophila_melanogaster - ps38
p-value
7e-03
1e-02
2e-02
4e-02
7e-02
Log-odds
0.5
1.0
1.5
2.0
2.5
3.0
3.5
4.0

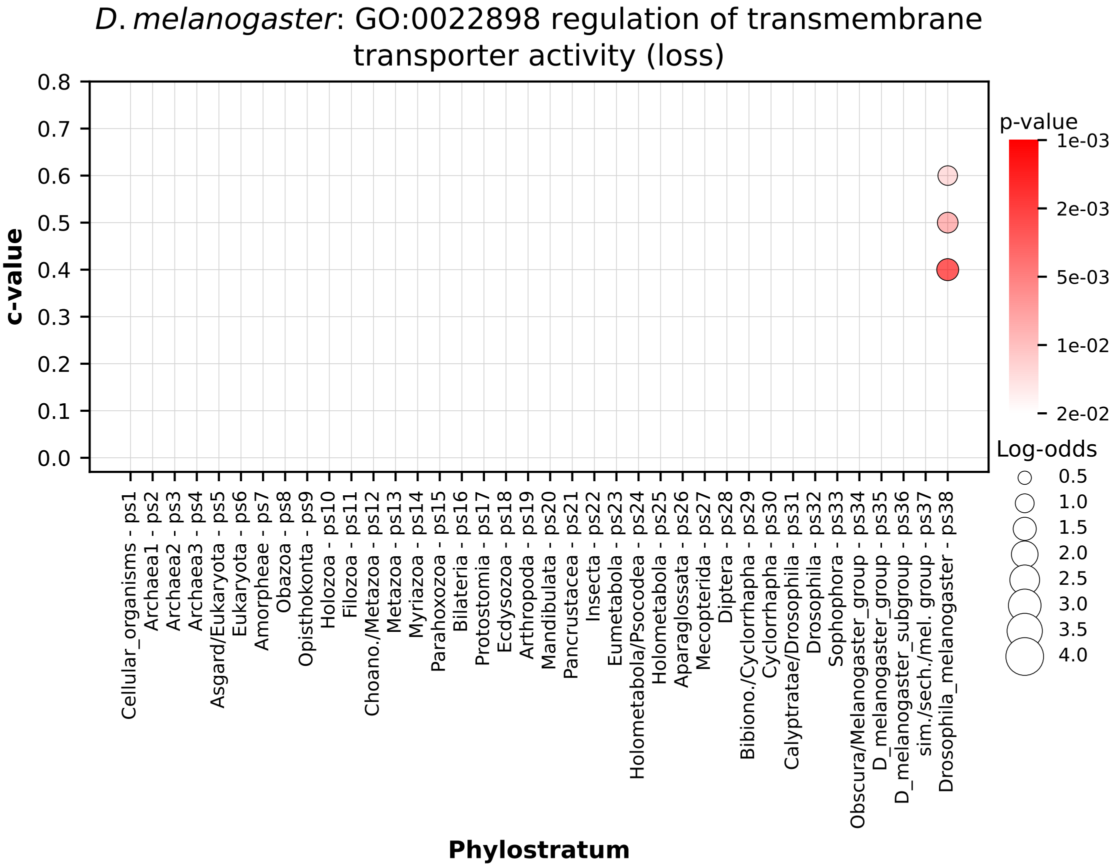

D. melanogaster: GO:0022898 regulation of transmembrane transporter activity (loss)
c-value
0.0
0.1
0.2
0.3
0.4
0.5
0.6
0.7
0.8
Phylostratum
Cellular_organisms - ps1
Archaea1 - ps2
Archaea2 - ps3
Archaea3 - ps4
Asgard/Eukaryota - ps5
Eukaryota - ps6
Amorpheae - ps7
Obazoa - ps8
Opisthokonta - ps9
Holozoa - ps10
Filozoa - ps11
Choano./Metazoa - ps12
Metazoa - ps13
Myriazoa - ps14
Parahoxozoa - ps15
Bilateria - ps16
Protostomia - ps17
Ecdysozoa - ps18
Arthropoda - ps19
Mandibulata - ps20
Pancrustacea - ps21
Insecta - ps22
Eumetabola - ps23
Holometabola/Psocodea - ps24
Holometabola - ps25
Aparaglossata - ps26
Mecopterida - ps27
Diptera - ps28
Bibiono./Cyclorrhapha - ps29
Cyclorrhapha - ps30
Calyptratae/Drosophila - ps31
Drosophila - ps32
Sophophora - ps33
Obscura/Melanogaster_group - ps34
D_melanogaster_group - ps35
D_melanogaster_subgroup - ps36
sim./sech./mel. group - ps37
Drosophila_melanogaster - ps38
p-value
1e-03
2e-03
5e-03
1e-02
2e-02
Log-odds
0.5
1.0
1.5
2.0
2.5
3.0
3.5
4.0

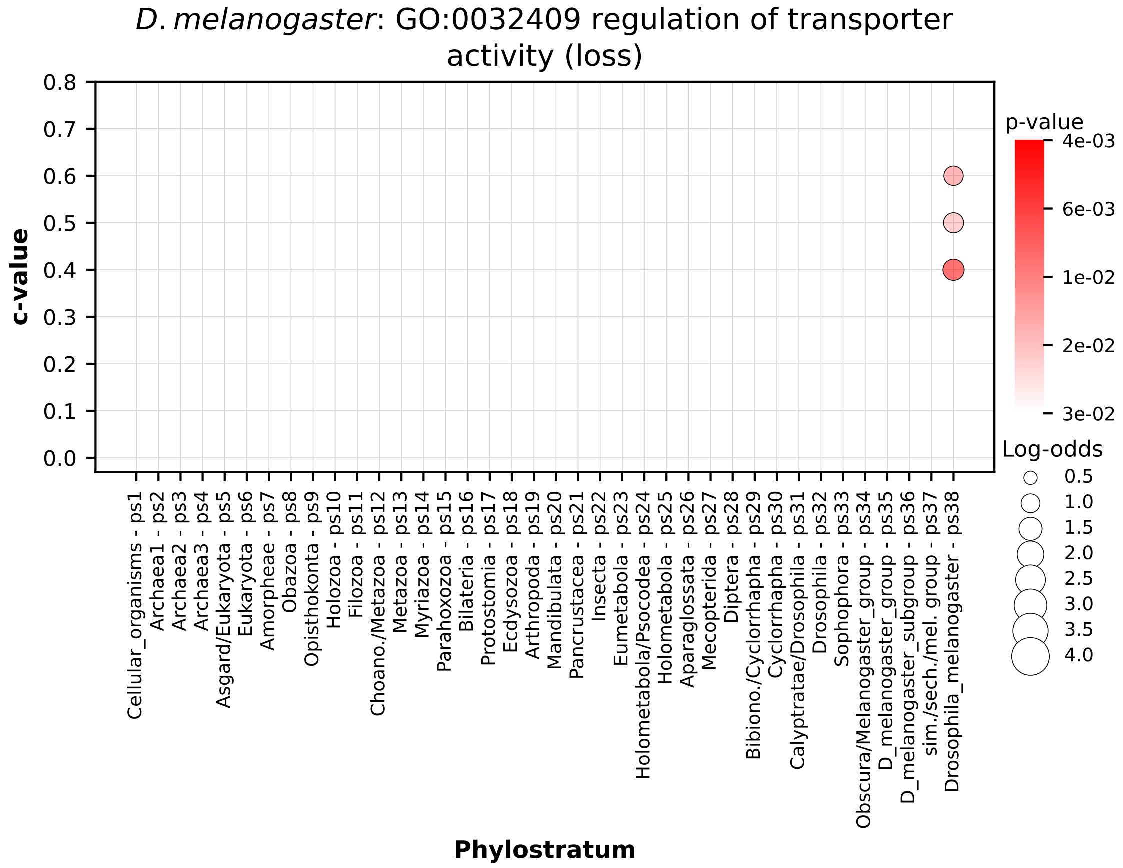

D. melanogaster: GO:0032409 regulation of transporter activity (loss)
c-value
0.8
0.7
0.6
0.5
0.4
0.3
0.2
0.1
0.0
p-value
4e-03
6e-03
1e-02
2e-02
3e-02
Log-odds
0.5
1.0
1.5
2.0
2.5
3.0
3.5
4.0
Cellular_organisms - ps1
Archaea1 - ps2
Archaea2 - ps3
Archaea3 - ps4
Asgard/Eukaryota - ps5
Eukaryota - ps6
Amorpheae - ps7
Obazoa - ps8
Opisthokonta - ps9
Holozoa - ps10
Filozoa - ps11
Choano./Metazoa - ps12
Metazoa - ps13
Myriazoa - ps14
Parahoxozoa - ps15
Bilateria - ps16
Protostomia - ps17
Ecdysozoa - ps18
Arthropoda - ps19
Mandibulata - ps20
Pancrustacea - ps21
Insecta - ps22
Eumetabola - ps23
Holometabola/Psocodea - ps24
Holometabola - ps25
Aparaglossata - ps26
Mecopterida - ps27
Diptera - ps28
Bibiono./Cyclorrhapha - ps29
Cyclorrhapha - ps30
Calyptratae/Drosophila - ps31
Drosophila - ps32
Sophophora - ps33
Obscura/Melanogaster_group - ps34
D_melanogaster_group - ps35
D_melanogaster_subgroup - ps36
sim./sech./mel. group - ps37
Drosophila_melanogaster - ps38
Phylostratum

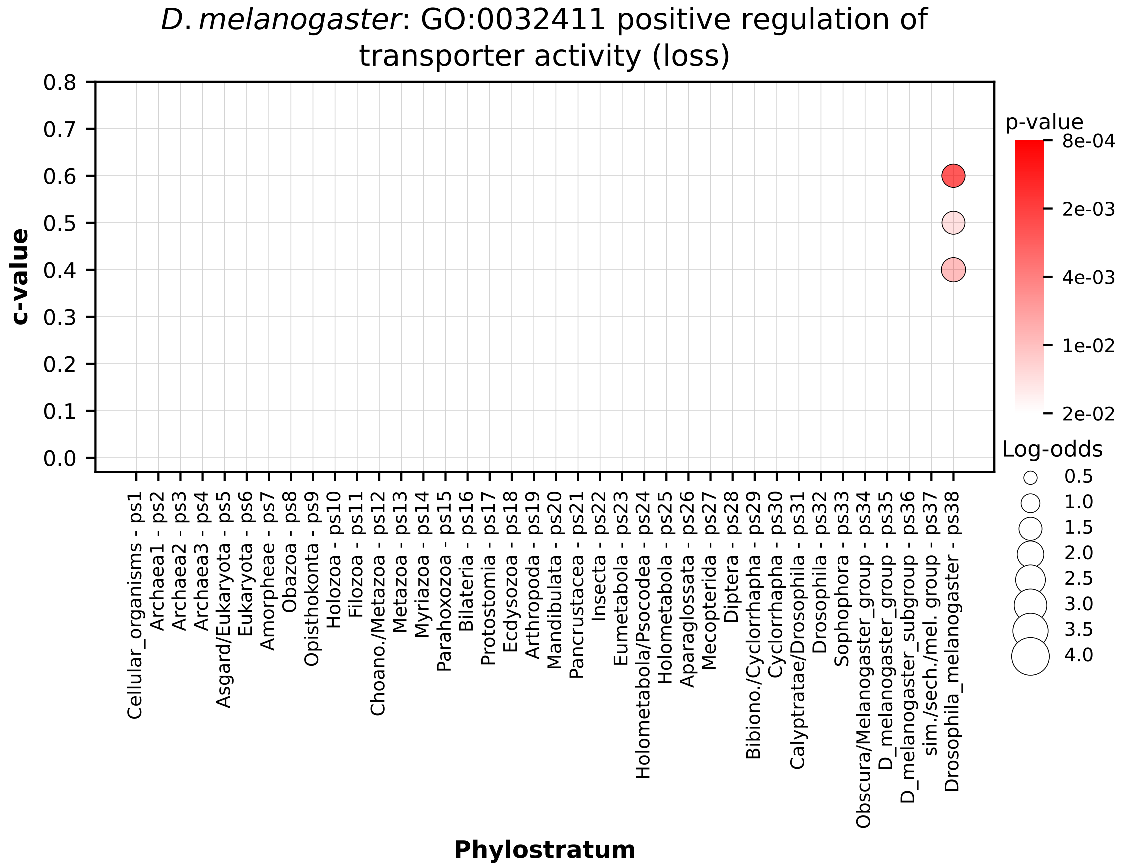

D. melanogaster: GO:0032411 positive regulation of transporter activity (loss)
c-value
0.8
0.7
0.6
0.5
0.4
0.3
0.2
0.1
0.0
p-value
8e-04
2e-03
4e-03
1e-02
2e-02
Log-odds
0.5
1.0
1.5
2.0
2.5
3.0
3.5
4.0
Cellular_organisms - ps1
Archaea1 - ps2
Archaea2 - ps3
Archaea3 - ps4
Asgard/Eukaryota - ps5
Eukaryota - ps6
Amorpheae - ps7
Obazoa - ps8
Opisthokonta - ps9
Holozoa - ps10
Filozoa - ps11
Choano./Metazoa - ps12
Metazoa - ps13
Myriazoa - ps14
Parahoxozoa - ps15
Bilateria - ps16
Protostomia - ps17
Ecdysozoa - ps18
Arthropoda - ps19
Mandibulata - ps20
Pancrustacea - ps21
Insecta - ps22
Eumetabola - ps23
Holometabola/Psocodea - ps24
Holometabola - ps25
Aparaglossata - ps26
Mecopterida - ps27
Diptera - ps28
Bibiono./Cyclorrhapha - ps29
Cyclorrhapha - ps30
Calyptratae/Drosophila - ps31
Drosophila - ps32
Sophophora - ps33
Obscura/Melanogaster_group - ps34
D_melanogaster_group - ps35
D_melanogaster_subgroup - ps36
sim./sech./mel. group - ps37
Drosophila_melanogaster - ps38
Phylostratum

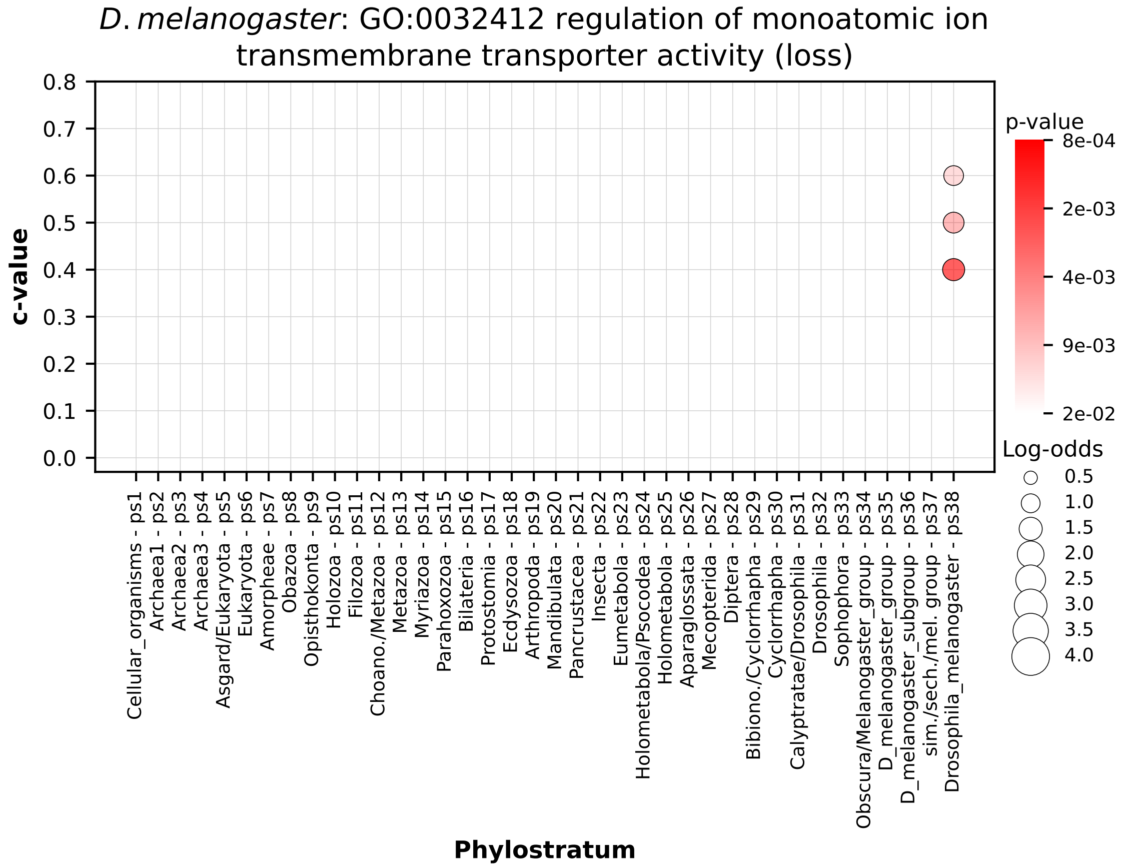
D. melanogaster: GO:0032412 regulation of monoatomic ion transmembrane transporter activity (loss)
c-value
0.0
0.1
0.2
0.3
0.4
0.5
0.6
0.7
0.8
Phylostratum
Cellular_organisms - ps1
Archaea1 - ps2
Archaea2 - ps3
Archaea3 - ps4
Asgard/Eukaryota - ps5
Eukaryota - ps6
Amorpheae - ps7
Obazoa - ps8
Opisthokonta - ps9
Holozoa - ps10
Filozoa - ps11
Choano./Metazoa - ps12
Metazoa - ps13
Myriazoa - ps14
Parahoxozoa - ps15
Bilateria - ps16
Protostomia - ps17
Ecdysozoa - ps18
Arthropoda - ps19
Mandibulata - ps20
Pancrustacea - ps21
Insecta - ps22
Eumetabola - ps23
Holometabola/Psocodea - ps24
Holometabola - ps25
Aparaglossata - ps26
Mecopterida - ps27
Diptera - ps28
Bibiono./Cyclorrhapha - ps29
Cyclorrhapha - ps30
Calyptratae/Drosophila - ps31
Drosophila - ps32
Sophophora - ps33
Obscura/Melanogaster_group - ps34
D_melanogaster_group - ps35
D_melanogaster_subgroup - ps36
sim./sech./mel. group - ps37
Drosophila_melanogaster - ps38
p-value
8e-04
2e-03
4e-03
9e-03
2e-02
Log-odds
0.5
1.0
1.5
2.0
2.5
3.0
3.5
4.0

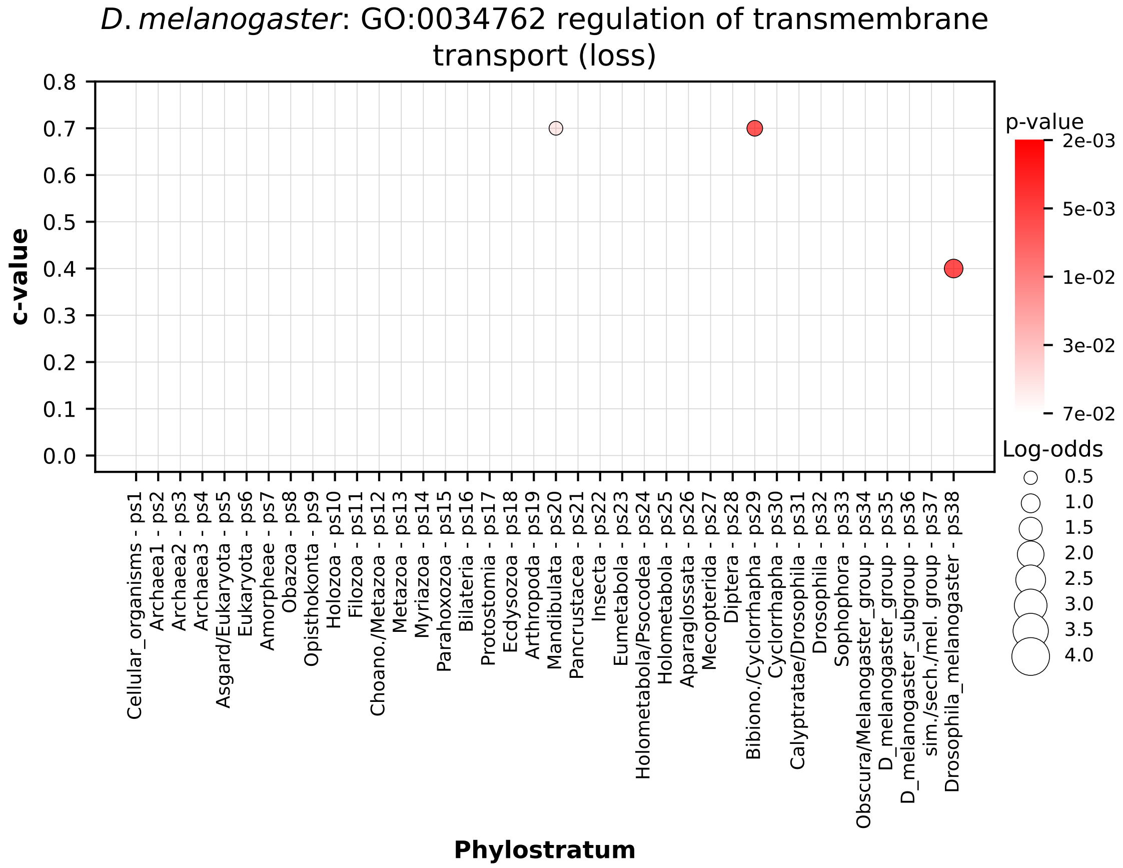

D. melanogaster: GO:0034762 regulation of transmembrane transport (loss)
c-value
0.8
0.7
0.6
0.5
0.4
0.3
0.2
0.1
0.0
p-value
2e-03
5e-03
1e-02
3e-02
7e-02
Log-odds
0.5
1.0
1.5
2.0
2.5
3.0
3.5
4.0
Cellular_organisms - ps1
Archaea1 - ps2
Archaea2 - ps3
Archaea3 - ps4
Asgard/Eukaryota - ps5
Eukaryota - ps6
Amorpheae - ps7
Obazoa - ps8
Opisthokonta - ps9
Holozoa - ps10
Filozoa - ps11
Choano./Metazoa - ps12
Metazoa - ps13
Myriazoa - ps14
Parahoxozoa - ps15
Bilateria - ps16
Protostomia - ps17
Ecdysozoa - ps18
Arthropoda - ps19
Mandibulata - ps20
Pancrustacea - ps21
Insecta - ps22
Eumetabola - ps23
Holometabola/Psocodea - ps24
Holometabola - ps25
Aparaglossata - ps26
Mecopterida - ps27
Diptera - ps28
Bibiono./Cyclorrhapha - ps29
Cyclorrhapha - ps30
Calyptratae/Drosophila - ps31
Drosophila - ps32
Sophophora - ps33
Obscura/Melanogaster_group - ps34
D_melanogaster_group - ps35
D_melanogaster_subgroup - ps36
sim./sech./mel. group - ps37
Drosophila_melanogaster - ps38
Phylostratum

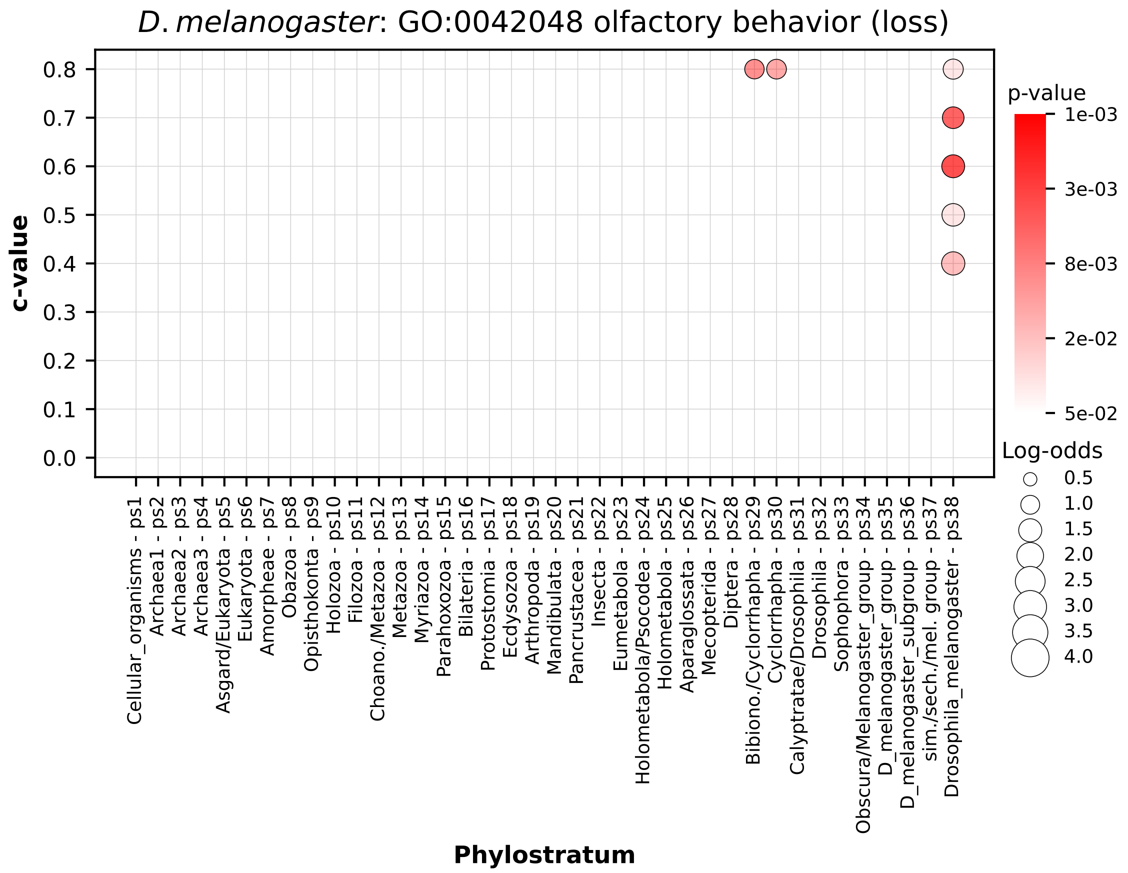

D. melanogaster: GO:0042048 olfactory behavior (loss)
c-value
0.0
0.1
0.2
0.3
0.4
0.5
0.6
0.7
0.8
Phylostratum
Cellular_organisms - ps1
Archaea1 - ps2
Archaea2 - ps3
Archaea3 - ps4
Asgard/Eukaryota - ps5
Eukaryota - ps6
Amorpheae - ps7
Obazoa - ps8
Opisthokonta - ps9
Holozoa - ps10
Filozoa - ps11
Choano./Metazoa - ps12
Metazoa - ps13
Myriazoa - ps14
Parahoxozoa - ps15
Bilateria - ps16
Protostomia - ps17
Ecdysozoa - ps18
Arthropoda - ps19
Mandibulata - ps20
Pancrustacea - ps21
Insecta - ps22
Eumetabola - ps23
Holometabola/Psocodea - ps24
Holometabola - ps25
Aparaglossata - ps26
Mecopterida - ps27
Diptera - ps28
Bibiono./Cyclorrhapha - ps29
Cyclorrhapha - ps30
Calyptratae/Drosophila - ps31
Drosophila - ps32
Sophophora - ps33
Obscura/Melanogaster_group - ps34
D_melanogaster_group - ps35
D_melanogaster_subgroup - ps36
sim./sech./mel. group - ps37
Drosophila_melanogaster - ps38
p-value
1e-03
3e-03
8e-03
2e-02
5e-02
Log-odds
0.5
1.0
1.5
2.0
2.5
3.0
3.5
4.0

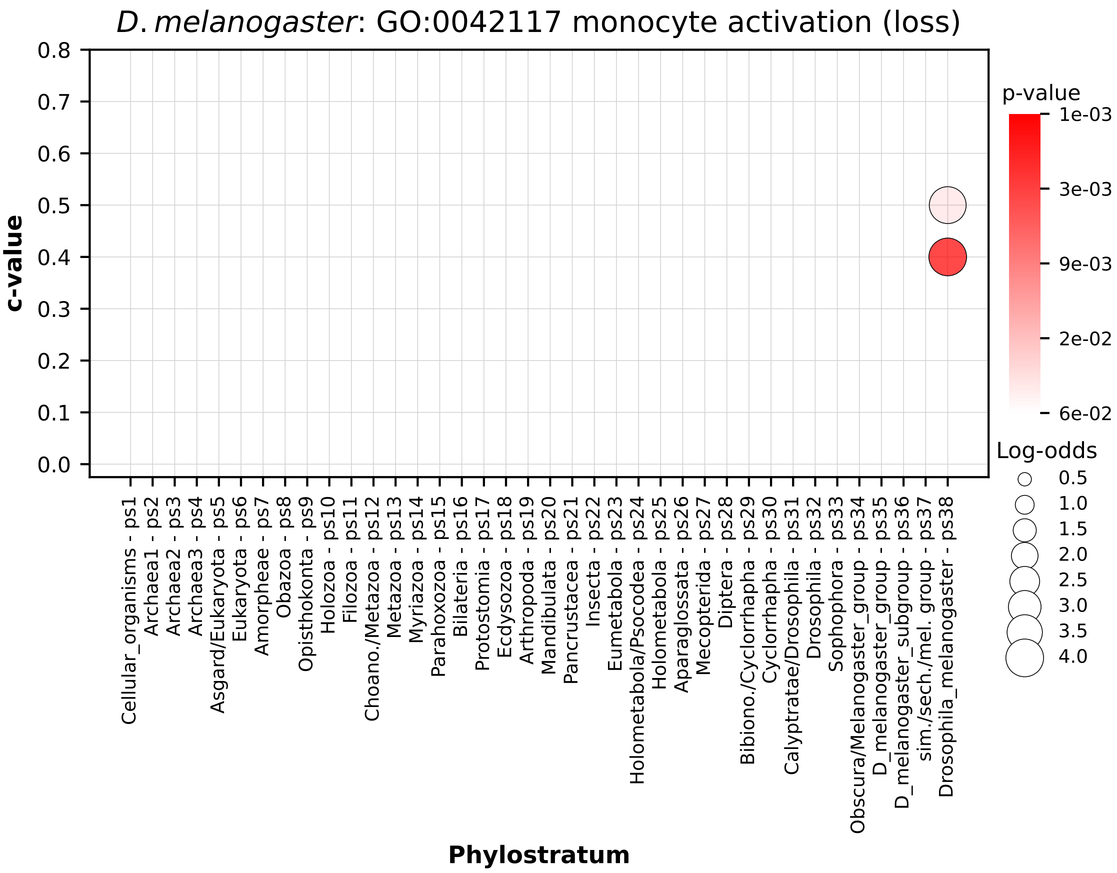

D. melanogaster: GO:0042117 monocyte activation (loss)
c-value
0.0
0.1
0.2
0.3
0.4
0.5
0.6
0.7
0.8
p-value
1e-03
3e-03
9e-03
2e-02
6e-02
Log-odds
0.5
1.0
1.5
2.0
2.5
3.0
3.5
4.0
Cellular_organisms - ps1
Archaea1 - ps2
Archaea2 - ps3
Archaea3 - ps4
Asgard/Eukaryota - ps5
Eukaryota - ps6
Amorpheae - ps7
Obazoa - ps8
Opisthokonta - ps9
Holozoa - ps10
Filozoa - ps11
Choano./Metazoa - ps12
Metazoa - ps13
Myriazoa - ps14
Parahoxozoa - ps15
Bilateria - ps16
Protostomia - ps17
Ecdysozoa - ps18
Arthropoda - ps19
Mandibulata - ps20
Pancrustacea - ps21
Insecta - ps22
Eumetabola - ps23
Holometabola/Psocodea - ps24
Holometabola - ps25
Aparaglossata - ps26
Mecopterida - ps27
Diptera - ps28
Bibiono./Cyclorrhapha - ps29
Cyclorrhapha - ps30
Calyptratae/Drosophila - ps31
Drosophila - ps32
Sophophora - ps33
Obscura/Melanogaster_group - ps34
D_melanogaster_group - ps35
D_melanogaster_subgroup - ps36
sim./sech./mel. group - ps37
Drosophila_melanogaster - ps38
Phylostratum

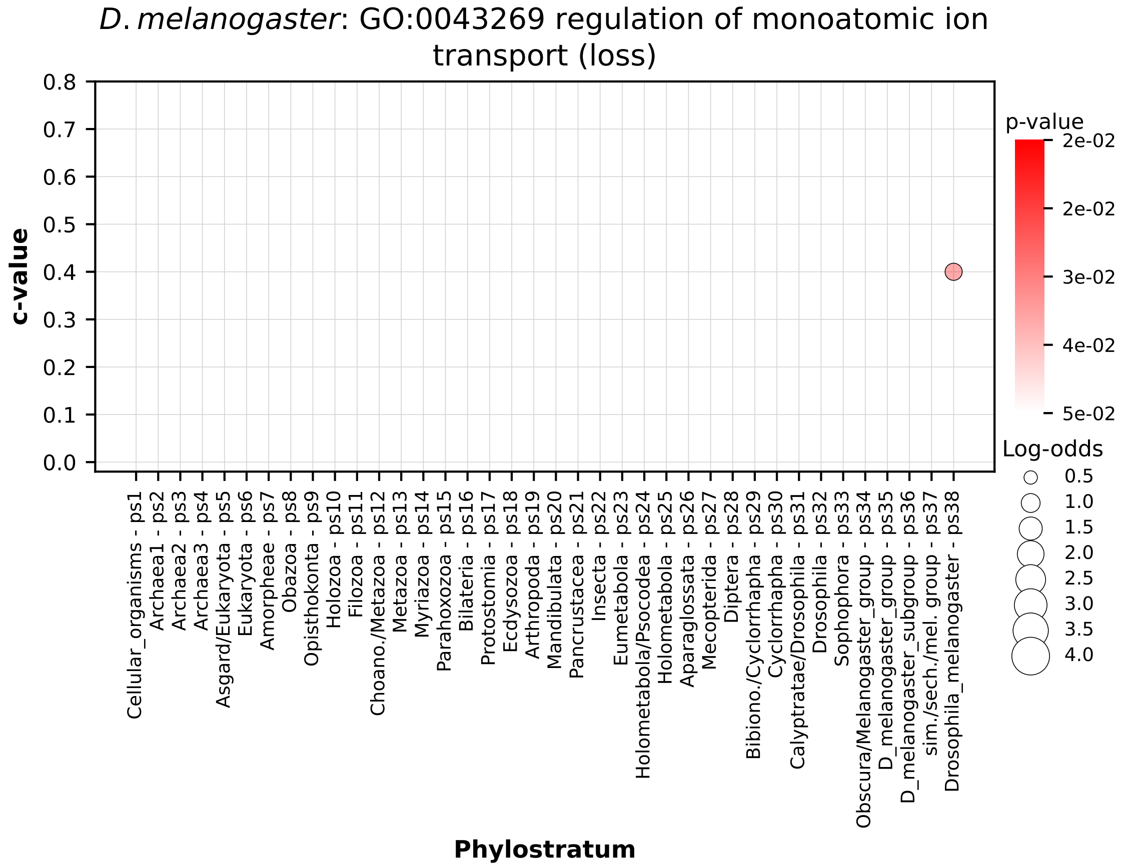

D. melanogaster: GO:0043269 regulation of monoatomic ion transport (loss)
c-value
0.8
0.7
0.6
0.5
0.4
0.3
0.2
0.1
0.0
p-value
2e-02
2e-02
3e-02
4e-02
5e-02
Log-odds
0.5
1.0
1.5
2.0
2.5
3.0
3.5
4.0
Cellular_organisms - ps1
Archaea1 - ps2
Archaea2 - ps3
Archaea3 - ps4
Asgard/Eukaryota - ps5
Eukaryota - ps6
Amorpheae - ps7
Obazoa - ps8
Opisthokonta - ps9
Holozoa - ps10
Filozoa - ps11
Choano./Metazoa - ps12
Metazoa - ps13
Myriazoa - ps14
Parahoxozoa - ps15
Bilateria - ps16
Protostomia - ps17
Ecdysozoa - ps18
Arthropoda - ps19
Mandibulata - ps20
Pancrustacea - ps21
Insecta - ps22
Eumetabola - ps23
Holometabola/Psocodea - ps24
Holometabola - ps25
Aparaglossata - ps26
Mecopterida - ps27
Diptera - ps28
Bibiono./Cyclorrhapha - ps29
Cyclorrhapha - ps30
Calyptratae/Drosophila - ps31
Drosophila - ps32
Sophophora - ps33
Obscura/Melanogaster_group - ps34
D_melanogaster_group - ps35
D_melanogaster_subgroup - ps36
sim./sech./mel. group - ps37
Drosophila_melanogaster - ps38
Phylostratum

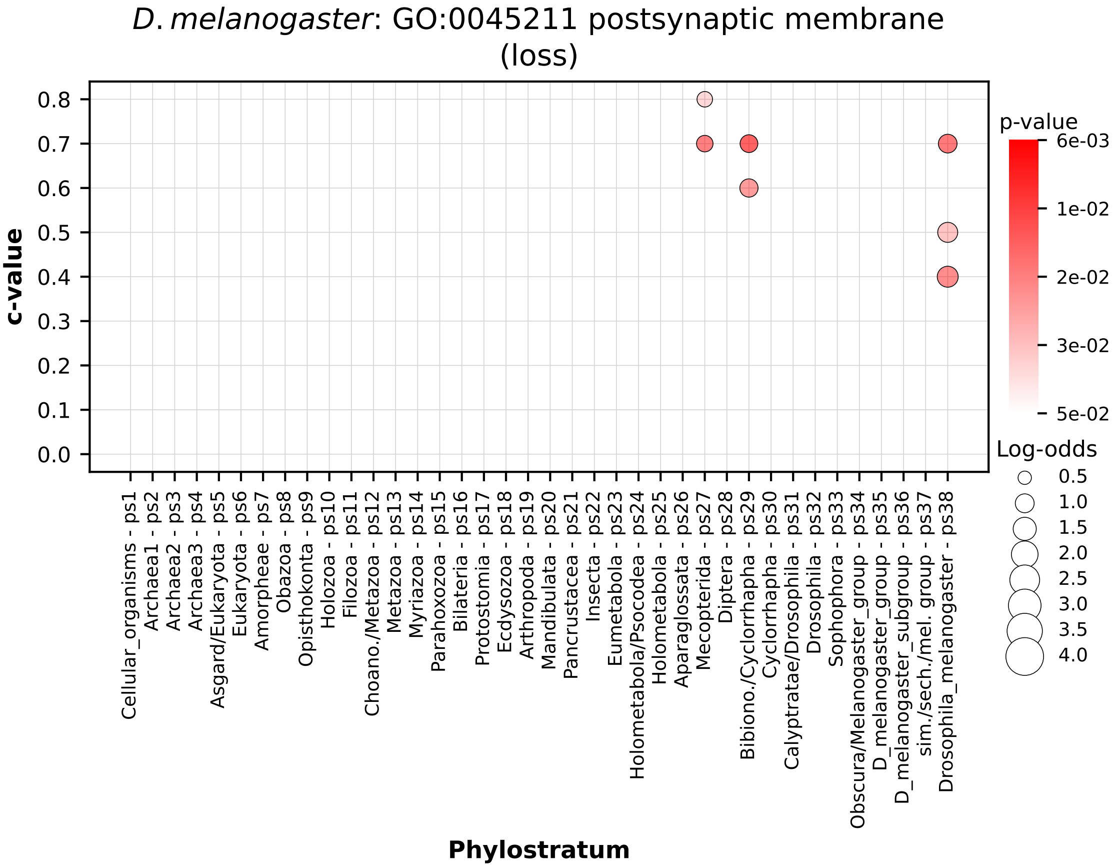

D. melanogaster: GO:0045211 postsynaptic membrane
(loss)
c-value
0.8
0.7
0.6
0.5
0.4
0.3
0.2
0.1
0.0
Phylostratum
Cellular_organisms - ps1
Archaea1 - ps2
Archaea2 - ps3
Archaea3 - ps4
Asgard/Eukaryota - ps5
Eukaryota - ps6
Amorpheae - ps7
Obazoa - ps8
Opisthokonta - ps9
Holozoa - ps10
Filozoa - ps11
Choano./Metazoa - ps12
Metazoa - ps13
Myriazoa - ps14
Parahoxozoa - ps15
Bilateria - ps16
Protostomia - ps17
Ecdysozoa - ps18
Arthropoda - ps19
Mandibulata - ps20
Pancrustacea - ps21
Insecta - ps22
Eumetabola - ps23
Holometabola/Psocodea - ps24
Holometabola - ps25
Aparaglossata - ps26
Mecopterida - ps27
Diptera - ps28
Bibiono./Cyclorrhapha - ps29
Cyclorrhapha - ps30
Calyptratae/Drosophila - ps31
Drosophila - ps32
Sophophora - ps33
Obscura/Melanogaster_group - ps34
D_melanogaster_group - ps35
D_melanogaster_subgroup - ps36
sim./sech./mel. group - ps37
Drosophila_melanogaster - ps38
p-value
6e-03
1e-02
2e-02
3e-02
5e-02
Log-odds
0.5
1.0
1.5
2.0
2.5
3.0
3.5
4.0

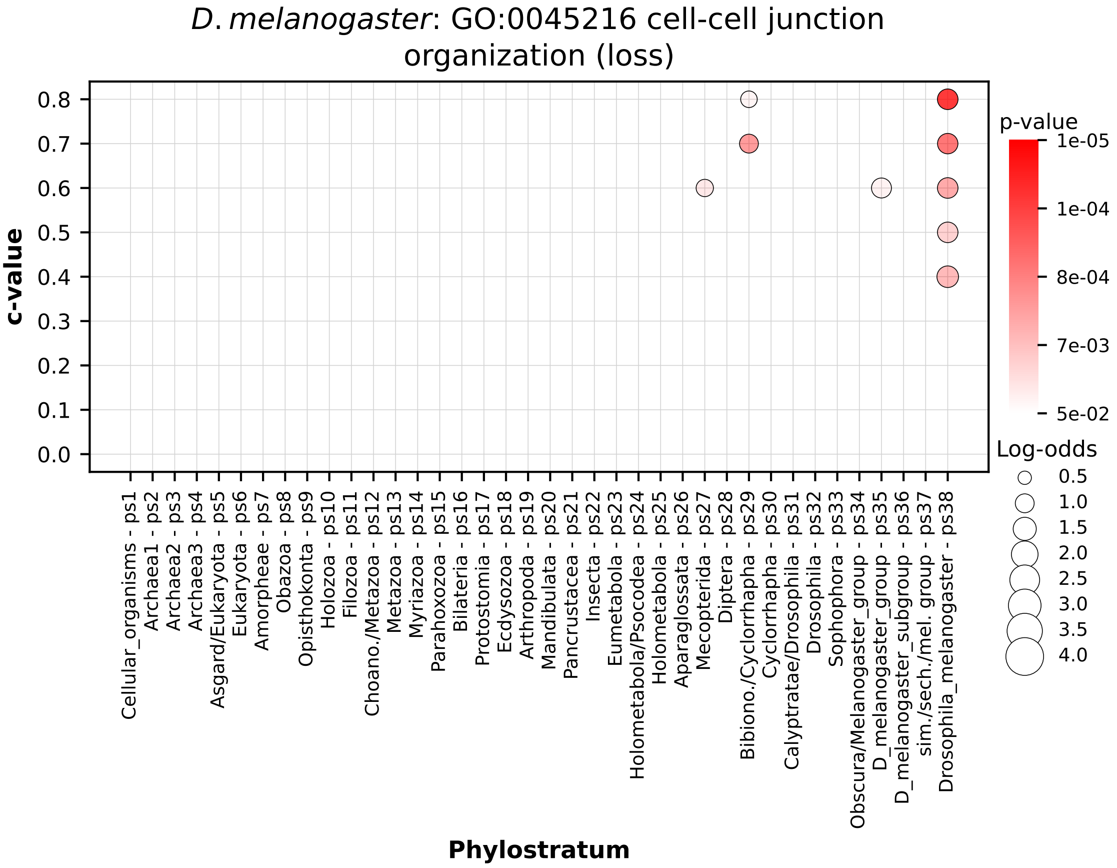

D. melanogaster: GO:0045216 cell-cell junction organization (loss)
c-value
0.8
0.7
0.6
0.5
0.4
0.3
0.2
0.1
0.0
p-value
1e-05
1e-04
8e-04
7e-03
5e-02
Log-odds
0.5
1.0
1.5
2.0
2.5
3.0
3.5
4.0
Cellular_organisms - ps1
Archaea1 - ps2
Archaea2 - ps3
Archaea3 - ps4
Asgard/Eukaryota - ps5
Eukaryota - ps6
Amorpheae - ps7
Obazoa - ps8
Opisthokonta - ps9
Holozoa - ps10
Filozoa - ps11
Choano./Metazoa - ps12
Metazoa - ps13
Myriazoa - ps14
Parahoxozoa - ps15
Bilateria - ps16
Protostomia - ps17
Ecdysozoa - ps18
Arthropoda - ps19
Mandibulata - ps20
Pancrustacea - ps21
Insecta - ps22
Eumetabola - ps23
Holometabola/Psocodea - ps24
Holometabola - ps25
Aparaglossata - ps26
Mecopterida - ps27
Diptera - ps28
Bibiono./Cyclorrhapha - ps29
Cyclorrhapha - ps30
Calyptratae/Drosophila - ps31
Drosophila - ps32
Sophophora - ps33
Obscura/Melanogaster_group - ps34
D_melanogaster_group - ps35
D_melanogaster_subgroup - ps36
sim./sech./mel. group - ps37
Drosophila_melanogaster - ps38
Phylostratum

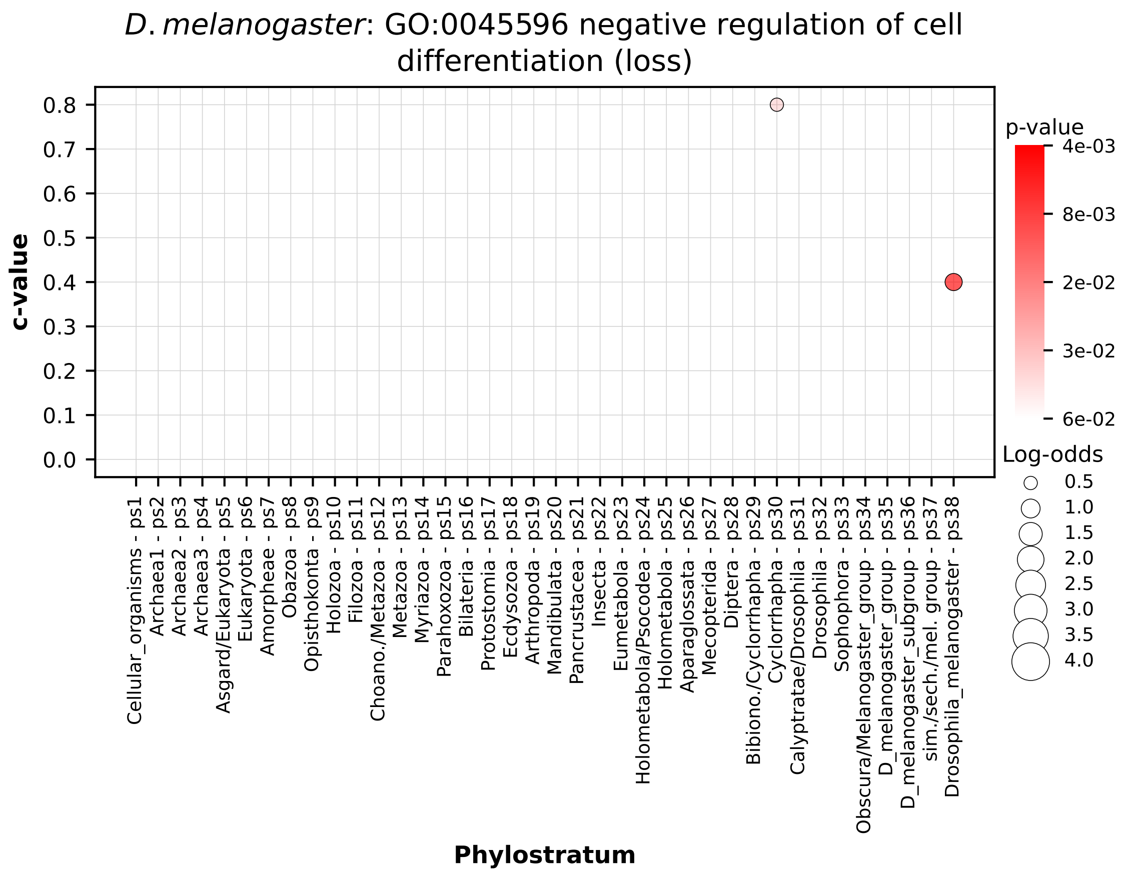

D. melanogaster: GO:0045596 negative regulation of cell differentiation (loss)
c-value
0.0
0.1
0.2
0.3
0.4
0.5
0.6
0.7
0.8
Phylostratum
Cellular_organisms - ps1
Archaea1 - ps2
Archaea2 - ps3
Archaea3 - ps4
Asgard/Eukaryota - ps5
Eukaryota - ps6
Amorpheae - ps7
Obazoa - ps8
Opisthokonta - ps9
Holozoa - ps10
Filozoa - ps11
Choano./Metazoa - ps12
Metazoa - ps13
Myriazoa - ps14
Parahoxozoa - ps15
Bilateria - ps16
Protostomia - ps17
Ecdysozoa - ps18
Arthropoda - ps19
Mandibulata - ps20
Pancrustacea - ps21
Insecta - ps22
Eumetabola - ps23
Holometabola/Psocodea - ps24
Holometabola - ps25
Aparaglossata - ps26
Mecopterida - ps27
Diptera - ps28
Bibiono./Cyclorrhapha - ps29
Cyclorrhapha - ps30
Calyptratae/Drosophila - ps31
Drosophila - ps32
Sophophora - ps33
Obscura/Melanogaster_group - ps34
D_melanogaster_group - ps35
D_melanogaster_subgroup - ps36
sim./sech./mel. group - ps37
Drosophila_melanogaster - ps38
p-value
4e-03
8e-03
2e-02
3e-02
6e-02
Log-odds
0.5
1.0
1.5
2.0
2.5
3.0
3.5
4.0

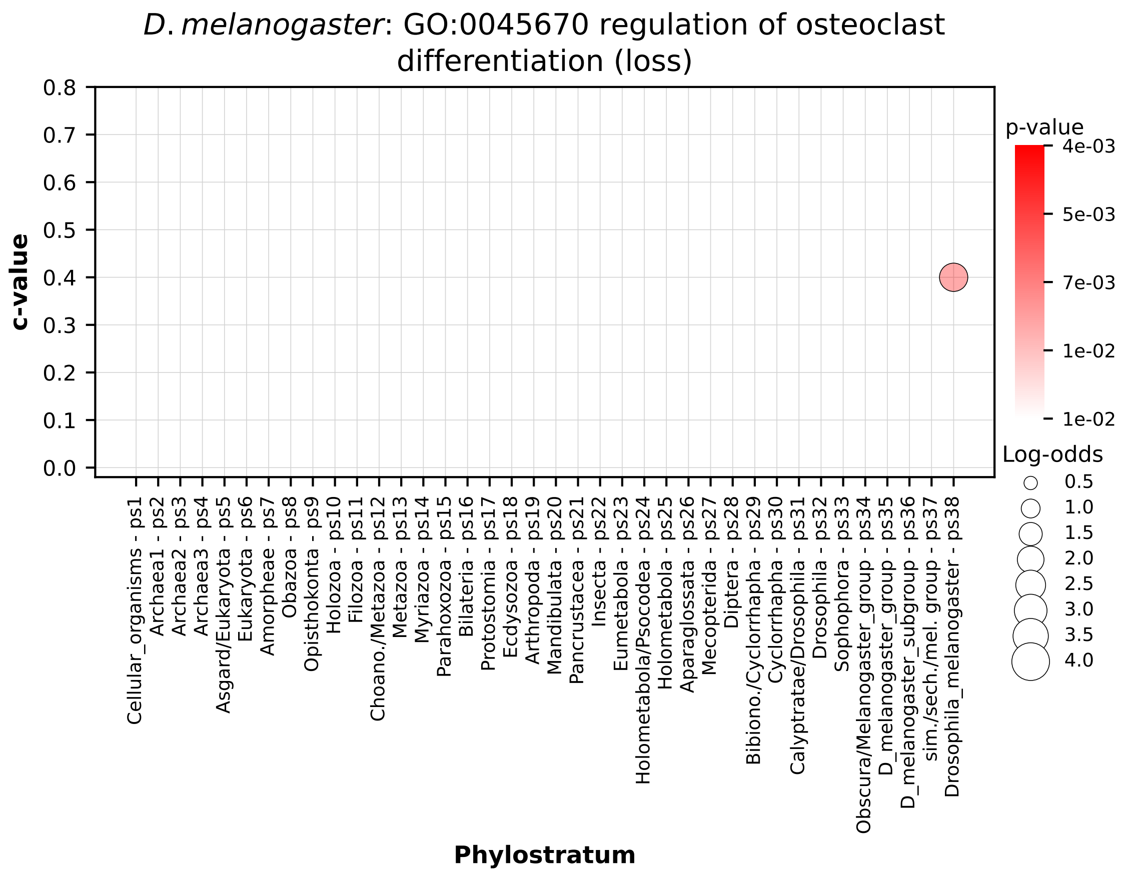

D. melanogaster: GO:0045670 regulation of osteoclast differentiation (loss)
c-value
0.8
0.7
0.6
0.5
0.4
0.3
0.2
0.1
0.0
Phylostratum
Cellular_organisms - ps1
Archaea1 - ps2
Archaea2 - ps3
Archaea3 - ps4
Asgard/Eukaryota - ps5
Eukaryota - ps6
Amorpheae - ps7
Obazoa - ps8
Opisthokonta - ps9
Holozoa - ps10
Filozoa - ps11
Choano./Metazoa - ps12
Metazoa - ps13
Myriazoa - ps14
Parahoxozoa - ps15
Bilateria - ps16
Protostomia - ps17
Ecdysozoa - ps18
Arthropoda - ps19
Mandibulata - ps20
Pancrustacea - ps21
Insecta - ps22
Eumetabola - ps23
Holometabola/Psocodea - ps24
Holometabola - ps25
Aparaglossata - ps26
Mecopterida - ps27
Diptera - ps28
Bibiono./Cyclorrhapha - ps29
Cyclorrhapha - ps30
Calyptratae/Drosophila - ps31
Drosophila - ps32
Sophophora - ps33
Obscura/Melanogaster_group - ps34
D_melanogaster_group - ps35
D_melanogaster_subgroup - ps36
sim./sech./mel. group - ps37
Drosophila_melanogaster - ps38
p-value
4e-03
5e-03
7e-03
1e-02
1e-02
Log-odds
0.5
1.0
1.5
2.0
2.5
3.0
3.5
4.0

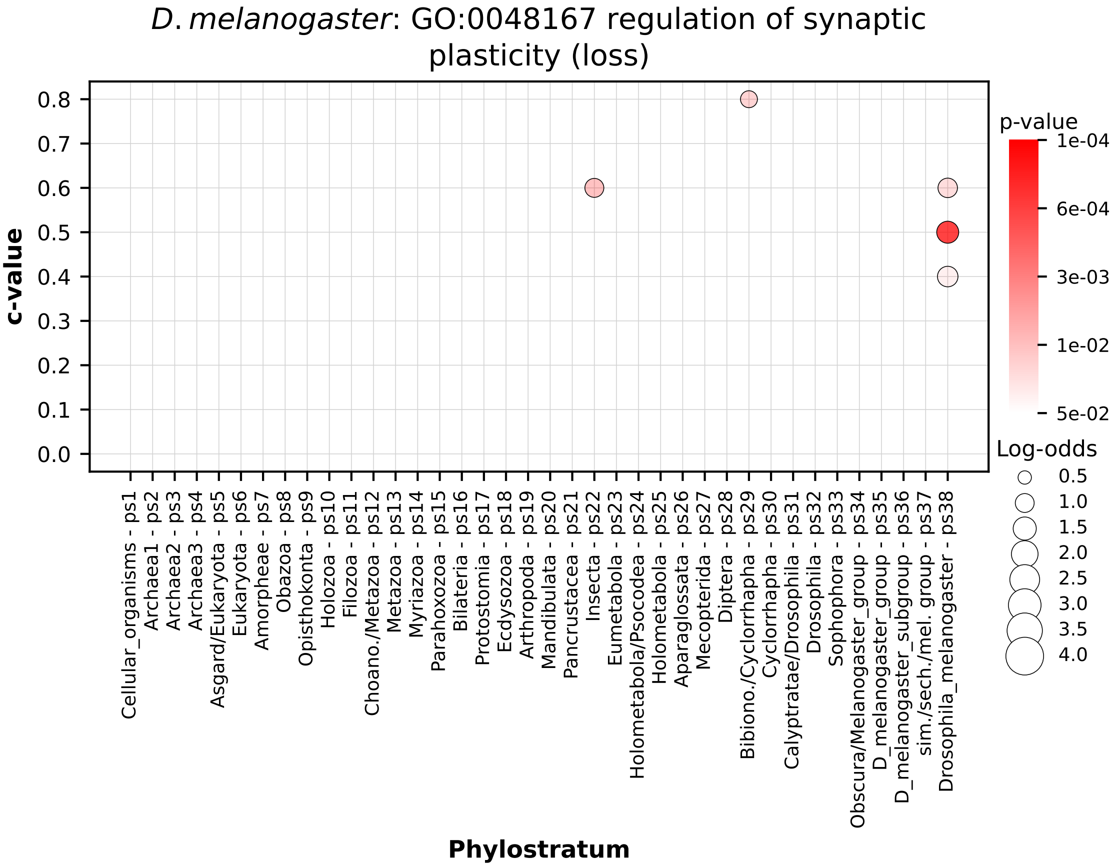

D. melanogaster: GO:0048167 regulation of synaptic plasticity (loss)
c-value
0.8
0.7
0.6
0.5
0.4
0.3
0.2
0.1
0.0
p-value
1e-04
6e-04
3e-03
1e-02
5e-02
Log-odds
0.5
1.0
1.5
2.0
2.5
3.0
3.5
4.0
Cellular_organisms - ps1
Archaea1 - ps2
Archaea2 - ps3
Archaea3 - ps4
Asgard/Eukaryota - ps5
Eukaryota - ps6
Amorpheae - ps7
Obazoa - ps8
Opisthokonta - ps9
Holozoa - ps10
Filozoa - ps11
Choano./Metazoa - ps12
Metazoa - ps13
Myriazoa - ps14
Parahoxozoa - ps15
Bilateria - ps16
Protostomia - ps17
Ecdysozoa - ps18
Arthropoda - ps19
Mandibulata - ps20
Pancrustacea - ps21
Insecta - ps22
Eumetabola - ps23
Holometabola/Psocodea - ps24
Holometabola - ps25
Aparaglossata - ps26
Mecopterida - ps27
Diptera - ps28
Bibiono./Cyclorrhapha - ps29
Cyclorrhapha - ps30
Calyptratae/Drosophila - ps31
Drosophila - ps32
Sophophora - ps33
Obscura/Melanogaster_group - ps34
D_melanogaster_group - ps35
D_melanogaster_subgroup - ps36
sim./sech./mel. group - ps37
Drosophila_melanogaster - ps38
Phylostratum

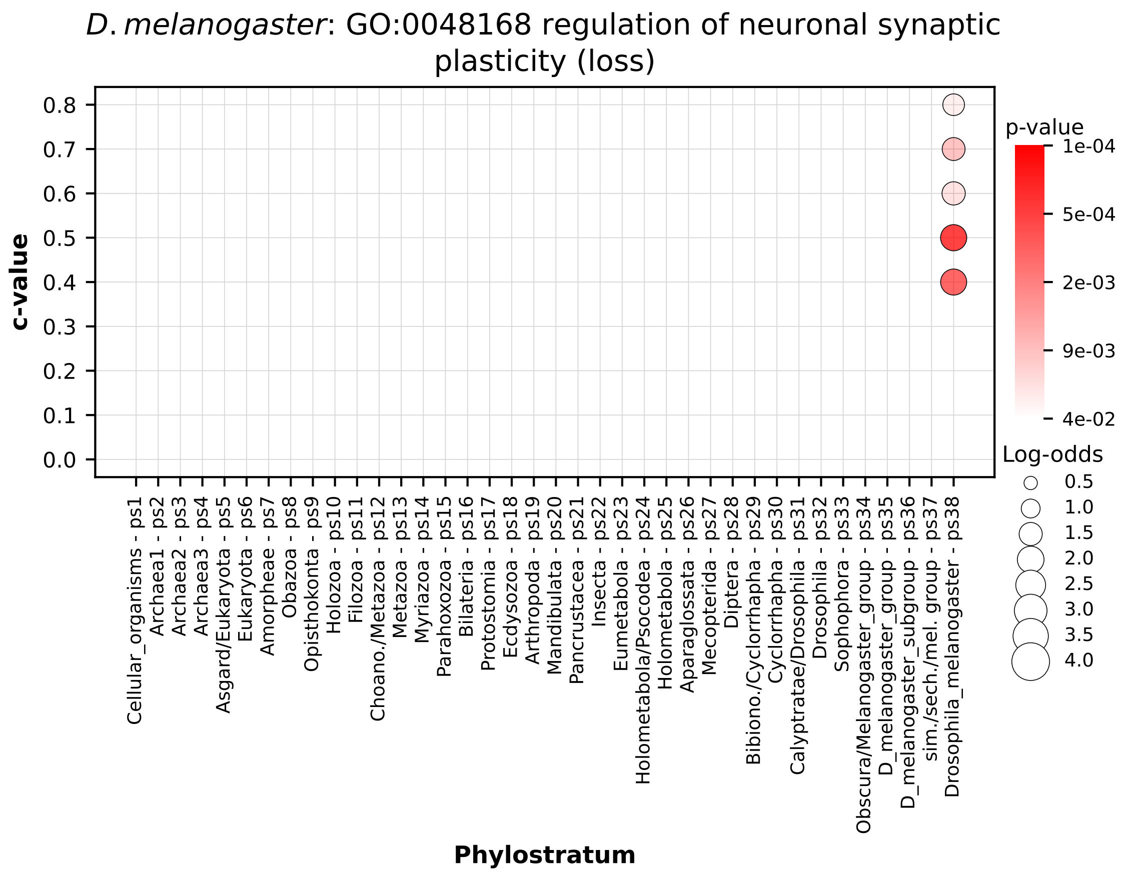

D. melanogaster: GO:0048168 regulation of neuronal synaptic plasticity (loss)
c-value
0.8
0.7
0.6
0.5
0.4
0.3
0.2
0.1
0.0
Phylostratum
Cellular_organisms - ps1
Archaea1 - ps2
Archaea2 - ps3
Archaea3 - ps4
Asgard/Eukaryota - ps5
Eukaryota - ps6
Amorpheae - ps7
Obazoa - ps8
Opisthokonta - ps9
Holozoa - ps10
Filozoa - ps11
Choano./Metazoa - ps12
Metazoa - ps13
Myriazoa - ps14
Parahoxozoa - ps15
Bilateria - ps16
Protostomia - ps17
Ecdysozoa - ps18
Arthropoda - ps19
Mandibulata - ps20
Pancrustacea - ps21
Insecta - ps22
Eumetabola - ps23
Holometabola/Psocodea - ps24
Holometabola - ps25
Aparaglossata - ps26
Mecopterida - ps27
Diptera - ps28
Bibiono./Cyclorrhapha - ps29
Cyclorrhapha - ps30
Calyptratae/Drosophila - ps31
Drosophila - ps32
Sophophora - ps33
Obscura/Melanogaster_group - ps34
D_melanogaster_group - ps35
D_melanogaster_subgroup - ps36
sim./sech./mel. group - ps37
Drosophila_melanogaster - ps38
p-value
1e-04
5e-04
2e-03
9e-03
4e-02
Log-odds
0.5
1.0
1.5
2.0
2.5
3.0
3.5
4.0

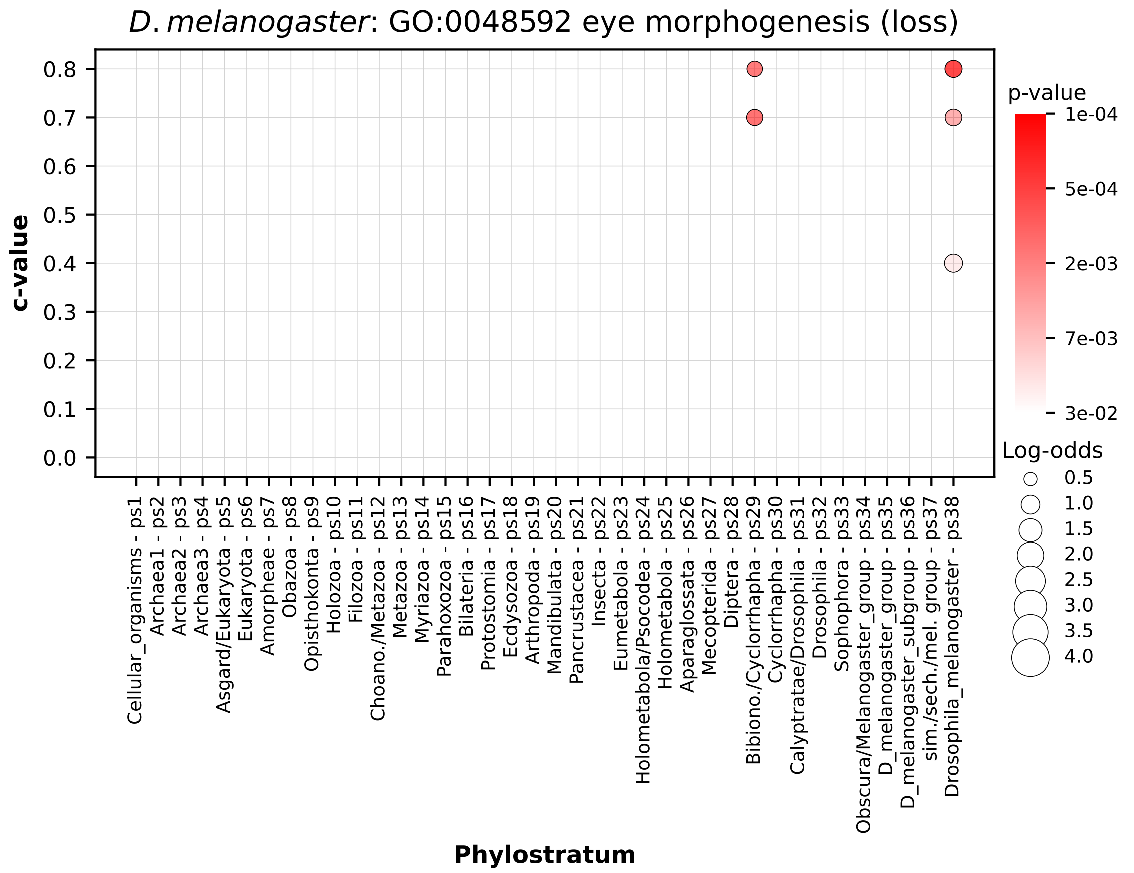

D. melanogaster: GO:0048592 eye morphogenesis (loss)
c-value
0.0
0.1
0.2
0.3
0.4
0.5
0.6
0.7
0.8
Phylostratum
Cellular_organisms - ps1
Archaea1 - ps2
Archaea2 - ps3
Archaea3 - ps4
Asgard/Eukaryota - ps5
Eukaryota - ps6
Amorpheae - ps7
Obazoa - ps8
Opisthokonta - ps9
Holozoa - ps10
Filozoa - ps11
Choano./Metazoa - ps12
Metazoa - ps13
Myriazoa - ps14
Parahoxozoa - ps15
Bilateria - ps16
Protostomia - ps17
Ecdysozoa - ps18
Arthropoda - ps19
Mandibulata - ps20
Pancrustacea - ps21
Insecta - ps22
Eumetabola - ps23
Holometabola/Psocodea - ps24
Holometabola - ps25
Aparaglossata - ps26
Mecopterida - ps27
Diptera - ps28
Bibiono./Cyclorrhapha - ps29
Cyclorrhapha - ps30
Calyptratae/Drosophila - ps31
Drosophila - ps32
Sophophora - ps33
Obscura/Melanogaster_group - ps34
D_melanogaster_group - ps35
D_melanogaster_subgroup - ps36
sim./sech./mel. group - ps37
Drosophila_melanogaster - ps38
p-value
1e-04
5e-04
2e-03
7e-03
3e-02
Log-odds
0.5
1.0
1.5
2.0
2.5
3.0
3.5
4.0

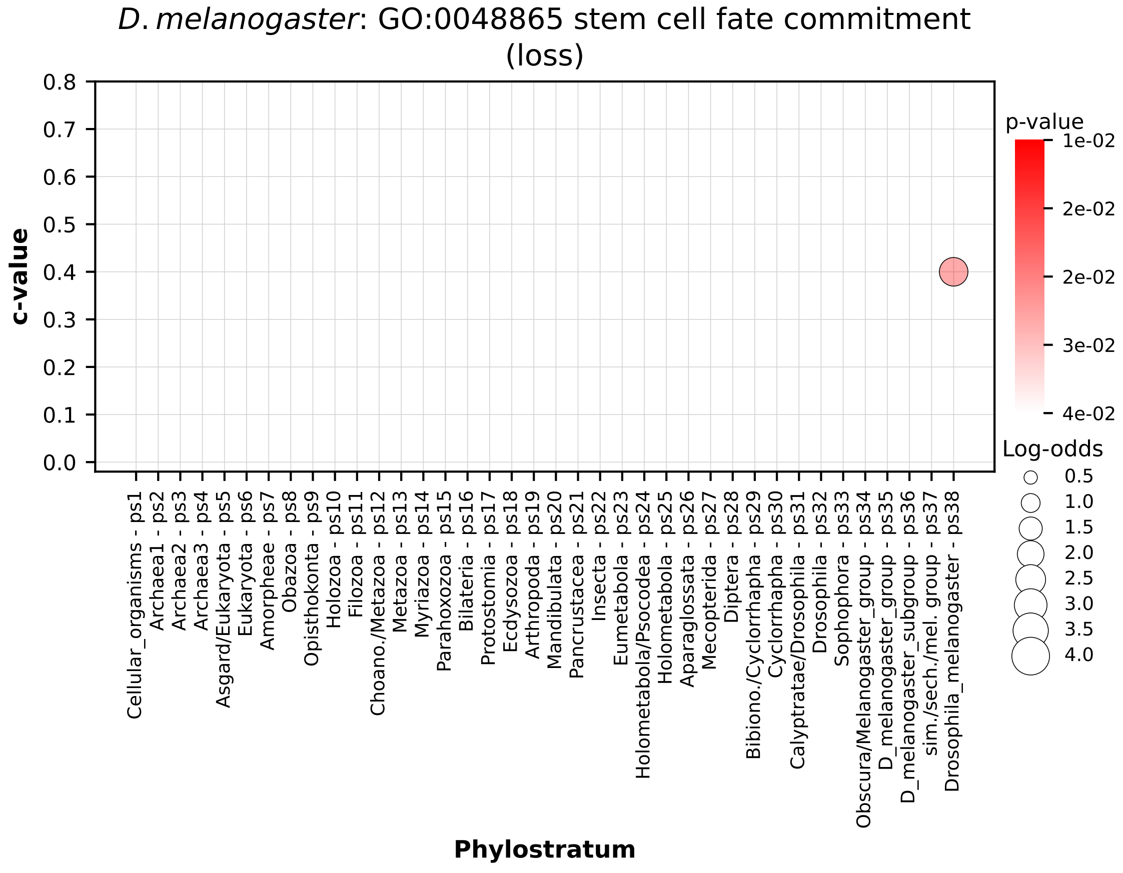

D. melanogaster: GO:0048865 stem cell fate commitment (loss)
c-value
0.8
0.7
0.6
0.5
0.4
0.3
0.2
0.1
0.0
p-value
1e-02
2e-02
2e-02
3e-02
4e-02
Log-odds
0.5
1.0
1.5
2.0
2.5
3.0
3.5
4.0
Cellular_organisms - ps1
Archaea1 - ps2
Archaea2 - ps3
Archaea3 - ps4
Asgard/Eukaryota - ps5
Eukaryota - ps6
Amorpheae - ps7
Obazoa - ps8
Opisthokonta - ps9
Holozoa - ps10
Filozoa - ps11
Choano./Metazoa - ps12
Metazoa - ps13
Myriazoa - ps14
Parahoxozoa - ps15
Bilateria - ps16
Protostomia - ps17
Ecdysozoa - ps18
Arthropoda - ps19
Mandibulata - ps20
Pancrustacea - ps21
Insecta - ps22
Eumetabola - ps23
Holometabola/Psocodea - ps24
Holometabola - ps25
Aparaglossata - ps26
Mecopterida - ps27
Diptera - ps28
Bibiono./Cyclorrhapha - ps29
Cyclorrhapha - ps30
Calyptratae/Drosophila - ps31
Drosophila - ps32
Sophophora - ps33
Obscura/Melanogaster_group - ps34
D_melanogaster_group - ps35
D_melanogaster_subgroup - ps36
sim./sech./mel. group - ps37
Drosophila_melanogaster - ps38
Phylostratum

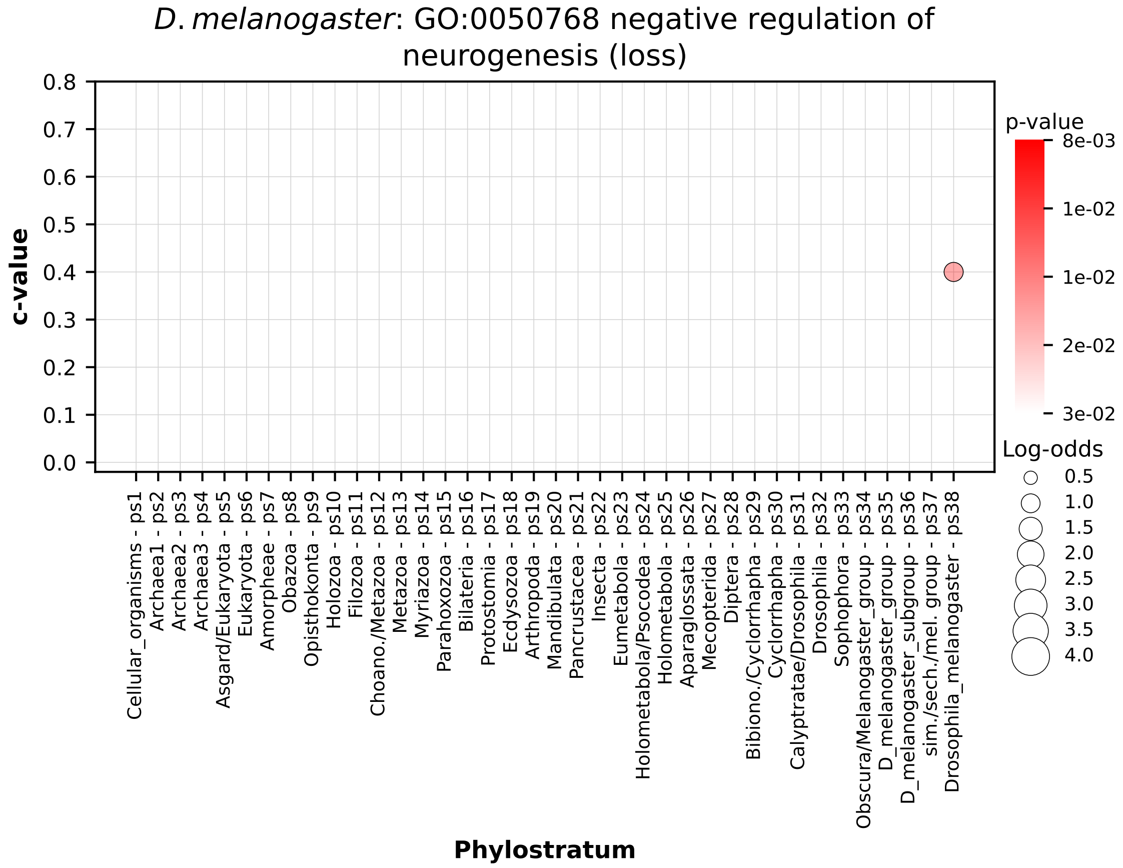

D. melanogaster: GO:0050768 negative regulation of neurogenesis (loss)
c-value
0.8
0.7
0.6
0.5
0.4
0.3
0.2
0.1
0.0
p-value
8e-03
1e-02
1e-02
2e-02
3e-02
Log-odds
0.5
1.0
1.5
2.0
2.5
3.0
3.5
4.0
Cellular_organisms - ps1
Archaea1 - ps2
Archaea2 - ps3
Archaea3 - ps4
Asgard/Eukaryota - ps5
Eukaryota - ps6
Amorpheae - ps7
Obazoa - ps8
Opisthokonta - ps9
Holozoa - ps10
Filozoa - ps11
Choano./Metazoa - ps12
Metazoa - ps13
Myriazoa - ps14
Parahoxozoa - ps15
Bilateria - ps16
Protostomia - ps17
Ecdysozoa - ps18
Arthropoda - ps19
Mandibulata - ps20
Pancrustacea - ps21
Insecta - ps22
Eumetabola - ps23
Holometabola/Psocodea - ps24
Holometabola - ps25
Aparaglossata - ps26
Mecopterida - ps27
Diptera - ps28
Bibiono./Cyclorrhapha - ps29
Cyclorrhapha - ps30
Calyptratae/Drosophila - ps31
Drosophila - ps32
Sophophora - ps33
Obscura/Melanogaster_group - ps34
D_melanogaster_group - ps35
D_melanogaster_subgroup - ps36
sim./sech./mel. group - ps37
Drosophila_melanogaster - ps38
Phylostratum

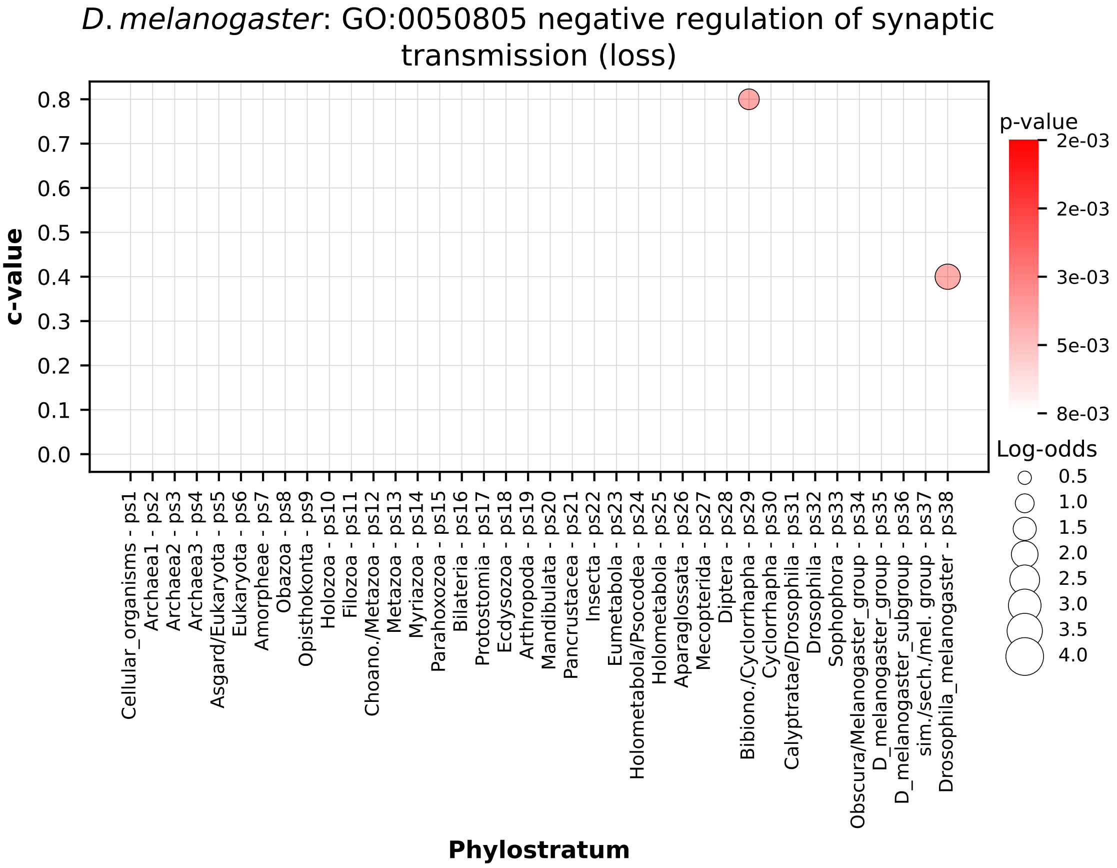
D. melanogaster: GO:0050805 negative regulation of synaptic transmission (loss)
c-value
0.8
0.7
0.6
0.5
0.4
0.3
0.2
0.1
0.0
p-value
2e-03
2e-03
3e-03
5e-03
8e-03
Log-odds
0.5
1.0
1.5
2.0
2.5
3.0
3.5
4.0
Cellular_organisms - ps1
Archaea1 - ps2
Archaea2 - ps3
Archaea3 - ps4
Asgard/Eukaryota - ps5
Eukaryota - ps6
Amorpheae - ps7
Obazoa - ps8
Opisthokonta - ps9
Holozoa - ps10
Filozoa - ps11
Choano./Metazoa - ps12
Metazoa - ps13
Myriazoa - ps14
Parahoxozoa - ps15
Bilateria - ps16
Protostomia - ps17
Ecdysozoa - ps18
Arthropoda - ps19
Mandibulata - ps20
Pancrustacea - ps21
Insecta - ps22
Eumetabola - ps23
Holometabola/Psocodea - ps24
Holometabola - ps25
Aparaglossata - ps26
Mecopterida - ps27
Diptera - ps28
Bibiono./Cyclorrhapha - ps29
Cyclorrhapha - ps30
Calyptratae/Drosophila - ps31
Drosophila - ps32
Sophophora - ps33
Obscura/Melanogaster_group - ps34
D_melanogaster_group - ps35
D_melanogaster_subgroup - ps36
sim./sech./mel. group - ps37
Drosophila_melanogaster - ps38
Phylostratum

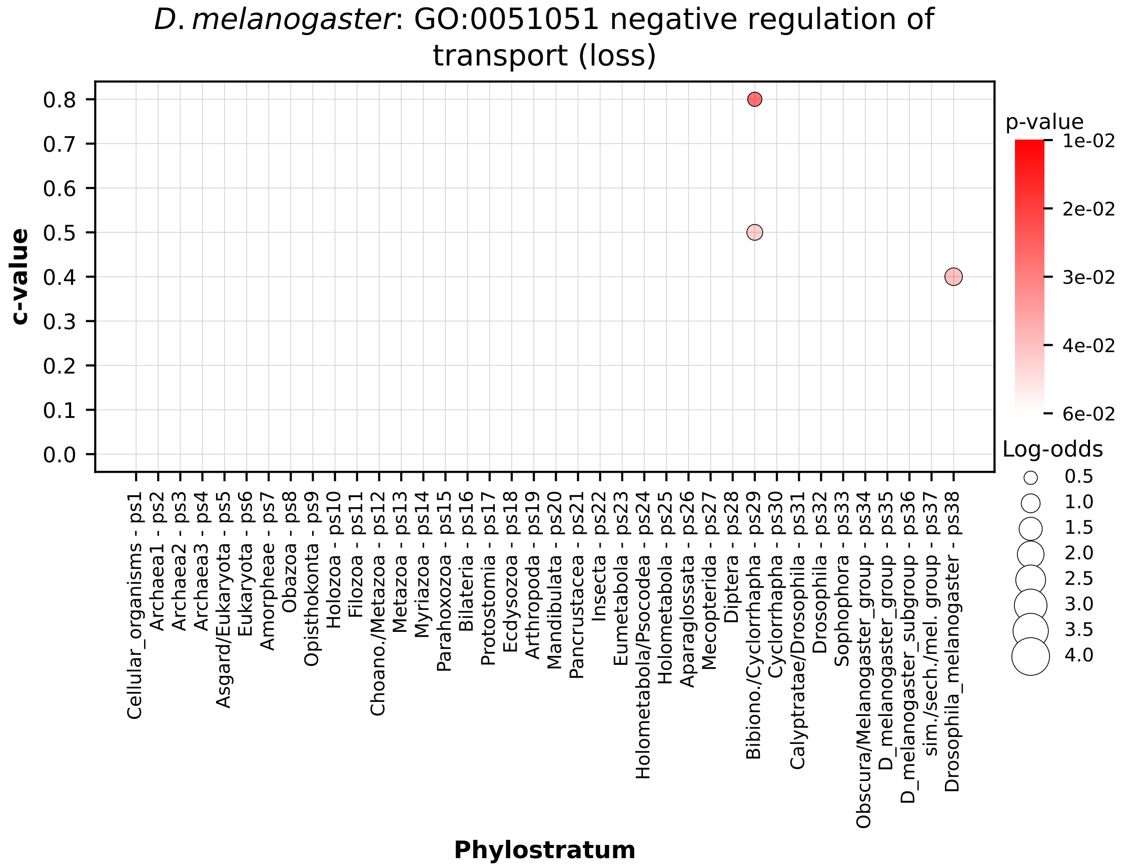

D. melanogaster: GO:0051051 negative regulation of transport (loss)
c-value
0.8
0.7
0.6
0.5
0.4
0.3
0.2
0.1
0.0
p-value
1e-02
2e-02
3e-02
4e-02
6e-02
Log-odds
0.5
1.0
1.5
2.0
2.5
3.0
3.5
4.0
Cellular_organisms - ps1
Archaea1 - ps2
Archaea2 - ps3
Archaea3 - ps4
Asgard/Eukaryota - ps5
Eukaryota - ps6
Amorpheae - ps7
Obazoa - ps8
Opisthokonta - ps9
Holozoa - ps10
Filozoa - ps11
Choano./Metazoa - ps12
Metazoa - ps13
Myriazoa - ps14
Parahoxozoa - ps15
Bilateria - ps16
Protostomia - ps17
Ecdysozoa - ps18
Arthropoda - ps19
Mandibulata - ps20
Pancrustacea - ps21
Insecta - ps22
Eumetabola - ps23
Holometabola/Psocodea - ps24
Holometabola - ps25
Aparaglossata - ps26
Mecopterida - ps27
Diptera - ps28
Bibiono./Cyclorrhapha - ps29
Cyclorrhapha - ps30
Calyptratae/Drosophila - ps31
Drosophila - ps32
Sophophora - ps33
Obscura/Melanogaster_group - ps34
D_melanogaster_group - ps35
D_melanogaster_subgroup - ps36
sim./sech./mel. group - ps37
Drosophila_melanogaster - ps38
Phylostratum

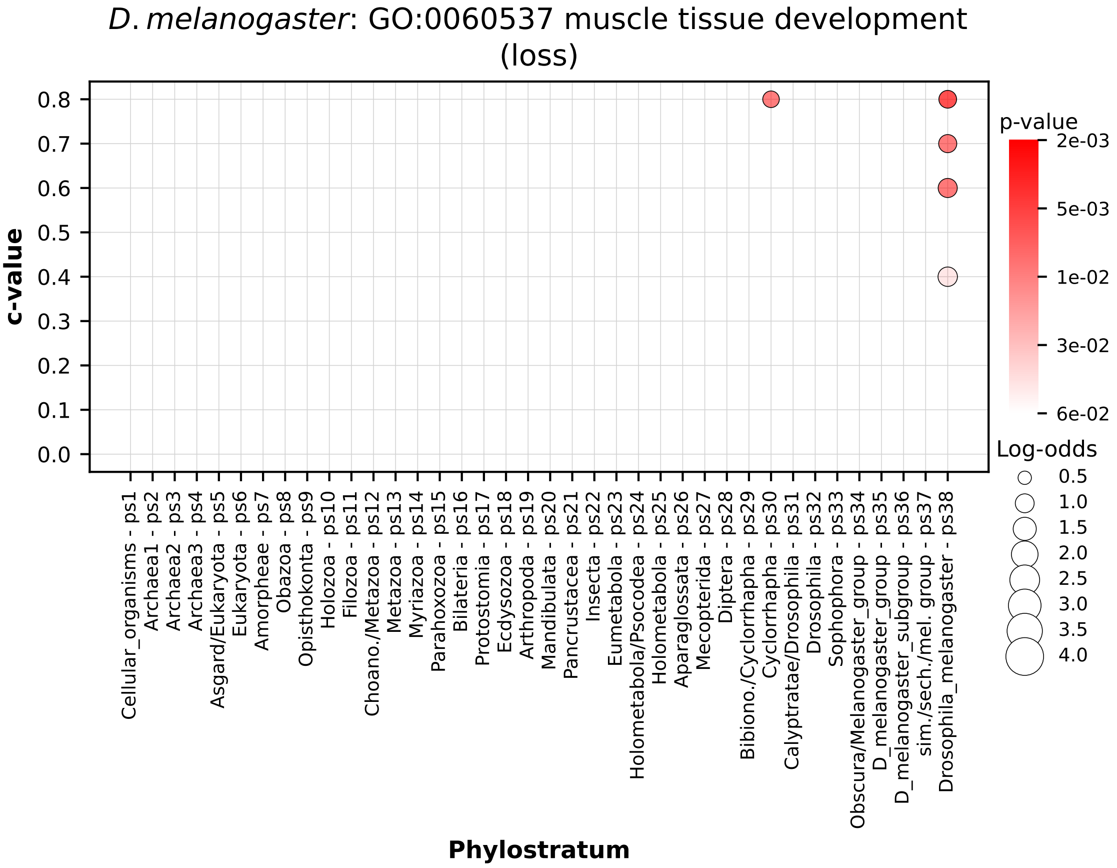
D. melanogaster: GO:0060537 muscle tissue development
(loss)
c-value
0.0
0.1
0.2
0.3
0.4
0.5
0.6
0.7
0.8
p-value
2e-03
5e-03
1e-02
3e-02
6e-02
Log-odds
0.5
1.0
1.5
2.0
2.5
3.0
3.5
4.0
Cellular_organisms - ps1
Archaea1 - ps2
Archaea2 - ps3
Archaea3 - ps4
Asgard/Eukaryota - ps5
Eukaryota - ps6
Amorpheae - ps7
Obazoa - ps8
Opisthokonta - ps9
Holozoa - ps10
Filozoa - ps11
Choano./Metazoa - ps12
Metazoa - ps13
Myriazoa - ps14
Parahoxozoa - ps15
Bilateria - ps16
Protostomia - ps17
Ecdysozoa - ps18
Arthropoda - ps19
Mandibulata - ps20
Pancrustacea - ps21
Insecta - ps22
Eumetabola - ps23
Holometabola/Psocodea - ps24
Holometabola - ps25
Aparaglossata - ps26
Mecopterida - ps27
Diptera - ps28
Bibiono./Cyclorrhapha - ps29
Cyclorrhapha - ps30
Calyptratae/Drosophila - ps31
Drosophila - ps32
Sophophora - ps33
Obscura/Melanogaster_group - ps34
D_melanogaster_group - ps35
D_melanogaster_subgroup - ps36
sim./sech./mel. group - ps37
Drosophila_melanogaster - ps38
Phylostratum

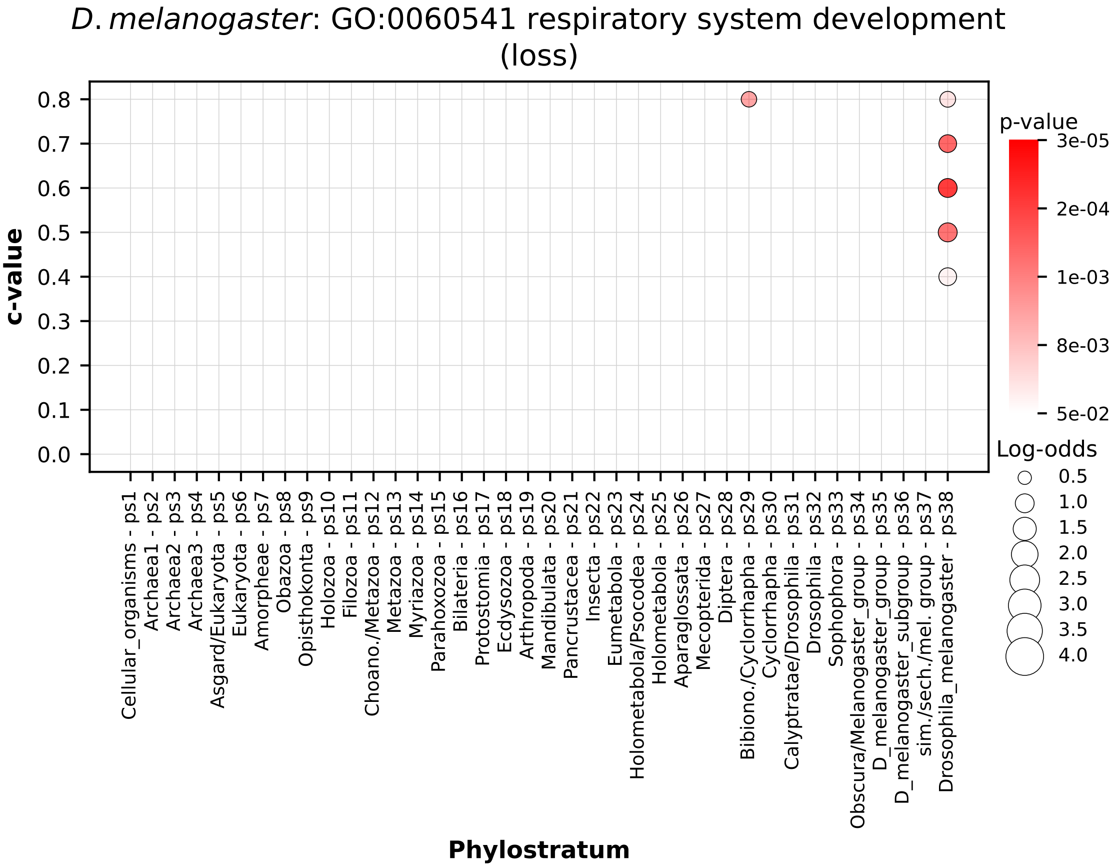

D. melanogaster: GO:0060541 respiratory system development
(loss)
c-value
0.0
0.1
0.2
0.3
0.4
0.5
0.6
0.7
0.8
Phylostratum
Cellular_organisms - ps1
Archaea1 - ps2
Archaea2 - ps3
Archaea3 - ps4
Asgard/Eukaryota - ps5
Eukaryota - ps6
Amorpheae - ps7
Obazoa - ps8
Opisthokonta - ps9
Holozoa - ps10
Filozoa - ps11
Choano./Metazoa - ps12
Metazoa - ps13
Myriazoa - ps14
Parahoxozoa - ps15
Bilateria - ps16
Protostomia - ps17
Ecdysozoa - ps18
Arthropoda - ps19
Mandibulata - ps20
Pancrustacea - ps21
Insecta - ps22
Eumetabola - ps23
Holometabola/Psocodea - ps24
Holometabola - ps25
Aparaglossata - ps26
Mecopterida - ps27
Diptera - ps28
Bibiono./Cyclorrhapha - ps29
Cyclorrhapha - ps30
Calyptratae/Drosophila - ps31
Drosophila - ps32
Sophophora - ps33
Obscura/Melanogaster_group - ps34
D_melanogaster_group - ps35
D_melanogaster_subgroup - ps36
sim./sech./mel. group - ps37
Drosophila_melanogaster - ps38
p-value
3e-05
2e-04
1e-03
8e-03
5e-02
Log-odds
0.5
1.0
1.5
2.0
2.5
3.0
3.5
4.0

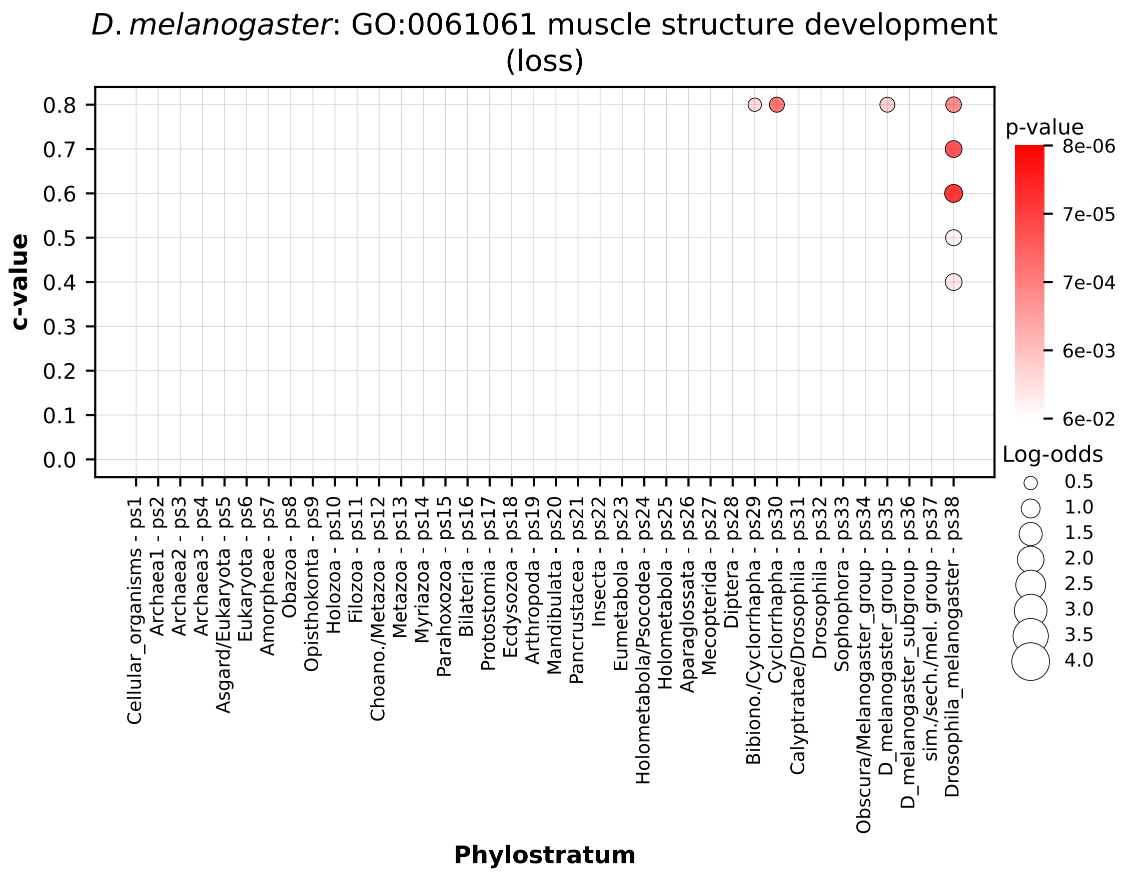

D. melanogaster: GO:0061061 muscle structure development (loss)
c-value
0.8
0.7
0.6
0.5
0.4
0.3
0.2
0.1
0.0
p-value
8e-06
7e-05
7e-04
6e-03
6e-02
Log-odds
0.5
1.0
1.5
2.0
2.5
3.0
3.5
4.0
Cellular_organisms - ps1
Archaea1 - ps2
Archaea2 - ps3
Archaea3 - ps4
Asgard/Eukaryota - ps5
Eukaryota - ps6
Amorpheae - ps7
Obazoa - ps8
Opisthokonta - ps9
Holozoa - ps10
Filozoa - ps11
Choano./Metazoa - ps12
Metazoa - ps13
Myriazoa - ps14
Parahoxozoa - ps15
Bilateria - ps16
Protostomia - ps17
Ecdysozoa - ps18
Arthropoda - ps19
Mandibulata - ps20
Pancrustacea - ps21
Insecta - ps22
Eumetabola - ps23
Holometabola/Psocodea - ps24
Holometabola - ps25
Aparaglossata - ps26
Mecopterida - ps27
Diptera - ps28
Bibiono./Cyclorrhapha - ps29
Cyclorrhapha - ps30
Calyptratae/Drosophila - ps31
Drosophila - ps32
Sophophora - ps33
Obscura/Melanogaster_group - ps34
D_melanogaster_group - ps35
D_melanogaster_subgroup - ps36
sim./sech./mel. group - ps37
Drosophila_melanogaster - ps38
Phylostratum

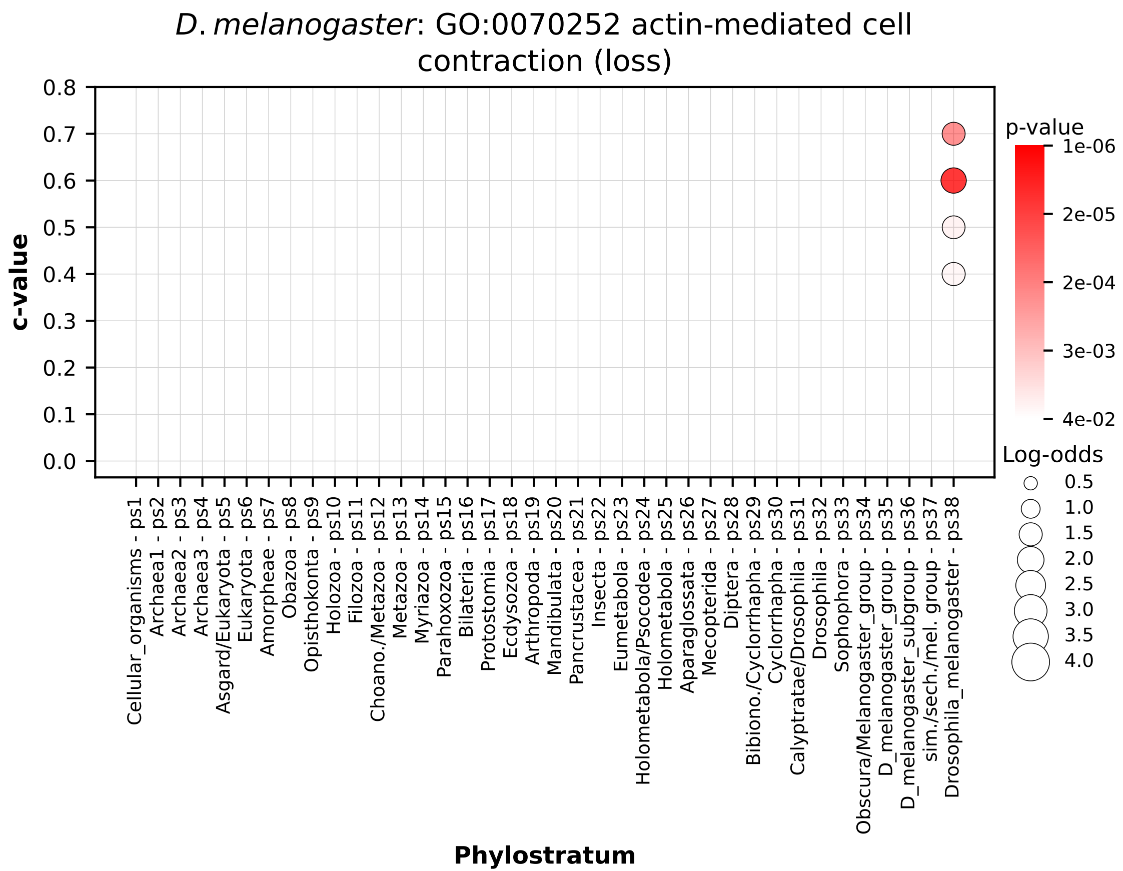

D. melanogaster: GO:0070252 actin-mediated cell contraction (loss)
c-value
0.8
0.7
0.6
0.5
0.4
0.3
0.2
0.1
0.0
Phylostratum
Cellular_organisms - ps1
Archaea1 - ps2
Archaea2 - ps3
Archaea3 - ps4
Asgard/Eukaryota - ps5
Eukaryota - ps6
Amorpheae - ps7
Obazoa - ps8
Opisthokonta - ps9
Holozoa - ps10
Filozoa - ps11
Choano./Metazoa - ps12
Metazoa - ps13
Myriazoa - ps14
Parahoxozoa - ps15
Bilateria - ps16
Protostomia - ps17
Ecdysozoa - ps18
Arthropoda - ps19
Mandibulata - ps20
Pancrustacea - ps21
Insecta - ps22
Eumetabola - ps23
Holometabola/Psocodea - ps24
Holometabola - ps25
Aparaglossata - ps26
Mecopterida - ps27
Diptera - ps28
Bibiono./Cyclorrhapha - ps29
Cyclorrhapha - ps30
Calyptratae/Drosophila - ps31
Drosophila - ps32
Sophophora - ps33
Obscura/Melanogaster_group - ps34
D_melanogaster_group - ps35
D_melanogaster_subgroup - ps36
sim./sech./mel. group - ps37
Drosophila_melanogaster - ps38
p-value
1e-06
2e-05
2e-04
3e-03
4e-02
Log-odds
0.5
1.0
1.5
2.0
2.5
3.0
3.5
4.0

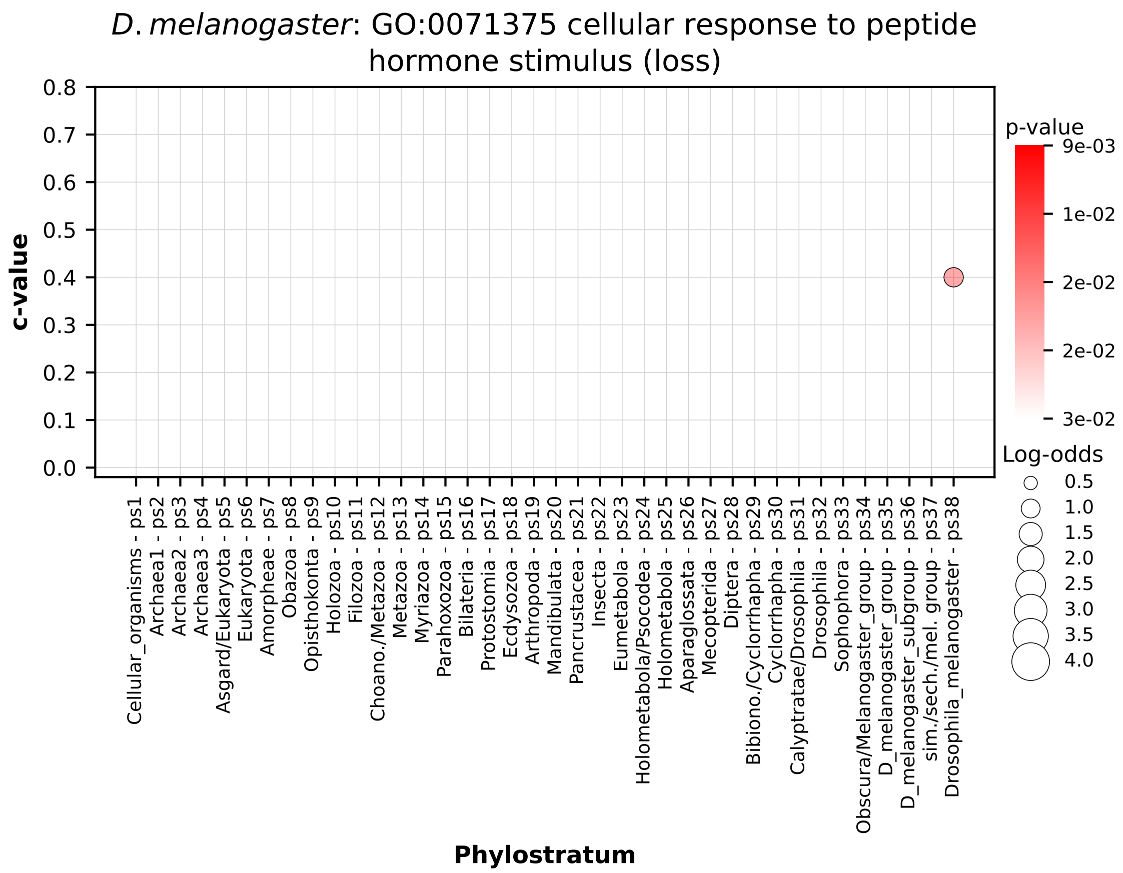

D. melanogaster: GO:0071375 cellular response to peptide hormone stimulus (loss)
c-value
0.8
0.7
0.6
0.5
0.4
0.3
0.2
0.1
0.0
p-value
9e-03
1e-02
2e-02
2e-02
3e-02
Log-odds
0.5
1.0
1.5
2.0
2.5
3.0
3.5
4.0
Cellular_organisms - ps1
Archaea1 - ps2
Archaea2 - ps3
Archaea3 - ps4
Asgard/Eukaryota - ps5
Eukaryota - ps6
Amorpheae - ps7
Obazoa - ps8
Opisthokonta - ps9
Holozoa - ps10
Filozoa - ps11
Choano./Metazoa - ps12
Metazoa - ps13
Myriazoa - ps14
Parahoxozoa - ps15
Bilateria - ps16
Protostomia - ps17
Ecdysozoa - ps18
Arthropoda - ps19
Mandibulata - ps20
Pancrustacea - ps21
Insecta - ps22
Eumetabola - ps23
Holometabola/Psocodea - ps24
Holometabola - ps25
Aparaglossata - ps26
Mecopterida - ps27
Diptera - ps28
Bibiono./Cyclorrhapha - ps29
Cyclorrhapha - ps30
Calyptratae/Drosophila - ps31
Drosophila - ps32
Sophophora - ps33
Obscura/Melanogaster_group - ps34
D_melanogaster_group - ps35
D_melanogaster_subgroup - ps36
sim./sech./mel. group - ps37
Drosophila_melanogaster - ps38
Phylostratum

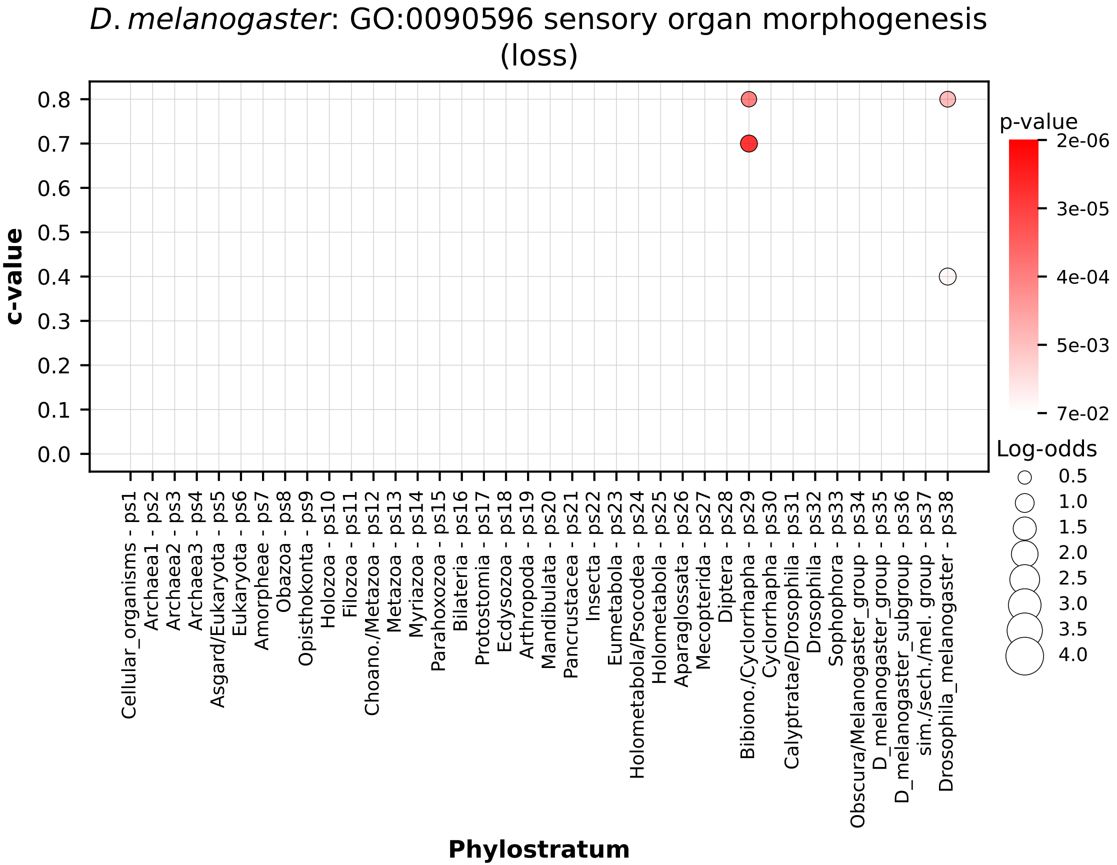

D. melanogaster: GO:0090596 sensory organ morphogenesis
(loss)
c-value
0.8
0.7
0.6
0.5
0.4
0.3
0.2
0.1
0.0
Phylostratum
Cellular_organisms - ps1
Archaea1 - ps2
Archaea2 - ps3
Archaea3 - ps4
Asgard/Eukaryota - ps5
Eukaryota - ps6
Amorpheae - ps7
Obazoa - ps8
Opisthokonta - ps9
Holozoa - ps10
Filozoa - ps11
Choano./Metazoa - ps12
Metazoa - ps13
Myriazoa - ps14
Parahoxozoa - ps15
Bilateria - ps16
Protostomia - ps17
Ecdysozoa - ps18
Arthropoda - ps19
Mandibulata - ps20
Pancrustacea - ps21
Insecta - ps22
Eumetabola - ps23
Holometabola/Psocodea - ps24
Holometabola - ps25
Aparaglossata - ps26
Mecopterida - ps27
Diptera - ps28
Bibiono./Cyclorrhapha - ps29
Cyclorrhapha - ps30
Calyptratae/Drosophila - ps31
Drosophila - ps32
Sophophora - ps33
Obscura/Melanogaster_group - ps34
D_melanogaster_group - ps35
D_melanogaster_subgroup - ps36
sim./sech./mel. group - ps37
Drosophila_melanogaster - ps38
p-value
2e-06
3e-05
4e-04
5e-03
7e-02
Log-odds
0.5
1.0
1.5
2.0
2.5
3.0
3.5
4.0

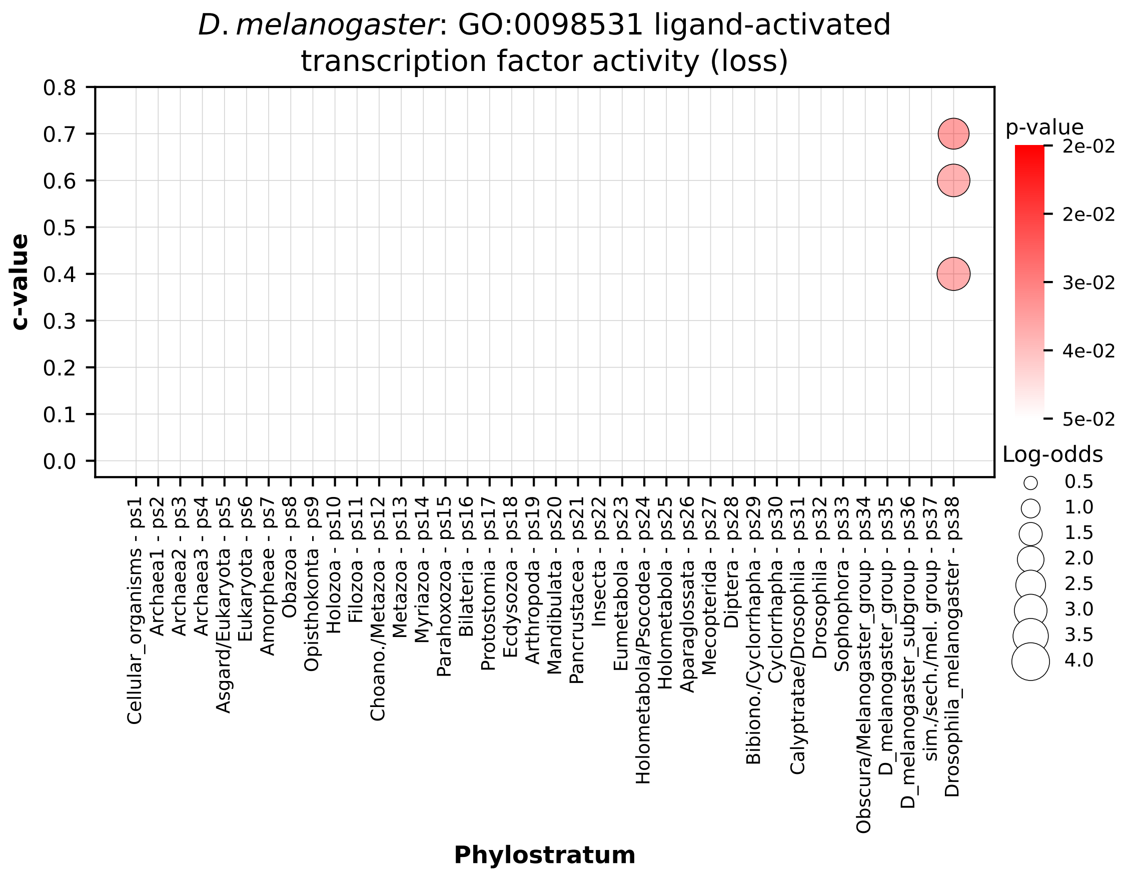

D. melanogaster: GO:0098531 ligand-activated transcription factor activity (loss)
c-value
0.0
0.1
0.2
0.3
0.4
0.5
0.6
0.7
0.8
p-value
2e-02
2e-02
3e-02
4e-02
5e-02
Log-odds
0.5
1.0
1.5
2.0
2.5
3.0
3.5
4.0
Cellular_organisms - ps1
Archaea1 - ps2
Archaea2 - ps3
Archaea3 - ps4
Asgard/Eukaryota - ps5
Eukaryota - ps6
Amorpheae - ps7
Obazoa - ps8
Opisthokonta - ps9
Holozoa - ps10
Filozoa - ps11
Choano./Metazoa - ps12
Metazoa - ps13
Myriazoa - ps14
Parahoxozoa - ps15
Bilateria - ps16
Protostomia - ps17
Ecdysozoa - ps18
Arthropoda - ps19
Mandibulata - ps20
Pancrustacea - ps21
Insecta - ps22
Eumetabola - ps23
Holometabola/Psocodea - ps24
Holometabola - ps25
Aparaglossata - ps26
Mecopterida - ps27
Diptera - ps28
Bibiono./Cyclorrhapha - ps29
Cyclorrhapha - ps30
Calyptratae/Drosophila - ps31
Drosophila - ps32
Sophophora - ps33
Obscura/Melanogaster_group - ps34
D_melanogaster_group - ps35
D_melanogaster_subgroup - ps36
sim./sech./mel. group - ps37
Drosophila_melanogaster - ps38
Phylostratum

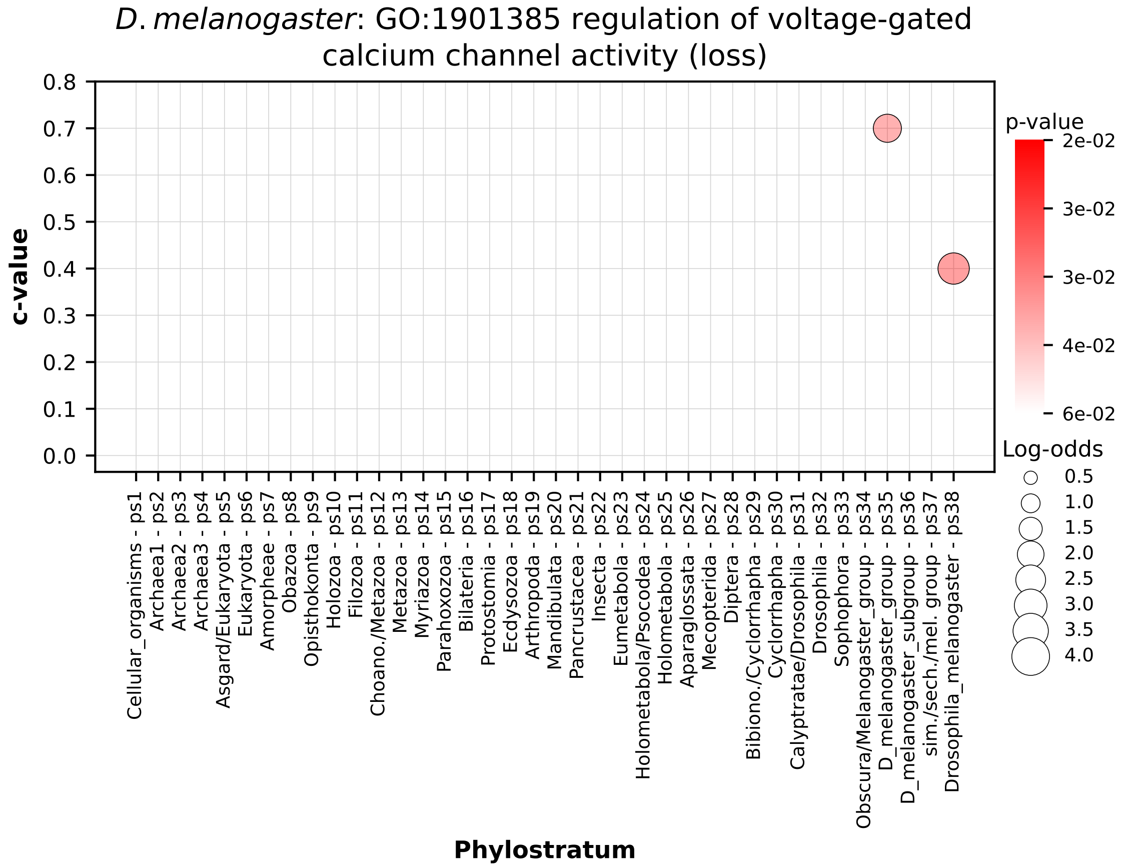

D. melanogaster: GO:1901385 regulation of voltage-gated calcium channel activity (loss)
c-value
0.0
0.1
0.2
0.3
0.4
0.5
0.6
0.7
0.8
Phylostratum
Cellular_organisms - ps1
Archaea1 - ps2
Archaea2 - ps3
Archaea3 - ps4
Asgard/Eukaryota - ps5
Eukaryota - ps6
Amorpheae - ps7
Obazoa - ps8
Opisthokonta - ps9
Holozoa - ps10
Filozoa - ps11
Choano./Metazoa - ps12
Metazoa - ps13
Myriazoa - ps14
Parahoxozoa - ps15
Bilateria - ps16
Protostomia - ps17
Ecdysozoa - ps18
Arthropoda - ps19
Mandibulata - ps20
Pancrustacea - ps21
Insecta - ps22
Eumetabola - ps23
Holometabola/Psocodea - ps24
Holometabola - ps25
Aparaglossata - ps26
Mecopterida - ps27
Diptera - ps28
Bibiono./Cyclorrhapha - ps29
Cyclorrhapha - ps30
Calyptratae/Drosophila - ps31
Drosophila - ps32
Sophophora - ps33
Obscura/Melanogaster_group - ps34
D_melanogaster_group - ps35
D_melanogaster_subgroup - ps36
sim./sech./mel. group - ps37
Drosophila_melanogaster - ps38
p-value
2e-02
3e-02
3e-02
4e-02
6e-02
Log-odds
0.5
1.0
1.5
2.0
2.5
3.0
3.5
4.0

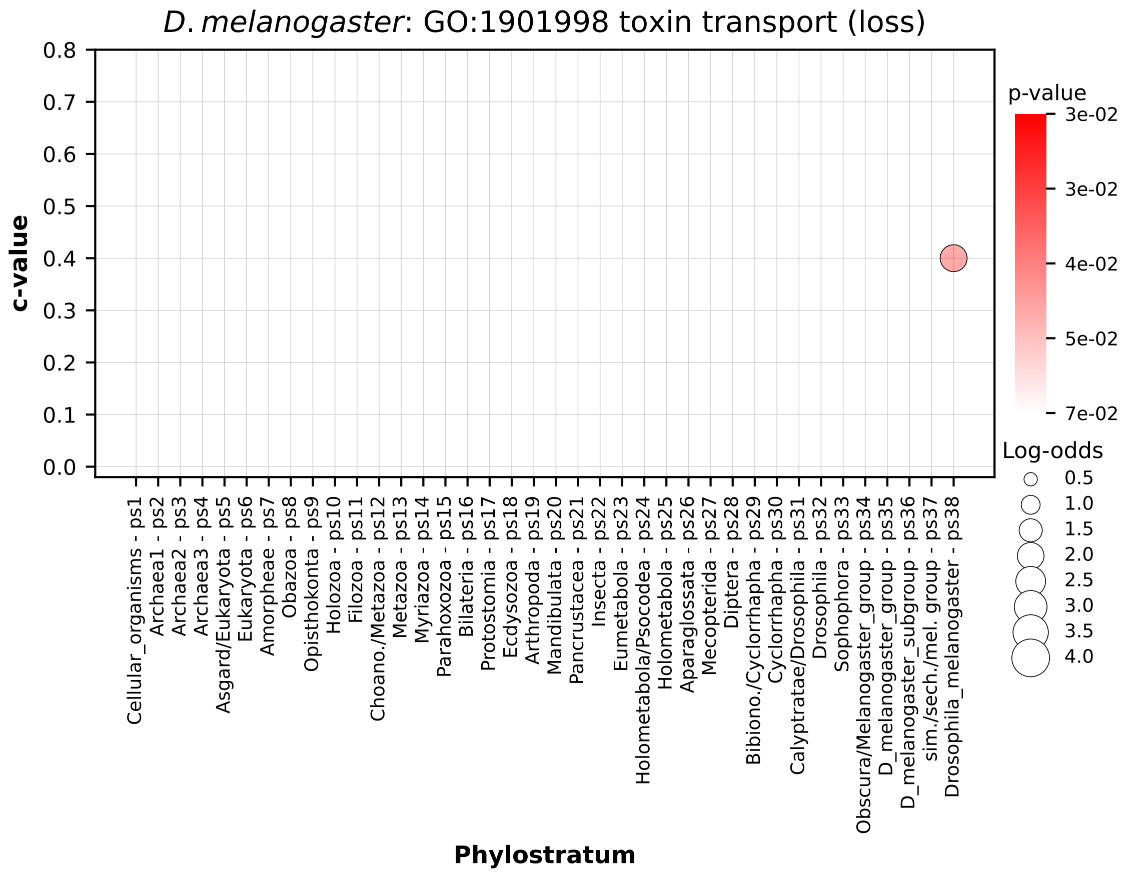

D. melanogaster: GO:1901998 toxin transport (loss)
c-value
0.8
0.7
0.6
0.5
0.4
0.3
0.2
0.1
0.0
p-value
3e-02
3e-02
4e-02
5e-02
7e-02
Log-odds
0.5
1.0
1.5
2.0
2.5
3.0
3.5
4.0
Cellular_organisms - ps1
Archaea1 - ps2
Archaea2 - ps3
Archaea3 - ps4
Asgard/Eukaryota - ps5
Eukaryota - ps6
Amorpheae - ps7
Obazoa - ps8
Opisthokonta - ps9
Holozoa - ps10
Filozoa - ps11
Choano./Metazoa - ps12
Metazoa - ps13
Myriazoa - ps14
Parahoxozoa - ps15
Bilateria - ps16
Protostomia - ps17
Ecdysozoa - ps18
Arthropoda - ps19
Mandibulata - ps20
Pancrustacea - ps21
Insecta - ps22
Eumetabola - ps23
Holometabola/Psocodea - ps24
Holometabola - ps25
Aparaglossata - ps26
Mecopterida - ps27
Diptera - ps28
Bibiono./Cyclorrhapha - ps29
Cyclorrhapha - ps30
Calyptratae/Drosophila - ps31
Drosophila - ps32
Sophophora - ps33
Obscura/Melanogaster_group - ps34
D_melanogaster_group - ps35
D_melanogaster_subgroup - ps36
sim./sech./mel. group - ps37
Drosophila_melanogaster - ps38
Phylostratum

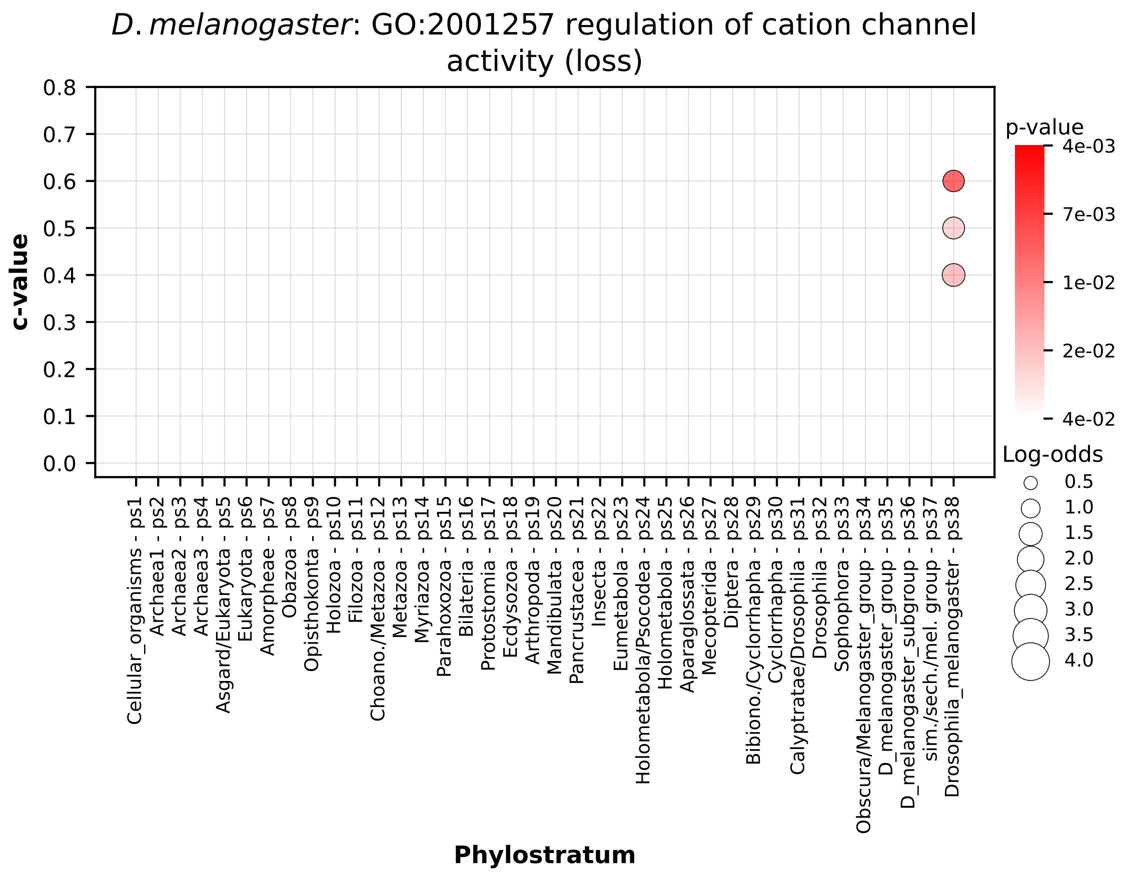

D. melanogaster: GO:2001257 regulation of cation channel activity (loss)
c-value
0.8
0.7
0.6
0.5
0.4
0.3
0.2
0.1
0.0
p-value
4e-03
7e-03
1e-02
2e-02
4e-02
Log-odds
0.5
1.0
1.5
2.0
2.5
3.0
3.5
4.0
Cellular_organisms - ps1
Archaea1 - ps2
Archaea2 - ps3
Archaea3 - ps4
Asgard/Eukaryota - ps5
Eukaryota - ps6
Amorpheae - ps7
Obazoa - ps8
Opisthokonta - ps9
Holozoa - ps10
Filozoa - ps11
Choano./Metazoa - ps12
Metazoa - ps13
Myriazoa - ps14
Parahoxozoa - ps15
Bilateria - ps16
Protostomia - ps17
Ecdysozoa - ps18
Arthropoda - ps19
Mandibulata - ps20
Pancrustacea - ps21
Insecta - ps22
Eumetabola - ps23
Holometabola/Psocodea - ps24
Holometabola - ps25
Aparaglossata - ps26
Mecopterida - ps27
Diptera - ps28
Bibiono./Cyclorrhapha - ps29
Cyclorrhapha - ps30
Calyptratae/Drosophila - ps31
Drosophila - ps32
Sophophora - ps33
Obscura/Melanogaster_group - ps34
D_melanogaster_group - ps35
D_melanogaster_subgroup - ps36
sim./sech./mel. group - ps37
Drosophila_melanogaster - ps38
Phylostratum

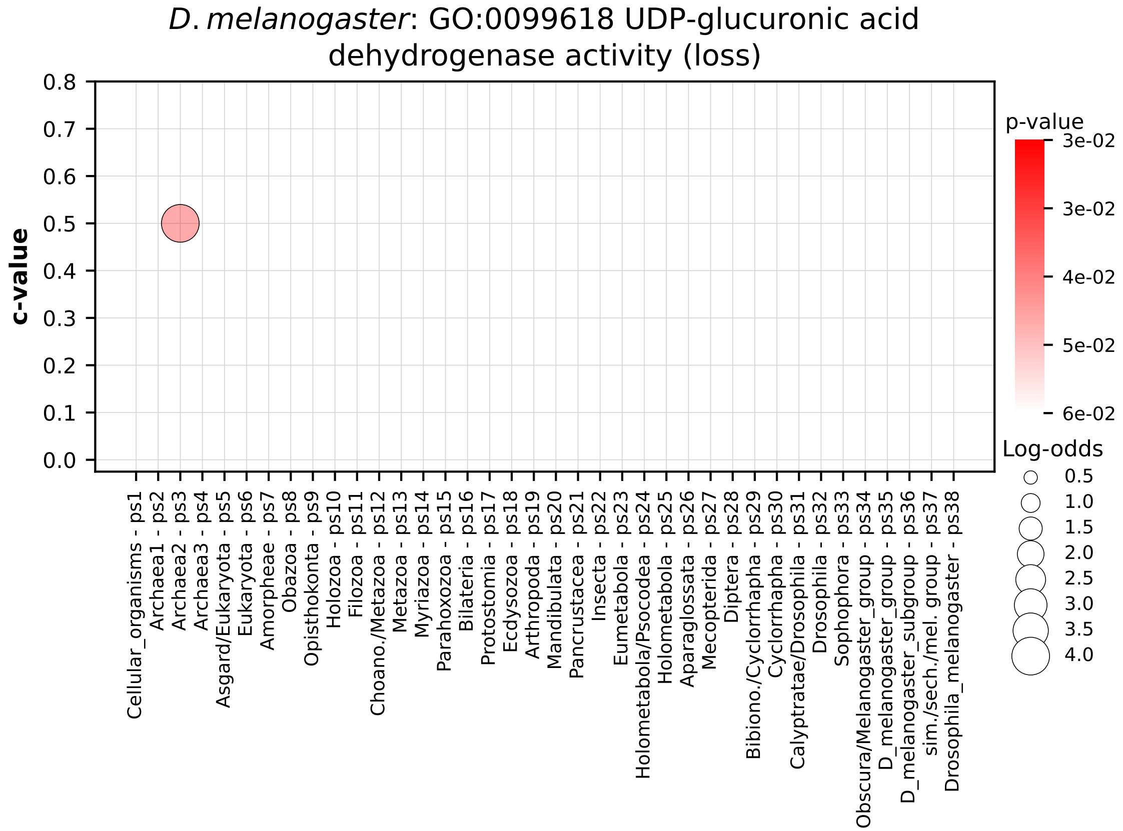
D. melanogaster: GO:0099618 UDP-glucuronic acid dehydrogenase activity (loss)
c-value
0.8
0.7
0.6
0.5
0.4
0.3
0.2
0.1
0.0
p-value
3e-02
3e-02
4e-02
5e-02
6e-02
Log-odds
0.5
1.0
1.5
2.0
2.5
3.0
3.5
4.0
Cellular_organisms - ps1
Archaea1 - ps2
Archaea2 - ps3
Archaea3 - ps4
Asgard/Eukaryota - ps5
Eukaryota - ps6
Amorpheae - ps7
Obazoa - ps8
Opisthokonta - ps9
Holozoa - ps10
Filozoa - ps11
Choano./Metazoa - ps12
Metazoa - ps13
Myriazoa - ps14
Parahoxozoa - ps15
Bilateria - ps16
Protostomia - ps17
Ecdysozoa - ps18
Arthropoda - ps19
Mandibulata - ps20
Pancrustacea - ps21
Insecta - ps22
Eumetabola - ps23
Holometabola/Psocodea - ps24
Holometabola - ps25
Aparaglossata - ps26
Mecopterida - ps27
Diptera - ps28
Bibiono./Cyclorrhapha - ps29
Cyclorrhapha - ps30
Calyptratae/Drosophila - ps31
Drosophila - ps32
Sophophora - ps33
Obscura/Melanogaster_group - ps34
D_melanogaster_group - ps35
D_melanogaster_subgroup - ps36
sim./sech./mel. group - ps37
Drosophila_melanogaster - ps38
Phylostratum

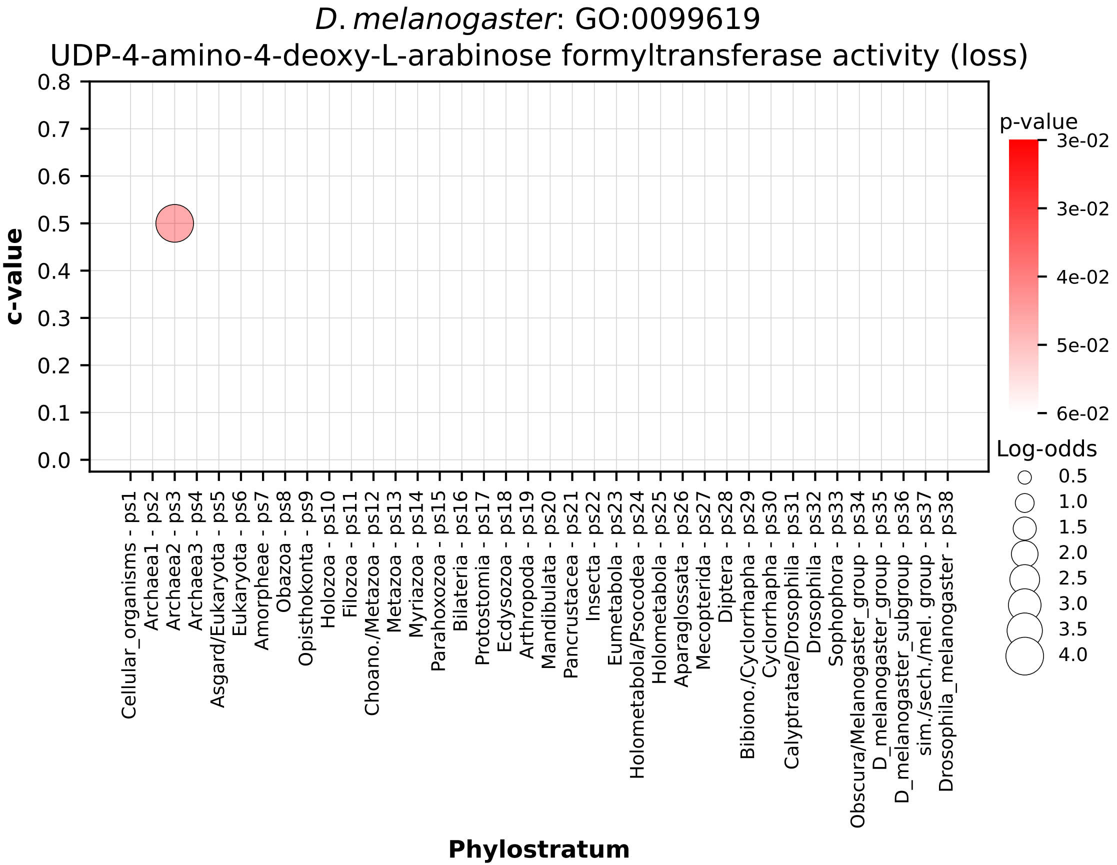

D. melanogaster: GO:0099619
UDP-4-amino-4-deoxy-L-arabinose formyltransferase activity (loss)
c-value
0.8
0.7
0.6
0.5
0.4
0.3
0.2
0.1
0.0
p-value
3e-02
3e-02
4e-02
5e-02
6e-02
Log-odds
0.5
1.0
1.5
2.0
2.5
3.0
3.5
4.0
Cellular_organisms - ps1
Archaea1 - ps2
Archaea2 - ps3
Archaea3 - ps4
Asgard/Eukaryota - ps5
Eukaryota - ps6
Amorpheae - ps7
Obazoa - ps8
Opisthokonta - ps9
Holozoa - ps10
Filozoa - ps11
Choano./Metazoa - ps12
Metazoa - ps13
Myriazoa - ps14
Parahoxozoa - ps15
Bilateria - ps16
Protostomia - ps17
Ecdysozoa - ps18
Arthropoda - ps19
Mandibulata - ps20
Pancrustacea - ps21
Insecta - ps22
Eumetabola - ps23
Holometabola/Psocodea - ps24
Holometabola - ps25
Aparaglossata - ps26
Mecopterida - ps27
Diptera - ps28
Bibiono./Cyclorrhapha - ps29
Cyclorrhapha - ps30
Calyptratae/Drosophila - ps31
Drosophila - ps32
Sophophora - ps33
Obscura/Melanogaster_group - ps34
D_melanogaster_group - ps35
D_melanogaster_subgroup - ps36
sim./sech./mel. group - ps37
Drosophila_melanogaster - ps38
Phylostratum

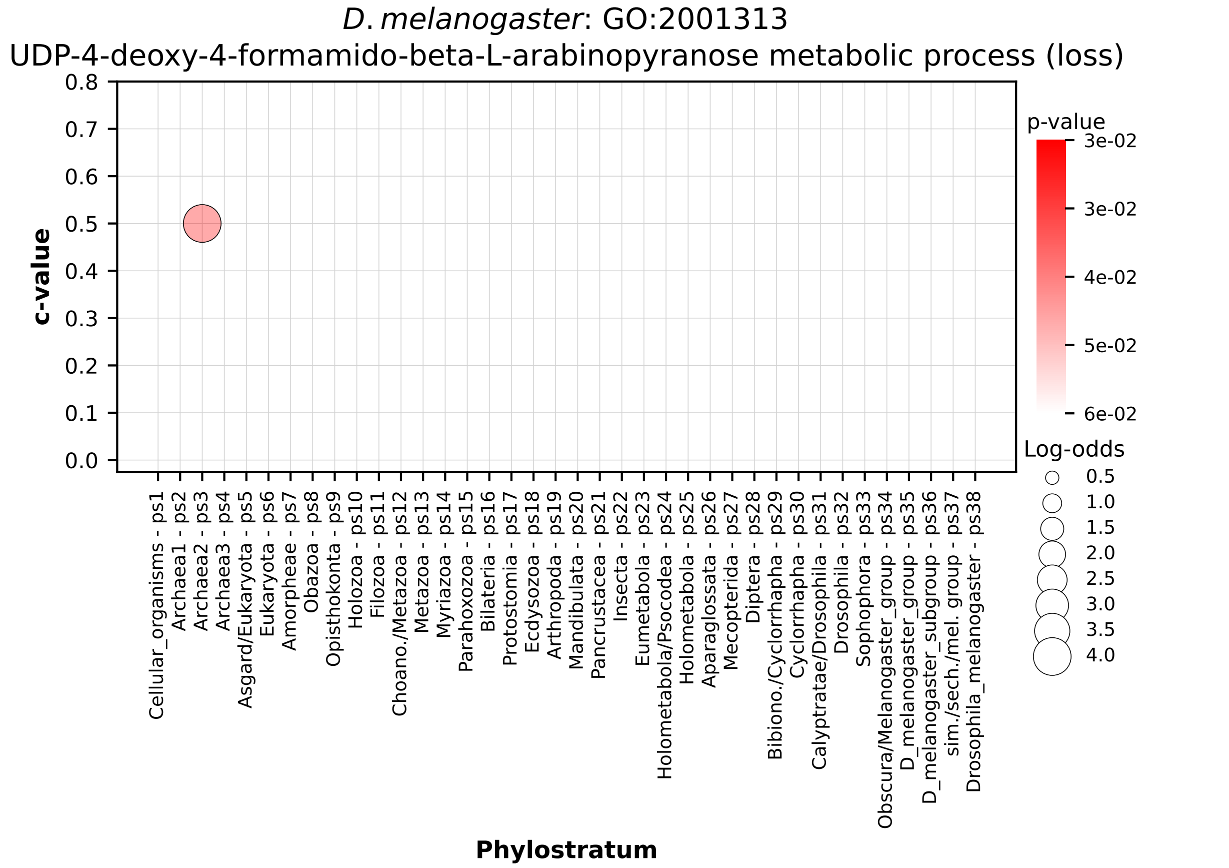

D. melanogaster: GO:2001313
UDP-4-deoxy-4-formamido-beta-L-arabinopyranose metabolic process (loss)
c-value
0.8
0.7
0.6
0.5
0.4
0.3
0.2
0.1
0.0
Phylostratum
Cellular_organisms - ps1
Archaea1 - ps2
Archaea2 - ps3
Archaea3 - ps4
Asgard/Eukaryota - ps5
Eukaryota - ps6
Amorpheae - ps7
Obazoa - ps8
Opisthokonta - ps9
Holozoa - ps10
Filozoa - ps11
Choano./Metazoa - ps12
Metazoa - ps13
Myriazoa - ps14
Parahoxozoa - ps15
Bilateria - ps16
Protostomia - ps17
Ecdysozoa - ps18
Arthropoda - ps19
Mandibulata - ps20
Pancrustacea - ps21
Insecta - ps22
Eumetabola - ps23
Holometabola/Psocodea - ps24
Holometabola - ps25
Aparaglossata - ps26
Mecopterida - ps27
Diptera - ps28
Bibiono./Cyclorrhapha - ps29
Cyclorrhapha - ps30
Calyptratae/Drosophila - ps31
Drosophila - ps32
Sophophora - ps33
Obscura/Melanogaster_group - ps34
D_melanogaster_group - ps35
D_melanogaster_subgroup - ps36
sim./sech./mel. group - ps37
Drosophila_melanogaster - ps38
p-value
3e-02
3e-02
4e-02
5e-02
6e-02
Log-odds
0.5
1.0
1.5
2.0
2.5
3.0
3.5
4.0

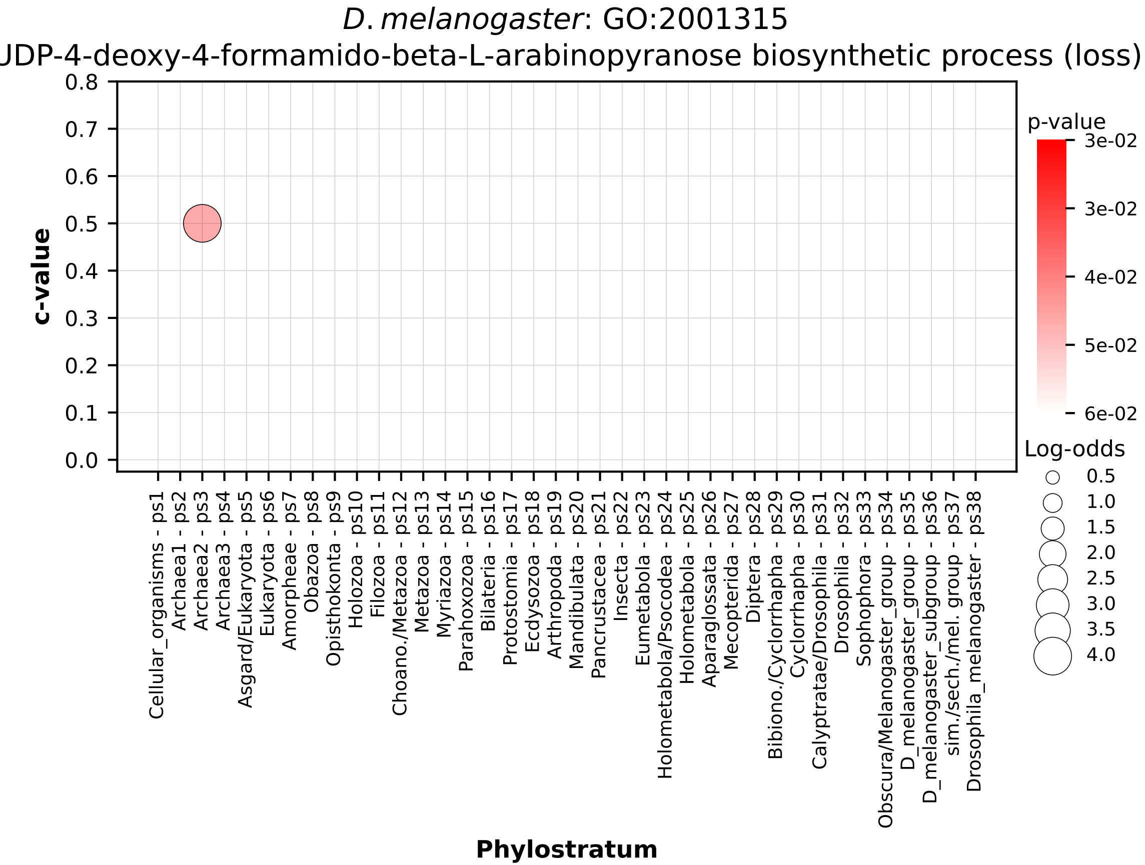

D. melanogaster: GO:2001315
UDP-4-deoxy-4-formamido-beta-L-arabinopyranose biosynthetic process (loss)
c-value
0.0
0.1
0.2
0.3
0.4
0.5
0.6
0.7
0.8
Phylostratum
Cellular_organisms - ps1
Archaea1 - ps2
Archaea2 - ps3
Archaea3 - ps4
Asgard/Eukaryota - ps5
Eukaryota - ps6
Amorpheae - ps7
Obazoa - ps8
Opisthokonta - ps9
Holozoa - ps10
Filozoa - ps11
Choano./Metazoa - ps12
Metazoa - ps13
Myriazoa - ps14
Parahoxozoa - ps15
Bilateria - ps16
Protostomia - ps17
Ecdysozoa - ps18
Arthropoda - ps19
Mandibulata - ps20
Pancrustacea - ps21
Insecta - ps22
Eumetabola - ps23
Holometabola/Psocodea - ps24
Holometabola - ps25
Aparaglossata - ps26
Mecopterida - ps27
Diptera - ps28
Bibiono./Cyclorrhapha - ps29
Cyclorrhapha - ps30
Calyptratae/Drosophila - ps31
Drosophila - ps32
Sophophora - ps33
Obscura/Melanogaster_group - ps34
D_melanogaster_group - ps35
D_melanogaster_subgroup - ps36
sim./sech./mel. group - ps37
Drosophila_melanogaster - ps38
p-value
3e-02
3e-02
4e-02
5e-02
6e-02
Log-odds
0.5
1.0
1.5
2.0
2.5
3.0
3.5
4.0

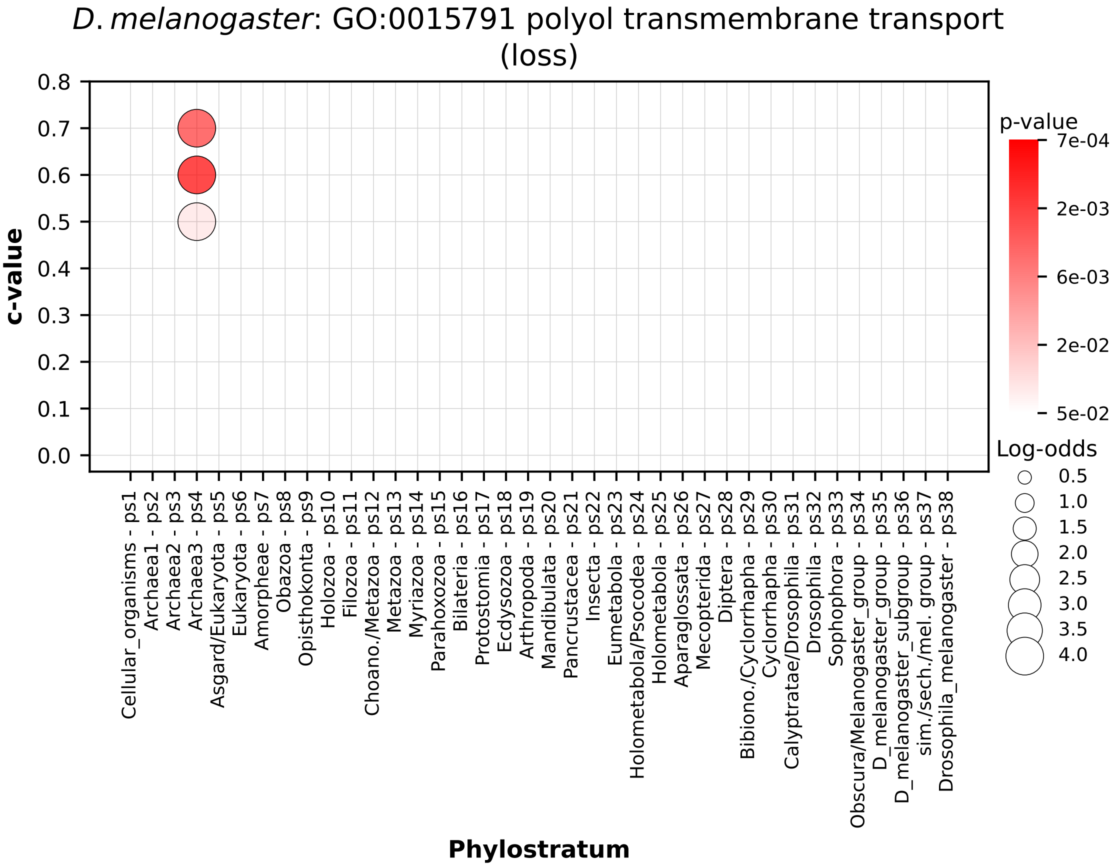

D. melanogaster: GO:0015791 polyol transmembrane transport (loss)
c-value
0.8
0.7
0.6
0.5
0.4
0.3
0.2
0.1
0.0
Phylostratum
Cellular_organisms - ps1
Archaea1 - ps2
Archaea2 - ps3
Archaea3 - ps4
Asgard/Eukaryota - ps5
Eukaryota - ps6
Amorpheae - ps7
Obazoa - ps8
Opisthokonta - ps9
Holozoa - ps10
Filozoa - ps11
Choano./Metazoa - ps12
Metazoa - ps13
Myriazoa - ps14
Parahoxozoa - ps15
Bilateria - ps16
Protostomia - ps17
Ecdysozoa - ps18
Arthropoda - ps19
Mandibulata - ps20
Pancrustacea - ps21
Insecta - ps22
Eumetabola - ps23
Holometabola/Psocodea - ps24
Holometabola - ps25
Aparaglossata - ps26
Mecopterida - ps27
Diptera - ps28
Bibiono./Cyclorrhapha - ps29
Cyclorrhapha - ps30
Calyptratae/Drosophila - ps31
Drosophila - ps32
Sophophora - ps33
Obscura/Melanogaster_group - ps34
D_melanogaster_group - ps35
D_melanogaster_subgroup - ps36
sim./sech./mel. group - ps37
Drosophila_melanogaster - ps38
p-value
7e-04
2e-03
6e-03
2e-02
5e-02
Log-odds
0.5
1.0
1.5
2.0
2.5
3.0
3.5
4.0

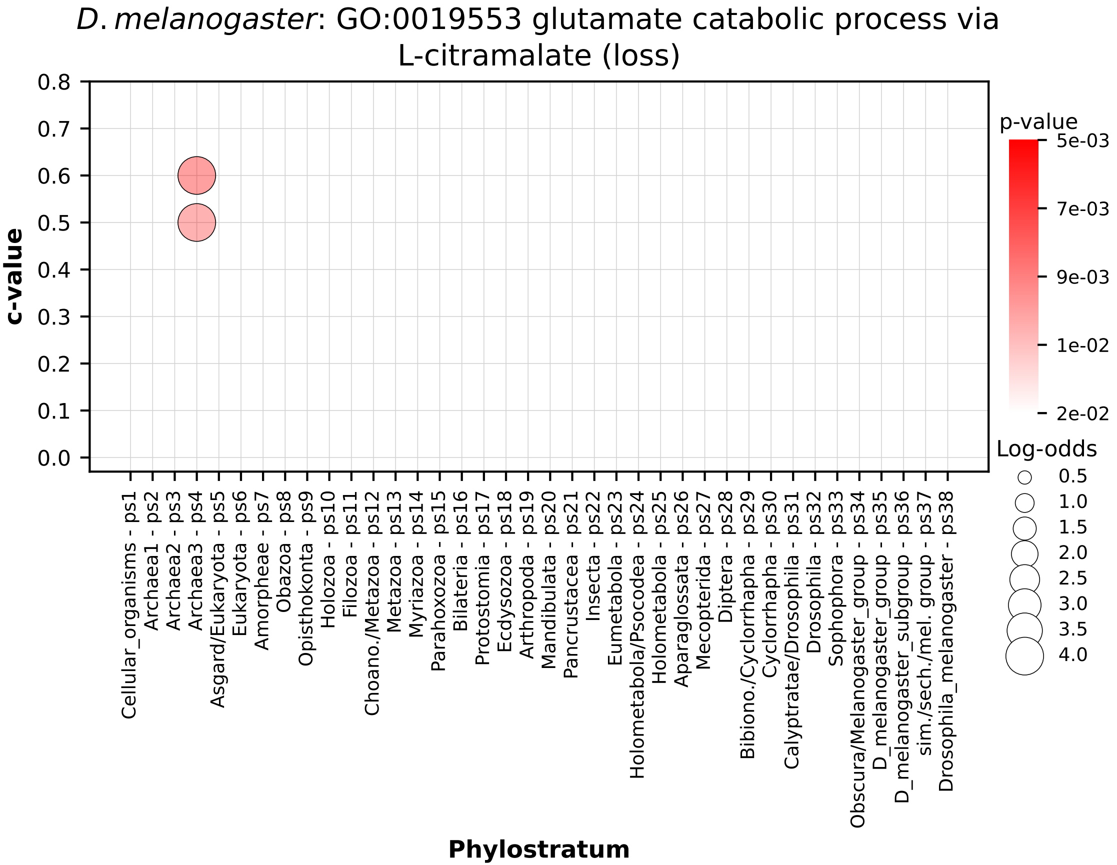

D. melanogaster: GO:0019553 glutamate catabolic process via L-citramalate (loss)
c-value
0.8
0.7
0.6
0.5
0.4
0.3
0.2
0.1
0.0
p-value
5e-03
7e-03
9e-03
1e-02
2e-02
Log-odds
0.5
1.0
1.5
2.0
2.5
3.0
3.5
4.0
Cellular_organisms - ps1
Archaea1 - ps2
Archaea2 - ps3
Archaea3 - ps4
Asgard/Eukaryota - ps5
Eukaryota - ps6
Amorpheae - ps7
Obazoa - ps8
Opisthokonta - ps9
Holozoa - ps10
Filozoa - ps11
Choano./Metazoa - ps12
Metazoa - ps13
Myriazoa - ps14
Parahoxozoa - ps15
Bilateria - ps16
Protostomia - ps17
Ecdysozoa - ps18
Arthropoda - ps19
Mandibulata - ps20
Pancrustacea - ps21
Insecta - ps22
Eumetabola - ps23
Holometabola/Psocodea - ps24
Holometabola - ps25
Aparaglossata - ps26
Mecopterida - ps27
Diptera - ps28
Bibiono./Cyclorrhapha - ps29
Cyclorrhapha - ps30
Calyptratae/Drosophila - ps31
Drosophila - ps32
Sophophora - ps33
Obscura/Melanogaster_group - ps34
D_melanogaster_group - ps35
D_melanogaster_subgroup - ps36
sim./sech./mel. group - ps37
Drosophila_melanogaster - ps38
Phylostratum

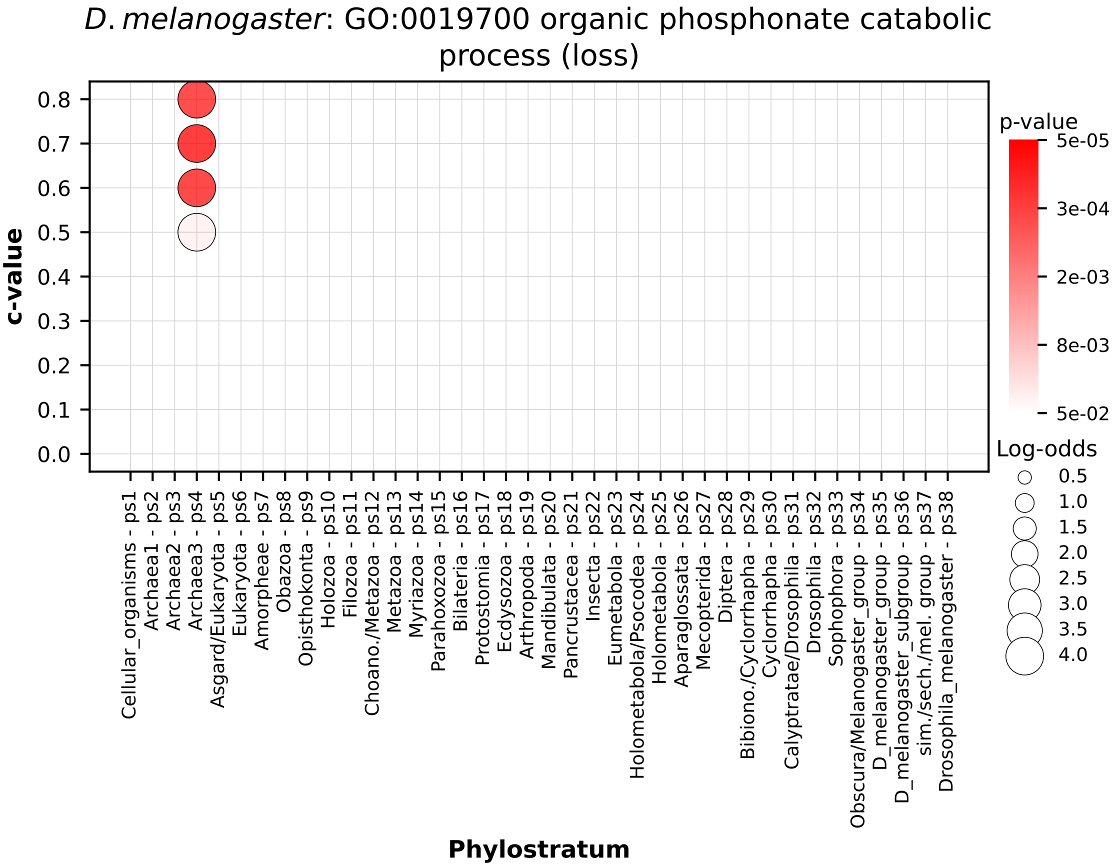
D. melanogaster: GO:0019700 organic phosphonate catabolic process (loss)
c-value
0.0
0.1
0.2
0.3
0.4
0.5
0.6
0.7
0.8
Phylostratum
Cellular_organisms - ps1
Archaea1 - ps2
Archaea2 - ps3
Archaea3 - ps4
Asgard/Eukaryota - ps5
Eukaryota - ps6
Amorpheae - ps7
Obazoa - ps8
Opisthokonta - ps9
Holozoa - ps10
Filozoa - ps11
Choano./Metazoa - ps12
Metazoa - ps13
Myriazoa - ps14
Parahoxozoa - ps15
Bilateria - ps16
Protostomia - ps17
Ecdysozoa - ps18
Arthropoda - ps19
Mandibulata - ps20
Pancrustacea - ps21
Insecta - ps22
Eumetabola - ps23
Holometabola/Psocodea - ps24
Holometabola - ps25
Aparaglossata - ps26
Mecopterida - ps27
Diptera - ps28
Bibiono./Cyclorrhapha - ps29
Cyclorrhapha - ps30
Calyptratae/Drosophila - ps31
Drosophila - ps32
Sophophora - ps33
Obscura/Melanogaster_group - ps34
D_melanogaster_group - ps35
D_melanogaster_subgroup - ps36
sim./sech./mel. group - ps37
Drosophila_melanogaster - ps38
p-value
5e-05
3e-04
2e-03
8e-03
5e-02
Log-odds
0.5
1.0
1.5
2.0
2.5
3.0
3.5
4.0

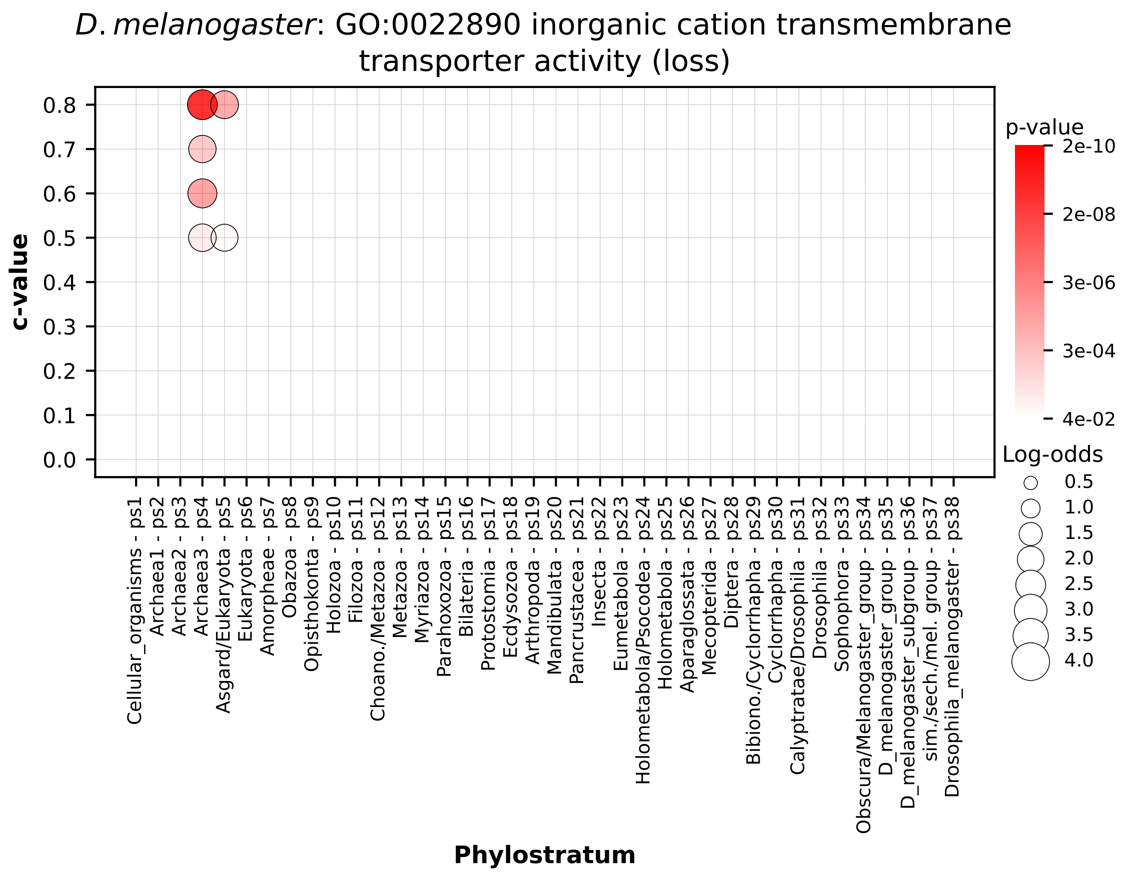

D. melanogaster: GO:0022890 inorganic cation transmembrane transporter activity (loss)
c-value
0.8
0.7
0.6
0.5
0.4
0.3
0.2
0.1
0.0
Phylostratum
Cellular_organisms - ps1
Archaea1 - ps2
Archaea2 - ps3
Archaea3 - ps4
Asgard/Eukaryota - ps5
Eukaryota - ps6
Amorpheae - ps7
Obazoa - ps8
Opisthokonta - ps9
Holozoa - ps10
Filozoa - ps11
Choano./Metazoa - ps12
Metazoa - ps13
Myriazoa - ps14
Parahoxozoa - ps15
Bilateria - ps16
Protostomia - ps17
Ecdysozoa - ps18
Arthropoda - ps19
Mandibulata - ps20
Pancrustacea - ps21
Insecta - ps22
Eumetabola - ps23
Holometabola/Psocodea - ps24
Holometabola - ps25
Aparaglossata - ps26
Mecopterida - ps27
Diptera - ps28
Bibiono./Cyclorrhapha - ps29
Cyclorrhapha - ps30
Calyptratae/Drosophila - ps31
Drosophila - ps32
Sophophora - ps33
Obscura/Melanogaster_group - ps34
D_melanogaster_group - ps35
D_melanogaster_subgroup - ps36
sim./sech./mel. group - ps37
Drosophila_melanogaster - ps38
p-value
2e-10
2e-08
3e-06
3e-04
4e-02
Log-odds
0.5
1.0
1.5
2.0
2.5
3.0
3.5
4.0

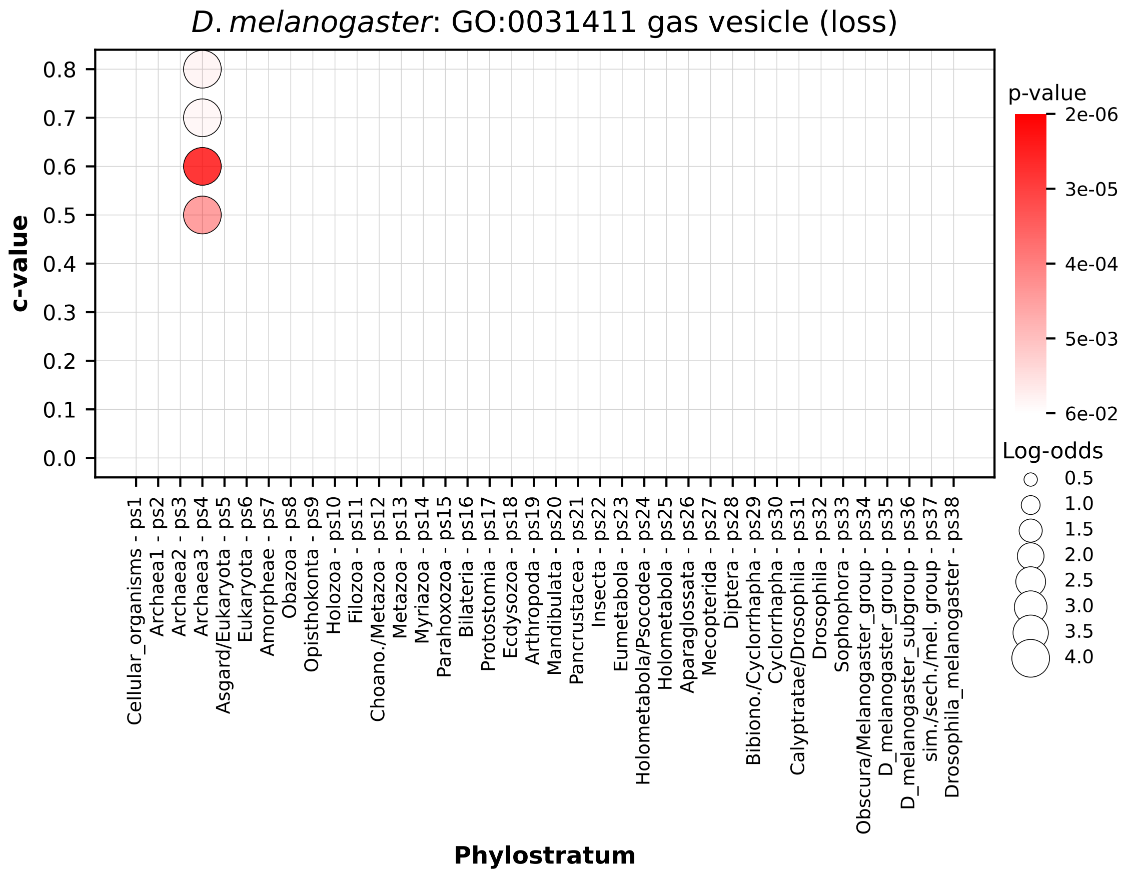

D. melanogaster: GO:0031411 gas vesicle (loss)
c-value
0.0
0.1
0.2
0.3
0.4
0.5
0.6
0.7
0.8
Phylostratum
Cellular_organisms - ps1
Archaea1 - ps2
Archaea2 - ps3
Archaea3 - ps4
Asgard/Eukaryota - ps5
Eukaryota - ps6
Amorpheae - ps7
Obazoa - ps8
Opisthokonta - ps9
Holozoa - ps10
Filozoa - ps11
Choano./Metazoa - ps12
Metazoa - ps13
Myriazoa - ps14
Parahoxozoa - ps15
Bilateria - ps16
Protostomia - ps17
Ecdysozoa - ps18
Arthropoda - ps19
Mandibulata - ps20
Pancrustacea - ps21
Insecta - ps22
Eumetabola - ps23
Holometabola/Psocodea - ps24
Holometabola - ps25
Aparaglossata - ps26
Mecopterida - ps27
Diptera - ps28
Bibiono./Cyclorrhapha - ps29
Cyclorrhapha - ps30
Calyptratae/Drosophila - ps31
Drosophila - ps32
Sophophora - ps33
Obscura/Melanogaster_group - ps34
D_melanogaster_group - ps35
D_melanogaster_subgroup - ps36
sim./sech./mel. group - ps37
Drosophila_melanogaster - ps38
p-value
2e-06
3e-05
4e-04
5e-03
6e-02
Log-odds
0.5
1.0
1.5
2.0
2.5
3.0
3.5
4.0

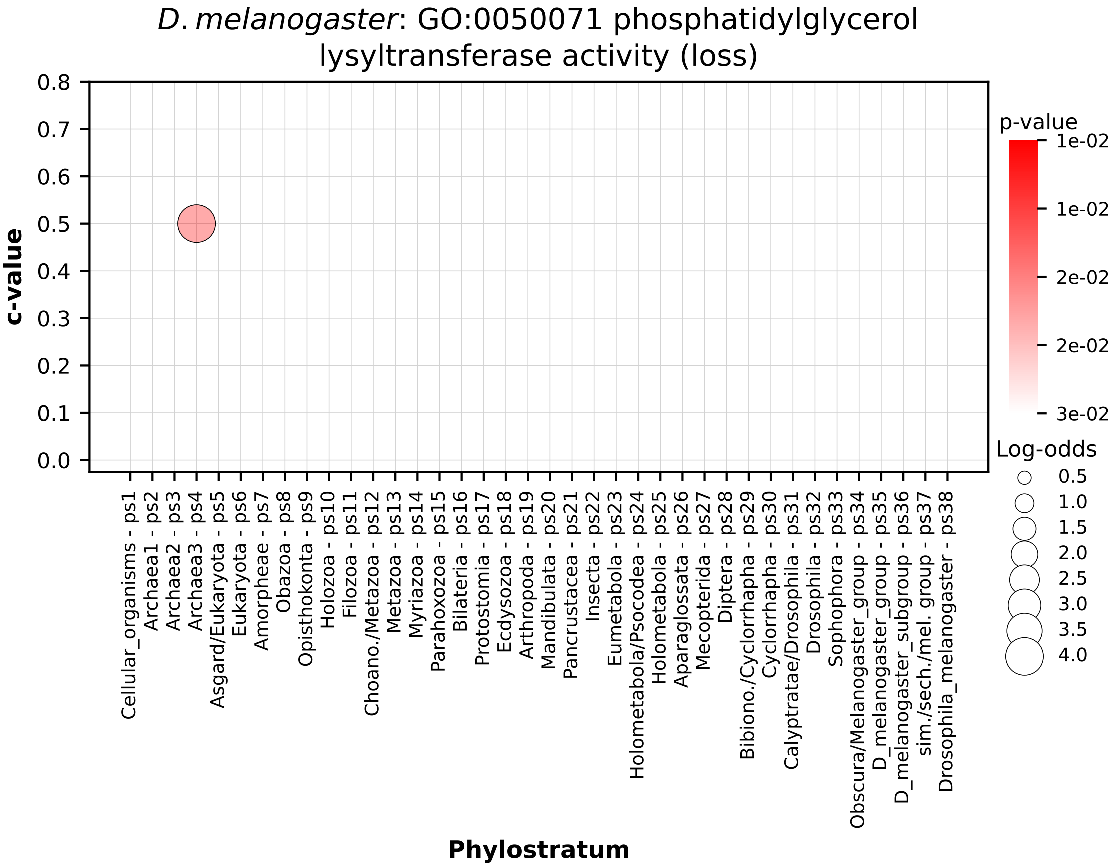

D. melanogaster: GO:0050071 phosphatidylglycerol lysyltransferase activity (loss)
c-value
0.8
0.7
0.6
0.5
0.4
0.3
0.2
0.1
0.0
p-value
1e-02
1e-02
2e-02
2e-02
3e-02
Log-odds
0.5
1.0
1.5
2.0
2.5
3.0
3.5
4.0
Cellular_organisms - ps1
Archaea1 - ps2
Archaea2 - ps3
Archaea3 - ps4
Asgard/Eukaryota - ps5
Eukaryota - ps6
Amorpheae - ps7
Obazoa - ps8
Opisthokonta - ps9
Holozoa - ps10
Filozoa - ps11
Choano./Metazoa - ps12
Metazoa - ps13
Myriazoa - ps14
Parahoxozoa - ps15
Bilateria - ps16
Protostomia - ps17
Ecdysozoa - ps18
Arthropoda - ps19
Mandibulata - ps20
Pancrustacea - ps21
Insecta - ps22
Eumetabola - ps23
Holometabola/Psocodea - ps24
Holometabola - ps25
Aparaglossata - ps26
Mecopterida - ps27
Diptera - ps28
Bibiono./Cyclorrhapha - ps29
Cyclorrhapha - ps30
Calyptratae/Drosophila - ps31
Drosophila - ps32
Sophophora - ps33
Obscura/Melanogaster_group - ps34
D_melanogaster_group - ps35
D_melanogaster_subgroup - ps36
sim./sech./mel. group - ps37
Drosophila_melanogaster - ps38
Phylostratum

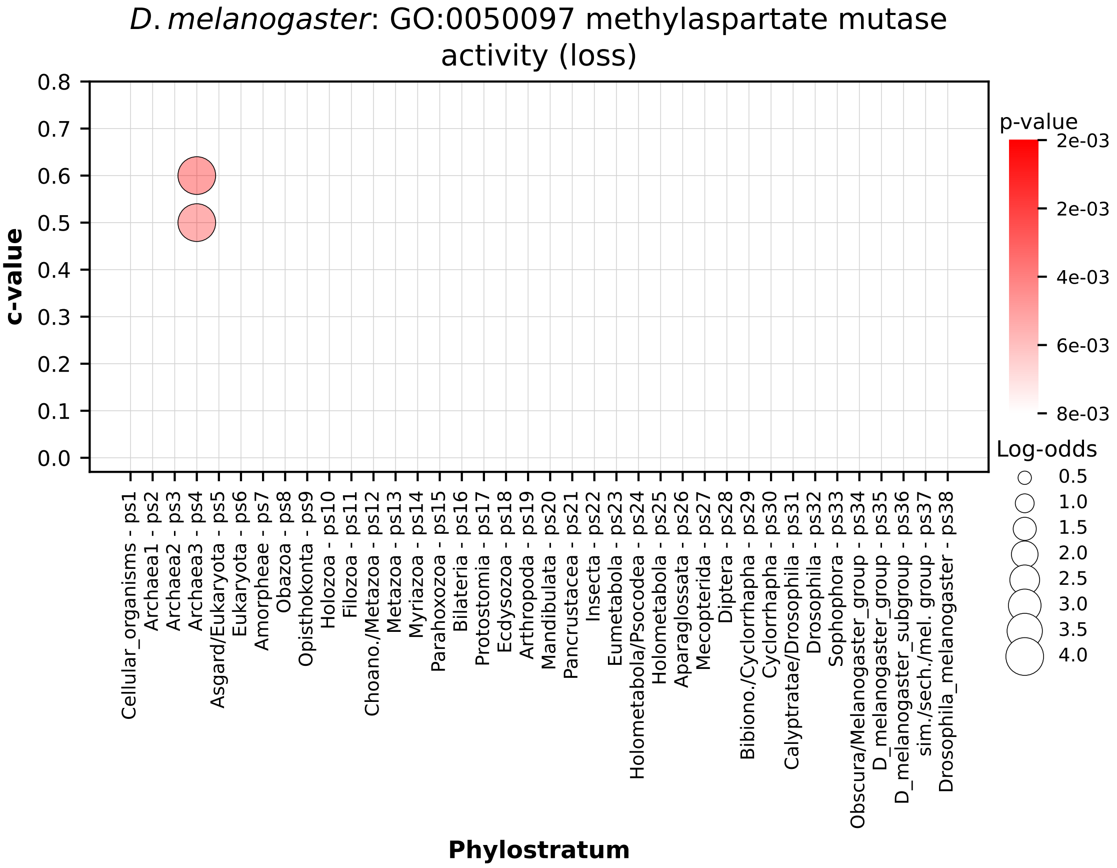

D. melanogaster: GO:0050097 methylaspartate mutase activity (loss)
c-value
0.8
0.7
0.6
0.5
0.4
0.3
0.2
0.1
0.0
p-value
2e-03
2e-03
4e-03
6e-03
8e-03
Log-odds
0.5
1.0
1.5
2.0
2.5
3.0
3.5
4.0
Cellular_organisms - ps1
Archaea1 - ps2
Archaea2 - ps3
Archaea3 - ps4
Asgard/Eukaryota - ps5
Eukaryota - ps6
Amorpheae - ps7
Obazoa - ps8
Opisthokonta - ps9
Holozoa - ps10
Filozoa - ps11
Choano./Metazoa - ps12
Metazoa - ps13
Myriazoa - ps14
Parahoxozoa - ps15
Bilateria - ps16
Protostomia - ps17
Ecdysozoa - ps18
Arthropoda - ps19
Mandibulata - ps20
Pancrustacea - ps21
Insecta - ps22
Eumetabola - ps23
Holometabola/Psocodea - ps24
Holometabola - ps25
Aparaglossata - ps26
Mecopterida - ps27
Diptera - ps28
Bibiono./Cyclorrhapha - ps29
Cyclorrhapha - ps30
Calyptratae/Drosophila - ps31
Drosophila - ps32
Sophophora - ps33
Obscura/Melanogaster_group - ps34
D_melanogaster_group - ps35
D_melanogaster_subgroup - ps36
sim./sech./mel. group - ps37
Drosophila_melanogaster - ps38
Phylostratum

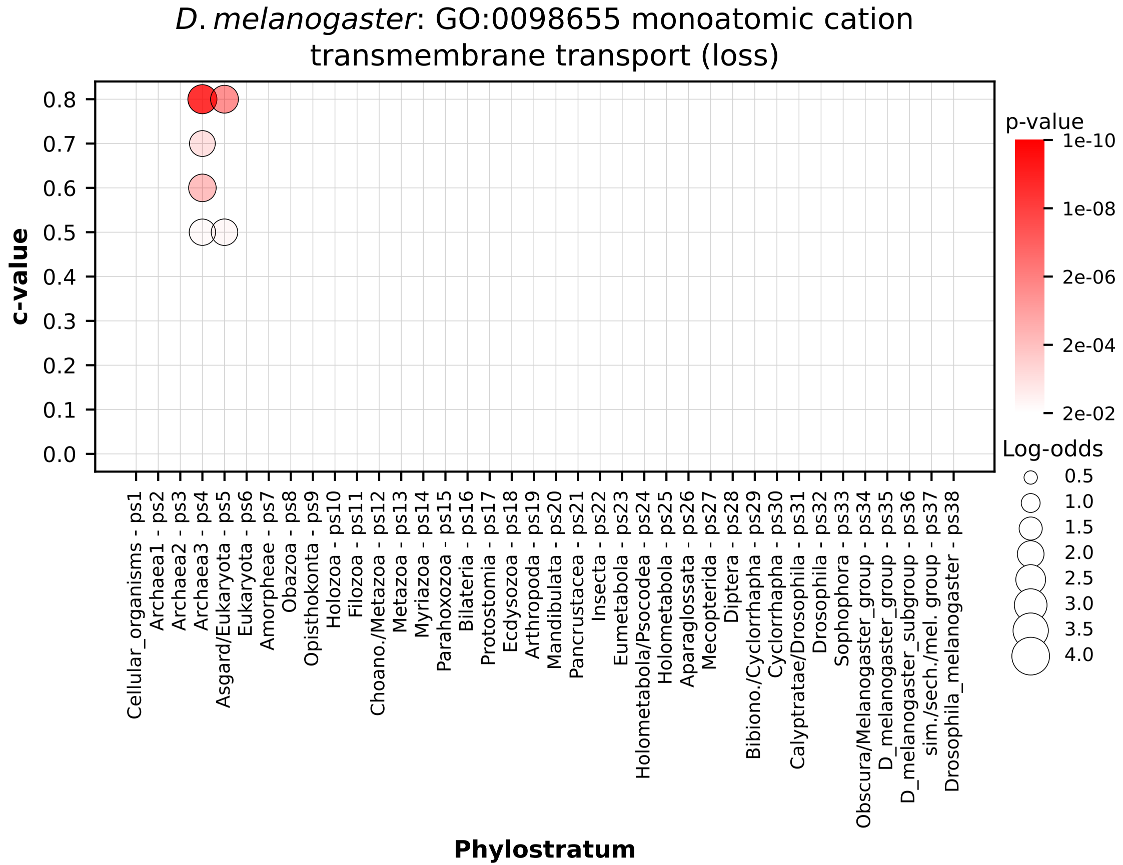

D. melanogaster: GO:0098655 monoatomic cation transmembrane transport (loss)
c-value
0.8
0.7
0.6
0.5
0.4
0.3
0.2
0.1
0.0
p-value
1e-10
1e-08
2e-06
2e-04
2e-02
Log-odds
0.5
1.0
1.5
2.0
2.5
3.0
3.5
4.0
Cellular_organisms - ps1
Archaea1 - ps2
Archaea2 - ps3
Archaea3 - ps4
Asgard/Eukaryota - ps5
Eukaryota - ps6
Amorpheae - ps7
Obazoa - ps8
Opisthokonta - ps9
Holozoa - ps10
Filozoa - ps11
Choano./Metazoa - ps12
Metazoa - ps13
Myriazoa - ps14
Parahoxozoa - ps15
Bilateria - ps16
Protostomia - ps17
Ecdysozoa - ps18
Arthropoda - ps19
Mandibulata - ps20
Pancrustacea - ps21
Insecta - ps22
Eumetabola - ps23
Holometabola/Psocodea - ps24
Holometabola - ps25
Aparaglossata - ps26
Mecopterida - ps27
Diptera - ps28
Bibiono./Cyclorrhapha - ps29
Cyclorrhapha - ps30
Calyptratae/Drosophila - ps31
Drosophila - ps32
Sophophora - ps33
Obscura/Melanogaster_group - ps34
D_melanogaster_group - ps35
D_melanogaster_subgroup - ps36
sim./sech./mel. group - ps37
Drosophila_melanogaster - ps38
Phylostratum

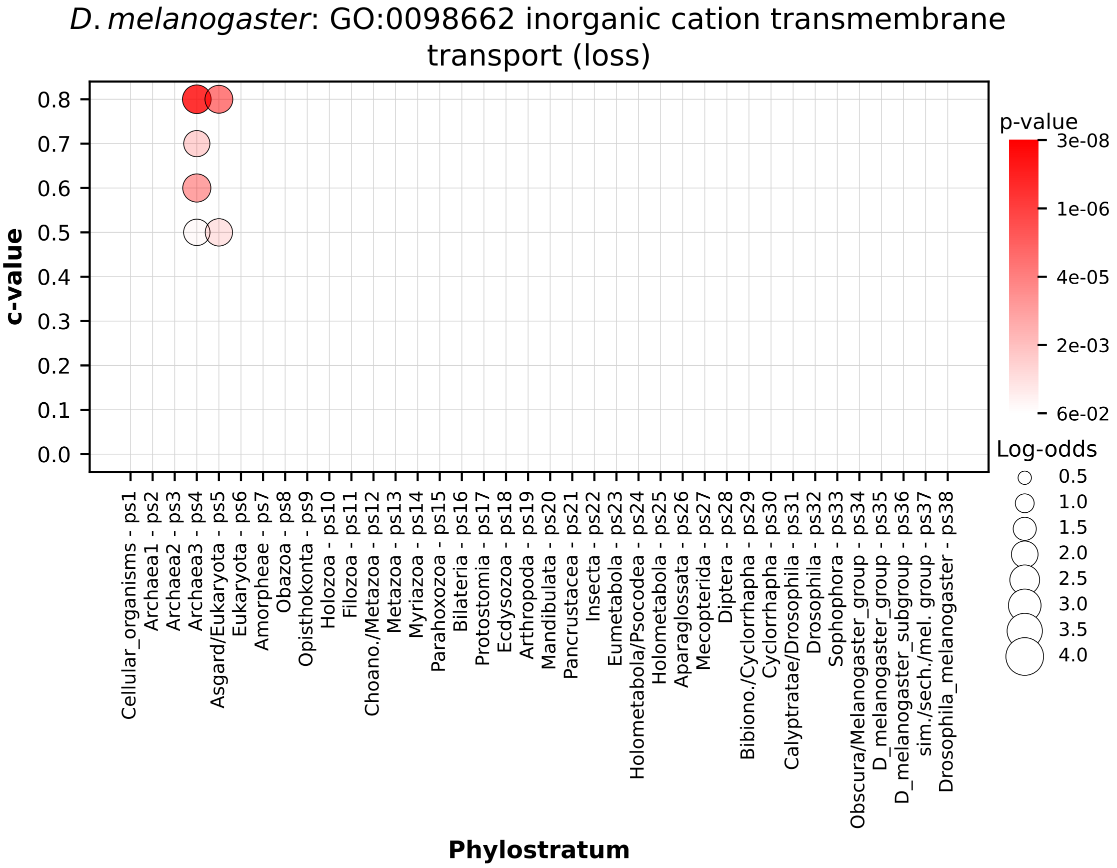
D. melanogaster: GO:0098662 inorganic cation transmembrane transport (loss)
c-value
0.0
0.1
0.2
0.3
0.4
0.5
0.6
0.7
0.8
Phylostratum
Cellular_organisms - ps1
Archaea1 - ps2
Archaea2 - ps3
Archaea3 - ps4
Asgard/Eukaryota - ps5
Eukaryota - ps6
Amorpheae - ps7
Obazoa - ps8
Opisthokonta - ps9
Holozoa - ps10
Filozoa - ps11
Choano./Metazoa - ps12
Metazoa - ps13
Myriazoa - ps14
Parahoxozoa - ps15
Bilateria - ps16
Protostomia - ps17
Ecdysozoa - ps18
Arthropoda - ps19
Mandibulata - ps20
Pancrustacea - ps21
Insecta - ps22
Eumetabola - ps23
Holometabola/Psocodea - ps24
Holometabola - ps25
Aparaglossata - ps26
Mecopterida - ps27
Diptera - ps28
Bibiono./Cyclorrhapha - ps29
Cyclorrhapha - ps30
Calyptratae/Drosophila - ps31
Drosophila - ps32
Sophophora - ps33
Obscura/Melanogaster_group - ps34
D_melanogaster_group - ps35
D_melanogaster_subgroup - ps36
sim./sech./mel. group - ps37
Drosophila_melanogaster - ps38
p-value
3e-08
1e-06
4e-05
2e-03
6e-02
Log-odds
0.5
1.0
1.5
2.0
2.5
3.0
3.5
4.0

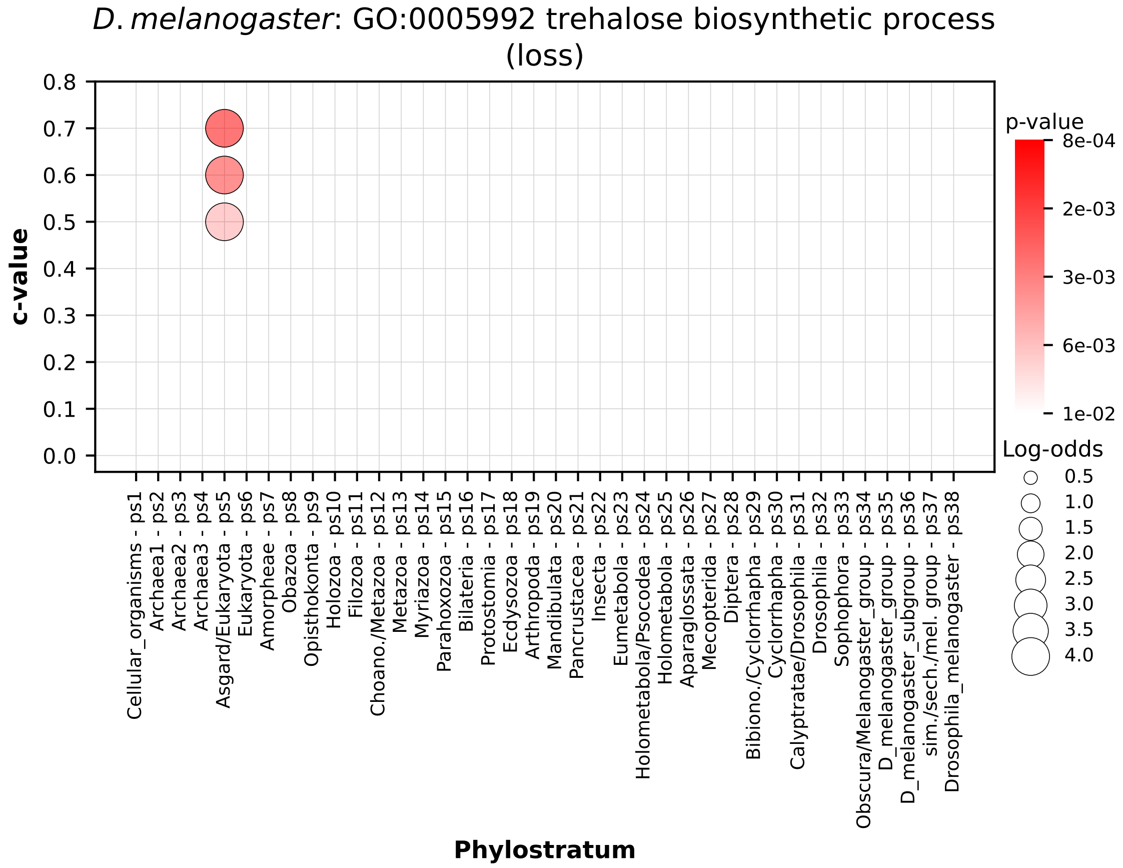
D. melanogaster: GO:0005992 trehalose biosynthetic process
(loss)
c-value
0.8
0.7
0.6
0.5
0.4
0.3
0.2
0.1
0.0
p-value
8e-04
2e-03
3e-03
6e-03
1e-02
Log-odds
0.5
1.0
1.5
2.0
2.5
3.0
3.5
4.0
Cellular_organisms - ps1
Archaea1 - ps2
Archaea2 - ps3
Archaea3 - ps4
Asgard/Eukaryota - ps5
Eukaryota - ps6
Amorpheae - ps7
Obazoa - ps8
Opisthokonta - ps9
Holozoa - ps10
Filozoa - ps11
Choano./Metazoa - ps12
Metazoa - ps13
Myriazoa - ps14
Parahoxozoa - ps15
Bilateria - ps16
Protostomia - ps17
Ecdysozoa - ps18
Arthropoda - ps19
Mandibulata - ps20
Pancrustacea - ps21
Insecta - ps22
Eumetabola - ps23
Holometabola/Psocodea - ps24
Holometabola - ps25
Aparaglossata - ps26
Mecopterida - ps27
Diptera - ps28
Bibiono./Cyclorrhapha - ps29
Cyclorrhapha - ps30
Calyptratae/Drosophila - ps31
Drosophila - ps32
Sophophora - ps33
Obscura/Melanogaster_group - ps34
D_melanogaster_group - ps35
D_melanogaster_subgroup - ps36
sim./sech./mel. group - ps37
Drosophila_melanogaster - ps38
Phylostratum

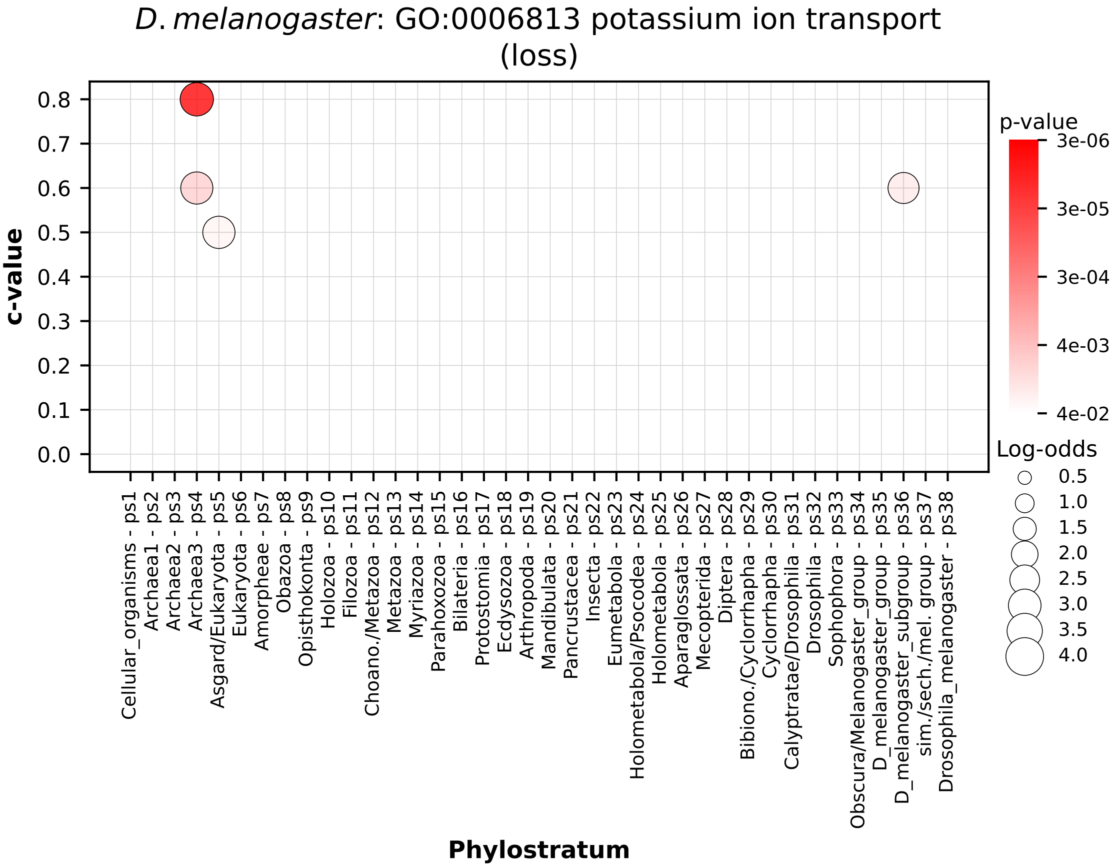

D. melanogaster: GO:0006813 potassium ion transport
(loss)
c-value
0.0
0.1
0.2
0.3
0.4
0.5
0.6
0.7
0.8
p-value
3e-06
3e-05
3e-04
4e-03
4e-02
Log-odds
0.5
1.0
1.5
2.0
2.5
3.0
3.5
4.0
Cellular_organisms - ps1
Archaea1 - ps2
Archaea2 - ps3
Archaea3 - ps4
Asgard/Eukaryota - ps5
Eukaryota - ps6
Amorpheae - ps7
Obazoa - ps8
Opisthokonta - ps9
Holozoa - ps10
Filozoa - ps11
Choano./Metazoa - ps12
Metazoa - ps13
Myriazoa - ps14
Parahoxozoa - ps15
Bilateria - ps16
Protostomia - ps17
Ecdysozoa - ps18
Arthropoda - ps19
Mandibulata - ps20
Pancrustacea - ps21
Insecta - ps22
Eumetabola - ps23
Holometabola/Psocodea - ps24
Holometabola - ps25
Aparaglossata - ps26
Mecopterida - ps27
Diptera - ps28
Bibiono./Cyclorrhapha - ps29
Cyclorrhapha - ps30
Calyptratae/Drosophila - ps31
Drosophila - ps32
Sophophora - ps33
Obscura/Melanogaster_group - ps34
D_melanogaster_group - ps35
D_melanogaster_subgroup - ps36
sim./sech./mel. group - ps37
Drosophila_melanogaster - ps38
Phylostratum

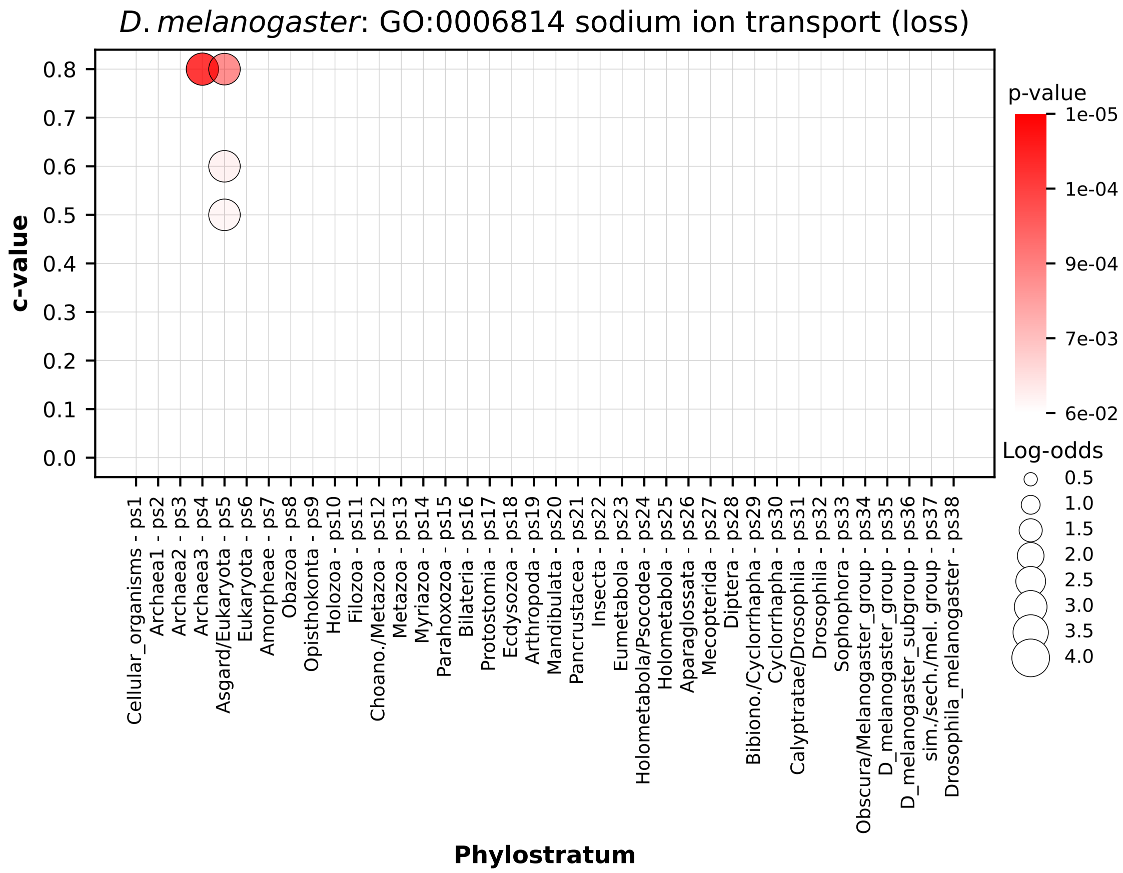

D. melanogaster: GO:0006814 sodium ion transport (loss)
c-value
0.8
0.7
0.6
0.5
0.4
0.3
0.2
0.1
0.0
Phylostratum
Cellular_organisms - ps1
Archaea1 - ps2
Archaea2 - ps3
Archaea3 - ps4
Asgard/Eukaryota - ps5
Eukaryota - ps6
Amorpheae - ps7
Obazoa - ps8
Opisthokonta - ps9
Holozoa - ps10
Filozoa - ps11
Choano./Metazoa - ps12
Metazoa - ps13
Myriazoa - ps14
Parahoxozoa - ps15
Bilateria - ps16
Protostomia - ps17
Ecdysozoa - ps18
Arthropoda - ps19
Mandibulata - ps20
Pancrustacea - ps21
Insecta - ps22
Eumetabola - ps23
Holometabola/Psocodea - ps24
Holometabola - ps25
Aparaglossata - ps26
Mecopterida - ps27
Diptera - ps28
Bibiono./Cyclorrhapha - ps29
Cyclorrhapha - ps30
Calyptratae/Drosophila - ps31
Drosophila - ps32
Sophophora - ps33
Obscura/Melanogaster_group - ps34
D_melanogaster_group - ps35
D_melanogaster_subgroup - ps36
sim./sech./mel. group - ps37
Drosophila_melanogaster - ps38
p-value
1e-05
1e-04
9e-04
7e-03
6e-02
Log-odds
0.5
1.0
1.5
2.0
2.5
3.0
3.5
4.0

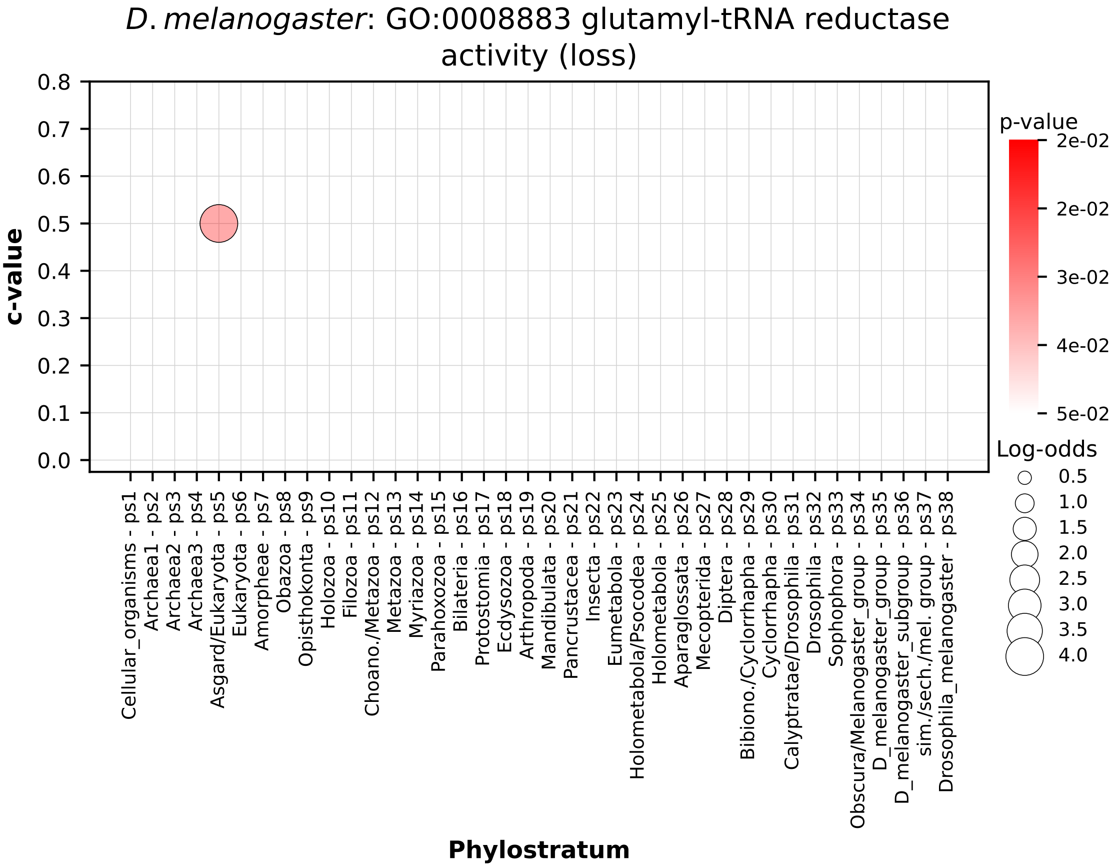
D. melanogaster: GO:0008883 glutamyl-tRNA reductase activity (loss)
c-value
0.8
0.7
0.6
0.5
0.4
0.3
0.2
0.1
0.0
p-value
2e-02
2e-02
3e-02
4e-02
5e-02
Log-odds
0.5
1.0
1.5
2.0
2.5
3.0
3.5
4.0
Cellular_organisms - ps1
Archaea1 - ps2
Archaea2 - ps3
Archaea3 - ps4
Asgard/Eukaryota - ps5
Eukaryota - ps6
Amorpheae - ps7
Obazoa - ps8
Opisthokonta - ps9
Holozoa - ps10
Filozoa - ps11
Choano./Metazoa - ps12
Metazoa - ps13
Myriazoa - ps14
Parahoxozoa - ps15
Bilateria - ps16
Protostomia - ps17
Ecdysozoa - ps18
Arthropoda - ps19
Mandibulata - ps20
Pancrustacea - ps21
Insecta - ps22
Eumetabola - ps23
Holometabola/Psocodea - ps24
Holometabola - ps25
Aparaglossata - ps26
Mecopterida - ps27
Diptera - ps28
Bibiono./Cyclorrhapha - ps29
Cyclorrhapha - ps30
Calyptratae/Drosophila - ps31
Drosophila - ps32
Sophophora - ps33
Obscura/Melanogaster_group - ps34
D_melanogaster_group - ps35
D_melanogaster_subgroup - ps36
sim./sech./mel. group - ps37
Drosophila_melanogaster - ps38
Phylostratum

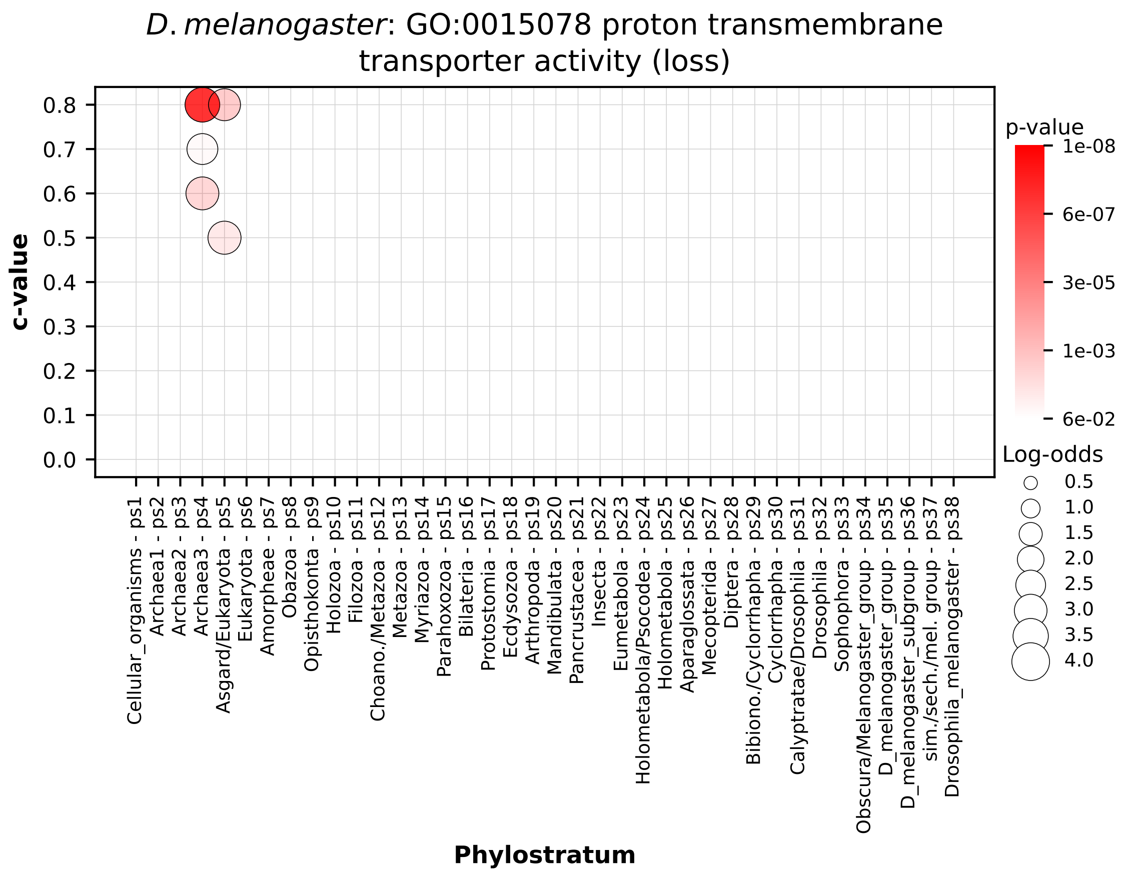

D. melanogaster: GO:0015078 proton transmembrane transporter activity (loss)
c-value
0.8
0.7
0.6
0.5
0.4
0.3
0.2
0.1
0.0
Phylostratum
Cellular_organisms - ps1
Archaea1 - ps2
Archaea2 - ps3
Archaea3 - ps4
Asgard/Eukaryota - ps5
Eukaryota - ps6
Amorpheae - ps7
Obazoa - ps8
Opisthokonta - ps9
Holozoa - ps10
Filozoa - ps11
Choano./Metazoa - ps12
Metazoa - ps13
Myriazoa - ps14
Parahoxozoa - ps15
Bilateria - ps16
Protostomia - ps17
Ecdysozoa - ps18
Arthropoda - ps19
Mandibulata - ps20
Pancrustacea - ps21
Insecta - ps22
Eumetabola - ps23
Holometabola/Psocodea - ps24
Holometabola - ps25
Aparaglossata - ps26
Mecopterida - ps27
Diptera - ps28
Bibiono./Cyclorrhapha - ps29
Cyclorrhapha - ps30
Calyptratae/Drosophila - ps31
Drosophila - ps32
Sophophora - ps33
Obscura/Melanogaster_group - ps34
D_melanogaster_group - ps35
D_melanogaster_subgroup - ps36
sim./sech./mel. group - ps37
Drosophila_melanogaster - ps38
p-value
1e-08
6e-07
3e-05
1e-03
6e-02
Log-odds
0.5
1.0
1.5
2.0
2.5
3.0
3.5
4.0

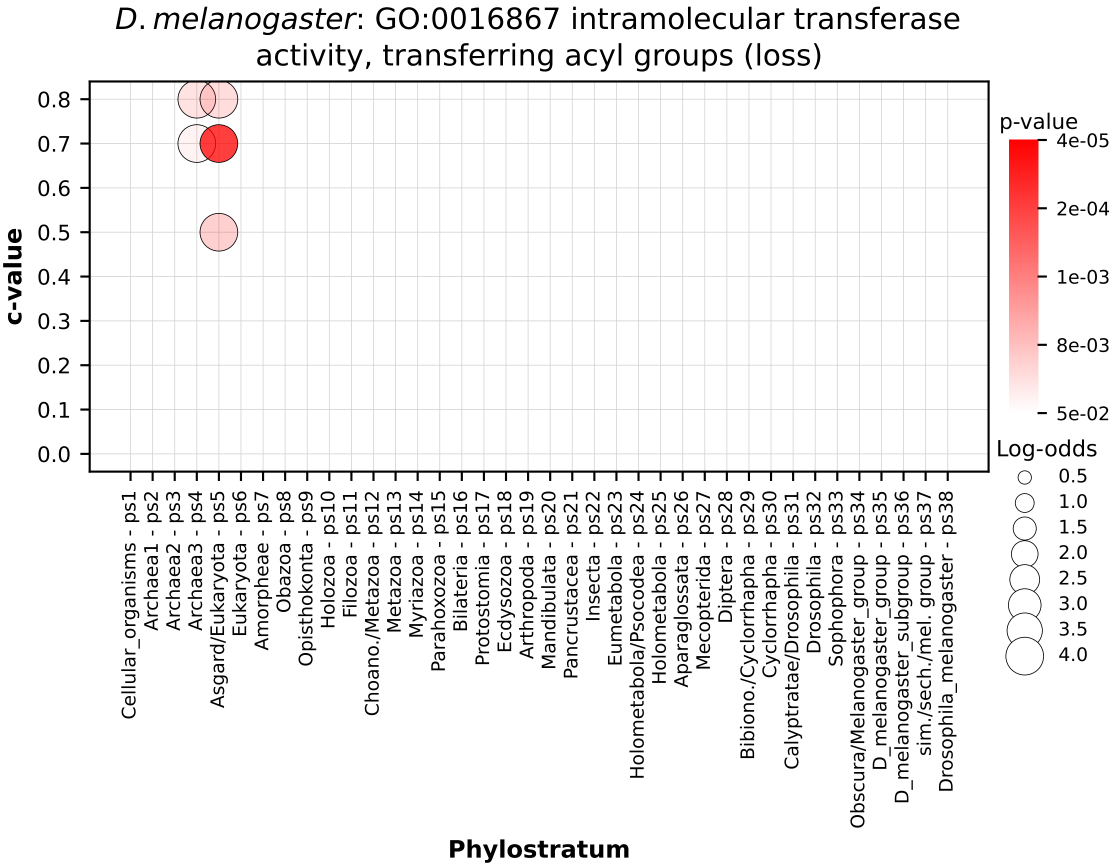

D. melanogaster: GO:0016867 intramolecular transferase activity, transferring acyl groups (loss)
c-value
0.0
0.1
0.2
0.3
0.4
0.5
0.6
0.7
0.8
Phylostratum
Cellular_organisms - ps1
Archaea1 - ps2
Archaea2 - ps3
Archaea3 - ps4
Asgard/Eukaryota - ps5
Eukaryota - ps6
Amorpheae - ps7
Obazoa - ps8
Opisthokonta - ps9
Holozoa - ps10
Filozoa - ps11
Choano./Metazoa - ps12
Metazoa - ps13
Myriazoa - ps14
Parahoxozoa - ps15
Bilateria - ps16
Protostomia - ps17
Ecdysozoa - ps18
Arthropoda - ps19
Mandibulata - ps20
Pancrustacea - ps21
Insecta - ps22
Eumetabola - ps23
Holometabola/Psocodea - ps24
Holometabola - ps25
Aparaglossata - ps26
Mecopterida - ps27
Diptera - ps28
Bibiono./Cyclorrhapha - ps29
Cyclorrhapha - ps30
Calyptratae/Drosophila - ps31
Drosophila - ps32
Sophophora - ps33
Obscura/Melanogaster_group - ps34
D_melanogaster_group - ps35
D_melanogaster_subgroup - ps36
sim./sech./mel. group - ps37
Drosophila_melanogaster - ps38
p-value
4e-05
2e-04
1e-03
8e-03
5e-02
Log-odds
0.5
1.0
1.5
2.0
2.5
3.0
3.5
4.0

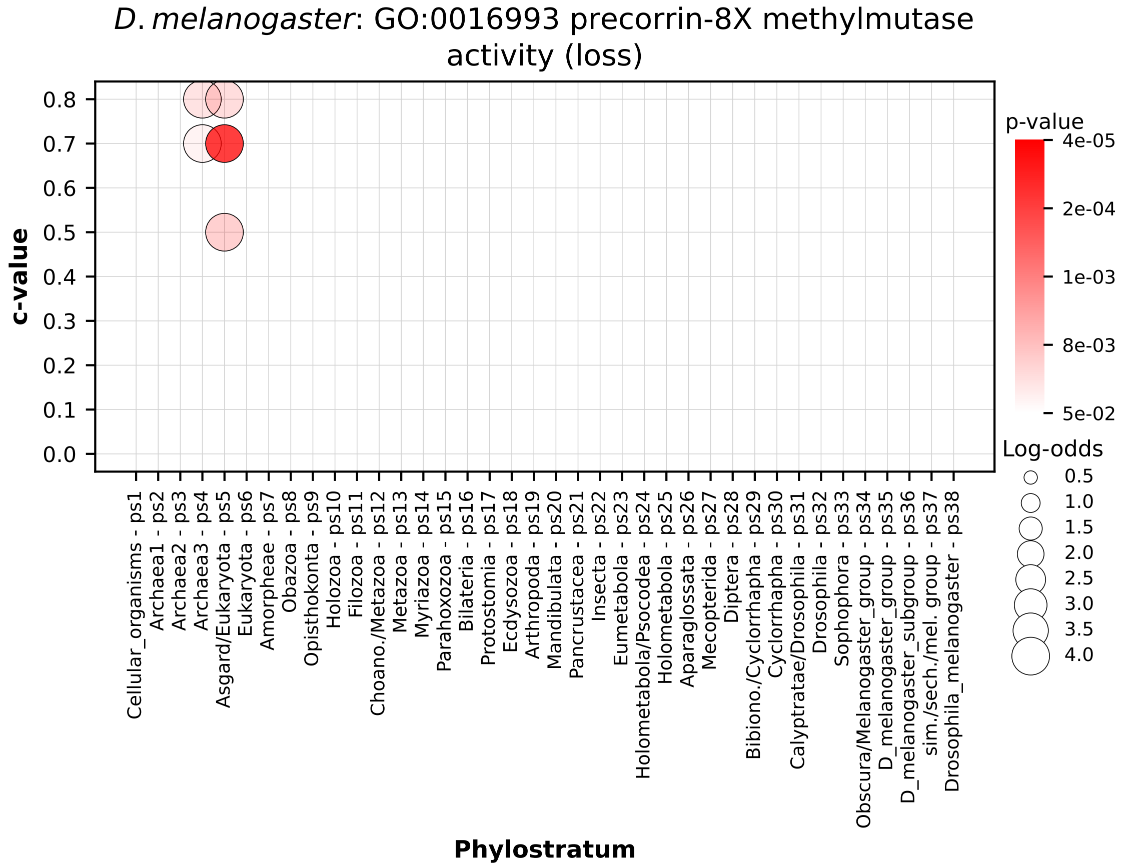

D. melanogaster: GO:0016993 precorrin-8X methylmutase activity (loss)
c-value
0.0
0.1
0.2
0.3
0.4
0.5
0.6
0.7
0.8
Phylostratum
Cellular_organisms - ps1
Archaea1 - ps2
Archaea2 - ps3
Archaea3 - ps4
Asgard/Eukaryota - ps5
Eukaryota - ps6
Amorpheae - ps7
Obazoa - ps8
Opisthokonta - ps9
Holozoa - ps10
Filozoa - ps11
Choano./Metazoa - ps12
Metazoa - ps13
Myriazoa - ps14
Parahoxozoa - ps15
Bilateria - ps16
Protostomia - ps17
Ecdysozoa - ps18
Arthropoda - ps19
Mandibulata - ps20
Pancrustacea - ps21
Insecta - ps22
Eumetabola - ps23
Holometabola/Psocodea - ps24
Holometabola - ps25
Aparaglossata - ps26
Mecopterida - ps27
Diptera - ps28
Bibiono./Cyclorrhapha - ps29
Cyclorrhapha - ps30
Calyptratae/Drosophila - ps31
Drosophila - ps32
Sophophora - ps33
Obscura/Melanogaster_group - ps34
D_melanogaster_group - ps35
D_melanogaster_subgroup - ps36
sim./sech./mel. group - ps37
Drosophila_melanogaster - ps38
p-value
4e-05
2e-04
1e-03
8e-03
5e-02
Log-odds
0.5
1.0
1.5
2.0
2.5
3.0
3.5
4.0

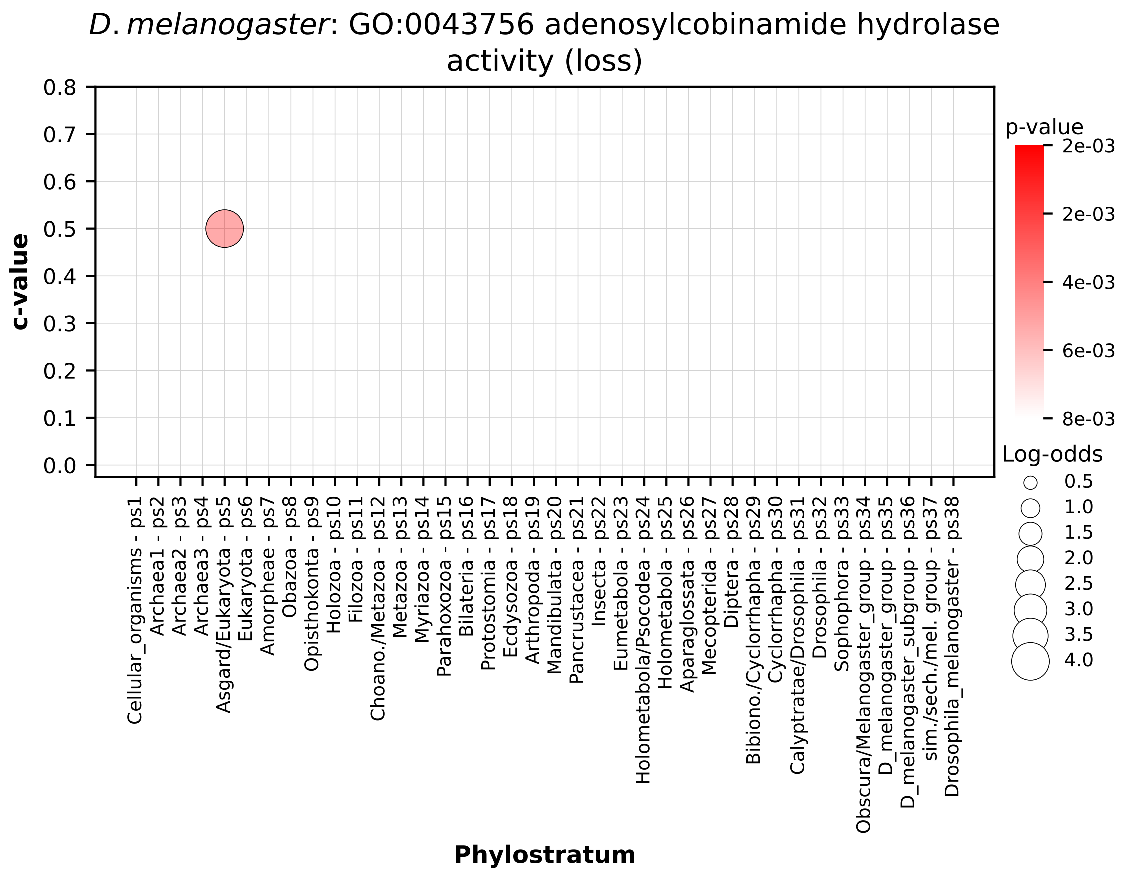

D. melanogaster: GO:0043756 adenosylcobinamide hydrolase activity (loss)
c-value
0.8
0.7
0.6
0.5
0.4
0.3
0.2
0.1
0.0
Phylostratum
Cellular_organisms - ps1
Archaea1 - ps2
Archaea2 - ps3
Archaea3 - ps4
Asgard/Eukaryota - ps5
Eukaryota - ps6
Amorpheae - ps7
Obazoa - ps8
Opisthokonta - ps9
Holozoa - ps10
Filozoa - ps11
Choano./Metazoa - ps12
Metazoa - ps13
Myriazoa - ps14
Parahoxozoa - ps15
Bilateria - ps16
Protostomia - ps17
Ecdysozoa - ps18
Arthropoda - ps19
Mandibulata - ps20
Pancrustacea - ps21
Insecta - ps22
Eumetabola - ps23
Holometabola/Psocodea - ps24
Holometabola - ps25
Aparaglossata - ps26
Mecopterida - ps27
Diptera - ps28
Bibiono./Cyclorrhapha - ps29
Cyclorrhapha - ps30
Calyptratae/Drosophila - ps31
Drosophila - ps32
Sophophora - ps33
Obscura/Melanogaster_group - ps34
D_melanogaster_group - ps35
D_melanogaster_subgroup - ps36
sim./sech./mel. group - ps37
Drosophila_melanogaster - ps38
p-value
2e-03
2e-03
4e-03
6e-03
8e-03
Log-odds
0.5
1.0
1.5
2.0
2.5
3.0
3.5
4.0

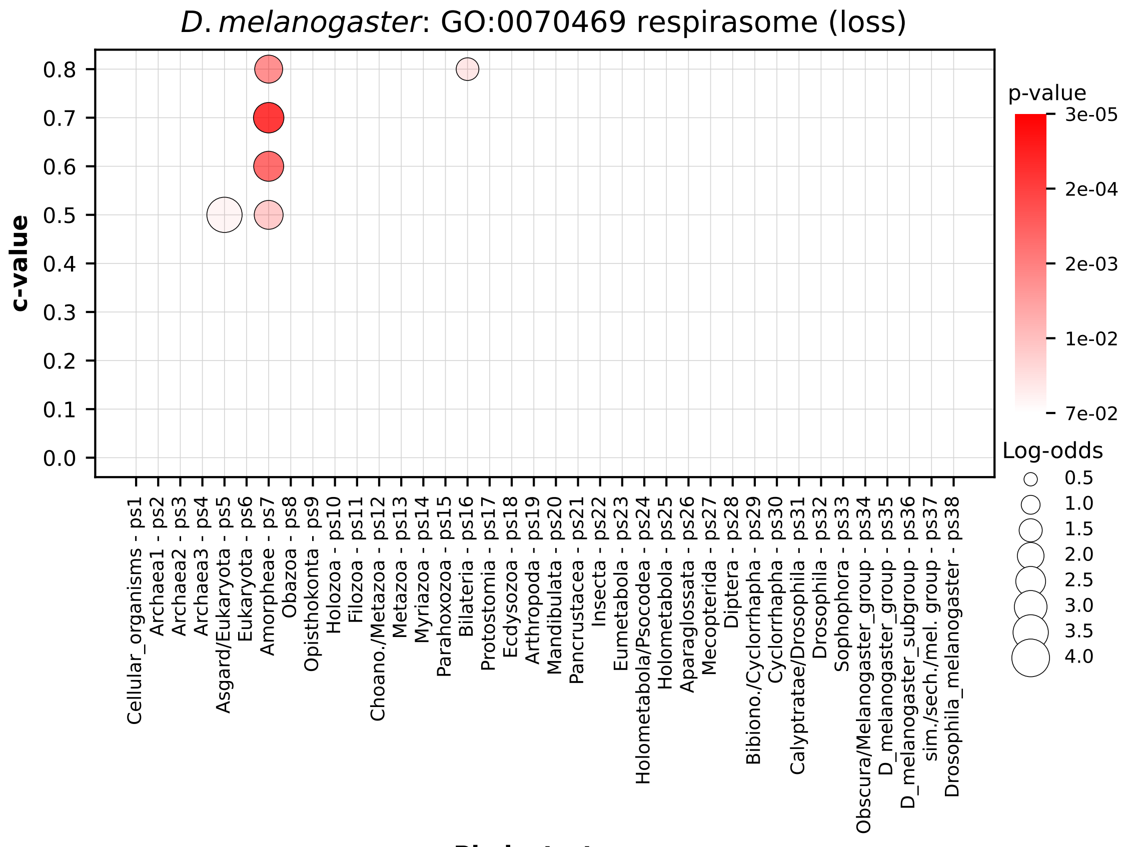

D. melanogaster: GO:0070469 respirasome (loss)
c-value
0.8
0.7
0.6
0.5
0.4
0.3
0.2
0.1
0.0
p-value
3e-05
2e-04
2e-03
1e-02
7e-02
Log-odds
0.5
1.0
1.5
2.0
2.5
3.0
3.5
4.0
Cellular_organisms - ps1
Archaea1 - ps2
Archaea2 - ps3
Archaea3 - ps4
Asgard/Eukaryota - ps5
Eukaryota - ps6
Amorpheae - ps7
Obazoa - ps8
Opisthokonta - ps9
Holozoa - ps10
Filozoa - ps11
Choano./Metazoa - ps12
Metazoa - ps13
Myriazoa - ps14
Parahoxozoa - ps15
Bilateria - ps16
Protostomia - ps17
Ecdysozoa - ps18
Arthropoda - ps19
Mandibulata - ps20
Pancrustacea - ps21
Insecta - ps22
Eumetabola - ps23
Holometabola/Psocodea - ps24
Holometabola - ps25
Aparaglossata - ps26
Mecopterida - ps27
Diptera - ps28
Bibiono./Cyclorrhapha - ps29
Cyclorrhapha - ps30
Calyptratae/Drosophila - ps31
Drosophila - ps32
Sophophora - ps33
Obscura/Melanogaster_group - ps34
D_melanogaster_group - ps35
D_melanogaster_subgroup - ps36
sim./sech./mel. group - ps37
Drosophila_melanogaster - ps38
Phylostratum

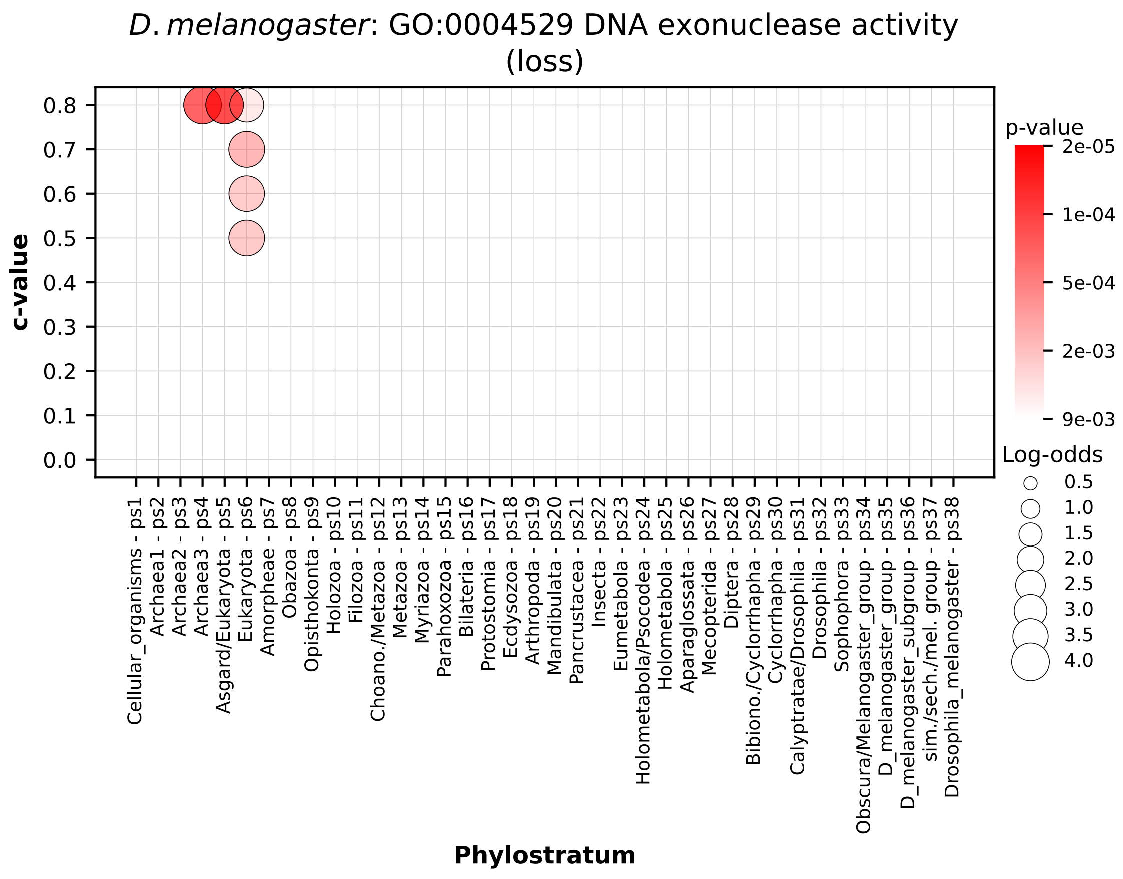

D. melanogaster: GO:0004529 DNA exonuclease activity (loss)
c-value
0.0
0.1
0.2
0.3
0.4
0.5
0.6
0.7
0.8
Phylostratum
Cellular_organisms - ps1
Archaea1 - ps2
Archaea2 - ps3
Archaea3 - ps4
Asgard/Eukaryota - ps5
Eukaryota - ps6
Amorpheae - ps7
Obazoa - ps8
Opisthokonta - ps9
Holozoa - ps10
Filozoa - ps11
Choano./Metazoa - ps12
Metazoa - ps13
Myriazoa - ps14
Parahoxozoa - ps15
Bilateria - ps16
Protostomia - ps17
Ecdysozoa - ps18
Arthropoda - ps19
Mandibulata - ps20
Pancrustacea - ps21
Insecta - ps22
Eumetabola - ps23
Holometabola/Psocodea - ps24
Holometabola - ps25
Aparaglossata - ps26
Mecopterida - ps27
Diptera - ps28
Bibiono./Cyclorrhapha - ps29
Cyclorrhapha - ps30
Calyptratae/Drosophila - ps31
Drosophila - ps32
Sophophora - ps33
Obscura/Melanogaster_group - ps34
D_melanogaster_group - ps35
D_melanogaster_subgroup - ps36
sim./sech./mel. group - ps37
Drosophila_melanogaster - ps38
p-value
2e-05
1e-04
5e-04
2e-03
9e-03
Log-odds
0.5
1.0
1.5
2.0
2.5
3.0
3.5
4.0

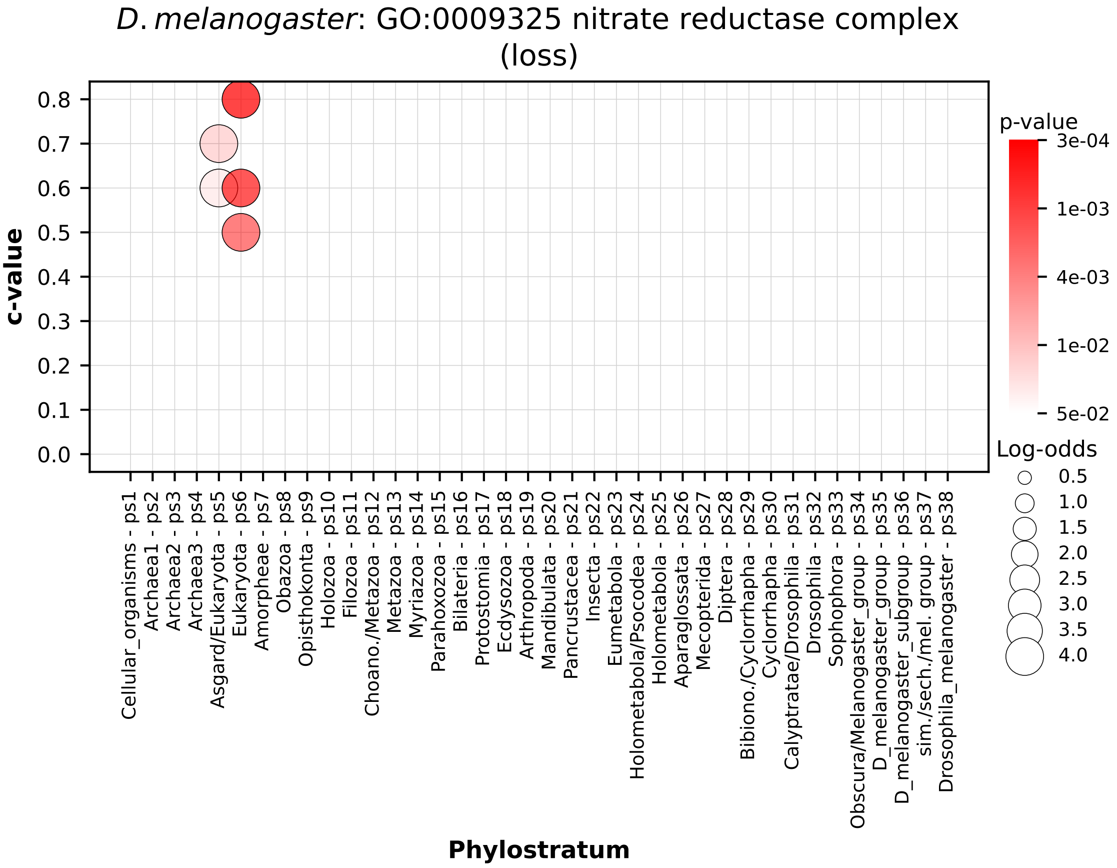

D. melanogaster: GO:0009325 nitrate reductase complex (loss)
c-value
0.0
0.1
0.2
0.3
0.4
0.5
0.6
0.7
0.8
Phylostratum
Cellular_organisms - ps1
Archaea1 - ps2
Archaea2 - ps3
Archaea3 - ps4
Asgard/Eukaryota - ps5
Eukaryota - ps6
Amorpheae - ps7
Obazoa - ps8
Opisthokonta - ps9
Holozoa - ps10
Filozoa - ps11
Choano./Metazoa - ps12
Metazoa - ps13
Myriazoa - ps14
Parahoxozoa - ps15
Bilateria - ps16
Protostomia - ps17
Ecdysozoa - ps18
Arthropoda - ps19
Mandibulata - ps20
Pancrustacea - ps21
Insecta - ps22
Eumetabola - ps23
Holometabola/Psocodea - ps24
Holometabola - ps25
Aparaglossata - ps26
Mecopterida - ps27
Diptera - ps28
Bibiono./Cyclorrhapha - ps29
Cyclorrhapha - ps30
Calyptratae/Drosophila - ps31
Drosophila - ps32
Sophophora - ps33
Obscura/Melanogaster_group - ps34
D_melanogaster_group - ps35
D_melanogaster_subgroup - ps36
sim./sech./mel. group - ps37
Drosophila_melanogaster - ps38
p-value
3e-04
1e-03
4e-03
1e-02
5e-02
Log-odds
0.5
1.0
1.5
2.0
2.5
3.0
3.5
4.0

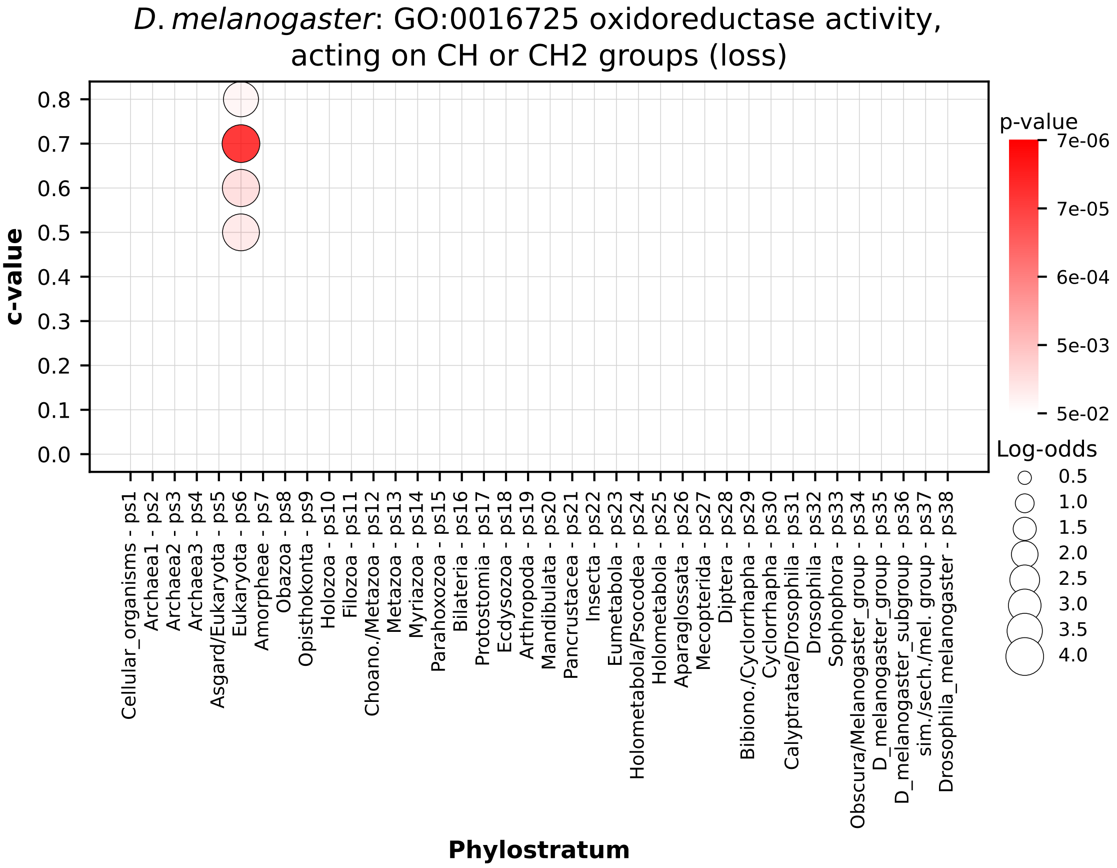

D. melanogaster: GO:0016725 oxidoreductase activity, acting on CH or CH2 groups (loss)
c-value
0.0
0.1
0.2
0.3
0.4
0.5
0.6
0.7
0.8
Phylostratum
Cellular_organisms - ps1
Archaea1 - ps2
Archaea2 - ps3
Archaea3 - ps4
Asgard/Eukaryota - ps5
Eukaryota - ps6
Amorpheae - ps7
Obazoa - ps8
Opisthokonta - ps9
Holozoa - ps10
Filozoa - ps11
Choano./Metazoa - ps12
Metazoa - ps13
Myriazoa - ps14
Parahoxozoa - ps15
Bilateria - ps16
Protostomia - ps17
Ecdysozoa - ps18
Arthropoda - ps19
Mandibulata - ps20
Pancrustacea - ps21
Insecta - ps22
Eumetabola - ps23
Holometabola/Psocodea - ps24
Holometabola - ps25
Aparaglossata - ps26
Mecopterida - ps27
Diptera - ps28
Bibiono./Cyclorrhapha - ps29
Cyclorrhapha - ps30
Calyptratae/Drosophila - ps31
Drosophila - ps32
Sophophora - ps33
Obscura/Melanogaster_group - ps34
D_melanogaster_group - ps35
D_melanogaster_subgroup - ps36
sim./sech./mel. group - ps37
Drosophila_melanogaster - ps38
p-value
7e-06
7e-05
6e-04
5e-03
5e-02
Log-odds
0.5
1.0
1.5
2.0
2.5
3.0
3.5
4.0

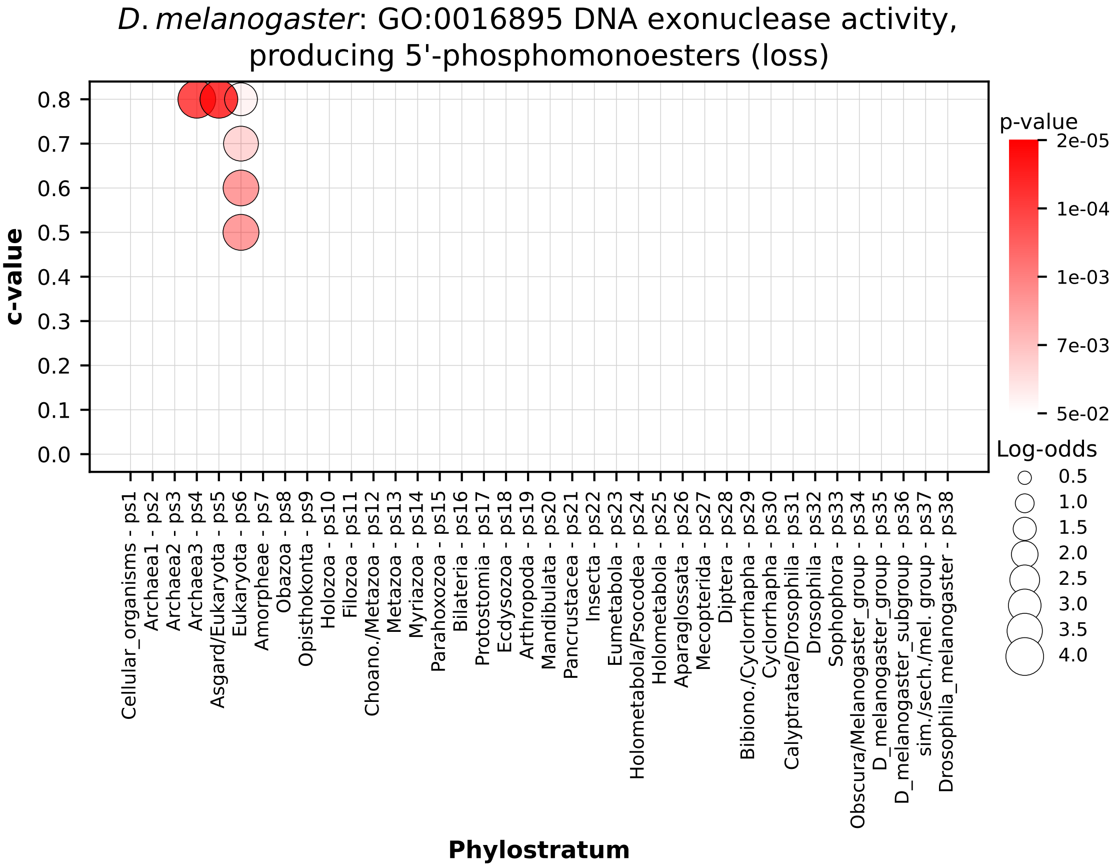

D. melanogaster: GO:0016895 DNA exonuclease activity, producing 5'-phosphomonoesters (loss)
c-value
0.0
0.1
0.2
0.3
0.4
0.5
0.6
0.7
0.8
Phylostratum
Cellular_organisms - ps1
Archaea1 - ps2
Archaea2 - ps3
Archaea3 - ps4
Asgard/Eukaryota - ps5
Eukaryota - ps6
Amorpheae - ps7
Obazoa - ps8
Opisthokonta - ps9
Holozoa - ps10
Filozoa - ps11
Choano./Metazoa - ps12
Metazoa - ps13
Myriazoa - ps14
Parahoxozoa - ps15
Bilateria - ps16
Protostomia - ps17
Ecdysozoa - ps18
Arthropoda - ps19
Mandibulata - ps20
Pancrustacea - ps21
Insecta - ps22
Eumetabola - ps23
Holometabola/Psocodea - ps24
Holometabola - ps25
Aparaglossata - ps26
Mecopterida - ps27
Diptera - ps28
Bibiono./Cyclorrhapha - ps29
Cyclorrhapha - ps30
Calyptratae/Drosophila - ps31
Drosophila - ps32
Sophophora - ps33
Obscura/Melanogaster_group - ps34
D_melanogaster_group - ps35
D_melanogaster_subgroup - ps36
sim./sech./mel. group - ps37
Drosophila_melanogaster - ps38
p-value
2e-05
1e-04
1e-03
7e-03
5e-02
Log-odds
0.5
1.0
1.5
2.0
2.5
3.0
3.5
4.0

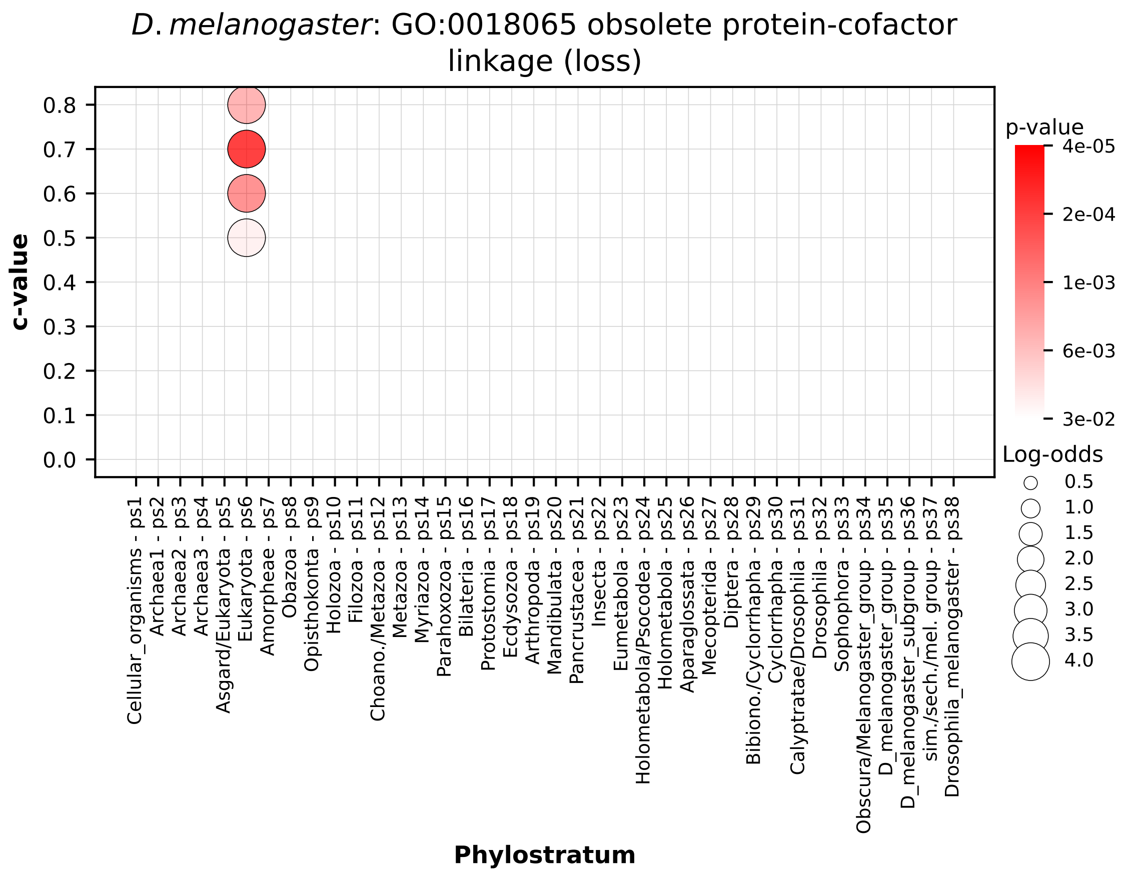

D. melanogaster: GO:0018065 obsolete protein-cofactor linkage (loss)
c-value
0.0
0.1
0.2
0.3
0.4
0.5
0.6
0.7
0.8
Phylostratum
Cellular_organisms - ps1
Archaea1 - ps2
Archaea2 - ps3
Archaea3 - ps4
Asgard/Eukaryota - ps5
Eukaryota - ps6
Amorpheae - ps7
Obazoa - ps8
Opisthokonta - ps9
Holozoa - ps10
Filozoa - ps11
Choano./Metazoa - ps12
Metazoa - ps13
Myriazoa - ps14
Parahoxozoa - ps15
Bilateria - ps16
Protostomia - ps17
Ecdysozoa - ps18
Arthropoda - ps19
Mandibulata - ps20
Pancrustacea - ps21
Insecta - ps22
Eumetabola - ps23
Holometabola/Psocodea - ps24
Holometabola - ps25
Aparaglossata - ps26
Mecopterida - ps27
Diptera - ps28
Bibiono./Cyclorrhapha - ps29
Cyclorrhapha - ps30
Calyptratae/Drosophila - ps31
Drosophila - ps32
Sophophora - ps33
Obscura/Melanogaster_group - ps34
D_melanogaster_group - ps35
D_melanogaster_subgroup - ps36
sim./sech./mel. group - ps37
Drosophila_melanogaster - ps38
p-value
4e-05
2e-04
1e-03
6e-03
3e-02
Log-odds
0.5
1.0
1.5
2.0
2.5
3.0
3.5
4.0

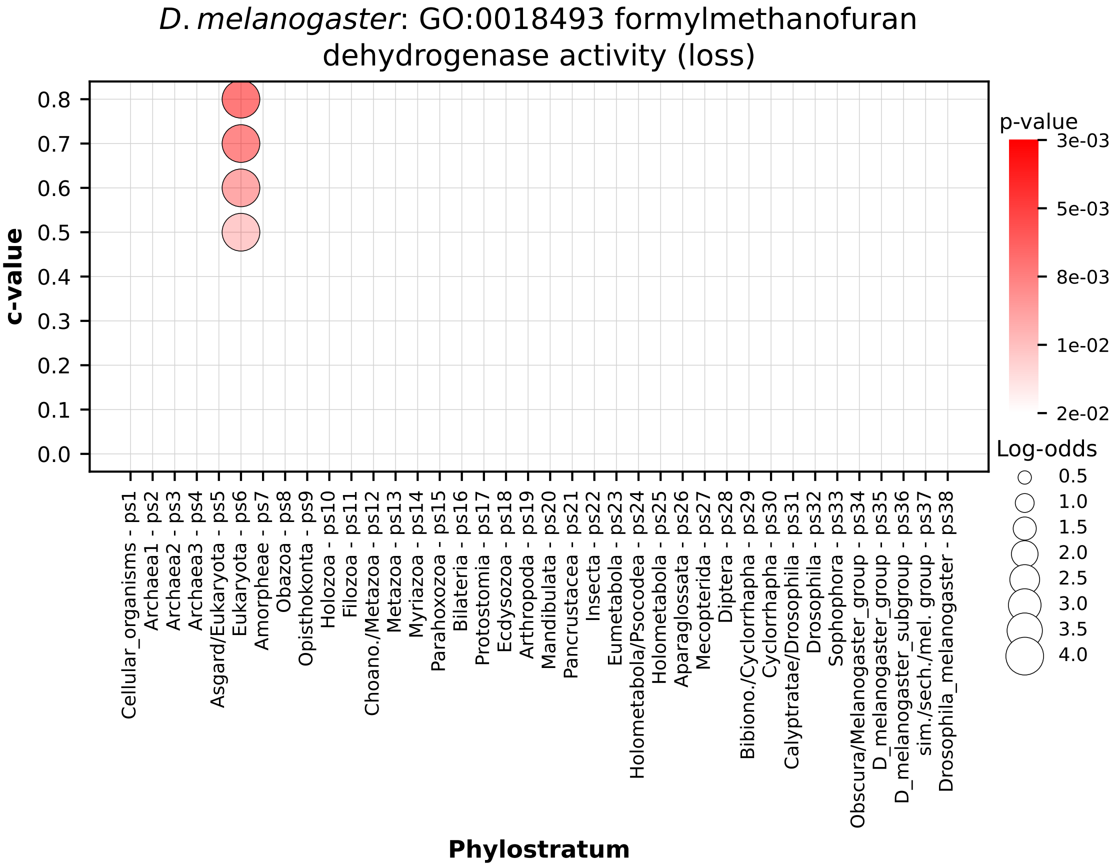

D. melanogaster: GO:0018493 formylmethanofuran dehydrogenase activity (loss)
c-value
0.8
0.7
0.6
0.5
0.4
0.3
0.2
0.1
0.0
p-value
3e-03
5e-03
8e-03
1e-02
2e-02
Log-odds
0.5
1.0
1.5
2.0
2.5
3.0
3.5
4.0
Cellular_organisms - ps1
Archaea1 - ps2
Archaea2 - ps3
Archaea3 - ps4
Asgard/Eukaryota - ps5
Eukaryota - ps6
Amorpheae - ps7
Obazoa - ps8
Opisthokonta - ps9
Holozoa - ps10
Filozoa - ps11
Choano./Metazoa - ps12
Metazoa - ps13
Myriazoa - ps14
Parahoxozoa - ps15
Bilateria - ps16
Protostomia - ps17
Ecdysozoa - ps18
Arthropoda - ps19
Mandibulata - ps20
Pancrustacea - ps21
Insecta - ps22
Eumetabola - ps23
Holometabola/Psocodea - ps24
Holometabola - ps25
Aparaglossata - ps26
Mecopterida - ps27
Diptera - ps28
Bibiono./Cyclorrhapha - ps29
Cyclorrhapha - ps30
Calyptratae/Drosophila - ps31
Drosophila - ps32
Sophophora - ps33
Obscura/Melanogaster_group - ps34
D_melanogaster_group - ps35
D_melanogaster_subgroup - ps36
sim./sech./mel. group - ps37
Drosophila_melanogaster - ps38
Phylostratum

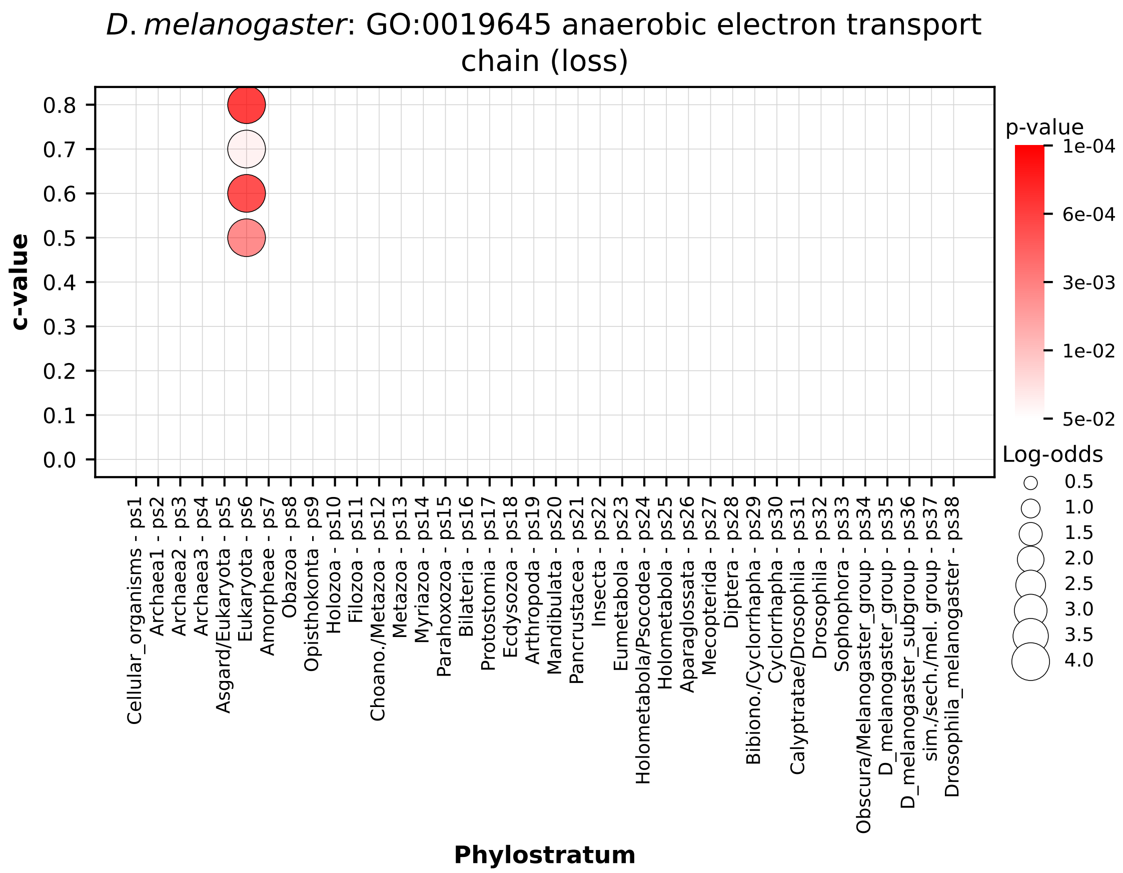
D. melanogaster: GO:0019645 anaerobic electron transport chain (loss)
c-value
0.0
0.1
0.2
0.3
0.4
0.5
0.6
0.7
0.8
Phylostratum
Cellular_organisms - ps1
Archaea1 - ps2
Archaea2 - ps3
Archaea3 - ps4
Asgard/Eukaryota - ps5
Eukaryota - ps6
Amorpheae - ps7
Obazoa - ps8
Opisthokonta - ps9
Holozoa - ps10
Filozoa - ps11
Choano./Metazoa - ps12
Metazoa - ps13
Myriazoa - ps14
Parahoxozoa - ps15
Bilateria - ps16
Protostomia - ps17
Ecdysozoa - ps18
Arthropoda - ps19
Mandibulata - ps20
Pancrustacea - ps21
Insecta - ps22
Eumetabola - ps23
Holometabola/Psocodea - ps24
Holometabola - ps25
Aparaglossata - ps26
Mecopterida - ps27
Diptera - ps28
Bibiono./Cyclorrhapha - ps29
Cyclorrhapha - ps30
Calyptratae/Drosophila - ps31
Drosophila - ps32
Sophophora - ps33
Obscura/Melanogaster_group - ps34
D_melanogaster_group - ps35
D_melanogaster_subgroup - ps36
sim./sech./mel. group - ps37
Drosophila_melanogaster - ps38
p-value
1e-04
6e-04
3e-03
1e-02
5e-02
Log-odds
0.5
1.0
1.5
2.0
2.5
3.0
3.5
4.0

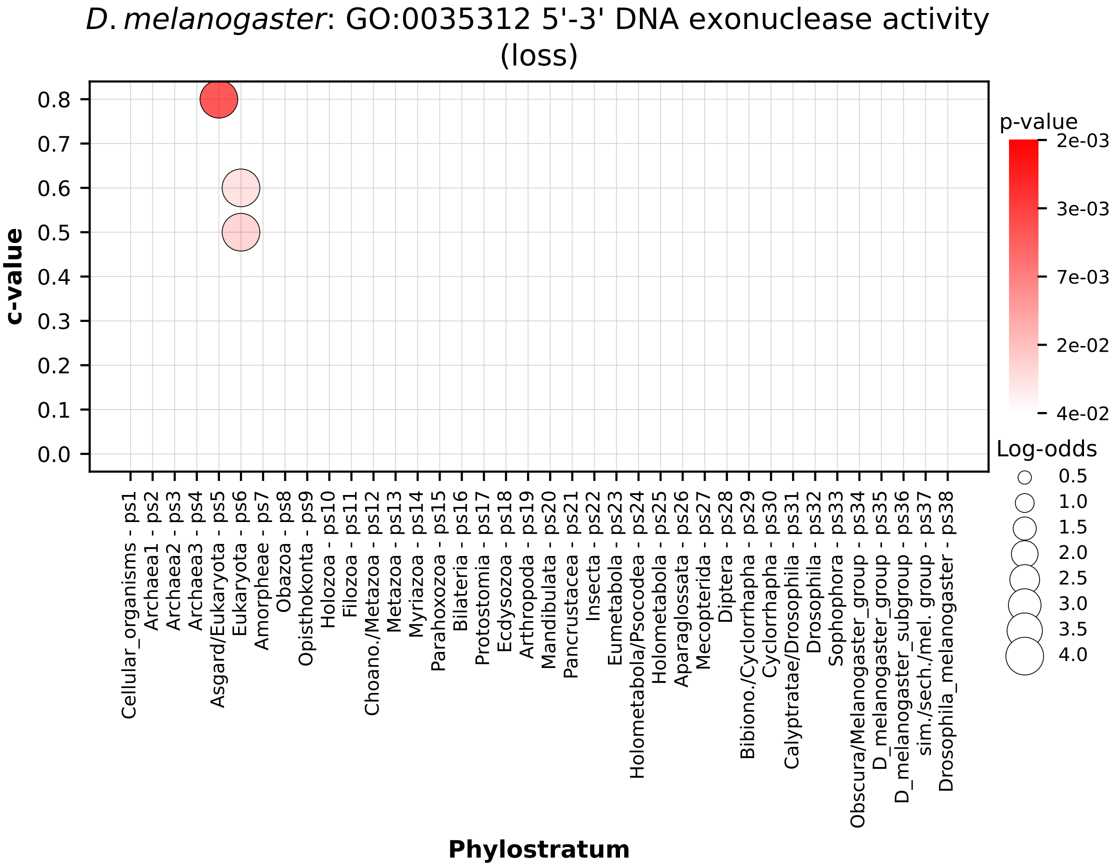

D. melanogaster: GO:0035312 5'-3' DNA exonuclease activity (loss)
c-value
0.0
0.1
0.2
0.3
0.4
0.5
0.6
0.7
0.8
Phylostratum
Cellular_organisms - ps1
Archaea1 - ps2
Archaea2 - ps3
Archaea3 - ps4
Asgard/Eukaryota - ps5
Eukaryota - ps6
Amorpheae - ps7
Obazoa - ps8
Opisthokonta - ps9
Holozoa - ps10
Filozoa - ps11
Choano./Metazoa - ps12
Metazoa - ps13
Myriazoa - ps14
Parahoxozoa - ps15
Bilateria - ps16
Protostomia - ps17
Ecdysozoa - ps18
Arthropoda - ps19
Mandibulata - ps20
Pancrustacea - ps21
Insecta - ps22
Eumetabola - ps23
Holometabola/Psocodea - ps24
Holometabola - ps25
Aparaglossata - ps26
Mecopterida - ps27
Diptera - ps28
Bibiono./Cyclorrhapha - ps29
Cyclorrhapha - ps30
Calyptratae/Drosophila - ps31
Drosophila - ps32
Sophophora - ps33
Obscura/Melanogaster_group - ps34
D_melanogaster_group - ps35
D_melanogaster_subgroup - ps36
sim./sech./mel. group - ps37
Drosophila_melanogaster - ps38
p-value
2e-03
3e-03
7e-03
2e-02
4e-02
Log-odds
0.5
1.0
1.5
2.0
2.5
3.0
3.5
4.0

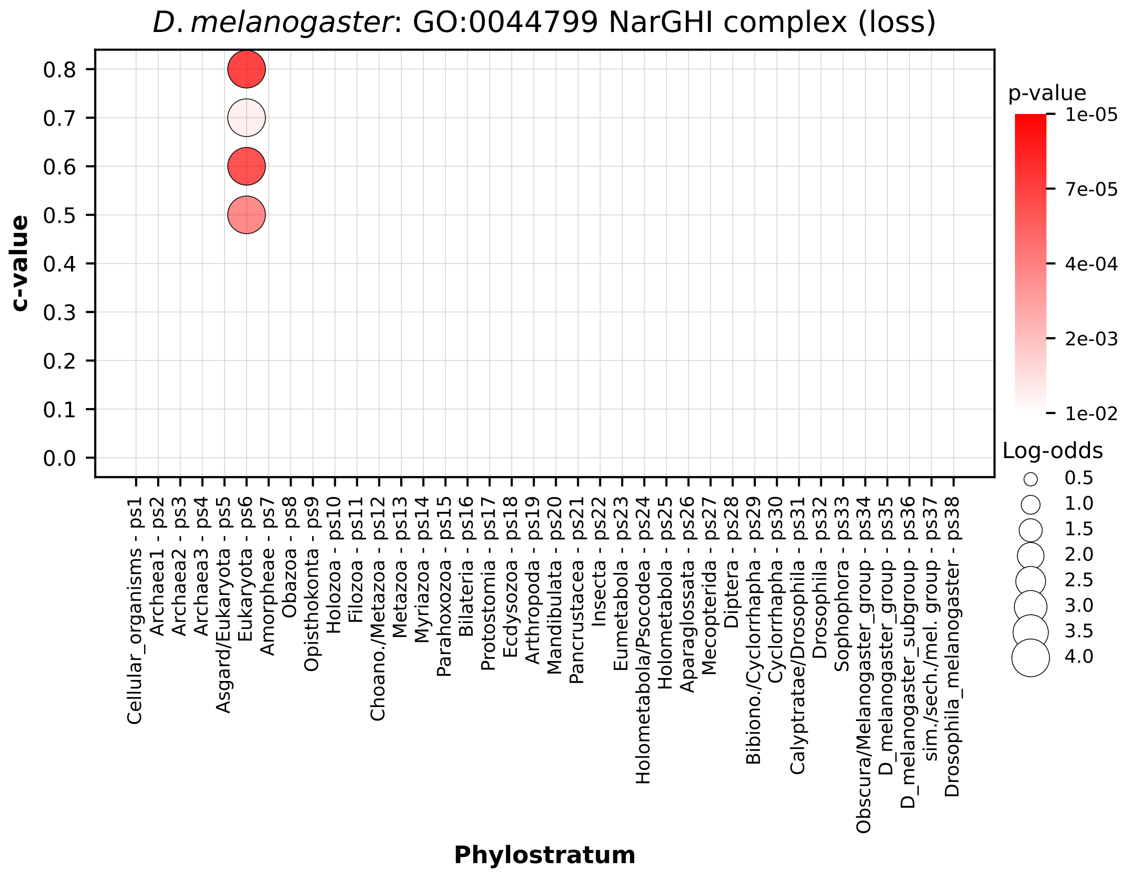

D. melanogaster: GO:0044799 NarGHI complex (loss)
c-value
0.8
0.7
0.6
0.5
0.4
0.3
0.2
0.1
0.0
Phylostratum
Cellular_organisms - ps1
Archaea1 - ps2
Archaea2 - ps3
Archaea3 - ps4
Asgard/Eukaryota - ps5
Eukaryota - ps6
Amorpheae - ps7
Obazoa - ps8
Opisthokonta - ps9
Holozoa - ps10
Filozoa - ps11
Choano./Metazoa - ps12
Metazoa - ps13
Myriazoa - ps14
Parahoxozoa - ps15
Bilateria - ps16
Protostomia - ps17
Ecdysozoa - ps18
Arthropoda - ps19
Mandibulata - ps20
Pancrustacea - ps21
Insecta - ps22
Eumetabola - ps23
Holometabola/Psocodea - ps24
Holometabola - ps25
Aparaglossata - ps26
Mecopterida - ps27
Diptera - ps28
Bibiono./Cyclorrhapha - ps29
Cyclorrhapha - ps30
Calyptratae/Drosophila - ps31
Drosophila - ps32
Sophophora - ps33
Obscura/Melanogaster_group - ps34
D_melanogaster_group - ps35
D_melanogaster_subgroup - ps36
sim./sech./mel. group - ps37
Drosophila_melanogaster - ps38
p-value
1e-05
7e-05
4e-04
2e-03
1e-02
Log-odds
0.5
1.0
1.5
2.0
2.5
3.0
3.5
4.0

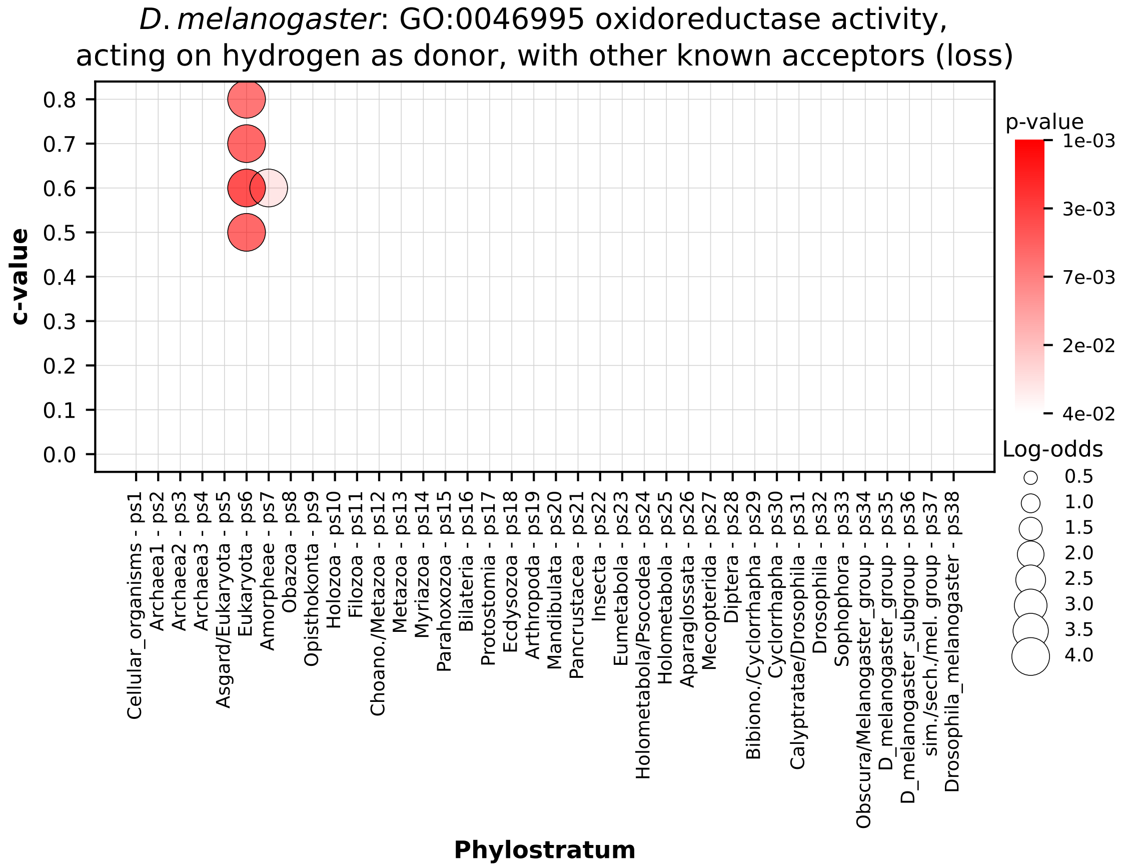

D. melanogaster: GO:0046995 oxidoreductase activity, acting on hydrogen as donor, with other known acceptors (loss)
c-value
0.8
0.7
0.6
0.5
0.4
0.3
0.2
0.1
0.0
Phylostratum
Cellular_organisms - ps1
Archaea1 - ps2
Archaea2 - ps3
Archaea3 - ps4
Asgard/Eukaryota - ps5
Eukaryota - ps6
Amorpheae - ps7
Obazoa - ps8
Opisthokonta - ps9
Holozoa - ps10
Filozoa - ps11
Choano./Metazoa - ps12
Metazoa - ps13
Myriazoa - ps14
Parahoxozoa - ps15
Bilateria - ps16
Protostomia - ps17
Ecdysozoa - ps18
Arthropoda - ps19
Mandibulata - ps20
Pancrustacea - ps21
Insecta - ps22
Eumetabola - ps23
Holometabola/Psocodea - ps24
Holometabola - ps25
Aparaglossata - ps26
Mecopterida - ps27
Diptera - ps28
Bibiono./Cyclorrhapha - ps29
Cyclorrhapha - ps30
Calyptratae/Drosophila - ps31
Drosophila - ps32
Sophophora - ps33
Obscura/Melanogaster_group - ps34
D_melanogaster_group - ps35
D_melanogaster_subgroup - ps36
sim./sech./mel. group - ps37
Drosophila_melanogaster - ps38
p-value
1e-03
3e-03
7e-03
2e-02
4e-02
Log-odds
0.5
1.0
1.5
2.0
2.5
3.0
3.5
4.0

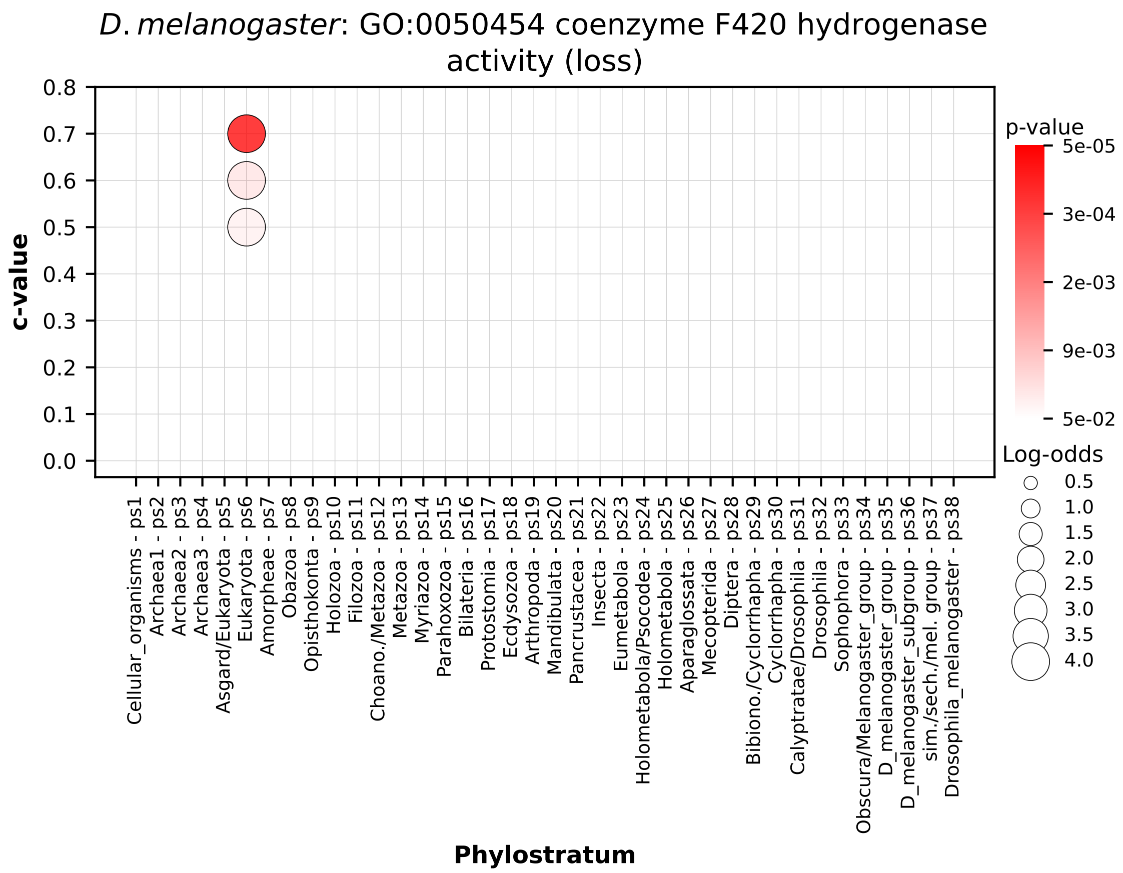

D. melanogaster: GO:0050454 coenzyme F420 hydrogenase activity (loss)
c-value
0.0
0.1
0.2
0.3
0.4
0.5
0.6
0.7
0.8
Phylostratum
Cellular_organisms - ps1
Archaea1 - ps2
Archaea2 - ps3
Archaea3 - ps4
Asgard/Eukaryota - ps5
Eukaryota - ps6
Amorpheae - ps7
Obazoa - ps8
Opisthokonta - ps9
Holozoa - ps10
Filozoa - ps11
Choano./Metazoa - ps12
Metazoa - ps13
Myriazoa - ps14
Parahoxozoa - ps15
Bilateria - ps16
Protostomia - ps17
Ecdysozoa - ps18
Arthropoda - ps19
Mandibulata - ps20
Pancrustacea - ps21
Insecta - ps22
Eumetabola - ps23
Holometabola/Psocodea - ps24
Holometabola - ps25
Aparaglossata - ps26
Mecopterida - ps27
Diptera - ps28
Bibiono./Cyclorrhapha - ps29
Cyclorrhapha - ps30
Calyptratae/Drosophila - ps31
Drosophila - ps32
Sophophora - ps33
Obscura/Melanogaster_group - ps34
D_melanogaster_group - ps35
D_melanogaster_subgroup - ps36
sim./sech./mel. group - ps37
Drosophila_melanogaster - ps38
p-value
5e-05
3e-04
2e-03
9e-03
5e-02
Log-odds
0.5
1.0
1.5
2.0
2.5
3.0
3.5
4.0

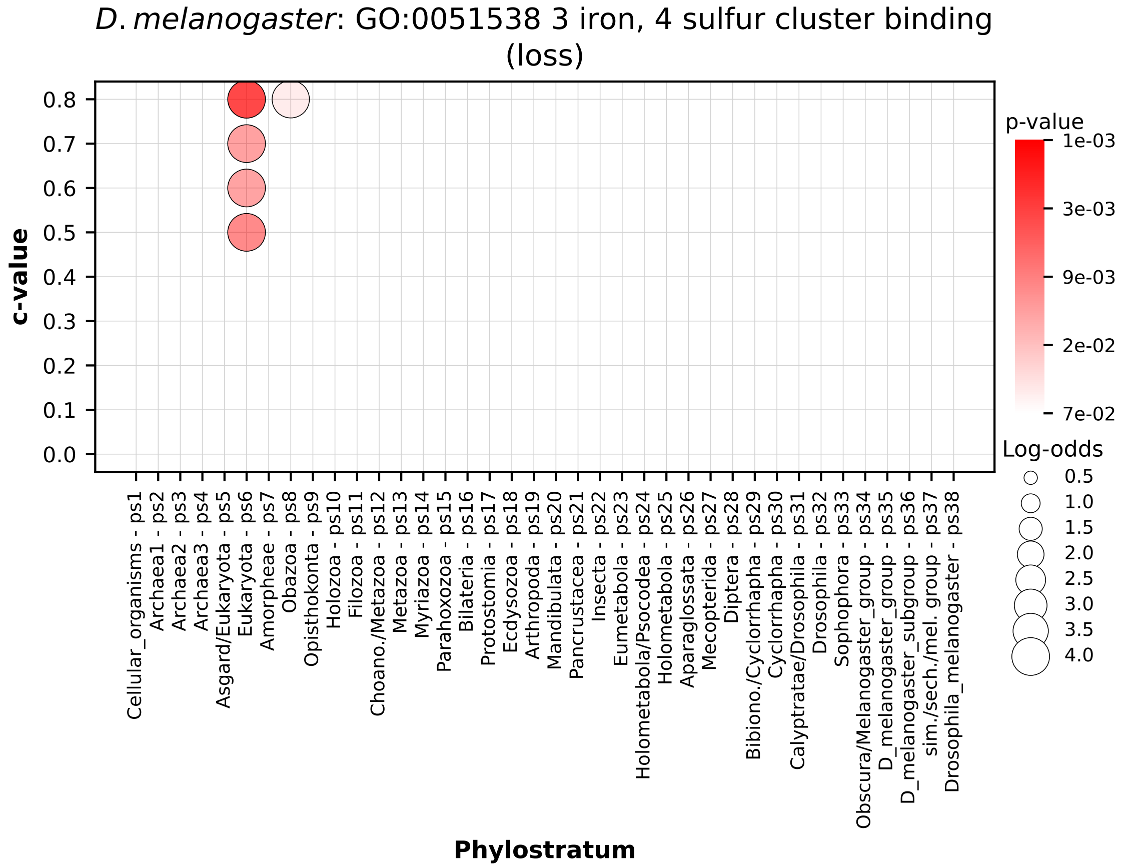
D. melanogaster: GO:0051538 3 iron, 4 sulfur cluster binding (loss)
c-value
0.8
0.7
0.6
0.5
0.4
0.3
0.2
0.1
0.0
Phylostratum
Cellular_organisms - ps1
Archaea1 - ps2
Archaea2 - ps3
Archaea3 - ps4
Asgard/Eukaryota - ps5
Eukaryota - ps6
Amorpheae - ps7
Obazoa - ps8
Opisthokonta - ps9
Holozoa - ps10
Filozoa - ps11
Choano./Metazoa - ps12
Metazoa - ps13
Myriazoa - ps14
Parahoxozoa - ps15
Bilateria - ps16
Protostomia - ps17
Ecdysozoa - ps18
Arthropoda - ps19
Mandibulata - ps20
Pancrustacea - ps21
Insecta - ps22
Eumetabola - ps23
Holometabola/Psocodea - ps24
Holometabola - ps25
Aparaglossata - ps26
Mecopterida - ps27
Diptera - ps28
Bibiono./Cyclorrhapha - ps29
Cyclorrhapha - ps30
Calyptratae/Drosophila - ps31
Drosophila - ps32
Sophophora - ps33
Obscura/Melanogaster_group - ps34
D_melanogaster_group - ps35
D_melanogaster_subgroup - ps36
sim./sech./mel. group - ps37
Drosophila_melanogaster - ps38
p-value
1e-03
3e-03
9e-03
2e-02
7e-02
Log-odds
0.5
1.0
1.5
2.0
2.5
3.0
3.5
4.0

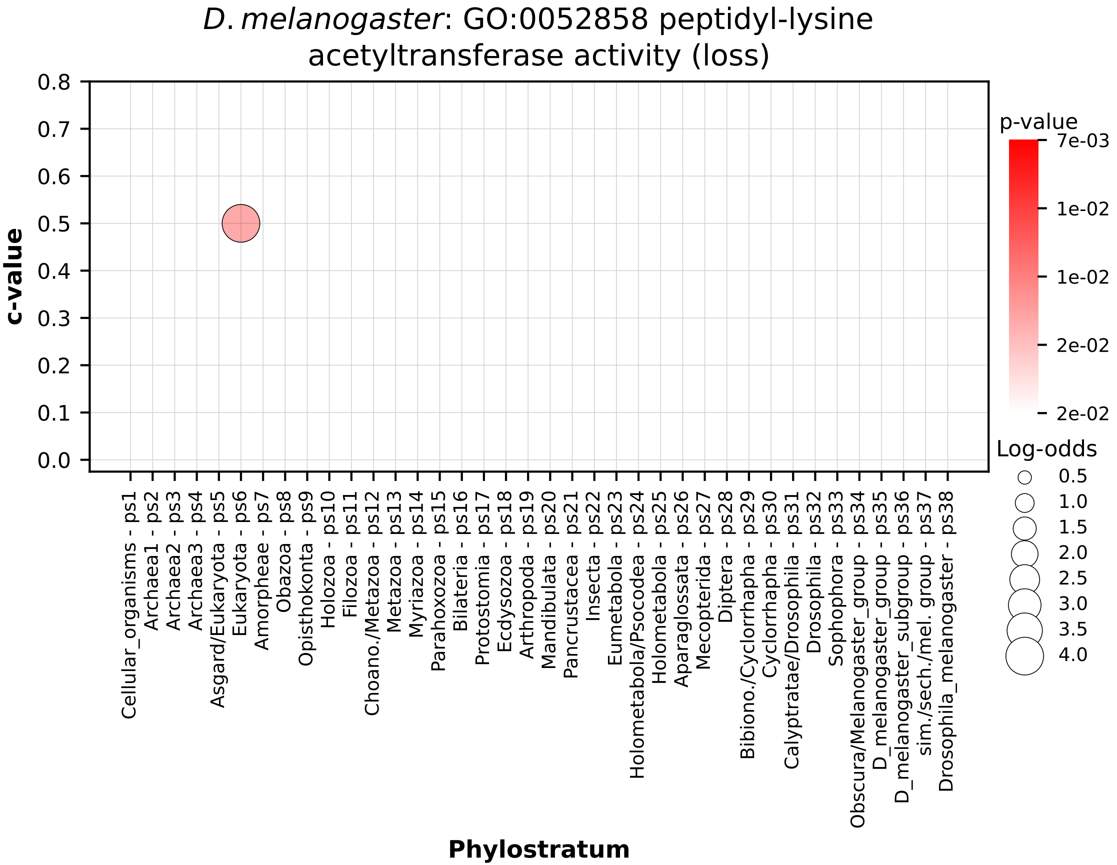

D. melanogaster: GO:0052858 peptidyl-lysine acetyltransferase activity (loss)
c-value
0.8
0.7
0.6
0.5
0.4
0.3
0.2
0.1
0.0
Phylostratum
Cellular_organisms - ps1
Archaea1 - ps2
Archaea2 - ps3
Archaea3 - ps4
Asgard/Eukaryota - ps5
Eukaryota - ps6
Amorpheae - ps7
Obazoa - ps8
Opisthokonta - ps9
Holozoa - ps10
Filozoa - ps11
Choano./Metazoa - ps12
Metazoa - ps13
Myriazoa - ps14
Parahoxozoa - ps15
Bilateria - ps16
Protostomia - ps17
Ecdysozoa - ps18
Arthropoda - ps19
Mandibulata - ps20
Pancrustacea - ps21
Insecta - ps22
Eumetabola - ps23
Holometabola/Psocodea - ps24
Holometabola - ps25
Aparaglossata - ps26
Mecopterida - ps27
Diptera - ps28
Bibiono./Cyclorrhapha - ps29
Cyclorrhapha - ps30
Calyptratae/Drosophila - ps31
Drosophila - ps32
Sophophora - ps33
Obscura/Melanogaster_group - ps34
D_melanogaster_group - ps35
D_melanogaster_subgroup - ps36
sim./sech./mel. group - ps37
Drosophila_melanogaster - ps38
p-value
7e-03
1e-02
1e-02
2e-02
2e-02
Log-odds
0.5
1.0
1.5
2.0
2.5
3.0
3.5
4.0

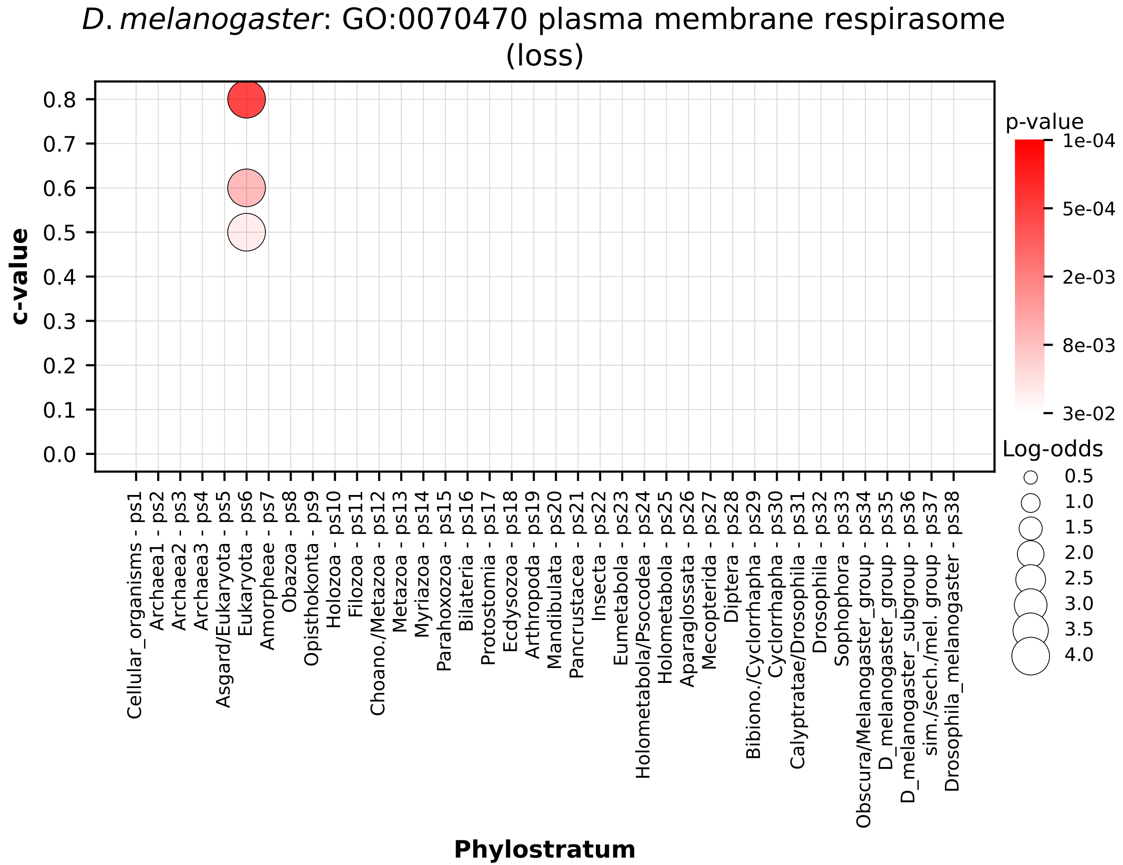

D. melanogaster: GO:0070470 plasma membrane respirasome (loss)
c-value
0.0
0.1
0.2
0.3
0.4
0.5
0.6
0.7
0.8
Phylostratum
Cellular_organisms - ps1
Archaea1 - ps2
Archaea2 - ps3
Archaea3 - ps4
Asgard/Eukaryota - ps5
Eukaryota - ps6
Amorpheae - ps7
Obazoa - ps8
Opisthokonta - ps9
Holozoa - ps10
Filozoa - ps11
Choano./Metazoa - ps12
Metazoa - ps13
Myriazoa - ps14
Parahoxozoa - ps15
Bilateria - ps16
Protostomia - ps17
Ecdysozoa - ps18
Arthropoda - ps19
Mandibulata - ps20
Pancrustacea - ps21
Insecta - ps22
Eumetabola - ps23
Holometabola/Psocodea - ps24
Holometabola - ps25
Aparaglossata - ps26
Mecopterida - ps27
Diptera - ps28
Bibiono./Cyclorrhapha - ps29
Cyclorrhapha - ps30
Calyptratae/Drosophila - ps31
Drosophila - ps32
Sophophora - ps33
Obscura/Melanogaster_group - ps34
D_melanogaster_group - ps35
D_melanogaster_subgroup - ps36
sim./sech./mel. group - ps37
Drosophila_melanogaster - ps38
p-value
1e-04
5e-04
2e-03
8e-03
3e-02
Log-odds
0.5
1.0
1.5
2.0
2.5
3.0
3.5
4.0

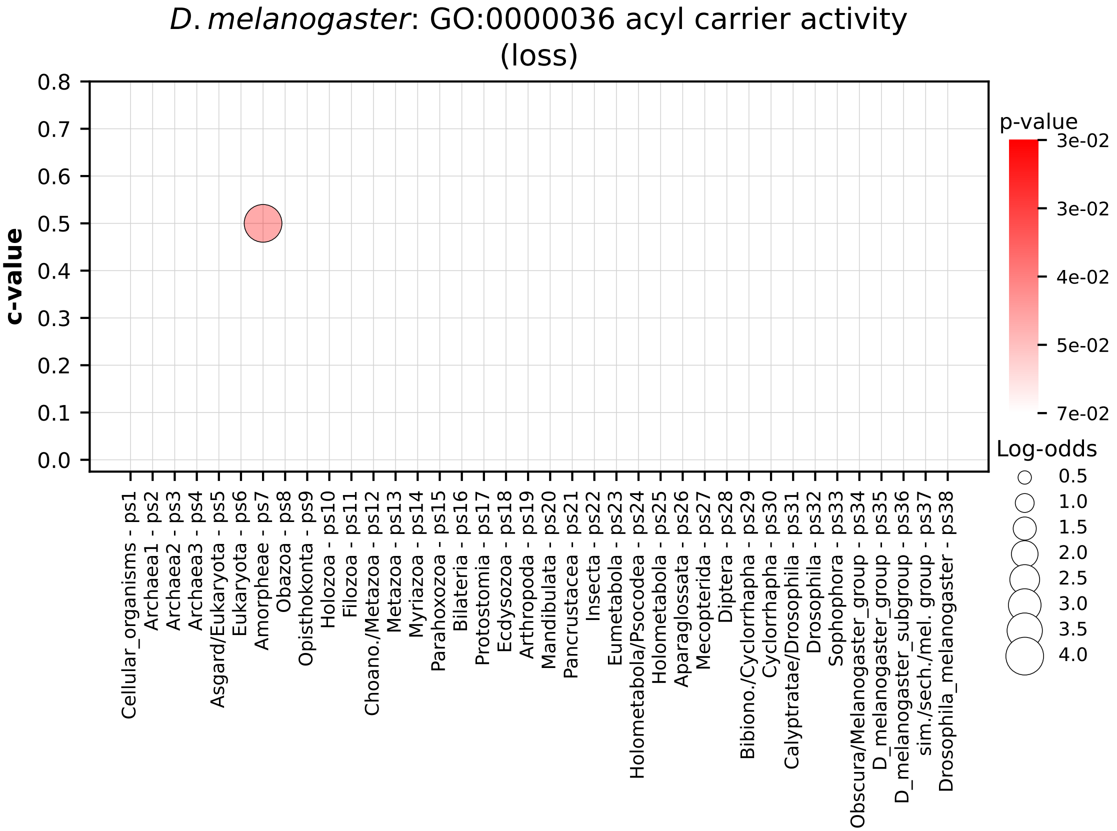

D. melanogaster: GO:0000036 acyl carrier activity
(loss)
c-value
0.8
0.7
0.6
0.5
0.4
0.3
0.2
0.1
0.0
Phylostratum
Cellular_organisms - ps1
Archaea1 - ps2
Archaea2 - ps3
Archaea3 - ps4
Asgard/Eukaryota - ps5
Eukaryota - ps6
Amorpheae - ps7
Obazoa - ps8
Opisthokonta - ps9
Holozoa - ps10
Filozoa - ps11
Choano./Metazoa - ps12
Metazoa - ps13
Myriazoa - ps14
Parahoxozoa - ps15
Bilateria - ps16
Protostomia - ps17
Ecdysozoa - ps18
Arthropoda - ps19
Mandibulata - ps20
Pancrustacea - ps21
Insecta - ps22
Eumetabola - ps23
Holometabola/Psocodea - ps24
Holometabola - ps25
Aparaglossata - ps26
Mecopterida - ps27
Diptera - ps28
Bibiono./Cyclorrhapha - ps29
Cyclorrhapha - ps30
Calyptratae/Drosophila - ps31
Drosophila - ps32
Sophophora - ps33
Obscura/Melanogaster_group - ps34
D_melanogaster_group - ps35
D_melanogaster_subgroup - ps36
sim./sech./mel. group - ps37
Drosophila_melanogaster - ps38
p-value
3e-02
3e-02
4e-02
5e-02
7e-02
Log-odds
0.5
1.0
1.5
2.0
2.5
3.0
3.5
4.0

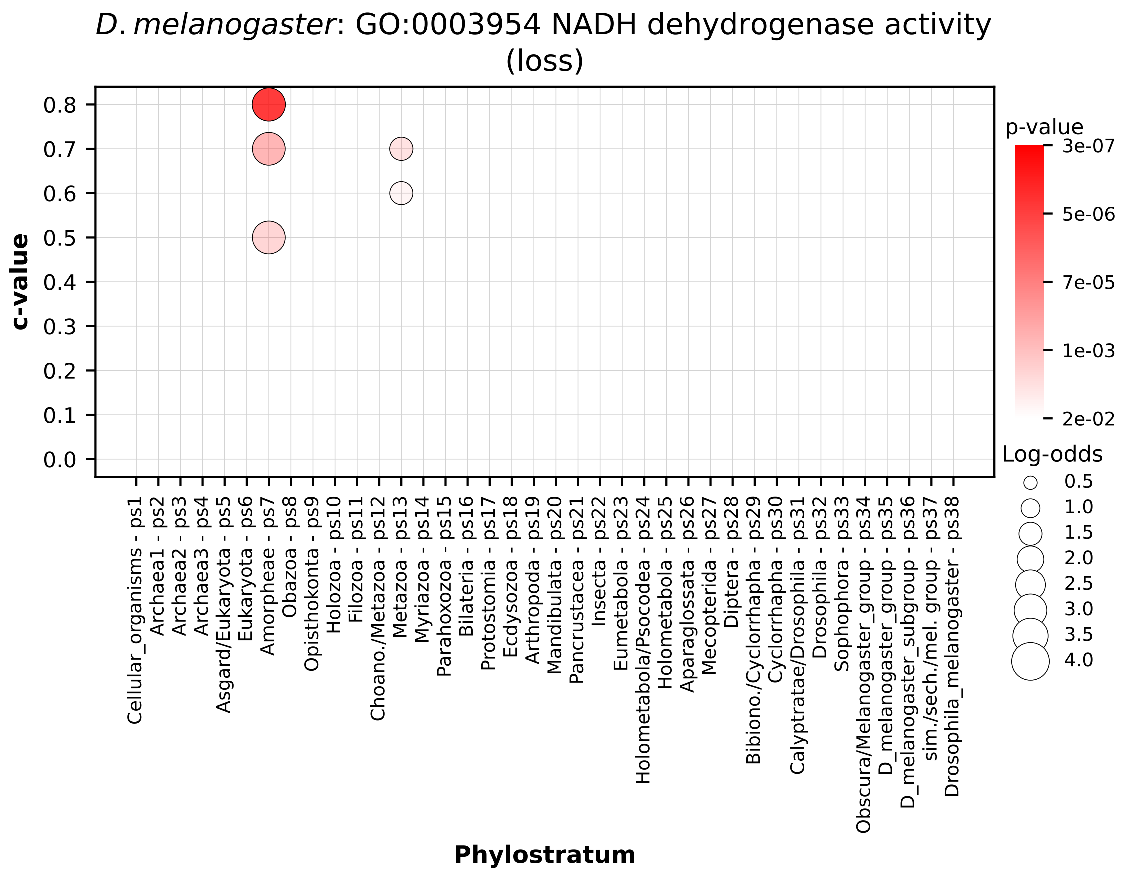

D. melanogaster: GO:0003954 NADH dehydrogenase activity
(loss)
c-value
0.8
0.7
0.6
0.5
0.4
0.3
0.2
0.1
0.0
Phylostratum
Cellular_organisms - ps1
Archaea1 - ps2
Archaea2 - ps3
Archaea3 - ps4
Asgard/Eukaryota - ps5
Eukaryota - ps6
Amorpheae - ps7
Obazoa - ps8
Opisthokonta - ps9
Holozoa - ps10
Filozoa - ps11
Choano./Metazoa - ps12
Metazoa - ps13
Myriazoa - ps14
Parahoxozoa - ps15
Bilateria - ps16
Protostomia - ps17
Ecdysozoa - ps18
Arthropoda - ps19
Mandibulata - ps20
Pancrustacea - ps21
Insecta - ps22
Eumetabola - ps23
Holometabola/Psocodea - ps24
Holometabola - ps25
Aparaglossata - ps26
Mecopterida - ps27
Diptera - ps28
Bibiono./Cyclorrhapha - ps29
Cyclorrhapha - ps30
Calyptratae/Drosophila - ps31
Drosophila - ps32
Sophophora - ps33
Obscura/Melanogaster_group - ps34
D_melanogaster_group - ps35
D_melanogaster_subgroup - ps36
sim./sech./mel. group - ps37
Drosophila_melanogaster - ps38
p-value
3e-07
5e-06
7e-05
1e-03
2e-02
Log-odds
0.5
1.0
1.5
2.0
2.5
3.0
3.5
4.0

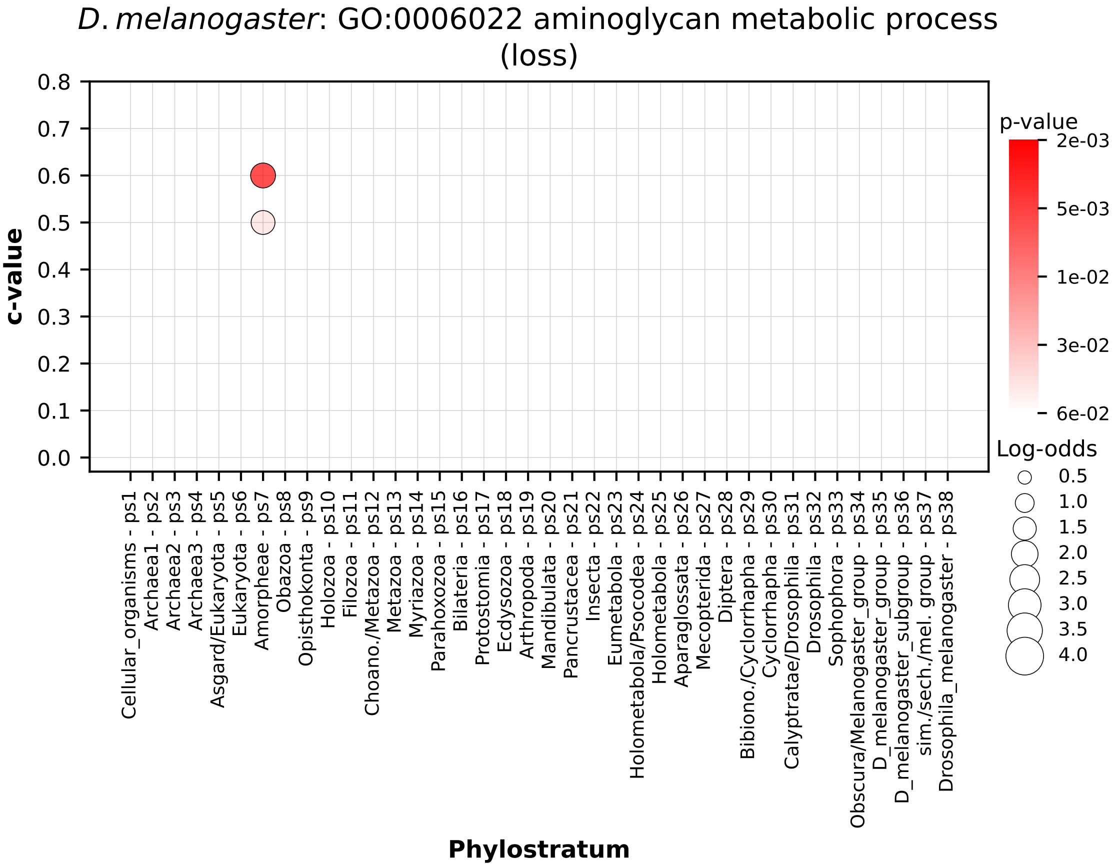

D. melanogaster: GO:0006022 aminoglycan metabolic process (loss)
c-value
0.8
0.7
0.6
0.5
0.4
0.3
0.2
0.1
0.0
Cellular_organisms - ps1
Archaea1 - ps2
Archaea2 - ps3
Archaea3 - ps4
Asgard/Eukaryota - ps5
Eukaryota - ps6
Amorpheae - ps7
Obazoa - ps8
Opisthokonta - ps9
Holozoa - ps10
Filozoa - ps11
Choano./Metazoa - ps12
Metazoa - ps13
Myriazoa - ps14
Parahoxozoa - ps15
Bilateria - ps16
Protostomia - ps17
Ecdysozoa - ps18
Arthropoda - ps19
Mandibulata - ps20
Pancrustacea - ps21
Insecta - ps22
Eumetabola - ps23
Holometabola/Psocodea - ps24
Holometabola - ps25
Aparaglossata - ps26
Mecopterida - ps27
Diptera - ps28
Bibiono./Cyclorrhapha - ps29
Cyclorrhapha - ps30
Calyptratae/Drosophila - ps31
Drosophila - ps32
Sophophora - ps33
Obscura/Melanogaster_group - ps34
D_melanogaster_group - ps35
D_melanogaster_subgroup - ps36
sim./sech./mel. group - ps37
Drosophila_melanogaster - ps38
Phylostratum
p-value
2e-03
5e-03
1e-02
3e-02
6e-02
Log-odds
0.5
1.0
1.5
2.0
2.5
3.0
3.5
4.0

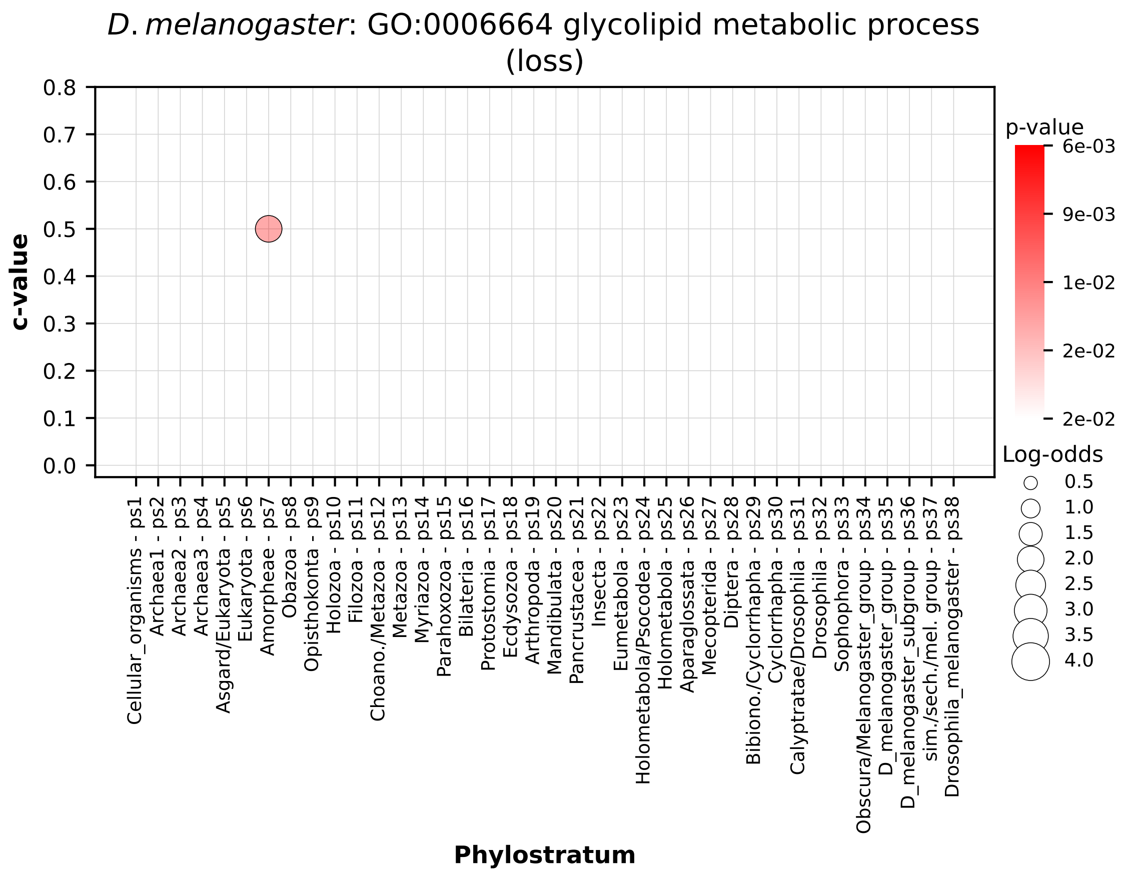

D. melanogaster: GO:0006664 glycolipid metabolic process (loss)
c-value
0.8
0.7
0.6
0.5
0.4
0.3
0.2
0.1
0.0
p-value
6e-03
9e-03
1e-02
2e-02
2e-02
Log-odds
0.5
1.0
1.5
2.0
2.5
3.0
3.5
4.0
Cellular_organisms - ps1
Archaea1 - ps2
Archaea2 - ps3
Archaea3 - ps4
Asgard/Eukaryota - ps5
Eukaryota - ps6
Amorpheae - ps7
Obazoa - ps8
Opisthokonta - ps9
Holozoa - ps10
Filozoa - ps11
Choano./Metazoa - ps12
Metazoa - ps13
Myriazoa - ps14
Parahoxozoa - ps15
Bilateria - ps16
Protostomia - ps17
Ecdysozoa - ps18
Arthropoda - ps19
Mandibulata - ps20
Pancrustacea - ps21
Insecta - ps22
Eumetabola - ps23
Holometabola/Psocodea - ps24
Holometabola - ps25
Aparaglossata - ps26
Mecopterida - ps27
Diptera - ps28
Bibiono./Cyclorrhapha - ps29
Cyclorrhapha - ps30
Calyptratae/Drosophila - ps31
Drosophila - ps32
Sophophora - ps33
Obscura/Melanogaster_group - ps34
D_melanogaster_group - ps35
D_melanogaster_subgroup - ps36
sim./sech./mel. group - ps37
Drosophila_melanogaster - ps38
Phylostratum

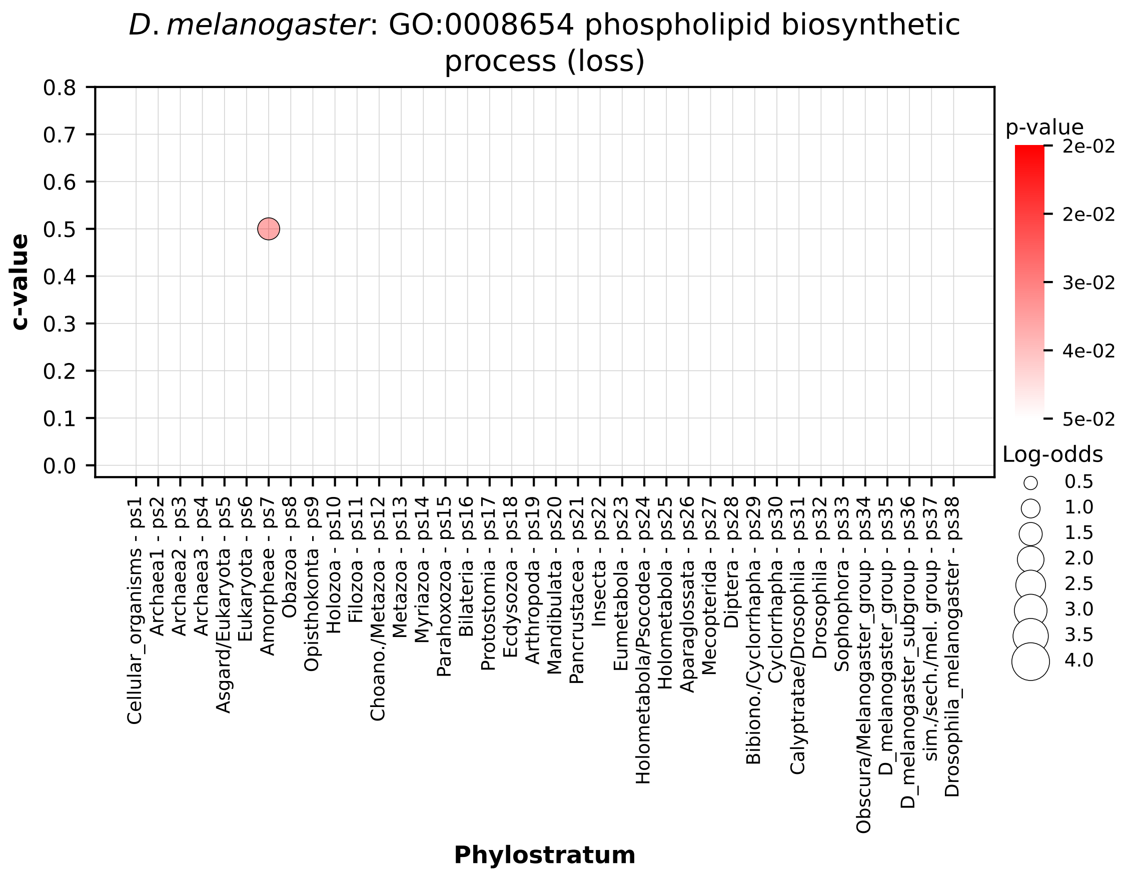

D. melanogaster: GO:0008654 phospholipid biosynthetic process (loss)
c-value
0.8
0.7
0.6
0.5
0.4
0.3
0.2
0.1
0.0
Phylostratum
Cellular_organisms - ps1
Archaea1 - ps2
Archaea2 - ps3
Archaea3 - ps4
Asgard/Eukaryota - ps5
Eukaryota - ps6
Amorpheae - ps7
Obazoa - ps8
Opisthokonta - ps9
Holozoa - ps10
Filozoa - ps11
Choano./Metazoa - ps12
Metazoa - ps13
Myriazoa - ps14
Parahoxozoa - ps15
Bilateria - ps16
Protostomia - ps17
Ecdysozoa - ps18
Arthropoda - ps19
Mandibulata - ps20
Pancrustacea - ps21
Insecta - ps22
Eumetabola - ps23
Holometabola/Psocodea - ps24
Holometabola - ps25
Aparaglossata - ps26
Mecopterida - ps27
Diptera - ps28
Bibiono./Cyclorrhapha - ps29
Cyclorrhapha - ps30
Calyptratae/Drosophila - ps31
Drosophila - ps32
Sophophora - ps33
Obscura/Melanogaster_group - ps34
D_melanogaster_group - ps35
D_melanogaster_subgroup - ps36
sim./sech./mel. group - ps37
Drosophila_melanogaster - ps38
p-value
2e-02
2e-02
3e-02
4e-02
5e-02
Log-odds
0.5
1.0
1.5
2.0
2.5
3.0
3.5
4.0

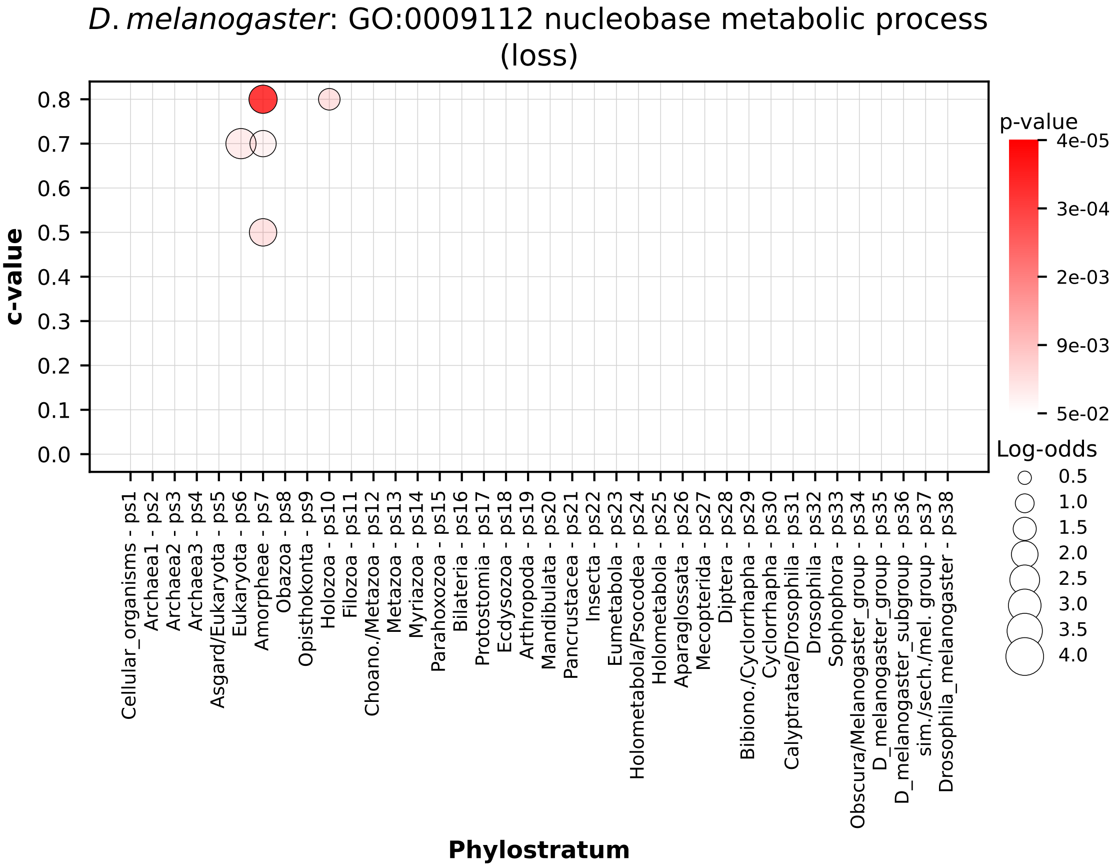

D. melanogaster: GO:0009112 nucleobase metabolic process
(loss)
c-value
0.8
0.7
0.6
0.5
0.4
0.3
0.2
0.1
0.0
p-value
4e-05
3e-04
2e-03
9e-03
5e-02
Log-odds
0.5
1.0
1.5
2.0
2.5
3.0
3.5
4.0
Cellular_organisms - ps1
Archaea1 - ps2
Archaea2 - ps3
Archaea3 - ps4
Asgard/Eukaryota - ps5
Eukaryota - ps6
Amorpheae - ps7
Obazoa - ps8
Opisthokonta - ps9
Holozoa - ps10
Filozoa - ps11
Choano./Metazoa - ps12
Metazoa - ps13
Myriazoa - ps14
Parahoxozoa - ps15
Bilateria - ps16
Protostomia - ps17
Ecdysozoa - ps18
Arthropoda - ps19
Mandibulata - ps20
Pancrustacea - ps21
Insecta - ps22
Eumetabola - ps23
Holometabola/Psocodea - ps24
Holometabola - ps25
Aparaglossata - ps26
Mecopterida - ps27
Diptera - ps28
Bibiono./Cyclorrhapha - ps29
Cyclorrhapha - ps30
Calyptratae/Drosophila - ps31
Drosophila - ps32
Sophophora - ps33
Obscura/Melanogaster_group - ps34
D_melanogaster_group - ps35
D_melanogaster_subgroup - ps36
sim./sech./mel. group - ps37
Drosophila_melanogaster - ps38
Phylostratum

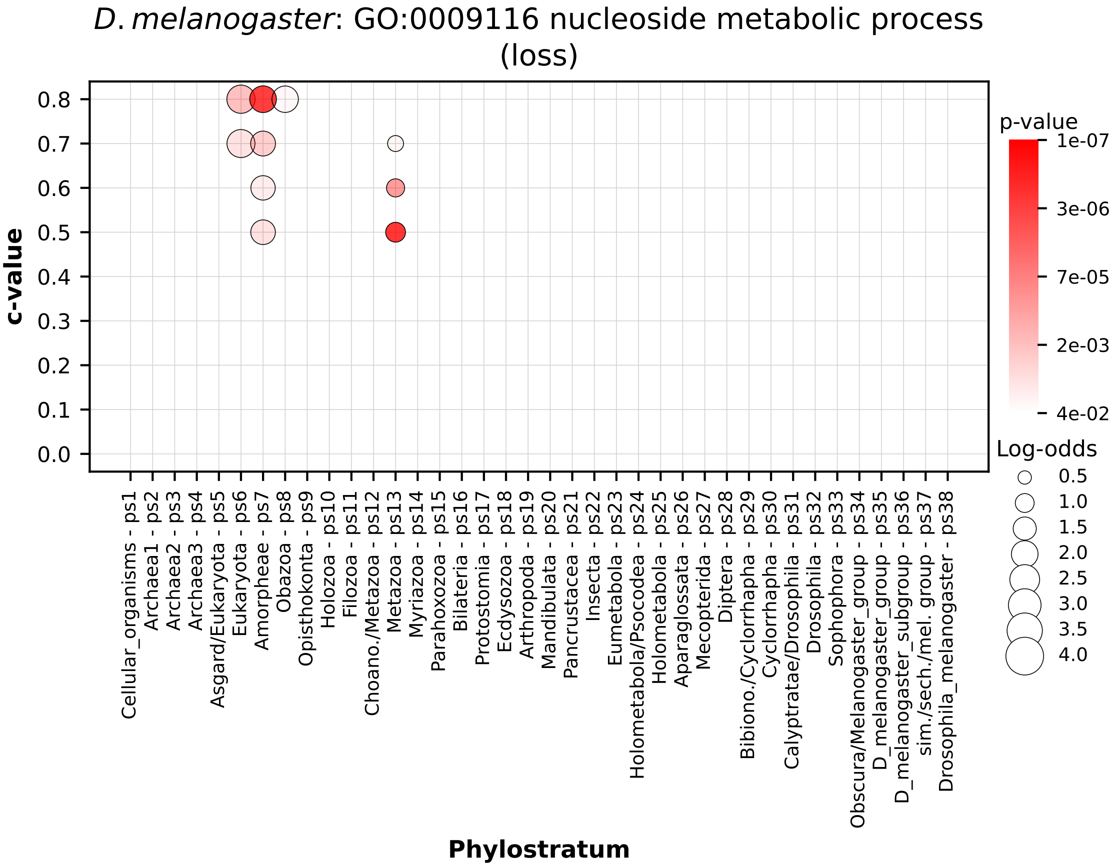

$D. melanogaster$: GO:0009116 nucleoside metabolic process (loss)
c-value
0.8
0.7
0.6
0.5
0.4
0.3
0.2
0.1
0.0
Phylostratum
Cellular_organisms - ps1
Archaea1 - ps2
Archaea2 - ps3
Archaea3 - ps4
Asgard/Eukaryota - ps5
Eukaryota - ps6
Amorpheae - ps7
Obazoa - ps8
Opisthokonta - ps9
Holozoa - ps10
Filozoa - ps11
Choano./Metazoa - ps12
Metazoa - ps13
Myriazoa - ps14
Parahoxozoa - ps15
Bilateria - ps16
Protostomia - ps17
Ecdysozoa - ps18
Arthropoda - ps19
Mandibulata - ps20
Pancrustacea - ps21
Insecta - ps22
Eumetabola - ps23
Holometabola/Psocodea - ps24
Holometabola - ps25
Aparaglossata - ps26
Mecopterida - ps27
Diptera - ps28
Bibiono./Cyclorrhapha - ps29
Cyclorrhapha - ps30
Calyptratae/Drosophila - ps31
Drosophila - ps32
Sophophora - ps33
Obscura/Melanogaster_group - ps34
D_melanogaster_group - ps35
D_melanogaster_subgroup - ps36
sim./sech./mel. group - ps37
Drosophila_melanogaster - ps38
p-value
1e-07
3e-06
7e-05
2e-03
4e-02
Log-odds
0.5
1.0
1.5
2.0
2.5
3.0
3.5
4.0

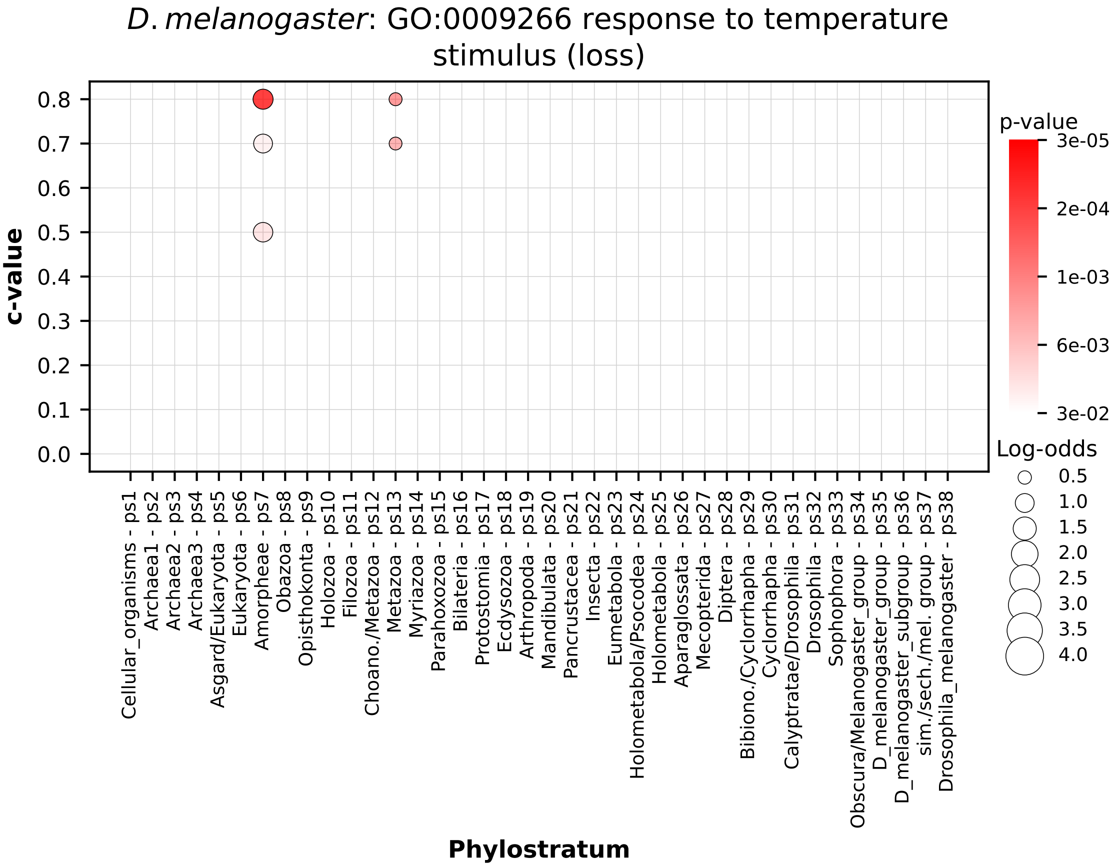
D. melanogaster: GO:0009266 response to temperature stimulus (loss)
c-value
0.8
0.7
0.6
0.5
0.4
0.3
0.2
0.1
0.0
Phylostratum
Cellular_organisms - ps1
Archaea1 - ps2
Archaea2 - ps3
Archaea3 - ps4
Asgard/Eukaryota - ps5
Eukaryota - ps6
Amorpheae - ps7
Obazoa - ps8
Opisthokonta - ps9
Holozoa - ps10
Filozoa - ps11
Choano./Metazoa - ps12
Metazoa - ps13
Myriazoa - ps14
Parahoxozoa - ps15
Bilateria - ps16
Protostomia - ps17
Ecdysozoa - ps18
Arthropoda - ps19
Mandibulata - ps20
Pancrustacea - ps21
Insecta - ps22
Eumetabola - ps23
Holometabola/Psocodea - ps24
Holometabola - ps25
Aparaglossata - ps26
Mecopterida - ps27
Diptera - ps28
Bibiono./Cyclorrhapha - ps29
Cyclorrhapha - ps30
Calyptratae/Drosophila - ps31
Drosophila - ps32
Sophophora - ps33
Obscura/Melanogaster_group - ps34
D_melanogaster_group - ps35
D_melanogaster_subgroup - ps36
sim./sech./mel. group - ps37
Drosophila_melanogaster - ps38
p-value
3e-05
2e-04
1e-03
6e-03
3e-02
Log-odds
0.5
1.0
1.5
2.0
2.5
3.0
3.5
4.0

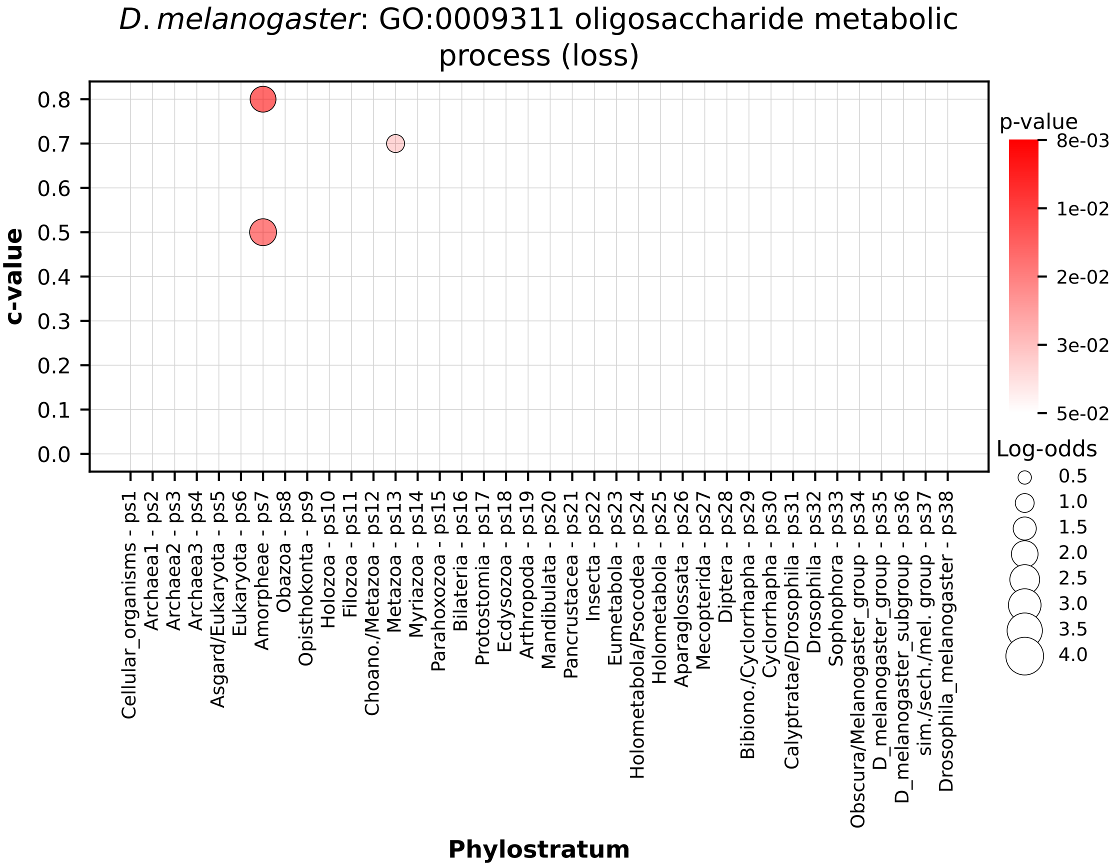

$D. melanogaster$: GO:0009311 oligosaccharide metabolic process (loss)
c-value
0.0
0.1
0.2
0.3
0.4
0.5
0.6
0.7
0.8
Phylostratum
Cellular_organisms - ps1
Archaea1 - ps2
Archaea2 - ps3
Archaea3 - ps4
Asgard/Eukaryota - ps5
Eukaryota - ps6
Amorpheae - ps7
Obazoa - ps8
Opisthokonta - ps9
Holozoa - ps10
Filozoa - ps11
Choano./Metazoa - ps12
Metazoa - ps13
Myriazoa - ps14
Parahoxozoa - ps15
Bilateria - ps16
Protostomia - ps17
Ecdysozoa - ps18
Arthropoda - ps19
Mandibulata - ps20
Pancrustacea - ps21
Insecta - ps22
Eumetabola - ps23
Holometabola/Psocodea - ps24
Holometabola - ps25
Aparaglossata - ps26
Mecopterida - ps27
Diptera - ps28
Bibiono./Cyclorrhapha - ps29
Cyclorrhapha - ps30
Calyptratae/Drosophila - ps31
Drosophila - ps32
Sophophora - ps33
Obscura/Melanogaster_group - ps34
D_melanogaster_group - ps35
D_melanogaster_subgroup - ps36
sim./sech./mel. group - ps37
Drosophila_melanogaster - ps38
p-value
8e-03
1e-02
2e-02
3e-02
5e-02
Log-odds
0.5
1.0
1.5
2.0
2.5
3.0
3.5
4.0

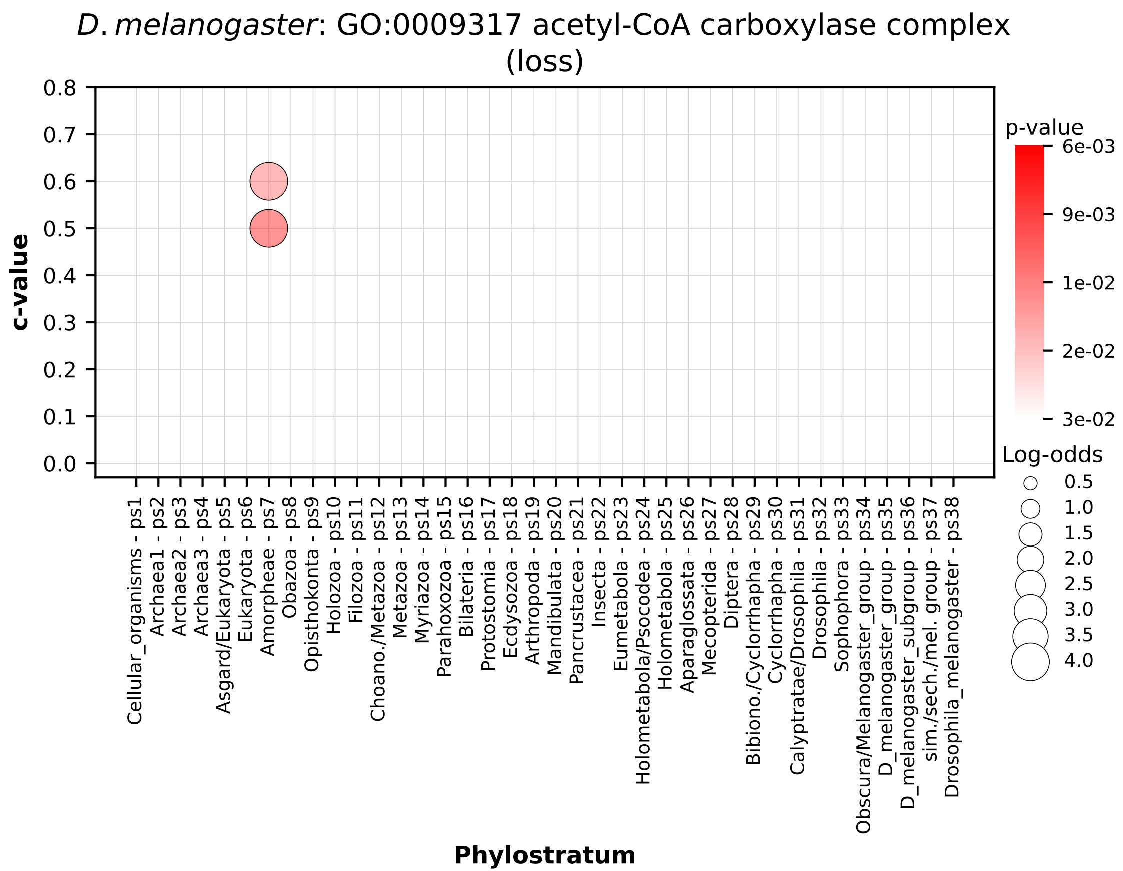

D. melanogaster: GO:0009317 acetyl-CoA carboxylase complex (loss)
c-value
0.8
0.7
0.6
0.5
0.4
0.3
0.2
0.1
0.0
Phylostratum
Cellular_organisms - ps1
Archaea1 - ps2
Archaea2 - ps3
Archaea3 - ps4
Asgard/Eukaryota - ps5
Eukaryota - ps6
Amorpheae - ps7
Obazoa - ps8
Opisthokonta - ps9
Holozoa - ps10
Filozoa - ps11
Choano./Metazoa - ps12
Metazoa - ps13
Myriazoa - ps14
Parahoxozoa - ps15
Bilateria - ps16
Protostomia - ps17
Ecdysozoa - ps18
Arthropoda - ps19
Mandibulata - ps20
Pancrustacea - ps21
Insecta - ps22
Eumetabola - ps23
Holometabola/Psocodea - ps24
Holometabola - ps25
Aparaglossata - ps26
Mecopterida - ps27
Diptera - ps28
Bibiono./Cyclorrhapha - ps29
Cyclorrhapha - ps30
Calyptratae/Drosophila - ps31
Drosophila - ps32
Sophophora - ps33
Obscura/Melanogaster_group - ps34
D_melanogaster_group - ps35
D_melanogaster_subgroup - ps36
sim./sech./mel. group - ps37
Drosophila_melanogaster - ps38
p-value
6e-03
9e-03
1e-02
2e-02
3e-02
Log-odds
0.5
1.0
1.5
2.0
2.5
3.0
3.5
4.0

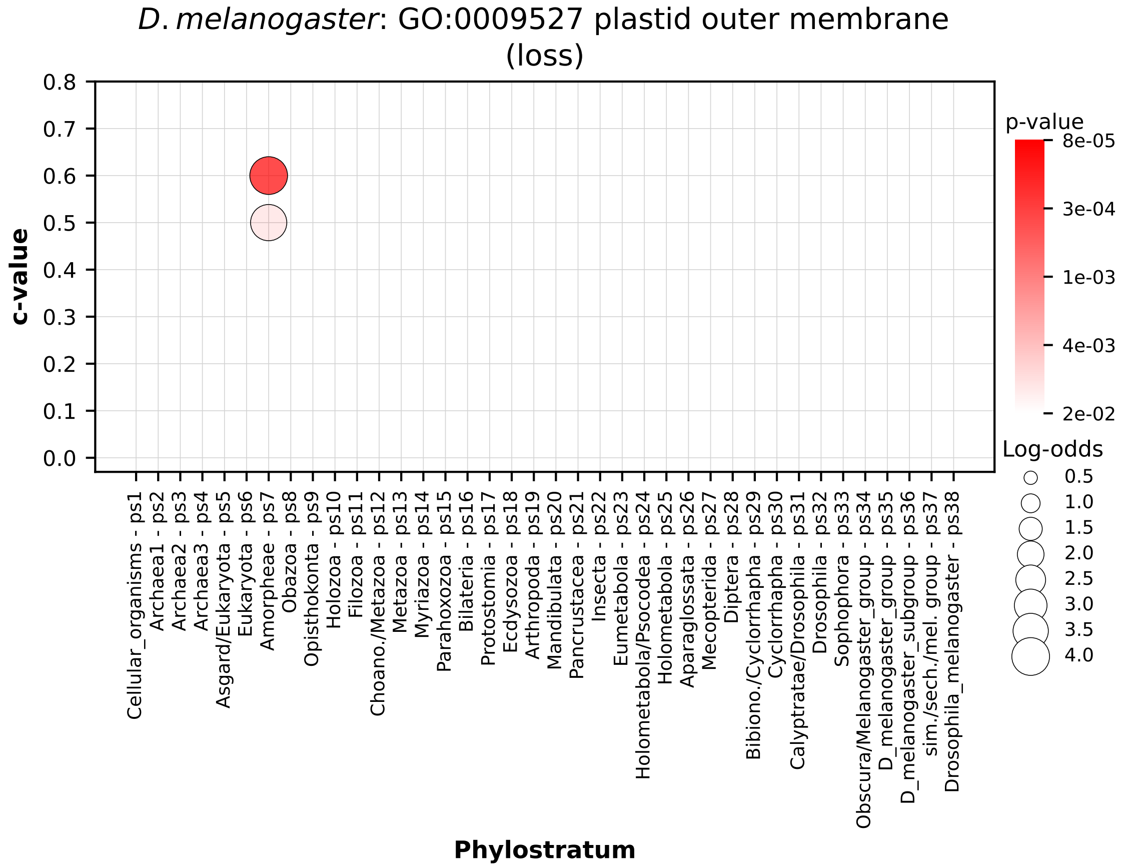

D. melanogaster: GO:0009527 plastid outer membrane
(loss)
c-value
0.8
0.7
0.6
0.5
0.4
0.3
0.2
0.1
0.0
p-value
8e-05
3e-04
1e-03
4e-03
2e-02
Log-odds
0.5
1.0
1.5
2.0
2.5
3.0
3.5
4.0
Cellular_organisms - ps1
Archaea1 - ps2
Archaea2 - ps3
Archaea3 - ps4
Asgard/Eukaryota - ps5
Eukaryota - ps6
Amorpheae - ps7
Obazoa - ps8
Opisthokonta - ps9
Holozoa - ps10
Filozoa - ps11
Choano./Metazoa - ps12
Metazoa - ps13
Myriazoa - ps14
Parahoxozoa - ps15
Bilateria - ps16
Protostomia - ps17
Ecdysozoa - ps18
Arthropoda - ps19
Mandibulata - ps20
Pancrustacea - ps21
Insecta - ps22
Eumetabola - ps23
Holometabola/Psocodea - ps24
Holometabola - ps25
Aparaglossata - ps26
Mecopterida - ps27
Diptera - ps28
Bibiono./Cyclorrhapha - ps29
Cyclorrhapha - ps30
Calyptratae/Drosophila - ps31
Drosophila - ps32
Sophophora - ps33
Obscura/Melanogaster_group - ps34
D_melanogaster_group - ps35
D_melanogaster_subgroup - ps36
sim./sech./mel. group - ps37
Drosophila_melanogaster - ps38
Phylostratum

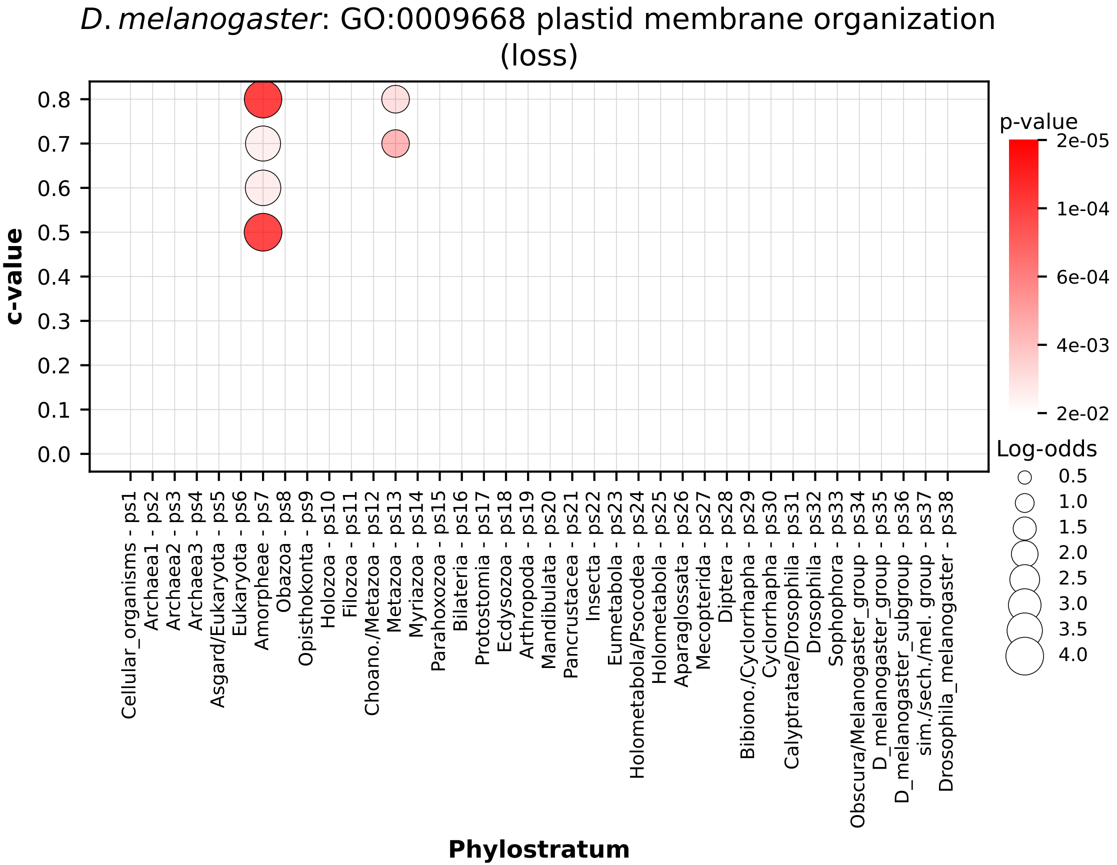

D. melanogaster: GO:0009668 plastid membrane organization (loss)
c-value
0.8
0.7
0.6
0.5
0.4
0.3
0.2
0.1
0.0
p-value
2e-05
1e-04
6e-04
4e-03
2e-02
Log-odds
0.5
1.0
1.5
2.0
2.5
3.0
3.5
4.0
Cellular_organisms - ps1
Archaea1 - ps2
Archaea2 - ps3
Archaea3 - ps4
Asgard/Eukaryota - ps5
Eukaryota - ps6
Amorpheae - ps7
Obazoa - ps8
Opisthokonta - ps9
Holozoa - ps10
Filozoa - ps11
Choano./Metazoa - ps12
Metazoa - ps13
Myriazoa - ps14
Parahoxozoa - ps15
Bilateria - ps16
Protostomia - ps17
Ecdysozoa - ps18
Arthropoda - ps19
Mandibulata - ps20
Pancrustacea - ps21
Insecta - ps22
Eumetabola - ps23
Holometabola/Psocodea - ps24
Holometabola - ps25
Aparaglossata - ps26
Mecopterida - ps27
Diptera - ps28
Bibiono./Cyclorrhapha - ps29
Cyclorrhapha - ps30
Calyptratae/Drosophila - ps31
Drosophila - ps32
Sophophora - ps33
Obscura/Melanogaster_group - ps34
D_melanogaster_group - ps35
D_melanogaster_subgroup - ps36
sim./sech./mel. group - ps37
Drosophila_melanogaster - ps38
Phylostratum

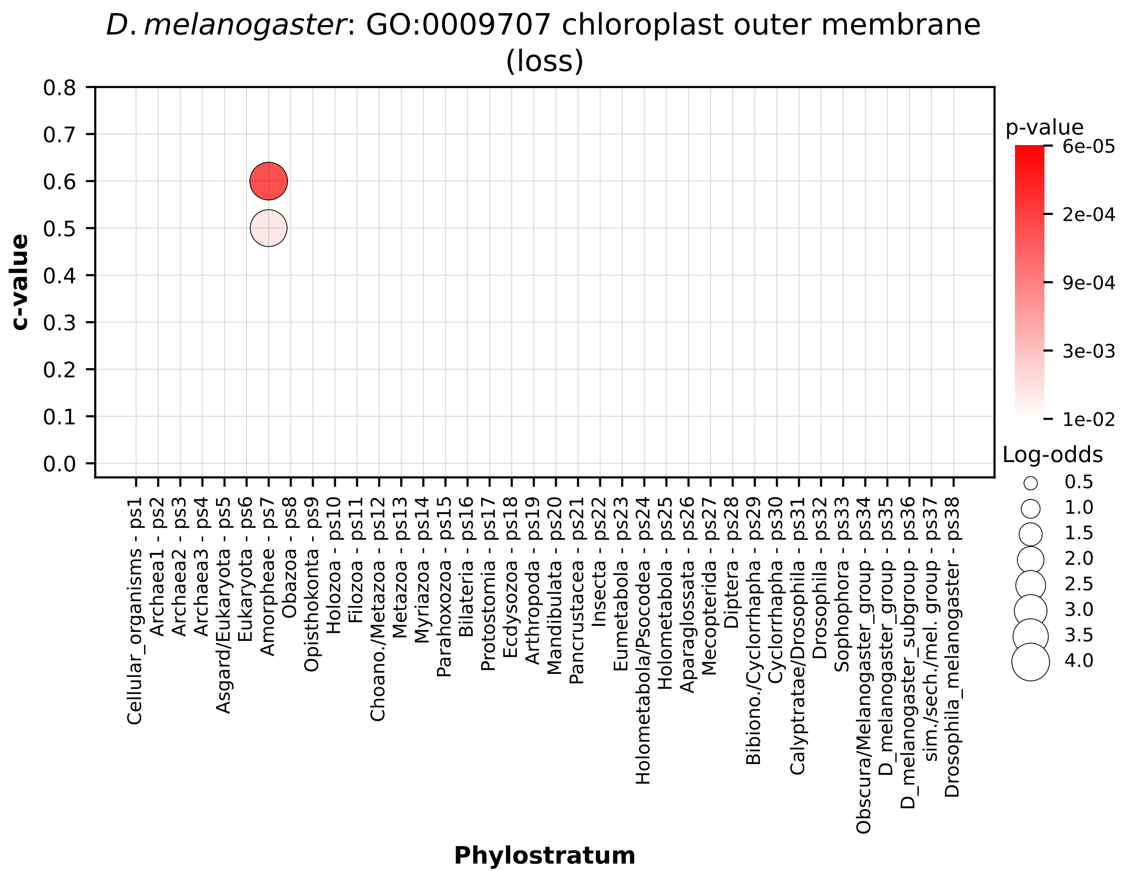

D. melanogaster: GO:0009707 chloroplast outer membrane (loss)
c-value
0.0
0.1
0.2
0.3
0.4
0.5
0.6
0.7
0.8
Phylostratum
Cellular_organisms - ps1
Archaea1 - ps2
Archaea2 - ps3
Archaea3 - ps4
Asgard/Eukaryota - ps5
Eukaryota - ps6
Amorpheae - ps7
Obazoa - ps8
Opisthokonta - ps9
Holozoa - ps10
Filozoa - ps11
Choano./Metazoa - ps12
Metazoa - ps13
Myriazoa - ps14
Parahoxozoa - ps15
Bilateria - ps16
Protostomia - ps17
Ecdysozoa - ps18
Arthropoda - ps19
Mandibulata - ps20
Pancrustacea - ps21
Insecta - ps22
Eumetabola - ps23
Holometabola/Psocodea - ps24
Holometabola - ps25
Aparaglossata - ps26
Mecopterida - ps27
Diptera - ps28
Bibiono./Cyclorrhapha - ps29
Cyclorrhapha - ps30
Calyptratae/Drosophila - ps31
Drosophila - ps32
Sophophora - ps33
Obscura/Melanogaster_group - ps34
D_melanogaster_group - ps35
D_melanogaster_subgroup - ps36
sim./sech./mel. group - ps37
Drosophila_melanogaster - ps38
p-value
6e-05
2e-04
9e-04
3e-03
1e-02
Log-odds
0.5
1.0
1.5
2.0
2.5
3.0
3.5
4.0

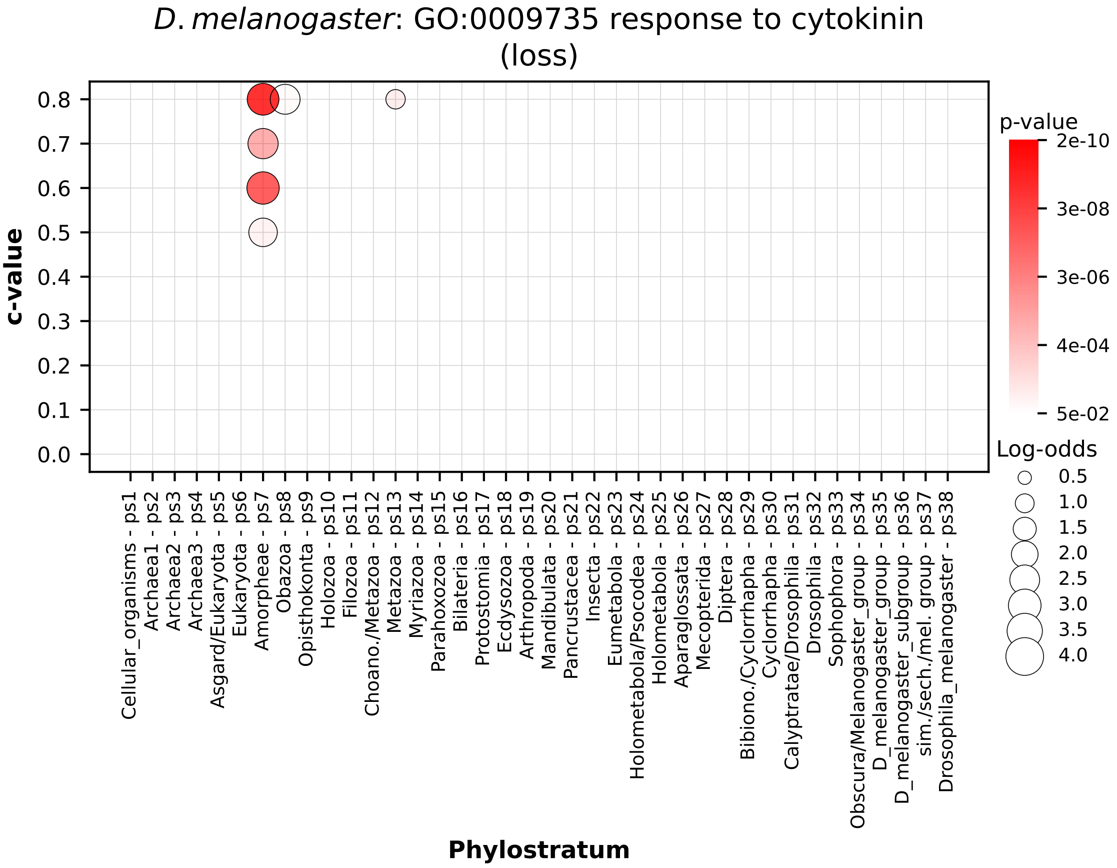

D. melanogaster: GO:0009735 response to cytokinin
(loss)
c-value
0.8
0.7
0.6
0.5
0.4
0.3
0.2
0.1
0.0
p-value
2e-10
3e-08
3e-06
4e-04
5e-02
Log-odds
0.5
1.0
1.5
2.0
2.5
3.0
3.5
4.0
Cellular_organisms - ps1
Archaea1 - ps2
Archaea2 - ps3
Archaea3 - ps4
Asgard/Eukaryota - ps5
Eukaryota - ps6
Amorpheae - ps7
Obazoa - ps8
Opisthokonta - ps9
Holozoa - ps10
Filozoa - ps11
Choano./Metazoa - ps12
Metazoa - ps13
Myriazoa - ps14
Parahoxozoa - ps15
Bilateria - ps16
Protostomia - ps17
Ecdysozoa - ps18
Arthropoda - ps19
Mandibulata - ps20
Pancrustacea - ps21
Insecta - ps22
Eumetabola - ps23
Holometabola/Psocodea - ps24
Holometabola - ps25
Aparaglossata - ps26
Mecopterida - ps27
Diptera - ps28
Bibiono./Cyclorrhapha - ps29
Cyclorrhapha - ps30
Calyptratae/Drosophila - ps31
Drosophila - ps32
Sophophora - ps33
Obscura/Melanogaster_group - ps34
D_melanogaster_group - ps35
D_melanogaster_subgroup - ps36
sim./sech./mel. group - ps37
Drosophila_melanogaster - ps38
Phylostratum

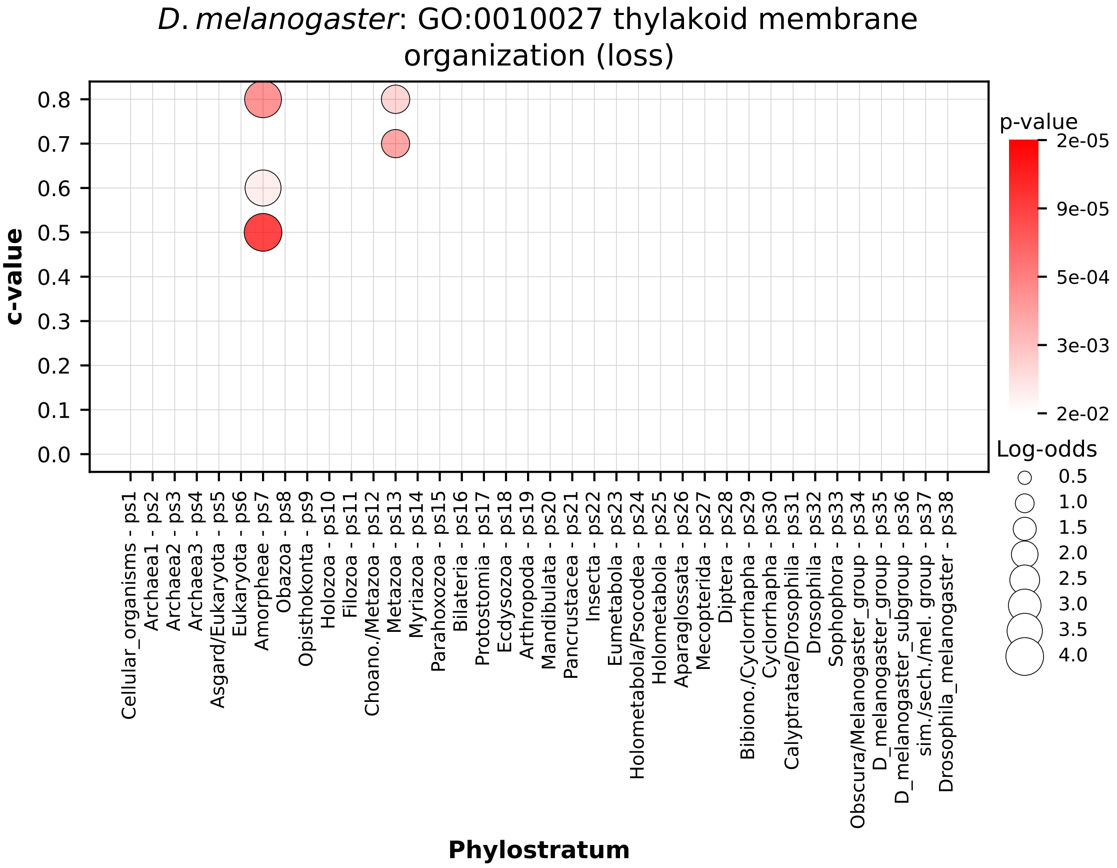

D. melanogaster: GO:0010027 thylakoid membrane organization (loss)
c-value
0.0
0.1
0.2
0.3
0.4
0.5
0.6
0.7
0.8
Phylostratum
Cellular_organisms - ps1
Archaea1 - ps2
Archaea2 - ps3
Archaea3 - ps4
Asgard/Eukaryota - ps5
Eukaryota - ps6
Amorpheae - ps7
Obazoa - ps8
Opisthokonta - ps9
Holozoa - ps10
Filozoa - ps11
Choano./Metazoa - ps12
Metazoa - ps13
Myriazoa - ps14
Parahoxozoa - ps15
Bilateria - ps16
Protostomia - ps17
Ecdysozoa - ps18
Arthropoda - ps19
Mandibulata - ps20
Pancrustacea - ps21
Insecta - ps22
Eumetabola - ps23
Holometabola/Psocodea - ps24
Holometabola - ps25
Aparaglossata - ps26
Mecopterida - ps27
Diptera - ps28
Bibiono./Cyclorrhapha - ps29
Cyclorrhapha - ps30
Calyptratae/Drosophila - ps31
Drosophila - ps32
Sophophora - ps33
Obscura/Melanogaster_group - ps34
D_melanogaster_group - ps35
D_melanogaster_subgroup - ps36
sim./sech./mel. group - ps37
Drosophila_melanogaster - ps38
p-value
2e-05
9e-05
5e-04
3e-03
2e-02
Log-odds
0.5
1.0
1.5
2.0
2.5
3.0
3.5
4.0

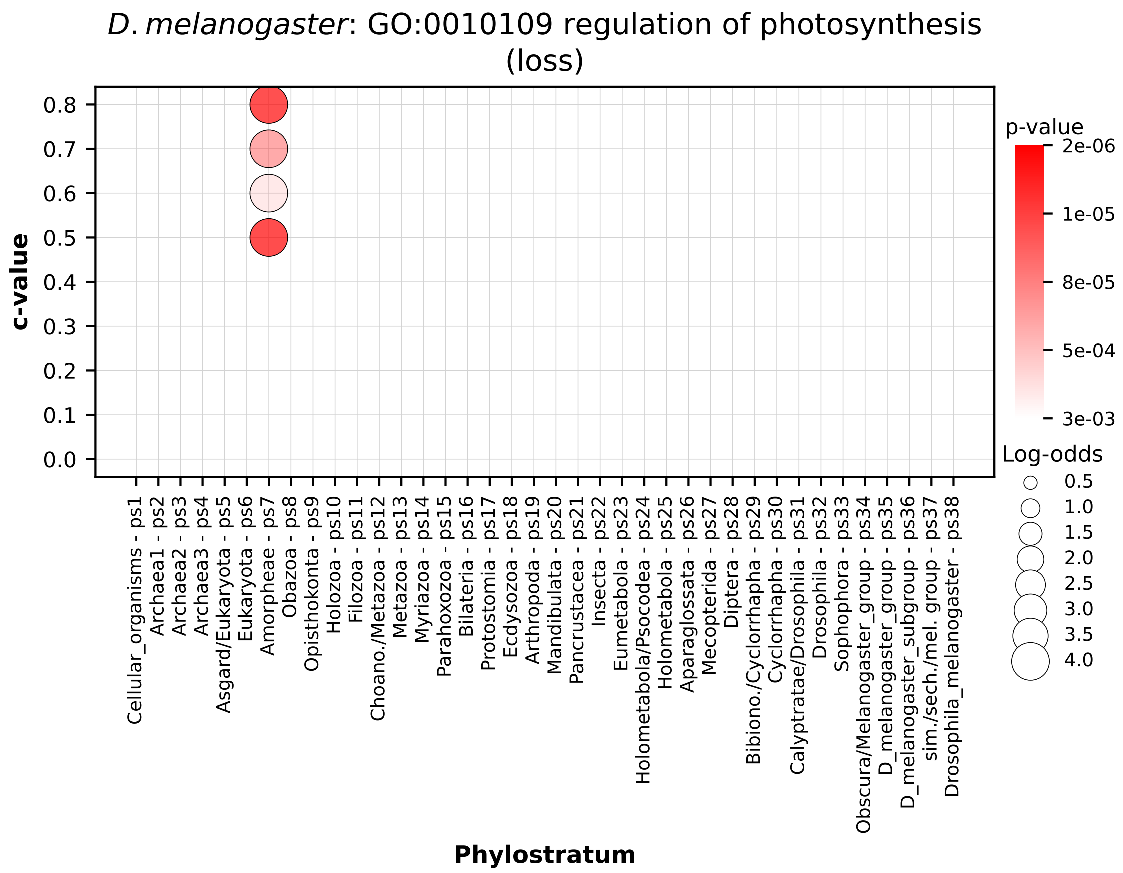

D. melanogaster: GO:0010109 regulation of photosynthesis (loss)
c-value
0.0
0.1
0.2
0.3
0.4
0.5
0.6
0.7
0.8
Phylostratum
Cellular_organisms - ps1
Archaea1 - ps2
Archaea2 - ps3
Archaea3 - ps4
Asgard/Eukaryota - ps5
Eukaryota - ps6
Amorpheae - ps7
Obazoa - ps8
Opisthokonta - ps9
Holozoa - ps10
Filozoa - ps11
Choano./Metazoa - ps12
Metazoa - ps13
Myriazoa - ps14
Parahoxozoa - ps15
Bilateria - ps16
Protostomia - ps17
Ecdysozoa - ps18
Arthropoda - ps19
Mandibulata - ps20
Pancrustacea - ps21
Insecta - ps22
Eumetabola - ps23
Holometabola/Psocodea - ps24
Holometabola - ps25
Aparaglossata - ps26
Mecopterida - ps27
Diptera - ps28
Bibiono./Cyclorrhapha - ps29
Cyclorrhapha - ps30
Calyptratae/Drosophila - ps31
Drosophila - ps32
Sophophora - ps33
Obscura/Melanogaster_group - ps34
D_melanogaster_group - ps35
D_melanogaster_subgroup - ps36
sim./sech./mel. group - ps37
Drosophila_melanogaster - ps38
p-value
2e-06
1e-05
8e-05
5e-04
3e-03
Log-odds
0.5
1.0
1.5
2.0
2.5
3.0
3.5
4.0

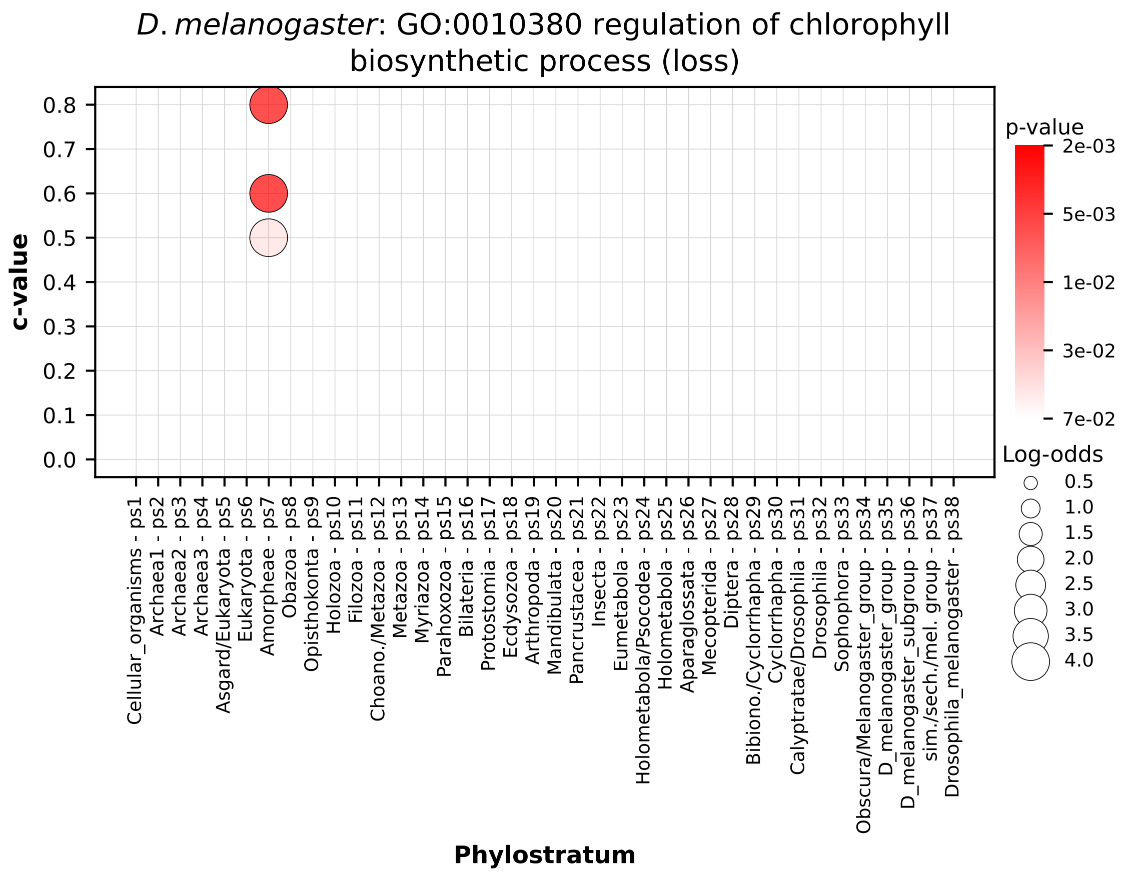

D. melanogaster: GO:0010380 regulation of chlorophyll biosynthetic process (loss)
c-value
0.0
0.1
0.2
0.3
0.4
0.5
0.6
0.7
0.8
Phylostratum
Cellular_organisms - ps1
Archaea1 - ps2
Archaea2 - ps3
Archaea3 - ps4
Asgard/Eukaryota - ps5
Eukaryota - ps6
Amorpheae - ps7
Obazoa - ps8
Opisthokonta - ps9
Holozoa - ps10
Filozoa - ps11
Choano./Metazoa - ps12
Metazoa - ps13
Myriazoa - ps14
Parahoxozoa - ps15
Bilateria - ps16
Protostomia - ps17
Ecdysozoa - ps18
Arthropoda - ps19
Mandibulata - ps20
Pancrustacea - ps21
Insecta - ps22
Eumetabola - ps23
Holometabola/Psocodea - ps24
Holometabola - ps25
Aparaglossata - ps26
Mecopterida - ps27
Diptera - ps28
Bibiono./Cyclorrhapha - ps29
Cyclorrhapha - ps30
Calyptratae/Drosophila - ps31
Drosophila - ps32
Sophophora - ps33
Obscura/Melanogaster_group - ps34
D_melanogaster_group - ps35
D_melanogaster_subgroup - ps36
sim./sech./mel. group - ps37
Drosophila_melanogaster - ps38
p-value
2e-03
5e-03
1e-02
3e-02
7e-02
Log-odds
0.5
1.0
1.5
2.0
2.5
3.0
3.5
4.0

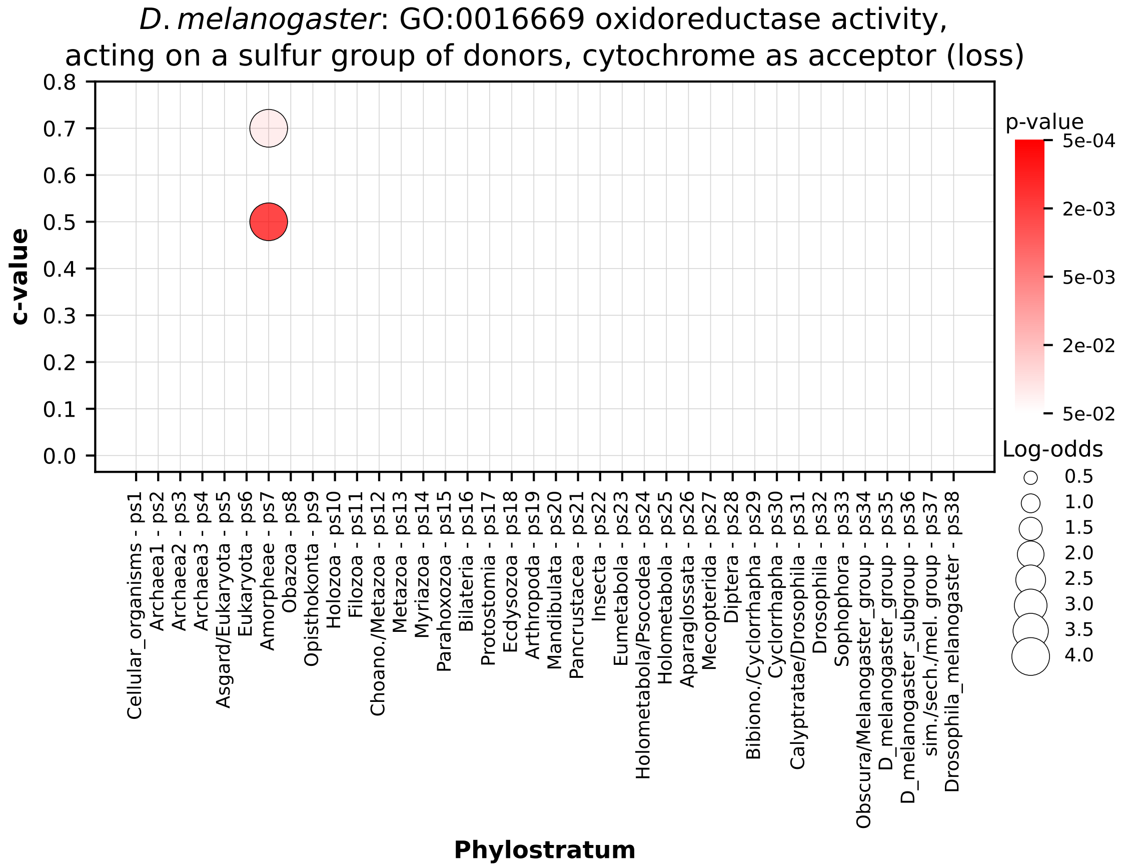

D. melanogaster: GO:0016669 oxidoreductase activity,
acting on a sulfur group of donors, cytochrome as acceptor (loss)
c-value
0.8
0.7
0.6
0.5
0.4
0.3
0.2
0.1
0.0
p-value
5e-04
2e-03
5e-03
2e-02
5e-02
Log-odds
0.5
1.0
1.5
2.0
2.5
3.0
3.5
4.0
Cellular_organisms - ps1
Archaea1 - ps2
Archaea2 - ps3
Archaea3 - ps4
Asgard/Eukaryota - ps5
Eukaryota - ps6
Amorpheae - ps7
Obazoa - ps8
Opisthokonta - ps9
Holozoa - ps10
Filozoa - ps11
Choano./Metazoa - ps12
Metazoa - ps13
Myriazoa - ps14
Parahoxozoa - ps15
Bilateria - ps16
Protostomia - ps17
Ecdysozoa - ps18
Arthropoda - ps19
Mandibulata - ps20
Pancrustacea - ps21
Insecta - ps22
Eumetabola - ps23
Holometabola/Psocodea - ps24
Holometabola - ps25
Aparaglossata - ps26
Mecopterida - ps27
Diptera - ps28
Bibiono./Cyclorrhapha - ps29
Cyclorrhapha - ps30
Calyptratae/Drosophila - ps31
Drosophila - ps32
Sophophora - ps33
Obscura/Melanogaster_group - ps34
D_melanogaster_group - ps35
D_melanogaster_subgroup - ps36
sim./sech./mel. group - ps37
Drosophila_melanogaster - ps38
Phylostratum

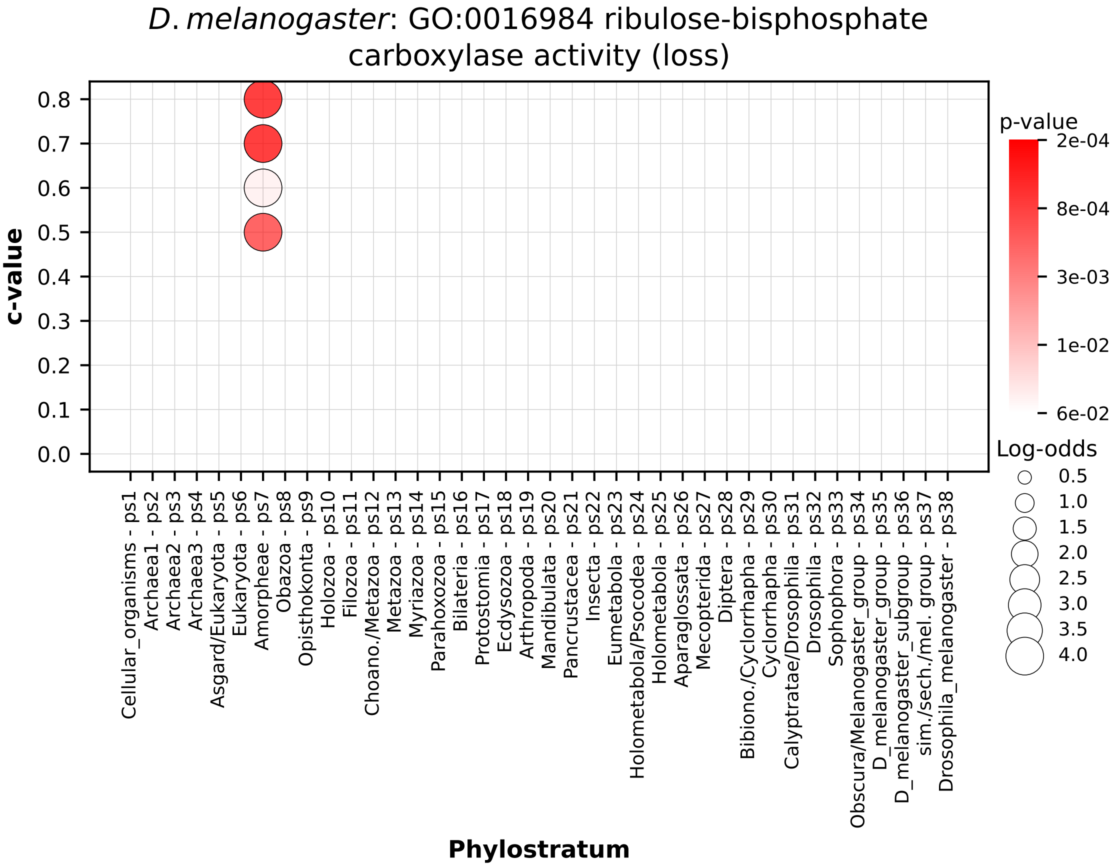
D. melanogaster: GO:0016984 ribulose-bisphosphate carboxylase activity (loss)
c-value
0.0
0.1
0.2
0.3
0.4
0.5
0.6
0.7
0.8
Phylostratum
Cellular_organisms - ps1
Archaea1 - ps2
Archaea2 - ps3
Archaea3 - ps4
Asgard/Eukaryota - ps5
Eukaryota - ps6
Amorpheae - ps7
Obazoa - ps8
Opisthokonta - ps9
Holozoa - ps10
Filozoa - ps11
Choano./Metazoa - ps12
Metazoa - ps13
Myriazoa - ps14
Parahoxozoa - ps15
Bilateria - ps16
Protostomia - ps17
Ecdysozoa - ps18
Arthropoda - ps19
Mandibulata - ps20
Pancrustacea - ps21
Insecta - ps22
Eumetabola - ps23
Holometabola/Psocodea - ps24
Holometabola - ps25
Aparaglossata - ps26
Mecopterida - ps27
Diptera - ps28
Bibiono./Cyclorrhapha - ps29
Cyclorrhapha - ps30
Calyptratae/Drosophila - ps31
Drosophila - ps32
Sophophora - ps33
Obscura/Melanogaster_group - ps34
D_melanogaster_group - ps35
D_melanogaster_subgroup - ps36
sim./sech./mel. group - ps37
Drosophila_melanogaster - ps38
p-value
2e-04
8e-04
3e-03
1e-02
6e-02
Log-odds
0.5
1.0
1.5
2.0
2.5
3.0
3.5
4.0

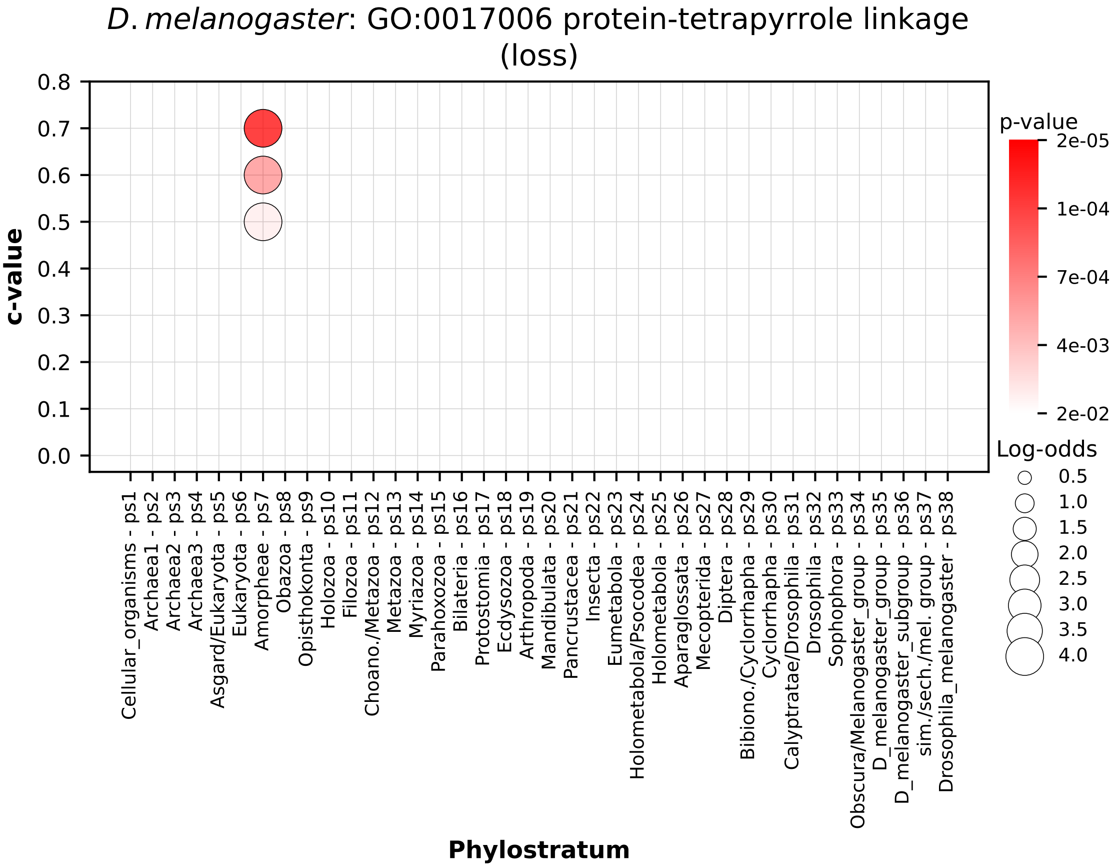

D. melanogaster: GO:0017006 protein-tetrapyrrole linkage (loss)
c-value
0.8
0.7
0.6
0.5
0.4
0.3
0.2
0.1
0.0
Phylostratum
Cellular_organisms - ps1
Archaea1 - ps2
Archaea2 - ps3
Archaea3 - ps4
Asgard/Eukaryota - ps5
Eukaryota - ps6
Amorpheae - ps7
Obazoa - ps8
Opisthokonta - ps9
Holozoa - ps10
Filozoa - ps11
Choano./Metazoa - ps12
Metazoa - ps13
Myriazoa - ps14
Parahoxozoa - ps15
Bilateria - ps16
Protostomia - ps17
Ecdysozoa - ps18
Arthropoda - ps19
Mandibulata - ps20
Pancrustacea - ps21
Insecta - ps22
Eumetabola - ps23
Holometabola/Psocodea - ps24
Holometabola - ps25
Aparaglossata - ps26
Mecopterida - ps27
Diptera - ps28
Bibiono./Cyclorrhapha - ps29
Cyclorrhapha - ps30
Calyptratae/Drosophila - ps31
Drosophila - ps32
Sophophora - ps33
Obscura/Melanogaster_group - ps34
D_melanogaster_group - ps35
D_melanogaster_subgroup - ps36
sim./sech./mel. group - ps37
Drosophila_melanogaster - ps38
p-value
2e-05
1e-04
7e-04
4e-03
2e-02
Log-odds
0.5
1.0
1.5
2.0
2.5
3.0
3.5
4.0

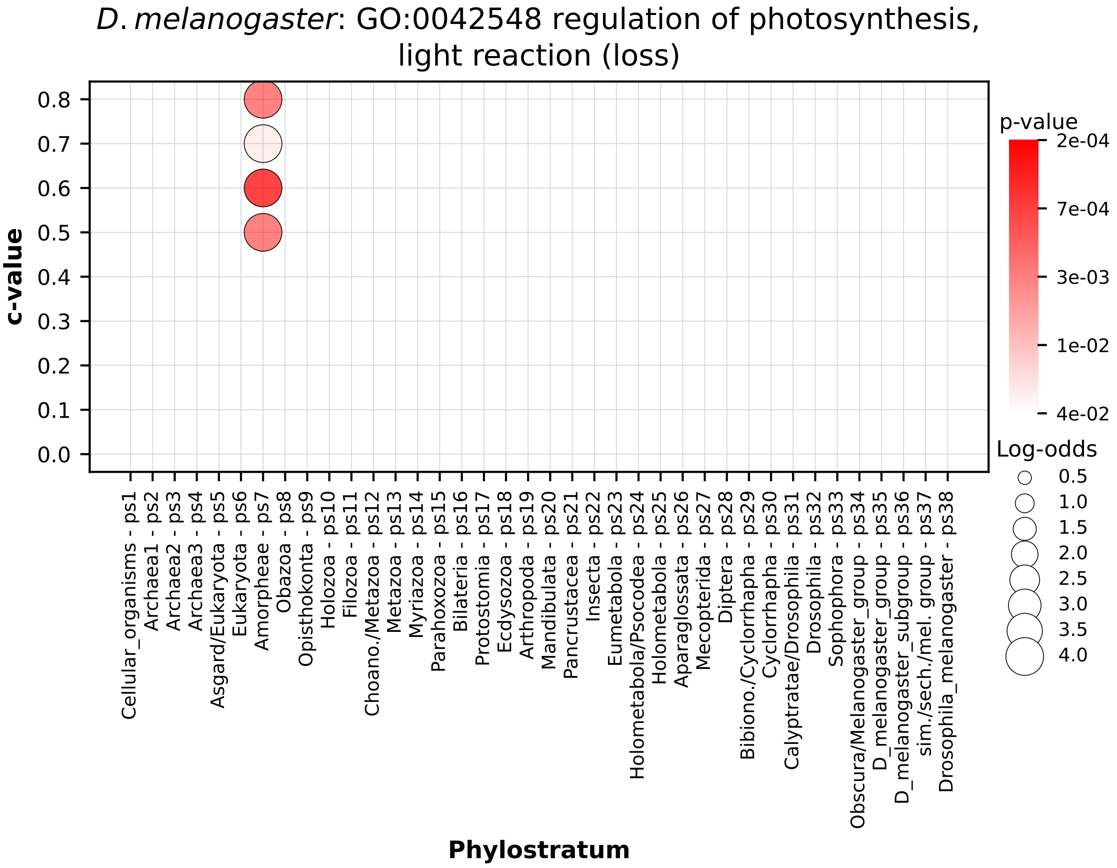
D. melanogaster: GO:0042548 regulation of photosynthesis, light reaction (loss)
c-value
0.0
0.1
0.2
0.3
0.4
0.5
0.6
0.7
0.8
p-value
2e-04
7e-04
3e-03
1e-02
4e-02
Log-odds
0.5
1.0
1.5
2.0
2.5
3.0
3.5
4.0
Cellular_organisms - ps1
Archaea1 - ps2
Archaea2 - ps3
Archaea3 - ps4
Asgard/Eukaryota - ps5
Eukaryota - ps6
Amorpheae - ps7
Obazoa - ps8
Opisthokonta - ps9
Holozoa - ps10
Filozoa - ps11
Choano./Metazoa - ps12
Metazoa - ps13
Myriazoa - ps14
Parahoxozoa - ps15
Bilateria - ps16
Protostomia - ps17
Ecdysozoa - ps18
Arthropoda - ps19
Mandibulata - ps20
Pancrustacea - ps21
Insecta - ps22
Eumetabola - ps23
Holometabola/Psocodea - ps24
Holometabola - ps25
Aparaglossata - ps26
Mecopterida - ps27
Diptera - ps28
Bibiono./Cyclorrhapha - ps29
Cyclorrhapha - ps30
Calyptratae/Drosophila - ps31
Drosophila - ps32
Sophophora - ps33
Obscura/Melanogaster_group - ps34
D_melanogaster_group - ps35
D_melanogaster_subgroup - ps36
sim./sech./mel. group - ps37
Drosophila_melanogaster - ps38
Phylostratum

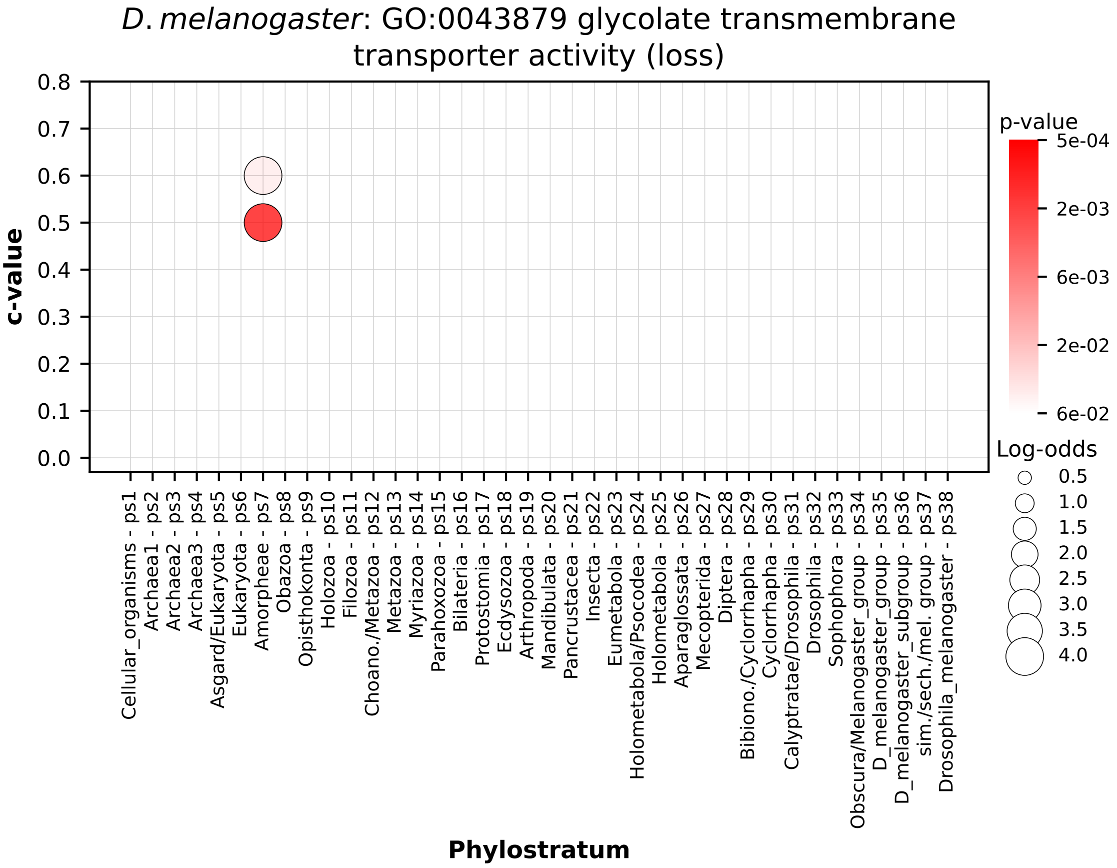
D. melanogaster: GO:0043879 glycolate transmembrane transporter activity (loss)
c-value
0.8
0.7
0.6
0.5
0.4
0.3
0.2
0.1
0.0
Phylostratum
Cellular_organisms - ps1
Archaea1 - ps2
Archaea2 - ps3
Archaea3 - ps4
Asgard/Eukaryota - ps5
Eukaryota - ps6
Amorpheae - ps7
Obazoa - ps8
Opisthokonta - ps9
Holozoa - ps10
Filozoa - ps11
Choano./Metazoa - ps12
Metazoa - ps13
Myriazoa - ps14
Parahoxozoa - ps15
Bilateria - ps16
Protostomia - ps17
Ecdysozoa - ps18
Arthropoda - ps19
Mandibulata - ps20
Pancrustacea - ps21
Insecta - ps22
Eumetabola - ps23
Holometabola/Psocodea - ps24
Holometabola - ps25
Aparaglossata - ps26
Mecopterida - ps27
Diptera - ps28
Bibiono./Cyclorrhapha - ps29
Cyclorrhapha - ps30
Calyptratae/Drosophila - ps31
Drosophila - ps32
Sophophora - ps33
Obscura/Melanogaster_group - ps34
D_melanogaster_group - ps35
D_melanogaster_subgroup - ps36
sim./sech./mel. group - ps37
Drosophila_melanogaster - ps38
p-value
5e-04
2e-03
6e-03
2e-02
6e-02
Log-odds
0.5
1.0
1.5
2.0
2.5
3.0
3.5
4.0

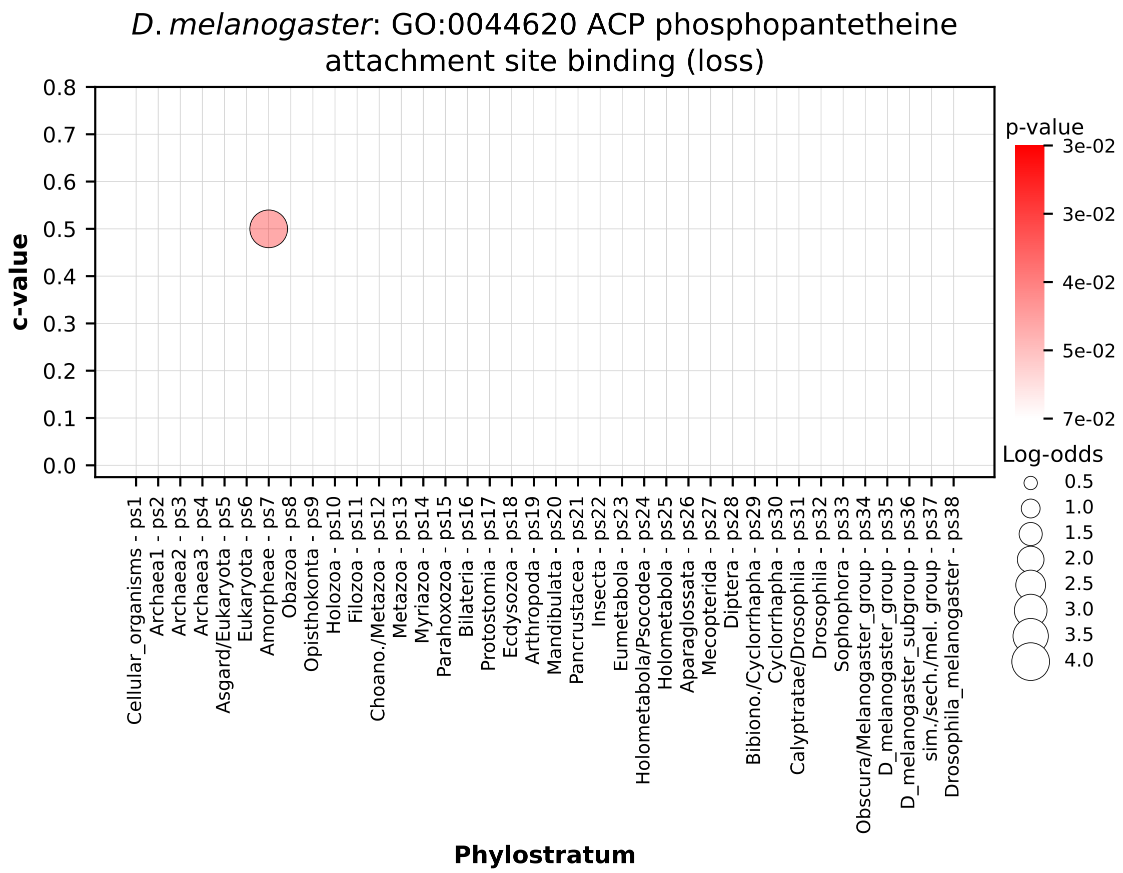
D. melanogaster: GO:0044620 ACP phosphopantetheine attachment site binding (loss)
c-value
0.0
0.1
0.2
0.3
0.4
0.5
0.6
0.7
0.8
Phylostratum
Cellular_organisms - ps1
Archaea1 - ps2
Archaea2 - ps3
Archaea3 - ps4
Asgard/Eukaryota - ps5
Eukaryota - ps6
Amorpheae - ps7
Obazoa - ps8
Opisthokonta - ps9
Holozoa - ps10
Filozoa - ps11
Choano./Metazoa - ps12
Metazoa - ps13
Myriazoa - ps14
Parahoxozoa - ps15
Bilateria - ps16
Protostomia - ps17
Ecdysozoa - ps18
Arthropoda - ps19
Mandibulata - ps20
Pancrustacea - ps21
Insecta - ps22
Eumetabola - ps23
Holometabola/Psocodea - ps24
Holometabola - ps25
Aparaglossata - ps26
Mecopterida - ps27
Diptera - ps28
Bibiono./Cyclorrhapha - ps29
Cyclorrhapha - ps30
Calyptratae/Drosophila - ps31
Drosophila - ps32
Sophophora - ps33
Obscura/Melanogaster_group - ps34
D_melanogaster_group - ps35
D_melanogaster_subgroup - ps36
sim./sech./mel. group - ps37
Drosophila_melanogaster - ps38
p-value
3e-02
3e-02
4e-02
5e-02
7e-02
Log-odds
0.5
1.0
1.5
2.0
2.5
3.0
3.5
4.0

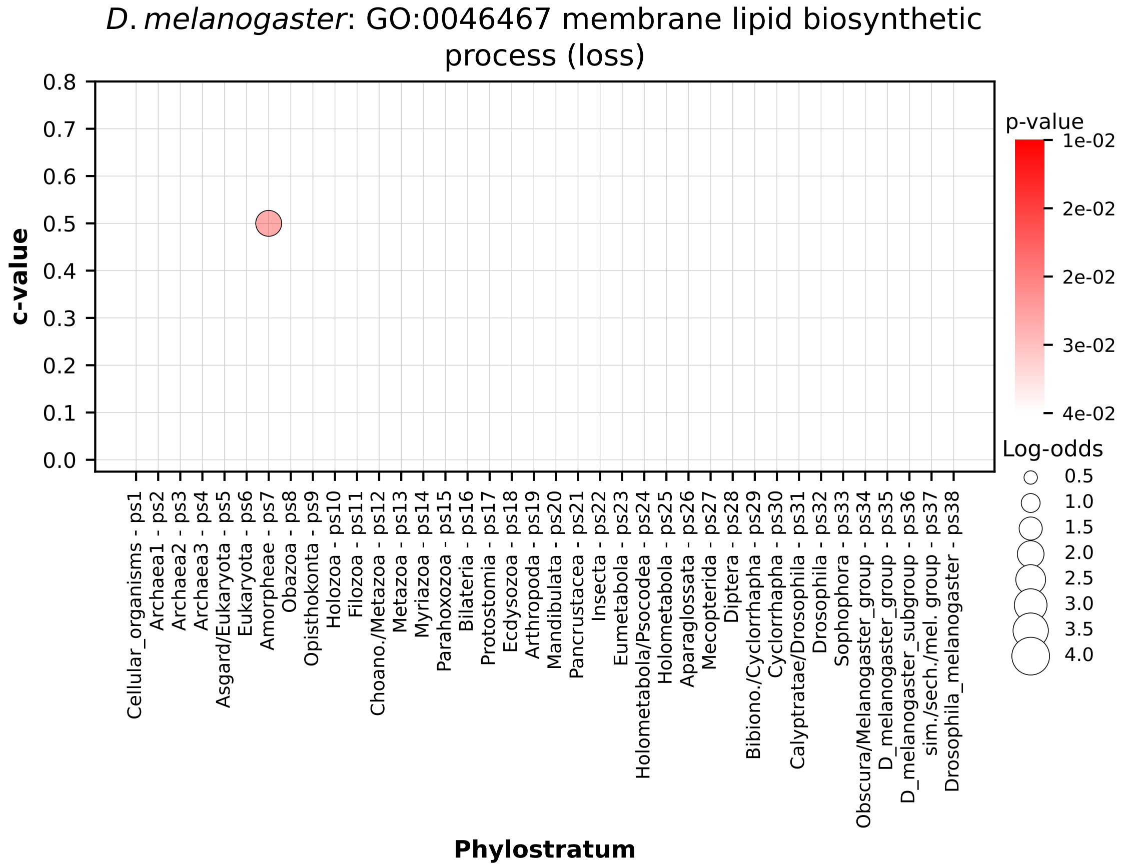

D. melanogaster: GO:0046467 membrane lipid biosynthetic process (loss)
c-value
0.8
0.7
0.6
0.5
0.4
0.3
0.2
0.1
0.0
Phylostratum
Cellular_organisms - ps1
Archaea1 - ps2
Archaea2 - ps3
Archaea3 - ps4
Asgard/Eukaryota - ps5
Eukaryota - ps6
Amorpheae - ps7
Obazoa - ps8
Opisthokonta - ps9
Holozoa - ps10
Filozoa - ps11
Choano./Metazoa - ps12
Metazoa - ps13
Myriazoa - ps14
Parahoxozoa - ps15
Bilateria - ps16
Protostomia - ps17
Ecdysozoa - ps18
Arthropoda - ps19
Mandibulata - ps20
Pancrustacea - ps21
Insecta - ps22
Eumetabola - ps23
Holometabola/Psocodea - ps24
Holometabola - ps25
Aparaglossata - ps26
Mecopterida - ps27
Diptera - ps28
Bibiono./Cyclorrhapha - ps29
Cyclorrhapha - ps30
Calyptratae/Drosophila - ps31
Drosophila - ps32
Sophophora - ps33
Obscura/Melanogaster_group - ps34
D_melanogaster_group - ps35
D_melanogaster_subgroup - ps36
sim./sech./mel. group - ps37
Drosophila_melanogaster - ps38
p-value
1e-02
2e-02
2e-02
3e-02
4e-02
Log-odds
0.5
1.0
1.5
2.0
2.5
3.0
3.5
4.0

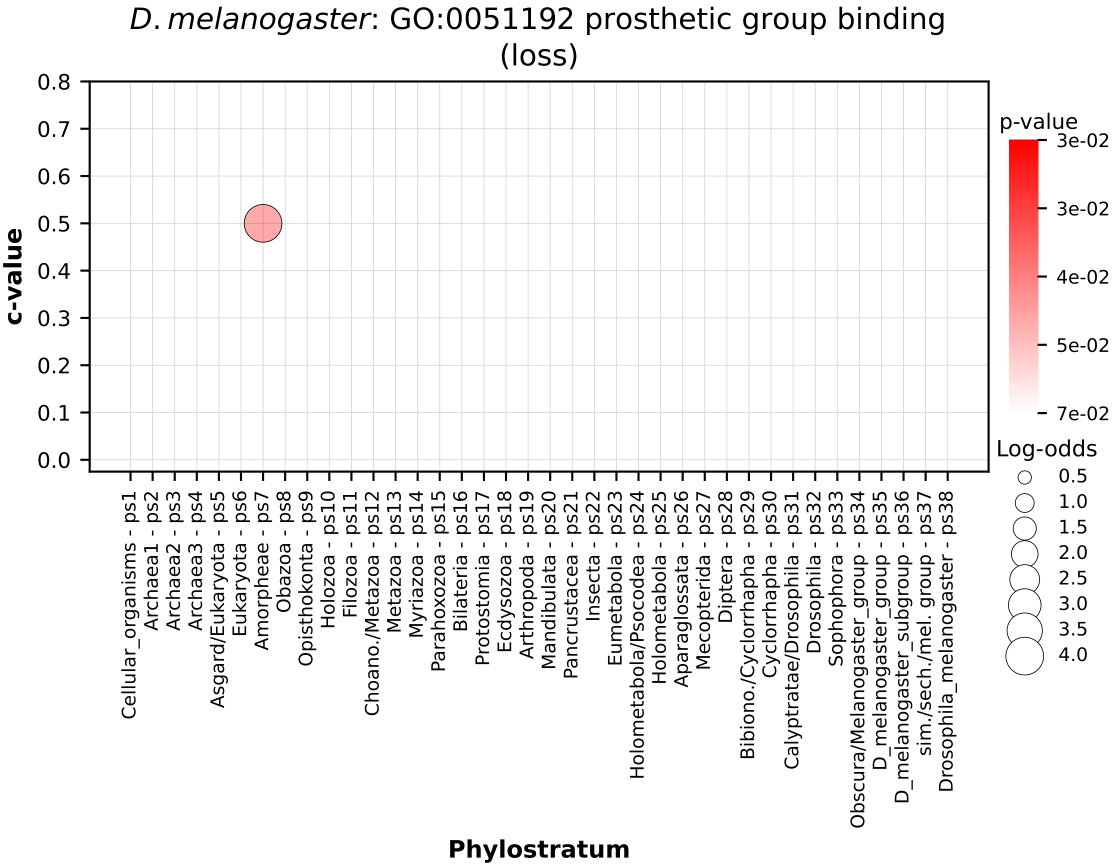
D. melanogaster: GO:0051192 prosthetic group binding (loss)
c-value
0.8
0.7
0.6
0.5
0.4
0.3
0.2
0.1
0.0
p-value
3e-02
3e-02
4e-02
5e-02
7e-02
Log-odds
0.5
1.0
1.5
2.0
2.5
3.0
3.5
4.0
Cellular_organisms - ps1
Archaea1 - ps2
Archaea2 - ps3
Archaea3 - ps4
Asgard/Eukaryota - ps5
Eukaryota - ps6
Amorpheae - ps7
Obazoa - ps8
Opisthokonta - ps9
Holozoa - ps10
Filozoa - ps11
Choano./Metazoa - ps12
Metazoa - ps13
Myriazoa - ps14
Parahoxozoa - ps15
Bilateria - ps16
Protostomia - ps17
Ecdysozoa - ps18
Arthropoda - ps19
Mandibulata - ps20
Pancrustacea - ps21
Insecta - ps22
Eumetabola - ps23
Holometabola/Psocodea - ps24
Holometabola - ps25
Aparaglossata - ps26
Mecopterida - ps27
Diptera - ps28
Bibiono./Cyclorrhapha - ps29
Cyclorrhapha - ps30
Calyptratae/Drosophila - ps31
Drosophila - ps32
Sophophora - ps33
Obscura/Melanogaster_group - ps34
D_melanogaster_group - ps35
D_melanogaster_subgroup - ps36
sim./sech./mel. group - ps37
Drosophila_melanogaster - ps38
Phylostratum

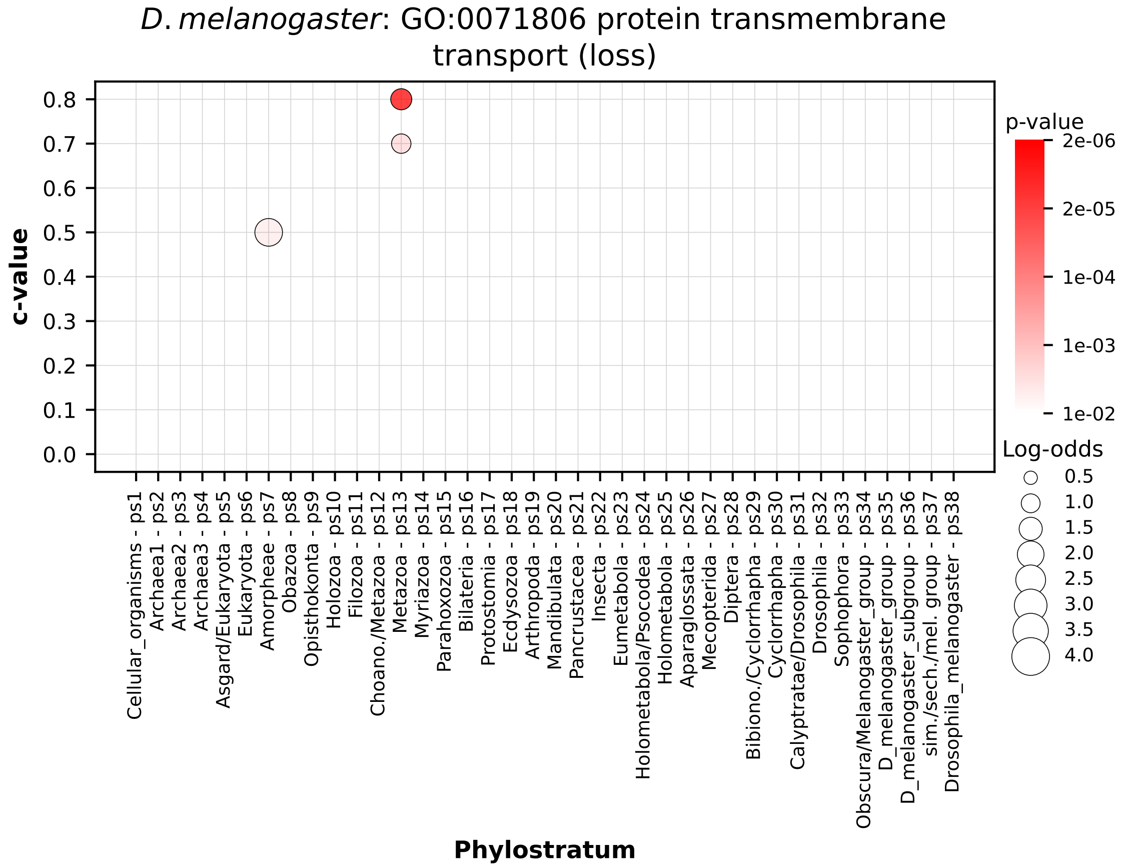
D. melanogaster: GO:0071806 protein transmembrane transport (loss)
c-value
0.8
0.7
0.6
0.5
0.4
0.3
0.2
0.1
0.0
p-value
2e-06
2e-05
1e-04
1e-03
1e-02
Log-odds
0.5
1.0
1.5
2.0
2.5
3.0
3.5
4.0
Cellular_organisms - ps1
Archaea1 - ps2
Archaea2 - ps3
Archaea3 - ps4
Asgard/Eukaryota - ps5
Eukaryota - ps6
Amorpheae - ps7
Obazoa - ps8
Opisthokonta - ps9
Holozoa - ps10
Filozoa - ps11
Choano./Metazoa - ps12
Metazoa - ps13
Myriazoa - ps14
Parahoxozoa - ps15
Bilateria - ps16
Protostomia - ps17
Ecdysozoa - ps18
Arthropoda - ps19
Mandibulata - ps20
Pancrustacea - ps21
Insecta - ps22
Eumetabola - ps23
Holometabola/Psocodea - ps24
Holometabola - ps25
Aparaglossata - ps26
Mecopterida - ps27
Diptera - ps28
Bibiono./Cyclorrhapha - ps29
Cyclorrhapha - ps30
Calyptratae/Drosophila - ps31
Drosophila - ps32
Sophophora - ps33
Obscura/Melanogaster_group - ps34
D_melanogaster_group - ps35
D_melanogaster_subgroup - ps36
sim./sech./mel. group - ps37
Drosophila_melanogaster - ps38
Phylostratum

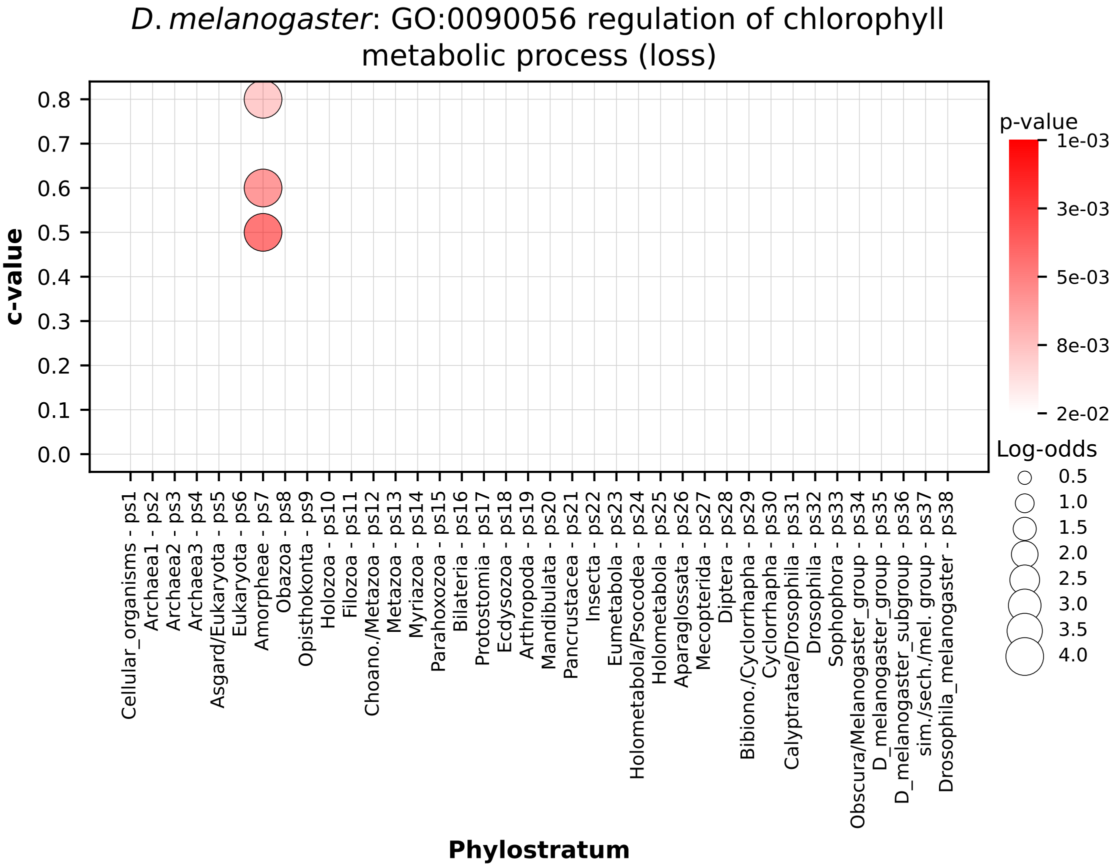

D. melanogaster: GO:0090056 regulation of chlorophyll metabolic process (loss)
c-value
0.8
0.7
0.6
0.5
0.4
0.3
0.2
0.1
0.0
p-value
1e-03
3e-03
5e-03
8e-03
2e-02
Log-odds
0.5
1.0
1.5
2.0
2.5
3.0
3.5
4.0
Cellular_organisms - ps1
Archaea1 - ps2
Archaea2 - ps3
Archaea3 - ps4
Asgard/Eukaryota - ps5
Eukaryota - ps6
Amorpheae - ps7
Obazoa - ps8
Opisthokonta - ps9
Holozoa - ps10
Filozoa - ps11
Choano./Metazoa - ps12
Metazoa - ps13
Myriazoa - ps14
Parahoxozoa - ps15
Bilateria - ps16
Protostomia - ps17
Ecdysozoa - ps18
Arthropoda - ps19
Mandibulata - ps20
Pancrustacea - ps21
Insecta - ps22
Eumetabola - ps23
Holometabola/Psocodea - ps24
Holometabola - ps25
Aparaglossata - ps26
Mecopterida - ps27
Diptera - ps28
Bibiono./Cyclorrhapha - ps29
Cyclorrhapha - ps30
Calyptratae/Drosophila - ps31
Drosophila - ps32
Sophophora - ps33
Obscura/Melanogaster_group - ps34
D_melanogaster_group - ps35
D_melanogaster_subgroup - ps36
sim./sech./mel. group - ps37
Drosophila_melanogaster - ps38
Phylostratum

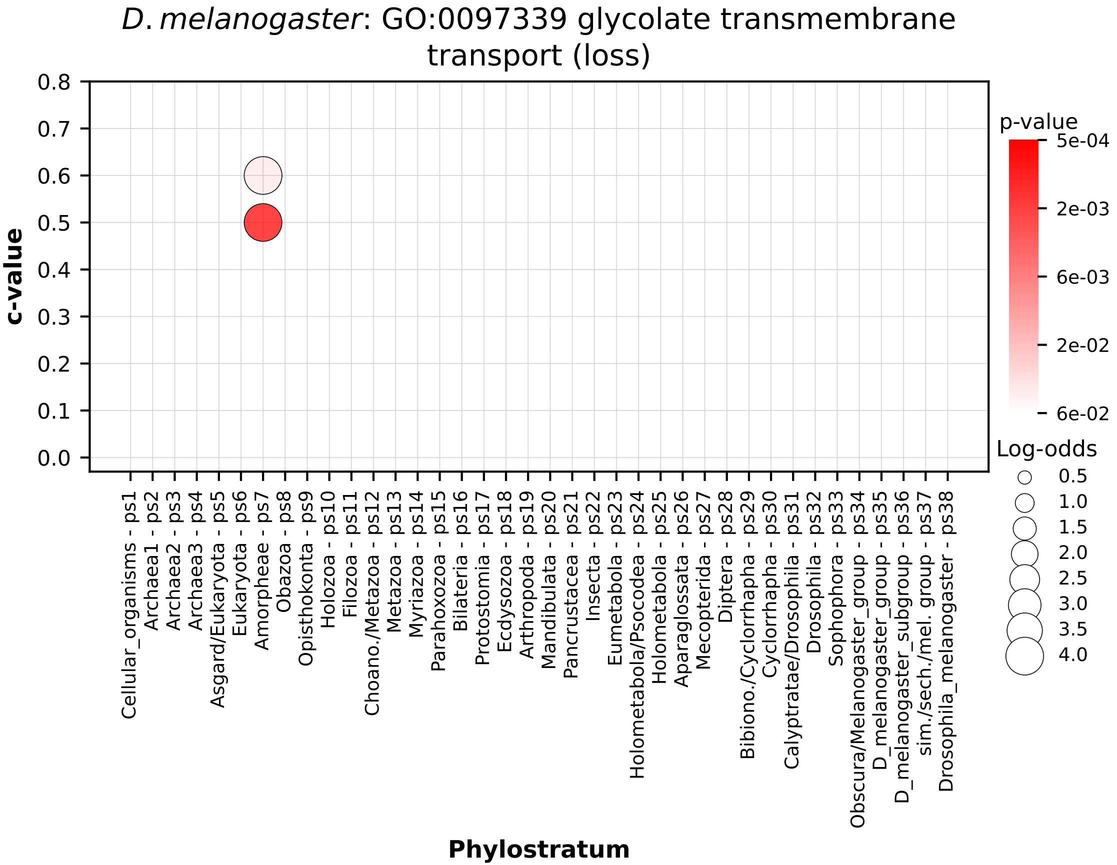

D. melanogaster: GO:0097339 glycolate transmembrane transport (loss)
c-value
0.8
0.7
0.6
0.5
0.4
0.3
0.2
0.1
0.0
Phylostratum
Cellular_organisms - ps1
Archaea1 - ps2
Archaea2 - ps3
Archaea3 - ps4
Asgard/Eukaryota - ps5
Eukaryota - ps6
Amorpheae - ps7
Obazoa - ps8
Opisthokonta - ps9
Holozoa - ps10
Filozoa - ps11
Choano./Metazoa - ps12
Metazoa - ps13
Myriazoa - ps14
Parahoxozoa - ps15
Bilateria - ps16
Protostomia - ps17
Ecdysozoa - ps18
Arthropoda - ps19
Mandibulata - ps20
Pancrustacea - ps21
Insecta - ps22
Eumetabola - ps23
Holometabola/Psocodea - ps24
Holometabola - ps25
Aparaglossata - ps26
Mecopterida - ps27
Diptera - ps28
Bibiono./Cyclorrhapha - ps29
Cyclorrhapha - ps30
Calyptratae/Drosophila - ps31
Drosophila - ps32
Sophophora - ps33
Obscura/Melanogaster_group - ps34
D_melanogaster_group - ps35
D_melanogaster_subgroup - ps36
sim./sech./mel. group - ps37
Drosophila_melanogaster - ps38
p-value
5e-04
2e-03
6e-03
2e-02
6e-02
Log-odds
0.5
1.0
1.5
2.0
2.5
3.0
3.5
4.0

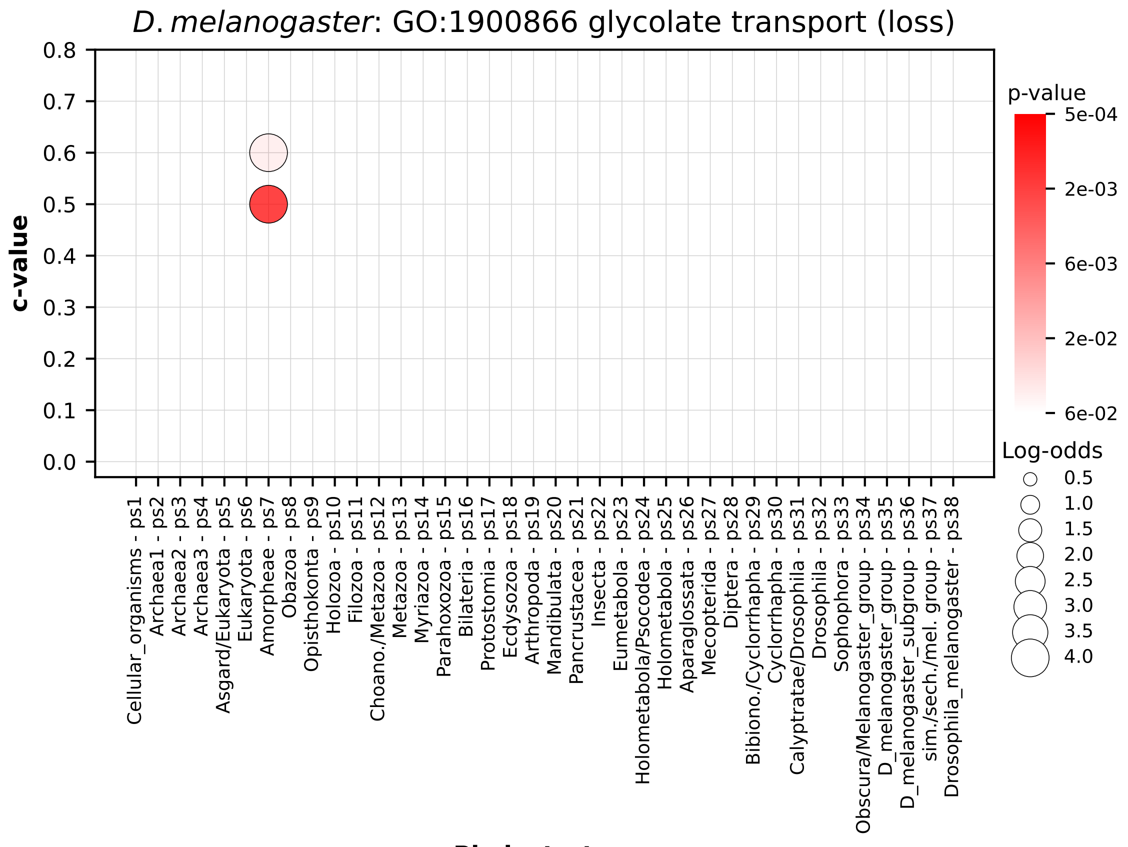
D. melanogaster: GO:1900866 glycolate transport (loss)
c-value
0.8
0.7
0.6
0.5
0.4
0.3
0.2
0.1
0.0
Phylostratum
Cellular_organisms - ps1
Archaea1 - ps2
Archaea2 - ps3
Archaea3 - ps4
Asgard/Eukaryota - ps5
Eukaryota - ps6
Amorpheae - ps7
Obazoa - ps8
Opisthokonta - ps9
Holozoa - ps10
Filozoa - ps11
Choano./Metazoa - ps12
Metazoa - ps13
Myriazoa - ps14
Parahoxozoa - ps15
Bilateria - ps16
Protostomia - ps17
Ecdysozoa - ps18
Arthropoda - ps19
Mandibulata - ps20
Pancrustacea - ps21
Insecta - ps22
Eumetabola - ps23
Holometabola/Psocodea - ps24
Holometabola - ps25
Aparaglossata - ps26
Mecopterida - ps27
Diptera - ps28
Bibiono./Cyclorrhapha - ps29
Cyclorrhapha - ps30
Calyptratae/Drosophila - ps31
Drosophila - ps32
Sophophora - ps33
Obscura/Melanogaster_group - ps34
D_melanogaster_group - ps35
D_melanogaster_subgroup - ps36
sim./sech./mel. group - ps37
Drosophila_melanogaster - ps38
p-value
5e-04
2e-03
6e-03
2e-02
6e-02
Log-odds
0.5
1.0
1.5
2.0
2.5
3.0
3.5
4.0

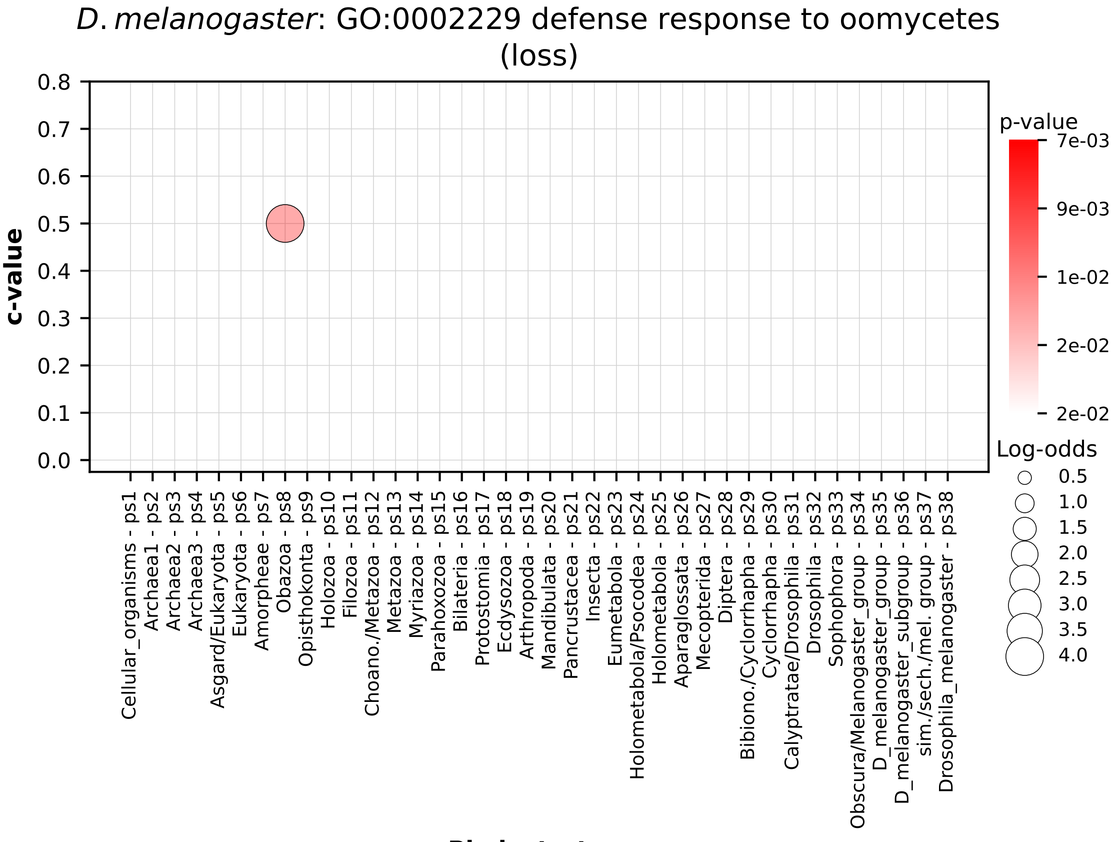

D. melanogaster: GO:0002229 defense response to oomycetes
(loss)
c-value
0.8
0.7
0.6
0.5
0.4
0.3
0.2
0.1
0.0
p-value
7e-03
9e-03
1e-02
2e-02
2e-02
Log-odds
0.5
1.0
1.5
2.0
2.5
3.0
3.5
4.0
Cellular_organisms - ps1
Archaea1 - ps2
Archaea2 - ps3
Archaea3 - ps4
Asgard/Eukaryota - ps5
Eukaryota - ps6
Amorpheae - ps7
Obazoa - ps8
Opisthokonta - ps9
Holozoa - ps10
Filozoa - ps11
Choano./Metazoa - ps12
Metazoa - ps13
Myriazoa - ps14
Parahoxozoa - ps15
Bilateria - ps16
Protostomia - ps17
Ecdysozoa - ps18
Arthropoda - ps19
Mandibulata - ps20
Pancrustacea - ps21
Insecta - ps22
Eumetabola - ps23
Holometabola/Psocodea - ps24
Holometabola - ps25
Aparaglossata - ps26
Mecopterida - ps27
Diptera - ps28
Bibiono./Cyclorrhapha - ps29
Cyclorrhapha - ps30
Calyptratae/Drosophila - ps31
Drosophila - ps32
Sophophora - ps33
Obscura/Melanogaster_group - ps34
D_melanogaster_group - ps35
D_melanogaster_subgroup - ps36
sim./sech./mel. group - ps37
Drosophila_melanogaster - ps38
Phylostratum

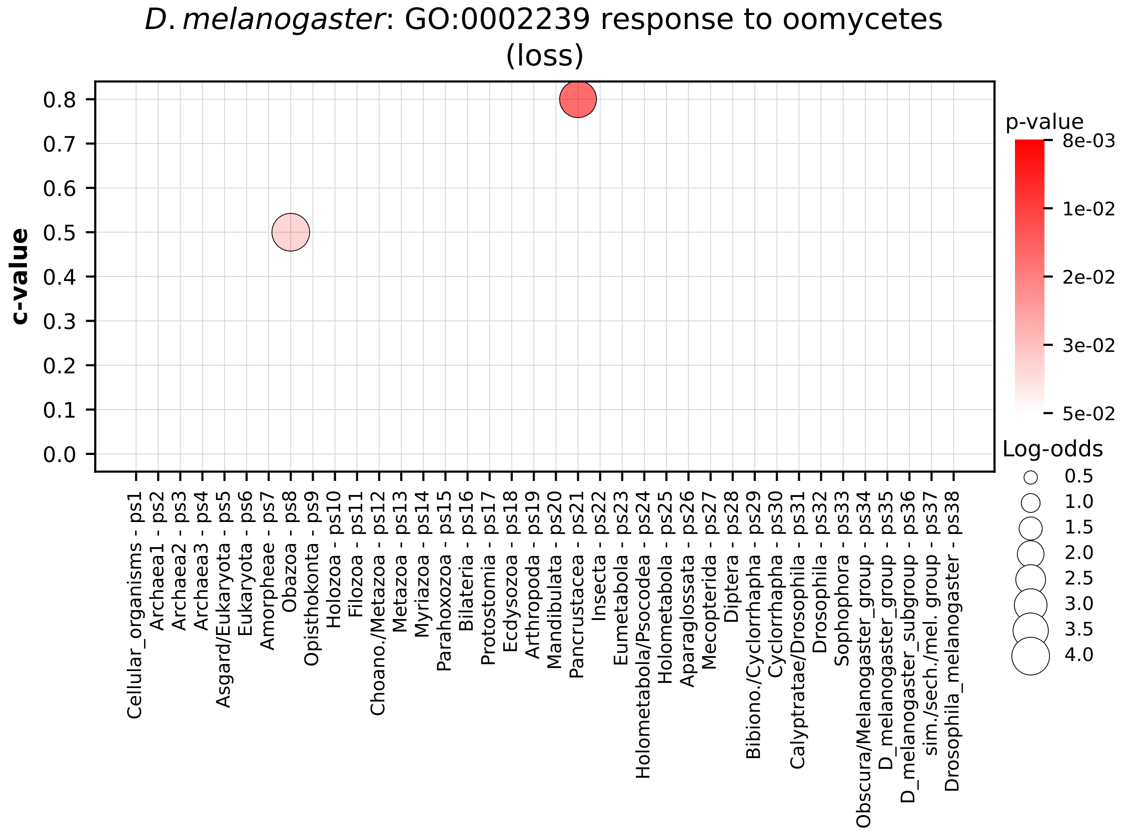
D. melanogaster: GO:0002239 response to oomycetes
(loss)
c-value
0.0
0.1
0.2
0.3
0.4
0.5
0.6
0.7
0.8
Phylostratum
Cellular_organisms - ps1
Archaea1 - ps2
Archaea2 - ps3
Archaea3 - ps4
Asgard/Eukaryota - ps5
Eukaryota - ps6
Amorpheae - ps7
Obazoa - ps8
Opisthokonta - ps9
Holozoa - ps10
Filozoa - ps11
Choano./Metazoa - ps12
Metazoa - ps13
Myriazoa - ps14
Parahoxozoa - ps15
Bilateria - ps16
Protostomia - ps17
Ecdysozoa - ps18
Arthropoda - ps19
Mandibulata - ps20
Pancrustacea - ps21
Insecta - ps22
Eumetabola - ps23
Holometabola/Psocodea - ps24
Holometabola - ps25
Aparaglossata - ps26
Mecopterida - ps27
Diptera - ps28
Bibiono./Cyclorrhapha - ps29
Cyclorrhapha - ps30
Calyptratae/Drosophila - ps31
Drosophila - ps32
Sophophora - ps33
Obscura/Melanogaster_group - ps34
D_melanogaster_group - ps35
D_melanogaster_subgroup - ps36
sim./sech./mel. group - ps37
Drosophila_melanogaster - ps38
p-value
8e-03
1e-02
2e-02
3e-02
5e-02
Log-odds
0.5
1.0
1.5
2.0
2.5
3.0
3.5
4.0

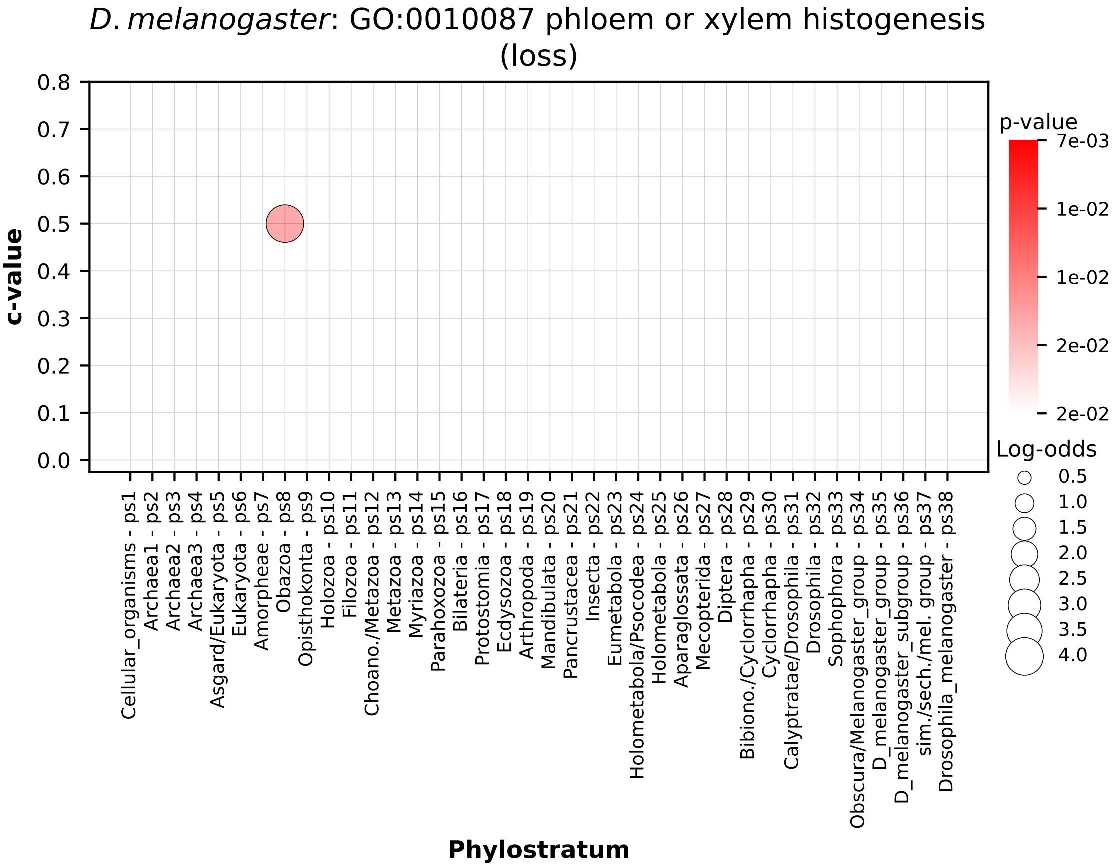

D. melanogaster: GO:0010087 phloem or xylem histogenesis
(loss)
c-value
0.8
0.7
0.6
0.5
0.4
0.3
0.2
0.1
0.0
p-value
7e-03
1e-02
1e-02
2e-02
2e-02
Log-odds
0.5
1.0
1.5
2.0
2.5
3.0
3.5
4.0
Cellular_organisms - ps1
Archaea1 - ps2
Archaea2 - ps3
Archaea3 - ps4
Asgard/Eukaryota - ps5
Eukaryota - ps6
Amorpheae - ps7
Obazoa - ps8
Opisthokonta - ps9
Holozoa - ps10
Filozoa - ps11
Choano./Metazoa - ps12
Metazoa - ps13
Myriazoa - ps14
Parahoxozoa - ps15
Bilateria - ps16
Protostomia - ps17
Ecdysozoa - ps18
Arthropoda - ps19
Mandibulata - ps20
Pancrustacea - ps21
Insecta - ps22
Eumetabola - ps23
Holometabola/Psocodea - ps24
Holometabola - ps25
Aparaglossata - ps26
Mecopterida - ps27
Diptera - ps28
Bibiono./Cyclorrhapha - ps29
Cyclorrhapha - ps30
Calyptratae/Drosophila - ps31
Drosophila - ps32
Sophophora - ps33
Obscura/Melanogaster_group - ps34
D_melanogaster_group - ps35
D_melanogaster_subgroup - ps36
sim./sech./mel. group - ps37
Drosophila_melanogaster - ps38
Phylostratum

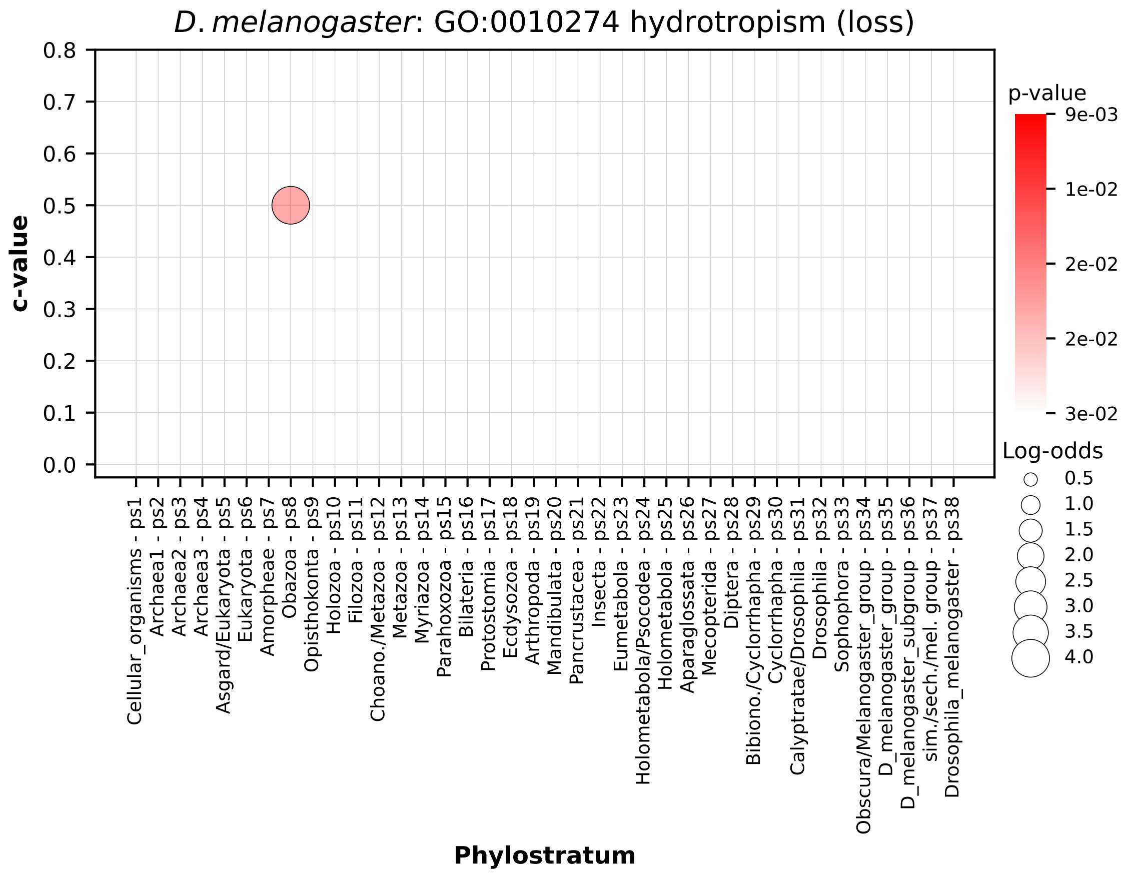

D. melanogaster: GO:0010274 hydrotropism (loss)
c-value
0.8
0.7
0.6
0.5
0.4
0.3
0.2
0.1
0.0
Phylostratum
Cellular_organisms - ps1
Archaea1 - ps2
Archaea2 - ps3
Archaea3 - ps4
Asgard/Eukaryota - ps5
Eukaryota - ps6
Amorpheae - ps7
Obazoa - ps8
Opisthokonta - ps9
Holozoa - ps10
Filozoa - ps11
Choano./Metazoa - ps12
Metazoa - ps13
Myriazoa - ps14
Parahoxozoa - ps15
Bilateria - ps16
Protostomia - ps17
Ecdysozoa - ps18
Arthropoda - ps19
Mandibulata - ps20
Pancrustacea - ps21
Insecta - ps22
Eumetabola - ps23
Holometabola/Psocodea - ps24
Holometabola - ps25
Aparaglossata - ps26
Mecopterida - ps27
Diptera - ps28
Bibiono./Cyclorrhapha - ps29
Cyclorrhapha - ps30
Calyptratae/Drosophila - ps31
Drosophila - ps32
Sophophora - ps33
Obscura/Melanogaster_group - ps34
D_melanogaster_group - ps35
D_melanogaster_subgroup - ps36
sim./sech./mel. group - ps37
Drosophila_melanogaster - ps38
p-value
9e-03
1e-02
2e-02
2e-02
3e-02
Log-odds
0.5
1.0
1.5
2.0
2.5
3.0
3.5
4.0

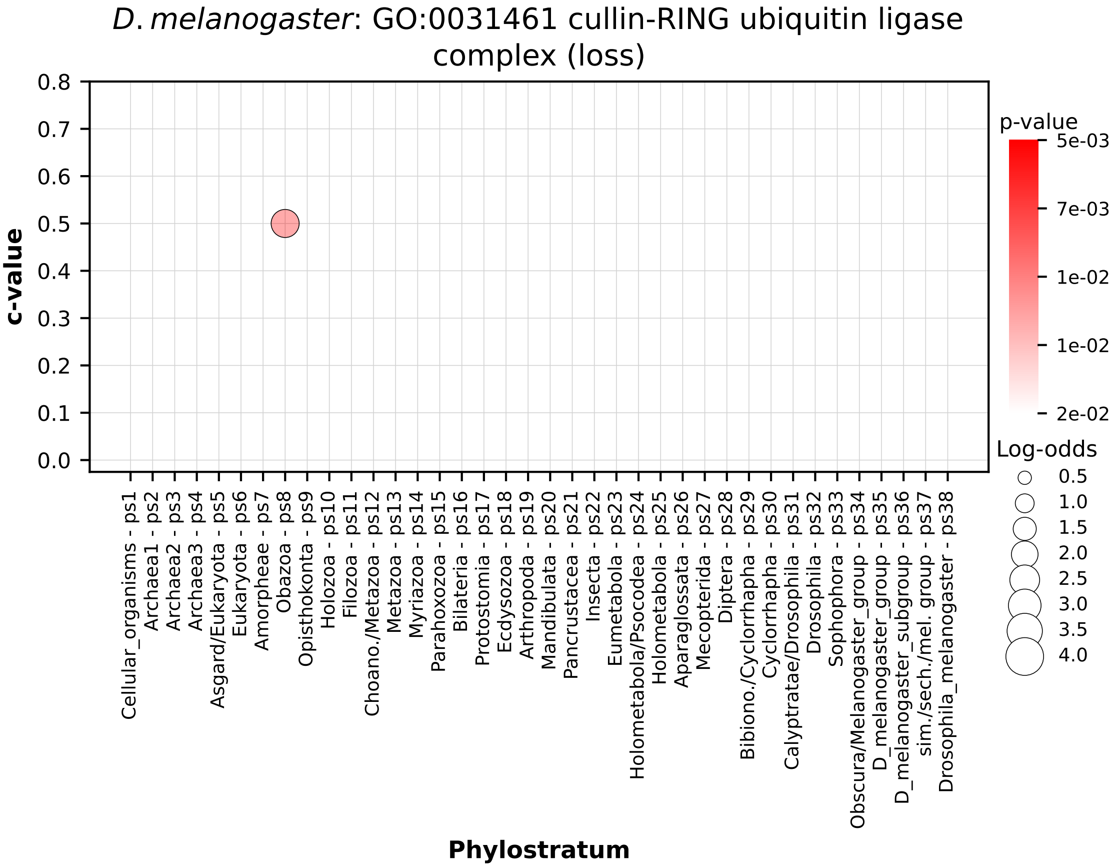
D. melanogaster: GO:0031461 cullin-RING ubiquitin ligase complex (loss)
c-value
0.8
0.7
0.6
0.5
0.4
0.3
0.2
0.1
0.0
Phylostratum
Cellular_organisms - ps1
Archaea1 - ps2
Archaea2 - ps3
Archaea3 - ps4
Asgard/Eukaryota - ps5
Eukaryota - ps6
Amorpheae - ps7
Obazoa - ps8
Opisthokonta - ps9
Holozoa - ps10
Filozoa - ps11
Choano./Metazoa - ps12
Metazoa - ps13
Myriazoa - ps14
Parahoxozoa - ps15
Bilateria - ps16
Protostomia - ps17
Ecdysozoa - ps18
Arthropoda - ps19
Mandibulata - ps20
Pancrustacea - ps21
Insecta - ps22
Eumetabola - ps23
Holometabola/Psocodea - ps24
Holometabola - ps25
Aparaglossata - ps26
Mecopterida - ps27
Diptera - ps28
Bibiono./Cyclorrhapha - ps29
Cyclorrhapha - ps30
Calyptratae/Drosophila - ps31
Drosophila - ps32
Sophophora - ps33
Obscura/Melanogaster_group - ps34
D_melanogaster_group - ps35
D_melanogaster_subgroup - ps36
sim./sech./mel. group - ps37
Drosophila_melanogaster - ps38
p-value
5e-03
7e-03
1e-02
1e-02
2e-02
Log-odds
0.5
1.0
1.5
2.0
2.5
3.0
3.5
4.0

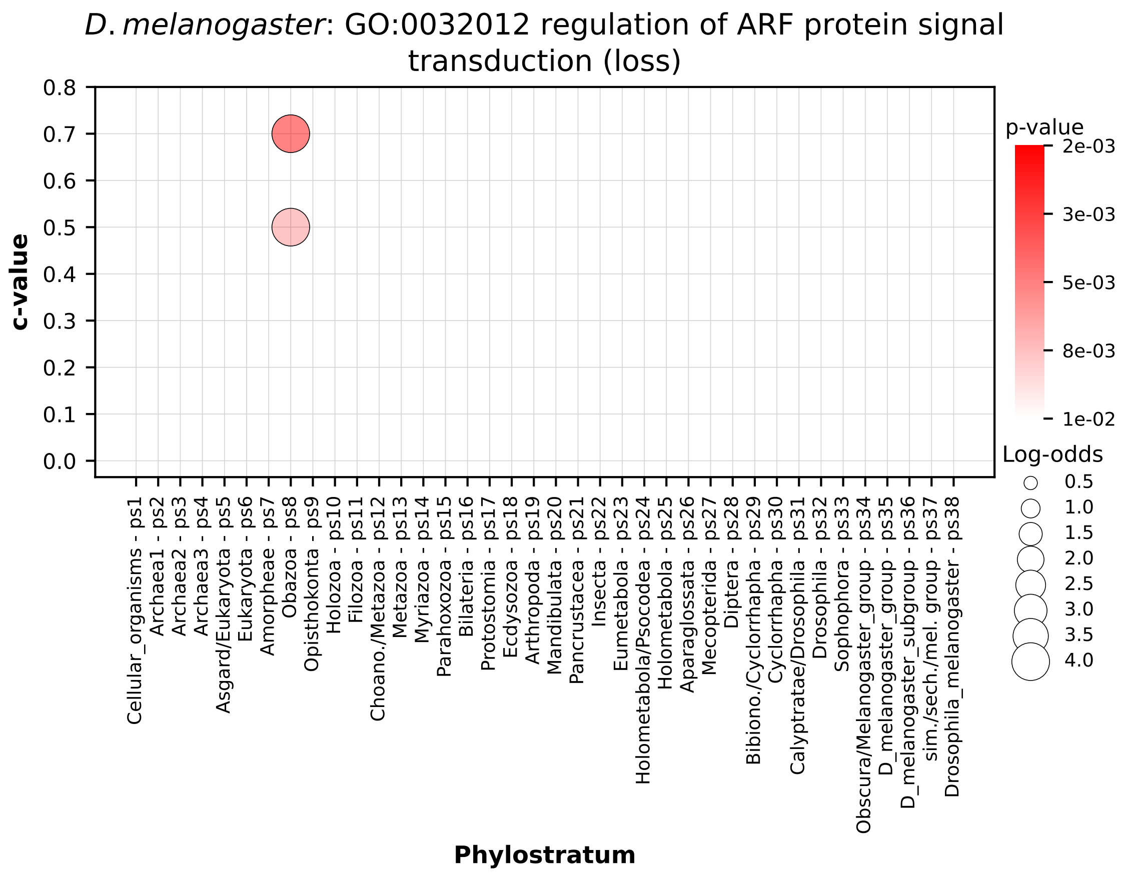

D. melanogaster: GO:0032012 regulation of ARF protein signal transduction (loss)
c-value
0.8
0.7
0.6
0.5
0.4
0.3
0.2
0.1
0.0
Phylostratum
Cellular_organisms - ps1
Archaea1 - ps2
Archaea2 - ps3
Archaea3 - ps4
Asgard/Eukaryota - ps5
Eukaryota - ps6
Amorpheae - ps7
Obazoa - ps8
Opisthokonta - ps9
Holozoa - ps10
Filozoa - ps11
Choano./Metazoa - ps12
Metazoa - ps13
Myriazoa - ps14
Parahoxozoa - ps15
Bilateria - ps16
Protostomia - ps17
Ecdysozoa - ps18
Arthropoda - ps19
Mandibulata - ps20
Pancrustacea - ps21
Insecta - ps22
Eumetabola - ps23
Holometabola/Psocodea - ps24
Holometabola - ps25
Aparaglossata - ps26
Mecopterida - ps27
Diptera - ps28
Bibiono./Cyclorrhapha - ps29
Cyclorrhapha - ps30
Calyptratae/Drosophila - ps31
Drosophila - ps32
Sophophora - ps33
Obscura/Melanogaster_group - ps34
D_melanogaster_group - ps35
D_melanogaster_subgroup - ps36
sim./sech./mel. group - ps37
Drosophila_melanogaster - ps38
p-value
2e-03
3e-03
5e-03
8e-03
1e-02
Log-odds
0.5
1.0
1.5
2.0
2.5
3.0
3.5
4.0

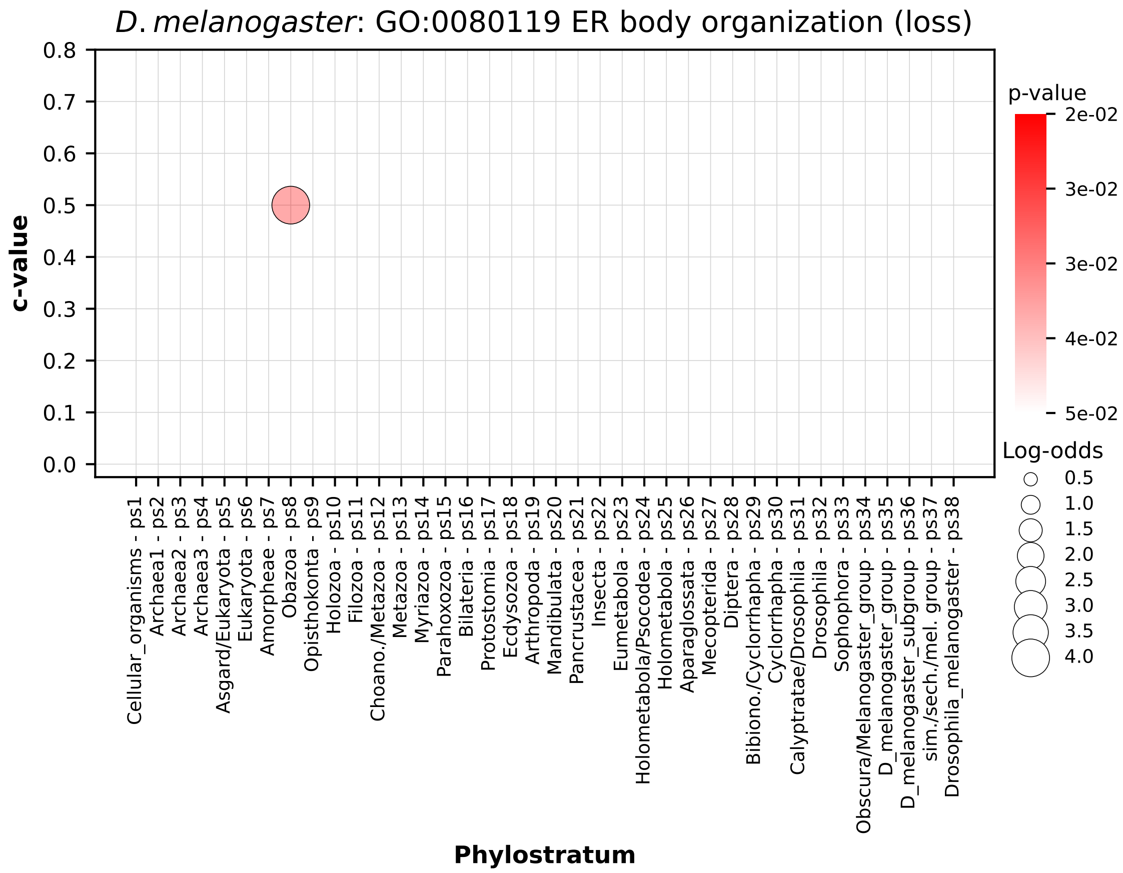

D. melanogaster: GO:0080119 ER body organization (loss)
c-value
0.8
0.7
0.6
0.5
0.4
0.3
0.2
0.1
0.0
p-value
2e-02
3e-02
3e-02
4e-02
5e-02
Log-odds
0.5
1.0
1.5
2.0
2.5
3.0
3.5
4.0
Cellular_organisms - ps1
Archaea1 - ps2
Archaea2 - ps3
Archaea3 - ps4
Asgard/Eukaryota - ps5
Eukaryota - ps6
Amorpheae - ps7
Obazoa - ps8
Opisthokonta - ps9
Holozoa - ps10
Filozoa - ps11
Choano./Metazoa - ps12
Metazoa - ps13
Myriazoa - ps14
Parahoxozoa - ps15
Bilateria - ps16
Protostomia - ps17
Ecdysozoa - ps18
Arthropoda - ps19
Mandibulata - ps20
Pancrustacea - ps21
Insecta - ps22
Eumetabola - ps23
Holometabola/Psocodea - ps24
Holometabola - ps25
Aparaglossata - ps26
Mecopterida - ps27
Diptera - ps28
Bibiono./Cyclorrhapha - ps29
Cyclorrhapha - ps30
Calyptratae/Drosophila - ps31
Drosophila - ps32
Sophophora - ps33
Obscura/Melanogaster_group - ps34
D_melanogaster_group - ps35
D_melanogaster_subgroup - ps36
sim./sech./mel. group - ps37
Drosophila_melanogaster - ps38
Phylostratum

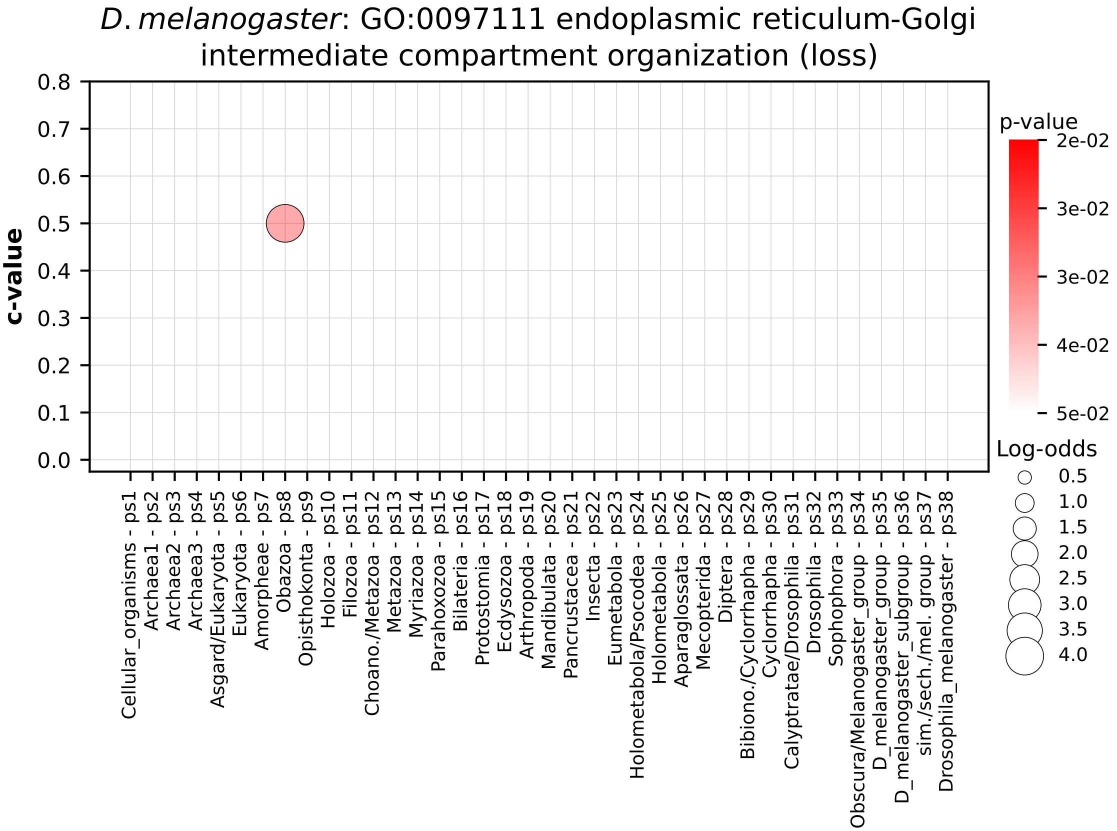

D. melanogaster: GO:0097111 endoplasmic reticulum-Golgi intermediate compartment organization (loss)
c-value
0.8
0.7
0.6
0.5
0.4
0.3
0.2
0.1
0.0
Phylostratum
Cellular_organisms - ps1
Archaea1 - ps2
Archaea2 - ps3
Archaea3 - ps4
Asgard/Eukaryota - ps5
Eukaryota - ps6
Amorpheae - ps7
Obazoa - ps8
Opisthokonta - ps9
Holozoa - ps10
Filozoa - ps11
Choano./Metazoa - ps12
Metazoa - ps13
Myriazoa - ps14
Parahoxozoa - ps15
Bilateria - ps16
Protostomia - ps17
Ecdysozoa - ps18
Arthropoda - ps19
Mandibulata - ps20
Pancrustacea - ps21
Insecta - ps22
Eumetabola - ps23
Holometabola/Psocodea - ps24
Holometabola - ps25
Aparaglossata - ps26
Mecopterida - ps27
Diptera - ps28
Bibiono./Cyclorrhapha - ps29
Cyclorrhapha - ps30
Calyptratae/Drosophila - ps31
Drosophila - ps32
Sophophora - ps33
Obscura/Melanogaster_group - ps34
D_melanogaster_group - ps35
D_melanogaster_subgroup - ps36
sim./sech./mel. group - ps37
Drosophila_melanogaster - ps38
p-value
2e-02
3e-02
3e-02
4e-02
5e-02
Log-odds
0.5
1.0
1.5
2.0
2.5
3.0
3.5
4.0

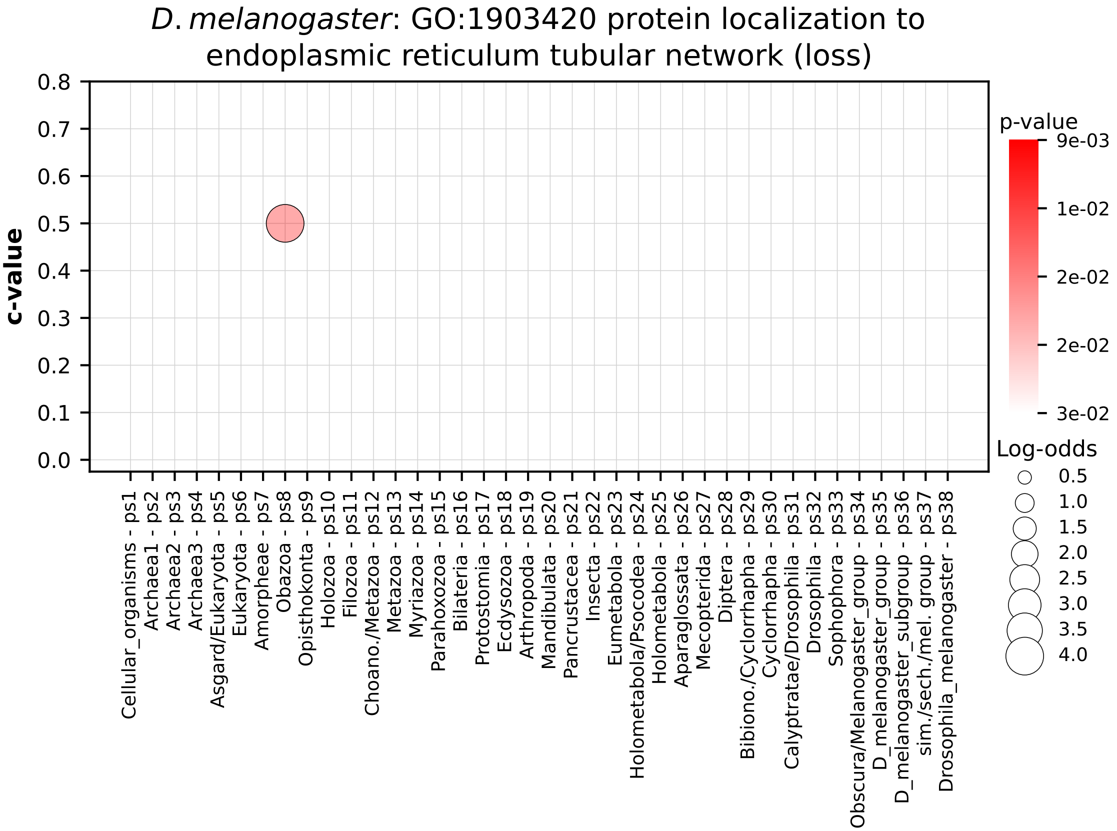

D. melanogaster: GO:1903420 protein localization to endoplasmic reticulum tubular network (loss)
c-value
0.0
0.1
0.2
0.3
0.4
0.5
0.6
0.7
0.8
Phylostratum
Cellular_organisms - ps1
Archaea1 - ps2
Archaea2 - ps3
Archaea3 - ps4
Asgard/Eukaryota - ps5
Eukaryota - ps6
Amorpheae - ps7
Obazoa - ps8
Opisthokonta - ps9
Holozoa - ps10
Filozoa - ps11
Choano./Metazoa - ps12
Metazoa - ps13
Myriazoa - ps14
Parahoxozoa - ps15
Bilateria - ps16
Protostomia - ps17
Ecdysozoa - ps18
Arthropoda - ps19
Mandibulata - ps20
Pancrustacea - ps21
Insecta - ps22
Eumetabola - ps23
Holometabola/Psocodea - ps24
Holometabola - ps25
Aparaglossata - ps26
Mecopterida - ps27
Diptera - ps28
Bibiono./Cyclorrhapha - ps29
Cyclorrhapha - ps30
Calyptratae/Drosophila - ps31
Drosophila - ps32
Sophophora - ps33
Obscura/Melanogaster_group - ps34
D_melanogaster_group - ps35
D_melanogaster_subgroup - ps36
sim./sech./mel. group - ps37
Drosophila_melanogaster - ps38
p-value
9e-03
1e-02
2e-02
2e-02
3e-02
Log-odds
0.5
1.0
1.5
2.0
2.5
3.0
3.5
4.0

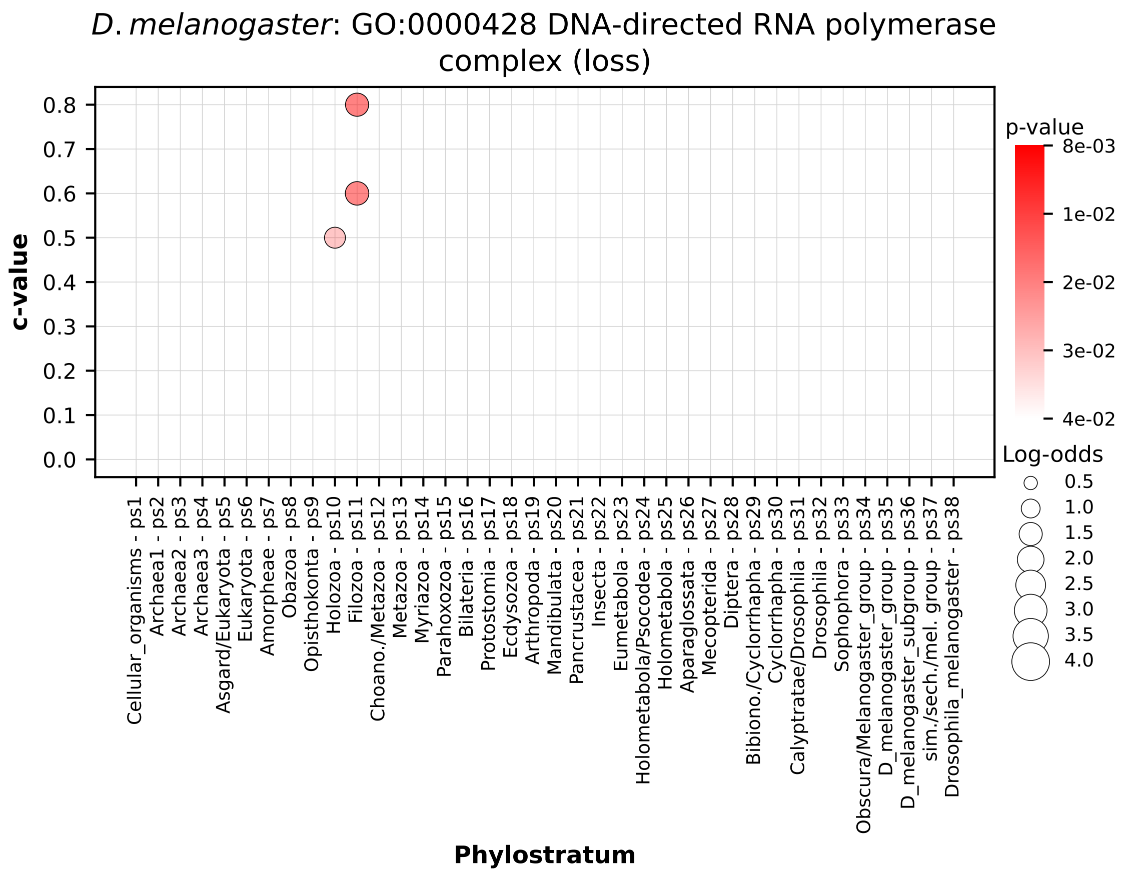

D. melanogaster: GO:0000428 DNA-directed RNA polymerase complex (loss)
c-value
0.0
0.1
0.2
0.3
0.4
0.5
0.6
0.7
0.8
Phylostratum
Cellular_organisms - ps1
Archaea1 - ps2
Archaea2 - ps3
Archaea3 - ps4
Asgard/Eukaryota - ps5
Eukaryota - ps6
Amorpheae - ps7
Obazoa - ps8
Opisthokonta - ps9
Holozoa - ps10
Filozoa - ps11
Choano./Metazoa - ps12
Metazoa - ps13
Myriazoa - ps14
Parahoxozoa - ps15
Bilateria - ps16
Protostomia - ps17
Ecdysozoa - ps18
Arthropoda - ps19
Mandibulata - ps20
Pancrustacea - ps21
Insecta - ps22
Eumetabola - ps23
Holometabola/Psocodea - ps24
Holometabola - ps25
Aparaglossata - ps26
Mecopterida - ps27
Diptera - ps28
Bibiono./Cyclorrhapha - ps29
Cyclorrhapha - ps30
Calyptratae/Drosophila - ps31
Drosophila - ps32
Sophophora - ps33
Obscura/Melanogaster_group - ps34
D_melanogaster_group - ps35
D_melanogaster_subgroup - ps36
sim./sech./mel. group - ps37
Drosophila_melanogaster - ps38
p-value
8e-03
1e-02
2e-02
3e-02
4e-02
Log-odds
0.5
1.0
1.5
2.0
2.5
3.0
3.5
4.0

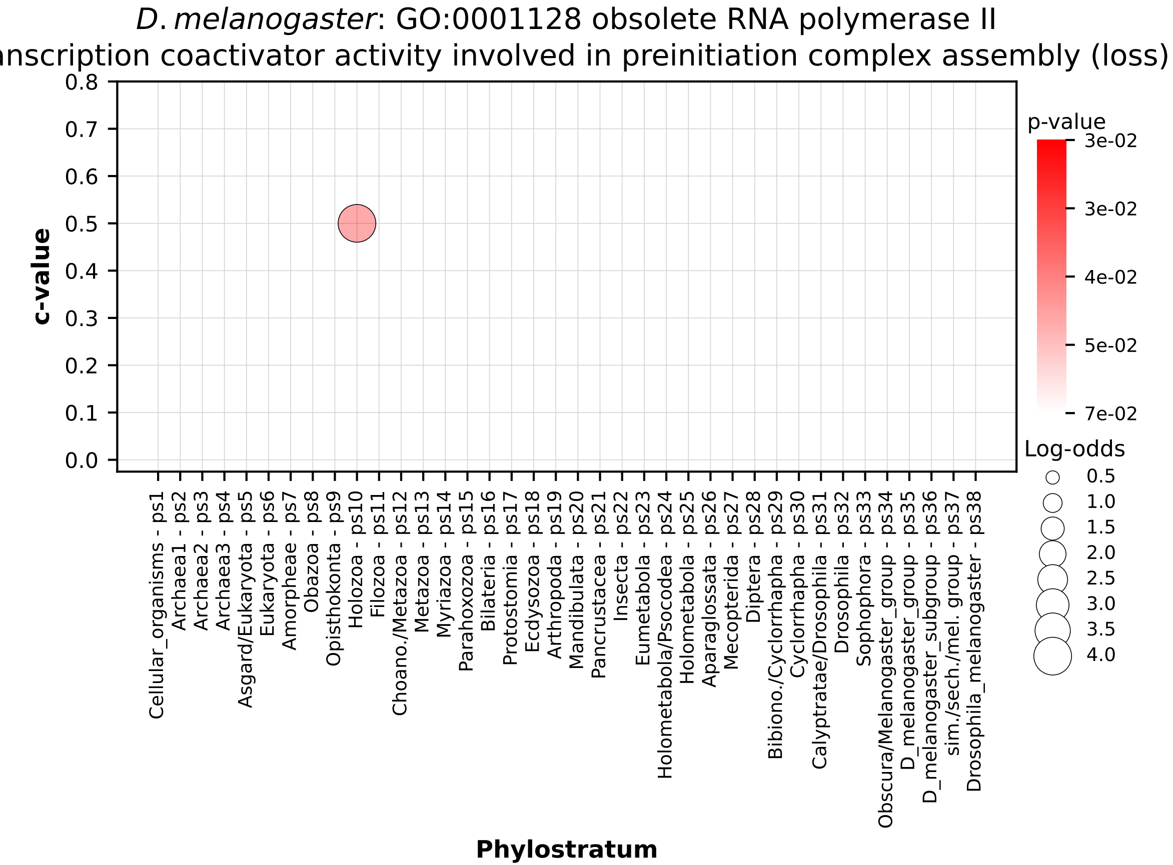

D. melanogaster: GO:0001128 obsolete RNA polymerase II
anscription coactivator activity involved in preinitiation complex assembly (loss)
c-value
0.8
0.7
0.6
0.5
0.4
0.3
0.2
0.1
0.0
p-value
3e-02
3e-02
4e-02
5e-02
7e-02
Log-odds
0.5
1.0
1.5
2.0
2.5
3.0
3.5
4.0
Cellular_organisms - ps1
Archaea1 - ps2
Archaea2 - ps3
Archaea3 - ps4
Asgard/Eukaryota - ps5
Eukaryota - ps6
Amorpheae - ps7
Obazoa - ps8
Opisthokonta - ps9
Holozoa - ps10
Filozoa - ps11
Choano./Metazoa - ps12
Metazoa - ps13
Myriazoa - ps14
Parahoxozoa - ps15
Bilateria - ps16
Protostomia - ps17
Ecdysozoa - ps18
Arthropoda - ps19
Mandibulata - ps20
Pancrustacea - ps21
Insecta - ps22
Eumetabola - ps23
Holometabola/Psocodea - ps24
Holometabola - ps25
Aparaglossata - ps26
Mecopterida - ps27
Diptera - ps28
Bibiono./Cyclorrhapha - ps29
Cyclorrhapha - ps30
Calyptratae/Drosophila - ps31
Drosophila - ps32
Sophophora - ps33
Obscura/Melanogaster_group - ps34
D_melanogaster_group - ps35
D_melanogaster_subgroup - ps36
sim./sech./mel. group - ps37
Drosophila_melanogaster - ps38
Phylostratum

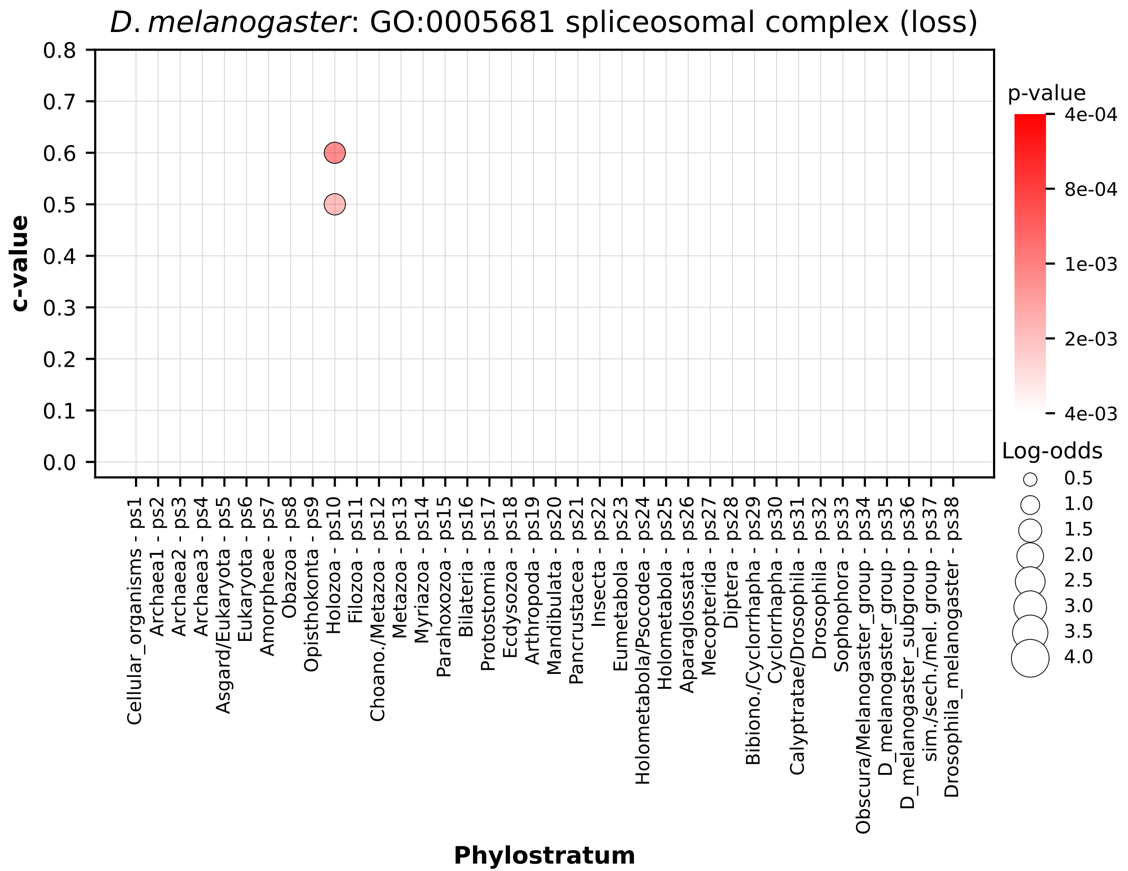

D. melanogaster: GO:0005681 spliceosomal complex (loss)
c-value
0.0
0.1
0.2
0.3
0.4
0.5
0.6
0.7
0.8
Phylostratum
Cellular_organisms - ps1
Archaea1 - ps2
Archaea2 - ps3
Archaea3 - ps4
Asgard/Eukaryota - ps5
Eukaryota - ps6
Amorpheae - ps7
Obazoa - ps8
Opisthokonta - ps9
Holozoa - ps10
Filozoa - ps11
Choano./Metazoa - ps12
Metazoa - ps13
Myriazoa - ps14
Parahoxozoa - ps15
Bilateria - ps16
Protostomia - ps17
Ecdysozoa - ps18
Arthropoda - ps19
Mandibulata - ps20
Pancrustacea - ps21
Insecta - ps22
Eumetabola - ps23
Holometabola/Psocodea - ps24
Holometabola - ps25
Aparaglossata - ps26
Mecopterida - ps27
Diptera - ps28
Bibiono./Cyclorrhapha - ps29
Cyclorrhapha - ps30
Calyptratae/Drosophila - ps31
Drosophila - ps32
Sophophora - ps33
Obscura/Melanogaster_group - ps34
D_melanogaster_group - ps35
D_melanogaster_subgroup - ps36
sim./sech./mel. group - ps37
Drosophila_melanogaster - ps38
p-value
4e-04
8e-04
1e-03
2e-03
4e-03
Log-odds
0.5
1.0
1.5
2.0
2.5
3.0
3.5
4.0

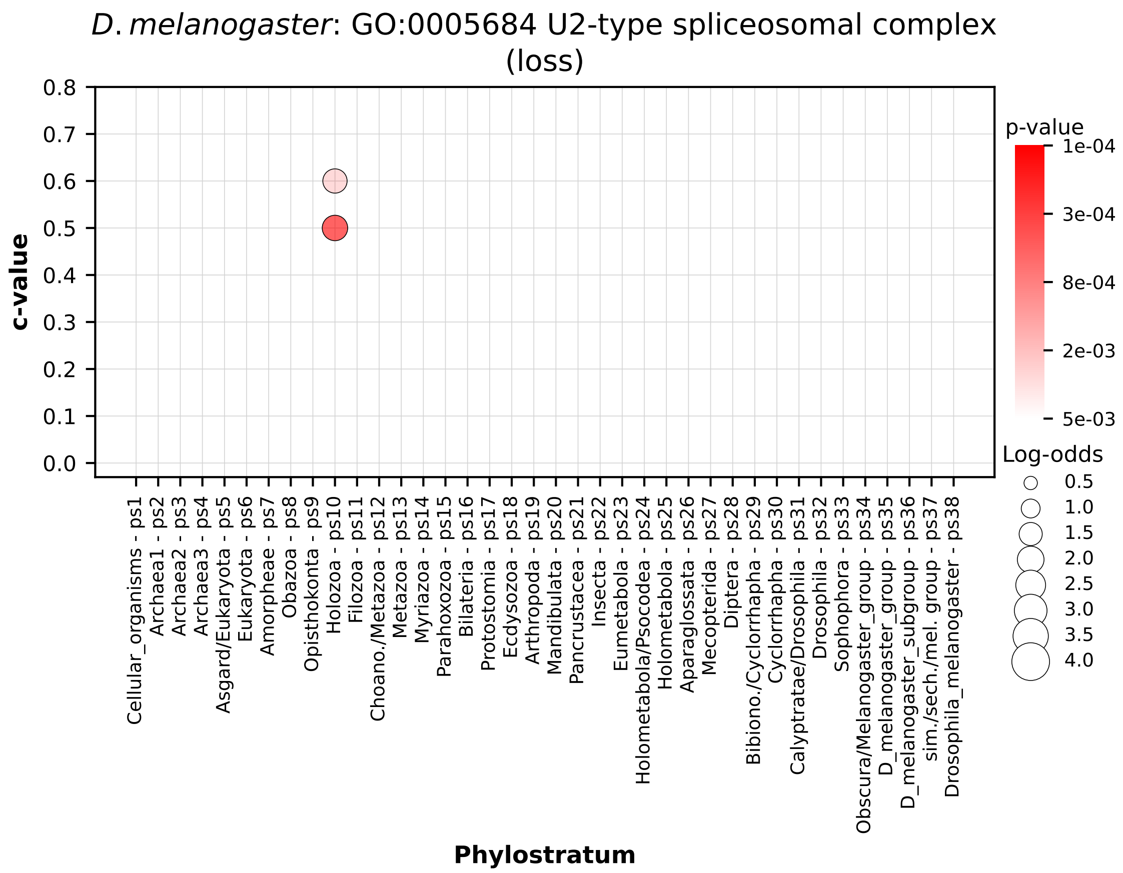

D. melanogaster: GO:0005684 U2-type spliceosomal complex (loss)
c-value
0.8
0.7
0.6
0.5
0.4
0.3
0.2
0.1
0.0
p-value
1e-04
3e-04
8e-04
2e-03
5e-03
Log-odds
0.5
1.0
1.5
2.0
2.5
3.0
3.5
4.0
Cellular_organisms - ps1
Archaea1 - ps2
Archaea2 - ps3
Archaea3 - ps4
Asgard/Eukaryota - ps5
Eukaryota - ps6
Amorpheae - ps7
Obazoa - ps8
Opisthokonta - ps9
Holozoa - ps10
Filozoa - ps11
Choano./Metazoa - ps12
Metazoa - ps13
Myriazoa - ps14
Parahoxozoa - ps15
Bilateria - ps16
Protostomia - ps17
Ecdysozoa - ps18
Arthropoda - ps19
Mandibulata - ps20
Pancrustacea - ps21
Insecta - ps22
Eumetabola - ps23
Holometabola/Psocodea - ps24
Holometabola - ps25
Aparaglossata - ps26
Mecopterida - ps27
Diptera - ps28
Bibiono./Cyclorrhapha - ps29
Cyclorrhapha - ps30
Calyptratae/Drosophila - ps31
Drosophila - ps32
Sophophora - ps33
Obscura/Melanogaster_group - ps34
D_melanogaster_group - ps35
D_melanogaster_subgroup - ps36
sim./sech./mel. group - ps37
Drosophila_melanogaster - ps38
Phylostratum

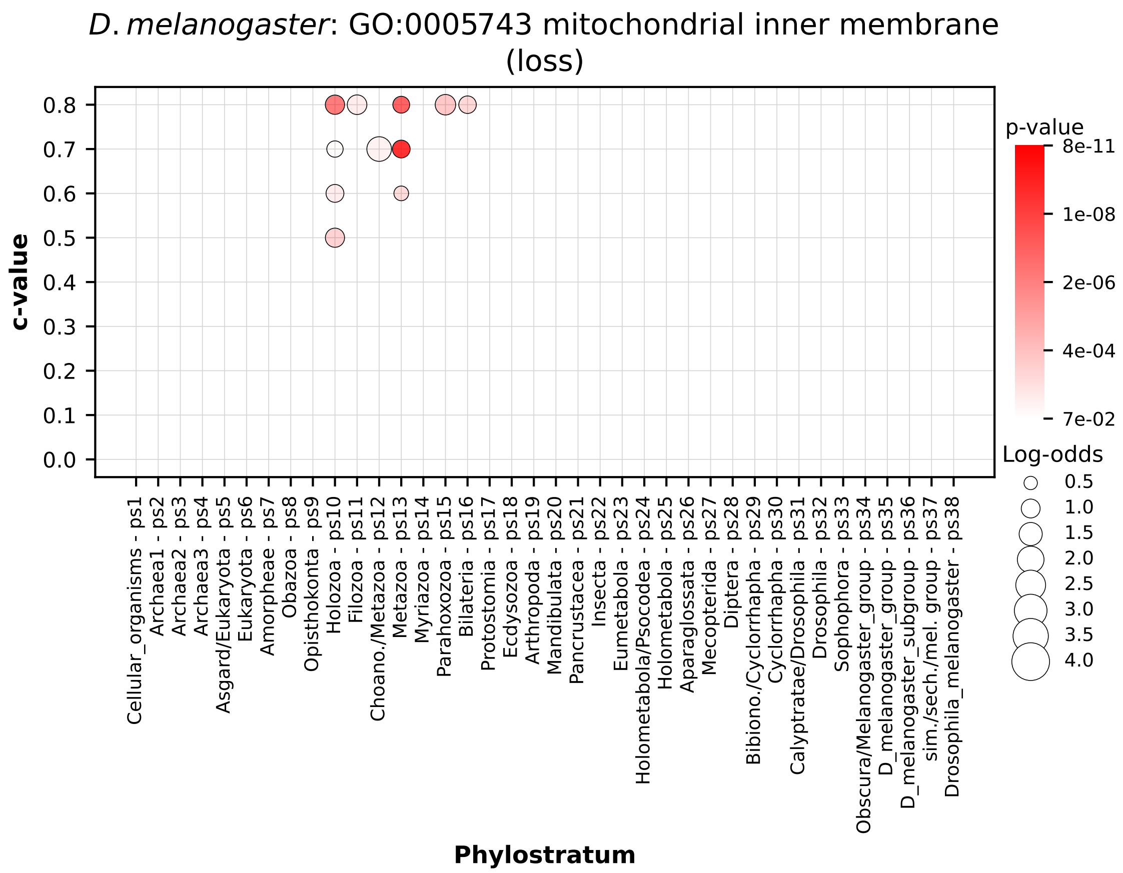

D. melanogaster: GO:0005743 mitochondrial inner membrane (loss)
c-value
0.8
0.7
0.6
0.5
0.4
0.3
0.2
0.1
0.0
Phylostratum
Cellular_organisms - ps1
Archaea1 - ps2
Archaea2 - ps3
Archaea3 - ps4
Asgard/Eukaryota - ps5
Eukaryota - ps6
Amorpheae - ps7
Obazoa - ps8
Opisthokonta - ps9
Holozoa - ps10
Filozoa - ps11
Choano./Metazoa - ps12
Metazoa - ps13
Myriazoa - ps14
Parahoxozoa - ps15
Bilateria - ps16
Protostomia - ps17
Ecdysozoa - ps18
Arthropoda - ps19
Mandibulata - ps20
Pancrustacea - ps21
Insecta - ps22
Eumetabola - ps23
Holometabola/Psocodea - ps24
Holometabola - ps25
Aparaglossata - ps26
Mecopterida - ps27
Diptera - ps28
Bibiono./Cyclorrhapha - ps29
Cyclorrhapha - ps30
Calyptratae/Drosophila - ps31
Drosophila - ps32
Sophophora - ps33
Obscura/Melanogaster_group - ps34
D_melanogaster_group - ps35
D_melanogaster_subgroup - ps36
sim./sech./mel. group - ps37
Drosophila_melanogaster - ps38
p-value
8e-11
1e-08
2e-06
4e-04
7e-02
Log-odds
0.5
1.0
1.5
2.0
2.5
3.0
3.5
4.0

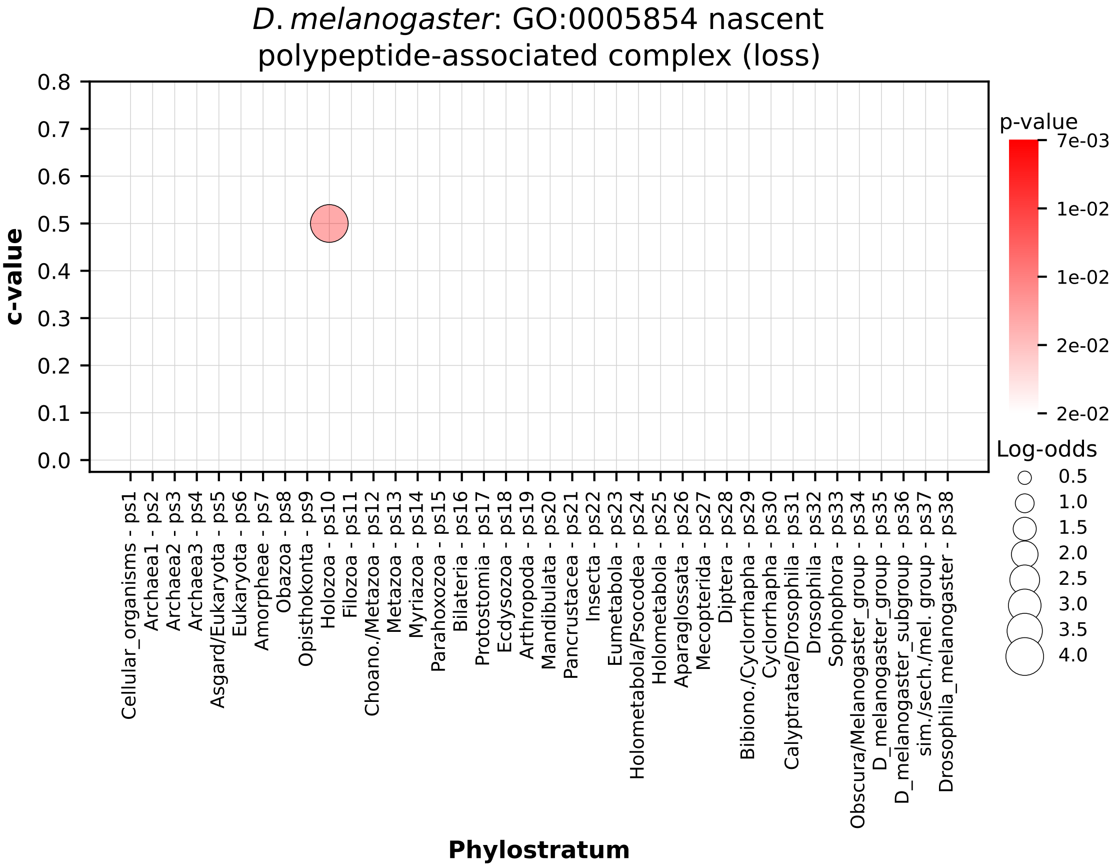

D. melanogaster: GO:0005854 nascent polypeptide-associated complex (loss)
c-value
0.8
0.7
0.6
0.5
0.4
0.3
0.2
0.1
0.0
Phylostratum
Cellular_organisms - ps1
Archaea1 - ps2
Archaea2 - ps3
Archaea3 - ps4
Asgard/Eukaryota - ps5
Eukaryota - ps6
Amorpheae - ps7
Obazoa - ps8
Opisthokonta - ps9
Holozoa - ps10
Filozoa - ps11
Choano./Metazoa - ps12
Metazoa - ps13
Myriazoa - ps14
Parahoxozoa - ps15
Bilateria - ps16
Protostomia - ps17
Ecdysozoa - ps18
Arthropoda - ps19
Mandibulata - ps20
Pancrustacea - ps21
Insecta - ps22
Eumetabola - ps23
Holometabola/Psocodea - ps24
Holometabola - ps25
Aparaglossata - ps26
Mecopterida - ps27
Diptera - ps28
Bibiono./Cyclorrhapha - ps29
Cyclorrhapha - ps30
Calyptratae/Drosophila - ps31
Drosophila - ps32
Sophophora - ps33
Obscura/Melanogaster_group - ps34
D_melanogaster_group - ps35
D_melanogaster_subgroup - ps36
sim./sech./mel. group - ps37
Drosophila_melanogaster - ps38
p-value
7e-03
1e-02
1e-02
2e-02
2e-02
Log-odds
0.5
1.0
1.5
2.0
2.5
3.0
3.5
4.0

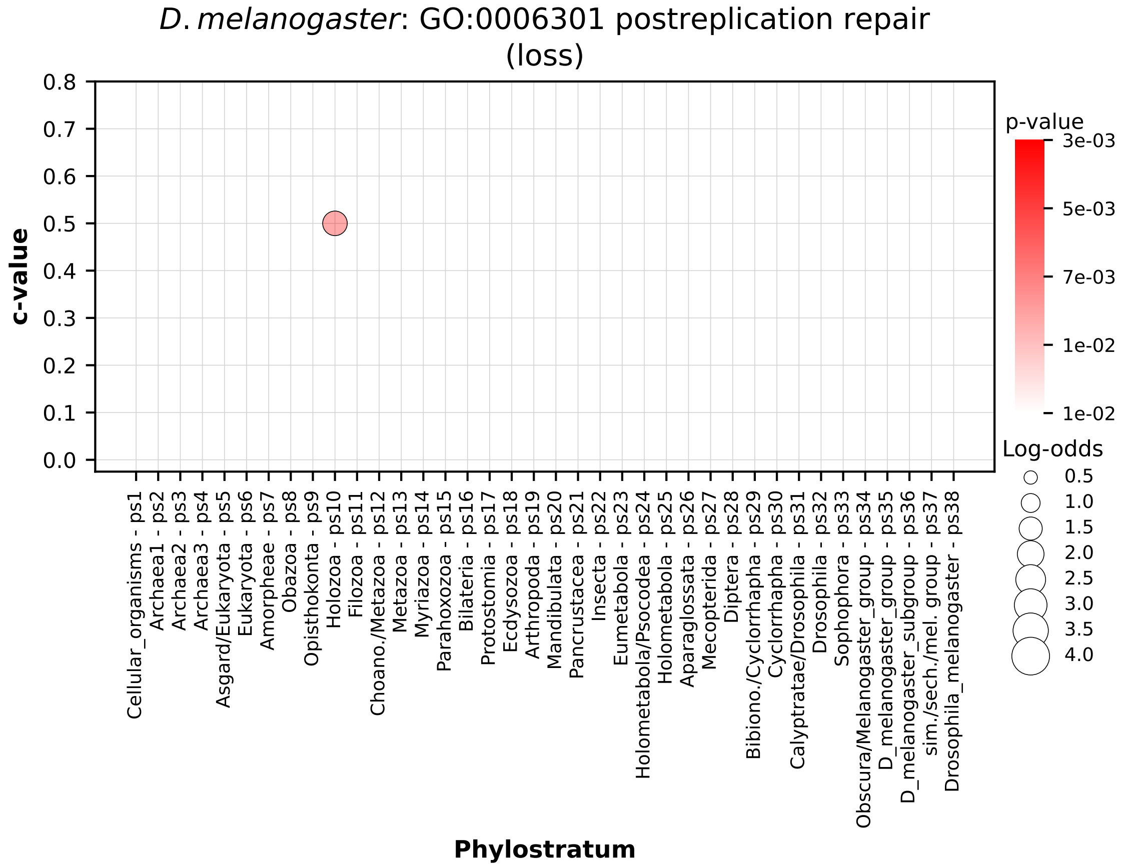

D. melanogaster: GO:0006301 postreplication repair
(loss)
c-value
0.0
0.1
0.2
0.3
0.4
0.5
0.6
0.7
0.8
Phylostratum
Cellular_organisms - ps1
Archaea1 - ps2
Archaea2 - ps3
Archaea3 - ps4
Asgard/Eukaryota - ps5
Eukaryota - ps6
Amorpheae - ps7
Obazoa - ps8
Opisthokonta - ps9
Holozoa - ps10
Filozoa - ps11
Choano./Metazoa - ps12
Metazoa - ps13
Myriazoa - ps14
Parahoxozoa - ps15
Bilateria - ps16
Protostomia - ps17
Ecdysozoa - ps18
Arthropoda - ps19
Mandibulata - ps20
Pancrustacea - ps21
Insecta - ps22
Eumetabola - ps23
Holometabola/Psocodea - ps24
Holometabola - ps25
Aparaglossata - ps26
Mecopterida - ps27
Diptera - ps28
Bibiono./Cyclorrhapha - ps29
Cyclorrhapha - ps30
Calyptratae/Drosophila - ps31
Drosophila - ps32
Sophophora - ps33
Obscura/Melanogaster_group - ps34
D_melanogaster_group - ps35
D_melanogaster_subgroup - ps36
sim./sech./mel. group - ps37
Drosophila_melanogaster - ps38
p-value
3e-03
5e-03
7e-03
1e-02
1e-02
Log-odds
0.5
1.0
1.5
2.0
2.5
3.0
3.5
4.0

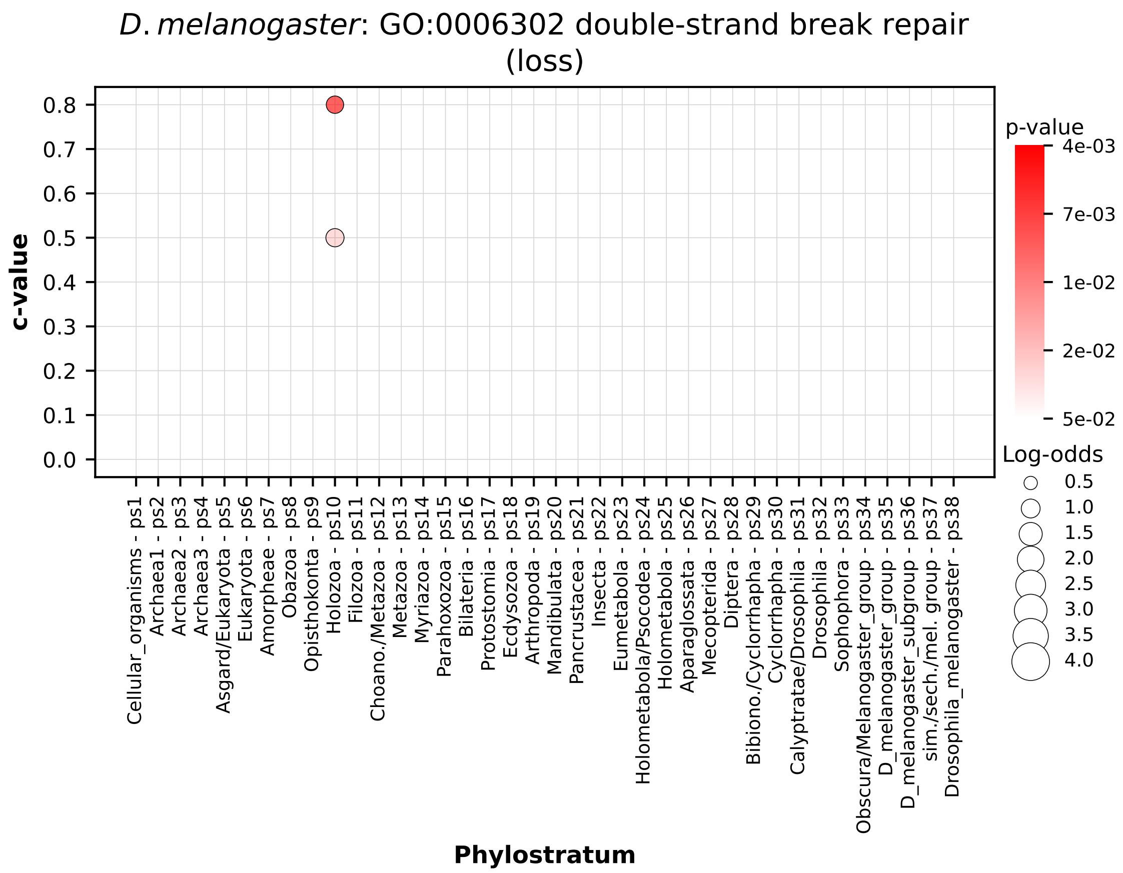
D. melanogaster: GO:0006302 double-strand break repair (loss)
c-value
0.8
0.7
0.6
0.5
0.4
0.3
0.2
0.1
0.0
Phylostratum
Cellular_organisms - ps1
Archaea1 - ps2
Archaea2 - ps3
Archaea3 - ps4
Asgard/Eukaryota - ps5
Eukaryota - ps6
Amorpheae - ps7
Obazoa - ps8
Opisthokonta - ps9
Holozoa - ps10
Filozoa - ps11
Choano./Metazoa - ps12
Metazoa - ps13
Myriazoa - ps14
Parahoxozoa - ps15
Bilateria - ps16
Protostomia - ps17
Ecdysozoa - ps18
Arthropoda - ps19
Mandibulata - ps20
Pancrustacea - ps21
Insecta - ps22
Eumetabola - ps23
Holometabola/Psocodea - ps24
Holometabola - ps25
Aparaglossata - ps26
Mecopterida - ps27
Diptera - ps28
Bibiono./Cyclorrhapha - ps29
Cyclorrhapha - ps30
Calyptratae/Drosophila - ps31
Drosophila - ps32
Sophophora - ps33
Obscura/Melanogaster_group - ps34
D_melanogaster_group - ps35
D_melanogaster_subgroup - ps36
sim./sech./mel. group - ps37
Drosophila_melanogaster - ps38
p-value
4e-03
7e-03
1e-02
2e-02
5e-02
Log-odds
0.5
1.0
1.5
2.0
2.5
3.0
3.5
4.0

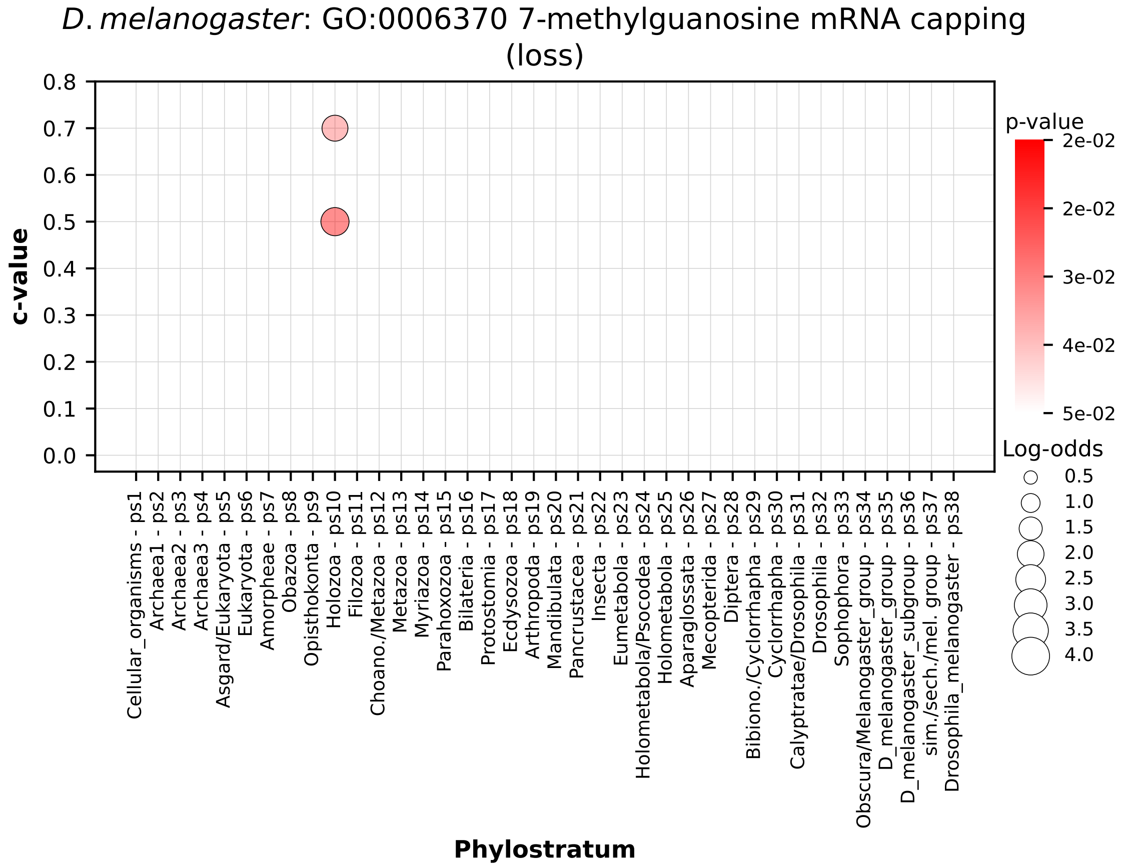

D. melanogaster: GO:0006370 7-methylguanosine mRNA capping (loss)
c-value
0.8
0.7
0.6
0.5
0.4
0.3
0.2
0.1
0.0
p-value
2e-02
2e-02
3e-02
4e-02
5e-02
Log-odds
0.5
1.0
1.5
2.0
2.5
3.0
3.5
4.0
Cellular_organisms - ps1
Archaea1 - ps2
Archaea2 - ps3
Archaea3 - ps4
Asgard/Eukaryota - ps5
Eukaryota - ps6
Amorpheae - ps7
Obazoa - ps8
Opisthokonta - ps9
Holozoa - ps10
Filozoa - ps11
Choano./Metazoa - ps12
Metazoa - ps13
Myriazoa - ps14
Parahoxozoa - ps15
Bilateria - ps16
Protostomia - ps17
Ecdysozoa - ps18
Arthropoda - ps19
Mandibulata - ps20
Pancrustacea - ps21
Insecta - ps22
Eumetabola - ps23
Holometabola/Psocodea - ps24
Holometabola - ps25
Aparaglossata - ps26
Mecopterida - ps27
Diptera - ps28
Bibiono./Cyclorrhapha - ps29
Cyclorrhapha - ps30
Calyptratae/Drosophila - ps31
Drosophila - ps32
Sophophora - ps33
Obscura/Melanogaster_group - ps34
D_melanogaster_group - ps35
D_melanogaster_subgroup - ps36
sim./sech./mel. group - ps37
Drosophila_melanogaster - ps38
Phylostratum

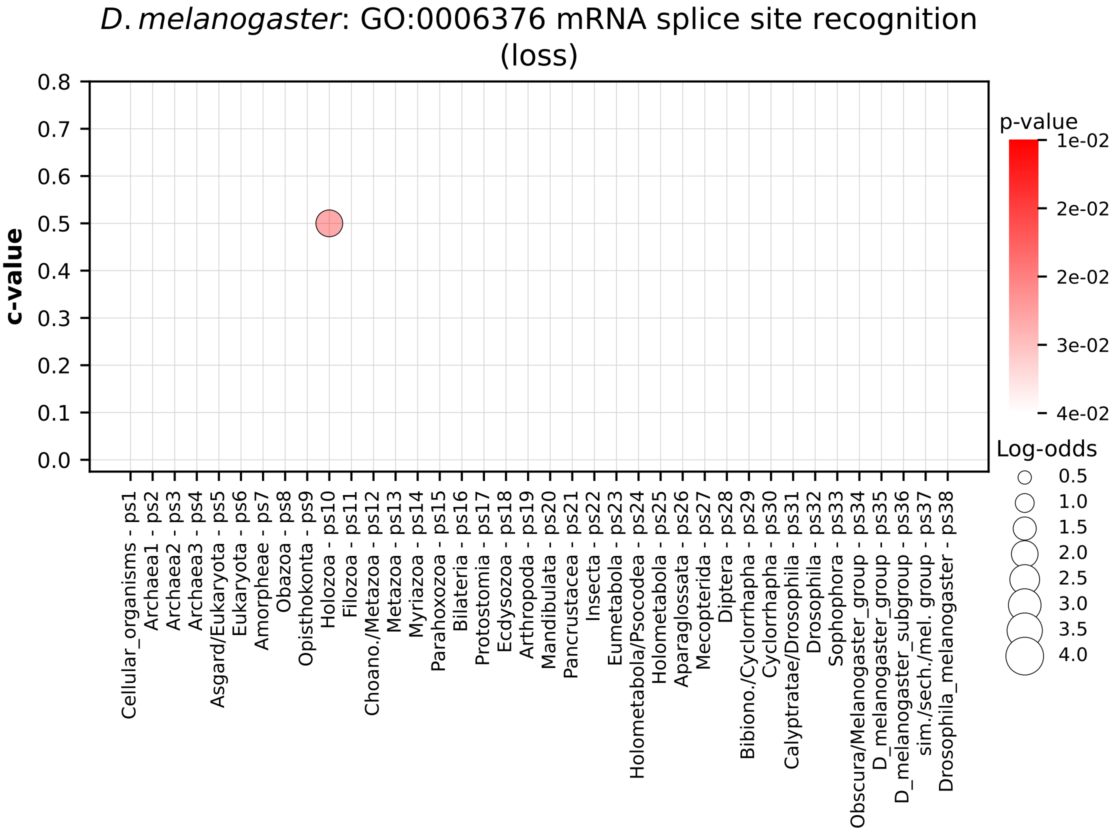

D. melanogaster: GO:0006376 mRNA splice site recognition (loss)
c-value
0.0
0.1
0.2
0.3
0.4
0.5
0.6
0.7
0.8
Phylostratum
Cellular_organisms - ps1
Archaea1 - ps2
Archaea2 - ps3
Archaea3 - ps4
Asgard/Eukaryota - ps5
Eukaryota - ps6
Amorpheae - ps7
Obazoa - ps8
Opisthokonta - ps9
Holozoa - ps10
Filozoa - ps11
Choano./Metazoa - ps12
Metazoa - ps13
Myriazoa - ps14
Parahoxozoa - ps15
Bilateria - ps16
Protostomia - ps17
Ecdysozoa - ps18
Arthropoda - ps19
Mandibulata - ps20
Pancrustacea - ps21
Insecta - ps22
Eumetabola - ps23
Holometabola/Psocodea - ps24
Holometabola - ps25
Aparaglossata - ps26
Mecopterida - ps27
Diptera - ps28
Bibiono./Cyclorrhapha - ps29
Cyclorrhapha - ps30
Calyptratae/Drosophila - ps31
Drosophila - ps32
Sophophora - ps33
Obscura/Melanogaster_group - ps34
D_melanogaster_group - ps35
D_melanogaster_subgroup - ps36
sim./sech./mel. group - ps37
Drosophila_melanogaster - ps38
p-value
1e-02
2e-02
2e-02
3e-02
4e-02
Log-odds
0.5
1.0
1.5
2.0
2.5
3.0
3.5
4.0

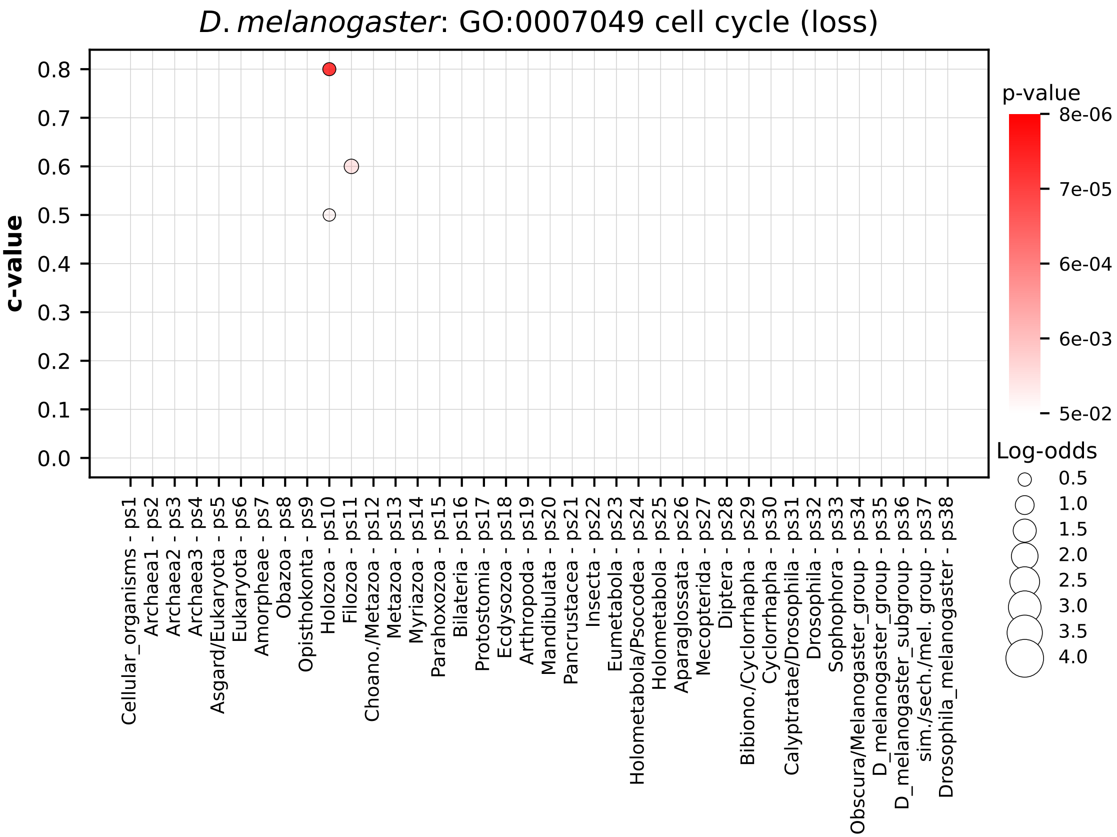

D. melanogaster: GO:0007049 cell cycle (loss)
c-value
0.0
0.1
0.2
0.3
0.4
0.5
0.6
0.7
0.8
Phylostratum
Cellular_organisms - ps1
Archaea1 - ps2
Archaea2 - ps3
Archaea3 - ps4
Asgard/Eukaryota - ps5
Eukaryota - ps6
Amorpheae - ps7
Obazoa - ps8
Opisthokonta - ps9
Holozoa - ps10
Filozoa - ps11
Choano./Metazoa - ps12
Metazoa - ps13
Myriazoa - ps14
Parahoxozoa - ps15
Bilateria - ps16
Protostomia - ps17
Ecdysozoa - ps18
Arthropoda - ps19
Mandibulata - ps20
Pancrustacea - ps21
Insecta - ps22
Eumetabola - ps23
Holometabola/Psocodea - ps24
Holometabola - ps25
Aparaglossata - ps26
Mecopterida - ps27
Diptera - ps28
Bibiono./Cyclorrhapha - ps29
Cyclorrhapha - ps30
Calyptratae/Drosophila - ps31
Drosophila - ps32
Sophophora - ps33
Obscura/Melanogaster_group - ps34
D_melanogaster_group - ps35
D_melanogaster_subgroup - ps36
sim./sech./mel. group - ps37
Drosophila_melanogaster - ps38
p-value
8e-06
7e-05
6e-04
6e-03
5e-02
Log-odds
0.5
1.0
1.5
2.0
2.5
3.0
3.5
4.0

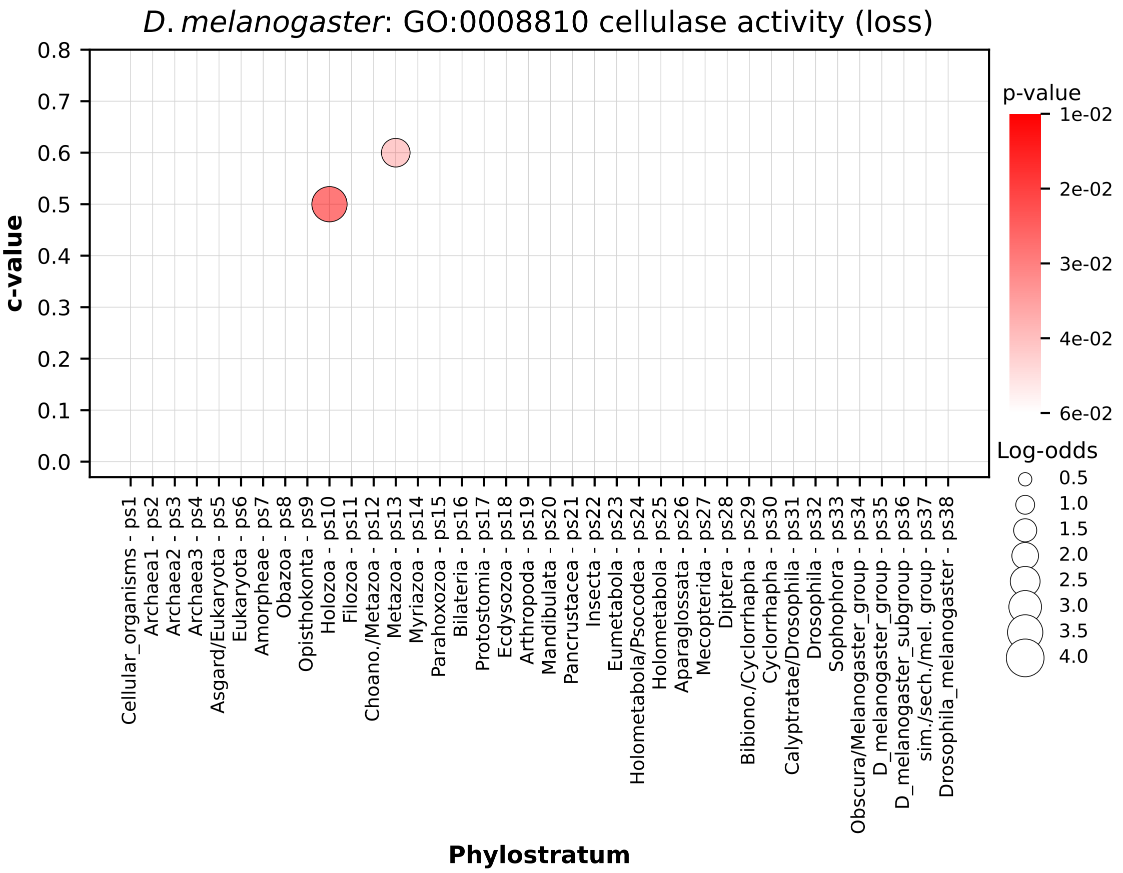
D. melanogaster: GO:0008810 cellulase activity (loss)
c-value
0.8
0.7
0.6
0.5
0.4
0.3
0.2
0.1
0.0
Phylostratum
Cellular_organisms - ps1
Archaea1 - ps2
Archaea2 - ps3
Archaea3 - ps4
Asgard/Eukaryota - ps5
Eukaryota - ps6
Amorpheae - ps7
Obazoa - ps8
Opisthokonta - ps9
Holozoa - ps10
Filozoa - ps11
Choano./Metazoa - ps12
Metazoa - ps13
Myriazoa - ps14
Parahoxozoa - ps15
Bilateria - ps16
Protostomia - ps17
Ecdysozoa - ps18
Arthropoda - ps19
Mandibulata - ps20
Pancrustacea - ps21
Insecta - ps22
Eumetabola - ps23
Holometabola/Psocodea - ps24
Holometabola - ps25
Aparaglossata - ps26
Mecopterida - ps27
Diptera - ps28
Bibiono./Cyclorrhapha - ps29
Cyclorrhapha - ps30
Calyptratae/Drosophila - ps31
Drosophila - ps32
Sophophora - ps33
Obscura/Melanogaster_group - ps34
D_melanogaster_group - ps35
D_melanogaster_subgroup - ps36
sim./sech./mel. group - ps37
Drosophila_melanogaster - ps38
p-value
1e-02
2e-02
3e-02
4e-02
6e-02
Log-odds
0.5
1.0
1.5
2.0
2.5
3.0
3.5
4.0

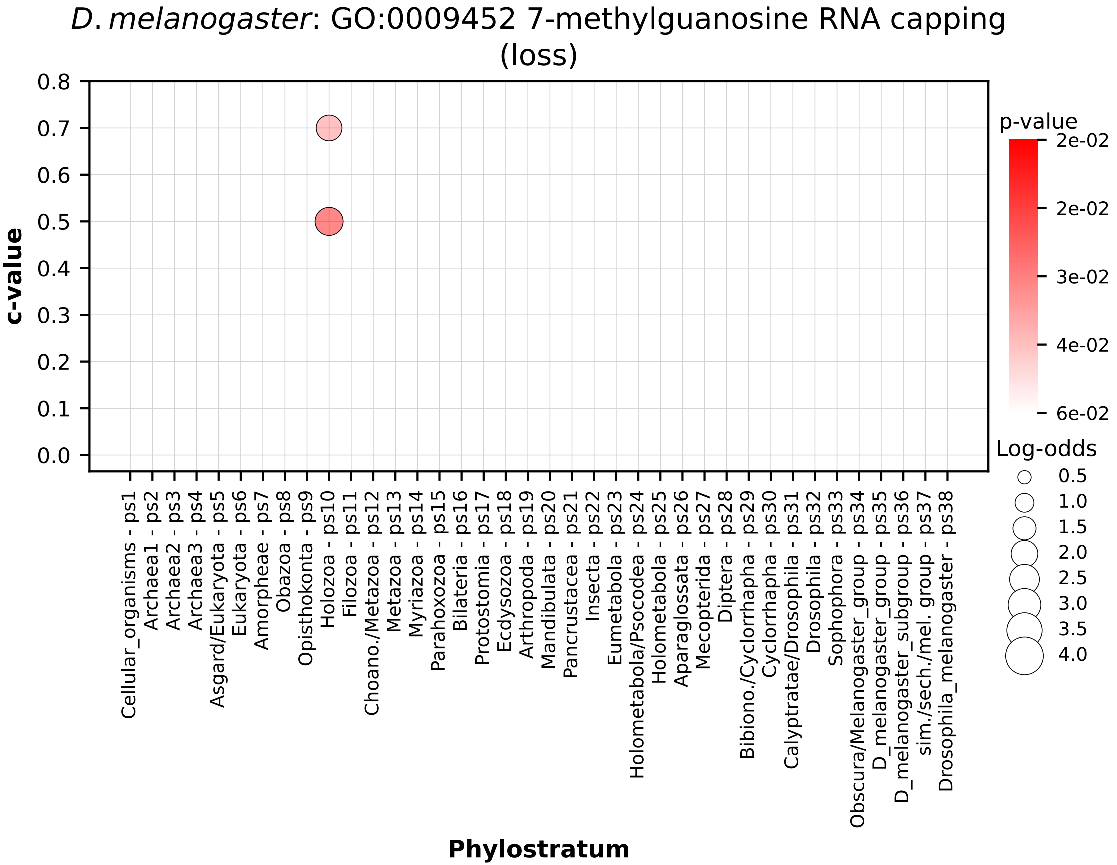

D. melanogaster: GO:0009452 7-methylguanosine RNA capping (loss)
c-value
0.8
0.7
0.6
0.5
0.4
0.3
0.2
0.1
0.0
p-value
2e-02
2e-02
3e-02
4e-02
6e-02
Log-odds
0.5
1.0
1.5
2.0
2.5
3.0
3.5
4.0
Cellular_organisms - ps1
Archaea1 - ps2
Archaea2 - ps3
Archaea3 - ps4
Asgard/Eukaryota - ps5
Eukaryota - ps6
Amorpheae - ps7
Obazoa - ps8
Opisthokonta - ps9
Holozoa - ps10
Filozoa - ps11
Choano./Metazoa - ps12
Metazoa - ps13
Myriazoa - ps14
Parahoxozoa - ps15
Bilateria - ps16
Protostomia - ps17
Ecdysozoa - ps18
Arthropoda - ps19
Mandibulata - ps20
Pancrustacea - ps21
Insecta - ps22
Eumetabola - ps23
Holometabola/Psocodea - ps24
Holometabola - ps25
Aparaglossata - ps26
Mecopterida - ps27
Diptera - ps28
Bibiono./Cyclorrhapha - ps29
Cyclorrhapha - ps30
Calyptratae/Drosophila - ps31
Drosophila - ps32
Sophophora - ps33
Obscura/Melanogaster_group - ps34
D_melanogaster_group - ps35
D_melanogaster_subgroup - ps36
sim./sech./mel. group - ps37
Drosophila_melanogaster - ps38
Phylostratum

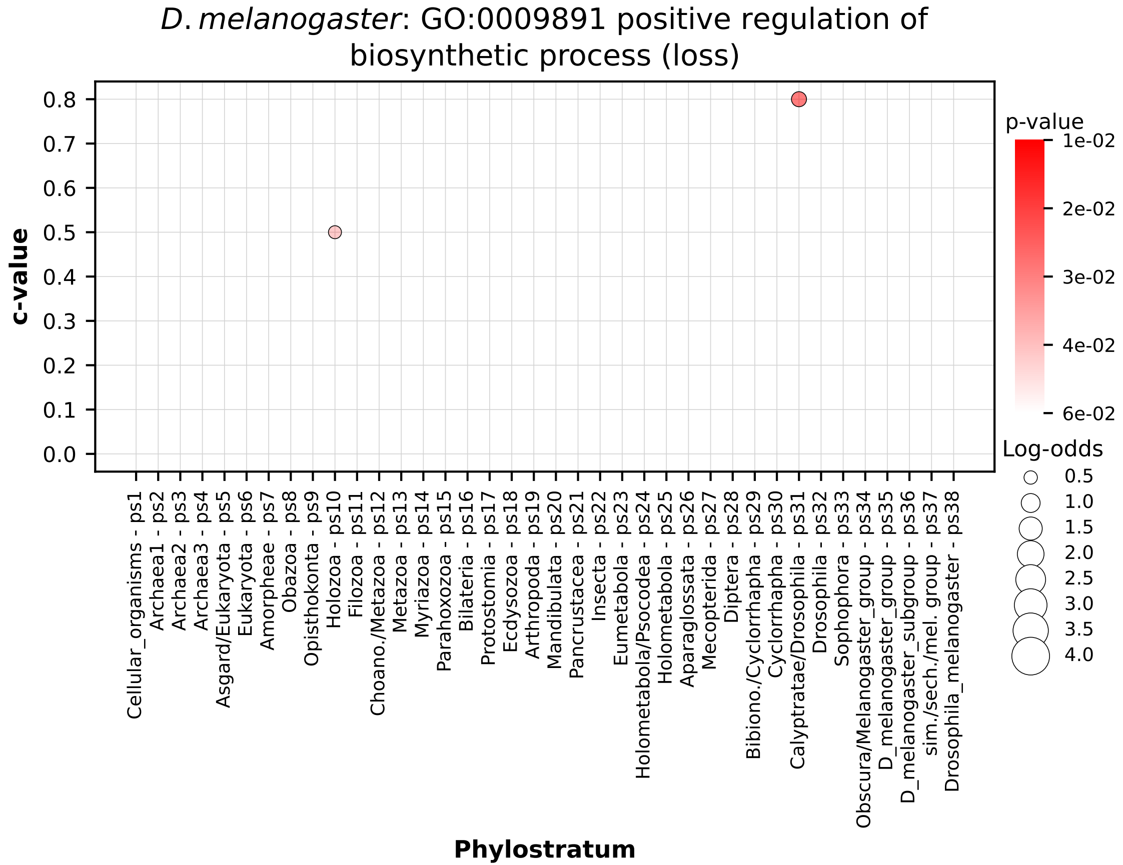

D. melanogaster: GO:0009891 positive regulation of biosynthetic process (loss)
c-value
0.0
0.1
0.2
0.3
0.4
0.5
0.6
0.7
0.8
p-value
1e-02
2e-02
3e-02
4e-02
6e-02
Log-odds
0.5
1.0
1.5
2.0
2.5
3.0
3.5
4.0
Cellular_organisms - ps1
Archaea1 - ps2
Archaea2 - ps3
Archaea3 - ps4
Asgard/Eukaryota - ps5
Eukaryota - ps6
Amorpheae - ps7
Obazoa - ps8
Opisthokonta - ps9
Holozoa - ps10
Filozoa - ps11
Choano./Metazoa - ps12
Metazoa - ps13
Myriazoa - ps14
Parahoxozoa - ps15
Bilateria - ps16
Protostomia - ps17
Ecdysozoa - ps18
Arthropoda - ps19
Mandibulata - ps20
Pancrustacea - ps21
Insecta - ps22
Eumetabola - ps23
Holometabola/Psocodea - ps24
Holometabola - ps25
Aparaglossata - ps26
Mecopterida - ps27
Diptera - ps28
Bibiono./Cyclorrhapha - ps29
Cyclorrhapha - ps30
Calyptratae/Drosophila - ps31
Drosophila - ps32
Sophophora - ps33
Obscura/Melanogaster_group - ps34
D_melanogaster_group - ps35
D_melanogaster_subgroup - ps36
sim./sech./mel. group - ps37
Drosophila_melanogaster - ps38
Phylostratum

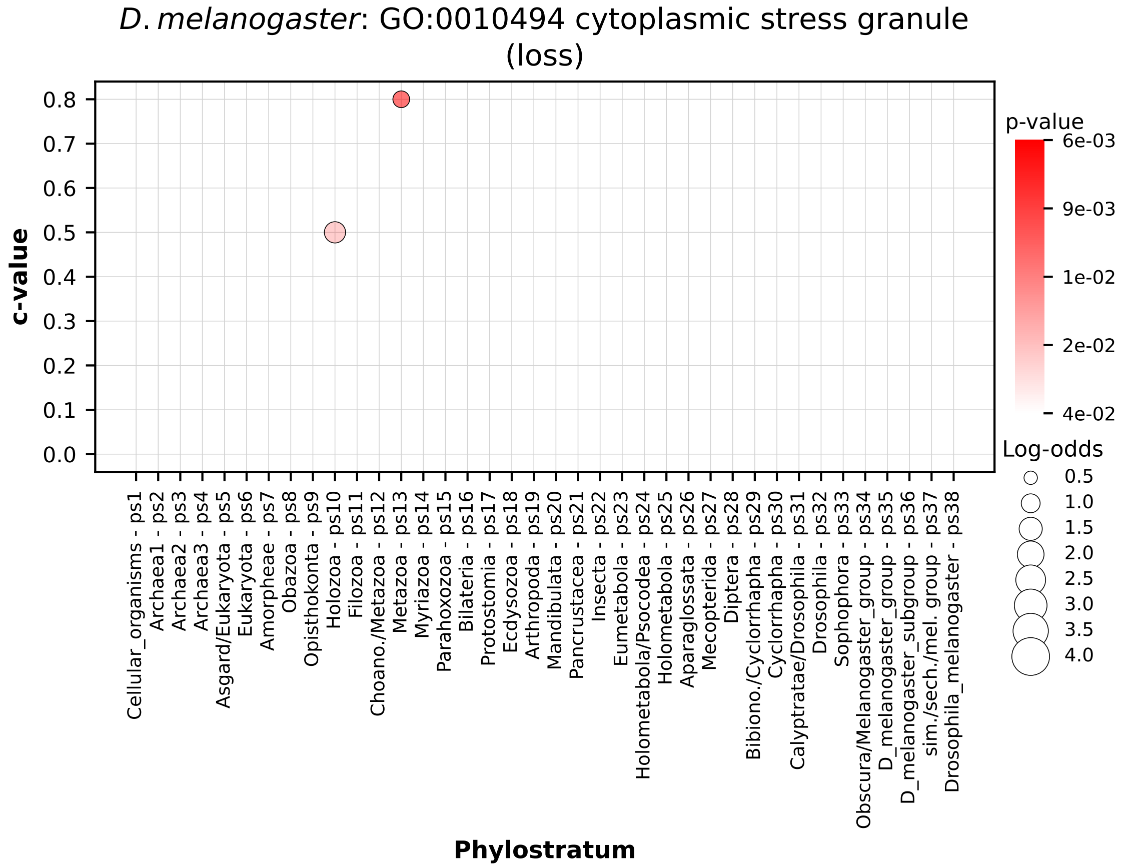
D. melanogaster: GO:0010494 cytoplasmic stress granule
(loss)
c-value
0.8
0.7
0.6
0.5
0.4
0.3
0.2
0.1
0.0
p-value
6e-03
9e-03
1e-02
2e-02
4e-02
Log-odds
0.5
1.0
1.5
2.0
2.5
3.0
3.5
4.0
Cellular_organisms - ps1
Archaea1 - ps2
Archaea2 - ps3
Archaea3 - ps4
Asgard/Eukaryota - ps5
Eukaryota - ps6
Amorpheae - ps7
Obazoa - ps8
Opisthokonta - ps9
Holozoa - ps10
Filozoa - ps11
Choano./Metazoa - ps12
Metazoa - ps13
Myriazoa - ps14
Parahoxozoa - ps15
Bilateria - ps16
Protostomia - ps17
Ecdysozoa - ps18
Arthropoda - ps19
Mandibulata - ps20
Pancrustacea - ps21
Insecta - ps22
Eumetabola - ps23
Holometabola/Psocodea - ps24
Holometabola - ps25
Aparaglossata - ps26
Mecopterida - ps27
Diptera - ps28
Bibiono./Cyclorrhapha - ps29
Cyclorrhapha - ps30
Calyptratae/Drosophila - ps31
Drosophila - ps32
Sophophora - ps33
Obscura/Melanogaster_group - ps34
D_melanogaster_group - ps35
D_melanogaster_subgroup - ps36
sim./sech./mel. group - ps37
Drosophila_melanogaster - ps38
Phylostratum

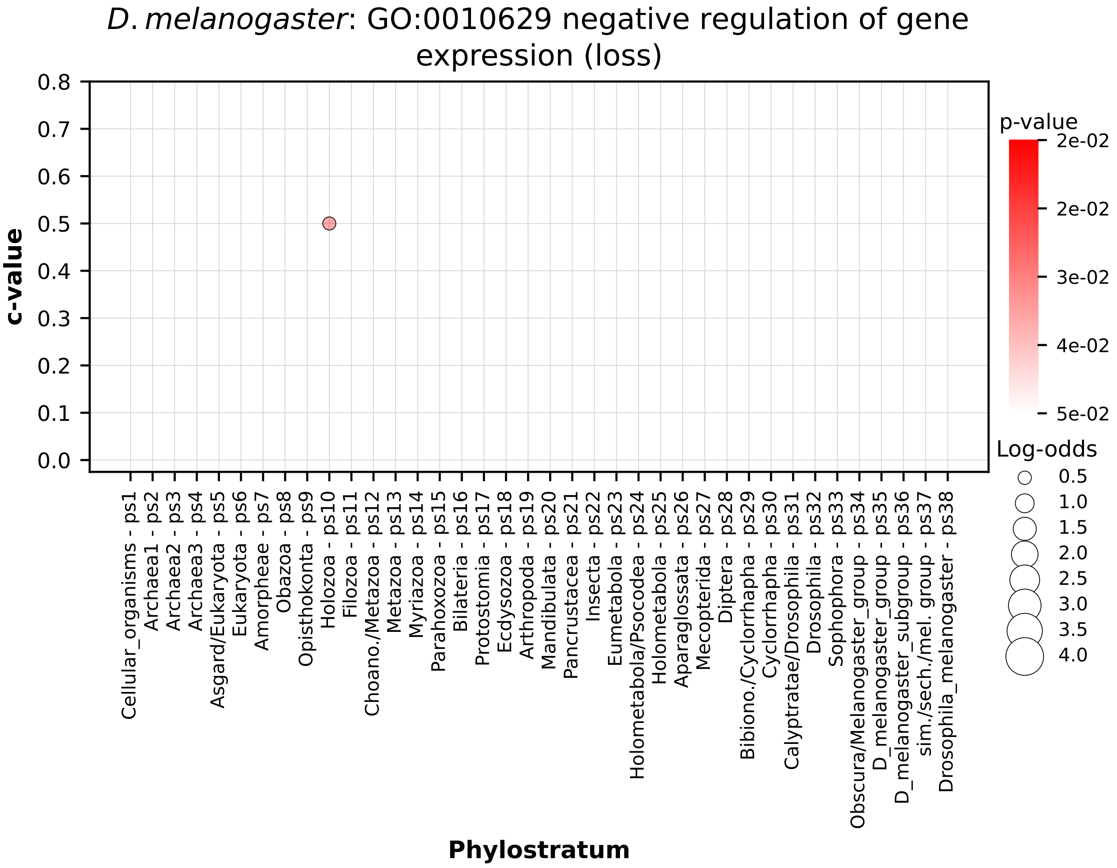
D. melanogaster: GO:0010629 negative regulation of gene expression (loss)
c-value
0.8
0.7
0.6
0.5
0.4
0.3
0.2
0.1
0.0
p-value
2e-02
2e-02
3e-02
4e-02
5e-02
Log-odds
0.5
1.0
1.5
2.0
2.5
3.0
3.5
4.0
Cellular_organisms - ps1
Archaea1 - ps2
Archaea2 - ps3
Archaea3 - ps4
Asgard/Eukaryota - ps5
Eukaryota - ps6
Amorpheae - ps7
Obazoa - ps8
Opisthokonta - ps9
Holozoa - ps10
Filozoa - ps11
Choano./Metazoa - ps12
Metazoa - ps13
Myriazoa - ps14
Parahoxozoa - ps15
Bilateria - ps16
Protostomia - ps17
Ecdysozoa - ps18
Arthropoda - ps19
Mandibulata - ps20
Pancrustacea - ps21
Insecta - ps22
Eumetabola - ps23
Holometabola/Psocodea - ps24
Holometabola - ps25
Aparaglossata - ps26
Mecopterida - ps27
Diptera - ps28
Bibiono./Cyclorrhapha - ps29
Cyclorrhapha - ps30
Calyptratae/Drosophila - ps31
Drosophila - ps32
Sophophora - ps33
Obscura/Melanogaster_group - ps34
D_melanogaster_group - ps35
D_melanogaster_subgroup - ps36
sim./sech./mel. group - ps37
Drosophila_melanogaster - ps38
Phylostratum

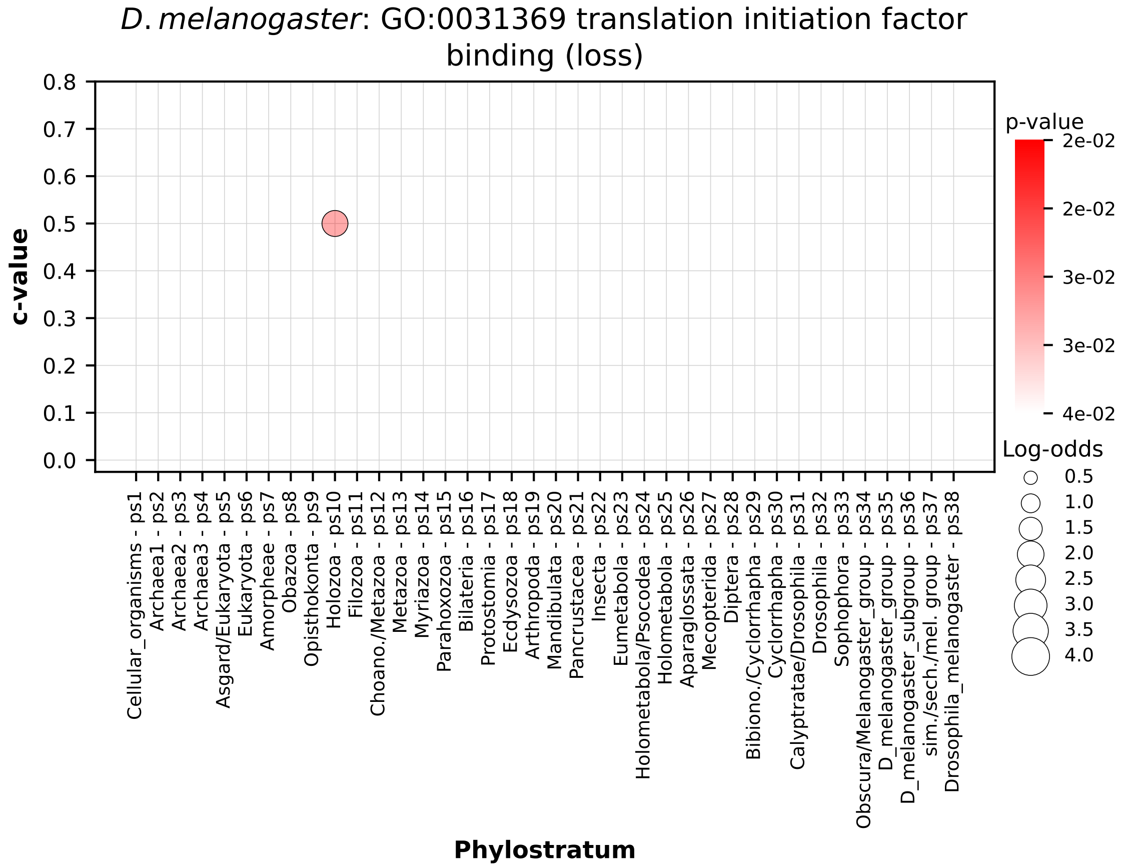

D. melanogaster: GO:0031369 translation initiation factor binding (loss)
c-value
0.8
0.7
0.6
0.5
0.4
0.3
0.2
0.1
0.0
Phylostratum
Cellular_organisms - ps1
Archaea1 - ps2
Archaea2 - ps3
Archaea3 - ps4
Asgard/Eukaryota - ps5
Eukaryota - ps6
Amorpheae - ps7
Obazoa - ps8
Opisthokonta - ps9
Holozoa - ps10
Filozoa - ps11
Choano./Metazoa - ps12
Metazoa - ps13
Myriazoa - ps14
Parahoxozoa - ps15
Bilateria - ps16
Protostomia - ps17
Ecdysozoa - ps18
Arthropoda - ps19
Mandibulata - ps20
Pancrustacea - ps21
Insecta - ps22
Eumetabola - ps23
Holometabola/Psocodea - ps24
Holometabola - ps25
Aparaglossata - ps26
Mecopterida - ps27
Diptera - ps28
Bibiono./Cyclorrhapha - ps29
Cyclorrhapha - ps30
Calyptratae/Drosophila - ps31
Drosophila - ps32
Sophophora - ps33
Obscura/Melanogaster_group - ps34
D_melanogaster_group - ps35
D_melanogaster_subgroup - ps36
sim./sech./mel. group - ps37
Drosophila_melanogaster - ps38
p-value
2e-02
2e-02
3e-02
3e-02
4e-02
Log-odds
0.5
1.0
1.5
2.0
2.5
3.0
3.5
4.0

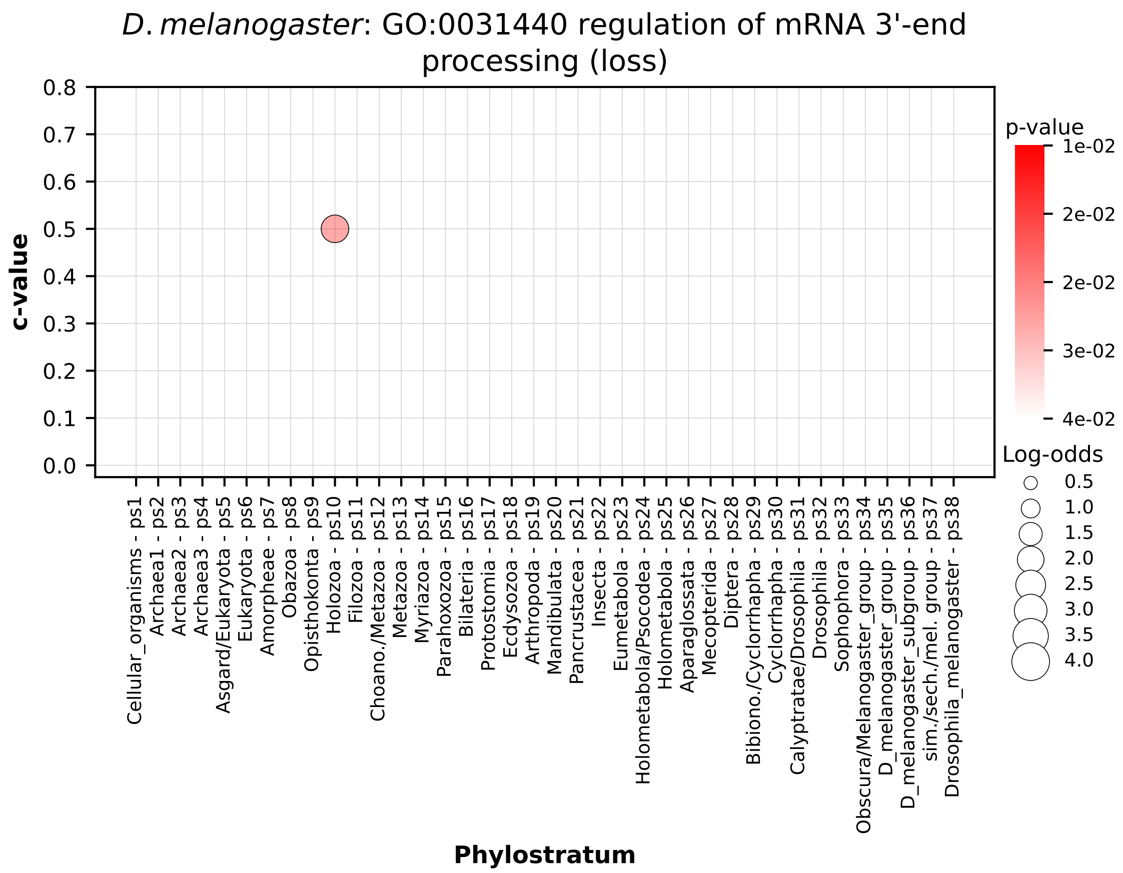
D. melanogaster: GO:0031440 regulation of mRNA 3'-end processing (loss)
c-value
0.8
0.7
0.6
0.5
0.4
0.3
0.2
0.1
0.0
Phylostratum
Cellular_organisms - ps1
Archaea1 - ps2
Archaea2 - ps3
Archaea3 - ps4
Asgard/Eukaryota - ps5
Eukaryota - ps6
Amorpheae - ps7
Obazoa - ps8
Opisthokonta - ps9
Holozoa - ps10
Filozoa - ps11
Choano./Metazoa - ps12
Metazoa - ps13
Myriazoa - ps14
Parahoxozoa - ps15
Bilateria - ps16
Protostomia - ps17
Ecdysozoa - ps18
Arthropoda - ps19
Mandibulata - ps20
Pancrustacea - ps21
Insecta - ps22
Eumetabola - ps23
Holometabola/Psocodea - ps24
Holometabola - ps25
Aparaglossata - ps26
Mecopterida - ps27
Diptera - ps28
Bibiono./Cyclorrhapha - ps29
Cyclorrhapha - ps30
Calyptratae/Drosophila - ps31
Drosophila - ps32
Sophophora - ps33
Obscura/Melanogaster_group - ps34
D_melanogaster_group - ps35
D_melanogaster_subgroup - ps36
sim./sech./mel. group - ps37
Drosophila_melanogaster - ps38
p-value
1e-02
2e-02
2e-02
3e-02
4e-02
Log-odds
0.5
1.0
1.5
2.0
2.5
3.0
3.5
4.0

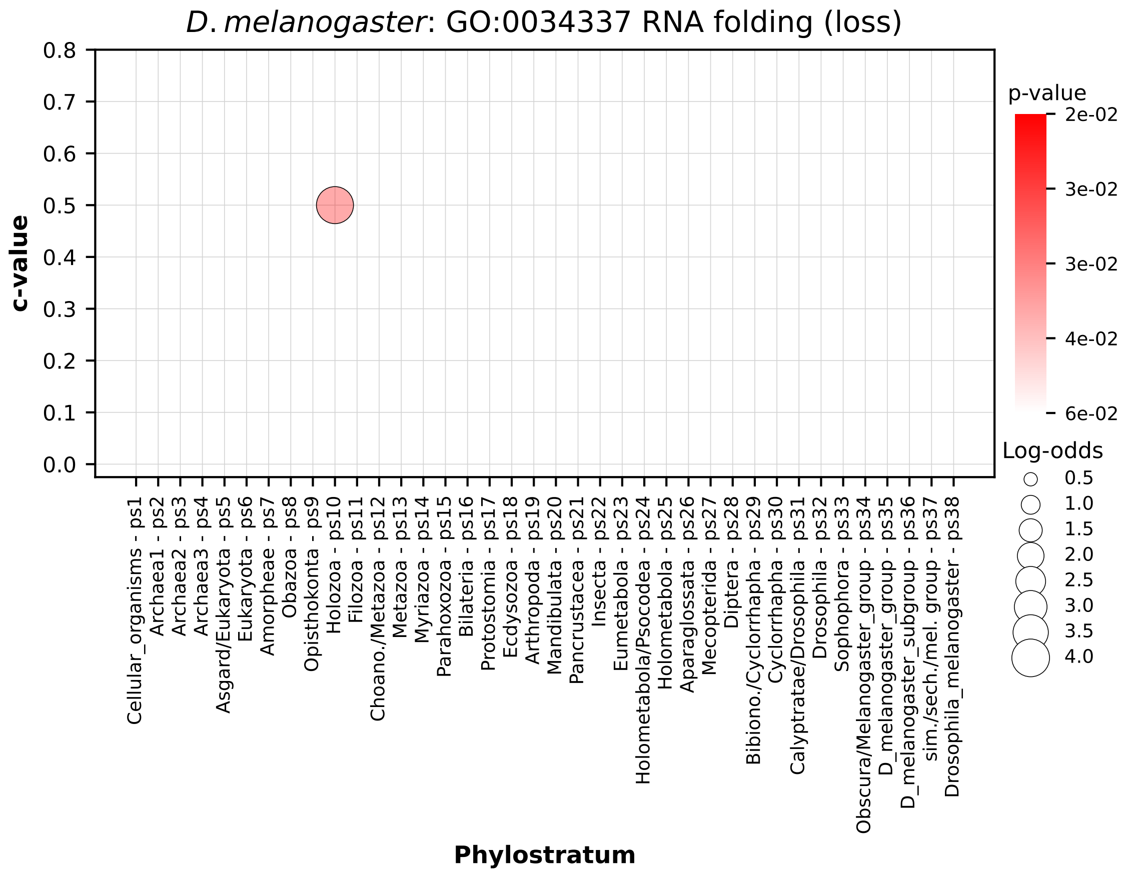

D. melanogaster: GO:0034337 RNA folding (loss)
c-value
0.8
0.7
0.6
0.5
0.4
0.3
0.2
0.1
0.0
p-value
2e-02
3e-02
3e-02
4e-02
6e-02
Log-odds
0.5
1.0
1.5
2.0
2.5
3.0
3.5
4.0
Cellular_organisms - ps1
Archaea1 - ps2
Archaea2 - ps3
Archaea3 - ps4
Asgard/Eukaryota - ps5
Eukaryota - ps6
Amorpheae - ps7
Obazoa - ps8
Opisthokonta - ps9
Holozoa - ps10
Filozoa - ps11
Choano./Metazoa - ps12
Metazoa - ps13
Myriazoa - ps14
Parahoxozoa - ps15
Bilateria - ps16
Protostomia - ps17
Ecdysozoa - ps18
Arthropoda - ps19
Mandibulata - ps20
Pancrustacea - ps21
Insecta - ps22
Eumetabola - ps23
Holometabola/Psocodea - ps24
Holometabola - ps25
Aparaglossata - ps26
Mecopterida - ps27
Diptera - ps28
Bibiono./Cyclorrhapha - ps29
Cyclorrhapha - ps30
Calyptratae/Drosophila - ps31
Drosophila - ps32
Sophophora - ps33
Obscura/Melanogaster_group - ps34
D_melanogaster_group - ps35
D_melanogaster_subgroup - ps36
sim./sech./mel. group - ps37
Drosophila_melanogaster - ps38
Phylostratum

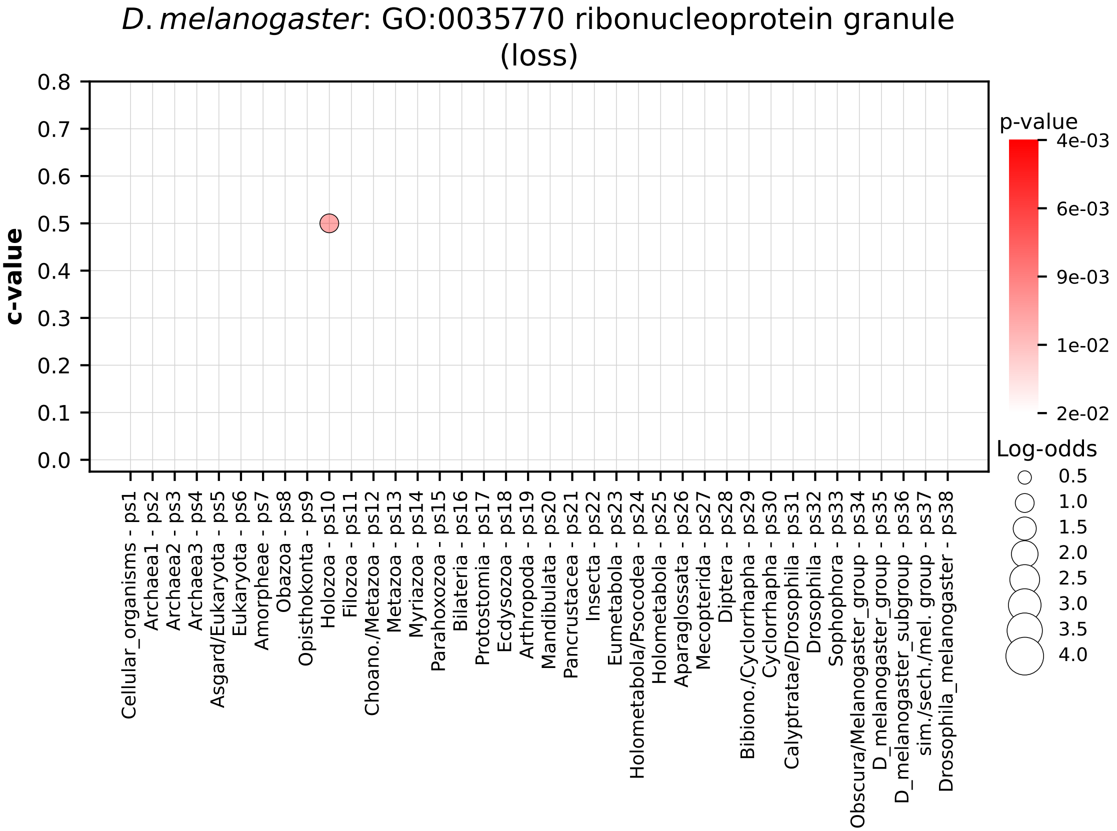

D. melanogaster: GO:0035770 ribonucleoprotein granule
(loss)
c-value
0.0
0.1
0.2
0.3
0.4
0.5
0.6
0.7
0.8
Phylostratum
Cellular_organisms - ps1
Archaea1 - ps2
Archaea2 - ps3
Archaea3 - ps4
Asgard/Eukaryota - ps5
Eukaryota - ps6
Amorpheae - ps7
Obazoa - ps8
Opisthokonta - ps9
Holozoa - ps10
Filozoa - ps11
Choano./Metazoa - ps12
Metazoa - ps13
Myriazoa - ps14
Parahoxozoa - ps15
Bilateria - ps16
Protostomia - ps17
Ecdysozoa - ps18
Arthropoda - ps19
Mandibulata - ps20
Pancrustacea - ps21
Insecta - ps22
Eumetabola - ps23
Holometabola/Psocodea - ps24
Holometabola - ps25
Aparaglossata - ps26
Mecopterida - ps27
Diptera - ps28
Bibiono./Cyclorrhapha - ps29
Cyclorrhapha - ps30
Calyptratae/Drosophila - ps31
Drosophila - ps32
Sophophora - ps33
Obscura/Melanogaster_group - ps34
D_melanogaster_group - ps35
D_melanogaster_subgroup - ps36
sim./sech./mel. group - ps37
Drosophila_melanogaster - ps38
p-value
4e-03
6e-03
9e-03
1e-02
2e-02
Log-odds
0.5
1.0
1.5
2.0
2.5
3.0
3.5
4.0

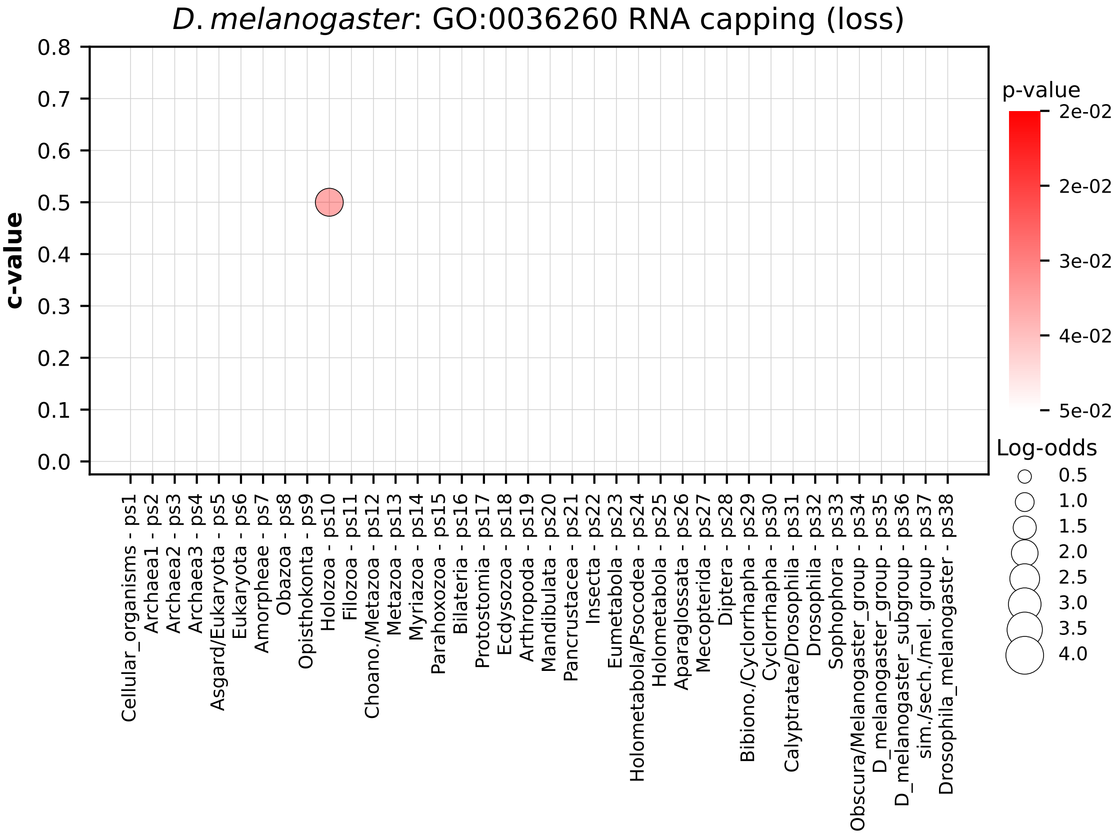
D. melanogaster: GO:0036260 RNA capping (loss)
c-value
0.0
0.1
0.2
0.3
0.4
0.5
0.6
0.7
0.8
Phylostratum
Cellular_organisms - ps1
Archaea1 - ps2
Archaea2 - ps3
Archaea3 - ps4
Asgard/Eukaryota - ps5
Eukaryota - ps6
Amorpheae - ps7
Obazoa - ps8
Opisthokonta - ps9
Holozoa - ps10
Filozoa - ps11
Choano./Metazoa - ps12
Metazoa - ps13
Myriazoa - ps14
Parahoxozoa - ps15
Bilateria - ps16
Protostomia - ps17
Ecdysozoa - ps18
Arthropoda - ps19
Mandibulata - ps20
Pancrustacea - ps21
Insecta - ps22
Eumetabola - ps23
Holometabola/Psocodea - ps24
Holometabola - ps25
Aparaglossata - ps26
Mecopterida - ps27
Diptera - ps28
Bibiono./Cyclorrhapha - ps29
Cyclorrhapha - ps30
Calyptratae/Drosophila - ps31
Drosophila - ps32
Sophophora - ps33
Obscura/Melanogaster_group - ps34
D_melanogaster_group - ps35
D_melanogaster_subgroup - ps36
sim./sech./mel. group - ps37
Drosophila_melanogaster - ps38
p-value
2e-02
2e-02
3e-02
4e-02
5e-02
Log-odds
0.5
1.0
1.5
2.0
2.5
3.0
3.5
4.0

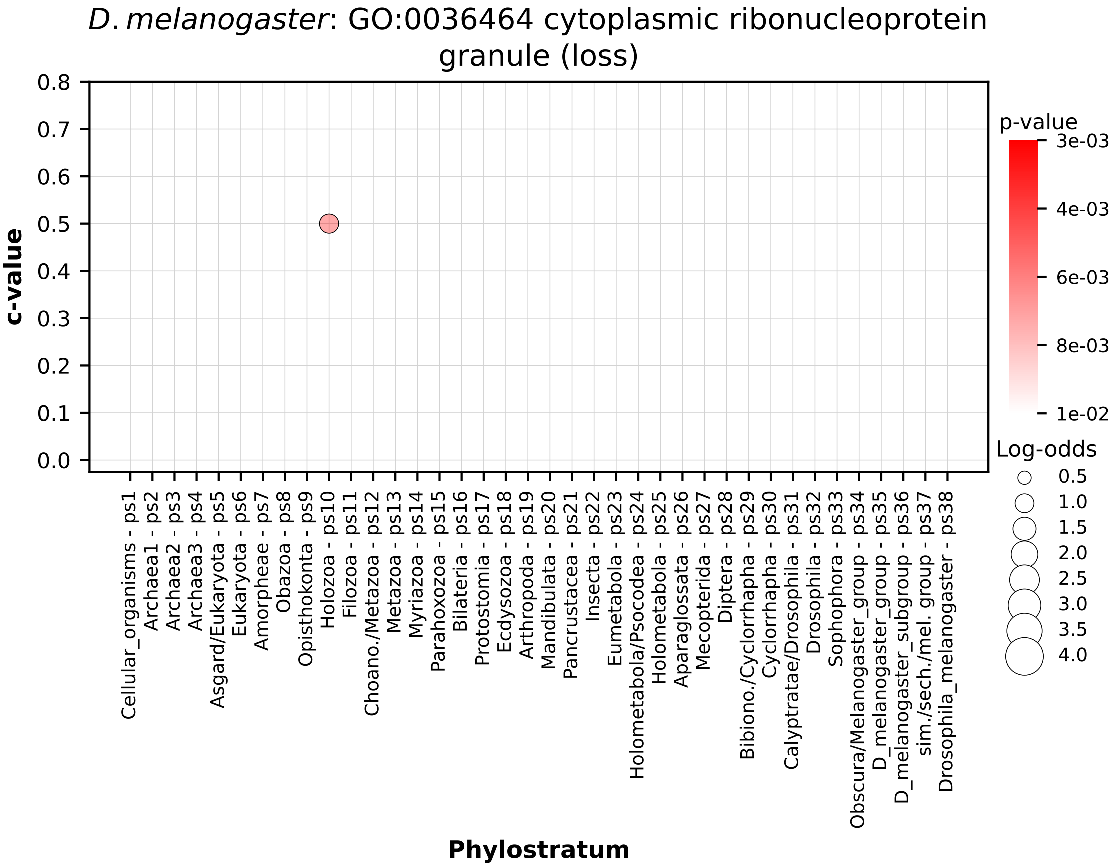

D. melanogaster: GO:0036464 cytoplasmic ribonucleoprotein granule (loss)
c-value
0.8
0.7
0.6
0.5
0.4
0.3
0.2
0.1
0.0
Phylostratum
Cellular_organisms - ps1
Archaea1 - ps2
Archaea2 - ps3
Archaea3 - ps4
Asgard/Eukaryota - ps5
Eukaryota - ps6
Amorpheae - ps7
Obazoa - ps8
Opisthokonta - ps9
Holozoa - ps10
Filozoa - ps11
Choano./Metazoa - ps12
Metazoa - ps13
Myriazoa - ps14
Parahoxozoa - ps15
Bilateria - ps16
Protostomia - ps17
Ecdysozoa - ps18
Arthropoda - ps19
Mandibulata - ps20
Pancrustacea - ps21
Insecta - ps22
Eumetabola - ps23
Holometabola/Psocodea - ps24
Holometabola - ps25
Aparaglossata - ps26
Mecopterida - ps27
Diptera - ps28
Bibiono./Cyclorrhapha - ps29
Cyclorrhapha - ps30
Calyptratae/Drosophila - ps31
Drosophila - ps32
Sophophora - ps33
Obscura/Melanogaster_group - ps34
D_melanogaster_group - ps35
D_melanogaster_subgroup - ps36
sim./sech./mel. group - ps37
Drosophila_melanogaster - ps38
p-value
3e-03
4e-03
6e-03
8e-03
1e-02
Log-odds
0.5
1.0
1.5
2.0
2.5
3.0
3.5
4.0

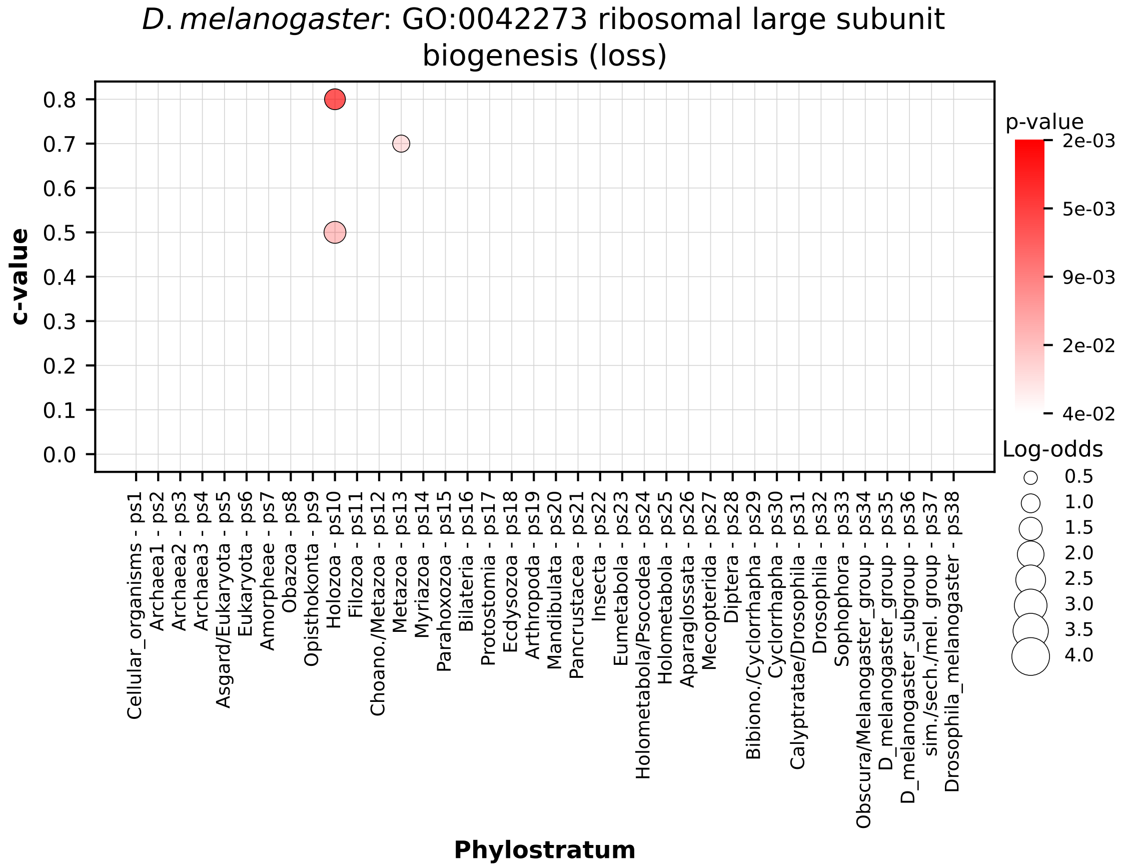

D. melanogaster: GO:0042273 ribosomal large subunit biogenesis (loss)
c-value
0.8
0.7
0.6
0.5
0.4
0.3
0.2
0.1
0.0
p-value
2e-03
5e-03
9e-03
2e-02
4e-02
Log-odds
0.5
1.0
1.5
2.0
2.5
3.0
3.5
4.0
Cellular_organisms - ps1
Archaea1 - ps2
Archaea2 - ps3
Archaea3 - ps4
Asgard/Eukaryota - ps5
Eukaryota - ps6
Amorpheae - ps7
Obazoa - ps8
Opisthokonta - ps9
Holozoa - ps10
Filozoa - ps11
Choano./Metazoa - ps12
Metazoa - ps13
Myriazoa - ps14
Parahoxozoa - ps15
Bilateria - ps16
Protostomia - ps17
Ecdysozoa - ps18
Arthropoda - ps19
Mandibulata - ps20
Pancrustacea - ps21
Insecta - ps22
Eumetabola - ps23
Holometabola/Psocodea - ps24
Holometabola - ps25
Aparaglossata - ps26
Mecopterida - ps27
Diptera - ps28
Bibiono./Cyclorrhapha - ps29
Cyclorrhapha - ps30
Calyptratae/Drosophila - ps31
Drosophila - ps32
Sophophora - ps33
Obscura/Melanogaster_group - ps34
D_melanogaster_group - ps35
D_melanogaster_subgroup - ps36
sim./sech./mel. group - ps37
Drosophila_melanogaster - ps38
Phylostratum

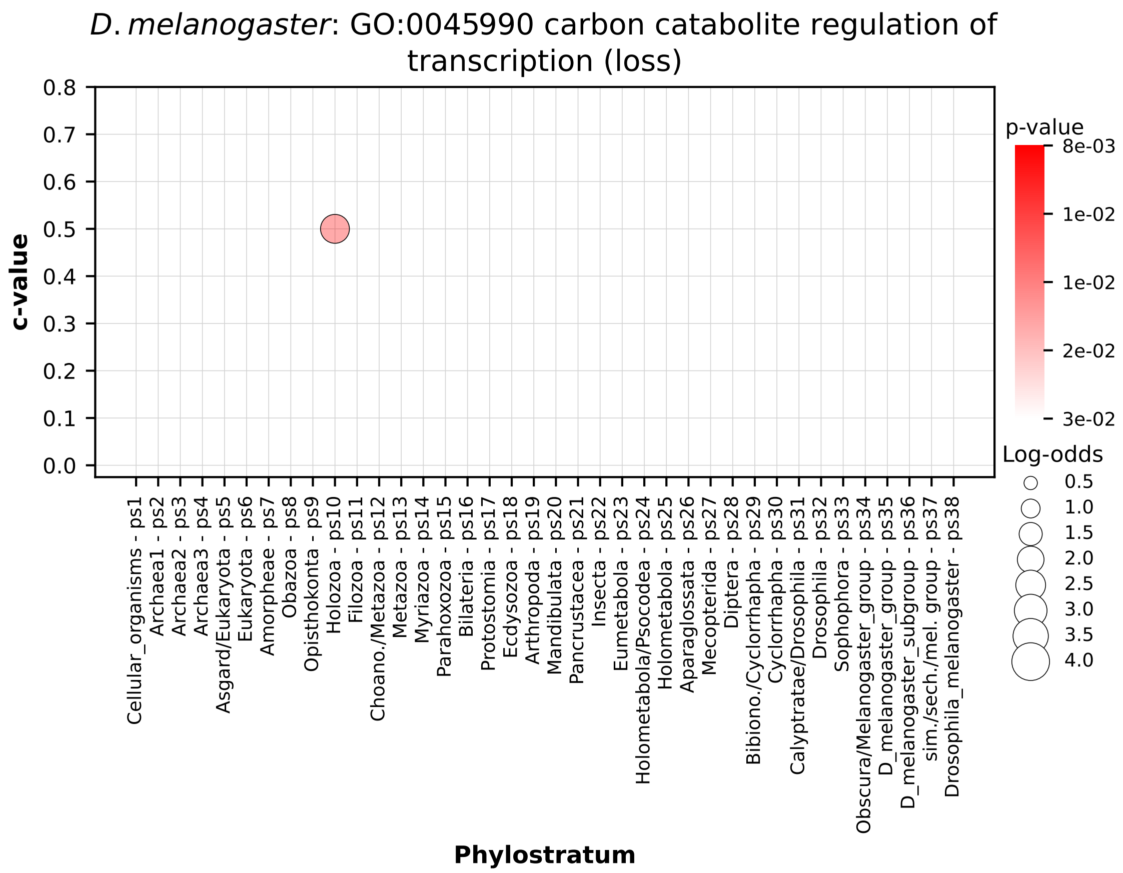

D. melanogaster: GO:0045990 carbon catabolite regulation of transcription (loss)
c-value
0.8
0.7
0.6
0.5
0.4
0.3
0.2
0.1
0.0
p-value
8e-03
1e-02
1e-02
2e-02
3e-02
Log-odds
0.5
1.0
1.5
2.0
2.5
3.0
3.5
4.0
Cellular_organisms - ps1
Archaea1 - ps2
Archaea2 - ps3
Archaea3 - ps4
Asgard/Eukaryota - ps5
Eukaryota - ps6
Amorpheae - ps7
Obazoa - ps8
Opisthokonta - ps9
Holozoa - ps10
Filozoa - ps11
Choano./Metazoa - ps12
Metazoa - ps13
Myriazoa - ps14
Parahoxozoa - ps15
Bilateria - ps16
Protostomia - ps17
Ecdysozoa - ps18
Arthropoda - ps19
Mandibulata - ps20
Pancrustacea - ps21
Insecta - ps22
Eumetabola - ps23
Holometabola/Psocodea - ps24
Holometabola - ps25
Aparaglossata - ps26
Mecopterida - ps27
Diptera - ps28
Bibiono./Cyclorrhapha - ps29
Cyclorrhapha - ps30
Calyptratae/Drosophila - ps31
Drosophila - ps32
Sophophora - ps33
Obscura/Melanogaster_group - ps34
D_melanogaster_group - ps35
D_melanogaster_subgroup - ps36
sim./sech./mel. group - ps37
Drosophila_melanogaster - ps38
Phylostratum

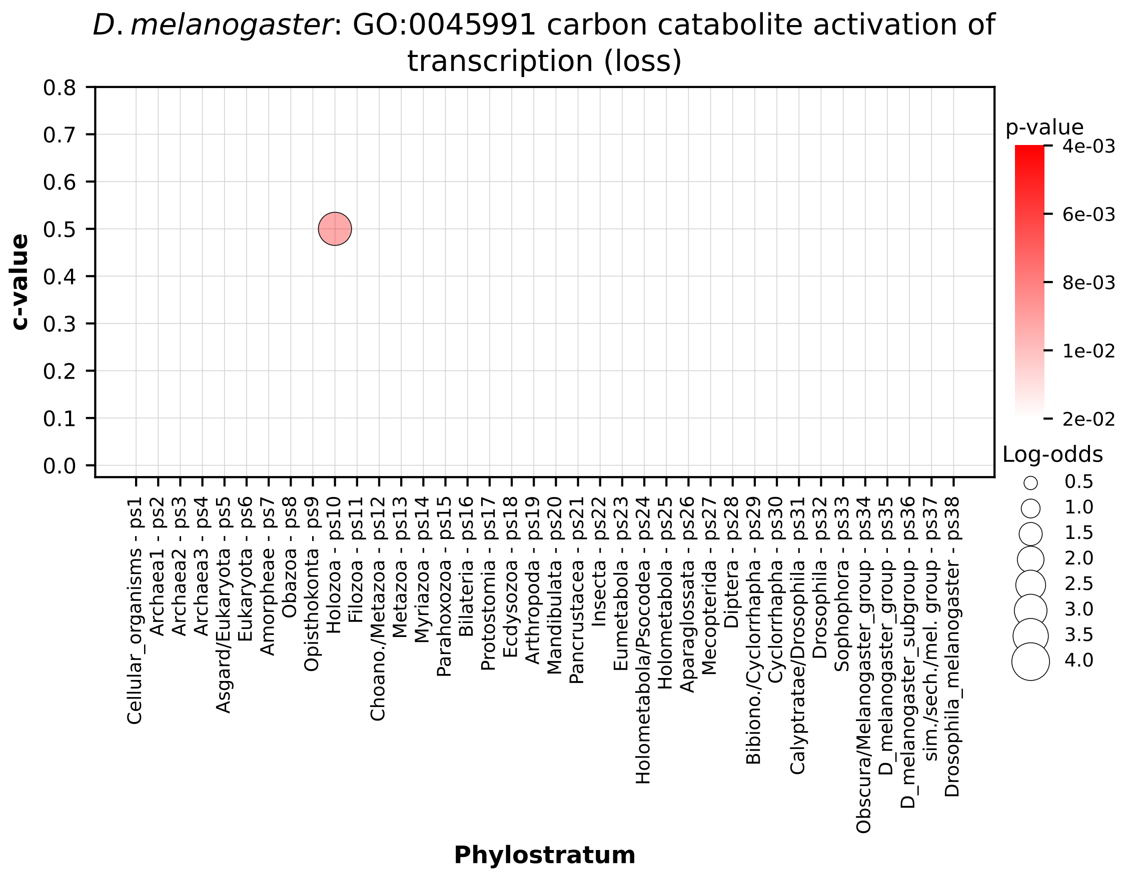
D. melanogaster: GO:0045991 carbon catabolite activation of transcription (loss)
c-value
0.8
0.7
0.6
0.5
0.4
0.3
0.2
0.1
0.0
Phylostratum
Cellular_organisms - ps1
Archaea1 - ps2
Archaea2 - ps3
Archaea3 - ps4
Asgard/Eukaryota - ps5
Eukaryota - ps6
Amorpheae - ps7
Obazoa - ps8
Opisthokonta - ps9
Holozoa - ps10
Filozoa - ps11
Choano./Metazoa - ps12
Metazoa - ps13
Myriazoa - ps14
Parahoxozoa - ps15
Bilateria - ps16
Protostomia - ps17
Ecdysozoa - ps18
Arthropoda - ps19
Mandibulata - ps20
Pancrustacea - ps21
Insecta - ps22
Eumetabola - ps23
Holometabola/Psocodea - ps24
Holometabola - ps25
Aparaglossata - ps26
Mecopterida - ps27
Diptera - ps28
Bibiono./Cyclorrhapha - ps29
Cyclorrhapha - ps30
Calyptratae/Drosophila - ps31
Drosophila - ps32
Sophophora - ps33
Obscura/Melanogaster_group - ps34
D_melanogaster_group - ps35
D_melanogaster_subgroup - ps36
sim./sech./mel. group - ps37
Drosophila_melanogaster - ps38
p-value
4e-03
6e-03
8e-03
1e-02
2e-02
Log-odds
0.5
1.0
1.5
2.0
2.5
3.0
3.5
4.0

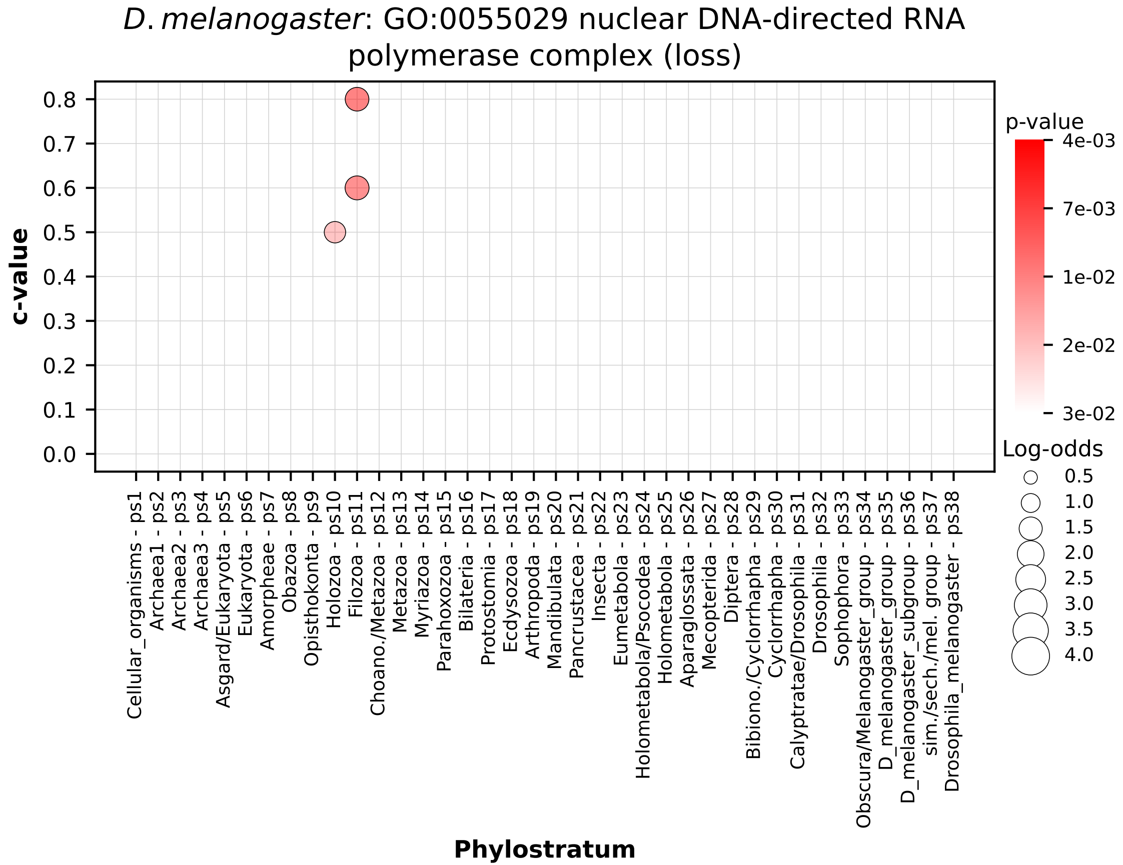

D. melanogaster: GO:0055029 nuclear DNA-directed RNA polymerase complex (loss)
c-value
0.0
0.1
0.2
0.3
0.4
0.5
0.6
0.7
0.8
Phylostratum
Cellular_organisms - ps1
Archaea1 - ps2
Archaea2 - ps3
Archaea3 - ps4
Asgard/Eukaryota - ps5
Eukaryota - ps6
Amorpheae - ps7
Obazoa - ps8
Opisthokonta - ps9
Holozoa - ps10
Filozoa - ps11
Choano./Metazoa - ps12
Metazoa - ps13
Myriazoa - ps14
Parahoxozoa - ps15
Bilateria - ps16
Protostomia - ps17
Ecdysozoa - ps18
Arthropoda - ps19
Mandibulata - ps20
Pancrustacea - ps21
Insecta - ps22
Eumetabola - ps23
Holometabola/Psocodea - ps24
Holometabola - ps25
Aparaglossata - ps26
Mecopterida - ps27
Diptera - ps28
Bibiono./Cyclorrhapha - ps29
Cyclorrhapha - ps30
Calyptratae/Drosophila - ps31
Drosophila - ps32
Sophophora - ps33
Obscura/Melanogaster_group - ps34
D_melanogaster_group - ps35
D_melanogaster_subgroup - ps36
sim./sech./mel. group - ps37
Drosophila_melanogaster - ps38
p-value
4e-03
7e-03
1e-02
2e-02
3e-02
Log-odds
0.5
1.0
1.5
2.0
2.5
3.0
3.5
4.0

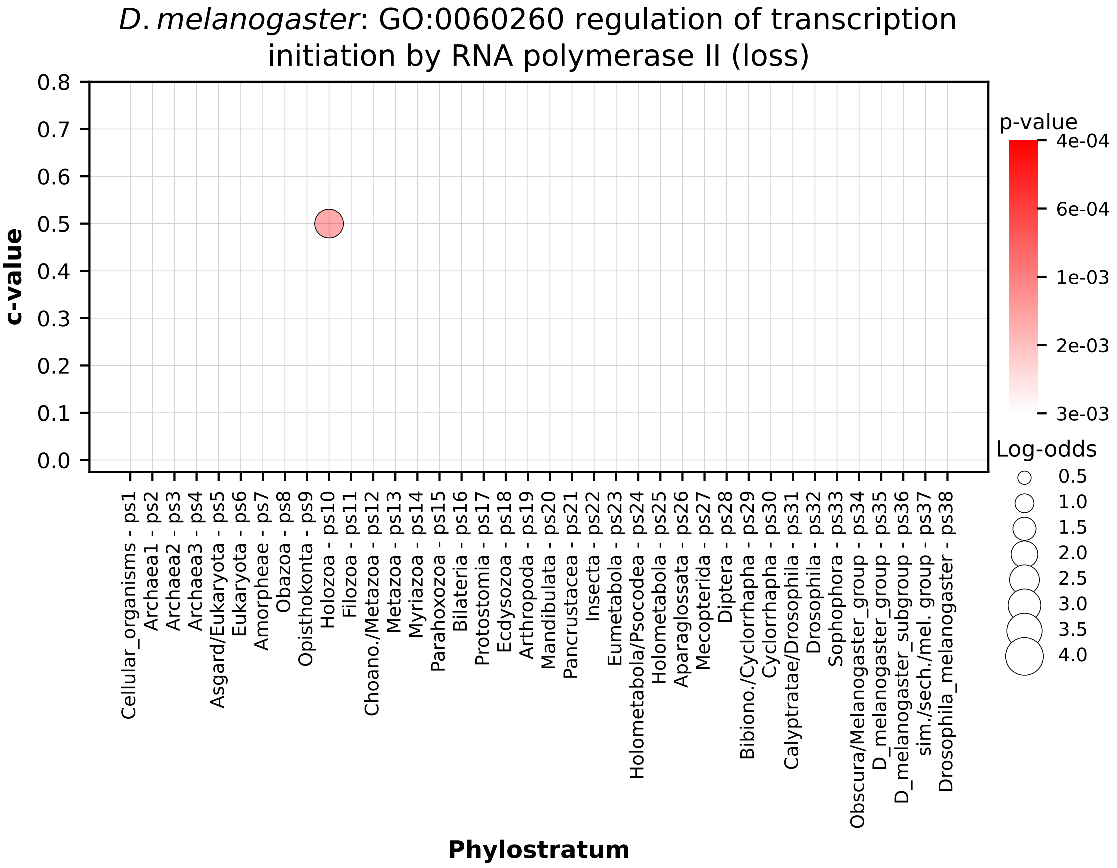

D. melanogaster: GO:0060260 regulation of transcription initiation by RNA polymerase II (loss)
c-value
0.0
0.1
0.2
0.3
0.4
0.5
0.6
0.7
0.8
Phylostratum
Cellular_organisms - ps1
Archaea1 - ps2
Archaea2 - ps3
Archaea3 - ps4
Asgard/Eukaryota - ps5
Eukaryota - ps6
Amorpheae - ps7
Obazoa - ps8
Opisthokonta - ps9
Holozoa - ps10
Filozoa - ps11
Choano./Metazoa - ps12
Metazoa - ps13
Myriazoa - ps14
Parahoxozoa - ps15
Bilateria - ps16
Protostomia - ps17
Ecdysozoa - ps18
Arthropoda - ps19
Mandibulata - ps20
Pancrustacea - ps21
Insecta - ps22
Eumetabola - ps23
Holometabola/Psocodea - ps24
Holometabola - ps25
Aparaglossata - ps26
Mecopterida - ps27
Diptera - ps28
Bibiono./Cyclorrhapha - ps29
Cyclorrhapha - ps30
Calyptratae/Drosophila - ps31
Drosophila - ps32
Sophophora - ps33
Obscura/Melanogaster_group - ps34
D_melanogaster_group - ps35
D_melanogaster_subgroup - ps36
sim./sech./mel. group - ps37
Drosophila_melanogaster - ps38
p-value
4e-04
6e-04
1e-03
2e-03
3e-03
Log-odds
0.5
1.0
1.5
2.0
2.5
3.0
3.5
4.0

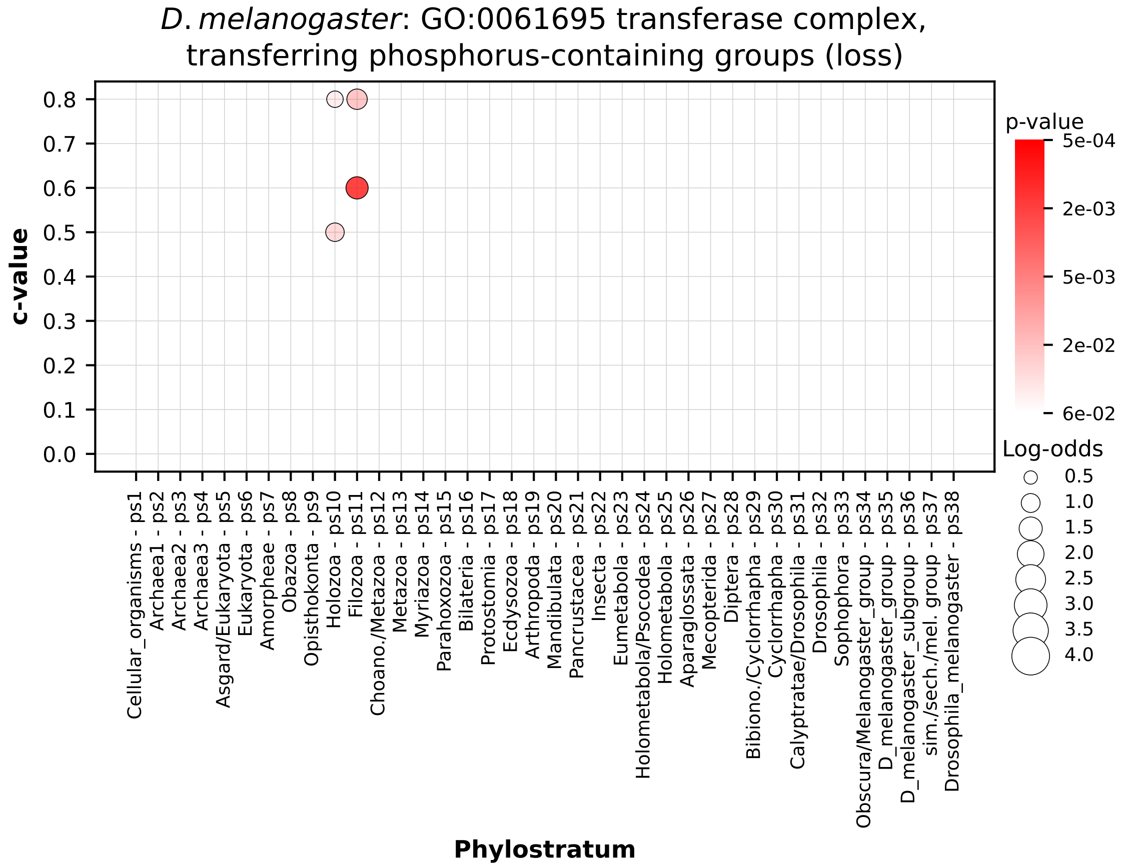

D. melanogaster: GO:0061695 transferase complex, transferring phosphorus-containing groups (loss)
c-value
0.8
0.7
0.6
0.5
0.4
0.3
0.2
0.1
0.0
Phylostratum
Cellular_organisms - ps1
Archaea1 - ps2
Archaea2 - ps3
Archaea3 - ps4
Asgard/Eukaryota - ps5
Eukaryota - ps6
Amorpheae - ps7
Obazoa - ps8
Opisthokonta - ps9
Holozoa - ps10
Filozoa - ps11
Choano./Metazoa - ps12
Metazoa - ps13
Myriazoa - ps14
Parahoxozoa - ps15
Bilateria - ps16
Protostomia - ps17
Ecdysozoa - ps18
Arthropoda - ps19
Mandibulata - ps20
Pancrustacea - ps21
Insecta - ps22
Eumetabola - ps23
Holometabola/Psocodea - ps24
Holometabola - ps25
Aparaglossata - ps26
Mecopterida - ps27
Diptera - ps28
Bibiono./Cyclorrhapha - ps29
Cyclorrhapha - ps30
Calyptratae/Drosophila - ps31
Drosophila - ps32
Sophophora - ps33
Obscura/Melanogaster_group - ps34
D_melanogaster_group - ps35
D_melanogaster_subgroup - ps36
sim./sech./mel. group - ps37
Drosophila_melanogaster - ps38
p-value
5e-04
2e-03
5e-03
2e-02
6e-02
Log-odds
0.5
1.0
1.5
2.0
2.5
3.0
3.5
4.0

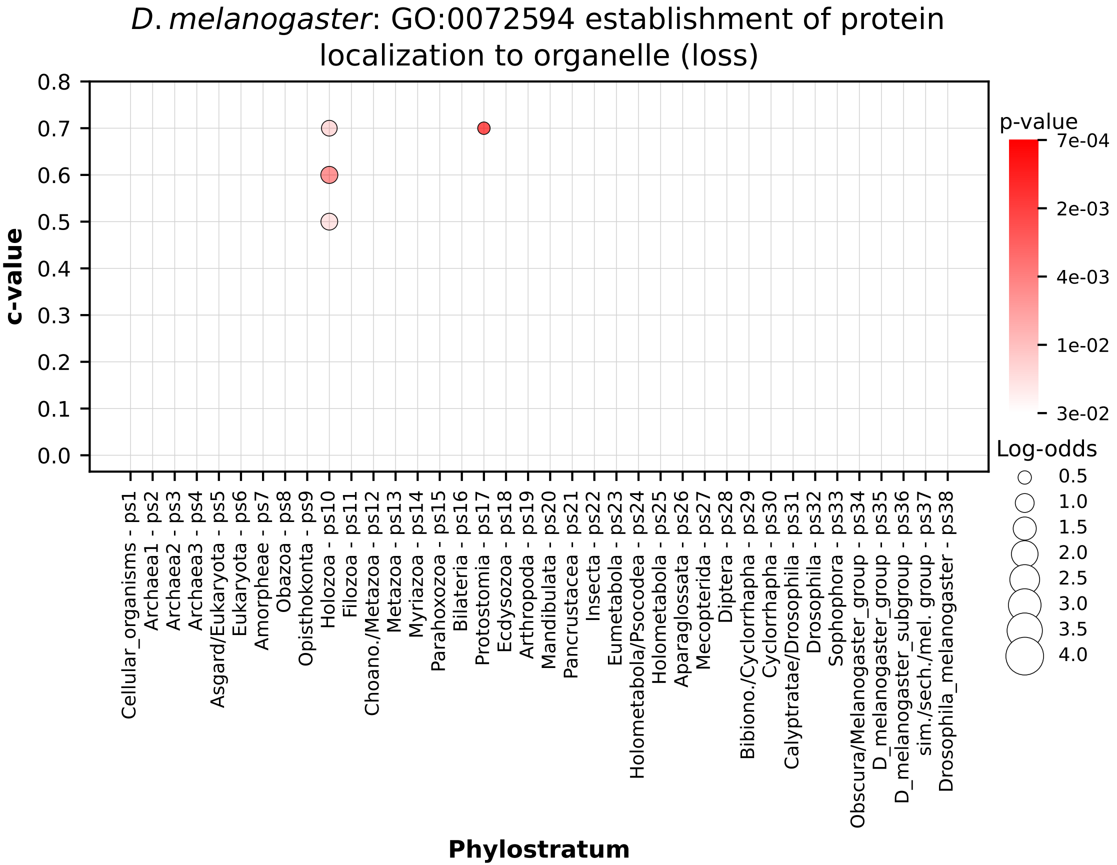

D. melanogaster: GO:0072594 establishment of protein localization to organelle (loss)
c-value
0.0
0.1
0.2
0.3
0.4
0.5
0.6
0.7
0.8
Phylostratum
Cellular_organisms - ps1
Archaea1 - ps2
Archaea2 - ps3
Archaea3 - ps4
Asgard/Eukaryota - ps5
Eukaryota - ps6
Amorpheae - ps7
Obazoa - ps8
Opisthokonta - ps9
Holozoa - ps10
Filozoa - ps11
Choano./Metazoa - ps12
Metazoa - ps13
Myriazoa - ps14
Parahoxozoa - ps15
Bilateria - ps16
Protostomia - ps17
Ecdysozoa - ps18
Arthropoda - ps19
Mandibulata - ps20
Pancrustacea - ps21
Insecta - ps22
Eumetabola - ps23
Holometabola/Psocodea - ps24
Holometabola - ps25
Aparaglossata - ps26
Mecopterida - ps27
Diptera - ps28
Bibiono./Cyclorrhapha - ps29
Cyclorrhapha - ps30
Calyptratae/Drosophila - ps31
Drosophila - ps32
Sophophora - ps33
Obscura/Melanogaster_group - ps34
D_melanogaster_group - ps35
D_melanogaster_subgroup - ps36
sim./sech./mel. group - ps37
Drosophila_melanogaster - ps38
p-value
7e-04
2e-03
4e-03
1e-02
3e-02
Log-odds
0.5
1.0
1.5
2.0
2.5
3.0
3.5
4.0

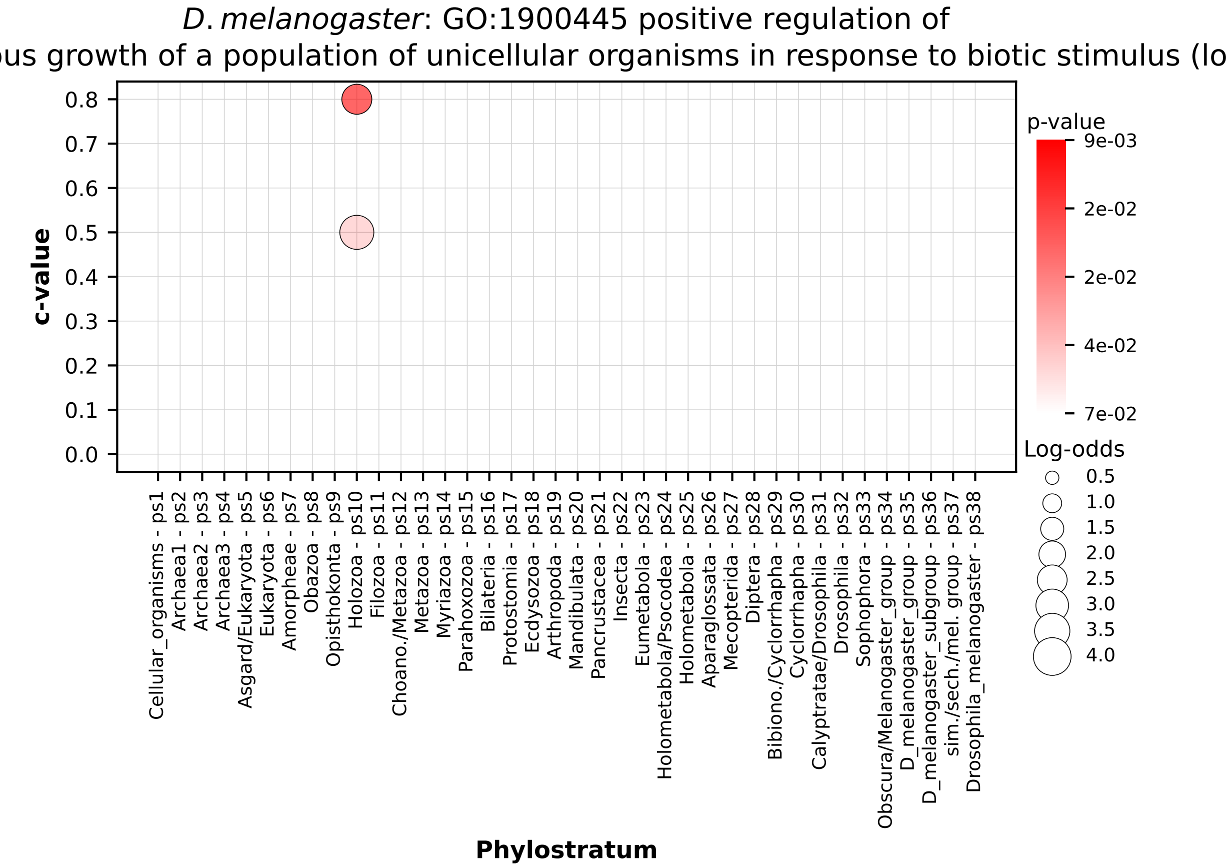

$D. melanogaster$: GO:1900445 positive regulation of
ous growth of a population of unicellular organisms in response to biotic stimulus (lo
c-value
0.8
0.7
0.6
0.5
0.4
0.3
0.2
0.1
0.0
p-value
9e-03
2e-02
2e-02
4e-02
7e-02
Log-odds
0.5
1.0
1.5
2.0
2.5
3.0
3.5
4.0
Cellular_organisms - ps1
Archaea1 - ps2
Archaea2 - ps3
Archaea3 - ps4
Asgard/Eukaryota - ps5
Eukaryota - ps6
Amorpheae - ps7
Obazoa - ps8
Opisthokonta - ps9
Holozoa - ps10
Filozoa - ps11
Choano./Metazoa - ps12
Metazoa - ps13
Myriazoa - ps14
Parahoxozoa - ps15
Bilateria - ps16
Protostomia - ps17
Ecdysozoa - ps18
Arthropoda - ps19
Mandibulata - ps20
Pancrustacea - ps21
Insecta - ps22
Eumetabola - ps23
Holometabola/Psocodea - ps24
Holometabola - ps25
Aparaglossata - ps26
Mecopterida - ps27
Diptera - ps28
Bibiono./Cyclorrhapha - ps29
Cyclorrhapha - ps30
Calyptratae/Drosophila - ps31
Drosophila - ps32
Sophophora - ps33
Obscura/Melanogaster_group - ps34
D_melanogaster_group - ps35
D_melanogaster_subgroup - ps36
sim./sech./mel. group - ps37
Drosophila_melanogaster - ps38
Phylostratum

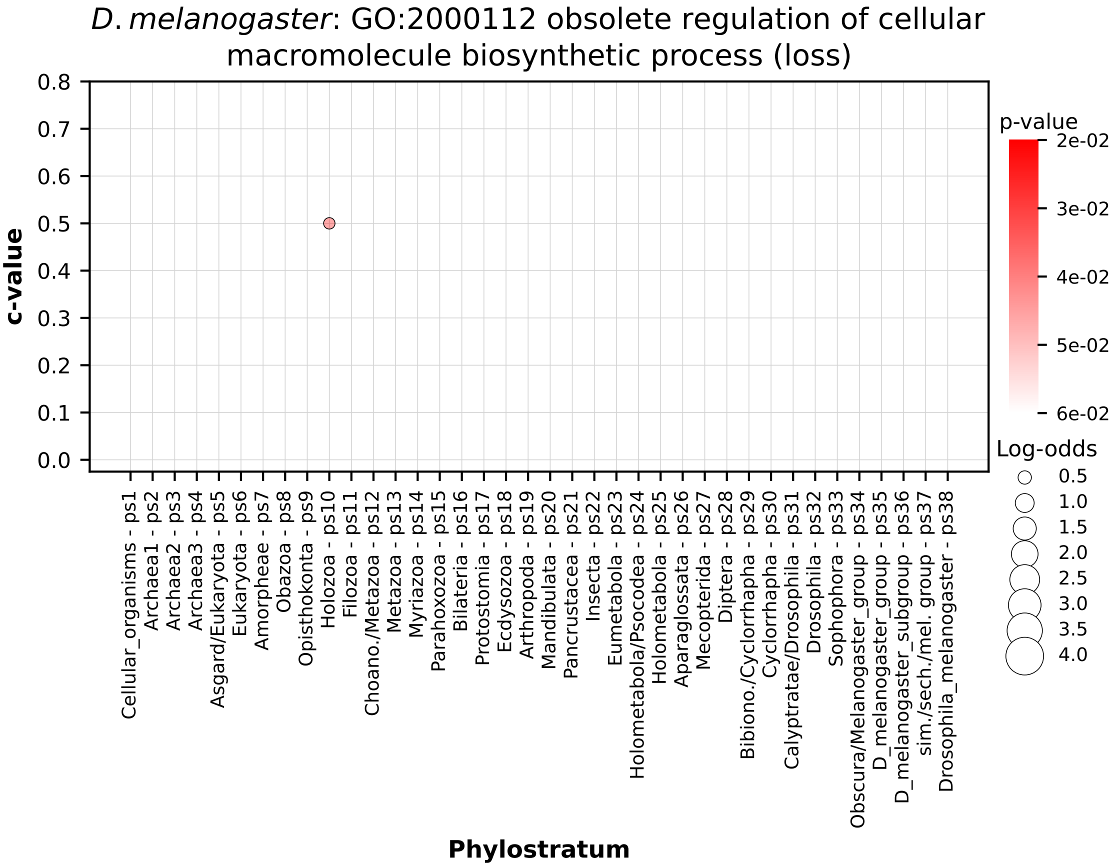

D. melanogaster: GO:2000112 obsolete regulation of cellular macromolecule biosynthetic process (loss)
c-value
0.8
0.7
0.6
0.5
0.4
0.3
0.2
0.1
0.0
Phylostratum
Cellular_organisms - ps1
Archaea1 - ps2
Archaea2 - ps3
Archaea3 - ps4
Asgard/Eukaryota - ps5
Eukaryota - ps6
Amorpheae - ps7
Obazoa - ps8
Opisthokonta - ps9
Holozoa - ps10
Filozoa - ps11
Choano./Metazoa - ps12
Metazoa - ps13
Myriazoa - ps14
Parahoxozoa - ps15
Bilateria - ps16
Protostomia - ps17
Ecdysozoa - ps18
Arthropoda - ps19
Mandibulata - ps20
Pancrustacea - ps21
Insecta - ps22
Eumetabola - ps23
Holometabola/Psocodea - ps24
Holometabola - ps25
Aparaglossata - ps26
Mecopterida - ps27
Diptera - ps28
Bibiono./Cyclorrhapha - ps29
Cyclorrhapha - ps30
Calyptratae/Drosophila - ps31
Drosophila - ps32
Sophophora - ps33
Obscura/Melanogaster_group - ps34
D_melanogaster_group - ps35
D_melanogaster_subgroup - ps36
sim./sech./mel. group - ps37
Drosophila_melanogaster - ps38
p-value
2e-02
3e-02
4e-02
5e-02
6e-02
Log-odds
0.5
1.0
1.5
2.0
2.5
3.0
3.5
4.0

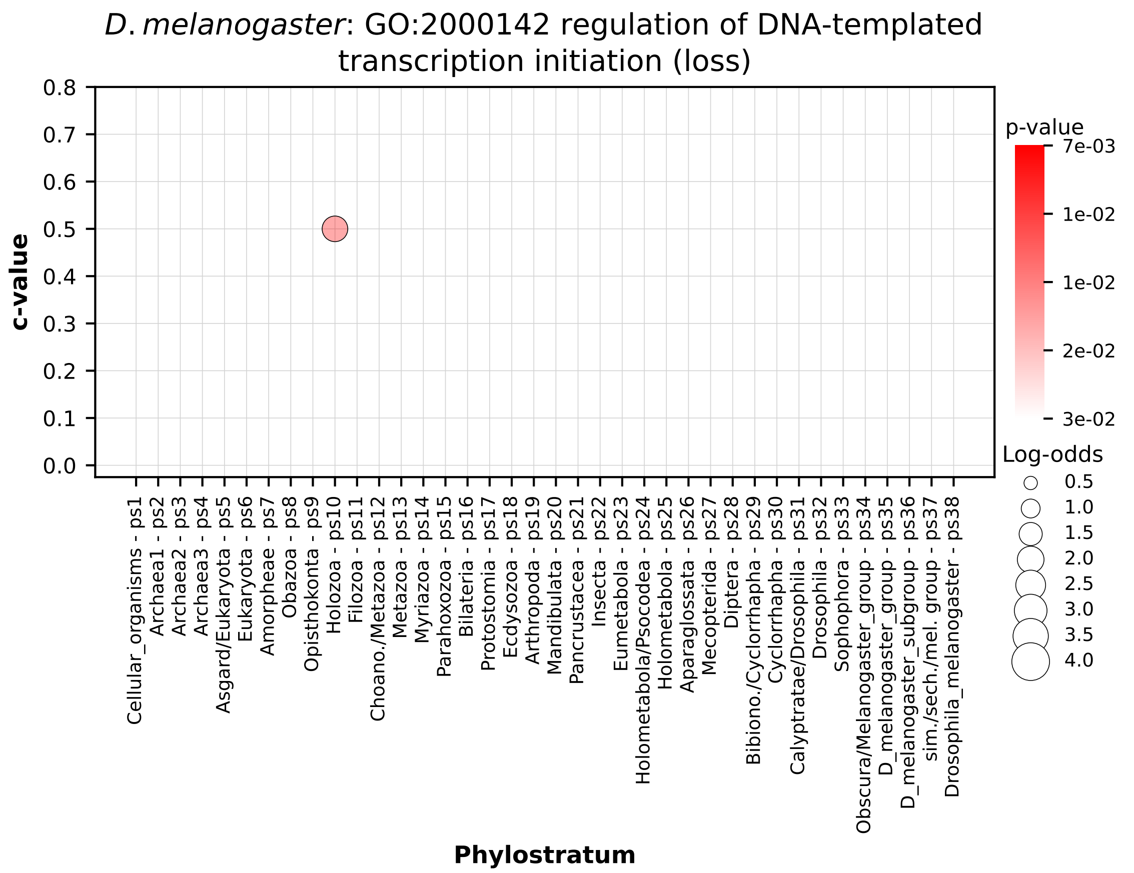

D. melanogaster: GO:2000142 regulation of DNA-templated transcription initiation (loss)
c-value
0.0
0.1
0.2
0.3
0.4
0.5
0.6
0.7
0.8
Phylostratum
Cellular_organisms - ps1
Archaea1 - ps2
Archaea2 - ps3
Archaea3 - ps4
Asgard/Eukaryota - ps5
Eukaryota - ps6
Amorpheae - ps7
Obazoa - ps8
Opisthokonta - ps9
Holozoa - ps10
Filozoa - ps11
Choano./Metazoa - ps12
Metazoa - ps13
Myriazoa - ps14
Parahoxozoa - ps15
Bilateria - ps16
Protostomia - ps17
Ecdysozoa - ps18
Arthropoda - ps19
Mandibulata - ps20
Pancrustacea - ps21
Insecta - ps22
Eumetabola - ps23
Holometabola/Psocodea - ps24
Holometabola - ps25
Aparaglossata - ps26
Mecopterida - ps27
Diptera - ps28
Bibiono./Cyclorrhapha - ps29
Cyclorrhapha - ps30
Calyptratae/Drosophila - ps31
Drosophila - ps32
Sophophora - ps33
Obscura/Melanogaster_group - ps34
D_melanogaster_group - ps35
D_melanogaster_subgroup - ps36
sim./sech./mel. group - ps37
Drosophila_melanogaster - ps38
p-value
7e-03
1e-02
1e-02
2e-02
3e-02
Log-odds
0.5
1.0
1.5
2.0
2.5
3.0
3.5
4.0

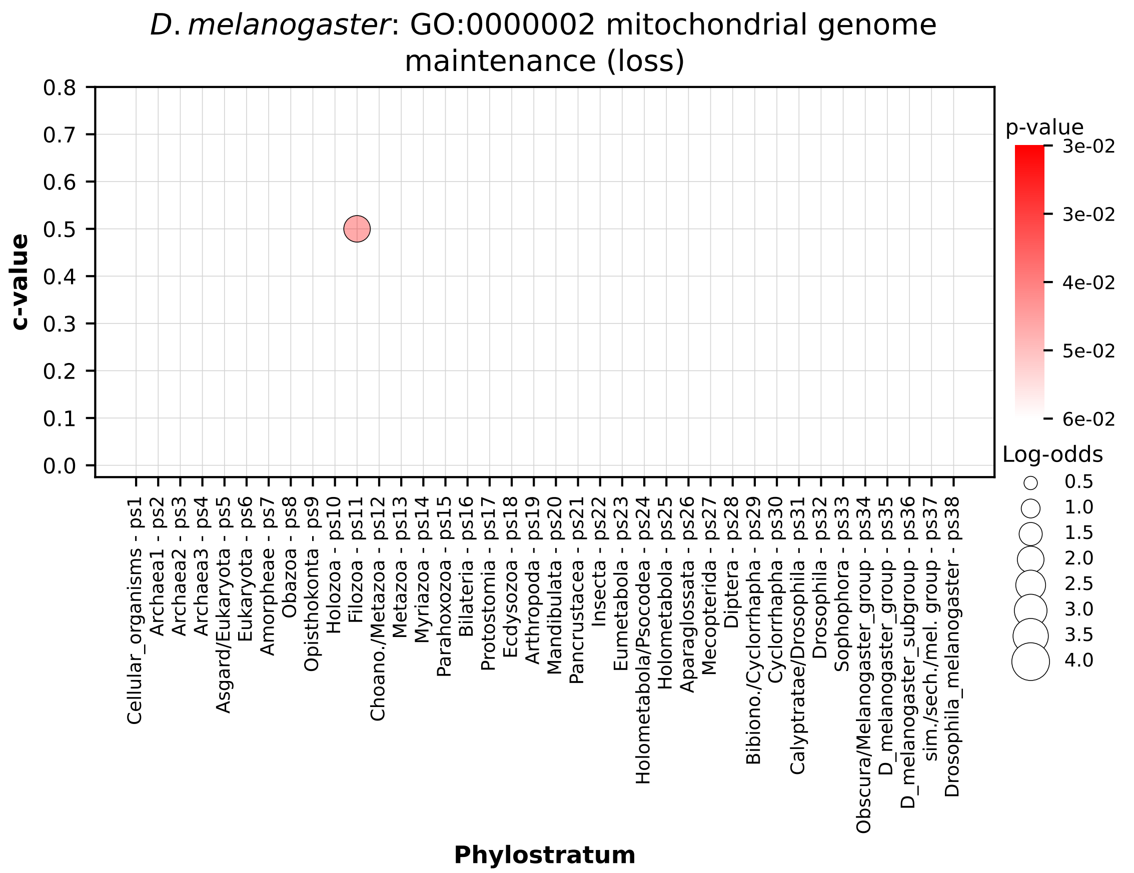
D. melanogaster: GO:0000002 mitochondrial genome maintenance (loss)
c-value
0.8
0.7
0.6
0.5
0.4
0.3
0.2
0.1
0.0
Phylostratum
Cellular_organisms - ps1
Archaea1 - ps2
Archaea2 - ps3
Archaea3 - ps4
Asgard/Eukaryota - ps5
Eukaryota - ps6
Amorpheae - ps7
Obazoa - ps8
Opisthokonta - ps9
Holozoa - ps10
Filozoa - ps11
Choano./Metazoa - ps12
Metazoa - ps13
Myriazoa - ps14
Parahoxozoa - ps15
Bilateria - ps16
Protostomia - ps17
Ecdysozoa - ps18
Arthropoda - ps19
Mandibulata - ps20
Pancrustacea - ps21
Insecta - ps22
Eumetabola - ps23
Holometabola/Psocodea - ps24
Holometabola - ps25
Aparaglossata - ps26
Mecopterida - ps27
Diptera - ps28
Bibiono./Cyclorrhapha - ps29
Cyclorrhapha - ps30
Calyptratae/Drosophila - ps31
Drosophila - ps32
Sophophora - ps33
Obscura/Melanogaster_group - ps34
D_melanogaster_group - ps35
D_melanogaster_subgroup - ps36
sim./sech./mel. group - ps37
Drosophila_melanogaster - ps38
p-value
3e-02
3e-02
4e-02
5e-02
6e-02
Log-odds
0.5
1.0
1.5
2.0
2.5
3.0
3.5
4.0

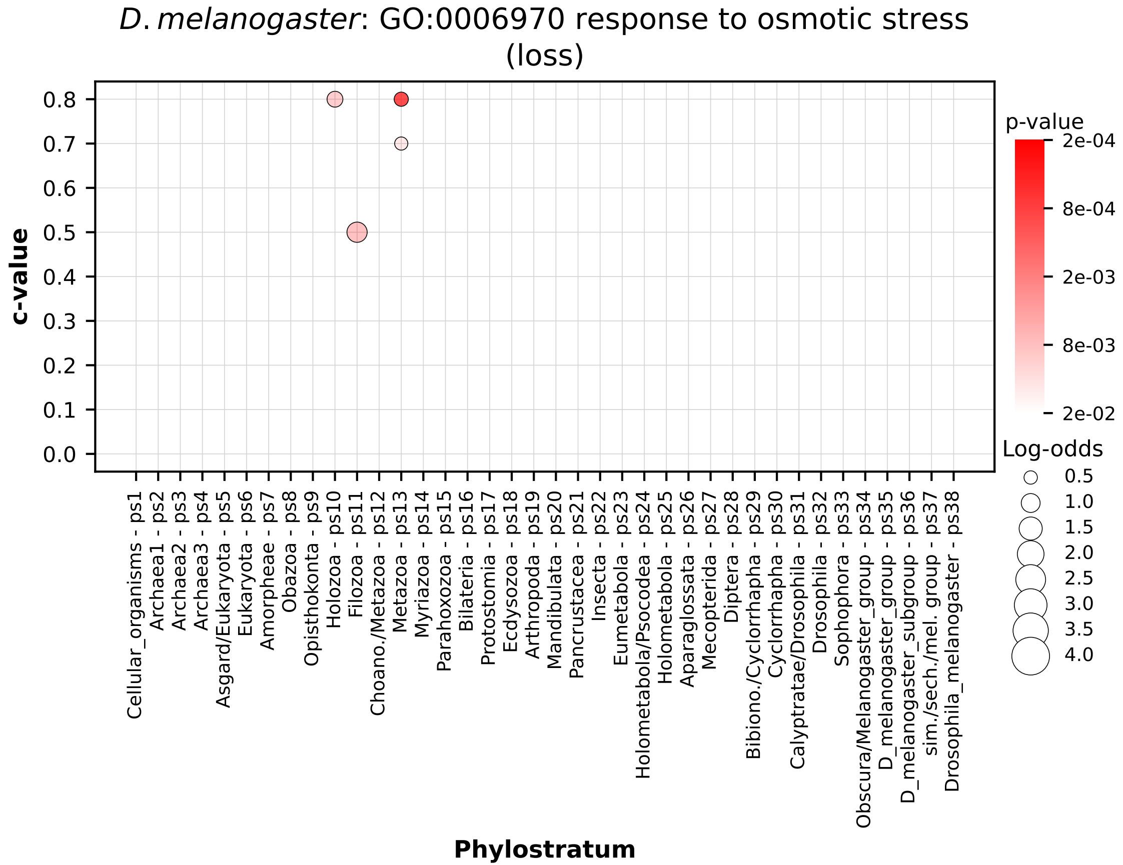

D. melanogaster: GO:0006970 response to osmotic stress
(loss)
c-value
0.0
0.1
0.2
0.3
0.4
0.5
0.6
0.7
0.8
p-value
2e-04
8e-04
2e-03
8e-03
2e-02
Log-odds
0.5
1.0
1.5
2.0
2.5
3.0
3.5
4.0
Cellular_organisms - ps1
Archaea1 - ps2
Archaea2 - ps3
Archaea3 - ps4
Asgard/Eukaryota - ps5
Eukaryota - ps6
Amorpheae - ps7
Obazoa - ps8
Opisthokonta - ps9
Holozoa - ps10
Filozoa - ps11
Choano./Metazoa - ps12
Metazoa - ps13
Myriazoa - ps14
Parahoxozoa - ps15
Bilateria - ps16
Protostomia - ps17
Ecdysozoa - ps18
Arthropoda - ps19
Mandibulata - ps20
Pancrustacea - ps21
Insecta - ps22
Eumetabola - ps23
Holometabola/Psocodea - ps24
Holometabola - ps25
Aparaglossata - ps26
Mecopterida - ps27
Diptera - ps28
Bibiono./Cyclorrhapha - ps29
Cyclorrhapha - ps30
Calyptratae/Drosophila - ps31
Drosophila - ps32
Sophophora - ps33
Obscura/Melanogaster_group - ps34
D_melanogaster_group - ps35
D_melanogaster_subgroup - ps36
sim./sech./mel. group - ps37
Drosophila_melanogaster - ps38
Phylostratum

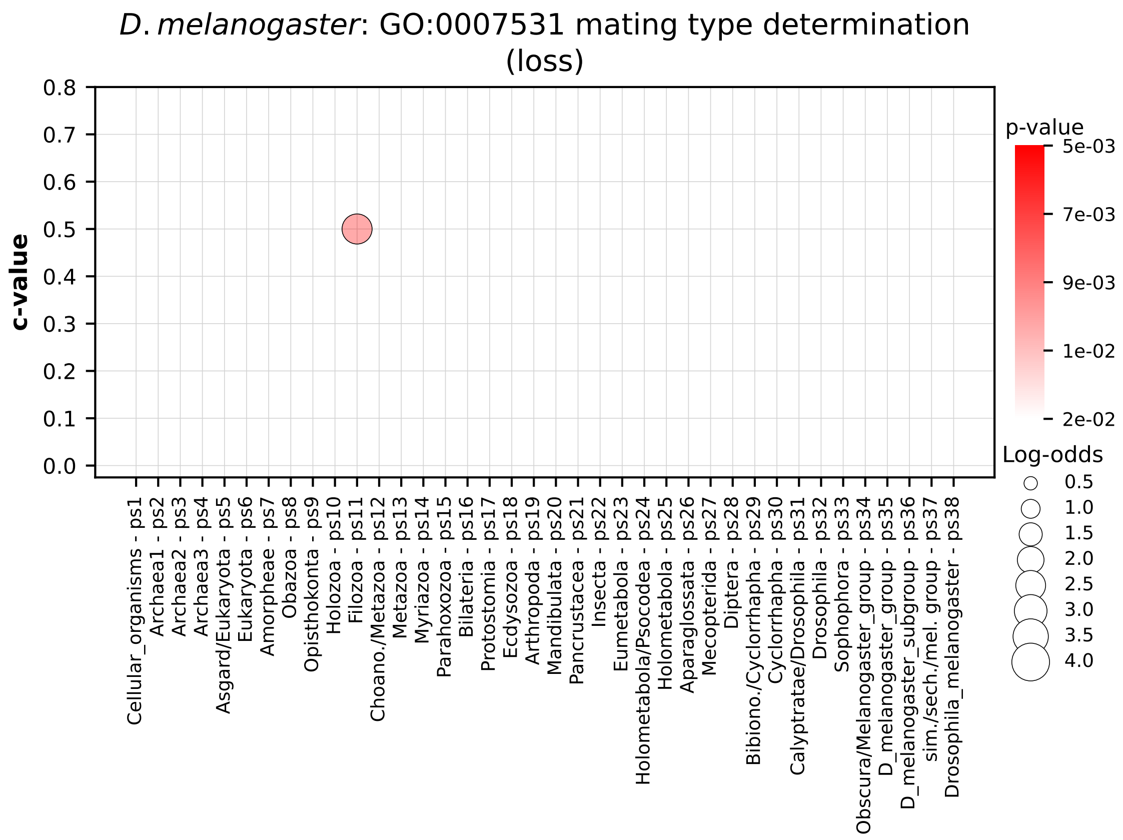

D. melanogaster: GO:0007531 mating type determination
(loss)
c-value
0.8
0.7
0.6
0.5
0.4
0.3
0.2
0.1
0.0
Phylostratum
Cellular_organisms - ps1
Archaea1 - ps2
Archaea2 - ps3
Archaea3 - ps4
Asgard/Eukaryota - ps5
Eukaryota - ps6
Amorpheae - ps7
Obazoa - ps8
Opisthokonta - ps9
Holozoa - ps10
Filozoa - ps11
Choano./Metazoa - ps12
Metazoa - ps13
Myriazoa - ps14
Parahoxozoa - ps15
Bilateria - ps16
Protostomia - ps17
Ecdysozoa - ps18
Arthropoda - ps19
Mandibulata - ps20
Pancrustacea - ps21
Insecta - ps22
Eumetabola - ps23
Holometabola/Psocodea - ps24
Holometabola - ps25
Aparaglossata - ps26
Mecopterida - ps27
Diptera - ps28
Bibiono./Cyclorrhapha - ps29
Cyclorrhapha - ps30
Calyptratae/Drosophila - ps31
Drosophila - ps32
Sophophora - ps33
Obscura/Melanogaster_group - ps34
D_melanogaster_group - ps35
D_melanogaster_subgroup - ps36
sim./sech./mel. group - ps37
Drosophila_melanogaster - ps38
p-value
5e-03
7e-03
9e-03
1e-02
2e-02
Log-odds
0.5
1.0
1.5
2.0
2.5
3.0
3.5
4.0

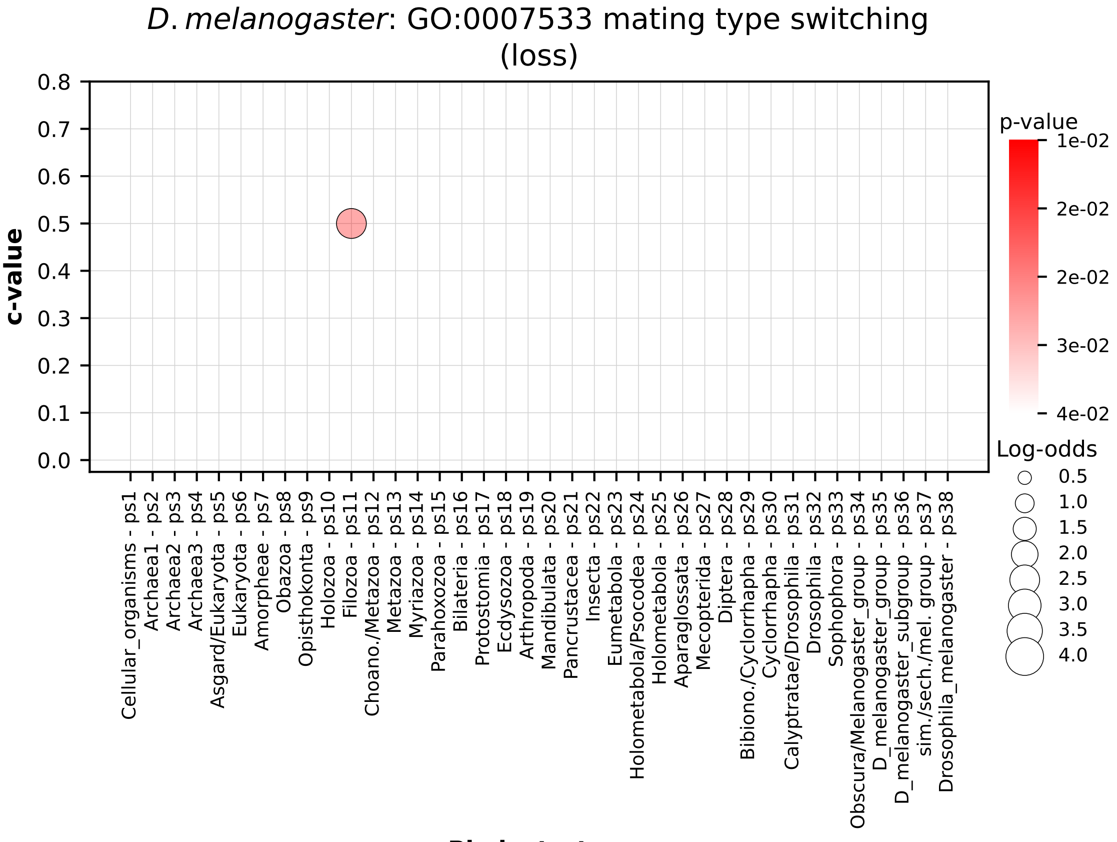

D. melanogaster: GO:0007533 mating type switching
(loss)
c-value
0.8
0.7
0.6
0.5
0.4
0.3
0.2
0.1
0.0
Phylostratum
Cellular_organisms - ps1
Archaea1 - ps2
Archaea2 - ps3
Archaea3 - ps4
Asgard/Eukaryota - ps5
Eukaryota - ps6
Amorpheae - ps7
Obazoa - ps8
Opisthokonta - ps9
Holozoa - ps10
Filozoa - ps11
Choano./Metazoa - ps12
Metazoa - ps13
Myriazoa - ps14
Parahoxozoa - ps15
Bilateria - ps16
Protostomia - ps17
Ecdysozoa - ps18
Arthropoda - ps19
Mandibulata - ps20
Pancrustacea - ps21
Insecta - ps22
Eumetabola - ps23
Holometabola/Psocodea - ps24
Holometabola - ps25
Aparaglossata - ps26
Mecopterida - ps27
Diptera - ps28
Bibiono./Cyclorrhapha - ps29
Cyclorrhapha - ps30
Calyptratae/Drosophila - ps31
Drosophila - ps32
Sophophora - ps33
Obscura/Melanogaster_group - ps34
D_melanogaster_group - ps35
D_melanogaster_subgroup - ps36
sim./sech./mel. group - ps37
Drosophila_melanogaster - ps38
p-value
1e-02
2e-02
2e-02
3e-02
4e-02
Log-odds
0.5
1.0
1.5
2.0
2.5
3.0
3.5
4.0

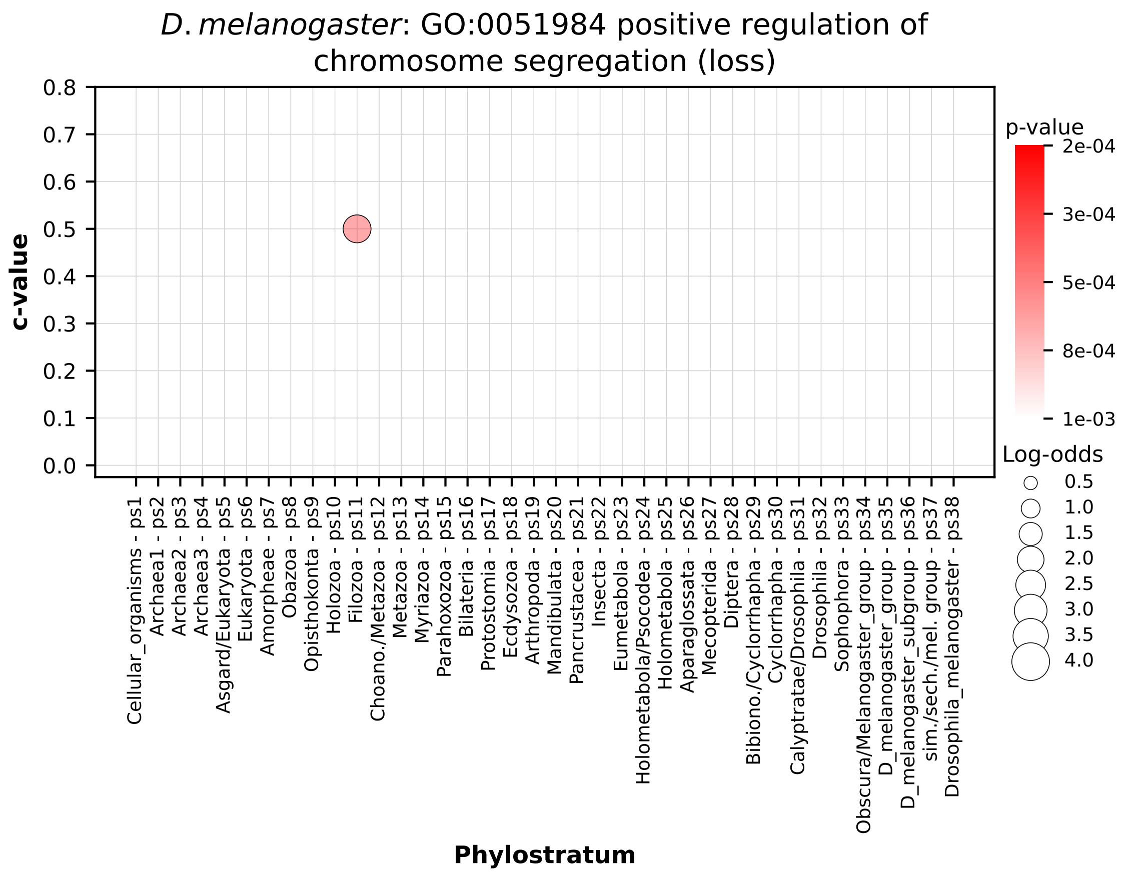
D. melanogaster: GO:0051984 positive regulation of chromosome segregation (loss)
c-value
0.0
0.1
0.2
0.3
0.4
0.5
0.6
0.7
0.8
Phylostratum
Cellular_organisms - ps1
Archaea1 - ps2
Archaea2 - ps3
Archaea3 - ps4
Asgard/Eukaryota - ps5
Eukaryota - ps6
Amorpheae - ps7
Obazoa - ps8
Opisthokonta - ps9
Holozoa - ps10
Filozoa - ps11
Choano./Metazoa - ps12
Metazoa - ps13
Myriazoa - ps14
Parahoxozoa - ps15
Bilateria - ps16
Protostomia - ps17
Ecdysozoa - ps18
Arthropoda - ps19
Mandibulata - ps20
Pancrustacea - ps21
Insecta - ps22
Eumetabola - ps23
Holometabola/Psocodea - ps24
Holometabola - ps25
Aparaglossata - ps26
Mecopterida - ps27
Diptera - ps28
Bibiono./Cyclorrhapha - ps29
Cyclorrhapha - ps30
Calyptratae/Drosophila - ps31
Drosophila - ps32
Sophophora - ps33
Obscura/Melanogaster_group - ps34
D_melanogaster_group - ps35
D_melanogaster_subgroup - ps36
sim./sech./mel. group - ps37
Drosophila_melanogaster - ps38
p-value
2e-04
3e-04
5e-04
8e-04
1e-03
Log-odds
0.5
1.0
1.5
2.0
2.5
3.0
3.5
4.0

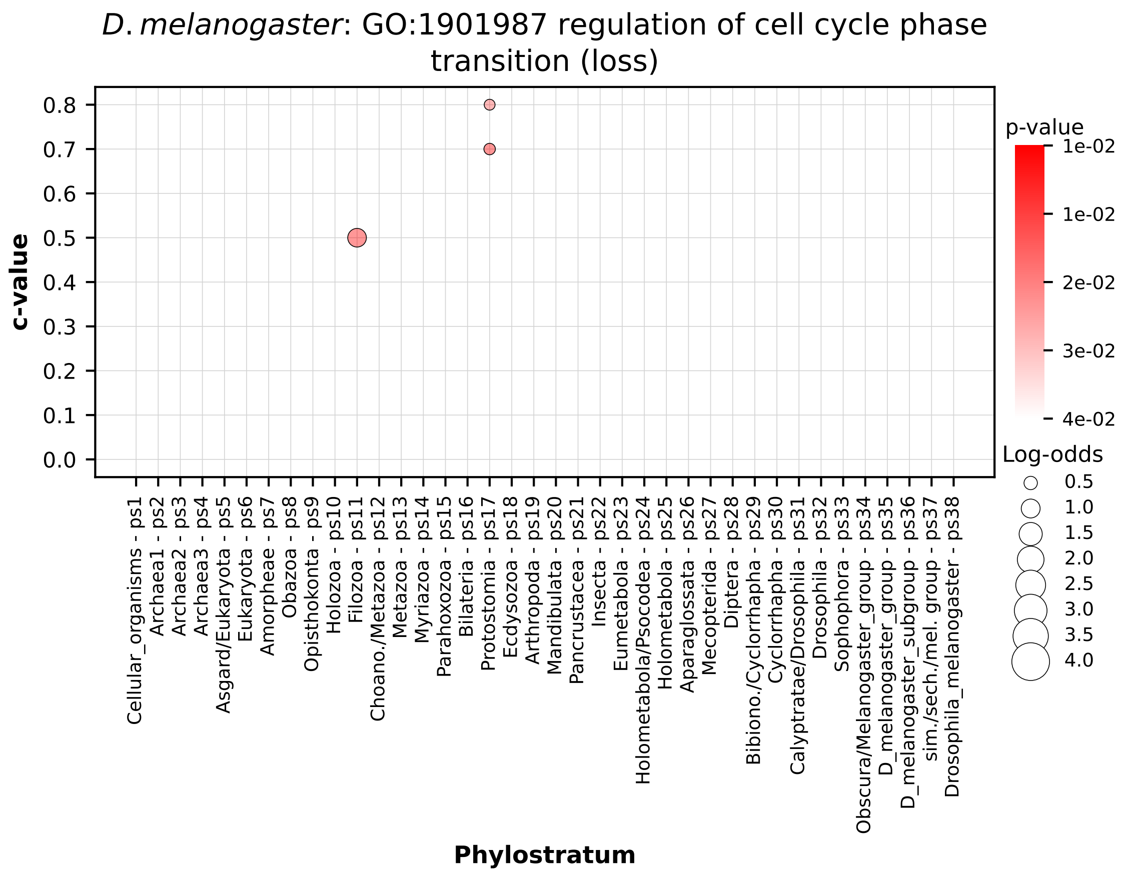

D. melanogaster: GO:1901987 regulation of cell cycle phase transition (loss)
c-value
0.0
0.1
0.2
0.3
0.4
0.5
0.6
0.7
0.8
Phylostratum
Cellular_organisms - ps1
Archaea1 - ps2
Archaea2 - ps3
Archaea3 - ps4
Asgard/Eukaryota - ps5
Eukaryota - ps6
Amorpheae - ps7
Obazoa - ps8
Opisthokonta - ps9
Holozoa - ps10
Filozoa - ps11
Choano./Metazoa - ps12
Metazoa - ps13
Myriazoa - ps14
Parahoxozoa - ps15
Bilateria - ps16
Protostomia - ps17
Ecdysozoa - ps18
Arthropoda - ps19
Mandibulata - ps20
Pancrustacea - ps21
Insecta - ps22
Eumetabola - ps23
Holometabola/Psocodea - ps24
Holometabola - ps25
Aparaglossata - ps26
Mecopterida - ps27
Diptera - ps28
Bibiono./Cyclorrhapha - ps29
Cyclorrhapha - ps30
Calyptratae/Drosophila - ps31
Drosophila - ps32
Sophophora - ps33
Obscura/Melanogaster_group - ps34
D_melanogaster_group - ps35
D_melanogaster_subgroup - ps36
sim./sech./mel. group - ps37
Drosophila_melanogaster - ps38
p-value
1e-02
1e-02
2e-02
3e-02
4e-02
Log-odds
0.5
1.0
1.5
2.0
2.5
3.0
3.5
4.0

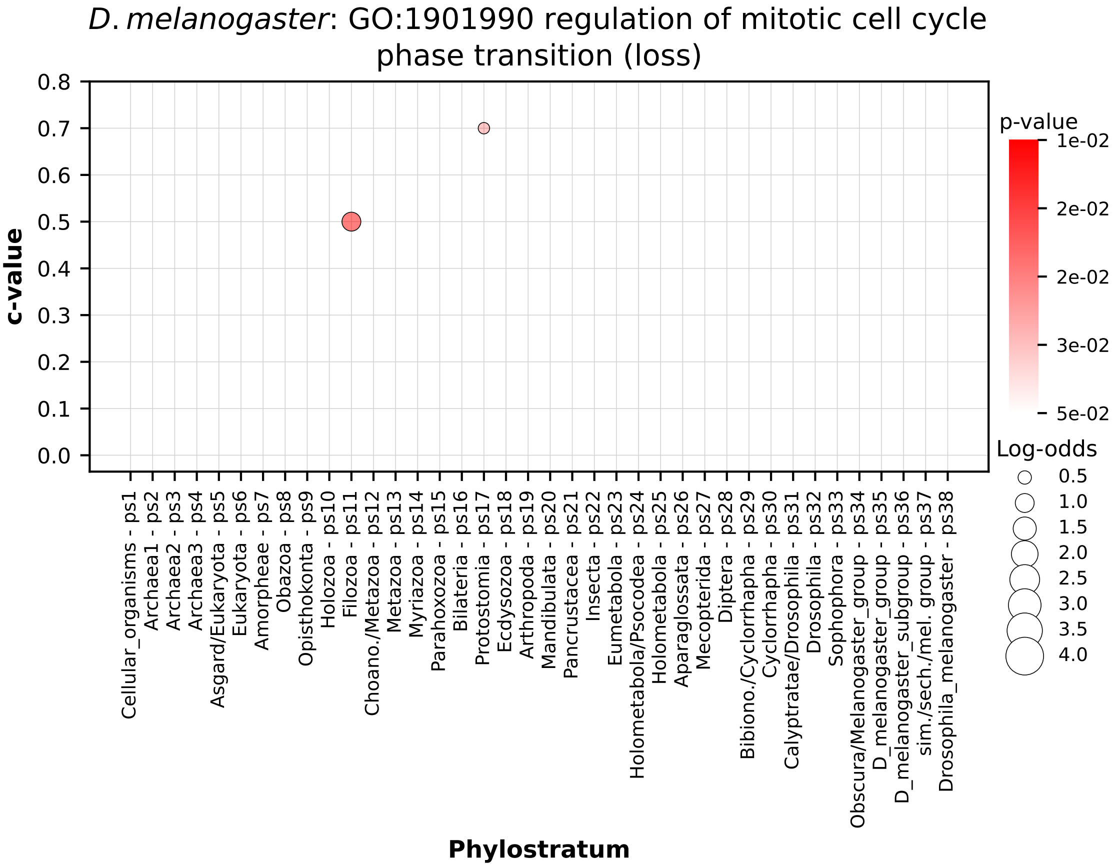

D. melanogaster: GO:1901990 regulation of mitotic cell cycle phase transition (loss)
c-value
0.8
0.7
0.6
0.5
0.4
0.3
0.2
0.1
0.0
Phylostratum
Cellular_organisms - ps1
Archaea1 - ps2
Archaea2 - ps3
Archaea3 - ps4
Asgard/Eukaryota - ps5
Eukaryota - ps6
Amorpheae - ps7
Obazoa - ps8
Opisthokonta - ps9
Holozoa - ps10
Filozoa - ps11
Choano./Metazoa - ps12
Metazoa - ps13
Myriazoa - ps14
Parahoxozoa - ps15
Bilateria - ps16
Protostomia - ps17
Ecdysozoa - ps18
Arthropoda - ps19
Mandibulata - ps20
Pancrustacea - ps21
Insecta - ps22
Eumetabola - ps23
Holometabola/Psocodea - ps24
Holometabola - ps25
Aparaglossata - ps26
Mecopterida - ps27
Diptera - ps28
Bibiono./Cyclorrhapha - ps29
Cyclorrhapha - ps30
Calyptratae/Drosophila - ps31
Drosophila - ps32
Sophophora - ps33
Obscura/Melanogaster_group - ps34
D_melanogaster_group - ps35
D_melanogaster_subgroup - ps36
sim./sech./mel. group - ps37
Drosophila_melanogaster - ps38
p-value
1e-02
2e-02
2e-02
3e-02
5e-02
Log-odds
0.5
1.0
1.5
2.0
2.5
3.0
3.5
4.0

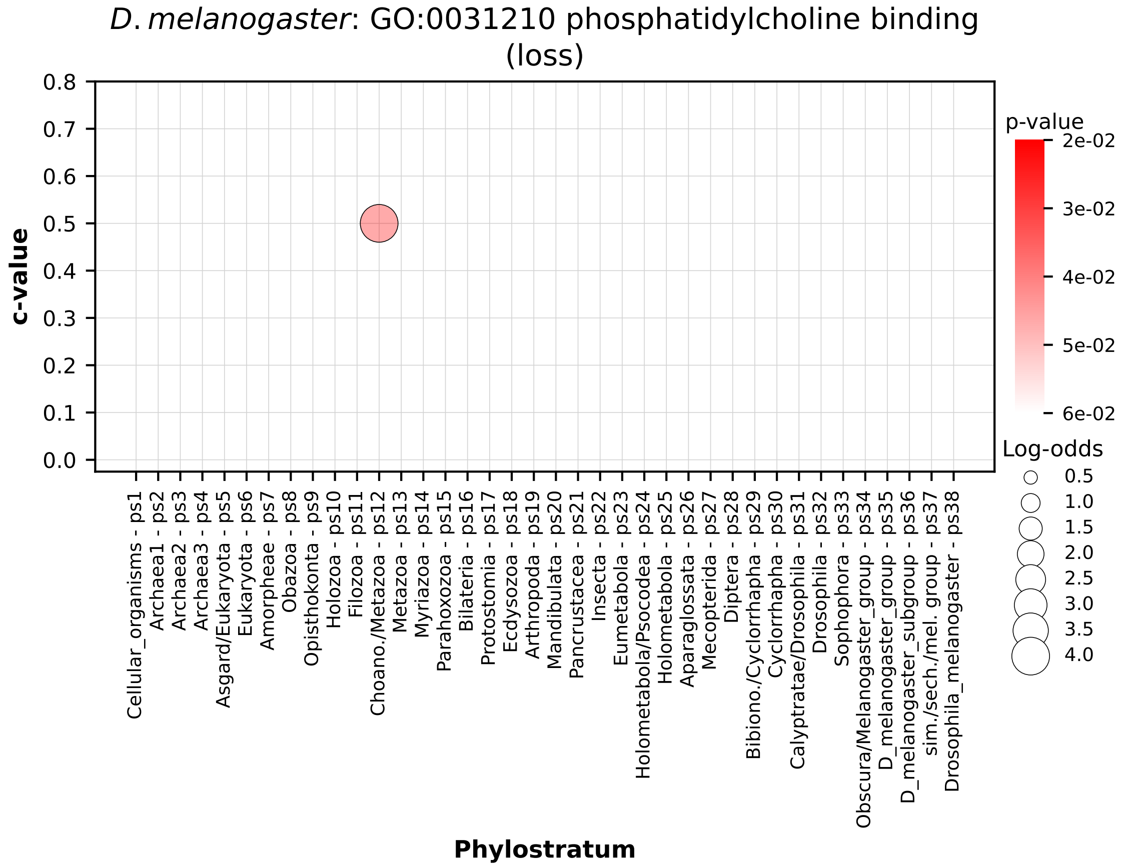

D. melanogaster: GO:0031210 phosphatidylcholine binding (loss)
c-value
0.8
0.7
0.6
0.5
0.4
0.3
0.2
0.1
0.0
Phylostratum
Cellular_organisms - ps1
Archaea1 - ps2
Archaea2 - ps3
Archaea3 - ps4
Asgard/Eukaryota - ps5
Eukaryota - ps6
Amorpheae - ps7
Obazoa - ps8
Opisthokonta - ps9
Holozoa - ps10
Filozoa - ps11
Choano./Metazoa - ps12
Metazoa - ps13
Myriazoa - ps14
Parahoxozoa - ps15
Bilateria - ps16
Protostomia - ps17
Ecdysozoa - ps18
Arthropoda - ps19
Mandibulata - ps20
Pancrustacea - ps21
Insecta - ps22
Eumetabola - ps23
Holometabola/Psocodea - ps24
Holometabola - ps25
Aparaglossata - ps26
Mecopterida - ps27
Diptera - ps28
Bibiono./Cyclorrhapha - ps29
Cyclorrhapha - ps30
Calyptratae/Drosophila - ps31
Drosophila - ps32
Sophophora - ps33
Obscura/Melanogaster_group - ps34
D_melanogaster_group - ps35
D_melanogaster_subgroup - ps36
sim./sech./mel. group - ps37
Drosophila_melanogaster - ps38
p-value
2e-02
3e-02
4e-02
5e-02
6e-02
Log-odds
0.5
1.0
1.5
2.0
2.5
3.0
3.5
4.0

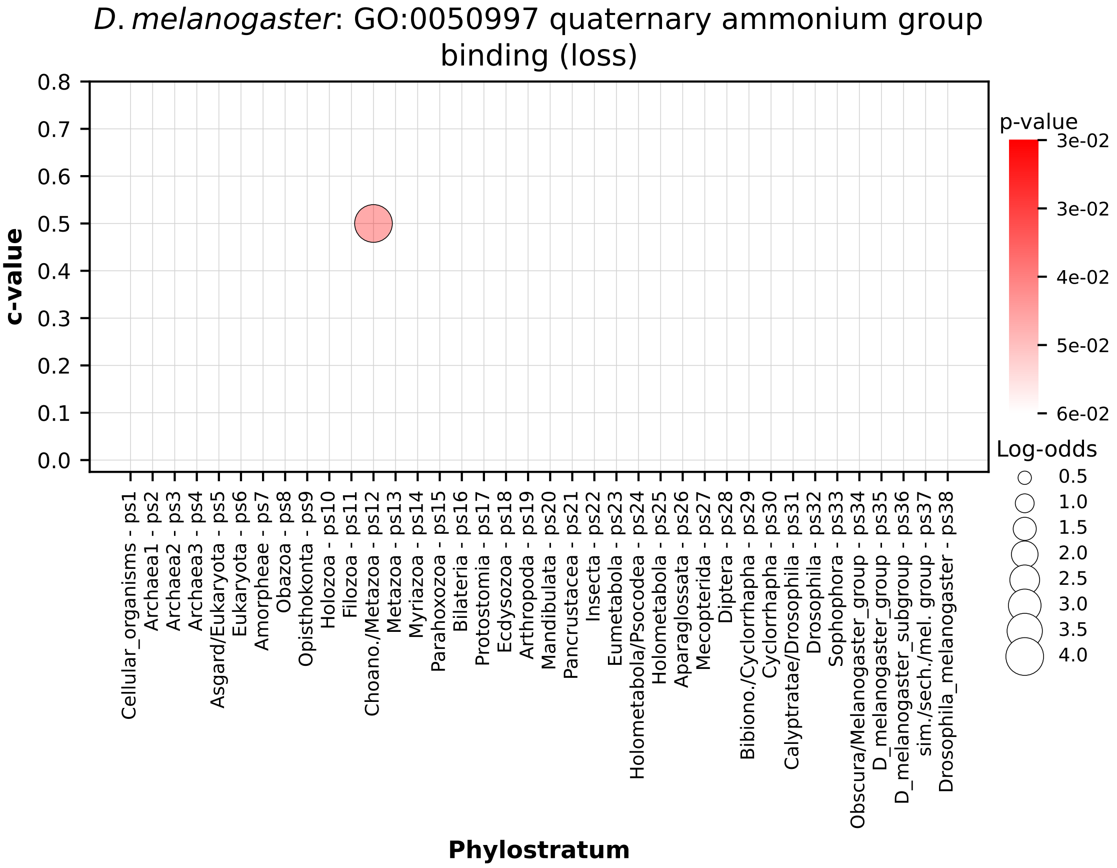

D. melanogaster: GO:0050997 quaternary ammonium group binding (loss)
c-value
0.8
0.7
0.6
0.5
0.4
0.3
0.2
0.1
0.0
Phylostratum
Cellular_organisms - ps1
Archaea1 - ps2
Archaea2 - ps3
Archaea3 - ps4
Asgard/Eukaryota - ps5
Eukaryota - ps6
Amorpheae - ps7
Obazoa - ps8
Opisthokonta - ps9
Holozoa - ps10
Filozoa - ps11
Choano./Metazoa - ps12
Metazoa - ps13
Myriazoa - ps14
Parahoxozoa - ps15
Bilateria - ps16
Protostomia - ps17
Ecdysozoa - ps18
Arthropoda - ps19
Mandibulata - ps20
Pancrustacea - ps21
Insecta - ps22
Eumetabola - ps23
Holometabola/Psocodea - ps24
Holometabola - ps25
Aparaglossata - ps26
Mecopterida - ps27
Diptera - ps28
Bibiono./Cyclorrhapha - ps29
Cyclorrhapha - ps30
Calyptratae/Drosophila - ps31
Drosophila - ps32
Sophophora - ps33
Obscura/Melanogaster_group - ps34
D_melanogaster_group - ps35
D_melanogaster_subgroup - ps36
sim./sech./mel. group - ps37
Drosophila_melanogaster - ps38
p-value
3e-02
3e-02
4e-02
5e-02
6e-02
Log-odds
0.5
1.0
1.5
2.0
2.5
3.0
3.5
4.0

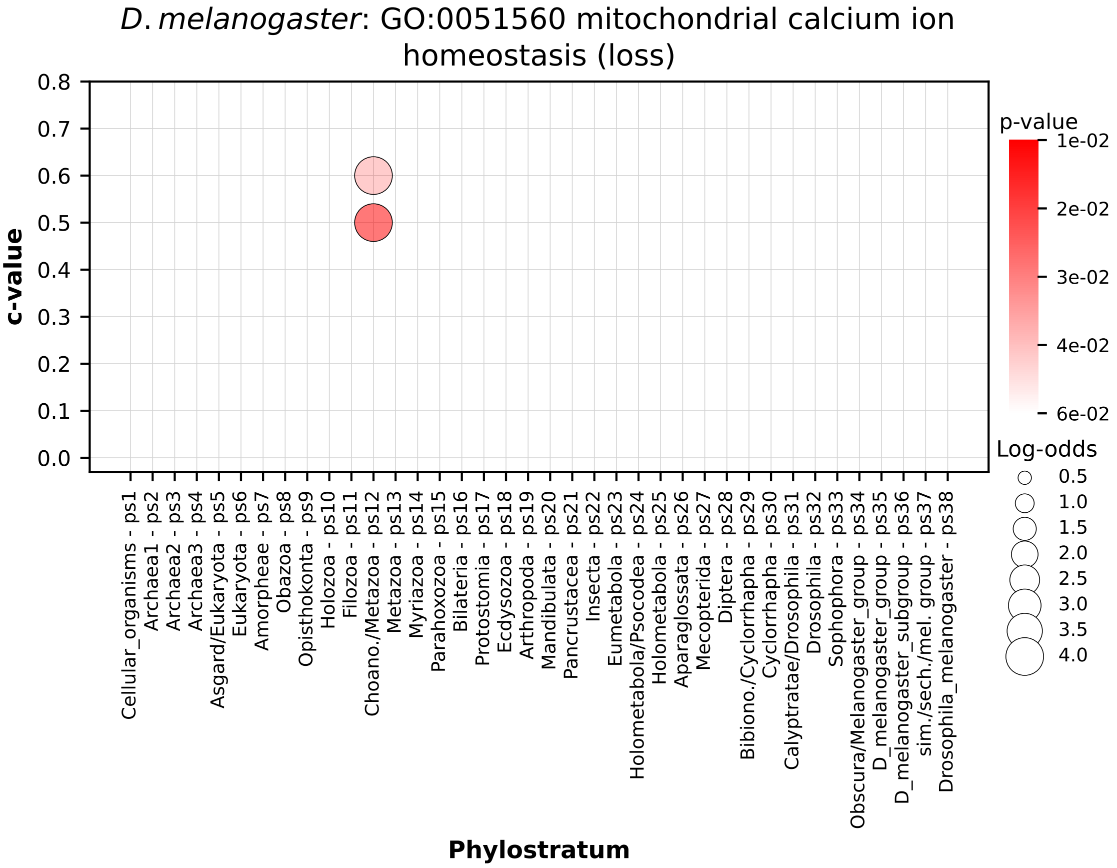

D. melanogaster: GO:0051560 mitochondrial calcium ion homeostasis (loss)
c-value
0.8
0.7
0.6
0.5
0.4
0.3
0.2
0.1
0.0
Phylostratum
Cellular_organisms - ps1
Archaea1 - ps2
Archaea2 - ps3
Archaea3 - ps4
Asgard/Eukaryota - ps5
Eukaryota - ps6
Amorpheae - ps7
Obazoa - ps8
Opisthokonta - ps9
Holozoa - ps10
Filozoa - ps11
Choano./Metazoa - ps12
Metazoa - ps13
Myriazoa - ps14
Parahoxozoa - ps15
Bilateria - ps16
Protostomia - ps17
Ecdysozoa - ps18
Arthropoda - ps19
Mandibulata - ps20
Pancrustacea - ps21
Insecta - ps22
Eumetabola - ps23
Holometabola/Psocodea - ps24
Holometabola - ps25
Aparaglossata - ps26
Mecopterida - ps27
Diptera - ps28
Bibiono./Cyclorrhapha - ps29
Cyclorrhapha - ps30
Calyptratae/Drosophila - ps31
Drosophila - ps32
Sophophora - ps33
Obscura/Melanogaster_group - ps34
D_melanogaster_group - ps35
D_melanogaster_subgroup - ps36
sim./sech./mel. group - ps37
Drosophila_melanogaster - ps38
p-value
1e-02
2e-02
3e-02
4e-02
6e-02
Log-odds
0.5
1.0
1.5
2.0
2.5
3.0
3.5
4.0

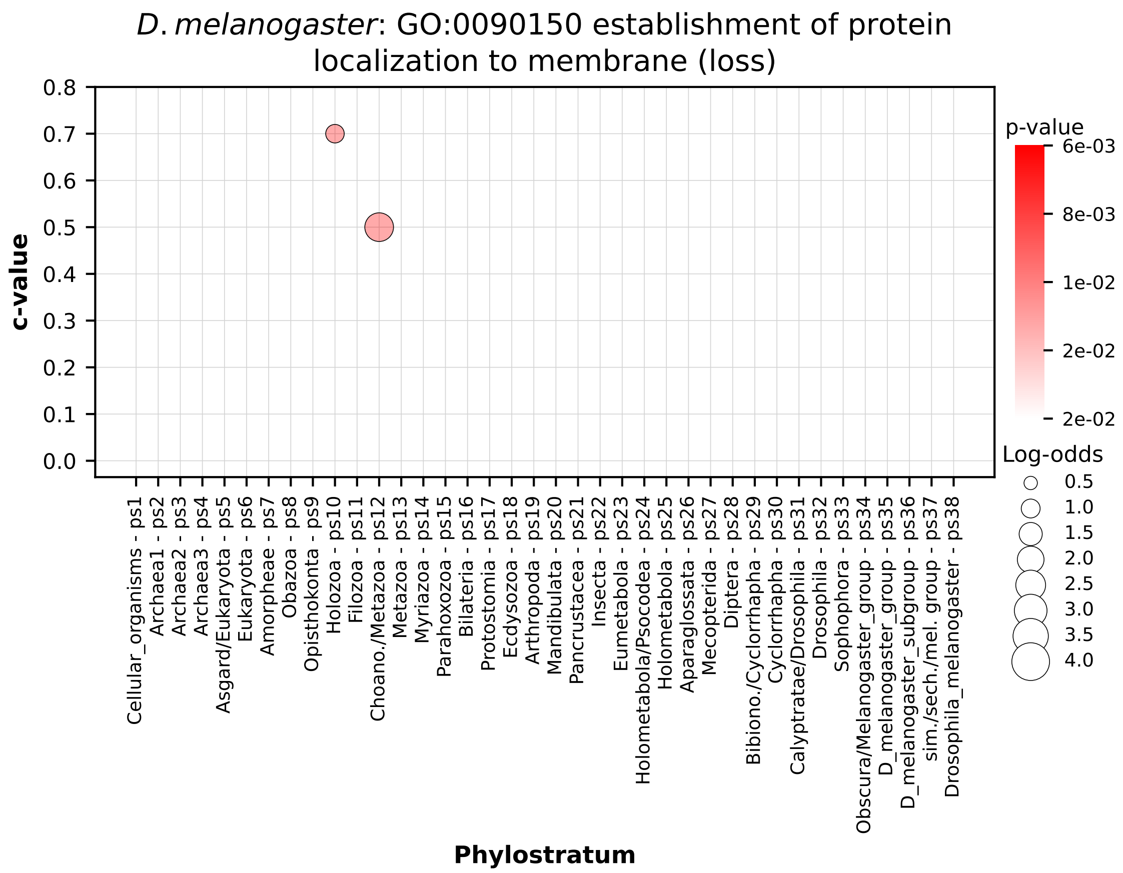
D. melanogaster: GO:0090150 establishment of protein localization to membrane (loss)
c-value
0.8
0.7
0.6
0.5
0.4
0.3
0.2
0.1
0.0
p-value
6e-03
8e-03
1e-02
2e-02
2e-02
Log-odds
0.5
1.0
1.5
2.0
2.5
3.0
3.5
4.0
Cellular_organisms - ps1
Archaea1 - ps2
Archaea2 - ps3
Archaea3 - ps4
Asgard/Eukaryota - ps5
Eukaryota - ps6
Amorpheae - ps7
Obazoa - ps8
Opisthokonta - ps9
Holozoa - ps10
Filozoa - ps11
Choano./Metazoa - ps12
Metazoa - ps13
Myriazoa - ps14
Parahoxozoa - ps15
Bilateria - ps16
Protostomia - ps17
Ecdysozoa - ps18
Arthropoda - ps19
Mandibulata - ps20
Pancrustacea - ps21
Insecta - ps22
Eumetabola - ps23
Holometabola/Psocodea - ps24
Holometabola - ps25
Aparaglossata - ps26
Mecopterida - ps27
Diptera - ps28
Bibiono./Cyclorrhapha - ps29
Cyclorrhapha - ps30
Calyptratae/Drosophila - ps31
Drosophila - ps32
Sophophora - ps33
Obscura/Melanogaster_group - ps34
D_melanogaster_group - ps35
D_melanogaster_subgroup - ps36
sim./sech./mel. group - ps37
Drosophila_melanogaster - ps38
Phylostratum

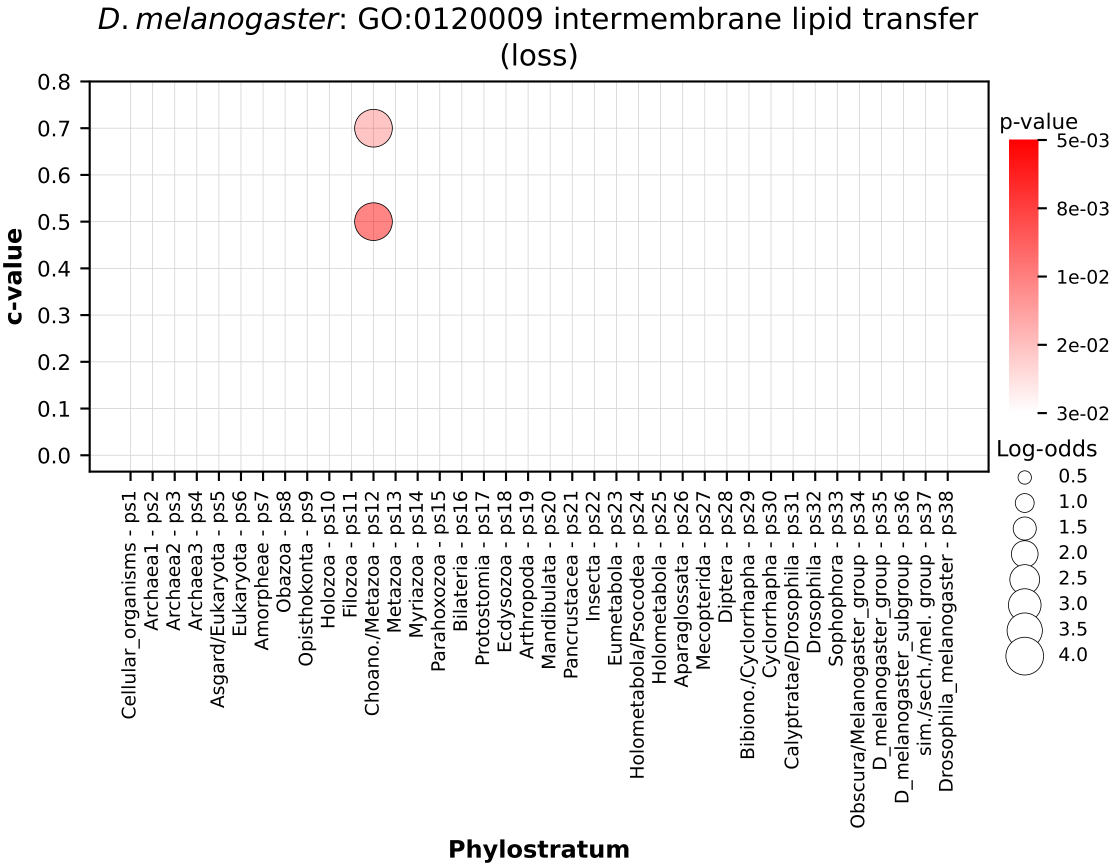

D. melanogaster: GO:0120009 intermembrane lipid transfer (loss)
c-value
0.8
0.7
0.6
0.5
0.4
0.3
0.2
0.1
0.0
Phylostratum
Cellular_organisms - ps1
Archaea1 - ps2
Archaea2 - ps3
Archaea3 - ps4
Asgard/Eukaryota - ps5
Eukaryota - ps6
Amorpheae - ps7
Obazoa - ps8
Opisthokonta - ps9
Holozoa - ps10
Filozoa - ps11
Choano./Metazoa - ps12
Metazoa - ps13
Myriazoa - ps14
Parahoxozoa - ps15
Bilateria - ps16
Protostomia - ps17
Ecdysozoa - ps18
Arthropoda - ps19
Mandibulata - ps20
Pancrustacea - ps21
Insecta - ps22
Eumetabola - ps23
Holometabola/Psocodea - ps24
Holometabola - ps25
Aparaglossata - ps26
Mecopterida - ps27
Diptera - ps28
Bibiono./Cyclorrhapha - ps29
Cyclorrhapha - ps30
Calyptratae/Drosophila - ps31
Drosophila - ps32
Sophophora - ps33
Obscura/Melanogaster_group - ps34
D_melanogaster_group - ps35
D_melanogaster_subgroup - ps36
sim./sech./mel. group - ps37
Drosophila_melanogaster - ps38
p-value
5e-03
8e-03
1e-02
2e-02
3e-02
Log-odds
0.5
1.0
1.5
2.0
2.5
3.0
3.5
4.0

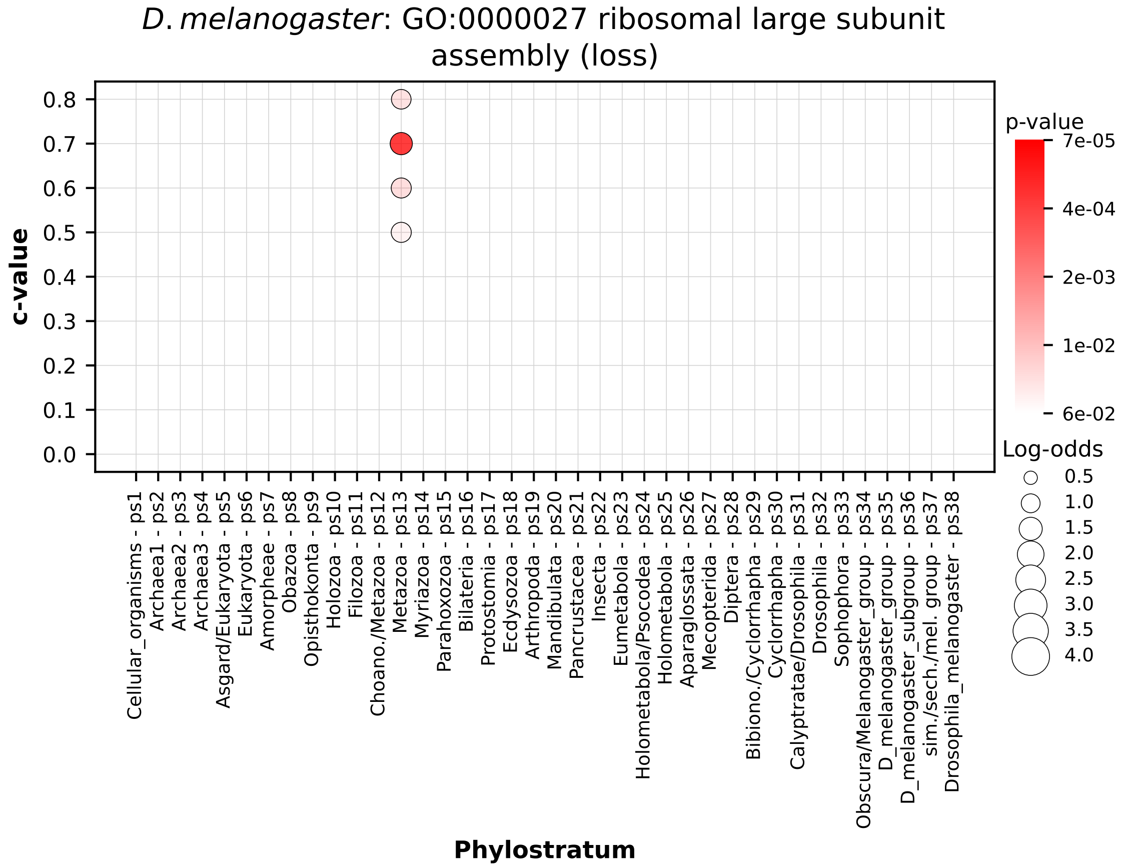

D. melanogaster: GO:0000027 ribosomal large subunit assembly (loss)
c-value
0.8
0.7
0.6
0.5
0.4
0.3
0.2
0.1
0.0
Cellular_organisms - ps1
Archaea1 - ps2
Archaea2 - ps3
Archaea3 - ps4
Asgard/Eukaryota - ps5
Eukaryota - ps6
Amorpheae - ps7
Obazoa - ps8
Opisthokonta - ps9
Holozoa - ps10
Filozoa - ps11
Choano./Metazoa - ps12
Metazoa - ps13
Myriazoa - ps14
Parahoxozoa - ps15
Bilateria - ps16
Protostomia - ps17
Ecdysozoa - ps18
Arthropoda - ps19
Mandibulata - ps20
Pancrustacea - ps21
Insecta - ps22
Eumetabola - ps23
Holometabola/Psocodea - ps24
Holometabola - ps25
Aparaglossata - ps26
Mecopterida - ps27
Diptera - ps28
Bibiono./Cyclorrhapha - ps29
Cyclorrhapha - ps30
Calyptratae/Drosophila - ps31
Drosophila - ps32
Sophophora - ps33
Obscura/Melanogaster_group - ps34
D_melanogaster_group - ps35
D_melanogaster_subgroup - ps36
sim./sech./mel. group - ps37
Drosophila_melanogaster - ps38
Phylostratum
p-value
7e-05
4e-04
2e-03
1e-02
6e-02
Log-odds
0.5
1.0
1.5
2.0
2.5
3.0
3.5
4.0

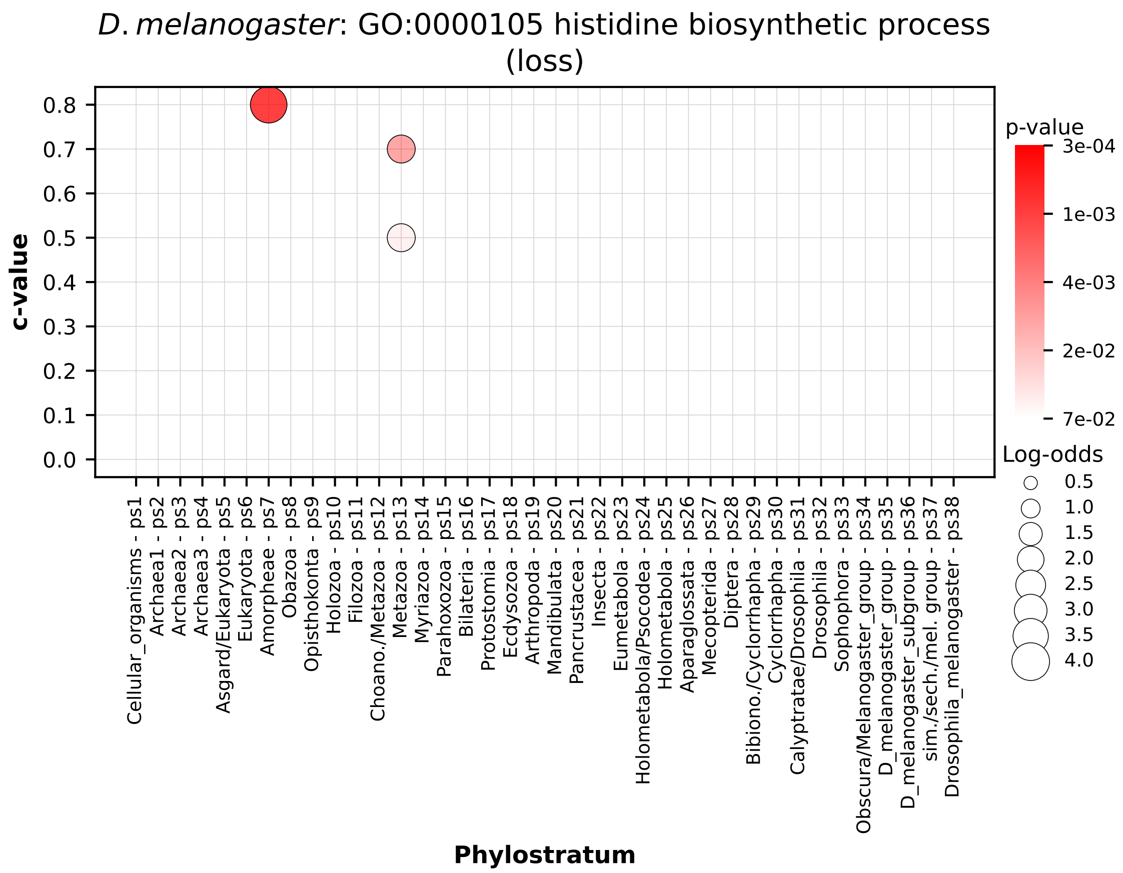

D. melanogaster: GO:0000105 histidine biosynthetic process
(loss)
c-value
0.8
0.7
0.6
0.5
0.4
0.3
0.2
0.1
0.0
p-value
3e-04
1e-03
4e-03
2e-02
7e-02
Log-odds
0.5
1.0
1.5
2.0
2.5
3.0
3.5
4.0
Cellular_organisms - ps1
Archaea1 - ps2
Archaea2 - ps3
Archaea3 - ps4
Asgard/Eukaryota - ps5
Eukaryota - ps6
Amorpheae - ps7
Obazoa - ps8
Opisthokonta - ps9
Holozoa - ps10
Filozoa - ps11
Choano./Metazoa - ps12
Metazoa - ps13
Myriazoa - ps14
Parahoxozoa - ps15
Bilateria - ps16
Protostomia - ps17
Ecdysozoa - ps18
Arthropoda - ps19
Mandibulata - ps20
Pancrustacea - ps21
Insecta - ps22
Eumetabola - ps23
Holometabola/Psocodea - ps24
Holometabola - ps25
Aparaglossata - ps26
Mecopterida - ps27
Diptera - ps28
Bibiono./Cyclorrhapha - ps29
Cyclorrhapha - ps30
Calyptratae/Drosophila - ps31
Drosophila - ps32
Sophophora - ps33
Obscura/Melanogaster_group - ps34
D_melanogaster_group - ps35
D_melanogaster_subgroup - ps36
sim./sech./mel. group - ps37
Drosophila_melanogaster - ps38
Phylostratum

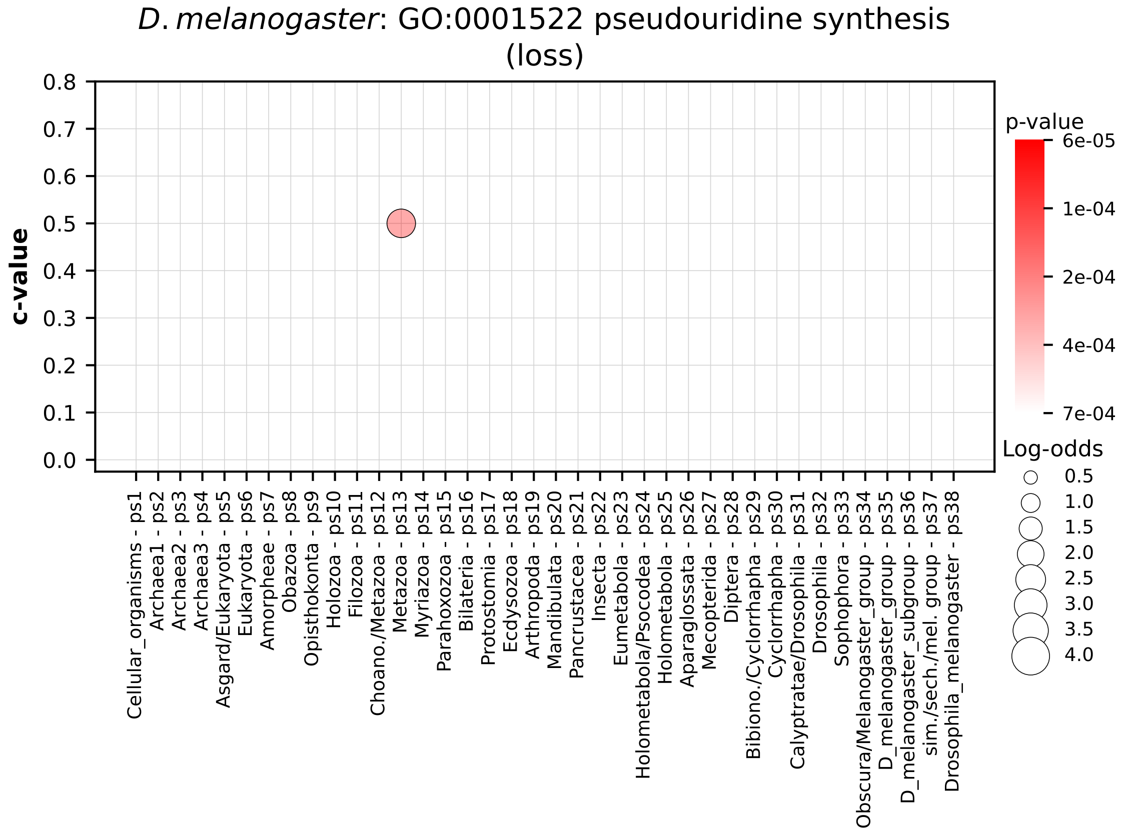

D. melanogaster: GO:0001522 pseudouridine synthesis (loss)
c-value
0.8
0.7
0.6
0.5
0.4
0.3
0.2
0.1
0.0
Phylostratum
Cellular_organisms - ps1
Archaea1 - ps2
Archaea2 - ps3
Archaea3 - ps4
Asgard/Eukaryota - ps5
Eukaryota - ps6
Amorpheae - ps7
Obazoa - ps8
Opisthokonta - ps9
Holozoa - ps10
Filozoa - ps11
Choano./Metazoa - ps12
Metazoa - ps13
Myriazoa - ps14
Parahoxozoa - ps15
Bilateria - ps16
Protostomia - ps17
Ecdysozoa - ps18
Arthropoda - ps19
Mandibulata - ps20
Pancrustacea - ps21
Insecta - ps22
Eumetabola - ps23
Holometabola/Psocodea - ps24
Holometabola - ps25
Aparaglossata - ps26
Mecopterida - ps27
Diptera - ps28
Bibiono./Cyclorrhapha - ps29
Cyclorrhapha - ps30
Calyptratae/Drosophila - ps31
Drosophila - ps32
Sophophora - ps33
Obscura/Melanogaster_group - ps34
D_melanogaster_group - ps35
D_melanogaster_subgroup - ps36
sim./sech./mel. group - ps37
Drosophila_melanogaster - ps38
p-value
6e-05
1e-04
2e-04
4e-04
7e-04
Log-odds
0.5
1.0
1.5
2.0
2.5
3.0
3.5
4.0

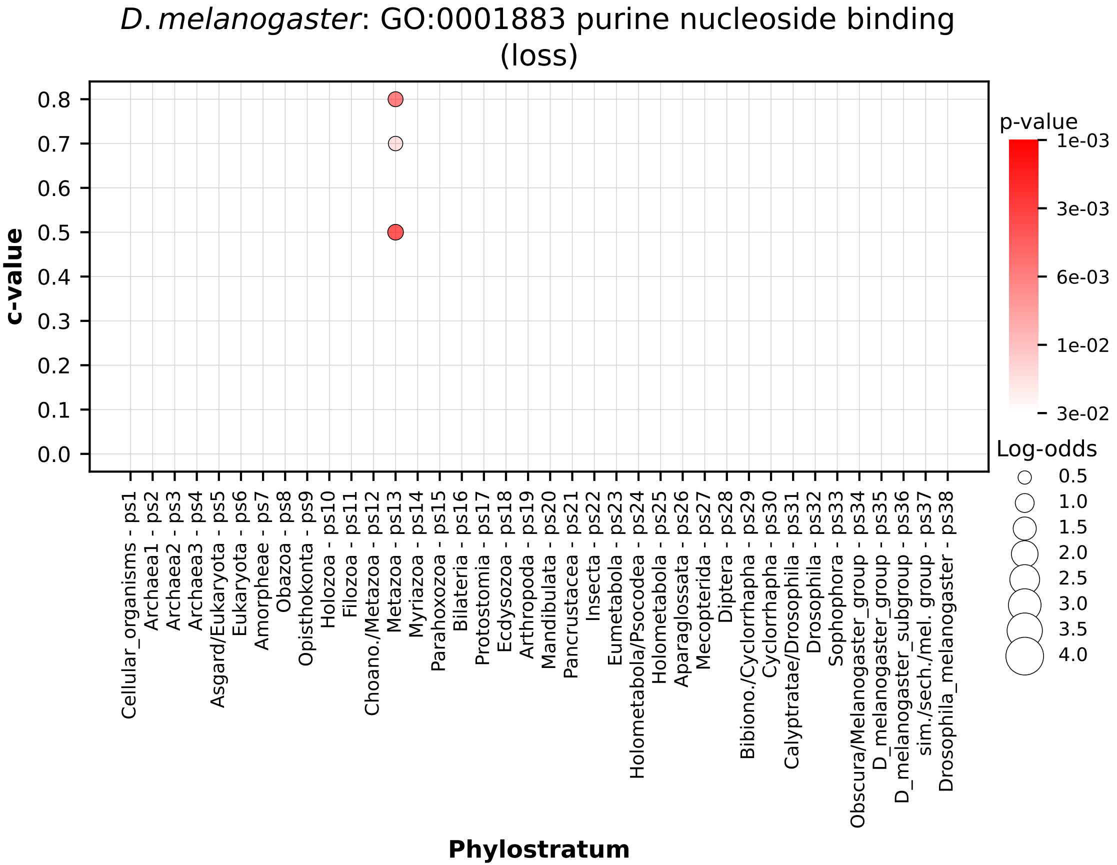

D. melanogaster: GO:0001883 purine nucleoside binding (loss)
c-value
0.0
0.1
0.2
0.3
0.4
0.5
0.6
0.7
0.8
Phylostratum
Cellular_organisms - ps1
Archaea1 - ps2
Archaea2 - ps3
Archaea3 - ps4
Asgard/Eukaryota - ps5
Eukaryota - ps6
Amorpheae - ps7
Obazoa - ps8
Opisthokonta - ps9
Holozoa - ps10
Filozoa - ps11
Choano./Metazoa - ps12
Metazoa - ps13
Myriazoa - ps14
Parahoxozoa - ps15
Bilateria - ps16
Protostomia - ps17
Ecdysozoa - ps18
Arthropoda - ps19
Mandibulata - ps20
Pancrustacea - ps21
Insecta - ps22
Eumetabola - ps23
Holometabola/Psocodea - ps24
Holometabola - ps25
Aparaglossata - ps26
Mecopterida - ps27
Diptera - ps28
Bibiono./Cyclorrhapha - ps29
Cyclorrhapha - ps30
Calyptratae/Drosophila - ps31
Drosophila - ps32
Sophophora - ps33
Obscura/Melanogaster_group - ps34
D_melanogaster_group - ps35
D_melanogaster_subgroup - ps36
sim./sech./mel. group - ps37
Drosophila_melanogaster - ps38
p-value
1e-03
3e-03
6e-03
1e-02
3e-02
Log-odds
0.5
1.0
1.5
2.0
2.5
3.0
3.5
4.0

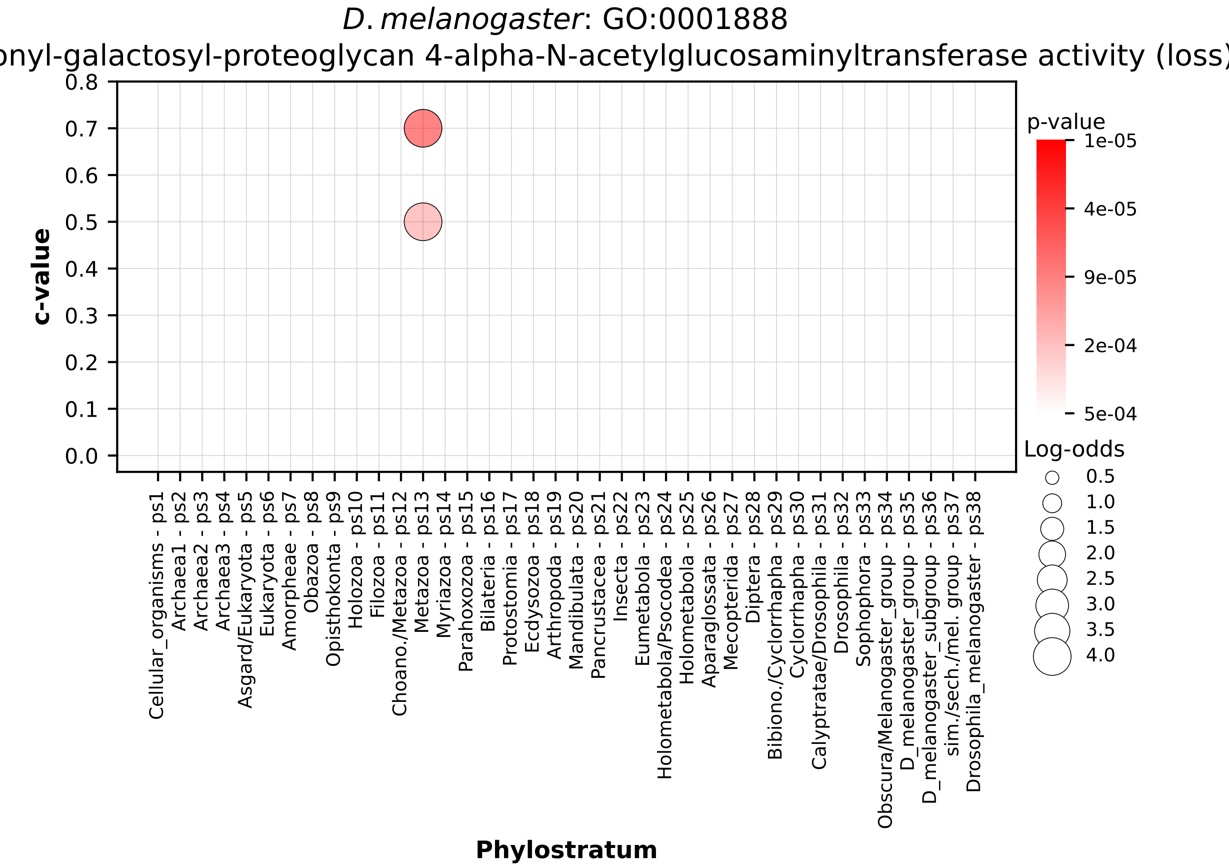

D. melanogaster: GOː0001888
onyl-galactosyl-proteoglycan 4-alpha-N-acetylglucosaminyltransferase activity (loss)
c-value
0.8
0.7
0.6
0.5
0.4
0.3
0.2
0.1
0.0
p-value
1e-05
4e-05
9e-05
2e-04
5e-04
Log-odds
0.5
1.0
1.5
2.0
2.5
3.0
3.5
4.0
Cellular_organisms - ps1
Archaea1 - ps2
Archaea2 - ps3
Archaea3 - ps4
Asgard/Eukaryota - ps5
Eukaryota - ps6
Amorpheae - ps7
Obazoa - ps8
Opisthokonta - ps9
Holozoa - ps10
Filozoa - ps11
Choano./Metazoa - ps12
Metazoa - ps13
Myriazoa - ps14
Parahoxozoa - ps15
Bilateria - ps16
Protostomia - ps17
Ecdysozoa - ps18
Arthropoda - ps19
Mandibulata - ps20
Pancrustacea - ps21
Insecta - ps22
Eumetabola - ps23
Holometabola/Psocodea - ps24
Holometabola - ps25
Aparaglossata - ps26
Mecopterida - ps27
Diptera - ps28
Bibiono./Cyclorrhapha - ps29
Cyclorrhapha - ps30
Calyptratae/Drosophila - ps31
Drosophila - ps32
Sophophora - ps33
Obscura/Melanogaster_group - ps34
D_melanogaster_group - ps35
D_melanogaster_subgroup - ps36
sim./sech./mel. group - ps37
Drosophila_melanogaster - ps38
Phylostratum

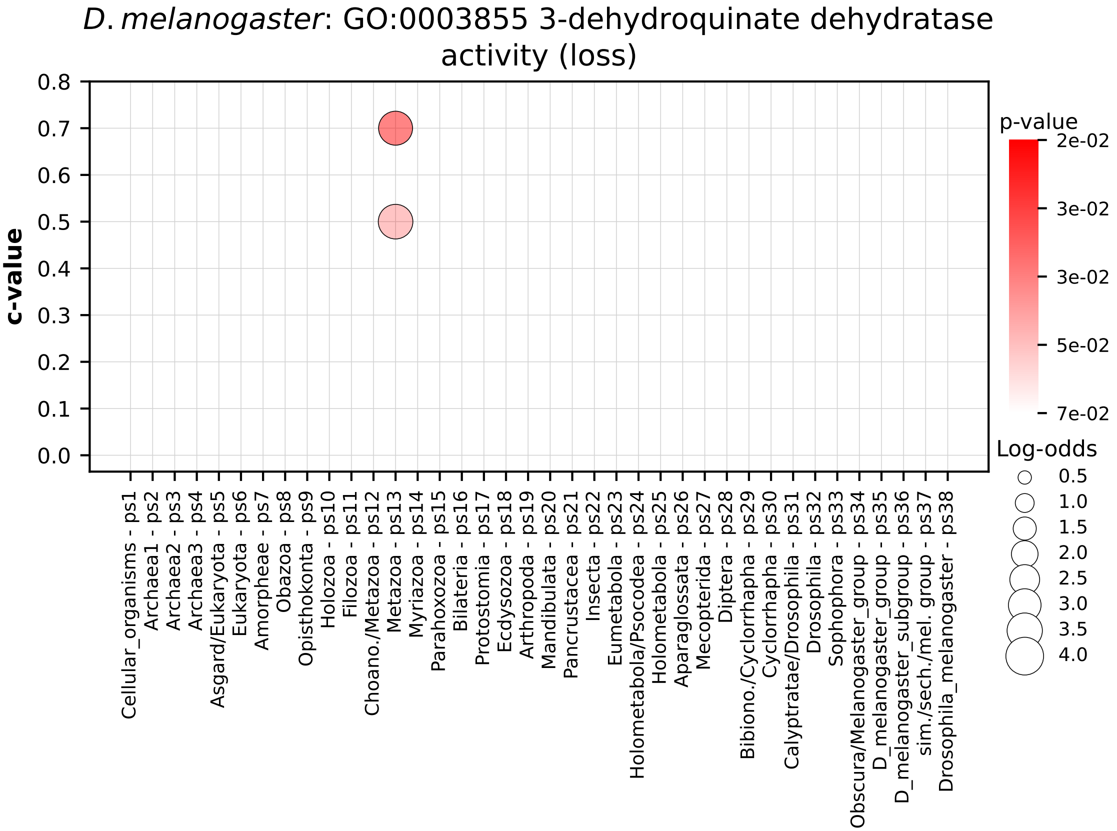

D. melanogaster: GO:0003855 3-dehydroquinate dehydratase activity (loss)
c-value
0.8
0.7
0.6
0.5
0.4
0.3
0.2
0.1
0.0
Phylostratum
Cellular_organisms - ps1
Archaea1 - ps2
Archaea2 - ps3
Archaea3 - ps4
Asgard/Eukaryota - ps5
Eukaryota - ps6
Amorpheae - ps7
Obazoa - ps8
Opisthokonta - ps9
Holozoa - ps10
Filozoa - ps11
Choano./Metazoa - ps12
Metazoa - ps13
Myriazoa - ps14
Parahoxozoa - ps15
Bilateria - ps16
Protostomia - ps17
Ecdysozoa - ps18
Arthropoda - ps19
Mandibulata - ps20
Pancrustacea - ps21
Insecta - ps22
Eumetabola - ps23
Holometabola/Psocodea - ps24
Holometabola - ps25
Aparaglossata - ps26
Mecopterida - ps27
Diptera - ps28
Bibiono./Cyclorrhapha - ps29
Cyclorrhapha - ps30
Calyptratae/Drosophila - ps31
Drosophila - ps32
Sophophora - ps33
Obscura/Melanogaster_group - ps34
D_melanogaster_group - ps35
D_melanogaster_subgroup - ps36
sim./sech./mel. group - ps37
Drosophila_melanogaster - ps38
p-value
2e-02
3e-02
3e-02
5e-02
7e-02
Log-odds
0.5
1.0
1.5
2.0
2.5
3.0
3.5
4.0

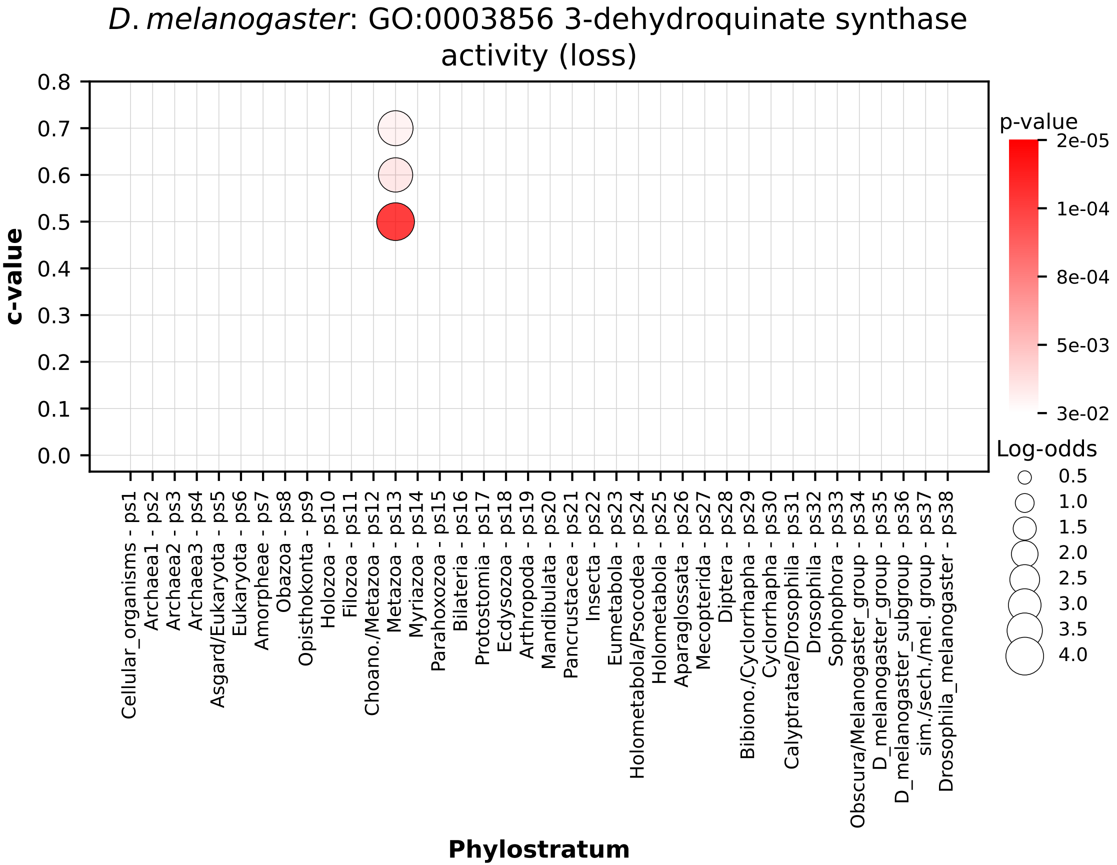
D. melanogaster: GO:0003856 3-dehydroquinate synthase activity (loss)
c-value
0.8
0.7
0.6
0.5
0.4
0.3
0.2
0.1
0.0
Phylostratum
Cellular_organisms - ps1
Archaea1 - ps2
Archaea2 - ps3
Archaea3 - ps4
Asgard/Eukaryota - ps5
Eukaryota - ps6
Amorpheae - ps7
Obazoa - ps8
Opisthokonta - ps9
Holozoa - ps10
Filozoa - ps11
Choano./Metazoa - ps12
Metazoa - ps13
Myriazoa - ps14
Parahoxozoa - ps15
Bilateria - ps16
Protostomia - ps17
Ecdysozoa - ps18
Arthropoda - ps19
Mandibulata - ps20
Pancrustacea - ps21
Insecta - ps22
Eumetabola - ps23
Holometabola/Psocodea - ps24
Holometabola - ps25
Aparaglossata - ps26
Mecopterida - ps27
Diptera - ps28
Bibiono./Cyclorrhapha - ps29
Cyclorrhapha - ps30
Calyptratae/Drosophila - ps31
Drosophila - ps32
Sophophora - ps33
Obscura/Melanogaster_group - ps34
D_melanogaster_group - ps35
D_melanogaster_subgroup - ps36
sim./sech./mel. group - ps37
Drosophila_melanogaster - ps38
p-value
2e-05
1e-04
8e-04
5e-03
3e-02
Log-odds
0.5
1.0
1.5
2.0
2.5
3.0
3.5
4.0

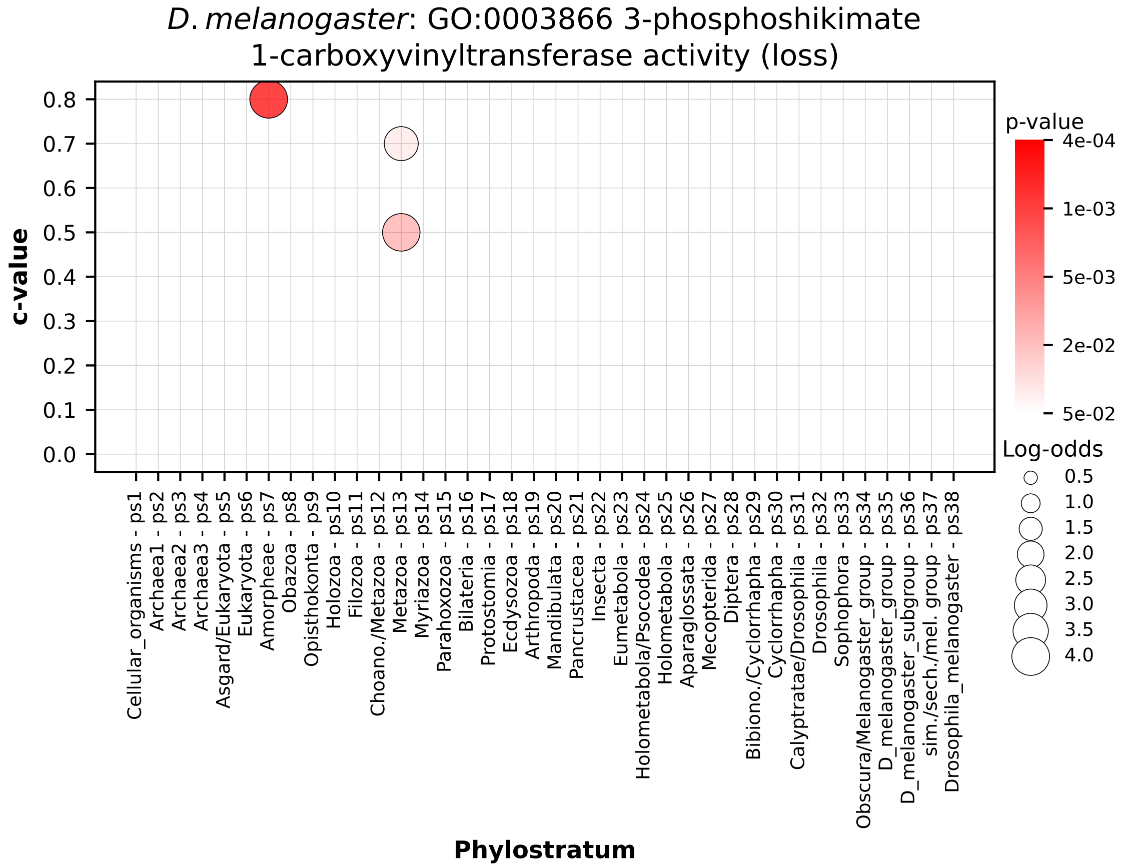

D. melanogaster: GO:0003866 3-phosphoshikimate 1-carboxyvinyltransferase activity (loss)
c-value
0.0
0.1
0.2
0.3
0.4
0.5
0.6
0.7
0.8
Phylostratum
Cellular_organisms - ps1
Archaea1 - ps2
Archaea2 - ps3
Archaea3 - ps4
Asgard/Eukaryota - ps5
Eukaryota - ps6
Amorpheae - ps7
Obazoa - ps8
Opisthokonta - ps9
Holozoa - ps10
Filozoa - ps11
Choano./Metazoa - ps12
Metazoa - ps13
Myriazoa - ps14
Parahoxozoa - ps15
Bilateria - ps16
Protostomia - ps17
Ecdysozoa - ps18
Arthropoda - ps19
Mandibulata - ps20
Pancrustacea - ps21
Insecta - ps22
Eumetabola - ps23
Holometabola/Psocodea - ps24
Holometabola - ps25
Aparaglossata - ps26
Mecopterida - ps27
Diptera - ps28
Bibiono./Cyclorrhapha - ps29
Cyclorrhapha - ps30
Calyptratae/Drosophila - ps31
Drosophila - ps32
Sophophora - ps33
Obscura/Melanogaster_group - ps34
D_melanogaster_group - ps35
D_melanogaster_subgroup - ps36
sim./sech./mel. group - ps37
Drosophila_melanogaster - ps38
p-value
4e-04
1e-03
5e-03
2e-02
5e-02
Log-odds
0.5
1.0
1.5
2.0
2.5
3.0
3.5
4.0

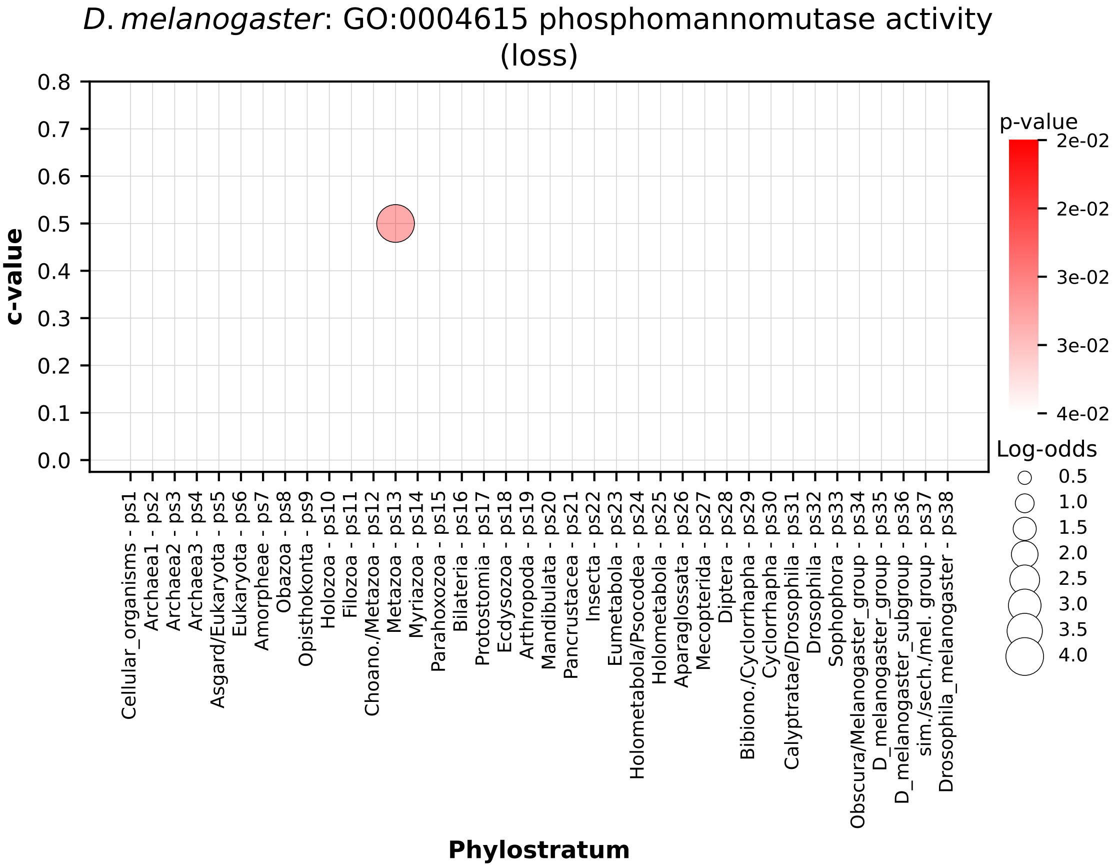

D. melanogaster: GO:0004615 phosphomannomutase activity (loss)
c-value
0.8
0.7
0.6
0.5
0.4
0.3
0.2
0.1
0.0
p-value
2e-02
2e-02
3e-02
3e-02
4e-02
Log-odds
0.5
1.0
1.5
2.0
2.5
3.0
3.5
4.0
Cellular_organisms - ps1
Archaea1 - ps2
Archaea2 - ps3
Archaea3 - ps4
Asgard/Eukaryota - ps5
Eukaryota - ps6
Amorpheae - ps7
Obazoa - ps8
Opisthokonta - ps9
Holozoa - ps10
Filozoa - ps11
Choano./Metazoa - ps12
Metazoa - ps13
Myriazoa - ps14
Parahoxozoa - ps15
Bilateria - ps16
Protostomia - ps17
Ecdysozoa - ps18
Arthropoda - ps19
Mandibulata - ps20
Pancrustacea - ps21
Insecta - ps22
Eumetabola - ps23
Holometabola/Psocodea - ps24
Holometabola - ps25
Aparaglossata - ps26
Mecopterida - ps27
Diptera - ps28
Bibiono./Cyclorrhapha - ps29
Cyclorrhapha - ps30
Calyptratae/Drosophila - ps31
Drosophila - ps32
Sophophora - ps33
Obscura/Melanogaster_group - ps34
D_melanogaster_group - ps35
D_melanogaster_subgroup - ps36
sim./sech./mel. group - ps37
Drosophila_melanogaster - ps38
Phylostratum

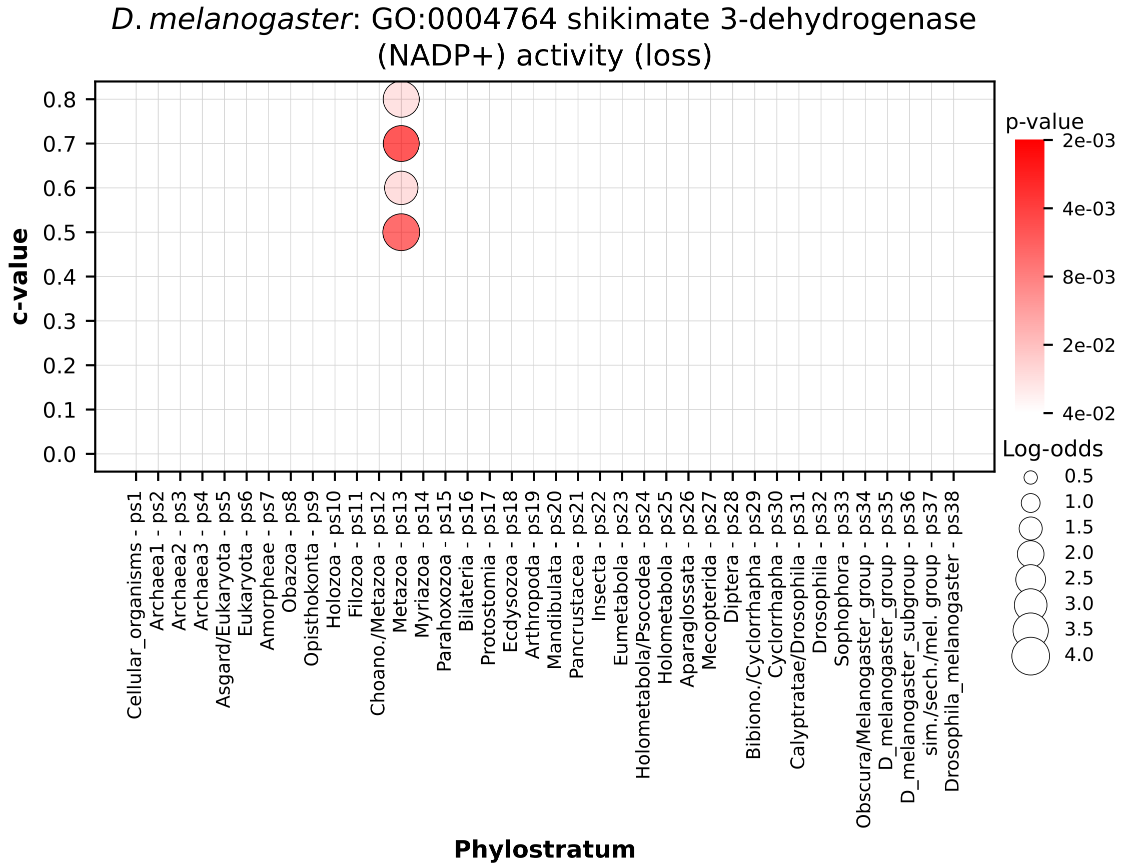
D. melanogaster: GO:0004764 shikimate 3-dehydrogenase (NADP+) activity (loss)
c-value
Phylostratum
p-value
2e-03
4e-03
8e-03
2e-02
4e-02
Log-odds
0.5
1.0
1.5
2.0
2.5
3.0
3.5
4.0
Cellular_organisms - ps1
Archaea1 - ps2
Archaea2 - ps3
Archaea3 - ps4
Asgard/Eukaryota - ps5
Eukaryota - ps6
Amorpheae - ps7
Obazoa - ps8
Opisthokonta - ps9
Holozoa - ps10
Filozoa - ps11
Choano./Metazoa - ps12
Metazoa - ps13
Myriazoa - ps14
Parahoxozoa - ps15
Bilateria - ps16
Protostomia - ps17
Ecdysozoa - ps18
Arthropoda - ps19
Mandibulata - ps20
Pancrustacea - ps21
Insecta - ps22
Eumetabola - ps23
Holometabola/Psocodea - ps24
Holometabola - ps25
Aparaglossata - ps26
Mecopterida - ps27
Diptera - ps28
Bibiono./Cyclorrhapha - ps29
Cyclorrhapha - ps30
Calyptratae/Drosophila - ps31
Drosophila - ps32
Sophophora - ps33
Obscura/Melanogaster_group - ps34
D_melanogaster_group - ps35
D_melanogaster_subgroup - ps36
sim./sech./mel. group - ps37
Drosophila_melanogaster - ps38

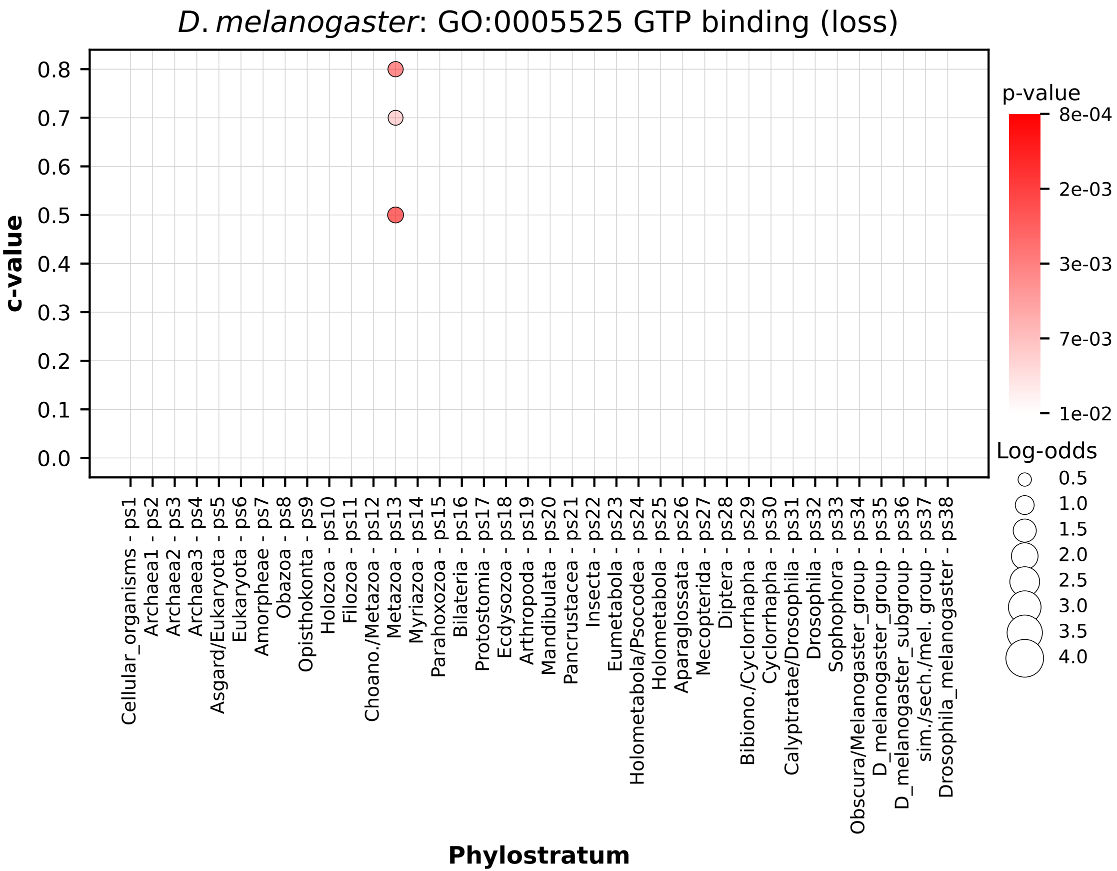

D. melanogaster: GO:0005525 GTP binding (loss)
c-value
0.8
0.7
0.6
0.5
0.4
0.3
0.2
0.1
0.0
Phylostratum
Cellular_organisms - ps1
Archaea1 - ps2
Archaea2 - ps3
Archaea3 - ps4
Asgard/Eukaryota - ps5
Eukaryota - ps6
Amorpheae - ps7
Obazoa - ps8
Opisthokonta - ps9
Holozoa - ps10
Filozoa - ps11
Choano./Metazoa - ps12
Metazoa - ps13
Myriazoa - ps14
Parahoxozoa - ps15
Bilateria - ps16
Protostomia - ps17
Ecdysozoa - ps18
Arthropoda - ps19
Mandibulata - ps20
Pancrustacea - ps21
Insecta - ps22
Eumetabola - ps23
Holometabola/Psocodea - ps24
Holometabola - ps25
Aparaglossata - ps26
Mecopterida - ps27
Diptera - ps28
Bibiono./Cyclorrhapha - ps29
Cyclorrhapha - ps30
Calyptratae/Drosophila - ps31
Drosophila - ps32
Sophophora - ps33
Obscura/Melanogaster_group - ps34
D_melanogaster_group - ps35
D_melanogaster_subgroup - ps36
sim./sech./mel. group - ps37
Drosophila_melanogaster - ps38
p-value
8e-04
2e-03
3e-03
7e-03
1e-02
Log-odds
0.5
1.0
1.5
2.0
2.5
3.0
3.5
4.0

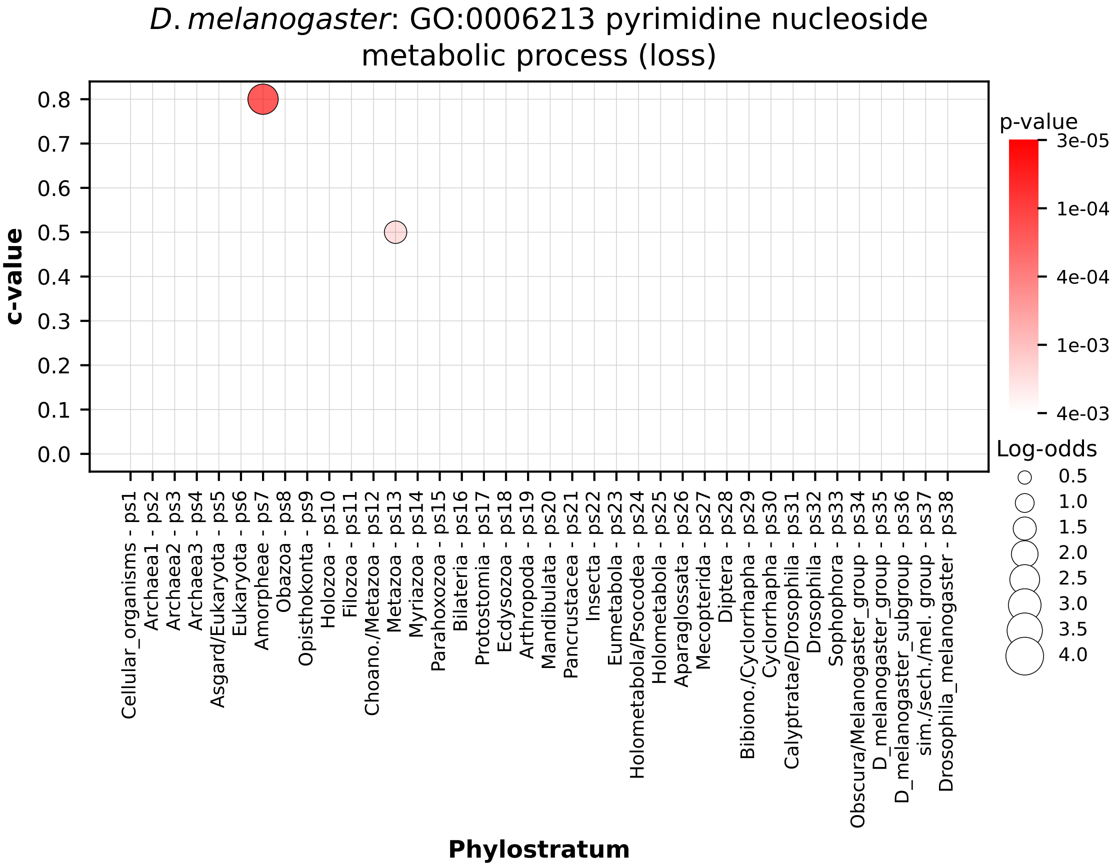
D. melanogaster: GO:0006213 pyrimidine nucleoside metabolic process (loss)
c-value
0.0
0.1
0.2
0.3
0.4
0.5
0.6
0.7
0.8
p-value
3e-05
1e-04
4e-04
1e-03
4e-03
Log-odds
0.5
1.0
1.5
2.0
2.5
3.0
3.5
4.0
Cellular_organisms - ps1
Archaea1 - ps2
Archaea2 - ps3
Archaea3 - ps4
Asgard/Eukaryota - ps5
Eukaryota - ps6
Amorpheae - ps7
Obazoa - ps8
Opisthokonta - ps9
Holozoa - ps10
Filozoa - ps11
Choano./Metazoa - ps12
Metazoa - ps13
Myriazoa - ps14
Parahoxozoa - ps15
Bilateria - ps16
Protostomia - ps17
Ecdysozoa - ps18
Arthropoda - ps19
Mandibulata - ps20
Pancrustacea - ps21
Insecta - ps22
Eumetabola - ps23
Holometabola/Psocodea - ps24
Holometabola - ps25
Aparaglossata - ps26
Mecopterida - ps27
Diptera - ps28
Bibiono./Cyclorrhapha - ps29
Cyclorrhapha - ps30
Calyptratae/Drosophila - ps31
Drosophila - ps32
Sophophora - ps33
Obscura/Melanogaster_group - ps34
D_melanogaster_group - ps35
D_melanogaster_subgroup - ps36
sim./sech./mel. group - ps37
Drosophila_melanogaster - ps38
Phylostratum

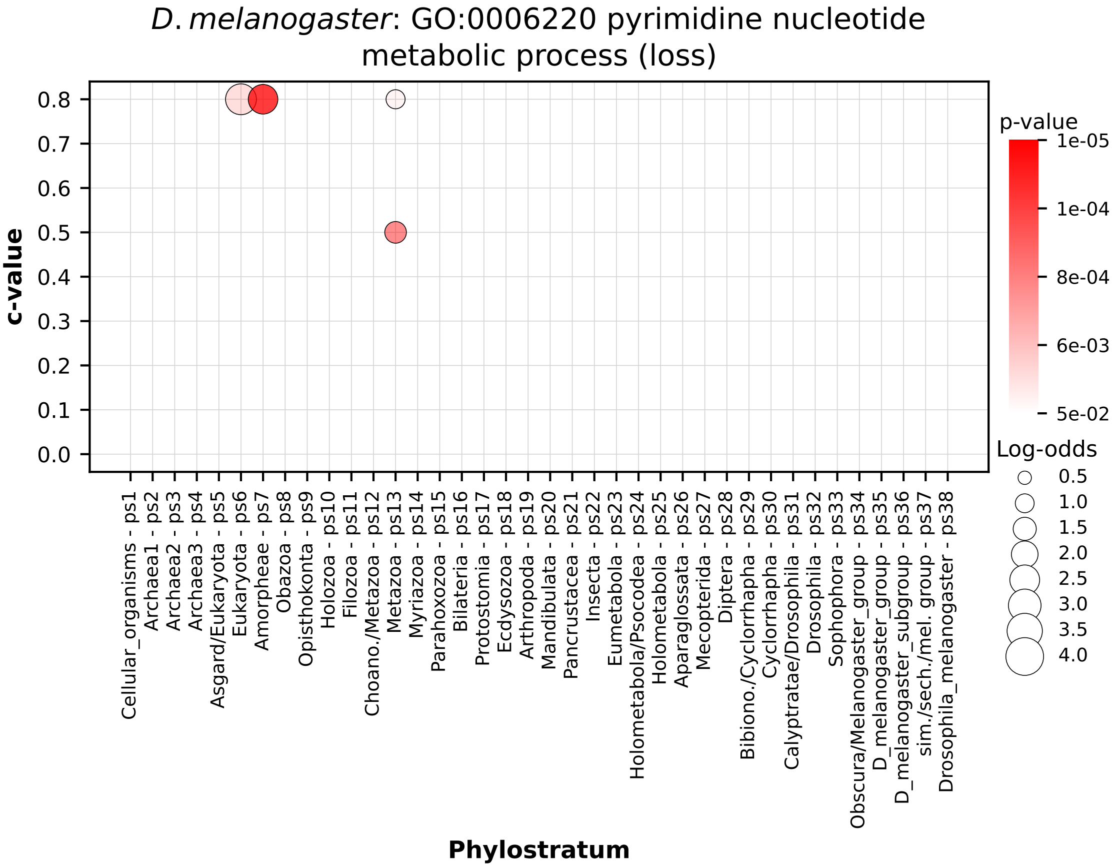

D. melanogaster: GO:0006220 pyrimidine nucleotide metabolic process (loss)
c-value
0.0
0.1
0.2
0.3
0.4
0.5
0.6
0.7
0.8
Phylostratum
Cellular_organisms - ps1
Archaea1 - ps2
Archaea2 - ps3
Archaea3 - ps4
Asgard/Eukaryota - ps5
Eukaryota - ps6
Amorpheae - ps7
Obazoa - ps8
Opisthokonta - ps9
Holozoa - ps10
Filozoa - ps11
Choano./Metazoa - ps12
Metazoa - ps13
Myriazoa - ps14
Parahoxozoa - ps15
Bilateria - ps16
Protostomia - ps17
Ecdysozoa - ps18
Arthropoda - ps19
Mandibulata - ps20
Pancrustacea - ps21
Insecta - ps22
Eumetabola - ps23
Holometabola/Psocodea - ps24
Holometabola - ps25
Aparaglossata - ps26
Mecopterida - ps27
Diptera - ps28
Bibiono./Cyclorrhapha - ps29
Cyclorrhapha - ps30
Calyptratae/Drosophila - ps31
Drosophila - ps32
Sophophora - ps33
Obscura/Melanogaster_group - ps34
D_melanogaster_group - ps35
D_melanogaster_subgroup - ps36
sim./sech./mel. group - ps37
Drosophila_melanogaster - ps38
p-value
1e-05
1e-04
8e-04
6e-03
5e-02
Log-odds
0.5
1.0
1.5
2.0
2.5
3.0
3.5
4.0

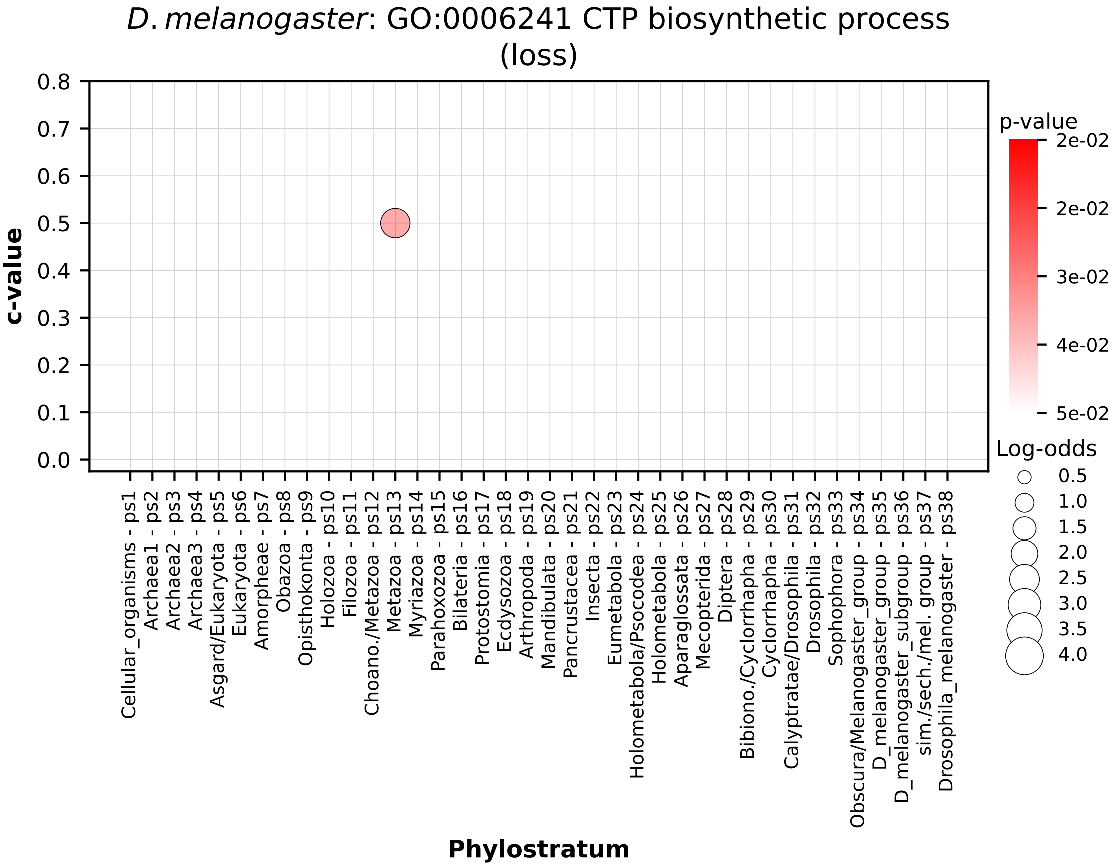

D. melanogaster: GO:0006241 CTP biosynthetic process
(loss)
c-value
0.8
0.7
0.6
0.5
0.4
0.3
0.2
0.1
0.0
p-value
2e-02
2e-02
3e-02
4e-02
5e-02
Log-odds
0.5
1.0
1.5
2.0
2.5
3.0
3.5
4.0
Cellular_organisms - ps1
Archaea1 - ps2
Archaea2 - ps3
Archaea3 - ps4
Asgard/Eukaryota - ps5
Eukaryota - ps6
Amorpheae - ps7
Obazoa - ps8
Opisthokonta - ps9
Holozoa - ps10
Filozoa - ps11
Choano./Metazoa - ps12
Metazoa - ps13
Myriazoa - ps14
Parahoxozoa - ps15
Bilateria - ps16
Protostomia - ps17
Ecdysozoa - ps18
Arthropoda - ps19
Mandibulata - ps20
Pancrustacea - ps21
Insecta - ps22
Eumetabola - ps23
Holometabola/Psocodea - ps24
Holometabola - ps25
Aparaglossata - ps26
Mecopterida - ps27
Diptera - ps28
Bibiono./Cyclorrhapha - ps29
Cyclorrhapha - ps30
Calyptratae/Drosophila - ps31
Drosophila - ps32
Sophophora - ps33
Obscura/Melanogaster_group - ps34
D_melanogaster_group - ps35
D_melanogaster_subgroup - ps36
sim./sech./mel. group - ps37
Drosophila_melanogaster - ps38
Phylostratum

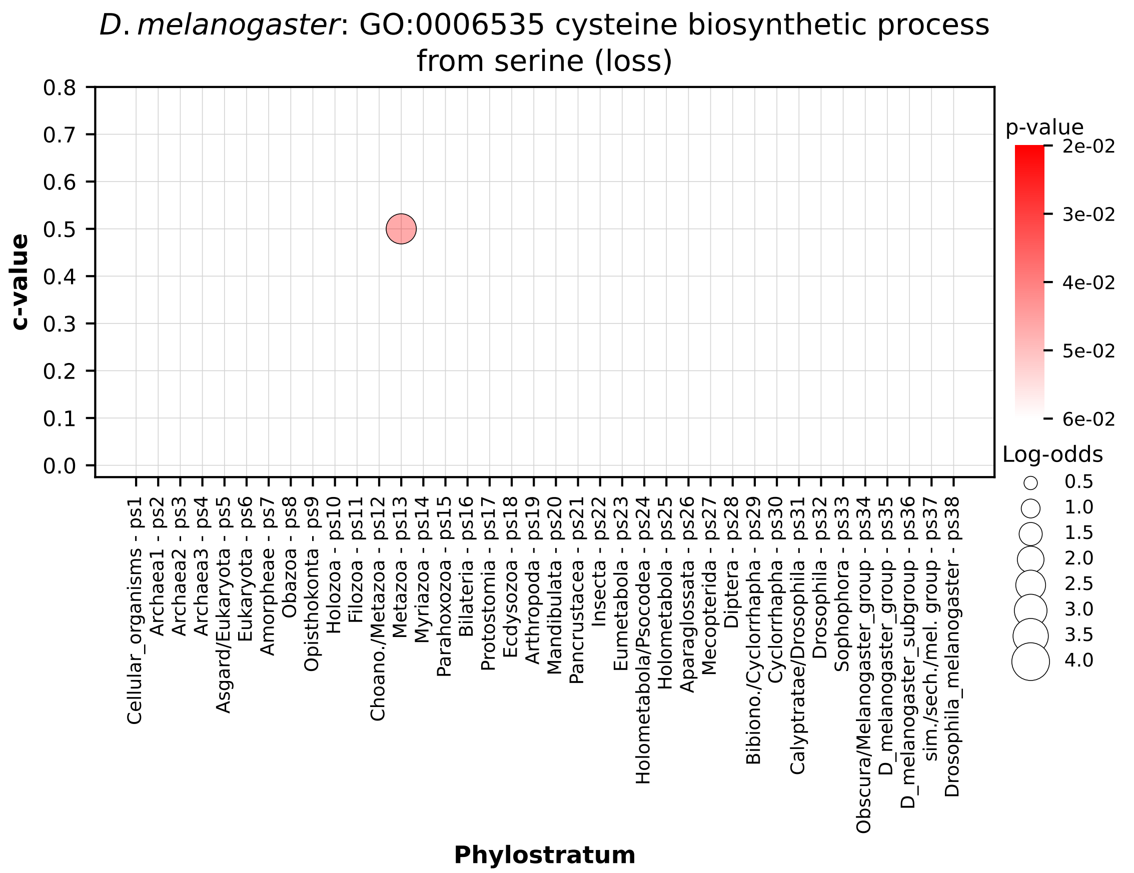

D. melanogaster: GO:0006535 cysteine biosynthetic process from serine (loss)
c-value
0.8
0.7
0.6
0.5
0.4
0.3
0.2
0.1
0.0
p-value
2e-02
3e-02
4e-02
5e-02
6e-02
Log-odds
0.5
1.0
1.5
2.0
2.5
3.0
3.5
4.0
Cellular_organisms - ps1
Archaea1 - ps2
Archaea2 - ps3
Archaea3 - ps4
Asgard/Eukaryota - ps5
Eukaryota - ps6
Amorpheae - ps7
Obazoa - ps8
Opisthokonta - ps9
Holozoa - ps10
Filozoa - ps11
Choano./Metazoa - ps12
Metazoa - ps13
Myriazoa - ps14
Parahoxozoa - ps15
Bilateria - ps16
Protostomia - ps17
Ecdysozoa - ps18
Arthropoda - ps19
Mandibulata - ps20
Pancrustacea - ps21
Insecta - ps22
Eumetabola - ps23
Holometabola/Psocodea - ps24
Holometabola - ps25
Aparaglossata - ps26
Mecopterida - ps27
Diptera - ps28
Bibiono./Cyclorrhapha - ps29
Cyclorrhapha - ps30
Calyptratae/Drosophila - ps31
Drosophila - ps32
Sophophora - ps33
Obscura/Melanogaster_group - ps34
D_melanogaster_group - ps35
D_melanogaster_subgroup - ps36
sim./sech./mel. group - ps37
Drosophila_melanogaster - ps38
Phylostratum

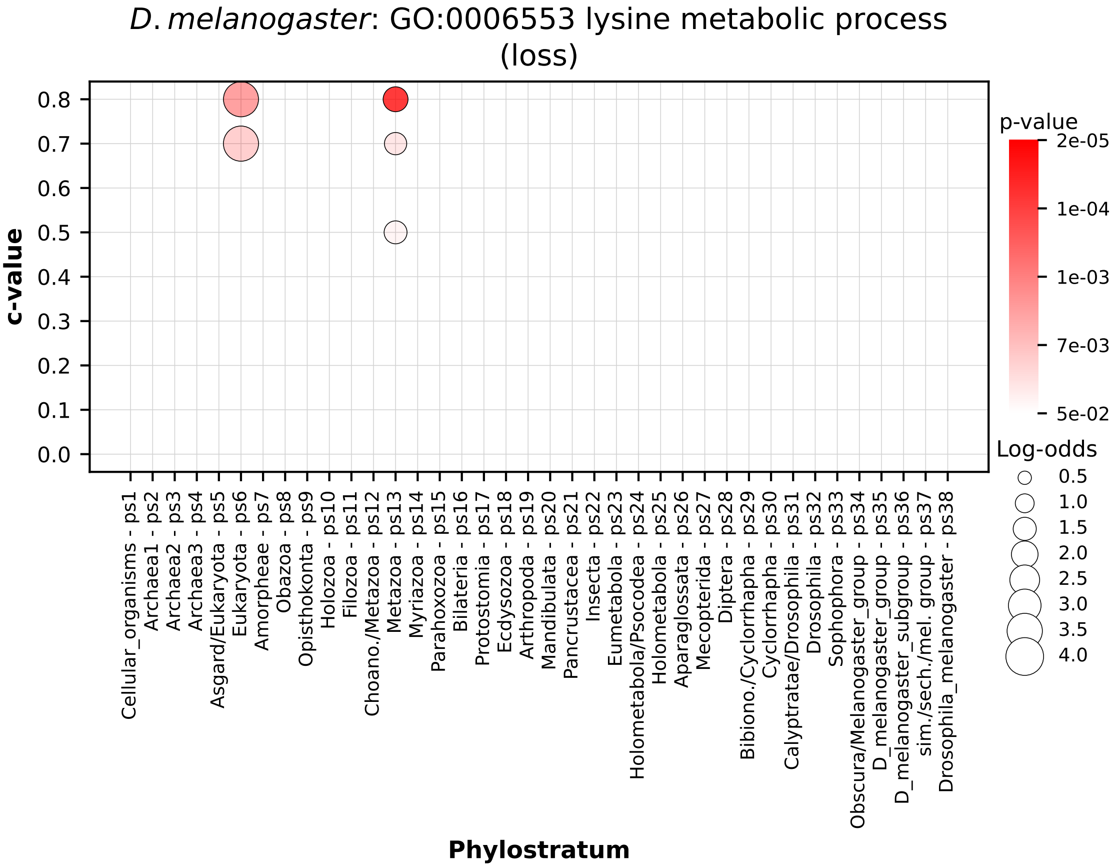

D. melanogaster: GO:0006553 lysine metabolic process
(loss)
c-value
0.8
0.7
0.6
0.5
0.4
0.3
0.2
0.1
0.0
p-value
2e-05
1e-04
1e-03
7e-03
5e-02
Log-odds
0.5
1.0
1.5
2.0
2.5
3.0
3.5
4.0
Cellular_organisms - ps1
Archaea1 - ps2
Archaea2 - ps3
Archaea3 - ps4
Asgard/Eukaryota - ps5
Eukaryota - ps6
Amorpheae - ps7
Obazoa - ps8
Opisthokonta - ps9
Holozoa - ps10
Filozoa - ps11
Choano./Metazoa - ps12
Metazoa - ps13
Myriazoa - ps14
Parahoxozoa - ps15
Bilateria - ps16
Protostomia - ps17
Ecdysozoa - ps18
Arthropoda - ps19
Mandibulata - ps20
Pancrustacea - ps21
Insecta - ps22
Eumetabola - ps23
Holometabola/Psocodea - ps24
Holometabola - ps25
Aparaglossata - ps26
Mecopterida - ps27
Diptera - ps28
Bibiono./Cyclorrhapha - ps29
Cyclorrhapha - ps30
Calyptratae/Drosophila - ps31
Drosophila - ps32
Sophophora - ps33
Obscura/Melanogaster_group - ps34
D_melanogaster_group - ps35
D_melanogaster_subgroup - ps36
sim./sech./mel. group - ps37
Drosophila_melanogaster - ps38
Phylostratum

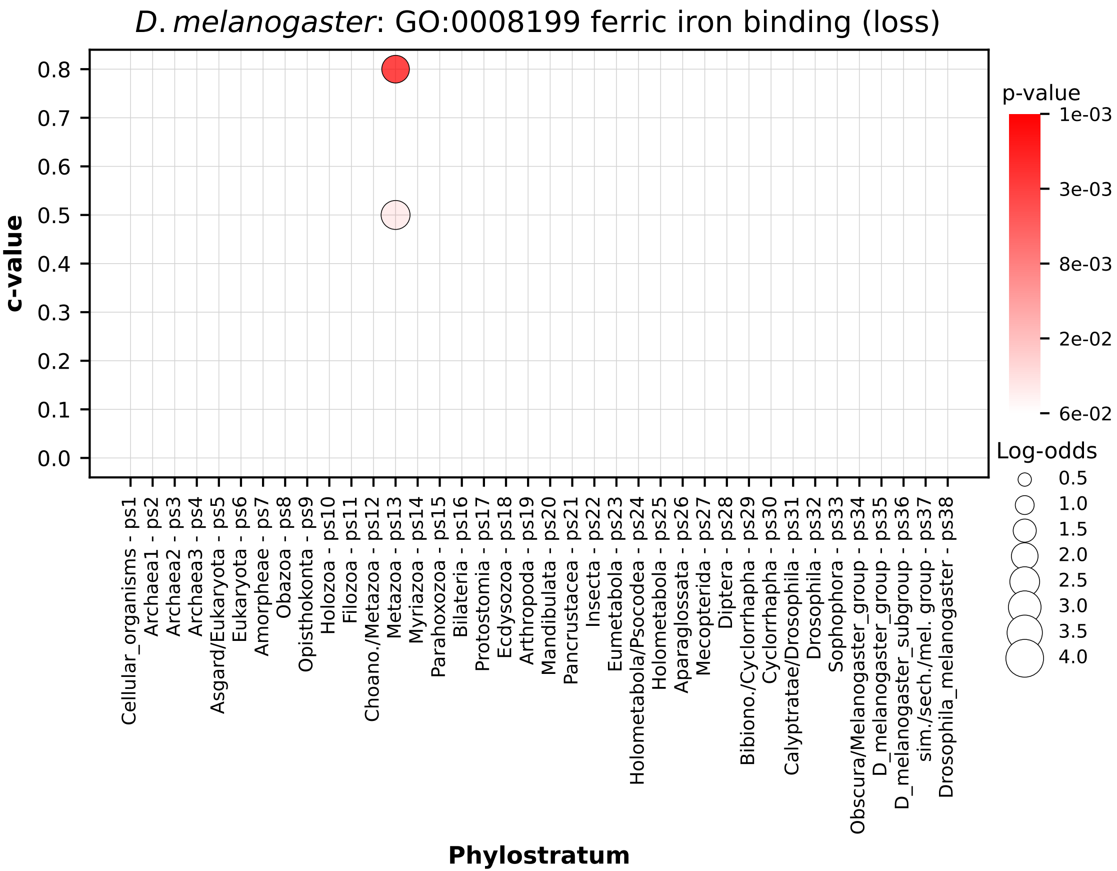

D. melanogaster: GO:0008199 ferric iron binding (loss)
c-value
0.0
0.1
0.2
0.3
0.4
0.5
0.6
0.7
0.8
Phylostratum
Cellular_organisms - ps1
Archaea1 - ps2
Archaea2 - ps3
Archaea3 - ps4
Asgard/Eukaryota - ps5
Eukaryota - ps6
Amorpheae - ps7
Obazoa - ps8
Opisthokonta - ps9
Holozoa - ps10
Filozoa - ps11
Choano./Metazoa - ps12
Metazoa - ps13
Myriazoa - ps14
Parahoxozoa - ps15
Bilateria - ps16
Protostomia - ps17
Ecdysozoa - ps18
Arthropoda - ps19
Mandibulata - ps20
Pancrustacea - ps21
Insecta - ps22
Eumetabola - ps23
Holometabola/Psocodea - ps24
Holometabola - ps25
Aparaglossata - ps26
Mecopterida - ps27
Diptera - ps28
Bibiono./Cyclorrhapha - ps29
Cyclorrhapha - ps30
Calyptratae/Drosophila - ps31
Drosophila - ps32
Sophophora - ps33
Obscura/Melanogaster_group - ps34
D_melanogaster_group - ps35
D_melanogaster_subgroup - ps36
sim./sech./mel. group - ps37
Drosophila_melanogaster - ps38
p-value
1e-03
3e-03
8e-03
2e-02
6e-02
Log-odds
0.5
1.0
1.5
2.0
2.5
3.0
3.5
4.0

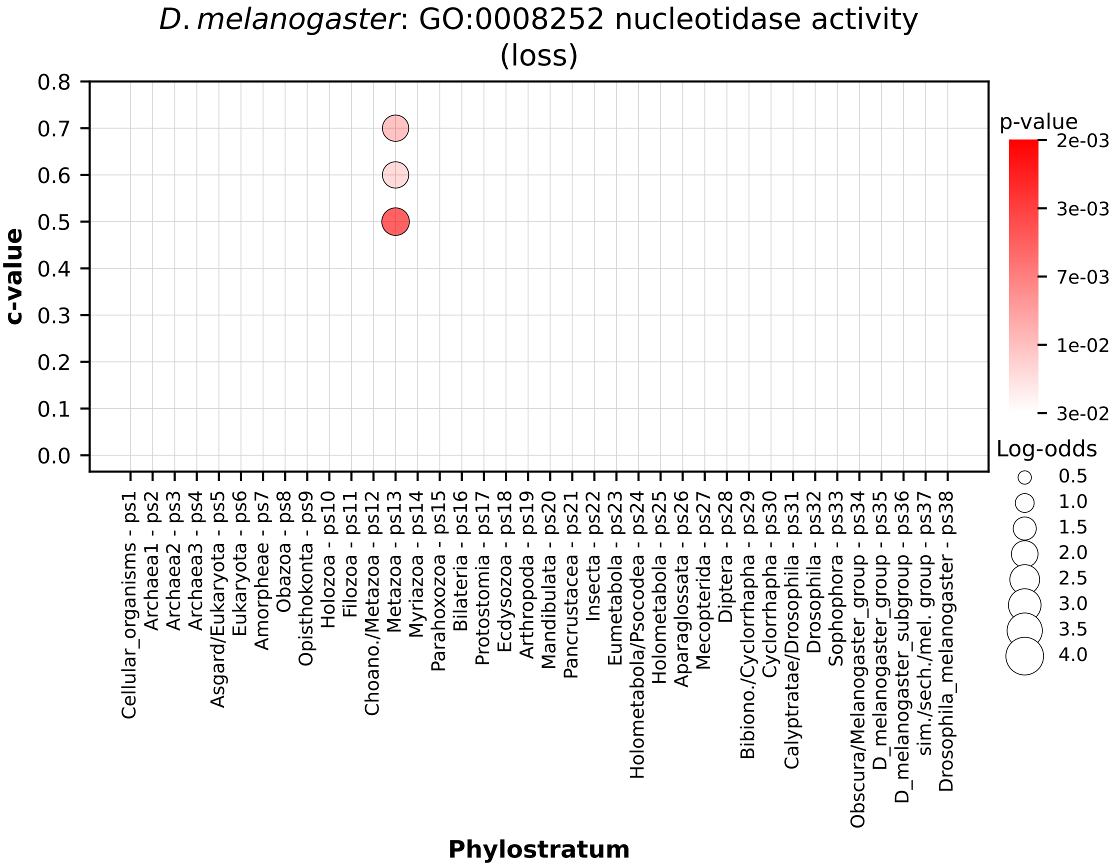

D. melanogaster: GO:0008252 nucleotidase activity
(loss)
c-value
0.8
0.7
0.6
0.5
0.4
0.3
0.2
0.1
0.0
p-value
2e-03
3e-03
7e-03
1e-02
3e-02
Log-odds
0.5
1.0
1.5
2.0
2.5
3.0
3.5
4.0
Cellular_organisms - ps1
Archaea1 - ps2
Archaea2 - ps3
Archaea3 - ps4
Asgard/Eukaryota - ps5
Eukaryota - ps6
Amorpheae - ps7
Obazoa - ps8
Opisthokonta - ps9
Holozoa - ps10
Filozoa - ps11
Choano./Metazoa - ps12
Metazoa - ps13
Myriazoa - ps14
Parahoxozoa - ps15
Bilateria - ps16
Protostomia - ps17
Ecdysozoa - ps18
Arthropoda - ps19
Mandibulata - ps20
Pancrustacea - ps21
Insecta - ps22
Eumetabola - ps23
Holometabola/Psocodea - ps24
Holometabola - ps25
Aparaglossata - ps26
Mecopterida - ps27
Diptera - ps28
Bibiono./Cyclorrhapha - ps29
Cyclorrhapha - ps30
Calyptratae/Drosophila - ps31
Drosophila - ps32
Sophophora - ps33
Obscura/Melanogaster_group - ps34
D_melanogaster_group - ps35
D_melanogaster_subgroup - ps36
sim./sech./mel. group - ps37
Drosophila_melanogaster - ps38
Phylostratum

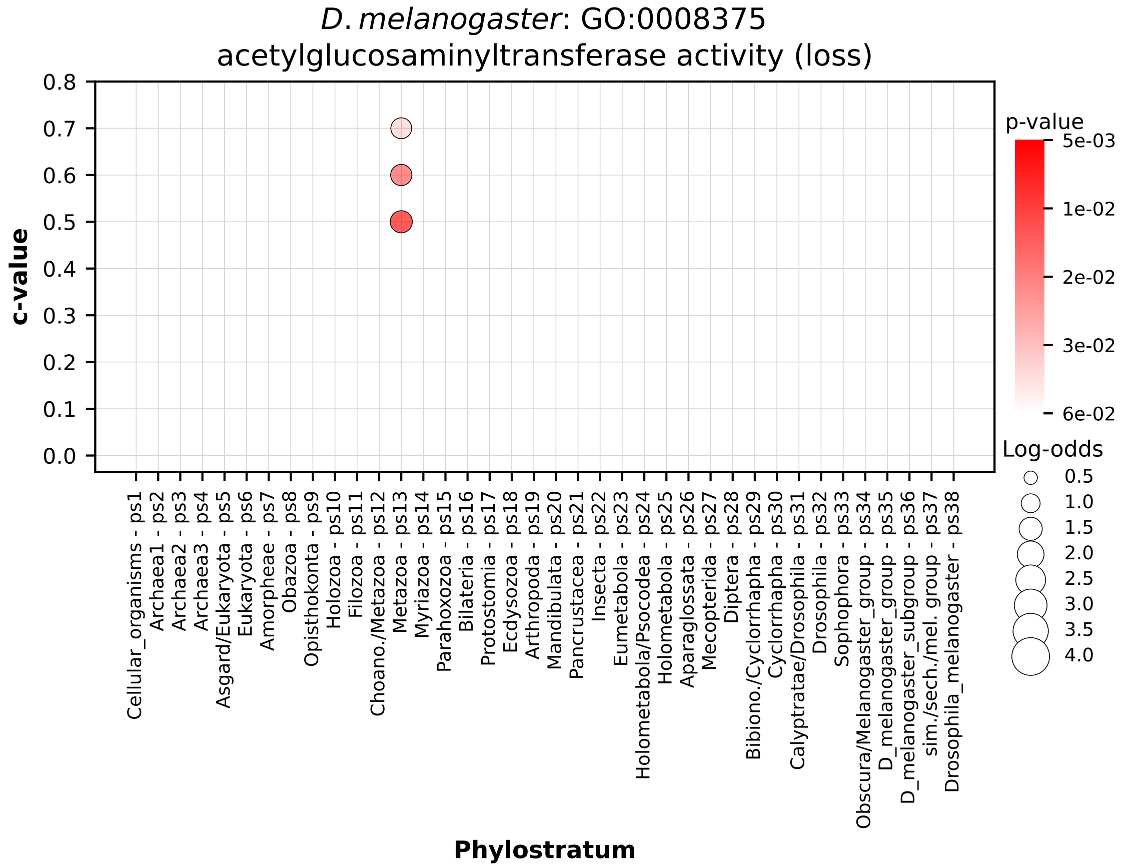
D. melanogaster: GO:0008375
acetylglucosaminyltransferase activity (loss)
c-value
0.8
0.7
0.6
0.5
0.4
0.3
0.2
0.1
0.0
Phylostratum
Cellular_organisms - ps1
Archaea1 - ps2
Archaea2 - ps3
Archaea3 - ps4
Asgard/Eukaryota - ps5
Eukaryota - ps6
Amorpheae - ps7
Obazoa - ps8
Opisthokonta - ps9
Holozoa - ps10
Filozoa - ps11
Choano./Metazoa - ps12
Metazoa - ps13
Myriazoa - ps14
Parahoxozoa - ps15
Bilateria - ps16
Protostomia - ps17
Ecdysozoa - ps18
Arthropoda - ps19
Mandibulata - ps20
Pancrustacea - ps21
Insecta - ps22
Eumetabola - ps23
Holometabola/Psocodea - ps24
Holometabola - ps25
Aparaglossata - ps26
Mecopterida - ps27
Diptera - ps28
Bibiono./Cyclorrhapha - ps29
Cyclorrhapha - ps30
Calyptratae/Drosophila - ps31
Drosophila - ps32
Sophophora - ps33
Obscura/Melanogaster_group - ps34
D_melanogaster_group - ps35
D_melanogaster_subgroup - ps36
sim./sech./mel. group - ps37
Drosophila_melanogaster - ps38
p-value
5e-03
1e-02
2e-02
3e-02
6e-02
Log-odds
0.5
1.0
1.5
2.0
2.5
3.0
3.5
4.0

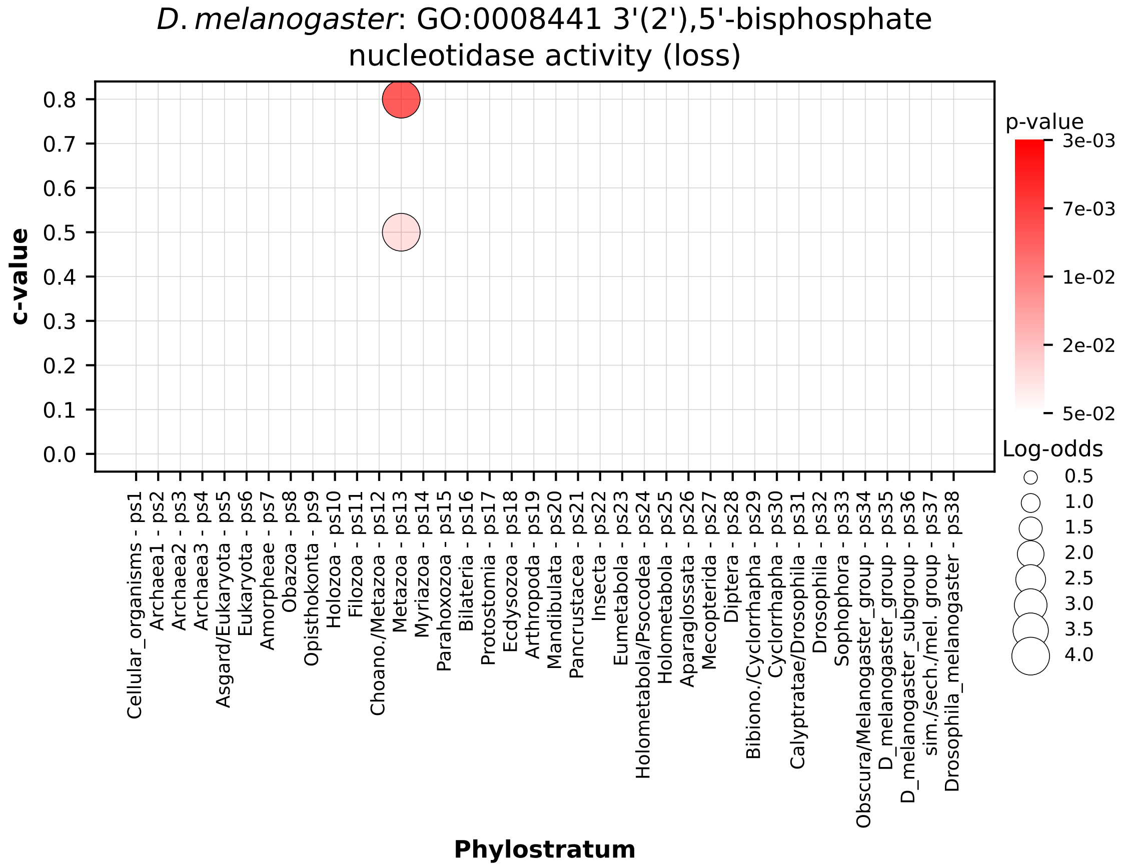

D. melanogaster: GO:0008441 3'(2'),5'-bisphosphate nucleotidase activity (loss)
c-value
0.0
0.1
0.2
0.3
0.4
0.5
0.6
0.7
0.8
p-value
3e-03
7e-03
1e-02
2e-02
5e-02
Log-odds
0.5
1.0
1.5
2.0
2.5
3.0
3.5
4.0
Cellular_organisms - ps1
Archaea1 - ps2
Archaea2 - ps3
Archaea3 - ps4
Asgard/Eukaryota - ps5
Eukaryota - ps6
Amorpheae - ps7
Obazoa - ps8
Opisthokonta - ps9
Holozoa - ps10
Filozoa - ps11
Choano./Metazoa - ps12
Metazoa - ps13
Myriazoa - ps14
Parahoxozoa - ps15
Bilateria - ps16
Protostomia - ps17
Ecdysozoa - ps18
Arthropoda - ps19
Mandibulata - ps20
Pancrustacea - ps21
Insecta - ps22
Eumetabola - ps23
Holometabola/Psocodea - ps24
Holometabola - ps25
Aparaglossata - ps26
Mecopterida - ps27
Diptera - ps28
Bibiono./Cyclorrhapha - ps29
Cyclorrhapha - ps30
Calyptratae/Drosophila - ps31
Drosophila - ps32
Sophophora - ps33
Obscura/Melanogaster_group - ps34
D_melanogaster_group - ps35
D_melanogaster_subgroup - ps36
sim./sech./mel. group - ps37
Drosophila_melanogaster - ps38
Phylostratum

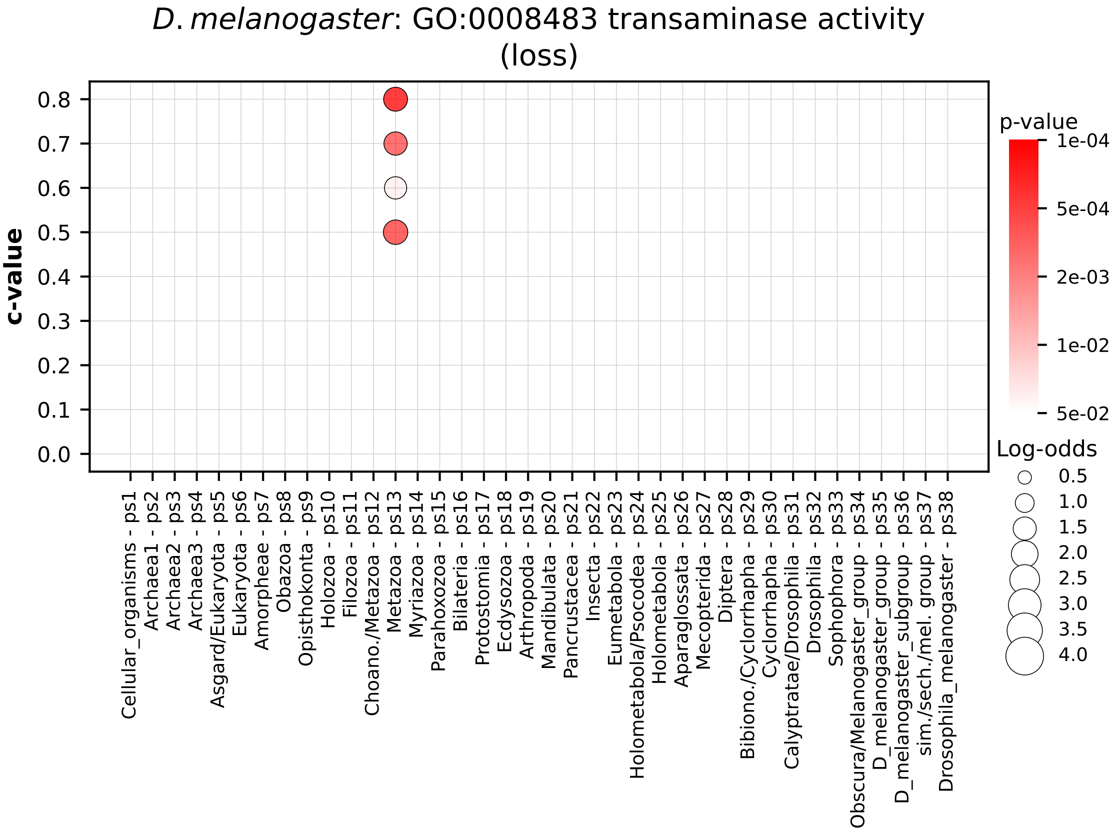

D. melanogaster: GO:0008483 transaminase activity
(loss)
c-value
0.8
0.7
0.6
0.5
0.4
0.3
0.2
0.1
0.0
Phylostratum
Cellular_organisms - ps1
Archaea1 - ps2
Archaea2 - ps3
Archaea3 - ps4
Asgard/Eukaryota - ps5
Eukaryota - ps6
Amorpheae - ps7
Obazoa - ps8
Opisthokonta - ps9
Holozoa - ps10
Filozoa - ps11
Choano./Metazoa - ps12
Metazoa - ps13
Myriazoa - ps14
Parahoxozoa - ps15
Bilateria - ps16
Protostomia - ps17
Ecdysozoa - ps18
Arthropoda - ps19
Mandibulata - ps20
Pancrustacea - ps21
Insecta - ps22
Eumetabola - ps23
Holometabola/Psocodea - ps24
Holometabola - ps25
Aparaglossata - ps26
Mecopterida - ps27
Diptera - ps28
Bibiono./Cyclorrhapha - ps29
Cyclorrhapha - ps30
Calyptratae/Drosophila - ps31
Drosophila - ps32
Sophophora - ps33
Obscura/Melanogaster_group - ps34
D_melanogaster_group - ps35
D_melanogaster_subgroup - ps36
sim./sech./mel. group - ps37
Drosophila_melanogaster - ps38
p-value
1e-04
5e-04
2e-03
1e-02
5e-02
Log-odds
0.5
1.0
1.5
2.0
2.5
3.0
3.5
4.0

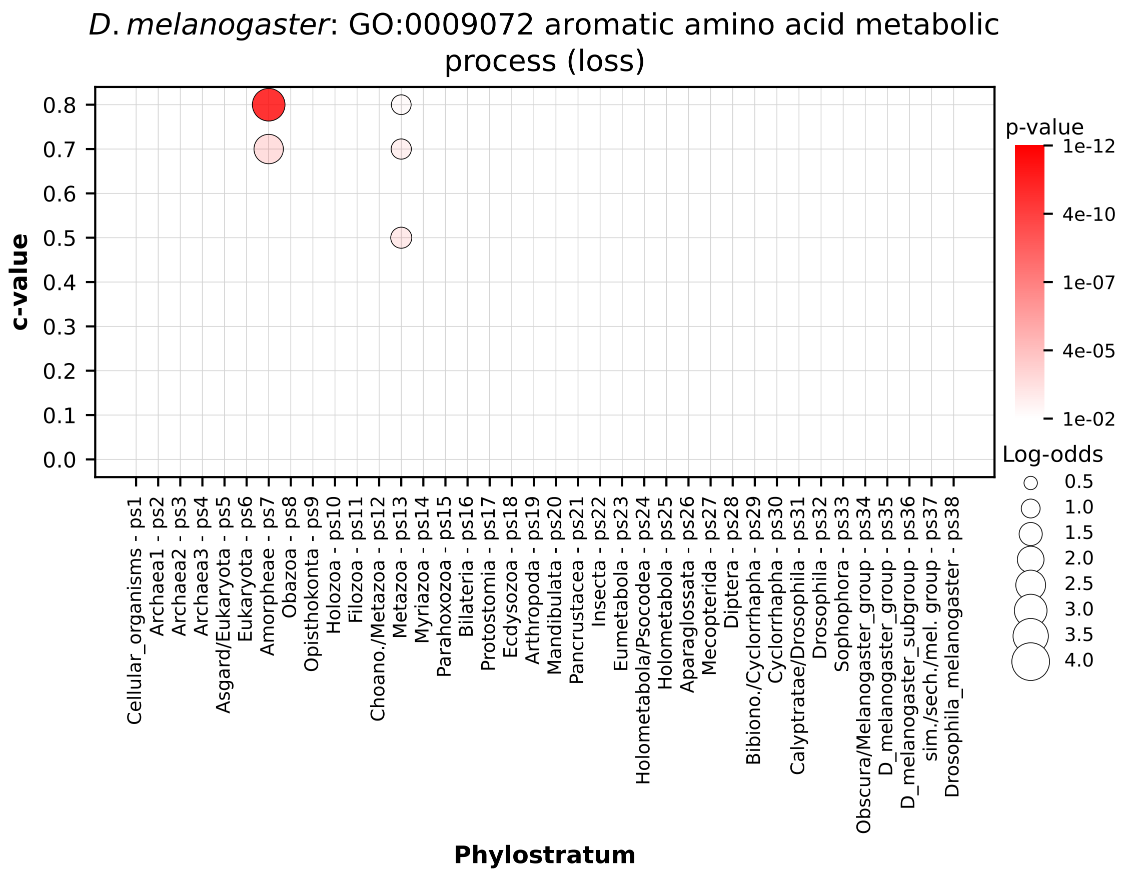

D. melanogaster: GO:0009072 aromatic amino acid metabolic process (loss)
c-value
0.8
0.7
0.6
0.5
0.4
0.3
0.2
0.1
0.0
Phylostratum
Cellular_organisms - ps1
Archaea1 - ps2
Archaea2 - ps3
Archaea3 - ps4
Asgard/Eukaryota - ps5
Eukaryota - ps6
Amorpheae - ps7
Obazoa - ps8
Opisthokonta - ps9
Holozoa - ps10
Filozoa - ps11
Choano./Metazoa - ps12
Metazoa - ps13
Myriazoa - ps14
Parahoxozoa - ps15
Bilateria - ps16
Protostomia - ps17
Ecdysozoa - ps18
Arthropoda - ps19
Mandibulata - ps20
Pancrustacea - ps21
Insecta - ps22
Eumetabola - ps23
Holometabola/Psocodea - ps24
Holometabola - ps25
Aparaglossata - ps26
Mecopterida - ps27
Diptera - ps28
Bibiono./Cyclorrhapha - ps29
Cyclorrhapha - ps30
Calyptratae/Drosophila - ps31
Drosophila - ps32
Sophophora - ps33
Obscura/Melanogaster_group - ps34
D_melanogaster_group - ps35
D_melanogaster_subgroup - ps36
sim./sech./mel. group - ps37
Drosophila_melanogaster - ps38
p-value
1e-12
4e-10
1e-07
4e-05
1e-02
Log-odds
0.5
1.0
1.5
2.0
2.5
3.0
3.5
4.0

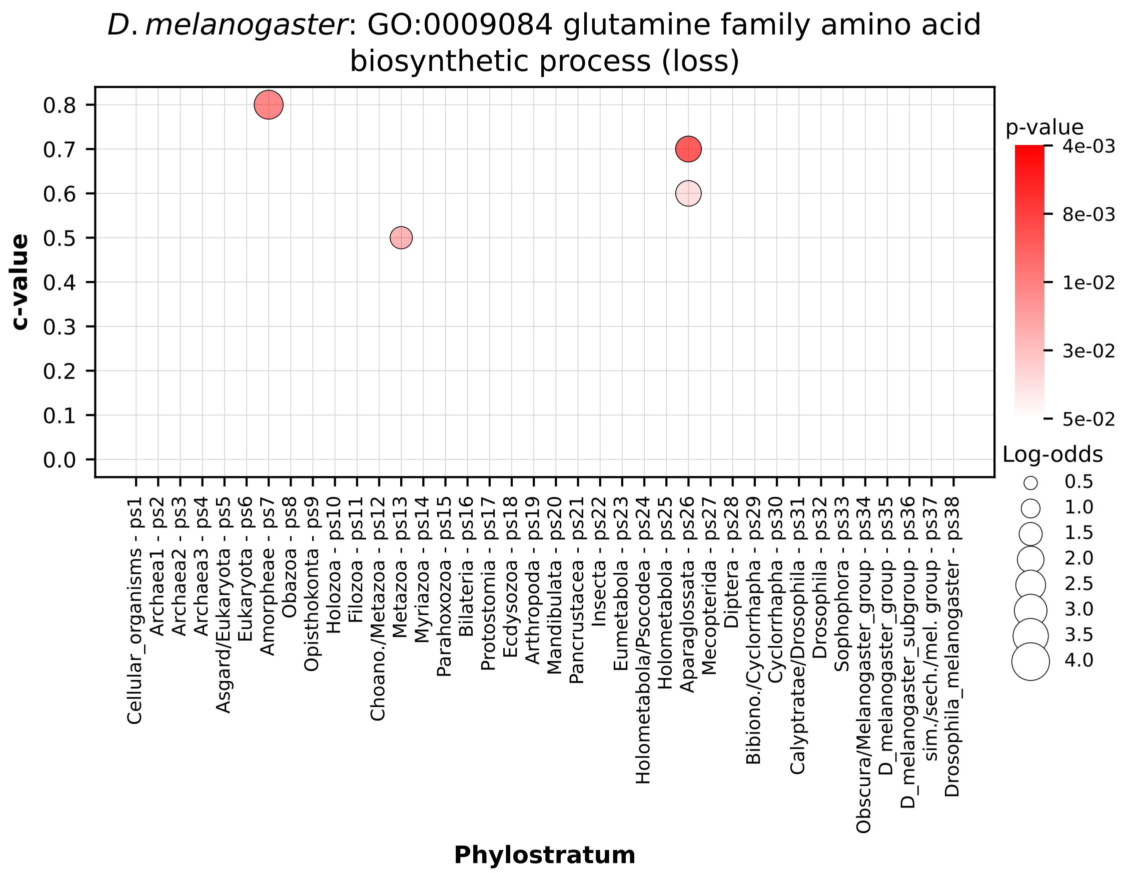
D. melanogaster: GO:0009084 glutamine family amino acid biosynthetic process (loss)
c-value
0.8
0.7
0.6
0.5
0.4
0.3
0.2
0.1
0.0
p-value
4e-03
8e-03
1e-02
3e-02
5e-02
Log-odds
0.5
1.0
1.5
2.0
2.5
3.0
3.5
4.0
Cellular_organisms - ps1
Archaea1 - ps2
Archaea2 - ps3
Archaea3 - ps4
Asgard/Eukaryota - ps5
Eukaryota - ps6
Amorpheae - ps7
Obazoa - ps8
Opisthokonta - ps9
Holozoa - ps10
Filozoa - ps11
Choano./Metazoa - ps12
Metazoa - ps13
Myriazoa - ps14
Parahoxozoa - ps15
Bilateria - ps16
Protostomia - ps17
Ecdysozoa - ps18
Arthropoda - ps19
Mandibulata - ps20
Pancrustacea - ps21
Insecta - ps22
Eumetabola - ps23
Holometabola/Psocodea - ps24
Holometabola - ps25
Aparaglossata - ps26
Mecopterida - ps27
Diptera - ps28
Bibiono./Cyclorrhapha - ps29
Cyclorrhapha - ps30
Calyptratae/Drosophila - ps31
Drosophila - ps32
Sophophora - ps33
Obscura/Melanogaster_group - ps34
D_melanogaster_group - ps35
D_melanogaster_subgroup - ps36
sim./sech./mel. group - ps37
Drosophila_melanogaster - ps38
Phylostratum

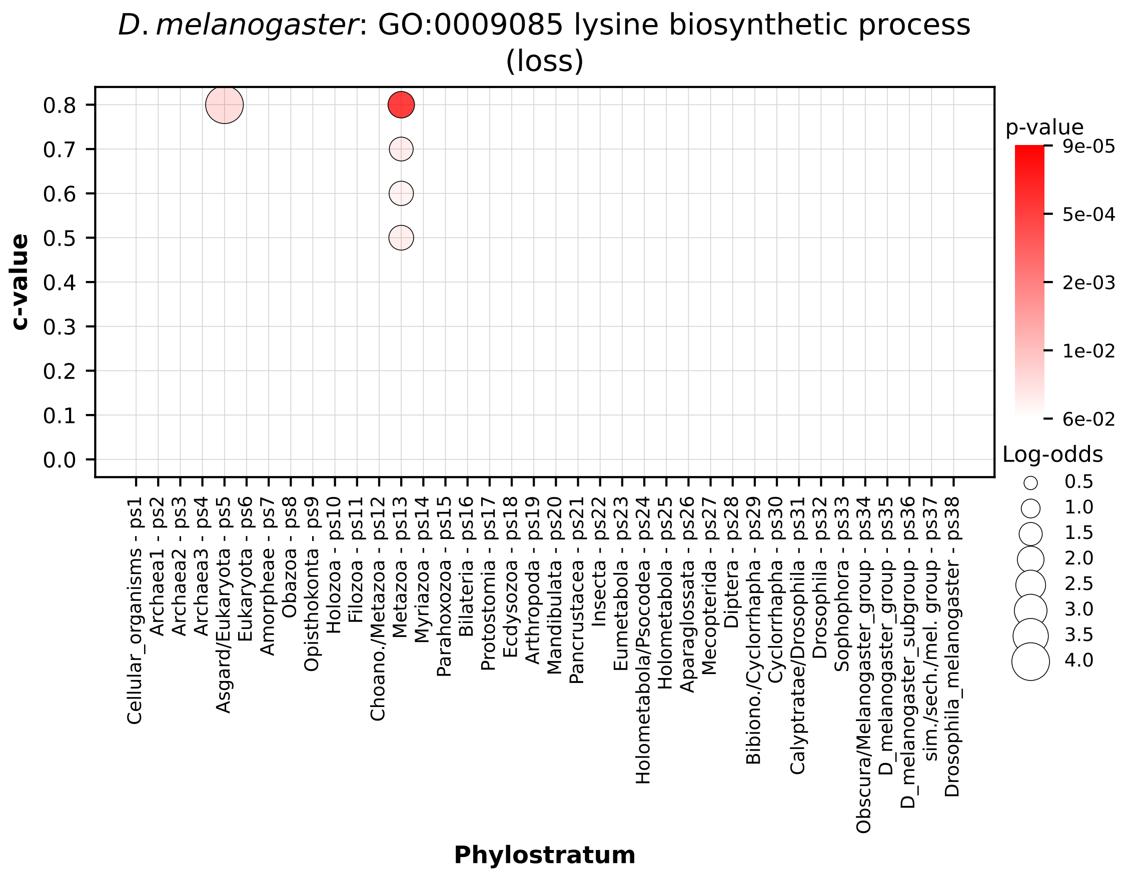

D. melanogaster: GO:0009085 lysine biosynthetic process (loss)
c-value
0.0
0.1
0.2
0.3
0.4
0.5
0.6
0.7
0.8
Phylostratum
Cellular_organisms - ps1
Archaea1 - ps2
Archaea2 - ps3
Archaea3 - ps4
Asgard/Eukaryota - ps5
Eukaryota - ps6
Amorpheae - ps7
Obazoa - ps8
Opisthokonta - ps9
Holozoa - ps10
Filozoa - ps11
Choano./Metazoa - ps12
Metazoa - ps13
Myriazoa - ps14
Parahoxozoa - ps15
Bilateria - ps16
Protostomia - ps17
Ecdysozoa - ps18
Arthropoda - ps19
Mandibulata - ps20
Pancrustacea - ps21
Insecta - ps22
Eumetabola - ps23
Holometabola/Psocodea - ps24
Holometabola - ps25
Aparaglossata - ps26
Mecopterida - ps27
Diptera - ps28
Bibiono./Cyclorrhapha - ps29
Cyclorrhapha - ps30
Calyptratae/Drosophila - ps31
Drosophila - ps32
Sophophora - ps33
Obscura/Melanogaster_group - ps34
D_melanogaster_group - ps35
D_melanogaster_subgroup - ps36
sim./sech./mel. group - ps37
Drosophila_melanogaster - ps38
p-value
9e-05
5e-04
2e-03
1e-02
6e-02
Log-odds
0.5
1.0
1.5
2.0
2.5
3.0
3.5
4.0

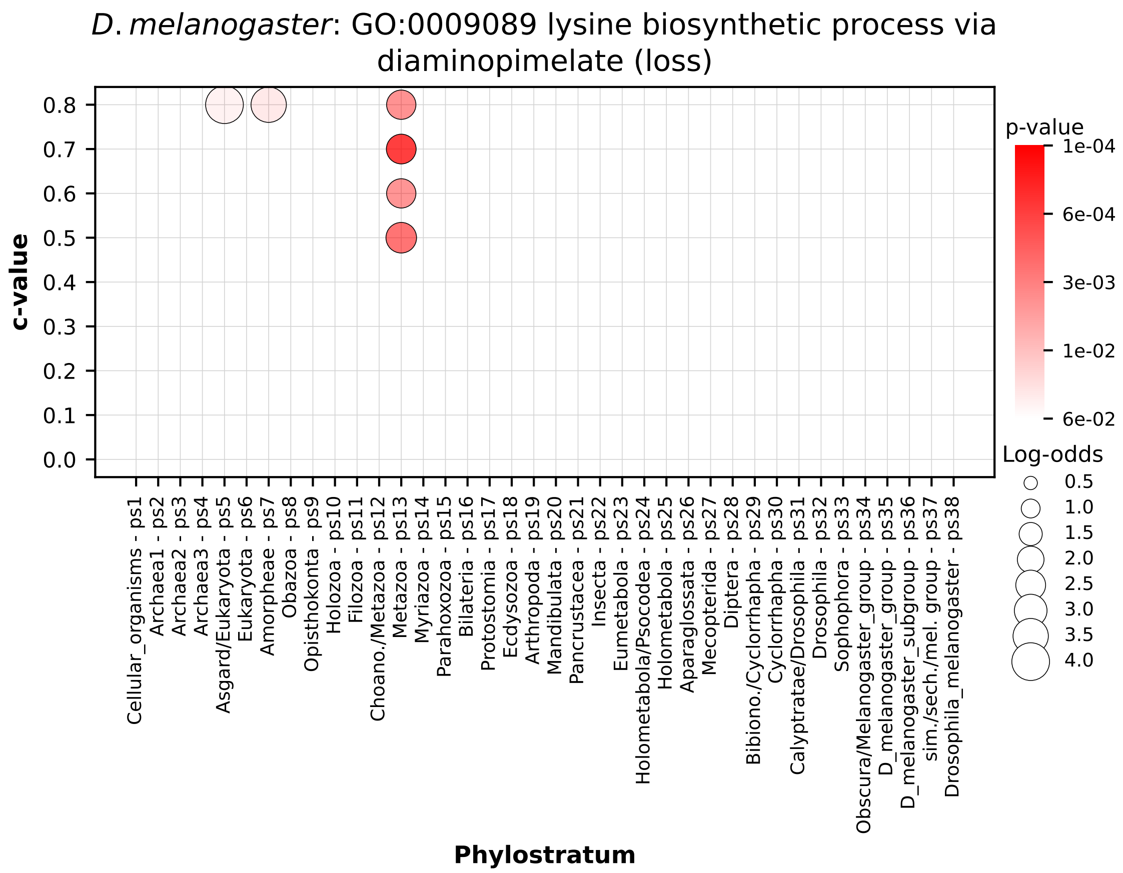

D. melanogaster: GO:0009089 lysine biosynthetic process via diaminopimelate (loss)
c-value
0.0
0.1
0.2
0.3
0.4
0.5
0.6
0.7
0.8
Phylostratum
Cellular_organisms - ps1
Archaea1 - ps2
Archaea2 - ps3
Archaea3 - ps4
Asgard/Eukaryota - ps5
Eukaryota - ps6
Amorpheae - ps7
Obazoa - ps8
Opisthokonta - ps9
Holozoa - ps10
Filozoa - ps11
Choano./Metazoa - ps12
Metazoa - ps13
Myriazoa - ps14
Parahoxozoa - ps15
Bilateria - ps16
Protostomia - ps17
Ecdysozoa - ps18
Arthropoda - ps19
Mandibulata - ps20
Pancrustacea - ps21
Insecta - ps22
Eumetabola - ps23
Holometabola/Psocodea - ps24
Holometabola - ps25
Aparaglossata - ps26
Mecopterida - ps27
Diptera - ps28
Bibiono./Cyclorrhapha - ps29
Cyclorrhapha - ps30
Calyptratae/Drosophila - ps31
Drosophila - ps32
Sophophora - ps33
Obscura/Melanogaster_group - ps34
D_melanogaster_group - ps35
D_melanogaster_subgroup - ps36
sim./sech./mel. group - ps37
Drosophila_melanogaster - ps38
p-value
1e-04
6e-04
3e-03
1e-02
6e-02
Log-odds
0.5
1.0
1.5
2.0
2.5
3.0
3.5
4.0

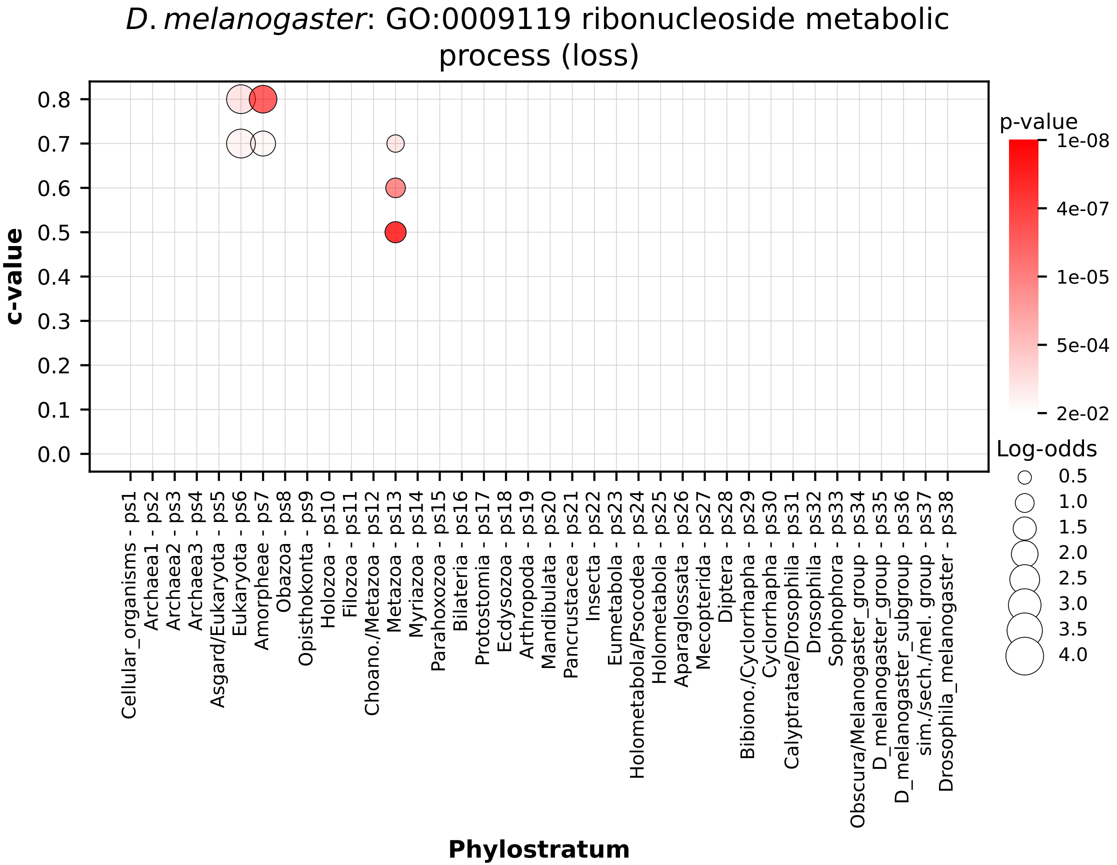
D. melanogaster: GO:0009119 ribonucleoside metabolic process (loss)
c-value
0.8
0.7
0.6
0.5
0.4
0.3
0.2
0.1
0.0
Phylostratum
Cellular_organisms - ps1
Archaea1 - ps2
Archaea2 - ps3
Archaea3 - ps4
Asgard/Eukaryota - ps5
Eukaryota - ps6
Amorpheae - ps7
Obazoa - ps8
Opisthokonta - ps9
Holozoa - ps10
Filozoa - ps11
Choano./Metazoa - ps12
Metazoa - ps13
Myriazoa - ps14
Parahoxozoa - ps15
Bilateria - ps16
Protostomia - ps17
Ecdysozoa - ps18
Arthropoda - ps19
Mandibulata - ps20
Pancrustacea - ps21
Insecta - ps22
Eumetabola - ps23
Holometabola/Psocodea - ps24
Holometabola - ps25
Aparaglossata - ps26
Mecopterida - ps27
Diptera - ps28
Bibiono./Cyclorrhapha - ps29
Cyclorrhapha - ps30
Calyptratae/Drosophila - ps31
Drosophila - ps32
Sophophora - ps33
Obscura/Melanogaster_group - ps34
D_melanogaster_group - ps35
D_melanogaster_subgroup - ps36
sim./sech./mel. group - ps37
Drosophila_melanogaster - ps38
p-value
1e-08
4e-07
1e-05
5e-04
2e-02
Log-odds
0.5
1.0
1.5
2.0
2.5
3.0
3.5
4.0

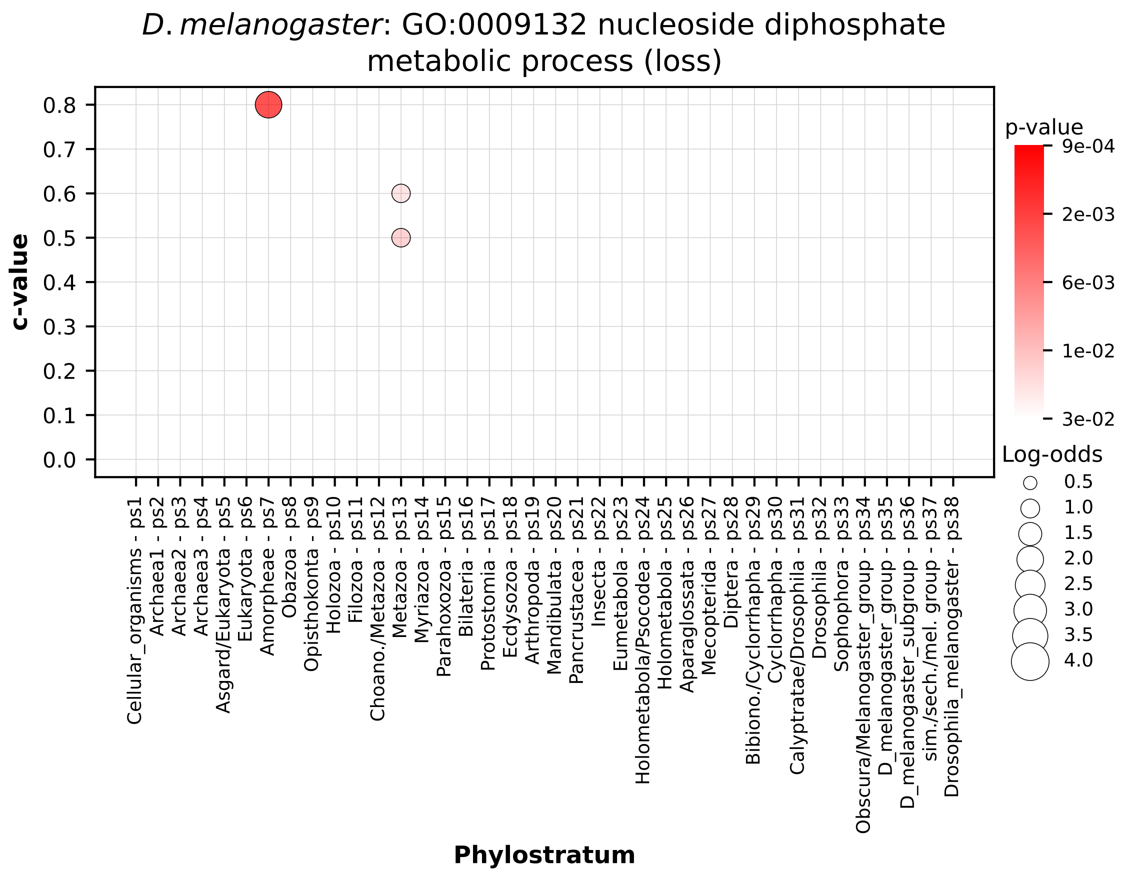

D. melanogaster: GO:0009132 nucleoside diphosphate metabolic process (loss)
c-value
0.8
0.7
0.6
0.5
0.4
0.3
0.2
0.1
0.0
p-value
9e-04
2e-03
6e-03
1e-02
3e-02
Log-odds
0.5
1.0
1.5
2.0
2.5
3.0
3.5
4.0
Cellular_organisms - ps1
Archaea1 - ps2
Archaea2 - ps3
Archaea3 - ps4
Asgard/Eukaryota - ps5
Eukaryota - ps6
Amorpheae - ps7
Obazoa - ps8
Opisthokonta - ps9
Holozoa - ps10
Filozoa - ps11
Choano./Metazoa - ps12
Metazoa - ps13
Myriazoa - ps14
Parahoxozoa - ps15
Bilateria - ps16
Protostomia - ps17
Ecdysozoa - ps18
Arthropoda - ps19
Mandibulata - ps20
Pancrustacea - ps21
Insecta - ps22
Eumetabola - ps23
Holometabola/Psocodea - ps24
Holometabola - ps25
Aparaglossata - ps26
Mecopterida - ps27
Diptera - ps28
Bibiono./Cyclorrhapha - ps29
Cyclorrhapha - ps30
Calyptratae/Drosophila - ps31
Drosophila - ps32
Sophophora - ps33
Obscura/Melanogaster_group - ps34
D_melanogaster_group - ps35
D_melanogaster_subgroup - ps36
sim./sech./mel. group - ps37
Drosophila_melanogaster - ps38
Phylostratum

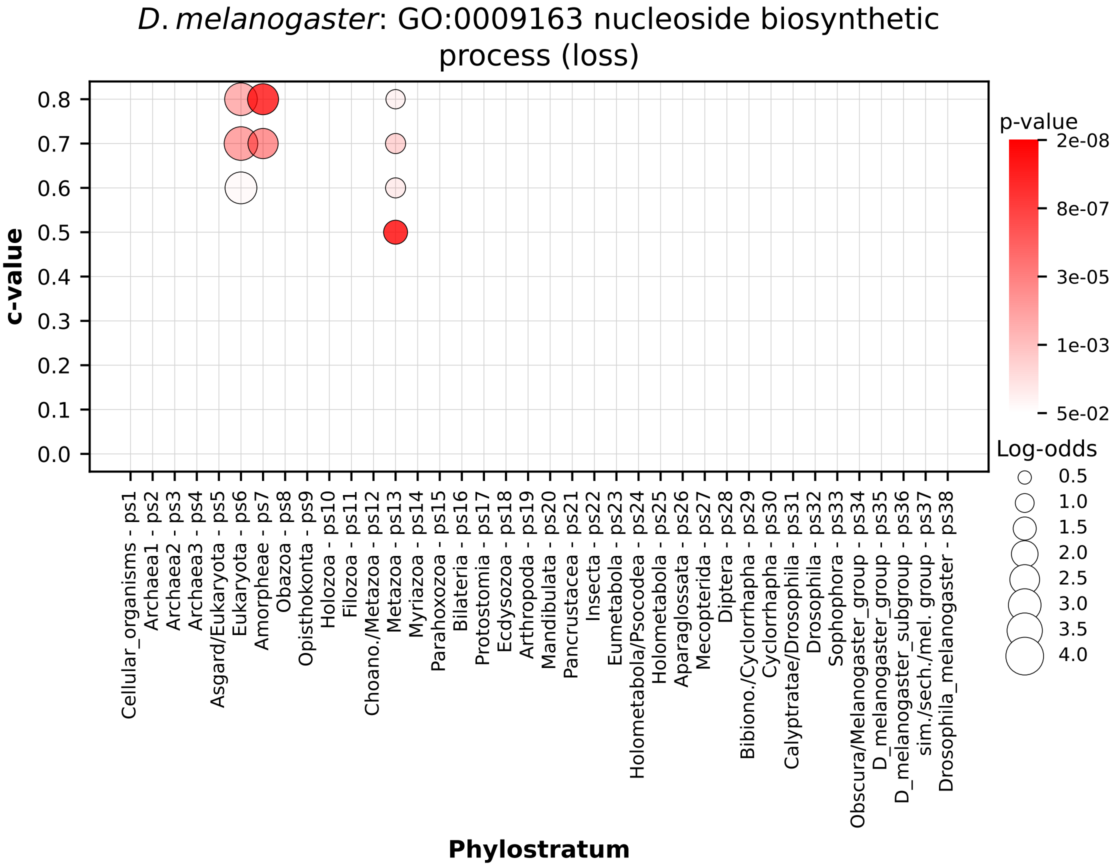

D. melanogaster: GO:0009163 nucleoside biosynthetic process (loss)
c-value
0.0
0.1
0.2
0.3
0.4
0.5
0.6
0.7
0.8
Phylostratum
Cellular_organisms - ps1
Archaea1 - ps2
Archaea2 - ps3
Archaea3 - ps4
Asgard/Eukaryota - ps5
Eukaryota - ps6
Amorpheae - ps7
Obazoa - ps8
Opisthokonta - ps9
Holozoa - ps10
Filozoa - ps11
Choano./Metazoa - ps12
Metazoa - ps13
Myriazoa - ps14
Parahoxozoa - ps15
Bilateria - ps16
Protostomia - ps17
Ecdysozoa - ps18
Arthropoda - ps19
Mandibulata - ps20
Pancrustacea - ps21
Insecta - ps22
Eumetabola - ps23
Holometabola/Psocodea - ps24
Holometabola - ps25
Aparaglossata - ps26
Mecopterida - ps27
Diptera - ps28
Bibiono./Cyclorrhapha - ps29
Cyclorrhapha - ps30
Calyptratae/Drosophila - ps31
Drosophila - ps32
Sophophora - ps33
Obscura/Melanogaster_group - ps34
D_melanogaster_group - ps35
D_melanogaster_subgroup - ps36
sim./sech./mel. group - ps37
Drosophila_melanogaster - ps38
p-value
2e-08
8e-07
3e-05
1e-03
5e-02
Log-odds
0.5
1.0
1.5
2.0
2.5
3.0
3.5
4.0

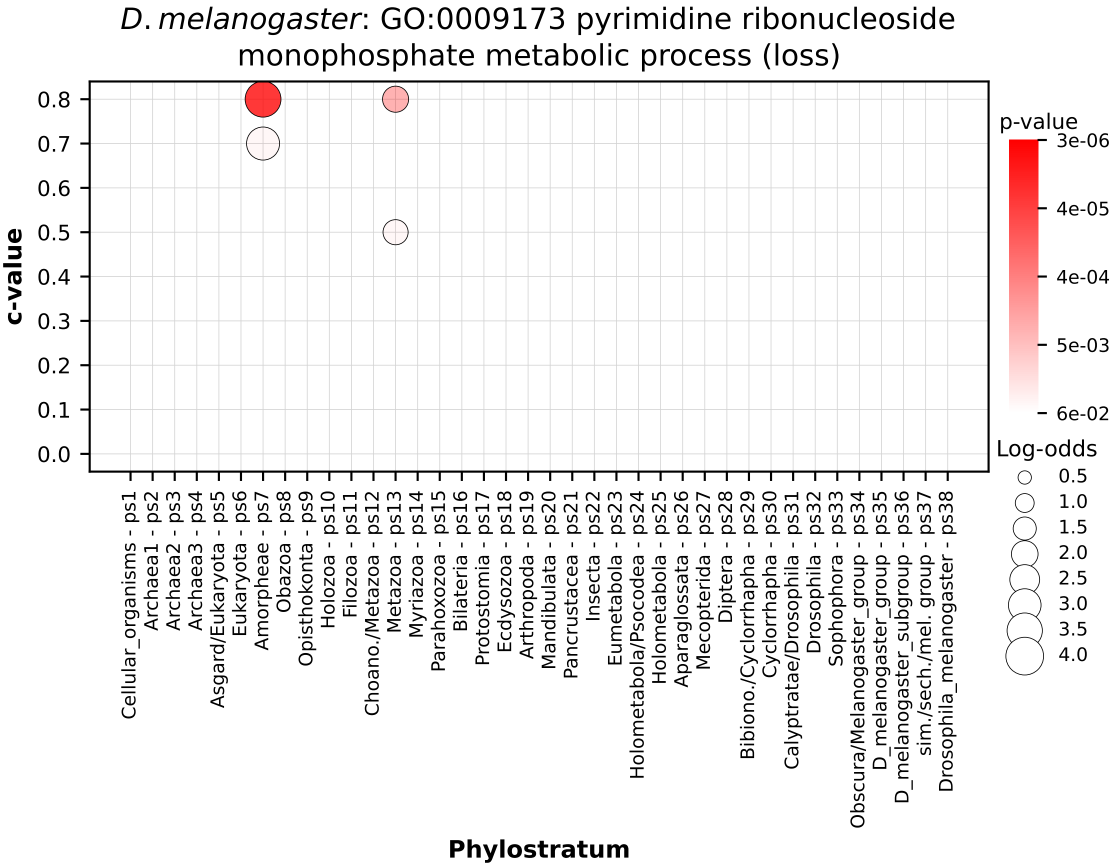

D. melanogaster: GO:0009173 pyrimidine ribonucleoside monophosphate metabolic process (loss)
c-value
0.0
0.1
0.2
0.3
0.4
0.5
0.6
0.7
0.8
p-value
3e-06
4e-05
4e-04
5e-03
6e-02
Log-odds
0.5
1.0
1.5
2.0
2.5
3.0
3.5
4.0
Cellular_organisms - ps1
Archaea1 - ps2
Archaea2 - ps3
Archaea3 - ps4
Asgard/Eukaryota - ps5
Eukaryota - ps6
Amorpheae - ps7
Obazoa - ps8
Opisthokonta - ps9
Holozoa - ps10
Filozoa - ps11
Choano./Metazoa - ps12
Metazoa - ps13
Myriazoa - ps14
Parahoxozoa - ps15
Bilateria - ps16
Protostomia - ps17
Ecdysozoa - ps18
Arthropoda - ps19
Mandibulata - ps20
Pancrustacea - ps21
Insecta - ps22
Eumetabola - ps23
Holometabola/Psocodea - ps24
Holometabola - ps25
Aparaglossata - ps26
Mecopterida - ps27
Diptera - ps28
Bibiono./Cyclorrhapha - ps29
Cyclorrhapha - ps30
Calyptratae/Drosophila - ps31
Drosophila - ps32
Sophophora - ps33
Obscura/Melanogaster_group - ps34
D_melanogaster_group - ps35
D_melanogaster_subgroup - ps36
sim./sech./mel. group - ps37
Drosophila_melanogaster - ps38
Phylostratum

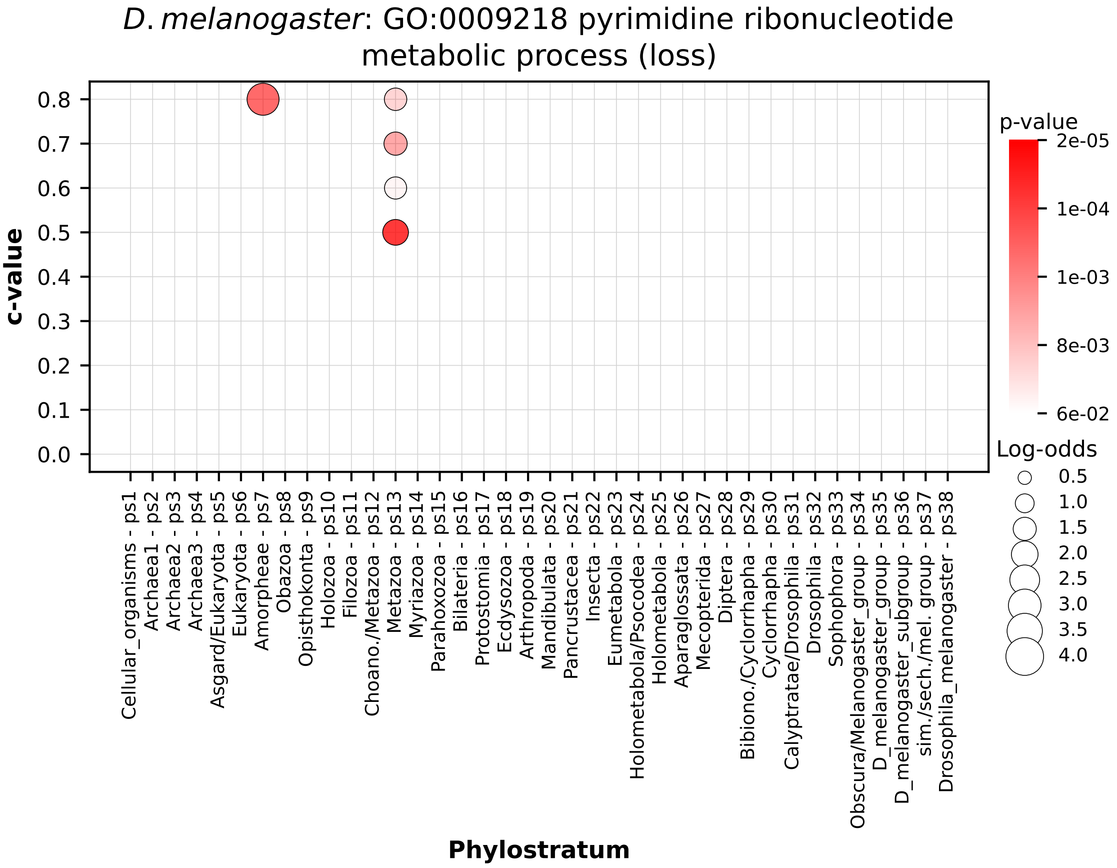

D. melanogaster: GO:0009218 pyrimidine ribonucleotide metabolic process (loss)
c-value
0.8
0.7
0.6
0.5
0.4
0.3
0.2
0.1
0.0
p-value
2e-05
1e-04
1e-03
8e-03
6e-02
Log-odds
0.5
1.0
1.5
2.0
2.5
3.0
3.5
4.0
Cellular_organisms - ps1
Archaea1 - ps2
Archaea2 - ps3
Archaea3 - ps4
Asgard/Eukaryota - ps5
Eukaryota - ps6
Amorpheae - ps7
Obazoa - ps8
Opisthokonta - ps9
Holozoa - ps10
Filozoa - ps11
Choano./Metazoa - ps12
Metazoa - ps13
Myriazoa - ps14
Parahoxozoa - ps15
Bilateria - ps16
Protostomia - ps17
Ecdysozoa - ps18
Arthropoda - ps19
Mandibulata - ps20
Pancrustacea - ps21
Insecta - ps22
Eumetabola - ps23
Holometabola/Psocodea - ps24
Holometabola - ps25
Aparaglossata - ps26
Mecopterida - ps27
Diptera - ps28
Bibiono./Cyclorrhapha - ps29
Cyclorrhapha - ps30
Calyptratae/Drosophila - ps31
Drosophila - ps32
Sophophora - ps33
Obscura/Melanogaster_group - ps34
D_melanogaster_group - ps35
D_melanogaster_subgroup - ps36
sim./sech./mel. group - ps37
Drosophila_melanogaster - ps38
Phylostratum

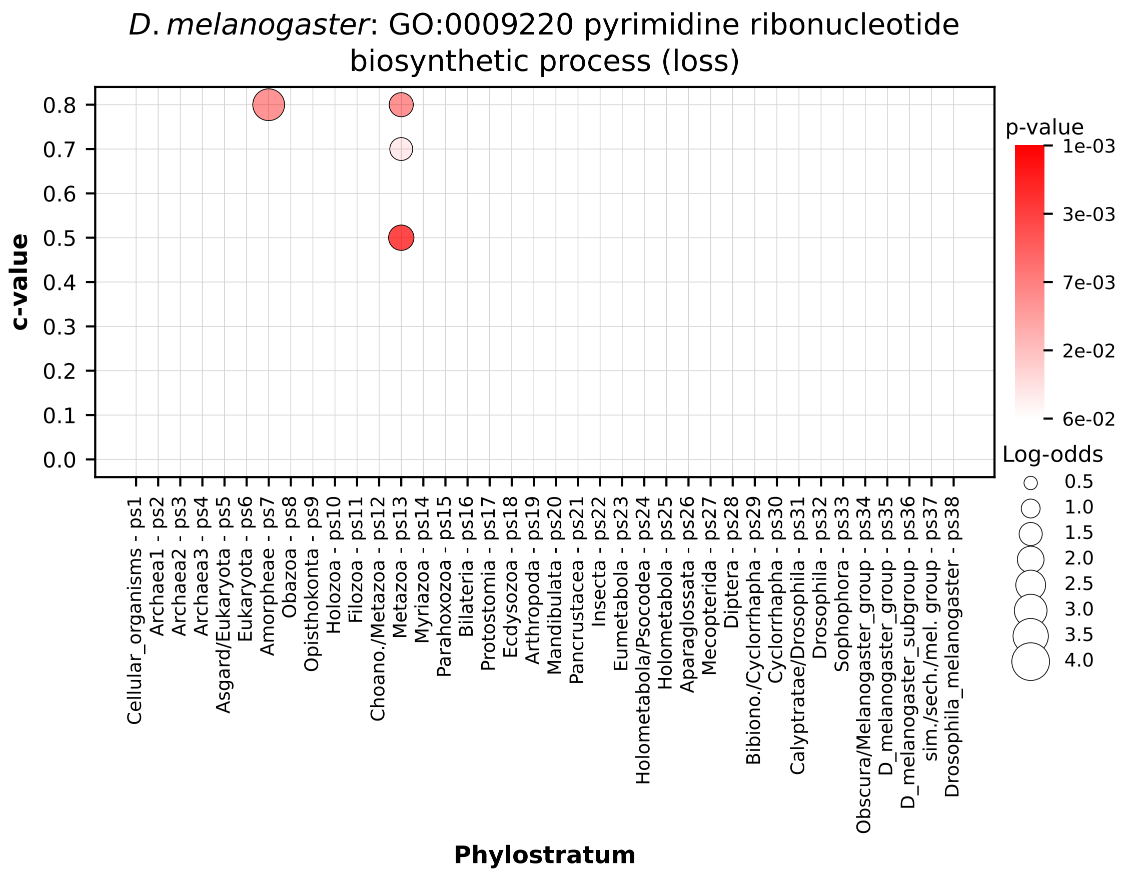

D. melanogaster: GO:0009220 pyrimidine ribonucleotide biosynthetic process (loss)
c-value
0.0
0.1
0.2
0.3
0.4
0.5
0.6
0.7
0.8
p-value
1e-03
3e-03
7e-03
2e-02
6e-02
Log-odds
0.5
1.0
1.5
2.0
2.5
3.0
3.5
4.0
Cellular_organisms - ps1
Archaea1 - ps2
Archaea2 - ps3
Archaea3 - ps4
Asgard/Eukaryota - ps5
Eukaryota - ps6
Amorpheae - ps7
Obazoa - ps8
Opisthokonta - ps9
Holozoa - ps10
Filozoa - ps11
Choano./Metazoa - ps12
Metazoa - ps13
Myriazoa - ps14
Parahoxozoa - ps15
Bilateria - ps16
Protostomia - ps17
Ecdysozoa - ps18
Arthropoda - ps19
Mandibulata - ps20
Pancrustacea - ps21
Insecta - ps22
Eumetabola - ps23
Holometabola/Psocodea - ps24
Holometabola - ps25
Aparaglossata - ps26
Mecopterida - ps27
Diptera - ps28
Bibiono./Cyclorrhapha - ps29
Cyclorrhapha - ps30
Calyptratae/Drosophila - ps31
Drosophila - ps32
Sophophora - ps33
Obscura/Melanogaster_group - ps34
D_melanogaster_group - ps35
D_melanogaster_subgroup - ps36
sim./sech./mel. group - ps37
Drosophila_melanogaster - ps38
Phylostratum

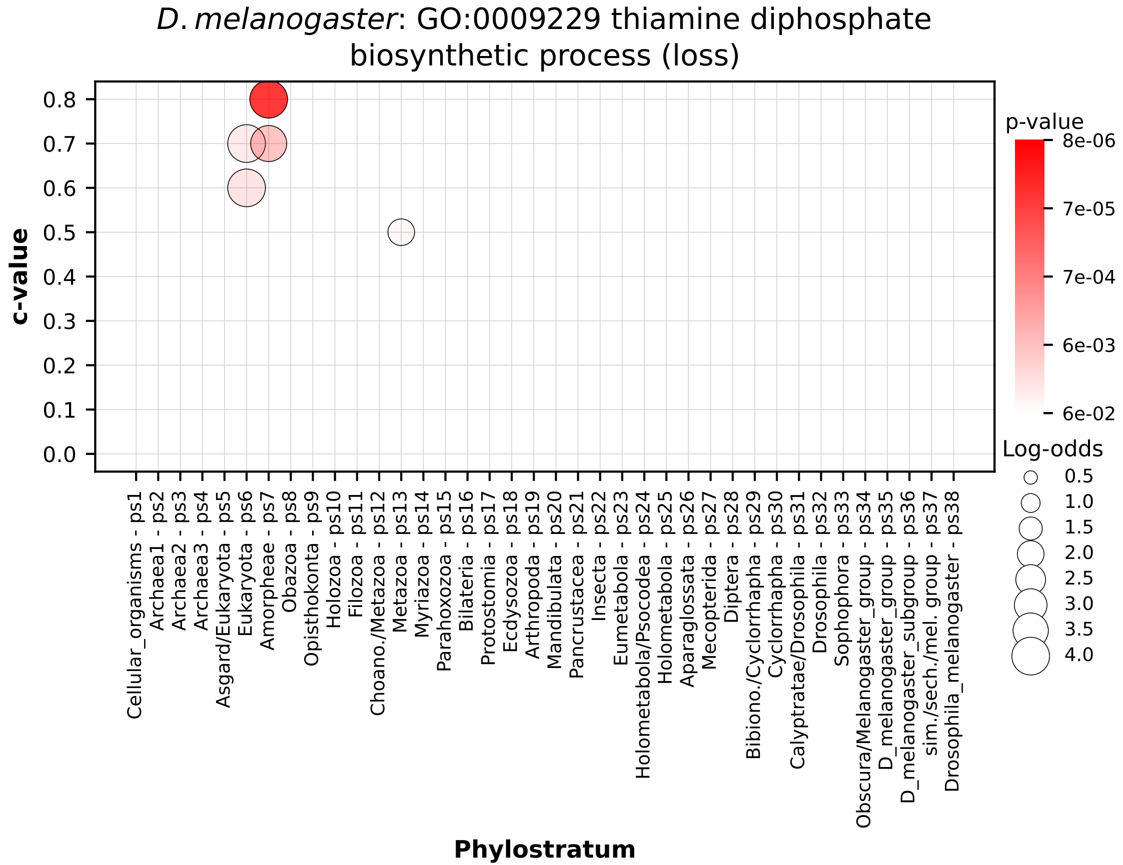

D. melanogaster: GO:0009229 thiamine diphosphate biosynthetic process (loss)
c-value
0.0
0.1
0.2
0.3
0.4
0.5
0.6
0.7
0.8
Phylostratum
Cellular_organisms - ps1
Archaea1 - ps2
Archaea2 - ps3
Archaea3 - ps4
Asgard/Eukaryota - ps5
Eukaryota - ps6
Amorpheae - ps7
Obazoa - ps8
Opisthokonta - ps9
Holozoa - ps10
Filozoa - ps11
Choano./Metazoa - ps12
Metazoa - ps13
Myriazoa - ps14
Parahoxozoa - ps15
Bilateria - ps16
Protostomia - ps17
Ecdysozoa - ps18
Arthropoda - ps19
Mandibulata - ps20
Pancrustacea - ps21
Insecta - ps22
Eumetabola - ps23
Holometabola/Psocodea - ps24
Holometabola - ps25
Aparaglossata - ps26
Mecopterida - ps27
Diptera - ps28
Bibiono./Cyclorrhapha - ps29
Cyclorrhapha - ps30
Calyptratae/Drosophila - ps31
Drosophila - ps32
Sophophora - ps33
Obscura/Melanogaster_group - ps34
D_melanogaster_group - ps35
D_melanogaster_subgroup - ps36
sim./sech./mel. group - ps37
Drosophila_melanogaster - ps38
p-value
8e-06
7e-05
7e-04
6e-03
6e-02
Log-odds
0.5
1.0
1.5
2.0
2.5
3.0
3.5
4.0

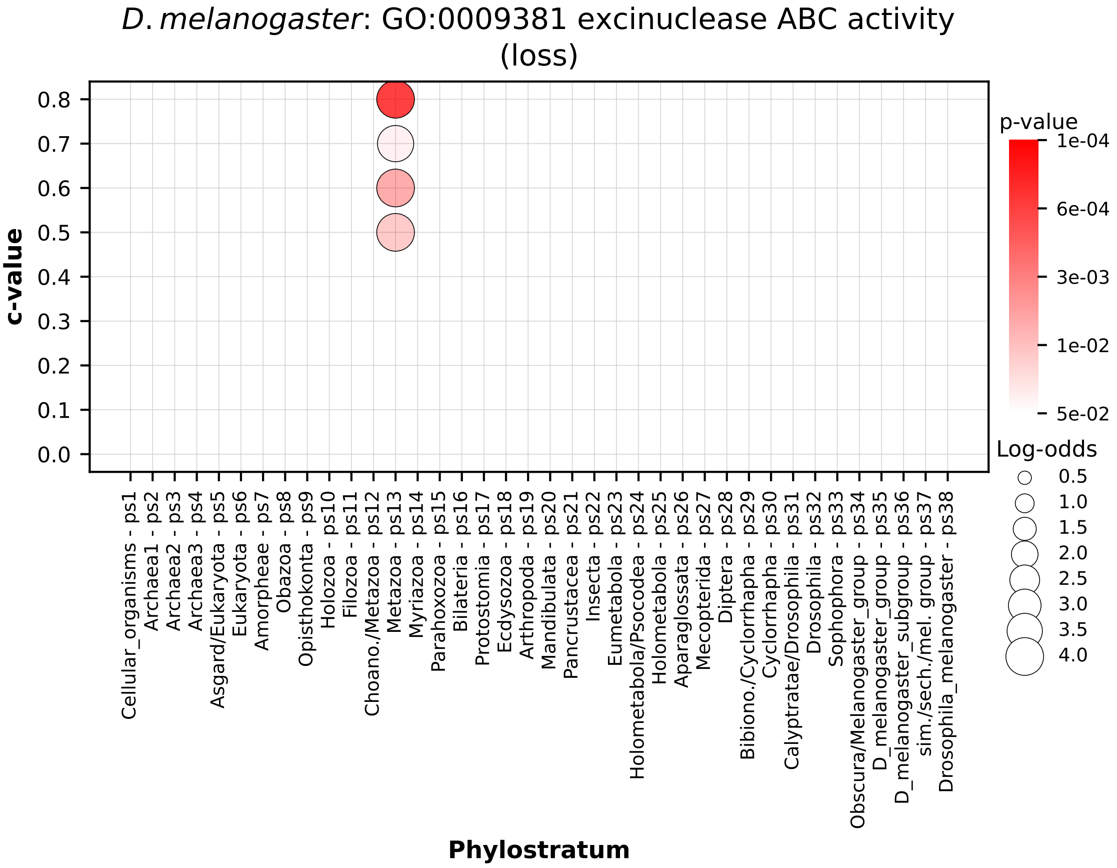
D. melanogaster: GO:0009381 excinuclease ABC activity
(loss)
c-value
0.0
0.1
0.2
0.3
0.4
0.5
0.6
0.7
0.8
p-value
1e-04
6e-04
3e-03
1e-02
5e-02
Log-odds
0.5
1.0
1.5
2.0
2.5
3.0
3.5
4.0
Cellular_organisms - ps1
Archaea1 - ps2
Archaea2 - ps3
Archaea3 - ps4
Asgard/Eukaryota - ps5
Eukaryota - ps6
Amorpheae - ps7
Obazoa - ps8
Opisthokonta - ps9
Holozoa - ps10
Filozoa - ps11
Choano./Metazoa - ps12
Metazoa - ps13
Myriazoa - ps14
Parahoxozoa - ps15
Bilateria - ps16
Protostomia - ps17
Ecdysozoa - ps18
Arthropoda - ps19
Mandibulata - ps20
Pancrustacea - ps21
Insecta - ps22
Eumetabola - ps23
Holometabola/Psocodea - ps24
Holometabola - ps25
Aparaglossata - ps26
Mecopterida - ps27
Diptera - ps28
Bibiono./Cyclorrhapha - ps29
Cyclorrhapha - ps30
Calyptratae/Drosophila - ps31
Drosophila - ps32
Sophophora - ps33
Obscura/Melanogaster_group - ps34
D_melanogaster_group - ps35
D_melanogaster_subgroup - ps36
sim./sech./mel. group - ps37
Drosophila_melanogaster - ps38
Phylostratum

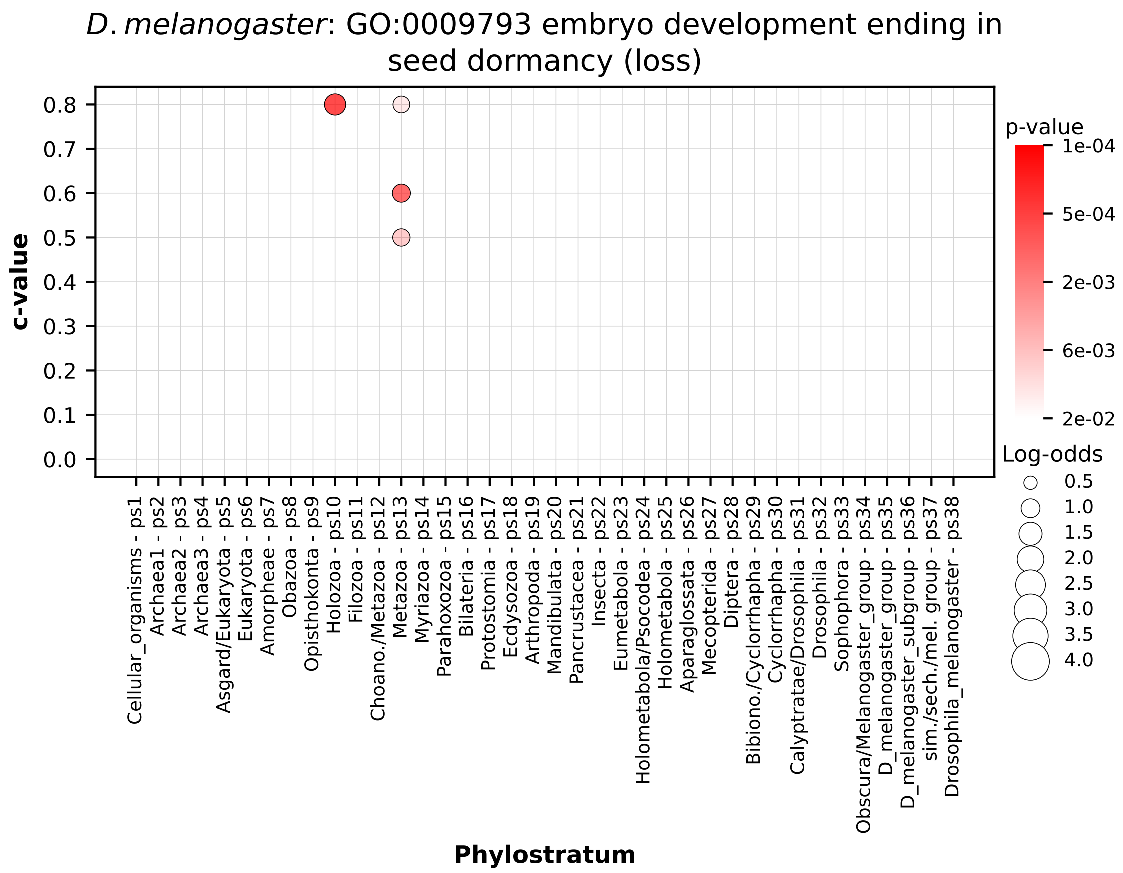
D. melanogaster: GO:0009793 embryo development ending in seed dormancy (loss)
c-value
0.0
0.1
0.2
0.3
0.4
0.5
0.6
0.7
0.8
Phylostratum
Cellular_organisms - ps1
Archaea1 - ps2
Archaea2 - ps3
Archaea3 - ps4
Asgard/Eukaryota - ps5
Eukaryota - ps6
Amorpheae - ps7
Obazoa - ps8
Opisthokonta - ps9
Holozoa - ps10
Filozoa - ps11
Choano./Metazoa - ps12
Metazoa - ps13
Myriazoa - ps14
Parahoxozoa - ps15
Bilateria - ps16
Protostomia - ps17
Ecdysozoa - ps18
Arthropoda - ps19
Mandibulata - ps20
Pancrustacea - ps21
Insecta - ps22
Eumetabola - ps23
Holometabola/Psocodea - ps24
Holometabola - ps25
Aparaglossata - ps26
Mecopterida - ps27
Diptera - ps28
Bibiono./Cyclorrhapha - ps29
Cyclorrhapha - ps30
Calyptratae/Drosophila - ps31
Drosophila - ps32
Sophophora - ps33
Obscura/Melanogaster_group - ps34
D_melanogaster_group - ps35
D_melanogaster_subgroup - ps36
sim./sech./mel. group - ps37
Drosophila_melanogaster - ps38
p-value
1e-04
5e-04
2e-03
6e-03
2e-02
Log-odds
0.5
1.0
1.5
2.0
2.5
3.0
3.5
4.0

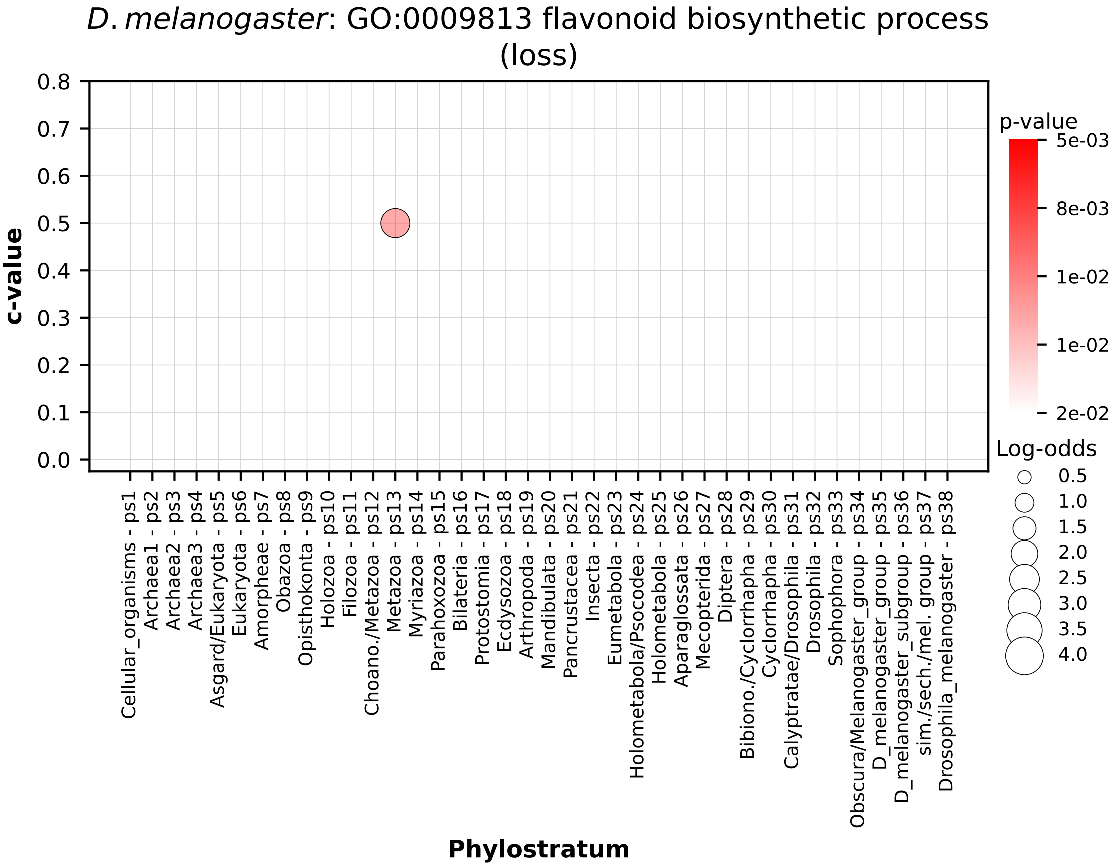

D. melanogaster: GO:0009813 flavonoid biosynthetic process (loss)
c-value
0.8
0.7
0.6
0.5
0.4
0.3
0.2
0.1
0.0
Phylostratum
Cellular_organisms - ps1
Archaea1 - ps2
Archaea2 - ps3
Archaea3 - ps4
Asgard/Eukaryota - ps5
Eukaryota - ps6
Amorpheae - ps7
Obazoa - ps8
Opisthokonta - ps9
Holozoa - ps10
Filozoa - ps11
Choano./Metazoa - ps12
Metazoa - ps13
Myriazoa - ps14
Parahoxozoa - ps15
Bilateria - ps16
Protostomia - ps17
Ecdysozoa - ps18
Arthropoda - ps19
Mandibulata - ps20
Pancrustacea - ps21
Insecta - ps22
Eumetabola - ps23
Holometabola/Psocodea - ps24
Holometabola - ps25
Aparaglossata - ps26
Mecopterida - ps27
Diptera - ps28
Bibiono./Cyclorrhapha - ps29
Cyclorrhapha - ps30
Calyptratae/Drosophila - ps31
Drosophila - ps32
Sophophora - ps33
Obscura/Melanogaster_group - ps34
D_melanogaster_group - ps35
D_melanogaster_subgroup - ps36
sim./sech./mel. group - ps37
Drosophila_melanogaster - ps38
p-value
5e-03
8e-03
1e-02
1e-02
2e-02
Log-odds
0.5
1.0
1.5
2.0
2.5
3.0
3.5
4.0

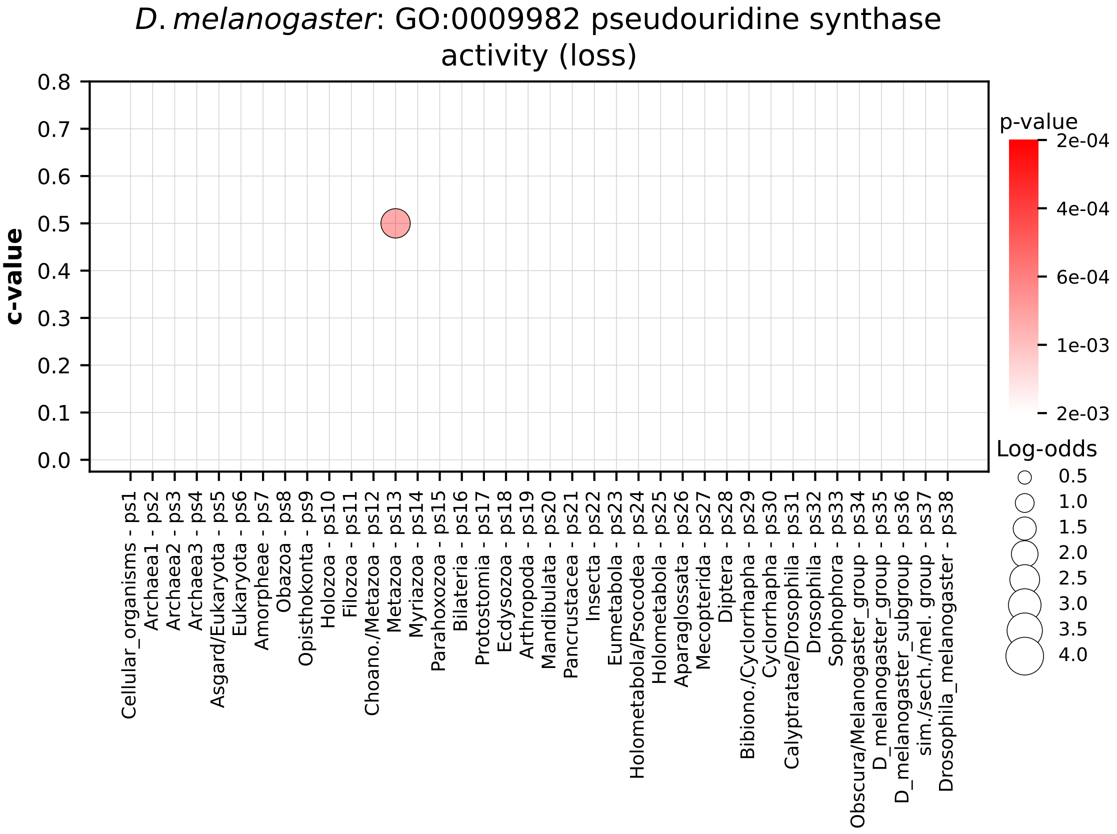

D. melanogaster: GO:0009982 pseudouridine synthase activity (loss)
c-value
0.8
0.7
0.6
0.5
0.4
0.3
0.2
0.1
0.0
p-value
2e-04
4e-04
6e-04
1e-03
2e-03
Log-odds
0.5
1.0
1.5
2.0
2.5
3.0
3.5
4.0
Cellular_organisms - ps1
Archaea1 - ps2
Archaea2 - ps3
Archaea3 - ps4
Asgard/Eukaryota - ps5
Eukaryota - ps6
Amorpheae - ps7
Obazoa - ps8
Opisthokonta - ps9
Holozoa - ps10
Filozoa - ps11
Choano./Metazoa - ps12
Metazoa - ps13
Myriazoa - ps14
Parahoxozoa - ps15
Bilateria - ps16
Protostomia - ps17
Ecdysozoa - ps18
Arthropoda - ps19
Mandibulata - ps20
Pancrustacea - ps21
Insecta - ps22
Eumetabola - ps23
Holometabola/Psocodea - ps24
Holometabola - ps25
Aparaglossata - ps26
Mecopterida - ps27
Diptera - ps28
Bibiono./Cyclorrhapha - ps29
Cyclorrhapha - ps30
Calyptratae/Drosophila - ps31
Drosophila - ps32
Sophophora - ps33
Obscura/Melanogaster_group - ps34
D_melanogaster_group - ps35
D_melanogaster_subgroup - ps36
sim./sech./mel. group - ps37
Drosophila_melanogaster - ps38
Phylostratum

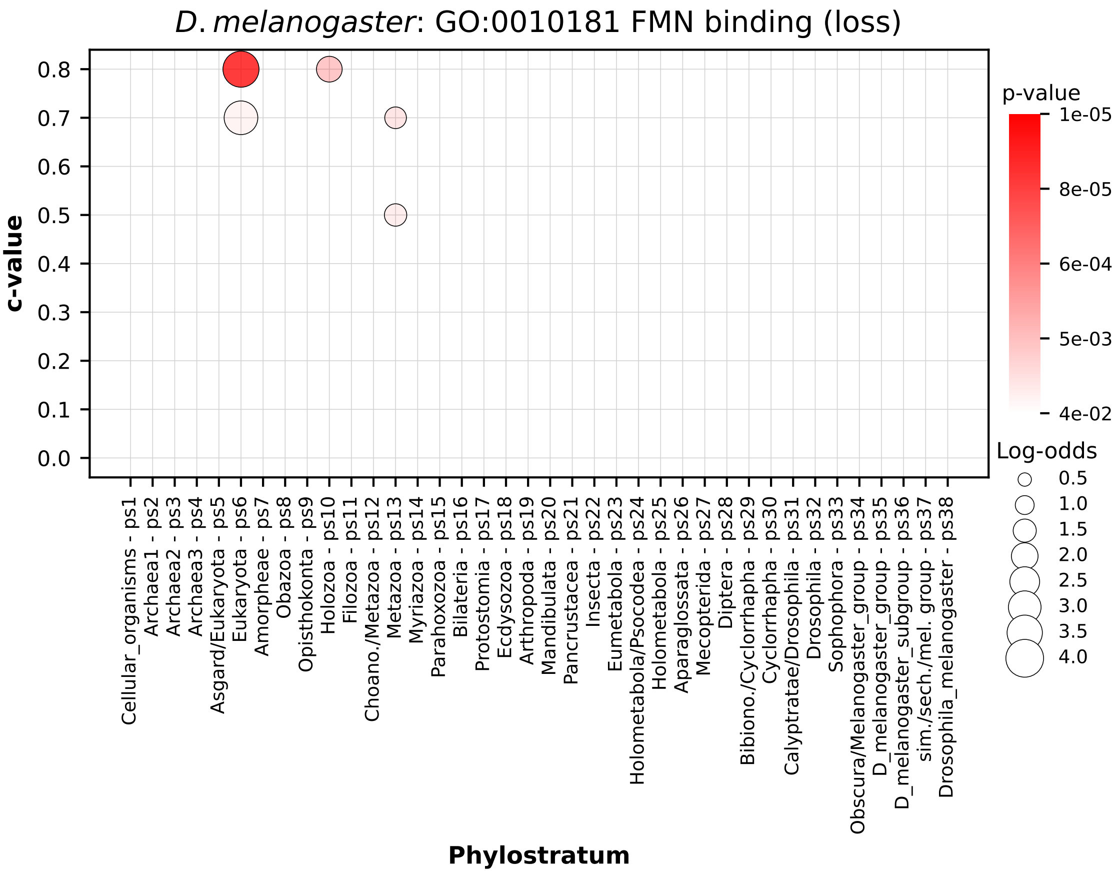

D. melanogaster: GO:0010181 FMN binding (loss)
c-value
0.8
0.7
0.6
0.5
0.4
0.3
0.2
0.1
0.0
Phylostratum
Cellular_organisms - ps1
Archaea1 - ps2
Archaea2 - ps3
Archaea3 - ps4
Asgard/Eukaryota - ps5
Eukaryota - ps6
Amorpheae - ps7
Obazoa - ps8
Opisthokonta - ps9
Holozoa - ps10
Filozoa - ps11
Choano./Metazoa - ps12
Metazoa - ps13
Myriazoa - ps14
Parahoxozoa - ps15
Bilateria - ps16
Protostomia - ps17
Ecdysozoa - ps18
Arthropoda - ps19
Mandibulata - ps20
Pancrustacea - ps21
Insecta - ps22
Eumetabola - ps23
Holometabola/Psocodea - ps24
Holometabola - ps25
Aparaglossata - ps26
Mecopterida - ps27
Diptera - ps28
Bibiono./Cyclorrhapha - ps29
Cyclorrhapha - ps30
Calyptratae/Drosophila - ps31
Drosophila - ps32
Sophophora - ps33
Obscura/Melanogaster_group - ps34
D_melanogaster_group - ps35
D_melanogaster_subgroup - ps36
sim./sech./mel. group - ps37
Drosophila_melanogaster - ps38
p-value
1e-05
8e-05
6e-04
5e-03
4e-02
Log-odds
0.5
1.0
1.5
2.0
2.5
3.0
3.5
4.0

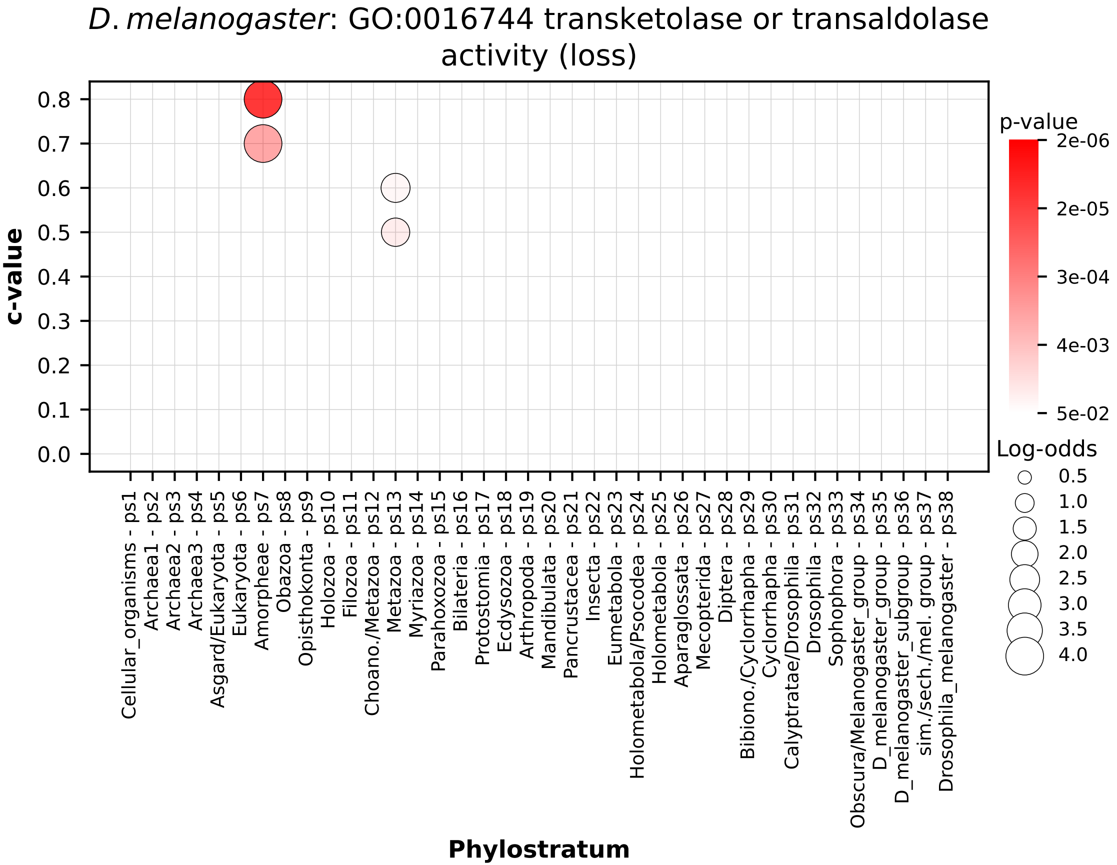

D. melanogaster: GO:0016744 transketolase or transaldolase activity (loss)
c-value
0.0
0.1
0.2
0.3
0.4
0.5
0.6
0.7
0.8
Phylostratum
Cellular_organisms - ps1
Archaea1 - ps2
Archaea2 - ps3
Archaea3 - ps4
Asgard/Eukaryota - ps5
Eukaryota - ps6
Amorpheae - ps7
Obazoa - ps8
Opisthokonta - ps9
Holozoa - ps10
Filozoa - ps11
Choano./Metazoa - ps12
Metazoa - ps13
Myriazoa - ps14
Parahoxozoa - ps15
Bilateria - ps16
Protostomia - ps17
Ecdysozoa - ps18
Arthropoda - ps19
Mandibulata - ps20
Pancrustacea - ps21
Insecta - ps22
Eumetabola - ps23
Holometabola/Psocodea - ps24
Holometabola - ps25
Aparaglossata - ps26
Mecopterida - ps27
Diptera - ps28
Bibiono./Cyclorrhapha - ps29
Cyclorrhapha - ps30
Calyptratae/Drosophila - ps31
Drosophila - ps32
Sophophora - ps33
Obscura/Melanogaster_group - ps34
D_melanogaster_group - ps35
D_melanogaster_subgroup - ps36
sim./sech./mel. group - ps37
Drosophila_melanogaster - ps38
p-value
2e-06
2e-05
3e-04
4e-03
5e-02
Log-odds
0.5
1.0
1.5
2.0
2.5
3.0
3.5
4.0

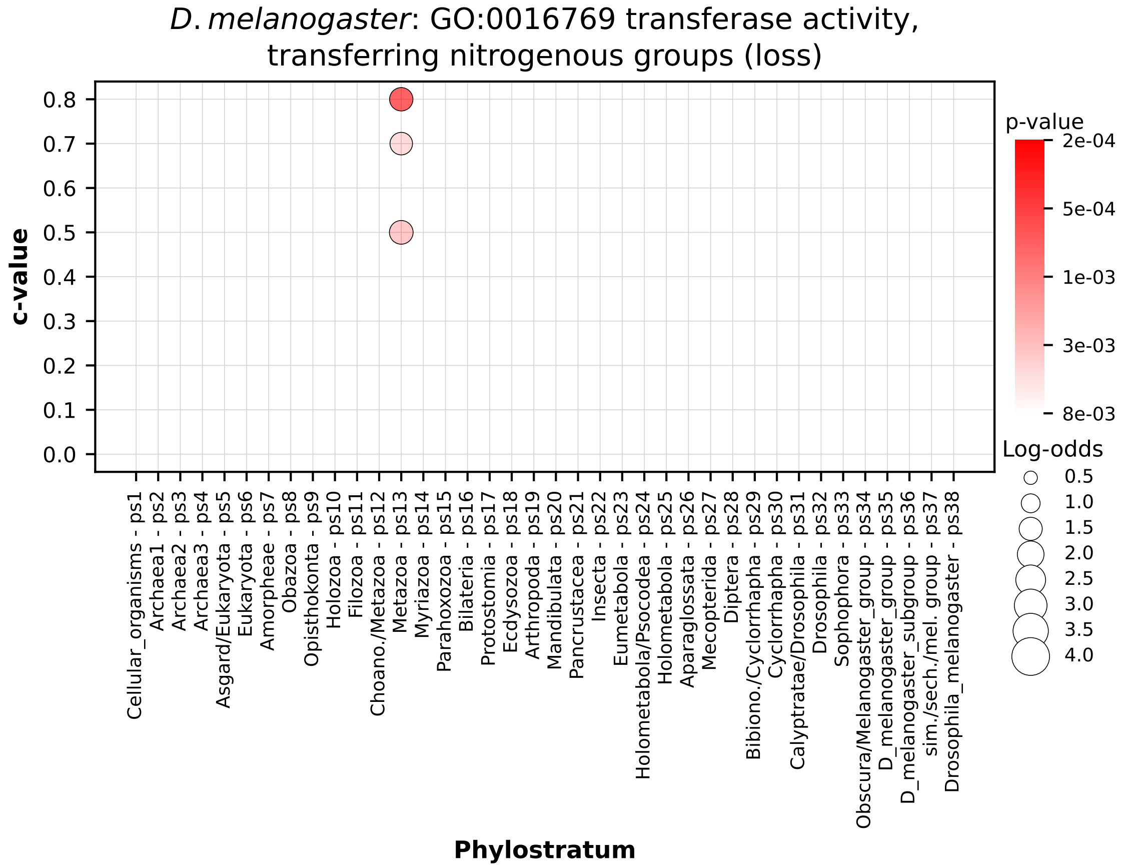
D. melanogaster: GO:0016769 transferase activity, transferring nitrogenous groups (loss)
c-value
0.0
0.1
0.2
0.3
0.4
0.5
0.6
0.7
0.8
Phylostratum
Cellular_organisms - ps1
Archaea1 - ps2
Archaea2 - ps3
Archaea3 - ps4
Asgard/Eukaryota - ps5
Eukaryota - ps6
Amorpheae - ps7
Obazoa - ps8
Opisthokonta - ps9
Holozoa - ps10
Filozoa - ps11
Choano./Metazoa - ps12
Metazoa - ps13
Myriazoa - ps14
Parahoxozoa - ps15
Bilateria - ps16
Protostomia - ps17
Ecdysozoa - ps18
Arthropoda - ps19
Mandibulata - ps20
Pancrustacea - ps21
Insecta - ps22
Eumetabola - ps23
Holometabola/Psocodea - ps24
Holometabola - ps25
Aparaglossata - ps26
Mecopterida - ps27
Diptera - ps28
Bibiono./Cyclorrhapha - ps29
Cyclorrhapha - ps30
Calyptratae/Drosophila - ps31
Drosophila - ps32
Sophophora - ps33
Obscura/Melanogaster_group - ps34
D_melanogaster_group - ps35
D_melanogaster_subgroup - ps36
sim./sech./mel. group - ps37
Drosophila_melanogaster - ps38
p-value
2e-04
5e-04
1e-03
3e-03
8e-03
Log-odds
0.5
1.0
1.5
2.0
2.5
3.0
3.5
4.0

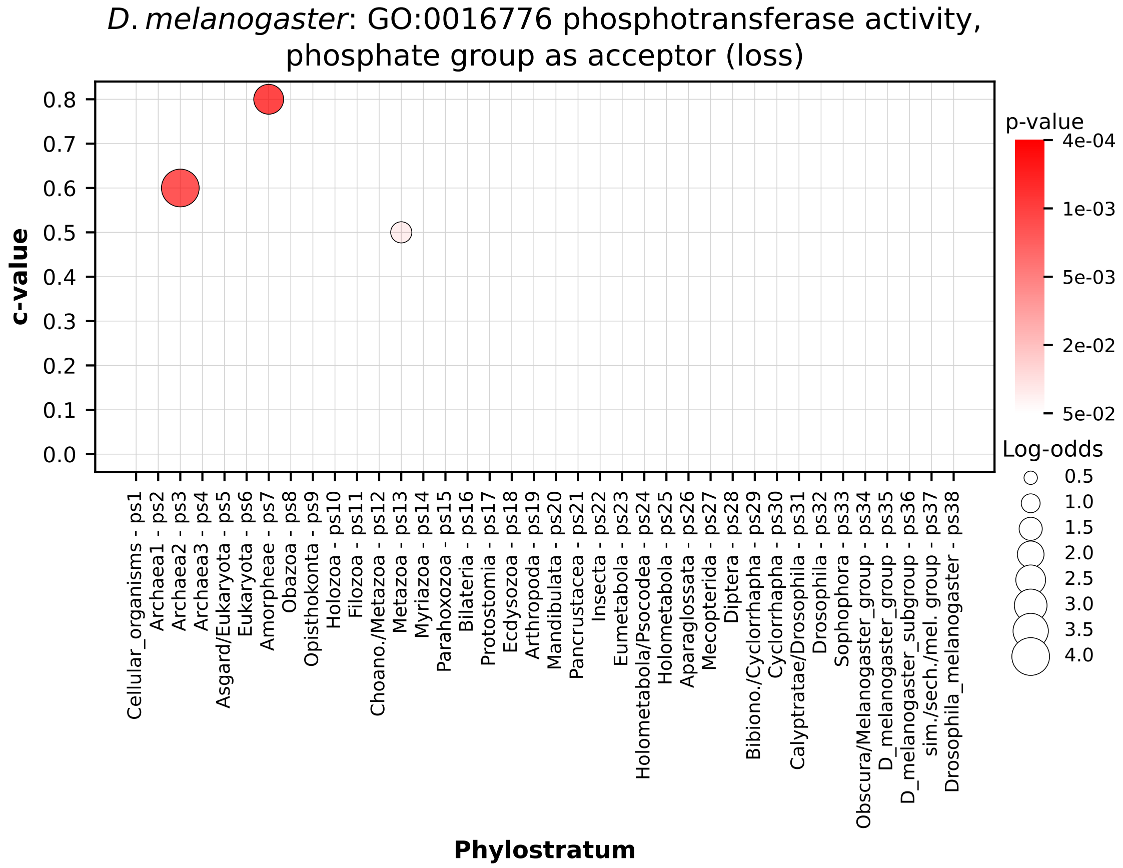

D. melanogaster: GO:0016776 phosphotransferase activity, phosphate group as acceptor (loss)
c-value
0.0
0.1
0.2
0.3
0.4
0.5
0.6
0.7
0.8
Phylostratum
Cellular_organisms - ps1
Archaea1 - ps2
Archaea2 - ps3
Archaea3 - ps4
Asgard/Eukaryota - ps5
Eukaryota - ps6
Amorpheae - ps7
Obazoa - ps8
Opisthokonta - ps9
Holozoa - ps10
Filozoa - ps11
Choano./Metazoa - ps12
Metazoa - ps13
Myriazoa - ps14
Parahoxozoa - ps15
Bilateria - ps16
Protostomia - ps17
Ecdysozoa - ps18
Arthropoda - ps19
Mandibulata - ps20
Pancrustacea - ps21
Insecta - ps22
Eumetabola - ps23
Holometabola/Psocodea - ps24
Holometabola - ps25
Aparaglossata - ps26
Mecopterida - ps27
Diptera - ps28
Bibiono./Cyclorrhapha - ps29
Cyclorrhapha - ps30
Calyptratae/Drosophila - ps31
Drosophila - ps32
Sophophora - ps33
Obscura/Melanogaster_group - ps34
D_melanogaster_group - ps35
D_melanogaster_subgroup - ps36
sim./sech./mel. group - ps37
Drosophila_melanogaster - ps38
p-value
4e-04
1e-03
5e-03
2e-02
5e-02
Log-odds
0.5
1.0
1.5
2.0
2.5
3.0
3.5
4.0

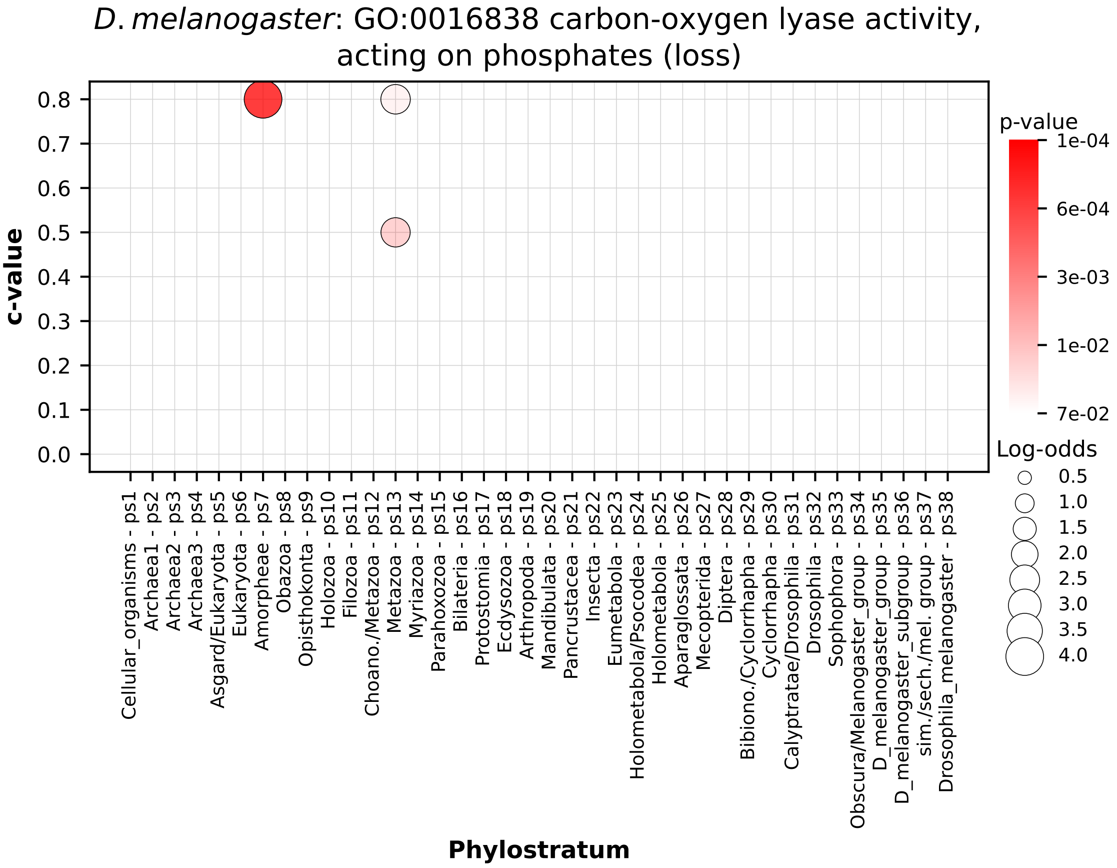

D. melanogaster: GO:0016838 carbon-oxygen lyase activity, acting on phosphates (loss)
c-value
0.8
0.7
0.6
0.5
0.4
0.3
0.2
0.1
0.0
p-value
1e-04
6e-04
3e-03
1e-02
7e-02
Log-odds
0.5
1.0
1.5
2.0
2.5
3.0
3.5
4.0
Cellular_organisms - ps1
Archaea1 - ps2
Archaea2 - ps3
Archaea3 - ps4
Asgard/Eukaryota - ps5
Eukaryota - ps6
Amorpheae - ps7
Obazoa - ps8
Opisthokonta - ps9
Holozoa - ps10
Filozoa - ps11
Choano./Metazoa - ps12
Metazoa - ps13
Myriazoa - ps14
Parahoxozoa - ps15
Bilateria - ps16
Protostomia - ps17
Ecdysozoa - ps18
Arthropoda - ps19
Mandibulata - ps20
Pancrustacea - ps21
Insecta - ps22
Eumetabola - ps23
Holometabola/Psocodea - ps24
Holometabola - ps25
Aparaglossata - ps26
Mecopterida - ps27
Diptera - ps28
Bibiono./Cyclorrhapha - ps29
Cyclorrhapha - ps30
Calyptratae/Drosophila - ps31
Drosophila - ps32
Sophophora - ps33
Obscura/Melanogaster_group - ps34
D_melanogaster_group - ps35
D_melanogaster_subgroup - ps36
sim./sech./mel. group - ps37
Drosophila_melanogaster - ps38
Phylostratum

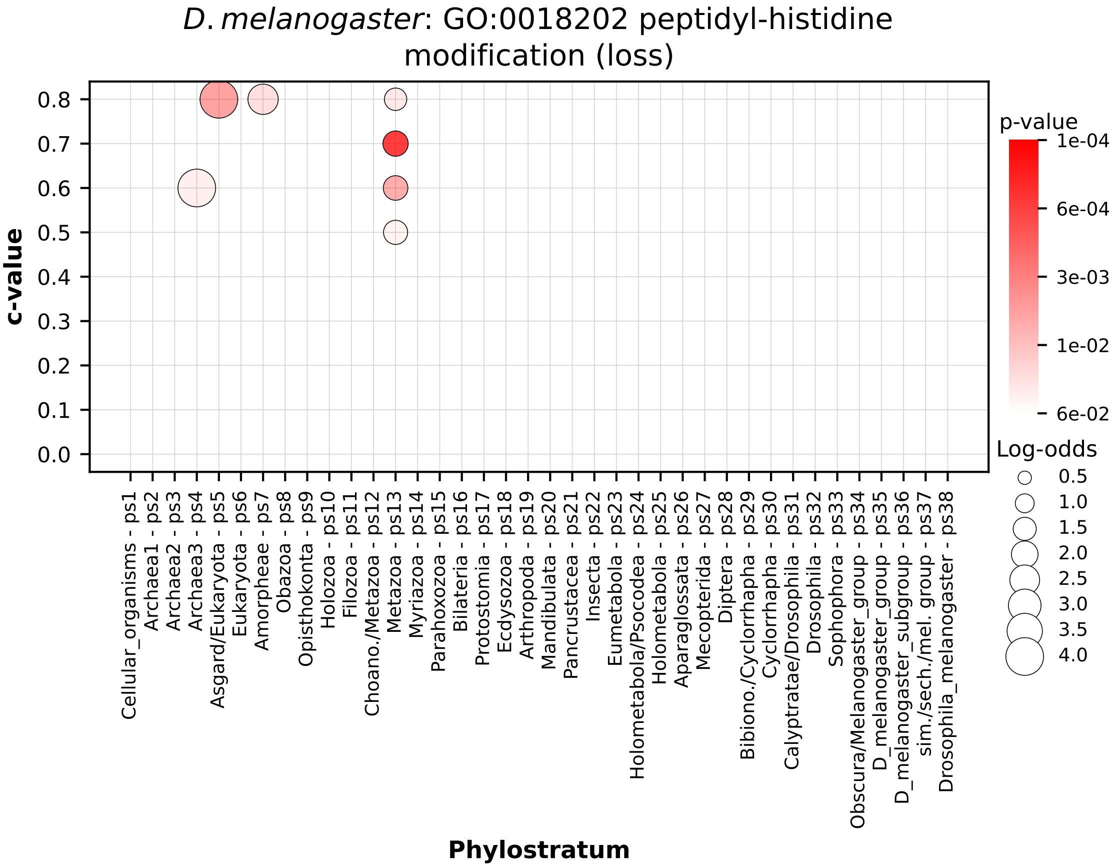

D. melanogaster: GO:0018202 peptidyl-histidine modification (loss)
c-value
0.8
0.7
0.6
0.5
0.4
0.3
0.2
0.1
0.0
Phylostratum
Cellular_organisms - ps1
Archaea1 - ps2
Archaea2 - ps3
Archaea3 - ps4
Asgard/Eukaryota - ps5
Eukaryota - ps6
Amorpheae - ps7
Obazoa - ps8
Opisthokonta - ps9
Holozoa - ps10
Filozoa - ps11
Choano./Metazoa - ps12
Metazoa - ps13
Myriazoa - ps14
Parahoxozoa - ps15
Bilateria - ps16
Protostomia - ps17
Ecdysozoa - ps18
Arthropoda - ps19
Mandibulata - ps20
Pancrustacea - ps21
Insecta - ps22
Eumetabola - ps23
Holometabola/Psocodea - ps24
Holometabola - ps25
Aparaglossata - ps26
Mecopterida - ps27
Diptera - ps28
Bibiono./Cyclorrhapha - ps29
Cyclorrhapha - ps30
Calyptratae/Drosophila - ps31
Drosophila - ps32
Sophophora - ps33
Obscura/Melanogaster_group - ps34
D_melanogaster_group - ps35
D_melanogaster_subgroup - ps36
sim./sech./mel. group - ps37
Drosophila_melanogaster - ps38
p-value
1e-04
6e-04
3e-03
1e-02
6e-02
Log-odds
0.5
1.0
1.5
2.0
2.5
3.0
3.5
4.0

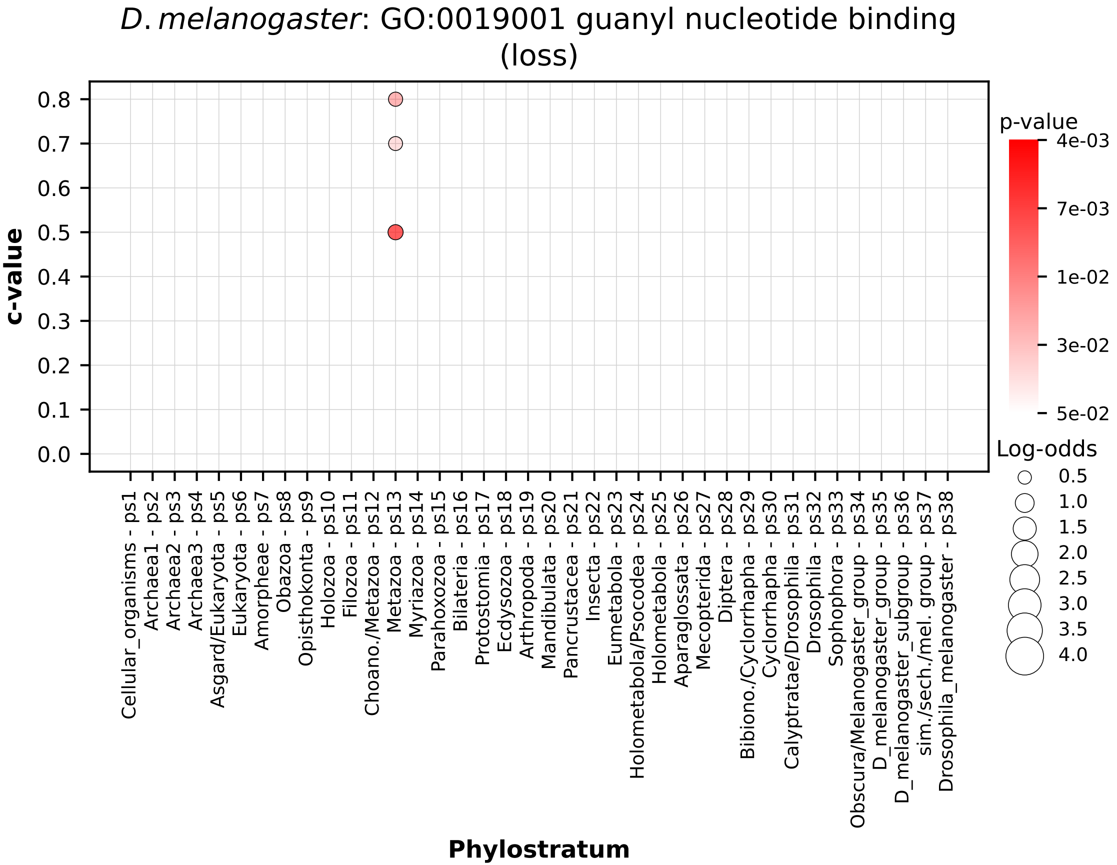

D. melanogaster: GO:0019001 guanyl nucleotide binding (loss)
c-value
0.8
0.7
0.6
0.5
0.4
0.3
0.2
0.1
0.0
p-value
4e-03
7e-03
1e-02
3e-02
5e-02
Log-odds
0.5
1.0
1.5
2.0
2.5
3.0
3.5
4.0
Cellular_organisms - ps1
Archaea1 - ps2
Archaea2 - ps3
Archaea3 - ps4
Asgard/Eukaryota - ps5
Eukaryota - ps6
Amorpheae - ps7
Obazoa - ps8
Opisthokonta - ps9
Holozoa - ps10
Filozoa - ps11
Choano./Metazoa - ps12
Metazoa - ps13
Myriazoa - ps14
Parahoxozoa - ps15
Bilateria - ps16
Protostomia - ps17
Ecdysozoa - ps18
Arthropoda - ps19
Mandibulata - ps20
Pancrustacea - ps21
Insecta - ps22
Eumetabola - ps23
Holometabola/Psocodea - ps24
Holometabola - ps25
Aparaglossata - ps26
Mecopterida - ps27
Diptera - ps28
Bibiono./Cyclorrhapha - ps29
Cyclorrhapha - ps30
Calyptratae/Drosophila - ps31
Drosophila - ps32
Sophophora - ps33
Obscura/Melanogaster_group - ps34
D_melanogaster_group - ps35
D_melanogaster_subgroup - ps36
sim./sech./mel. group - ps37
Drosophila_melanogaster - ps38
Phylostratum

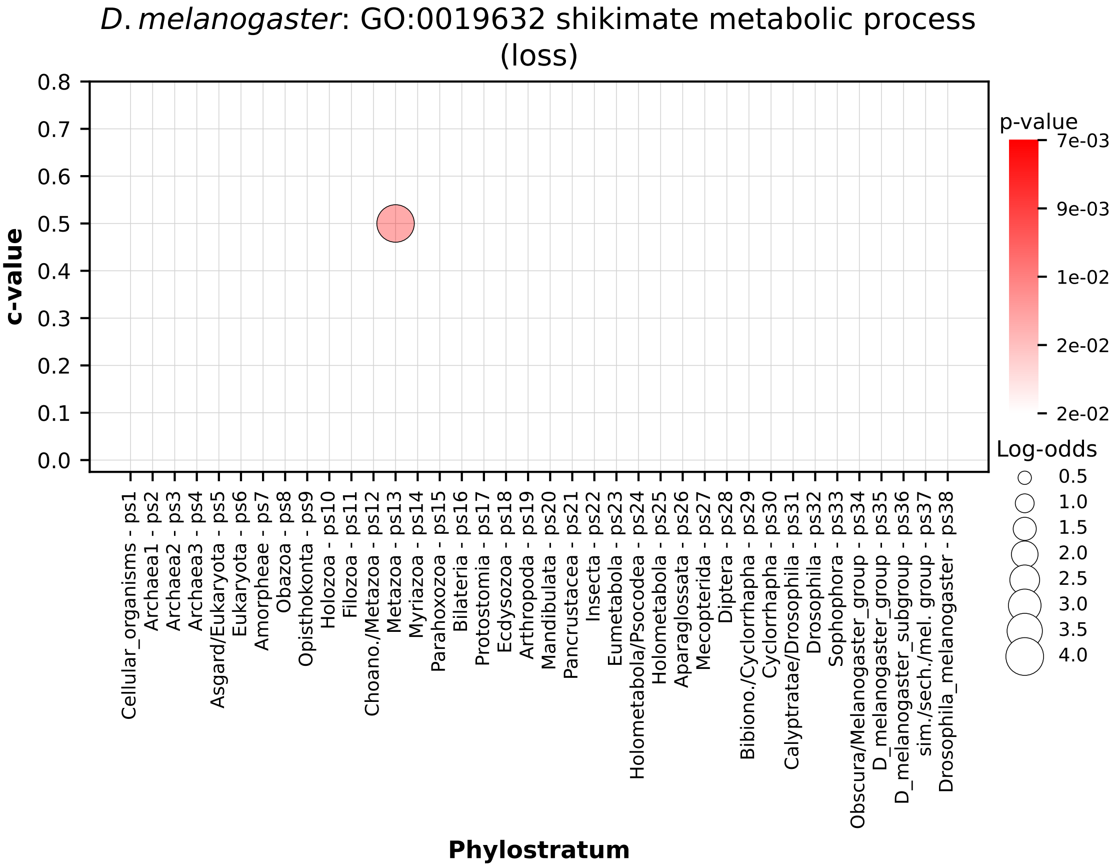
D. melanogaster: GO:0019632 shikimate metabolic process
(loss)
c-value
0.8
0.7
0.6
0.5
0.4
0.3
0.2
0.1
0.0
p-value
7e-03
9e-03
1e-02
2e-02
2e-02
Log-odds
0.5
1.0
1.5
2.0
2.5
3.0
3.5
4.0
Cellular_organisms - ps1
Archaea1 - ps2
Archaea2 - ps3
Archaea3 - ps4
Asgard/Eukaryota - ps5
Eukaryota - ps6
Amorpheae - ps7
Obazoa - ps8
Opisthokonta - ps9
Holozoa - ps10
Filozoa - ps11
Choano./Metazoa - ps12
Metazoa - ps13
Myriazoa - ps14
Parahoxozoa - ps15
Bilateria - ps16
Protostomia - ps17
Ecdysozoa - ps18
Arthropoda - ps19
Mandibulata - ps20
Pancrustacea - ps21
Insecta - ps22
Eumetabola - ps23
Holometabola/Psocodea - ps24
Holometabola - ps25
Aparaglossata - ps26
Mecopterida - ps27
Diptera - ps28
Bibiono./Cyclorrhapha - ps29
Cyclorrhapha - ps30
Calyptratae/Drosophila - ps31
Drosophila - ps32
Sophophora - ps33
Obscura/Melanogaster_group - ps34
D_melanogaster_group - ps35
D_melanogaster_subgroup - ps36
sim./sech./mel. group - ps37
Drosophila_melanogaster - ps38
Phylostratum

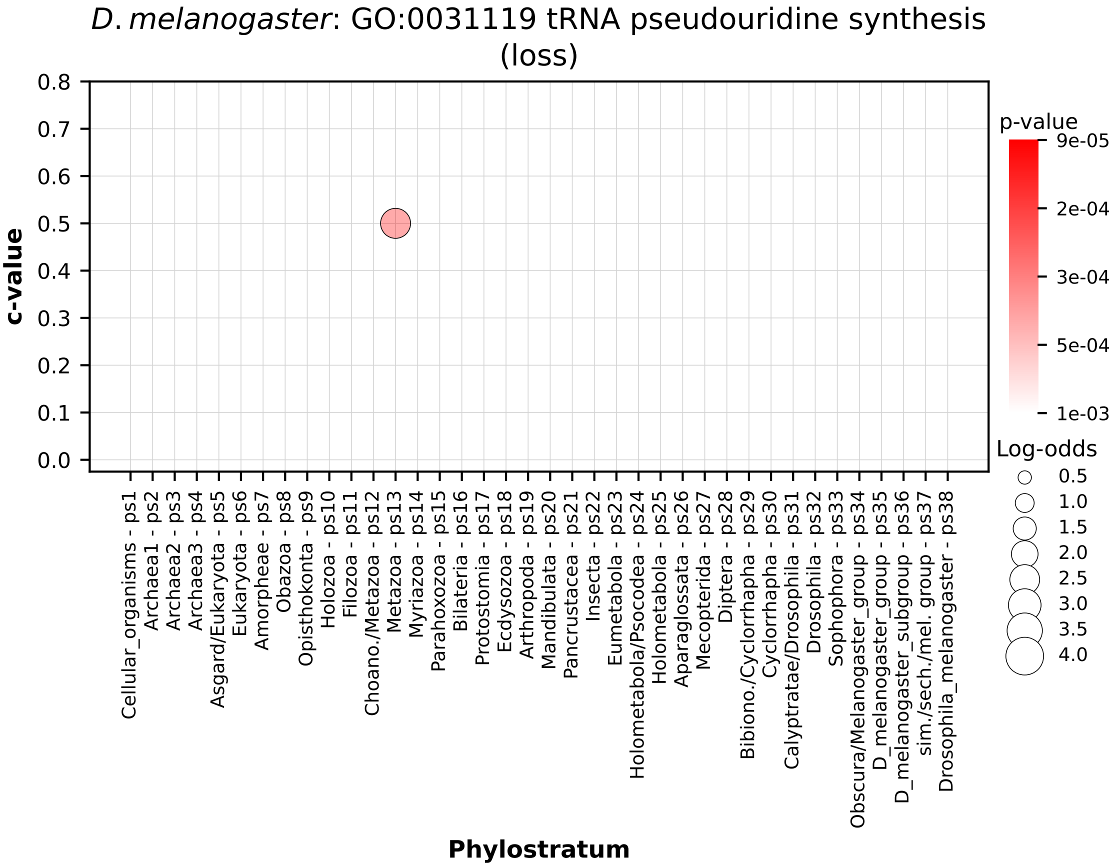

D. melanogaster: GO:0031119 tRNA pseudouridine synthesis (loss)
c-value
0.0
0.1
0.2
0.3
0.4
0.5
0.6
0.7
0.8
Phylostratum
Cellular_organisms - ps1
Archaea1 - ps2
Archaea2 - ps3
Archaea3 - ps4
Asgard/Eukaryota - ps5
Eukaryota - ps6
Amorpheae - ps7
Obazoa - ps8
Opisthokonta - ps9
Holozoa - ps10
Filozoa - ps11
Choano./Metazoa - ps12
Metazoa - ps13
Myriazoa - ps14
Parahoxozoa - ps15
Bilateria - ps16
Protostomia - ps17
Ecdysozoa - ps18
Arthropoda - ps19
Mandibulata - ps20
Pancrustacea - ps21
Insecta - ps22
Eumetabola - ps23
Holometabola/Psocodea - ps24
Holometabola - ps25
Aparaglossata - ps26
Mecopterida - ps27
Diptera - ps28
Bibiono./Cyclorrhapha - ps29
Cyclorrhapha - ps30
Calyptratae/Drosophila - ps31
Drosophila - ps32
Sophophora - ps33
Obscura/Melanogaster_group - ps34
D_melanogaster_group - ps35
D_melanogaster_subgroup - ps36
sim./sech./mel. group - ps37
Drosophila_melanogaster - ps38
p-value
9e-05
2e-04
3e-04
5e-04
1e-03
Log-odds
0.5
1.0
1.5
2.0
2.5
3.0
3.5
4.0

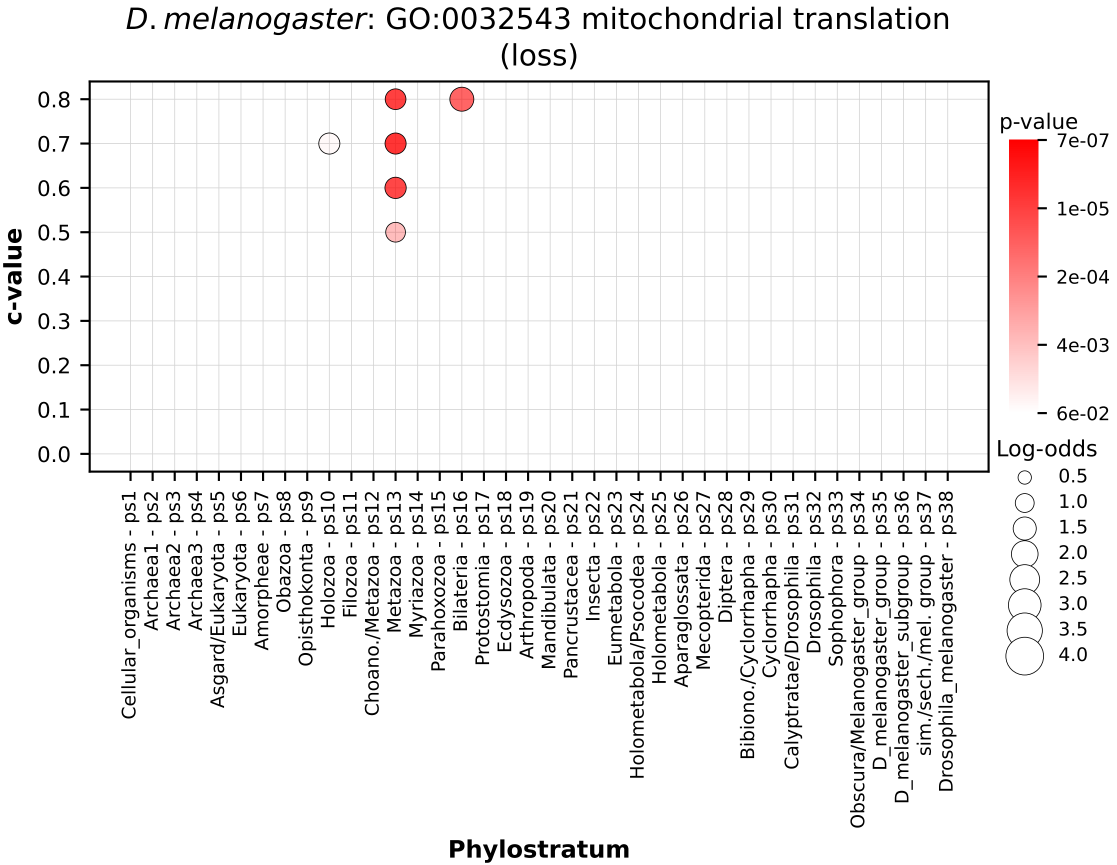

D. melanogaster: GO:0032543 mitochondrial translation (loss)
c-value
0.8
0.7
0.6
0.5
0.4
0.3
0.2
0.1
0.0
p-value
7e-07
1e-05
2e-04
4e-03
6e-02
Log-odds
0.5
1.0
1.5
2.0
2.5
3.0
3.5
4.0
Cellular_organisms - ps1
Archaea1 - ps2
Archaea2 - ps3
Archaea3 - ps4
Asgard/Eukaryota - ps5
Eukaryota - ps6
Amorpheae - ps7
Obazoa - ps8
Opisthokonta - ps9
Holozoa - ps10
Filozoa - ps11
Choano./Metazoa - ps12
Metazoa - ps13
Myriazoa - ps14
Parahoxozoa - ps15
Bilateria - ps16
Protostomia - ps17
Ecdysozoa - ps18
Arthropoda - ps19
Mandibulata - ps20
Pancrustacea - ps21
Insecta - ps22
Eumetabola - ps23
Holometabola/Psocodea - ps24
Holometabola - ps25
Aparaglossata - ps26
Mecopterida - ps27
Diptera - ps28
Bibiono./Cyclorrhapha - ps29
Cyclorrhapha - ps30
Calyptratae/Drosophila - ps31
Drosophila - ps32
Sophophora - ps33
Obscura/Melanogaster_group - ps34
D_melanogaster_group - ps35
D_melanogaster_subgroup - ps36
sim./sech./mel. group - ps37
Drosophila_melanogaster - ps38
Phylostratum

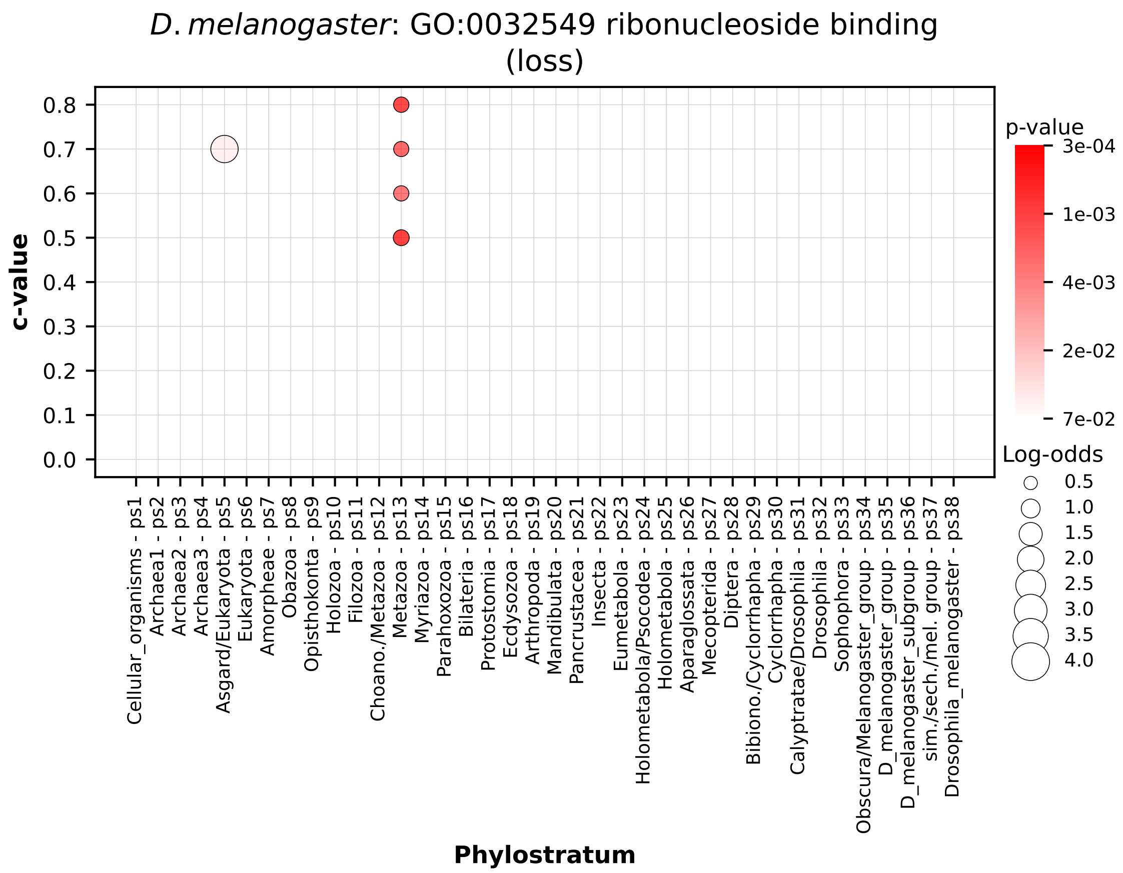

D. melanogaster: GO:0032549 ribonucleoside binding
(loss)
c-value
0.0
0.1
0.2
0.3
0.4
0.5
0.6
0.7
0.8
p-value
3e-04
1e-03
4e-03
2e-02
7e-02
Log-odds
0.5
1.0
1.5
2.0
2.5
3.0
3.5
4.0
Cellular_organisms - ps1
Archaea1 - ps2
Archaea2 - ps3
Archaea3 - ps4
Asgard/Eukaryota - ps5
Eukaryota - ps6
Amorpheae - ps7
Obazoa - ps8
Opisthokonta - ps9
Holozoa - ps10
Filozoa - ps11
Choano./Metazoa - ps12
Metazoa - ps13
Myriazoa - ps14
Parahoxozoa - ps15
Bilateria - ps16
Protostomia - ps17
Ecdysozoa - ps18
Arthropoda - ps19
Mandibulata - ps20
Pancrustacea - ps21
Insecta - ps22
Eumetabola - ps23
Holometabola/Psocodea - ps24
Holometabola - ps25
Aparaglossata - ps26
Mecopterida - ps27
Diptera - ps28
Bibiono./Cyclorrhapha - ps29
Cyclorrhapha - ps30
Calyptratae/Drosophila - ps31
Drosophila - ps32
Sophophora - ps33
Obscura/Melanogaster_group - ps34
D_melanogaster_group - ps35
D_melanogaster_subgroup - ps36
sim./sech./mel. group - ps37
Drosophila_melanogaster - ps38
Phylostratum

D. melanogaster: GO:0032550 purine ribonucleoside binding
(loss)

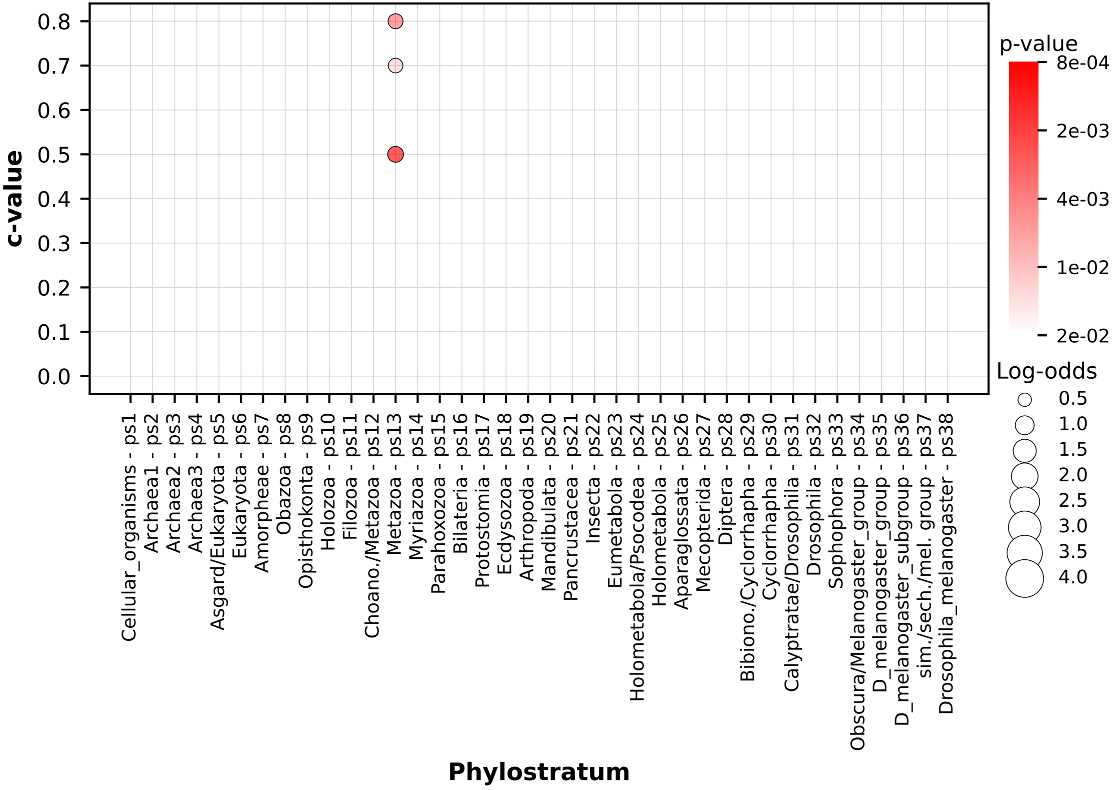

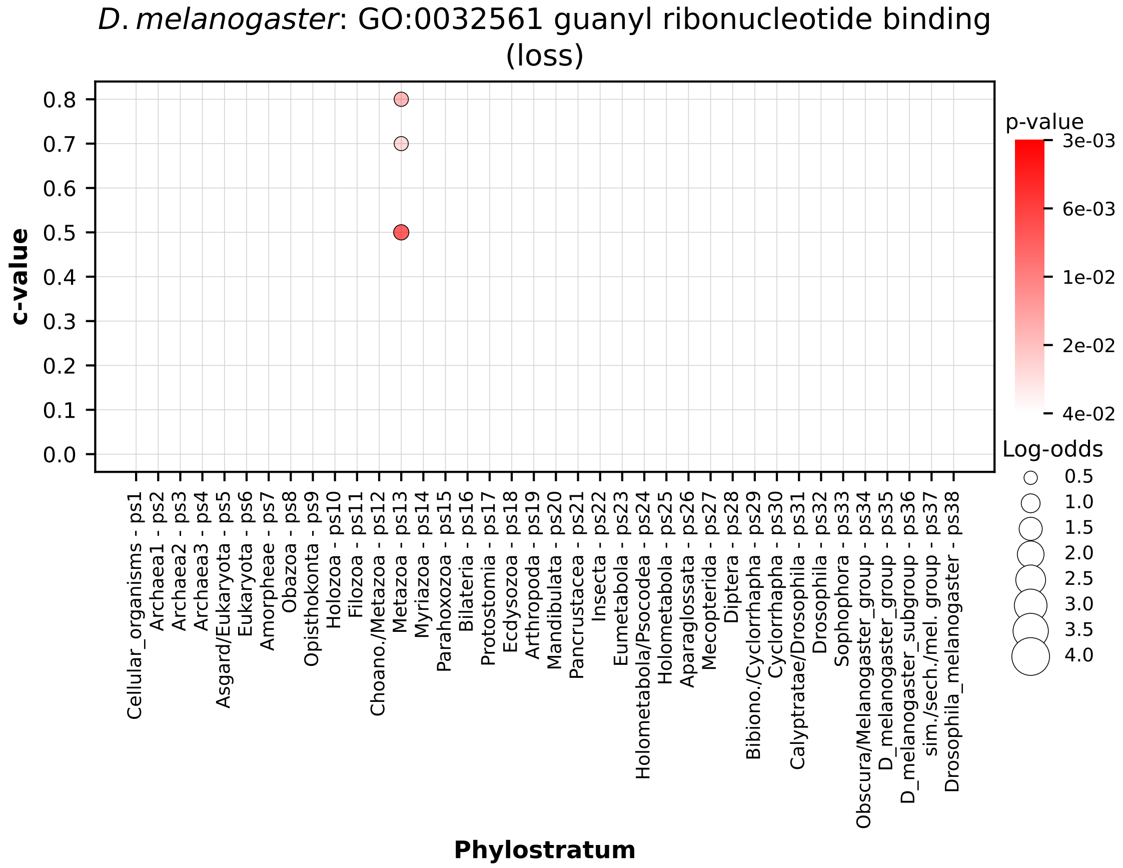

D. melanogaster: GO:0032561 guanyl ribonucleotide binding
(loss)
c-value
0.8
0.7
0.6
0.5
0.4
0.3
0.2
0.1
0.0
Phylostratum
Cellular_organisms - ps1
Archaea1 - ps2
Archaea2 - ps3
Archaea3 - ps4
Asgard/Eukaryota - ps5
Eukaryota - ps6
Amorpheae - ps7
Obazoa - ps8
Opisthokonta - ps9
Holozoa - ps10
Filozoa - ps11
Choano./Metazoa - ps12
Metazoa - ps13
Myriazoa - ps14
Parahoxozoa - ps15
Bilateria - ps16
Protostomia - ps17
Ecdysozoa - ps18
Arthropoda - ps19
Mandibulata - ps20
Pancrustacea - ps21
Insecta - ps22
Eumetabola - ps23
Holometabola/Psocodea - ps24
Holometabola - ps25
Aparaglossata - ps26
Mecopterida - ps27
Diptera - ps28
Bibiono./Cyclorrhapha - ps29
Cyclorrhapha - ps30
Calyptratae/Drosophila - ps31
Drosophila - ps32
Sophophora - ps33
Obscura/Melanogaster_group - ps34
D_melanogaster_group - ps35
D_melanogaster_subgroup - ps36
sim./sech./mel. group - ps37
Drosophila_melanogaster - ps38
p-value
3e-03
6e-03
1e-02
2e-02
4e-02
Log-odds
0.5
1.0
1.5
2.0
2.5
3.0
3.5
4.0

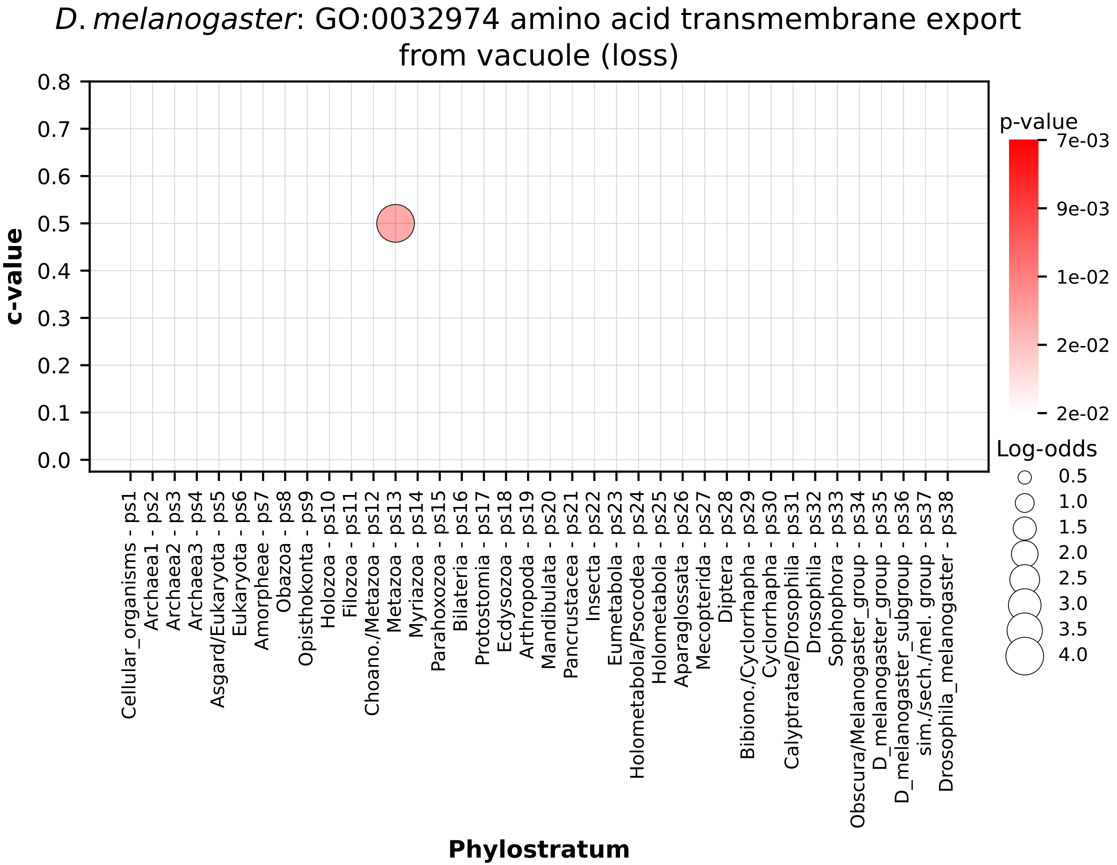
D. melanogaster: GO:0032974 amino acid transmembrane export from vacuole (loss)
c-value
0.8
0.7
0.6
0.5
0.4
0.3
0.2
0.1
0.0
Phylostratum
Cellular_organisms - ps1
Archaea1 - ps2
Archaea2 - ps3
Archaea3 - ps4
Asgard/Eukaryota - ps5
Eukaryota - ps6
Amorpheae - ps7
Obazoa - ps8
Opisthokonta - ps9
Holozoa - ps10
Filozoa - ps11
Choano./Metazoa - ps12
Metazoa - ps13
Myriazoa - ps14
Parahoxozoa - ps15
Bilateria - ps16
Protostomia - ps17
Ecdysozoa - ps18
Arthropoda - ps19
Mandibulata - ps20
Pancrustacea - ps21
Insecta - ps22
Eumetabola - ps23
Holometabola/Psocodea - ps24
Holometabola - ps25
Aparaglossata - ps26
Mecopterida - ps27
Diptera - ps28
Bibiono./Cyclorrhapha - ps29
Cyclorrhapha - ps30
Calyptratae/Drosophila - ps31
Drosophila - ps32
Sophophora - ps33
Obscura/Melanogaster_group - ps34
D_melanogaster_group - ps35
D_melanogaster_subgroup - ps36
sim./sech./mel. group - ps37
Drosophila_melanogaster - ps38
p-value
7e-03
9e-03
1e-02
2e-02
2e-02
Log-odds
0.5
1.0
1.5
2.0
2.5
3.0
3.5
4.0

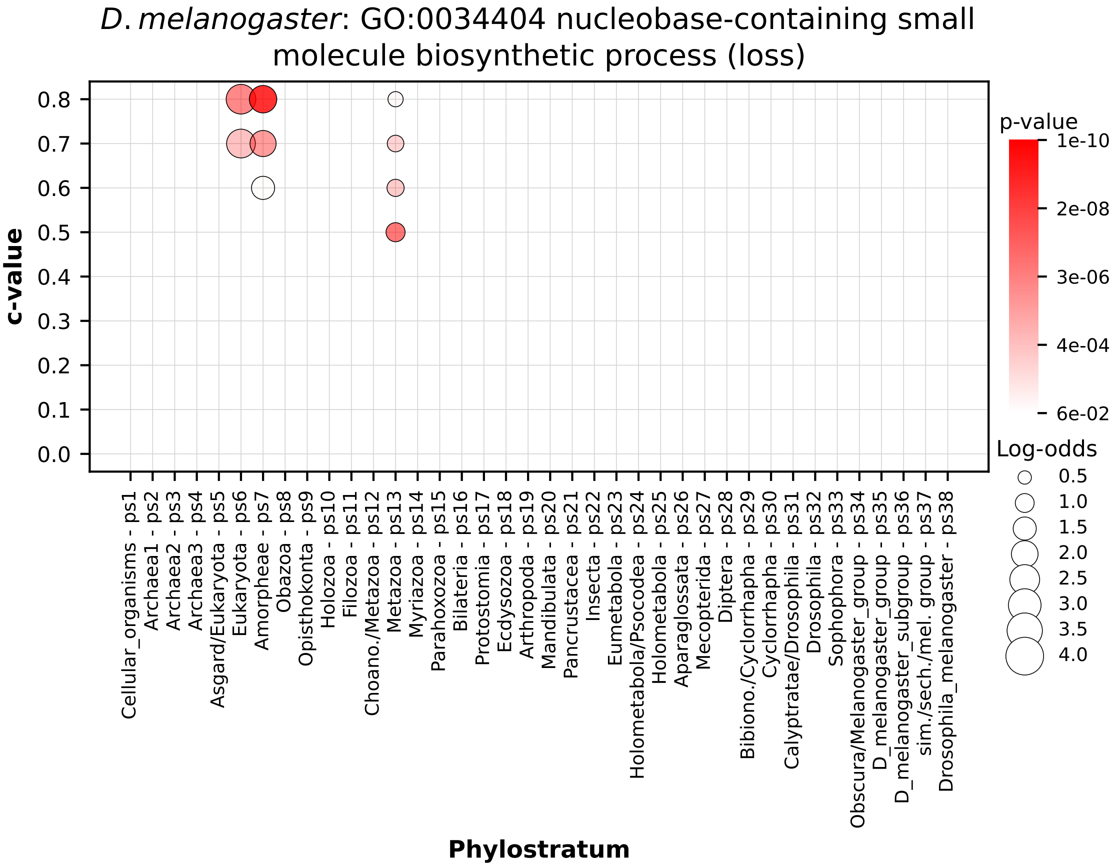

D. melanogaster: GO:0034404 nucleobase-containing small molecule biosynthetic process (loss)
c-value
0.8
0.7
0.6
0.5
0.4
0.3
0.2
0.1
0.0
p-value
1e-10
2e-08
3e-06
4e-04
6e-02
Log-odds
0.5
1.0
1.5
2.0
2.5
3.0
3.5
4.0
Cellular_organisms - ps1
Archaea1 - ps2
Archaea2 - ps3
Archaea3 - ps4
Asgard/Eukaryota - ps5
Eukaryota - ps6
Amorpheae - ps7
Obazoa - ps8
Opisthokonta - ps9
Holozoa - ps10
Filozoa - ps11
Choano./Metazoa - ps12
Metazoa - ps13
Myriazoa - ps14
Parahoxozoa - ps15
Bilateria - ps16
Protostomia - ps17
Ecdysozoa - ps18
Arthropoda - ps19
Mandibulata - ps20
Pancrustacea - ps21
Insecta - ps22
Eumetabola - ps23
Holometabola/Psocodea - ps24
Holometabola - ps25
Aparaglossata - ps26
Mecopterida - ps27
Diptera - ps28
Bibiono./Cyclorrhapha - ps29
Cyclorrhapha - ps30
Calyptratae/Drosophila - ps31
Drosophila - ps32
Sophophora - ps33
Obscura/Melanogaster_group - ps34
D_melanogaster_group - ps35
D_melanogaster_subgroup - ps36
sim./sech./mel. group - ps37
Drosophila_melanogaster - ps38
Phylostratum

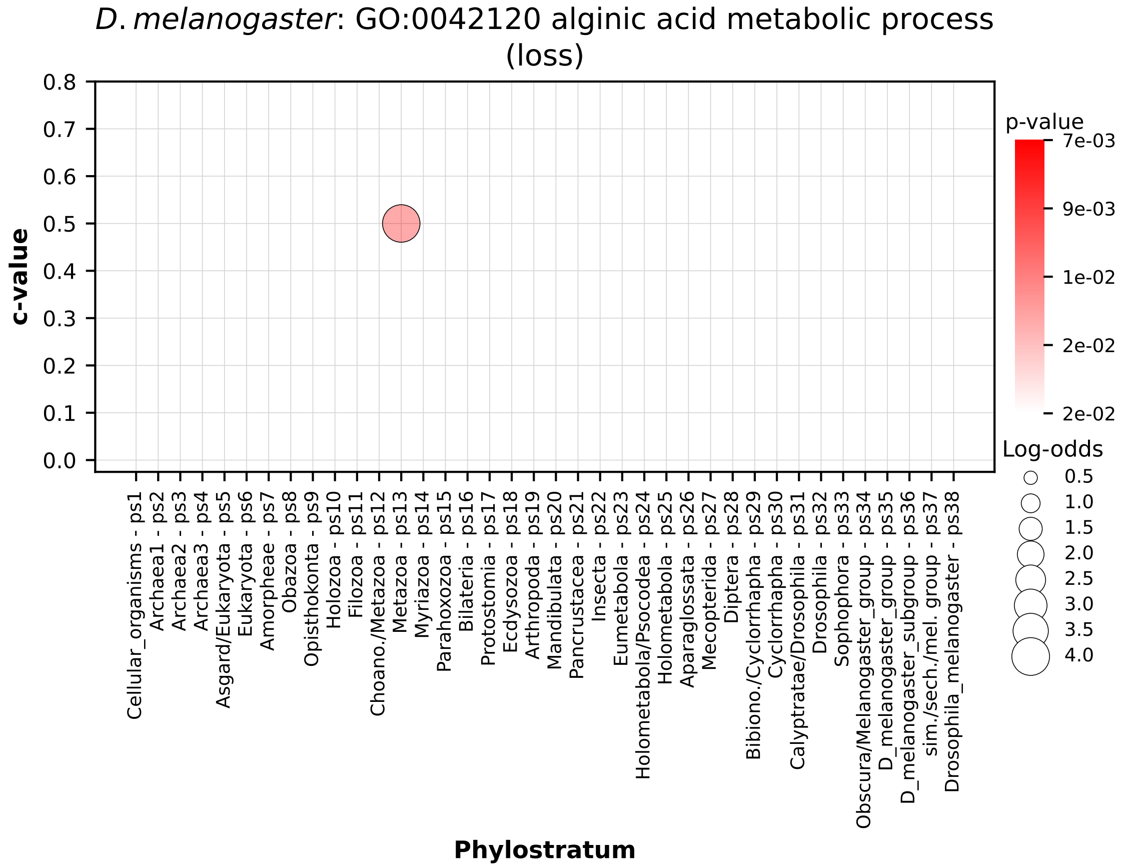

D. melanogaster: GO:0042120 alginic acid metabolic process
(loss)
c-value
0.0
0.1
0.2
0.3
0.4
0.5
0.6
0.7
0.8
Phylostratum
Cellular_organisms - ps1
Archaea1 - ps2
Archaea2 - ps3
Archaea3 - ps4
Asgard/Eukaryota - ps5
Eukaryota - ps6
Amorpheae - ps7
Obazoa - ps8
Opisthokonta - ps9
Holozoa - ps10
Filozoa - ps11
Choano./Metazoa - ps12
Metazoa - ps13
Myriazoa - ps14
Parahoxozoa - ps15
Bilateria - ps16
Protostomia - ps17
Ecdysozoa - ps18
Arthropoda - ps19
Mandibulata - ps20
Pancrustacea - ps21
Insecta - ps22
Eumetabola - ps23
Holometabola/Psocodea - ps24
Holometabola - ps25
Aparaglossata - ps26
Mecopterida - ps27
Diptera - ps28
Bibiono./Cyclorrhapha - ps29
Cyclorrhapha - ps30
Calyptratae/Drosophila - ps31
Drosophila - ps32
Sophophora - ps33
Obscura/Melanogaster_group - ps34
D_melanogaster_group - ps35
D_melanogaster_subgroup - ps36
sim./sech./mel. group - ps37
Drosophila_melanogaster - ps38
p-value
7e-03
9e-03
1e-02
2e-02
2e-02
Log-odds
0.5
1.0
1.5
2.0
2.5
3.0
3.5
4.0

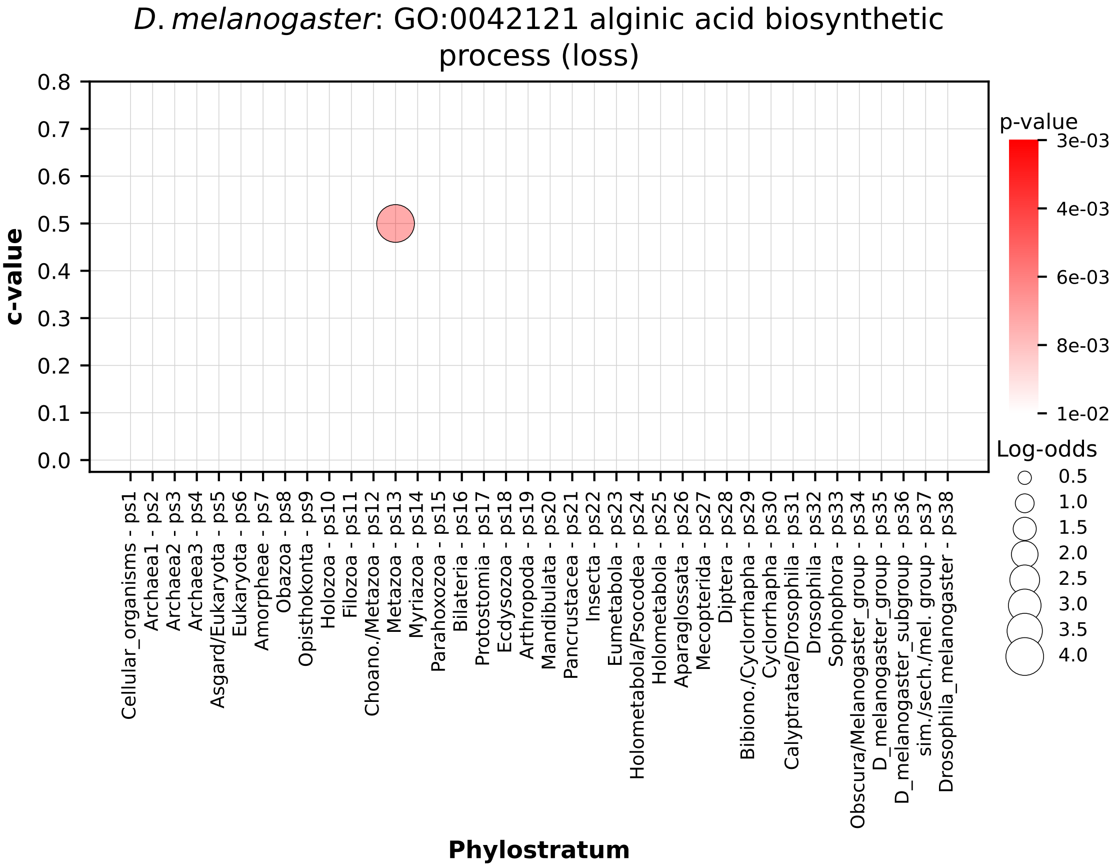

D. melanogaster: GO:0042121 alginic acid biosynthetic process (loss)
c-value
0.8
0.7
0.6
0.5
0.4
0.3
0.2
0.1
0.0
p-value
3e-03
4e-03
6e-03
8e-03
1e-02
Log-odds
0.5
1.0
1.5
2.0
2.5
3.0
3.5
4.0
Cellular_organisms - ps1
Archaea1 - ps2
Archaea2 - ps3
Archaea3 - ps4
Asgard/Eukaryota - ps5
Eukaryota - ps6
Amorpheae - ps7
Obazoa - ps8
Opisthokonta - ps9
Holozoa - ps10
Filozoa - ps11
Choano./Metazoa - ps12
Metazoa - ps13
Myriazoa - ps14
Parahoxozoa - ps15
Bilateria - ps16
Protostomia - ps17
Ecdysozoa - ps18
Arthropoda - ps19
Mandibulata - ps20
Pancrustacea - ps21
Insecta - ps22
Eumetabola - ps23
Holometabola/Psocodea - ps24
Holometabola - ps25
Aparaglossata - ps26
Mecopterida - ps27
Diptera - ps28
Bibiono./Cyclorrhapha - ps29
Cyclorrhapha - ps30
Calyptratae/Drosophila - ps31
Drosophila - ps32
Sophophora - ps33
Obscura/Melanogaster_group - ps34
D_melanogaster_group - ps35
D_melanogaster_subgroup - ps36
sim./sech./mel. group - ps37
Drosophila_melanogaster - ps38
Phylostratum

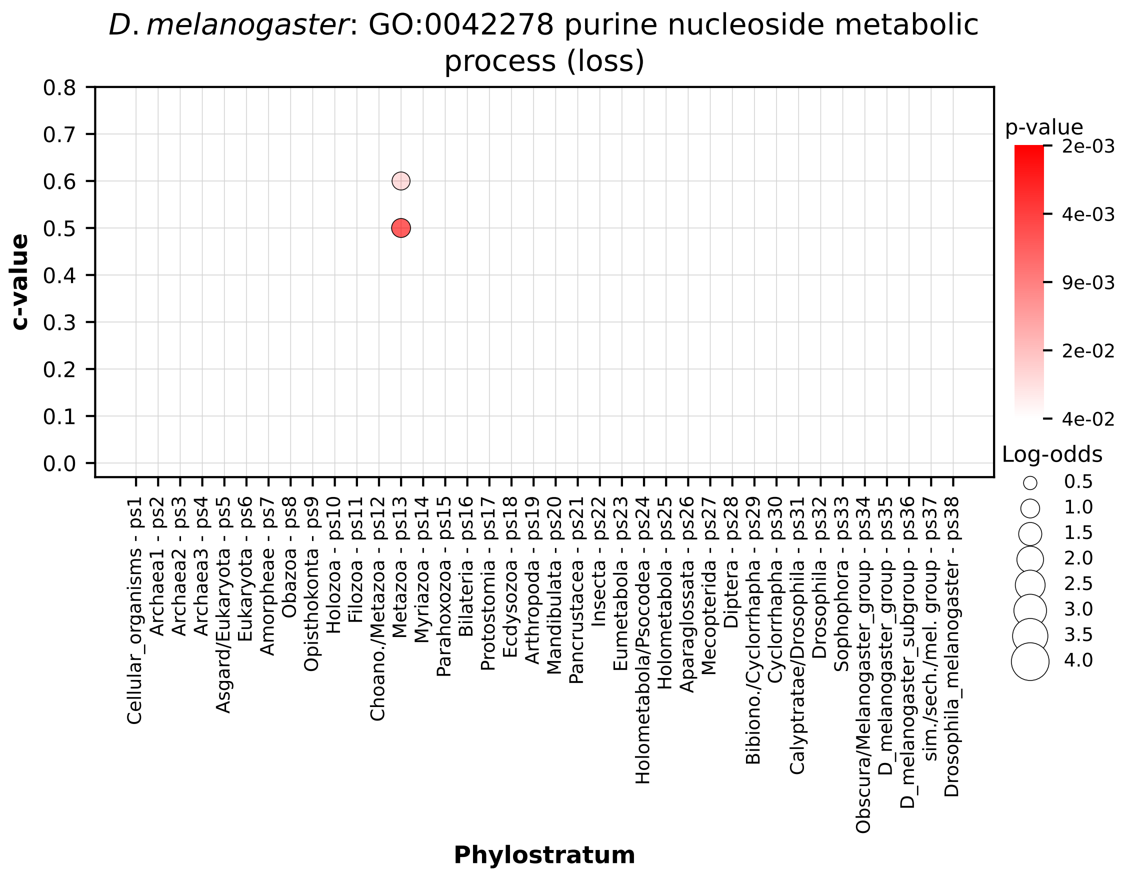
D. melanogaster: GO:0042278 purine nucleoside metabolic process (loss)
c-value
0.0
0.1
0.2
0.3
0.4
0.5
0.6
0.7
0.8
p-value
2e-03
4e-03
9e-03
2e-02
4e-02
Log-odds
0.5
1.0
1.5
2.0
2.5
3.0
3.5
4.0
Cellular_organisms - ps1
Archaea1 - ps2
Archaea2 - ps3
Archaea3 - ps4
Asgard/Eukaryota - ps5
Eukaryota - ps6
Amorpheae - ps7
Obazoa - ps8
Opisthokonta - ps9
Holozoa - ps10
Filozoa - ps11
Choano./Metazoa - ps12
Metazoa - ps13
Myriazoa - ps14
Parahoxozoa - ps15
Bilateria - ps16
Protostomia - ps17
Ecdysozoa - ps18
Arthropoda - ps19
Mandibulata - ps20
Pancrustacea - ps21
Insecta - ps22
Eumetabola - ps23
Holometabola/Psocodea - ps24
Holometabola - ps25
Aparaglossata - ps26
Mecopterida - ps27
Diptera - ps28
Bibiono./Cyclorrhapha - ps29
Cyclorrhapha - ps30
Calyptratae/Drosophila - ps31
Drosophila - ps32
Sophophora - ps33
Obscura/Melanogaster_group - ps34
D_melanogaster_group - ps35
D_melanogaster_subgroup - ps36
sim./sech./mel. group - ps37
Drosophila_melanogaster - ps38
Phylostratum

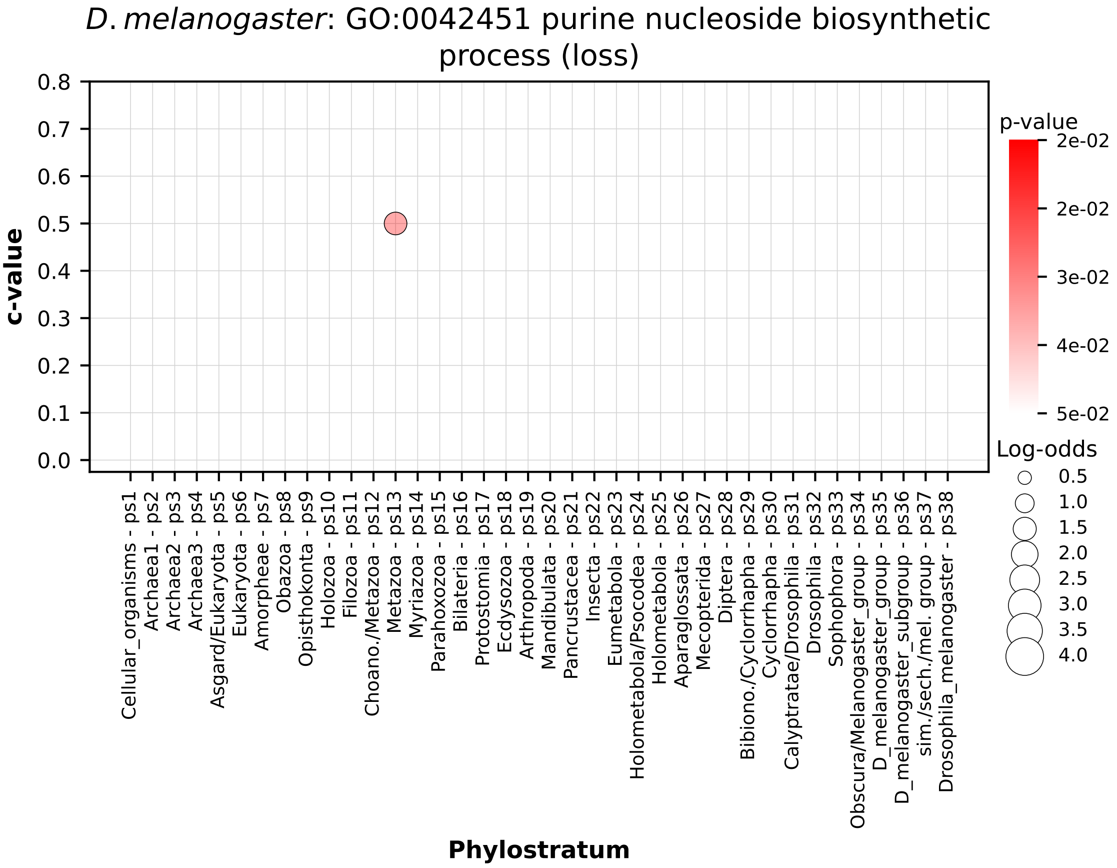

D. melanogaster: GO:0042451 purine nucleoside biosynthetic process (loss)
c-value
0.0
0.1
0.2
0.3
0.4
0.5
0.6
0.7
0.8
Phylostratum
Cellular_organisms - ps1
Archaea1 - ps2
Archaea2 - ps3
Archaea3 - ps4
Asgard/Eukaryota - ps5
Eukaryota - ps6
Amorpheae - ps7
Obazoa - ps8
Opisthokonta - ps9
Holozoa - ps10
Filozoa - ps11
Choano./Metazoa - ps12
Metazoa - ps13
Myriazoa - ps14
Parahoxozoa - ps15
Bilateria - ps16
Protostomia - ps17
Ecdysozoa - ps18
Arthropoda - ps19
Mandibulata - ps20
Pancrustacea - ps21
Insecta - ps22
Eumetabola - ps23
Holometabola/Psocodea - ps24
Holometabola - ps25
Aparaglossata - ps26
Mecopterida - ps27
Diptera - ps28
Bibiono./Cyclorrhapha - ps29
Cyclorrhapha - ps30
Calyptratae/Drosophila - ps31
Drosophila - ps32
Sophophora - ps33
Obscura/Melanogaster_group - ps34
D_melanogaster_group - ps35
D_melanogaster_subgroup - ps36
sim./sech./mel. group - ps37
Drosophila_melanogaster - ps38
p-value
2e-02
2e-02
3e-02
4e-02
5e-02
Log-odds
0.5
1.0
1.5
2.0
2.5
3.0
3.5
4.0

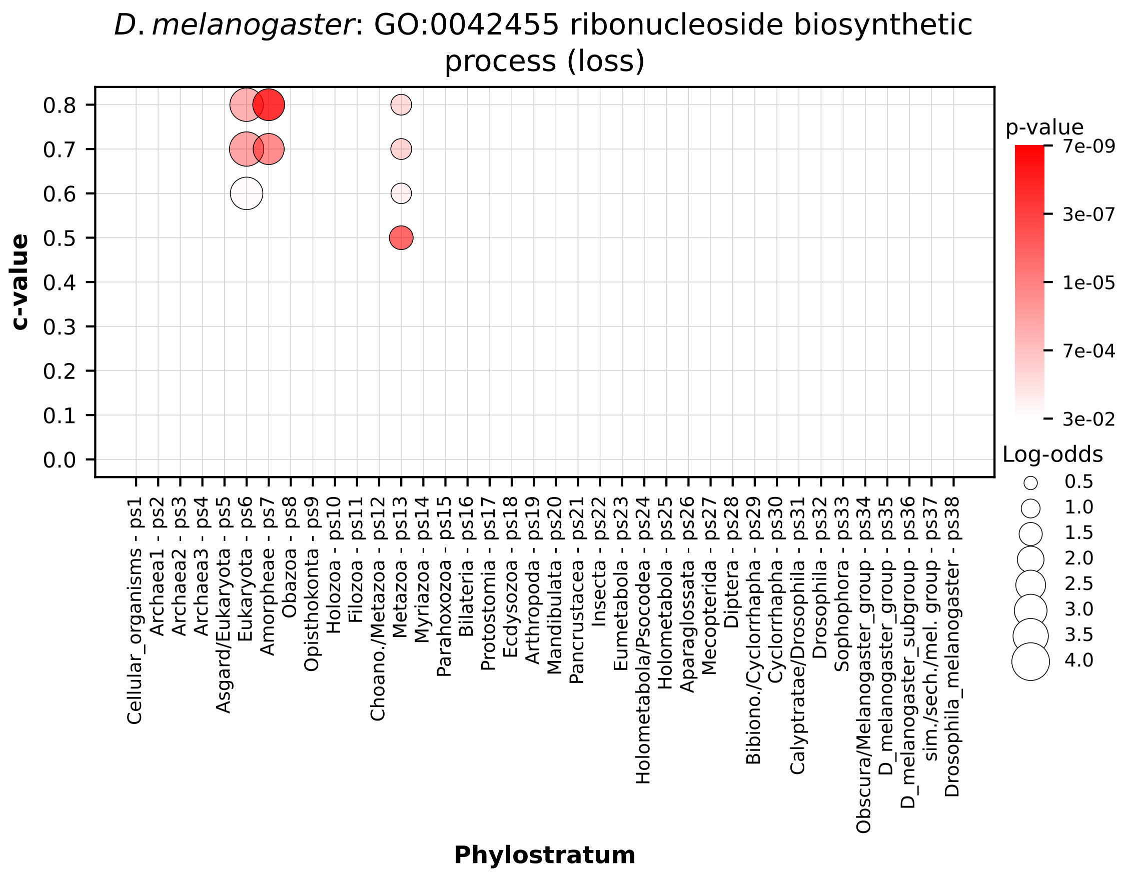

$D. melanogaster$: GO:0042455 ribonucleoside biosynthetic process (loss)
c-value
0.0
0.1
0.2
0.3
0.4
0.5
0.6
0.7
0.8
Phylostratum
Cellular_organisms - ps1
Archaea1 - ps2
Archaea2 - ps3
Archaea3 - ps4
Asgard/Eukaryota - ps5
Eukaryota - ps6
Amorpheae - ps7
Obazoa - ps8
Opisthokonta - ps9
Holozoa - ps10
Filozoa - ps11
Choano./Metazoa - ps12
Metazoa - ps13
Myriazoa - ps14
Parahoxozoa - ps15
Bilateria - ps16
Protostomia - ps17
Ecdysozoa - ps18
Arthropoda - ps19
Mandibulata - ps20
Pancrustacea - ps21
Insecta - ps22
Eumetabola - ps23
Holometabola/Psocodea - ps24
Holometabola - ps25
Aparaglossata - ps26
Mecopterida - ps27
Diptera - ps28
Bibiono./Cyclorrhapha - ps29
Cyclorrhapha - ps30
Calyptratae/Drosophila - ps31
Drosophila - ps32
Sophophora - ps33
Obscura/Melanogaster_group - ps34
D_melanogaster_group - ps35
D_melanogaster_subgroup - ps36
sim./sech./mel. group - ps37
Drosophila_melanogaster - ps38
p-value
7e-09
3e-07
1e-05
7e-04
3e-02
Log-odds
0.5
1.0
1.5
2.0
2.5
3.0
3.5
4.0

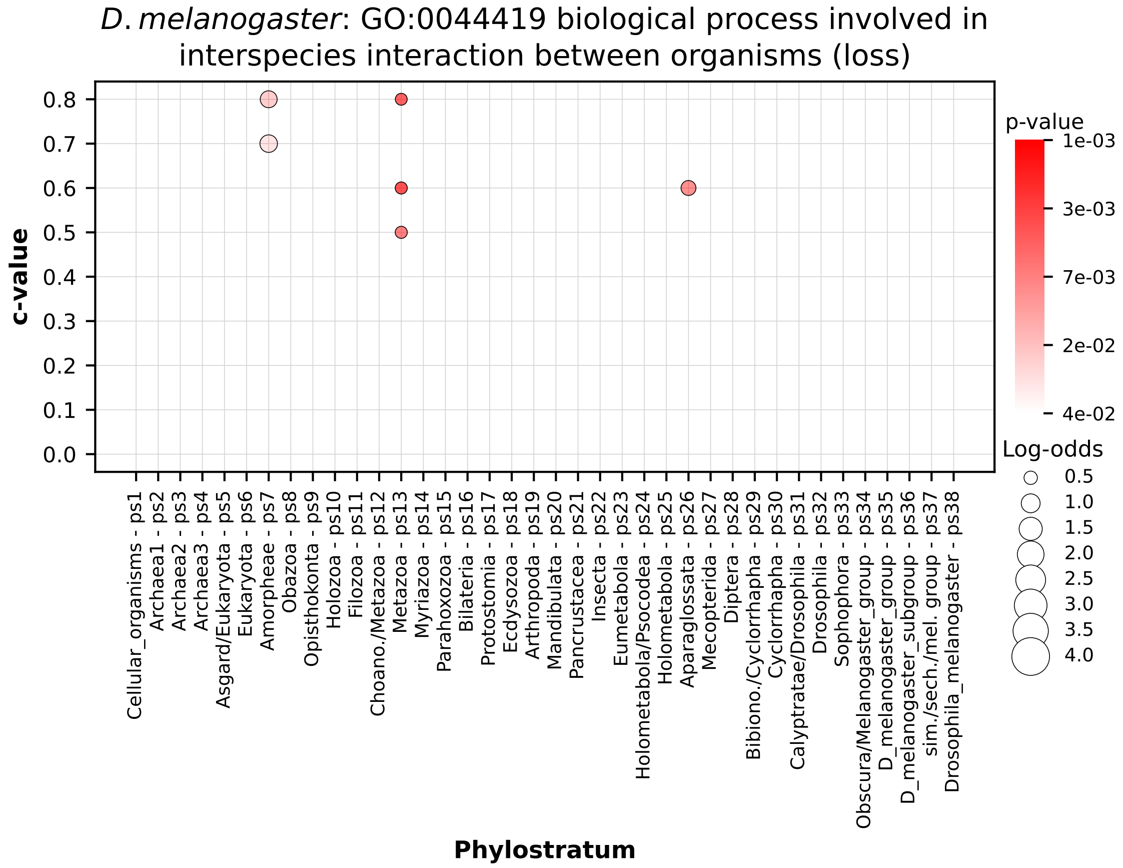
D. melanogaster: GO:0044419 biological process involved in interspecies interaction between organisms (loss)
c-value
0.8
0.7
0.6
0.5
0.4
0.3
0.2
0.1
0.0
p-value
1e-03
3e-03
7e-03
2e-02
4e-02
Log-odds
0.5
1.0
1.5
2.0
2.5
3.0
3.5
4.0
Cellular_organisms - ps1
Archaea1 - ps2
Archaea2 - ps3
Archaea3 - ps4
Asgard/Eukaryota - ps5
Eukaryota - ps6
Amorpheae - ps7
Obazoa - ps8
Opisthokonta - ps9
Holozoa - ps10
Filozoa - ps11
Choano./Metazoa - ps12
Metazoa - ps13
Myriazoa - ps14
Parahoxozoa - ps15
Bilateria - ps16
Protostomia - ps17
Ecdysozoa - ps18
Arthropoda - ps19
Mandibulata - ps20
Pancrustacea - ps21
Insecta - ps22
Eumetabola - ps23
Holometabola/Psocodea - ps24
Holometabola - ps25
Aparaglossata - ps26
Mecopterida - ps27
Diptera - ps28
Bibiono./Cyclorrhapha - ps29
Cyclorrhapha - ps30
Calyptratae/Drosophila - ps31
Drosophila - ps32
Sophophora - ps33
Obscura/Melanogaster_group - ps34
D_melanogaster_group - ps35
D_melanogaster_subgroup - ps36
sim./sech./mel. group - ps37
Drosophila_melanogaster - ps38
Phylostratum

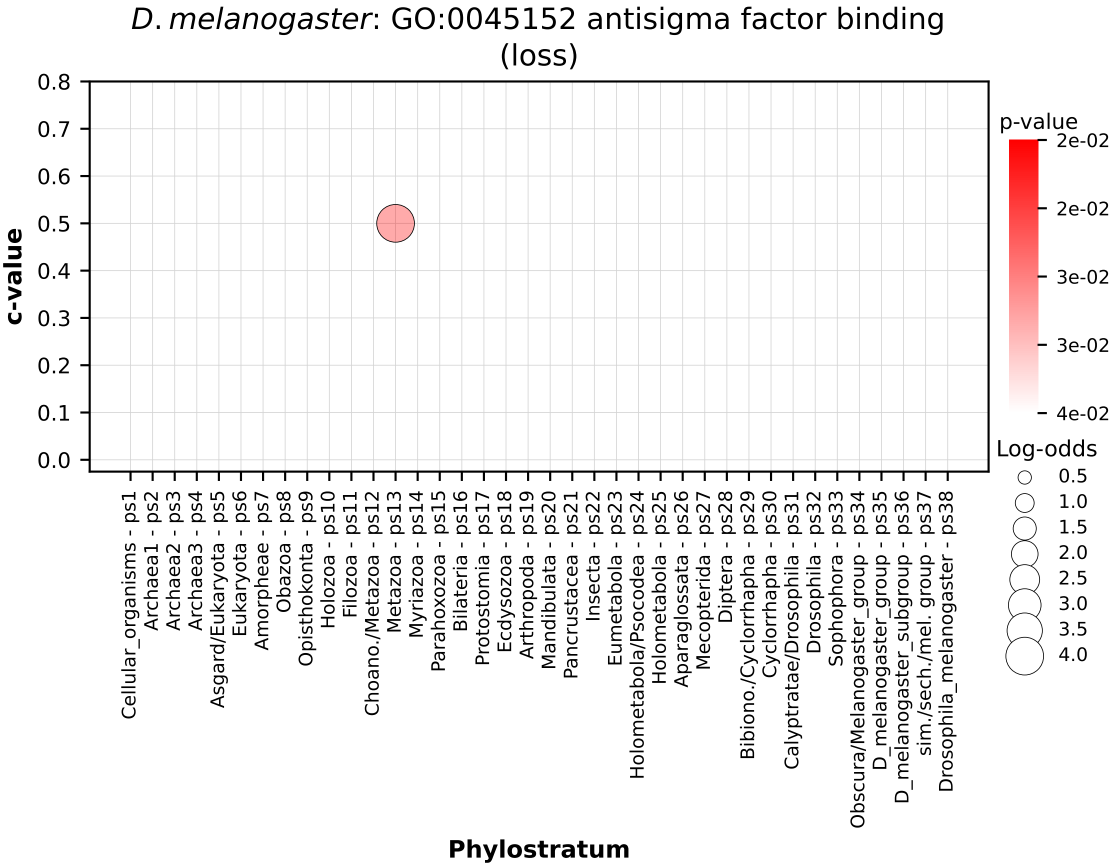
D. melanogaster: GO:0045152 antisigma factor binding
(loss)
c-value
0.8
0.7
0.6
0.5
0.4
0.3
0.2
0.1
0.0
Phylostratum
Cellular_organisms - ps1
Archaea1 - ps2
Archaea2 - ps3
Archaea3 - ps4
Asgard/Eukaryota - ps5
Eukaryota - ps6
Amorpheae - ps7
Obazoa - ps8
Opisthokonta - ps9
Holozoa - ps10
Filozoa - ps11
Choano./Metazoa - ps12
Metazoa - ps13
Myriazoa - ps14
Parahoxozoa - ps15
Bilateria - ps16
Protostomia - ps17
Ecdysozoa - ps18
Arthropoda - ps19
Mandibulata - ps20
Pancrustacea - ps21
Insecta - ps22
Eumetabola - ps23
Holometabola/Psocodea - ps24
Holometabola - ps25
Aparaglossata - ps26
Mecopterida - ps27
Diptera - ps28
Bibiono./Cyclorrhapha - ps29
Cyclorrhapha - ps30
Calyptratae/Drosophila - ps31
Drosophila - ps32
Sophophora - ps33
Obscura/Melanogaster_group - ps34
D_melanogaster_group - ps35
D_melanogaster_subgroup - ps36
sim./sech./mel. group - ps37
Drosophila_melanogaster - ps38
p-value
2e-02
2e-02
3e-02
3e-02
4e-02
Log-odds
0.5
1.0
1.5
2.0
2.5
3.0
3.5
4.0

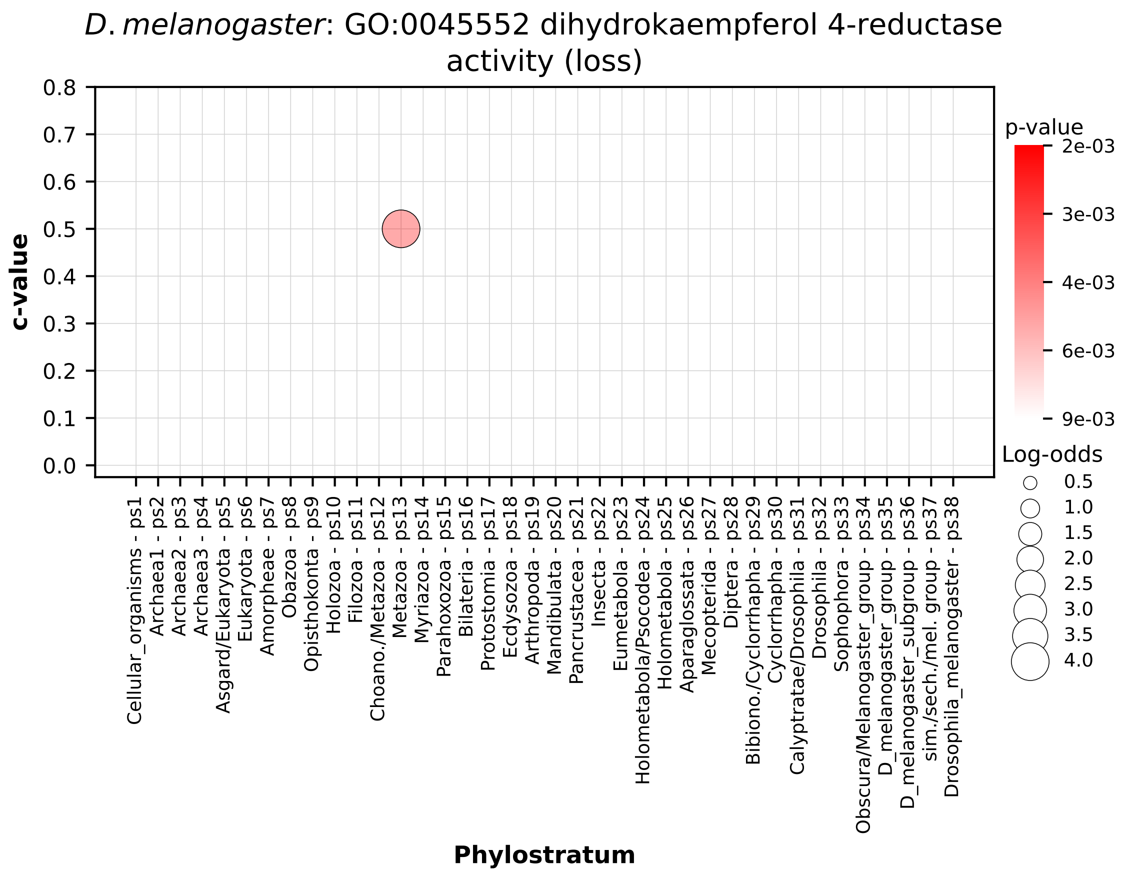

D. melanogaster: GO:0045552 dihydrokaempferol 4-reductase activity (loss)
c-value
0.0
0.1
0.2
0.3
0.4
0.5
0.6
0.7
0.8
Phylostratum
Cellular_organisms - ps1
Archaea1 - ps2
Archaea2 - ps3
Archaea3 - ps4
Asgard/Eukaryota - ps5
Eukaryota - ps6
Amorpheae - ps7
Obazoa - ps8
Opisthokonta - ps9
Holozoa - ps10
Filozoa - ps11
Choano./Metazoa - ps12
Metazoa - ps13
Myriazoa - ps14
Parahoxozoa - ps15
Bilateria - ps16
Protostomia - ps17
Ecdysozoa - ps18
Arthropoda - ps19
Mandibulata - ps20
Pancrustacea - ps21
Insecta - ps22
Eumetabola - ps23
Holometabola/Psocodea - ps24
Holometabola - ps25
Aparaglossata - ps26
Mecopterida - ps27
Diptera - ps28
Bibiono./Cyclorrhapha - ps29
Cyclorrhapha - ps30
Calyptratae/Drosophila - ps31
Drosophila - ps32
Sophophora - ps33
Obscura/Melanogaster_group - ps34
D_melanogaster_group - ps35
D_melanogaster_subgroup - ps36
sim./sech./mel. group - ps37
Drosophila_melanogaster - ps38
p-value
2e-03
3e-03
4e-03
6e-03
9e-03
Log-odds
0.5
1.0
1.5
2.0
2.5
3.0
3.5
4.0

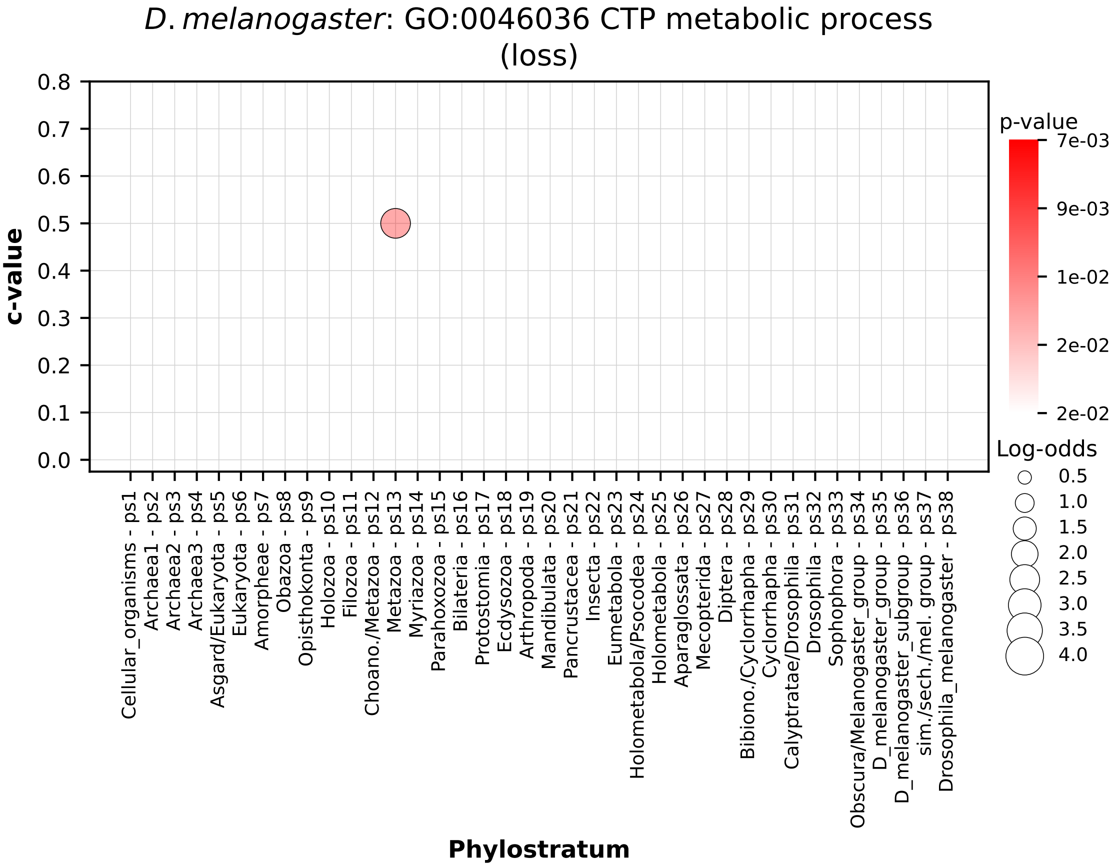

D. melanogaster: GO:0046036 CTP metabolic process
(loss)
c-value
0.0
0.1
0.2
0.3
0.4
0.5
0.6
0.7
0.8
p-value
7e-03
9e-03
1e-02
2e-02
2e-02
Log-odds
0.5
1.0
1.5
2.0
2.5
3.0
3.5
4.0
Cellular_organisms - ps1
Archaea1 - ps2
Archaea2 - ps3
Archaea3 - ps4
Asgard/Eukaryota - ps5
Eukaryota - ps6
Amorpheae - ps7
Obazoa - ps8
Opisthokonta - ps9
Holozoa - ps10
Filozoa - ps11
Choano./Metazoa - ps12
Metazoa - ps13
Myriazoa - ps14
Parahoxozoa - ps15
Bilateria - ps16
Protostomia - ps17
Ecdysozoa - ps18
Arthropoda - ps19
Mandibulata - ps20
Pancrustacea - ps21
Insecta - ps22
Eumetabola - ps23
Holometabola/Psocodea - ps24
Holometabola - ps25
Aparaglossata - ps26
Mecopterida - ps27
Diptera - ps28
Bibiono./Cyclorrhapha - ps29
Cyclorrhapha - ps30
Calyptratae/Drosophila - ps31
Drosophila - ps32
Sophophora - ps33
Obscura/Melanogaster_group - ps34
D_melanogaster_group - ps35
D_melanogaster_subgroup - ps36
sim./sech./mel. group - ps37
Drosophila_melanogaster - ps38
Phylostratum

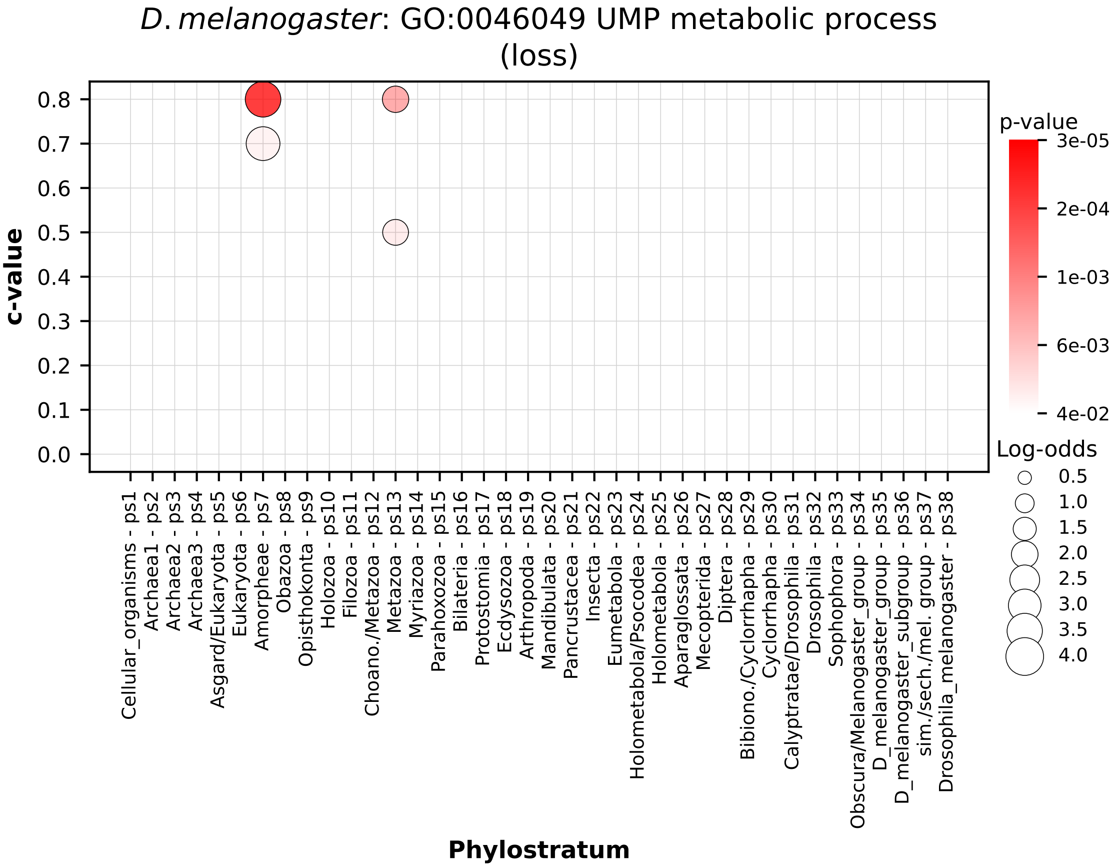

D. melanogaster: GO:0046049 UMP metabolic process
(loss)
c-value
0.0
0.1
0.2
0.3
0.4
0.5
0.6
0.7
0.8
Phylostratum
Cellular_organisms - ps1
Archaea1 - ps2
Archaea2 - ps3
Archaea3 - ps4
Asgard/Eukaryota - ps5
Eukaryota - ps6
Amorpheae - ps7
Obazoa - ps8
Opisthokonta - ps9
Holozoa - ps10
Filozoa - ps11
Choano./Metazoa - ps12
Metazoa - ps13
Myriazoa - ps14
Parahoxozoa - ps15
Bilateria - ps16
Protostomia - ps17
Ecdysozoa - ps18
Arthropoda - ps19
Mandibulata - ps20
Pancrustacea - ps21
Insecta - ps22
Eumetabola - ps23
Holometabola/Psocodea - ps24
Holometabola - ps25
Aparaglossata - ps26
Mecopterida - ps27
Diptera - ps28
Bibiono./Cyclorrhapha - ps29
Cyclorrhapha - ps30
Calyptratae/Drosophila - ps31
Drosophila - ps32
Sophophora - ps33
Obscura/Melanogaster_group - ps34
D_melanogaster_group - ps35
D_melanogaster_subgroup - ps36
sim./sech./mel. group - ps37
Drosophila_melanogaster - ps38
p-value
3e-05
2e-04
1e-03
6e-03
4e-02
Log-odds
0.5
1.0
1.5
2.0
2.5
3.0
3.5
4.0

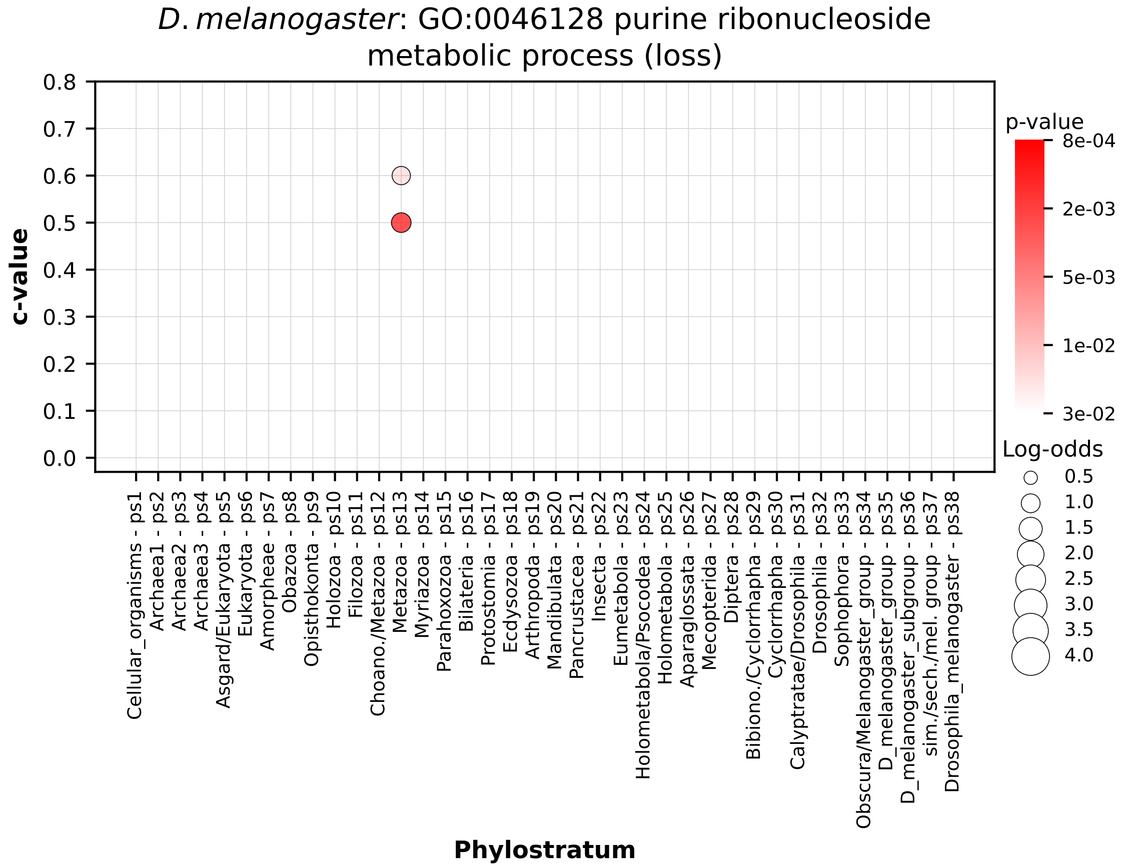

D. melanogaster: GO:0046128 purine ribonucleoside metabolic process (loss)
c-value
0.8
0.7
0.6
0.5
0.4
0.3
0.2
0.1
0.0
Phylostratum
Cellular_organisms - ps1
Archaea1 - ps2
Archaea2 - ps3
Archaea3 - ps4
Asgard/Eukaryota - ps5
Eukaryota - ps6
Amorpheae - ps7
Obazoa - ps8
Opisthokonta - ps9
Holozoa - ps10
Filozoa - ps11
Choano./Metazoa - ps12
Metazoa - ps13
Myriazoa - ps14
Parahoxozoa - ps15
Bilateria - ps16
Protostomia - ps17
Ecdysozoa - ps18
Arthropoda - ps19
Mandibulata - ps20
Pancrustacea - ps21
Insecta - ps22
Eumetabola - ps23
Holometabola/Psocodea - ps24
Holometabola - ps25
Aparaglossata - ps26
Mecopterida - ps27
Diptera - ps28
Bibiono./Cyclorrhapha - ps29
Cyclorrhapha - ps30
Calyptratae/Drosophila - ps31
Drosophila - ps32
Sophophora - ps33
Obscura/Melanogaster_group - ps34
D_melanogaster_group - ps35
D_melanogaster_subgroup - ps36
sim./sech./mel. group - ps37
Drosophila_melanogaster - ps38
p-value
8e-04
2e-03
5e-03
1e-02
3e-02
Log-odds
0.5
1.0
1.5
2.0
2.5
3.0
3.5
4.0

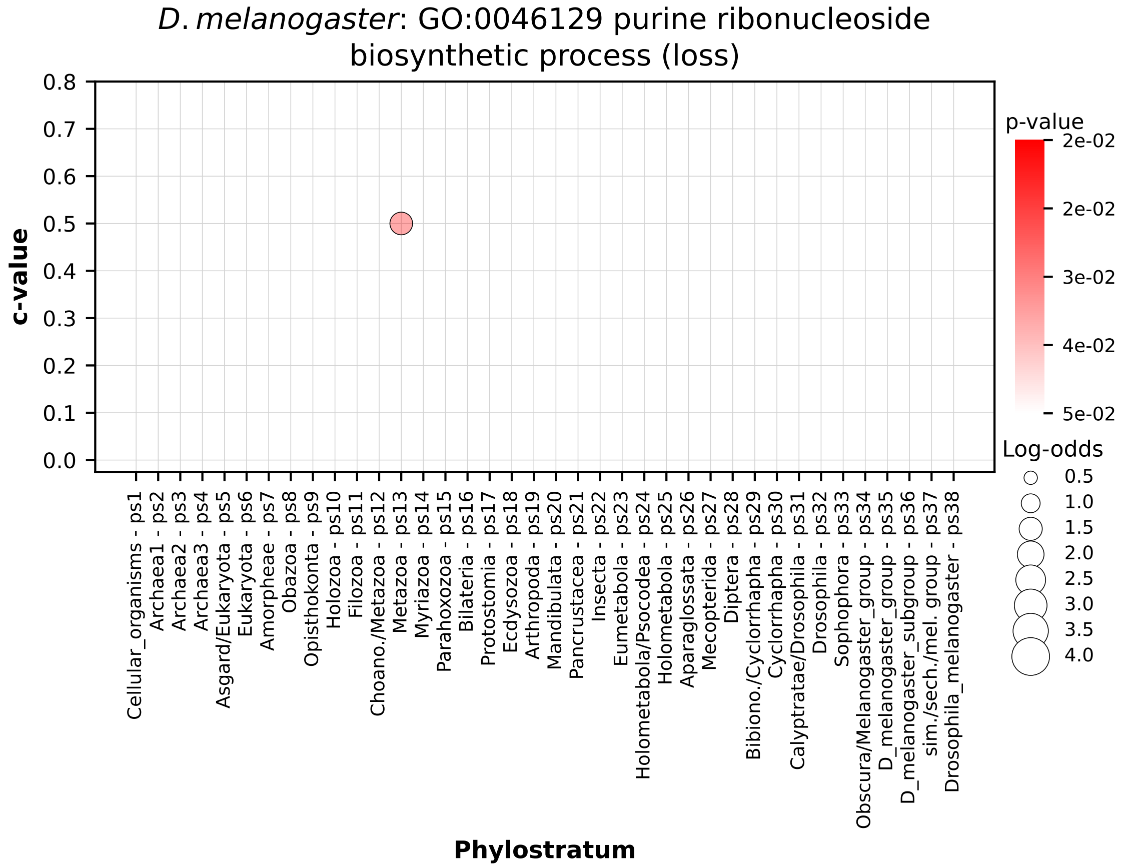

D. melanogaster: GO:0046129 purine ribonucleoside biosynthetic process (loss)
c-value
0.8
0.7
0.6
0.5
0.4
0.3
0.2
0.1
0.0
p-value
2e-02
2e-02
3e-02
4e-02
5e-02
Log-odds
0.5
1.0
1.5
2.0
2.5
3.0
3.5
4.0
Cellular_organisms - ps1
Archaea1 - ps2
Archaea2 - ps3
Archaea3 - ps4
Asgard/Eukaryota - ps5
Eukaryota - ps6
Amorpheae - ps7
Obazoa - ps8
Opisthokonta - ps9
Holozoa - ps10
Filozoa - ps11
Choano./Metazoa - ps12
Metazoa - ps13
Myriazoa - ps14
Parahoxozoa - ps15
Bilateria - ps16
Protostomia - ps17
Ecdysozoa - ps18
Arthropoda - ps19
Mandibulata - ps20
Pancrustacea - ps21
Insecta - ps22
Eumetabola - ps23
Holometabola/Psocodea - ps24
Holometabola - ps25
Aparaglossata - ps26
Mecopterida - ps27
Diptera - ps28
Bibiono./Cyclorrhapha - ps29
Cyclorrhapha - ps30
Calyptratae/Drosophila - ps31
Drosophila - ps32
Sophophora - ps33
Obscura/Melanogaster_group - ps34
D_melanogaster_group - ps35
D_melanogaster_subgroup - ps36
sim./sech./mel. group - ps37
Drosophila_melanogaster - ps38
Phylostratum

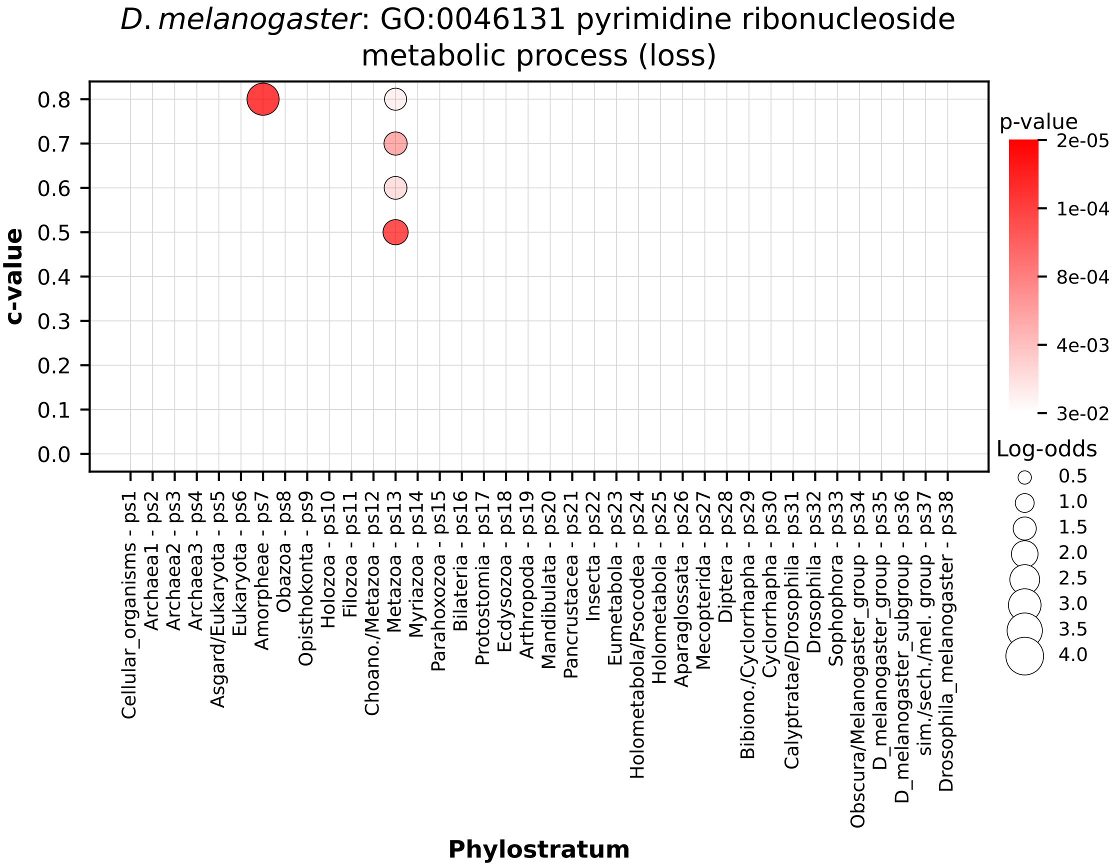

D. melanogaster: GO:0046131 pyrimidine ribonucleoside metabolic process (loss)
c-value
0.8
0.7
0.6
0.5
0.4
0.3
0.2
0.1
0.0
p-value
2e-05
1e-04
8e-04
4e-03
3e-02
Log-odds
0.5
1.0
1.5
2.0
2.5
3.0
3.5
4.0
Cellular_organisms - ps1
Archaea1 - ps2
Archaea2 - ps3
Archaea3 - ps4
Asgard/Eukaryota - ps5
Eukaryota - ps6
Amorpheae - ps7
Obazoa - ps8
Opisthokonta - ps9
Holozoa - ps10
Filozoa - ps11
Choano./Metazoa - ps12
Metazoa - ps13
Myriazoa - ps14
Parahoxozoa - ps15
Bilateria - ps16
Protostomia - ps17
Ecdysozoa - ps18
Arthropoda - ps19
Mandibulata - ps20
Pancrustacea - ps21
Insecta - ps22
Eumetabola - ps23
Holometabola/Psocodea - ps24
Holometabola - ps25
Aparaglossata - ps26
Mecopterida - ps27
Diptera - ps28
Bibiono./Cyclorrhapha - ps29
Cyclorrhapha - ps30
Calyptratae/Drosophila - ps31
Drosophila - ps32
Sophophora - ps33
Obscura/Melanogaster_group - ps34
D_melanogaster_group - ps35
D_melanogaster_subgroup - ps36
sim./sech./mel. group - ps37
Drosophila_melanogaster - ps38
Phylostratum

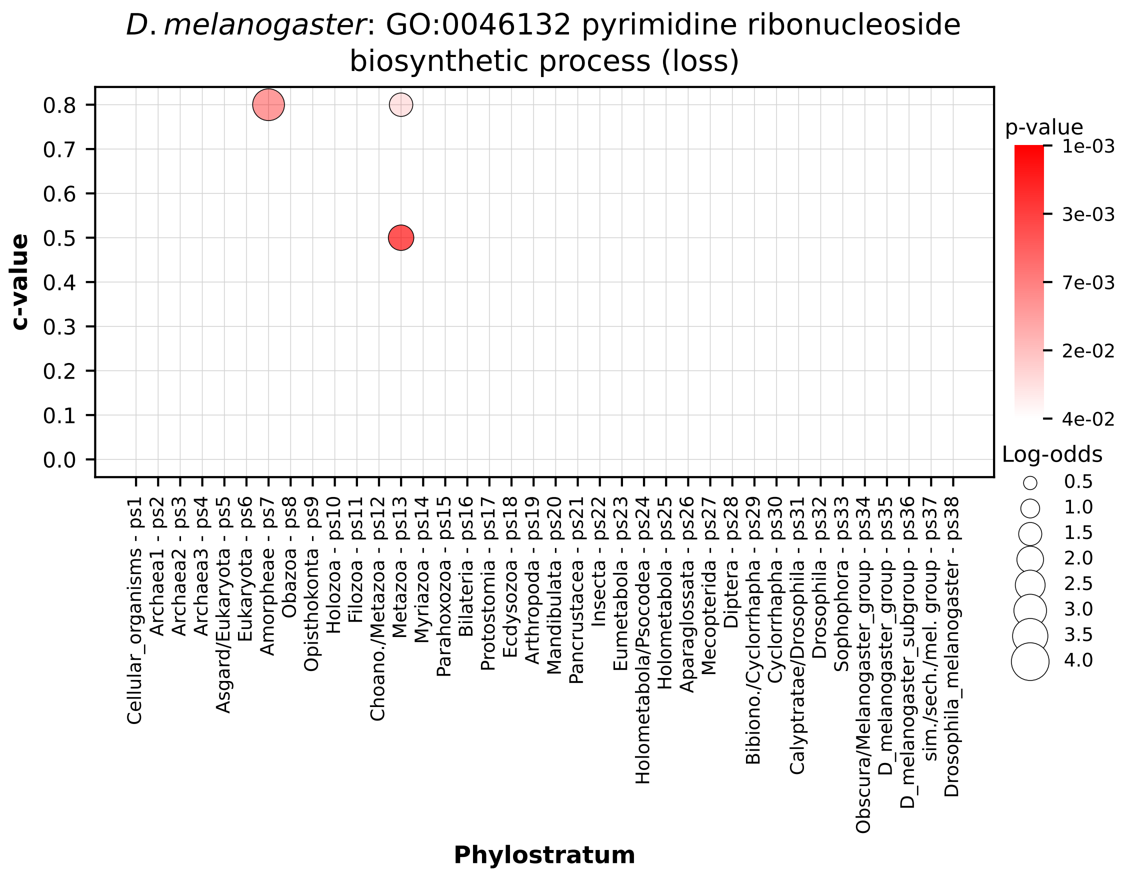

D. melanogaster: GO:0046132 pyrimidine ribonucleoside biosynthetic process (loss)
c-value
0.8
0.7
0.6
0.5
0.4
0.3
0.2
0.1
0.0
Phylostratum
Cellular_organisms - ps1
Archaea1 - ps2
Archaea2 - ps3
Archaea3 - ps4
Asgard/Eukaryota - ps5
Eukaryota - ps6
Amorpheae - ps7
Obazoa - ps8
Opisthokonta - ps9
Holozoa - ps10
Filozoa - ps11
Choano./Metazoa - ps12
Metazoa - ps13
Myriazoa - ps14
Parahoxozoa - ps15
Bilateria - ps16
Protostomia - ps17
Ecdysozoa - ps18
Arthropoda - ps19
Mandibulata - ps20
Pancrustacea - ps21
Insecta - ps22
Eumetabola - ps23
Holometabola/Psocodea - ps24
Holometabola - ps25
Aparaglossata - ps26
Mecopterida - ps27
Diptera - ps28
Bibiono./Cyclorrhapha - ps29
Cyclorrhapha - ps30
Calyptratae/Drosophila - ps31
Drosophila - ps32
Sophophora - ps33
Obscura/Melanogaster_group - ps34
D_melanogaster_group - ps35
D_melanogaster_subgroup - ps36
sim./sech./mel. group - ps37
Drosophila_melanogaster - ps38
p-value
1e-03
3e-03
7e-03
2e-02
4e-02
Log-odds
0.5
1.0
1.5
2.0
2.5
3.0
3.5
4.0

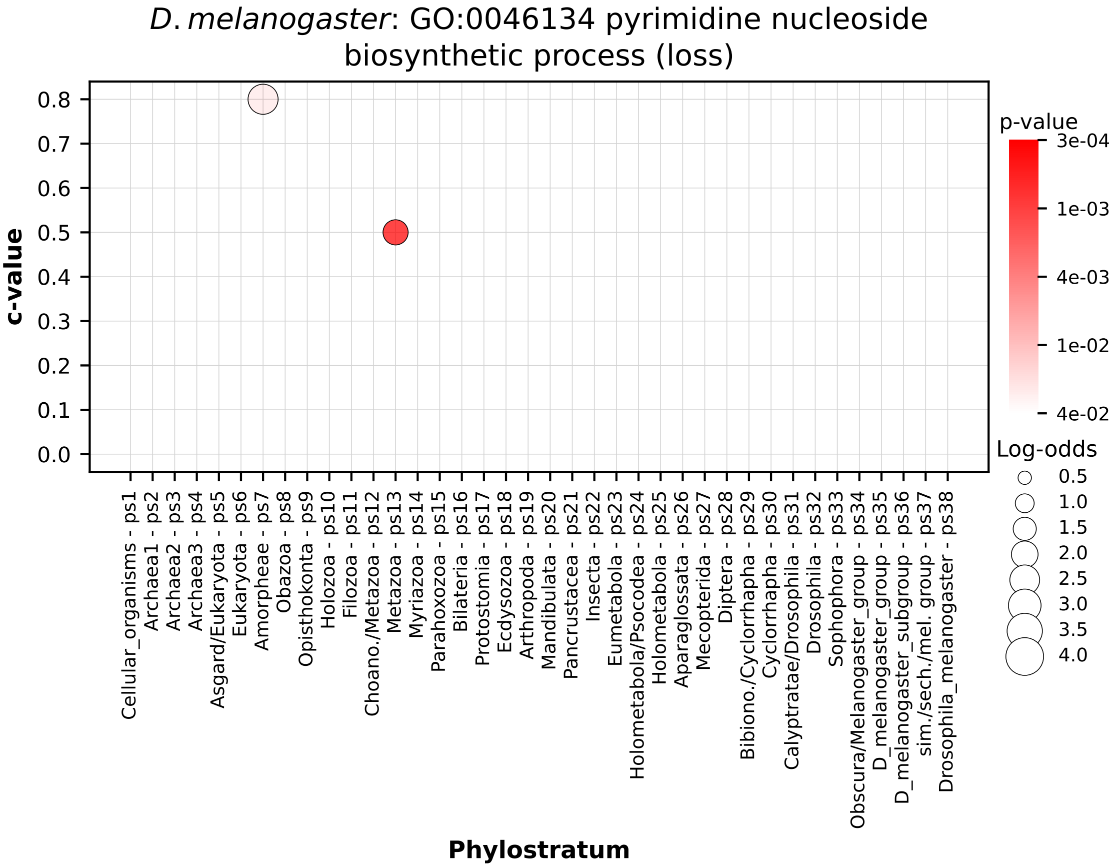

D. melanogaster: GO:0046134 pyrimidine nucleoside biosynthetic process (loss)
c-value
0.0
0.1
0.2
0.3
0.4
0.5
0.6
0.7
0.8
Phylostratum
Cellular_organisms - ps1
Archaea1 - ps2
Archaea2 - ps3
Archaea3 - ps4
Asgard/Eukaryota - ps5
Eukaryota - ps6
Amorpheae - ps7
Obazoa - ps8
Opisthokonta - ps9
Holozoa - ps10
Filozoa - ps11
Choano./Metazoa - ps12
Metazoa - ps13
Myriazoa - ps14
Parahoxozoa - ps15
Bilateria - ps16
Protostomia - ps17
Ecdysozoa - ps18
Arthropoda - ps19
Mandibulata - ps20
Pancrustacea - ps21
Insecta - ps22
Eumetabola - ps23
Holometabola/Psocodea - ps24
Holometabola - ps25
Aparaglossata - ps26
Mecopterida - ps27
Diptera - ps28
Bibiono./Cyclorrhapha - ps29
Cyclorrhapha - ps30
Calyptratae/Drosophila - ps31
Drosophila - ps32
Sophophora - ps33
Obscura/Melanogaster_group - ps34
D_melanogaster_group - ps35
D_melanogaster_subgroup - ps36
sim./sech./mel. group - ps37
Drosophila_melanogaster - ps38
p-value
3e-04
1e-03
4e-03
1e-02
4e-02
Log-odds
0.5
1.0
1.5
2.0
2.5
3.0
3.5
4.0

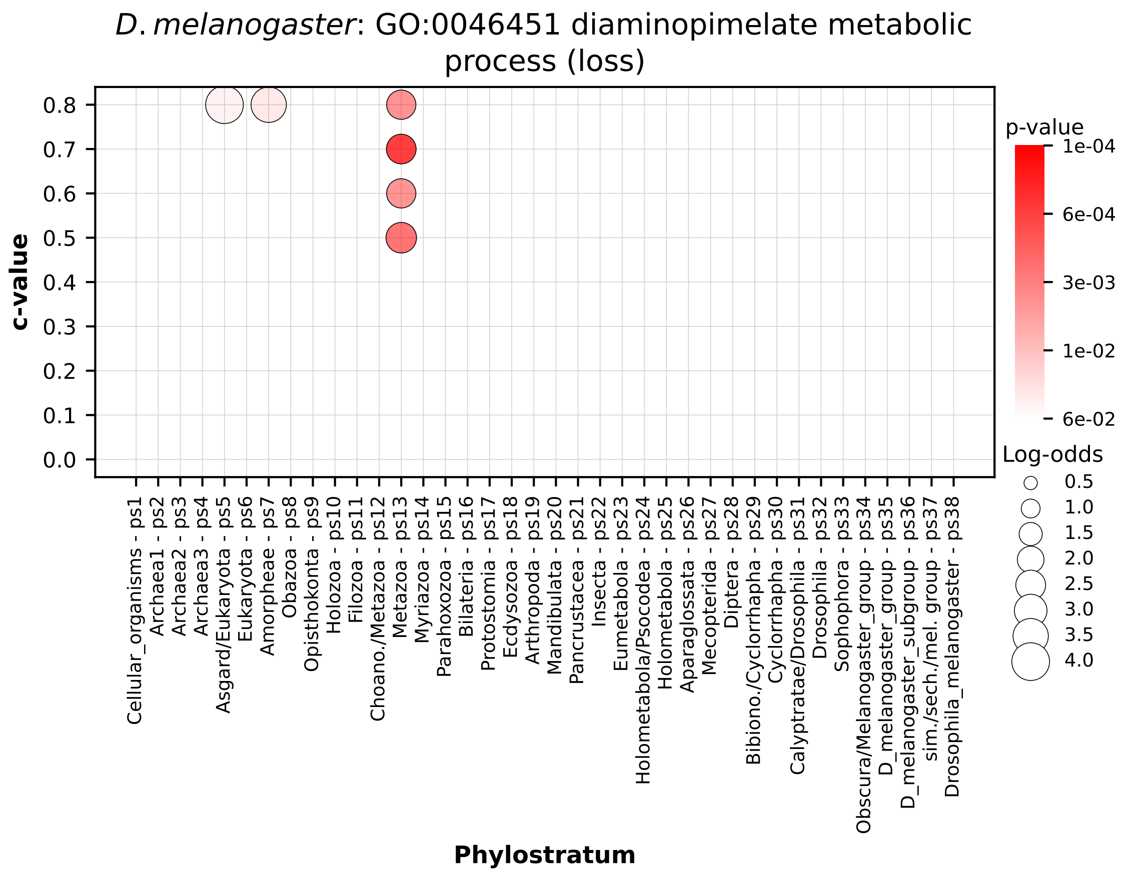

D. melanogaster: GO:0046451 diaminopimelate metabolic process (loss)
c-value
0.0
0.1
0.2
0.3
0.4
0.5
0.6
0.7
0.8
Phylostratum
Cellular_organisms - ps1
Archaea1 - ps2
Archaea2 - ps3
Archaea3 - ps4
Asgard/Eukaryota - ps5
Eukaryota - ps6
Amorpheae - ps7
Obazoa - ps8
Opisthokonta - ps9
Holozoa - ps10
Filozoa - ps11
Choano./Metazoa - ps12
Metazoa - ps13
Myriazoa - ps14
Parahoxozoa - ps15
Bilateria - ps16
Protostomia - ps17
Ecdysozoa - ps18
Arthropoda - ps19
Mandibulata - ps20
Pancrustacea - ps21
Insecta - ps22
Eumetabola - ps23
Holometabola/Psocodea - ps24
Holometabola - ps25
Aparaglossata - ps26
Mecopterida - ps27
Diptera - ps28
Bibiono./Cyclorrhapha - ps29
Cyclorrhapha - ps30
Calyptratae/Drosophila - ps31
Drosophila - ps32
Sophophora - ps33
Obscura/Melanogaster_group - ps34
D_melanogaster_group - ps35
D_melanogaster_subgroup - ps36
sim./sech./mel. group - ps37
Drosophila_melanogaster - ps38
p-value
1e-04
6e-04
3e-03
1e-02
6e-02
Log-odds
0.5
1.0
1.5
2.0
2.5
3.0
3.5
4.0

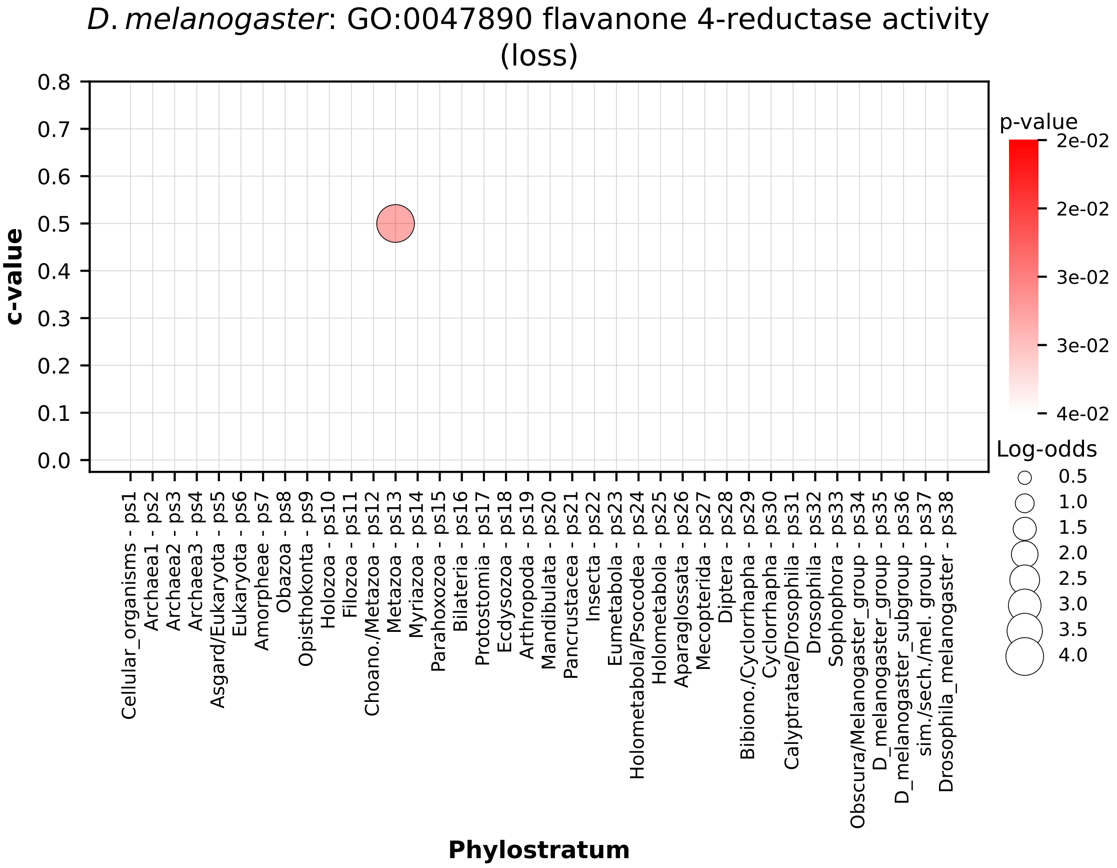

D. melanogaster: GO:0047890 flavanone 4-reductase activity (loss)
c-value
0.8
0.7
0.6
0.5
0.4
0.3
0.2
0.1
0.0
p-value
2e-02
2e-02
3e-02
3e-02
4e-02
Log-odds
0.5
1.0
1.5
2.0
2.5
3.0
3.5
4.0
Cellular_organisms - ps1
Archaea1 - ps2
Archaea2 - ps3
Archaea3 - ps4
Asgard/Eukaryota - ps5
Eukaryota - ps6
Amorpheae - ps7
Obazoa - ps8
Opisthokonta - ps9
Holozoa - ps10
Filozoa - ps11
Choano./Metazoa - ps12
Metazoa - ps13
Myriazoa - ps14
Parahoxozoa - ps15
Bilateria - ps16
Protostomia - ps17
Ecdysozoa - ps18
Arthropoda - ps19
Mandibulata - ps20
Pancrustacea - ps21
Insecta - ps22
Eumetabola - ps23
Holometabola/Psocodea - ps24
Holometabola - ps25
Aparaglossata - ps26
Mecopterida - ps27
Diptera - ps28
Bibiono./Cyclorrhapha - ps29
Cyclorrhapha - ps30
Calyptratae/Drosophila - ps31
Drosophila - ps32
Sophophora - ps33
Obscura/Melanogaster_group - ps34
D_melanogaster_group - ps35
D_melanogaster_subgroup - ps36
sim./sech./mel. group - ps37
Drosophila_melanogaster - ps38
Phylostratum

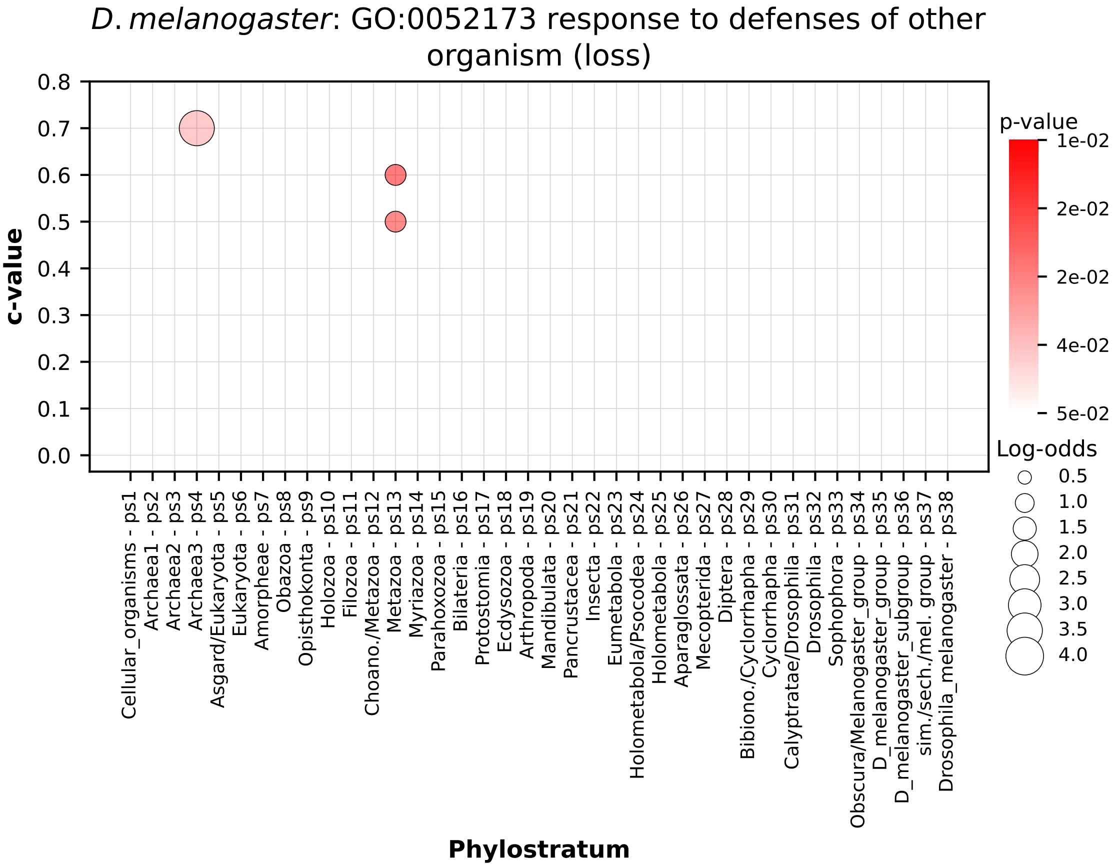

D. melanogaster: GO:0052173 response to defenses of other organism (loss)
c-value
0.8
0.7
0.6
0.5
0.4
0.3
0.2
0.1
0.0
Phylostratum
Cellular_organisms - ps1
Archaea1 - ps2
Archaea2 - ps3
Archaea3 - ps4
Asgard/Eukaryota - ps5
Eukaryota - ps6
Amorpheae - ps7
Obazoa - ps8
Opisthokonta - ps9
Holozoa - ps10
Filozoa - ps11
Choano./Metazoa - ps12
Metazoa - ps13
Myriazoa - ps14
Parahoxozoa - ps15
Bilateria - ps16
Protostomia - ps17
Ecdysozoa - ps18
Arthropoda - ps19
Mandibulata - ps20
Pancrustacea - ps21
Insecta - ps22
Eumetabola - ps23
Holometabola/Psocodea - ps24
Holometabola - ps25
Aparaglossata - ps26
Mecopterida - ps27
Diptera - ps28
Bibiono./Cyclorrhapha - ps29
Cyclorrhapha - ps30
Calyptratae/Drosophila - ps31
Drosophila - ps32
Sophophora - ps33
Obscura/Melanogaster_group - ps34
D_melanogaster_group - ps35
D_melanogaster_subgroup - ps36
sim./sech./mel. group - ps37
Drosophila_melanogaster - ps38
p-value
1e-02
2e-02
2e-02
4e-02
5e-02
Log-odds
0.5
1.0
1.5
2.0
2.5
3.0
3.5
4.0

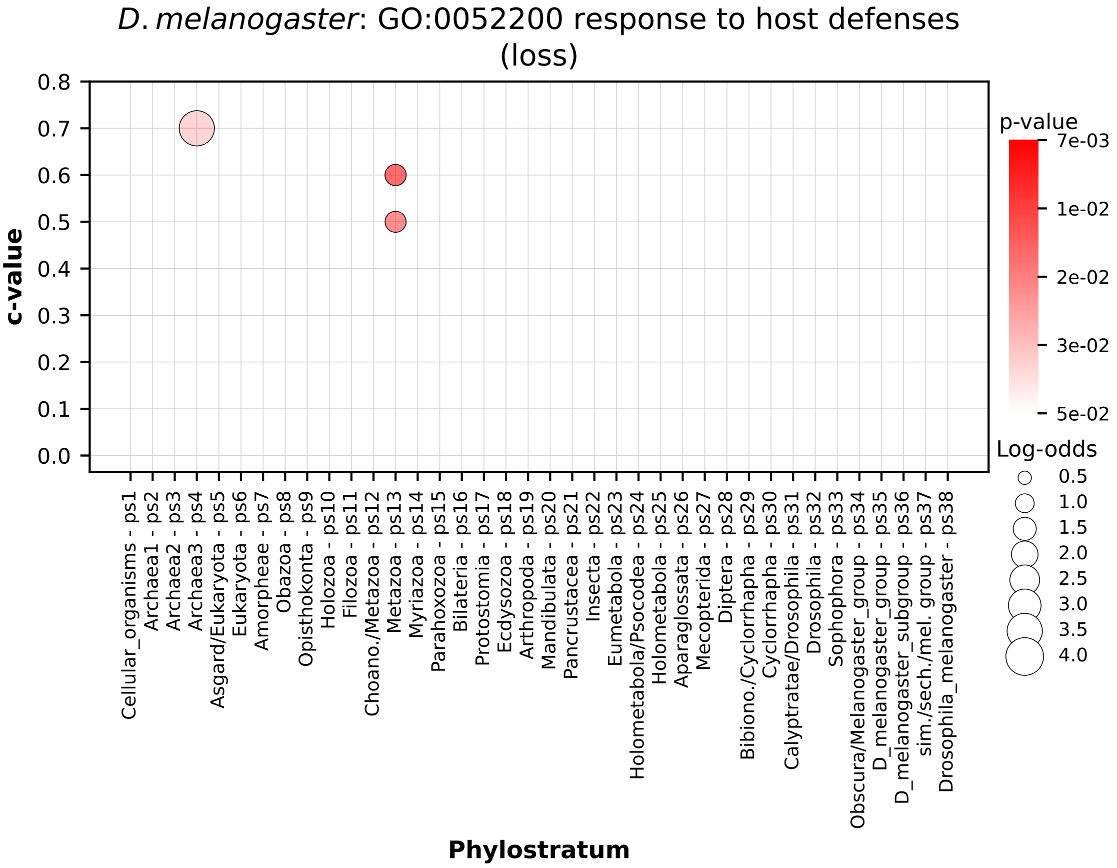
D. melanogaster: GO:0052200 response to host defenses
(loss)
c-value
0.0
0.1
0.2
0.3
0.4
0.5
0.6
0.7
0.8
p-value
7e-03
1e-02
2e-02
3e-02
5e-02
Log-odds
0.5
1.0
1.5
2.0
2.5
3.0
3.5
4.0
Cellular_organisms - ps1
Archaea1 - ps2
Archaea2 - ps3
Archaea3 - ps4
Asgard/Eukaryota - ps5
Eukaryota - ps6
Amorpheae - ps7
Obazoa - ps8
Opisthokonta - ps9
Holozoa - ps10
Filozoa - ps11
Choano./Metazoa - ps12
Metazoa - ps13
Myriazoa - ps14
Parahoxozoa - ps15
Bilateria - ps16
Protostomia - ps17
Ecdysozoa - ps18
Arthropoda - ps19
Mandibulata - ps20
Pancrustacea - ps21
Insecta - ps22
Eumetabola - ps23
Holometabola/Psocodea - ps24
Holometabola - ps25
Aparaglossata - ps26
Mecopterida - ps27
Diptera - ps28
Bibiono./Cyclorrhapha - ps29
Cyclorrhapha - ps30
Calyptratae/Drosophila - ps31
Drosophila - ps32
Sophophora - ps33
Obscura/Melanogaster_group - ps34
D_melanogaster_group - ps35
D_melanogaster_subgroup - ps36
sim./sech./mel. group - ps37
Drosophila_melanogaster - ps38
Phylostratum

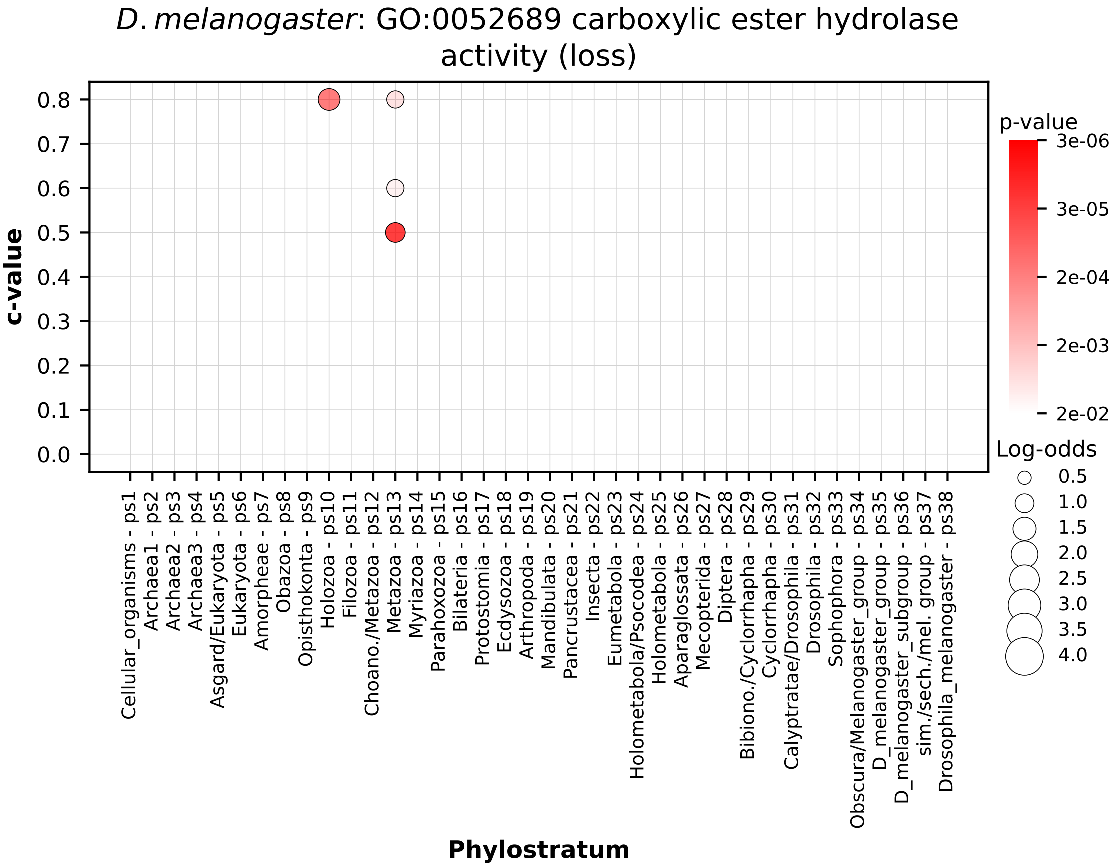

D. melanogaster: GO:0052689 carboxylic ester hydrolase activity (loss)
c-value
0.0
0.1
0.2
0.3
0.4
0.5
0.6
0.7
0.8
Phylostratum
Cellular_organisms - ps1
Archaea1 - ps2
Archaea2 - ps3
Archaea3 - ps4
Asgard/Eukaryota - ps5
Eukaryota - ps6
Amorpheae - ps7
Obazoa - ps8
Opisthokonta - ps9
Holozoa - ps10
Filozoa - ps11
Choano./Metazoa - ps12
Metazoa - ps13
Myriazoa - ps14
Parahoxozoa - ps15
Bilateria - ps16
Protostomia - ps17
Ecdysozoa - ps18
Arthropoda - ps19
Mandibulata - ps20
Pancrustacea - ps21
Insecta - ps22
Eumetabola - ps23
Holometabola/Psocodea - ps24
Holometabola - ps25
Aparaglossata - ps26
Mecopterida - ps27
Diptera - ps28
Bibiono./Cyclorrhapha - ps29
Cyclorrhapha - ps30
Calyptratae/Drosophila - ps31
Drosophila - ps32
Sophophora - ps33
Obscura/Melanogaster_group - ps34
D_melanogaster_group - ps35
D_melanogaster_subgroup - ps36
sim./sech./mel. group - ps37
Drosophila_melanogaster - ps38
p-value
3e-06
3e-05
2e-04
2e-03
2e-02
Log-odds
0.5
1.0
1.5
2.0
2.5
3.0
3.5
4.0

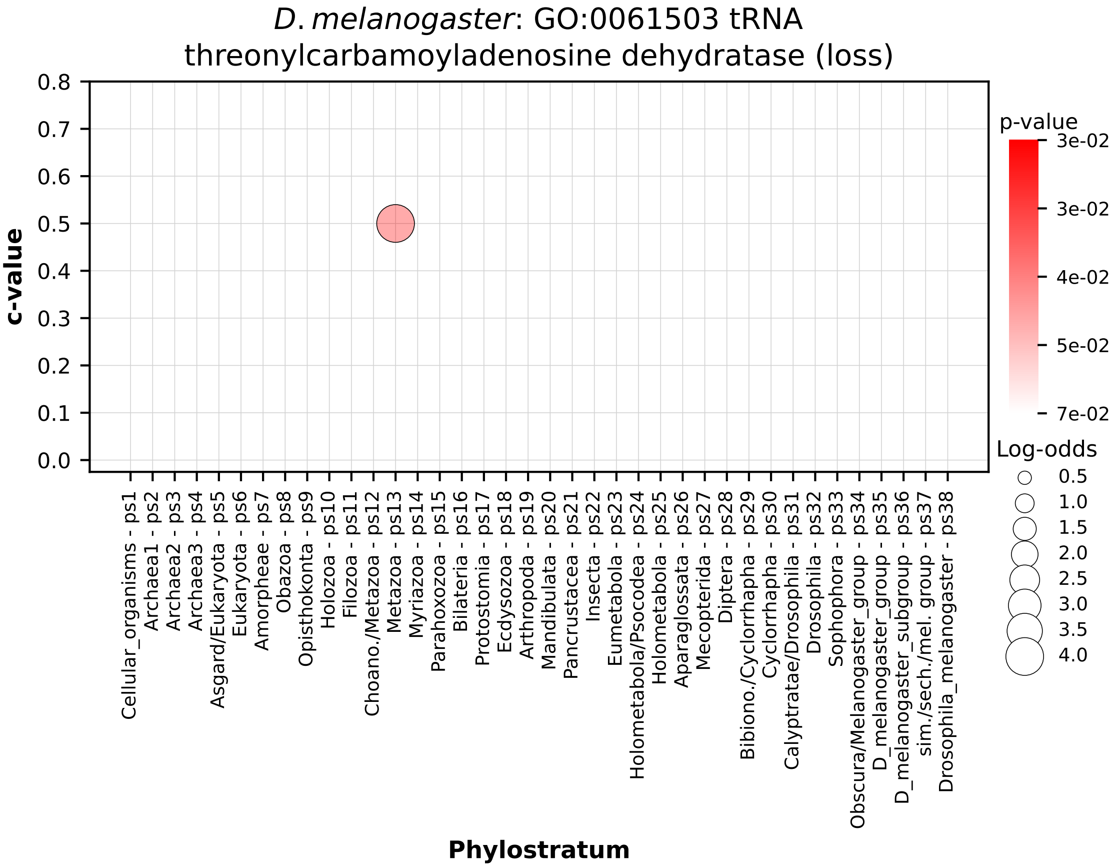
D. melanogaster: GO:0061503 tRNA threonylcarbamoyladenosine dehydratase (loss)
c-value
0.8
0.7
0.6
0.5
0.4
0.3
0.2
0.1
0.0
Phylostratum
Cellular_organisms - ps1
Archaea1 - ps2
Archaea2 - ps3
Archaea3 - ps4
Asgard/Eukaryota - ps5
Eukaryota - ps6
Amorpheae - ps7
Obazoa - ps8
Opisthokonta - ps9
Holozoa - ps10
Filozoa - ps11
Choano./Metazoa - ps12
Metazoa - ps13
Myriazoa - ps14
Parahoxozoa - ps15
Bilateria - ps16
Protostomia - ps17
Ecdysozoa - ps18
Arthropoda - ps19
Mandibulata - ps20
Pancrustacea - ps21
Insecta - ps22
Eumetabola - ps23
Holometabola/Psocodea - ps24
Holometabola - ps25
Aparaglossata - ps26
Mecopterida - ps27
Diptera - ps28
Bibiono./Cyclorrhapha - ps29
Cyclorrhapha - ps30
Calyptratae/Drosophila - ps31
Drosophila - ps32
Sophophora - ps33
Obscura/Melanogaster_group - ps34
D_melanogaster_group - ps35
D_melanogaster_subgroup - ps36
sim./sech./mel. group - ps37
Drosophila_melanogaster - ps38
p-value
3e-02
3e-02
4e-02
5e-02
7e-02
Log-odds
0.5
1.0
1.5
2.0
2.5
3.0
3.5
4.0

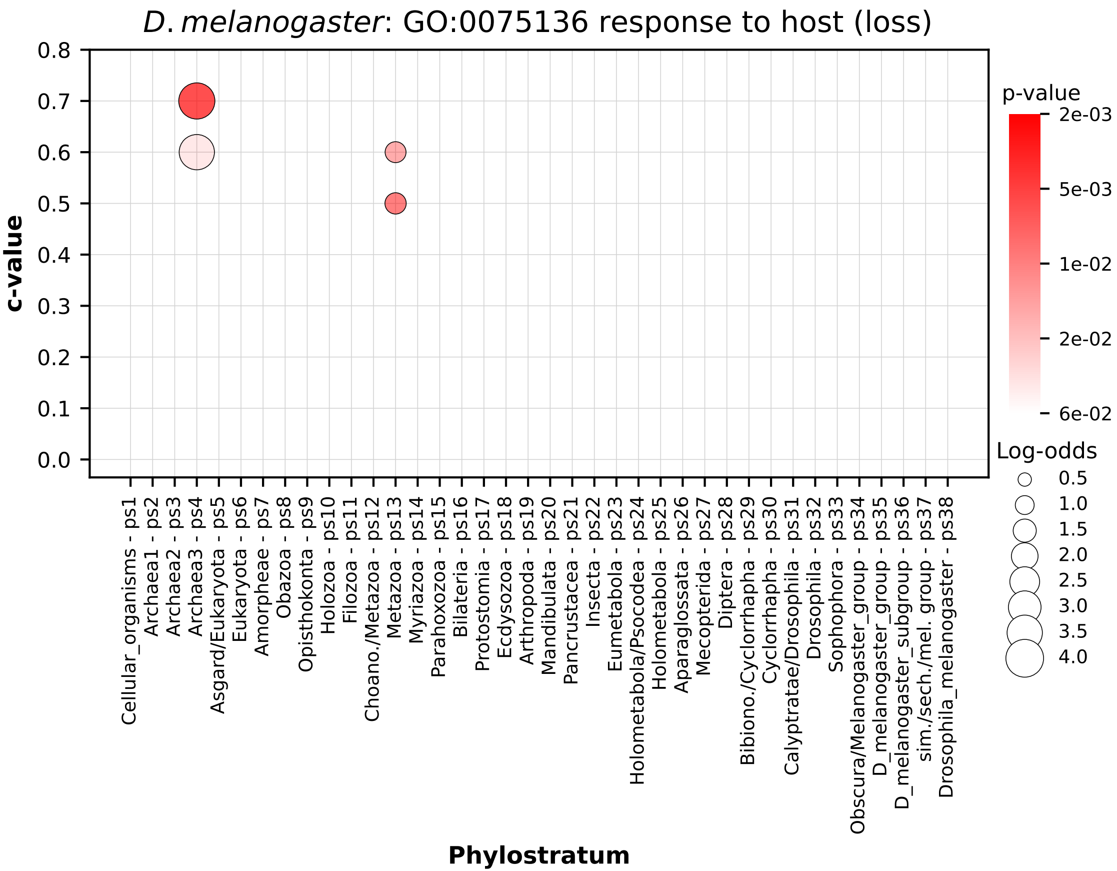

D. melanogaster: GO:0075136 response to host (loss)
c-value
0.8
0.7
0.6
0.5
0.4
0.3
0.2
0.1
0.0
Phylostratum
Cellular_organisms - ps1
Archaea1 - ps2
Archaea2 - ps3
Archaea3 - ps4
Asgard/Eukaryota - ps5
Eukaryota - ps6
Amorpheae - ps7
Obazoa - ps8
Opisthokonta - ps9
Holozoa - ps10
Filozoa - ps11
Choano./Metazoa - ps12
Metazoa - ps13
Myriazoa - ps14
Parahoxozoa - ps15
Bilateria - ps16
Protostomia - ps17
Ecdysozoa - ps18
Arthropoda - ps19
Mandibulata - ps20
Pancrustacea - ps21
Insecta - ps22
Eumetabola - ps23
Holometabola/Psocodea - ps24
Holometabola - ps25
Aparaglossata - ps26
Mecopterida - ps27
Diptera - ps28
Bibiono./Cyclorrhapha - ps29
Cyclorrhapha - ps30
Calyptratae/Drosophila - ps31
Drosophila - ps32
Sophophora - ps33
Obscura/Melanogaster_group - ps34
D_melanogaster_group - ps35
D_melanogaster_subgroup - ps36
sim./sech./mel. group - ps37
Drosophila_melanogaster - ps38
p-value
2e-03
5e-03
1e-02
2e-02
6e-02
Log-odds
0.5
1.0
1.5
2.0
2.5
3.0
3.5
4.0

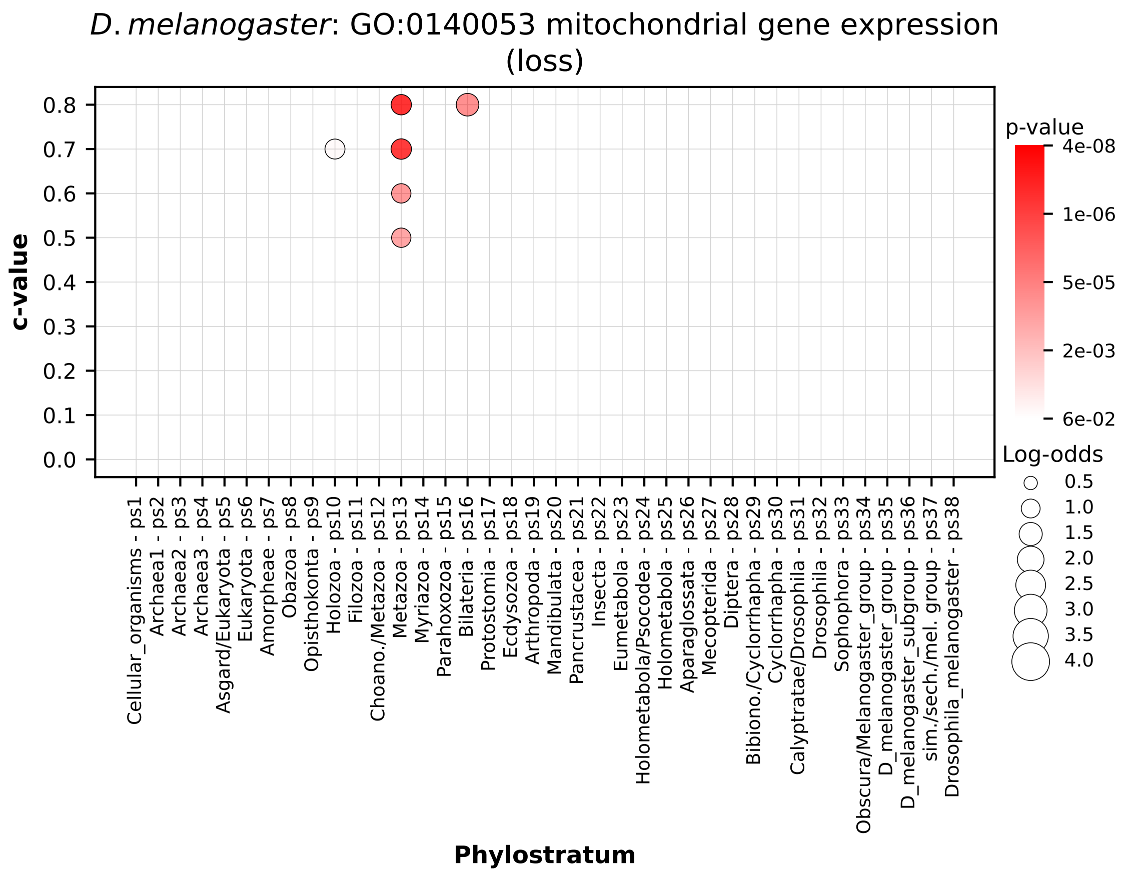

D. melanogaster: GO:0140053 mitochondrial gene expression (loss)
c-value
0.0
0.1
0.2
0.3
0.4
0.5
0.6
0.7
0.8
Phylostratum
Cellular_organisms - ps1
Archaea1 - ps2
Archaea2 - ps3
Archaea3 - ps4
Asgard/Eukaryota - ps5
Eukaryota - ps6
Amorpheae - ps7
Obazoa - ps8
Opisthokonta - ps9
Holozoa - ps10
Filozoa - ps11
Choano./Metazoa - ps12
Metazoa - ps13
Myriazoa - ps14
Parahoxozoa - ps15
Bilateria - ps16
Protostomia - ps17
Ecdysozoa - ps18
Arthropoda - ps19
Mandibulata - ps20
Pancrustacea - ps21
Insecta - ps22
Eumetabola - ps23
Holometabola/Psocodea - ps24
Holometabola - ps25
Aparaglossata - ps26
Mecopterida - ps27
Diptera - ps28
Bibiono./Cyclorrhapha - ps29
Cyclorrhapha - ps30
Calyptratae/Drosophila - ps31
Drosophila - ps32
Sophophora - ps33
Obscura/Melanogaster_group - ps34
D_melanogaster_group - ps35
D_melanogaster_subgroup - ps36
sim./sech./mel. group - ps37
Drosophila_melanogaster - ps38
p-value
4e-08
1e-06
5e-05
2e-03
6e-02
Log-odds
0.5
1.0
1.5
2.0
2.5
3.0
3.5
4.0

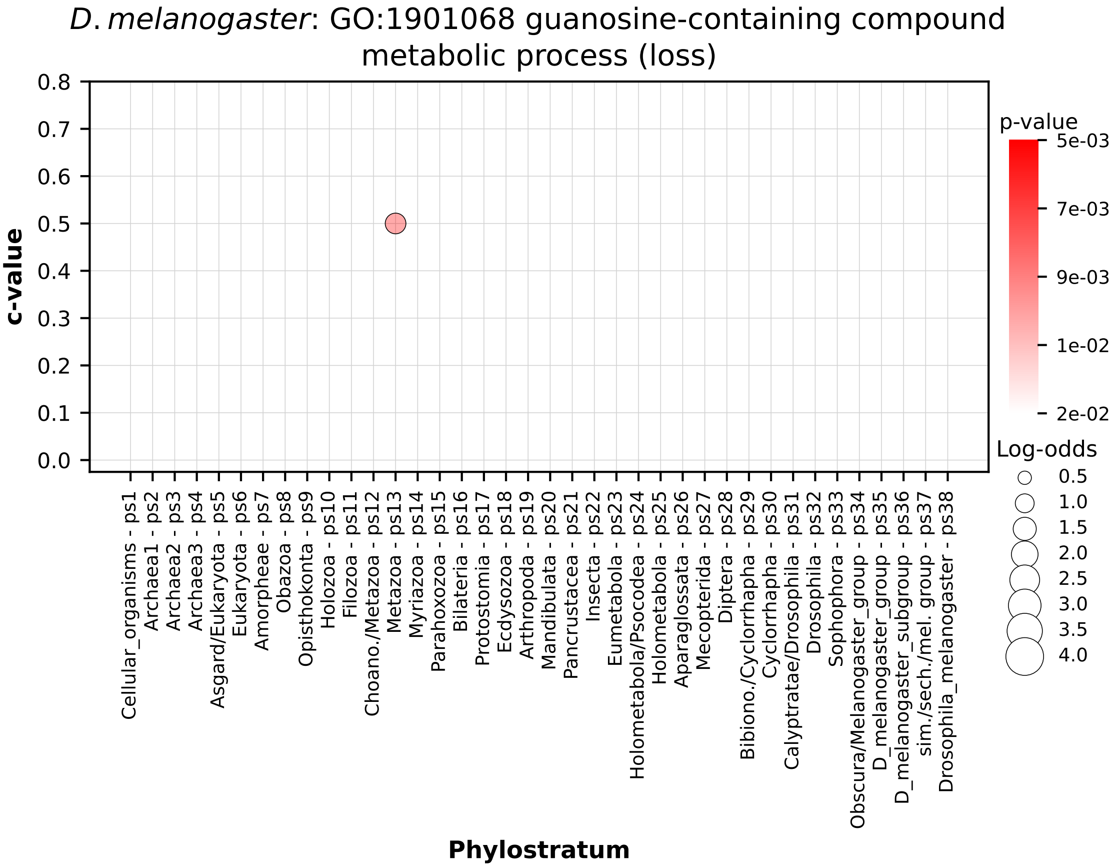
D. melanogaster: GO:1901068 guanosine-containing compound metabolic process (loss)
c-value
0.8
0.7
0.6
0.5
0.4
0.3
0.2
0.1
0.0
Phylostratum
Cellular_organisms - ps1
Archaea1 - ps2
Archaea2 - ps3
Archaea3 - ps4
Asgard/Eukaryota - ps5
Eukaryota - ps6
Amorpheae - ps7
Obazoa - ps8
Opisthokonta - ps9
Holozoa - ps10
Filozoa - ps11
Choano./Metazoa - ps12
Metazoa - ps13
Myriazoa - ps14
Parahoxozoa - ps15
Bilateria - ps16
Protostomia - ps17
Ecdysozoa - ps18
Arthropoda - ps19
Mandibulata - ps20
Pancrustacea - ps21
Insecta - ps22
Eumetabola - ps23
Holometabola/Psocodea - ps24
Holometabola - ps25
Aparaglossata - ps26
Mecopterida - ps27
Diptera - ps28
Bibiono./Cyclorrhapha - ps29
Cyclorrhapha - ps30
Calyptratae/Drosophila - ps31
Drosophila - ps32
Sophophora - ps33
Obscura/Melanogaster_group - ps34
D_melanogaster_group - ps35
D_melanogaster_subgroup - ps36
sim./sech./mel. group - ps37
Drosophila_melanogaster - ps38
p-value
5e-03
7e-03
9e-03
1e-02
2e-02
Log-odds
0.5
1.0
1.5
2.0
2.5
3.0
3.5
4.0

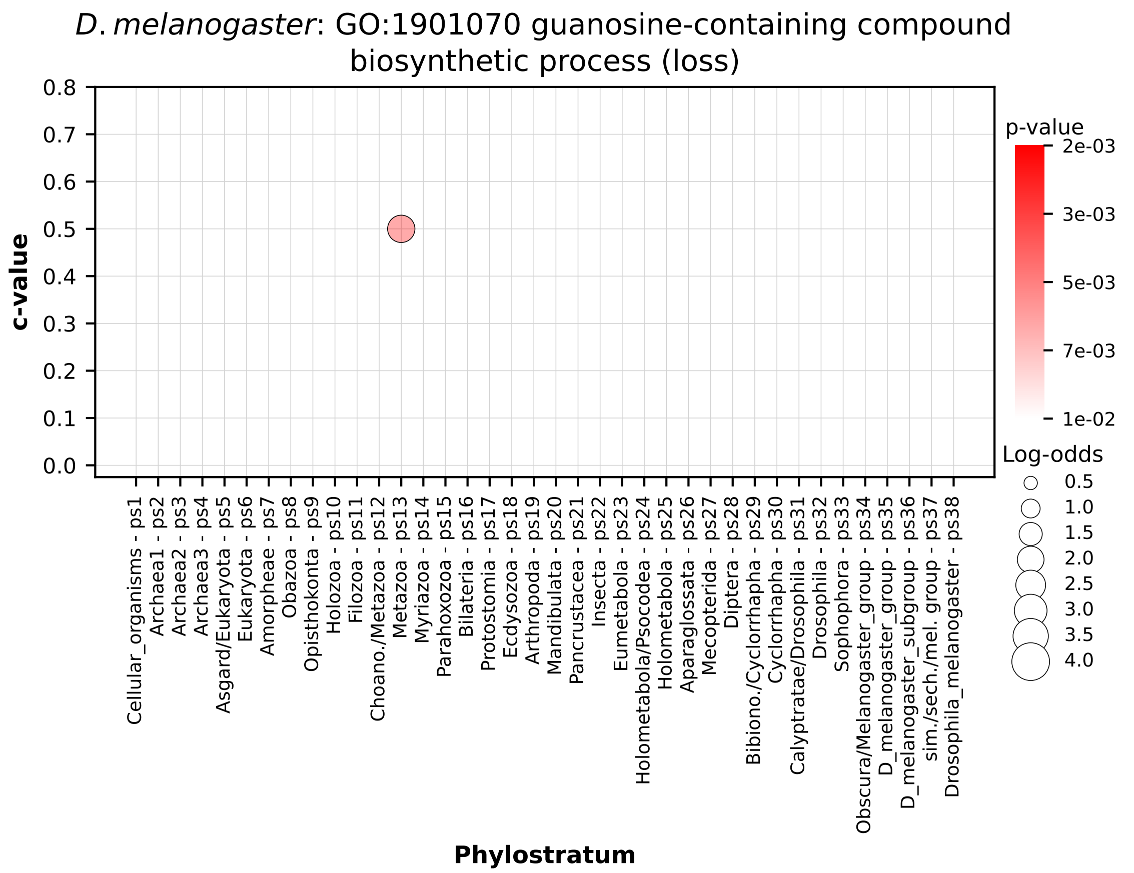

D. melanogaster: GO:1901070 guanosine-containing compound biosynthetic process (loss)
c-value
0.8
0.7
0.6
0.5
0.4
0.3
0.2
0.1
0.0
p-value
2e-03
3e-03
5e-03
7e-03
1e-02
Log-odds
0.5
1.0
1.5
2.0
2.5
3.0
3.5
4.0
Cellular_organisms - ps1
Archaea1 - ps2
Archaea2 - ps3
Archaea3 - ps4
Asgard/Eukaryota - ps5
Eukaryota - ps6
Amorpheae - ps7
Obazoa - ps8
Opisthokonta - ps9
Holozoa - ps10
Filozoa - ps11
Choano./Metazoa - ps12
Metazoa - ps13
Myriazoa - ps14
Parahoxozoa - ps15
Bilateria - ps16
Protostomia - ps17
Ecdysozoa - ps18
Arthropoda - ps19
Mandibulata - ps20
Pancrustacea - ps21
Insecta - ps22
Eumetabola - ps23
Holometabola/Psocodea - ps24
Holometabola - ps25
Aparaglossata - ps26
Mecopterida - ps27
Diptera - ps28
Bibiono./Cyclorrhapha - ps29
Cyclorrhapha - ps30
Calyptratae/Drosophila - ps31
Drosophila - ps32
Sophophora - ps33
Obscura/Melanogaster_group - ps34
D_melanogaster_group - ps35
D_melanogaster_subgroup - ps36
sim./sech./mel. group - ps37
Drosophila_melanogaster - ps38
Phylostratum

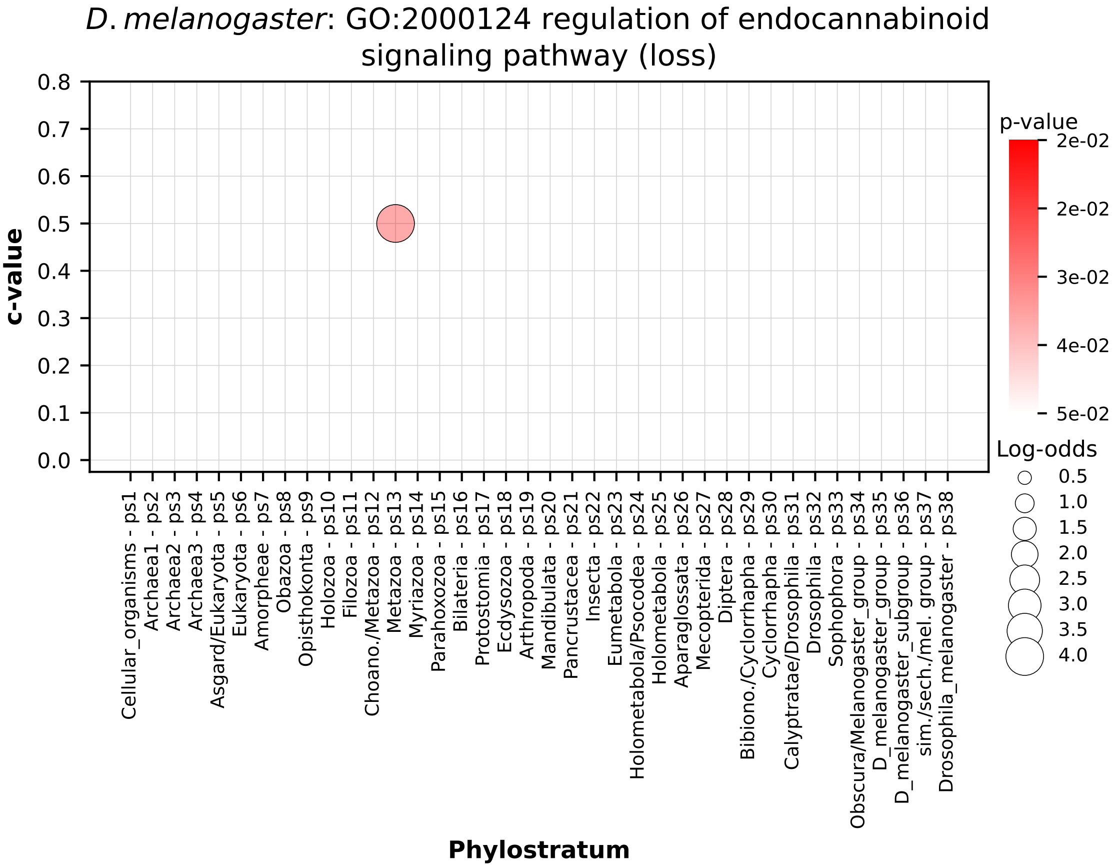

D. melanogaster: GO:2000124 regulation of endocannabinoid signaling pathway (loss)
c-value
0.8
0.7
0.6
0.5
0.4
0.3
0.2
0.1
0.0
p-value
2e-02
2e-02
3e-02
4e-02
5e-02
Log-odds
0.5
1.0
1.5
2.0
2.5
3.0
3.5
4.0
Cellular_organisms - ps1
Archaea1 - ps2
Archaea2 - ps3
Archaea3 - ps4
Asgard/Eukaryota - ps5
Eukaryota - ps6
Amorpheae - ps7
Obazoa - ps8
Opisthokonta - ps9
Holozoa - ps10
Filozoa - ps11
Choano./Metazoa - ps12
Metazoa - ps13
Myriazoa - ps14
Parahoxozoa - ps15
Bilateria - ps16
Protostomia - ps17
Ecdysozoa - ps18
Arthropoda - ps19
Mandibulata - ps20
Pancrustacea - ps21
Insecta - ps22
Eumetabola - ps23
Holometabola/Psocodea - ps24
Holometabola - ps25
Aparaglossata - ps26
Mecopterida - ps27
Diptera - ps28
Bibiono./Cyclorrhapha - ps29
Cyclorrhapha - ps30
Calyptratae/Drosophila - ps31
Drosophila - ps32
Sophophora - ps33
Obscura/Melanogaster_group - ps34
D_melanogaster_group - ps35
D_melanogaster_subgroup - ps36
sim./sech./mel. group - ps37
Drosophila_melanogaster - ps38
Phylostratum

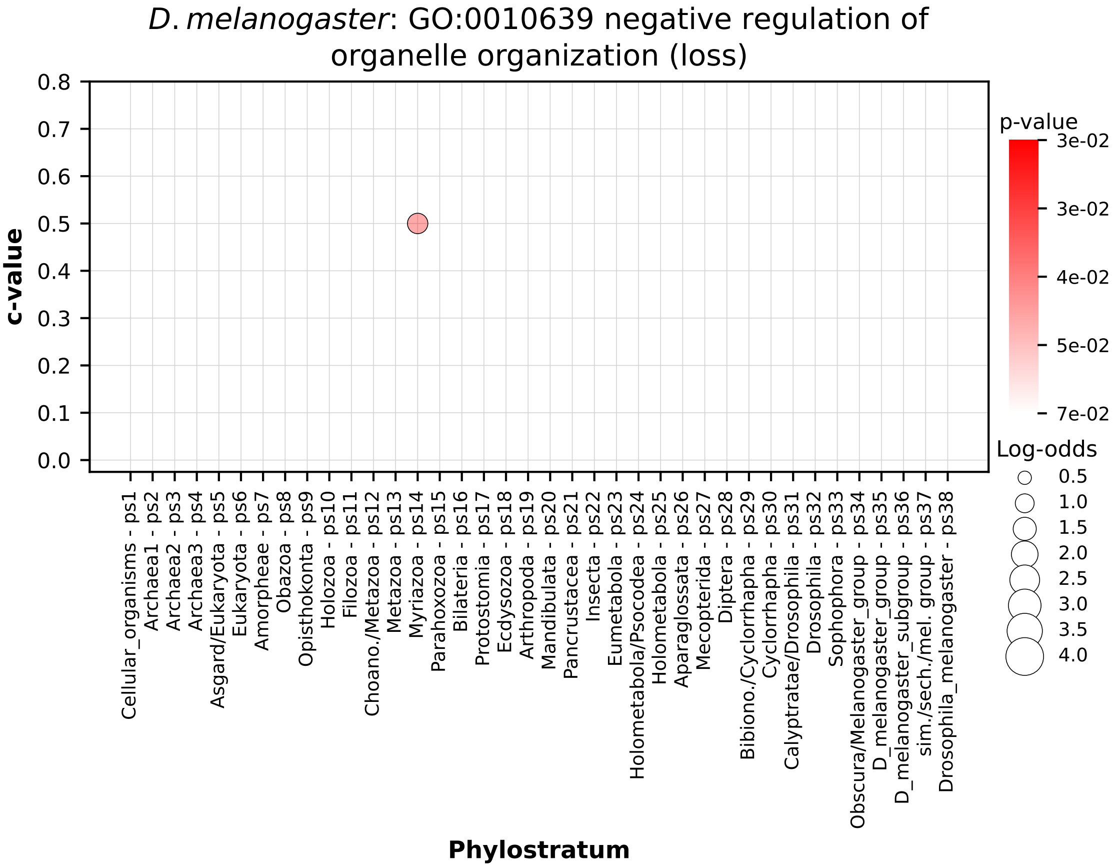

D. melanogaster: GO:0010639 negative regulation of organelle organization (loss)
c-value
0.8
0.7
0.6
0.5
0.4
0.3
0.2
0.1
0.0
p-value
3e-02
3e-02
4e-02
5e-02
7e-02
Log-odds
0.5
1.0
1.5
2.0
2.5
3.0
3.5
4.0
Cellular_organisms - ps1
Archaea1 - ps2
Archaea2 - ps3
Archaea3 - ps4
Asgard/Eukaryota - ps5
Eukaryota - ps6
Amorpheae - ps7
Obazoa - ps8
Opisthokonta - ps9
Holozoa - ps10
Filozoa - ps11
Choano./Metazoa - ps12
Metazoa - ps13
Myriazoa - ps14
Parahoxozoa - ps15
Bilateria - ps16
Protostomia - ps17
Ecdysozoa - ps18
Arthropoda - ps19
Mandibulata - ps20
Pancrustacea - ps21
Insecta - ps22
Eumetabola - ps23
Holometabola/Psocodea - ps24
Holometabola - ps25
Aparaglossata - ps26
Mecopterida - ps27
Diptera - ps28
Bibiono./Cyclorrhapha - ps29
Cyclorrhapha - ps30
Calyptratae/Drosophila - ps31
Drosophila - ps32
Sophophora - ps33
Obscura/Melanogaster_group - ps34
D_melanogaster_group - ps35
D_melanogaster_subgroup - ps36
sim./sech./mel. group - ps37
Drosophila_melanogaster - ps38
Phylostratum

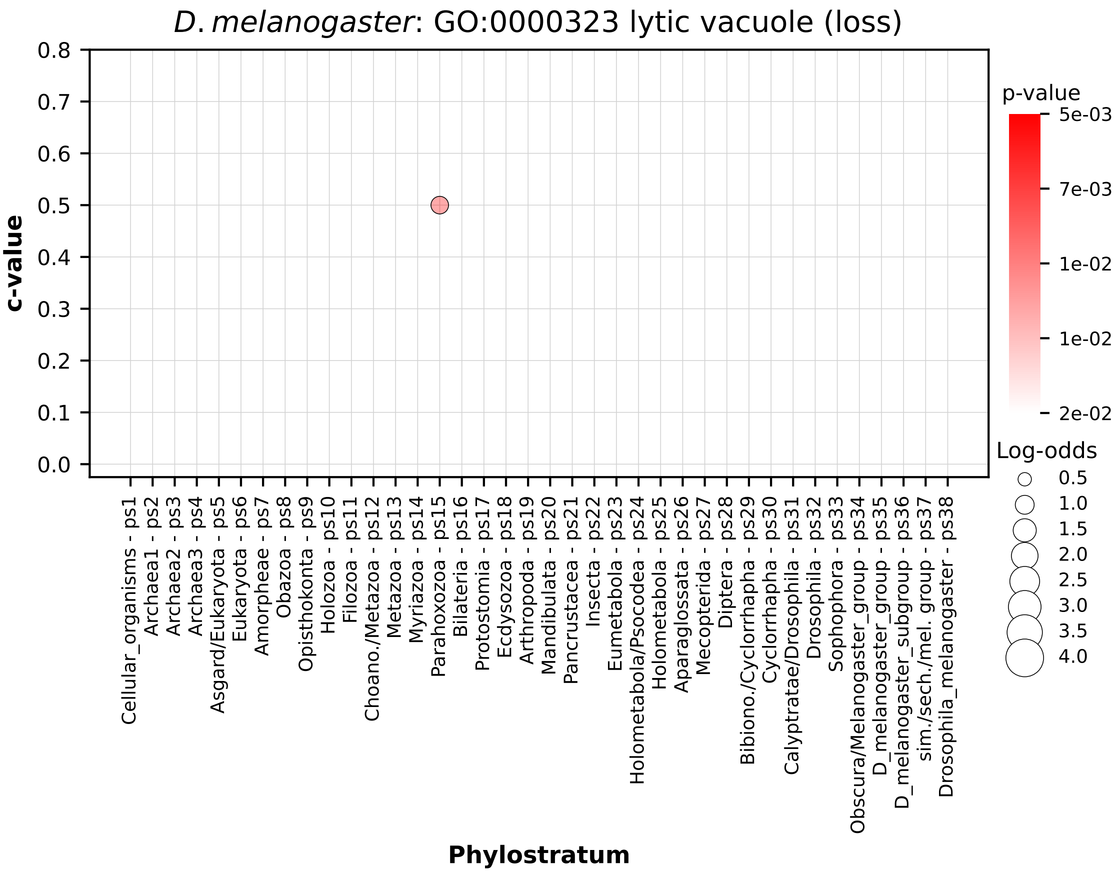

D. melanogaster: GO:0000323 lytic vacuole (loss)
c-value
0.8
0.7
0.6
0.5
0.4
0.3
0.2
0.1
0.0
p-value
5e-03
7e-03
1e-02
1e-02
2e-02
Log-odds
0.5
1.0
1.5
2.0
2.5
3.0
3.5
4.0
Cellular_organisms - ps1
Archaea1 - ps2
Archaea2 - ps3
Archaea3 - ps4
Asgard/Eukaryota - ps5
Eukaryota - ps6
Amorpheae - ps7
Obazoa - ps8
Opisthokonta - ps9
Holozoa - ps10
Filozoa - ps11
Choano./Metazoa - ps12
Metazoa - ps13
Myriazoa - ps14
Parahoxozoa - ps15
Bilateria - ps16
Protostomia - ps17
Ecdysozoa - ps18
Arthropoda - ps19
Mandibulata - ps20
Pancrustacea - ps21
Insecta - ps22
Eumetabola - ps23
Holometabola/Psocodea - ps24
Holometabola - ps25
Aparaglossata - ps26
Mecopterida - ps27
Diptera - ps28
Bibiono./Cyclorrhapha - ps29
Cyclorrhapha - ps30
Calyptratae/Drosophila - ps31
Drosophila - ps32
Sophophora - ps33
Obscura/Melanogaster_group - ps34
D_melanogaster_group - ps35
D_melanogaster_subgroup - ps36
sim./sech./mel. group - ps37
Drosophila_melanogaster - ps38
Phylostratum

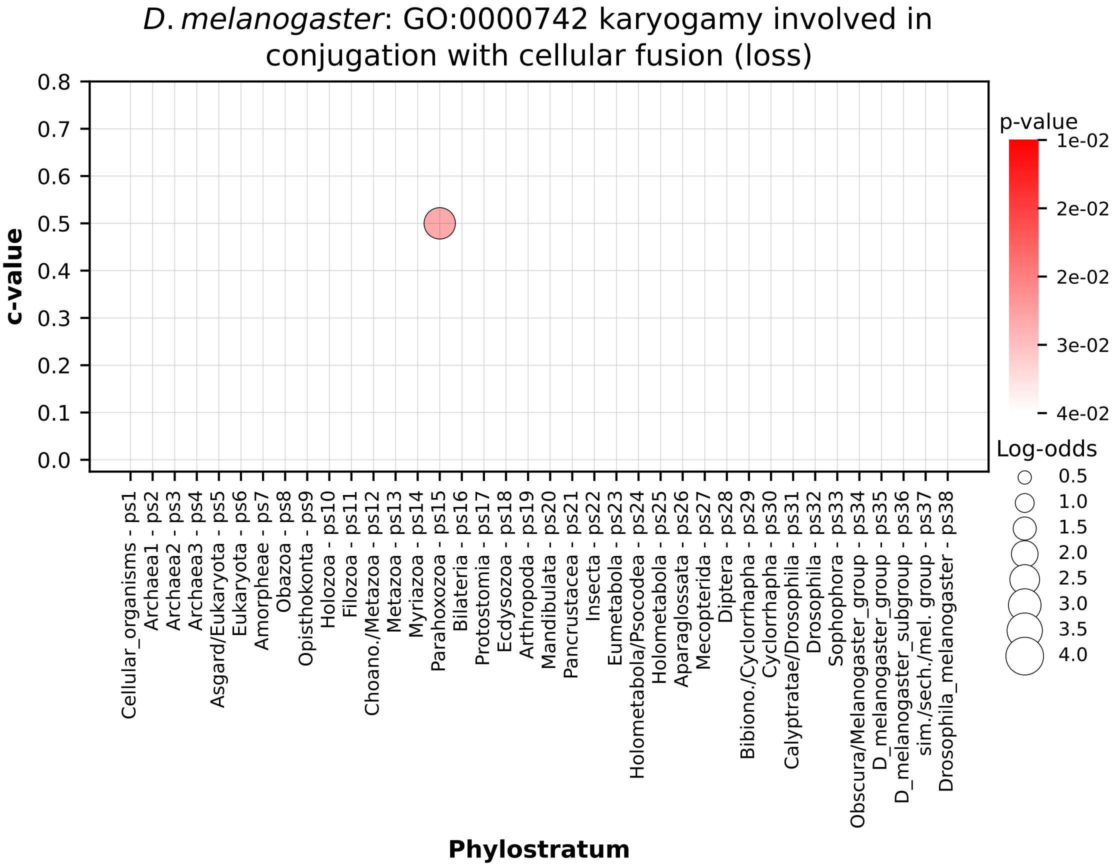

D. melanogaster: GO:0000742 karyogamy involved in conjugation with cellular fusion (loss)
c-value
0.8
0.7
0.6
0.5
0.4
0.3
0.2
0.1
0.0
p-value
1e-02
2e-02
2e-02
3e-02
4e-02
Log-odds
0.5
1.0
1.5
2.0
2.5
3.0
3.5
4.0
Cellular_organisms - ps1
Archaea1 - ps2
Archaea2 - ps3
Archaea3 - ps4
Asgard/Eukaryota - ps5
Eukaryota - ps6
Amorpheae - ps7
Obazoa - ps8
Opisthokonta - ps9
Holozoa - ps10
Filozoa - ps11
Choano./Metazoa - ps12
Metazoa - ps13
Myriazoa - ps14
Parahoxozoa - ps15
Bilateria - ps16
Protostomia - ps17
Ecdysozoa - ps18
Arthropoda - ps19
Mandibulata - ps20
Pancrustacea - ps21
Insecta - ps22
Eumetabola - ps23
Holometabola/Psocodea - ps24
Holometabola - ps25
Aparaglossata - ps26
Mecopterida - ps27
Diptera - ps28
Bibiono./Cyclorrhapha - ps29
Cyclorrhapha - ps30
Calyptratae/Drosophila - ps31
Drosophila - ps32
Sophophora - ps33
Obscura/Melanogaster_group - ps34
D_melanogaster_group - ps35
D_melanogaster_subgroup - ps36
sim./sech./mel. group - ps37
Drosophila_melanogaster - ps38
Phylostratum

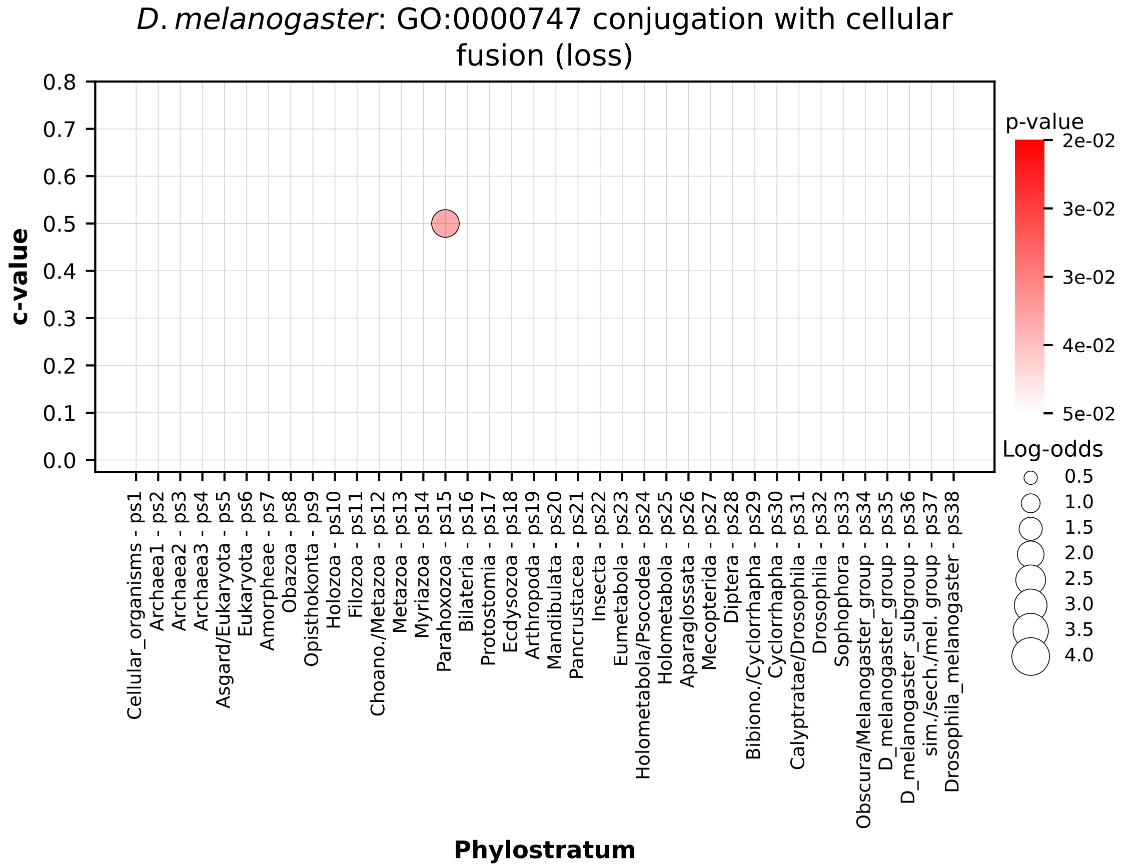

D. melanogaster: GO:0000747 conjugation with cellular fusion (loss)
c-value
0.8
0.7
0.6
0.5
0.4
0.3
0.2
0.1
0.0
Phylostratum
Cellular_organisms - ps1
Archaea1 - ps2
Archaea2 - ps3
Archaea3 - ps4
Asgard/Eukaryota - ps5
Eukaryota - ps6
Amorpheae - ps7
Obazoa - ps8
Opisthokonta - ps9
Holozoa - ps10
Filozoa - ps11
Choano./Metazoa - ps12
Metazoa - ps13
Myriazoa - ps14
Parahoxozoa - ps15
Bilateria - ps16
Protostomia - ps17
Ecdysozoa - ps18
Arthropoda - ps19
Mandibulata - ps20
Pancrustacea - ps21
Insecta - ps22
Eumetabola - ps23
Holometabola/Psocodea - ps24
Holometabola - ps25
Aparaglossata - ps26
Mecopterida - ps27
Diptera - ps28
Bibiono./Cyclorrhapha - ps29
Cyclorrhapha - ps30
Calyptratae/Drosophila - ps31
Drosophila - ps32
Sophophora - ps33
Obscura/Melanogaster_group - ps34
D_melanogaster_group - ps35
D_melanogaster_subgroup - ps36
sim./sech./mel. group - ps37
Drosophila_melanogaster - ps38
p-value
2e-02
3e-02
3e-02
4e-02
5e-02
Log-odds
0.5
1.0
1.5
2.0
2.5
3.0
3.5
4.0

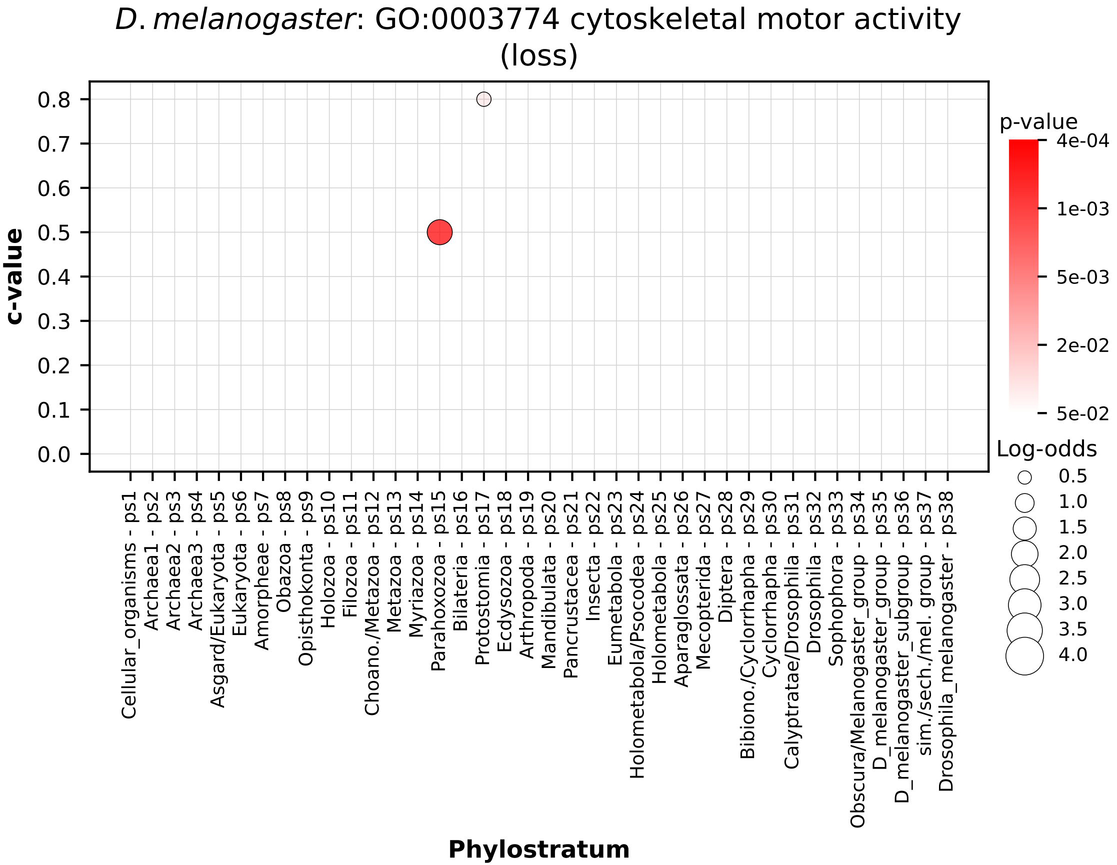

D. melanogaster: GO:0003774 cytoskeletal motor activity
(loss)
c-value
0.0
0.1
0.2
0.3
0.4
0.5
0.6
0.7
0.8
Phylostratum
Cellular_organisms - ps1
Archaea1 - ps2
Archaea2 - ps3
Archaea3 - ps4
Asgard/Eukaryota - ps5
Eukaryota - ps6
Amorpheae - ps7
Obazoa - ps8
Opisthokonta - ps9
Holozoa - ps10
Filozoa - ps11
Choano./Metazoa - ps12
Metazoa - ps13
Myriazoa - ps14
Parahoxozoa - ps15
Bilateria - ps16
Protostomia - ps17
Ecdysozoa - ps18
Arthropoda - ps19
Mandibulata - ps20
Pancrustacea - ps21
Insecta - ps22
Eumetabola - ps23
Holometabola/Psocodea - ps24
Holometabola - ps25
Aparaglossata - ps26
Mecopterida - ps27
Diptera - ps28
Bibiono./Cyclorrhapha - ps29
Cyclorrhapha - ps30
Calyptratae/Drosophila - ps31
Drosophila - ps32
Sophophora - ps33
Obscura/Melanogaster_group - ps34
D_melanogaster_group - ps35
D_melanogaster_subgroup - ps36
sim./sech./mel. group - ps37
Drosophila_melanogaster - ps38
p-value
4e-04
1e-03
5e-03
2e-02
5e-02
Log-odds
0.5
1.0
1.5
2.0
2.5
3.0
3.5
4.0

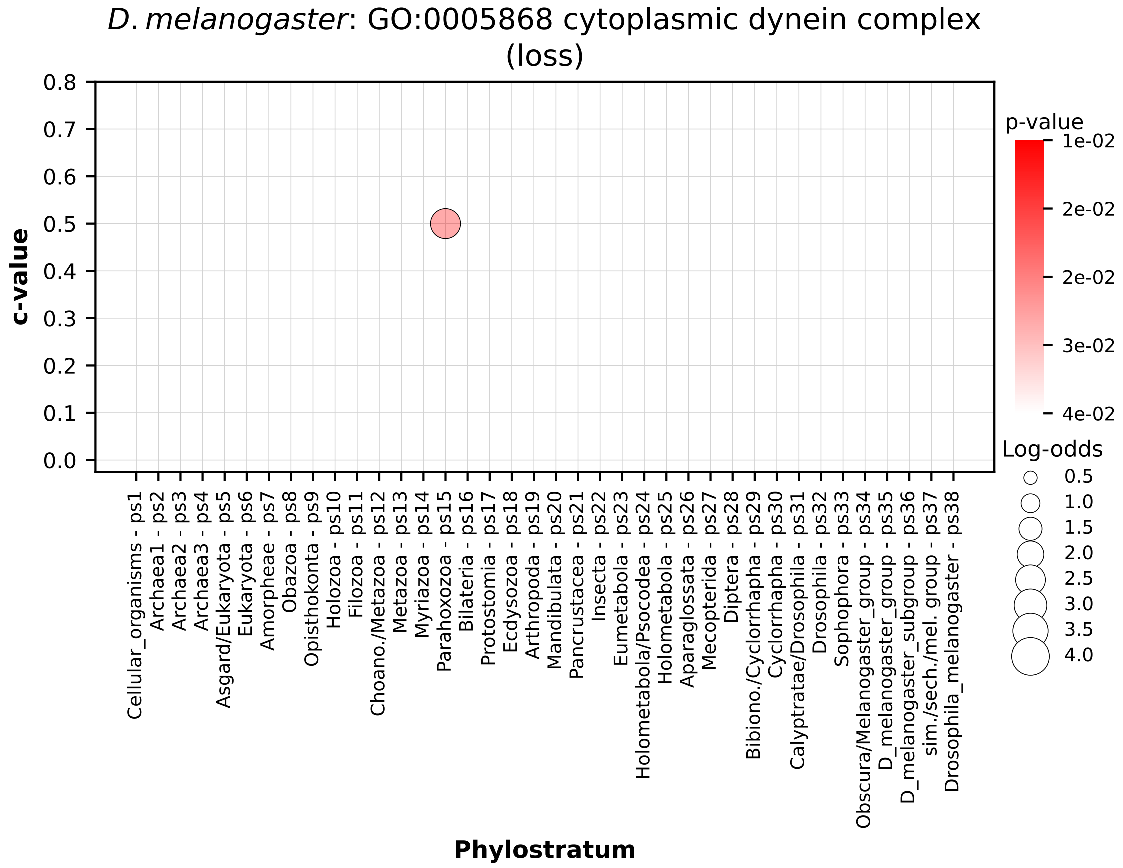

D. melanogaster: GO:0005868 cytoplasmic dynein complex (loss)
c-value
0.8
0.7
0.6
0.5
0.4
0.3
0.2
0.1
0.0
p-value
1e-02
2e-02
2e-02
3e-02
4e-02
Log-odds
0.5
1.0
1.5
2.0
2.5
3.0
3.5
4.0
Cellular_organisms - ps1
Archaea1 - ps2
Archaea2 - ps3
Archaea3 - ps4
Asgard/Eukaryota - ps5
Eukaryota - ps6
Amorpheae - ps7
Obazoa - ps8
Opisthokonta - ps9
Holozoa - ps10
Filozoa - ps11
Choano./Metazoa - ps12
Metazoa - ps13
Myriazoa - ps14
Parahoxozoa - ps15
Bilateria - ps16
Protostomia - ps17
Ecdysozoa - ps18
Arthropoda - ps19
Mandibulata - ps20
Pancrustacea - ps21
Insecta - ps22
Eumetabola - ps23
Holometabola/Psocodea - ps24
Holometabola - ps25
Aparaglossata - ps26
Mecopterida - ps27
Diptera - ps28
Bibiono./Cyclorrhapha - ps29
Cyclorrhapha - ps30
Calyptratae/Drosophila - ps31
Drosophila - ps32
Sophophora - ps33
Obscura/Melanogaster_group - ps34
D_melanogaster_group - ps35
D_melanogaster_subgroup - ps36
sim./sech./mel. group - ps37
Drosophila_melanogaster - ps38
Phylostratum

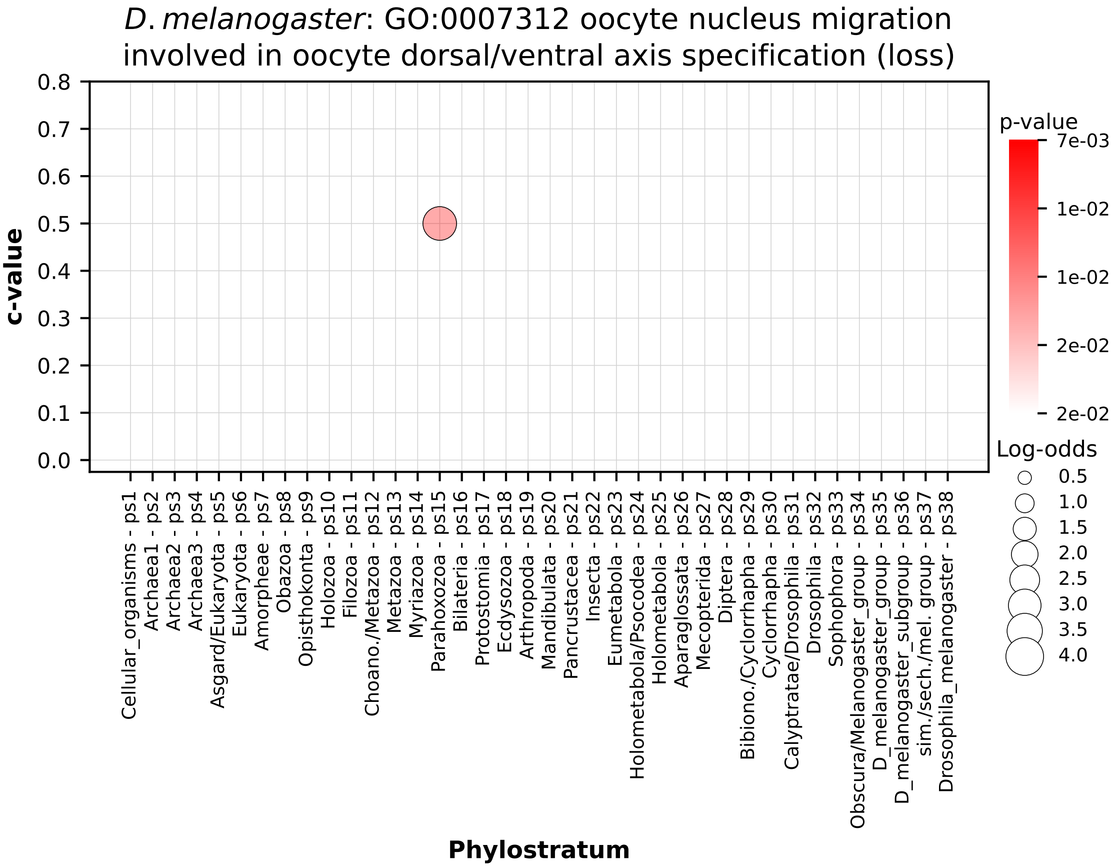

D. melanogaster: GO:0007312 oocyte nucleus migration involved in oocyte dorsal/ventral axis specification (loss)
c-value
0.8
0.7
0.6
0.5
0.4
0.3
0.2
0.1
0.0
p-value
7e-03
1e-02
1e-02
2e-02
2e-02
Log-odds
0.5
1.0
1.5
2.0
2.5
3.0
3.5
4.0
Cellular_organisms - ps1
Archaea1 - ps2
Archaea2 - ps3
Archaea3 - ps4
Asgard/Eukaryota - ps5
Eukaryota - ps6
Amorpheae - ps7
Obazoa - ps8
Opisthokonta - ps9
Holozoa - ps10
Filozoa - ps11
Choano./Metazoa - ps12
Metazoa - ps13
Myriazoa - ps14
Parahoxozoa - ps15
Bilateria - ps16
Protostomia - ps17
Ecdysozoa - ps18
Arthropoda - ps19
Mandibulata - ps20
Pancrustacea - ps21
Insecta - ps22
Eumetabola - ps23
Holometabola/Psocodea - ps24
Holometabola - ps25
Aparaglossata - ps26
Mecopterida - ps27
Diptera - ps28
Bibiono./Cyclorrhapha - ps29
Cyclorrhapha - ps30
Calyptratae/Drosophila - ps31
Drosophila - ps32
Sophophora - ps33
Obscura/Melanogaster_group - ps34
D_melanogaster_group - ps35
D_melanogaster_subgroup - ps36
sim./sech./mel. group - ps37
Drosophila_melanogaster - ps38
Phylostratum

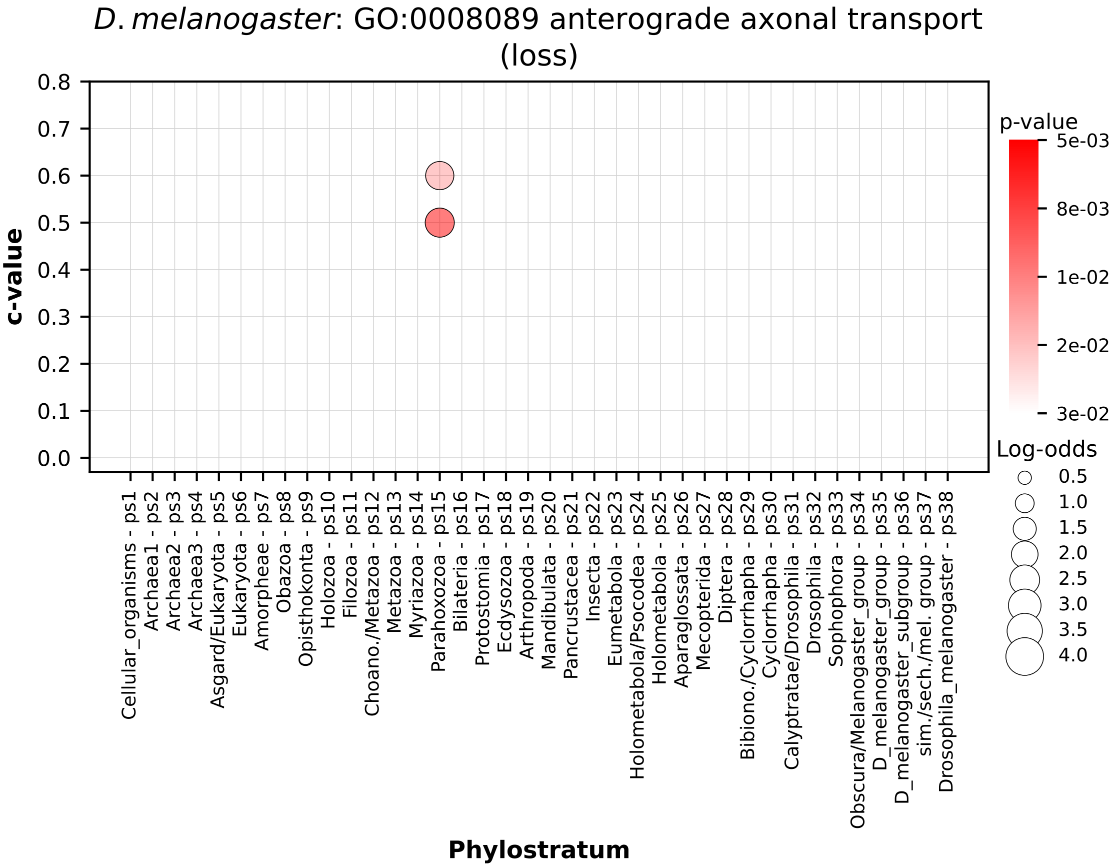

D. melanogaster: GO:0008089 anterograde axonal transport (loss)
c-value
0.8
0.7
0.6
0.5
0.4
0.3
0.2
0.1
0.0
p-value
5e-03
8e-03
1e-02
2e-02
3e-02
Log-odds
0.5
1.0
1.5
2.0
2.5
3.0
3.5
4.0
Cellular_organisms - ps1
Archaea1 - ps2
Archaea2 - ps3
Archaea3 - ps4
Asgard/Eukaryota - ps5
Eukaryota - ps6
Amorpheae - ps7
Obazoa - ps8
Opisthokonta - ps9
Holozoa - ps10
Filozoa - ps11
Choano./Metazoa - ps12
Metazoa - ps13
Myriazoa - ps14
Parahoxozoa - ps15
Bilateria - ps16
Protostomia - ps17
Ecdysozoa - ps18
Arthropoda - ps19
Mandibulata - ps20
Pancrustacea - ps21
Insecta - ps22
Eumetabola - ps23
Holometabola/Psocodea - ps24
Holometabola - ps25
Aparaglossata - ps26
Mecopterida - ps27
Diptera - ps28
Bibiono./Cyclorrhapha - ps29
Cyclorrhapha - ps30
Calyptratae/Drosophila - ps31
Drosophila - ps32
Sophophora - ps33
Obscura/Melanogaster_group - ps34
D_melanogaster_group - ps35
D_melanogaster_subgroup - ps36
sim./sech./mel. group - ps37
Drosophila_melanogaster - ps38
Phylostratum

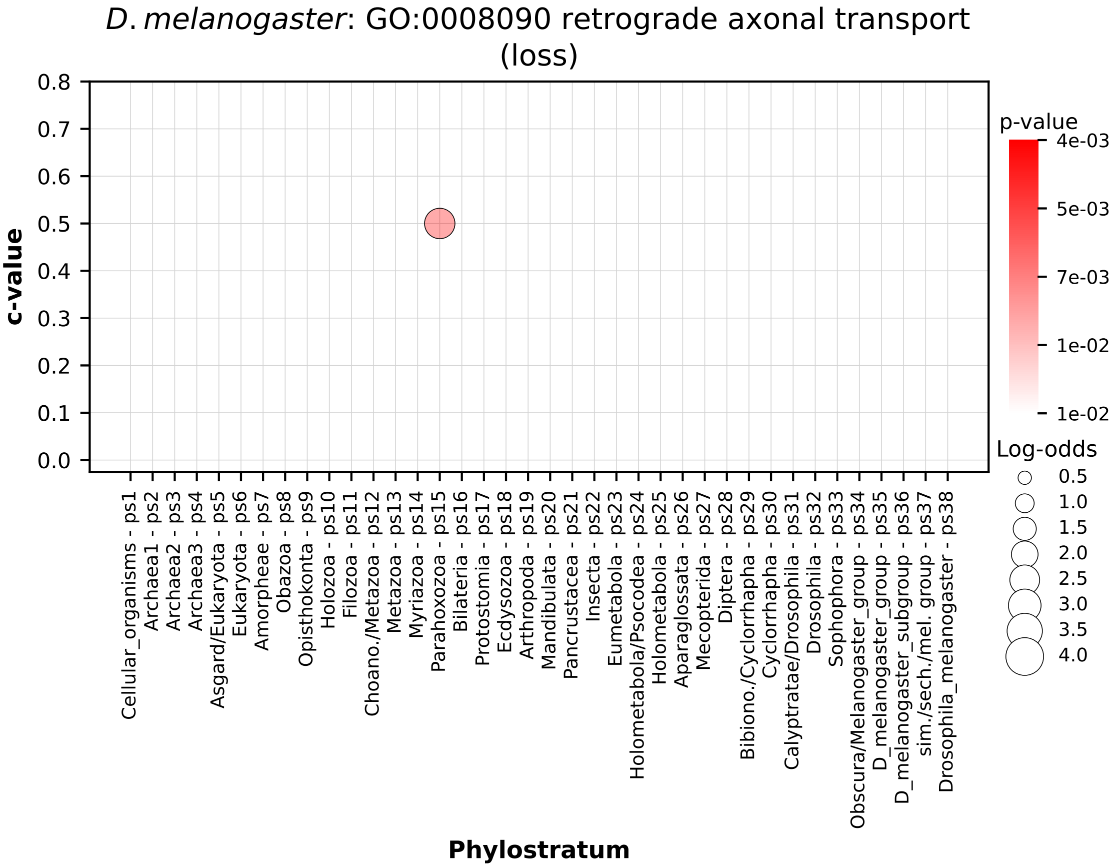

D. melanogaster: GO:0008090 retrograde axonal transport
(loss)
c-value
0.0
0.1
0.2
0.3
0.4
0.5
0.6
0.7
0.8
p-value
4e-03
5e-03
7e-03
1e-02
1e-02
Log-odds
0.5
1.0
1.5
2.0
2.5
3.0
3.5
4.0
Cellular_organisms - ps1
Archaea1 - ps2
Archaea2 - ps3
Archaea3 - ps4
Asgard/Eukaryota - ps5
Eukaryota - ps6
Amorpheae - ps7
Obazoa - ps8
Opisthokonta - ps9
Holozoa - ps10
Filozoa - ps11
Choano./Metazoa - ps12
Metazoa - ps13
Myriazoa - ps14
Parahoxozoa - ps15
Bilateria - ps16
Protostomia - ps17
Ecdysozoa - ps18
Arthropoda - ps19
Mandibulata - ps20
Pancrustacea - ps21
Insecta - ps22
Eumetabola - ps23
Holometabola/Psocodea - ps24
Holometabola - ps25
Aparaglossata - ps26
Mecopterida - ps27
Diptera - ps28
Bibiono./Cyclorrhapha - ps29
Cyclorrhapha - ps30
Calyptratae/Drosophila - ps31
Drosophila - ps32
Sophophora - ps33
Obscura/Melanogaster_group - ps34
D_melanogaster_group - ps35
D_melanogaster_subgroup - ps36
sim./sech./mel. group - ps37
Drosophila_melanogaster - ps38
Phylostratum

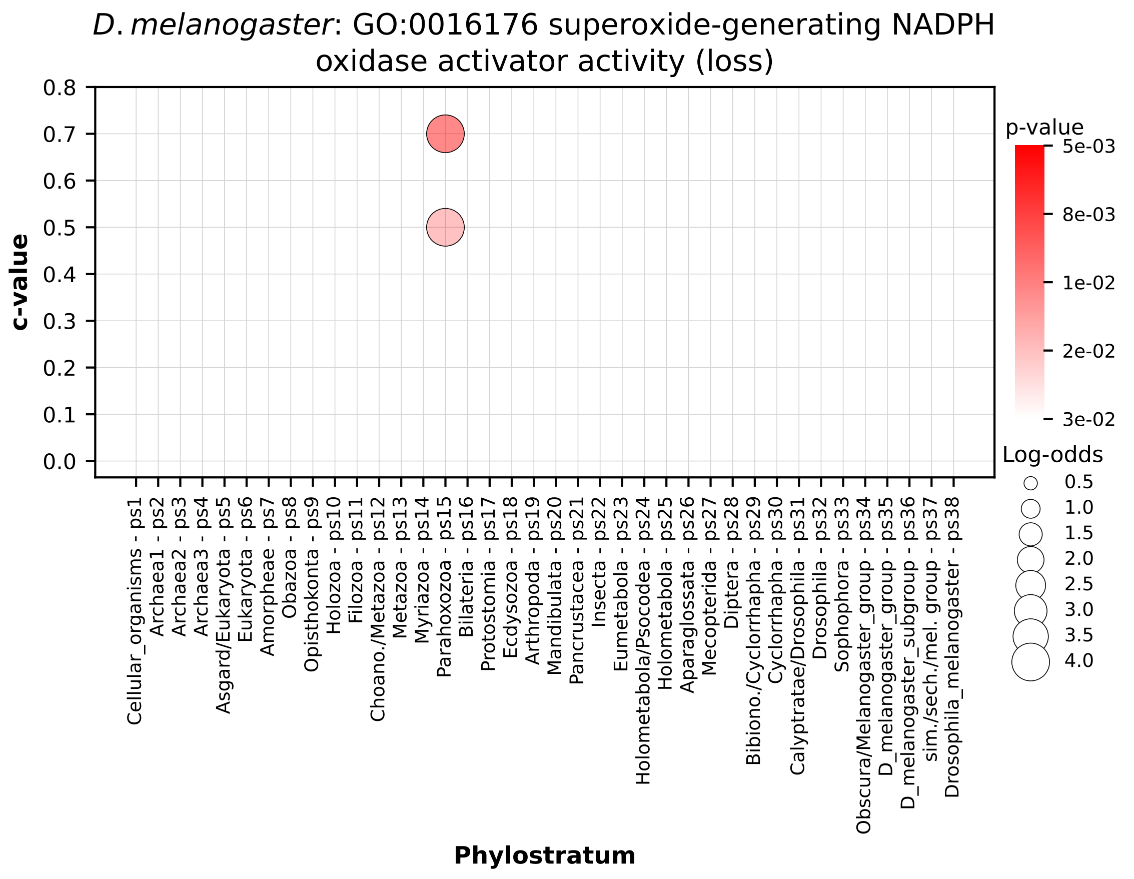
D. melanogaster: GO:0016176 superoxide-generating NADPH oxidase activator activity (loss)
c-value
0.8
0.7
0.6
0.5
0.4
0.3
0.2
0.1
0.0
Phylostratum
Cellular_organisms - ps1
Archaea1 - ps2
Archaea2 - ps3
Archaea3 - ps4
Asgard/Eukaryota - ps5
Eukaryota - ps6
Amorpheae - ps7
Obazoa - ps8
Opisthokonta - ps9
Holozoa - ps10
Filozoa - ps11
Choano./Metazoa - ps12
Metazoa - ps13
Myriazoa - ps14
Parahoxozoa - ps15
Bilateria - ps16
Protostomia - ps17
Ecdysozoa - ps18
Arthropoda - ps19
Mandibulata - ps20
Pancrustacea - ps21
Insecta - ps22
Eumetabola - ps23
Holometabola/Psocodea - ps24
Holometabola - ps25
Aparaglossata - ps26
Mecopterida - ps27
Diptera - ps28
Bibiono./Cyclorrhapha - ps29
Cyclorrhapha - ps30
Calyptratae/Drosophila - ps31
Drosophila - ps32
Sophophora - ps33
Obscura/Melanogaster_group - ps34
D_melanogaster_group - ps35
D_melanogaster_subgroup - ps36
sim./sech./mel. group - ps37
Drosophila_melanogaster - ps38
p-value
5e-03
8e-03
1e-02
2e-02
3e-02
Log-odds
0.5
1.0
1.5
2.0
2.5
3.0
3.5
4.0

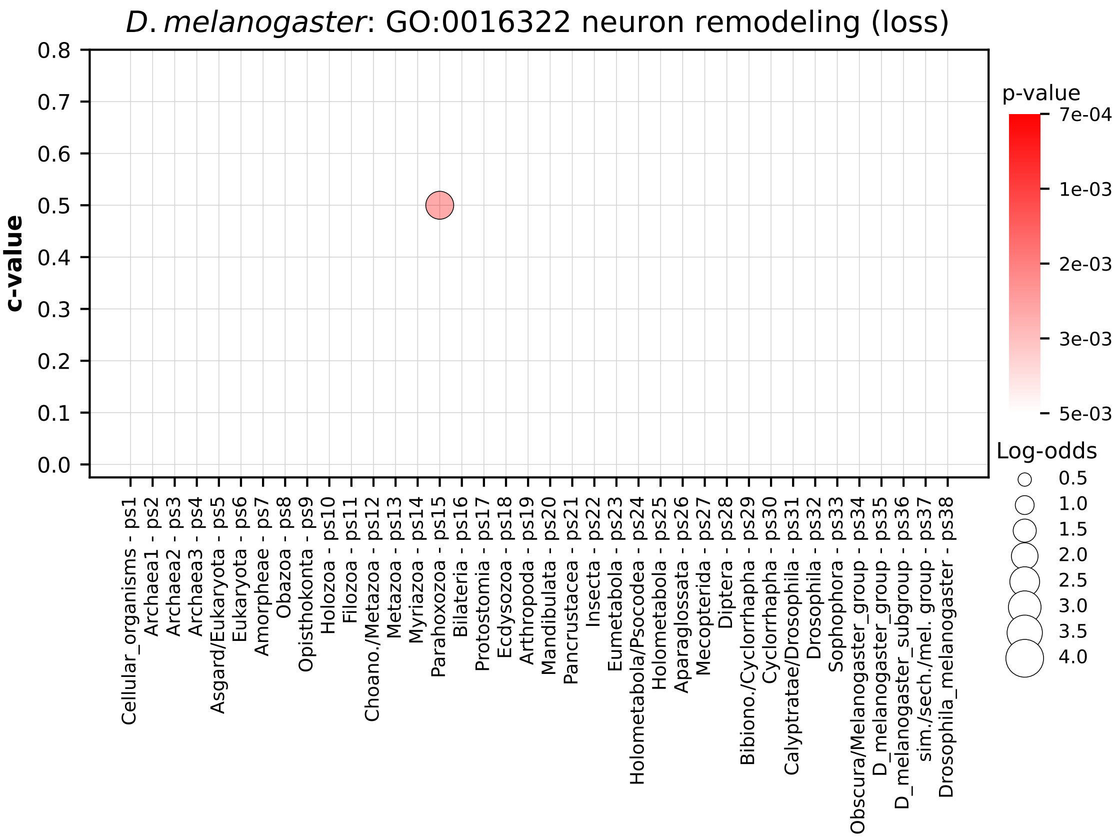
D. melanogaster: GO:0016322 neuron remodeling (loss)
c-value
0.0
0.1
0.2
0.3
0.4
0.5
0.6
0.7
0.8
Phylostratum
Cellular_organisms - ps1
Archaea1 - ps2
Archaea2 - ps3
Archaea3 - ps4
Asgard/Eukaryota - ps5
Eukaryota - ps6
Amorpheae - ps7
Obazoa - ps8
Opisthokonta - ps9
Holozoa - ps10
Filozoa - ps11
Choano./Metazoa - ps12
Metazoa - ps13
Myriazoa - ps14
Parahoxozoa - ps15
Bilateria - ps16
Protostomia - ps17
Ecdysozoa - ps18
Arthropoda - ps19
Mandibulata - ps20
Pancrustacea - ps21
Insecta - ps22
Eumetabola - ps23
Holometabola/Psocodea - ps24
Holometabola - ps25
Aparaglossata - ps26
Mecopterida - ps27
Diptera - ps28
Bibiono./Cyclorrhapha - ps29
Cyclorrhapha - ps30
Calyptratae/Drosophila - ps31
Drosophila - ps32
Sophophora - ps33
Obscura/Melanogaster_group - ps34
D_melanogaster_group - ps35
D_melanogaster_subgroup - ps36
sim./sech./mel. group - ps37
Drosophila_melanogaster - ps38
p-value
7e-04
1e-03
2e-03
3e-03
5e-03
Log-odds
0.5
1.0
1.5
2.0
2.5
3.0
3.5
4.0

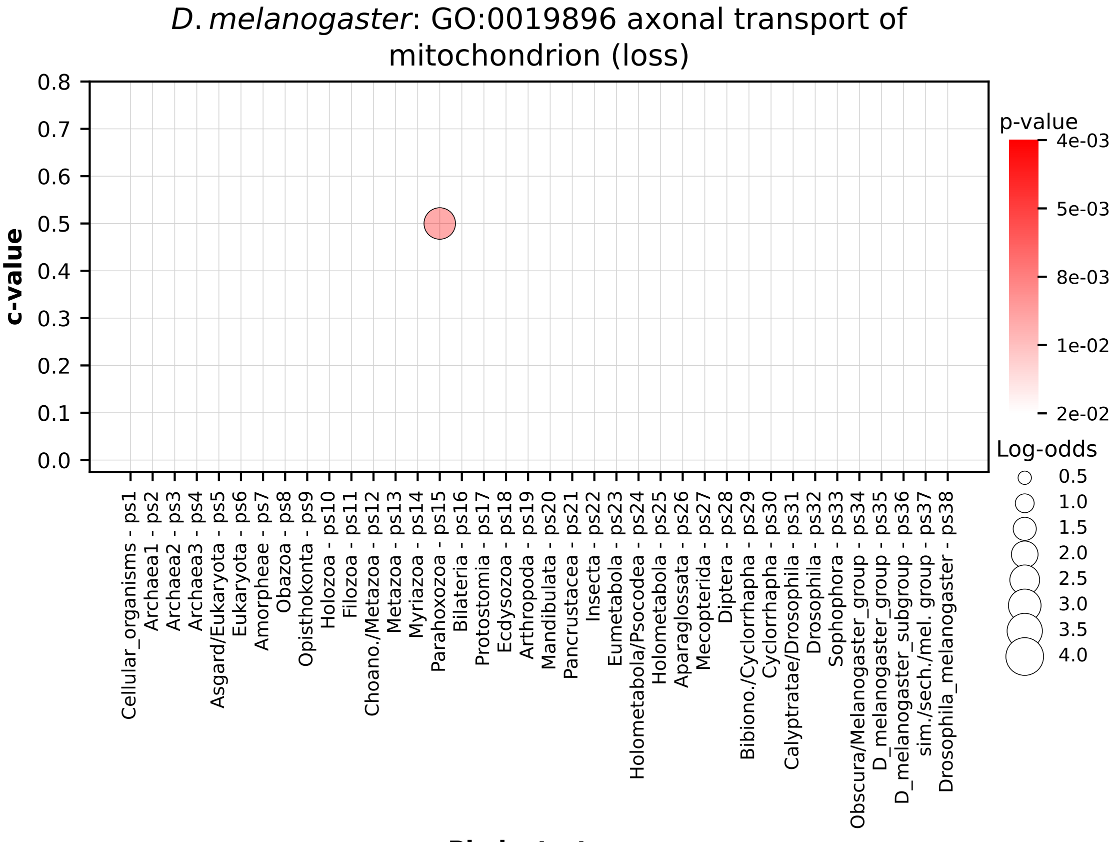

D. melanogaster: GO:0019896 axonal transport of mitochondrion (loss)
c-value
0.8
0.7
0.6
0.5
0.4
0.3
0.2
0.1
0.0
p-value
4e-03
5e-03
8e-03
1e-02
2e-02
Log-odds
0.5
1.0
1.5
2.0
2.5
3.0
3.5
4.0
Cellular_organisms - ps1
Archaea1 - ps2
Archaea2 - ps3
Archaea3 - ps4
Asgard/Eukaryota - ps5
Eukaryota - ps6
Amorpheae - ps7
Obazoa - ps8
Opisthokonta - ps9
Holozoa - ps10
Filozoa - ps11
Choano./Metazoa - ps12
Metazoa - ps13
Myriazoa - ps14
Parahoxozoa - ps15
Bilateria - ps16
Protostomia - ps17
Ecdysozoa - ps18
Arthropoda - ps19
Mandibulata - ps20
Pancrustacea - ps21
Insecta - ps22
Eumetabola - ps23
Holometabola/Psocodea - ps24
Holometabola - ps25
Aparaglossata - ps26
Mecopterida - ps27
Diptera - ps28
Bibiono./Cyclorrhapha - ps29
Cyclorrhapha - ps30
Calyptratae/Drosophila - ps31
Drosophila - ps32
Sophophora - ps33
Obscura/Melanogaster_group - ps34
D_melanogaster_group - ps35
D_melanogaster_subgroup - ps36
sim./sech./mel. group - ps37
Drosophila_melanogaster - ps38
Phylostratum

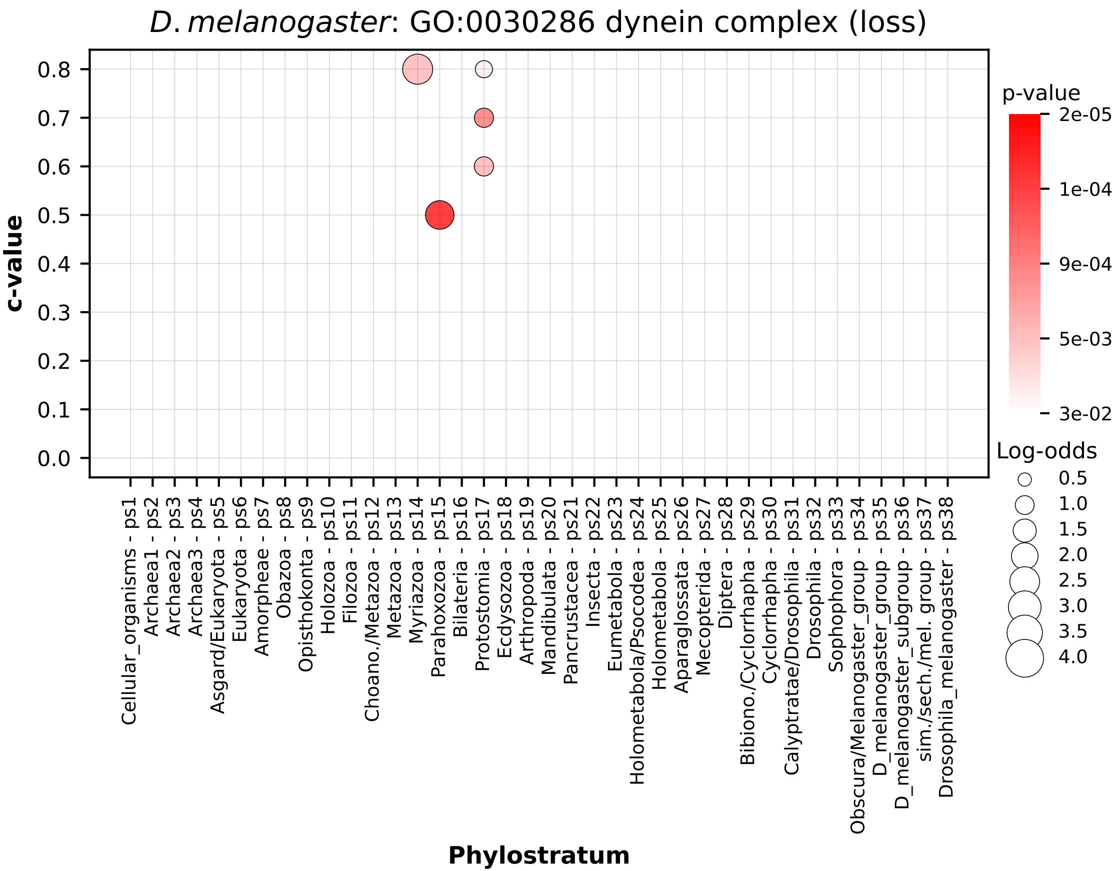
D. melanogaster: GO:0030286 dynein complex (loss)
c-value
Phylostratum
p-value
2e-05
1e-04
9e-04
5e-03
3e-02
Log-odds
0.5
1.0
1.5
2.0
2.5
3.0
3.5
4.0
Cellular_organisms - ps1
Archaea1 - ps2
Archaea2 - ps3
Archaea3 - ps4
Asgard/Eukaryota - ps5
Eukaryota - ps6
Amorpheae - ps7
Obazoa - ps8
Opisthokonta - ps9
Holozoa - ps10
Filozoa - ps11
Choano./Metazoa - ps12
Metazoa - ps13
Myriazoa - ps14
Parahoxozoa - ps15
Bilateria - ps16
Protostomia - ps17
Ecdysozoa - ps18
Arthropoda - ps19
Mandibulata - ps20
Pancrustacea - ps21
Insecta - ps22
Eumetabola - ps23
Holometabola/Psocodea - ps24
Holometabola - ps25
Aparaglossata - ps26
Mecopterida - ps27
Diptera - ps28
Bibiono./Cyclorrhapha - ps29
Cyclorrhapha - ps30
Calyptratae/Drosophila - ps31
Drosophila - ps32
Sophophora - ps33
Obscura/Melanogaster_group - ps34
D_melanogaster_group - ps35
D_melanogaster_subgroup - ps36
sim./sech./mel. group - ps37
Drosophila_melanogaster - ps38

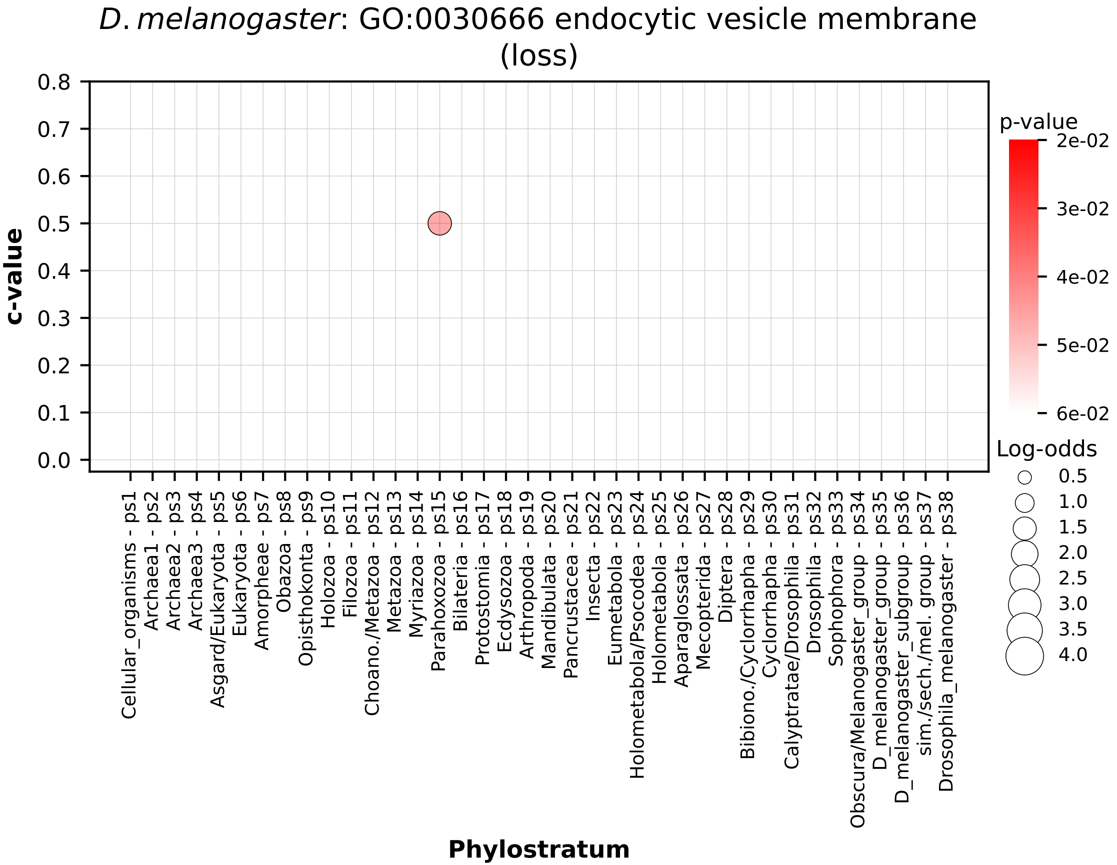

D. melanogaster: GO:0030666 endocytic vesicle membrane (loss)
c-value
0.8
0.7
0.6
0.5
0.4
0.3
0.2
0.1
0.0
p-value
2e-02
3e-02
4e-02
5e-02
6e-02
Log-odds
0.5
1.0
1.5
2.0
2.5
3.0
3.5
4.0
Cellular_organisms - ps1
Archaea1 - ps2
Archaea2 - ps3
Archaea3 - ps4
Asgard/Eukaryota - ps5
Eukaryota - ps6
Amorpheae - ps7
Obazoa - ps8
Opisthokonta - ps9
Holozoa - ps10
Filozoa - ps11
Choano./Metazoa - ps12
Metazoa - ps13
Myriazoa - ps14
Parahoxozoa - ps15
Bilateria - ps16
Protostomia - ps17
Ecdysozoa - ps18
Arthropoda - ps19
Mandibulata - ps20
Pancrustacea - ps21
Insecta - ps22
Eumetabola - ps23
Holometabola/Psocodea - ps24
Holometabola - ps25
Aparaglossata - ps26
Mecopterida - ps27
Diptera - ps28
Bibiono./Cyclorrhapha - ps29
Cyclorrhapha - ps30
Calyptratae/Drosophila - ps31
Drosophila - ps32
Sophophora - ps33
Obscura/Melanogaster_group - ps34
D_melanogaster_group - ps35
D_melanogaster_subgroup - ps36
sim./sech./mel. group - ps37
Drosophila_melanogaster - ps38
Phylostratum

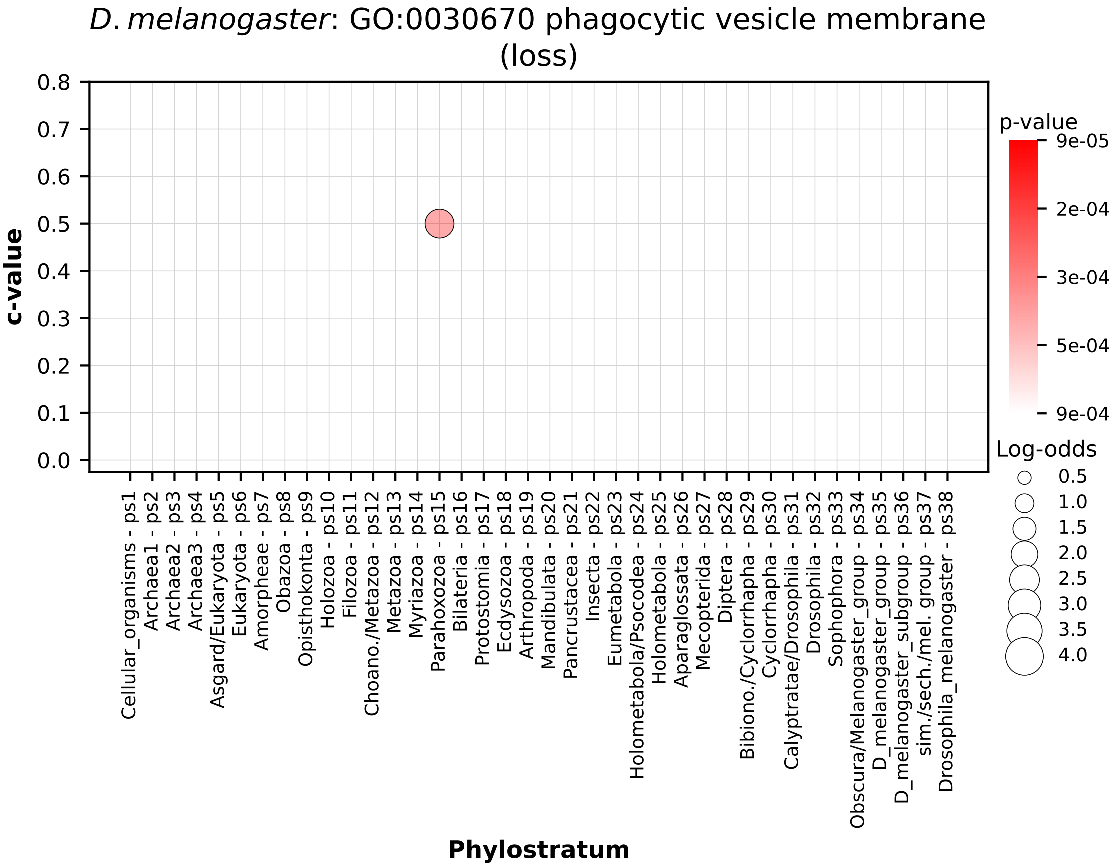
D. melanogaster: GO:0030670 phagocytic vesicle membrane
(loss)
c-value
0.0
0.1
0.2
0.3
0.4
0.5
0.6
0.7
0.8
Phylostratum
Cellular_organisms - ps1
Archaea1 - ps2
Archaea2 - ps3
Archaea3 - ps4
Asgard/Eukaryota - ps5
Eukaryota - ps6
Amorpheae - ps7
Obazoa - ps8
Opisthokonta - ps9
Holozoa - ps10
Filozoa - ps11
Choano./Metazoa - ps12
Metazoa - ps13
Myriazoa - ps14
Parahoxozoa - ps15
Bilateria - ps16
Protostomia - ps17
Ecdysozoa - ps18
Arthropoda - ps19
Mandibulata - ps20
Pancrustacea - ps21
Insecta - ps22
Eumetabola - ps23
Holometabola/Psocodea - ps24
Holometabola - ps25
Aparaglossata - ps26
Mecopterida - ps27
Diptera - ps28
Bibiono./Cyclorrhapha - ps29
Cyclorrhapha - ps30
Calyptratae/Drosophila - ps31
Drosophila - ps32
Sophophora - ps33
Obscura/Melanogaster_group - ps34
D_melanogaster_group - ps35
D_melanogaster_subgroup - ps36
sim./sech./mel. group - ps37
Drosophila_melanogaster - ps38
p-value
9e-05
2e-04
3e-04
5e-04
9e-04
Log-odds
0.5
1.0
1.5
2.0
2.5
3.0
3.5
4.0

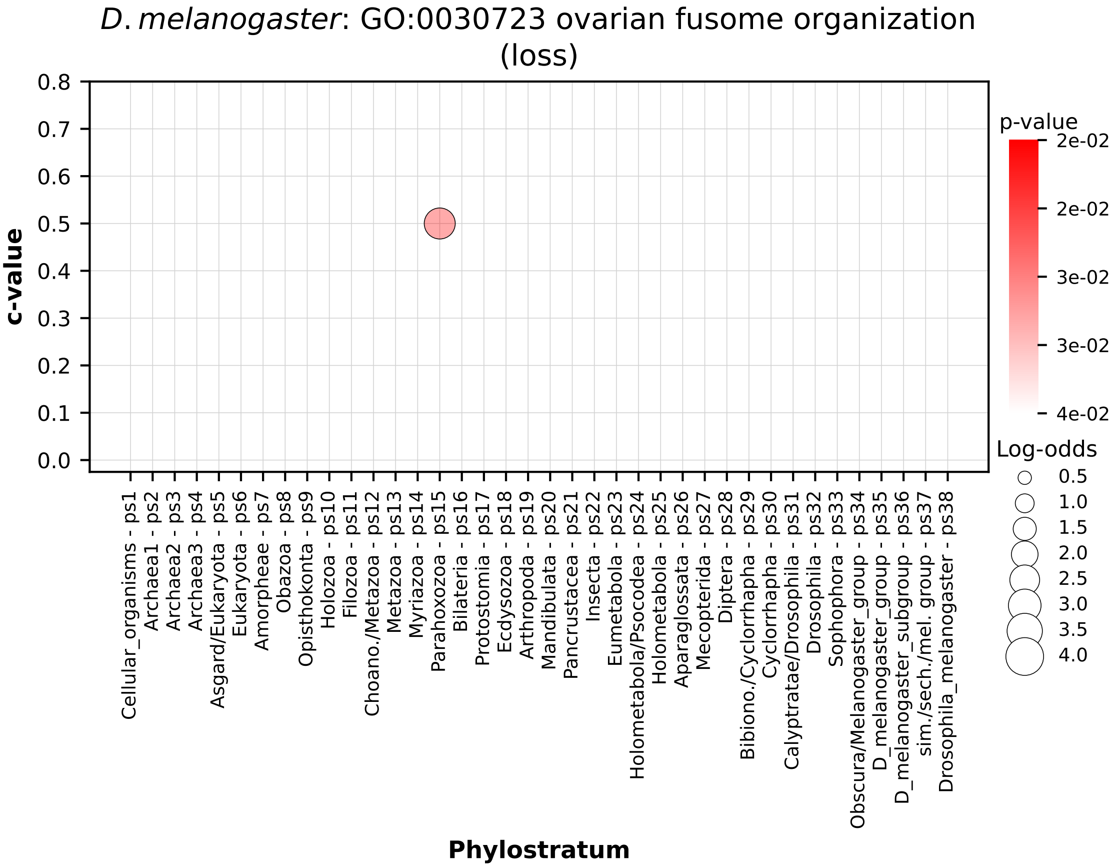
D. melanogaster: GO:0030723 ovarian fusome organization
(loss)
c-value
0.0
0.1
0.2
0.3
0.4
0.5
0.6
0.7
0.8
p-value
2e-02
2e-02
3e-02
3e-02
4e-02
Log-odds
0.5
1.0
1.5
2.0
2.5
3.0
3.5
4.0
Cellular_organisms - ps1
Archaea1 - ps2
Archaea2 - ps3
Archaea3 - ps4
Asgard/Eukaryota - ps5
Eukaryota - ps6
Amorpheae - ps7
Obazoa - ps8
Opisthokonta - ps9
Holozoa - ps10
Filozoa - ps11
Choano./Metazoa - ps12
Metazoa - ps13
Myriazoa - ps14
Parahoxozoa - ps15
Bilateria - ps16
Protostomia - ps17
Ecdysozoa - ps18
Arthropoda - ps19
Mandibulata - ps20
Pancrustacea - ps21
Insecta - ps22
Eumetabola - ps23
Holometabola/Psocodea - ps24
Holometabola - ps25
Aparaglossata - ps26
Mecopterida - ps27
Diptera - ps28
Bibiono./Cyclorrhapha - ps29
Cyclorrhapha - ps30
Calyptratae/Drosophila - ps31
Drosophila - ps32
Sophophora - ps33
Obscura/Melanogaster_group - ps34
D_melanogaster_group - ps35
D_melanogaster_subgroup - ps36
sim./sech./mel. group - ps37
Drosophila_melanogaster - ps38
Phylostratum

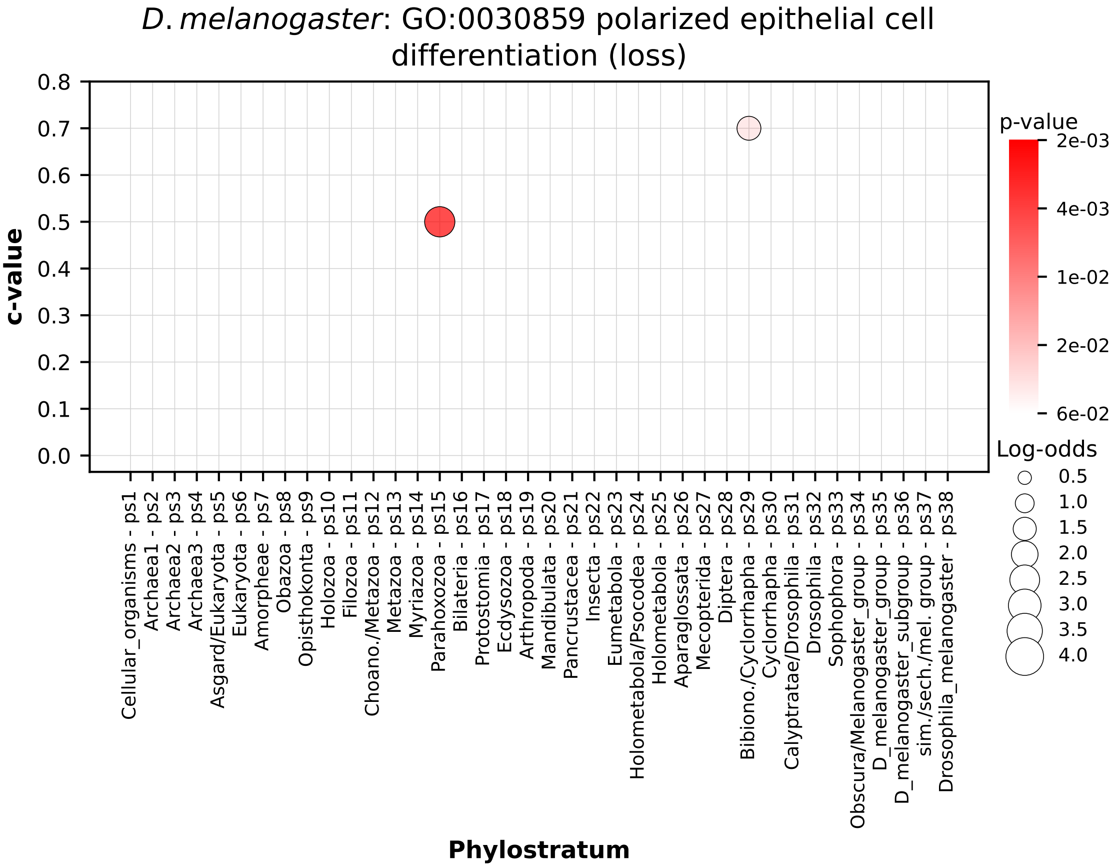

D. melanogaster: GO:0030859 polarized epithelial cell differentiation (loss)
c-value
0.8
0.7
0.6
0.5
0.4
0.3
0.2
0.1
0.0
p-value
2e-03
4e-03
1e-02
2e-02
6e-02
Log-odds
0.5
1.0
1.5
2.0
2.5
3.0
3.5
4.0
Cellular_organisms - ps1
Archaea1 - ps2
Archaea2 - ps3
Archaea3 - ps4
Asgard/Eukaryota - ps5
Eukaryota - ps6
Amorpheae - ps7
Obazoa - ps8
Opisthokonta - ps9
Holozoa - ps10
Filozoa - ps11
Choano./Metazoa - ps12
Metazoa - ps13
Myriazoa - ps14
Parahoxozoa - ps15
Bilateria - ps16
Protostomia - ps17
Ecdysozoa - ps18
Arthropoda - ps19
Mandibulata - ps20
Pancrustacea - ps21
Insecta - ps22
Eumetabola - ps23
Holometabola/Psocodea - ps24
Holometabola - ps25
Aparaglossata - ps26
Mecopterida - ps27
Diptera - ps28
Bibiono./Cyclorrhapha - ps29
Cyclorrhapha - ps30
Calyptratae/Drosophila - ps31
Drosophila - ps32
Sophophora - ps33
Obscura/Melanogaster_group - ps34
D_melanogaster_group - ps35
D_melanogaster_subgroup - ps36
sim./sech./mel. group - ps37
Drosophila_melanogaster - ps38
Phylostratum

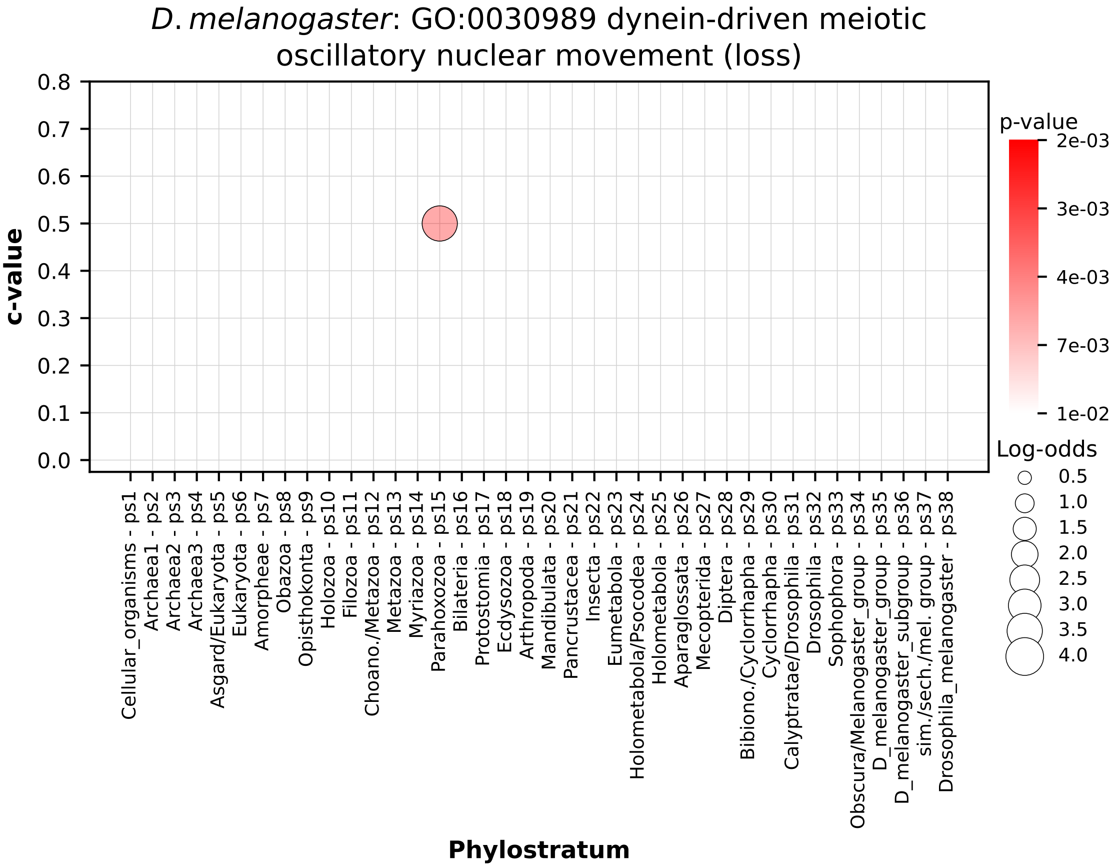
D. melanogaster: GO:0030989 dynein-driven meiotic oscillatory nuclear movement (loss)
c-value
0.8
0.7
0.6
0.5
0.4
0.3
0.2
0.1
0.0
Phylostratum
Cellular_organisms - ps1
Archaea1 - ps2
Archaea2 - ps3
Archaea3 - ps4
Asgard/Eukaryota - ps5
Eukaryota - ps6
Amorpheae - ps7
Obazoa - ps8
Opisthokonta - ps9
Holozoa - ps10
Filozoa - ps11
Choano./Metazoa - ps12
Metazoa - ps13
Myriazoa - ps14
Parahoxozoa - ps15
Bilateria - ps16
Protostomia - ps17
Ecdysozoa - ps18
Arthropoda - ps19
Mandibulata - ps20
Pancrustacea - ps21
Insecta - ps22
Eumetabola - ps23
Holometabola/Psocodea - ps24
Holometabola - ps25
Aparaglossata - ps26
Mecopterida - ps27
Diptera - ps28
Bibiono./Cyclorrhapha - ps29
Cyclorrhapha - ps30
Calyptratae/Drosophila - ps31
Drosophila - ps32
Sophophora - ps33
Obscura/Melanogaster_group - ps34
D_melanogaster_group - ps35
D_melanogaster_subgroup - ps36
sim./sech./mel. group - ps37
Drosophila_melanogaster - ps38
p-value
2e-03
3e-03
4e-03
7e-03
1e-02
Log-odds
0.5
1.0
1.5
2.0
2.5
3.0
3.5
4.0

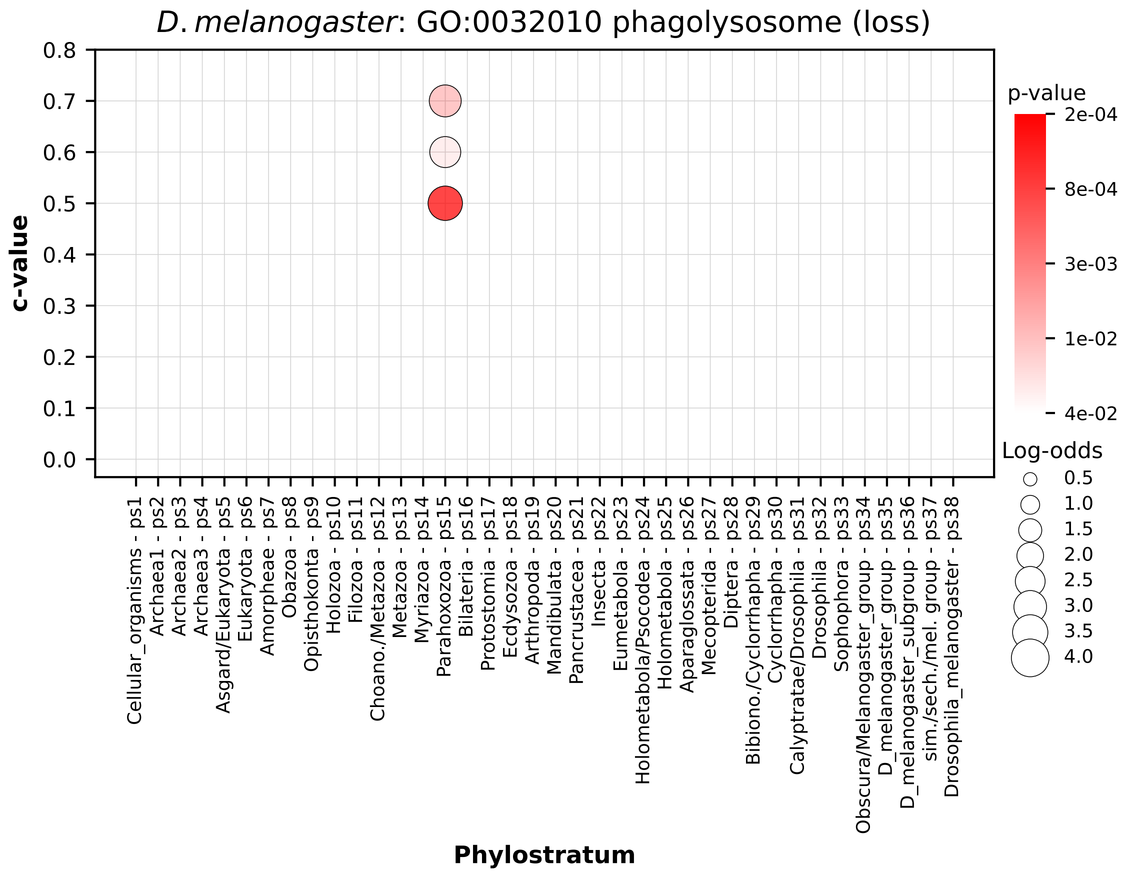

D. melanogaster: GO:0032010 phagolysosome (loss)
c-value
0.8
0.7
0.6
0.5
0.4
0.3
0.2
0.1
0.0
Phylostratum
Cellular_organisms - ps1
Archaea1 - ps2
Archaea2 - ps3
Archaea3 - ps4
Asgard/Eukaryota - ps5
Eukaryota - ps6
Amorpheae - ps7
Obazoa - ps8
Opisthokonta - ps9
Holozoa - ps10
Filozoa - ps11
Choano./Metazoa - ps12
Metazoa - ps13
Myriazoa - ps14
Parahoxozoa - ps15
Bilateria - ps16
Protostomia - ps17
Ecdysozoa - ps18
Arthropoda - ps19
Mandibulata - ps20
Pancrustacea - ps21
Insecta - ps22
Eumetabola - ps23
Holometabola/Psocodea - ps24
Holometabola - ps25
Aparaglossata - ps26
Mecopterida - ps27
Diptera - ps28
Bibiono./Cyclorrhapha - ps29
Cyclorrhapha - ps30
Calyptratae/Drosophila - ps31
Drosophila - ps32
Sophophora - ps33
Obscura/Melanogaster_group - ps34
D_melanogaster_group - ps35
D_melanogaster_subgroup - ps36
sim./sech./mel. group - ps37
Drosophila_melanogaster - ps38
p-value
2e-04
8e-04
3e-03
1e-02
4e-02
Log-odds
0.5
1.0
1.5
2.0
2.5
3.0
3.5
4.0

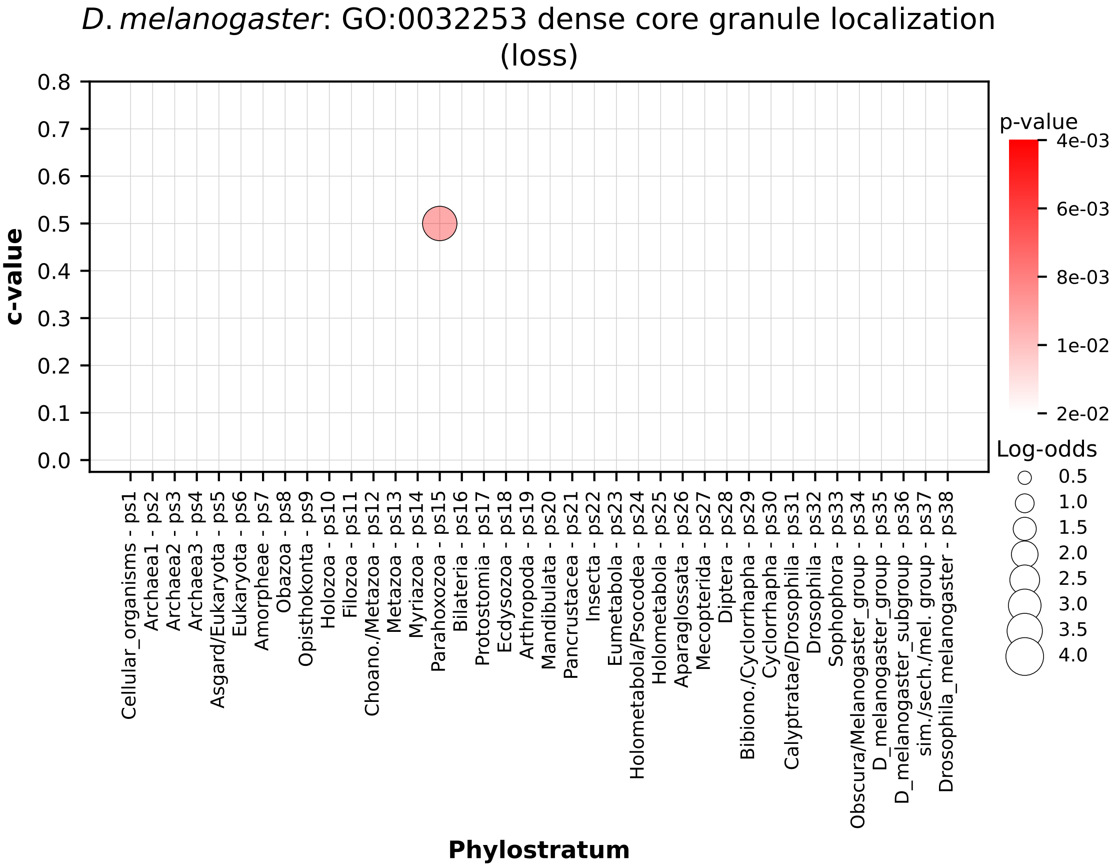

D. melanogaster: GO:0032253 dense core granule localization (loss)
c-value
0.8
0.7
0.6
0.5
0.4
0.3
0.2
0.1
0.0
p-value
4e-03
6e-03
8e-03
1e-02
2e-02
Log-odds
0.5
1.0
1.5
2.0
2.5
3.0
3.5
4.0
Cellular_organisms - ps1
Archaea1 - ps2
Archaea2 - ps3
Archaea3 - ps4
Asgard/Eukaryota - ps5
Eukaryota - ps6
Amorpheae - ps7
Obazoa - ps8
Opisthokonta - ps9
Holozoa - ps10
Filozoa - ps11
Choano./Metazoa - ps12
Metazoa - ps13
Myriazoa - ps14
Parahoxozoa - ps15
Bilateria - ps16
Protostomia - ps17
Ecdysozoa - ps18
Arthropoda - ps19
Mandibulata - ps20
Pancrustacea - ps21
Insecta - ps22
Eumetabola - ps23
Holometabola/Psocodea - ps24
Holometabola - ps25
Aparaglossata - ps26
Mecopterida - ps27
Diptera - ps28
Bibiono./Cyclorrhapha - ps29
Cyclorrhapha - ps30
Calyptratae/Drosophila - ps31
Drosophila - ps32
Sophophora - ps33
Obscura/Melanogaster_group - ps34
D_melanogaster_group - ps35
D_melanogaster_subgroup - ps36
sim./sech./mel. group - ps37
Drosophila_melanogaster - ps38
Phylostratum

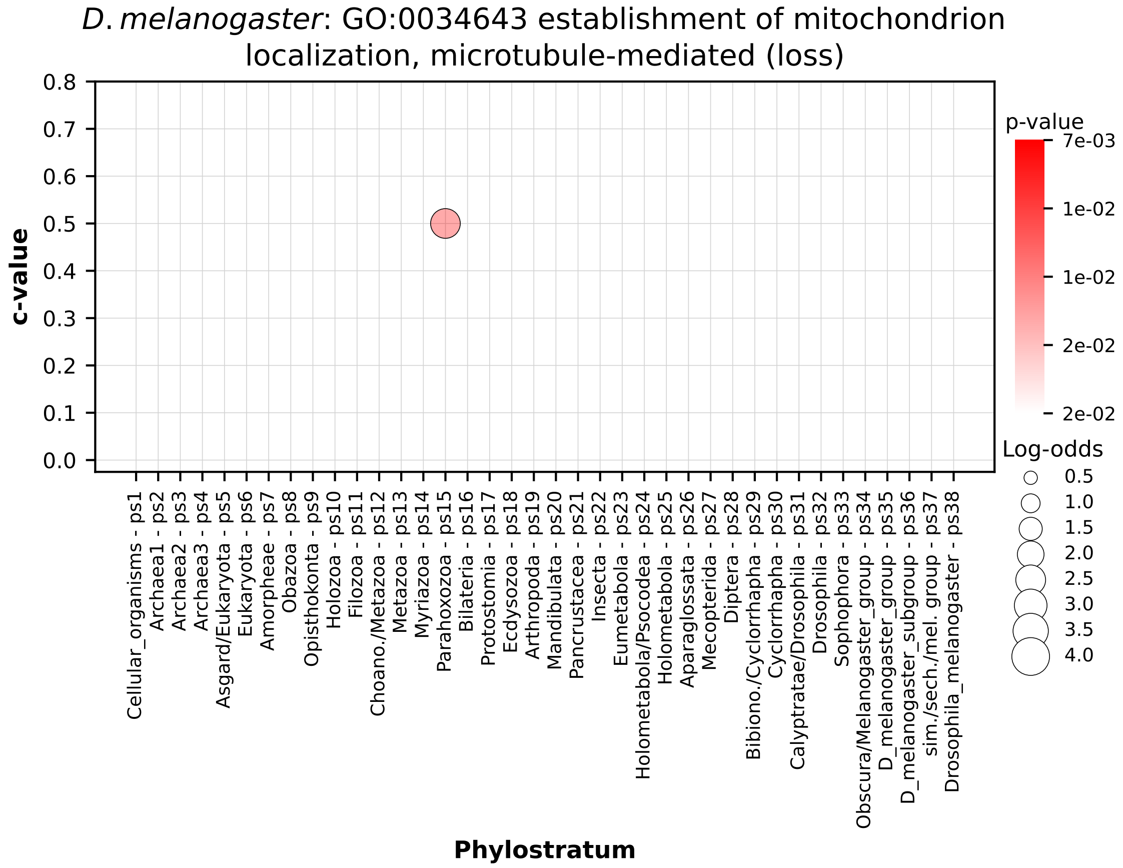

D. melanogaster: GO:0034643 establishment of mitochondrion localization, microtubule-mediated (loss)
c-value
0.8
0.7
0.6
0.5
0.4
0.3
0.2
0.1
0.0
Phylostratum
Cellular_organisms - ps1
Archaea1 - ps2
Archaea2 - ps3
Archaea3 - ps4
Asgard/Eukaryota - ps5
Eukaryota - ps6
Amorpheae - ps7
Obazoa - ps8
Opisthokonta - ps9
Holozoa - ps10
Filozoa - ps11
Choano./Metazoa - ps12
Metazoa - ps13
Myriazoa - ps14
Parahoxozoa - ps15
Bilateria - ps16
Protostomia - ps17
Ecdysozoa - ps18
Arthropoda - ps19
Mandibulata - ps20
Pancrustacea - ps21
Insecta - ps22
Eumetabola - ps23
Holometabola/Psocodea - ps24
Holometabola - ps25
Aparaglossata - ps26
Mecopterida - ps27
Diptera - ps28
Bibiono./Cyclorrhapha - ps29
Cyclorrhapha - ps30
Calyptratae/Drosophila - ps31
Drosophila - ps32
Sophophora - ps33
Obscura/Melanogaster_group - ps34
D_melanogaster_group - ps35
D_melanogaster_subgroup - ps36
sim./sech./mel. group - ps37
Drosophila_melanogaster - ps38
p-value
7e-03
1e-02
1e-02
2e-02
2e-02
Log-odds
0.5
1.0
1.5
2.0
2.5
3.0
3.5
4.0

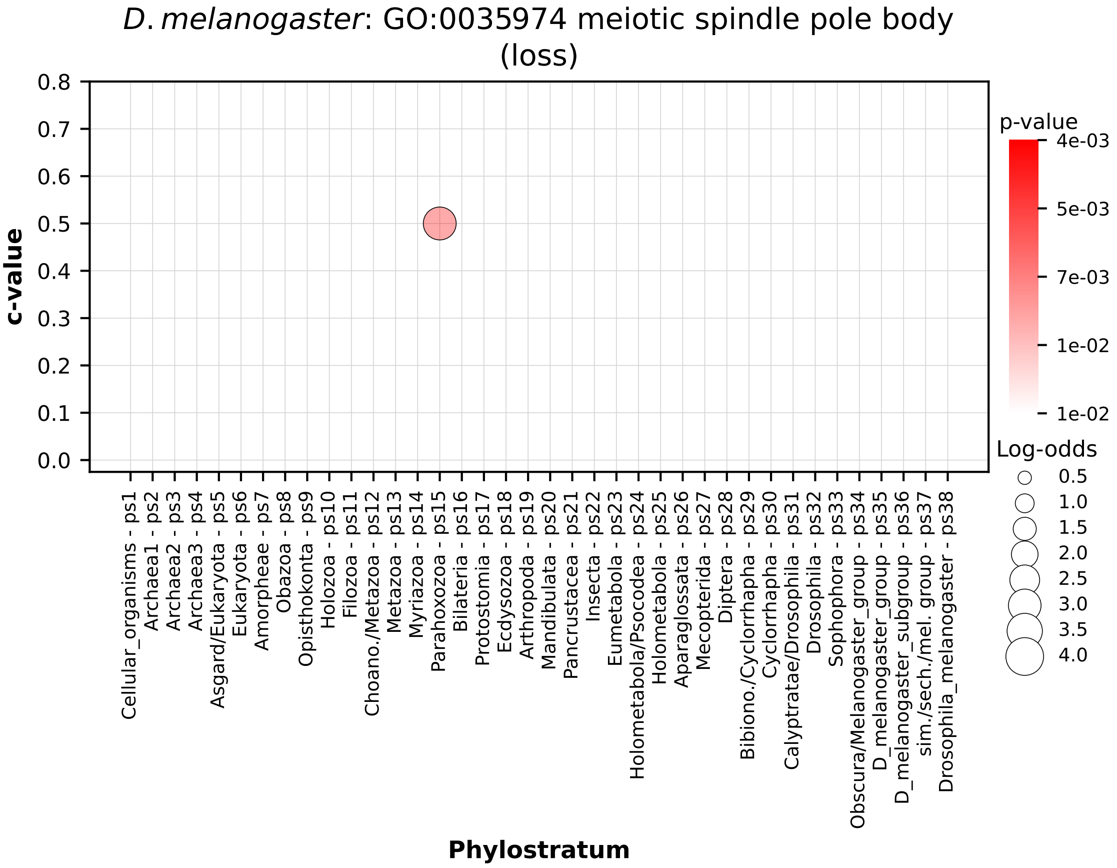

D. melanogaster: GO:0035974 meiotic spindle pole body
(loss)
c-value
0.8
0.7
0.6
0.5
0.4
0.3
0.2
0.1
0.0
Phylostratum
Cellular_organisms - ps1
Archaea1 - ps2
Archaea2 - ps3
Archaea3 - ps4
Asgard/Eukaryota - ps5
Eukaryota - ps6
Amorpheae - ps7
Obazoa - ps8
Opisthokonta - ps9
Holozoa - ps10
Filozoa - ps11
Choano./Metazoa - ps12
Metazoa - ps13
Myriazoa - ps14
Parahoxozoa - ps15
Bilateria - ps16
Protostomia - ps17
Ecdysozoa - ps18
Arthropoda - ps19
Mandibulata - ps20
Pancrustacea - ps21
Insecta - ps22
Eumetabola - ps23
Holometabola/Psocodea - ps24
Holometabola - ps25
Aparaglossata - ps26
Mecopterida - ps27
Diptera - ps28
Bibiono./Cyclorrhapha - ps29
Cyclorrhapha - ps30
Calyptratae/Drosophila - ps31
Drosophila - ps32
Sophophora - ps33
Obscura/Melanogaster_group - ps34
D_melanogaster_group - ps35
D_melanogaster_subgroup - ps36
sim./sech./mel. group - ps37
Drosophila_melanogaster - ps38
p-value
4e-03
5e-03
7e-03
1e-02
1e-02
Log-odds
0.5
1.0
1.5
2.0
2.5
3.0
3.5
4.0

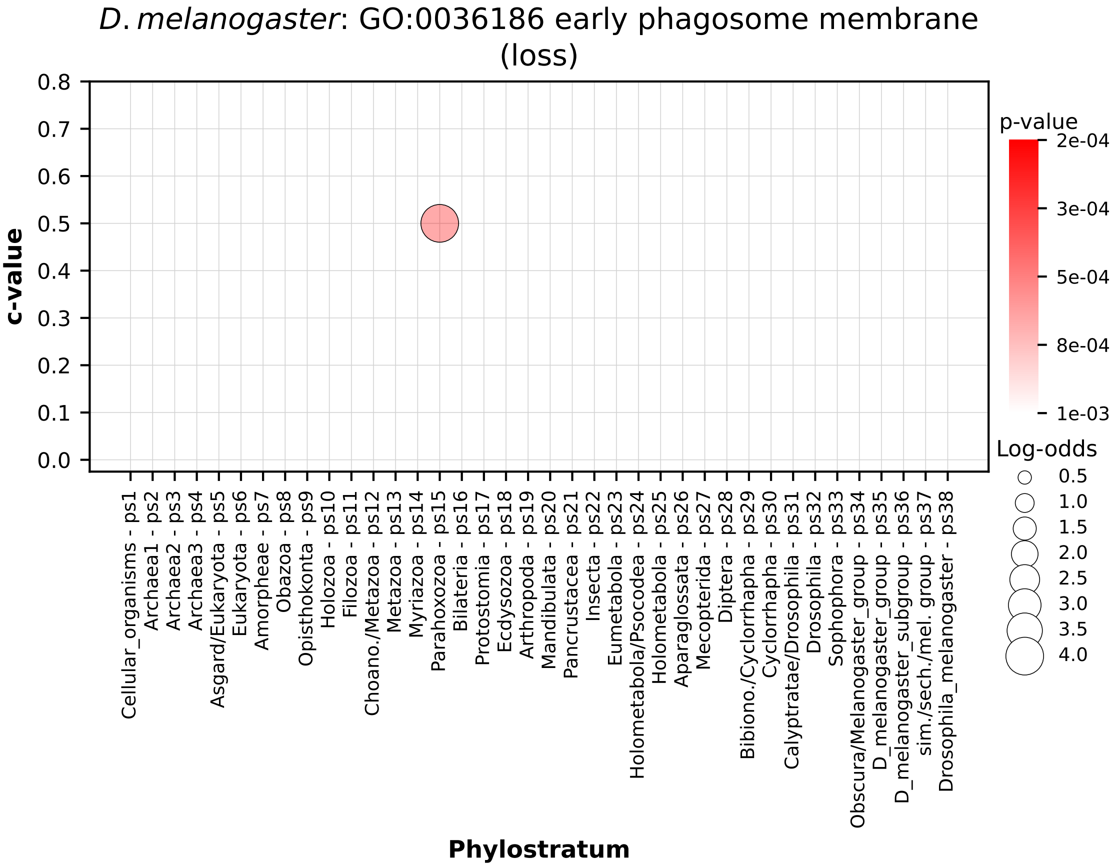

D. melanogaster: GO:0036186 early phagosome membrane (loss)
c-value
0.8
0.7
0.6
0.5
0.4
0.3
0.2
0.1
0.0
Phylostratum
Cellular_organisms - ps1
Archaea1 - ps2
Archaea2 - ps3
Archaea3 - ps4
Asgard/Eukaryota - ps5
Eukaryota - ps6
Amorpheae - ps7
Obazoa - ps8
Opisthokonta - ps9
Holozoa - ps10
Filozoa - ps11
Choano./Metazoa - ps12
Metazoa - ps13
Myriazoa - ps14
Parahoxozoa - ps15
Bilateria - ps16
Protostomia - ps17
Ecdysozoa - ps18
Arthropoda - ps19
Mandibulata - ps20
Pancrustacea - ps21
Insecta - ps22
Eumetabola - ps23
Holometabola/Psocodea - ps24
Holometabola - ps25
Aparaglossata - ps26
Mecopterida - ps27
Diptera - ps28
Bibiono./Cyclorrhapha - ps29
Cyclorrhapha - ps30
Calyptratae/Drosophila - ps31
Drosophila - ps32
Sophophora - ps33
Obscura/Melanogaster_group - ps34
D_melanogaster_group - ps35
D_melanogaster_subgroup - ps36
sim./sech./mel. group - ps37
Drosophila_melanogaster - ps38
p-value
2e-04
3e-04
5e-04
8e-04
1e-03
Log-odds
0.5
1.0
1.5
2.0
2.5
3.0
3.5
4.0

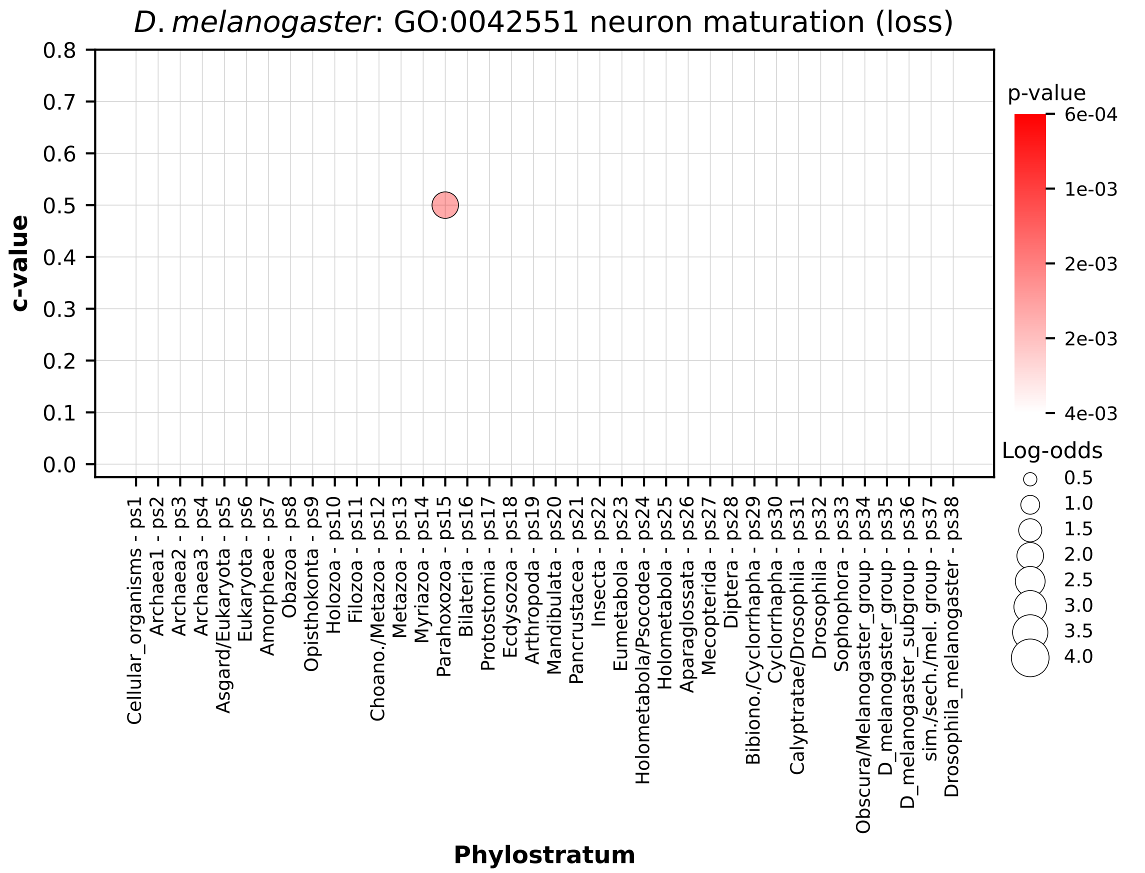
D. melanogaster: GO:0042551 neuron maturation (loss)
c-value
0.0
0.1
0.2
0.3
0.4
0.5
0.6
0.7
0.8
Phylostratum
Cellular_organisms - ps1
Archaea1 - ps2
Archaea2 - ps3
Archaea3 - ps4
Asgard/Eukaryota - ps5
Eukaryota - ps6
Amorpheae - ps7
Obazoa - ps8
Opisthokonta - ps9
Holozoa - ps10
Filozoa - ps11
Choano./Metazoa - ps12
Metazoa - ps13
Myriazoa - ps14
Parahoxozoa - ps15
Bilateria - ps16
Protostomia - ps17
Ecdysozoa - ps18
Arthropoda - ps19
Mandibulata - ps20
Pancrustacea - ps21
Insecta - ps22
Eumetabola - ps23
Holometabola/Psocodea - ps24
Holometabola - ps25
Aparaglossata - ps26
Mecopterida - ps27
Diptera - ps28
Bibiono./Cyclorrhapha - ps29
Cyclorrhapha - ps30
Calyptratae/Drosophila - ps31
Drosophila - ps32
Sophophora - ps33
Obscura/Melanogaster_group - ps34
D_melanogaster_group - ps35
D_melanogaster_subgroup - ps36
sim./sech./mel. group - ps37
Drosophila_melanogaster - ps38
p-value
6e-04
1e-03
2e-03
2e-03
4e-03
Log-odds
0.5
1.0
1.5
2.0
2.5
3.0
3.5
4.0

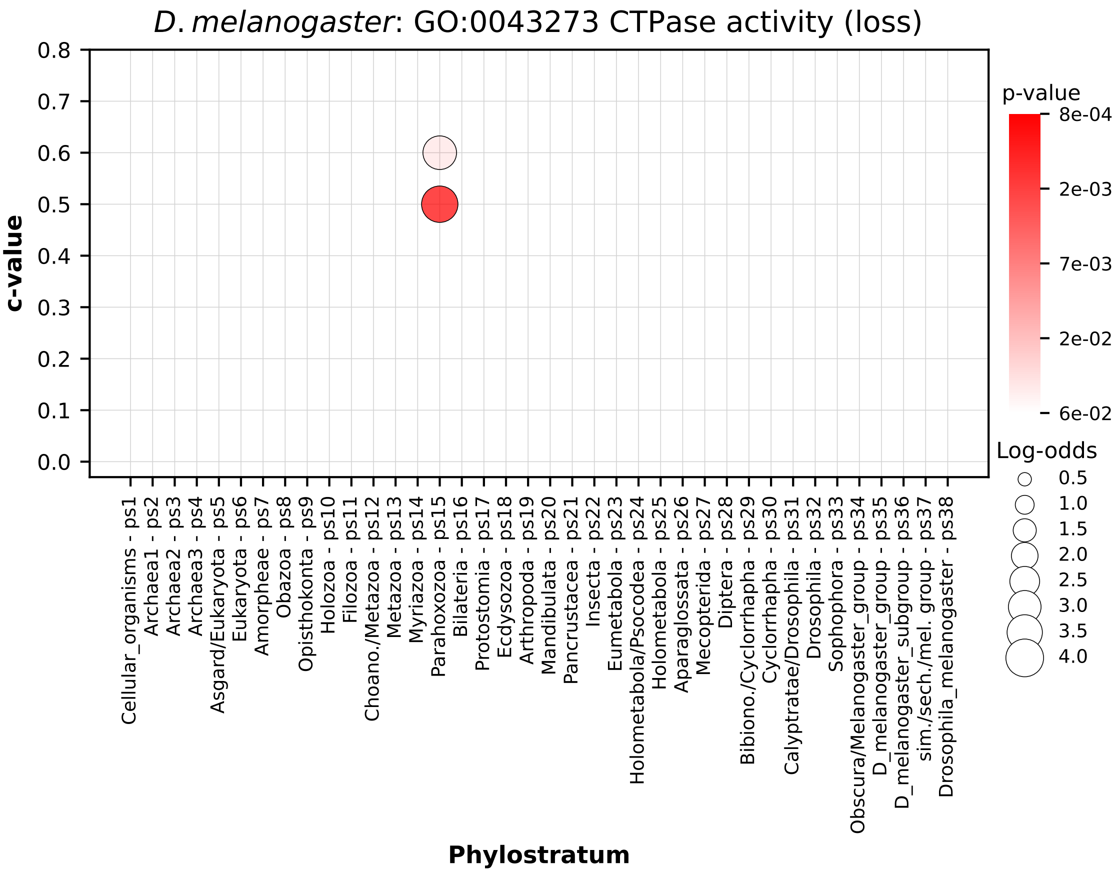
D. melanogaster: GO:0043273 CTPase activity (loss)
c-value
0.8
0.7
0.6
0.5
0.4
0.3
0.2
0.1
0.0
p-value
8e-04
2e-03
7e-03
2e-02
6e-02
Log-odds
0.5
1.0
1.5
2.0
2.5
3.0
3.5
4.0
Cellular_organisms - ps1
Archaea1 - ps2
Archaea2 - ps3
Archaea3 - ps4
Asgard/Eukaryota - ps5
Eukaryota - ps6
Amorpheae - ps7
Obazoa - ps8
Opisthokonta - ps9
Holozoa - ps10
Filozoa - ps11
Choano./Metazoa - ps12
Metazoa - ps13
Myriazoa - ps14
Parahoxozoa - ps15
Bilateria - ps16
Protostomia - ps17
Ecdysozoa - ps18
Arthropoda - ps19
Mandibulata - ps20
Pancrustacea - ps21
Insecta - ps22
Eumetabola - ps23
Holometabola/Psocodea - ps24
Holometabola - ps25
Aparaglossata - ps26
Mecopterida - ps27
Diptera - ps28
Bibiono./Cyclorrhapha - ps29
Cyclorrhapha - ps30
Calyptratae/Drosophila - ps31
Drosophila - ps32
Sophophora - ps33
Obscura/Melanogaster_group - ps34
D_melanogaster_group - ps35
D_melanogaster_subgroup - ps36
sim./sech./mel. group - ps37
Drosophila_melanogaster - ps38
Phylostratum

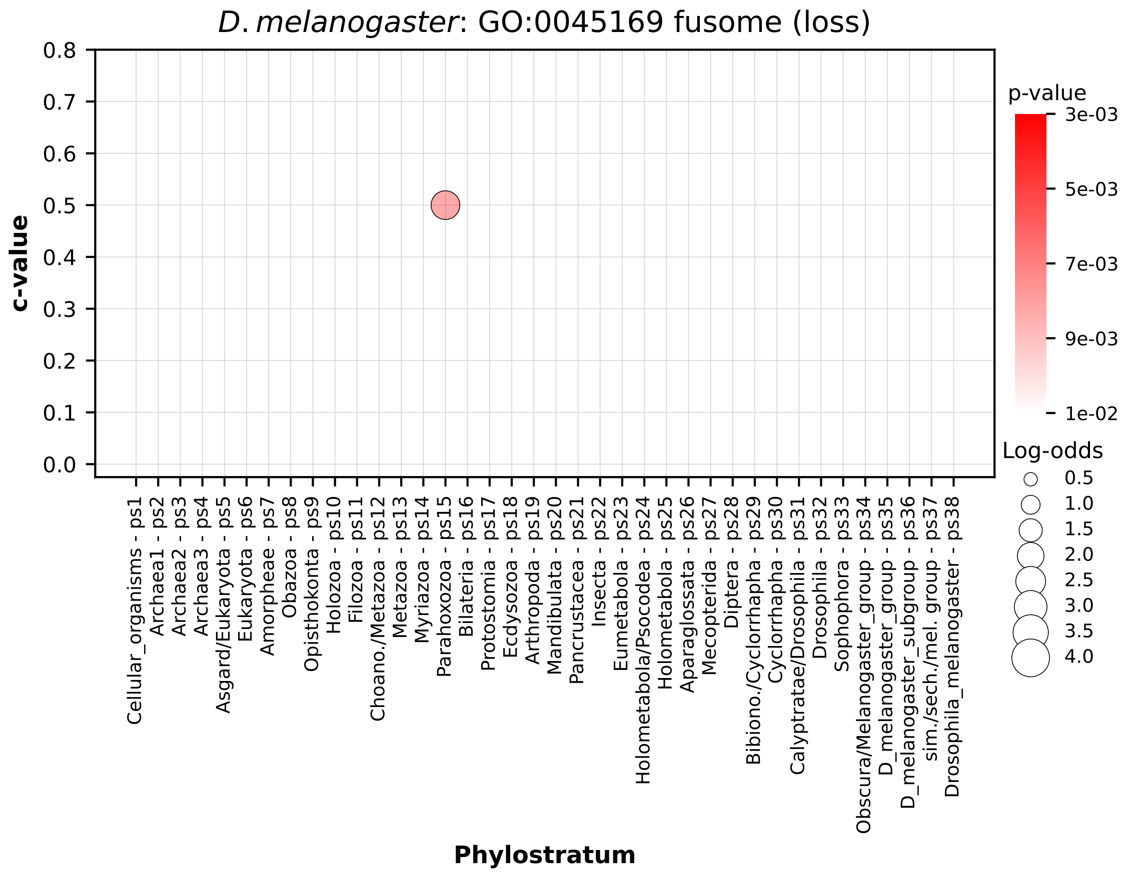
D. melanogaster: GO:0045169 fusome (loss)
c-value
0.8
0.7
0.6
0.5
0.4
0.3
0.2
0.1
0.0
p-value
3e-03
5e-03
7e-03
9e-03
1e-02
Log-odds
0.5
1.0
1.5
2.0
2.5
3.0
3.5
4.0
Cellular_organisms - ps1
Archaea1 - ps2
Archaea2 - ps3
Archaea3 - ps4
Asgard/Eukaryota - ps5
Eukaryota - ps6
Amorpheae - ps7
Obazoa - ps8
Opisthokonta - ps9
Holozoa - ps10
Filozoa - ps11
Choano./Metazoa - ps12
Metazoa - ps13
Myriazoa - ps14
Parahoxozoa - ps15
Bilateria - ps16
Protostomia - ps17
Ecdysozoa - ps18
Arthropoda - ps19
Mandibulata - ps20
Pancrustacea - ps21
Insecta - ps22
Eumetabola - ps23
Holometabola/Psocodea - ps24
Holometabola - ps25
Aparaglossata - ps26
Mecopterida - ps27
Diptera - ps28
Bibiono./Cyclorrhapha - ps29
Cyclorrhapha - ps30
Calyptratae/Drosophila - ps31
Drosophila - ps32
Sophophora - ps33
Obscura/Melanogaster_group - ps34
D_melanogaster_group - ps35
D_melanogaster_subgroup - ps36
sim./sech./mel. group - ps37
Drosophila_melanogaster - ps38
Phylostratum

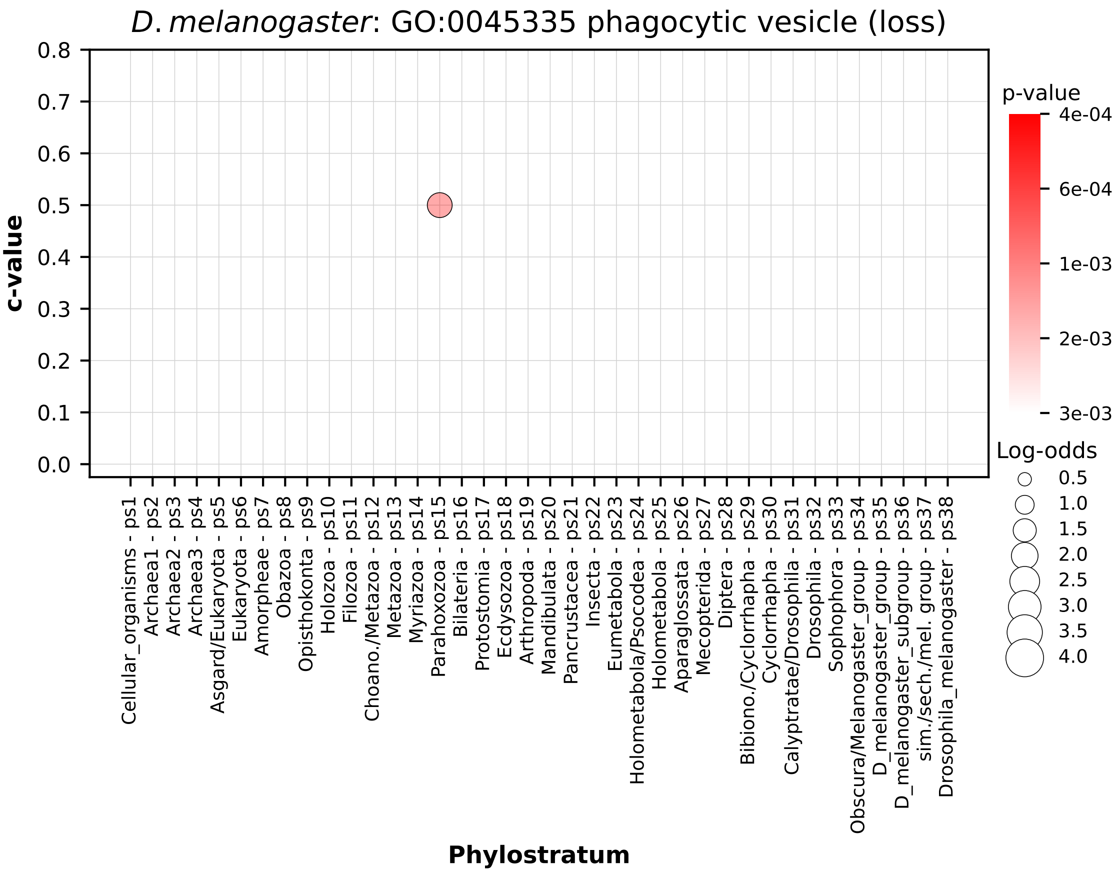
D. melanogaster: GO:0045335 phagocytic vesicle (loss)
c-value
0.8
0.7
0.6
0.5
0.4
0.3
0.2
0.1
0.0
Phylostratum
Cellular_organisms - ps1
Archaea1 - ps2
Archaea2 - ps3
Archaea3 - ps4
Asgard/Eukaryota - ps5
Eukaryota - ps6
Amorpheae - ps7
Obazoa - ps8
Opisthokonta - ps9
Holozoa - ps10
Filozoa - ps11
Choano./Metazoa - ps12
Metazoa - ps13
Myriazoa - ps14
Parahoxozoa - ps15
Bilateria - ps16
Protostomia - ps17
Ecdysozoa - ps18
Arthropoda - ps19
Mandibulata - ps20
Pancrustacea - ps21
Insecta - ps22
Eumetabola - ps23
Holometabola/Psocodea - ps24
Holometabola - ps25
Aparaglossata - ps26
Mecopterida - ps27
Diptera - ps28
Bibiono./Cyclorrhapha - ps29
Cyclorrhapha - ps30
Calyptratae/Drosophila - ps31
Drosophila - ps32
Sophophora - ps33
Obscura/Melanogaster_group - ps34
D_melanogaster_group - ps35
D_melanogaster_subgroup - ps36
sim./sech./mel. group - ps37
Drosophila_melanogaster - ps38
p-value
4e-04
6e-04
1e-03
2e-03
3e-03
Log-odds
0.5
1.0
1.5
2.0
2.5
3.0
3.5
4.0

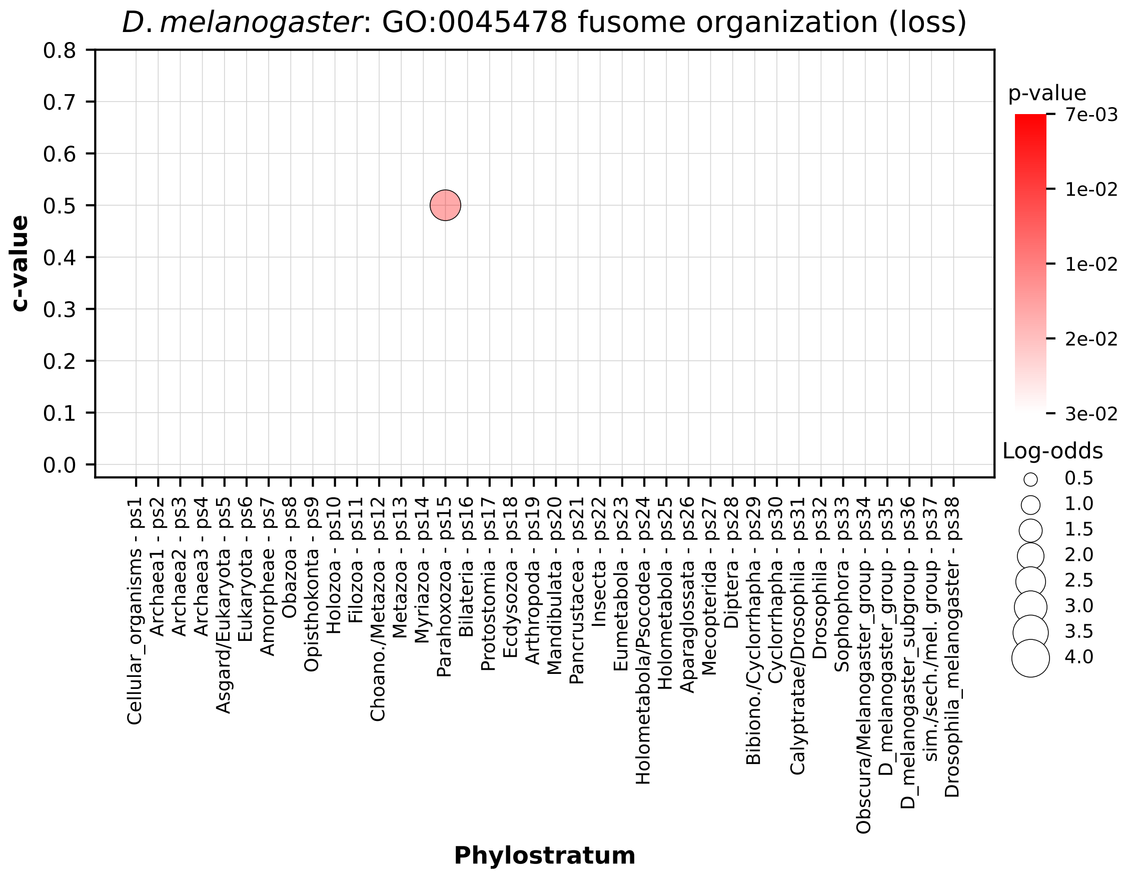

D. melanogaster: GO:0045478 fusome organization (loss)
c-value
0.8
0.7
0.6
0.5
0.4
0.3
0.2
0.1
0.0
p-value
7e-03
1e-02
1e-02
2e-02
3e-02
Log-odds
0.5
1.0
1.5
2.0
2.5
3.0
3.5
4.0
Cellular_organisms - ps1
Archaea1 - ps2
Archaea2 - ps3
Archaea3 - ps4
Asgard/Eukaryota - ps5
Eukaryota - ps6
Amorpheae - ps7
Obazoa - ps8
Opisthokonta - ps9
Holozoa - ps10
Filozoa - ps11
Choano./Metazoa - ps12
Metazoa - ps13
Myriazoa - ps14
Parahoxozoa - ps15
Bilateria - ps16
Protostomia - ps17
Ecdysozoa - ps18
Arthropoda - ps19
Mandibulata - ps20
Pancrustacea - ps21
Insecta - ps22
Eumetabola - ps23
Holometabola/Psocodea - ps24
Holometabola - ps25
Aparaglossata - ps26
Mecopterida - ps27
Diptera - ps28
Bibiono./Cyclorrhapha - ps29
Cyclorrhapha - ps30
Calyptratae/Drosophila - ps31
Drosophila - ps32
Sophophora - ps33
Obscura/Melanogaster_group - ps34
D_melanogaster_group - ps35
D_melanogaster_subgroup - ps36
sim./sech./mel. group - ps37
Drosophila_melanogaster - ps38
Phylostratum

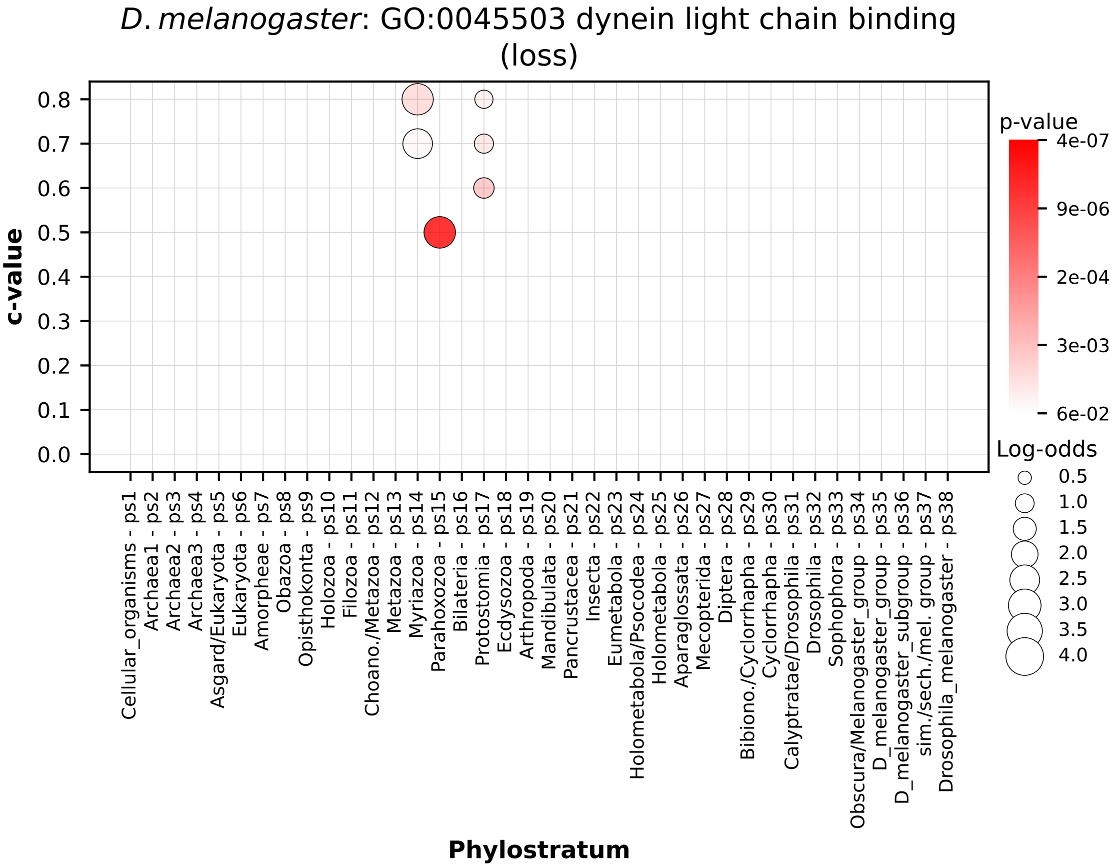

D. melanogaster: GO:0045503 dynein light chain binding (loss)
c-value
0.8
0.7
0.6
0.5
0.4
0.3
0.2
0.1
0.0
p-value
4e-07
9e-06
2e-04
3e-03
6e-02
Log-odds
0.5
1.0
1.5
2.0
2.5
3.0
3.5
4.0
Cellular_organisms - ps1
Archaea1 - ps2
Archaea2 - ps3
Archaea3 - ps4
Asgard/Eukaryota - ps5
Eukaryota - ps6
Amorpheae - ps7
Obazoa - ps8
Opisthokonta - ps9
Holozoa - ps10
Filozoa - ps11
Choano./Metazoa - ps12
Metazoa - ps13
Myriazoa - ps14
Parahoxozoa - ps15
Bilateria - ps16
Protostomia - ps17
Ecdysozoa - ps18
Arthropoda - ps19
Mandibulata - ps20
Pancrustacea - ps21
Insecta - ps22
Eumetabola - ps23
Holometabola/Psocodea - ps24
Holometabola - ps25
Aparaglossata - ps26
Mecopterida - ps27
Diptera - ps28
Bibiono./Cyclorrhapha - ps29
Cyclorrhapha - ps30
Calyptratae/Drosophila - ps31
Drosophila - ps32
Sophophora - ps33
Obscura/Melanogaster_group - ps34
D_melanogaster_group - ps35
D_melanogaster_subgroup - ps36
sim./sech./mel. group - ps37
Drosophila_melanogaster - ps38
Phylostratum

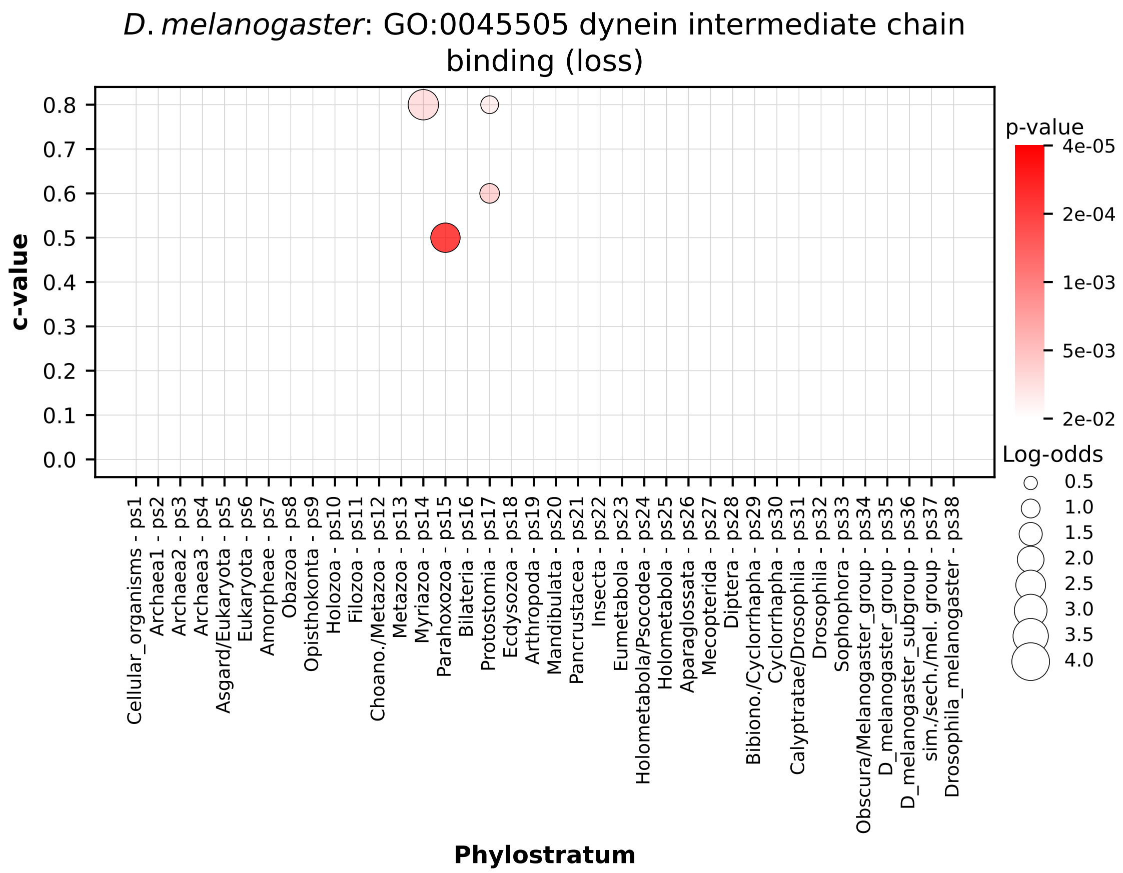

D. melanogaster: GO:0045505 dynein intermediate chain binding (loss)
c-value
0.8
0.7
0.6
0.5
0.4
0.3
0.2
0.1
0.0
p-value
4e-05
2e-04
1e-03
5e-03
2e-02
Log-odds
0.5
1.0
1.5
2.0
2.5
3.0
3.5
4.0
Cellular_organisms - ps1
Archaea1 - ps2
Archaea2 - ps3
Archaea3 - ps4
Asgard/Eukaryota - ps5
Eukaryota - ps6
Amorpheae - ps7
Obazoa - ps8
Opisthokonta - ps9
Holozoa - ps10
Filozoa - ps11
Choano./Metazoa - ps12
Metazoa - ps13
Myriazoa - ps14
Parahoxozoa - ps15
Bilateria - ps16
Protostomia - ps17
Ecdysozoa - ps18
Arthropoda - ps19
Mandibulata - ps20
Pancrustacea - ps21
Insecta - ps22
Eumetabola - ps23
Holometabola/Psocodea - ps24
Holometabola - ps25
Aparaglossata - ps26
Mecopterida - ps27
Diptera - ps28
Bibiono./Cyclorrhapha - ps29
Cyclorrhapha - ps30
Calyptratae/Drosophila - ps31
Drosophila - ps32
Sophophora - ps33
Obscura/Melanogaster_group - ps34
D_melanogaster_group - ps35
D_melanogaster_subgroup - ps36
sim./sech./mel. group - ps37
Drosophila_melanogaster - ps38
Phylostratum

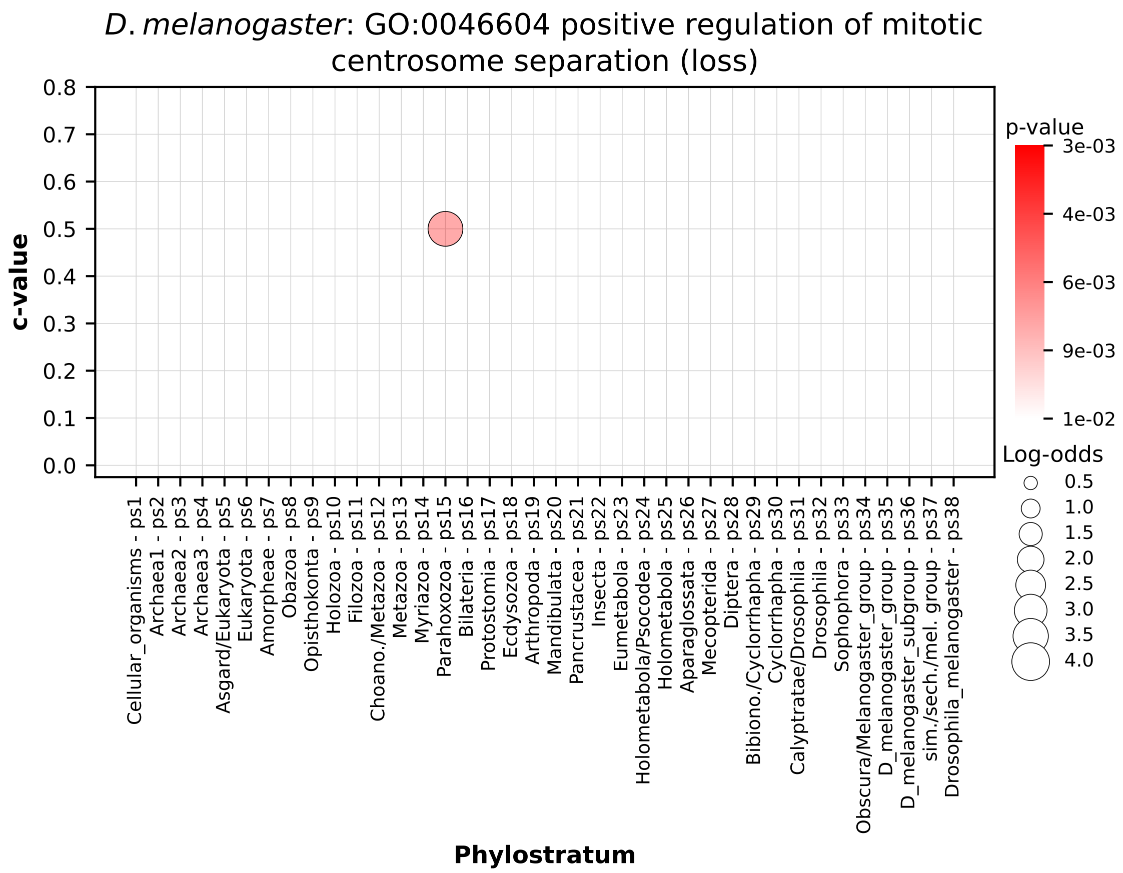

D. melanogaster: GO:0046604 positive regulation of mitotic centrosome separation (loss)
c-value
0.8
0.7
0.6
0.5
0.4
0.3
0.2
0.1
0.0
Phylostratum
Cellular_organisms - ps1
Archaea1 - ps2
Archaea2 - ps3
Archaea3 - ps4
Asgard/Eukaryota - ps5
Eukaryota - ps6
Amorpheae - ps7
Obazoa - ps8
Opisthokonta - ps9
Holozoa - ps10
Filozoa - ps11
Choano./Metazoa - ps12
Metazoa - ps13
Myriazoa - ps14
Parahoxozoa - ps15
Bilateria - ps16
Protostomia - ps17
Ecdysozoa - ps18
Arthropoda - ps19
Mandibulata - ps20
Pancrustacea - ps21
Insecta - ps22
Eumetabola - ps23
Holometabola/Psocodea - ps24
Holometabola - ps25
Aparaglossata - ps26
Mecopterida - ps27
Diptera - ps28
Bibiono./Cyclorrhapha - ps29
Cyclorrhapha - ps30
Calyptratae/Drosophila - ps31
Drosophila - ps32
Sophophora - ps33
Obscura/Melanogaster_group - ps34
D_melanogaster_group - ps35
D_melanogaster_subgroup - ps36
sim./sech./mel. group - ps37
Drosophila_melanogaster - ps38
p-value
3e-03
4e-03
6e-03
9e-03
1e-02
Log-odds
0.5
1.0
1.5
2.0
2.5
3.0
3.5
4.0

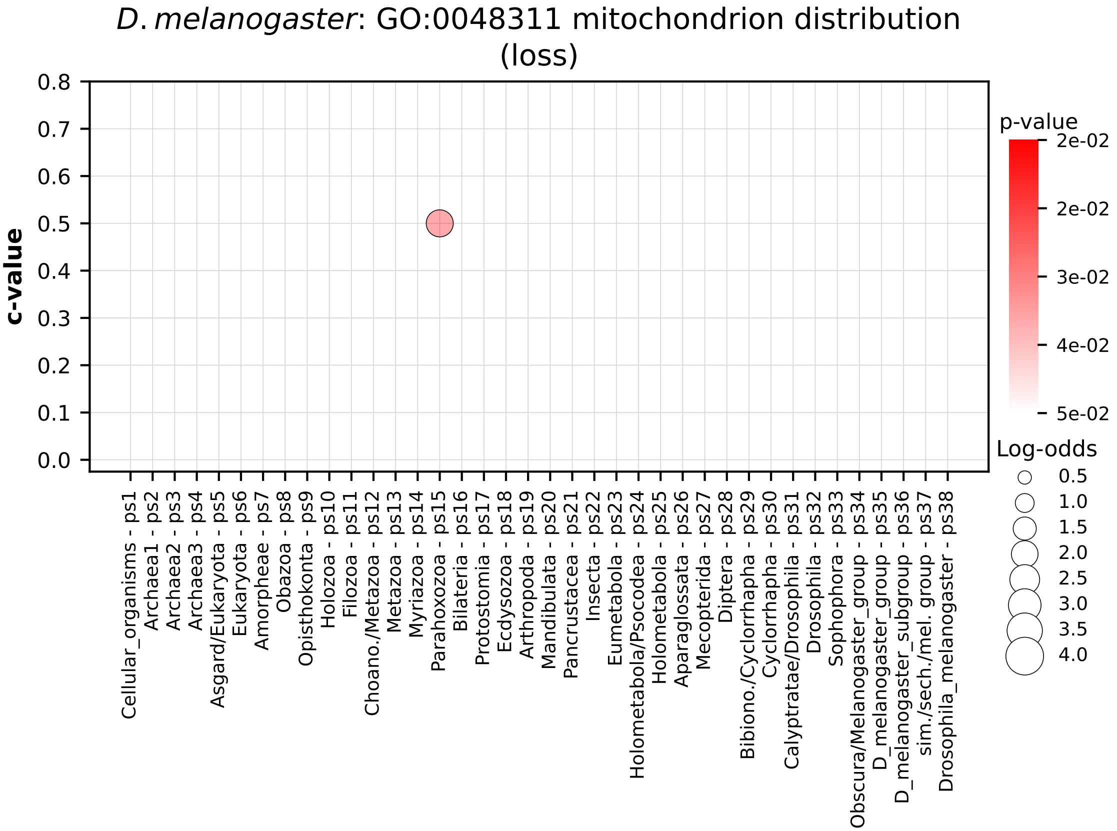

D. melanogaster: GO:0048311 mitochondrion distribution
(loss)
c-value
0.8
0.7
0.6
0.5
0.4
0.3
0.2
0.1
0.0
Phylostratum
Cellular_organisms - ps1
Archaea1 - ps2
Archaea2 - ps3
Archaea3 - ps4
Asgard/Eukaryota - ps5
Eukaryota - ps6
Amorpheae - ps7
Obazoa - ps8
Opisthokonta - ps9
Holozoa - ps10
Filozoa - ps11
Choano./Metazoa - ps12
Metazoa - ps13
Myriazoa - ps14
Parahoxozoa - ps15
Bilateria - ps16
Protostomia - ps17
Ecdysozoa - ps18
Arthropoda - ps19
Mandibulata - ps20
Pancrustacea - ps21
Insecta - ps22
Eumetabola - ps23
Holometabola/Psocodea - ps24
Holometabola - ps25
Aparaglossata - ps26
Mecopterida - ps27
Diptera - ps28
Bibiono./Cyclorrhapha - ps29
Cyclorrhapha - ps30
Calyptratae/Drosophila - ps31
Drosophila - ps32
Sophophora - ps33
Obscura/Melanogaster_group - ps34
D_melanogaster_group - ps35
D_melanogaster_subgroup - ps36
sim./sech./mel. group - ps37
Drosophila_melanogaster - ps38
p-value
2e-02
2e-02
3e-02
4e-02
5e-02
Log-odds
0.5
1.0
1.5
2.0
2.5
3.0
3.5
4.0

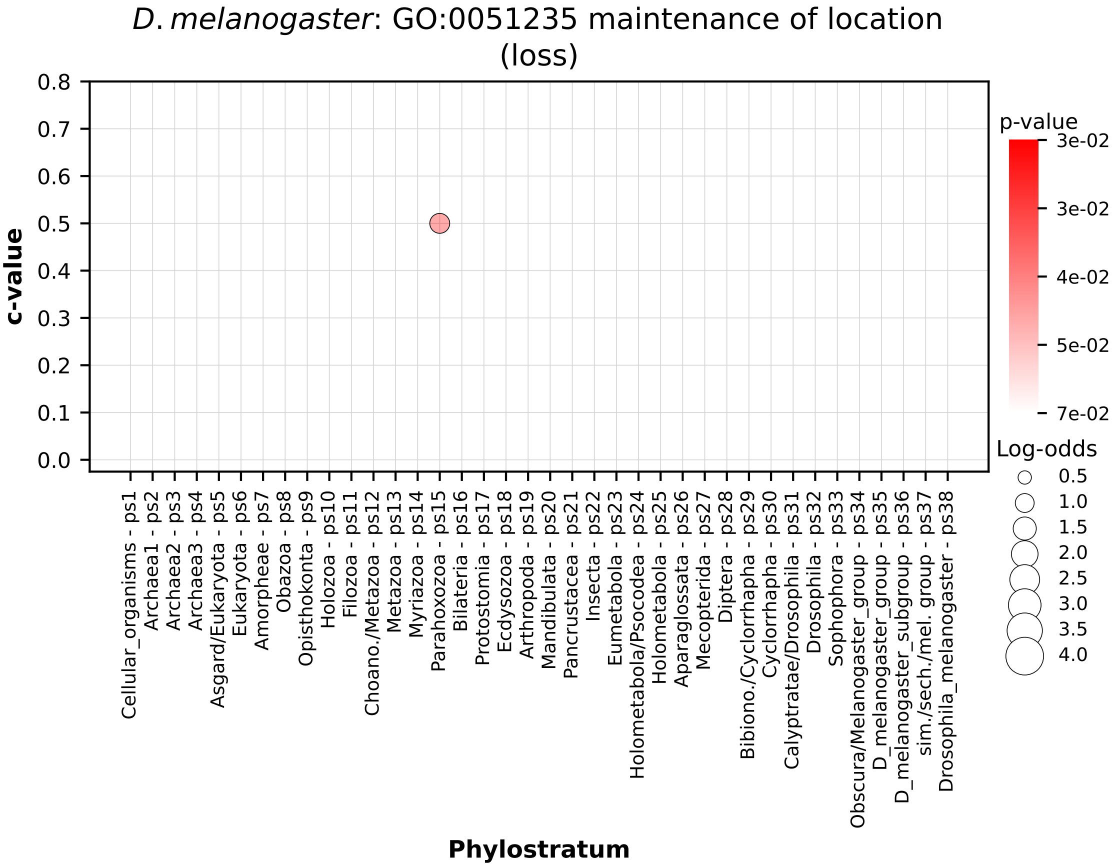
D. melanogaster: GO:0051235 maintenance of location
(loss)
c-value
0.0
0.1
0.2
0.3
0.4
0.5
0.6
0.7
0.8
Phylostratum
Cellular_organisms - ps1
Archaea1 - ps2
Archaea2 - ps3
Archaea3 - ps4
Asgard/Eukaryota - ps5
Eukaryota - ps6
Amorpheae - ps7
Obazoa - ps8
Opisthokonta - ps9
Holozoa - ps10
Filozoa - ps11
Choano./Metazoa - ps12
Metazoa - ps13
Myriazoa - ps14
Parahoxozoa - ps15
Bilateria - ps16
Protostomia - ps17
Ecdysozoa - ps18
Arthropoda - ps19
Mandibulata - ps20
Pancrustacea - ps21
Insecta - ps22
Eumetabola - ps23
Holometabola/Psocodea - ps24
Holometabola - ps25
Aparaglossata - ps26
Mecopterida - ps27
Diptera - ps28
Bibiono./Cyclorrhapha - ps29
Cyclorrhapha - ps30
Calyptratae/Drosophila - ps31
Drosophila - ps32
Sophophora - ps33
Obscura/Melanogaster_group - ps34
D_melanogaster_group - ps35
D_melanogaster_subgroup - ps36
sim./sech./mel. group - ps37
Drosophila_melanogaster - ps38
p-value
3e-02
3e-02
4e-02
5e-02
7e-02
Log-odds
0.5
1.0
1.5
2.0
2.5
3.0
3.5
4.0

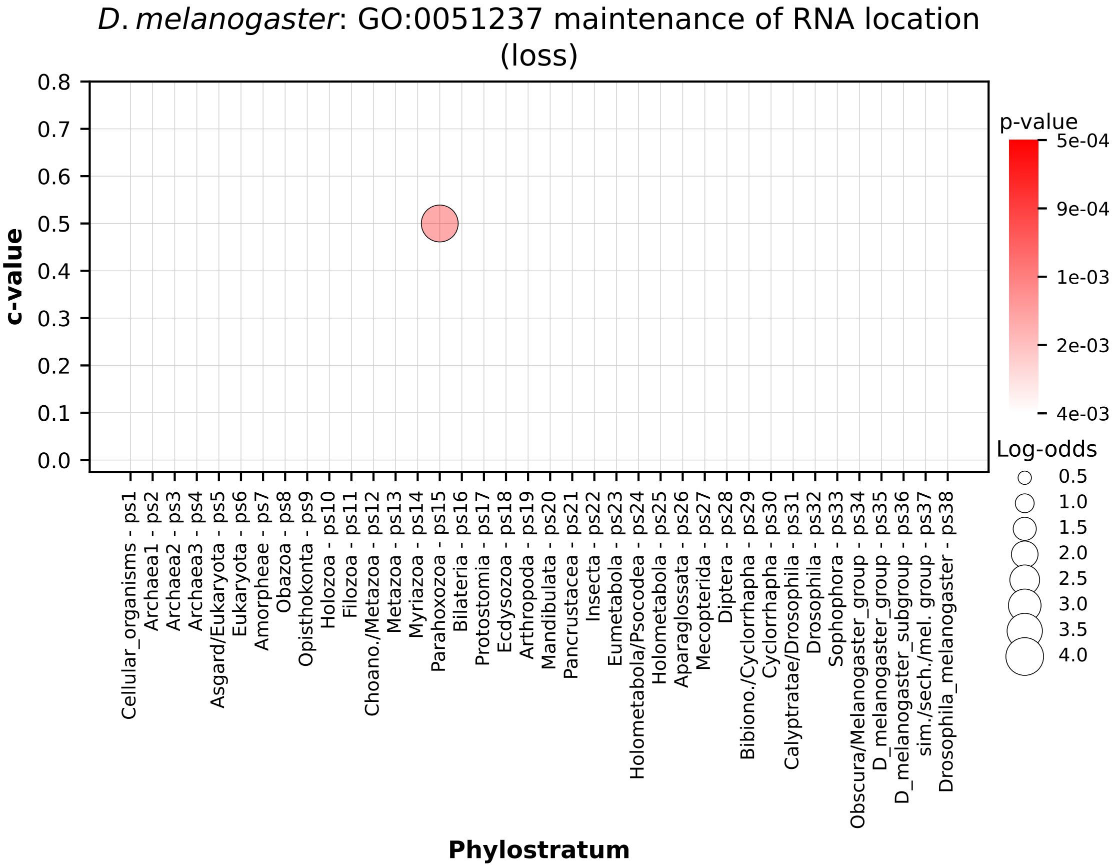
D. melanogaster: GO:0051237 maintenance of RNA location
(loss)
c-value
0.0
0.1
0.2
0.3
0.4
0.5
0.6
0.7
0.8
Phylostratum
Cellular_organisms - ps1
Archaea1 - ps2
Archaea2 - ps3
Archaea3 - ps4
Asgard/Eukaryota - ps5
Eukaryota - ps6
Amorpheae - ps7
Obazoa - ps8
Opisthokonta - ps9
Holozoa - ps10
Filozoa - ps11
Choano./Metazoa - ps12
Metazoa - ps13
Myriazoa - ps14
Parahoxozoa - ps15
Bilateria - ps16
Protostomia - ps17
Ecdysozoa - ps18
Arthropoda - ps19
Mandibulata - ps20
Pancrustacea - ps21
Insecta - ps22
Eumetabola - ps23
Holometabola/Psocodea - ps24
Holometabola - ps25
Aparaglossata - ps26
Mecopterida - ps27
Diptera - ps28
Bibiono./Cyclorrhapha - ps29
Cyclorrhapha - ps30
Calyptratae/Drosophila - ps31
Drosophila - ps32
Sophophora - ps33
Obscura/Melanogaster_group - ps34
D_melanogaster_group - ps35
D_melanogaster_subgroup - ps36
sim./sech./mel. group - ps37
Drosophila_melanogaster - ps38
p-value
5e-04
9e-04
1e-03
2e-03
4e-03
Log-odds
0.5
1.0
1.5
2.0
2.5
3.0
3.5
4.0

D. melanogaster: GO:0051296 establishment of meiotic spindle orientation (loss)

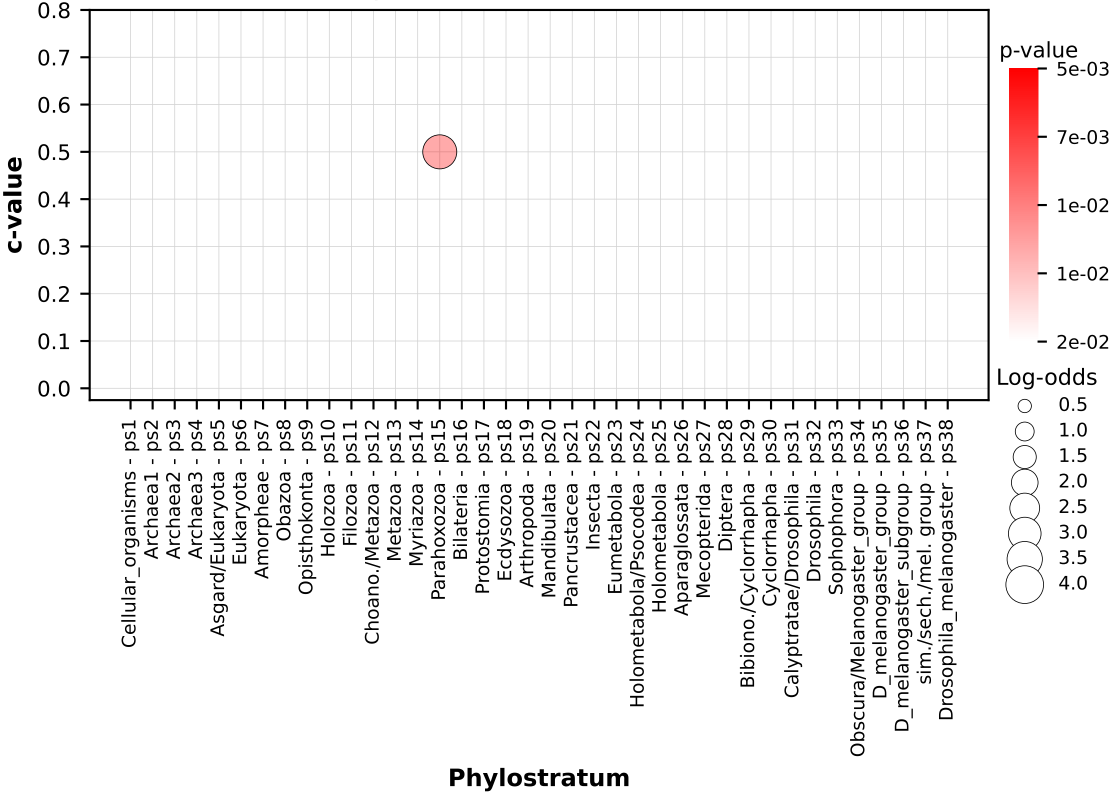

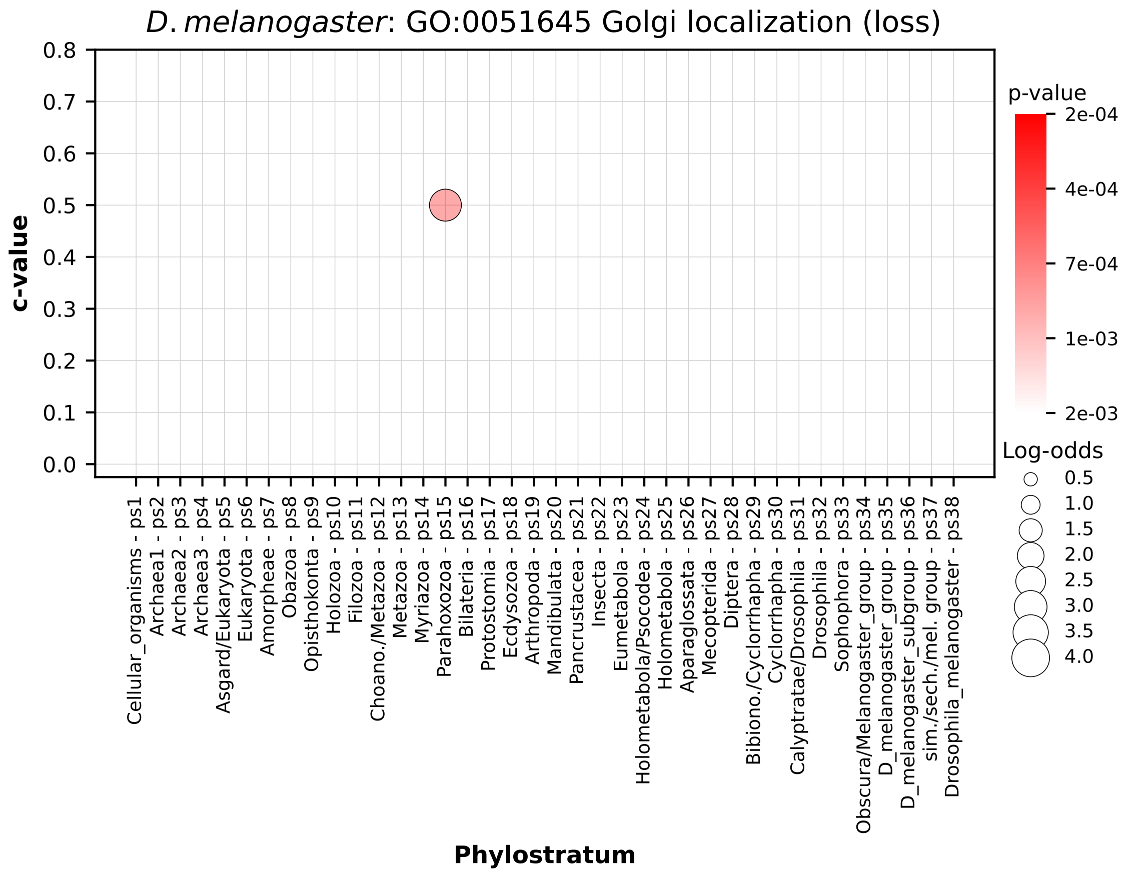

D. melanogaster: GO:0051645 Golgi localization (loss)
c-value
0.8
0.7
0.6
0.5
0.4
0.3
0.2
0.1
0.0
Phylostratum
Cellular_organisms - ps1
Archaea1 - ps2
Archaea2 - ps3
Archaea3 - ps4
Asgard/Eukaryota - ps5
Eukaryota - ps6
Amorpheae - ps7
Obazoa - ps8
Opisthokonta - ps9
Holozoa - ps10
Filozoa - ps11
Choano./Metazoa - ps12
Metazoa - ps13
Myriazoa - ps14
Parahoxozoa - ps15
Bilateria - ps16
Protostomia - ps17
Ecdysozoa - ps18
Arthropoda - ps19
Mandibulata - ps20
Pancrustacea - ps21
Insecta - ps22
Eumetabola - ps23
Holometabola/Psocodea - ps24
Holometabola - ps25
Aparaglossata - ps26
Mecopterida - ps27
Diptera - ps28
Bibiono./Cyclorrhapha - ps29
Cyclorrhapha - ps30
Calyptratae/Drosophila - ps31
Drosophila - ps32
Sophophora - ps33
Obscura/Melanogaster_group - ps34
D_melanogaster_group - ps35
D_melanogaster_subgroup - ps36
sim./sech./mel. group - ps37
Drosophila_melanogaster - ps38
p-value
2e-04
4e-04
7e-04
1e-03
2e-03
Log-odds
0.5
1.0
1.5
2.0
2.5
3.0
3.5
4.0

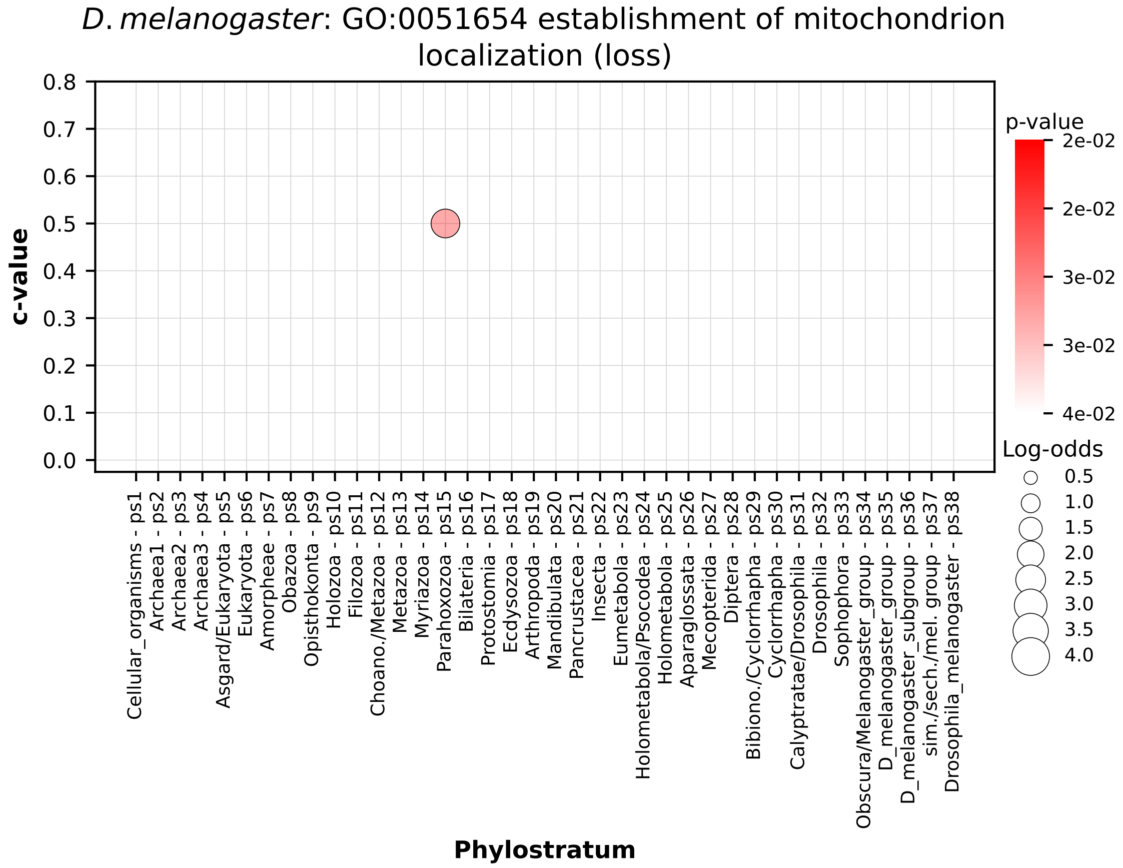

D. melanogaster: GO:0051654 establishment of mitochondrion localization (loss)
c-value
0.8
0.7
0.6
0.5
0.4
0.3
0.2
0.1
0.0
Phylostratum
Cellular_organisms - ps1
Archaea1 - ps2
Archaea2 - ps3
Archaea3 - ps4
Asgard/Eukaryota - ps5
Eukaryota - ps6
Amorpheae - ps7
Obazoa - ps8
Opisthokonta - ps9
Holozoa - ps10
Filozoa - ps11
Choano./Metazoa - ps12
Metazoa - ps13
Myriazoa - ps14
Parahoxozoa - ps15
Bilateria - ps16
Protostomia - ps17
Ecdysozoa - ps18
Arthropoda - ps19
Mandibulata - ps20
Pancrustacea - ps21
Insecta - ps22
Eumetabola - ps23
Holometabola/Psocodea - ps24
Holometabola - ps25
Aparaglossata - ps26
Mecopterida - ps27
Diptera - ps28
Bibiono./Cyclorrhapha - ps29
Cyclorrhapha - ps30
Calyptratae/Drosophila - ps31
Drosophila - ps32
Sophophora - ps33
Obscura/Melanogaster_group - ps34
D_melanogaster_group - ps35
D_melanogaster_subgroup - ps36
sim./sech./mel. group - ps37
Drosophila_melanogaster - ps38
p-value
2e-02
2e-02
3e-02
3e-02
4e-02
Log-odds
0.5
1.0
1.5
2.0
2.5
3.0
3.5
4.0

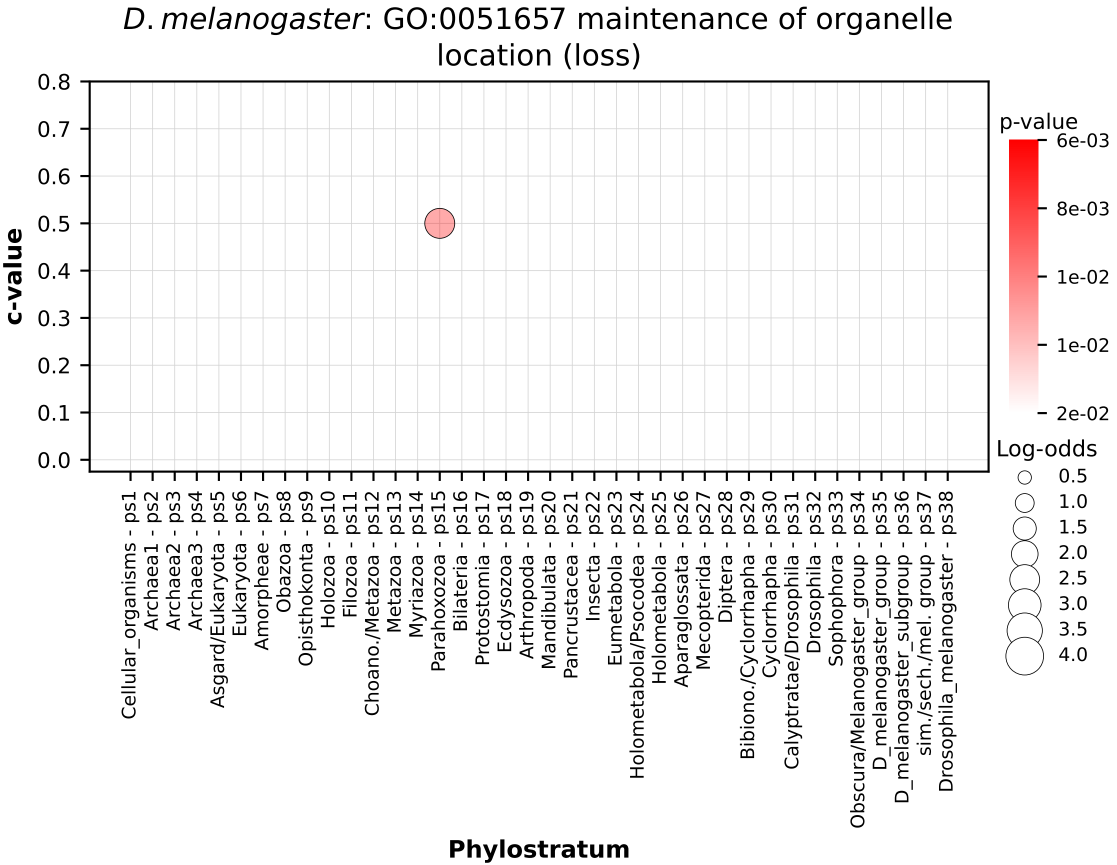
D. melanogaster: GO:0051657 maintenance of organelle location (loss)
c-value
0.0
0.1
0.2
0.3
0.4
0.5
0.6
0.7
0.8
Phylostratum
Cellular_organisms - ps1
Archaea1 - ps2
Archaea2 - ps3
Archaea3 - ps4
Asgard/Eukaryota - ps5
Eukaryota - ps6
Amorpheae - ps7
Obazoa - ps8
Opisthokonta - ps9
Holozoa - ps10
Filozoa - ps11
Choano./Metazoa - ps12
Metazoa - ps13
Myriazoa - ps14
Parahoxozoa - ps15
Bilateria - ps16
Protostomia - ps17
Ecdysozoa - ps18
Arthropoda - ps19
Mandibulata - ps20
Pancrustacea - ps21
Insecta - ps22
Eumetabola - ps23
Holometabola/Psocodea - ps24
Holometabola - ps25
Aparaglossata - ps26
Mecopterida - ps27
Diptera - ps28
Bibiono./Cyclorrhapha - ps29
Cyclorrhapha - ps30
Calyptratae/Drosophila - ps31
Drosophila - ps32
Sophophora - ps33
Obscura/Melanogaster_group - ps34
D_melanogaster_group - ps35
D_melanogaster_subgroup - ps36
sim./sech./mel. group - ps37
Drosophila_melanogaster - ps38
p-value
6e-03
8e-03
1e-02
1e-02
2e-02
Log-odds
0.5
1.0
1.5
2.0
2.5
3.0
3.5
4.0

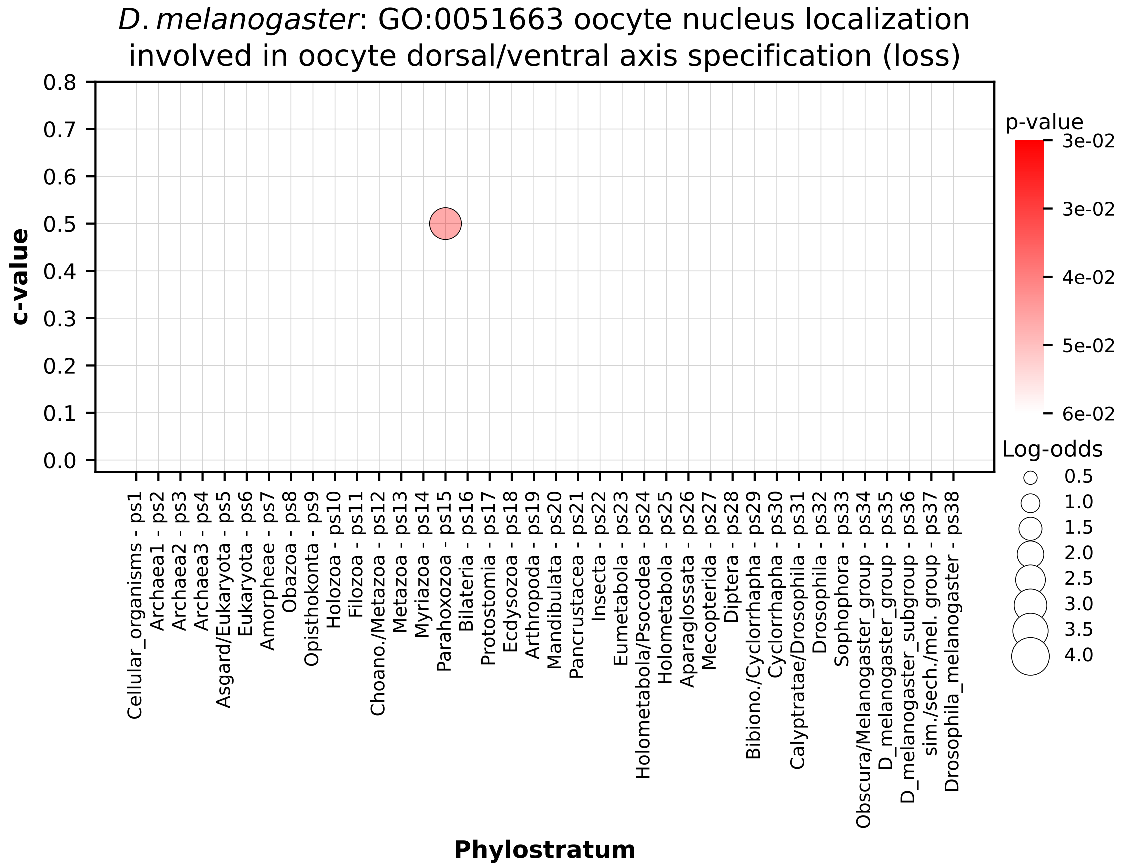

D. melanogaster: GO:0051663 oocyte nucleus localization involved in oocyte dorsal/ventral axis specification (loss)
c-value
0.8
0.7
0.6
0.5
0.4
0.3
0.2
0.1
0.0
p-value
3e-02
3e-02
4e-02
5e-02
6e-02
Log-odds
0.5
1.0
1.5
2.0
2.5
3.0
3.5
4.0
Cellular_organisms - ps1
Archaea1 - ps2
Archaea2 - ps3
Archaea3 - ps4
Asgard/Eukaryota - ps5
Eukaryota - ps6
Amorpheae - ps7
Obazoa - ps8
Opisthokonta - ps9
Holozoa - ps10
Filozoa - ps11
Choano./Metazoa - ps12
Metazoa - ps13
Myriazoa - ps14
Parahoxozoa - ps15
Bilateria - ps16
Protostomia - ps17
Ecdysozoa - ps18
Arthropoda - ps19
Mandibulata - ps20
Pancrustacea - ps21
Insecta - ps22
Eumetabola - ps23
Holometabola/Psocodea - ps24
Holometabola - ps25
Aparaglossata - ps26
Mecopterida - ps27
Diptera - ps28
Bibiono./Cyclorrhapha - ps29
Cyclorrhapha - ps30
Calyptratae/Drosophila - ps31
Drosophila - ps32
Sophophora - ps33
Obscura/Melanogaster_group - ps34
D_melanogaster_group - ps35
D_melanogaster_subgroup - ps36
sim./sech./mel. group - ps37
Drosophila_melanogaster - ps38
Phylostratum

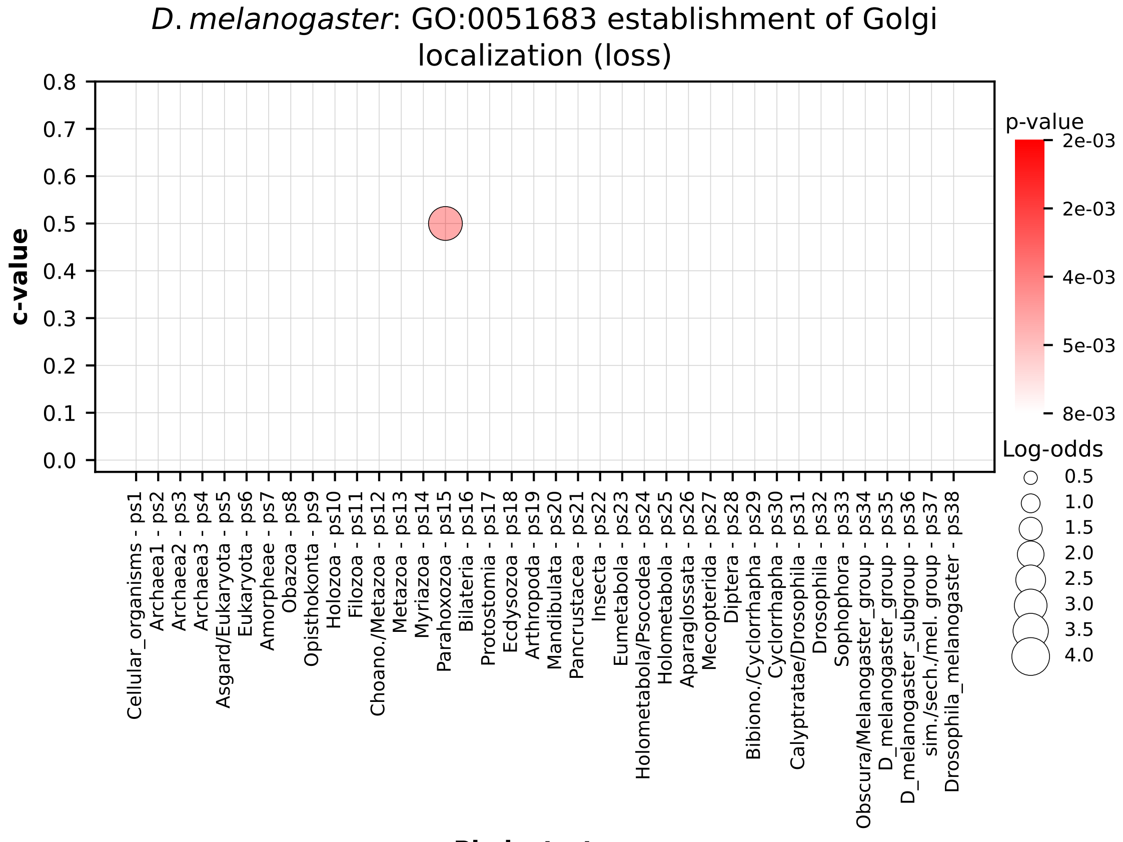

D. melanogaster: GO:0051683 establishment of Golgi localization (loss)
c-value
0.8
0.7
0.6
0.5
0.4
0.3
0.2
0.1
0.0
Phylostratum
Cellular_organisms - ps1
Archaea1 - ps2
Archaea2 - ps3
Archaea3 - ps4
Asgard/Eukaryota - ps5
Eukaryota - ps6
Amorpheae - ps7
Obazoa - ps8
Opisthokonta - ps9
Holozoa - ps10
Filozoa - ps11
Choano./Metazoa - ps12
Metazoa - ps13
Myriazoa - ps14
Parahoxozoa - ps15
Bilateria - ps16
Protostomia - ps17
Ecdysozoa - ps18
Arthropoda - ps19
Mandibulata - ps20
Pancrustacea - ps21
Insecta - ps22
Eumetabola - ps23
Holometabola/Psocodea - ps24
Holometabola - ps25
Aparaglossata - ps26
Mecopterida - ps27
Diptera - ps28
Bibiono./Cyclorrhapha - ps29
Cyclorrhapha - ps30
Calyptratae/Drosophila - ps31
Drosophila - ps32
Sophophora - ps33
Obscura/Melanogaster_group - ps34
D_melanogaster_group - ps35
D_melanogaster_subgroup - ps36
sim./sech./mel. group - ps37
Drosophila_melanogaster - ps38
p-value
2e-03
2e-03
4e-03
5e-03
8e-03
Log-odds
0.5
1.0
1.5
2.0
2.5
3.0
3.5
4.0

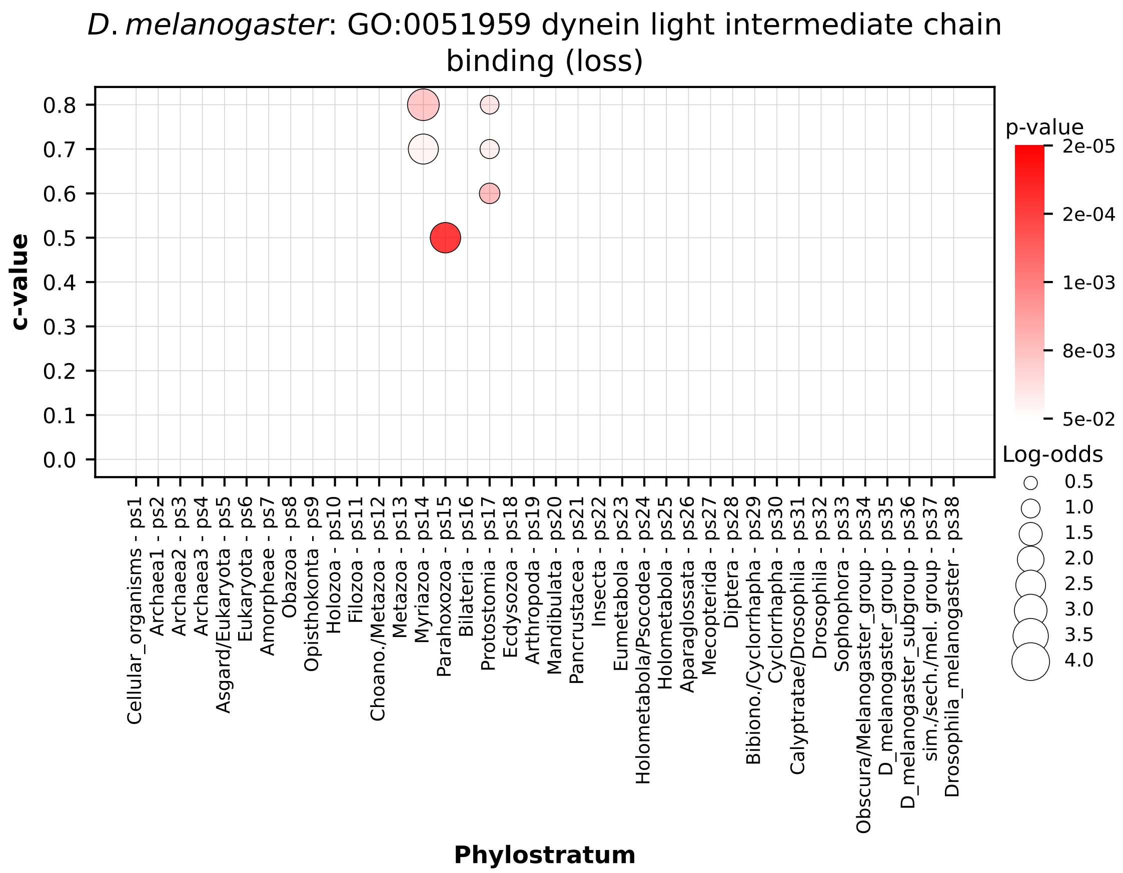

D. melanogaster: GO:0051959 dynein light intermediate chain binding (loss)
c-value
0.0
0.1
0.2
0.3
0.4
0.5
0.6
0.7
0.8
Phylostratum
Cellular_organisms - ps1
Archaea1 - ps2
Archaea2 - ps3
Archaea3 - ps4
Asgard/Eukaryota - ps5
Eukaryota - ps6
Amorpheae - ps7
Obazoa - ps8
Opisthokonta - ps9
Holozoa - ps10
Filozoa - ps11
Choano./Metazoa - ps12
Metazoa - ps13
Myriazoa - ps14
Parahoxozoa - ps15
Bilateria - ps16
Protostomia - ps17
Ecdysozoa - ps18
Arthropoda - ps19
Mandibulata - ps20
Pancrustacea - ps21
Insecta - ps22
Eumetabola - ps23
Holometabola/Psocodea - ps24
Holometabola - ps25
Aparaglossata - ps26
Mecopterida - ps27
Diptera - ps28
Bibiono./Cyclorrhapha - ps29
Cyclorrhapha - ps30
Calyptratae/Drosophila - ps31
Drosophila - ps32
Sophophora - ps33
Obscura/Melanogaster_group - ps34
D_melanogaster_group - ps35
D_melanogaster_subgroup - ps36
sim./sech./mel. group - ps37
Drosophila_melanogaster - ps38
p-value
2e-05
2e-04
1e-03
8e-03
5e-02
Log-odds
0.5
1.0
1.5
2.0
2.5
3.0
3.5
4.0

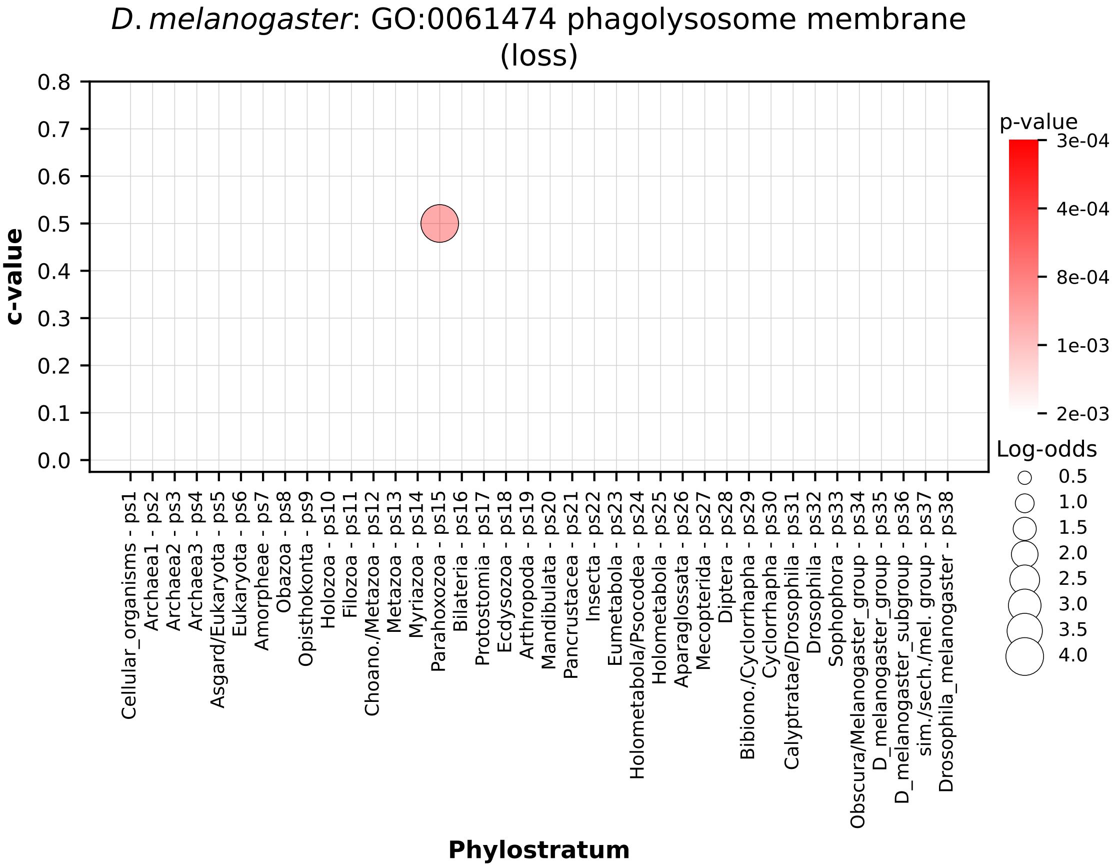

D. melanogaster: GO:0061474 phagolysosome membrane (loss)
c-value
0.8
0.7
0.6
0.5
0.4
0.3
0.2
0.1
0.0
Phylostratum
Cellular_organisms - ps1
Archaea1 - ps2
Archaea2 - ps3
Archaea3 - ps4
Asgard/Eukaryota - ps5
Eukaryota - ps6
Amorpheae - ps7
Obazoa - ps8
Opisthokonta - ps9
Holozoa - ps10
Filozoa - ps11
Choano./Metazoa - ps12
Metazoa - ps13
Myriazoa - ps14
Parahoxozoa - ps15
Bilateria - ps16
Protostomia - ps17
Ecdysozoa - ps18
Arthropoda - ps19
Mandibulata - ps20
Pancrustacea - ps21
Insecta - ps22
Eumetabola - ps23
Holometabola/Psocodea - ps24
Holometabola - ps25
Aparaglossata - ps26
Mecopterida - ps27
Diptera - ps28
Bibiono./Cyclorrhapha - ps29
Cyclorrhapha - ps30
Calyptratae/Drosophila - ps31
Drosophila - ps32
Sophophora - ps33
Obscura/Melanogaster_group - ps34
D_melanogaster_group - ps35
D_melanogaster_subgroup - ps36
sim./sech./mel. group - ps37
Drosophila_melanogaster - ps38
p-value
3e-04
4e-04
8e-04
1e-03
2e-03
Log-odds
0.5
1.0
1.5
2.0
2.5
3.0
3.5
4.0

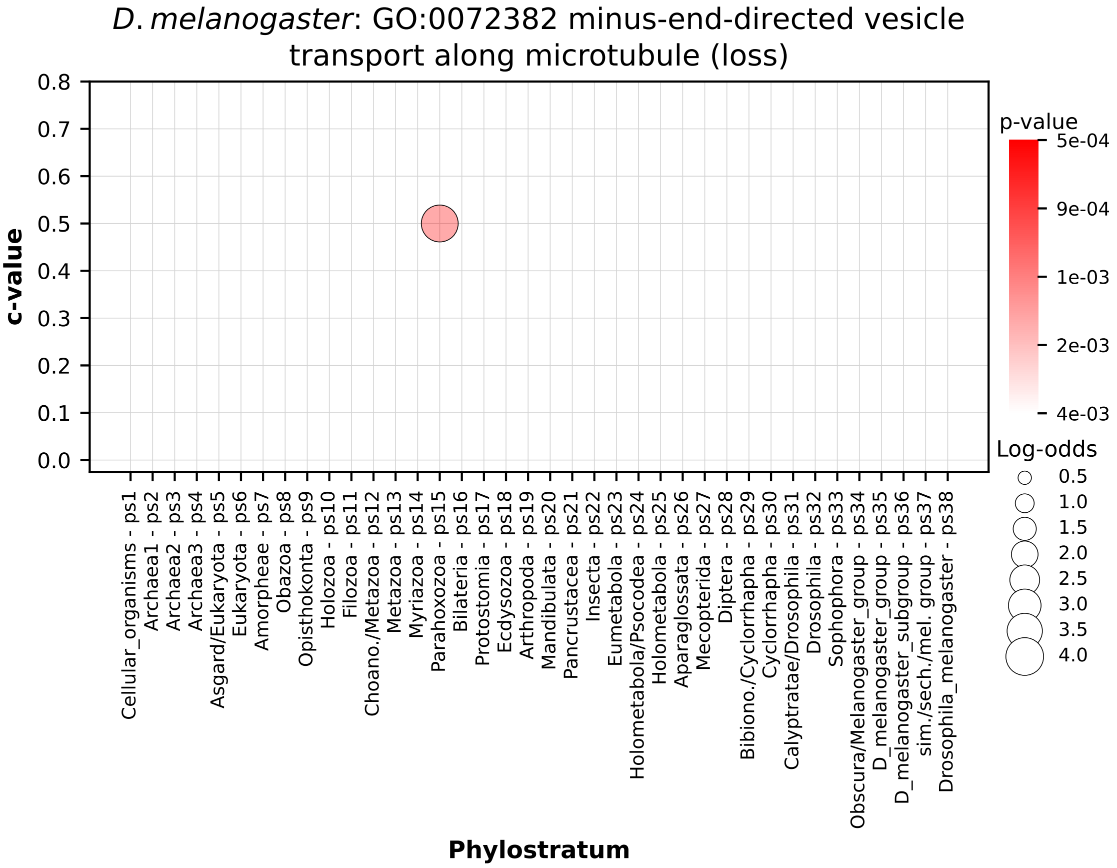
D. melanogaster: GO:0072382 minus-end-directed vesicle transport along microtubule (loss)
c-value
0.8
0.7
0.6
0.5
0.4
0.3
0.2
0.1
0.0
Phylostratum
Cellular_organisms - ps1
Archaea1 - ps2
Archaea2 - ps3
Archaea3 - ps4
Asgard/Eukaryota - ps5
Eukaryota - ps6
Amorpheae - ps7
Obazoa - ps8
Opisthokonta - ps9
Holozoa - ps10
Filozoa - ps11
Choano./Metazoa - ps12
Metazoa - ps13
Myriazoa - ps14
Parahoxozoa - ps15
Bilateria - ps16
Protostomia - ps17
Ecdysozoa - ps18
Arthropoda - ps19
Mandibulata - ps20
Pancrustacea - ps21
Insecta - ps22
Eumetabola - ps23
Holometabola/Psocodea - ps24
Holometabola - ps25
Aparaglossata - ps26
Mecopterida - ps27
Diptera - ps28
Bibiono./Cyclorrhapha - ps29
Cyclorrhapha - ps30
Calyptratae/Drosophila - ps31
Drosophila - ps32
Sophophora - ps33
Obscura/Melanogaster_group - ps34
D_melanogaster_group - ps35
D_melanogaster_subgroup - ps36
sim./sech./mel. group - ps37
Drosophila_melanogaster - ps38
p-value
5e-04
9e-04
1e-03
2e-03
4e-03
Log-odds
0.5
1.0
1.5
2.0
2.5
3.0
3.5
4.0

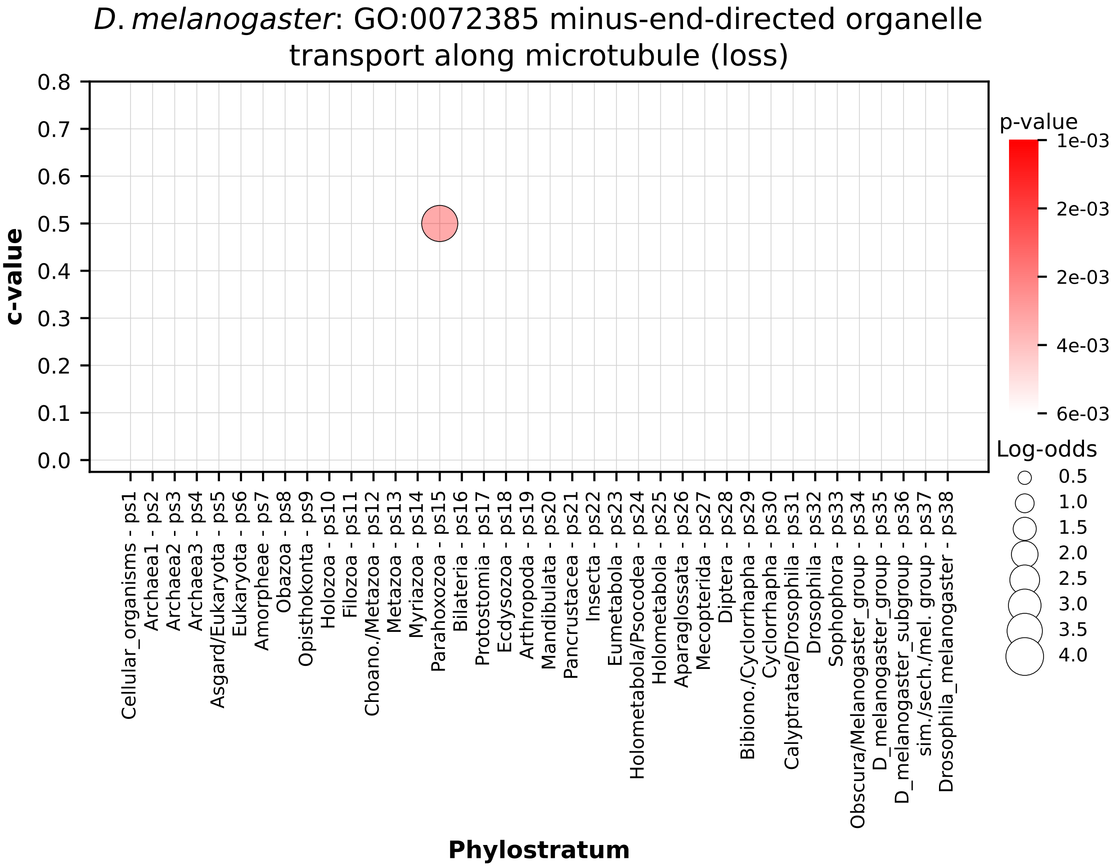

D. melanogaster: GO:0072385 minus-end-directed organelle transport along microtubule (loss)
c-value
0.8
0.7
0.6
0.5
0.4
0.3
0.2
0.1
0.0
Phylostratum
Cellular_organisms - ps1
Archaea1 - ps2
Archaea2 - ps3
Archaea3 - ps4
Asgard/Eukaryota - ps5
Eukaryota - ps6
Amorpheae - ps7
Obazoa - ps8
Opisthokonta - ps9
Holozoa - ps10
Filozoa - ps11
Choano./Metazoa - ps12
Metazoa - ps13
Myriazoa - ps14
Parahoxozoa - ps15
Bilateria - ps16
Protostomia - ps17
Ecdysozoa - ps18
Arthropoda - ps19
Mandibulata - ps20
Pancrustacea - ps21
Insecta - ps22
Eumetabola - ps23
Holometabola/Psocodea - ps24
Holometabola - ps25
Aparaglossata - ps26
Mecopterida - ps27
Diptera - ps28
Bibiono./Cyclorrhapha - ps29
Cyclorrhapha - ps30
Calyptratae/Drosophila - ps31
Drosophila - ps32
Sophophora - ps33
Obscura/Melanogaster_group - ps34
D_melanogaster_group - ps35
D_melanogaster_subgroup - ps36
sim./sech./mel. group - ps37
Drosophila_melanogaster - ps38
p-value
1e-03
2e-03
2e-03
4e-03
6e-03
Log-odds
0.5
1.0
1.5
2.0
2.5
3.0
3.5
4.0

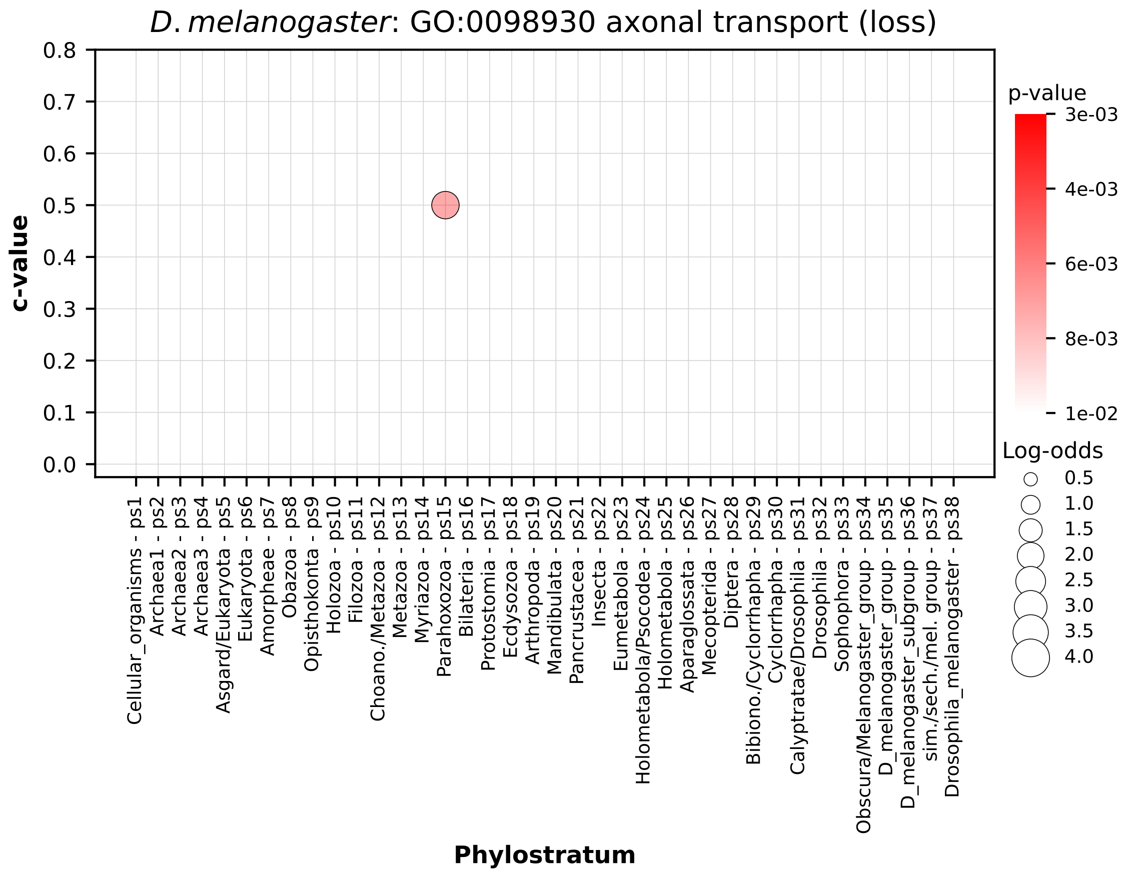

D. melanogaster: GO:0098930 axonal transport (loss)
c-value
0.8
0.7
0.6
0.5
0.4
0.3
0.2
0.1
0.0
Phylostratum
Cellular_organisms - ps1
Archaea1 - ps2
Archaea2 - ps3
Archaea3 - ps4
Asgard/Eukaryota - ps5
Eukaryota - ps6
Amorpheae - ps7
Obazoa - ps8
Opisthokonta - ps9
Holozoa - ps10
Filozoa - ps11
Choano./Metazoa - ps12
Metazoa - ps13
Myriazoa - ps14
Parahoxozoa - ps15
Bilateria - ps16
Protostomia - ps17
Ecdysozoa - ps18
Arthropoda - ps19
Mandibulata - ps20
Pancrustacea - ps21
Insecta - ps22
Eumetabola - ps23
Holometabola/Psocodea - ps24
Holometabola - ps25
Aparaglossata - ps26
Mecopterida - ps27
Diptera - ps28
Bibiono./Cyclorrhapha - ps29
Cyclorrhapha - ps30
Calyptratae/Drosophila - ps31
Drosophila - ps32
Sophophora - ps33
Obscura/Melanogaster_group - ps34
D_melanogaster_group - ps35
D_melanogaster_subgroup - ps36
sim./sech./mel. group - ps37
Drosophila_melanogaster - ps38
p-value
3e-03
4e-03
6e-03
8e-03
1e-02
Log-odds
0.5
1.0
1.5
2.0
2.5
3.0
3.5
4.0

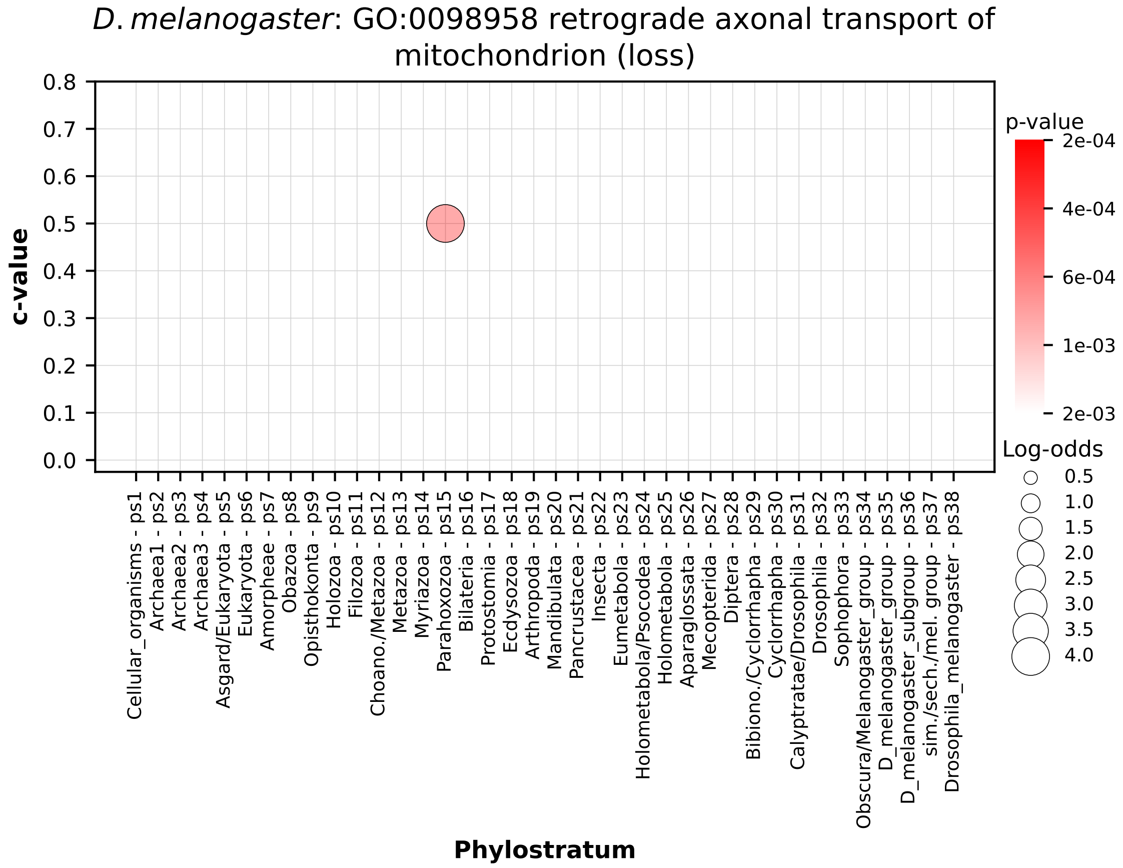

D. melanogaster: GO:0098958 retrograde axonal transport of mitochondrion (loss)
c-value
0.8
0.7
0.6
0.5
0.4
0.3
0.2
0.1
0.0
p-value
2e-04
4e-04
6e-04
1e-03
2e-03
Log-odds
0.5
1.0
1.5
2.0
2.5
3.0
3.5
4.0
Cellular_organisms - ps1
Archaea1 - ps2
Archaea2 - ps3
Archaea3 - ps4
Asgard/Eukaryota - ps5
Eukaryota - ps6
Amorpheae - ps7
Obazoa - ps8
Opisthokonta - ps9
Holozoa - ps10
Filozoa - ps11
Choano./Metazoa - ps12
Metazoa - ps13
Myriazoa - ps14
Parahoxozoa - ps15
Bilateria - ps16
Protostomia - ps17
Ecdysozoa - ps18
Arthropoda - ps19
Mandibulata - ps20
Pancrustacea - ps21
Insecta - ps22
Eumetabola - ps23
Holometabola/Psocodea - ps24
Holometabola - ps25
Aparaglossata - ps26
Mecopterida - ps27
Diptera - ps28
Bibiono./Cyclorrhapha - ps29
Cyclorrhapha - ps30
Calyptratae/Drosophila - ps31
Drosophila - ps32
Sophophora - ps33
Obscura/Melanogaster_group - ps34
D_melanogaster_group - ps35
D_melanogaster_subgroup - ps36
sim./sech./mel. group - ps37
Drosophila_melanogaster - ps38
Phylostratum

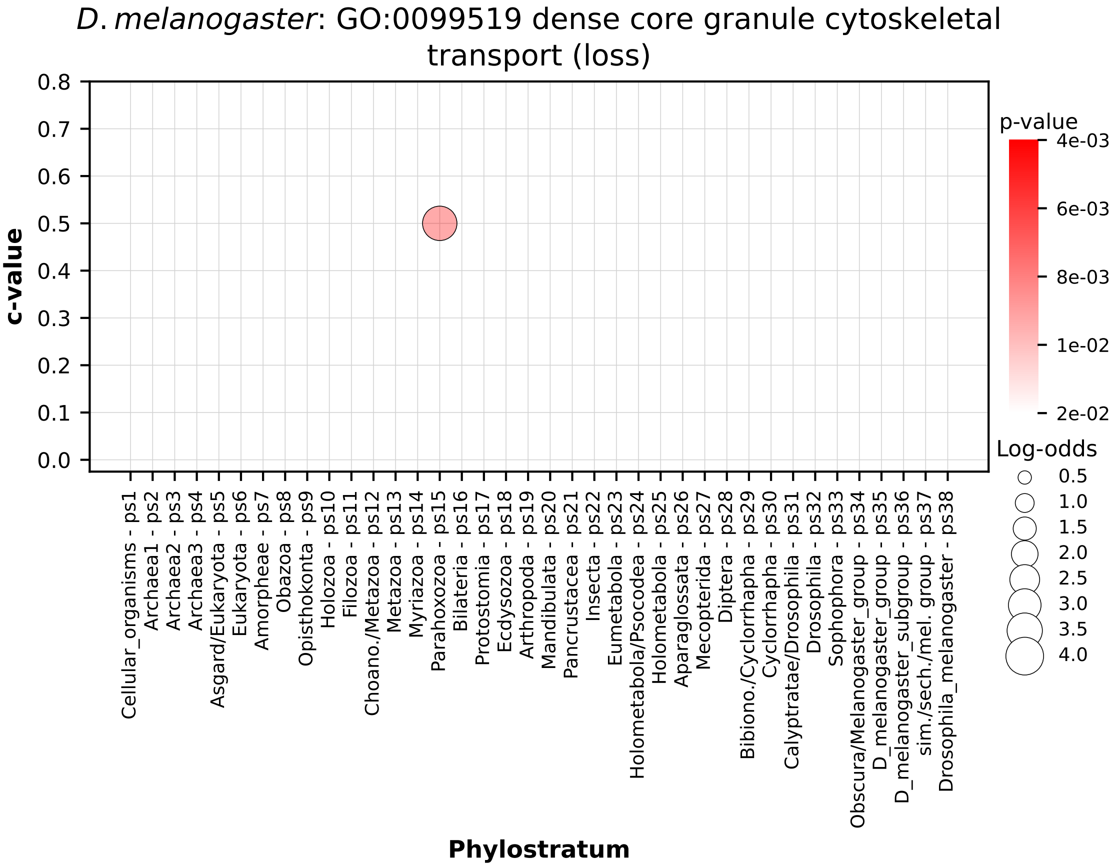

D. melanogaster: GO:0099519 dense core granule cytoskeletal transport (loss)
c-value
0.8
0.7
0.6
0.5
0.4
0.3
0.2
0.1
0.0
Phylostratum
Cellular_organisms - ps1
Archaea1 - ps2
Archaea2 - ps3
Archaea3 - ps4
Asgard/Eukaryota - ps5
Eukaryota - ps6
Amorpheae - ps7
Obazoa - ps8
Opisthokonta - ps9
Holozoa - ps10
Filozoa - ps11
Choano./Metazoa - ps12
Metazoa - ps13
Myriazoa - ps14
Parahoxozoa - ps15
Bilateria - ps16
Protostomia - ps17
Ecdysozoa - ps18
Arthropoda - ps19
Mandibulata - ps20
Pancrustacea - ps21
Insecta - ps22
Eumetabola - ps23
Holometabola/Psocodea - ps24
Holometabola - ps25
Aparaglossata - ps26
Mecopterida - ps27
Diptera - ps28
Bibiono./Cyclorrhapha - ps29
Cyclorrhapha - ps30
Calyptratae/Drosophila - ps31
Drosophila - ps32
Sophophora - ps33
Obscura/Melanogaster_group - ps34
D_melanogaster_group - ps35
D_melanogaster_subgroup - ps36
sim./sech./mel. group - ps37
Drosophila_melanogaster - ps38
p-value
4e-03
6e-03
8e-03
1e-02
2e-02
Log-odds
0.5
1.0
1.5
2.0
2.5
3.0
3.5
4.0

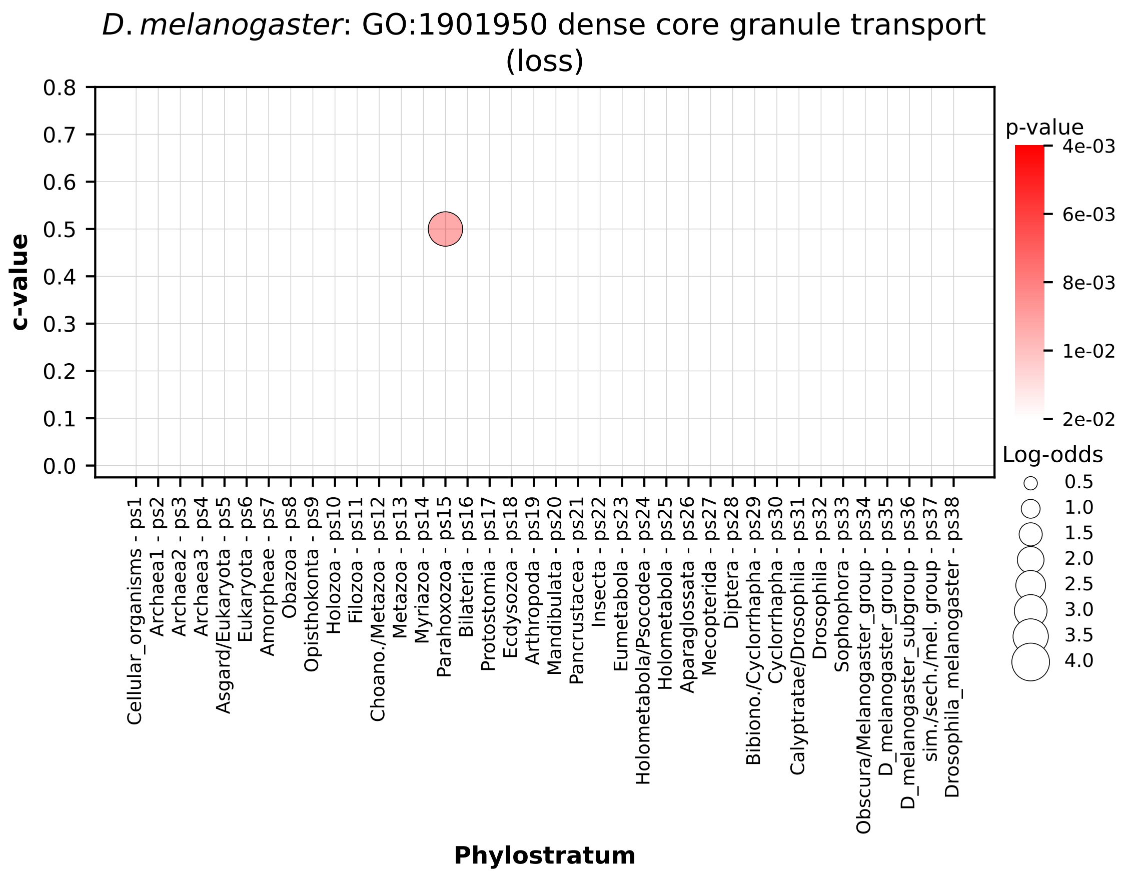

D. melanogaster: GO:1901950 dense core granule transport (loss)
c-value
0.0
0.1
0.2
0.3
0.4
0.5
0.6
0.7
0.8
Phylostratum
Cellular_organisms - ps1
Archaea1 - ps2
Archaea2 - ps3
Archaea3 - ps4
Asgard/Eukaryota - ps5
Eukaryota - ps6
Amorpheae - ps7
Obazoa - ps8
Opisthokonta - ps9
Holozoa - ps10
Filozoa - ps11
Choano./Metazoa - ps12
Metazoa - ps13
Myriazoa - ps14
Parahoxozoa - ps15
Bilateria - ps16
Protostomia - ps17
Ecdysozoa - ps18
Arthropoda - ps19
Mandibulata - ps20
Pancrustacea - ps21
Insecta - ps22
Eumetabola - ps23
Holometabola/Psocodea - ps24
Holometabola - ps25
Aparaglossata - ps26
Mecopterida - ps27
Diptera - ps28
Bibiono./Cyclorrhapha - ps29
Cyclorrhapha - ps30
Calyptratae/Drosophila - ps31
Drosophila - ps32
Sophophora - ps33
Obscura/Melanogaster_group - ps34
D_melanogaster_group - ps35
D_melanogaster_subgroup - ps36
sim./sech./mel. group - ps37
Drosophila_melanogaster - ps38
p-value
4e-03
6e-03
8e-03
1e-02
2e-02
Log-odds
0.5
1.0
1.5
2.0
2.5
3.0
3.5
4.0

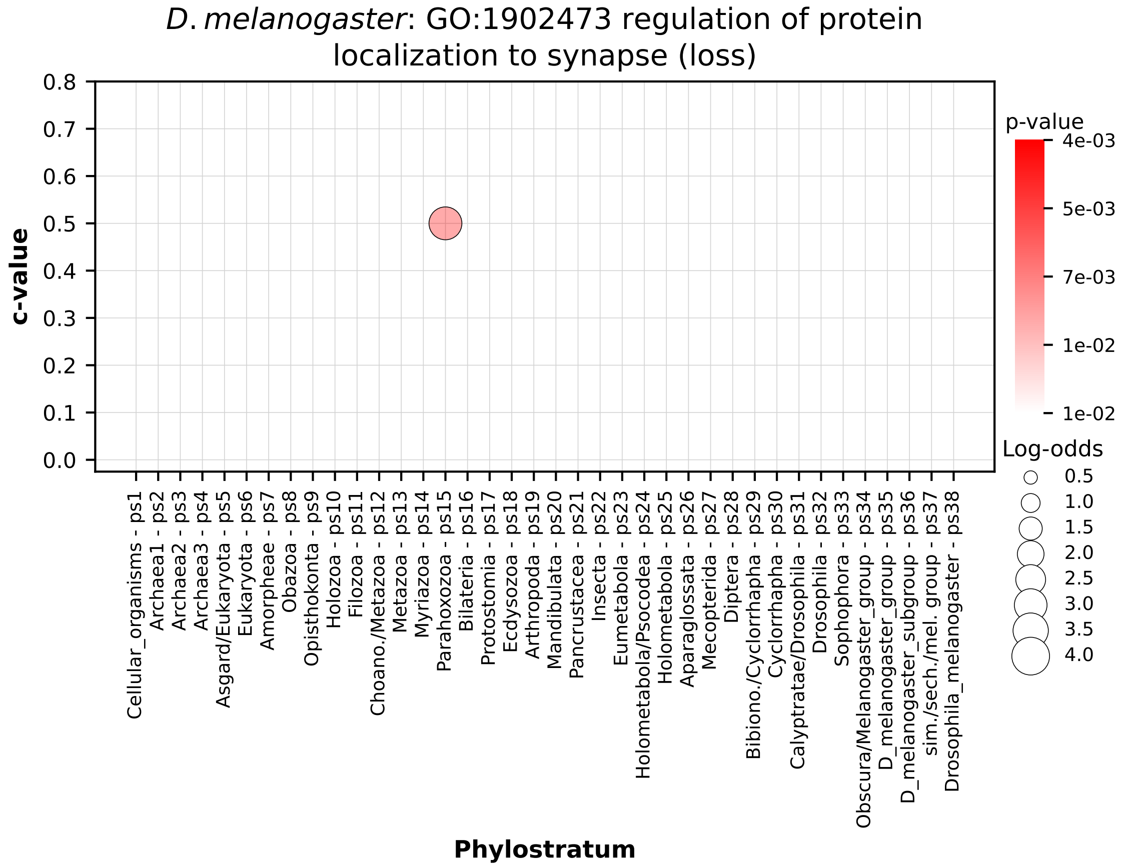

D. melanogaster: GO:1902473 regulation of protein localization to synapse (loss)
c-value
0.8
0.7
0.6
0.5
0.4
0.3
0.2
0.1
0.0
Phylostratum
Cellular_organisms - ps1
Archaea1 - ps2
Archaea2 - ps3
Archaea3 - ps4
Asgard/Eukaryota - ps5
Eukaryota - ps6
Amorpheae - ps7
Obazoa - ps8
Opisthokonta - ps9
Holozoa - ps10
Filozoa - ps11
Choano./Metazoa - ps12
Metazoa - ps13
Myriazoa - ps14
Parahoxozoa - ps15
Bilateria - ps16
Protostomia - ps17
Ecdysozoa - ps18
Arthropoda - ps19
Mandibulata - ps20
Pancrustacea - ps21
Insecta - ps22
Eumetabola - ps23
Holometabola/Psocodea - ps24
Holometabola - ps25
Aparaglossata - ps26
Mecopterida - ps27
Diptera - ps28
Bibiono./Cyclorrhapha - ps29
Cyclorrhapha - ps30
Calyptratae/Drosophila - ps31
Drosophila - ps32
Sophophora - ps33
Obscura/Melanogaster_group - ps34
D_melanogaster_group - ps35
D_melanogaster_subgroup - ps36
sim./sech./mel. group - ps37
Drosophila_melanogaster - ps38
p-value
4e-03
5e-03
7e-03
1e-02
1e-02
Log-odds
0.5
1.0
1.5
2.0
2.5
3.0
3.5
4.0

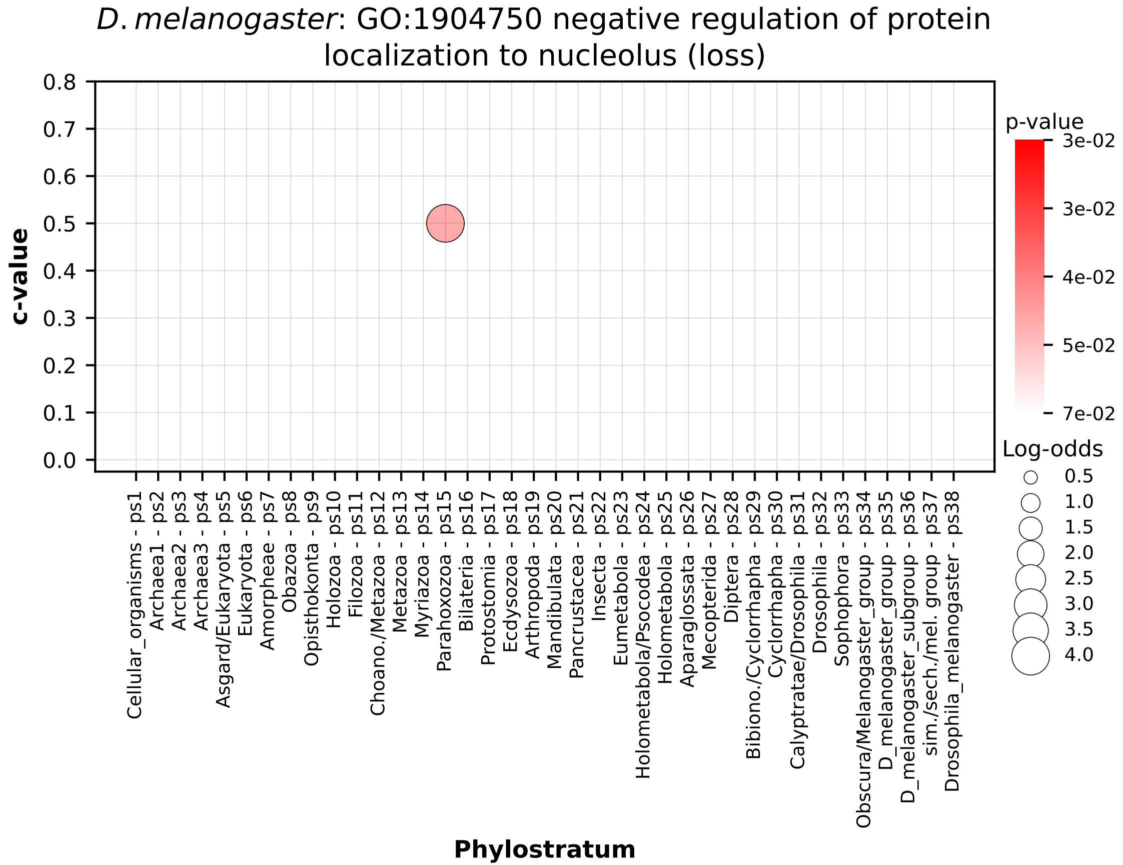

D. melanogaster: GO:1904750 negative regulation of protein localization to nucleolus (loss)
c-value
0.8
0.7
0.6
0.5
0.4
0.3
0.2
0.1
0.0
Phylostratum
Cellular_organisms - ps1
Archaea1 - ps2
Archaea2 - ps3
Archaea3 - ps4
Asgard/Eukaryota - ps5
Eukaryota - ps6
Amorpheae - ps7
Obazoa - ps8
Opisthokonta - ps9
Holozoa - ps10
Filozoa - ps11
Choano./Metazoa - ps12
Metazoa - ps13
Myriazoa - ps14
Parahoxozoa - ps15
Bilateria - ps16
Protostomia - ps17
Ecdysozoa - ps18
Arthropoda - ps19
Mandibulata - ps20
Pancrustacea - ps21
Insecta - ps22
Eumetabola - ps23
Holometabola/Psocodea - ps24
Holometabola - ps25
Aparaglossata - ps26
Mecopterida - ps27
Diptera - ps28
Bibiono./Cyclorrhapha - ps29
Cyclorrhapha - ps30
Calyptratae/Drosophila - ps31
Drosophila - ps32
Sophophora - ps33
Obscura/Melanogaster_group - ps34
D_melanogaster_group - ps35
D_melanogaster_subgroup - ps36
sim./sech./mel. group - ps37
Drosophila_melanogaster - ps38
p-value
3e-02
3e-02
4e-02
5e-02
7e-02
Log-odds
0.5
1.0
1.5
2.0
2.5
3.0
3.5
4.0

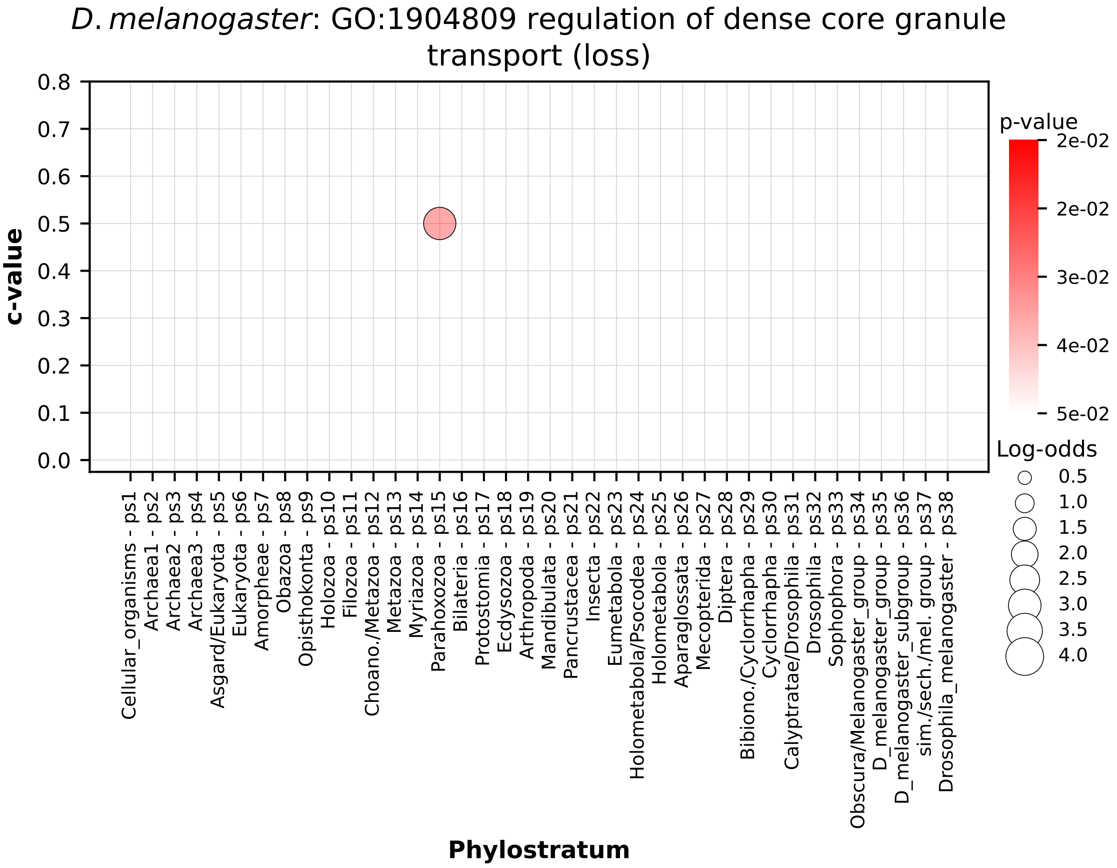

D. melanogaster: GO:1904809 regulation of dense core granule transport (loss)
c-value
0.8
0.7
0.6
0.5
0.4
0.3
0.2
0.1
0.0
p-value
2e-02
2e-02
3e-02
4e-02
5e-02
Log-odds
0.5
1.0
1.5
2.0
2.5
3.0
3.5
4.0
Cellular_organisms - ps1
Archaea1 - ps2
Archaea2 - ps3
Archaea3 - ps4
Asgard/Eukaryota - ps5
Eukaryota - ps6
Amorpheae - ps7
Obazoa - ps8
Opisthokonta - ps9
Holozoa - ps10
Filozoa - ps11
Choano./Metazoa - ps12
Metazoa - ps13
Myriazoa - ps14
Parahoxozoa - ps15
Bilateria - ps16
Protostomia - ps17
Ecdysozoa - ps18
Arthropoda - ps19
Mandibulata - ps20
Pancrustacea - ps21
Insecta - ps22
Eumetabola - ps23
Holometabola/Psocodea - ps24
Holometabola - ps25
Aparaglossata - ps26
Mecopterida - ps27
Diptera - ps28
Bibiono./Cyclorrhapha - ps29
Cyclorrhapha - ps30
Calyptratae/Drosophila - ps31
Drosophila - ps32
Sophophora - ps33
Obscura/Melanogaster_group - ps34
D_melanogaster_group - ps35
D_melanogaster_subgroup - ps36
sim./sech./mel. group - ps37
Drosophila_melanogaster - ps38
Phylostratum

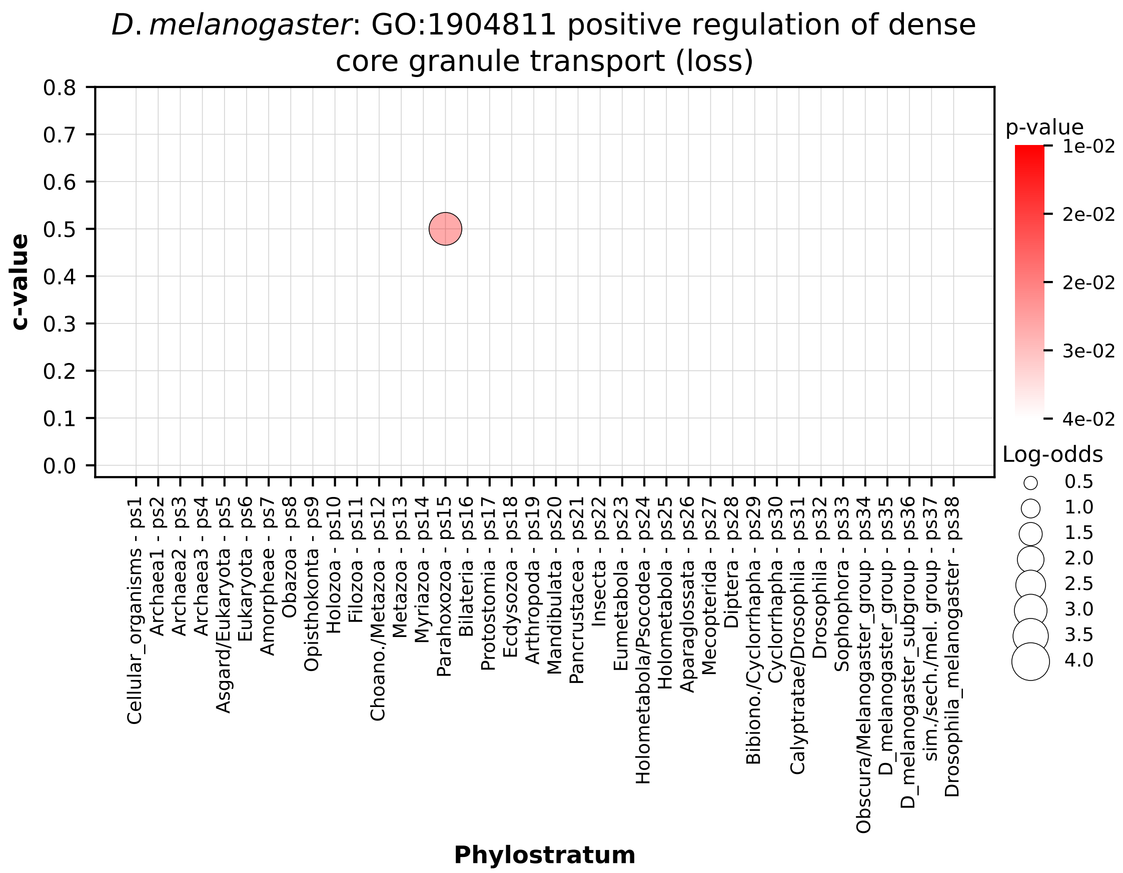

$D. melanogaster$: GO:1904811 positive regulation of dense core granule transport (loss)
c-value
0.8
0.7
0.6
0.5
0.4
0.3
0.2
0.1
0.0
Phylostratum
Cellular_organisms - ps1
Archaea1 - ps2
Archaea2 - ps3
Archaea3 - ps4
Asgard/Eukaryota - ps5
Eukaryota - ps6
Amorpheae - ps7
Obazoa - ps8
Opisthokonta - ps9
Holozoa - ps10
Filozoa - ps11
Choano./Metazoa - ps12
Metazoa - ps13
Myriazoa - ps14
Parahoxozoa - ps15
Bilateria - ps16
Protostomia - ps17
Ecdysozoa - ps18
Arthropoda - ps19
Mandibulata - ps20
Pancrustacea - ps21
Insecta - ps22
Eumetabola - ps23
Holometabola/Psocodea - ps24
Holometabola - ps25
Aparaglossata - ps26
Mecopterida - ps27
Diptera - ps28
Bibiono./Cyclorrhapha - ps29
Cyclorrhapha - ps30
Calyptratae/Drosophila - ps31
Drosophila - ps32
Sophophora - ps33
Obscura/Melanogaster_group - ps34
D_melanogaster_group - ps35
D_melanogaster_subgroup - ps36
sim./sech./mel. group - ps37
Drosophila_melanogaster - ps38
p-value
1e-02
2e-02
2e-02
3e-02
4e-02
Log-odds
0.5
1.0
1.5
2.0
2.5
3.0
3.5
4.0

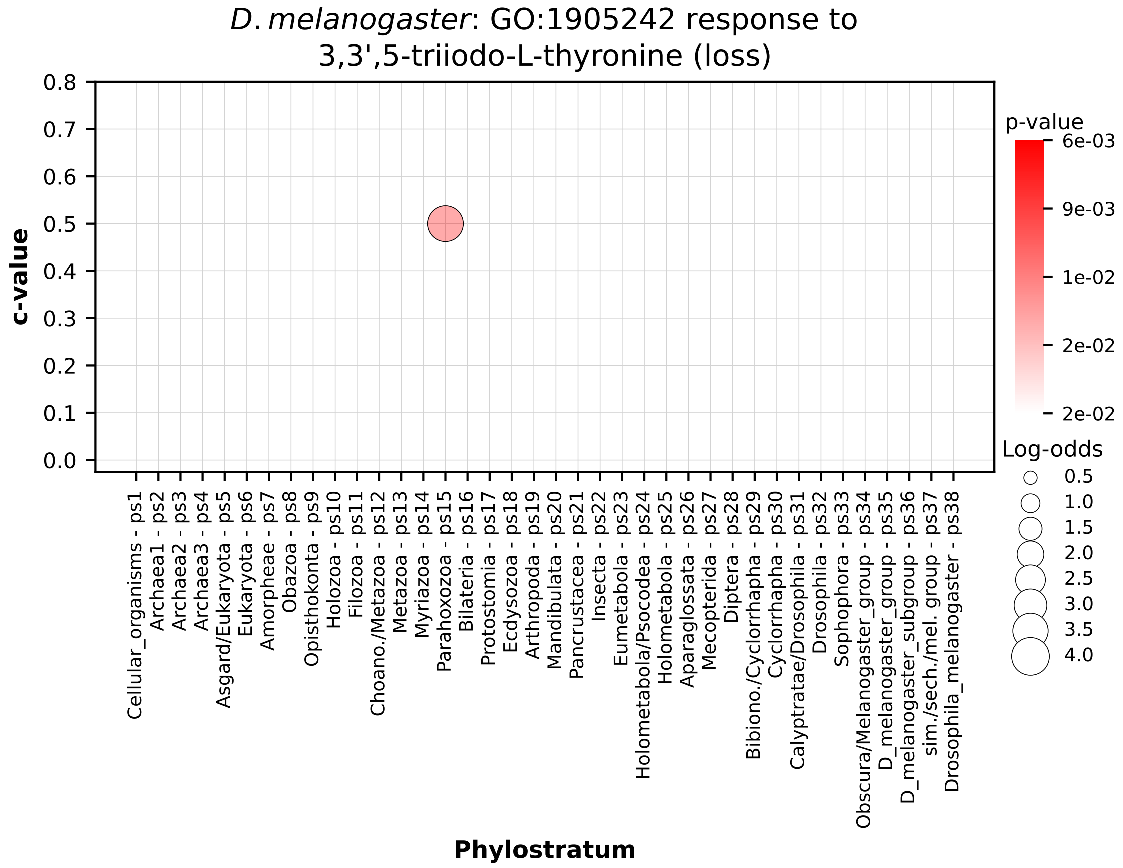

D. melanogaster: GO:1905242 response to 3,3',5-triiodo-L-thyronine (loss)
c-value
0.0
0.1
0.2
0.3
0.4
0.5
0.6
0.7
0.8
Phylostratum
Cellular_organisms - ps1
Archaea1 - ps2
Archaea2 - ps3
Archaea3 - ps4
Asgard/Eukaryota - ps5
Eukaryota - ps6
Amorpheae - ps7
Obazoa - ps8
Opisthokonta - ps9
Holozoa - ps10
Filozoa - ps11
Choano./Metazoa - ps12
Metazoa - ps13
Myriazoa - ps14
Parahoxozoa - ps15
Bilateria - ps16
Protostomia - ps17
Ecdysozoa - ps18
Arthropoda - ps19
Mandibulata - ps20
Pancrustacea - ps21
Insecta - ps22
Eumetabola - ps23
Holometabola/Psocodea - ps24
Holometabola - ps25
Aparaglossata - ps26
Mecopterida - ps27
Diptera - ps28
Bibiono./Cyclorrhapha - ps29
Cyclorrhapha - ps30
Calyptratae/Drosophila - ps31
Drosophila - ps32
Sophophora - ps33
Obscura/Melanogaster_group - ps34
D_melanogaster_group - ps35
D_melanogaster_subgroup - ps36
sim./sech./mel. group - ps37
Drosophila_melanogaster - ps38
p-value
6e-03
9e-03
1e-02
2e-02
2e-02
Log-odds
0.5
1.0
1.5
2.0
2.5
3.0
3.5
4.0

D. melanogaster: GO:1905243 cellular response to 3,3',5-triiodo-L-thyronine (loss)

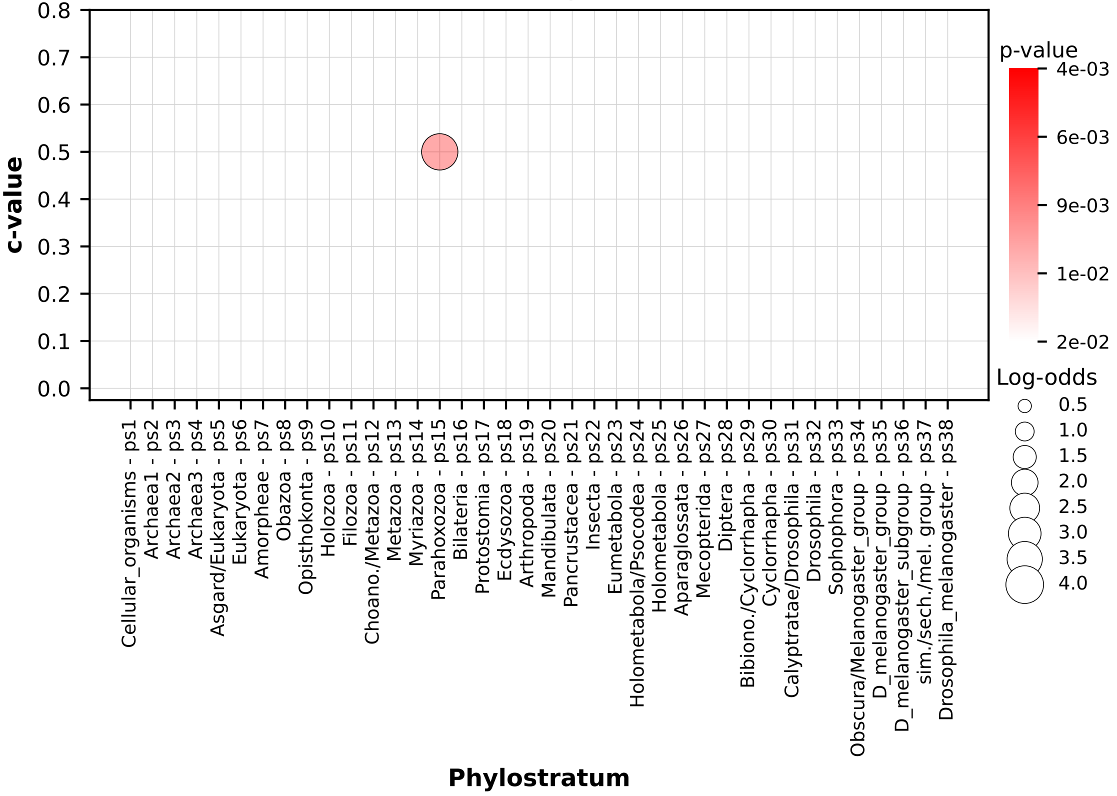

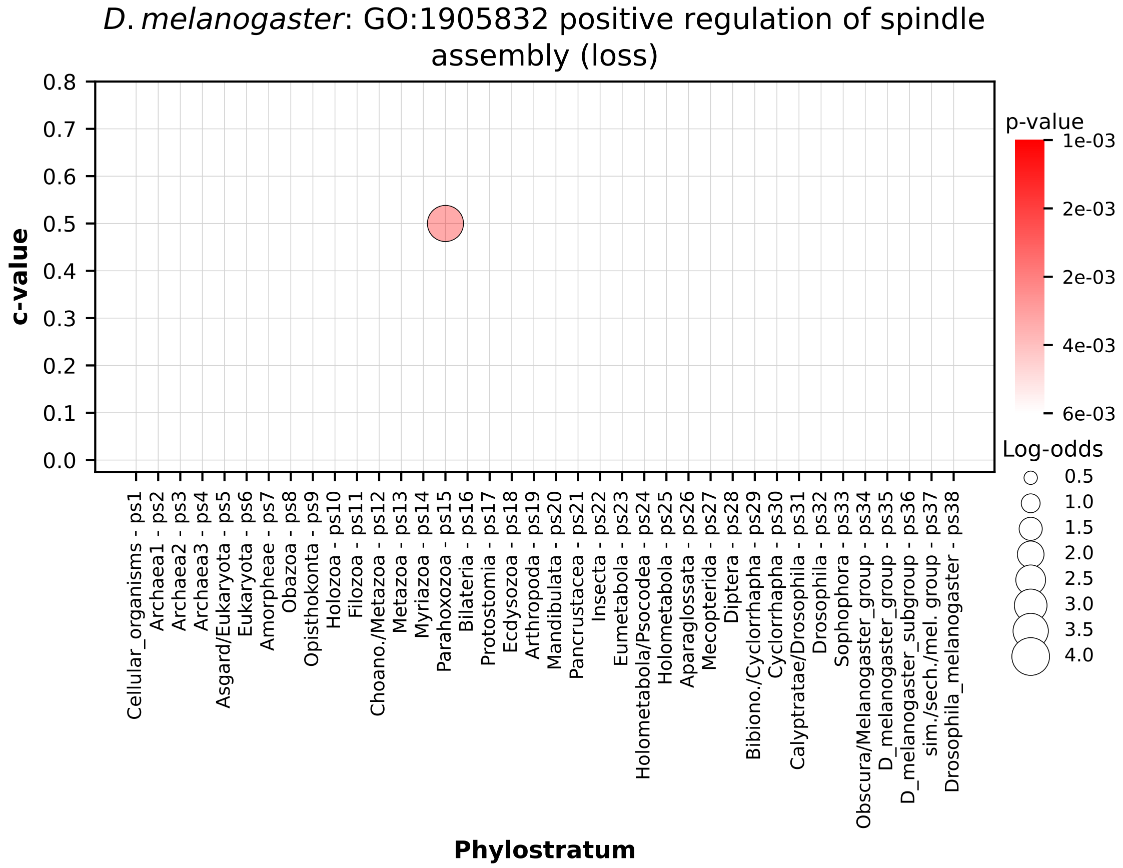
D. melanogaster: GO:1905832 positive regulation of spindle assembly (loss)
c-value
0.8
0.7
0.6
0.5
0.4
0.3
0.2
0.1
0.0
p-value
1e-03
2e-03
2e-03
4e-03
6e-03
Log-odds
0.5
1.0
1.5
2.0
2.5
3.0
3.5
4.0
Cellular_organisms - ps1
Archaea1 - ps2
Archaea2 - ps3
Archaea3 - ps4
Asgard/Eukaryota - ps5
Eukaryota - ps6
Amorpheae - ps7
Obazoa - ps8
Opisthokonta - ps9
Holozoa - ps10
Filozoa - ps11
Choano./Metazoa - ps12
Metazoa - ps13
Myriazoa - ps14
Parahoxozoa - ps15
Bilateria - ps16
Protostomia - ps17
Ecdysozoa - ps18
Arthropoda - ps19
Mandibulata - ps20
Pancrustacea - ps21
Insecta - ps22
Eumetabola - ps23
Holometabola/Psocodea - ps24
Holometabola - ps25
Aparaglossata - ps26
Mecopterida - ps27
Diptera - ps28
Bibiono./Cyclorrhapha - ps29
Cyclorrhapha - ps30
Calyptratae/Drosophila - ps31
Drosophila - ps32
Sophophora - ps33
Obscura/Melanogaster_group - ps34
D_melanogaster_group - ps35
D_melanogaster_subgroup - ps36
sim./sech./mel. group - ps37
Drosophila_melanogaster - ps38
Phylostratum

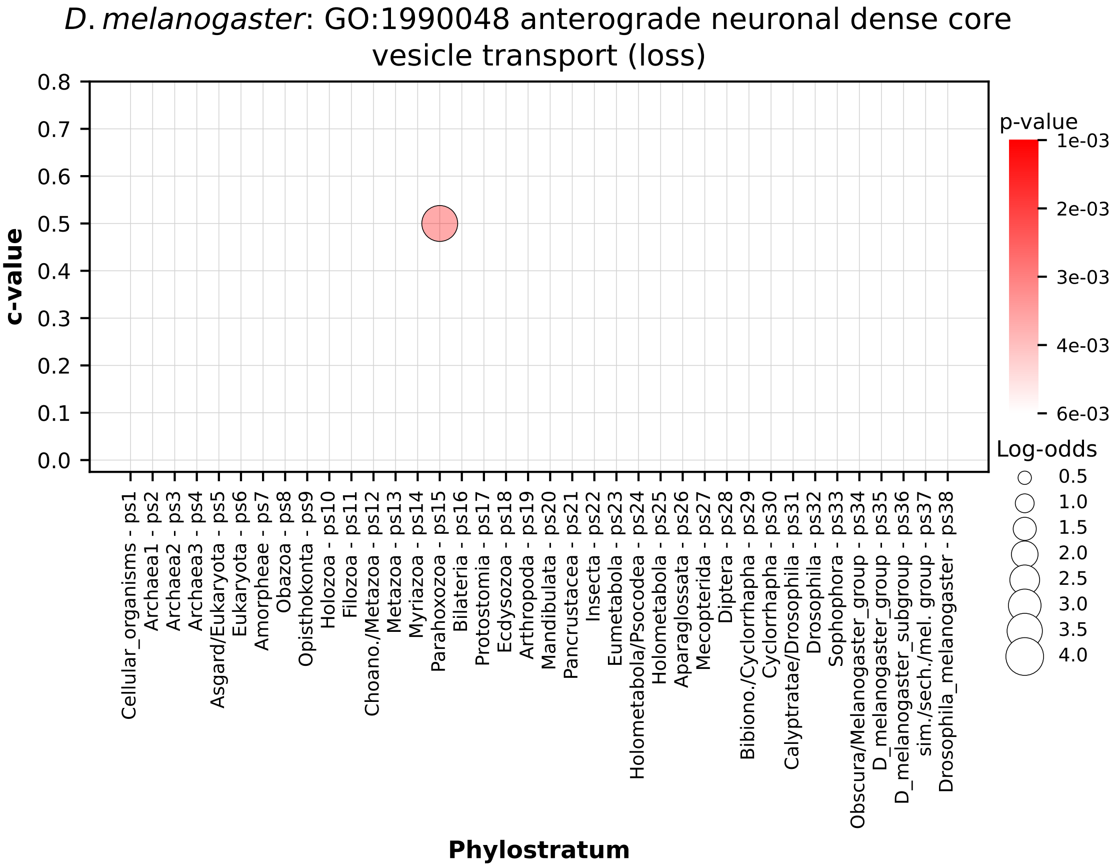

D. melanogaster: GO:1990048 anterograde neuronal dense core vesicle transport (loss)
c-value
0.8
0.7
0.6
0.5
0.4
0.3
0.2
0.1
0.0
p-value
1e-03
2e-03
3e-03
4e-03
6e-03
Log-odds
0.5
1.0
1.5
2.0
2.5
3.0
3.5
4.0
Cellular_organisms - ps1
Archaea1 - ps2
Archaea2 - ps3
Archaea3 - ps4
Asgard/Eukaryota - ps5
Eukaryota - ps6
Amorpheae - ps7
Obazoa - ps8
Opisthokonta - ps9
Holozoa - ps10
Filozoa - ps11
Choano./Metazoa - ps12
Metazoa - ps13
Myriazoa - ps14
Parahoxozoa - ps15
Bilateria - ps16
Protostomia - ps17
Ecdysozoa - ps18
Arthropoda - ps19
Mandibulata - ps20
Pancrustacea - ps21
Insecta - ps22
Eumetabola - ps23
Holometabola/Psocodea - ps24
Holometabola - ps25
Aparaglossata - ps26
Mecopterida - ps27
Diptera - ps28
Bibiono./Cyclorrhapha - ps29
Cyclorrhapha - ps30
Calyptratae/Drosophila - ps31
Drosophila - ps32
Sophophora - ps33
Obscura/Melanogaster_group - ps34
D_melanogaster_group - ps35
D_melanogaster_subgroup - ps36
sim./sech./mel. group - ps37
Drosophila_melanogaster - ps38
Phylostratum

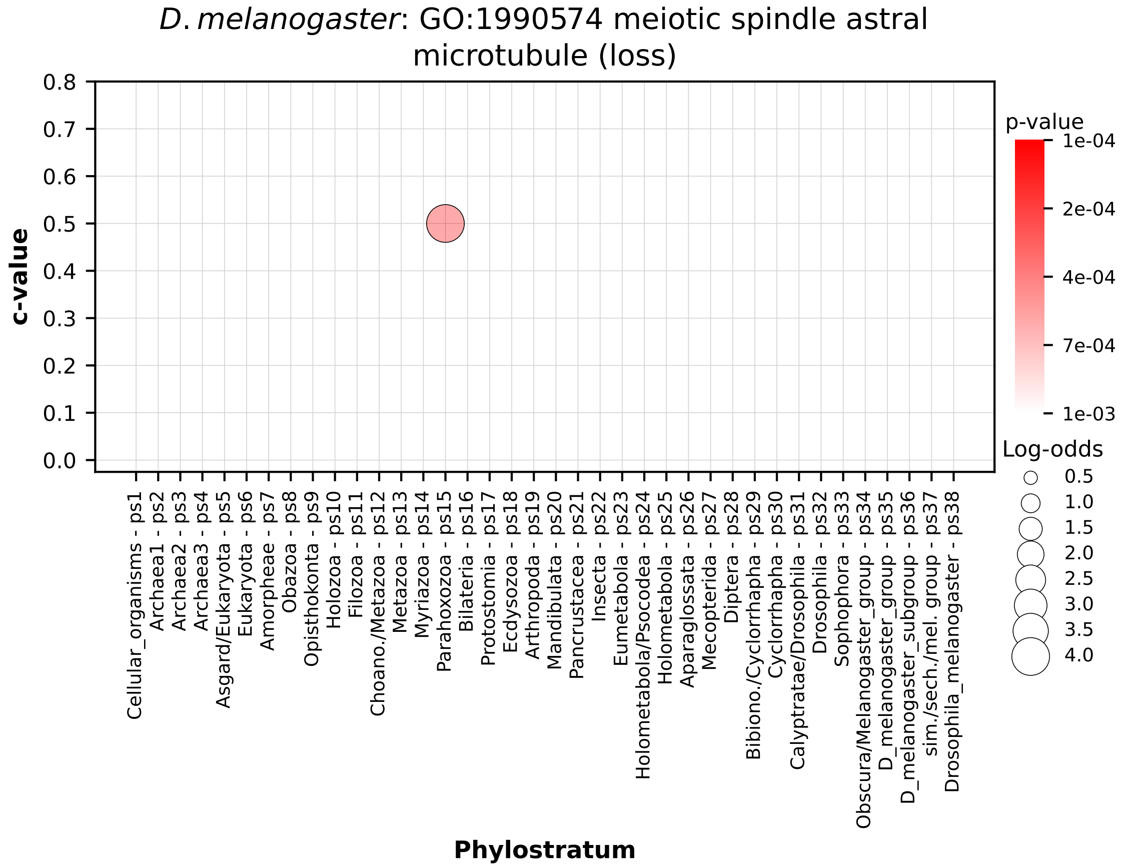

D. melanogaster: GO:1990574 meiotic spindle astral microtubule (loss)
c-value
0.8
0.7
0.6
0.5
0.4
0.3
0.2
0.1
0.0
Phylostratum
Cellular_organisms - ps1
Archaea1 - ps2
Archaea2 - ps3
Archaea3 - ps4
Asgard/Eukaryota - ps5
Eukaryota - ps6
Amorpheae - ps7
Obazoa - ps8
Opisthokonta - ps9
Holozoa - ps10
Filozoa - ps11
Choano./Metazoa - ps12
Metazoa - ps13
Myriazoa - ps14
Parahoxozoa - ps15
Bilateria - ps16
Protostomia - ps17
Ecdysozoa - ps18
Arthropoda - ps19
Mandibulata - ps20
Pancrustacea - ps21
Insecta - ps22
Eumetabola - ps23
Holometabola/Psocodea - ps24
Holometabola - ps25
Aparaglossata - ps26
Mecopterida - ps27
Diptera - ps28
Bibiono./Cyclorrhapha - ps29
Cyclorrhapha - ps30
Calyptratae/Drosophila - ps31
Drosophila - ps32
Sophophora - ps33
Obscura/Melanogaster_group - ps34
D_melanogaster_group - ps35
D_melanogaster_subgroup - ps36
sim./sech./mel. group - ps37
Drosophila_melanogaster - ps38
p-value
1e-04
2e-04
4e-04
7e-04
1e-03
Log-odds
0.5
1.0
1.5
2.0
2.5
3.0
3.5
4.0

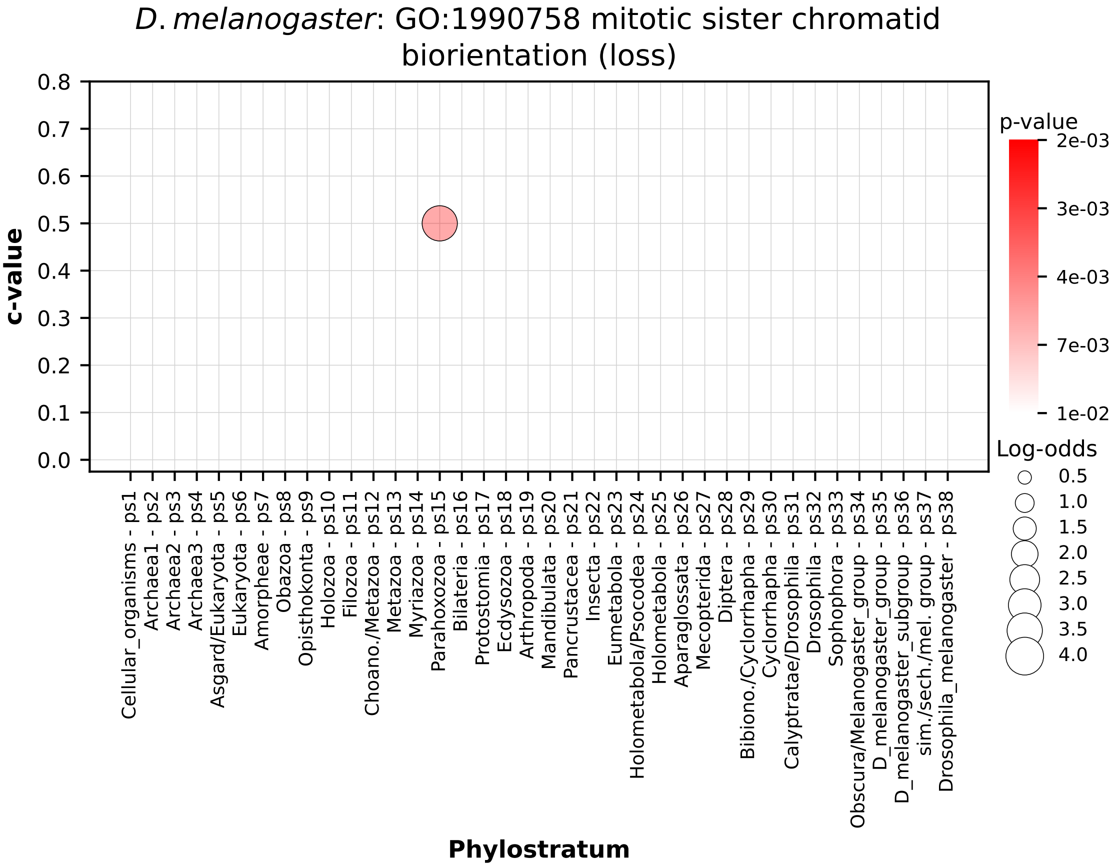

D. melanogaster: GO:1990758 mitotic sister chromatid biorientation (loss)
c-value
0.0
0.1
0.2
0.3
0.4
0.5
0.6
0.7
0.8
p-value
2e-03
3e-03
4e-03
7e-03
1e-02
Log-odds
0.5
1.0
1.5
2.0
2.5
3.0
3.5
4.0
Cellular_organisms - ps1
Archaea1 - ps2
Archaea2 - ps3
Archaea3 - ps4
Asgard/Eukaryota - ps5
Eukaryota - ps6
Amorpheae - ps7
Obazoa - ps8
Opisthokonta - ps9
Holozoa - ps10
Filozoa - ps11
Choano./Metazoa - ps12
Metazoa - ps13
Myriazoa - ps14
Parahoxozoa - ps15
Bilateria - ps16
Protostomia - ps17
Ecdysozoa - ps18
Arthropoda - ps19
Mandibulata - ps20
Pancrustacea - ps21
Insecta - ps22
Eumetabola - ps23
Holometabola/Psocodea - ps24
Holometabola - ps25
Aparaglossata - ps26
Mecopterida - ps27
Diptera - ps28
Bibiono./Cyclorrhapha - ps29
Cyclorrhapha - ps30
Calyptratae/Drosophila - ps31
Drosophila - ps32
Sophophora - ps33
Obscura/Melanogaster_group - ps34
D_melanogaster_group - ps35
D_melanogaster_subgroup - ps36
sim./sech./mel. group - ps37
Drosophila_melanogaster - ps38
Phylostratum

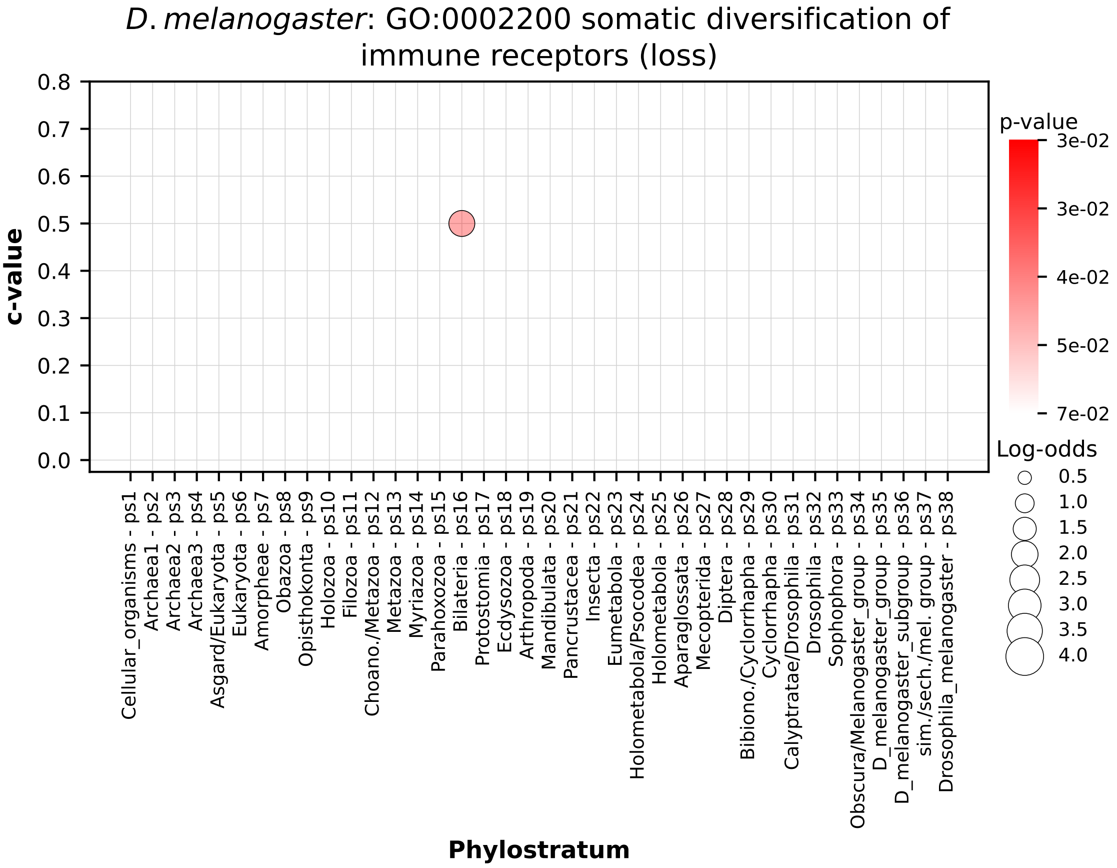

D. melanogaster: GO:0002200 somatic diversification of immune receptors (loss)
c-value
0.8
0.7
0.6
0.5
0.4
0.3
0.2
0.1
0.0
p-value
3e-02
3e-02
4e-02
5e-02
7e-02
Log-odds
0.5
1.0
1.5
2.0
2.5
3.0
3.5
4.0
Cellular_organisms - ps1
Archaea1 - ps2
Archaea2 - ps3
Archaea3 - ps4
Asgard/Eukaryota - ps5
Eukaryota - ps6
Amorpheae - ps7
Obazoa - ps8
Opisthokonta - ps9
Holozoa - ps10
Filozoa - ps11
Choano./Metazoa - ps12
Metazoa - ps13
Myriazoa - ps14
Parahoxozoa - ps15
Bilateria - ps16
Protostomia - ps17
Ecdysozoa - ps18
Arthropoda - ps19
Mandibulata - ps20
Pancrustacea - ps21
Insecta - ps22
Eumetabola - ps23
Holometabola/Psocodea - ps24
Holometabola - ps25
Aparaglossata - ps26
Mecopterida - ps27
Diptera - ps28
Bibiono./Cyclorrhapha - ps29
Cyclorrhapha - ps30
Calyptratae/Drosophila - ps31
Drosophila - ps32
Sophophora - ps33
Obscura/Melanogaster_group - ps34
D_melanogaster_group - ps35
D_melanogaster_subgroup - ps36
sim./sech./mel. group - ps37
Drosophila_melanogaster - ps38
Phylostratum

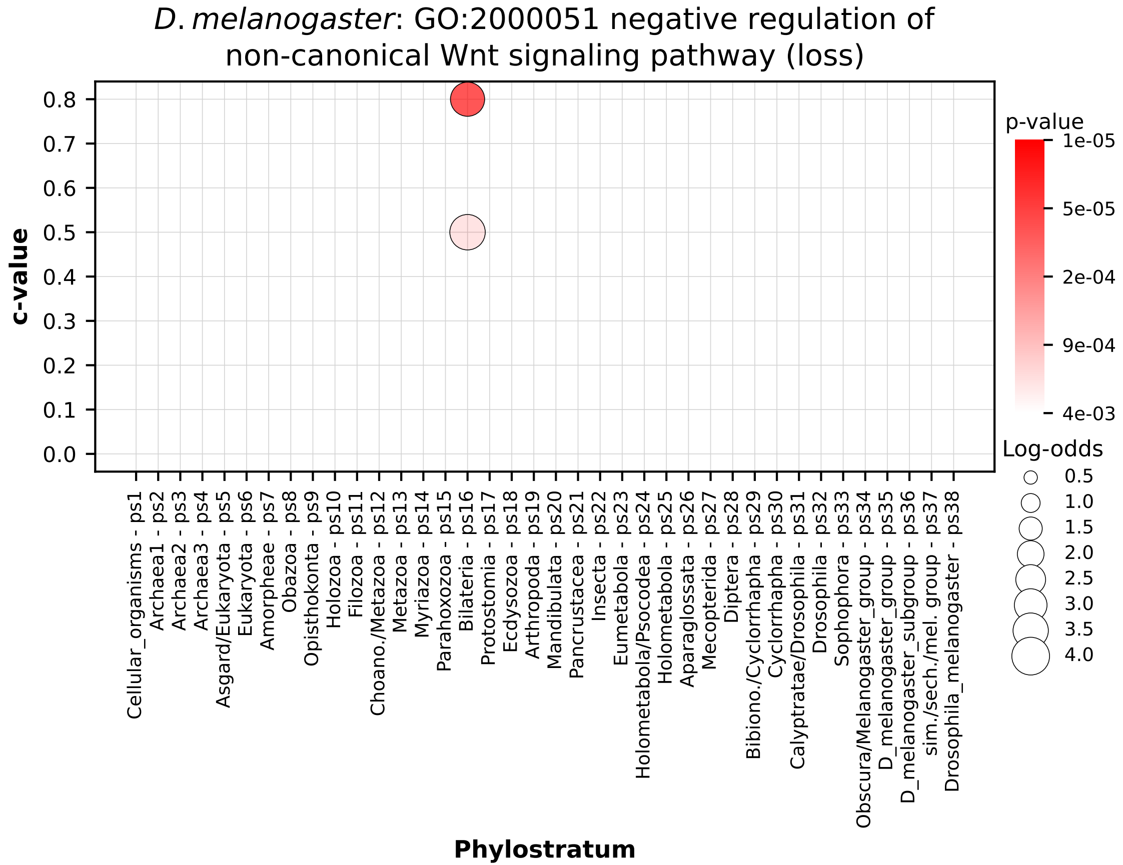

D. melanogaster: GO:2000051 negative regulation of non-canonical Wnt signaling pathway (loss)
c-value
0.8
0.7
0.6
0.5
0.4
0.3
0.2
0.1
0.0
Phylostratum
Cellular_organisms - ps1
Archaea1 - ps2
Archaea2 - ps3
Archaea3 - ps4
Asgard/Eukaryota - ps5
Eukaryota - ps6
Amorpheae - ps7
Obazoa - ps8
Opisthokonta - ps9
Holozoa - ps10
Filozoa - ps11
Choano./Metazoa - ps12
Metazoa - ps13
Myriazoa - ps14
Parahoxozoa - ps15
Bilateria - ps16
Protostomia - ps17
Ecdysozoa - ps18
Arthropoda - ps19
Mandibulata - ps20
Pancrustacea - ps21
Insecta - ps22
Eumetabola - ps23
Holometabola/Psocodea - ps24
Holometabola - ps25
Aparaglossata - ps26
Mecopterida - ps27
Diptera - ps28
Bibiono./Cyclorrhapha - ps29
Cyclorrhapha - ps30
Calyptratae/Drosophila - ps31
Drosophila - ps32
Sophophora - ps33
Obscura/Melanogaster_group - ps34
D_melanogaster_group - ps35
D_melanogaster_subgroup - ps36
sim./sech./mel. group - ps37
Drosophila_melanogaster - ps38
p-value
1e-05
5e-05
2e-04
9e-04
4e-03
Log-odds
0.5
1.0
1.5
2.0
2.5
3.0
3.5
4.0

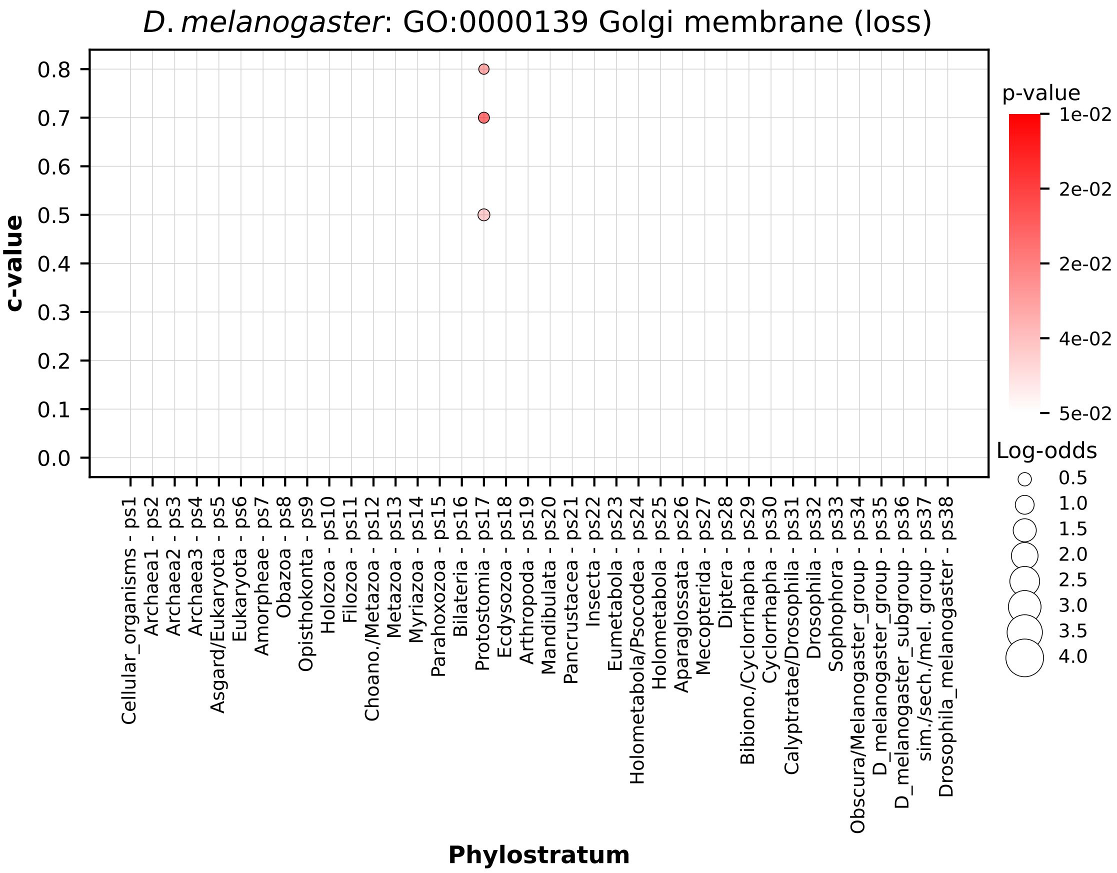

D. melanogaster: GO:0000139 Golgi membrane (loss)
c-value
0.0
0.1
0.2
0.3
0.4
0.5
0.6
0.7
0.8
Phylostratum
Cellular_organisms - ps1
Archaea1 - ps2
Archaea2 - ps3
Archaea3 - ps4
Asgard/Eukaryota - ps5
Eukaryota - ps6
Amorpheae - ps7
Obazoa - ps8
Opisthokonta - ps9
Holozoa - ps10
Filozoa - ps11
Choano./Metazoa - ps12
Metazoa - ps13
Myriazoa - ps14
Parahoxozoa - ps15
Bilateria - ps16
Protostomia - ps17
Ecdysozoa - ps18
Arthropoda - ps19
Mandibulata - ps20
Pancrustacea - ps21
Insecta - ps22
Eumetabola - ps23
Holometabola/Psocodea - ps24
Holometabola - ps25
Aparaglossata - ps26
Mecopterida - ps27
Diptera - ps28
Bibiono./Cyclorrhapha - ps29
Cyclorrhapha - ps30
Calyptratae/Drosophila - ps31
Drosophila - ps32
Sophophora - ps33
Obscura/Melanogaster_group - ps34
D_melanogaster_group - ps35
D_melanogaster_subgroup - ps36
sim./sech./mel. group - ps37
Drosophila_melanogaster - ps38
p-value
1e-02
2e-02
2e-02
4e-02
5e-02
Log-odds
0.5
1.0
1.5
2.0
2.5
3.0
3.5
4.0

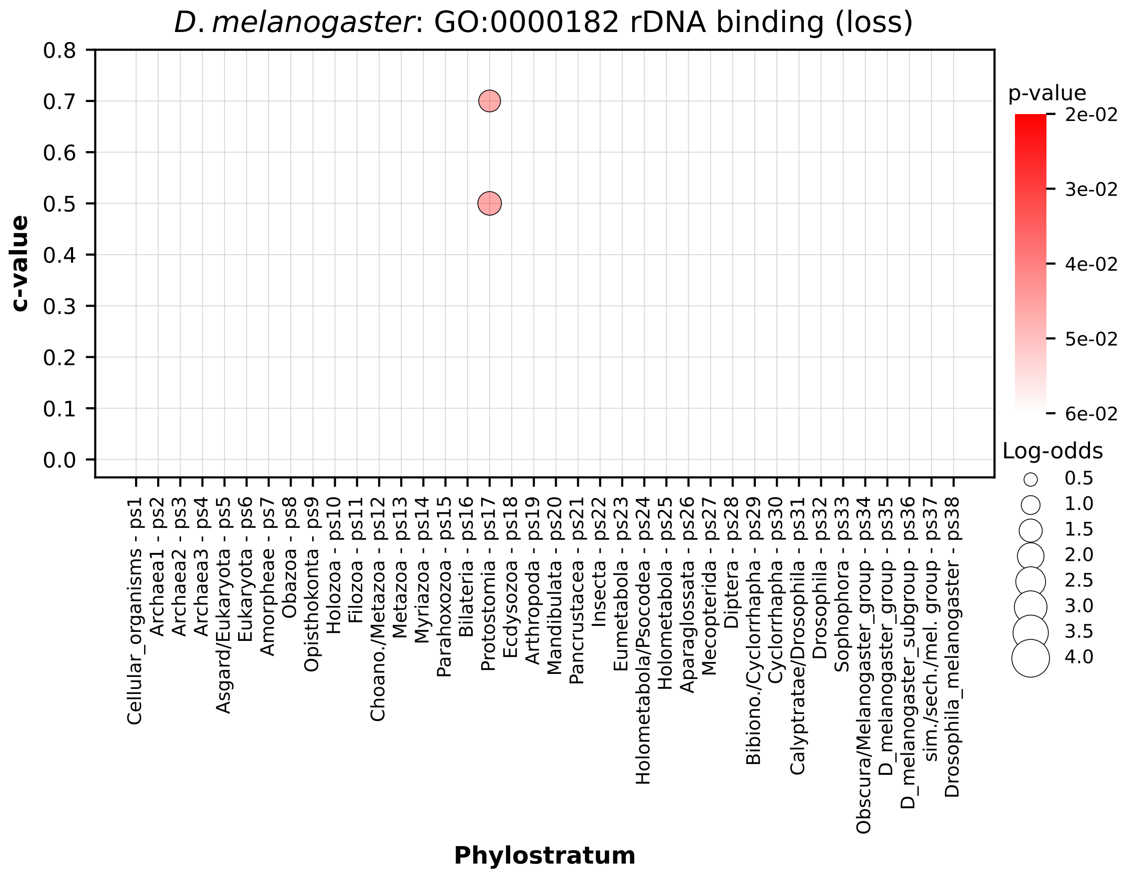
D. melanogaster: GO:0000182 rDNA binding (loss)
c-value
0.8
0.7
0.6
0.5
0.4
0.3
0.2
0.1
0.0
p-value
2e-02
3e-02
4e-02
5e-02
6e-02
Log-odds
0.5
1.0
1.5
2.0
2.5
3.0
3.5
4.0
Cellular_organisms - ps1
Archaea1 - ps2
Archaea2 - ps3
Archaea3 - ps4
Asgard/Eukaryota - ps5
Eukaryota - ps6
Amorpheae - ps7
Obazoa - ps8
Opisthokonta - ps9
Holozoa - ps10
Filozoa - ps11
Choano./Metazoa - ps12
Metazoa - ps13
Myriazoa - ps14
Parahoxozoa - ps15
Bilateria - ps16
Protostomia - ps17
Ecdysozoa - ps18
Arthropoda - ps19
Mandibulata - ps20
Pancrustacea - ps21
Insecta - ps22
Eumetabola - ps23
Holometabola/Psocodea - ps24
Holometabola - ps25
Aparaglossata - ps26
Mecopterida - ps27
Diptera - ps28
Bibiono./Cyclorrhapha - ps29
Cyclorrhapha - ps30
Calyptratae/Drosophila - ps31
Drosophila - ps32
Sophophora - ps33
Obscura/Melanogaster_group - ps34
D_melanogaster_group - ps35
D_melanogaster_subgroup - ps36
sim./sech./mel. group - ps37
Drosophila_melanogaster - ps38
Phylostratum

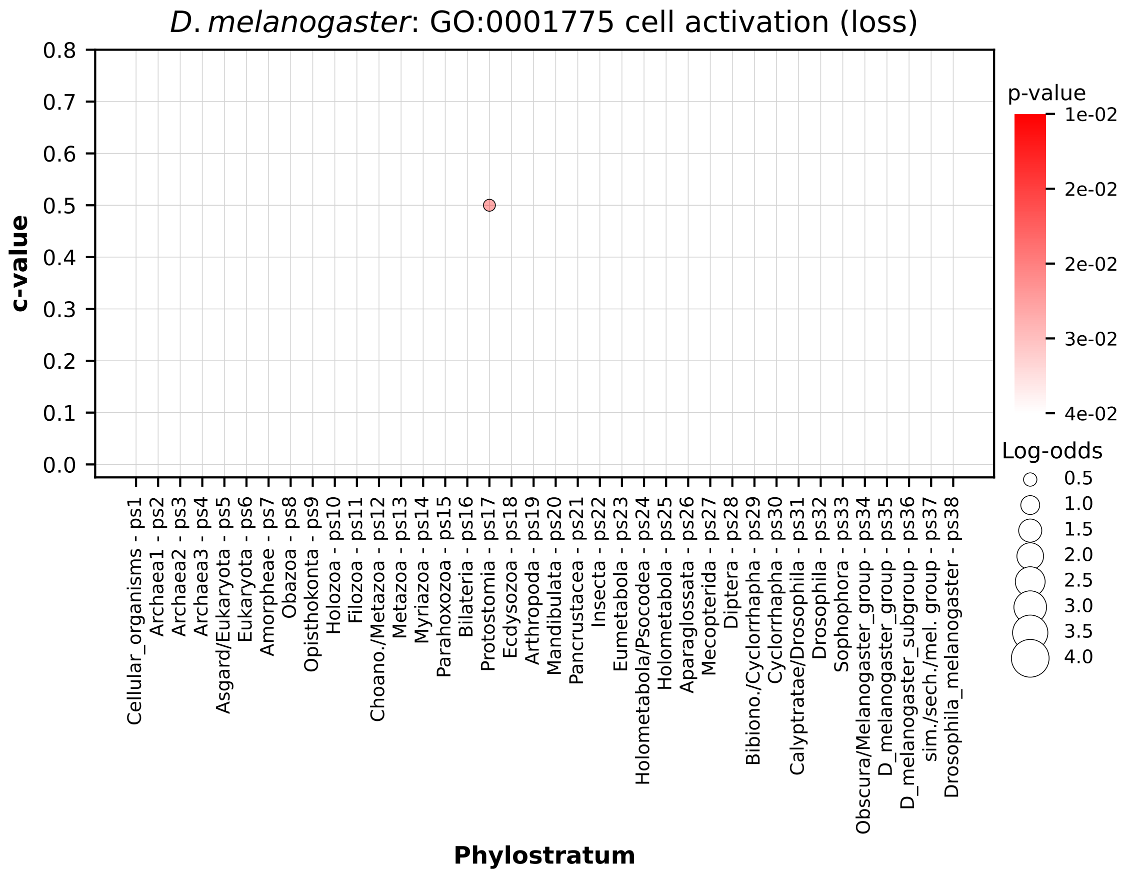

D. melanogaster: GO:0001775 cell activation (loss)
c-value
0.8
0.7
0.6
0.5
0.4
0.3
0.2
0.1
0.0
Phylostratum
Cellular_organisms - ps1
Archaea1 - ps2
Archaea2 - ps3
Archaea3 - ps4
Asgard/Eukaryota - ps5
Eukaryota - ps6
Amorpheae - ps7
Obazoa - ps8
Opisthokonta - ps9
Holozoa - ps10
Filozoa - ps11
Choano./Metazoa - ps12
Metazoa - ps13
Myriazoa - ps14
Parahoxozoa - ps15
Bilateria - ps16
Protostomia - ps17
Ecdysozoa - ps18
Arthropoda - ps19
Mandibulata - ps20
Pancrustacea - ps21
Insecta - ps22
Eumetabola - ps23
Holometabola/Psocodea - ps24
Holometabola - ps25
Aparaglossata - ps26
Mecopterida - ps27
Diptera - ps28
Bibiono./Cyclorrhapha - ps29
Cyclorrhapha - ps30
Calyptratae/Drosophila - ps31
Drosophila - ps32
Sophophora - ps33
Obscura/Melanogaster_group - ps34
D_melanogaster_group - ps35
D_melanogaster_subgroup - ps36
sim./sech./mel. group - ps37
Drosophila_melanogaster - ps38
p-value
1e-02
2e-02
2e-02
3e-02
4e-02
Log-odds
0.5
1.0
1.5
2.0
2.5
3.0
3.5
4.0

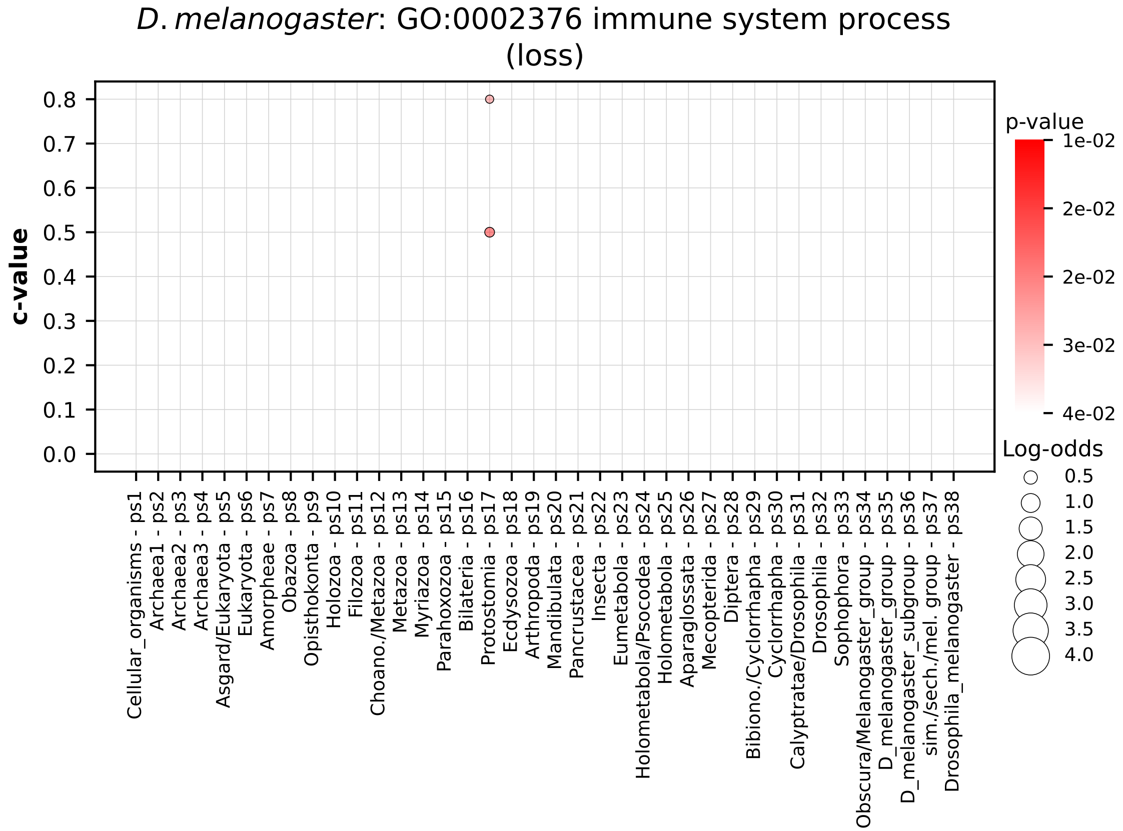

D. melanogaster: GO:0002376 immune system process
(loss)
c-value
0.0
0.1
0.2
0.3
0.4
0.5
0.6
0.7
0.8
Phylostratum
Cellular_organisms - ps1
Archaea1 - ps2
Archaea2 - ps3
Archaea3 - ps4
Asgard/Eukaryota - ps5
Eukaryota - ps6
Amorpheae - ps7
Obazoa - ps8
Opisthokonta - ps9
Holozoa - ps10
Filozoa - ps11
Choano./Metazoa - ps12
Metazoa - ps13
Myriazoa - ps14
Parahoxozoa - ps15
Bilateria - ps16
Protostomia - ps17
Ecdysozoa - ps18
Arthropoda - ps19
Mandibulata - ps20
Pancrustacea - ps21
Insecta - ps22
Eumetabola - ps23
Holometabola/Psocodea - ps24
Holometabola - ps25
Aparaglossata - ps26
Mecopterida - ps27
Diptera - ps28
Bibiono./Cyclorrhapha - ps29
Cyclorrhapha - ps30
Calyptratae/Drosophila - ps31
Drosophila - ps32
Sophophora - ps33
Obscura/Melanogaster_group - ps34
D_melanogaster_group - ps35
D_melanogaster_subgroup - ps36
sim./sech./mel. group - ps37
Drosophila_melanogaster - ps38
p-value
1e-02
2e-02
2e-02
3e-02
4e-02
Log-odds
0.5
1.0
1.5
2.0
2.5
3.0
3.5
4.0

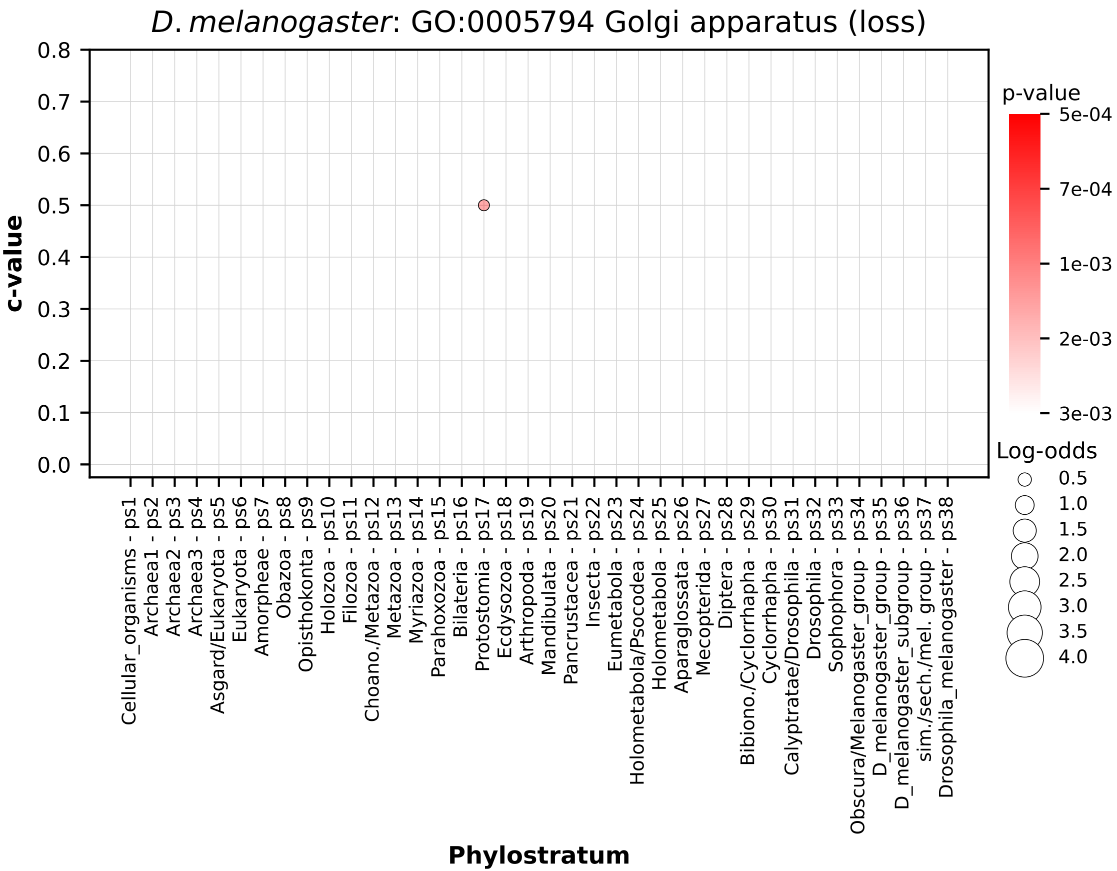

D. melanogaster: GO:0005794 Golgi apparatus (loss)
c-value
0.8
0.7
0.6
0.5
0.4
0.3
0.2
0.1
0.0
Phylostratum
Cellular_organisms - ps1
Archaea1 - ps2
Archaea2 - ps3
Archaea3 - ps4
Asgard/Eukaryota - ps5
Eukaryota - ps6
Amorpheae - ps7
Obazoa - ps8
Opisthokonta - ps9
Holozoa - ps10
Filozoa - ps11
Choano./Metazoa - ps12
Metazoa - ps13
Myriazoa - ps14
Parahoxozoa - ps15
Bilateria - ps16
Protostomia - ps17
Ecdysozoa - ps18
Arthropoda - ps19
Mandibulata - ps20
Pancrustacea - ps21
Insecta - ps22
Eumetabola - ps23
Holometabola/Psocodea - ps24
Holometabola - ps25
Aparaglossata - ps26
Mecopterida - ps27
Diptera - ps28
Bibiono./Cyclorrhapha - ps29
Cyclorrhapha - ps30
Calyptratae/Drosophila - ps31
Drosophila - ps32
Sophophora - ps33
Obscura/Melanogaster_group - ps34
D_melanogaster_group - ps35
D_melanogaster_subgroup - ps36
sim./sech./mel. group - ps37
Drosophila_melanogaster - ps38
p-value
5e-04
7e-04
1e-03
2e-03
3e-03
Log-odds
0.5
1.0
1.5
2.0
2.5
3.0
3.5
4.0

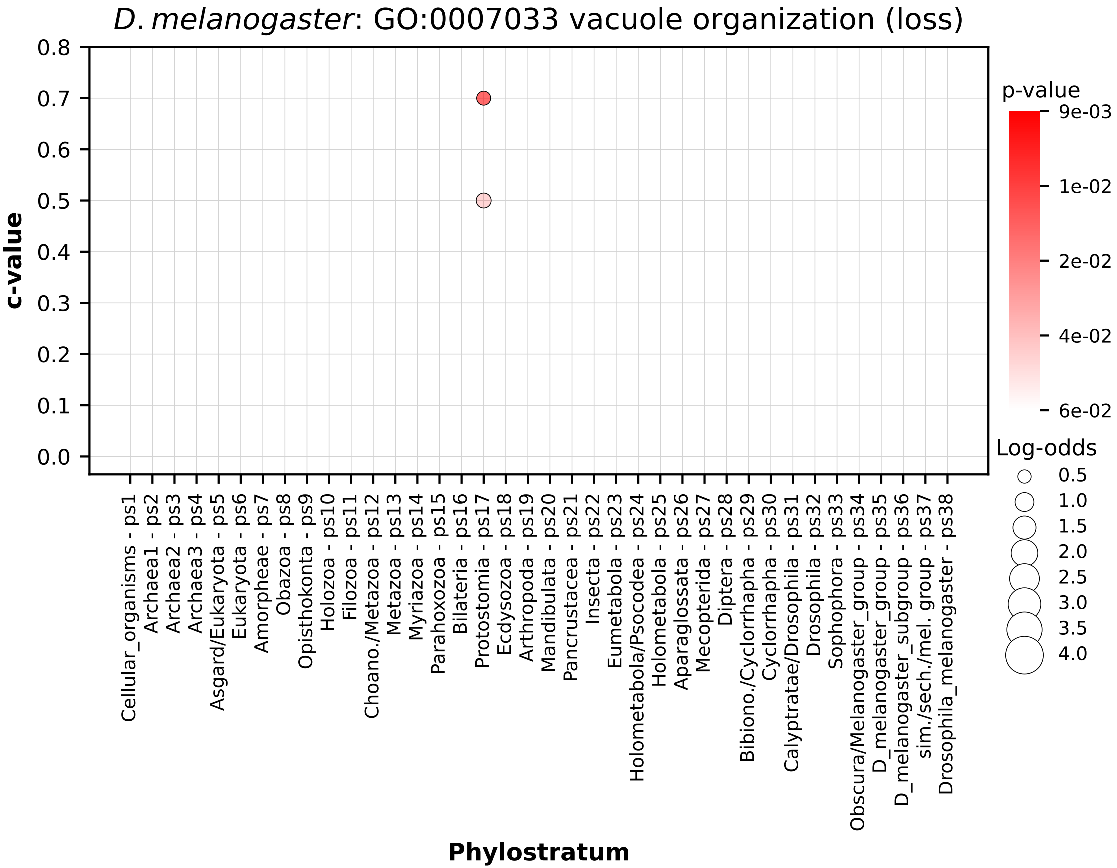

D. melanogaster: GO:0007033 vacuole organization (loss)
c-value
0.8
0.7
0.6
0.5
0.4
0.3
0.2
0.1
0.0
p-value
9e-03
1e-02
2e-02
4e-02
6e-02
Log-odds
0.5
1.0
1.5
2.0
2.5
3.0
3.5
4.0
Cellular_organisms - ps1
Archaea1 - ps2
Archaea2 - ps3
Archaea3 - ps4
Asgard/Eukaryota - ps5
Eukaryota - ps6
Amorpheae - ps7
Obazoa - ps8
Opisthokonta - ps9
Holozoa - ps10
Filozoa - ps11
Choano./Metazoa - ps12
Metazoa - ps13
Myriazoa - ps14
Parahoxozoa - ps15
Bilateria - ps16
Protostomia - ps17
Ecdysozoa - ps18
Arthropoda - ps19
Mandibulata - ps20
Pancrustacea - ps21
Insecta - ps22
Eumetabola - ps23
Holometabola/Psocodea - ps24
Holometabola - ps25
Aparaglossata - ps26
Mecopterida - ps27
Diptera - ps28
Bibiono./Cyclorrhapha - ps29
Cyclorrhapha - ps30
Calyptratae/Drosophila - ps31
Drosophila - ps32
Sophophora - ps33
Obscura/Melanogaster_group - ps34
D_melanogaster_group - ps35
D_melanogaster_subgroup - ps36
sim./sech./mel. group - ps37
Drosophila_melanogaster - ps38
Phylostratum

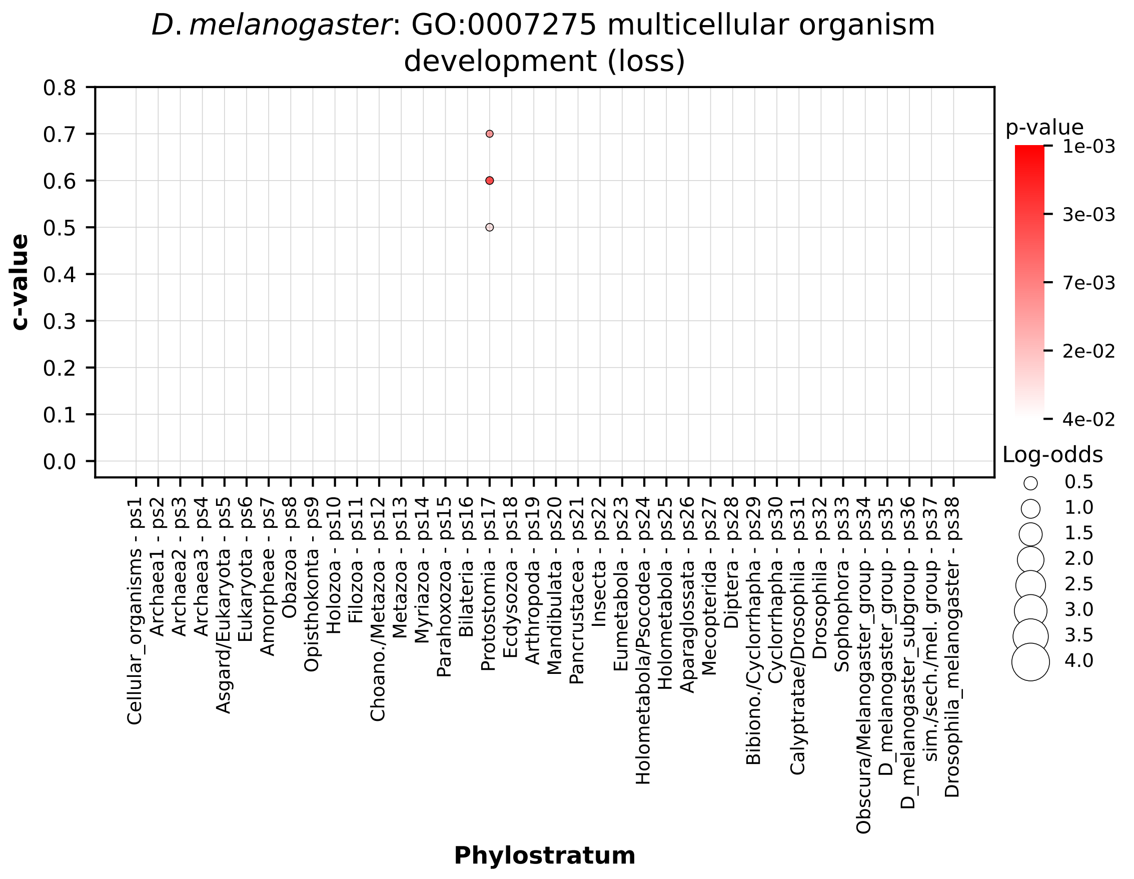

D. melanogaster: GO:0007275 multicellular organism development (loss)
c-value
0.8
0.7
0.6
0.5
0.4
0.3
0.2
0.1
0.0
p-value
1e-03
3e-03
7e-03
2e-02
4e-02
Log-odds
0.5
1.0
1.5
2.0
2.5
3.0
3.5
4.0
Cellular_organisms - ps1
Archaea1 - ps2
Archaea2 - ps3
Archaea3 - ps4
Asgard/Eukaryota - ps5
Eukaryota - ps6
Amorpheae - ps7
Obazoa - ps8
Opisthokonta - ps9
Holozoa - ps10
Filozoa - ps11
Choano./Metazoa - ps12
Metazoa - ps13
Myriazoa - ps14
Parahoxozoa - ps15
Bilateria - ps16
Protostomia - ps17
Ecdysozoa - ps18
Arthropoda - ps19
Mandibulata - ps20
Pancrustacea - ps21
Insecta - ps22
Eumetabola - ps23
Holometabola/Psocodea - ps24
Holometabola - ps25
Aparaglossata - ps26
Mecopterida - ps27
Diptera - ps28
Bibiono./Cyclorrhapha - ps29
Cyclorrhapha - ps30
Calyptratae/Drosophila - ps31
Drosophila - ps32
Sophophora - ps33
Obscura/Melanogaster_group - ps34
D_melanogaster_group - ps35
D_melanogaster_subgroup - ps36
sim./sech./mel. group - ps37
Drosophila_melanogaster - ps38
Phylostratum

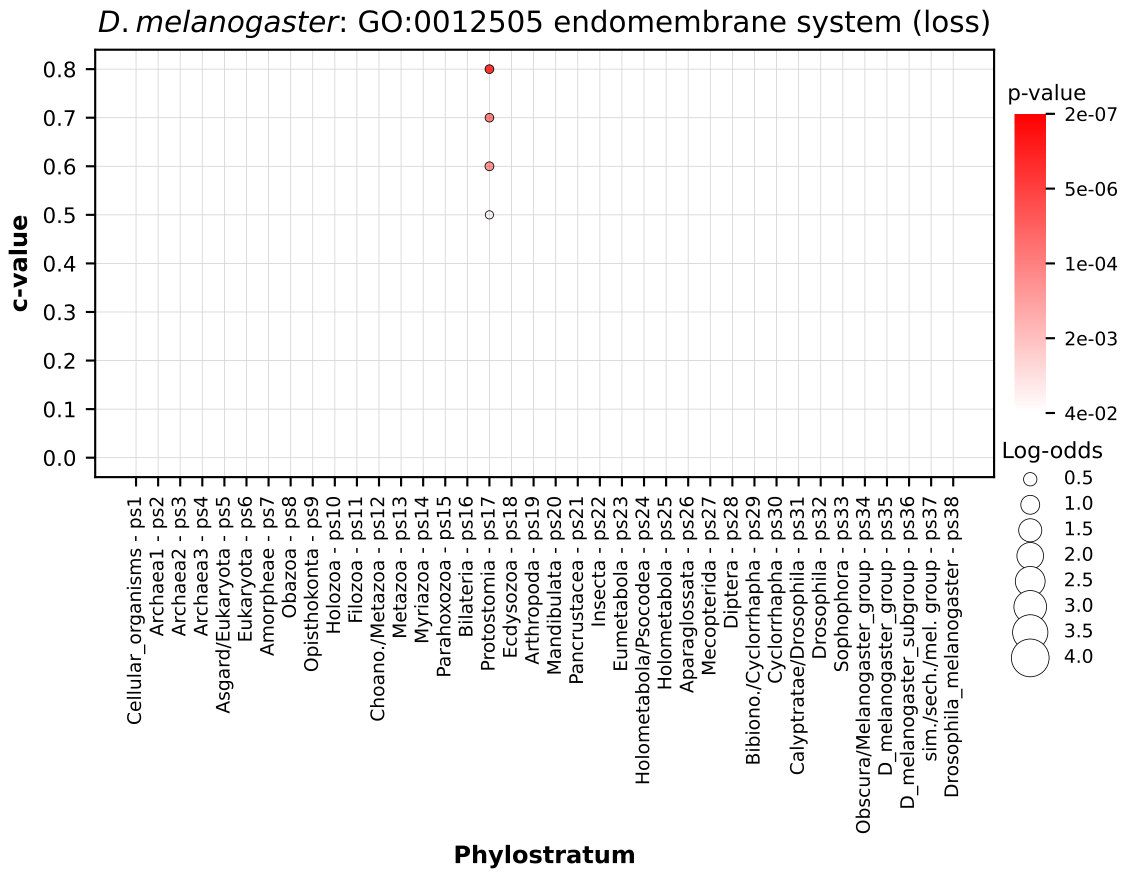
D. melanogaster: GO:0012505 endomembrane system (loss)
c-value
0.0
0.1
0.2
0.3
0.4
0.5
0.6
0.7
0.8
Phylostratum
Cellular_organisms - ps1
Archaea1 - ps2
Archaea2 - ps3
Archaea3 - ps4
Asgard/Eukaryota - ps5
Eukaryota - ps6
Amorpheae - ps7
Obazoa - ps8
Opisthokonta - ps9
Holozoa - ps10
Filozoa - ps11
Choano./Metazoa - ps12
Metazoa - ps13
Myriazoa - ps14
Parahoxozoa - ps15
Bilateria - ps16
Protostomia - ps17
Ecdysozoa - ps18
Arthropoda - ps19
Mandibulata - ps20
Pancrustacea - ps21
Insecta - ps22
Eumetabola - ps23
Holometabola/Psocodea - ps24
Holometabola - ps25
Aparaglossata - ps26
Mecopterida - ps27
Diptera - ps28
Bibiono./Cyclorrhapha - ps29
Cyclorrhapha - ps30
Calyptratae/Drosophila - ps31
Drosophila - ps32
Sophophora - ps33
Obscura/Melanogaster_group - ps34
D_melanogaster_group - ps35
D_melanogaster_subgroup - ps36
sim./sech./mel. group - ps37
Drosophila_melanogaster - ps38
p-value
2e-07
5e-06
1e-04
2e-03
4e-02
Log-odds
0.5
1.0
1.5
2.0
2.5
3.0
3.5
4.0

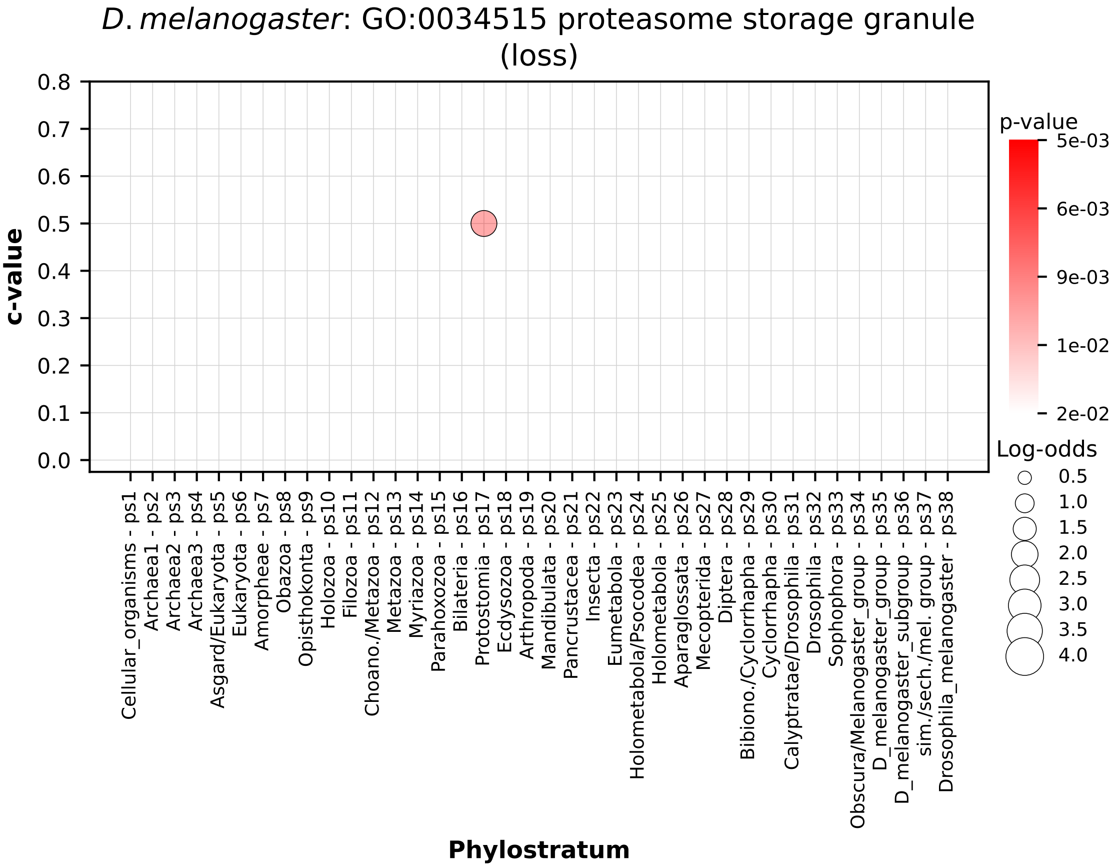

D. melanogaster: GO:0034515 proteasome storage granule (loss)
c-value
0.0
0.1
0.2
0.3
0.4
0.5
0.6
0.7
0.8
Phylostratum
Cellular_organisms - ps1
Archaea1 - ps2
Archaea2 - ps3
Archaea3 - ps4
Asgard/Eukaryota - ps5
Eukaryota - ps6
Amorpheae - ps7
Obazoa - ps8
Opisthokonta - ps9
Holozoa - ps10
Filozoa - ps11
Choano./Metazoa - ps12
Metazoa - ps13
Myriazoa - ps14
Parahoxozoa - ps15
Bilateria - ps16
Protostomia - ps17
Ecdysozoa - ps18
Arthropoda - ps19
Mandibulata - ps20
Pancrustacea - ps21
Insecta - ps22
Eumetabola - ps23
Holometabola/Psocodea - ps24
Holometabola - ps25
Aparaglossata - ps26
Mecopterida - ps27
Diptera - ps28
Bibiono./Cyclorrhapha - ps29
Cyclorrhapha - ps30
Calyptratae/Drosophila - ps31
Drosophila - ps32
Sophophora - ps33
Obscura/Melanogaster_group - ps34
D_melanogaster_group - ps35
D_melanogaster_subgroup - ps36
sim./sech./mel. group - ps37
Drosophila_melanogaster - ps38
p-value
5e-03
6e-03
9e-03
1e-02
2e-02
Log-odds
0.5
1.0
1.5
2.0
2.5
3.0
3.5
4.0

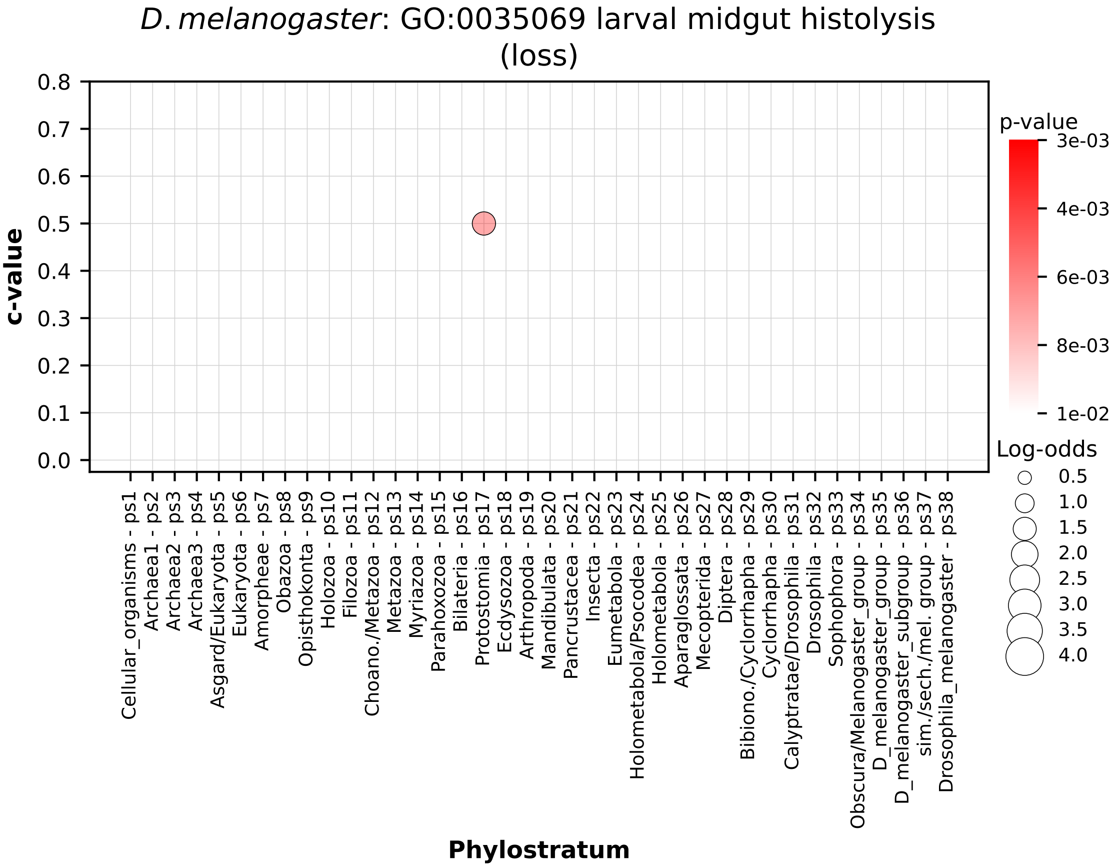

D. melanogaster: GO:0035069 larval midgut histolysis (loss)
c-value
0.8
0.7
0.6
0.5
0.4
0.3
0.2
0.1
0.0
p-value
3e-03
4e-03
6e-03
8e-03
1e-02
Log-odds
0.5
1.0
1.5
2.0
2.5
3.0
3.5
4.0
Cellular_organisms - ps1
Archaea1 - ps2
Archaea2 - ps3
Archaea3 - ps4
Asgard/Eukaryota - ps5
Eukaryota - ps6
Amorpheae - ps7
Obazoa - ps8
Opisthokonta - ps9
Holozoa - ps10
Filozoa - ps11
Choano./Metazoa - ps12
Metazoa - ps13
Myriazoa - ps14
Parahoxozoa - ps15
Bilateria - ps16
Protostomia - ps17
Ecdysozoa - ps18
Arthropoda - ps19
Mandibulata - ps20
Pancrustacea - ps21
Insecta - ps22
Eumetabola - ps23
Holometabola/Psocodea - ps24
Holometabola - ps25
Aparaglossata - ps26
Mecopterida - ps27
Diptera - ps28
Bibiono./Cyclorrhapha - ps29
Cyclorrhapha - ps30
Calyptratae/Drosophila - ps31
Drosophila - ps32
Sophophora - ps33
Obscura/Melanogaster_group - ps34
D_melanogaster_group - ps35
D_melanogaster_subgroup - ps36
sim./sech./mel. group - ps37
Drosophila_melanogaster - ps38
Phylostratum

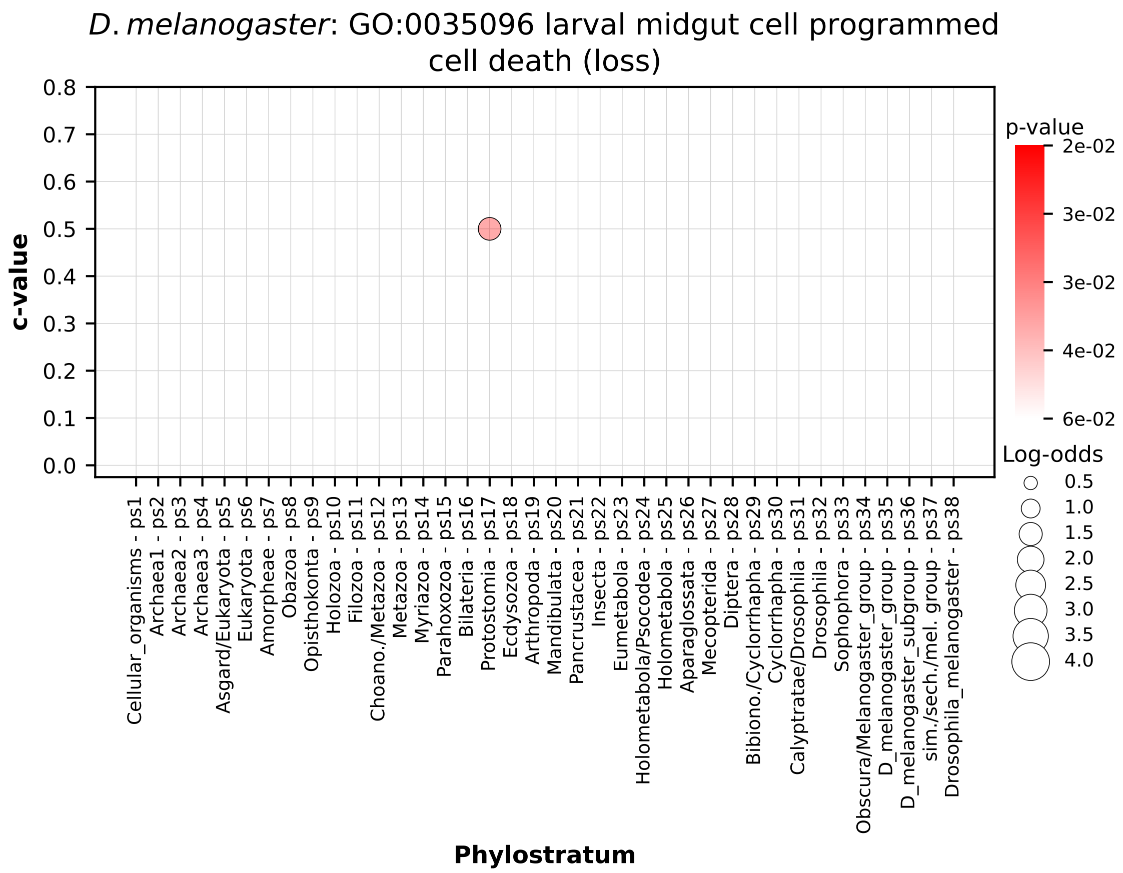

D. melanogaster: GO:0035096 larval midgut cell programmed cell death (loss)
c-value
0.8
0.7
0.6
0.5
0.4
0.3
0.2
0.1
0.0
p-value
2e-02
3e-02
3e-02
4e-02
6e-02
Log-odds
0.5
1.0
1.5
2.0
2.5
3.0
3.5
4.0
Cellular_organisms - ps1
Archaea1 - ps2
Archaea2 - ps3
Archaea3 - ps4
Asgard/Eukaryota - ps5
Eukaryota - ps6
Amorpheae - ps7
Obazoa - ps8
Opisthokonta - ps9
Holozoa - ps10
Filozoa - ps11
Choano./Metazoa - ps12
Metazoa - ps13
Myriazoa - ps14
Parahoxozoa - ps15
Bilateria - ps16
Protostomia - ps17
Ecdysozoa - ps18
Arthropoda - ps19
Mandibulata - ps20
Pancrustacea - ps21
Insecta - ps22
Eumetabola - ps23
Holometabola/Psocodea - ps24
Holometabola - ps25
Aparaglossata - ps26
Mecopterida - ps27
Diptera - ps28
Bibiono./Cyclorrhapha - ps29
Cyclorrhapha - ps30
Calyptratae/Drosophila - ps31
Drosophila - ps32
Sophophora - ps33
Obscura/Melanogaster_group - ps34
D_melanogaster_group - ps35
D_melanogaster_subgroup - ps36
sim./sech./mel. group - ps37
Drosophila_melanogaster - ps38
Phylostratum

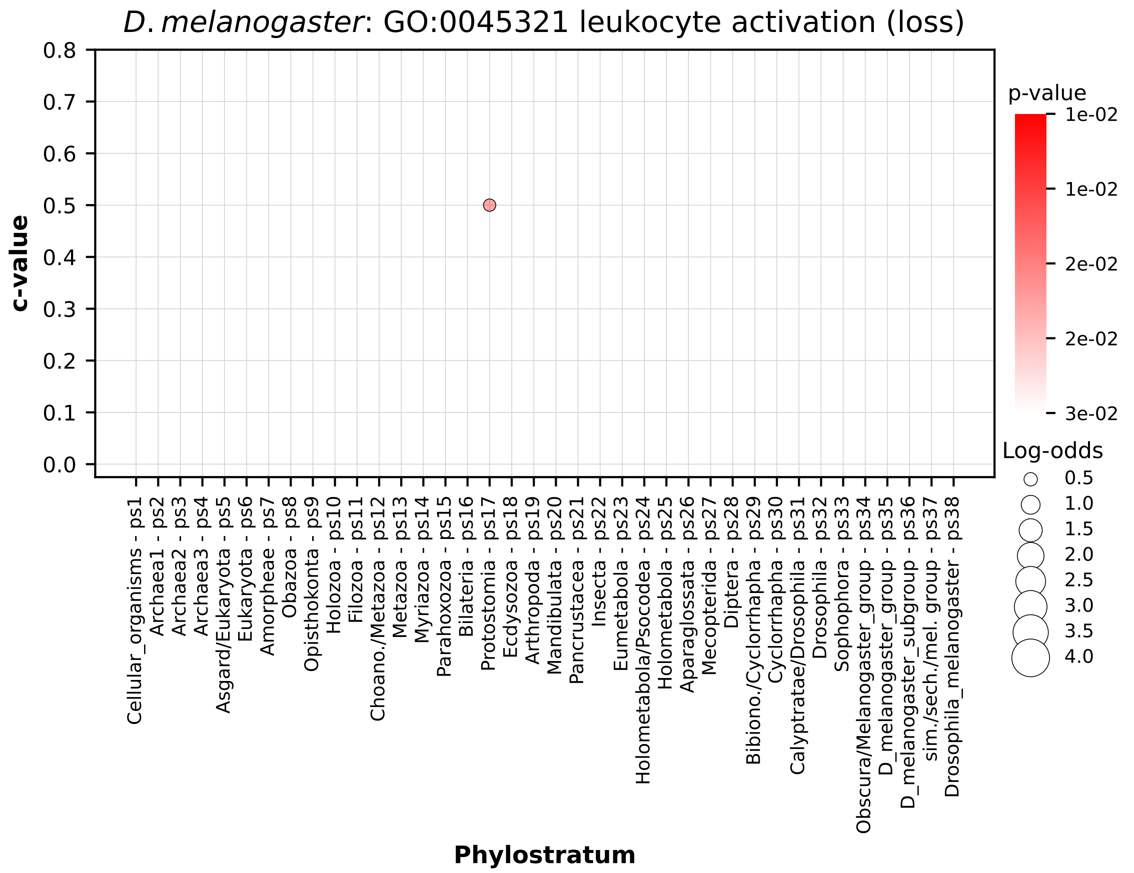
D. melanogaster: GO:0045321 leukocyte activation (loss)
c-value
0.0
0.1
0.2
0.3
0.4
0.5
0.6
0.7
0.8
Phylostratum
Cellular_organisms - ps1
Archaea1 - ps2
Archaea2 - ps3
Archaea3 - ps4
Asgard/Eukaryota - ps5
Eukaryota - ps6
Amorpheae - ps7
Obazoa - ps8
Opisthokonta - ps9
Holozoa - ps10
Filozoa - ps11
Choano./Metazoa - ps12
Metazoa - ps13
Myriazoa - ps14
Parahoxozoa - ps15
Bilateria - ps16
Protostomia - ps17
Ecdysozoa - ps18
Arthropoda - ps19
Mandibulata - ps20
Pancrustacea - ps21
Insecta - ps22
Eumetabola - ps23
Holometabola/Psocodea - ps24
Holometabola - ps25
Aparaglossata - ps26
Mecopterida - ps27
Diptera - ps28
Bibiono./Cyclorrhapha - ps29
Cyclorrhapha - ps30
Calyptratae/Drosophila - ps31
Drosophila - ps32
Sophophora - ps33
Obscura/Melanogaster_group - ps34
D_melanogaster_group - ps35
D_melanogaster_subgroup - ps36
sim./sech./mel. group - ps37
Drosophila_melanogaster - ps38
p-value
1e-02
1e-02
2e-02
2e-02
3e-02
Log-odds
0.5
1.0
1.5
2.0
2.5
3.0
3.5
4.0

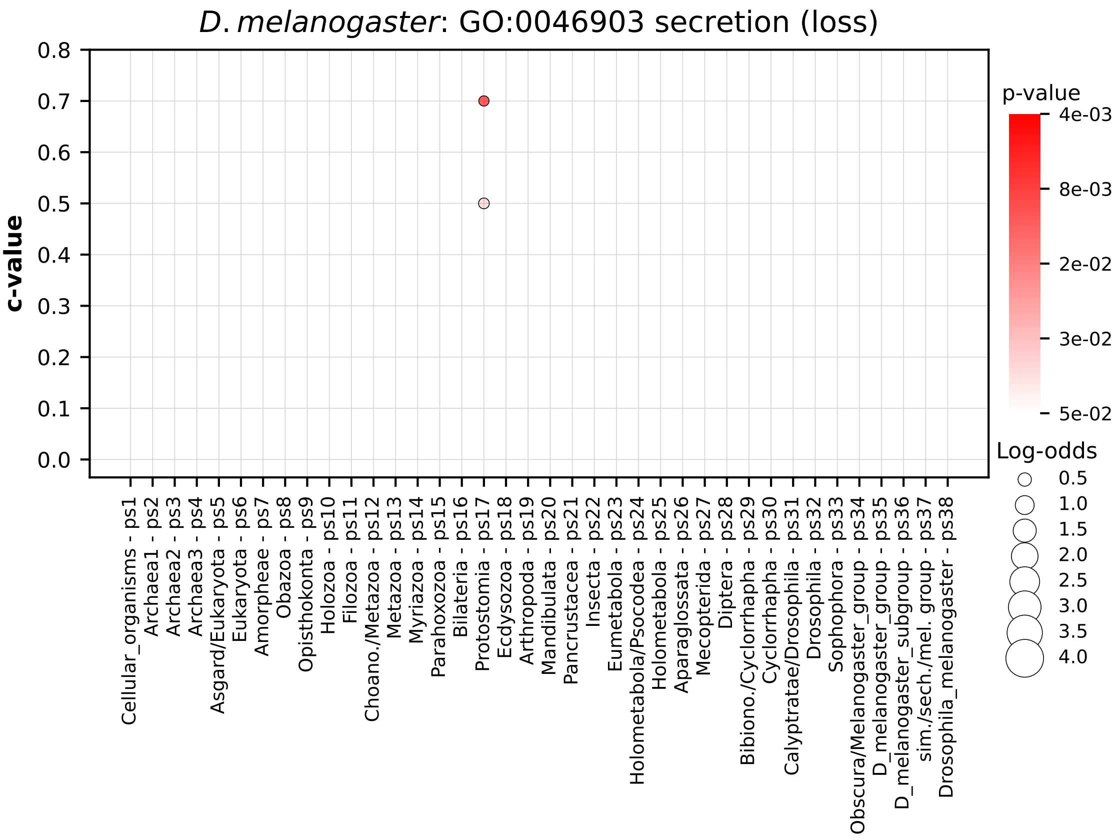
D. melanogaster: GO:0046903 secretion (loss)
c-value
0.8
0.7
0.6
0.5
0.4
0.3
0.2
0.1
0.0
Phylostratum
Cellular_organisms - ps1
Archaea1 - ps2
Archaea2 - ps3
Archaea3 - ps4
Asgard/Eukaryota - ps5
Eukaryota - ps6
Amorpheae - ps7
Obazoa - ps8
Opisthokonta - ps9
Holozoa - ps10
Filozoa - ps11
Choano./Metazoa - ps12
Metazoa - ps13
Myriazoa - ps14
Parahoxozoa - ps15
Bilateria - ps16
Protostomia - ps17
Ecdysozoa - ps18
Arthropoda - ps19
Mandibulata - ps20
Pancrustacea - ps21
Insecta - ps22
Eumetabola - ps23
Holometabola/Psocodea - ps24
Holometabola - ps25
Aparaglossata - ps26
Mecopterida - ps27
Diptera - ps28
Bibiono./Cyclorrhapha - ps29
Cyclorrhapha - ps30
Calyptratae/Drosophila - ps31
Drosophila - ps32
Sophophora - ps33
Obscura/Melanogaster_group - ps34
D_melanogaster_group - ps35
D_melanogaster_subgroup - ps36
sim./sech./mel. group - ps37
Drosophila_melanogaster - ps38
p-value
4e-03
8e-03
2e-02
3e-02
5e-02
Log-odds
0.5
1.0
1.5
2.0
2.5
3.0
3.5
4.0

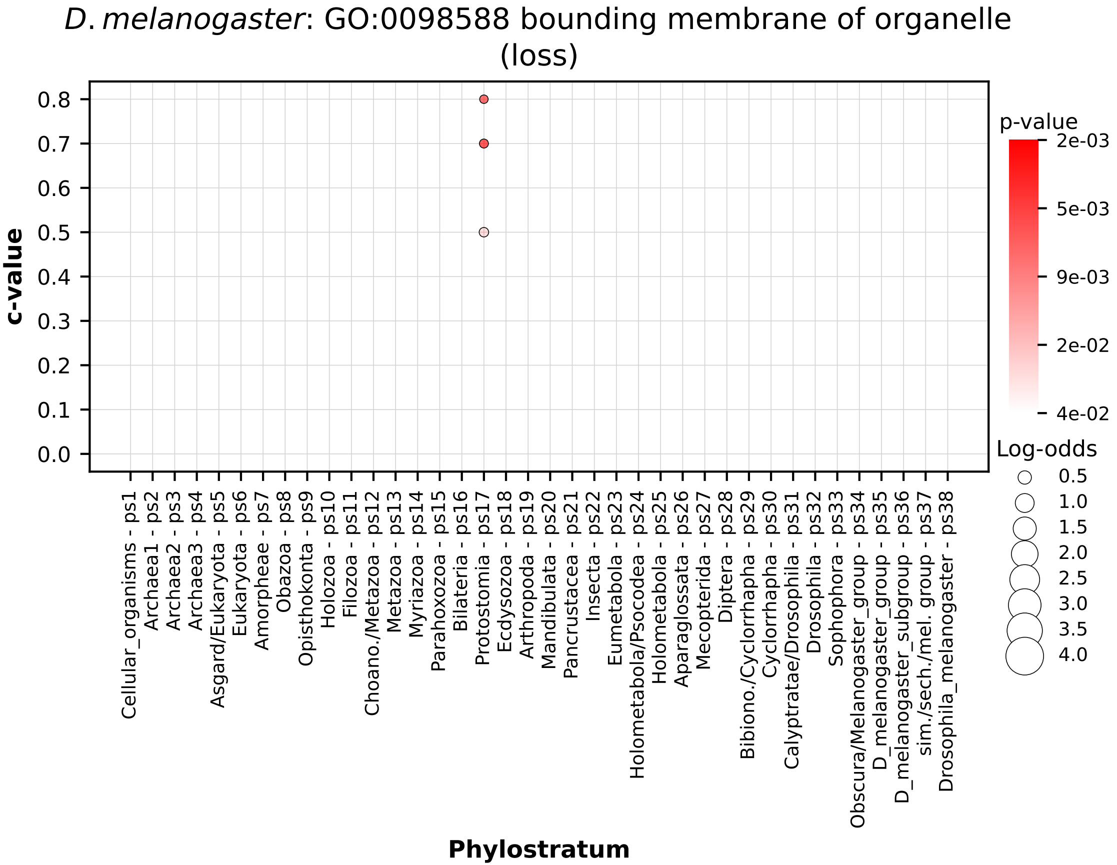

D. melanogaster: GO:0098588 bounding membrane of organelle (loss)
c-value
0.0
0.1
0.2
0.3
0.4
0.5
0.6
0.7
0.8
p-value
2e-03
5e-03
9e-03
2e-02
4e-02
Log-odds
0.5
1.0
1.5
2.0
2.5
3.0
3.5
4.0
Cellular_organisms - ps1
Archaea1 - ps2
Archaea2 - ps3
Archaea3 - ps4
Asgard/Eukaryota - ps5
Eukaryota - ps6
Amorpheae - ps7
Obazoa - ps8
Opisthokonta - ps9
Holozoa - ps10
Filozoa - ps11
Choano./Metazoa - ps12
Metazoa - ps13
Myriazoa - ps14
Parahoxozoa - ps15
Bilateria - ps16
Protostomia - ps17
Ecdysozoa - ps18
Arthropoda - ps19
Mandibulata - ps20
Pancrustacea - ps21
Insecta - ps22
Eumetabola - ps23
Holometabola/Psocodea - ps24
Holometabola - ps25
Aparaglossata - ps26
Mecopterida - ps27
Diptera - ps28
Bibiono./Cyclorrhapha - ps29
Cyclorrhapha - ps30
Calyptratae/Drosophila - ps31
Drosophila - ps32
Sophophora - ps33
Obscura/Melanogaster_group - ps34
D_melanogaster_group - ps35
D_melanogaster_subgroup - ps36
sim./sech./mel. group - ps37
Drosophila_melanogaster - ps38
Phylostratum

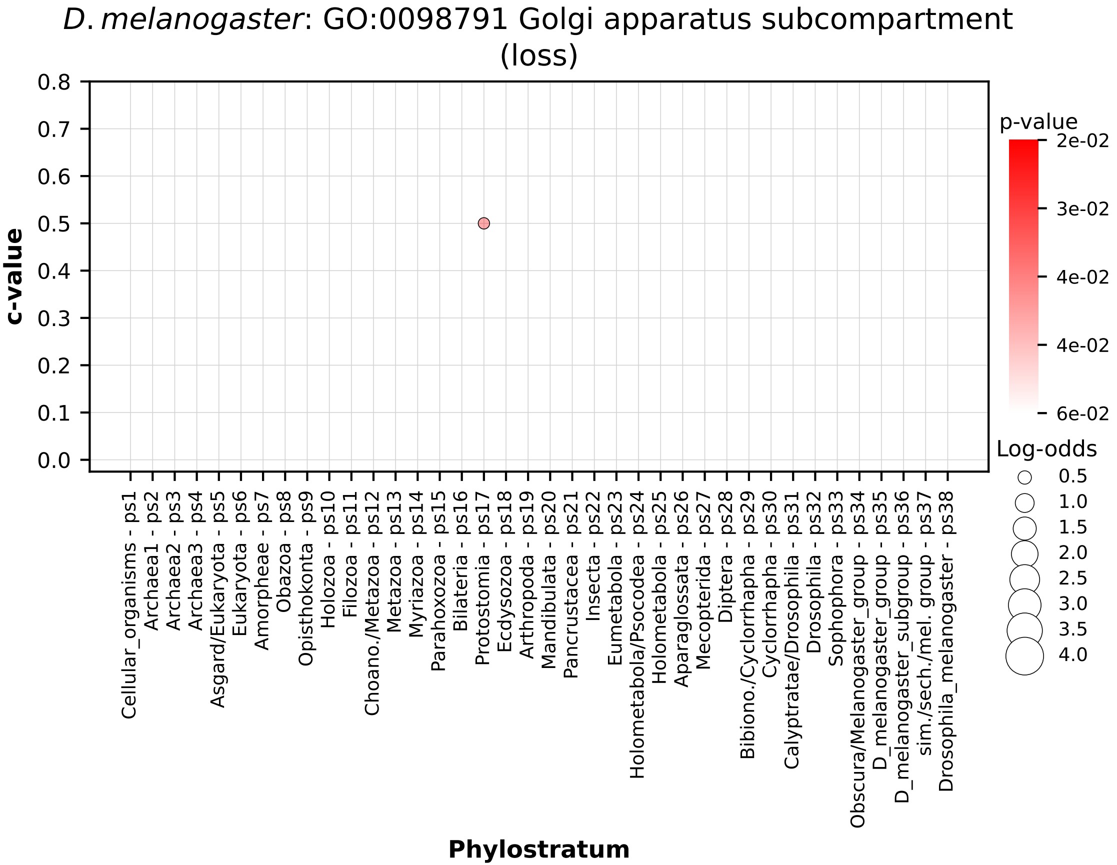

D. melanogaster: GO:0098791 Golgi apparatus subcompartment (loss)
c-value
0.8
0.7
0.6
0.5
0.4
0.3
0.2
0.1
0.0
Phylostratum
Cellular_organisms - ps1
Archaea1 - ps2
Archaea2 - ps3
Archaea3 - ps4
Asgard/Eukaryota - ps5
Eukaryota - ps6
Amorpheae - ps7
Obazoa - ps8
Opisthokonta - ps9
Holozoa - ps10
Filozoa - ps11
Choano./Metazoa - ps12
Metazoa - ps13
Myriazoa - ps14
Parahoxozoa - ps15
Bilateria - ps16
Protostomia - ps17
Ecdysozoa - ps18
Arthropoda - ps19
Mandibulata - ps20
Pancrustacea - ps21
Insecta - ps22
Eumetabola - ps23
Holometabola/Psocodea - ps24
Holometabola - ps25
Aparaglossata - ps26
Mecopterida - ps27
Diptera - ps28
Bibiono./Cyclorrhapha - ps29
Cyclorrhapha - ps30
Calyptratae/Drosophila - ps31
Drosophila - ps32
Sophophora - ps33
Obscura/Melanogaster_group - ps34
D_melanogaster_group - ps35
D_melanogaster_subgroup - ps36
sim./sech./mel. group - ps37
Drosophila_melanogaster - ps38
p-value
2e-02
3e-02
4e-02
4e-02
6e-02
Log-odds
0.5
1.0
1.5
2.0
2.5
3.0
3.5
4.0

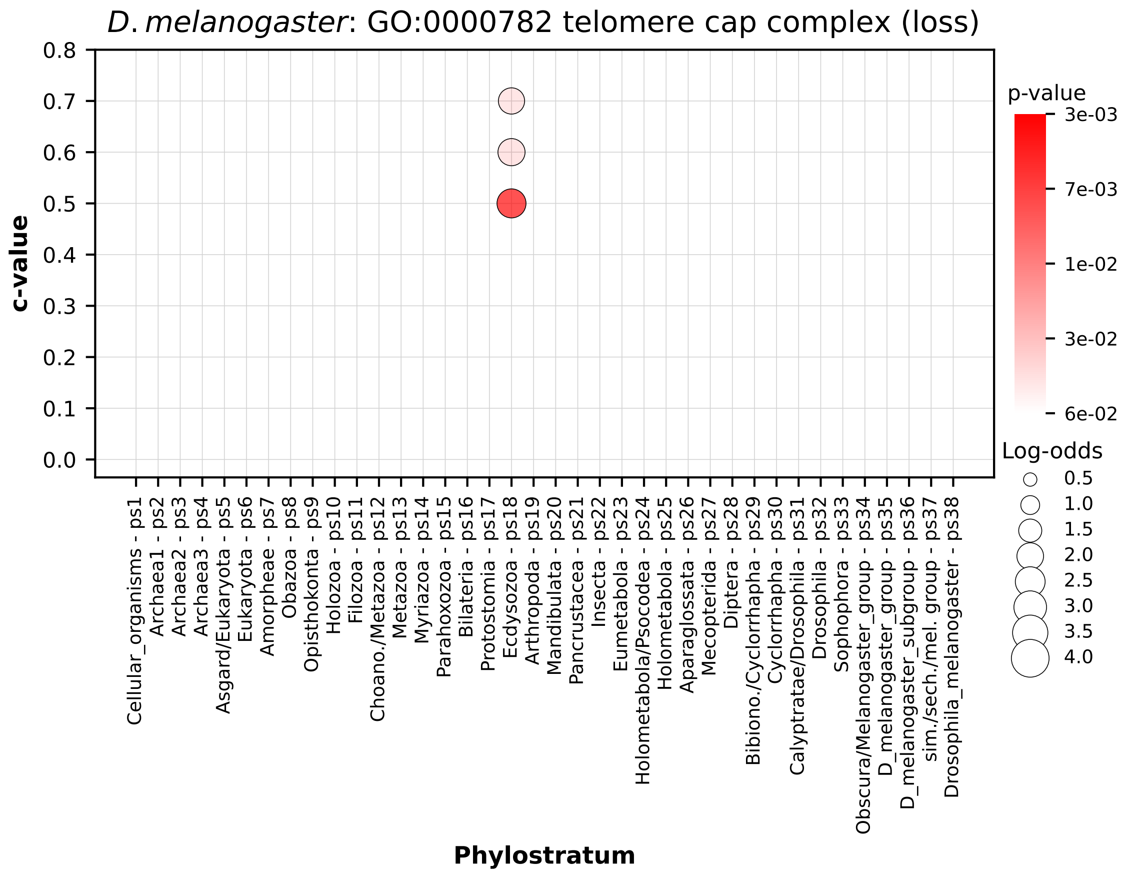

D. melanogaster: GO:0000782 telomere cap complex (loss)
c-value
0.8
0.7
0.6
0.5
0.4
0.3
0.2
0.1
0.0
p-value
3e-03
7e-03
1e-02
3e-02
6e-02
Log-odds
0.5
1.0
1.5
2.0
2.5
3.0
3.5
4.0
Cellular_organisms - ps1
Archaea1 - ps2
Archaea2 - ps3
Archaea3 - ps4
Asgard/Eukaryota - ps5
Eukaryota - ps6
Amorpheae - ps7
Obazoa - ps8
Opisthokonta - ps9
Holozoa - ps10
Filozoa - ps11
Choano./Metazoa - ps12
Metazoa - ps13
Myriazoa - ps14
Parahoxozoa - ps15
Bilateria - ps16
Protostomia - ps17
Ecdysozoa - ps18
Arthropoda - ps19
Mandibulata - ps20
Pancrustacea - ps21
Insecta - ps22
Eumetabola - ps23
Holometabola/Psocodea - ps24
Holometabola - ps25
Aparaglossata - ps26
Mecopterida - ps27
Diptera - ps28
Bibiono./Cyclorrhapha - ps29
Cyclorrhapha - ps30
Calyptratae/Drosophila - ps31
Drosophila - ps32
Sophophora - ps33
Obscura/Melanogaster_group - ps34
D_melanogaster_group - ps35
D_melanogaster_subgroup - ps36
sim./sech./mel. group - ps37
Drosophila_melanogaster - ps38
Phylostratum

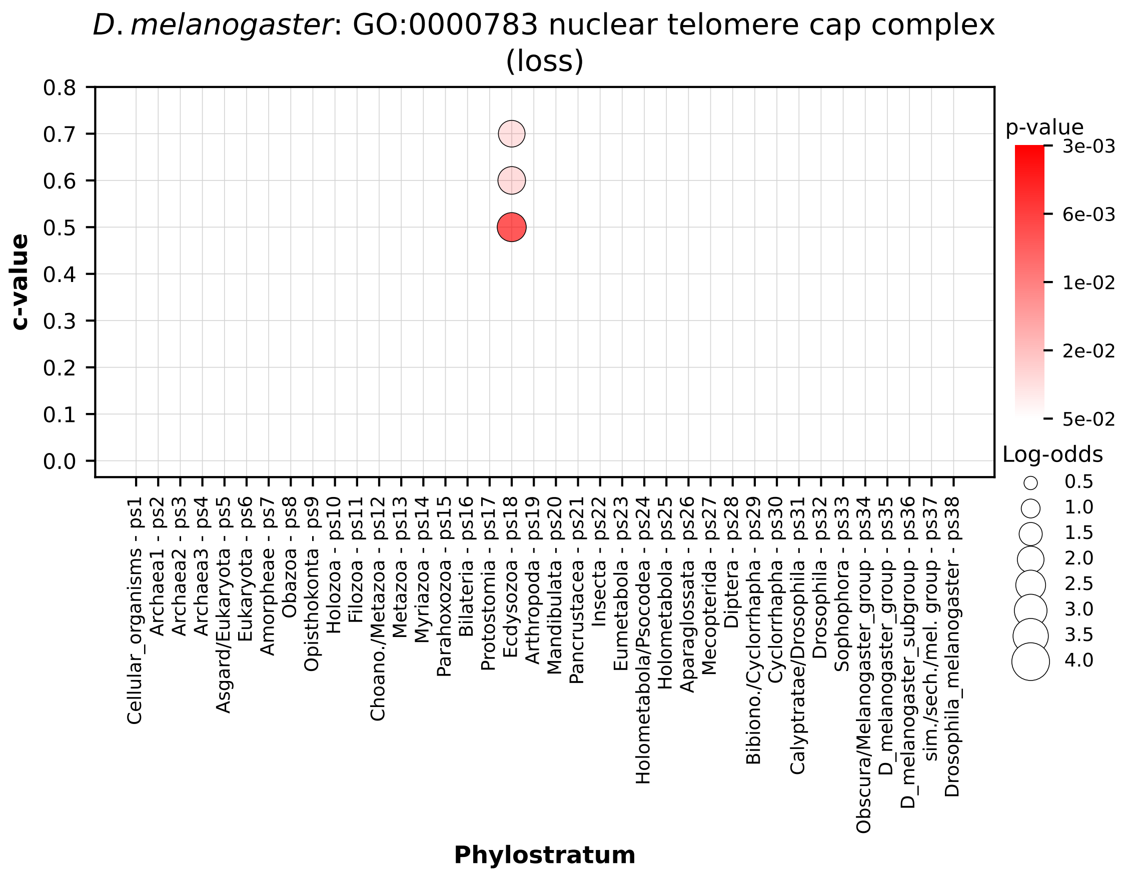

D. melanogaster: GO:0000783 nuclear telomere cap complex
(loss)
c-value
0.8
0.7
0.6
0.5
0.4
0.3
0.2
0.1
0.0
Phylostratum
Cellular_organisms - ps1
Archaea1 - ps2
Archaea2 - ps3
Archaea3 - ps4
Asgard/Eukaryota - ps5
Eukaryota - ps6
Amorpheae - ps7
Obazoa - ps8
Opisthokonta - ps9
Holozoa - ps10
Filozoa - ps11
Choano./Metazoa - ps12
Metazoa - ps13
Myriazoa - ps14
Parahoxozoa - ps15
Bilateria - ps16
Protostomia - ps17
Ecdysozoa - ps18
Arthropoda - ps19
Mandibulata - ps20
Pancrustacea - ps21
Insecta - ps22
Eumetabola - ps23
Holometabola/Psocodea - ps24
Holometabola - ps25
Aparaglossata - ps26
Mecopterida - ps27
Diptera - ps28
Bibiono./Cyclorrhapha - ps29
Cyclorrhapha - ps30
Calyptratae/Drosophila - ps31
Drosophila - ps32
Sophophora - ps33
Obscura/Melanogaster_group - ps34
D_melanogaster_group - ps35
D_melanogaster_subgroup - ps36
sim./sech./mel. group - ps37
Drosophila_melanogaster - ps38
p-value
3e-03
6e-03
1e-02
2e-02
5e-02
Log-odds
0.5
1.0
1.5
2.0
2.5
3.0
3.5
4.0

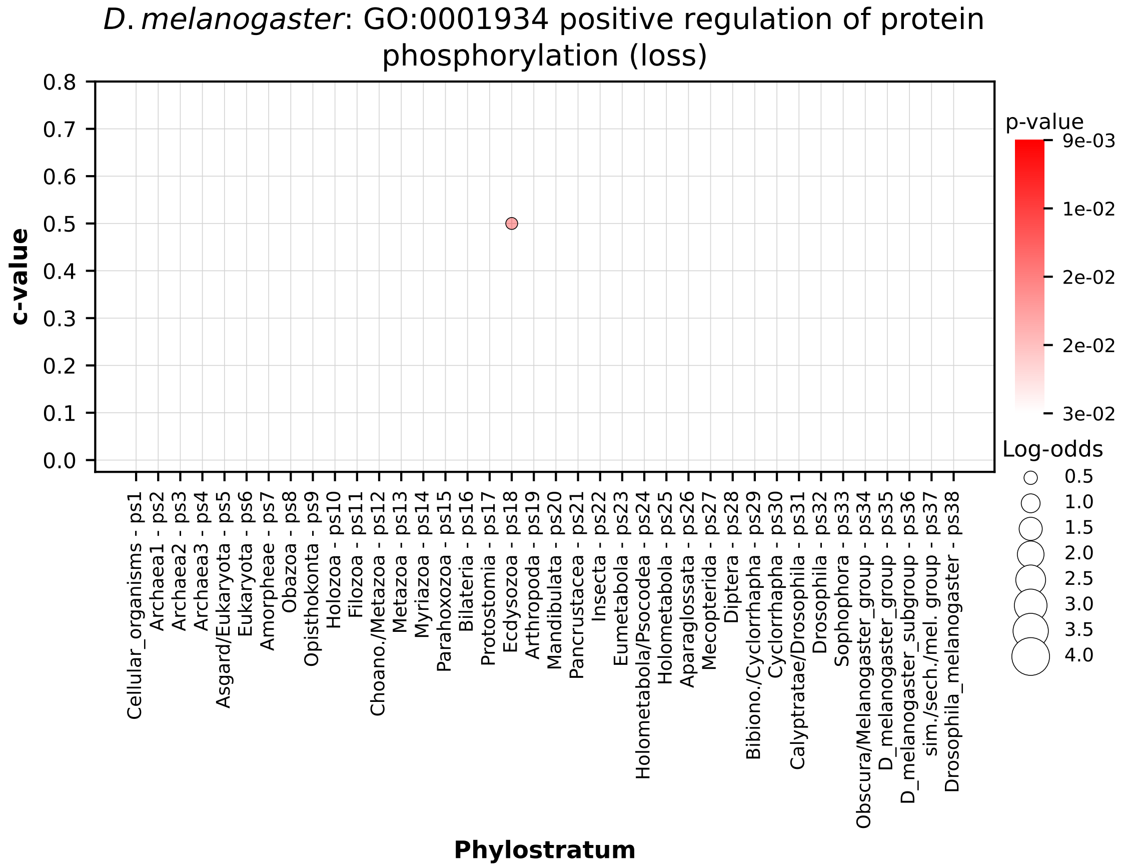
D. melanogaster: GO:0001934 positive regulation of protein phosphorylation (loss)
c-value
0.8
0.7
0.6
0.5
0.4
0.3
0.2
0.1
0.0
Phylostratum
Cellular_organisms - ps1
Archaea1 - ps2
Archaea2 - ps3
Archaea3 - ps4
Asgard/Eukaryota - ps5
Eukaryota - ps6
Amorpheae - ps7
Obazoa - ps8
Opisthokonta - ps9
Holozoa - ps10
Filozoa - ps11
Choano./Metazoa - ps12
Metazoa - ps13
Myriazoa - ps14
Parahoxozoa - ps15
Bilateria - ps16
Protostomia - ps17
Ecdysozoa - ps18
Arthropoda - ps19
Mandibulata - ps20
Pancrustacea - ps21
Insecta - ps22
Eumetabola - ps23
Holometabola/Psocodea - ps24
Holometabola - ps25
Aparaglossata - ps26
Mecopterida - ps27
Diptera - ps28
Bibiono./Cyclorrhapha - ps29
Cyclorrhapha - ps30
Calyptratae/Drosophila - ps31
Drosophila - ps32
Sophophora - ps33
Obscura/Melanogaster_group - ps34
D_melanogaster_group - ps35
D_melanogaster_subgroup - ps36
sim./sech./mel. group - ps37
Drosophila_melanogaster - ps38
p-value
9e-03
1e-02
2e-02
2e-02
3e-02
Log-odds
0.5
1.0
1.5
2.0
2.5
3.0
3.5
4.0

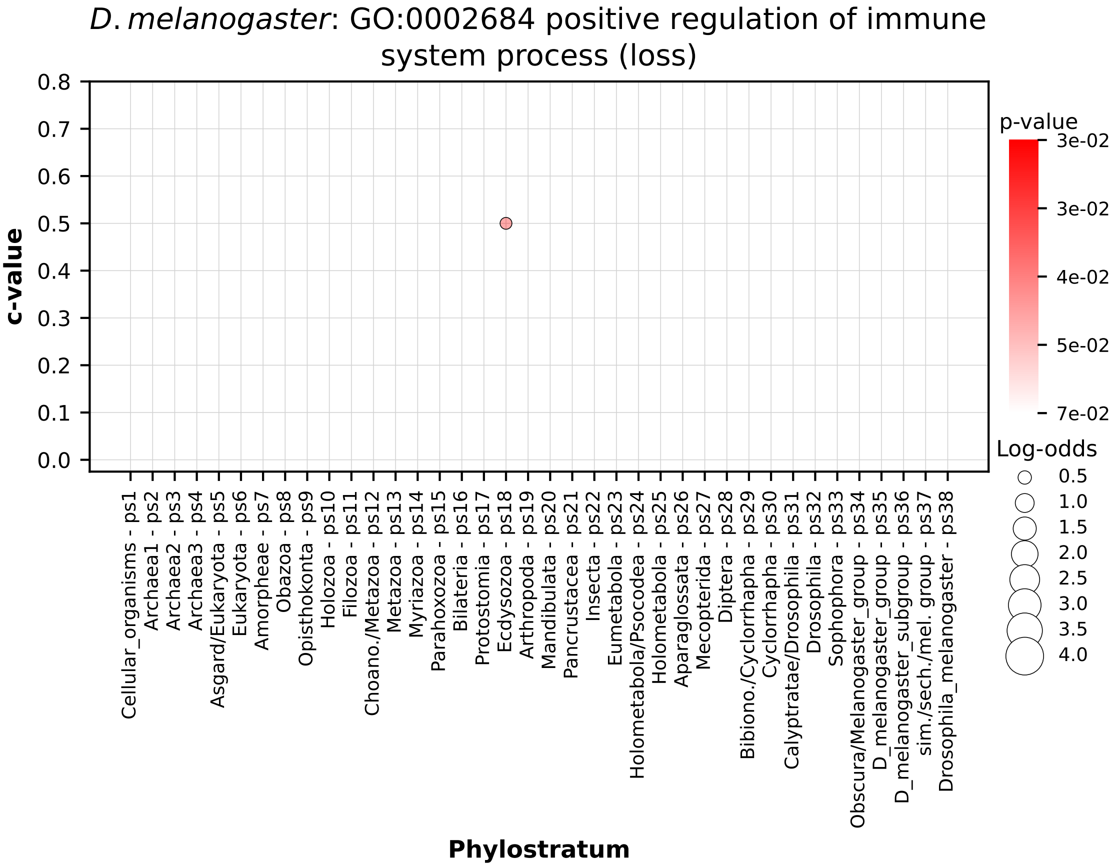

D. melanogaster: GO:0002684 positive regulation of immune system process (loss)
c-value
0.8
0.7
0.6
0.5
0.4
0.3
0.2
0.1
0.0
p-value
3e-02
3e-02
4e-02
5e-02
7e-02
Log-odds
0.5
1.0
1.5
2.0
2.5
3.0
3.5
4.0
Cellular_organisms - ps1
Archaea1 - ps2
Archaea2 - ps3
Archaea3 - ps4
Asgard/Eukaryota - ps5
Eukaryota - ps6
Amorpheae - ps7
Obazoa - ps8
Opisthokonta - ps9
Holozoa - ps10
Filozoa - ps11
Choano./Metazoa - ps12
Metazoa - ps13
Myriazoa - ps14
Parahoxozoa - ps15
Bilateria - ps16
Protostomia - ps17
Ecdysozoa - ps18
Arthropoda - ps19
Mandibulata - ps20
Pancrustacea - ps21
Insecta - ps22
Eumetabola - ps23
Holometabola/Psocodea - ps24
Holometabola - ps25
Aparaglossata - ps26
Mecopterida - ps27
Diptera - ps28
Bibiono./Cyclorrhapha - ps29
Cyclorrhapha - ps30
Calyptratae/Drosophila - ps31
Drosophila - ps32
Sophophora - ps33
Obscura/Melanogaster_group - ps34
D_melanogaster_group - ps35
D_melanogaster_subgroup - ps36
sim./sech./mel. group - ps37
Drosophila_melanogaster - ps38
Phylostratum

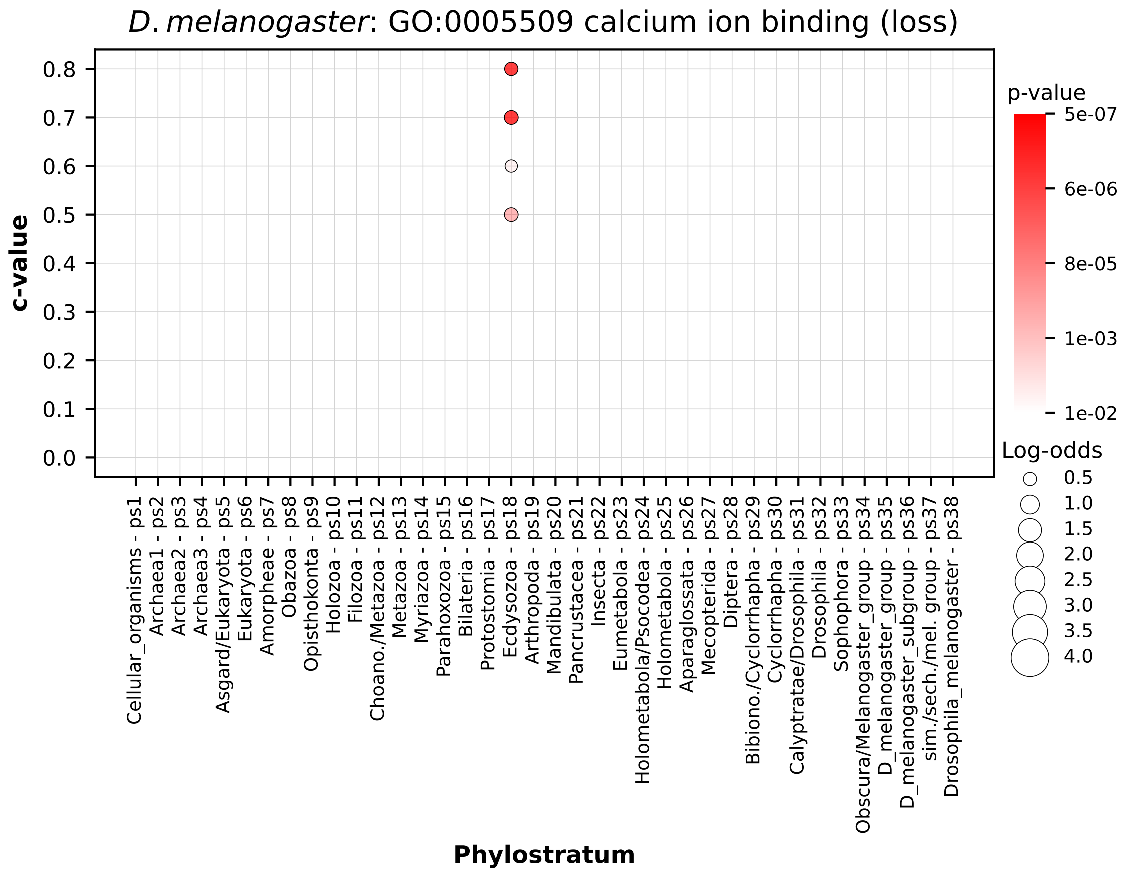

D. melanogaster: GO:0005509 calcium ion binding (loss)
c-value
0.0
0.1
0.2
0.3
0.4
0.5
0.6
0.7
0.8
Phylostratum
Cellular_organisms - ps1
Archaea1 - ps2
Archaea2 - ps3
Archaea3 - ps4
Asgard/Eukaryota - ps5
Eukaryota - ps6
Amorpheae - ps7
Obazoa - ps8
Opisthokonta - ps9
Holozoa - ps10
Filozoa - ps11
Choano./Metazoa - ps12
Metazoa - ps13
Myriazoa - ps14
Parahoxozoa - ps15
Bilateria - ps16
Protostomia - ps17
Ecdysozoa - ps18
Arthropoda - ps19
Mandibulata - ps20
Pancrustacea - ps21
Insecta - ps22
Eumetabola - ps23
Holometabola/Psocodea - ps24
Holometabola - ps25
Aparaglossata - ps26
Mecopterida - ps27
Diptera - ps28
Bibiono./Cyclorrhapha - ps29
Cyclorrhapha - ps30
Calyptratae/Drosophila - ps31
Drosophila - ps32
Sophophora - ps33
Obscura/Melanogaster_group - ps34
D_melanogaster_group - ps35
D_melanogaster_subgroup - ps36
sim./sech./mel. group - ps37
Drosophila_melanogaster - ps38
p-value
5e-07
6e-06
8e-05
1e-03
1e-02
Log-odds
0.5
1.0
1.5
2.0
2.5
3.0
3.5
4.0

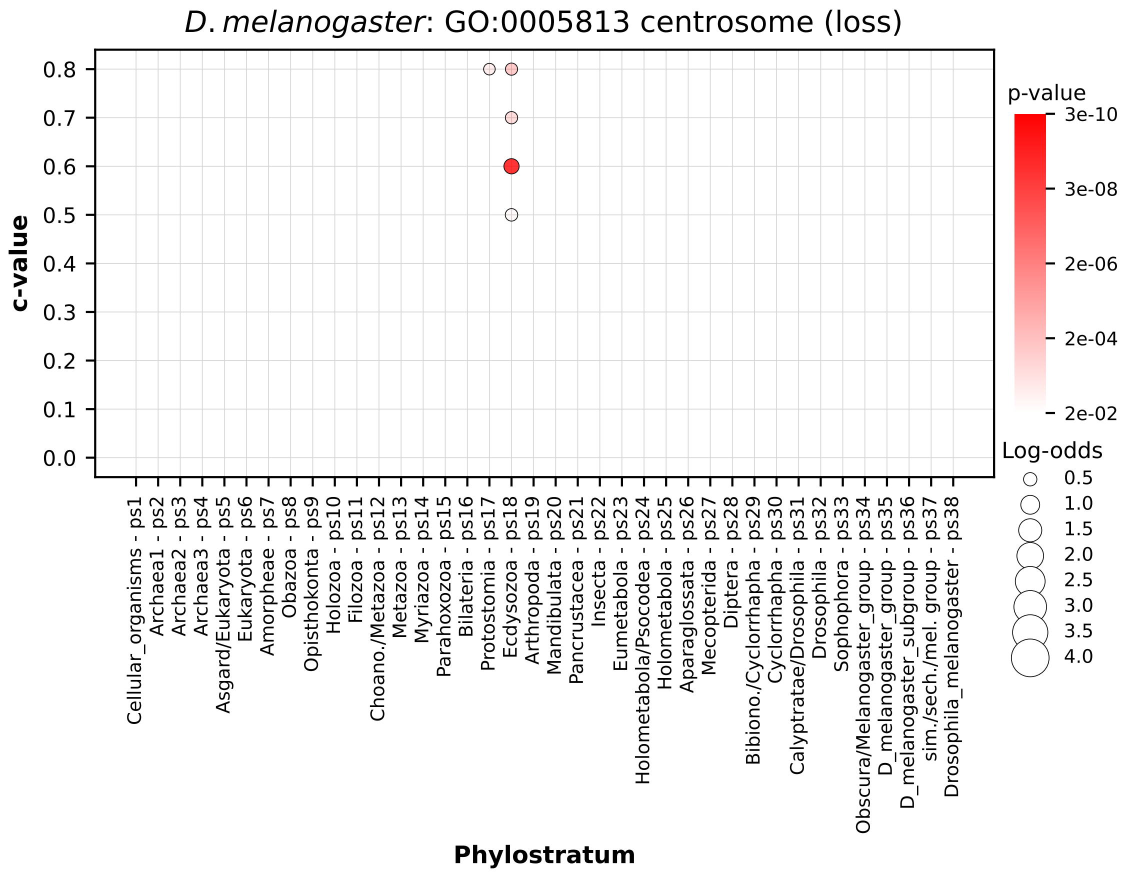

D. melanogaster: GO:0005813 centrosome (loss)
c-value
0.0
0.1
0.2
0.3
0.4
0.5
0.6
0.7
0.8
Phylostratum
Cellular_organisms - ps1
Archaea1 - ps2
Archaea2 - ps3
Archaea3 - ps4
Asgard/Eukaryota - ps5
Eukaryota - ps6
Amorpheae - ps7
Obazoa - ps8
Opisthokonta - ps9
Holozoa - ps10
Filozoa - ps11
Choano./Metazoa - ps12
Metazoa - ps13
Myriazoa - ps14
Parahoxozoa - ps15
Bilateria - ps16
Protostomia - ps17
Ecdysozoa - ps18
Arthropoda - ps19
Mandibulata - ps20
Pancrustacea - ps21
Insecta - ps22
Eumetabola - ps23
Holometabola/Psocodea - ps24
Holometabola - ps25
Aparaglossata - ps26
Mecopterida - ps27
Diptera - ps28
Bibiono./Cyclorrhapha - ps29
Cyclorrhapha - ps30
Calyptratae/Drosophila - ps31
Drosophila - ps32
Sophophora - ps33
Obscura/Melanogaster_group - ps34
D_melanogaster_group - ps35
D_melanogaster_subgroup - ps36
sim./sech./mel. group - ps37
Drosophila_melanogaster - ps38
p-value
3e-10
3e-08
2e-06
2e-04
2e-02
Log-odds
0.5
1.0
1.5
2.0
2.5
3.0
3.5
4.0

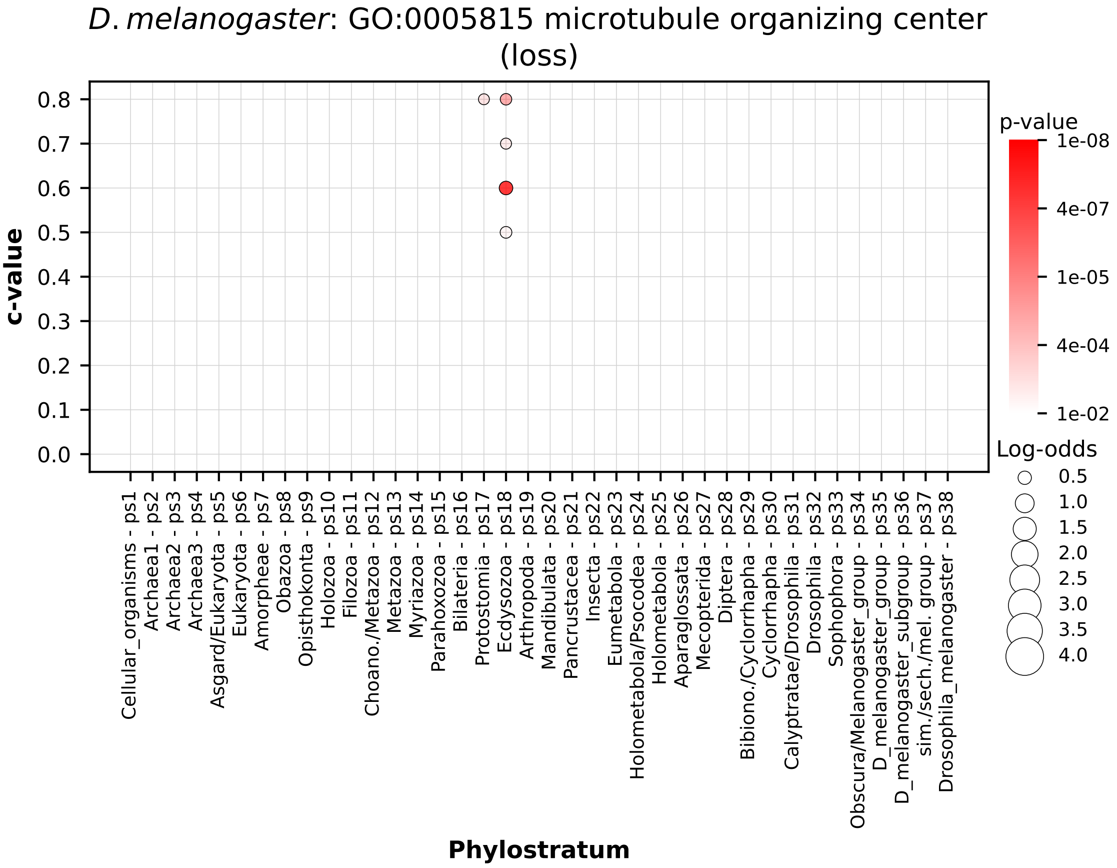

D. melanogaster: GO:0005815 microtubule organizing center (loss)
c-value
0.0
0.1
0.2
0.3
0.4
0.5
0.6
0.7
0.8
Phylostratum
Cellular_organisms - ps1
Archaea1 - ps2
Archaea2 - ps3
Archaea3 - ps4
Asgard/Eukaryota - ps5
Eukaryota - ps6
Amorpheae - ps7
Obazoa - ps8
Opisthokonta - ps9
Holozoa - ps10
Filozoa - ps11
Choano./Metazoa - ps12
Metazoa - ps13
Myriazoa - ps14
Parahoxozoa - ps15
Bilateria - ps16
Protostomia - ps17
Ecdysozoa - ps18
Arthropoda - ps19
Mandibulata - ps20
Pancrustacea - ps21
Insecta - ps22
Eumetabola - ps23
Holometabola/Psocodea - ps24
Holometabola - ps25
Aparaglossata - ps26
Mecopterida - ps27
Diptera - ps28
Bibiono./Cyclorrhapha - ps29
Cyclorrhapha - ps30
Calyptratae/Drosophila - ps31
Drosophila - ps32
Sophophora - ps33
Obscura/Melanogaster_group - ps34
D_melanogaster_group - ps35
D_melanogaster_subgroup - ps36
sim./sech./mel. group - ps37
Drosophila_melanogaster - ps38
p-value
1e-08
4e-07
1e-05
4e-04
1e-02
Log-odds
0.5
1.0
1.5
2.0
2.5
3.0
3.5
4.0

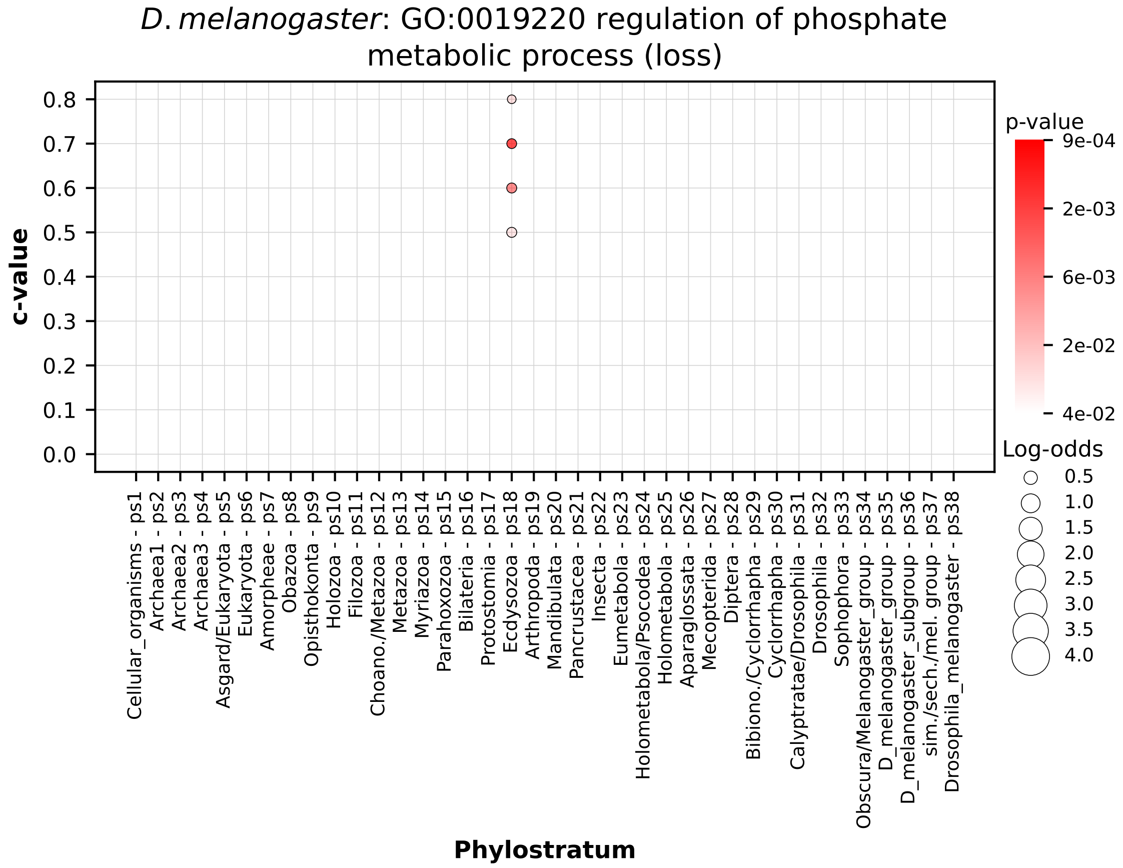
D. melanogaster: GO:0019220 regulation of phosphate metabolic process (loss)
c-value
0.8
0.7
0.6
0.5
0.4
0.3
0.2
0.1
0.0
p-value
9e-04
2e-03
6e-03
2e-02
4e-02
Log-odds
0.5
1.0
1.5
2.0
2.5
3.0
3.5
4.0
Cellular_organisms - ps1
Archaea1 - ps2
Archaea2 - ps3
Archaea3 - ps4
Asgard/Eukaryota - ps5
Eukaryota - ps6
Amorpheae - ps7
Obazoa - ps8
Opisthokonta - ps9
Holozoa - ps10
Filozoa - ps11
Choano./Metazoa - ps12
Metazoa - ps13
Myriazoa - ps14
Parahoxozoa - ps15
Bilateria - ps16
Protostomia - ps17
Ecdysozoa - ps18
Arthropoda - ps19
Mandibulata - ps20
Pancrustacea - ps21
Insecta - ps22
Eumetabola - ps23
Holometabola/Psocodea - ps24
Holometabola - ps25
Aparaglossata - ps26
Mecopterida - ps27
Diptera - ps28
Bibiono./Cyclorrhapha - ps29
Cyclorrhapha - ps30
Calyptratae/Drosophila - ps31
Drosophila - ps32
Sophophora - ps33
Obscura/Melanogaster_group - ps34
D_melanogaster_group - ps35
D_melanogaster_subgroup - ps36
sim./sech./mel. group - ps37
Drosophila_melanogaster - ps38
Phylostratum

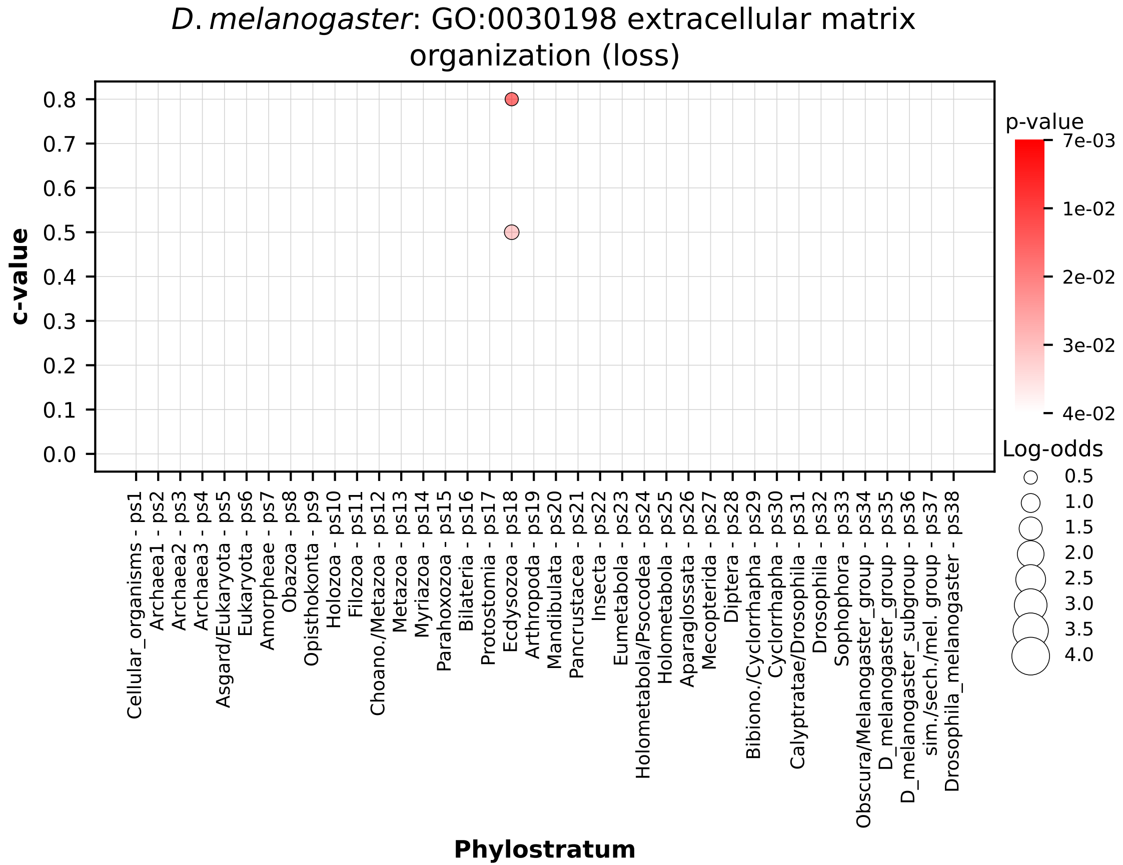
D. melanogaster: GO:0030198 extracellular matrix organization (loss)
c-value
0.8
0.7
0.6
0.5
0.4
0.3
0.2
0.1
0.0
p-value
7e-03
1e-02
2e-02
3e-02
4e-02
Log-odds
0.5
1.0
1.5
2.0
2.5
3.0
3.5
4.0
Cellular_organisms - ps1
Archaea1 - ps2
Archaea2 - ps3
Archaea3 - ps4
Asgard/Eukaryota - ps5
Eukaryota - ps6
Amorpheae - ps7
Obazoa - ps8
Opisthokonta - ps9
Holozoa - ps10
Filozoa - ps11
Choano./Metazoa - ps12
Metazoa - ps13
Myriazoa - ps14
Parahoxozoa - ps15
Bilateria - ps16
Protostomia - ps17
Ecdysozoa - ps18
Arthropoda - ps19
Mandibulata - ps20
Pancrustacea - ps21
Insecta - ps22
Eumetabola - ps23
Holometabola/Psocodea - ps24
Holometabola - ps25
Aparaglossata - ps26
Mecopterida - ps27
Diptera - ps28
Bibiono./Cyclorrhapha - ps29
Cyclorrhapha - ps30
Calyptratae/Drosophila - ps31
Drosophila - ps32
Sophophora - ps33
Obscura/Melanogaster_group - ps34
D_melanogaster_group - ps35
D_melanogaster_subgroup - ps36
sim./sech./mel. group - ps37
Drosophila_melanogaster - ps38
Phylostratum

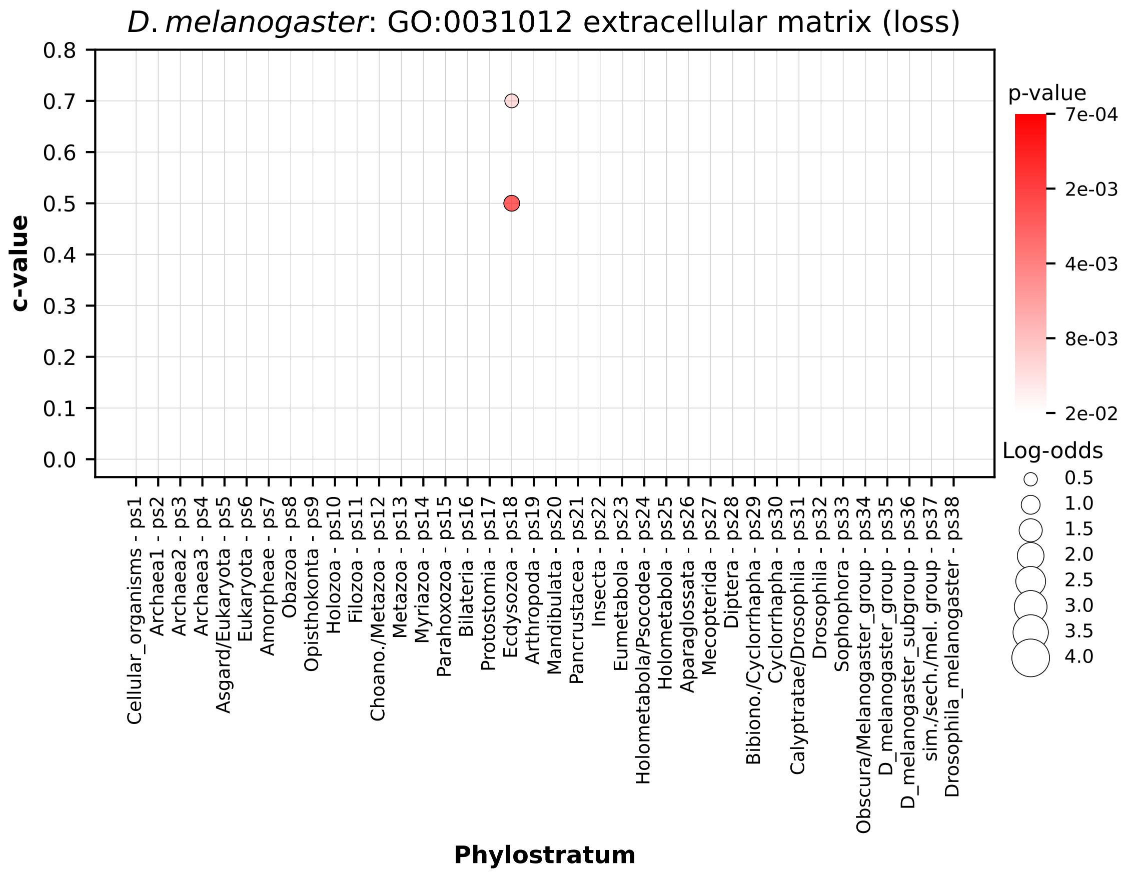

D. melanogaster: GO:0031012 extracellular matrix (loss)
c-value
0.8
0.7
0.6
0.5
0.4
0.3
0.2
0.1
0.0
Phylostratum
Cellular_organisms - ps1
Archaea1 - ps2
Archaea2 - ps3
Archaea3 - ps4
Asgard/Eukaryota - ps5
Eukaryota - ps6
Amorpheae - ps7
Obazoa - ps8
Opisthokonta - ps9
Holozoa - ps10
Filozoa - ps11
Choano./Metazoa - ps12
Metazoa - ps13
Myriazoa - ps14
Parahoxozoa - ps15
Bilateria - ps16
Protostomia - ps17
Ecdysozoa - ps18
Arthropoda - ps19
Mandibulata - ps20
Pancrustacea - ps21
Insecta - ps22
Eumetabola - ps23
Holometabola/Psocodea - ps24
Holometabola - ps25
Aparaglossata - ps26
Mecopterida - ps27
Diptera - ps28
Bibiono./Cyclorrhapha - ps29
Cyclorrhapha - ps30
Calyptratae/Drosophila - ps31
Drosophila - ps32
Sophophora - ps33
Obscura/Melanogaster_group - ps34
D_melanogaster_group - ps35
D_melanogaster_subgroup - ps36
sim./sech./mel. group - ps37
Drosophila_melanogaster - ps38
p-value
7e-04
2e-03
4e-03
8e-03
2e-02
Log-odds
0.5
1.0
1.5
2.0
2.5
3.0
3.5
4.0

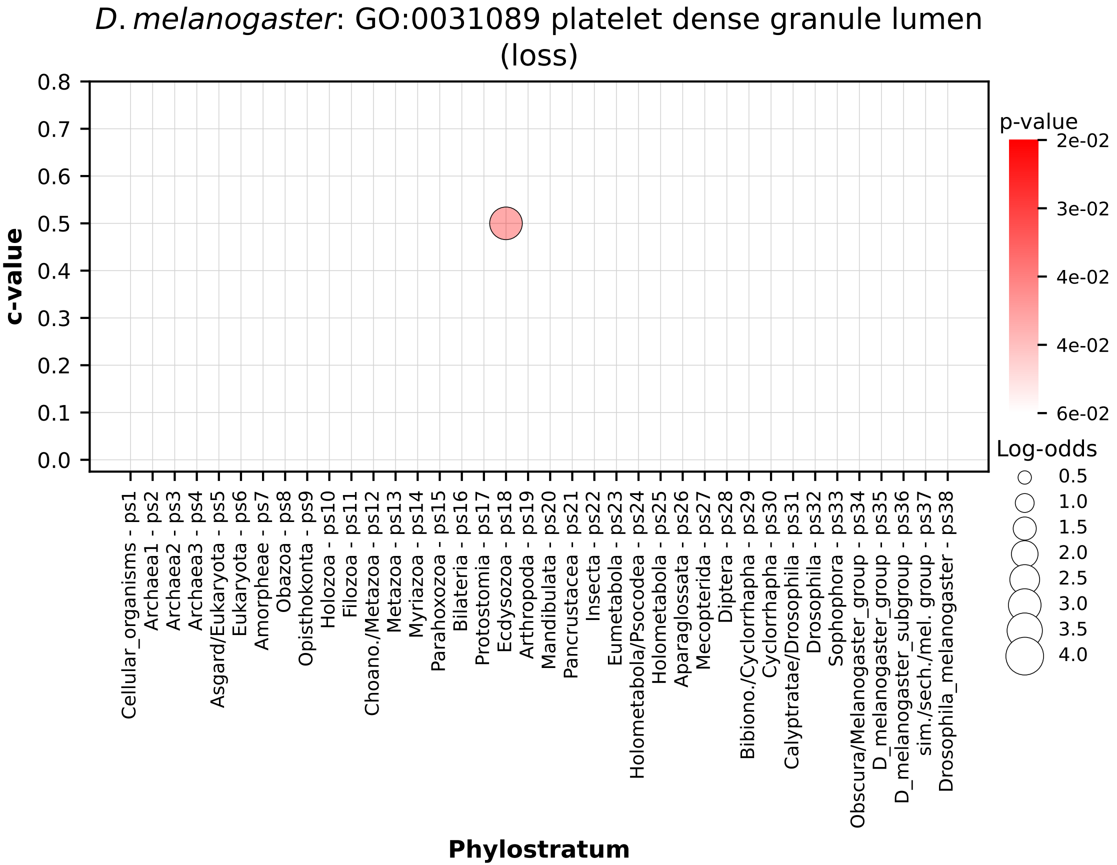

D. melanogaster: GO:0031089 platelet dense granule lumen (loss)
c-value
0.8
0.7
0.6
0.5
0.4
0.3
0.2
0.1
0.0
Phylostratum
Cellular_organisms - ps1
Archaea1 - ps2
Archaea2 - ps3
Archaea3 - ps4
Asgard/Eukaryota - ps5
Eukaryota - ps6
Amorpheae - ps7
Obazoa - ps8
Opisthokonta - ps9
Holozoa - ps10
Filozoa - ps11
Choano./Metazoa - ps12
Metazoa - ps13
Myriazoa - ps14
Parahoxozoa - ps15
Bilateria - ps16
Protostomia - ps17
Ecdysozoa - ps18
Arthropoda - ps19
Mandibulata - ps20
Pancrustacea - ps21
Insecta - ps22
Eumetabola - ps23
Holometabola/Psocodea - ps24
Holometabola - ps25
Aparaglossata - ps26
Mecopterida - ps27
Diptera - ps28
Bibiono./Cyclorrhapha - ps29
Cyclorrhapha - ps30
Calyptratae/Drosophila - ps31
Drosophila - ps32
Sophophora - ps33
Obscura/Melanogaster_group - ps34
D_melanogaster_group - ps35
D_melanogaster_subgroup - ps36
sim./sech./mel. group - ps37
Drosophila_melanogaster - ps38
p-value
2e-02
3e-02
4e-02
4e-02
6e-02
Log-odds
0.5
1.0
1.5
2.0
2.5
3.0
3.5
4.0

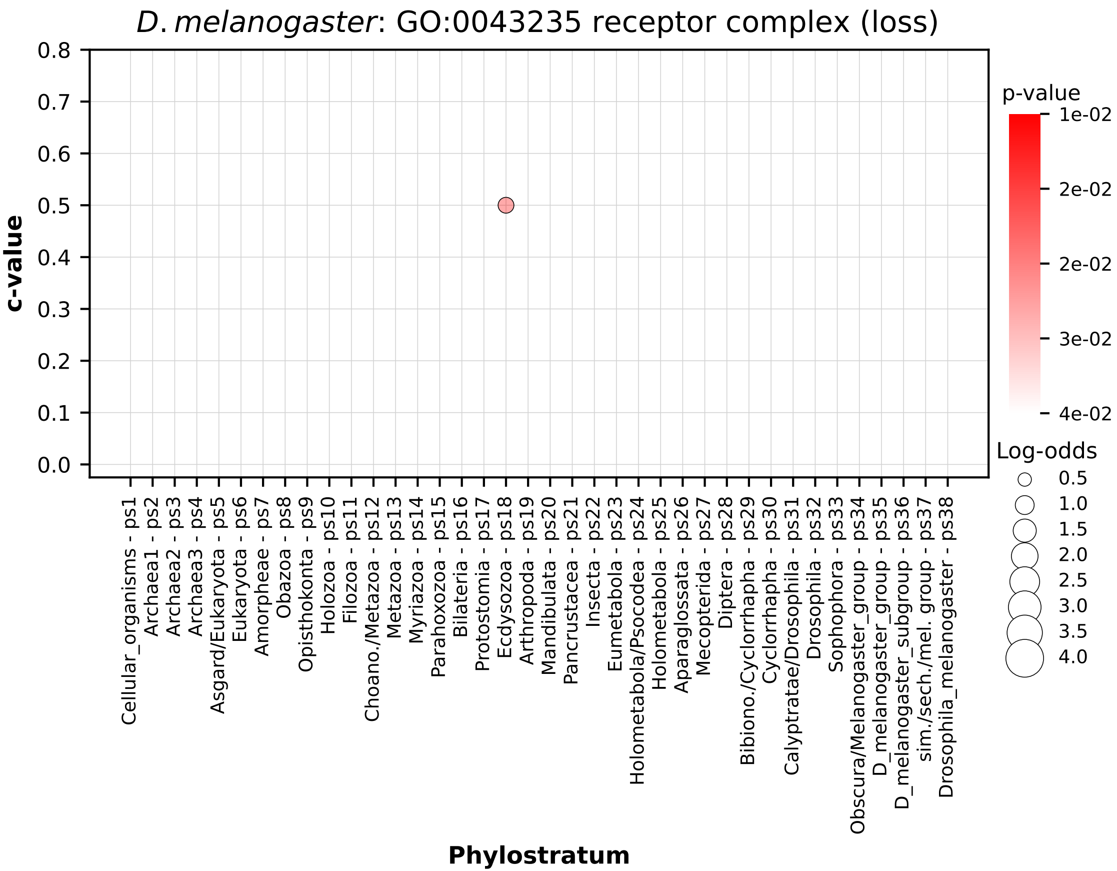
D. melanogaster: GO:0043235 receptor complex (loss)
c-value
0.0
0.1
0.2
0.3
0.4
0.5
0.6
0.7
0.8
Phylostratum
Cellular_organisms - ps1
Archaea1 - ps2
Archaea2 - ps3
Archaea3 - ps4
Asgard/Eukaryota - ps5
Eukaryota - ps6
Amorpheae - ps7
Obazoa - ps8
Opisthokonta - ps9
Holozoa - ps10
Filozoa - ps11
Choano./Metazoa - ps12
Metazoa - ps13
Myriazoa - ps14
Parahoxozoa - ps15
Bilateria - ps16
Protostomia - ps17
Ecdysozoa - ps18
Arthropoda - ps19
Mandibulata - ps20
Pancrustacea - ps21
Insecta - ps22
Eumetabola - ps23
Holometabola/Psocodea - ps24
Holometabola - ps25
Aparaglossata - ps26
Mecopterida - ps27
Diptera - ps28
Bibiono./Cyclorrhapha - ps29
Cyclorrhapha - ps30
Calyptratae/Drosophila - ps31
Drosophila - ps32
Sophophora - ps33
Obscura/Melanogaster_group - ps34
D_melanogaster_group - ps35
D_melanogaster_subgroup - ps36
sim./sech./mel. group - ps37
Drosophila_melanogaster - ps38
p-value
1e-02
2e-02
2e-02
3e-02
4e-02
Log-odds
0.5
1.0
1.5
2.0
2.5
3.0
3.5
4.0

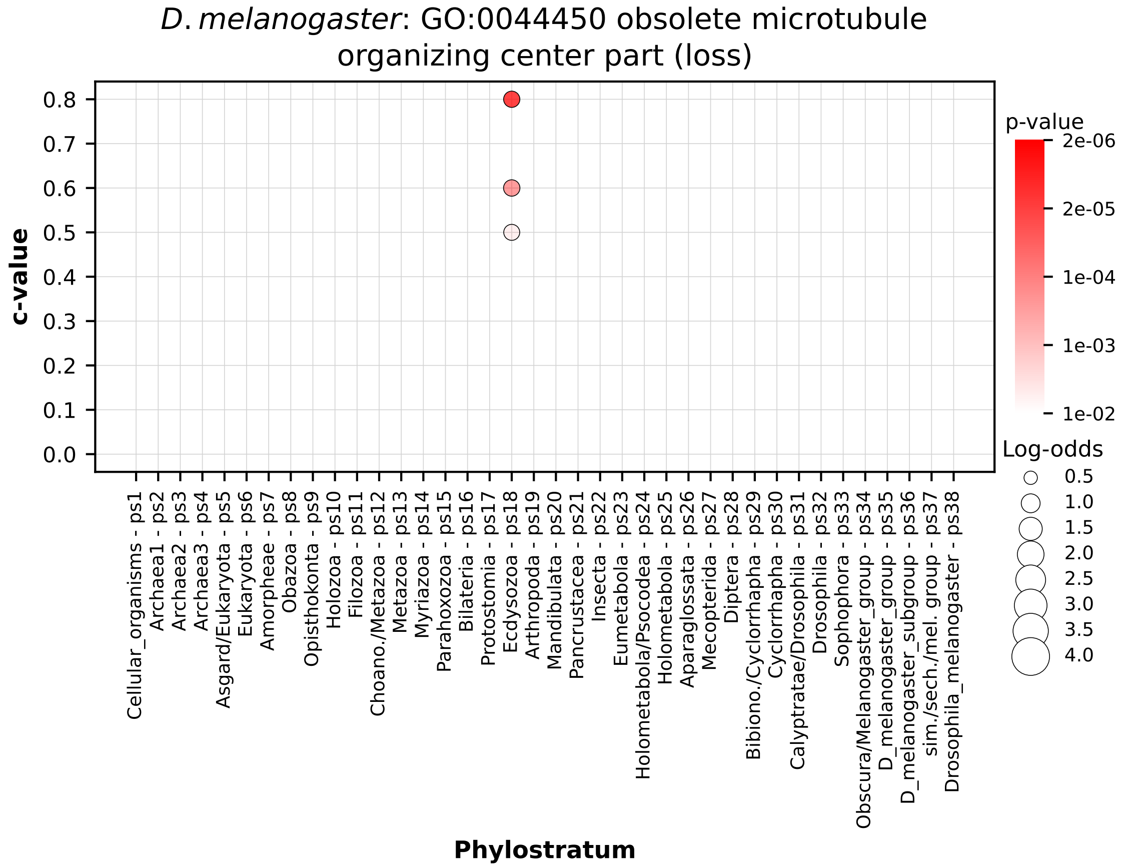

D. melanogaster: GO:0044450 obsolete microtubule organizing center part (loss)
c-value
0.8
0.7
0.6
0.5
0.4
0.3
0.2
0.1
0.0
p-value
2e-06
2e-05
1e-04
1e-03
1e-02
Log-odds
0.5
1.0
1.5
2.0
2.5
3.0
3.5
4.0
Cellular_organisms - ps1
Archaea1 - ps2
Archaea2 - ps3
Archaea3 - ps4
Asgard/Eukaryota - ps5
Eukaryota - ps6
Amorpheae - ps7
Obazoa - ps8
Opisthokonta - ps9
Holozoa - ps10
Filozoa - ps11
Choano./Metazoa - ps12
Metazoa - ps13
Myriazoa - ps14
Parahoxozoa - ps15
Bilateria - ps16
Protostomia - ps17
Ecdysozoa - ps18
Arthropoda - ps19
Mandibulata - ps20
Pancrustacea - ps21
Insecta - ps22
Eumetabola - ps23
Holometabola/Psocodea - ps24
Holometabola - ps25
Aparaglossata - ps26
Mecopterida - ps27
Diptera - ps28
Bibiono./Cyclorrhapha - ps29
Cyclorrhapha - ps30
Calyptratae/Drosophila - ps31
Drosophila - ps32
Sophophora - ps33
Obscura/Melanogaster_group - ps34
D_melanogaster_group - ps35
D_melanogaster_subgroup - ps36
sim./sech./mel. group - ps37
Drosophila_melanogaster - ps38
Phylostratum

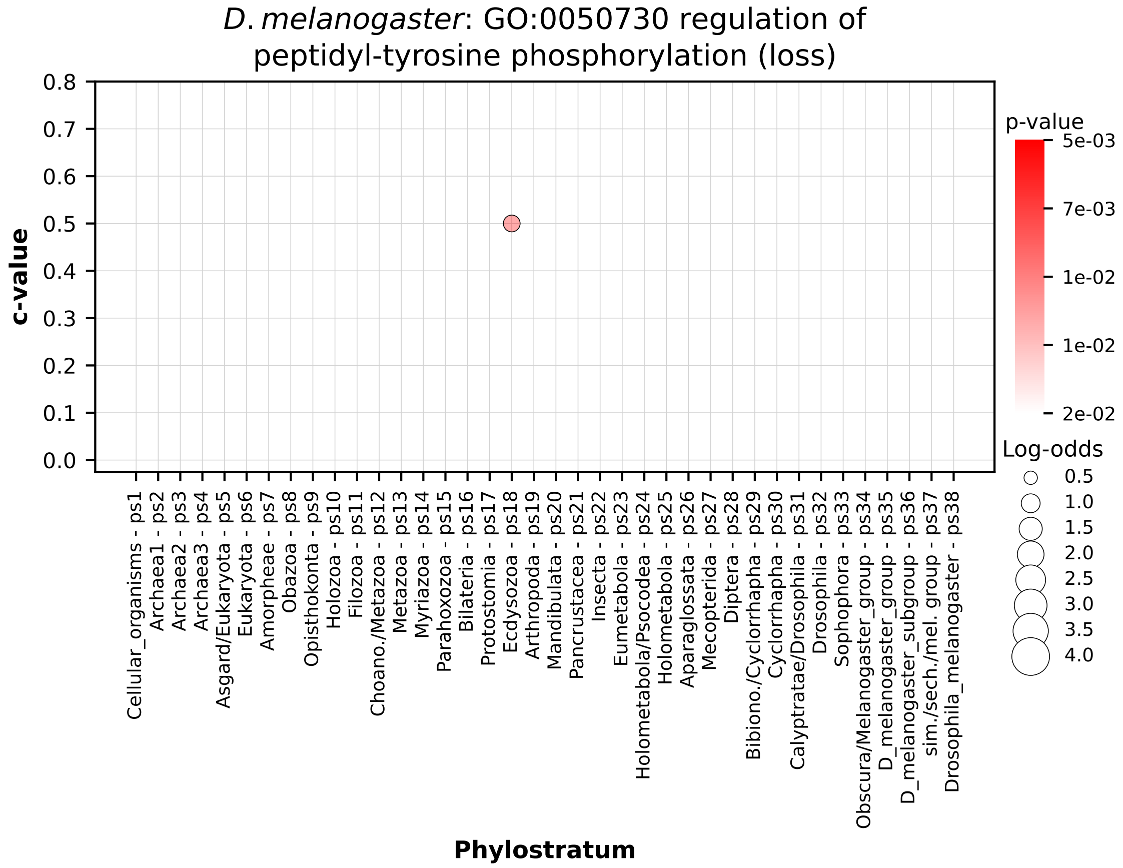
D. melanogaster: GO:0050730 regulation of
peptidyl-tyrosine phosphorylation (loss)
c-value
0.8
0.7
0.6
0.5
0.4
0.3
0.2
0.1
0.0
p-value
5e-03
7e-03
1e-02
1e-02
2e-02
Log-odds
0.5
1.0
1.5
2.0
2.5
3.0
3.5
4.0
Cellular_organisms - ps1
Archaea1 - ps2
Archaea2 - ps3
Archaea3 - ps4
Asgard/Eukaryota - ps5
Eukaryota - ps6
Amorpheae - ps7
Obazoa - ps8
Opisthokonta - ps9
Holozoa - ps10
Filozoa - ps11
Choano./Metazoa - ps12
Metazoa - ps13
Myriazoa - ps14
Parahoxozoa - ps15
Bilateria - ps16
Protostomia - ps17
Ecdysozoa - ps18
Arthropoda - ps19
Mandibulata - ps20
Pancrustacea - ps21
Insecta - ps22
Eumetabola - ps23
Holometabola/Psocodea - ps24
Holometabola - ps25
Aparaglossata - ps26
Mecopterida - ps27
Diptera - ps28
Bibiono./Cyclorrhapha - ps29
Cyclorrhapha - ps30
Calyptratae/Drosophila - ps31
Drosophila - ps32
Sophophora - ps33
Obscura/Melanogaster_group - ps34
D_melanogaster_group - ps35
D_melanogaster_subgroup - ps36
sim./sech./mel. group - ps37
Drosophila_melanogaster - ps38
Phylostratum

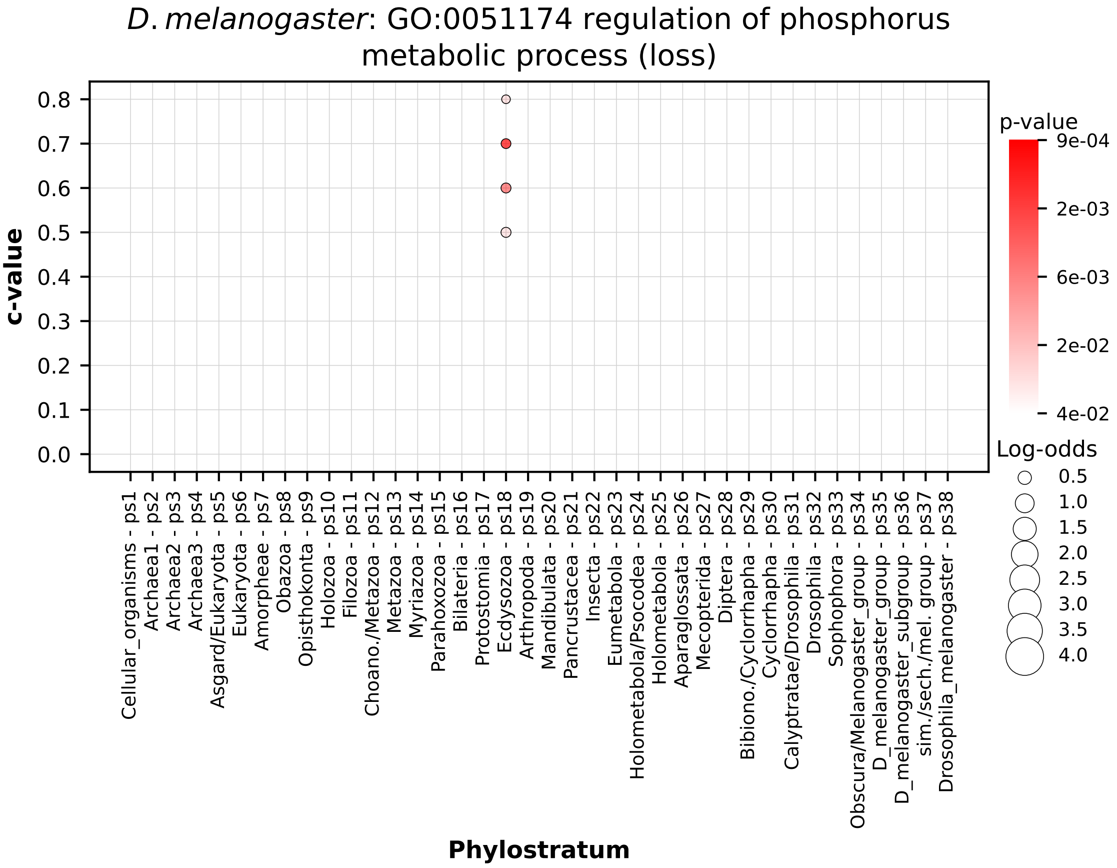

D. melanogaster: GO:0051174 regulation of phosphorus metabolic process (loss)
c-value
0.0
0.1
0.2
0.3
0.4
0.5
0.6
0.7
0.8
Phylostratum
Cellular_organisms - ps1
Archaea1 - ps2
Archaea2 - ps3
Archaea3 - ps4
Asgard/Eukaryota - ps5
Eukaryota - ps6
Amorpheae - ps7
Obazoa - ps8
Opisthokonta - ps9
Holozoa - ps10
Filozoa - ps11
Choano./Metazoa - ps12
Metazoa - ps13
Myriazoa - ps14
Parahoxozoa - ps15
Bilateria - ps16
Protostomia - ps17
Ecdysozoa - ps18
Arthropoda - ps19
Mandibulata - ps20
Pancrustacea - ps21
Insecta - ps22
Eumetabola - ps23
Holometabola/Psocodea - ps24
Holometabola - ps25
Aparaglossata - ps26
Mecopterida - ps27
Diptera - ps28
Bibiono./Cyclorrhapha - ps29
Cyclorrhapha - ps30
Calyptratae/Drosophila - ps31
Drosophila - ps32
Sophophora - ps33
Obscura/Melanogaster_group - ps34
D_melanogaster_group - ps35
D_melanogaster_subgroup - ps36
sim./sech./mel. group - ps37
Drosophila_melanogaster - ps38
p-value
9e-04
2e-03
6e-03
2e-02
4e-02
Log-odds
0.5
1.0
1.5
2.0
2.5
3.0
3.5
4.0

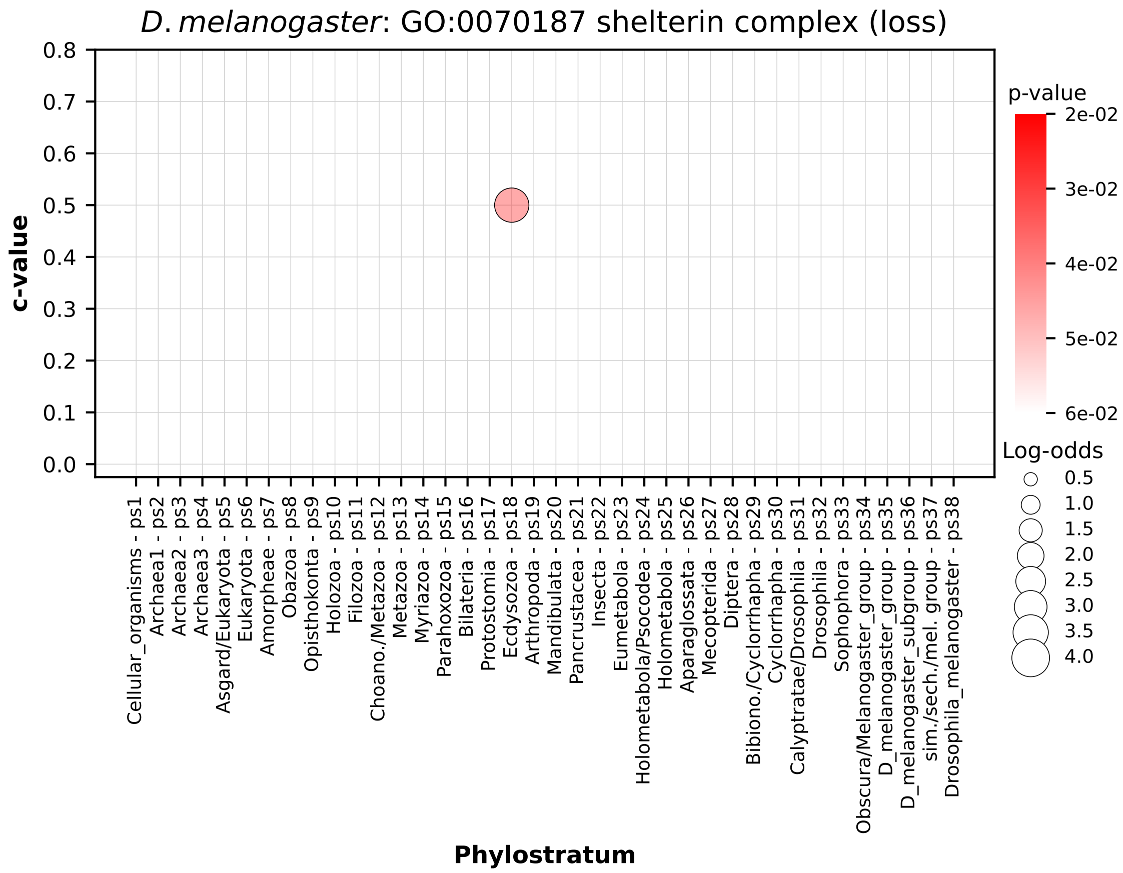

D. melanogaster: GO:0070187 shelterin complex (loss)
c-value
0.8
0.7
0.6
0.5
0.4
0.3
0.2
0.1
0.0
Phylostratum
Cellular_organisms - ps1
Archaea1 - ps2
Archaea2 - ps3
Archaea3 - ps4
Asgard/Eukaryota - ps5
Eukaryota - ps6
Amorpheae - ps7
Obazoa - ps8
Opisthokonta - ps9
Holozoa - ps10
Filozoa - ps11
Choano./Metazoa - ps12
Metazoa - ps13
Myriazoa - ps14
Parahoxozoa - ps15
Bilateria - ps16
Protostomia - ps17
Ecdysozoa - ps18
Arthropoda - ps19
Mandibulata - ps20
Pancrustacea - ps21
Insecta - ps22
Eumetabola - ps23
Holometabola/Psocodea - ps24
Holometabola - ps25
Aparaglossata - ps26
Mecopterida - ps27
Diptera - ps28
Bibiono./Cyclorrhapha - ps29
Cyclorrhapha - ps30
Calyptratae/Drosophila - ps31
Drosophila - ps32
Sophophora - ps33
Obscura/Melanogaster_group - ps34
D_melanogaster_group - ps35
D_melanogaster_subgroup - ps36
sim./sech./mel. group - ps37
Drosophila_melanogaster - ps38
p-value
2e-02
3e-02
4e-02
5e-02
6e-02
Log-odds
0.5
1.0
1.5
2.0
2.5
3.0
3.5
4.0

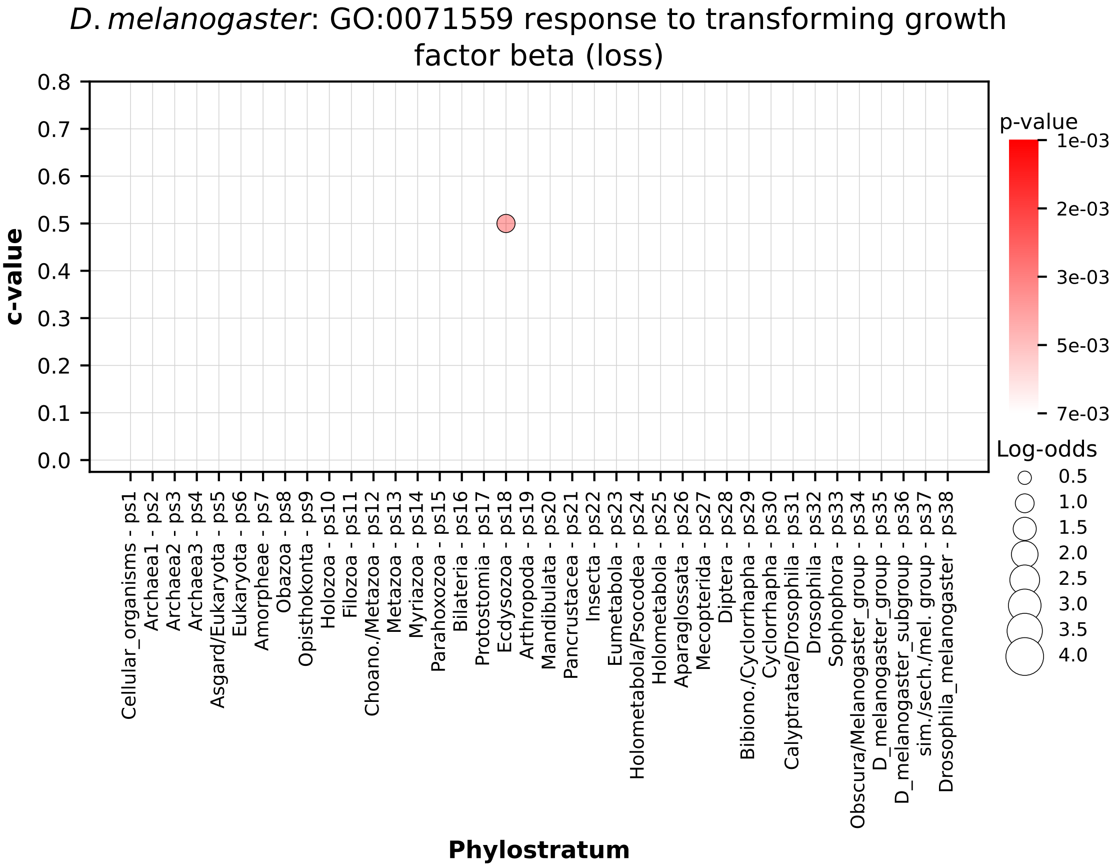

D. melanogaster: GO:0071559 response to transforming growth factor beta (loss)
c-value
0.8
0.7
0.6
0.5
0.4
0.3
0.2
0.1
0.0
p-value
1e-03
2e-03
3e-03
5e-03
7e-03
Log-odds
0.5
1.0
1.5
2.0
2.5
3.0
3.5
4.0
Cellular_organisms - ps1
Archaea1 - ps2
Archaea2 - ps3
Archaea3 - ps4
Asgard/Eukaryota - ps5
Eukaryota - ps6
Amorpheae - ps7
Obazoa - ps8
Opisthokonta - ps9
Holozoa - ps10
Filozoa - ps11
Choano./Metazoa - ps12
Metazoa - ps13
Myriazoa - ps14
Parahoxozoa - ps15
Bilateria - ps16
Protostomia - ps17
Ecdysozoa - ps18
Arthropoda - ps19
Mandibulata - ps20
Pancrustacea - ps21
Insecta - ps22
Eumetabola - ps23
Holometabola/Psocodea - ps24
Holometabola - ps25
Aparaglossata - ps26
Mecopterida - ps27
Diptera - ps28
Bibiono./Cyclorrhapha - ps29
Cyclorrhapha - ps30
Calyptratae/Drosophila - ps31
Drosophila - ps32
Sophophora - ps33
Obscura/Melanogaster_group - ps34
D_melanogaster_group - ps35
D_melanogaster_subgroup - ps36
sim./sech./mel. group - ps37
Drosophila_melanogaster - ps38
Phylostratum

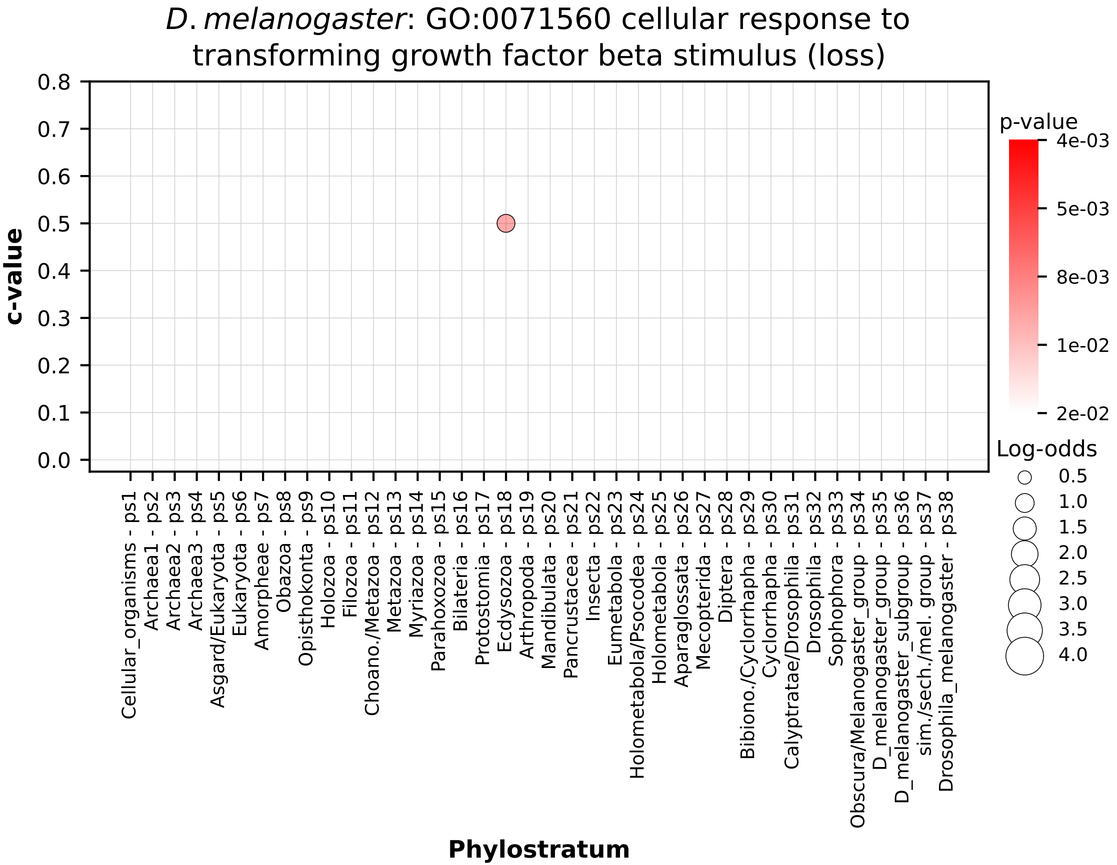

D. melanogaster: GO:0071560 cellular response to transforming growth factor beta stimulus (loss)
c-value
0.8
0.7
0.6
0.5
0.4
0.3
0.2
0.1
0.0
Phylostratum
Cellular_organisms - ps1
Archaea1 - ps2
Archaea2 - ps3
Archaea3 - ps4
Asgard/Eukaryota - ps5
Eukaryota - ps6
Amorpheae - ps7
Obazoa - ps8
Opisthokonta - ps9
Holozoa - ps10
Filozoa - ps11
Choano./Metazoa - ps12
Metazoa - ps13
Myriazoa - ps14
Parahoxozoa - ps15
Bilateria - ps16
Protostomia - ps17
Ecdysozoa - ps18
Arthropoda - ps19
Mandibulata - ps20
Pancrustacea - ps21
Insecta - ps22
Eumetabola - ps23
Holometabola/Psocodea - ps24
Holometabola - ps25
Aparaglossata - ps26
Mecopterida - ps27
Diptera - ps28
Bibiono./Cyclorrhapha - ps29
Cyclorrhapha - ps30
Calyptratae/Drosophila - ps31
Drosophila - ps32
Sophophora - ps33
Obscura/Melanogaster_group - ps34
D_melanogaster_group - ps35
D_melanogaster_subgroup - ps36
sim./sech./mel. group - ps37
Drosophila_melanogaster - ps38
p-value
4e-03
5e-03
8e-03
1e-02
2e-02
Log-odds
0.5
1.0
1.5
2.0
2.5
3.0
3.5
4.0

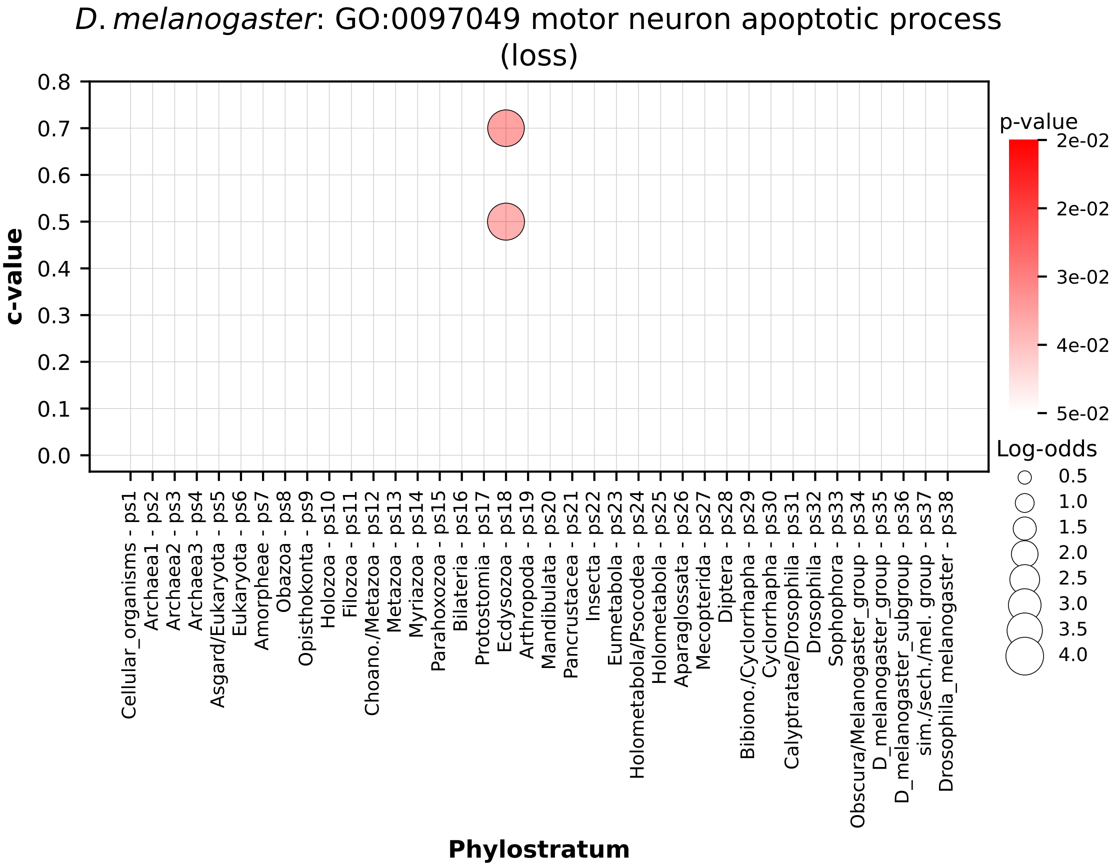
D. melanogaster: GO:0097049 motor neuron apoptotic process
(loss)
c-value
0.8
0.7
0.6
0.5
0.4
0.3
0.2
0.1
0.0
Phylostratum
Cellular_organisms - ps1
Archaea1 - ps2
Archaea2 - ps3
Archaea3 - ps4
Asgard/Eukaryota - ps5
Eukaryota - ps6
Amorpheae - ps7
Obazoa - ps8
Opisthokonta - ps9
Holozoa - ps10
Filozoa - ps11
Choano./Metazoa - ps12
Metazoa - ps13
Myriazoa - ps14
Parahoxozoa - ps15
Bilateria - ps16
Protostomia - ps17
Ecdysozoa - ps18
Arthropoda - ps19
Mandibulata - ps20
Pancrustacea - ps21
Insecta - ps22
Eumetabola - ps23
Holometabola/Psocodea - ps24
Holometabola - ps25
Aparaglossata - ps26
Mecopterida - ps27
Diptera - ps28
Bibiono./Cyclorrhapha - ps29
Cyclorrhapha - ps30
Calyptratae/Drosophila - ps31
Drosophila - ps32
Sophophora - ps33
Obscura/Melanogaster_group - ps34
D_melanogaster_group - ps35
D_melanogaster_subgroup - ps36
sim./sech./mel. group - ps37
Drosophila_melanogaster - ps38
p-value
2e-02
2e-02
3e-02
4e-02
5e-02
Log-odds
0.5
1.0
1.5
2.0
2.5
3.0
3.5
4.0

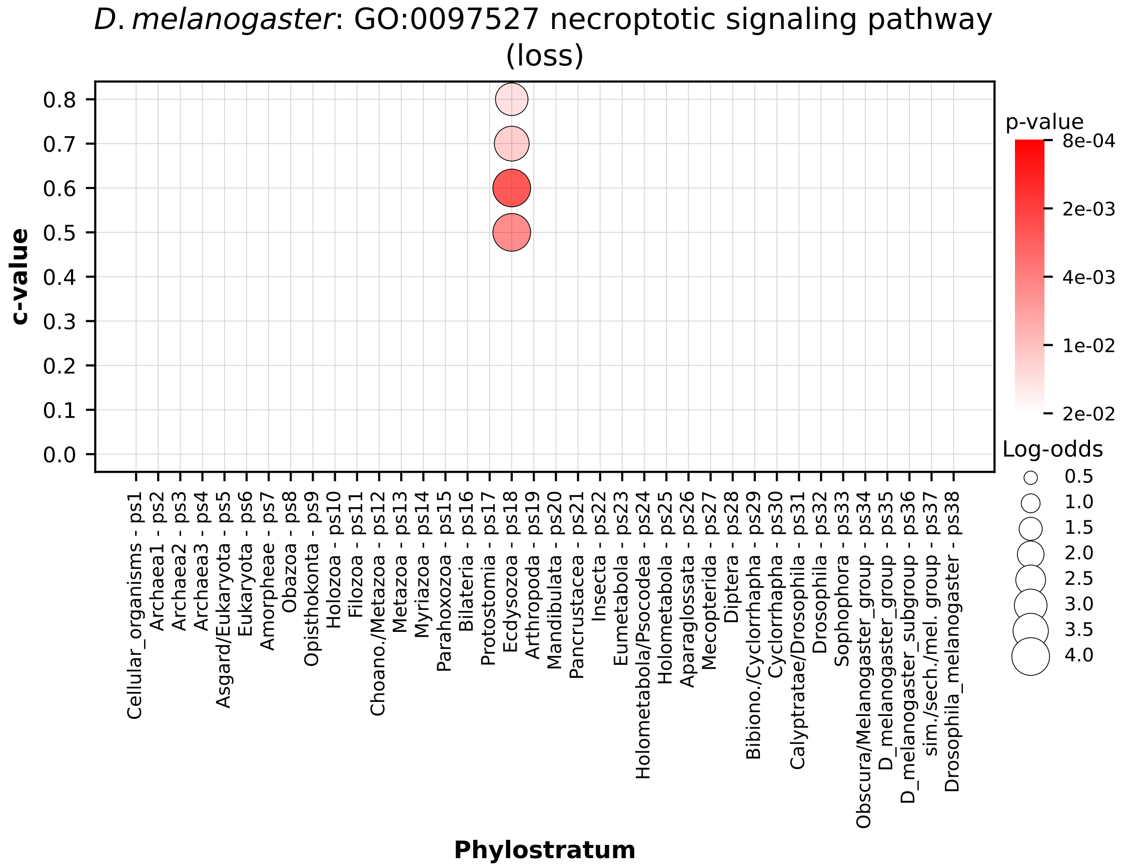

D. melanogaster: GO:0097527 necroptotic signaling pathway (loss)
c-value
0.8
0.7
0.6
0.5
0.4
0.3
0.2
0.1
0.0
p-value
8e-04
2e-03
4e-03
1e-02
2e-02
Log-odds
0.5
1.0
1.5
2.0
2.5
3.0
3.5
4.0
Cellular_organisms - ps1
Archaea1 - ps2
Archaea2 - ps3
Archaea3 - ps4
Asgard/Eukaryota - ps5
Eukaryota - ps6
Amorpheae - ps7
Obazoa - ps8
Opisthokonta - ps9
Holozoa - ps10
Filozoa - ps11
Choano./Metazoa - ps12
Metazoa - ps13
Myriazoa - ps14
Parahoxozoa - ps15
Bilateria - ps16
Protostomia - ps17
Ecdysozoa - ps18
Arthropoda - ps19
Mandibulata - ps20
Pancrustacea - ps21
Insecta - ps22
Eumetabola - ps23
Holometabola/Psocodea - ps24
Holometabola - ps25
Aparaglossata - ps26
Mecopterida - ps27
Diptera - ps28
Bibiono./Cyclorrhapha - ps29
Cyclorrhapha - ps30
Calyptratae/Drosophila - ps31
Drosophila - ps32
Sophophora - ps33
Obscura/Melanogaster_group - ps34
D_melanogaster_group - ps35
D_melanogaster_subgroup - ps36
sim./sech./mel. group - ps37
Drosophila_melanogaster - ps38
Phylostratum

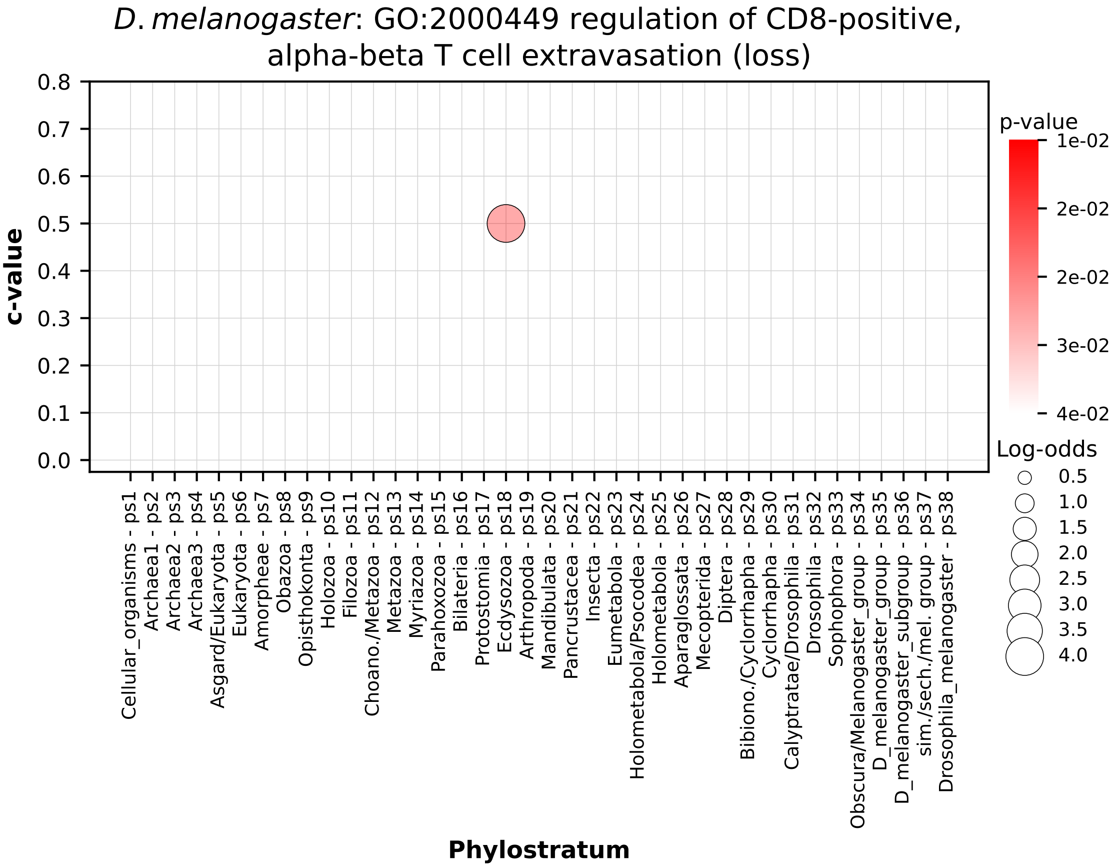

D. melanogaster: GO:2000449 regulation of CD8-positive, alpha-beta T cell extravasation (loss)
c-value
0.8
0.7
0.6
0.5
0.4
0.3
0.2
0.1
0.0
Phylostratum
Cellular_organisms - ps1
Archaea1 - ps2
Archaea2 - ps3
Archaea3 - ps4
Asgard/Eukaryota - ps5
Eukaryota - ps6
Amorpheae - ps7
Obazoa - ps8
Opisthokonta - ps9
Holozoa - ps10
Filozoa - ps11
Choano./Metazoa - ps12
Metazoa - ps13
Myriazoa - ps14
Parahoxozoa - ps15
Bilateria - ps16
Protostomia - ps17
Ecdysozoa - ps18
Arthropoda - ps19
Mandibulata - ps20
Pancrustacea - ps21
Insecta - ps22
Eumetabola - ps23
Holometabola/Psocodea - ps24
Holometabola - ps25
Aparaglossata - ps26
Mecopterida - ps27
Diptera - ps28
Bibiono./Cyclorrhapha - ps29
Cyclorrhapha - ps30
Calyptratae/Drosophila - ps31
Drosophila - ps32
Sophophora - ps33
Obscura/Melanogaster_group - ps34
D_melanogaster_group - ps35
D_melanogaster_subgroup - ps36
sim./sech./mel. group - ps37
Drosophila_melanogaster - ps38
p-value
1e-02
2e-02
2e-02
3e-02
4e-02
Log-odds
0.5
1.0
1.5
2.0
2.5
3.0
3.5
4.0

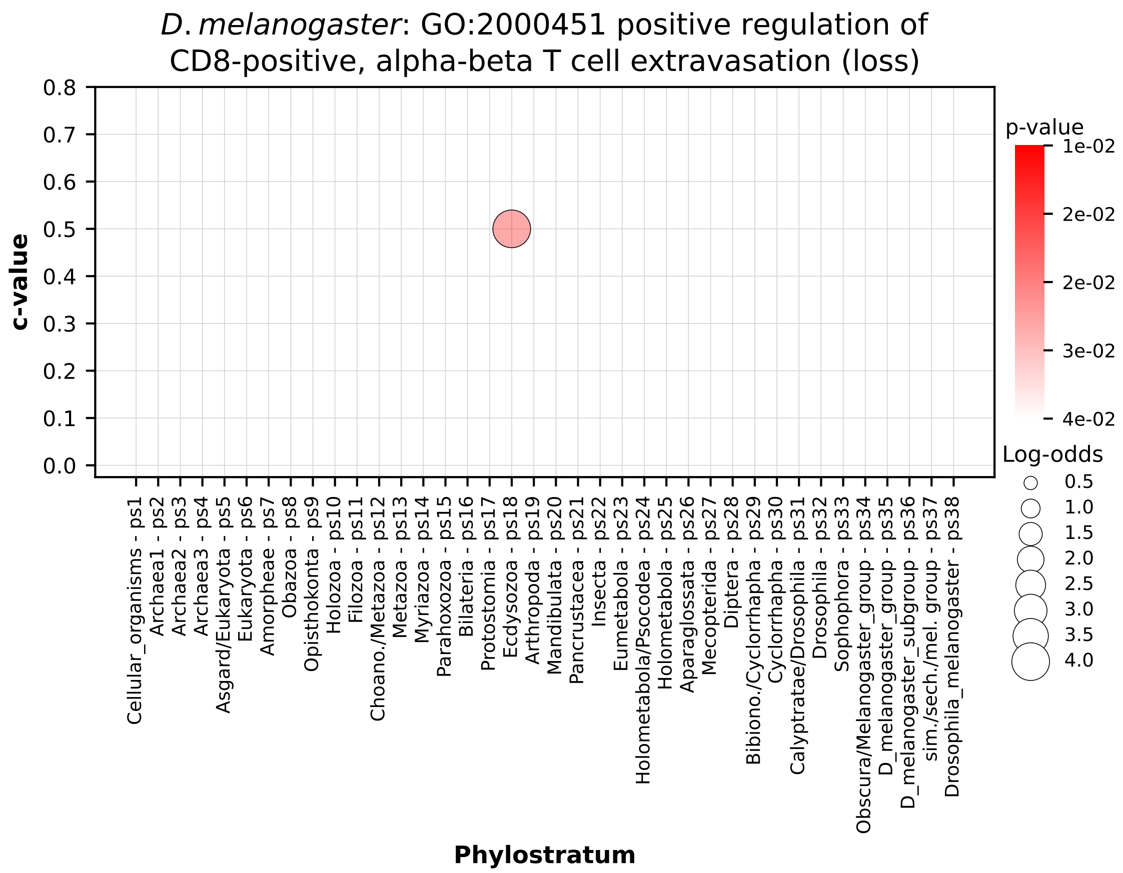

D. melanogaster: GO:2000451 positive regulation of CD8-positive, alpha-beta T cell extravasation (loss)
c-value
0.8
0.7
0.6
0.5
0.4
0.3
0.2
0.1
0.0
p-value
1e-02
2e-02
2e-02
3e-02
4e-02
Log-odds
0.5
1.0
1.5
2.0
2.5
3.0
3.5
4.0
Cellular_organisms - ps1
Archaea1 - ps2
Archaea2 - ps3
Archaea3 - ps4
Asgard/Eukaryota - ps5
Eukaryota - ps6
Amorpheae - ps7
Obazoa - ps8
Opisthokonta - ps9
Holozoa - ps10
Filozoa - ps11
Choano./Metazoa - ps12
Metazoa - ps13
Myriazoa - ps14
Parahoxozoa - ps15
Bilateria - ps16
Protostomia - ps17
Ecdysozoa - ps18
Arthropoda - ps19
Mandibulata - ps20
Pancrustacea - ps21
Insecta - ps22
Eumetabola - ps23
Holometabola/Psocodea - ps24
Holometabola - ps25
Aparaglossata - ps26
Mecopterida - ps27
Diptera - ps28
Bibiono./Cyclorrhapha - ps29
Cyclorrhapha - ps30
Calyptratae/Drosophila - ps31
Drosophila - ps32
Sophophora - ps33
Obscura/Melanogaster_group - ps34
D_melanogaster_group - ps35
D_melanogaster_subgroup - ps36
sim./sech./mel. group - ps37
Drosophila_melanogaster - ps38
Phylostratum

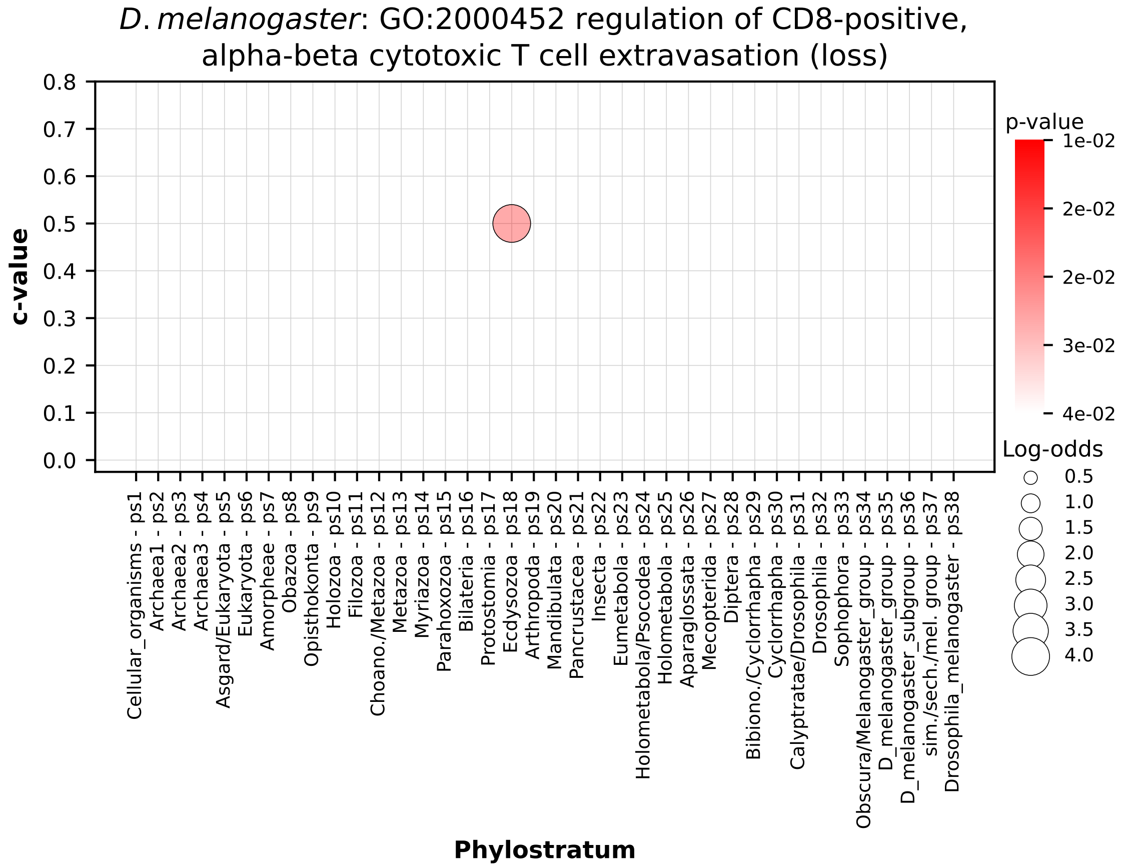
D. melanogaster: GO:2000452 regulation of CD8-positive, alpha-beta cytotoxic T cell extravasation (loss)
c-value
0.8
0.7
0.6
0.5
0.4
0.3
0.2
0.1
0.0
p-value
1e-02
2e-02
2e-02
3e-02
4e-02
Log-odds
0.5
1.0
1.5
2.0
2.5
3.0
3.5
4.0
Cellular_organisms - ps1
Archaea1 - ps2
Archaea2 - ps3
Archaea3 - ps4
Asgard/Eukaryota - ps5
Eukaryota - ps6
Amorpheae - ps7
Obazoa - ps8
Opisthokonta - ps9
Holozoa - ps10
Filozoa - ps11
Choano./Metazoa - ps12
Metazoa - ps13
Myriazoa - ps14
Parahoxozoa - ps15
Bilateria - ps16
Protostomia - ps17
Ecdysozoa - ps18
Arthropoda - ps19
Mandibulata - ps20
Pancrustacea - ps21
Insecta - ps22
Eumetabola - ps23
Holometabola/Psocodea - ps24
Holometabola - ps25
Aparaglossata - ps26
Mecopterida - ps27
Diptera - ps28
Bibiono./Cyclorrhapha - ps29
Cyclorrhapha - ps30
Calyptratae/Drosophila - ps31
Drosophila - ps32
Sophophora - ps33
Obscura/Melanogaster_group - ps34
D_melanogaster_group - ps35
D_melanogaster_subgroup - ps36
sim./sech./mel. group - ps37
Drosophila_melanogaster - ps38
Phylostratum

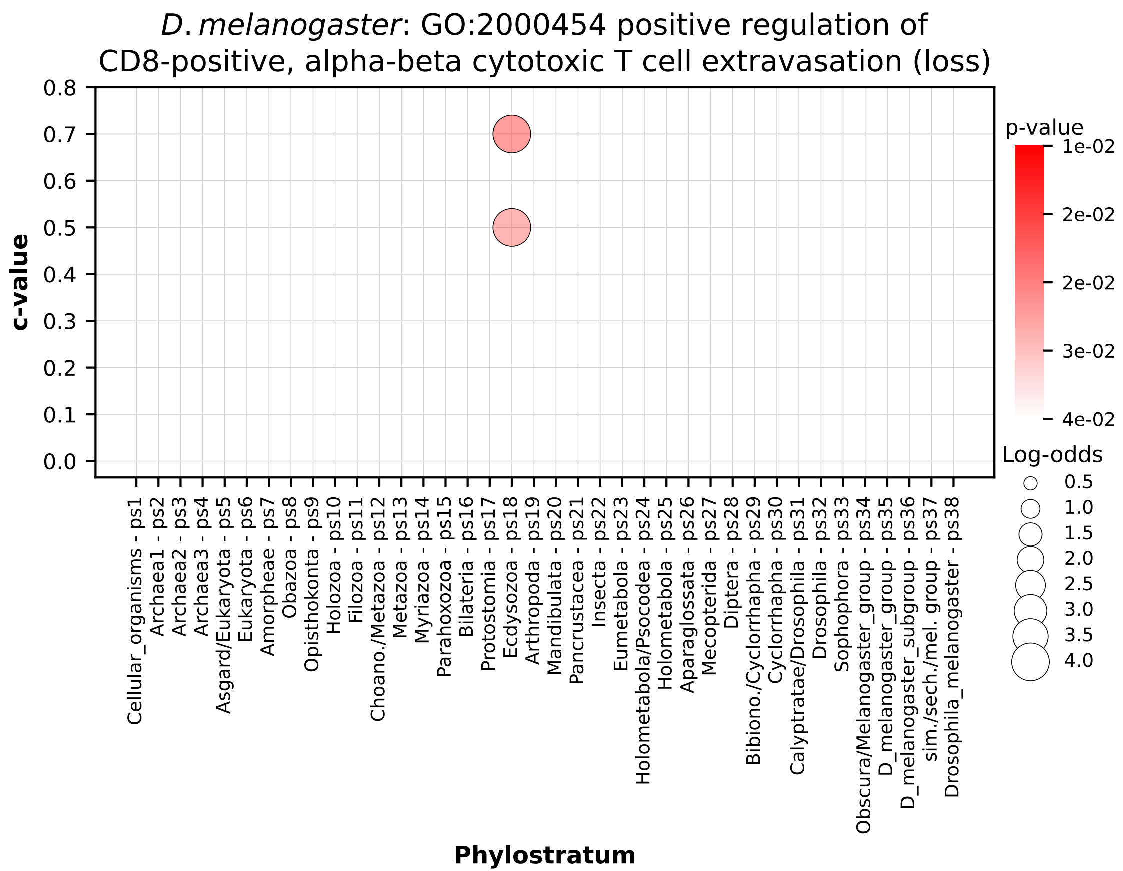

D. melanogaster: GO:2000454 positive regulation of
CD8-positive, alpha-beta cytotoxic T cell extravasation (loss)
c-value
0.8
0.7
0.6
0.5
0.4
0.3
0.2
0.1
0.0
Phylostratum
Cellular_organisms - ps1
Archaea1 - ps2
Archaea2 - ps3
Archaea3 - ps4
Asgard/Eukaryota - ps5
Eukaryota - ps6
Amorpheae - ps7
Obazoa - ps8
Opisthokonta - ps9
Holozoa - ps10
Filozoa - ps11
Choano./Metazoa - ps12
Metazoa - ps13
Myriazoa - ps14
Parahoxozoa - ps15
Bilateria - ps16
Protostomia - ps17
Ecdysozoa - ps18
Arthropoda - ps19
Mandibulata - ps20
Pancrustacea - ps21
Insecta - ps22
Eumetabola - ps23
Holometabola/Psocodea - ps24
Holometabola - ps25
Aparaglossata - ps26
Mecopterida - ps27
Diptera - ps28
Bibiono./Cyclorrhapha - ps29
Cyclorrhapha - ps30
Calyptratae/Drosophila - ps31
Drosophila - ps32
Sophophora - ps33
Obscura/Melanogaster_group - ps34
D_melanogaster_group - ps35
D_melanogaster_subgroup - ps36
sim./sech./mel. group - ps37
Drosophila_melanogaster - ps38
p-value
1e-02
2e-02
2e-02
3e-02
4e-02
Log-odds
0.5
1.0
1.5
2.0
2.5
3.0
3.5
4.0

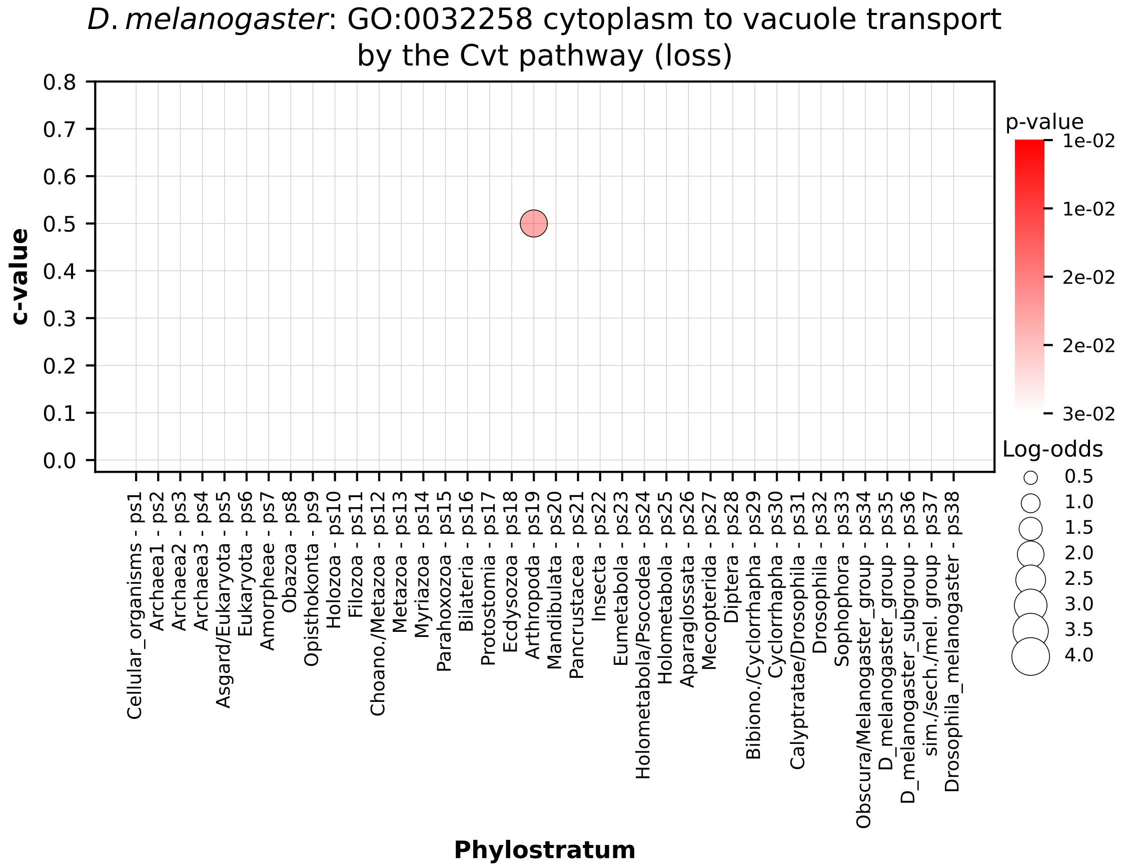

D. melanogaster: GO:0032258 cytoplasm to vacuole transport by the Cvt pathway (loss)
c-value
0.8
0.7
0.6
0.5
0.4
0.3
0.2
0.1
0.0
Phylostratum
Cellular_organisms - ps1
Archaea1 - ps2
Archaea2 - ps3
Archaea3 - ps4
Asgard/Eukaryota - ps5
Eukaryota - ps6
Amorpheae - ps7
Obazoa - ps8
Opisthokonta - ps9
Holozoa - ps10
Filozoa - ps11
Choano./Metazoa - ps12
Metazoa - ps13
Myriazoa - ps14
Parahoxozoa - ps15
Bilateria - ps16
Protostomia - ps17
Ecdysozoa - ps18
Arthropoda - ps19
Mandibulata - ps20
Pancrustacea - ps21
Insecta - ps22
Eumetabola - ps23
Holometabola/Psocodea - ps24
Holometabola - ps25
Aparaglossata - ps26
Mecopterida - ps27
Diptera - ps28
Bibiono./Cyclorrhapha - ps29
Cyclorrhapha - ps30
Calyptratae/Drosophila - ps31
Drosophila - ps32
Sophophora - ps33
Obscura/Melanogaster_group - ps34
D_melanogaster_group - ps35
D_melanogaster_subgroup - ps36
sim./sech./mel. group - ps37
Drosophila_melanogaster - ps38
p-value
1e-02
1e-02
2e-02
2e-02
3e-02
Log-odds
0.5
1.0
1.5
2.0
2.5
3.0
3.5
4.0

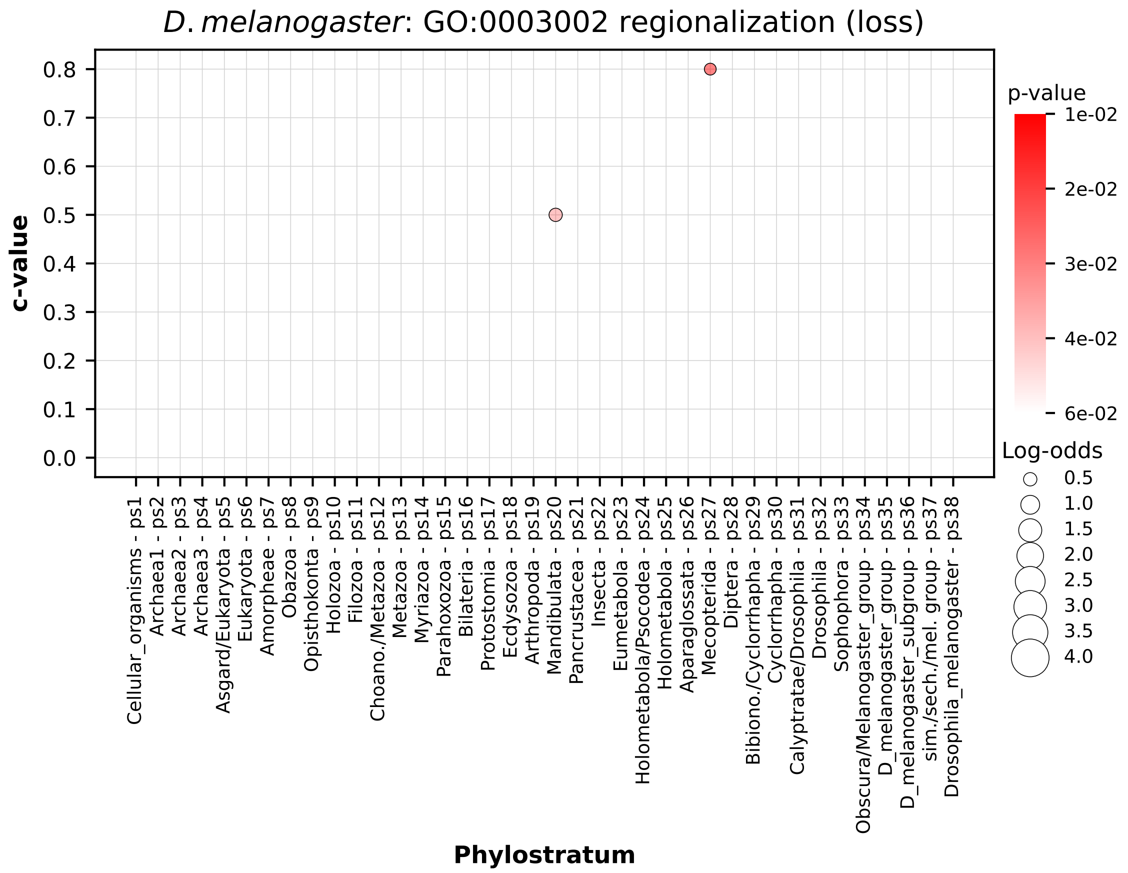

D. melanogaster: GO:0003002 regionalization (loss)
c-value
0.8
0.7
0.6
0.5
0.4
0.3
0.2
0.1
0.0
p-value
1e-02
2e-02
3e-02
4e-02
6e-02
Log-odds
0.5
1.0
1.5
2.0
2.5
3.0
3.5
4.0
Cellular_organisms - ps1
Archaea1 - ps2
Archaea2 - ps3
Archaea3 - ps4
Asgard/Eukaryota - ps5
Eukaryota - ps6
Amorpheae - ps7
Obazoa - ps8
Opisthokonta - ps9
Holozoa - ps10
Filozoa - ps11
Choano./Metazoa - ps12
Metazoa - ps13
Myriazoa - ps14
Parahoxozoa - ps15
Bilateria - ps16
Protostomia - ps17
Ecdysozoa - ps18
Arthropoda - ps19
Mandibulata - ps20
Pancrustacea - ps21
Insecta - ps22
Eumetabola - ps23
Holometabola/Psocodea - ps24
Holometabola - ps25
Aparaglossata - ps26
Mecopterida - ps27
Diptera - ps28
Bibiono./Cyclorrhapha - ps29
Cyclorrhapha - ps30
Calyptratae/Drosophila - ps31
Drosophila - ps32
Sophophora - ps33
Obscura/Melanogaster_group - ps34
D_melanogaster_group - ps35
D_melanogaster_subgroup - ps36
sim./sech./mel. group - ps37
Drosophila_melanogaster - ps38
Phylostratum

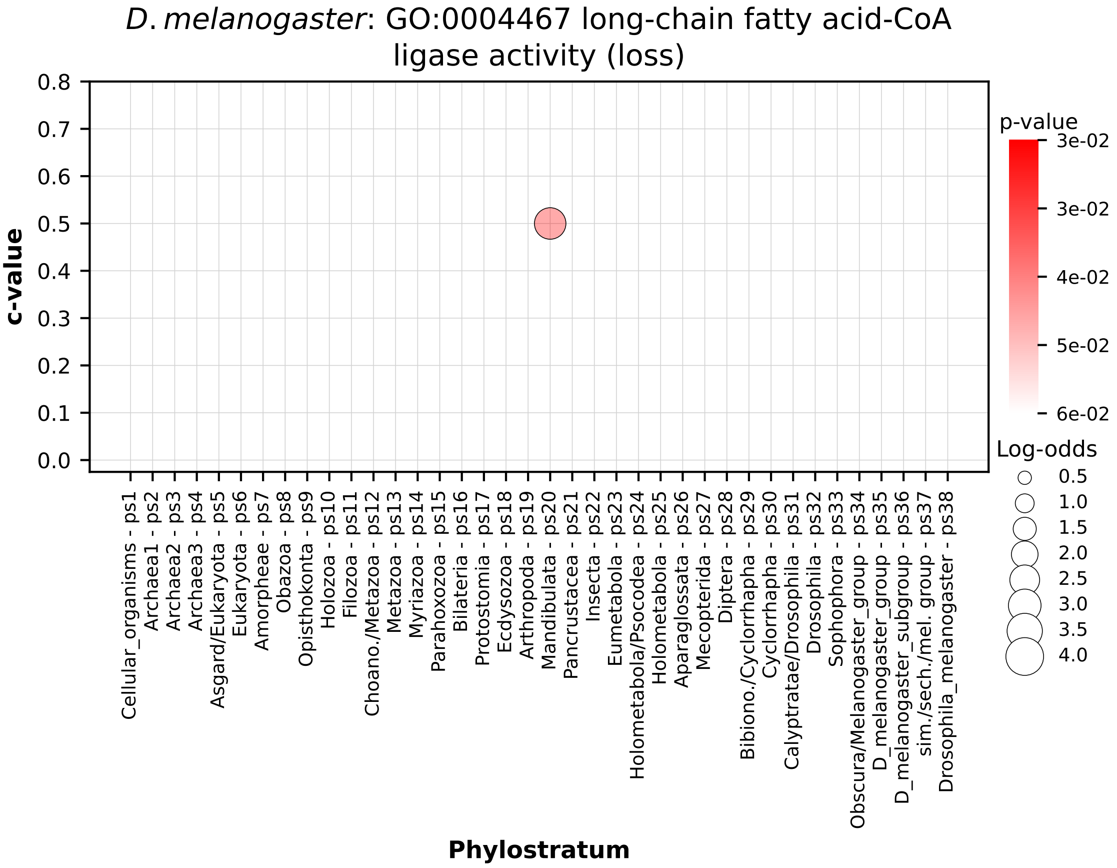

D. melanogaster: GO:0004467 long-chain fatty acid-CoA ligase activity (loss)
c-value
0.8
0.7
0.6
0.5
0.4
0.3
0.2
0.1
0.0
p-value
3e-02
3e-02
4e-02
5e-02
6e-02
Log-odds
0.5
1.0
1.5
2.0
2.5
3.0
3.5
4.0
Cellular_organisms - ps1
Archaea1 - ps2
Archaea2 - ps3
Archaea3 - ps4
Asgard/Eukaryota - ps5
Eukaryota - ps6
Amorpheae - ps7
Obazoa - ps8
Opisthokonta - ps9
Holozoa - ps10
Filozoa - ps11
Choano./Metazoa - ps12
Metazoa - ps13
Myriazoa - ps14
Parahoxozoa - ps15
Bilateria - ps16
Protostomia - ps17
Ecdysozoa - ps18
Arthropoda - ps19
Mandibulata - ps20
Pancrustacea - ps21
Insecta - ps22
Eumetabola - ps23
Holometabola/Psocodea - ps24
Holometabola - ps25
Aparaglossata - ps26
Mecopterida - ps27
Diptera - ps28
Bibiono./Cyclorrhapha - ps29
Cyclorrhapha - ps30
Calyptratae/Drosophila - ps31
Drosophila - ps32
Sophophora - ps33
Obscura/Melanogaster_group - ps34
D_melanogaster_group - ps35
D_melanogaster_subgroup - ps36
sim./sech./mel. group - ps37
Drosophila_melanogaster - ps38
Phylostratum

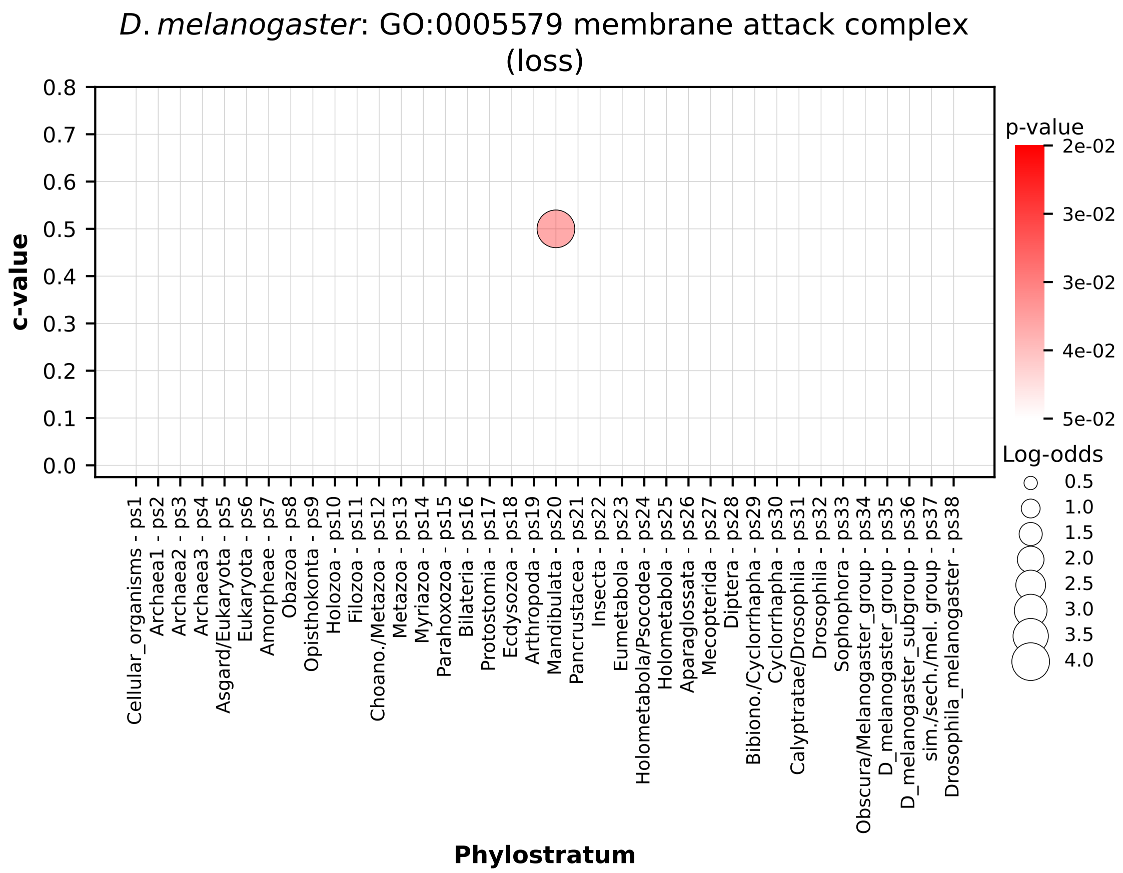

D. melanogaster: GO:0005579 membrane attack complex
(loss)
c-value
0.0
0.1
0.2
0.3
0.4
0.5
0.6
0.7
0.8
Phylostratum
Cellular_organisms - ps1
Archaea1 - ps2
Archaea2 - ps3
Archaea3 - ps4
Asgard/Eukaryota - ps5
Eukaryota - ps6
Amorpheae - ps7
Obazoa - ps8
Opisthokonta - ps9
Holozoa - ps10
Filozoa - ps11
Choano./Metazoa - ps12
Metazoa - ps13
Myriazoa - ps14
Parahoxozoa - ps15
Bilateria - ps16
Protostomia - ps17
Ecdysozoa - ps18
Arthropoda - ps19
Mandibulata - ps20
Pancrustacea - ps21
Insecta - ps22
Eumetabola - ps23
Holometabola/Psocodea - ps24
Holometabola - ps25
Aparaglossata - ps26
Mecopterida - ps27
Diptera - ps28
Bibiono./Cyclorrhapha - ps29
Cyclorrhapha - ps30
Calyptratae/Drosophila - ps31
Drosophila - ps32
Sophophora - ps33
Obscura/Melanogaster_group - ps34
D_melanogaster_group - ps35
D_melanogaster_subgroup - ps36
sim./sech./mel. group - ps37
Drosophila_melanogaster - ps38
p-value
2e-02
3e-02
3e-02
4e-02
5e-02
Log-odds
0.5
1.0
1.5
2.0
2.5
3.0
3.5
4.0

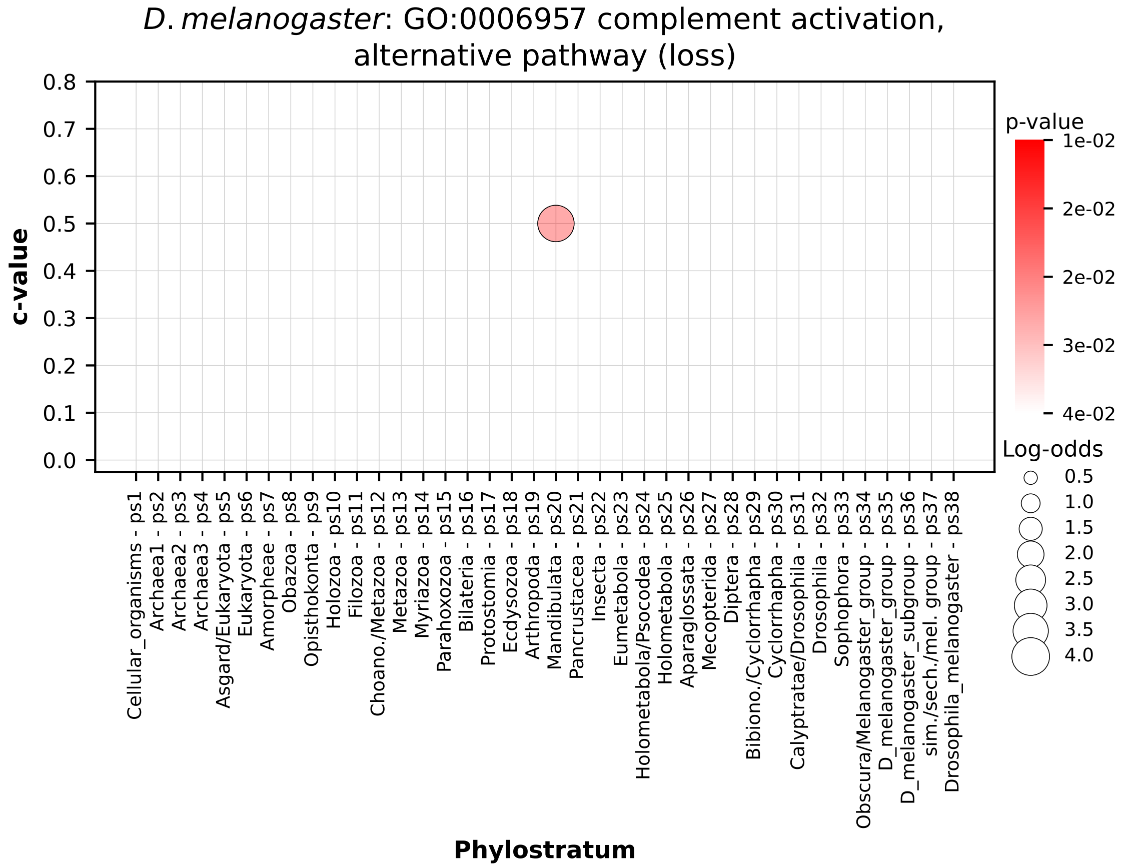

D. melanogaster: GO:0006957 complement activation, alternative pathway (loss)
c-value
0.8
0.7
0.6
0.5
0.4
0.3
0.2
0.1
0.0
p-value
1e-02
2e-02
2e-02
3e-02
4e-02
Log-odds
0.5
1.0
1.5
2.0
2.5
3.0
3.5
4.0
Cellular_organisms - ps1
Archaea1 - ps2
Archaea2 - ps3
Archaea3 - ps4
Asgard/Eukaryota - ps5
Eukaryota - ps6
Amorpheae - ps7
Obazoa - ps8
Opisthokonta - ps9
Holozoa - ps10
Filozoa - ps11
Choano./Metazoa - ps12
Metazoa - ps13
Myriazoa - ps14
Parahoxozoa - ps15
Bilateria - ps16
Protostomia - ps17
Ecdysozoa - ps18
Arthropoda - ps19
Mandibulata - ps20
Pancrustacea - ps21
Insecta - ps22
Eumetabola - ps23
Holometabola/Psocodea - ps24
Holometabola - ps25
Aparaglossata - ps26
Mecopterida - ps27
Diptera - ps28
Bibiono./Cyclorrhapha - ps29
Cyclorrhapha - ps30
Calyptratae/Drosophila - ps31
Drosophila - ps32
Sophophora - ps33
Obscura/Melanogaster_group - ps34
D_melanogaster_group - ps35
D_melanogaster_subgroup - ps36
sim./sech./mel. group - ps37
Drosophila_melanogaster - ps38
Phylostratum

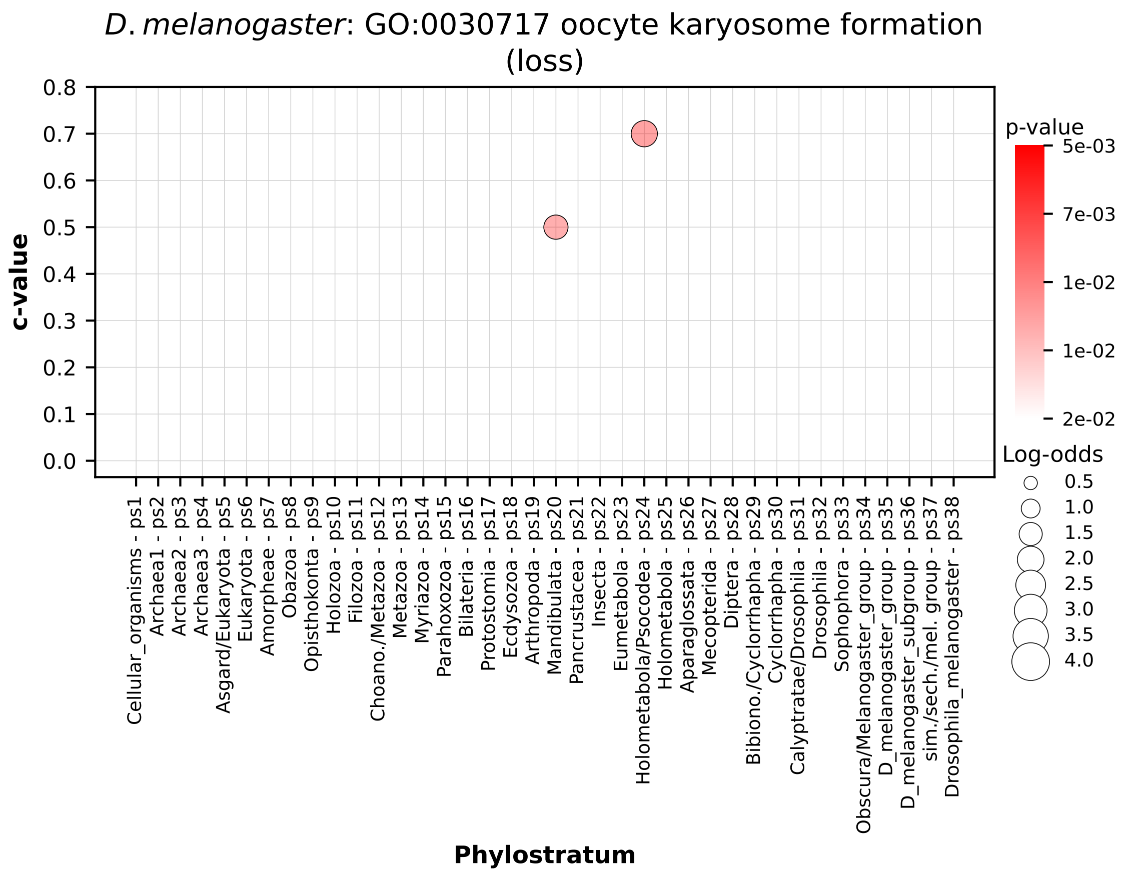

D. melanogaster: GO:0030717 oocyte karyosome formation (loss)
c-value
0.0
0.1
0.2
0.3
0.4
0.5
0.6
0.7
0.8
Phylostratum
Cellular_organisms - ps1
Archaea1 - ps2
Archaea2 - ps3
Archaea3 - ps4
Asgard/Eukaryota - ps5
Eukaryota - ps6
Amorpheae - ps7
Obazoa - ps8
Opisthokonta - ps9
Holozoa - ps10
Filozoa - ps11
Choano./Metazoa - ps12
Metazoa - ps13
Myriazoa - ps14
Parahoxozoa - ps15
Bilateria - ps16
Protostomia - ps17
Ecdysozoa - ps18
Arthropoda - ps19
Mandibulata - ps20
Pancrustacea - ps21
Insecta - ps22
Eumetabola - ps23
Holometabola/Psocodea - ps24
Holometabola - ps25
Aparaglossata - ps26
Mecopterida - ps27
Diptera - ps28
Bibiono./Cyclorrhapha - ps29
Cyclorrhapha - ps30
Calyptratae/Drosophila - ps31
Drosophila - ps32
Sophophora - ps33
Obscura/Melanogaster_group - ps34
D_melanogaster_group - ps35
D_melanogaster_subgroup - ps36
sim./sech./mel. group - ps37
Drosophila_melanogaster - ps38
p-value
5e-03
7e-03
1e-02
1e-02
2e-02
Log-odds
0.5
1.0
1.5
2.0
2.5
3.0
3.5
4.0

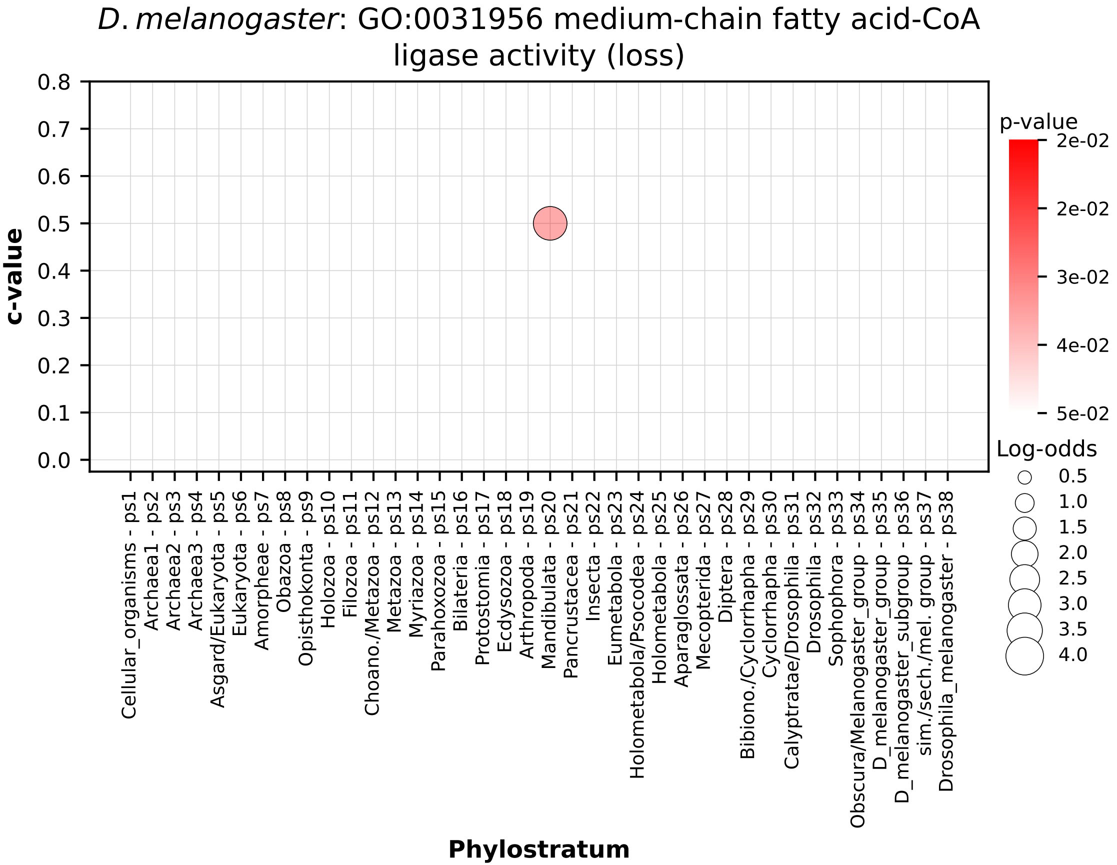
D. melanogaster: GO:0031956 medium-chain fatty acid-CoA ligase activity (loss)
c-value
0.8
0.7
0.6
0.5
0.4
0.3
0.2
0.1
0.0
p-value
2e-02
2e-02
3e-02
4e-02
5e-02
Log-odds
0.5
1.0
1.5
2.0
2.5
3.0
3.5
4.0
Cellular_organisms - ps1
Archaea1 - ps2
Archaea2 - ps3
Archaea3 - ps4
Asgard/Eukaryota - ps5
Eukaryota - ps6
Amorpheae - ps7
Obazoa - ps8
Opisthokonta - ps9
Holozoa - ps10
Filozoa - ps11
Choano./Metazoa - ps12
Metazoa - ps13
Myriazoa - ps14
Parahoxozoa - ps15
Bilateria - ps16
Protostomia - ps17
Ecdysozoa - ps18
Arthropoda - ps19
Mandibulata - ps20
Pancrustacea - ps21
Insecta - ps22
Eumetabola - ps23
Holometabola/Psocodea - ps24
Holometabola - ps25
Aparaglossata - ps26
Mecopterida - ps27
Diptera - ps28
Bibiono./Cyclorrhapha - ps29
Cyclorrhapha - ps30
Calyptratae/Drosophila - ps31
Drosophila - ps32
Sophophora - ps33
Obscura/Melanogaster_group - ps34
D_melanogaster_group - ps35
D_melanogaster_subgroup - ps36
sim./sech./mel. group - ps37
Drosophila_melanogaster - ps38
Phylostratum

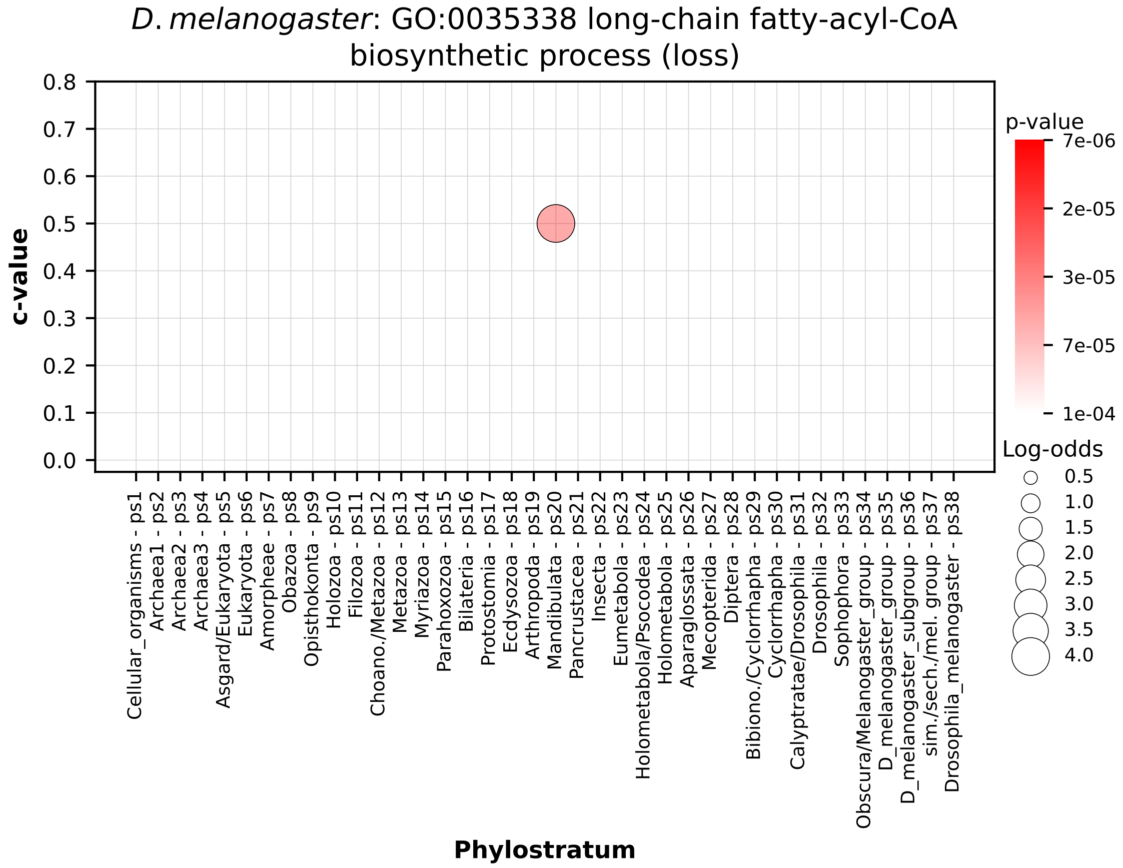
D. melanogaster: GO:0035338 long-chain fatty-acyl-CoA biosynthetic process (loss)
c-value
0.8
0.7
0.6
0.5
0.4
0.3
0.2
0.1
0.0
p-value
7e-06
2e-05
3e-05
7e-05
1e-04
Log-odds
0.5
1.0
1.5
2.0
2.5
3.0
3.5
4.0
Cellular_organisms - ps1
Archaea1 - ps2
Archaea2 - ps3
Archaea3 - ps4
Asgard/Eukaryota - ps5
Eukaryota - ps6
Amorpheae - ps7
Obazoa - ps8
Opisthokonta - ps9
Holozoa - ps10
Filozoa - ps11
Choano./Metazoa - ps12
Metazoa - ps13
Myriazoa - ps14
Parahoxozoa - ps15
Bilateria - ps16
Protostomia - ps17
Ecdysozoa - ps18
Arthropoda - ps19
Mandibulata - ps20
Pancrustacea - ps21
Insecta - ps22
Eumetabola - ps23
Holometabola/Psocodea - ps24
Holometabola - ps25
Aparaglossata - ps26
Mecopterida - ps27
Diptera - ps28
Bibiono./Cyclorrhapha - ps29
Cyclorrhapha - ps30
Calyptratae/Drosophila - ps31
Drosophila - ps32
Sophophora - ps33
Obscura/Melanogaster_group - ps34
D_melanogaster_group - ps35
D_melanogaster_subgroup - ps36
sim./sech./mel. group - ps37
Drosophila_melanogaster - ps38
Phylostratum

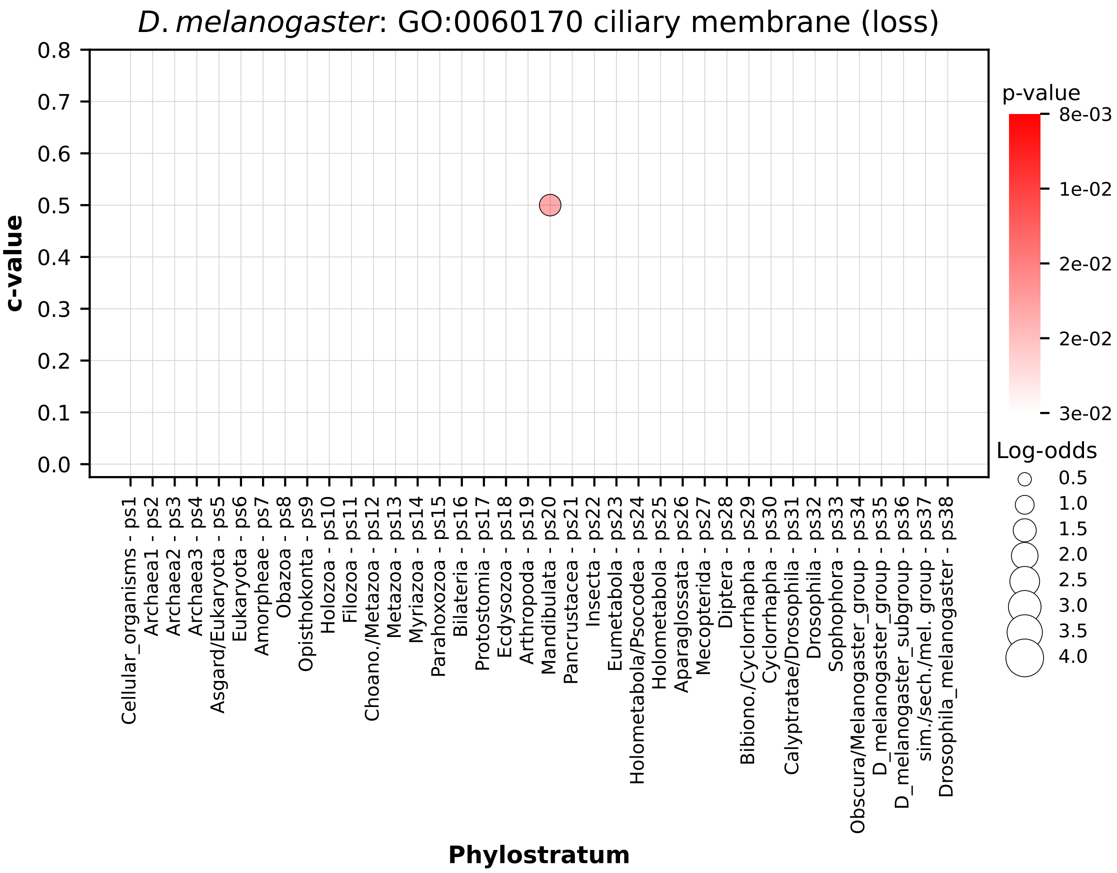

D. melanogaster: GO:0060170 ciliary membrane (loss)
c-value
0.0
0.1
0.2
0.3
0.4
0.5
0.6
0.7
0.8
Phylostratum
Cellular_organisms - ps1
Archaea1 - ps2
Archaea2 - ps3
Archaea3 - ps4
Asgard/Eukaryota - ps5
Eukaryota - ps6
Amorpheae - ps7
Obazoa - ps8
Opisthokonta - ps9
Holozoa - ps10
Filozoa - ps11
Choano./Metazoa - ps12
Metazoa - ps13
Myriazoa - ps14
Parahoxozoa - ps15
Bilateria - ps16
Protostomia - ps17
Ecdysozoa - ps18
Arthropoda - ps19
Mandibulata - ps20
Pancrustacea - ps21
Insecta - ps22
Eumetabola - ps23
Holometabola/Psocodea - ps24
Holometabola - ps25
Aparaglossata - ps26
Mecopterida - ps27
Diptera - ps28
Bibiono./Cyclorrhapha - ps29
Cyclorrhapha - ps30
Calyptratae/Drosophila - ps31
Drosophila - ps32
Sophophora - ps33
Obscura/Melanogaster_group - ps34
D_melanogaster_group - ps35
D_melanogaster_subgroup - ps36
sim./sech./mel. group - ps37
Drosophila_melanogaster - ps38
p-value
8e-03
1e-02
2e-02
2e-02
3e-02
Log-odds
0.5
1.0
1.5
2.0
2.5
3.0
3.5
4.0

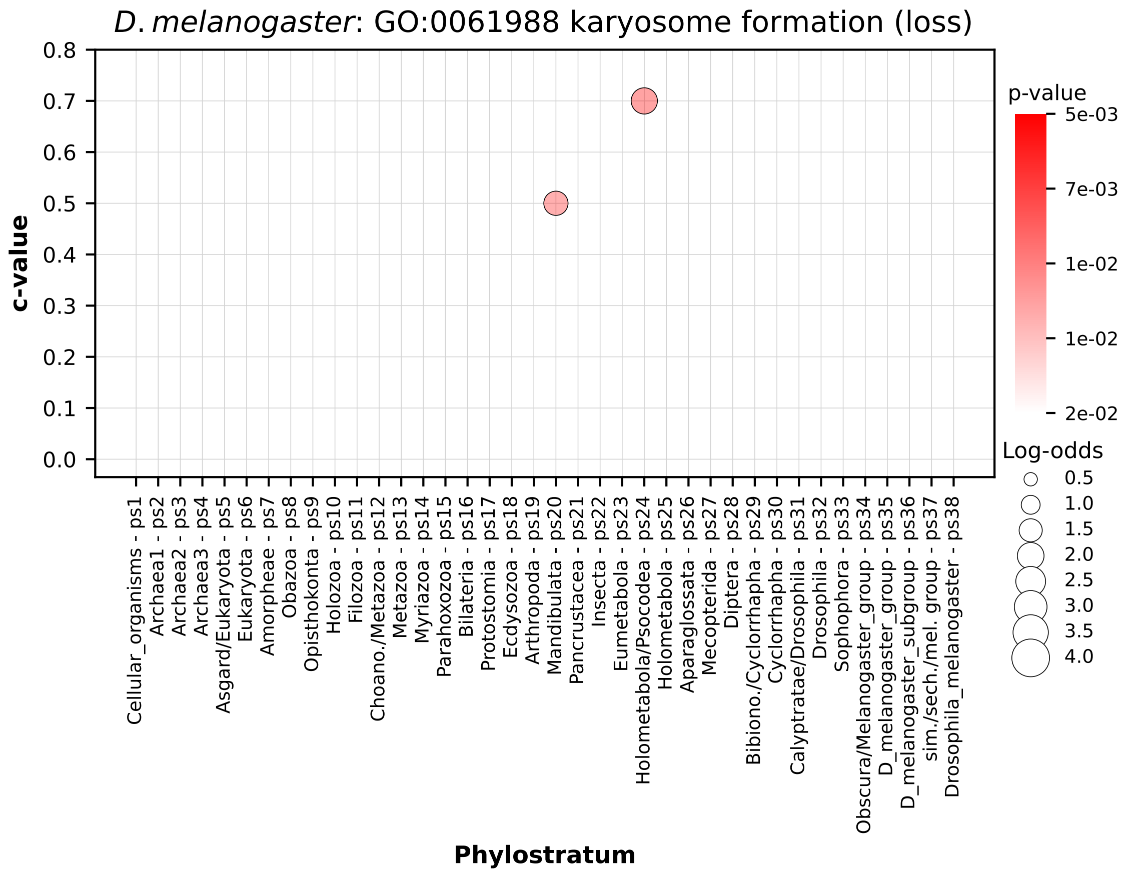

D. melanogaster: GO:0061988 karyosome formation (loss)
c-value
0.8
0.7
0.6
0.5
0.4
0.3
0.2
0.1
0.0
Phylostratum
Cellular_organisms - ps1
Archaea1 - ps2
Archaea2 - ps3
Archaea3 - ps4
Asgard/Eukaryota - ps5
Eukaryota - ps6
Amorpheae - ps7
Obazoa - ps8
Opisthokonta - ps9
Holozoa - ps10
Filozoa - ps11
Choano./Metazoa - ps12
Metazoa - ps13
Myriazoa - ps14
Parahoxozoa - ps15
Bilateria - ps16
Protostomia - ps17
Ecdysozoa - ps18
Arthropoda - ps19
Mandibulata - ps20
Pancrustacea - ps21
Insecta - ps22
Eumetabola - ps23
Holometabola/Psocodea - ps24
Holometabola - ps25
Aparaglossata - ps26
Mecopterida - ps27
Diptera - ps28
Bibiono./Cyclorrhapha - ps29
Cyclorrhapha - ps30
Calyptratae/Drosophila - ps31
Drosophila - ps32
Sophophora - ps33
Obscura/Melanogaster_group - ps34
D_melanogaster_group - ps35
D_melanogaster_subgroup - ps36
sim./sech./mel. group - ps37
Drosophila_melanogaster - ps38
p-value
5e-03
7e-03
1e-02
1e-02
2e-02
Log-odds
0.5
1.0
1.5
2.0
2.5
3.0
3.5
4.0

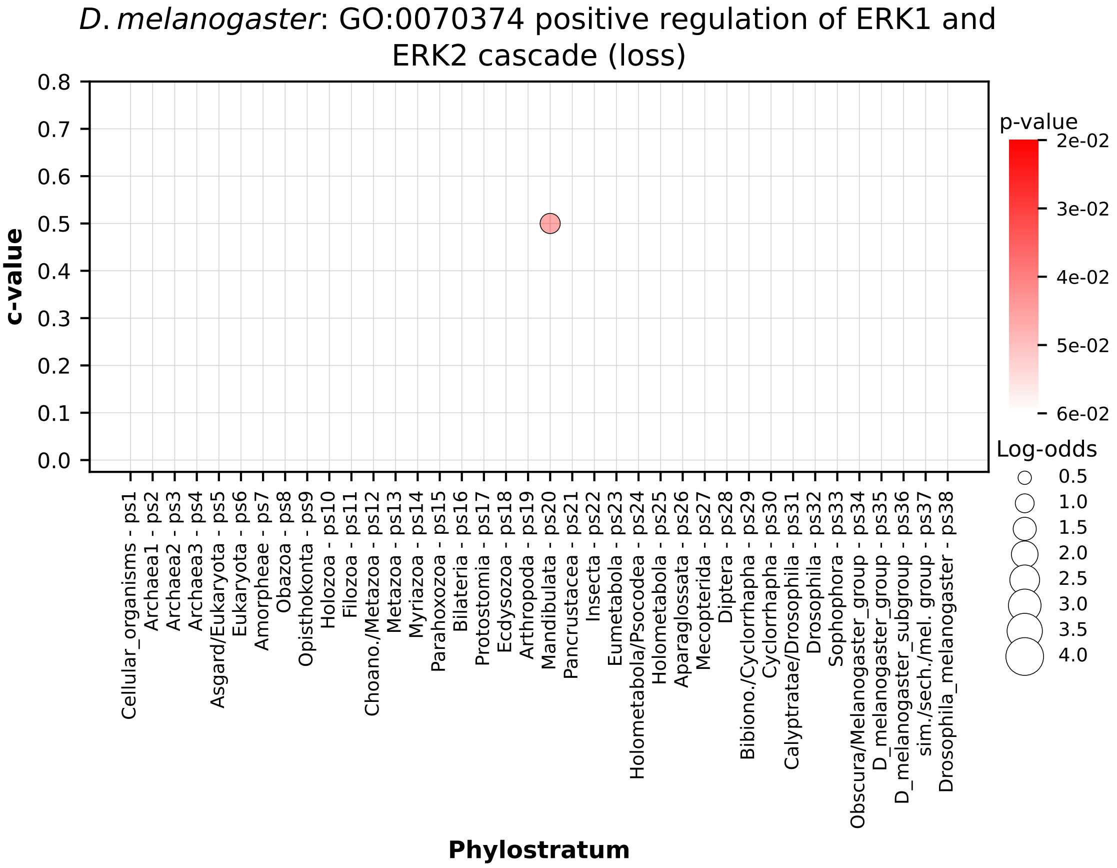

D. melanogaster: GO:0070374 positive regulation of ERK1 and ERK2 cascade (loss)
c-value
0.8
0.7
0.6
0.5
0.4
0.3
0.2
0.1
0.0
Cellular_organisms - ps1
Archaea1 - ps2
Archaea2 - ps3
Archaea3 - ps4
Asgard/Eukaryota - ps5
Eukaryota - ps6
Amorpheae - ps7
Obazoa - ps8
Opisthokonta - ps9
Holozoa - ps10
Filozoa - ps11
Choano./Metazoa - ps12
Metazoa - ps13
Myriazoa - ps14
Parahoxozoa - ps15
Bilateria - ps16
Protostomia - ps17
Ecdysozoa - ps18
Arthropoda - ps19
Mandibulata - ps20
Pancrustacea - ps21
Insecta - ps22
Eumetabola - ps23
Holometabola/Psocodea - ps24
Holometabola - ps25
Aparaglossata - ps26
Mecopterida - ps27
Diptera - ps28
Bibiono./Cyclorrhapha - ps29
Cyclorrhapha - ps30
Calyptratae/Drosophila - ps31
Drosophila - ps32
Sophophora - ps33
Obscura/Melanogaster_group - ps34
D_melanogaster_group - ps35
D_melanogaster_subgroup - ps36
sim./sech./mel. group - ps37
Drosophila_melanogaster - ps38
Phylostratum
p-value
2e-02
3e-02
4e-02
5e-02
6e-02
Log-odds
0.5
1.0
1.5
2.0
2.5
3.0
3.5
4.0

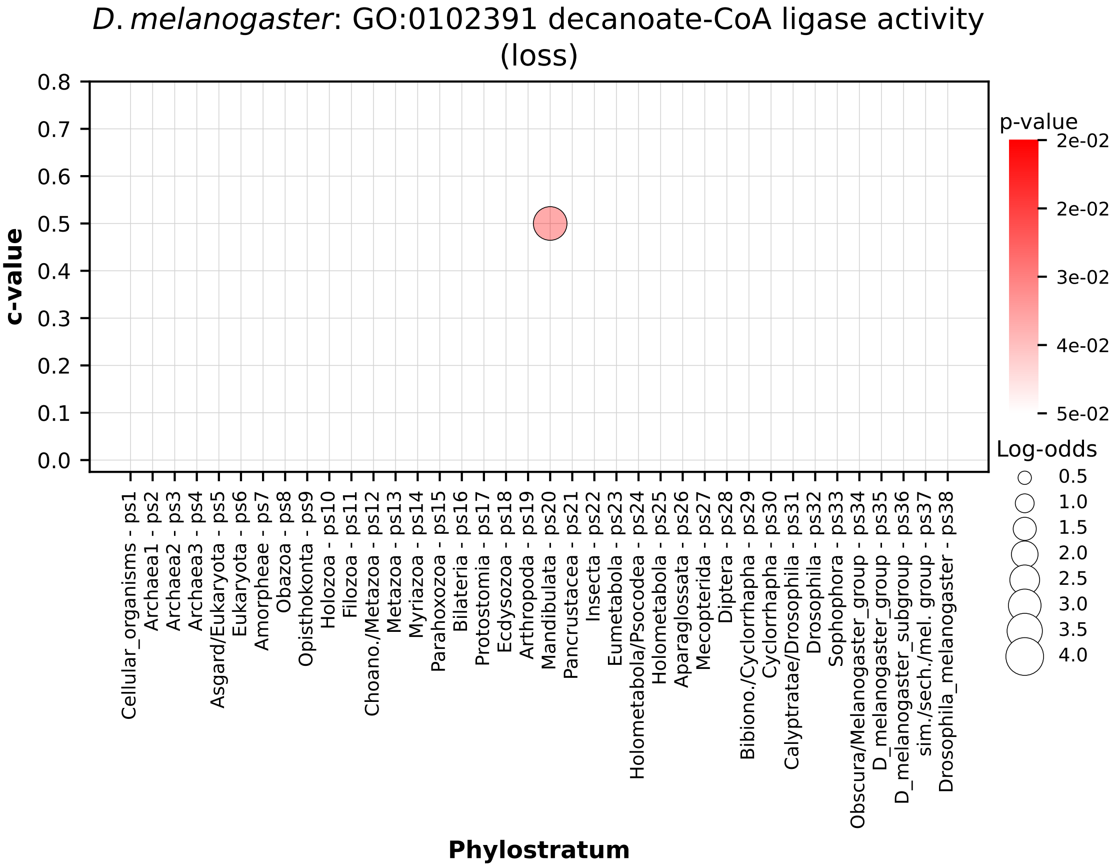

D. melanogaster: GO:0102391 decanoate-CoA ligase activity (loss)
c-value
0.8
0.7
0.6
0.5
0.4
0.3
0.2
0.1
0.0
p-value
2e-02
2e-02
3e-02
4e-02
5e-02
Log-odds
0.5
1.0
1.5
2.0
2.5
3.0
3.5
4.0
Cellular_organisms - ps1
Archaea1 - ps2
Archaea2 - ps3
Archaea3 - ps4
Asgard/Eukaryota - ps5
Eukaryota - ps6
Amorpheae - ps7
Obazoa - ps8
Opisthokonta - ps9
Holozoa - ps10
Filozoa - ps11
Choano./Metazoa - ps12
Metazoa - ps13
Myriazoa - ps14
Parahoxozoa - ps15
Bilateria - ps16
Protostomia - ps17
Ecdysozoa - ps18
Arthropoda - ps19
Mandibulata - ps20
Pancrustacea - ps21
Insecta - ps22
Eumetabola - ps23
Holometabola/Psocodea - ps24
Holometabola - ps25
Aparaglossata - ps26
Mecopterida - ps27
Diptera - ps28
Bibiono./Cyclorrhapha - ps29
Cyclorrhapha - ps30
Calyptratae/Drosophila - ps31
Drosophila - ps32
Sophophora - ps33
Obscura/Melanogaster_group - ps34
D_melanogaster_group - ps35
D_melanogaster_subgroup - ps36
sim./sech./mel. group - ps37
Drosophila_melanogaster - ps38
Phylostratum

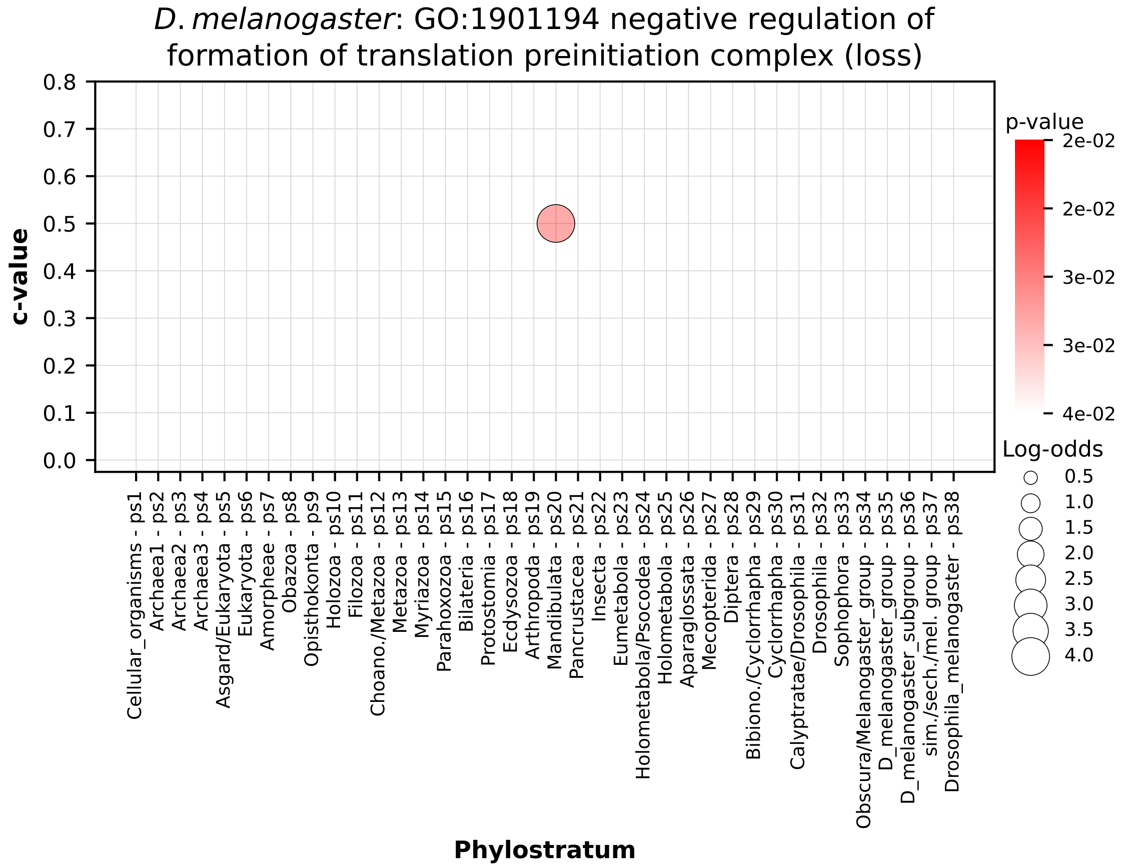
D. melanogaster: GO:1901194 negative regulation of formation of translation preinitiation complex (loss)
c-value
0.8
0.7
0.6
0.5
0.4
0.3
0.2
0.1
0.0
p-value
2e-02
2e-02
3e-02
3e-02
4e-02
Log-odds
0.5
1.0
1.5
2.0
2.5
3.0
3.5
4.0
Cellular_organisms - ps1
Archaea1 - ps2
Archaea2 - ps3
Archaea3 - ps4
Asgard/Eukaryota - ps5
Eukaryota - ps6
Amorpheae - ps7
Obazoa - ps8
Opisthokonta - ps9
Holozoa - ps10
Filozoa - ps11
Choano./Metazoa - ps12
Metazoa - ps13
Myriazoa - ps14
Parahoxozoa - ps15
Bilateria - ps16
Protostomia - ps17
Ecdysozoa - ps18
Arthropoda - ps19
Mandibulata - ps20
Pancrustacea - ps21
Insecta - ps22
Eumetabola - ps23
Holometabola/Psocodea - ps24
Holometabola - ps25
Aparaglossata - ps26
Mecopterida - ps27
Diptera - ps28
Bibiono./Cyclorrhapha - ps29
Cyclorrhapha - ps30
Calyptratae/Drosophila - ps31
Drosophila - ps32
Sophophora - ps33
Obscura/Melanogaster_group - ps34
D_melanogaster_group - ps35
D_melanogaster_subgroup - ps36
sim./sech./mel. group - ps37
Drosophila_melanogaster - ps38
Phylostratum

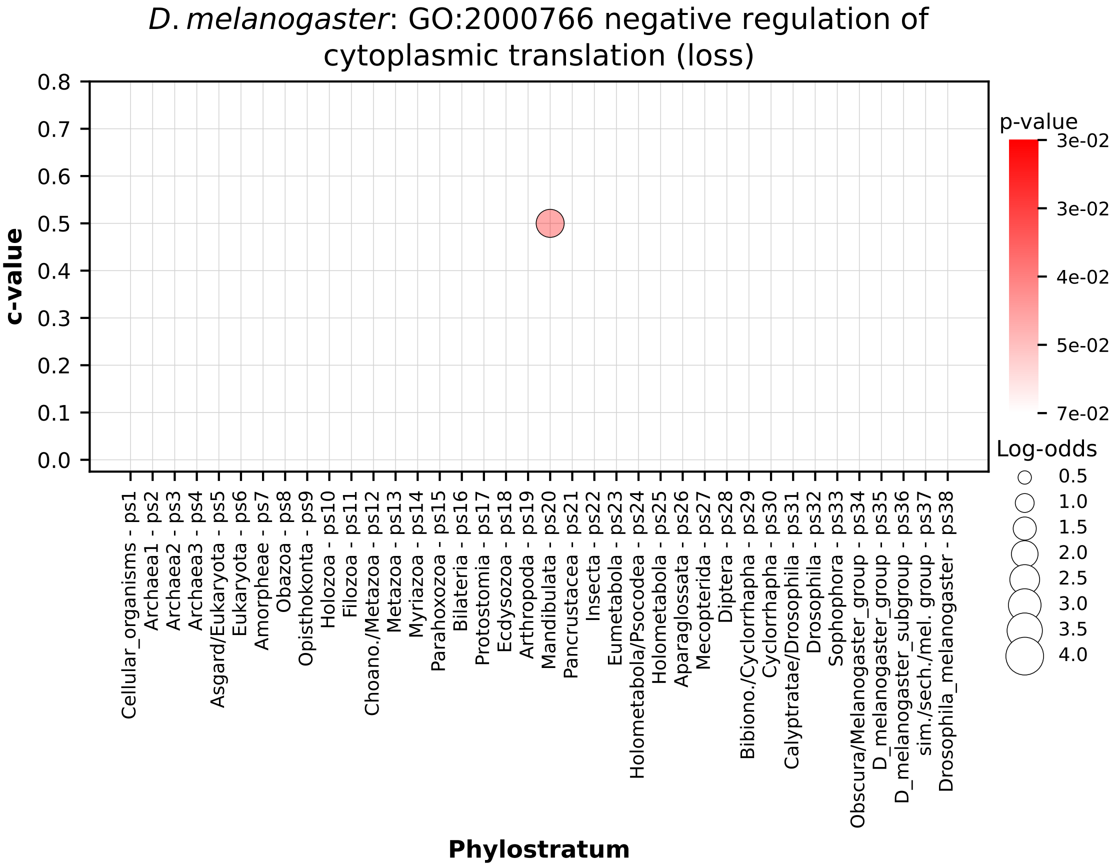

D. melanogaster: GO:2000766 negative regulation of cytoplasmic translation (loss)
c-value
0.8
0.7
0.6
0.5
0.4
0.3
0.2
0.1
0.0
Phylostratum
Cellular_organisms - ps1
Archaea1 - ps2
Archaea2 - ps3
Archaea3 - ps4
Asgard/Eukaryota - ps5
Eukaryota - ps6
Amorpheae - ps7
Obazoa - ps8
Opisthokonta - ps9
Holozoa - ps10
Filozoa - ps11
Choano./Metazoa - ps12
Metazoa - ps13
Myriazoa - ps14
Parahoxozoa - ps15
Bilateria - ps16
Protostomia - ps17
Ecdysozoa - ps18
Arthropoda - ps19
Mandibulata - ps20
Pancrustacea - ps21
Insecta - ps22
Eumetabola - ps23
Holometabola/Psocodea - ps24
Holometabola - ps25
Aparaglossata - ps26
Mecopterida - ps27
Diptera - ps28
Bibiono./Cyclorrhapha - ps29
Cyclorrhapha - ps30
Calyptratae/Drosophila - ps31
Drosophila - ps32
Sophophora - ps33
Obscura/Melanogaster_group - ps34
D_melanogaster_group - ps35
D_melanogaster_subgroup - ps36
sim./sech./mel. group - ps37
Drosophila_melanogaster - ps38
p-value
3e-02
3e-02
4e-02
5e-02
7e-02
Log-odds
0.5
1.0
1.5
2.0
2.5
3.0
3.5
4.0

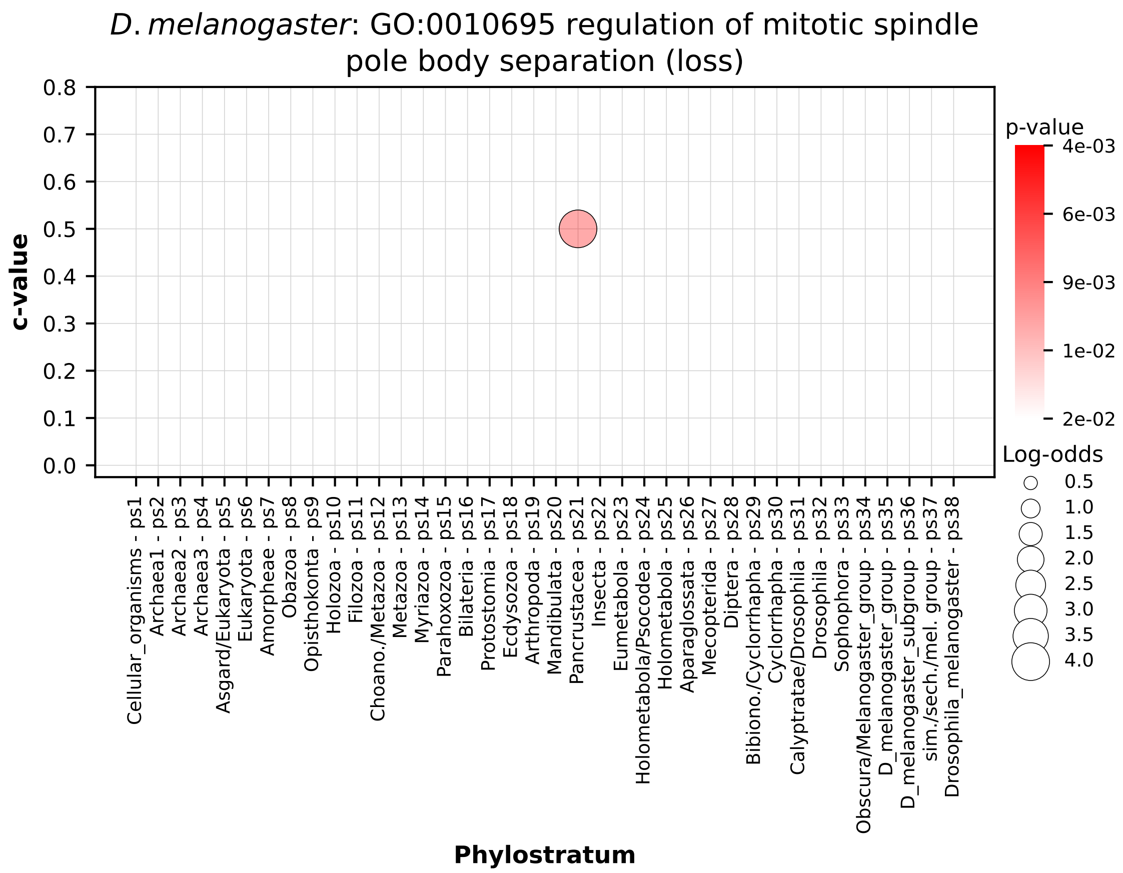

D. melanogaster: GO:0010695 regulation of mitotic spindle pole body separation (loss)
c-value
0.8
0.7
0.6
0.5
0.4
0.3
0.2
0.1
0.0
Phylostratum
Cellular_organisms - ps1
Archaea1 - ps2
Archaea2 - ps3
Archaea3 - ps4
Asgard/Eukaryota - ps5
Eukaryota - ps6
Amorpheae - ps7
Obazoa - ps8
Opisthokonta - ps9
Holozoa - ps10
Filozoa - ps11
Choano./Metazoa - ps12
Metazoa - ps13
Myriazoa - ps14
Parahoxozoa - ps15
Bilateria - ps16
Protostomia - ps17
Ecdysozoa - ps18
Arthropoda - ps19
Mandibulata - ps20
Pancrustacea - ps21
Insecta - ps22
Eumetabola - ps23
Holometabola/Psocodea - ps24
Holometabola - ps25
Aparaglossata - ps26
Mecopterida - ps27
Diptera - ps28
Bibiono./Cyclorrhapha - ps29
Cyclorrhapha - ps30
Calyptratae/Drosophila - ps31
Drosophila - ps32
Sophophora - ps33
Obscura/Melanogaster_group - ps34
D_melanogaster_group - ps35
D_melanogaster_subgroup - ps36
sim./sech./mel. group - ps37
Drosophila_melanogaster - ps38
p-value
4e-03
6e-03
9e-03
1e-02
2e-02
Log-odds
0.5
1.0
1.5
2.0
2.5
3.0
3.5
4.0

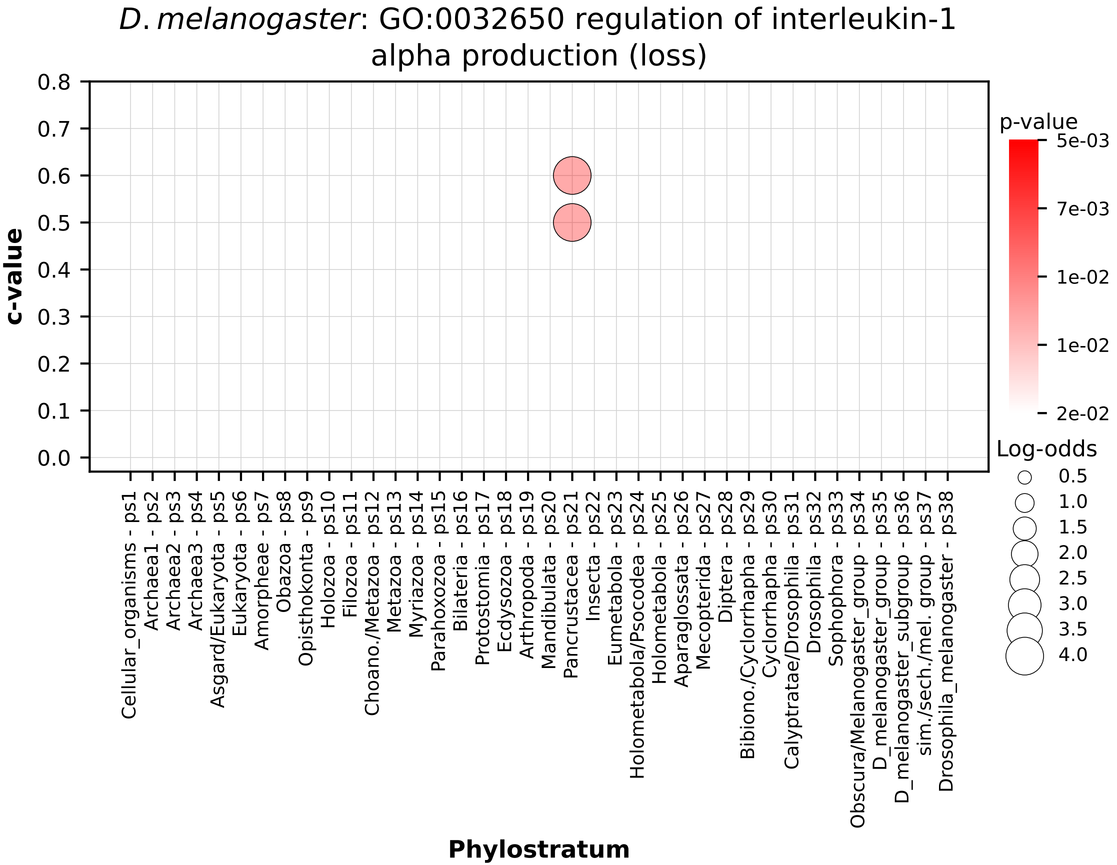

D. melanogaster: GO:0032650 regulation of interleukin-1 alpha production (loss)
c-value
0.8
0.7
0.6
0.5
0.4
0.3
0.2
0.1
0.0
Phylostratum
Cellular_organisms - ps1
Archaea1 - ps2
Archaea2 - ps3
Archaea3 - ps4
Asgard/Eukaryota - ps5
Eukaryota - ps6
Amorpheae - ps7
Obazoa - ps8
Opisthokonta - ps9
Holozoa - ps10
Filozoa - ps11
Choano./Metazoa - ps12
Metazoa - ps13
Myriazoa - ps14
Parahoxozoa - ps15
Bilateria - ps16
Protostomia - ps17
Ecdysozoa - ps18
Arthropoda - ps19
Mandibulata - ps20
Pancrustacea - ps21
Insecta - ps22
Eumetabola - ps23
Holometabola/Psocodea - ps24
Holometabola - ps25
Aparaglossata - ps26
Mecopterida - ps27
Diptera - ps28
Bibiono./Cyclorrhapha - ps29
Cyclorrhapha - ps30
Calyptratae/Drosophila - ps31
Drosophila - ps32
Sophophora - ps33
Obscura/Melanogaster_group - ps34
D_melanogaster_group - ps35
D_melanogaster_subgroup - ps36
sim./sech./mel. group - ps37
Drosophila_melanogaster - ps38
p-value
5e-03
7e-03
1e-02
1e-02
2e-02
Log-odds
0.5
1.0
1.5
2.0
2.5
3.0
3.5
4.0

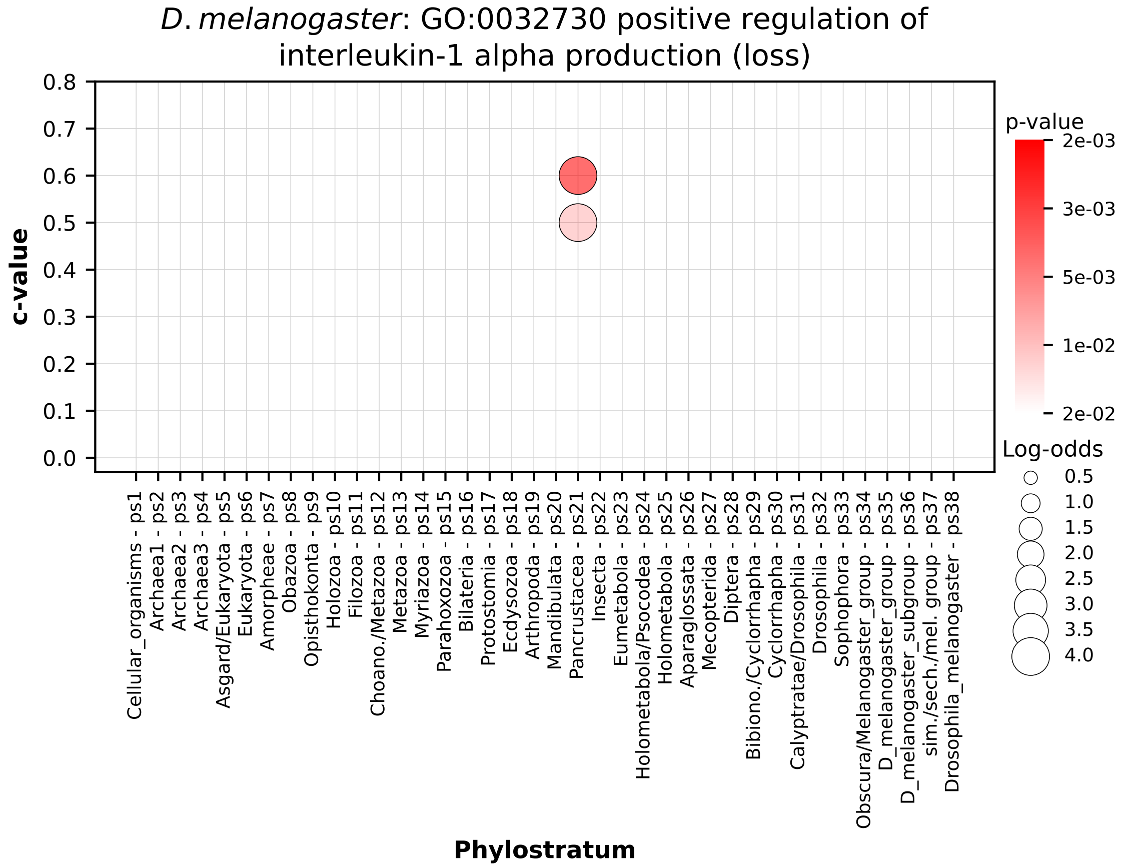

D. melanogaster: GO:0032730 positive regulation of interleukin-1 alpha production (loss)
c-value
0.8
0.7
0.6
0.5
0.4
0.3
0.2
0.1
0.0
Phylostratum
Cellular_organisms - ps1
Archaea1 - ps2
Archaea2 - ps3
Archaea3 - ps4
Asgard/Eukaryota - ps5
Eukaryota - ps6
Amorpheae - ps7
Obazoa - ps8
Opisthokonta - ps9
Holozoa - ps10
Filozoa - ps11
Choano./Metazoa - ps12
Metazoa - ps13
Myriazoa - ps14
Parahoxozoa - ps15
Bilateria - ps16
Protostomia - ps17
Ecdysozoa - ps18
Arthropoda - ps19
Mandibulata - ps20
Pancrustacea - ps21
Insecta - ps22
Eumetabola - ps23
Holometabola/Psocodea - ps24
Holometabola - ps25
Aparaglossata - ps26
Mecopterida - ps27
Diptera - ps28
Bibiono./Cyclorrhapha - ps29
Cyclorrhapha - ps30
Calyptratae/Drosophila - ps31
Drosophila - ps32
Sophophora - ps33
Obscura/Melanogaster_group - ps34
D_melanogaster_group - ps35
D_melanogaster_subgroup - ps36
sim./sech./mel. group - ps37
Drosophila_melanogaster - ps38
p-value
2e-03
3e-03
5e-03
1e-02
2e-02
Log-odds
0.5
1.0
1.5
2.0
2.5
3.0
3.5
4.0

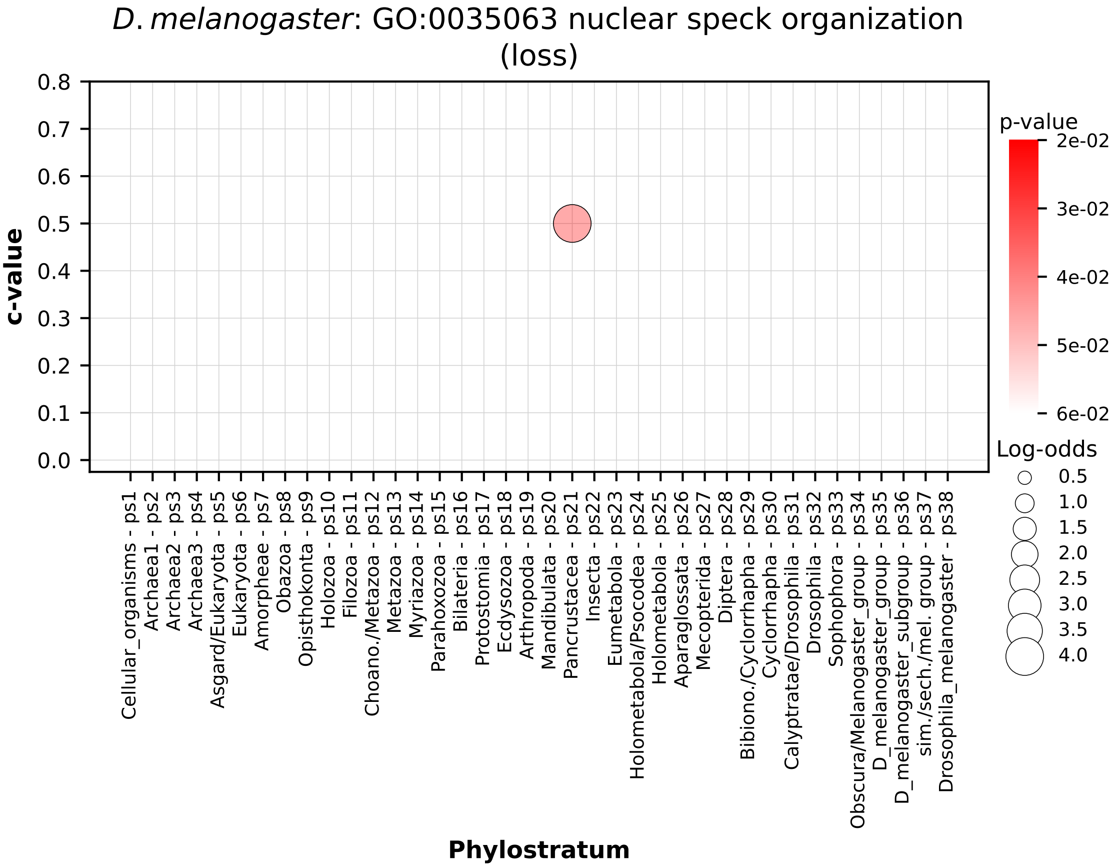
D. melanogaster: GO:0035063 nuclear speck organization (loss)
c-value
0.0
0.1
0.2
0.3
0.4
0.5
0.6
0.7
0.8
Phylostratum
Cellular_organisms - ps1
Archaea1 - ps2
Archaea2 - ps3
Archaea3 - ps4
Asgard/Eukaryota - ps5
Eukaryota - ps6
Amorpheae - ps7
Obazoa - ps8
Opisthokonta - ps9
Holozoa - ps10
Filozoa - ps11
Choano./Metazoa - ps12
Metazoa - ps13
Myriazoa - ps14
Parahoxozoa - ps15
Bilateria - ps16
Protostomia - ps17
Ecdysozoa - ps18
Arthropoda - ps19
Mandibulata - ps20
Pancrustacea - ps21
Insecta - ps22
Eumetabola - ps23
Holometabola/Psocodea - ps24
Holometabola - ps25
Aparaglossata - ps26
Mecopterida - ps27
Diptera - ps28
Bibiono./Cyclorrhapha - ps29
Cyclorrhapha - ps30
Calyptratae/Drosophila - ps31
Drosophila - ps32
Sophophora - ps33
Obscura/Melanogaster_group - ps34
D_melanogaster_group - ps35
D_melanogaster_subgroup - ps36
sim./sech./mel. group - ps37
Drosophila_melanogaster - ps38
p-value
2e-02
3e-02
4e-02
5e-02
6e-02
Log-odds
0.5
1.0
1.5
2.0
2.5
3.0
3.5
4.0

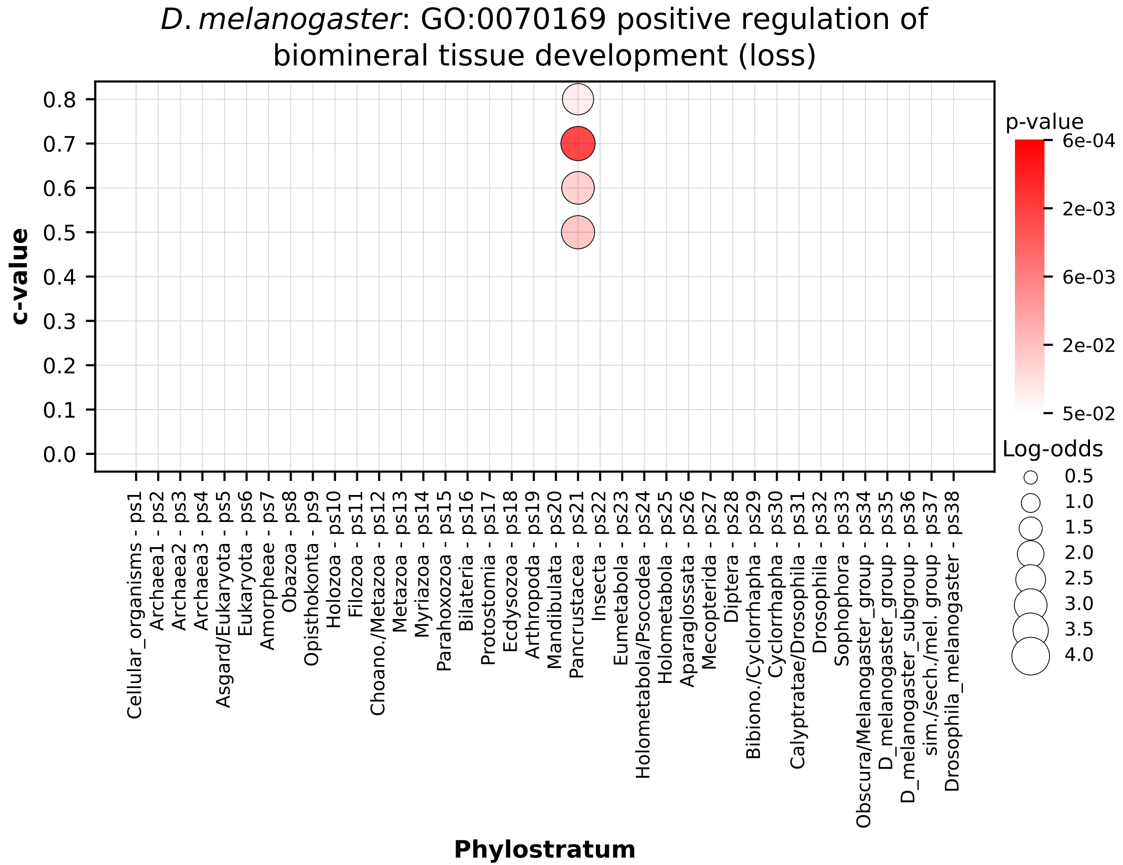

D. melanogaster: GO:0070169 positive regulation of biomineral tissue development (loss)
c-value
0.8
0.7
0.6
0.5
0.4
0.3
0.2
0.1
0.0
Phylostratum
Cellular_organisms - ps1
Archaea1 - ps2
Archaea2 - ps3
Archaea3 - ps4
Asgard/Eukaryota - ps5
Eukaryota - ps6
Amorpheae - ps7
Obazoa - ps8
Opisthokonta - ps9
Holozoa - ps10
Filozoa - ps11
Choano./Metazoa - ps12
Metazoa - ps13
Myriazoa - ps14
Parahoxozoa - ps15
Bilateria - ps16
Protostomia - ps17
Ecdysozoa - ps18
Arthropoda - ps19
Mandibulata - ps20
Pancrustacea - ps21
Insecta - ps22
Eumetabola - ps23
Holometabola/Psocodea - ps24
Holometabola - ps25
Aparaglossata - ps26
Mecopterida - ps27
Diptera - ps28
Bibiono./Cyclorrhapha - ps29
Cyclorrhapha - ps30
Calyptratae/Drosophila - ps31
Drosophila - ps32
Sophophora - ps33
Obscura/Melanogaster_group - ps34
D_melanogaster_group - ps35
D_melanogaster_subgroup - ps36
sim./sech./mel. group - ps37
Drosophila_melanogaster - ps38
p-value
6e-04
2e-03
6e-03
2e-02
5e-02
Log-odds
0.5
1.0
1.5
2.0
2.5
3.0
3.5
4.0

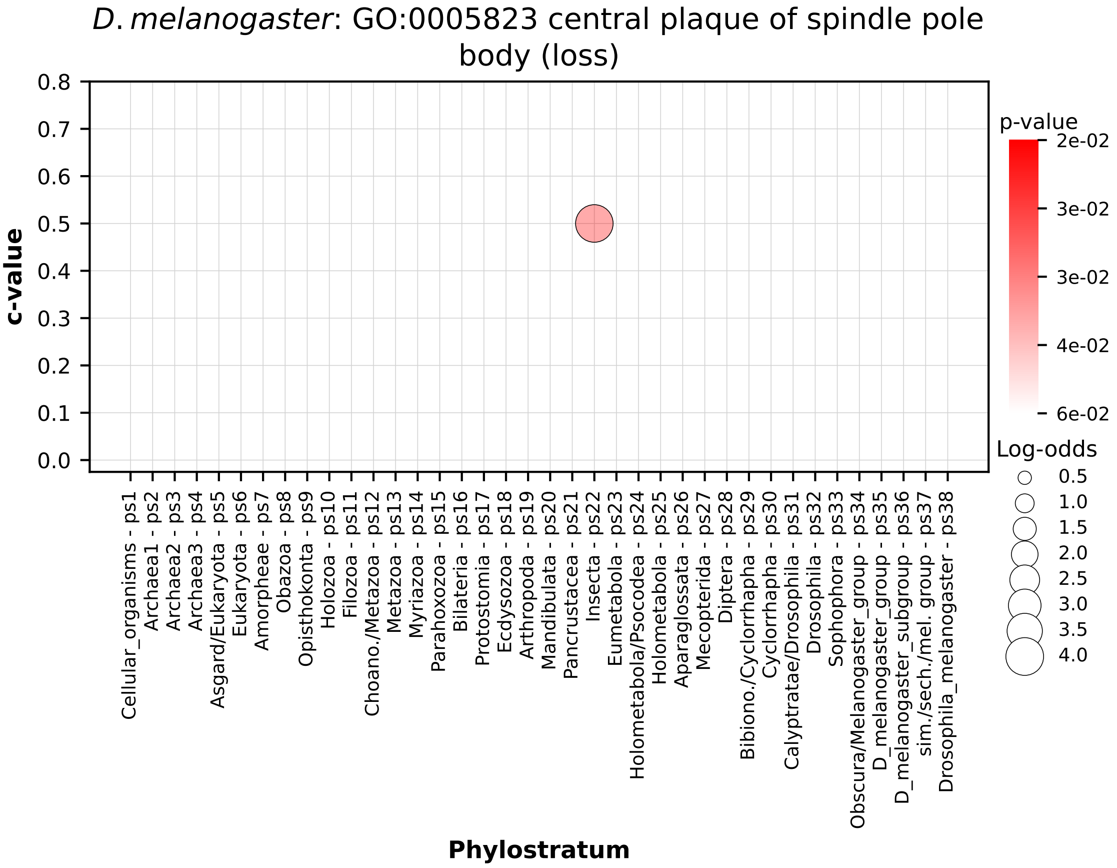

D. melanogaster: GO:0005823 central plaque of spindle pole body (loss)
c-value
0.8
0.7
0.6
0.5
0.4
0.3
0.2
0.1
0.0
p-value
2e-02
3e-02
3e-02
4e-02
6e-02
Log-odds
0.5
1.0
1.5
2.0
2.5
3.0
3.5
4.0
Cellular_organisms - ps1
Archaea1 - ps2
Archaea2 - ps3
Archaea3 - ps4
Asgard/Eukaryota - ps5
Eukaryota - ps6
Amorpheae - ps7
Obazoa - ps8
Opisthokonta - ps9
Holozoa - ps10
Filozoa - ps11
Choano./Metazoa - ps12
Metazoa - ps13
Myriazoa - ps14
Parahoxozoa - ps15
Bilateria - ps16
Protostomia - ps17
Ecdysozoa - ps18
Arthropoda - ps19
Mandibulata - ps20
Pancrustacea - ps21
Insecta - ps22
Eumetabola - ps23
Holometabola/Psocodea - ps24
Holometabola - ps25
Aparaglossata - ps26
Mecopterida - ps27
Diptera - ps28
Bibiono./Cyclorrhapha - ps29
Cyclorrhapha - ps30
Calyptratae/Drosophila - ps31
Drosophila - ps32
Sophophora - ps33
Obscura/Melanogaster_group - ps34
D_melanogaster_group - ps35
D_melanogaster_subgroup - ps36
sim./sech./mel. group - ps37
Drosophila_melanogaster - ps38
Phylostratum

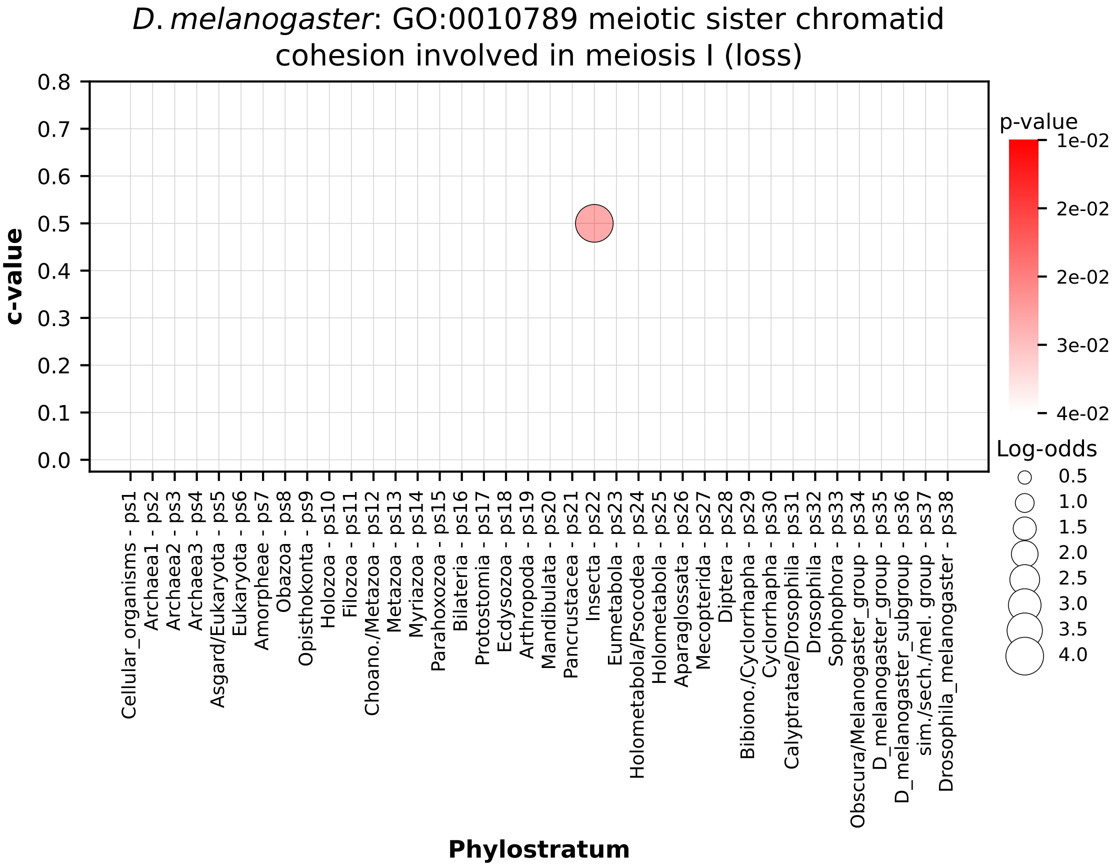

D. melanogaster: GO:0010789 meiotic sister chromatid cohesion involved in meiosis I (loss)
c-value
0.8
0.7
0.6
0.5
0.4
0.3
0.2
0.1
0.0
p-value
1e-02
2e-02
2e-02
3e-02
4e-02
Log-odds
0.5
1.0
1.5
2.0
2.5
3.0
3.5
4.0
Cellular_organisms - ps1
Archaea1 - ps2
Archaea2 - ps3
Archaea3 - ps4
Asgard/Eukaryota - ps5
Eukaryota - ps6
Amorpheae - ps7
Obazoa - ps8
Opisthokonta - ps9
Holozoa - ps10
Filozoa - ps11
Choano./Metazoa - ps12
Metazoa - ps13
Myriazoa - ps14
Parahoxozoa - ps15
Bilateria - ps16
Protostomia - ps17
Ecdysozoa - ps18
Arthropoda - ps19
Mandibulata - ps20
Pancrustacea - ps21
Insecta - ps22
Eumetabola - ps23
Holometabola/Psocodea - ps24
Holometabola - ps25
Aparaglossata - ps26
Mecopterida - ps27
Diptera - ps28
Bibiono./Cyclorrhapha - ps29
Cyclorrhapha - ps30
Calyptratae/Drosophila - ps31
Drosophila - ps32
Sophophora - ps33
Obscura/Melanogaster_group - ps34
D_melanogaster_group - ps35
D_melanogaster_subgroup - ps36
sim./sech./mel. group - ps37
Drosophila_melanogaster - ps38
Phylostratum

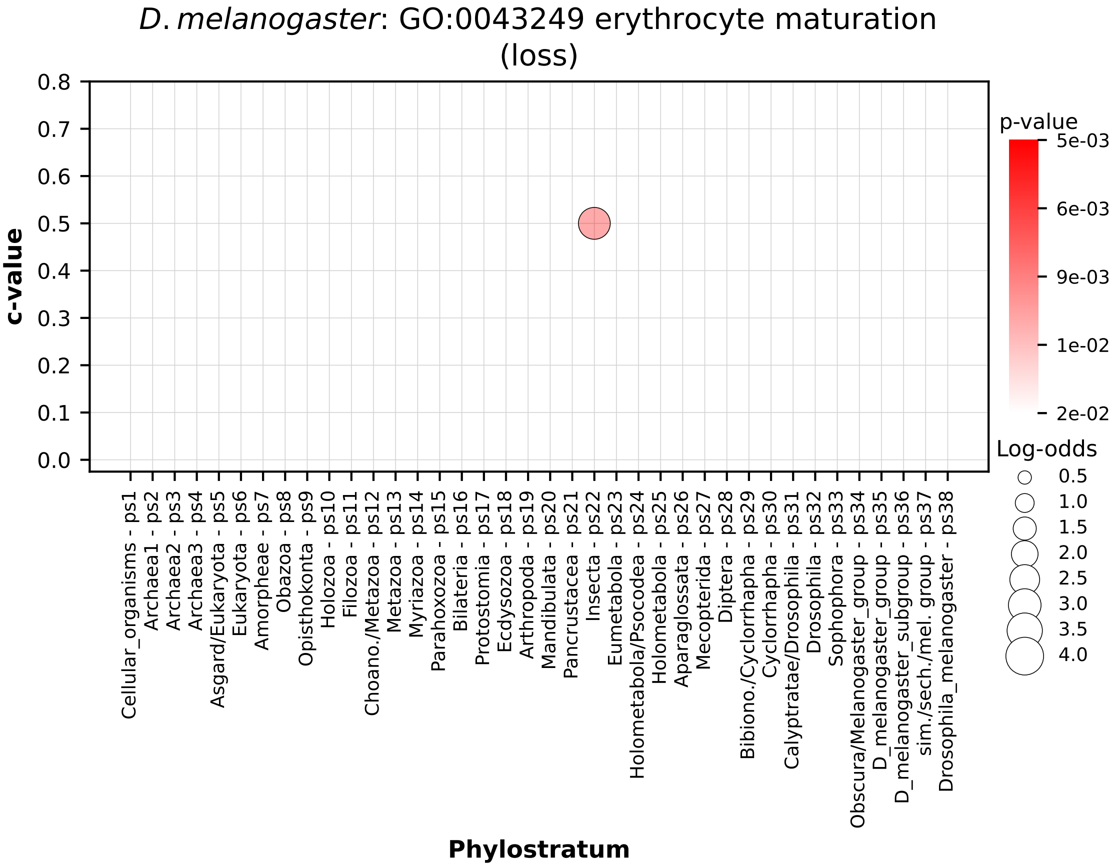

D. melanogaster: GO:0043249 erythrocyte maturation
(loss)
c-value
0.0
0.1
0.2
0.3
0.4
0.5
0.6
0.7
0.8
p-value
5e-03
6e-03
9e-03
1e-02
2e-02
Log-odds
0.5
1.0
1.5
2.0
2.5
3.0
3.5
4.0
Cellular_organisms - ps1
Archaea1 - ps2
Archaea2 - ps3
Archaea3 - ps4
Asgard/Eukaryota - ps5
Eukaryota - ps6
Amorpheae - ps7
Obazoa - ps8
Opisthokonta - ps9
Holozoa - ps10
Filozoa - ps11
Choano./Metazoa - ps12
Metazoa - ps13
Myriazoa - ps14
Parahoxozoa - ps15
Bilateria - ps16
Protostomia - ps17
Ecdysozoa - ps18
Arthropoda - ps19
Mandibulata - ps20
Pancrustacea - ps21
Insecta - ps22
Eumetabola - ps23
Holometabola/Psocodea - ps24
Holometabola - ps25
Aparaglossata - ps26
Mecopterida - ps27
Diptera - ps28
Bibiono./Cyclorrhapha - ps29
Cyclorrhapha - ps30
Calyptratae/Drosophila - ps31
Drosophila - ps32
Sophophora - ps33
Obscura/Melanogaster_group - ps34
D_melanogaster_group - ps35
D_melanogaster_subgroup - ps36
sim./sech./mel. group - ps37
Drosophila_melanogaster - ps38
Phylostratum

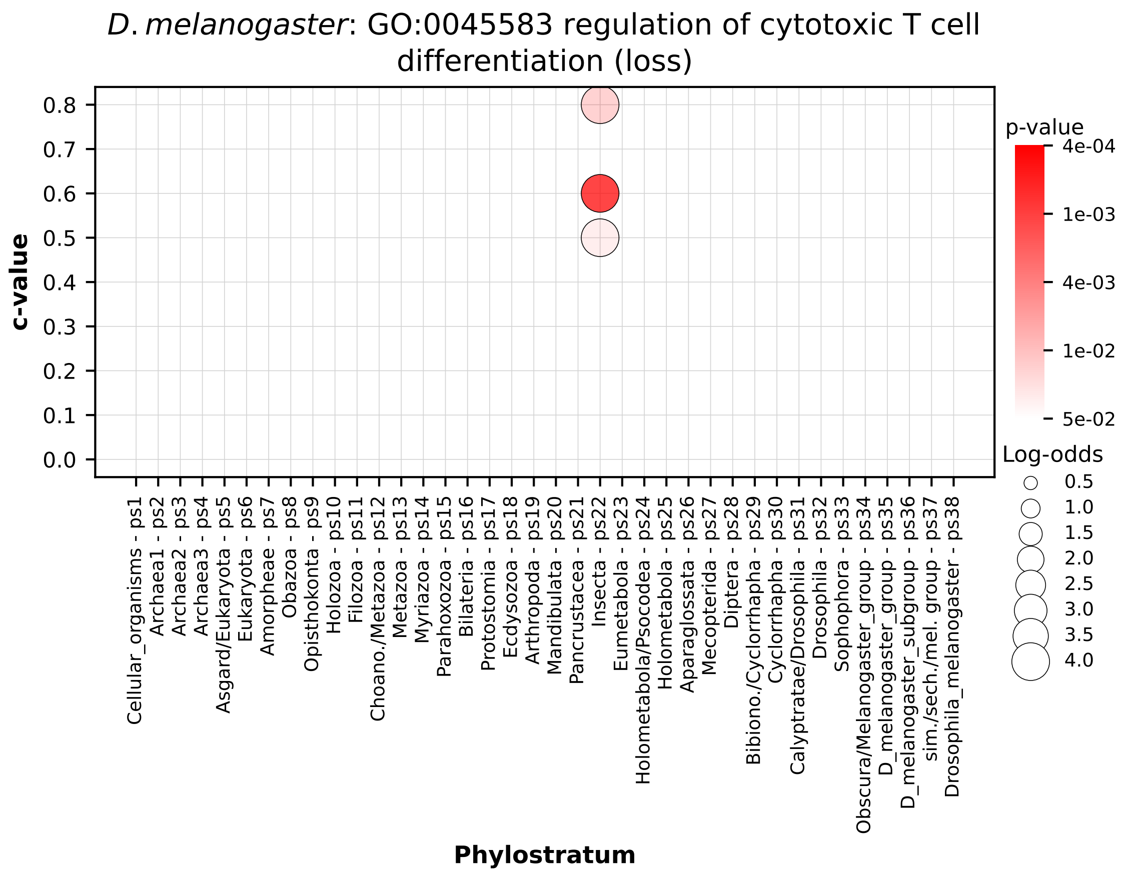

D. melanogaster: GO:0045583 regulation of cytotoxic T cell differentiation (loss)
c-value
0.0
0.1
0.2
0.3
0.4
0.5
0.6
0.7
0.8
Phylostratum
Cellular_organisms - ps1
Archaea1 - ps2
Archaea2 - ps3
Archaea3 - ps4
Asgard/Eukaryota - ps5
Eukaryota - ps6
Amorpheae - ps7
Obazoa - ps8
Opisthokonta - ps9
Holozoa - ps10
Filozoa - ps11
Choano./Metazoa - ps12
Metazoa - ps13
Myriazoa - ps14
Parahoxozoa - ps15
Bilateria - ps16
Protostomia - ps17
Ecdysozoa - ps18
Arthropoda - ps19
Mandibulata - ps20
Pancrustacea - ps21
Insecta - ps22
Eumetabola - ps23
Holometabola/Psocodea - ps24
Holometabola - ps25
Aparaglossata - ps26
Mecopterida - ps27
Diptera - ps28
Bibiono./Cyclorrhapha - ps29
Cyclorrhapha - ps30
Calyptratae/Drosophila - ps31
Drosophila - ps32
Sophophora - ps33
Obscura/Melanogaster_group - ps34
D_melanogaster_group - ps35
D_melanogaster_subgroup - ps36
sim./sech./mel. group - ps37
Drosophila_melanogaster - ps38
p-value
4e-04
1e-03
4e-03
1e-02
5e-02
Log-odds
0.5
1.0
1.5
2.0
2.5
3.0
3.5
4.0

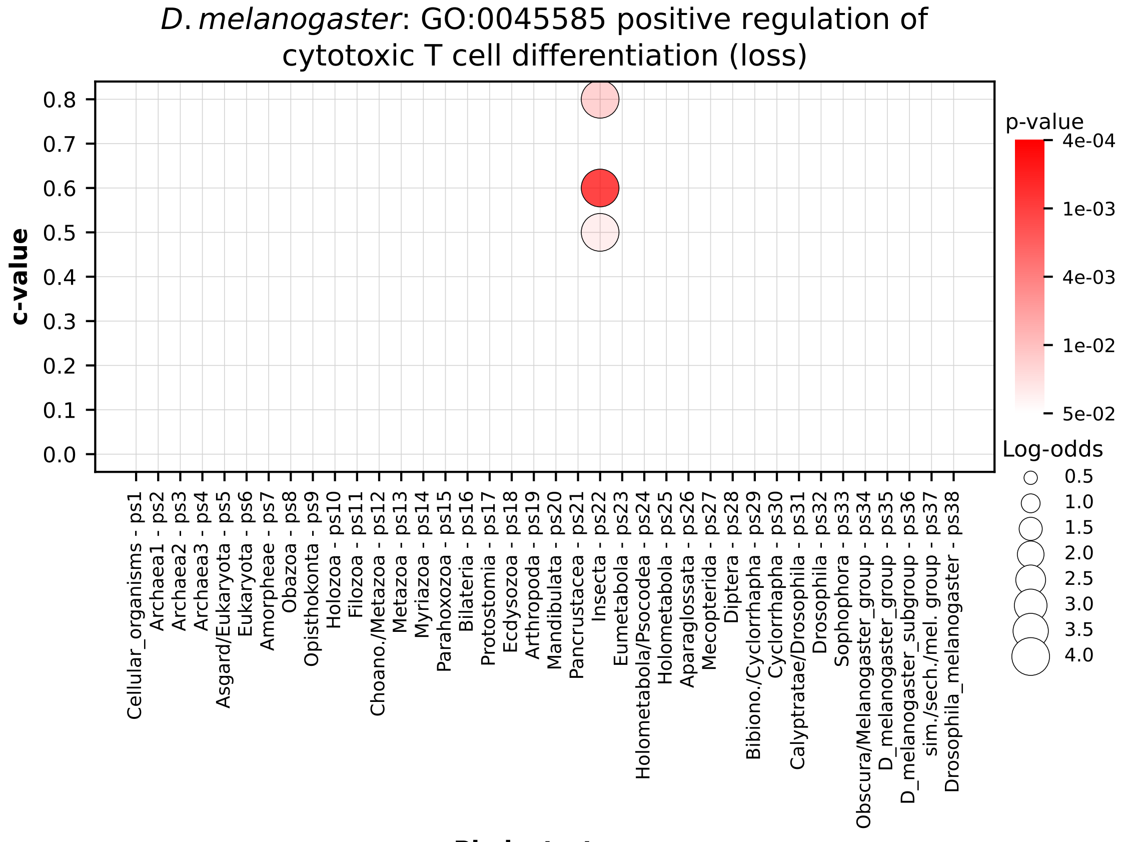

D. melanogaster: GO:0045585 positive regulation of cytotoxic T cell differentiation (loss)
c-value
0.8
0.7
0.6
0.5
0.4
0.3
0.2
0.1
0.0
Phylostratum
Cellular_organisms - ps1
Archaea1 - ps2
Archaea2 - ps3
Archaea3 - ps4
Asgard/Eukaryota - ps5
Eukaryota - ps6
Amorpheae - ps7
Obazoa - ps8
Opisthokonta - ps9
Holozoa - ps10
Filozoa - ps11
Choano./Metazoa - ps12
Metazoa - ps13
Myriazoa - ps14
Parahoxozoa - ps15
Bilateria - ps16
Protostomia - ps17
Ecdysozoa - ps18
Arthropoda - ps19
Mandibulata - ps20
Pancrustacea - ps21
Insecta - ps22
Eumetabola - ps23
Holometabola/Psocodea - ps24
Holometabola - ps25
Aparaglossata - ps26
Mecopterida - ps27
Diptera - ps28
Bibiono./Cyclorrhapha - ps29
Cyclorrhapha - ps30
Calyptratae/Drosophila - ps31
Drosophila - ps32
Sophophora - ps33
Obscura/Melanogaster_group - ps34
D_melanogaster_group - ps35
D_melanogaster_subgroup - ps36
sim./sech./mel. group - ps37
Drosophila_melanogaster - ps38
p-value
4e-04
1e-03
4e-03
1e-02
5e-02
Log-odds
0.5
1.0
1.5
2.0
2.5
3.0
3.5
4.0

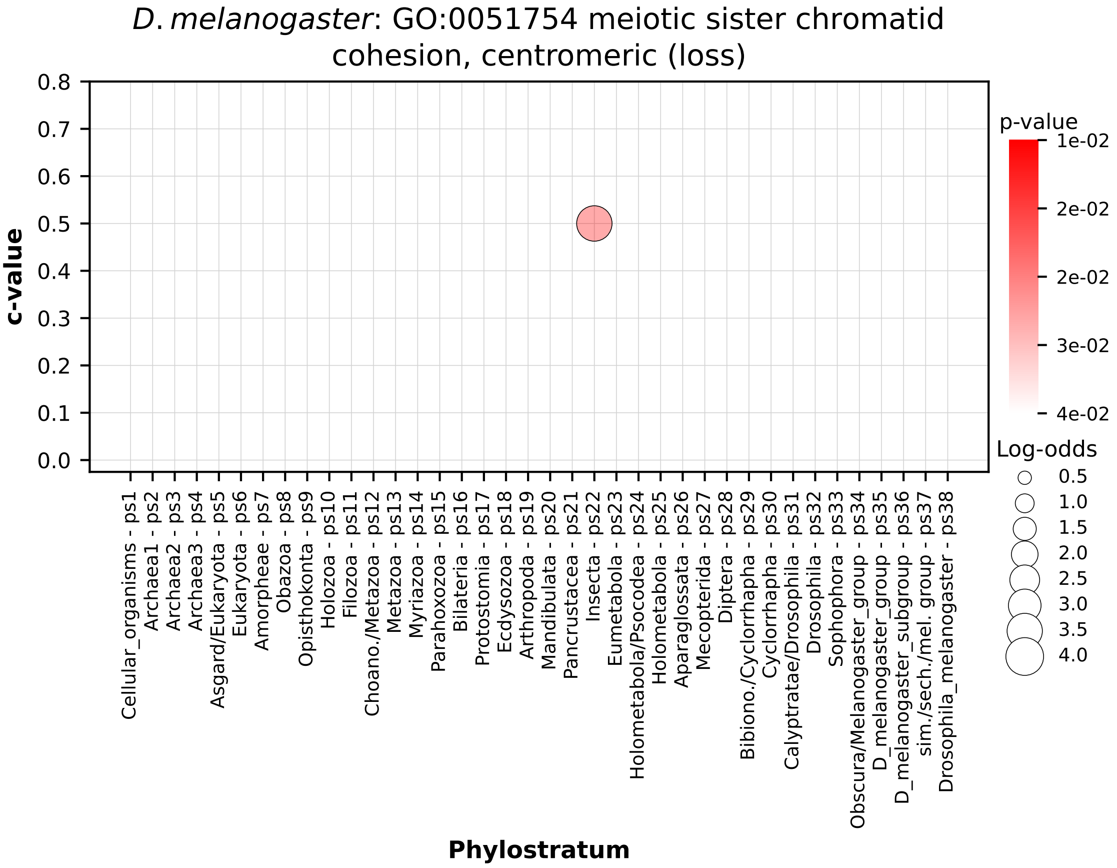

D. melanogaster: GO:0051754 meiotic sister chromatid cohesion, centromeric (loss)
c-value
0.8
0.7
0.6
0.5
0.4
0.3
0.2
0.1
0.0
p-value
1e-02
2e-02
2e-02
3e-02
4e-02
Log-odds
0.5
1.0
1.5
2.0
2.5
3.0
3.5
4.0
Cellular_organisms - ps1
Archaea1 - ps2
Archaea2 - ps3
Archaea3 - ps4
Asgard/Eukaryota - ps5
Eukaryota - ps6
Amorpheae - ps7
Obazoa - ps8
Opisthokonta - ps9
Holozoa - ps10
Filozoa - ps11
Choano./Metazoa - ps12
Metazoa - ps13
Myriazoa - ps14
Parahoxozoa - ps15
Bilateria - ps16
Protostomia - ps17
Ecdysozoa - ps18
Arthropoda - ps19
Mandibulata - ps20
Pancrustacea - ps21
Insecta - ps22
Eumetabola - ps23
Holometabola/Psocodea - ps24
Holometabola - ps25
Aparaglossata - ps26
Mecopterida - ps27
Diptera - ps28
Bibiono./Cyclorrhapha - ps29
Cyclorrhapha - ps30
Calyptratae/Drosophila - ps31
Drosophila - ps32
Sophophora - ps33
Obscura/Melanogaster_group - ps34
D_melanogaster_group - ps35
D_melanogaster_subgroup - ps36
sim./sech./mel. group - ps37
Drosophila_melanogaster - ps38
Phylostratum

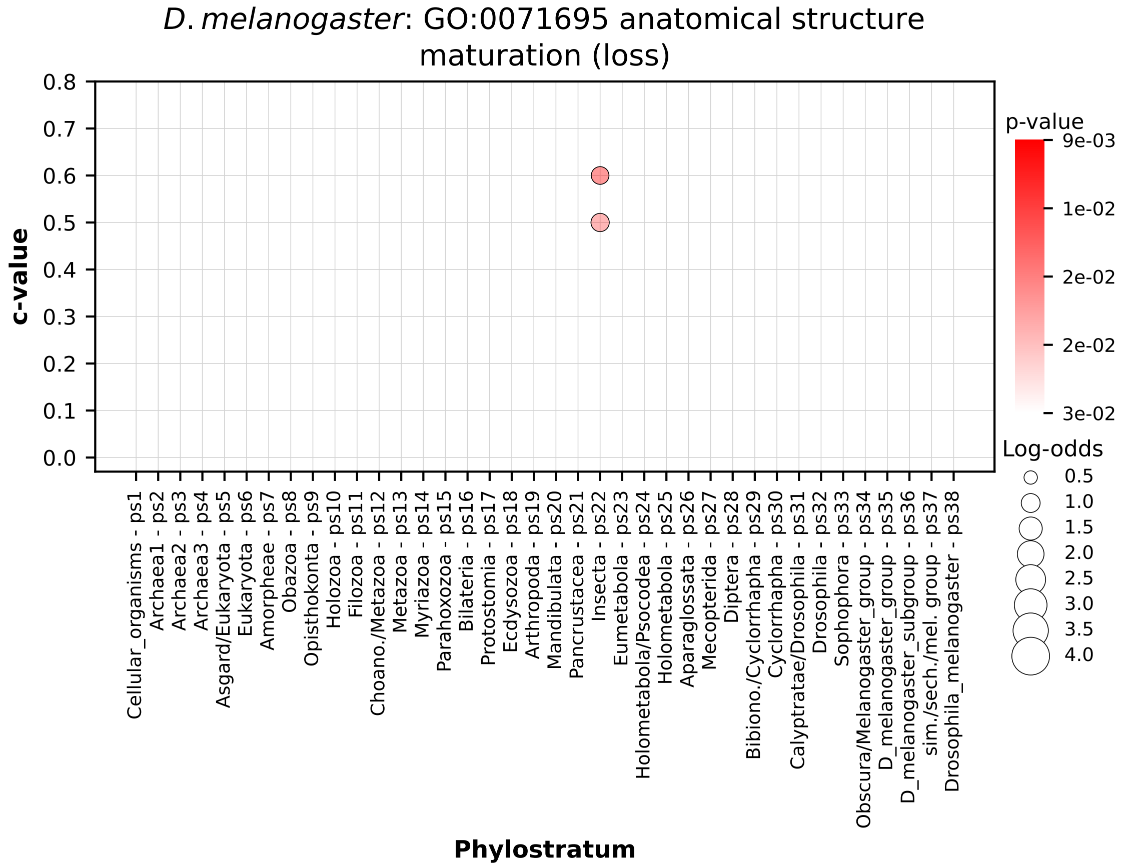

D. melanogaster: GO:0071695 anatomical structure maturation (loss)
c-value
0.8
0.7
0.6
0.5
0.4
0.3
0.2
0.1
0.0
Phylostratum
Cellular_organisms - ps1
Archaea1 - ps2
Archaea2 - ps3
Archaea3 - ps4
Asgard/Eukaryota - ps5
Eukaryota - ps6
Amorpheae - ps7
Obazoa - ps8
Opisthokonta - ps9
Holozoa - ps10
Filozoa - ps11
Choano./Metazoa - ps12
Metazoa - ps13
Myriazoa - ps14
Parahoxozoa - ps15
Bilateria - ps16
Protostomia - ps17
Ecdysozoa - ps18
Arthropoda - ps19
Mandibulata - ps20
Pancrustacea - ps21
Insecta - ps22
Eumetabola - ps23
Holometabola/Psocodea - ps24
Holometabola - ps25
Aparaglossata - ps26
Mecopterida - ps27
Diptera - ps28
Bibiono./Cyclorrhapha - ps29
Cyclorrhapha - ps30
Calyptratae/Drosophila - ps31
Drosophila - ps32
Sophophora - ps33
Obscura/Melanogaster_group - ps34
D_melanogaster_group - ps35
D_melanogaster_subgroup - ps36
sim./sech./mel. group - ps37
Drosophila_melanogaster - ps38
p-value
9e-03
1e-02
2e-02
2e-02
3e-02
Log-odds
0.5
1.0
1.5
2.0
2.5
3.0
3.5
4.0

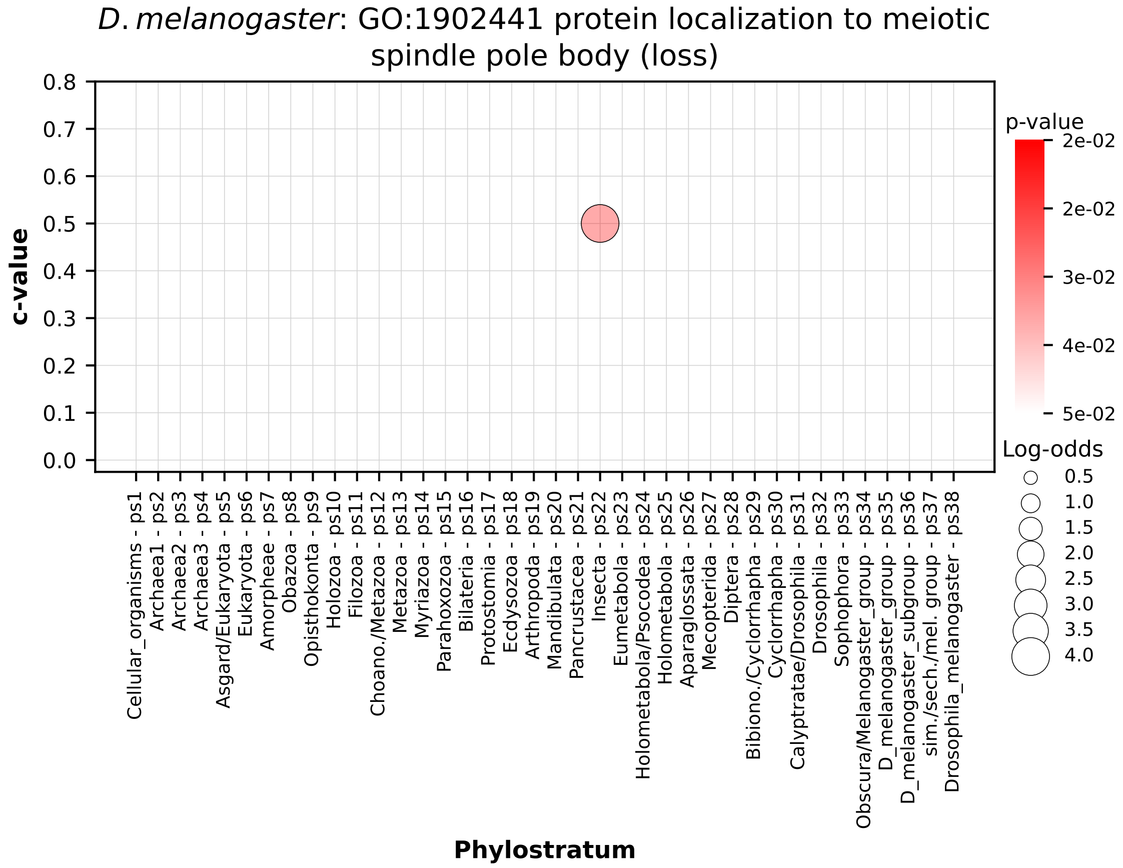

D. melanogaster: GO:1902441 protein localization to meiotic spindle pole body (loss)
c-value
0.8
0.7
0.6
0.5
0.4
0.3
0.2
0.1
0.0
Phylostratum
Cellular_organisms - ps1
Archaea1 - ps2
Archaea2 - ps3
Archaea3 - ps4
Asgard/Eukaryota - ps5
Eukaryota - ps6
Amorpheae - ps7
Obazoa - ps8
Opisthokonta - ps9
Holozoa - ps10
Filozoa - ps11
Choano./Metazoa - ps12
Metazoa - ps13
Myriazoa - ps14
Parahoxozoa - ps15
Bilateria - ps16
Protostomia - ps17
Ecdysozoa - ps18
Arthropoda - ps19
Mandibulata - ps20
Pancrustacea - ps21
Insecta - ps22
Eumetabola - ps23
Holometabola/Psocodea - ps24
Holometabola - ps25
Aparaglossata - ps26
Mecopterida - ps27
Diptera - ps28
Bibiono./Cyclorrhapha - ps29
Cyclorrhapha - ps30
Calyptratae/Drosophila - ps31
Drosophila - ps32
Sophophora - ps33
Obscura/Melanogaster_group - ps34
D_melanogaster_group - ps35
D_melanogaster_subgroup - ps36
sim./sech./mel. group - ps37
Drosophila_melanogaster - ps38
p-value
2e-02
2e-02
3e-02
4e-02
5e-02
Log-odds
0.5
1.0
1.5
2.0
2.5
3.0
3.5
4.0

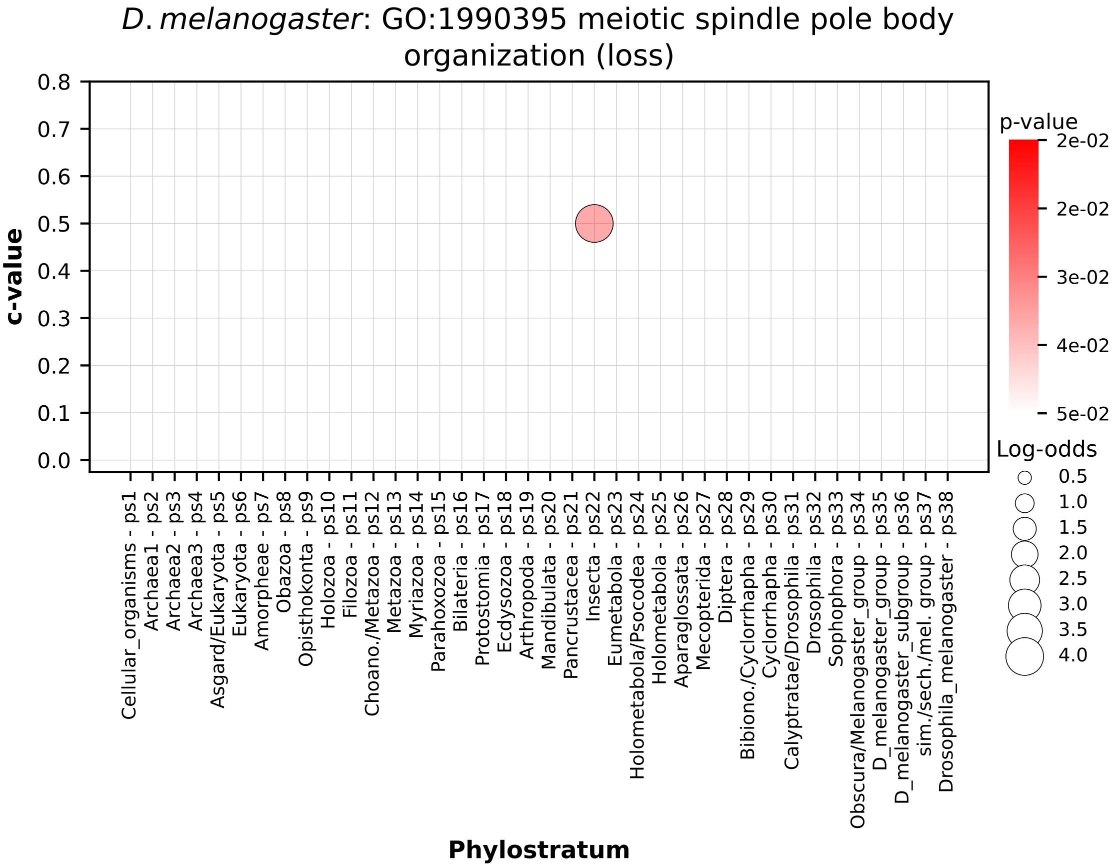

D. melanogaster: GO:1990395 meiotic spindle pole body organization (loss)
c-value
0.8
0.7
0.6
0.5
0.4
0.3
0.2
0.1
0.0
Phylostratum
Cellular_organisms - ps1
Archaea1 - ps2
Archaea2 - ps3
Archaea3 - ps4
Asgard/Eukaryota - ps5
Eukaryota - ps6
Amorpheae - ps7
Obazoa - ps8
Opisthokonta - ps9
Holozoa - ps10
Filozoa - ps11
Choano./Metazoa - ps12
Metazoa - ps13
Myriazoa - ps14
Parahoxozoa - ps15
Bilateria - ps16
Protostomia - ps17
Ecdysozoa - ps18
Arthropoda - ps19
Mandibulata - ps20
Pancrustacea - ps21
Insecta - ps22
Eumetabola - ps23
Holometabola/Psocodea - ps24
Holometabola - ps25
Aparaglossata - ps26
Mecopterida - ps27
Diptera - ps28
Bibiono./Cyclorrhapha - ps29
Cyclorrhapha - ps30
Calyptratae/Drosophila - ps31
Drosophila - ps32
Sophophora - ps33
Obscura/Melanogaster_group - ps34
D_melanogaster_group - ps35
D_melanogaster_subgroup - ps36
sim./sech./mel. group - ps37
Drosophila_melanogaster - ps38
p-value
2e-02
2e-02
3e-02
4e-02
5e-02
Log-odds
0.5
1.0
1.5
2.0
2.5
3.0
3.5
4.0

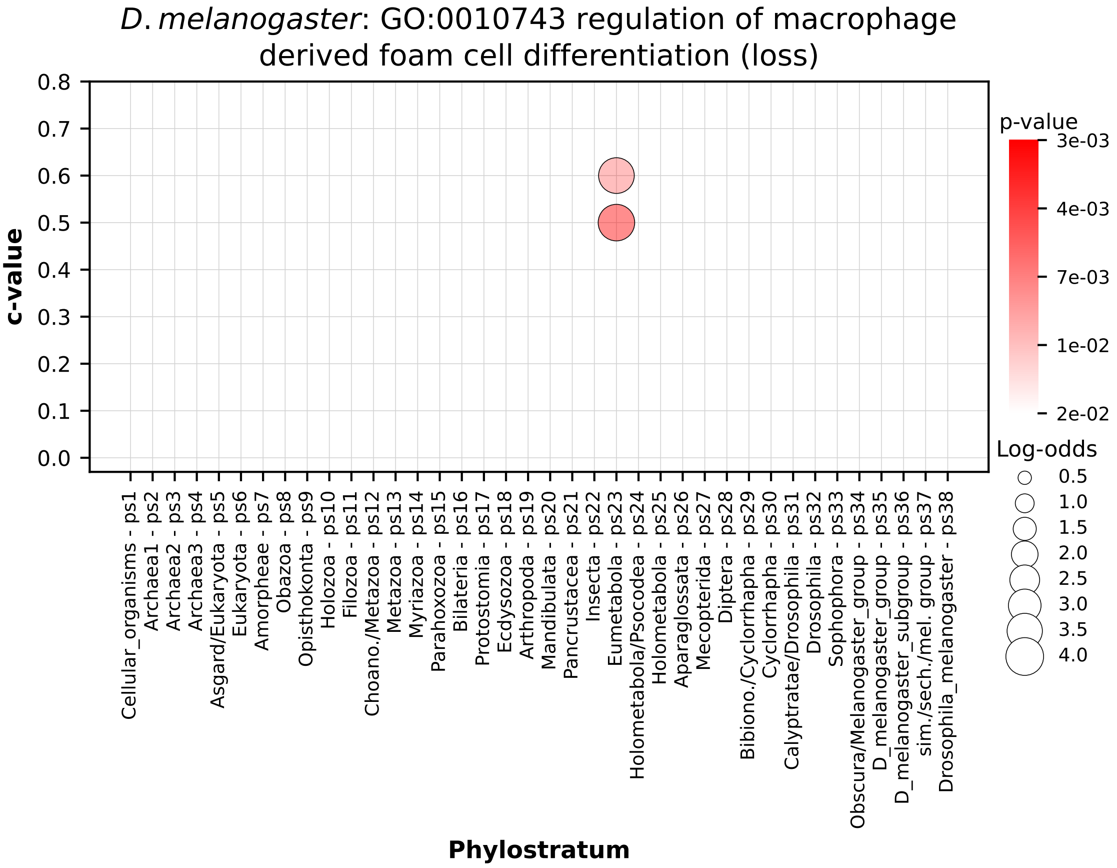
D. melanogaster: GO:0010743 regulation of macrophage derived foam cell differentiation (loss)
c-value
0.0
0.1
0.2
0.3
0.4
0.5
0.6
0.7
0.8
p-value
3e-03
4e-03
7e-03
1e-02
2e-02
Log-odds
0.5
1.0
1.5
2.0
2.5
3.0
3.5
4.0
Cellular_organisms - ps1
Archaea1 - ps2
Archaea2 - ps3
Archaea3 - ps4
Asgard/Eukaryota - ps5
Eukaryota - ps6
Amorpheae - ps7
Obazoa - ps8
Opisthokonta - ps9
Holozoa - ps10
Filozoa - ps11
Choano./Metazoa - ps12
Metazoa - ps13
Myriazoa - ps14
Parahoxozoa - ps15
Bilateria - ps16
Protostomia - ps17
Ecdysozoa - ps18
Arthropoda - ps19
Mandibulata - ps20
Pancrustacea - ps21
Insecta - ps22
Eumetabola - ps23
Holometabola/Psocodea - ps24
Holometabola - ps25
Aparaglossata - ps26
Mecopterida - ps27
Diptera - ps28
Bibiono./Cyclorrhapha - ps29
Cyclorrhapha - ps30
Calyptratae/Drosophila - ps31
Drosophila - ps32
Sophophora - ps33
Obscura/Melanogaster_group - ps34
D_melanogaster_group - ps35
D_melanogaster_subgroup - ps36
sim./sech./mel. group - ps37
Drosophila_melanogaster - ps38
Phylostratum

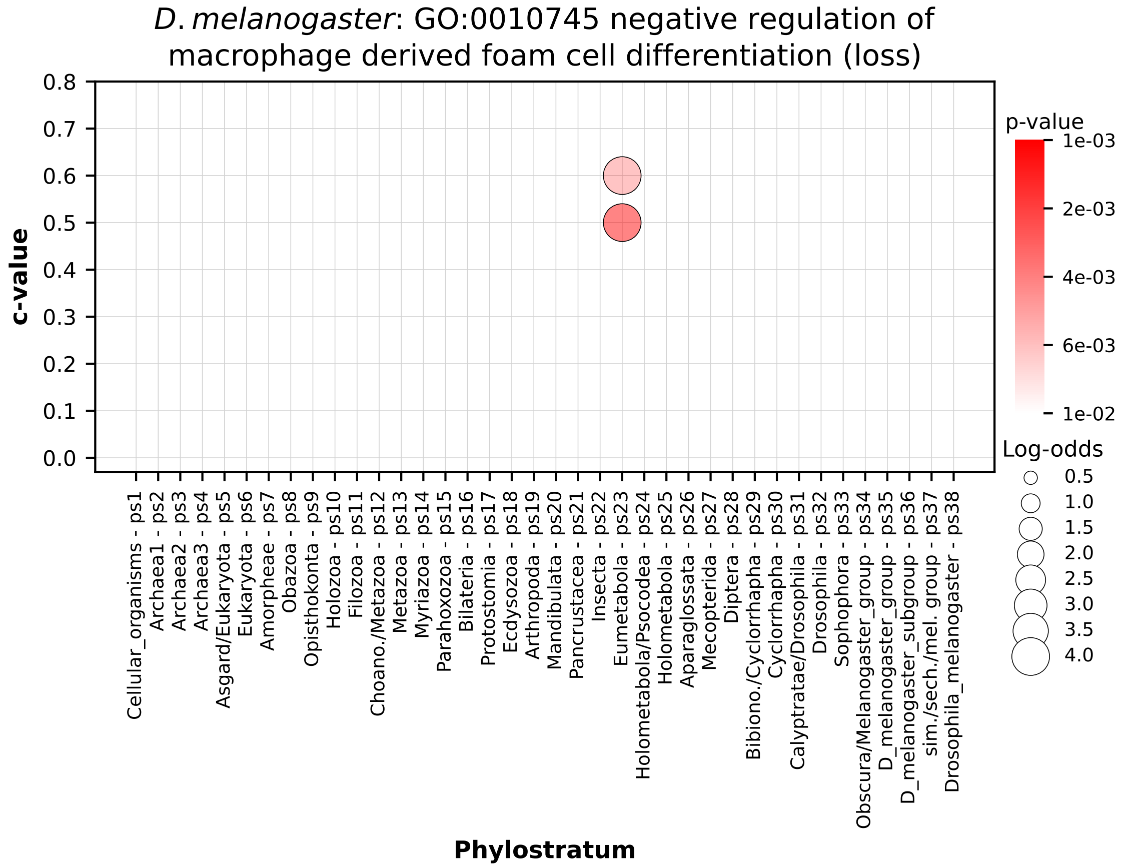

D. melanogaster: GO:0010745 negative regulation of macrophage derived foam cell differentiation (loss)
c-value
0.8
0.7
0.6
0.5
0.4
0.3
0.2
0.1
0.0
Phylostratum
Cellular_organisms - ps1
Archaea1 - ps2
Archaea2 - ps3
Archaea3 - ps4
Asgard/Eukaryota - ps5
Eukaryota - ps6
Amorpheae - ps7
Obazoa - ps8
Opisthokonta - ps9
Holozoa - ps10
Filozoa - ps11
Choano./Metazoa - ps12
Metazoa - ps13
Myriazoa - ps14
Parahoxozoa - ps15
Bilateria - ps16
Protostomia - ps17
Ecdysozoa - ps18
Arthropoda - ps19
Mandibulata - ps20
Pancrustacea - ps21
Insecta - ps22
Eumetabola - ps23
Holometabola/Psocodea - ps24
Holometabola - ps25
Aparaglossata - ps26
Mecopterida - ps27
Diptera - ps28
Bibiono./Cyclorrhapha - ps29
Cyclorrhapha - ps30
Calyptratae/Drosophila - ps31
Drosophila - ps32
Sophophora - ps33
Obscura/Melanogaster_group - ps34
D_melanogaster_group - ps35
D_melanogaster_subgroup - ps36
sim./sech./mel. group - ps37
Drosophila_melanogaster - ps38
p-value
1e-03
2e-03
4e-03
6e-03
1e-02
Log-odds
0.5
1.0
1.5
2.0
2.5
3.0
3.5
4.0

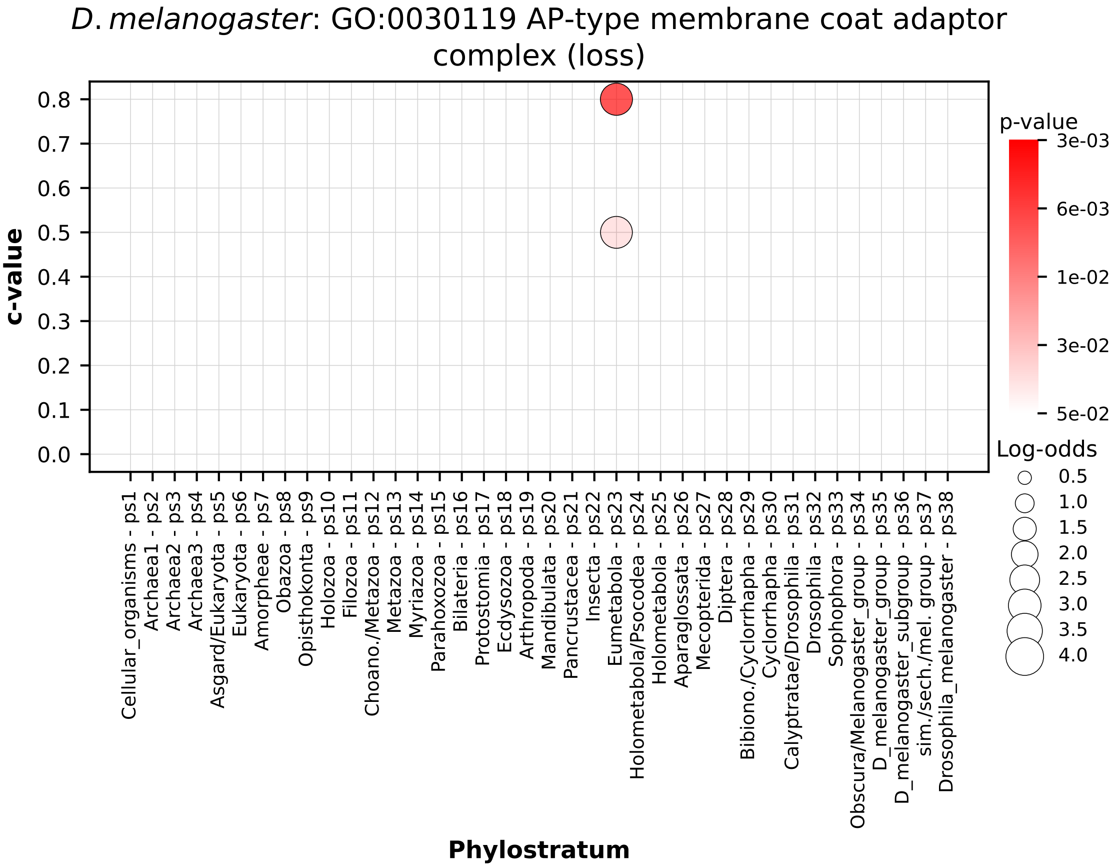

D. melanogaster: GO:0030119 AP-type membrane coat adaptor complex (loss)
c-value
0.8
0.7
0.6
0.5
0.4
0.3
0.2
0.1
0.0
Phylostratum
Cellular_organisms - ps1
Archaea1 - ps2
Archaea2 - ps3
Archaea3 - ps4
Asgard/Eukaryota - ps5
Eukaryota - ps6
Amorpheae - ps7
Obazoa - ps8
Opisthokonta - ps9
Holozoa - ps10
Filozoa - ps11
Choano./Metazoa - ps12
Metazoa - ps13
Myriazoa - ps14
Parahoxozoa - ps15
Bilateria - ps16
Protostomia - ps17
Ecdysozoa - ps18
Arthropoda - ps19
Mandibulata - ps20
Pancrustacea - ps21
Insecta - ps22
Eumetabola - ps23
Holometabola/Psocodea - ps24
Holometabola - ps25
Aparaglossata - ps26
Mecopterida - ps27
Diptera - ps28
Bibiono./Cyclorrhapha - ps29
Cyclorrhapha - ps30
Calyptratae/Drosophila - ps31
Drosophila - ps32
Sophophora - ps33
Obscura/Melanogaster_group - ps34
D_melanogaster_group - ps35
D_melanogaster_subgroup - ps36
sim./sech./mel. group - ps37
Drosophila_melanogaster - ps38
p-value
3e-03
6e-03
1e-02
3e-02
5e-02
Log-odds
0.5
1.0
1.5
2.0
2.5
3.0
3.5
4.0

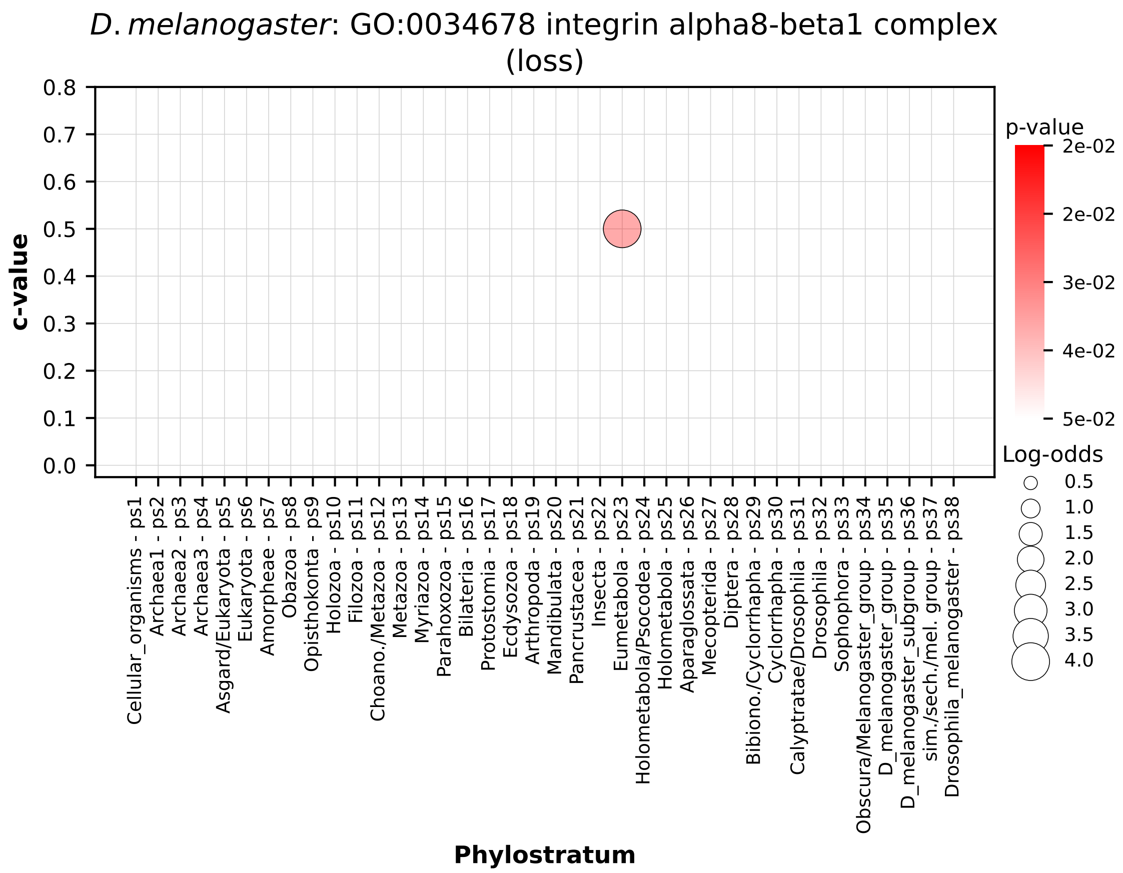

D. melanogaster: GO:0034678 integrin alpha8-beta1 complex
(loss)
c-value
0.0
0.1
0.2
0.3
0.4
0.5
0.6
0.7
0.8
p-value
2e-02
2e-02
3e-02
4e-02
5e-02
Log-odds
0.5
1.0
1.5
2.0
2.5
3.0
3.5
4.0
Cellular_organisms - ps1
Archaea1 - ps2
Archaea2 - ps3
Archaea3 - ps4
Asgard/Eukaryota - ps5
Eukaryota - ps6
Amorpheae - ps7
Obazoa - ps8
Opisthokonta - ps9
Holozoa - ps10
Filozoa - ps11
Choano./Metazoa - ps12
Metazoa - ps13
Myriazoa - ps14
Parahoxozoa - ps15
Bilateria - ps16
Protostomia - ps17
Ecdysozoa - ps18
Arthropoda - ps19
Mandibulata - ps20
Pancrustacea - ps21
Insecta - ps22
Eumetabola - ps23
Holometabola/Psocodea - ps24
Holometabola - ps25
Aparaglossata - ps26
Mecopterida - ps27
Diptera - ps28
Bibiono./Cyclorrhapha - ps29
Cyclorrhapha - ps30
Calyptratae/Drosophila - ps31
Drosophila - ps32
Sophophora - ps33
Obscura/Melanogaster_group - ps34
D_melanogaster_group - ps35
D_melanogaster_subgroup - ps36
sim./sech./mel. group - ps37
Drosophila_melanogaster - ps38
Phylostratum

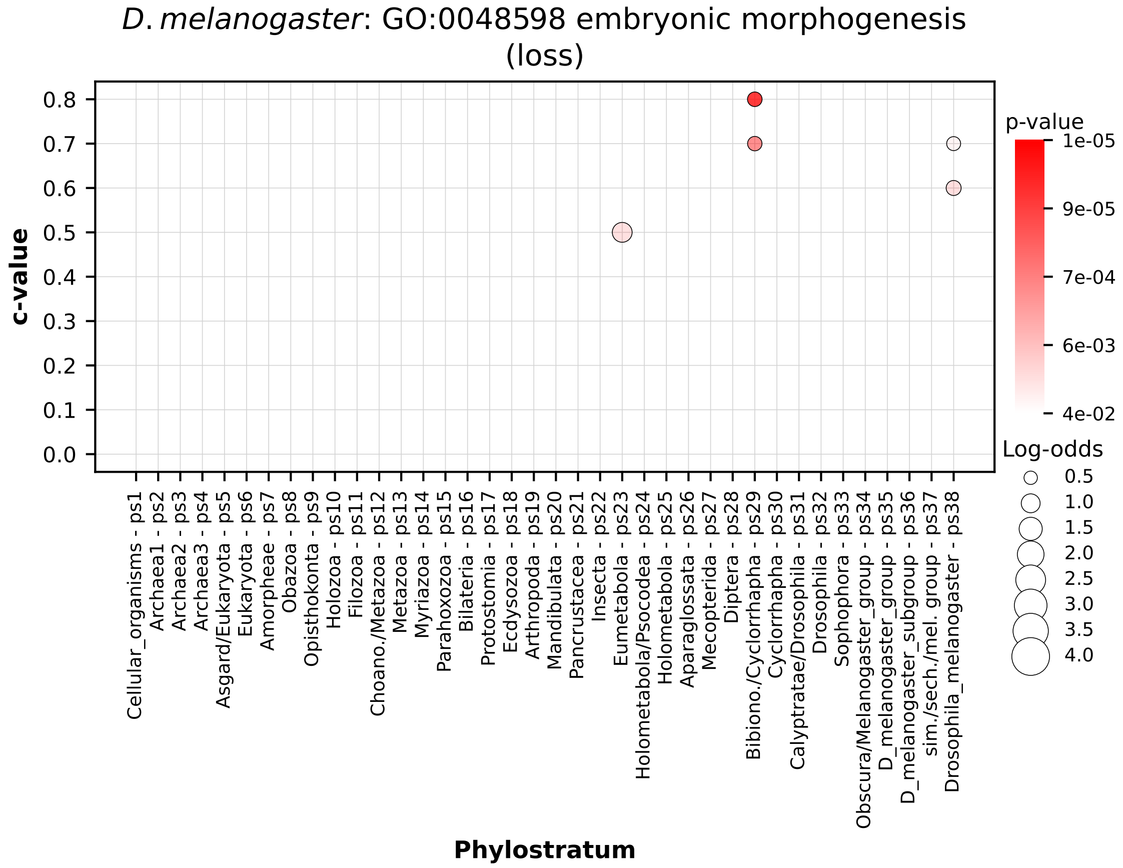
D. melanogaster: GO:0048598 embryonic morphogenesis (loss)
c-value
0.0
0.1
0.2
0.3
0.4
0.5
0.6
0.7
0.8
p-value
1e-05
9e-05
7e-04
6e-03
4e-02
Log-odds
0.5
1.0
1.5
2.0
2.5
3.0
3.5
4.0
Cellular_organisms - ps1
Archaea1 - ps2
Archaea2 - ps3
Archaea3 - ps4
Asgard/Eukaryota - ps5
Eukaryota - ps6
Amorpheae - ps7
Obazoa - ps8
Opisthokonta - ps9
Holozoa - ps10
Filozoa - ps11
Choano./Metazoa - ps12
Metazoa - ps13
Myriazoa - ps14
Parahoxozoa - ps15
Bilateria - ps16
Protostomia - ps17
Ecdysozoa - ps18
Arthropoda - ps19
Mandibulata - ps20
Pancrustacea - ps21
Insecta - ps22
Eumetabola - ps23
Holometabola/Psocodea - ps24
Holometabola - ps25
Aparaglossata - ps26
Mecopterida - ps27
Diptera - ps28
Bibiono./Cyclorrhapha - ps29
Cyclorrhapha - ps30
Calyptratae/Drosophila - ps31
Drosophila - ps32
Sophophora - ps33
Obscura/Melanogaster_group - ps34
D_melanogaster_group - ps35
D_melanogaster_subgroup - ps36
sim./sech./mel. group - ps37
Drosophila_melanogaster - ps38
Phylostratum

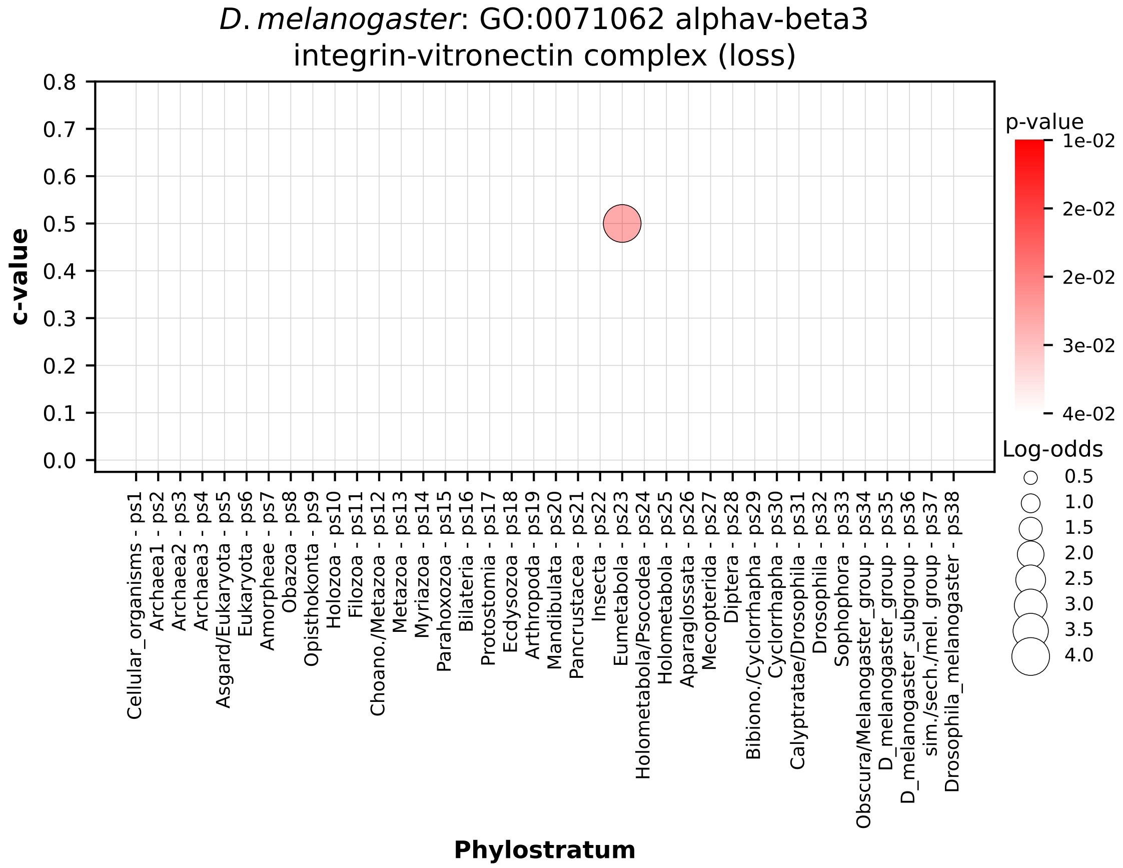

D. melanogaster: GO:0071062 alphav-beta3 integrin-vitronectin complex (loss)
c-value
0.8
0.7
0.6
0.5
0.4
0.3
0.2
0.1
0.0
p-value
1e-02
2e-02
2e-02
3e-02
4e-02
Log-odds
0.5
1.0
1.5
2.0
2.5
3.0
3.5
4.0
Cellular_organisms - ps1
Archaea1 - ps2
Archaea2 - ps3
Archaea3 - ps4
Asgard/Eukaryota - ps5
Eukaryota - ps6
Amorpheae - ps7
Obazoa - ps8
Opisthokonta - ps9
Holozoa - ps10
Filozoa - ps11
Choano./Metazoa - ps12
Metazoa - ps13
Myriazoa - ps14
Parahoxozoa - ps15
Bilateria - ps16
Protostomia - ps17
Ecdysozoa - ps18
Arthropoda - ps19
Mandibulata - ps20
Pancrustacea - ps21
Insecta - ps22
Eumetabola - ps23
Holometabola/Psocodea - ps24
Holometabola - ps25
Aparaglossata - ps26
Mecopterida - ps27
Diptera - ps28
Bibiono./Cyclorrhapha - ps29
Cyclorrhapha - ps30
Calyptratae/Drosophila - ps31
Drosophila - ps32
Sophophora - ps33
Obscura/Melanogaster_group - ps34
D_melanogaster_group - ps35
D_melanogaster_subgroup - ps36
sim./sech./mel. group - ps37
Drosophila_melanogaster - ps38
Phylostratum

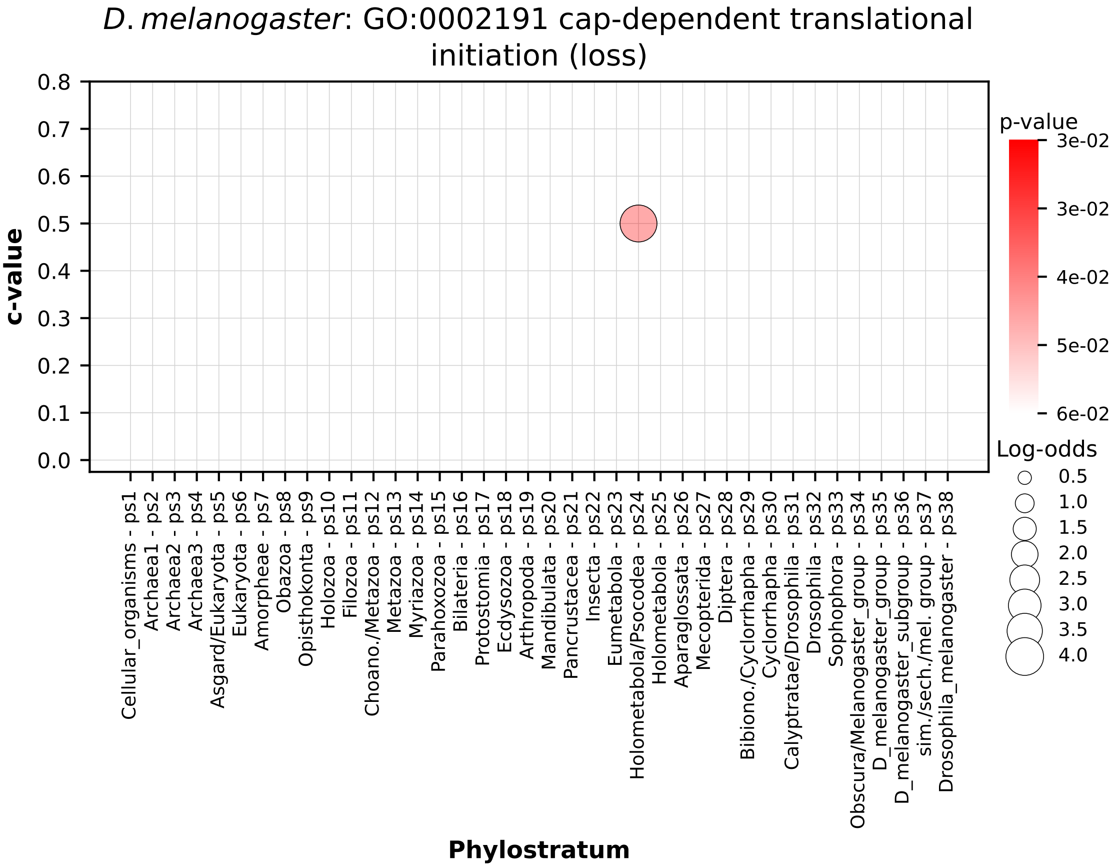
D. melanogaster: GO:0002191 cap-dependent translational initiation (loss)
c-value
0.8
0.7
0.6
0.5
0.4
0.3
0.2
0.1
0.0
Phylostratum
Cellular_organisms - ps1
Archaea1 - ps2
Archaea2 - ps3
Archaea3 - ps4
Asgard/Eukaryota - ps5
Eukaryota - ps6
Amorpheae - ps7
Obazoa - ps8
Opisthokonta - ps9
Holozoa - ps10
Filozoa - ps11
Choano./Metazoa - ps12
Metazoa - ps13
Myriazoa - ps14
Parahoxozoa - ps15
Bilateria - ps16
Protostomia - ps17
Ecdysozoa - ps18
Arthropoda - ps19
Mandibulata - ps20
Pancrustacea - ps21
Insecta - ps22
Eumetabola - ps23
Holometabola/Psocodea - ps24
Holometabola - ps25
Aparaglossata - ps26
Mecopterida - ps27
Diptera - ps28
Bibiono./Cyclorrhapha - ps29
Cyclorrhapha - ps30
Calyptratae/Drosophila - ps31
Drosophila - ps32
Sophophora - ps33
Obscura/Melanogaster_group - ps34
D_melanogaster_group - ps35
D_melanogaster_subgroup - ps36
sim./sech./mel. group - ps37
Drosophila_melanogaster - ps38
p-value
3e-02
3e-02
4e-02
5e-02
6e-02
Log-odds
0.5
1.0
1.5
2.0
2.5
3.0
3.5
4.0

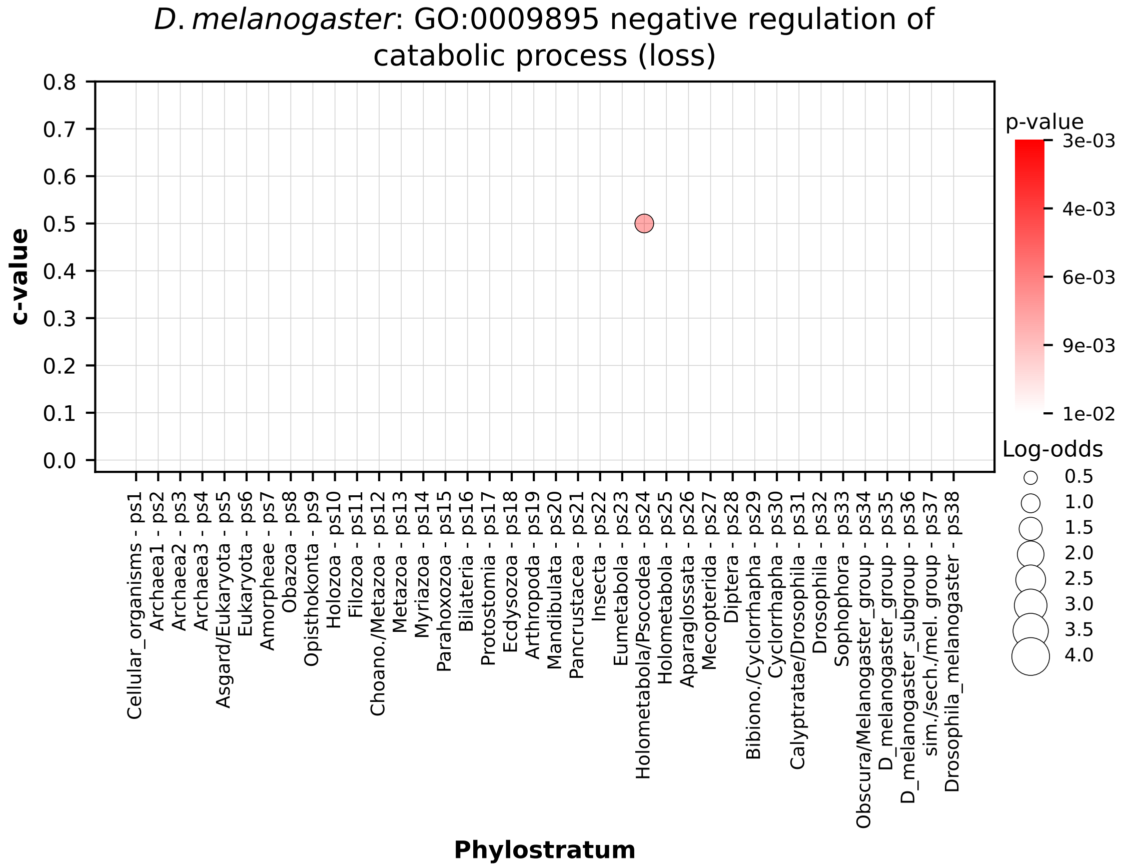

D. melanogaster: GO:0009895 negative regulation of catabolic process (loss)
c-value
0.8
0.7
0.6
0.5
0.4
0.3
0.2
0.1
0.0
p-value
3e-03
4e-03
6e-03
9e-03
1e-02
Log-odds
0.5
1.0
1.5
2.0
2.5
3.0
3.5
4.0
Cellular_organisms - ps1
Archaea1 - ps2
Archaea2 - ps3
Archaea3 - ps4
Asgard/Eukaryota - ps5
Eukaryota - ps6
Amorpheae - ps7
Obazoa - ps8
Opisthokonta - ps9
Holozoa - ps10
Filozoa - ps11
Choano./Metazoa - ps12
Metazoa - ps13
Myriazoa - ps14
Parahoxozoa - ps15
Bilateria - ps16
Protostomia - ps17
Ecdysozoa - ps18
Arthropoda - ps19
Mandibulata - ps20
Pancrustacea - ps21
Insecta - ps22
Eumetabola - ps23
Holometabola/Psocodea - ps24
Holometabola - ps25
Aparaglossata - ps26
Mecopterida - ps27
Diptera - ps28
Bibiono./Cyclorrhapha - ps29
Cyclorrhapha - ps30
Calyptratae/Drosophila - ps31
Drosophila - ps32
Sophophora - ps33
Obscura/Melanogaster_group - ps34
D_melanogaster_group - ps35
D_melanogaster_subgroup - ps36
sim./sech./mel. group - ps37
Drosophila_melanogaster - ps38
Phylostratum

D. melanogaster: GO:0031330 negative regulation of cellular catabolic process (loss)

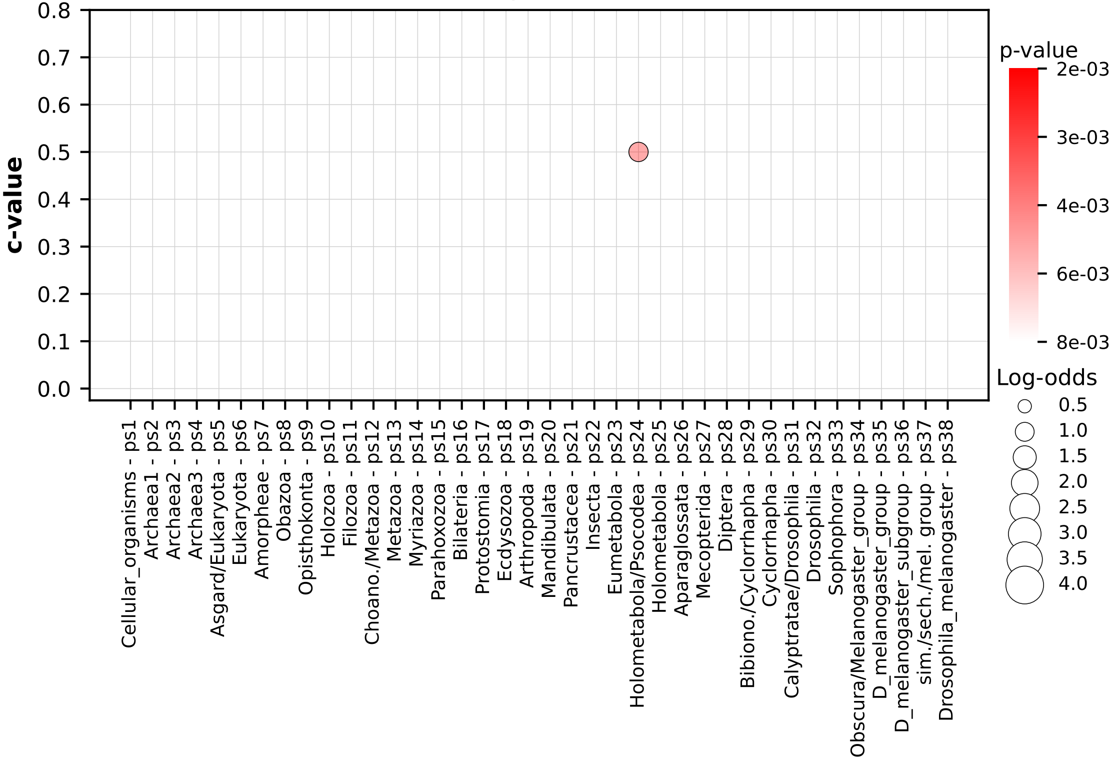

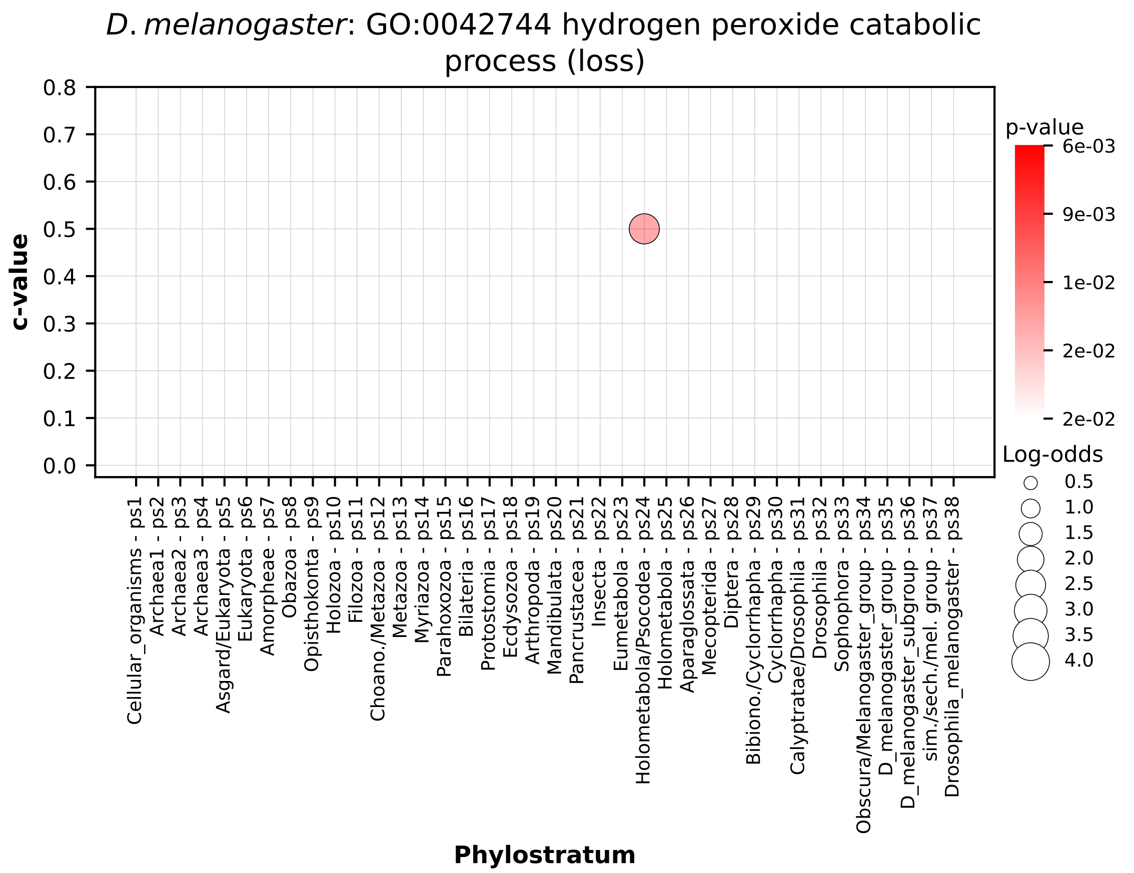

D. melanogaster: GO:0042744 hydrogen peroxide catabolic process (loss)
c-value
0.8
0.7
0.6
0.5
0.4
0.3
0.2
0.1
0.0
Phylostratum
Cellular_organisms - ps1
Archaea1 - ps2
Archaea2 - ps3
Archaea3 - ps4
Asgard/Eukaryota - ps5
Eukaryota - ps6
Amorpheae - ps7
Obazoa - ps8
Opisthokonta - ps9
Holozoa - ps10
Filozoa - ps11
Choano./Metazoa - ps12
Metazoa - ps13
Myriazoa - ps14
Parahoxozoa - ps15
Bilateria - ps16
Protostomia - ps17
Ecdysozoa - ps18
Arthropoda - ps19
Mandibulata - ps20
Pancrustacea - ps21
Insecta - ps22
Eumetabola - ps23
Holometabola/Psocodea - ps24
Holometabola - ps25
Aparaglossata - ps26
Mecopterida - ps27
Diptera - ps28
Bibiono./Cyclorrhapha - ps29
Cyclorrhapha - ps30
Calyptratae/Drosophila - ps31
Drosophila - ps32
Sophophora - ps33
Obscura/Melanogaster_group - ps34
D_melanogaster_group - ps35
D_melanogaster_subgroup - ps36
sim./sech./mel. group - ps37
Drosophila_melanogaster - ps38
p-value
6e-03
9e-03
1e-02
2e-02
2e-02
Log-odds
0.5
1.0
1.5
2.0
2.5
3.0
3.5
4.0

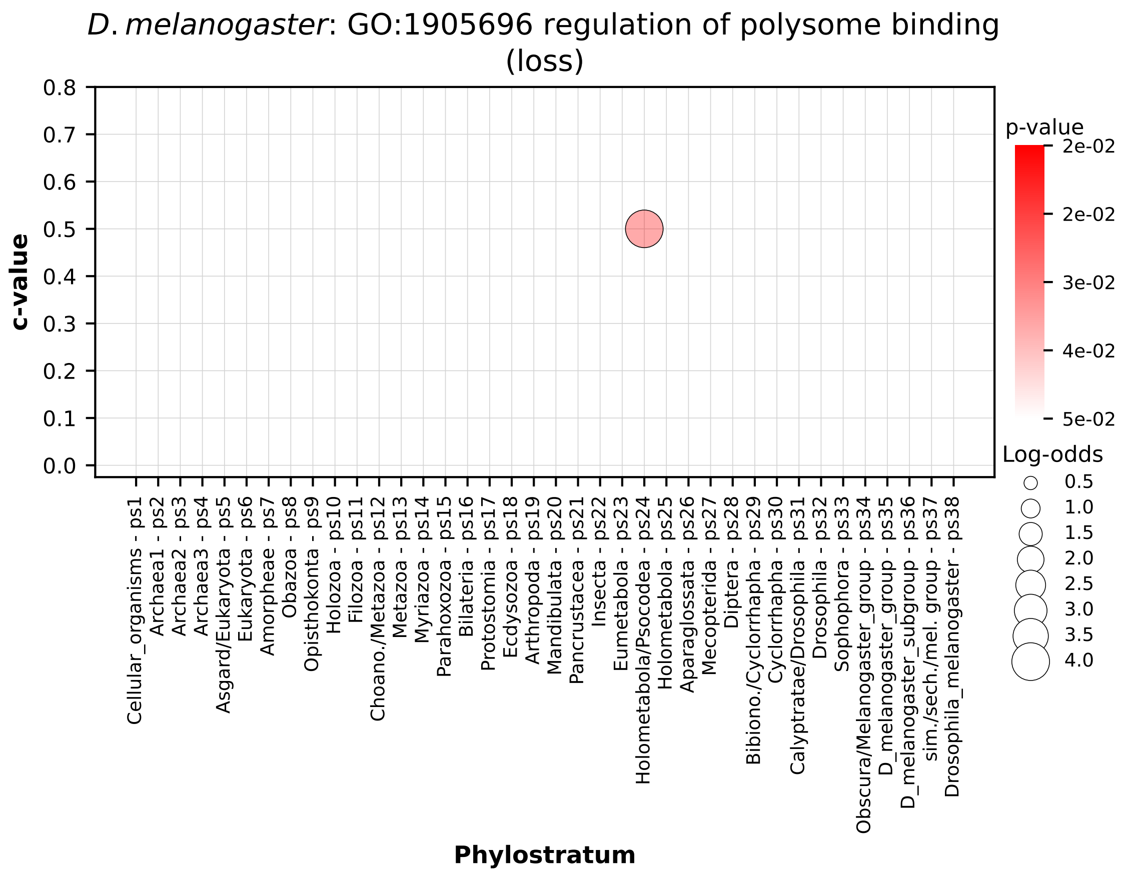

D. melanogaster: GO:1905696 regulation of polysome binding
(loss)
c-value
0.8
0.7
0.6
0.5
0.4
0.3
0.2
0.1
0.0
p-value
2e-02
2e-02
3e-02
4e-02
5e-02
Log-odds
0.5
1.0
1.5
2.0
2.5
3.0
3.5
4.0
Cellular_organisms - ps1
Archaea1 - ps2
Archaea2 - ps3
Archaea3 - ps4
Asgard/Eukaryota - ps5
Eukaryota - ps6
Amorpheae - ps7
Obazoa - ps8
Opisthokonta - ps9
Holozoa - ps10
Filozoa - ps11
Choano./Metazoa - ps12
Metazoa - ps13
Myriazoa - ps14
Parahoxozoa - ps15
Bilateria - ps16
Protostomia - ps17
Ecdysozoa - ps18
Arthropoda - ps19
Mandibulata - ps20
Pancrustacea - ps21
Insecta - ps22
Eumetabola - ps23
Holometabola/Psocodea - ps24
Holometabola - ps25
Aparaglossata - ps26
Mecopterida - ps27
Diptera - ps28
Bibiono./Cyclorrhapha - ps29
Cyclorrhapha - ps30
Calyptratae/Drosophila - ps31
Drosophila - ps32
Sophophora - ps33
Obscura/Melanogaster_group - ps34
D_melanogaster_group - ps35
D_melanogaster_subgroup - ps36
sim./sech./mel. group - ps37
Drosophila_melanogaster - ps38
Phylostratum

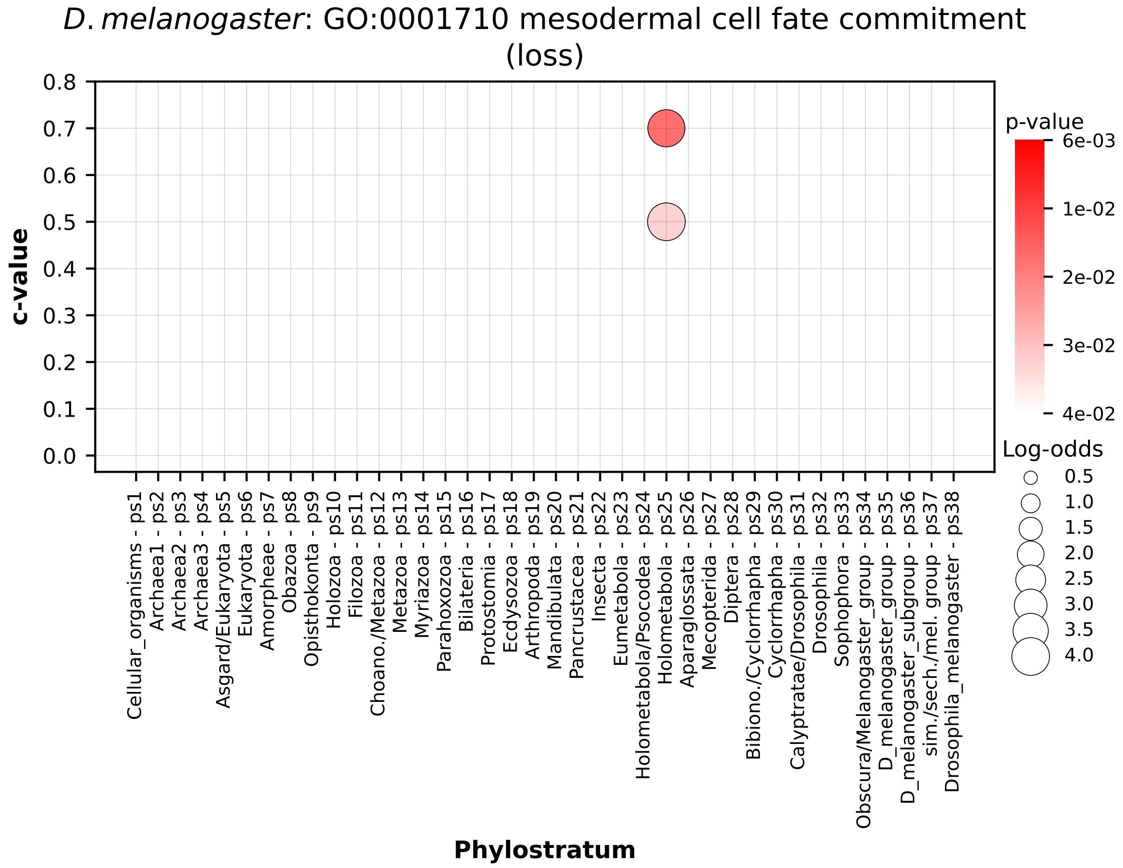

D. melanogaster: GO:0001710 mesodermal cell fate commitment (loss)
c-value
0.8
0.7
0.6
0.5
0.4
0.3
0.2
0.1
0.0
Phylostratum
Cellular_organisms - ps1
Archaea1 - ps2
Archaea2 - ps3
Archaea3 - ps4
Asgard/Eukaryota - ps5
Eukaryota - ps6
Amorpheae - ps7
Obazoa - ps8
Opisthokonta - ps9
Holozoa - ps10
Filozoa - ps11
Choano./Metazoa - ps12
Metazoa - ps13
Myriazoa - ps14
Parahoxozoa - ps15
Bilateria - ps16
Protostomia - ps17
Ecdysozoa - ps18
Arthropoda - ps19
Mandibulata - ps20
Pancrustacea - ps21
Insecta - ps22
Eumetabola - ps23
Holometabola/Psocodea - ps24
Holometabola - ps25
Aparaglossata - ps26
Mecopterida - ps27
Diptera - ps28
Bibiono./Cyclorrhapha - ps29
Cyclorrhapha - ps30
Calyptratae/Drosophila - ps31
Drosophila - ps32
Sophophora - ps33
Obscura/Melanogaster_group - ps34
D_melanogaster_group - ps35
D_melanogaster_subgroup - ps36
sim./sech./mel. group - ps37
Drosophila_melanogaster - ps38
p-value
6e-03
1e-02
2e-02
3e-02
4e-02
Log-odds
0.5
1.0
1.5
2.0
2.5
3.0
3.5
4.0

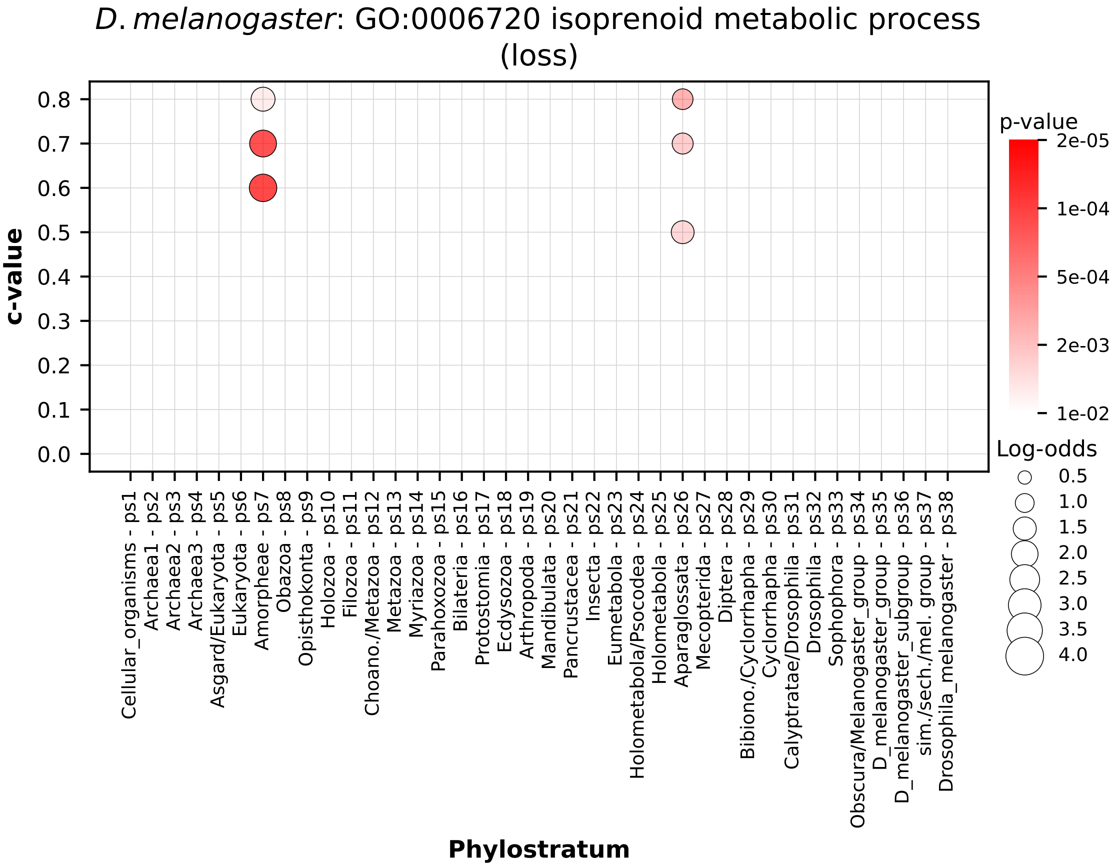

D. melanogaster: GO:0006720 isoprenoid metabolic process (loss)
c-value
0.8
0.7
0.6
0.5
0.4
0.3
0.2
0.1
0.0
Phylostratum
Cellular_organisms - ps1
Archaea1 - ps2
Archaea2 - ps3
Archaea3 - ps4
Asgard/Eukaryota - ps5
Eukaryota - ps6
Amorpheae - ps7
Obazoa - ps8
Opisthokonta - ps9
Holozoa - ps10
Filozoa - ps11
Choano./Metazoa - ps12
Metazoa - ps13
Myriazoa - ps14
Parahoxozoa - ps15
Bilateria - ps16
Protostomia - ps17
Ecdysozoa - ps18
Arthropoda - ps19
Mandibulata - ps20
Pancrustacea - ps21
Insecta - ps22
Eumetabola - ps23
Holometabola/Psocodea - ps24
Holometabola - ps25
Aparaglossata - ps26
Mecopterida - ps27
Diptera - ps28
Bibiono./Cyclorrhapha - ps29
Cyclorrhapha - ps30
Calyptratae/Drosophila - ps31
Drosophila - ps32
Sophophora - ps33
Obscura/Melanogaster_group - ps34
D_melanogaster_group - ps35
D_melanogaster_subgroup - ps36
sim./sech./mel. group - ps37
Drosophila_melanogaster - ps38
p-value
2e-05
1e-04
5e-04
2e-03
1e-02
Log-odds
0.5
1.0
1.5
2.0
2.5
3.0
3.5
4.0

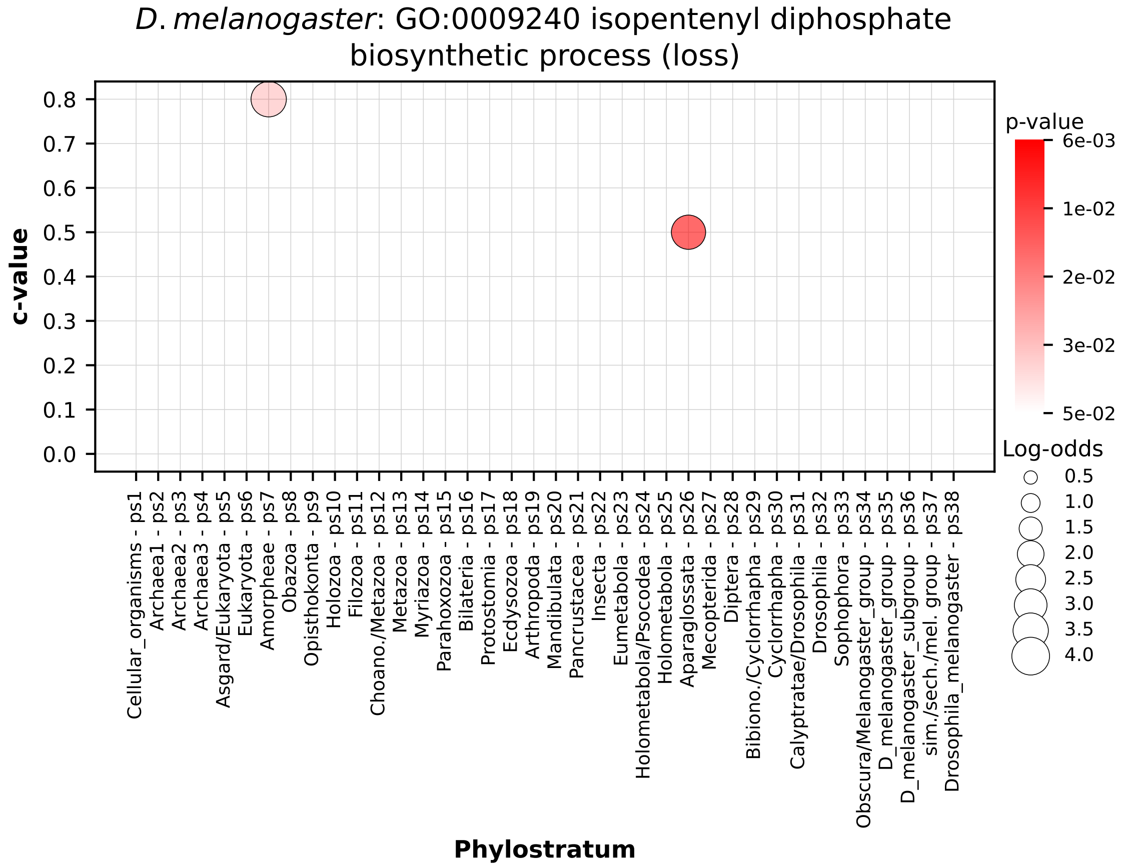

D. melanogaster: GO:0009240 isopentenyl diphosphate biosynthetic process (loss)
c-value
0.0
0.1
0.2
0.3
0.4
0.5
0.6
0.7
0.8
Phylostratum
Cellular_organisms - ps1
Archaea1 - ps2
Archaea2 - ps3
Archaea3 - ps4
Asgard/Eukaryota - ps5
Eukaryota - ps6
Amorpheae - ps7
Obazoa - ps8
Opisthokonta - ps9
Holozoa - ps10
Filozoa - ps11
Choano./Metazoa - ps12
Metazoa - ps13
Myriazoa - ps14
Parahoxozoa - ps15
Bilateria - ps16
Protostomia - ps17
Ecdysozoa - ps18
Arthropoda - ps19
Mandibulata - ps20
Pancrustacea - ps21
Insecta - ps22
Eumetabola - ps23
Holometabola/Psocodea - ps24
Holometabola - ps25
Aparaglossata - ps26
Mecopterida - ps27
Diptera - ps28
Bibiono./Cyclorrhapha - ps29
Cyclorrhapha - ps30
Calyptratae/Drosophila - ps31
Drosophila - ps32
Sophophora - ps33
Obscura/Melanogaster_group - ps34
D_melanogaster_group - ps35
D_melanogaster_subgroup - ps36
sim./sech./mel. group - ps37
Drosophila_melanogaster - ps38
p-value
6e-03
1e-02
2e-02
3e-02
5e-02
Log-odds
0.5
1.0
1.5
2.0
2.5
3.0
3.5
4.0

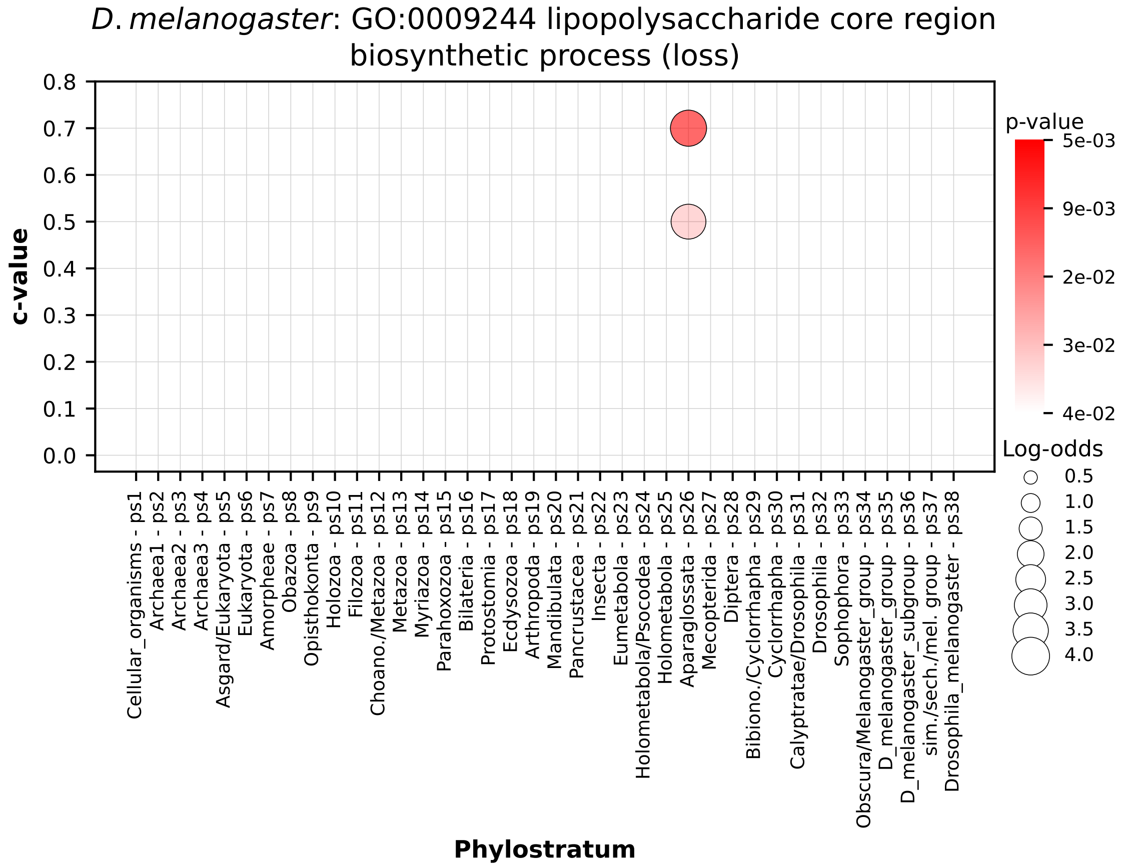

D. melanogaster: GO:0009244 lipopolysaccharide core region biosynthetic process (loss)
c-value
0.8
0.7
0.6
0.5
0.4
0.3
0.2
0.1
0.0
p-value
5e-03
9e-03
2e-02
3e-02
4e-02
Log-odds
0.5
1.0
1.5
2.0
2.5
3.0
3.5
4.0
Cellular_organisms - ps1
Archaea1 - ps2
Archaea2 - ps3
Archaea3 - ps4
Asgard/Eukaryota - ps5
Eukaryota - ps6
Amorpheae - ps7
Obazoa - ps8
Opisthokonta - ps9
Holozoa - ps10
Filozoa - ps11
Choano./Metazoa - ps12
Metazoa - ps13
Myriazoa - ps14
Parahoxozoa - ps15
Bilateria - ps16
Protostomia - ps17
Ecdysozoa - ps18
Arthropoda - ps19
Mandibulata - ps20
Pancrustacea - ps21
Insecta - ps22
Eumetabola - ps23
Holometabola/Psocodea - ps24
Holometabola - ps25
Aparaglossata - ps26
Mecopterida - ps27
Diptera - ps28
Bibiono./Cyclorrhapha - ps29
Cyclorrhapha - ps30
Calyptratae/Drosophila - ps31
Drosophila - ps32
Sophophora - ps33
Obscura/Melanogaster_group - ps34
D_melanogaster_group - ps35
D_melanogaster_subgroup - ps36
sim./sech./mel. group - ps37
Drosophila_melanogaster - ps38
Phylostratum

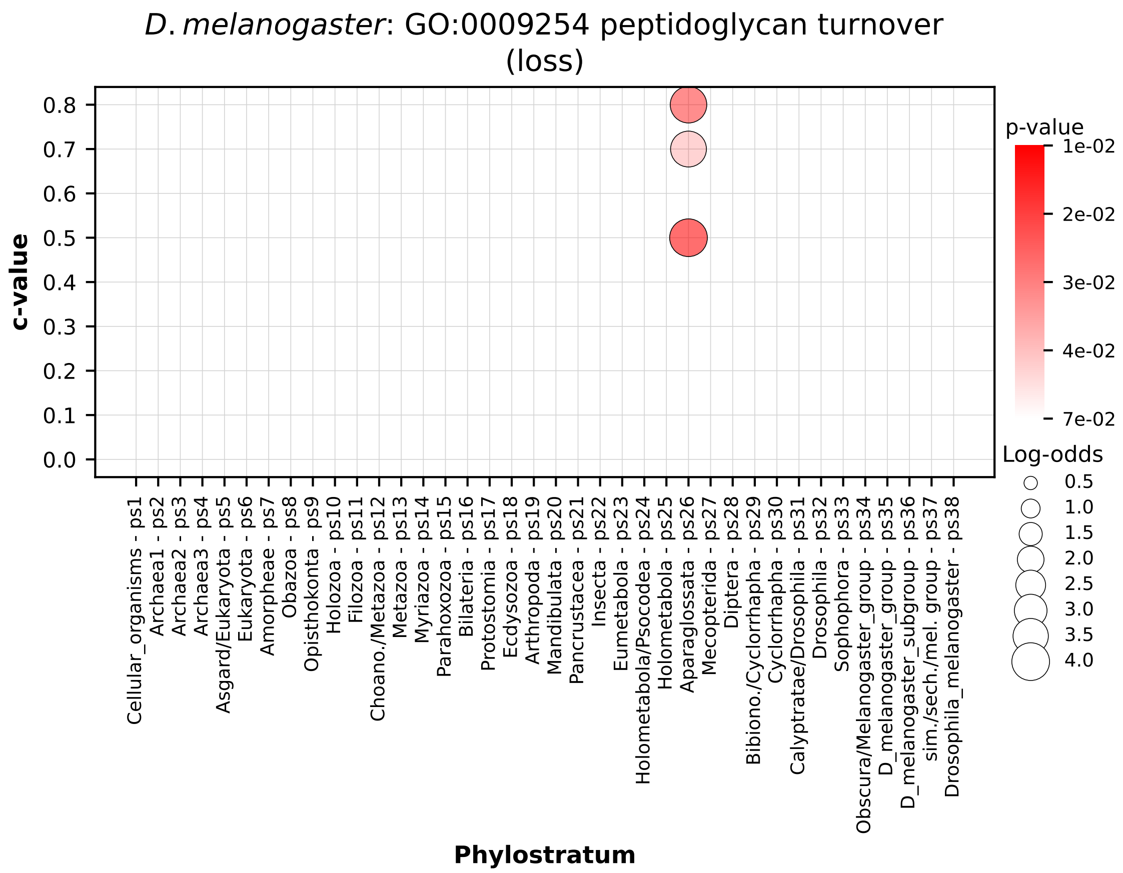

D. melanogaster: GO:0009254 peptidoglycan turnover
(loss)
c-value
Phylostratum
p-value
1e-02
2e-02
3e-02
4e-02
7e-02
Log-odds
0.5
1.0
1.5
2.0
2.5
3.0
3.5
4.0
Cellular_organisms - ps1
Archaea1 - ps2
Archaea2 - ps3
Archaea3 - ps4
Asgard/Eukaryota - ps5
Eukaryota - ps6
Amorpheae - ps7
Obazoa - ps8
Opisthokonta - ps9
Holozoa - ps10
Filozoa - ps11
Choano./Metazoa - ps12
Metazoa - ps13
Myriazoa - ps14
Parahoxozoa - ps15
Bilateria - ps16
Protostomia - ps17
Ecdysozoa - ps18
Arthropoda - ps19
Mandibulata - ps20
Pancrustacea - ps21
Insecta - ps22
Eumetabola - ps23
Holometabola/Psocodea - ps24
Holometabola - ps25
Aparaglossata - ps26
Mecopterida - ps27
Diptera - ps28
Bibiono./Cyclorrhapha - ps29
Cyclorrhapha - ps30
Calyptratae/Drosophila - ps31
Drosophila - ps32
Sophophora - ps33
Obscura/Melanogaster_group - ps34
D_melanogaster_group - ps35
D_melanogaster_subgroup - ps36
sim./sech./mel. group - ps37
Drosophila_melanogaster - ps38

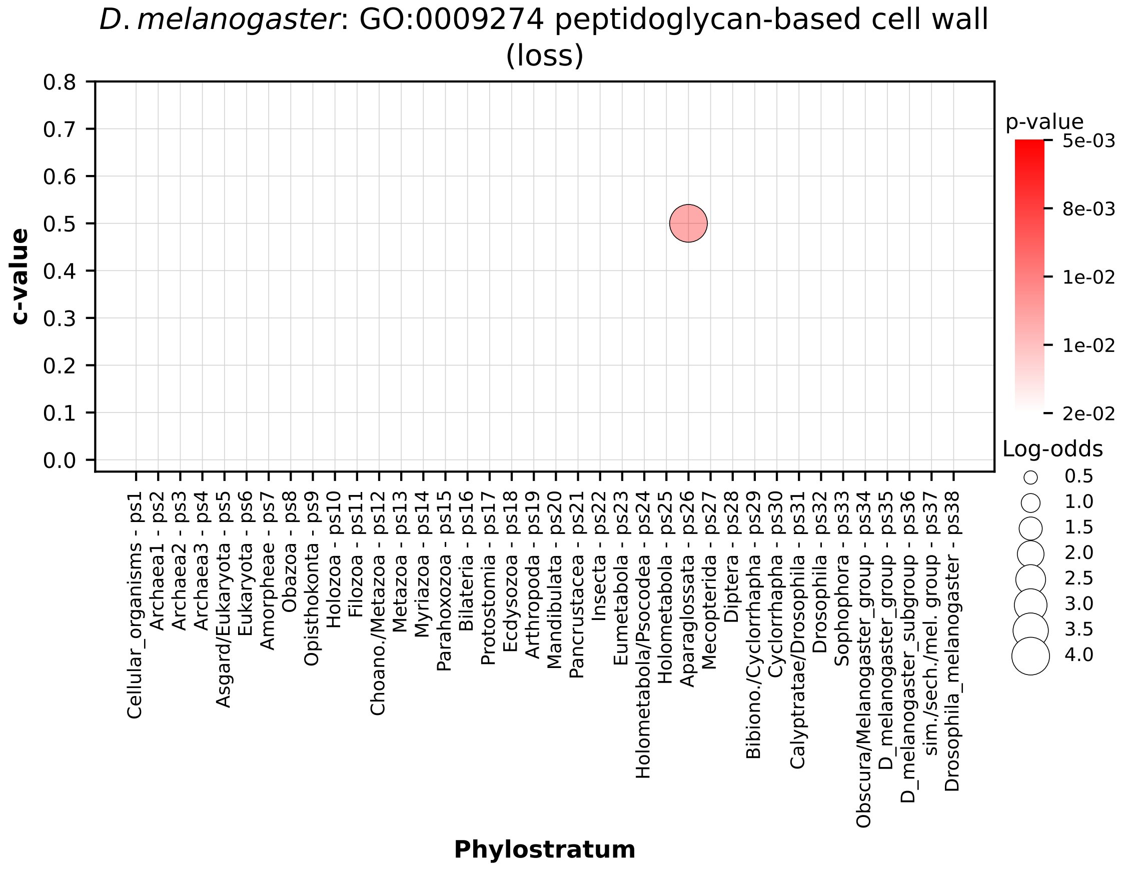

D. melanogaster: GO:0009274 peptidoglycan-based cell wall (loss)
c-value
0.8
0.7
0.6
0.5
0.4
0.3
0.2
0.1
0.0
Phylostratum
Cellular_organisms - ps1
Archaea1 - ps2
Archaea2 - ps3
Archaea3 - ps4
Asgard/Eukaryota - ps5
Eukaryota - ps6
Amorpheae - ps7
Obazoa - ps8
Opisthokonta - ps9
Holozoa - ps10
Filozoa - ps11
Choano./Metazoa - ps12
Metazoa - ps13
Myriazoa - ps14
Parahoxozoa - ps15
Bilateria - ps16
Protostomia - ps17
Ecdysozoa - ps18
Arthropoda - ps19
Mandibulata - ps20
Pancrustacea - ps21
Insecta - ps22
Eumetabola - ps23
Holometabola/Psocodea - ps24
Holometabola - ps25
Aparaglossata - ps26
Mecopterida - ps27
Diptera - ps28
Bibiono./Cyclorrhapha - ps29
Cyclorrhapha - ps30
Calyptratae/Drosophila - ps31
Drosophila - ps32
Sophophora - ps33
Obscura/Melanogaster_group - ps34
D_melanogaster_group - ps35
D_melanogaster_subgroup - ps36
sim./sech./mel. group - ps37
Drosophila_melanogaster - ps38
p-value
5e-03
8e-03
1e-02
1e-02
2e-02
Log-odds
0.5
1.0
1.5
2.0
2.5
3.0
3.5
4.0

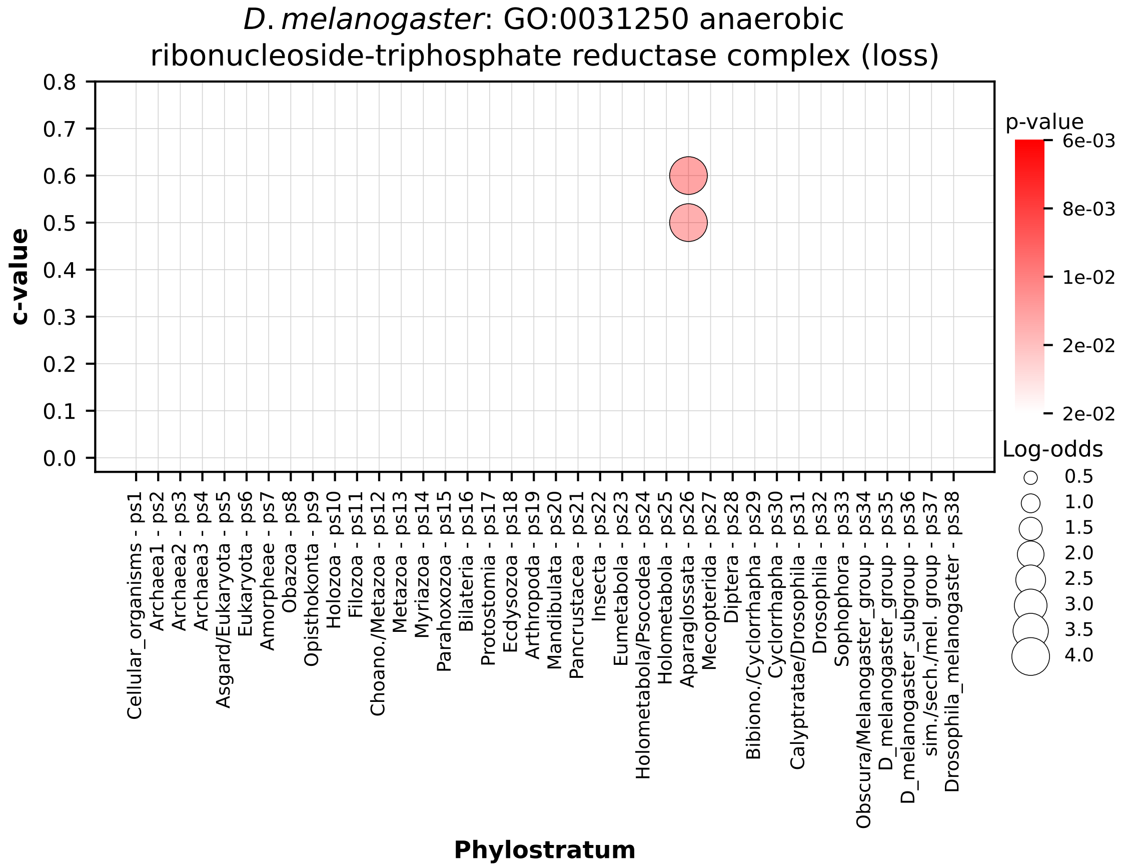

D. melanogaster: GO:0031250 anaerobic ribonucleoside-triphosphate reductase complex (loss)
c-value
0.8
0.7
0.6
0.5
0.4
0.3
0.2
0.1
0.0
p-value
6e-03
8e-03
1e-02
2e-02
2e-02
Log-odds
0.5
1.0
1.5
2.0
2.5
3.0
3.5
4.0
Cellular_organisms - ps1
Archaea1 - ps2
Archaea2 - ps3
Archaea3 - ps4
Asgard/Eukaryota - ps5
Eukaryota - ps6
Amorpheae - ps7
Obazoa - ps8
Opisthokonta - ps9
Holozoa - ps10
Filozoa - ps11
Choano./Metazoa - ps12
Metazoa - ps13
Myriazoa - ps14
Parahoxozoa - ps15
Bilateria - ps16
Protostomia - ps17
Ecdysozoa - ps18
Arthropoda - ps19
Mandibulata - ps20
Pancrustacea - ps21
Insecta - ps22
Eumetabola - ps23
Holometabola/Psocodea - ps24
Holometabola - ps25
Aparaglossata - ps26
Mecopterida - ps27
Diptera - ps28
Bibiono./Cyclorrhapha - ps29
Cyclorrhapha - ps30
Calyptratae/Drosophila - ps31
Drosophila - ps32
Sophophora - ps33
Obscura/Melanogaster_group - ps34
D_melanogaster_group - ps35
D_melanogaster_subgroup - ps36
sim./sech./mel. group - ps37
Drosophila_melanogaster - ps38
Phylostratum

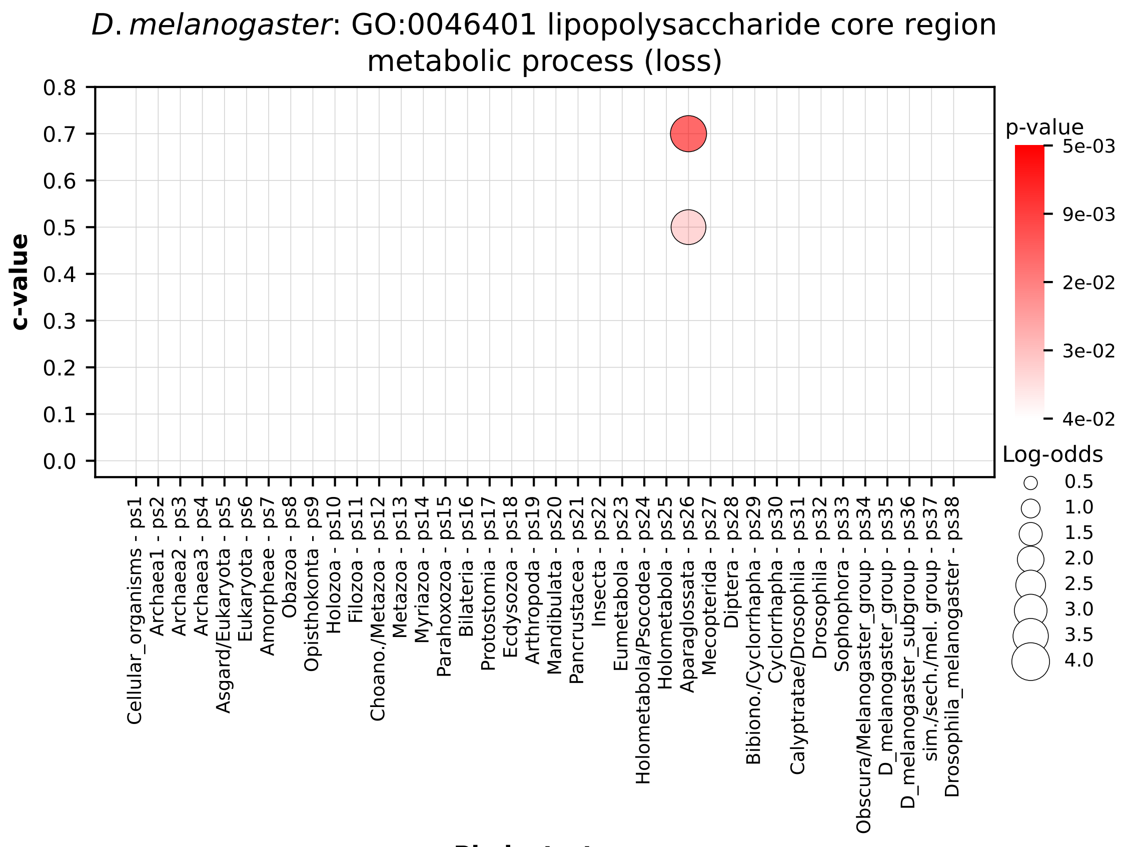
D. melanogaster: GO:0046401 lipopolysaccharide core region metabolic process (loss)
c-value
0.8
0.7
0.6
0.5
0.4
0.3
0.2
0.1
0.0
Phylostratum
Cellular_organisms - ps1
Archaea1 - ps2
Archaea2 - ps3
Archaea3 - ps4
Asgard/Eukaryota - ps5
Eukaryota - ps6
Amorpheae - ps7
Obazoa - ps8
Opisthokonta - ps9
Holozoa - ps10
Filozoa - ps11
Choano./Metazoa - ps12
Metazoa - ps13
Myriazoa - ps14
Parahoxozoa - ps15
Bilateria - ps16
Protostomia - ps17
Ecdysozoa - ps18
Arthropoda - ps19
Mandibulata - ps20
Pancrustacea - ps21
Insecta - ps22
Eumetabola - ps23
Holometabola/Psocodea - ps24
Holometabola - ps25
Aparaglossata - ps26
Mecopterida - ps27
Diptera - ps28
Bibiono./Cyclorrhapha - ps29
Cyclorrhapha - ps30
Calyptratae/Drosophila - ps31
Drosophila - ps32
Sophophora - ps33
Obscura/Melanogaster_group - ps34
D_melanogaster_group - ps35
D_melanogaster_subgroup - ps36
sim./sech./mel. group - ps37
Drosophila_melanogaster - ps38
p-value
5e-03
9e-03
2e-02
3e-02
4e-02
Log-odds
0.5
1.0
1.5
2.0
2.5
3.0
3.5
4.0

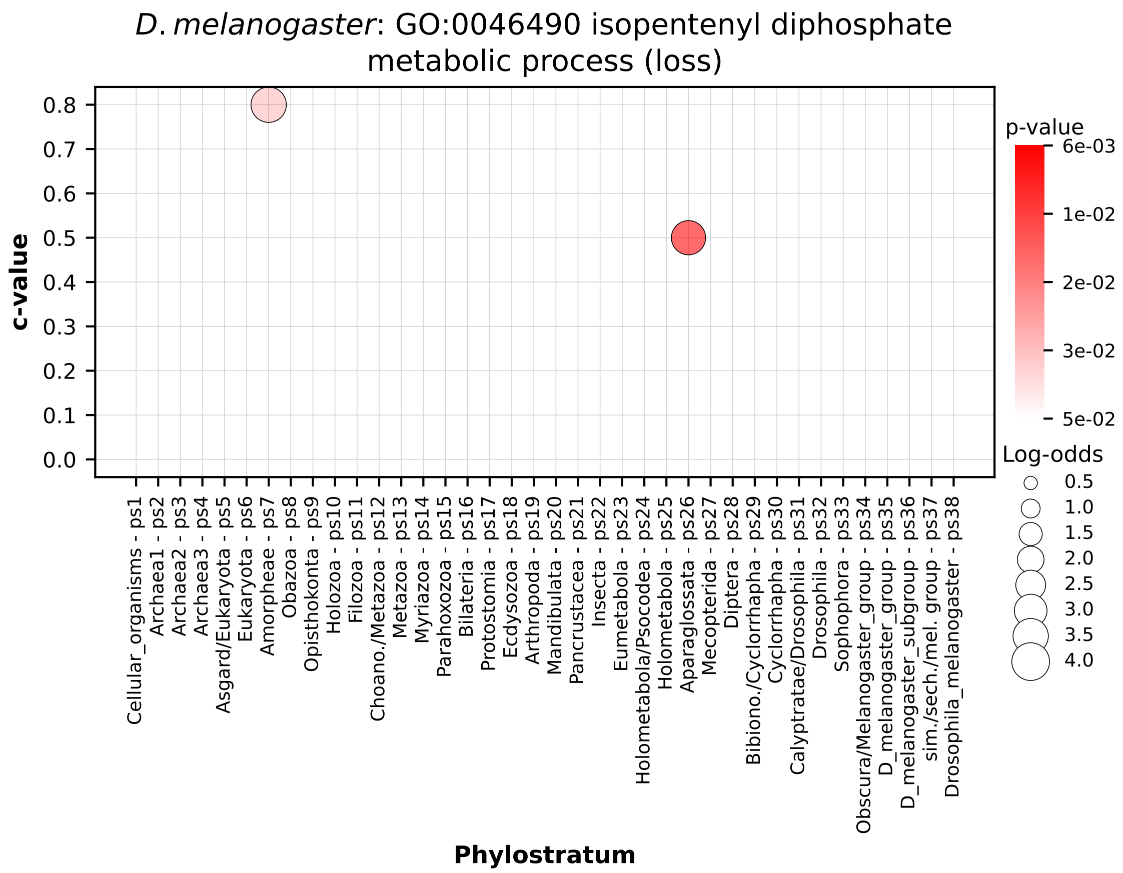

D. melanogaster: GO:0046490 isopentenyl diphosphate metabolic process (loss)
c-value
0.0
0.1
0.2
0.3
0.4
0.5
0.6
0.7
0.8
p-value
6e-03
1e-02
2e-02
3e-02
5e-02
Log-odds
0.5
1.0
1.5
2.0
2.5
3.0
3.5
4.0
Cellular_organisms - ps1
Archaea1 - ps2
Archaea2 - ps3
Archaea3 - ps4
Asgard/Eukaryota - ps5
Eukaryota - ps6
Amorpheae - ps7
Obazoa - ps8
Opisthokonta - ps9
Holozoa - ps10
Filozoa - ps11
Choano./Metazoa - ps12
Metazoa - ps13
Myriazoa - ps14
Parahoxozoa - ps15
Bilateria - ps16
Protostomia - ps17
Ecdysozoa - ps18
Arthropoda - ps19
Mandibulata - ps20
Pancrustacea - ps21
Insecta - ps22
Eumetabola - ps23
Holometabola/Psocodea - ps24
Holometabola - ps25
Aparaglossata - ps26
Mecopterida - ps27
Diptera - ps28
Bibiono./Cyclorrhapha - ps29
Cyclorrhapha - ps30
Calyptratae/Drosophila - ps31
Drosophila - ps32
Sophophora - ps33
Obscura/Melanogaster_group - ps34
D_melanogaster_group - ps35
D_melanogaster_subgroup - ps36
sim./sech./mel. group - ps37
Drosophila_melanogaster - ps38
Phylostratum

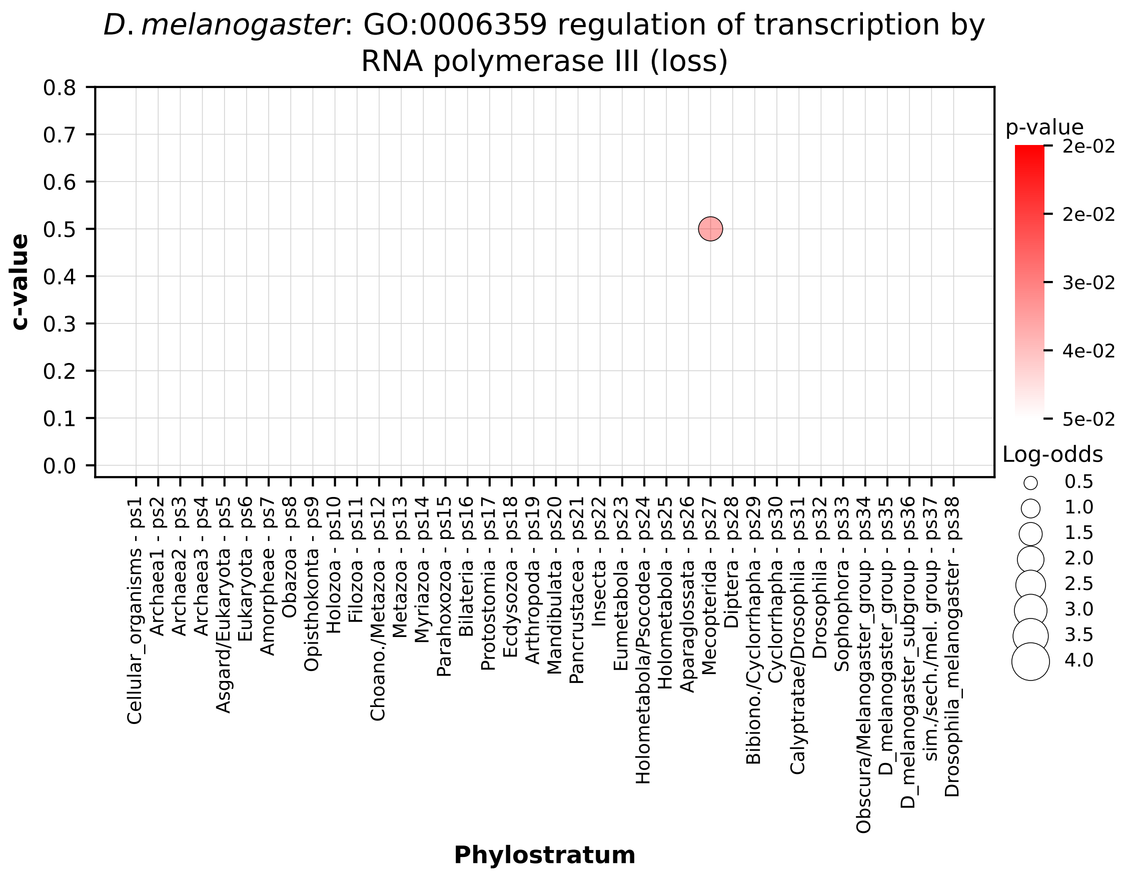
D. melanogaster: GO:0006359 regulation of transcription by RNA polymerase III (loss)
c-value
0.8
0.7
0.6
0.5
0.4
0.3
0.2
0.1
0.0
p-value
2e-02
2e-02
3e-02
4e-02
5e-02
Log-odds
0.5
1.0
1.5
2.0
2.5
3.0
3.5
4.0
Cellular_organisms - ps1
Archaea1 - ps2
Archaea2 - ps3
Archaea3 - ps4
Asgard/Eukaryota - ps5
Eukaryota - ps6
Amorpheae - ps7
Obazoa - ps8
Opisthokonta - ps9
Holozoa - ps10
Filozoa - ps11
Choano./Metazoa - ps12
Metazoa - ps13
Myriazoa - ps14
Parahoxozoa - ps15
Bilateria - ps16
Protostomia - ps17
Ecdysozoa - ps18
Arthropoda - ps19
Mandibulata - ps20
Pancrustacea - ps21
Insecta - ps22
Eumetabola - ps23
Holometabola/Psocodea - ps24
Holometabola - ps25
Aparaglossata - ps26
Mecopterida - ps27
Diptera - ps28
Bibiono./Cyclorrhapha - ps29
Cyclorrhapha - ps30
Calyptratae/Drosophila - ps31
Drosophila - ps32
Sophophora - ps33
Obscura/Melanogaster_group - ps34
D_melanogaster_group - ps35
D_melanogaster_subgroup - ps36
sim./sech./mel. group - ps37
Drosophila_melanogaster - ps38
Phylostratum

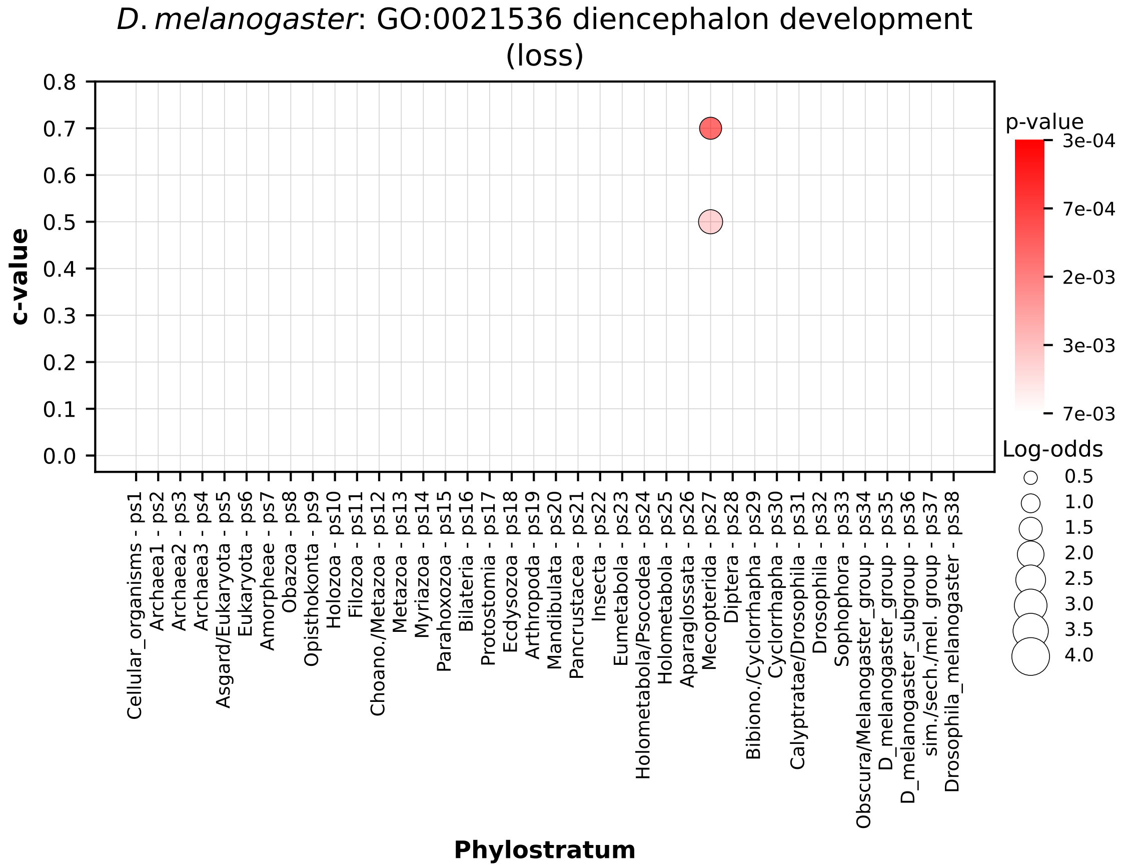

D. melanogaster: GO:0021536 diencephalon development
(loss)
c-value
0.8
0.7
0.6
0.5
0.4
0.3
0.2
0.1
0.0
Phylostratum
Cellular_organisms - ps1
Archaea1 - ps2
Archaea2 - ps3
Archaea3 - ps4
Asgard/Eukaryota - ps5
Eukaryota - ps6
Amorpheae - ps7
Obazoa - ps8
Opisthokonta - ps9
Holozoa - ps10
Filozoa - ps11
Choano./Metazoa - ps12
Metazoa - ps13
Myriazoa - ps14
Parahoxozoa - ps15
Bilateria - ps16
Protostomia - ps17
Ecdysozoa - ps18
Arthropoda - ps19
Mandibulata - ps20
Pancrustacea - ps21
Insecta - ps22
Eumetabola - ps23
Holometabola/Psocodea - ps24
Holometabola - ps25
Aparaglossata - ps26
Mecopterida - ps27
Diptera - ps28
Bibiono./Cyclorrhapha - ps29
Cyclorrhapha - ps30
Calyptratae/Drosophila - ps31
Drosophila - ps32
Sophophora - ps33
Obscura/Melanogaster_group - ps34
D_melanogaster_group - ps35
D_melanogaster_subgroup - ps36
sim./sech./mel. group - ps37
Drosophila_melanogaster - ps38
p-value
3e-04
7e-04
2e-03
3e-03
7e-03
Log-odds
0.5
1.0
1.5
2.0
2.5
3.0
3.5
4.0

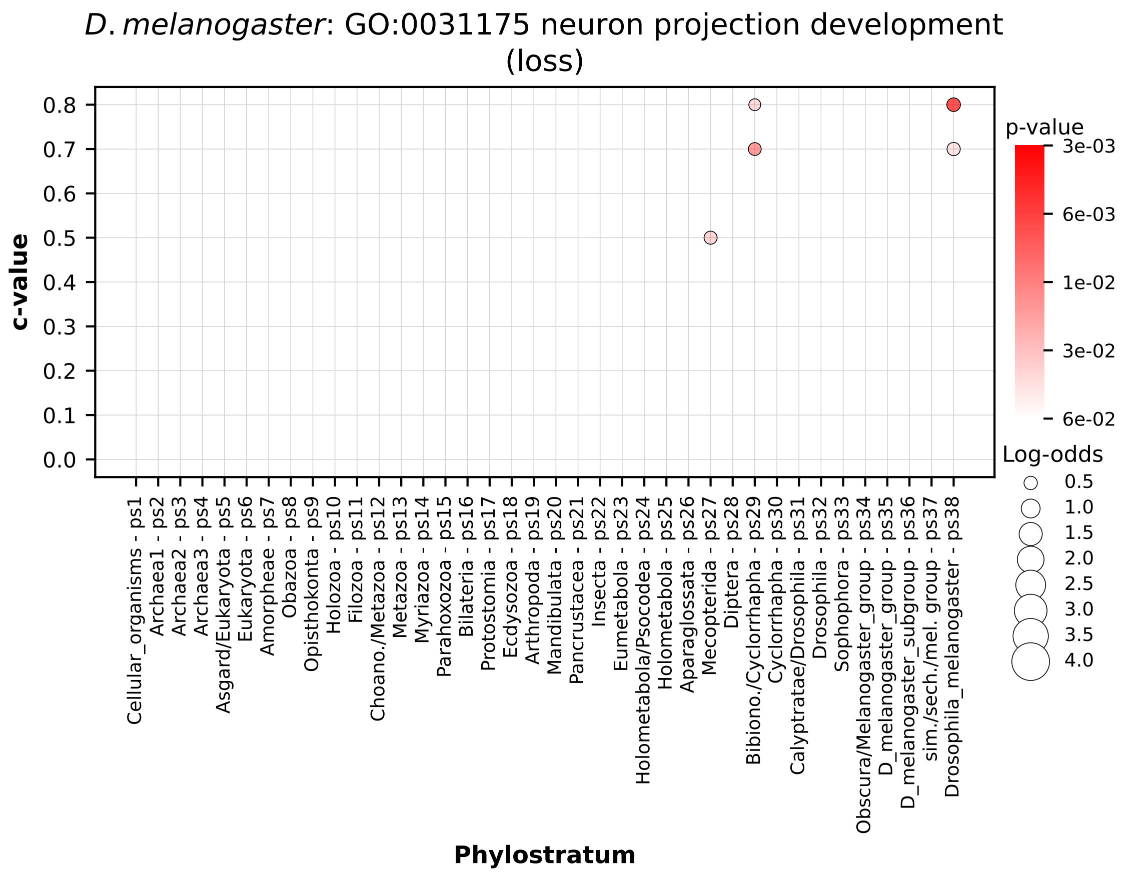

D. melanogaster: GO:0031175 neuron projection development (loss)
c-value
0.0
0.1
0.2
0.3
0.4
0.5
0.6
0.7
0.8
p-value
3e-03
6e-03
1e-02
3e-02
6e-02
Log-odds
0.5
1.0
1.5
2.0
2.5
3.0
3.5
4.0
Cellular_organisms - ps1
Archaea1 - ps2
Archaea2 - ps3
Archaea3 - ps4
Asgard/Eukaryota - ps5
Eukaryota - ps6
Amorpheae - ps7
Obazoa - ps8
Opisthokonta - ps9
Holozoa - ps10
Filozoa - ps11
Choano./Metazoa - ps12
Metazoa - ps13
Myriazoa - ps14
Parahoxozoa - ps15
Bilateria - ps16
Protostomia - ps17
Ecdysozoa - ps18
Arthropoda - ps19
Mandibulata - ps20
Pancrustacea - ps21
Insecta - ps22
Eumetabola - ps23
Holometabola/Psocodea - ps24
Holometabola - ps25
Aparaglossata - ps26
Mecopterida - ps27
Diptera - ps28
Bibiono./Cyclorrhapha - ps29
Cyclorrhapha - ps30
Calyptratae/Drosophila - ps31
Drosophila - ps32
Sophophora - ps33
Obscura/Melanogaster_group - ps34
D_melanogaster_group - ps35
D_melanogaster_subgroup - ps36
sim./sech./mel. group - ps37
Drosophila_melanogaster - ps38
Phylostratum

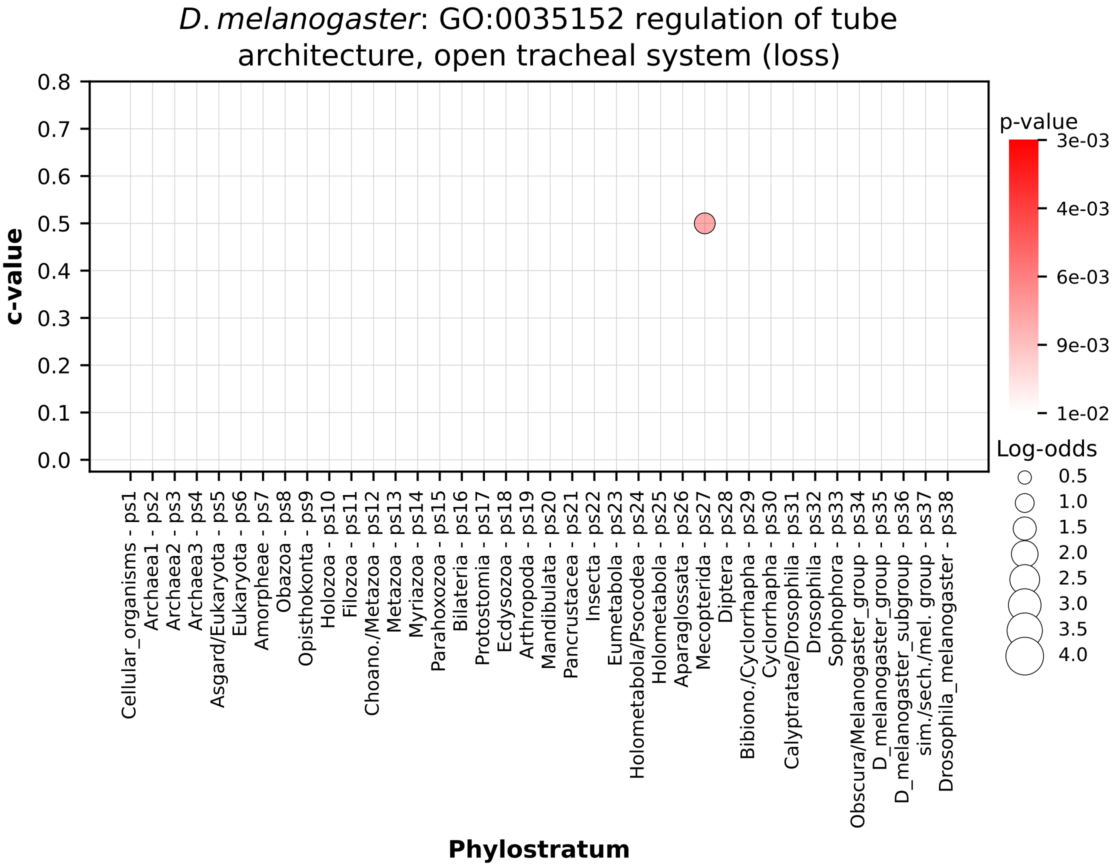
D. melanogaster: GO:0035152 regulation of tube architecture, open tracheal system (loss)
c-value
0.8
0.7
0.6
0.5
0.4
0.3
0.2
0.1
0.0
Phylostratum
Cellular_organisms - ps1
Archaea1 - ps2
Archaea2 - ps3
Archaea3 - ps4
Asgard/Eukaryota - ps5
Eukaryota - ps6
Amorpheae - ps7
Obazoa - ps8
Opisthokonta - ps9
Holozoa - ps10
Filozoa - ps11
Choano./Metazoa - ps12
Metazoa - ps13
Myriazoa - ps14
Parahoxozoa - ps15
Bilateria - ps16
Protostomia - ps17
Ecdysozoa - ps18
Arthropoda - ps19
Mandibulata - ps20
Pancrustacea - ps21
Insecta - ps22
Eumetabola - ps23
Holometabola/Psocodea - ps24
Holometabola - ps25
Aparaglossata - ps26
Mecopterida - ps27
Diptera - ps28
Bibiono./Cyclorrhapha - ps29
Cyclorrhapha - ps30
Calyptratae/Drosophila - ps31
Drosophila - ps32
Sophophora - ps33
Obscura/Melanogaster_group - ps34
D_melanogaster_group - ps35
D_melanogaster_subgroup - ps36
sim./sech./mel. group - ps37
Drosophila_melanogaster - ps38
p-value
3e-03
4e-03
6e-03
9e-03
1e-02
Log-odds
0.5
1.0
1.5
2.0
2.5
3.0
3.5
4.0

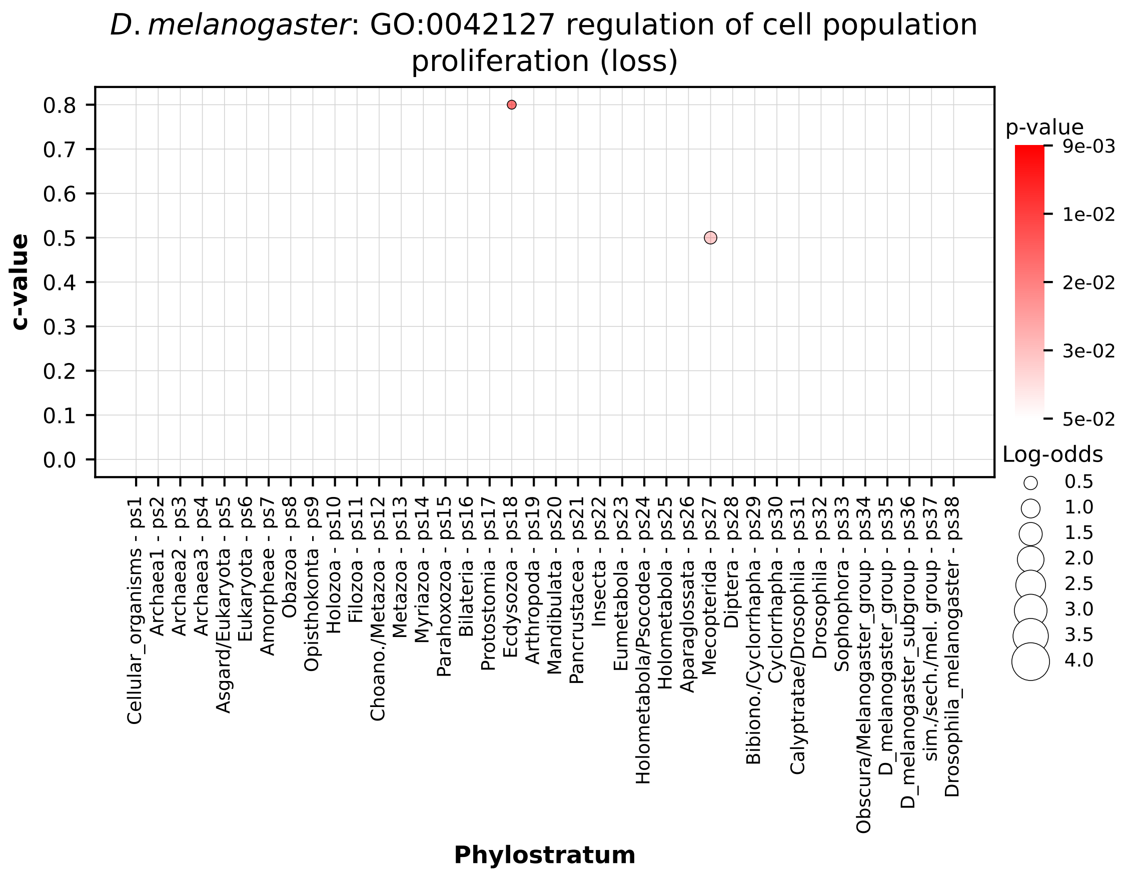
D. melanogaster: GO:0042127 regulation of cell population proliferation (loss)
c-value
0.0
0.1
0.2
0.3
0.4
0.5
0.6
0.7
0.8
Phylostratum
Cellular_organisms - ps1
Archaea1 - ps2
Archaea2 - ps3
Archaea3 - ps4
Asgard/Eukaryota - ps5
Eukaryota - ps6
Amorpheae - ps7
Obazoa - ps8
Opisthokonta - ps9
Holozoa - ps10
Filozoa - ps11
Choano./Metazoa - ps12
Metazoa - ps13
Myriazoa - ps14
Parahoxozoa - ps15
Bilateria - ps16
Protostomia - ps17
Ecdysozoa - ps18
Arthropoda - ps19
Mandibulata - ps20
Pancrustacea - ps21
Insecta - ps22
Eumetabola - ps23
Holometabola/Psocodea - ps24
Holometabola - ps25
Aparaglossata - ps26
Mecopterida - ps27
Diptera - ps28
Bibiono./Cyclorrhapha - ps29
Cyclorrhapha - ps30
Calyptratae/Drosophila - ps31
Drosophila - ps32
Sophophora - ps33
Obscura/Melanogaster_group - ps34
D_melanogaster_group - ps35
D_melanogaster_subgroup - ps36
sim./sech./mel. group - ps37
Drosophila_melanogaster - ps38
p-value
9e-03
1e-02
2e-02
3e-02
5e-02
Log-odds
0.5
1.0
1.5
2.0
2.5
3.0
3.5
4.0

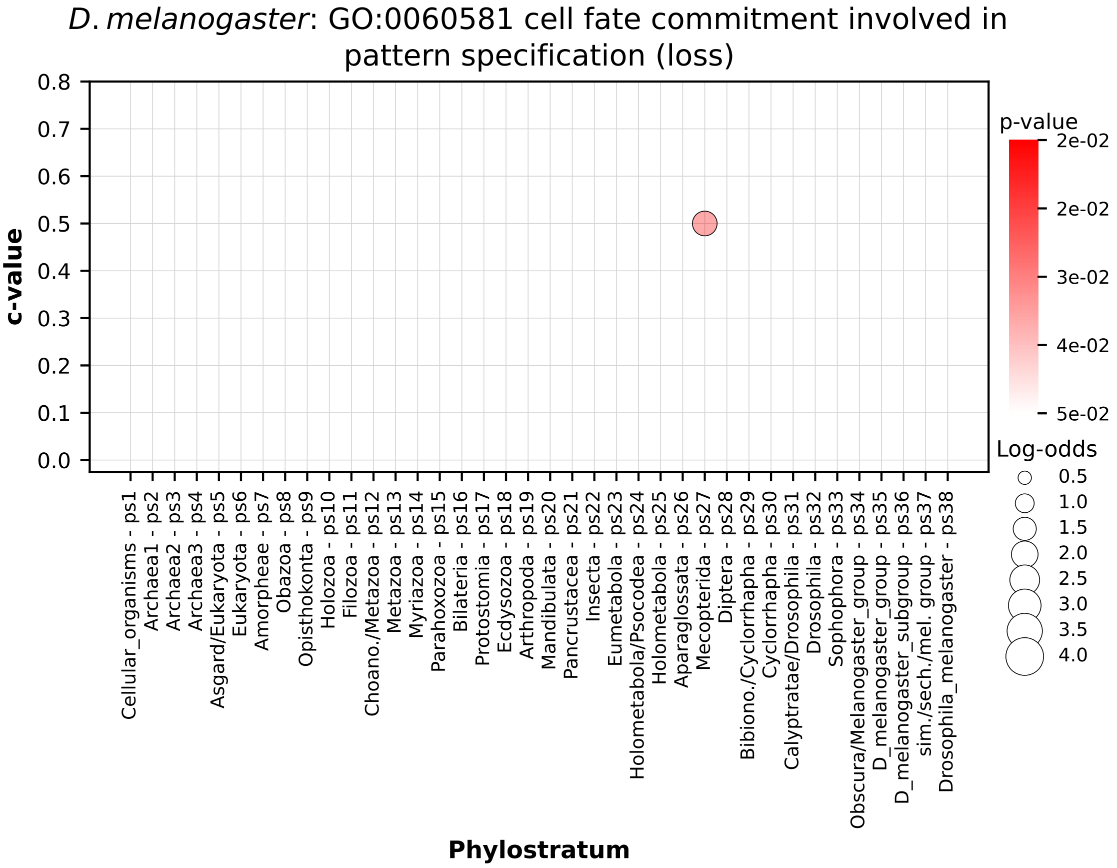

D. melanogaster: GO:0060581 cell fate commitment involved in pattern specification (loss)
c-value
0.8
0.7
0.6
0.5
0.4
0.3
0.2
0.1
0.0
p-value
2e-02
2e-02
3e-02
4e-02
5e-02
Log-odds
0.5
1.0
1.5
2.0
2.5
3.0
3.5
4.0
Cellular_organisms - ps1
Archaea1 - ps2
Archaea2 - ps3
Archaea3 - ps4
Asgard/Eukaryota - ps5
Eukaryota - ps6
Amorpheae - ps7
Obazoa - ps8
Opisthokonta - ps9
Holozoa - ps10
Filozoa - ps11
Choano./Metazoa - ps12
Metazoa - ps13
Myriazoa - ps14
Parahoxozoa - ps15
Bilateria - ps16
Protostomia - ps17
Ecdysozoa - ps18
Arthropoda - ps19
Mandibulata - ps20
Pancrustacea - ps21
Insecta - ps22
Eumetabola - ps23
Holometabola/Psocodea - ps24
Holometabola - ps25
Aparaglossata - ps26
Mecopterida - ps27
Diptera - ps28
Bibiono./Cyclorrhapha - ps29
Cyclorrhapha - ps30
Calyptratae/Drosophila - ps31
Drosophila - ps32
Sophophora - ps33
Obscura/Melanogaster_group - ps34
D_melanogaster_group - ps35
D_melanogaster_subgroup - ps36
sim./sech./mel. group - ps37
Drosophila_melanogaster - ps38
Phylostratum

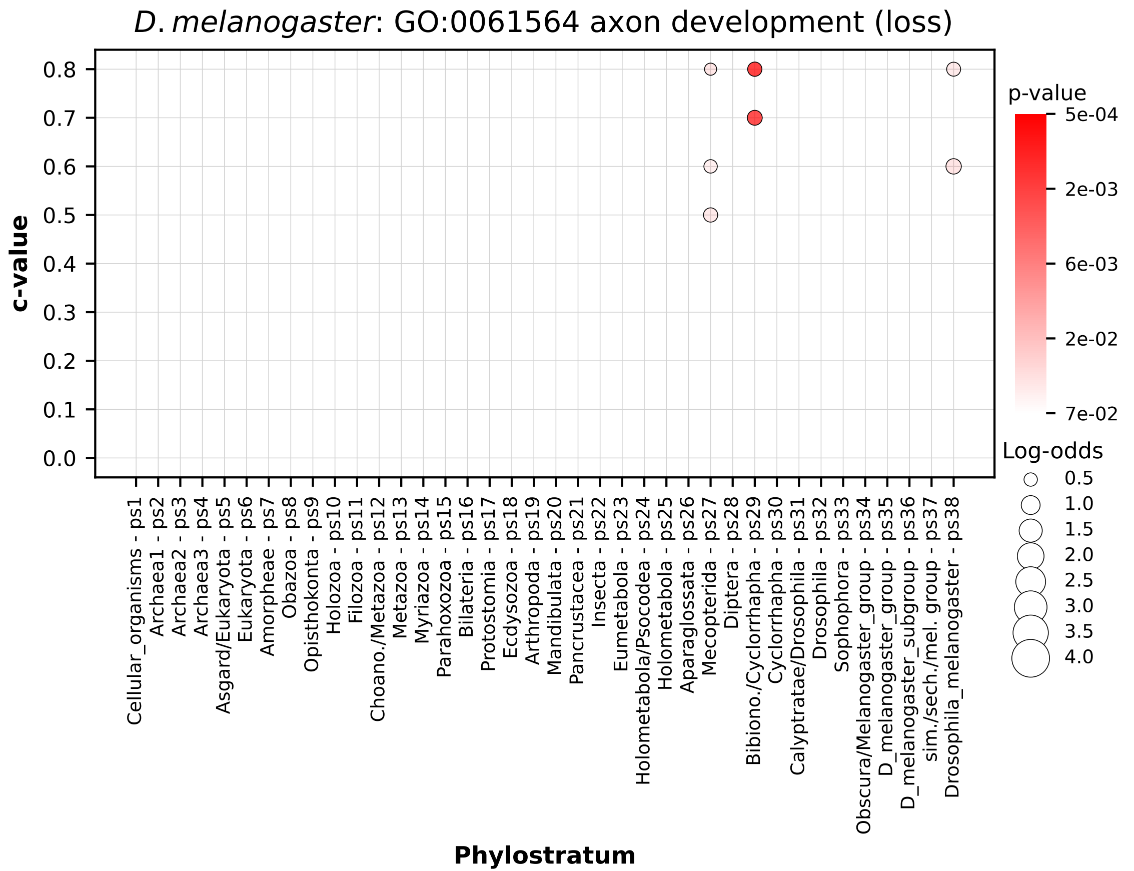

D. melanogaster: GO:0061564 axon development (loss)
c-value
0.0
0.1
0.2
0.3
0.4
0.5
0.6
0.7
0.8
Phylostratum
Cellular_organisms - ps1
Archaea1 - ps2
Archaea2 - ps3
Archaea3 - ps4
Asgard/Eukaryota - ps5
Eukaryota - ps6
Amorpheae - ps7
Obazoa - ps8
Opisthokonta - ps9
Holozoa - ps10
Filozoa - ps11
Choano./Metazoa - ps12
Metazoa - ps13
Myriazoa - ps14
Parahoxozoa - ps15
Bilateria - ps16
Protostomia - ps17
Ecdysozoa - ps18
Arthropoda - ps19
Mandibulata - ps20
Pancrustacea - ps21
Insecta - ps22
Eumetabola - ps23
Holometabola/Psocodea - ps24
Holometabola - ps25
Aparaglossata - ps26
Mecopterida - ps27
Diptera - ps28
Bibiono./Cyclorrhapha - ps29
Cyclorrhapha - ps30
Calyptratae/Drosophila - ps31
Drosophila - ps32
Sophophora - ps33
Obscura/Melanogaster_group - ps34
D_melanogaster_group - ps35
D_melanogaster_subgroup - ps36
sim./sech./mel. group - ps37
Drosophila_melanogaster - ps38
p-value
5e-04
2e-03
6e-03
2e-02
7e-02
Log-odds
0.5
1.0
1.5
2.0
2.5
3.0
3.5
4.0

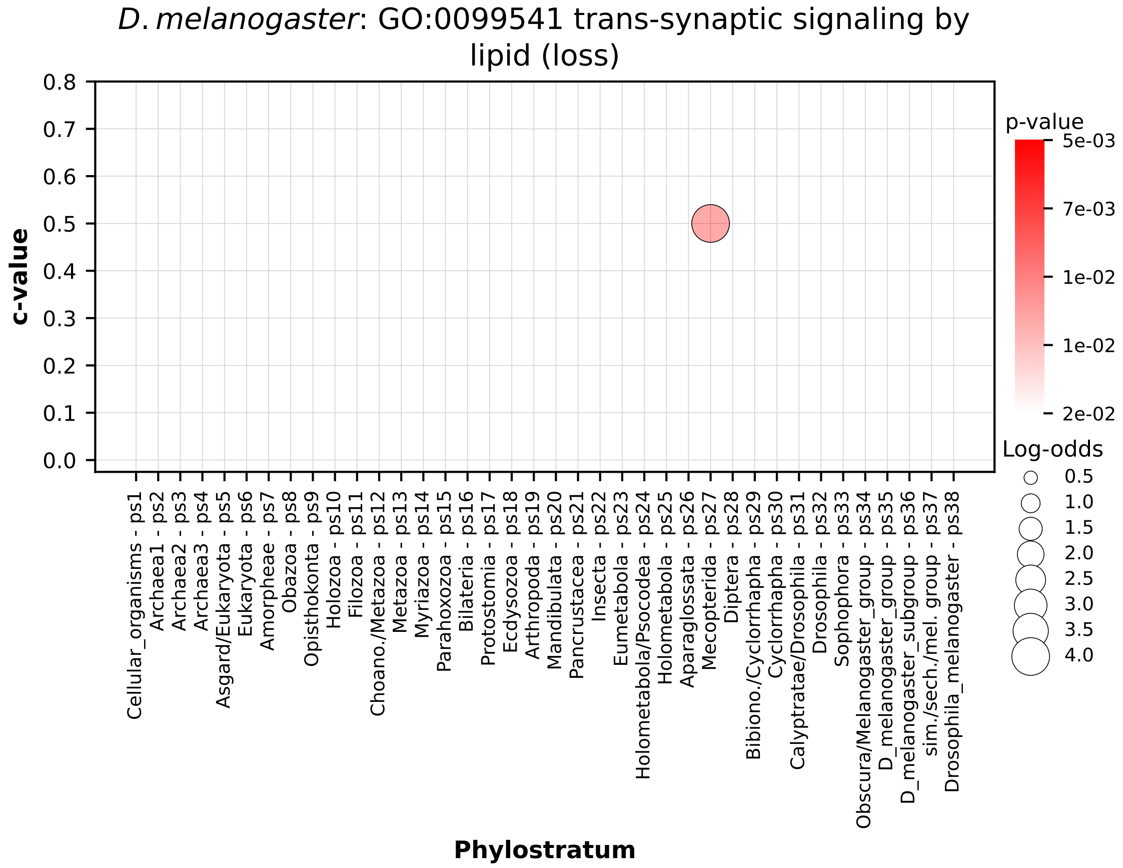

D. melanogaster: GO:0099541 trans-synaptic signaling by lipid (loss)
c-value
0.8
0.7
0.6
0.5
0.4
0.3
0.2
0.1
0.0
p-value
5e-03
7e-03
1e-02
1e-02
2e-02
Log-odds
0.5
1.0
1.5
2.0
2.5
3.0
3.5
4.0
Cellular_organisms - ps1
Archaea1 - ps2
Archaea2 - ps3
Archaea3 - ps4
Asgard/Eukaryota - ps5
Eukaryota - ps6
Amorpheae - ps7
Obazoa - ps8
Opisthokonta - ps9
Holozoa - ps10
Filozoa - ps11
Choano./Metazoa - ps12
Metazoa - ps13
Myriazoa - ps14
Parahoxozoa - ps15
Bilateria - ps16
Protostomia - ps17
Ecdysozoa - ps18
Arthropoda - ps19
Mandibulata - ps20
Pancrustacea - ps21
Insecta - ps22
Eumetabola - ps23
Holometabola/Psocodea - ps24
Holometabola - ps25
Aparaglossata - ps26
Mecopterida - ps27
Diptera - ps28
Bibiono./Cyclorrhapha - ps29
Cyclorrhapha - ps30
Calyptratae/Drosophila - ps31
Drosophila - ps32
Sophophora - ps33
Obscura/Melanogaster_group - ps34
D_melanogaster_group - ps35
D_melanogaster_subgroup - ps36
sim./sech./mel. group - ps37
Drosophila_melanogaster - ps38
Phylostratum

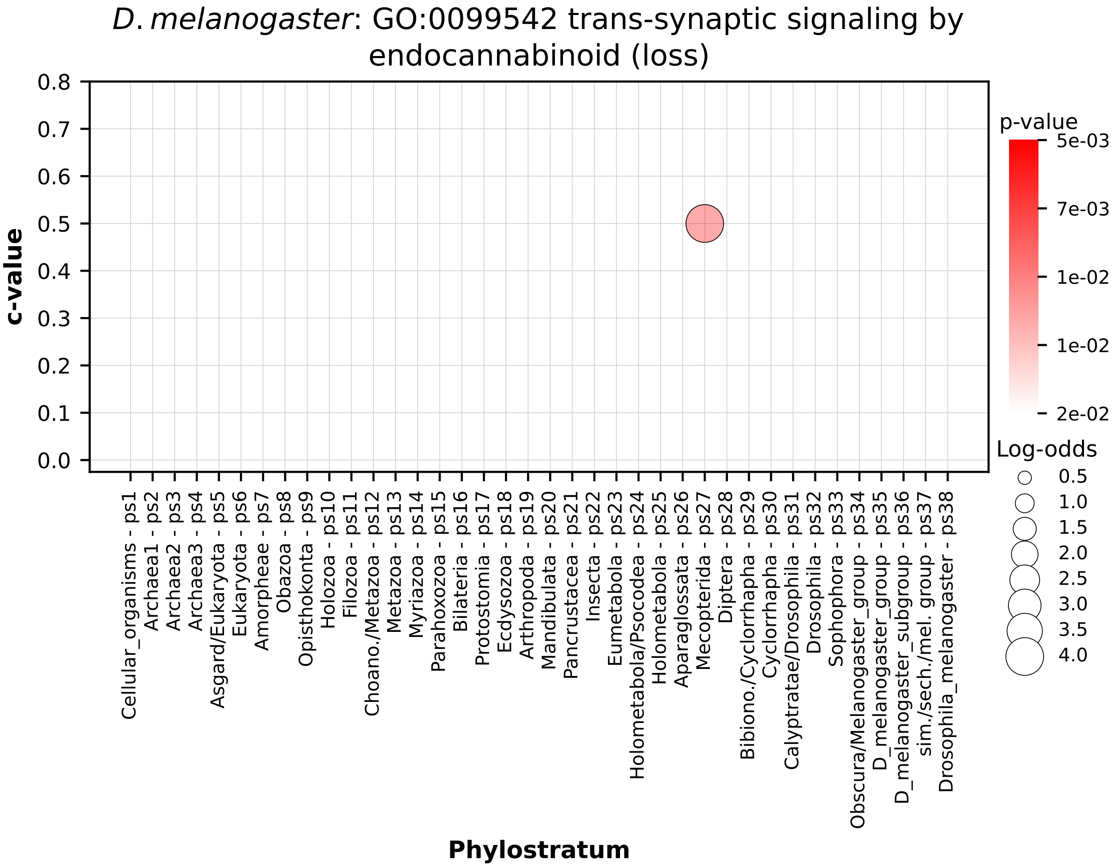

D. melanogaster: GO:0099542 trans-synaptic signaling by endocannabinoid (loss)
c-value
0.0
0.1
0.2
0.3
0.4
0.5
0.6
0.7
0.8
Phylostratum
Cellular_organisms - ps1
Archaea1 - ps2
Archaea2 - ps3
Archaea3 - ps4
Asgard/Eukaryota - ps5
Eukaryota - ps6
Amorpheae - ps7
Obazoa - ps8
Opisthokonta - ps9
Holozoa - ps10
Filozoa - ps11
Choano./Metazoa - ps12
Metazoa - ps13
Myriazoa - ps14
Parahoxozoa - ps15
Bilateria - ps16
Protostomia - ps17
Ecdysozoa - ps18
Arthropoda - ps19
Mandibulata - ps20
Pancrustacea - ps21
Insecta - ps22
Eumetabola - ps23
Holometabola/Psocodea - ps24
Holometabola - ps25
Aparaglossata - ps26
Mecopterida - ps27
Diptera - ps28
Bibiono./Cyclorrhapha - ps29
Cyclorrhapha - ps30
Calyptratae/Drosophila - ps31
Drosophila - ps32
Sophophora - ps33
Obscura/Melanogaster_group - ps34
D_melanogaster_group - ps35
D_melanogaster_subgroup - ps36
sim./sech./mel. group - ps37
Drosophila_melanogaster - ps38
p-value
5e-03
7e-03
1e-02
1e-02
2e-02
Log-odds
0.5
1.0
1.5
2.0
2.5
3.0
3.5
4.0

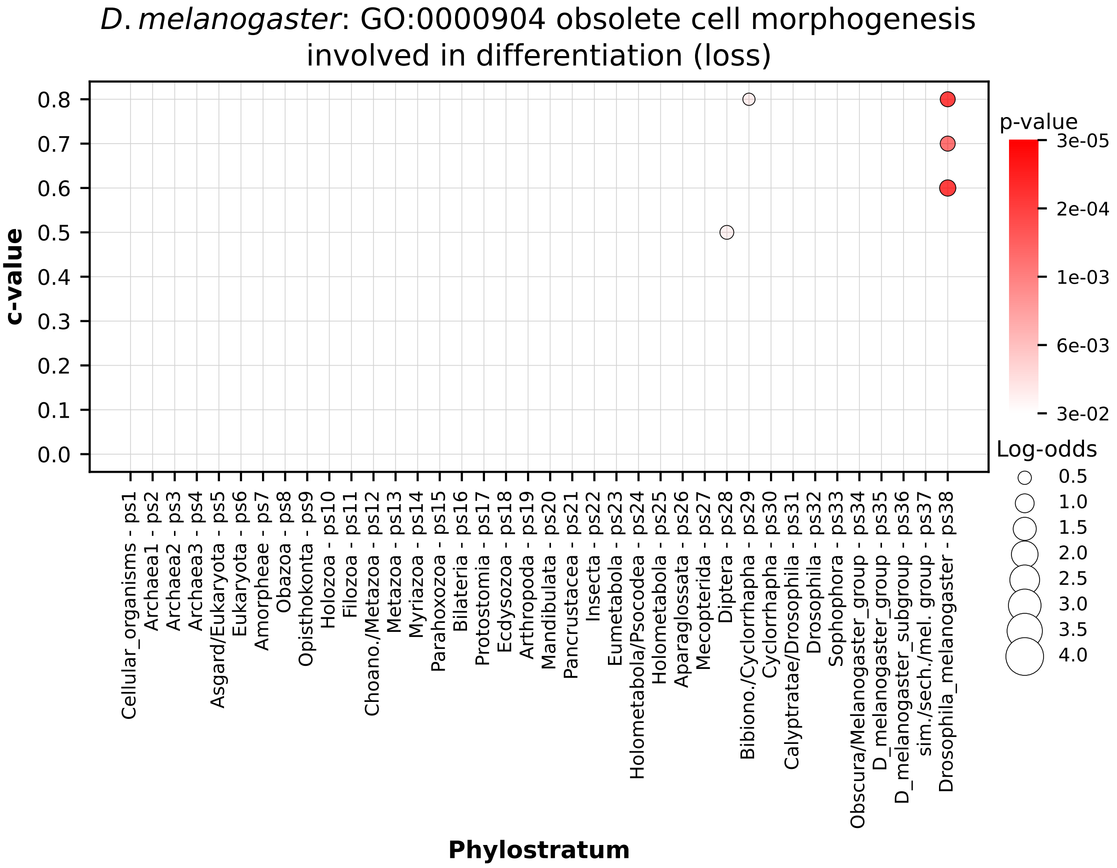

D. melanogaster: GO:0000904 obsolete cell morphogenesis involved in differentiation (loss)
c-value
0.8
0.7
0.6
0.5
0.4
0.3
0.2
0.1
0.0
Phylostratum
Cellular_organisms - ps1
Archaea1 - ps2
Archaea2 - ps3
Archaea3 - ps4
Asgard/Eukaryota - ps5
Eukaryota - ps6
Amorpheae - ps7
Obazoa - ps8
Opisthokonta - ps9
Holozoa - ps10
Filozoa - ps11
Choano./Metazoa - ps12
Metazoa - ps13
Myriazoa - ps14
Parahoxozoa - ps15
Bilateria - ps16
Protostomia - ps17
Ecdysozoa - ps18
Arthropoda - ps19
Mandibulata - ps20
Pancrustacea - ps21
Insecta - ps22
Eumetabola - ps23
Holometabola/Psocodea - ps24
Holometabola - ps25
Aparaglossata - ps26
Mecopterida - ps27
Diptera - ps28
Bibiono./Cyclorrhapha - ps29
Cyclorrhapha - ps30
Calyptratae/Drosophila - ps31
Drosophila - ps32
Sophophora - ps33
Obscura/Melanogaster_group - ps34
D_melanogaster_group - ps35
D_melanogaster_subgroup - ps36
sim./sech./mel. group - ps37
Drosophila_melanogaster - ps38
p-value
3e-05
2e-04
1e-03
6e-03
3e-02
Log-odds
0.5
1.0
1.5
2.0
2.5
3.0
3.5
4.0

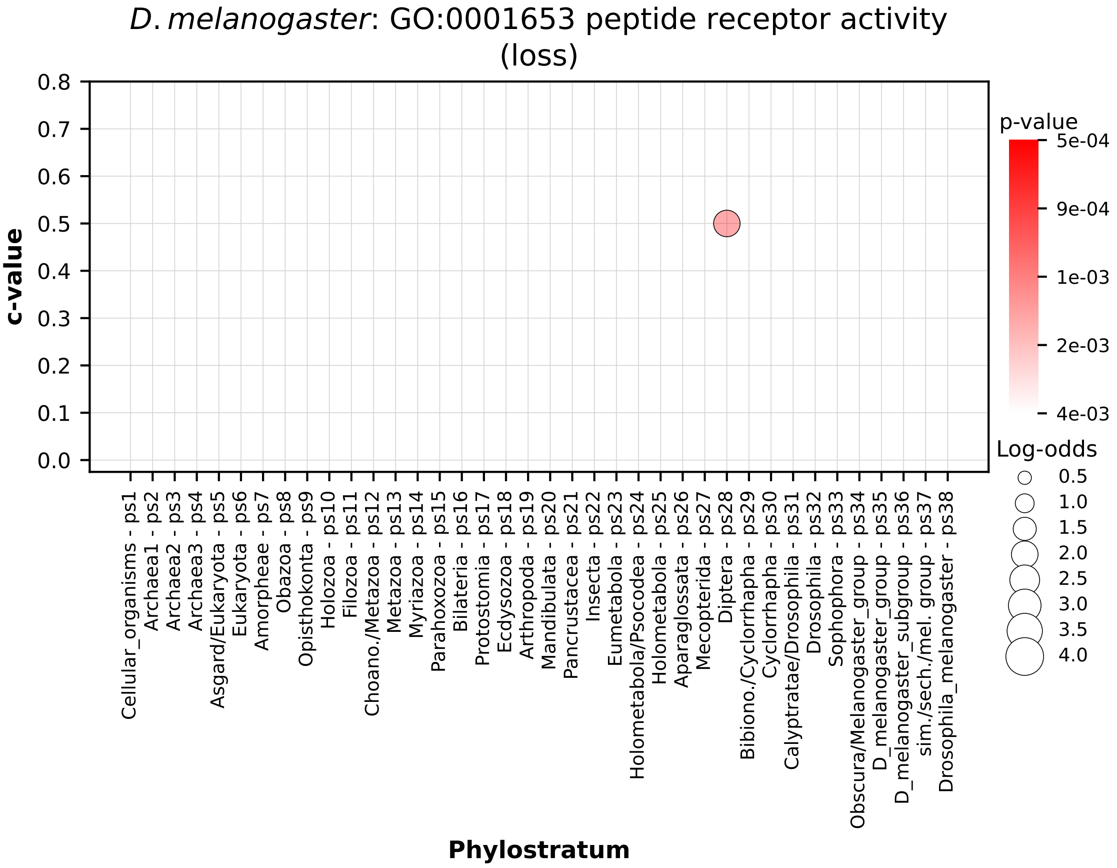

D. melanogaster: GO:0001653 peptide receptor activity
(loss)
c-value
0.0
0.1
0.2
0.3
0.4
0.5
0.6
0.7
0.8
p-value
5e-04
9e-04
1e-03
2e-03
4e-03
Log-odds
0.5
1.0
1.5
2.0
2.5
3.0
3.5
4.0
Cellular_organisms - ps1
Archaea1 - ps2
Archaea2 - ps3
Archaea3 - ps4
Asgard/Eukaryota - ps5
Eukaryota - ps6
Amorpheae - ps7
Obazoa - ps8
Opisthokonta - ps9
Holozoa - ps10
Filozoa - ps11
Choano./Metazoa - ps12
Metazoa - ps13
Myriazoa - ps14
Parahoxozoa - ps15
Bilateria - ps16
Protostomia - ps17
Ecdysozoa - ps18
Arthropoda - ps19
Mandibulata - ps20
Pancrustacea - ps21
Insecta - ps22
Eumetabola - ps23
Holometabola/Psocodea - ps24
Holometabola - ps25
Aparaglossata - ps26
Mecopterida - ps27
Diptera - ps28
Bibiono./Cyclorrhapha - ps29
Cyclorrhapha - ps30
Calyptratae/Drosophila - ps31
Drosophila - ps32
Sophophora - ps33
Obscura/Melanogaster_group - ps34
D_melanogaster_group - ps35
D_melanogaster_subgroup - ps36
sim./sech./mel. group - ps37
Drosophila_melanogaster - ps38
Phylostratum

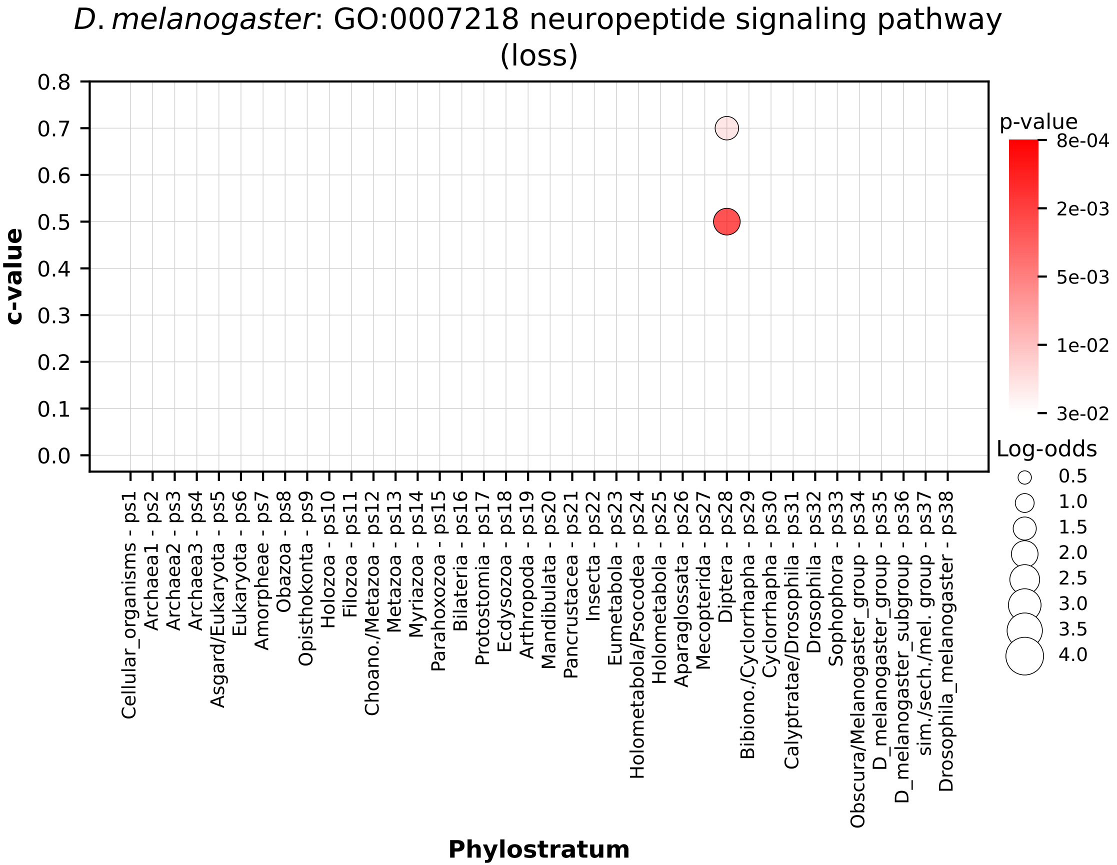

D. melanogaster: GO:0007218 neuropeptide signaling pathway (loss)
c-value
0.8
0.7
0.6
0.5
0.4
0.3
0.2
0.1
0.0
Phylostratum
Cellular_organisms - ps1
Archaea1 - ps2
Archaea2 - ps3
Archaea3 - ps4
Asgard/Eukaryota - ps5
Eukaryota - ps6
Amorpheae - ps7
Obazoa - ps8
Opisthokonta - ps9
Holozoa - ps10
Filozoa - ps11
Choano./Metazoa - ps12
Metazoa - ps13
Myriazoa - ps14
Parahoxozoa - ps15
Bilateria - ps16
Protostomia - ps17
Ecdysozoa - ps18
Arthropoda - ps19
Mandibulata - ps20
Pancrustacea - ps21
Insecta - ps22
Eumetabola - ps23
Holometabola/Psocodea - ps24
Holometabola - ps25
Aparaglossata - ps26
Mecopterida - ps27
Diptera - ps28
Bibiono./Cyclorrhapha - ps29
Cyclorrhapha - ps30
Calyptratae/Drosophila - ps31
Drosophila - ps32
Sophophora - ps33
Obscura/Melanogaster_group - ps34
D_melanogaster_group - ps35
D_melanogaster_subgroup - ps36
sim./sech./mel. group - ps37
Drosophila_melanogaster - ps38
p-value
8e-04
2e-03
5e-03
1e-02
3e-02
Log-odds
0.5
1.0
1.5
2.0
2.5
3.0
3.5
4.0

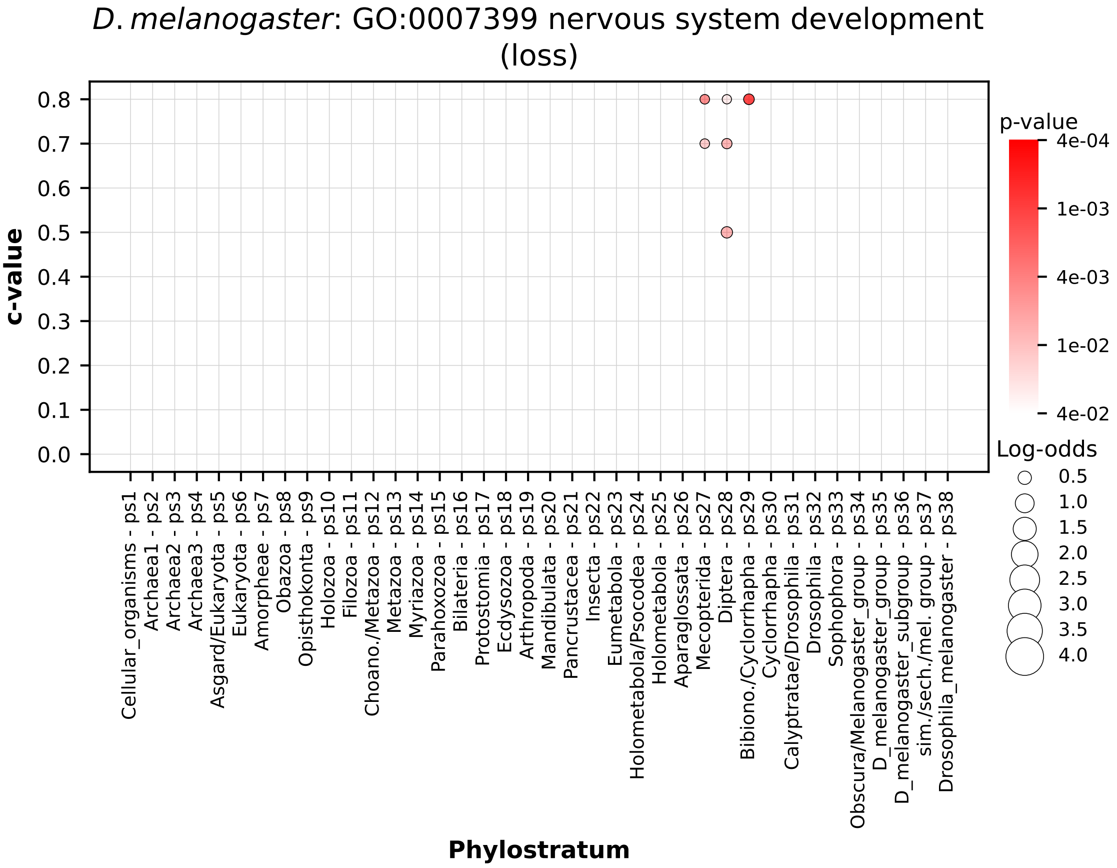

D. melanogaster: GO:0007399 nervous system development
(loss)
c-value
0.0
0.1
0.2
0.3
0.4
0.5
0.6
0.7
0.8
p-value
4e-04
1e-03
4e-03
1e-02
4e-02
Log-odds
0.5
1.0
1.5
2.0
2.5
3.0
3.5
4.0
Cellular_organisms - ps1
Archaea1 - ps2
Archaea2 - ps3
Archaea3 - ps4
Asgard/Eukaryota - ps5
Eukaryota - ps6
Amorpheae - ps7
Obazoa - ps8
Opisthokonta - ps9
Holozoa - ps10
Filozoa - ps11
Choano./Metazoa - ps12
Metazoa - ps13
Myriazoa - ps14
Parahoxozoa - ps15
Bilateria - ps16
Protostomia - ps17
Ecdysozoa - ps18
Arthropoda - ps19
Mandibulata - ps20
Pancrustacea - ps21
Insecta - ps22
Eumetabola - ps23
Holometabola/Psocodea - ps24
Holometabola - ps25
Aparaglossata - ps26
Mecopterida - ps27
Diptera - ps28
Bibiono./Cyclorrhapha - ps29
Cyclorrhapha - ps30
Calyptratae/Drosophila - ps31
Drosophila - ps32
Sophophora - ps33
Obscura/Melanogaster_group - ps34
D_melanogaster_group - ps35
D_melanogaster_subgroup - ps36
sim./sech./mel. group - ps37
Drosophila_melanogaster - ps38
Phylostratum

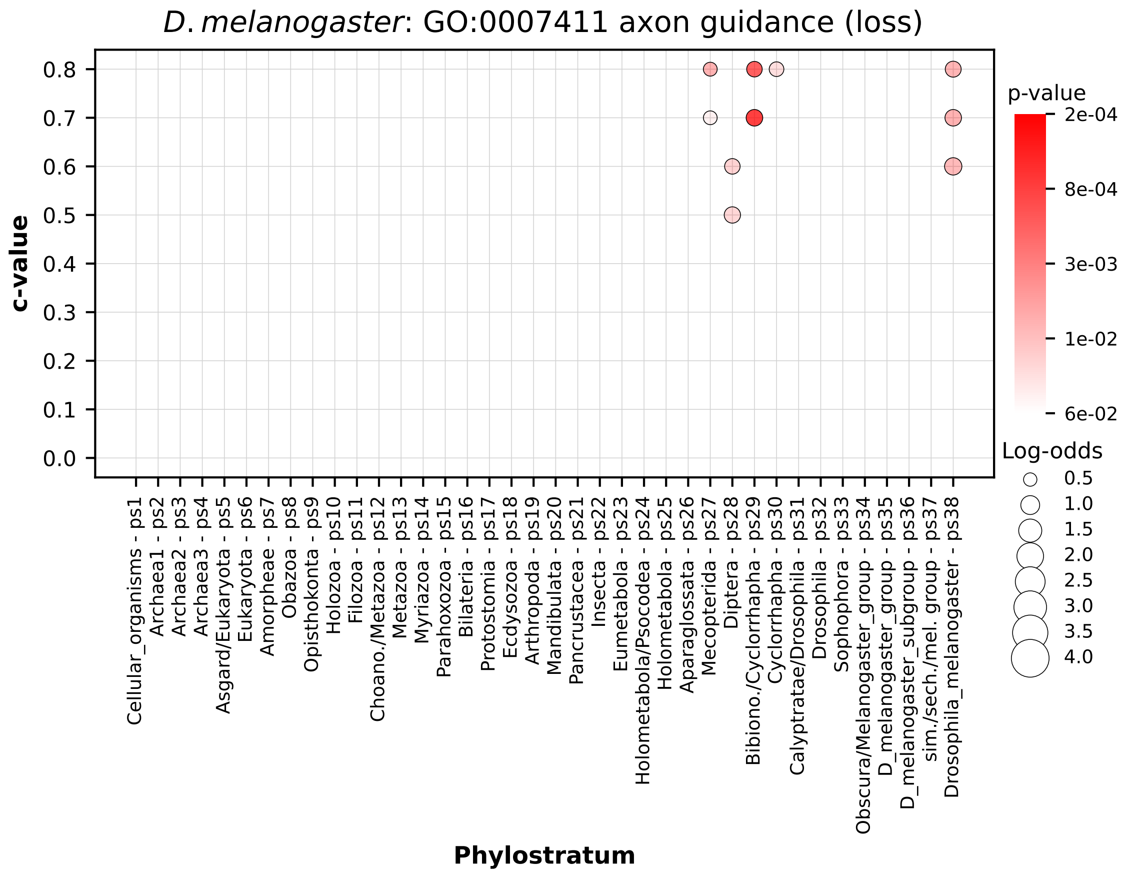

D. melanogaster: GO:0007411 axon guidance (loss)
c-value
0.0
0.1
0.2
0.3
0.4
0.5
0.6
0.7
0.8
Phylostratum
Cellular_organisms - ps1
Archaea1 - ps2
Archaea2 - ps3
Archaea3 - ps4
Asgard/Eukaryota - ps5
Eukaryota - ps6
Amorpheae - ps7
Obazoa - ps8
Opisthokonta - ps9
Holozoa - ps10
Filozoa - ps11
Choano./Metazoa - ps12
Metazoa - ps13
Myriazoa - ps14
Parahoxozoa - ps15
Bilateria - ps16
Protostomia - ps17
Ecdysozoa - ps18
Arthropoda - ps19
Mandibulata - ps20
Pancrustacea - ps21
Insecta - ps22
Eumetabola - ps23
Holometabola/Psocodea - ps24
Holometabola - ps25
Aparaglossata - ps26
Mecopterida - ps27
Diptera - ps28
Bibiono./Cyclorrhapha - ps29
Cyclorrhapha - ps30
Calyptratae/Drosophila - ps31
Drosophila - ps32
Sophophora - ps33
Obscura/Melanogaster_group - ps34
D_melanogaster_group - ps35
D_melanogaster_subgroup - ps36
sim./sech./mel. group - ps37
Drosophila_melanogaster - ps38
p-value
2e-04
8e-04
3e-03
1e-02
6e-02
Log-odds
0.5
1.0
1.5
2.0
2.5
3.0
3.5
4.0

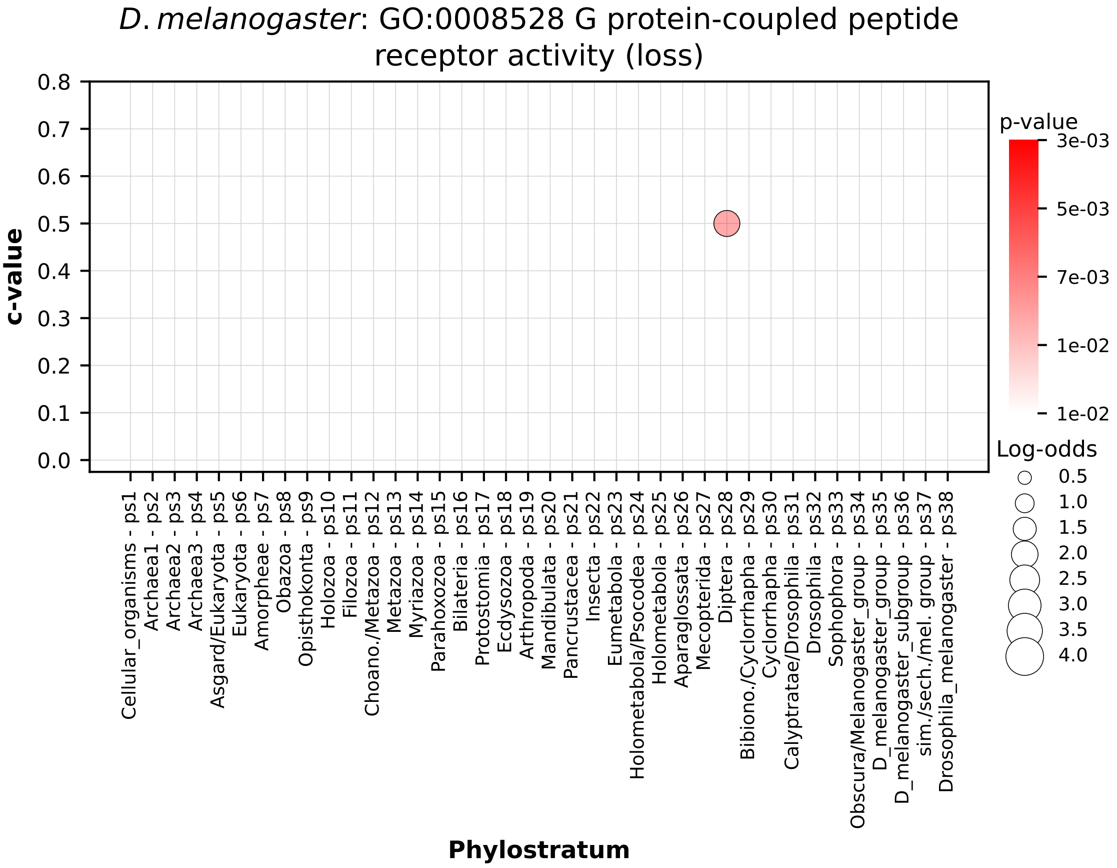
D. melanogaster: GO:0008528 G protein-coupled peptide receptor activity (loss)
c-value
0.8
0.7
0.6
0.5
0.4
0.3
0.2
0.1
0.0
Phylostratum
Cellular_organisms - ps1
Archaea1 - ps2
Archaea2 - ps3
Archaea3 - ps4
Asgard/Eukaryota - ps5
Eukaryota - ps6
Amorpheae - ps7
Obazoa - ps8
Opisthokonta - ps9
Holozoa - ps10
Filozoa - ps11
Choano./Metazoa - ps12
Metazoa - ps13
Myriazoa - ps14
Parahoxozoa - ps15
Bilateria - ps16
Protostomia - ps17
Ecdysozoa - ps18
Arthropoda - ps19
Mandibulata - ps20
Pancrustacea - ps21
Insecta - ps22
Eumetabola - ps23
Holometabola/Psocodea - ps24
Holometabola - ps25
Aparaglossata - ps26
Mecopterida - ps27
Diptera - ps28
Bibiono./Cyclorrhapha - ps29
Cyclorrhapha - ps30
Calyptratae/Drosophila - ps31
Drosophila - ps32
Sophophora - ps33
Obscura/Melanogaster_group - ps34
D_melanogaster_group - ps35
D_melanogaster_subgroup - ps36
sim./sech./mel. group - ps37
Drosophila_melanogaster - ps38
p-value
3e-03
5e-03
7e-03
1e-02
1e-02
Log-odds
0.5
1.0
1.5
2.0
2.5
3.0
3.5
4.0

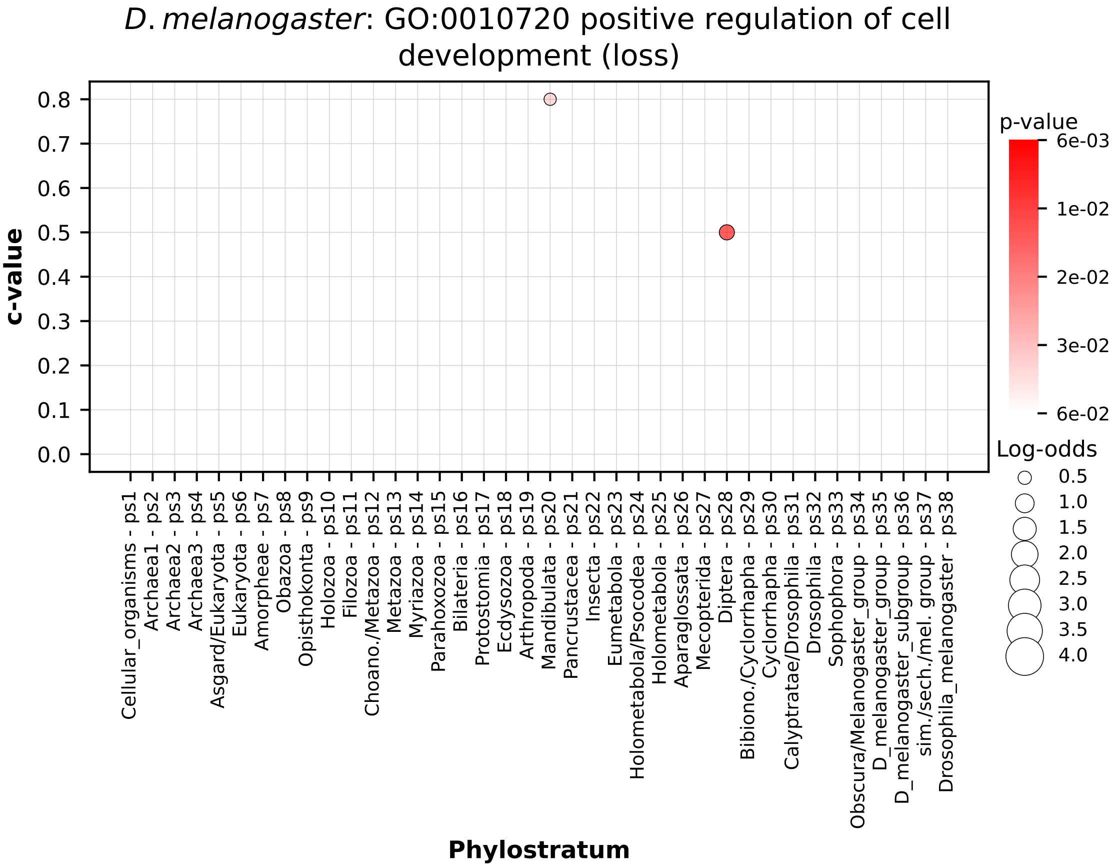

D. melanogaster: GO:0010720 positive regulation of cell development (loss)
c-value
0.8
0.7
0.6
0.5
0.4
0.3
0.2
0.1
0.0
Phylostratum
Cellular_organisms - ps1
Archaea1 - ps2
Archaea2 - ps3
Archaea3 - ps4
Asgard/Eukaryota - ps5
Eukaryota - ps6
Amorpheae - ps7
Obazoa - ps8
Opisthokonta - ps9
Holozoa - ps10
Filozoa - ps11
Choano./Metazoa - ps12
Metazoa - ps13
Myriazoa - ps14
Parahoxozoa - ps15
Bilateria - ps16
Protostomia - ps17
Ecdysozoa - ps18
Arthropoda - ps19
Mandibulata - ps20
Pancrustacea - ps21
Insecta - ps22
Eumetabola - ps23
Holometabola/Psocodea - ps24
Holometabola - ps25
Aparaglossata - ps26
Mecopterida - ps27
Diptera - ps28
Bibiono./Cyclorrhapha - ps29
Cyclorrhapha - ps30
Calyptratae/Drosophila - ps31
Drosophila - ps32
Sophophora - ps33
Obscura/Melanogaster_group - ps34
D_melanogaster_group - ps35
D_melanogaster_subgroup - ps36
sim./sech./mel. group - ps37
Drosophila_melanogaster - ps38
p-value
6e-03
1e-02
2e-02
3e-02
6e-02
Log-odds
0.5
1.0
1.5
2.0
2.5
3.0
3.5
4.0

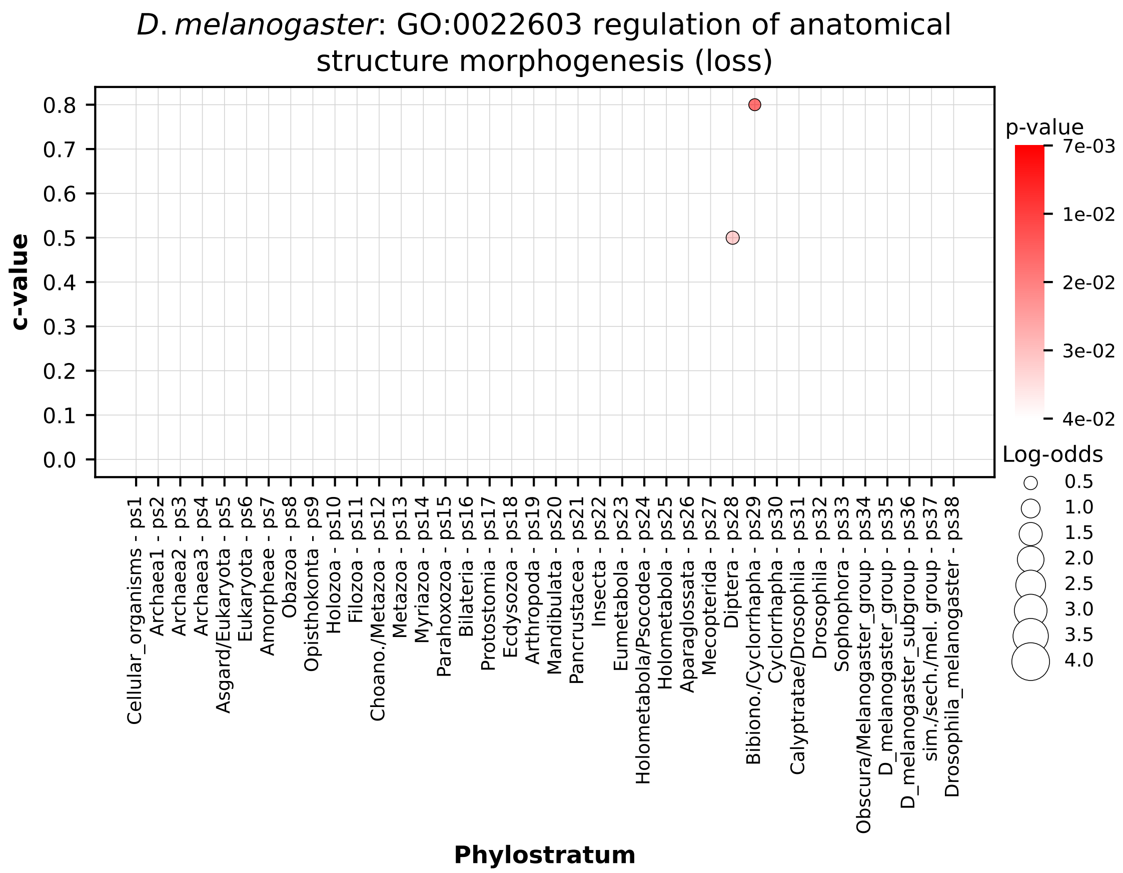

D. melanogaster: GO:0022603 regulation of anatomical structure morphogenesis (loss)
c-value
0.8
0.7
0.6
0.5
0.4
0.3
0.2
0.1
0.0
p-value
7e-03
1e-02
2e-02
3e-02
4e-02
Log-odds
0.5
1.0
1.5
2.0
2.5
3.0
3.5
4.0
Cellular_organisms - ps1
Archaea1 - ps2
Archaea2 - ps3
Archaea3 - ps4
Asgard/Eukaryota - ps5
Eukaryota - ps6
Amorpheae - ps7
Obazoa - ps8
Opisthokonta - ps9
Holozoa - ps10
Filozoa - ps11
Choano./Metazoa - ps12
Metazoa - ps13
Myriazoa - ps14
Parahoxozoa - ps15
Bilateria - ps16
Protostomia - ps17
Ecdysozoa - ps18
Arthropoda - ps19
Mandibulata - ps20
Pancrustacea - ps21
Insecta - ps22
Eumetabola - ps23
Holometabola/Psocodea - ps24
Holometabola - ps25
Aparaglossata - ps26
Mecopterida - ps27
Diptera - ps28
Bibiono./Cyclorrhapha - ps29
Cyclorrhapha - ps30
Calyptratae/Drosophila - ps31
Drosophila - ps32
Sophophora - ps33
Obscura/Melanogaster_group - ps34
D_melanogaster_group - ps35
D_melanogaster_subgroup - ps36
sim./sech./mel. group - ps37
Drosophila_melanogaster - ps38
Phylostratum

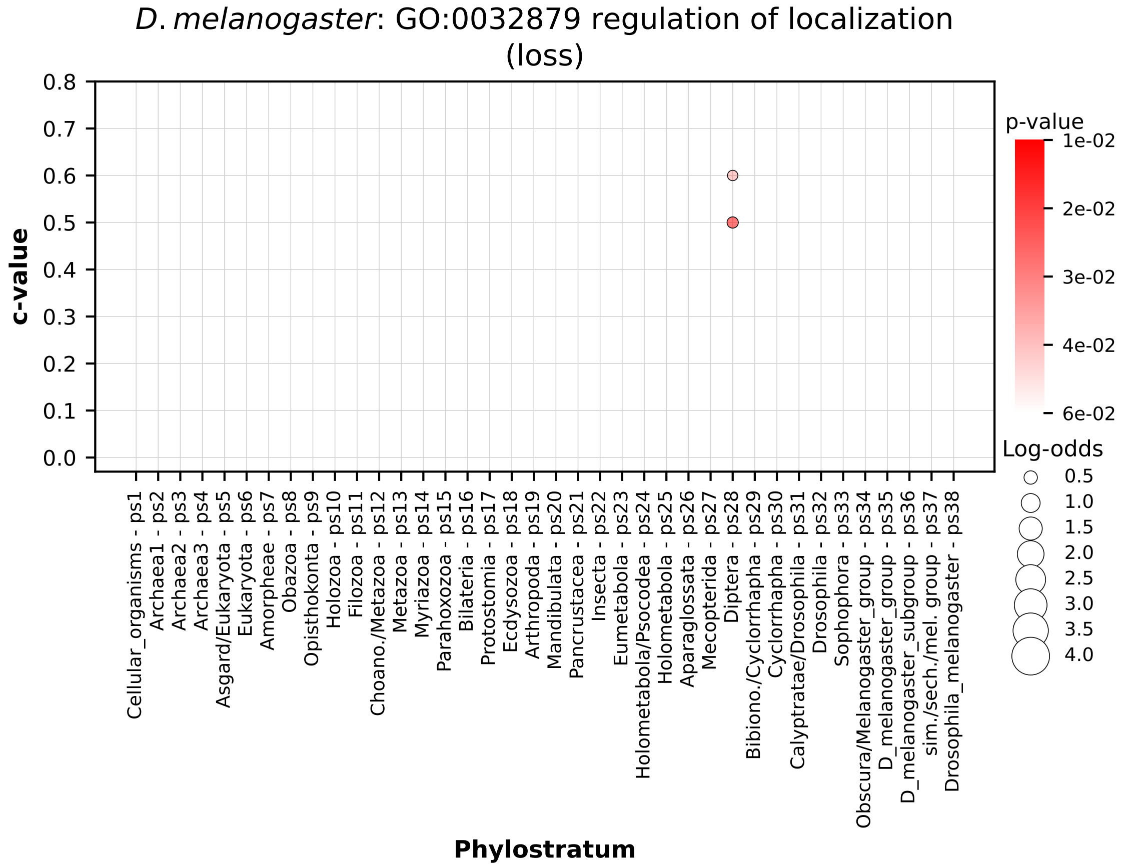

D. melanogaster: GO:0032879 regulation of localization
(loss)
c-value
0.8
0.7
0.6
0.5
0.4
0.3
0.2
0.1
0.0
Phylostratum
Cellular_organisms - ps1
Archaea1 - ps2
Archaea2 - ps3
Archaea3 - ps4
Asgard/Eukaryota - ps5
Eukaryota - ps6
Amorpheae - ps7
Obazoa - ps8
Opisthokonta - ps9
Holozoa - ps10
Filozoa - ps11
Choano./Metazoa - ps12
Metazoa - ps13
Myriazoa - ps14
Parahoxozoa - ps15
Bilateria - ps16
Protostomia - ps17
Ecdysozoa - ps18
Arthropoda - ps19
Mandibulata - ps20
Pancrustacea - ps21
Insecta - ps22
Eumetabola - ps23
Holometabola/Psocodea - ps24
Holometabola - ps25
Aparaglossata - ps26
Mecopterida - ps27
Diptera - ps28
Bibiono./Cyclorrhapha - ps29
Cyclorrhapha - ps30
Calyptratae/Drosophila - ps31
Drosophila - ps32
Sophophora - ps33
Obscura/Melanogaster_group - ps34
D_melanogaster_group - ps35
D_melanogaster_subgroup - ps36
sim./sech./mel. group - ps37
Drosophila_melanogaster - ps38
p-value
1e-02
2e-02
3e-02
4e-02
6e-02
Log-odds
0.5
1.0
1.5
2.0
2.5
3.0
3.5
4.0

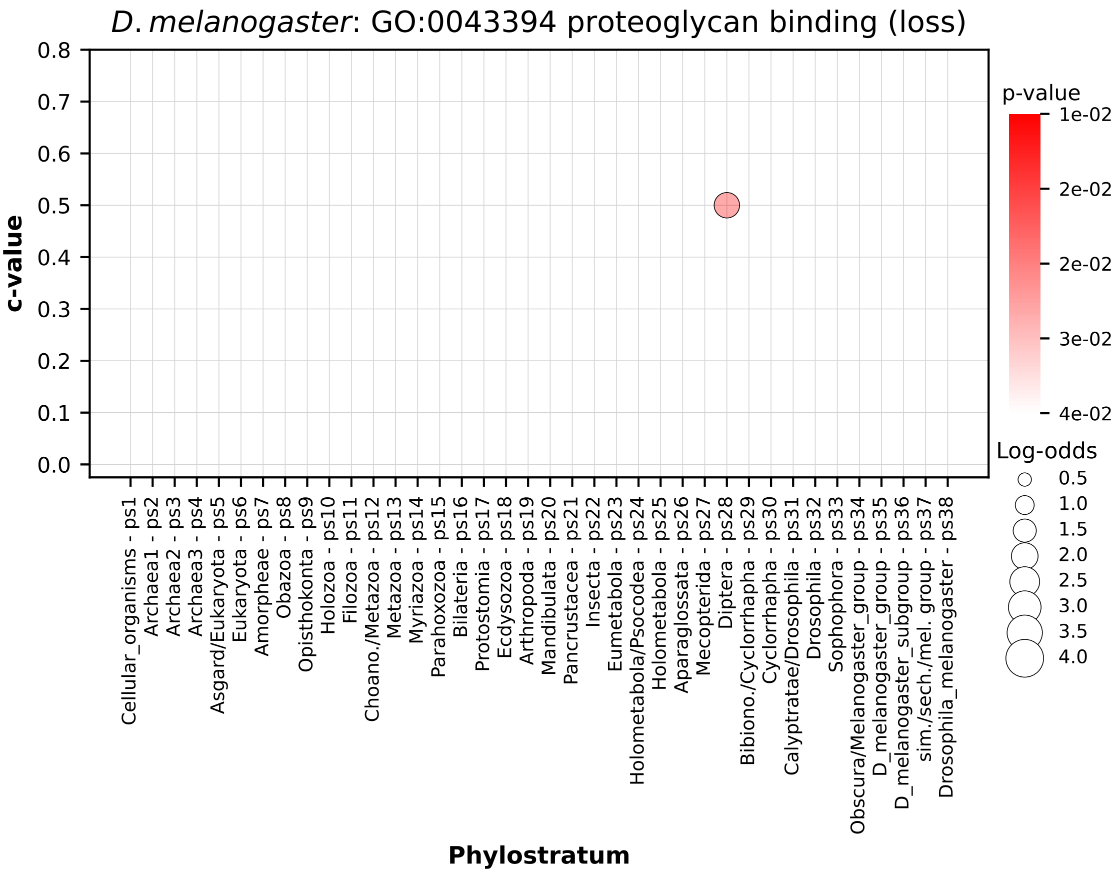

D. melanogaster: GO:0043394 proteoglycan binding (loss)
c-value
0.8
0.7
0.6
0.5
0.4
0.3
0.2
0.1
0.0
p-value
1e-02
2e-02
2e-02
3e-02
4e-02
Log-odds
0.5
1.0
1.5
2.0
2.5
3.0
3.5
4.0
Cellular_organisms - ps1
Archaea1 - ps2
Archaea2 - ps3
Archaea3 - ps4
Asgard/Eukaryota - ps5
Eukaryota - ps6
Amorpheae - ps7
Obazoa - ps8
Opisthokonta - ps9
Holozoa - ps10
Filozoa - ps11
Choano./Metazoa - ps12
Metazoa - ps13
Myriazoa - ps14
Parahoxozoa - ps15
Bilateria - ps16
Protostomia - ps17
Ecdysozoa - ps18
Arthropoda - ps19
Mandibulata - ps20
Pancrustacea - ps21
Insecta - ps22
Eumetabola - ps23
Holometabola/Psocodea - ps24
Holometabola - ps25
Aparaglossata - ps26
Mecopterida - ps27
Diptera - ps28
Bibiono./Cyclorrhapha - ps29
Cyclorrhapha - ps30
Calyptratae/Drosophila - ps31
Drosophila - ps32
Sophophora - ps33
Obscura/Melanogaster_group - ps34
D_melanogaster_group - ps35
D_melanogaster_subgroup - ps36
sim./sech./mel. group - ps37
Drosophila_melanogaster - ps38
Phylostratum

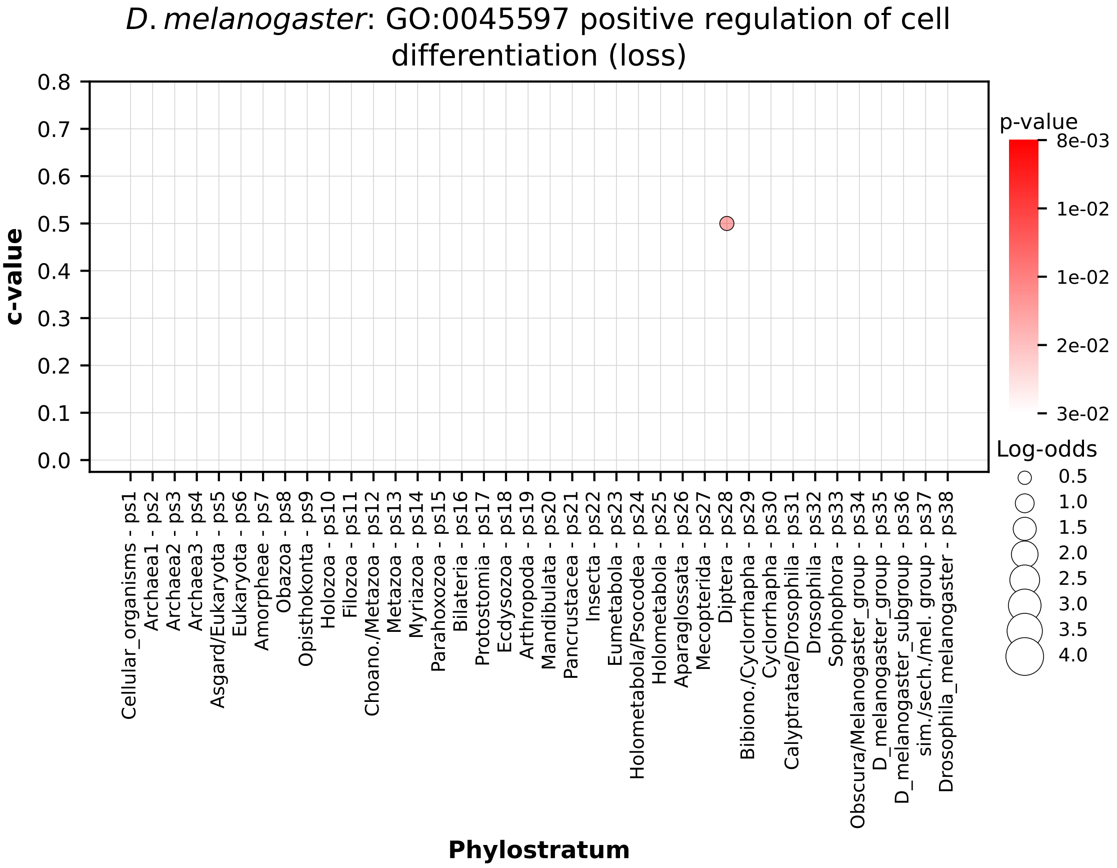

D. melanogaster: GO:0045597 positive regulation of cell differentiation (loss)
c-value
0.0
0.1
0.2
0.3
0.4
0.5
0.6
0.7
0.8
Phylostratum
Cellular_organisms - ps1
Archaea1 - ps2
Archaea2 - ps3
Archaea3 - ps4
Asgard/Eukaryota - ps5
Eukaryota - ps6
Amorpheae - ps7
Obazoa - ps8
Opisthokonta - ps9
Holozoa - ps10
Filozoa - ps11
Choano./Metazoa - ps12
Metazoa - ps13
Myriazoa - ps14
Parahoxozoa - ps15
Bilateria - ps16
Protostomia - ps17
Ecdysozoa - ps18
Arthropoda - ps19
Mandibulata - ps20
Pancrustacea - ps21
Insecta - ps22
Eumetabola - ps23
Holometabola/Psocodea - ps24
Holometabola - ps25
Aparaglossata - ps26
Mecopterida - ps27
Diptera - ps28
Bibiono./Cyclorrhapha - ps29
Cyclorrhapha - ps30
Calyptratae/Drosophila - ps31
Drosophila - ps32
Sophophora - ps33
Obscura/Melanogaster_group - ps34
D_melanogaster_group - ps35
D_melanogaster_subgroup - ps36
sim./sech./mel. group - ps37
Drosophila_melanogaster - ps38
p-value
8e-03
1e-02
1e-02
2e-02
3e-02
Log-odds
0.5
1.0
1.5
2.0
2.5
3.0
3.5
4.0

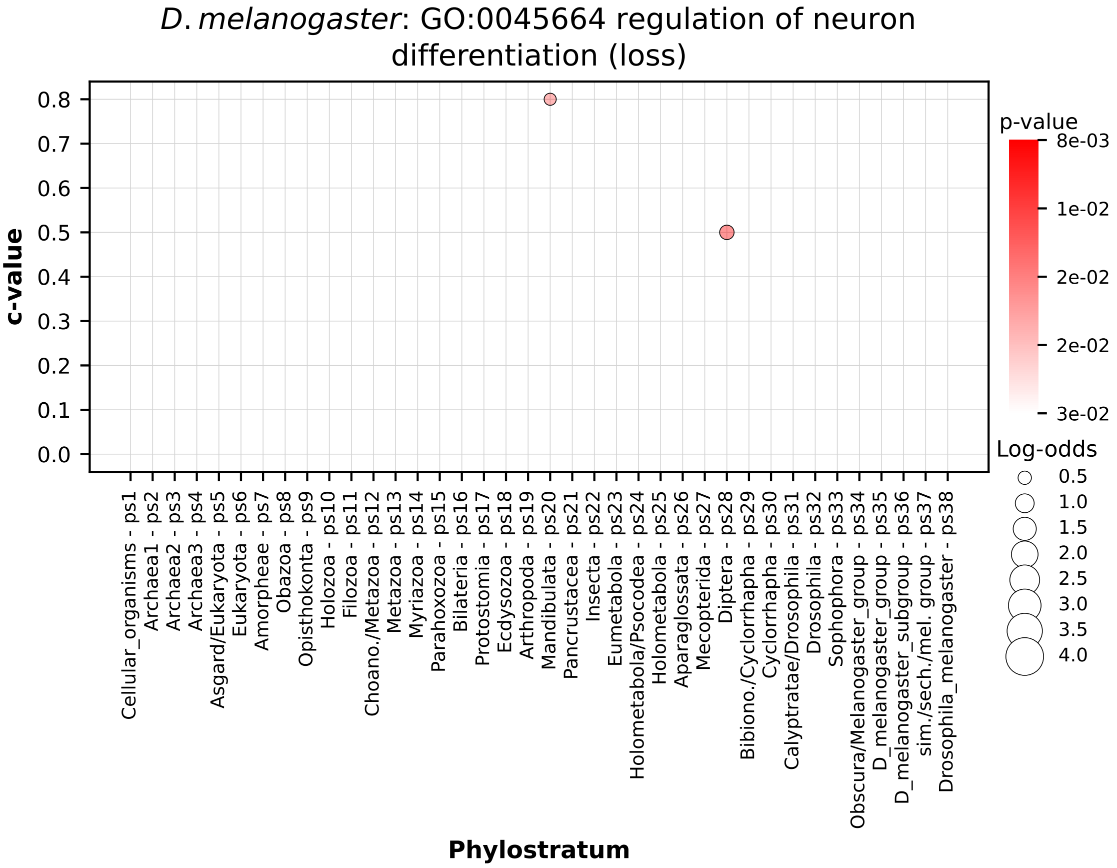
D. melanogaster: GO:0045664 regulation of neuron differentiation (loss)
c-value
0.0
0.1
0.2
0.3
0.4
0.5
0.6
0.7
0.8
Phylostratum
Cellular_organisms - ps1
Archaea1 - ps2
Archaea2 - ps3
Archaea3 - ps4
Asgard/Eukaryota - ps5
Eukaryota - ps6
Amorpheae - ps7
Obazoa - ps8
Opisthokonta - ps9
Holozoa - ps10
Filozoa - ps11
Choano./Metazoa - ps12
Metazoa - ps13
Myriazoa - ps14
Parahoxozoa - ps15
Bilateria - ps16
Protostomia - ps17
Ecdysozoa - ps18
Arthropoda - ps19
Mandibulata - ps20
Pancrustacea - ps21
Insecta - ps22
Eumetabola - ps23
Holometabola/Psocodea - ps24
Holometabola - ps25
Aparaglossata - ps26
Mecopterida - ps27
Diptera - ps28
Bibiono./Cyclorrhapha - ps29
Cyclorrhapha - ps30
Calyptratae/Drosophila - ps31
Drosophila - ps32
Sophophora - ps33
Obscura/Melanogaster_group - ps34
D_melanogaster_group - ps35
D_melanogaster_subgroup - ps36
sim./sech./mel. group - ps37
Drosophila_melanogaster - ps38
p-value
8e-03
1e-02
2e-02
2e-02
3e-02
Log-odds
0.5
1.0
1.5
2.0
2.5
3.0
3.5
4.0

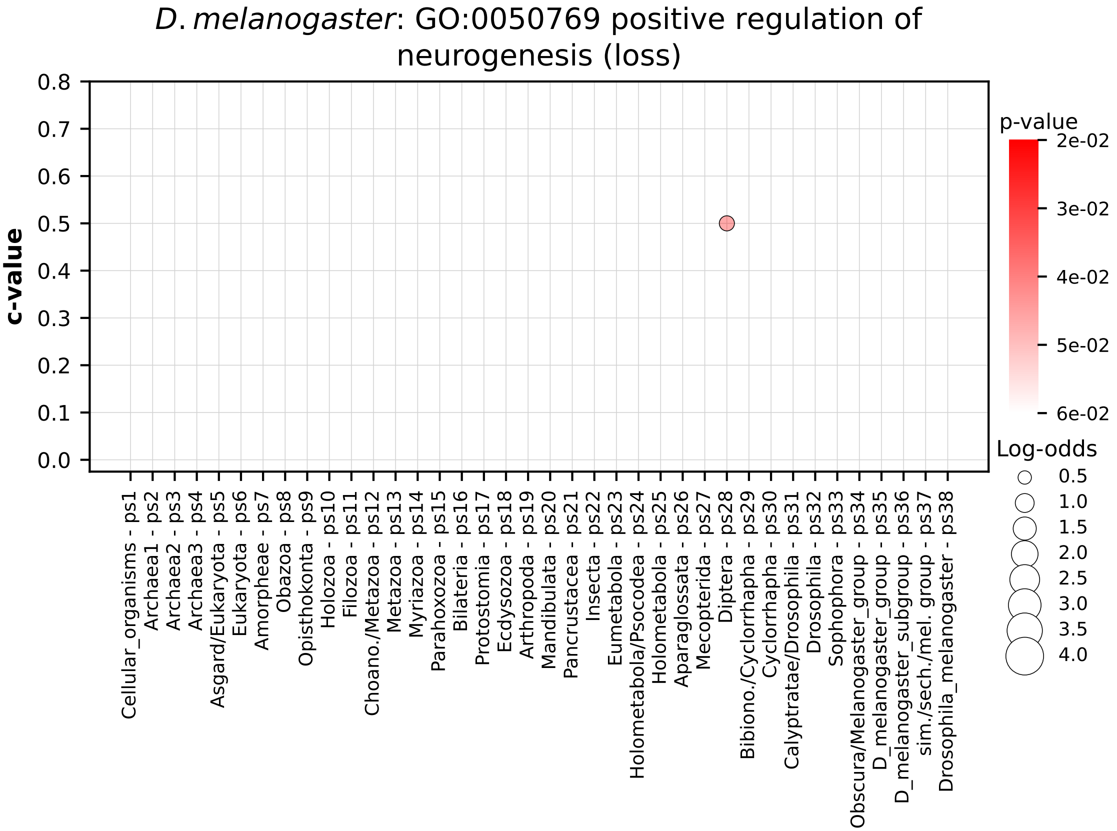

D. melanogaster: GO:0050769 positive regulation of neurogenesis (loss)
c-value
0.8
0.7
0.6
0.5
0.4
0.3
0.2
0.1
0.0
Phylostratum
Cellular_organisms - ps1
Archaea1 - ps2
Archaea2 - ps3
Archaea3 - ps4
Asgard/Eukaryota - ps5
Eukaryota - ps6
Amorpheae - ps7
Obazoa - ps8
Opisthokonta - ps9
Holozoa - ps10
Filozoa - ps11
Choano./Metazoa - ps12
Metazoa - ps13
Myriazoa - ps14
Parahoxozoa - ps15
Bilateria - ps16
Protostomia - ps17
Ecdysozoa - ps18
Arthropoda - ps19
Mandibulata - ps20
Pancrustacea - ps21
Insecta - ps22
Eumetabola - ps23
Holometabola/Psocodea - ps24
Holometabola - ps25
Aparaglossata - ps26
Mecopterida - ps27
Diptera - ps28
Bibiono./Cyclorrhapha - ps29
Cyclorrhapha - ps30
Calyptratae/Drosophila - ps31
Drosophila - ps32
Sophophora - ps33
Obscura/Melanogaster_group - ps34
D_melanogaster_group - ps35
D_melanogaster_subgroup - ps36
sim./sech./mel. group - ps37
Drosophila_melanogaster - ps38
p-value
2e-02
3e-02
4e-02
5e-02
6e-02
Log-odds
0.5
1.0
1.5
2.0
2.5
3.0
3.5
4.0

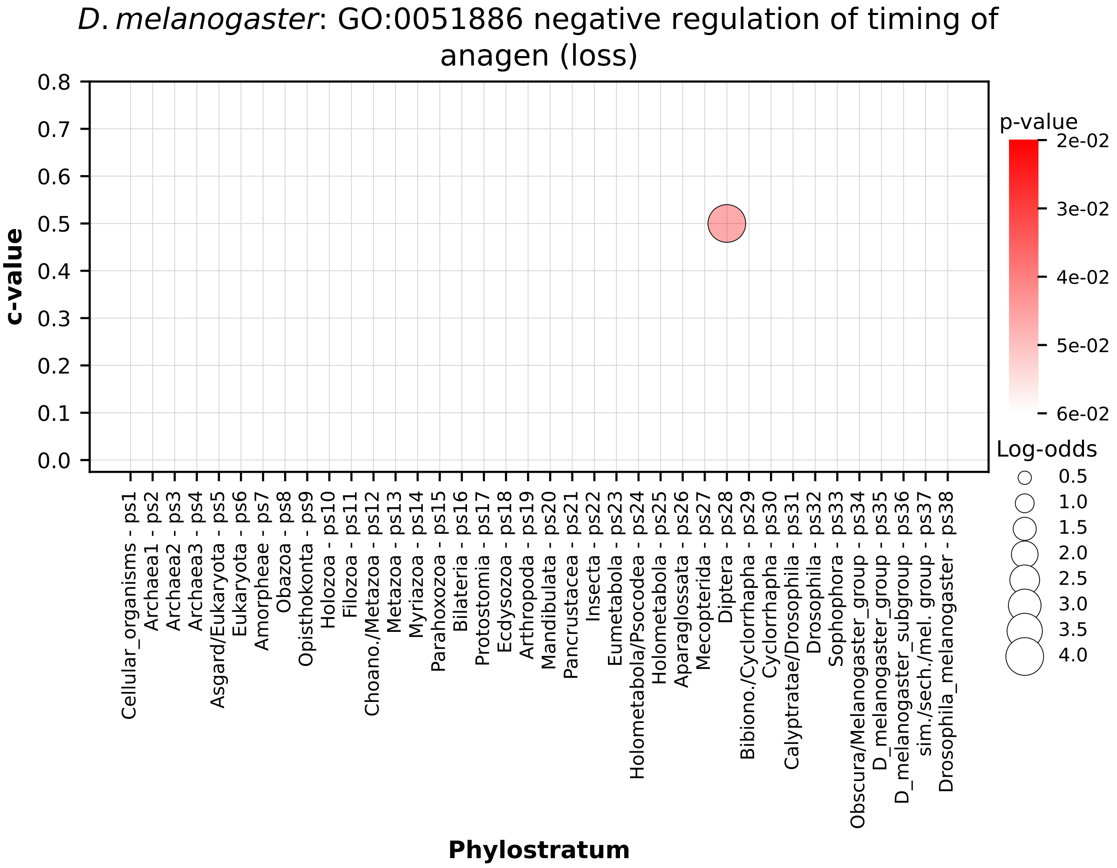

D. melanogaster: GO:0051886 negative regulation of timing of anagen (loss)
c-value
0.8
0.7
0.6
0.5
0.4
0.3
0.2
0.1
0.0
p-value
2e-02
3e-02
4e-02
5e-02
6e-02
Log-odds
0.5
1.0
1.5
2.0
2.5
3.0
3.5
4.0
Cellular_organisms - ps1
Archaea1 - ps2
Archaea2 - ps3
Archaea3 - ps4
Asgard/Eukaryota - ps5
Eukaryota - ps6
Amorpheae - ps7
Obazoa - ps8
Opisthokonta - ps9
Holozoa - ps10
Filozoa - ps11
Choano./Metazoa - ps12
Metazoa - ps13
Myriazoa - ps14
Parahoxozoa - ps15
Bilateria - ps16
Protostomia - ps17
Ecdysozoa - ps18
Arthropoda - ps19
Mandibulata - ps20
Pancrustacea - ps21
Insecta - ps22
Eumetabola - ps23
Holometabola/Psocodea - ps24
Holometabola - ps25
Aparaglossata - ps26
Mecopterida - ps27
Diptera - ps28
Bibiono./Cyclorrhapha - ps29
Cyclorrhapha - ps30
Calyptratae/Drosophila - ps31
Drosophila - ps32
Sophophora - ps33
Obscura/Melanogaster_group - ps34
D_melanogaster_group - ps35
D_melanogaster_subgroup - ps36
sim./sech./mel. group - ps37
Drosophila_melanogaster - ps38
Phylostratum

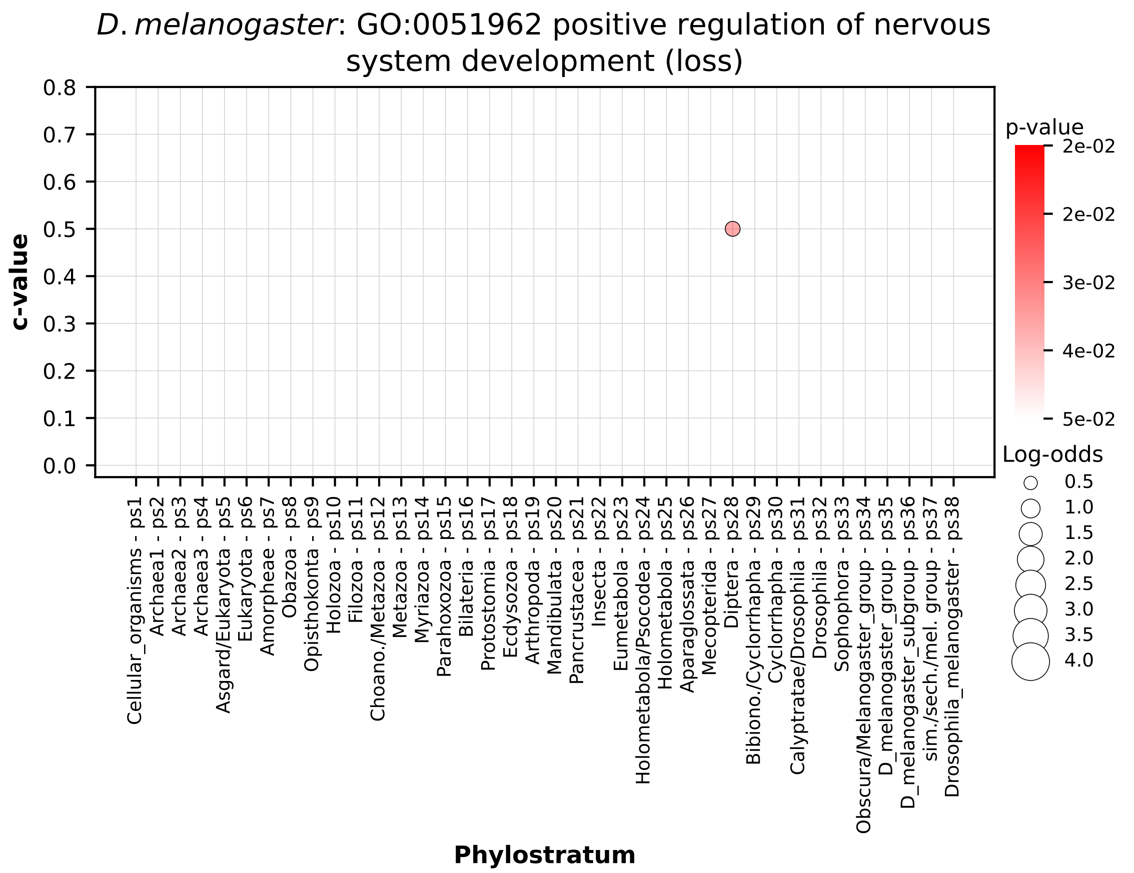
D. melanogaster: GO:0051962 positive regulation of nervous system development (loss)
c-value
0.8
0.7
0.6
0.5
0.4
0.3
0.2
0.1
0.0
Phylostratum
Cellular_organisms - ps1
Archaea1 - ps2
Archaea2 - ps3
Archaea3 - ps4
Asgard/Eukaryota - ps5
Eukaryota - ps6
Amorpheae - ps7
Obazoa - ps8
Opisthokonta - ps9
Holozoa - ps10
Filozoa - ps11
Choano./Metazoa - ps12
Metazoa - ps13
Myriazoa - ps14
Parahoxozoa - ps15
Bilateria - ps16
Protostomia - ps17
Ecdysozoa - ps18
Arthropoda - ps19
Mandibulata - ps20
Pancrustacea - ps21
Insecta - ps22
Eumetabola - ps23
Holometabola/Psocodea - ps24
Holometabola - ps25
Aparaglossata - ps26
Mecopterida - ps27
Diptera - ps28
Bibiono./Cyclorrhapha - ps29
Cyclorrhapha - ps30
Calyptratae/Drosophila - ps31
Drosophila - ps32
Sophophora - ps33
Obscura/Melanogaster_group - ps34
D_melanogaster_group - ps35
D_melanogaster_subgroup - ps36
sim./sech./mel. group - ps37
Drosophila_melanogaster - ps38
p-value
2e-02
2e-02
3e-02
4e-02
5e-02
Log-odds
0.5
1.0
1.5
2.0
2.5
3.0
3.5
4.0

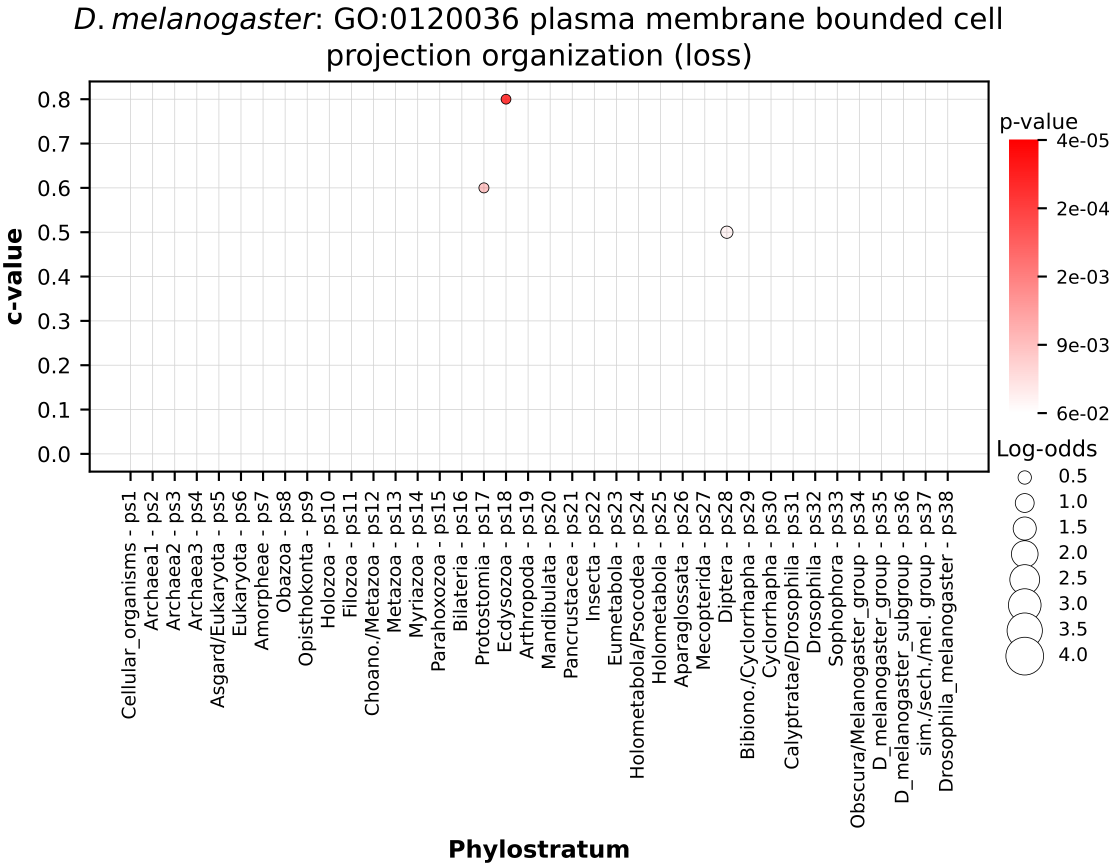
D. melanogaster: GO:0120036 plasma membrane bounded cell projection organization (loss)
c-value
Phylostratum
p-value
4e-05
2e-04
2e-03
9e-03
6e-02
Log-odds
0.5
1.0
1.5
2.0
2.5
3.0
3.5
4.0
Cellular_organisms - ps1
Archaea1 - ps2
Archaea2 - ps3
Archaea3 - ps4
Asgard/Eukaryota - ps5
Eukaryota - ps6
Amorpheae - ps7
Obazoa - ps8
Opisthokonta - ps9
Holozoa - ps10
Filozoa - ps11
Choano./Metazoa - ps12
Metazoa - ps13
Myriazoa - ps14
Parahoxozoa - ps15
Bilateria - ps16
Protostomia - ps17
Ecdysozoa - ps18
Arthropoda - ps19
Mandibulata - ps20
Pancrustacea - ps21
Insecta - ps22
Eumetabola - ps23
Holometabola/Psocodea - ps24
Holometabola - ps25
Aparaglossata - ps26
Mecopterida - ps27
Diptera - ps28
Bibiono./Cyclorrhapha - ps29
Cyclorrhapha - ps30
Calyptratae/Drosophila - ps31
Drosophila - ps32
Sophophora - ps33
Obscura/Melanogaster_group - ps34
D_melanogaster_group - ps35
D_melanogaster_subgroup - ps36
sim./sech./mel. group - ps37
Drosophila_melanogaster - ps38

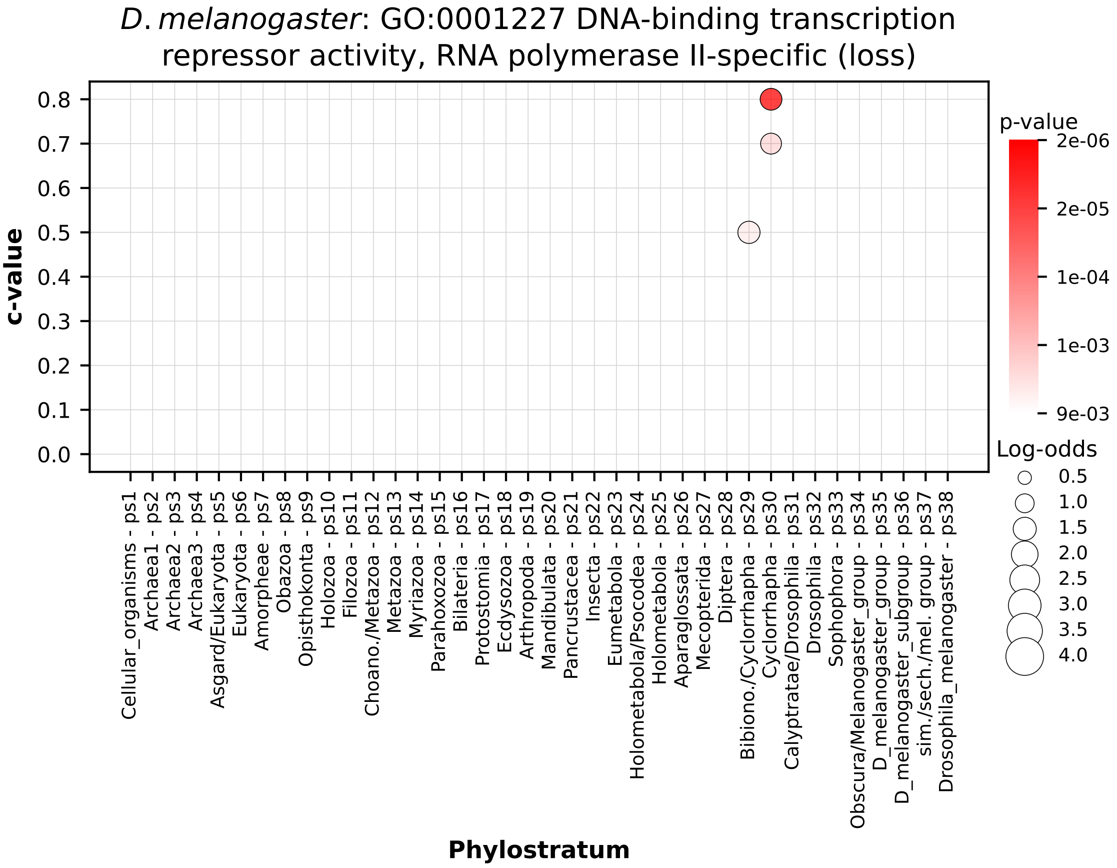

D. melanogaster: GO:0001227 DNA-binding transcription repressor activity, RNA polymerase II-specific (loss)
c-value
0.8
0.7
0.6
0.5
0.4
0.3
0.2
0.1
0.0
p-value
2e-06
2e-05
1e-04
1e-03
9e-03
Log-odds
0.5
1.0
1.5
2.0
2.5
3.0
3.5
4.0
Cellular_organisms - ps1
Archaea1 - ps2
Archaea2 - ps3
Archaea3 - ps4
Asgard/Eukaryota - ps5
Eukaryota - ps6
Amorpheae - ps7
Obazoa - ps8
Opisthokonta - ps9
Holozoa - ps10
Filozoa - ps11
Choano./Metazoa - ps12
Metazoa - ps13
Myriazoa - ps14
Parahoxozoa - ps15
Bilateria - ps16
Protostomia - ps17
Ecdysozoa - ps18
Arthropoda - ps19
Mandibulata - ps20
Pancrustacea - ps21
Insecta - ps22
Eumetabola - ps23
Holometabola/Psocodea - ps24
Holometabola - ps25
Aparaglossata - ps26
Mecopterida - ps27
Diptera - ps28
Bibiono./Cyclorrhapha - ps29
Cyclorrhapha - ps30
Calyptratae/Drosophila - ps31
Drosophila - ps32
Sophophora - ps33
Obscura/Melanogaster_group - ps34
D_melanogaster_group - ps35
D_melanogaster_subgroup - ps36
sim./sech./mel. group - ps37
Drosophila_melanogaster - ps38
Phylostratum

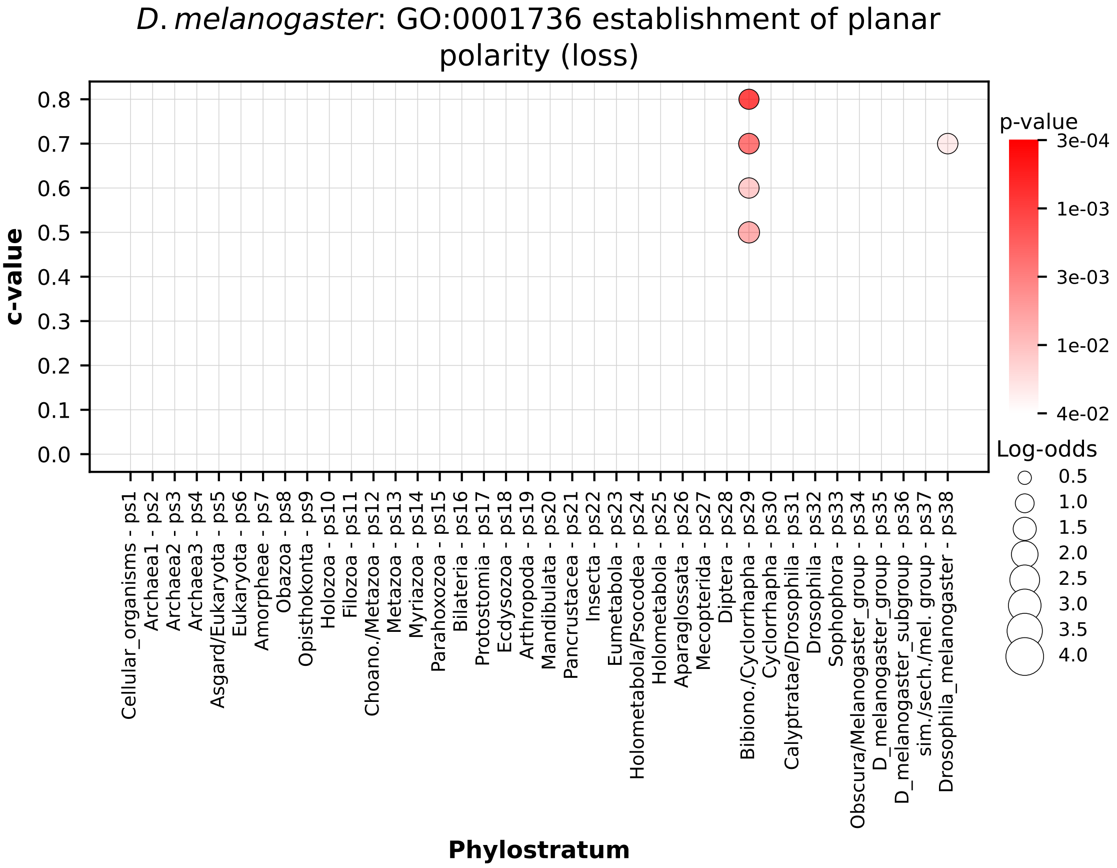
D. melanogaster: GO:0001736 establishment of planar polarity (loss)
c-value
0.0
0.1
0.2
0.3
0.4
0.5
0.6
0.7
0.8
Phylostratum
Cellular_organisms - ps1
Archaea1 - ps2
Archaea2 - ps3
Archaea3 - ps4
Asgard/Eukaryota - ps5
Eukaryota - ps6
Amorpheae - ps7
Obazoa - ps8
Opisthokonta - ps9
Holozoa - ps10
Filozoa - ps11
Choano./Metazoa - ps12
Metazoa - ps13
Myriazoa - ps14
Parahoxozoa - ps15
Bilateria - ps16
Protostomia - ps17
Ecdysozoa - ps18
Arthropoda - ps19
Mandibulata - ps20
Pancrustacea - ps21
Insecta - ps22
Eumetabola - ps23
Holometabola/Psocodea - ps24
Holometabola - ps25
Aparaglossata - ps26
Mecopterida - ps27
Diptera - ps28
Bibiono./Cyclorrhapha - ps29
Cyclorrhapha - ps30
Calyptratae/Drosophila - ps31
Drosophila - ps32
Sophophora - ps33
Obscura/Melanogaster_group - ps34
D_melanogaster_group - ps35
D_melanogaster_subgroup - ps36
sim./sech./mel. group - ps37
Drosophila_melanogaster - ps38
p-value
3e-04
1e-03
3e-03
1e-02
4e-02
Log-odds
0.5
1.0
1.5
2.0
2.5
3.0
3.5
4.0

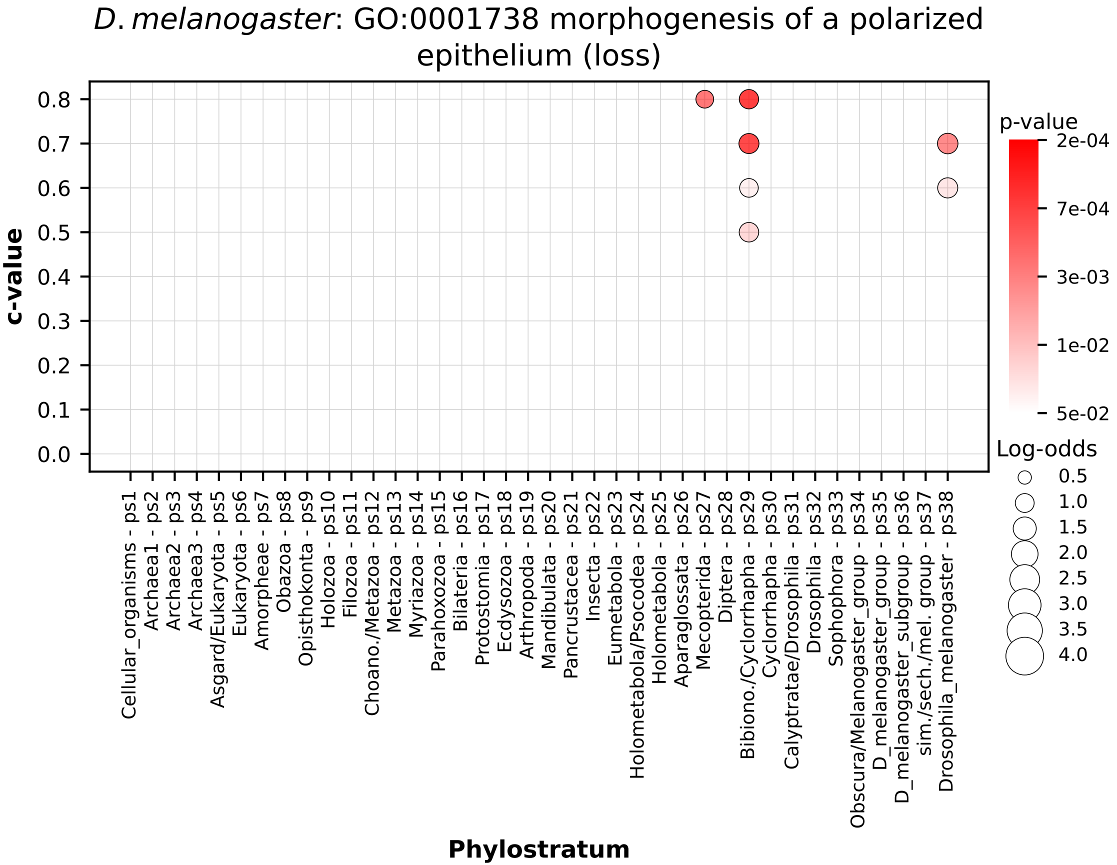

D. melanogaster: GO:0001738 morphogenesis of a polarized epithelium (loss)
c-value
0.0
0.1
0.2
0.3
0.4
0.5
0.6
0.7
0.8
p-value
2e-04
7e-04
3e-03
1e-02
5e-02
Log-odds
0.5
1.0
1.5
2.0
2.5
3.0
3.5
4.0
Cellular_organisms - ps1
Archaea1 - ps2
Archaea2 - ps3
Archaea3 - ps4
Asgard/Eukaryota - ps5
Eukaryota - ps6
Amorpheae - ps7
Obazoa - ps8
Opisthokonta - ps9
Holozoa - ps10
Filozoa - ps11
Choano./Metazoa - ps12
Metazoa - ps13
Myriazoa - ps14
Parahoxozoa - ps15
Bilateria - ps16
Protostomia - ps17
Ecdysozoa - ps18
Arthropoda - ps19
Mandibulata - ps20
Pancrustacea - ps21
Insecta - ps22
Eumetabola - ps23
Holometabola/Psocodea - ps24
Holometabola - ps25
Aparaglossata - ps26
Mecopterida - ps27
Diptera - ps28
Bibiono./Cyclorrhapha - ps29
Cyclorrhapha - ps30
Calyptratae/Drosophila - ps31
Drosophila - ps32
Sophophora - ps33
Obscura/Melanogaster_group - ps34
D_melanogaster_group - ps35
D_melanogaster_subgroup - ps36
sim./sech./mel. group - ps37
Drosophila_melanogaster - ps38
Phylostratum

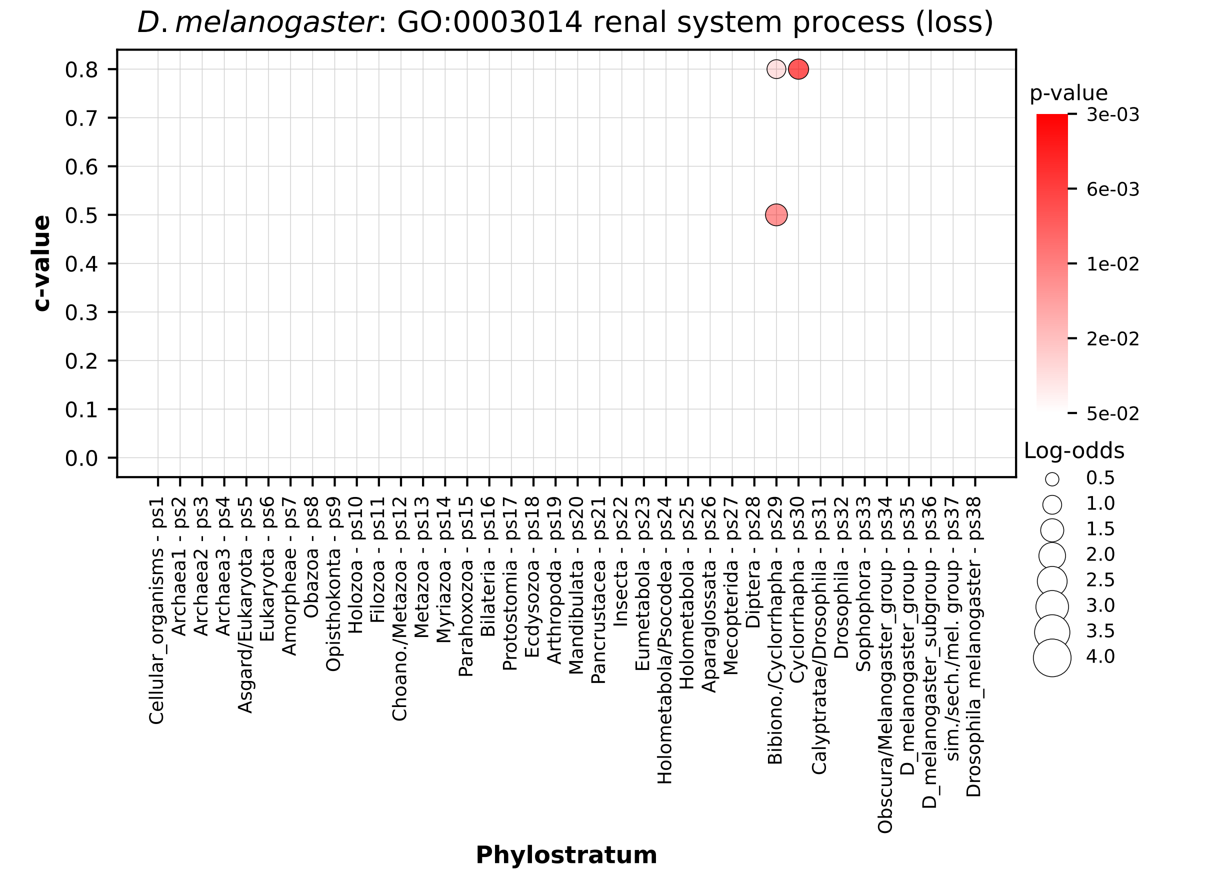

D. melanogaster: GO:0003014 renal system process (loss)
c-value
0.0
0.1
0.2
0.3
0.4
0.5
0.6
0.7
0.8
Phylostratum
Cellular_organisms - ps1
Archaea1 - ps2
Archaea2 - ps3
Archaea3 - ps4
Asgard/Eukaryota - ps5
Eukaryota - ps6
Amorpheae - ps7
Obazoa - ps8
Opisthokonta - ps9
Holozoa - ps10
Filozoa - ps11
Choano./Metazoa - ps12
Metazoa - ps13
Myriazoa - ps14
Parahoxozoa - ps15
Bilateria - ps16
Protostomia - ps17
Ecdysozoa - ps18
Arthropoda - ps19
Mandibulata - ps20
Pancrustacea - ps21
Insecta - ps22
Eumetabola - ps23
Holometabola/Psocodea - ps24
Holometabola - ps25
Aparaglossata - ps26
Mecopterida - ps27
Diptera - ps28
Bibiono./Cyclorrhapha - ps29
Cyclorrhapha - ps30
Calyptratae/Drosophila - ps31
Drosophila - ps32
Sophophora - ps33
Obscura/Melanogaster_group - ps34
D_melanogaster_group - ps35
D_melanogaster_subgroup - ps36
sim./sech./mel. group - ps37
Drosophila_melanogaster - ps38
p-value
3e-03
6e-03
1e-02
2e-02
5e-02
Log-odds
0.5
1.0
1.5
2.0
2.5
3.0
3.5
4.0

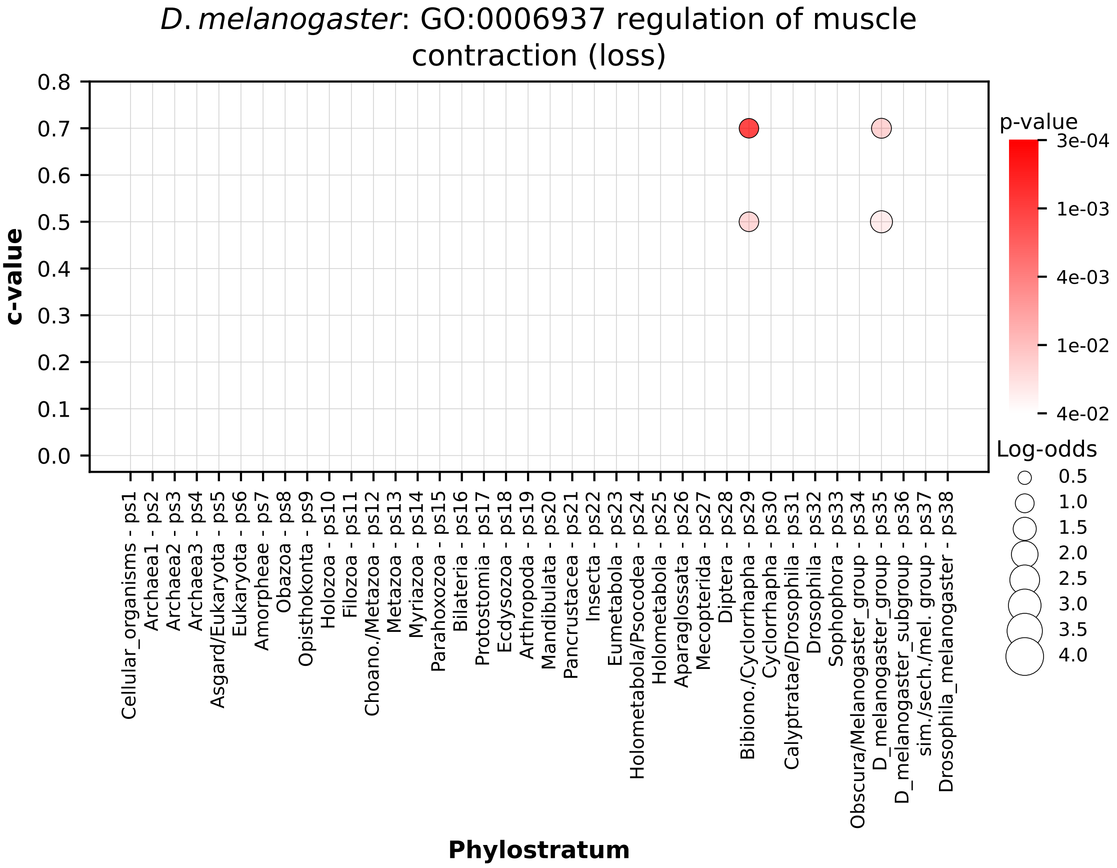
D. melanogaster: GO:0006937 regulation of muscle contraction (loss)
c-value
0.8
0.7
0.6
0.5
0.4
0.3
0.2
0.1
0.0
p-value
3e-04
1e-03
4e-03
1e-02
4e-02
Log-odds
0.5
1.0
1.5
2.0
2.5
3.0
3.5
4.0
Cellular_organisms - ps1
Archaea1 - ps2
Archaea2 - ps3
Archaea3 - ps4
Asgard/Eukaryota - ps5
Eukaryota - ps6
Amorpheae - ps7
Obazoa - ps8
Opisthokonta - ps9
Holozoa - ps10
Filozoa - ps11
Choano./Metazoa - ps12
Metazoa - ps13
Myriazoa - ps14
Parahoxozoa - ps15
Bilateria - ps16
Protostomia - ps17
Ecdysozoa - ps18
Arthropoda - ps19
Mandibulata - ps20
Pancrustacea - ps21
Insecta - ps22
Eumetabola - ps23
Holometabola/Psocodea - ps24
Holometabola - ps25
Aparaglossata - ps26
Mecopterida - ps27
Diptera - ps28
Bibiono./Cyclorrhapha - ps29
Cyclorrhapha - ps30
Calyptratae/Drosophila - ps31
Drosophila - ps32
Sophophora - ps33
Obscura/Melanogaster_group - ps34
D_melanogaster_group - ps35
D_melanogaster_subgroup - ps36
sim./sech./mel. group - ps37
Drosophila_melanogaster - ps38
Phylostratum

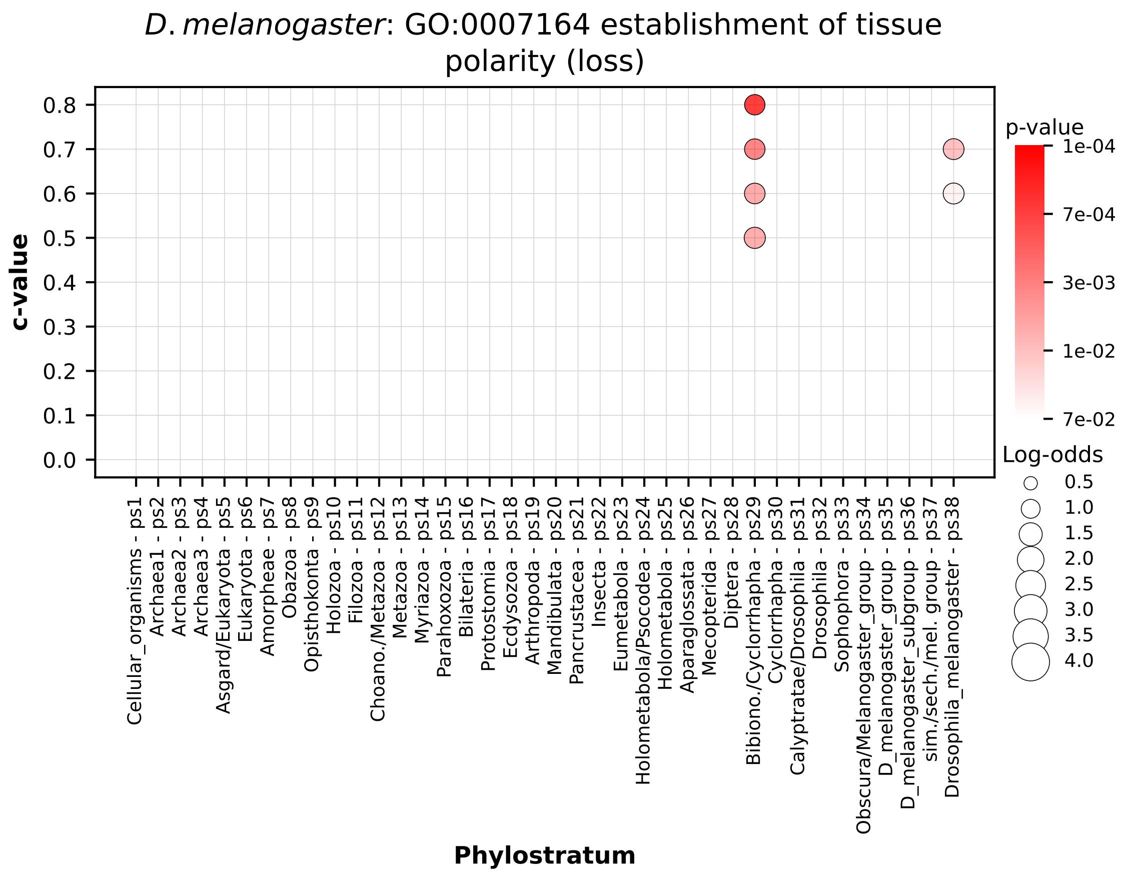

D. melanogaster: GO:0007164 establishment of tissue polarity (loss)
c-value
0.0
0.1
0.2
0.3
0.4
0.5
0.6
0.7
0.8
Phylostratum
Cellular_organisms - ps1
Archaea1 - ps2
Archaea2 - ps3
Archaea3 - ps4
Asgard/Eukaryota - ps5
Eukaryota - ps6
Amorpheae - ps7
Obazoa - ps8
Opisthokonta - ps9
Holozoa - ps10
Filozoa - ps11
Choano./Metazoa - ps12
Metazoa - ps13
Myriazoa - ps14
Parahoxozoa - ps15
Bilateria - ps16
Protostomia - ps17
Ecdysozoa - ps18
Arthropoda - ps19
Mandibulata - ps20
Pancrustacea - ps21
Insecta - ps22
Eumetabola - ps23
Holometabola/Psocodea - ps24
Holometabola - ps25
Aparaglossata - ps26
Mecopterida - ps27
Diptera - ps28
Bibiono./Cyclorrhapha - ps29
Cyclorrhapha - ps30
Calyptratae/Drosophila - ps31
Drosophila - ps32
Sophophora - ps33
Obscura/Melanogaster_group - ps34
D_melanogaster_group - ps35
D_melanogaster_subgroup - ps36
sim./sech./mel. group - ps37
Drosophila_melanogaster - ps38
p-value
1e-04
7e-04
3e-03
1e-02
7e-02
Log-odds
0.5
1.0
1.5
2.0
2.5
3.0
3.5
4.0

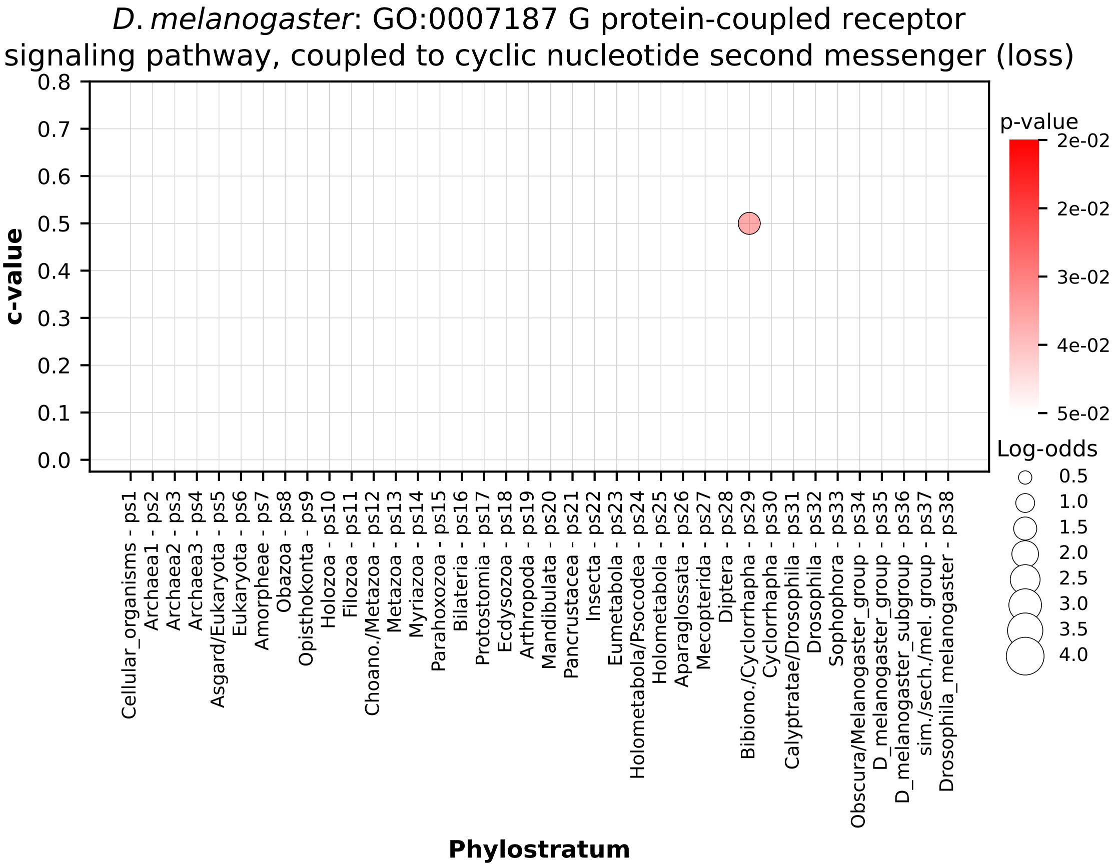

D. melanogaster: GO:0007187 G protein-coupled receptor signaling pathway, coupled to cyclic nucleotide second messenger (loss)
c-value
0.8
0.7
0.6
0.5
0.4
0.3
0.2
0.1
0.0
p-value
2e-02
2e-02
3e-02
4e-02
5e-02
Log-odds
0.5
1.0
1.5
2.0
2.5
3.0
3.5
4.0
Cellular_organisms - ps1
Archaea1 - ps2
Archaea2 - ps3
Archaea3 - ps4
Asgard/Eukaryota - ps5
Eukaryota - ps6
Amorpheae - ps7
Obazoa - ps8
Opisthokonta - ps9
Holozoa - ps10
Filozoa - ps11
Choano./Metazoa - ps12
Metazoa - ps13
Myriazoa - ps14
Parahoxozoa - ps15
Bilateria - ps16
Protostomia - ps17
Ecdysozoa - ps18
Arthropoda - ps19
Mandibulata - ps20
Pancrustacea - ps21
Insecta - ps22
Eumetabola - ps23
Holometabola/Psocodea - ps24
Holometabola - ps25
Aparaglossata - ps26
Mecopterida - ps27
Diptera - ps28
Bibiono./Cyclorrhapha - ps29
Cyclorrhapha - ps30
Calyptratae/Drosophila - ps31
Drosophila - ps32
Sophophora - ps33
Obscura/Melanogaster_group - ps34
D_melanogaster_group - ps35
D_melanogaster_subgroup - ps36
sim./sech./mel. group - ps37
Drosophila_melanogaster - ps38
Phylostratum

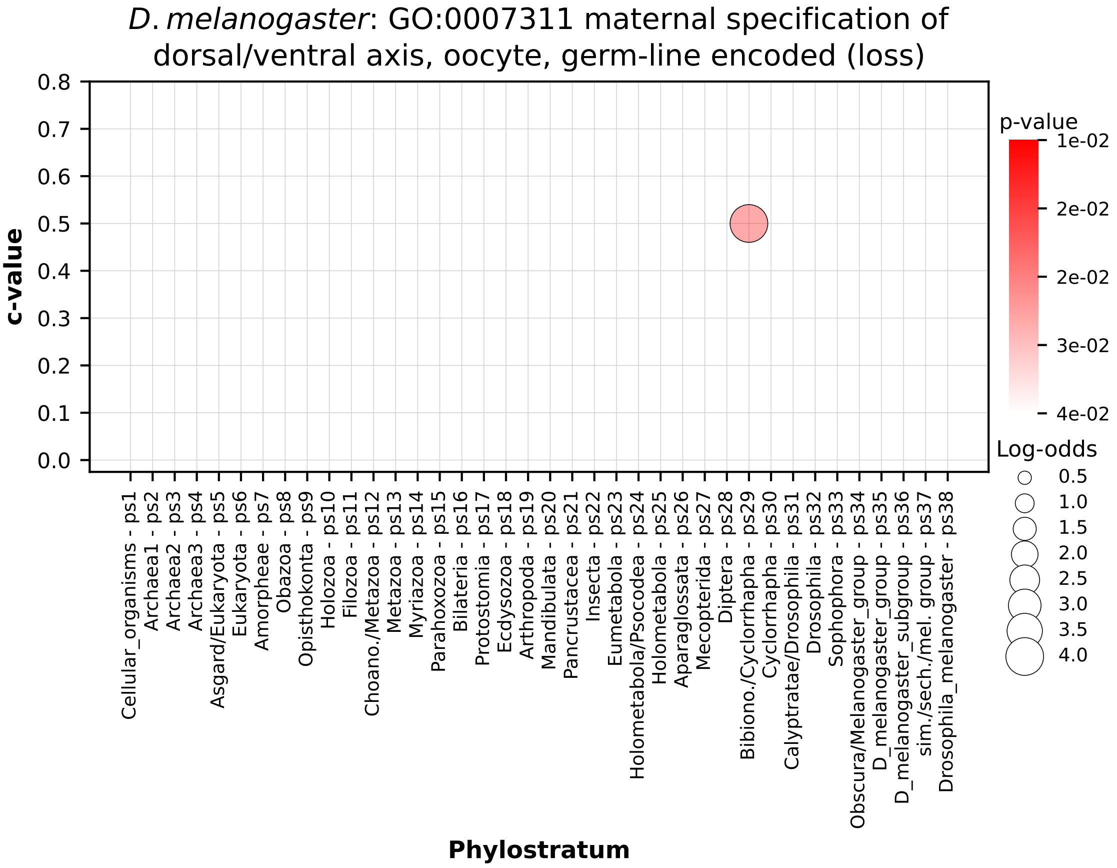

D. melanogaster: GO:0007311 maternal specification of dorsal/ventral axis, oocyte, germ-line encoded (loss)
c-value
0.8
0.7
0.6
0.5
0.4
0.3
0.2
0.1
0.0
Phylostratum
Cellular_organisms - ps1
Archaea1 - ps2
Archaea2 - ps3
Archaea3 - ps4
Asgard/Eukaryota - ps5
Eukaryota - ps6
Amorpheae - ps7
Obazoa - ps8
Opisthokonta - ps9
Holozoa - ps10
Filozoa - ps11
Choano./Metazoa - ps12
Metazoa - ps13
Myriazoa - ps14
Parahoxozoa - ps15
Bilateria - ps16
Protostomia - ps17
Ecdysozoa - ps18
Arthropoda - ps19
Mandibulata - ps20
Pancrustacea - ps21
Insecta - ps22
Eumetabola - ps23
Holometabola/Psocodea - ps24
Holometabola - ps25
Aparaglossata - ps26
Mecopterida - ps27
Diptera - ps28
Bibiono./Cyclorrhapha - ps29
Cyclorrhapha - ps30
Calyptratae/Drosophila - ps31
Drosophila - ps32
Sophophora - ps33
Obscura/Melanogaster_group - ps34
D_melanogaster_group - ps35
D_melanogaster_subgroup - ps36
sim./sech./mel. group - ps37
Drosophila_melanogaster - ps38
p-value
1e-02
2e-02
2e-02
3e-02
4e-02
Log-odds
0.5
1.0
1.5
2.0
2.5
3.0
3.5
4.0

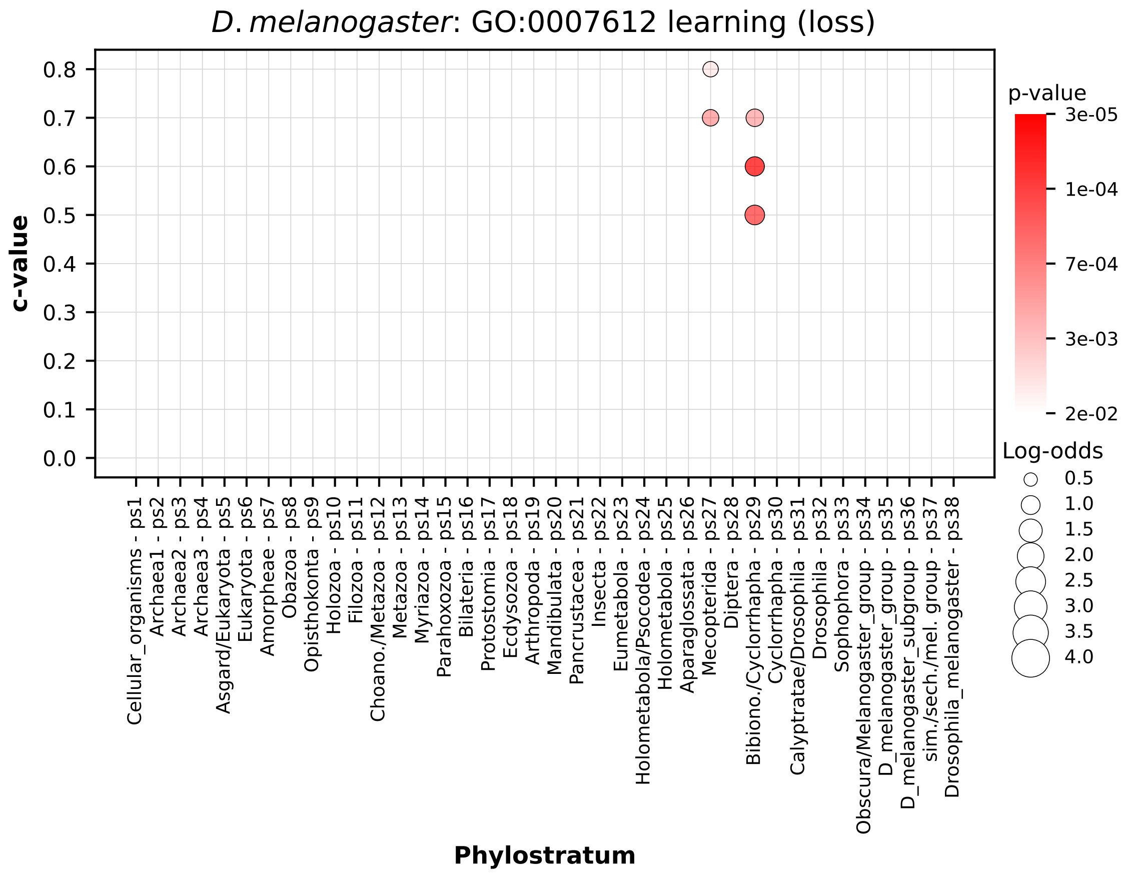
D. melanogaster: GO:0007612 learning (loss)
c-value
0.8
0.7
0.6
0.5
0.4
0.3
0.2
0.1
0.0
Phylostratum
Cellular_organisms - ps1
Archaea1 - ps2
Archaea2 - ps3
Archaea3 - ps4
Asgard/Eukaryota - ps5
Eukaryota - ps6
Amorpheae - ps7
Obazoa - ps8
Opisthokonta - ps9
Holozoa - ps10
Filozoa - ps11
Choano./Metazoa - ps12
Metazoa - ps13
Myriazoa - ps14
Parahoxozoa - ps15
Bilateria - ps16
Protostomia - ps17
Ecdysozoa - ps18
Arthropoda - ps19
Mandibulata - ps20
Pancrustacea - ps21
Insecta - ps22
Eumetabola - ps23
Holometabola/Psocodea - ps24
Holometabola - ps25
Aparaglossata - ps26
Mecopterida - ps27
Diptera - ps28
Bibiono./Cyclorrhapha - ps29
Cyclorrhapha - ps30
Calyptratae/Drosophila - ps31
Drosophila - ps32
Sophophora - ps33
Obscura/Melanogaster_group - ps34
D_melanogaster_group - ps35
D_melanogaster_subgroup - ps36
sim./sech./mel. group - ps37
Drosophila_melanogaster - ps38
p-value
3e-05
1e-04
7e-04
3e-03
2e-02
Log-odds
0.5
1.0
1.5
2.0
2.5
3.0
3.5
4.0

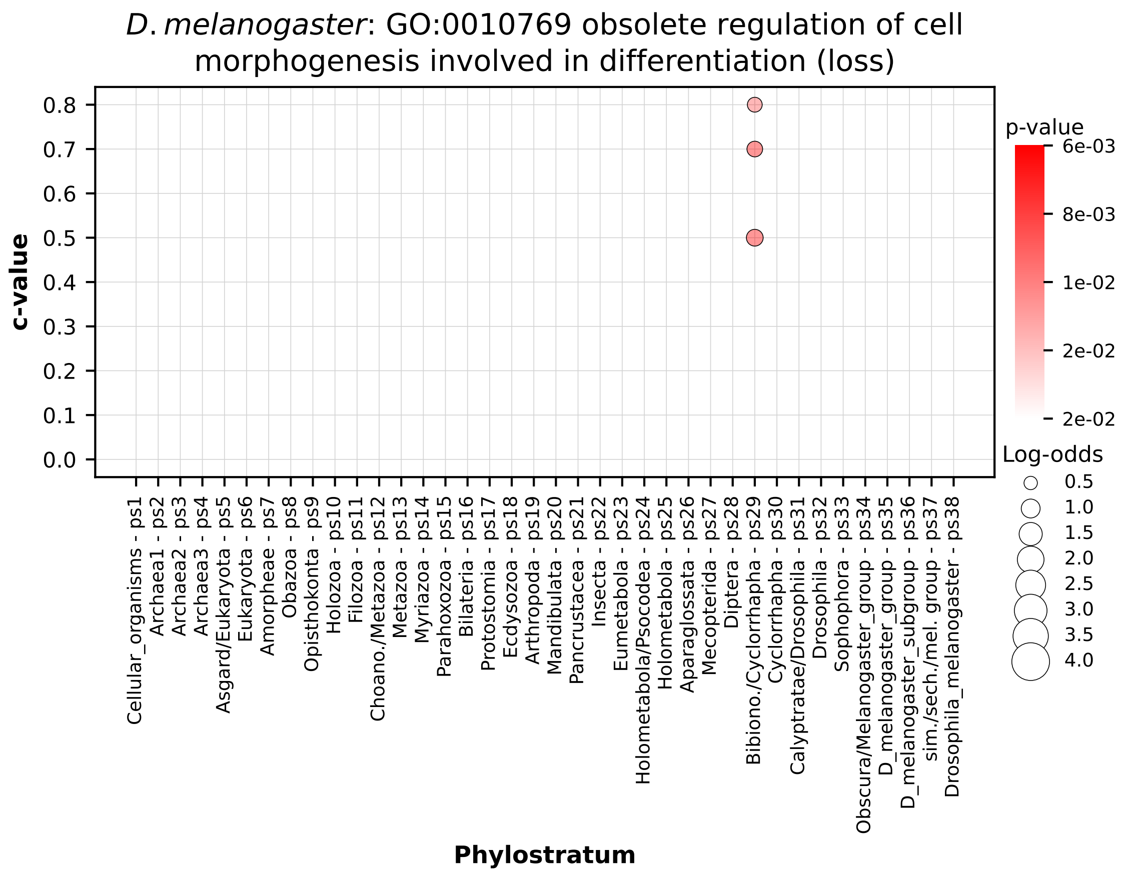

D. melanogaster: GO:0010769 obsolete regulation of cell morphogenesis involved in differentiation (loss)
c-value
0.8
0.7
0.6
0.5
0.4
0.3
0.2
0.1
0.0
p-value
6e-03
8e-03
1e-02
2e-02
2e-02
Log-odds
0.5
1.0
1.5
2.0
2.5
3.0
3.5
4.0
Cellular_organisms - ps1
Archaea1 - ps2
Archaea2 - ps3
Archaea3 - ps4
Asgard/Eukaryota - ps5
Eukaryota - ps6
Amorpheae - ps7
Obazoa - ps8
Opisthokonta - ps9
Holozoa - ps10
Filozoa - ps11
Choano./Metazoa - ps12
Metazoa - ps13
Myriazoa - ps14
Parahoxozoa - ps15
Bilateria - ps16
Protostomia - ps17
Ecdysozoa - ps18
Arthropoda - ps19
Mandibulata - ps20
Pancrustacea - ps21
Insecta - ps22
Eumetabola - ps23
Holometabola/Psocodea - ps24
Holometabola - ps25
Aparaglossata - ps26
Mecopterida - ps27
Diptera - ps28
Bibiono./Cyclorrhapha - ps29
Cyclorrhapha - ps30
Calyptratae/Drosophila - ps31
Drosophila - ps32
Sophophora - ps33
Obscura/Melanogaster_group - ps34
D_melanogaster_group - ps35
D_melanogaster_subgroup - ps36
sim./sech./mel. group - ps37
Drosophila_melanogaster - ps38
Phylostratum

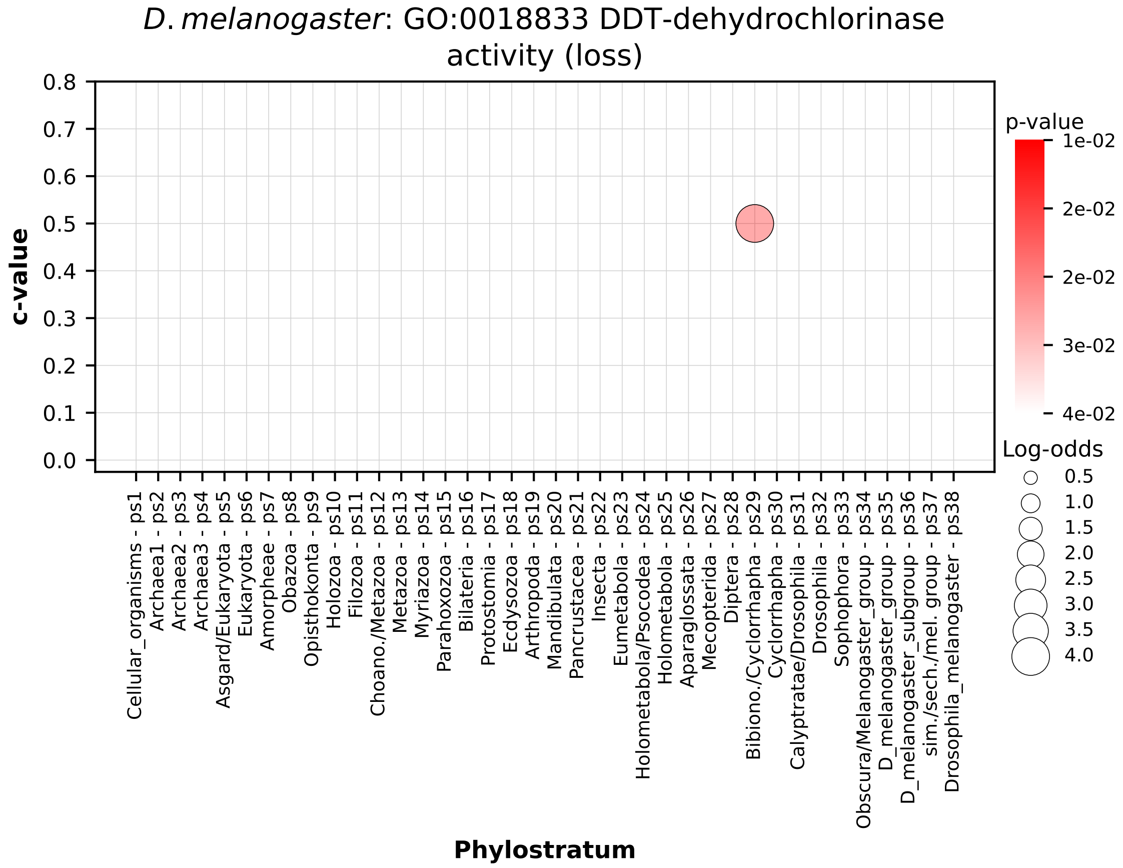

D. melanogaster: GO:0018833 DDT-dehydrochlorinase activity (loss)
c-value
0.8
0.7
0.6
0.5
0.4
0.3
0.2
0.1
0.0
Phylostratum
Cellular_organisms - ps1
Archaea1 - ps2
Archaea2 - ps3
Archaea3 - ps4
Asgard/Eukaryota - ps5
Eukaryota - ps6
Amorpheae - ps7
Obazoa - ps8
Opisthokonta - ps9
Holozoa - ps10
Filozoa - ps11
Choano./Metazoa - ps12
Metazoa - ps13
Myriazoa - ps14
Parahoxozoa - ps15
Bilateria - ps16
Protostomia - ps17
Ecdysozoa - ps18
Arthropoda - ps19
Mandibulata - ps20
Pancrustacea - ps21
Insecta - ps22
Eumetabola - ps23
Holometabola/Psocodea - ps24
Holometabola - ps25
Aparaglossata - ps26
Mecopterida - ps27
Diptera - ps28
Bibiono./Cyclorrhapha - ps29
Cyclorrhapha - ps30
Calyptratae/Drosophila - ps31
Drosophila - ps32
Sophophora - ps33
Obscura/Melanogaster_group - ps34
D_melanogaster_group - ps35
D_melanogaster_subgroup - ps36
sim./sech./mel. group - ps37
Drosophila_melanogaster - ps38
p-value
1e-02
2e-02
2e-02
3e-02
4e-02
Log-odds
0.5
1.0
1.5
2.0
2.5
3.0
3.5
4.0

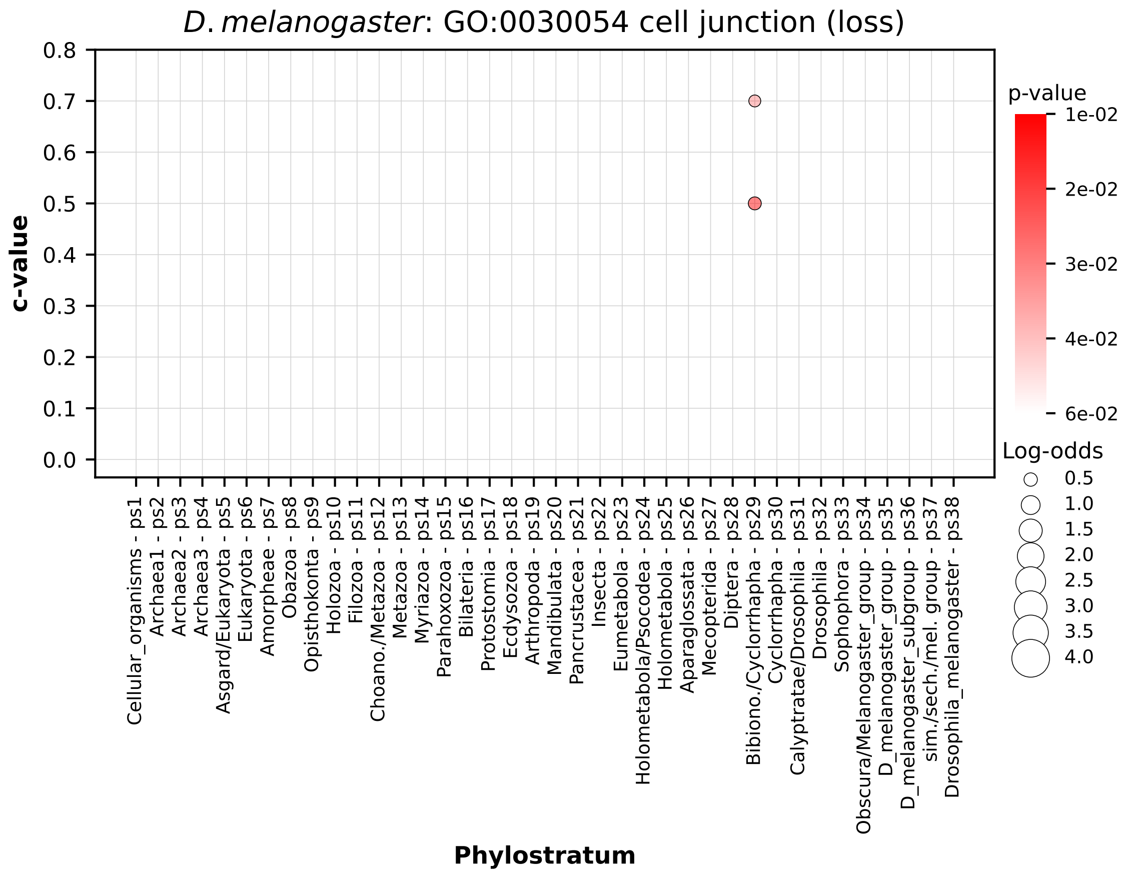

D. melanogaster: GO:0030054 cell junction (loss)
c-value
0.8
0.7
0.6
0.5
0.4
0.3
0.2
0.1
0.0
p-value
1e-02
2e-02
3e-02
4e-02
6e-02
Log-odds
0.5
1.0
1.5
2.0
2.5
3.0
3.5
4.0
Cellular_organisms - ps1
Archaea1 - ps2
Archaea2 - ps3
Archaea3 - ps4
Asgard/Eukaryota - ps5
Eukaryota - ps6
Amorpheae - ps7
Obazoa - ps8
Opisthokonta - ps9
Holozoa - ps10
Filozoa - ps11
Choano./Metazoa - ps12
Metazoa - ps13
Myriazoa - ps14
Parahoxozoa - ps15
Bilateria - ps16
Protostomia - ps17
Ecdysozoa - ps18
Arthropoda - ps19
Mandibulata - ps20
Pancrustacea - ps21
Insecta - ps22
Eumetabola - ps23
Holometabola/Psocodea - ps24
Holometabola - ps25
Aparaglossata - ps26
Mecopterida - ps27
Diptera - ps28
Bibiono./Cyclorrhapha - ps29
Cyclorrhapha - ps30
Calyptratae/Drosophila - ps31
Drosophila - ps32
Sophophora - ps33
Obscura/Melanogaster_group - ps34
D_melanogaster_group - ps35
D_melanogaster_subgroup - ps36
sim./sech./mel. group - ps37
Drosophila_melanogaster - ps38
Phylostratum

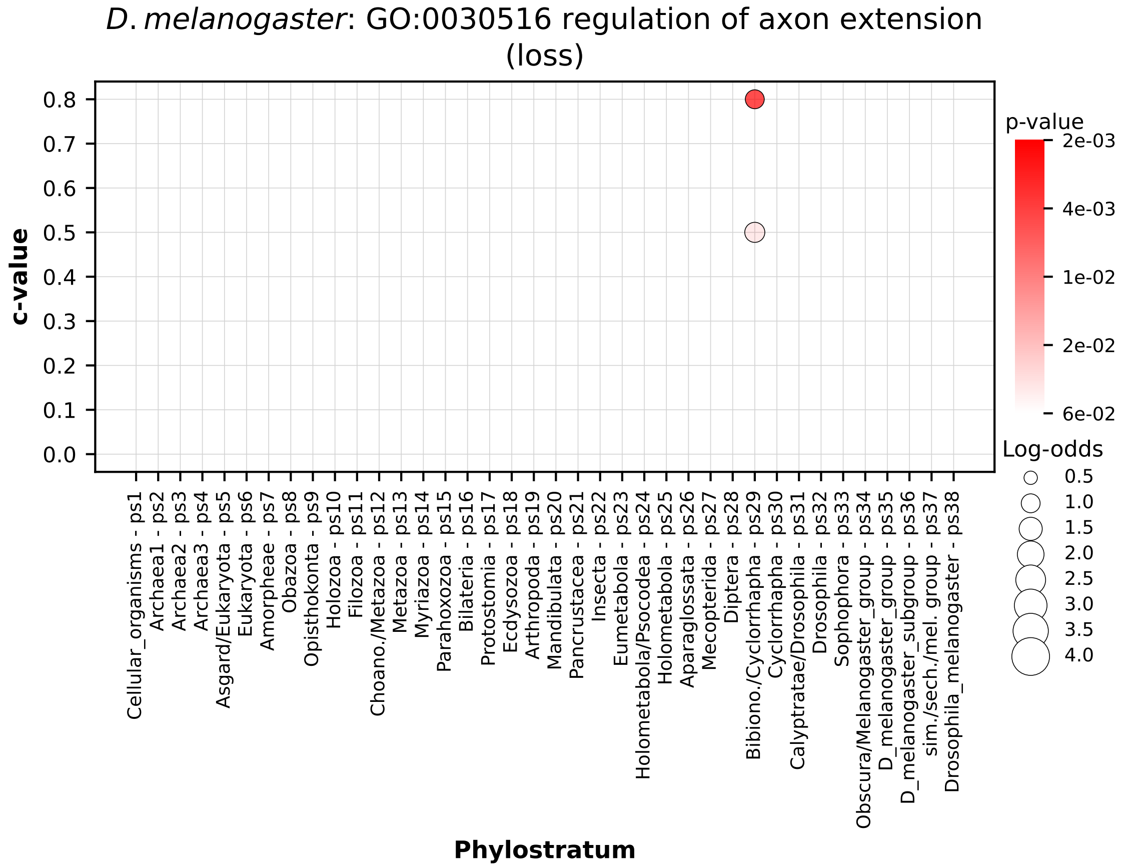

D. melanogaster: GO:0030516 regulation of axon extension (loss)
c-value
0.0
0.1
0.2
0.3
0.4
0.5
0.6
0.7
0.8
Phylostratum
Cellular_organisms - ps1
Archaea1 - ps2
Archaea2 - ps3
Archaea3 - ps4
Asgard/Eukaryota - ps5
Eukaryota - ps6
Amorpheae - ps7
Obazoa - ps8
Opisthokonta - ps9
Holozoa - ps10
Filozoa - ps11
Choano./Metazoa - ps12
Metazoa - ps13
Myriazoa - ps14
Parahoxozoa - ps15
Bilateria - ps16
Protostomia - ps17
Ecdysozoa - ps18
Arthropoda - ps19
Mandibulata - ps20
Pancrustacea - ps21
Insecta - ps22
Eumetabola - ps23
Holometabola/Psocodea - ps24
Holometabola - ps25
Aparaglossata - ps26
Mecopterida - ps27
Diptera - ps28
Bibiono./Cyclorrhapha - ps29
Cyclorrhapha - ps30
Calyptratae/Drosophila - ps31
Drosophila - ps32
Sophophora - ps33
Obscura/Melanogaster_group - ps34
D_melanogaster_group - ps35
D_melanogaster_subgroup - ps36
sim./sech./mel. group - ps37
Drosophila_melanogaster - ps38
p-value
2e-03
4e-03
1e-02
2e-02
6e-02
Log-odds
0.5
1.0
1.5
2.0
2.5
3.0
3.5
4.0

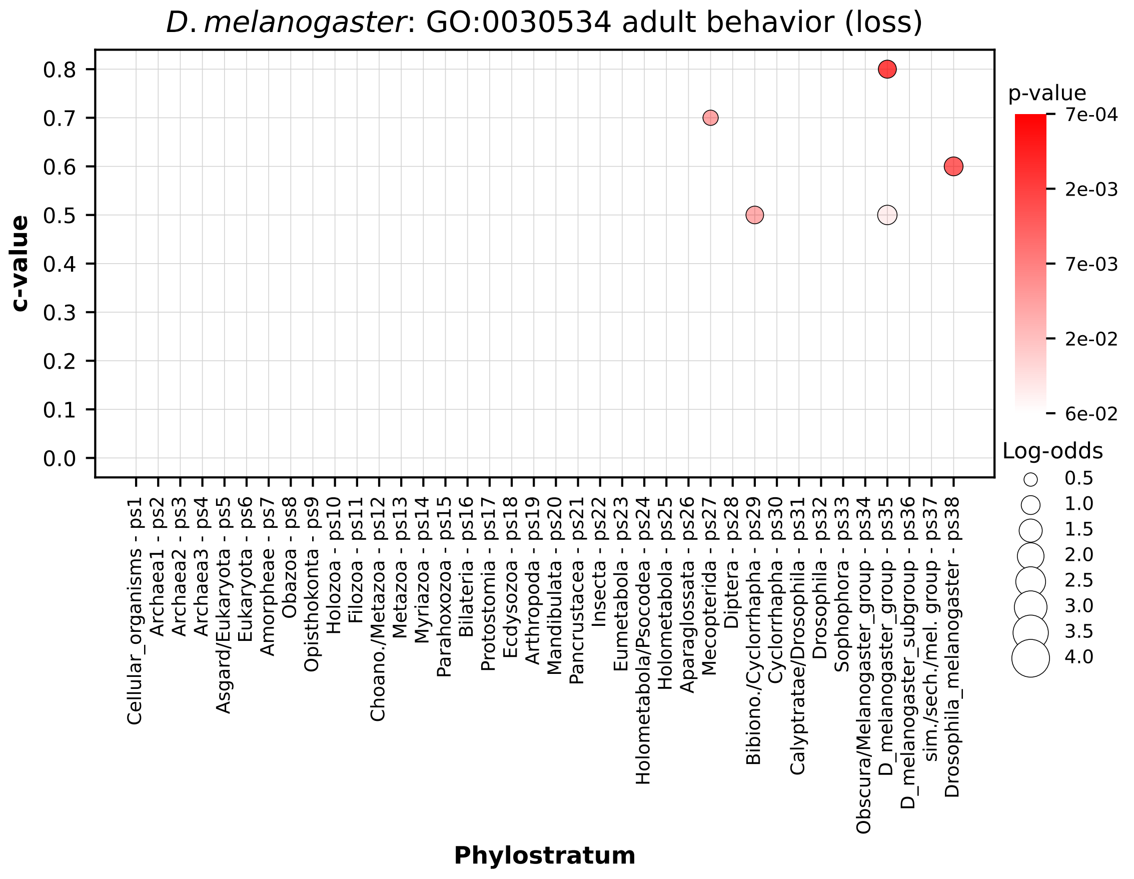

D. melanogaster: GO:0030534 adult behavior (loss)
c-value
0.0
0.1
0.2
0.3
0.4
0.5
0.6
0.7
0.8
Phylostratum
Cellular_organisms - ps1
Archaea1 - ps2
Archaea2 - ps3
Archaea3 - ps4
Asgard/Eukaryota - ps5
Eukaryota - ps6
Amorpheae - ps7
Obazoa - ps8
Opisthokonta - ps9
Holozoa - ps10
Filozoa - ps11
Choano./Metazoa - ps12
Metazoa - ps13
Myriazoa - ps14
Parahoxozoa - ps15
Bilateria - ps16
Protostomia - ps17
Ecdysozoa - ps18
Arthropoda - ps19
Mandibulata - ps20
Pancrustacea - ps21
Insecta - ps22
Eumetabola - ps23
Holometabola/Psocodea - ps24
Holometabola - ps25
Aparaglossata - ps26
Mecopterida - ps27
Diptera - ps28
Bibiono./Cyclorrhapha - ps29
Cyclorrhapha - ps30
Calyptratae/Drosophila - ps31
Drosophila - ps32
Sophophora - ps33
Obscura/Melanogaster_group - ps34
D_melanogaster_group - ps35
D_melanogaster_subgroup - ps36
sim./sech./mel. group - ps37
Drosophila_melanogaster - ps38
p-value
7e-04
2e-03
7e-03
2e-02
6e-02
Log-odds
0.5
1.0
1.5
2.0
2.5
3.0
3.5
4.0

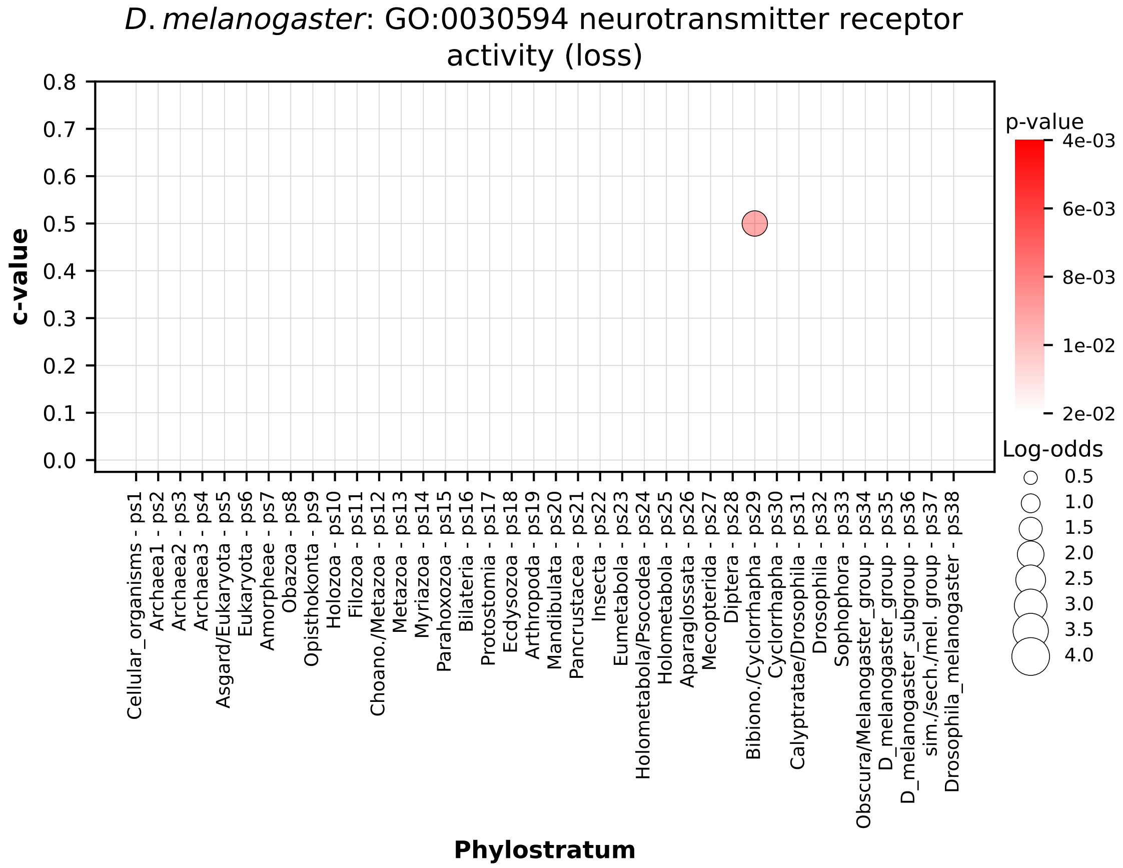

$D. melanogaster$: GO:0030594 neurotransmitter receptor activity (loss)
c-value
0.8
0.7
0.6
0.5
0.4
0.3
0.2
0.1
0.0
p-value
4e-03
6e-03
8e-03
1e-02
2e-02
Log-odds
0.5
1.0
1.5
2.0
2.5
3.0
3.5
4.0
Cellular_organisms - ps1
Archaea1 - ps2
Archaea2 - ps3
Archaea3 - ps4
Asgard/Eukaryota - ps5
Eukaryota - ps6
Amorpheae - ps7
Obazoa - ps8
Opisthokonta - ps9
Holozoa - ps10
Filozoa - ps11
Choano./Metazoa - ps12
Metazoa - ps13
Myriazoa - ps14
Parahoxozoa - ps15
Bilateria - ps16
Protostomia - ps17
Ecdysozoa - ps18
Arthropoda - ps19
Mandibulata - ps20
Pancrustacea - ps21
Insecta - ps22
Eumetabola - ps23
Holometabola/Psocodea - ps24
Holometabola - ps25
Aparaglossata - ps26
Mecopterida - ps27
Diptera - ps28
Bibiono./Cyclorrhapha - ps29
Cyclorrhapha - ps30
Calyptratae/Drosophila - ps31
Drosophila - ps32
Sophophora - ps33
Obscura/Melanogaster_group - ps34
D_melanogaster_group - ps35
D_melanogaster_subgroup - ps36
sim./sech./mel. group - ps37
Drosophila_melanogaster - ps38
Phylostratum

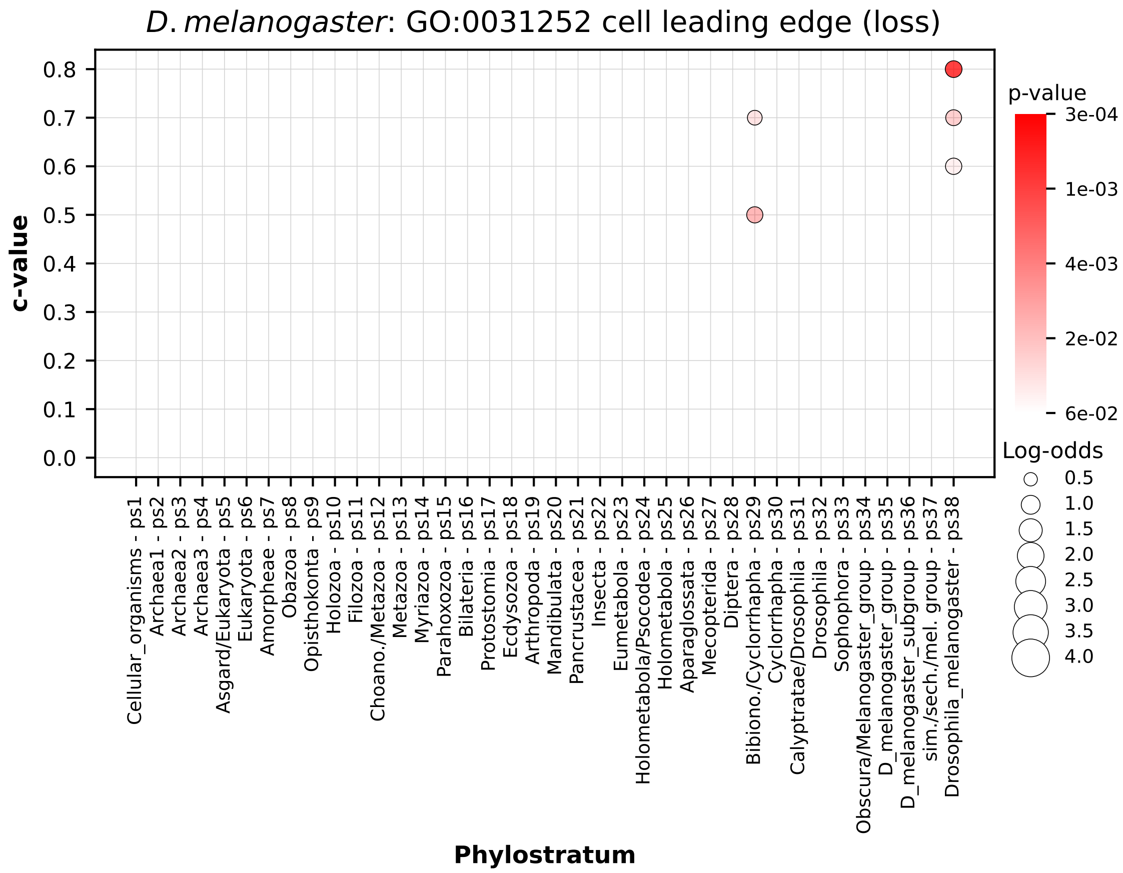

D. melanogaster: GO:0031252 cell leading edge (loss)
c-value
0.0
0.1
0.2
0.3
0.4
0.5
0.6
0.7
0.8
Phylostratum
Cellular_organisms - ps1
Archaea1 - ps2
Archaea2 - ps3
Archaea3 - ps4
Asgard/Eukaryota - ps5
Eukaryota - ps6
Amorpheae - ps7
Obazoa - ps8
Opisthokonta - ps9
Holozoa - ps10
Filozoa - ps11
Choano./Metazoa - ps12
Metazoa - ps13
Myriazoa - ps14
Parahoxozoa - ps15
Bilateria - ps16
Protostomia - ps17
Ecdysozoa - ps18
Arthropoda - ps19
Mandibulata - ps20
Pancrustacea - ps21
Insecta - ps22
Eumetabola - ps23
Holometabola/Psocodea - ps24
Holometabola - ps25
Aparaglossata - ps26
Mecopterida - ps27
Diptera - ps28
Bibiono./Cyclorrhapha - ps29
Cyclorrhapha - ps30
Calyptratae/Drosophila - ps31
Drosophila - ps32
Sophophora - ps33
Obscura/Melanogaster_group - ps34
D_melanogaster_group - ps35
D_melanogaster_subgroup - ps36
sim./sech./mel. group - ps37
Drosophila_melanogaster - ps38
p-value
3e-04
1e-03
4e-03
2e-02
6e-02
Log-odds
0.5
1.0
1.5
2.0
2.5
3.0
3.5
4.0

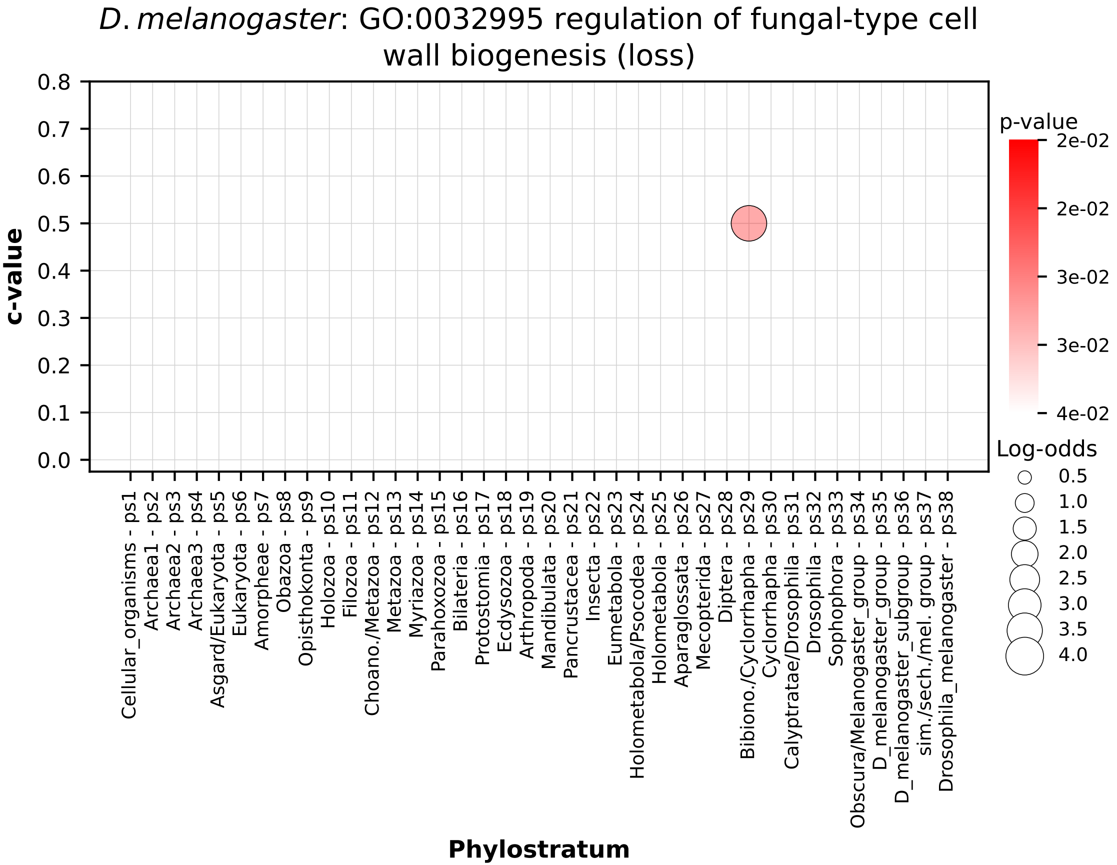
D. melanogaster: GO:0032995 regulation of fungal-type cell wall biogenesis (loss)
c-value
0.8
0.7
0.6
0.5
0.4
0.3
0.2
0.1
0.0
Phylostratum
Cellular_organisms - ps1
Archaea1 - ps2
Archaea2 - ps3
Archaea3 - ps4
Asgard/Eukaryota - ps5
Eukaryota - ps6
Amorpheae - ps7
Obazoa - ps8
Opisthokonta - ps9
Holozoa - ps10
Filozoa - ps11
Choano./Metazoa - ps12
Metazoa - ps13
Myriazoa - ps14
Parahoxozoa - ps15
Bilateria - ps16
Protostomia - ps17
Ecdysozoa - ps18
Arthropoda - ps19
Mandibulata - ps20
Pancrustacea - ps21
Insecta - ps22
Eumetabola - ps23
Holometabola/Psocodea - ps24
Holometabola - ps25
Aparaglossata - ps26
Mecopterida - ps27
Diptera - ps28
Bibiono./Cyclorrhapha - ps29
Cyclorrhapha - ps30
Calyptratae/Drosophila - ps31
Drosophila - ps32
Sophophora - ps33
Obscura/Melanogaster_group - ps34
D_melanogaster_group - ps35
D_melanogaster_subgroup - ps36
sim./sech./mel. group - ps37
Drosophila_melanogaster - ps38
p-value
2e-02
2e-02
3e-02
3e-02
4e-02
Log-odds
0.5
1.0
1.5
2.0
2.5
3.0
3.5
4.0

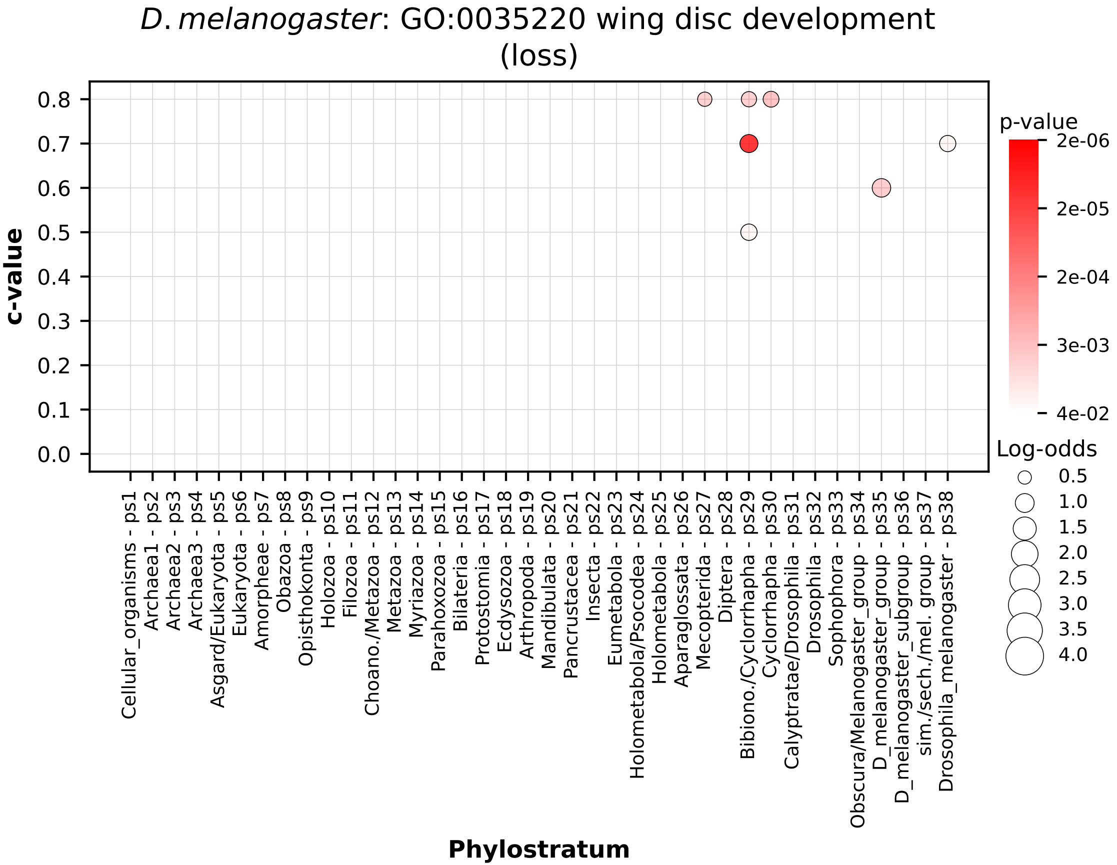

D. melanogaster: GO:0035220 wing disc development
(loss)
c-value
0.0
0.1
0.2
0.3
0.4
0.5
0.6
0.7
0.8
p-value
2e-06
2e-05
2e-04
3e-03
4e-02
Log-odds
0.5
1.0
1.5
2.0
2.5
3.0
3.5
4.0
Cellular_organisms - ps1
Archaea1 - ps2
Archaea2 - ps3
Archaea3 - ps4
Asgard/Eukaryota - ps5
Eukaryota - ps6
Amorpheae - ps7
Obazoa - ps8
Opisthokonta - ps9
Holozoa - ps10
Filozoa - ps11
Choano./Metazoa - ps12
Metazoa - ps13
Myriazoa - ps14
Parahoxozoa - ps15
Bilateria - ps16
Protostomia - ps17
Ecdysozoa - ps18
Arthropoda - ps19
Mandibulata - ps20
Pancrustacea - ps21
Insecta - ps22
Eumetabola - ps23
Holometabola/Psocodea - ps24
Holometabola - ps25
Aparaglossata - ps26
Mecopterida - ps27
Diptera - ps28
Bibiono./Cyclorrhapha - ps29
Cyclorrhapha - ps30
Calyptratae/Drosophila - ps31
Drosophila - ps32
Sophophora - ps33
Obscura/Melanogaster_group - ps34
D_melanogaster_group - ps35
D_melanogaster_subgroup - ps36
sim./sech./mel. group - ps37
Drosophila_melanogaster - ps38
Phylostratum

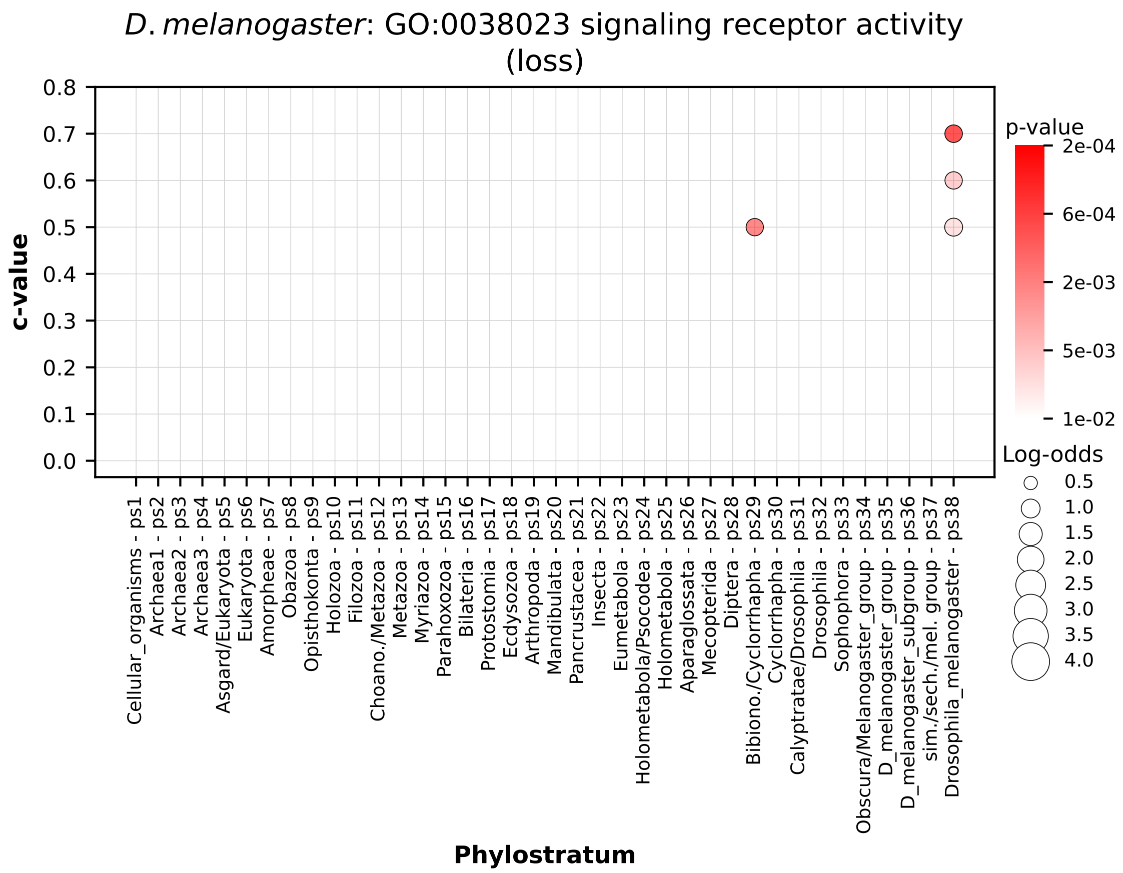
D. melanogaster: GO:0038023 signaling receptor activity
(loss)
c-value
0.8
0.7
0.6
0.5
0.4
0.3
0.2
0.1
0.0
Phylostratum
Cellular_organisms - ps1
Archaea1 - ps2
Archaea2 - ps3
Archaea3 - ps4
Asgard/Eukaryota - ps5
Eukaryota - ps6
Amorpheae - ps7
Obazoa - ps8
Opisthokonta - ps9
Holozoa - ps10
Filozoa - ps11
Choano./Metazoa - ps12
Metazoa - ps13
Myriazoa - ps14
Parahoxozoa - ps15
Bilateria - ps16
Protostomia - ps17
Ecdysozoa - ps18
Arthropoda - ps19
Mandibulata - ps20
Pancrustacea - ps21
Insecta - ps22
Eumetabola - ps23
Holometabola/Psocodea - ps24
Holometabola - ps25
Aparaglossata - ps26
Mecopterida - ps27
Diptera - ps28
Bibiono./Cyclorrhapha - ps29
Cyclorrhapha - ps30
Calyptratae/Drosophila - ps31
Drosophila - ps32
Sophophora - ps33
Obscura/Melanogaster_group - ps34
D_melanogaster_group - ps35
D_melanogaster_subgroup - ps36
sim./sech./mel. group - ps37
Drosophila_melanogaster - ps38
p-value
2e-04
6e-04
2e-03
5e-03
1e-02
Log-odds
0.5
1.0
1.5
2.0
2.5
3.0
3.5
4.0

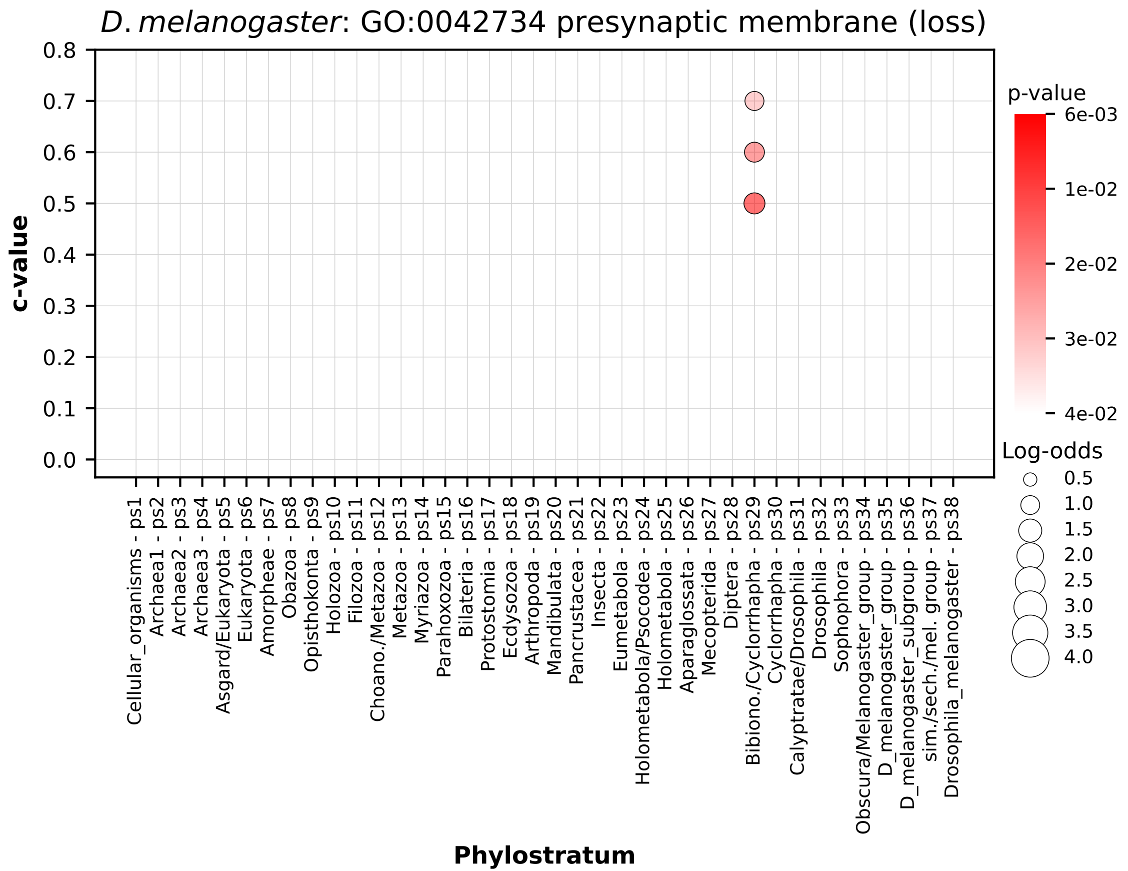

D. melanogaster: GO:0042734 presynaptic membrane (loss)
c-value
0.0
0.1
0.2
0.3
0.4
0.5
0.6
0.7
0.8
Phylostratum
Cellular_organisms - ps1
Archaea1 - ps2
Archaea2 - ps3
Archaea3 - ps4
Asgard/Eukaryota - ps5
Eukaryota - ps6
Amorpheae - ps7
Obazoa - ps8
Opisthokonta - ps9
Holozoa - ps10
Filozoa - ps11
Choano./Metazoa - ps12
Metazoa - ps13
Myriazoa - ps14
Parahoxozoa - ps15
Bilateria - ps16
Protostomia - ps17
Ecdysozoa - ps18
Arthropoda - ps19
Mandibulata - ps20
Pancrustacea - ps21
Insecta - ps22
Eumetabola - ps23
Holometabola/Psocodea - ps24
Holometabola - ps25
Aparaglossata - ps26
Mecopterida - ps27
Diptera - ps28
Bibiono./Cyclorrhapha - ps29
Cyclorrhapha - ps30
Calyptratae/Drosophila - ps31
Drosophila - ps32
Sophophora - ps33
Obscura/Melanogaster_group - ps34
D_melanogaster_group - ps35
D_melanogaster_subgroup - ps36
sim./sech./mel. group - ps37
Drosophila_melanogaster - ps38
p-value
6e-03
1e-02
2e-02
3e-02
4e-02
Log-odds
0.5
1.0
1.5
2.0
2.5
3.0
3.5
4.0

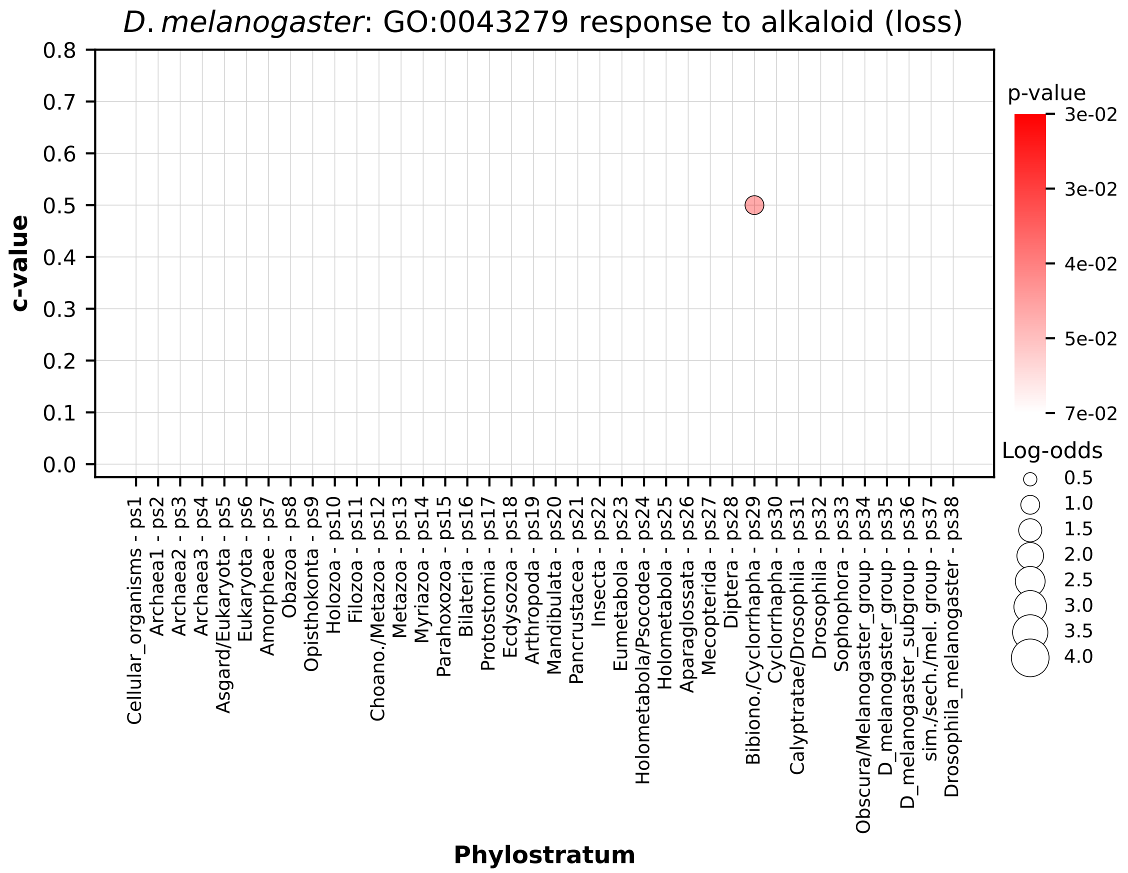

D. melanogaster: GO:0043279 response to alkaloid (loss)
c-value
0.0
0.1
0.2
0.3
0.4
0.5
0.6
0.7
0.8
Phylostratum
Cellular_organisms - ps1
Archaea1 - ps2
Archaea2 - ps3
Archaea3 - ps4
Asgard/Eukaryota - ps5
Eukaryota - ps6
Amorpheae - ps7
Obazoa - ps8
Opisthokonta - ps9
Holozoa - ps10
Filozoa - ps11
Choano./Metazoa - ps12
Metazoa - ps13
Myriazoa - ps14
Parahoxozoa - ps15
Bilateria - ps16
Protostomia - ps17
Ecdysozoa - ps18
Arthropoda - ps19
Mandibulata - ps20
Pancrustacea - ps21
Insecta - ps22
Eumetabola - ps23
Holometabola/Psocodea - ps24
Holometabola - ps25
Aparaglossata - ps26
Mecopterida - ps27
Diptera - ps28
Bibiono./Cyclorrhapha - ps29
Cyclorrhapha - ps30
Calyptratae/Drosophila - ps31
Drosophila - ps32
Sophophora - ps33
Obscura/Melanogaster_group - ps34
D_melanogaster_group - ps35
D_melanogaster_subgroup - ps36
sim./sech./mel. group - ps37
Drosophila_melanogaster - ps38
p-value
3e-02
3e-02
4e-02
5e-02
7e-02
Log-odds
0.5
1.0
1.5
2.0
2.5
3.0
3.5
4.0

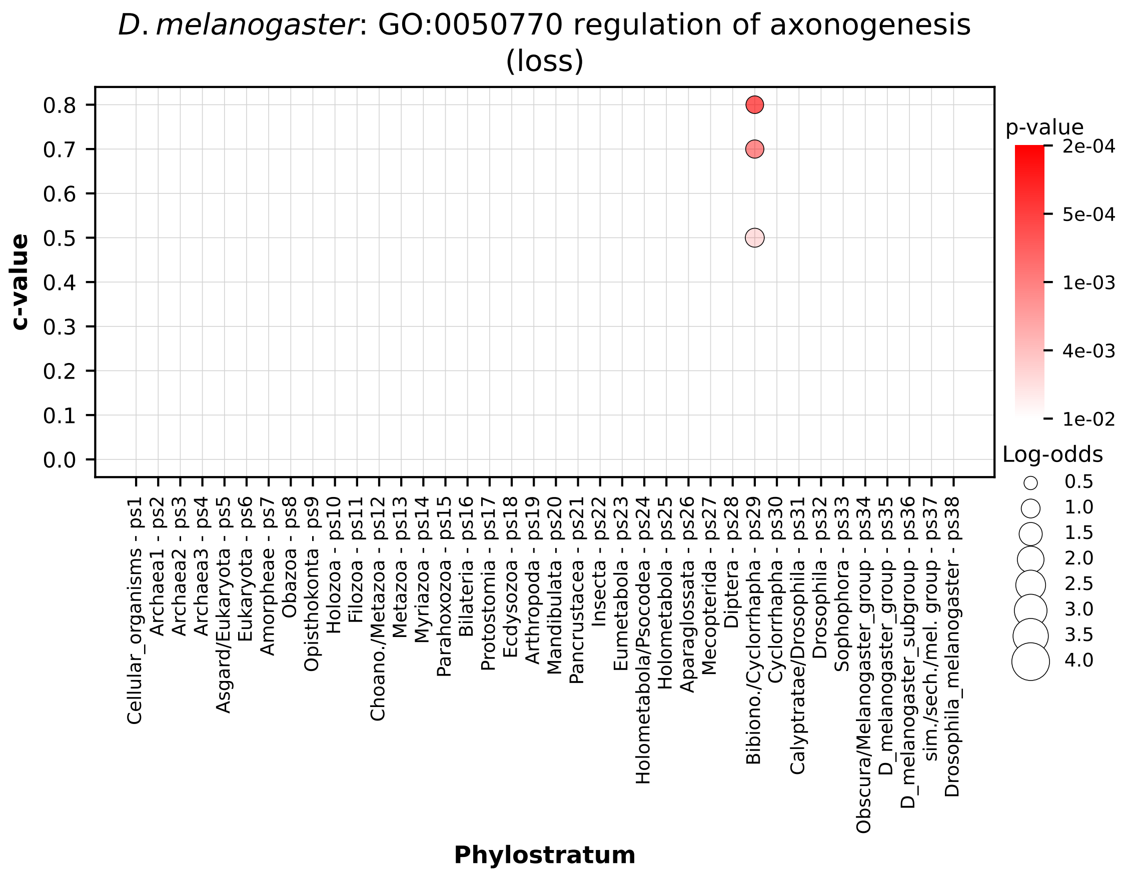

D. melanogaster: GO:0050770 regulation of axonogenesis
(loss)
c-value
0.8
0.7
0.6
0.5
0.4
0.3
0.2
0.1
0.0
p-value
2e-04
5e-04
1e-03
4e-03
1e-02
Log-odds
0.5
1.0
1.5
2.0
2.5
3.0
3.5
4.0
Cellular_organisms - ps1
Archaea1 - ps2
Archaea2 - ps3
Archaea3 - ps4
Asgard/Eukaryota - ps5
Eukaryota - ps6
Amorpheae - ps7
Obazoa - ps8
Opisthokonta - ps9
Holozoa - ps10
Filozoa - ps11
Choano./Metazoa - ps12
Metazoa - ps13
Myriazoa - ps14
Parahoxozoa - ps15
Bilateria - ps16
Protostomia - ps17
Ecdysozoa - ps18
Arthropoda - ps19
Mandibulata - ps20
Pancrustacea - ps21
Insecta - ps22
Eumetabola - ps23
Holometabola/Psocodea - ps24
Holometabola - ps25
Aparaglossata - ps26
Mecopterida - ps27
Diptera - ps28
Bibiono./Cyclorrhapha - ps29
Cyclorrhapha - ps30
Calyptratae/Drosophila - ps31
Drosophila - ps32
Sophophora - ps33
Obscura/Melanogaster_group - ps34
D_melanogaster_group - ps35
D_melanogaster_subgroup - ps36
sim./sech./mel. group - ps37
Drosophila_melanogaster - ps38
Phylostratum

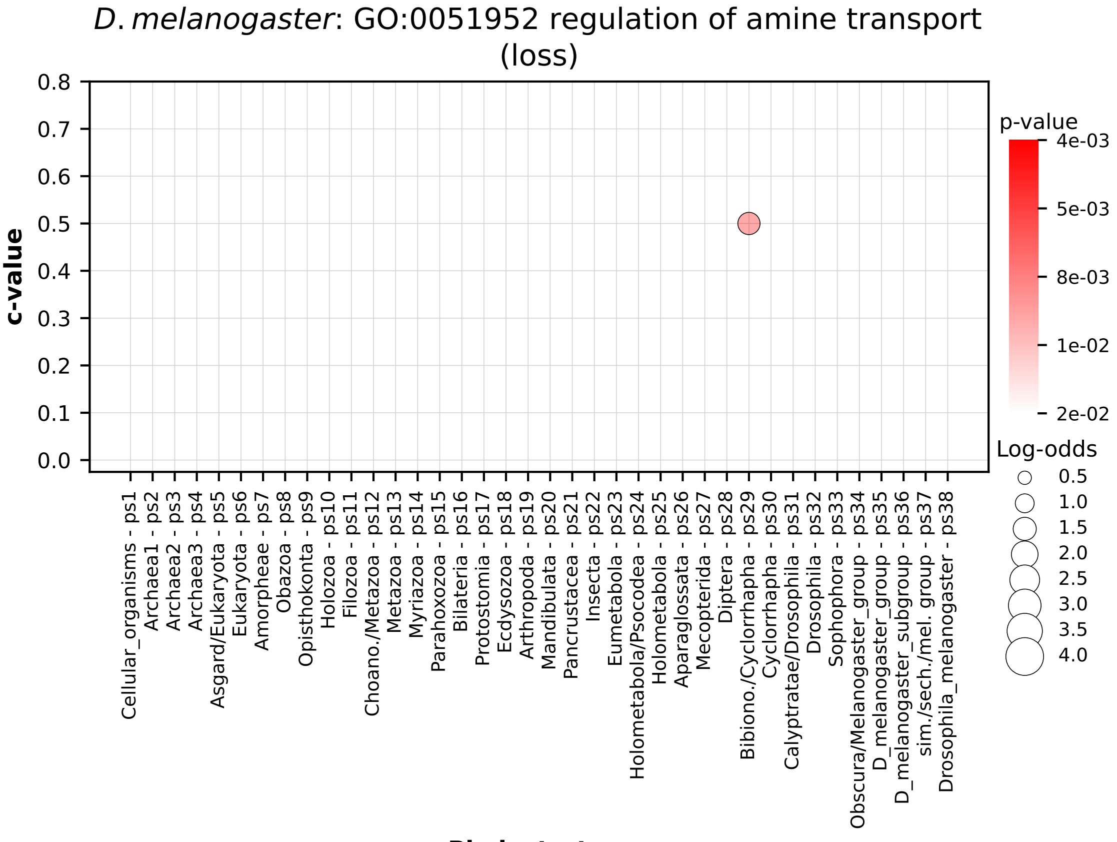

D. melanogaster: GO:0051952 regulation of amine transport (loss)
c-value
0.0
0.1
0.2
0.3
0.4
0.5
0.6
0.7
0.8
Phylostratum
Cellular_organisms - ps1
Archaea1 - ps2
Archaea2 - ps3
Archaea3 - ps4
Asgard/Eukaryota - ps5
Eukaryota - ps6
Amorpheae - ps7
Obazoa - ps8
Opisthokonta - ps9
Holozoa - ps10
Filozoa - ps11
Choano./Metazoa - ps12
Metazoa - ps13
Myriazoa - ps14
Parahoxozoa - ps15
Bilateria - ps16
Protostomia - ps17
Ecdysozoa - ps18
Arthropoda - ps19
Mandibulata - ps20
Pancrustacea - ps21
Insecta - ps22
Eumetabola - ps23
Holometabola/Psocodea - ps24
Holometabola - ps25
Aparaglossata - ps26
Mecopterida - ps27
Diptera - ps28
Bibiono./Cyclorrhapha - ps29
Cyclorrhapha - ps30
Calyptratae/Drosophila - ps31
Drosophila - ps32
Sophophora - ps33
Obscura/Melanogaster_group - ps34
D_melanogaster_group - ps35
D_melanogaster_subgroup - ps36
sim./sech./mel. group - ps37
Drosophila_melanogaster - ps38
p-value
4e-03
5e-03
8e-03
1e-02
2e-02
Log-odds
0.5
1.0
1.5
2.0
2.5
3.0
3.5
4.0

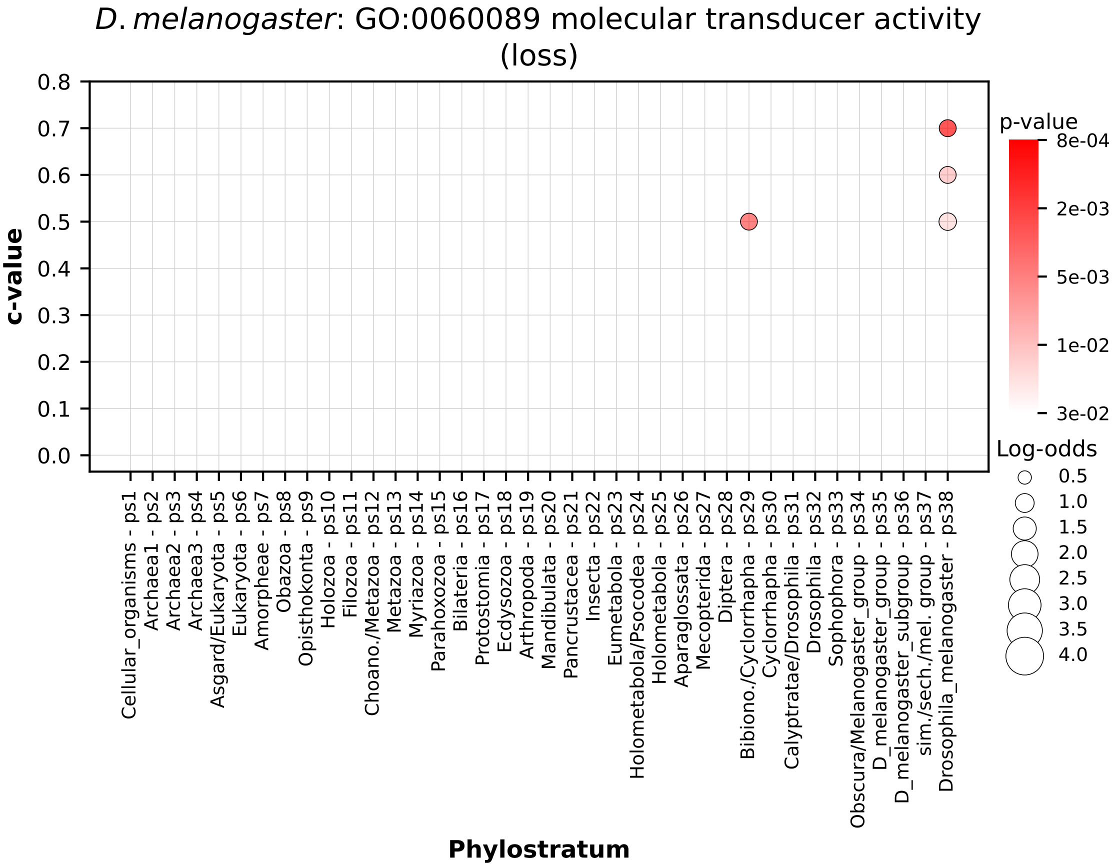
D. melanogaster: GO:0060089 molecular transducer activity (loss)
c-value
Phylostratum
p-value
8e-04
2e-03
5e-03
1e-02
3e-02
Log-odds
0.5
1.0
1.5
2.0
2.5
3.0
3.5
4.0
Cellular_organisms - ps1
Archaea1 - ps2
Archaea2 - ps3
Archaea3 - ps4
Asgard/Eukaryota - ps5
Eukaryota - ps6
Amorpheae - ps7
Obazoa - ps8
Opisthokonta - ps9
Holozoa - ps10
Filozoa - ps11
Choano./Metazoa - ps12
Metazoa - ps13
Myriazoa - ps14
Parahoxozoa - ps15
Bilateria - ps16
Protostomia - ps17
Ecdysozoa - ps18
Arthropoda - ps19
Mandibulata - ps20
Pancrustacea - ps21
Insecta - ps22
Eumetabola - ps23
Holometabola/Psocodea - ps24
Holometabola - ps25
Aparaglossata - ps26
Mecopterida - ps27
Diptera - ps28
Bibiono./Cyclorrhapha - ps29
Cyclorrhapha - ps30
Calyptratae/Drosophila - ps31
Drosophila - ps32
Sophophora - ps33
Obscura/Melanogaster_group - ps34
D_melanogaster_group - ps35
D_melanogaster_subgroup - ps36
sim./sech./mel. group - ps37
Drosophila_melanogaster - ps38

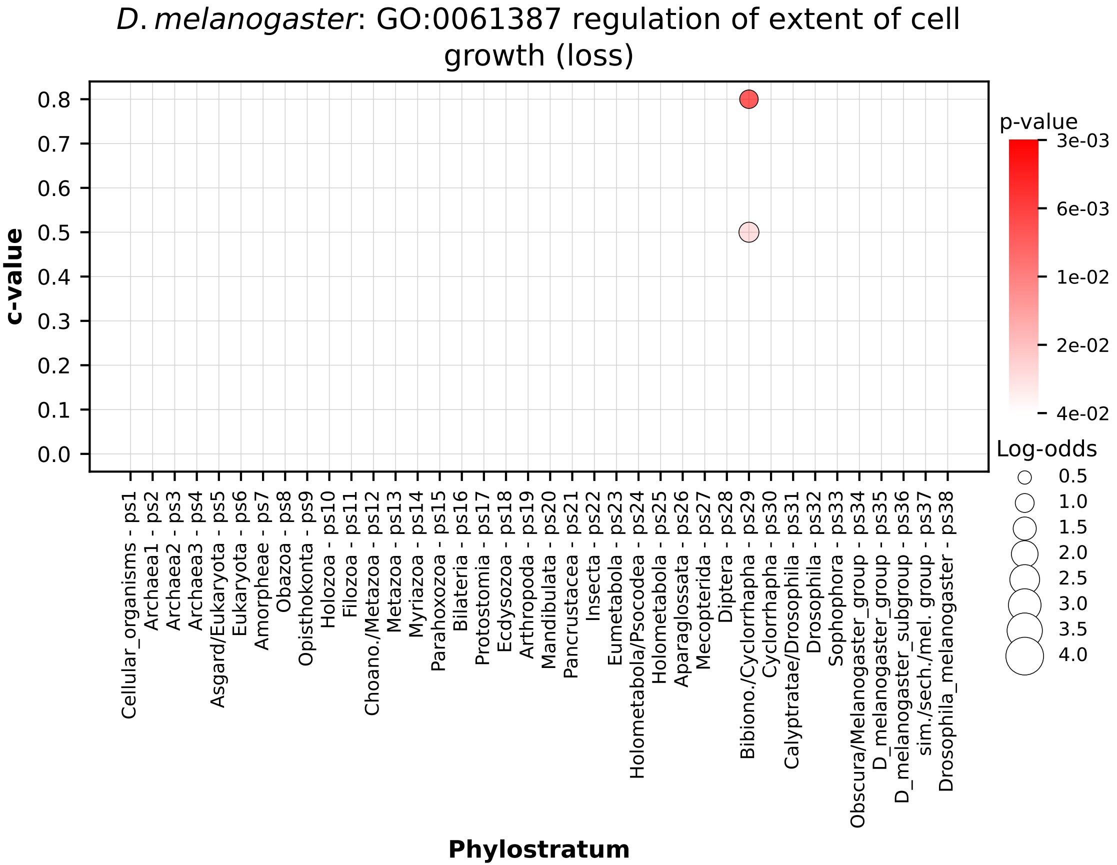

$D. melanogaster$: GO:0061387 regulation of extent of cell growth (loss)
c-value
0.8
0.7
0.6
0.5
0.4
0.3
0.2
0.1
0.0
Phylostratum
Cellular_organisms - ps1
Archaea1 - ps2
Archaea2 - ps3
Archaea3 - ps4
Asgard/Eukaryota - ps5
Eukaryota - ps6
Amorpheae - ps7
Obazoa - ps8
Opisthokonta - ps9
Holozoa - ps10
Filozoa - ps11
Choano./Metazoa - ps12
Metazoa - ps13
Myriazoa - ps14
Parahoxozoa - ps15
Bilateria - ps16
Protostomia - ps17
Ecdysozoa - ps18
Arthropoda - ps19
Mandibulata - ps20
Pancrustacea - ps21
Insecta - ps22
Eumetabola - ps23
Holometabola/Psocodea - ps24
Holometabola - ps25
Aparaglossata - ps26
Mecopterida - ps27
Diptera - ps28
Bibiono./Cyclorrhapha - ps29
Cyclorrhapha - ps30
Calyptratae/Drosophila - ps31
Drosophila - ps32
Sophophora - ps33
Obscura/Melanogaster_group - ps34
D_melanogaster_group - ps35
D_melanogaster_subgroup - ps36
sim./sech./mel. group - ps37
Drosophila_melanogaster - ps38
p-value
3e-03
6e-03
1e-02
2e-02
4e-02
Log-odds
0.5
1.0
1.5
2.0
2.5
3.0
3.5
4.0

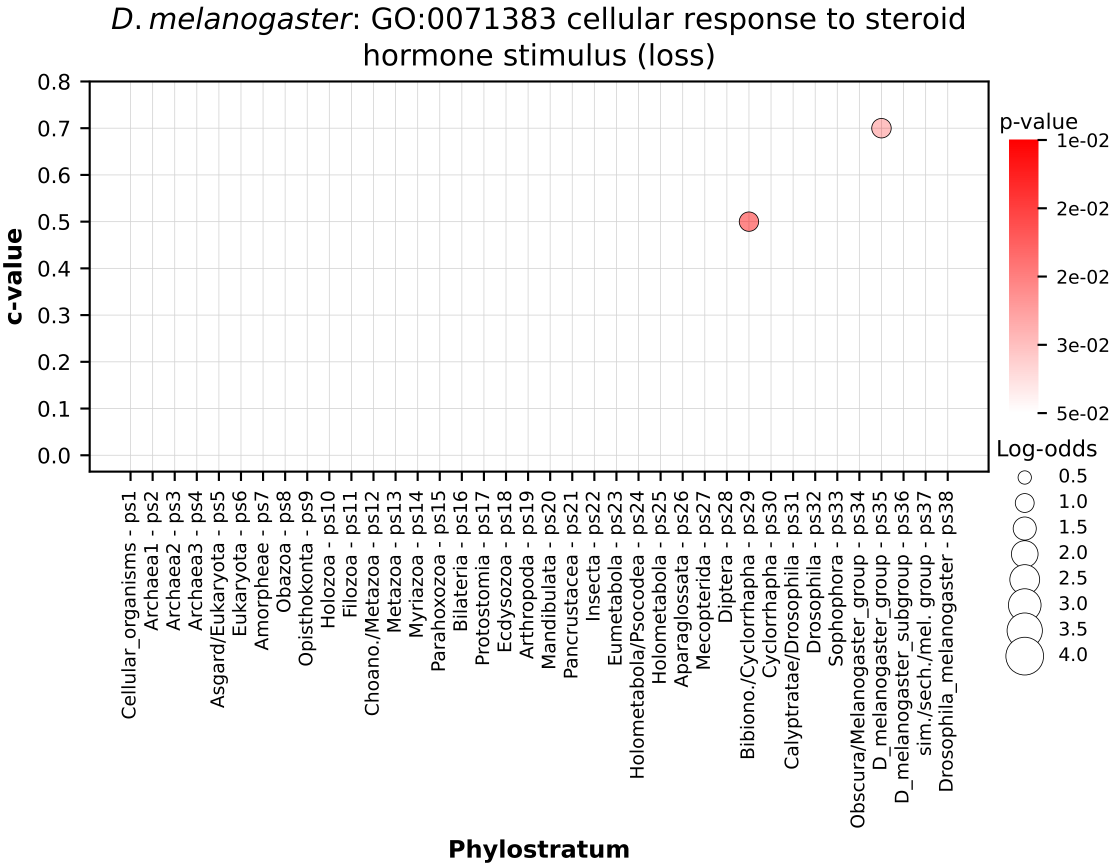

D. melanogaster: GO:0071383 cellular response to steroid hormone stimulus (loss)
c-value
0.8
0.7
0.6
0.5
0.4
0.3
0.2
0.1
0.0
p-value
1e-02
2e-02
2e-02
3e-02
5e-02
Log-odds
0.5
1.0
1.5
2.0
2.5
3.0
3.5
4.0
Cellular_organisms - ps1
Archaea1 - ps2
Archaea2 - ps3
Archaea3 - ps4
Asgard/Eukaryota - ps5
Eukaryota - ps6
Amorpheae - ps7
Obazoa - ps8
Opisthokonta - ps9
Holozoa - ps10
Filozoa - ps11
Choano./Metazoa - ps12
Metazoa - ps13
Myriazoa - ps14
Parahoxozoa - ps15
Bilateria - ps16
Protostomia - ps17
Ecdysozoa - ps18
Arthropoda - ps19
Mandibulata - ps20
Pancrustacea - ps21
Insecta - ps22
Eumetabola - ps23
Holometabola/Psocodea - ps24
Holometabola - ps25
Aparaglossata - ps26
Mecopterida - ps27
Diptera - ps28
Bibiono./Cyclorrhapha - ps29
Cyclorrhapha - ps30
Calyptratae/Drosophila - ps31
Drosophila - ps32
Sophophora - ps33
Obscura/Melanogaster_group - ps34
D_melanogaster_group - ps35
D_melanogaster_subgroup - ps36
sim./sech./mel. group - ps37
Drosophila_melanogaster - ps38
Phylostratum

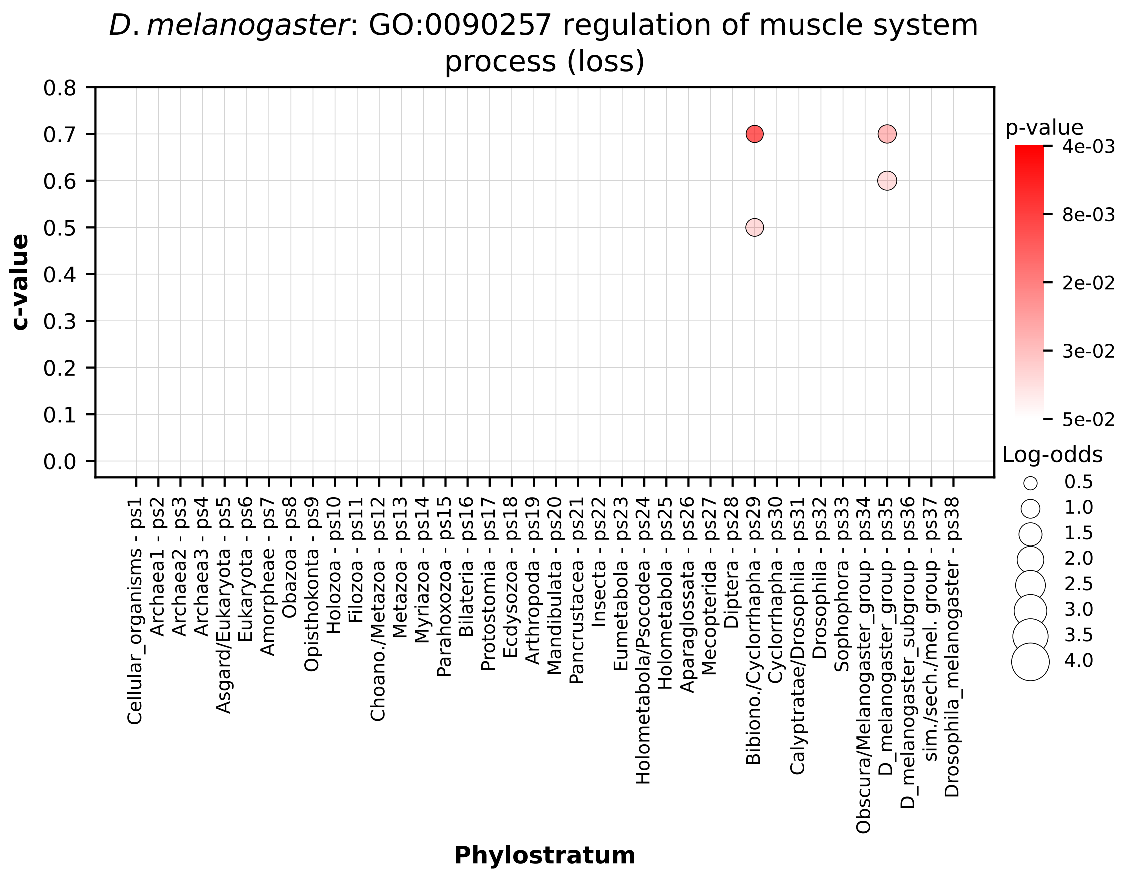

D. melanogaster: GO:0090257 regulation of muscle system process (loss)
c-value
0.8
0.7
0.6
0.5
0.4
0.3
0.2
0.1
0.0
p-value
4e-03
8e-03
2e-02
3e-02
5e-02
Log-odds
0.5
1.0
1.5
2.0
2.5
3.0
3.5
4.0
Cellular_organisms - ps1
Archaea1 - ps2
Archaea2 - ps3
Archaea3 - ps4
Asgard/Eukaryota - ps5
Eukaryota - ps6
Amorpheae - ps7
Obazoa - ps8
Opisthokonta - ps9
Holozoa - ps10
Filozoa - ps11
Choano./Metazoa - ps12
Metazoa - ps13
Myriazoa - ps14
Parahoxozoa - ps15
Bilateria - ps16
Protostomia - ps17
Ecdysozoa - ps18
Arthropoda - ps19
Mandibulata - ps20
Pancrustacea - ps21
Insecta - ps22
Eumetabola - ps23
Holometabola/Psocodea - ps24
Holometabola - ps25
Aparaglossata - ps26
Mecopterida - ps27
Diptera - ps28
Bibiono./Cyclorrhapha - ps29
Cyclorrhapha - ps30
Calyptratae/Drosophila - ps31
Drosophila - ps32
Sophophora - ps33
Obscura/Melanogaster_group - ps34
D_melanogaster_group - ps35
D_melanogaster_subgroup - ps36
sim./sech./mel. group - ps37
Drosophila_melanogaster - ps38
Phylostratum

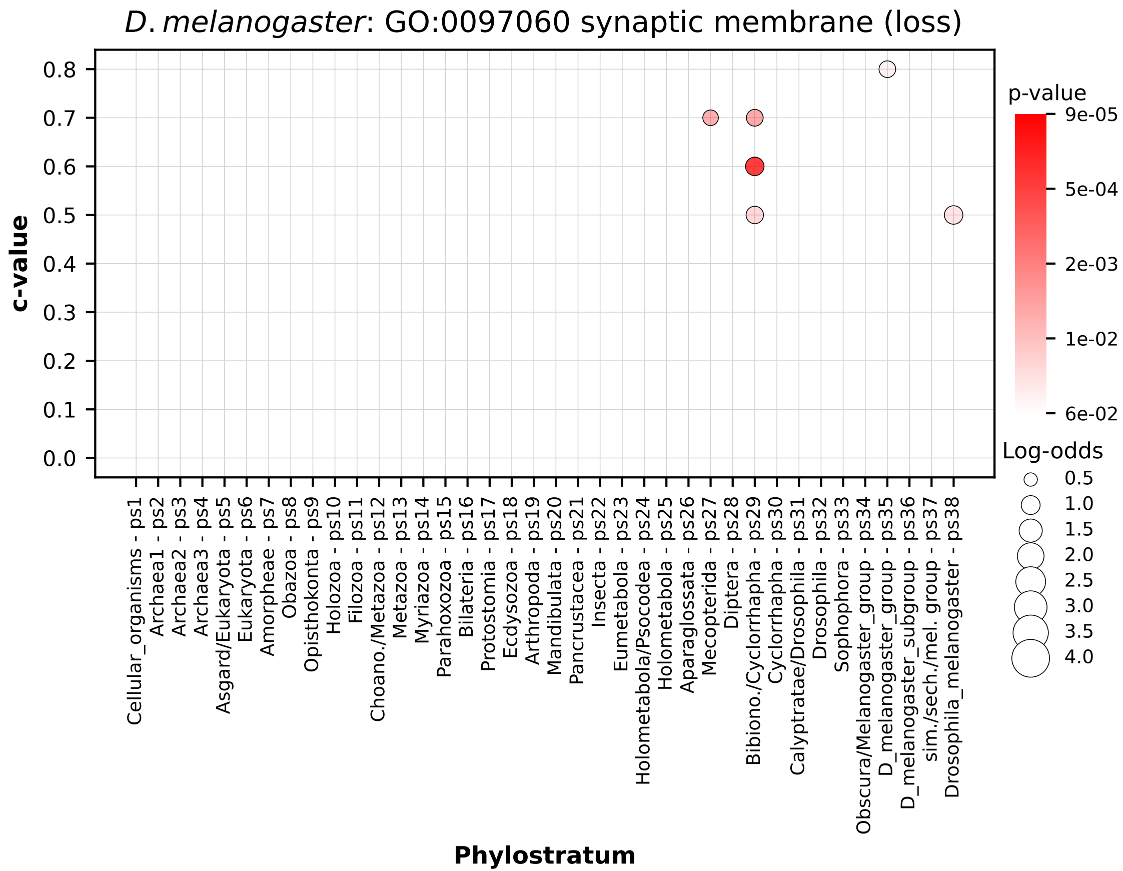

D. melanogaster: GO:0097060 synaptic membrane (loss)
c-value
0.8
0.7
0.6
0.5
0.4
0.3
0.2
0.1
0.0
Phylostratum
Cellular_organisms - ps1
Archaea1 - ps2
Archaea2 - ps3
Archaea3 - ps4
Asgard/Eukaryota - ps5
Eukaryota - ps6
Amorpheae - ps7
Obazoa - ps8
Opisthokonta - ps9
Holozoa - ps10
Filozoa - ps11
Choano./Metazoa - ps12
Metazoa - ps13
Myriazoa - ps14
Parahoxozoa - ps15
Bilateria - ps16
Protostomia - ps17
Ecdysozoa - ps18
Arthropoda - ps19
Mandibulata - ps20
Pancrustacea - ps21
Insecta - ps22
Eumetabola - ps23
Holometabola/Psocodea - ps24
Holometabola - ps25
Aparaglossata - ps26
Mecopterida - ps27
Diptera - ps28
Bibiono./Cyclorrhapha - ps29
Cyclorrhapha - ps30
Calyptratae/Drosophila - ps31
Drosophila - ps32
Sophophora - ps33
Obscura/Melanogaster_group - ps34
D_melanogaster_group - ps35
D_melanogaster_subgroup - ps36
sim./sech./mel. group - ps37
Drosophila_melanogaster - ps38
p-value
9e-05
5e-04
2e-03
1e-02
6e-02
Log-odds
0.5
1.0
1.5
2.0
2.5
3.0
3.5
4.0

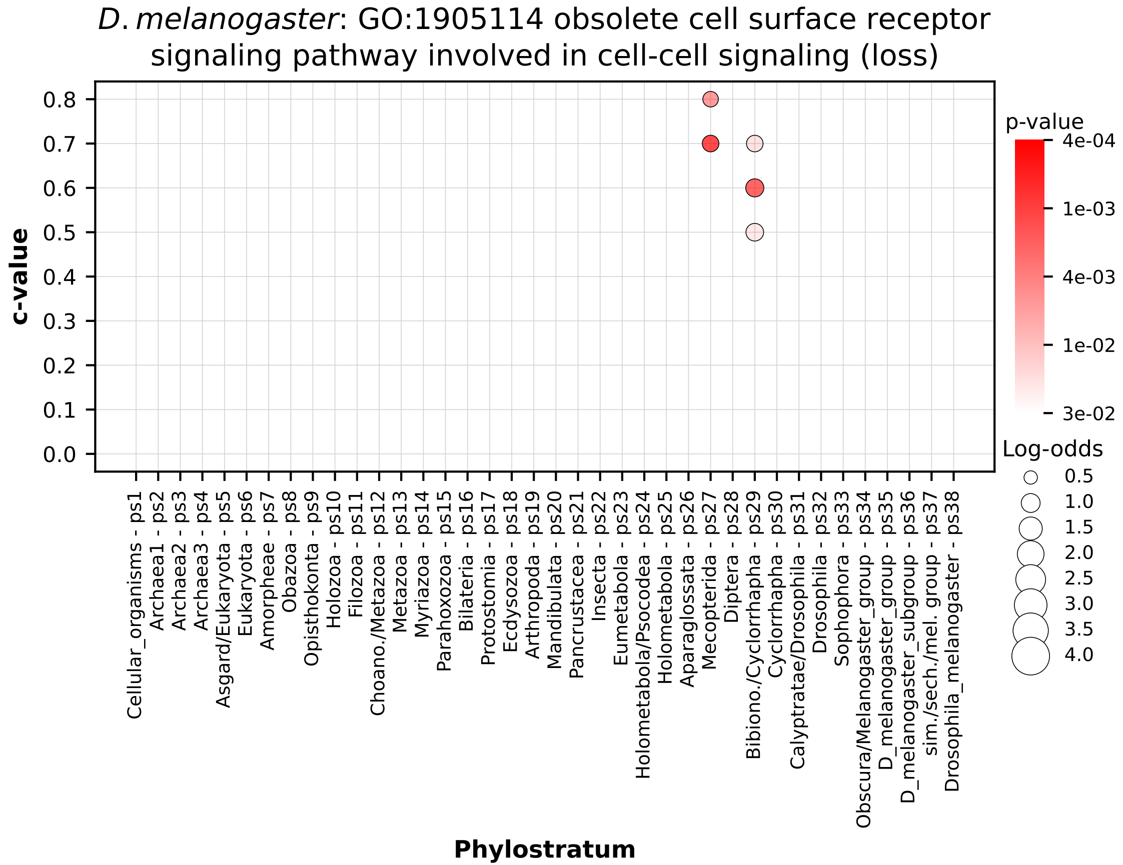

D. melanogaster: GO:1905114 obsolete cell surface receptor signaling pathway involved in cell-cell signaling (loss)
c-value
0.8
0.7
0.6
0.5
0.4
0.3
0.2
0.1
0.0
Phylostratum
Cellular_organisms - ps1
Archaea1 - ps2
Archaea2 - ps3
Archaea3 - ps4
Asgard/Eukaryota - ps5
Eukaryota - ps6
Amorpheae - ps7
Obazoa - ps8
Opisthokonta - ps9
Holozoa - ps10
Filozoa - ps11
Choano./Metazoa - ps12
Metazoa - ps13
Myriazoa - ps14
Parahoxozoa - ps15
Bilateria - ps16
Protostomia - ps17
Ecdysozoa - ps18
Arthropoda - ps19
Mandibulata - ps20
Pancrustacea - ps21
Insecta - ps22
Eumetabola - ps23
Holometabola/Psocodea - ps24
Holometabola - ps25
Aparaglossata - ps26
Mecopterida - ps27
Diptera - ps28
Bibiono./Cyclorrhapha - ps29
Cyclorrhapha - ps30
Calyptratae/Drosophila - ps31
Drosophila - ps32
Sophophora - ps33
Obscura/Melanogaster_group - ps34
D_melanogaster_group - ps35
D_melanogaster_subgroup - ps36
sim./sech./mel. group - ps37
Drosophila_melanogaster - ps38
p-value
4e-04
1e-03
4e-03
1e-02
3e-02
Log-odds
0.5
1.0
1.5
2.0
2.5
3.0
3.5
4.0

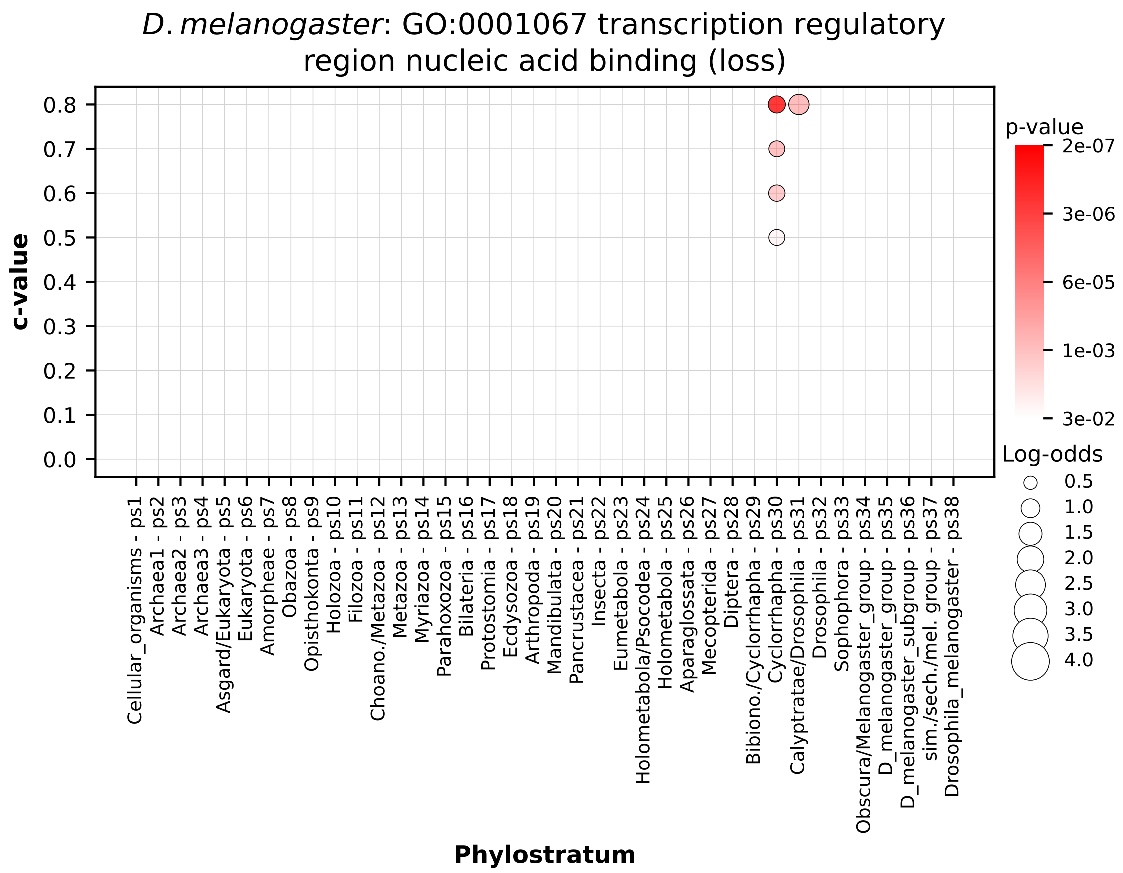

D. melanogaster: GO:0001067 transcription regulatory region nucleic acid binding (loss)
c-value
0.8
0.7
0.6
0.5
0.4
0.3
0.2
0.1
0.0
p-value
2e-07
3e-06
6e-05
1e-03
3e-02
Log-odds
0.5
1.0
1.5
2.0
2.5
3.0
3.5
4.0
Cellular_organisms - ps1
Archaea1 - ps2
Archaea2 - ps3
Archaea3 - ps4
Asgard/Eukaryota - ps5
Eukaryota - ps6
Amorpheae - ps7
Obazoa - ps8
Opisthokonta - ps9
Holozoa - ps10
Filozoa - ps11
Choano./Metazoa - ps12
Metazoa - ps13
Myriazoa - ps14
Parahoxozoa - ps15
Bilateria - ps16
Protostomia - ps17
Ecdysozoa - ps18
Arthropoda - ps19
Mandibulata - ps20
Pancrustacea - ps21
Insecta - ps22
Eumetabola - ps23
Holometabola/Psocodea - ps24
Holometabola - ps25
Aparaglossata - ps26
Mecopterida - ps27
Diptera - ps28
Bibiono./Cyclorrhapha - ps29
Cyclorrhapha - ps30
Calyptratae/Drosophila - ps31
Drosophila - ps32
Sophophora - ps33
Obscura/Melanogaster_group - ps34
D_melanogaster_group - ps35
D_melanogaster_subgroup - ps36
sim./sech./mel. group - ps37
Drosophila_melanogaster - ps38
Phylostratum

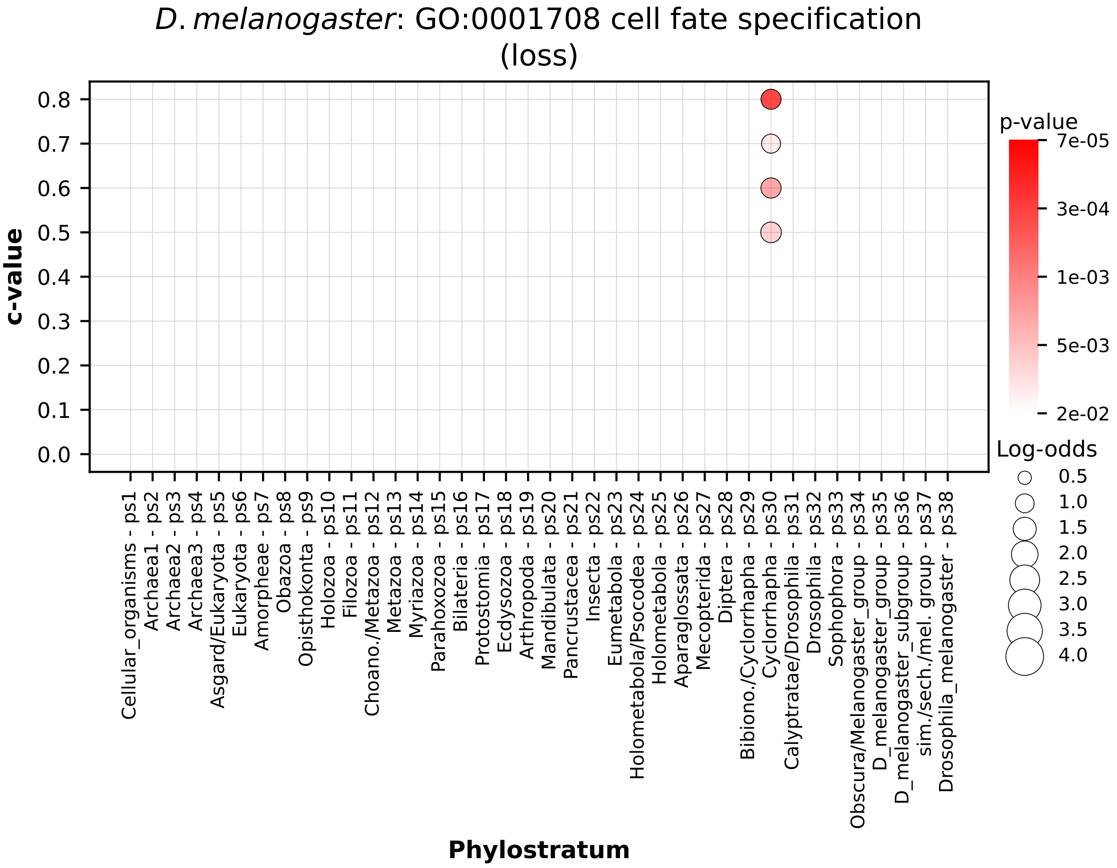

D. melanogaster: GO:0001708 cell fate specification
(loss)
c-value
0.0
0.1
0.2
0.3
0.4
0.5
0.6
0.7
0.8
p-value
7e-05
3e-04
1e-03
5e-03
2e-02
Log-odds
0.5
1.0
1.5
2.0
2.5
3.0
3.5
4.0
Cellular_organisms - ps1
Archaea1 - ps2
Archaea2 - ps3
Archaea3 - ps4
Asgard/Eukaryota - ps5
Eukaryota - ps6
Amorpheae - ps7
Obazoa - ps8
Opisthokonta - ps9
Holozoa - ps10
Filozoa - ps11
Choano./Metazoa - ps12
Metazoa - ps13
Myriazoa - ps14
Parahoxozoa - ps15
Bilateria - ps16
Protostomia - ps17
Ecdysozoa - ps18
Arthropoda - ps19
Mandibulata - ps20
Pancrustacea - ps21
Insecta - ps22
Eumetabola - ps23
Holometabola/Psocodea - ps24
Holometabola - ps25
Aparaglossata - ps26
Mecopterida - ps27
Diptera - ps28
Bibiono./Cyclorrhapha - ps29
Cyclorrhapha - ps30
Calyptratae/Drosophila - ps31
Drosophila - ps32
Sophophora - ps33
Obscura/Melanogaster_group - ps34
D_melanogaster_group - ps35
D_melanogaster_subgroup - ps36
sim./sech./mel. group - ps37
Drosophila_melanogaster - ps38
Phylostratum

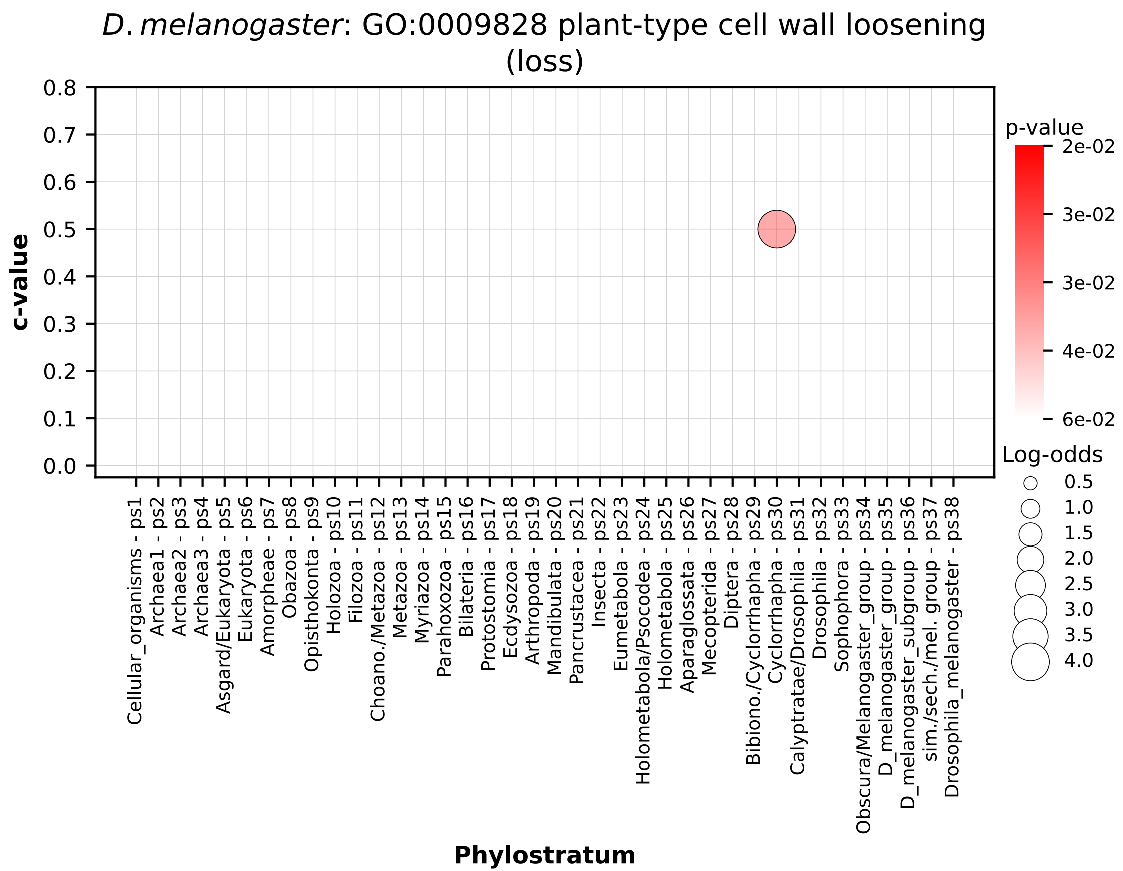

D. melanogaster: GO:0009828 plant-type cell wall loosening (loss)
c-value
0.8
0.7
0.6
0.5
0.4
0.3
0.2
0.1
0.0
p-value
2e-02
3e-02
3e-02
4e-02
6e-02
Log-odds
0.5
1.0
1.5
2.0
2.5
3.0
3.5
4.0
Cellular_organisms - ps1
Archaea1 - ps2
Archaea2 - ps3
Archaea3 - ps4
Asgard/Eukaryota - ps5
Eukaryota - ps6
Amorpheae - ps7
Obazoa - ps8
Opisthokonta - ps9
Holozoa - ps10
Filozoa - ps11
Choano./Metazoa - ps12
Metazoa - ps13
Myriazoa - ps14
Parahoxozoa - ps15
Bilateria - ps16
Protostomia - ps17
Ecdysozoa - ps18
Arthropoda - ps19
Mandibulata - ps20
Pancrustacea - ps21
Insecta - ps22
Eumetabola - ps23
Holometabola/Psocodea - ps24
Holometabola - ps25
Aparaglossata - ps26
Mecopterida - ps27
Diptera - ps28
Bibiono./Cyclorrhapha - ps29
Cyclorrhapha - ps30
Calyptratae/Drosophila - ps31
Drosophila - ps32
Sophophora - ps33
Obscura/Melanogaster_group - ps34
D_melanogaster_group - ps35
D_melanogaster_subgroup - ps36
sim./sech./mel. group - ps37
Drosophila_melanogaster - ps38
Phylostratum

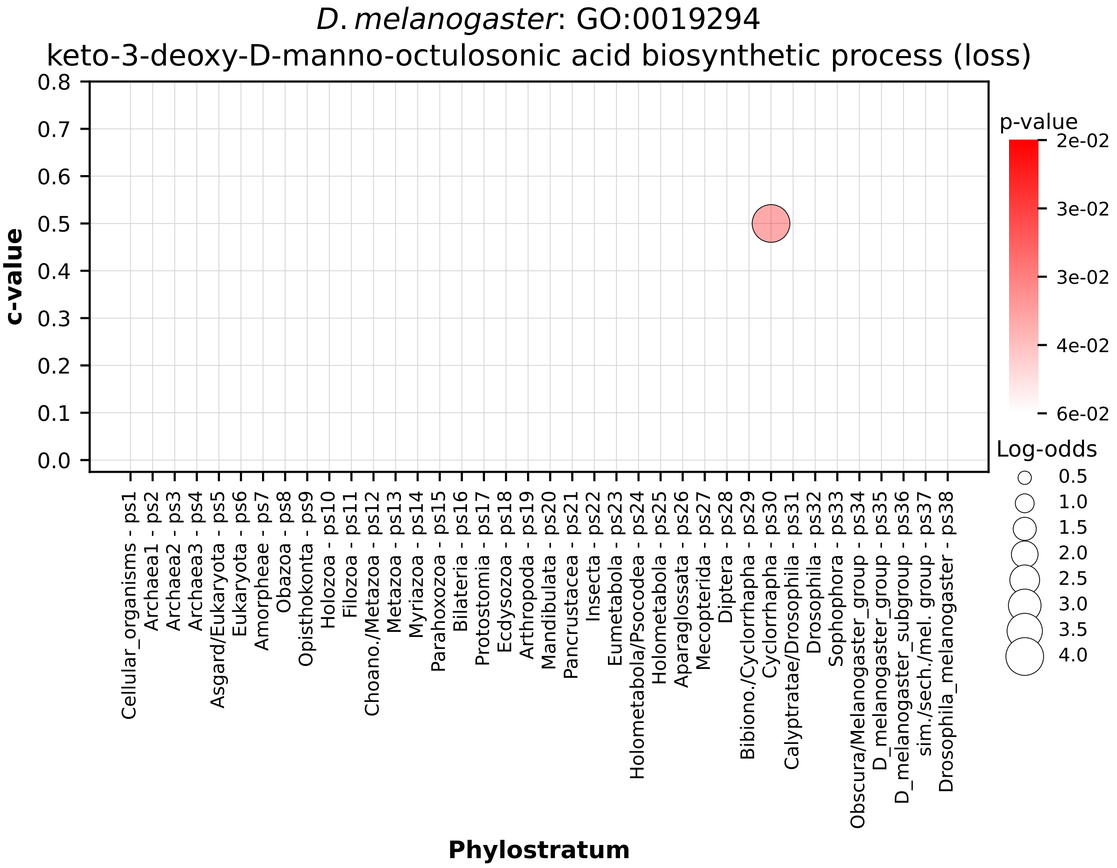

D. melanogaster: GO:0019294
keto-3-deoxy-D-manno-octulosonic acid biosynthetic process (loss)
c-value
0.8
0.7
0.6
0.5
0.4
0.3
0.2
0.1
0.0
p-value
2e-02
3e-02
3e-02
4e-02
6e-02
Log-odds
0.5
1.0
1.5
2.0
2.5
3.0
3.5
4.0
Cellular_organisms - ps1
Archaea1 - ps2
Archaea2 - ps3
Archaea3 - ps4
Asgard/Eukaryota - ps5
Eukaryota - ps6
Amorpheae - ps7
Obazoa - ps8
Opisthokonta - ps9
Holozoa - ps10
Filozoa - ps11
Choano./Metazoa - ps12
Metazoa - ps13
Myriazoa - ps14
Parahoxozoa - ps15
Bilateria - ps16
Protostomia - ps17
Ecdysozoa - ps18
Arthropoda - ps19
Mandibulata - ps20
Pancrustacea - ps21
Insecta - ps22
Eumetabola - ps23
Holometabola/Psocodea - ps24
Holometabola - ps25
Aparaglossata - ps26
Mecopterida - ps27
Diptera - ps28
Bibiono./Cyclorrhapha - ps29
Cyclorrhapha - ps30
Calyptratae/Drosophila - ps31
Drosophila - ps32
Sophophora - ps33
Obscura/Melanogaster_group - ps34
D_melanogaster_group - ps35
D_melanogaster_subgroup - ps36
sim./sech./mel. group - ps37
Drosophila_melanogaster - ps38
Phylostratum

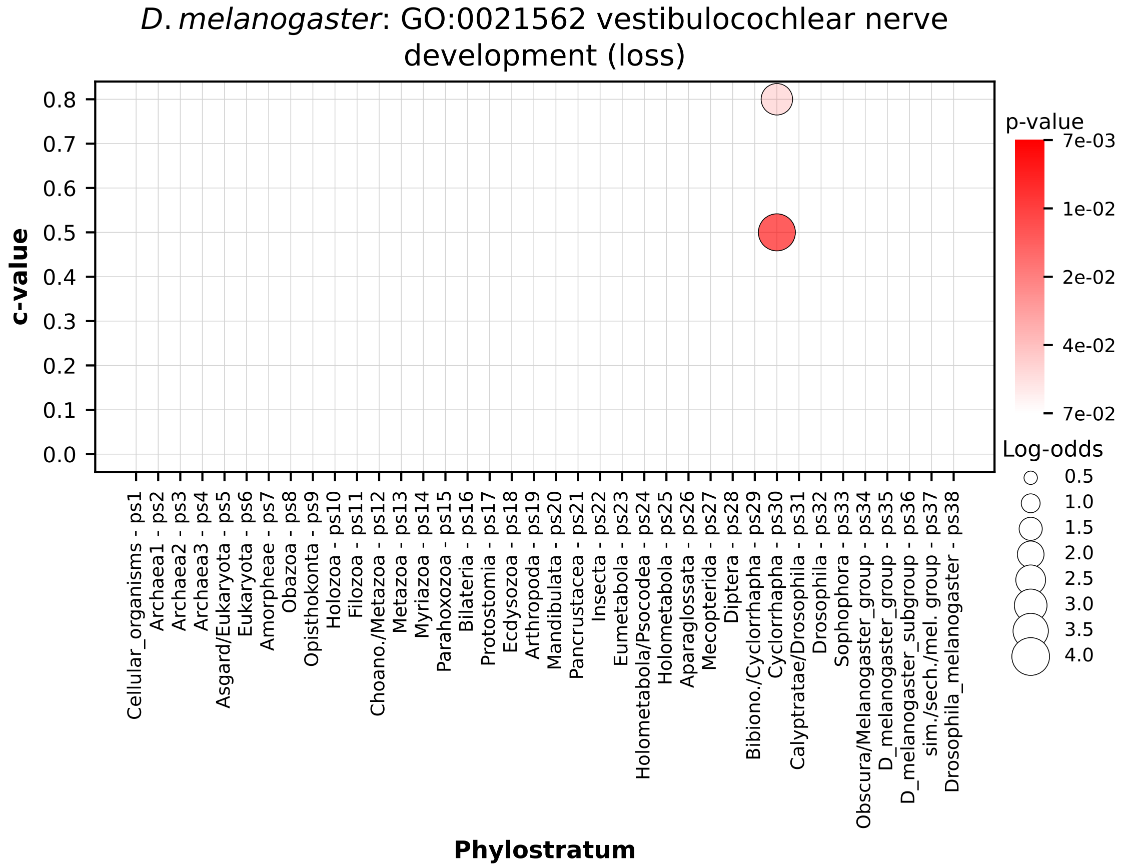

D. melanogaster: GO:0021562 vestibulocochlear nerve development (loss)
c-value
0.0
0.1
0.2
0.3
0.4
0.5
0.6
0.7
0.8
Phylostratum
Cellular_organisms - ps1
Archaea1 - ps2
Archaea2 - ps3
Archaea3 - ps4
Asgard/Eukaryota - ps5
Eukaryota - ps6
Amorpheae - ps7
Obazoa - ps8
Opisthokonta - ps9
Holozoa - ps10
Filozoa - ps11
Choano./Metazoa - ps12
Metazoa - ps13
Myriazoa - ps14
Parahoxozoa - ps15
Bilateria - ps16
Protostomia - ps17
Ecdysozoa - ps18
Arthropoda - ps19
Mandibulata - ps20
Pancrustacea - ps21
Insecta - ps22
Eumetabola - ps23
Holometabola/Psocodea - ps24
Holometabola - ps25
Aparaglossata - ps26
Mecopterida - ps27
Diptera - ps28
Bibiono./Cyclorrhapha - ps29
Cyclorrhapha - ps30
Calyptratae/Drosophila - ps31
Drosophila - ps32
Sophophora - ps33
Obscura/Melanogaster_group - ps34
D_melanogaster_group - ps35
D_melanogaster_subgroup - ps36
sim./sech./mel. group - ps37
Drosophila_melanogaster - ps38
p-value
7e-03
1e-02
2e-02
4e-02
7e-02
Log-odds
0.5
1.0
1.5
2.0
2.5
3.0
3.5
4.0

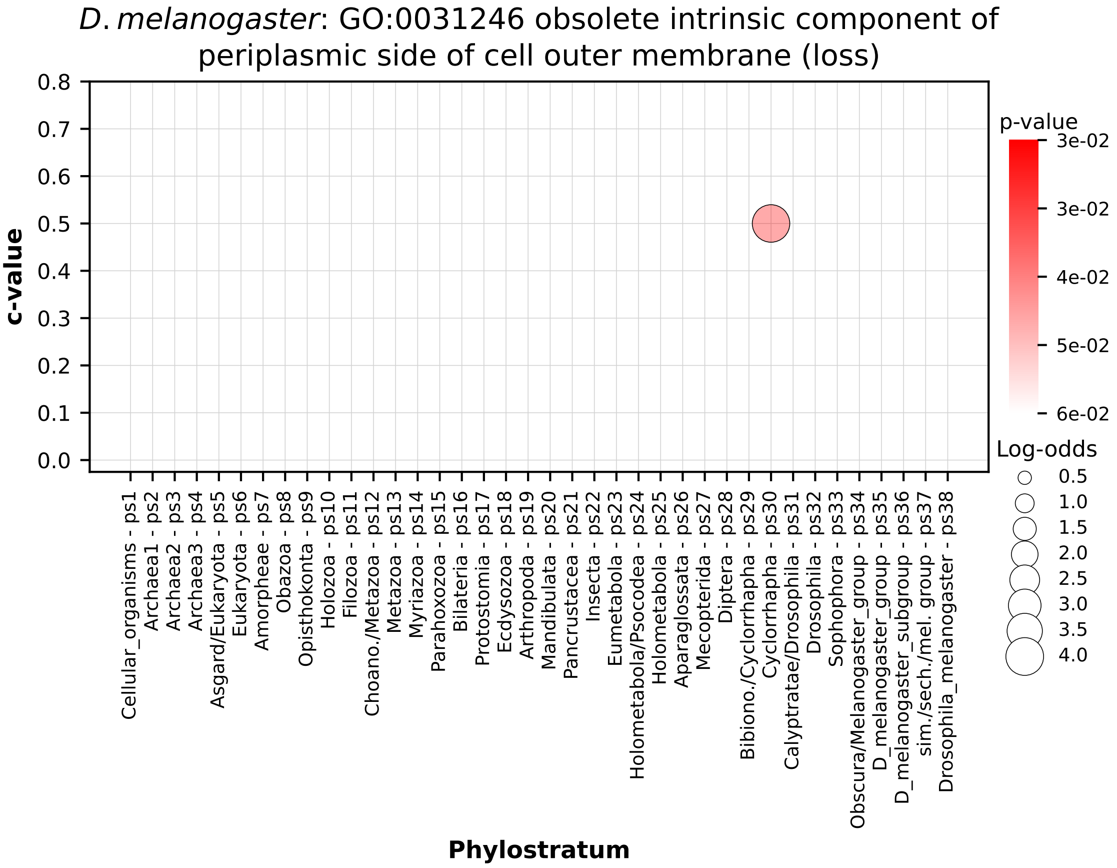

D. melanogaster: GO:0031246 obsolete intrinsic component of periplasmic side of cell outer membrane (loss)
c-value
0.8
0.7
0.6
0.5
0.4
0.3
0.2
0.1
0.0
p-value
3e-02
3e-02
4e-02
5e-02
6e-02
Log-odds
0.5
1.0
1.5
2.0
2.5
3.0
3.5
4.0
Cellular_organisms - ps1
Archaea1 - ps2
Archaea2 - ps3
Archaea3 - ps4
Asgard/Eukaryota - ps5
Eukaryota - ps6
Amorpheae - ps7
Obazoa - ps8
Opisthokonta - ps9
Holozoa - ps10
Filozoa - ps11
Choano./Metazoa - ps12
Metazoa - ps13
Myriazoa - ps14
Parahoxozoa - ps15
Bilateria - ps16
Protostomia - ps17
Ecdysozoa - ps18
Arthropoda - ps19
Mandibulata - ps20
Pancrustacea - ps21
Insecta - ps22
Eumetabola - ps23
Holometabola/Psocodea - ps24
Holometabola - ps25
Aparaglossata - ps26
Mecopterida - ps27
Diptera - ps28
Bibiono./Cyclorrhapha - ps29
Cyclorrhapha - ps30
Calyptratae/Drosophila - ps31
Drosophila - ps32
Sophophora - ps33
Obscura/Melanogaster_group - ps34
D_melanogaster_group - ps35
D_melanogaster_subgroup - ps36
sim./sech./mel. group - ps37
Drosophila_melanogaster - ps38
Phylostratum

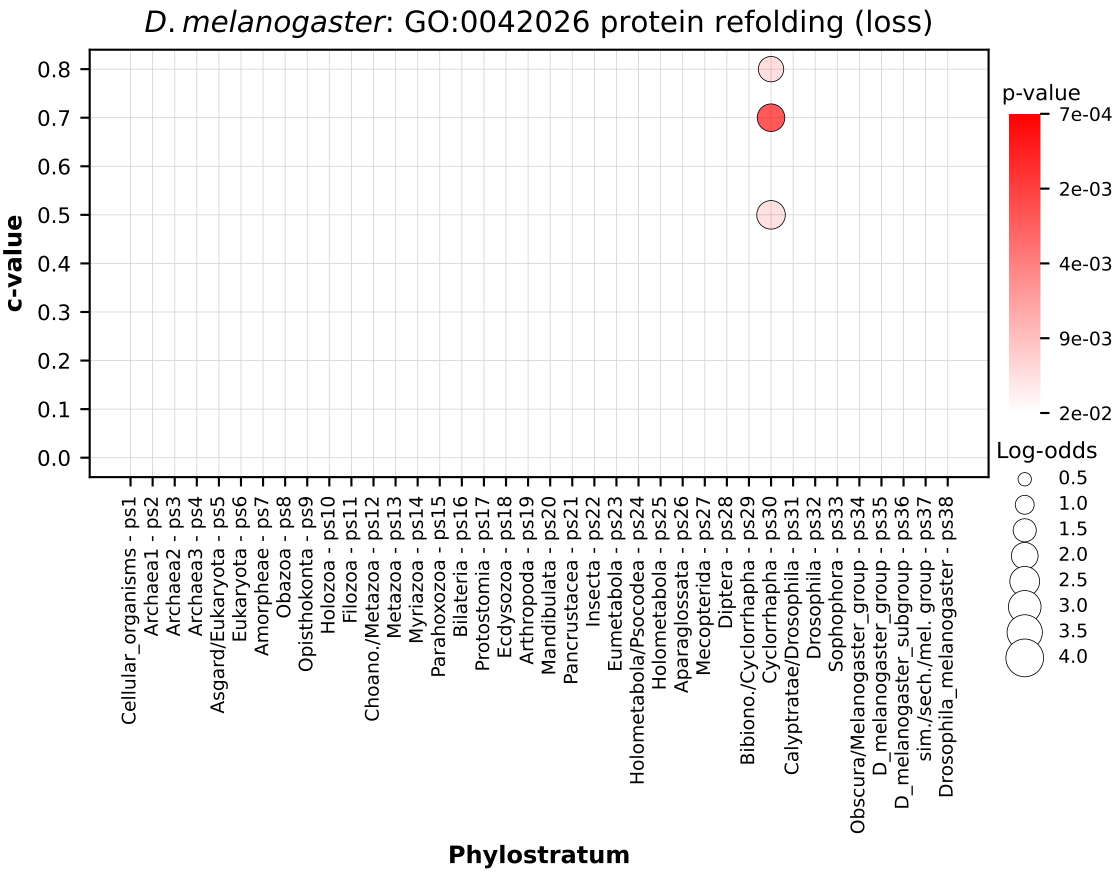

D. melanogaster: GO:0042026 protein refolding (loss)
c-value
0.8
0.7
0.6
0.5
0.4
0.3
0.2
0.1
0.0
Phylostratum
Cellular_organisms - ps1
Archaea1 - ps2
Archaea2 - ps3
Archaea3 - ps4
Asgard/Eukaryota - ps5
Eukaryota - ps6
Amorpheae - ps7
Obazoa - ps8
Opisthokonta - ps9
Holozoa - ps10
Filozoa - ps11
Choano./Metazoa - ps12
Metazoa - ps13
Myriazoa - ps14
Parahoxozoa - ps15
Bilateria - ps16
Protostomia - ps17
Ecdysozoa - ps18
Arthropoda - ps19
Mandibulata - ps20
Pancrustacea - ps21
Insecta - ps22
Eumetabola - ps23
Holometabola/Psocodea - ps24
Holometabola - ps25
Aparaglossata - ps26
Mecopterida - ps27
Diptera - ps28
Bibiono./Cyclorrhapha - ps29
Cyclorrhapha - ps30
Calyptratae/Drosophila - ps31
Drosophila - ps32
Sophophora - ps33
Obscura/Melanogaster_group - ps34
D_melanogaster_group - ps35
D_melanogaster_subgroup - ps36
sim./sech./mel. group - ps37
Drosophila_melanogaster - ps38
p-value
7e-04
2e-03
4e-03
9e-03
2e-02
Log-odds
0.5
1.0
1.5
2.0
2.5
3.0
3.5
4.0

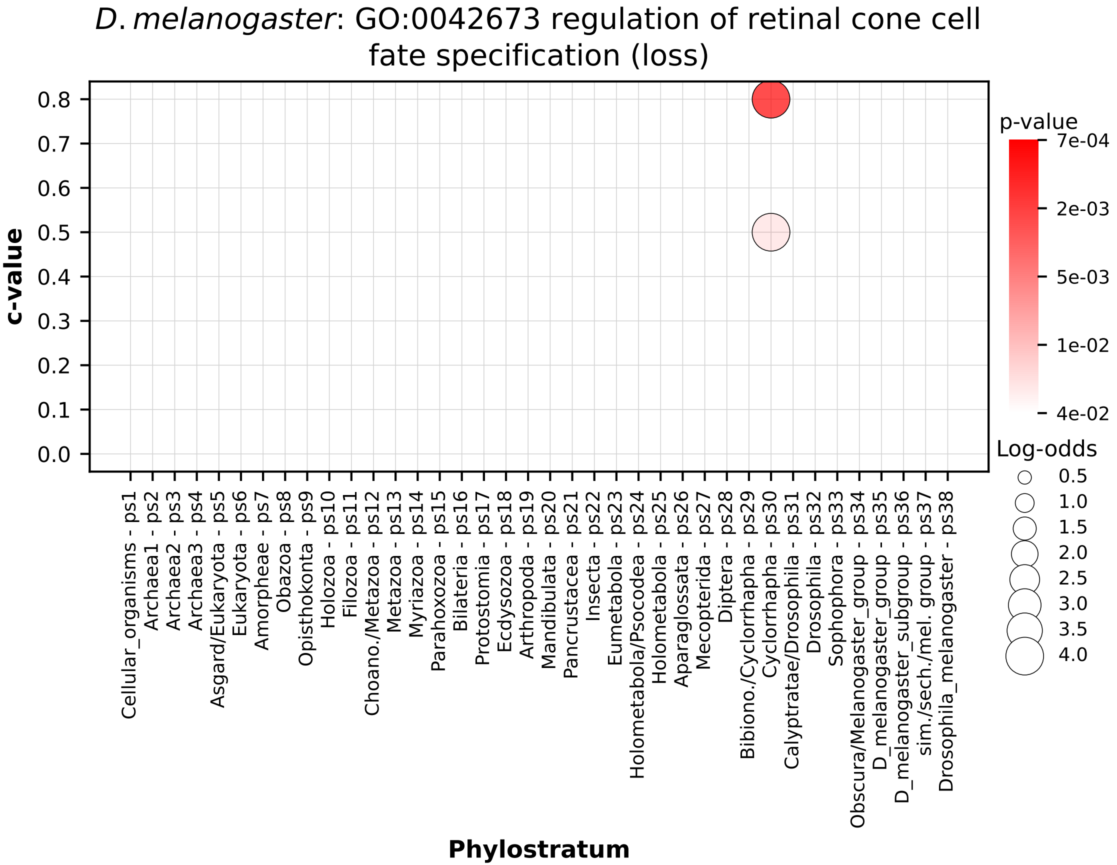

$D. melanogaster$: GO:0042673 regulation of retinal cone cell fate specification (loss)
c-value
0.8
0.7
0.6
0.5
0.4
0.3
0.2
0.1
0.0
p-value
7e-04
2e-03
5e-03
1e-02
4e-02
Log-odds
0.5
1.0
1.5
2.0
2.5
3.0
3.5
4.0
Cellular_organisms - ps1
Archaea1 - ps2
Archaea2 - ps3
Archaea3 - ps4
Asgard/Eukaryota - ps5
Eukaryota - ps6
Amorpheae - ps7
Obazoa - ps8
Opisthokonta - ps9
Holozoa - ps10
Filozoa - ps11
Choano./Metazoa - ps12
Metazoa - ps13
Myriazoa - ps14
Parahoxozoa - ps15
Bilateria - ps16
Protostomia - ps17
Ecdysozoa - ps18
Arthropoda - ps19
Mandibulata - ps20
Pancrustacea - ps21
Insecta - ps22
Eumetabola - ps23
Holometabola/Psocodea - ps24
Holometabola - ps25
Aparaglossata - ps26
Mecopterida - ps27
Diptera - ps28
Bibiono./Cyclorrhapha - ps29
Cyclorrhapha - ps30
Calyptratae/Drosophila - ps31
Drosophila - ps32
Sophophora - ps33
Obscura/Melanogaster_group - ps34
D_melanogaster_group - ps35
D_melanogaster_subgroup - ps36
sim./sech./mel. group - ps37
Drosophila_melanogaster - ps38
Phylostratum

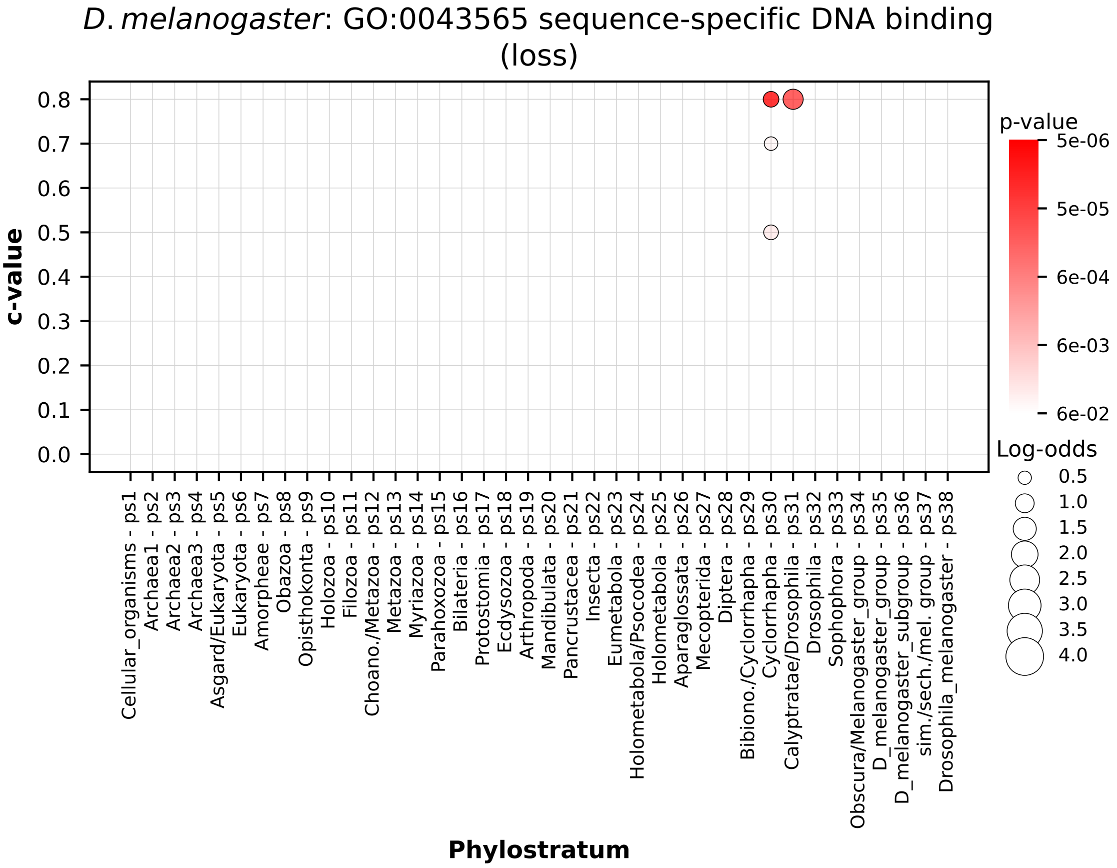

D. melanogaster: GO:0043565 sequence-specific DNA binding (loss)
c-value
0.8
0.7
0.6
0.5
0.4
0.3
0.2
0.1
0.0
p-value
5e-06
5e-05
6e-04
6e-03
6e-02
Log-odds
0.5
1.0
1.5
2.0
2.5
3.0
3.5
4.0
Cellular_organisms - ps1
Archaea1 - ps2
Archaea2 - ps3
Archaea3 - ps4
Asgard/Eukaryota - ps5
Eukaryota - ps6
Amorpheae - ps7
Obazoa - ps8
Opisthokonta - ps9
Holozoa - ps10
Filozoa - ps11
Choano./Metazoa - ps12
Metazoa - ps13
Myriazoa - ps14
Parahoxozoa - ps15
Bilateria - ps16
Protostomia - ps17
Ecdysozoa - ps18
Arthropoda - ps19
Mandibulata - ps20
Pancrustacea - ps21
Insecta - ps22
Eumetabola - ps23
Holometabola/Psocodea - ps24
Holometabola - ps25
Aparaglossata - ps26
Mecopterida - ps27
Diptera - ps28
Bibiono./Cyclorrhapha - ps29
Cyclorrhapha - ps30
Calyptratae/Drosophila - ps31
Drosophila - ps32
Sophophora - ps33
Obscura/Melanogaster_group - ps34
D_melanogaster_group - ps35
D_melanogaster_subgroup - ps36
sim./sech./mel. group - ps37
Drosophila_melanogaster - ps38
Phylostratum

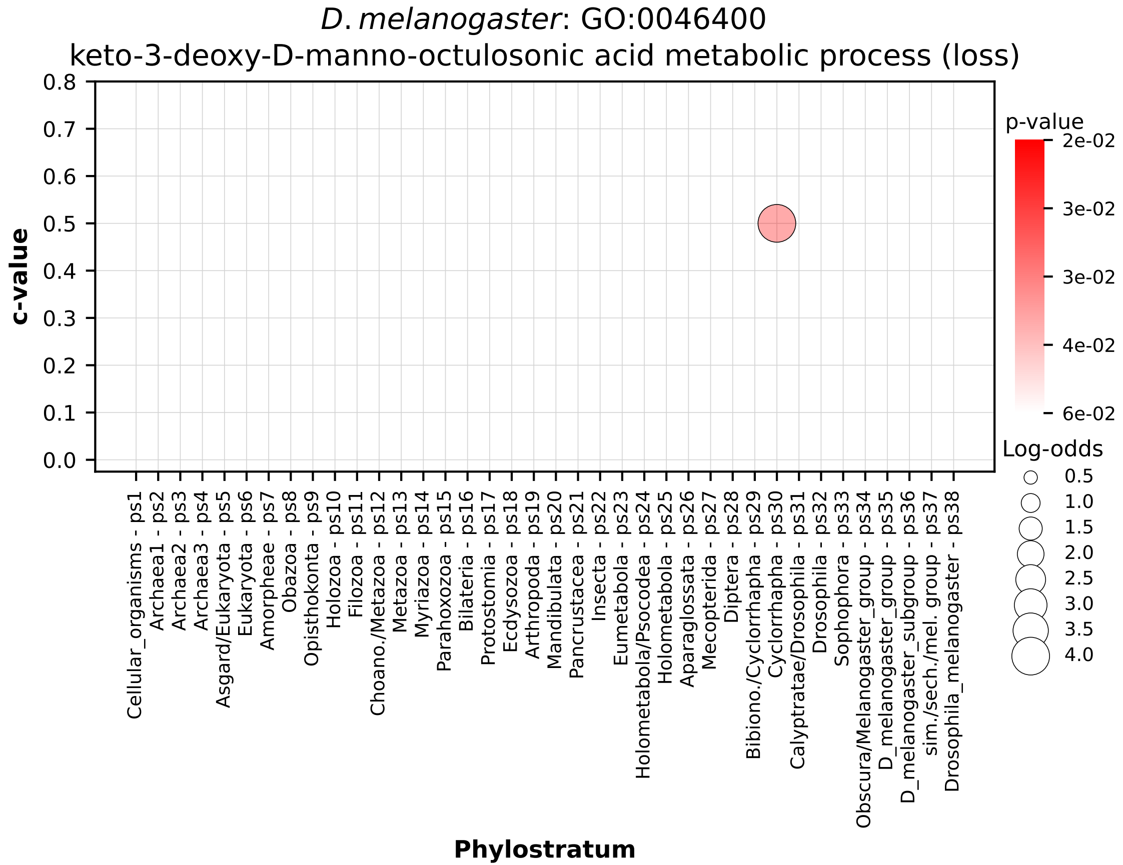

D. melanogaster: GO:0046400
keto-3-deoxy-D-manno-octulosonic acid metabolic process (loss)
c-value
0.0
0.1
0.2
0.3
0.4
0.5
0.6
0.7
0.8
Phylostratum
Cellular_organisms - ps1
Archaea1 - ps2
Archaea2 - ps3
Archaea3 - ps4
Asgard/Eukaryota - ps5
Eukaryota - ps6
Amorpheae - ps7
Obazoa - ps8
Opisthokonta - ps9
Holozoa - ps10
Filozoa - ps11
Choano./Metazoa - ps12
Metazoa - ps13
Myriazoa - ps14
Parahoxozoa - ps15
Bilateria - ps16
Protostomia - ps17
Ecdysozoa - ps18
Arthropoda - ps19
Mandibulata - ps20
Pancrustacea - ps21
Insecta - ps22
Eumetabola - ps23
Holometabola/Psocodea - ps24
Holometabola - ps25
Aparaglossata - ps26
Mecopterida - ps27
Diptera - ps28
Bibiono./Cyclorrhapha - ps29
Cyclorrhapha - ps30
Calyptratae/Drosophila - ps31
Drosophila - ps32
Sophophora - ps33
Obscura/Melanogaster_group - ps34
D_melanogaster_group - ps35
D_melanogaster_subgroup - ps36
sim./sech./mel. group - ps37
Drosophila_melanogaster - ps38
p-value
2e-02
3e-02
3e-02
4e-02
6e-02
Log-odds
0.5
1.0
1.5
2.0
2.5
3.0
3.5
4.0

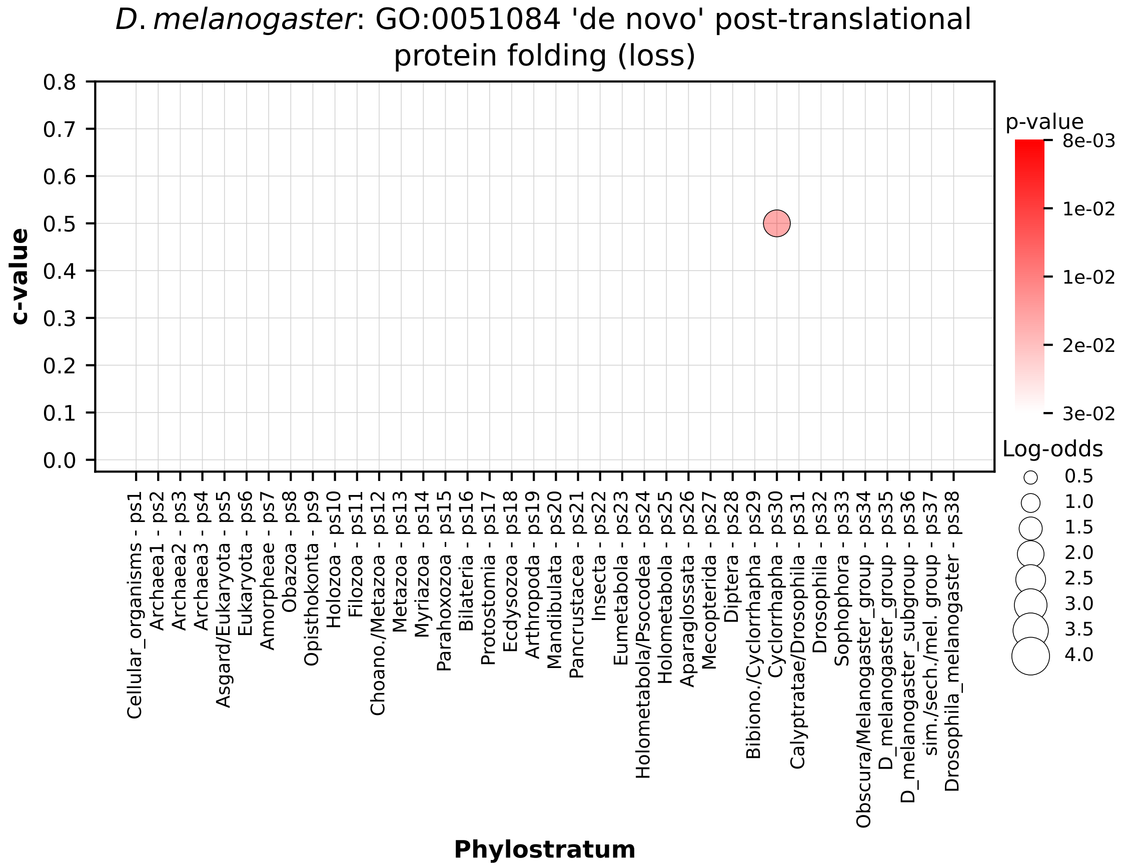
$D. melanogaster$: GO:0051084 'de novo' post-translational protein folding (loss)
c-value
0.8
0.7
0.6
0.5
0.4
0.3
0.2
0.1
0.0
Phylostratum
Cellular_organisms - ps1
Archaea1 - ps2
Archaea2 - ps3
Archaea3 - ps4
Asgard/Eukaryota - ps5
Eukaryota - ps6
Amorpheae - ps7
Obazoa - ps8
Opisthokonta - ps9
Holozoa - ps10
Filozoa - ps11
Choano./Metazoa - ps12
Metazoa - ps13
Myriazoa - ps14
Parahoxozoa - ps15
Bilateria - ps16
Protostomia - ps17
Ecdysozoa - ps18
Arthropoda - ps19
Mandibulata - ps20
Pancrustacea - ps21
Insecta - ps22
Eumetabola - ps23
Holometabola/Psocodea - ps24
Holometabola - ps25
Aparaglossata - ps26
Mecopterida - ps27
Diptera - ps28
Bibiono./Cyclorrhapha - ps29
Cyclorrhapha - ps30
Calyptratae/Drosophila - ps31
Drosophila - ps32
Sophophora - ps33
Obscura/Melanogaster_group - ps34
D_melanogaster_group - ps35
D_melanogaster_subgroup - ps36
sim./sech./mel. group - ps37
Drosophila_melanogaster - ps38
p-value
8e-03
1e-02
1e-02
2e-02
3e-02
Log-odds
0.5
1.0
1.5
2.0
2.5
3.0
3.5
4.0

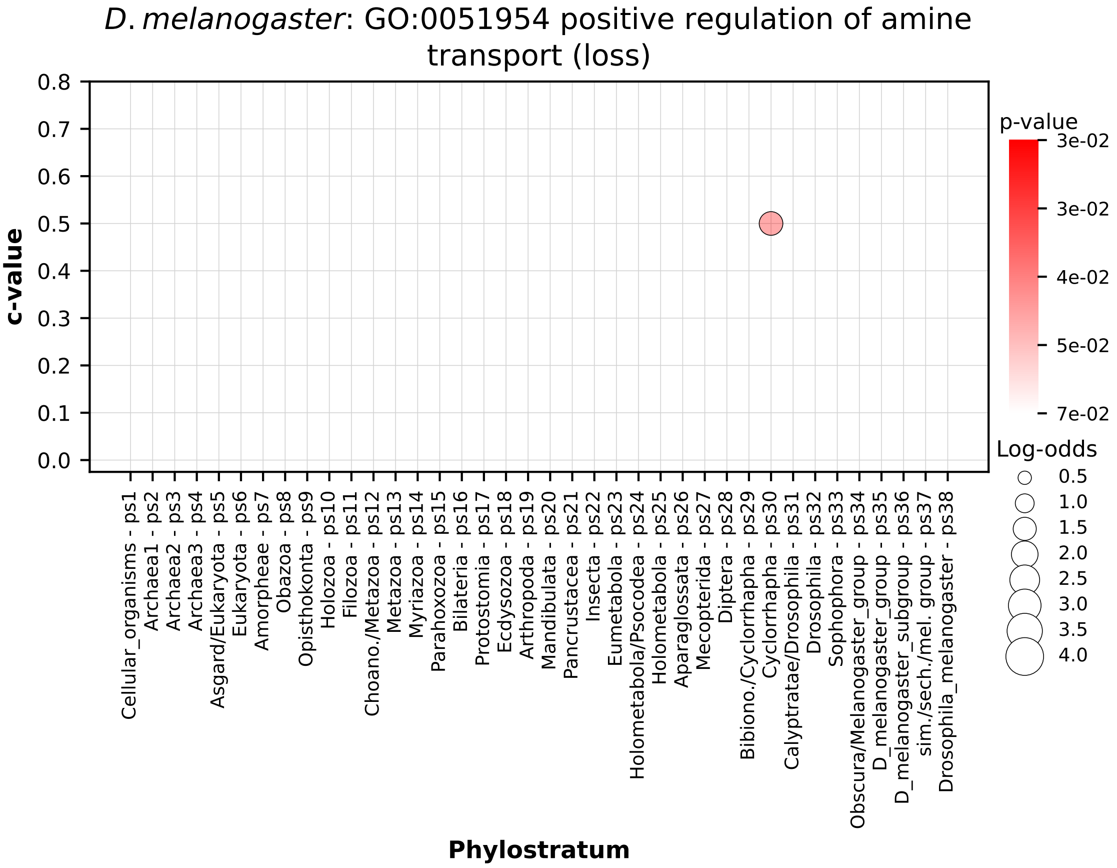

D. melanogaster: GO:0051954 positive regulation of amine transport (loss)
c-value
0.8
0.7
0.6
0.5
0.4
0.3
0.2
0.1
0.0
p-value
3e-02
3e-02
4e-02
5e-02
7e-02
Log-odds
0.5
1.0
1.5
2.0
2.5
3.0
3.5
4.0
Cellular_organisms - ps1
Archaea1 - ps2
Archaea2 - ps3
Archaea3 - ps4
Asgard/Eukaryota - ps5
Eukaryota - ps6
Amorpheae - ps7
Obazoa - ps8
Opisthokonta - ps9
Holozoa - ps10
Filozoa - ps11
Choano./Metazoa - ps12
Metazoa - ps13
Myriazoa - ps14
Parahoxozoa - ps15
Bilateria - ps16
Protostomia - ps17
Ecdysozoa - ps18
Arthropoda - ps19
Mandibulata - ps20
Pancrustacea - ps21
Insecta - ps22
Eumetabola - ps23
Holometabola/Psocodea - ps24
Holometabola - ps25
Aparaglossata - ps26
Mecopterida - ps27
Diptera - ps28
Bibiono./Cyclorrhapha - ps29
Cyclorrhapha - ps30
Calyptratae/Drosophila - ps31
Drosophila - ps32
Sophophora - ps33
Obscura/Melanogaster_group - ps34
D_melanogaster_group - ps35
D_melanogaster_subgroup - ps36
sim./sech./mel. group - ps37
Drosophila_melanogaster - ps38
Phylostratum

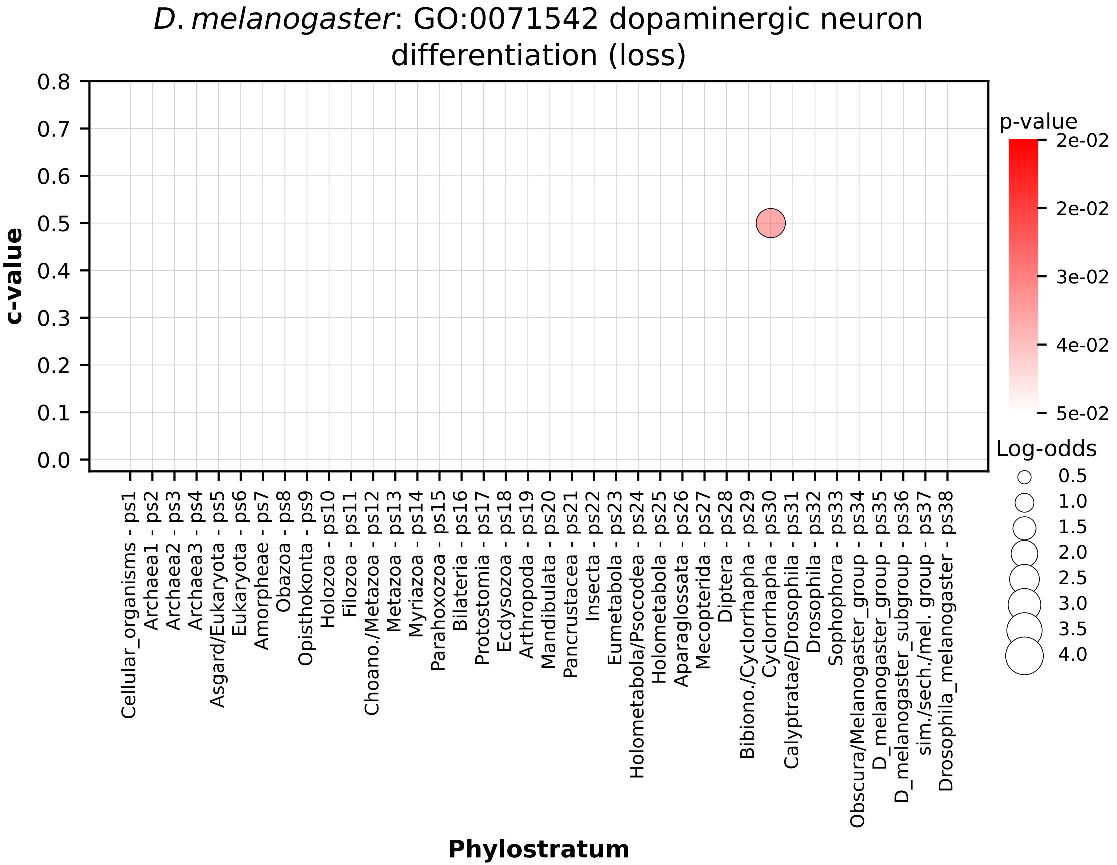

D. melanogaster: GO:0071542 dopaminergic neuron differentiation (loss)
c-value
0.8
0.7
0.6
0.5
0.4
0.3
0.2
0.1
0.0
Phylostratum
Cellular_organisms - ps1
Archaea1 - ps2
Archaea2 - ps3
Archaea3 - ps4
Asgard/Eukaryota - ps5
Eukaryota - ps6
Amorpheae - ps7
Obazoa - ps8
Opisthokonta - ps9
Holozoa - ps10
Filozoa - ps11
Choano./Metazoa - ps12
Metazoa - ps13
Myriazoa - ps14
Parahoxozoa - ps15
Bilateria - ps16
Protostomia - ps17
Ecdysozoa - ps18
Arthropoda - ps19
Mandibulata - ps20
Pancrustacea - ps21
Insecta - ps22
Eumetabola - ps23
Holometabola/Psocodea - ps24
Holometabola - ps25
Aparaglossata - ps26
Mecopterida - ps27
Diptera - ps28
Bibiono./Cyclorrhapha - ps29
Cyclorrhapha - ps30
Calyptratae/Drosophila - ps31
Drosophila - ps32
Sophophora - ps33
Obscura/Melanogaster_group - ps34
D_melanogaster_group - ps35
D_melanogaster_subgroup - ps36
sim./sech./mel. group - ps37
Drosophila_melanogaster - ps38
p-value
2e-02
2e-02
3e-02
4e-02
5e-02
Log-odds
0.5
1.0
1.5
2.0
2.5
3.0
3.5
4.0

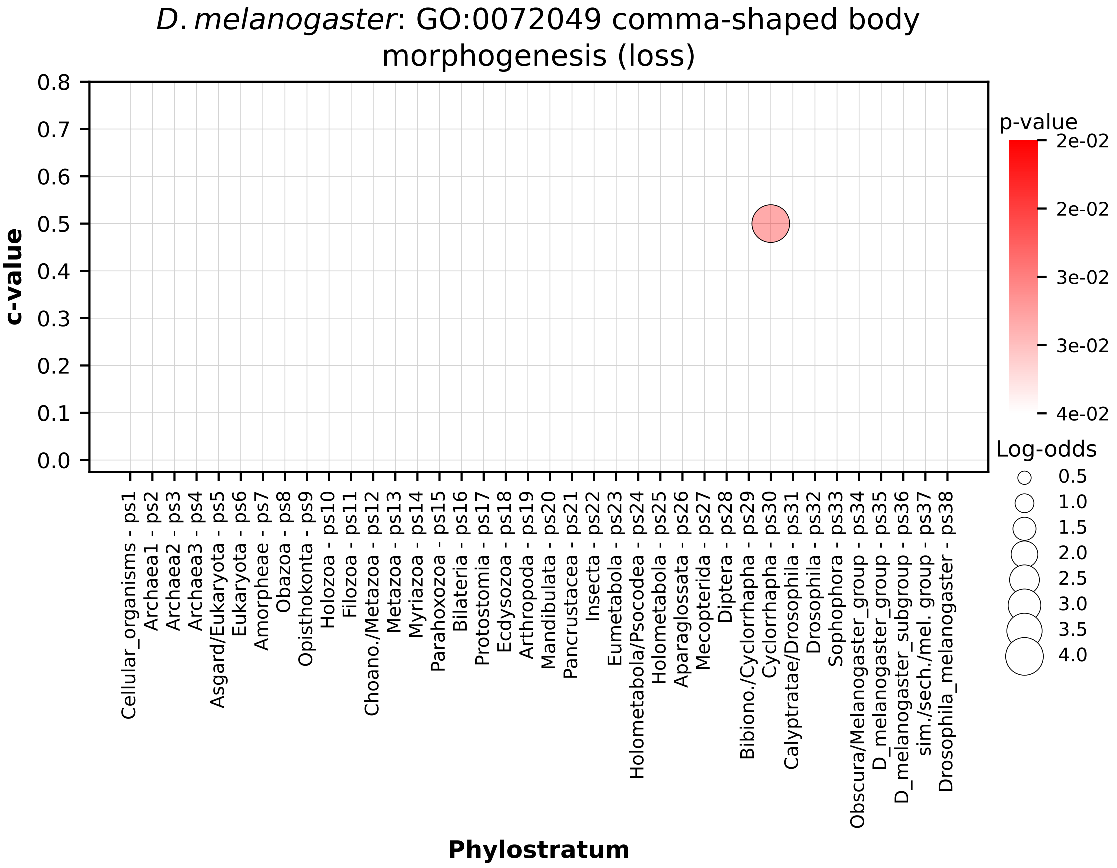

D. melanogaster: GO:0072049 comma-shaped body morphogenesis (loss)
c-value
0.8
0.7
0.6
0.5
0.4
0.3
0.2
0.1
0.0
Phylostratum
Cellular_organisms - ps1
Archaea1 - ps2
Archaea2 - ps3
Archaea3 - ps4
Asgard/Eukaryota - ps5
Eukaryota - ps6
Amorpheae - ps7
Obazoa - ps8
Opisthokonta - ps9
Holozoa - ps10
Filozoa - ps11
Choano./Metazoa - ps12
Metazoa - ps13
Myriazoa - ps14
Parahoxozoa - ps15
Bilateria - ps16
Protostomia - ps17
Ecdysozoa - ps18
Arthropoda - ps19
Mandibulata - ps20
Pancrustacea - ps21
Insecta - ps22
Eumetabola - ps23
Holometabola/Psocodea - ps24
Holometabola - ps25
Aparaglossata - ps26
Mecopterida - ps27
Diptera - ps28
Bibiono./Cyclorrhapha - ps29
Cyclorrhapha - ps30
Calyptratae/Drosophila - ps31
Drosophila - ps32
Sophophora - ps33
Obscura/Melanogaster_group - ps34
D_melanogaster_group - ps35
D_melanogaster_subgroup - ps36
sim./sech./mel. group - ps37
Drosophila_melanogaster - ps38
p-value
2e-02
2e-02
3e-02
3e-02
4e-02
Log-odds
0.5
1.0
1.5
2.0
2.5
3.0
3.5
4.0

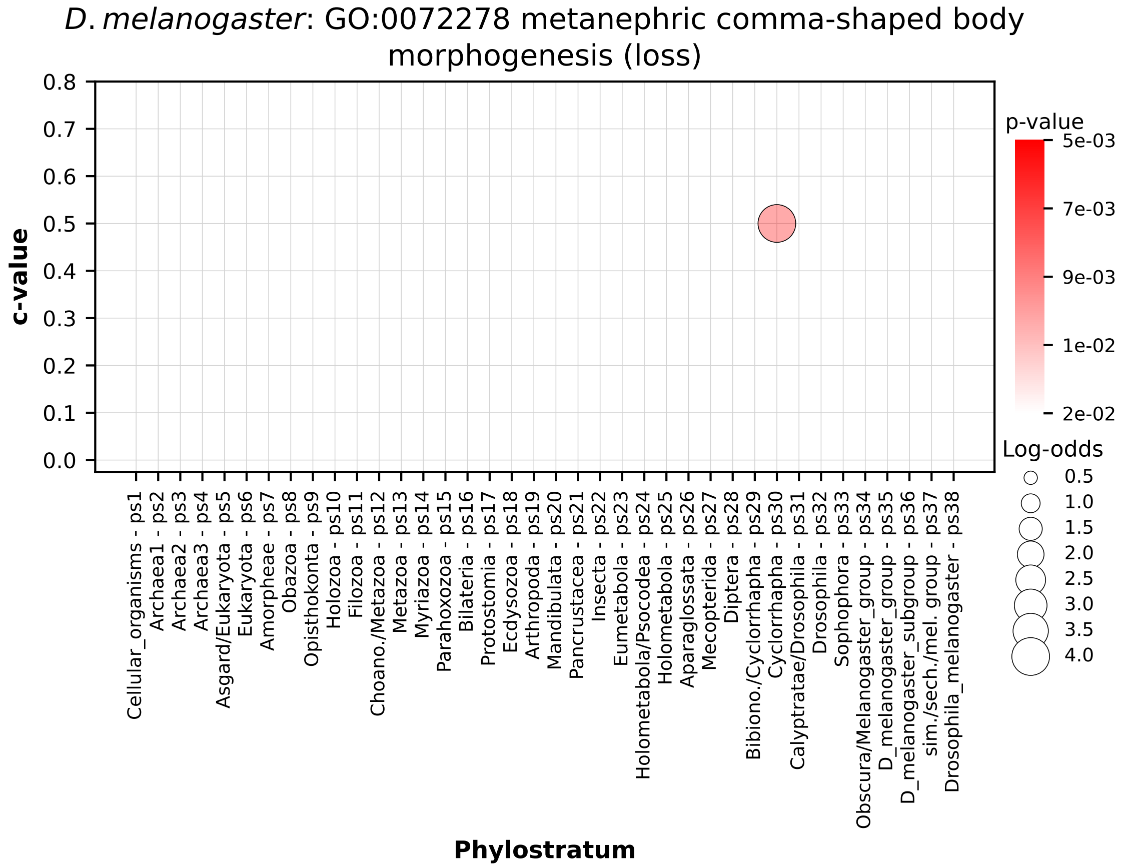

D. melanogaster: GO:0072278 metanephric comma-shaped body morphogenesis (loss)
c-value
0.8
0.7
0.6
0.5
0.4
0.3
0.2
0.1
0.0
Phylostratum
Cellular_organisms - ps1
Archaea1 - ps2
Archaea2 - ps3
Archaea3 - ps4
Asgard/Eukaryota - ps5
Eukaryota - ps6
Amorpheae - ps7
Obazoa - ps8
Opisthokonta - ps9
Holozoa - ps10
Filozoa - ps11
Choano./Metazoa - ps12
Metazoa - ps13
Myriazoa - ps14
Parahoxozoa - ps15
Bilateria - ps16
Protostomia - ps17
Ecdysozoa - ps18
Arthropoda - ps19
Mandibulata - ps20
Pancrustacea - ps21
Insecta - ps22
Eumetabola - ps23
Holometabola/Psocodea - ps24
Holometabola - ps25
Aparaglossata - ps26
Mecopterida - ps27
Diptera - ps28
Bibiono./Cyclorrhapha - ps29
Cyclorrhapha - ps30
Calyptratae/Drosophila - ps31
Drosophila - ps32
Sophophora - ps33
Obscura/Melanogaster_group - ps34
D_melanogaster_group - ps35
D_melanogaster_subgroup - ps36
sim./sech./mel. group - ps37
Drosophila_melanogaster - ps38
p-value
5e-03
7e-03
9e-03
1e-02
2e-02
Log-odds
0.5
1.0
1.5
2.0
2.5
3.0
3.5
4.0

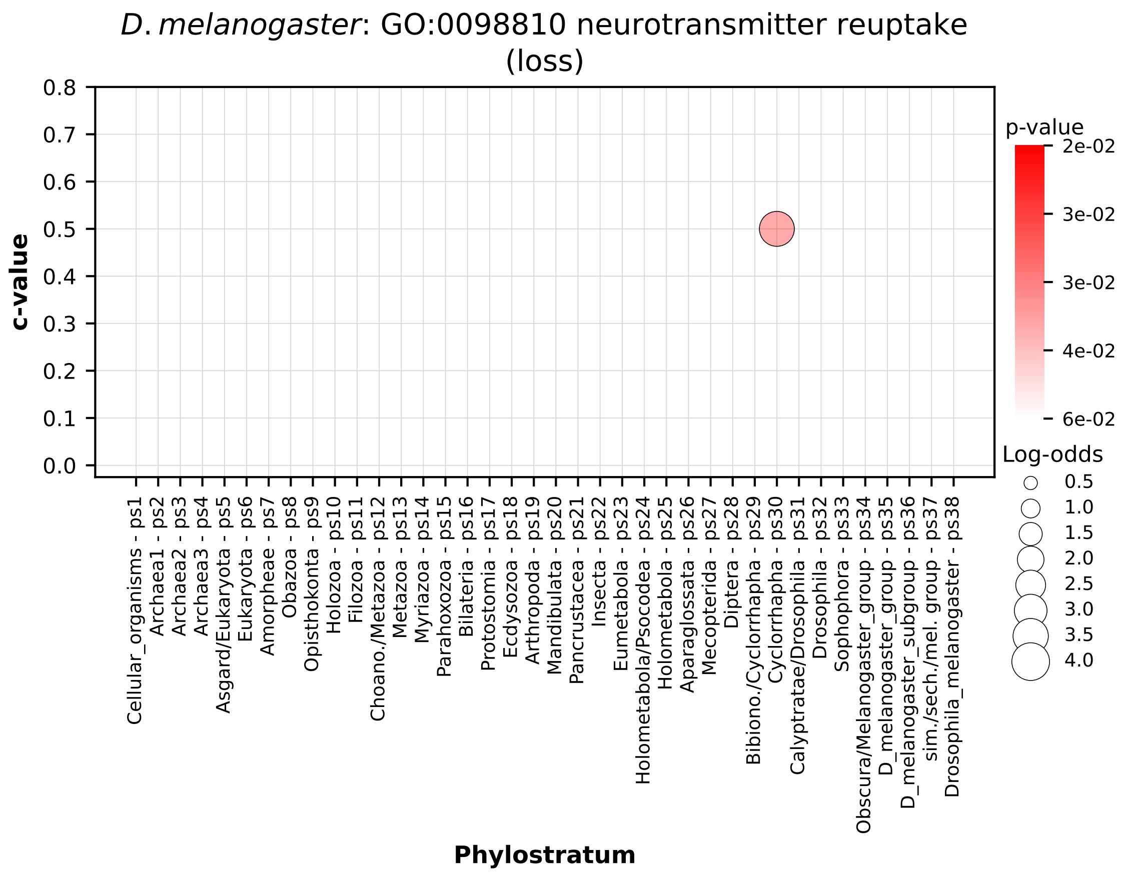

D. melanogaster: GO:0098810 neurotransmitter reuptake
(loss)
c-value
0.8
0.7
0.6
0.5
0.4
0.3
0.2
0.1
0.0
Phylostratum
Cellular_organisms - ps1
Archaea1 - ps2
Archaea2 - ps3
Archaea3 - ps4
Asgard/Eukaryota - ps5
Eukaryota - ps6
Amorpheae - ps7
Obazoa - ps8
Opisthokonta - ps9
Holozoa - ps10
Filozoa - ps11
Choano./Metazoa - ps12
Metazoa - ps13
Myriazoa - ps14
Parahoxozoa - ps15
Bilateria - ps16
Protostomia - ps17
Ecdysozoa - ps18
Arthropoda - ps19
Mandibulata - ps20
Pancrustacea - ps21
Insecta - ps22
Eumetabola - ps23
Holometabola/Psocodea - ps24
Holometabola - ps25
Aparaglossata - ps26
Mecopterida - ps27
Diptera - ps28
Bibiono./Cyclorrhapha - ps29
Cyclorrhapha - ps30
Calyptratae/Drosophila - ps31
Drosophila - ps32
Sophophora - ps33
Obscura/Melanogaster_group - ps34
D_melanogaster_group - ps35
D_melanogaster_subgroup - ps36
sim./sech./mel. group - ps37
Drosophila_melanogaster - ps38
p-value
2e-02
3e-02
3e-02
4e-02
6e-02
Log-odds
0.5
1.0
1.5
2.0
2.5
3.0
3.5
4.0

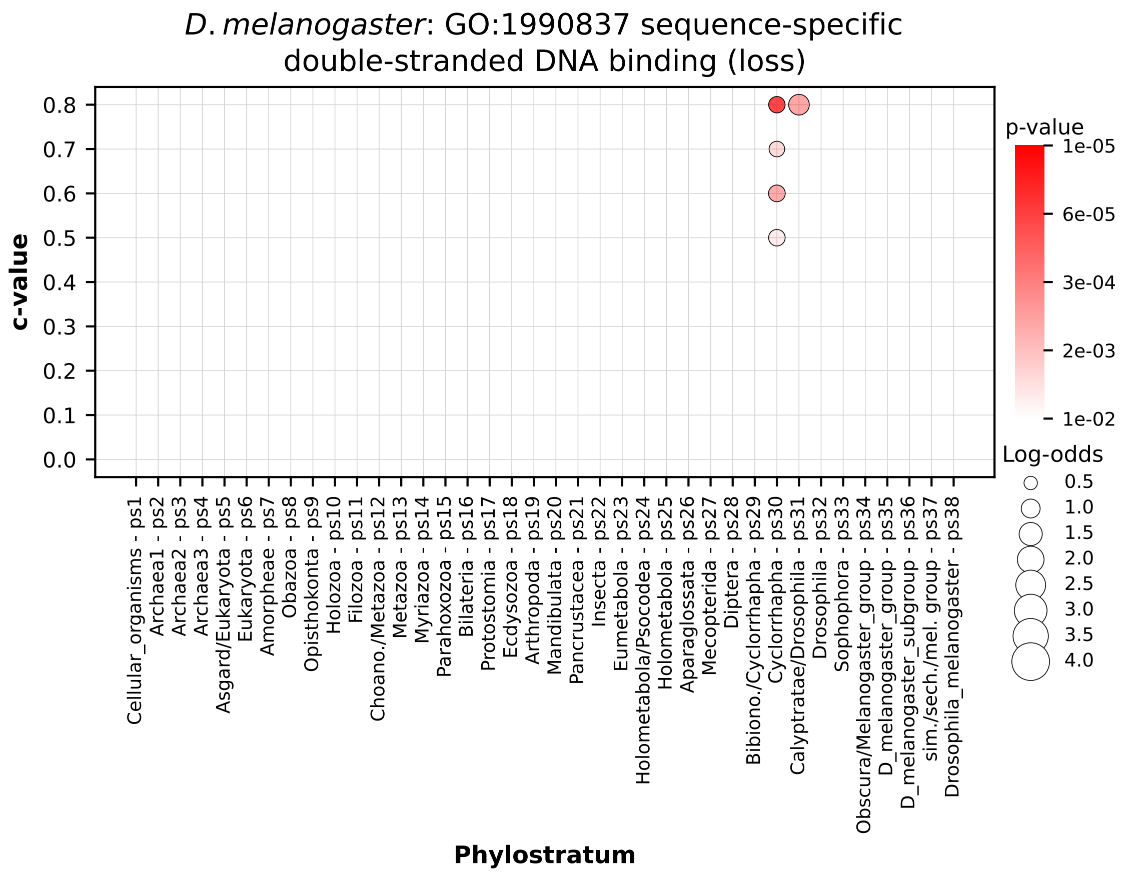

D. melanogaster: GO:1990837 sequence-specific double-stranded DNA binding (loss)
c-value
0.8
0.7
0.6
0.5
0.4
0.3
0.2
0.1
0.0
Phylostratum
Cellular_organisms - ps1
Archaea1 - ps2
Archaea2 - ps3
Archaea3 - ps4
Asgard/Eukaryota - ps5
Eukaryota - ps6
Amorpheae - ps7
Obazoa - ps8
Opisthokonta - ps9
Holozoa - ps10
Filozoa - ps11
Choano./Metazoa - ps12
Metazoa - ps13
Myriazoa - ps14
Parahoxozoa - ps15
Bilateria - ps16
Protostomia - ps17
Ecdysozoa - ps18
Arthropoda - ps19
Mandibulata - ps20
Pancrustacea - ps21
Insecta - ps22
Eumetabola - ps23
Holometabola/Psocodea - ps24
Holometabola - ps25
Aparaglossata - ps26
Mecopterida - ps27
Diptera - ps28
Bibiono./Cyclorrhapha - ps29
Cyclorrhapha - ps30
Calyptratae/Drosophila - ps31
Drosophila - ps32
Sophophora - ps33
Obscura/Melanogaster_group - ps34
D_melanogaster_group - ps35
D_melanogaster_subgroup - ps36
sim./sech./mel. group - ps37
Drosophila_melanogaster - ps38
p-value
1e-05
6e-05
3e-04
2e-03
1e-02
Log-odds
0.5
1.0
1.5
2.0
2.5
3.0
3.5
4.0

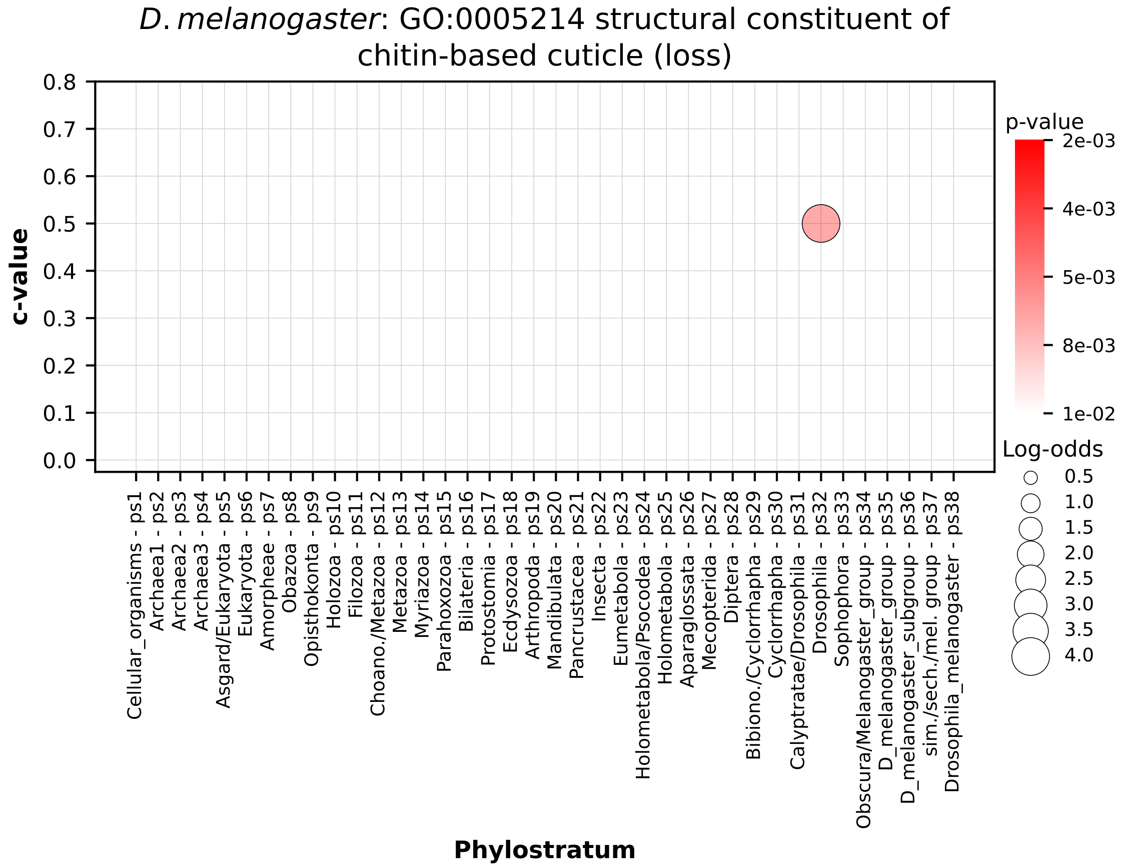

D. melanogaster: GO:0005214 structural constituent of chitin-based cuticle (loss)
c-value
0.0
0.1
0.2
0.3
0.4
0.5
0.6
0.7
0.8
Phylostratum
Cellular_organisms - ps1
Archaea1 - ps2
Archaea2 - ps3
Archaea3 - ps4
Asgard/Eukaryota - ps5
Eukaryota - ps6
Amorpheae - ps7
Obazoa - ps8
Opisthokonta - ps9
Holozoa - ps10
Filozoa - ps11
Choano./Metazoa - ps12
Metazoa - ps13
Myriazoa - ps14
Parahoxozoa - ps15
Bilateria - ps16
Protostomia - ps17
Ecdysozoa - ps18
Arthropoda - ps19
Mandibulata - ps20
Pancrustacea - ps21
Insecta - ps22
Eumetabola - ps23
Holometabola/Psocodea - ps24
Holometabola - ps25
Aparaglossata - ps26
Mecopterida - ps27
Diptera - ps28
Bibiono./Cyclorrhapha - ps29
Cyclorrhapha - ps30
Calyptratae/Drosophila - ps31
Drosophila - ps32
Sophophora - ps33
Obscura/Melanogaster_group - ps34
D_melanogaster_group - ps35
D_melanogaster_subgroup - ps36
sim./sech./mel. group - ps37
Drosophila_melanogaster - ps38
p-value
2e-03
4e-03
5e-03
8e-03
1e-02
Log-odds
0.5
1.0
1.5
2.0
2.5
3.0
3.5
4.0

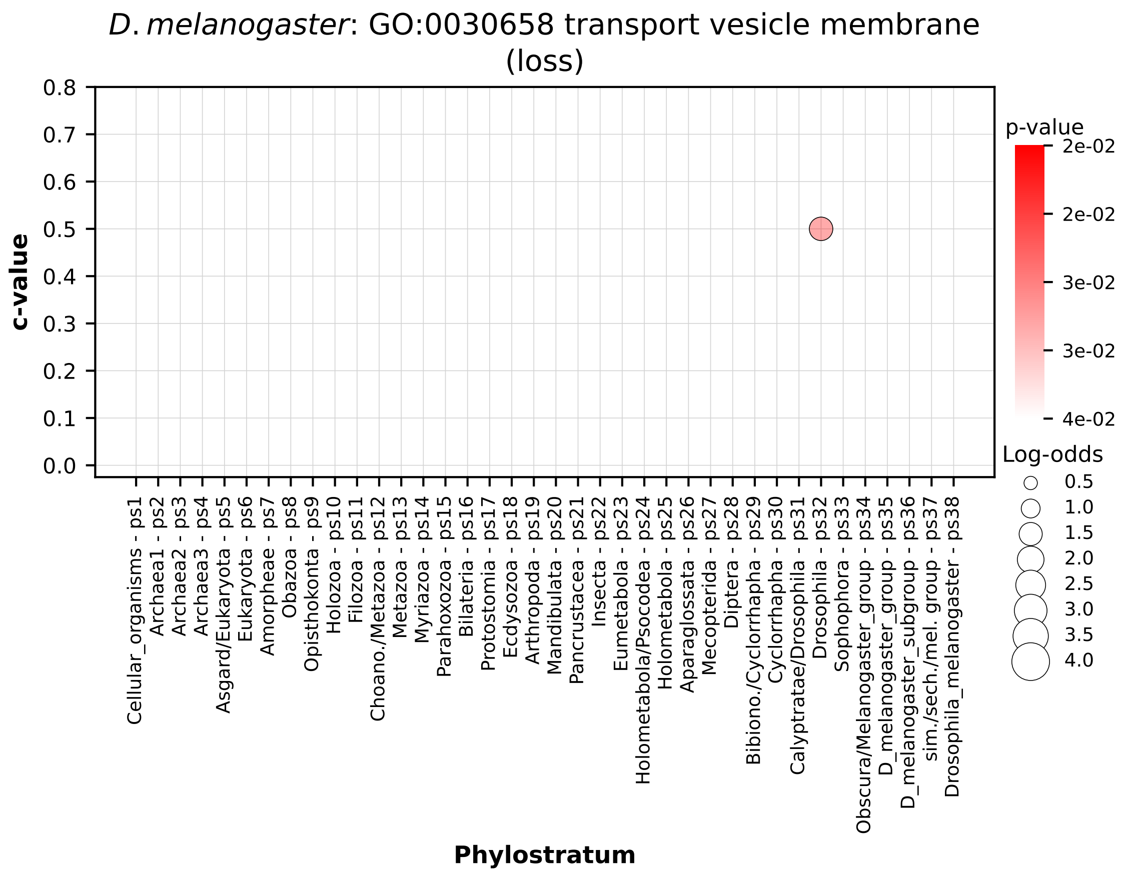

D. melanogaster: GO:0030658 transport vesicle membrane (loss)
c-value
0.0
0.1
0.2
0.3
0.4
0.5
0.6
0.7
0.8
Phylostratum
Cellular_organisms - ps1
Archaea1 - ps2
Archaea2 - ps3
Archaea3 - ps4
Asgard/Eukaryota - ps5
Eukaryota - ps6
Amorpheae - ps7
Obazoa - ps8
Opisthokonta - ps9
Holozoa - ps10
Filozoa - ps11
Choano./Metazoa - ps12
Metazoa - ps13
Myriazoa - ps14
Parahoxozoa - ps15
Bilateria - ps16
Protostomia - ps17
Ecdysozoa - ps18
Arthropoda - ps19
Mandibulata - ps20
Pancrustacea - ps21
Insecta - ps22
Eumetabola - ps23
Holometabola/Psocodea - ps24
Holometabola - ps25
Aparaglossata - ps26
Mecopterida - ps27
Diptera - ps28
Bibiono./Cyclorrhapha - ps29
Cyclorrhapha - ps30
Calyptratae/Drosophila - ps31
Drosophila - ps32
Sophophora - ps33
Obscura/Melanogaster_group - ps34
D_melanogaster_group - ps35
D_melanogaster_subgroup - ps36
sim./sech./mel. group - ps37
Drosophila_melanogaster - ps38
p-value
2e-02
2e-02
3e-02
3e-02
4e-02
Log-odds
0.5
1.0
1.5
2.0
2.5
3.0
3.5
4.0

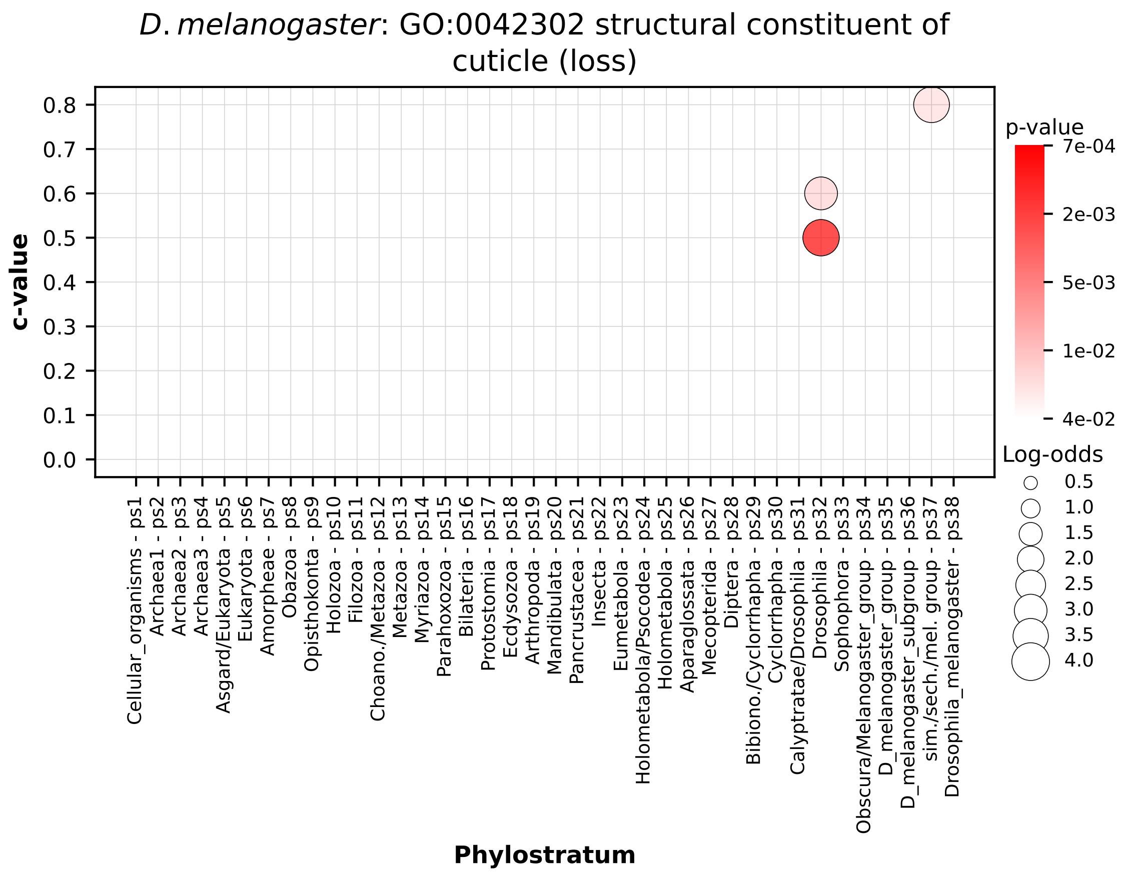

D. melanogaster: GO:0042302 structural constituent of cuticle (loss)
c-value
0.0
0.1
0.2
0.3
0.4
0.5
0.6
0.7
0.8
Phylostratum
Cellular_organisms - ps1
Archaea1 - ps2
Archaea2 - ps3
Archaea3 - ps4
Asgard/Eukaryota - ps5
Eukaryota - ps6
Amorpheae - ps7
Obazoa - ps8
Opisthokonta - ps9
Holozoa - ps10
Filozoa - ps11
Choano./Metazoa - ps12
Metazoa - ps13
Myriazoa - ps14
Parahoxozoa - ps15
Bilateria - ps16
Protostomia - ps17
Ecdysozoa - ps18
Arthropoda - ps19
Mandibulata - ps20
Pancrustacea - ps21
Insecta - ps22
Eumetabola - ps23
Holometabola/Psocodea - ps24
Holometabola - ps25
Aparaglossata - ps26
Mecopterida - ps27
Diptera - ps28
Bibiono./Cyclorrhapha - ps29
Cyclorrhapha - ps30
Calyptratae/Drosophila - ps31
Drosophila - ps32
Sophophora - ps33
Obscura/Melanogaster_group - ps34
D_melanogaster_group - ps35
D_melanogaster_subgroup - ps36
sim./sech./mel. group - ps37
Drosophila_melanogaster - ps38
p-value
7e-04
2e-03
5e-03
1e-02
4e-02
Log-odds
0.5
1.0
1.5
2.0
2.5
3.0
3.5
4.0

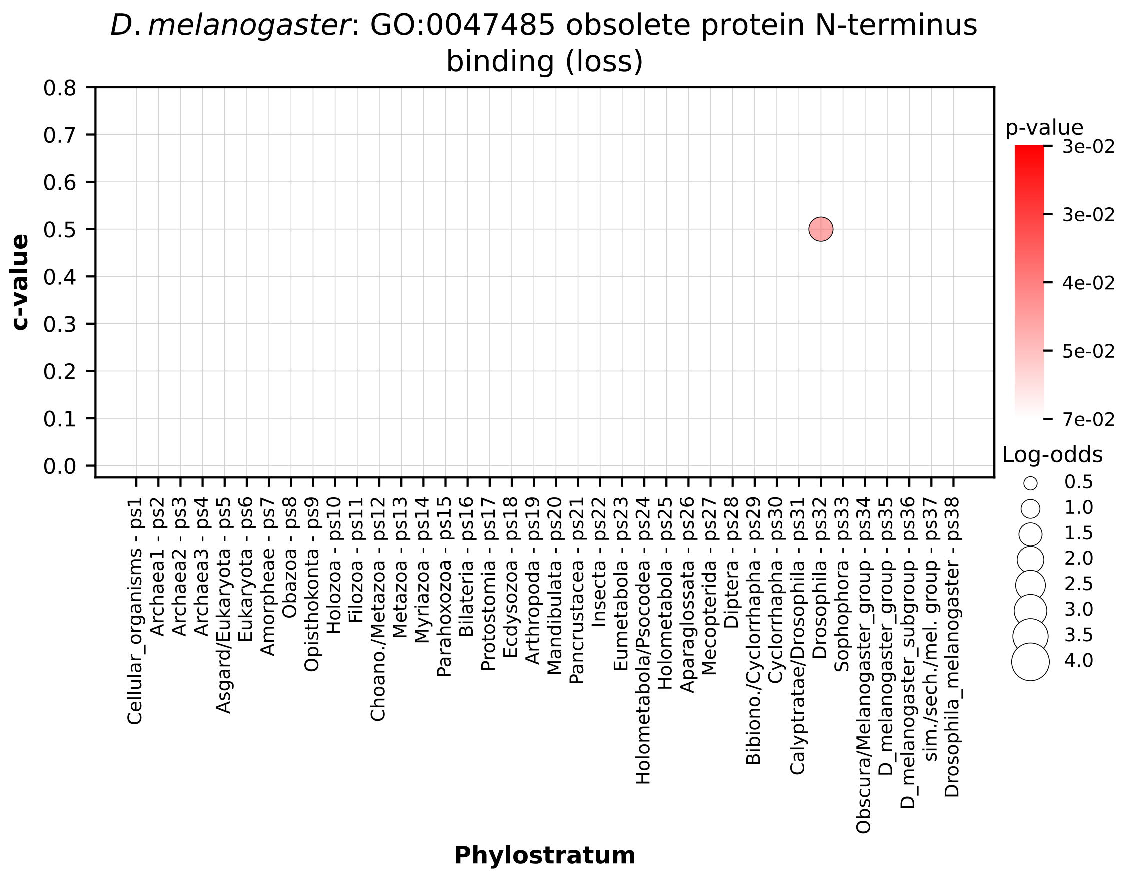
D. melanogaster: GO:0047485 obsolete protein N-terminus binding (loss)
c-value
0.8
0.7
0.6
0.5
0.4
0.3
0.2
0.1
0.0
Phylostratum
Cellular_organisms - ps1
Archaea1 - ps2
Archaea2 - ps3
Archaea3 - ps4
Asgard/Eukaryota - ps5
Eukaryota - ps6
Amorpheae - ps7
Obazoa - ps8
Opisthokonta - ps9
Holozoa - ps10
Filozoa - ps11
Choano./Metazoa - ps12
Metazoa - ps13
Myriazoa - ps14
Parahoxozoa - ps15
Bilateria - ps16
Protostomia - ps17
Ecdysozoa - ps18
Arthropoda - ps19
Mandibulata - ps20
Pancrustacea - ps21
Insecta - ps22
Eumetabola - ps23
Holometabola/Psocodea - ps24
Holometabola - ps25
Aparaglossata - ps26
Mecopterida - ps27
Diptera - ps28
Bibiono./Cyclorrhapha - ps29
Cyclorrhapha - ps30
Calyptratae/Drosophila - ps31
Drosophila - ps32
Sophophora - ps33
Obscura/Melanogaster_group - ps34
D_melanogaster_group - ps35
D_melanogaster_subgroup - ps36
sim./sech./mel. group - ps37
Drosophila_melanogaster - ps38
p-value
3e-02
3e-02
4e-02
5e-02
7e-02
Log-odds
0.5
1.0
1.5
2.0
2.5
3.0
3.5
4.0

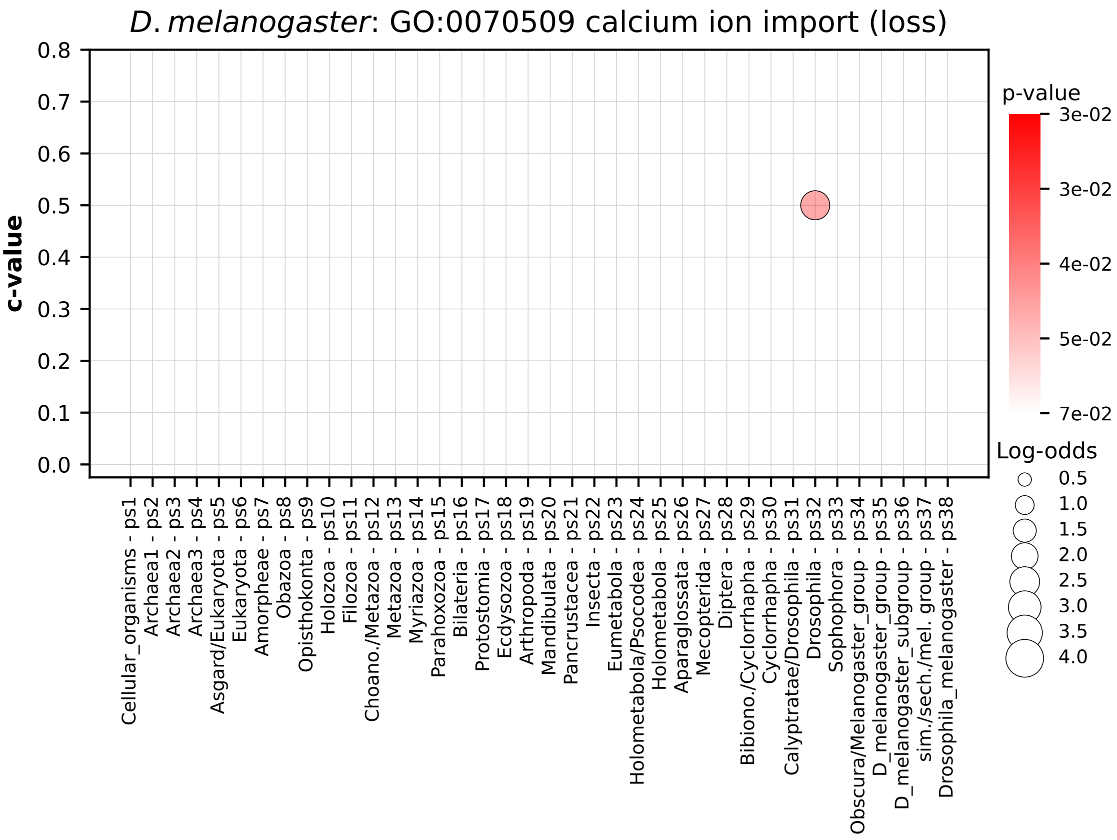

D. melanogaster: GO:0070509 calcium ion import (loss)
c-value
0.0
0.1
0.2
0.3
0.4
0.5
0.6
0.7
0.8
Phylostratum
Cellular_organisms - ps1
Archaea1 - ps2
Archaea2 - ps3
Archaea3 - ps4
Asgard/Eukaryota - ps5
Eukaryota - ps6
Amorpheae - ps7
Obazoa - ps8
Opisthokonta - ps9
Holozoa - ps10
Filozoa - ps11
Choano./Metazoa - ps12
Metazoa - ps13
Myriazoa - ps14
Parahoxozoa - ps15
Bilateria - ps16
Protostomia - ps17
Ecdysozoa - ps18
Arthropoda - ps19
Mandibulata - ps20
Pancrustacea - ps21
Insecta - ps22
Eumetabola - ps23
Holometabola/Psocodea - ps24
Holometabola - ps25
Aparaglossata - ps26
Mecopterida - ps27
Diptera - ps28
Bibiono./Cyclorrhapha - ps29
Cyclorrhapha - ps30
Calyptratae/Drosophila - ps31
Drosophila - ps32
Sophophora - ps33
Obscura/Melanogaster_group - ps34
D_melanogaster_group - ps35
D_melanogaster_subgroup - ps36
sim./sech./mel. group - ps37
Drosophila_melanogaster - ps38
p-value
3e-02
3e-02
4e-02
5e-02
7e-02
Log-odds
0.5
1.0
1.5
2.0
2.5
3.0
3.5
4.0

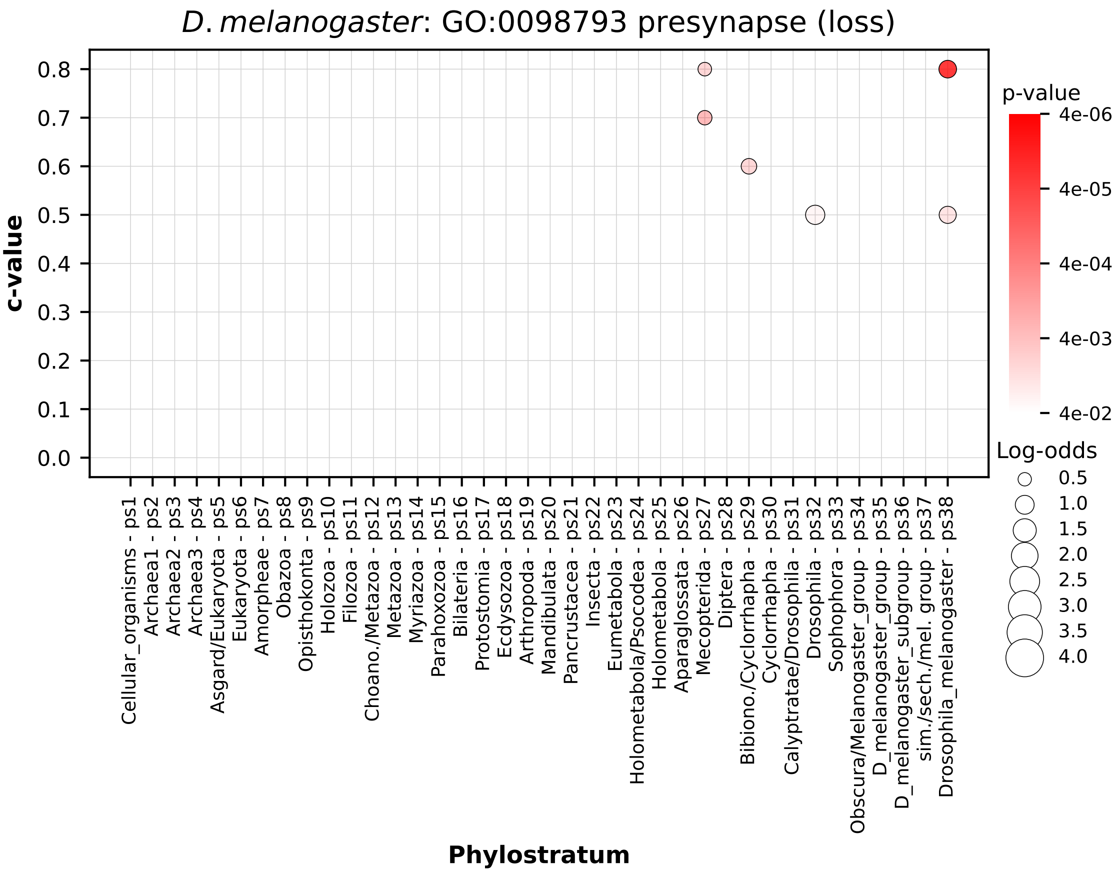
D. melanogaster: GO:0098793 presynapse (loss)
c-value
0.8
0.7
0.6
0.5
0.4
0.3
0.2
0.1
0.0
Phylostratum
Cellular_organisms - ps1
Archaea1 - ps2
Archaea2 - ps3
Archaea3 - ps4
Asgard/Eukaryota - ps5
Eukaryota - ps6
Amorpheae - ps7
Obazoa - ps8
Opisthokonta - ps9
Holozoa - ps10
Filozoa - ps11
Choano./Metazoa - ps12
Metazoa - ps13
Myriazoa - ps14
Parahoxozoa - ps15
Bilateria - ps16
Protostomia - ps17
Ecdysozoa - ps18
Arthropoda - ps19
Mandibulata - ps20
Pancrustacea - ps21
Insecta - ps22
Eumetabola - ps23
Holometabola/Psocodea - ps24
Holometabola - ps25
Aparaglossata - ps26
Mecopterida - ps27
Diptera - ps28
Bibiono./Cyclorrhapha - ps29
Cyclorrhapha - ps30
Calyptratae/Drosophila - ps31
Drosophila - ps32
Sophophora - ps33
Obscura/Melanogaster_group - ps34
D_melanogaster_group - ps35
D_melanogaster_subgroup - ps36
sim./sech./mel. group - ps37
Drosophila_melanogaster - ps38
p-value
4e-06
4e-05
4e-04
4e-03
4e-02
Log-odds
0.5
1.0
1.5
2.0
2.5
3.0
3.5
4.0

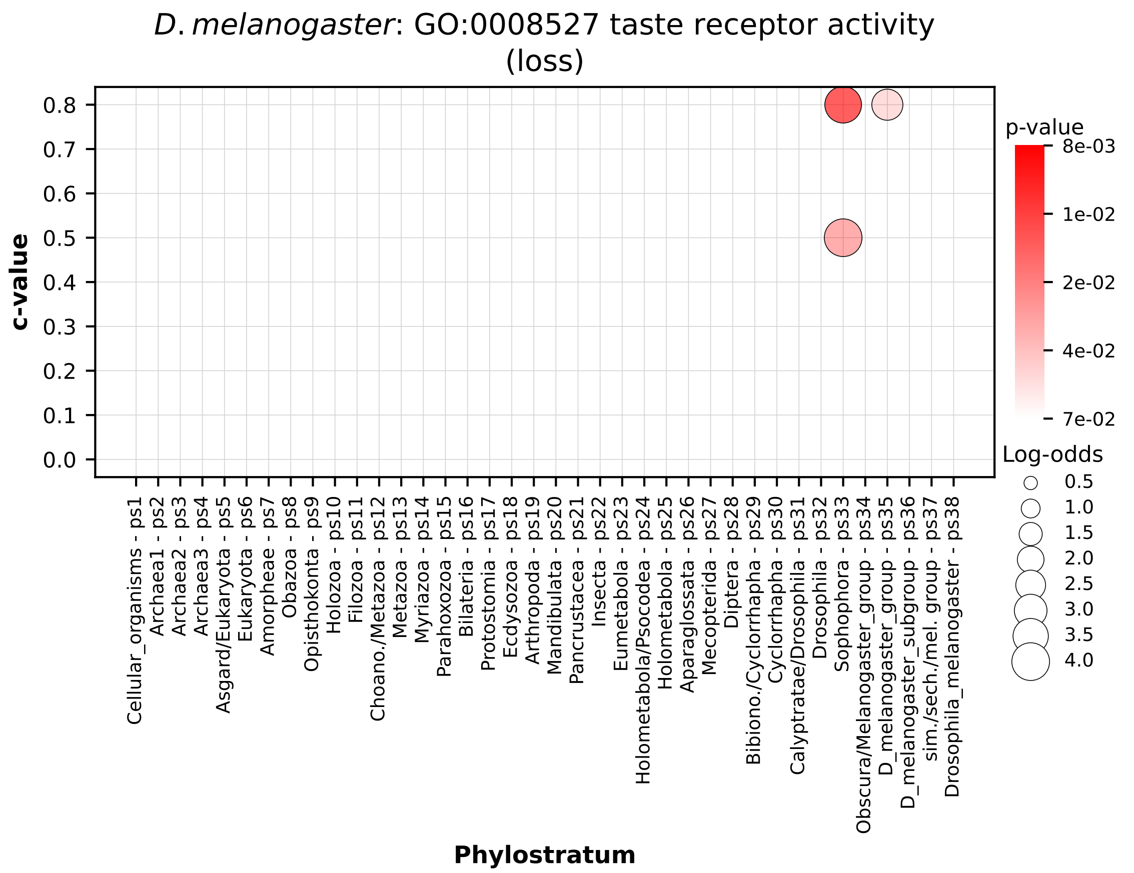

D. melanogaster: GO:0008527 taste receptor activity
(loss)
c-value
0.0
0.1
0.2
0.3
0.4
0.5
0.6
0.7
0.8
p-value
8e-03
1e-02
2e-02
4e-02
7e-02
Log-odds
0.5
1.0
1.5
2.0
2.5
3.0
3.5
4.0
Cellular_organisms - ps1
Archaea1 - ps2
Archaea2 - ps3
Archaea3 - ps4
Asgard/Eukaryota - ps5
Eukaryota - ps6
Amorpheae - ps7
Obazoa - ps8
Opisthokonta - ps9
Holozoa - ps10
Filozoa - ps11
Choano./Metazoa - ps12
Metazoa - ps13
Myriazoa - ps14
Parahoxozoa - ps15
Bilateria - ps16
Protostomia - ps17
Ecdysozoa - ps18
Arthropoda - ps19
Mandibulata - ps20
Pancrustacea - ps21
Insecta - ps22
Eumetabola - ps23
Holometabola/Psocodea - ps24
Holometabola - ps25
Aparaglossata - ps26
Mecopterida - ps27
Diptera - ps28
Bibiono./Cyclorrhapha - ps29
Cyclorrhapha - ps30
Calyptratae/Drosophila - ps31
Drosophila - ps32
Sophophora - ps33
Obscura/Melanogaster_group - ps34
D_melanogaster_group - ps35
D_melanogaster_subgroup - ps36
sim./sech./mel. group - ps37
Drosophila_melanogaster - ps38
Phylostratum

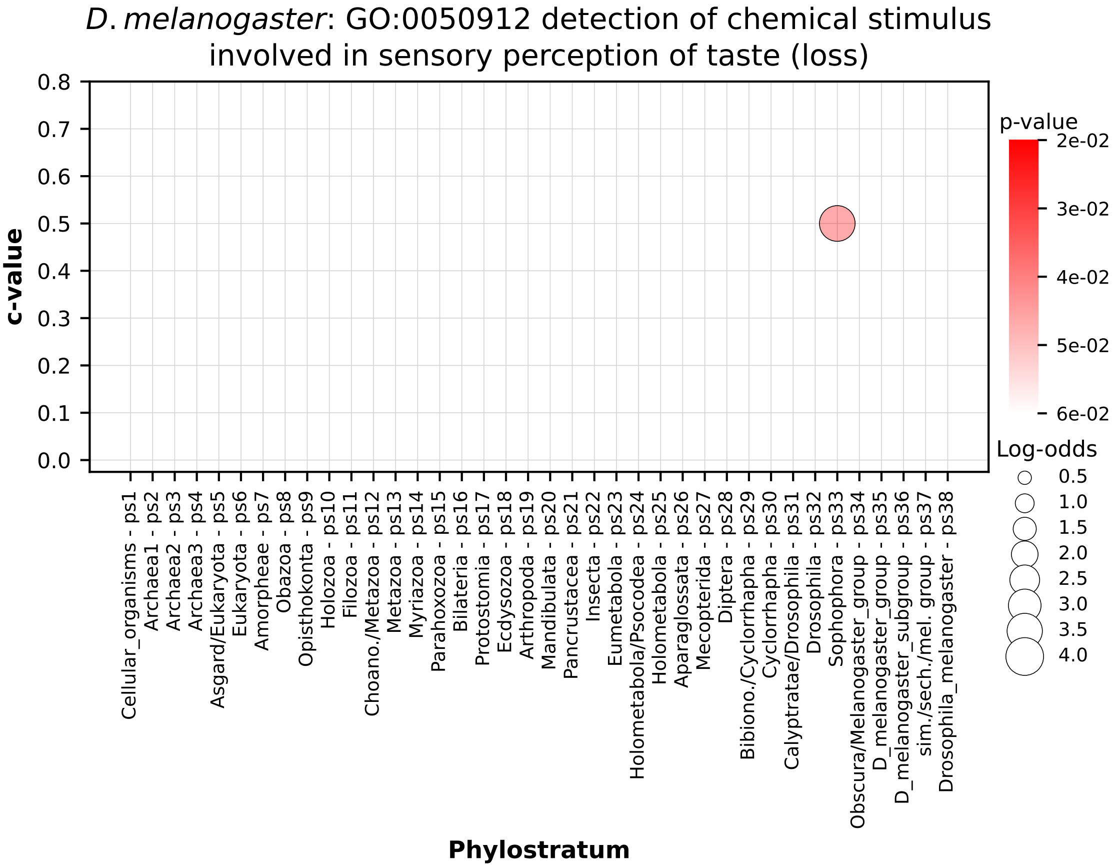

D. melanogaster: GO:0050912 detection of chemical stimulus involved in sensory perception of taste (loss)
c-value
0.8
0.7
0.6
0.5
0.4
0.3
0.2
0.1
0.0
Phylostratum
Cellular_organisms - ps1
Archaea1 - ps2
Archaea2 - ps3
Archaea3 - ps4
Asgard/Eukaryota - ps5
Eukaryota - ps6
Amorpheae - ps7
Obazoa - ps8
Opisthokonta - ps9
Holozoa - ps10
Filozoa - ps11
Choano./Metazoa - ps12
Metazoa - ps13
Myriazoa - ps14
Parahoxozoa - ps15
Bilateria - ps16
Protostomia - ps17
Ecdysozoa - ps18
Arthropoda - ps19
Mandibulata - ps20
Pancrustacea - ps21
Insecta - ps22
Eumetabola - ps23
Holometabola/Psocodea - ps24
Holometabola - ps25
Aparaglossata - ps26
Mecopterida - ps27
Diptera - ps28
Bibiono./Cyclorrhapha - ps29
Cyclorrhapha - ps30
Calyptratae/Drosophila - ps31
Drosophila - ps32
Sophophora - ps33
Obscura/Melanogaster_group - ps34
D_melanogaster_group - ps35
D_melanogaster_subgroup - ps36
sim./sech./mel. group - ps37
Drosophila_melanogaster - ps38
p-value
2e-02
3e-02
4e-02
5e-02
6e-02
Log-odds
0.5
1.0
1.5
2.0
2.5
3.0
3.5
4.0

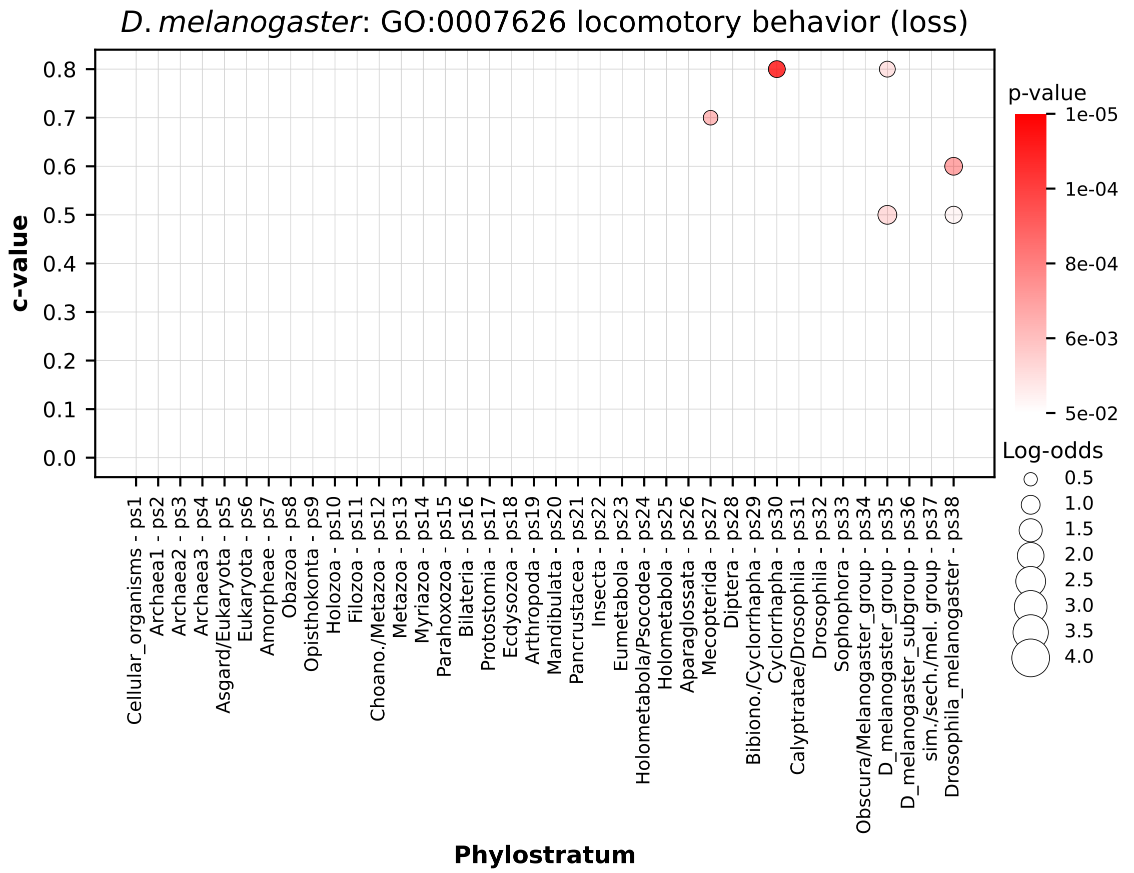

D. melanogaster: GO:0007626 locomotory behavior (loss)
c-value
0.0
0.1
0.2
0.3
0.4
0.5
0.6
0.7
0.8
Phylostratum
Cellular_organisms - ps1
Archaea1 - ps2
Archaea2 - ps3
Archaea3 - ps4
Asgard/Eukaryota - ps5
Eukaryota - ps6
Amorpheae - ps7
Obazoa - ps8
Opisthokonta - ps9
Holozoa - ps10
Filozoa - ps11
Choano./Metazoa - ps12
Metazoa - ps13
Myriazoa - ps14
Parahoxozoa - ps15
Bilateria - ps16
Protostomia - ps17
Ecdysozoa - ps18
Arthropoda - ps19
Mandibulata - ps20
Pancrustacea - ps21
Insecta - ps22
Eumetabola - ps23
Holometabola/Psocodea - ps24
Holometabola - ps25
Aparaglossata - ps26
Mecopterida - ps27
Diptera - ps28
Bibiono./Cyclorrhapha - ps29
Cyclorrhapha - ps30
Calyptratae/Drosophila - ps31
Drosophila - ps32
Sophophora - ps33
Obscura/Melanogaster_group - ps34
D_melanogaster_group - ps35
D_melanogaster_subgroup - ps36
sim./sech./mel. group - ps37
Drosophila_melanogaster - ps38
p-value
1e-05
1e-04
8e-04
6e-03
5e-02
Log-odds
0.5
1.0
1.5
2.0
2.5
3.0
3.5
4.0

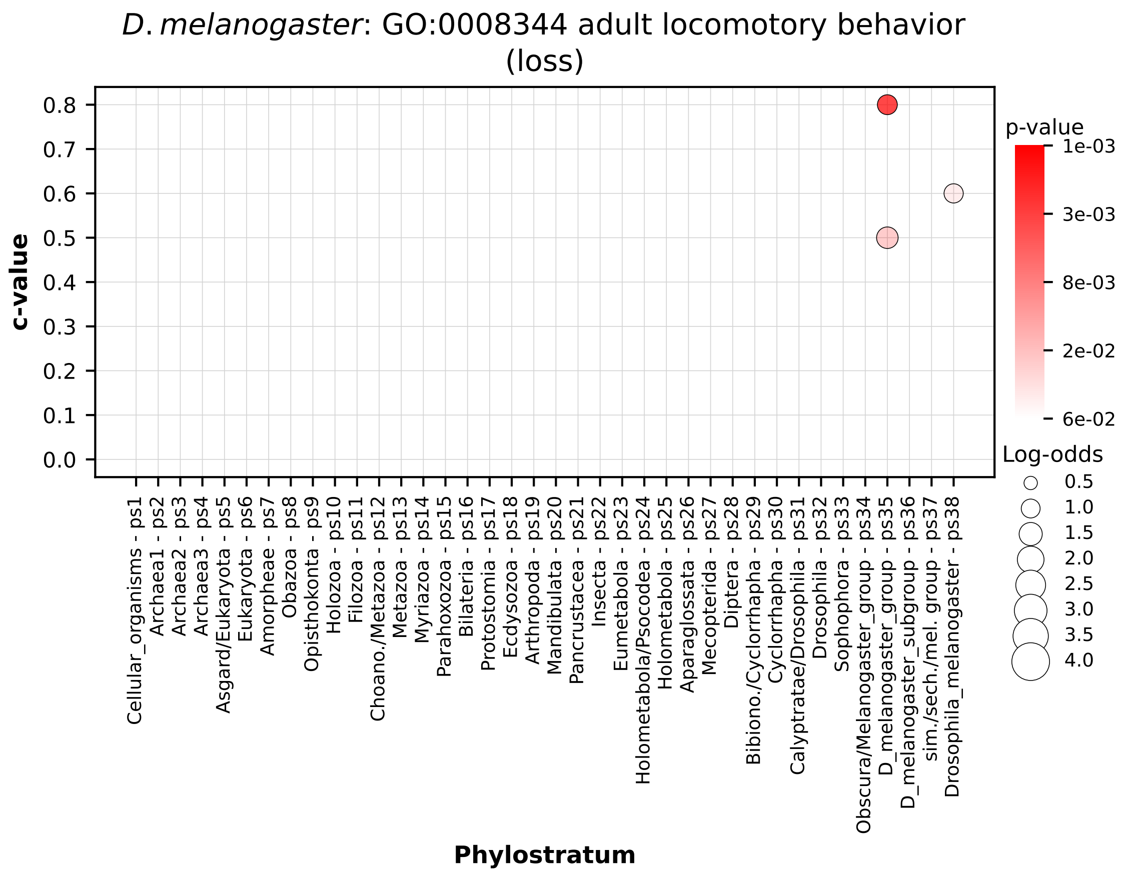

D. melanogaster: GO:0008344 adult locomotory behavior
(loss)
c-value
0.8
0.7
0.6
0.5
0.4
0.3
0.2
0.1
0.0
p-value
1e-03
3e-03
8e-03
2e-02
6e-02
Log-odds
0.5
1.0
1.5
2.0
2.5
3.0
3.5
4.0
Cellular_organisms - ps1
Archaea1 - ps2
Archaea2 - ps3
Archaea3 - ps4
Asgard/Eukaryota - ps5
Eukaryota - ps6
Amorpheae - ps7
Obazoa - ps8
Opisthokonta - ps9
Holozoa - ps10
Filozoa - ps11
Choano./Metazoa - ps12
Metazoa - ps13
Myriazoa - ps14
Parahoxozoa - ps15
Bilateria - ps16
Protostomia - ps17
Ecdysozoa - ps18
Arthropoda - ps19
Mandibulata - ps20
Pancrustacea - ps21
Insecta - ps22
Eumetabola - ps23
Holometabola/Psocodea - ps24
Holometabola - ps25
Aparaglossata - ps26
Mecopterida - ps27
Diptera - ps28
Bibiono./Cyclorrhapha - ps29
Cyclorrhapha - ps30
Calyptratae/Drosophila - ps31
Drosophila - ps32
Sophophora - ps33
Obscura/Melanogaster_group - ps34
D_melanogaster_group - ps35
D_melanogaster_subgroup - ps36
sim./sech./mel. group - ps37
Drosophila_melanogaster - ps38
Phylostratum

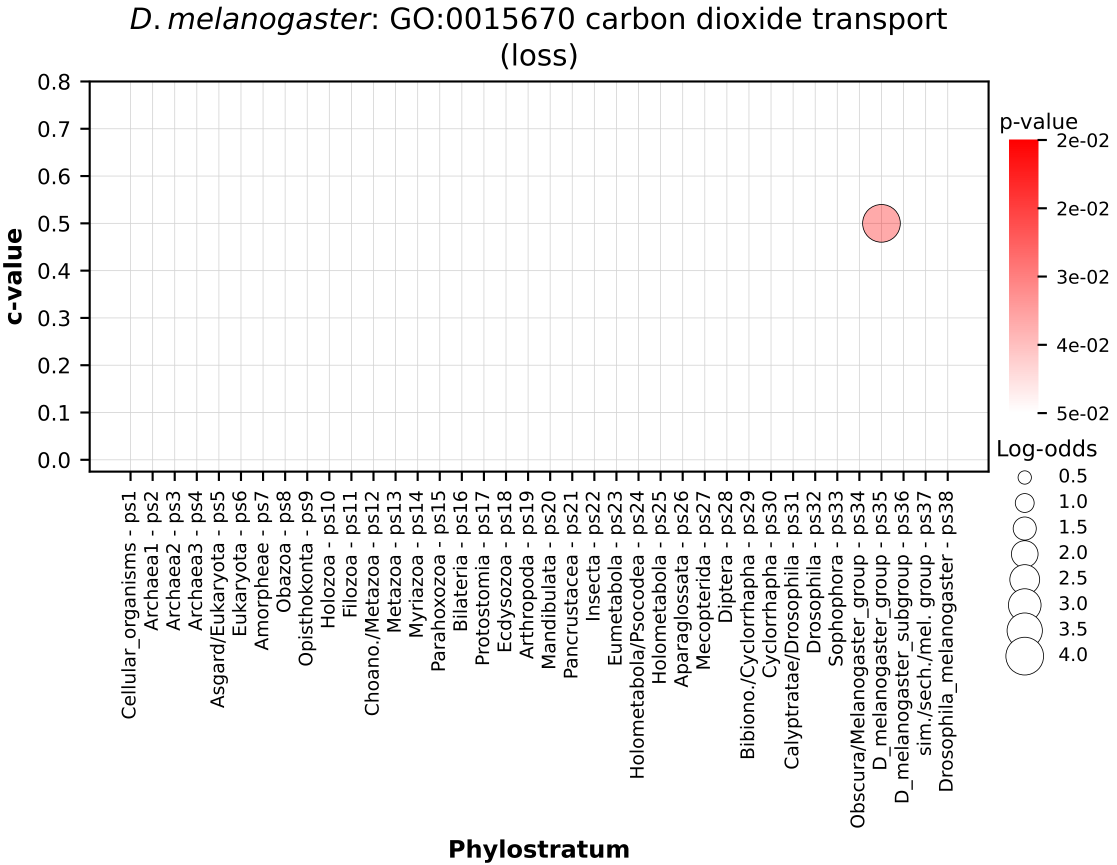

D. melanogaster: GO:0015670 carbon dioxide transport
(loss)
c-value
0.8
0.7
0.6
0.5
0.4
0.3
0.2
0.1
0.0
p-value
2e-02
2e-02
3e-02
4e-02
5e-02
Log-odds
0.5
1.0
1.5
2.0
2.5
3.0
3.5
4.0
Cellular_organisms - ps1
Archaea1 - ps2
Archaea2 - ps3
Archaea3 - ps4
Asgard/Eukaryota - ps5
Eukaryota - ps6
Amorpheae - ps7
Obazoa - ps8
Opisthokonta - ps9
Holozoa - ps10
Filozoa - ps11
Choano./Metazoa - ps12
Metazoa - ps13
Myriazoa - ps14
Parahoxozoa - ps15
Bilateria - ps16
Protostomia - ps17
Ecdysozoa - ps18
Arthropoda - ps19
Mandibulata - ps20
Pancrustacea - ps21
Insecta - ps22
Eumetabola - ps23
Holometabola/Psocodea - ps24
Holometabola - ps25
Aparaglossata - ps26
Mecopterida - ps27
Diptera - ps28
Bibiono./Cyclorrhapha - ps29
Cyclorrhapha - ps30
Calyptratae/Drosophila - ps31
Drosophila - ps32
Sophophora - ps33
Obscura/Melanogaster_group - ps34
D_melanogaster_group - ps35
D_melanogaster_subgroup - ps36
sim./sech./mel. group - ps37
Drosophila_melanogaster - ps38
Phylostratum

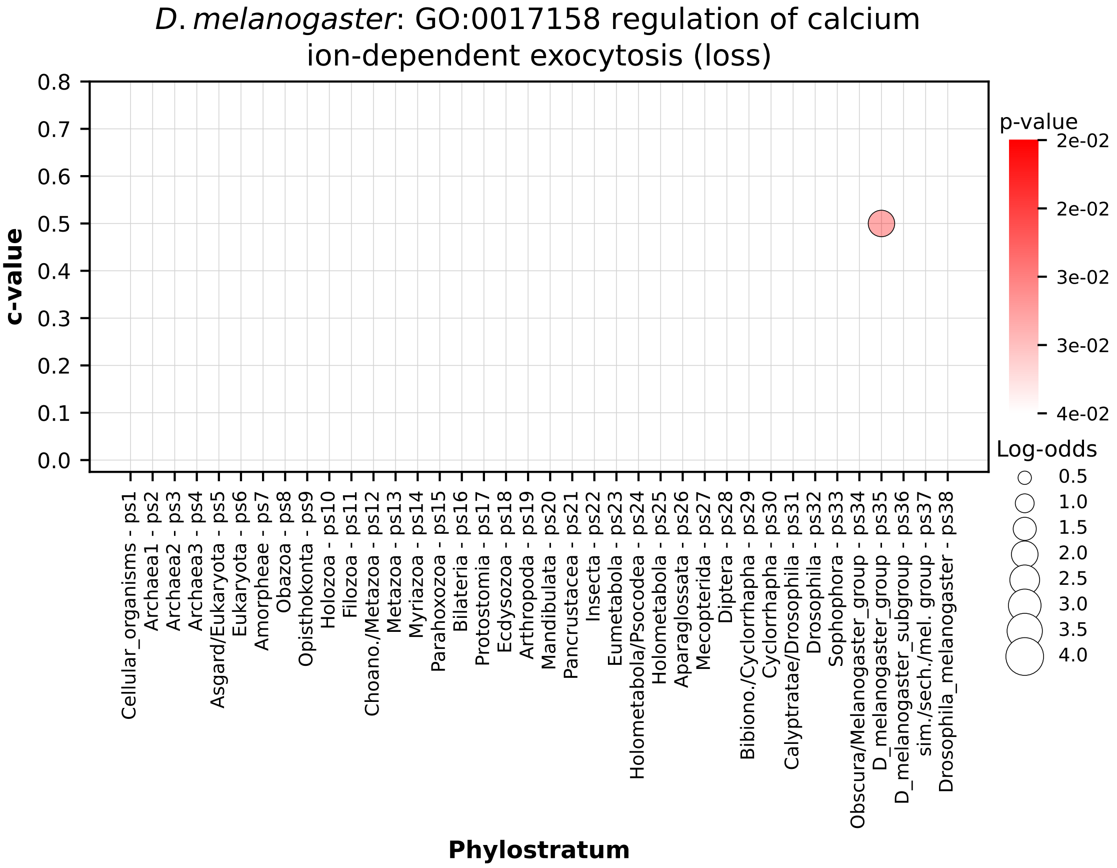
D. melanogaster: GO:0017158 regulation of calcium ion-dependent exocytosis (loss)
c-value
0.8
0.7
0.6
0.5
0.4
0.3
0.2
0.1
0.0
p-value
2e-02
2e-02
3e-02
3e-02
4e-02
Log-odds
0.5
1.0
1.5
2.0
2.5
3.0
3.5
4.0
Cellular_organisms - ps1
Archaea1 - ps2
Archaea2 - ps3
Archaea3 - ps4
Asgard/Eukaryota - ps5
Eukaryota - ps6
Amorpheae - ps7
Obazoa - ps8
Opisthokonta - ps9
Holozoa - ps10
Filozoa - ps11
Choano./Metazoa - ps12
Metazoa - ps13
Myriazoa - ps14
Parahoxozoa - ps15
Bilateria - ps16
Protostomia - ps17
Ecdysozoa - ps18
Arthropoda - ps19
Mandibulata - ps20
Pancrustacea - ps21
Insecta - ps22
Eumetabola - ps23
Holometabola/Psocodea - ps24
Holometabola - ps25
Aparaglossata - ps26
Mecopterida - ps27
Diptera - ps28
Bibiono./Cyclorrhapha - ps29
Cyclorrhapha - ps30
Calyptratae/Drosophila - ps31
Drosophila - ps32
Sophophora - ps33
Obscura/Melanogaster_group - ps34
D_melanogaster_group - ps35
D_melanogaster_subgroup - ps36
sim./sech./mel. group - ps37
Drosophila_melanogaster - ps38
Phylostratum

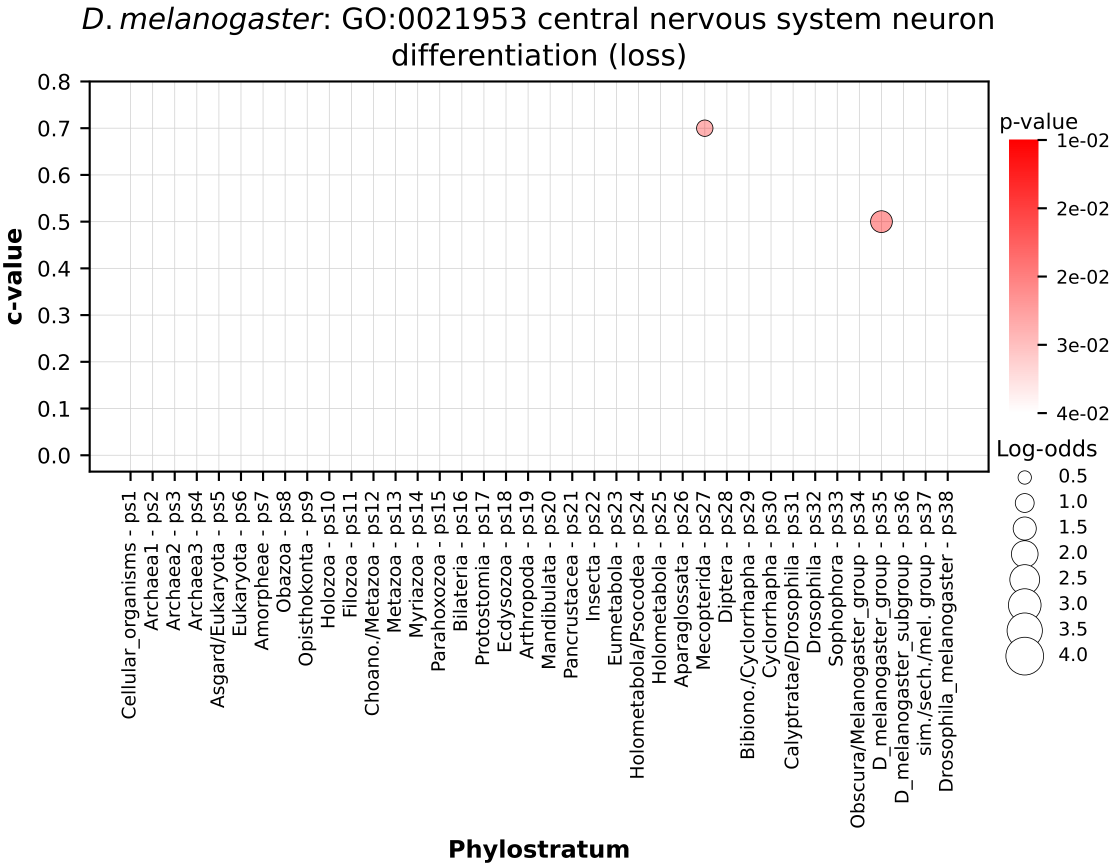

D. melanogaster: GO:0021953 central nervous system neuron differentiation (loss)
c-value
0.8
0.7
0.6
0.5
0.4
0.3
0.2
0.1
0.0
Phylostratum
Cellular_organisms - ps1
Archaea1 - ps2
Archaea2 - ps3
Archaea3 - ps4
Asgard/Eukaryota - ps5
Eukaryota - ps6
Amorpheae - ps7
Obazoa - ps8
Opisthokonta - ps9
Holozoa - ps10
Filozoa - ps11
Choano./Metazoa - ps12
Metazoa - ps13
Myriazoa - ps14
Parahoxozoa - ps15
Bilateria - ps16
Protostomia - ps17
Ecdysozoa - ps18
Arthropoda - ps19
Mandibulata - ps20
Pancrustacea - ps21
Insecta - ps22
Eumetabola - ps23
Holometabola/Psocodea - ps24
Holometabola - ps25
Aparaglossata - ps26
Mecopterida - ps27
Diptera - ps28
Bibiono./Cyclorrhapha - ps29
Cyclorrhapha - ps30
Calyptratae/Drosophila - ps31
Drosophila - ps32
Sophophora - ps33
Obscura/Melanogaster_group - ps34
D_melanogaster_group - ps35
D_melanogaster_subgroup - ps36
sim./sech./mel. group - ps37
Drosophila_melanogaster - ps38
p-value
1e-02
2e-02
2e-02
3e-02
4e-02
Log-odds
0.5
1.0
1.5
2.0
2.5
3.0
3.5
4.0

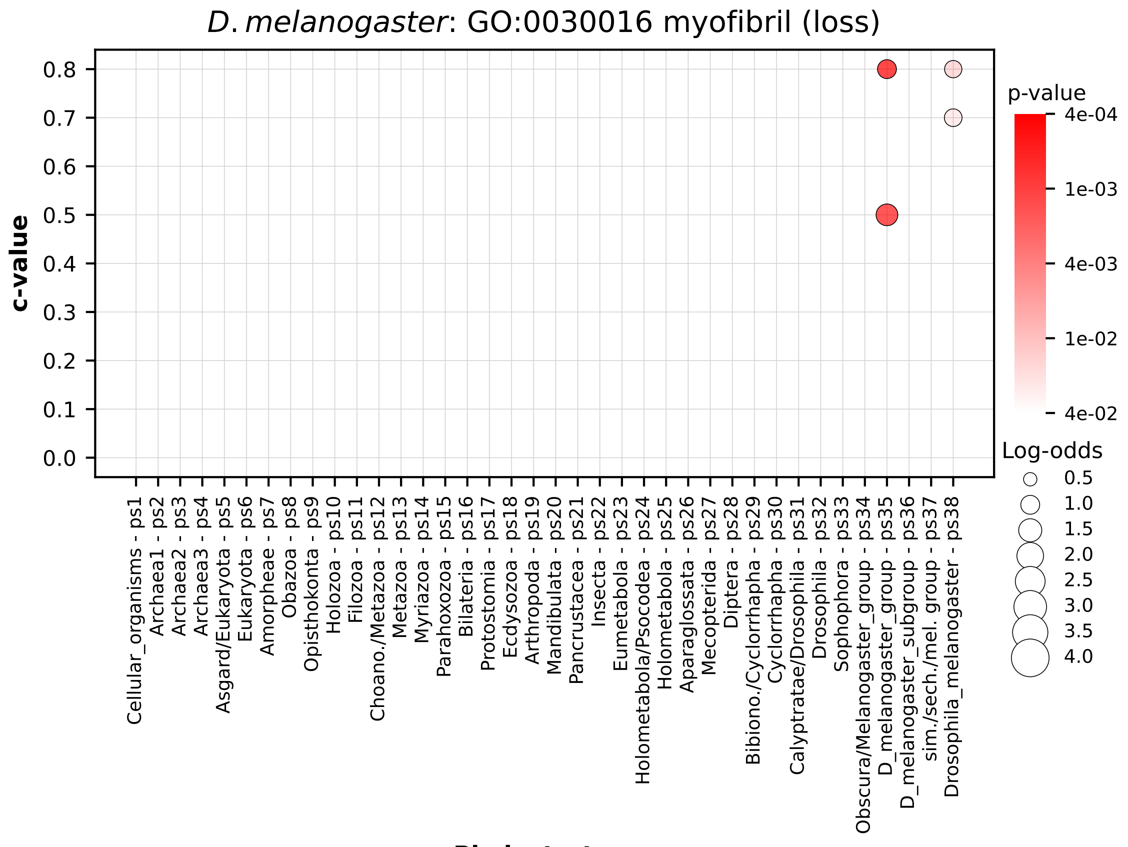

D. melanogaster: GO:0030016 myofibril (loss)
c-value
0.0
0.1
0.2
0.3
0.4
0.5
0.6
0.7
0.8
Phylostratum
Cellular_organisms - ps1
Archaea1 - ps2
Archaea2 - ps3
Archaea3 - ps4
Asgard/Eukaryota - ps5
Eukaryota - ps6
Amorpheae - ps7
Obazoa - ps8
Opisthokonta - ps9
Holozoa - ps10
Filozoa - ps11
Choano./Metazoa - ps12
Metazoa - ps13
Myriazoa - ps14
Parahoxozoa - ps15
Bilateria - ps16
Protostomia - ps17
Ecdysozoa - ps18
Arthropoda - ps19
Mandibulata - ps20
Pancrustacea - ps21
Insecta - ps22
Eumetabola - ps23
Holometabola/Psocodea - ps24
Holometabola - ps25
Aparaglossata - ps26
Mecopterida - ps27
Diptera - ps28
Bibiono./Cyclorrhapha - ps29
Cyclorrhapha - ps30
Calyptratae/Drosophila - ps31
Drosophila - ps32
Sophophora - ps33
Obscura/Melanogaster_group - ps34
D_melanogaster_group - ps35
D_melanogaster_subgroup - ps36
sim./sech./mel. group - ps37
Drosophila_melanogaster - ps38
p-value
4e-04
1e-03
4e-03
1e-02
4e-02
Log-odds
0.5
1.0
1.5
2.0
2.5
3.0
3.5
4.0

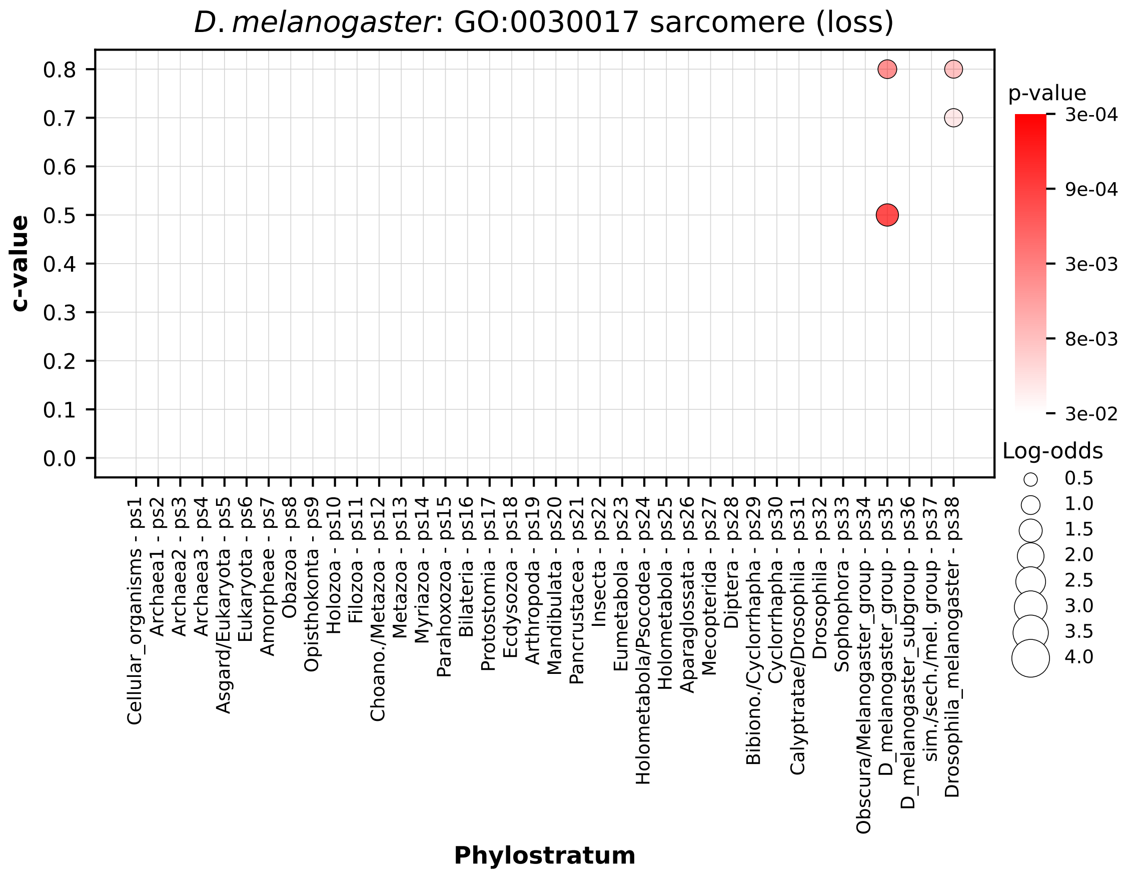

D. melanogaster: GO:0030017 sarcomere (loss)
c-value
0.0
0.1
0.2
0.3
0.4
0.5
0.6
0.7
0.8
Phylostratum
Cellular_organisms - ps1
Archaea1 - ps2
Archaea2 - ps3
Archaea3 - ps4
Asgard/Eukaryota - ps5
Eukaryota - ps6
Amorpheae - ps7
Obazoa - ps8
Opisthokonta - ps9
Holozoa - ps10
Filozoa - ps11
Choano./Metazoa - ps12
Metazoa - ps13
Myriazoa - ps14
Parahoxozoa - ps15
Bilateria - ps16
Protostomia - ps17
Ecdysozoa - ps18
Arthropoda - ps19
Mandibulata - ps20
Pancrustacea - ps21
Insecta - ps22
Eumetabola - ps23
Holometabola/Psocodea - ps24
Holometabola - ps25
Aparaglossata - ps26
Mecopterida - ps27
Diptera - ps28
Bibiono./Cyclorrhapha - ps29
Cyclorrhapha - ps30
Calyptratae/Drosophila - ps31
Drosophila - ps32
Sophophora - ps33
Obscura/Melanogaster_group - ps34
D_melanogaster_group - ps35
D_melanogaster_subgroup - ps36
sim./sech./mel. group - ps37
Drosophila_melanogaster - ps38
p-value
3e-04
9e-04
3e-03
8e-03
3e-02
Log-odds
0.5
1.0
1.5
2.0
2.5
3.0
3.5
4.0

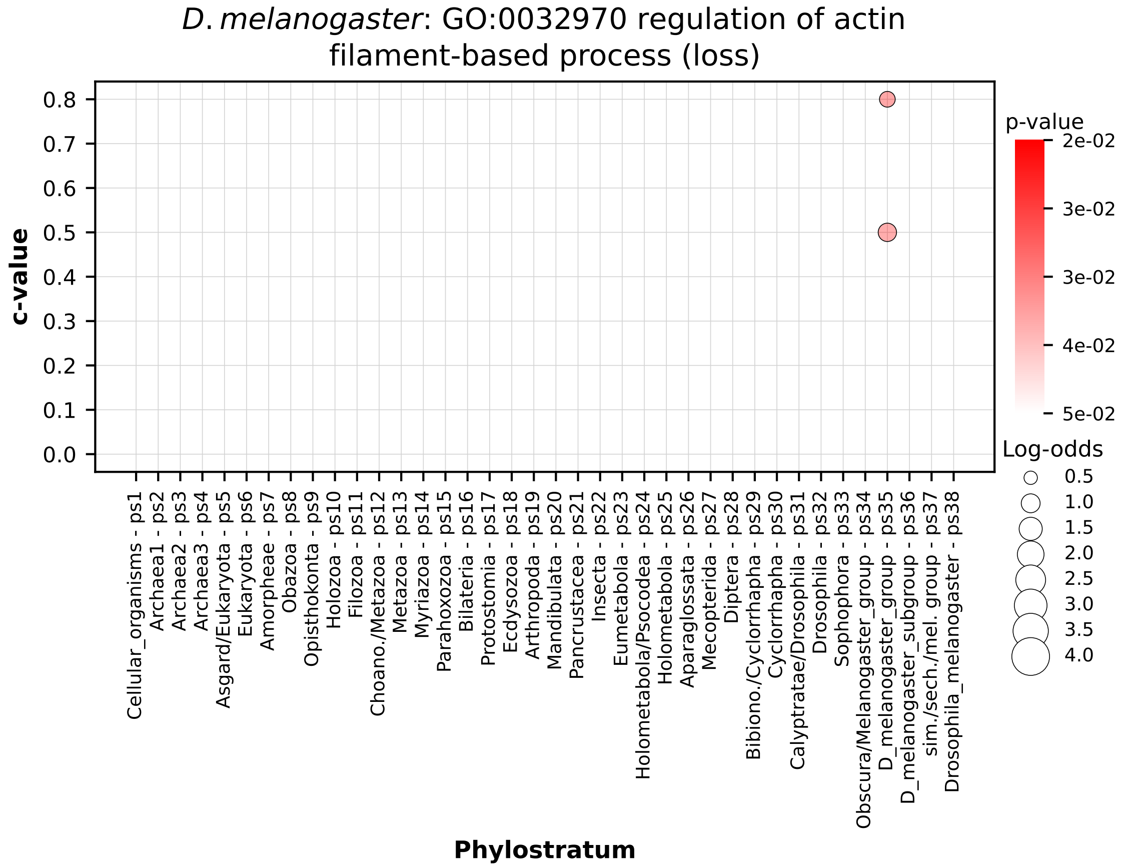

D. melanogaster: GO:0032970 regulation of actin filament-based process (loss)
c-value
0.8
0.7
0.6
0.5
0.4
0.3
0.2
0.1
0.0
p-value
2e-02
3e-02
3e-02
4e-02
5e-02
Log-odds
0.5
1.0
1.5
2.0
2.5
3.0
3.5
4.0
Cellular_organisms - ps1
Archaea1 - ps2
Archaea2 - ps3
Archaea3 - ps4
Asgard/Eukaryota - ps5
Eukaryota - ps6
Amorpheae - ps7
Obazoa - ps8
Opisthokonta - ps9
Holozoa - ps10
Filozoa - ps11
Choano./Metazoa - ps12
Metazoa - ps13
Myriazoa - ps14
Parahoxozoa - ps15
Bilateria - ps16
Protostomia - ps17
Ecdysozoa - ps18
Arthropoda - ps19
Mandibulata - ps20
Pancrustacea - ps21
Insecta - ps22
Eumetabola - ps23
Holometabola/Psocodea - ps24
Holometabola - ps25
Aparaglossata - ps26
Mecopterida - ps27
Diptera - ps28
Bibiono./Cyclorrhapha - ps29
Cyclorrhapha - ps30
Calyptratae/Drosophila - ps31
Drosophila - ps32
Sophophora - ps33
Obscura/Melanogaster_group - ps34
D_melanogaster_group - ps35
D_melanogaster_subgroup - ps36
sim./sech./mel. group - ps37
Drosophila_melanogaster - ps38
Phylostratum

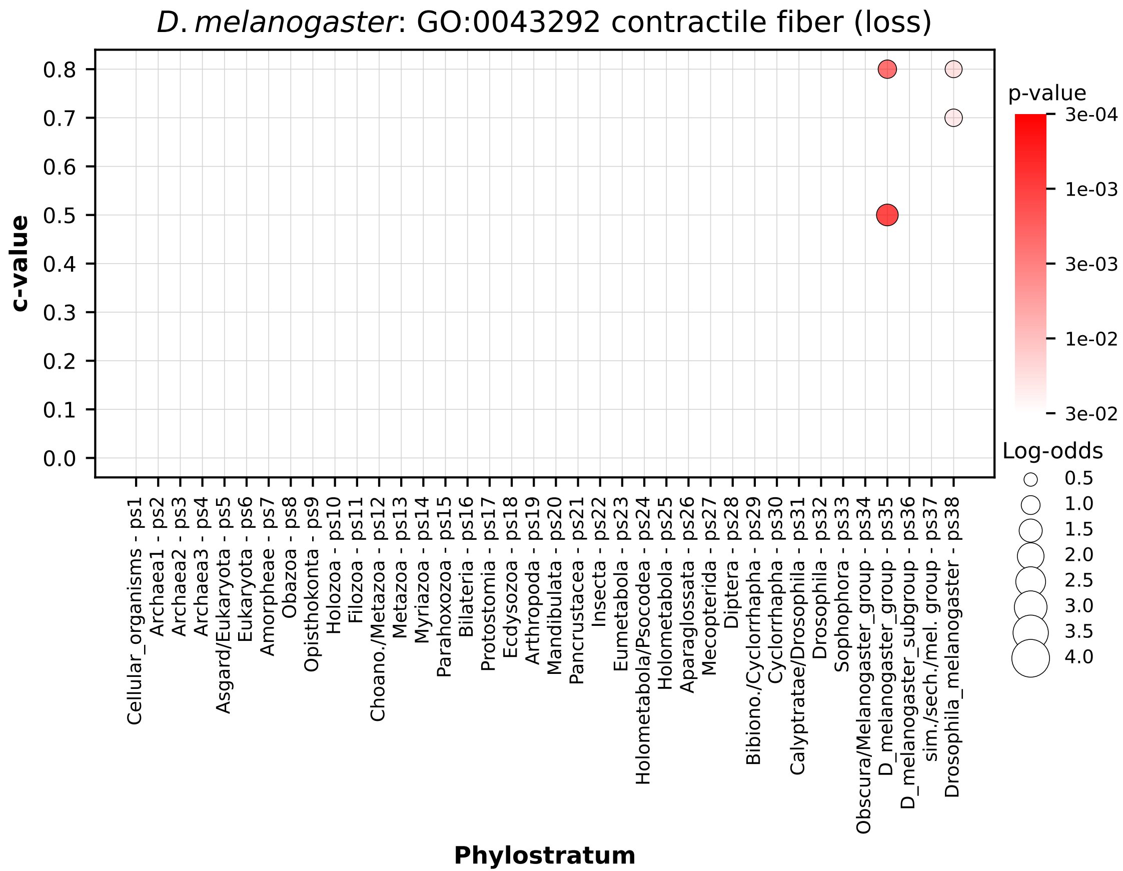
D. melanogaster: GO:0043292 contractile fiber (loss)
c-value
0.8
0.7
0.6
0.5
0.4
0.3
0.2
0.1
0.0
Phylostratum
Cellular_organisms - ps1
Archaea1 - ps2
Archaea2 - ps3
Archaea3 - ps4
Asgard/Eukaryota - ps5
Eukaryota - ps6
Amorpheae - ps7
Obazoa - ps8
Opisthokonta - ps9
Holozoa - ps10
Filozoa - ps11
Choano./Metazoa - ps12
Metazoa - ps13
Myriazoa - ps14
Parahoxozoa - ps15
Bilateria - ps16
Protostomia - ps17
Ecdysozoa - ps18
Arthropoda - ps19
Mandibulata - ps20
Pancrustacea - ps21
Insecta - ps22
Eumetabola - ps23
Holometabola/Psocodea - ps24
Holometabola - ps25
Aparaglossata - ps26
Mecopterida - ps27
Diptera - ps28
Bibiono./Cyclorrhapha - ps29
Cyclorrhapha - ps30
Calyptratae/Drosophila - ps31
Drosophila - ps32
Sophophora - ps33
Obscura/Melanogaster_group - ps34
D_melanogaster_group - ps35
D_melanogaster_subgroup - ps36
sim./sech./mel. group - ps37
Drosophila_melanogaster - ps38
p-value
3e-04
1e-03
3e-03
1e-02
3e-02
Log-odds
0.5
1.0
1.5
2.0
2.5
3.0
3.5
4.0

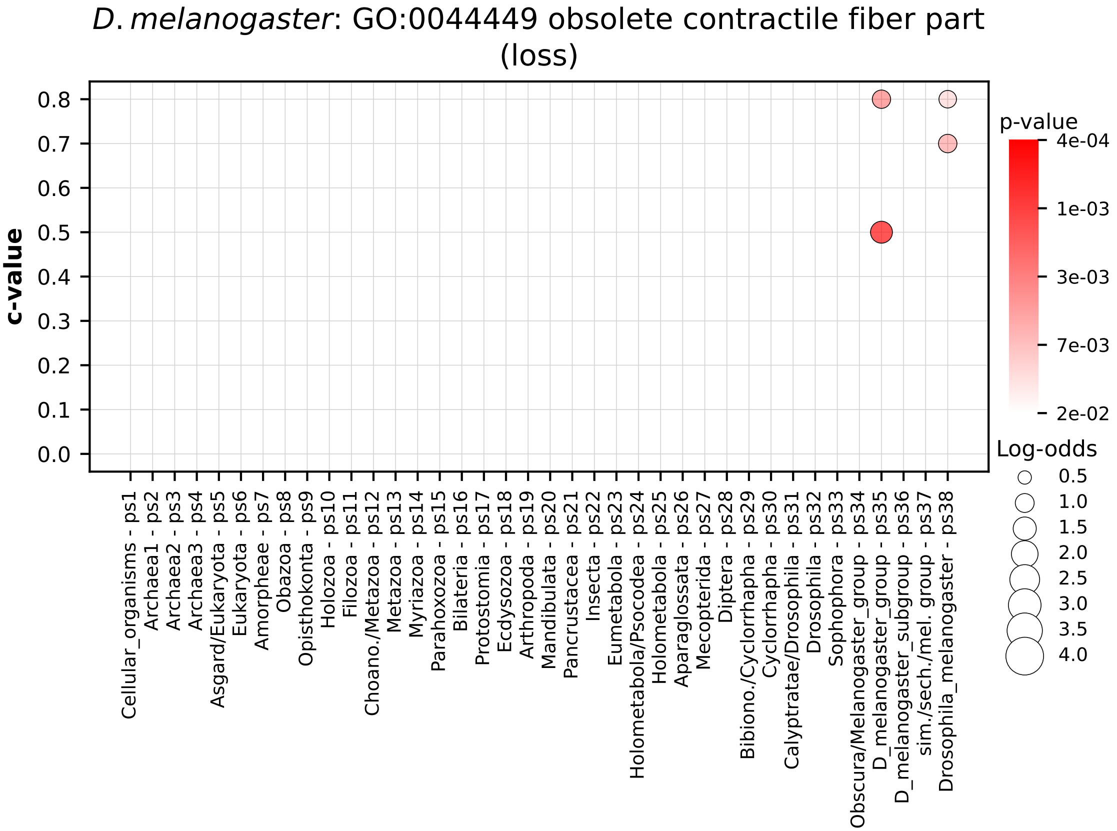

D. melanogaster: GO:0044449 obsolete contractile fiber part (loss)
c-value
0.8
0.7
0.6
0.5
0.4
0.3
0.2
0.1
0.0
p-value
4e-04
1e-03
3e-03
7e-03
2e-02
Log-odds
0.5
1.0
1.5
2.0
2.5
3.0
3.5
4.0
Cellular_organisms - ps1
Archaea1 - ps2
Archaea2 - ps3
Archaea3 - ps4
Asgard/Eukaryota - ps5
Eukaryota - ps6
Amorpheae - ps7
Obazoa - ps8
Opisthokonta - ps9
Holozoa - ps10
Filozoa - ps11
Choano./Metazoa - ps12
Metazoa - ps13
Myriazoa - ps14
Parahoxozoa - ps15
Bilateria - ps16
Protostomia - ps17
Ecdysozoa - ps18
Arthropoda - ps19
Mandibulata - ps20
Pancrustacea - ps21
Insecta - ps22
Eumetabola - ps23
Holometabola/Psocodea - ps24
Holometabola - ps25
Aparaglossata - ps26
Mecopterida - ps27
Diptera - ps28
Bibiono./Cyclorrhapha - ps29
Cyclorrhapha - ps30
Calyptratae/Drosophila - ps31
Drosophila - ps32
Sophophora - ps33
Obscura/Melanogaster_group - ps34
D_melanogaster_group - ps35
D_melanogaster_subgroup - ps36
sim./sech./mel. group - ps37
Drosophila_melanogaster - ps38
Phylostratum

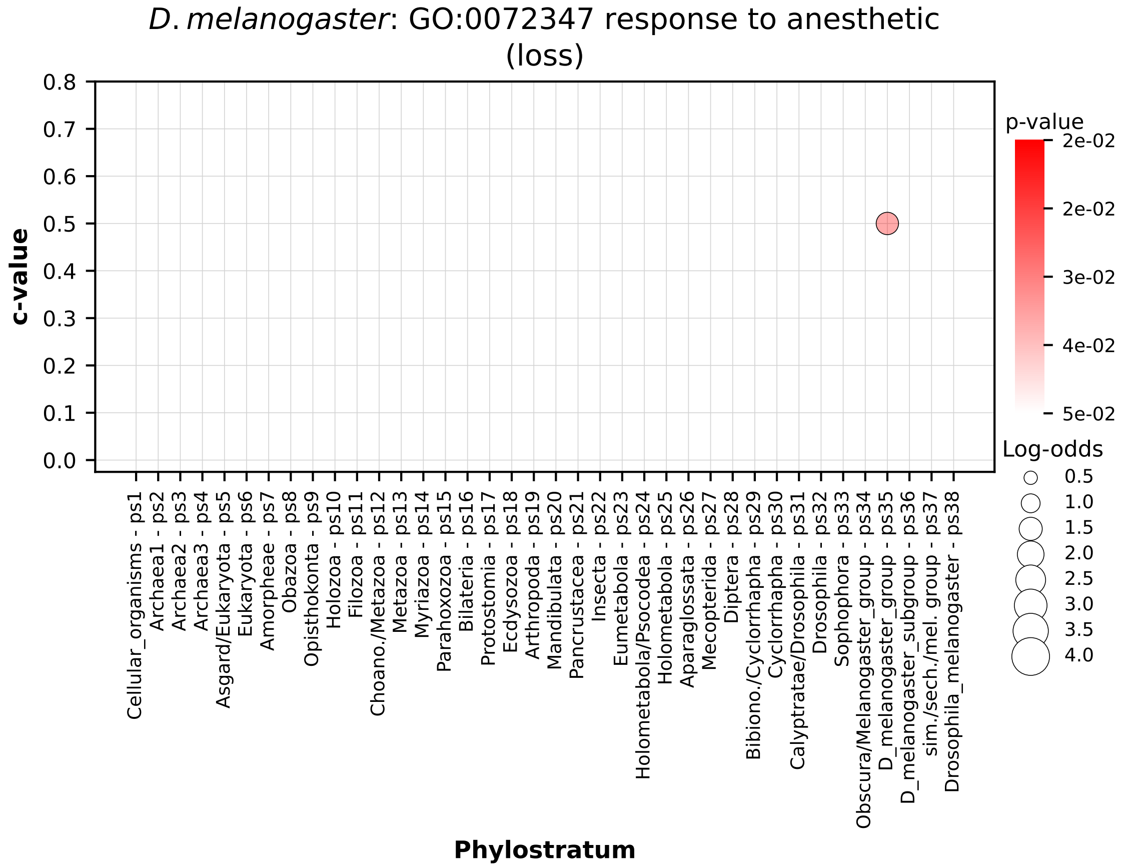

D. melanogaster: GO:0072347 response to anesthetic
(loss)
c-value
0.8
0.7
0.6
0.5
0.4
0.3
0.2
0.1
0.0
p-value
2e-02
2e-02
3e-02
4e-02
5e-02
Log-odds
0.5
1.0
1.5
2.0
2.5
3.0
3.5
4.0
Cellular_organisms - ps1
Archaea1 - ps2
Archaea2 - ps3
Archaea3 - ps4
Asgard/Eukaryota - ps5
Eukaryota - ps6
Amorpheae - ps7
Obazoa - ps8
Opisthokonta - ps9
Holozoa - ps10
Filozoa - ps11
Choano./Metazoa - ps12
Metazoa - ps13
Myriazoa - ps14
Parahoxozoa - ps15
Bilateria - ps16
Protostomia - ps17
Ecdysozoa - ps18
Arthropoda - ps19
Mandibulata - ps20
Pancrustacea - ps21
Insecta - ps22
Eumetabola - ps23
Holometabola/Psocodea - ps24
Holometabola - ps25
Aparaglossata - ps26
Mecopterida - ps27
Diptera - ps28
Bibiono./Cyclorrhapha - ps29
Cyclorrhapha - ps30
Calyptratae/Drosophila - ps31
Drosophila - ps32
Sophophora - ps33
Obscura/Melanogaster_group - ps34
D_melanogaster_group - ps35
D_melanogaster_subgroup - ps36
sim./sech./mel. group - ps37
Drosophila_melanogaster - ps38
Phylostratum

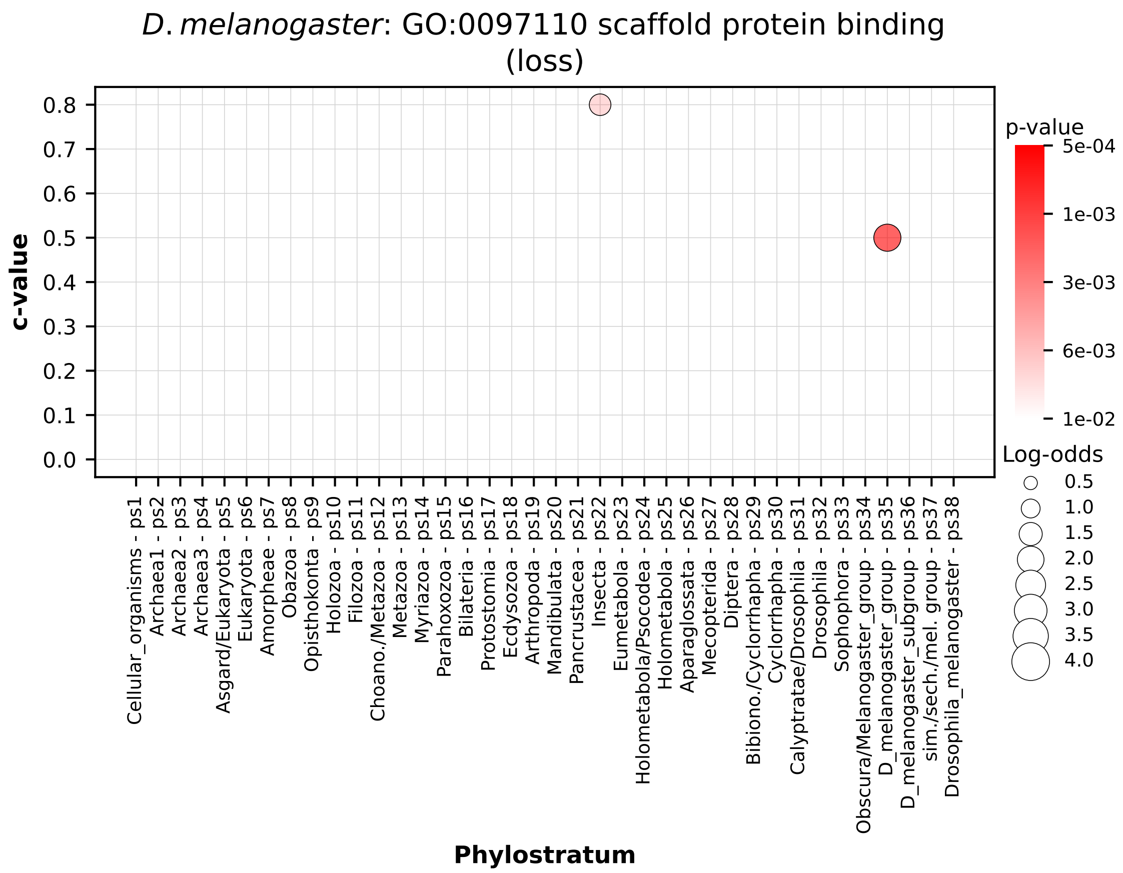

D. melanogaster: GO:0097110 scaffold protein binding
(loss)
c-value
0.0
0.1
0.2
0.3
0.4
0.5
0.6
0.7
0.8
Phylostratum
Cellular_organisms - ps1
Archaea1 - ps2
Archaea2 - ps3
Archaea3 - ps4
Asgard/Eukaryota - ps5
Eukaryota - ps6
Amorpheae - ps7
Obazoa - ps8
Opisthokonta - ps9
Holozoa - ps10
Filozoa - ps11
Choano./Metazoa - ps12
Metazoa - ps13
Myriazoa - ps14
Parahoxozoa - ps15
Bilateria - ps16
Protostomia - ps17
Ecdysozoa - ps18
Arthropoda - ps19
Mandibulata - ps20
Pancrustacea - ps21
Insecta - ps22
Eumetabola - ps23
Holometabola/Psocodea - ps24
Holometabola - ps25
Aparaglossata - ps26
Mecopterida - ps27
Diptera - ps28
Bibiono./Cyclorrhapha - ps29
Cyclorrhapha - ps30
Calyptratae/Drosophila - ps31
Drosophila - ps32
Sophophora - ps33
Obscura/Melanogaster_group - ps34
D_melanogaster_group - ps35
D_melanogaster_subgroup - ps36
sim./sech./mel. group - ps37
Drosophila_melanogaster - ps38
p-value
5e-04
1e-03
3e-03
6e-03
1e-02
Log-odds
0.5
1.0
1.5
2.0
2.5
3.0
3.5
4.0

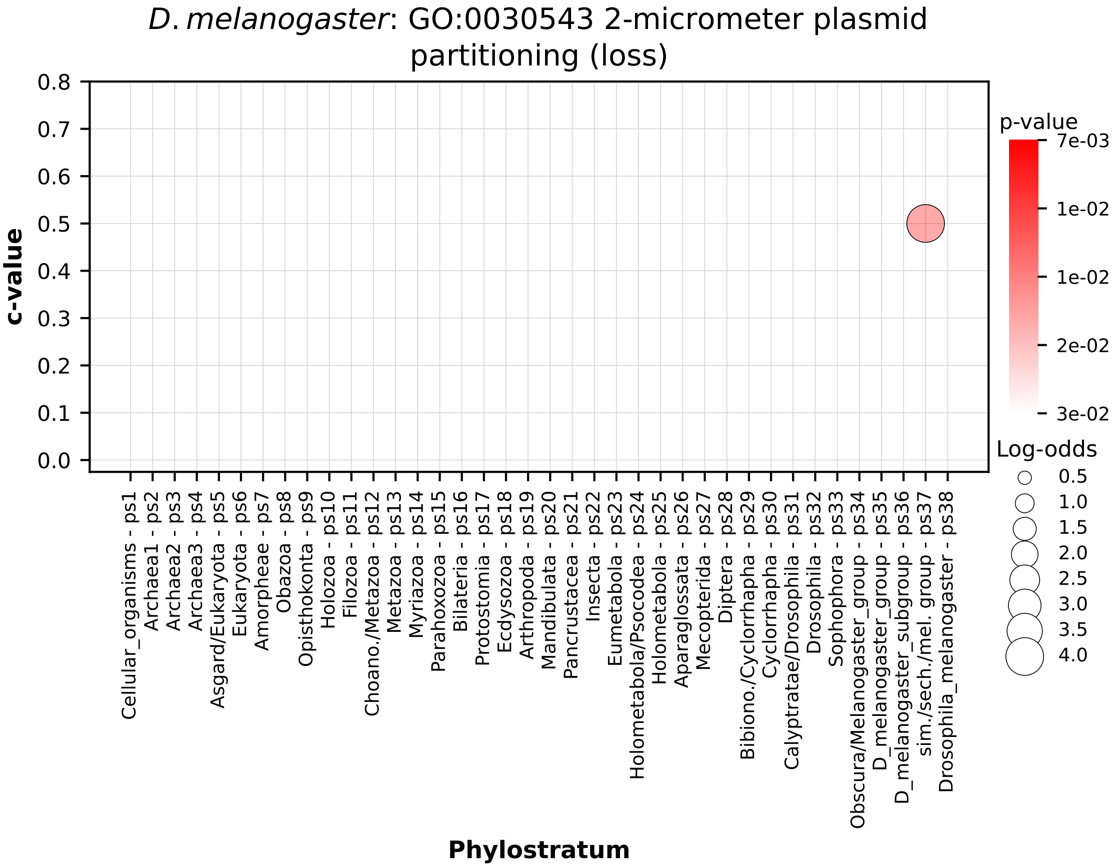
D. melanogaster: GO:0030543 2-micrometer plasmid partitioning (loss)
c-value
0.8
0.7
0.6
0.5
0.4
0.3
0.2
0.1
0.0
p-value
7e-03
1e-02
1e-02
2e-02
3e-02
Log-odds
0.5
1.0
1.5
2.0
2.5
3.0
3.5
4.0
Cellular_organisms - ps1
Archaea1 - ps2
Archaea2 - ps3
Archaea3 - ps4
Asgard/Eukaryota - ps5
Eukaryota - ps6
Amorpheae - ps7
Obazoa - ps8
Opisthokonta - ps9
Holozoa - ps10
Filozoa - ps11
Choano./Metazoa - ps12
Metazoa - ps13
Myriazoa - ps14
Parahoxozoa - ps15
Bilateria - ps16
Protostomia - ps17
Ecdysozoa - ps18
Arthropoda - ps19
Mandibulata - ps20
Pancrustacea - ps21
Insecta - ps22
Eumetabola - ps23
Holometabola/Psocodea - ps24
Holometabola - ps25
Aparaglossata - ps26
Mecopterida - ps27
Diptera - ps28
Bibiono./Cyclorrhapha - ps29
Cyclorrhapha - ps30
Calyptratae/Drosophila - ps31
Drosophila - ps32
Sophophora - ps33
Obscura/Melanogaster_group - ps34
D_melanogaster_group - ps35
D_melanogaster_subgroup - ps36
sim./sech./mel. group - ps37
Drosophila_melanogaster - ps38
Phylostratum

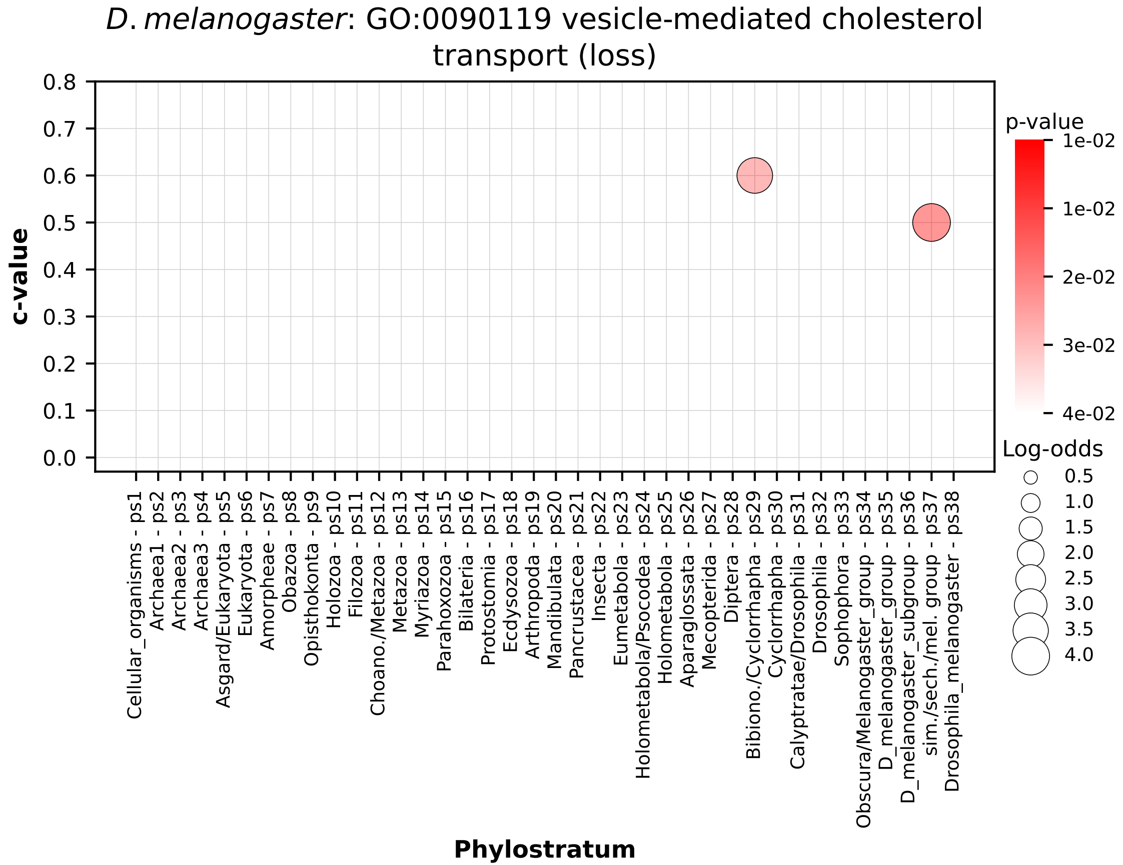

D. melanogaster: GO:0090119 vesicle-mediated cholesterol transport (loss)
c-value
0.8
0.7
0.6
0.5
0.4
0.3
0.2
0.1
0.0
Phylostratum
Cellular_organisms - ps1
Archaea1 - ps2
Archaea2 - ps3
Archaea3 - ps4
Asgard/Eukaryota - ps5
Eukaryota - ps6
Amorpheae - ps7
Obazoa - ps8
Opisthokonta - ps9
Holozoa - ps10
Filozoa - ps11
Choano./Metazoa - ps12
Metazoa - ps13
Myriazoa - ps14
Parahoxozoa - ps15
Bilateria - ps16
Protostomia - ps17
Ecdysozoa - ps18
Arthropoda - ps19
Mandibulata - ps20
Pancrustacea - ps21
Insecta - ps22
Eumetabola - ps23
Holometabola/Psocodea - ps24
Holometabola - ps25
Aparaglossata - ps26
Mecopterida - ps27
Diptera - ps28
Bibiono./Cyclorrhapha - ps29
Cyclorrhapha - ps30
Calyptratae/Drosophila - ps31
Drosophila - ps32
Sophophora - ps33
Obscura/Melanogaster_group - ps34
D_melanogaster_group - ps35
D_melanogaster_subgroup - ps36
sim./sech./mel. group - ps37
Drosophila_melanogaster - ps38
p-value
1e-02
1e-02
2e-02
3e-02
4e-02
Log-odds
0.5
1.0
1.5
2.0
2.5
3.0
3.5
4.0

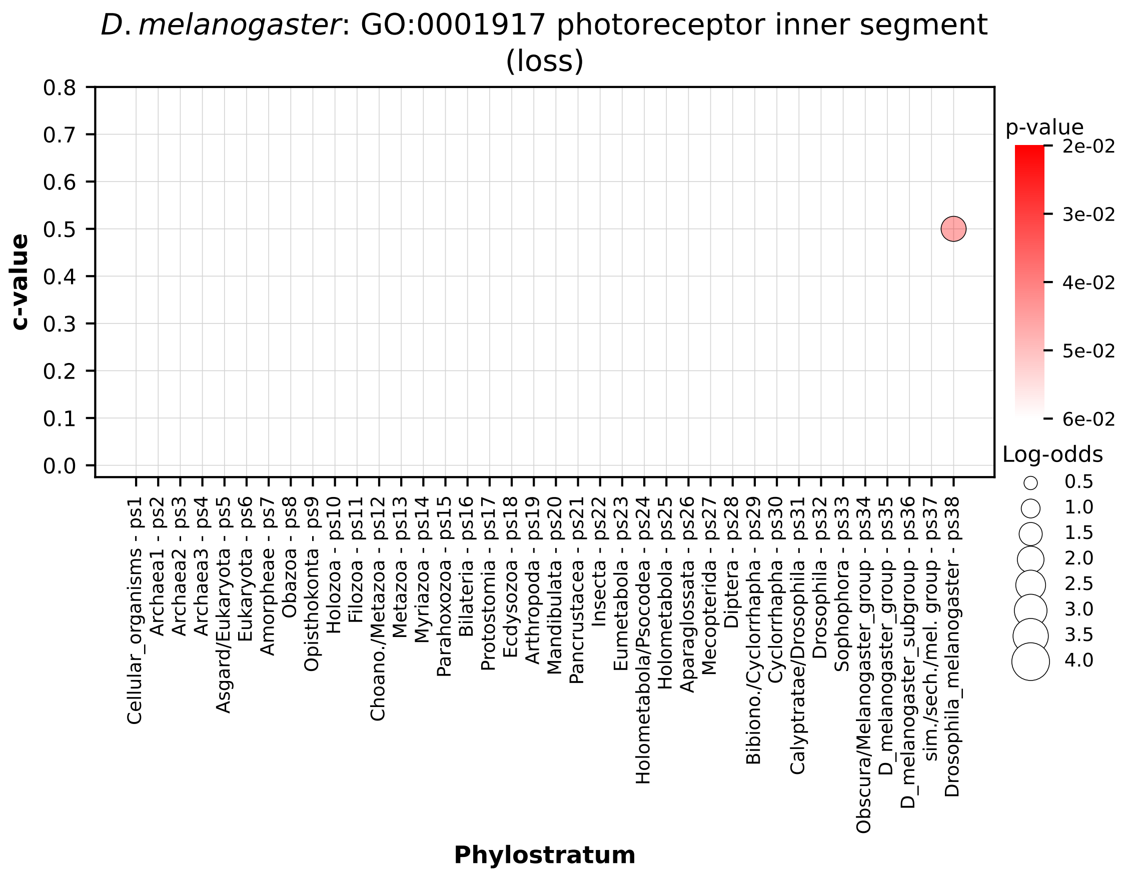

D. melanogaster: GO:0001917 photoreceptor inner segment (loss)
c-value
0.8
0.7
0.6
0.5
0.4
0.3
0.2
0.1
0.0
p-value
2e-02
3e-02
4e-02
5e-02
6e-02
Log-odds
0.5
1.0
1.5
2.0
2.5
3.0
3.5
4.0
Cellular_organisms - ps1
Archaea1 - ps2
Archaea2 - ps3
Archaea3 - ps4
Asgard/Eukaryota - ps5
Eukaryota - ps6
Amorpheae - ps7
Obazoa - ps8
Opisthokonta - ps9
Holozoa - ps10
Filozoa - ps11
Choano./Metazoa - ps12
Metazoa - ps13
Myriazoa - ps14
Parahoxozoa - ps15
Bilateria - ps16
Protostomia - ps17
Ecdysozoa - ps18
Arthropoda - ps19
Mandibulata - ps20
Pancrustacea - ps21
Insecta - ps22
Eumetabola - ps23
Holometabola/Psocodea - ps24
Holometabola - ps25
Aparaglossata - ps26
Mecopterida - ps27
Diptera - ps28
Bibiono./Cyclorrhapha - ps29
Cyclorrhapha - ps30
Calyptratae/Drosophila - ps31
Drosophila - ps32
Sophophora - ps33
Obscura/Melanogaster_group - ps34
D_melanogaster_group - ps35
D_melanogaster_subgroup - ps36
sim./sech./mel. group - ps37
Drosophila_melanogaster - ps38
Phylostratum

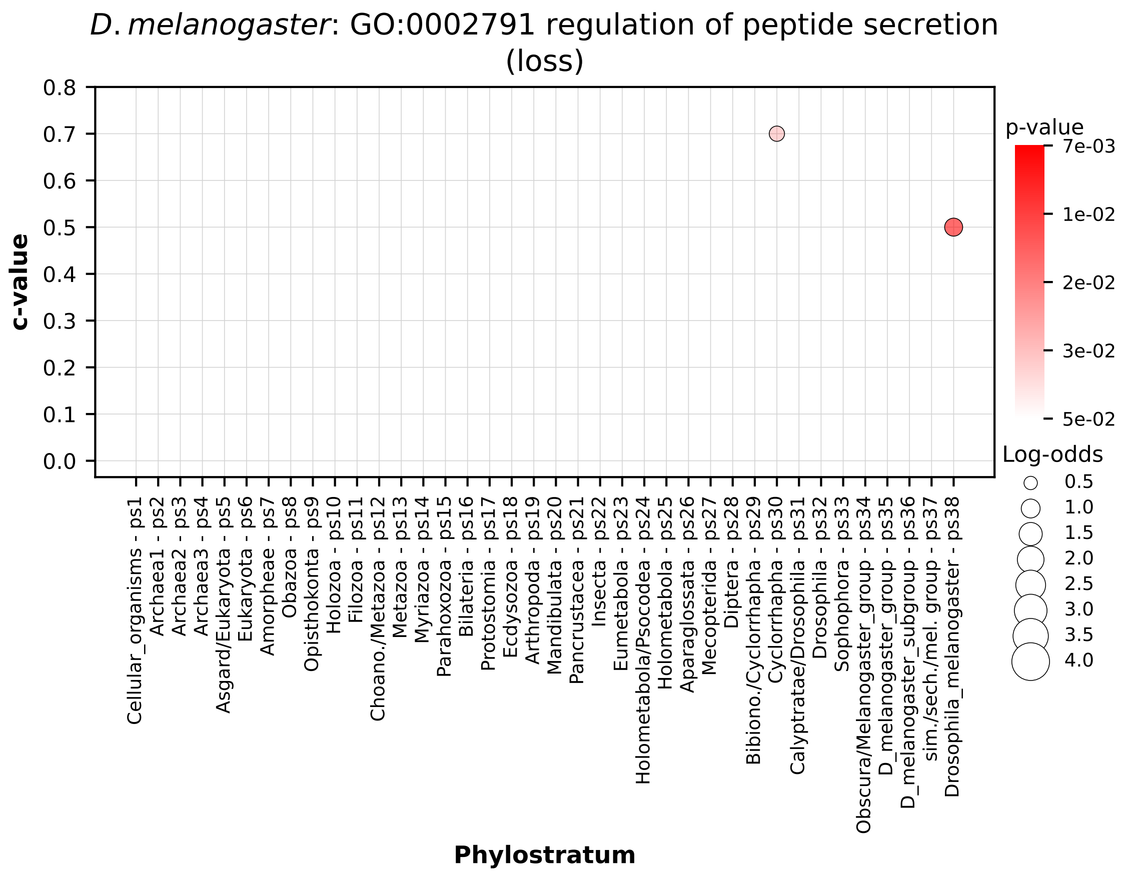

D. melanogaster: GO:0002791 regulation of peptide secretion (loss)
c-value
0.8
0.7
0.6
0.5
0.4
0.3
0.2
0.1
0.0
p-value
7e-03
1e-02
2e-02
3e-02
5e-02
Log-odds
0.5
1.0
1.5
2.0
2.5
3.0
3.5
4.0
Cellular_organisms - ps1
Archaea1 - ps2
Archaea2 - ps3
Archaea3 - ps4
Asgard/Eukaryota - ps5
Eukaryota - ps6
Amorpheae - ps7
Obazoa - ps8
Opisthokonta - ps9
Holozoa - ps10
Filozoa - ps11
Choano./Metazoa - ps12
Metazoa - ps13
Myriazoa - ps14
Parahoxozoa - ps15
Bilateria - ps16
Protostomia - ps17
Ecdysozoa - ps18
Arthropoda - ps19
Mandibulata - ps20
Pancrustacea - ps21
Insecta - ps22
Eumetabola - ps23
Holometabola/Psocodea - ps24
Holometabola - ps25
Aparaglossata - ps26
Mecopterida - ps27
Diptera - ps28
Bibiono./Cyclorrhapha - ps29
Cyclorrhapha - ps30
Calyptratae/Drosophila - ps31
Drosophila - ps32
Sophophora - ps33
Obscura/Melanogaster_group - ps34
D_melanogaster_group - ps35
D_melanogaster_subgroup - ps36
sim./sech./mel. group - ps37
Drosophila_melanogaster - ps38
Phylostratum

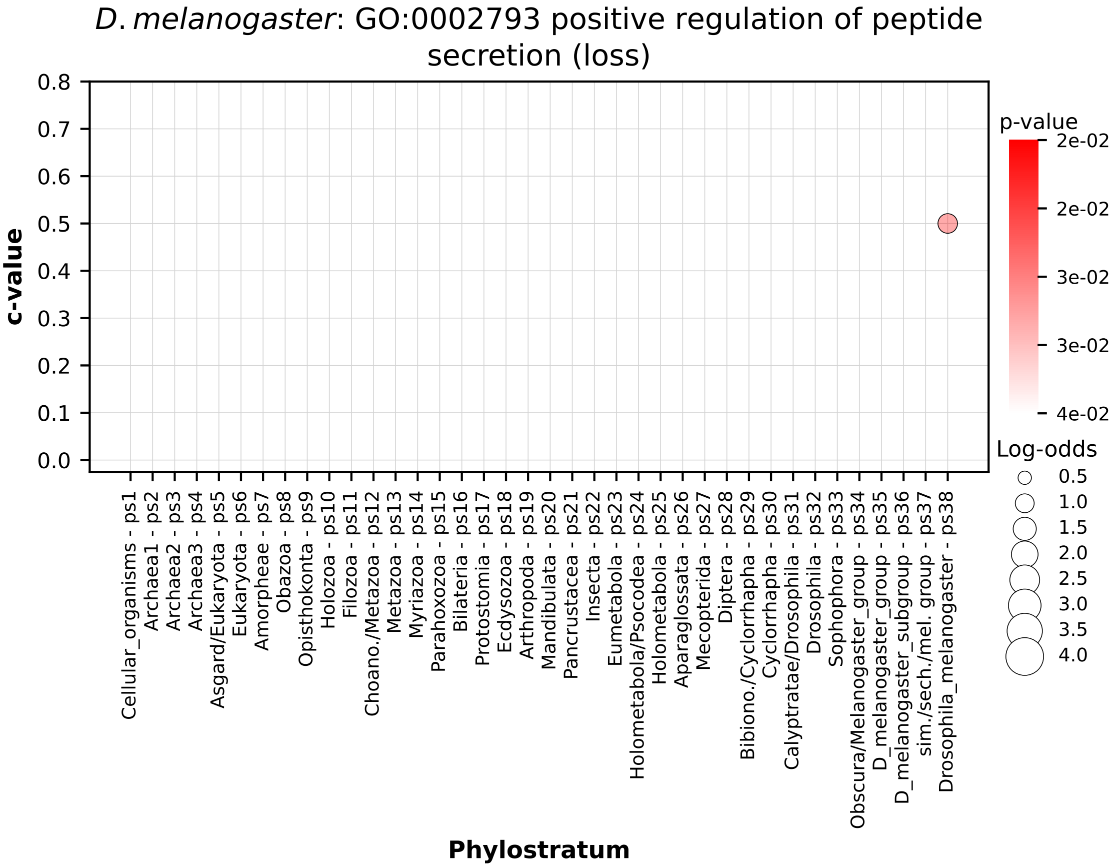

D. melanogaster: GO:0002793 positive regulation of peptide secretion (loss)
c-value
0.8
0.7
0.6
0.5
0.4
0.3
0.2
0.1
0.0
p-value
2e-02
2e-02
3e-02
3e-02
4e-02
Log-odds
0.5
1.0
1.5
2.0
2.5
3.0
3.5
4.0
Cellular_organisms - ps1
Archaea1 - ps2
Archaea2 - ps3
Archaea3 - ps4
Asgard/Eukaryota - ps5
Eukaryota - ps6
Amorpheae - ps7
Obazoa - ps8
Opisthokonta - ps9
Holozoa - ps10
Filozoa - ps11
Choano./Metazoa - ps12
Metazoa - ps13
Myriazoa - ps14
Parahoxozoa - ps15
Bilateria - ps16
Protostomia - ps17
Ecdysozoa - ps18
Arthropoda - ps19
Mandibulata - ps20
Pancrustacea - ps21
Insecta - ps22
Eumetabola - ps23
Holometabola/Psocodea - ps24
Holometabola - ps25
Aparaglossata - ps26
Mecopterida - ps27
Diptera - ps28
Bibiono./Cyclorrhapha - ps29
Cyclorrhapha - ps30
Calyptratae/Drosophila - ps31
Drosophila - ps32
Sophophora - ps33
Obscura/Melanogaster_group - ps34
D_melanogaster_group - ps35
D_melanogaster_subgroup - ps36
sim./sech./mel. group - ps37
Drosophila_melanogaster - ps38
Phylostratum

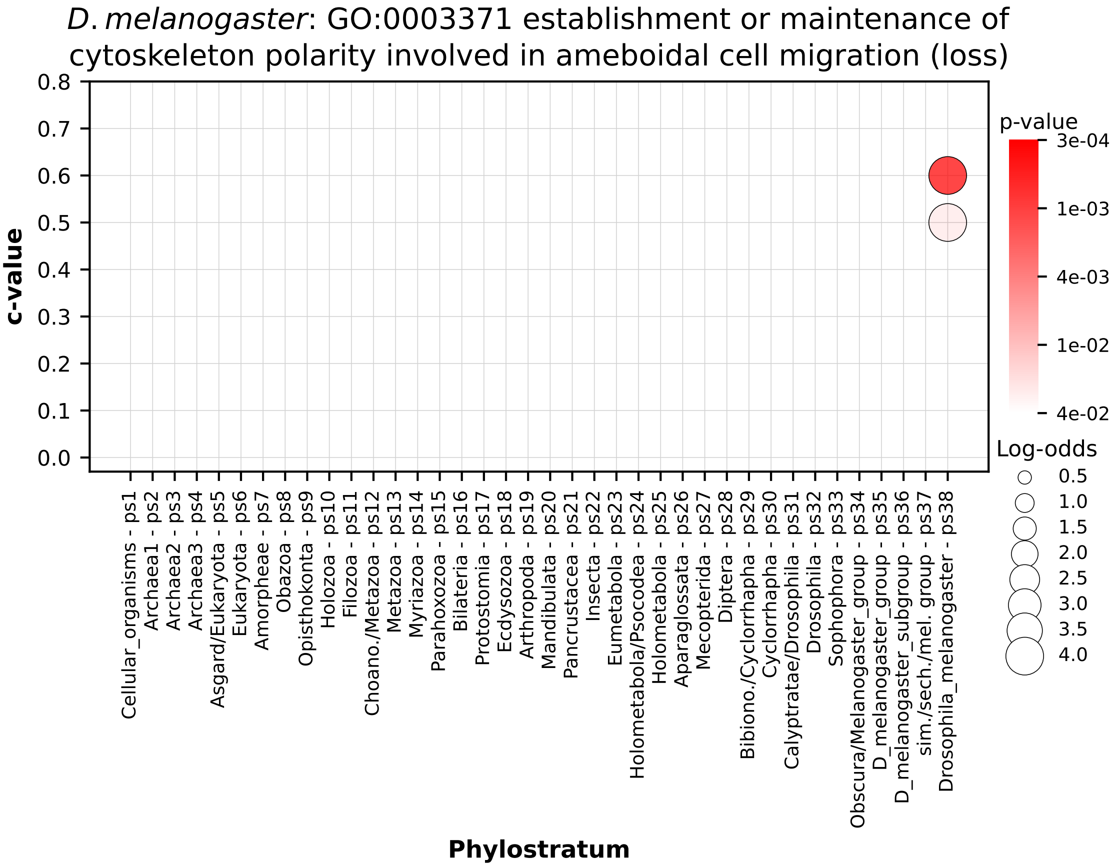

D. melanogaster: GO:0003371 establishment or maintenance of cytoskeleton polarity involved in ameboidal cell migration (loss)
c-value
0.0
0.1
0.2
0.3
0.4
0.5
0.6
0.7
0.8
p-value
3e-04
1e-03
4e-03
1e-02
4e-02
Log-odds
0.5
1.0
1.5
2.0
2.5
3.0
3.5
4.0
Cellular_organisms - ps1
Archaea1 - ps2
Archaea2 - ps3
Archaea3 - ps4
Asgard/Eukaryota - ps5
Eukaryota - ps6
Amorpheae - ps7
Obazoa - ps8
Opisthokonta - ps9
Holozoa - ps10
Filozoa - ps11
Choano./Metazoa - ps12
Metazoa - ps13
Myriazoa - ps14
Parahoxozoa - ps15
Bilateria - ps16
Protostomia - ps17
Ecdysozoa - ps18
Arthropoda - ps19
Mandibulata - ps20
Pancrustacea - ps21
Insecta - ps22
Eumetabola - ps23
Holometabola/Psocodea - ps24
Holometabola - ps25
Aparaglossata - ps26
Mecopterida - ps27
Diptera - ps28
Bibiono./Cyclorrhapha - ps29
Cyclorrhapha - ps30
Calyptratae/Drosophila - ps31
Drosophila - ps32
Sophophora - ps33
Obscura/Melanogaster_group - ps34
D_melanogaster_group - ps35
D_melanogaster_subgroup - ps36
sim./sech./mel. group - ps37
Drosophila_melanogaster - ps38
Phylostratum

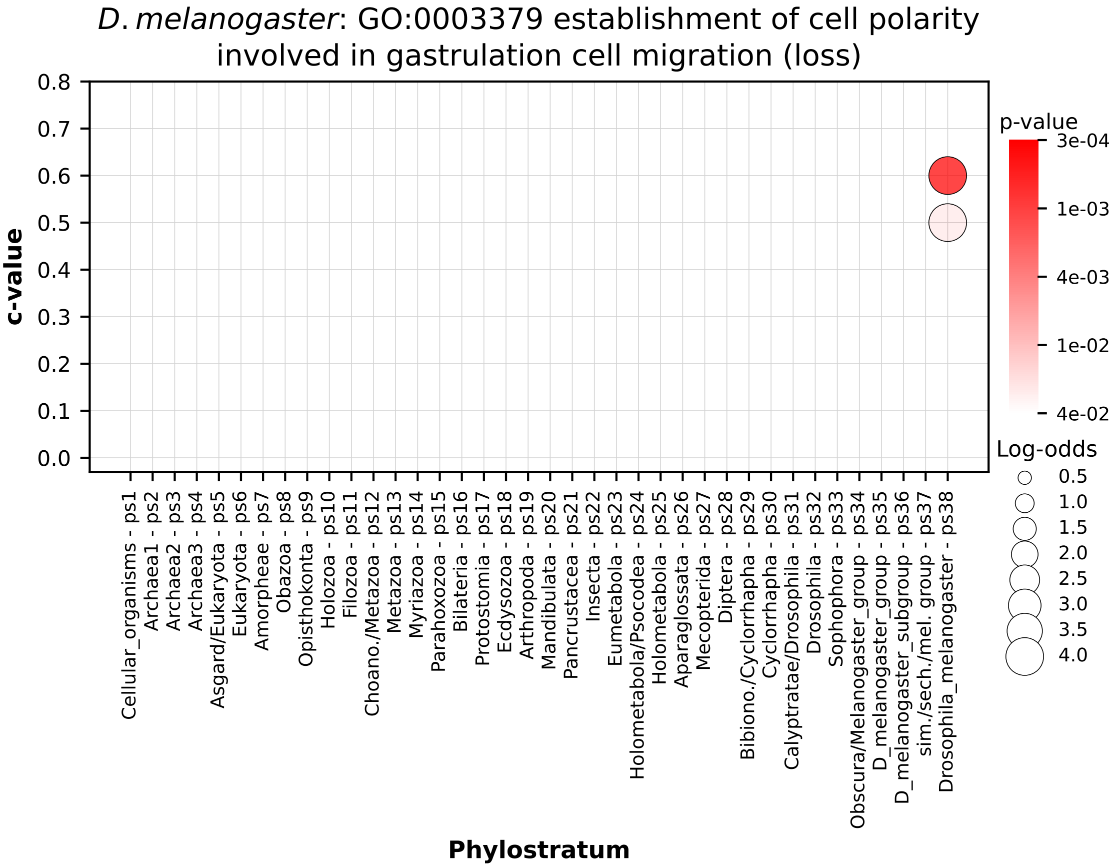
D. melanogaster: GO:0003379 establishment of cell polarity involved in gastrulation cell migration (loss)
c-value
0.8
0.7
0.6
0.5
0.4
0.3
0.2
0.1
0.0
Phylostratum
Cellular_organisms - ps1
Archaea1 - ps2
Archaea2 - ps3
Archaea3 - ps4
Asgard/Eukaryota - ps5
Eukaryota - ps6
Amorpheae - ps7
Obazoa - ps8
Opisthokonta - ps9
Holozoa - ps10
Filozoa - ps11
Choano./Metazoa - ps12
Metazoa - ps13
Myriazoa - ps14
Parahoxozoa - ps15
Bilateria - ps16
Protostomia - ps17
Ecdysozoa - ps18
Arthropoda - ps19
Mandibulata - ps20
Pancrustacea - ps21
Insecta - ps22
Eumetabola - ps23
Holometabola/Psocodea - ps24
Holometabola - ps25
Aparaglossata - ps26
Mecopterida - ps27
Diptera - ps28
Bibiono./Cyclorrhapha - ps29
Cyclorrhapha - ps30
Calyptratae/Drosophila - ps31
Drosophila - ps32
Sophophora - ps33
Obscura/Melanogaster_group - ps34
D_melanogaster_group - ps35
D_melanogaster_subgroup - ps36
sim./sech./mel. group - ps37
Drosophila_melanogaster - ps38
p-value
3e-04
1e-03
4e-03
1e-02
4e-02
Log-odds
0.5
1.0
1.5
2.0
2.5
3.0
3.5
4.0

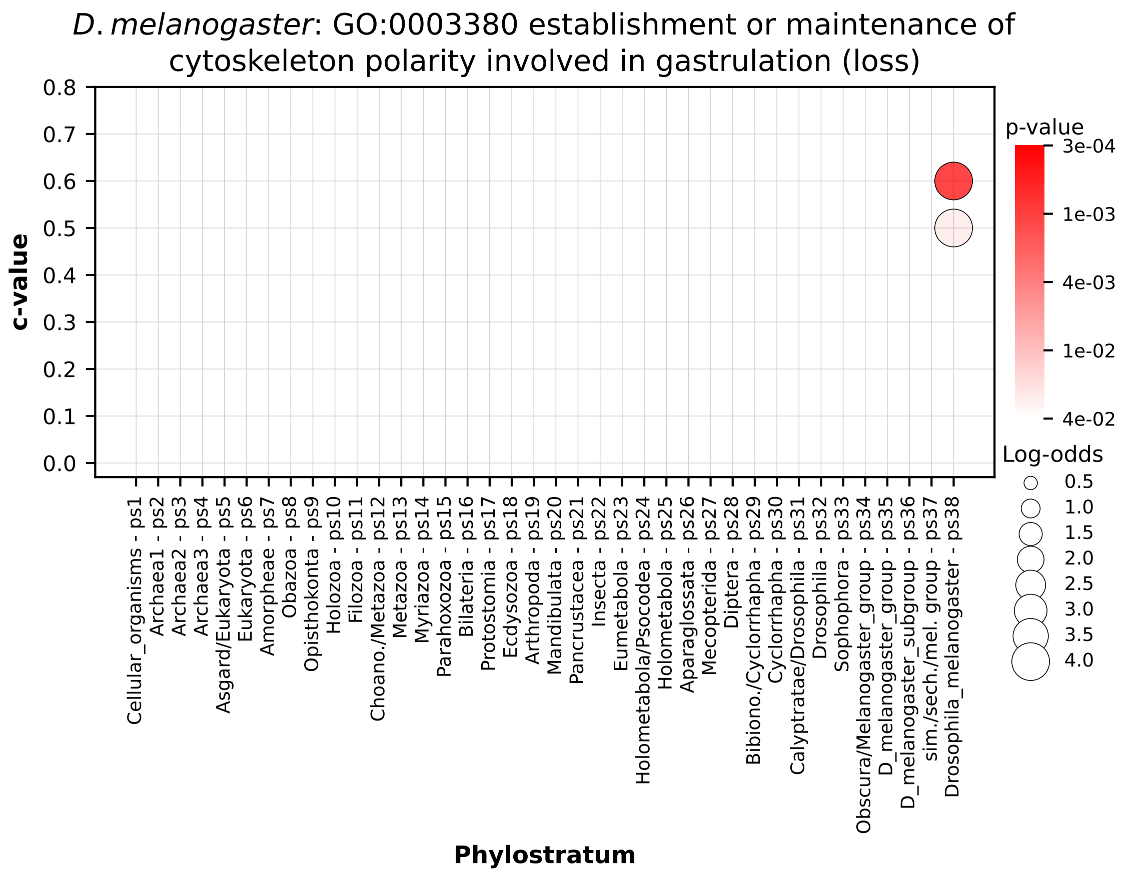

D. melanogaster: GO:0003380 establishment or maintenance of cytoskeleton polarity involved in gastrulation (loss)
c-value
0.8
0.7
0.6
0.5
0.4
0.3
0.2
0.1
0.0
p-value
3e-04
1e-03
4e-03
1e-02
4e-02
Log-odds
0.5
1.0
1.5
2.0
2.5
3.0
3.5
4.0
Cellular_organisms - ps1
Archaea1 - ps2
Archaea2 - ps3
Archaea3 - ps4
Asgard/Eukaryota - ps5
Eukaryota - ps6
Amorpheae - ps7
Obazoa - ps8
Opisthokonta - ps9
Holozoa - ps10
Filozoa - ps11
Choano./Metazoa - ps12
Metazoa - ps13
Myriazoa - ps14
Parahoxozoa - ps15
Bilateria - ps16
Protostomia - ps17
Ecdysozoa - ps18
Arthropoda - ps19
Mandibulata - ps20
Pancrustacea - ps21
Insecta - ps22
Eumetabola - ps23
Holometabola/Psocodea - ps24
Holometabola - ps25
Aparaglossata - ps26
Mecopterida - ps27
Diptera - ps28
Bibiono./Cyclorrhapha - ps29
Cyclorrhapha - ps30
Calyptratae/Drosophila - ps31
Drosophila - ps32
Sophophora - ps33
Obscura/Melanogaster_group - ps34
D_melanogaster_group - ps35
D_melanogaster_subgroup - ps36
sim./sech./mel. group - ps37
Drosophila_melanogaster - ps38
Phylostratum

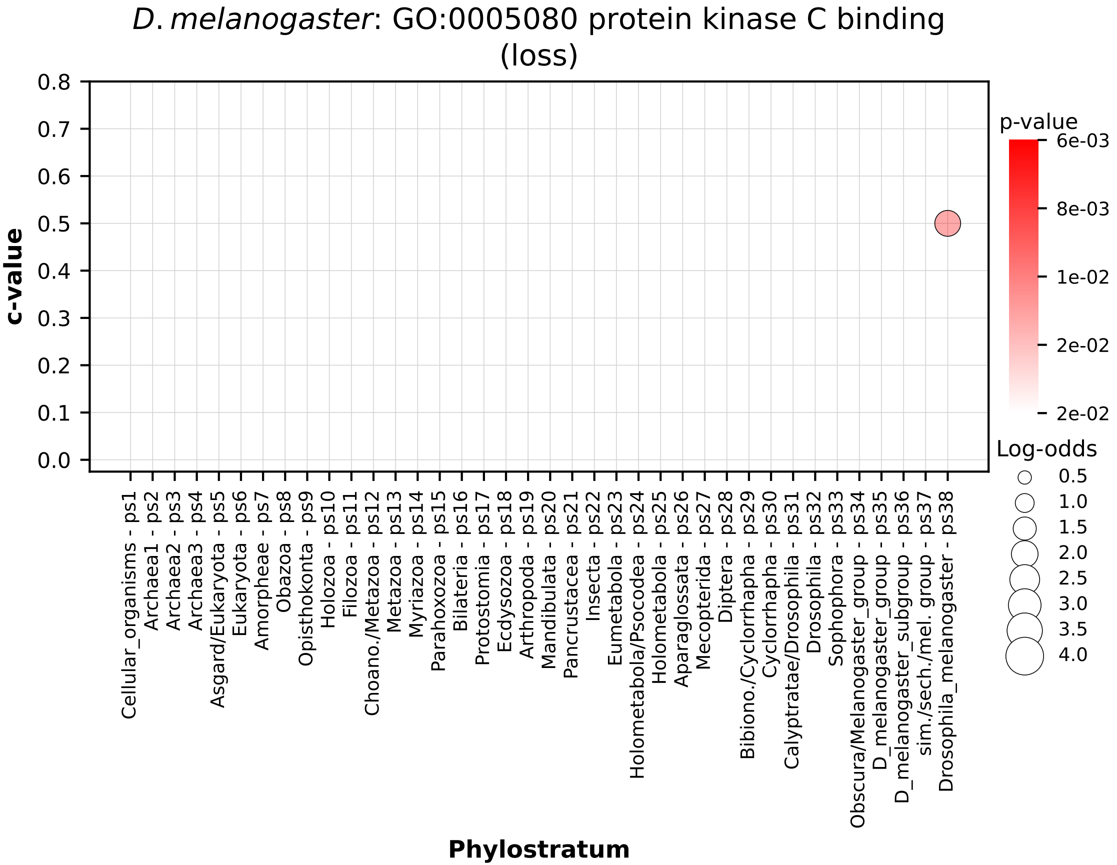

D. melanogaster: GO:0005080 protein kinase C binding (loss)
c-value
0.8
0.7
0.6
0.5
0.4
0.3
0.2
0.1
0.0
p-value
6e-03
8e-03
1e-02
2e-02
2e-02
Log-odds
0.5
1.0
1.5
2.0
2.5
3.0
3.5
4.0
Cellular_organisms - ps1
Archaea1 - ps2
Archaea2 - ps3
Archaea3 - ps4
Asgard/Eukaryota - ps5
Eukaryota - ps6
Amorpheae - ps7
Obazoa - ps8
Opisthokonta - ps9
Holozoa - ps10
Filozoa - ps11
Choano./Metazoa - ps12
Metazoa - ps13
Myriazoa - ps14
Parahoxozoa - ps15
Bilateria - ps16
Protostomia - ps17
Ecdysozoa - ps18
Arthropoda - ps19
Mandibulata - ps20
Pancrustacea - ps21
Insecta - ps22
Eumetabola - ps23
Holometabola/Psocodea - ps24
Holometabola - ps25
Aparaglossata - ps26
Mecopterida - ps27
Diptera - ps28
Bibiono./Cyclorrhapha - ps29
Cyclorrhapha - ps30
Calyptratae/Drosophila - ps31
Drosophila - ps32
Sophophora - ps33
Obscura/Melanogaster_group - ps34
D_melanogaster_group - ps35
D_melanogaster_subgroup - ps36
sim./sech./mel. group - ps37
Drosophila_melanogaster - ps38
Phylostratum

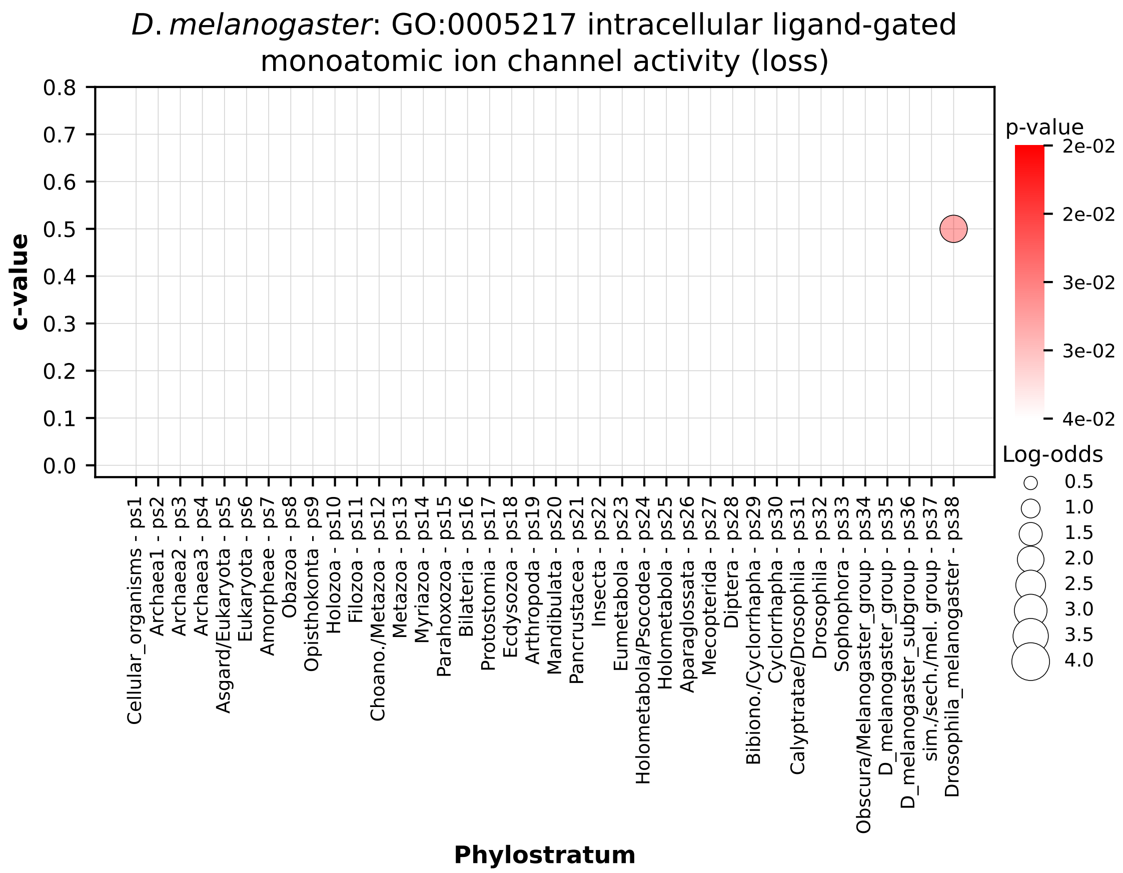

D. melanogaster: GO:0005217 intracellular ligand-gated monoatomic ion channel activity (loss)
c-value
0.0
0.1
0.2
0.3
0.4
0.5
0.6
0.7
0.8
p-value
2e-02
2e-02
3e-02
3e-02
4e-02
Log-odds
0.5
1.0
1.5
2.0
2.5
3.0
3.5
4.0
Cellular_organisms - ps1
Archaea1 - ps2
Archaea2 - ps3
Archaea3 - ps4
Asgard/Eukaryota - ps5
Eukaryota - ps6
Amorpheae - ps7
Obazoa - ps8
Opisthokonta - ps9
Holozoa - ps10
Filozoa - ps11
Choano./Metazoa - ps12
Metazoa - ps13
Myriazoa - ps14
Parahoxozoa - ps15
Bilateria - ps16
Protostomia - ps17
Ecdysozoa - ps18
Arthropoda - ps19
Mandibulata - ps20
Pancrustacea - ps21
Insecta - ps22
Eumetabola - ps23
Holometabola/Psocodea - ps24
Holometabola - ps25
Aparaglossata - ps26
Mecopterida - ps27
Diptera - ps28
Bibiono./Cyclorrhapha - ps29
Cyclorrhapha - ps30
Calyptratae/Drosophila - ps31
Drosophila - ps32
Sophophora - ps33
Obscura/Melanogaster_group - ps34
D_melanogaster_group - ps35
D_melanogaster_subgroup - ps36
sim./sech./mel. group - ps37
Drosophila_melanogaster - ps38
Phylostratum

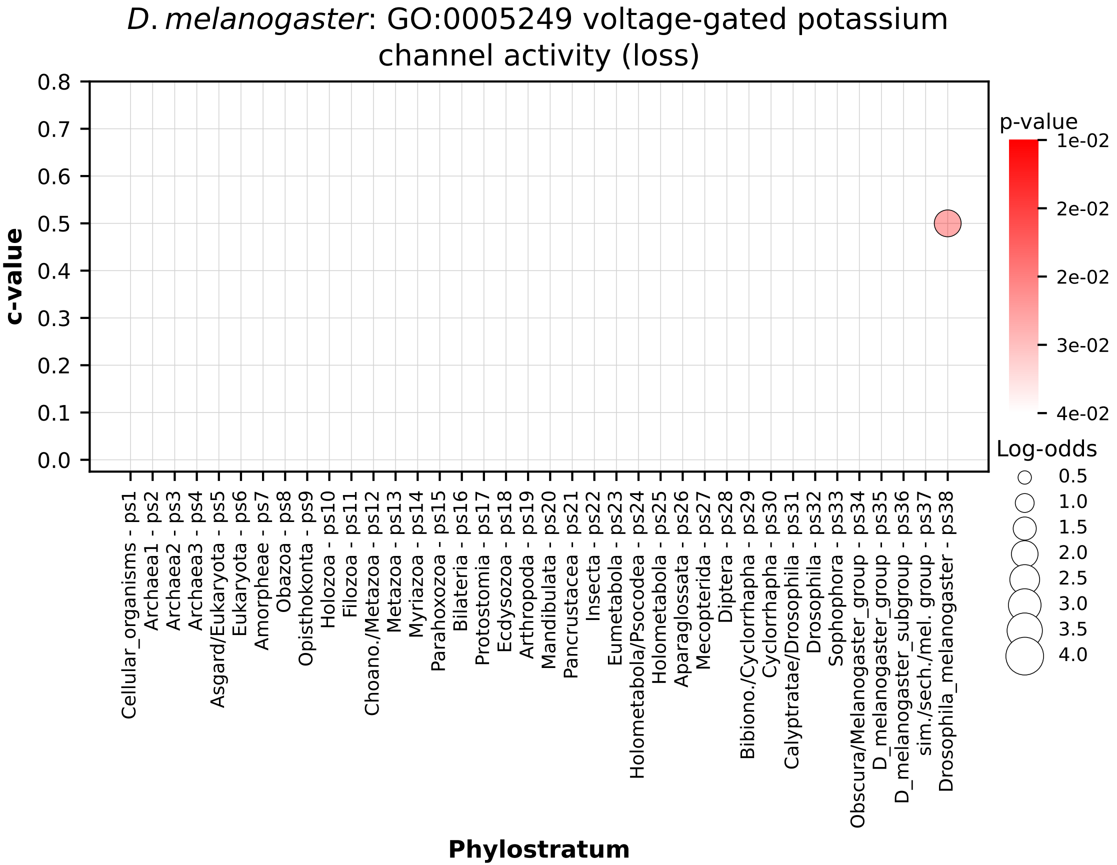

D. melanogaster: GO:0005249 voltage-gated potassium channel activity (loss)
c-value
0.8
0.7
0.6
0.5
0.4
0.3
0.2
0.1
0.0
p-value
1e-02
2e-02
2e-02
3e-02
4e-02
Log-odds
0.5
1.0
1.5
2.0
2.5
3.0
3.5
4.0
Cellular_organisms - ps1
Archaea1 - ps2
Archaea2 - ps3
Archaea3 - ps4
Asgard/Eukaryota - ps5
Eukaryota - ps6
Amorpheae - ps7
Obazoa - ps8
Opisthokonta - ps9
Holozoa - ps10
Filozoa - ps11
Choano./Metazoa - ps12
Metazoa - ps13
Myriazoa - ps14
Parahoxozoa - ps15
Bilateria - ps16
Protostomia - ps17
Ecdysozoa - ps18
Arthropoda - ps19
Mandibulata - ps20
Pancrustacea - ps21
Insecta - ps22
Eumetabola - ps23
Holometabola/Psocodea - ps24
Holometabola - ps25
Aparaglossata - ps26
Mecopterida - ps27
Diptera - ps28
Bibiono./Cyclorrhapha - ps29
Cyclorrhapha - ps30
Calyptratae/Drosophila - ps31
Drosophila - ps32
Sophophora - ps33
Obscura/Melanogaster_group - ps34
D_melanogaster_group - ps35
D_melanogaster_subgroup - ps36
sim./sech./mel. group - ps37
Drosophila_melanogaster - ps38
Phylostratum

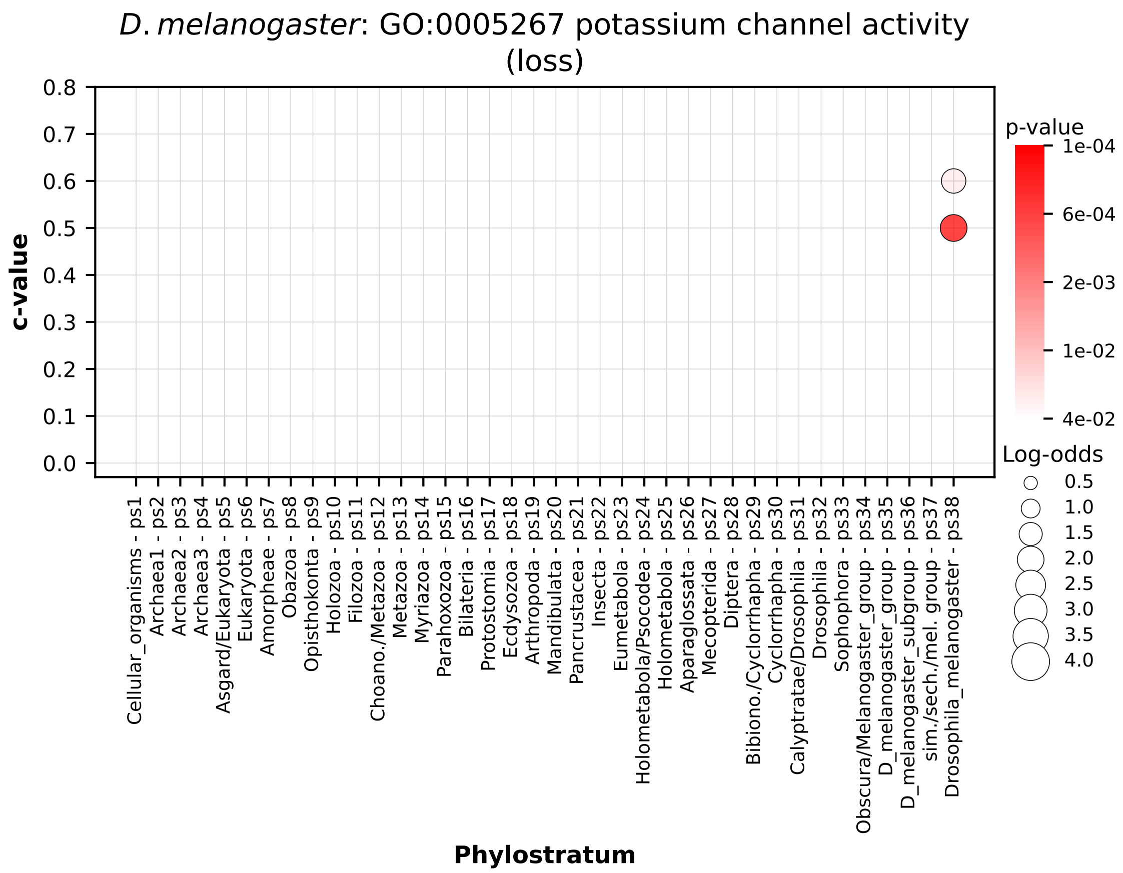
D. melanogaster: GO:0005267 potassium channel activity
(loss)
c-value
0.0
0.1
0.2
0.3
0.4
0.5
0.6
0.7
0.8
Phylostratum
Cellular_organisms - ps1
Archaea1 - ps2
Archaea2 - ps3
Archaea3 - ps4
Asgard/Eukaryota - ps5
Eukaryota - ps6
Amorpheae - ps7
Obazoa - ps8
Opisthokonta - ps9
Holozoa - ps10
Filozoa - ps11
Choano./Metazoa - ps12
Metazoa - ps13
Myriazoa - ps14
Parahoxozoa - ps15
Bilateria - ps16
Protostomia - ps17
Ecdysozoa - ps18
Arthropoda - ps19
Mandibulata - ps20
Pancrustacea - ps21
Insecta - ps22
Eumetabola - ps23
Holometabola/Psocodea - ps24
Holometabola - ps25
Aparaglossata - ps26
Mecopterida - ps27
Diptera - ps28
Bibiono./Cyclorrhapha - ps29
Cyclorrhapha - ps30
Calyptratae/Drosophila - ps31
Drosophila - ps32
Sophophora - ps33
Obscura/Melanogaster_group - ps34
D_melanogaster_group - ps35
D_melanogaster_subgroup - ps36
sim./sech./mel. group - ps37
Drosophila_melanogaster - ps38
p-value
1e-04
6e-04
2e-03
1e-02
4e-02
Log-odds
0.5
1.0
1.5
2.0
2.5
3.0
3.5
4.0

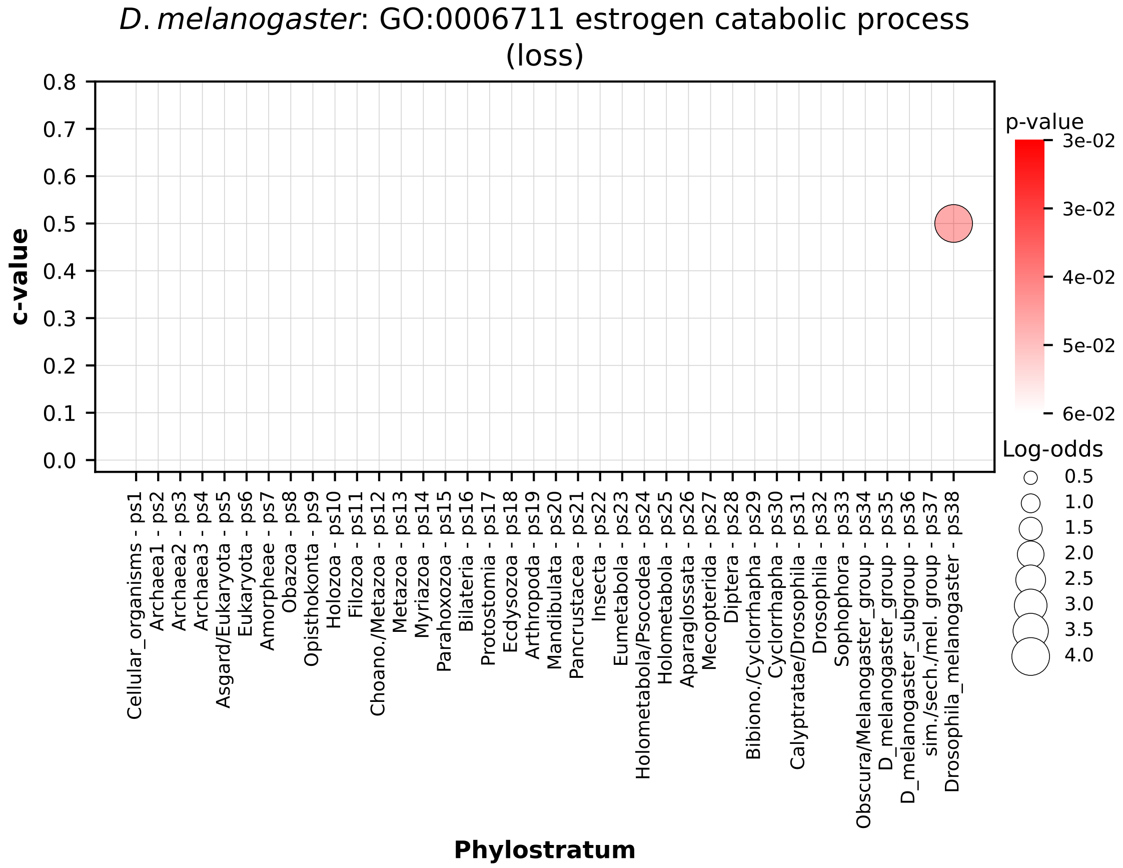
D. melanogaster: GO:0006711 estrogen catabolic process
(loss)
c-value
0.0
0.1
0.2
0.3
0.4
0.5
0.6
0.7
0.8
Phylostratum
Cellular_organisms - ps1
Archaea1 - ps2
Archaea2 - ps3
Archaea3 - ps4
Asgard/Eukaryota - ps5
Eukaryota - ps6
Amorpheae - ps7
Obazoa - ps8
Opisthokonta - ps9
Holozoa - ps10
Filozoa - ps11
Choano./Metazoa - ps12
Metazoa - ps13
Myriazoa - ps14
Parahoxozoa - ps15
Bilateria - ps16
Protostomia - ps17
Ecdysozoa - ps18
Arthropoda - ps19
Mandibulata - ps20
Pancrustacea - ps21
Insecta - ps22
Eumetabola - ps23
Holometabola/Psocodea - ps24
Holometabola - ps25
Aparaglossata - ps26
Mecopterida - ps27
Diptera - ps28
Bibiono./Cyclorrhapha - ps29
Cyclorrhapha - ps30
Calyptratae/Drosophila - ps31
Drosophila - ps32
Sophophora - ps33
Obscura/Melanogaster_group - ps34
D_melanogaster_group - ps35
D_melanogaster_subgroup - ps36
sim./sech./mel. group - ps37
Drosophila_melanogaster - ps38
p-value
3e-02
3e-02
4e-02
5e-02
6e-02
Log-odds
0.5
1.0
1.5
2.0
2.5
3.0
3.5
4.0

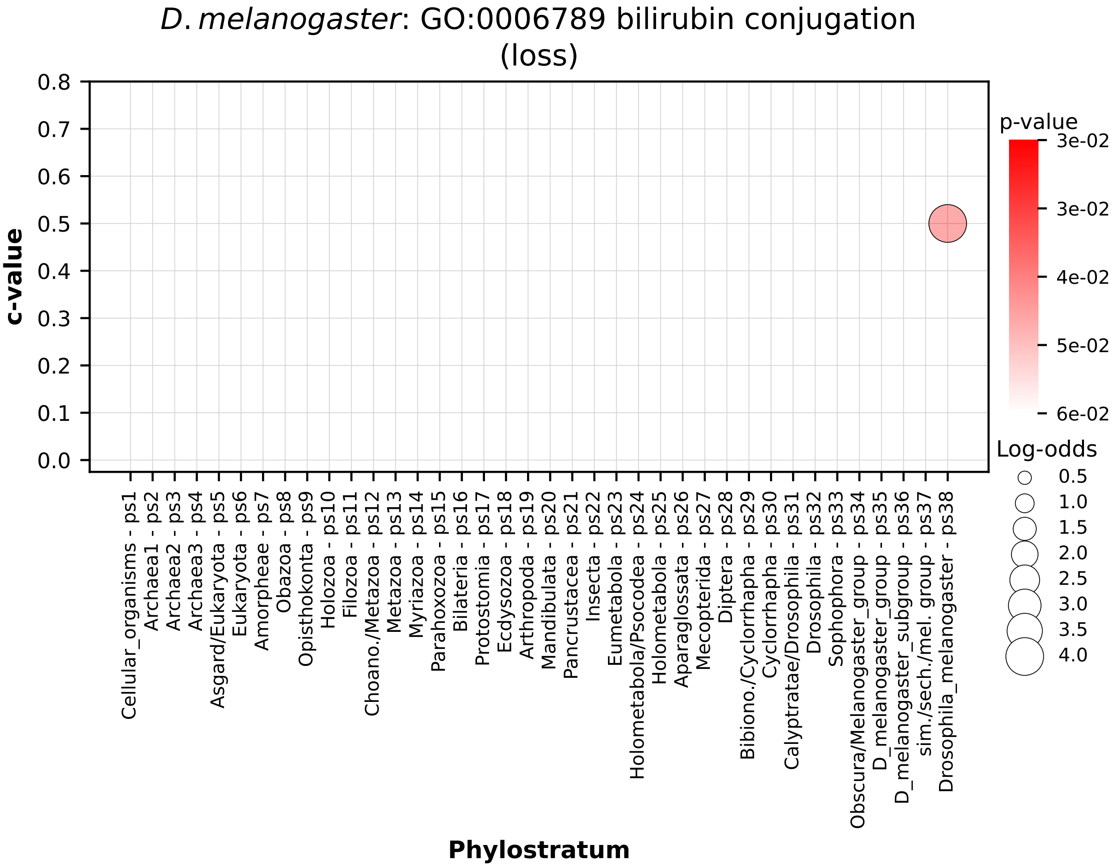

D. melanogaster: GO:0006789 bilirubin conjugation
(loss)
c-value
0.8
0.7
0.6
0.5
0.4
0.3
0.2
0.1
0.0
p-value
3e-02
3e-02
4e-02
5e-02
6e-02
Log-odds
0.5
1.0
1.5
2.0
2.5
3.0
3.5
4.0
Cellular_organisms - ps1
Archaea1 - ps2
Archaea2 - ps3
Archaea3 - ps4
Asgard/Eukaryota - ps5
Eukaryota - ps6
Amorpheae - ps7
Obazoa - ps8
Opisthokonta - ps9
Holozoa - ps10
Filozoa - ps11
Choano./Metazoa - ps12
Metazoa - ps13
Myriazoa - ps14
Parahoxozoa - ps15
Bilateria - ps16
Protostomia - ps17
Ecdysozoa - ps18
Arthropoda - ps19
Mandibulata - ps20
Pancrustacea - ps21
Insecta - ps22
Eumetabola - ps23
Holometabola/Psocodea - ps24
Holometabola - ps25
Aparaglossata - ps26
Mecopterida - ps27
Diptera - ps28
Bibiono./Cyclorrhapha - ps29
Cyclorrhapha - ps30
Calyptratae/Drosophila - ps31
Drosophila - ps32
Sophophora - ps33
Obscura/Melanogaster_group - ps34
D_melanogaster_group - ps35
D_melanogaster_subgroup - ps36
sim./sech./mel. group - ps37
Drosophila_melanogaster - ps38
Phylostratum

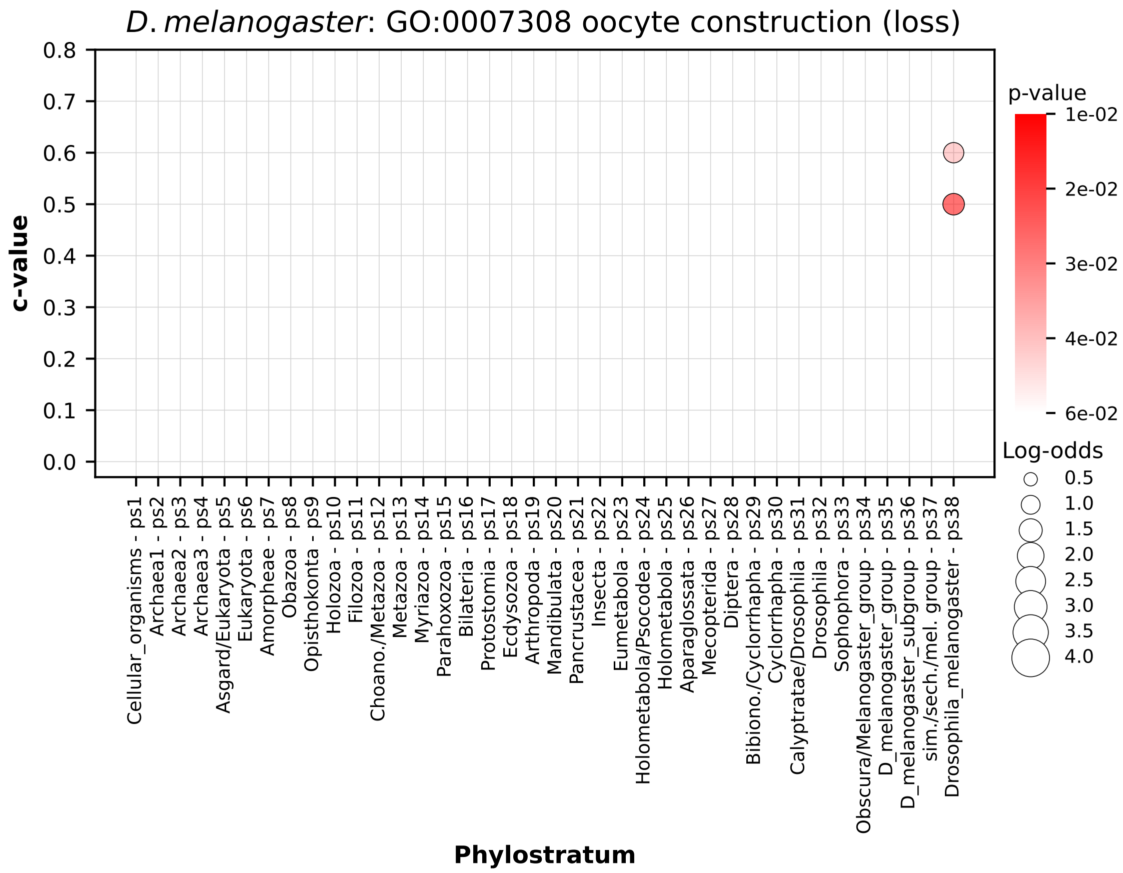

D. melanogaster: GO:0007308 oocyte construction (loss)
c-value
0.0
0.1
0.2
0.3
0.4
0.5
0.6
0.7
0.8
Phylostratum
Cellular_organisms - ps1
Archaea1 - ps2
Archaea2 - ps3
Archaea3 - ps4
Asgard/Eukaryota - ps5
Eukaryota - ps6
Amorpheae - ps7
Obazoa - ps8
Opisthokonta - ps9
Holozoa - ps10
Filozoa - ps11
Choano./Metazoa - ps12
Metazoa - ps13
Myriazoa - ps14
Parahoxozoa - ps15
Bilateria - ps16
Protostomia - ps17
Ecdysozoa - ps18
Arthropoda - ps19
Mandibulata - ps20
Pancrustacea - ps21
Insecta - ps22
Eumetabola - ps23
Holometabola/Psocodea - ps24
Holometabola - ps25
Aparaglossata - ps26
Mecopterida - ps27
Diptera - ps28
Bibiono./Cyclorrhapha - ps29
Cyclorrhapha - ps30
Calyptratae/Drosophila - ps31
Drosophila - ps32
Sophophora - ps33
Obscura/Melanogaster_group - ps34
D_melanogaster_group - ps35
D_melanogaster_subgroup - ps36
sim./sech./mel. group - ps37
Drosophila_melanogaster - ps38
p-value
1e-02
2e-02
3e-02
4e-02
6e-02
Log-odds
0.5
1.0
1.5
2.0
2.5
3.0
3.5
4.0

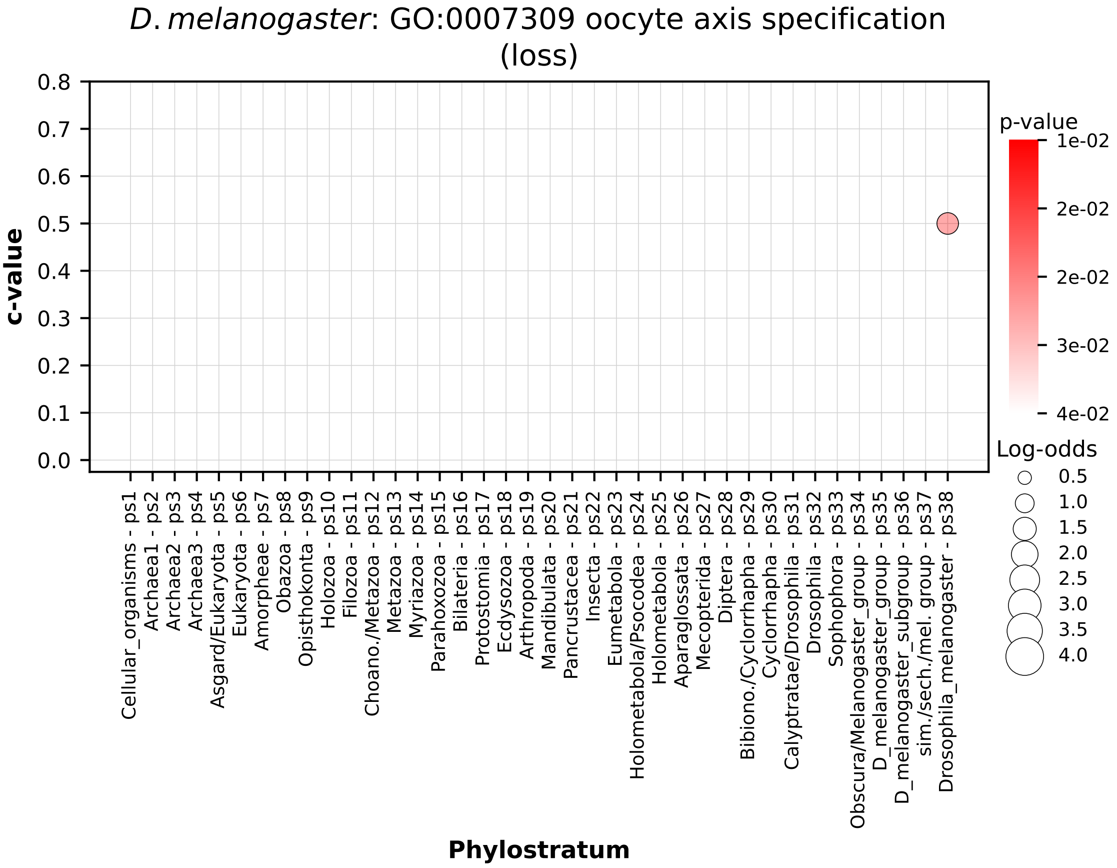

D. melanogaster: GO:0007309 oocyte axis specification
(loss)
c-value
0.8
0.7
0.6
0.5
0.4
0.3
0.2
0.1
0.0
p-value
1e-02
2e-02
2e-02
3e-02
4e-02
Log-odds
0.5
1.0
1.5
2.0
2.5
3.0
3.5
4.0
Cellular_organisms - ps1
Archaea1 - ps2
Archaea2 - ps3
Archaea3 - ps4
Asgard/Eukaryota - ps5
Eukaryota - ps6
Amorpheae - ps7
Obazoa - ps8
Opisthokonta - ps9
Holozoa - ps10
Filozoa - ps11
Choano./Metazoa - ps12
Metazoa - ps13
Myriazoa - ps14
Parahoxozoa - ps15
Bilateria - ps16
Protostomia - ps17
Ecdysozoa - ps18
Arthropoda - ps19
Mandibulata - ps20
Pancrustacea - ps21
Insecta - ps22
Eumetabola - ps23
Holometabola/Psocodea - ps24
Holometabola - ps25
Aparaglossata - ps26
Mecopterida - ps27
Diptera - ps28
Bibiono./Cyclorrhapha - ps29
Cyclorrhapha - ps30
Calyptratae/Drosophila - ps31
Drosophila - ps32
Sophophora - ps33
Obscura/Melanogaster_group - ps34
D_melanogaster_group - ps35
D_melanogaster_subgroup - ps36
sim./sech./mel. group - ps37
Drosophila_melanogaster - ps38
Phylostratum

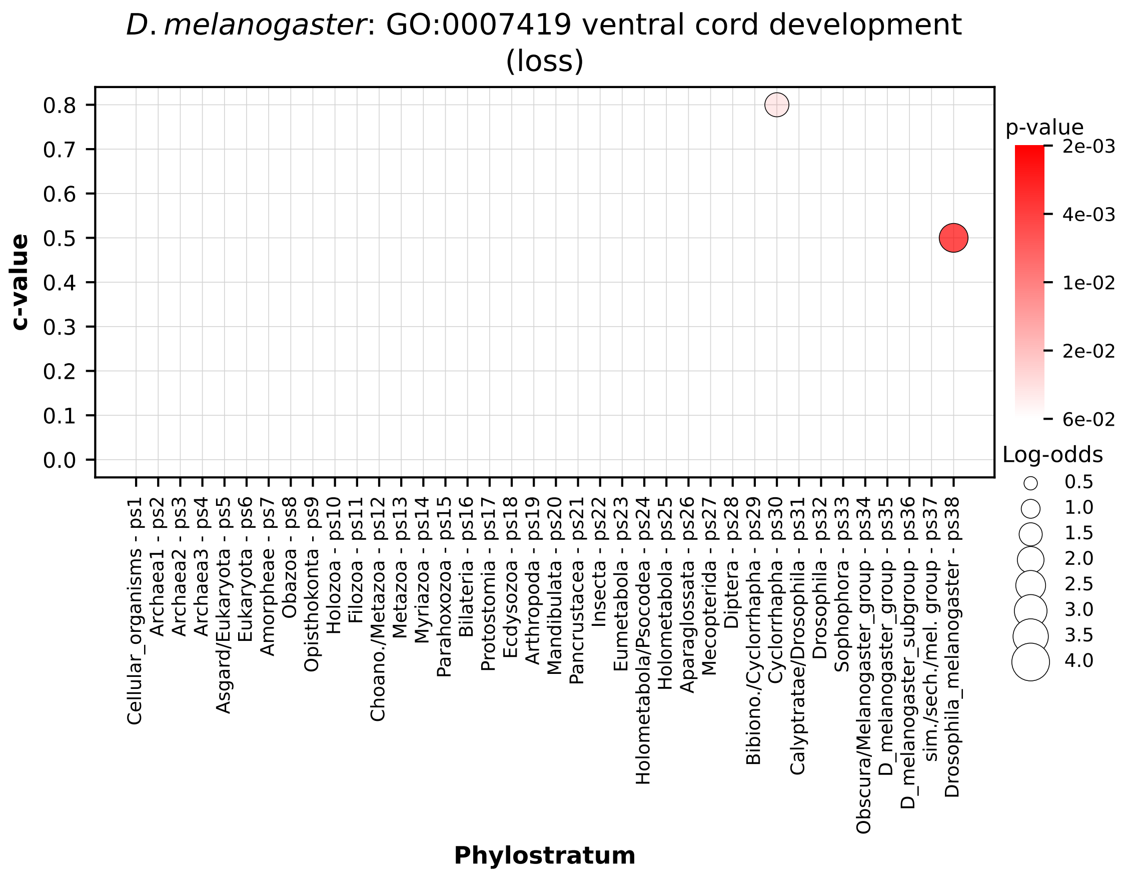

D. melanogaster: GO:0007419 ventral cord development
(loss)
c-value
0.0
0.1
0.2
0.3
0.4
0.5
0.6
0.7
0.8
Phylostratum
Cellular_organisms - ps1
Archaea1 - ps2
Archaea2 - ps3
Archaea3 - ps4
Asgard/Eukaryota - ps5
Eukaryota - ps6
Amorpheae - ps7
Obazoa - ps8
Opisthokonta - ps9
Holozoa - ps10
Filozoa - ps11
Choano./Metazoa - ps12
Metazoa - ps13
Myriazoa - ps14
Parahoxozoa - ps15
Bilateria - ps16
Protostomia - ps17
Ecdysozoa - ps18
Arthropoda - ps19
Mandibulata - ps20
Pancrustacea - ps21
Insecta - ps22
Eumetabola - ps23
Holometabola/Psocodea - ps24
Holometabola - ps25
Aparaglossata - ps26
Mecopterida - ps27
Diptera - ps28
Bibiono./Cyclorrhapha - ps29
Cyclorrhapha - ps30
Calyptratae/Drosophila - ps31
Drosophila - ps32
Sophophora - ps33
Obscura/Melanogaster_group - ps34
D_melanogaster_group - ps35
D_melanogaster_subgroup - ps36
sim./sech./mel. group - ps37
Drosophila_melanogaster - ps38
p-value
2e-03
4e-03
1e-02
2e-02
6e-02
Log-odds
0.5
1.0
1.5
2.0
2.5
3.0
3.5
4.0

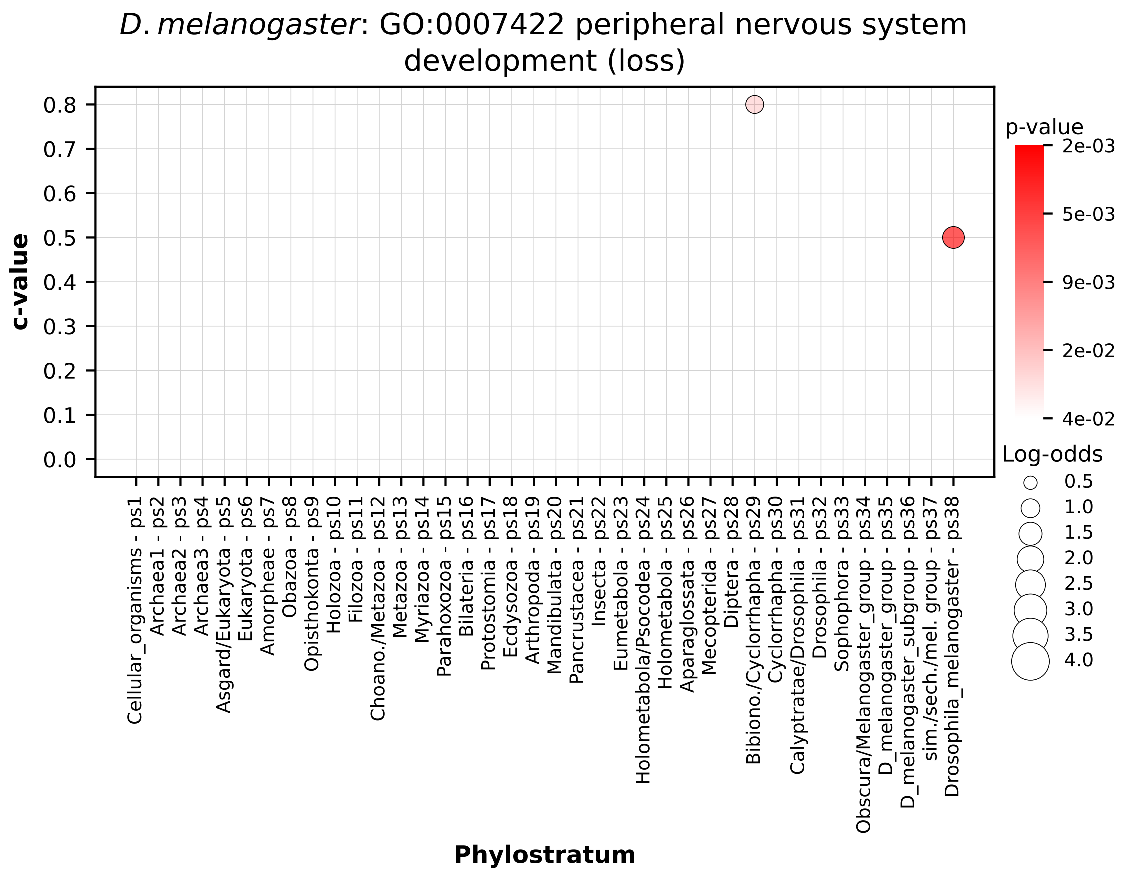
D. melanogaster: GO:0007422 peripheral nervous system development (loss)
c-value
0.0
0.1
0.2
0.3
0.4
0.5
0.6
0.7
0.8
Phylostratum
Cellular_organisms - ps1
Archaea1 - ps2
Archaea2 - ps3
Archaea3 - ps4
Asgard/Eukaryota - ps5
Eukaryota - ps6
Amorpheae - ps7
Obazoa - ps8
Opisthokonta - ps9
Holozoa - ps10
Filozoa - ps11
Choano./Metazoa - ps12
Metazoa - ps13
Myriazoa - ps14
Parahoxozoa - ps15
Bilateria - ps16
Protostomia - ps17
Ecdysozoa - ps18
Arthropoda - ps19
Mandibulata - ps20
Pancrustacea - ps21
Insecta - ps22
Eumetabola - ps23
Holometabola/Psocodea - ps24
Holometabola - ps25
Aparaglossata - ps26
Mecopterida - ps27
Diptera - ps28
Bibiono./Cyclorrhapha - ps29
Cyclorrhapha - ps30
Calyptratae/Drosophila - ps31
Drosophila - ps32
Sophophora - ps33
Obscura/Melanogaster_group - ps34
D_melanogaster_group - ps35
D_melanogaster_subgroup - ps36
sim./sech./mel. group - ps37
Drosophila_melanogaster - ps38
p-value
2e-03
5e-03
9e-03
2e-02
4e-02
Log-odds
0.5
1.0
1.5
2.0
2.5
3.0
3.5
4.0

D. melanogaster: GO:0007424 open tracheal system development (loss)

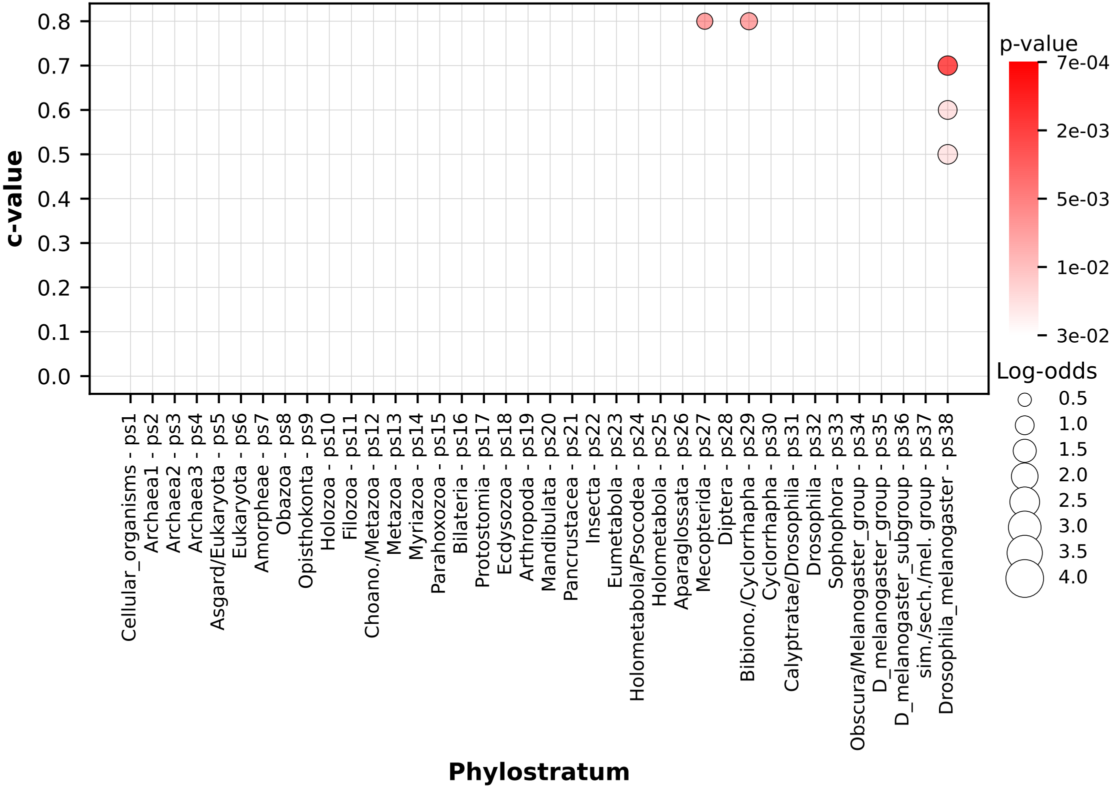

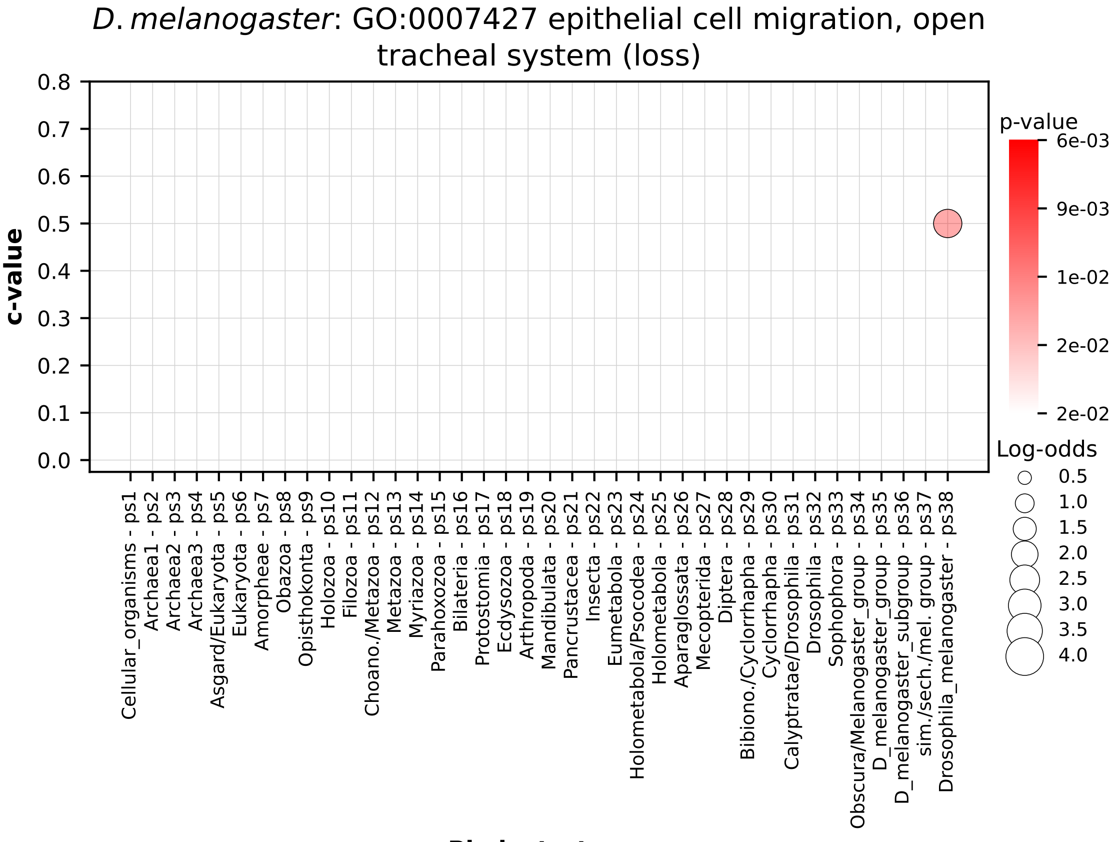

D. melanogaster: GO:0007427 epithelial cell migration, open tracheal system (loss)
c-value
0.8
0.7
0.6
0.5
0.4
0.3
0.2
0.1
0.0
p-value
6e-03
9e-03
1e-02
2e-02
2e-02
Log-odds
0.5
1.0
1.5
2.0
2.5
3.0
3.5
4.0
Cellular_organisms - ps1
Archaea1 - ps2
Archaea2 - ps3
Archaea3 - ps4
Asgard/Eukaryota - ps5
Eukaryota - ps6
Amorpheae - ps7
Obazoa - ps8
Opisthokonta - ps9
Holozoa - ps10
Filozoa - ps11
Choano./Metazoa - ps12
Metazoa - ps13
Myriazoa - ps14
Parahoxozoa - ps15
Bilateria - ps16
Protostomia - ps17
Ecdysozoa - ps18
Arthropoda - ps19
Mandibulata - ps20
Pancrustacea - ps21
Insecta - ps22
Eumetabola - ps23
Holometabola/Psocodea - ps24
Holometabola - ps25
Aparaglossata - ps26
Mecopterida - ps27
Diptera - ps28
Bibiono./Cyclorrhapha - ps29
Cyclorrhapha - ps30
Calyptratae/Drosophila - ps31
Drosophila - ps32
Sophophora - ps33
Obscura/Melanogaster_group - ps34
D_melanogaster_group - ps35
D_melanogaster_subgroup - ps36
sim./sech./mel. group - ps37
Drosophila_melanogaster - ps38
Phylostratum

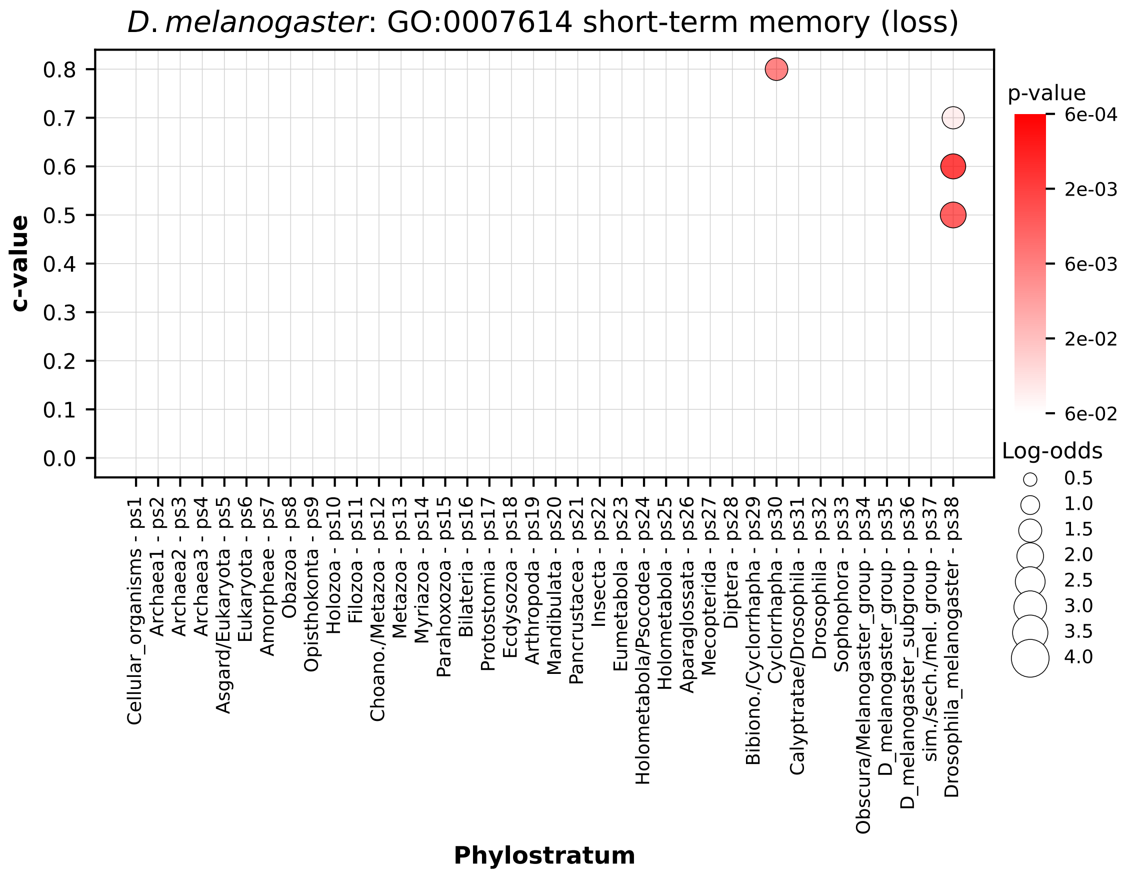
D. melanogaster: GO:0007614 short-term memory (loss)
c-value
0.8
0.7
0.6
0.5
0.4
0.3
0.2
0.1
0.0
Phylostratum
Cellular_organisms - ps1
Archaea1 - ps2
Archaea2 - ps3
Archaea3 - ps4
Asgard/Eukaryota - ps5
Eukaryota - ps6
Amorpheae - ps7
Obazoa - ps8
Opisthokonta - ps9
Holozoa - ps10
Filozoa - ps11
Choano./Metazoa - ps12
Metazoa - ps13
Myriazoa - ps14
Parahoxozoa - ps15
Bilateria - ps16
Protostomia - ps17
Ecdysozoa - ps18
Arthropoda - ps19
Mandibulata - ps20
Pancrustacea - ps21
Insecta - ps22
Eumetabola - ps23
Holometabola/Psocodea - ps24
Holometabola - ps25
Aparaglossata - ps26
Mecopterida - ps27
Diptera - ps28
Bibiono./Cyclorrhapha - ps29
Cyclorrhapha - ps30
Calyptratae/Drosophila - ps31
Drosophila - ps32
Sophophora - ps33
Obscura/Melanogaster_group - ps34
D_melanogaster_group - ps35
D_melanogaster_subgroup - ps36
sim./sech./mel. group - ps37
Drosophila_melanogaster - ps38
p-value
6e-04
2e-03
6e-03
2e-02
6e-02
Log-odds
0.5
1.0
1.5
2.0
2.5
3.0
3.5
4.0

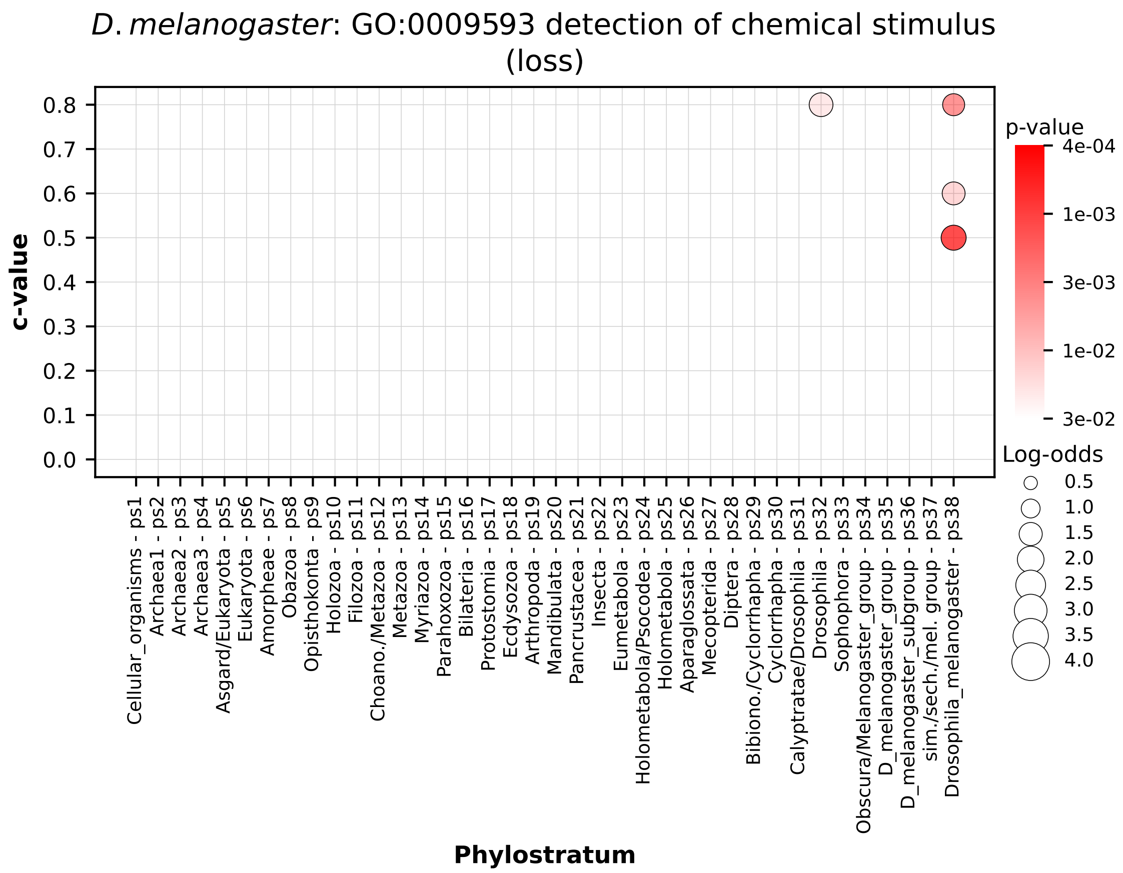

D. melanogaster: GO:0009593 detection of chemical stimulus (loss)
c-value
0.0
0.1
0.2
0.3
0.4
0.5
0.6
0.7
0.8
p-value
4e-04
1e-03
3e-03
1e-02
3e-02
Log-odds
0.5
1.0
1.5
2.0
2.5
3.0
3.5
4.0
Cellular_organisms - ps1
Archaea1 - ps2
Archaea2 - ps3
Archaea3 - ps4
Asgard/Eukaryota - ps5
Eukaryota - ps6
Amorpheae - ps7
Obazoa - ps8
Opisthokonta - ps9
Holozoa - ps10
Filozoa - ps11
Choano./Metazoa - ps12
Metazoa - ps13
Myriazoa - ps14
Parahoxozoa - ps15
Bilateria - ps16
Protostomia - ps17
Ecdysozoa - ps18
Arthropoda - ps19
Mandibulata - ps20
Pancrustacea - ps21
Insecta - ps22
Eumetabola - ps23
Holometabola/Psocodea - ps24
Holometabola - ps25
Aparaglossata - ps26
Mecopterida - ps27
Diptera - ps28
Bibiono./Cyclorrhapha - ps29
Cyclorrhapha - ps30
Calyptratae/Drosophila - ps31
Drosophila - ps32
Sophophora - ps33
Obscura/Melanogaster_group - ps34
D_melanogaster_group - ps35
D_melanogaster_subgroup - ps36
sim./sech./mel. group - ps37
Drosophila_melanogaster - ps38
Phylostratum

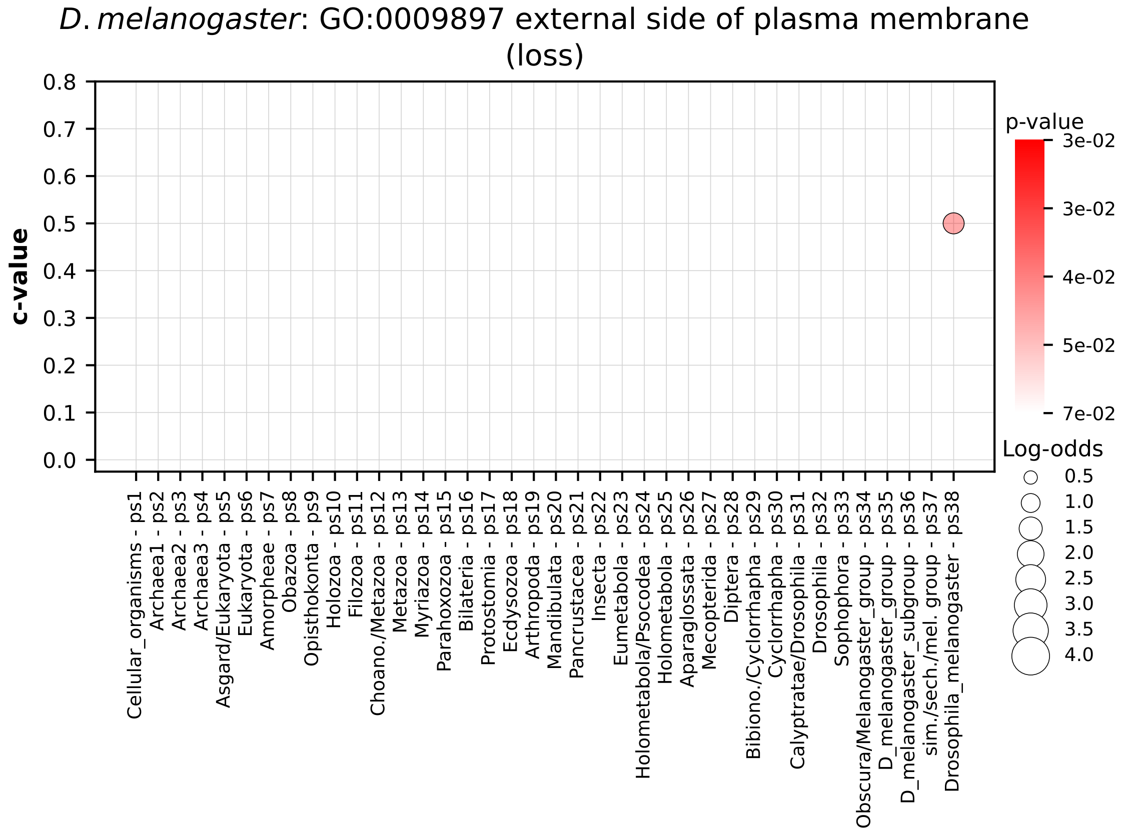
D. melanogaster: GO:0009897 external side of plasma membrane (loss)
c-value
0.8
0.7
0.6
0.5
0.4
0.3
0.2
0.1
0.0
Phylostratum
Cellular_organisms - ps1
Archaea1 - ps2
Archaea2 - ps3
Archaea3 - ps4
Asgard/Eukaryota - ps5
Eukaryota - ps6
Amorpheae - ps7
Obazoa - ps8
Opisthokonta - ps9
Holozoa - ps10
Filozoa - ps11
Choano./Metazoa - ps12
Metazoa - ps13
Myriazoa - ps14
Parahoxozoa - ps15
Bilateria - ps16
Protostomia - ps17
Ecdysozoa - ps18
Arthropoda - ps19
Mandibulata - ps20
Pancrustacea - ps21
Insecta - ps22
Eumetabola - ps23
Holometabola/Psocodea - ps24
Holometabola - ps25
Aparaglossata - ps26
Mecopterida - ps27
Diptera - ps28
Bibiono./Cyclorrhapha - ps29
Cyclorrhapha - ps30
Calyptratae/Drosophila - ps31
Drosophila - ps32
Sophophora - ps33
Obscura/Melanogaster_group - ps34
D_melanogaster_group - ps35
D_melanogaster_subgroup - ps36
sim./sech./mel. group - ps37
Drosophila_melanogaster - ps38
p-value
3e-02
3e-02
4e-02
5e-02
7e-02
Log-odds
0.5
1.0
1.5
2.0
2.5
3.0
3.5
4.0

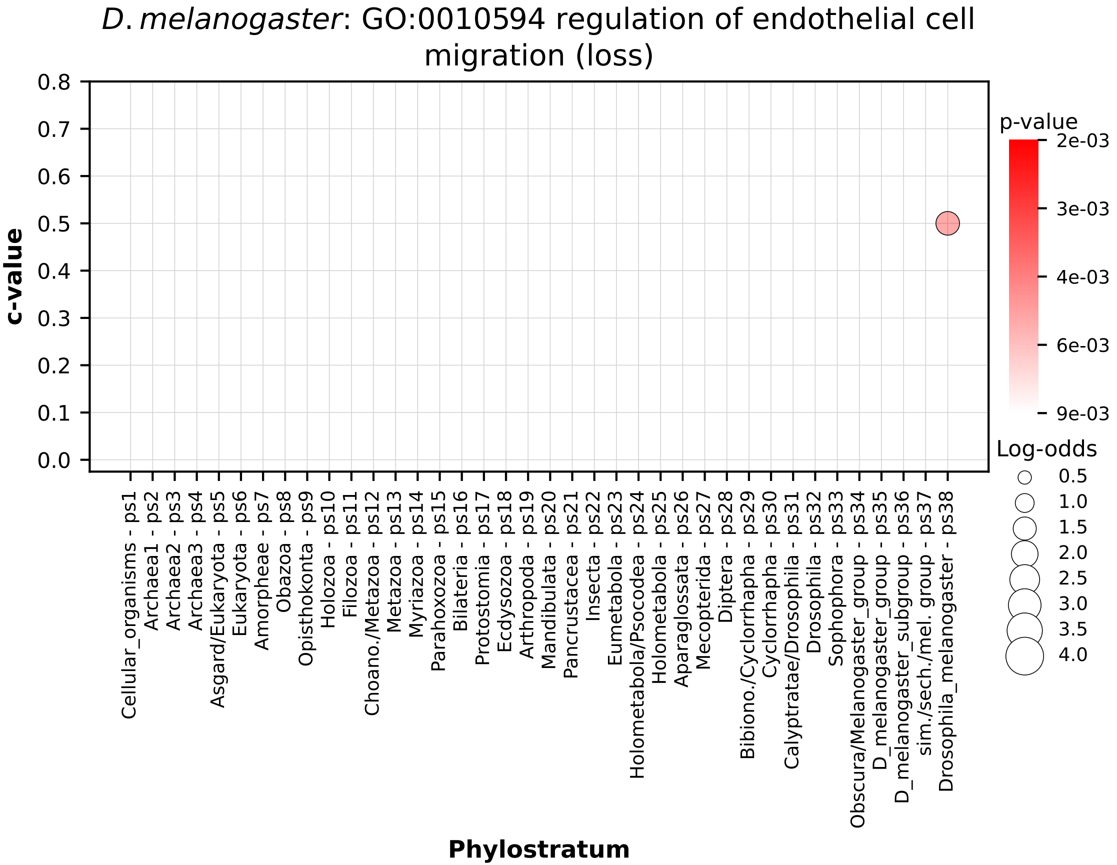

D. melanogaster: GO:0010594 regulation of endothelial cell migration (loss)
c-value
0.8
0.7
0.6
0.5
0.4
0.3
0.2
0.1
0.0
p-value
2e-03
3e-03
4e-03
6e-03
9e-03
Log-odds
0.5
1.0
1.5
2.0
2.5
3.0
3.5
4.0
Cellular_organisms - ps1
Archaea1 - ps2
Archaea2 - ps3
Archaea3 - ps4
Asgard/Eukaryota - ps5
Eukaryota - ps6
Amorpheae - ps7
Obazoa - ps8
Opisthokonta - ps9
Holozoa - ps10
Filozoa - ps11
Choano./Metazoa - ps12
Metazoa - ps13
Myriazoa - ps14
Parahoxozoa - ps15
Bilateria - ps16
Protostomia - ps17
Ecdysozoa - ps18
Arthropoda - ps19
Mandibulata - ps20
Pancrustacea - ps21
Insecta - ps22
Eumetabola - ps23
Holometabola/Psocodea - ps24
Holometabola - ps25
Aparaglossata - ps26
Mecopterida - ps27
Diptera - ps28
Bibiono./Cyclorrhapha - ps29
Cyclorrhapha - ps30
Calyptratae/Drosophila - ps31
Drosophila - ps32
Sophophora - ps33
Obscura/Melanogaster_group - ps34
D_melanogaster_group - ps35
D_melanogaster_subgroup - ps36
sim./sech./mel. group - ps37
Drosophila_melanogaster - ps38
Phylostratum

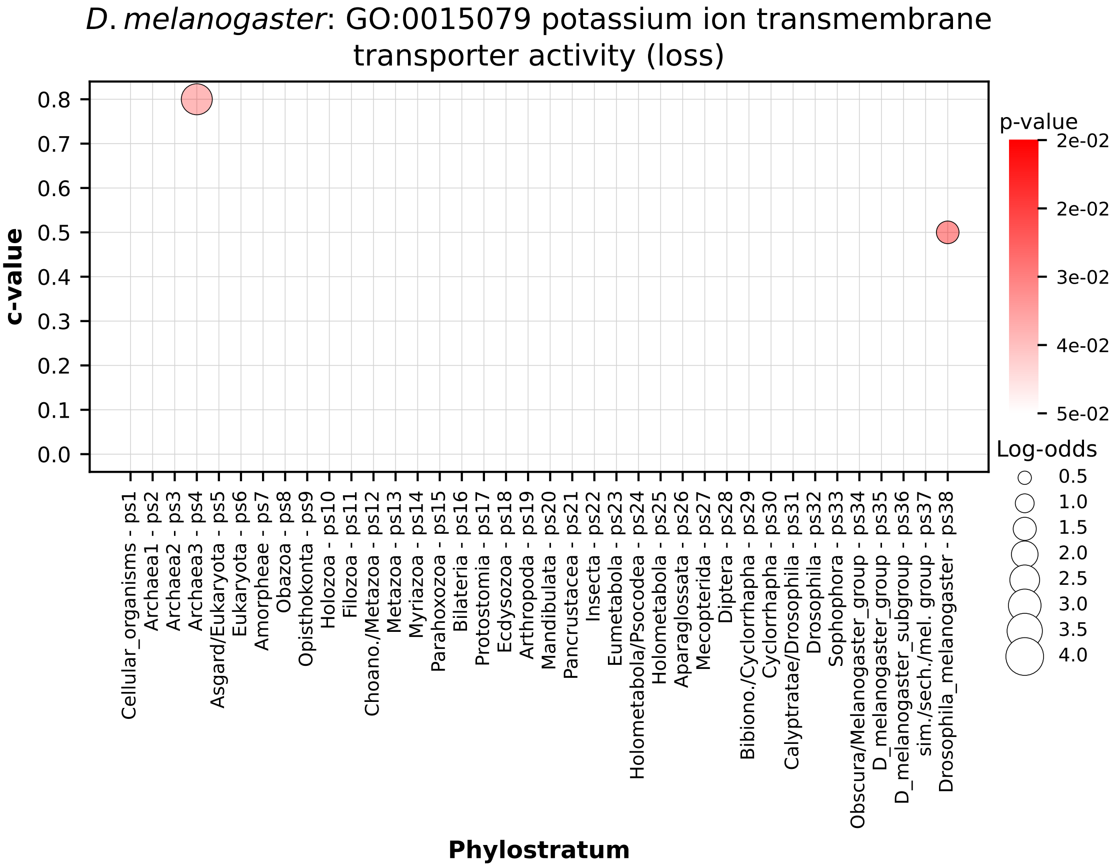

D. melanogaster: GO:0015079 potassium ion transmembrane transporter activity (loss)
c-value
0.8
0.7
0.6
0.5
0.4
[truncated: 353,966 more chars]
