# Supplementary material for: Macroevolutionary dynamics of gene family gain and loss along multicellular eukaryotic lineages
Source: Nat Commun. 2024 Mar 26;15:2663. doi: 10.1038/s41467-024-47017-w (PMC10966110; doi:10.1038/s41467-024-47017-w)
Supplement: Supplementary file 17 — Supplementary Dataset 12 [file 41467_2024_47017_MOESM17_ESM.pdf]

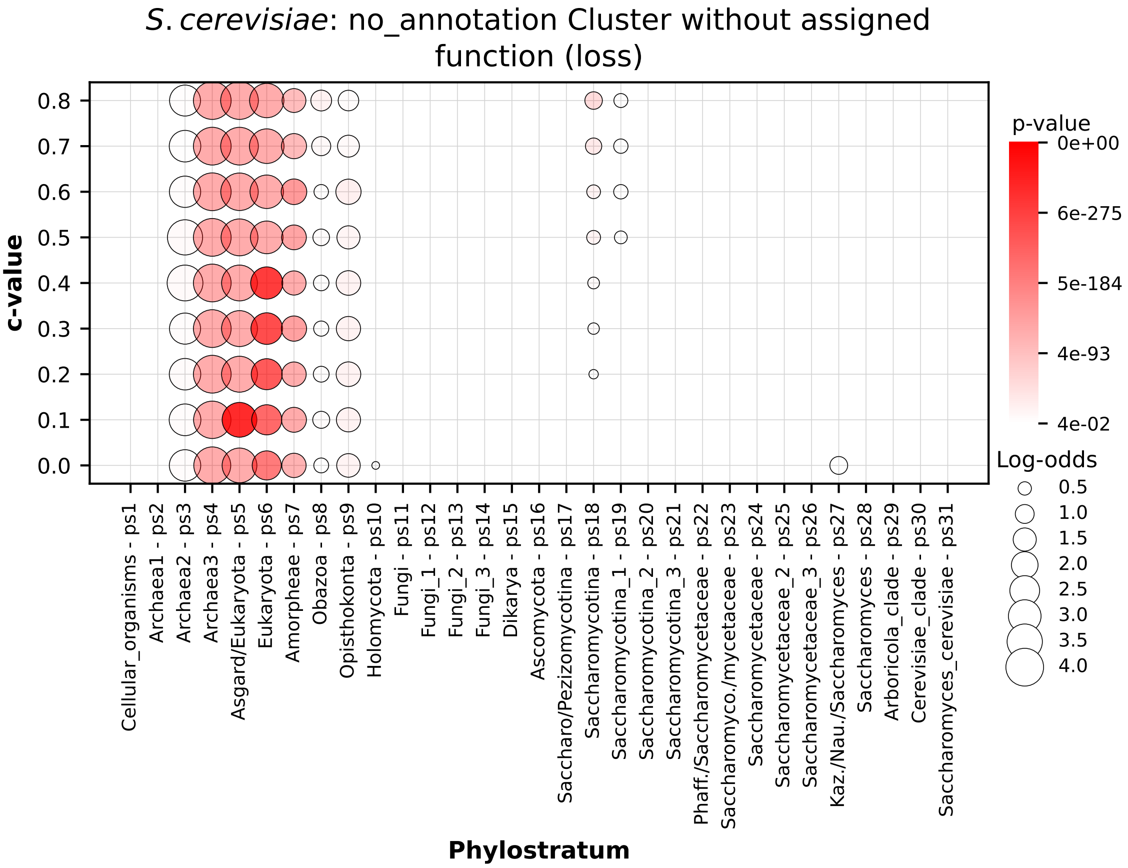

S. cerevisiae: no_annotation Cluster without assigned function (loss)
c-value
0.8
0.7
0.6
0.5
0.4
0.3
0.2
0.1
0.0
p-value
0e+00
6e-275
5e-184
4e-93
4e-02
Log-odds
0.5
1.0
1.5
2.0
2.5
3.0
3.5
4.0
Cellular_organisms - ps1
Archaea1 - ps2
Archaea2 - ps3
Archaea3 - ps4
Asgard/Eukaryota - ps5
Eukaryota - ps6
Amorpheae - ps7
Obazoa - ps8
Opisthokonta - ps9
Holomycota - ps10
Fungi - ps11
Fungi_1 - ps12
Fungi_2 - ps13
Fungi_3 - ps14
Dikarya - ps15
Ascomycota - ps16
Saccharo/Pezizomycotina - ps17
Saccharomycotina - ps18
Saccharomycotina_1 - ps19
Saccharomycotina_2 - ps20
Saccharomycotina_3 - ps21
Phaff./Saccharomycetaceae - ps22
Saccharomyco./mycetaceae - ps23
Saccharomycetaceae - ps24
Saccharomycetaceae_2 - ps25
Saccharomycetaceae_3 - ps26
Kaz./Nau./Saccharomyces - ps27
Saccharomyces - ps28
Arboricola_clade - ps29
Cerevisiae_clade - ps30
Saccharomyces_cerevisiae - ps31
Phylostratum

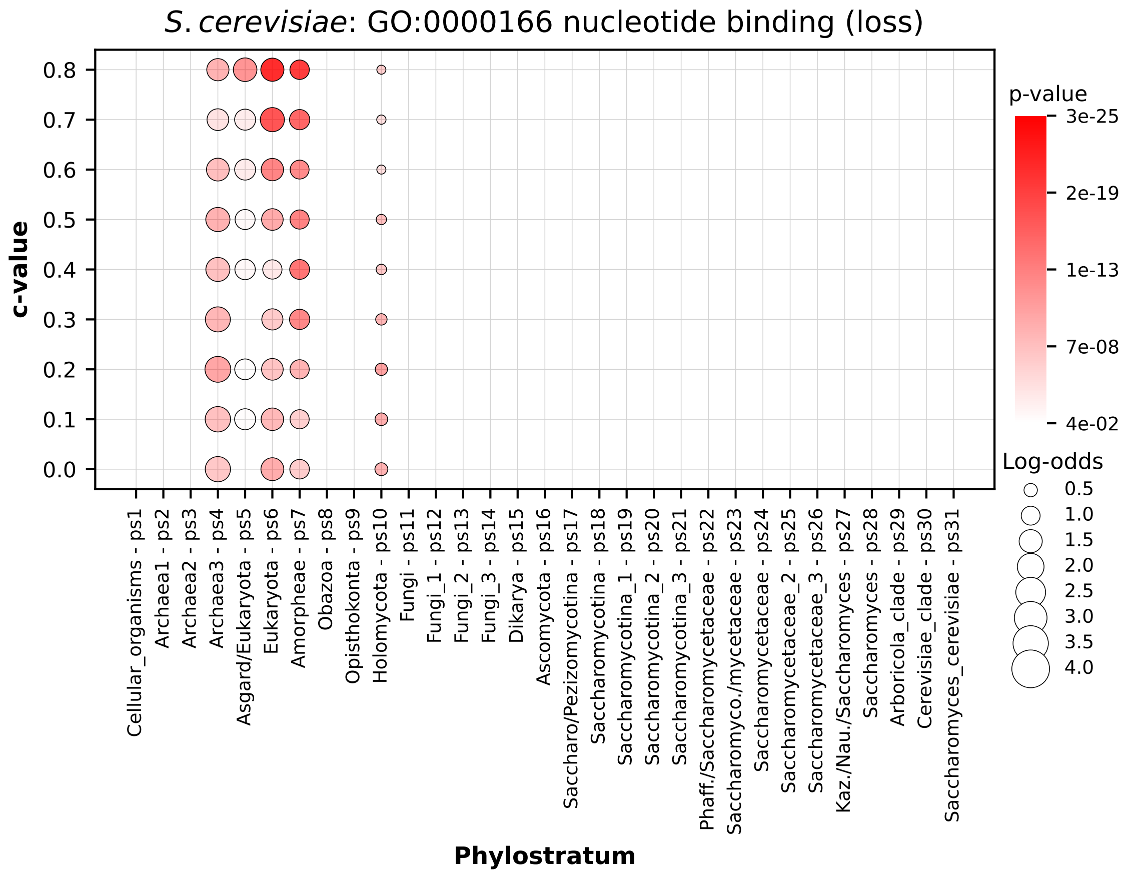
S. cerevisiae: GO:0000166 nucleotide binding (loss)
c-value
0.8
0.7
0.6
0.5
0.4
0.3
0.2
0.1
0.0
p-value
3e-25
2e-19
1e-13
7e-08
4e-02
Log-odds
0.5
1.0
1.5
2.0
2.5
3.0
3.5
4.0
Cellular_organisms - ps1
Archaea1 - ps2
Archaea2 - ps3
Archaea3 - ps4
Asgard/Eukaryota - ps5
Eukaryota - ps6
Amorpheae - ps7
Obazoa - ps8
Opisthokonta - ps9
Holomycota - ps10
Fungi - ps11
Fungi_1 - ps12
Fungi_2 - ps13
Fungi_3 - ps14
Dikarya - ps15
Ascomycota - ps16
Saccharo/Pezizomycotina - ps17
Saccharomycotina - ps18
Saccharomycotina_1 - ps19
Saccharomycotina_2 - ps20
Saccharomycotina_3 - ps21
Phaff./Saccharomycetaceae - ps22
Saccharomyco./mycetaceae - ps23
Saccharomycetaceae - ps24
Saccharomycetaceae_2 - ps25
Saccharomycetaceae_3 - ps26
Kaz./Nau./Saccharomyces - ps27
Saccharomyces - ps28
Arboricola_clade - ps29
Cerevisiae_clade - ps30
Saccharomyces_cerevisiae - ps31
Phylostratum

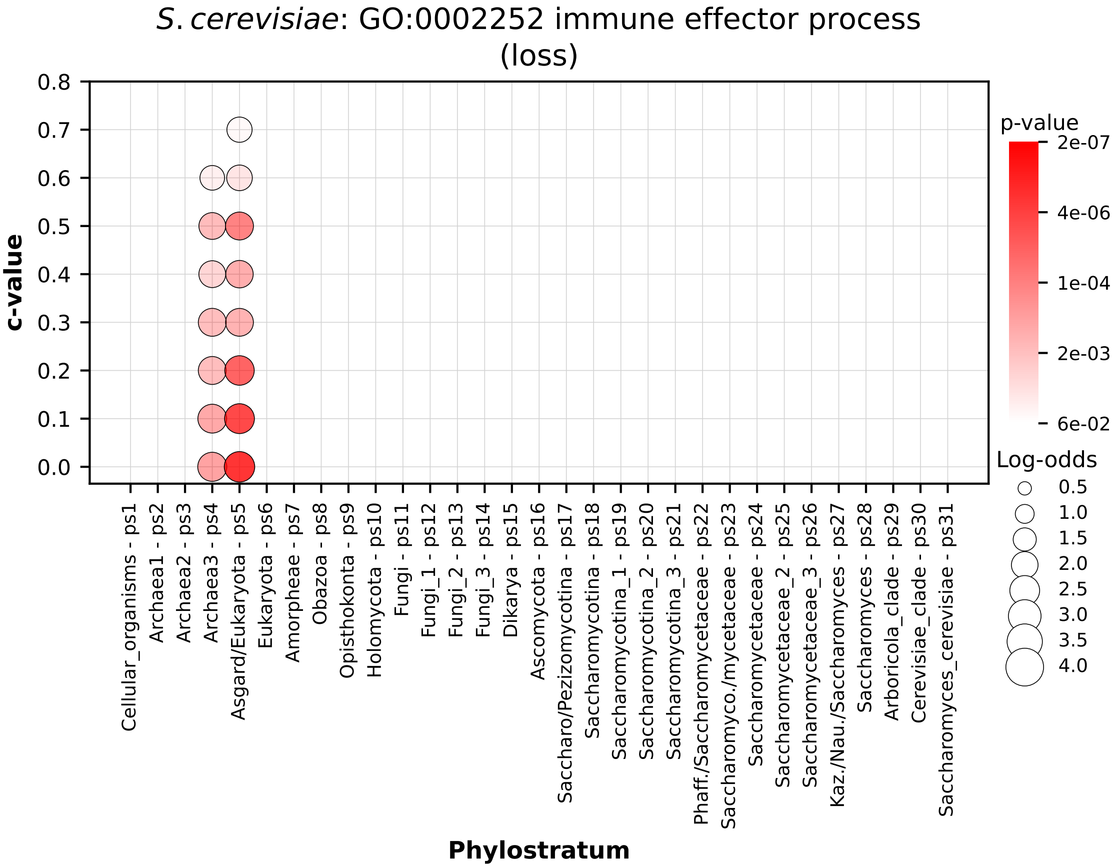
S. cerevisiae: GO:0002252 immune effector process
(loss)
c-value
0.8
0.7
0.6
0.5
0.4
0.3
0.2
0.1
0.0
p-value
2e-07
4e-06
1e-04
2e-03
6e-02
Log-odds
0.5
1.0
1.5
2.0
2.5
3.0
3.5
4.0
Cellular_organisms - ps1
Archaea1 - ps2
Archaea2 - ps3
Archaea3 - ps4
Asgard/Eukaryota - ps5
Eukaryota - ps6
Amorpheae - ps7
Obazoa - ps8
Opisthokonta - ps9
Holomycota - ps10
Fungi - ps11
Fungi_1 - ps12
Fungi_2 - ps13
Fungi_3 - ps14
Dikarya - ps15
Ascomycota - ps16
Saccharo/Pezizomycotina - ps17
Saccharomycotina - ps18
Saccharomycotina_1 - ps19
Saccharomycotina_2 - ps20
Saccharomycotina_3 - ps21
Phaff./Saccharomycetaceae - ps22
Saccharomyco./mycetaceae - ps23
Saccharomycetaceae - ps24
Saccharomycetaceae_2 - ps25
Saccharomycetaceae_3 - ps26
Kaz./Nau./Saccharomyces - ps27
Saccharomyces - ps28
Arboricola_clade - ps29
Cerevisiae_clade - ps30
Saccharomyces_cerevisiae - ps31
Phylostratum

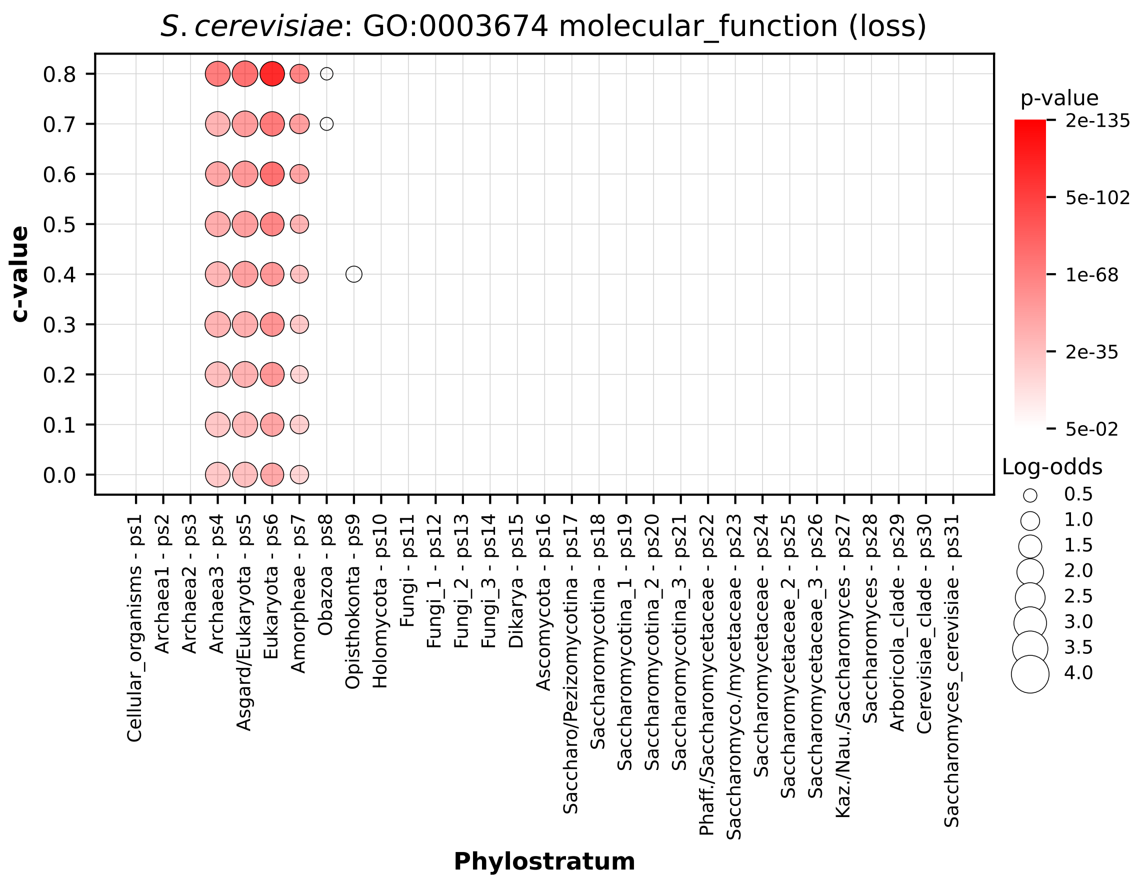

S. cerevisiae: GO:0003674 molecular_function (loss)
c-value
0.8
0.7
0.6
0.5
0.4
0.3
0.2
0.1
0.0
p-value
2e-135
5e-102
1e-68
2e-35
5e-02
Log-odds
0.5
1.0
1.5
2.0
2.5
3.0
3.5
4.0
Cellular_organisms - ps1
Archaea1 - ps2
Archaea2 - ps3
Archaea3 - ps4
Asgard/Eukaryota - ps5
Eukaryota - ps6
Amorpheae - ps7
Obazoa - ps8
Opisthokonta - ps9
Holomycota - ps10
Fungi - ps11
Fungi_1 - ps12
Fungi_2 - ps13
Fungi_3 - ps14
Dikarya - ps15
Ascomycota - ps16
Saccharo/Pezizomycotina - ps17
Saccharomycotina - ps18
Saccharomycotina_1 - ps19
Saccharomycotina_2 - ps20
Saccharomycotina_3 - ps21
Phaff./Saccharomycetaceae - ps22
Saccharomyco./mycetaceae - ps23
Saccharomycetaceae - ps24
Saccharomycetaceae_2 - ps25
Saccharomycetaceae_3 - ps26
Kaz./Nau./Saccharomyces - ps27
Saccharomyces - ps28
Arboricola_clade - ps29
Cerevisiae_clade - ps30
Saccharomyces_cerevisiae - ps31
Phylostratum

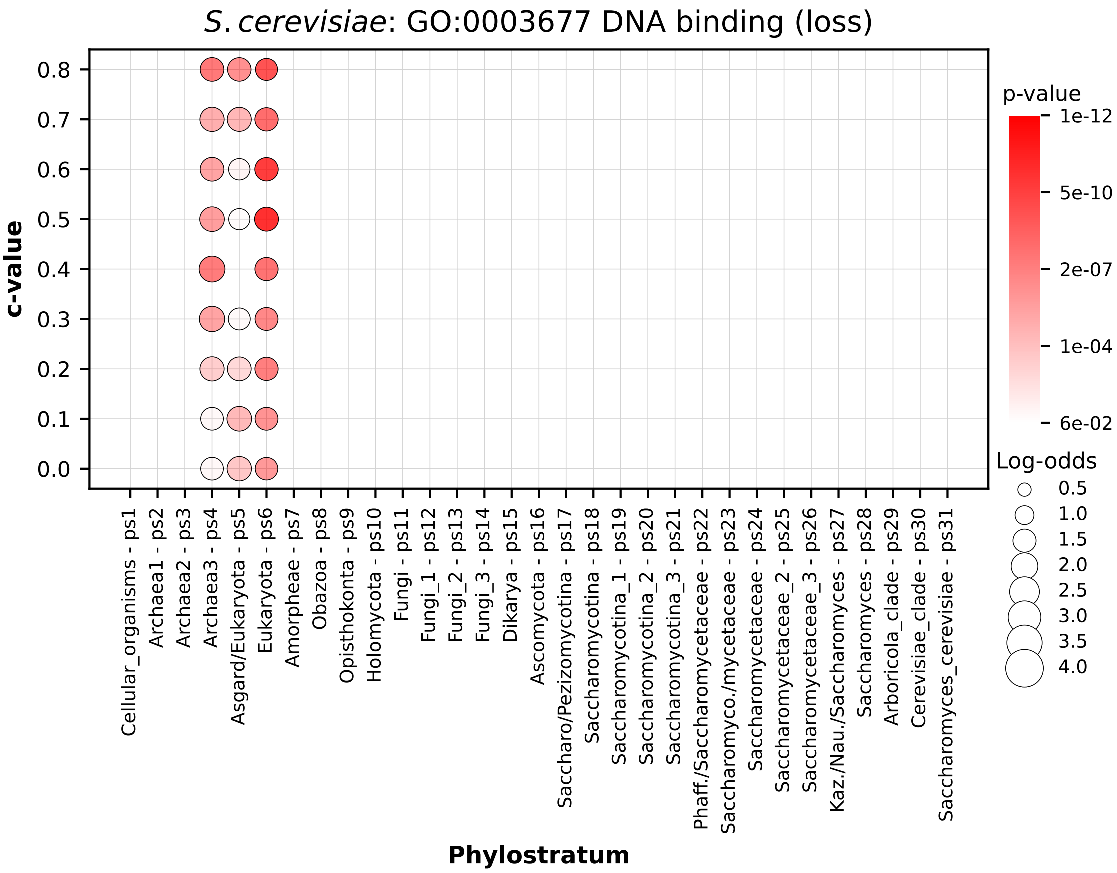

S. cerevisiae: GO:0003677 DNA binding (loss)
c-value
0.0
0.1
0.2
0.3
0.4
0.5
0.6
0.7
0.8
p-value
1e-12
5e-10
2e-07
1e-04
6e-02
Log-odds
0.5
1.0
1.5
2.0
2.5
3.0
3.5
4.0
Cellular_organisms - ps1
Archaea1 - ps2
Archaea2 - ps3
Archaea3 - ps4
Asgard/Eukaryota - ps5
Eukaryota - ps6
Amorpheae - ps7
Obazoa - ps8
Opisthokonta - ps9
Holomycota - ps10
Fungi - ps11
Fungi_1 - ps12
Fungi_2 - ps13
Fungi_3 - ps14
Dikarya - ps15
Ascomycota - ps16
Saccharo/Pezizomycotina - ps17
Saccharomycotina - ps18
Saccharomycotina_1 - ps19
Saccharomycotina_2 - ps20
Saccharomycotina_3 - ps21
Phaff./Saccharomycetaceae - ps22
Saccharomyco./mycetaceae - ps23
Saccharomycetaceae - ps24
Saccharomycetaceae_2 - ps25
Saccharomycetaceae_3 - ps26
Kaz./Nau./Saccharomyces - ps27
Saccharomyces - ps28
Arboricola_clade - ps29
Cerevisiae_clade - ps30
Saccharomyces_cerevisiae - ps31
Phylostratum

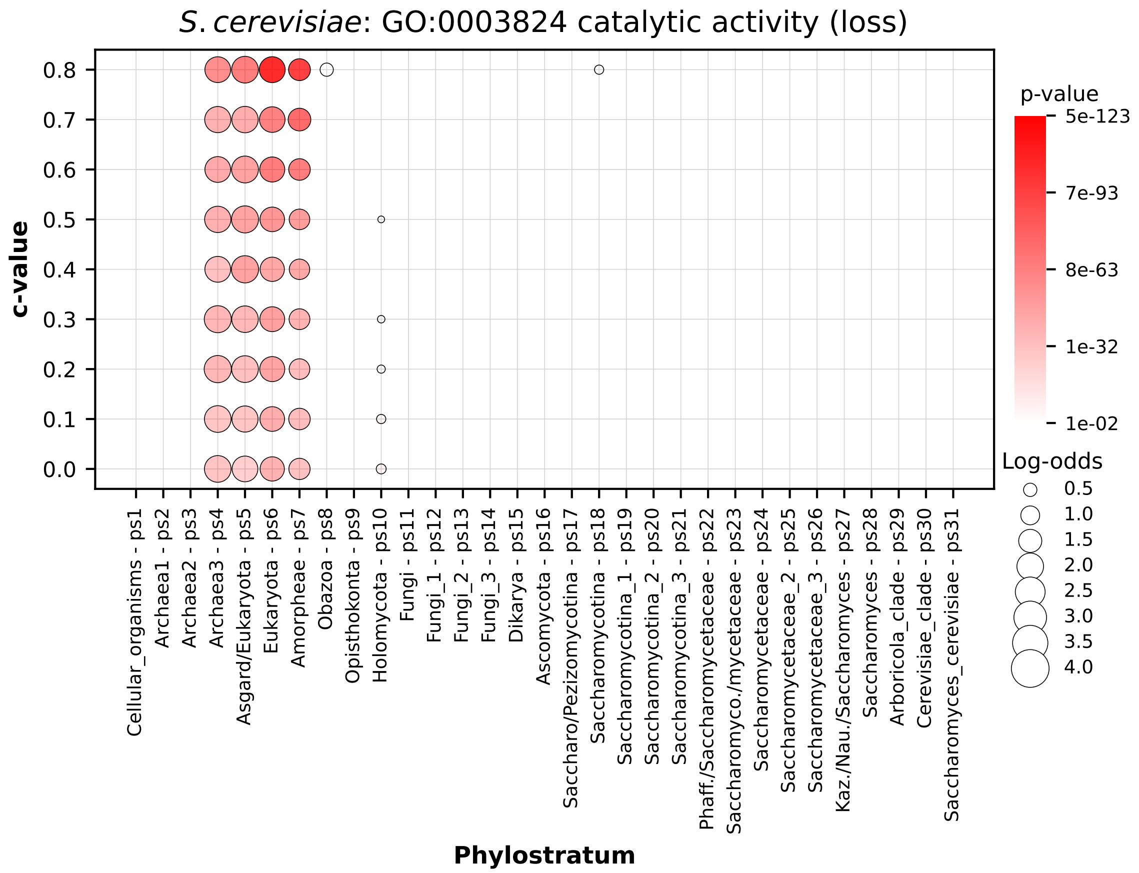

S. cerevisiae: GO:0003824 catalytic activity (loss)
c-value
0.8
0.7
0.6
0.5
0.4
0.3
0.2
0.1
0.0
p-value
5e-123
7e-93
8e-63
1e-32
1e-02
Log-odds
0.5
1.0
1.5
2.0
2.5
3.0
3.5
4.0
Cellular_organisms - ps1
Archaea1 - ps2
Archaea2 - ps3
Archaea3 - ps4
Asgard/Eukaryota - ps5
Eukaryota - ps6
Amorpheae - ps7
Obazoa - ps8
Opisthokonta - ps9
Holomycota - ps10
Fungi - ps11
Fungi_1 - ps12
Fungi_2 - ps13
Fungi_3 - ps14
Dikarya - ps15
Ascomycota - ps16
Saccharo/Pezizomycotina - ps17
Saccharomycotina - ps18
Saccharomycotina_1 - ps19
Saccharomycotina_2 - ps20
Saccharomycotina_3 - ps21
Phaff./Saccharomycetaceae - ps22
Saccharomyco./mycetaceae - ps23
Saccharomycetaceae - ps24
Saccharomycetaceae_2 - ps25
Saccharomycetaceae_3 - ps26
Kaz./Nau./Saccharomyces - ps27
Saccharomyces - ps28
Arboricola_clade - ps29
Cerevisiae_clade - ps30
Saccharomyces_cerevisiae - ps31
Phylostratum

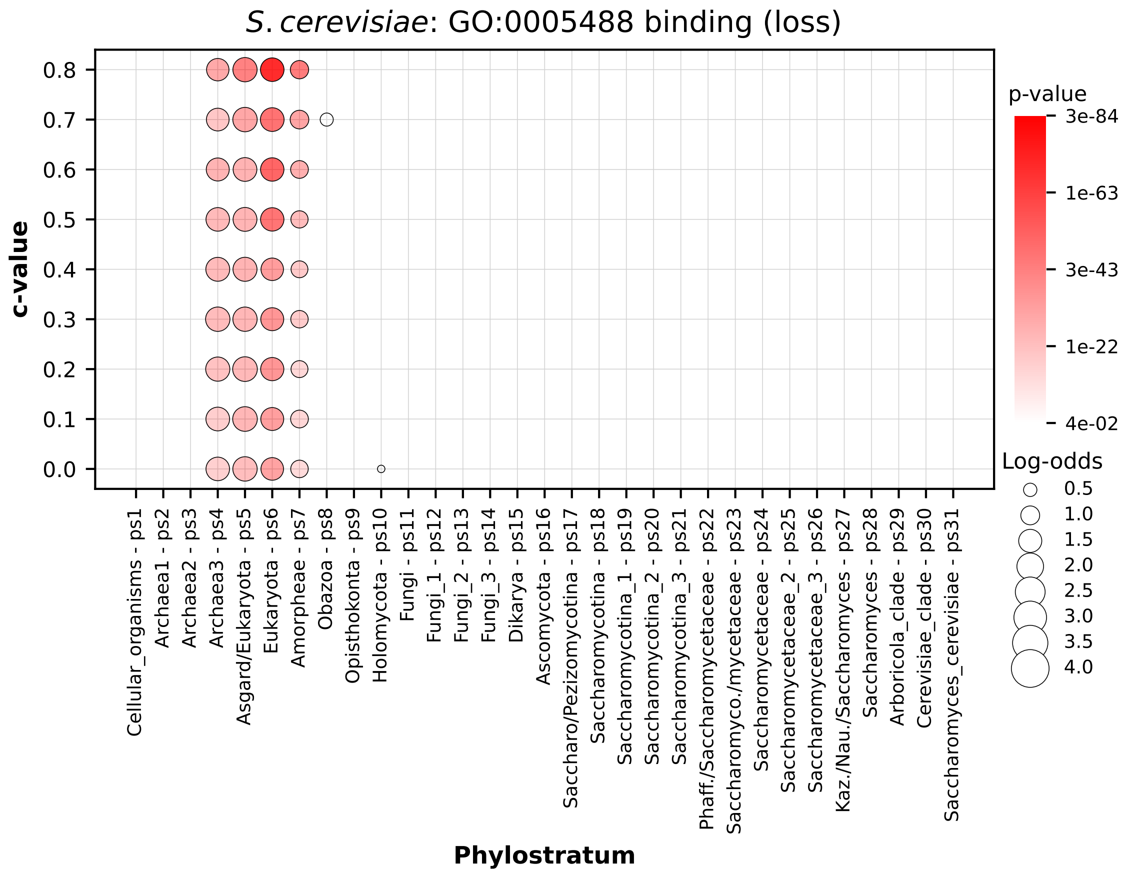
S. cerevisiae: GO:0005488 binding (loss)
c-value
0.8
0.7
0.6
0.5
0.4
0.3
0.2
0.1
0.0
p-value
3e-84
1e-63
3e-43
1e-22
4e-02
Log-odds
0.5
1.0
1.5
2.0
2.5
3.0
3.5
4.0
Cellular_organisms - ps1
Archaea1 - ps2
Archaea2 - ps3
Archaea3 - ps4
Asgard/Eukaryota - ps5
Eukaryota - ps6
Amorpheae - ps7
Obazoa - ps8
Opisthokonta - ps9
Holomycota - ps10
Fungi - ps11
Fungi_1 - ps12
Fungi_2 - ps13
Fungi_3 - ps14
Dikarya - ps15
Ascomycota - ps16
Saccharo/Pezizomycotina - ps17
Saccharomycotina - ps18
Saccharomycotina_1 - ps19
Saccharomycotina_2 - ps20
Saccharomycotina_3 - ps21
Phaff./Saccharomycetaceae - ps22
Saccharomyco./mycetaceae - ps23
Saccharomycetaceae - ps24
Saccharomycetaceae_2 - ps25
Saccharomycetaceae_3 - ps26
Kaz./Nau./Saccharomyces - ps27
Saccharomyces - ps28
Arboricola_clade - ps29
Cerevisiae_clade - ps30
Saccharomyces_cerevisiae - ps31
Phylostratum

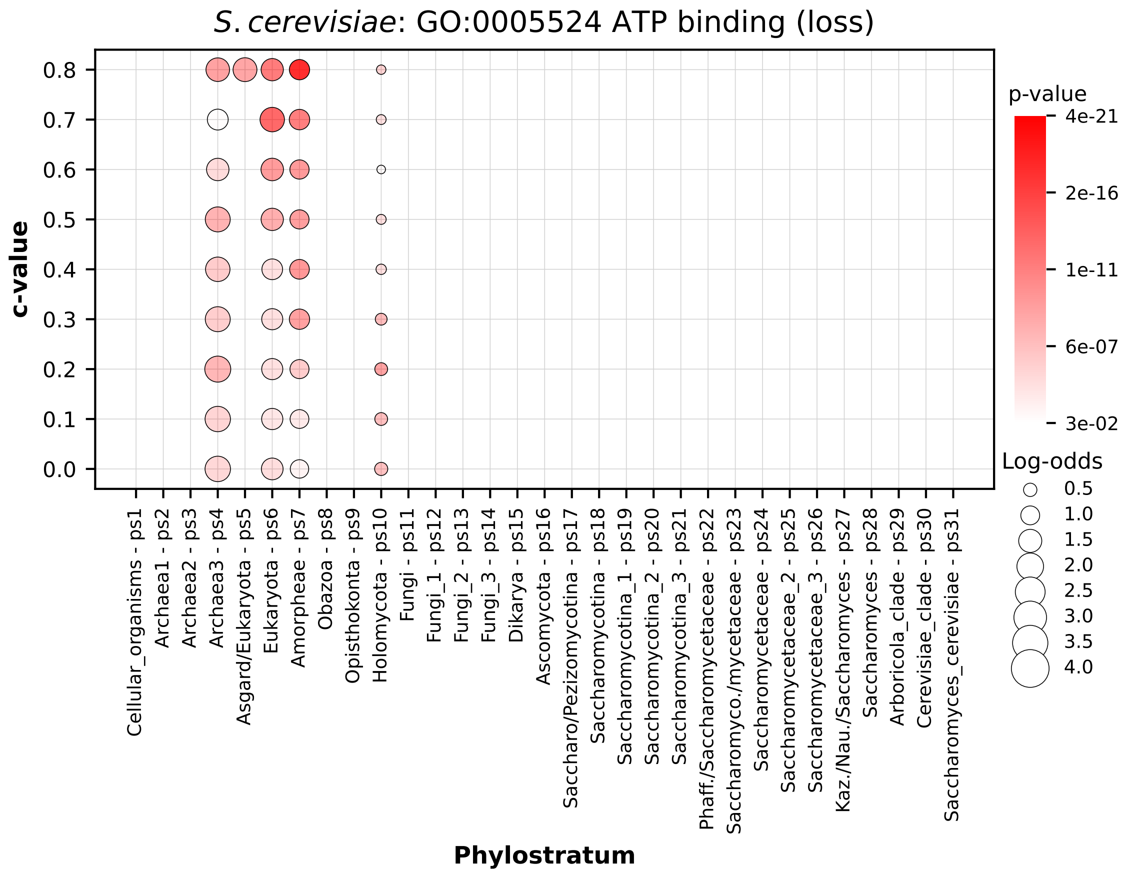

S. cerevisiae: GO:0005524 ATP binding (loss)
c-value
0.8
0.7
0.6
0.5
0.4
0.3
0.2
0.1
0.0
Phylostratum
Cellular_organisms - ps1
Archaea1 - ps2
Archaea2 - ps3
Archaea3 - ps4
Asgard/Eukaryota - ps5
Eukaryota - ps6
Amorpheae - ps7
Obazoa - ps8
Opisthokonta - ps9
Holomycota - ps10
Fungi - ps11
Fungi_1 - ps12
Fungi_2 - ps13
Fungi_3 - ps14
Dikarya - ps15
Ascomycota - ps16
Saccharo/Pezizomycotina - ps17
Saccharomycotina - ps18
Saccharomycotina_1 - ps19
Saccharomycotina_2 - ps20
Saccharomycotina_3 - ps21
Phaff./Saccharomycetaceae - ps22
Saccharomyco./mycetaceae - ps23
Saccharomycetaceae - ps24
Saccharomycetaceae_2 - ps25
Saccharomycetaceae_3 - ps26
Kaz./Nau./Saccharomyces - ps27
Saccharomyces - ps28
Arboricola_clade - ps29
Cerevisiae_clade - ps30
Saccharomyces_cerevisiae - ps31
p-value
4e-21
2e-16
1e-11
6e-07
3e-02
Log-odds
0.5
1.0
1.5
2.0
2.5
3.0
3.5
4.0

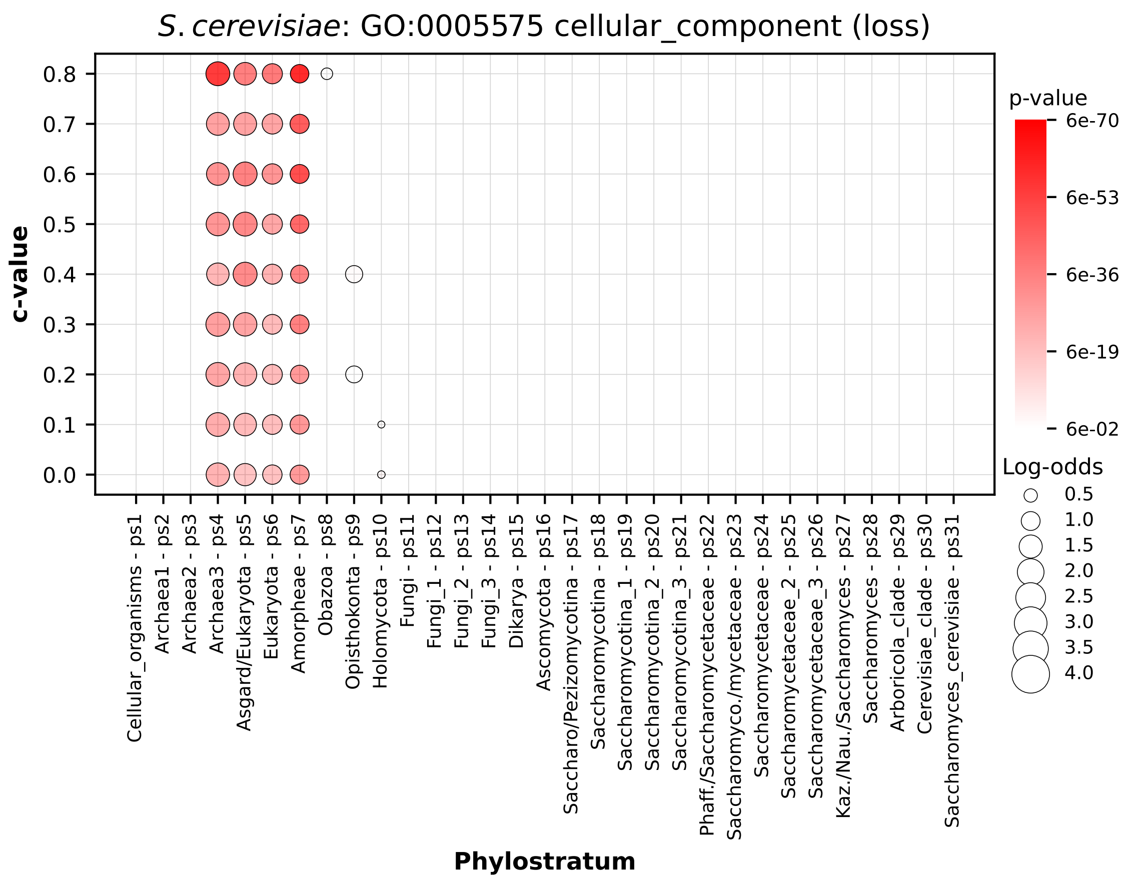

S. cerevisiae: GO:0005575 cellular_component (loss)
c-value
0.0
0.1
0.2
0.3
0.4
0.5
0.6
0.7
0.8
Phylostratum
Cellular_organisms - ps1
Archaea1 - ps2
Archaea2 - ps3
Archaea3 - ps4
Asgard/Eukaryota - ps5
Eukaryota - ps6
Amorpheae - ps7
Obazoa - ps8
Opisthokonta - ps9
Holomycota - ps10
Fungi - ps11
Fungi_1 - ps12
Fungi_2 - ps13
Fungi_3 - ps14
Dikarya - ps15
Ascomycota - ps16
Saccharo/Pezizomycotina - ps17
Saccharomycotina - ps18
Saccharomycotina_1 - ps19
Saccharomycotina_2 - ps20
Saccharomycotina_3 - ps21
Phaff./Saccharomycetaceae - ps22
Saccharomyco./mycetaceae - ps23
Saccharomycetaceae - ps24
Saccharomycetaceae_2 - ps25
Saccharomycetaceae_3 - ps26
Kaz./Nau./Saccharomyces - ps27
Saccharomyces - ps28
Arboricola_clade - ps29
Cerevisiae_clade - ps30
Saccharomyces_cerevisiae - ps31
p-value
6e-70
6e-53
6e-36
6e-19
6e-02
Log-odds
0.5
1.0
1.5
2.0
2.5
3.0
3.5
4.0

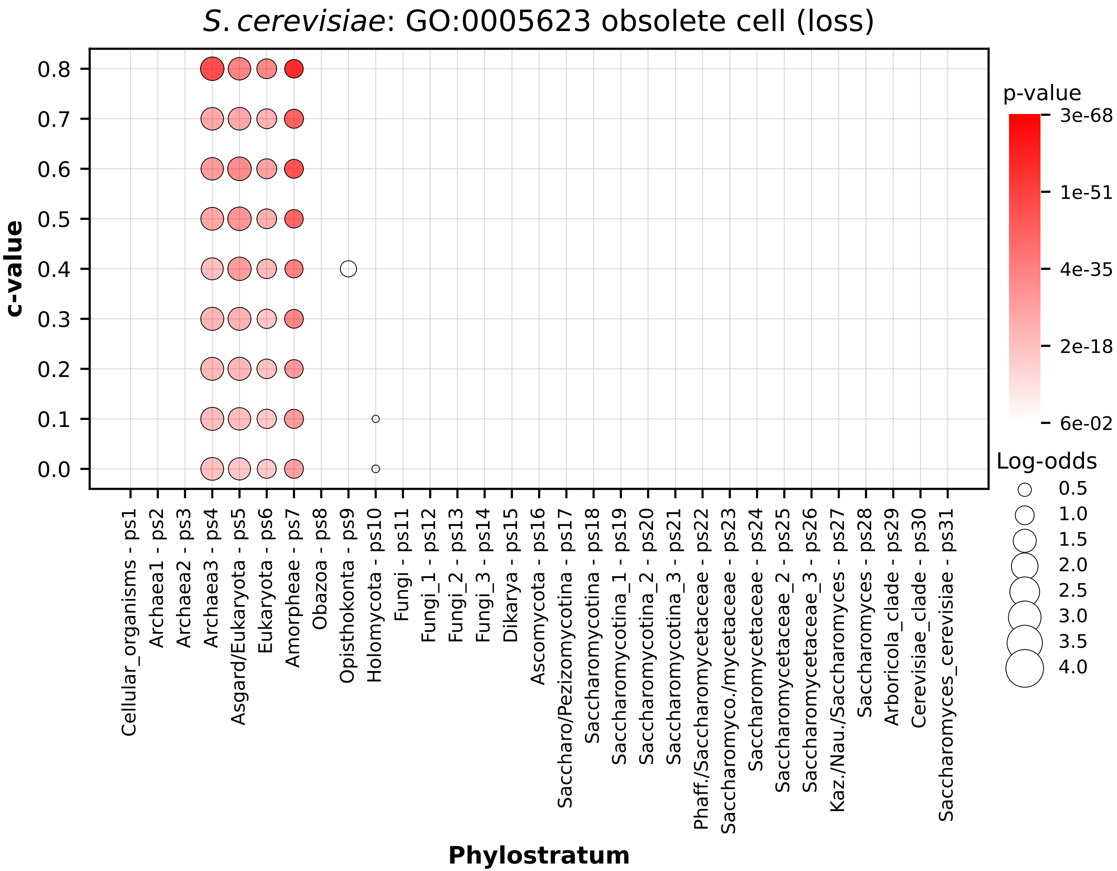
S. cerevisiae: GO:0005623 obsolete cell (loss)
c-value
0.8
0.7
0.6
0.5
0.4
0.3
0.2
0.1
0.0
p-value
3e-68
1e-51
4e-35
2e-18
6e-02
Log-odds
0.5
1.0
1.5
2.0
2.5
3.0
3.5
4.0
Cellular_organisms - ps1
Archaea1 - ps2
Archaea2 - ps3
Archaea3 - ps4
Asgard/Eukaryota - ps5
Eukaryota - ps6
Amorpheae - ps7
Obazoa - ps8
Opisthokonta - ps9
Holomycota - ps10
Fungi - ps11
Fungi_1 - ps12
Fungi_2 - ps13
Fungi_3 - ps14
Dikarya - ps15
Ascomycota - ps16
Saccharo/Pezizomycotina - ps17
Saccharomycotina - ps18
Saccharomycotina_1 - ps19
Saccharomycotina_2 - ps20
Saccharomycotina_3 - ps21
Phaff./Saccharomycetaceae - ps22
Saccharomyco./mycetaceae - ps23
Saccharomycetaceae - ps24
Saccharomycetaceae_2 - ps25
Saccharomycetaceae_3 - ps26
Kaz./Nau./Saccharomyces - ps27
Saccharomyces - ps28
Arboricola_clade - ps29
Cerevisiae_clade - ps30
Saccharomyces_cerevisiae - ps31
Phylostratum

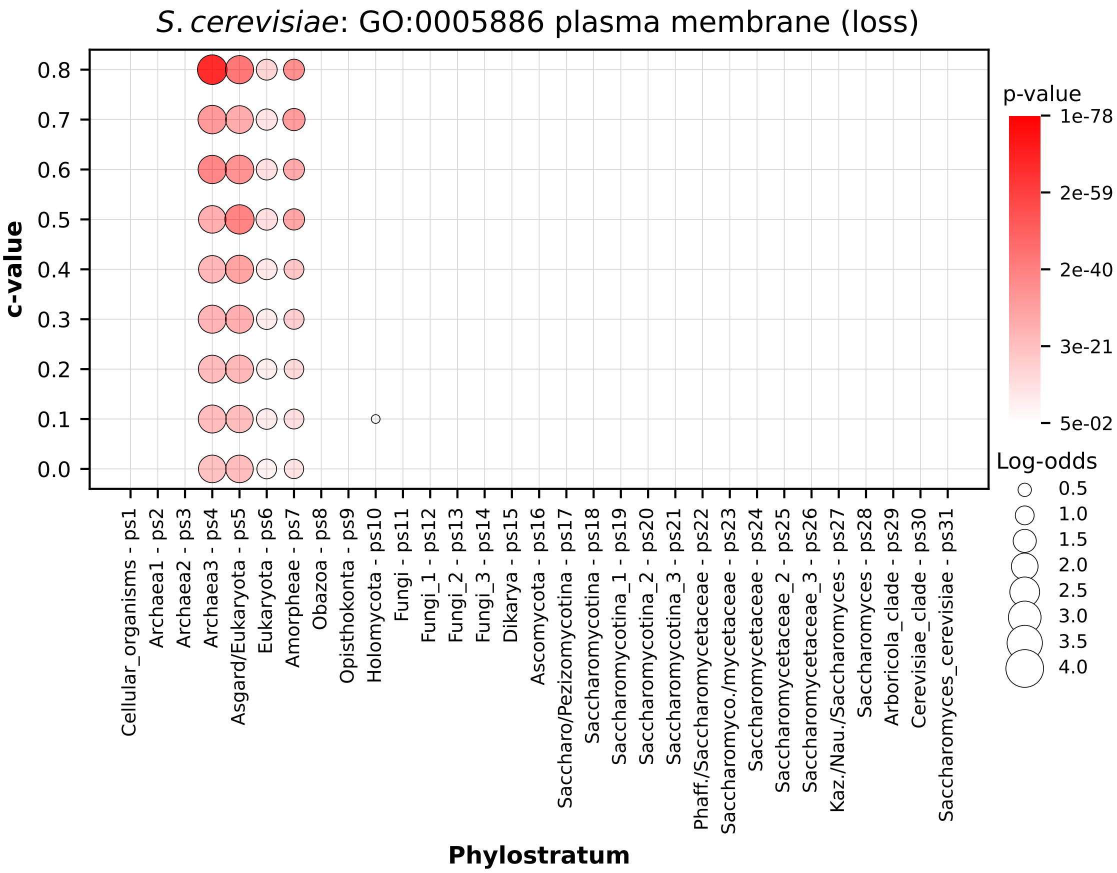

S. cerevisiae: GO:0005886 plasma membrane (loss)
c-value
0.8
0.7
0.6
0.5
0.4
0.3
0.2
0.1
0.0
Phylostratum
Cellular_organisms - ps1
Archaea1 - ps2
Archaea2 - ps3
Archaea3 - ps4
Asgard/Eukaryota - ps5
Eukaryota - ps6
Amorpheae - ps7
Obazoa - ps8
Opisthokonta - ps9
Holomycota - ps10
Fungi - ps11
Fungi_1 - ps12
Fungi_2 - ps13
Fungi_3 - ps14
Dikarya - ps15
Ascomycota - ps16
Saccharo/Pezizomycotina - ps17
Saccharomycotina - ps18
Saccharomycotina_1 - ps19
Saccharomycotina_2 - ps20
Saccharomycotina_3 - ps21
Phaff./Saccharomycetaceae - ps22
Saccharomyco./mycetaceae - ps23
Saccharomycetaceae - ps24
Saccharomycetaceae_2 - ps25
Saccharomycetaceae_3 - ps26
Kaz./Nau./Saccharomyces - ps27
Saccharomyces - ps28
Arboricola_clade - ps29
Cerevisiae_clade - ps30
Saccharomyces_cerevisiae - ps31
p-value
1e-78
2e-59
2e-40
3e-21
5e-02
Log-odds
0.5
1.0
1.5
2.0
2.5
3.0
3.5
4.0

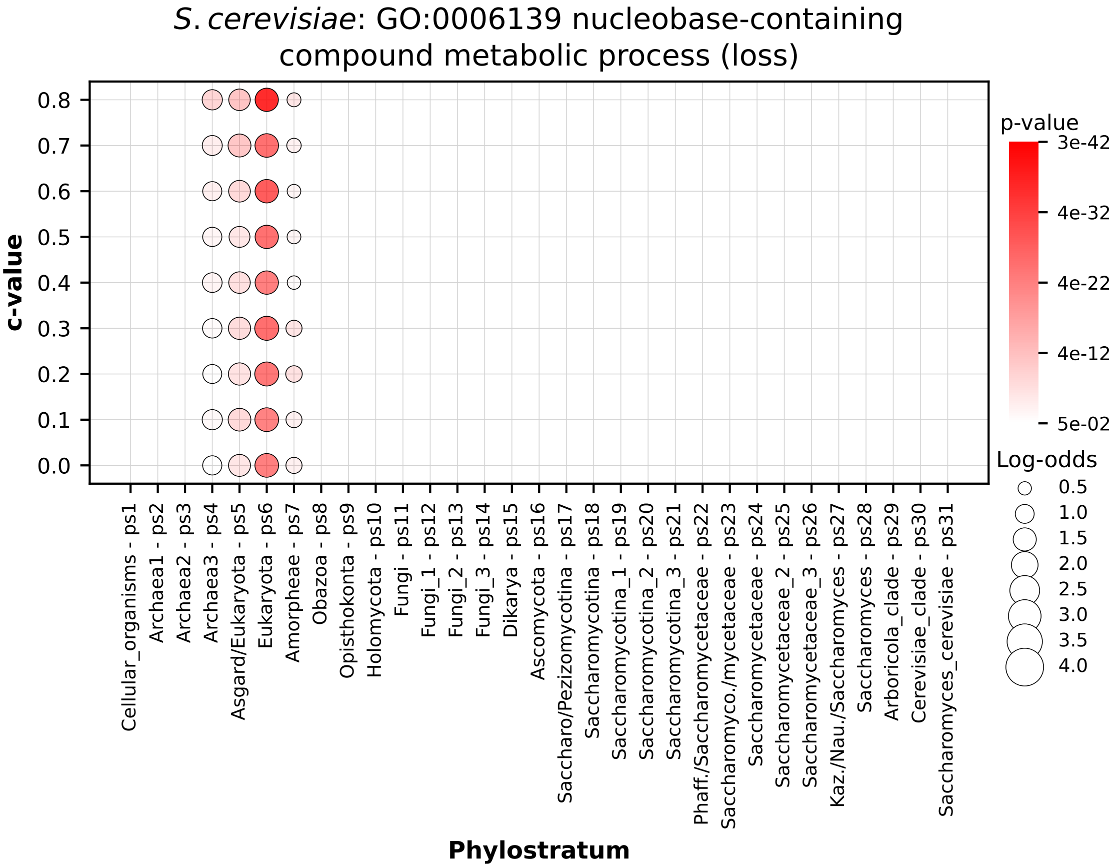

S. cerevisiae: GO:0006139 nucleobase-containing compound metabolic process (loss)
c-value
0.8
0.7
0.6
0.5
0.4
0.3
0.2
0.1
0.0
p-value
3e-42
4e-32
4e-22
4e-12
5e-02
Log-odds
0.5
1.0
1.5
2.0
2.5
3.0
3.5
4.0
Cellular_organisms - ps1
Archaea1 - ps2
Archaea2 - ps3
Archaea3 - ps4
Asgard/Eukaryota - ps5
Eukaryota - ps6
Amorpheae - ps7
Obazoa - ps8
Opisthokonta - ps9
Holomycota - ps10
Fungi - ps11
Fungi_1 - ps12
Fungi_2 - ps13
Fungi_3 - ps14
Dikarya - ps15
Ascomycota - ps16
Saccharo/Pezizomycotina - ps17
Saccharomycotina - ps18
Saccharomycotina_1 - ps19
Saccharomycotina_2 - ps20
Saccharomycotina_3 - ps21
Phaff./Saccharomycetaceae - ps22
Saccharomyco./mycetaceae - ps23
Saccharomycetaceae - ps24
Saccharomycetaceae_2 - ps25
Saccharomycetaceae_3 - ps26
Kaz./Nau./Saccharomyces - ps27
Saccharomyces - ps28
Arboricola_clade - ps29
Cerevisiae_clade - ps30
Saccharomyces_cerevisiae - ps31
Phylostratum

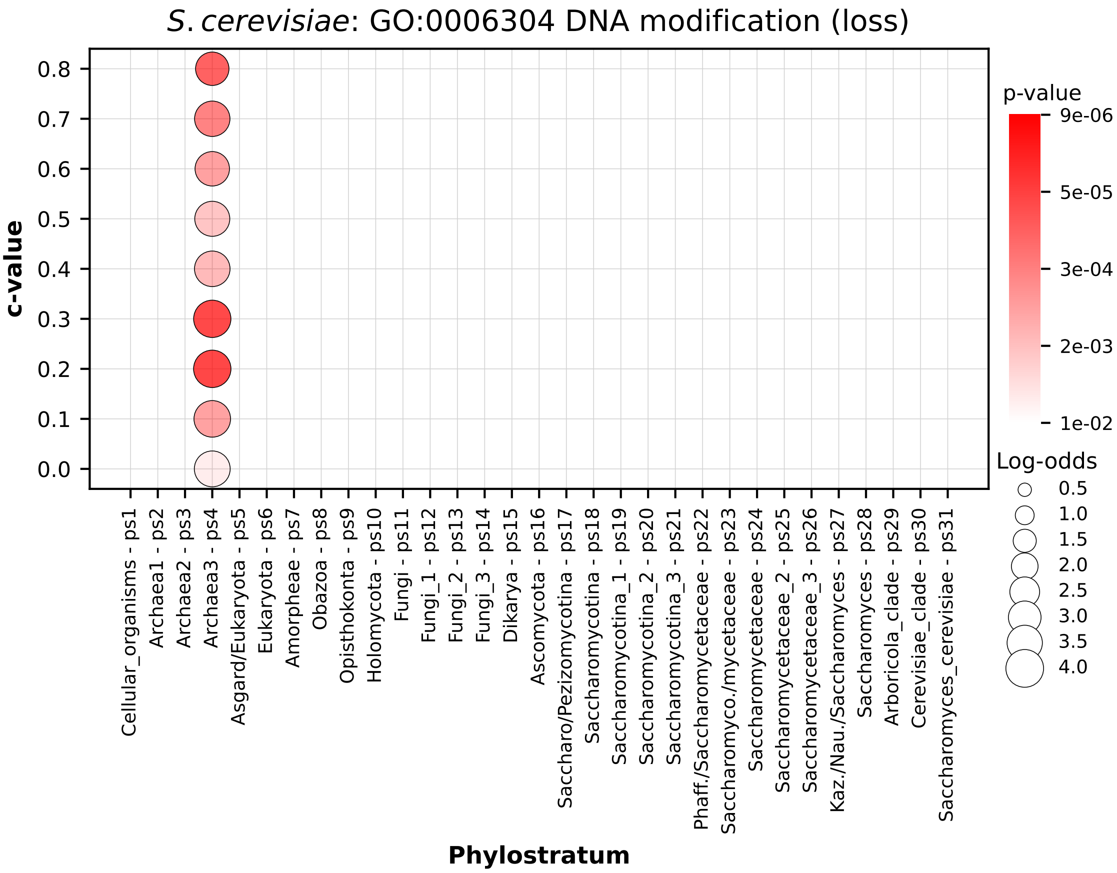
S. cerevisiae: GO:0006304 DNA modification (loss)
c-value
0.8
0.7
0.6
0.5
0.4
0.3
0.2
0.1
0.0
Phylostratum
Cellular_organisms - ps1
Archaea1 - ps2
Archaea2 - ps3
Archaea3 - ps4
Asgard/Eukaryota - ps5
Eukaryota - ps6
Amorpheae - ps7
Obazoa - ps8
Opisthokonta - ps9
Holomycota - ps10
Fungi - ps11
Fungi_1 - ps12
Fungi_2 - ps13
Fungi_3 - ps14
Dikarya - ps15
Ascomycota - ps16
Saccharo/Pezizomycotina - ps17
Saccharomycotina - ps18
Saccharomycotina_1 - ps19
Saccharomycotina_2 - ps20
Saccharomycotina_3 - ps21
Phaff./Saccharomycetaceae - ps22
Saccharomyco./mycetaceae - ps23
Saccharomycetaceae - ps24
Saccharomycetaceae_2 - ps25
Saccharomycetaceae_3 - ps26
Kaz./Nau./Saccharomyces - ps27
Saccharomyces - ps28
Arboricola_clade - ps29
Cerevisiae_clade - ps30
Saccharomyces_cerevisiae - ps31
p-value
9e-06
5e-05
3e-04
2e-03
1e-02
Log-odds
0.5
1.0
1.5
2.0
2.5
3.0
3.5
4.0

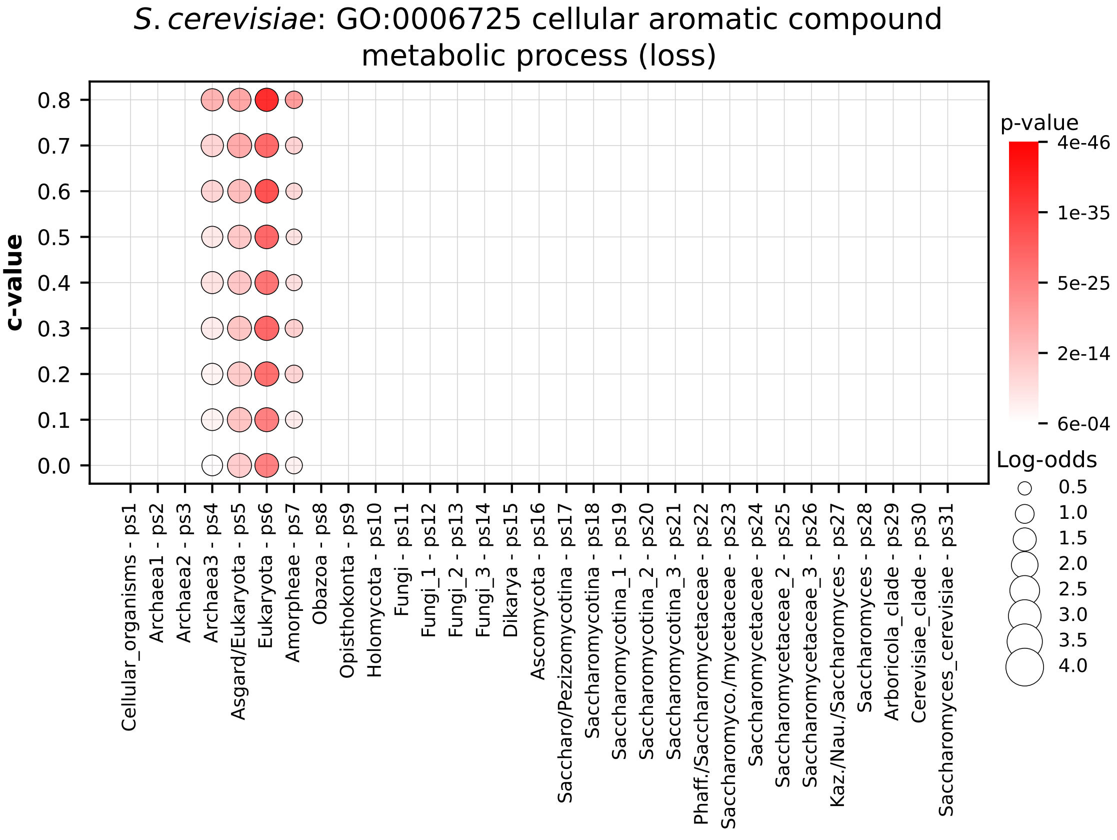

S. cerevisiae: GO:0006725 cellular aromatic compound metabolic process (loss)
c-value
0.0
0.1
0.2
0.3
0.4
0.5
0.6
0.7
0.8
Phylostratum
Cellular_organisms - ps1
Archaea1 - ps2
Archaea2 - ps3
Archaea3 - ps4
Asgard/Eukaryota - ps5
Eukaryota - ps6
Amorpheae - ps7
Obazoa - ps8
Opisthokonta - ps9
Holomycota - ps10
Fungi - ps11
Fungi_1 - ps12
Fungi_2 - ps13
Fungi_3 - ps14
Dikarya - ps15
Ascomycota - ps16
Saccharo/Pezizomycotina - ps17
Saccharomycotina - ps18
Saccharomycotina_1 - ps19
Saccharomycotina_2 - ps20
Saccharomycotina_3 - ps21
Phaff./Saccharomycetaceae - ps22
Saccharomyco./mycetaceae - ps23
Saccharomycetaceae - ps24
Saccharomycetaceae_2 - ps25
Saccharomycetaceae_3 - ps26
Kaz./Nau./Saccharomyces - ps27
Saccharomyces - ps28
Arboricola_clade - ps29
Cerevisiae_clade - ps30
Saccharomyces_cerevisiae - ps31
p-value
4e-46
1e-35
5e-25
2e-14
6e-04
Log-odds
0.5
1.0
1.5
2.0
2.5
3.0
3.5
4.0

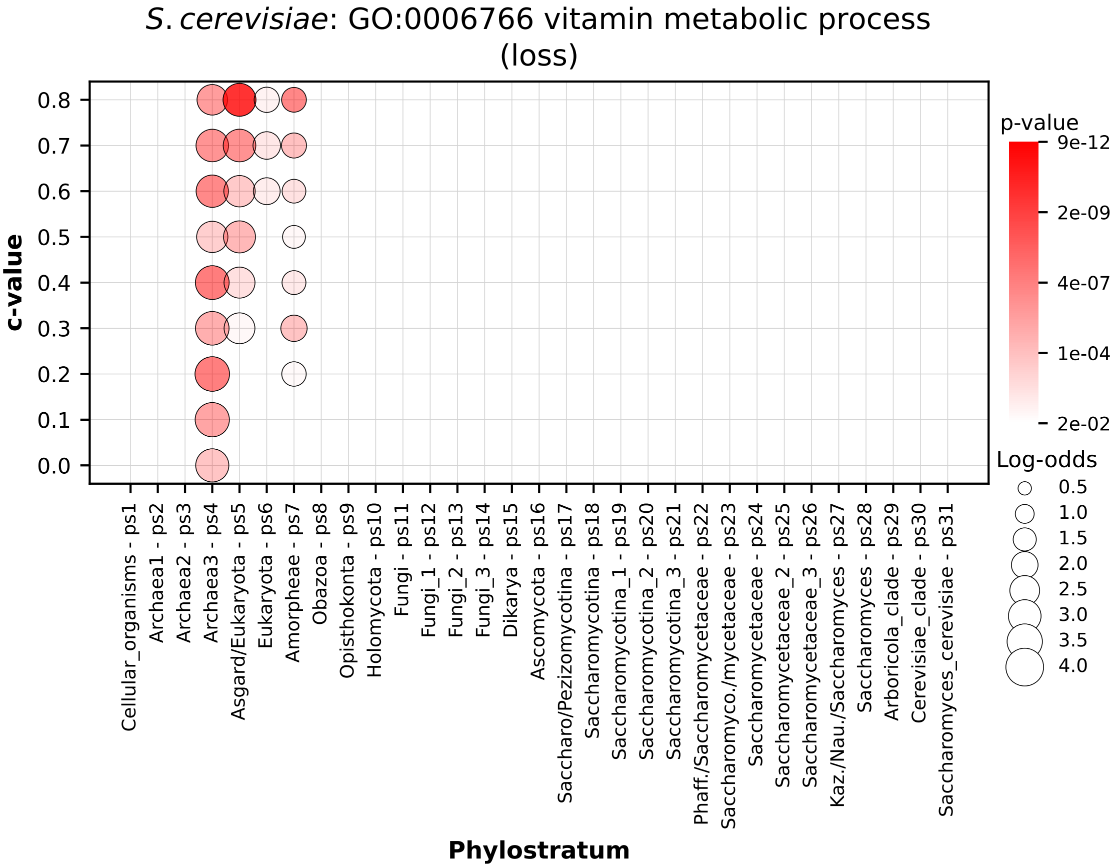

S. cerevisiae: GO:0006766 vitamin metabolic process
(loss)
c-value
0.8
0.7
0.6
0.5
0.4
0.3
0.2
0.1
0.0
Phylostratum
Cellular_organisms - ps1
Archaea1 - ps2
Archaea2 - ps3
Archaea3 - ps4
Asgard/Eukaryota - ps5
Eukaryota - ps6
Amorpheae - ps7
Obazoa - ps8
Opisthokonta - ps9
Holomycota - ps10
Fungi - ps11
Fungi_1 - ps12
Fungi_2 - ps13
Fungi_3 - ps14
Dikarya - ps15
Ascomycota - ps16
Saccharo/Pezizomycotina - ps17
Saccharomycotina - ps18
Saccharomycotina_1 - ps19
Saccharomycotina_2 - ps20
Saccharomycotina_3 - ps21
Phaff./Saccharomycetaceae - ps22
Saccharomyco./mycetaceae - ps23
Saccharomycetaceae - ps24
Saccharomycetaceae_2 - ps25
Saccharomycetaceae_3 - ps26
Kaz./Nau./Saccharomyces - ps27
Saccharomyces - ps28
Arboricola_clade - ps29
Cerevisiae_clade - ps30
Saccharomyces_cerevisiae - ps31
p-value
9e-12
2e-09
4e-07
1e-04
2e-02
Log-odds
0.5
1.0
1.5
2.0
2.5
3.0
3.5
4.0

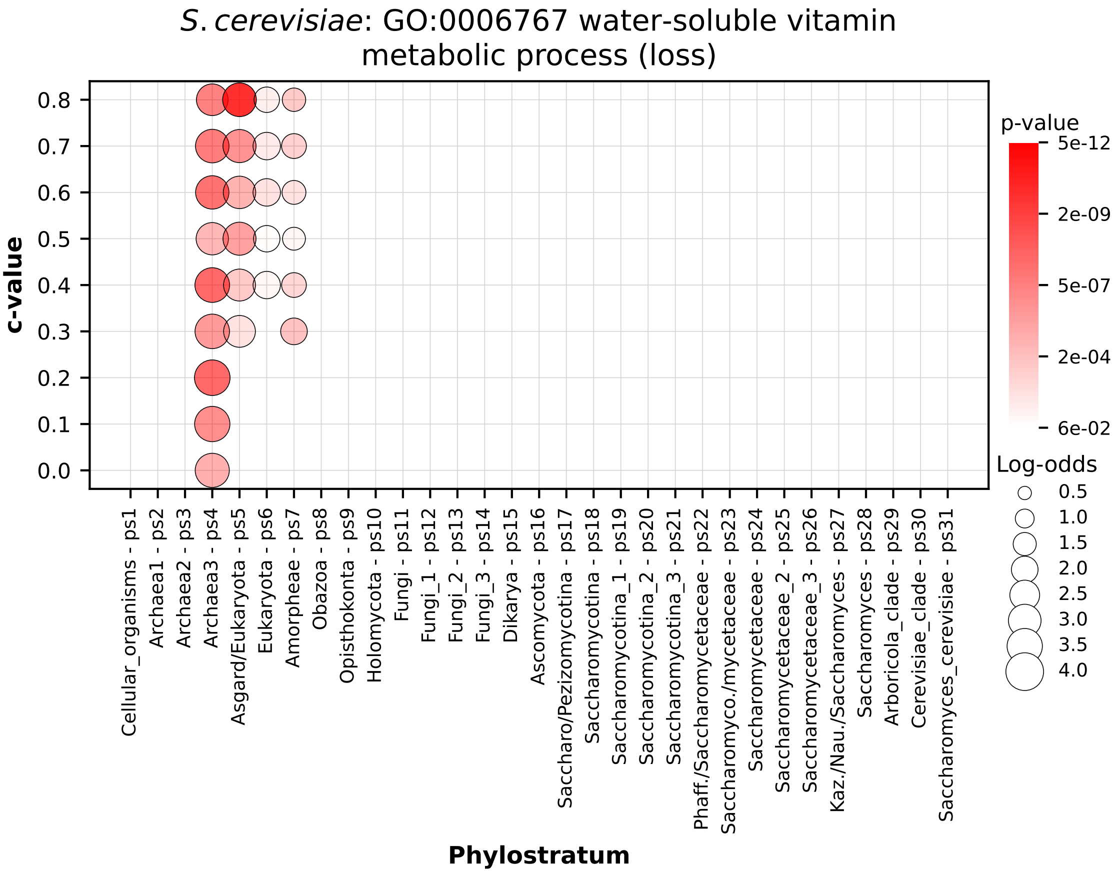

S. cerevisiae: GO:0006767 water-soluble vitamin metabolic process (loss)
c-value
0.8
0.7
0.6
0.5
0.4
0.3
0.2
0.1
0.0
p-value
5e-12
2e-09
5e-07
2e-04
6e-02
Log-odds
0.5
1.0
1.5
2.0
2.5
3.0
3.5
4.0
Cellular_organisms - ps1
Archaea1 - ps2
Archaea2 - ps3
Archaea3 - ps4
Asgard/Eukaryota - ps5
Eukaryota - ps6
Amorpheae - ps7
Obazoa - ps8
Opisthokonta - ps9
Holomycota - ps10
Fungi - ps11
Fungi_1 - ps12
Fungi_2 - ps13
Fungi_3 - ps14
Dikarya - ps15
Ascomycota - ps16
Saccharo/Pezizomycotina - ps17
Saccharomycotina - ps18
Saccharomycotina_1 - ps19
Saccharomycotina_2 - ps20
Saccharomycotina_3 - ps21
Phaff./Saccharomycetaceae - ps22
Saccharomyco./mycetaceae - ps23
Saccharomycetaceae - ps24
Saccharomycetaceae_2 - ps25
Saccharomycetaceae_3 - ps26
Kaz./Nau./Saccharomyces - ps27
Saccharomyces - ps28
Arboricola_clade - ps29
Cerevisiae_clade - ps30
Saccharomyces_cerevisiae - ps31
Phylostratum

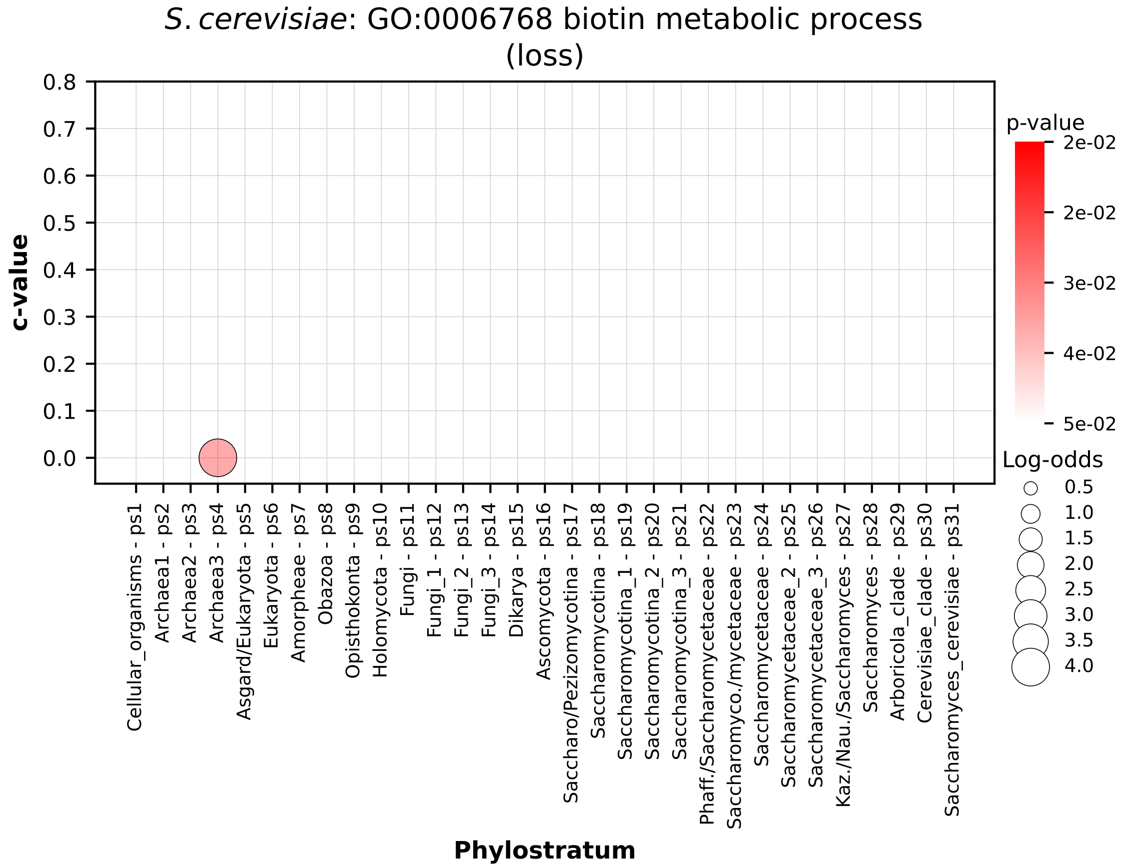

S. cerevisiae: GO:0006768 biotin metabolic process
(loss)
c-value
0.8
0.7
0.6
0.5
0.4
0.3
0.2
0.1
0.0
p-value
2e-02
2e-02
3e-02
4e-02
5e-02
Log-odds
0.5
1.0
1.5
2.0
2.5
3.0
3.5
4.0
Cellular_organisms - ps1
Archaea1 - ps2
Archaea2 - ps3
Archaea3 - ps4
Asgard/Eukaryota - ps5
Eukaryota - ps6
Amorpheae - ps7
Obazoa - ps8
Opisthokonta - ps9
Holomycota - ps10
Fungi - ps11
Fungi_1 - ps12
Fungi_2 - ps13
Fungi_3 - ps14
Dikarya - ps15
Ascomycota - ps16
Saccharo/Pezizomycotina - ps17
Saccharomycotina - ps18
Saccharomycotina_1 - ps19
Saccharomycotina_2 - ps20
Saccharomycotina_3 - ps21
Phaff./Saccharomycetaceae - ps22
Saccharomyco./mycetaceae - ps23
Saccharomycetaceae - ps24
Saccharomycetaceae_2 - ps25
Saccharomycetaceae_3 - ps26
Kaz./Nau./Saccharomyces - ps27
Saccharomyces - ps28
Arboricola_clade - ps29
Cerevisiae_clade - ps30
Saccharomyces_cerevisiae - ps31
Phylostratum

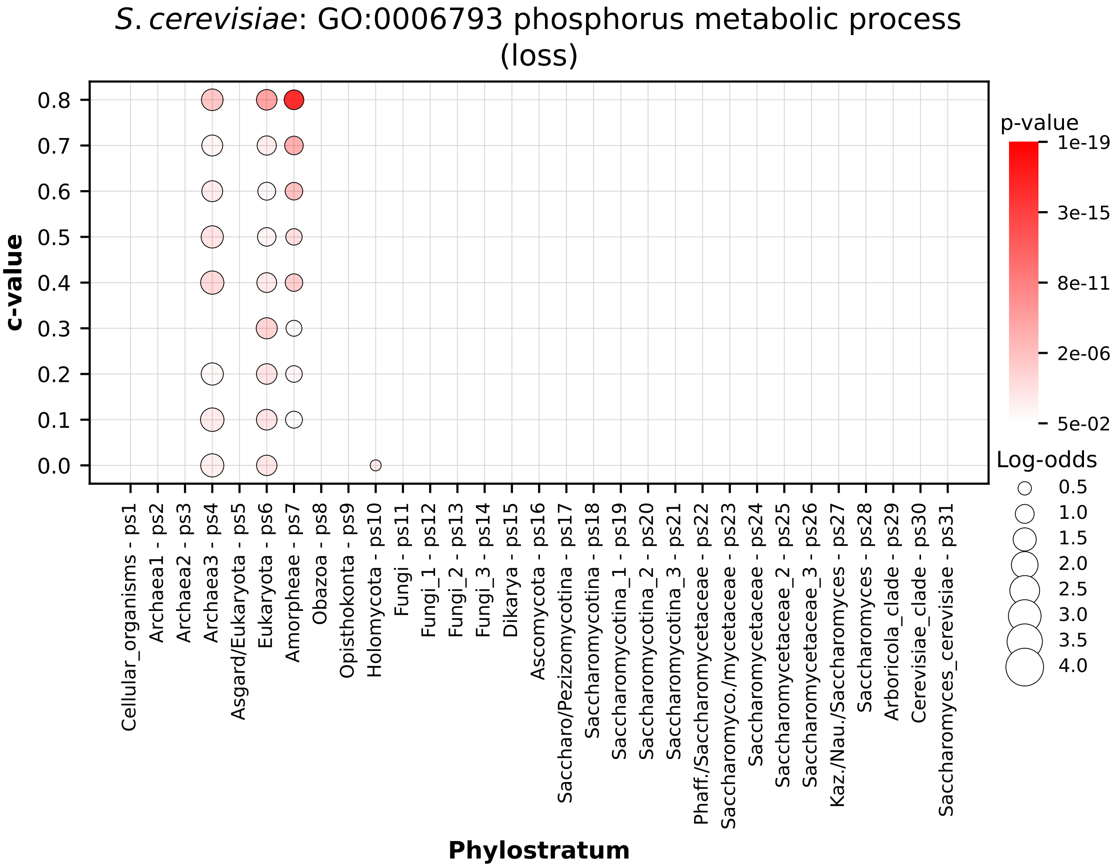
S. cerevisiae: GO:0006793 phosphorus metabolic process
(loss)
c-value
0.8
0.7
0.6
0.5
0.4
0.3
0.2
0.1
0.0
Phylostratum
Cellular_organisms - ps1
Archaea1 - ps2
Archaea2 - ps3
Archaea3 - ps4
Asgard/Eukaryota - ps5
Eukaryota - ps6
Amorpheae - ps7
Obazoa - ps8
Opisthokonta - ps9
Holomycota - ps10
Fungi - ps11
Fungi_1 - ps12
Fungi_2 - ps13
Fungi_3 - ps14
Dikarya - ps15
Ascomycota - ps16
Saccharo/Pezizomycotina - ps17
Saccharomycotina - ps18
Saccharomycotina_1 - ps19
Saccharomycotina_2 - ps20
Saccharomycotina_3 - ps21
Phaff./Saccharomycetaceae - ps22
Saccharomyco./mycetaceae - ps23
Saccharomycetaceae - ps24
Saccharomycetaceae_2 - ps25
Saccharomycetaceae_3 - ps26
Kaz./Nau./Saccharomyces - ps27
Saccharomyces - ps28
Arboricola_clade - ps29
Cerevisiae_clade - ps30
Saccharomyces_cerevisiae - ps31
p-value
1e-19
3e-15
8e-11
2e-06
5e-02
Log-odds
0.5
1.0
1.5
2.0
2.5
3.0
3.5
4.0

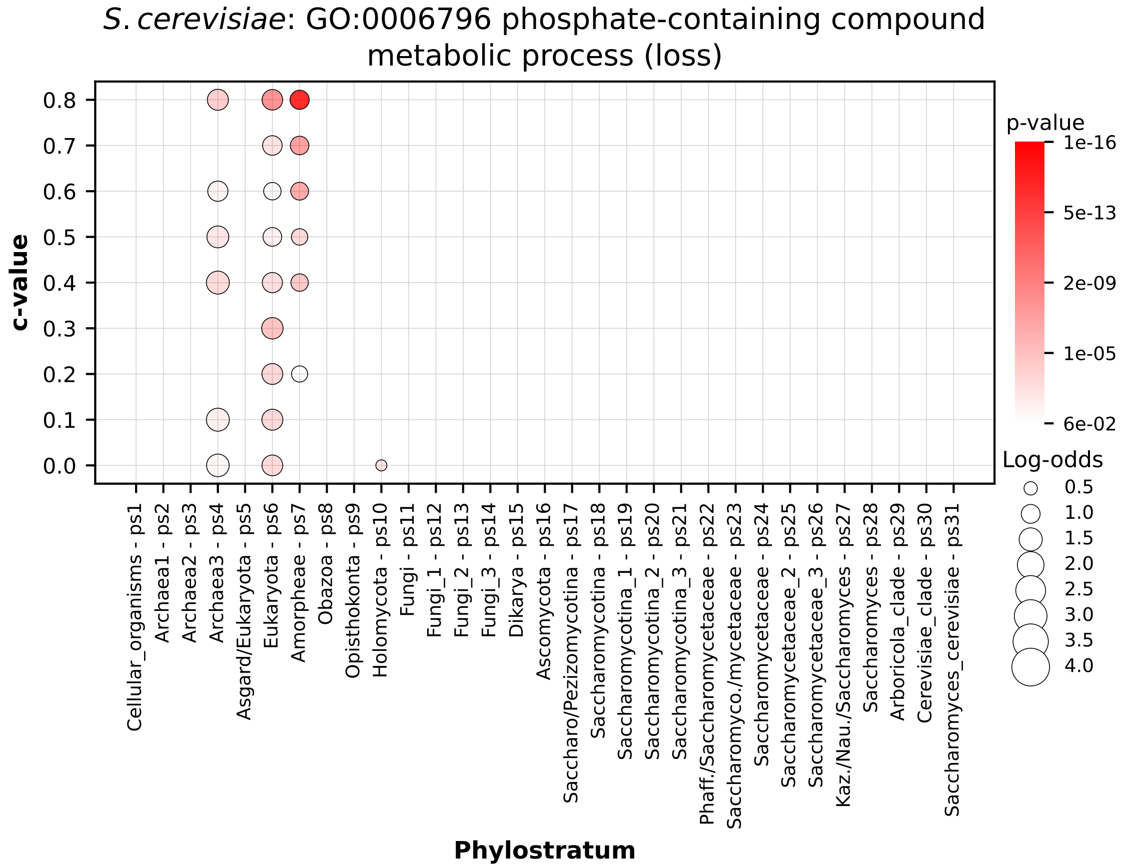
S. cerevisiae: GO:0006796 phosphate-containing compound metabolic process (loss)
c-value
0.8
0.7
0.6
0.5
0.4
0.3
0.2
0.1
0.0
p-value
1e-16
5e-13
2e-09
1e-05
6e-02
Log-odds
0.5
1.0
1.5
2.0
2.5
3.0
3.5
4.0
Cellular_organisms - ps1
Archaea1 - ps2
Archaea2 - ps3
Archaea3 - ps4
Asgard/Eukaryota - ps5
Eukaryota - ps6
Amorpheae - ps7
Obazoa - ps8
Opisthokonta - ps9
Holomycota - ps10
Fungi - ps11
Fungi_1 - ps12
Fungi_2 - ps13
Fungi_3 - ps14
Dikarya - ps15
Ascomycota - ps16
Saccharo/Pezizomycotina - ps17
Saccharomycotina - ps18
Saccharomycotina_1 - ps19
Saccharomycotina_2 - ps20
Saccharomycotina_3 - ps21
Phaff./Saccharomycetaceae - ps22
Saccharomyco./mycetaceae - ps23
Saccharomycetaceae - ps24
Saccharomycetaceae_2 - ps25
Saccharomycetaceae_3 - ps26
Kaz./Nau./Saccharomyces - ps27
Saccharomyces - ps28
Arboricola_clade - ps29
Cerevisiae_clade - ps30
Saccharomyces_cerevisiae - ps31
Phylostratum

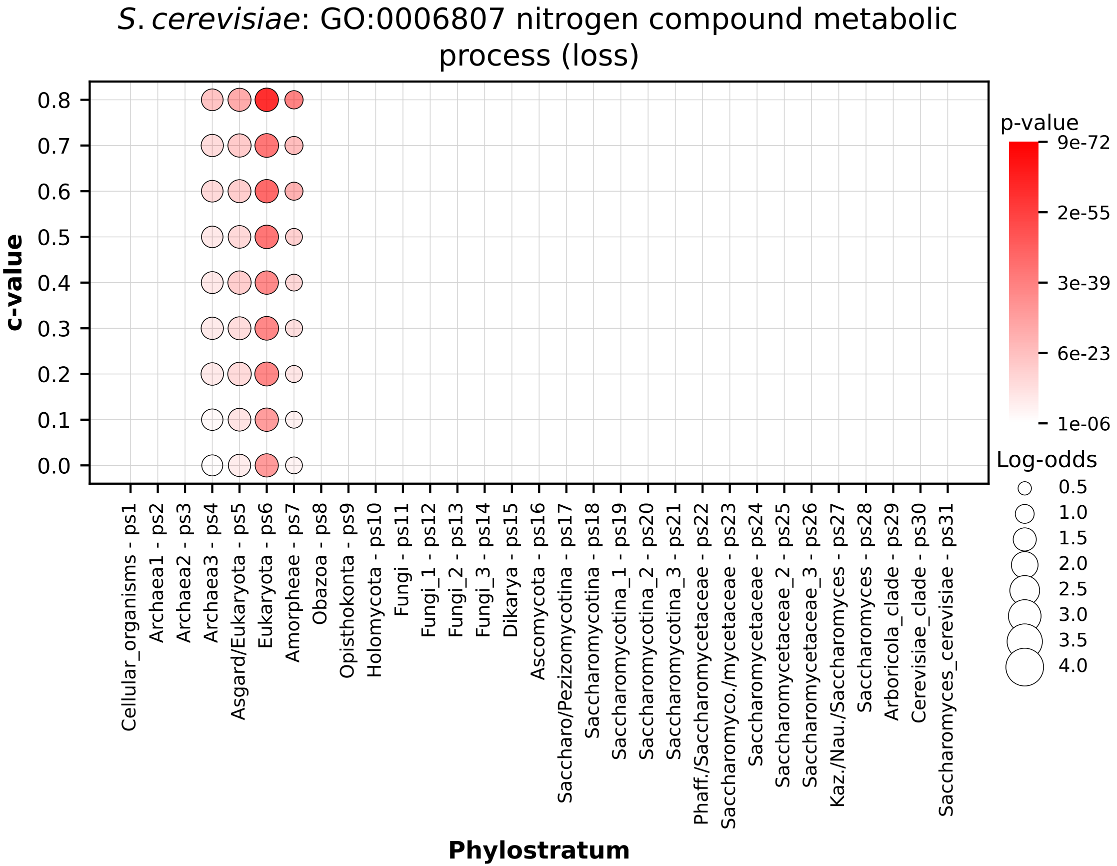

S. cerevisiae: GO:0006807 nitrogen compound metabolic process (loss)
c-value
0.8
0.7
0.6
0.5
0.4
0.3
0.2
0.1
0.0
p-value
9e-72
2e-55
3e-39
6e-23
1e-06
Log-odds
0.5
1.0
1.5
2.0
2.5
3.0
3.5
4.0
Cellular_organisms - ps1
Archaea1 - ps2
Archaea2 - ps3
Archaea3 - ps4
Asgard/Eukaryota - ps5
Eukaryota - ps6
Amorpheae - ps7
Obazoa - ps8
Opisthokonta - ps9
Holomycota - ps10
Fungi - ps11
Fungi_1 - ps12
Fungi_2 - ps13
Fungi_3 - ps14
Dikarya - ps15
Ascomycota - ps16
Saccharo/Pezizomycotina - ps17
Saccharomycotina - ps18
Saccharomycotina_1 - ps19
Saccharomycotina_2 - ps20
Saccharomycotina_3 - ps21
Phaff./Saccharomycetaceae - ps22
Saccharomyco./mycetaceae - ps23
Saccharomycetaceae - ps24
Saccharomycetaceae_2 - ps25
Saccharomycetaceae_3 - ps26
Kaz./Nau./Saccharomyces - ps27
Saccharomyces - ps28
Arboricola_clade - ps29
Cerevisiae_clade - ps30
Saccharomyces_cerevisiae - ps31
Phylostratum

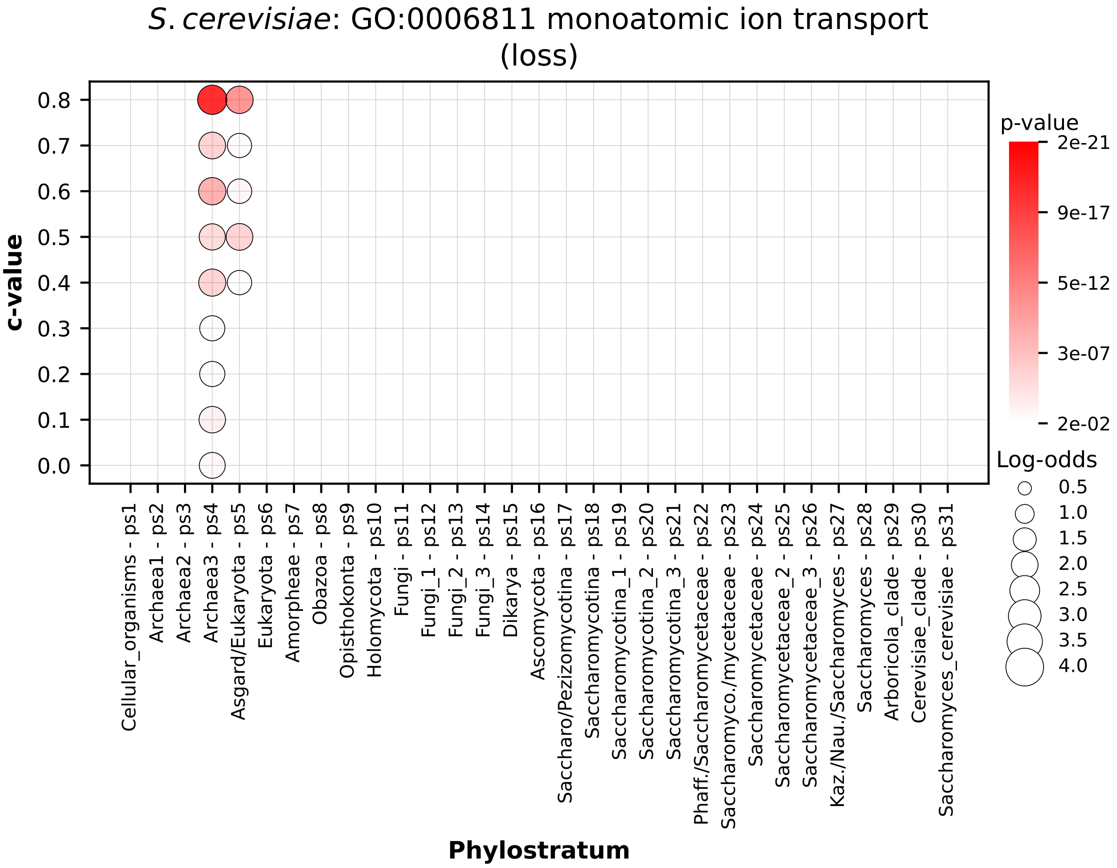

S. cerevisiae: GO:0006811 monoatomic ion transport
(loss)
c-value
0.8
0.7
0.6
0.5
0.4
0.3
0.2
0.1
0.0
Phylostratum
Cellular_organisms - ps1
Archaea1 - ps2
Archaea2 - ps3
Archaea3 - ps4
Asgard/Eukaryota - ps5
Eukaryota - ps6
Amorpheae - ps7
Obazoa - ps8
Opisthokonta - ps9
Holomycota - ps10
Fungi - ps11
Fungi_1 - ps12
Fungi_2 - ps13
Fungi_3 - ps14
Dikarya - ps15
Ascomycota - ps16
Saccharo/Pezizomycotina - ps17
Saccharomycotina - ps18
Saccharomycotina_1 - ps19
Saccharomycotina_2 - ps20
Saccharomycotina_3 - ps21
Phaff./Saccharomycetaceae - ps22
Saccharomyco./mycetaceae - ps23
Saccharomycetaceae - ps24
Saccharomycetaceae_2 - ps25
Saccharomycetaceae_3 - ps26
Kaz./Nau./Saccharomyces - ps27
Saccharomyces - ps28
Arboricola_clade - ps29
Cerevisiae_clade - ps30
Saccharomyces_cerevisiae - ps31
p-value
2e-21
9e-17
5e-12
3e-07
2e-02
Log-odds
0.5
1.0
1.5
2.0
2.5
3.0
3.5
4.0

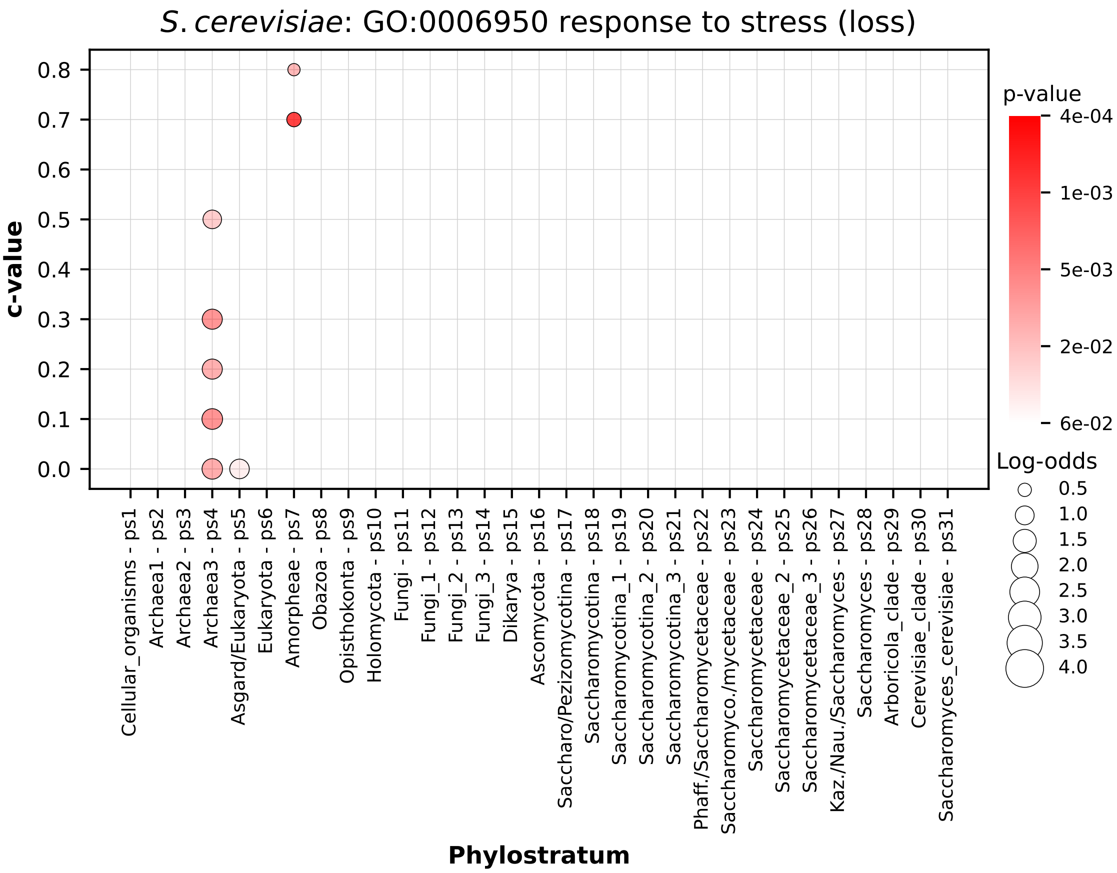
S. cerevisiae: GO:0006950 response to stress (loss)
c-value
0.8
0.7
0.6
0.5
0.4
0.3
0.2
0.1
0.0
p-value
4e-04
1e-03
5e-03
2e-02
6e-02
Log-odds
0.5
1.0
1.5
2.0
2.5
3.0
3.5
4.0
Cellular_organisms - ps1
Archaea1 - ps2
Archaea2 - ps3
Archaea3 - ps4
Asgard/Eukaryota - ps5
Eukaryota - ps6
Amorpheae - ps7
Obazoa - ps8
Opisthokonta - ps9
Holomycota - ps10
Fungi - ps11
Fungi_1 - ps12
Fungi_2 - ps13
Fungi_3 - ps14
Dikarya - ps15
Ascomycota - ps16
Saccharo/Pezizomycotina - ps17
Saccharomycotina - ps18
Saccharomycotina_1 - ps19
Saccharomycotina_2 - ps20
Saccharomycotina_3 - ps21
Phaff./Saccharomycetaceae - ps22
Saccharomyco./mycetaceae - ps23
Saccharomycetaceae - ps24
Saccharomycetaceae_2 - ps25
Saccharomycetaceae_3 - ps26
Kaz./Nau./Saccharomyces - ps27
Saccharomyces - ps28
Arboricola_clade - ps29
Cerevisiae_clade - ps30
Saccharomyces_cerevisiae - ps31
Phylostratum

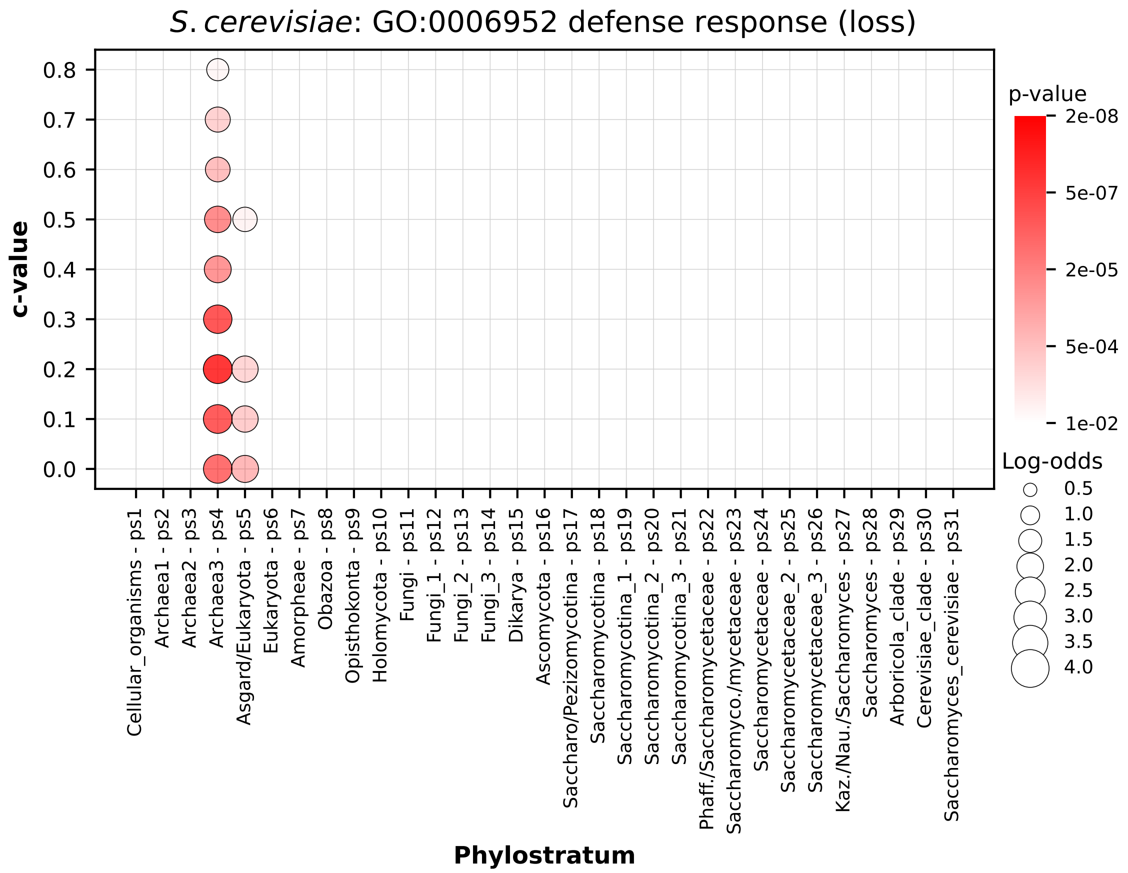

S. cerevisiae: GO:0006952 defense response (loss)
c-value
0.8
0.7
0.6
0.5
0.4
0.3
0.2
0.1
0.0
Phylostratum
Cellular_organisms - ps1
Archaea1 - ps2
Archaea2 - ps3
Archaea3 - ps4
Asgard/Eukaryota - ps5
Eukaryota - ps6
Amorpheae - ps7
Obazoa - ps8
Opisthokonta - ps9
Holomycota - ps10
Fungi - ps11
Fungi_1 - ps12
Fungi_2 - ps13
Fungi_3 - ps14
Dikarya - ps15
Ascomycota - ps16
Saccharo/Pezizomycotina - ps17
Saccharomycotina - ps18
Saccharomycotina_1 - ps19
Saccharomycotina_2 - ps20
Saccharomycotina_3 - ps21
Phaff./Saccharomycetaceae - ps22
Saccharomyco./mycetaceae - ps23
Saccharomycetaceae - ps24
Saccharomycetaceae_2 - ps25
Saccharomycetaceae_3 - ps26
Kaz./Nau./Saccharomyces - ps27
Saccharomyces - ps28
Arboricola_clade - ps29
Cerevisiae_clade - ps30
Saccharomyces_cerevisiae - ps31
p-value
2e-08
5e-07
2e-05
5e-04
1e-02
Log-odds
0.5
1.0
1.5
2.0
2.5
3.0
3.5
4.0

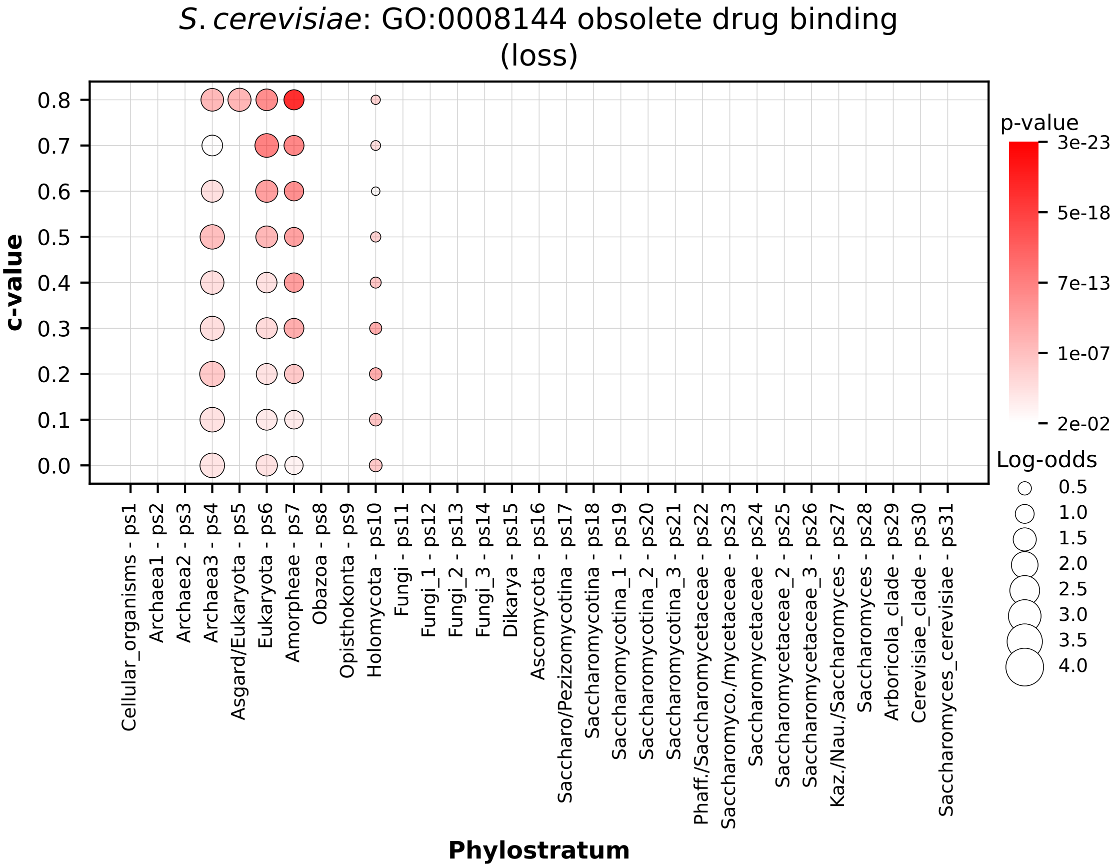

S. cerevisiae: GO:0008144 obsolete drug binding
(loss)
c-value
0.8
0.7
0.6
0.5
0.4
0.3
0.2
0.1
0.0
Phylostratum
Cellular_organisms - ps1
Archaea1 - ps2
Archaea2 - ps3
Archaea3 - ps4
Asgard/Eukaryota - ps5
Eukaryota - ps6
Amorpheae - ps7
Obazoa - ps8
Opisthokonta - ps9
Holomycota - ps10
Fungi - ps11
Fungi_1 - ps12
Fungi_2 - ps13
Fungi_3 - ps14
Dikarya - ps15
Ascomycota - ps16
Saccharo/Pezizomycotina - ps17
Saccharomycotina - ps18
Saccharomycotina_1 - ps19
Saccharomycotina_2 - ps20
Saccharomycotina_3 - ps21
Phaff./Saccharomycetaceae - ps22
Saccharomyco./mycetaceae - ps23
Saccharomycetaceae - ps24
Saccharomycetaceae_2 - ps25
Saccharomycetaceae_3 - ps26
Kaz./Nau./Saccharomyces - ps27
Saccharomyces - ps28
Arboricola_clade - ps29
Cerevisiae_clade - ps30
Saccharomyces_cerevisiae - ps31
p-value
3e-23
5e-18
7e-13
1e-07
2e-02
Log-odds
0.5
1.0
1.5
2.0
2.5
3.0
3.5
4.0

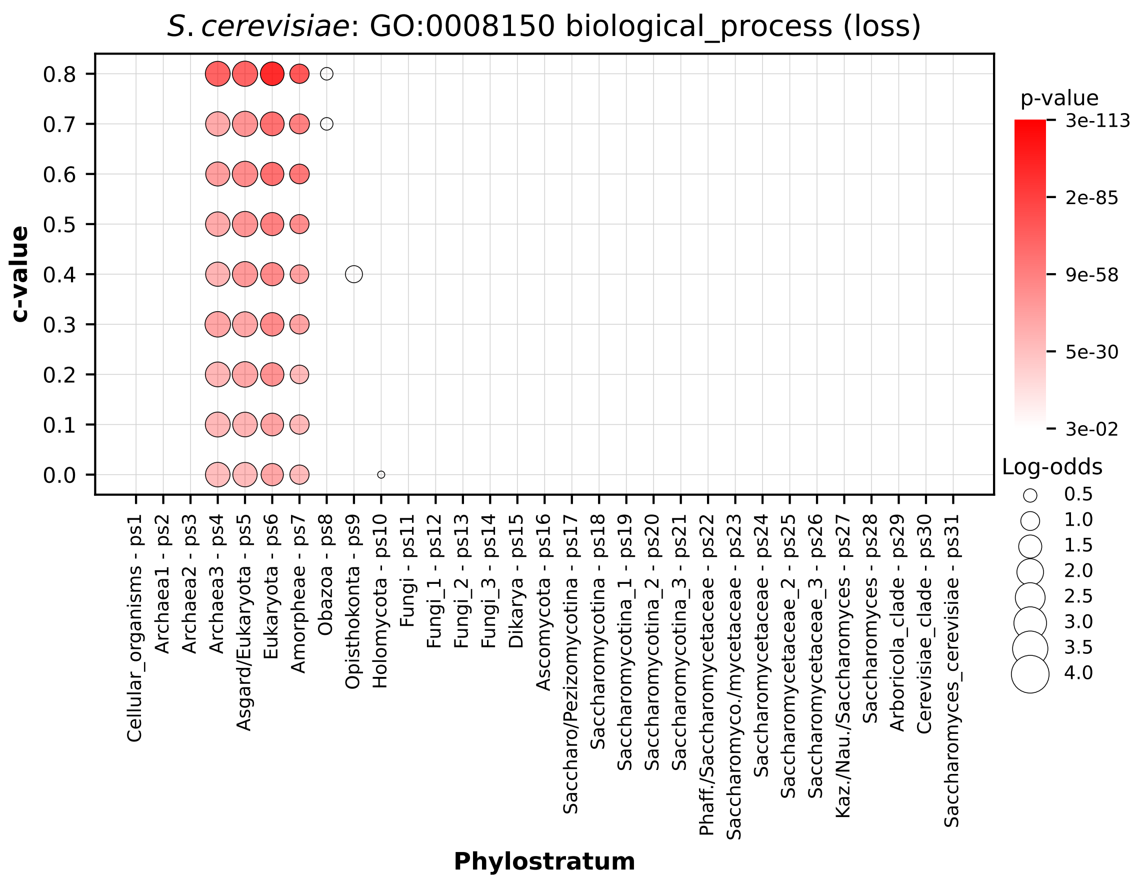
S. cerevisiae: GO:0008150 biological_process (loss)
c-value
0.8
0.7
0.6
0.5
0.4
0.3
0.2
0.1
0.0
p-value
3e-113
2e-85
9e-58
5e-30
3e-02
Log-odds
0.5
1.0
1.5
2.0
2.5
3.0
3.5
4.0
Cellular_organisms - ps1
Archaea1 - ps2
Archaea2 - ps3
Archaea3 - ps4
Asgard/Eukaryota - ps5
Eukaryota - ps6
Amorpheae - ps7
Obazoa - ps8
Opisthokonta - ps9
Holomycota - ps10
Fungi - ps11
Fungi_1 - ps12
Fungi_2 - ps13
Fungi_3 - ps14
Dikarya - ps15
Ascomycota - ps16
Saccharo/Pezizomycotina - ps17
Saccharomycotina - ps18
Saccharomycotina_1 - ps19
Saccharomycotina_2 - ps20
Saccharomycotina_3 - ps21
Phaff./Saccharomycetaceae - ps22
Saccharomyco./mycetaceae - ps23
Saccharomycetaceae - ps24
Saccharomycetaceae_2 - ps25
Saccharomycetaceae_3 - ps26
Kaz./Nau./Saccharomyces - ps27
Saccharomyces - ps28
Arboricola_clade - ps29
Cerevisiae_clade - ps30
Saccharomyces_cerevisiae - ps31
Phylostratum

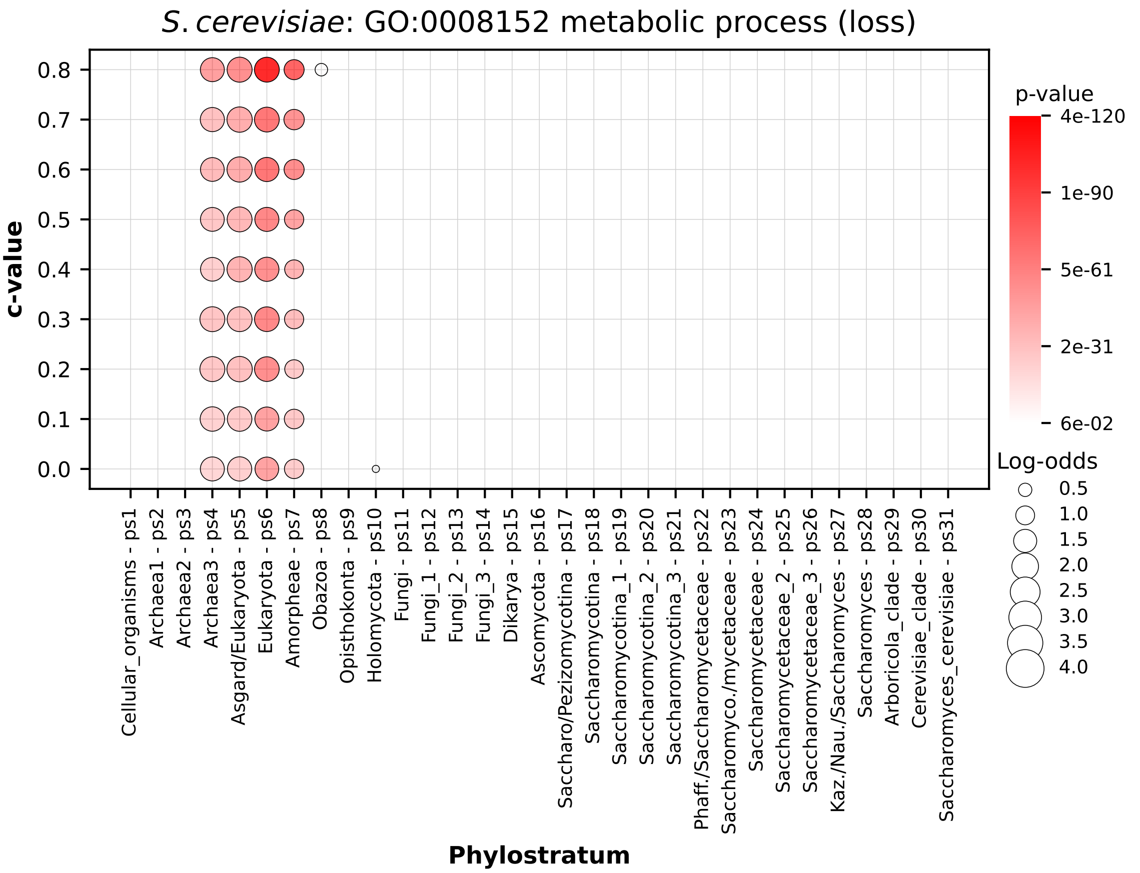

S. cerevisiae: GO:0008152 metabolic process (loss)
c-value
0.8
0.7
0.6
0.5
0.4
0.3
0.2
0.1
0.0
p-value
4e-120
1e-90
5e-61
2e-31
6e-02
Log-odds
0.5
1.0
1.5
2.0
2.5
3.0
3.5
4.0
Cellular_organisms - ps1
Archaea1 - ps2
Archaea2 - ps3
Archaea3 - ps4
Asgard/Eukaryota - ps5
Eukaryota - ps6
Amorpheae - ps7
Obazoa - ps8
Opisthokonta - ps9
Holomycota - ps10
Fungi - ps11
Fungi_1 - ps12
Fungi_2 - ps13
Fungi_3 - ps14
Dikarya - ps15
Ascomycota - ps16
Saccharo/Pezizomycotina - ps17
Saccharomycotina - ps18
Saccharomycotina_1 - ps19
Saccharomycotina_2 - ps20
Saccharomycotina_3 - ps21
Phaff./Saccharomycetaceae - ps22
Saccharomyco./mycetaceae - ps23
Saccharomycetaceae - ps24
Saccharomycetaceae_2 - ps25
Saccharomycetaceae_3 - ps26
Kaz./Nau./Saccharomyces - ps27
Saccharomyces - ps28
Arboricola_clade - ps29
Cerevisiae_clade - ps30
Saccharomyces_cerevisiae - ps31
Phylostratum

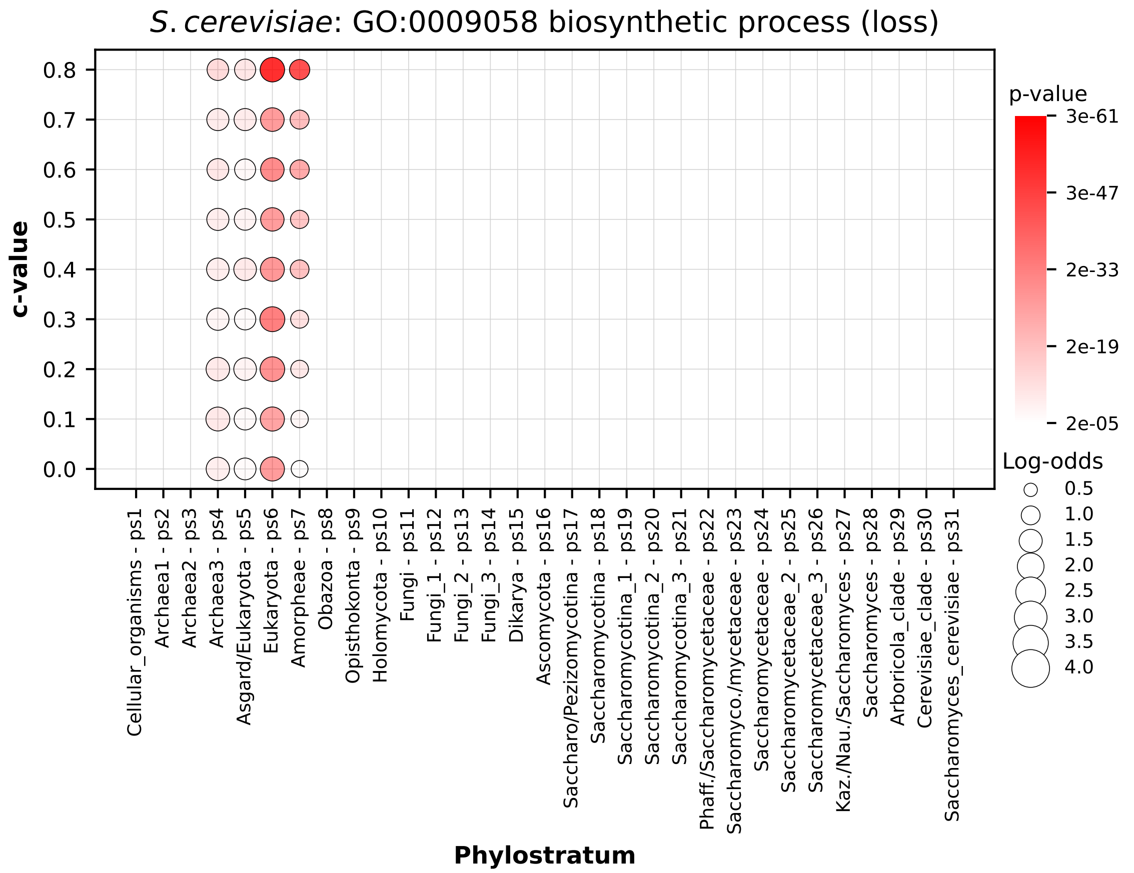

S. cerevisiae: GO:0009058 biosynthetic process (loss)
c-value
0.8
0.7
0.6
0.5
0.4
0.3
0.2
0.1
0.0
p-value
3e-61
3e-47
2e-33
2e-19
2e-05
Log-odds
0.5
1.0
1.5
2.0
2.5
3.0
3.5
4.0
Cellular_organisms - ps1
Archaea1 - ps2
Archaea2 - ps3
Archaea3 - ps4
Asgard/Eukaryota - ps5
Eukaryota - ps6
Amorpheae - ps7
Obazoa - ps8
Opisthokonta - ps9
Holomycota - ps10
Fungi - ps11
Fungi_1 - ps12
Fungi_2 - ps13
Fungi_3 - ps14
Dikarya - ps15
Ascomycota - ps16
Saccharo/Pezizomycotina - ps17
Saccharomycotina - ps18
Saccharomycotina_1 - ps19
Saccharomycotina_2 - ps20
Saccharomycotina_3 - ps21
Phaff./Saccharomycetaceae - ps22
Saccharomyco./mycetaceae - ps23
Saccharomycetaceae - ps24
Saccharomycetaceae_2 - ps25
Saccharomycetaceae_3 - ps26
Kaz./Nau./Saccharomyces - ps27
Saccharomyces - ps28
Arboricola_clade - ps29
Cerevisiae_clade - ps30
Saccharomyces_cerevisiae - ps31
Phylostratum

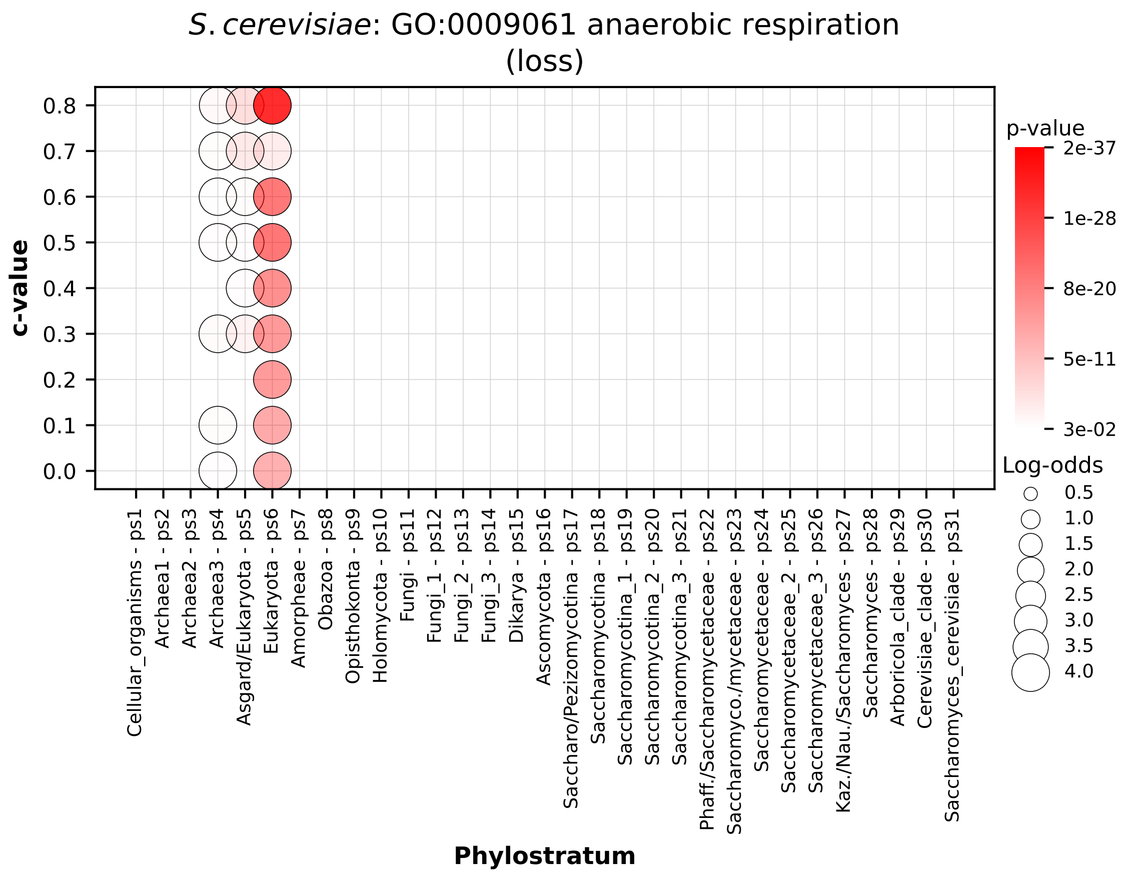
S. cerevisiae: GO:0009061 anaerobic respiration
(loss)
c-value
0.8
0.7
0.6
0.5
0.4
0.3
0.2
0.1
0.0
Phylostratum
Cellular_organisms - ps1
Archaea1 - ps2
Archaea2 - ps3
Archaea3 - ps4
Asgard/Eukaryota - ps5
Eukaryota - ps6
Amorpheae - ps7
Obazoa - ps8
Opisthokonta - ps9
Holomycota - ps10
Fungi - ps11
Fungi_1 - ps12
Fungi_2 - ps13
Fungi_3 - ps14
Dikarya - ps15
Ascomycota - ps16
Saccharo/Pezizomycotina - ps17
Saccharomycotina - ps18
Saccharomycotina_1 - ps19
Saccharomycotina_2 - ps20
Saccharomycotina_3 - ps21
Phaff./Saccharomycetaceae - ps22
Saccharomyco./mycetaceae - ps23
Saccharomycetaceae - ps24
Saccharomycetaceae_2 - ps25
Saccharomycetaceae_3 - ps26
Kaz./Nau./Saccharomyces - ps27
Saccharomyces - ps28
Arboricola_clade - ps29
Cerevisiae_clade - ps30
Saccharomyces_cerevisiae - ps31
p-value
2e-37
1e-28
8e-20
5e-11
3e-02
Log-odds
0.5
1.0
1.5
2.0
2.5
3.0
3.5
4.0

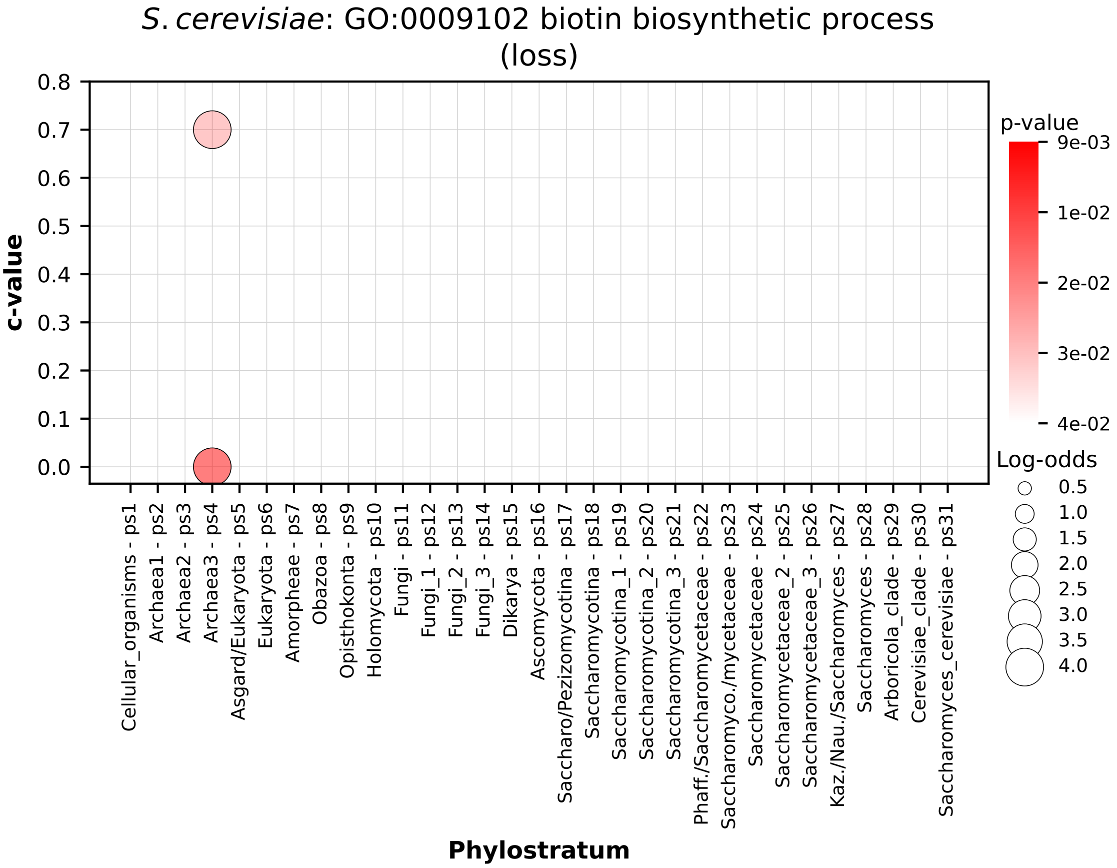
S. cerevisiae: GO:0009102 biotin biosynthetic process
(loss)
c-value
0.8
0.7
0.6
0.5
0.4
0.3
0.2
0.1
0.0
p-value
9e-03
1e-02
2e-02
3e-02
4e-02
Log-odds
0.5
1.0
1.5
2.0
2.5
3.0
3.5
4.0
Cellular_organisms - ps1
Archaea1 - ps2
Archaea2 - ps3
Archaea3 - ps4
Asgard/Eukaryota - ps5
Eukaryota - ps6
Amorpheae - ps7
Obazoa - ps8
Opisthokonta - ps9
Holomycota - ps10
Fungi - ps11
Fungi_1 - ps12
Fungi_2 - ps13
Fungi_3 - ps14
Dikarya - ps15
Ascomycota - ps16
Saccharo/Pezizomycotina - ps17
Saccharomycotina - ps18
Saccharomycotina_1 - ps19
Saccharomycotina_2 - ps20
Saccharomycotina_3 - ps21
Phaff./Saccharomycetaceae - ps22
Saccharomyco./mycetaceae - ps23
Saccharomycetaceae - ps24
Saccharomycetaceae_2 - ps25
Saccharomycetaceae_3 - ps26
Kaz./Nau./Saccharomyces - ps27
Saccharomyces - ps28
Arboricola_clade - ps29
Cerevisiae_clade - ps30
Saccharomyces_cerevisiae - ps31
Phylostratum

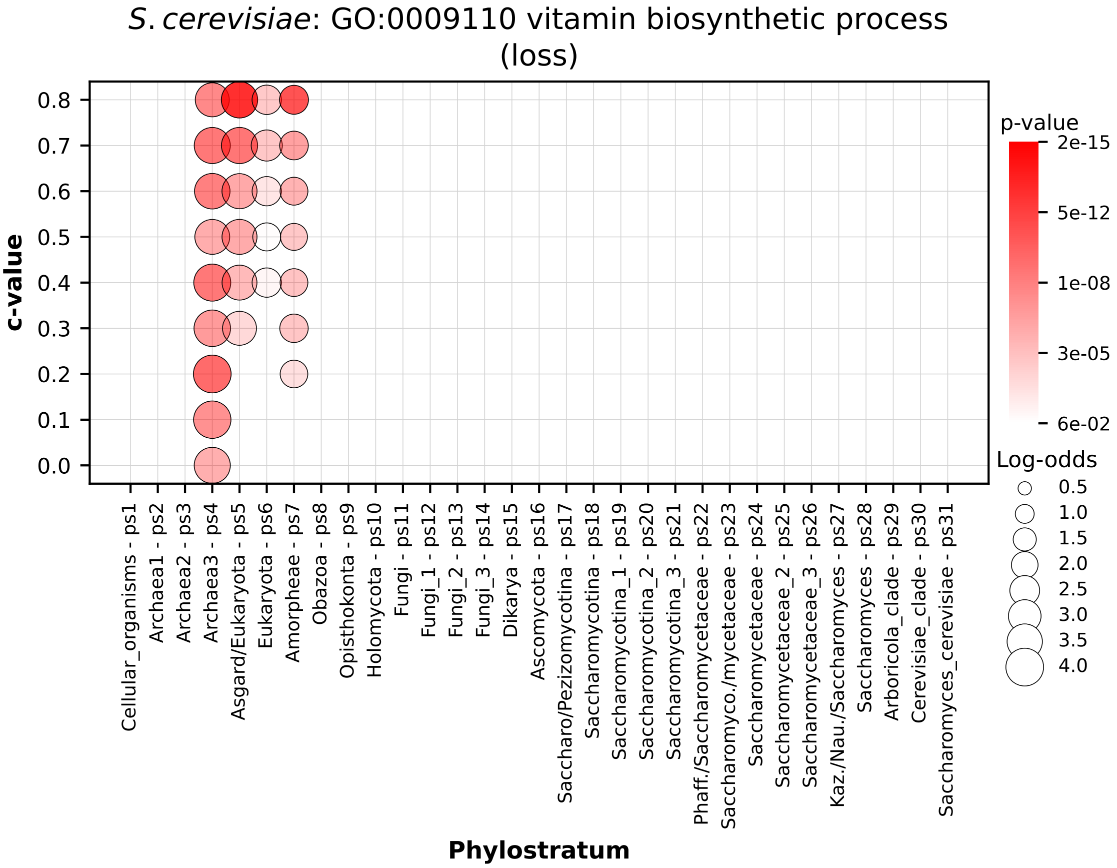

S. cerevisiae: GO:0009110 vitamin biosynthetic process
(loss)
c-value
0.8
0.7
0.6
0.5
0.4
0.3
0.2
0.1
0.0
p-value
2e-15
5e-12
1e-08
3e-05
6e-02
Log-odds
0.5
1.0
1.5
2.0
2.5
3.0
3.5
4.0
Cellular_organisms - ps1
Archaea1 - ps2
Archaea2 - ps3
Archaea3 - ps4
Asgard/Eukaryota - ps5
Eukaryota - ps6
Amorpheae - ps7
Obazoa - ps8
Opisthokonta - ps9
Holomycota - ps10
Fungi - ps11
Fungi_1 - ps12
Fungi_2 - ps13
Fungi_3 - ps14
Dikarya - ps15
Ascomycota - ps16
Saccharo/Pezizomycotina - ps17
Saccharomycotina - ps18
Saccharomycotina_1 - ps19
Saccharomycotina_2 - ps20
Saccharomycotina_3 - ps21
Phaff./Saccharomycetaceae - ps22
Saccharomyco./mycetaceae - ps23
Saccharomycetaceae - ps24
Saccharomycetaceae_2 - ps25
Saccharomycetaceae_3 - ps26
Kaz./Nau./Saccharomyces - ps27
Saccharomyces - ps28
Arboricola_clade - ps29
Cerevisiae_clade - ps30
Saccharomyces_cerevisiae - ps31
Phylostratum

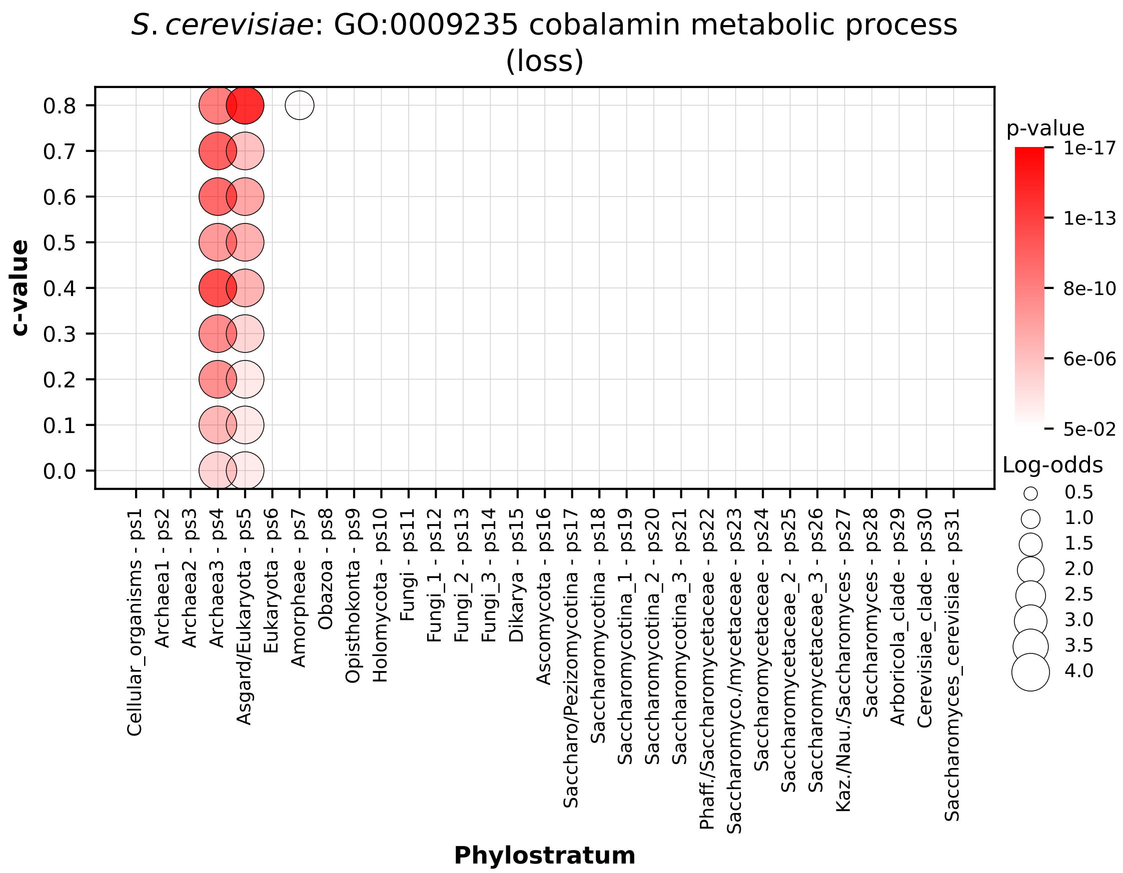

$S. cerevisiae$: GO:0009235 cobalamin metabolic process
(loss)
c-value
0.8
0.7
0.6
0.5
0.4
0.3
0.2
0.1
0.0
p-value
1e-17
1e-13
8e-10
6e-06
5e-02
Log-odds
0.5
1.0
1.5
2.0
2.5
3.0
3.5
4.0
Cellular_organisms - ps1
Archaea1 - ps2
Archaea2 - ps3
Archaea3 - ps4
Asgard/Eukaryota - ps5
Eukaryota - ps6
Amorpheae - ps7
Obazoa - ps8
Opisthokonta - ps9
Holomycota - ps10
Fungi - ps11
Fungi_1 - ps12
Fungi_2 - ps13
Fungi_3 - ps14
Dikarya - ps15
Ascomycota - ps16
Saccharo/Pezizomycotina - ps17
Saccharomycotina - ps18
Saccharomycotina_1 - ps19
Saccharomycotina_2 - ps20
Saccharomycotina_3 - ps21
Phaff./Saccharomycetaceae - ps22
Saccharomyco./mycetaceae - ps23
Saccharomycetaceae - ps24
Saccharomycetaceae_2 - ps25
Saccharomycetaceae_3 - ps26
Kaz./Nau./Saccharomyces - ps27
Saccharomyces - ps28
Arboricola_clade - ps29
Cerevisiae_clade - ps30
Saccharomyces_cerevisiae - ps31
Phylostratum

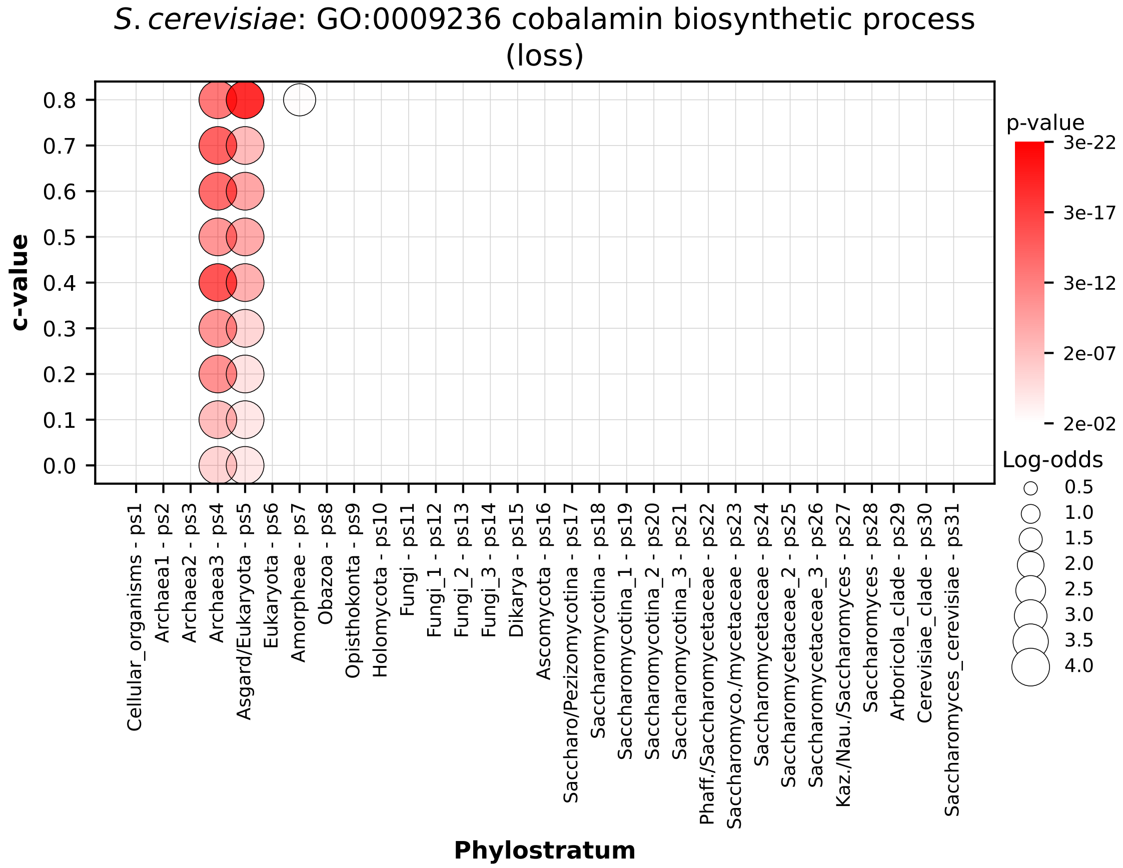
S. cerevisiae: GO:0009236 cobalamin biosynthetic process
(loss)
c-value
0.8
0.7
0.6
0.5
0.4
0.3
0.2
0.1
0.0
p-value
3e-22
3e-17
3e-12
2e-07
2e-02
Log-odds
0.5
1.0
1.5
2.0
2.5
3.0
3.5
4.0
Cellular_organisms - ps1
Archaea1 - ps2
Archaea2 - ps3
Archaea3 - ps4
Asgard/Eukaryota - ps5
Eukaryota - ps6
Amorpheae - ps7
Obazoa - ps8
Opisthokonta - ps9
Holomycota - ps10
Fungi - ps11
Fungi_1 - ps12
Fungi_2 - ps13
Fungi_3 - ps14
Dikarya - ps15
Ascomycota - ps16
Saccharo/Pezizomycotina - ps17
Saccharomycotina - ps18
Saccharomycotina_1 - ps19
Saccharomycotina_2 - ps20
Saccharomycotina_3 - ps21
Phaff./Saccharomycetaceae - ps22
Saccharomyco./mycetaceae - ps23
Saccharomycetaceae - ps24
Saccharomycetaceae_2 - ps25
Saccharomycetaceae_3 - ps26
Kaz./Nau./Saccharomyces - ps27
Saccharomyces - ps28
Arboricola_clade - ps29
Cerevisiae_clade - ps30
Saccharomyces_cerevisiae - ps31
Phylostratum

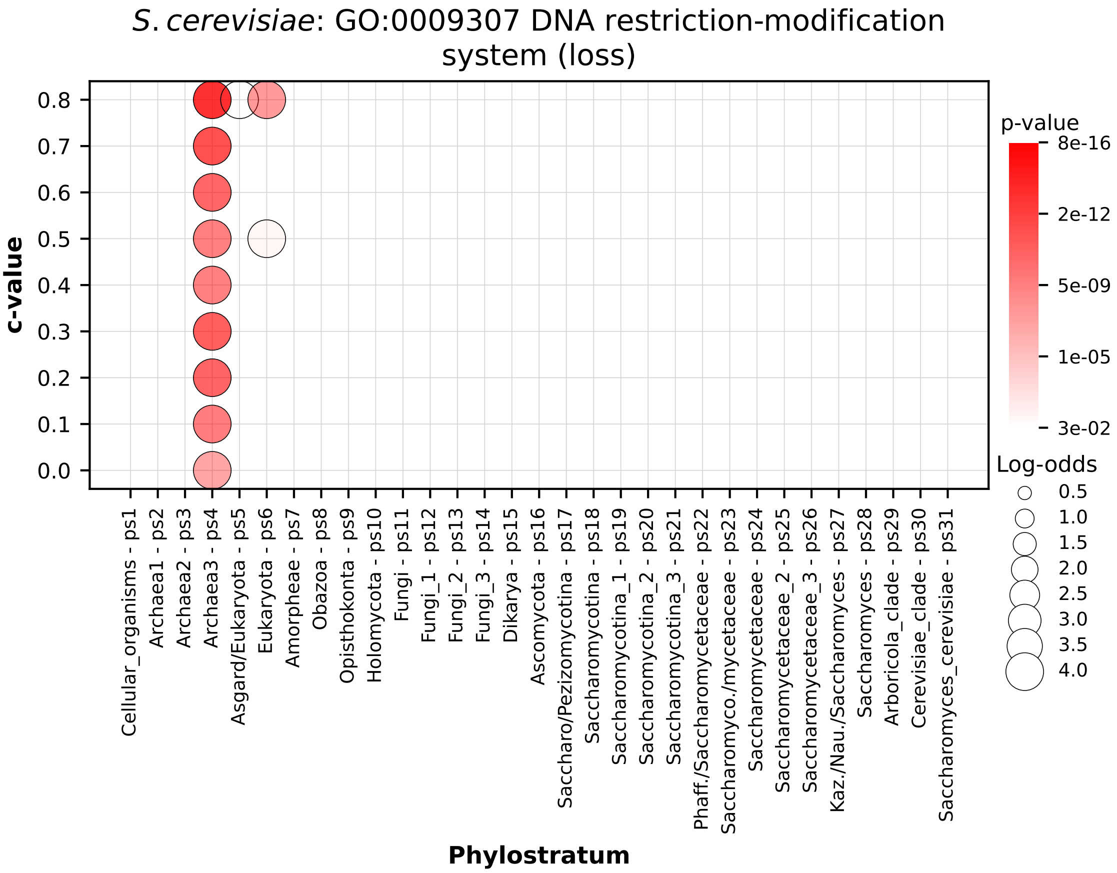

S. cerevisiae: GO:0009307 DNA restriction-modification system (loss)
c-value
0.8
0.7
0.6
0.5
0.4
0.3
0.2
0.1
0.0
Phylostratum
Cellular_organisms - ps1
Archaea1 - ps2
Archaea2 - ps3
Archaea3 - ps4
Asgard/Eukaryota - ps5
Eukaryota - ps6
Amorpheae - ps7
Obazoa - ps8
Opisthokonta - ps9
Holomycota - ps10
Fungi - ps11
Fungi_1 - ps12
Fungi_2 - ps13
Fungi_3 - ps14
Dikarya - ps15
Ascomycota - ps16
Saccharo/Pezizomycotina - ps17
Saccharomycotina - ps18
Saccharomycotina_1 - ps19
Saccharomycotina_2 - ps20
Saccharomycotina_3 - ps21
Phaff./Saccharomycetaceae - ps22
Saccharomyco./mycetaceae - ps23
Saccharomycetaceae - ps24
Saccharomycetaceae_2 - ps25
Saccharomycetaceae_3 - ps26
Kaz./Nau./Saccharomyces - ps27
Saccharomyces - ps28
Arboricola_clade - ps29
Cerevisiae_clade - ps30
Saccharomyces_cerevisiae - ps31
p-value
8e-16
2e-12
5e-09
1e-05
3e-02
Log-odds
0.5
1.0
1.5
2.0
2.5
3.0
3.5
4.0

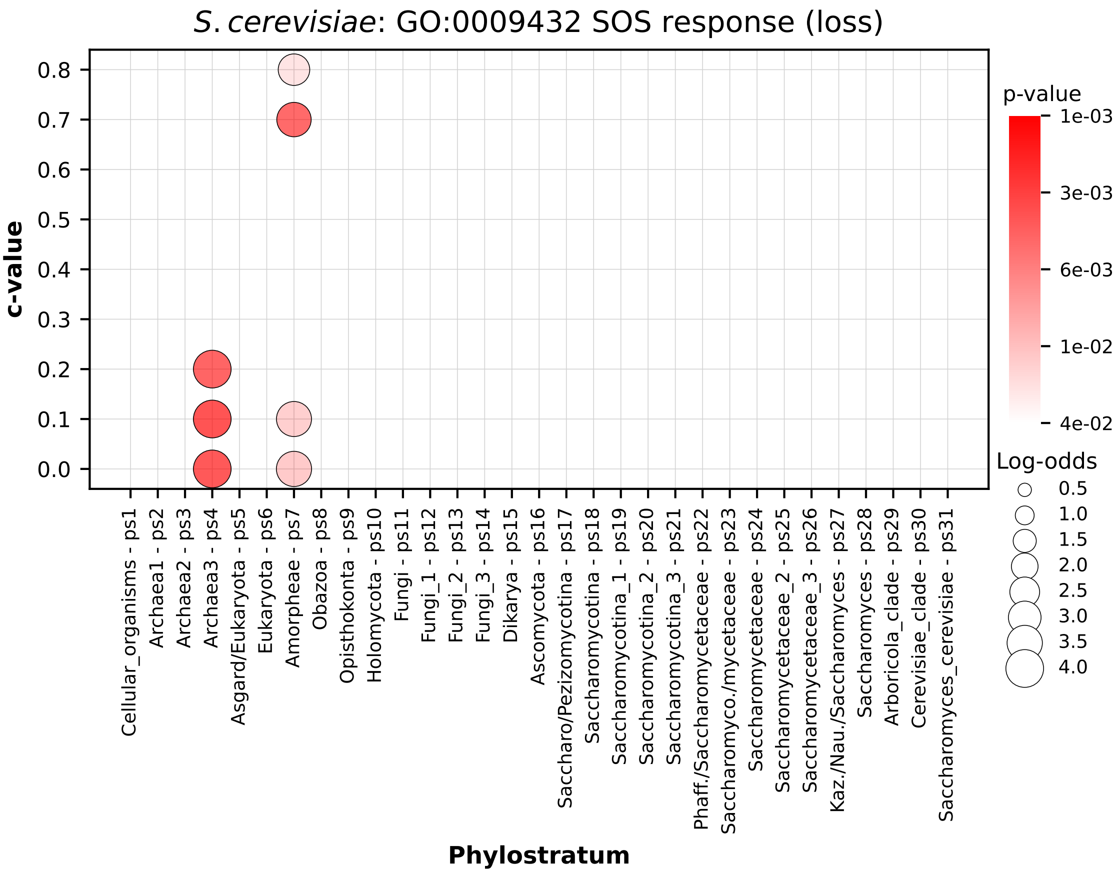

S. cerevisiae: GO:0009432 SOS response (loss)
c-value
0.8
0.7
0.6
0.5
0.4
0.3
0.2
0.1
0.0
p-value
1e-03
3e-03
6e-03
1e-02
4e-02
Log-odds
0.5
1.0
1.5
2.0
2.5
3.0
3.5
4.0
Cellular_organisms - ps1
Archaea1 - ps2
Archaea2 - ps3
Archaea3 - ps4
Asgard/Eukaryota - ps5
Eukaryota - ps6
Amorpheae - ps7
Obazoa - ps8
Opisthokonta - ps9
Holomycota - ps10
Fungi - ps11
Fungi_1 - ps12
Fungi_2 - ps13
Fungi_3 - ps14
Dikarya - ps15
Ascomycota - ps16
Saccharo/Pezizomycotina - ps17
Saccharomycotina - ps18
Saccharomycotina_1 - ps19
Saccharomycotina_2 - ps20
Saccharomycotina_3 - ps21
Phaff./Saccharomycetaceae - ps22
Saccharomyco./mycetaceae - ps23
Saccharomycetaceae - ps24
Saccharomycetaceae_2 - ps25
Saccharomycetaceae_3 - ps26
Kaz./Nau./Saccharomyces - ps27
Saccharomyces - ps28
Arboricola_clade - ps29
Cerevisiae_clade - ps30
Saccharomyces_cerevisiae - ps31
Phylostratum

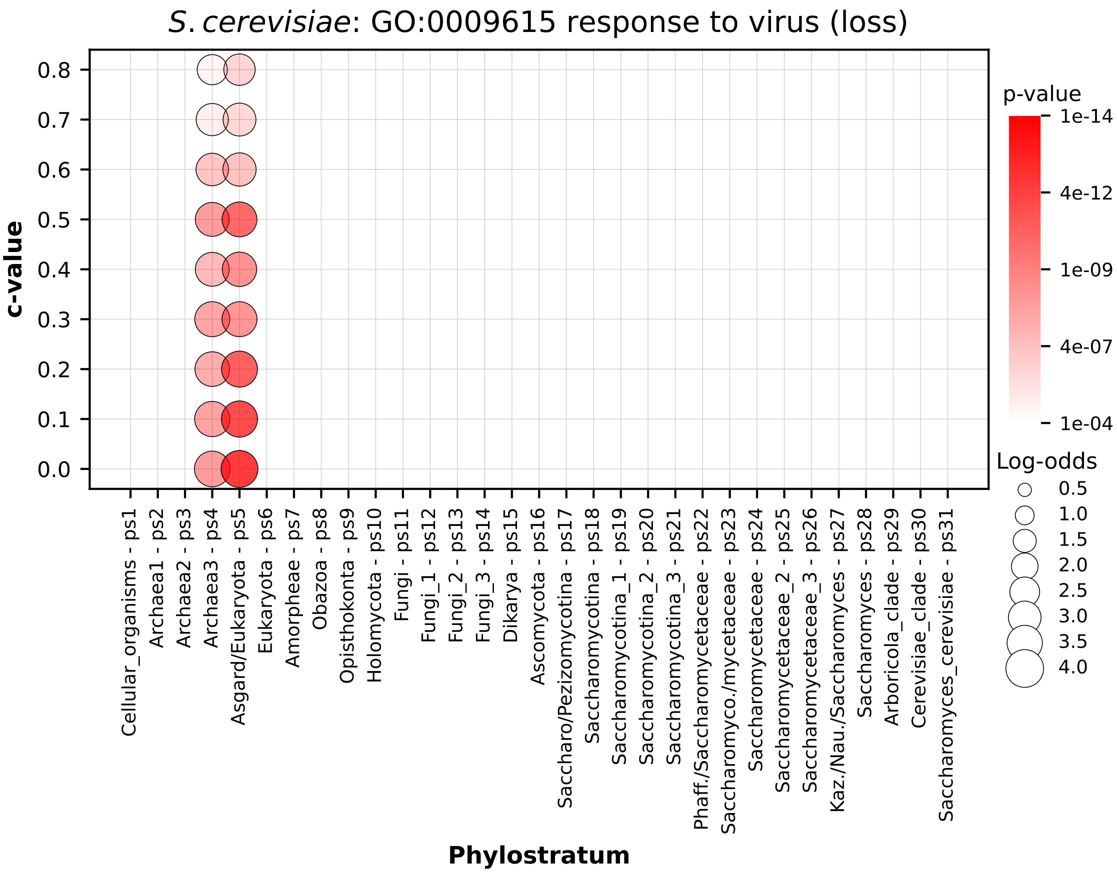
S. cerevisiae: GO:0009615 response to virus (loss)
c-value
0.8
0.7
0.6
0.5
0.4
0.3
0.2
0.1
0.0
p-value
1e-14
4e-12
1e-09
4e-07
1e-04
Log-odds
0.5
1.0
1.5
2.0
2.5
3.0
3.5
4.0
Cellular_organisms - ps1
Archaea1 - ps2
Archaea2 - ps3
Archaea3 - ps4
Asgard/Eukaryota - ps5
Eukaryota - ps6
Amorpheae - ps7
Obazoa - ps8
Opisthokonta - ps9
Holomycota - ps10
Fungi - ps11
Fungi_1 - ps12
Fungi_2 - ps13
Fungi_3 - ps14
Dikarya - ps15
Ascomycota - ps16
Saccharo/Pezizomycotina - ps17
Saccharomycotina - ps18
Saccharomycotina_1 - ps19
Saccharomycotina_2 - ps20
Saccharomycotina_3 - ps21
Phaff./Saccharomycetaceae - ps22
Saccharomyco./mycetaceae - ps23
Saccharomycetaceae - ps24
Saccharomycetaceae_2 - ps25
Saccharomycetaceae_3 - ps26
Kaz./Nau./Saccharomyces - ps27
Saccharomyces - ps28
Arboricola_clade - ps29
Cerevisiae_clade - ps30
Saccharomyces_cerevisiae - ps31
Phylostratum

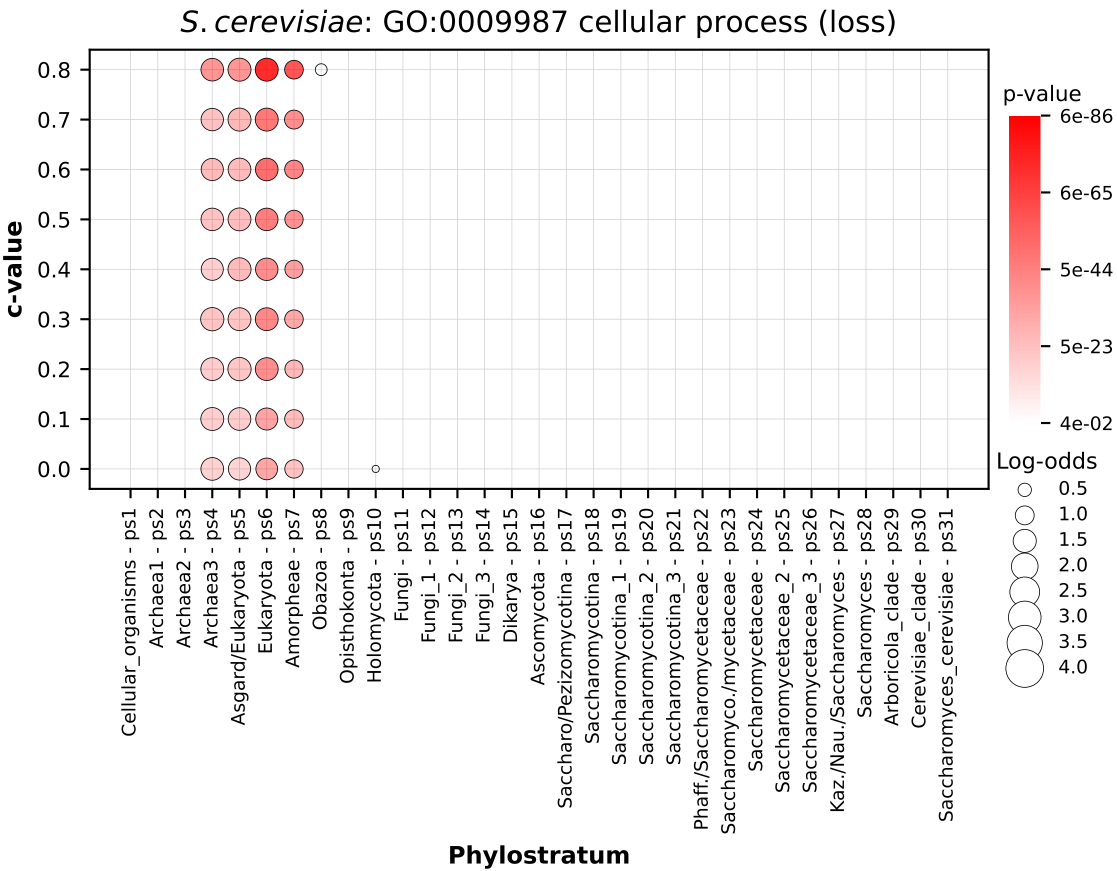
S. cerevisiae: GO:0009987 cellular process (loss)
c-value
0.8
0.7
0.6
0.5
0.4
0.3
0.2
0.1
0.0
Cellular_organisms - ps1
Archaea1 - ps2
Archaea2 - ps3
Archaea3 - ps4
Asgard/Eukaryota - ps5
Eukaryota - ps6
Amorpheae - ps7
Obazoa - ps8
Opisthokonta - ps9
Holomycota - ps10
Fungi - ps11
Fungi_1 - ps12
Fungi_2 - ps13
Fungi_3 - ps14
Dikarya - ps15
Ascomycota - ps16
Saccharo/Pezizomycotina - ps17
Saccharomycotina - ps18
Saccharomycotina_1 - ps19
Saccharomycotina_2 - ps20
Saccharomycotina_3 - ps21
Phaff./Saccharomycetaceae - ps22
Saccharomyco./mycetaceae - ps23
Saccharomycetaceae - ps24
Saccharomycetaceae_2 - ps25
Saccharomycetaceae_3 - ps26
Kaz./Nau./Saccharomyces - ps27
Saccharomyces - ps28
Arboricola_clade - ps29
Cerevisiae_clade - ps30
Saccharomyces_cerevisiae - ps31
Phylostratum
p-value
6e-86
6e-65
5e-44
5e-23
4e-02
Log-odds
0.5
1.0
1.5
2.0
2.5
3.0
3.5
4.0

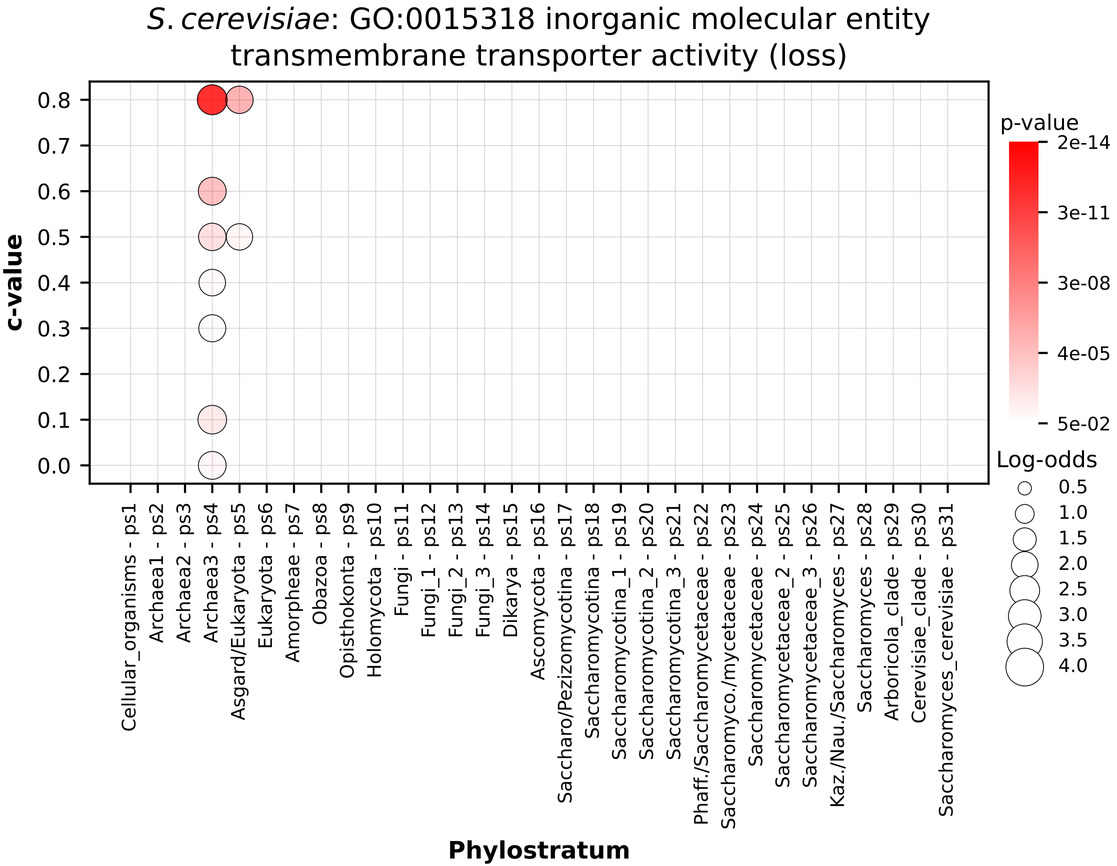
S. cerevisiae: GO:0015318 inorganic molecular entity transmembrane transporter activity (loss)
c-value
0.8
0.7
0.6
0.5
0.4
0.3
0.2
0.1
0.0
Phylostratum
Cellular_organisms - ps1
Archaea1 - ps2
Archaea2 - ps3
Archaea3 - ps4
Asgard/Eukaryota - ps5
Eukaryota - ps6
Amorpheae - ps7
Obazoa - ps8
Opisthokonta - ps9
Holomycota - ps10
Fungi - ps11
Fungi_1 - ps12
Fungi_2 - ps13
Fungi_3 - ps14
Dikarya - ps15
Ascomycota - ps16
Saccharo/Pezizomycotina - ps17
Saccharomycotina - ps18
Saccharomycotina_1 - ps19
Saccharomycotina_2 - ps20
Saccharomycotina_3 - ps21
Phaff./Saccharomycetaceae - ps22
Saccharomyco./mycetaceae - ps23
Saccharomycetaceae - ps24
Saccharomycetaceae_2 - ps25
Saccharomycetaceae_3 - ps26
Kaz./Nau./Saccharomyces - ps27
Saccharomyces - ps28
Arboricola_clade - ps29
Cerevisiae_clade - ps30
Saccharomyces_cerevisiae - ps31
p-value
2e-14
3e-11
3e-08
4e-05
5e-02
Log-odds
0.5
1.0
1.5
2.0
2.5
3.0
3.5
4.0

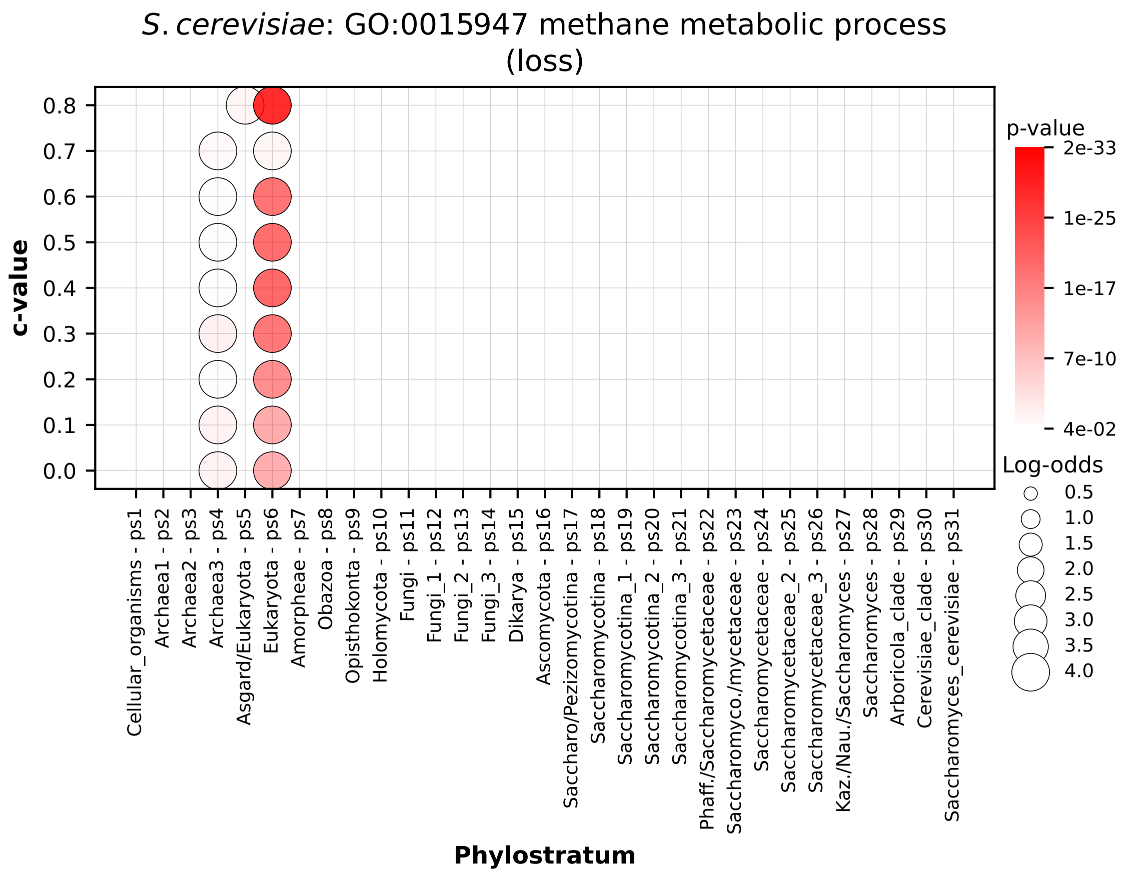

S. cerevisiae: GO:0015947 methane metabolic process
(loss)
c-value
0.8
0.7
0.6
0.5
0.4
0.3
0.2
0.1
0.0
p-value
2e-33
1e-25
1e-17
7e-10
4e-02
Log-odds
0.5
1.0
1.5
2.0
2.5
3.0
3.5
4.0
Cellular_organisms - ps1
Archaea1 - ps2
Archaea2 - ps3
Archaea3 - ps4
Asgard/Eukaryota - ps5
Eukaryota - ps6
Amorpheae - ps7
Obazoa - ps8
Opisthokonta - ps9
Holomycota - ps10
Fungi - ps11
Fungi_1 - ps12
Fungi_2 - ps13
Fungi_3 - ps14
Dikarya - ps15
Ascomycota - ps16
Saccharo/Pezizomycotina - ps17
Saccharomycotina - ps18
Saccharomycotina_1 - ps19
Saccharomycotina_2 - ps20
Saccharomycotina_3 - ps21
Phaff./Saccharomycetaceae - ps22
Saccharomyco./mycetaceae - ps23
Saccharomycetaceae - ps24
Saccharomycetaceae_2 - ps25
Saccharomycetaceae_3 - ps26
Kaz./Nau./Saccharomyces - ps27
Saccharomyces - ps28
Arboricola_clade - ps29
Cerevisiae_clade - ps30
Saccharomyces_cerevisiae - ps31
Phylostratum

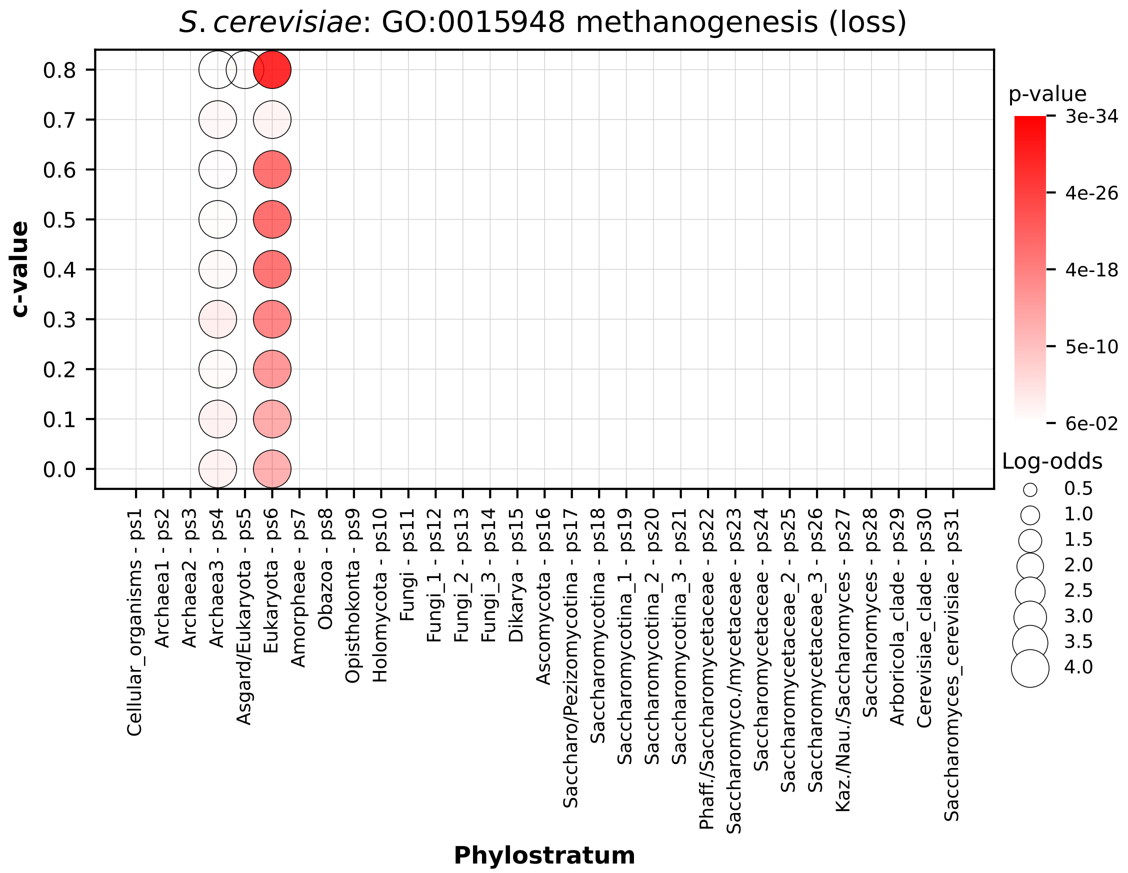

S. cerevisiae: GO:0015948 methanogenesis (loss)
c-value
0.8
0.7
0.6
0.5
0.4
0.3
0.2
0.1
0.0
p-value
3e-34
4e-26
4e-18
5e-10
6e-02
Log-odds
0.5
1.0
1.5
2.0
2.5
3.0
3.5
4.0
Cellular_organisms - ps1
Archaea1 - ps2
Archaea2 - ps3
Archaea3 - ps4
Asgard/Eukaryota - ps5
Eukaryota - ps6
Amorpheae - ps7
Obazoa - ps8
Opisthokonta - ps9
Holomycota - ps10
Fungi - ps11
Fungi_1 - ps12
Fungi_2 - ps13
Fungi_3 - ps14
Dikarya - ps15
Ascomycota - ps16
Saccharo/Pezizomycotina - ps17
Saccharomycotina - ps18
Saccharomycotina_1 - ps19
Saccharomycotina_2 - ps20
Saccharomycotina_3 - ps21
Phaff./Saccharomycetaceae - ps22
Saccharomyco./mycetaceae - ps23
Saccharomycetaceae - ps24
Saccharomycetaceae_2 - ps25
Saccharomycetaceae_3 - ps26
Kaz./Nau./Saccharomyces - ps27
Saccharomyces - ps28
Arboricola_clade - ps29
Cerevisiae_clade - ps30
Saccharomyces_cerevisiae - ps31
Phylostratum

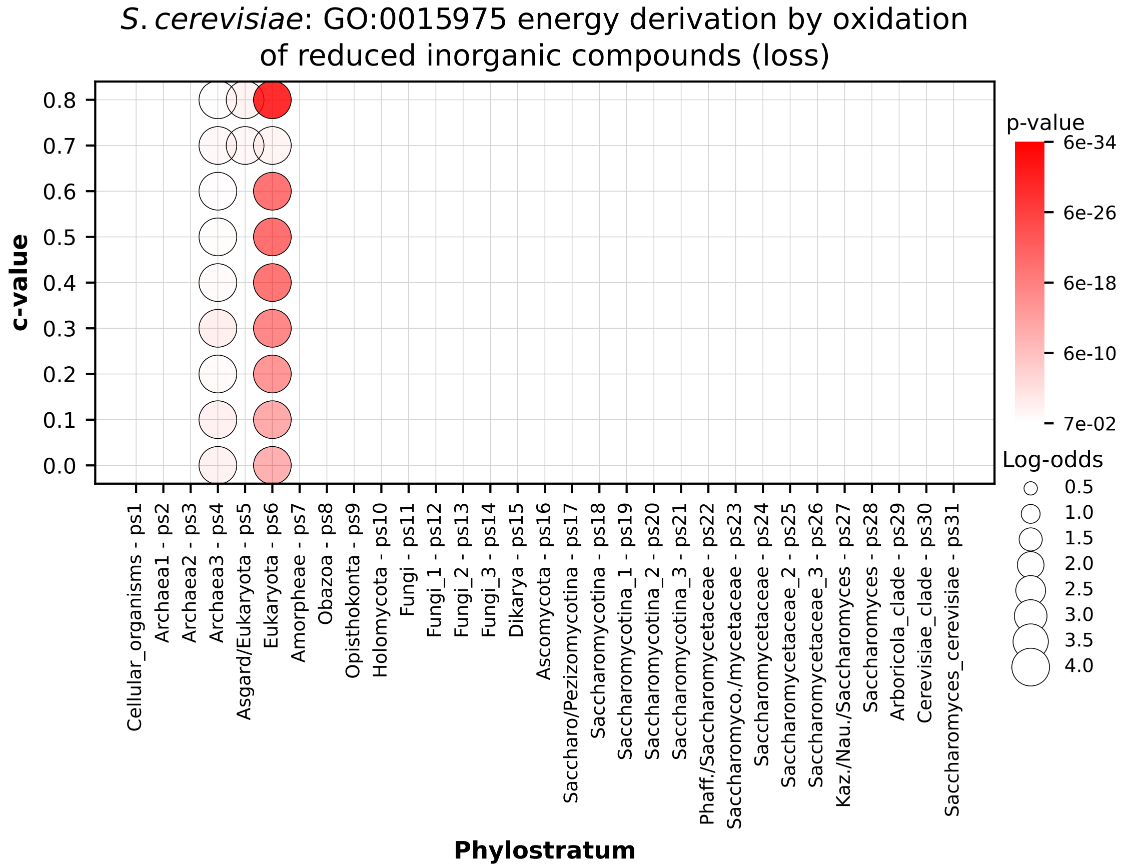

S. cerevisiae: GO:0015975 energy derivation by oxidation of reduced inorganic compounds (loss)
c-value
0.8
0.7
0.6
0.5
0.4
0.3
0.2
0.1
0.0
p-value
6e-34
6e-26
6e-18
6e-10
7e-02
Log-odds
0.5
1.0
1.5
2.0
2.5
3.0
3.5
4.0
Cellular_organisms - ps1
Archaea1 - ps2
Archaea2 - ps3
Archaea3 - ps4
Asgard/Eukaryota - ps5
Eukaryota - ps6
Amorpheae - ps7
Obazoa - ps8
Opisthokonta - ps9
Holomycota - ps10
Fungi - ps11
Fungi_1 - ps12
Fungi_2 - ps13
Fungi_3 - ps14
Dikarya - ps15
Ascomycota - ps16
Saccharo/Pezizomycotina - ps17
Saccharomycotina - ps18
Saccharomycotina_1 - ps19
Saccharomycotina_2 - ps20
Saccharomycotina_3 - ps21
Phaff./Saccharomycetaceae - ps22
Saccharomyco./mycetaceae - ps23
Saccharomycetaceae - ps24
Saccharomycetaceae_2 - ps25
Saccharomycetaceae_3 - ps26
Kaz./Nau./Saccharomyces - ps27
Saccharomyces - ps28
Arboricola_clade - ps29
Cerevisiae_clade - ps30
Saccharomyces_cerevisiae - ps31
Phylostratum

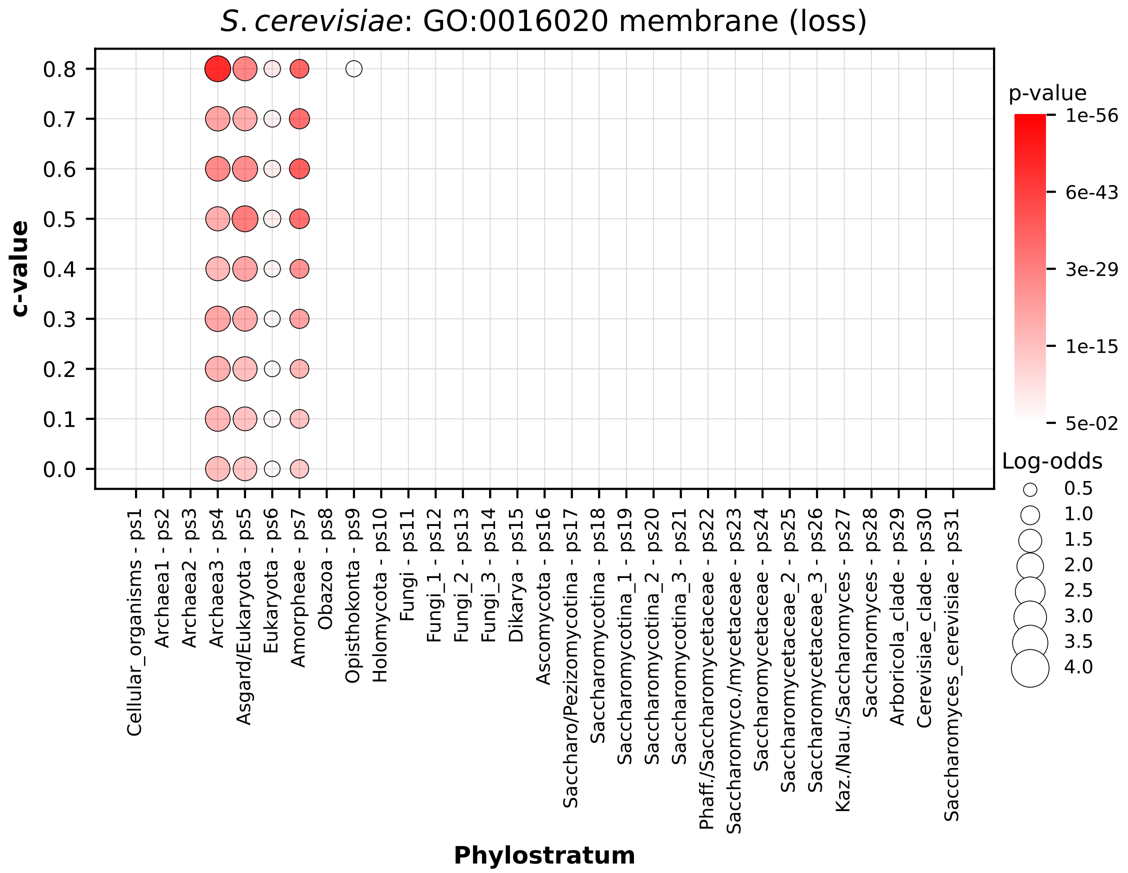
S. cerevisiae: GO:0016020 membrane (loss)
c-value
0.8
0.7
0.6
0.5
0.4
0.3
0.2
0.1
0.0
p-value
1e-56
6e-43
3e-29
1e-15
5e-02
Log-odds
0.5
1.0
1.5
2.0
2.5
3.0
3.5
4.0
Cellular_organisms - ps1
Archaea1 - ps2
Archaea2 - ps3
Archaea3 - ps4
Asgard/Eukaryota - ps5
Eukaryota - ps6
Amorpheae - ps7
Obazoa - ps8
Opisthokonta - ps9
Holomycota - ps10
Fungi - ps11
Fungi_1 - ps12
Fungi_2 - ps13
Fungi_3 - ps14
Dikarya - ps15
Ascomycota - ps16
Saccharo/Pezizomycotina - ps17
Saccharomycotina - ps18
Saccharomycotina_1 - ps19
Saccharomycotina_2 - ps20
Saccharomycotina_3 - ps21
Phaff./Saccharomycetaceae - ps22
Saccharomyco./mycetaceae - ps23
Saccharomycetaceae - ps24
Saccharomycetaceae_2 - ps25
Saccharomycetaceae_3 - ps26
Kaz./Nau./Saccharomyces - ps27
Saccharomyces - ps28
Arboricola_clade - ps29
Cerevisiae_clade - ps30
Saccharomyces_cerevisiae - ps31
Phylostratum

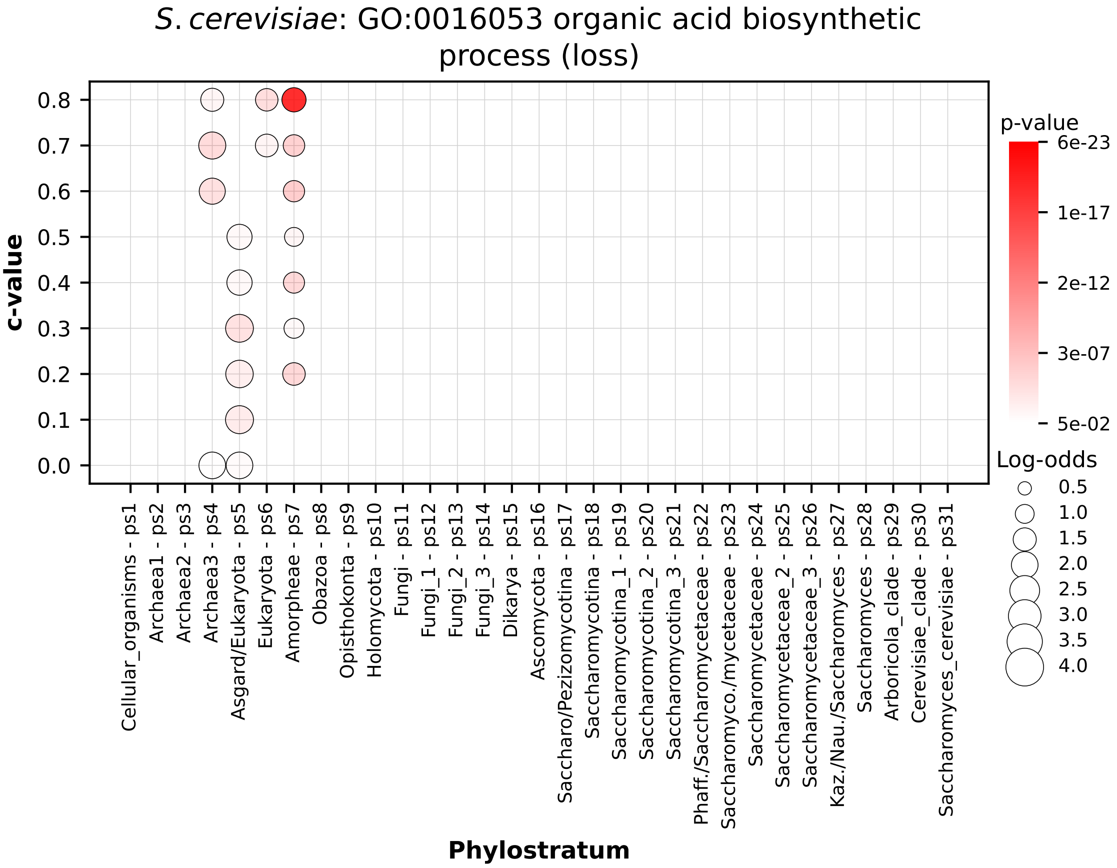

S. cerevisiae: GO:0016053 organic acid biosynthetic process (loss)
c-value
0.8
0.7
0.6
0.5
0.4
0.3
0.2
0.1
0.0
p-value
6e-23
1e-17
2e-12
3e-07
5e-02
Log-odds
0.5
1.0
1.5
2.0
2.5
3.0
3.5
4.0
Cellular_organisms - ps1
Archaea1 - ps2
Archaea2 - ps3
Archaea3 - ps4
Asgard/Eukaryota - ps5
Eukaryota - ps6
Amorpheae - ps7
Obazoa - ps8
Opisthokonta - ps9
Holomycota - ps10
Fungi - ps11
Fungi_1 - ps12
Fungi_2 - ps13
Fungi_3 - ps14
Dikarya - ps15
Ascomycota - ps16
Saccharo/Pezizomycotina - ps17
Saccharomycotina - ps18
Saccharomycotina_1 - ps19
Saccharomycotina_2 - ps20
Saccharomycotina_3 - ps21
Phaff./Saccharomycetaceae - ps22
Saccharomyco./mycetaceae - ps23
Saccharomycetaceae - ps24
Saccharomycetaceae_2 - ps25
Saccharomycetaceae_3 - ps26
Kaz./Nau./Saccharomyces - ps27
Saccharomyces - ps28
Arboricola_clade - ps29
Cerevisiae_clade - ps30
Saccharomyces_cerevisiae - ps31
Phylostratum

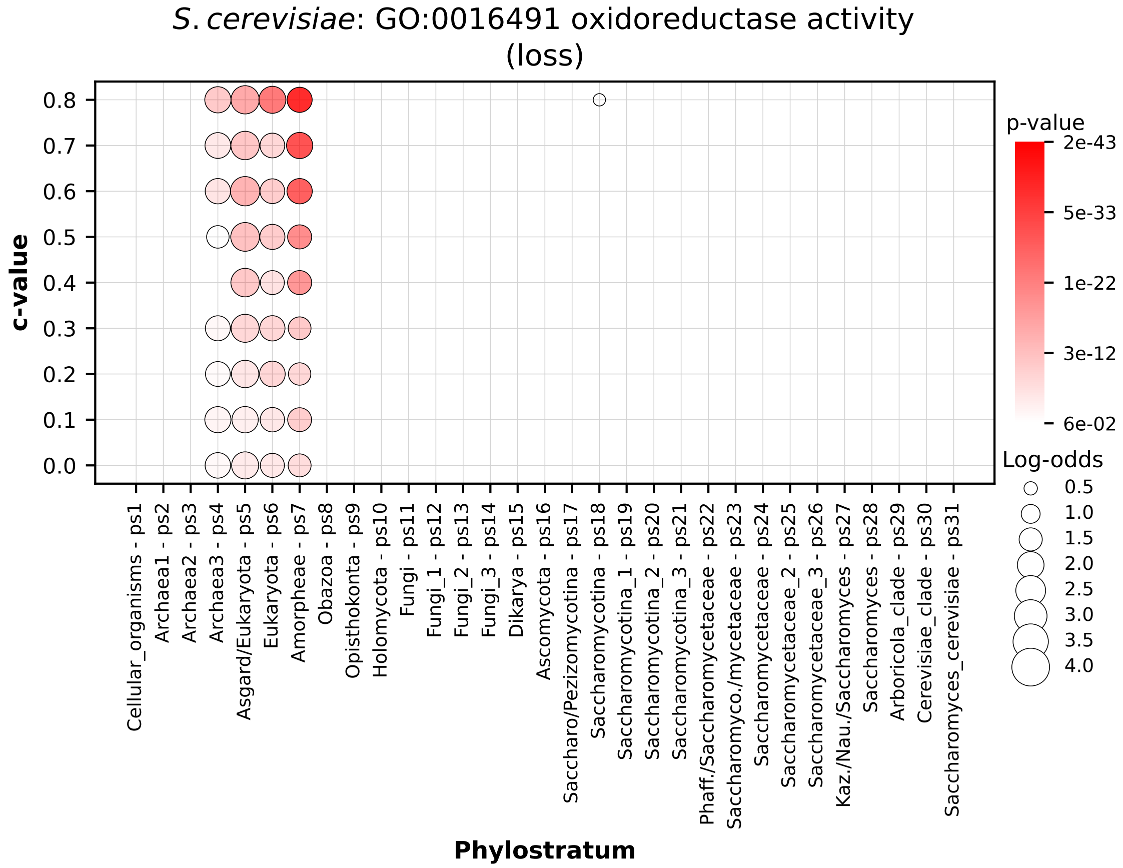
S. cerevisiae: GO:0016491 oxidoreductase activity
(loss)
c-value
0.8
0.7
0.6
0.5
0.4
0.3
0.2
0.1
0.0
p-value
2e-43
5e-33
1e-22
3e-12
6e-02
Log-odds
0.5
1.0
1.5
2.0
2.5
3.0
3.5
4.0
Cellular_organisms - ps1
Archaea1 - ps2
Archaea2 - ps3
Archaea3 - ps4
Asgard/Eukaryota - ps5
Eukaryota - ps6
Amorpheae - ps7
Obazoa - ps8
Opisthokonta - ps9
Holomycota - ps10
Fungi - ps11
Fungi_1 - ps12
Fungi_2 - ps13
Fungi_3 - ps14
Dikarya - ps15
Ascomycota - ps16
Saccharo/Pezizomycotina - ps17
Saccharomycotina - ps18
Saccharomycotina_1 - ps19
Saccharomycotina_2 - ps20
Saccharomycotina_3 - ps21
Phaff./Saccharomycetaceae - ps22
Saccharomyco./mycetaceae - ps23
Saccharomycetaceae - ps24
Saccharomycetaceae_2 - ps25
Saccharomycetaceae_3 - ps26
Kaz./Nau./Saccharomyces - ps27
Saccharomyces - ps28
Arboricola_clade - ps29
Cerevisiae_clade - ps30
Saccharomyces_cerevisiae - ps31
Phylostratum

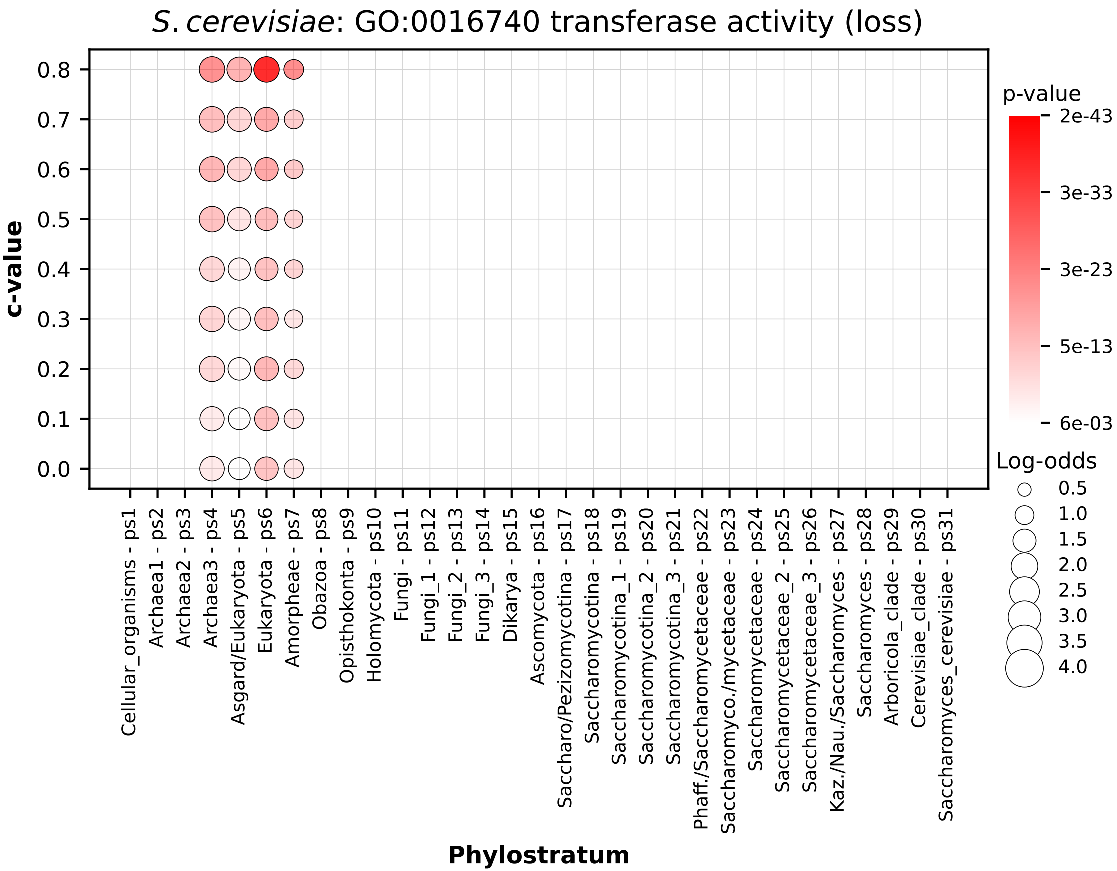

S. cerevisiae: GO:0016740 transferase activity (loss)
c-value
0.8
0.7
0.6
0.5
0.4
0.3
0.2
0.1
0.0
p-value
2e-43
3e-33
3e-23
5e-13
6e-03
Log-odds
0.5
1.0
1.5
2.0
2.5
3.0
3.5
4.0
Cellular_organisms - ps1
Archaea1 - ps2
Archaea2 - ps3
Archaea3 - ps4
Asgard/Eukaryota - ps5
Eukaryota - ps6
Amorpheae - ps7
Obazoa - ps8
Opisthokonta - ps9
Holomycota - ps10
Fungi - ps11
Fungi_1 - ps12
Fungi_2 - ps13
Fungi_3 - ps14
Dikarya - ps15
Ascomycota - ps16
Saccharo/Pezizomycotina - ps17
Saccharomycotina - ps18
Saccharomycotina_1 - ps19
Saccharomycotina_2 - ps20
Saccharomycotina_3 - ps21
Phaff./Saccharomycetaceae - ps22
Saccharomyco./mycetaceae - ps23
Saccharomycetaceae - ps24
Saccharomycetaceae_2 - ps25
Saccharomycetaceae_3 - ps26
Kaz./Nau./Saccharomyces - ps27
Saccharomyces - ps28
Arboricola_clade - ps29
Cerevisiae_clade - ps30
Saccharomyces_cerevisiae - ps31
Phylostratum

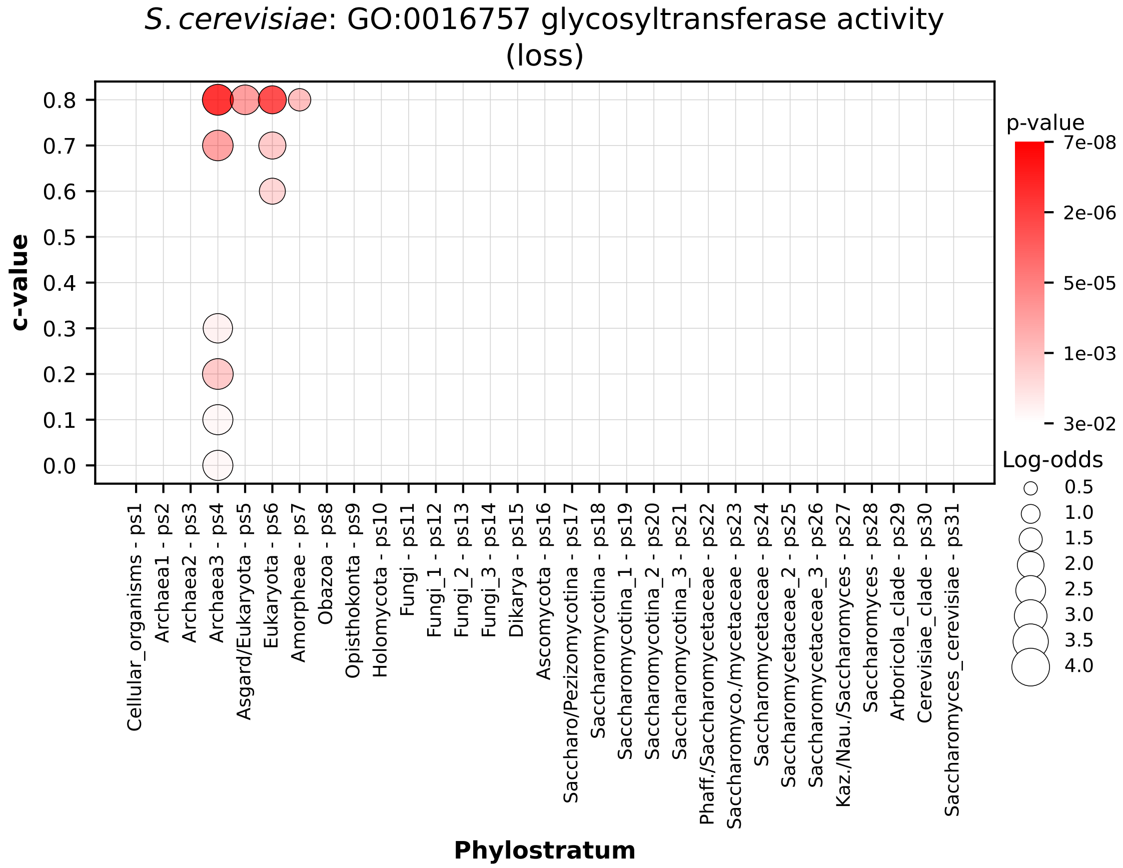

S. cerevisiae: GO:0016757 glycosyltransferase activity (loss)
c-value
0.8
0.7
0.6
0.5
0.4
0.3
0.2
0.1
0.0
p-value
7e-08
2e-06
5e-05
1e-03
3e-02
Log-odds
0.5
1.0
1.5
2.0
2.5
3.0
3.5
4.0
Cellular_organisms - ps1
Archaea1 - ps2
Archaea2 - ps3
Archaea3 - ps4
Asgard/Eukaryota - ps5
Eukaryota - ps6
Amorpheae - ps7
Obazoa - ps8
Opisthokonta - ps9
Holomycota - ps10
Fungi - ps11
Fungi_1 - ps12
Fungi_2 - ps13
Fungi_3 - ps14
Dikarya - ps15
Ascomycota - ps16
Saccharo/Pezizomycotina - ps17
Saccharomycotina - ps18
Saccharomycotina_1 - ps19
Saccharomycotina_2 - ps20
Saccharomycotina_3 - ps21
Phaff./Saccharomycetaceae - ps22
Saccharomyco./mycetaceae - ps23
Saccharomycetaceae - ps24
Saccharomycetaceae_2 - ps25
Saccharomycetaceae_3 - ps26
Kaz./Nau./Saccharomyces - ps27
Saccharomyces - ps28
Arboricola_clade - ps29
Cerevisiae_clade - ps30
Saccharomyces_cerevisiae - ps31
Phylostratum

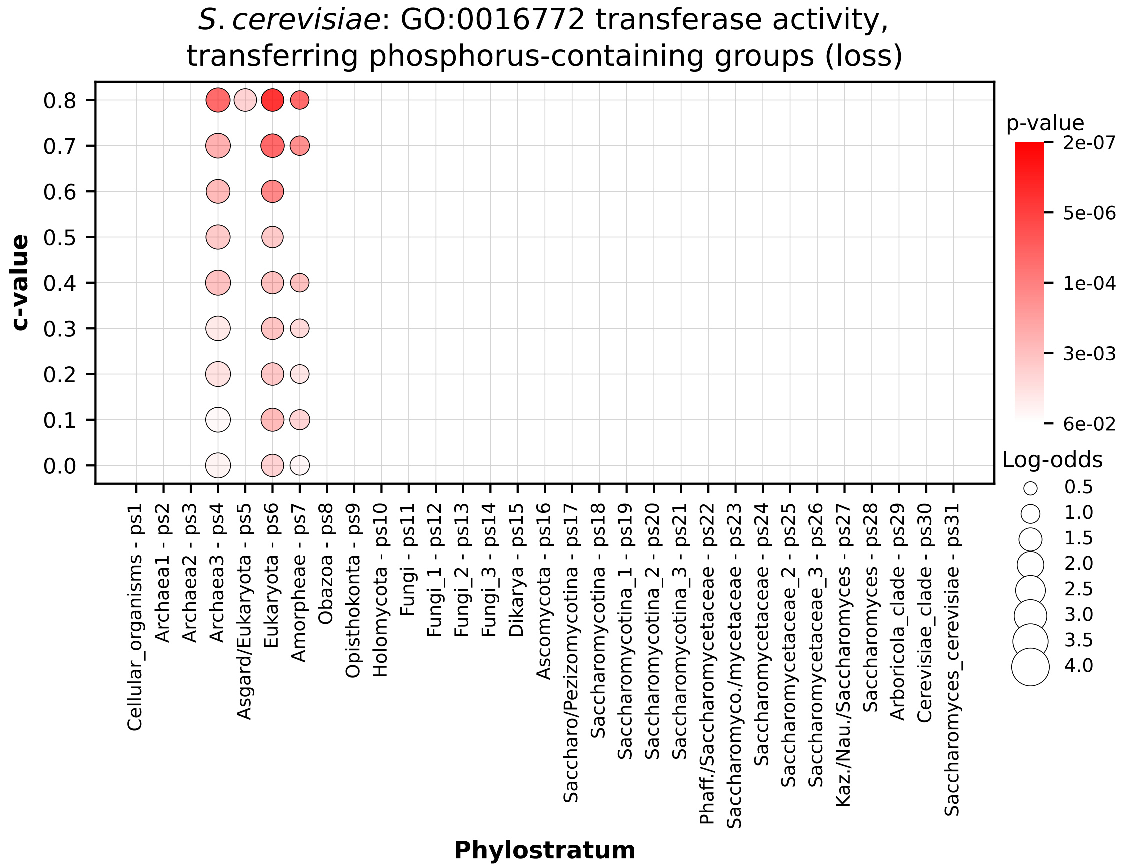
S. cerevisiae: GO:0016772 transferase activity,
transferring phosphorus-containing groups (loss)
c-value
0.8
0.7
0.6
0.5
0.4
0.3
0.2
0.1
0.0
p-value
2e-07
5e-06
1e-04
3e-03
6e-02
Log-odds
0.5
1.0
1.5
2.0
2.5
3.0
3.5
4.0
Cellular_organisms - ps1
Archaea1 - ps2
Archaea2 - ps3
Archaea3 - ps4
Asgard/Eukaryota - ps5
Eukaryota - ps6
Amorpheae - ps7
Obazoa - ps8
Opisthokonta - ps9
Holomycota - ps10
Fungi - ps11
Fungi_1 - ps12
Fungi_2 - ps13
Fungi_3 - ps14
Dikarya - ps15
Ascomycota - ps16
Saccharo/Pezizomycotina - ps17
Saccharomycotina - ps18
Saccharomycotina_1 - ps19
Saccharomycotina_2 - ps20
Saccharomycotina_3 - ps21
Phaff./Saccharomycetaceae - ps22
Saccharomyco./mycetaceae - ps23
Saccharomycetaceae - ps24
Saccharomycetaceae_2 - ps25
Saccharomycetaceae_3 - ps26
Kaz./Nau./Saccharomyces - ps27
Saccharomyces - ps28
Arboricola_clade - ps29
Cerevisiae_clade - ps30
Saccharomyces_cerevisiae - ps31
Phylostratum

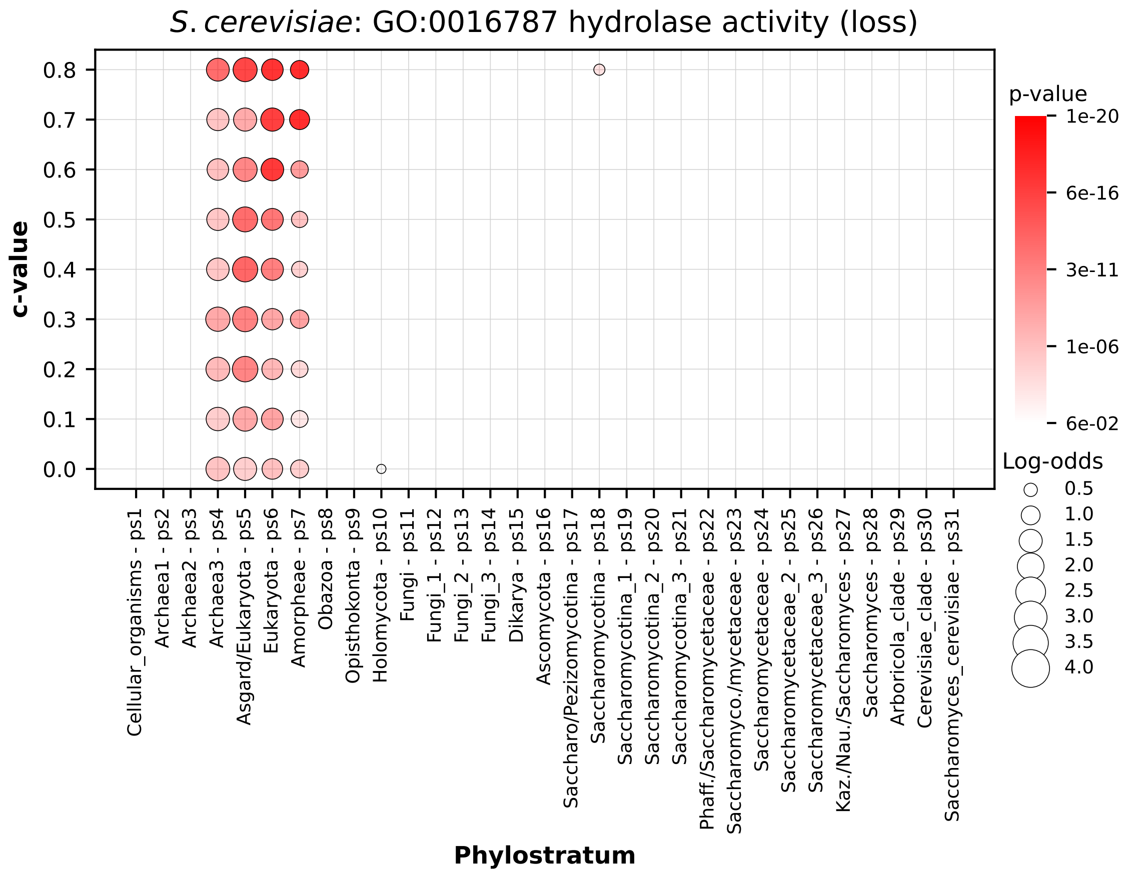
S. cerevisiae: GO:0016787 hydrolase activity (loss)
c-value
0.8
0.7
0.6
0.5
0.4
0.3
0.2
0.1
0.0
Phylostratum
Cellular_organisms - ps1
Archaea1 - ps2
Archaea2 - ps3
Archaea3 - ps4
Asgard/Eukaryota - ps5
Eukaryota - ps6
Amorpheae - ps7
Obazoa - ps8
Opisthokonta - ps9
Holomycota - ps10
Fungi - ps11
Fungi_1 - ps12
Fungi_2 - ps13
Fungi_3 - ps14
Dikarya - ps15
Ascomycota - ps16
Saccharo/Pezizomycotina - ps17
Saccharomycotina - ps18
Saccharomycotina_1 - ps19
Saccharomycotina_2 - ps20
Saccharomycotina_3 - ps21
Phaff./Saccharomycetaceae - ps22
Saccharomyco./mycetaceae - ps23
Saccharomycetaceae - ps24
Saccharomycetaceae_2 - ps25
Saccharomycetaceae_3 - ps26
Kaz./Nau./Saccharomyces - ps27
Saccharomyces - ps28
Arboricola_clade - ps29
Cerevisiae_clade - ps30
Saccharomyces_cerevisiae - ps31
p-value
1e-20
6e-16
3e-11
1e-06
6e-02
Log-odds
0.5
1.0
1.5
2.0
2.5
3.0
3.5
4.0

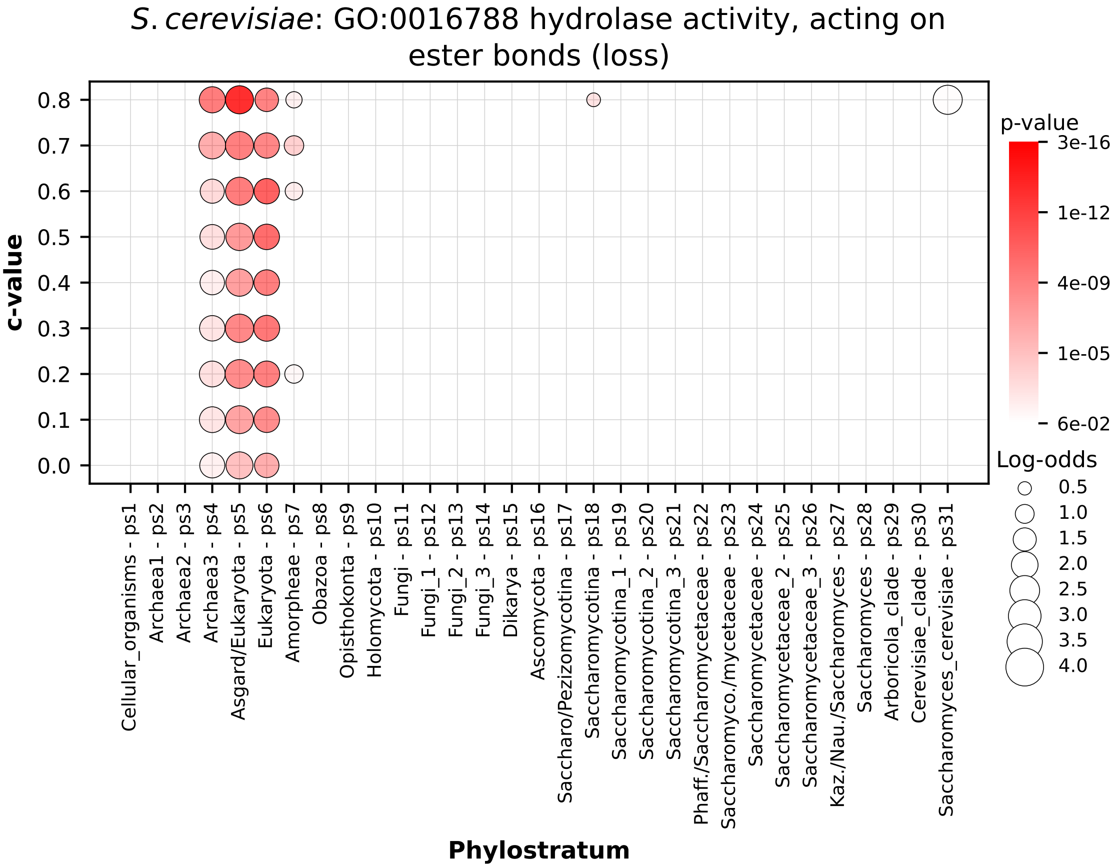

S. cerevisiae: GO:0016788 hydrolase activity, acting on ester bonds (loss)
c-value
0.8
0.7
0.6
0.5
0.4
0.3
0.2
0.1
0.0
p-value
3e-16
1e-12
4e-09
1e-05
6e-02
Log-odds
0.5
1.0
1.5
2.0
2.5
3.0
3.5
4.0
Cellular_organisms - ps1
Archaea1 - ps2
Archaea2 - ps3
Archaea3 - ps4
Asgard/Eukaryota - ps5
Eukaryota - ps6
Amorpheae - ps7
Obazoa - ps8
Opisthokonta - ps9
Holomycota - ps10
Fungi - ps11
Fungi_1 - ps12
Fungi_2 - ps13
Fungi_3 - ps14
Dikarya - ps15
Ascomycota - ps16
Saccharo/Pezizomycotina - ps17
Saccharomycotina - ps18
Saccharomycotina_1 - ps19
Saccharomycotina_2 - ps20
Saccharomycotina_3 - ps21
Phaff./Saccharomycetaceae - ps22
Saccharomyco./mycetaceae - ps23
Saccharomycetaceae - ps24
Saccharomycetaceae_2 - ps25
Saccharomycetaceae_3 - ps26
Kaz./Nau./Saccharomyces - ps27
Saccharomyces - ps28
Arboricola_clade - ps29
Cerevisiae_clade - ps30
Saccharomyces_cerevisiae - ps31
Phylostratum

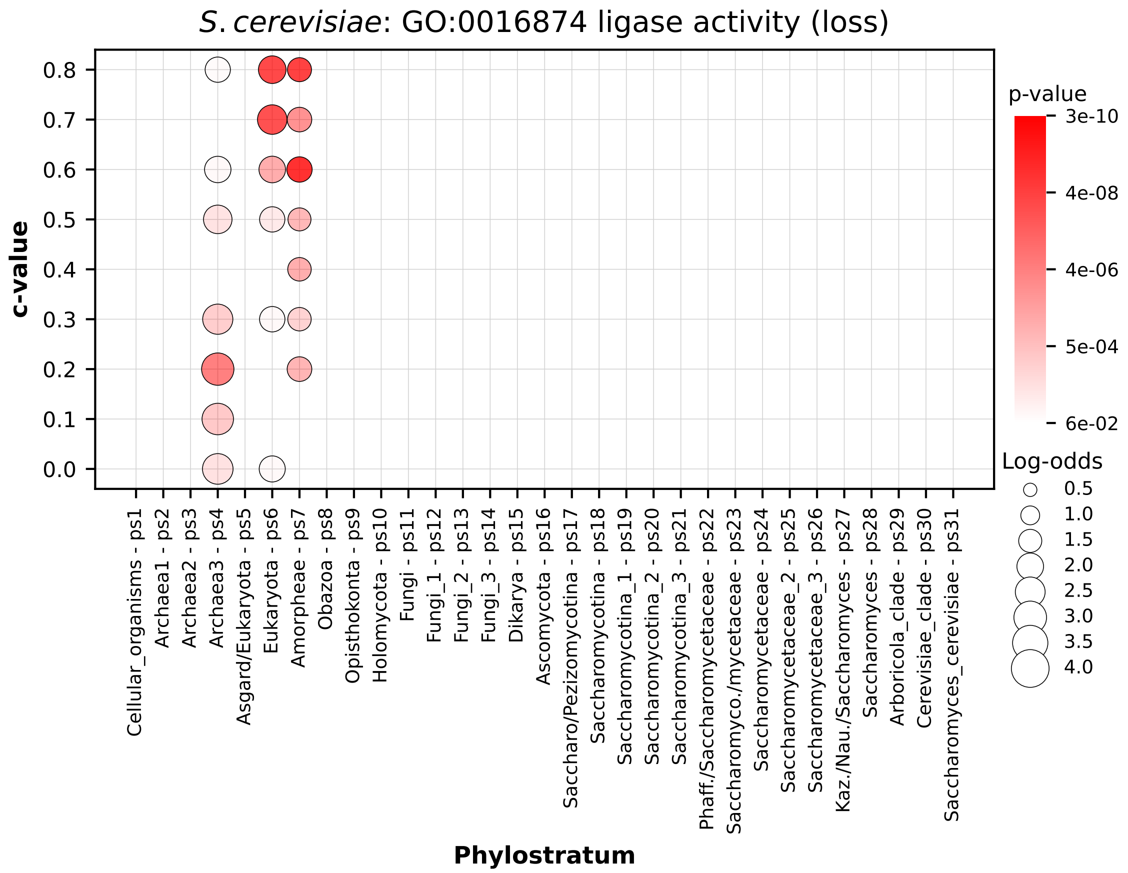

S. cerevisiae: GO:0016874 ligase activity (loss)
c-value
0.8
0.7
0.6
0.5
0.4
0.3
0.2
0.1
0.0
p-value
3e-10
4e-08
4e-06
5e-04
6e-02
Log-odds
0.5
1.0
1.5
2.0
2.5
3.0
3.5
4.0
Cellular_organisms - ps1
Archaea1 - ps2
Archaea2 - ps3
Archaea3 - ps4
Asgard/Eukaryota - ps5
Eukaryota - ps6
Amorpheae - ps7
Obazoa - ps8
Opisthokonta - ps9
Holomycota - ps10
Fungi - ps11
Fungi_1 - ps12
Fungi_2 - ps13
Fungi_3 - ps14
Dikarya - ps15
Ascomycota - ps16
Saccharo/Pezizomycotina - ps17
Saccharomycotina - ps18
Saccharomycotina_1 - ps19
Saccharomycotina_2 - ps20
Saccharomycotina_3 - ps21
Phaff./Saccharomycetaceae - ps22
Saccharomyco./mycetaceae - ps23
Saccharomycetaceae - ps24
Saccharomycetaceae_2 - ps25
Saccharomycetaceae_3 - ps26
Kaz./Nau./Saccharomyces - ps27
Saccharomyces - ps28
Arboricola_clade - ps29
Cerevisiae_clade - ps30
Saccharomyces_cerevisiae - ps31
Phylostratum

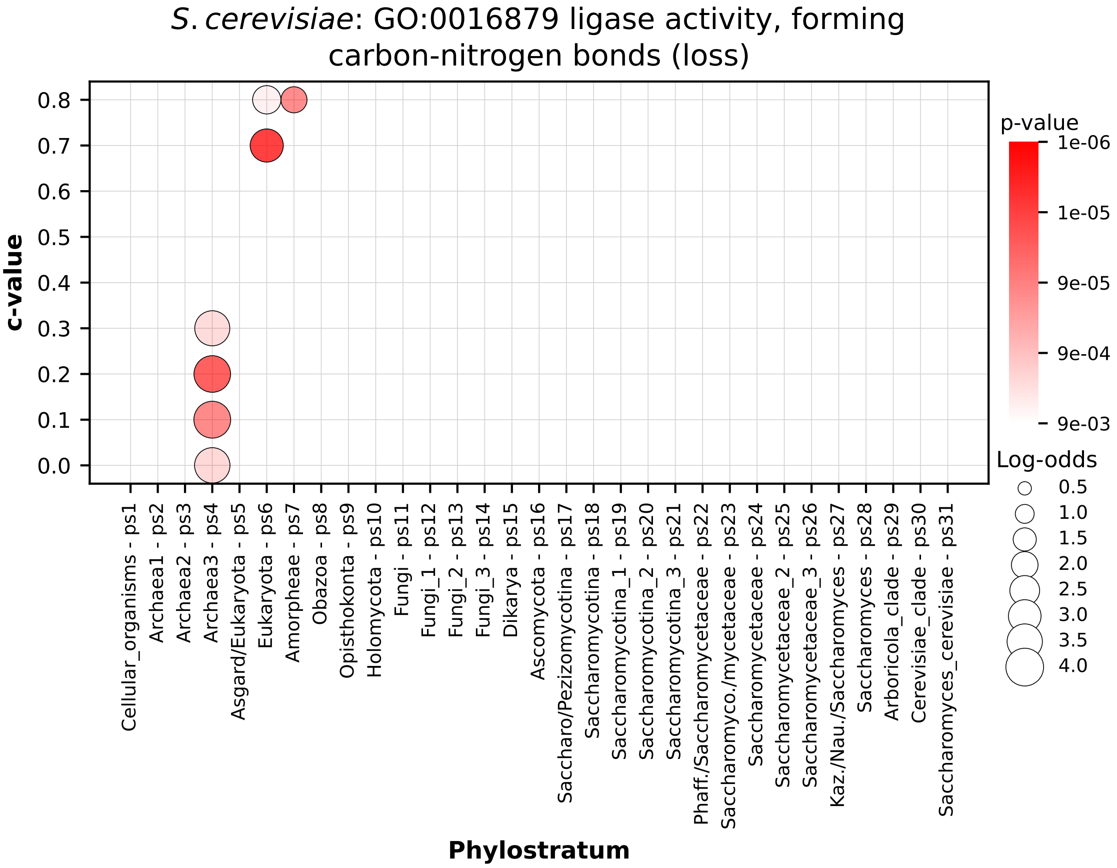

S. cerevisiae: GO:0016879 ligase activity, forming carbon-nitrogen bonds (loss)
c-value
0.8
0.7
0.6
0.5
0.4
0.3
0.2
0.1
0.0
p-value
1e-06
1e-05
9e-05
9e-04
9e-03
Log-odds
0.5
1.0
1.5
2.0
2.5
3.0
3.5
4.0
Cellular_organisms - ps1
Archaea1 - ps2
Archaea2 - ps3
Archaea3 - ps4
Asgard/Eukaryota - ps5
Eukaryota - ps6
Amorpheae - ps7
Obazoa - ps8
Opisthokonta - ps9
Holomycota - ps10
Fungi - ps11
Fungi_1 - ps12
Fungi_2 - ps13
Fungi_3 - ps14
Dikarya - ps15
Ascomycota - ps16
Saccharo/Pezizomycotina - ps17
Saccharomycotina - ps18
Saccharomycotina_1 - ps19
Saccharomycotina_2 - ps20
Saccharomycotina_3 - ps21
Phaff./Saccharomycetaceae - ps22
Saccharomyco./mycetaceae - ps23
Saccharomycetaceae - ps24
Saccharomycetaceae_2 - ps25
Saccharomycetaceae_3 - ps26
Kaz./Nau./Saccharomyces - ps27
Saccharomyces - ps28
Arboricola_clade - ps29
Cerevisiae_clade - ps30
Saccharomyces_cerevisiae - ps31
Phylostratum

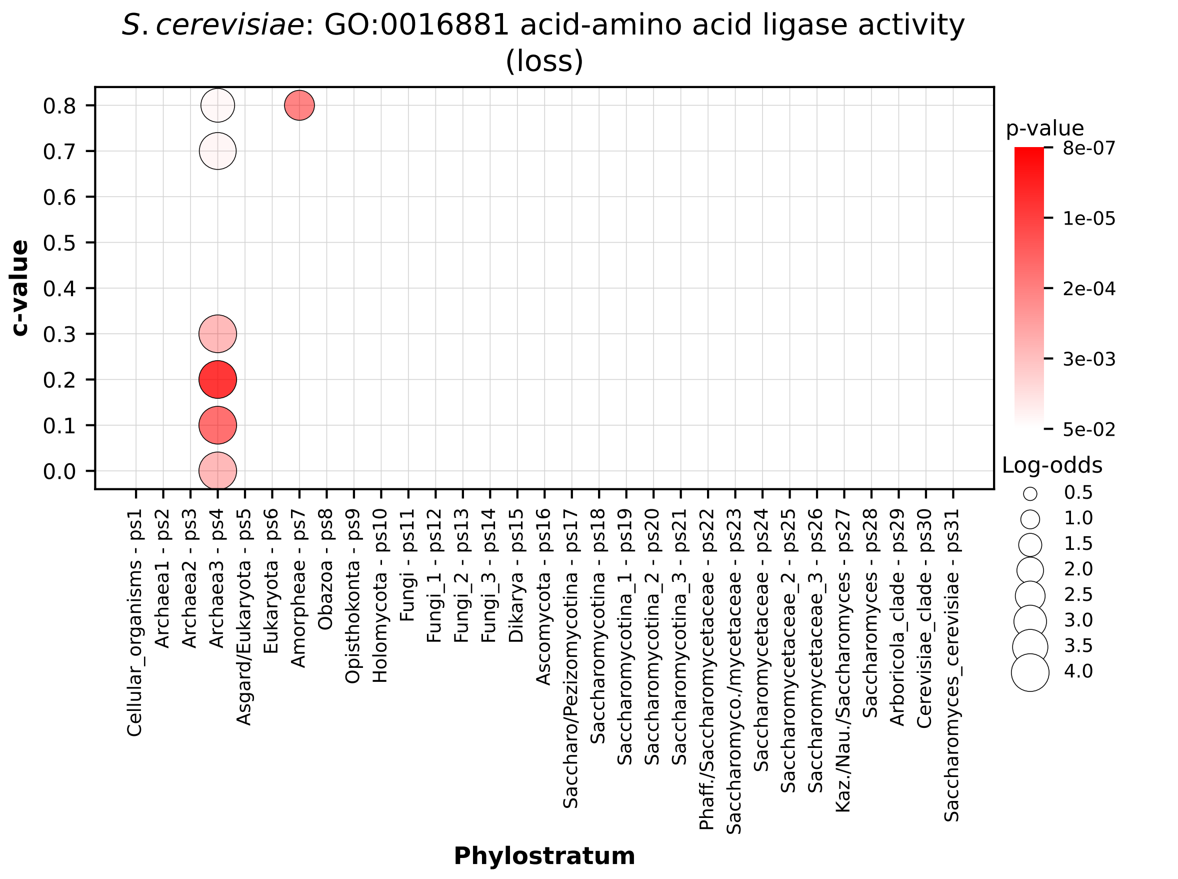

S. cerevisiae: GO:0016881 acid-amino acid ligase activity (loss)
c-value
0.0
0.1
0.2
0.3
0.4
0.5
0.6
0.7
0.8
Phylostratum
Cellular_organisms - ps1
Archaea1 - ps2
Archaea2 - ps3
Archaea3 - ps4
Asgard/Eukaryota - ps5
Eukaryota - ps6
Amorpheae - ps7
Obazoa - ps8
Opisthokonta - ps9
Holomycota - ps10
Fungi - ps11
Fungi_1 - ps12
Fungi_2 - ps13
Fungi_3 - ps14
Dikarya - ps15
Ascomycota - ps16
Saccharo/Pezizomycotina - ps17
Saccharomycotina - ps18
Saccharomycotina_1 - ps19
Saccharomycotina_2 - ps20
Saccharomycotina_3 - ps21
Phaff./Saccharomycetaceae - ps22
Saccharomyco./mycetaceae - ps23
Saccharomycetaceae - ps24
Saccharomycetaceae_2 - ps25
Saccharomycetaceae_3 - ps26
Kaz./Nau./Saccharomyces - ps27
Saccharomyces - ps28
Arboricola_clade - ps29
Cerevisiae_clade - ps30
Saccharomyces_cerevisiae - ps31
p-value
8e-07
1e-05
2e-04
3e-03
5e-02
Log-odds
0.5
1.0
1.5
2.0
2.5
3.0
3.5
4.0

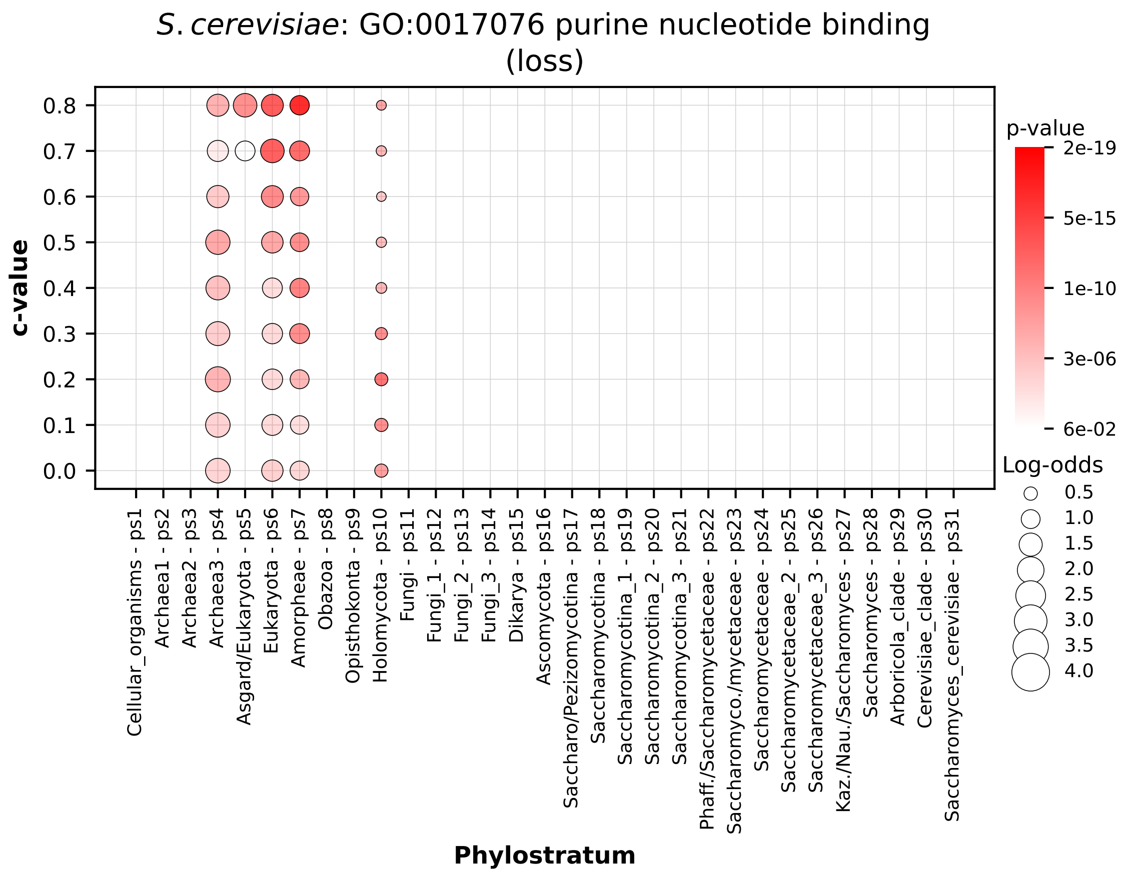

S. cerevisiae: GO:0017076 purine nucleotide binding
(loss)
c-value
0.8
0.7
0.6
0.5
0.4
0.3
0.2
0.1
0.0
p-value
2e-19
5e-15
1e-10
3e-06
6e-02
Log-odds
0.5
1.0
1.5
2.0
2.5
3.0
3.5
4.0
Cellular_organisms - ps1
Archaea1 - ps2
Archaea2 - ps3
Archaea3 - ps4
Asgard/Eukaryota - ps5
Eukaryota - ps6
Amorpheae - ps7
Obazoa - ps8
Opisthokonta - ps9
Holomycota - ps10
Fungi - ps11
Fungi_1 - ps12
Fungi_2 - ps13
Fungi_3 - ps14
Dikarya - ps15
Ascomycota - ps16
Saccharo/Pezizomycotina - ps17
Saccharomycotina - ps18
Saccharomycotina_1 - ps19
Saccharomycotina_2 - ps20
Saccharomycotina_3 - ps21
Phaff./Saccharomycetaceae - ps22
Saccharomyco./mycetaceae - ps23
Saccharomycetaceae - ps24
Saccharomycetaceae_2 - ps25
Saccharomycetaceae_3 - ps26
Kaz./Nau./Saccharomyces - ps27
Saccharomyces - ps28
Arboricola_clade - ps29
Cerevisiae_clade - ps30
Saccharomyces_cerevisiae - ps31
Phylostratum

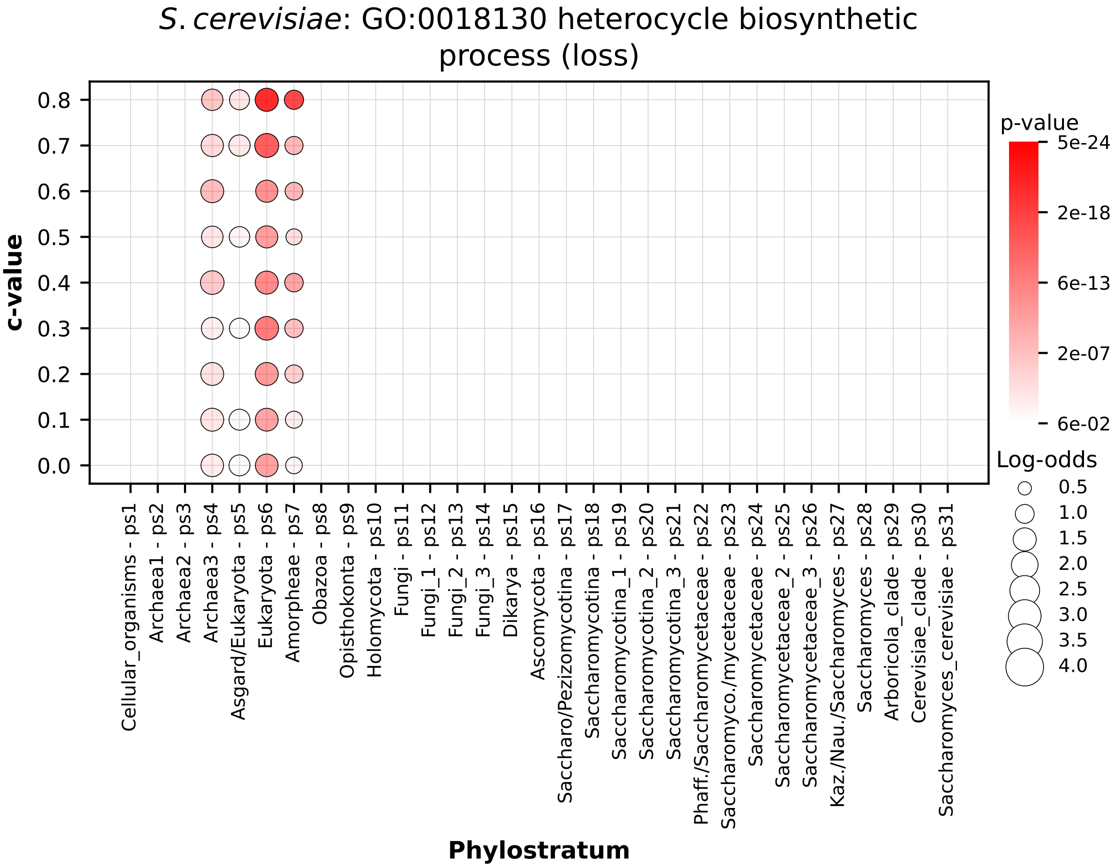
S. cerevisiae: GO:0018130 heterocycle biosynthetic process (loss)
c-value
0.8
0.7
0.6
0.5
0.4
0.3
0.2
0.1
0.0
p-value
5e-24
2e-18
6e-13
2e-07
6e-02
Log-odds
0.5
1.0
1.5
2.0
2.5
3.0
3.5
4.0
Cellular_organisms - ps1
Archaea1 - ps2
Archaea2 - ps3
Archaea3 - ps4
Asgard/Eukaryota - ps5
Eukaryota - ps6
Amorpheae - ps7
Obazoa - ps8
Opisthokonta - ps9
Holomycota - ps10
Fungi - ps11
Fungi_1 - ps12
Fungi_2 - ps13
Fungi_3 - ps14
Dikarya - ps15
Ascomycota - ps16
Saccharo/Pezizomycotina - ps17
Saccharomycotina - ps18
Saccharomycotina_1 - ps19
Saccharomycotina_2 - ps20
Saccharomycotina_3 - ps21
Phaff./Saccharomycetaceae - ps22
Saccharomyco./mycetaceae - ps23
Saccharomycetaceae - ps24
Saccharomycetaceae_2 - ps25
Saccharomycetaceae_3 - ps26
Kaz./Nau./Saccharomyces - ps27
Saccharomyces - ps28
Arboricola_clade - ps29
Cerevisiae_clade - ps30
Saccharomyces_cerevisiae - ps31
Phylostratum

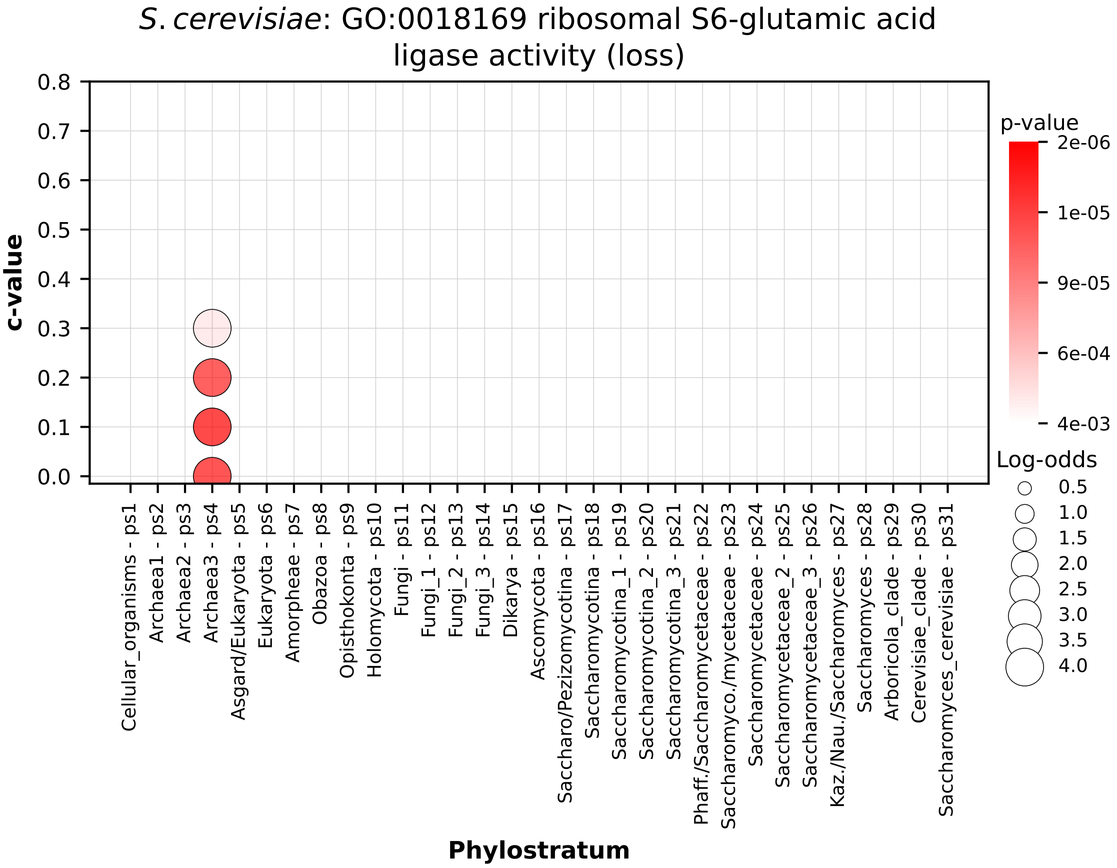

S. cerevisiae: GO:0018169 ribosomal S6-glutamic acid ligase activity (loss)
c-value
0.8
0.7
0.6
0.5
0.4
0.3
0.2
0.1
0.0
Phylostratum
Cellular_organisms - ps1
Archaea1 - ps2
Archaea2 - ps3
Archaea3 - ps4
Asgard/Eukaryota - ps5
Eukaryota - ps6
Amorpheae - ps7
Obazoa - ps8
Opisthokonta - ps9
Holomycota - ps10
Fungi - ps11
Fungi_1 - ps12
Fungi_2 - ps13
Fungi_3 - ps14
Dikarya - ps15
Ascomycota - ps16
Saccharo/Pezizomycotina - ps17
Saccharomycotina - ps18
Saccharomycotina_1 - ps19
Saccharomycotina_2 - ps20
Saccharomycotina_3 - ps21
Phaff./Saccharomycetaceae - ps22
Saccharomyco./mycetaceae - ps23
Saccharomycetaceae - ps24
Saccharomycetaceae_2 - ps25
Saccharomycetaceae_3 - ps26
Kaz./Nau./Saccharomyces - ps27
Saccharomyces - ps28
Arboricola_clade - ps29
Cerevisiae_clade - ps30
Saccharomyces_cerevisiae - ps31
p-value
2e-06
1e-05
9e-05
6e-04
4e-03
Log-odds
0.5
1.0
1.5
2.0
2.5
3.0
3.5
4.0

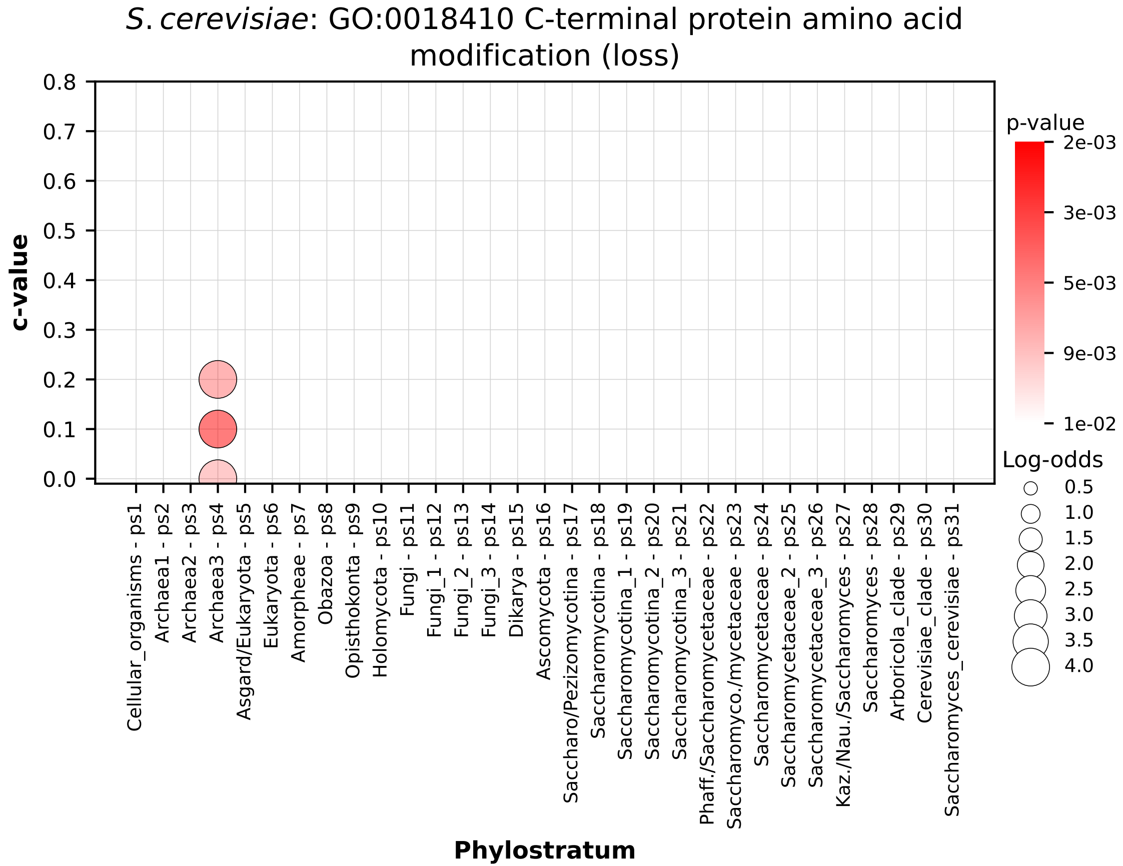

S. cerevisiae: GO:0018410 C-terminal protein amino acid modification (loss)
c-value
0.8
0.7
0.6
0.5
0.4
0.3
0.2
0.1
0.0
Cellular_organisms - ps1
Archaea1 - ps2
Archaea2 - ps3
Archaea3 - ps4
Asgard/Eukaryota - ps5
Eukaryota - ps6
Amorpheae - ps7
Obazoa - ps8
Opisthokonta - ps9
Holomycota - ps10
Fungi - ps11
Fungi_1 - ps12
Fungi_2 - ps13
Fungi_3 - ps14
Dikarya - ps15
Ascomycota - ps16
Saccharo/Pezizomycotina - ps17
Saccharomycotina - ps18
Saccharomycotina_1 - ps19
Saccharomycotina_2 - ps20
Saccharomycotina_3 - ps21
Phaff./Saccharomycetaceae - ps22
Saccharomyco./mycetaceae - ps23
Saccharomycetaceae - ps24
Saccharomycetaceae_2 - ps25
Saccharomycetaceae_3 - ps26
Kaz./Nau./Saccharomyces - ps27
Saccharomyces - ps28
Arboricola_clade - ps29
Cerevisiae_clade - ps30
Saccharomyces_cerevisiae - ps31
Phylostratum
p-value
2e-03
3e-03
5e-03
9e-03
1e-02
Log-odds
0.5
1.0
1.5
2.0
2.5
3.0
3.5
4.0

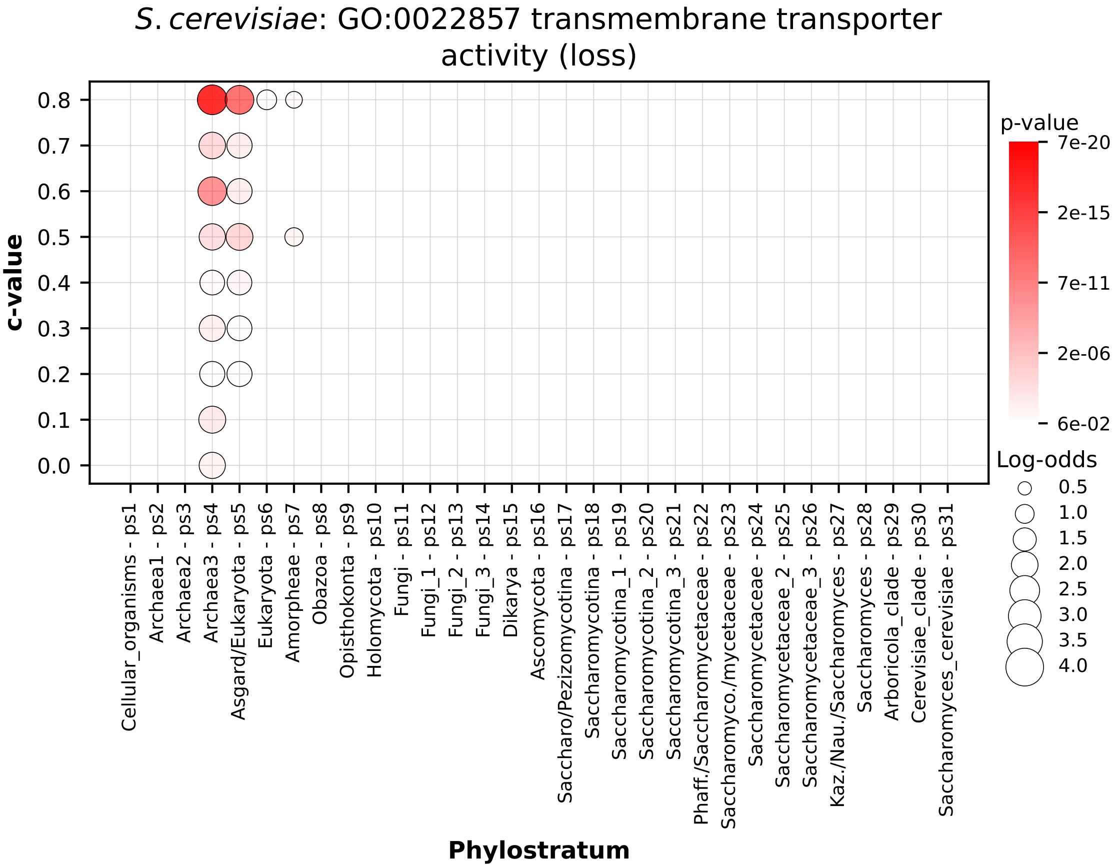
S. cerevisiae: GO:0022857 transmembrane transporter activity (loss)
c-value
0.8
0.7
0.6
0.5
0.4
0.3
0.2
0.1
0.0
Phylostratum
Cellular_organisms - ps1
Archaea1 - ps2
Archaea2 - ps3
Archaea3 - ps4
Asgard/Eukaryota - ps5
Eukaryota - ps6
Amorpheae - ps7
Obazoa - ps8
Opisthokonta - ps9
Holomycota - ps10
Fungi - ps11
Fungi_1 - ps12
Fungi_2 - ps13
Fungi_3 - ps14
Dikarya - ps15
Ascomycota - ps16
Saccharo/Pezizomycotina - ps17
Saccharomycotina - ps18
Saccharomycotina_1 - ps19
Saccharomycotina_2 - ps20
Saccharomycotina_3 - ps21
Phaff./Saccharomycetaceae - ps22
Saccharomyco./mycetaceae - ps23
Saccharomycetaceae - ps24
Saccharomycetaceae_2 - ps25
Saccharomycetaceae_3 - ps26
Kaz./Nau./Saccharomyces - ps27
Saccharomyces - ps28
Arboricola_clade - ps29
Cerevisiae_clade - ps30
Saccharomyces_cerevisiae - ps31
p-value
7e-20
2e-15
7e-11
2e-06
6e-02
Log-odds
0.5
1.0
1.5
2.0
2.5
3.0
3.5
4.0

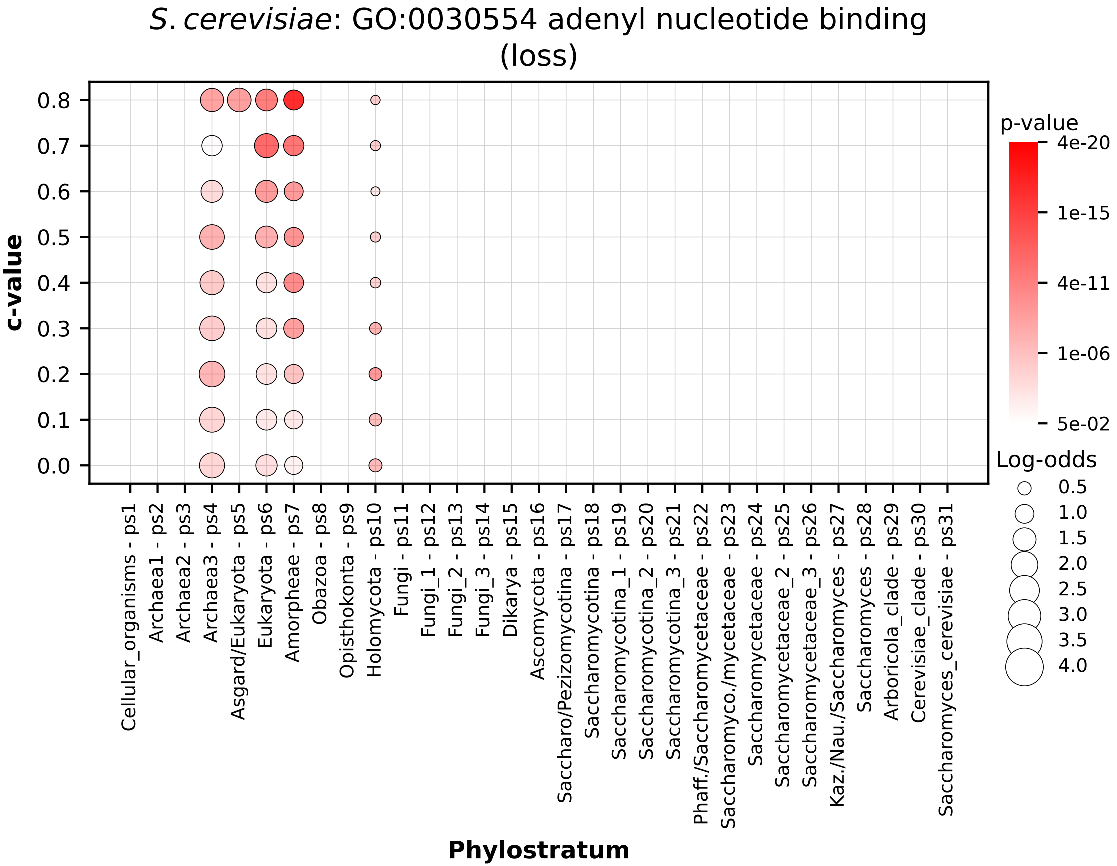

S. cerevisiae: GO:0030554 adenyl nucleotide binding (loss)
c-value
0.8
0.7
0.6
0.5
0.4
0.3
0.2
0.1
0.0
p-value
4e-20
1e-15
4e-11
1e-06
5e-02
Log-odds
0.5
1.0
1.5
2.0
2.5
3.0
3.5
4.0
Cellular_organisms - ps1
Archaea1 - ps2
Archaea2 - ps3
Archaea3 - ps4
Asgard/Eukaryota - ps5
Eukaryota - ps6
Amorpheae - ps7
Obazoa - ps8
Opisthokonta - ps9
Holomycota - ps10
Fungi - ps11
Fungi_1 - ps12
Fungi_2 - ps13
Fungi_3 - ps14
Dikarya - ps15
Ascomycota - ps16
Saccharo/Pezizomycotina - ps17
Saccharomycotina - ps18
Saccharomycotina_1 - ps19
Saccharomycotina_2 - ps20
Saccharomycotina_3 - ps21
Phaff./Saccharomycetaceae - ps22
Saccharomyco./mycetaceae - ps23
Saccharomycetaceae - ps24
Saccharomycetaceae_2 - ps25
Saccharomycetaceae_3 - ps26
Kaz./Nau./Saccharomyces - ps27
Saccharomyces - ps28
Arboricola_clade - ps29
Cerevisiae_clade - ps30
Saccharomyces_cerevisiae - ps31
Phylostratum

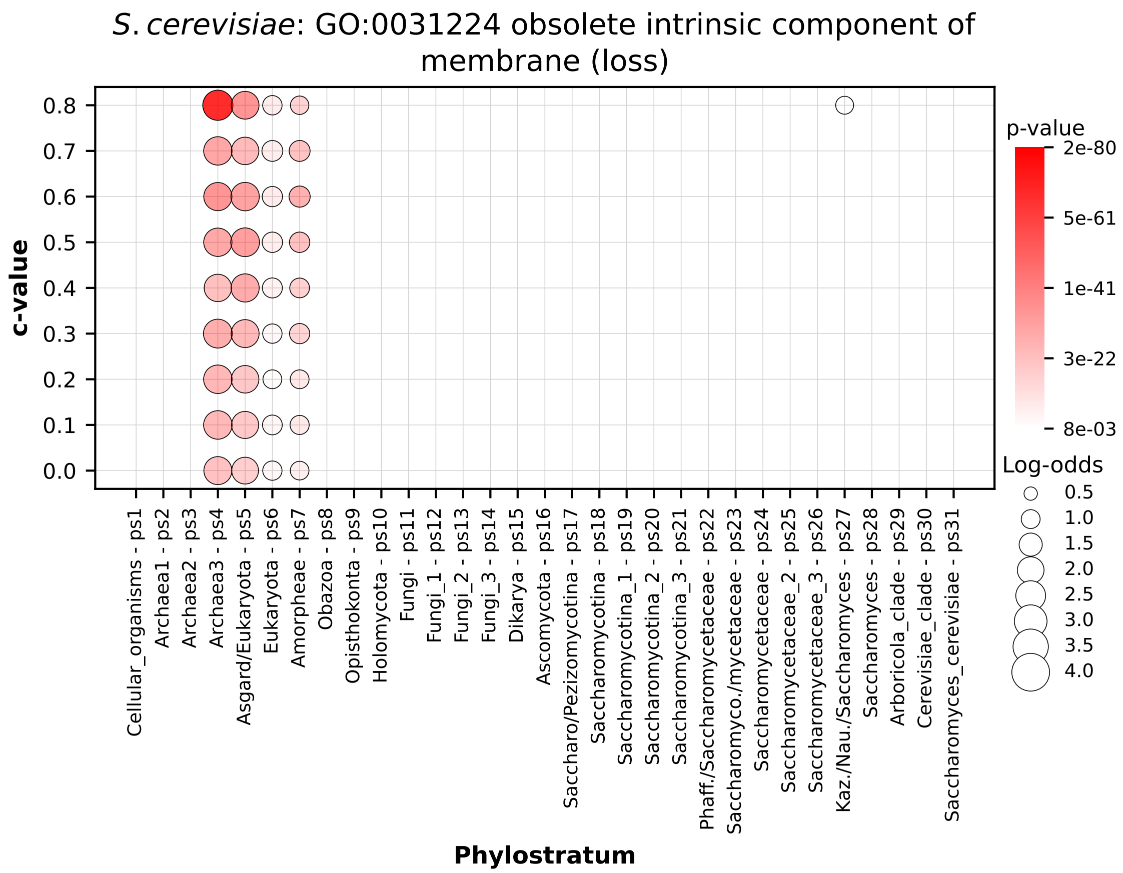

S. cerevisiae: GO:0031224 obsolete intrinsic component of membrane (loss)
c-value
0.8
0.7
0.6
0.5
0.4
0.3
0.2
0.1
0.0
p-value
2e-80
5e-61
1e-41
3e-22
8e-03
Log-odds
0.5
1.0
1.5
2.0
2.5
3.0
3.5
4.0
Cellular_organisms - ps1
Archaea1 - ps2
Archaea2 - ps3
Archaea3 - ps4
Asgard/Eukaryota - ps5
Eukaryota - ps6
Amorpheae - ps7
Obazoa - ps8
Opisthokonta - ps9
Holomycota - ps10
Fungi - ps11
Fungi_1 - ps12
Fungi_2 - ps13
Fungi_3 - ps14
Dikarya - ps15
Ascomycota - ps16
Saccharo/Pezizomycotina - ps17
Saccharomycotina - ps18
Saccharomycotina_1 - ps19
Saccharomycotina_2 - ps20
Saccharomycotina_3 - ps21
Phaff./Saccharomycetaceae - ps22
Saccharomyco./mycetaceae - ps23
Saccharomycetaceae - ps24
Saccharomycetaceae_2 - ps25
Saccharomycetaceae_3 - ps26
Kaz./Nau./Saccharomyces - ps27
Saccharomyces - ps28
Arboricola_clade - ps29
Cerevisiae_clade - ps30
Saccharomyces_cerevisiae - ps31
Phylostratum

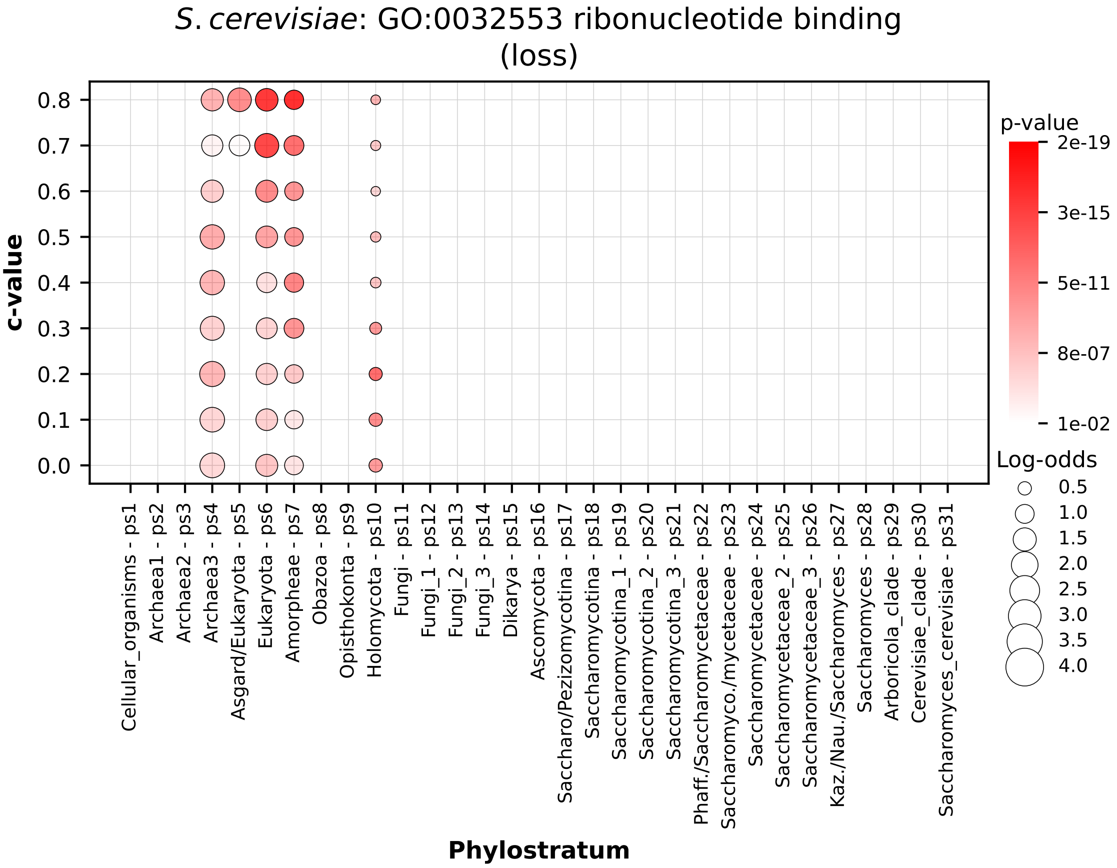
S. cerevisiae: GO:0032553 ribonucleotide binding
(loss)
c-value
0.8
0.7
0.6
0.5
0.4
0.3
0.2
0.1
0.0
p-value
2e-19
3e-15
5e-11
8e-07
1e-02
Log-odds
0.5
1.0
1.5
2.0
2.5
3.0
3.5
4.0
Cellular_organisms - ps1
Archaea1 - ps2
Archaea2 - ps3
Archaea3 - ps4
Asgard/Eukaryota - ps5
Eukaryota - ps6
Amorpheae - ps7
Obazoa - ps8
Opisthokonta - ps9
Holomycota - ps10
Fungi - ps11
Fungi_1 - ps12
Fungi_2 - ps13
Fungi_3 - ps14
Dikarya - ps15
Ascomycota - ps16
Saccharo/Pezizomycotina - ps17
Saccharomycotina - ps18
Saccharomycotina_1 - ps19
Saccharomycotina_2 - ps20
Saccharomycotina_3 - ps21
Phaff./Saccharomycetaceae - ps22
Saccharomyco./mycetaceae - ps23
Saccharomycetaceae - ps24
Saccharomycetaceae_2 - ps25
Saccharomycetaceae_3 - ps26
Kaz./Nau./Saccharomyces - ps27
Saccharomyces - ps28
Arboricola_clade - ps29
Cerevisiae_clade - ps30
Saccharomyces_cerevisiae - ps31
Phylostratum

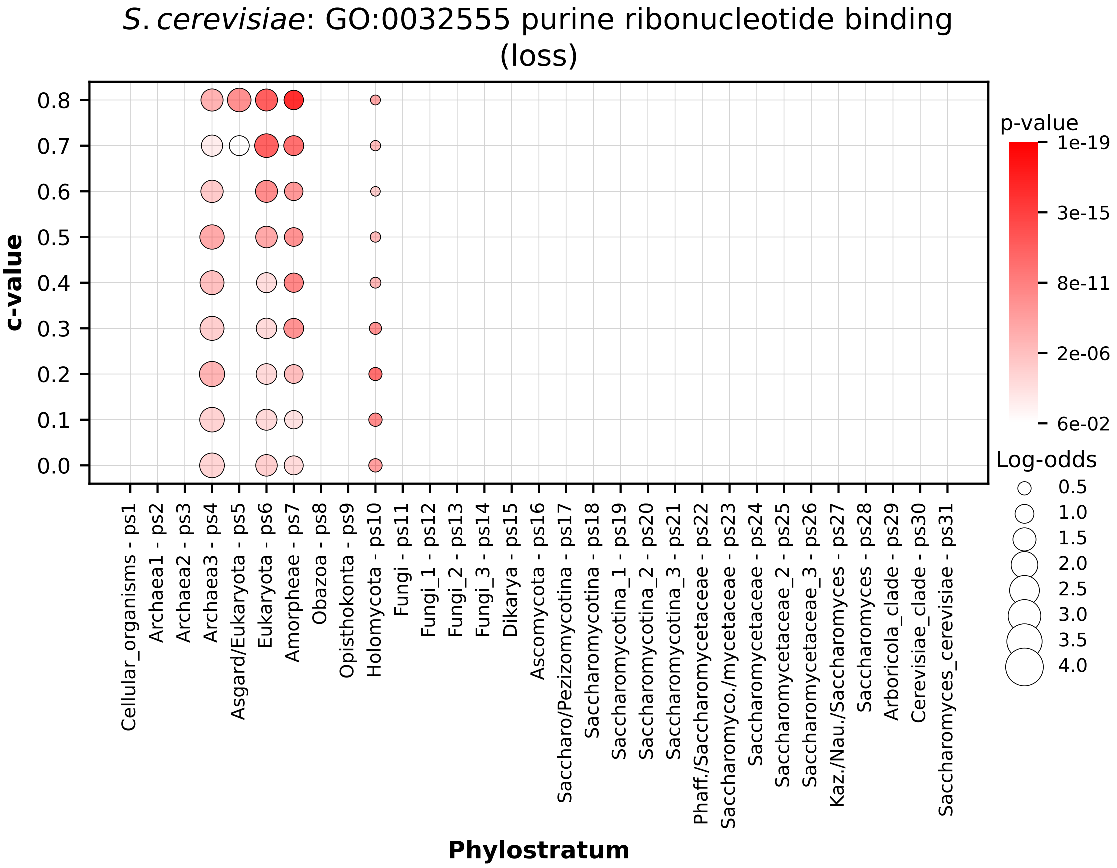
S. cerevisiae: GO:0032555 purine ribonucleotide binding
(loss)
c-value
0.8
0.7
0.6
0.5
0.4
0.3
0.2
0.1
0.0
p-value
1e-19
3e-15
8e-11
2e-06
6e-02
Log-odds
0.5
1.0
1.5
2.0
2.5
3.0
3.5
4.0
Cellular_organisms - ps1
Archaea1 - ps2
Archaea2 - ps3
Archaea3 - ps4
Asgard/Eukaryota - ps5
Eukaryota - ps6
Amorpheae - ps7
Obazoa - ps8
Opisthokonta - ps9
Holomycota - ps10
Fungi - ps11
Fungi_1 - ps12
Fungi_2 - ps13
Fungi_3 - ps14
Dikarya - ps15
Ascomycota - ps16
Saccharo/Pezizomycotina - ps17
Saccharomycotina - ps18
Saccharomycotina_1 - ps19
Saccharomycotina_2 - ps20
Saccharomycotina_3 - ps21
Phaff./Saccharomycetaceae - ps22
Saccharomyco./mycetaceae - ps23
Saccharomycetaceae - ps24
Saccharomycetaceae_2 - ps25
Saccharomycetaceae_3 - ps26
Kaz./Nau./Saccharomyces - ps27
Saccharomyces - ps28
Arboricola_clade - ps29
Cerevisiae_clade - ps30
Saccharomyces_cerevisiae - ps31
Phylostratum

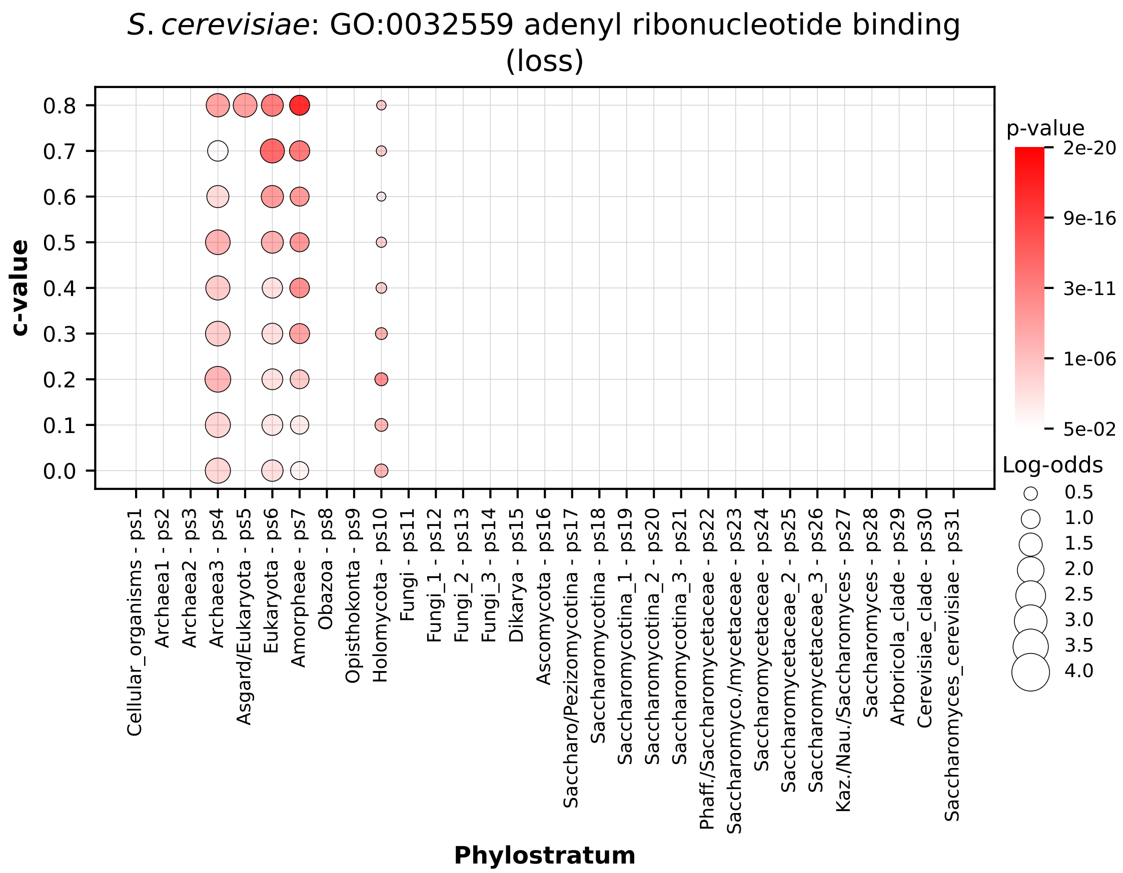

S. cerevisiae: GO:0032559 adenyl ribonucleotide binding
(loss)
c-value
0.8
0.7
0.6
0.5
0.4
0.3
0.2
0.1
0.0
Phylostratum
Cellular_organisms - ps1
Archaea1 - ps2
Archaea2 - ps3
Archaea3 - ps4
Asgard/Eukaryota - ps5
Eukaryota - ps6
Amorpheae - ps7
Obazoa - ps8
Opisthokonta - ps9
Holomycota - ps10
Fungi - ps11
Fungi_1 - ps12
Fungi_2 - ps13
Fungi_3 - ps14
Dikarya - ps15
Ascomycota - ps16
Saccharo/Pezizomycotina - ps17
Saccharomycotina - ps18
Saccharomycotina_1 - ps19
Saccharomycotina_2 - ps20
Saccharomycotina_3 - ps21
Phaff./Saccharomycetaceae - ps22
Saccharomyco./mycetaceae - ps23
Saccharomycetaceae - ps24
Saccharomycetaceae_2 - ps25
Saccharomycetaceae_3 - ps26
Kaz./Nau./Saccharomyces - ps27
Saccharomyces - ps28
Arboricola_clade - ps29
Cerevisiae_clade - ps30
Saccharomyces_cerevisiae - ps31
p-value
2e-20
9e-16
3e-11
1e-06
5e-02
Log-odds
0.5
1.0
1.5
2.0
2.5
3.0
3.5
4.0

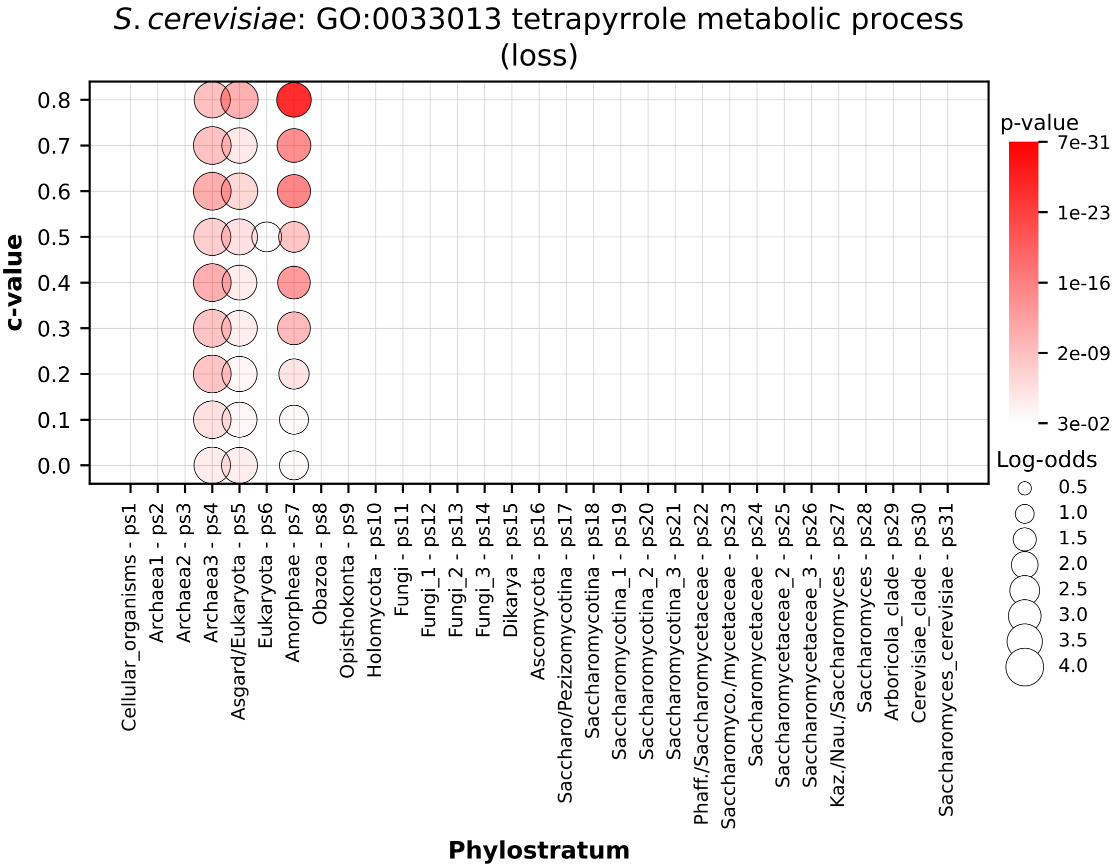

S. cerevisiae: GO:0033013 tetrapyrrole metabolic process
(loss)
c-value
0.8
0.7
0.6
0.5
0.4
0.3
0.2
0.1
0.0
p-value
7e-31
1e-23
1e-16
2e-09
3e-02
Log-odds
0.5
1.0
1.5
2.0
2.5
3.0
3.5
4.0
Cellular_organisms - ps1
Archaea1 - ps2
Archaea2 - ps3
Archaea3 - ps4
Asgard/Eukaryota - ps5
Eukaryota - ps6
Amorpheae - ps7
Obazoa - ps8
Opisthokonta - ps9
Holomycota - ps10
Fungi - ps11
Fungi_1 - ps12
Fungi_2 - ps13
Fungi_3 - ps14
Dikarya - ps15
Ascomycota - ps16
Saccharo/Pezizomycotina - ps17
Saccharomycotina - ps18
Saccharomycotina_1 - ps19
Saccharomycotina_2 - ps20
Saccharomycotina_3 - ps21
Phaff./Saccharomycetaceae - ps22
Saccharomyco./mycetaceae - ps23
Saccharomycetaceae - ps24
Saccharomycetaceae_2 - ps25
Saccharomycetaceae_3 - ps26
Kaz./Nau./Saccharomyces - ps27
Saccharomyces - ps28
Arboricola_clade - ps29
Cerevisiae_clade - ps30
Saccharomyces_cerevisiae - ps31
Phylostratum

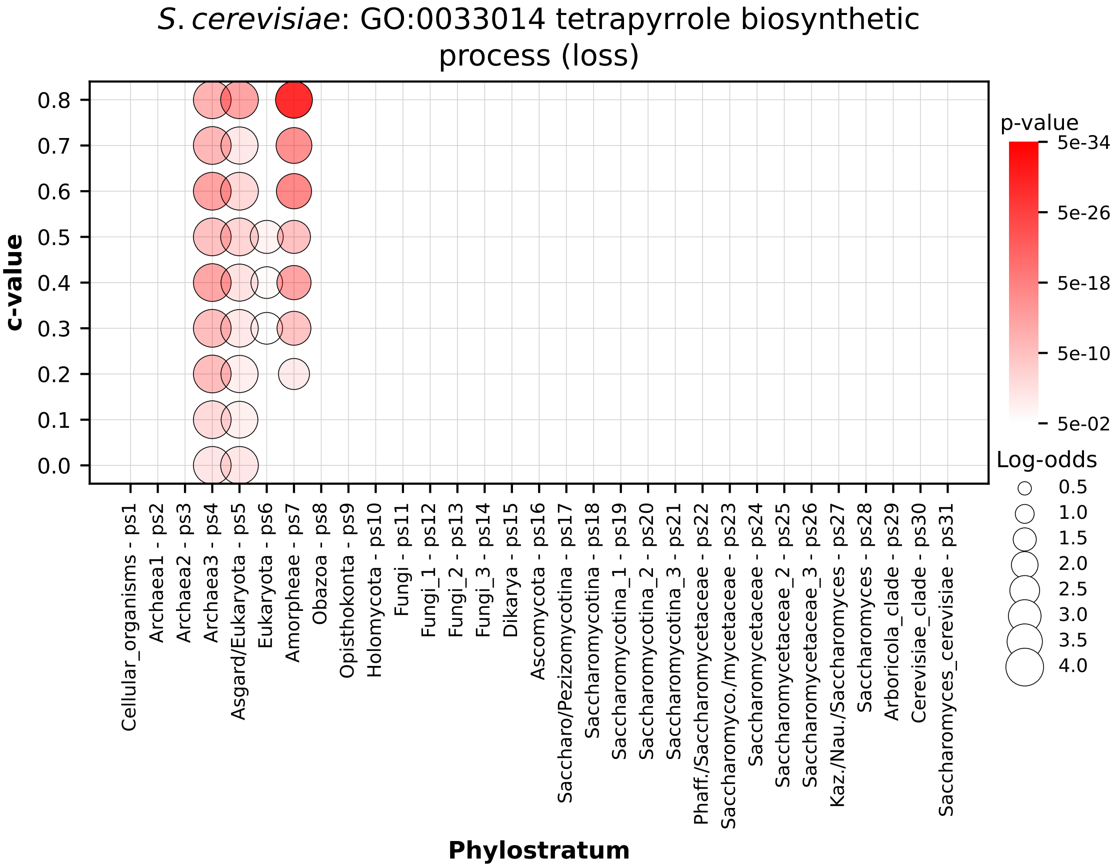

S. cerevisiae: GO:0033014 tetrapyrrole biosynthetic process (loss)
c-value
0.8
0.7
0.6
0.5
0.4
0.3
0.2
0.1
0.0
p-value
5e-34
5e-26
5e-18
5e-10
5e-02
Log-odds
0.5
1.0
1.5
2.0
2.5
3.0
3.5
4.0
Cellular_organisms - ps1
Archaea1 - ps2
Archaea2 - ps3
Archaea3 - ps4
Asgard/Eukaryota - ps5
Eukaryota - ps6
Amorpheae - ps7
Obazoa - ps8
Opisthokonta - ps9
Holomycota - ps10
Fungi - ps11
Fungi_1 - ps12
Fungi_2 - ps13
Fungi_3 - ps14
Dikarya - ps15
Ascomycota - ps16
Saccharo/Pezizomycotina - ps17
Saccharomycotina - ps18
Saccharomycotina_1 - ps19
Saccharomycotina_2 - ps20
Saccharomycotina_3 - ps21
Phaff./Saccharomycetaceae - ps22
Saccharomyco./mycetaceae - ps23
Saccharomycetaceae - ps24
Saccharomycetaceae_2 - ps25
Saccharomycetaceae_3 - ps26
Kaz./Nau./Saccharomyces - ps27
Saccharomyces - ps28
Arboricola_clade - ps29
Cerevisiae_clade - ps30
Saccharomyces_cerevisiae - ps31
Phylostratum

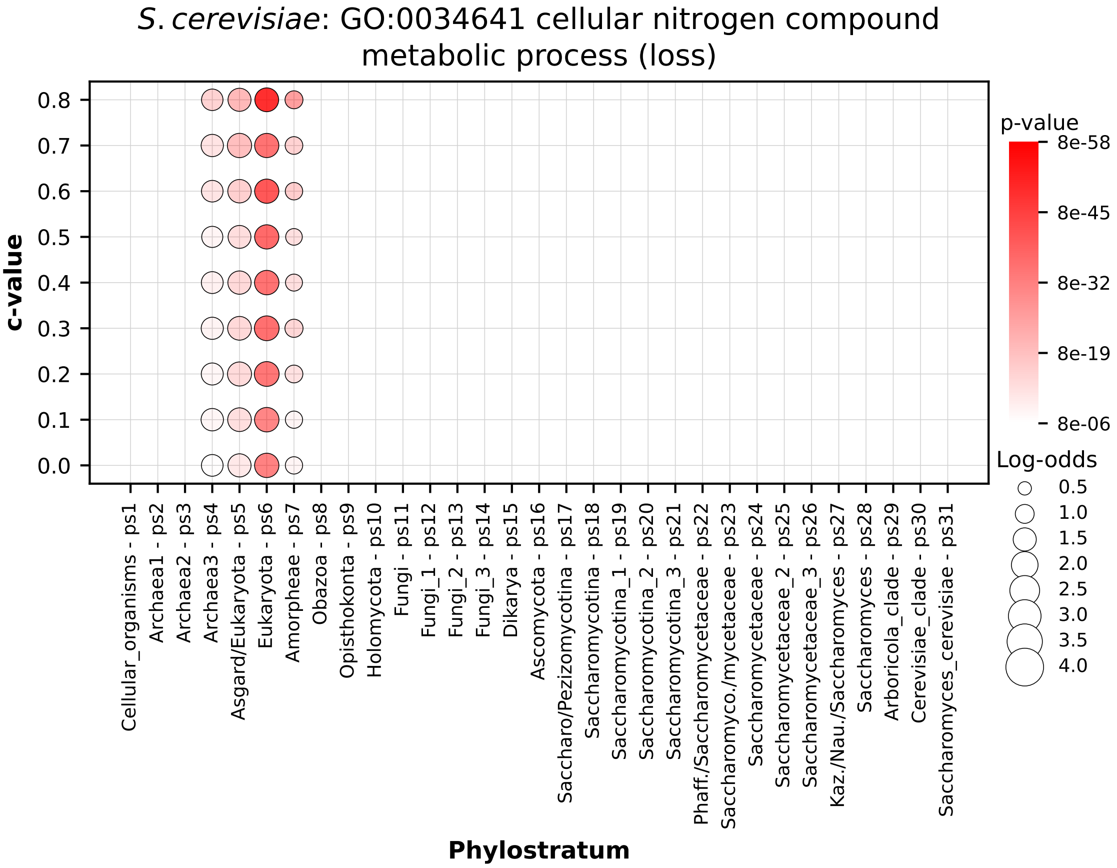
S. cerevisiae: GO:0034641 cellular nitrogen compound metabolic process (loss)
c-value
0.8
0.7
0.6
0.5
0.4
0.3
0.2
0.1
0.0
p-value
8e-58
8e-45
8e-32
8e-19
8e-06
Log-odds
0.5
1.0
1.5
2.0
2.5
3.0
3.5
4.0
Cellular_organisms - ps1
Archaea1 - ps2
Archaea2 - ps3
Archaea3 - ps4
Asgard/Eukaryota - ps5
Eukaryota - ps6
Amorpheae - ps7
Obazoa - ps8
Opisthokonta - ps9
Holomycota - ps10
Fungi - ps11
Fungi_1 - ps12
Fungi_2 - ps13
Fungi_3 - ps14
Dikarya - ps15
Ascomycota - ps16
Saccharo/Pezizomycotina - ps17
Saccharomycotina - ps18
Saccharomycotina_1 - ps19
Saccharomycotina_2 - ps20
Saccharomycotina_3 - ps21
Phaff./Saccharomycetaceae - ps22
Saccharomyco./mycetaceae - ps23
Saccharomycetaceae - ps24
Saccharomycetaceae_2 - ps25
Saccharomycetaceae_3 - ps26
Kaz./Nau./Saccharomyces - ps27
Saccharomyces - ps28
Arboricola_clade - ps29
Cerevisiae_clade - ps30
Saccharomyces_cerevisiae - ps31
Phylostratum

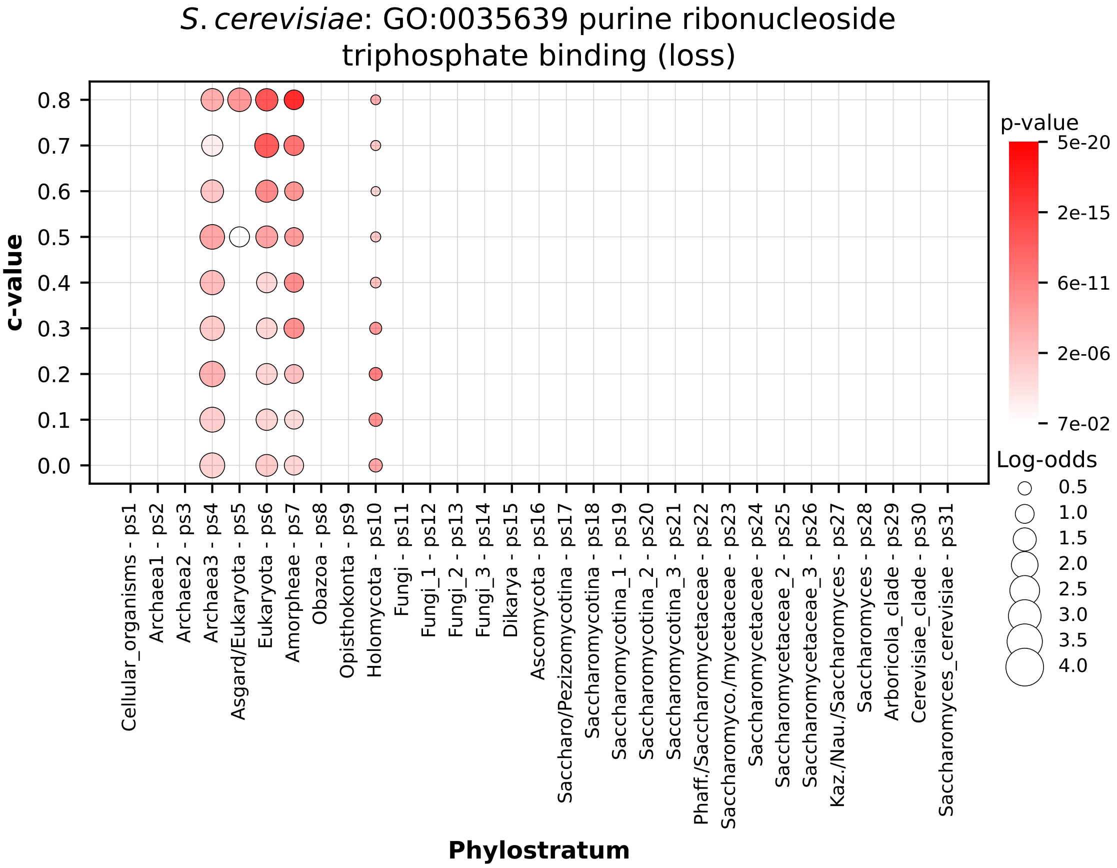

S. cerevisiae: GO:0035639 purine ribonucleoside triphosphate binding (loss)
c-value
0.8
0.7
0.6
0.5
0.4
0.3
0.2
0.1
0.0
p-value
5e-20
2e-15
6e-11
2e-06
7e-02
Log-odds
0.5
1.0
1.5
2.0
2.5
3.0
3.5
4.0
Cellular_organisms - ps1
Archaea1 - ps2
Archaea2 - ps3
Archaea3 - ps4
Asgard/Eukaryota - ps5
Eukaryota - ps6
Amorpheae - ps7
Obazoa - ps8
Opisthokonta - ps9
Holomycota - ps10
Fungi - ps11
Fungi_1 - ps12
Fungi_2 - ps13
Fungi_3 - ps14
Dikarya - ps15
Ascomycota - ps16
Saccharo/Pezizomycotina - ps17
Saccharomycotina - ps18
Saccharomycotina_1 - ps19
Saccharomycotina_2 - ps20
Saccharomycotina_3 - ps21
Phaff./Saccharomycetaceae - ps22
Saccharomyco./mycetaceae - ps23
Saccharomycetaceae - ps24
Saccharomycetaceae_2 - ps25
Saccharomycetaceae_3 - ps26
Kaz./Nau./Saccharomyces - ps27
Saccharomyces - ps28
Arboricola_clade - ps29
Cerevisiae_clade - ps30
Saccharomyces_cerevisiae - ps31
Phylostratum

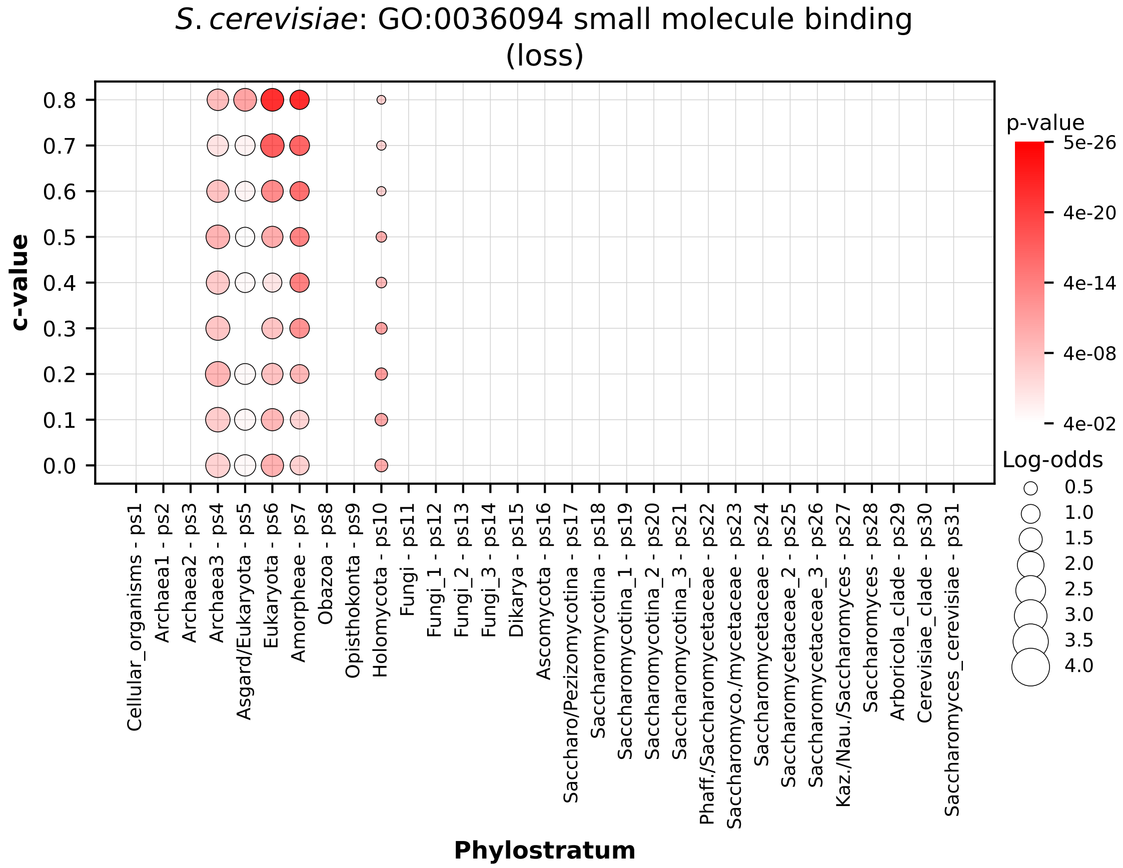

S. cerevisiae: GO:0036094 small molecule binding
(loss)
c-value
0.8
0.7
0.6
0.5
0.4
0.3
0.2
0.1
0.0
p-value
5e-26
4e-20
4e-14
4e-08
4e-02
Log-odds
0.5
1.0
1.5
2.0
2.5
3.0
3.5
4.0
Cellular_organisms - ps1
Archaea1 - ps2
Archaea2 - ps3
Archaea3 - ps4
Asgard/Eukaryota - ps5
Eukaryota - ps6
Amorpheae - ps7
Obazoa - ps8
Opisthokonta - ps9
Holomycota - ps10
Fungi - ps11
Fungi_1 - ps12
Fungi_2 - ps13
Fungi_3 - ps14
Dikarya - ps15
Ascomycota - ps16
Saccharo/Pezizomycotina - ps17
Saccharomycotina - ps18
Saccharomycotina_1 - ps19
Saccharomycotina_2 - ps20
Saccharomycotina_3 - ps21
Phaff./Saccharomycetaceae - ps22
Saccharomyco./mycetaceae - ps23
Saccharomycetaceae - ps24
Saccharomycetaceae_2 - ps25
Saccharomycetaceae_3 - ps26
Kaz./Nau./Saccharomyces - ps27
Saccharomyces - ps28
Arboricola_clade - ps29
Cerevisiae_clade - ps30
Saccharomyces_cerevisiae - ps31
Phylostratum

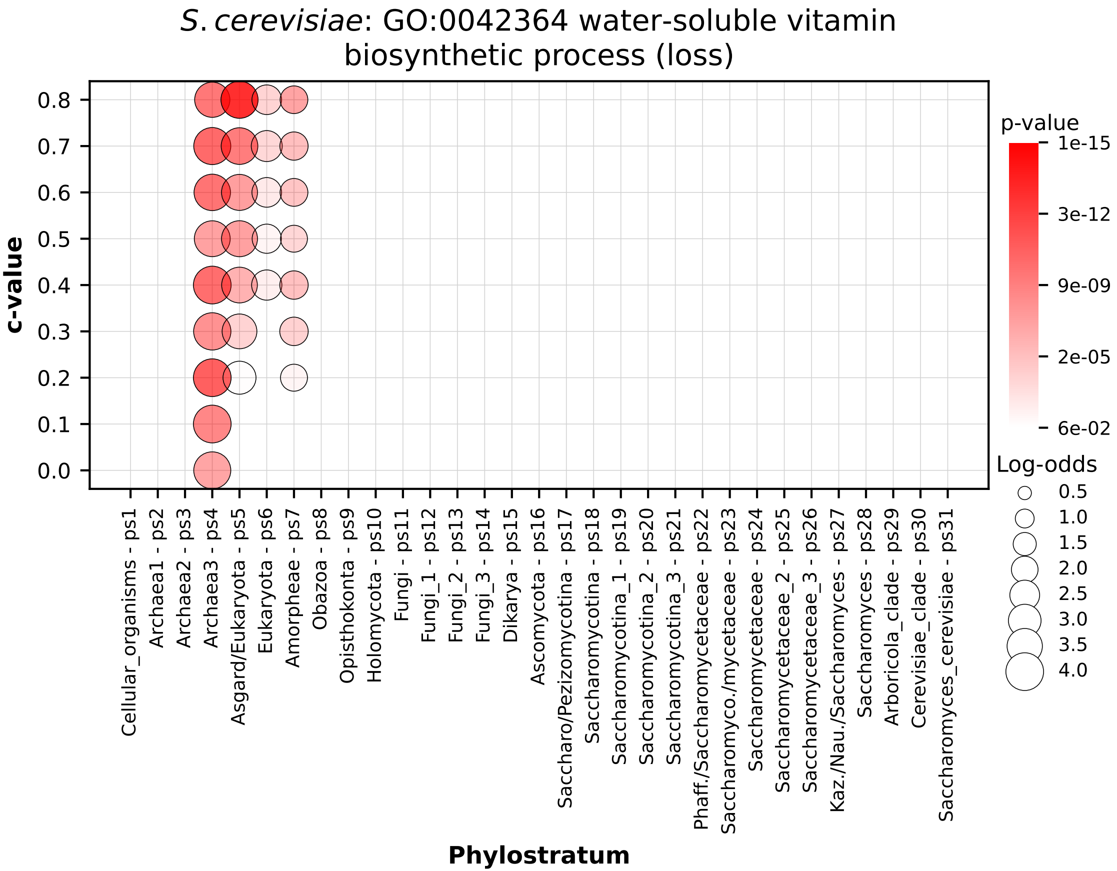

S. cerevisiae: GO:0042364 water-soluble vitamin biosynthetic process (loss)
c-value
0.8
0.7
0.6
0.5
0.4
0.3
0.2
0.1
0.0
p-value
1e-15
3e-12
9e-09
2e-05
6e-02
Log-odds
0.5
1.0
1.5
2.0
2.5
3.0
3.5
4.0
Cellular_organisms - ps1
Archaea1 - ps2
Archaea2 - ps3
Archaea3 - ps4
Asgard/Eukaryota - ps5
Eukaryota - ps6
Amorpheae - ps7
Obazoa - ps8
Opisthokonta - ps9
Holomycota - ps10
Fungi - ps11
Fungi_1 - ps12
Fungi_2 - ps13
Fungi_3 - ps14
Dikarya - ps15
Ascomycota - ps16
Saccharo/Pezizomycotina - ps17
Saccharomycotina - ps18
Saccharomycotina_1 - ps19
Saccharomycotina_2 - ps20
Saccharomycotina_3 - ps21
Phaff./Saccharomycetaceae - ps22
Saccharomyco./mycetaceae - ps23
Saccharomycetaceae - ps24
Saccharomycetaceae_2 - ps25
Saccharomycetaceae_3 - ps26
Kaz./Nau./Saccharomyces - ps27
Saccharomyces - ps28
Arboricola_clade - ps29
Cerevisiae_clade - ps30
Saccharomyces_cerevisiae - ps31
Phylostratum

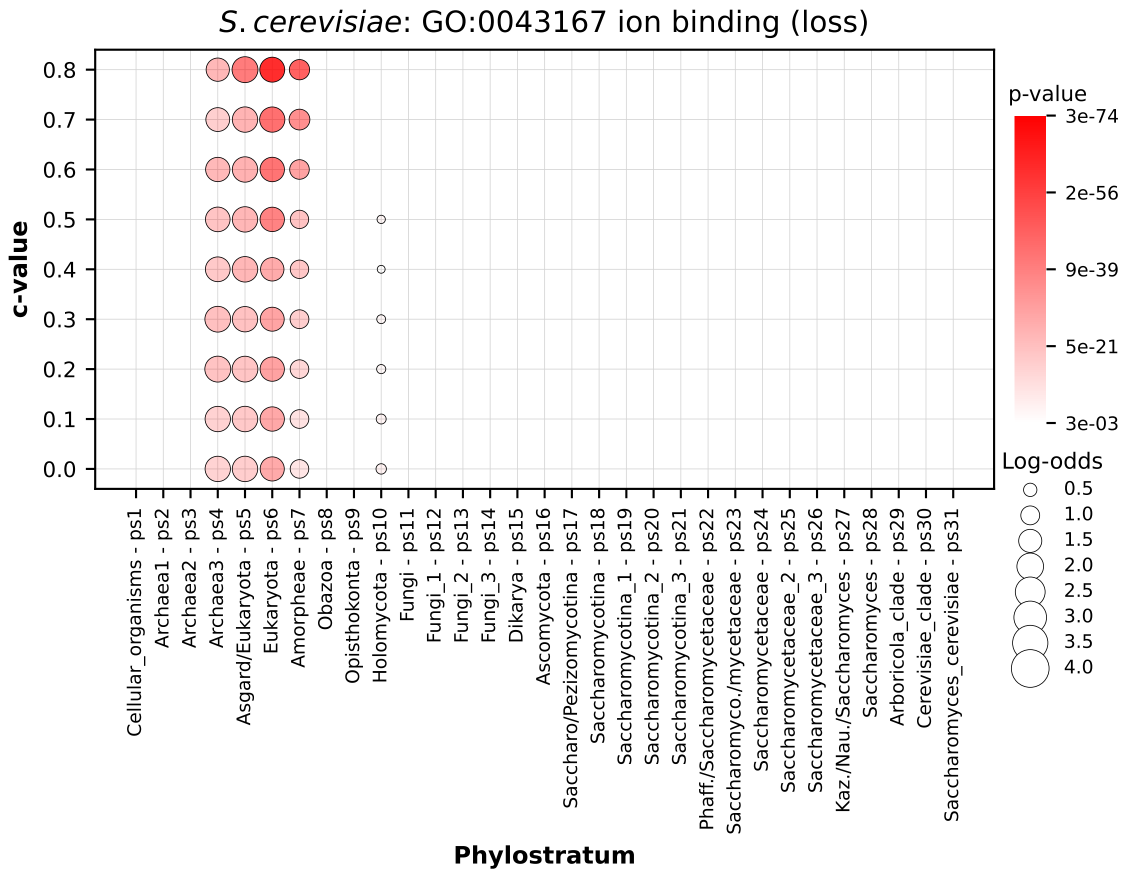

S. cerevisiae: GO:0043167 ion binding (loss)
c-value
0.0
0.1
0.2
0.3
0.4
0.5
0.6
0.7
0.8
Phylostratum
Cellular_organisms - ps1
Archaea1 - ps2
Archaea2 - ps3
Archaea3 - ps4
Asgard/Eukaryota - ps5
Eukaryota - ps6
Amorpheae - ps7
Obazoa - ps8
Opisthokonta - ps9
Holomycota - ps10
Fungi - ps11
Fungi_1 - ps12
Fungi_2 - ps13
Fungi_3 - ps14
Dikarya - ps15
Ascomycota - ps16
Saccharo/Pezizomycotina - ps17
Saccharomycotina - ps18
Saccharomycotina_1 - ps19
Saccharomycotina_2 - ps20
Saccharomycotina_3 - ps21
Phaff./Saccharomycetaceae - ps22
Saccharomyco./mycetaceae - ps23
Saccharomycetaceae - ps24
Saccharomycetaceae_2 - ps25
Saccharomycetaceae_3 - ps26
Kaz./Nau./Saccharomyces - ps27
Saccharomyces - ps28
Arboricola_clade - ps29
Cerevisiae_clade - ps30
Saccharomyces_cerevisiae - ps31
p-value
3e-74
2e-56
9e-39
5e-21
3e-03
Log-odds
0.5
1.0
1.5
2.0
2.5
3.0
3.5
4.0

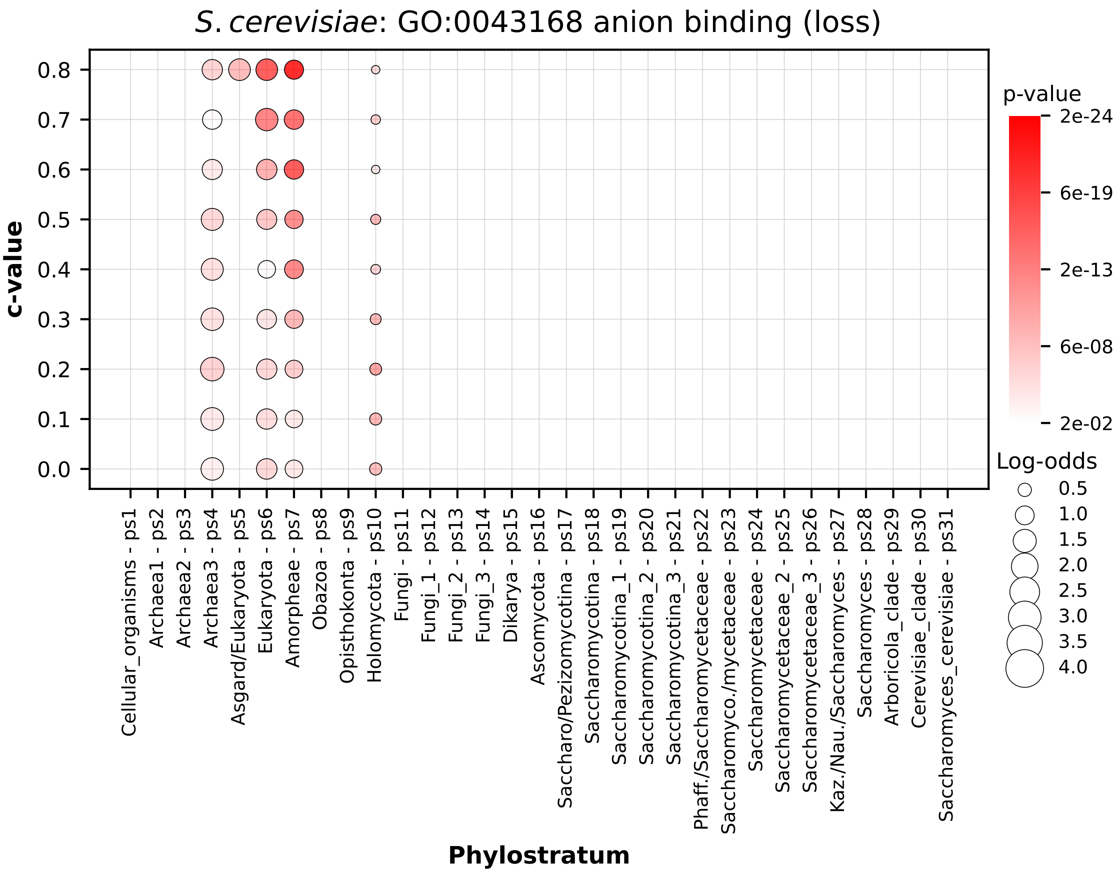

S. cerevisiae: GO:0043168 anion binding (loss)
c-value
0.8
0.7
0.6
0.5
0.4
0.3
0.2
0.1
0.0
Phylostratum
Cellular_organisms - ps1
Archaea1 - ps2
Archaea2 - ps3
Archaea3 - ps4
Asgard/Eukaryota - ps5
Eukaryota - ps6
Amorpheae - ps7
Obazoa - ps8
Opisthokonta - ps9
Holomycota - ps10
Fungi - ps11
Fungi_1 - ps12
Fungi_2 - ps13
Fungi_3 - ps14
Dikarya - ps15
Ascomycota - ps16
Saccharo/Pezizomycotina - ps17
Saccharomycotina - ps18
Saccharomycotina_1 - ps19
Saccharomycotina_2 - ps20
Saccharomycotina_3 - ps21
Phaff./Saccharomycetaceae - ps22
Saccharomyco./mycetaceae - ps23
Saccharomycetaceae - ps24
Saccharomycetaceae_2 - ps25
Saccharomycetaceae_3 - ps26
Kaz./Nau./Saccharomyces - ps27
Saccharomyces - ps28
Arboricola_clade - ps29
Cerevisiae_clade - ps30
Saccharomyces_cerevisiae - ps31
p-value
2e-24
6e-19
2e-13
6e-08
2e-02
Log-odds
0.5
1.0
1.5
2.0
2.5
3.0
3.5
4.0

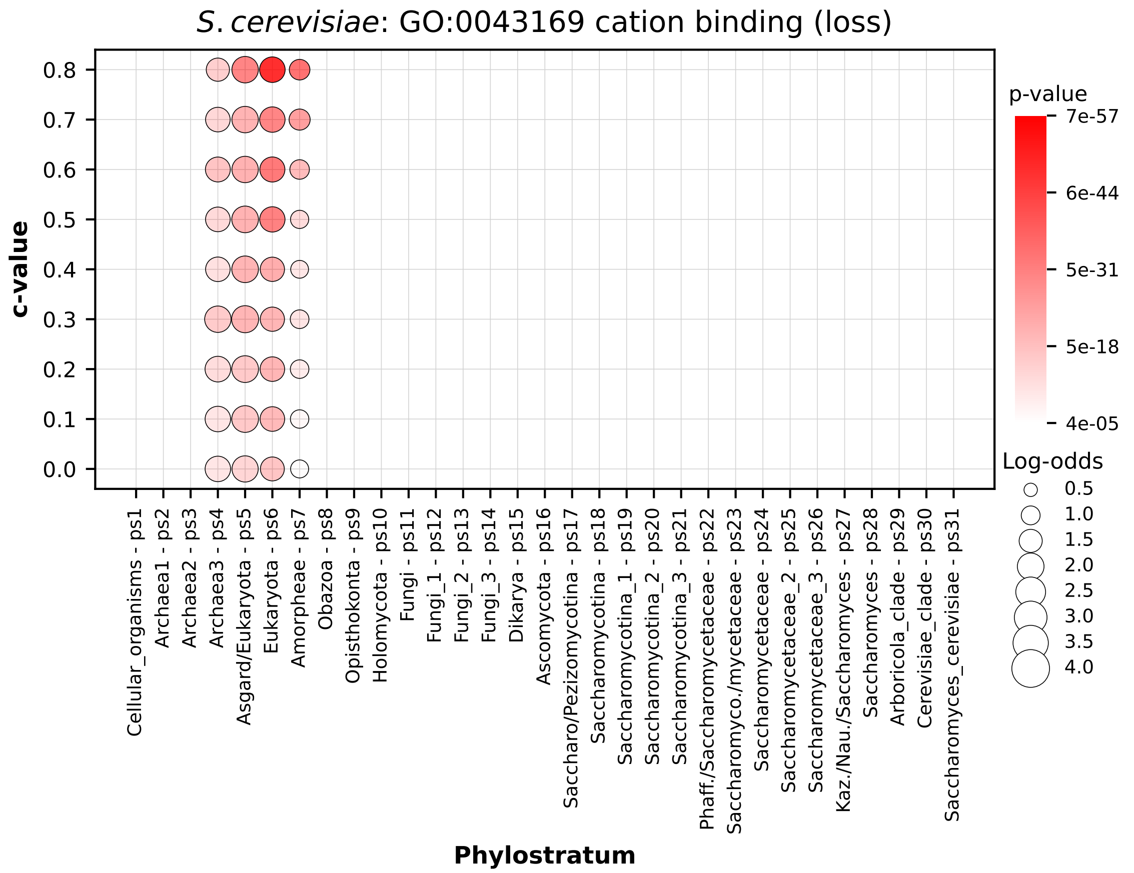
S. cerevisiae: GO:0043169 cation binding (loss)
c-value
0.8
0.7
0.6
0.5
0.4
0.3
0.2
0.1
0.0
p-value
7e-57
6e-44
5e-31
5e-18
4e-05
Log-odds
0.5
1.0
1.5
2.0
2.5
3.0
3.5
4.0
Cellular_organisms - ps1
Archaea1 - ps2
Archaea2 - ps3
Archaea3 - ps4
Asgard/Eukaryota - ps5
Eukaryota - ps6
Amorpheae - ps7
Obazoa - ps8
Opisthokonta - ps9
Holomycota - ps10
Fungi - ps11
Fungi_1 - ps12
Fungi_2 - ps13
Fungi_3 - ps14
Dikarya - ps15
Ascomycota - ps16
Saccharo/Pezizomycotina - ps17
Saccharomycotina - ps18
Saccharomycotina_1 - ps19
Saccharomycotina_2 - ps20
Saccharomycotina_3 - ps21
Phaff./Saccharomycetaceae - ps22
Saccharomyco./mycetaceae - ps23
Saccharomycetaceae - ps24
Saccharomycetaceae_2 - ps25
Saccharomycetaceae_3 - ps26
Kaz./Nau./Saccharomyces - ps27
Saccharomyces - ps28
Arboricola_clade - ps29
Cerevisiae_clade - ps30
Saccharomyces_cerevisiae - ps31
Phylostratum

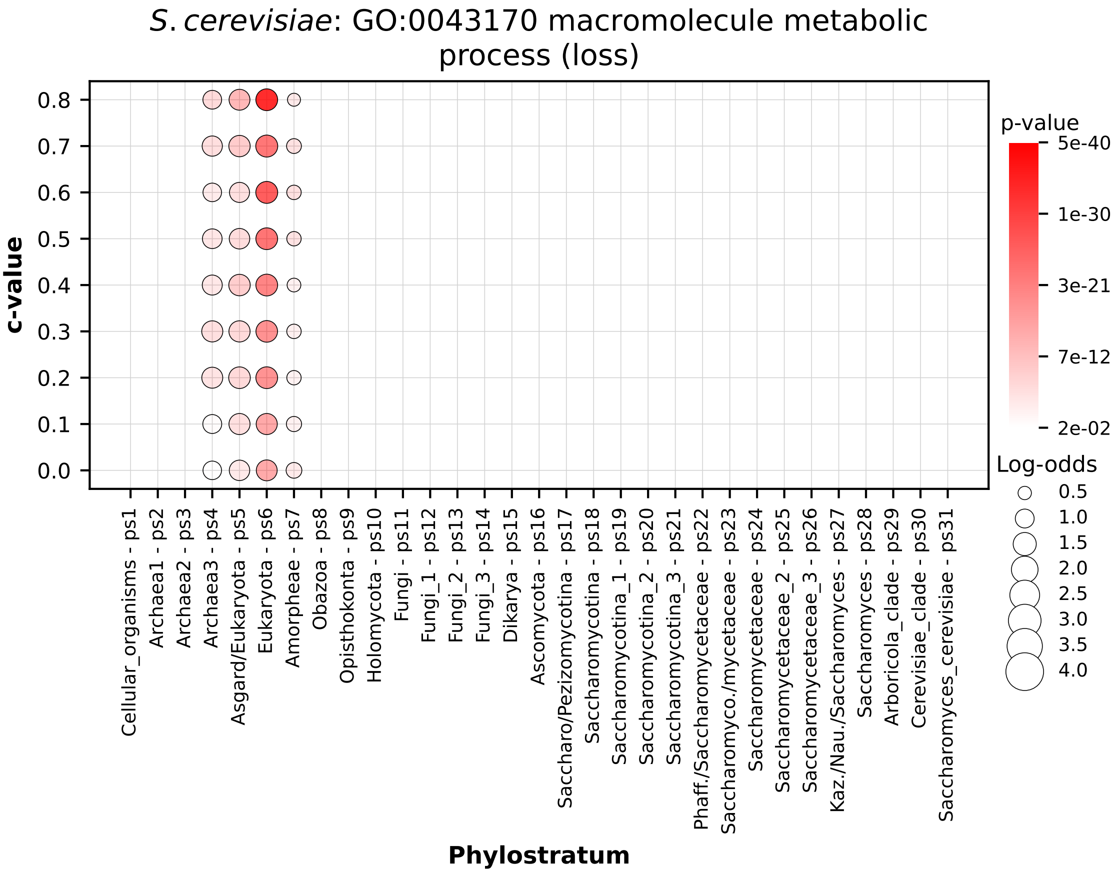
S. cerevisiae: GO:0043170 macromolecule metabolic process (loss)
c-value
0.8
0.7
0.6
0.5
0.4
0.3
0.2
0.1
0.0
p-value
5e-40
1e-30
3e-21
7e-12
2e-02
Log-odds
0.5
1.0
1.5
2.0
2.5
3.0
3.5
4.0
Cellular_organisms - ps1
Archaea1 - ps2
Archaea2 - ps3
Archaea3 - ps4
Asgard/Eukaryota - ps5
Eukaryota - ps6
Amorpheae - ps7
Obazoa - ps8
Opisthokonta - ps9
Holomycota - ps10
Fungi - ps11
Fungi_1 - ps12
Fungi_2 - ps13
Fungi_3 - ps14
Dikarya - ps15
Ascomycota - ps16
Saccharo/Pezizomycotina - ps17
Saccharomycotina - ps18
Saccharomycotina_1 - ps19
Saccharomycotina_2 - ps20
Saccharomycotina_3 - ps21
Phaff./Saccharomycetaceae - ps22
Saccharomyco./mycetaceae - ps23
Saccharomycetaceae - ps24
Saccharomycetaceae_2 - ps25
Saccharomycetaceae_3 - ps26
Kaz./Nau./Saccharomyces - ps27
Saccharomyces - ps28
Arboricola_clade - ps29
Cerevisiae_clade - ps30
Saccharomyces_cerevisiae - ps31
Phylostratum

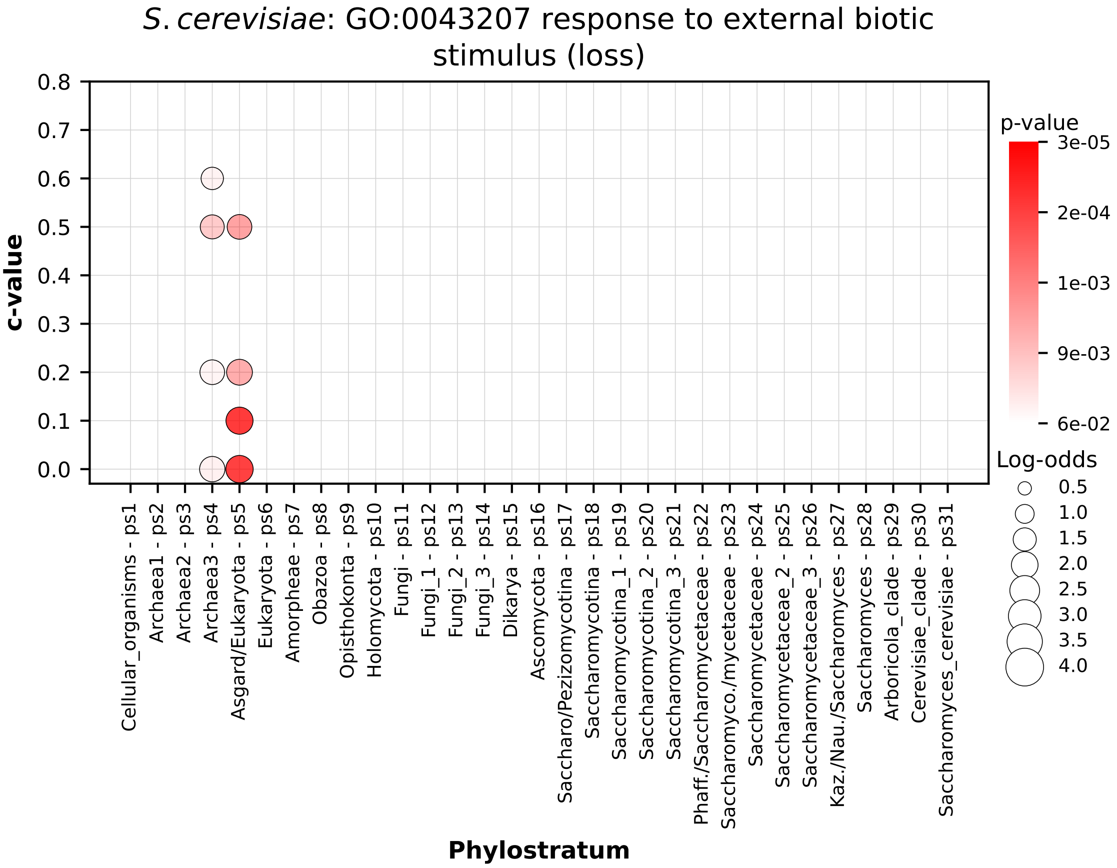

S. cerevisiae: GO:0043207 response to external biotic stimulus (loss)
c-value
0.8
0.7
0.6
0.5
0.4
0.3
0.2
0.1
0.0
Phylostratum
Cellular_organisms - ps1
Archaea1 - ps2
Archaea2 - ps3
Archaea3 - ps4
Asgard/Eukaryota - ps5
Eukaryota - ps6
Amorpheae - ps7
Obazoa - ps8
Opisthokonta - ps9
Holomycota - ps10
Fungi - ps11
Fungi_1 - ps12
Fungi_2 - ps13
Fungi_3 - ps14
Dikarya - ps15
Ascomycota - ps16
Saccharo/Pezizomycotina - ps17
Saccharomycotina - ps18
Saccharomycotina_1 - ps19
Saccharomycotina_2 - ps20
Saccharomycotina_3 - ps21
Phaff./Saccharomycetaceae - ps22
Saccharomyco./mycetaceae - ps23
Saccharomycetaceae - ps24
Saccharomycetaceae_2 - ps25
Saccharomycetaceae_3 - ps26
Kaz./Nau./Saccharomyces - ps27
Saccharomyces - ps28
Arboricola_clade - ps29
Cerevisiae_clade - ps30
Saccharomyces_cerevisiae - ps31
p-value
3e-05
2e-04
1e-03
9e-03
6e-02
Log-odds
0.5
1.0
1.5
2.0
2.5
3.0
3.5
4.0

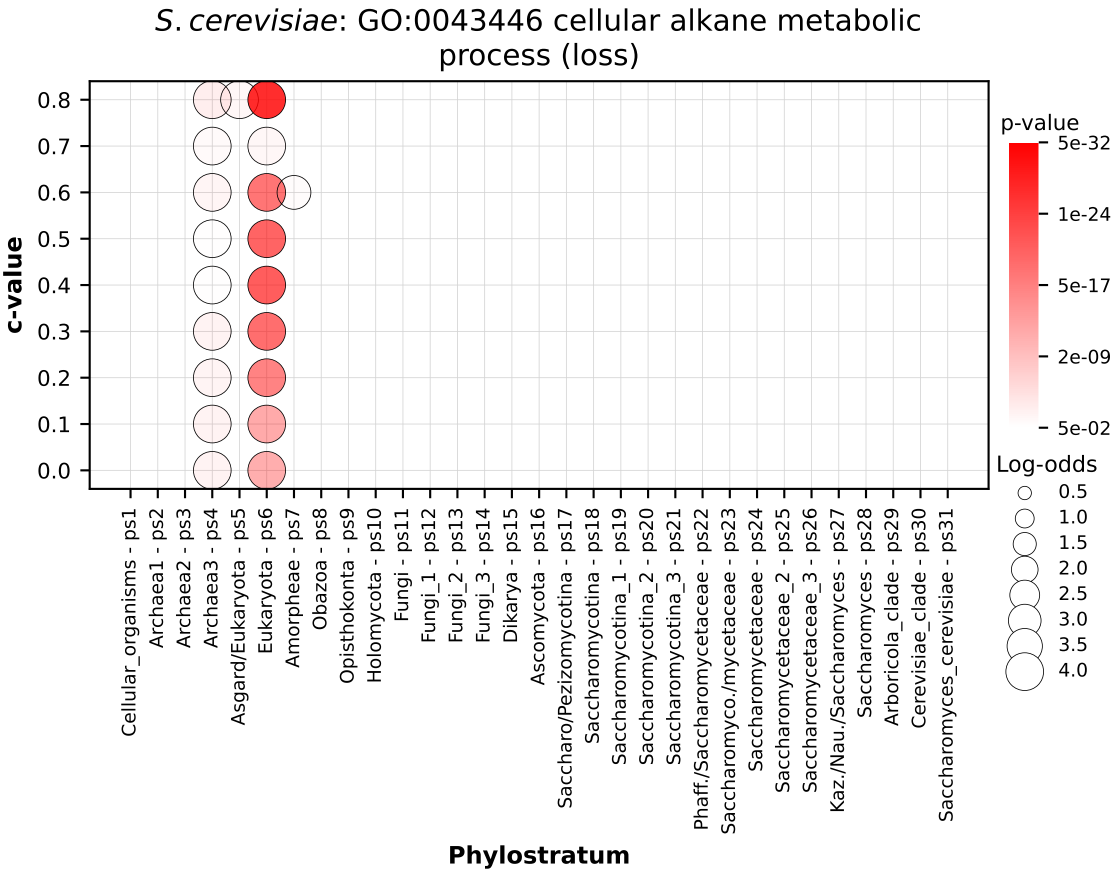

S. cerevisiae: GO:0043446 cellular alkane metabolic process (loss)
c-value
0.8
0.7
0.6
0.5
0.4
0.3
0.2
0.1
0.0
p-value
5e-32
1e-24
5e-17
2e-09
5e-02
Log-odds
0.5
1.0
1.5
2.0
2.5
3.0
3.5
4.0
Cellular_organisms - ps1
Archaea1 - ps2
Archaea2 - ps3
Archaea3 - ps4
Asgard/Eukaryota - ps5
Eukaryota - ps6
Amorpheae - ps7
Obazoa - ps8
Opisthokonta - ps9
Holomycota - ps10
Fungi - ps11
Fungi_1 - ps12
Fungi_2 - ps13
Fungi_3 - ps14
Dikarya - ps15
Ascomycota - ps16
Saccharo/Pezizomycotina - ps17
Saccharomycotina - ps18
Saccharomycotina_1 - ps19
Saccharomycotina_2 - ps20
Saccharomycotina_3 - ps21
Phaff./Saccharomycetaceae - ps22
Saccharomyco./mycetaceae - ps23
Saccharomycetaceae - ps24
Saccharomycetaceae_2 - ps25
Saccharomycetaceae_3 - ps26
Kaz./Nau./Saccharomyces - ps27
Saccharomyces - ps28
Arboricola_clade - ps29
Cerevisiae_clade - ps30
Saccharomyces_cerevisiae - ps31
Phylostratum

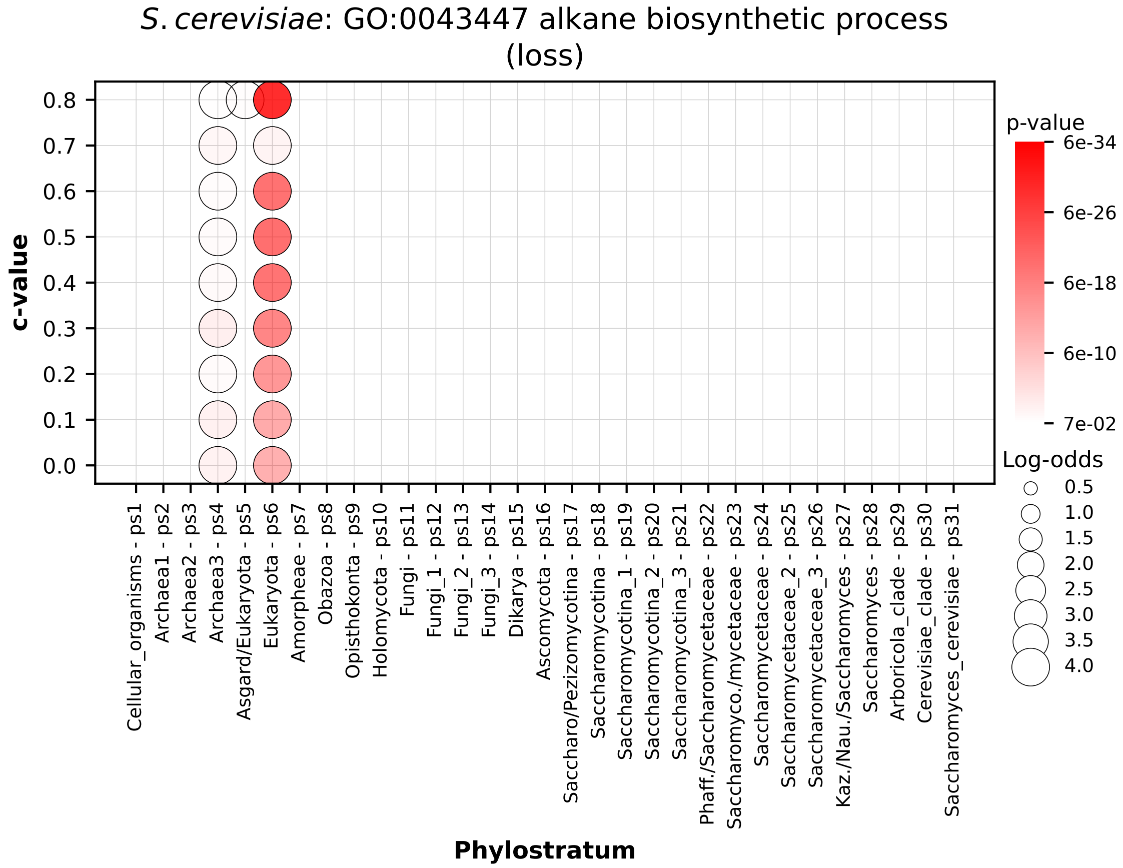
S. cerevisiae: GO:0043447 alkane biosynthetic process
(loss)
c-value
0.8
0.7
0.6
0.5
0.4
0.3
0.2
0.1
0.0
p-value
6e-34
6e-26
6e-18
6e-10
7e-02
Log-odds
0.5
1.0
1.5
2.0
2.5
3.0
3.5
4.0
Cellular_organisms - ps1
Archaea1 - ps2
Archaea2 - ps3
Archaea3 - ps4
Asgard/Eukaryota - ps5
Eukaryota - ps6
Amorpheae - ps7
Obazoa - ps8
Opisthokonta - ps9
Holomycota - ps10
Fungi - ps11
Fungi_1 - ps12
Fungi_2 - ps13
Fungi_3 - ps14
Dikarya - ps15
Ascomycota - ps16
Saccharo/Pezizomycotina - ps17
Saccharomycotina - ps18
Saccharomycotina_1 - ps19
Saccharomycotina_2 - ps20
Saccharomycotina_3 - ps21
Phaff./Saccharomycetaceae - ps22
Saccharomyco./mycetaceae - ps23
Saccharomycetaceae - ps24
Saccharomycetaceae_2 - ps25
Saccharomycetaceae_3 - ps26
Kaz./Nau./Saccharomyces - ps27
Saccharomyces - ps28
Arboricola_clade - ps29
Cerevisiae_clade - ps30
Saccharomyces_cerevisiae - ps31
Phylostratum

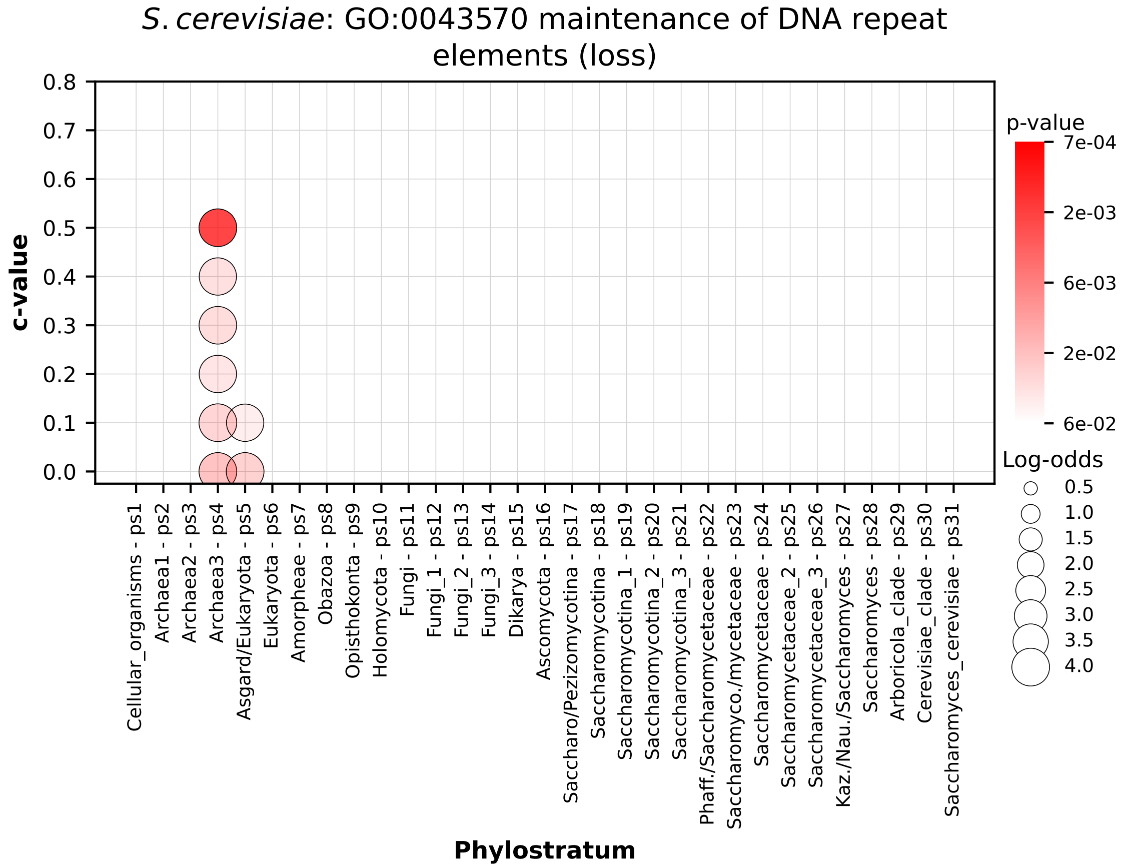
S. cerevisiae: GO:0043570 maintenance of DNA repeat elements (loss)
c-value
0.8
0.7
0.6
0.5
0.4
0.3
0.2
0.1
0.0
p-value
7e-04
2e-03
6e-03
2e-02
6e-02
Log-odds
0.5
1.0
1.5
2.0
2.5
3.0
3.5
4.0
Cellular_organisms - ps1
Archaea1 - ps2
Archaea2 - ps3
Archaea3 - ps4
Asgard/Eukaryota - ps5
Eukaryota - ps6
Amorpheae - ps7
Obazoa - ps8
Opisthokonta - ps9
Holomycota - ps10
Fungi - ps11
Fungi_1 - ps12
Fungi_2 - ps13
Fungi_3 - ps14
Dikarya - ps15
Ascomycota - ps16
Saccharo/Pezizomycotina - ps17
Saccharomycotina - ps18
Saccharomycotina_1 - ps19
Saccharomycotina_2 - ps20
Saccharomycotina_3 - ps21
Phaff./Saccharomycetaceae - ps22
Saccharomyco./mycetaceae - ps23
Saccharomycetaceae - ps24
Saccharomycetaceae_2 - ps25
Saccharomycetaceae_3 - ps26
Kaz./Nau./Saccharomyces - ps27
Saccharomyces - ps28
Arboricola_clade - ps29
Cerevisiae_clade - ps30
Saccharomyces_cerevisiae - ps31
Phylostratum

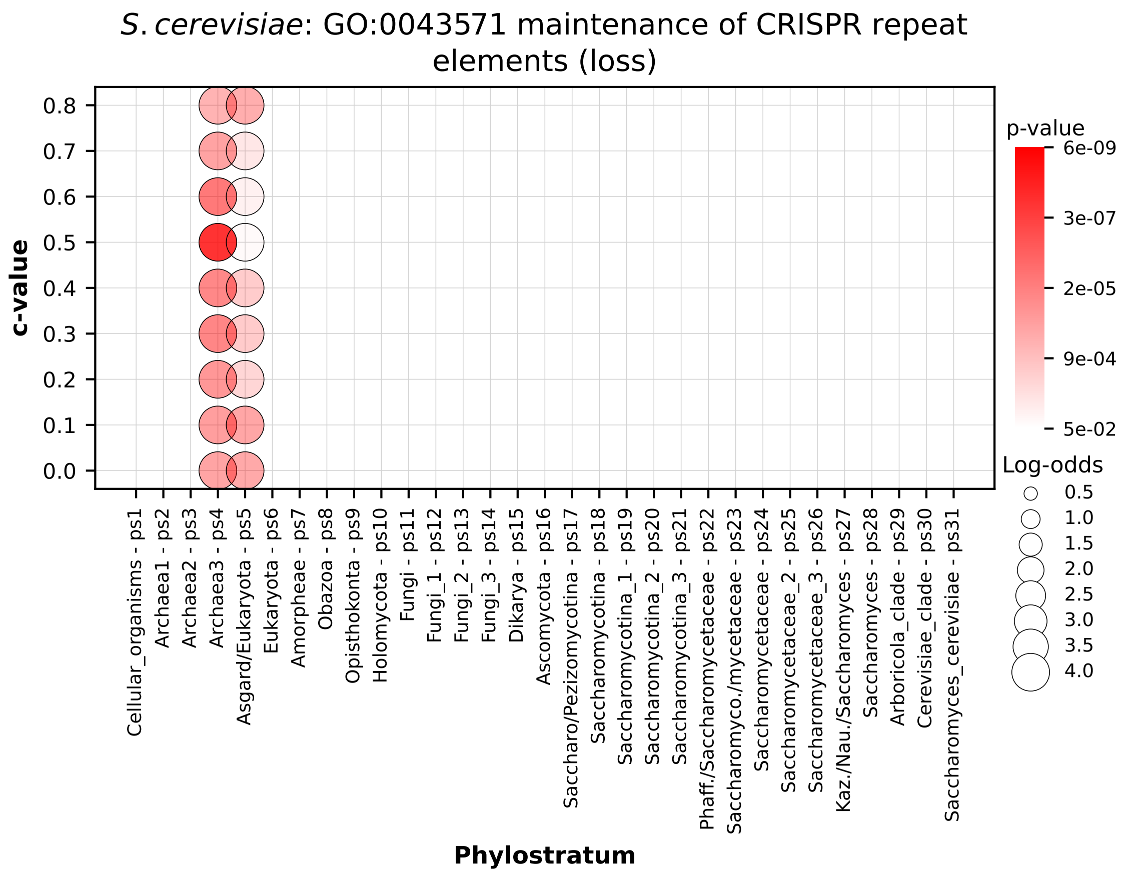
S. cerevisiae: GO:0043571 maintenance of CRISPR repeat elements (loss)
c-value
0.8
0.7
0.6
0.5
0.4
0.3
0.2
0.1
0.0
p-value
6e-09
3e-07
2e-05
9e-04
5e-02
Log-odds
0.5
1.0
1.5
2.0
2.5
3.0
3.5
4.0
Cellular_organisms - ps1
Archaea1 - ps2
Archaea2 - ps3
Archaea3 - ps4
Asgard/Eukaryota - ps5
Eukaryota - ps6
Amorpheae - ps7
Obazoa - ps8
Opisthokonta - ps9
Holomycota - ps10
Fungi - ps11
Fungi_1 - ps12
Fungi_2 - ps13
Fungi_3 - ps14
Dikarya - ps15
Ascomycota - ps16
Saccharo/Pezizomycotina - ps17
Saccharomycotina - ps18
Saccharomycotina_1 - ps19
Saccharomycotina_2 - ps20
Saccharomycotina_3 - ps21
Phaff./Saccharomycetaceae - ps22
Saccharomyco./mycetaceae - ps23
Saccharomycetaceae - ps24
Saccharomycetaceae_2 - ps25
Saccharomycetaceae_3 - ps26
Kaz./Nau./Saccharomyces - ps27
Saccharomyces - ps28
Arboricola_clade - ps29
Cerevisiae_clade - ps30
Saccharomyces_cerevisiae - ps31
Phylostratum

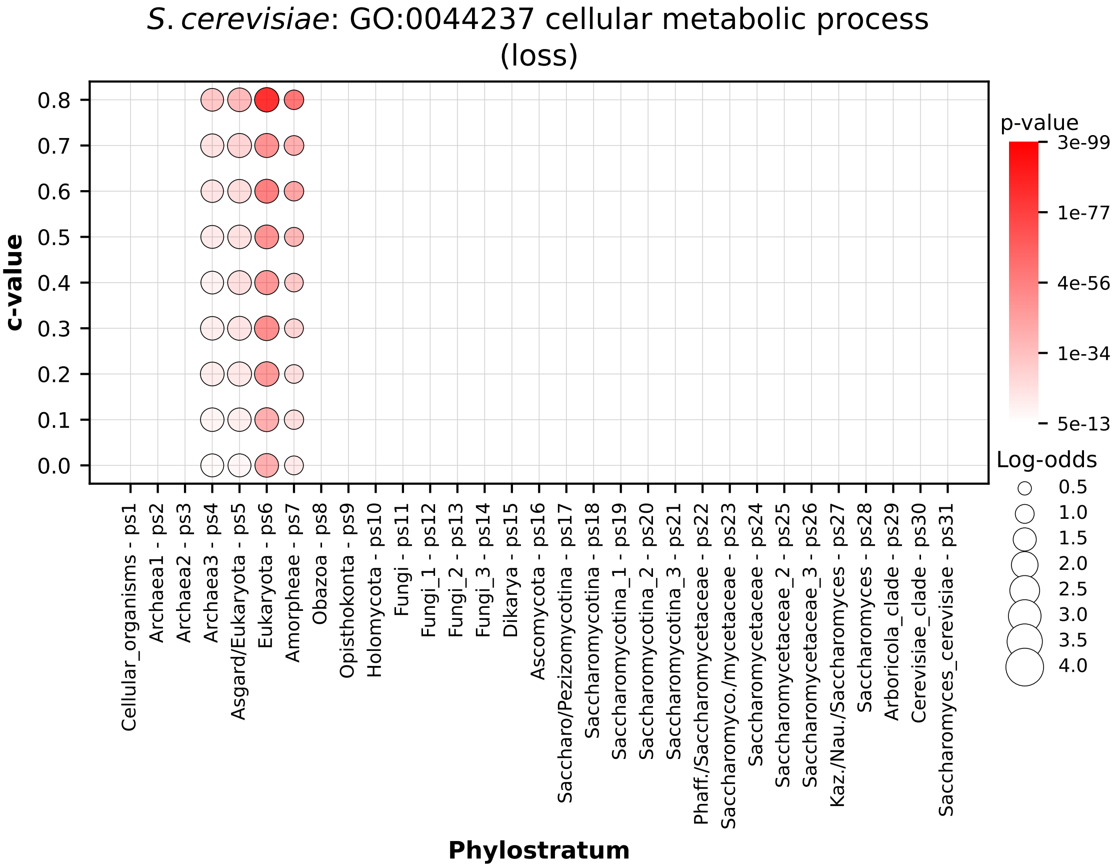

S. cerevisiae: GO:0044237 cellular metabolic process (loss)
c-value
0.8
0.7
0.6
0.5
0.4
0.3
0.2
0.1
0.0
p-value
3e-99
1e-77
4e-56
1e-34
5e-13
Log-odds
0.5
1.0
1.5
2.0
2.5
3.0
3.5
4.0
Cellular_organisms - ps1
Archaea1 - ps2
Archaea2 - ps3
Archaea3 - ps4
Asgard/Eukaryota - ps5
Eukaryota - ps6
Amorpheae - ps7
Obazoa - ps8
Opisthokonta - ps9
Holomycota - ps10
Fungi - ps11
Fungi_1 - ps12
Fungi_2 - ps13
Fungi_3 - ps14
Dikarya - ps15
Ascomycota - ps16
Saccharo/Pezizomycotina - ps17
Saccharomycotina - ps18
Saccharomycotina_1 - ps19
Saccharomycotina_2 - ps20
Saccharomycotina_3 - ps21
Phaff./Saccharomycetaceae - ps22
Saccharomyco./mycetaceae - ps23
Saccharomycetaceae - ps24
Saccharomycetaceae_2 - ps25
Saccharomycetaceae_3 - ps26
Kaz./Nau./Saccharomyces - ps27
Saccharomyces - ps28
Arboricola_clade - ps29
Cerevisiae_clade - ps30
Saccharomyces_cerevisiae - ps31
Phylostratum

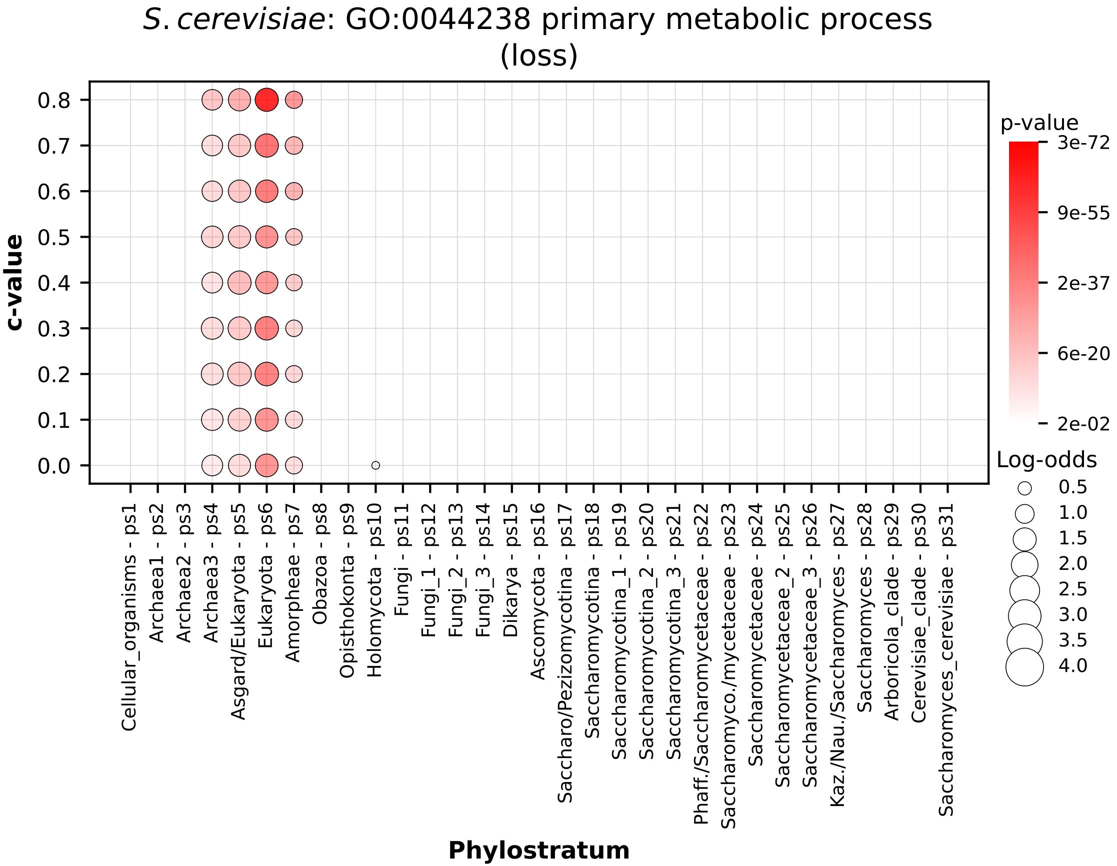
S. cerevisiae: GO:0044238 primary metabolic process
(loss)
c-value
0.8
0.7
0.6
0.5
0.4
0.3
0.2
0.1
0.0
p-value
3e-72
9e-55
2e-37
6e-20
2e-02
Log-odds
0.5
1.0
1.5
2.0
2.5
3.0
3.5
4.0
Cellular_organisms - ps1
Archaea1 - ps2
Archaea2 - ps3
Archaea3 - ps4
Asgard/Eukaryota - ps5
Eukaryota - ps6
Amorpheae - ps7
Obazoa - ps8
Opisthokonta - ps9
Holomycota - ps10
Fungi - ps11
Fungi_1 - ps12
Fungi_2 - ps13
Fungi_3 - ps14
Dikarya - ps15
Ascomycota - ps16
Saccharo/Pezizomycotina - ps17
Saccharomycotina - ps18
Saccharomycotina_1 - ps19
Saccharomycotina_2 - ps20
Saccharomycotina_3 - ps21
Phaff./Saccharomycetaceae - ps22
Saccharomyco./mycetaceae - ps23
Saccharomycetaceae - ps24
Saccharomycetaceae_2 - ps25
Saccharomycetaceae_3 - ps26
Kaz./Nau./Saccharomyces - ps27
Saccharomyces - ps28
Arboricola_clade - ps29
Cerevisiae_clade - ps30
Saccharomyces_cerevisiae - ps31
Phylostratum

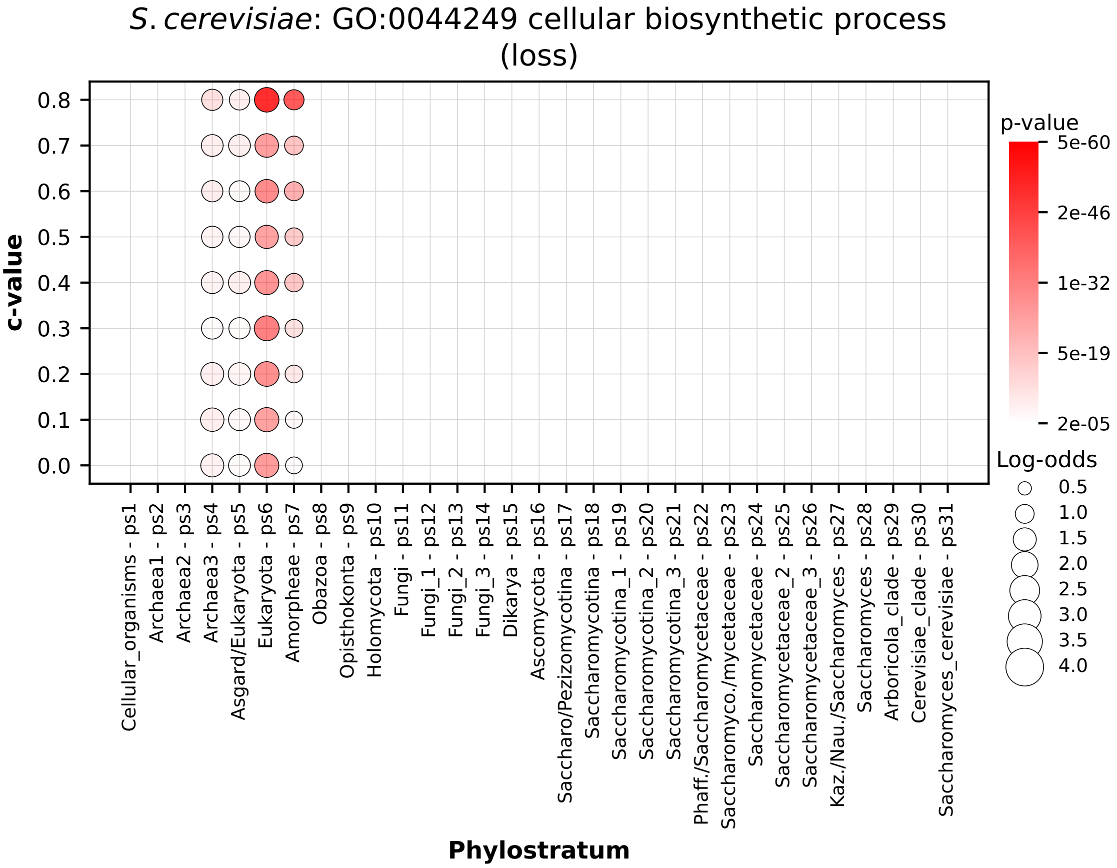
S. cerevisiae: GO:0044249 cellular biosynthetic process
(loss)
c-value
0.8
0.7
0.6
0.5
0.4
0.3
0.2
0.1
0.0
p-value
5e-60
2e-46
1e-32
5e-19
2e-05
Log-odds
0.5
1.0
1.5
2.0
2.5
3.0
3.5
4.0
Cellular_organisms - ps1
Archaea1 - ps2
Archaea2 - ps3
Archaea3 - ps4
Asgard/Eukaryota - ps5
Eukaryota - ps6
Amorpheae - ps7
Obazoa - ps8
Opisthokonta - ps9
Holomycota - ps10
Fungi - ps11
Fungi_1 - ps12
Fungi_2 - ps13
Fungi_3 - ps14
Dikarya - ps15
Ascomycota - ps16
Saccharo/Pezizomycotina - ps17
Saccharomycotina - ps18
Saccharomycotina_1 - ps19
Saccharomycotina_2 - ps20
Saccharomycotina_3 - ps21
Phaff./Saccharomycetaceae - ps22
Saccharomyco./mycetaceae - ps23
Saccharomycetaceae - ps24
Saccharomycetaceae_2 - ps25
Saccharomycetaceae_3 - ps26
Kaz./Nau./Saccharomyces - ps27
Saccharomyces - ps28
Arboricola_clade - ps29
Cerevisiae_clade - ps30
Saccharomyces_cerevisiae - ps31
Phylostratum

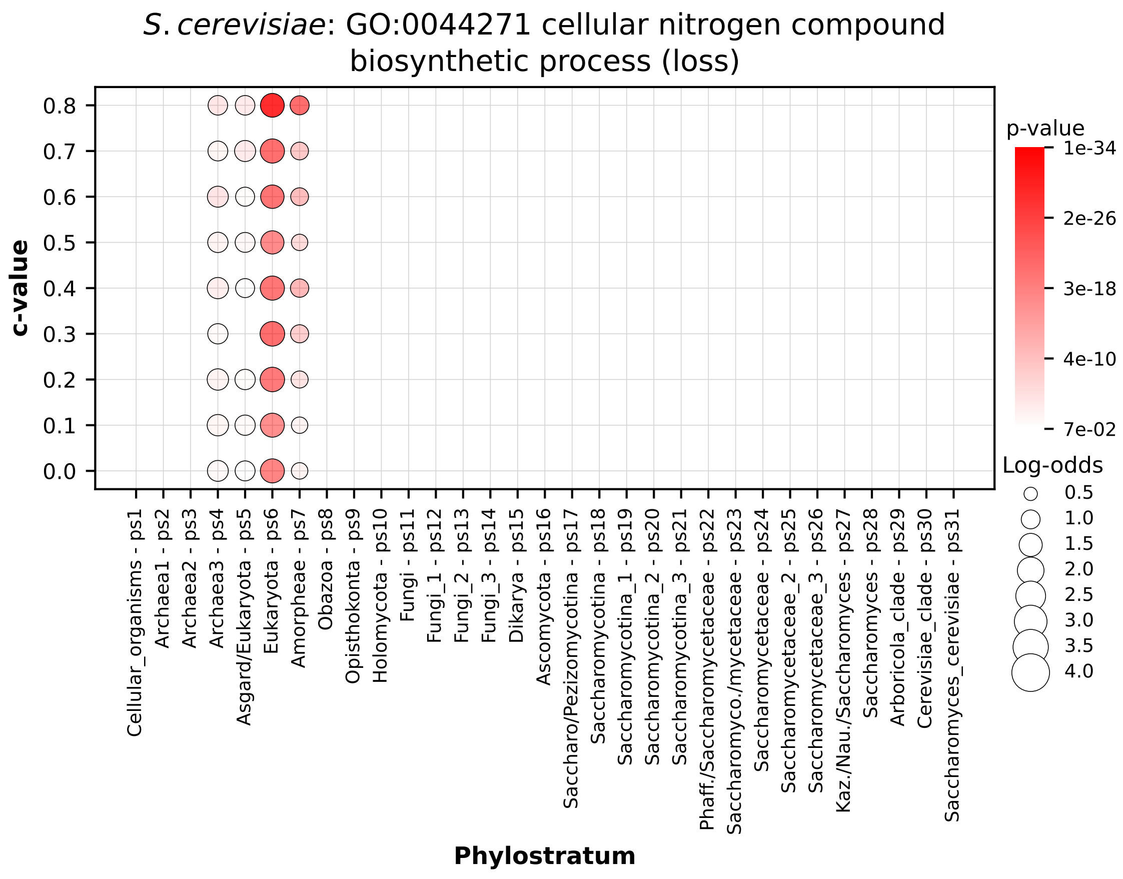
S. cerevisiae: GO:0044271 cellular nitrogen compound biosynthetic process (loss)
c-value
0.8
0.7
0.6
0.5
0.4
0.3
0.2
0.1
0.0
Phylostratum
Cellular_organisms - ps1
Archaea1 - ps2
Archaea2 - ps3
Archaea3 - ps4
Asgard/Eukaryota - ps5
Eukaryota - ps6
Amorpheae - ps7
Obazoa - ps8
Opisthokonta - ps9
Holomycota - ps10
Fungi - ps11
Fungi_1 - ps12
Fungi_2 - ps13
Fungi_3 - ps14
Dikarya - ps15
Ascomycota - ps16
Saccharo/Pezizomycotina - ps17
Saccharomycotina - ps18
Saccharomycotina_1 - ps19
Saccharomycotina_2 - ps20
Saccharomycotina_3 - ps21
Phaff./Saccharomycetaceae - ps22
Saccharomyco./mycetaceae - ps23
Saccharomycetaceae - ps24
Saccharomycetaceae_2 - ps25
Saccharomycetaceae_3 - ps26
Kaz./Nau./Saccharomyces - ps27
Saccharomyces - ps28
Arboricola_clade - ps29
Cerevisiae_clade - ps30
Saccharomyces_cerevisiae - ps31
p-value
1e-34
2e-26
3e-18
4e-10
7e-02
Log-odds
0.5
1.0
1.5
2.0
2.5
3.0
3.5
4.0

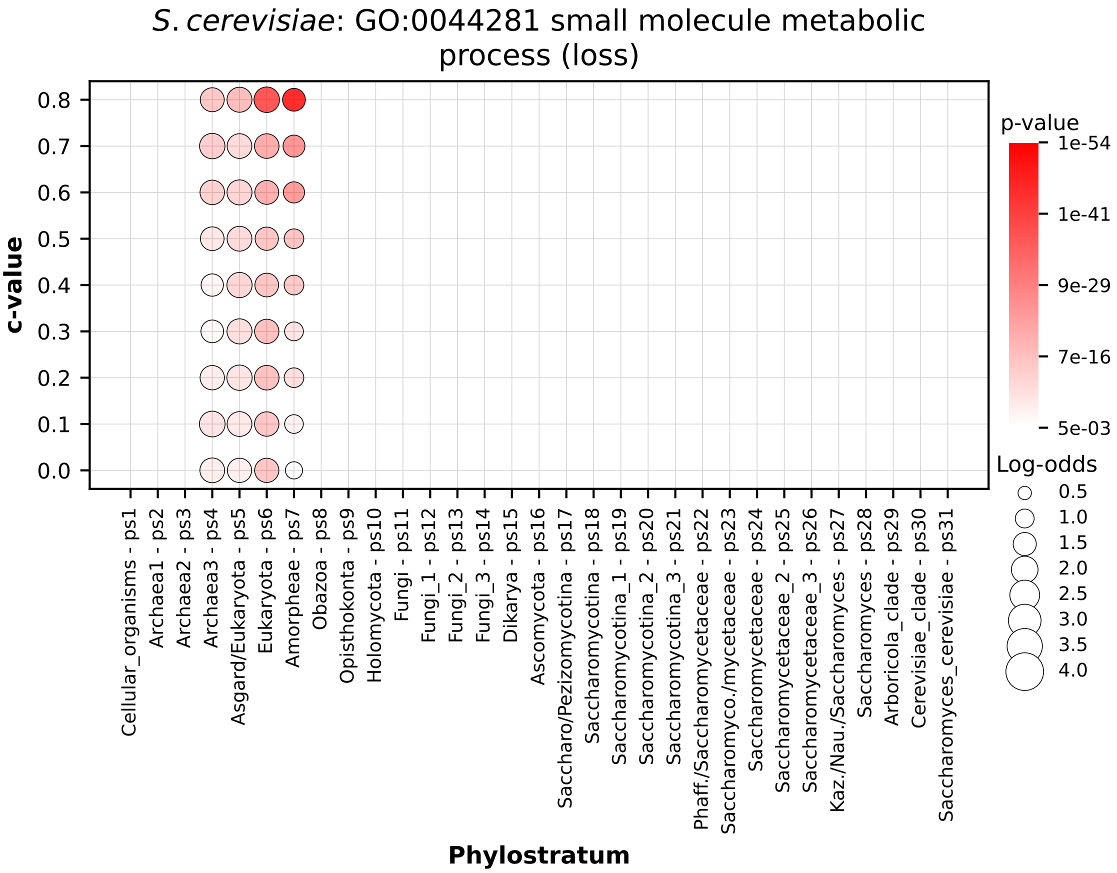

S. cerevisiae: GO:0044281 small molecule metabolic process (loss)
c-value
0.8
0.7
0.6
0.5
0.4
0.3
0.2
0.1
0.0
p-value
1e-54
1e-41
9e-29
7e-16
5e-03
Log-odds
0.5
1.0
1.5
2.0
2.5
3.0
3.5
4.0
Cellular_organisms - ps1
Archaea1 - ps2
Archaea2 - ps3
Archaea3 - ps4
Asgard/Eukaryota - ps5
Eukaryota - ps6
Amorpheae - ps7
Obazoa - ps8
Opisthokonta - ps9
Holomycota - ps10
Fungi - ps11
Fungi_1 - ps12
Fungi_2 - ps13
Fungi_3 - ps14
Dikarya - ps15
Ascomycota - ps16
Saccharo/Pezizomycotina - ps17
Saccharomycotina - ps18
Saccharomycotina_1 - ps19
Saccharomycotina_2 - ps20
Saccharomycotina_3 - ps21
Phaff./Saccharomycetaceae - ps22
Saccharomyco./mycetaceae - ps23
Saccharomycetaceae - ps24
Saccharomycetaceae_2 - ps25
Saccharomycetaceae_3 - ps26
Kaz./Nau./Saccharomyces - ps27
Saccharomyces - ps28
Arboricola_clade - ps29
Cerevisiae_clade - ps30
Saccharomyces_cerevisiae - ps31
Phylostratum

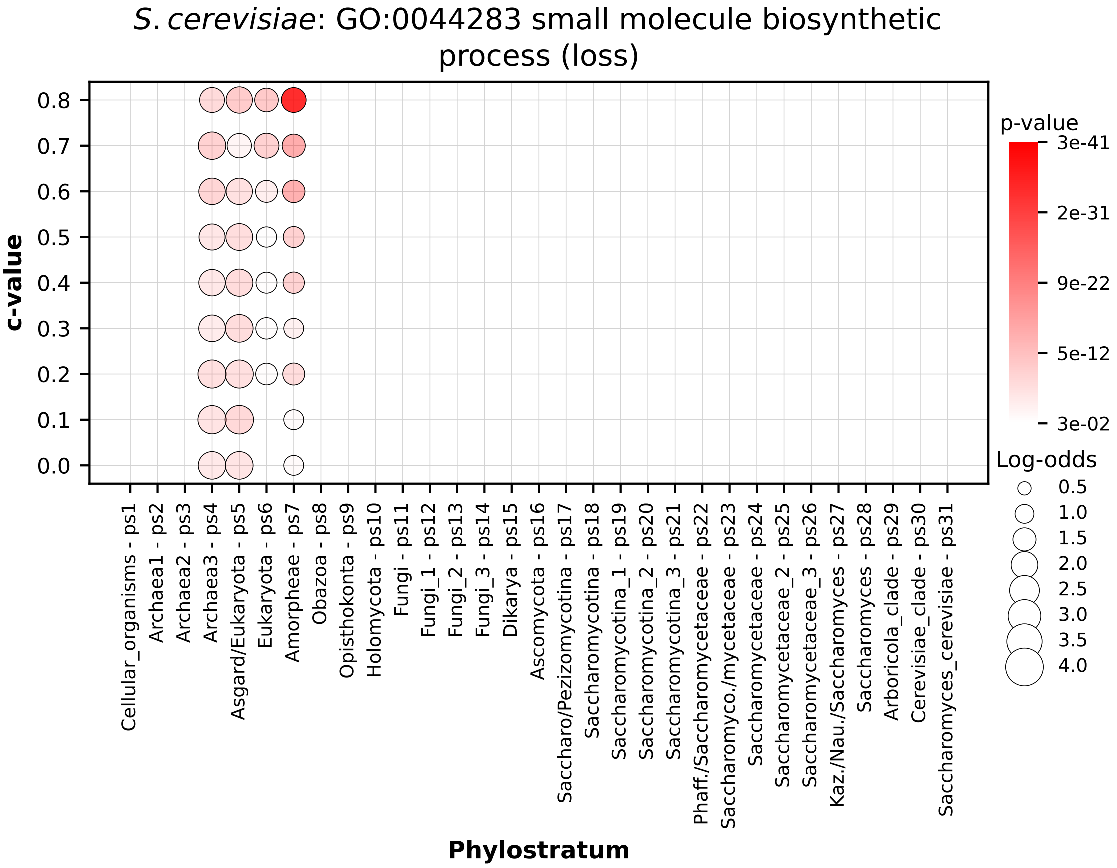

S. cerevisiae: GO:0044283 small molecule biosynthetic process (loss)
c-value
0.8
0.7
0.6
0.5
0.4
0.3
0.2
0.1
0.0
p-value
3e-41
2e-31
9e-22
5e-12
3e-02
Log-odds
0.5
1.0
1.5
2.0
2.5
3.0
3.5
4.0
Cellular_organisms - ps1
Archaea1 - ps2
Archaea2 - ps3
Archaea3 - ps4
Asgard/Eukaryota - ps5
Eukaryota - ps6
Amorpheae - ps7
Obazoa - ps8
Opisthokonta - ps9
Holomycota - ps10
Fungi - ps11
Fungi_1 - ps12
Fungi_2 - ps13
Fungi_3 - ps14
Dikarya - ps15
Ascomycota - ps16
Saccharo/Pezizomycotina - ps17
Saccharomycotina - ps18
Saccharomycotina_1 - ps19
Saccharomycotina_2 - ps20
Saccharomycotina_3 - ps21
Phaff./Saccharomycetaceae - ps22
Saccharomyco./mycetaceae - ps23
Saccharomycetaceae - ps24
Saccharomycetaceae_2 - ps25
Saccharomycetaceae_3 - ps26
Kaz./Nau./Saccharomyces - ps27
Saccharomyces - ps28
Arboricola_clade - ps29
Cerevisiae_clade - ps30
Saccharomyces_cerevisiae - ps31
Phylostratum

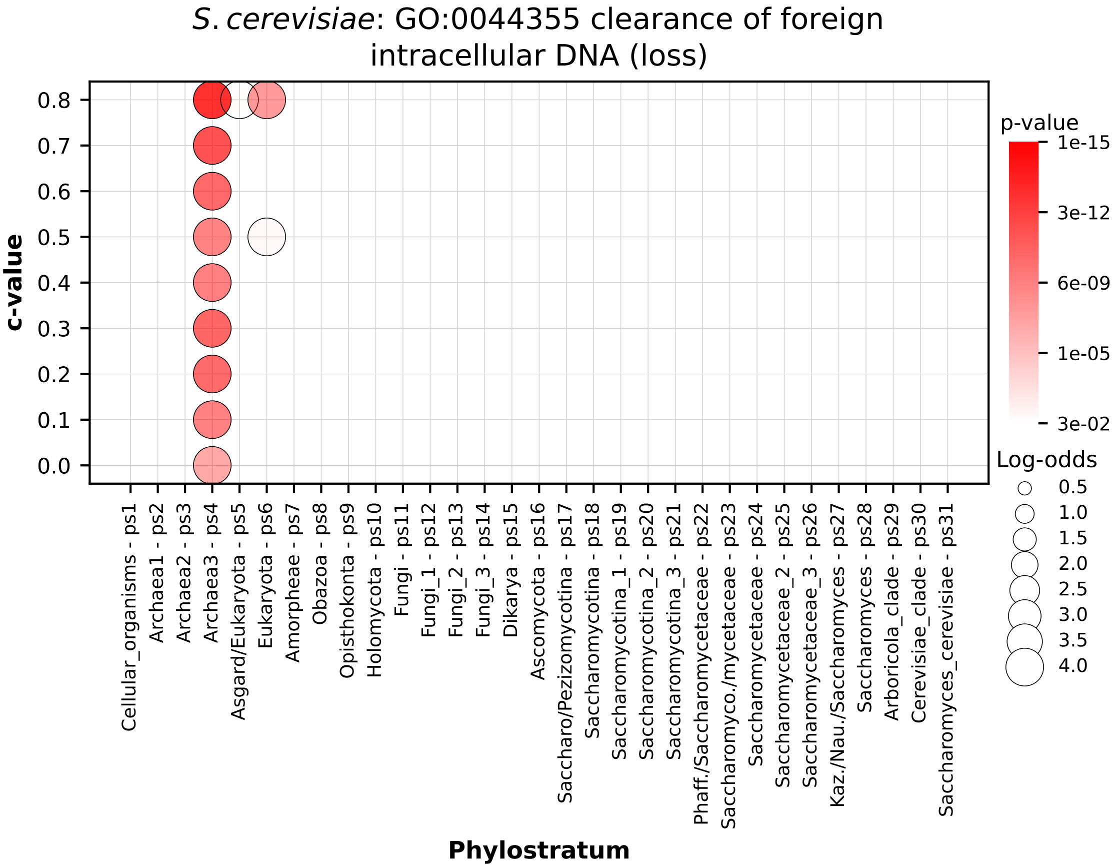

S. cerevisiae: GO:0044355 clearance of foreign intracellular DNA (loss)
c-value
0.8
0.7
0.6
0.5
0.4
0.3
0.2
0.1
0.0
p-value
1e-15
3e-12
6e-09
1e-05
3e-02
Log-odds
0.5
1.0
1.5
2.0
2.5
3.0
3.5
4.0
Cellular_organisms - ps1
Archaea1 - ps2
Archaea2 - ps3
Archaea3 - ps4
Asgard/Eukaryota - ps5
Eukaryota - ps6
Amorpheae - ps7
Obazoa - ps8
Opisthokonta - ps9
Holomycota - ps10
Fungi - ps11
Fungi_1 - ps12
Fungi_2 - ps13
Fungi_3 - ps14
Dikarya - ps15
Ascomycota - ps16
Saccharo/Pezizomycotina - ps17
Saccharomycotina - ps18
Saccharomycotina_1 - ps19
Saccharomycotina_2 - ps20
Saccharomycotina_3 - ps21
Phaff./Saccharomycetaceae - ps22
Saccharomyco./mycetaceae - ps23
Saccharomycetaceae - ps24
Saccharomycetaceae_2 - ps25
Saccharomycetaceae_3 - ps26
Kaz./Nau./Saccharomyces - ps27
Saccharomyces - ps28
Arboricola_clade - ps29
Cerevisiae_clade - ps30
Saccharomyces_cerevisiae - ps31
Phylostratum

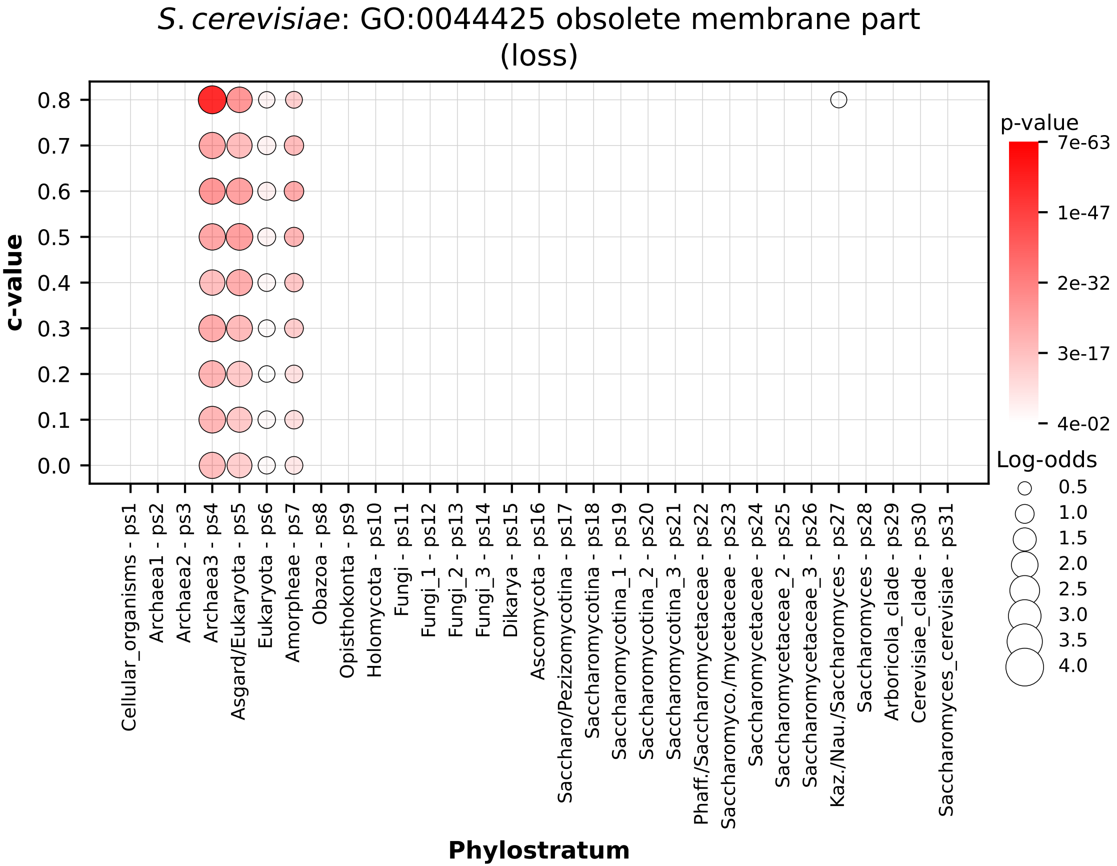

S. cerevisiae: GO:0044425 obsolete membrane part (loss)
c-value
0.8
0.7
0.6
0.5
0.4
0.3
0.2
0.1
0.0
p-value
7e-63
1e-47
2e-32
3e-17
4e-02
Log-odds
0.5
1.0
1.5
2.0
2.5
3.0
3.5
4.0
Cellular_organisms - ps1
Archaea1 - ps2
Archaea2 - ps3
Archaea3 - ps4
Asgard/Eukaryota - ps5
Eukaryota - ps6
Amorpheae - ps7
Obazoa - ps8
Opisthokonta - ps9
Holomycota - ps10
Fungi - ps11
Fungi_1 - ps12
Fungi_2 - ps13
Fungi_3 - ps14
Dikarya - ps15
Ascomycota - ps16
Saccharo/Pezizomycotina - ps17
Saccharomycotina - ps18
Saccharomycotina_1 - ps19
Saccharomycotina_2 - ps20
Saccharomycotina_3 - ps21
Phaff./Saccharomycetaceae - ps22
Saccharomyco./mycetaceae - ps23
Saccharomycetaceae - ps24
Saccharomycetaceae_2 - ps25
Saccharomycetaceae_3 - ps26
Kaz./Nau./Saccharomyces - ps27
Saccharomyces - ps28
Arboricola_clade - ps29
Cerevisiae_clade - ps30
Saccharomyces_cerevisiae - ps31
Phylostratum

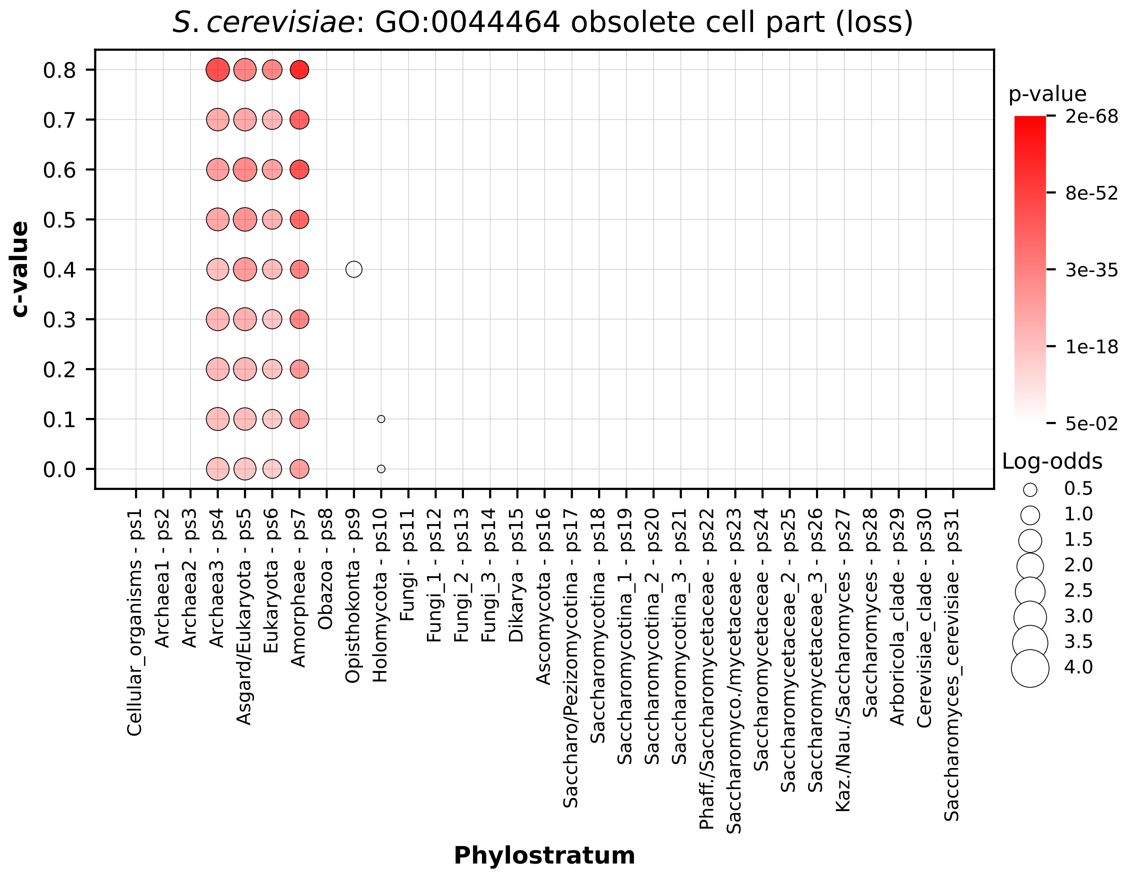
S. cerevisiae: GO:0044464 obsolete cell part (loss)
c-value
0.8
0.7
0.6
0.5
0.4
0.3
0.2
0.1
0.0
Phylostratum
Cellular_organisms - ps1
Archaea1 - ps2
Archaea2 - ps3
Archaea3 - ps4
Asgard/Eukaryota - ps5
Eukaryota - ps6
Amorpheae - ps7
Obazoa - ps8
Opisthokonta - ps9
Holomycota - ps10
Fungi - ps11
Fungi_1 - ps12
Fungi_2 - ps13
Fungi_3 - ps14
Dikarya - ps15
Ascomycota - ps16
Saccharo/Pezizomycotina - ps17
Saccharomycotina - ps18
Saccharomycotina_1 - ps19
Saccharomycotina_2 - ps20
Saccharomycotina_3 - ps21
Phaff./Saccharomycetaceae - ps22
Saccharomyco./mycetaceae - ps23
Saccharomycetaceae - ps24
Saccharomycetaceae_2 - ps25
Saccharomycetaceae_3 - ps26
Kaz./Nau./Saccharomyces - ps27
Saccharomyces - ps28
Arboricola_clade - ps29
Cerevisiae_clade - ps30
Saccharomyces_cerevisiae - ps31
p-value
2e-68
8e-52
3e-35
1e-18
5e-02
Log-odds
0.5
1.0
1.5
2.0
2.5
3.0
3.5
4.0

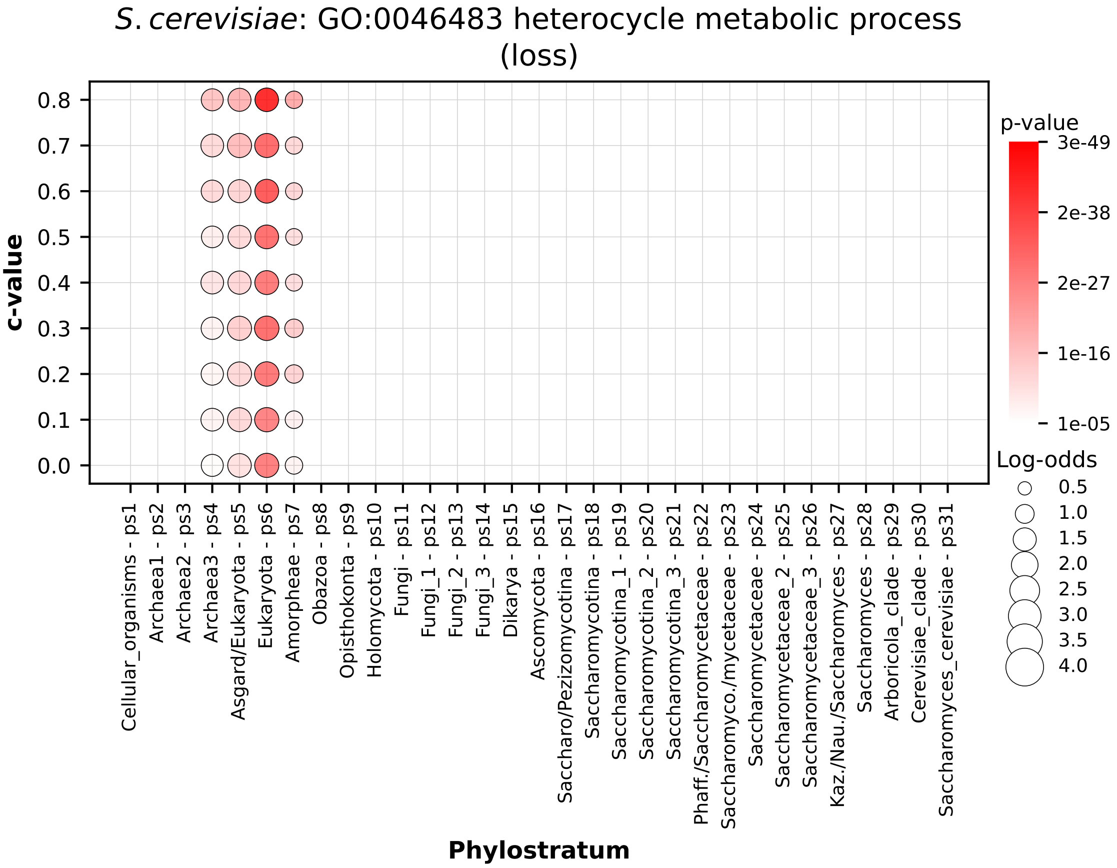

S. cerevisiae: GO:0046483 heterocycle metabolic process
(loss)
c-value
0.8
0.7
0.6
0.5
0.4
0.3
0.2
0.1
0.0
p-value
3e-49
2e-38
2e-27
1e-16
1e-05
Log-odds
0.5
1.0
1.5
2.0
2.5
3.0
3.5
4.0
Cellular_organisms - ps1
Archaea1 - ps2
Archaea2 - ps3
Archaea3 - ps4
Asgard/Eukaryota - ps5
Eukaryota - ps6
Amorpheae - ps7
Obazoa - ps8
Opisthokonta - ps9
Holomycota - ps10
Fungi - ps11
Fungi_1 - ps12
Fungi_2 - ps13
Fungi_3 - ps14
Dikarya - ps15
Ascomycota - ps16
Saccharo/Pezizomycotina - ps17
Saccharomycotina - ps18
Saccharomycotina_1 - ps19
Saccharomycotina_2 - ps20
Saccharomycotina_3 - ps21
Phaff./Saccharomycetaceae - ps22
Saccharomyco./mycetaceae - ps23
Saccharomycetaceae - ps24
Saccharomycetaceae_2 - ps25
Saccharomycetaceae_3 - ps26
Kaz./Nau./Saccharomyces - ps27
Saccharomyces - ps28
Arboricola_clade - ps29
Cerevisiae_clade - ps30
Saccharomyces_cerevisiae - ps31
Phylostratum

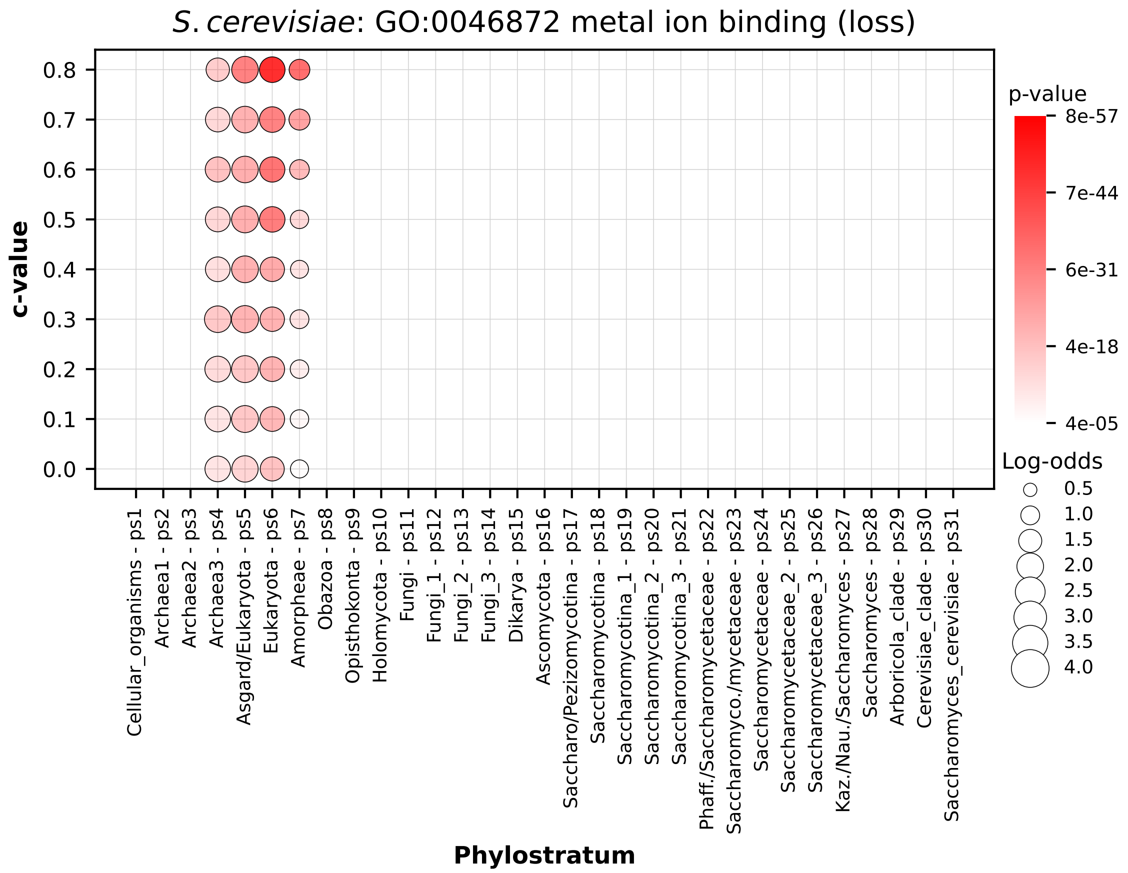
S. cerevisiae: GO:0046872 metal ion binding (loss)
c-value
0.0
0.1
0.2
0.3
0.4
0.5
0.6
0.7
0.8
p-value
8e-57
7e-44
6e-31
4e-18
4e-05
Log-odds
0.5
1.0
1.5
2.0
2.5
3.0
3.5
4.0
Cellular_organisms - ps1
Archaea1 - ps2
Archaea2 - ps3
Archaea3 - ps4
Asgard/Eukaryota - ps5
Eukaryota - ps6
Amorpheae - ps7
Obazoa - ps8
Opisthokonta - ps9
Holomycota - ps10
Fungi - ps11
Fungi_1 - ps12
Fungi_2 - ps13
Fungi_3 - ps14
Dikarya - ps15
Ascomycota - ps16
Saccharo/Pezizomycotina - ps17
Saccharomycotina - ps18
Saccharomycotina_1 - ps19
Saccharomycotina_2 - ps20
Saccharomycotina_3 - ps21
Phaff./Saccharomycetaceae - ps22
Saccharomyco./mycetaceae - ps23
Saccharomycetaceae - ps24
Saccharomycetaceae_2 - ps25
Saccharomycetaceae_3 - ps26
Kaz./Nau./Saccharomyces - ps27
Saccharomyces - ps28
Arboricola_clade - ps29
Cerevisiae_clade - ps30
Saccharomyces_cerevisiae - ps31
Phylostratum

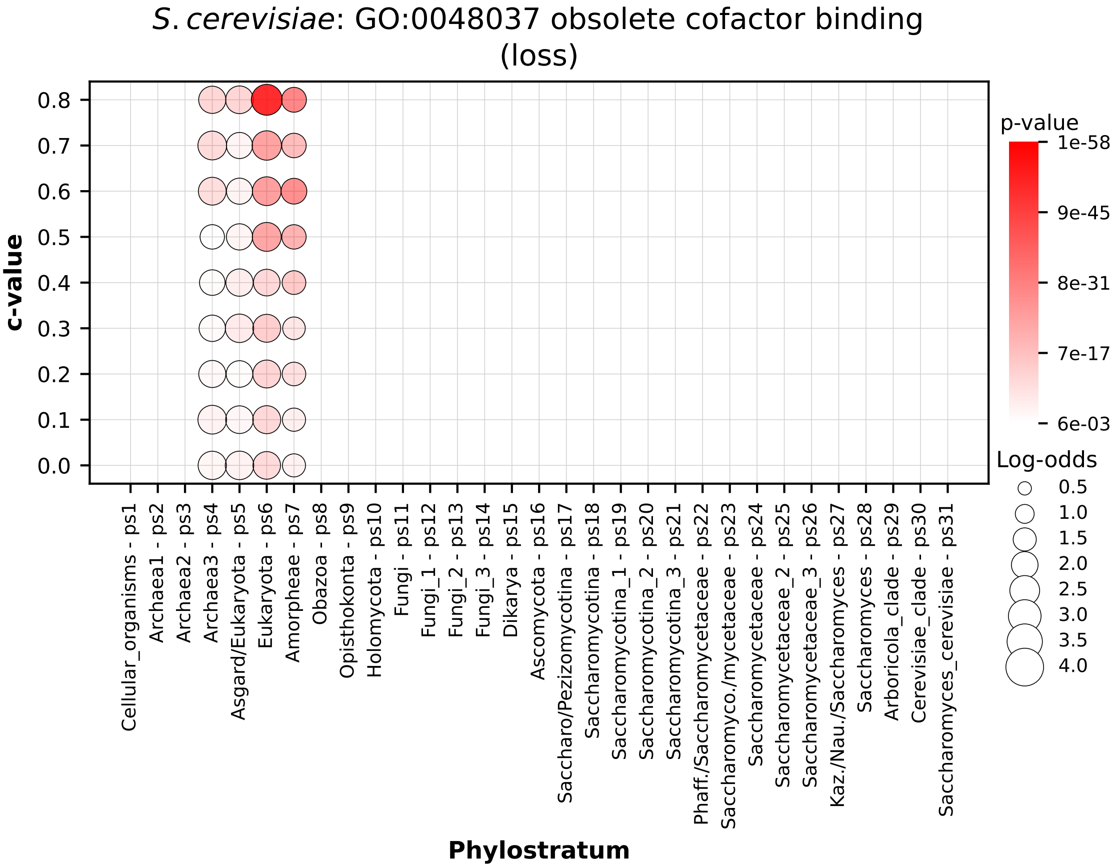
S. cerevisiae: GO:0048037 obsolete cofactor binding
(loss)
c-value
0.8
0.7
0.6
0.5
0.4
0.3
0.2
0.1
0.0
p-value
1e-58
9e-45
8e-31
7e-17
6e-03
Log-odds
0.5
1.0
1.5
2.0
2.5
3.0
3.5
4.0
Cellular_organisms - ps1
Archaea1 - ps2
Archaea2 - ps3
Archaea3 - ps4
Asgard/Eukaryota - ps5
Eukaryota - ps6
Amorpheae - ps7
Obazoa - ps8
Opisthokonta - ps9
Holomycota - ps10
Fungi - ps11
Fungi_1 - ps12
Fungi_2 - ps13
Fungi_3 - ps14
Dikarya - ps15
Ascomycota - ps16
Saccharo/Pezizomycotina - ps17
Saccharomycotina - ps18
Saccharomycotina_1 - ps19
Saccharomycotina_2 - ps20
Saccharomycotina_3 - ps21
Phaff./Saccharomycetaceae - ps22
Saccharomyco./mycetaceae - ps23
Saccharomycetaceae - ps24
Saccharomycetaceae_2 - ps25
Saccharomycetaceae_3 - ps26
Kaz./Nau./Saccharomyces - ps27
Saccharomyces - ps28
Arboricola_clade - ps29
Cerevisiae_clade - ps30
Saccharomyces_cerevisiae - ps31
Phylostratum

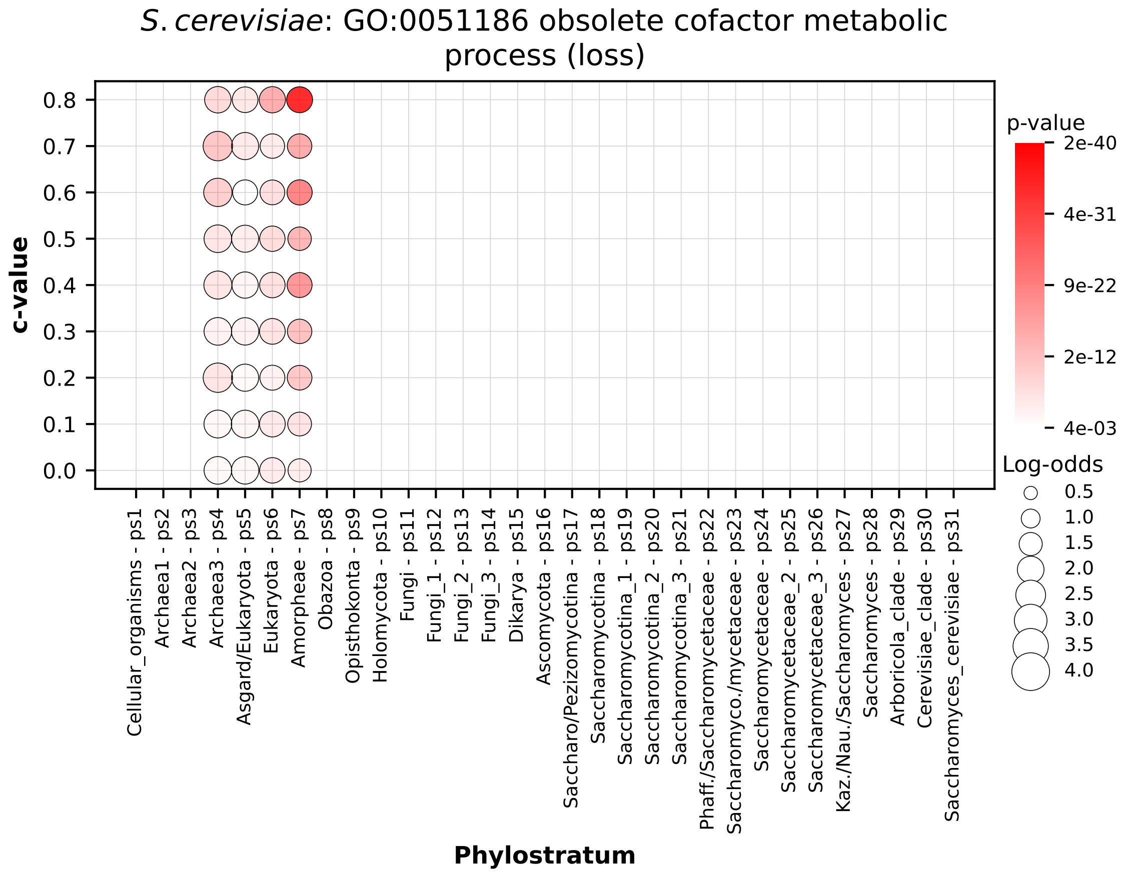

S. cerevisiae: GO:0051186 obsolete cofactor metabolic process (loss)
c-value
0.8
0.7
0.6
0.5
0.4
0.3
0.2
0.1
0.0
p-value
2e-40
4e-31
9e-22
2e-12
4e-03
Log-odds
0.5
1.0
1.5
2.0
2.5
3.0
3.5
4.0
Cellular_organisms - ps1
Archaea1 - ps2
Archaea2 - ps3
Archaea3 - ps4
Asgard/Eukaryota - ps5
Eukaryota - ps6
Amorpheae - ps7
Obazoa - ps8
Opisthokonta - ps9
Holomycota - ps10
Fungi - ps11
Fungi_1 - ps12
Fungi_2 - ps13
Fungi_3 - ps14
Dikarya - ps15
Ascomycota - ps16
Saccharo/Pezizomycotina - ps17
Saccharomycotina - ps18
Saccharomycotina_1 - ps19
Saccharomycotina_2 - ps20
Saccharomycotina_3 - ps21
Phaff./Saccharomycetaceae - ps22
Saccharomyco./mycetaceae - ps23
Saccharomycetaceae - ps24
Saccharomycetaceae_2 - ps25
Saccharomycetaceae_3 - ps26
Kaz./Nau./Saccharomyces - ps27
Saccharomyces - ps28
Arboricola_clade - ps29
Cerevisiae_clade - ps30
Saccharomyces_cerevisiae - ps31
Phylostratum

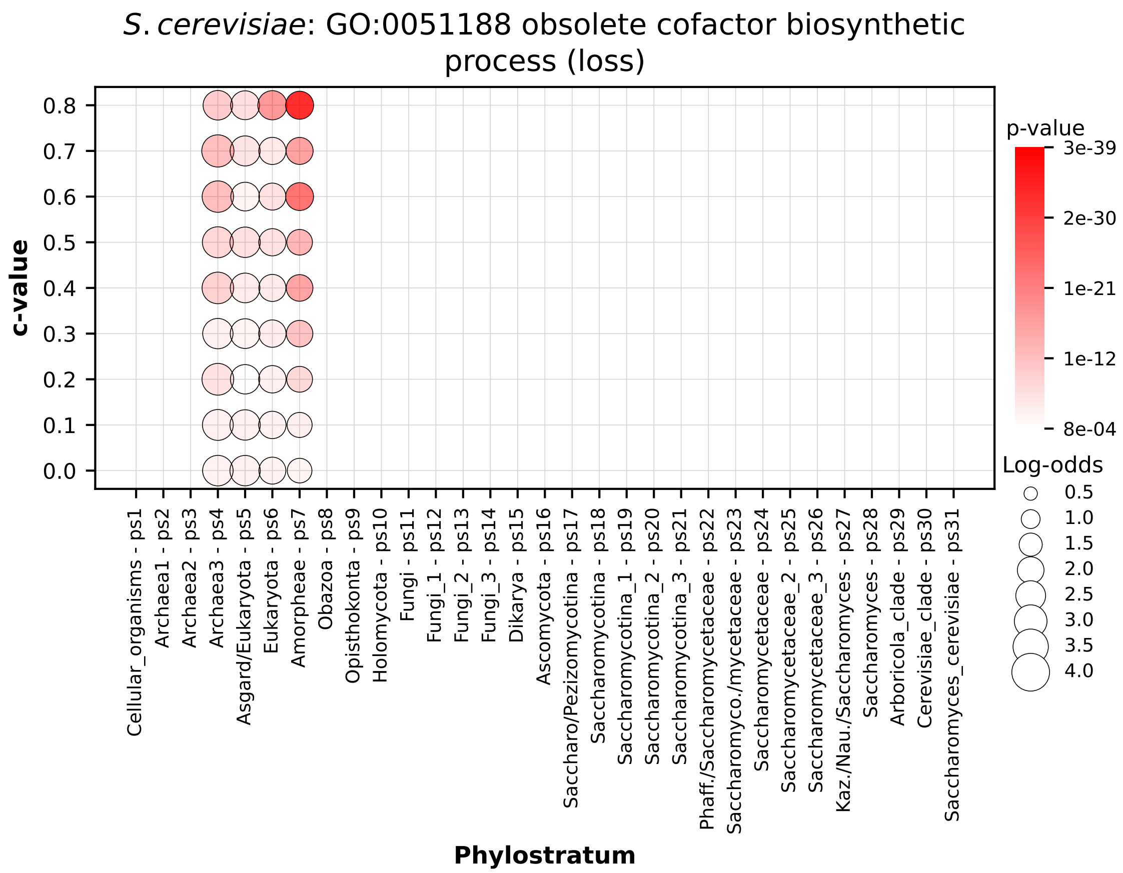

S. cerevisiae: GO:0051188 obsolete cofactor biosynthetic process (loss)
c-value
0.8
0.7
0.6
0.5
0.4
0.3
0.2
0.1
0.0
p-value
3e-39
2e-30
1e-21
1e-12
8e-04
Log-odds
0.5
1.0
1.5
2.0
2.5
3.0
3.5
4.0
Cellular_organisms - ps1
Archaea1 - ps2
Archaea2 - ps3
Archaea3 - ps4
Asgard/Eukaryota - ps5
Eukaryota - ps6
Amorpheae - ps7
Obazoa - ps8
Opisthokonta - ps9
Holomycota - ps10
Fungi - ps11
Fungi_1 - ps12
Fungi_2 - ps13
Fungi_3 - ps14
Dikarya - ps15
Ascomycota - ps16
Saccharo/Pezizomycotina - ps17
Saccharomycotina - ps18
Saccharomycotina_1 - ps19
Saccharomycotina_2 - ps20
Saccharomycotina_3 - ps21
Phaff./Saccharomycetaceae - ps22
Saccharomyco./mycetaceae - ps23
Saccharomycetaceae - ps24
Saccharomycetaceae_2 - ps25
Saccharomycetaceae_3 - ps26
Kaz./Nau./Saccharomyces - ps27
Saccharomyces - ps28
Arboricola_clade - ps29
Cerevisiae_clade - ps30
Saccharomyces_cerevisiae - ps31
Phylostratum

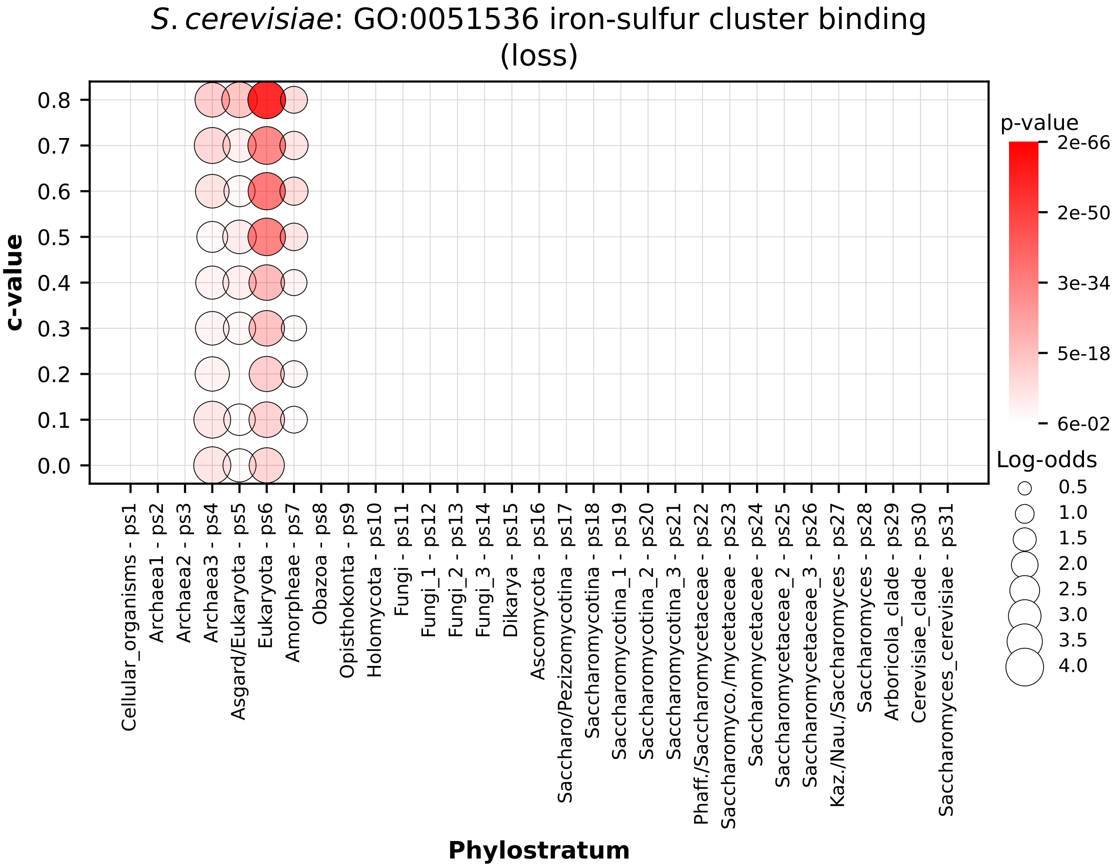

S. cerevisiae: GO:0051536 iron-sulfur cluster binding
(loss)
c-value
0.8
0.7
0.6
0.5
0.4
0.3
0.2
0.1
0.0
p-value
2e-66
2e-50
3e-34
5e-18
6e-02
Log-odds
0.5
1.0
1.5
2.0
2.5
3.0
3.5
4.0
Cellular_organisms - ps1
Archaea1 - ps2
Archaea2 - ps3
Archaea3 - ps4
Asgard/Eukaryota - ps5
Eukaryota - ps6
Amorpheae - ps7
Obazoa - ps8
Opisthokonta - ps9
Holomycota - ps10
Fungi - ps11
Fungi_1 - ps12
Fungi_2 - ps13
Fungi_3 - ps14
Dikarya - ps15
Ascomycota - ps16
Saccharo/Pezizomycotina - ps17
Saccharomycotina - ps18
Saccharomycotina_1 - ps19
Saccharomycotina_2 - ps20
Saccharomycotina_3 - ps21
Phaff./Saccharomycetaceae - ps22
Saccharomyco./mycetaceae - ps23
Saccharomycetaceae - ps24
Saccharomycetaceae_2 - ps25
Saccharomycetaceae_3 - ps26
Kaz./Nau./Saccharomyces - ps27
Saccharomyces - ps28
Arboricola_clade - ps29
Cerevisiae_clade - ps30
Saccharomyces_cerevisiae - ps31
Phylostratum

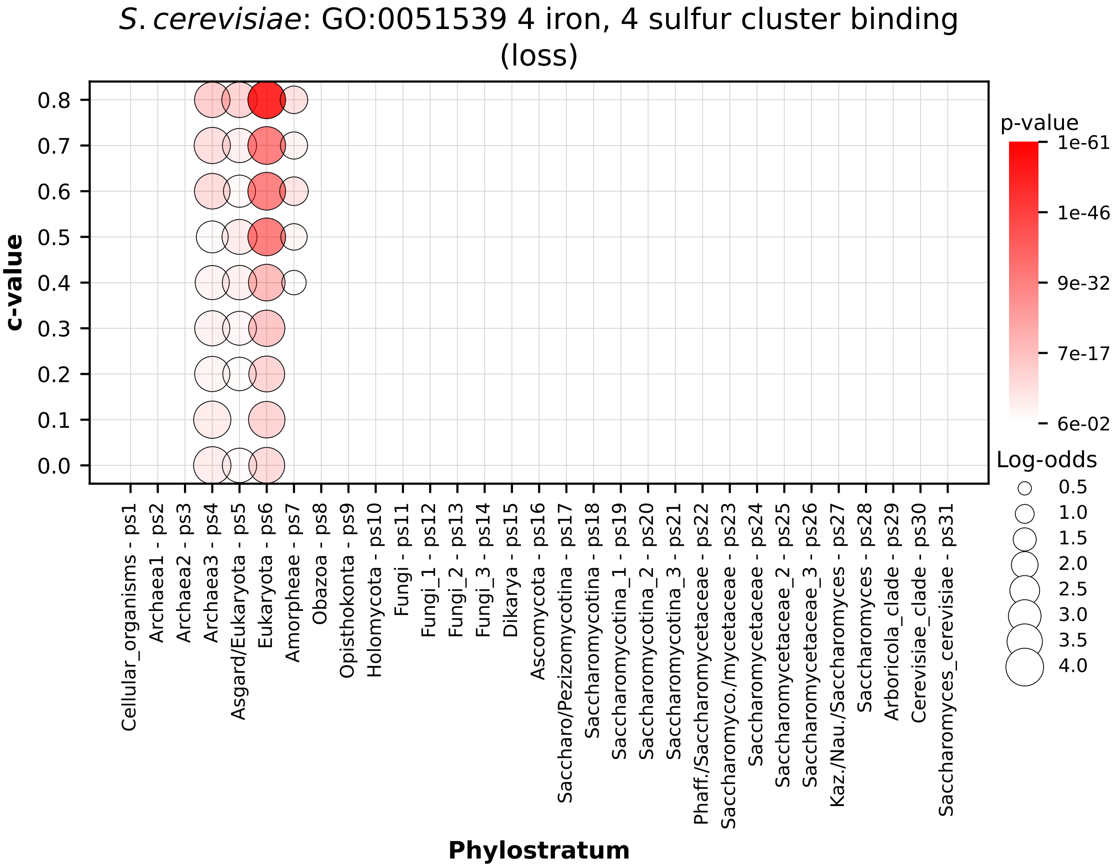
S. cerevisiae: GO:0051539 4 iron, 4 sulfur cluster binding
(loss)
c-value
0.8
0.7
0.6
0.5
0.4
0.3
0.2
0.1
0.0
p-value
1e-61
1e-46
9e-32
7e-17
6e-02
Log-odds
0.5
1.0
1.5
2.0
2.5
3.0
3.5
4.0
Cellular_organisms - ps1
Archaea1 - ps2
Archaea2 - ps3
Archaea3 - ps4
Asgard/Eukaryota - ps5
Eukaryota - ps6
Amorpheae - ps7
Obazoa - ps8
Opisthokonta - ps9
Holomycota - ps10
Fungi - ps11
Fungi_1 - ps12
Fungi_2 - ps13
Fungi_3 - ps14
Dikarya - ps15
Ascomycota - ps16
Saccharo/Pezizomycotina - ps17
Saccharomycotina - ps18
Saccharomycotina_1 - ps19
Saccharomycotina_2 - ps20
Saccharomycotina_3 - ps21
Phaff./Saccharomycetaceae - ps22
Saccharomyco./mycetaceae - ps23
Saccharomycetaceae - ps24
Saccharomycetaceae_2 - ps25
Saccharomycetaceae_3 - ps26
Kaz./Nau./Saccharomyces - ps27
Saccharomyces - ps28
Arboricola_clade - ps29
Cerevisiae_clade - ps30
Saccharomyces_cerevisiae - ps31
Phylostratum

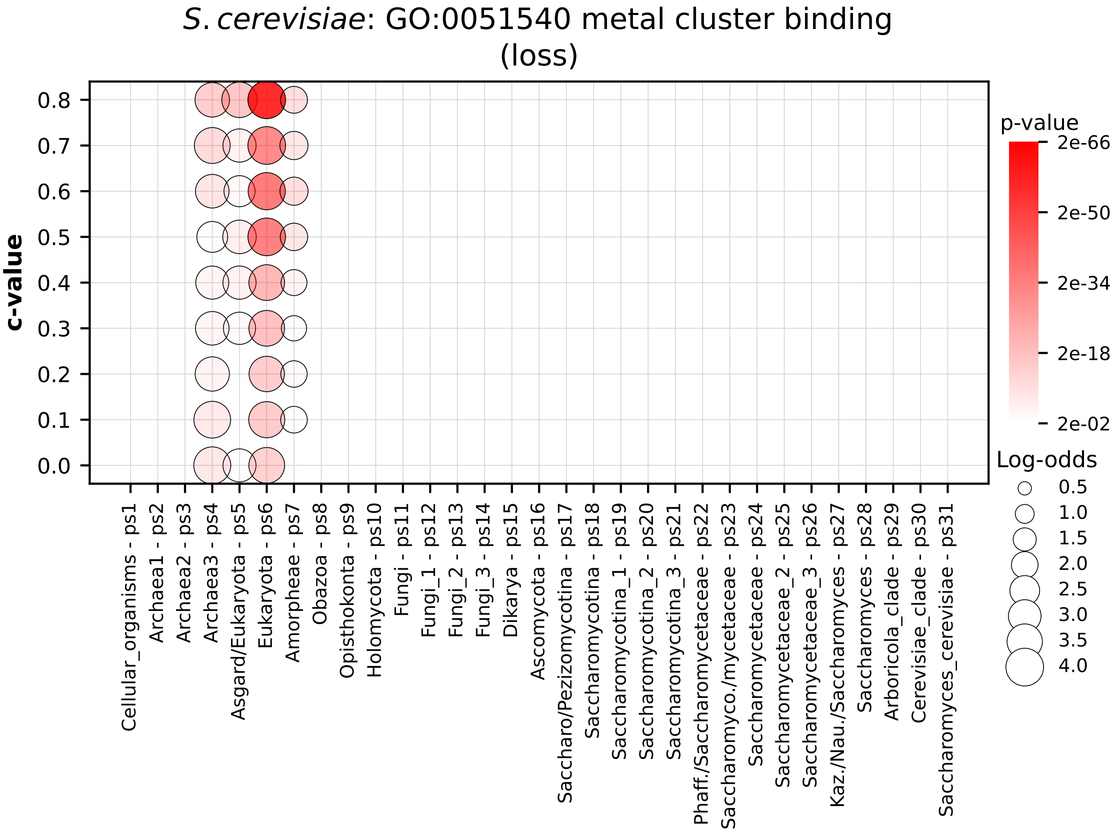

S. cerevisiae: GO:0051540 metal cluster binding (loss)
c-value
0.8
0.7
0.6
0.5
0.4
0.3
0.2
0.1
0.0
Phylostratum
Cellular_organisms - ps1
Archaea1 - ps2
Archaea2 - ps3
Archaea3 - ps4
Asgard/Eukaryota - ps5
Eukaryota - ps6
Amorpheae - ps7
Obazoa - ps8
Opisthokonta - ps9
Holomycota - ps10
Fungi - ps11
Fungi_1 - ps12
Fungi_2 - ps13
Fungi_3 - ps14
Dikarya - ps15
Ascomycota - ps16
Saccharo/Pezizomycotina - ps17
Saccharomycotina - ps18
Saccharomycotina_1 - ps19
Saccharomycotina_2 - ps20
Saccharomycotina_3 - ps21
Phaff./Saccharomycetaceae - ps22
Saccharomyco./mycetaceae - ps23
Saccharomycetaceae - ps24
Saccharomycetaceae_2 - ps25
Saccharomycetaceae_3 - ps26
Kaz./Nau./Saccharomyces - ps27
Saccharomyces - ps28
Arboricola_clade - ps29
Cerevisiae_clade - ps30
Saccharomyces_cerevisiae - ps31
p-value
2e-66
2e-50
2e-34
2e-18
2e-02
Log-odds
0.5
1.0
1.5
2.0
2.5
3.0
3.5
4.0

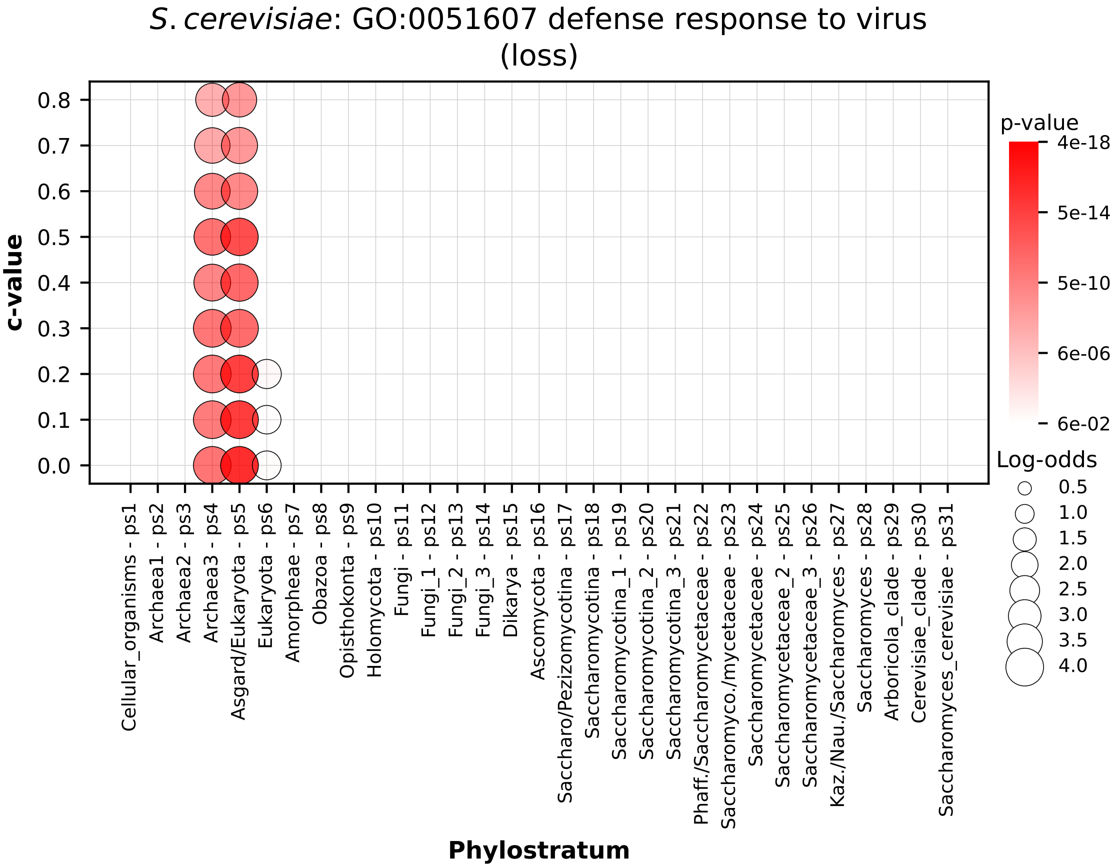

S. cerevisiae: GO:0051607 defense response to virus
(loss)
c-value
0.8
0.7
0.6
0.5
0.4
0.3
0.2
0.1
0.0
p-value
4e-18
5e-14
5e-10
6e-06
6e-02
Log-odds
0.5
1.0
1.5
2.0
2.5
3.0
3.5
4.0
Cellular_organisms - ps1
Archaea1 - ps2
Archaea2 - ps3
Archaea3 - ps4
Asgard/Eukaryota - ps5
Eukaryota - ps6
Amorpheae - ps7
Obazoa - ps8
Opisthokonta - ps9
Holomycota - ps10
Fungi - ps11
Fungi_1 - ps12
Fungi_2 - ps13
Fungi_3 - ps14
Dikarya - ps15
Ascomycota - ps16
Saccharo/Pezizomycotina - ps17
Saccharomycotina - ps18
Saccharomycotina_1 - ps19
Saccharomycotina_2 - ps20
Saccharomycotina_3 - ps21
Phaff./Saccharomycetaceae - ps22
Saccharomyco./mycetaceae - ps23
Saccharomycetaceae - ps24
Saccharomycetaceae_2 - ps25
Saccharomycetaceae_3 - ps26
Kaz./Nau./Saccharomyces - ps27
Saccharomyces - ps28
Arboricola_clade - ps29
Cerevisiae_clade - ps30
Saccharomyces_cerevisiae - ps31
Phylostratum

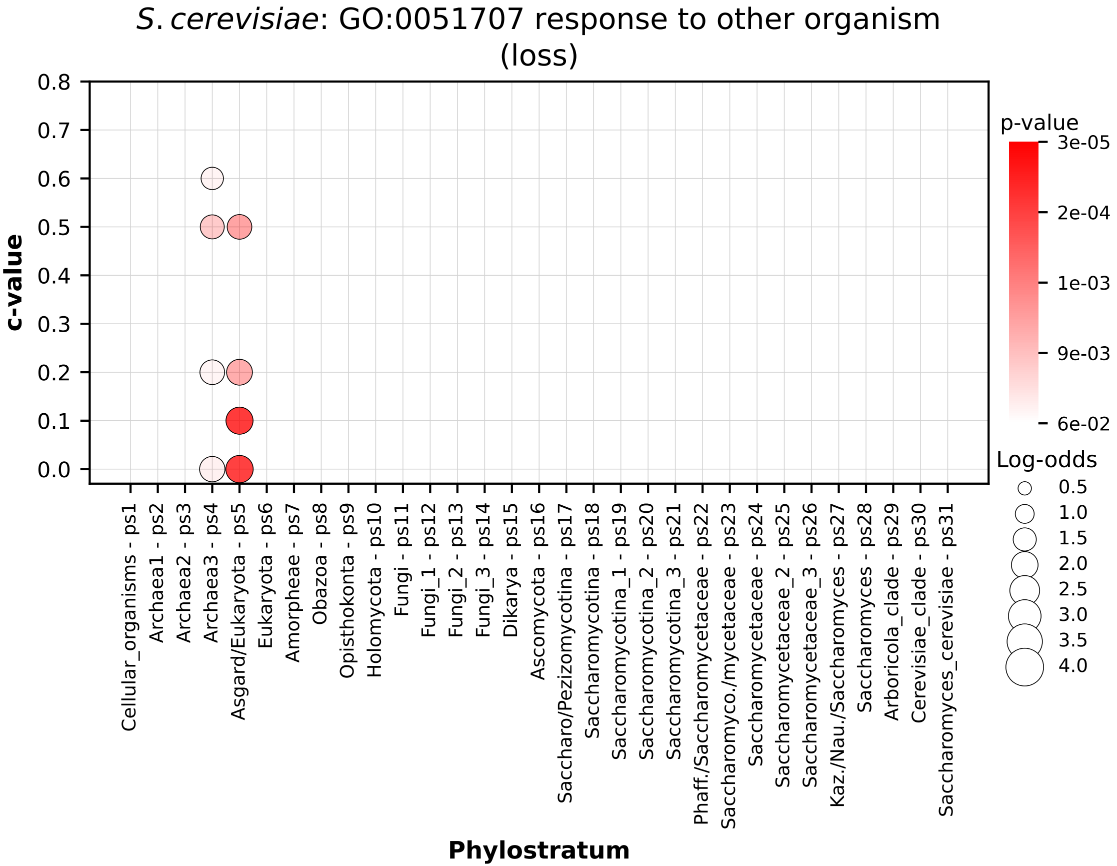

S. cerevisiae: GO:0051707 response to other organism
(loss)
c-value
0.0
0.1
0.2
0.3
0.4
0.5
0.6
0.7
0.8
Phylostratum
Cellular_organisms - ps1
Archaea1 - ps2
Archaea2 - ps3
Archaea3 - ps4
Asgard/Eukaryota - ps5
Eukaryota - ps6
Amorpheae - ps7
Obazoa - ps8
Opisthokonta - ps9
Holomycota - ps10
Fungi - ps11
Fungi_1 - ps12
Fungi_2 - ps13
Fungi_3 - ps14
Dikarya - ps15
Ascomycota - ps16
Saccharo/Pezizomycotina - ps17
Saccharomycotina - ps18
Saccharomycotina_1 - ps19
Saccharomycotina_2 - ps20
Saccharomycotina_3 - ps21
Phaff./Saccharomycetaceae - ps22
Saccharomyco./mycetaceae - ps23
Saccharomycetaceae - ps24
Saccharomycetaceae_2 - ps25
Saccharomycetaceae_3 - ps26
Kaz./Nau./Saccharomyces - ps27
Saccharomyces - ps28
Arboricola_clade - ps29
Cerevisiae_clade - ps30
Saccharomyces_cerevisiae - ps31
p-value
3e-05
2e-04
1e-03
9e-03
6e-02
Log-odds
0.5
1.0
1.5
2.0
2.5
3.0
3.5
4.0

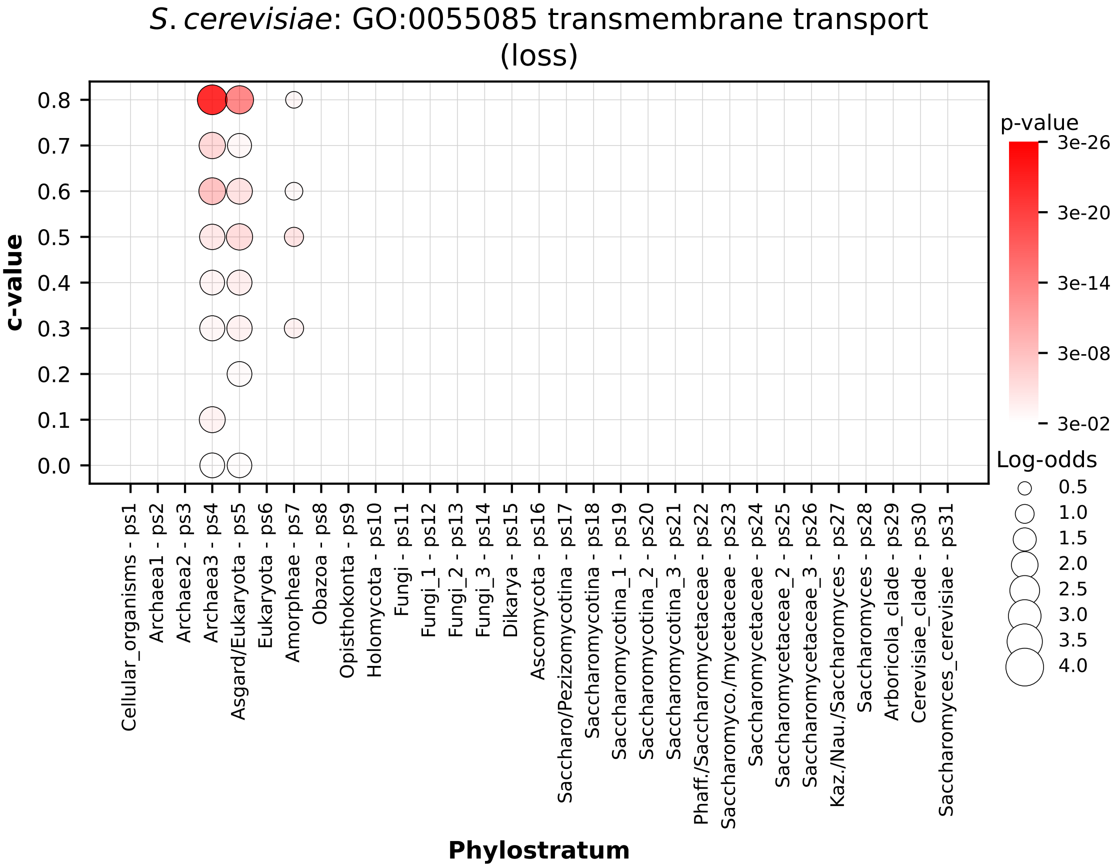

S. cerevisiae: GO:0055085 transmembrane transport
(loss)
c-value
0.8
0.7
0.6
0.5
0.4
0.3
0.2
0.1
0.0
p-value
3e-26
3e-20
3e-14
3e-08
3e-02
Log-odds
0.5
1.0
1.5
2.0
2.5
3.0
3.5
4.0
Cellular_organisms - ps1
Archaea1 - ps2
Archaea2 - ps3
Archaea3 - ps4
Asgard/Eukaryota - ps5
Eukaryota - ps6
Amorpheae - ps7
Obazoa - ps8
Opisthokonta - ps9
Holomycota - ps10
Fungi - ps11
Fungi_1 - ps12
Fungi_2 - ps13
Fungi_3 - ps14
Dikarya - ps15
Ascomycota - ps16
Saccharo/Pezizomycotina - ps17
Saccharomycotina - ps18
Saccharomycotina_1 - ps19
Saccharomycotina_2 - ps20
Saccharomycotina_3 - ps21
Phaff./Saccharomycetaceae - ps22
Saccharomyco./mycetaceae - ps23
Saccharomycetaceae - ps24
Saccharomycetaceae_2 - ps25
Saccharomycetaceae_3 - ps26
Kaz./Nau./Saccharomyces - ps27
Saccharomyces - ps28
Arboricola_clade - ps29
Cerevisiae_clade - ps30
Saccharomyces_cerevisiae - ps31
Phylostratum

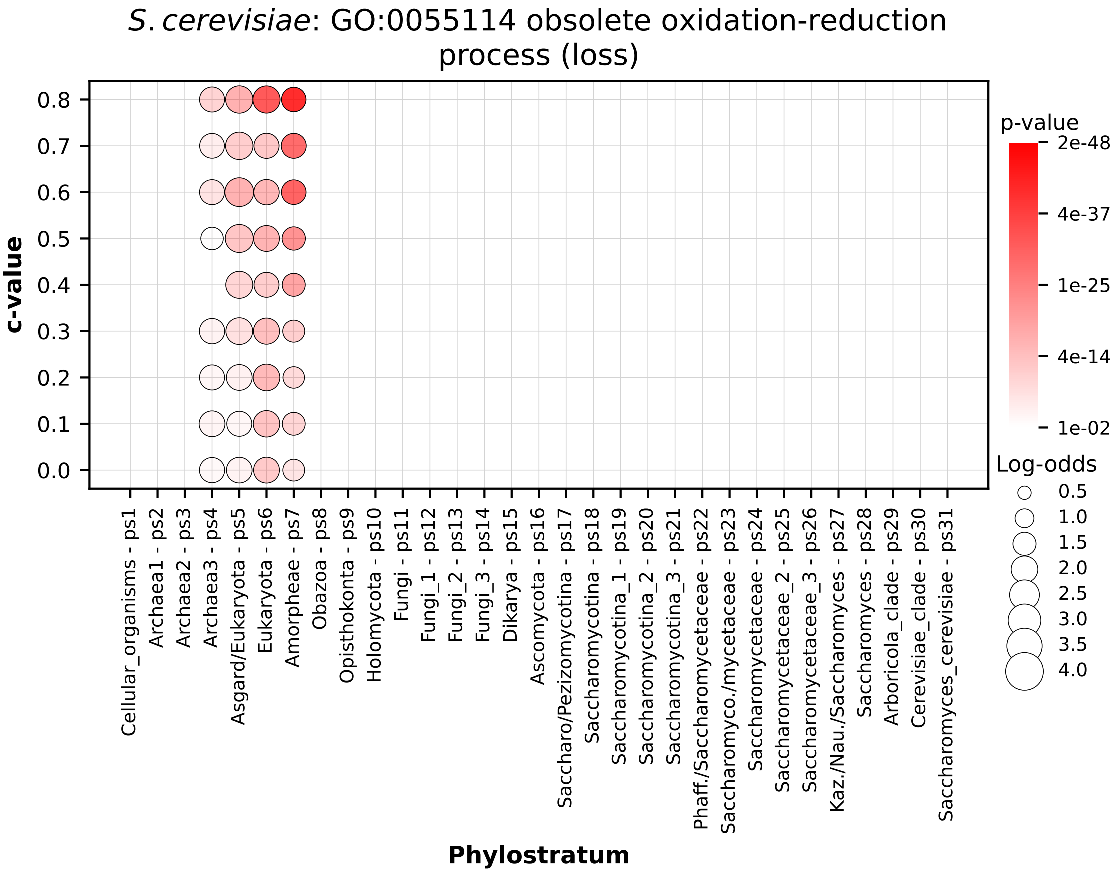

S. cerevisiae: GO:0055114 obsolete oxidation-reduction process (loss)
c-value
0.8
0.7
0.6
0.5
0.4
0.3
0.2
0.1
0.0
p-value
2e-48
4e-37
1e-25
4e-14
1e-02
Log-odds
0.5
1.0
1.5
2.0
2.5
3.0
3.5
4.0
Cellular_organisms - ps1
Archaea1 - ps2
Archaea2 - ps3
Archaea3 - ps4
Asgard/Eukaryota - ps5
Eukaryota - ps6
Amorpheae - ps7
Obazoa - ps8
Opisthokonta - ps9
Holomycota - ps10
Fungi - ps11
Fungi_1 - ps12
Fungi_2 - ps13
Fungi_3 - ps14
Dikarya - ps15
Ascomycota - ps16
Saccharo/Pezizomycotina - ps17
Saccharomycotina - ps18
Saccharomycotina_1 - ps19
Saccharomycotina_2 - ps20
Saccharomycotina_3 - ps21
Phaff./Saccharomycetaceae - ps22
Saccharomyco./mycetaceae - ps23
Saccharomycetaceae - ps24
Saccharomycetaceae_2 - ps25
Saccharomycetaceae_3 - ps26
Kaz./Nau./Saccharomyces - ps27
Saccharomyces - ps28
Arboricola_clade - ps29
Cerevisiae_clade - ps30
Saccharomyces_cerevisiae - ps31
Phylostratum

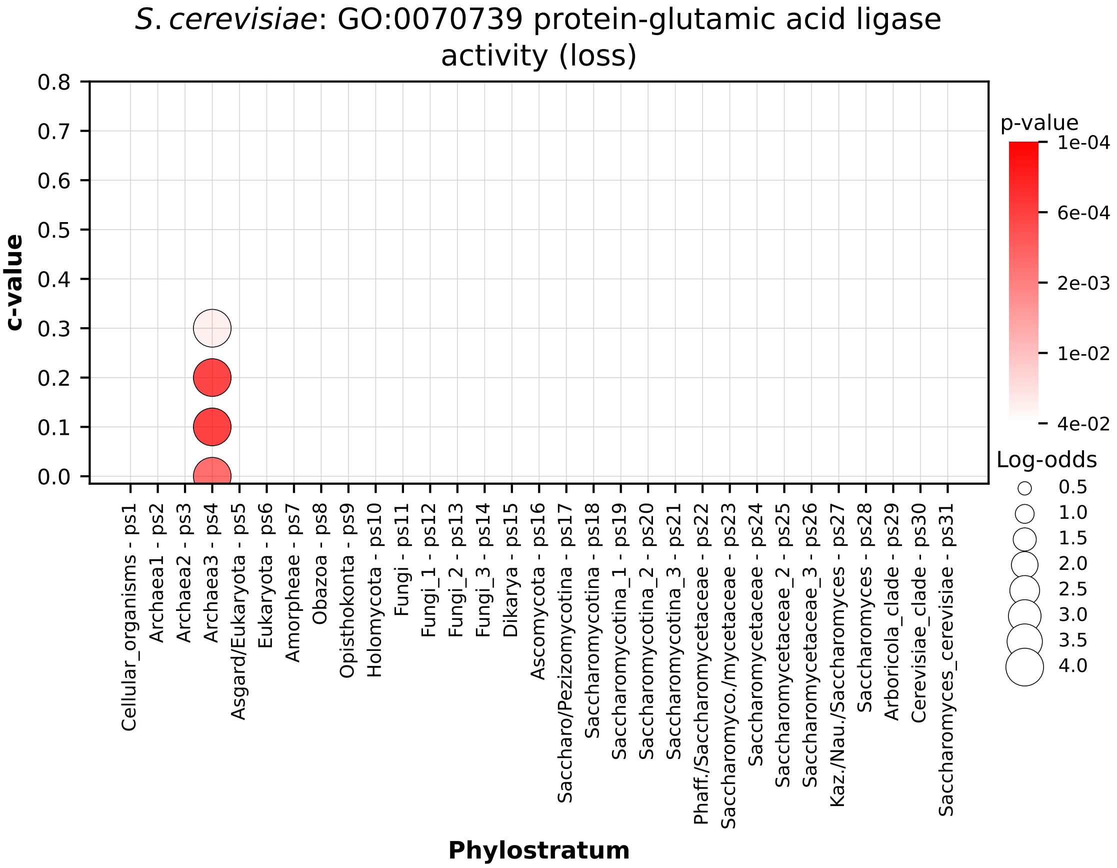

S. cerevisiae: GO:0070739 protein-glutamic acid ligase activity (loss)
c-value
0.8
0.7
0.6
0.5
0.4
0.3
0.2
0.1
0.0
Phylostratum
Cellular_organisms - ps1
Archaea1 - ps2
Archaea2 - ps3
Archaea3 - ps4
Asgard/Eukaryota - ps5
Eukaryota - ps6
Amorpheae - ps7
Obazoa - ps8
Opisthokonta - ps9
Holomycota - ps10
Fungi - ps11
Fungi_1 - ps12
Fungi_2 - ps13
Fungi_3 - ps14
Dikarya - ps15
Ascomycota - ps16
Saccharo/Pezizomycotina - ps17
Saccharomycotina - ps18
Saccharomycotina_1 - ps19
Saccharomycotina_2 - ps20
Saccharomycotina_3 - ps21
Phaff./Saccharomycetaceae - ps22
Saccharomyco./mycetaceae - ps23
Saccharomycetaceae - ps24
Saccharomycetaceae_2 - ps25
Saccharomycetaceae_3 - ps26
Kaz./Nau./Saccharomyces - ps27
Saccharomyces - ps28
Arboricola_clade - ps29
Cerevisiae_clade - ps30
Saccharomyces_cerevisiae - ps31
p-value
1e-04
6e-04
2e-03
1e-02
4e-02
Log-odds
0.5
1.0
1.5
2.0
2.5
3.0
3.5
4.0

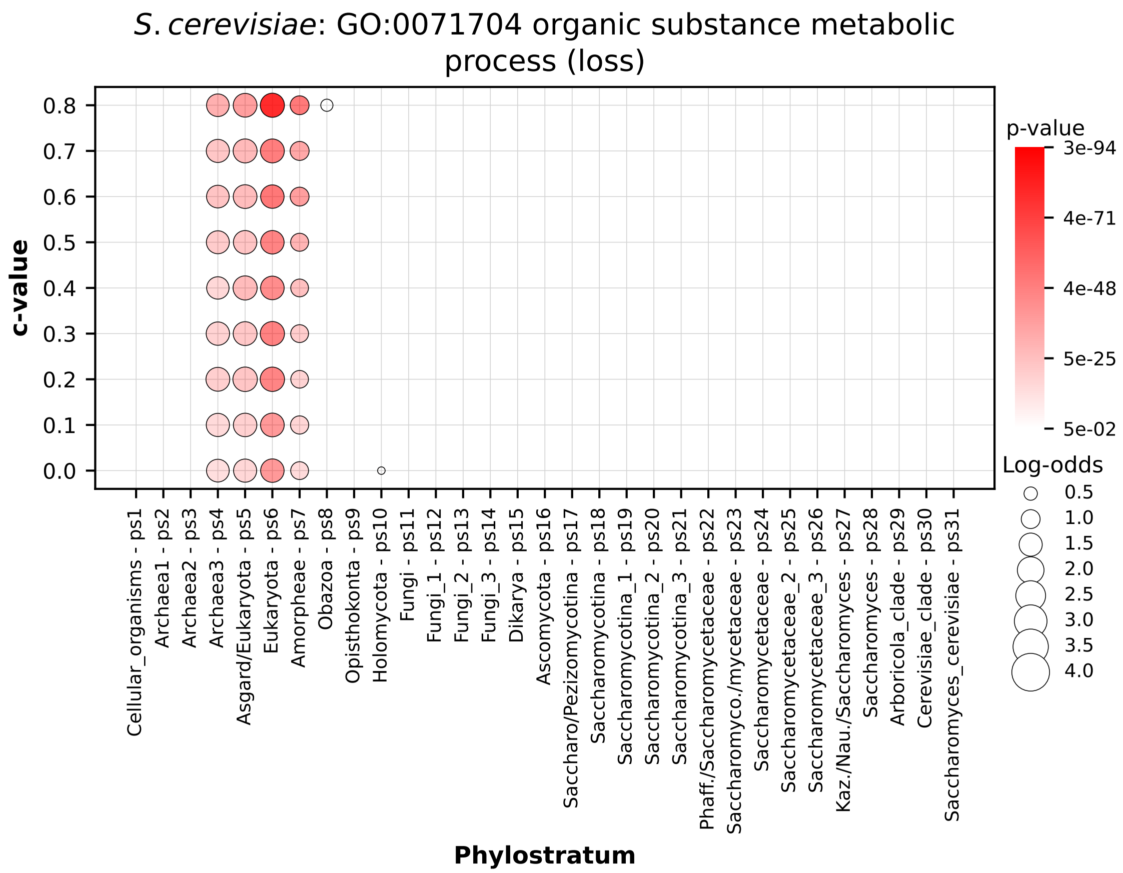
S. cerevisiae: GO:0071704 organic substance metabolic process (loss)
c-value
0.8
0.7
0.6
0.5
0.4
0.3
0.2
0.1
0.0
p-value
3e-94
4e-71
4e-48
5e-25
5e-02
Log-odds
0.5
1.0
1.5
2.0
2.5
3.0
3.5
4.0
Cellular_organisms - ps1
Archaea1 - ps2
Archaea2 - ps3
Archaea3 - ps4
Asgard/Eukaryota - ps5
Eukaryota - ps6
Amorpheae - ps7
Obazoa - ps8
Opisthokonta - ps9
Holomycota - ps10
Fungi - ps11
Fungi_1 - ps12
Fungi_2 - ps13
Fungi_3 - ps14
Dikarya - ps15
Ascomycota - ps16
Saccharo/Pezizomycotina - ps17
Saccharomycotina - ps18
Saccharomycotina_1 - ps19
Saccharomycotina_2 - ps20
Saccharomycotina_3 - ps21
Phaff./Saccharomycetaceae - ps22
Saccharomyco./mycetaceae - ps23
Saccharomycetaceae - ps24
Saccharomycetaceae_2 - ps25
Saccharomycetaceae_3 - ps26
Kaz./Nau./Saccharomyces - ps27
Saccharomyces - ps28
Arboricola_clade - ps29
Cerevisiae_clade - ps30
Saccharomyces_cerevisiae - ps31
Phylostratum

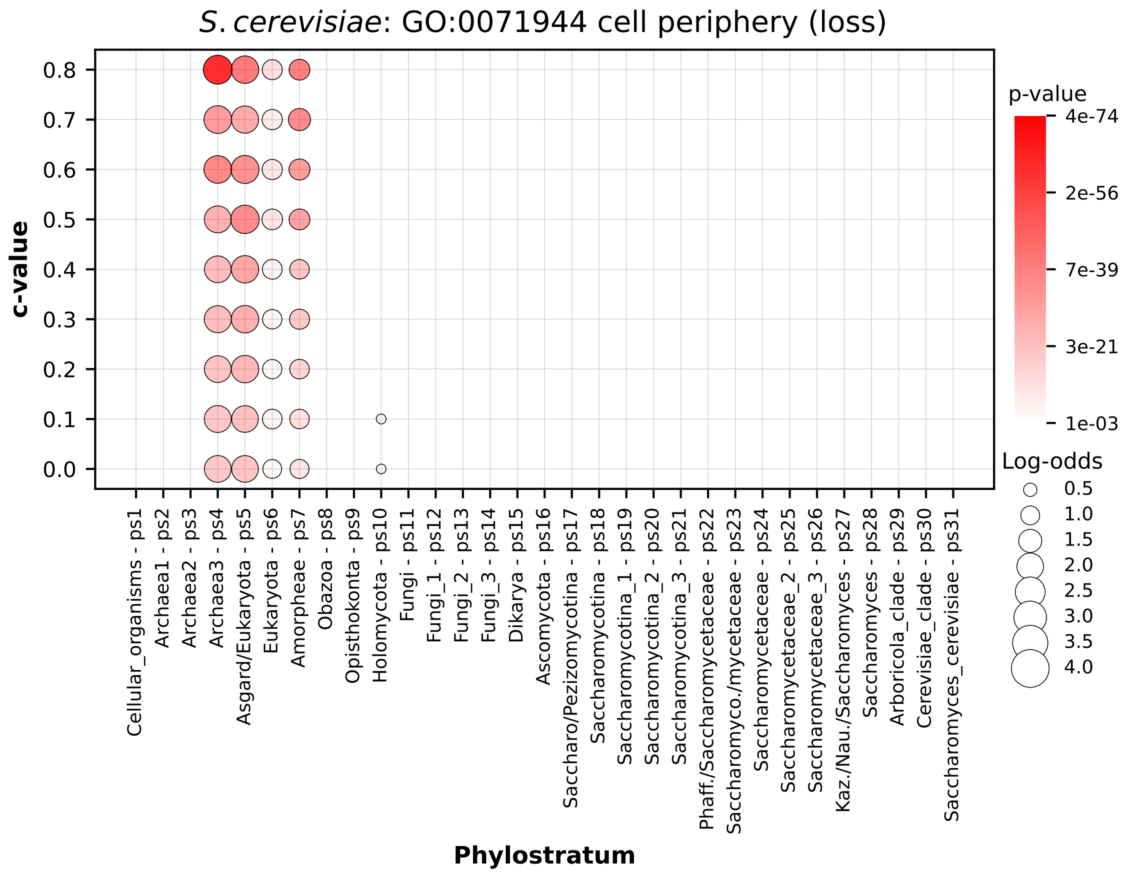

S. cerevisiae: GO:0071944 cell periphery (loss)
c-value
0.8
0.7
0.6
0.5
0.4
0.3
0.2
0.1
0.0
p-value
4e-74
2e-56
7e-39
3e-21
1e-03
Log-odds
0.5
1.0
1.5
2.0
2.5
3.0
3.5
4.0
Cellular_organisms - ps1
Archaea1 - ps2
Archaea2 - ps3
Archaea3 - ps4
Asgard/Eukaryota - ps5
Eukaryota - ps6
Amorpheae - ps7
Obazoa - ps8
Opisthokonta - ps9
Holomycota - ps10
Fungi - ps11
Fungi_1 - ps12
Fungi_2 - ps13
Fungi_3 - ps14
Dikarya - ps15
Ascomycota - ps16
Saccharo/Pezizomycotina - ps17
Saccharomycotina - ps18
Saccharomycotina_1 - ps19
Saccharomycotina_2 - ps20
Saccharomycotina_3 - ps21
Phaff./Saccharomycetaceae - ps22
Saccharomyco./mycetaceae - ps23
Saccharomycetaceae - ps24
Saccharomycetaceae_2 - ps25
Saccharomycetaceae_3 - ps26
Kaz./Nau./Saccharomyces - ps27
Saccharomyces - ps28
Arboricola_clade - ps29
Cerevisiae_clade - ps30
Saccharomyces_cerevisiae - ps31
Phylostratum

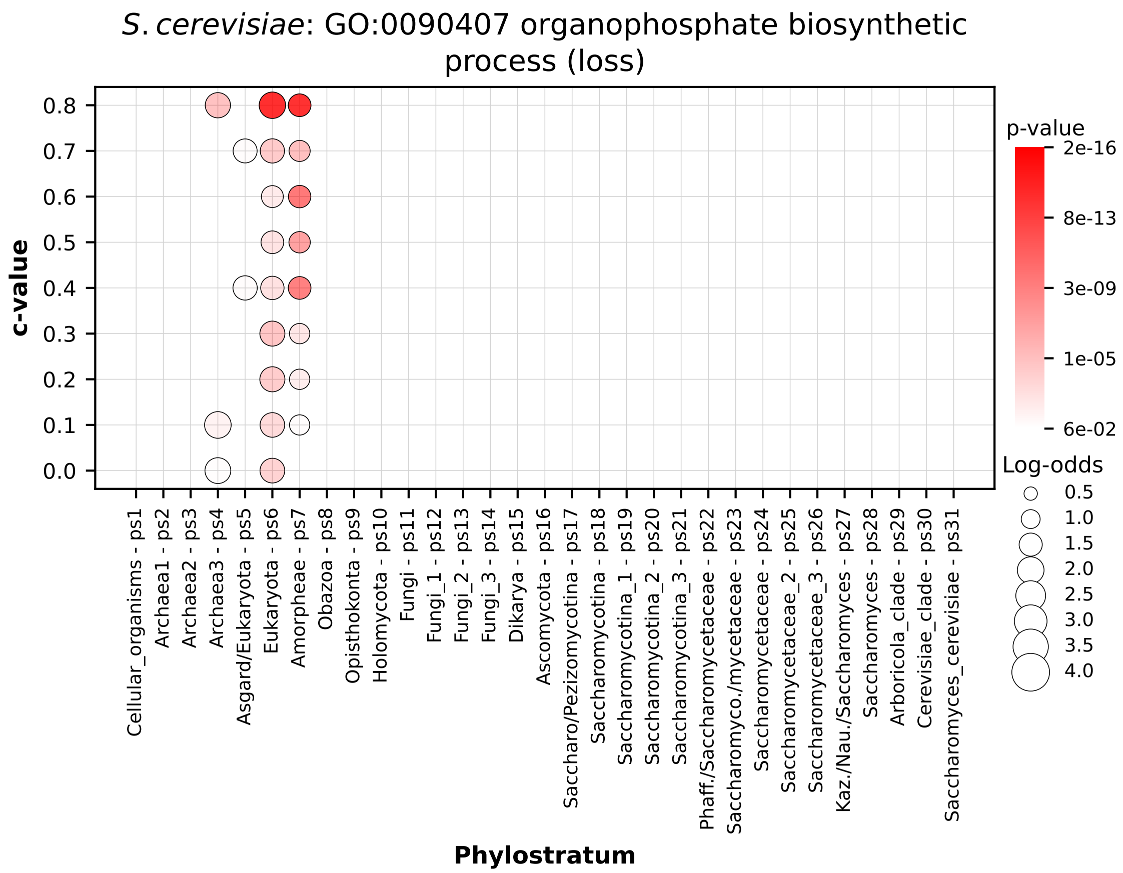
S. cerevisiae: GO:0090407 organophosphate biosynthetic process (loss)
c-value
0.8
0.7
0.6
0.5
0.4
0.3
0.2
0.1
0.0
p-value
2e-16
8e-13
3e-09
1e-05
6e-02
Log-odds
0.5
1.0
1.5
2.0
2.5
3.0
3.5
4.0
Cellular_organisms - ps1
Archaea1 - ps2
Archaea2 - ps3
Archaea3 - ps4
Asgard/Eukaryota - ps5
Eukaryota - ps6
Amorpheae - ps7
Obazoa - ps8
Opisthokonta - ps9
Holomycota - ps10
Fungi - ps11
Fungi_1 - ps12
Fungi_2 - ps13
Fungi_3 - ps14
Dikarya - ps15
Ascomycota - ps16
Saccharo/Pezizomycotina - ps17
Saccharomycotina - ps18
Saccharomycotina_1 - ps19
Saccharomycotina_2 - ps20
Saccharomycotina_3 - ps21
Phaff./Saccharomycetaceae - ps22
Saccharomyco./mycetaceae - ps23
Saccharomycetaceae - ps24
Saccharomycetaceae_2 - ps25
Saccharomycetaceae_3 - ps26
Kaz./Nau./Saccharomyces - ps27
Saccharomyces - ps28
Arboricola_clade - ps29
Cerevisiae_clade - ps30
Saccharomyces_cerevisiae - ps31
Phylostratum

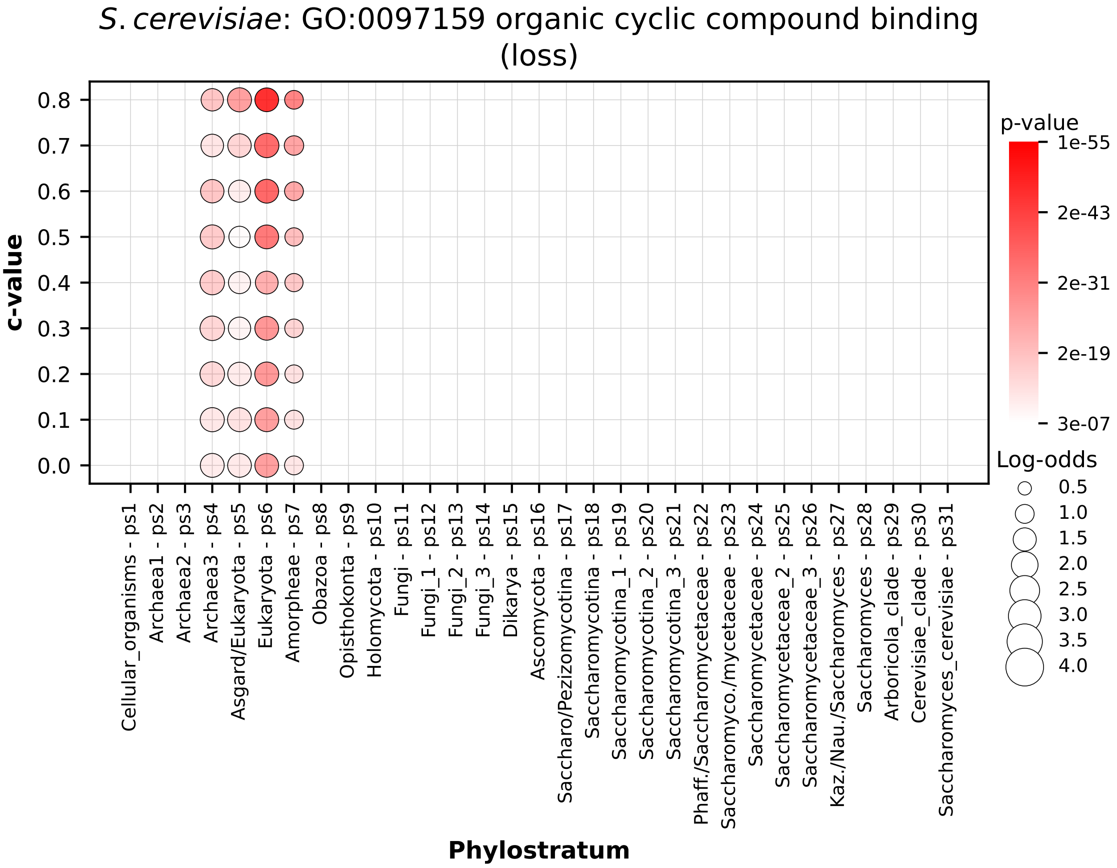

S. cerevisiae: GO:0097159 organic cyclic compound binding
(loss)
c-value
0.8
0.7
0.6
0.5
0.4
0.3
0.2
0.1
0.0
p-value
1e-55
2e-43
2e-31
2e-19
3e-07
Log-odds
0.5
1.0
1.5
2.0
2.5
3.0
3.5
4.0
Cellular_organisms - ps1
Archaea1 - ps2
Archaea2 - ps3
Archaea3 - ps4
Asgard/Eukaryota - ps5
Eukaryota - ps6
Amorpheae - ps7
Obazoa - ps8
Opisthokonta - ps9
Holomycota - ps10
Fungi - ps11
Fungi_1 - ps12
Fungi_2 - ps13
Fungi_3 - ps14
Dikarya - ps15
Ascomycota - ps16
Saccharo/Pezizomycotina - ps17
Saccharomycotina - ps18
Saccharomycotina_1 - ps19
Saccharomycotina_2 - ps20
Saccharomycotina_3 - ps21
Phaff./Saccharomycetaceae - ps22
Saccharomyco./mycetaceae - ps23
Saccharomycetaceae - ps24
Saccharomycetaceae_2 - ps25
Saccharomycetaceae_3 - ps26
Kaz./Nau./Saccharomyces - ps27
Saccharomyces - ps28
Arboricola_clade - ps29
Cerevisiae_clade - ps30
Saccharomyces_cerevisiae - ps31
Phylostratum

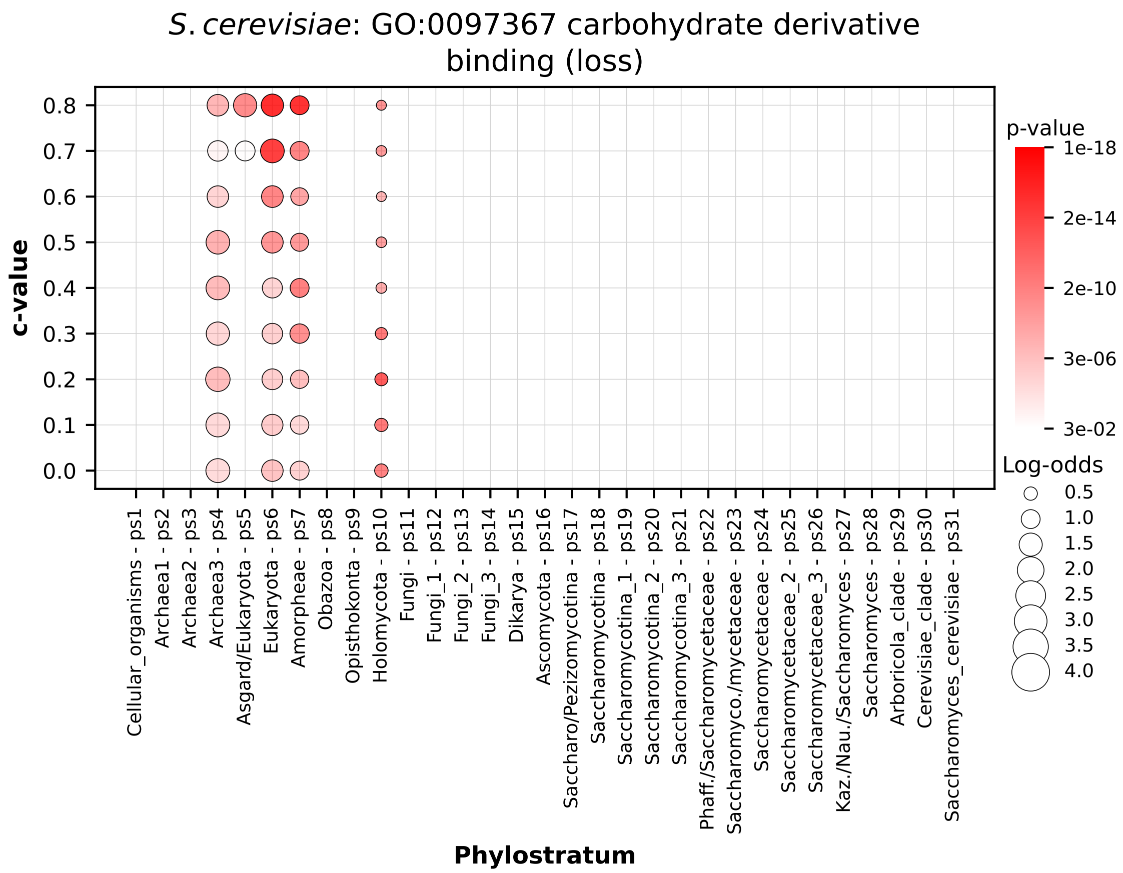

S. cerevisiae: GO:0097367 carbohydrate derivative binding (loss)
c-value
0.8
0.7
0.6
0.5
0.4
0.3
0.2
0.1
0.0
p-value
1e-18
2e-14
2e-10
3e-06
3e-02
Log-odds
0.5
1.0
1.5
2.0
2.5
3.0
3.5
4.0
Cellular_organisms - ps1
Archaea1 - ps2
Archaea2 - ps3
Archaea3 - ps4
Asgard/Eukaryota - ps5
Eukaryota - ps6
Amorpheae - ps7
Obazoa - ps8
Opisthokonta - ps9
Holomycota - ps10
Fungi - ps11
Fungi_1 - ps12
Fungi_2 - ps13
Fungi_3 - ps14
Dikarya - ps15
Ascomycota - ps16
Saccharo/Pezizomycotina - ps17
Saccharomycotina - ps18
Saccharomycotina_1 - ps19
Saccharomycotina_2 - ps20
Saccharomycotina_3 - ps21
Phaff./Saccharomycetaceae - ps22
Saccharomyco./mycetaceae - ps23
Saccharomycetaceae - ps24
Saccharomycetaceae_2 - ps25
Saccharomycetaceae_3 - ps26
Kaz./Nau./Saccharomyces - ps27
Saccharomyces - ps28
Arboricola_clade - ps29
Cerevisiae_clade - ps30
Saccharomyces_cerevisiae - ps31
Phylostratum

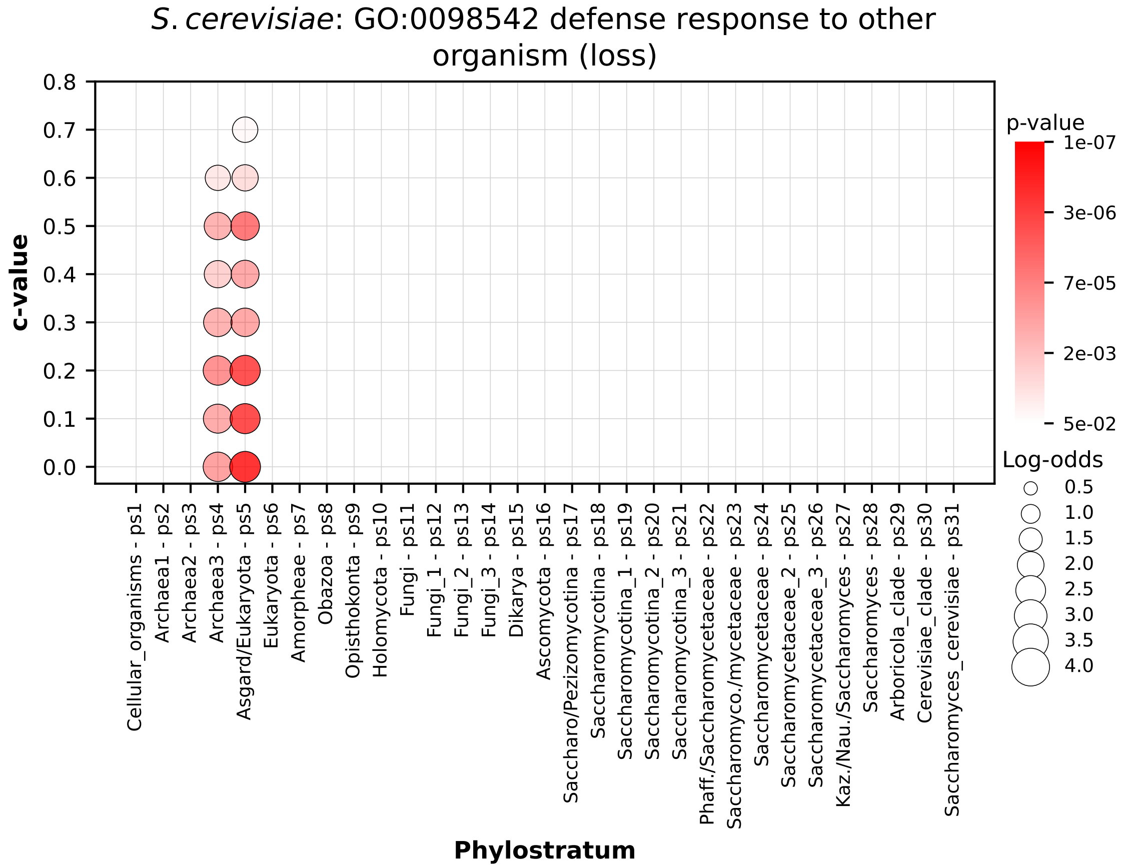

S. cerevisiae: GO:0098542 defense response to other organism (loss)
c-value
0.8
0.7
0.6
0.5
0.4
0.3
0.2
0.1
0.0
p-value
1e-07
3e-06
7e-05
2e-03
5e-02
Log-odds
0.5
1.0
1.5
2.0
2.5
3.0
3.5
4.0
Cellular_organisms - ps1
Archaea1 - ps2
Archaea2 - ps3
Archaea3 - ps4
Asgard/Eukaryota - ps5
Eukaryota - ps6
Amorpheae - ps7
Obazoa - ps8
Opisthokonta - ps9
Holomycota - ps10
Fungi - ps11
Fungi_1 - ps12
Fungi_2 - ps13
Fungi_3 - ps14
Dikarya - ps15
Ascomycota - ps16
Saccharo/Pezizomycotina - ps17
Saccharomycotina - ps18
Saccharomycotina_1 - ps19
Saccharomycotina_2 - ps20
Saccharomycotina_3 - ps21
Phaff./Saccharomycetaceae - ps22
Saccharomyco./mycetaceae - ps23
Saccharomycetaceae - ps24
Saccharomycetaceae_2 - ps25
Saccharomycetaceae_3 - ps26
Kaz./Nau./Saccharomyces - ps27
Saccharomyces - ps28
Arboricola_clade - ps29
Cerevisiae_clade - ps30
Saccharomyces_cerevisiae - ps31
Phylostratum

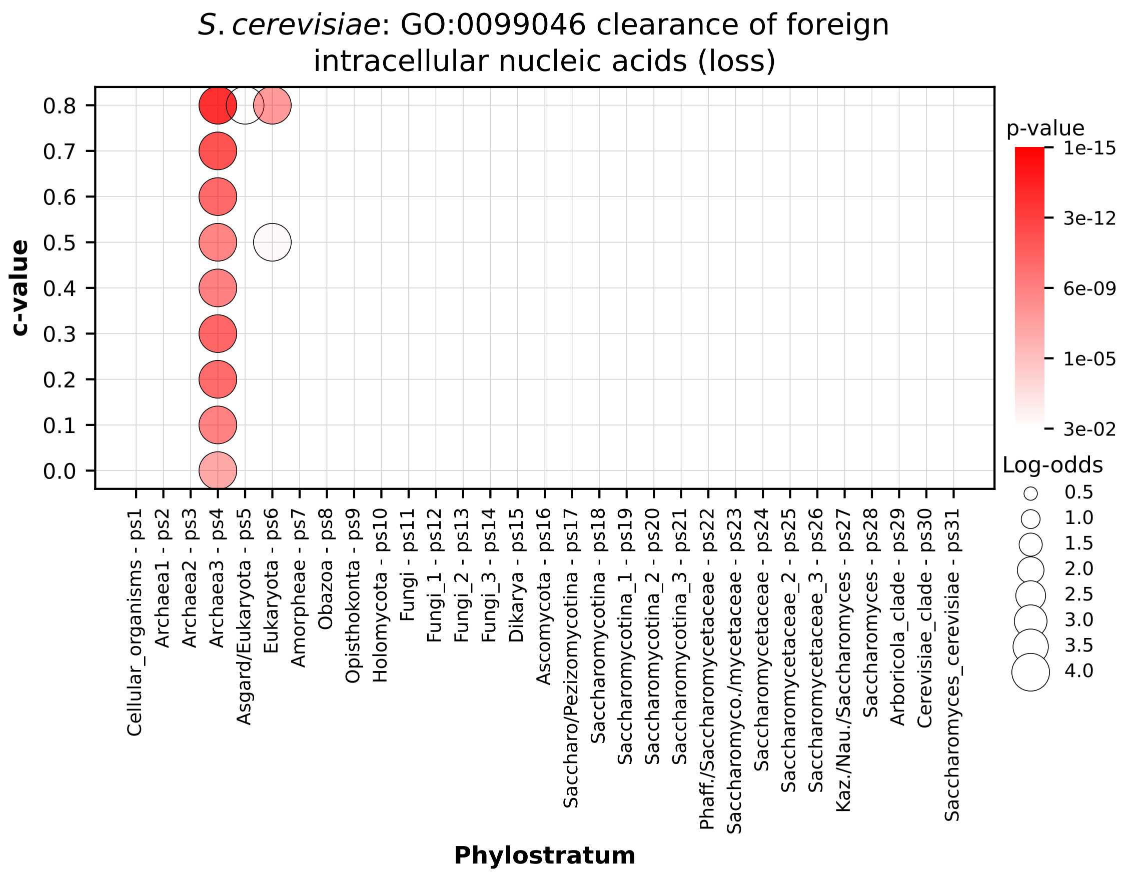
S. cerevisiae: GO:0099046 clearance of foreign intracellular nucleic acids (loss)
c-value
0.0
0.1
0.2
0.3
0.4
0.5
0.6
0.7
0.8
p-value
1e-15
3e-12
6e-09
1e-05
3e-02
Log-odds
0.5
1.0
1.5
2.0
2.5
3.0
3.5
4.0
Cellular_organisms - ps1
Archaea1 - ps2
Archaea2 - ps3
Archaea3 - ps4
Asgard/Eukaryota - ps5
Eukaryota - ps6
Amorpheae - ps7
Obazoa - ps8
Opisthokonta - ps9
Holomycota - ps10
Fungi - ps11
Fungi_1 - ps12
Fungi_2 - ps13
Fungi_3 - ps14
Dikarya - ps15
Ascomycota - ps16
Saccharo/Pezizomycotina - ps17
Saccharomycotina - ps18
Saccharomycotina_1 - ps19
Saccharomycotina_2 - ps20
Saccharomycotina_3 - ps21
Phaff./Saccharomycetaceae - ps22
Saccharomyco./mycetaceae - ps23
Saccharomycetaceae - ps24
Saccharomycetaceae_2 - ps25
Saccharomycetaceae_3 - ps26
Kaz./Nau./Saccharomyces - ps27
Saccharomyces - ps28
Arboricola_clade - ps29
Cerevisiae_clade - ps30
Saccharomyces_cerevisiae - ps31
Phylostratum

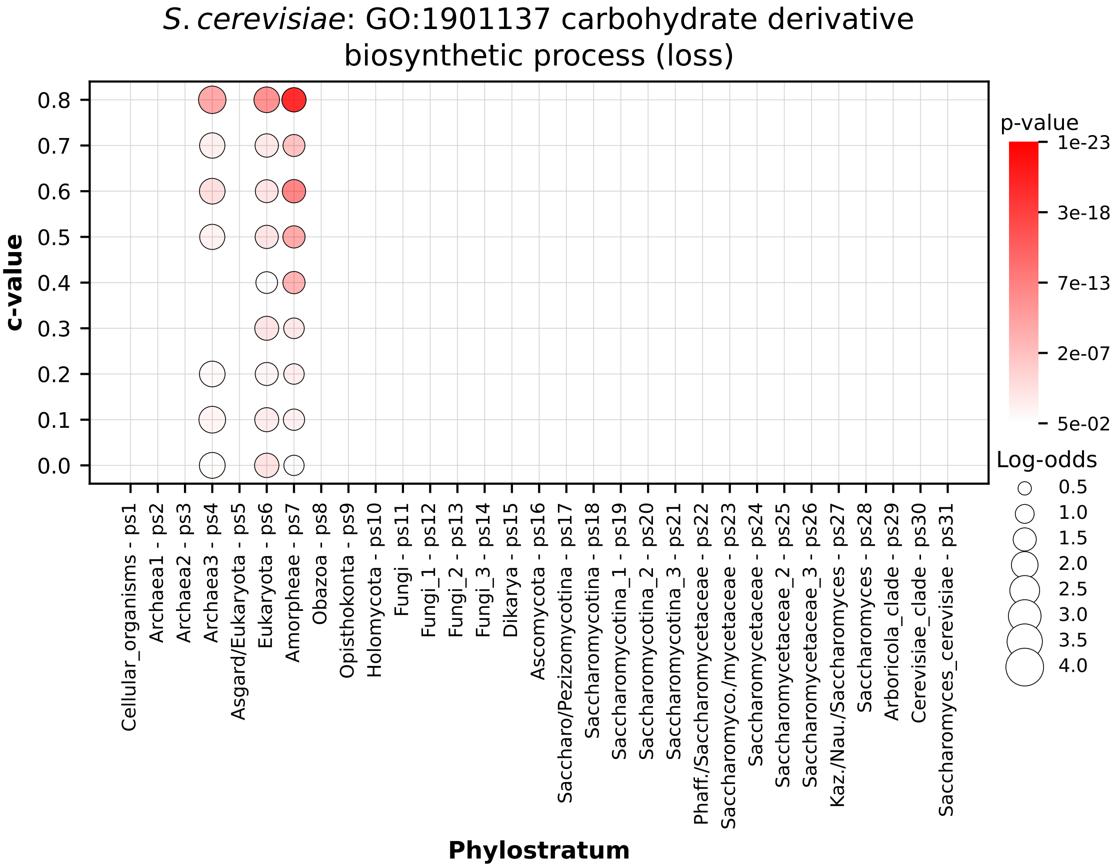

S. cerevisiae: GO:1901137 carbohydrate derivative biosynthetic process (loss)
c-value
0.8
0.7
0.6
0.5
0.4
0.3
0.2
0.1
0.0
Phylostratum
Cellular_organisms - ps1
Archaea1 - ps2
Archaea2 - ps3
Archaea3 - ps4
Asgard/Eukaryota - ps5
Eukaryota - ps6
Amorpheae - ps7
Obazoa - ps8
Opisthokonta - ps9
Holomycota - ps10
Fungi - ps11
Fungi_1 - ps12
Fungi_2 - ps13
Fungi_3 - ps14
Dikarya - ps15
Ascomycota - ps16
Saccharo/Pezizomycotina - ps17
Saccharomycotina - ps18
Saccharomycotina_1 - ps19
Saccharomycotina_2 - ps20
Saccharomycotina_3 - ps21
Phaff./Saccharomycetaceae - ps22
Saccharomyco./mycetaceae - ps23
Saccharomycetaceae - ps24
Saccharomycetaceae_2 - ps25
Saccharomycetaceae_3 - ps26
Kaz./Nau./Saccharomyces - ps27
Saccharomyces - ps28
Arboricola_clade - ps29
Cerevisiae_clade - ps30
Saccharomyces_cerevisiae - ps31
p-value
1e-23
3e-18
7e-13
2e-07
5e-02
Log-odds
0.5
1.0
1.5
2.0
2.5
3.0
3.5
4.0

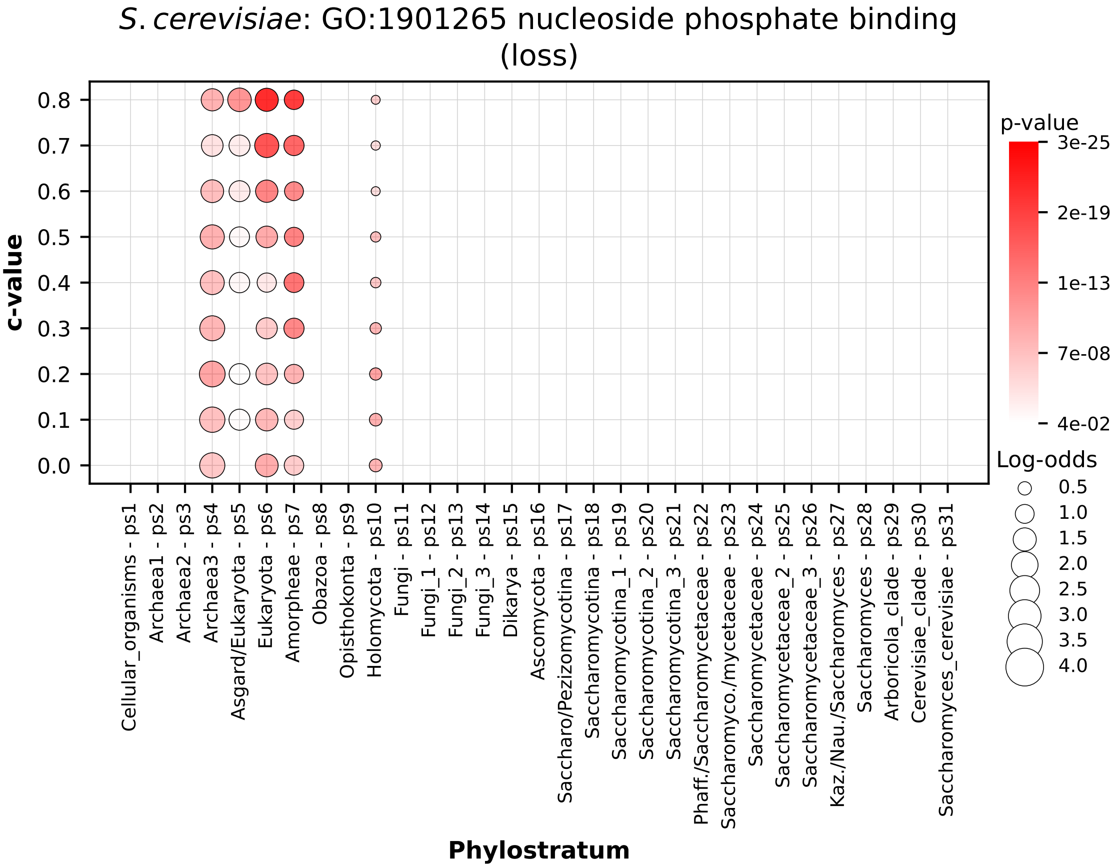
S. cerevisiae: GO:1901265 nucleoside phosphate binding
(loss)
c-value
0.8
0.7
0.6
0.5
0.4
0.3
0.2
0.1
0.0
p-value
3e-25
2e-19
1e-13
7e-08
4e-02
Log-odds
0.5
1.0
1.5
2.0
2.5
3.0
3.5
4.0
Cellular_organisms - ps1
Archaea1 - ps2
Archaea2 - ps3
Archaea3 - ps4
Asgard/Eukaryota - ps5
Eukaryota - ps6
Amorpheae - ps7
Obazoa - ps8
Opisthokonta - ps9
Holomycota - ps10
Fungi - ps11
Fungi_1 - ps12
Fungi_2 - ps13
Fungi_3 - ps14
Dikarya - ps15
Ascomycota - ps16
Saccharo/Pezizomycotina - ps17
Saccharomycotina - ps18
Saccharomycotina_1 - ps19
Saccharomycotina_2 - ps20
Saccharomycotina_3 - ps21
Phaff./Saccharomycetaceae - ps22
Saccharomyco./mycetaceae - ps23
Saccharomycetaceae - ps24
Saccharomycetaceae_2 - ps25
Saccharomycetaceae_3 - ps26
Kaz./Nau./Saccharomyces - ps27
Saccharomyces - ps28
Arboricola_clade - ps29
Cerevisiae_clade - ps30
Saccharomyces_cerevisiae - ps31
Phylostratum

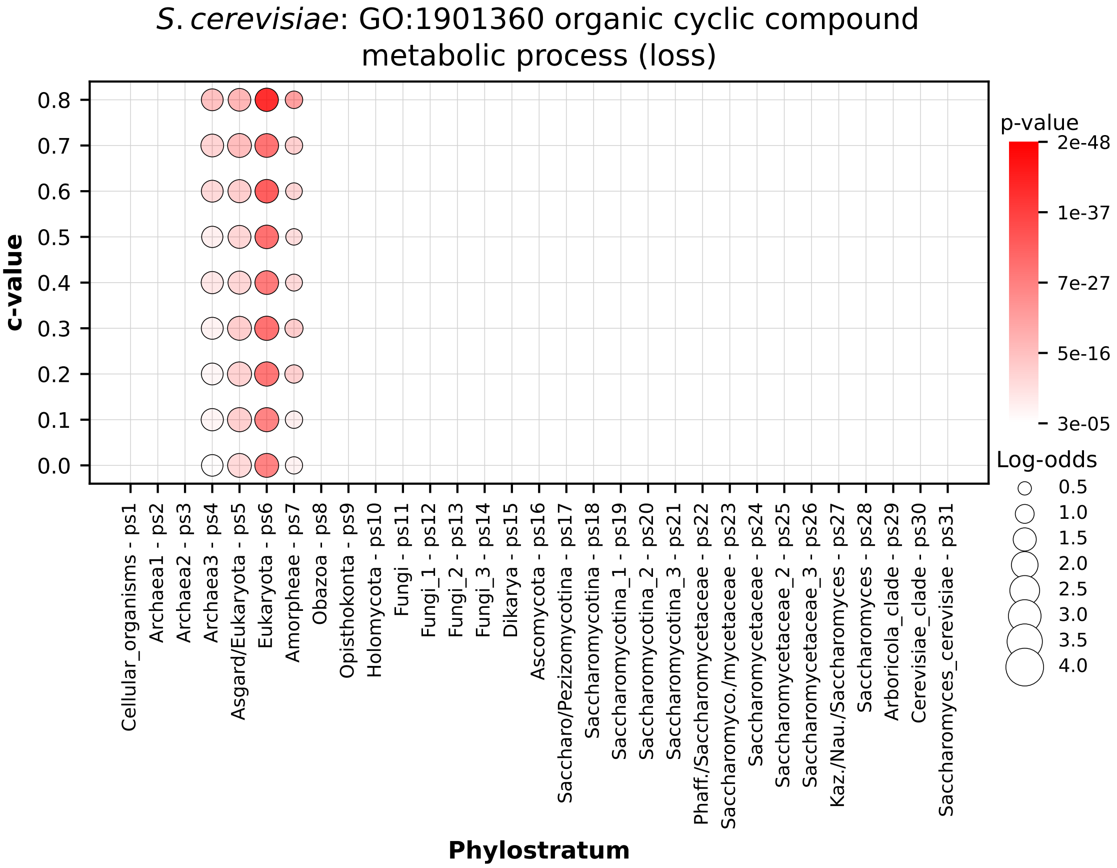

S. cerevisiae: GO:1901360 organic cyclic compound metabolic process (loss)
c-value
0.8
0.7
0.6
0.5
0.4
0.3
0.2
0.1
0.0
Phylostratum
Cellular_organisms - ps1
Archaea1 - ps2
Archaea2 - ps3
Archaea3 - ps4
Asgard/Eukaryota - ps5
Eukaryota - ps6
Amorpheae - ps7
Obazoa - ps8
Opisthokonta - ps9
Holomycota - ps10
Fungi - ps11
Fungi_1 - ps12
Fungi_2 - ps13
Fungi_3 - ps14
Dikarya - ps15
Ascomycota - ps16
Saccharo/Pezizomycotina - ps17
Saccharomycotina - ps18
Saccharomycotina_1 - ps19
Saccharomycotina_2 - ps20
Saccharomycotina_3 - ps21
Phaff./Saccharomycetaceae - ps22
Saccharomyco./mycetaceae - ps23
Saccharomycetaceae - ps24
Saccharomycetaceae_2 - ps25
Saccharomycetaceae_3 - ps26
Kaz./Nau./Saccharomyces - ps27
Saccharomyces - ps28
Arboricola_clade - ps29
Cerevisiae_clade - ps30
Saccharomyces_cerevisiae - ps31
p-value
2e-48
1e-37
7e-27
5e-16
3e-05
Log-odds
0.5
1.0
1.5
2.0
2.5
3.0
3.5
4.0

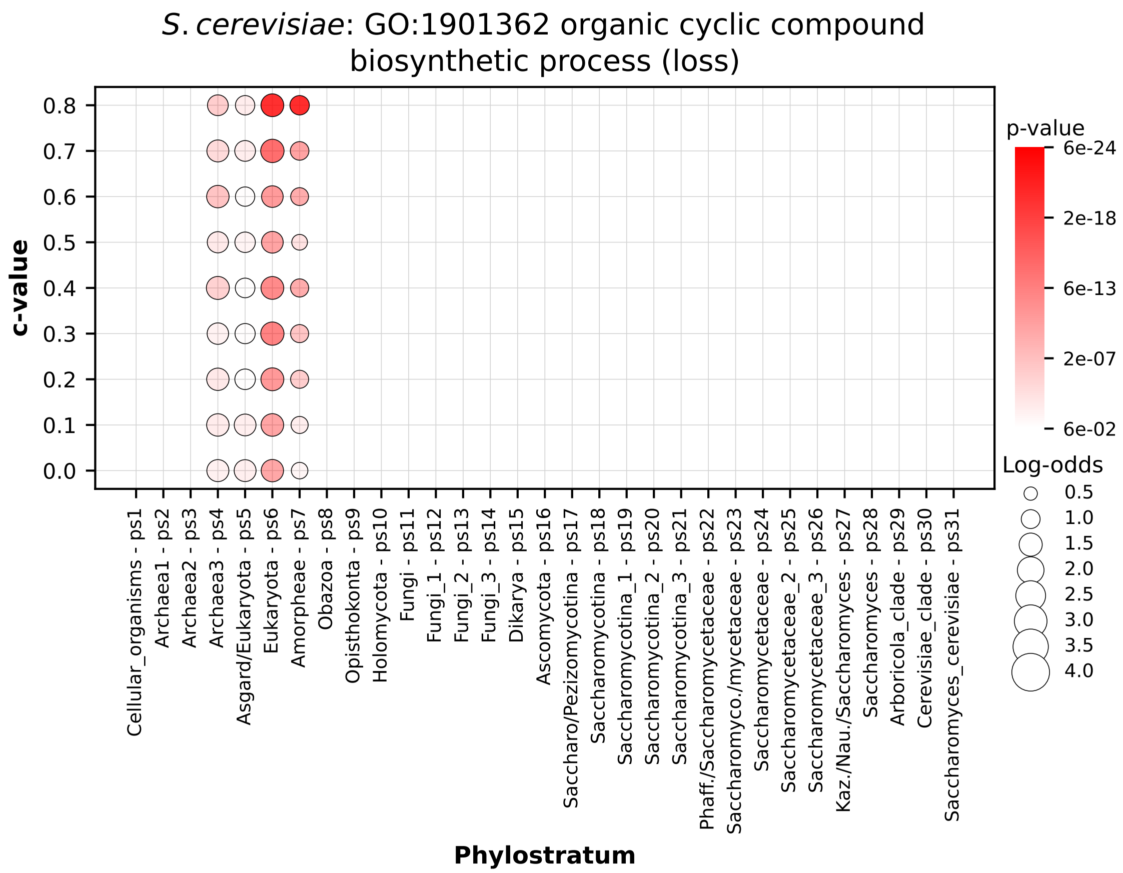

S. cerevisiae: GO:1901362 organic cyclic compound biosynthetic process (loss)
c-value
0.8
0.7
0.6
0.5
0.4
0.3
0.2
0.1
0.0
p-value
6e-24
2e-18
6e-13
2e-07
6e-02
Log-odds
0.5
1.0
1.5
2.0
2.5
3.0
3.5
4.0
Cellular_organisms - ps1
Archaea1 - ps2
Archaea2 - ps3
Archaea3 - ps4
Asgard/Eukaryota - ps5
Eukaryota - ps6
Amorpheae - ps7
Obazoa - ps8
Opisthokonta - ps9
Holomycota - ps10
Fungi - ps11
Fungi_1 - ps12
Fungi_2 - ps13
Fungi_3 - ps14
Dikarya - ps15
Ascomycota - ps16
Saccharo/Pezizomycotina - ps17
Saccharomycotina - ps18
Saccharomycotina_1 - ps19
Saccharomycotina_2 - ps20
Saccharomycotina_3 - ps21
Phaff./Saccharomycetaceae - ps22
Saccharomyco./mycetaceae - ps23
Saccharomycetaceae - ps24
Saccharomycetaceae_2 - ps25
Saccharomycetaceae_3 - ps26
Kaz./Nau./Saccharomyces - ps27
Saccharomyces - ps28
Arboricola_clade - ps29
Cerevisiae_clade - ps30
Saccharomyces_cerevisiae - ps31
Phylostratum

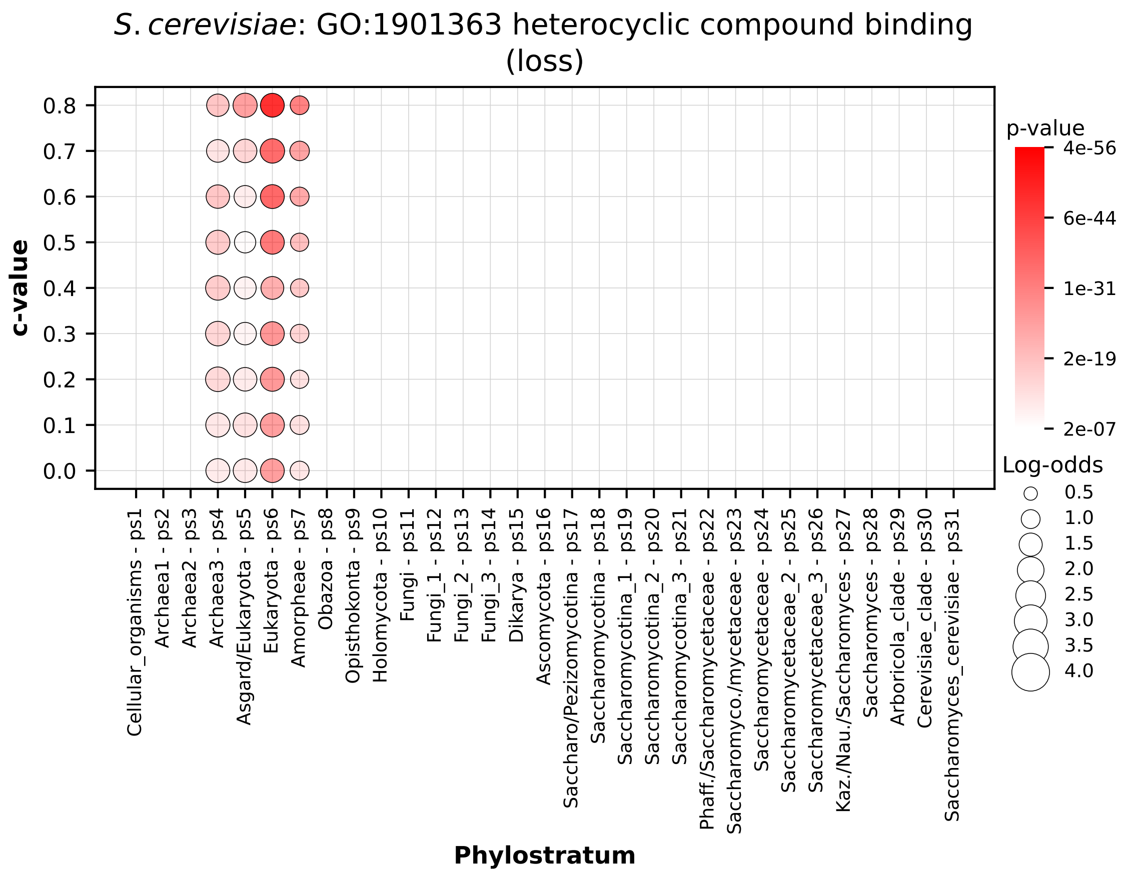

S. cerevisiae: GO:1901363 heterocyclic compound binding
(loss)
c-value
0.8
0.7
0.6
0.5
0.4
0.3
0.2
0.1
0.0
p-value
4e-56
6e-44
1e-31
2e-19
2e-07
Log-odds
0.5
1.0
1.5
2.0
2.5
3.0
3.5
4.0
Cellular_organisms - ps1
Archaea1 - ps2
Archaea2 - ps3
Archaea3 - ps4
Asgard/Eukaryota - ps5
Eukaryota - ps6
Amorpheae - ps7
Obazoa - ps8
Opisthokonta - ps9
Holomycota - ps10
Fungi - ps11
Fungi_1 - ps12
Fungi_2 - ps13
Fungi_3 - ps14
Dikarya - ps15
Ascomycota - ps16
Saccharo/Pezizomycotina - ps17
Saccharomycotina - ps18
Saccharomycotina_1 - ps19
Saccharomycotina_2 - ps20
Saccharomycotina_3 - ps21
Phaff./Saccharomycetaceae - ps22
Saccharomyco./mycetaceae - ps23
Saccharomycetaceae - ps24
Saccharomycetaceae_2 - ps25
Saccharomycetaceae_3 - ps26
Kaz./Nau./Saccharomyces - ps27
Saccharomyces - ps28
Arboricola_clade - ps29
Cerevisiae_clade - ps30
Saccharomyces_cerevisiae - ps31
Phylostratum

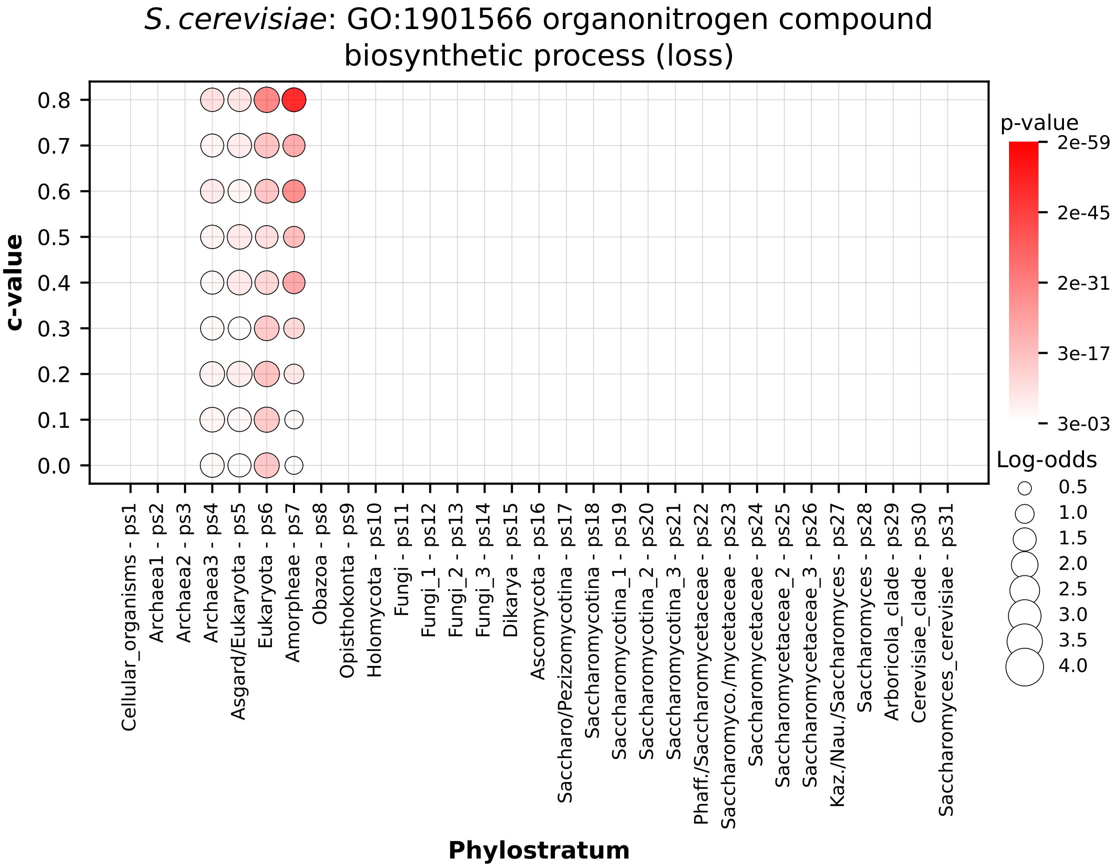
S. cerevisiae: GO:1901566 organonitrogen compound biosynthetic process (loss)
c-value
0.8
0.7
0.6
0.5
0.4
0.3
0.2
0.1
0.0
p-value
2e-59
2e-45
2e-31
3e-17
3e-03
Log-odds
0.5
1.0
1.5
2.0
2.5
3.0
3.5
4.0
Cellular_organisms - ps1
Archaea1 - ps2
Archaea2 - ps3
Archaea3 - ps4
Asgard/Eukaryota - ps5
Eukaryota - ps6
Amorpheae - ps7
Obazoa - ps8
Opisthokonta - ps9
Holomycota - ps10
Fungi - ps11
Fungi_1 - ps12
Fungi_2 - ps13
Fungi_3 - ps14
Dikarya - ps15
Ascomycota - ps16
Saccharo/Pezizomycotina - ps17
Saccharomycotina - ps18
Saccharomycotina_1 - ps19
Saccharomycotina_2 - ps20
Saccharomycotina_3 - ps21
Phaff./Saccharomycetaceae - ps22
Saccharomyco./mycetaceae - ps23
Saccharomycetaceae - ps24
Saccharomycetaceae_2 - ps25
Saccharomycetaceae_3 - ps26
Kaz./Nau./Saccharomyces - ps27
Saccharomyces - ps28
Arboricola_clade - ps29
Cerevisiae_clade - ps30
Saccharomyces_cerevisiae - ps31
Phylostratum

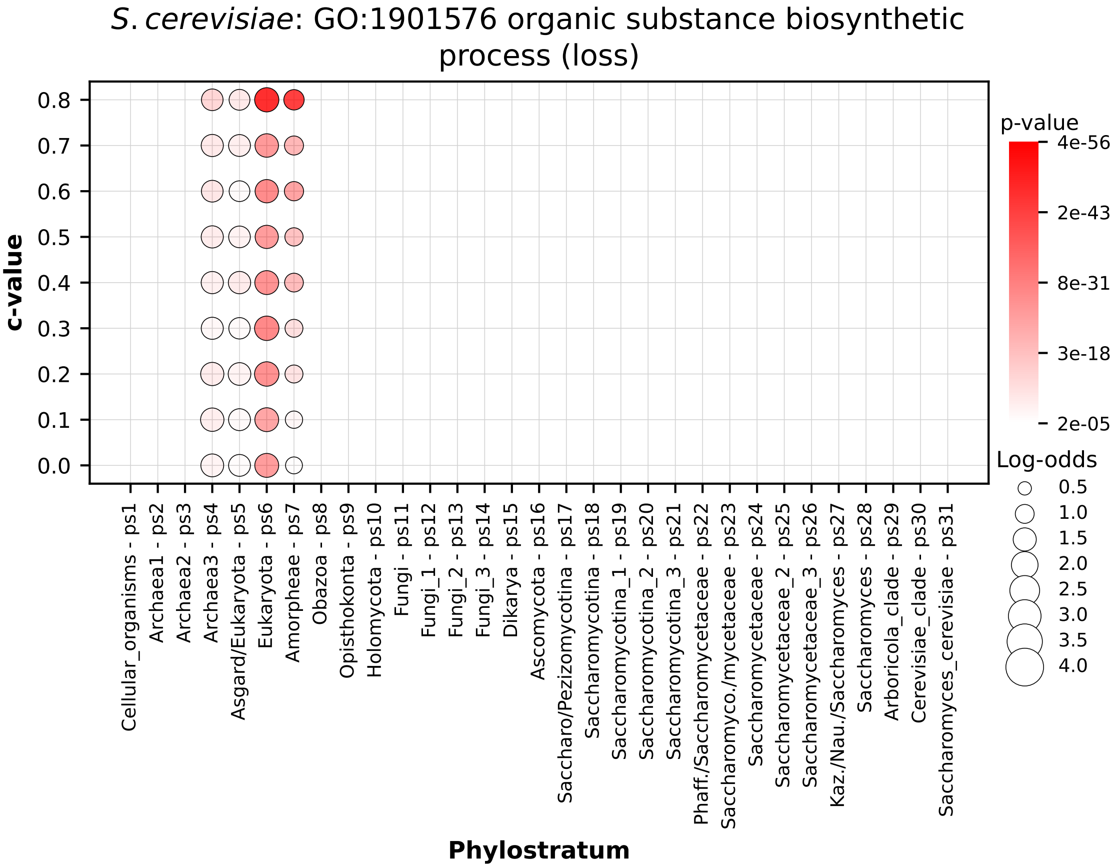

S. cerevisiae: GO:1901576 organic substance biosynthetic process (loss)
c-value
0.8
0.7
0.6
0.5
0.4
0.3
0.2
0.1
0.0
p-value
4e-56
2e-43
8e-31
3e-18
2e-05
Log-odds
0.5
1.0
1.5
2.0
2.5
3.0
3.5
4.0
Cellular_organisms - ps1
Archaea1 - ps2
Archaea2 - ps3
Archaea3 - ps4
Asgard/Eukaryota - ps5
Eukaryota - ps6
Amorpheae - ps7
Obazoa - ps8
Opisthokonta - ps9
Holomycota - ps10
Fungi - ps11
Fungi_1 - ps12
Fungi_2 - ps13
Fungi_3 - ps14
Dikarya - ps15
Ascomycota - ps16
Saccharo/Pezizomycotina - ps17
Saccharomycotina - ps18
Saccharomycotina_1 - ps19
Saccharomycotina_2 - ps20
Saccharomycotina_3 - ps21
Phaff./Saccharomycetaceae - ps22
Saccharomyco./mycetaceae - ps23
Saccharomycetaceae - ps24
Saccharomycetaceae_2 - ps25
Saccharomycetaceae_3 - ps26
Kaz./Nau./Saccharomyces - ps27
Saccharomyces - ps28
Arboricola_clade - ps29
Cerevisiae_clade - ps30
Saccharomyces_cerevisiae - ps31
Phylostratum

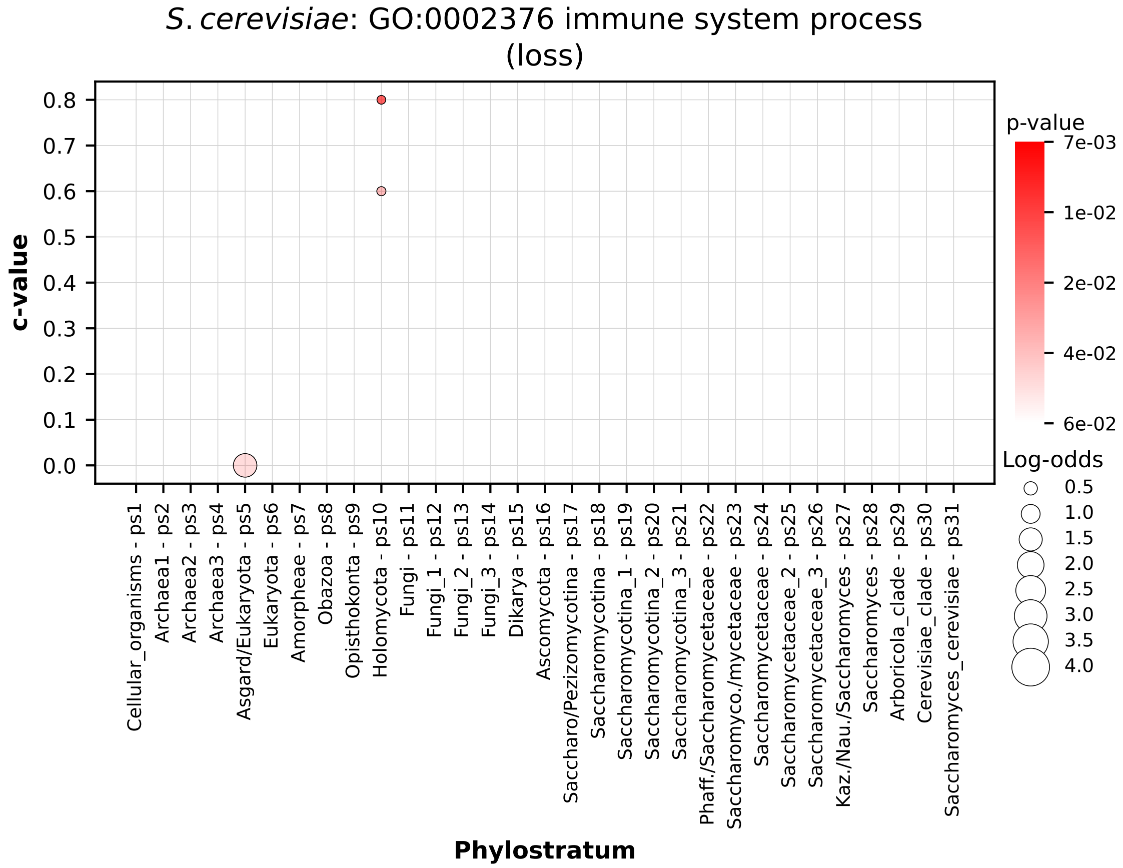
S. cerevisiae: GO:0002376 immune system process
(loss)
c-value
0.0
0.1
0.2
0.3
0.4
0.5
0.6
0.7
0.8
p-value
7e-03
1e-02
2e-02
4e-02
6e-02
Log-odds
0.5
1.0
1.5
2.0
2.5
3.0
3.5
4.0
Cellular_organisms - ps1
Archaea1 - ps2
Archaea2 - ps3
Archaea3 - ps4
Asgard/Eukaryota - ps5
Eukaryota - ps6
Amorpheae - ps7
Obazoa - ps8
Opisthokonta - ps9
Holomycota - ps10
Fungi - ps11
Fungi_1 - ps12
Fungi_2 - ps13
Fungi_3 - ps14
Dikarya - ps15
Ascomycota - ps16
Saccharo/Pezizomycotina - ps17
Saccharomycotina - ps18
Saccharomycotina_1 - ps19
Saccharomycotina_2 - ps20
Saccharomycotina_3 - ps21
Phaff./Saccharomycetaceae - ps22
Saccharomyco./mycetaceae - ps23
Saccharomycetaceae - ps24
Saccharomycetaceae_2 - ps25
Saccharomycetaceae_3 - ps26
Kaz./Nau./Saccharomyces - ps27
Saccharomyces - ps28
Arboricola_clade - ps29
Cerevisiae_clade - ps30
Saccharomyces_cerevisiae - ps31
Phylostratum

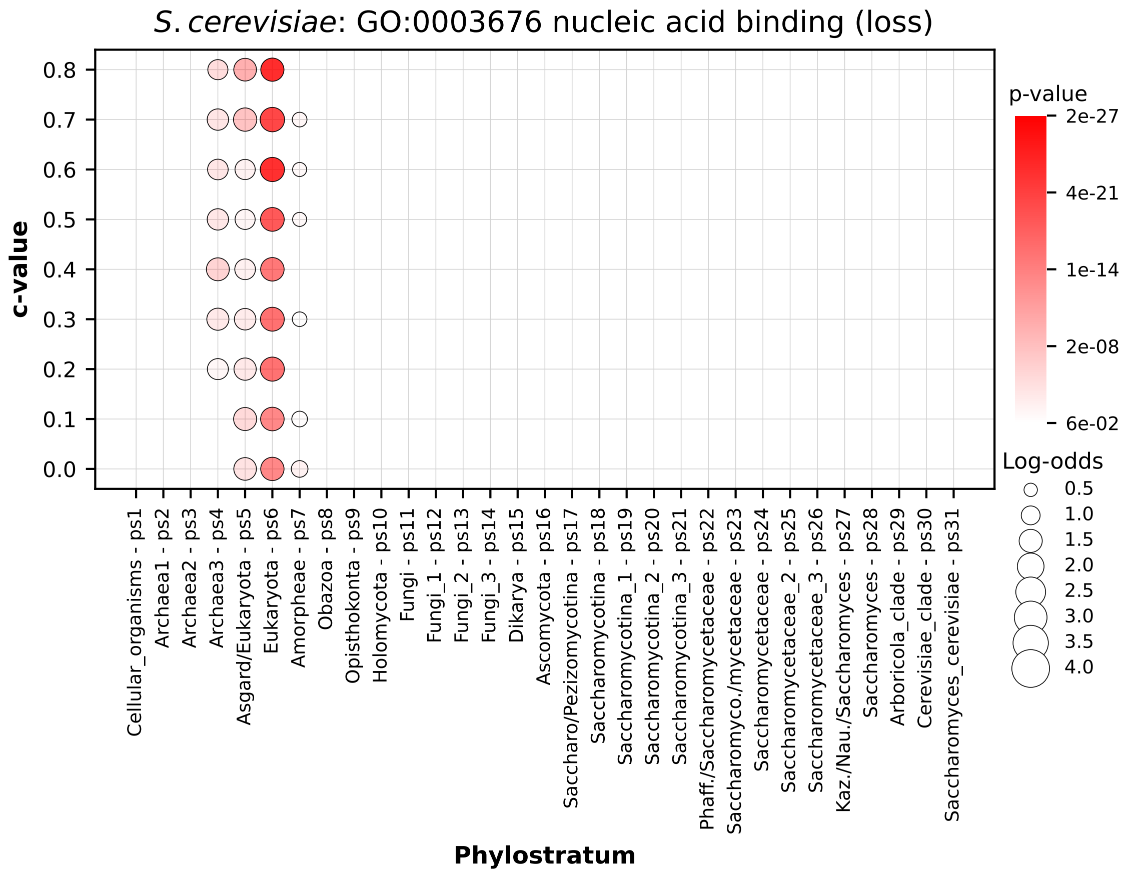
S. cerevisiae: GO:0003676 nucleic acid binding (loss)
c-value
0.8
0.7
0.6
0.5
0.4
0.3
0.2
0.1
0.0
p-value
2e-27
4e-21
1e-14
2e-08
6e-02
Log-odds
0.5
1.0
1.5
2.0
2.5
3.0
3.5
4.0
Cellular_organisms - ps1
Archaea1 - ps2
Archaea2 - ps3
Archaea3 - ps4
Asgard/Eukaryota - ps5
Eukaryota - ps6
Amorpheae - ps7
Obazoa - ps8
Opisthokonta - ps9
Holomycota - ps10
Fungi - ps11
Fungi_1 - ps12
Fungi_2 - ps13
Fungi_3 - ps14
Dikarya - ps15
Ascomycota - ps16
Saccharo/Pezizomycotina - ps17
Saccharomycotina - ps18
Saccharomycotina_1 - ps19
Saccharomycotina_2 - ps20
Saccharomycotina_3 - ps21
Phaff./Saccharomycetaceae - ps22
Saccharomyco./mycetaceae - ps23
Saccharomycetaceae - ps24
Saccharomycetaceae_2 - ps25
Saccharomycetaceae_3 - ps26
Kaz./Nau./Saccharomyces - ps27
Saccharomyces - ps28
Arboricola_clade - ps29
Cerevisiae_clade - ps30
Saccharomyces_cerevisiae - ps31
Phylostratum

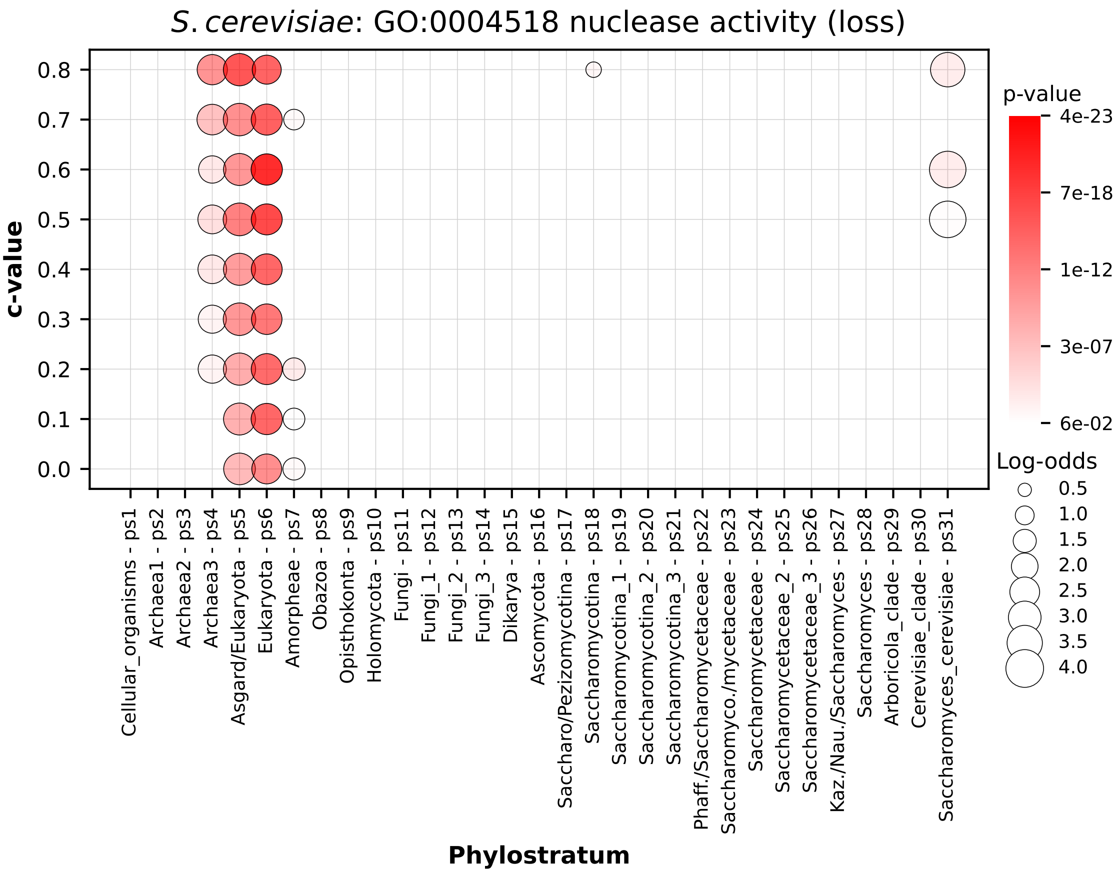
S. cerevisiae: GO:0004518 nuclease activity (loss)
c-value
0.8
0.7
0.6
0.5
0.4
0.3
0.2
0.1
0.0
p-value
4e-23
7e-18
1e-12
3e-07
6e-02
Log-odds
0.5
1.0
1.5
2.0
2.5
3.0
3.5
4.0
Cellular_organisms - ps1
Archaea1 - ps2
Archaea2 - ps3
Archaea3 - ps4
Asgard/Eukaryota - ps5
Eukaryota - ps6
Amorpheae - ps7
Obazoa - ps8
Opisthokonta - ps9
Holomycota - ps10
Fungi - ps11
Fungi_1 - ps12
Fungi_2 - ps13
Fungi_3 - ps14
Dikarya - ps15
Ascomycota - ps16
Saccharo/Pezizomycotina - ps17
Saccharomycotina - ps18
Saccharomycotina_1 - ps19
Saccharomycotina_2 - ps20
Saccharomycotina_3 - ps21
Phaff./Saccharomycetaceae - ps22
Saccharomyco./mycetaceae - ps23
Saccharomycetaceae - ps24
Saccharomycetaceae_2 - ps25
Saccharomycetaceae_3 - ps26
Kaz./Nau./Saccharomyces - ps27
Saccharomyces - ps28
Arboricola_clade - ps29
Cerevisiae_clade - ps30
Saccharomyces_cerevisiae - ps31
Phylostratum

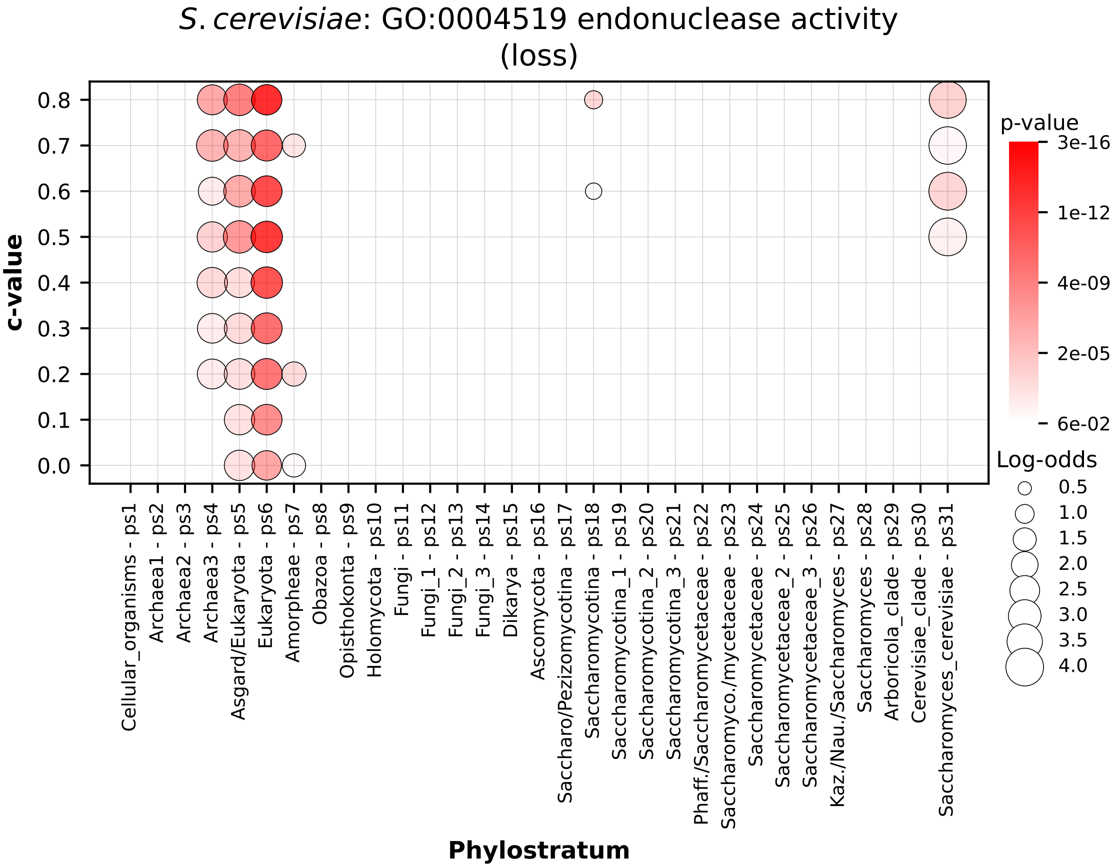

S. cerevisiae: GO:0004519 endonuclease activity
(loss)
c-value
0.8
0.7
0.6
0.5
0.4
0.3
0.2
0.1
0.0
p-value
3e-16
1e-12
4e-09
2e-05
6e-02
Log-odds
0.5
1.0
1.5
2.0
2.5
3.0
3.5
4.0
Cellular_organisms - ps1
Archaea1 - ps2
Archaea2 - ps3
Archaea3 - ps4
Asgard/Eukaryota - ps5
Eukaryota - ps6
Amorpheae - ps7
Obazoa - ps8
Opisthokonta - ps9
Holomycota - ps10
Fungi - ps11
Fungi_1 - ps12
Fungi_2 - ps13
Fungi_3 - ps14
Dikarya - ps15
Ascomycota - ps16
Saccharo/Pezizomycotina - ps17
Saccharomycotina - ps18
Saccharomycotina_1 - ps19
Saccharomycotina_2 - ps20
Saccharomycotina_3 - ps21
Phaff./Saccharomycetaceae - ps22
Saccharomyco./mycetaceae - ps23
Saccharomycetaceae - ps24
Saccharomycetaceae_2 - ps25
Saccharomycetaceae_3 - ps26
Kaz./Nau./Saccharomyces - ps27
Saccharomyces - ps28
Arboricola_clade - ps29
Cerevisiae_clade - ps30
Saccharomyces_cerevisiae - ps31
Phylostratum

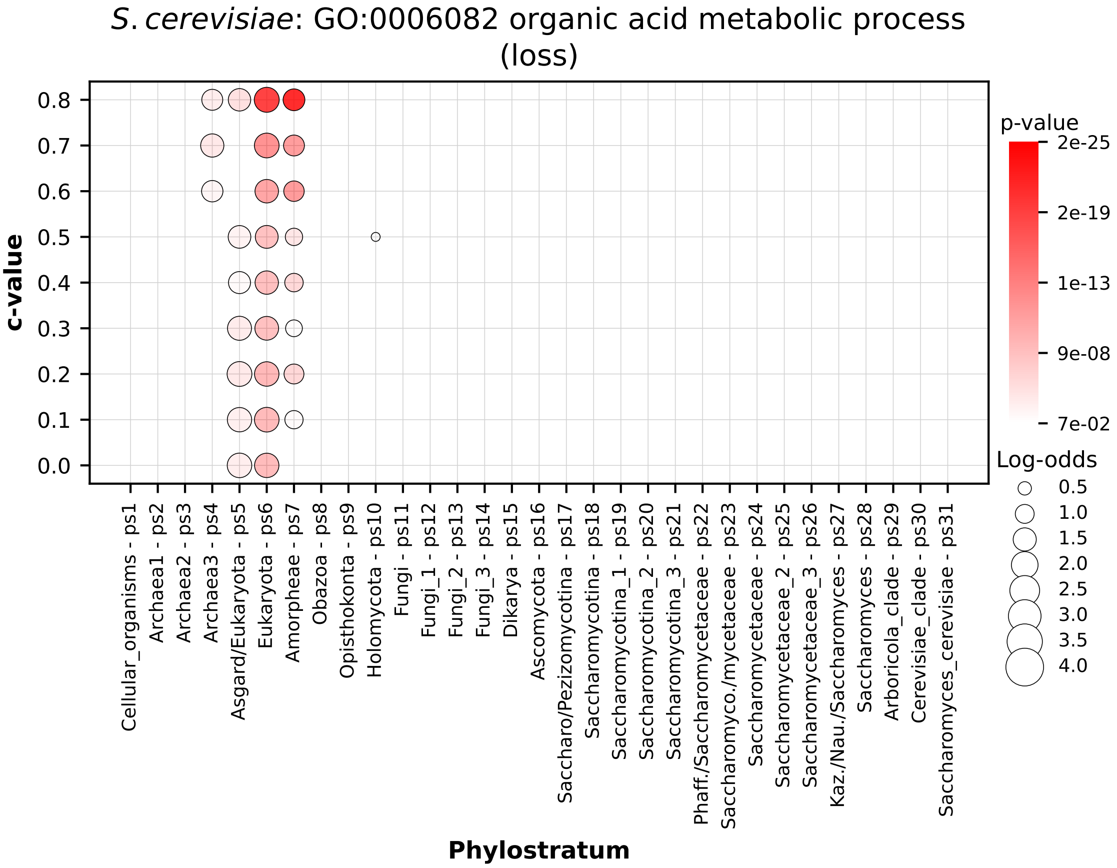

S. cerevisiae: GO:0006082 organic acid metabolic process
(loss)
c-value
0.8
0.7
0.6
0.5
0.4
0.3
0.2
0.1
0.0
p-value
2e-25
2e-19
1e-13
9e-08
7e-02
Log-odds
0.5
1.0
1.5
2.0
2.5
3.0
3.5
4.0
Cellular_organisms - ps1
Archaea1 - ps2
Archaea2 - ps3
Archaea3 - ps4
Asgard/Eukaryota - ps5
Eukaryota - ps6
Amorpheae - ps7
Obazoa - ps8
Opisthokonta - ps9
Holomycota - ps10
Fungi - ps11
Fungi_1 - ps12
Fungi_2 - ps13
Fungi_3 - ps14
Dikarya - ps15
Ascomycota - ps16
Saccharo/Pezizomycotina - ps17
Saccharomycotina - ps18
Saccharomycotina_1 - ps19
Saccharomycotina_2 - ps20
Saccharomycotina_3 - ps21
Phaff./Saccharomycetaceae - ps22
Saccharomyco./mycetaceae - ps23
Saccharomycetaceae - ps24
Saccharomycetaceae_2 - ps25
Saccharomycetaceae_3 - ps26
Kaz./Nau./Saccharomyces - ps27
Saccharomyces - ps28
Arboricola_clade - ps29
Cerevisiae_clade - ps30
Saccharomyces_cerevisiae - ps31
Phylostratum

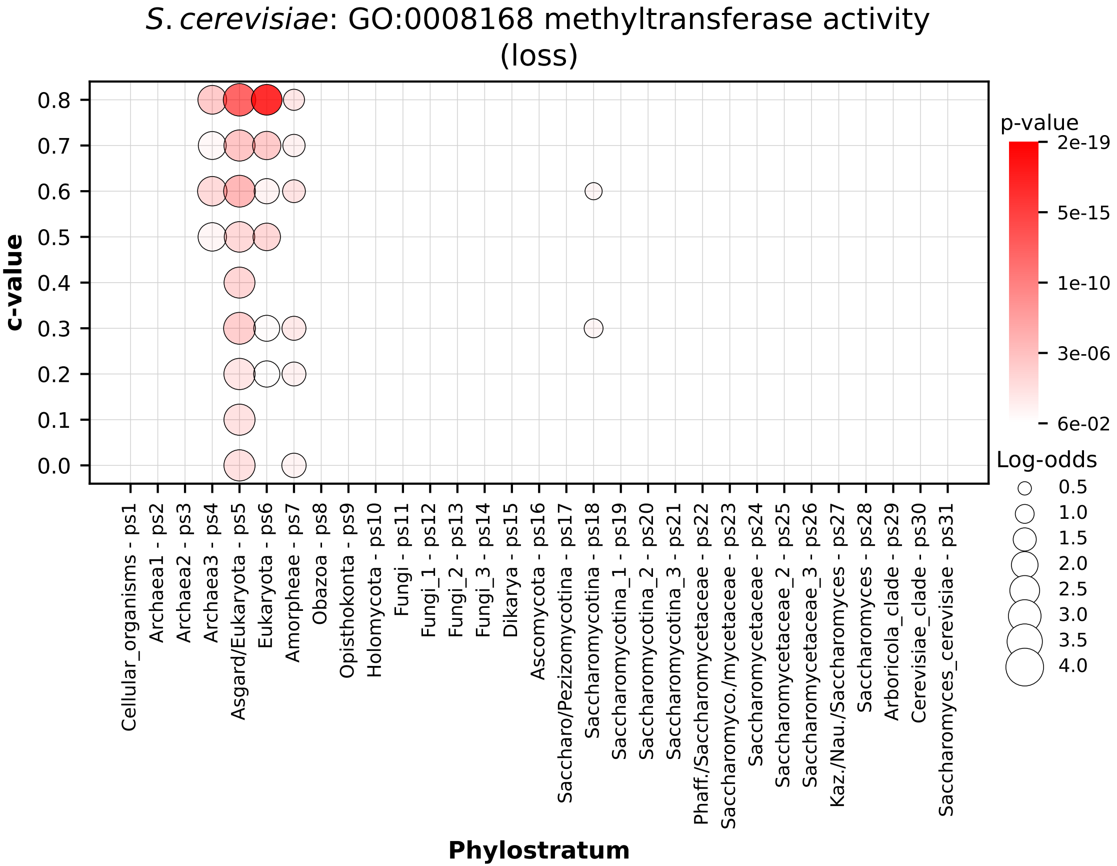
S. cerevisiae: GO:0008168 methyltransferase activity
(loss)
c-value
0.8
0.7
0.6
0.5
0.4
0.3
0.2
0.1
0.0
p-value
2e-19
5e-15
1e-10
3e-06
6e-02
Log-odds
0.5
1.0
1.5
2.0
2.5
3.0
3.5
4.0
Cellular_organisms - ps1
Archaea1 - ps2
Archaea2 - ps3
Archaea3 - ps4
Asgard/Eukaryota - ps5
Eukaryota - ps6
Amorpheae - ps7
Obazoa - ps8
Opisthokonta - ps9
Holomycota - ps10
Fungi - ps11
Fungi_1 - ps12
Fungi_2 - ps13
Fungi_3 - ps14
Dikarya - ps15
Ascomycota - ps16
Saccharo/Pezizomycotina - ps17
Saccharomycotina - ps18
Saccharomycotina_1 - ps19
Saccharomycotina_2 - ps20
Saccharomycotina_3 - ps21
Phaff./Saccharomycetaceae - ps22
Saccharomyco./mycetaceae - ps23
Saccharomycetaceae - ps24
Saccharomycetaceae_2 - ps25
Saccharomycetaceae_3 - ps26
Kaz./Nau./Saccharomyces - ps27
Saccharomyces - ps28
Arboricola_clade - ps29
Cerevisiae_clade - ps30
Saccharomyces_cerevisiae - ps31
Phylostratum

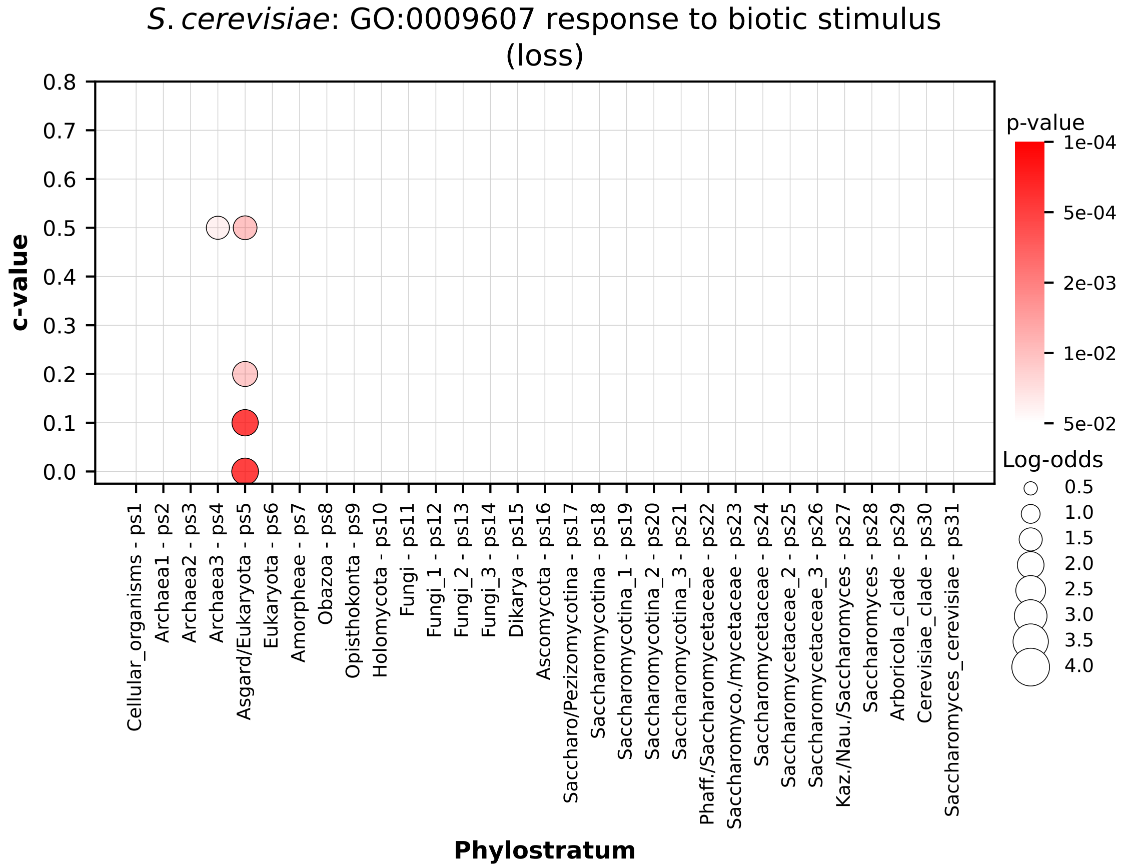
S. cerevisiae: GO:0009607 response to biotic stimulus (loss)
c-value
0.8
0.7
0.6
0.5
0.4
0.3
0.2
0.1
0.0
p-value
1e-04
5e-04
2e-03
1e-02
5e-02
Log-odds
0.5
1.0
1.5
2.0
2.5
3.0
3.5
4.0
Cellular_organisms - ps1
Archaea1 - ps2
Archaea2 - ps3
Archaea3 - ps4
Asgard/Eukaryota - ps5
Eukaryota - ps6
Amorpheae - ps7
Obazoa - ps8
Opisthokonta - ps9
Holomycota - ps10
Fungi - ps11
Fungi_1 - ps12
Fungi_2 - ps13
Fungi_3 - ps14
Dikarya - ps15
Ascomycota - ps16
Saccharo/Pezizomycotina - ps17
Saccharomycotina - ps18
Saccharomycotina_1 - ps19
Saccharomycotina_2 - ps20
Saccharomycotina_3 - ps21
Phaff./Saccharomycetaceae - ps22
Saccharomyco./mycetaceae - ps23
Saccharomycetaceae - ps24
Saccharomycetaceae_2 - ps25
Saccharomycetaceae_3 - ps26
Kaz./Nau./Saccharomyces - ps27
Saccharomyces - ps28
Arboricola_clade - ps29
Cerevisiae_clade - ps30
Saccharomyces_cerevisiae - ps31
Phylostratum

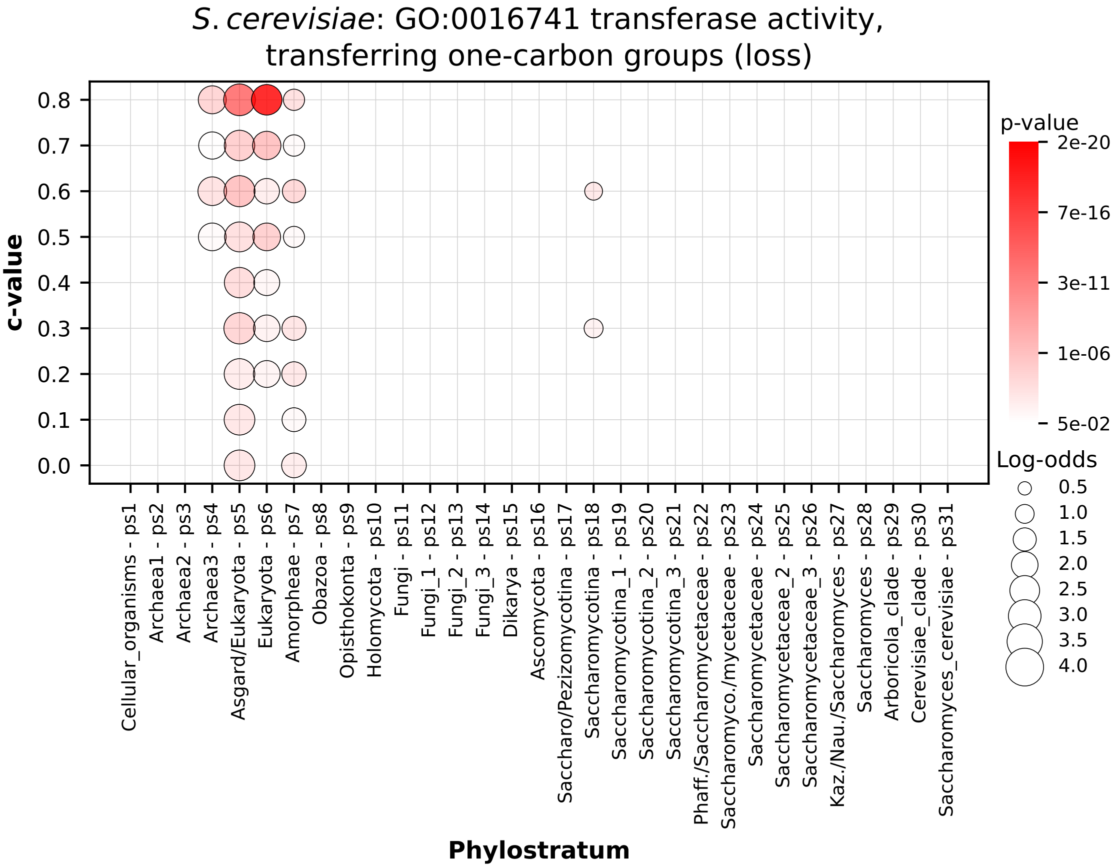

S. cerevisiae: GO:0016741 transferase activity, transferring one-carbon groups (loss)
c-value
0.8
0.7
0.6
0.5
0.4
0.3
0.2
0.1
0.0
p-value
2e-20
7e-16
3e-11
1e-06
5e-02
Log-odds
0.5
1.0
1.5
2.0
2.5
3.0
3.5
4.0
Cellular_organisms - ps1
Archaea1 - ps2
Archaea2 - ps3
Archaea3 - ps4
Asgard/Eukaryota - ps5
Eukaryota - ps6
Amorpheae - ps7
Obazoa - ps8
Opisthokonta - ps9
Holomycota - ps10
Fungi - ps11
Fungi_1 - ps12
Fungi_2 - ps13
Fungi_3 - ps14
Dikarya - ps15
Ascomycota - ps16
Saccharo/Pezizomycotina - ps17
Saccharomycotina - ps18
Saccharomycotina_1 - ps19
Saccharomycotina_2 - ps20
Saccharomycotina_3 - ps21
Phaff./Saccharomycetaceae - ps22
Saccharomyco./mycetaceae - ps23
Saccharomycetaceae - ps24
Saccharomycetaceae_2 - ps25
Saccharomycetaceae_3 - ps26
Kaz./Nau./Saccharomyces - ps27
Saccharomyces - ps28
Arboricola_clade - ps29
Cerevisiae_clade - ps30
Saccharomyces_cerevisiae - ps31
Phylostratum

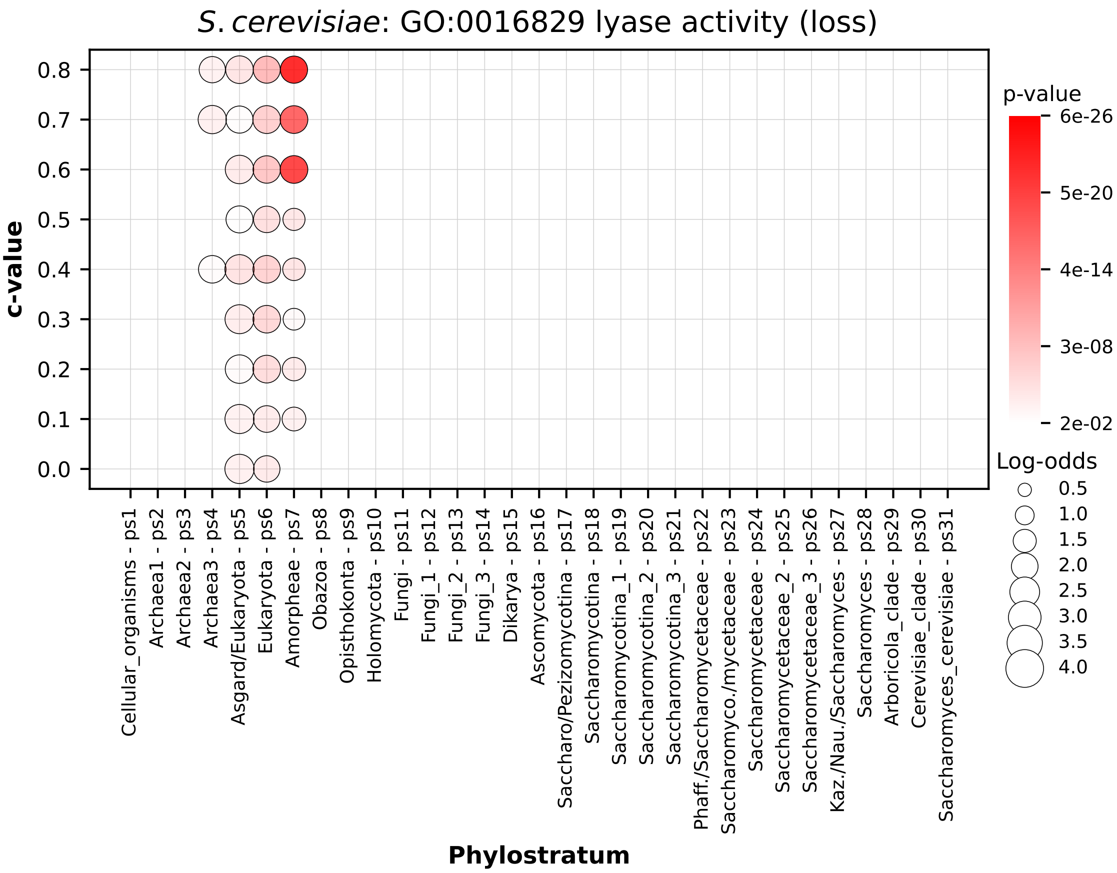

S. cerevisiae: GO:0016829 lyase activity (loss)
c-value
0.8
0.7
0.6
0.5
0.4
0.3
0.2
0.1
0.0
p-value
6e-26
5e-20
4e-14
3e-08
2e-02
Log-odds
0.5
1.0
1.5
2.0
2.5
3.0
3.5
4.0
Cellular_organisms - ps1
Archaea1 - ps2
Archaea2 - ps3
Archaea3 - ps4
Asgard/Eukaryota - ps5
Eukaryota - ps6
Amorpheae - ps7
Obazoa - ps8
Opisthokonta - ps9
Holomycota - ps10
Fungi - ps11
Fungi_1 - ps12
Fungi_2 - ps13
Fungi_3 - ps14
Dikarya - ps15
Ascomycota - ps16
Saccharo/Pezizomycotina - ps17
Saccharomycotina - ps18
Saccharomycotina_1 - ps19
Saccharomycotina_2 - ps20
Saccharomycotina_3 - ps21
Phaff./Saccharomycetaceae - ps22
Saccharomyco./mycetaceae - ps23
Saccharomycetaceae - ps24
Saccharomycetaceae_2 - ps25
Saccharomycetaceae_3 - ps26
Kaz./Nau./Saccharomyces - ps27
Saccharomyces - ps28
Arboricola_clade - ps29
Cerevisiae_clade - ps30
Saccharomyces_cerevisiae - ps31
Phylostratum

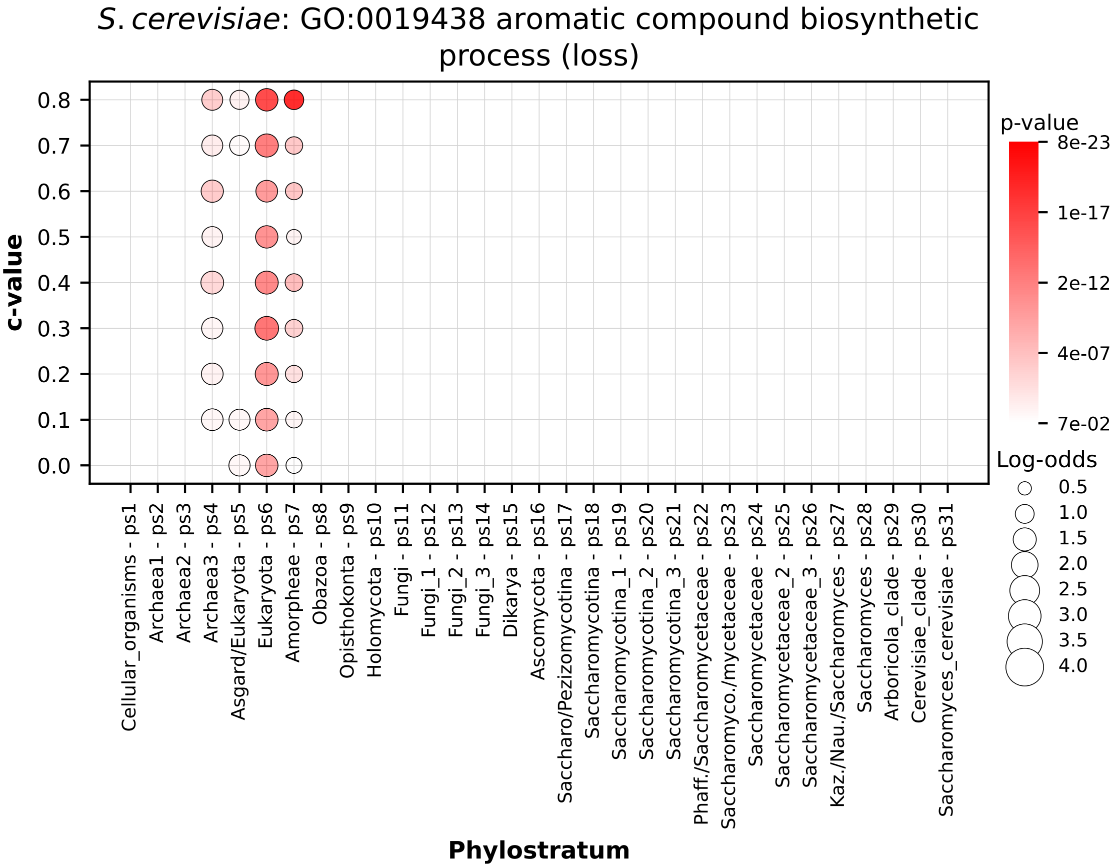
S. cerevisiae: GO:0019438 aromatic compound biosynthetic process (loss)
c-value
0.8
0.7
0.6
0.5
0.4
0.3
0.2
0.1
0.0
p-value
8e-23
1e-17
2e-12
4e-07
7e-02
Log-odds
0.5
1.0
1.5
2.0
2.5
3.0
3.5
4.0
Cellular_organisms - ps1
Archaea1 - ps2
Archaea2 - ps3
Archaea3 - ps4
Asgard/Eukaryota - ps5
Eukaryota - ps6
Amorpheae - ps7
Obazoa - ps8
Opisthokonta - ps9
Holomycota - ps10
Fungi - ps11
Fungi_1 - ps12
Fungi_2 - ps13
Fungi_3 - ps14
Dikarya - ps15
Ascomycota - ps16
Saccharo/Pezizomycotina - ps17
Saccharomycotina - ps18
Saccharomycotina_1 - ps19
Saccharomycotina_2 - ps20
Saccharomycotina_3 - ps21
Phaff./Saccharomycetaceae - ps22
Saccharomyco./mycetaceae - ps23
Saccharomycetaceae - ps24
Saccharomycetaceae_2 - ps25
Saccharomycetaceae_3 - ps26
Kaz./Nau./Saccharomyces - ps27
Saccharomyces - ps28
Arboricola_clade - ps29
Cerevisiae_clade - ps30
Saccharomyces_cerevisiae - ps31
Phylostratum

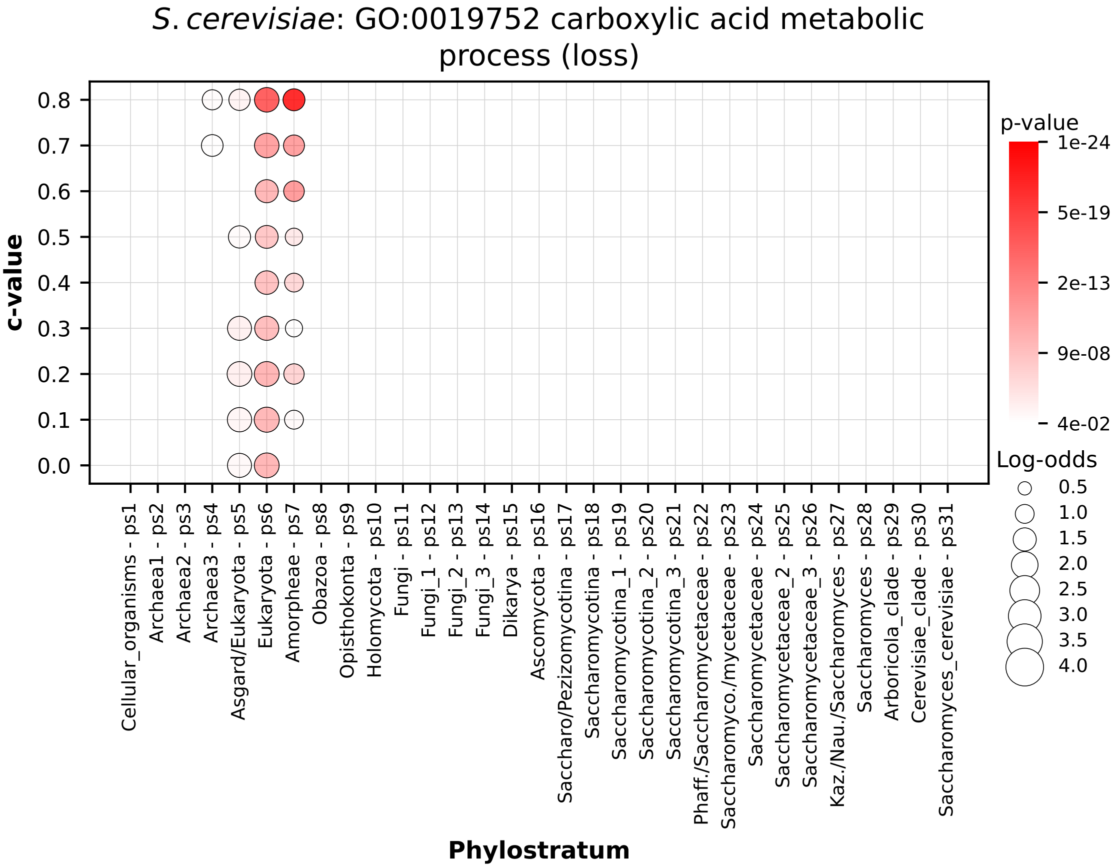

S. cerevisiae: GO:0019752 carboxylic acid metabolic process (loss)
c-value
0.8
0.7
0.6
0.5
0.4
0.3
0.2
0.1
0.0
Phylostratum
Cellular_organisms - ps1
Archaea1 - ps2
Archaea2 - ps3
Archaea3 - ps4
Asgard/Eukaryota - ps5
Eukaryota - ps6
Amorpheae - ps7
Obazoa - ps8
Opisthokonta - ps9
Holomycota - ps10
Fungi - ps11
Fungi_1 - ps12
Fungi_2 - ps13
Fungi_3 - ps14
Dikarya - ps15
Ascomycota - ps16
Saccharo/Pezizomycotina - ps17
Saccharomycotina - ps18
Saccharomycotina_1 - ps19
Saccharomycotina_2 - ps20
Saccharomycotina_3 - ps21
Phaff./Saccharomycetaceae - ps22
Saccharomyco./mycetaceae - ps23
Saccharomycetaceae - ps24
Saccharomycetaceae_2 - ps25
Saccharomycetaceae_3 - ps26
Kaz./Nau./Saccharomyces - ps27
Saccharomyces - ps28
Arboricola_clade - ps29
Cerevisiae_clade - ps30
Saccharomyces_cerevisiae - ps31
p-value
1e-24
5e-19
2e-13
9e-08
4e-02
Log-odds
0.5
1.0
1.5
2.0
2.5
3.0
3.5
4.0

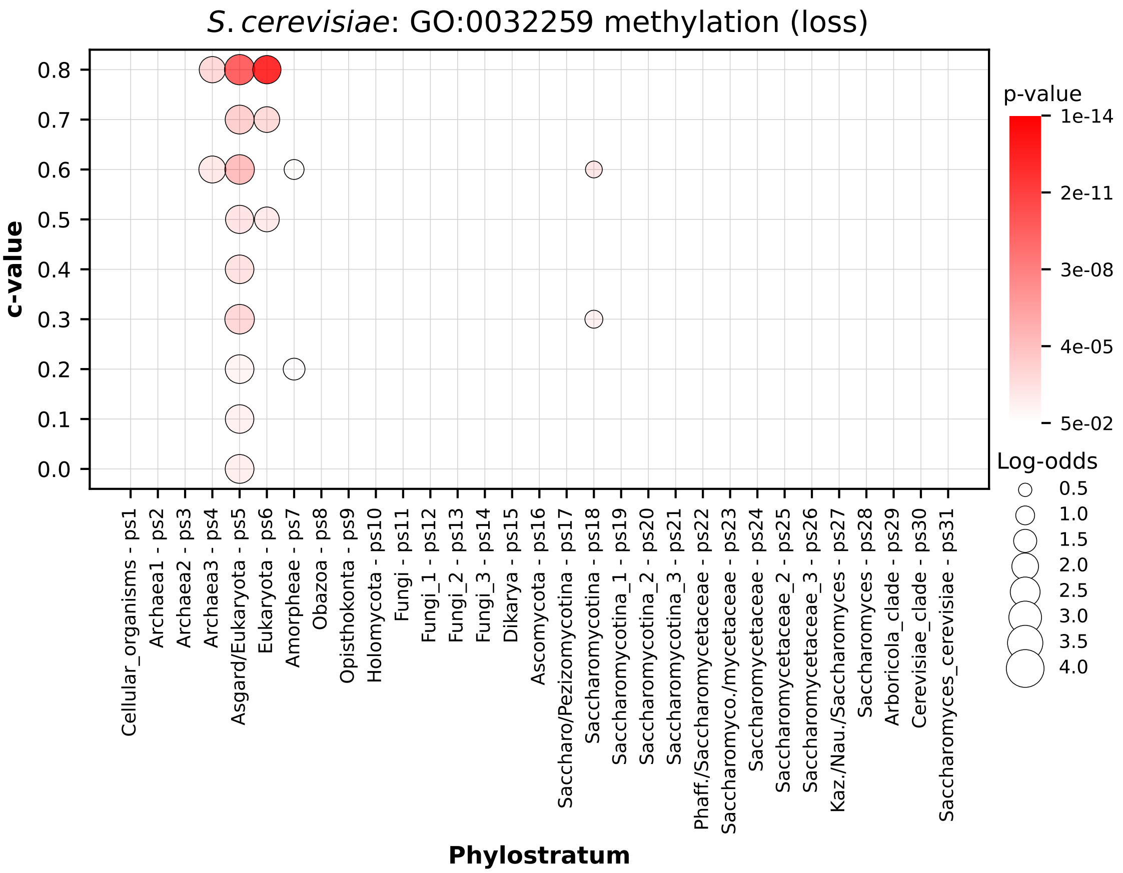

S. cerevisiae: GO:0032259 methylation (loss)
c-value
0.8
0.7
0.6
0.5
0.4
0.3
0.2
0.1
0.0
Phylostratum
Cellular_organisms - ps1
Archaea1 - ps2
Archaea2 - ps3
Archaea3 - ps4
Asgard/Eukaryota - ps5
Eukaryota - ps6
Amorpheae - ps7
Obazoa - ps8
Opisthokonta - ps9
Holomycota - ps10
Fungi - ps11
Fungi_1 - ps12
Fungi_2 - ps13
Fungi_3 - ps14
Dikarya - ps15
Ascomycota - ps16
Saccharo/Pezizomycotina - ps17
Saccharomycotina - ps18
Saccharomycotina_1 - ps19
Saccharomycotina_2 - ps20
Saccharomycotina_3 - ps21
Phaff./Saccharomycetaceae - ps22
Saccharomyco./mycetaceae - ps23
Saccharomycetaceae - ps24
Saccharomycetaceae_2 - ps25
Saccharomycetaceae_3 - ps26
Kaz./Nau./Saccharomyces - ps27
Saccharomyces - ps28
Arboricola_clade - ps29
Cerevisiae_clade - ps30
Saccharomyces_cerevisiae - ps31
p-value
1e-14
2e-11
3e-08
4e-05
5e-02
Log-odds
0.5
1.0
1.5
2.0
2.5
3.0
3.5
4.0

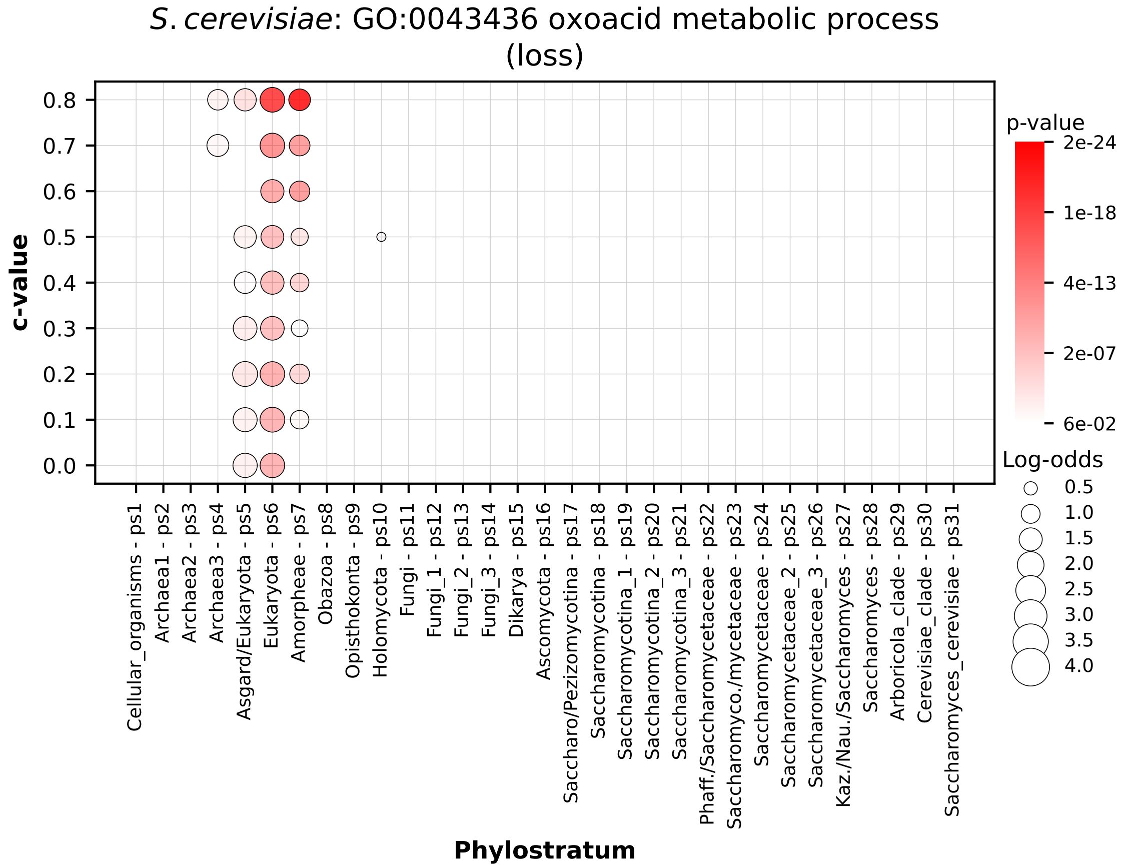

$S. cerevisiae$: GO:0043436 oxoacid metabolic process (loss)
c-value
0.8
0.7
0.6
0.5
0.4
0.3
0.2
0.1
0.0
p-value
2e-24
1e-18
4e-13
2e-07
6e-02
Log-odds
0.5
1.0
1.5
2.0
2.5
3.0
3.5
4.0
Cellular_organisms - ps1
Archaea1 - ps2
Archaea2 - ps3
Archaea3 - ps4
Asgard/Eukaryota - ps5
Eukaryota - ps6
Amorpheae - ps7
Obazoa - ps8
Opisthokonta - ps9
Holomycota - ps10
Fungi - ps11
Fungi_1 - ps12
Fungi_2 - ps13
Fungi_3 - ps14
Dikarya - ps15
Ascomycota - ps16
Saccharo/Pezizomycotina - ps17
Saccharomycotina - ps18
Saccharomycotina_1 - ps19
Saccharomycotina_2 - ps20
Saccharomycotina_3 - ps21
Phaff./Saccharomycetaceae - ps22
Saccharomyco./mycetaceae - ps23
Saccharomycetaceae - ps24
Saccharomycetaceae_2 - ps25
Saccharomycetaceae_3 - ps26
Kaz./Nau./Saccharomyces - ps27
Saccharomyces - ps28
Arboricola_clade - ps29
Cerevisiae_clade - ps30
Saccharomyces_cerevisiae - ps31
Phylostratum

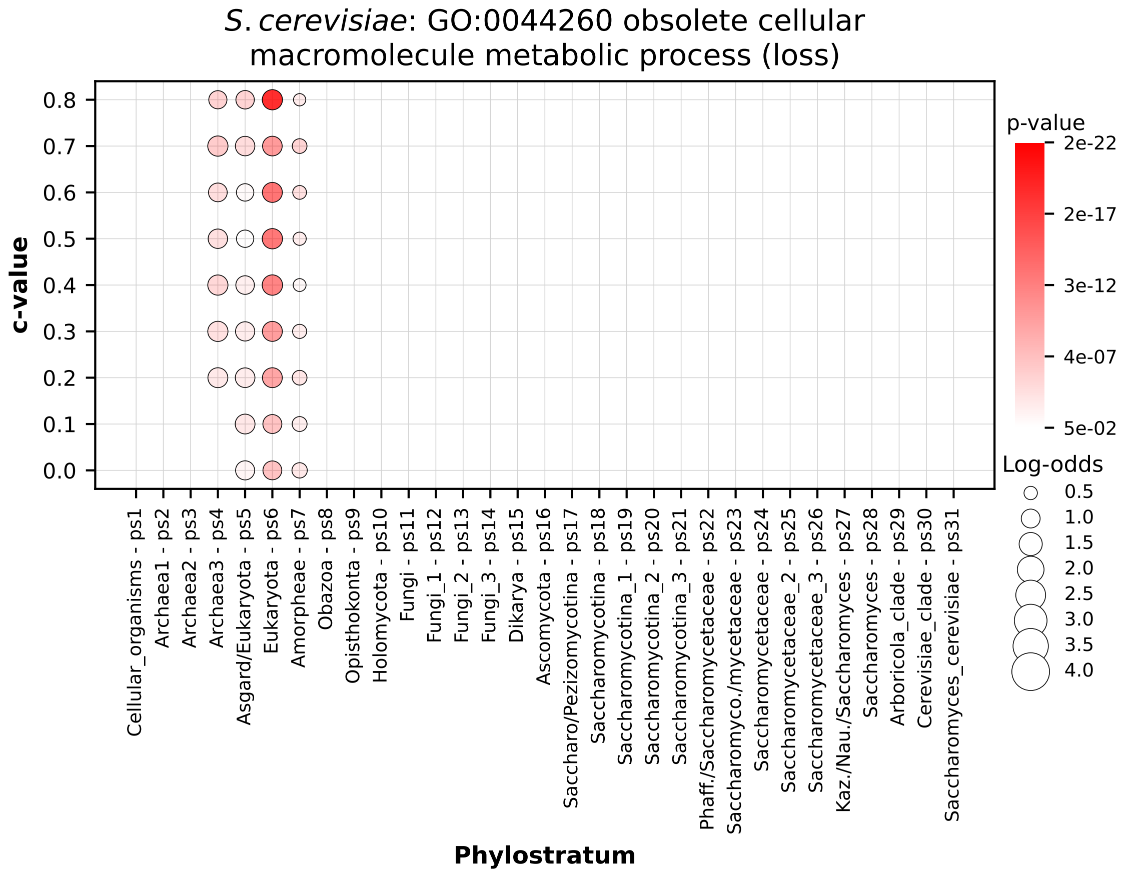
S. cerevisiae: GO:0044260 obsolete cellular macromolecule metabolic process (loss)
c-value
0.8
0.7
0.6
0.5
0.4
0.3
0.2
0.1
0.0
p-value
2e-22
2e-17
3e-12
4e-07
5e-02
Log-odds
0.5
1.0
1.5
2.0
2.5
3.0
3.5
4.0
Cellular_organisms - ps1
Archaea1 - ps2
Archaea2 - ps3
Archaea3 - ps4
Asgard/Eukaryota - ps5
Eukaryota - ps6
Amorpheae - ps7
Obazoa - ps8
Opisthokonta - ps9
Holomycota - ps10
Fungi - ps11
Fungi_1 - ps12
Fungi_2 - ps13
Fungi_3 - ps14
Dikarya - ps15
Ascomycota - ps16
Saccharo/Pezizomycotina - ps17
Saccharomycotina - ps18
Saccharomycotina_1 - ps19
Saccharomycotina_2 - ps20
Saccharomycotina_3 - ps21
Phaff./Saccharomycetaceae - ps22
Saccharomyco./mycetaceae - ps23
Saccharomycetaceae - ps24
Saccharomycetaceae_2 - ps25
Saccharomycetaceae_3 - ps26
Kaz./Nau./Saccharomyces - ps27
Saccharomyces - ps28
Arboricola_clade - ps29
Cerevisiae_clade - ps30
Saccharomyces_cerevisiae - ps31
Phylostratum

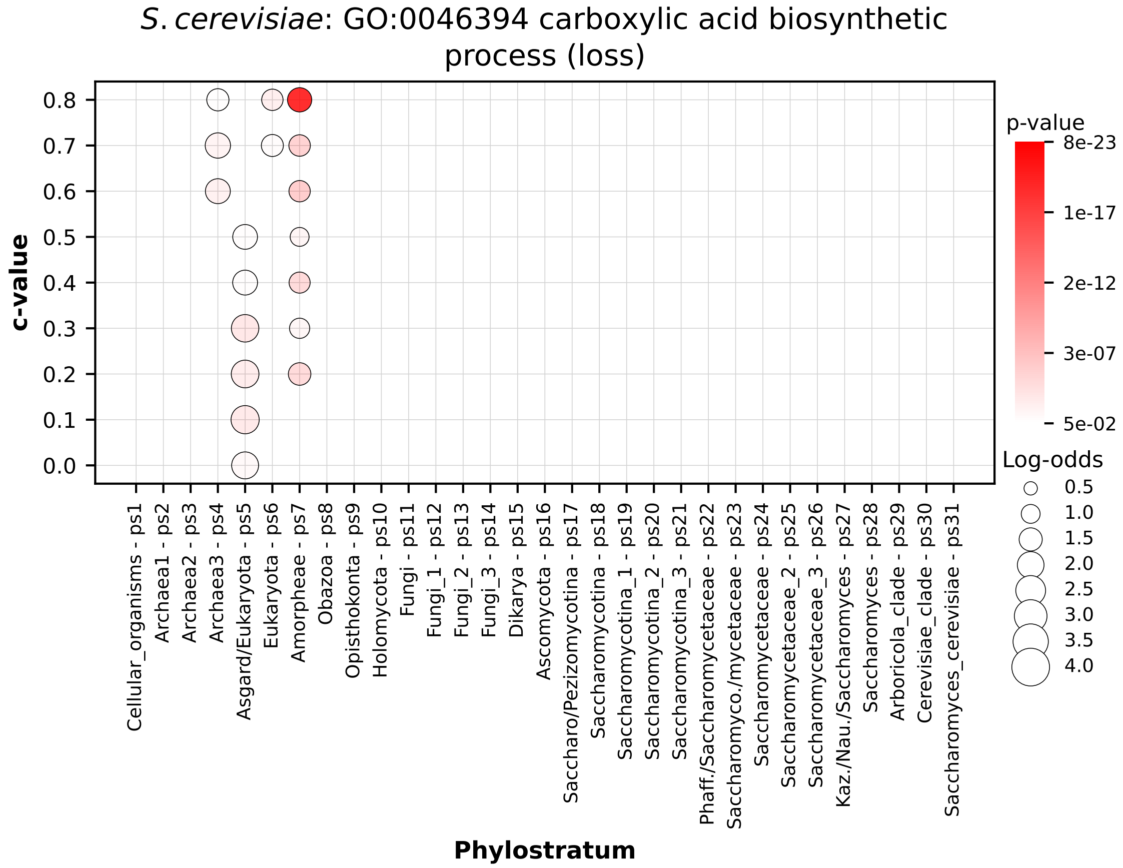
S. cerevisiae: GO:0046394 carboxylic acid biosynthetic process (loss)
c-value
0.8
0.7
0.6
0.5
0.4
0.3
0.2
0.1
0.0
Phylostratum
Cellular_organisms - ps1
Archaea1 - ps2
Archaea2 - ps3
Archaea3 - ps4
Asgard/Eukaryota - ps5
Eukaryota - ps6
Amorpheae - ps7
Obazoa - ps8
Opisthokonta - ps9
Holomycota - ps10
Fungi - ps11
Fungi_1 - ps12
Fungi_2 - ps13
Fungi_3 - ps14
Dikarya - ps15
Ascomycota - ps16
Saccharo/Pezizomycotina - ps17
Saccharomycotina - ps18
Saccharomycotina_1 - ps19
Saccharomycotina_2 - ps20
Saccharomycotina_3 - ps21
Phaff./Saccharomycetaceae - ps22
Saccharomyco./mycetaceae - ps23
Saccharomycetaceae - ps24
Saccharomycetaceae_2 - ps25
Saccharomycetaceae_3 - ps26
Kaz./Nau./Saccharomyces - ps27
Saccharomyces - ps28
Arboricola_clade - ps29
Cerevisiae_clade - ps30
Saccharomyces_cerevisiae - ps31
p-value
8e-23
1e-17
2e-12
3e-07
5e-02
Log-odds
0.5
1.0
1.5
2.0
2.5
3.0
3.5
4.0

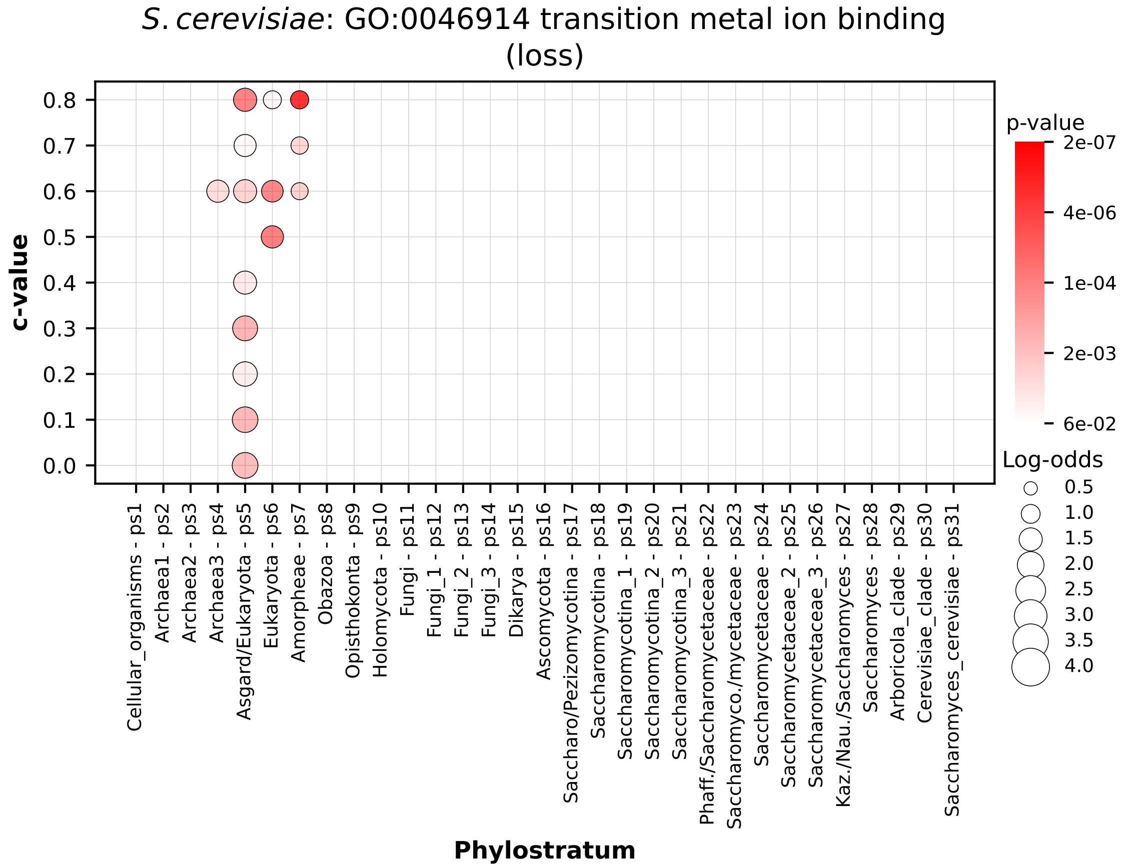

S. cerevisiae: GO:0046914 transition metal ion binding
(loss)
c-value
0.8
0.7
0.6
0.5
0.4
0.3
0.2
0.1
0.0
p-value
2e-07
4e-06
1e-04
2e-03
6e-02
Log-odds
0.5
1.0
1.5
2.0
2.5
3.0
3.5
4.0
Cellular_organisms - ps1
Archaea1 - ps2
Archaea2 - ps3
Archaea3 - ps4
Asgard/Eukaryota - ps5
Eukaryota - ps6
Amorpheae - ps7
Obazoa - ps8
Opisthokonta - ps9
Holomycota - ps10
Fungi - ps11
Fungi_1 - ps12
Fungi_2 - ps13
Fungi_3 - ps14
Dikarya - ps15
Ascomycota - ps16
Saccharo/Pezizomycotina - ps17
Saccharomycotina - ps18
Saccharomycotina_1 - ps19
Saccharomycotina_2 - ps20
Saccharomycotina_3 - ps21
Phaff./Saccharomycetaceae - ps22
Saccharomyco./mycetaceae - ps23
Saccharomycetaceae - ps24
Saccharomycetaceae_2 - ps25
Saccharomycetaceae_3 - ps26
Kaz./Nau./Saccharomyces - ps27
Saccharomyces - ps28
Arboricola_clade - ps29
Cerevisiae_clade - ps30
Saccharomyces_cerevisiae - ps31
Phylostratum

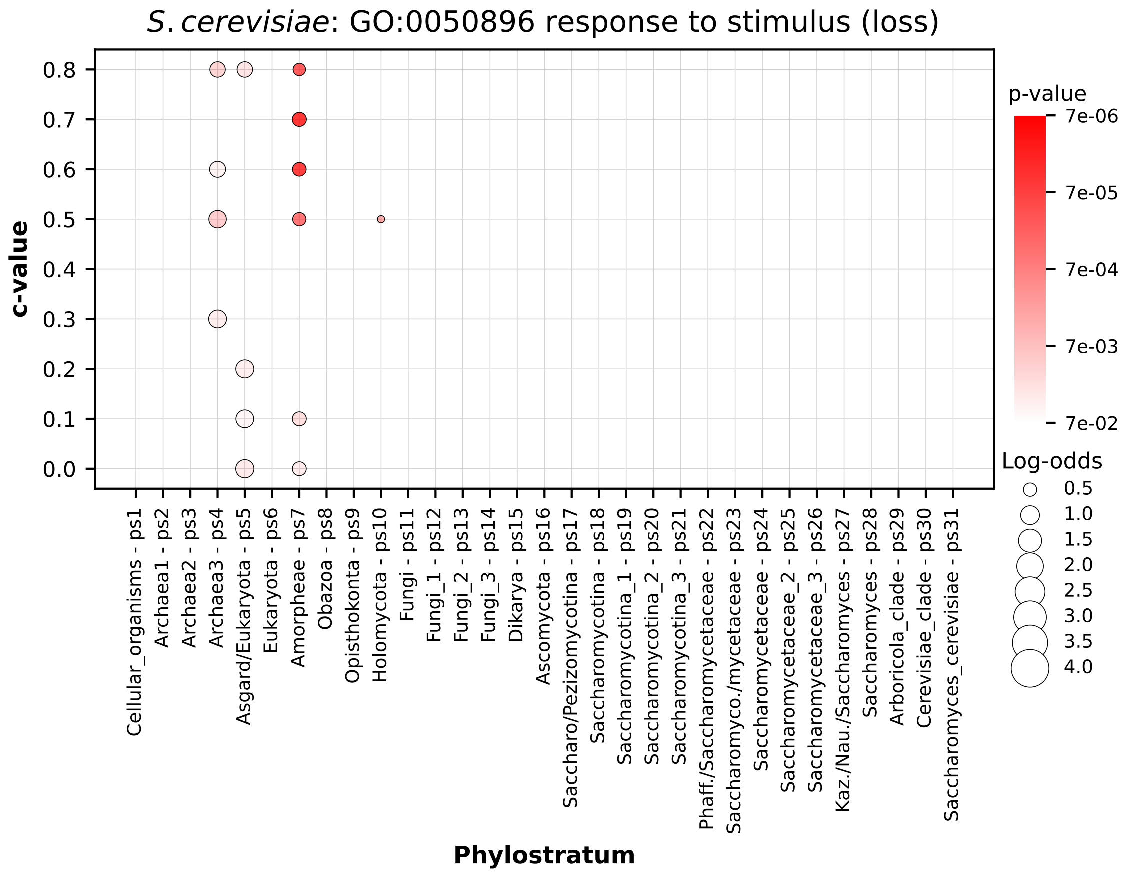
S. cerevisiae: GO:0050896 response to stimulus (loss)
c-value
0.8
0.7
0.6
0.5
0.4
0.3
0.2
0.1
0.0
Phylostratum
Cellular_organisms - ps1
Archaea1 - ps2
Archaea2 - ps3
Archaea3 - ps4
Asgard/Eukaryota - ps5
Eukaryota - ps6
Amorpheae - ps7
Obazoa - ps8
Opisthokonta - ps9
Holomycota - ps10
Fungi - ps11
Fungi_1 - ps12
Fungi_2 - ps13
Fungi_3 - ps14
Dikarya - ps15
Ascomycota - ps16
Saccharo/Pezizomycotina - ps17
Saccharomycotina - ps18
Saccharomycotina_1 - ps19
Saccharomycotina_2 - ps20
Saccharomycotina_3 - ps21
Phaff./Saccharomycetaceae - ps22
Saccharomyco./mycetaceae - ps23
Saccharomycetaceae - ps24
Saccharomycetaceae_2 - ps25
Saccharomycetaceae_3 - ps26
Kaz./Nau./Saccharomyces - ps27
Saccharomyces - ps28
Arboricola_clade - ps29
Cerevisiae_clade - ps30
Saccharomyces_cerevisiae - ps31
p-value
7e-06
7e-05
7e-04
7e-03
7e-02
Log-odds
0.5
1.0
1.5
2.0
2.5
3.0
3.5
4.0

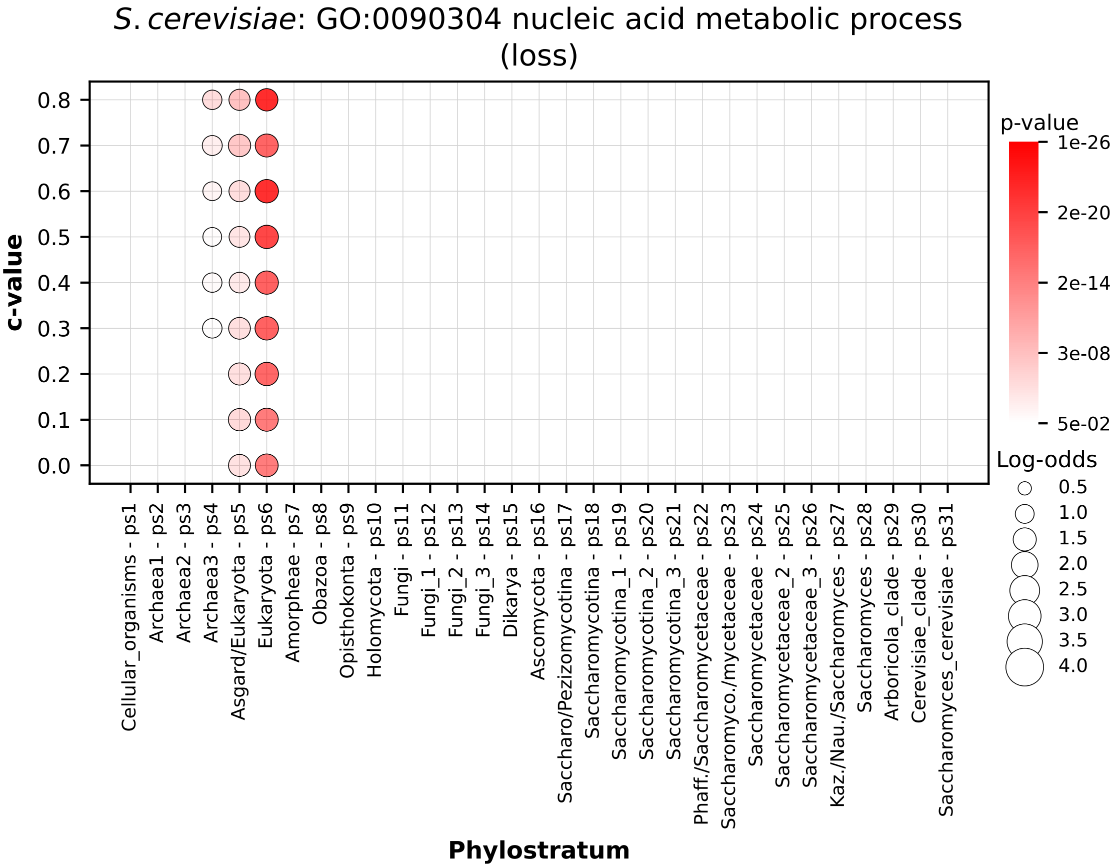

S. cerevisiae: GO:0090304 nucleic acid metabolic process
(loss)
c-value
0.8
0.7
0.6
0.5
0.4
0.3
0.2
0.1
0.0
Phylostratum
Cellular_organisms - ps1
Archaea1 - ps2
Archaea2 - ps3
Archaea3 - ps4
Asgard/Eukaryota - ps5
Eukaryota - ps6
Amorpheae - ps7
Obazoa - ps8
Opisthokonta - ps9
Holomycota - ps10
Fungi - ps11
Fungi_1 - ps12
Fungi_2 - ps13
Fungi_3 - ps14
Dikarya - ps15
Ascomycota - ps16
Saccharo/Pezizomycotina - ps17
Saccharomycotina - ps18
Saccharomycotina_1 - ps19
Saccharomycotina_2 - ps20
Saccharomycotina_3 - ps21
Phaff./Saccharomycetaceae - ps22
Saccharomyco./mycetaceae - ps23
Saccharomycetaceae - ps24
Saccharomycetaceae_2 - ps25
Saccharomycetaceae_3 - ps26
Kaz./Nau./Saccharomyces - ps27
Saccharomyces - ps28
Arboricola_clade - ps29
Cerevisiae_clade - ps30
Saccharomyces_cerevisiae - ps31
p-value
1e-26
2e-20
2e-14
3e-08
5e-02
Log-odds
0.5
1.0
1.5
2.0
2.5
3.0
3.5
4.0

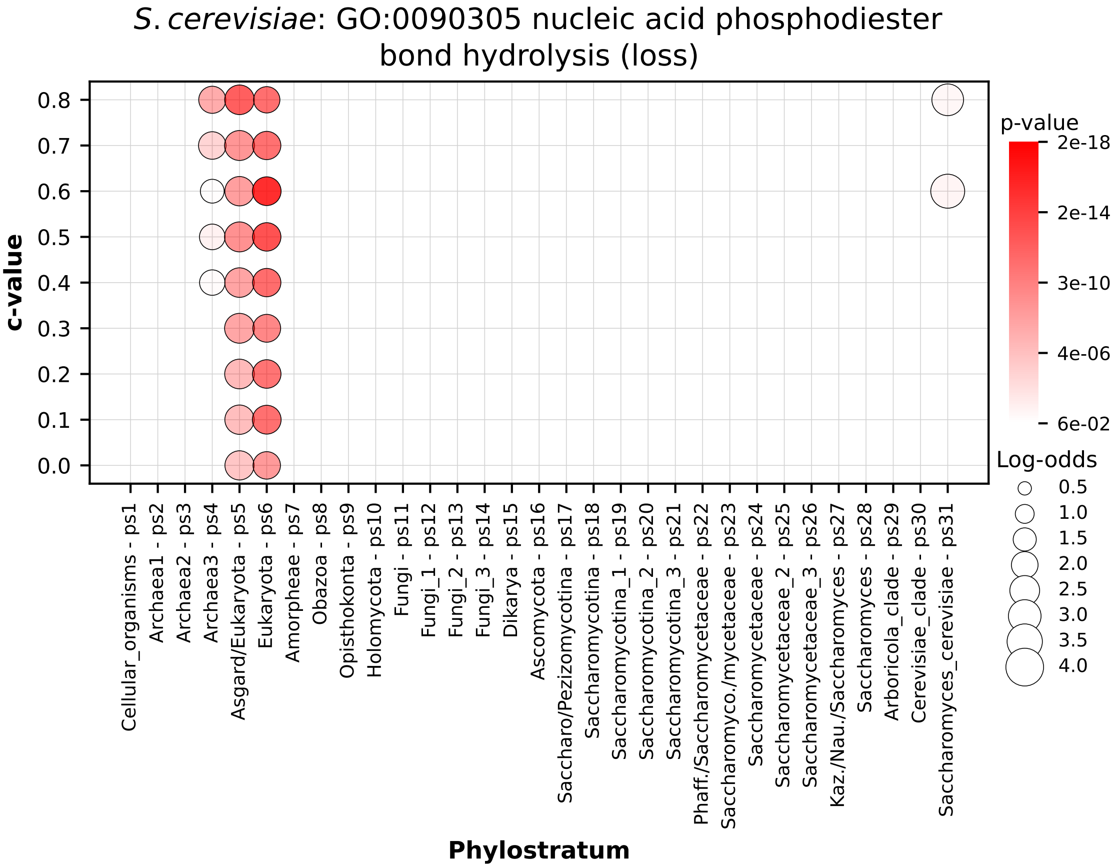

S. cerevisiae: GO:0090305 nucleic acid phosphodiester bond hydrolysis (loss)
c-value
0.8
0.7
0.6
0.5
0.4
0.3
0.2
0.1
0.0
p-value
2e-18
2e-14
3e-10
4e-06
6e-02
Log-odds
0.5
1.0
1.5
2.0
2.5
3.0
3.5
4.0
Cellular_organisms - ps1
Archaea1 - ps2
Archaea2 - ps3
Archaea3 - ps4
Asgard/Eukaryota - ps5
Eukaryota - ps6
Amorpheae - ps7
Obazoa - ps8
Opisthokonta - ps9
Holomycota - ps10
Fungi - ps11
Fungi_1 - ps12
Fungi_2 - ps13
Fungi_3 - ps14
Dikarya - ps15
Ascomycota - ps16
Saccharo/Pezizomycotina - ps17
Saccharomycotina - ps18
Saccharomycotina_1 - ps19
Saccharomycotina_2 - ps20
Saccharomycotina_3 - ps21
Phaff./Saccharomycetaceae - ps22
Saccharomyco./mycetaceae - ps23
Saccharomycetaceae - ps24
Saccharomycetaceae_2 - ps25
Saccharomycetaceae_3 - ps26
Kaz./Nau./Saccharomyces - ps27
Saccharomyces - ps28
Arboricola_clade - ps29
Cerevisiae_clade - ps30
Saccharomyces_cerevisiae - ps31
Phylostratum

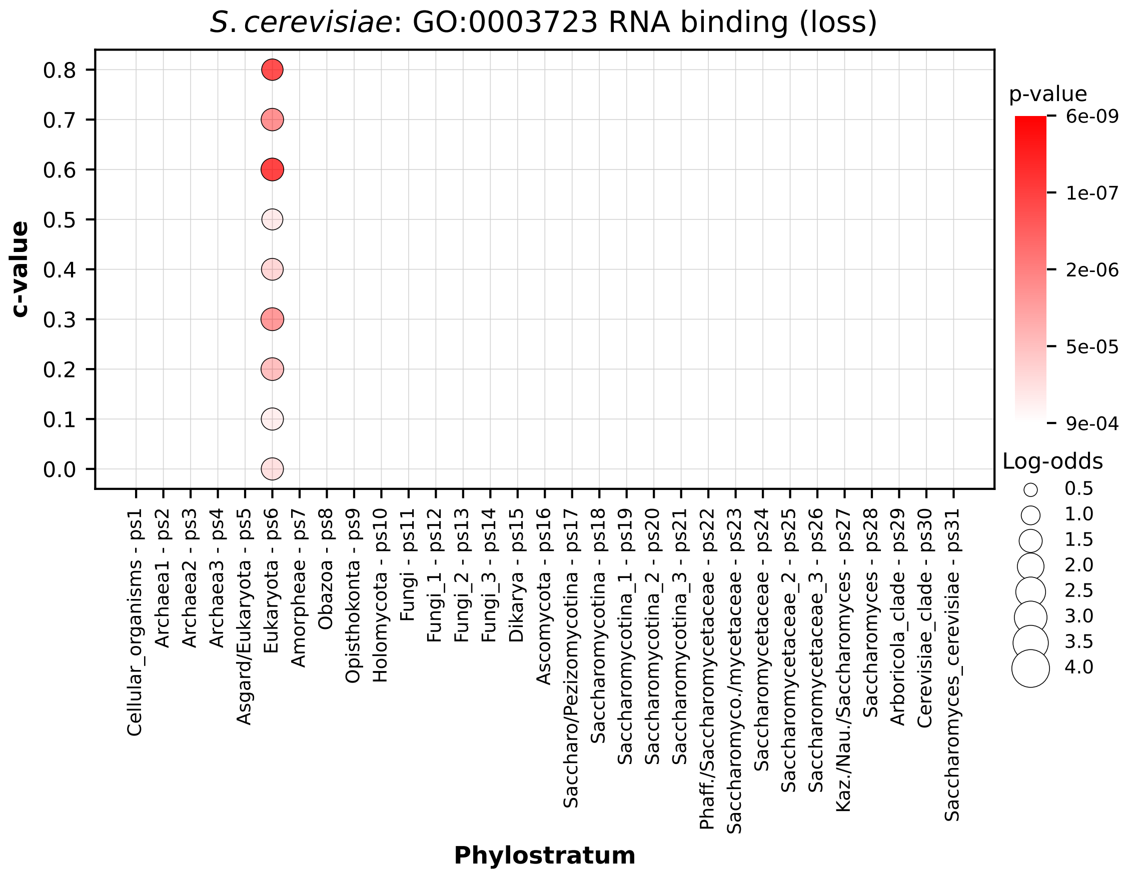

S. cerevisiae: GO:0003723 RNA binding (loss)
c-value
0.8
0.7
0.6
0.5
0.4
0.3
0.2
0.1
0.0
p-value
6e-09
1e-07
2e-06
5e-05
9e-04
Log-odds
0.5
1.0
1.5
2.0
2.5
3.0
3.5
4.0
Cellular_organisms - ps1
Archaea1 - ps2
Archaea2 - ps3
Archaea3 - ps4
Asgard/Eukaryota - ps5
Eukaryota - ps6
Amorpheae - ps7
Obazoa - ps8
Opisthokonta - ps9
Holomycota - ps10
Fungi - ps11
Fungi_1 - ps12
Fungi_2 - ps13
Fungi_3 - ps14
Dikarya - ps15
Ascomycota - ps16
Saccharo/Pezizomycotina - ps17
Saccharomycotina - ps18
Saccharomycotina_1 - ps19
Saccharomycotina_2 - ps20
Saccharomycotina_3 - ps21
Phaff./Saccharomycetaceae - ps22
Saccharomyco./mycetaceae - ps23
Saccharomycetaceae - ps24
Saccharomycetaceae_2 - ps25
Saccharomycetaceae_3 - ps26
Kaz./Nau./Saccharomyces - ps27
Saccharomyces - ps28
Arboricola_clade - ps29
Cerevisiae_clade - ps30
Saccharomyces_cerevisiae - ps31
Phylostratum

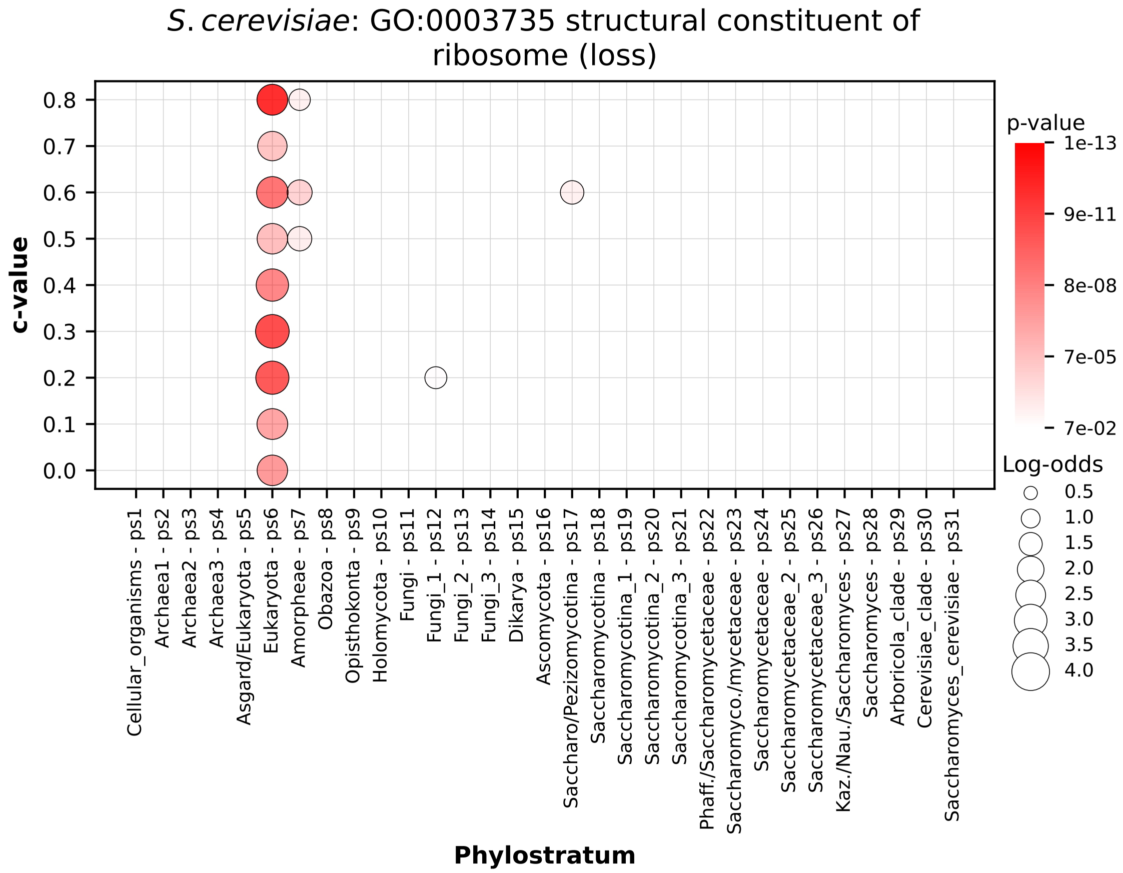
S. cerevisiae: GO:0003735 structural constituent of ribosome (loss)
c-value
0.8
0.7
0.6
0.5
0.4
0.3
0.2
0.1
0.0
Phylostratum
Cellular_organisms - ps1
Archaea1 - ps2
Archaea2 - ps3
Archaea3 - ps4
Asgard/Eukaryota - ps5
Eukaryota - ps6
Amorpheae - ps7
Obazoa - ps8
Opisthokonta - ps9
Holomycota - ps10
Fungi - ps11
Fungi_1 - ps12
Fungi_2 - ps13
Fungi_3 - ps14
Dikarya - ps15
Ascomycota - ps16
Saccharo/Pezizomycotina - ps17
Saccharomycotina - ps18
Saccharomycotina_1 - ps19
Saccharomycotina_2 - ps20
Saccharomycotina_3 - ps21
Phaff./Saccharomycetaceae - ps22
Saccharomyco./mycetaceae - ps23
Saccharomycetaceae - ps24
Saccharomycetaceae_2 - ps25
Saccharomycetaceae_3 - ps26
Kaz./Nau./Saccharomyces - ps27
Saccharomyces - ps28
Arboricola_clade - ps29
Cerevisiae_clade - ps30
Saccharomyces_cerevisiae - ps31
p-value
1e-13
9e-11
8e-08
7e-05
7e-02
Log-odds
0.5
1.0
1.5
2.0
2.5
3.0
3.5
4.0

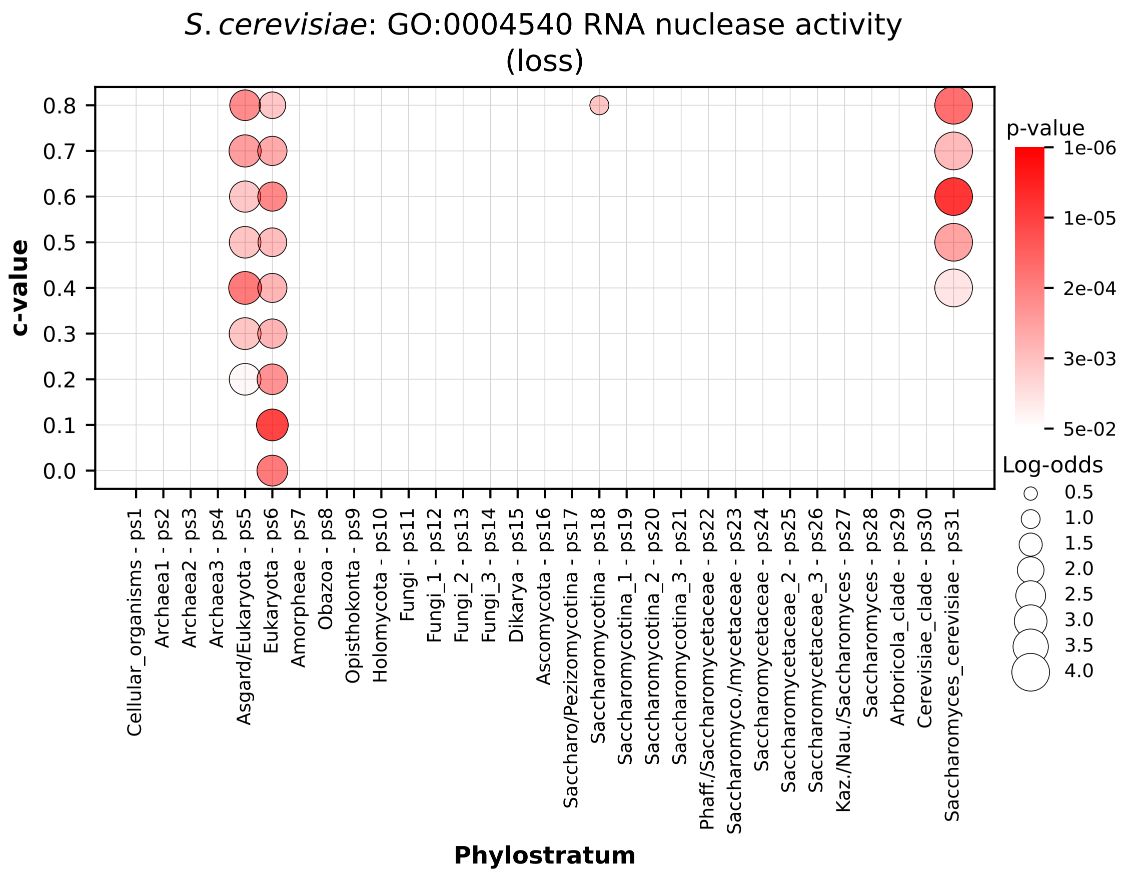

S. cerevisiae: GO:0004540 RNA nuclease activity (loss)
c-value
0.0
0.1
0.2
0.3
0.4
0.5
0.6
0.7
0.8
Phylostratum
Cellular_organisms - ps1
Archaea1 - ps2
Archaea2 - ps3
Archaea3 - ps4
Asgard/Eukaryota - ps5
Eukaryota - ps6
Amorpheae - ps7
Obazoa - ps8
Opisthokonta - ps9
Holomycota - ps10
Fungi - ps11
Fungi_1 - ps12
Fungi_2 - ps13
Fungi_3 - ps14
Dikarya - ps15
Ascomycota - ps16
Saccharo/Pezizomycotina - ps17
Saccharomycotina - ps18
Saccharomycotina_1 - ps19
Saccharomycotina_2 - ps20
Saccharomycotina_3 - ps21
Phaff./Saccharomycetaceae - ps22
Saccharomyco./mycetaceae - ps23
Saccharomycetaceae - ps24
Saccharomycetaceae_2 - ps25
Saccharomycetaceae_3 - ps26
Kaz./Nau./Saccharomyces - ps27
Saccharomyces - ps28
Arboricola_clade - ps29
Cerevisiae_clade - ps30
Saccharomyces_cerevisiae - ps31
p-value
1e-06
1e-05
2e-04
3e-03
5e-02
Log-odds
0.5
1.0
1.5
2.0
2.5
3.0
3.5
4.0

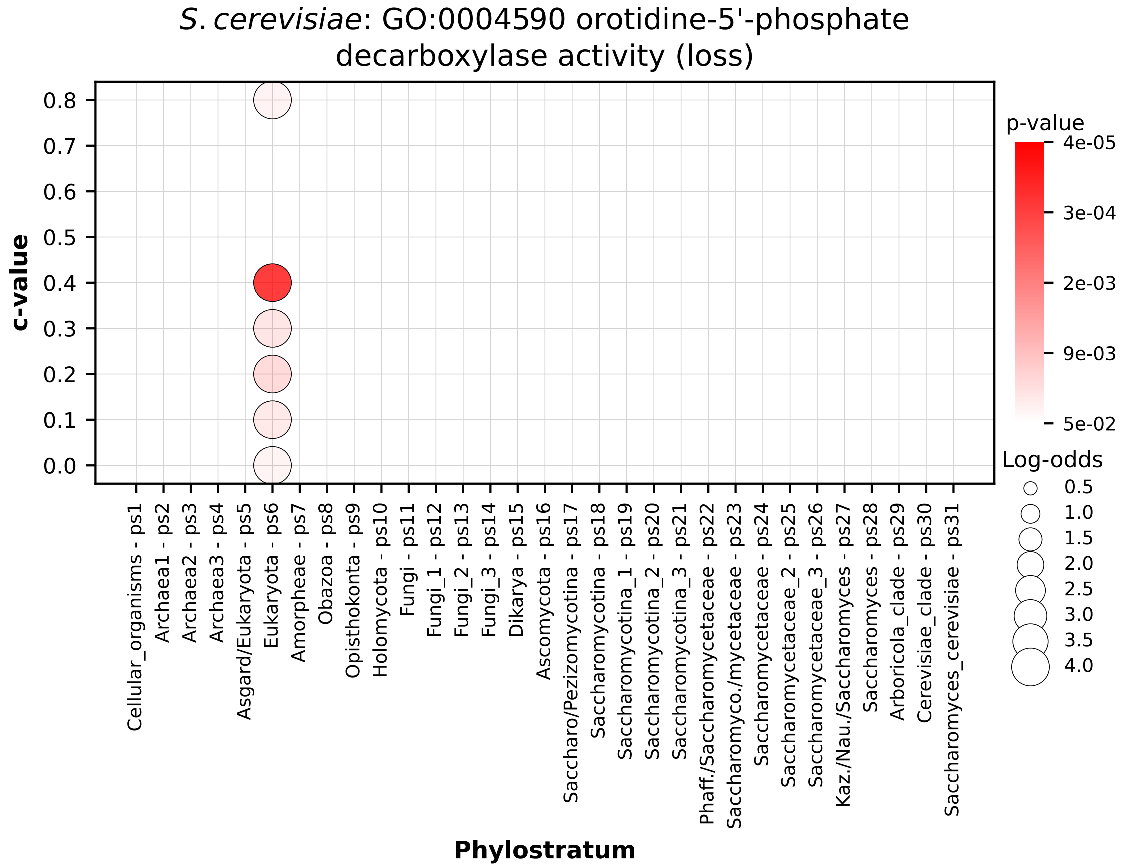
S. cerevisiae: GO:0004590 orotidine-5'-phosphate decarboxylase activity (loss)
c-value
0.8
0.7
0.6
0.5
0.4
0.3
0.2
0.1
0.0
p-value
4e-05
3e-04
2e-03
9e-03
5e-02
Log-odds
0.5
1.0
1.5
2.0
2.5
3.0
3.5
4.0
Cellular_organisms - ps1
Archaea1 - ps2
Archaea2 - ps3
Archaea3 - ps4
Asgard/Eukaryota - ps5
Eukaryota - ps6
Amorpheae - ps7
Obazoa - ps8
Opisthokonta - ps9
Holomycota - ps10
Fungi - ps11
Fungi_1 - ps12
Fungi_2 - ps13
Fungi_3 - ps14
Dikarya - ps15
Ascomycota - ps16
Saccharo/Pezizomycotina - ps17
Saccharomycotina - ps18
Saccharomycotina_1 - ps19
Saccharomycotina_2 - ps20
Saccharomycotina_3 - ps21
Phaff./Saccharomycetaceae - ps22
Saccharomyco./mycetaceae - ps23
Saccharomycetaceae - ps24
Saccharomycetaceae_2 - ps25
Saccharomycetaceae_3 - ps26
Kaz./Nau./Saccharomyces - ps27
Saccharomyces - ps28
Arboricola_clade - ps29
Cerevisiae_clade - ps30
Saccharomyces_cerevisiae - ps31
Phylostratum

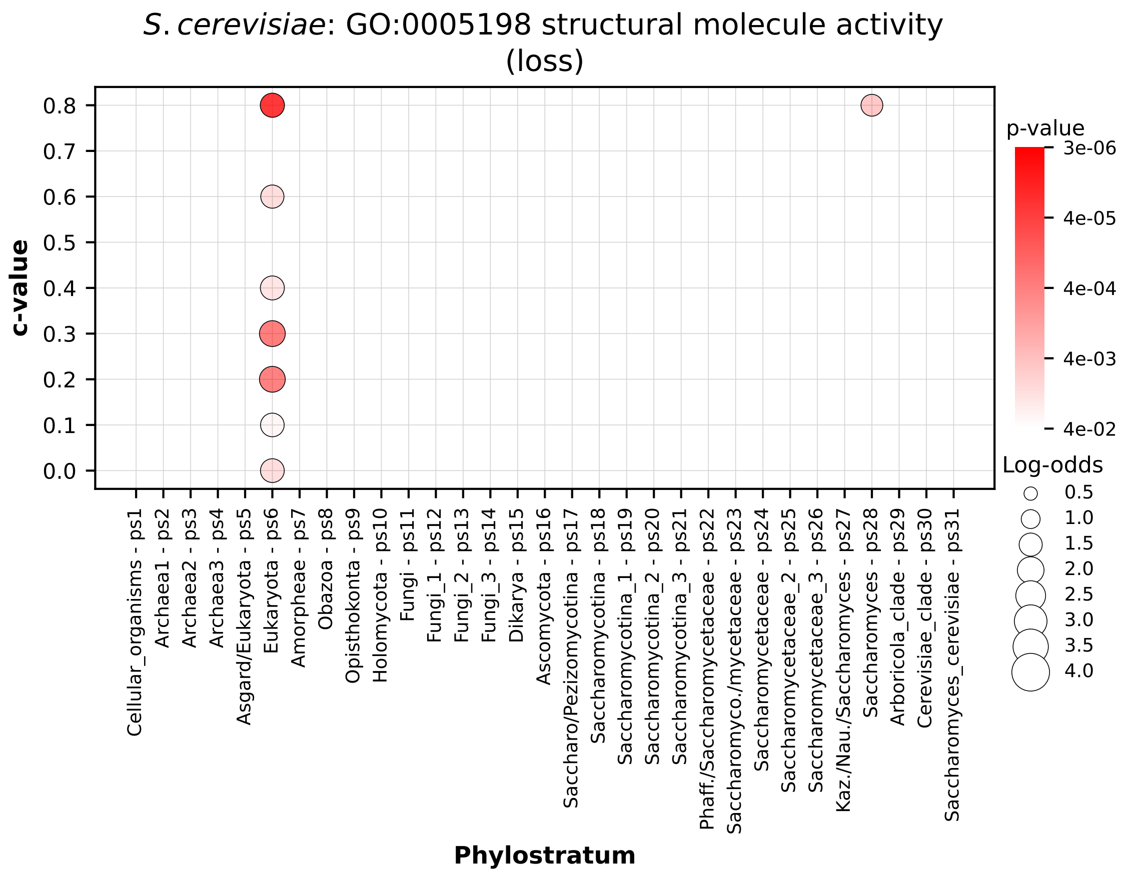

S. cerevisiae: GO:0005198 structural molecule activity (loss)
c-value
0.8
0.7
0.6
0.5
0.4
0.3
0.2
0.1
0.0
p-value
3e-06
4e-05
4e-04
4e-03
4e-02
Log-odds
0.5
1.0
1.5
2.0
2.5
3.0
3.5
4.0
Cellular_organisms - ps1
Archaea1 - ps2
Archaea2 - ps3
Archaea3 - ps4
Asgard/Eukaryota - ps5
Eukaryota - ps6
Amorpheae - ps7
Obazoa - ps8
Opisthokonta - ps9
Holomycota - ps10
Fungi - ps11
Fungi_1 - ps12
Fungi_2 - ps13
Fungi_3 - ps14
Dikarya - ps15
Ascomycota - ps16
Saccharo/Pezizomycotina - ps17
Saccharomycotina - ps18
Saccharomycotina_1 - ps19
Saccharomycotina_2 - ps20
Saccharomycotina_3 - ps21
Phaff./Saccharomycetaceae - ps22
Saccharomyco./mycetaceae - ps23
Saccharomycetaceae - ps24
Saccharomycetaceae_2 - ps25
Saccharomycetaceae_3 - ps26
Kaz./Nau./Saccharomyces - ps27
Saccharomyces - ps28
Arboricola_clade - ps29
Cerevisiae_clade - ps30
Saccharomyces_cerevisiae - ps31
Phylostratum

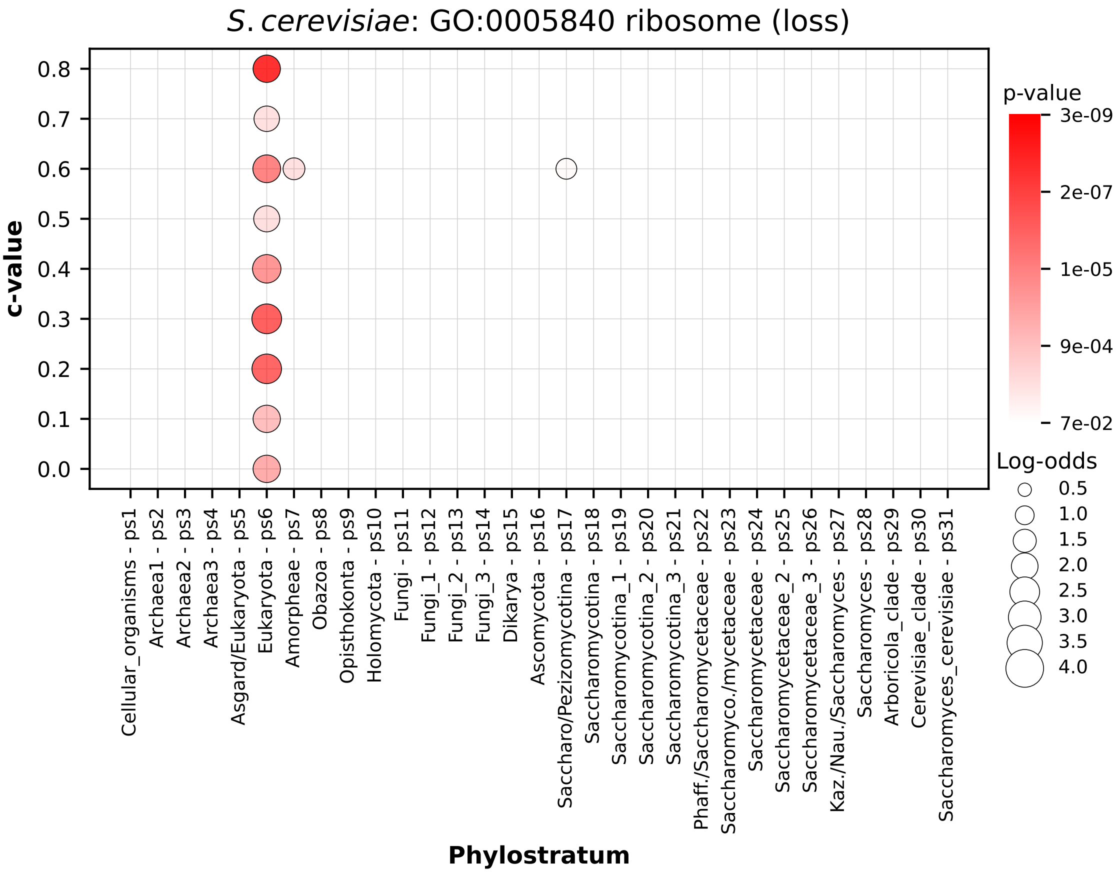

S. cerevisiae: GO:0005840 ribosome (loss)
c-value
0.8
0.7
0.6
0.5
0.4
0.3
0.2
0.1
0.0
p-value
3e-09
2e-07
1e-05
9e-04
7e-02
Log-odds
0.5
1.0
1.5
2.0
2.5
3.0
3.5
4.0
Cellular_organisms - ps1
Archaea1 - ps2
Archaea2 - ps3
Archaea3 - ps4
Asgard/Eukaryota - ps5
Eukaryota - ps6
Amorpheae - ps7
Obazoa - ps8
Opisthokonta - ps9
Holomycota - ps10
Fungi - ps11
Fungi_1 - ps12
Fungi_2 - ps13
Fungi_3 - ps14
Dikarya - ps15
Ascomycota - ps16
Saccharo/Pezizomycotina - ps17
Saccharomycotina - ps18
Saccharomycotina_1 - ps19
Saccharomycotina_2 - ps20
Saccharomycotina_3 - ps21
Phaff./Saccharomycetaceae - ps22
Saccharomyco./mycetaceae - ps23
Saccharomycetaceae - ps24
Saccharomycetaceae_2 - ps25
Saccharomycetaceae_3 - ps26
Kaz./Nau./Saccharomyces - ps27
Saccharomyces - ps28
Arboricola_clade - ps29
Cerevisiae_clade - ps30
Saccharomyces_cerevisiae - ps31
Phylostratum

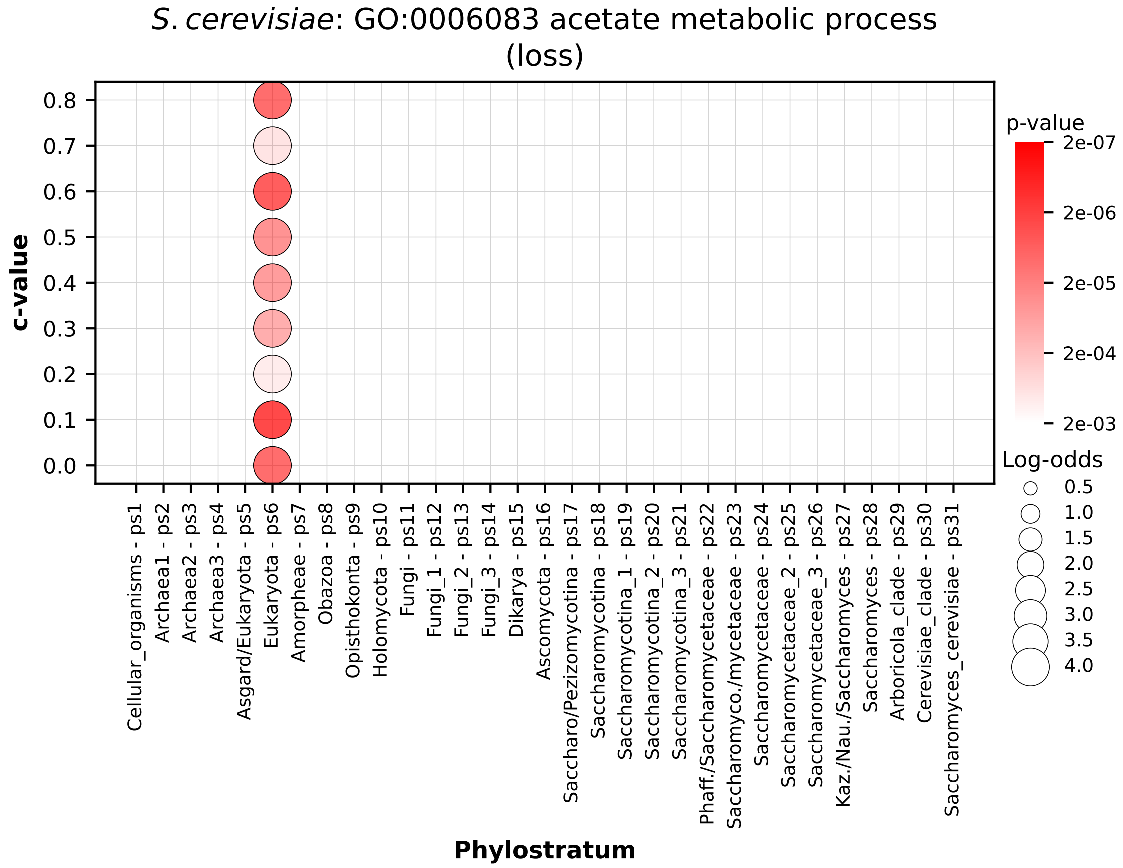
S. cerevisiae: GO:0006083 acetate metabolic process
(loss)
c-value
0.8
0.7
0.6
0.5
0.4
0.3
0.2
0.1
0.0
p-value
2e-07
2e-06
2e-05
2e-04
2e-03
Log-odds
0.5
1.0
1.5
2.0
2.5
3.0
3.5
4.0
Cellular_organisms - ps1
Archaea1 - ps2
Archaea2 - ps3
Archaea3 - ps4
Asgard/Eukaryota - ps5
Eukaryota - ps6
Amorpheae - ps7
Obazoa - ps8
Opisthokonta - ps9
Holomycota - ps10
Fungi - ps11
Fungi_1 - ps12
Fungi_2 - ps13
Fungi_3 - ps14
Dikarya - ps15
Ascomycota - ps16
Saccharo/Pezizomycotina - ps17
Saccharomycotina - ps18
Saccharomycotina_1 - ps19
Saccharomycotina_2 - ps20
Saccharomycotina_3 - ps21
Phaff./Saccharomycetaceae - ps22
Saccharomyco./mycetaceae - ps23
Saccharomycetaceae - ps24
Saccharomycetaceae_2 - ps25
Saccharomycetaceae_3 - ps26
Kaz./Nau./Saccharomyces - ps27
Saccharomyces - ps28
Arboricola_clade - ps29
Cerevisiae_clade - ps30
Saccharomyces_cerevisiae - ps31
Phylostratum

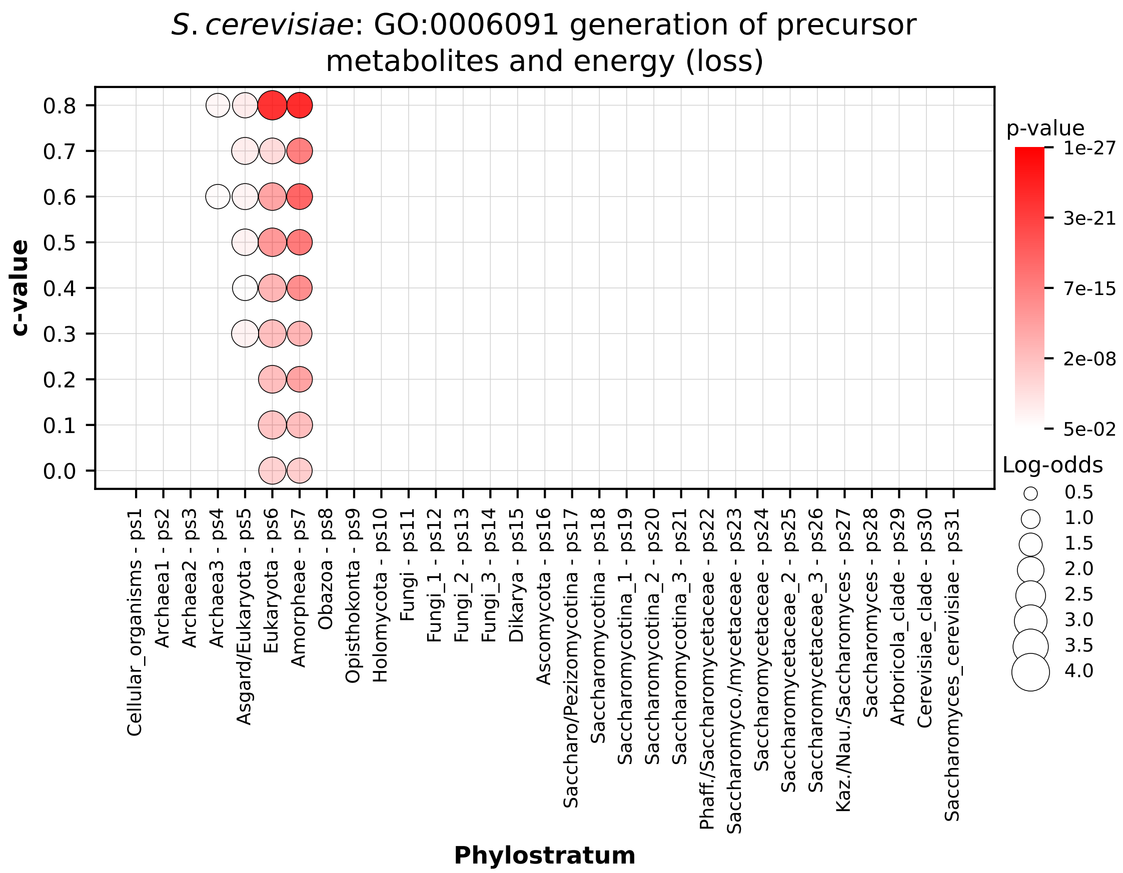
S. cerevisiae: GO:0006091 generation of precursor metabolites and energy (loss)
c-value
0.8
0.7
0.6
0.5
0.4
0.3
0.2
0.1
0.0
p-value
1e-27
3e-21
7e-15
2e-08
5e-02
Log-odds
0.5
1.0
1.5
2.0
2.5
3.0
3.5
4.0
Cellular_organisms - ps1
Archaea1 - ps2
Archaea2 - ps3
Archaea3 - ps4
Asgard/Eukaryota - ps5
Eukaryota - ps6
Amorpheae - ps7
Obazoa - ps8
Opisthokonta - ps9
Holomycota - ps10
Fungi - ps11
Fungi_1 - ps12
Fungi_2 - ps13
Fungi_3 - ps14
Dikarya - ps15
Ascomycota - ps16
Saccharo/Pezizomycotina - ps17
Saccharomycotina - ps18
Saccharomycotina_1 - ps19
Saccharomycotina_2 - ps20
Saccharomycotina_3 - ps21
Phaff./Saccharomycetaceae - ps22
Saccharomyco./mycetaceae - ps23
Saccharomycetaceae - ps24
Saccharomycetaceae_2 - ps25
Saccharomycetaceae_3 - ps26
Kaz./Nau./Saccharomyces - ps27
Saccharomyces - ps28
Arboricola_clade - ps29
Cerevisiae_clade - ps30
Saccharomyces_cerevisiae - ps31
Phylostratum

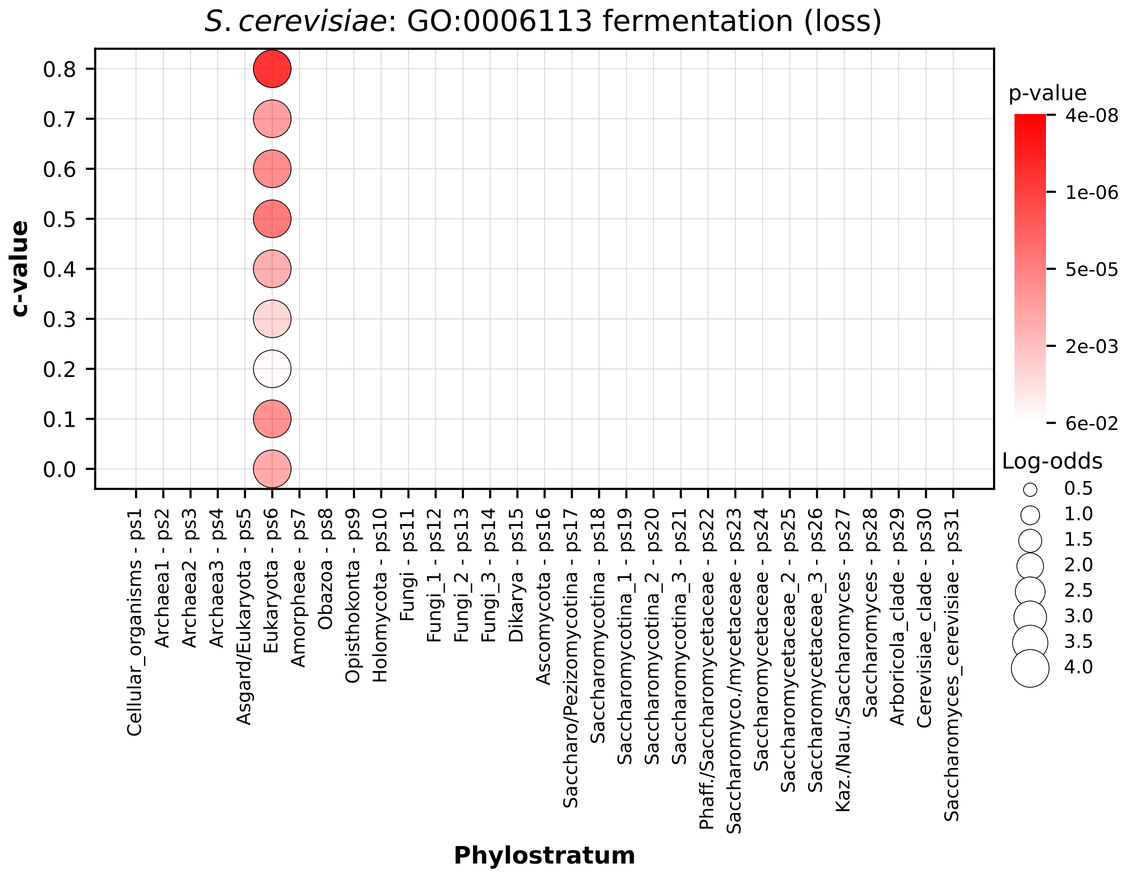
S. cerevisiae: GO:0006113 fermentation (loss)
c-value
0.8
0.7
0.6
0.5
0.4
0.3
0.2
0.1
0.0
p-value
4e-08
1e-06
5e-05
2e-03
6e-02
Log-odds
0.5
1.0
1.5
2.0
2.5
3.0
3.5
4.0
Cellular_organisms - ps1
Archaea1 - ps2
Archaea2 - ps3
Archaea3 - ps4
Asgard/Eukaryota - ps5
Eukaryota - ps6
Amorpheae - ps7
Obazoa - ps8
Opisthokonta - ps9
Holomycota - ps10
Fungi - ps11
Fungi_1 - ps12
Fungi_2 - ps13
Fungi_3 - ps14
Dikarya - ps15
Ascomycota - ps16
Saccharo/Pezizomycotina - ps17
Saccharomycotina - ps18
Saccharomycotina_1 - ps19
Saccharomycotina_2 - ps20
Saccharomycotina_3 - ps21
Phaff./Saccharomycetaceae - ps22
Saccharomyco./mycetaceae - ps23
Saccharomycetaceae - ps24
Saccharomycetaceae_2 - ps25
Saccharomycetaceae_3 - ps26
Kaz./Nau./Saccharomyces - ps27
Saccharomyces - ps28
Arboricola_clade - ps29
Cerevisiae_clade - ps30
Saccharomyces_cerevisiae - ps31
Phylostratum

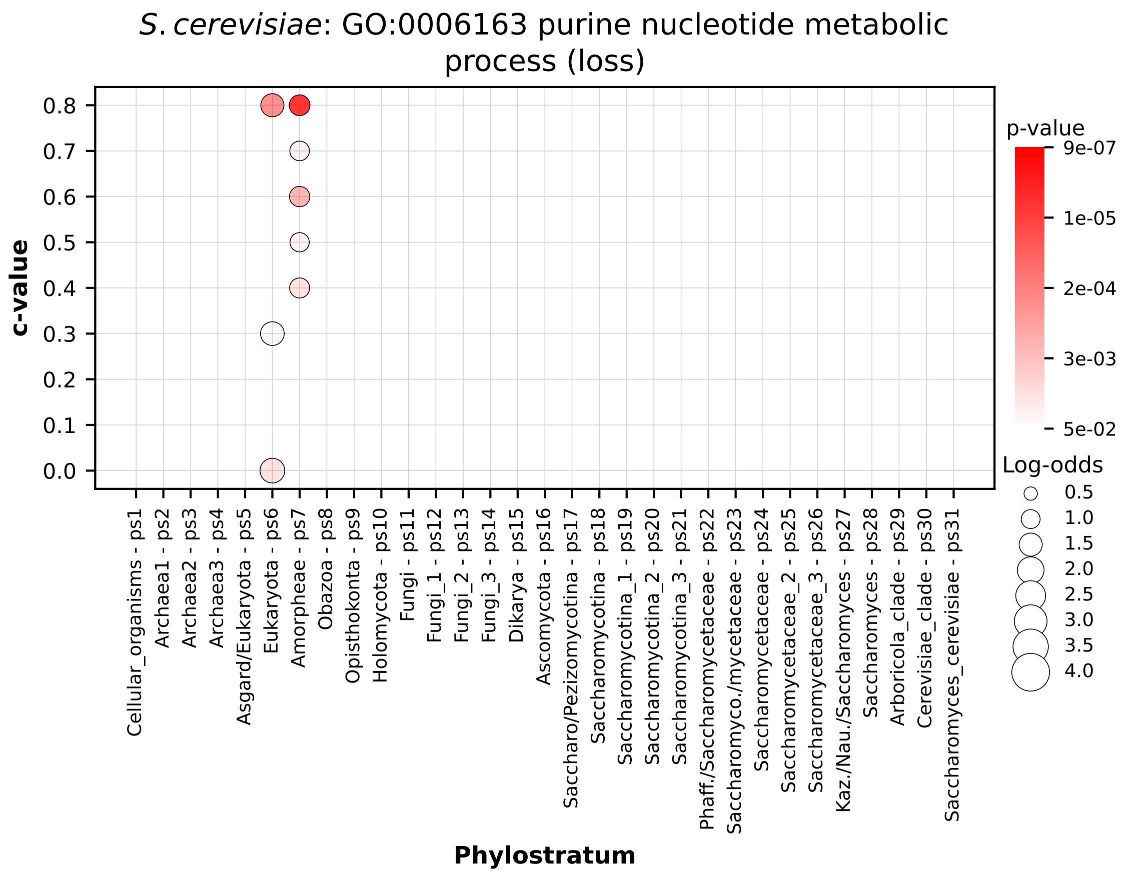
S. cerevisiae: GO:0006163 purine nucleotide metabolic process (loss)
c-value
0.0
0.1
0.2
0.3
0.4
0.5
0.6
0.7
0.8
p-value
9e-07
1e-05
2e-04
3e-03
5e-02
Log-odds
0.5
1.0
1.5
2.0
2.5
3.0
3.5
4.0
Cellular_organisms - ps1
Archaea1 - ps2
Archaea2 - ps3
Archaea3 - ps4
Asgard/Eukaryota - ps5
Eukaryota - ps6
Amorpheae - ps7
Obazoa - ps8
Opisthokonta - ps9
Holomycota - ps10
Fungi - ps11
Fungi_1 - ps12
Fungi_2 - ps13
Fungi_3 - ps14
Dikarya - ps15
Ascomycota - ps16
Saccharo/Pezizomycotina - ps17
Saccharomycotina - ps18
Saccharomycotina_1 - ps19
Saccharomycotina_2 - ps20
Saccharomycotina_3 - ps21
Phaff./Saccharomycetaceae - ps22
Saccharomyco./mycetaceae - ps23
Saccharomycetaceae - ps24
Saccharomycetaceae_2 - ps25
Saccharomycetaceae_3 - ps26
Kaz./Nau./Saccharomyces - ps27
Saccharomyces - ps28
Arboricola_clade - ps29
Cerevisiae_clade - ps30
Saccharomyces_cerevisiae - ps31
Phylostratum

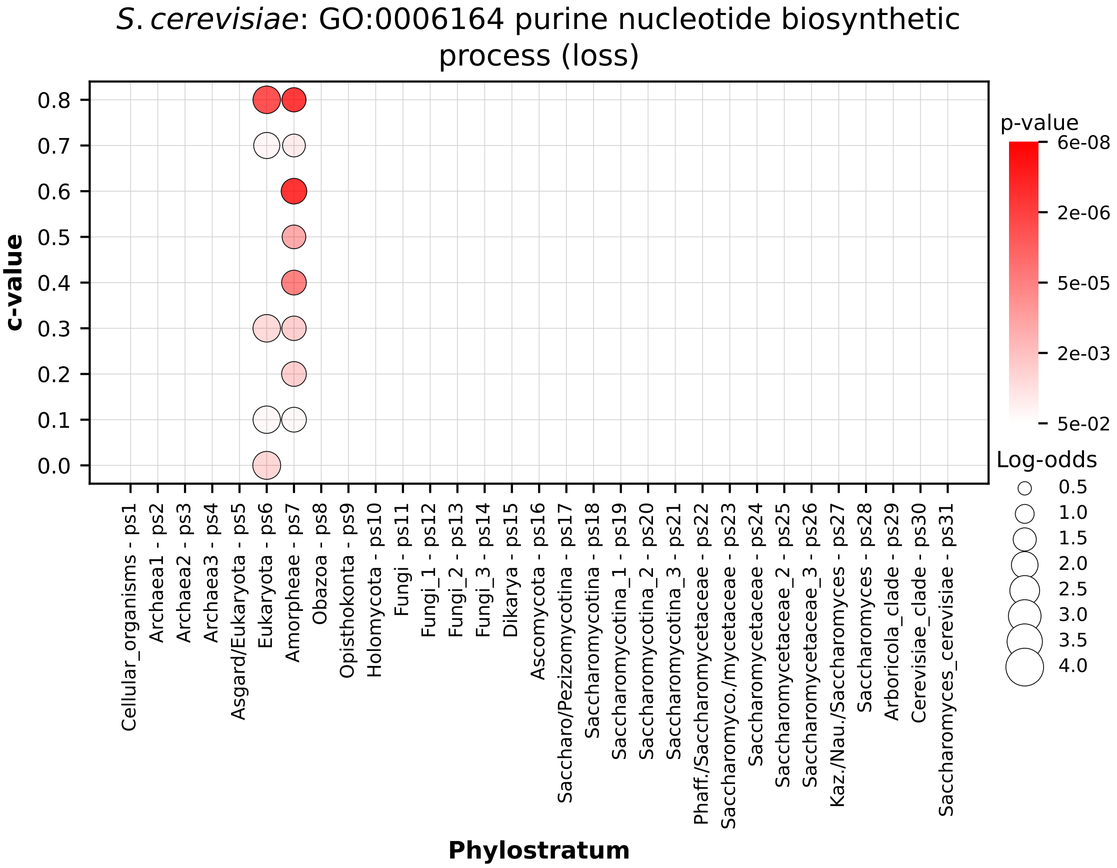

S. cerevisiae: GO:0006164 purine nucleotide biosynthetic process (loss)
c-value
0.8
0.7
0.6
0.5
0.4
0.3
0.2
0.1
0.0
Phylostratum
Cellular_organisms - ps1
Archaea1 - ps2
Archaea2 - ps3
Archaea3 - ps4
Asgard/Eukaryota - ps5
Eukaryota - ps6
Amorpheae - ps7
Obazoa - ps8
Opisthokonta - ps9
Holomycota - ps10
Fungi - ps11
Fungi_1 - ps12
Fungi_2 - ps13
Fungi_3 - ps14
Dikarya - ps15
Ascomycota - ps16
Saccharo/Pezizomycotina - ps17
Saccharomycotina - ps18
Saccharomycotina_1 - ps19
Saccharomycotina_2 - ps20
Saccharomycotina_3 - ps21
Phaff./Saccharomycetaceae - ps22
Saccharomyco./mycetaceae - ps23
Saccharomycetaceae - ps24
Saccharomycetaceae_2 - ps25
Saccharomycetaceae_3 - ps26
Kaz./Nau./Saccharomyces - ps27
Saccharomyces - ps28
Arboricola_clade - ps29
Cerevisiae_clade - ps30
Saccharomyces_cerevisiae - ps31
p-value
6e-08
2e-06
5e-05
2e-03
5e-02
Log-odds
0.5
1.0
1.5
2.0
2.5
3.0
3.5
4.0

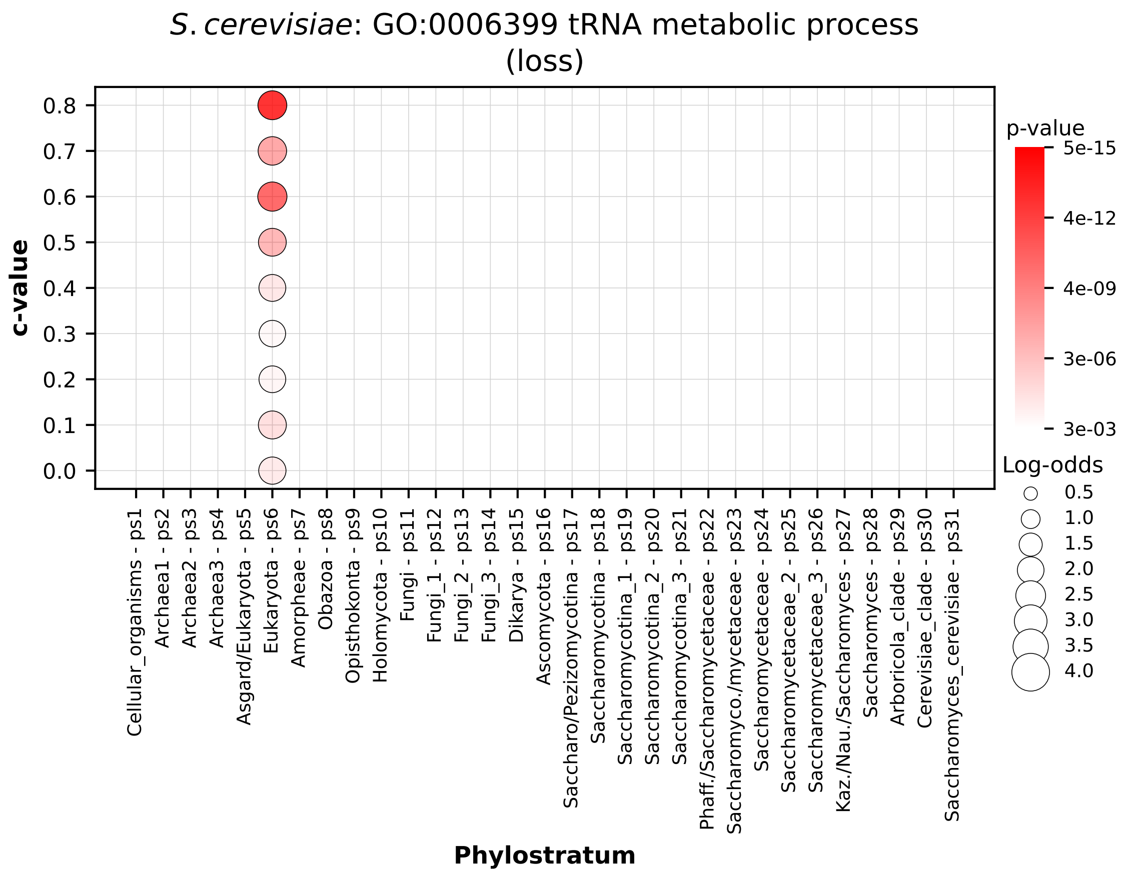
S. cerevisiae: GO:0006399 tRNA metabolic process
(loss)
c-value
0.8
0.7
0.6
0.5
0.4
0.3
0.2
0.1
0.0
p-value
5e-15
4e-12
4e-09
3e-06
3e-03
Log-odds
0.5
1.0
1.5
2.0
2.5
3.0
3.5
4.0
Cellular_organisms - ps1
Archaea1 - ps2
Archaea2 - ps3
Archaea3 - ps4
Asgard/Eukaryota - ps5
Eukaryota - ps6
Amorpheae - ps7
Obazoa - ps8
Opisthokonta - ps9
Holomycota - ps10
Fungi - ps11
Fungi_1 - ps12
Fungi_2 - ps13
Fungi_3 - ps14
Dikarya - ps15
Ascomycota - ps16
Saccharo/Pezizomycotina - ps17
Saccharomycotina - ps18
Saccharomycotina_1 - ps19
Saccharomycotina_2 - ps20
Saccharomycotina_3 - ps21
Phaff./Saccharomycetaceae - ps22
Saccharomyco./mycetaceae - ps23
Saccharomycetaceae - ps24
Saccharomycetaceae_2 - ps25
Saccharomycetaceae_3 - ps26
Kaz./Nau./Saccharomyces - ps27
Saccharomyces - ps28
Arboricola_clade - ps29
Cerevisiae_clade - ps30
Saccharomyces_cerevisiae - ps31
Phylostratum

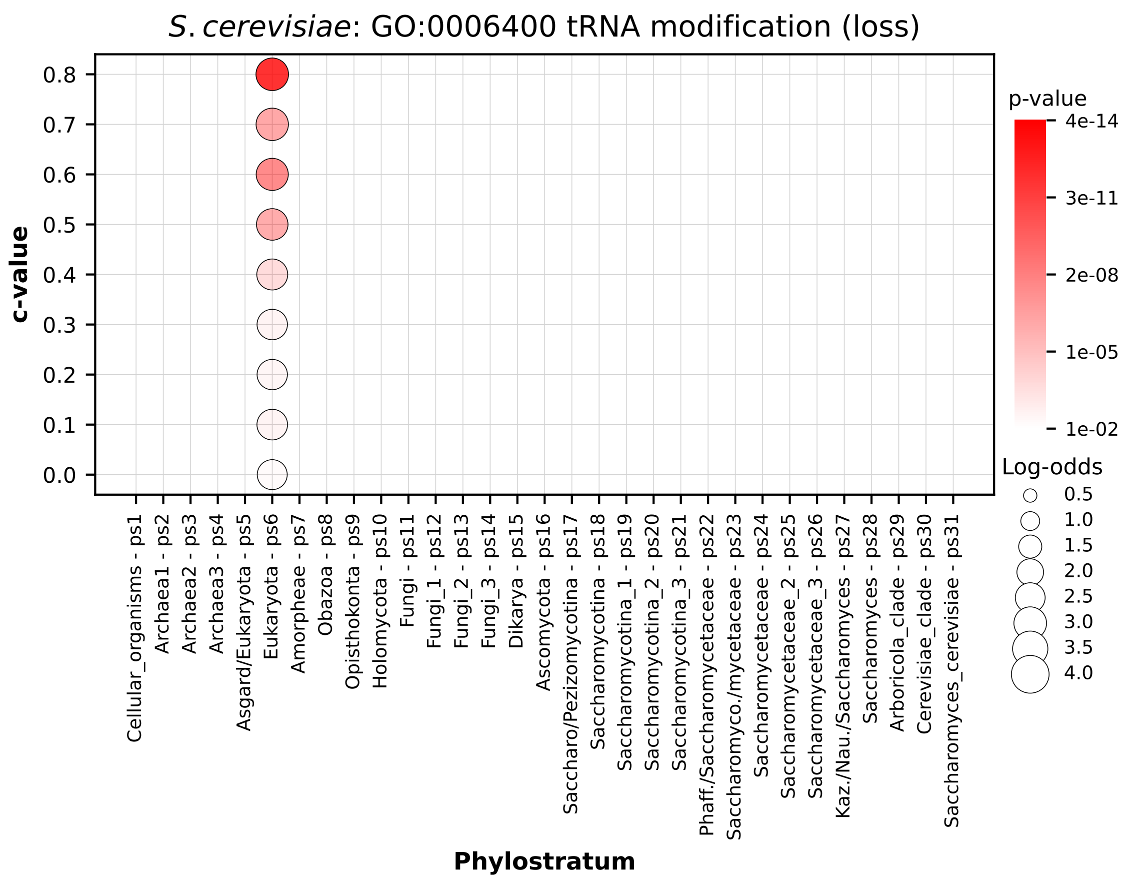

S. cerevisiae: GO:0006400 tRNA modification (loss)
c-value
0.8
0.7
0.6
0.5
0.4
0.3
0.2
0.1
0.0
p-value
4e-14
3e-11
2e-08
1e-05
1e-02
Log-odds
0.5
1.0
1.5
2.0
2.5
3.0
3.5
4.0
Cellular_organisms - ps1
Archaea1 - ps2
Archaea2 - ps3
Archaea3 - ps4
Asgard/Eukaryota - ps5
Eukaryota - ps6
Amorpheae - ps7
Obazoa - ps8
Opisthokonta - ps9
Holomycota - ps10
Fungi - ps11
Fungi_1 - ps12
Fungi_2 - ps13
Fungi_3 - ps14
Dikarya - ps15
Ascomycota - ps16
Saccharo/Pezizomycotina - ps17
Saccharomycotina - ps18
Saccharomycotina_1 - ps19
Saccharomycotina_2 - ps20
Saccharomycotina_3 - ps21
Phaff./Saccharomycetaceae - ps22
Saccharomyco./mycetaceae - ps23
Saccharomycetaceae - ps24
Saccharomycetaceae_2 - ps25
Saccharomycetaceae_3 - ps26
Kaz./Nau./Saccharomyces - ps27
Saccharomyces - ps28
Arboricola_clade - ps29
Cerevisiae_clade - ps30
Saccharomyces_cerevisiae - ps31
Phylostratum

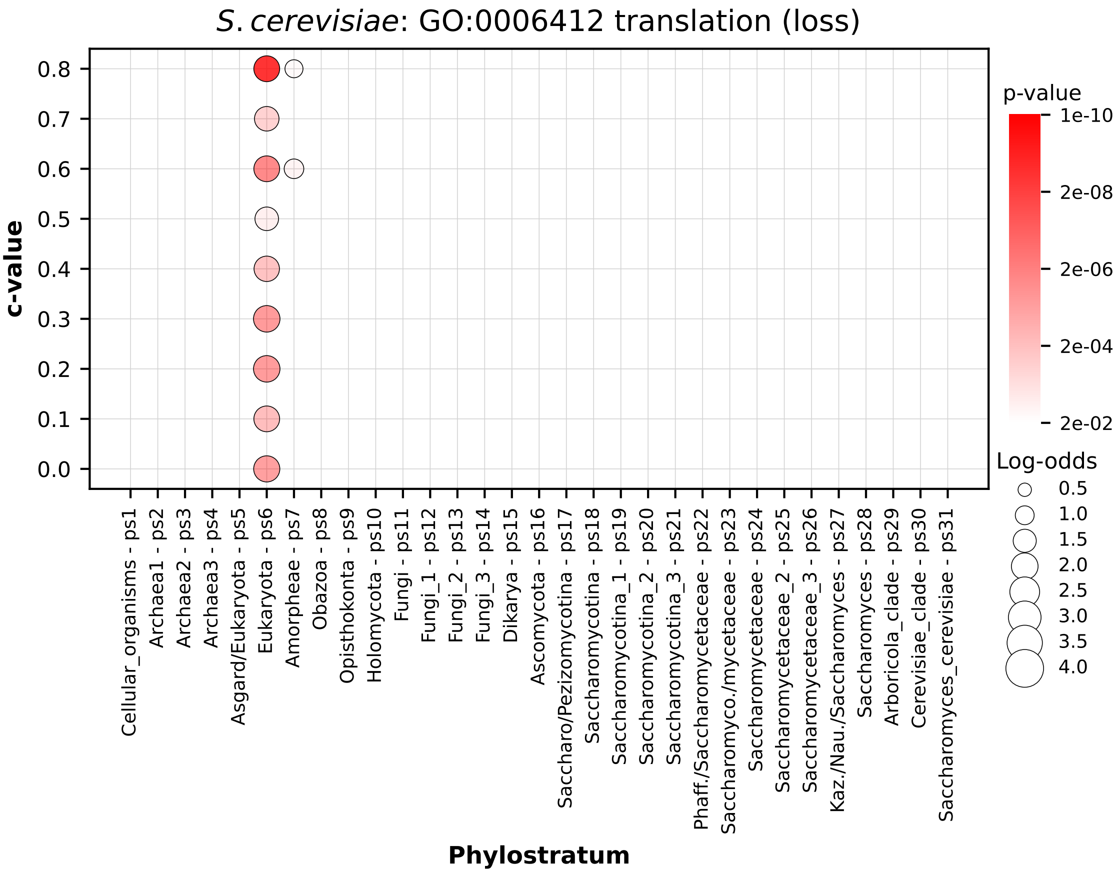

S. cerevisiae: GO:0006412 translation (loss)
c-value
0.0
0.1
0.2
0.3
0.4
0.5
0.6
0.7
0.8
p-value
1e-10
2e-08
2e-06
2e-04
2e-02
Log-odds
0.5
1.0
1.5
2.0
2.5
3.0
3.5
4.0
Cellular_organisms - ps1
Archaea1 - ps2
Archaea2 - ps3
Archaea3 - ps4
Asgard/Eukaryota - ps5
Eukaryota - ps6
Amorpheae - ps7
Obazoa - ps8
Opisthokonta - ps9
Holomycota - ps10
Fungi - ps11
Fungi_1 - ps12
Fungi_2 - ps13
Fungi_3 - ps14
Dikarya - ps15
Ascomycota - ps16
Saccharo/Pezizomycotina - ps17
Saccharomycotina - ps18
Saccharomycotina_1 - ps19
Saccharomycotina_2 - ps20
Saccharomycotina_3 - ps21
Phaff./Saccharomycetaceae - ps22
Saccharomyco./mycetaceae - ps23
Saccharomycetaceae - ps24
Saccharomycetaceae_2 - ps25
Saccharomycetaceae_3 - ps26
Kaz./Nau./Saccharomyces - ps27
Saccharomyces - ps28
Arboricola_clade - ps29
Cerevisiae_clade - ps30
Saccharomyces_cerevisiae - ps31
Phylostratum

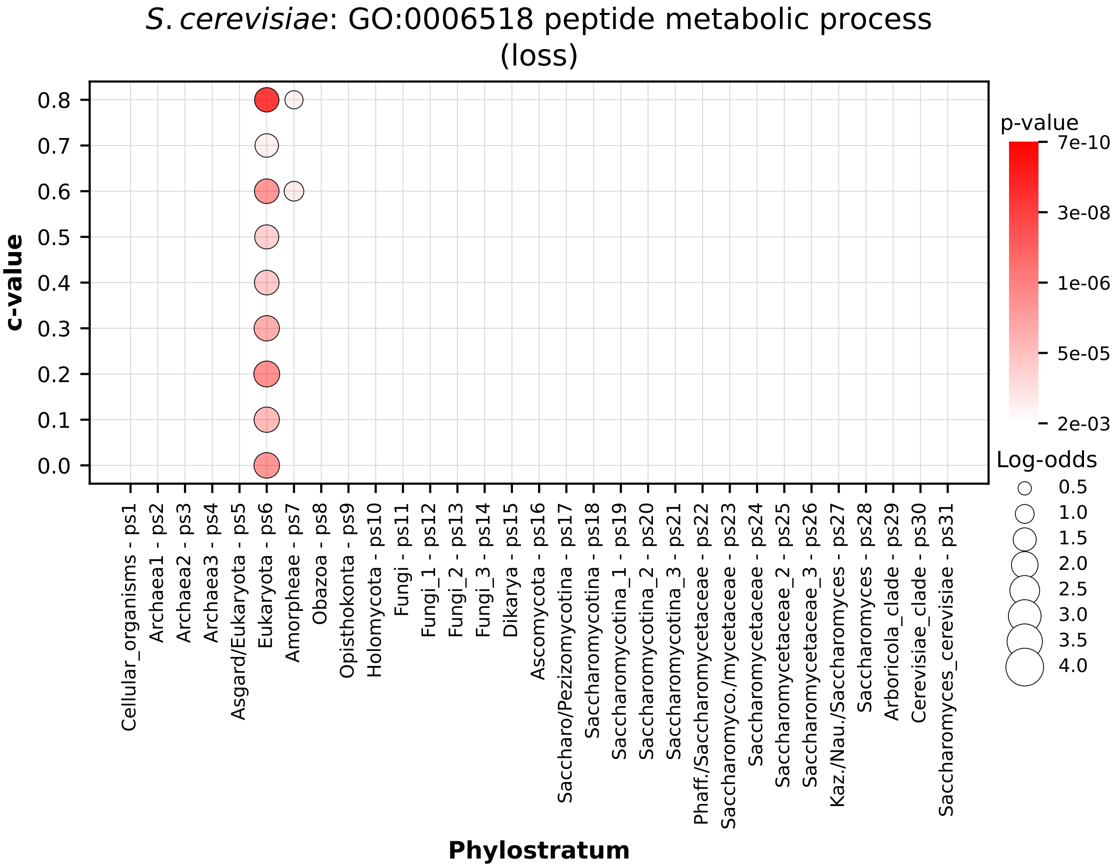

S. cerevisiae: GO:0006518 peptide metabolic process
(loss)
c-value
0.8
0.7
0.6
0.5
0.4
0.3
0.2
0.1
0.0
p-value
7e-10
3e-08
1e-06
5e-05
2e-03
Log-odds
0.5
1.0
1.5
2.0
2.5
3.0
3.5
4.0
Cellular_organisms - ps1
Archaea1 - ps2
Archaea2 - ps3
Archaea3 - ps4
Asgard/Eukaryota - ps5
Eukaryota - ps6
Amorpheae - ps7
Obazoa - ps8
Opisthokonta - ps9
Holomycota - ps10
Fungi - ps11
Fungi_1 - ps12
Fungi_2 - ps13
Fungi_3 - ps14
Dikarya - ps15
Ascomycota - ps16
Saccharo/Pezizomycotina - ps17
Saccharomycotina - ps18
Saccharomycotina_1 - ps19
Saccharomycotina_2 - ps20
Saccharomycotina_3 - ps21
Phaff./Saccharomycetaceae - ps22
Saccharomyco./mycetaceae - ps23
Saccharomycetaceae - ps24
Saccharomycetaceae_2 - ps25
Saccharomycetaceae_3 - ps26
Kaz./Nau./Saccharomyces - ps27
Saccharomyces - ps28
Arboricola_clade - ps29
Cerevisiae_clade - ps30
Saccharomyces_cerevisiae - ps31
Phylostratum

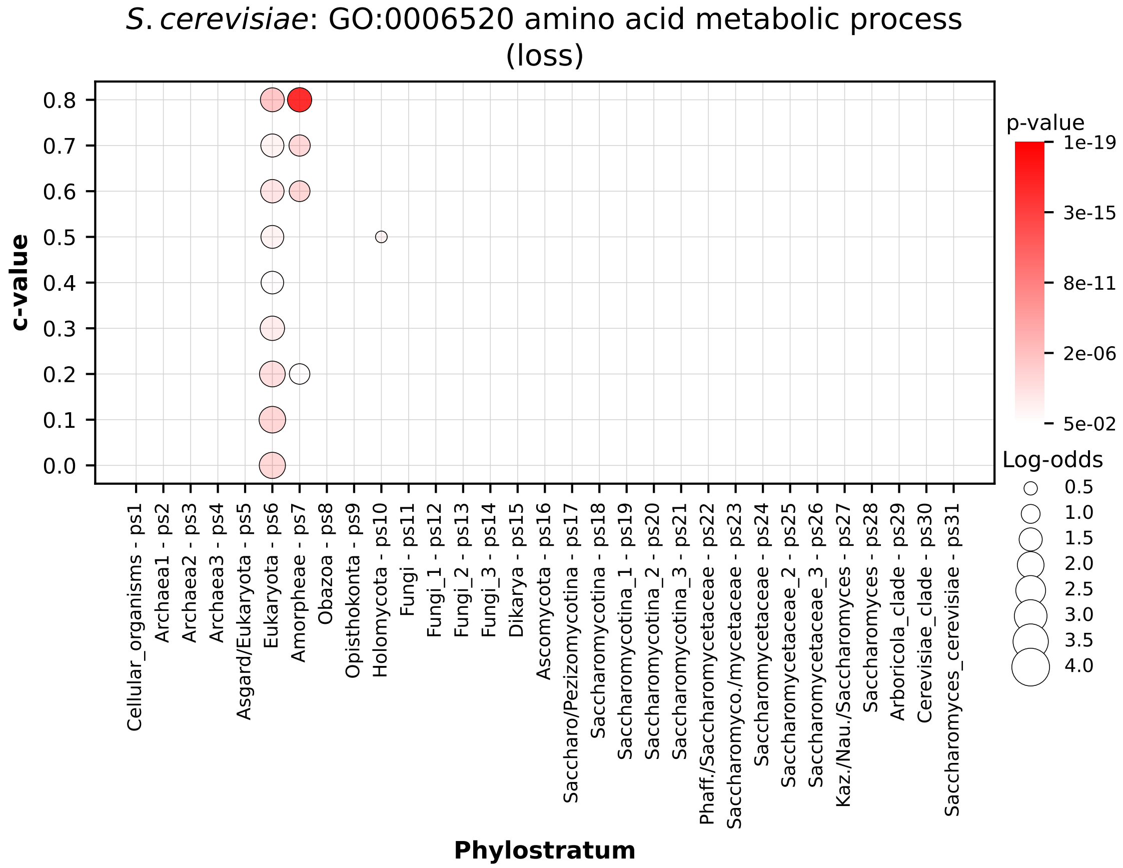
S. cerevisiae: GO:0006520 amino acid metabolic process
(loss)
c-value
0.8
0.7
0.6
0.5
0.4
0.3
0.2
0.1
0.0
p-value
1e-19
3e-15
8e-11
2e-06
5e-02
Log-odds
0.5
1.0
1.5
2.0
2.5
3.0
3.5
4.0
Cellular_organisms - ps1
Archaea1 - ps2
Archaea2 - ps3
Archaea3 - ps4
Asgard/Eukaryota - ps5
Eukaryota - ps6
Amorpheae - ps7
Obazoa - ps8
Opisthokonta - ps9
Holomycota - ps10
Fungi - ps11
Fungi_1 - ps12
Fungi_2 - ps13
Fungi_3 - ps14
Dikarya - ps15
Ascomycota - ps16
Saccharo/Pezizomycotina - ps17
Saccharomycotina - ps18
Saccharomycotina_1 - ps19
Saccharomycotina_2 - ps20
Saccharomycotina_3 - ps21
Phaff./Saccharomycetaceae - ps22
Saccharomyco./mycetaceae - ps23
Saccharomycetaceae - ps24
Saccharomycetaceae_2 - ps25
Saccharomycetaceae_3 - ps26
Kaz./Nau./Saccharomyces - ps27
Saccharomyces - ps28
Arboricola_clade - ps29
Cerevisiae_clade - ps30
Saccharomyces_cerevisiae - ps31
Phylostratum

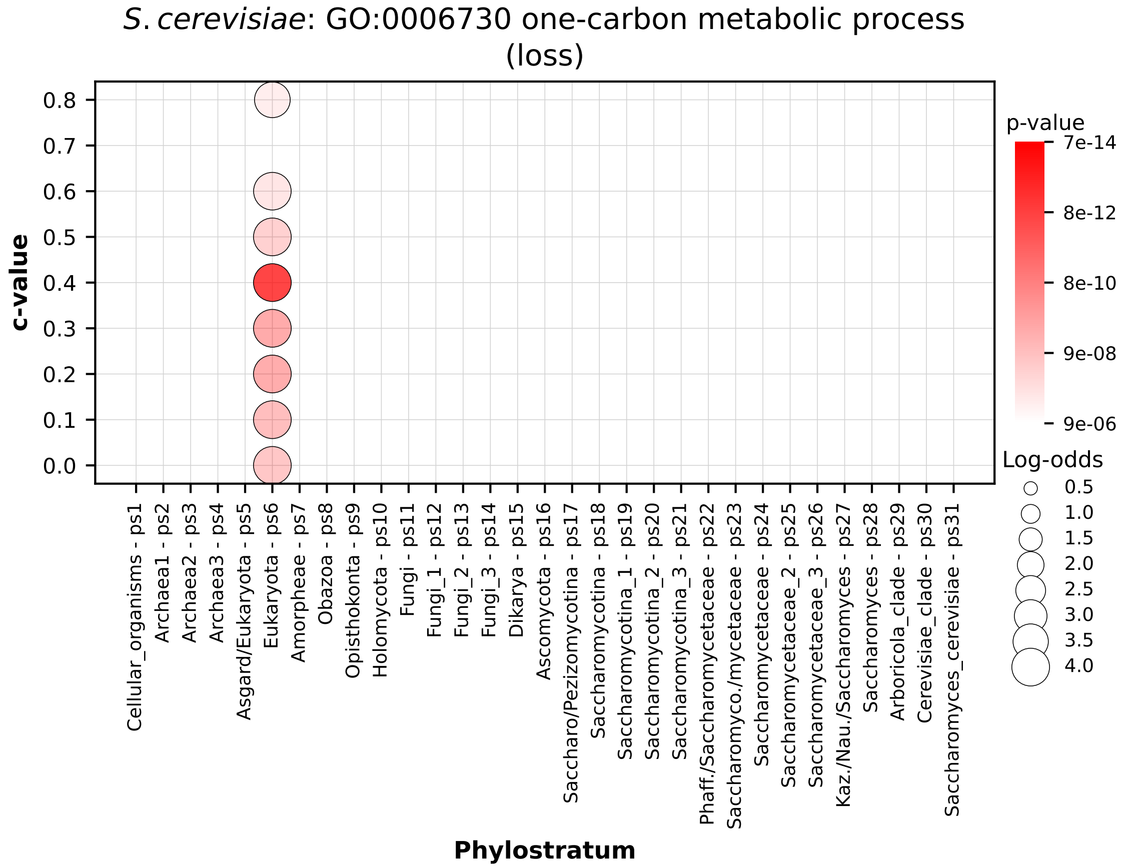

S. cerevisiae: GO:0006730 one-carbon metabolic process (loss)
c-value
0.8
0.7
0.6
0.5
0.4
0.3
0.2
0.1
0.0
p-value
7e-14
8e-12
8e-10
9e-08
9e-06
Log-odds
0.5
1.0
1.5
2.0
2.5
3.0
3.5
4.0
Cellular_organisms - ps1
Archaea1 - ps2
Archaea2 - ps3
Archaea3 - ps4
Asgard/Eukaryota - ps5
Eukaryota - ps6
Amorpheae - ps7
Obazoa - ps8
Opisthokonta - ps9
Holomycota - ps10
Fungi - ps11
Fungi_1 - ps12
Fungi_2 - ps13
Fungi_3 - ps14
Dikarya - ps15
Ascomycota - ps16
Saccharo/Pezizomycotina - ps17
Saccharomycotina - ps18
Saccharomycotina_1 - ps19
Saccharomycotina_2 - ps20
Saccharomycotina_3 - ps21
Phaff./Saccharomycetaceae - ps22
Saccharomyco./mycetaceae - ps23
Saccharomycetaceae - ps24
Saccharomycetaceae_2 - ps25
Saccharomycetaceae_3 - ps26
Kaz./Nau./Saccharomyces - ps27
Saccharomyces - ps28
Arboricola_clade - ps29
Cerevisiae_clade - ps30
Saccharomyces_cerevisiae - ps31
Phylostratum

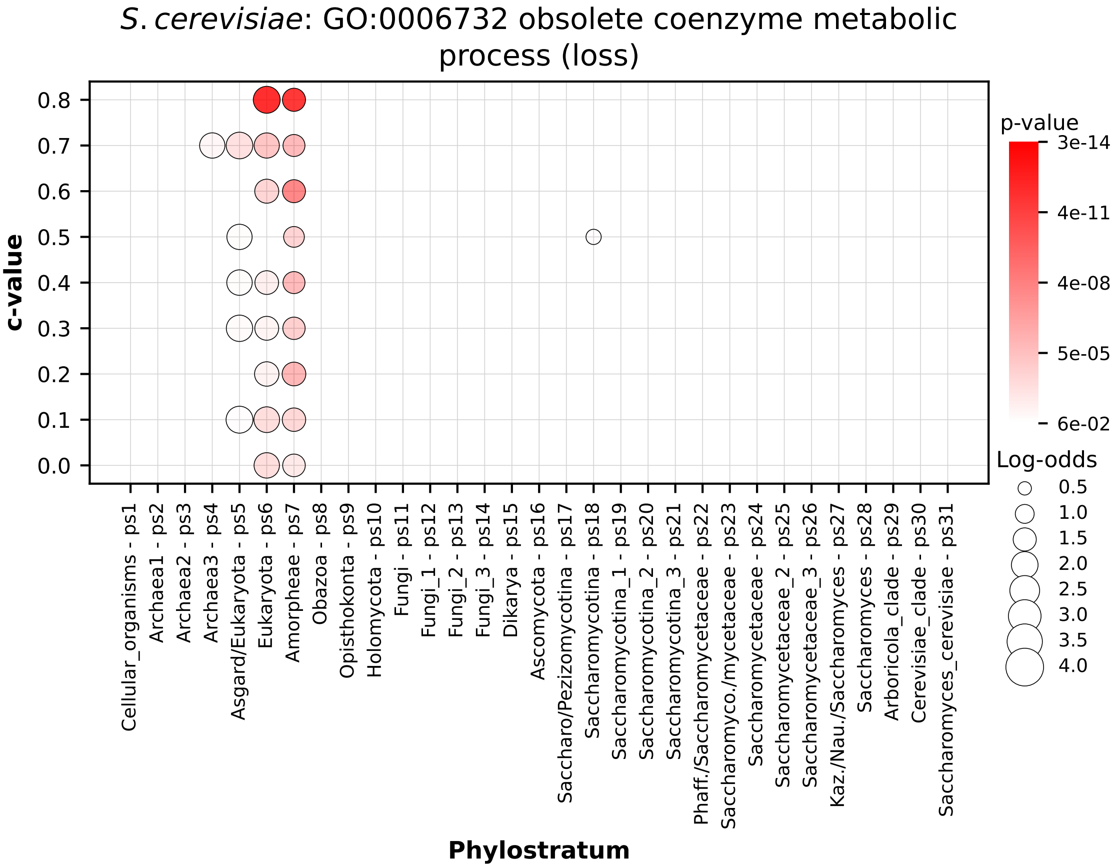

S. cerevisiae: GO:0006732 obsolete coenzyme metabolic process (loss)
c-value
0.8
0.7
0.6
0.5
0.4
0.3
0.2
0.1
0.0
Phylostratum
Cellular_organisms - ps1
Archaea1 - ps2
Archaea2 - ps3
Archaea3 - ps4
Asgard/Eukaryota - ps5
Eukaryota - ps6
Amorpheae - ps7
Obazoa - ps8
Opisthokonta - ps9
Holomycota - ps10
Fungi - ps11
Fungi_1 - ps12
Fungi_2 - ps13
Fungi_3 - ps14
Dikarya - ps15
Ascomycota - ps16
Saccharo/Pezizomycotina - ps17
Saccharomycotina - ps18
Saccharomycotina_1 - ps19
Saccharomycotina_2 - ps20
Saccharomycotina_3 - ps21
Phaff./Saccharomycetaceae - ps22
Saccharomyco./mycetaceae - ps23
Saccharomycetaceae - ps24
Saccharomycetaceae_2 - ps25
Saccharomycetaceae_3 - ps26
Kaz./Nau./Saccharomyces - ps27
Saccharomyces - ps28
Arboricola_clade - ps29
Cerevisiae_clade - ps30
Saccharomyces_cerevisiae - ps31
p-value
3e-14
4e-11
4e-08
5e-05
6e-02
Log-odds
0.5
1.0
1.5
2.0
2.5
3.0
3.5
4.0

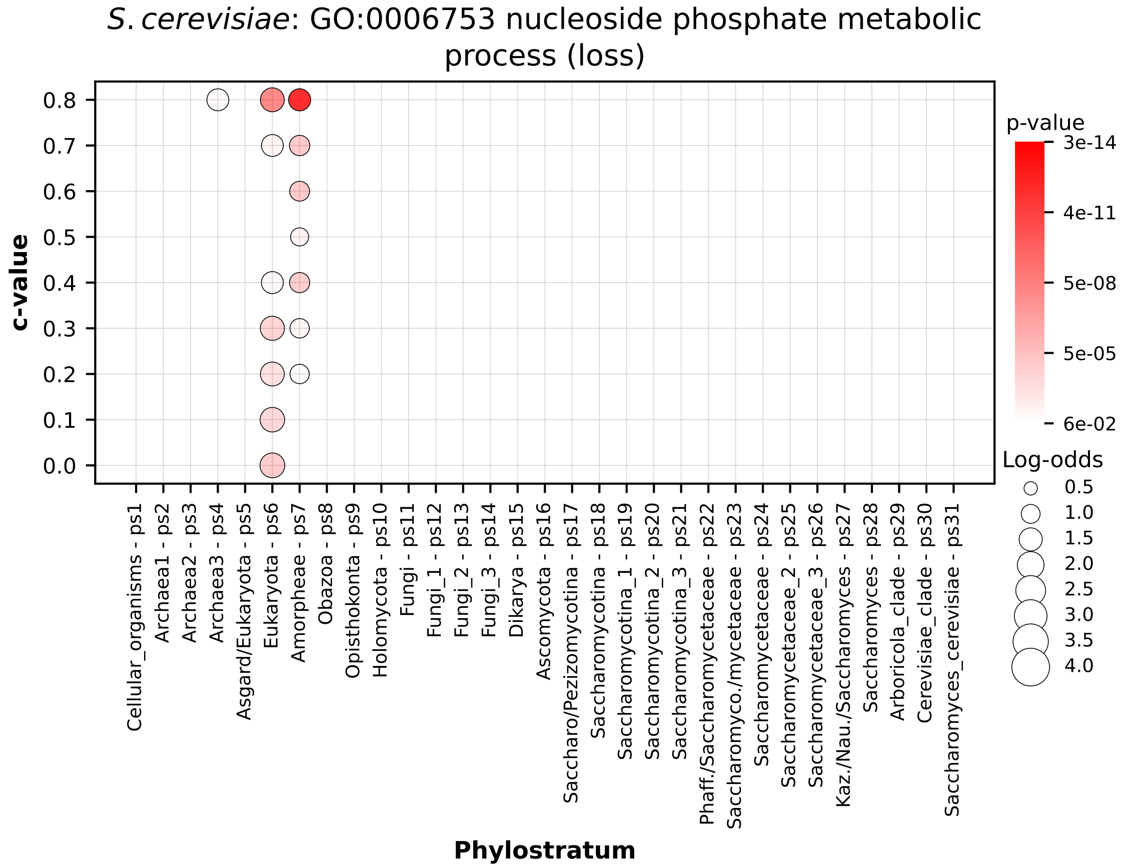
S. cerevisiae: GO:0006753 nucleoside phosphate metabolic process (loss)
c-value
0.8
0.7
0.6
0.5
0.4
0.3
0.2
0.1
0.0
p-value
3e-14
4e-11
5e-08
5e-05
6e-02
Log-odds
0.5
1.0
1.5
2.0
2.5
3.0
3.5
4.0
Cellular_organisms - ps1
Archaea1 - ps2
Archaea2 - ps3
Archaea3 - ps4
Asgard/Eukaryota - ps5
Eukaryota - ps6
Amorpheae - ps7
Obazoa - ps8
Opisthokonta - ps9
Holomycota - ps10
Fungi - ps11
Fungi_1 - ps12
Fungi_2 - ps13
Fungi_3 - ps14
Dikarya - ps15
Ascomycota - ps16
Saccharo/Pezizomycotina - ps17
Saccharomycotina - ps18
Saccharomycotina_1 - ps19
Saccharomycotina_2 - ps20
Saccharomycotina_3 - ps21
Phaff./Saccharomycetaceae - ps22
Saccharomyco./mycetaceae - ps23
Saccharomycetaceae - ps24
Saccharomycetaceae_2 - ps25
Saccharomycetaceae_3 - ps26
Kaz./Nau./Saccharomyces - ps27
Saccharomyces - ps28
Arboricola_clade - ps29
Cerevisiae_clade - ps30
Saccharomyces_cerevisiae - ps31
Phylostratum

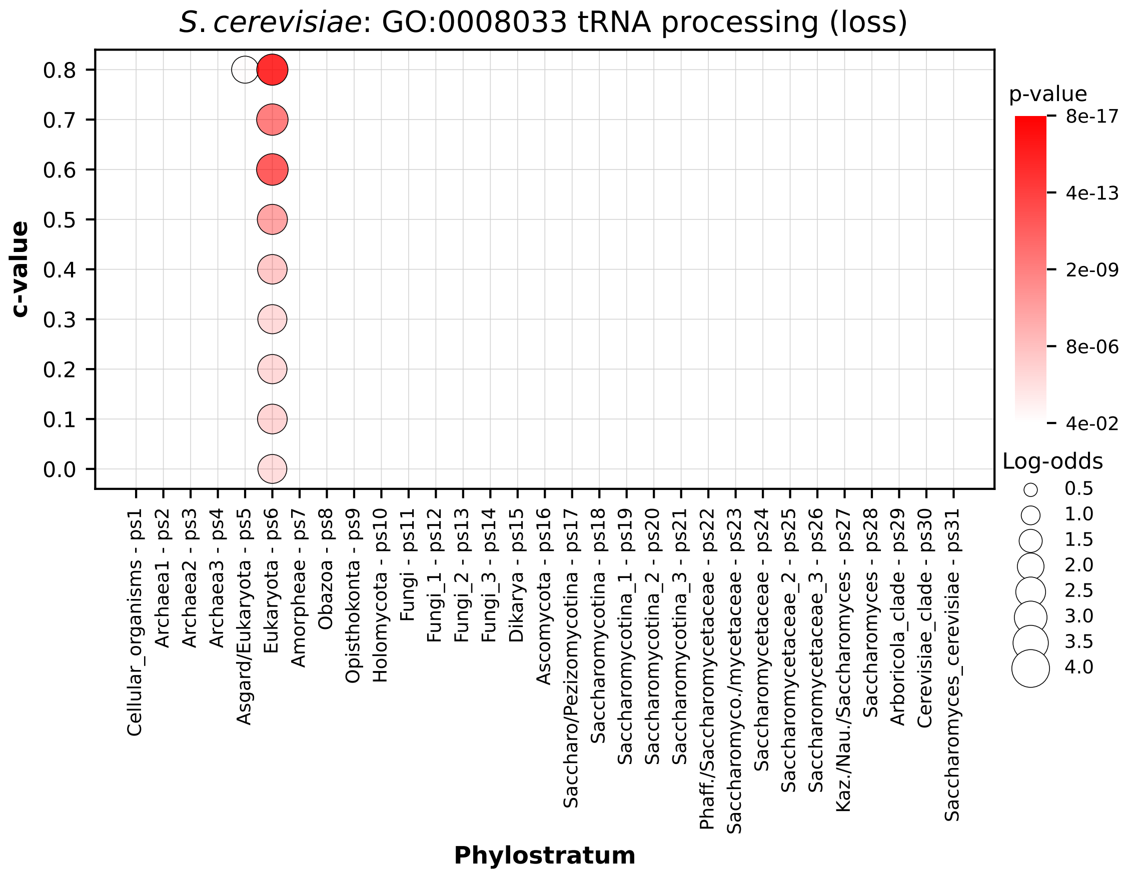

S. cerevisiae: GO:0008033 tRNA processing (loss)
c-value
0.8
0.7
0.6
0.5
0.4
0.3
0.2
0.1
0.0
p-value
8e-17
4e-13
2e-09
8e-06
4e-02
Log-odds
0.5
1.0
1.5
2.0
2.5
3.0
3.5
4.0
Cellular_organisms - ps1
Archaea1 - ps2
Archaea2 - ps3
Archaea3 - ps4
Asgard/Eukaryota - ps5
Eukaryota - ps6
Amorpheae - ps7
Obazoa - ps8
Opisthokonta - ps9
Holomycota - ps10
Fungi - ps11
Fungi_1 - ps12
Fungi_2 - ps13
Fungi_3 - ps14
Dikarya - ps15
Ascomycota - ps16
Saccharo/Pezizomycotina - ps17
Saccharomycotina - ps18
Saccharomycotina_1 - ps19
Saccharomycotina_2 - ps20
Saccharomycotina_3 - ps21
Phaff./Saccharomycetaceae - ps22
Saccharomyco./mycetaceae - ps23
Saccharomycetaceae - ps24
Saccharomycetaceae_2 - ps25
Saccharomycetaceae_3 - ps26
Kaz./Nau./Saccharomyces - ps27
Saccharomyces - ps28
Arboricola_clade - ps29
Cerevisiae_clade - ps30
Saccharomyces_cerevisiae - ps31
Phylostratum

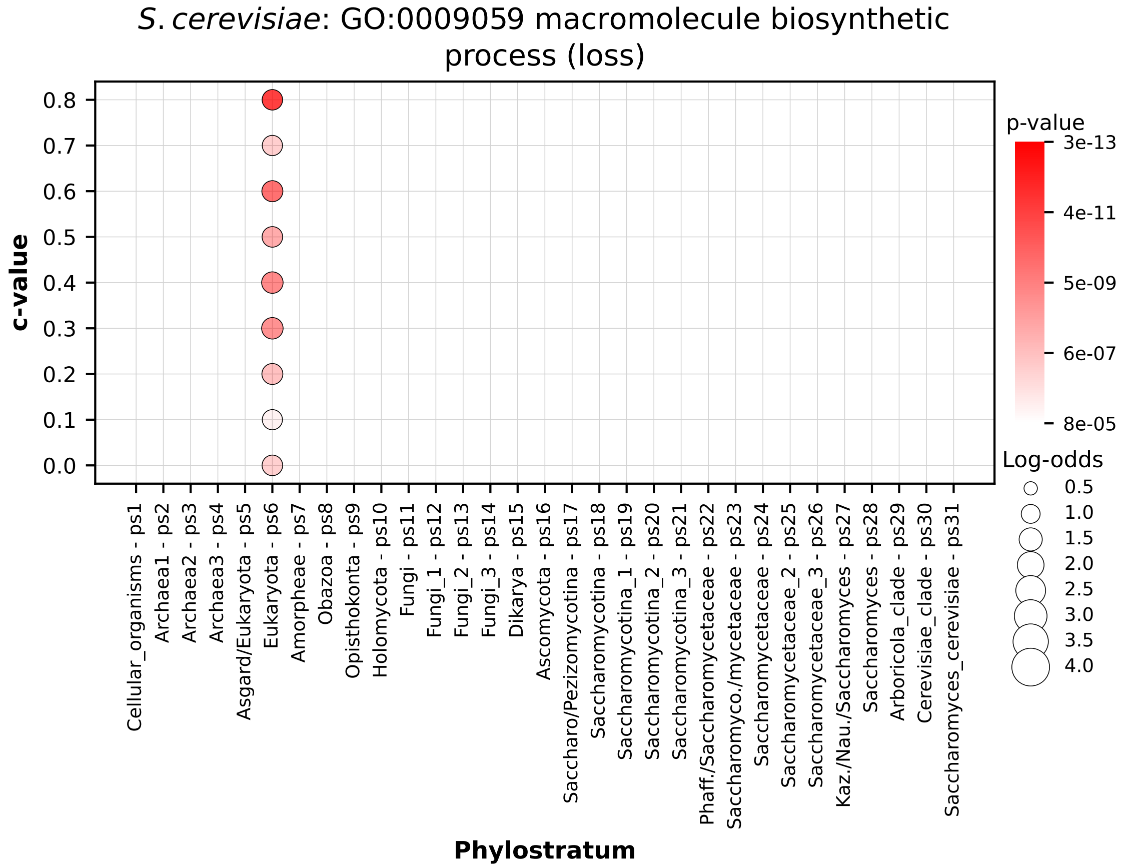

S. cerevisiae: GO:0009059 macromolecule biosynthetic process (loss)
c-value
0.8
0.7
0.6
0.5
0.4
0.3
0.2
0.1
0.0
p-value
3e-13
4e-11
5e-09
6e-07
8e-05
Log-odds
0.5
1.0
1.5
2.0
2.5
3.0
3.5
4.0
Cellular_organisms - ps1
Archaea1 - ps2
Archaea2 - ps3
Archaea3 - ps4
Asgard/Eukaryota - ps5
Eukaryota - ps6
Amorpheae - ps7
Obazoa - ps8
Opisthokonta - ps9
Holomycota - ps10
Fungi - ps11
Fungi_1 - ps12
Fungi_2 - ps13
Fungi_3 - ps14
Dikarya - ps15
Ascomycota - ps16
Saccharo/Pezizomycotina - ps17
Saccharomycotina - ps18
Saccharomycotina_1 - ps19
Saccharomycotina_2 - ps20
Saccharomycotina_3 - ps21
Phaff./Saccharomycetaceae - ps22
Saccharomyco./mycetaceae - ps23
Saccharomycetaceae - ps24
Saccharomycetaceae_2 - ps25
Saccharomycetaceae_3 - ps26
Kaz./Nau./Saccharomyces - ps27
Saccharomyces - ps28
Arboricola_clade - ps29
Cerevisiae_clade - ps30
Saccharomyces_cerevisiae - ps31
Phylostratum

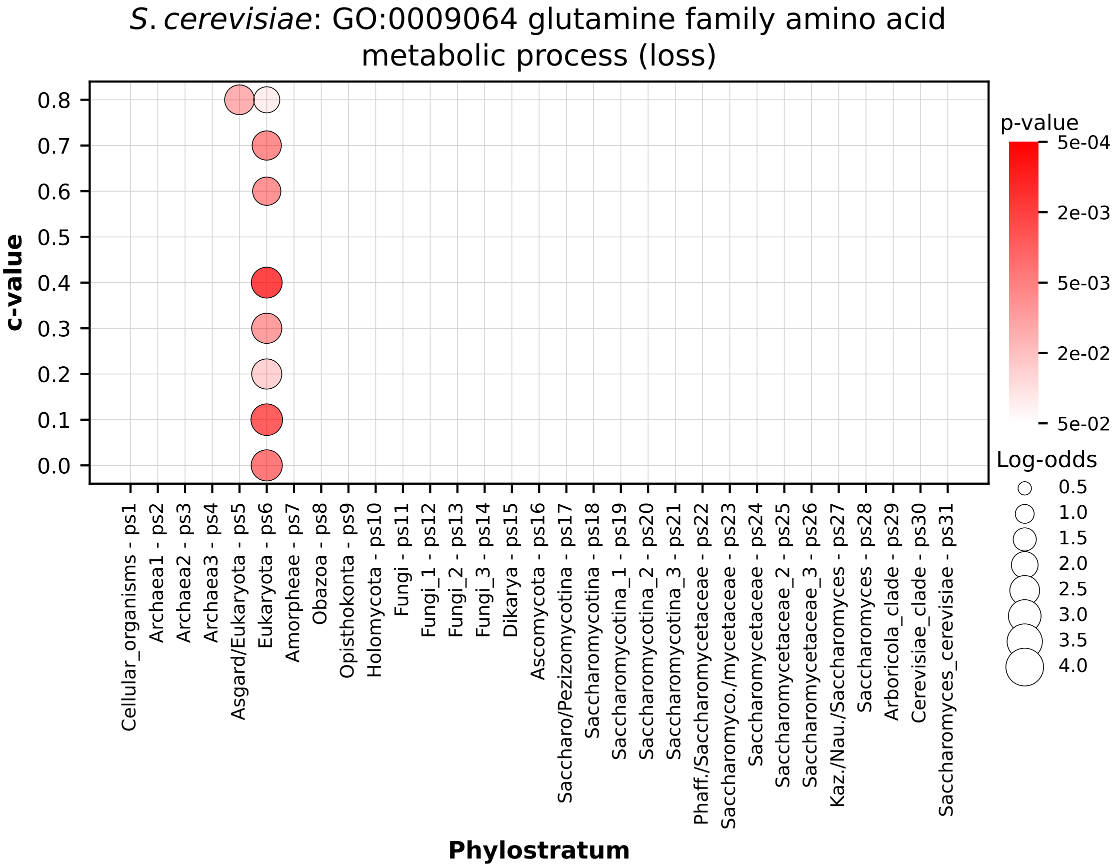

S. cerevisiae: GO:0009064 glutamine family amino acid metabolic process (loss)
c-value
0.8
0.7
0.6
0.5
0.4
0.3
0.2
0.1
0.0
p-value
5e-04
2e-03
5e-03
2e-02
5e-02
Log-odds
0.5
1.0
1.5
2.0
2.5
3.0
3.5
4.0
Cellular_organisms - ps1
Archaea1 - ps2
Archaea2 - ps3
Archaea3 - ps4
Asgard/Eukaryota - ps5
Eukaryota - ps6
Amorpheae - ps7
Obazoa - ps8
Opisthokonta - ps9
Holomycota - ps10
Fungi - ps11
Fungi_1 - ps12
Fungi_2 - ps13
Fungi_3 - ps14
Dikarya - ps15
Ascomycota - ps16
Saccharo/Pezizomycotina - ps17
Saccharomycotina - ps18
Saccharomycotina_1 - ps19
Saccharomycotina_2 - ps20
Saccharomycotina_3 - ps21
Phaff./Saccharomycetaceae - ps22
Saccharomyco./mycetaceae - ps23
Saccharomycetaceae - ps24
Saccharomycetaceae_2 - ps25
Saccharomycetaceae_3 - ps26
Kaz./Nau./Saccharomyces - ps27
Saccharomyces - ps28
Arboricola_clade - ps29
Cerevisiae_clade - ps30
Saccharomyces_cerevisiae - ps31
Phylostratum

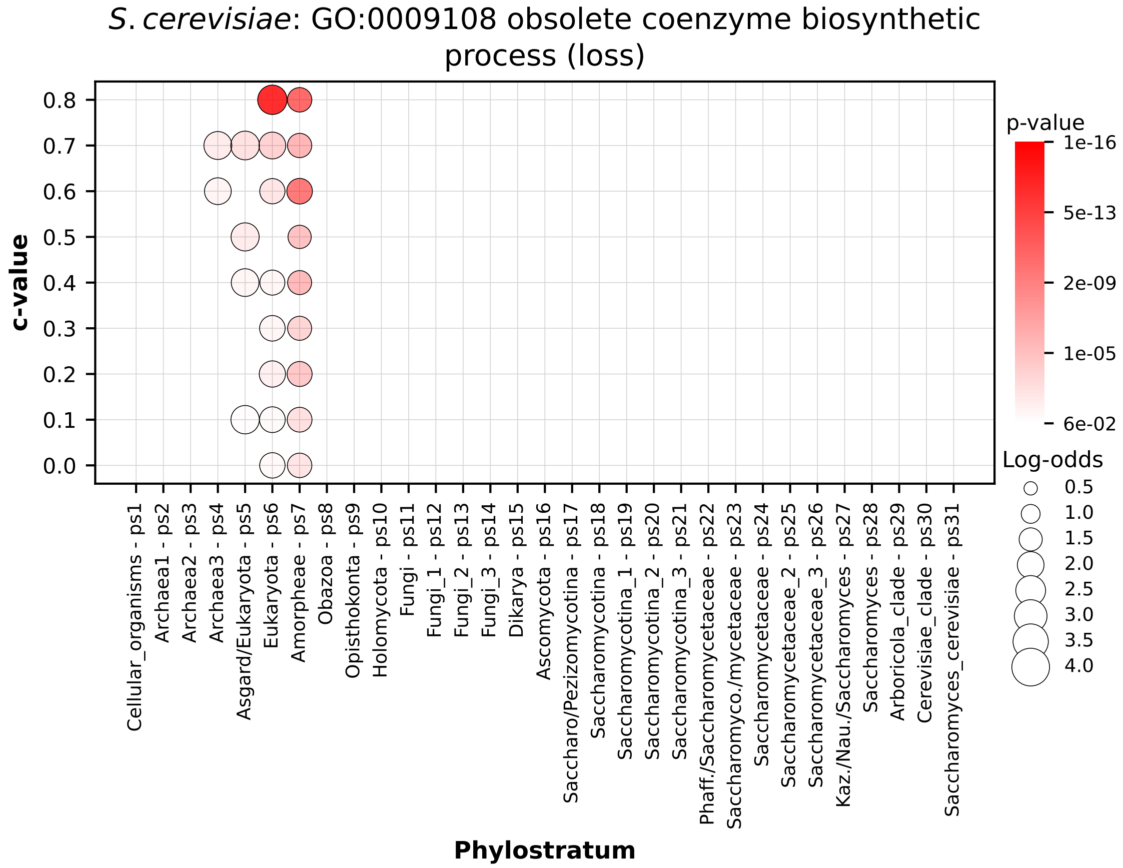

S. cerevisiae: GO:0009108 obsolete coenzyme biosynthetic process (loss)
c-value
0.8
0.7
0.6
0.5
0.4
0.3
0.2
0.1
0.0
p-value
1e-16
5e-13
2e-09
1e-05
6e-02
Log-odds
0.5
1.0
1.5
2.0
2.5
3.0
3.5
4.0
Cellular_organisms - ps1
Archaea1 - ps2
Archaea2 - ps3
Archaea3 - ps4
Asgard/Eukaryota - ps5
Eukaryota - ps6
Amorpheae - ps7
Obazoa - ps8
Opisthokonta - ps9
Holomycota - ps10
Fungi - ps11
Fungi_1 - ps12
Fungi_2 - ps13
Fungi_3 - ps14
Dikarya - ps15
Ascomycota - ps16
Saccharo/Pezizomycotina - ps17
Saccharomycotina - ps18
Saccharomycotina_1 - ps19
Saccharomycotina_2 - ps20
Saccharomycotina_3 - ps21
Phaff./Saccharomycetaceae - ps22
Saccharomyco./mycetaceae - ps23
Saccharomycetaceae - ps24
Saccharomycetaceae_2 - ps25
Saccharomycetaceae_3 - ps26
Kaz./Nau./Saccharomyces - ps27
Saccharomyces - ps28
Arboricola_clade - ps29
Cerevisiae_clade - ps30
Saccharomyces_cerevisiae - ps31
Phylostratum

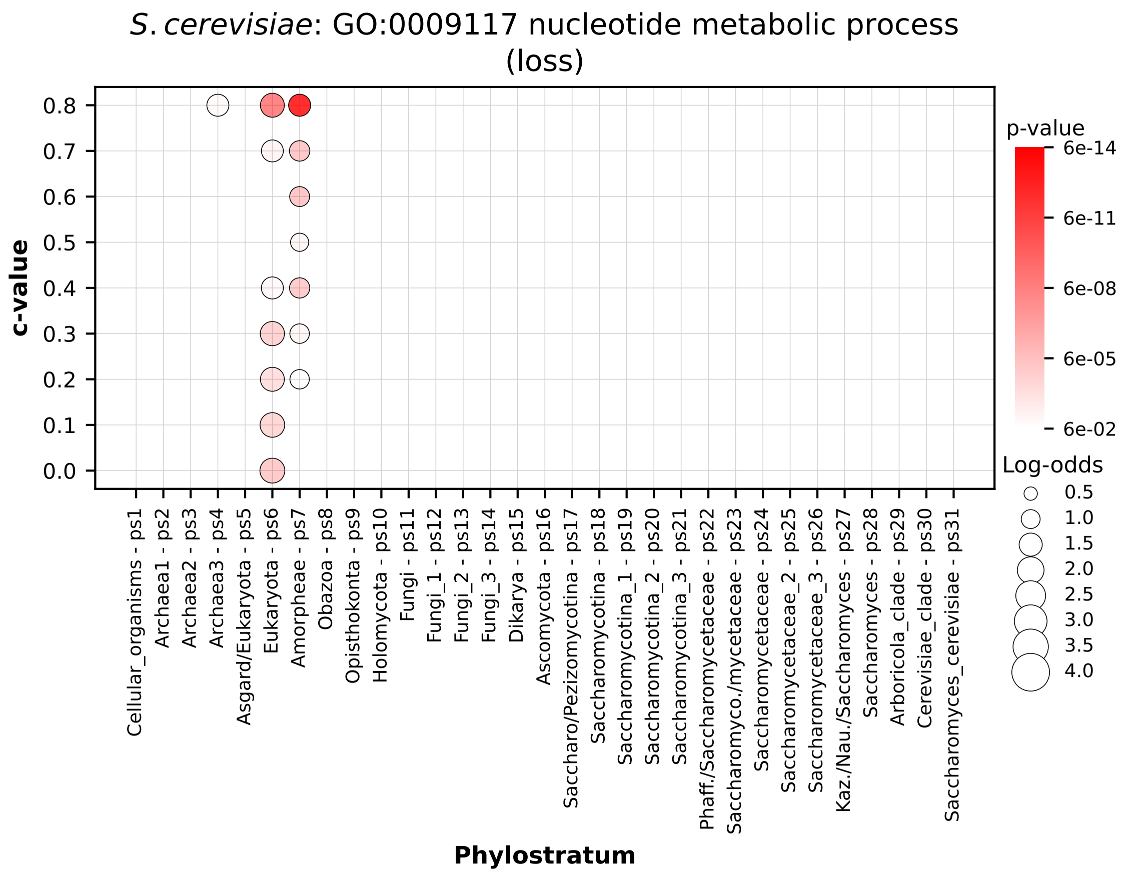
S. cerevisiae: GO:0009117 nucleotide metabolic process
(loss)
c-value
0.8
0.7
0.6
0.5
0.4
0.3
0.2
0.1
0.0
p-value
6e-14
6e-11
6e-08
6e-05
6e-02
Log-odds
0.5
1.0
1.5
2.0
2.5
3.0
3.5
4.0
Cellular_organisms - ps1
Archaea1 - ps2
Archaea2 - ps3
Archaea3 - ps4
Asgard/Eukaryota - ps5
Eukaryota - ps6
Amorpheae - ps7
Obazoa - ps8
Opisthokonta - ps9
Holomycota - ps10
Fungi - ps11
Fungi_1 - ps12
Fungi_2 - ps13
Fungi_3 - ps14
Dikarya - ps15
Ascomycota - ps16
Saccharo/Pezizomycotina - ps17
Saccharomycotina - ps18
Saccharomycotina_1 - ps19
Saccharomycotina_2 - ps20
Saccharomycotina_3 - ps21
Phaff./Saccharomycetaceae - ps22
Saccharomyco./mycetaceae - ps23
Saccharomycetaceae - ps24
Saccharomycetaceae_2 - ps25
Saccharomycetaceae_3 - ps26
Kaz./Nau./Saccharomyces - ps27
Saccharomyces - ps28
Arboricola_clade - ps29
Cerevisiae_clade - ps30
Saccharomyces_cerevisiae - ps31
Phylostratum

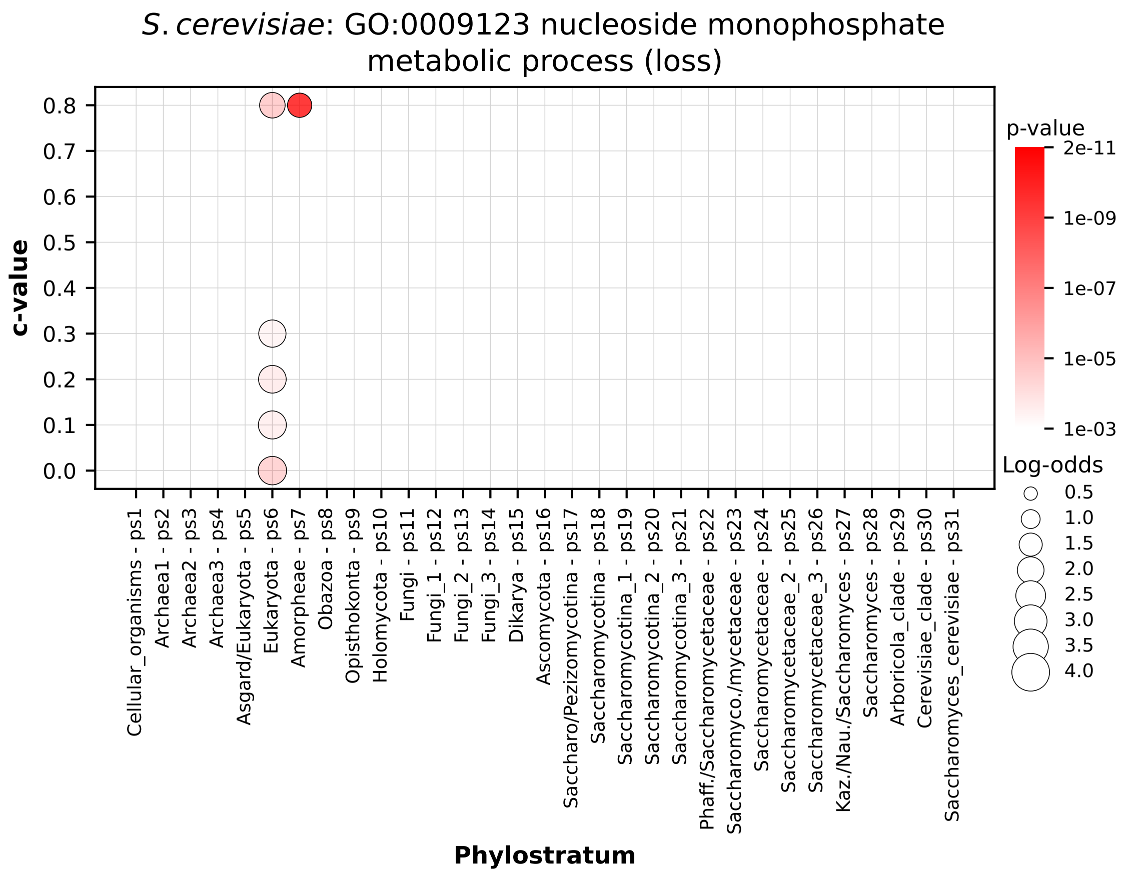
S. cerevisiae: GO:0009123 nucleoside monophosphate metabolic process (loss)
c-value
0.8
0.7
0.6
0.5
0.4
0.3
0.2
0.1
0.0
p-value
2e-11
1e-09
1e-07
1e-05
1e-03
Log-odds
0.5
1.0
1.5
2.0
2.5
3.0
3.5
4.0
Cellular_organisms - ps1
Archaea1 - ps2
Archaea2 - ps3
Archaea3 - ps4
Asgard/Eukaryota - ps5
Eukaryota - ps6
Amorpheae - ps7
Obazoa - ps8
Opisthokonta - ps9
Holomycota - ps10
Fungi - ps11
Fungi_1 - ps12
Fungi_2 - ps13
Fungi_3 - ps14
Dikarya - ps15
Ascomycota - ps16
Saccharo/Pezizomycotina - ps17
Saccharomycotina - ps18
Saccharomycotina_1 - ps19
Saccharomycotina_2 - ps20
Saccharomycotina_3 - ps21
Phaff./Saccharomycetaceae - ps22
Saccharomyco./mycetaceae - ps23
Saccharomycetaceae - ps24
Saccharomycetaceae_2 - ps25
Saccharomycetaceae_3 - ps26
Kaz./Nau./Saccharomyces - ps27
Saccharomyces - ps28
Arboricola_clade - ps29
Cerevisiae_clade - ps30
Saccharomyces_cerevisiae - ps31
Phylostratum

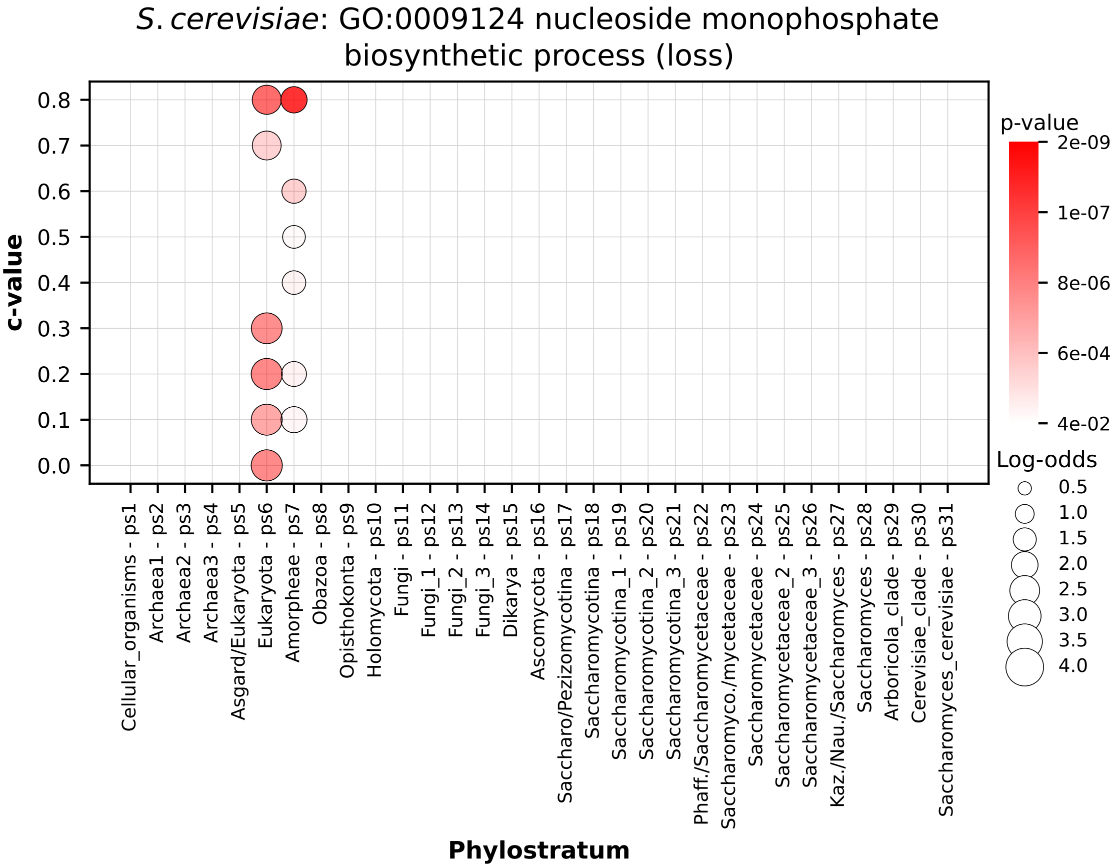

S. cerevisiae: GO:0009124 nucleoside monophosphate biosynthetic process (loss)
c-value
0.8
0.7
0.6
0.5
0.4
0.3
0.2
0.1
0.0
p-value
2e-09
1e-07
8e-06
6e-04
4e-02
Log-odds
0.5
1.0
1.5
2.0
2.5
3.0
3.5
4.0
Cellular_organisms - ps1
Archaea1 - ps2
Archaea2 - ps3
Archaea3 - ps4
Asgard/Eukaryota - ps5
Eukaryota - ps6
Amorpheae - ps7
Obazoa - ps8
Opisthokonta - ps9
Holomycota - ps10
Fungi - ps11
Fungi_1 - ps12
Fungi_2 - ps13
Fungi_3 - ps14
Dikarya - ps15
Ascomycota - ps16
Saccharo/Pezizomycotina - ps17
Saccharomycotina - ps18
Saccharomycotina_1 - ps19
Saccharomycotina_2 - ps20
Saccharomycotina_3 - ps21
Phaff./Saccharomycetaceae - ps22
Saccharomyco./mycetaceae - ps23
Saccharomycetaceae - ps24
Saccharomycetaceae_2 - ps25
Saccharomycetaceae_3 - ps26
Kaz./Nau./Saccharomyces - ps27
Saccharomyces - ps28
Arboricola_clade - ps29
Cerevisiae_clade - ps30
Saccharomyces_cerevisiae - ps31
Phylostratum

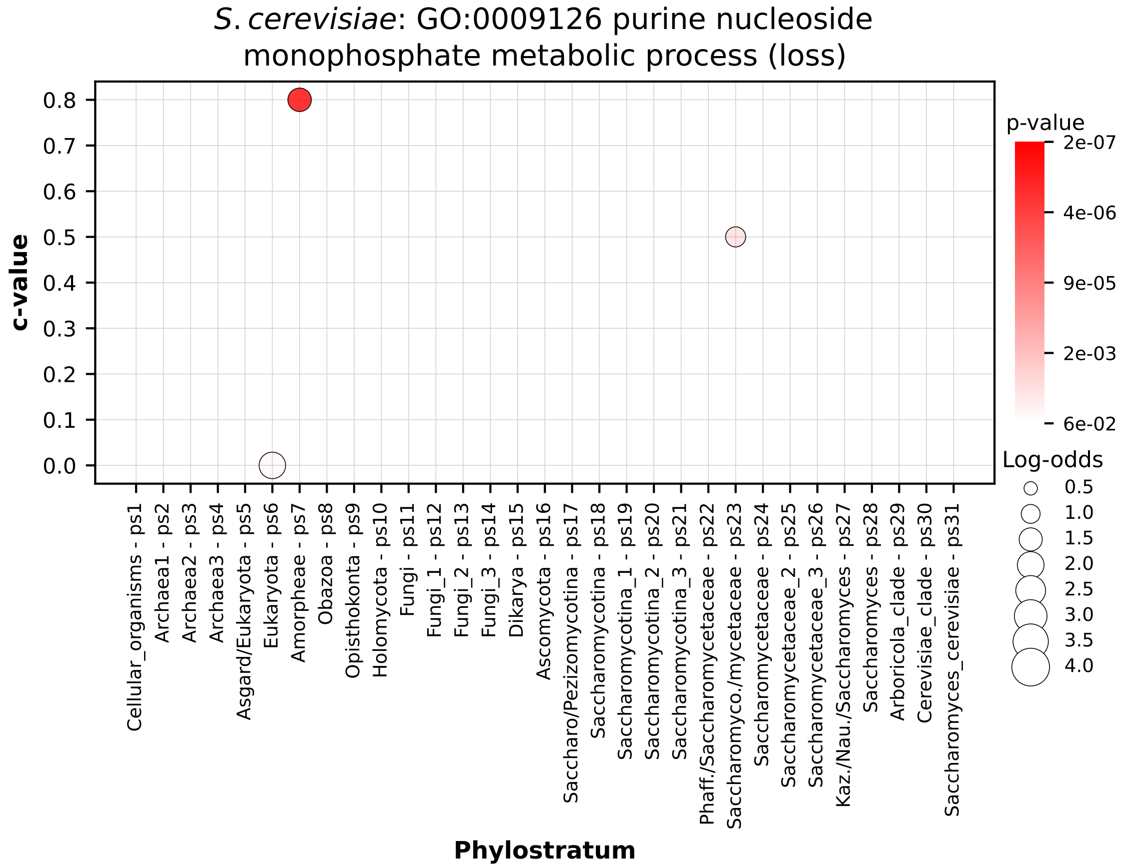

S. cerevisiae: GO:0009126 purine nucleoside monophosphate metabolic process (loss)
c-value
0.8
0.7
0.6
0.5
0.4
0.3
0.2
0.1
0.0
p-value
2e-07
4e-06
9e-05
2e-03
6e-02
Log-odds
0.5
1.0
1.5
2.0
2.5
3.0
3.5
4.0
Cellular_organisms - ps1
Archaea1 - ps2
Archaea2 - ps3
Archaea3 - ps4
Asgard/Eukaryota - ps5
Eukaryota - ps6
Amorpheae - ps7
Obazoa - ps8
Opisthokonta - ps9
Holomycota - ps10
Fungi - ps11
Fungi_1 - ps12
Fungi_2 - ps13
Fungi_3 - ps14
Dikarya - ps15
Ascomycota - ps16
Saccharo/Pezizomycotina - ps17
Saccharomycotina - ps18
Saccharomycotina_1 - ps19
Saccharomycotina_2 - ps20
Saccharomycotina_3 - ps21
Phaff./Saccharomycetaceae - ps22
Saccharomyco./mycetaceae - ps23
Saccharomycetaceae - ps24
Saccharomycetaceae_2 - ps25
Saccharomycetaceae_3 - ps26
Kaz./Nau./Saccharomyces - ps27
Saccharomyces - ps28
Arboricola_clade - ps29
Cerevisiae_clade - ps30
Saccharomyces_cerevisiae - ps31
Phylostratum

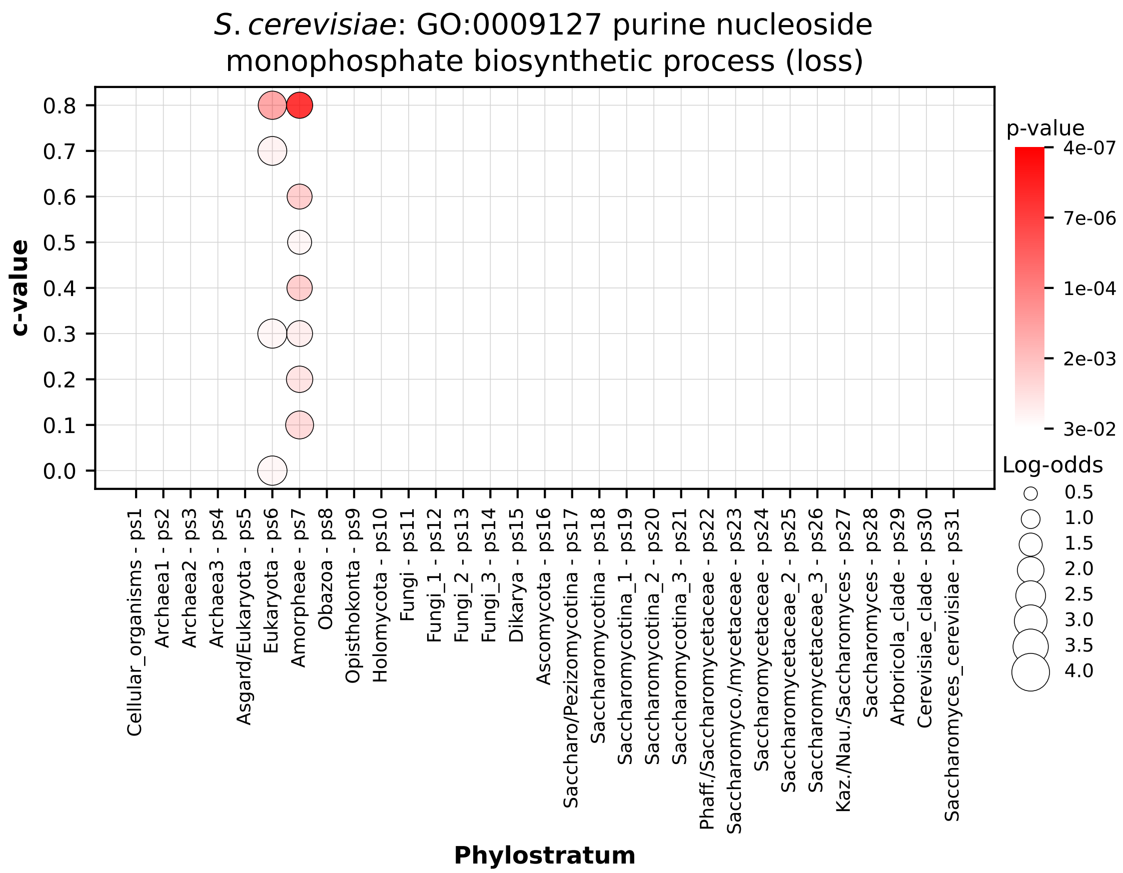

S. cerevisiae: GO:0009127 purine nucleoside monophosphate biosynthetic process (loss)
c-value
0.8
0.7
0.6
0.5
0.4
0.3
0.2
0.1
0.0
Phylostratum
Cellular_organisms - ps1
Archaea1 - ps2
Archaea2 - ps3
Archaea3 - ps4
Asgard/Eukaryota - ps5
Eukaryota - ps6
Amorpheae - ps7
Obazoa - ps8
Opisthokonta - ps9
Holomycota - ps10
Fungi - ps11
Fungi_1 - ps12
Fungi_2 - ps13
Fungi_3 - ps14
Dikarya - ps15
Ascomycota - ps16
Saccharo/Pezizomycotina - ps17
Saccharomycotina - ps18
Saccharomycotina_1 - ps19
Saccharomycotina_2 - ps20
Saccharomycotina_3 - ps21
Phaff./Saccharomycetaceae - ps22
Saccharomyco./mycetaceae - ps23
Saccharomycetaceae - ps24
Saccharomycetaceae_2 - ps25
Saccharomycetaceae_3 - ps26
Kaz./Nau./Saccharomyces - ps27
Saccharomyces - ps28
Arboricola_clade - ps29
Cerevisiae_clade - ps30
Saccharomyces_cerevisiae - ps31
p-value
4e-07
7e-06
1e-04
2e-03
3e-02
Log-odds
0.5
1.0
1.5
2.0
2.5
3.0
3.5
4.0

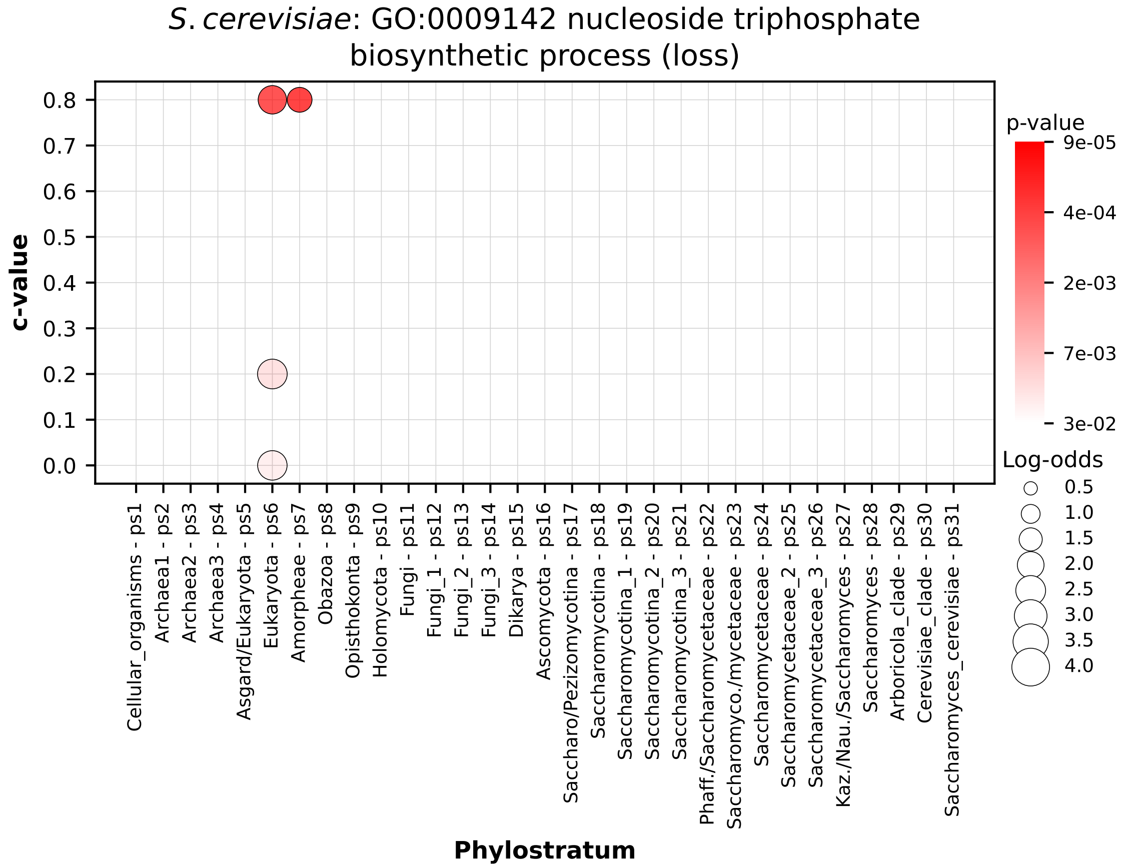

S. cerevisiae: GO:0009142 nucleoside triphosphate biosynthetic process (loss)
c-value
0.0
0.1
0.2
0.3
0.4
0.5
0.6
0.7
0.8
p-value
9e-05
4e-04
2e-03
7e-03
3e-02
Log-odds
0.5
1.0
1.5
2.0
2.5
3.0
3.5
4.0
Cellular_organisms - ps1
Archaea1 - ps2
Archaea2 - ps3
Archaea3 - ps4
Asgard/Eukaryota - ps5
Eukaryota - ps6
Amorpheae - ps7
Obazoa - ps8
Opisthokonta - ps9
Holomycota - ps10
Fungi - ps11
Fungi_1 - ps12
Fungi_2 - ps13
Fungi_3 - ps14
Dikarya - ps15
Ascomycota - ps16
Saccharo/Pezizomycotina - ps17
Saccharomycotina - ps18
Saccharomycotina_1 - ps19
Saccharomycotina_2 - ps20
Saccharomycotina_3 - ps21
Phaff./Saccharomycetaceae - ps22
Saccharomyco./mycetaceae - ps23
Saccharomycetaceae - ps24
Saccharomycetaceae_2 - ps25
Saccharomycetaceae_3 - ps26
Kaz./Nau./Saccharomyces - ps27
Saccharomyces - ps28
Arboricola_clade - ps29
Cerevisiae_clade - ps30
Saccharomyces_cerevisiae - ps31
Phylostratum

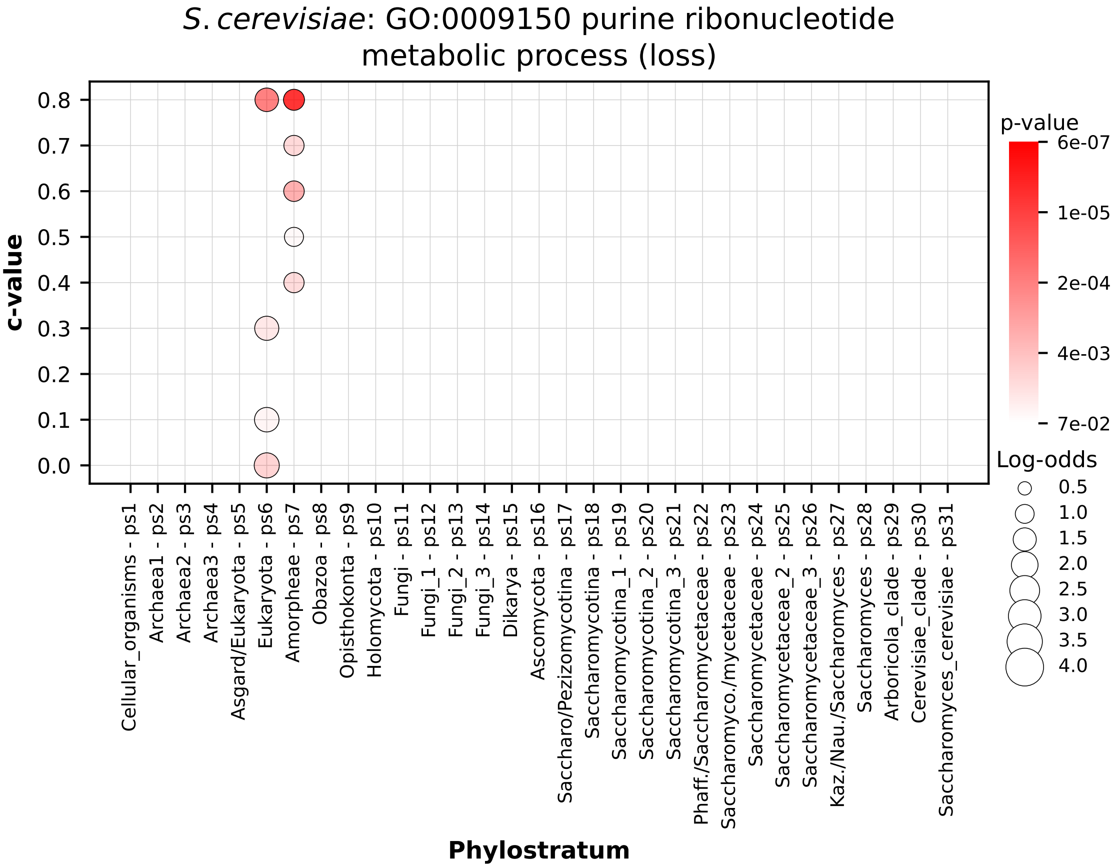
S. cerevisiae: GO:0009150 purine ribonucleotide metabolic process (loss)
c-value
0.8
0.7
0.6
0.5
0.4
0.3
0.2
0.1
0.0
p-value
6e-07
1e-05
2e-04
4e-03
7e-02
Log-odds
0.5
1.0
1.5
2.0
2.5
3.0
3.5
4.0
Cellular_organisms - ps1
Archaea1 - ps2
Archaea2 - ps3
Archaea3 - ps4
Asgard/Eukaryota - ps5
Eukaryota - ps6
Amorpheae - ps7
Obazoa - ps8
Opisthokonta - ps9
Holomycota - ps10
Fungi - ps11
Fungi_1 - ps12
Fungi_2 - ps13
Fungi_3 - ps14
Dikarya - ps15
Ascomycota - ps16
Saccharo/Pezizomycotina - ps17
Saccharomycotina - ps18
Saccharomycotina_1 - ps19
Saccharomycotina_2 - ps20
Saccharomycotina_3 - ps21
Phaff./Saccharomycetaceae - ps22
Saccharomyco./mycetaceae - ps23
Saccharomycetaceae - ps24
Saccharomycetaceae_2 - ps25
Saccharomycetaceae_3 - ps26
Kaz./Nau./Saccharomyces - ps27
Saccharomyces - ps28
Arboricola_clade - ps29
Cerevisiae_clade - ps30
Saccharomyces_cerevisiae - ps31
Phylostratum

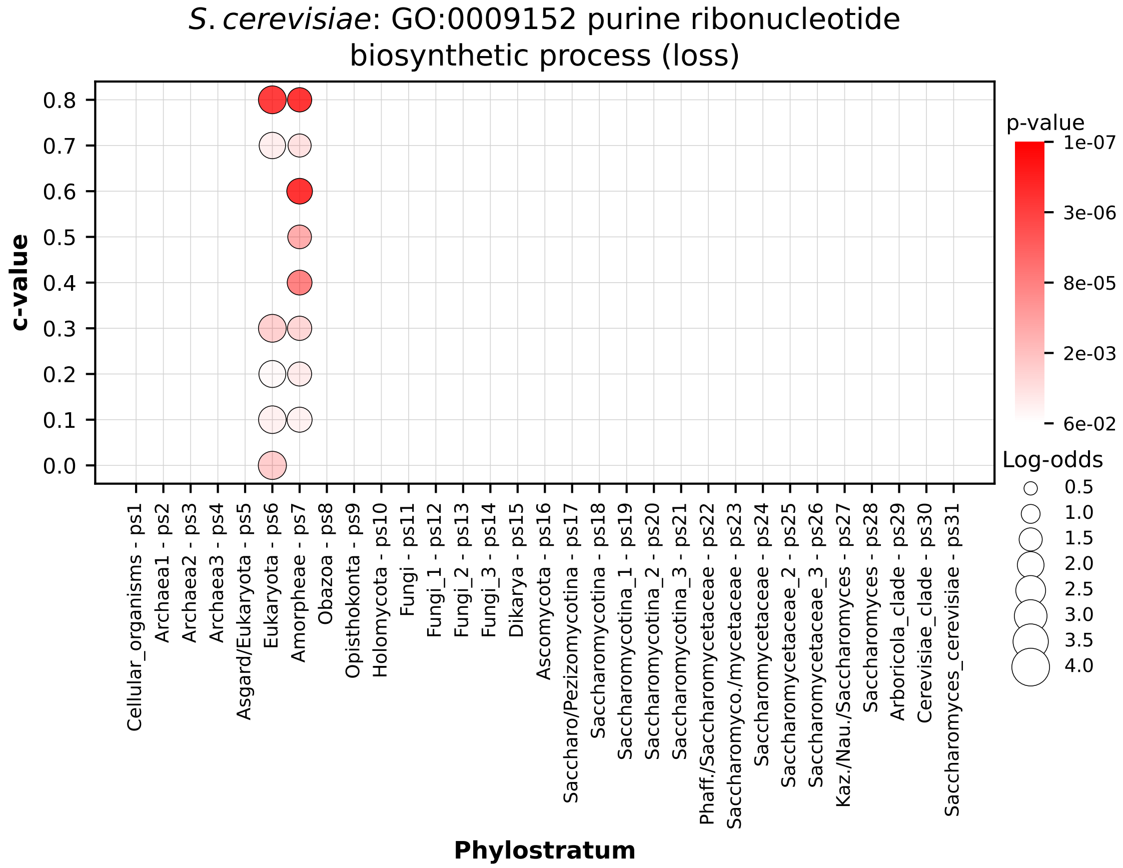
S. cerevisiae: GO:0009152 purine ribonucleotide biosynthetic process (loss)
c-value
0.8
0.7
0.6
0.5
0.4
0.3
0.2
0.1
0.0
p-value
1e-07
3e-06
8e-05
2e-03
6e-02
Log-odds
0.5
1.0
1.5
2.0
2.5
3.0
3.5
4.0
Cellular_organisms - ps1
Archaea1 - ps2
Archaea2 - ps3
Archaea3 - ps4
Asgard/Eukaryota - ps5
Eukaryota - ps6
Amorpheae - ps7
Obazoa - ps8
Opisthokonta - ps9
Holomycota - ps10
Fungi - ps11
Fungi_1 - ps12
Fungi_2 - ps13
Fungi_3 - ps14
Dikarya - ps15
Ascomycota - ps16
Saccharo/Pezizomycotina - ps17
Saccharomycotina - ps18
Saccharomycotina_1 - ps19
Saccharomycotina_2 - ps20
Saccharomycotina_3 - ps21
Phaff./Saccharomycetaceae - ps22
Saccharomyco./mycetaceae - ps23
Saccharomycetaceae - ps24
Saccharomycetaceae_2 - ps25
Saccharomycetaceae_3 - ps26
Kaz./Nau./Saccharomyces - ps27
Saccharomyces - ps28
Arboricola_clade - ps29
Cerevisiae_clade - ps30
Saccharomyces_cerevisiae - ps31
Phylostratum

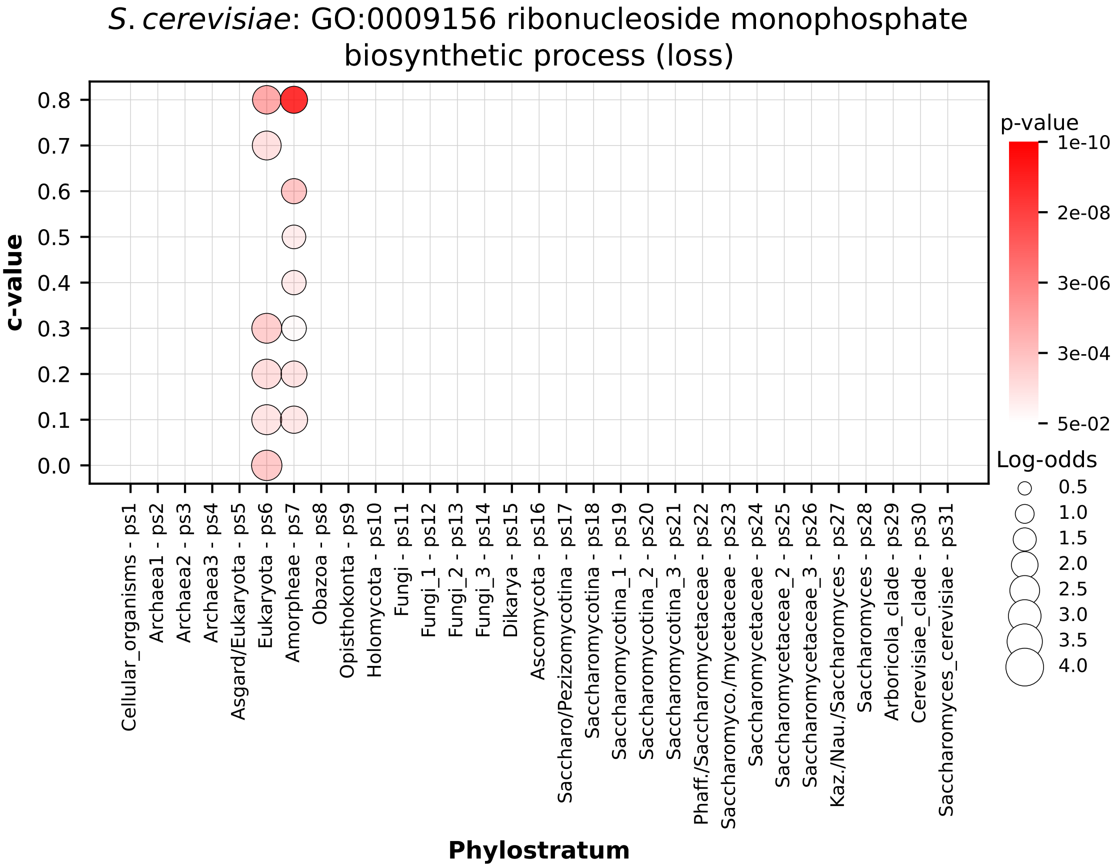

S. cerevisiae: GO:0009156 ribonucleoside monophosphate biosynthetic process (loss)
c-value
0.8
0.7
0.6
0.5
0.4
0.3
0.2
0.1
0.0
p-value
1e-10
2e-08
3e-06
3e-04
5e-02
Log-odds
0.5
1.0
1.5
2.0
2.5
3.0
3.5
4.0
Cellular_organisms - ps1
Archaea1 - ps2
Archaea2 - ps3
Archaea3 - ps4
Asgard/Eukaryota - ps5
Eukaryota - ps6
Amorpheae - ps7
Obazoa - ps8
Opisthokonta - ps9
Holomycota - ps10
Fungi - ps11
Fungi_1 - ps12
Fungi_2 - ps13
Fungi_3 - ps14
Dikarya - ps15
Ascomycota - ps16
Saccharo/Pezizomycotina - ps17
Saccharomycotina - ps18
Saccharomycotina_1 - ps19
Saccharomycotina_2 - ps20
Saccharomycotina_3 - ps21
Phaff./Saccharomycetaceae - ps22
Saccharomyco./mycetaceae - ps23
Saccharomycetaceae - ps24
Saccharomycetaceae_2 - ps25
Saccharomycetaceae_3 - ps26
Kaz./Nau./Saccharomyces - ps27
Saccharomyces - ps28
Arboricola_clade - ps29
Cerevisiae_clade - ps30
Saccharomyces_cerevisiae - ps31
Phylostratum

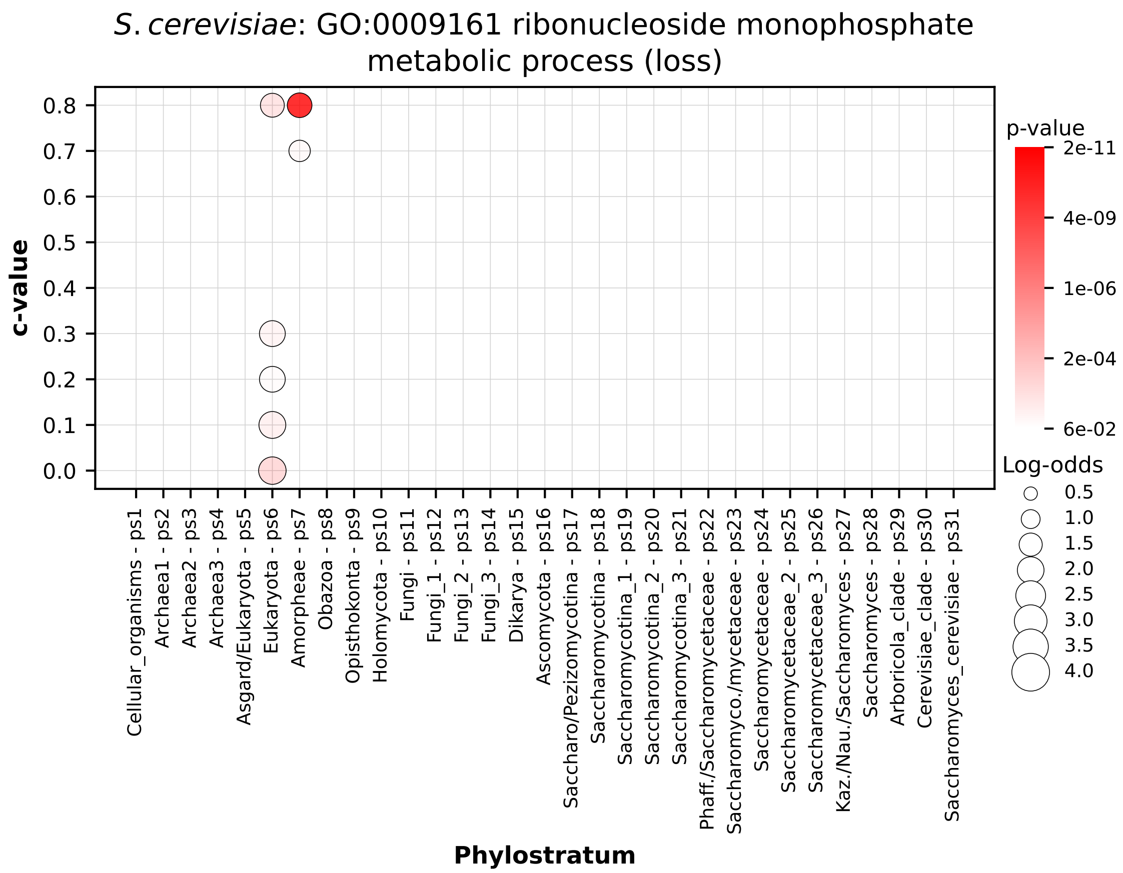

S. cerevisiae: GO:0009161 ribonucleoside monophosphate metabolic process (loss)
c-value
0.8
0.7
0.6
0.5
0.4
0.3
0.2
0.1
0.0
Phylostratum
Cellular_organisms - ps1
Archaea1 - ps2
Archaea2 - ps3
Archaea3 - ps4
Asgard/Eukaryota - ps5
Eukaryota - ps6
Amorpheae - ps7
Obazoa - ps8
Opisthokonta - ps9
Holomycota - ps10
Fungi - ps11
Fungi_1 - ps12
Fungi_2 - ps13
Fungi_3 - ps14
Dikarya - ps15
Ascomycota - ps16
Saccharo/Pezizomycotina - ps17
Saccharomycotina - ps18
Saccharomycotina_1 - ps19
Saccharomycotina_2 - ps20
Saccharomycotina_3 - ps21
Phaff./Saccharomycetaceae - ps22
Saccharomyco./mycetaceae - ps23
Saccharomycetaceae - ps24
Saccharomycetaceae_2 - ps25
Saccharomycetaceae_3 - ps26
Kaz./Nau./Saccharomyces - ps27
Saccharomyces - ps28
Arboricola_clade - ps29
Cerevisiae_clade - ps30
Saccharomyces_cerevisiae - ps31
p-value
2e-11
4e-09
1e-06
2e-04
6e-02
Log-odds
0.5
1.0
1.5
2.0
2.5
3.0
3.5
4.0

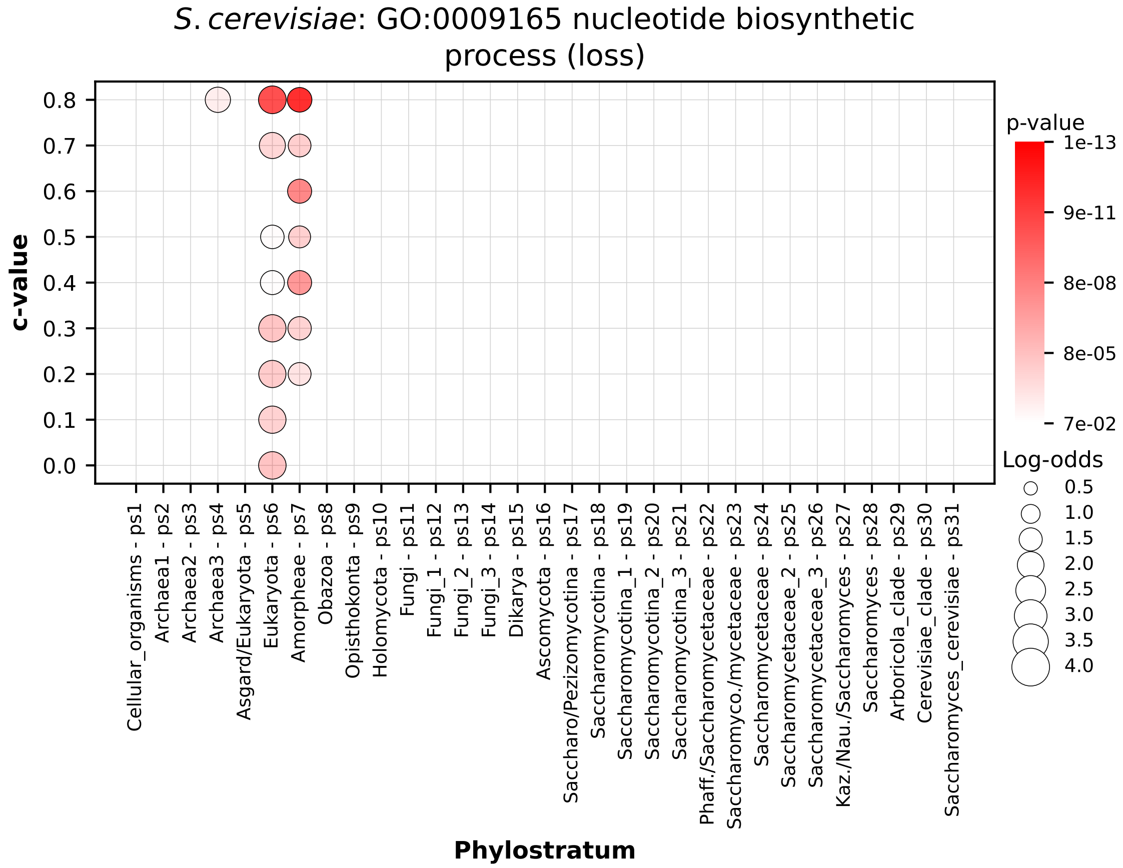

S. cerevisiae: GO:0009165 nucleotide biosynthetic process (loss)
c-value
0.8
0.7
0.6
0.5
0.4
0.3
0.2
0.1
0.0
Phylostratum
Cellular_organisms - ps1
Archaea1 - ps2
Archaea2 - ps3
Archaea3 - ps4
Asgard/Eukaryota - ps5
Eukaryota - ps6
Amorpheae - ps7
Obazoa - ps8
Opisthokonta - ps9
Holomycota - ps10
Fungi - ps11
Fungi_1 - ps12
Fungi_2 - ps13
Fungi_3 - ps14
Dikarya - ps15
Ascomycota - ps16
Saccharo/Pezizomycotina - ps17
Saccharomycotina - ps18
Saccharomycotina_1 - ps19
Saccharomycotina_2 - ps20
Saccharomycotina_3 - ps21
Phaff./Saccharomycetaceae - ps22
Saccharomyco./mycetaceae - ps23
Saccharomycetaceae - ps24
Saccharomycetaceae_2 - ps25
Saccharomycetaceae_3 - ps26
Kaz./Nau./Saccharomyces - ps27
Saccharomyces - ps28
Arboricola_clade - ps29
Cerevisiae_clade - ps30
Saccharomyces_cerevisiae - ps31
p-value
1e-13
9e-11
8e-08
8e-05
7e-02
Log-odds
0.5
1.0
1.5
2.0
2.5
3.0
3.5
4.0

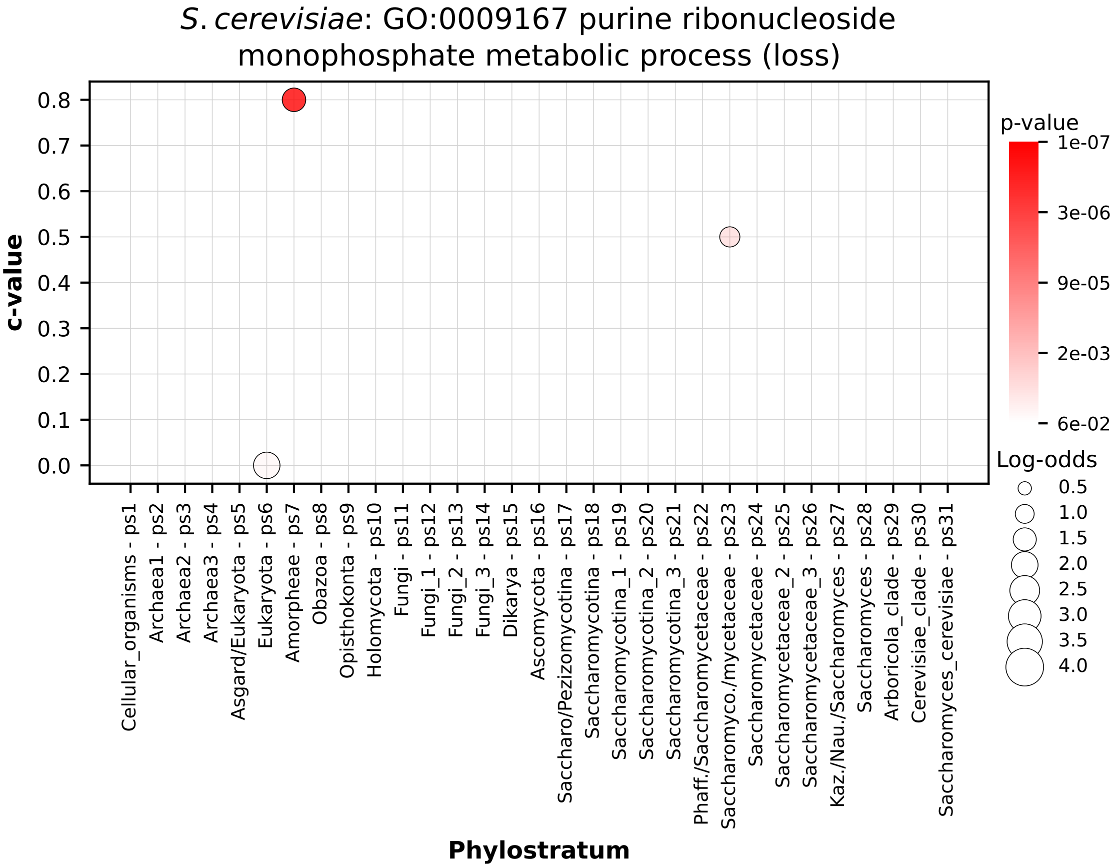
S. cerevisiae: GO:0009167 purine ribonucleoside monophosphate metabolic process (loss)
c-value
0.0
0.1
0.2
0.3
0.4
0.5
0.6
0.7
0.8
Phylostratum
Cellular_organisms - ps1
Archaea1 - ps2
Archaea2 - ps3
Archaea3 - ps4
Asgard/Eukaryota - ps5
Eukaryota - ps6
Amorpheae - ps7
Obazoa - ps8
Opisthokonta - ps9
Holomycota - ps10
Fungi - ps11
Fungi_1 - ps12
Fungi_2 - ps13
Fungi_3 - ps14
Dikarya - ps15
Ascomycota - ps16
Saccharo/Pezizomycotina - ps17
Saccharomycotina - ps18
Saccharomycotina_1 - ps19
Saccharomycotina_2 - ps20
Saccharomycotina_3 - ps21
Phaff./Saccharomycetaceae - ps22
Saccharomyco./mycetaceae - ps23
Saccharomycetaceae - ps24
Saccharomycetaceae_2 - ps25
Saccharomycetaceae_3 - ps26
Kaz./Nau./Saccharomyces - ps27
Saccharomyces - ps28
Arboricola_clade - ps29
Cerevisiae_clade - ps30
Saccharomyces_cerevisiae - ps31
p-value
1e-07
3e-06
9e-05
2e-03
6e-02
Log-odds
0.5
1.0
1.5
2.0
2.5
3.0
3.5
4.0

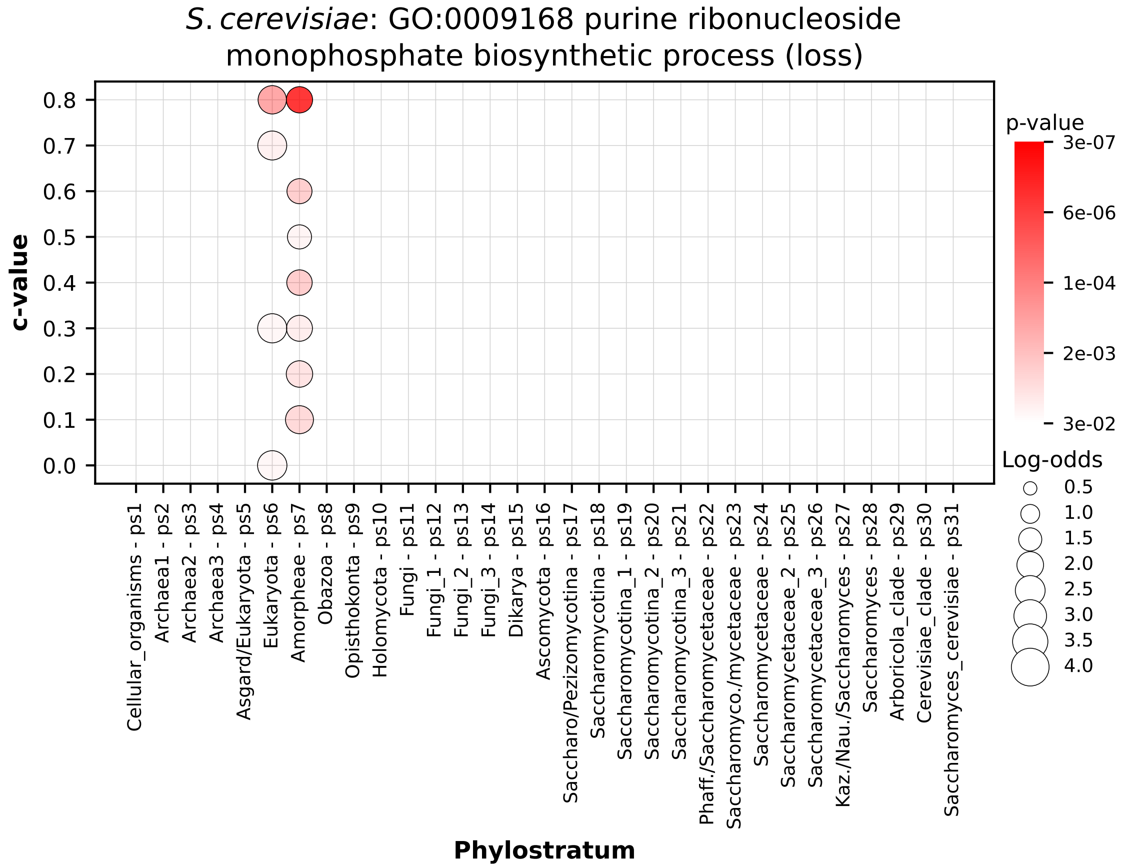

S. cerevisiae: GO:0009168 purine ribonucleoside monophosphate biosynthetic process (loss)
c-value
0.8
0.7
0.6
0.5
0.4
0.3
0.2
0.1
0.0
Phylostratum
Cellular_organisms - ps1
Archaea1 - ps2
Archaea2 - ps3
Archaea3 - ps4
Asgard/Eukaryota - ps5
Eukaryota - ps6
Amorpheae - ps7
Obazoa - ps8
Opisthokonta - ps9
Holomycota - ps10
Fungi - ps11
Fungi_1 - ps12
Fungi_2 - ps13
Fungi_3 - ps14
Dikarya - ps15
Ascomycota - ps16
Saccharo/Pezizomycotina - ps17
Saccharomycotina - ps18
Saccharomycotina_1 - ps19
Saccharomycotina_2 - ps20
Saccharomycotina_3 - ps21
Phaff./Saccharomycetaceae - ps22
Saccharomyco./mycetaceae - ps23
Saccharomycetaceae - ps24
Saccharomycetaceae_2 - ps25
Saccharomycetaceae_3 - ps26
Kaz./Nau./Saccharomyces - ps27
Saccharomyces - ps28
Arboricola_clade - ps29
Cerevisiae_clade - ps30
Saccharomyces_cerevisiae - ps31
p-value
3e-07
6e-06
1e-04
2e-03
3e-02
Log-odds
0.5
1.0
1.5
2.0
2.5
3.0
3.5
4.0

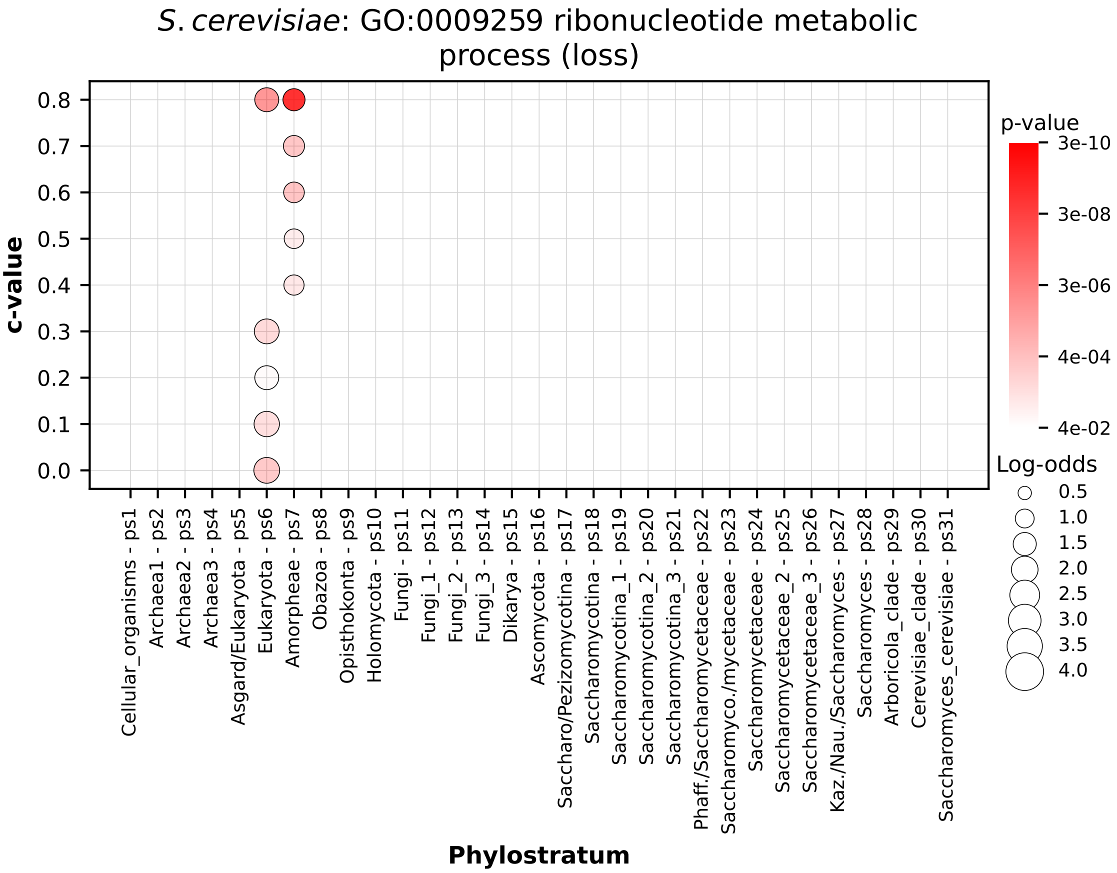

$S. cerevisiae$: GO:0009259 ribonucleotide metabolic process (loss)
c-value
0.8
0.7
0.6
0.5
0.4
0.3
0.2
0.1
0.0
p-value
3e-10
3e-08
3e-06
4e-04
4e-02
Log-odds
0.5
1.0
1.5
2.0
2.5
3.0
3.5
4.0
Cellular_organisms - ps1
Archaea1 - ps2
Archaea2 - ps3
Archaea3 - ps4
Asgard/Eukaryota - ps5
Eukaryota - ps6
Amorpheae - ps7
Obazoa - ps8
Opisthokonta - ps9
Holomycota - ps10
Fungi - ps11
Fungi_1 - ps12
Fungi_2 - ps13
Fungi_3 - ps14
Dikarya - ps15
Ascomycota - ps16
Saccharo/Pezizomycotina - ps17
Saccharomycotina - ps18
Saccharomycotina_1 - ps19
Saccharomycotina_2 - ps20
Saccharomycotina_3 - ps21
Phaff./Saccharomycetaceae - ps22
Saccharomyco./mycetaceae - ps23
Saccharomycetaceae - ps24
Saccharomycetaceae_2 - ps25
Saccharomycetaceae_3 - ps26
Kaz./Nau./Saccharomyces - ps27
Saccharomyces - ps28
Arboricola_clade - ps29
Cerevisiae_clade - ps30
Saccharomyces_cerevisiae - ps31
Phylostratum

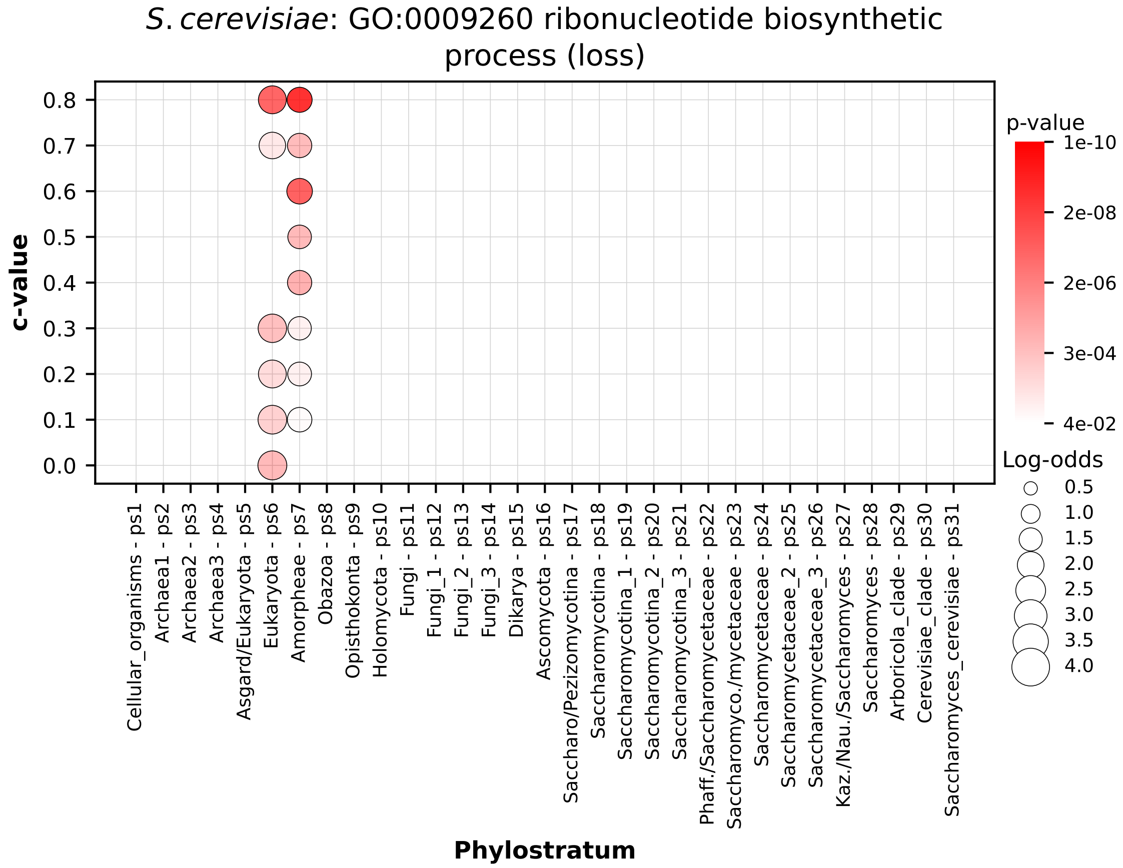
S. cerevisiae: GO:0009260 ribonucleotide biosynthetic process (loss)
c-value
0.8
0.7
0.6
0.5
0.4
0.3
0.2
0.1
0.0
p-value
1e-10
2e-08
2e-06
3e-04
4e-02
Log-odds
0.5
1.0
1.5
2.0
2.5
3.0
3.5
4.0
Cellular_organisms - ps1
Archaea1 - ps2
Archaea2 - ps3
Archaea3 - ps4
Asgard/Eukaryota - ps5
Eukaryota - ps6
Amorpheae - ps7
Obazoa - ps8
Opisthokonta - ps9
Holomycota - ps10
Fungi - ps11
Fungi_1 - ps12
Fungi_2 - ps13
Fungi_3 - ps14
Dikarya - ps15
Ascomycota - ps16
Saccharo/Pezizomycotina - ps17
Saccharomycotina - ps18
Saccharomycotina_1 - ps19
Saccharomycotina_2 - ps20
Saccharomycotina_3 - ps21
Phaff./Saccharomycetaceae - ps22
Saccharomyco./mycetaceae - ps23
Saccharomycetaceae - ps24
Saccharomycetaceae_2 - ps25
Saccharomycetaceae_3 - ps26
Kaz./Nau./Saccharomyces - ps27
Saccharomyces - ps28
Arboricola_clade - ps29
Cerevisiae_clade - ps30
Saccharomyces_cerevisiae - ps31
Phylostratum

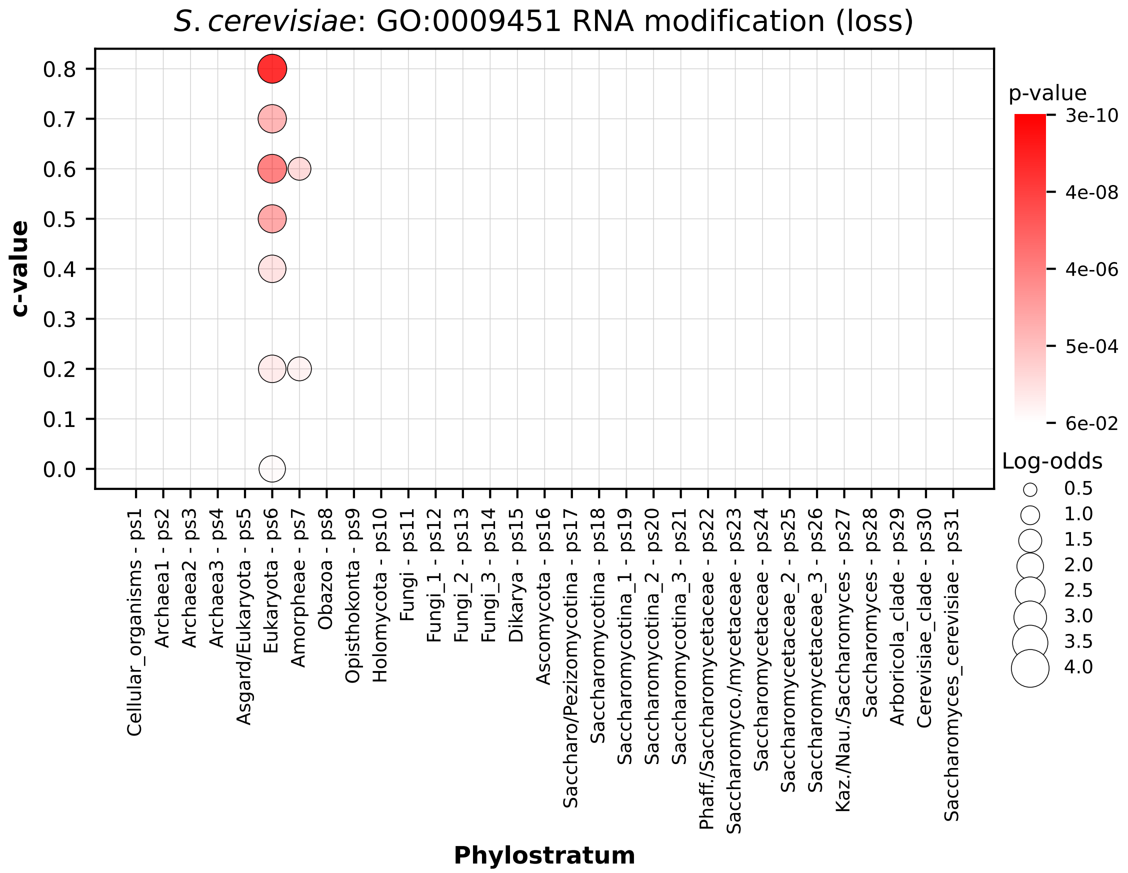
S. cerevisiae: GO:0009451 RNA modification (loss)
c-value
0.0
0.1
0.2
0.3
0.4
0.5
0.6
0.7
0.8
Phylostratum
Cellular_organisms - ps1
Archaea1 - ps2
Archaea2 - ps3
Archaea3 - ps4
Asgard/Eukaryota - ps5
Eukaryota - ps6
Amorpheae - ps7
Obazoa - ps8
Opisthokonta - ps9
Holomycota - ps10
Fungi - ps11
Fungi_1 - ps12
Fungi_2 - ps13
Fungi_3 - ps14
Dikarya - ps15
Ascomycota - ps16
Saccharo/Pezizomycotina - ps17
Saccharomycotina - ps18
Saccharomycotina_1 - ps19
Saccharomycotina_2 - ps20
Saccharomycotina_3 - ps21
Phaff./Saccharomycetaceae - ps22
Saccharomyco./mycetaceae - ps23
Saccharomycetaceae - ps24
Saccharomycetaceae_2 - ps25
Saccharomycetaceae_3 - ps26
Kaz./Nau./Saccharomyces - ps27
Saccharomyces - ps28
Arboricola_clade - ps29
Cerevisiae_clade - ps30
Saccharomyces_cerevisiae - ps31
p-value
3e-10
4e-08
4e-06
5e-04
6e-02
Log-odds
0.5
1.0
1.5
2.0
2.5
3.0
3.5
4.0

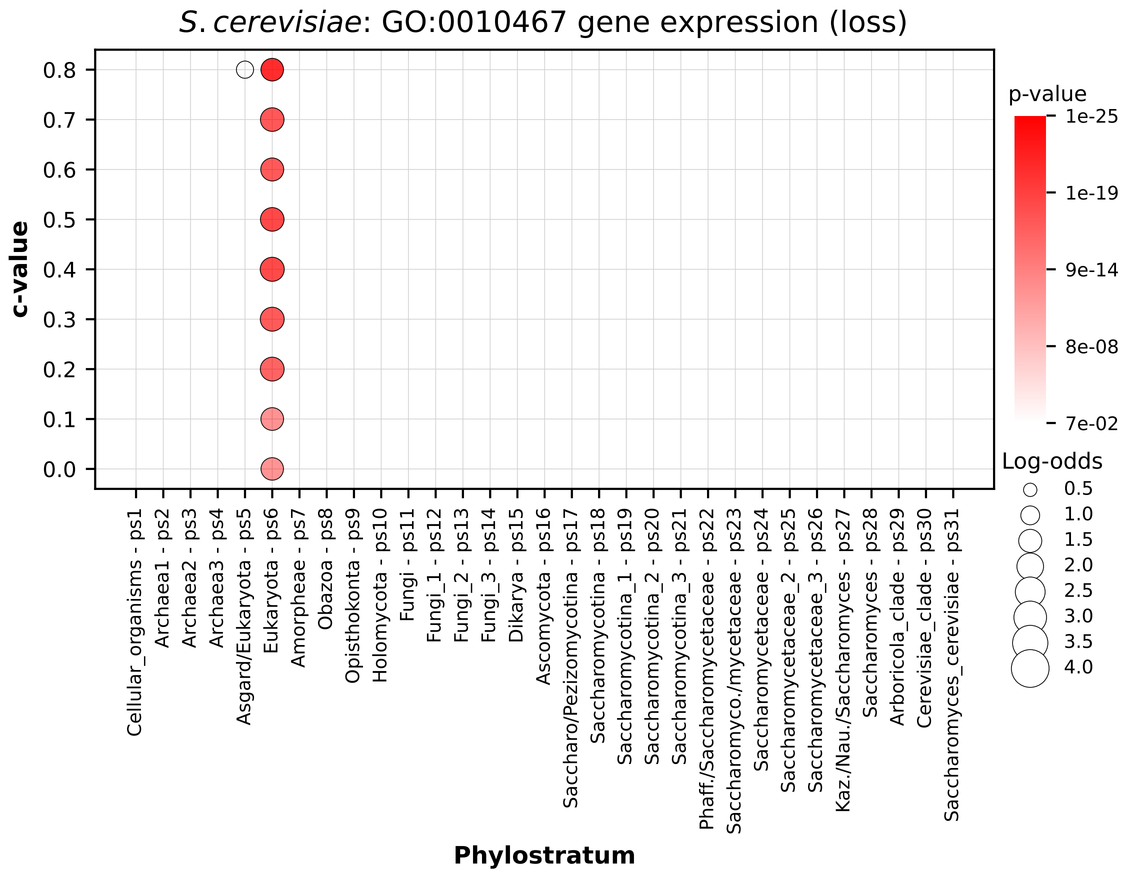

S. cerevisiae: GO:0010467 gene expression (loss)
c-value
0.8
0.7
0.6
0.5
0.4
0.3
0.2
0.1
0.0
p-value
1e-25
1e-19
9e-14
8e-08
7e-02
Log-odds
0.5
1.0
1.5
2.0
2.5
3.0
3.5
4.0
Cellular_organisms - ps1
Archaea1 - ps2
Archaea2 - ps3
Archaea3 - ps4
Asgard/Eukaryota - ps5
Eukaryota - ps6
Amorpheae - ps7
Obazoa - ps8
Opisthokonta - ps9
Holomycota - ps10
Fungi - ps11
Fungi_1 - ps12
Fungi_2 - ps13
Fungi_3 - ps14
Dikarya - ps15
Ascomycota - ps16
Saccharo/Pezizomycotina - ps17
Saccharomycotina - ps18
Saccharomycotina_1 - ps19
Saccharomycotina_2 - ps20
Saccharomycotina_3 - ps21
Phaff./Saccharomycetaceae - ps22
Saccharomyco./mycetaceae - ps23
Saccharomycetaceae - ps24
Saccharomycetaceae_2 - ps25
Saccharomycetaceae_3 - ps26
Kaz./Nau./Saccharomyces - ps27
Saccharomyces - ps28
Arboricola_clade - ps29
Cerevisiae_clade - ps30
Saccharomyces_cerevisiae - ps31
Phylostratum

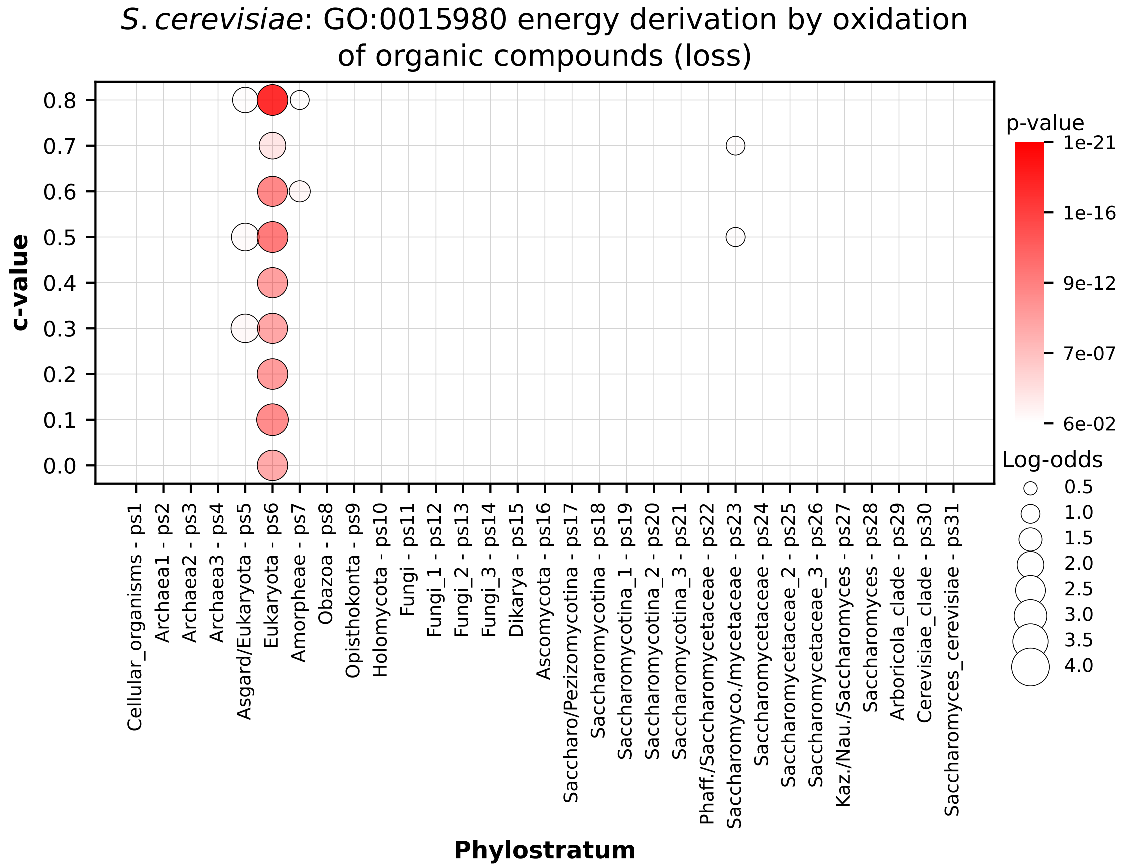
S. cerevisiae: GO:0015980 energy derivation by oxidation of organic compounds (loss)
c-value
0.8
0.7
0.6
0.5
0.4
0.3
0.2
0.1
0.0
p-value
1e-21
1e-16
9e-12
7e-07
6e-02
Log-odds
0.5
1.0
1.5
2.0
2.5
3.0
3.5
4.0
Cellular_organisms - ps1
Archaea1 - ps2
Archaea2 - ps3
Archaea3 - ps4
Asgard/Eukaryota - ps5
Eukaryota - ps6
Amorpheae - ps7
Obazoa - ps8
Opisthokonta - ps9
Holomycota - ps10
Fungi - ps11
Fungi_1 - ps12
Fungi_2 - ps13
Fungi_3 - ps14
Dikarya - ps15
Ascomycota - ps16
Saccharo/Pezizomycotina - ps17
Saccharomycotina - ps18
Saccharomycotina_1 - ps19
Saccharomycotina_2 - ps20
Saccharomycotina_3 - ps21
Phaff./Saccharomycetaceae - ps22
Saccharomyco./mycetaceae - ps23
Saccharomycetaceae - ps24
Saccharomycetaceae_2 - ps25
Saccharomycetaceae_3 - ps26
Kaz./Nau./Saccharomyces - ps27
Saccharomyces - ps28
Arboricola_clade - ps29
Cerevisiae_clade - ps30
Saccharomyces_cerevisiae - ps31
Phylostratum

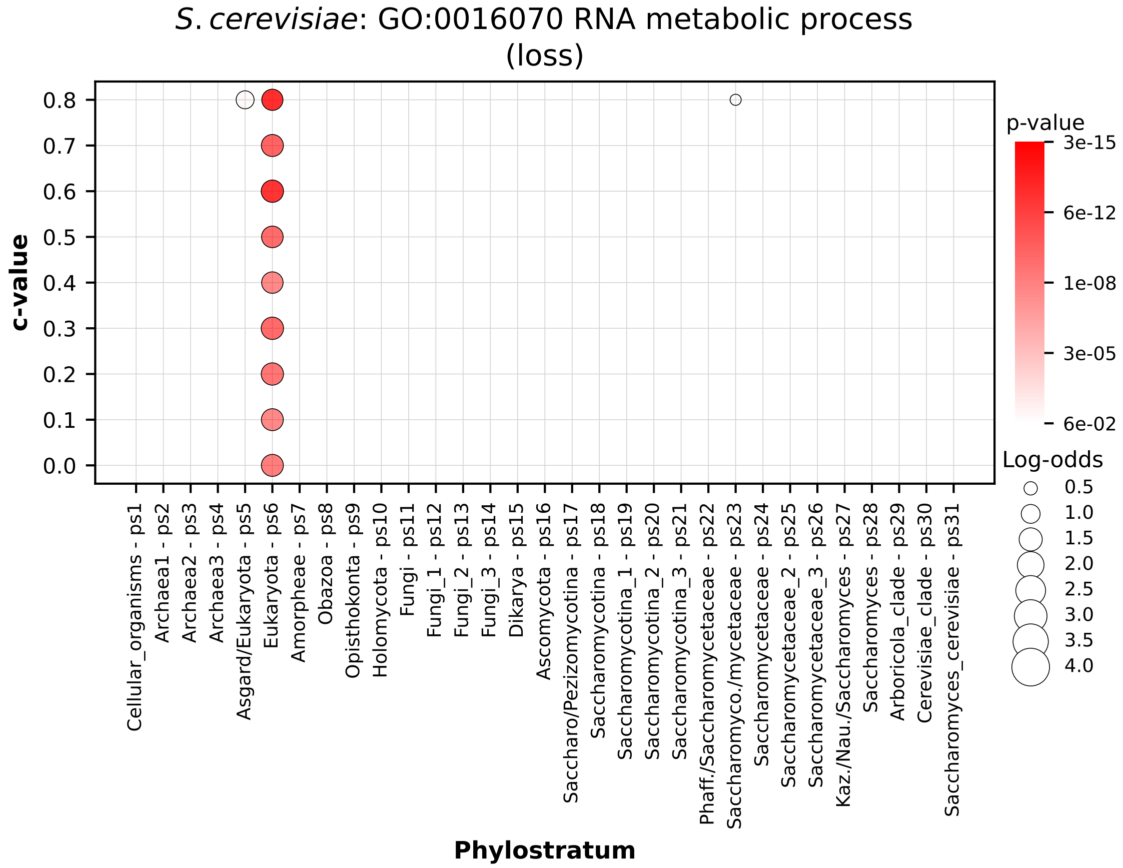
S. cerevisiae: GO:0016070 RNA metabolic process
(loss)
c-value
0.8
0.7
0.6
0.5
0.4
0.3
0.2
0.1
0.0
p-value
3e-15
6e-12
1e-08
3e-05
6e-02
Log-odds
0.5
1.0
1.5
2.0
2.5
3.0
3.5
4.0
Cellular_organisms - ps1
Archaea1 - ps2
Archaea2 - ps3
Archaea3 - ps4
Asgard/Eukaryota - ps5
Eukaryota - ps6
Amorpheae - ps7
Obazoa - ps8
Opisthokonta - ps9
Holomycota - ps10
Fungi - ps11
Fungi_1 - ps12
Fungi_2 - ps13
Fungi_3 - ps14
Dikarya - ps15
Ascomycota - ps16
Saccharo/Pezizomycotina - ps17
Saccharomycotina - ps18
Saccharomycotina_1 - ps19
Saccharomycotina_2 - ps20
Saccharomycotina_3 - ps21
Phaff./Saccharomycetaceae - ps22
Saccharomyco./mycetaceae - ps23
Saccharomycetaceae - ps24
Saccharomycetaceae_2 - ps25
Saccharomycetaceae_3 - ps26
Kaz./Nau./Saccharomyces - ps27
Saccharomyces - ps28
Arboricola_clade - ps29
Cerevisiae_clade - ps30
Saccharomyces_cerevisiae - ps31
Phylostratum

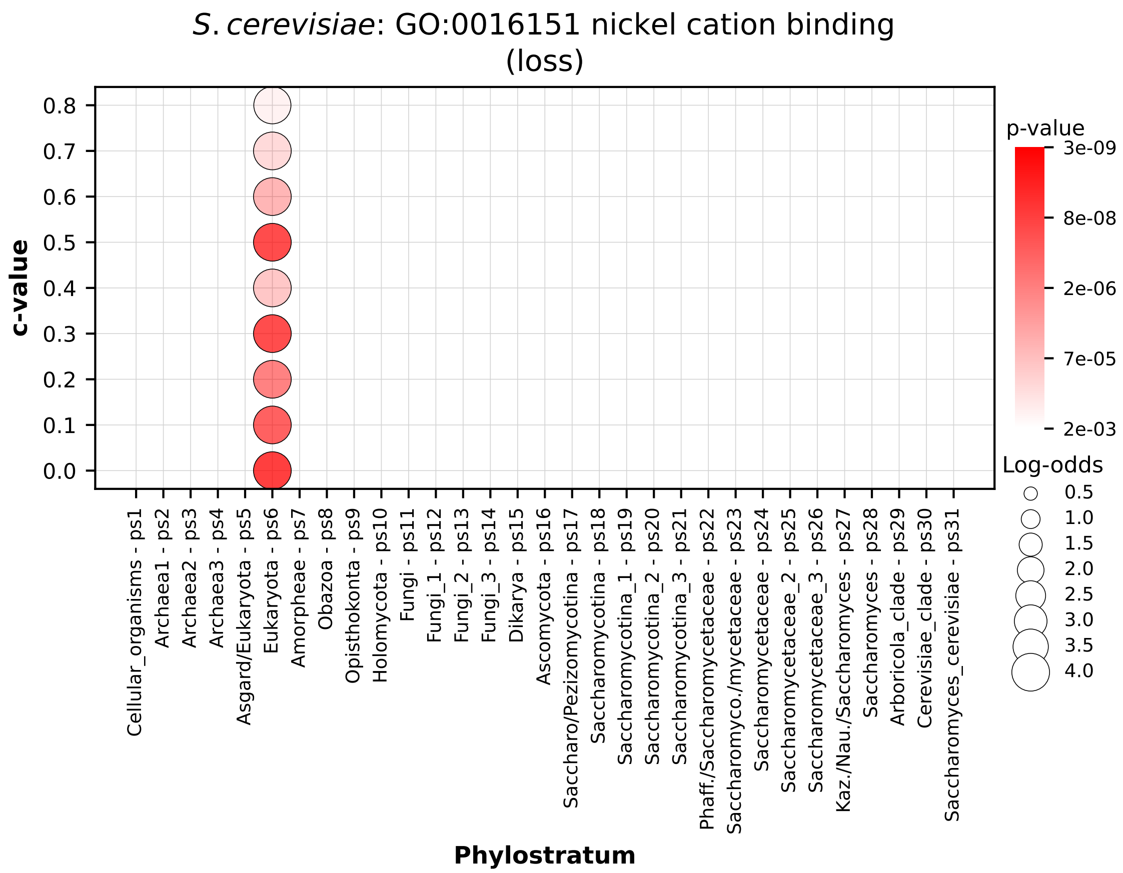

S. cerevisiae: GO:0016151 nickel cation binding
(loss)
c-value
0.8
0.7
0.6
0.5
0.4
0.3
0.2
0.1
0.0
p-value
3e-09
8e-08
2e-06
7e-05
2e-03
Log-odds
0.5
1.0
1.5
2.0
2.5
3.0
3.5
4.0
Cellular_organisms - ps1
Archaea1 - ps2
Archaea2 - ps3
Archaea3 - ps4
Asgard/Eukaryota - ps5
Eukaryota - ps6
Amorpheae - ps7
Obazoa - ps8
Opisthokonta - ps9
Holomycota - ps10
Fungi - ps11
Fungi_1 - ps12
Fungi_2 - ps13
Fungi_3 - ps14
Dikarya - ps15
Ascomycota - ps16
Saccharo/Pezizomycotina - ps17
Saccharomycotina - ps18
Saccharomycotina_1 - ps19
Saccharomycotina_2 - ps20
Saccharomycotina_3 - ps21
Phaff./Saccharomycetaceae - ps22
Saccharomyco./mycetaceae - ps23
Saccharomycetaceae - ps24
Saccharomycetaceae_2 - ps25
Saccharomycetaceae_3 - ps26
Kaz./Nau./Saccharomyces - ps27
Saccharomyces - ps28
Arboricola_clade - ps29
Cerevisiae_clade - ps30
Saccharomyces_cerevisiae - ps31
Phylostratum

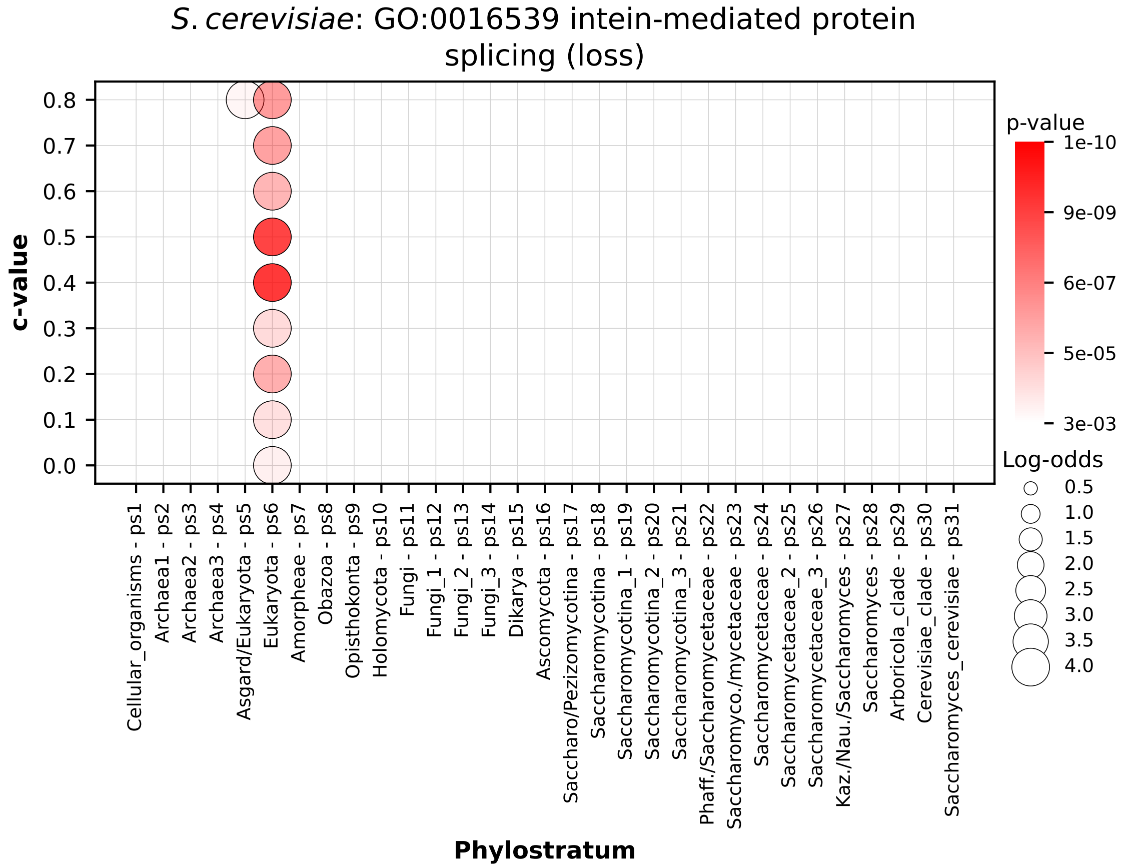
S. cerevisiae: GO:0016539 intein-mediated protein splicing (loss)
c-value
0.8
0.7
0.6
0.5
0.4
0.3
0.2
0.1
0.0
p-value
1e-10
9e-09
6e-07
5e-05
3e-03
Log-odds
0.5
1.0
1.5
2.0
2.5
3.0
3.5
4.0
Cellular_organisms - ps1
Archaea1 - ps2
Archaea2 - ps3
Archaea3 - ps4
Asgard/Eukaryota - ps5
Eukaryota - ps6
Amorpheae - ps7
Obazoa - ps8
Opisthokonta - ps9
Holomycota - ps10
Fungi - ps11
Fungi_1 - ps12
Fungi_2 - ps13
Fungi_3 - ps14
Dikarya - ps15
Ascomycota - ps16
Saccharo/Pezizomycotina - ps17
Saccharomycotina - ps18
Saccharomycotina_1 - ps19
Saccharomycotina_2 - ps20
Saccharomycotina_3 - ps21
Phaff./Saccharomycetaceae - ps22
Saccharomyco./mycetaceae - ps23
Saccharomycetaceae - ps24
Saccharomycetaceae_2 - ps25
Saccharomycetaceae_3 - ps26
Kaz./Nau./Saccharomyces - ps27
Saccharomyces - ps28
Arboricola_clade - ps29
Cerevisiae_clade - ps30
Saccharomyces_cerevisiae - ps31
Phylostratum

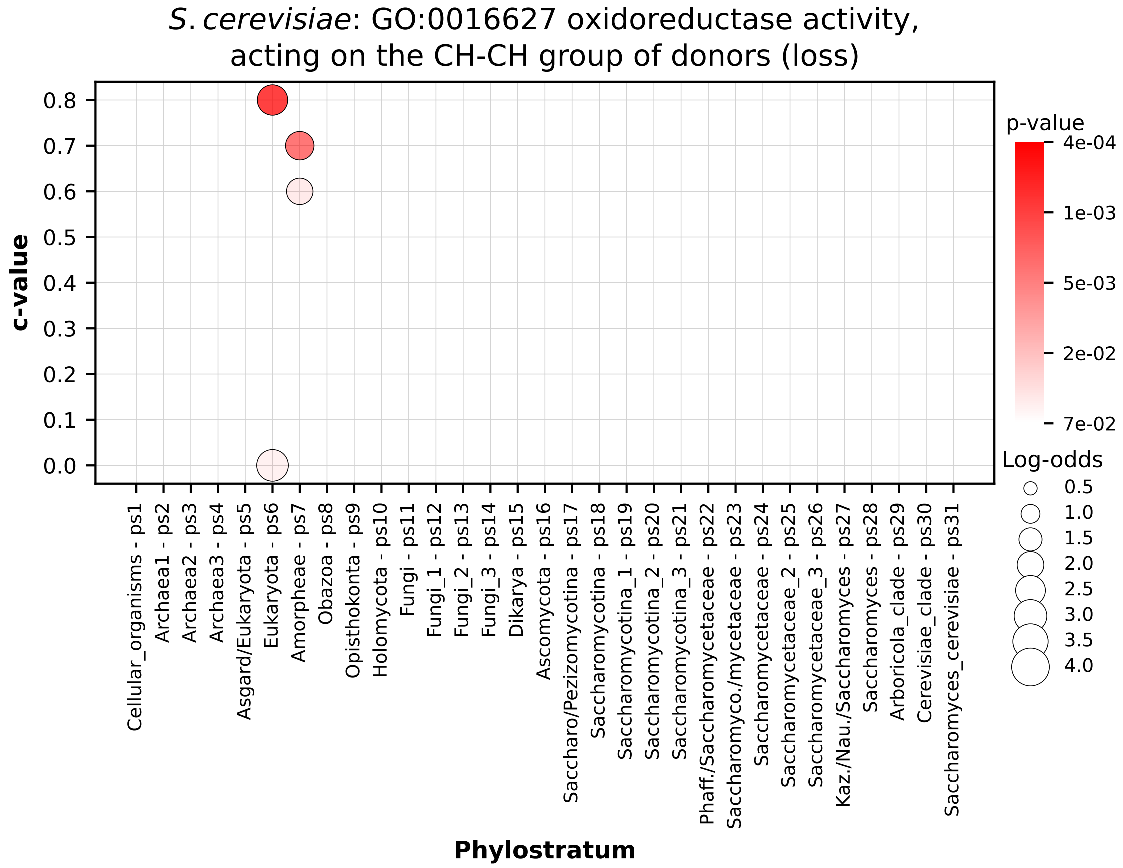
S. cerevisiae: GO:0016627 oxidoreductase activity, acting on the CH-CH group of donors (loss)
c-value
0.0
0.1
0.2
0.3
0.4
0.5
0.6
0.7
0.8
p-value
4e-04
1e-03
5e-03
2e-02
7e-02
Log-odds
0.5
1.0
1.5
2.0
2.5
3.0
3.5
4.0
Cellular_organisms - ps1
Archaea1 - ps2
Archaea2 - ps3
Archaea3 - ps4
Asgard/Eukaryota - ps5
Eukaryota - ps6
Amorpheae - ps7
Obazoa - ps8
Opisthokonta - ps9
Holomycota - ps10
Fungi - ps11
Fungi_1 - ps12
Fungi_2 - ps13
Fungi_3 - ps14
Dikarya - ps15
Ascomycota - ps16
Saccharo/Pezizomycotina - ps17
Saccharomycotina - ps18
Saccharomycotina_1 - ps19
Saccharomycotina_2 - ps20
Saccharomycotina_3 - ps21
Phaff./Saccharomycetaceae - ps22
Saccharomyco./mycetaceae - ps23
Saccharomycetaceae - ps24
Saccharomycetaceae_2 - ps25
Saccharomycetaceae_3 - ps26
Kaz./Nau./Saccharomyces - ps27
Saccharomyces - ps28
Arboricola_clade - ps29
Cerevisiae_clade - ps30
Saccharomyces_cerevisiae - ps31
Phylostratum

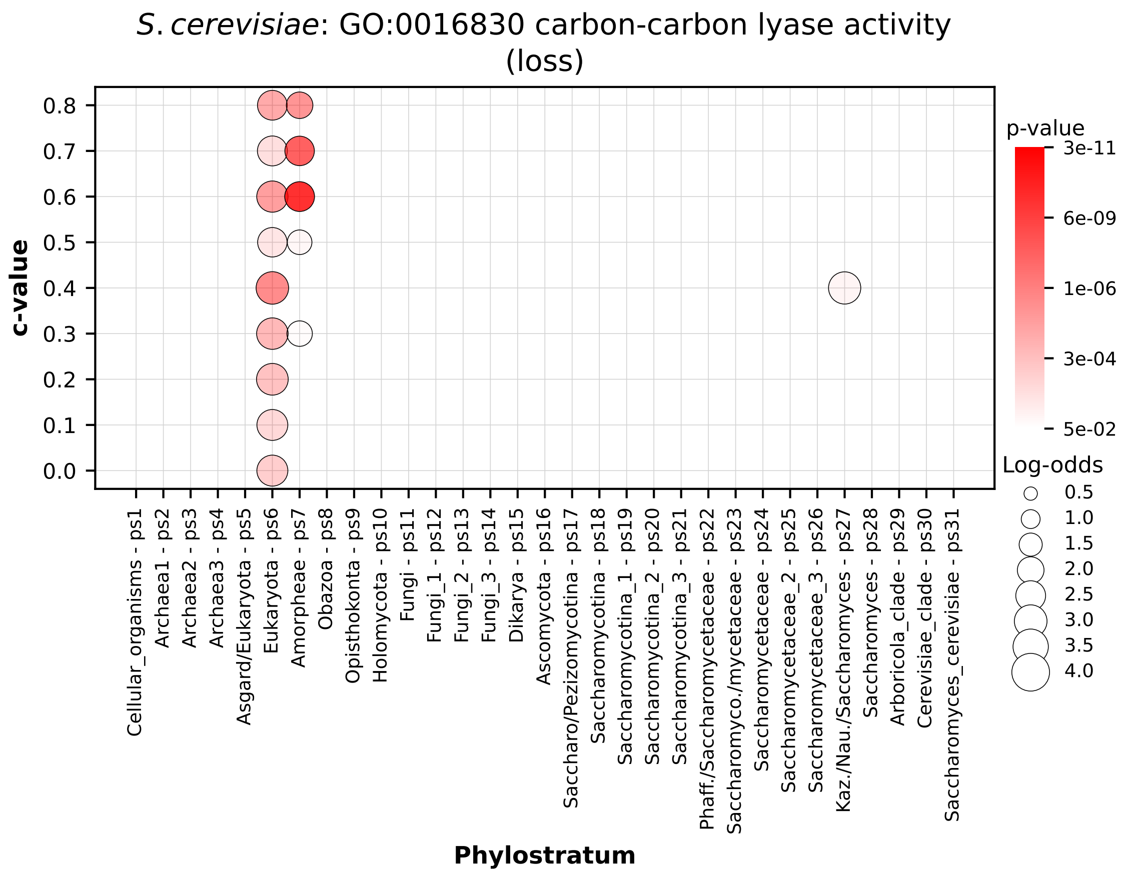
S. cerevisiae: GO:0016830 carbon-carbon lyase activity
(loss)
c-value
0.0
0.1
0.2
0.3
0.4
0.5
0.6
0.7
0.8
p-value
3e-11
6e-09
1e-06
3e-04
5e-02
Log-odds
0.5
1.0
1.5
2.0
2.5
3.0
3.5
4.0
Cellular_organisms - ps1
Archaea1 - ps2
Archaea2 - ps3
Archaea3 - ps4
Asgard/Eukaryota - ps5
Eukaryota - ps6
Amorpheae - ps7
Obazoa - ps8
Opisthokonta - ps9
Holomycota - ps10
Fungi - ps11
Fungi_1 - ps12
Fungi_2 - ps13
Fungi_3 - ps14
Dikarya - ps15
Ascomycota - ps16
Saccharo/Pezizomycotina - ps17
Saccharomycotina - ps18
Saccharomycotina_1 - ps19
Saccharomycotina_2 - ps20
Saccharomycotina_3 - ps21
Phaff./Saccharomycetaceae - ps22
Saccharomyco./mycetaceae - ps23
Saccharomycetaceae - ps24
Saccharomycetaceae_2 - ps25
Saccharomycetaceae_3 - ps26
Kaz./Nau./Saccharomyces - ps27
Saccharomyces - ps28
Arboricola_clade - ps29
Cerevisiae_clade - ps30
Saccharomyces_cerevisiae - ps31
Phylostratum

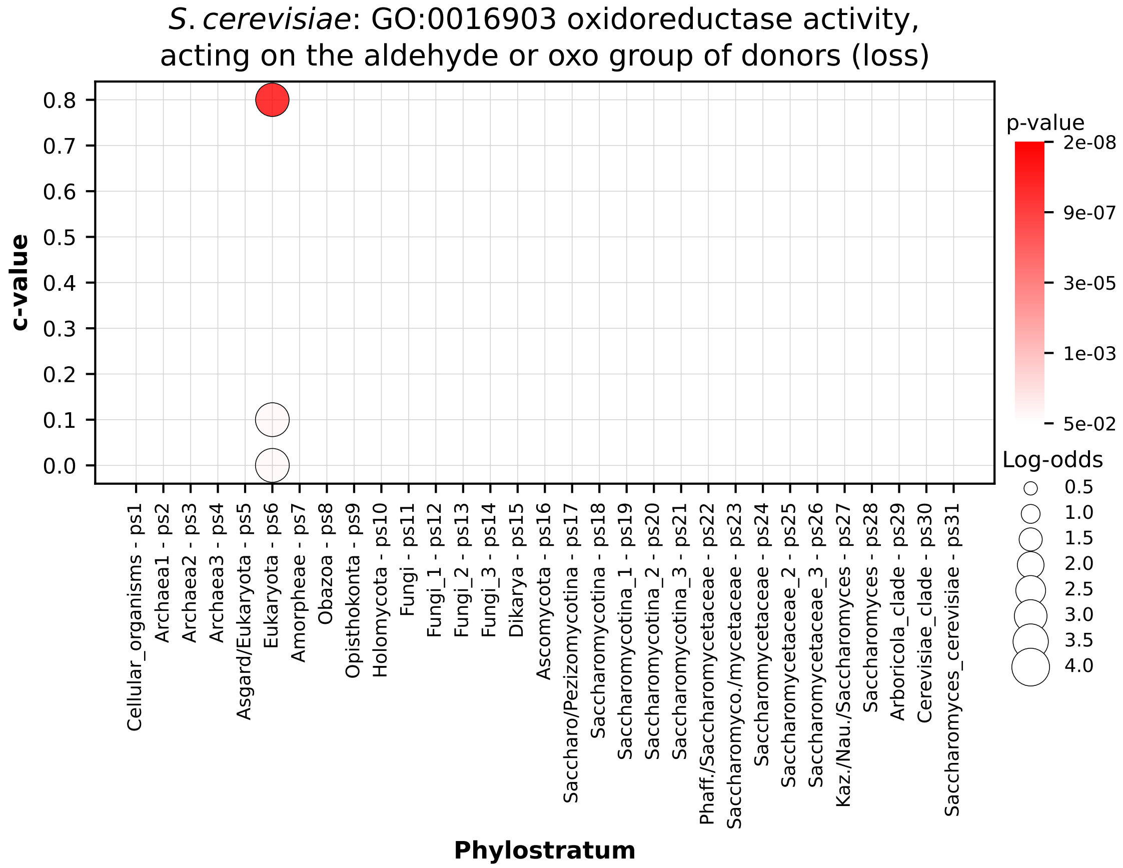
S. cerevisiae: GO:0016903 oxidoreductase activity,
acting on the aldehyde or oxo group of donors (loss)
c-value
0.8
0.7
0.6
0.5
0.4
0.3
0.2
0.1
0.0
p-value
2e-08
9e-07
3e-05
1e-03
5e-02
Log-odds
0.5
1.0
1.5
2.0
2.5
3.0
3.5
4.0
Cellular_organisms - ps1
Archaea1 - ps2
Archaea2 - ps3
Archaea3 - ps4
Asgard/Eukaryota - ps5
Eukaryota - ps6
Amorpheae - ps7
Obazoa - ps8
Opisthokonta - ps9
Holomycota - ps10
Fungi - ps11
Fungi_1 - ps12
Fungi_2 - ps13
Fungi_3 - ps14
Dikarya - ps15
Ascomycota - ps16
Saccharo/Pezizomycotina - ps17
Saccharomycotina - ps18
Saccharomycotina_1 - ps19
Saccharomycotina_2 - ps20
Saccharomycotina_3 - ps21
Phaff./Saccharomycetaceae - ps22
Saccharomyco./mycetaceae - ps23
Saccharomycetaceae - ps24
Saccharomycetaceae_2 - ps25
Saccharomycetaceae_3 - ps26
Kaz./Nau./Saccharomyces - ps27
Saccharomyces - ps28
Arboricola_clade - ps29
Cerevisiae_clade - ps30
Saccharomyces_cerevisiae - ps31
Phylostratum

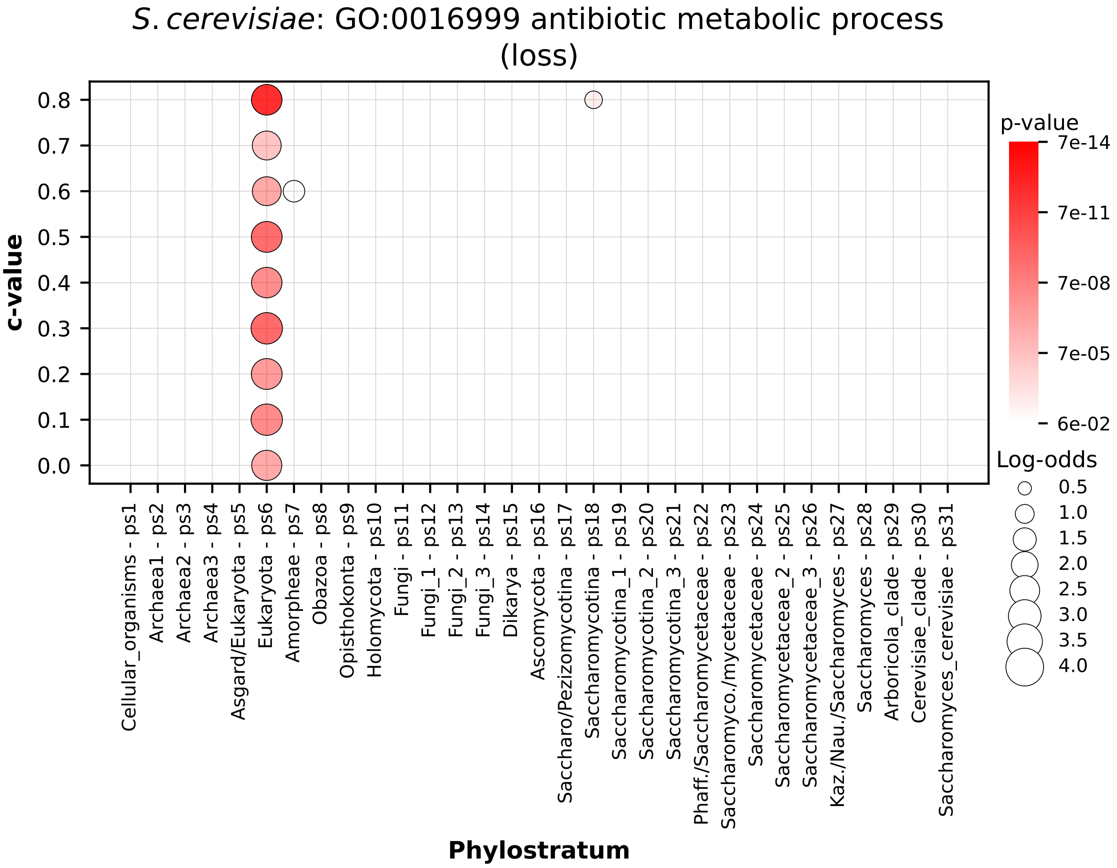

S. cerevisiae: GO:0016999 antibiotic metabolic process (loss)
c-value
0.0
0.1
0.2
0.3
0.4
0.5
0.6
0.7
0.8
Phylostratum
Cellular_organisms - ps1
Archaea1 - ps2
Archaea2 - ps3
Archaea3 - ps4
Asgard/Eukaryota - ps5
Eukaryota - ps6
Amorpheae - ps7
Obazoa - ps8
Opisthokonta - ps9
Holomycota - ps10
Fungi - ps11
Fungi_1 - ps12
Fungi_2 - ps13
Fungi_3 - ps14
Dikarya - ps15
Ascomycota - ps16
Saccharo/Pezizomycotina - ps17
Saccharomycotina - ps18
Saccharomycotina_1 - ps19
Saccharomycotina_2 - ps20
Saccharomycotina_3 - ps21
Phaff./Saccharomycetaceae - ps22
Saccharomyco./mycetaceae - ps23
Saccharomycetaceae - ps24
Saccharomycetaceae_2 - ps25
Saccharomycetaceae_3 - ps26
Kaz./Nau./Saccharomyces - ps27
Saccharomyces - ps28
Arboricola_clade - ps29
Cerevisiae_clade - ps30
Saccharomyces_cerevisiae - ps31
p-value
7e-14
7e-11
7e-08
7e-05
6e-02
Log-odds
0.5
1.0
1.5
2.0
2.5
3.0
3.5
4.0

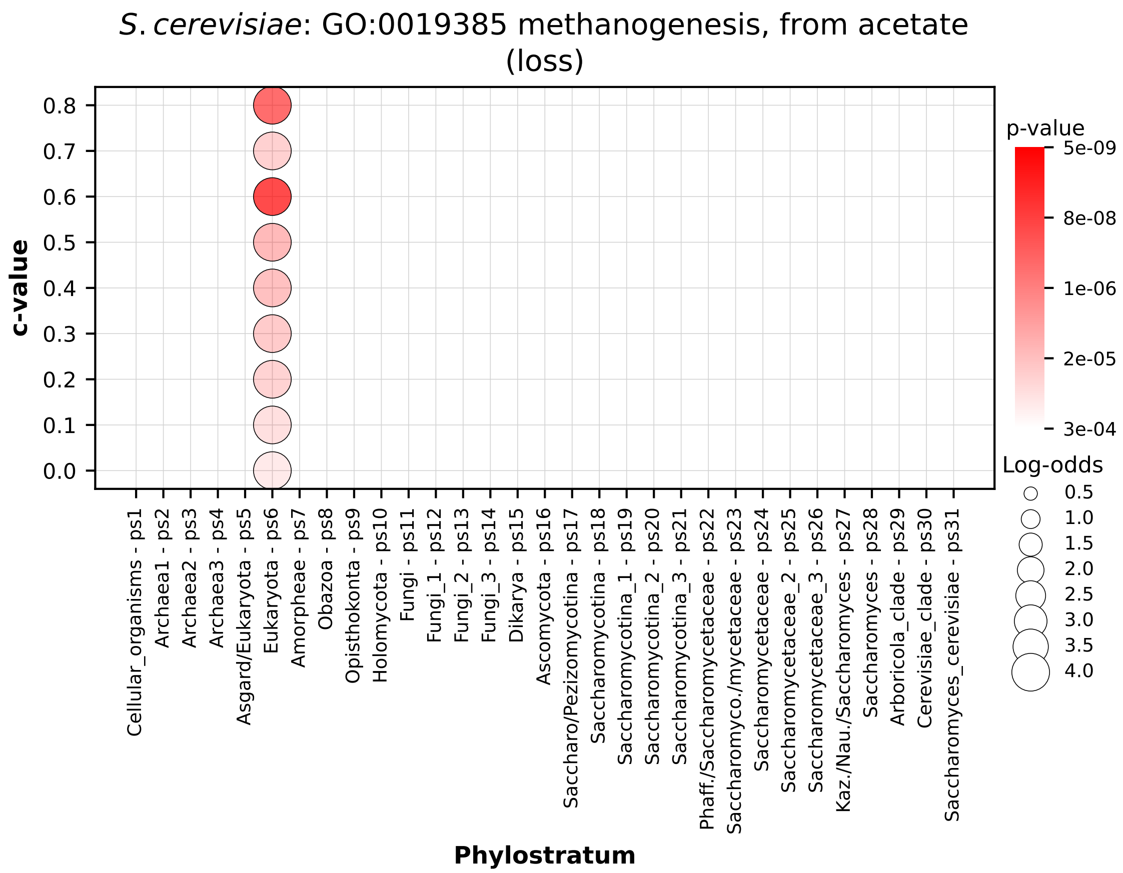

S. cerevisiae: GO:0019385 methanogenesis, from acetate
(loss)
c-value
0.8
0.7
0.6
0.5
0.4
0.3
0.2
0.1
0.0
p-value
5e-09
8e-08
1e-06
2e-05
3e-04
Log-odds
0.5
1.0
1.5
2.0
2.5
3.0
3.5
4.0
Cellular_organisms - ps1
Archaea1 - ps2
Archaea2 - ps3
Archaea3 - ps4
Asgard/Eukaryota - ps5
Eukaryota - ps6
Amorpheae - ps7
Obazoa - ps8
Opisthokonta - ps9
Holomycota - ps10
Fungi - ps11
Fungi_1 - ps12
Fungi_2 - ps13
Fungi_3 - ps14
Dikarya - ps15
Ascomycota - ps16
Saccharo/Pezizomycotina - ps17
Saccharomycotina - ps18
Saccharomycotina_1 - ps19
Saccharomycotina_2 - ps20
Saccharomycotina_3 - ps21
Phaff./Saccharomycetaceae - ps22
Saccharomyco./mycetaceae - ps23
Saccharomycetaceae - ps24
Saccharomycetaceae_2 - ps25
Saccharomycetaceae_3 - ps26
Kaz./Nau./Saccharomyces - ps27
Saccharomyces - ps28
Arboricola_clade - ps29
Cerevisiae_clade - ps30
Saccharomyces_cerevisiae - ps31
Phylostratum

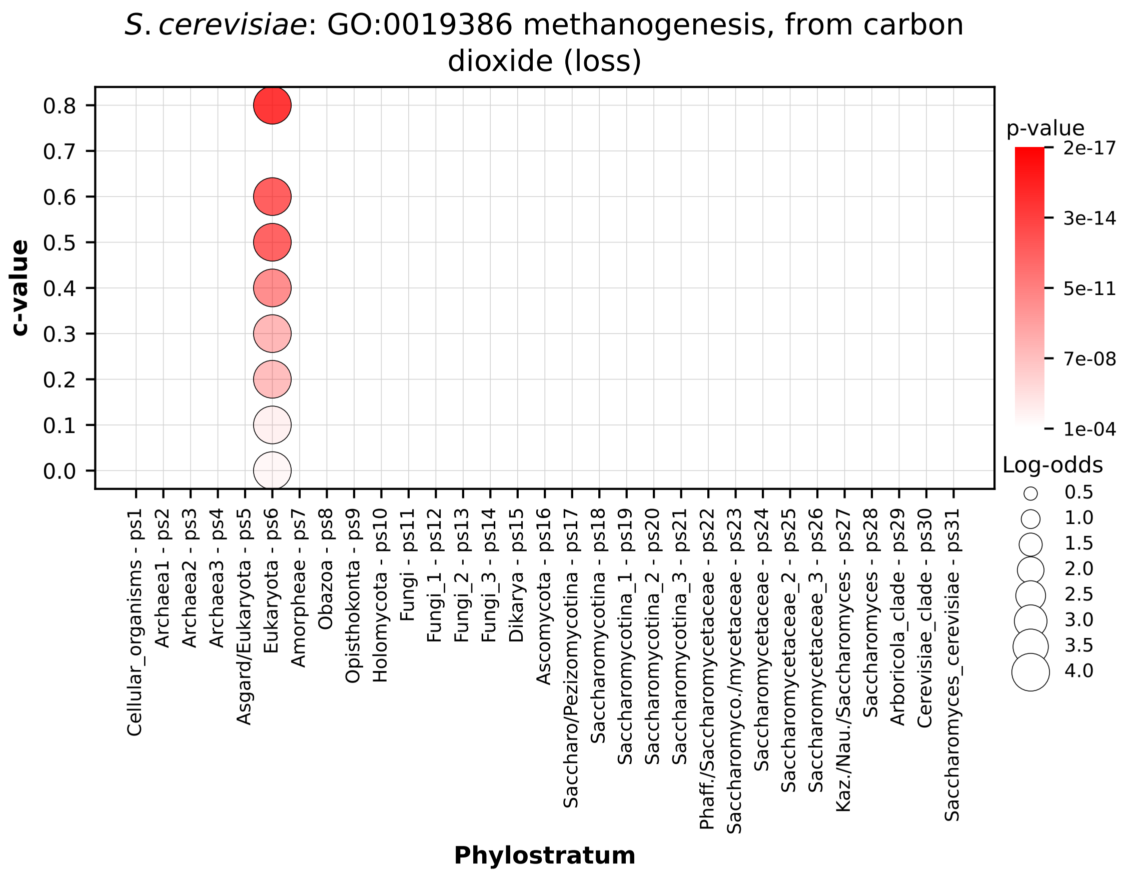

S. cerevisiae: GO:0019386 methanogenesis, from carbon dioxide (loss)
c-value
0.0
0.1
0.2
0.3
0.4
0.5
0.6
0.7
0.8
Phylostratum
Cellular_organisms - ps1
Archaea1 - ps2
Archaea2 - ps3
Archaea3 - ps4
Asgard/Eukaryota - ps5
Eukaryota - ps6
Amorpheae - ps7
Obazoa - ps8
Opisthokonta - ps9
Holomycota - ps10
Fungi - ps11
Fungi_1 - ps12
Fungi_2 - ps13
Fungi_3 - ps14
Dikarya - ps15
Ascomycota - ps16
Saccharo/Pezizomycotina - ps17
Saccharomycotina - ps18
Saccharomycotina_1 - ps19
Saccharomycotina_2 - ps20
Saccharomycotina_3 - ps21
Phaff./Saccharomycetaceae - ps22
Saccharomyco./mycetaceae - ps23
Saccharomycetaceae - ps24
Saccharomycetaceae_2 - ps25
Saccharomycetaceae_3 - ps26
Kaz./Nau./Saccharomyces - ps27
Saccharomyces - ps28
Arboricola_clade - ps29
Cerevisiae_clade - ps30
Saccharomyces_cerevisiae - ps31
p-value
2e-17
3e-14
5e-11
7e-08
1e-04
Log-odds
0.5
1.0
1.5
2.0
2.5
3.0
3.5
4.0

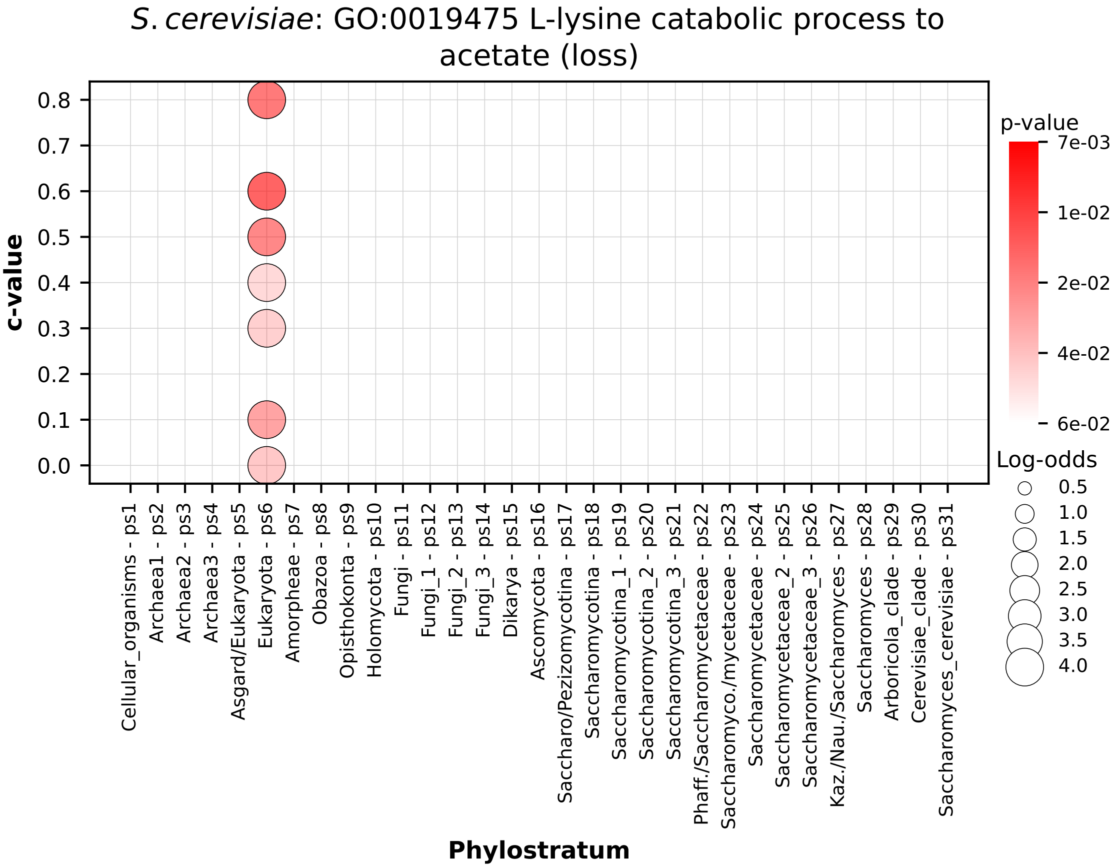
S. cerevisiae: GO:0019475 L-lysine catabolic process to acetate (loss)
c-value
0.0
0.1
0.2
0.3
0.4
0.5
0.6
0.7
0.8
p-value
7e-03
1e-02
2e-02
4e-02
6e-02
Log-odds
0.5
1.0
1.5
2.0
2.5
3.0
3.5
4.0
Cellular_organisms - ps1
Archaea1 - ps2
Archaea2 - ps3
Archaea3 - ps4
Asgard/Eukaryota - ps5
Eukaryota - ps6
Amorpheae - ps7
Obazoa - ps8
Opisthokonta - ps9
Holomycota - ps10
Fungi - ps11
Fungi_1 - ps12
Fungi_2 - ps13
Fungi_3 - ps14
Dikarya - ps15
Ascomycota - ps16
Saccharo/Pezizomycotina - ps17
Saccharomycotina - ps18
Saccharomycotina_1 - ps19
Saccharomycotina_2 - ps20
Saccharomycotina_3 - ps21
Phaff./Saccharomycetaceae - ps22
Saccharomyco./mycetaceae - ps23
Saccharomycetaceae - ps24
Saccharomycetaceae_2 - ps25
Saccharomycetaceae_3 - ps26
Kaz./Nau./Saccharomyces - ps27
Saccharomyces - ps28
Arboricola_clade - ps29
Cerevisiae_clade - ps30
Saccharomyces_cerevisiae - ps31
Phylostratum

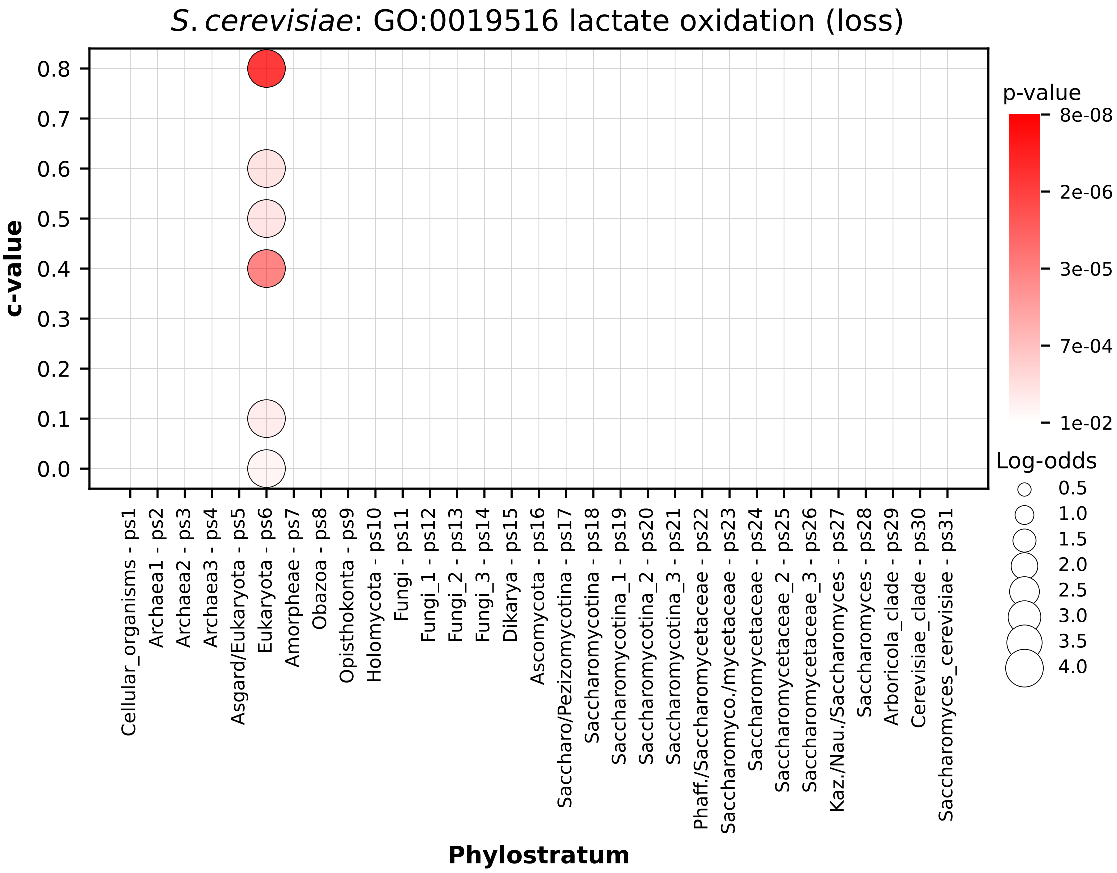

S. cerevisiae: GO:0019516 lactate oxidation (loss)
c-value
0.0
0.1
0.2
0.3
0.4
0.5
0.6
0.7
0.8
p-value
8e-08
2e-06
3e-05
7e-04
1e-02
Log-odds
0.5
1.0
1.5
2.0
2.5
3.0
3.5
4.0
Cellular_organisms - ps1
Archaea1 - ps2
Archaea2 - ps3
Archaea3 - ps4
Asgard/Eukaryota - ps5
Eukaryota - ps6
Amorpheae - ps7
Obazoa - ps8
Opisthokonta - ps9
Holomycota - ps10
Fungi - ps11
Fungi_1 - ps12
Fungi_2 - ps13
Fungi_3 - ps14
Dikarya - ps15
Ascomycota - ps16
Saccharo/Pezizomycotina - ps17
Saccharomycotina - ps18
Saccharomycotina_1 - ps19
Saccharomycotina_2 - ps20
Saccharomycotina_3 - ps21
Phaff./Saccharomycetaceae - ps22
Saccharomyco./mycetaceae - ps23
Saccharomycetaceae - ps24
Saccharomycetaceae_2 - ps25
Saccharomycetaceae_3 - ps26
Kaz./Nau./Saccharomyces - ps27
Saccharomyces - ps28
Arboricola_clade - ps29
Cerevisiae_clade - ps30
Saccharomyces_cerevisiae - ps31
Phylostratum

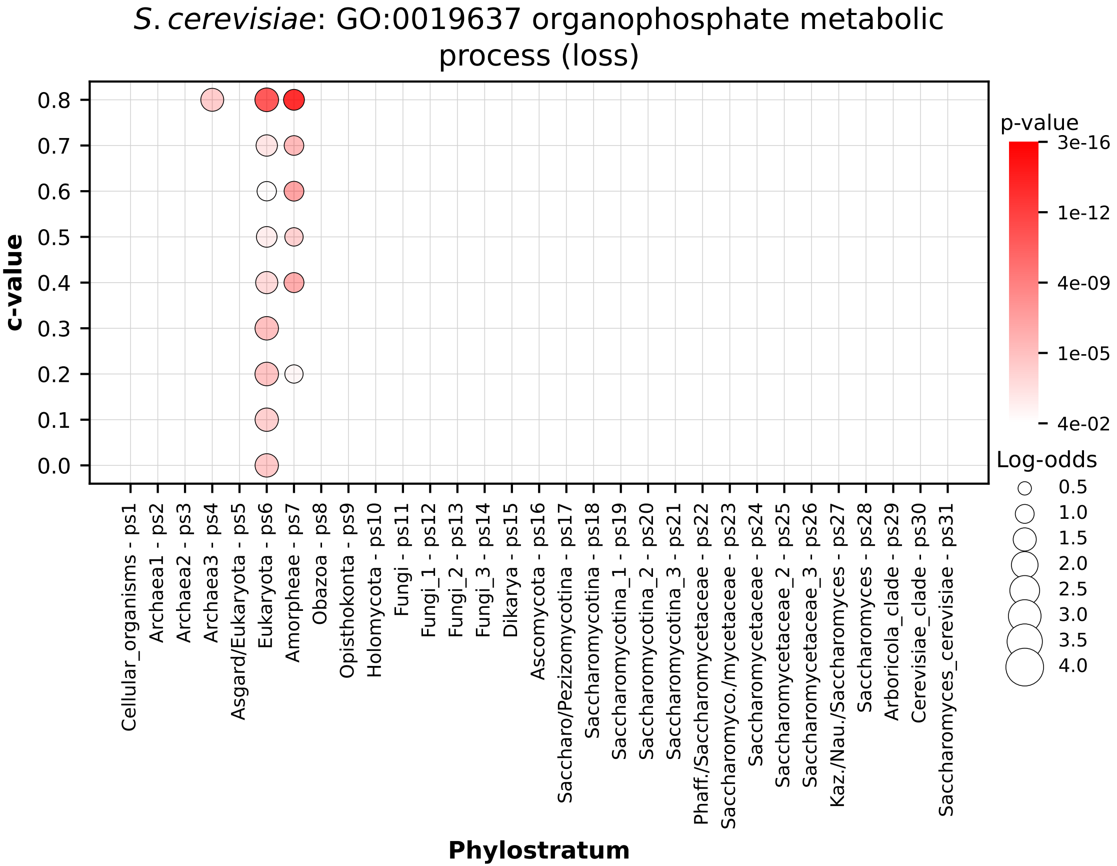

S. cerevisiae: GO:0019637 organophosphate metabolic process (loss)
c-value
0.8
0.7
0.6
0.5
0.4
0.3
0.2
0.1
0.0
Cellular_organisms - ps1
Archaea1 - ps2
Archaea2 - ps3
Archaea3 - ps4
Asgard/Eukaryota - ps5
Eukaryota - ps6
Amorpheae - ps7
Obazoa - ps8
Opisthokonta - ps9
Holomycota - ps10
Fungi - ps11
Fungi_1 - ps12
Fungi_2 - ps13
Fungi_3 - ps14
Dikarya - ps15
Ascomycota - ps16
Saccharo/Pezizomycotina - ps17
Saccharomycotina - ps18
Saccharomycotina_1 - ps19
Saccharomycotina_2 - ps20
Saccharomycotina_3 - ps21
Phaff./Saccharomycetaceae - ps22
Saccharomyco./mycetaceae - ps23
Saccharomycetaceae - ps24
Saccharomycetaceae_2 - ps25
Saccharomycetaceae_3 - ps26
Kaz./Nau./Saccharomyces - ps27
Saccharomyces - ps28
Arboricola_clade - ps29
Cerevisiae_clade - ps30
Saccharomyces_cerevisiae - ps31
Phylostratum
p-value
3e-16
1e-12
4e-09
1e-05
4e-02
Log-odds
0.5
1.0
1.5
2.0
2.5
3.0
3.5
4.0

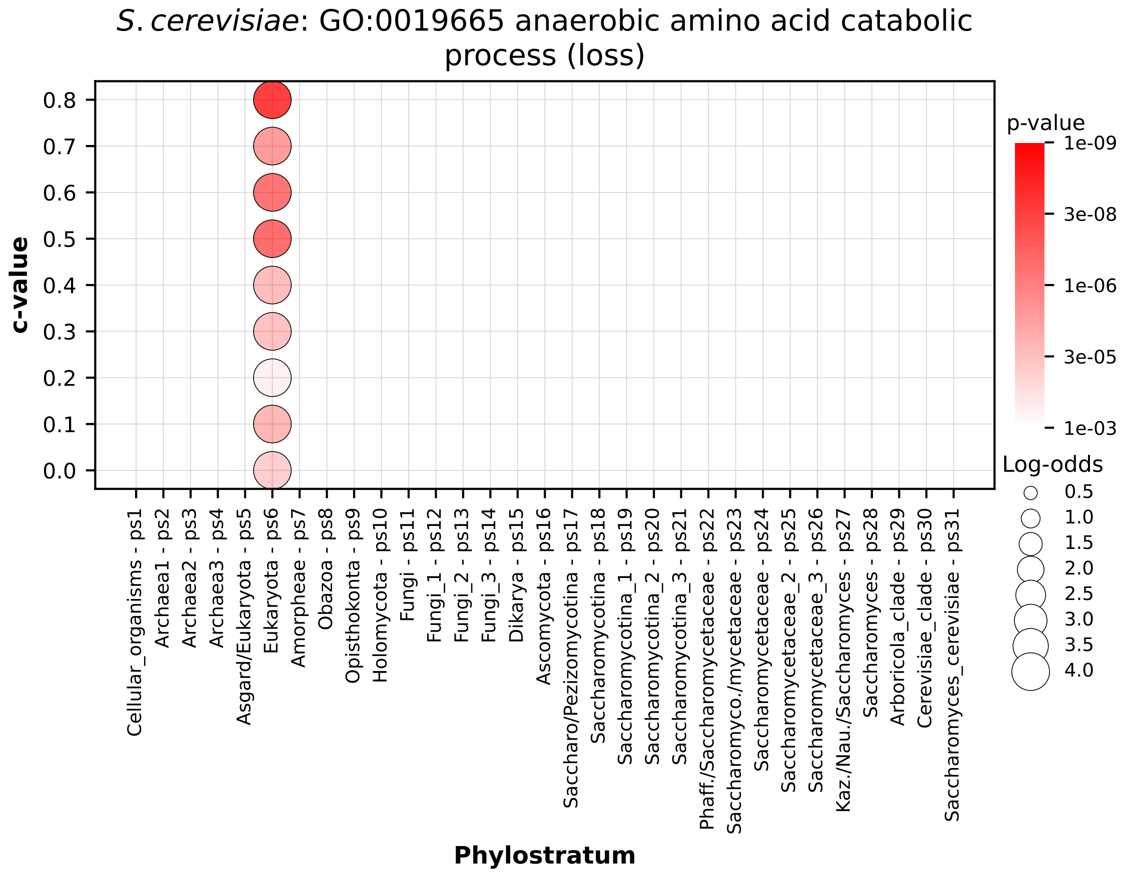

S. cerevisiae: GO:0019665 anaerobic amino acid catabolic process (loss)
c-value
0.8
0.7
0.6
0.5
0.4
0.3
0.2
0.1
0.0
p-value
1e-09
3e-08
1e-06
3e-05
1e-03
Log-odds
0.5
1.0
1.5
2.0
2.5
3.0
3.5
4.0
Cellular_organisms - ps1
Archaea1 - ps2
Archaea2 - ps3
Archaea3 - ps4
Asgard/Eukaryota - ps5
Eukaryota - ps6
Amorpheae - ps7
Obazoa - ps8
Opisthokonta - ps9
Holomycota - ps10
Fungi - ps11
Fungi_1 - ps12
Fungi_2 - ps13
Fungi_3 - ps14
Dikarya - ps15
Ascomycota - ps16
Saccharo/Pezizomycotina - ps17
Saccharomycotina - ps18
Saccharomycotina_1 - ps19
Saccharomycotina_2 - ps20
Saccharomycotina_3 - ps21
Phaff./Saccharomycetaceae - ps22
Saccharomyco./mycetaceae - ps23
Saccharomycetaceae - ps24
Saccharomycetaceae_2 - ps25
Saccharomycetaceae_3 - ps26
Kaz./Nau./Saccharomyces - ps27
Saccharomyces - ps28
Arboricola_clade - ps29
Cerevisiae_clade - ps30
Saccharomyces_cerevisiae - ps31
Phylostratum

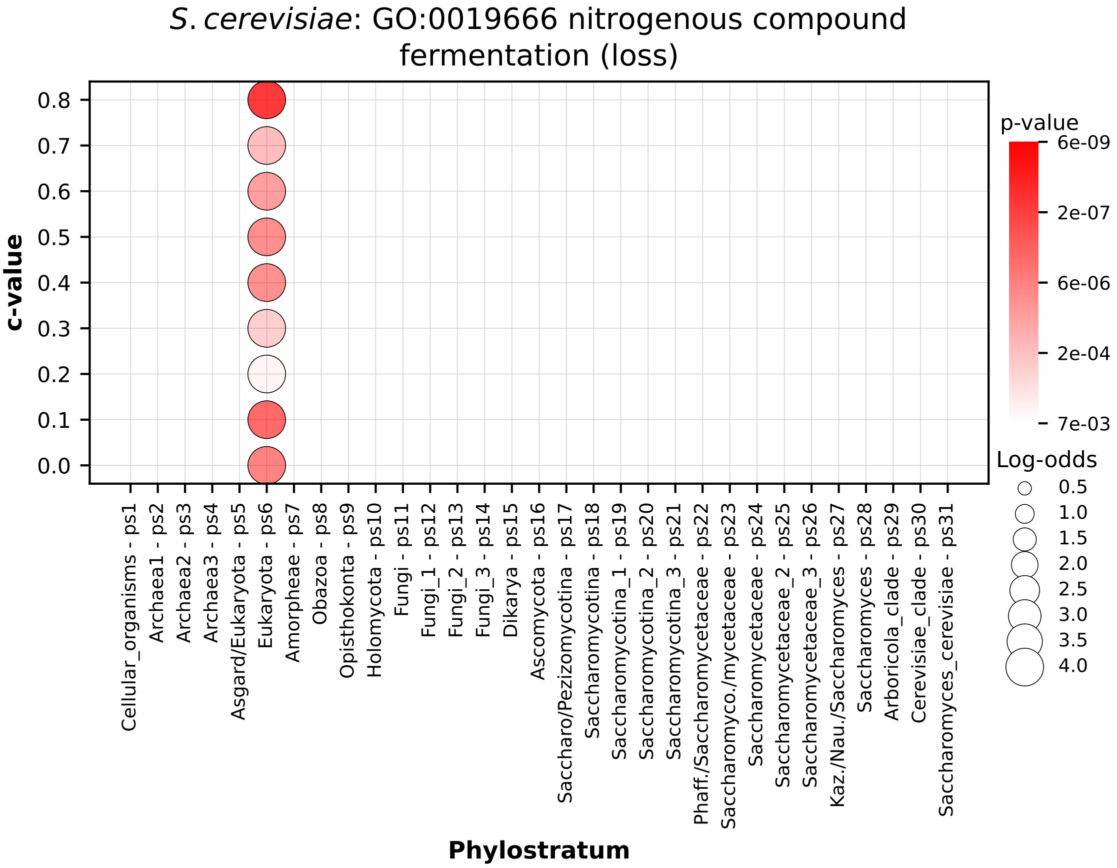
S. cerevisiae: GO:0019666 nitrogenous compound fermentation (loss)
c-value
0.8
0.7
0.6
0.5
0.4
0.3
0.2
0.1
0.0
Phylostratum
Cellular_organisms - ps1
Archaea1 - ps2
Archaea2 - ps3
Archaea3 - ps4
Asgard/Eukaryota - ps5
Eukaryota - ps6
Amorpheae - ps7
Obazoa - ps8
Opisthokonta - ps9
Holomycota - ps10
Fungi - ps11
Fungi_1 - ps12
Fungi_2 - ps13
Fungi_3 - ps14
Dikarya - ps15
Ascomycota - ps16
Saccharo/Pezizomycotina - ps17
Saccharomycotina - ps18
Saccharomycotina_1 - ps19
Saccharomycotina_2 - ps20
Saccharomycotina_3 - ps21
Phaff./Saccharomycetaceae - ps22
Saccharomyco./mycetaceae - ps23
Saccharomycetaceae - ps24
Saccharomycetaceae_2 - ps25
Saccharomycetaceae_3 - ps26
Kaz./Nau./Saccharomyces - ps27
Saccharomyces - ps28
Arboricola_clade - ps29
Cerevisiae_clade - ps30
Saccharomyces_cerevisiae - ps31
p-value
6e-09
2e-07
6e-06
2e-04
7e-03
Log-odds
0.5
1.0
1.5
2.0
2.5
3.0
3.5
4.0

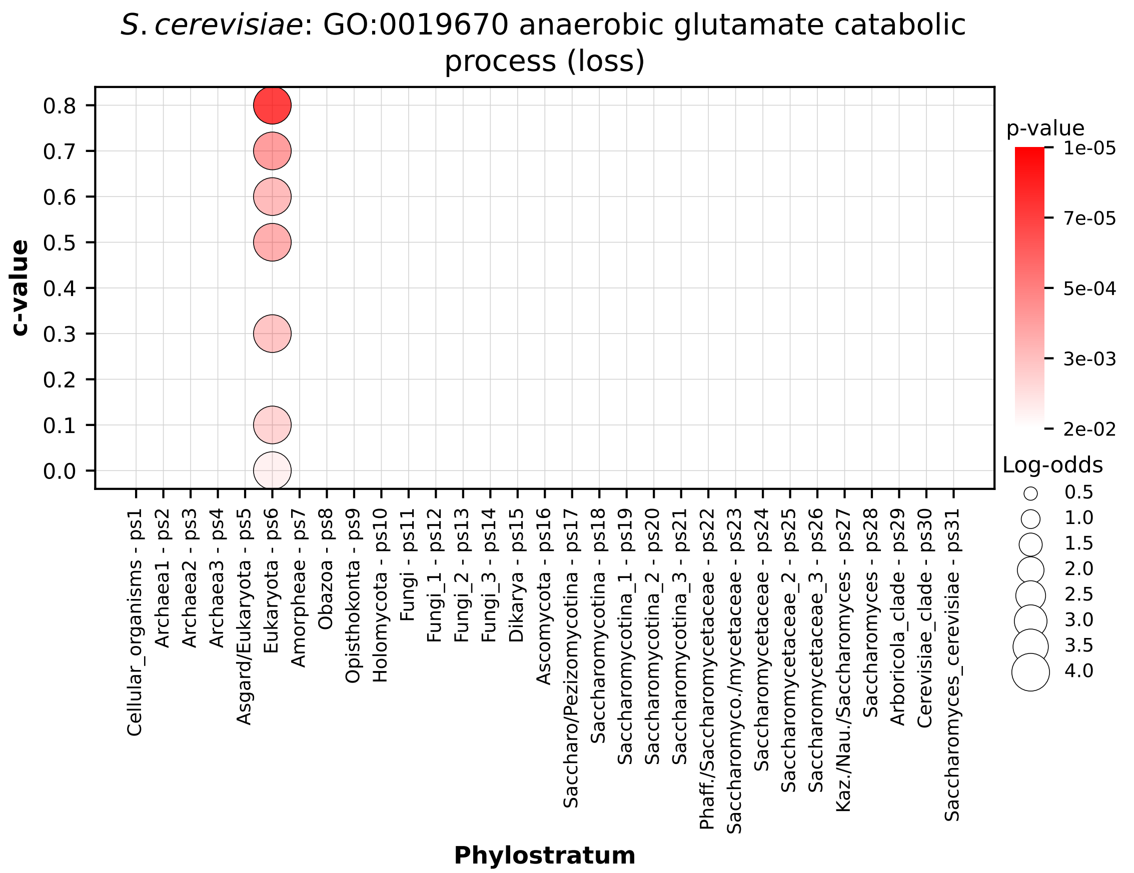

S. cerevisiae: GO:0019670 anaerobic glutamate catabolic process (loss)
c-value
0.8
0.7
0.6
0.5
0.4
0.3
0.2
0.1
0.0
p-value
1e-05
7e-05
5e-04
3e-03
2e-02
Log-odds
0.5
1.0
1.5
2.0
2.5
3.0
3.5
4.0
Cellular_organisms - ps1
Archaea1 - ps2
Archaea2 - ps3
Archaea3 - ps4
Asgard/Eukaryota - ps5
Eukaryota - ps6
Amorpheae - ps7
Obazoa - ps8
Opisthokonta - ps9
Holomycota - ps10
Fungi - ps11
Fungi_1 - ps12
Fungi_2 - ps13
Fungi_3 - ps14
Dikarya - ps15
Ascomycota - ps16
Saccharo/Pezizomycotina - ps17
Saccharomycotina - ps18
Saccharomycotina_1 - ps19
Saccharomycotina_2 - ps20
Saccharomycotina_3 - ps21
Phaff./Saccharomycetaceae - ps22
Saccharomyco./mycetaceae - ps23
Saccharomycetaceae - ps24
Saccharomycetaceae_2 - ps25
Saccharomycetaceae_3 - ps26
Kaz./Nau./Saccharomyces - ps27
Saccharomyces - ps28
Arboricola_clade - ps29
Cerevisiae_clade - ps30
Saccharomyces_cerevisiae - ps31
Phylostratum

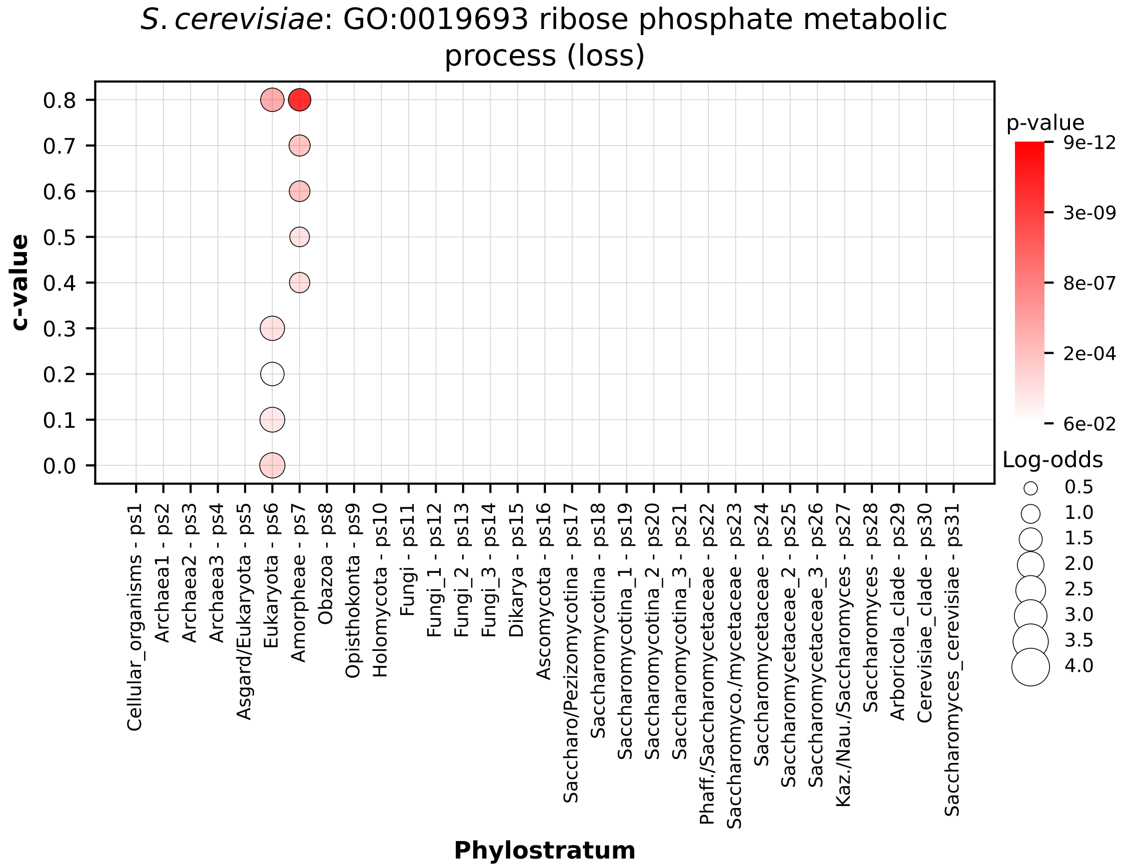
S. cerevisiae: GO:0019693 ribose phosphate metabolic process (loss)
c-value
0.8
0.7
0.6
0.5
0.4
0.3
0.2
0.1
0.0
p-value
9e-12
3e-09
8e-07
2e-04
6e-02
Log-odds
0.5
1.0
1.5
2.0
2.5
3.0
3.5
4.0
Cellular_organisms - ps1
Archaea1 - ps2
Archaea2 - ps3
Archaea3 - ps4
Asgard/Eukaryota - ps5
Eukaryota - ps6
Amorpheae - ps7
Obazoa - ps8
Opisthokonta - ps9
Holomycota - ps10
Fungi - ps11
Fungi_1 - ps12
Fungi_2 - ps13
Fungi_3 - ps14
Dikarya - ps15
Ascomycota - ps16
Saccharo/Pezizomycotina - ps17
Saccharomycotina - ps18
Saccharomycotina_1 - ps19
Saccharomycotina_2 - ps20
Saccharomycotina_3 - ps21
Phaff./Saccharomycetaceae - ps22
Saccharomyco./mycetaceae - ps23
Saccharomycetaceae - ps24
Saccharomycetaceae_2 - ps25
Saccharomycetaceae_3 - ps26
Kaz./Nau./Saccharomyces - ps27
Saccharomyces - ps28
Arboricola_clade - ps29
Cerevisiae_clade - ps30
Saccharomyces_cerevisiae - ps31
Phylostratum

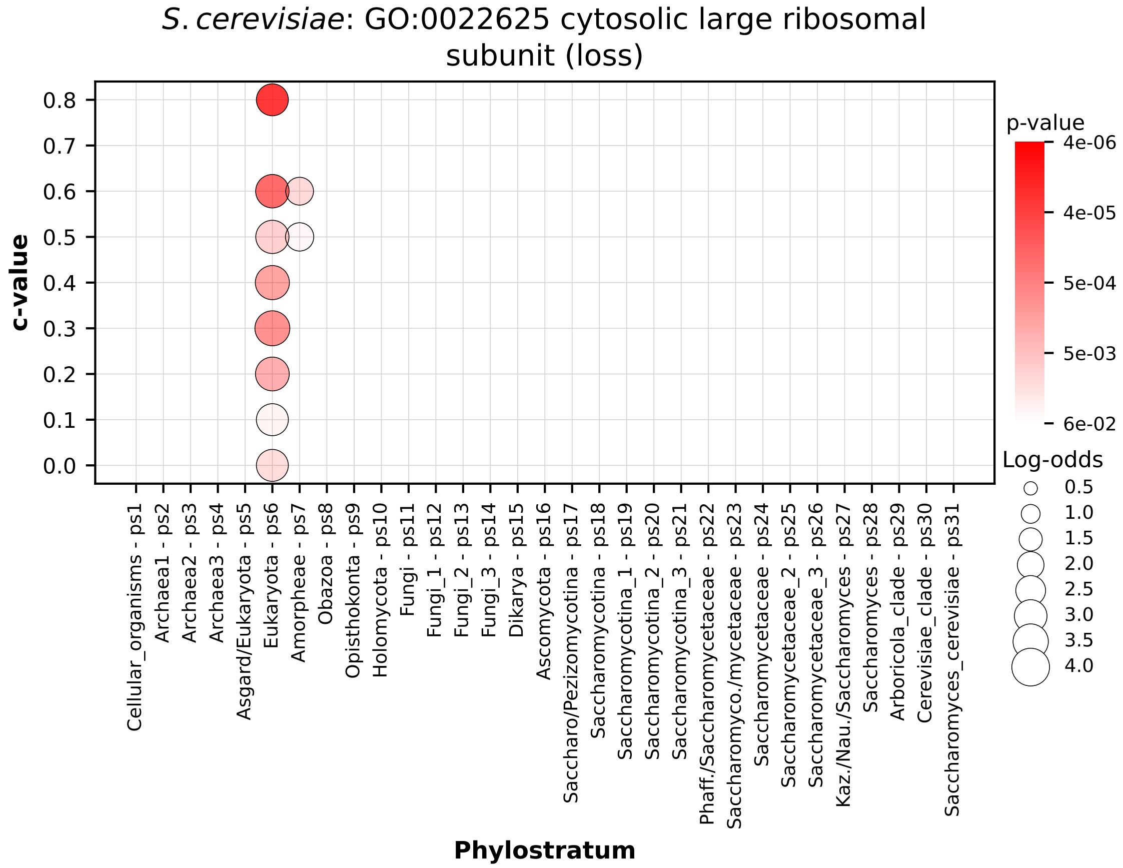
S. cerevisiae: GO:0022625 cytosolic large ribosomal subunit (loss)
c-value
0.8
0.7
0.6
0.5
0.4
0.3
0.2
0.1
0.0
p-value
4e-06
4e-05
5e-04
5e-03
6e-02
Log-odds
0.5
1.0
1.5
2.0
2.5
3.0
3.5
4.0
Cellular_organisms - ps1
Archaea1 - ps2
Archaea2 - ps3
Archaea3 - ps4
Asgard/Eukaryota - ps5
Eukaryota - ps6
Amorpheae - ps7
Obazoa - ps8
Opisthokonta - ps9
Holomycota - ps10
Fungi - ps11
Fungi_1 - ps12
Fungi_2 - ps13
Fungi_3 - ps14
Dikarya - ps15
Ascomycota - ps16
Saccharo/Pezizomycotina - ps17
Saccharomycotina - ps18
Saccharomycotina_1 - ps19
Saccharomycotina_2 - ps20
Saccharomycotina_3 - ps21
Phaff./Saccharomycetaceae - ps22
Saccharomyco./mycetaceae - ps23
Saccharomycetaceae - ps24
Saccharomycetaceae_2 - ps25
Saccharomycetaceae_3 - ps26
Kaz./Nau./Saccharomyces - ps27
Saccharomyces - ps28
Arboricola_clade - ps29
Cerevisiae_clade - ps30
Saccharomyces_cerevisiae - ps31
Phylostratum

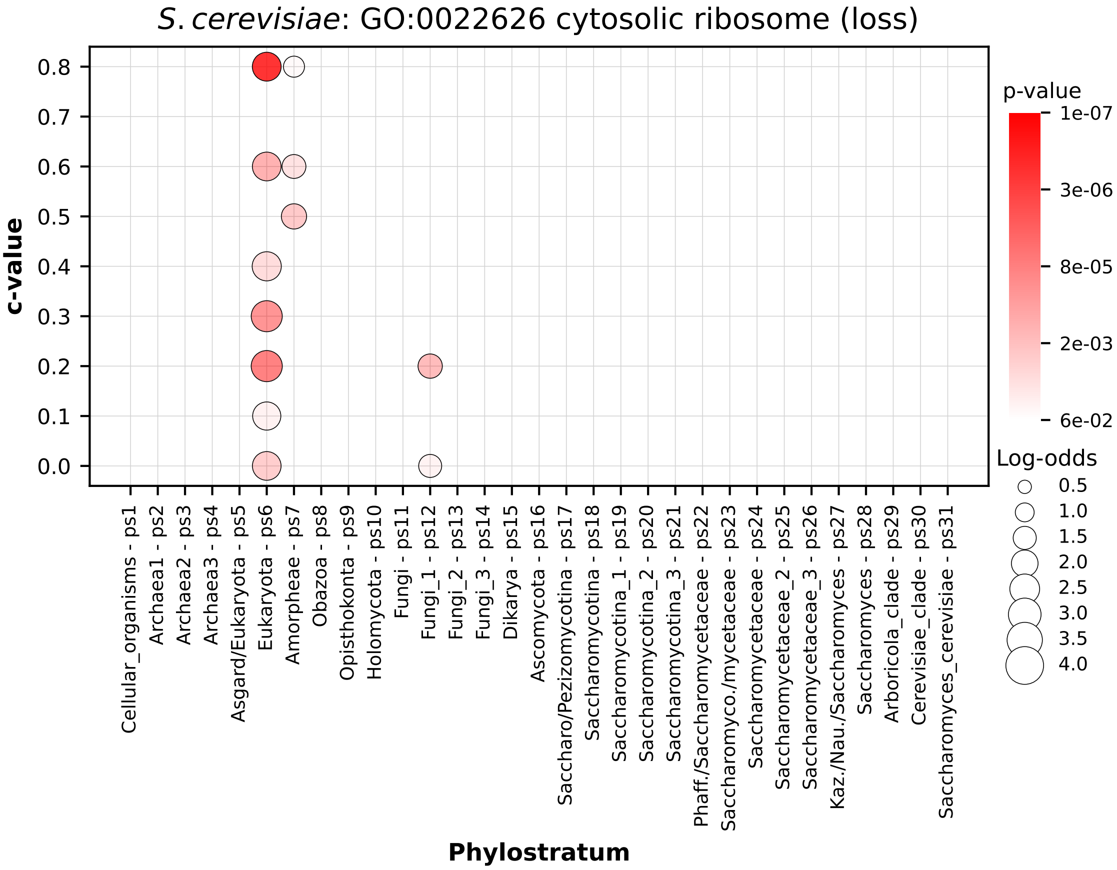

S. cerevisiae: GO:0022626 cytosolic ribosome (loss)
c-value
0.0
0.1
0.2
0.3
0.4
0.5
0.6
0.7
0.8
p-value
1e-07
3e-06
8e-05
2e-03
6e-02
Log-odds
0.5
1.0
1.5
2.0
2.5
3.0
3.5
4.0
Cellular_organisms - ps1
Archaea1 - ps2
Archaea2 - ps3
Archaea3 - ps4
Asgard/Eukaryota - ps5
Eukaryota - ps6
Amorpheae - ps7
Obazoa - ps8
Opisthokonta - ps9
Holomycota - ps10
Fungi - ps11
Fungi_1 - ps12
Fungi_2 - ps13
Fungi_3 - ps14
Dikarya - ps15
Ascomycota - ps16
Saccharo/Pezizomycotina - ps17
Saccharomycotina - ps18
Saccharomycotina_1 - ps19
Saccharomycotina_2 - ps20
Saccharomycotina_3 - ps21
Phaff./Saccharomycetaceae - ps22
Saccharomyco./mycetaceae - ps23
Saccharomycetaceae - ps24
Saccharomycetaceae_2 - ps25
Saccharomycetaceae_3 - ps26
Kaz./Nau./Saccharomyces - ps27
Saccharomyces - ps28
Arboricola_clade - ps29
Cerevisiae_clade - ps30
Saccharomyces_cerevisiae - ps31
Phylostratum

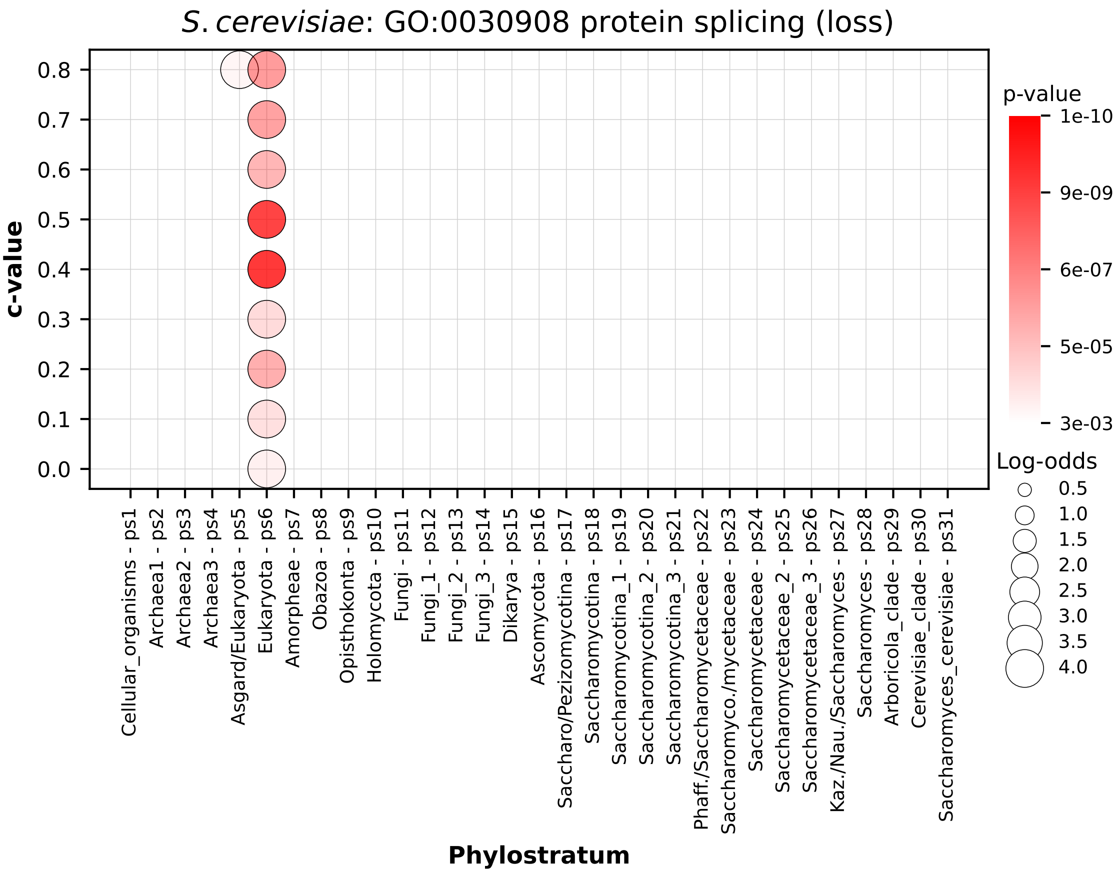

S. cerevisiae: GO:0030908 protein splicing (loss)
c-value
0.8
0.7
0.6
0.5
0.4
0.3
0.2
0.1
0.0
p-value
1e-10
9e-09
6e-07
5e-05
3e-03
Log-odds
0.5
1.0
1.5
2.0
2.5
3.0
3.5
4.0
Cellular_organisms - ps1
Archaea1 - ps2
Archaea2 - ps3
Archaea3 - ps4
Asgard/Eukaryota - ps5
Eukaryota - ps6
Amorpheae - ps7
Obazoa - ps8
Opisthokonta - ps9
Holomycota - ps10
Fungi - ps11
Fungi_1 - ps12
Fungi_2 - ps13
Fungi_3 - ps14
Dikarya - ps15
Ascomycota - ps16
Saccharo/Pezizomycotina - ps17
Saccharomycotina - ps18
Saccharomycotina_1 - ps19
Saccharomycotina_2 - ps20
Saccharomycotina_3 - ps21
Phaff./Saccharomycetaceae - ps22
Saccharomyco./mycetaceae - ps23
Saccharomycetaceae - ps24
Saccharomycetaceae_2 - ps25
Saccharomycetaceae_3 - ps26
Kaz./Nau./Saccharomyces - ps27
Saccharomyces - ps28
Arboricola_clade - ps29
Cerevisiae_clade - ps30
Saccharomyces_cerevisiae - ps31
Phylostratum

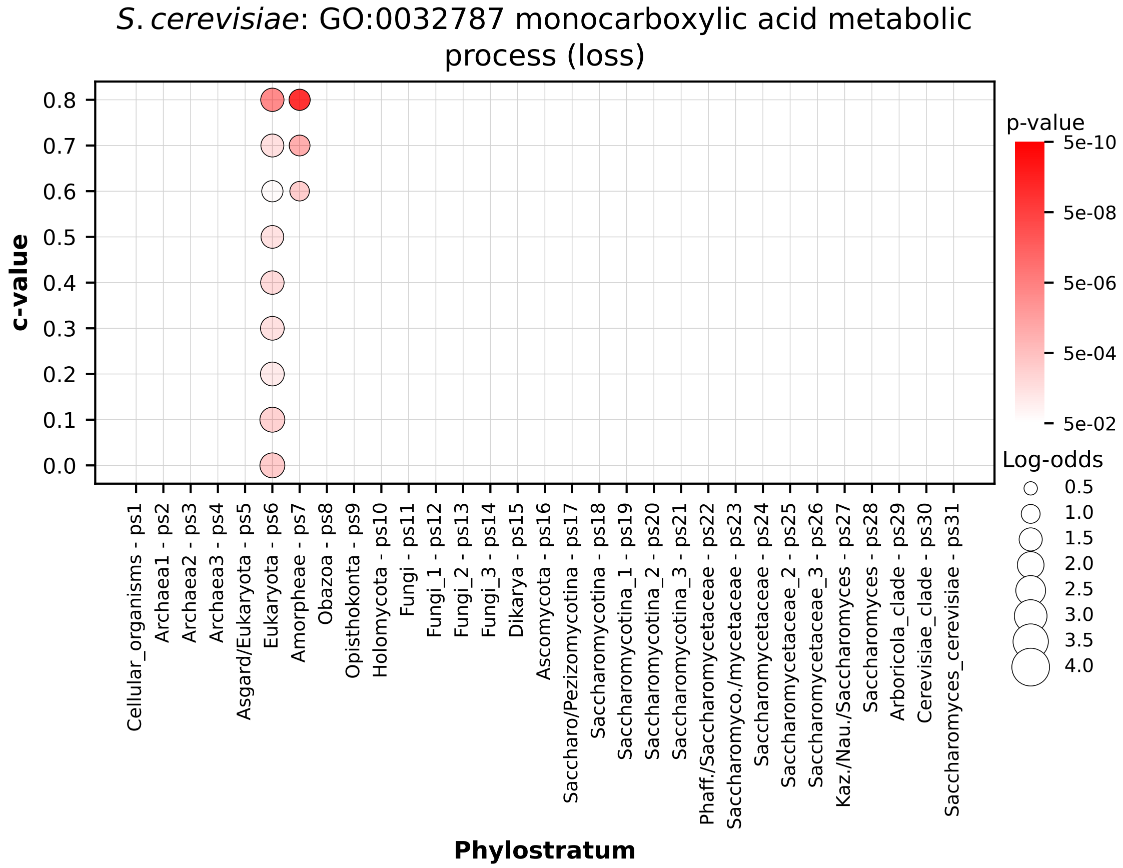

S. cerevisiae: GO:0032787 monocarboxylic acid metabolic process (loss)
c-value
0.8
0.7
0.6
0.5
0.4
0.3
0.2
0.1
0.0
p-value
5e-10
5e-08
5e-06
5e-04
5e-02
Log-odds
0.5
1.0
1.5
2.0
2.5
3.0
3.5
4.0
Cellular_organisms - ps1
Archaea1 - ps2
Archaea2 - ps3
Archaea3 - ps4
Asgard/Eukaryota - ps5
Eukaryota - ps6
Amorpheae - ps7
Obazoa - ps8
Opisthokonta - ps9
Holomycota - ps10
Fungi - ps11
Fungi_1 - ps12
Fungi_2 - ps13
Fungi_3 - ps14
Dikarya - ps15
Ascomycota - ps16
Saccharo/Pezizomycotina - ps17
Saccharomycotina - ps18
Saccharomycotina_1 - ps19
Saccharomycotina_2 - ps20
Saccharomycotina_3 - ps21
Phaff./Saccharomycetaceae - ps22
Saccharomyco./mycetaceae - ps23
Saccharomycetaceae - ps24
Saccharomycetaceae_2 - ps25
Saccharomycetaceae_3 - ps26
Kaz./Nau./Saccharomyces - ps27
Saccharomyces - ps28
Arboricola_clade - ps29
Cerevisiae_clade - ps30
Saccharomyces_cerevisiae - ps31
Phylostratum

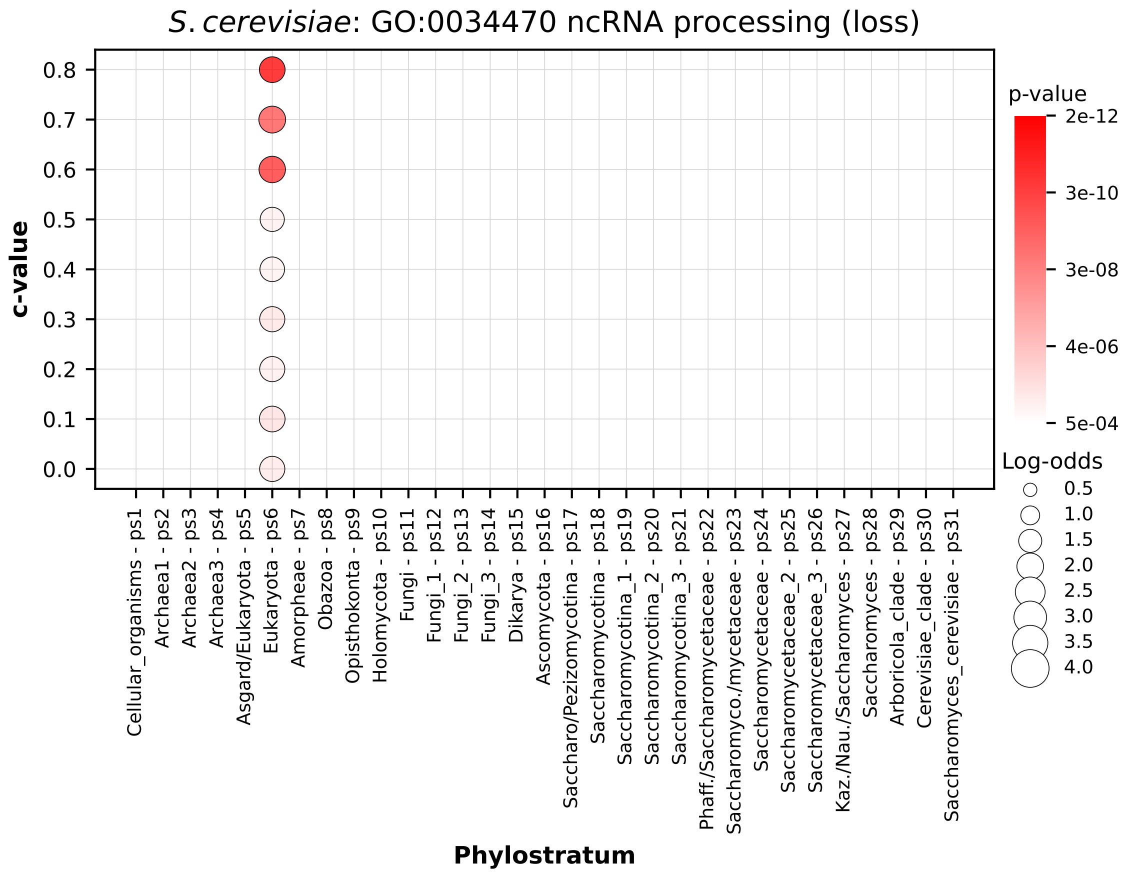
S. cerevisiae: GO:0034470 ncRNA processing (loss)
c-value
0.0
0.1
0.2
0.3
0.4
0.5
0.6
0.7
0.8
p-value
2e-12
3e-10
3e-08
4e-06
5e-04
Log-odds
0.5
1.0
1.5
2.0
2.5
3.0
3.5
4.0
Cellular_organisms - ps1
Archaea1 - ps2
Archaea2 - ps3
Archaea3 - ps4
Asgard/Eukaryota - ps5
Eukaryota - ps6
Amorpheae - ps7
Obazoa - ps8
Opisthokonta - ps9
Holomycota - ps10
Fungi - ps11
Fungi_1 - ps12
Fungi_2 - ps13
Fungi_3 - ps14
Dikarya - ps15
Ascomycota - ps16
Saccharo/Pezizomycotina - ps17
Saccharomycotina - ps18
Saccharomycotina_1 - ps19
Saccharomycotina_2 - ps20
Saccharomycotina_3 - ps21
Phaff./Saccharomycetaceae - ps22
Saccharomyco./mycetaceae - ps23
Saccharomycetaceae - ps24
Saccharomycetaceae_2 - ps25
Saccharomycetaceae_3 - ps26
Kaz./Nau./Saccharomyces - ps27
Saccharomyces - ps28
Arboricola_clade - ps29
Cerevisiae_clade - ps30
Saccharomyces_cerevisiae - ps31
Phylostratum

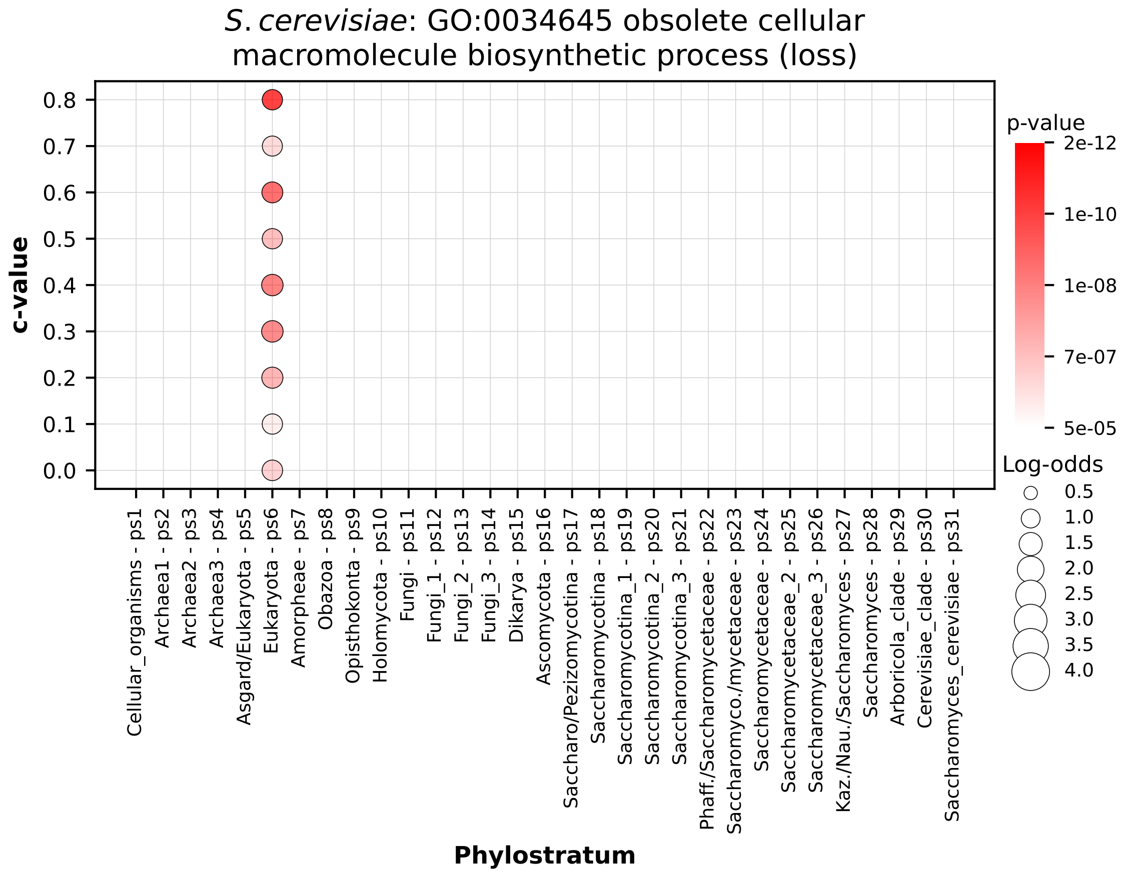

S. cerevisiae: GO:0034645 obsolete cellular macromolecule biosynthetic process (loss)
c-value
0.0
0.1
0.2
0.3
0.4
0.5
0.6
0.7
0.8
p-value
2e-12
1e-10
1e-08
7e-07
5e-05
Log-odds
0.5
1.0
1.5
2.0
2.5
3.0
3.5
4.0
Cellular_organisms - ps1
Archaea1 - ps2
Archaea2 - ps3
Archaea3 - ps4
Asgard/Eukaryota - ps5
Eukaryota - ps6
Amorpheae - ps7
Obazoa - ps8
Opisthokonta - ps9
Holomycota - ps10
Fungi - ps11
Fungi_1 - ps12
Fungi_2 - ps13
Fungi_3 - ps14
Dikarya - ps15
Ascomycota - ps16
Saccharo/Pezizomycotina - ps17
Saccharomycotina - ps18
Saccharomycotina_1 - ps19
Saccharomycotina_2 - ps20
Saccharomycotina_3 - ps21
Phaff./Saccharomycetaceae - ps22
Saccharomyco./mycetaceae - ps23
Saccharomycetaceae - ps24
Saccharomycetaceae_2 - ps25
Saccharomycetaceae_3 - ps26
Kaz./Nau./Saccharomyces - ps27
Saccharomyces - ps28
Arboricola_clade - ps29
Cerevisiae_clade - ps30
Saccharomyces_cerevisiae - ps31
Phylostratum

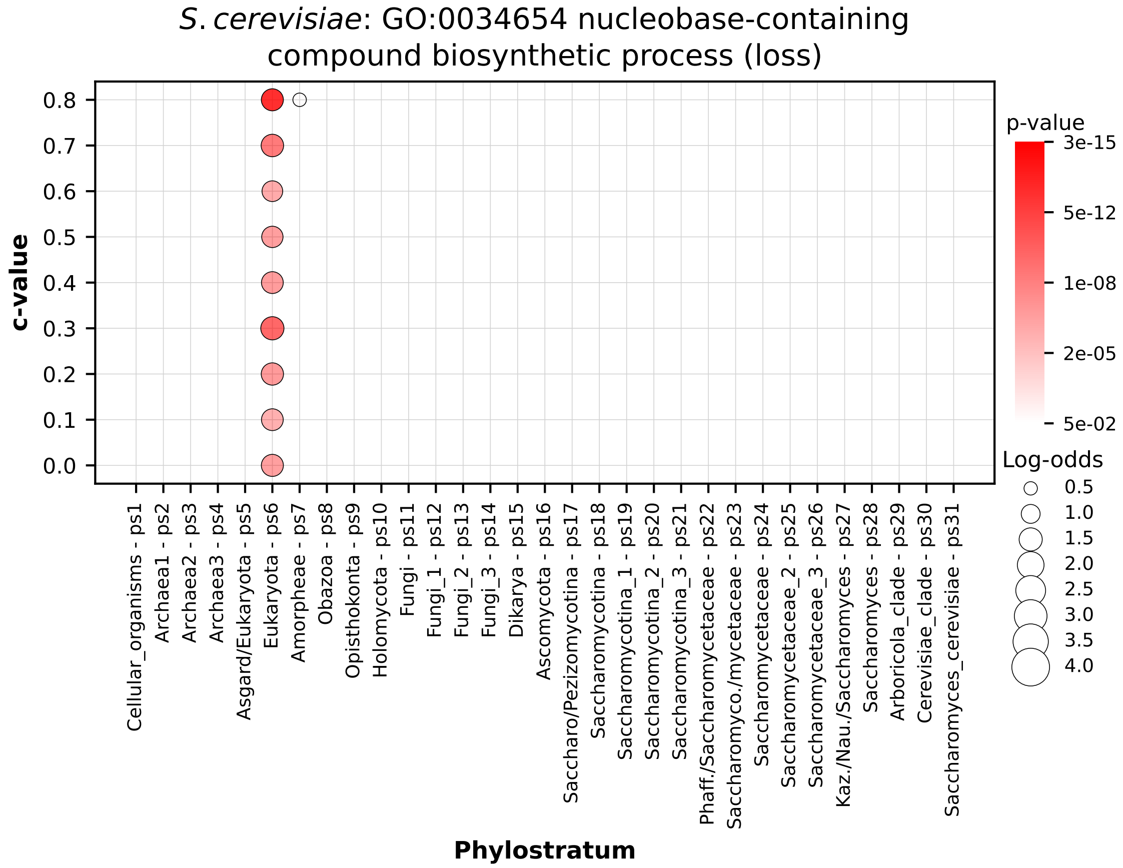
S. cerevisiae: GO:0034654 nucleobase-containing compound biosynthetic process (loss)
c-value
0.8
0.7
0.6
0.5
0.4
0.3
0.2
0.1
0.0
p-value
3e-15
5e-12
1e-08
2e-05
5e-02
Log-odds
0.5
1.0
1.5
2.0
2.5
3.0
3.5
4.0
Cellular_organisms - ps1
Archaea1 - ps2
Archaea2 - ps3
Archaea3 - ps4
Asgard/Eukaryota - ps5
Eukaryota - ps6
Amorpheae - ps7
Obazoa - ps8
Opisthokonta - ps9
Holomycota - ps10
Fungi - ps11
Fungi_1 - ps12
Fungi_2 - ps13
Fungi_3 - ps14
Dikarya - ps15
Ascomycota - ps16
Saccharo/Pezizomycotina - ps17
Saccharomycotina - ps18
Saccharomycotina_1 - ps19
Saccharomycotina_2 - ps20
Saccharomycotina_3 - ps21
Phaff./Saccharomycetaceae - ps22
Saccharomyco./mycetaceae - ps23
Saccharomycetaceae - ps24
Saccharomycetaceae_2 - ps25
Saccharomycetaceae_3 - ps26
Kaz./Nau./Saccharomyces - ps27
Saccharomyces - ps28
Arboricola_clade - ps29
Cerevisiae_clade - ps30
Saccharomyces_cerevisiae - ps31
Phylostratum

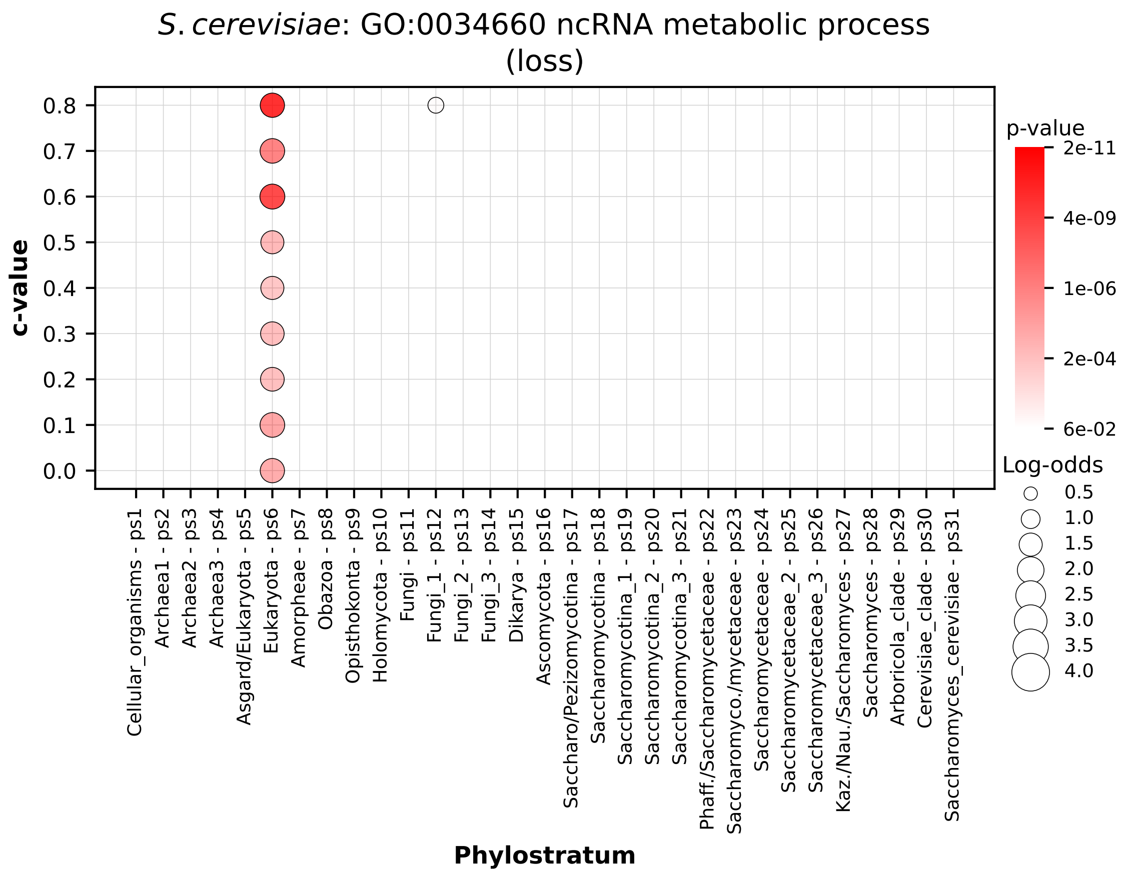

S. cerevisiae: GO:0034660 ncRNA metabolic process
(loss)
c-value
0.8
0.7
0.6
0.5
0.4
0.3
0.2
0.1
0.0
p-value
2e-11
4e-09
1e-06
2e-04
6e-02
Log-odds
0.5
1.0
1.5
2.0
2.5
3.0
3.5
4.0
Cellular_organisms - ps1
Archaea1 - ps2
Archaea2 - ps3
Archaea3 - ps4
Asgard/Eukaryota - ps5
Eukaryota - ps6
Amorpheae - ps7
Obazoa - ps8
Opisthokonta - ps9
Holomycota - ps10
Fungi - ps11
Fungi_1 - ps12
Fungi_2 - ps13
Fungi_3 - ps14
Dikarya - ps15
Ascomycota - ps16
Saccharo/Pezizomycotina - ps17
Saccharomycotina - ps18
Saccharomycotina_1 - ps19
Saccharomycotina_2 - ps20
Saccharomycotina_3 - ps21
Phaff./Saccharomycetaceae - ps22
Saccharomyco./mycetaceae - ps23
Saccharomycetaceae - ps24
Saccharomycetaceae_2 - ps25
Saccharomycetaceae_3 - ps26
Kaz./Nau./Saccharomyces - ps27
Saccharomyces - ps28
Arboricola_clade - ps29
Cerevisiae_clade - ps30
Saccharomyces_cerevisiae - ps31
Phylostratum

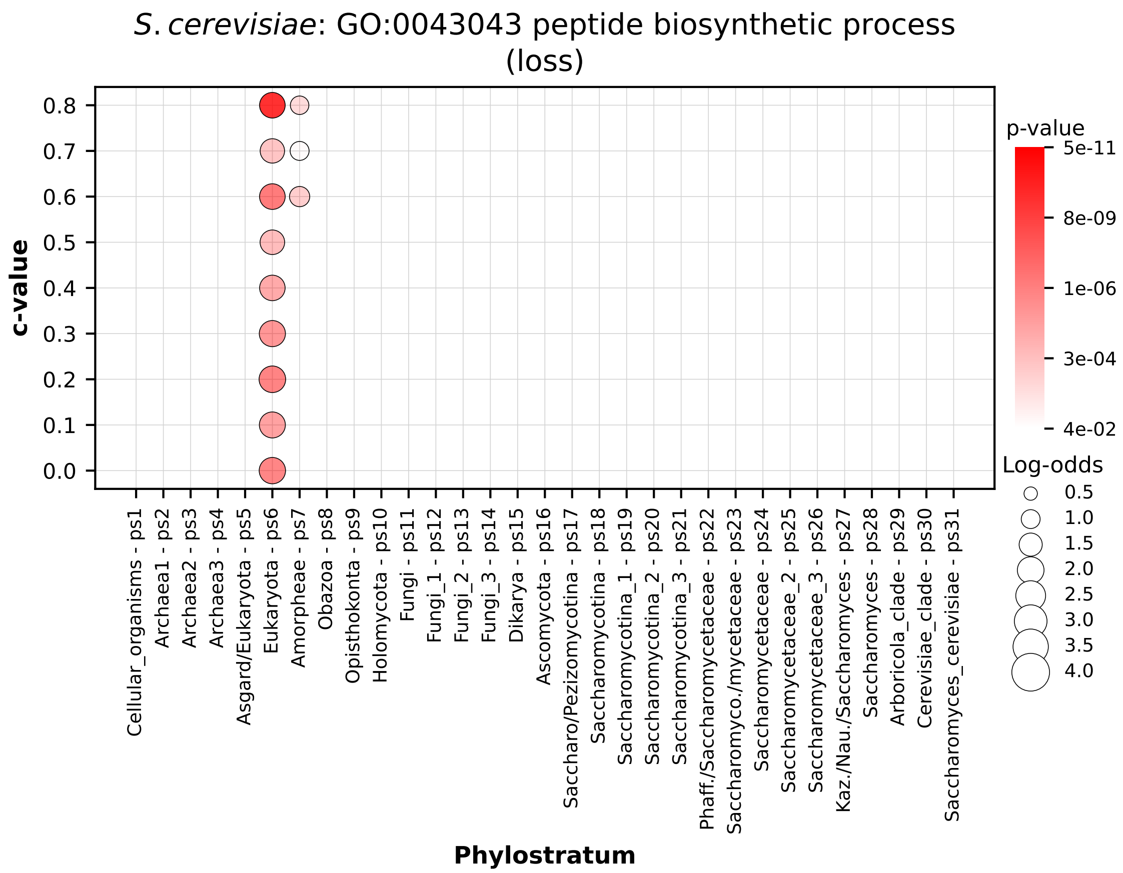

S. cerevisiae: GO:0043043 peptide biosynthetic process
(loss)
c-value
0.8
0.7
0.6
0.5
0.4
0.3
0.2
0.1
0.0
p-value
5e-11
8e-09
1e-06
3e-04
4e-02
Log-odds
0.5
1.0
1.5
2.0
2.5
3.0
3.5
4.0
Cellular_organisms - ps1
Archaea1 - ps2
Archaea2 - ps3
Archaea3 - ps4
Asgard/Eukaryota - ps5
Eukaryota - ps6
Amorpheae - ps7
Obazoa - ps8
Opisthokonta - ps9
Holomycota - ps10
Fungi - ps11
Fungi_1 - ps12
Fungi_2 - ps13
Fungi_3 - ps14
Dikarya - ps15
Ascomycota - ps16
Saccharo/Pezizomycotina - ps17
Saccharomycotina - ps18
Saccharomycotina_1 - ps19
Saccharomycotina_2 - ps20
Saccharomycotina_3 - ps21
Phaff./Saccharomycetaceae - ps22
Saccharomyco./mycetaceae - ps23
Saccharomycetaceae - ps24
Saccharomycetaceae_2 - ps25
Saccharomycetaceae_3 - ps26
Kaz./Nau./Saccharomyces - ps27
Saccharomyces - ps28
Arboricola_clade - ps29
Cerevisiae_clade - ps30
Saccharomyces_cerevisiae - ps31
Phylostratum

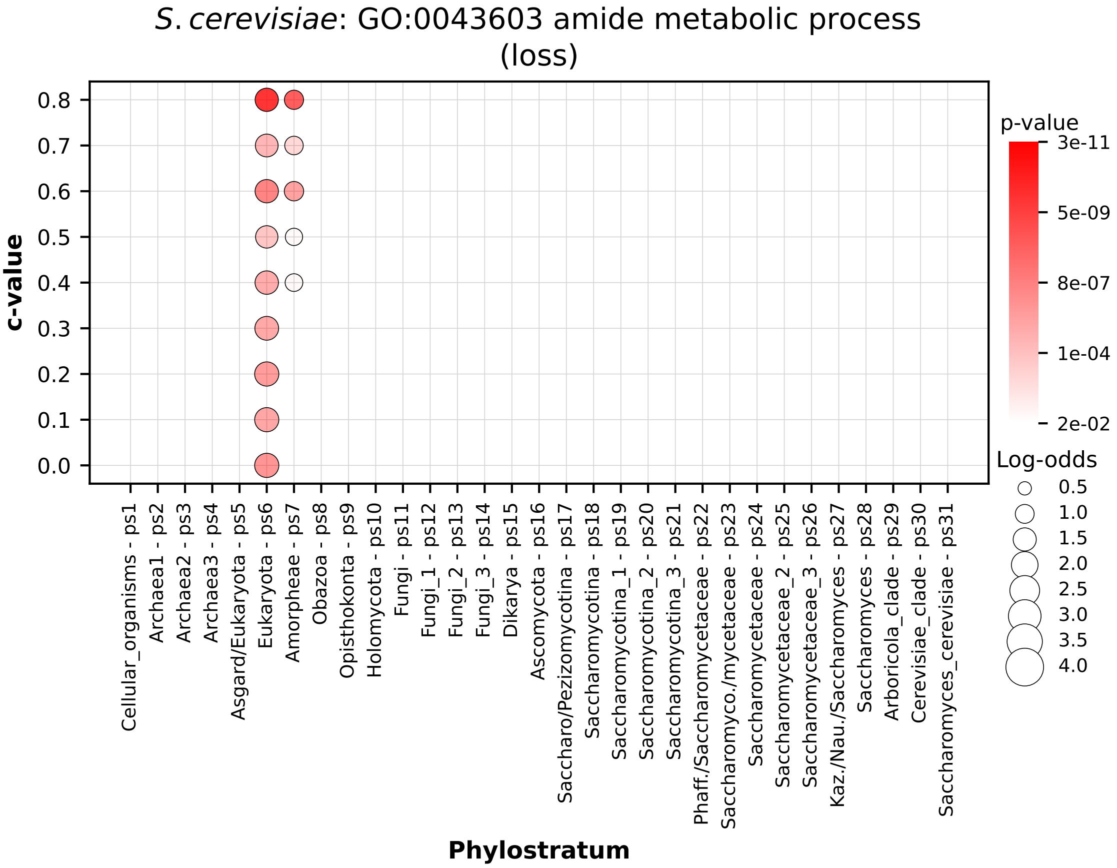

S. cerevisiae: GO:0043603 amide metabolic process
(loss)
c-value
0.0
0.1
0.2
0.3
0.4
0.5
0.6
0.7
0.8
p-value
3e-11
5e-09
8e-07
1e-04
2e-02
Log-odds
0.5
1.0
1.5
2.0
2.5
3.0
3.5
4.0
Cellular_organisms - ps1
Archaea1 - ps2
Archaea2 - ps3
Archaea3 - ps4
Asgard/Eukaryota - ps5
Eukaryota - ps6
Amorpheae - ps7
Obazoa - ps8
Opisthokonta - ps9
Holomycota - ps10
Fungi - ps11
Fungi_1 - ps12
Fungi_2 - ps13
Fungi_3 - ps14
Dikarya - ps15
Ascomycota - ps16
Saccharo/Pezizomycotina - ps17
Saccharomycotina - ps18
Saccharomycotina_1 - ps19
Saccharomycotina_2 - ps20
Saccharomycotina_3 - ps21
Phaff./Saccharomycetaceae - ps22
Saccharomyco./mycetaceae - ps23
Saccharomycetaceae - ps24
Saccharomycetaceae_2 - ps25
Saccharomycetaceae_3 - ps26
Kaz./Nau./Saccharomyces - ps27
Saccharomyces - ps28
Arboricola_clade - ps29
Cerevisiae_clade - ps30
Saccharomyces_cerevisiae - ps31
Phylostratum

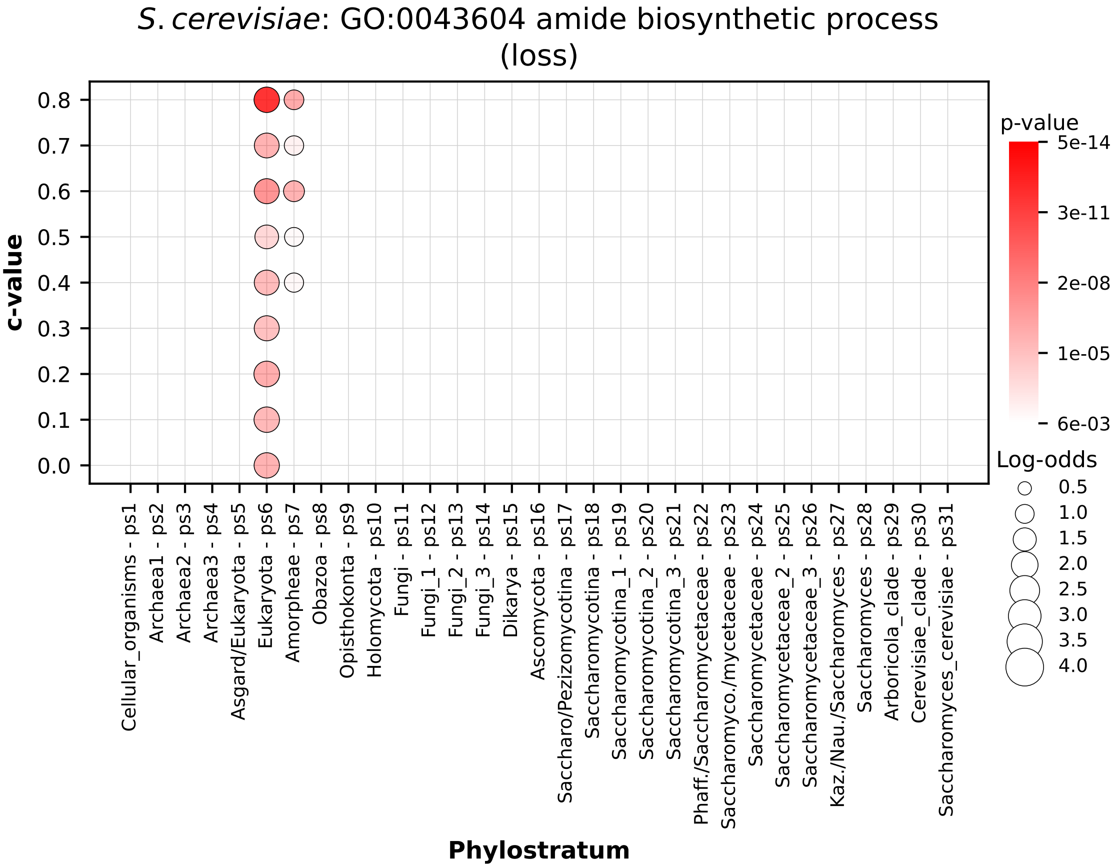
S. cerevisiae: GO:0043604 amide biosynthetic process
(loss)
c-value
0.8
0.7
0.6
0.5
0.4
0.3
0.2
0.1
0.0
p-value
5e-14
3e-11
2e-08
1e-05
6e-03
Log-odds
0.5
1.0
1.5
2.0
2.5
3.0
3.5
4.0
Cellular_organisms - ps1
Archaea1 - ps2
Archaea2 - ps3
Archaea3 - ps4
Asgard/Eukaryota - ps5
Eukaryota - ps6
Amorpheae - ps7
Obazoa - ps8
Opisthokonta - ps9
Holomycota - ps10
Fungi - ps11
Fungi_1 - ps12
Fungi_2 - ps13
Fungi_3 - ps14
Dikarya - ps15
Ascomycota - ps16
Saccharo/Pezizomycotina - ps17
Saccharomycotina - ps18
Saccharomycotina_1 - ps19
Saccharomycotina_2 - ps20
Saccharomycotina_3 - ps21
Phaff./Saccharomycetaceae - ps22
Saccharomyco./mycetaceae - ps23
Saccharomycetaceae - ps24
Saccharomycetaceae_2 - ps25
Saccharomycetaceae_3 - ps26
Kaz./Nau./Saccharomyces - ps27
Saccharomyces - ps28
Arboricola_clade - ps29
Cerevisiae_clade - ps30
Saccharomyces_cerevisiae - ps31
Phylostratum

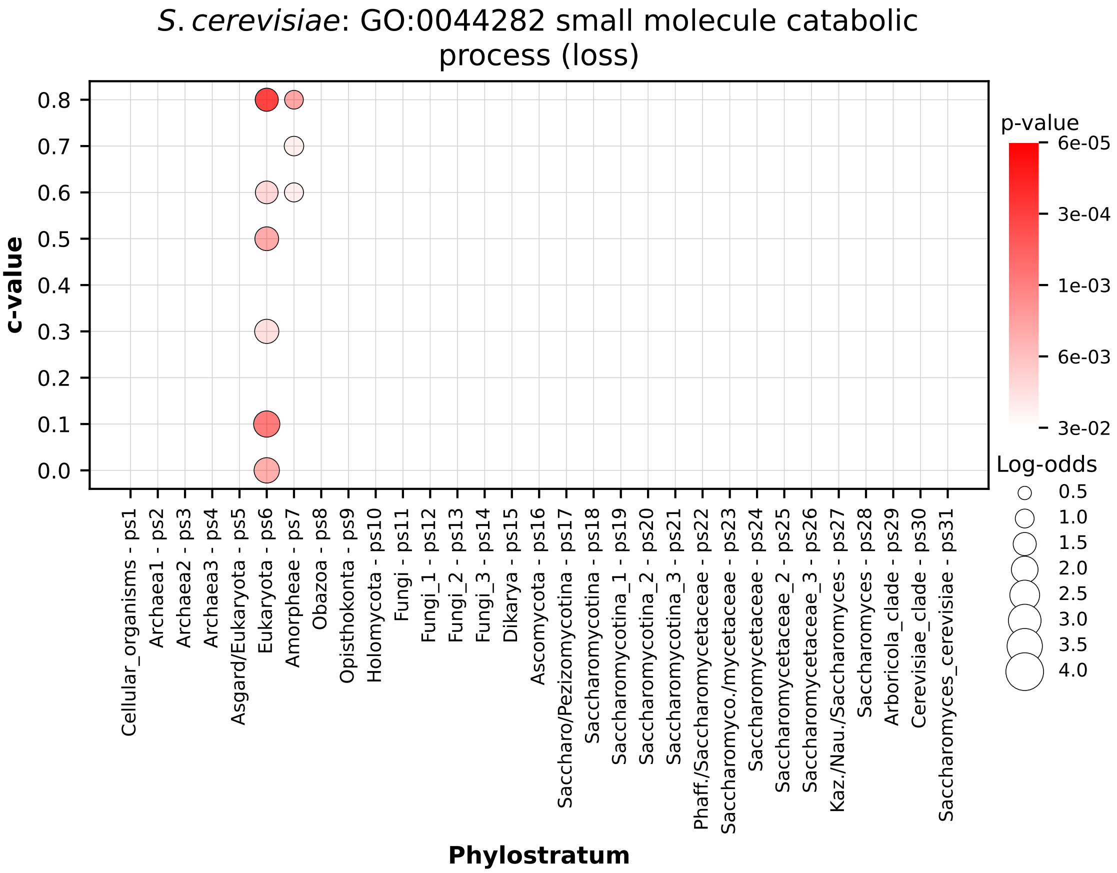
S. cerevisiae: GO:0044282 small molecule catabolic process (loss)
c-value
0.8
0.7
0.6
0.5
0.4
0.3
0.2
0.1
0.0
Phylostratum
Cellular_organisms - ps1
Archaea1 - ps2
Archaea2 - ps3
Archaea3 - ps4
Asgard/Eukaryota - ps5
Eukaryota - ps6
Amorpheae - ps7
Obazoa - ps8
Opisthokonta - ps9
Holomycota - ps10
Fungi - ps11
Fungi_1 - ps12
Fungi_2 - ps13
Fungi_3 - ps14
Dikarya - ps15
Ascomycota - ps16
Saccharo/Pezizomycotina - ps17
Saccharomycotina - ps18
Saccharomycotina_1 - ps19
Saccharomycotina_2 - ps20
Saccharomycotina_3 - ps21
Phaff./Saccharomycetaceae - ps22
Saccharomyco./mycetaceae - ps23
Saccharomycetaceae - ps24
Saccharomycetaceae_2 - ps25
Saccharomycetaceae_3 - ps26
Kaz./Nau./Saccharomyces - ps27
Saccharomyces - ps28
Arboricola_clade - ps29
Cerevisiae_clade - ps30
Saccharomyces_cerevisiae - ps31
p-value
6e-05
3e-04
1e-03
6e-03
3e-02
Log-odds
0.5
1.0
1.5
2.0
2.5
3.0
3.5
4.0

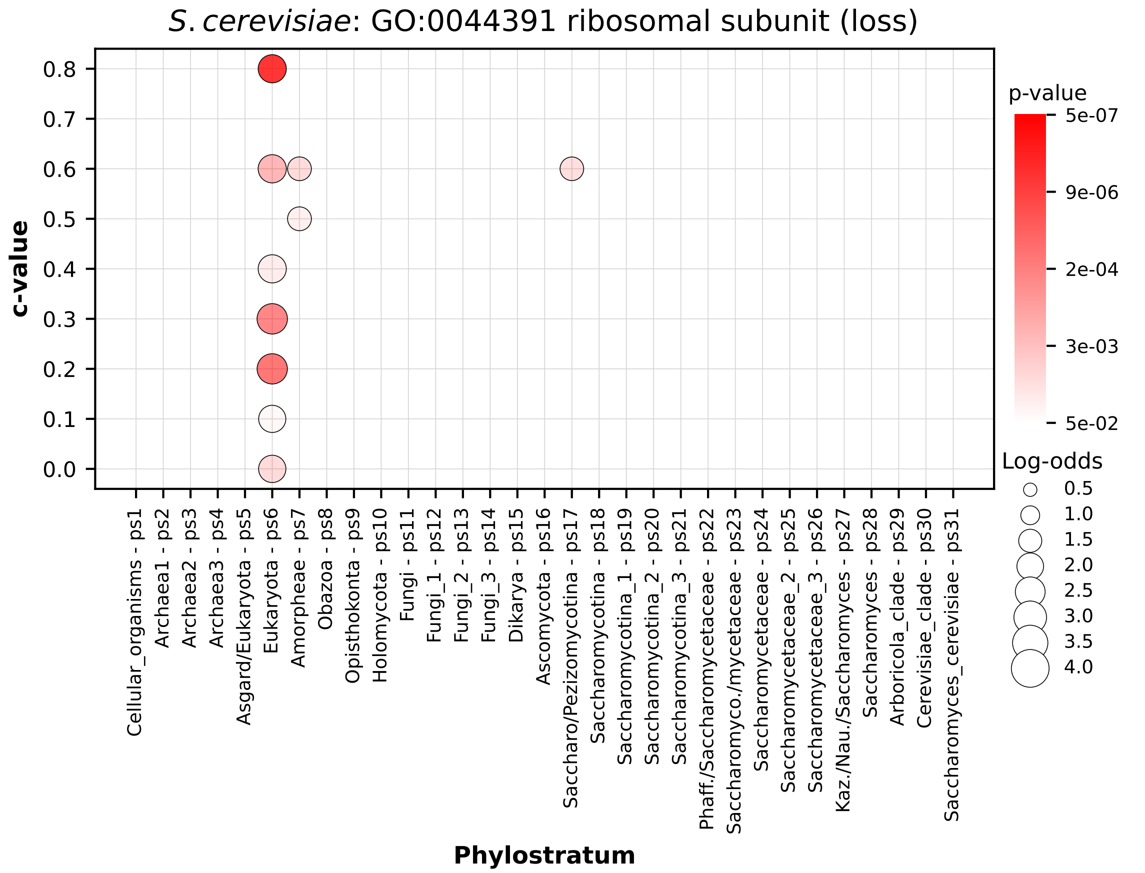
S. cerevisiae: GO:0044391 ribosomal subunit (loss)
c-value
0.8
0.7
0.6
0.5
0.4
0.3
0.2
0.1
0.0
p-value
5e-07
9e-06
2e-04
3e-03
5e-02
Log-odds
0.5
1.0
1.5
2.0
2.5
3.0
3.5
4.0
Cellular_organisms - ps1
Archaea1 - ps2
Archaea2 - ps3
Archaea3 - ps4
Asgard/Eukaryota - ps5
Eukaryota - ps6
Amorpheae - ps7
Obazoa - ps8
Opisthokonta - ps9
Holomycota - ps10
Fungi - ps11
Fungi_1 - ps12
Fungi_2 - ps13
Fungi_3 - ps14
Dikarya - ps15
Ascomycota - ps16
Saccharo/Pezizomycotina - ps17
Saccharomycotina - ps18
Saccharomycotina_1 - ps19
Saccharomycotina_2 - ps20
Saccharomycotina_3 - ps21
Phaff./Saccharomycetaceae - ps22
Saccharomyco./mycetaceae - ps23
Saccharomycetaceae - ps24
Saccharomycetaceae_2 - ps25
Saccharomycetaceae_3 - ps26
Kaz./Nau./Saccharomyces - ps27
Saccharomyces - ps28
Arboricola_clade - ps29
Cerevisiae_clade - ps30
Saccharomyces_cerevisiae - ps31
Phylostratum

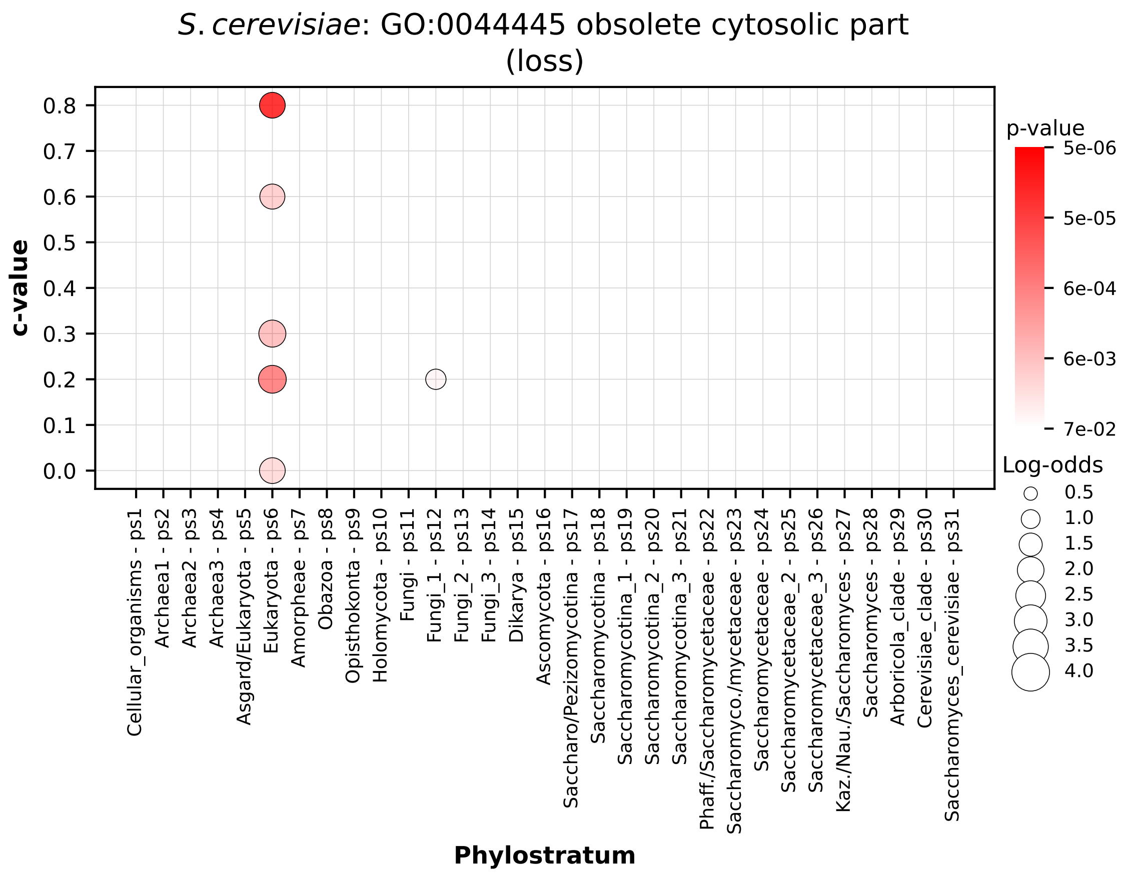

S. cerevisiae: GO:0044445 obsolete cytosolic part
(loss)
c-value
0.8
0.7
0.6
0.5
0.4
0.3
0.2
0.1
0.0
p-value
5e-06
5e-05
6e-04
6e-03
7e-02
Log-odds
0.5
1.0
1.5
2.0
2.5
3.0
3.5
4.0
Cellular_organisms - ps1
Archaea1 - ps2
Archaea2 - ps3
Archaea3 - ps4
Asgard/Eukaryota - ps5
Eukaryota - ps6
Amorpheae - ps7
Obazoa - ps8
Opisthokonta - ps9
Holomycota - ps10
Fungi - ps11
Fungi_1 - ps12
Fungi_2 - ps13
Fungi_3 - ps14
Dikarya - ps15
Ascomycota - ps16
Saccharo/Pezizomycotina - ps17
Saccharomycotina - ps18
Saccharomycotina_1 - ps19
Saccharomycotina_2 - ps20
Saccharomycotina_3 - ps21
Phaff./Saccharomycetaceae - ps22
Saccharomyco./mycetaceae - ps23
Saccharomycetaceae - ps24
Saccharomycetaceae_2 - ps25
Saccharomycetaceae_3 - ps26
Kaz./Nau./Saccharomyces - ps27
Saccharomyces - ps28
Arboricola_clade - ps29
Cerevisiae_clade - ps30
Saccharomyces_cerevisiae - ps31
Phylostratum

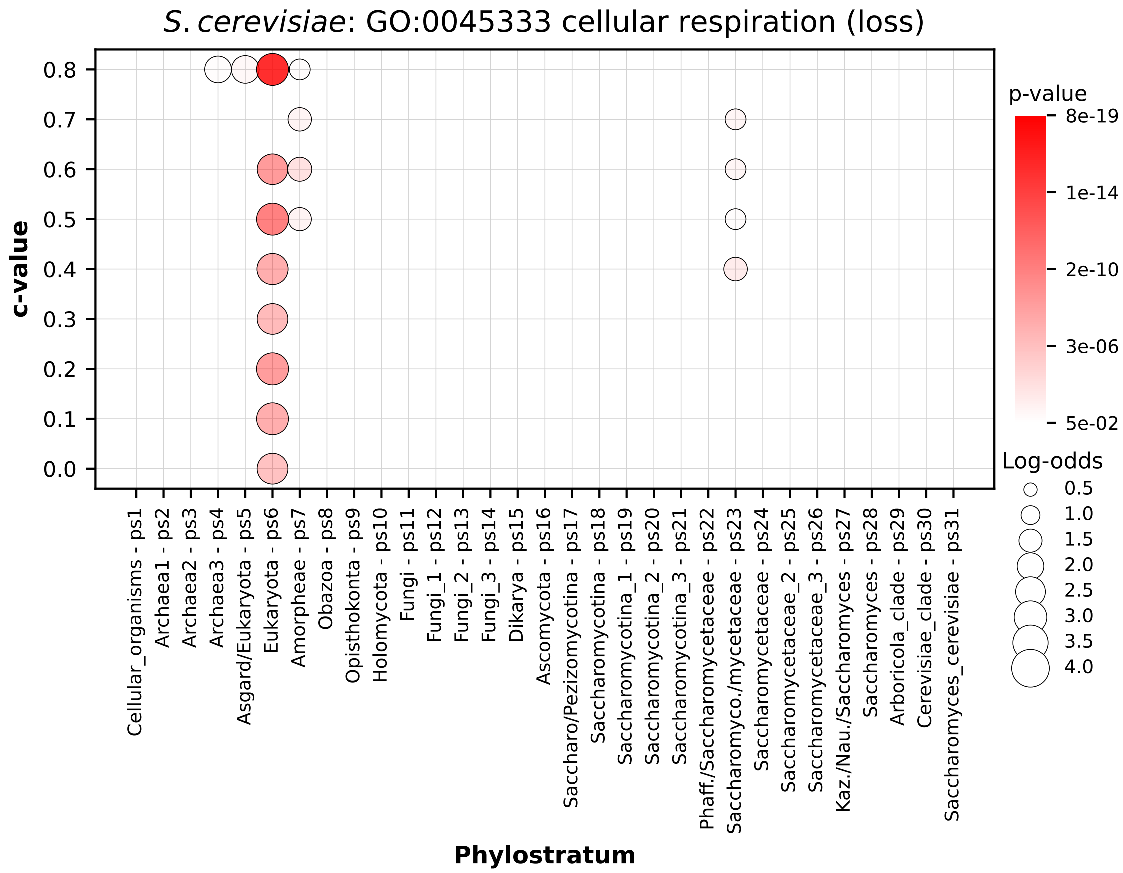
S. cerevisiae: GO:0045333 cellular respiration (loss)
c-value
0.8
0.7
0.6
0.5
0.4
0.3
0.2
0.1
0.0
p-value
8e-19
1e-14
2e-10
3e-06
5e-02
Log-odds
0.5
1.0
1.5
2.0
2.5
3.0
3.5
4.0
Cellular_organisms - ps1
Archaea1 - ps2
Archaea2 - ps3
Archaea3 - ps4
Asgard/Eukaryota - ps5
Eukaryota - ps6
Amorpheae - ps7
Obazoa - ps8
Opisthokonta - ps9
Holomycota - ps10
Fungi - ps11
Fungi_1 - ps12
Fungi_2 - ps13
Fungi_3 - ps14
Dikarya - ps15
Ascomycota - ps16
Saccharo/Pezizomycotina - ps17
Saccharomycotina - ps18
Saccharomycotina_1 - ps19
Saccharomycotina_2 - ps20
Saccharomycotina_3 - ps21
Phaff./Saccharomycetaceae - ps22
Saccharomyco./mycetaceae - ps23
Saccharomycetaceae - ps24
Saccharomycetaceae_2 - ps25
Saccharomycetaceae_3 - ps26
Kaz./Nau./Saccharomyces - ps27
Saccharomyces - ps28
Arboricola_clade - ps29
Cerevisiae_clade - ps30
Saccharomyces_cerevisiae - ps31
Phylostratum

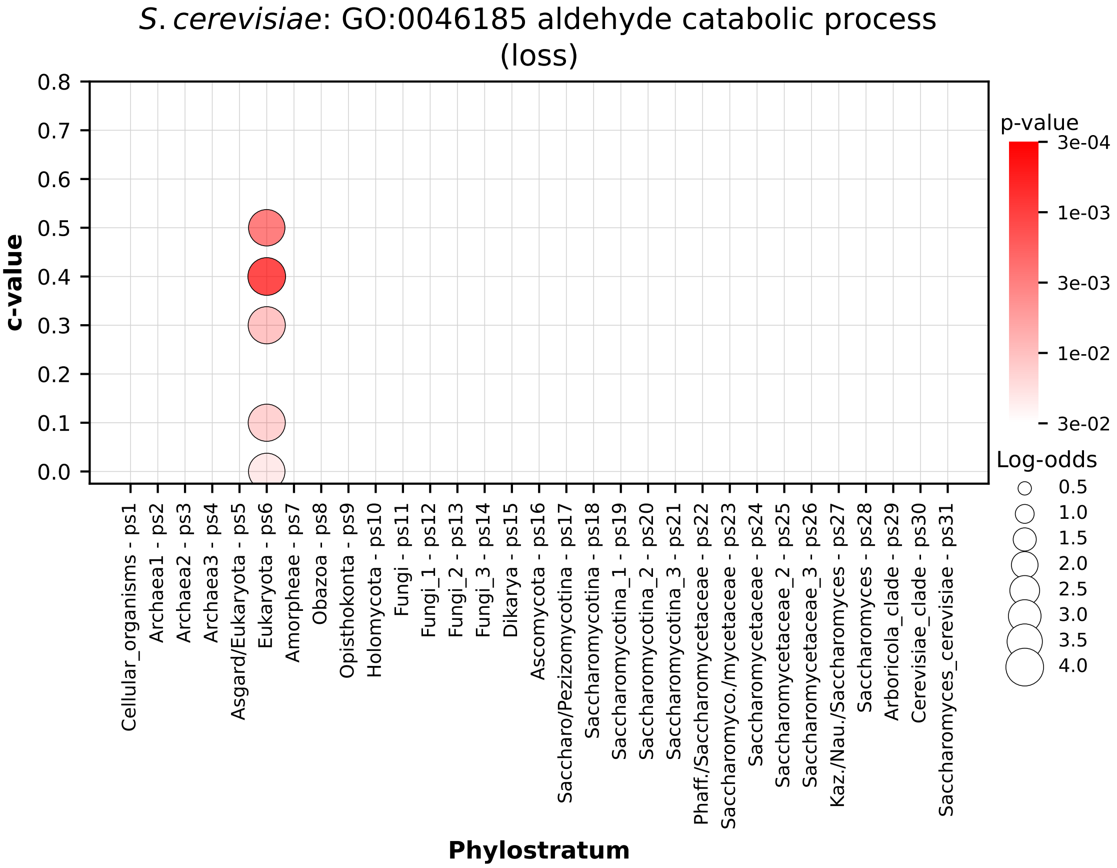

S. cerevisiae: GO:0046185 aldehyde catabolic process
(loss)
c-value
0.8
0.7
0.6
0.5
0.4
0.3
0.2
0.1
0.0
Phylostratum
Cellular_organisms - ps1
Archaea1 - ps2
Archaea2 - ps3
Archaea3 - ps4
Asgard/Eukaryota - ps5
Eukaryota - ps6
Amorpheae - ps7
Obazoa - ps8
Opisthokonta - ps9
Holomycota - ps10
Fungi - ps11
Fungi_1 - ps12
Fungi_2 - ps13
Fungi_3 - ps14
Dikarya - ps15
Ascomycota - ps16
Saccharo/Pezizomycotina - ps17
Saccharomycotina - ps18
Saccharomycotina_1 - ps19
Saccharomycotina_2 - ps20
Saccharomycotina_3 - ps21
Phaff./Saccharomycetaceae - ps22
Saccharomyco./mycetaceae - ps23
Saccharomycetaceae - ps24
Saccharomycetaceae_2 - ps25
Saccharomycetaceae_3 - ps26
Kaz./Nau./Saccharomyces - ps27
Saccharomyces - ps28
Arboricola_clade - ps29
Cerevisiae_clade - ps30
Saccharomyces_cerevisiae - ps31
p-value
3e-04
1e-03
3e-03
1e-02
3e-02
Log-odds
0.5
1.0
1.5
2.0
2.5
3.0
3.5
4.0

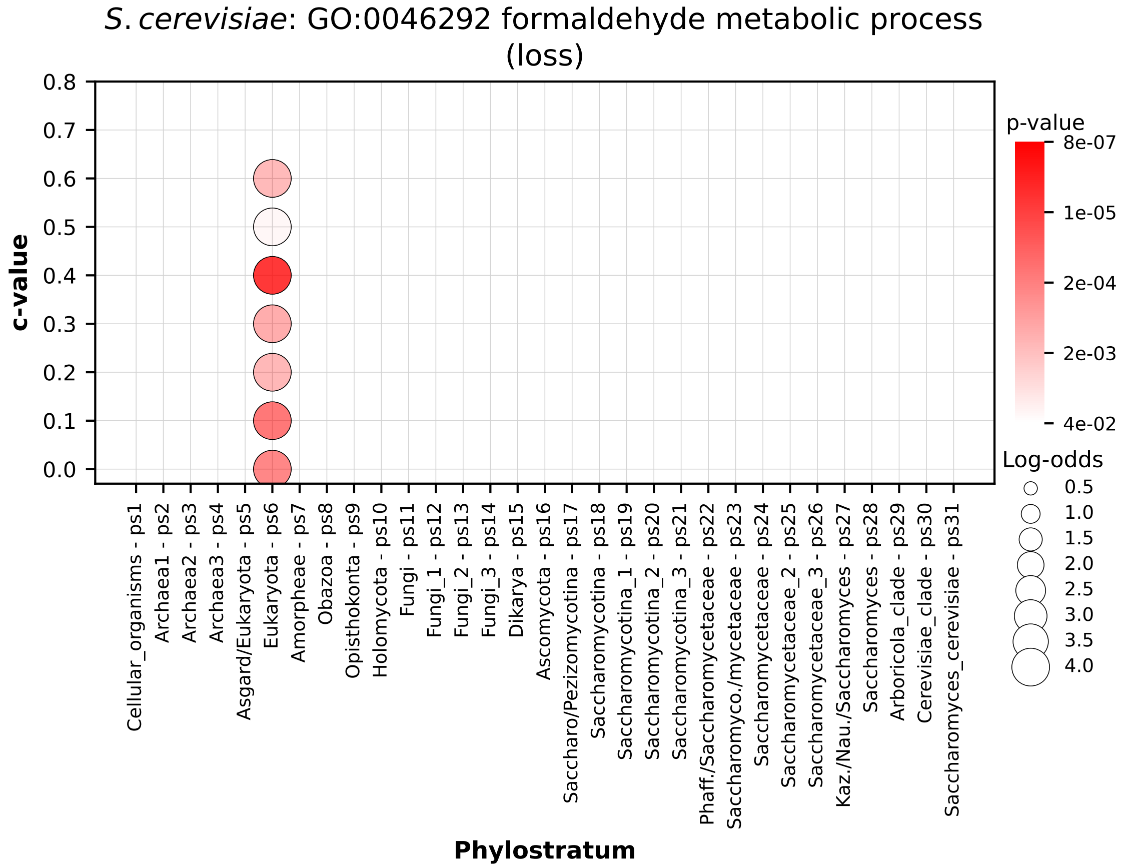

S. cerevisiae: GO:0046292 formaldehyde metabolic process
(loss)
c-value
0.0
0.1
0.2
0.3
0.4
0.5
0.6
0.7
0.8
p-value
8e-07
1e-05
2e-04
2e-03
4e-02
Log-odds
0.5
1.0
1.5
2.0
2.5
3.0
3.5
4.0
Cellular_organisms - ps1
Archaea1 - ps2
Archaea2 - ps3
Archaea3 - ps4
Asgard/Eukaryota - ps5
Eukaryota - ps6
Amorpheae - ps7
Obazoa - ps8
Opisthokonta - ps9
Holomycota - ps10
Fungi - ps11
Fungi_1 - ps12
Fungi_2 - ps13
Fungi_3 - ps14
Dikarya - ps15
Ascomycota - ps16
Saccharo/Pezizomycotina - ps17
Saccharomycotina - ps18
Saccharomycotina_1 - ps19
Saccharomycotina_2 - ps20
Saccharomycotina_3 - ps21
Phaff./Saccharomycetaceae - ps22
Saccharomyco./mycetaceae - ps23
Saccharomycetaceae - ps24
Saccharomycetaceae_2 - ps25
Saccharomycetaceae_3 - ps26
Kaz./Nau./Saccharomyces - ps27
Saccharomyces - ps28
Arboricola_clade - ps29
Cerevisiae_clade - ps30
Saccharomyces_cerevisiae - ps31
Phylostratum

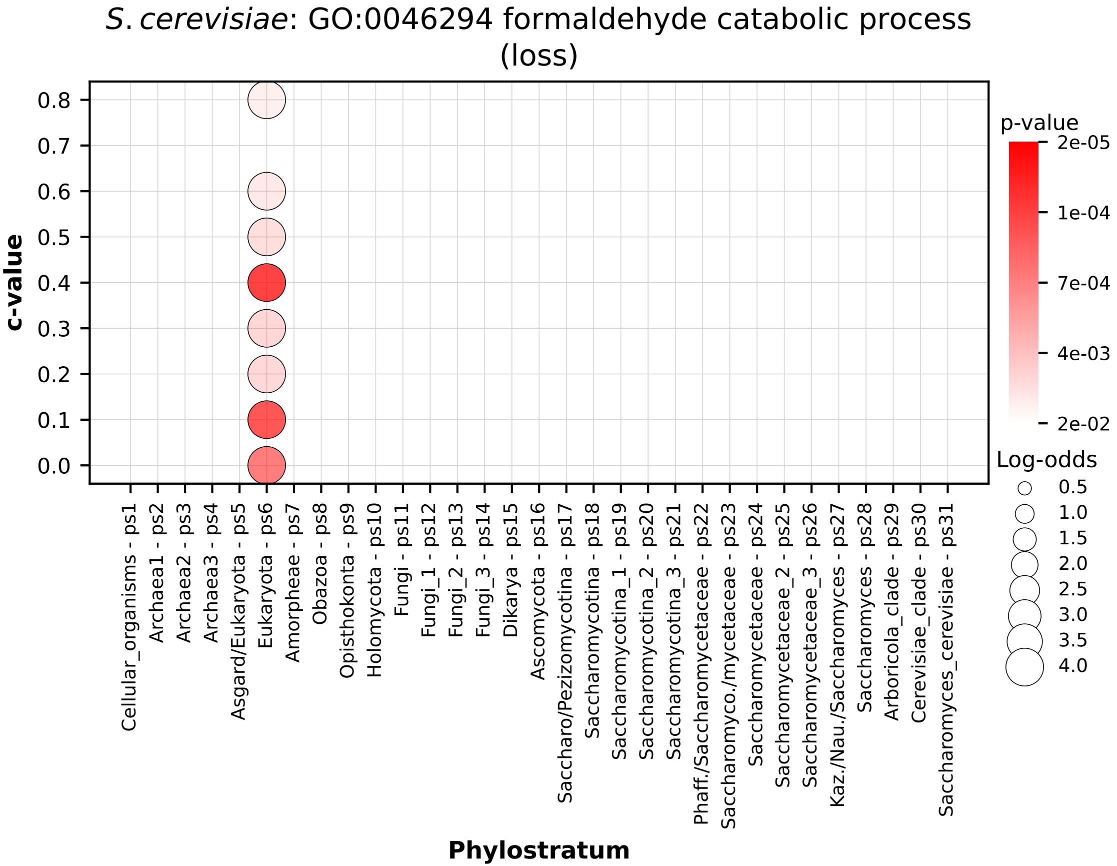

S. cerevisiae: GO:0046294 formaldehyde catabolic process
(loss)
c-value
0.8
0.7
0.6
0.5
0.4
0.3
0.2
0.1
0.0
p-value
2e-05
1e-04
7e-04
4e-03
2e-02
Log-odds
0.5
1.0
1.5
2.0
2.5
3.0
3.5
4.0
Cellular_organisms - ps1
Archaea1 - ps2
Archaea2 - ps3
Archaea3 - ps4
Asgard/Eukaryota - ps5
Eukaryota - ps6
Amorpheae - ps7
Obazoa - ps8
Opisthokonta - ps9
Holomycota - ps10
Fungi - ps11
Fungi_1 - ps12
Fungi_2 - ps13
Fungi_3 - ps14
Dikarya - ps15
Ascomycota - ps16
Saccharo/Pezizomycotina - ps17
Saccharomycotina - ps18
Saccharomycotina_1 - ps19
Saccharomycotina_2 - ps20
Saccharomycotina_3 - ps21
Phaff./Saccharomycetaceae - ps22
Saccharomyco./mycetaceae - ps23
Saccharomycetaceae - ps24
Saccharomycetaceae_2 - ps25
Saccharomycetaceae_3 - ps26
Kaz./Nau./Saccharomyces - ps27
Saccharomyces - ps28
Arboricola_clade - ps29
Cerevisiae_clade - ps30
Saccharomyces_cerevisiae - ps31
Phylostratum

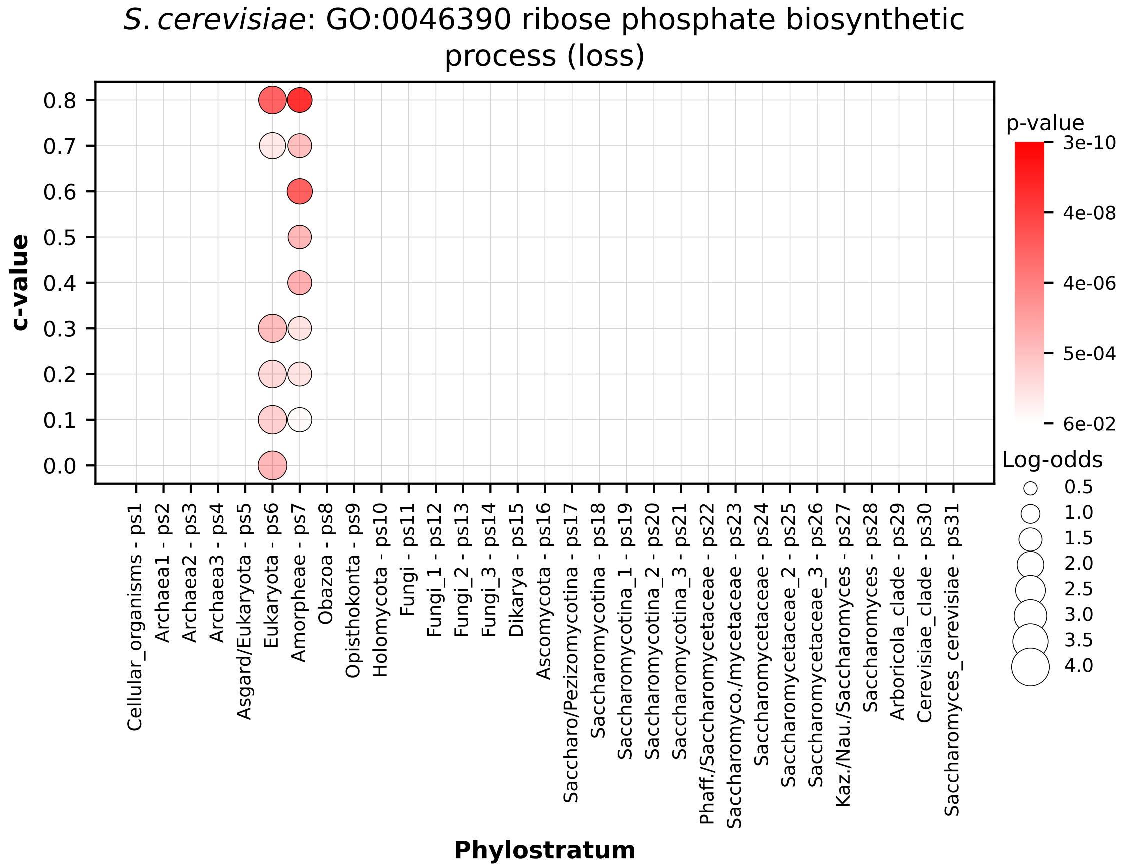

S. cerevisiae: GO:0046390 ribose phosphate biosynthetic process (loss)
c-value
0.8
0.7
0.6
0.5
0.4
0.3
0.2
0.1
0.0
p-value
3e-10
4e-08
4e-06
5e-04
6e-02
Log-odds
0.5
1.0
1.5
2.0
2.5
3.0
3.5
4.0
Cellular_organisms - ps1
Archaea1 - ps2
Archaea2 - ps3
Archaea3 - ps4
Asgard/Eukaryota - ps5
Eukaryota - ps6
Amorpheae - ps7
Obazoa - ps8
Opisthokonta - ps9
Holomycota - ps10
Fungi - ps11
Fungi_1 - ps12
Fungi_2 - ps13
Fungi_3 - ps14
Dikarya - ps15
Ascomycota - ps16
Saccharo/Pezizomycotina - ps17
Saccharomycotina - ps18
Saccharomycotina_1 - ps19
Saccharomycotina_2 - ps20
Saccharomycotina_3 - ps21
Phaff./Saccharomycetaceae - ps22
Saccharomyco./mycetaceae - ps23
Saccharomycetaceae - ps24
Saccharomycetaceae_2 - ps25
Saccharomycetaceae_3 - ps26
Kaz./Nau./Saccharomyces - ps27
Saccharomyces - ps28
Arboricola_clade - ps29
Cerevisiae_clade - ps30
Saccharomyces_cerevisiae - ps31
Phylostratum

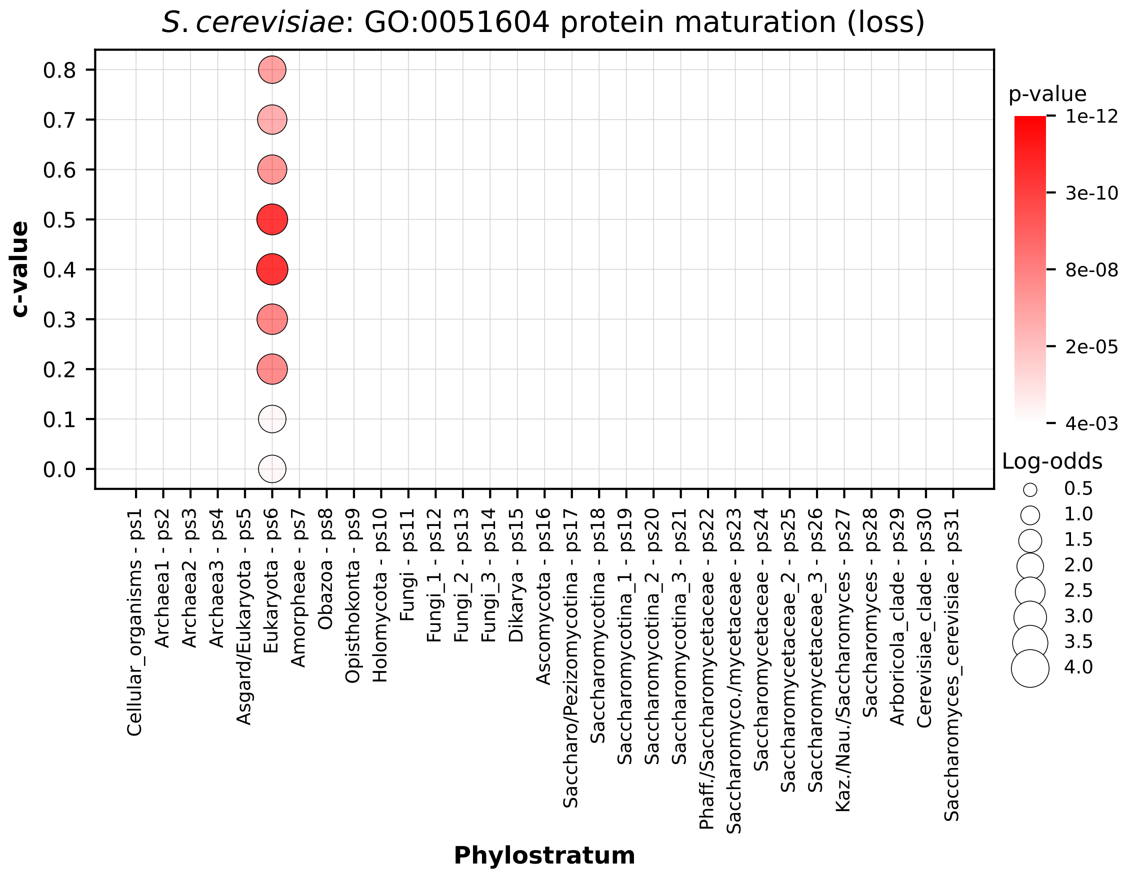

S. cerevisiae: GO:0051604 protein maturation (loss)
c-value
0.8
0.7
0.6
0.5
0.4
0.3
0.2
0.1
0.0
Phylostratum
Cellular_organisms - ps1
Archaea1 - ps2
Archaea2 - ps3
Archaea3 - ps4
Asgard/Eukaryota - ps5
Eukaryota - ps6
Amorpheae - ps7
Obazoa - ps8
Opisthokonta - ps9
Holomycota - ps10
Fungi - ps11
Fungi_1 - ps12
Fungi_2 - ps13
Fungi_3 - ps14
Dikarya - ps15
Ascomycota - ps16
Saccharo/Pezizomycotina - ps17
Saccharomycotina - ps18
Saccharomycotina_1 - ps19
Saccharomycotina_2 - ps20
Saccharomycotina_3 - ps21
Phaff./Saccharomycetaceae - ps22
Saccharomyco./mycetaceae - ps23
Saccharomycetaceae - ps24
Saccharomycetaceae_2 - ps25
Saccharomycetaceae_3 - ps26
Kaz./Nau./Saccharomyces - ps27
Saccharomyces - ps28
Arboricola_clade - ps29
Cerevisiae_clade - ps30
Saccharomyces_cerevisiae - ps31
p-value
1e-12
3e-10
8e-08
2e-05
4e-03
Log-odds
0.5
1.0
1.5
2.0
2.5
3.0
3.5
4.0

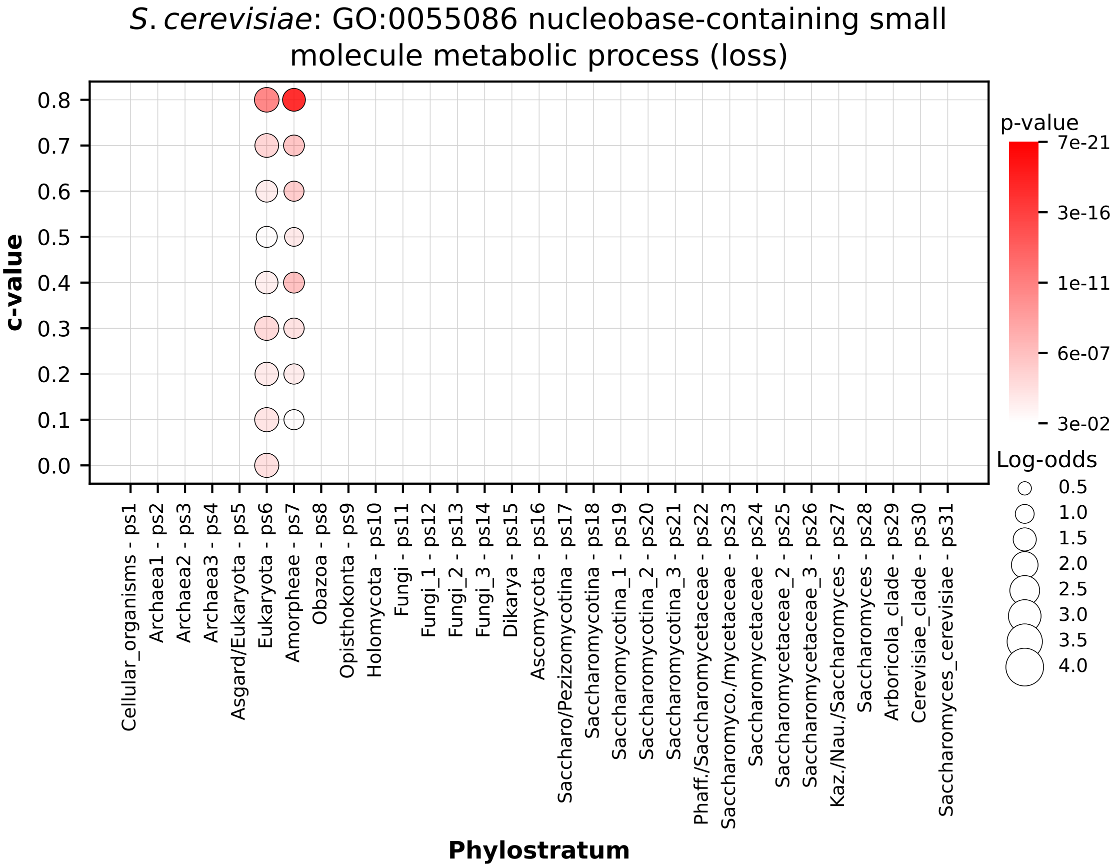

S. cerevisiae: GO:0055086 nucleobase-containing small molecule metabolic process (loss)
c-value
0.8
0.7
0.6
0.5
0.4
0.3
0.2
0.1
0.0
p-value
7e-21
3e-16
1e-11
6e-07
3e-02
Log-odds
0.5
1.0
1.5
2.0
2.5
3.0
3.5
4.0
Cellular_organisms - ps1
Archaea1 - ps2
Archaea2 - ps3
Archaea3 - ps4
Asgard/Eukaryota - ps5
Eukaryota - ps6
Amorpheae - ps7
Obazoa - ps8
Opisthokonta - ps9
Holomycota - ps10
Fungi - ps11
Fungi_1 - ps12
Fungi_2 - ps13
Fungi_3 - ps14
Dikarya - ps15
Ascomycota - ps16
Saccharo/Pezizomycotina - ps17
Saccharomycotina - ps18
Saccharomycotina_1 - ps19
Saccharomycotina_2 - ps20
Saccharomycotina_3 - ps21
Phaff./Saccharomycetaceae - ps22
Saccharomyco./mycetaceae - ps23
Saccharomycetaceae - ps24
Saccharomycetaceae_2 - ps25
Saccharomycetaceae_3 - ps26
Kaz./Nau./Saccharomyces - ps27
Saccharomyces - ps28
Arboricola_clade - ps29
Cerevisiae_clade - ps30
Saccharomyces_cerevisiae - ps31
Phylostratum

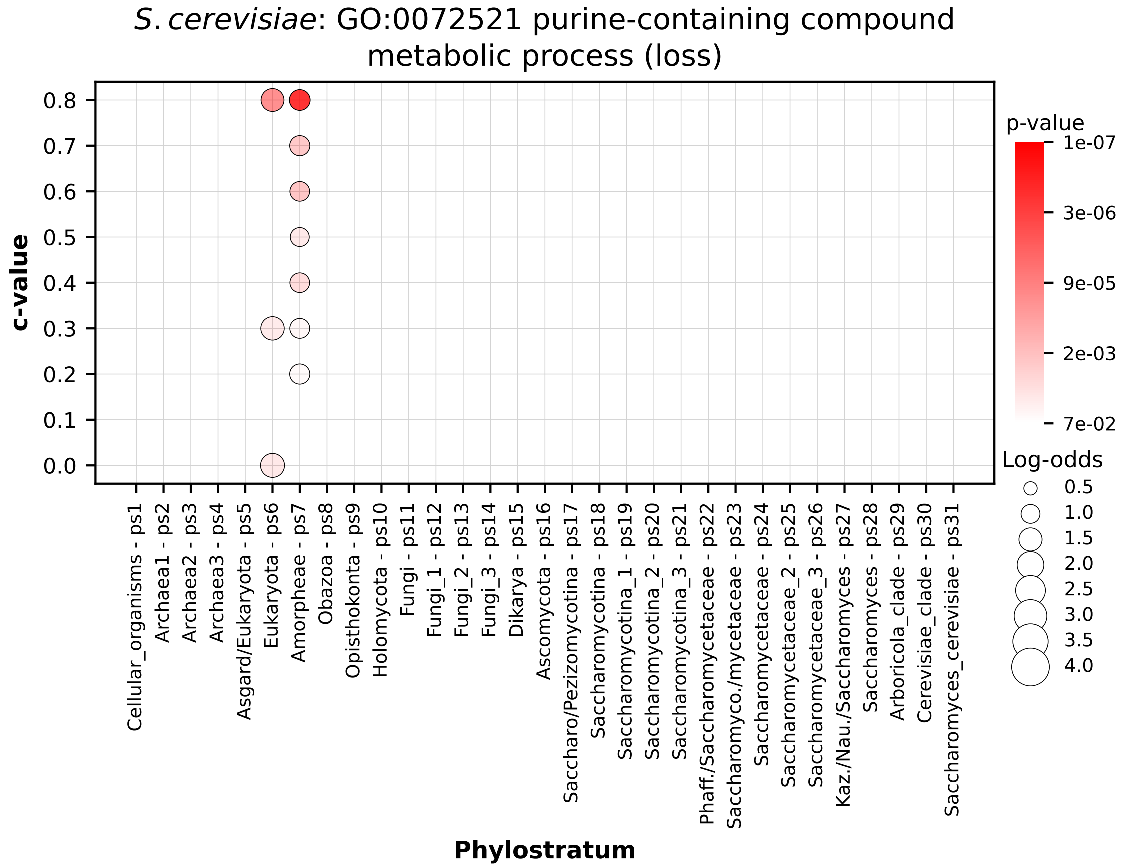
S. cerevisiae: GO:0072521 purine-containing compound metabolic process (loss)
c-value
0.0
0.1
0.2
0.3
0.4
0.5
0.6
0.7
0.8
p-value
1e-07
3e-06
9e-05
2e-03
7e-02
Log-odds
0.5
1.0
1.5
2.0
2.5
3.0
3.5
4.0
Cellular_organisms - ps1
Archaea1 - ps2
Archaea2 - ps3
Archaea3 - ps4
Asgard/Eukaryota - ps5
Eukaryota - ps6
Amorpheae - ps7
Obazoa - ps8
Opisthokonta - ps9
Holomycota - ps10
Fungi - ps11
Fungi_1 - ps12
Fungi_2 - ps13
Fungi_3 - ps14
Dikarya - ps15
Ascomycota - ps16
Saccharo/Pezizomycotina - ps17
Saccharomycotina - ps18
Saccharomycotina_1 - ps19
Saccharomycotina_2 - ps20
Saccharomycotina_3 - ps21
Phaff./Saccharomycetaceae - ps22
Saccharomyco./mycetaceae - ps23
Saccharomycetaceae - ps24
Saccharomycetaceae_2 - ps25
Saccharomycetaceae_3 - ps26
Kaz./Nau./Saccharomyces - ps27
Saccharomyces - ps28
Arboricola_clade - ps29
Cerevisiae_clade - ps30
Saccharomyces_cerevisiae - ps31
Phylostratum

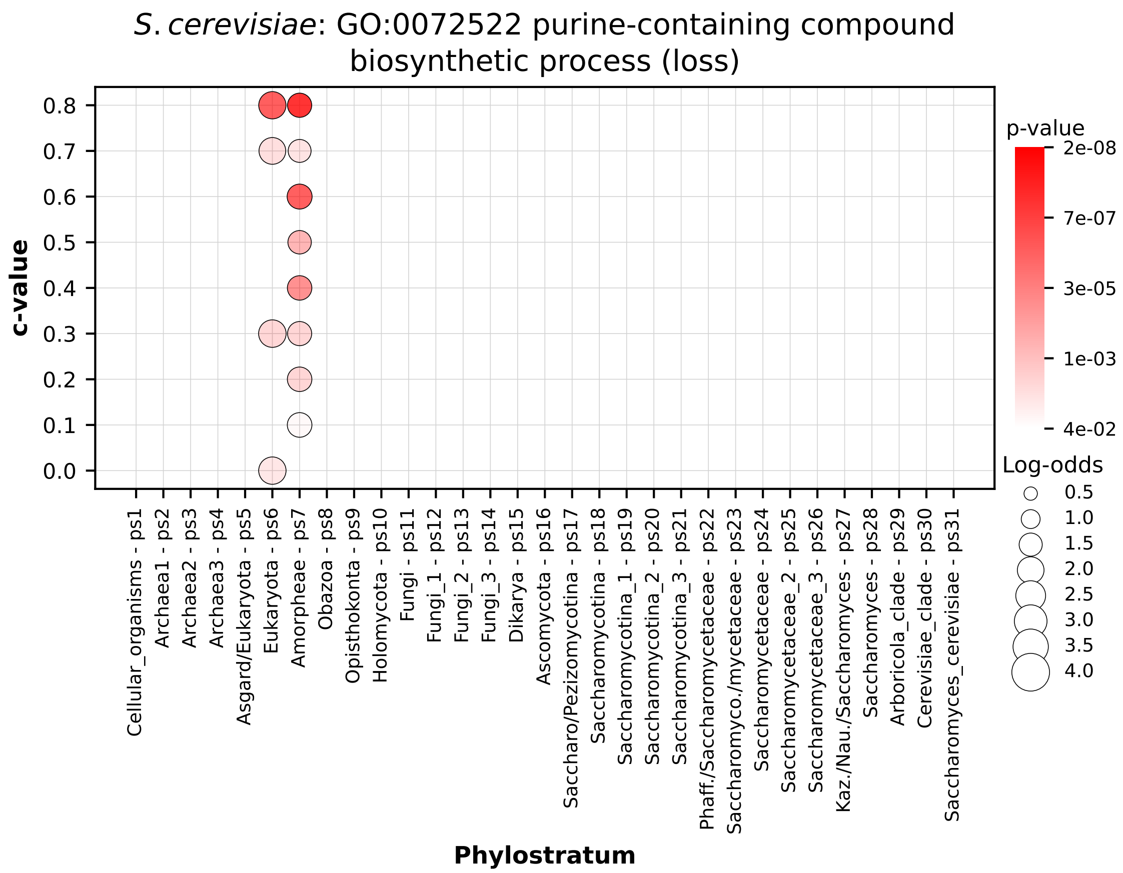
S. cerevisiae: GO:0072522 purine-containing compound biosynthetic process (loss)
c-value
0.8
0.7
0.6
0.5
0.4
0.3
0.2
0.1
0.0
p-value
2e-08
7e-07
3e-05
1e-03
4e-02
Log-odds
0.5
1.0
1.5
2.0
2.5
3.0
3.5
4.0
Cellular_organisms - ps1
Archaea1 - ps2
Archaea2 - ps3
Archaea3 - ps4
Asgard/Eukaryota - ps5
Eukaryota - ps6
Amorpheae - ps7
Obazoa - ps8
Opisthokonta - ps9
Holomycota - ps10
Fungi - ps11
Fungi_1 - ps12
Fungi_2 - ps13
Fungi_3 - ps14
Dikarya - ps15
Ascomycota - ps16
Saccharo/Pezizomycotina - ps17
Saccharomycotina - ps18
Saccharomycotina_1 - ps19
Saccharomycotina_2 - ps20
Saccharomycotina_3 - ps21
Phaff./Saccharomycetaceae - ps22
Saccharomyco./mycetaceae - ps23
Saccharomycetaceae - ps24
Saccharomycetaceae_2 - ps25
Saccharomycetaceae_3 - ps26
Kaz./Nau./Saccharomyces - ps27
Saccharomyces - ps28
Arboricola_clade - ps29
Cerevisiae_clade - ps30
Saccharomyces_cerevisiae - ps31
Phylostratum

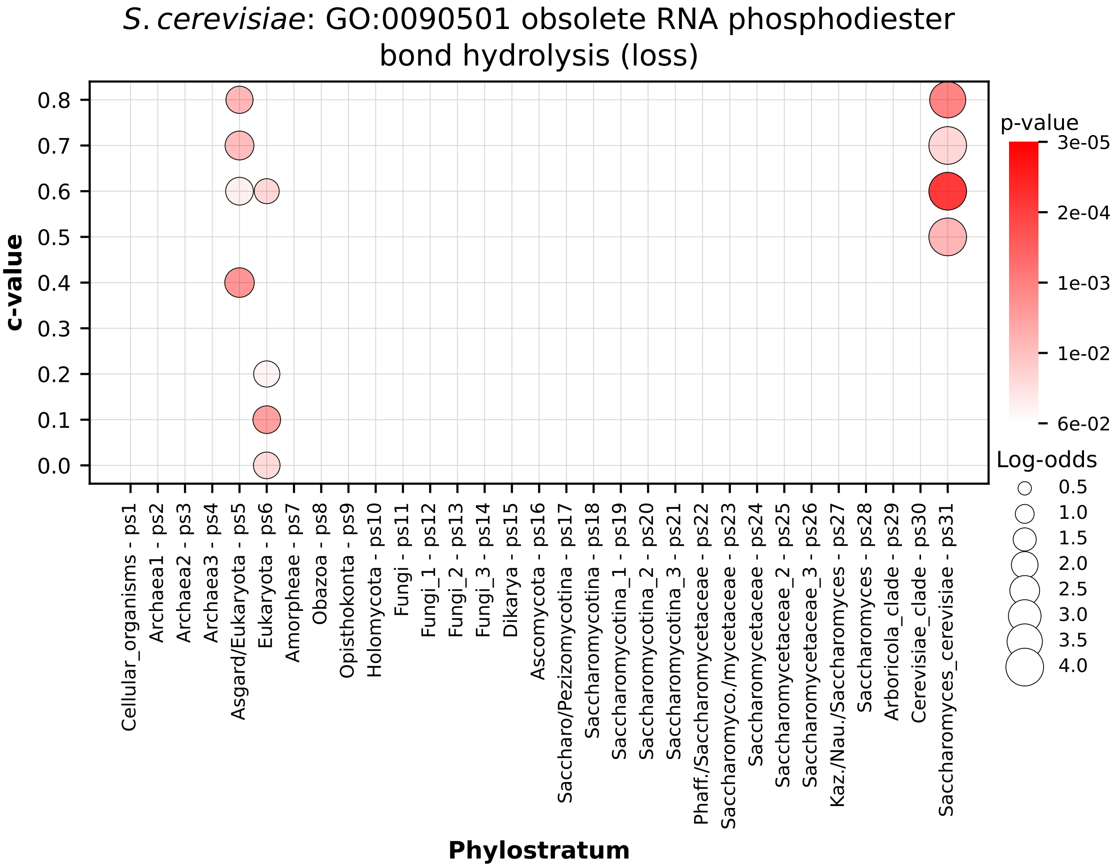

S. cerevisiae: GO:0090501 obsolete RNA phosphodiester bond hydrolysis (loss)
c-value
0.0
0.1
0.2
0.3
0.4
0.5
0.6
0.7
0.8
p-value
3e-05
2e-04
1e-03
1e-02
6e-02
Log-odds
0.5
1.0
1.5
2.0
2.5
3.0
3.5
4.0
Cellular_organisms - ps1
Archaea1 - ps2
Archaea2 - ps3
Archaea3 - ps4
Asgard/Eukaryota - ps5
Eukaryota - ps6
Amorpheae - ps7
Obazoa - ps8
Opisthokonta - ps9
Holomycota - ps10
Fungi - ps11
Fungi_1 - ps12
Fungi_2 - ps13
Fungi_3 - ps14
Dikarya - ps15
Ascomycota - ps16
Saccharo/Pezizomycotina - ps17
Saccharomycotina - ps18
Saccharomycotina_1 - ps19
Saccharomycotina_2 - ps20
Saccharomycotina_3 - ps21
Phaff./Saccharomycetaceae - ps22
Saccharomyco./mycetaceae - ps23
Saccharomycetaceae - ps24
Saccharomycetaceae_2 - ps25
Saccharomycetaceae_3 - ps26
Kaz./Nau./Saccharomyces - ps27
Saccharomyces - ps28
Arboricola_clade - ps29
Cerevisiae_clade - ps30
Saccharomyces_cerevisiae - ps31
Phylostratum

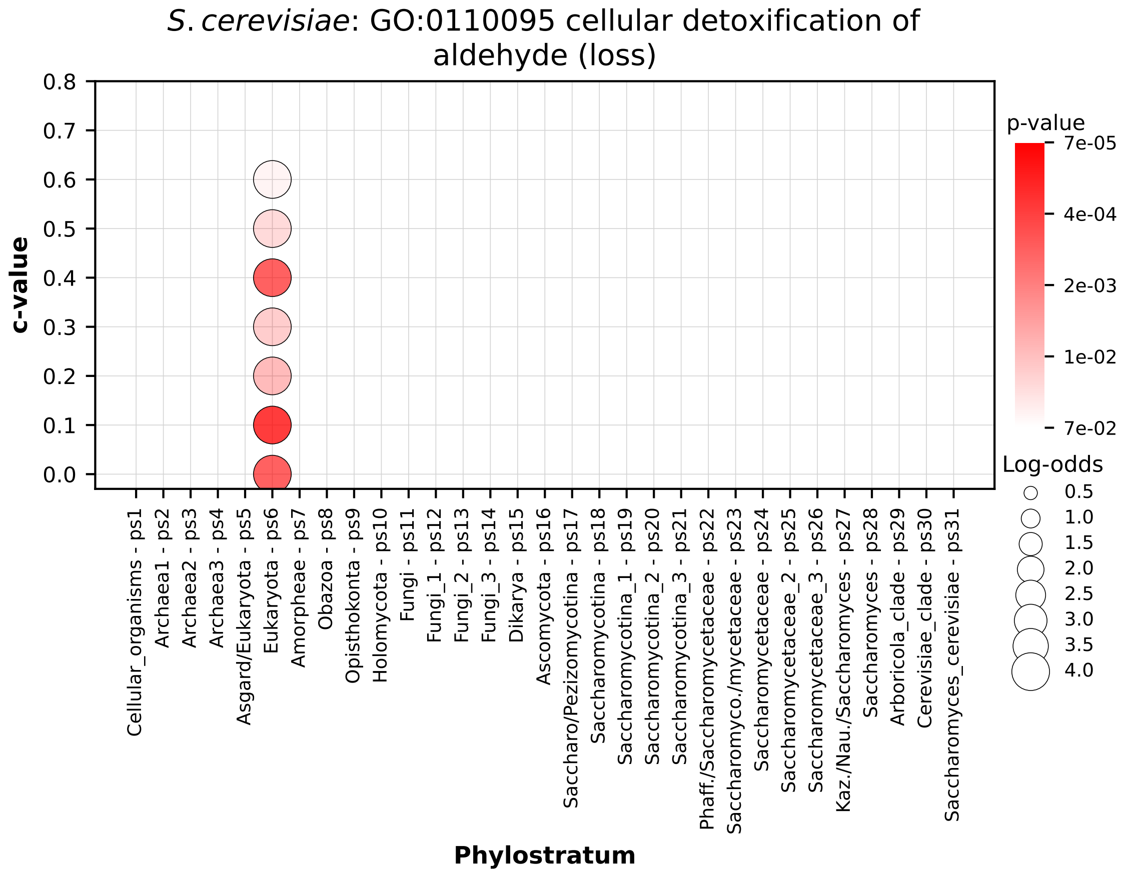

S. cerevisiae: GO:0110095 cellular detoxification of aldehyde (loss)
c-value
0.0
0.1
0.2
0.3
0.4
0.5
0.6
0.7
0.8
Phylostratum
Cellular_organisms - ps1
Archaea1 - ps2
Archaea2 - ps3
Archaea3 - ps4
Asgard/Eukaryota - ps5
Eukaryota - ps6
Amorpheae - ps7
Obazoa - ps8
Opisthokonta - ps9
Holomycota - ps10
Fungi - ps11
Fungi_1 - ps12
Fungi_2 - ps13
Fungi_3 - ps14
Dikarya - ps15
Ascomycota - ps16
Saccharo/Pezizomycotina - ps17
Saccharomycotina - ps18
Saccharomycotina_1 - ps19
Saccharomycotina_2 - ps20
Saccharomycotina_3 - ps21
Phaff./Saccharomycetaceae - ps22
Saccharomyco./mycetaceae - ps23
Saccharomycetaceae - ps24
Saccharomycetaceae_2 - ps25
Saccharomycetaceae_3 - ps26
Kaz./Nau./Saccharomyces - ps27
Saccharomyces - ps28
Arboricola_clade - ps29
Cerevisiae_clade - ps30
Saccharomyces_cerevisiae - ps31
p-value
7e-05
4e-04
2e-03
1e-02
7e-02
Log-odds
0.5
1.0
1.5
2.0
2.5
3.0
3.5
4.0

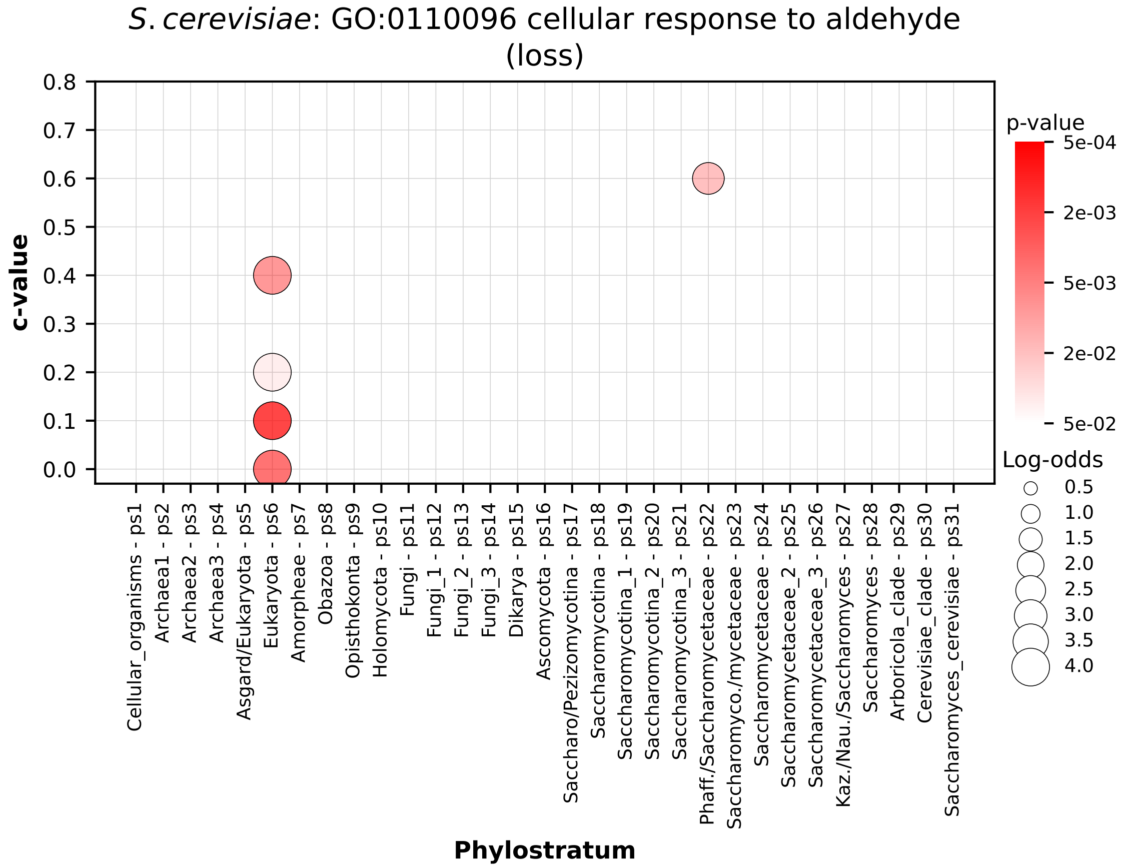

S. cerevisiae: GO:0110096 cellular response to aldehyde (loss)
c-value
0.8
0.7
0.6
0.5
0.4
0.3
0.2
0.1
0.0
Phylostratum
Cellular_organisms - ps1
Archaea1 - ps2
Archaea2 - ps3
Archaea3 - ps4
Asgard/Eukaryota - ps5
Eukaryota - ps6
Amorpheae - ps7
Obazoa - ps8
Opisthokonta - ps9
Holomycota - ps10
Fungi - ps11
Fungi_1 - ps12
Fungi_2 - ps13
Fungi_3 - ps14
Dikarya - ps15
Ascomycota - ps16
Saccharo/Pezizomycotina - ps17
Saccharomycotina - ps18
Saccharomycotina_1 - ps19
Saccharomycotina_2 - ps20
Saccharomycotina_3 - ps21
Phaff./Saccharomycetaceae - ps22
Saccharomyco./mycetaceae - ps23
Saccharomycetaceae - ps24
Saccharomycetaceae_2 - ps25
Saccharomycetaceae_3 - ps26
Kaz./Nau./Saccharomyces - ps27
Saccharomyces - ps28
Arboricola_clade - ps29
Cerevisiae_clade - ps30
Saccharomyces_cerevisiae - ps31
p-value
5e-04
2e-03
5e-03
2e-02
5e-02
Log-odds
0.5
1.0
1.5
2.0
2.5
3.0
3.5
4.0

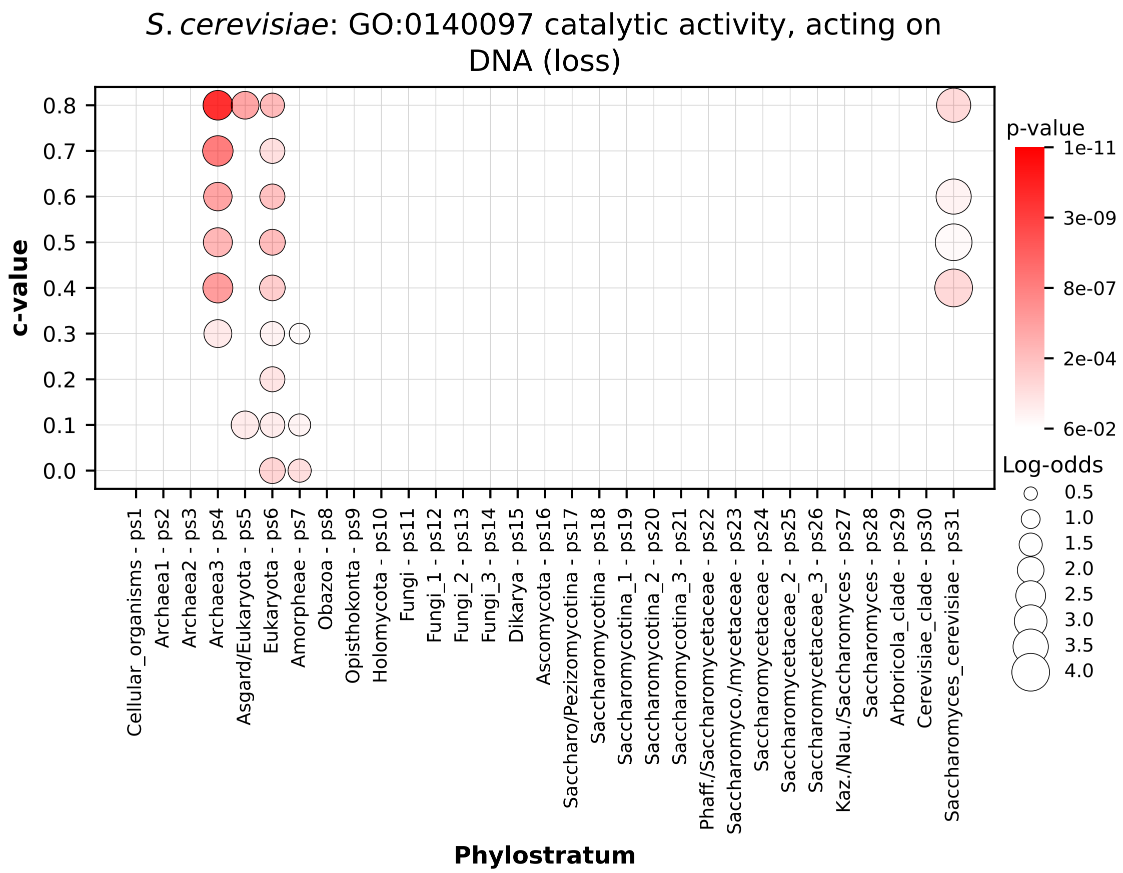
S. cerevisiae: GO:0140097 catalytic activity, acting on DNA (loss)
c-value
0.8
0.7
0.6
0.5
0.4
0.3
0.2
0.1
0.0
p-value
1e-11
3e-09
8e-07
2e-04
6e-02
Log-odds
0.5
1.0
1.5
2.0
2.5
3.0
3.5
4.0
Cellular_organisms - ps1
Archaea1 - ps2
Archaea2 - ps3
Archaea3 - ps4
Asgard/Eukaryota - ps5
Eukaryota - ps6
Amorpheae - ps7
Obazoa - ps8
Opisthokonta - ps9
Holomycota - ps10
Fungi - ps11
Fungi_1 - ps12
Fungi_2 - ps13
Fungi_3 - ps14
Dikarya - ps15
Ascomycota - ps16
Saccharo/Pezizomycotina - ps17
Saccharomycotina - ps18
Saccharomycotina_1 - ps19
Saccharomycotina_2 - ps20
Saccharomycotina_3 - ps21
Phaff./Saccharomycetaceae - ps22
Saccharomyco./mycetaceae - ps23
Saccharomycetaceae - ps24
Saccharomycetaceae_2 - ps25
Saccharomycetaceae_3 - ps26
Kaz./Nau./Saccharomyces - ps27
Saccharomyces - ps28
Arboricola_clade - ps29
Cerevisiae_clade - ps30
Saccharomyces_cerevisiae - ps31
Phylostratum

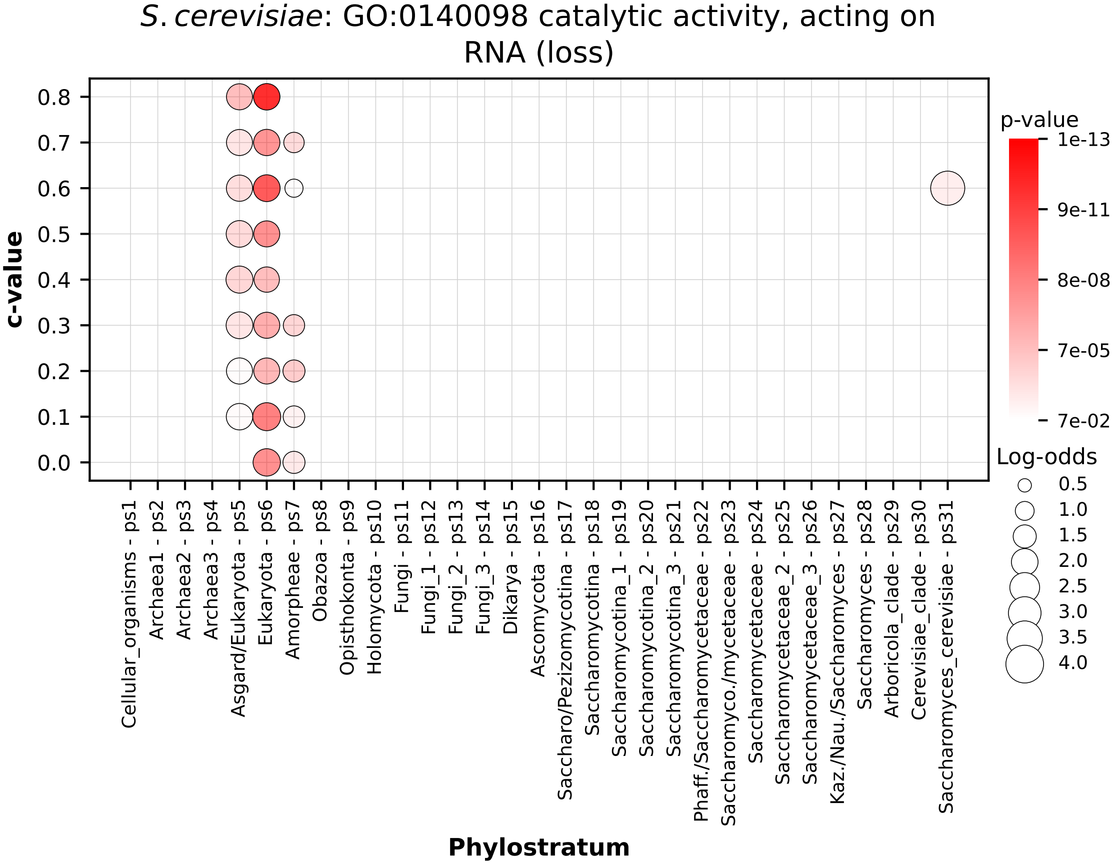
S. cerevisiae: GO:0140098 catalytic activity, acting on RNA (loss)
c-value
0.8
0.7
0.6
0.5
0.4
0.3
0.2
0.1
0.0
p-value
1e-13
9e-11
8e-08
7e-05
7e-02
Log-odds
0.5
1.0
1.5
2.0
2.5
3.0
3.5
4.0
Cellular_organisms - ps1
Archaea1 - ps2
Archaea2 - ps3
Archaea3 - ps4
Asgard/Eukaryota - ps5
Eukaryota - ps6
Amorpheae - ps7
Obazoa - ps8
Opisthokonta - ps9
Holomycota - ps10
Fungi - ps11
Fungi_1 - ps12
Fungi_2 - ps13
Fungi_3 - ps14
Dikarya - ps15
Ascomycota - ps16
Saccharo/Pezizomycotina - ps17
Saccharomycotina - ps18
Saccharomycotina_1 - ps19
Saccharomycotina_2 - ps20
Saccharomycotina_3 - ps21
Phaff./Saccharomycetaceae - ps22
Saccharomyco./mycetaceae - ps23
Saccharomycetaceae - ps24
Saccharomycetaceae_2 - ps25
Saccharomycetaceae_3 - ps26
Kaz./Nau./Saccharomyces - ps27
Saccharomyces - ps28
Arboricola_clade - ps29
Cerevisiae_clade - ps30
Saccharomyces_cerevisiae - ps31
Phylostratum

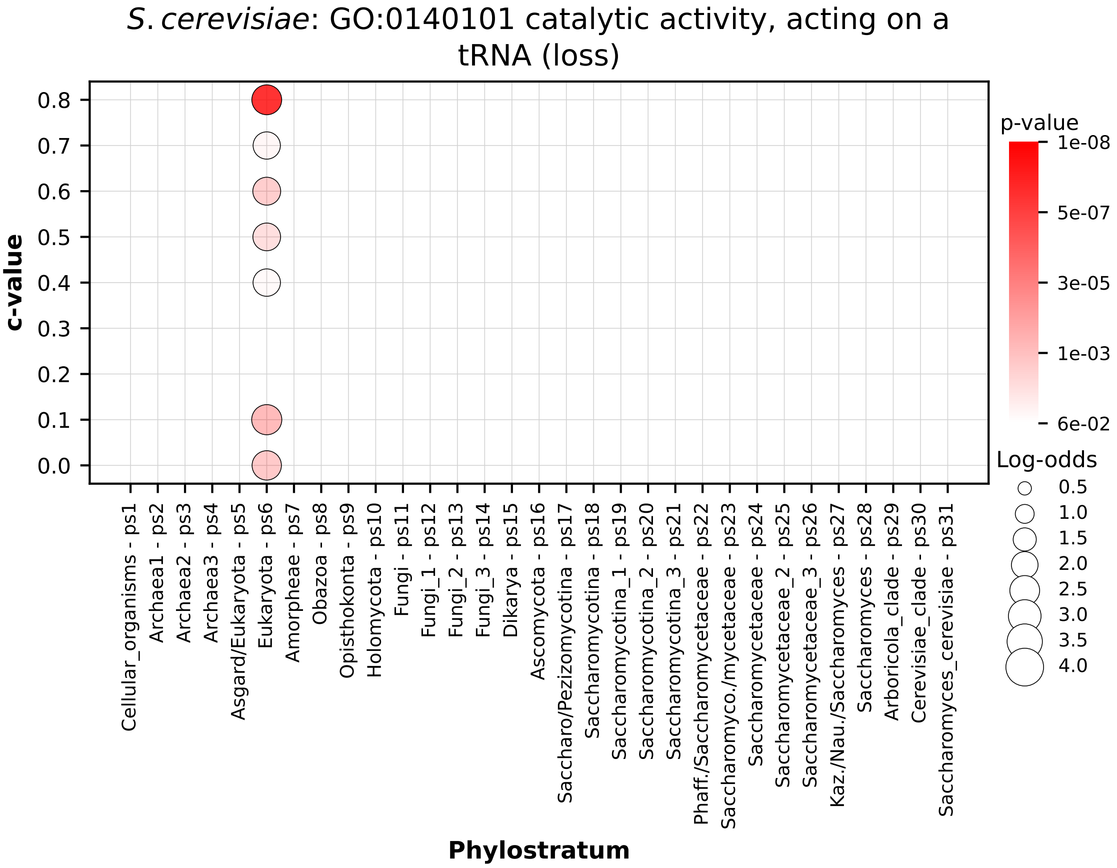

S. cerevisiae: GO:0140101 catalytic activity, acting on a tRNA (loss)
c-value
0.8
0.7
0.6
0.5
0.4
0.3
0.2
0.1
0.0
p-value
1e-08
5e-07
3e-05
1e-03
6e-02
Log-odds
0.5
1.0
1.5
2.0
2.5
3.0
3.5
4.0
Cellular_organisms - ps1
Archaea1 - ps2
Archaea2 - ps3
Archaea3 - ps4
Asgard/Eukaryota - ps5
Eukaryota - ps6
Amorpheae - ps7
Obazoa - ps8
Opisthokonta - ps9
Holomycota - ps10
Fungi - ps11
Fungi_1 - ps12
Fungi_2 - ps13
Fungi_3 - ps14
Dikarya - ps15
Ascomycota - ps16
Saccharo/Pezizomycotina - ps17
Saccharomycotina - ps18
Saccharomycotina_1 - ps19
Saccharomycotina_2 - ps20
Saccharomycotina_3 - ps21
Phaff./Saccharomycetaceae - ps22
Saccharomyco./mycetaceae - ps23
Saccharomycetaceae - ps24
Saccharomycetaceae_2 - ps25
Saccharomycetaceae_3 - ps26
Kaz./Nau./Saccharomyces - ps27
Saccharomyces - ps28
Arboricola_clade - ps29
Cerevisiae_clade - ps30
Saccharomyces_cerevisiae - ps31
Phylostratum

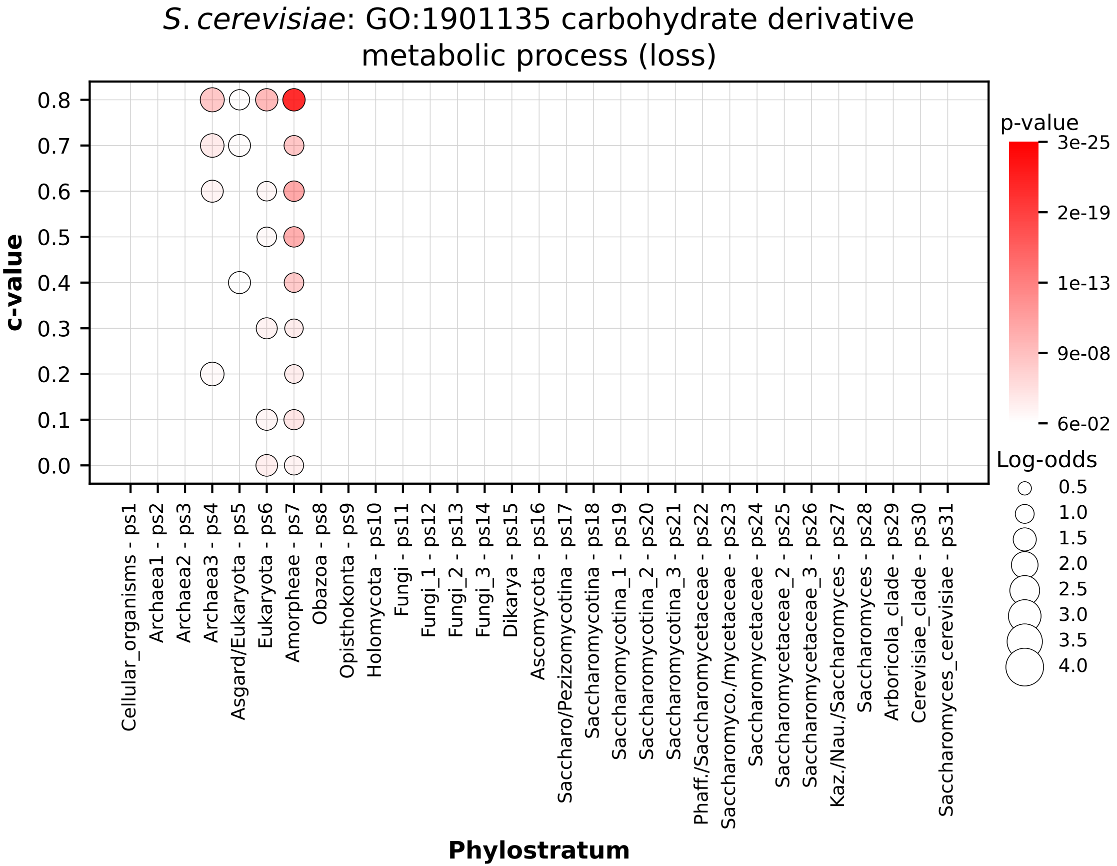
S. cerevisiae: GO:1901135 carbohydrate derivative metabolic process (loss)
c-value
0.8
0.7
0.6
0.5
0.4
0.3
0.2
0.1
0.0
Phylostratum
Cellular_organisms - ps1
Archaea1 - ps2
Archaea2 - ps3
Archaea3 - ps4
Asgard/Eukaryota - ps5
Eukaryota - ps6
Amorpheae - ps7
Obazoa - ps8
Opisthokonta - ps9
Holomycota - ps10
Fungi - ps11
Fungi_1 - ps12
Fungi_2 - ps13
Fungi_3 - ps14
Dikarya - ps15
Ascomycota - ps16
Saccharo/Pezizomycotina - ps17
Saccharomycotina - ps18
Saccharomycotina_1 - ps19
Saccharomycotina_2 - ps20
Saccharomycotina_3 - ps21
Phaff./Saccharomycetaceae - ps22
Saccharomyco./mycetaceae - ps23
Saccharomycetaceae - ps24
Saccharomycetaceae_2 - ps25
Saccharomycetaceae_3 - ps26
Kaz./Nau./Saccharomyces - ps27
Saccharomyces - ps28
Arboricola_clade - ps29
Cerevisiae_clade - ps30
Saccharomyces_cerevisiae - ps31
p-value
3e-25
2e-19
1e-13
9e-08
6e-02
Log-odds
0.5
1.0
1.5
2.0
2.5
3.0
3.5
4.0

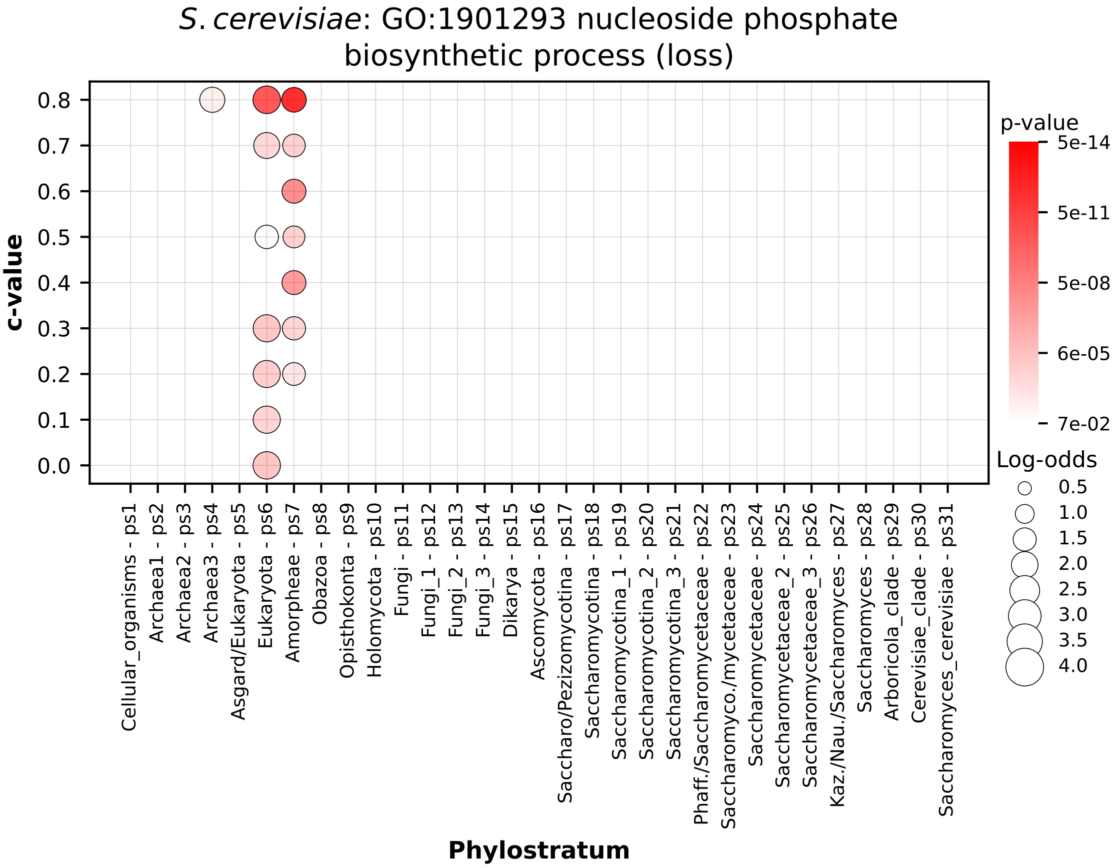

S. cerevisiae: GO:1901293 nucleoside phosphate biosynthetic process (loss)
c-value
0.8
0.7
0.6
0.5
0.4
0.3
0.2
0.1
0.0
Phylostratum
Cellular_organisms - ps1
Archaea1 - ps2
Archaea2 - ps3
Archaea3 - ps4
Asgard/Eukaryota - ps5
Eukaryota - ps6
Amorpheae - ps7
Obazoa - ps8
Opisthokonta - ps9
Holomycota - ps10
Fungi - ps11
Fungi_1 - ps12
Fungi_2 - ps13
Fungi_3 - ps14
Dikarya - ps15
Ascomycota - ps16
Saccharo/Pezizomycotina - ps17
Saccharomycotina - ps18
Saccharomycotina_1 - ps19
Saccharomycotina_2 - ps20
Saccharomycotina_3 - ps21
Phaff./Saccharomycetaceae - ps22
Saccharomyco./mycetaceae - ps23
Saccharomycetaceae - ps24
Saccharomycetaceae_2 - ps25
Saccharomycetaceae_3 - ps26
Kaz./Nau./Saccharomyces - ps27
Saccharomyces - ps28
Arboricola_clade - ps29
Cerevisiae_clade - ps30
Saccharomyces_cerevisiae - ps31
p-value
5e-14
5e-11
5e-08
6e-05
7e-02
Log-odds
0.5
1.0
1.5
2.0
2.5
3.0
3.5
4.0

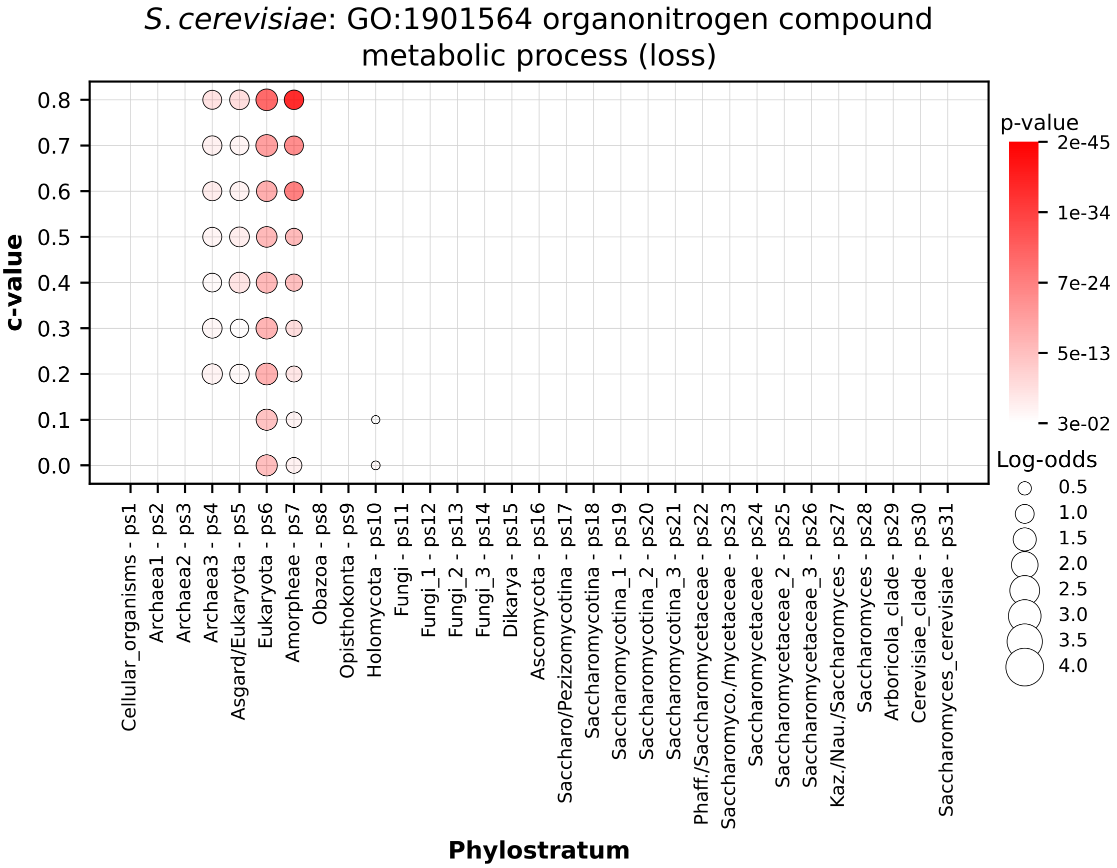
S. cerevisiae: GO:1901564 organonitrogen compound metabolic process (loss)
c-value
0.8
0.7
0.6
0.5
0.4
0.3
0.2
0.1
0.0
p-value
2e-45
1e-34
7e-24
5e-13
3e-02
Log-odds
0.5
1.0
1.5
2.0
2.5
3.0
3.5
4.0
Cellular_organisms - ps1
Archaea1 - ps2
Archaea2 - ps3
Archaea3 - ps4
Asgard/Eukaryota - ps5
Eukaryota - ps6
Amorpheae - ps7
Obazoa - ps8
Opisthokonta - ps9
Holomycota - ps10
Fungi - ps11
Fungi_1 - ps12
Fungi_2 - ps13
Fungi_3 - ps14
Dikarya - ps15
Ascomycota - ps16
Saccharo/Pezizomycotina - ps17
Saccharomycotina - ps18
Saccharomycotina_1 - ps19
Saccharomycotina_2 - ps20
Saccharomycotina_3 - ps21
Phaff./Saccharomycetaceae - ps22
Saccharomyco./mycetaceae - ps23
Saccharomycetaceae - ps24
Saccharomycetaceae_2 - ps25
Saccharomycetaceae_3 - ps26
Kaz./Nau./Saccharomyces - ps27
Saccharomyces - ps28
Arboricola_clade - ps29
Cerevisiae_clade - ps30
Saccharomyces_cerevisiae - ps31
Phylostratum

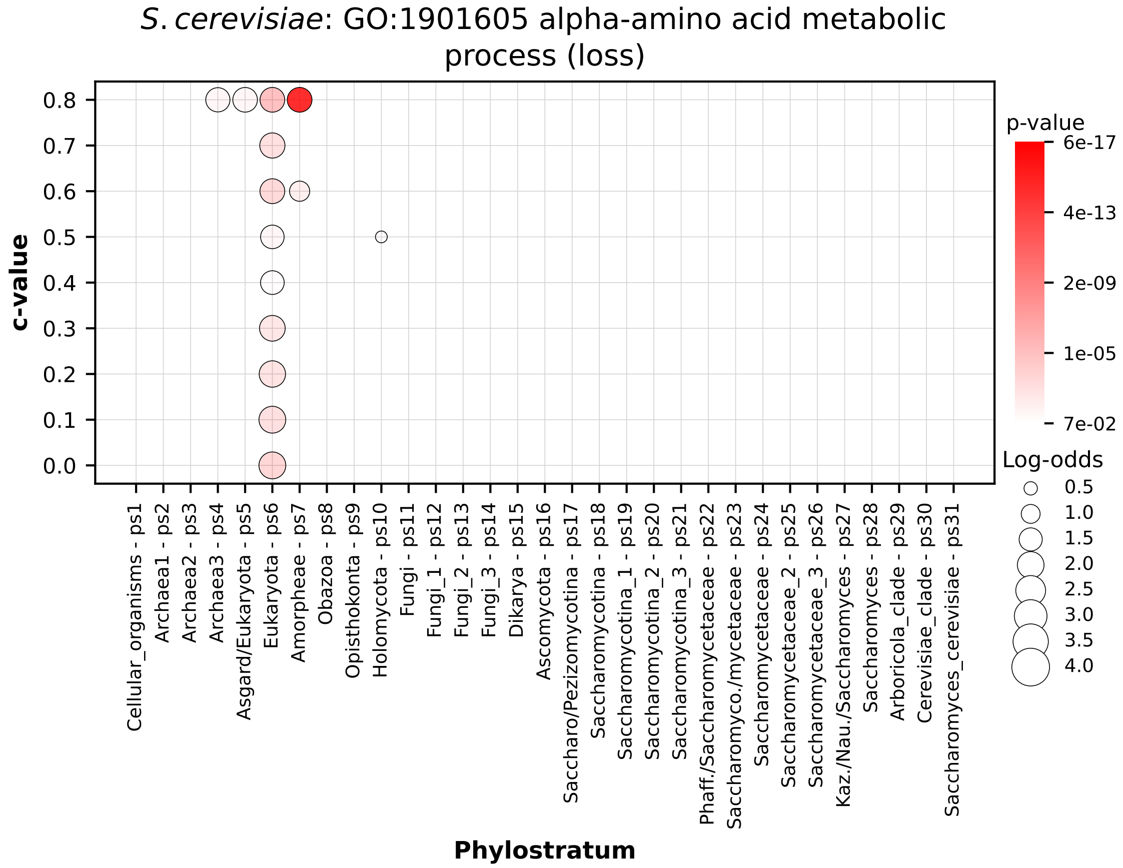

S. cerevisiae: GO:1901605 alpha-amino acid metabolic process (loss)
c-value
0.8
0.7
0.6
0.5
0.4
0.3
0.2
0.1
0.0
Phylostratum
Cellular_organisms - ps1
Archaea1 - ps2
Archaea2 - ps3
Archaea3 - ps4
Asgard/Eukaryota - ps5
Eukaryota - ps6
Amorpheae - ps7
Obazoa - ps8
Opisthokonta - ps9
Holomycota - ps10
Fungi - ps11
Fungi_1 - ps12
Fungi_2 - ps13
Fungi_3 - ps14
Dikarya - ps15
Ascomycota - ps16
Saccharo/Pezizomycotina - ps17
Saccharomycotina - ps18
Saccharomycotina_1 - ps19
Saccharomycotina_2 - ps20
Saccharomycotina_3 - ps21
Phaff./Saccharomycetaceae - ps22
Saccharomyco./mycetaceae - ps23
Saccharomycetaceae - ps24
Saccharomycetaceae_2 - ps25
Saccharomycetaceae_3 - ps26
Kaz./Nau./Saccharomyces - ps27
Saccharomyces - ps28
Arboricola_clade - ps29
Cerevisiae_clade - ps30
Saccharomyces_cerevisiae - ps31
p-value
6e-17
4e-13
2e-09
1e-05
7e-02
Log-odds
0.5
1.0
1.5
2.0
2.5
3.0
3.5
4.0

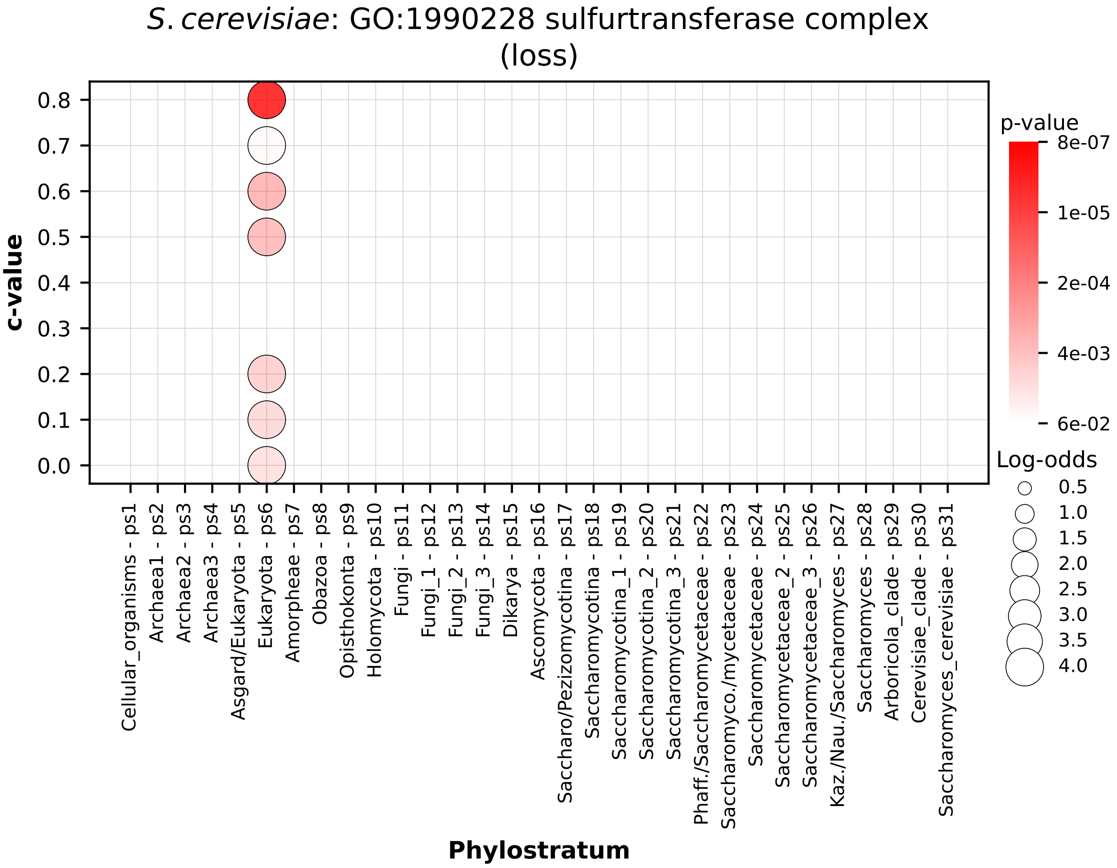
S. cerevisiae: GO:1990228 sulfurtransferase complex
(loss)
c-value
0.8
0.7
0.6
0.5
0.4
0.3
0.2
0.1
0.0
p-value
8e-07
1e-05
2e-04
4e-03
6e-02
Log-odds
0.5
1.0
1.5
2.0
2.5
3.0
3.5
4.0
Cellular_organisms - ps1
Archaea1 - ps2
Archaea2 - ps3
Archaea3 - ps4
Asgard/Eukaryota - ps5
Eukaryota - ps6
Amorpheae - ps7
Obazoa - ps8
Opisthokonta - ps9
Holomycota - ps10
Fungi - ps11
Fungi_1 - ps12
Fungi_2 - ps13
Fungi_3 - ps14
Dikarya - ps15
Ascomycota - ps16
Saccharo/Pezizomycotina - ps17
Saccharomycotina - ps18
Saccharomycotina_1 - ps19
Saccharomycotina_2 - ps20
Saccharomycotina_3 - ps21
Phaff./Saccharomycetaceae - ps22
Saccharomyco./mycetaceae - ps23
Saccharomycetaceae - ps24
Saccharomycetaceae_2 - ps25
Saccharomycetaceae_3 - ps26
Kaz./Nau./Saccharomyces - ps27
Saccharomyces - ps28
Arboricola_clade - ps29
Cerevisiae_clade - ps30
Saccharomyces_cerevisiae - ps31
Phylostratum

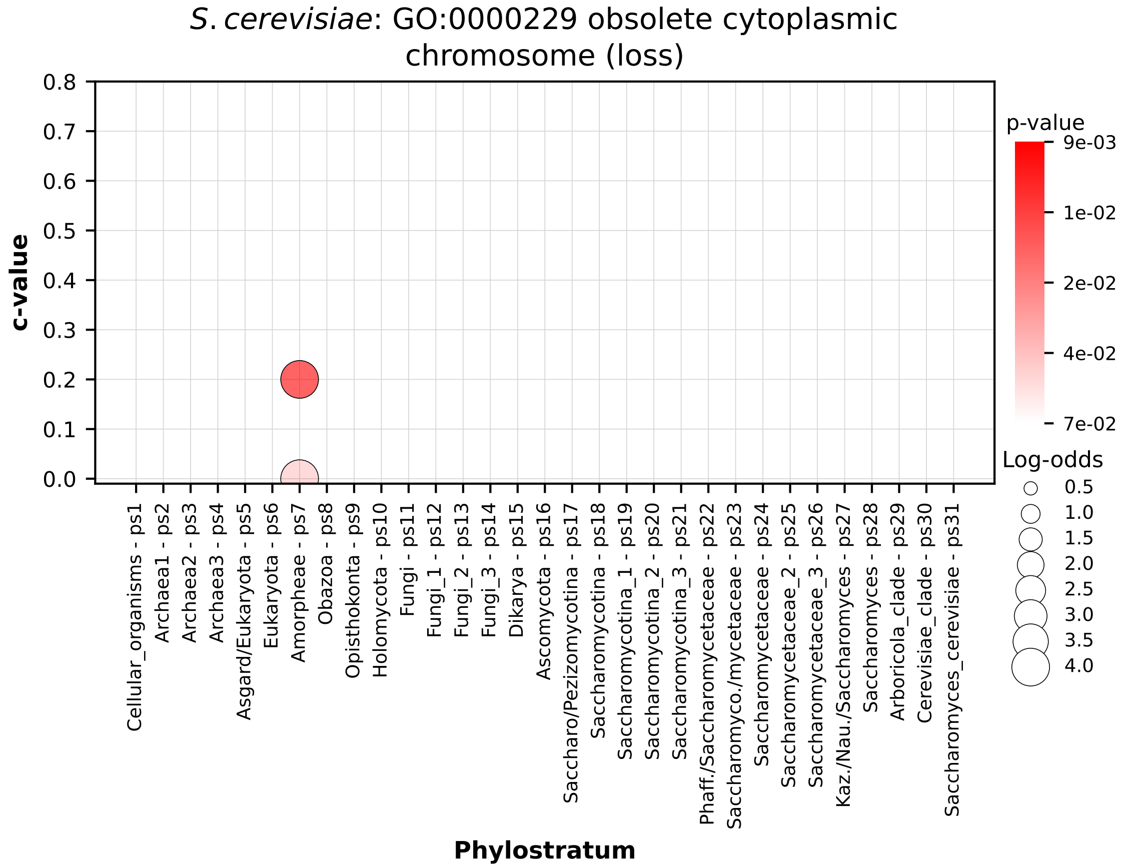

S. cerevisiae: GO:0000229 obsolete cytoplasmic chromosome (loss)
c-value
0.8
0.7
0.6
0.5
0.4
0.3
0.2
0.1
0.0
p-value
9e-03
1e-02
2e-02
4e-02
7e-02
Log-odds
0.5
1.0
1.5
2.0
2.5
3.0
3.5
4.0
Cellular_organisms - ps1
Archaea1 - ps2
Archaea2 - ps3
Archaea3 - ps4
Asgard/Eukaryota - ps5
Eukaryota - ps6
Amorpheae - ps7
Obazoa - ps8
Opisthokonta - ps9
Holomycota - ps10
Fungi - ps11
Fungi_1 - ps12
Fungi_2 - ps13
Fungi_3 - ps14
Dikarya - ps15
Ascomycota - ps16
Saccharo/Pezizomycotina - ps17
Saccharomycotina - ps18
Saccharomycotina_1 - ps19
Saccharomycotina_2 - ps20
Saccharomycotina_3 - ps21
Phaff./Saccharomycetaceae - ps22
Saccharomyco./mycetaceae - ps23
Saccharomycetaceae - ps24
Saccharomycetaceae_2 - ps25
Saccharomycetaceae_3 - ps26
Kaz./Nau./Saccharomyces - ps27
Saccharomyces - ps28
Arboricola_clade - ps29
Cerevisiae_clade - ps30
Saccharomyces_cerevisiae - ps31
Phylostratum

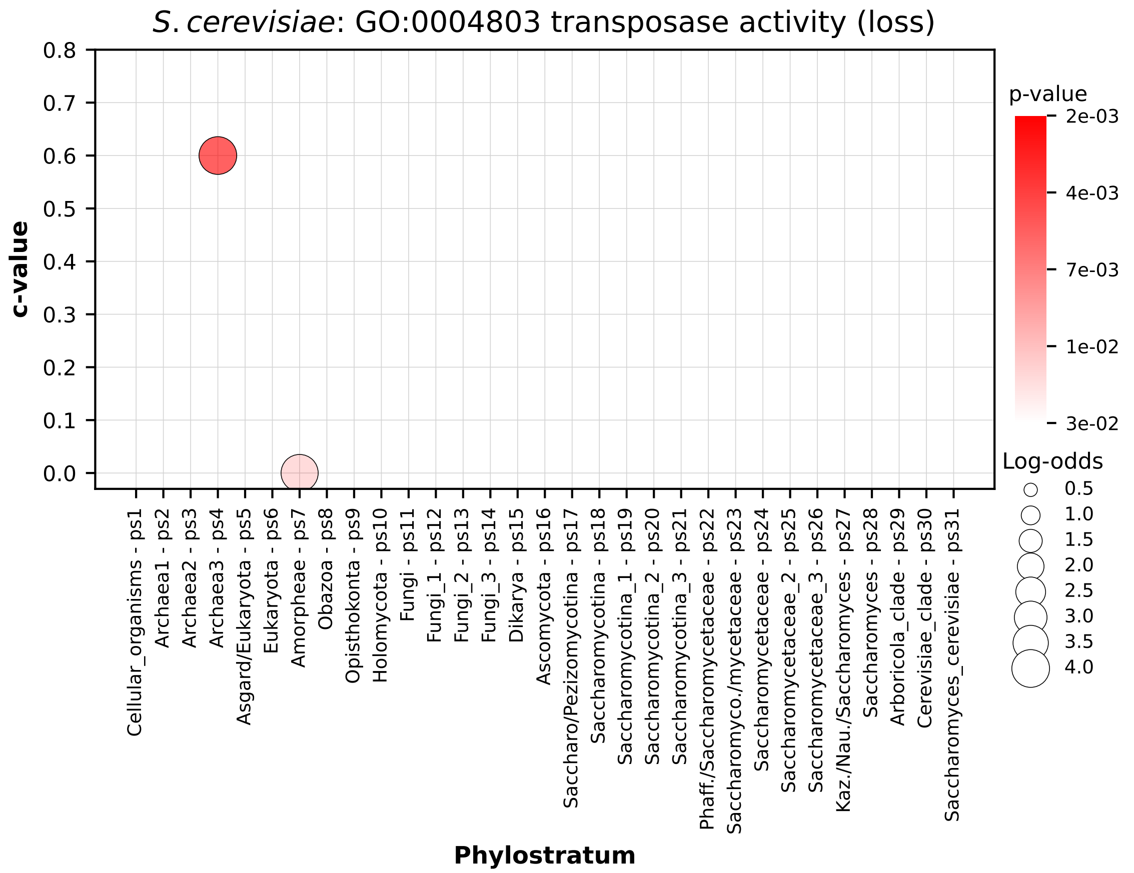

S. cerevisiae: GO:0004803 transposase activity (loss)
c-value
0.8
0.7
0.6
0.5
0.4
0.3
0.2
0.1
0.0
Phylostratum
Cellular_organisms - ps1
Archaea1 - ps2
Archaea2 - ps3
Archaea3 - ps4
Asgard/Eukaryota - ps5
Eukaryota - ps6
Amorpheae - ps7
Obazoa - ps8
Opisthokonta - ps9
Holomycota - ps10
Fungi - ps11
Fungi_1 - ps12
Fungi_2 - ps13
Fungi_3 - ps14
Dikarya - ps15
Ascomycota - ps16
Saccharo/Pezizomycotina - ps17
Saccharomycotina - ps18
Saccharomycotina_1 - ps19
Saccharomycotina_2 - ps20
Saccharomycotina_3 - ps21
Phaff./Saccharomycetaceae - ps22
Saccharomyco./mycetaceae - ps23
Saccharomycetaceae - ps24
Saccharomycetaceae_2 - ps25
Saccharomycetaceae_3 - ps26
Kaz./Nau./Saccharomyces - ps27
Saccharomyces - ps28
Arboricola_clade - ps29
Cerevisiae_clade - ps30
Saccharomyces_cerevisiae - ps31
p-value
2e-03
4e-03
7e-03
1e-02
3e-02
Log-odds
0.5
1.0
1.5
2.0
2.5
3.0
3.5
4.0

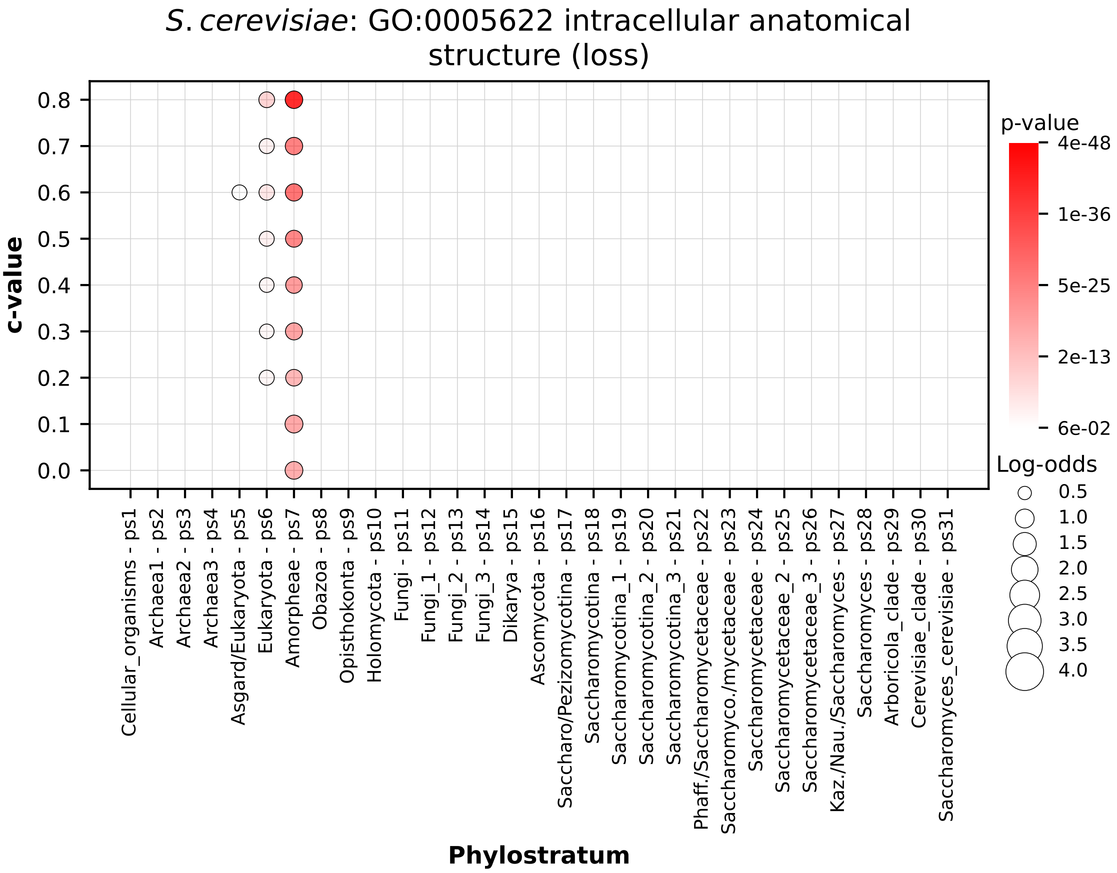

S. cerevisiae: GO:0005622 intracellular anatomical structure (loss)
c-value
0.8
0.7
0.6
0.5
0.4
0.3
0.2
0.1
0.0
p-value
4e-48
1e-36
5e-25
2e-13
6e-02
Log-odds
0.5
1.0
1.5
2.0
2.5
3.0
3.5
4.0
Cellular_organisms - ps1
Archaea1 - ps2
Archaea2 - ps3
Archaea3 - ps4
Asgard/Eukaryota - ps5
Eukaryota - ps6
Amorpheae - ps7
Obazoa - ps8
Opisthokonta - ps9
Holomycota - ps10
Fungi - ps11
Fungi_1 - ps12
Fungi_2 - ps13
Fungi_3 - ps14
Dikarya - ps15
Ascomycota - ps16
Saccharo/Pezizomycotina - ps17
Saccharomycotina - ps18
Saccharomycotina_1 - ps19
Saccharomycotina_2 - ps20
Saccharomycotina_3 - ps21
Phaff./Saccharomycetaceae - ps22
Saccharomyco./mycetaceae - ps23
Saccharomycetaceae - ps24
Saccharomycetaceae_2 - ps25
Saccharomycetaceae_3 - ps26
Kaz./Nau./Saccharomyces - ps27
Saccharomyces - ps28
Arboricola_clade - ps29
Cerevisiae_clade - ps30
Saccharomyces_cerevisiae - ps31
Phylostratum

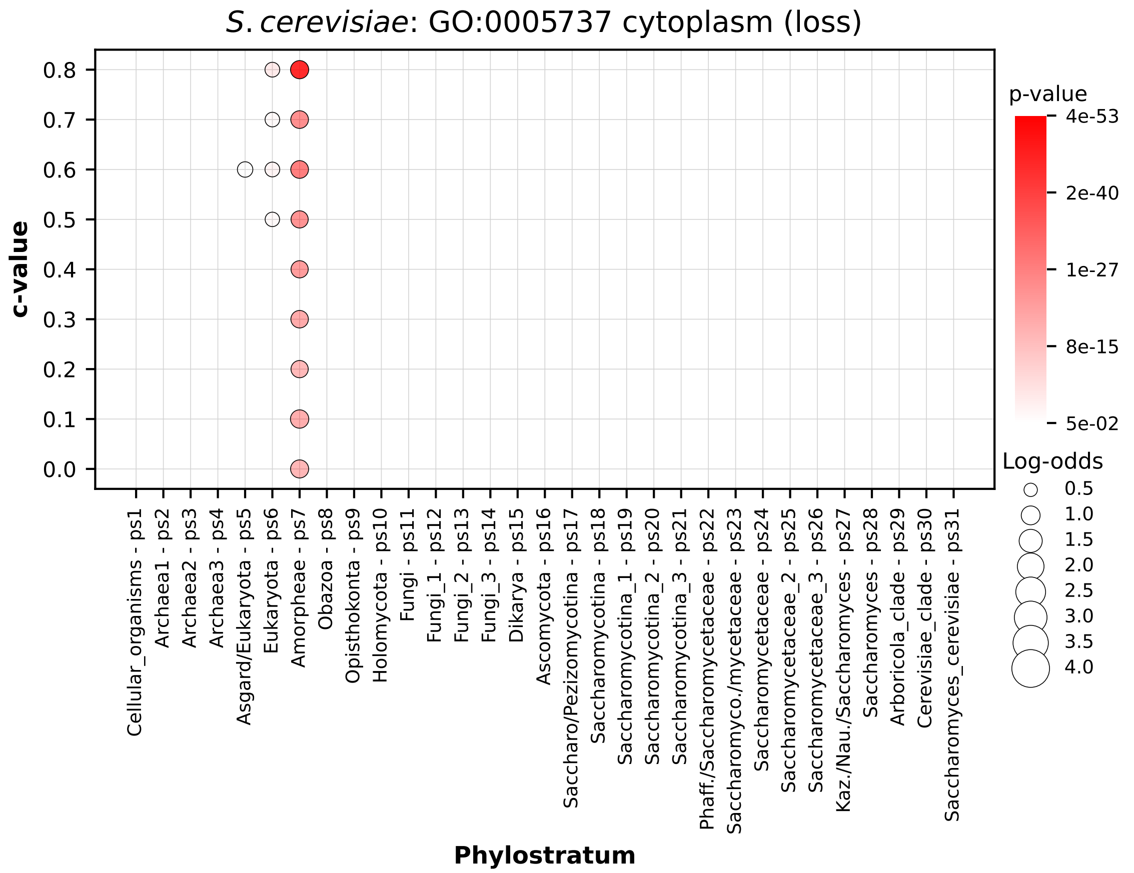

S. cerevisiae: GO:0005737 cytoplasm (loss)
c-value
0.8
0.7
0.6
0.5
0.4
0.3
0.2
0.1
0.0
p-value
4e-53
2e-40
1e-27
8e-15
5e-02
Log-odds
0.5
1.0
1.5
2.0
2.5
3.0
3.5
4.0
Cellular_organisms - ps1
Archaea1 - ps2
Archaea2 - ps3
Archaea3 - ps4
Asgard/Eukaryota - ps5
Eukaryota - ps6
Amorpheae - ps7
Obazoa - ps8
Opisthokonta - ps9
Holomycota - ps10
Fungi - ps11
Fungi_1 - ps12
Fungi_2 - ps13
Fungi_3 - ps14
Dikarya - ps15
Ascomycota - ps16
Saccharo/Pezizomycotina - ps17
Saccharomycotina - ps18
Saccharomycotina_1 - ps19
Saccharomycotina_2 - ps20
Saccharomycotina_3 - ps21
Phaff./Saccharomycetaceae - ps22
Saccharomyco./mycetaceae - ps23
Saccharomycetaceae - ps24
Saccharomycetaceae_2 - ps25
Saccharomycetaceae_3 - ps26
Kaz./Nau./Saccharomyces - ps27
Saccharomyces - ps28
Arboricola_clade - ps29
Cerevisiae_clade - ps30
Saccharomyces_cerevisiae - ps31
Phylostratum

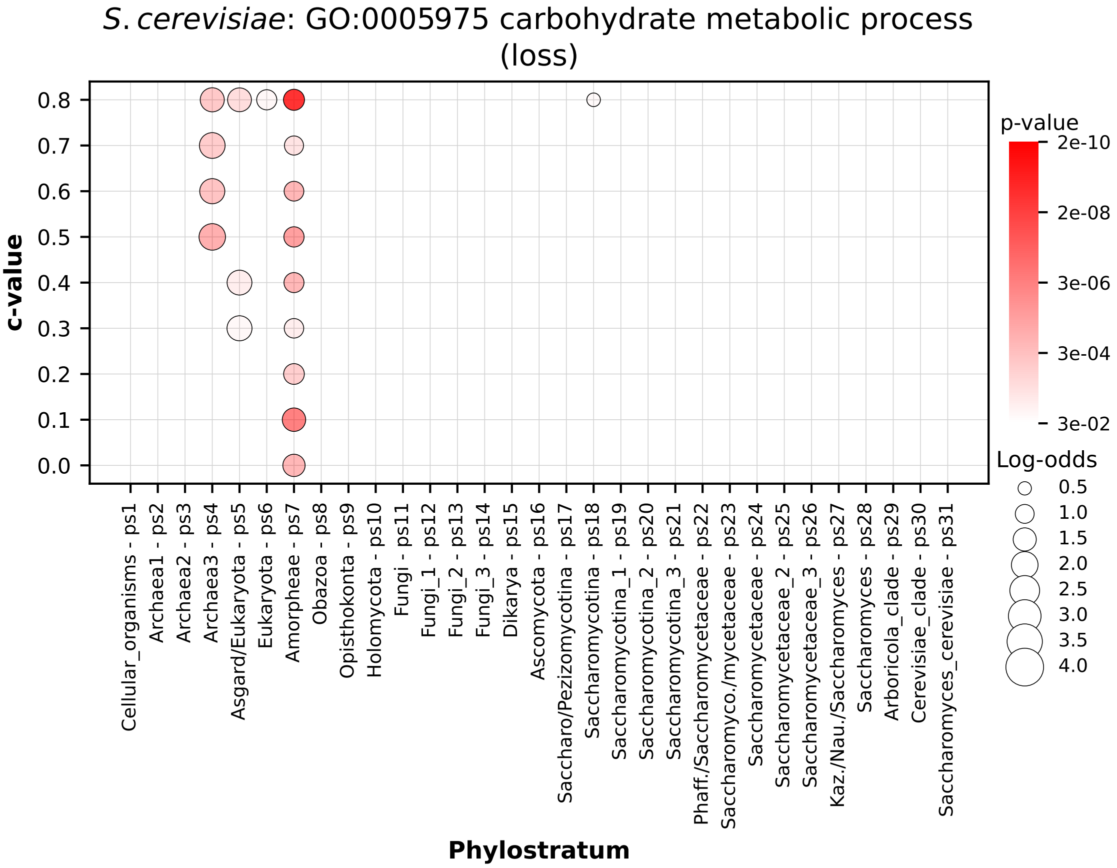

S. cerevisiae: GO:0005975 carbohydrate metabolic process (loss)
c-value
0.8
0.7
0.6
0.5
0.4
0.3
0.2
0.1
0.0
Phylostratum
Cellular_organisms - ps1
Archaea1 - ps2
Archaea2 - ps3
Archaea3 - ps4
Asgard/Eukaryota - ps5
Eukaryota - ps6
Amorpheae - ps7
Obazoa - ps8
Opisthokonta - ps9
Holomycota - ps10
Fungi - ps11
Fungi_1 - ps12
Fungi_2 - ps13
Fungi_3 - ps14
Dikarya - ps15
Ascomycota - ps16
Saccharo/Pezizomycotina - ps17
Saccharomycotina - ps18
Saccharomycotina_1 - ps19
Saccharomycotina_2 - ps20
Saccharomycotina_3 - ps21
Phaff./Saccharomycetaceae - ps22
Saccharomyco./mycetaceae - ps23
Saccharomycetaceae - ps24
Saccharomycetaceae_2 - ps25
Saccharomycetaceae_3 - ps26
Kaz./Nau./Saccharomyces - ps27
Saccharomyces - ps28
Arboricola_clade - ps29
Cerevisiae_clade - ps30
Saccharomyces_cerevisiae - ps31
p-value
2e-10
2e-08
3e-06
3e-04
3e-02
Log-odds
0.5
1.0
1.5
2.0
2.5
3.0
3.5
4.0

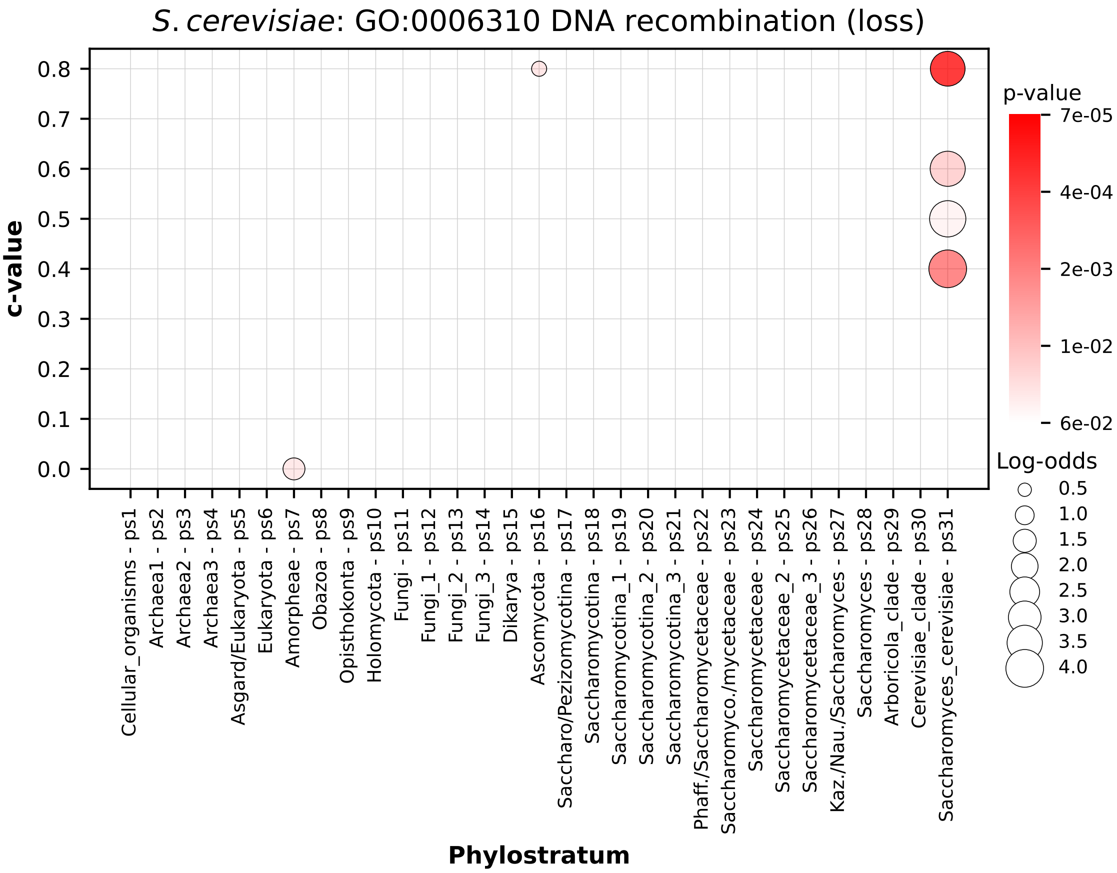
S. cerevisiae: GO:0006310 DNA recombination (loss)
c-value
0.0
0.1
0.2
0.3
0.4
0.5
0.6
0.7
0.8
Phylostratum
Cellular_organisms - ps1
Archaea1 - ps2
Archaea2 - ps3
Archaea3 - ps4
Asgard/Eukaryota - ps5
Eukaryota - ps6
Amorpheae - ps7
Obazoa - ps8
Opisthokonta - ps9
Holomycota - ps10
Fungi - ps11
Fungi_1 - ps12
Fungi_2 - ps13
Fungi_3 - ps14
Dikarya - ps15
Ascomycota - ps16
Saccharo/Pezizomycotina - ps17
Saccharomycotina - ps18
Saccharomycotina_1 - ps19
Saccharomycotina_2 - ps20
Saccharomycotina_3 - ps21
Phaff./Saccharomycetaceae - ps22
Saccharomyco./mycetaceae - ps23
Saccharomycetaceae - ps24
Saccharomycetaceae_2 - ps25
Saccharomycetaceae_3 - ps26
Kaz./Nau./Saccharomyces - ps27
Saccharomyces - ps28
Arboricola_clade - ps29
Cerevisiae_clade - ps30
Saccharomyces_cerevisiae - ps31
p-value
7e-05
4e-04
2e-03
1e-02
6e-02
Log-odds
0.5
1.0
1.5
2.0
2.5
3.0
3.5
4.0

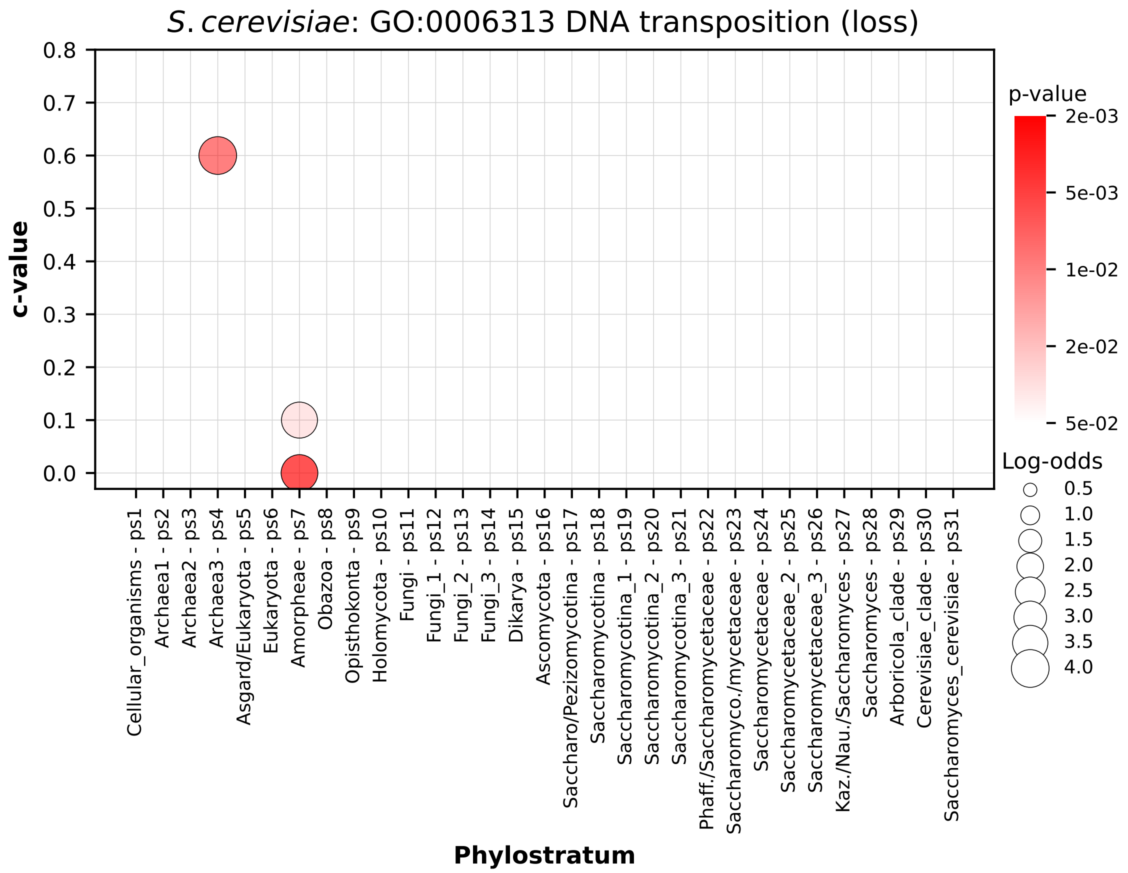

S. cerevisiae: GO:0006313 DNA transposition (loss)
c-value
0.8
0.7
0.6
0.5
0.4
0.3
0.2
0.1
0.0
Phylostratum
Cellular_organisms - ps1
Archaea1 - ps2
Archaea2 - ps3
Archaea3 - ps4
Asgard/Eukaryota - ps5
Eukaryota - ps6
Amorpheae - ps7
Obazoa - ps8
Opisthokonta - ps9
Holomycota - ps10
Fungi - ps11
Fungi_1 - ps12
Fungi_2 - ps13
Fungi_3 - ps14
Dikarya - ps15
Ascomycota - ps16
Saccharo/Pezizomycotina - ps17
Saccharomycotina - ps18
Saccharomycotina_1 - ps19
Saccharomycotina_2 - ps20
Saccharomycotina_3 - ps21
Phaff./Saccharomycetaceae - ps22
Saccharomyco./mycetaceae - ps23
Saccharomycetaceae - ps24
Saccharomycetaceae_2 - ps25
Saccharomycetaceae_3 - ps26
Kaz./Nau./Saccharomyces - ps27
Saccharomyces - ps28
Arboricola_clade - ps29
Cerevisiae_clade - ps30
Saccharomyces_cerevisiae - ps31
p-value
2e-03
5e-03
1e-02
2e-02
5e-02
Log-odds
0.5
1.0
1.5
2.0
2.5
3.0
3.5
4.0

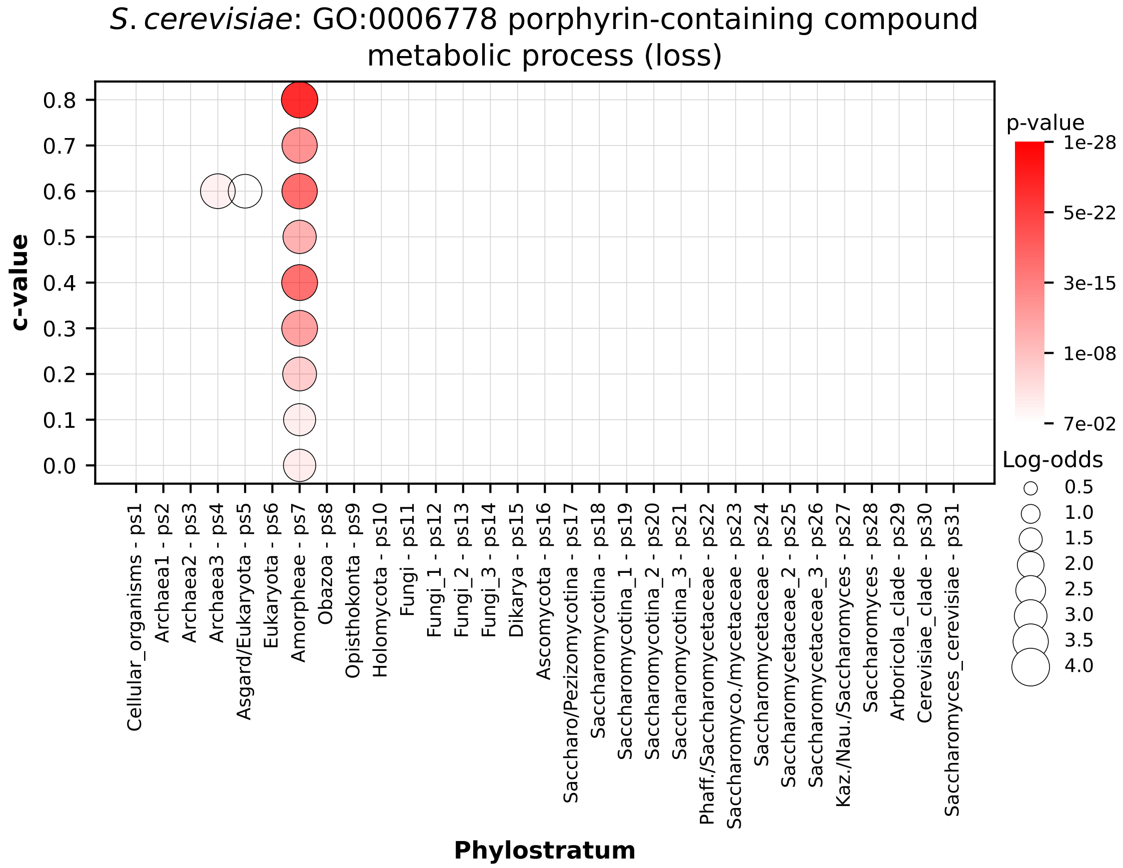

S. cerevisiae: GO:0006778 porphyrin-containing compound metabolic process (loss)
c-value
0.0
0.1
0.2
0.3
0.4
0.5
0.6
0.7
0.8
p-value
1e-28
5e-22
3e-15
1e-08
7e-02
Log-odds
0.5
1.0
1.5
2.0
2.5
3.0
3.5
4.0
Cellular_organisms - ps1
Archaea1 - ps2
Archaea2 - ps3
Archaea3 - ps4
Asgard/Eukaryota - ps5
Eukaryota - ps6
Amorpheae - ps7
Obazoa - ps8
Opisthokonta - ps9
Holomycota - ps10
Fungi - ps11
Fungi_1 - ps12
Fungi_2 - ps13
Fungi_3 - ps14
Dikarya - ps15
Ascomycota - ps16
Saccharo/Pezizomycotina - ps17
Saccharomycotina - ps18
Saccharomycotina_1 - ps19
Saccharomycotina_2 - ps20
Saccharomycotina_3 - ps21
Phaff./Saccharomycetaceae - ps22
Saccharomyco./mycetaceae - ps23
Saccharomycetaceae - ps24
Saccharomycetaceae_2 - ps25
Saccharomycetaceae_3 - ps26
Kaz./Nau./Saccharomyces - ps27
Saccharomyces - ps28
Arboricola_clade - ps29
Cerevisiae_clade - ps30
Saccharomyces_cerevisiae - ps31
Phylostratum

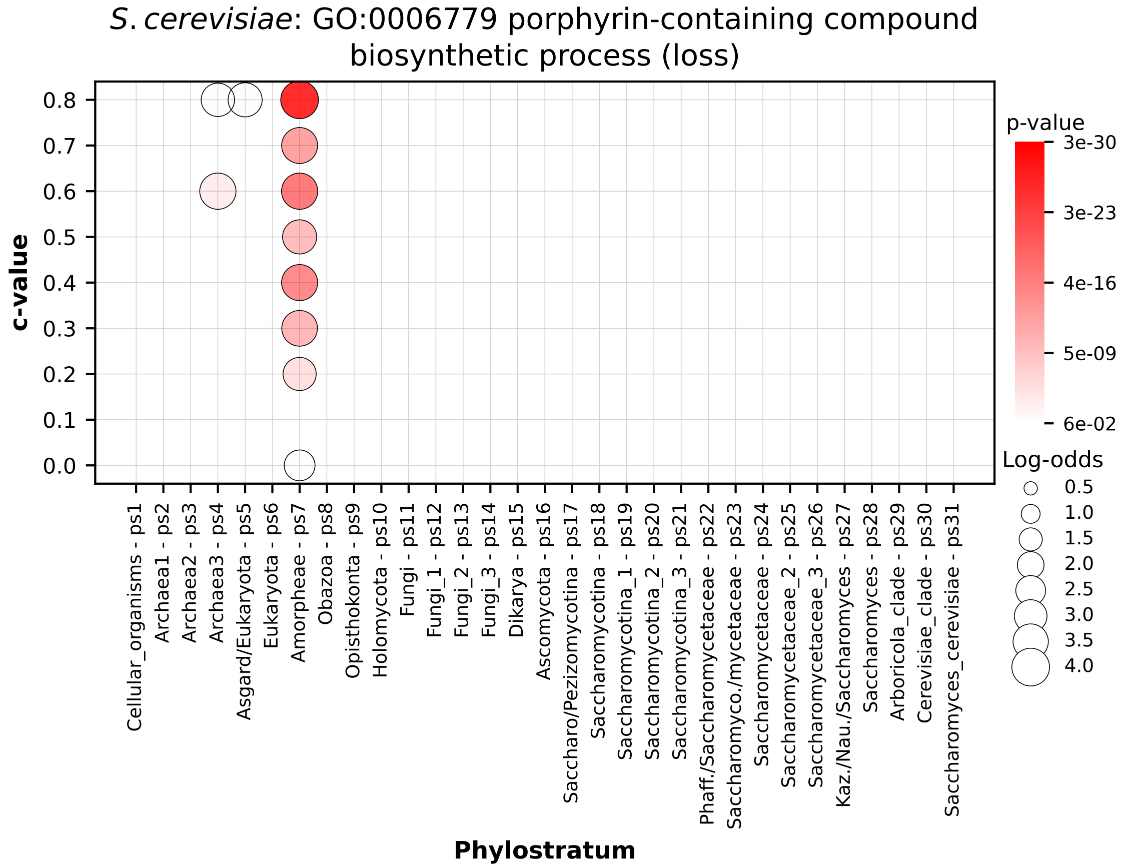

S. cerevisiae: GO:0006779 porphyrin-containing compound biosynthetic process (loss)
c-value
0.8
0.7
0.6
0.5
0.4
0.3
0.2
0.1
0.0
p-value
3e-30
3e-23
4e-16
5e-09
6e-02
Log-odds
0.5
1.0
1.5
2.0
2.5
3.0
3.5
4.0
Cellular_organisms - ps1
Archaea1 - ps2
Archaea2 - ps3
Archaea3 - ps4
Asgard/Eukaryota - ps5
Eukaryota - ps6
Amorpheae - ps7
Obazoa - ps8
Opisthokonta - ps9
Holomycota - ps10
Fungi - ps11
Fungi_1 - ps12
Fungi_2 - ps13
Fungi_3 - ps14
Dikarya - ps15
Ascomycota - ps16
Saccharo/Pezizomycotina - ps17
Saccharomycotina - ps18
Saccharomycotina_1 - ps19
Saccharomycotina_2 - ps20
Saccharomycotina_3 - ps21
Phaff./Saccharomycetaceae - ps22
Saccharomyco./mycetaceae - ps23
Saccharomycetaceae - ps24
Saccharomycetaceae_2 - ps25
Saccharomycetaceae_3 - ps26
Kaz./Nau./Saccharomyces - ps27
Saccharomyces - ps28
Arboricola_clade - ps29
Cerevisiae_clade - ps30
Saccharomyces_cerevisiae - ps31
Phylostratum

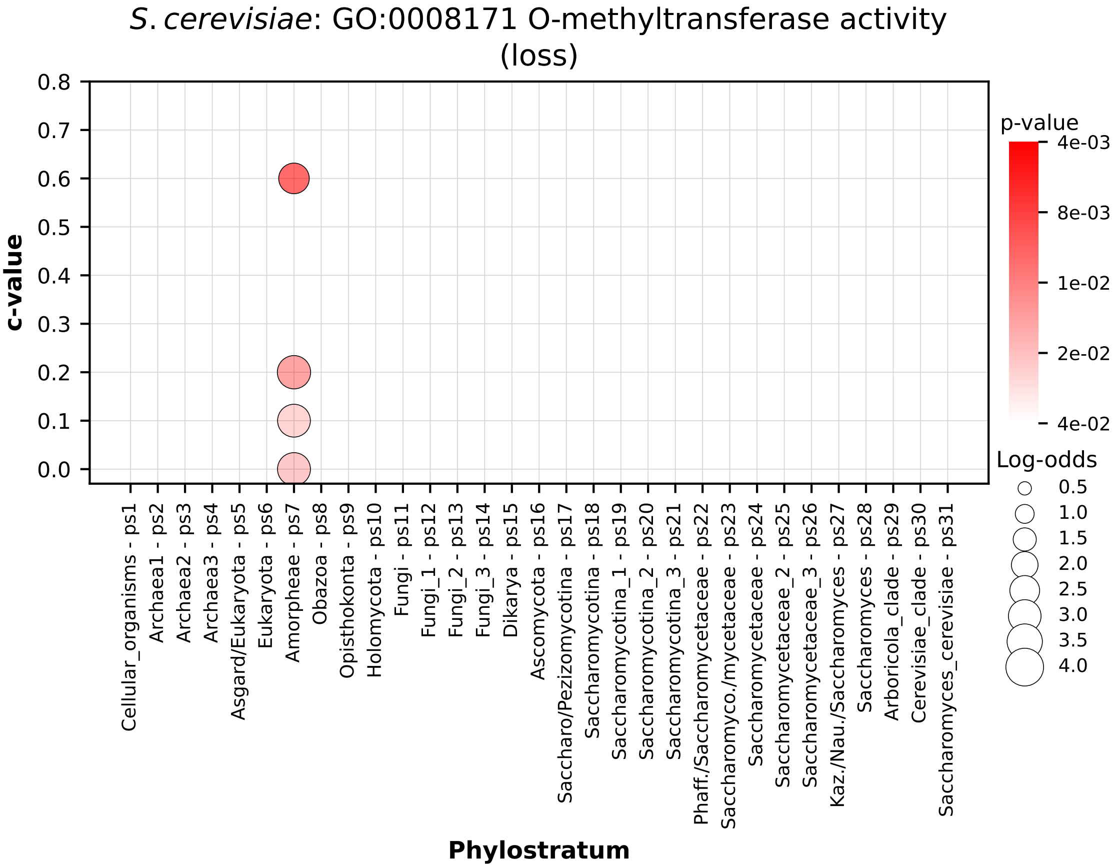

S. cerevisiae: GO:0008171 O-methyltransferase activity (loss)
c-value
0.8
0.7
0.6
0.5
0.4
0.3
0.2
0.1
0.0
Phylostratum
Cellular_organisms - ps1
Archaea1 - ps2
Archaea2 - ps3
Archaea3 - ps4
Asgard/Eukaryota - ps5
Eukaryota - ps6
Amorpheae - ps7
Obazoa - ps8
Opisthokonta - ps9
Holomycota - ps10
Fungi - ps11
Fungi_1 - ps12
Fungi_2 - ps13
Fungi_3 - ps14
Dikarya - ps15
Ascomycota - ps16
Saccharo/Pezizomycotina - ps17
Saccharomycotina - ps18
Saccharomycotina_1 - ps19
Saccharomycotina_2 - ps20
Saccharomycotina_3 - ps21
Phaff./Saccharomycetaceae - ps22
Saccharomyco./mycetaceae - ps23
Saccharomycetaceae - ps24
Saccharomycetaceae_2 - ps25
Saccharomycetaceae_3 - ps26
Kaz./Nau./Saccharomyces - ps27
Saccharomyces - ps28
Arboricola_clade - ps29
Cerevisiae_clade - ps30
Saccharomyces_cerevisiae - ps31
p-value
4e-03
8e-03
1e-02
2e-02
4e-02
Log-odds
0.5
1.0
1.5
2.0
2.5
3.0
3.5
4.0

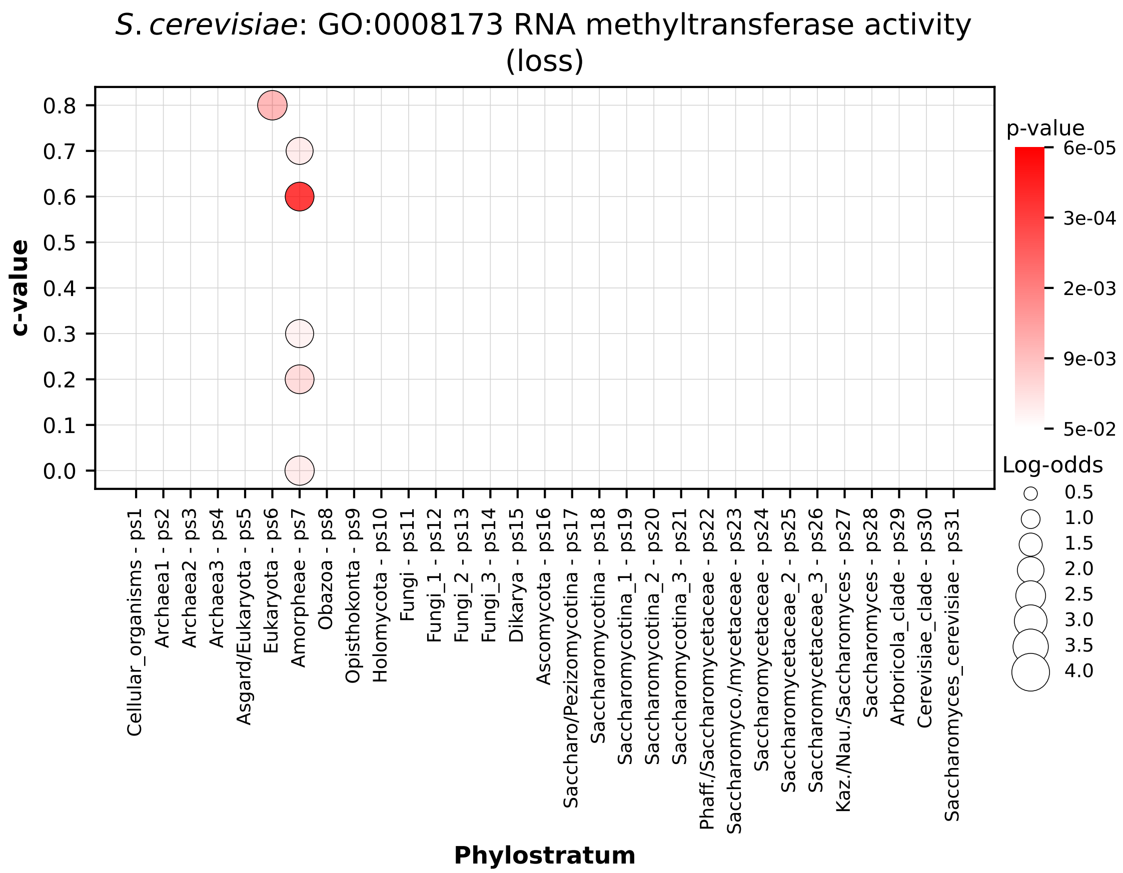

S. cerevisiae: GO:0008173 RNA methyltransferase activity
(loss)
c-value
0.8
0.7
0.6
0.5
0.4
0.3
0.2
0.1
0.0
p-value
6e-05
3e-04
2e-03
9e-03
5e-02
Log-odds
0.5
1.0
1.5
2.0
2.5
3.0
3.5
4.0
Cellular_organisms - ps1
Archaea1 - ps2
Archaea2 - ps3
Archaea3 - ps4
Asgard/Eukaryota - ps5
Eukaryota - ps6
Amorpheae - ps7
Obazoa - ps8
Opisthokonta - ps9
Holomycota - ps10
Fungi - ps11
Fungi_1 - ps12
Fungi_2 - ps13
Fungi_3 - ps14
Dikarya - ps15
Ascomycota - ps16
Saccharo/Pezizomycotina - ps17
Saccharomycotina - ps18
Saccharomycotina_1 - ps19
Saccharomycotina_2 - ps20
Saccharomycotina_3 - ps21
Phaff./Saccharomycetaceae - ps22
Saccharomyco./mycetaceae - ps23
Saccharomycetaceae - ps24
Saccharomycetaceae_2 - ps25
Saccharomycetaceae_3 - ps26
Kaz./Nau./Saccharomyces - ps27
Saccharomyces - ps28
Arboricola_clade - ps29
Cerevisiae_clade - ps30
Saccharomyces_cerevisiae - ps31
Phylostratum

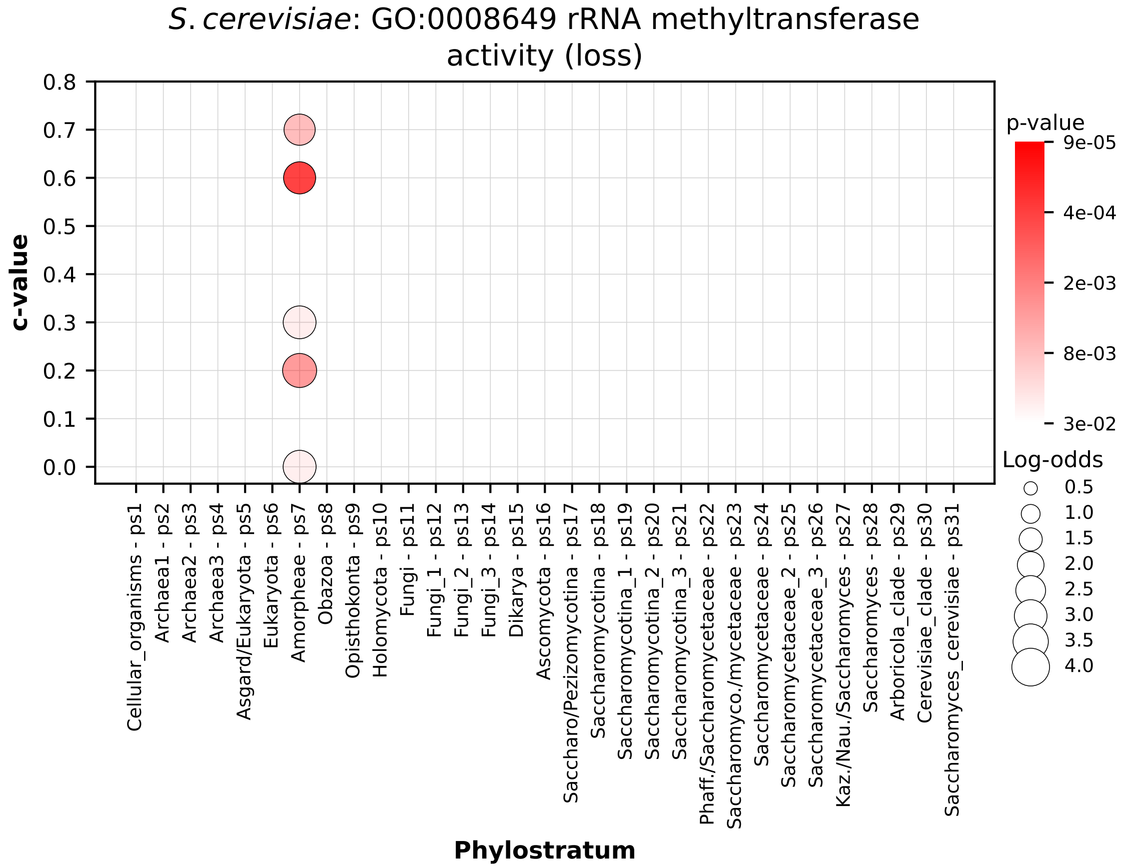
S. cerevisiae: GO:0008649 rRNA methyltransferase activity (loss)
c-value
0.8
0.7
0.6
0.5
0.4
0.3
0.2
0.1
0.0
Phylostratum
Cellular_organisms - ps1
Archaea1 - ps2
Archaea2 - ps3
Archaea3 - ps4
Asgard/Eukaryota - ps5
Eukaryota - ps6
Amorpheae - ps7
Obazoa - ps8
Opisthokonta - ps9
Holomycota - ps10
Fungi - ps11
Fungi_1 - ps12
Fungi_2 - ps13
Fungi_3 - ps14
Dikarya - ps15
Ascomycota - ps16
Saccharo/Pezizomycotina - ps17
Saccharomycotina - ps18
Saccharomycotina_1 - ps19
Saccharomycotina_2 - ps20
Saccharomycotina_3 - ps21
Phaff./Saccharomycetaceae - ps22
Saccharomyco./mycetaceae - ps23
Saccharomycetaceae - ps24
Saccharomycetaceae_2 - ps25
Saccharomycetaceae_3 - ps26
Kaz./Nau./Saccharomyces - ps27
Saccharomyces - ps28
Arboricola_clade - ps29
Cerevisiae_clade - ps30
Saccharomyces_cerevisiae - ps31
p-value
9e-05
4e-04
2e-03
8e-03
3e-02
Log-odds
0.5
1.0
1.5
2.0
2.5
3.0
3.5
4.0

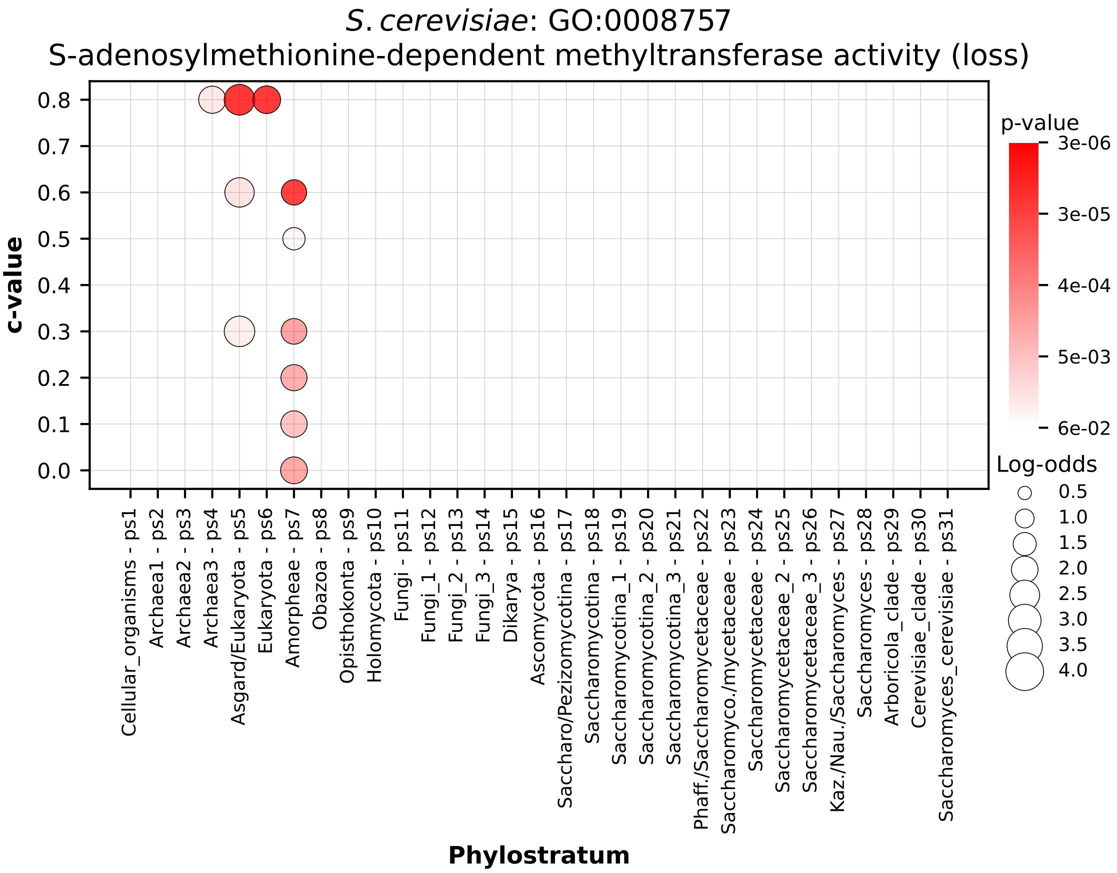
S. cerevisiae: GO:0008757
S-adenosylmethionine-dependent methyltransferase activity (loss)
c-value
0.8
0.7
0.6
0.5
0.4
0.3
0.2
0.1
0.0
p-value
3e-06
3e-05
4e-04
5e-03
6e-02
Log-odds
0.5
1.0
1.5
2.0
2.5
3.0
3.5
4.0
Cellular_organisms - ps1
Archaea1 - ps2
Archaea2 - ps3
Archaea3 - ps4
Asgard/Eukaryota - ps5
Eukaryota - ps6
Amorpheae - ps7
Obazoa - ps8
Opisthokonta - ps9
Holomycota - ps10
Fungi - ps11
Fungi_1 - ps12
Fungi_2 - ps13
Fungi_3 - ps14
Dikarya - ps15
Ascomycota - ps16
Saccharo/Pezizomycotina - ps17
Saccharomycotina - ps18
Saccharomycotina_1 - ps19
Saccharomycotina_2 - ps20
Saccharomycotina_3 - ps21
Phaff./Saccharomycetaceae - ps22
Saccharomyco./mycetaceae - ps23
Saccharomycetaceae - ps24
Saccharomycetaceae_2 - ps25
Saccharomycetaceae_3 - ps26
Kaz./Nau./Saccharomyces - ps27
Saccharomyces - ps28
Arboricola_clade - ps29
Cerevisiae_clade - ps30
Saccharomyces_cerevisiae - ps31
Phylostratum

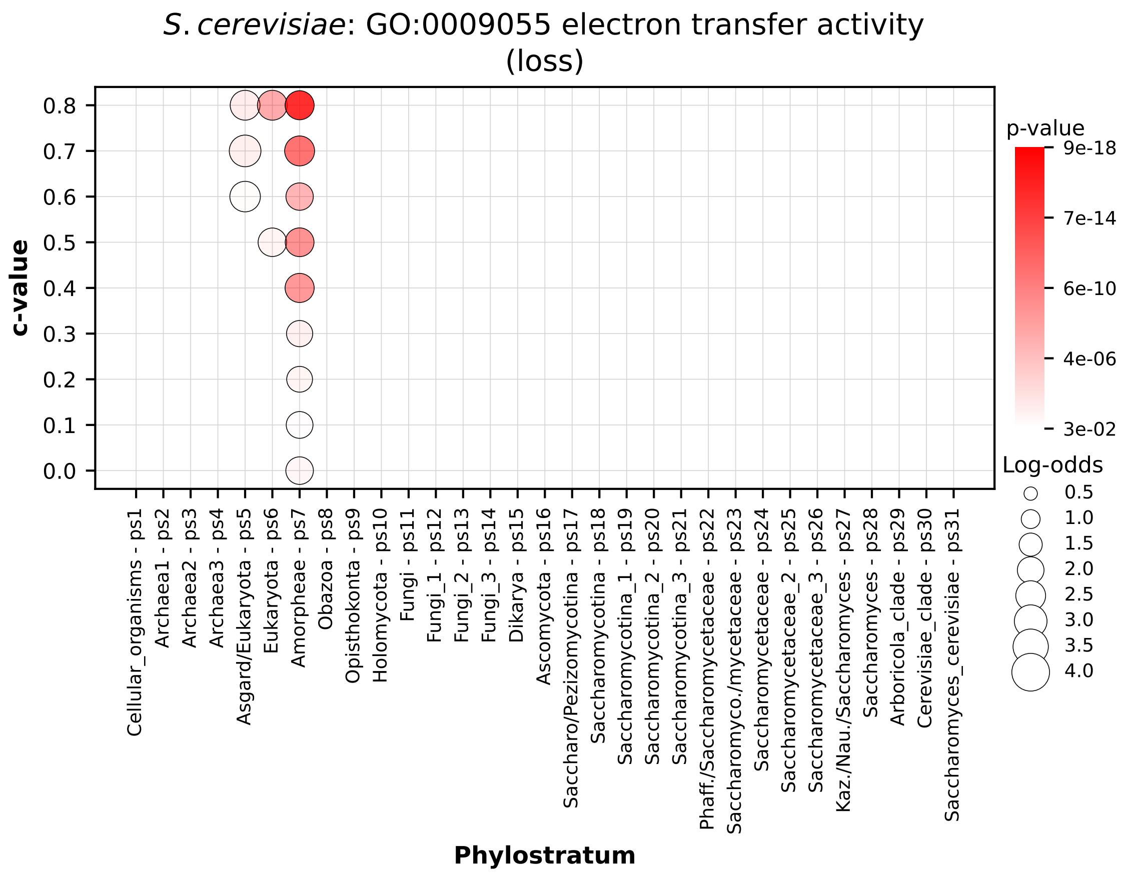

S. cerevisiae: GO:0009055 electron transfer activity
(loss)
c-value
0.8
0.7
0.6
0.5
0.4
0.3
0.2
0.1
0.0
p-value
9e-18
7e-14
6e-10
4e-06
3e-02
Log-odds
0.5
1.0
1.5
2.0
2.5
3.0
3.5
4.0
Cellular_organisms - ps1
Archaea1 - ps2
Archaea2 - ps3
Archaea3 - ps4
Asgard/Eukaryota - ps5
Eukaryota - ps6
Amorpheae - ps7
Obazoa - ps8
Opisthokonta - ps9
Holomycota - ps10
Fungi - ps11
Fungi_1 - ps12
Fungi_2 - ps13
Fungi_3 - ps14
Dikarya - ps15
Ascomycota - ps16
Saccharo/Pezizomycotina - ps17
Saccharomycotina - ps18
Saccharomycotina_1 - ps19
Saccharomycotina_2 - ps20
Saccharomycotina_3 - ps21
Phaff./Saccharomycetaceae - ps22
Saccharomyco./mycetaceae - ps23
Saccharomycetaceae - ps24
Saccharomycetaceae_2 - ps25
Saccharomycetaceae_3 - ps26
Kaz./Nau./Saccharomyces - ps27
Saccharomyces - ps28
Arboricola_clade - ps29
Cerevisiae_clade - ps30
Saccharomyces_cerevisiae - ps31
Phylostratum

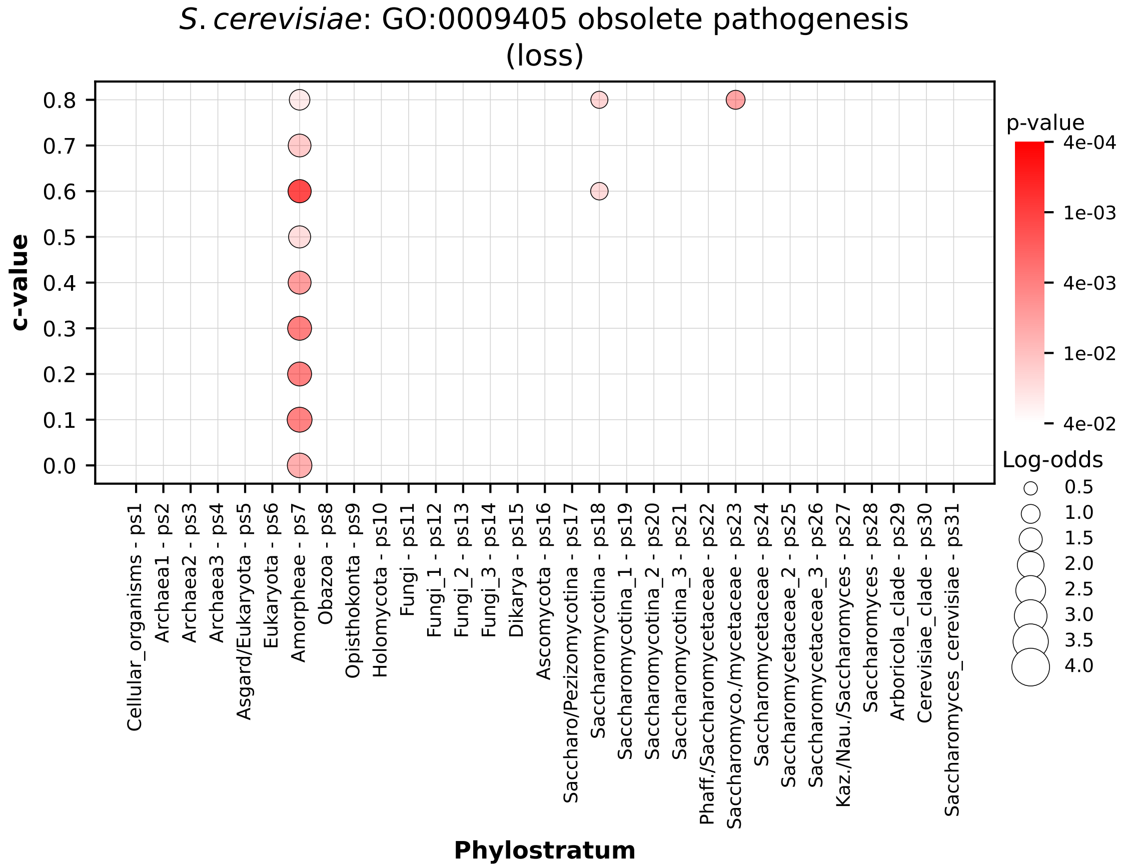

$S. cerevisiae$: GO:0009405 obsolete pathogenesis
(loss)
c-value
0.8
0.7
0.6
0.5
0.4
0.3
0.2
0.1
0.0
Phylostratum
Cellular_organisms - ps1
Archaea1 - ps2
Archaea2 - ps3
Archaea3 - ps4
Asgard/Eukaryota - ps5
Eukaryota - ps6
Amorpheae - ps7
Obazoa - ps8
Opisthokonta - ps9
Holomycota - ps10
Fungi - ps11
Fungi_1 - ps12
Fungi_2 - ps13
Fungi_3 - ps14
Dikarya - ps15
Ascomycota - ps16
Saccharo/Pezizomycotina - ps17
Saccharomycotina - ps18
Saccharomycotina_1 - ps19
Saccharomycotina_2 - ps20
Saccharomycotina_3 - ps21
Phaff./Saccharomycetaceae - ps22
Saccharomyco./mycetaceae - ps23
Saccharomycetaceae - ps24
Saccharomycetaceae_2 - ps25
Saccharomycetaceae_3 - ps26
Kaz./Nau./Saccharomyces - ps27
Saccharomyces - ps28
Arboricola_clade - ps29
Cerevisiae_clade - ps30
Saccharomyces_cerevisiae - ps31
p-value
4e-04
1e-03
4e-03
1e-02
4e-02
Log-odds
0.5
1.0
1.5
2.0
2.5
3.0
3.5
4.0

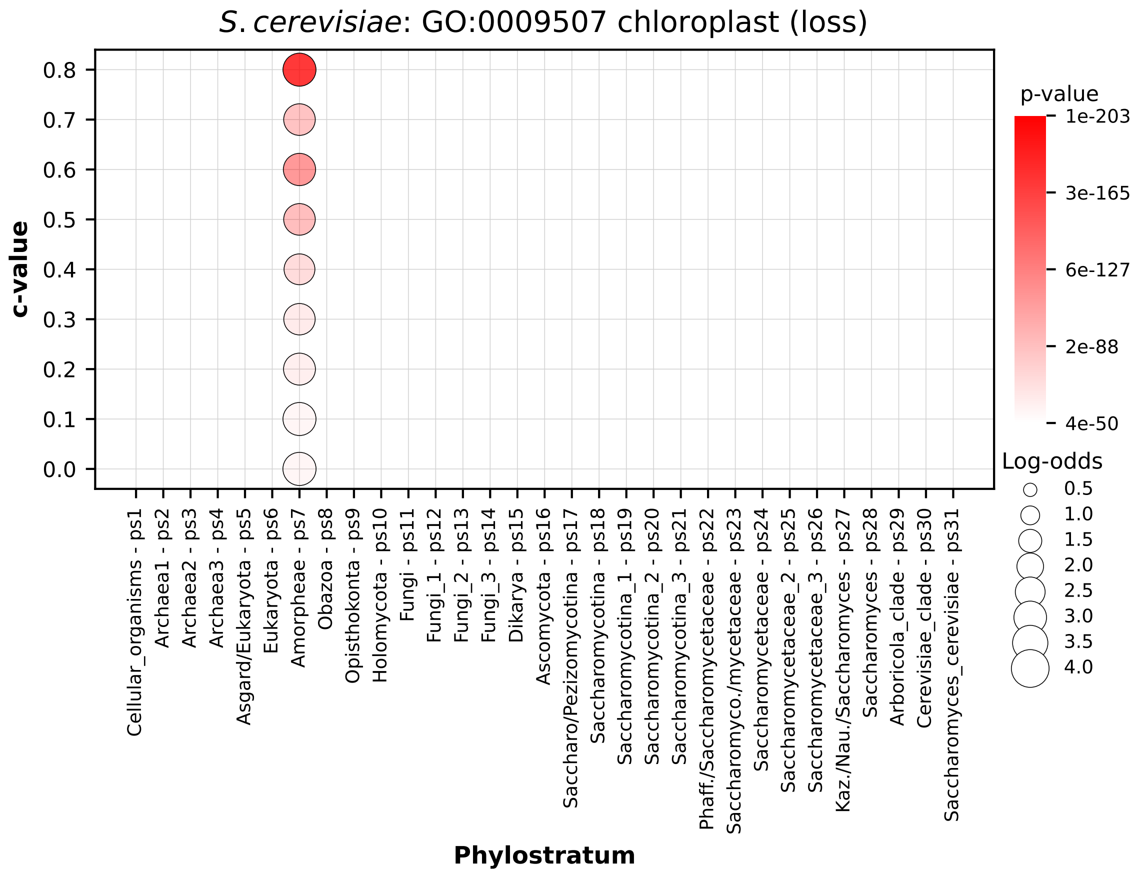

S. cerevisiae: GO:0009507 chloroplast (loss)
c-value
0.8
0.7
0.6
0.5
0.4
0.3
0.2
0.1
0.0
p-value
1e-203
3e-165
6e-127
2e-88
4e-50
Log-odds
0.5
1.0
1.5
2.0
2.5
3.0
3.5
4.0
Cellular_organisms - ps1
Archaea1 - ps2
Archaea2 - ps3
Archaea3 - ps4
Asgard/Eukaryota - ps5
Eukaryota - ps6
Amorpheae - ps7
Obazoa - ps8
Opisthokonta - ps9
Holomycota - ps10
Fungi - ps11
Fungi_1 - ps12
Fungi_2 - ps13
Fungi_3 - ps14
Dikarya - ps15
Ascomycota - ps16
Saccharo/Pezizomycotina - ps17
Saccharomycotina - ps18
Saccharomycotina_1 - ps19
Saccharomycotina_2 - ps20
Saccharomycotina_3 - ps21
Phaff./Saccharomycetaceae - ps22
Saccharomyco./mycetaceae - ps23
Saccharomycetaceae - ps24
Saccharomycetaceae_2 - ps25
Saccharomycetaceae_3 - ps26
Kaz./Nau./Saccharomyces - ps27
Saccharomyces - ps28
Arboricola_clade - ps29
Cerevisiae_clade - ps30
Saccharomyces_cerevisiae - ps31
Phylostratum

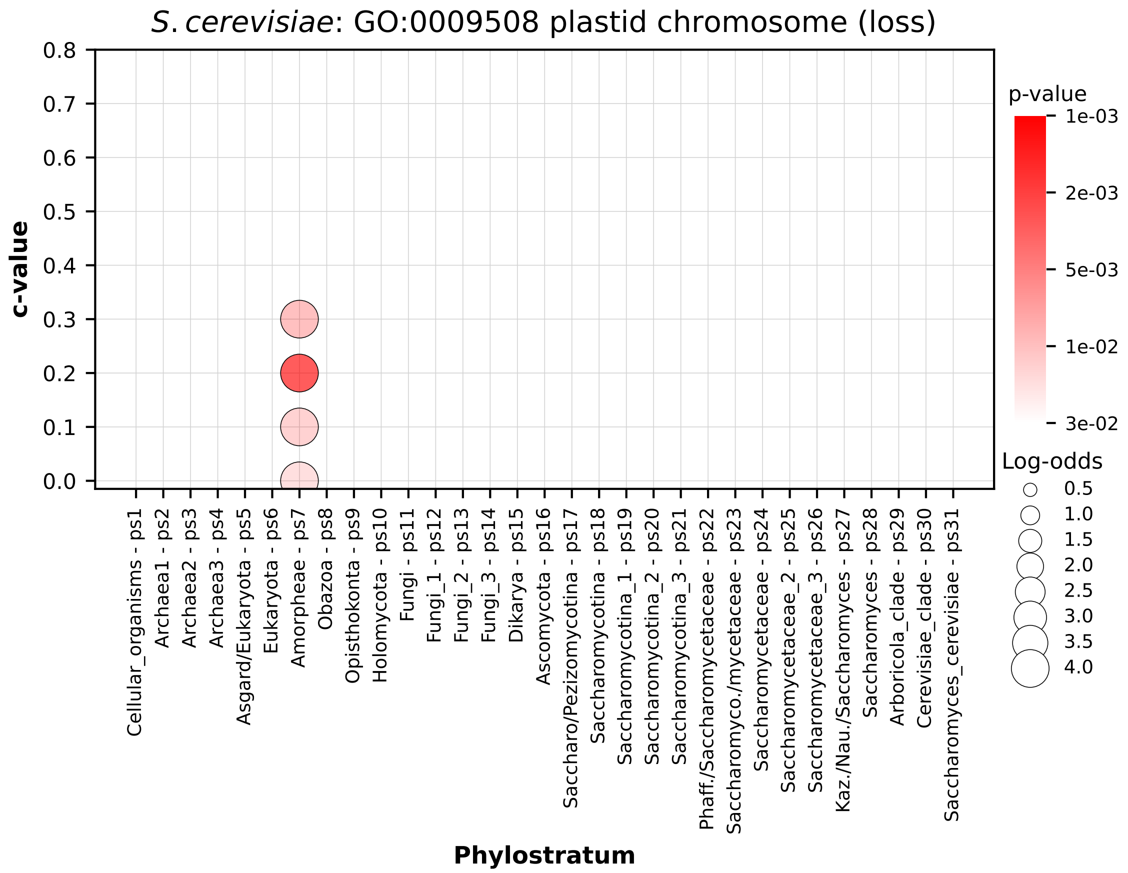

S. cerevisiae: GO:0009508 plastid chromosome (loss)
c-value
0.0
0.1
0.2
0.3
0.4
0.5
0.6
0.7
0.8
Phylostratum
Cellular_organisms - ps1
Archaea1 - ps2
Archaea2 - ps3
Archaea3 - ps4
Asgard/Eukaryota - ps5
Eukaryota - ps6
Amorpheae - ps7
Obazoa - ps8
Opisthokonta - ps9
Holomycota - ps10
Fungi - ps11
Fungi_1 - ps12
Fungi_2 - ps13
Fungi_3 - ps14
Dikarya - ps15
Ascomycota - ps16
Saccharo/Pezizomycotina - ps17
Saccharomycotina - ps18
Saccharomycotina_1 - ps19
Saccharomycotina_2 - ps20
Saccharomycotina_3 - ps21
Phaff./Saccharomycetaceae - ps22
Saccharomyco./mycetaceae - ps23
Saccharomycetaceae - ps24
Saccharomycetaceae_2 - ps25
Saccharomycetaceae_3 - ps26
Kaz./Nau./Saccharomyces - ps27
Saccharomyces - ps28
Arboricola_clade - ps29
Cerevisiae_clade - ps30
Saccharomyces_cerevisiae - ps31
p-value
1e-03
2e-03
5e-03
1e-02
3e-02
Log-odds
0.5
1.0
1.5
2.0
2.5
3.0
3.5
4.0

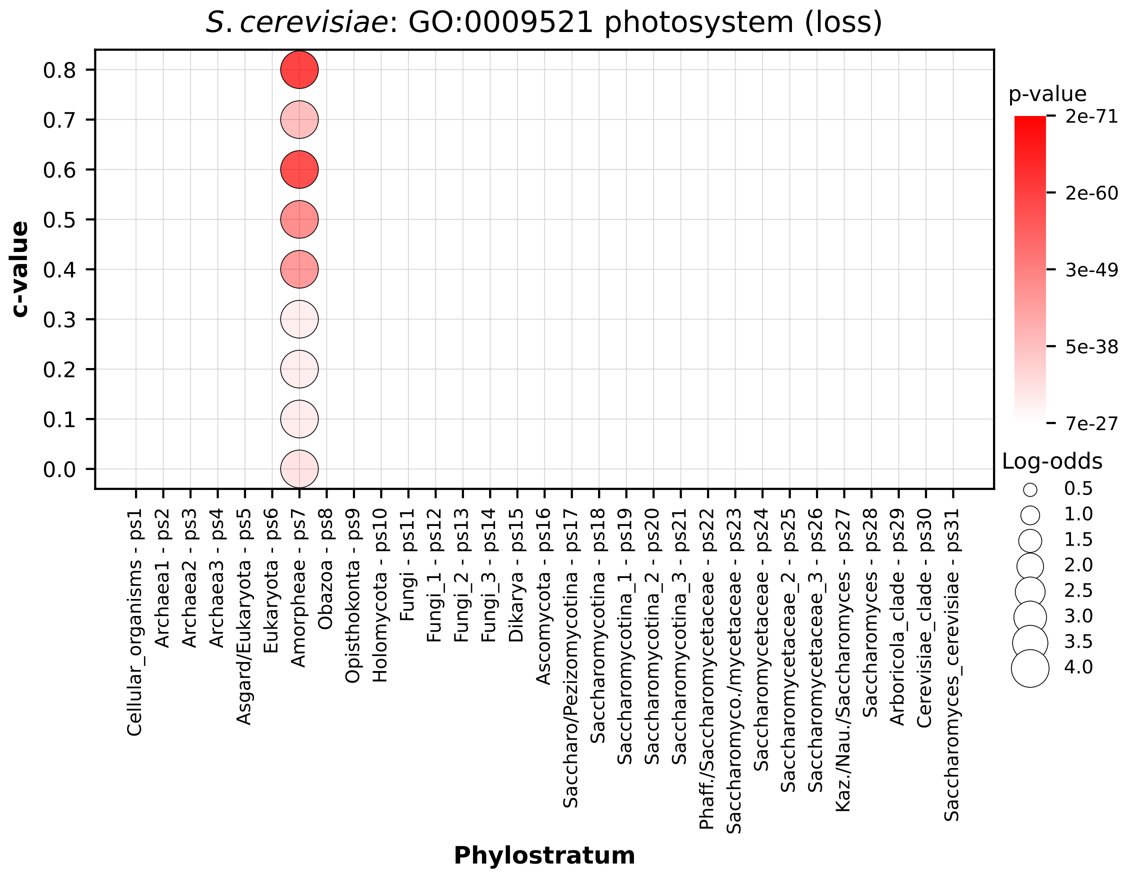

S. cerevisiae: GO:0009521 photosystem (loss)
c-value
0.8
0.7
0.6
0.5
0.4
0.3
0.2
0.1
0.0
p-value
2e-71
2e-60
3e-49
5e-38
7e-27
Log-odds
0.5
1.0
1.5
2.0
2.5
3.0
3.5
4.0
Cellular_organisms - ps1
Archaea1 - ps2
Archaea2 - ps3
Archaea3 - ps4
Asgard/Eukaryota - ps5
Eukaryota - ps6
Amorpheae - ps7
Obazoa - ps8
Opisthokonta - ps9
Holomycota - ps10
Fungi - ps11
Fungi_1 - ps12
Fungi_2 - ps13
Fungi_3 - ps14
Dikarya - ps15
Ascomycota - ps16
Saccharo/Pezizomycotina - ps17
Saccharomycotina - ps18
Saccharomycotina_1 - ps19
Saccharomycotina_2 - ps20
Saccharomycotina_3 - ps21
Phaff./Saccharomycetaceae - ps22
Saccharomyco./mycetaceae - ps23
Saccharomycetaceae - ps24
Saccharomycetaceae_2 - ps25
Saccharomycetaceae_3 - ps26
Kaz./Nau./Saccharomyces - ps27
Saccharomyces - ps28
Arboricola_clade - ps29
Cerevisiae_clade - ps30
Saccharomyces_cerevisiae - ps31
Phylostratum

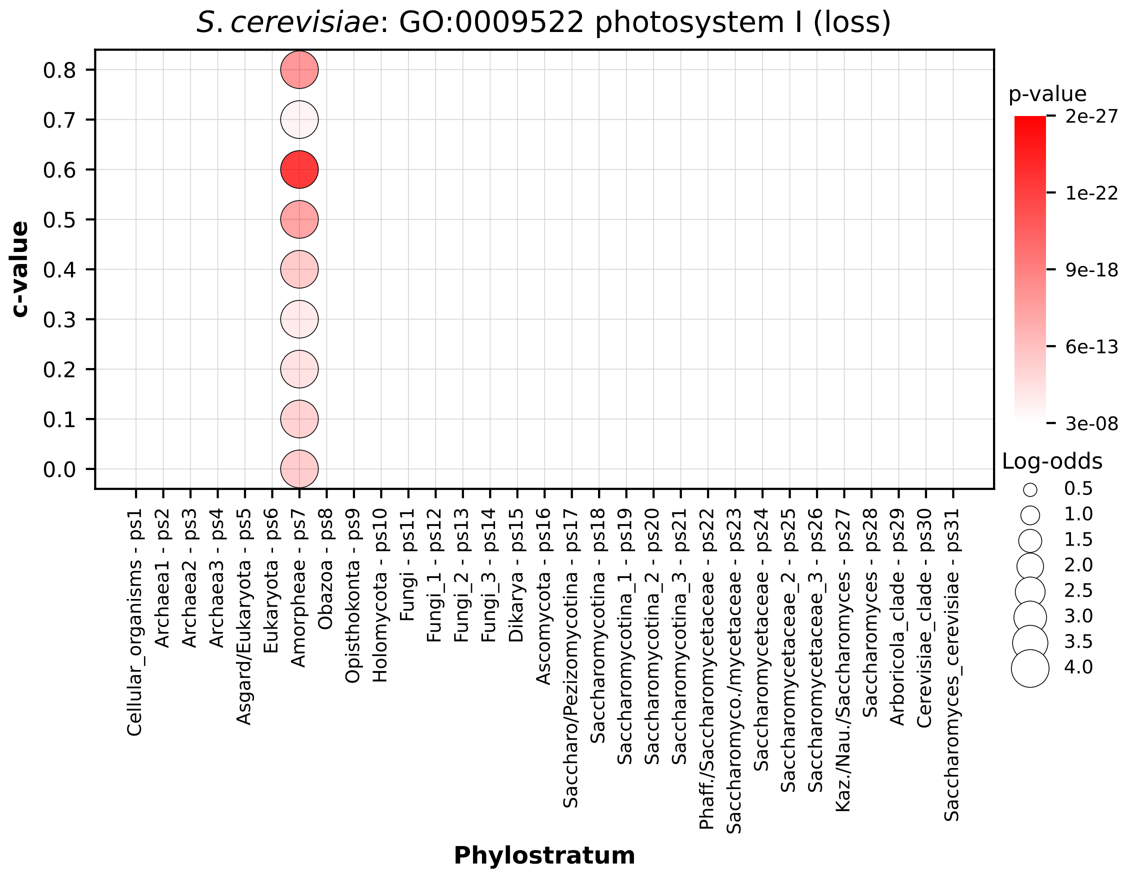
S. cerevisiae: GO:0009522 photosystem I (loss)
c-value
0.0
0.1
0.2
0.3
0.4
0.5
0.6
0.7
0.8
Phylostratum
Cellular_organisms - ps1
Archaea1 - ps2
Archaea2 - ps3
Archaea3 - ps4
Asgard/Eukaryota - ps5
Eukaryota - ps6
Amorpheae - ps7
Obazoa - ps8
Opisthokonta - ps9
Holomycota - ps10
Fungi - ps11
Fungi_1 - ps12
Fungi_2 - ps13
Fungi_3 - ps14
Dikarya - ps15
Ascomycota - ps16
Saccharo/Pezizomycotina - ps17
Saccharomycotina - ps18
Saccharomycotina_1 - ps19
Saccharomycotina_2 - ps20
Saccharomycotina_3 - ps21
Phaff./Saccharomycetaceae - ps22
Saccharomyco./mycetaceae - ps23
Saccharomycetaceae - ps24
Saccharomycetaceae_2 - ps25
Saccharomycetaceae_3 - ps26
Kaz./Nau./Saccharomyces - ps27
Saccharomyces - ps28
Arboricola_clade - ps29
Cerevisiae_clade - ps30
Saccharomyces_cerevisiae - ps31
p-value
2e-27
1e-22
9e-18
6e-13
3e-08
Log-odds
0.5
1.0
1.5
2.0
2.5
3.0
3.5
4.0

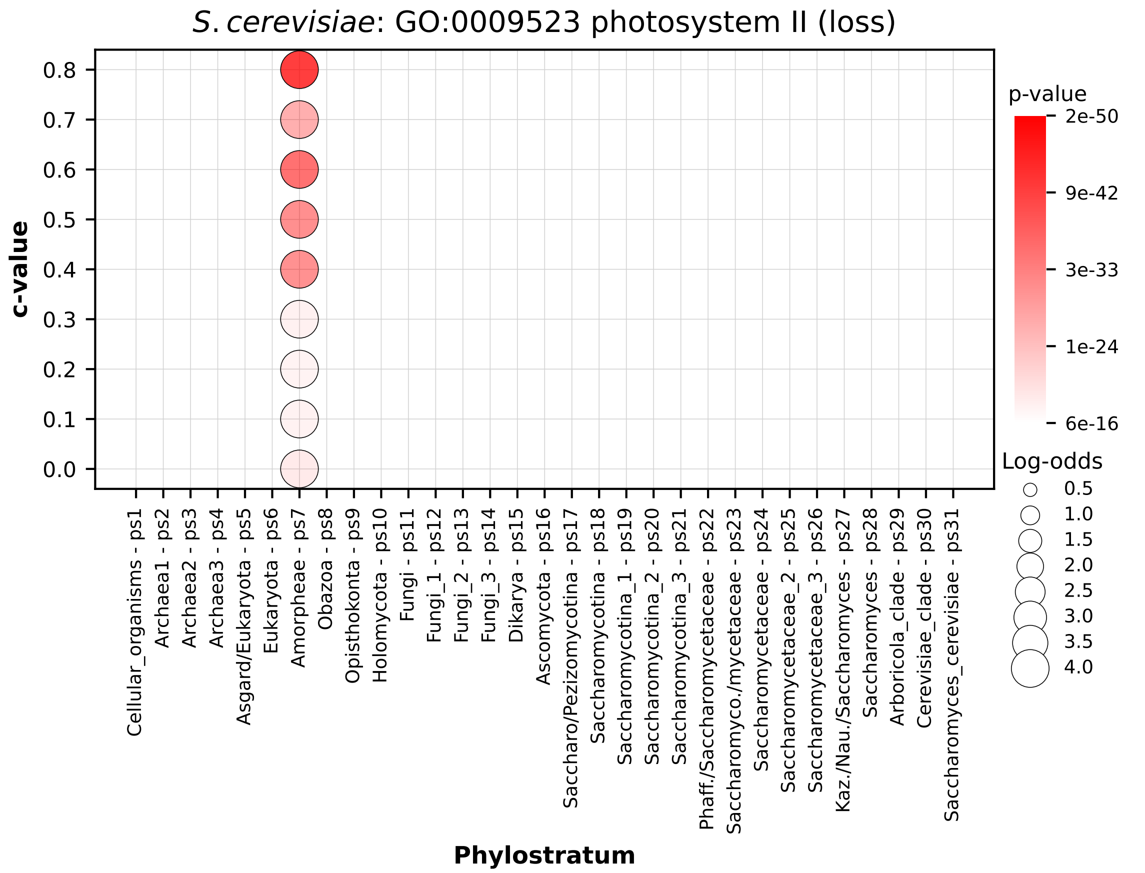
S. cerevisiae: GO:0009523 photosystem II (loss)
c-value
0.8
0.7
0.6
0.5
0.4
0.3
0.2
0.1
0.0
p-value
2e-50
9e-42
3e-33
1e-24
6e-16
Log-odds
0.5
1.0
1.5
2.0
2.5
3.0
3.5
4.0
Cellular_organisms - ps1
Archaea1 - ps2
Archaea2 - ps3
Archaea3 - ps4
Asgard/Eukaryota - ps5
Eukaryota - ps6
Amorpheae - ps7
Obazoa - ps8
Opisthokonta - ps9
Holomycota - ps10
Fungi - ps11
Fungi_1 - ps12
Fungi_2 - ps13
Fungi_3 - ps14
Dikarya - ps15
Ascomycota - ps16
Saccharo/Pezizomycotina - ps17
Saccharomycotina - ps18
Saccharomycotina_1 - ps19
Saccharomycotina_2 - ps20
Saccharomycotina_3 - ps21
Phaff./Saccharomycetaceae - ps22
Saccharomyco./mycetaceae - ps23
Saccharomycetaceae - ps24
Saccharomycetaceae_2 - ps25
Saccharomycetaceae_3 - ps26
Kaz./Nau./Saccharomyces - ps27
Saccharomyces - ps28
Arboricola_clade - ps29
Cerevisiae_clade - ps30
Saccharomyces_cerevisiae - ps31
Phylostratum

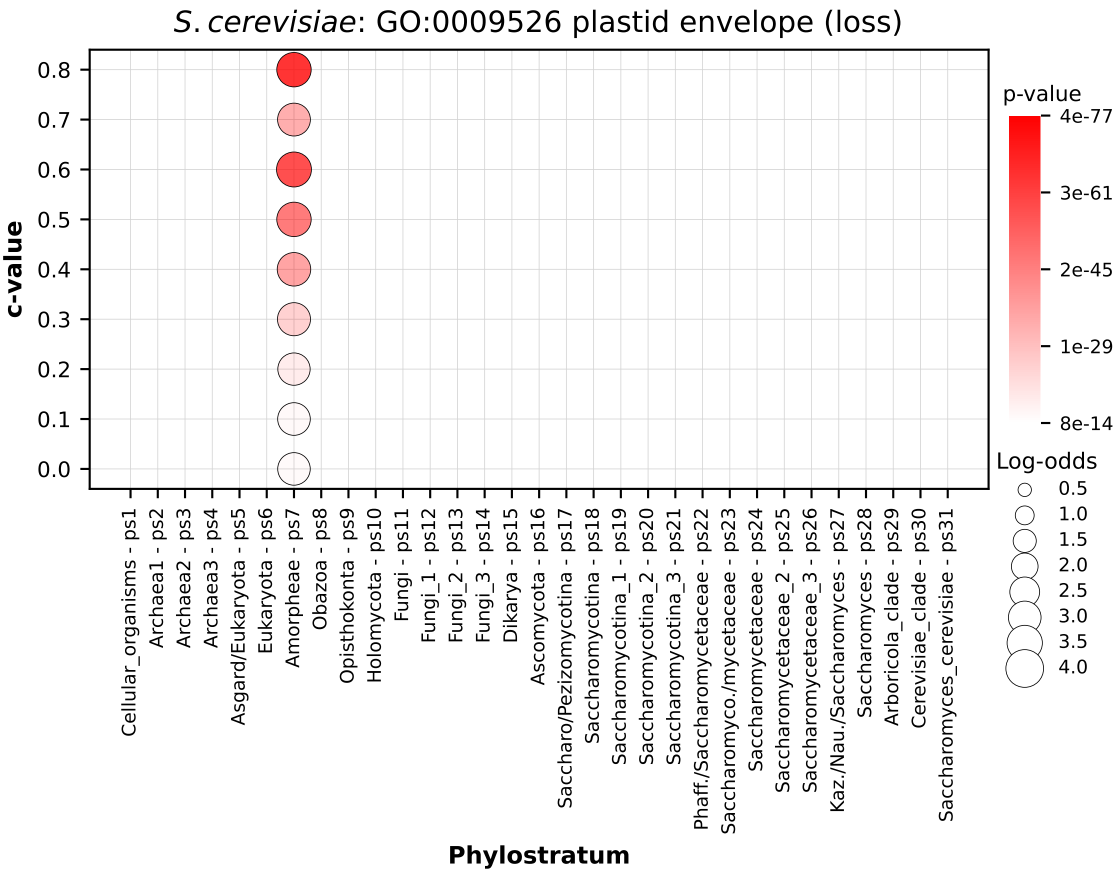

S. cerevisiae: GO:0009526 plastid envelope (loss)
c-value
0.8
0.7
0.6
0.5
0.4
0.3
0.2
0.1
0.0
p-value
4e-77
3e-61
2e-45
1e-29
8e-14
Log-odds
0.5
1.0
1.5
2.0
2.5
3.0
3.5
4.0
Cellular_organisms - ps1
Archaea1 - ps2
Archaea2 - ps3
Archaea3 - ps4
Asgard/Eukaryota - ps5
Eukaryota - ps6
Amorpheae - ps7
Obazoa - ps8
Opisthokonta - ps9
Holomycota - ps10
Fungi - ps11
Fungi_1 - ps12
Fungi_2 - ps13
Fungi_3 - ps14
Dikarya - ps15
Ascomycota - ps16
Saccharo/Pezizomycotina - ps17
Saccharomycotina - ps18
Saccharomycotina_1 - ps19
Saccharomycotina_2 - ps20
Saccharomycotina_3 - ps21
Phaff./Saccharomycetaceae - ps22
Saccharomyco./mycetaceae - ps23
Saccharomycetaceae - ps24
Saccharomycetaceae_2 - ps25
Saccharomycetaceae_3 - ps26
Kaz./Nau./Saccharomyces - ps27
Saccharomyces - ps28
Arboricola_clade - ps29
Cerevisiae_clade - ps30
Saccharomyces_cerevisiae - ps31
Phylostratum

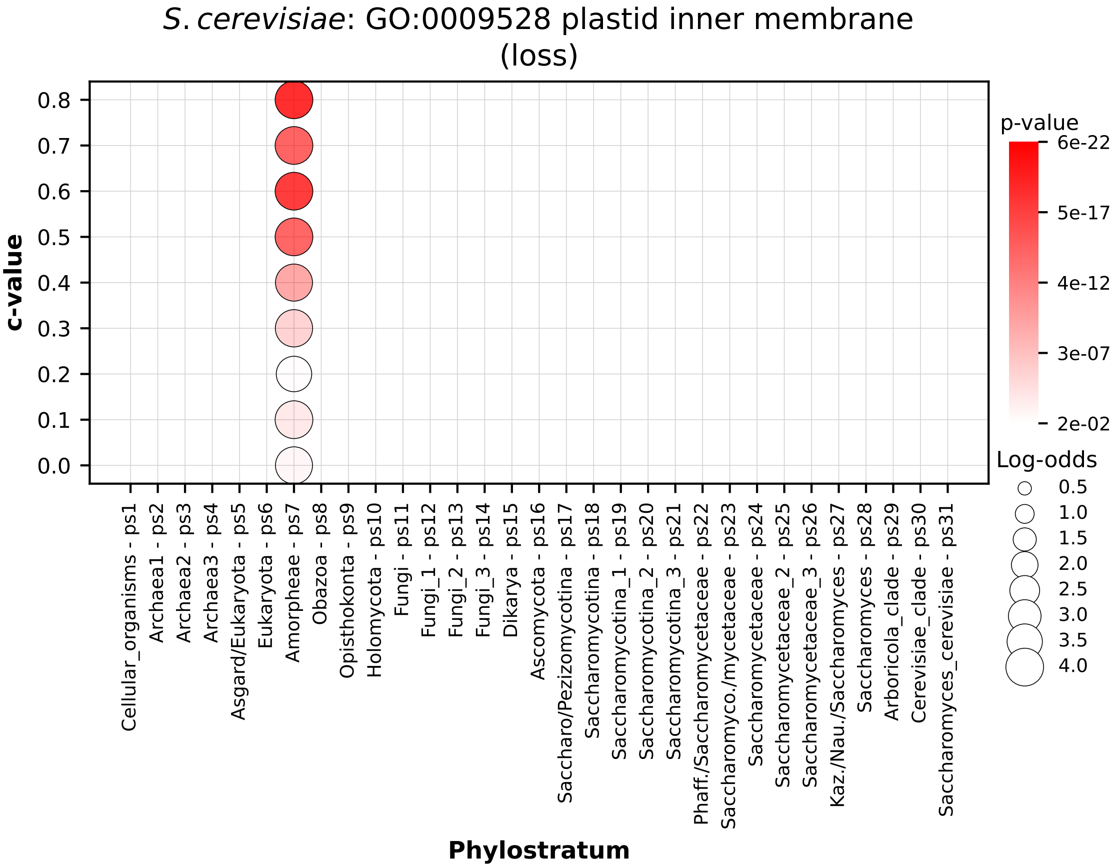

S. cerevisiae: GO:0009528 plastid inner membrane
(loss)
c-value
0.8
0.7
0.6
0.5
0.4
0.3
0.2
0.1
0.0
p-value
6e-22
5e-17
4e-12
3e-07
2e-02
Log-odds
0.5
1.0
1.5
2.0
2.5
3.0
3.5
4.0
Cellular_organisms - ps1
Archaea1 - ps2
Archaea2 - ps3
Archaea3 - ps4
Asgard/Eukaryota - ps5
Eukaryota - ps6
Amorpheae - ps7
Obazoa - ps8
Opisthokonta - ps9
Holomycota - ps10
Fungi - ps11
Fungi_1 - ps12
Fungi_2 - ps13
Fungi_3 - ps14
Dikarya - ps15
Ascomycota - ps16
Saccharo/Pezizomycotina - ps17
Saccharomycotina - ps18
Saccharomycotina_1 - ps19
Saccharomycotina_2 - ps20
Saccharomycotina_3 - ps21
Phaff./Saccharomycetaceae - ps22
Saccharomyco./mycetaceae - ps23
Saccharomycetaceae - ps24
Saccharomycetaceae_2 - ps25
Saccharomycetaceae_3 - ps26
Kaz./Nau./Saccharomyces - ps27
Saccharomyces - ps28
Arboricola_clade - ps29
Cerevisiae_clade - ps30
Saccharomyces_cerevisiae - ps31
Phylostratum

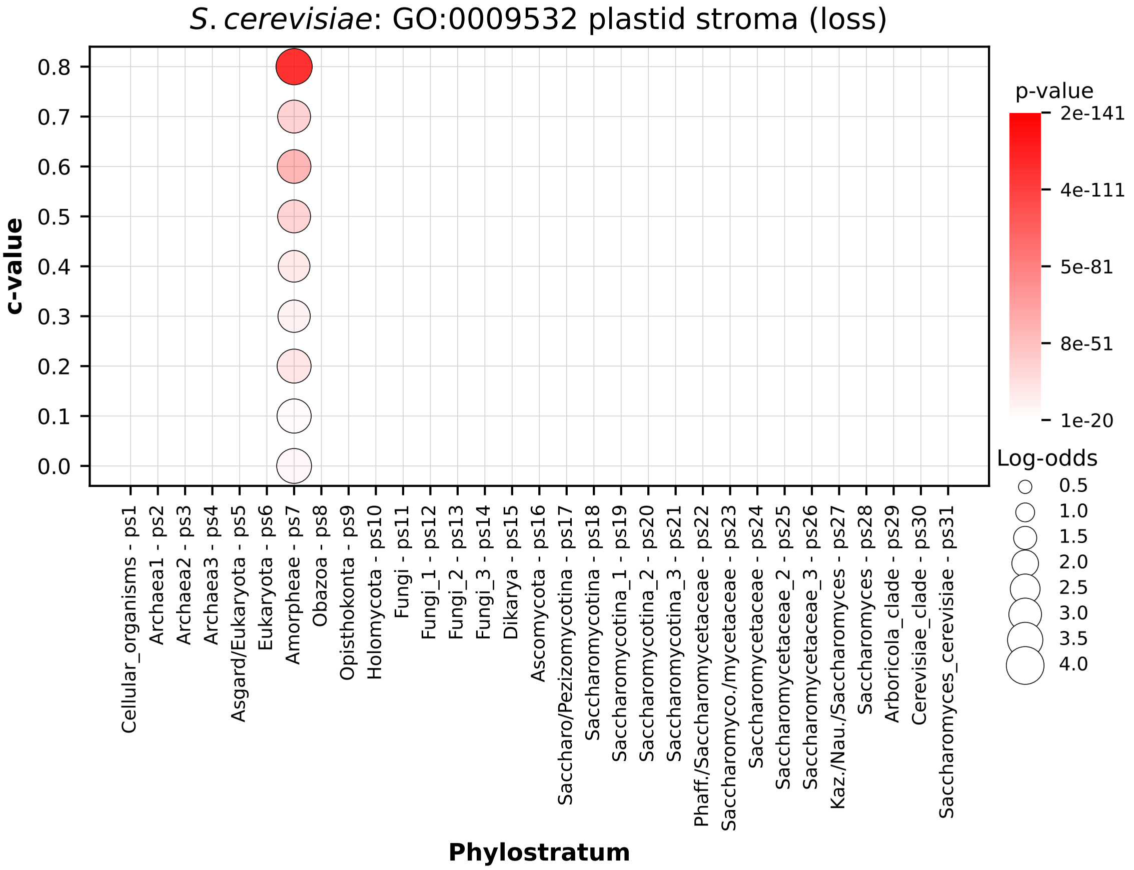
S. cerevisiae: GO:0009532 plastid stroma (loss)
c-value
0.8
0.7
0.6
0.5
0.4
0.3
0.2
0.1
0.0
p-value
2e-141
4e-111
5e-81
8e-51
1e-20
Log-odds
0.5
1.0
1.5
2.0
2.5
3.0
3.5
4.0
Cellular_organisms - ps1
Archaea1 - ps2
Archaea2 - ps3
Archaea3 - ps4
Asgard/Eukaryota - ps5
Eukaryota - ps6
Amorpheae - ps7
Obazoa - ps8
Opisthokonta - ps9
Holomycota - ps10
Fungi - ps11
Fungi_1 - ps12
Fungi_2 - ps13
Fungi_3 - ps14
Dikarya - ps15
Ascomycota - ps16
Saccharo/Pezizomycotina - ps17
Saccharomycotina - ps18
Saccharomycotina_1 - ps19
Saccharomycotina_2 - ps20
Saccharomycotina_3 - ps21
Phaff./Saccharomycetaceae - ps22
Saccharomyco./mycetaceae - ps23
Saccharomycetaceae - ps24
Saccharomycetaceae_2 - ps25
Saccharomycetaceae_3 - ps26
Kaz./Nau./Saccharomyces - ps27
Saccharomyces - ps28
Arboricola_clade - ps29
Cerevisiae_clade - ps30
Saccharomyces_cerevisiae - ps31
Phylostratum

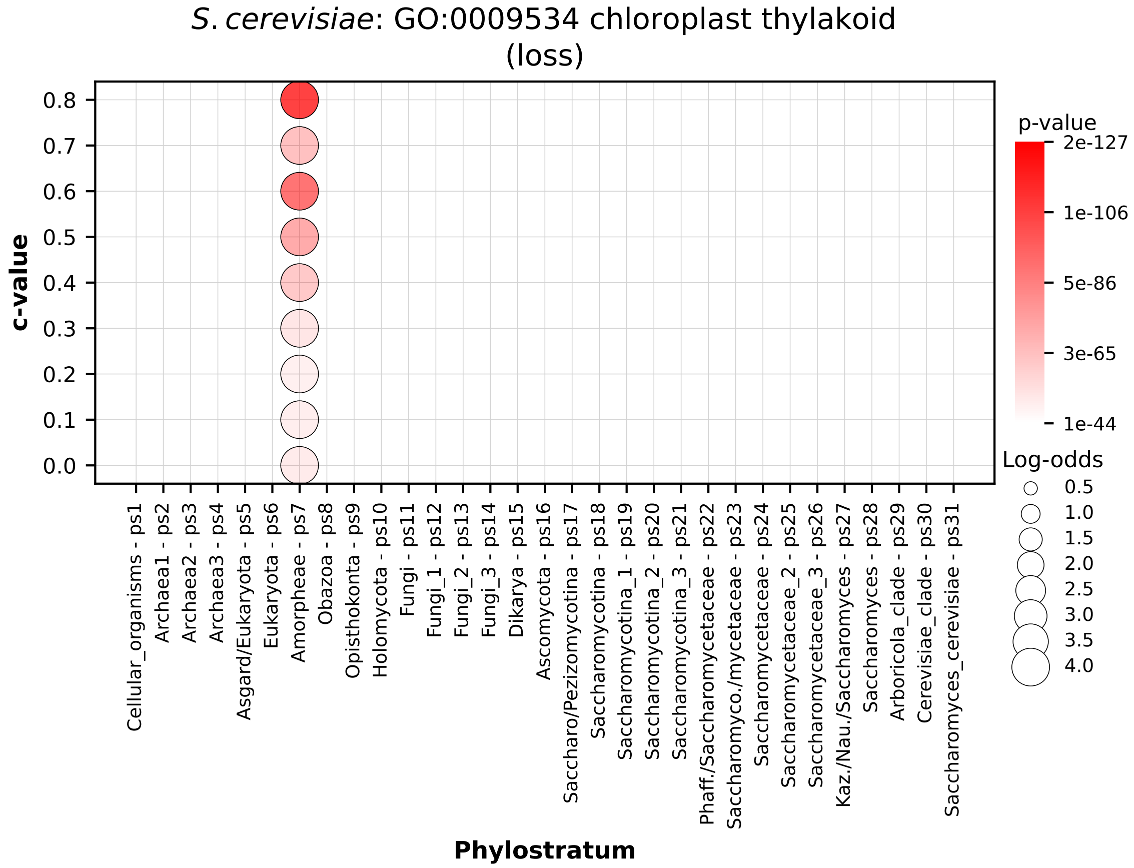

S. cerevisiae: GO:0009534 chloroplast thylakoid
(loss)
c-value
0.8
0.7
0.6
0.5
0.4
0.3
0.2
0.1
0.0
p-value
2e-127
1e-106
5e-86
3e-65
1e-44
Log-odds
0.5
1.0
1.5
2.0
2.5
3.0
3.5
4.0
Cellular_organisms - ps1
Archaea1 - ps2
Archaea2 - ps3
Archaea3 - ps4
Asgard/Eukaryota - ps5
Eukaryota - ps6
Amorpheae - ps7
Obazoa - ps8
Opisthokonta - ps9
Holomycota - ps10
Fungi - ps11
Fungi_1 - ps12
Fungi_2 - ps13
Fungi_3 - ps14
Dikarya - ps15
Ascomycota - ps16
Saccharo/Pezizomycotina - ps17
Saccharomycotina - ps18
Saccharomycotina_1 - ps19
Saccharomycotina_2 - ps20
Saccharomycotina_3 - ps21
Phaff./Saccharomycetaceae - ps22
Saccharomyco./mycetaceae - ps23
Saccharomycetaceae - ps24
Saccharomycetaceae_2 - ps25
Saccharomycetaceae_3 - ps26
Kaz./Nau./Saccharomyces - ps27
Saccharomyces - ps28
Arboricola_clade - ps29
Cerevisiae_clade - ps30
Saccharomyces_cerevisiae - ps31
Phylostratum

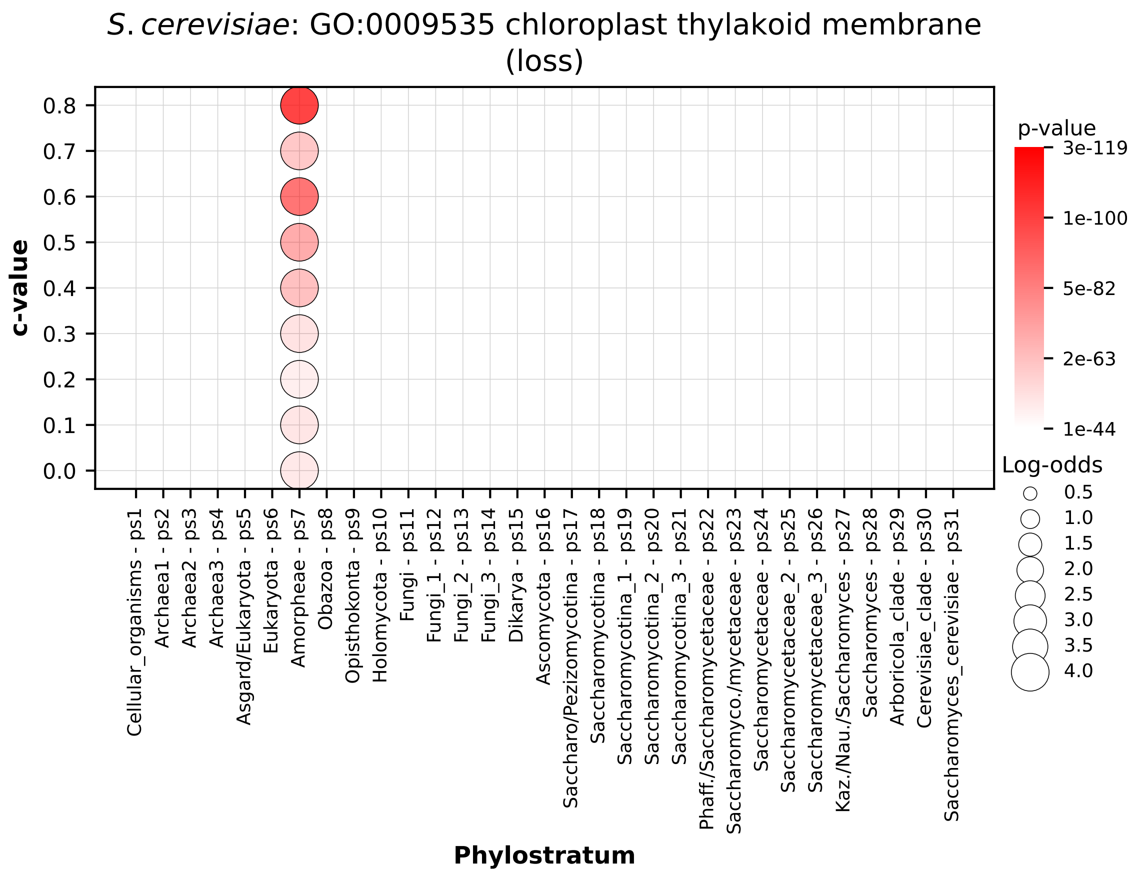
S. cerevisiae: GO:0009535 chloroplast thylakoid membrane
(loss)
c-value
0.0
0.1
0.2
0.3
0.4
0.5
0.6
0.7
0.8
p-value
3e-119
1e-100
5e-82
2e-63
1e-44
Log-odds
0.5
1.0
1.5
2.0
2.5
3.0
3.5
4.0
Cellular_organisms - ps1
Archaea1 - ps2
Archaea2 - ps3
Archaea3 - ps4
Asgard/Eukaryota - ps5
Eukaryota - ps6
Amorpheae - ps7
Obazoa - ps8
Opisthokonta - ps9
Holomycota - ps10
Fungi - ps11
Fungi_1 - ps12
Fungi_2 - ps13
Fungi_3 - ps14
Dikarya - ps15
Ascomycota - ps16
Saccharo/Pezizomycotina - ps17
Saccharomycotina - ps18
Saccharomycotina_1 - ps19
Saccharomycotina_2 - ps20
Saccharomycotina_3 - ps21
Phaff./Saccharomycetaceae - ps22
Saccharomyco./mycetaceae - ps23
Saccharomycetaceae - ps24
Saccharomycetaceae_2 - ps25
Saccharomycetaceae_3 - ps26
Kaz./Nau./Saccharomyces - ps27
Saccharomyces - ps28
Arboricola_clade - ps29
Cerevisiae_clade - ps30
Saccharomyces_cerevisiae - ps31
Phylostratum

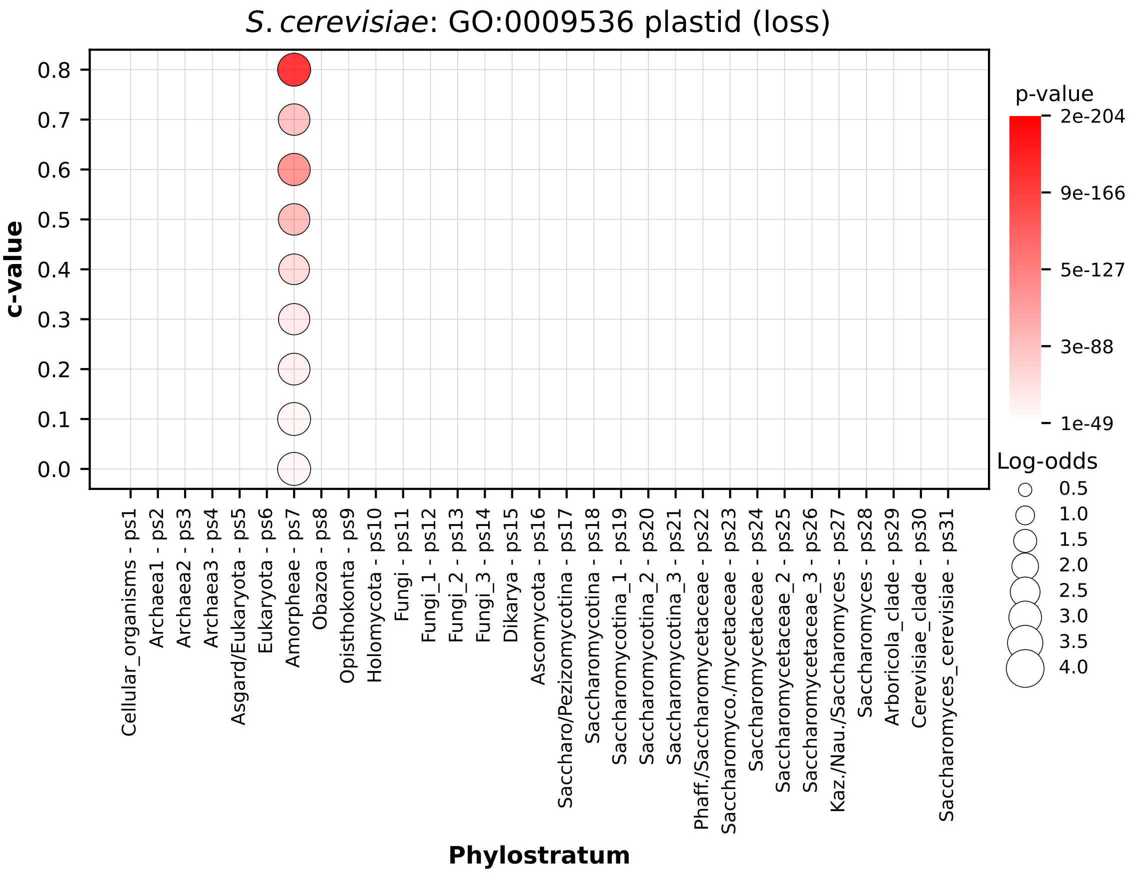

S. cerevisiae: GO:0009536 plastid (loss)
c-value
0.8
0.7
0.6
0.5
0.4
0.3
0.2
0.1
0.0
p-value
2e-204
9e-166
5e-127
3e-88
1e-49
Log-odds
0.5
1.0
1.5
2.0
2.5
3.0
3.5
4.0
Cellular_organisms - ps1
Archaea1 - ps2
Archaea2 - ps3
Archaea3 - ps4
Asgard/Eukaryota - ps5
Eukaryota - ps6
Amorpheae - ps7
Obazoa - ps8
Opisthokonta - ps9
Holomycota - ps10
Fungi - ps11
Fungi_1 - ps12
Fungi_2 - ps13
Fungi_3 - ps14
Dikarya - ps15
Ascomycota - ps16
Saccharo/Pezizomycotina - ps17
Saccharomycotina - ps18
Saccharomycotina_1 - ps19
Saccharomycotina_2 - ps20
Saccharomycotina_3 - ps21
Phaff./Saccharomycetaceae - ps22
Saccharomyco./mycetaceae - ps23
Saccharomycetaceae - ps24
Saccharomycetaceae_2 - ps25
Saccharomycetaceae_3 - ps26
Kaz./Nau./Saccharomyces - ps27
Saccharomyces - ps28
Arboricola_clade - ps29
Cerevisiae_clade - ps30
Saccharomyces_cerevisiae - ps31
Phylostratum

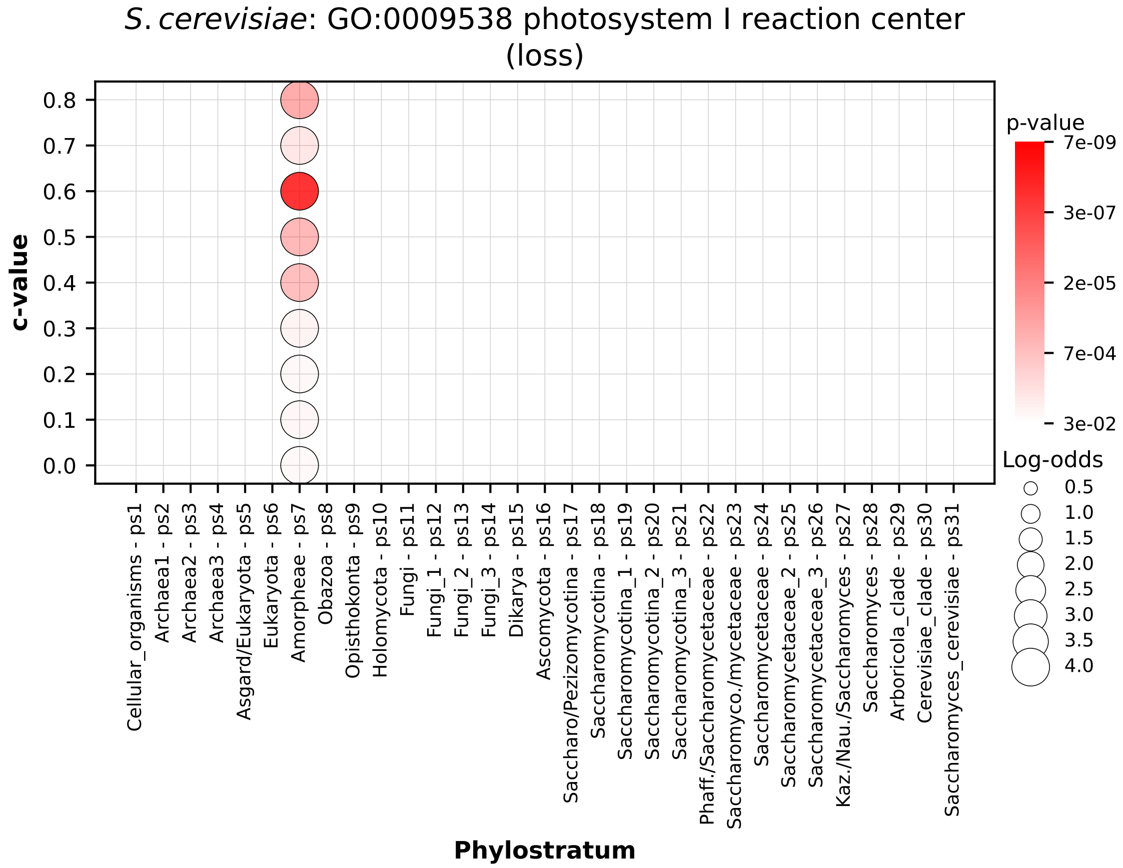

S. cerevisiae: GO:0009538 photosystem I reaction center
(loss)
c-value
0.8
0.7
0.6
0.5
0.4
0.3
0.2
0.1
0.0
p-value
7e-09
3e-07
2e-05
7e-04
3e-02
Log-odds
0.5
1.0
1.5
2.0
2.5
3.0
3.5
4.0
Cellular_organisms - ps1
Archaea1 - ps2
Archaea2 - ps3
Archaea3 - ps4
Asgard/Eukaryota - ps5
Eukaryota - ps6
Amorpheae - ps7
Obazoa - ps8
Opisthokonta - ps9
Holomycota - ps10
Fungi - ps11
Fungi_1 - ps12
Fungi_2 - ps13
Fungi_3 - ps14
Dikarya - ps15
Ascomycota - ps16
Saccharo/Pezizomycotina - ps17
Saccharomycotina - ps18
Saccharomycotina_1 - ps19
Saccharomycotina_2 - ps20
Saccharomycotina_3 - ps21
Phaff./Saccharomycetaceae - ps22
Saccharomyco./mycetaceae - ps23
Saccharomycetaceae - ps24
Saccharomycetaceae_2 - ps25
Saccharomycetaceae_3 - ps26
Kaz./Nau./Saccharomyces - ps27
Saccharomyces - ps28
Arboricola_clade - ps29
Cerevisiae_clade - ps30
Saccharomyces_cerevisiae - ps31
Phylostratum

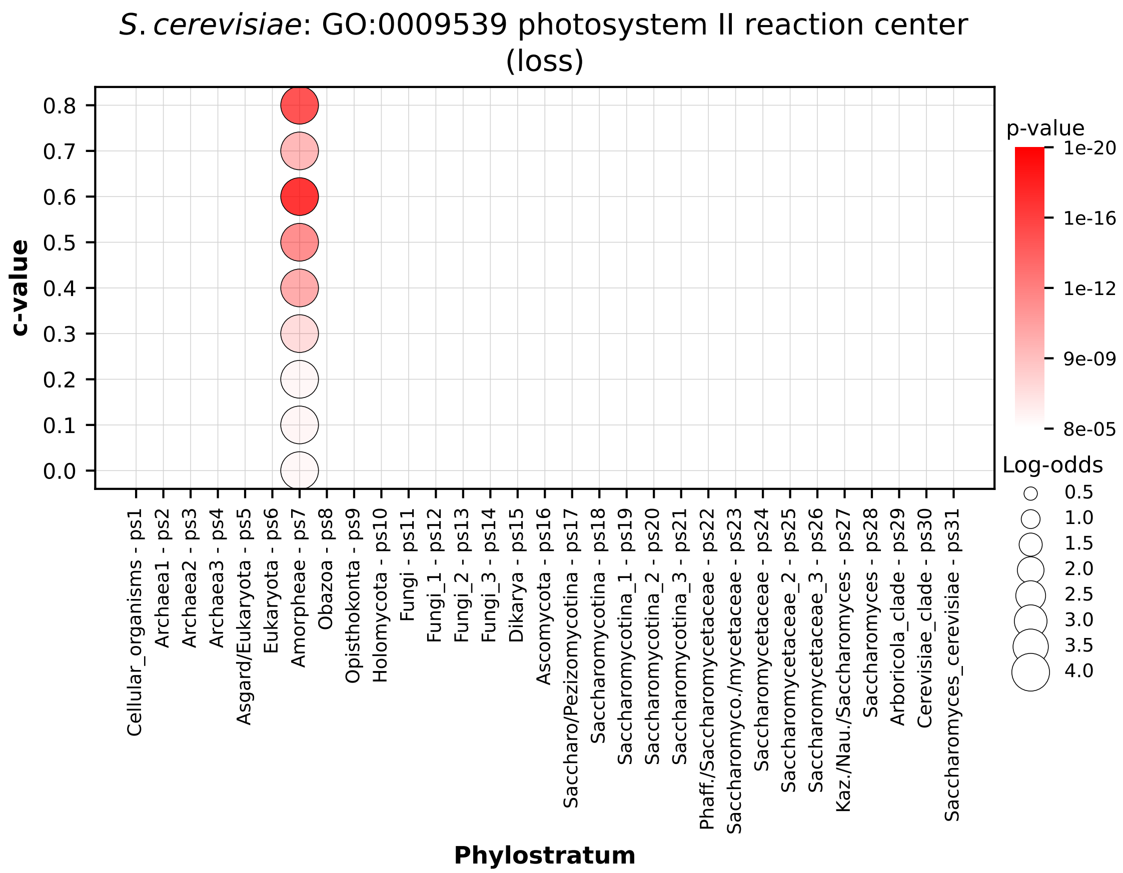
S. cerevisiae: GO:0009539 photosystem II reaction center (loss)
c-value
0.8
0.7
0.6
0.5
0.4
0.3
0.2
0.1
0.0
p-value
1e-20
1e-16
1e-12
9e-09
8e-05
Log-odds
0.5
1.0
1.5
2.0
2.5
3.0
3.5
4.0
Cellular_organisms - ps1
Archaea1 - ps2
Archaea2 - ps3
Archaea3 - ps4
Asgard/Eukaryota - ps5
Eukaryota - ps6
Amorpheae - ps7
Obazoa - ps8
Opisthokonta - ps9
Holomycota - ps10
Fungi - ps11
Fungi_1 - ps12
Fungi_2 - ps13
Fungi_3 - ps14
Dikarya - ps15
Ascomycota - ps16
Saccharo/Pezizomycotina - ps17
Saccharomycotina - ps18
Saccharomycotina_1 - ps19
Saccharomycotina_2 - ps20
Saccharomycotina_3 - ps21
Phaff./Saccharomycetaceae - ps22
Saccharomyco./mycetaceae - ps23
Saccharomycetaceae - ps24
Saccharomycetaceae_2 - ps25
Saccharomycetaceae_3 - ps26
Kaz./Nau./Saccharomyces - ps27
Saccharomyces - ps28
Arboricola_clade - ps29
Cerevisiae_clade - ps30
Saccharomyces_cerevisiae - ps31
Phylostratum

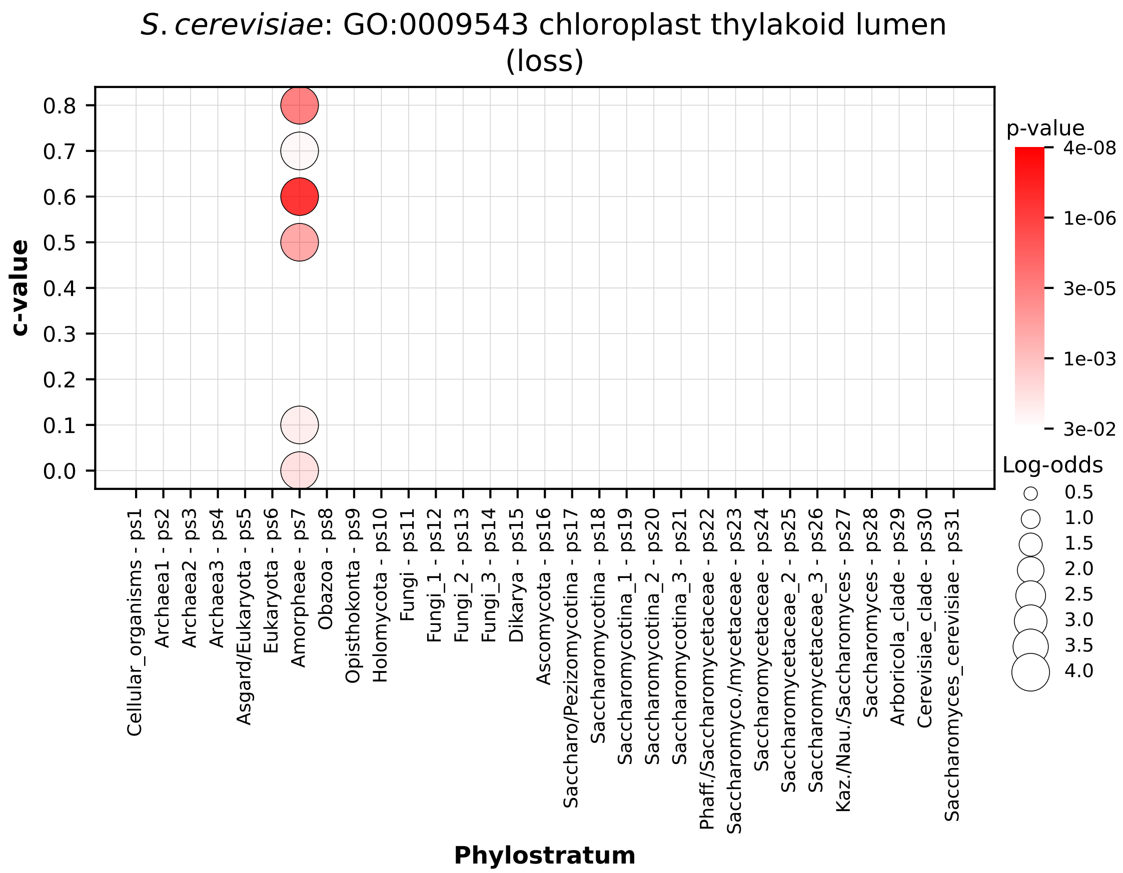
S. cerevisiae: GO:0009543 chloroplast thylakoid lumen
(loss)
c-value
0.8
0.7
0.6
0.5
0.4
0.3
0.2
0.1
0.0
p-value
4e-08
1e-06
3e-05
1e-03
3e-02
Log-odds
0.5
1.0
1.5
2.0
2.5
3.0
3.5
4.0
Cellular_organisms - ps1
Archaea1 - ps2
Archaea2 - ps3
Archaea3 - ps4
Asgard/Eukaryota - ps5
Eukaryota - ps6
Amorpheae - ps7
Obazoa - ps8
Opisthokonta - ps9
Holomycota - ps10
Fungi - ps11
Fungi_1 - ps12
Fungi_2 - ps13
Fungi_3 - ps14
Dikarya - ps15
Ascomycota - ps16
Saccharo/Pezizomycotina - ps17
Saccharomycotina - ps18
Saccharomycotina_1 - ps19
Saccharomycotina_2 - ps20
Saccharomycotina_3 - ps21
Phaff./Saccharomycetaceae - ps22
Saccharomyco./mycetaceae - ps23
Saccharomycetaceae - ps24
Saccharomycetaceae_2 - ps25
Saccharomycetaceae_3 - ps26
Kaz./Nau./Saccharomyces - ps27
Saccharomyces - ps28
Arboricola_clade - ps29
Cerevisiae_clade - ps30
Saccharomyces_cerevisiae - ps31
Phylostratum

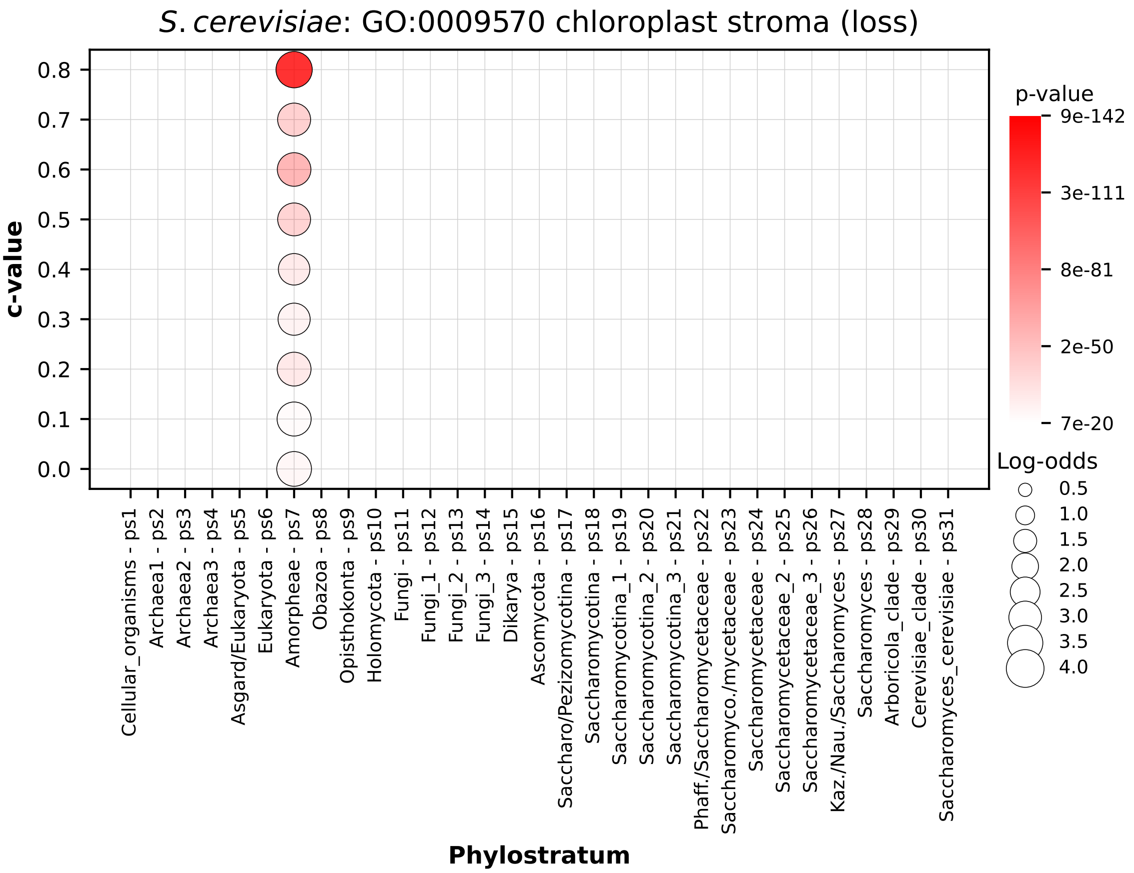

S. cerevisiae: GO:0009570 chloroplast stroma (loss)
c-value
0.0
0.1
0.2
0.3
0.4
0.5
0.6
0.7
0.8
p-value
9e-142
3e-111
8e-81
2e-50
7e-20
Log-odds
0.5
1.0
1.5
2.0
2.5
3.0
3.5
4.0
Cellular_organisms - ps1
Archaea1 - ps2
Archaea2 - ps3
Archaea3 - ps4
Asgard/Eukaryota - ps5
Eukaryota - ps6
Amorpheae - ps7
Obazoa - ps8
Opisthokonta - ps9
Holomycota - ps10
Fungi - ps11
Fungi_1 - ps12
Fungi_2 - ps13
Fungi_3 - ps14
Dikarya - ps15
Ascomycota - ps16
Saccharo/Pezizomycotina - ps17
Saccharomycotina - ps18
Saccharomycotina_1 - ps19
Saccharomycotina_2 - ps20
Saccharomycotina_3 - ps21
Phaff./Saccharomycetaceae - ps22
Saccharomyco./mycetaceae - ps23
Saccharomycetaceae - ps24
Saccharomycetaceae_2 - ps25
Saccharomycetaceae_3 - ps26
Kaz./Nau./Saccharomyces - ps27
Saccharomyces - ps28
Arboricola_clade - ps29
Cerevisiae_clade - ps30
Saccharomyces_cerevisiae - ps31
Phylostratum

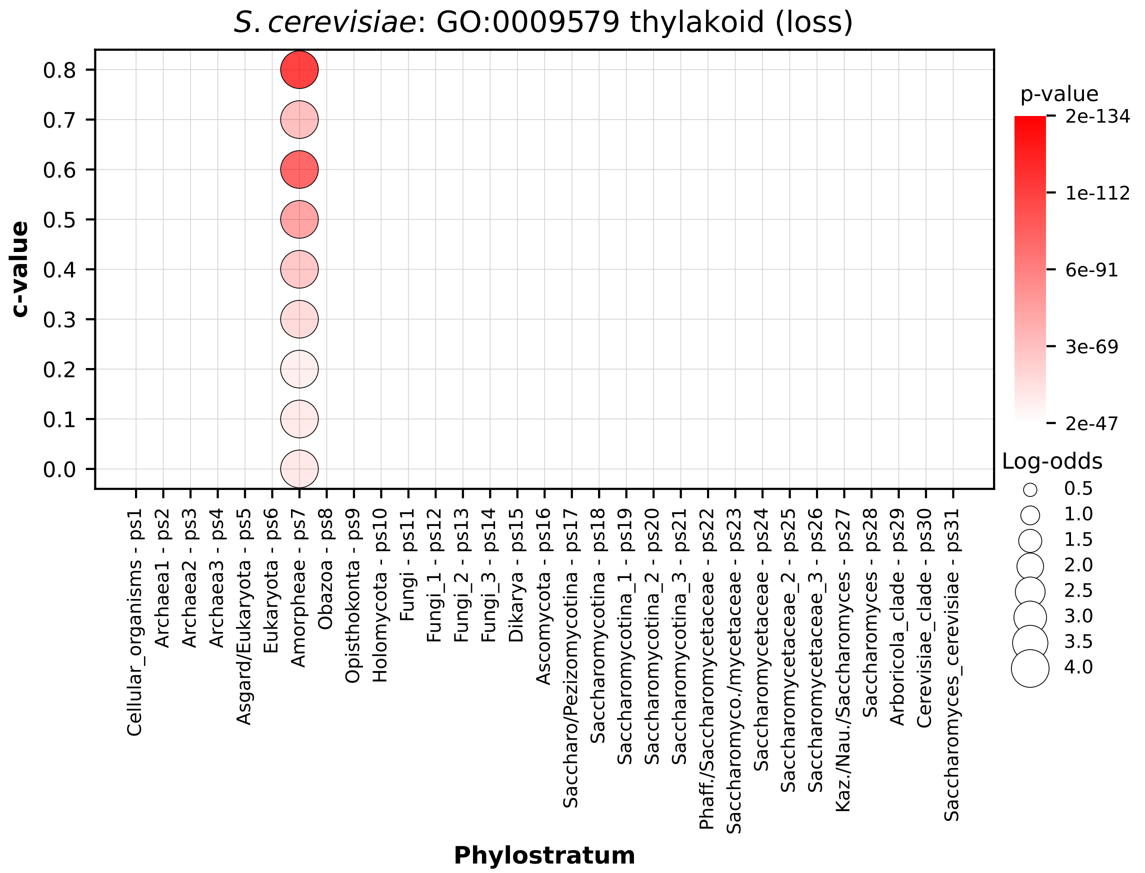

S. cerevisiae: GO:0009579 thylakoid (loss)
c-value
0.8
0.7
0.6
0.5
0.4
0.3
0.2
0.1
0.0
p-value
2e-134
1e-112
6e-91
3e-69
2e-47
Log-odds
0.5
1.0
1.5
2.0
2.5
3.0
3.5
4.0
Cellular_organisms - ps1
Archaea1 - ps2
Archaea2 - ps3
Archaea3 - ps4
Asgard/Eukaryota - ps5
Eukaryota - ps6
Amorpheae - ps7
Obazoa - ps8
Opisthokonta - ps9
Holomycota - ps10
Fungi - ps11
Fungi_1 - ps12
Fungi_2 - ps13
Fungi_3 - ps14
Dikarya - ps15
Ascomycota - ps16
Saccharo/Pezizomycotina - ps17
Saccharomycotina - ps18
Saccharomycotina_1 - ps19
Saccharomycotina_2 - ps20
Saccharomycotina_3 - ps21
Phaff./Saccharomycetaceae - ps22
Saccharomyco./mycetaceae - ps23
Saccharomycetaceae - ps24
Saccharomycetaceae_2 - ps25
Saccharomycetaceae_3 - ps26
Kaz./Nau./Saccharomyces - ps27
Saccharomyces - ps28
Arboricola_clade - ps29
Cerevisiae_clade - ps30
Saccharomyces_cerevisiae - ps31
Phylostratum

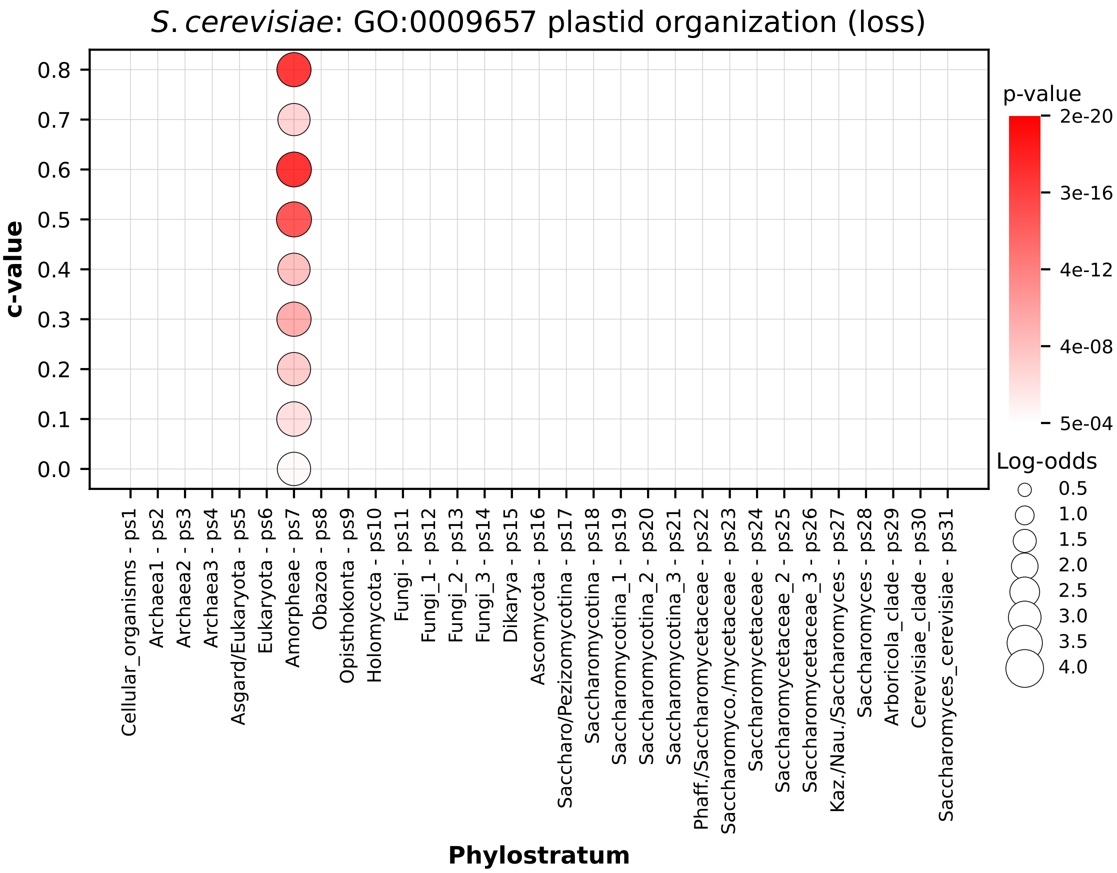
S. cerevisiae: GO:0009657 plastid organization (loss)
c-value
0.8
0.7
0.6
0.5
0.4
0.3
0.2
0.1
0.0
p-value
2e-20
3e-16
4e-12
4e-08
5e-04
Log-odds
0.5
1.0
1.5
2.0
2.5
3.0
3.5
4.0
Cellular_organisms - ps1
Archaea1 - ps2
Archaea2 - ps3
Archaea3 - ps4
Asgard/Eukaryota - ps5
Eukaryota - ps6
Amorpheae - ps7
Obazoa - ps8
Opisthokonta - ps9
Holomycota - ps10
Fungi - ps11
Fungi_1 - ps12
Fungi_2 - ps13
Fungi_3 - ps14
Dikarya - ps15
Ascomycota - ps16
Saccharo/Pezizomycotina - ps17
Saccharomycotina - ps18
Saccharomycotina_1 - ps19
Saccharomycotina_2 - ps20
Saccharomycotina_3 - ps21
Phaff./Saccharomycetaceae - ps22
Saccharomyco./mycetaceae - ps23
Saccharomycetaceae - ps24
Saccharomycetaceae_2 - ps25
Saccharomycetaceae_3 - ps26
Kaz./Nau./Saccharomyces - ps27
Saccharomyces - ps28
Arboricola_clade - ps29
Cerevisiae_clade - ps30
Saccharomyces_cerevisiae - ps31
Phylostratum

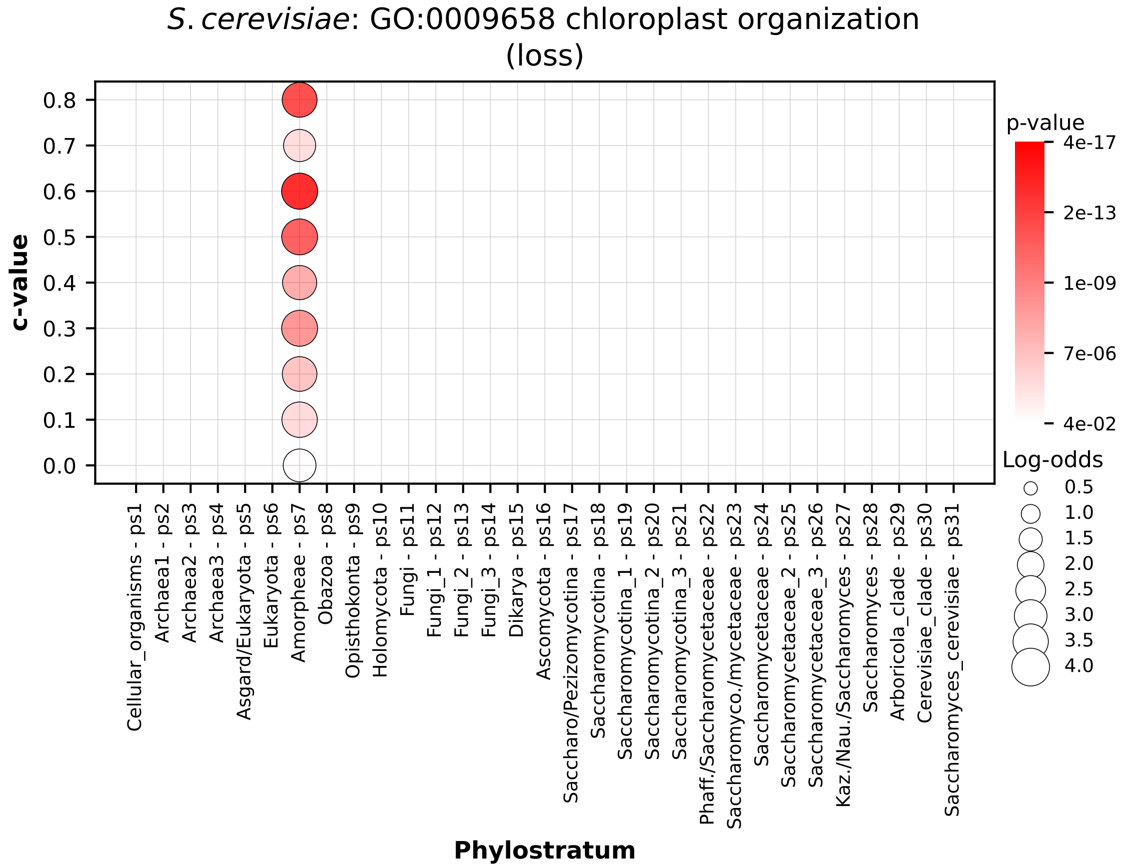
S. cerevisiae: GO:0009658 chloroplast organization
(loss)
c-value
0.8
0.7
0.6
0.5
0.4
0.3
0.2
0.1
0.0
p-value
4e-17
2e-13
1e-09
7e-06
4e-02
Log-odds
0.5
1.0
1.5
2.0
2.5
3.0
3.5
4.0
Cellular_organisms - ps1
Archaea1 - ps2
Archaea2 - ps3
Archaea3 - ps4
Asgard/Eukaryota - ps5
Eukaryota - ps6
Amorpheae - ps7
Obazoa - ps8
Opisthokonta - ps9
Holomycota - ps10
Fungi - ps11
Fungi_1 - ps12
Fungi_2 - ps13
Fungi_3 - ps14
Dikarya - ps15
Ascomycota - ps16
Saccharo/Pezizomycotina - ps17
Saccharomycotina - ps18
Saccharomycotina_1 - ps19
Saccharomycotina_2 - ps20
Saccharomycotina_3 - ps21
Phaff./Saccharomycetaceae - ps22
Saccharomyco./mycetaceae - ps23
Saccharomycetaceae - ps24
Saccharomycetaceae_2 - ps25
Saccharomycetaceae_3 - ps26
Kaz./Nau./Saccharomyces - ps27
Saccharomyces - ps28
Arboricola_clade - ps29
Cerevisiae_clade - ps30
Saccharomyces_cerevisiae - ps31
Phylostratum

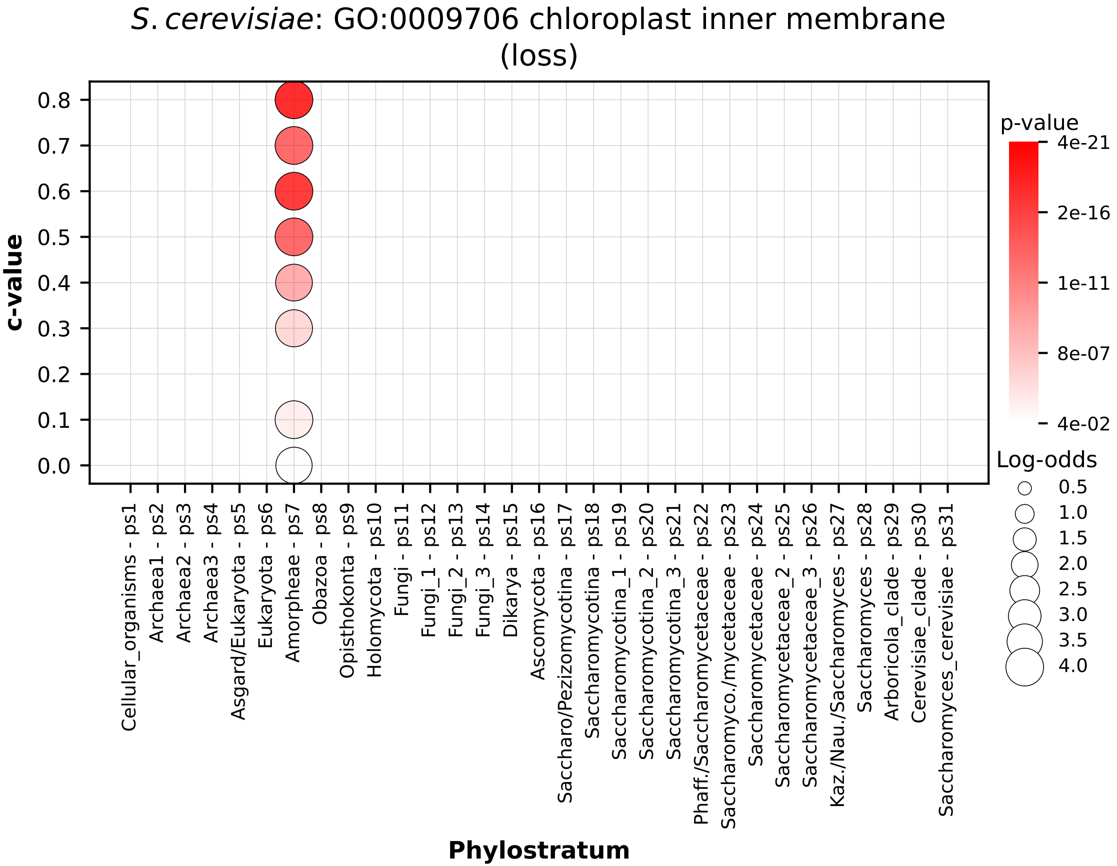
S. cerevisiae: GO:0009706 chloroplast inner membrane
(loss)
c-value
0.0
0.1
0.2
0.3
0.4
0.5
0.6
0.7
0.8
Phylostratum
Cellular_organisms - ps1
Archaea1 - ps2
Archaea2 - ps3
Archaea3 - ps4
Asgard/Eukaryota - ps5
Eukaryota - ps6
Amorpheae - ps7
Obazoa - ps8
Opisthokonta - ps9
Holomycota - ps10
Fungi - ps11
Fungi_1 - ps12
Fungi_2 - ps13
Fungi_3 - ps14
Dikarya - ps15
Ascomycota - ps16
Saccharo/Pezizomycotina - ps17
Saccharomycotina - ps18
Saccharomycotina_1 - ps19
Saccharomycotina_2 - ps20
Saccharomycotina_3 - ps21
Phaff./Saccharomycetaceae - ps22
Saccharomyco./mycetaceae - ps23
Saccharomycetaceae - ps24
Saccharomycetaceae_2 - ps25
Saccharomycetaceae_3 - ps26
Kaz./Nau./Saccharomyces - ps27
Saccharomyces - ps28
Arboricola_clade - ps29
Cerevisiae_clade - ps30
Saccharomyces_cerevisiae - ps31
p-value
4e-21
2e-16
1e-11
8e-07
4e-02
Log-odds
0.5
1.0
1.5
2.0
2.5
3.0
3.5
4.0

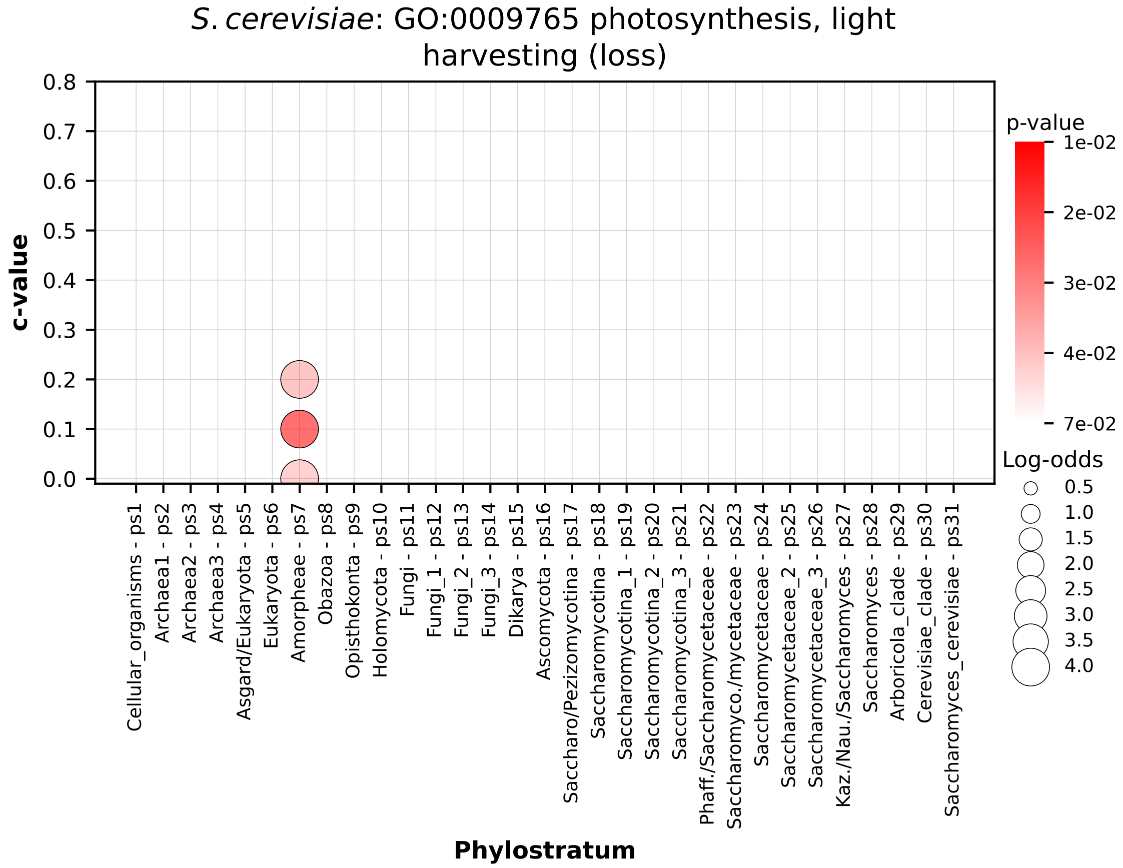
S. cerevisiae: GO:0009765 photosynthesis, light harvesting (loss)
c-value
0.8
0.7
0.6
0.5
0.4
0.3
0.2
0.1
0.0
p-value
1e-02
2e-02
3e-02
4e-02
7e-02
Log-odds
0.5
1.0
1.5
2.0
2.5
3.0
3.5
4.0
Cellular_organisms - ps1
Archaea1 - ps2
Archaea2 - ps3
Archaea3 - ps4
Asgard/Eukaryota - ps5
Eukaryota - ps6
Amorpheae - ps7
Obazoa - ps8
Opisthokonta - ps9
Holomycota - ps10
Fungi - ps11
Fungi_1 - ps12
Fungi_2 - ps13
Fungi_3 - ps14
Dikarya - ps15
Ascomycota - ps16
Saccharo/Pezizomycotina - ps17
Saccharomycotina - ps18
Saccharomycotina_1 - ps19
Saccharomycotina_2 - ps20
Saccharomycotina_3 - ps21
Phaff./Saccharomycetaceae - ps22
Saccharomyco./mycetaceae - ps23
Saccharomycetaceae - ps24
Saccharomycetaceae_2 - ps25
Saccharomycetaceae_3 - ps26
Kaz./Nau./Saccharomyces - ps27
Saccharomyces - ps28
Arboricola_clade - ps29
Cerevisiae_clade - ps30
Saccharomyces_cerevisiae - ps31
Phylostratum

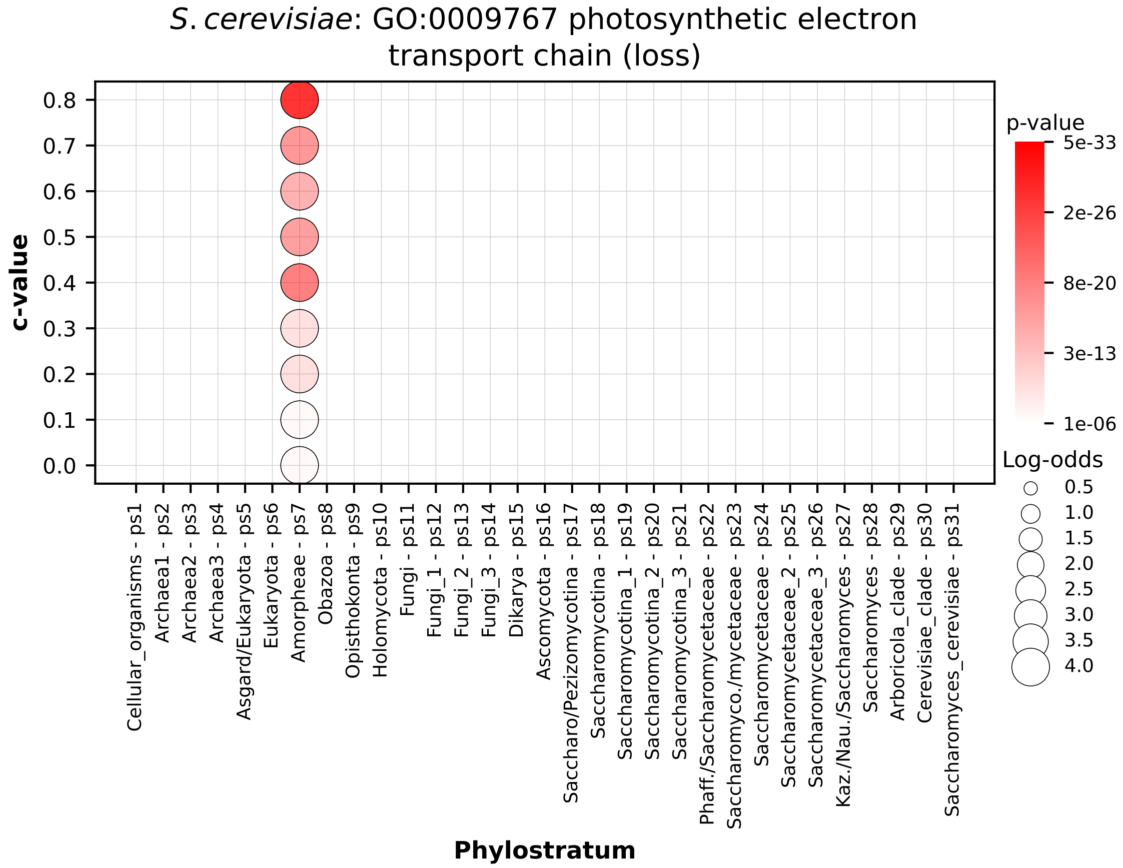

S. cerevisiae: GO:0009767 photosynthetic electron transport chain (loss)
c-value
0.8
0.7
0.6
0.5
0.4
0.3
0.2
0.1
0.0
p-value
5e-33
2e-26
8e-20
3e-13
1e-06
Log-odds
0.5
1.0
1.5
2.0
2.5
3.0
3.5
4.0
Cellular_organisms - ps1
Archaea1 - ps2
Archaea2 - ps3
Archaea3 - ps4
Asgard/Eukaryota - ps5
Eukaryota - ps6
Amorpheae - ps7
Obazoa - ps8
Opisthokonta - ps9
Holomycota - ps10
Fungi - ps11
Fungi_1 - ps12
Fungi_2 - ps13
Fungi_3 - ps14
Dikarya - ps15
Ascomycota - ps16
Saccharo/Pezizomycotina - ps17
Saccharomycotina - ps18
Saccharomycotina_1 - ps19
Saccharomycotina_2 - ps20
Saccharomycotina_3 - ps21
Phaff./Saccharomycetaceae - ps22
Saccharomyco./mycetaceae - ps23
Saccharomycetaceae - ps24
Saccharomycetaceae_2 - ps25
Saccharomycetaceae_3 - ps26
Kaz./Nau./Saccharomyces - ps27
Saccharomyces - ps28
Arboricola_clade - ps29
Cerevisiae_clade - ps30
Saccharomyces_cerevisiae - ps31
Phylostratum

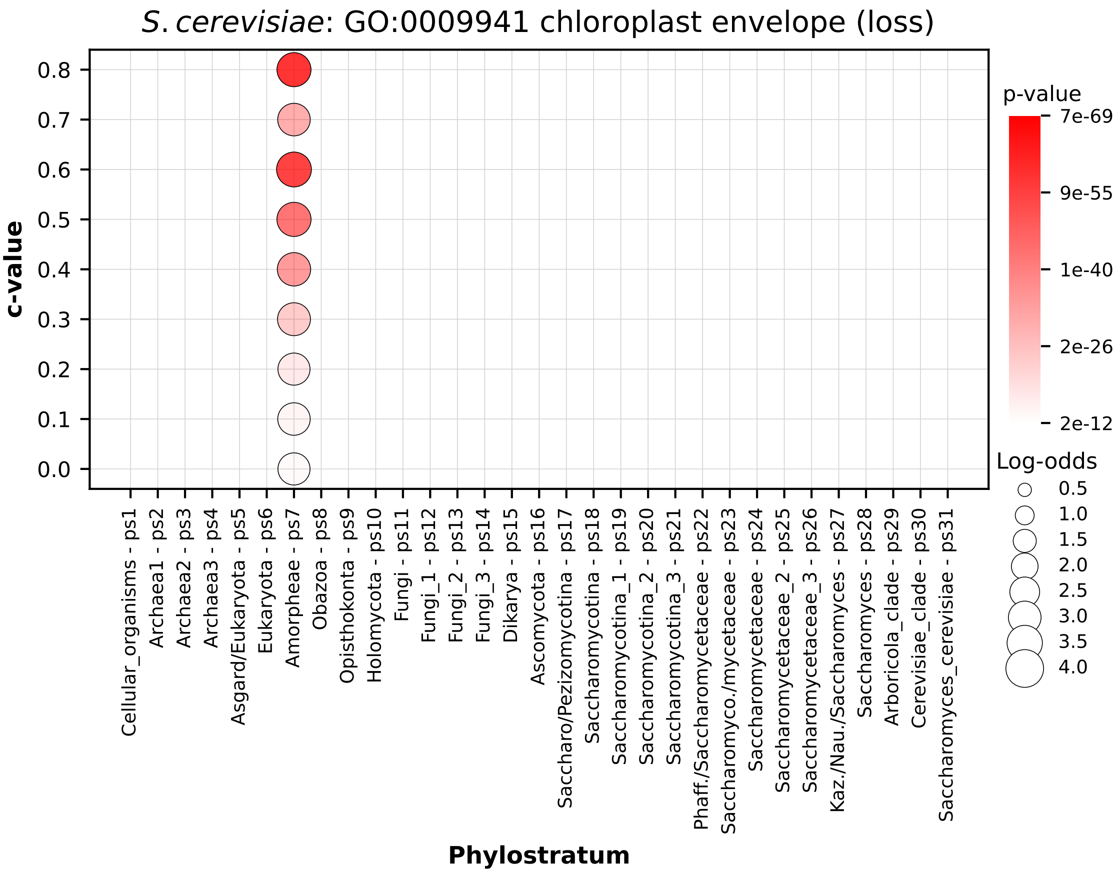

S. cerevisiae: GO:0009941 chloroplast envelope (loss)
c-value
0.8
0.7
0.6
0.5
0.4
0.3
0.2
0.1
0.0
p-value
7e-69
9e-55
1e-40
2e-26
2e-12
Log-odds
0.5
1.0
1.5
2.0
2.5
3.0
3.5
4.0
Cellular_organisms - ps1
Archaea1 - ps2
Archaea2 - ps3
Archaea3 - ps4
Asgard/Eukaryota - ps5
Eukaryota - ps6
Amorpheae - ps7
Obazoa - ps8
Opisthokonta - ps9
Holomycota - ps10
Fungi - ps11
Fungi_1 - ps12
Fungi_2 - ps13
Fungi_3 - ps14
Dikarya - ps15
Ascomycota - ps16
Saccharo/Pezizomycotina - ps17
Saccharomycotina - ps18
Saccharomycotina_1 - ps19
Saccharomycotina_2 - ps20
Saccharomycotina_3 - ps21
Phaff./Saccharomycetaceae - ps22
Saccharomyco./mycetaceae - ps23
Saccharomycetaceae - ps24
Saccharomycetaceae_2 - ps25
Saccharomycetaceae_3 - ps26
Kaz./Nau./Saccharomyces - ps27
Saccharomyces - ps28
Arboricola_clade - ps29
Cerevisiae_clade - ps30
Saccharomyces_cerevisiae - ps31
Phylostratum

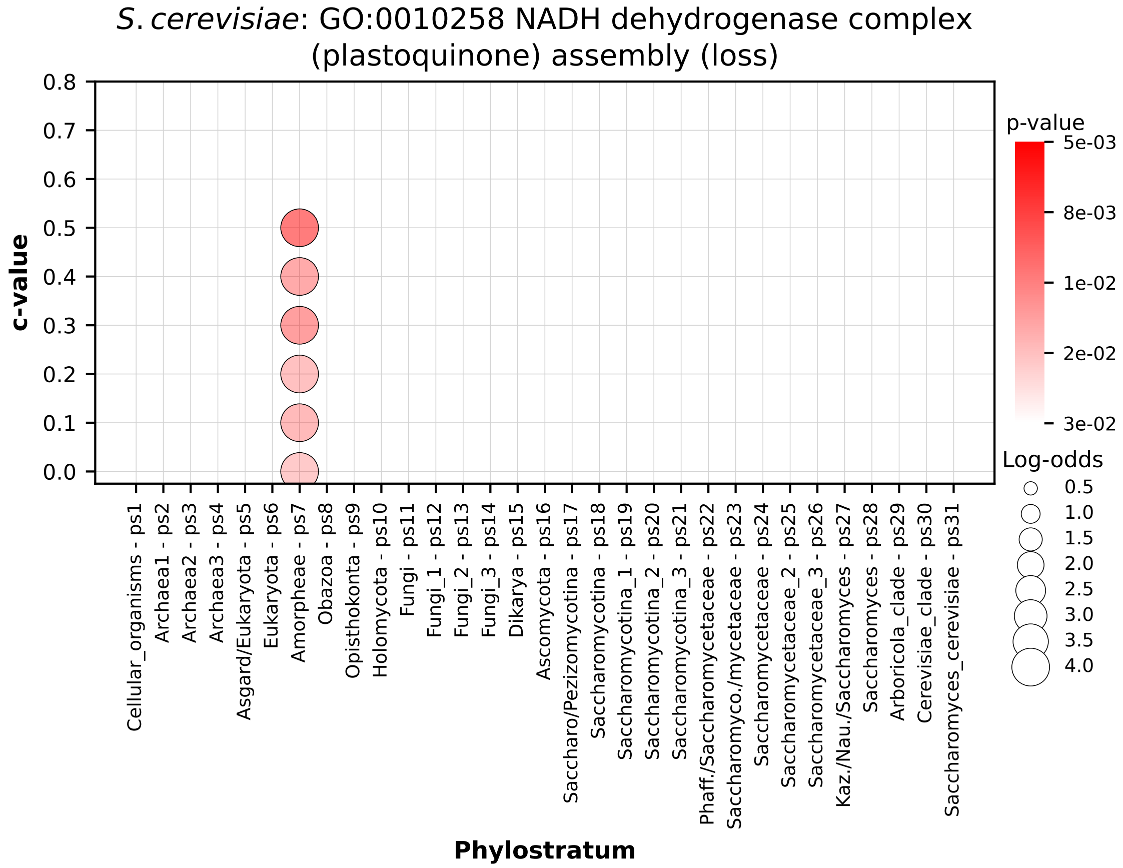

S. cerevisiae: GO:0010258 NADH dehydrogenase complex (plastoquinone) assembly (loss)
c-value
0.8
0.7
0.6
0.5
0.4
0.3
0.2
0.1
0.0
Phylostratum
Cellular_organisms - ps1
Archaea1 - ps2
Archaea2 - ps3
Archaea3 - ps4
Asgard/Eukaryota - ps5
Eukaryota - ps6
Amorpheae - ps7
Obazoa - ps8
Opisthokonta - ps9
Holomycota - ps10
Fungi - ps11
Fungi_1 - ps12
Fungi_2 - ps13
Fungi_3 - ps14
Dikarya - ps15
Ascomycota - ps16
Saccharo/Pezizomycotina - ps17
Saccharomycotina - ps18
Saccharomycotina_1 - ps19
Saccharomycotina_2 - ps20
Saccharomycotina_3 - ps21
Phaff./Saccharomycetaceae - ps22
Saccharomyco./mycetaceae - ps23
Saccharomycetaceae - ps24
Saccharomycetaceae_2 - ps25
Saccharomycetaceae_3 - ps26
Kaz./Nau./Saccharomyces - ps27
Saccharomyces - ps28
Arboricola_clade - ps29
Cerevisiae_clade - ps30
Saccharomyces_cerevisiae - ps31
p-value
5e-03
8e-03
1e-02
2e-02
3e-02
Log-odds
0.5
1.0
1.5
2.0
2.5
3.0
3.5
4.0

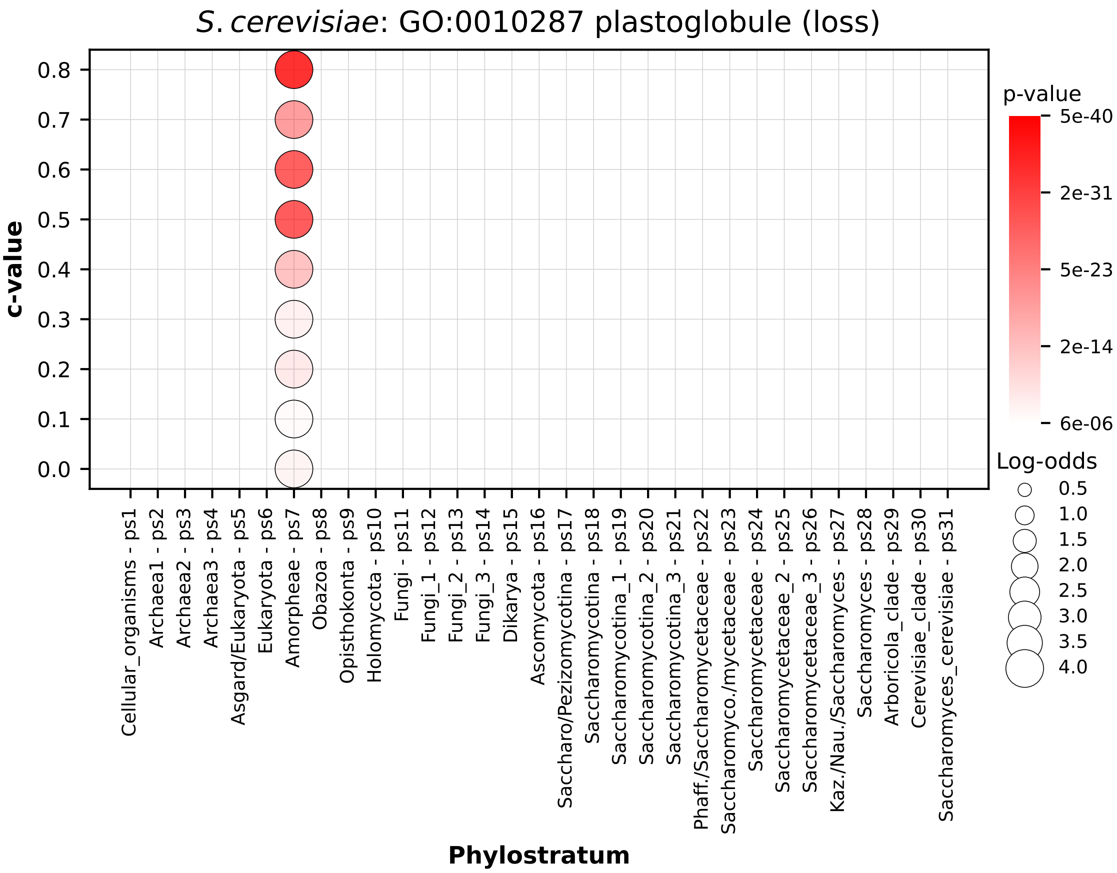

S. cerevisiae: GO:0010287 plastoglobule (loss)
c-value
0.8
0.7
0.6
0.5
0.4
0.3
0.2
0.1
0.0
p-value
5e-40
2e-31
5e-23
2e-14
6e-06
Log-odds
0.5
1.0
1.5
2.0
2.5
3.0
3.5
4.0
Cellular_organisms - ps1
Archaea1 - ps2
Archaea2 - ps3
Archaea3 - ps4
Asgard/Eukaryota - ps5
Eukaryota - ps6
Amorpheae - ps7
Obazoa - ps8
Opisthokonta - ps9
Holomycota - ps10
Fungi - ps11
Fungi_1 - ps12
Fungi_2 - ps13
Fungi_3 - ps14
Dikarya - ps15
Ascomycota - ps16
Saccharo/Pezizomycotina - ps17
Saccharomycotina - ps18
Saccharomycotina_1 - ps19
Saccharomycotina_2 - ps20
Saccharomycotina_3 - ps21
Phaff./Saccharomycetaceae - ps22
Saccharomyco./mycetaceae - ps23
Saccharomycetaceae - ps24
Saccharomycetaceae_2 - ps25
Saccharomycetaceae_3 - ps26
Kaz./Nau./Saccharomyces - ps27
Saccharomyces - ps28
Arboricola_clade - ps29
Cerevisiae_clade - ps30
Saccharomyces_cerevisiae - ps31
Phylostratum

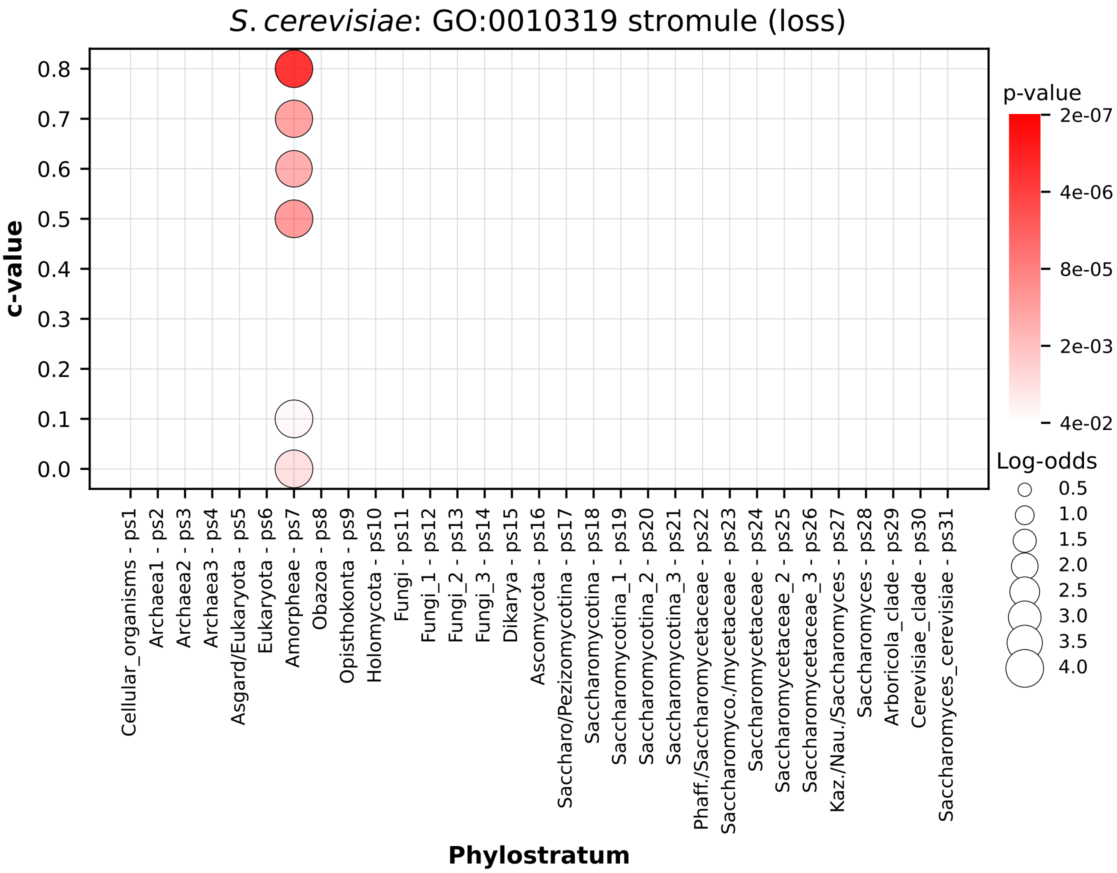

S. cerevisiae: GO:0010319 stromule (loss)
c-value
0.8
0.7
0.6
0.5
0.4
0.3
0.2
0.1
0.0
p-value
2e-07
4e-06
8e-05
2e-03
4e-02
Log-odds
0.5
1.0
1.5
2.0
2.5
3.0
3.5
4.0
Cellular_organisms - ps1
Archaea1 - ps2
Archaea2 - ps3
Archaea3 - ps4
Asgard/Eukaryota - ps5
Eukaryota - ps6
Amorpheae - ps7
Obazoa - ps8
Opisthokonta - ps9
Holomycota - ps10
Fungi - ps11
Fungi_1 - ps12
Fungi_2 - ps13
Fungi_3 - ps14
Dikarya - ps15
Ascomycota - ps16
Saccharo/Pezizomycotina - ps17
Saccharomycotina - ps18
Saccharomycotina_1 - ps19
Saccharomycotina_2 - ps20
Saccharomycotina_3 - ps21
Phaff./Saccharomycetaceae - ps22
Saccharomyco./mycetaceae - ps23
Saccharomycetaceae - ps24
Saccharomycetaceae_2 - ps25
Saccharomycetaceae_3 - ps26
Kaz./Nau./Saccharomyces - ps27
Saccharomyces - ps28
Arboricola_clade - ps29
Cerevisiae_clade - ps30
Saccharomyces_cerevisiae - ps31
Phylostratum

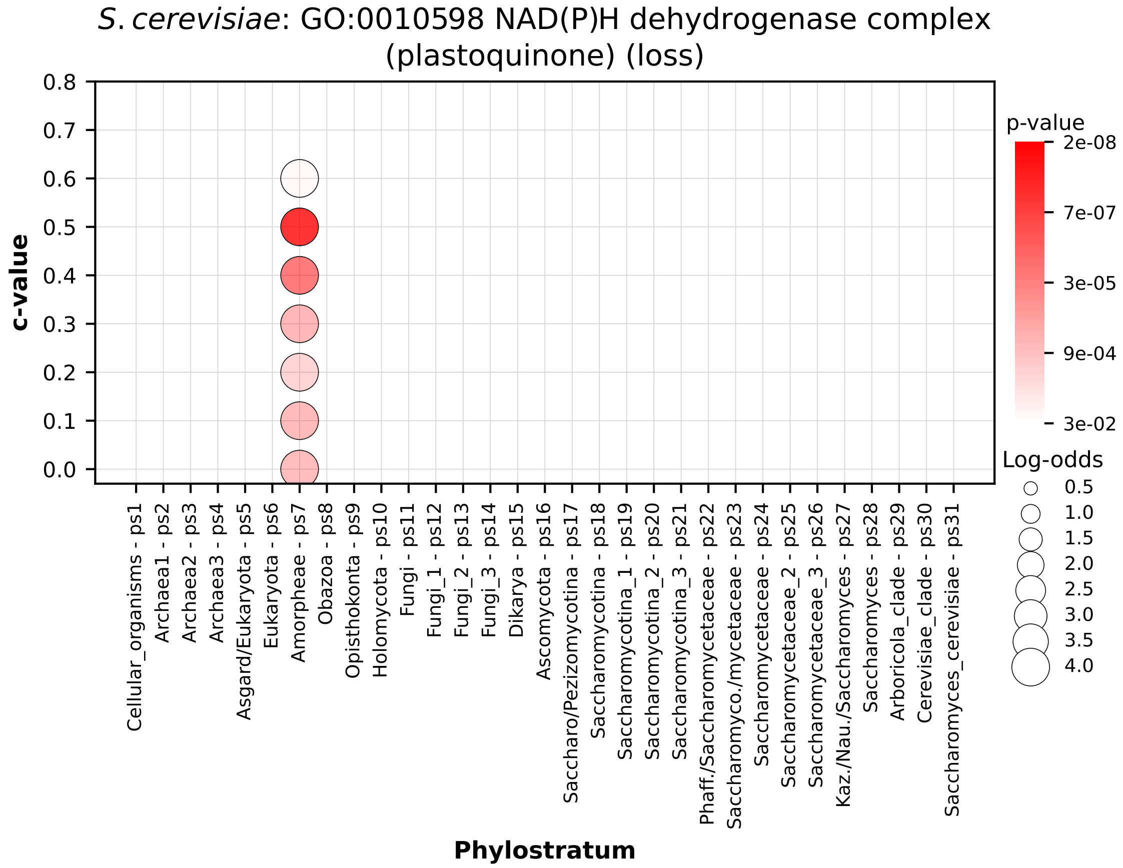

S. cerevisiae: GO:0010598 NAD(P)H dehydrogenase complex (plastoquinone) (loss)
c-value
0.8
0.7
0.6
0.5
0.4
0.3
0.2
0.1
0.0
p-value
2e-08
7e-07
3e-05
9e-04
3e-02
Log-odds
0.5
1.0
1.5
2.0
2.5
3.0
3.5
4.0
Cellular_organisms - ps1
Archaea1 - ps2
Archaea2 - ps3
Archaea3 - ps4
Asgard/Eukaryota - ps5
Eukaryota - ps6
Amorpheae - ps7
Obazoa - ps8
Opisthokonta - ps9
Holomycota - ps10
Fungi - ps11
Fungi_1 - ps12
Fungi_2 - ps13
Fungi_3 - ps14
Dikarya - ps15
Ascomycota - ps16
Saccharo/Pezizomycotina - ps17
Saccharomycotina - ps18
Saccharomycotina_1 - ps19
Saccharomycotina_2 - ps20
Saccharomycotina_3 - ps21
Phaff./Saccharomycetaceae - ps22
Saccharomyco./mycetaceae - ps23
Saccharomycetaceae - ps24
Saccharomycetaceae_2 - ps25
Saccharomycetaceae_3 - ps26
Kaz./Nau./Saccharomyces - ps27
Saccharomyces - ps28
Arboricola_clade - ps29
Cerevisiae_clade - ps30
Saccharomyces_cerevisiae - ps31
Phylostratum

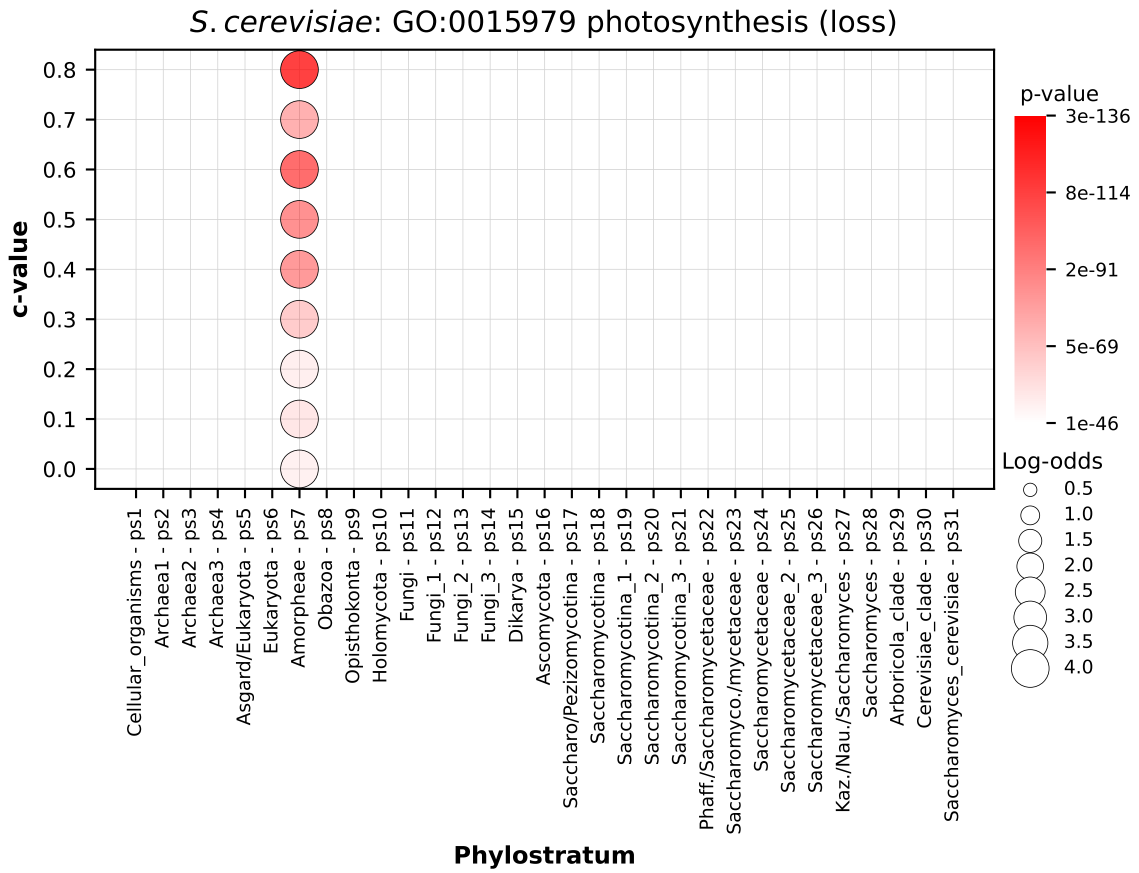

S. cerevisiae: GO:0015979 photosynthesis (loss)
c-value
0.0
0.1
0.2
0.3
0.4
0.5
0.6
0.7
0.8
p-value
3e-136
8e-114
2e-91
5e-69
1e-46
Log-odds
0.5
1.0
1.5
2.0
2.5
3.0
3.5
4.0
Cellular_organisms - ps1
Archaea1 - ps2
Archaea2 - ps3
Archaea3 - ps4
Asgard/Eukaryota - ps5
Eukaryota - ps6
Amorpheae - ps7
Obazoa - ps8
Opisthokonta - ps9
Holomycota - ps10
Fungi - ps11
Fungi_1 - ps12
Fungi_2 - ps13
Fungi_3 - ps14
Dikarya - ps15
Ascomycota - ps16
Saccharo/Pezizomycotina - ps17
Saccharomycotina - ps18
Saccharomycotina_1 - ps19
Saccharomycotina_2 - ps20
Saccharomycotina_3 - ps21
Phaff./Saccharomycetaceae - ps22
Saccharomyco./mycetaceae - ps23
Saccharomycetaceae - ps24
Saccharomycetaceae_2 - ps25
Saccharomycetaceae_3 - ps26
Kaz./Nau./Saccharomyces - ps27
Saccharomyces - ps28
Arboricola_clade - ps29
Cerevisiae_clade - ps30
Saccharomyces_cerevisiae - ps31
Phylostratum

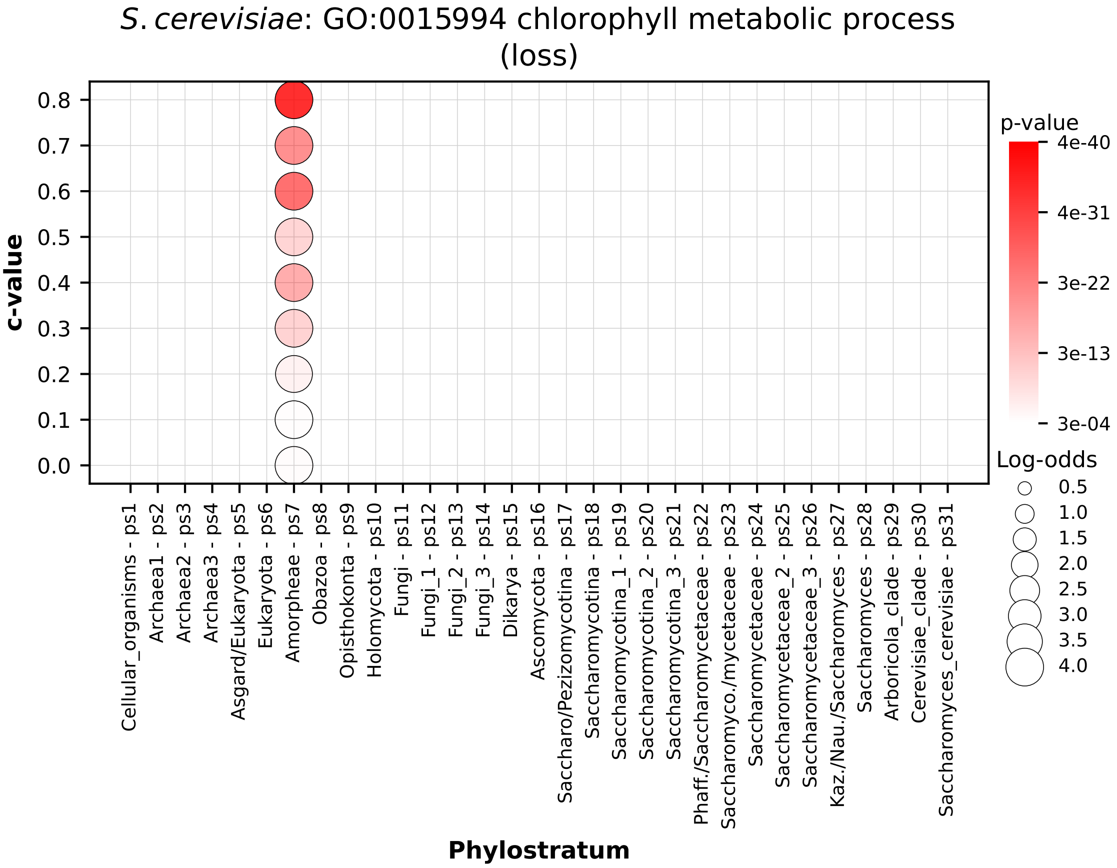

S. cerevisiae: GO:0015994 chlorophyll metabolic process
(loss)
c-value
0.8
0.7
0.6
0.5
0.4
0.3
0.2
0.1
0.0
p-value
4e-40
4e-31
3e-22
3e-13
3e-04
Log-odds
0.5
1.0
1.5
2.0
2.5
3.0
3.5
4.0
Cellular_organisms - ps1
Archaea1 - ps2
Archaea2 - ps3
Archaea3 - ps4
Asgard/Eukaryota - ps5
Eukaryota - ps6
Amorpheae - ps7
Obazoa - ps8
Opisthokonta - ps9
Holomycota - ps10
Fungi - ps11
Fungi_1 - ps12
Fungi_2 - ps13
Fungi_3 - ps14
Dikarya - ps15
Ascomycota - ps16
Saccharo/Pezizomycotina - ps17
Saccharomycotina - ps18
Saccharomycotina_1 - ps19
Saccharomycotina_2 - ps20
Saccharomycotina_3 - ps21
Phaff./Saccharomycetaceae - ps22
Saccharomyco./mycetaceae - ps23
Saccharomycetaceae - ps24
Saccharomycetaceae_2 - ps25
Saccharomycetaceae_3 - ps26
Kaz./Nau./Saccharomyces - ps27
Saccharomyces - ps28
Arboricola_clade - ps29
Cerevisiae_clade - ps30
Saccharomyces_cerevisiae - ps31
Phylostratum

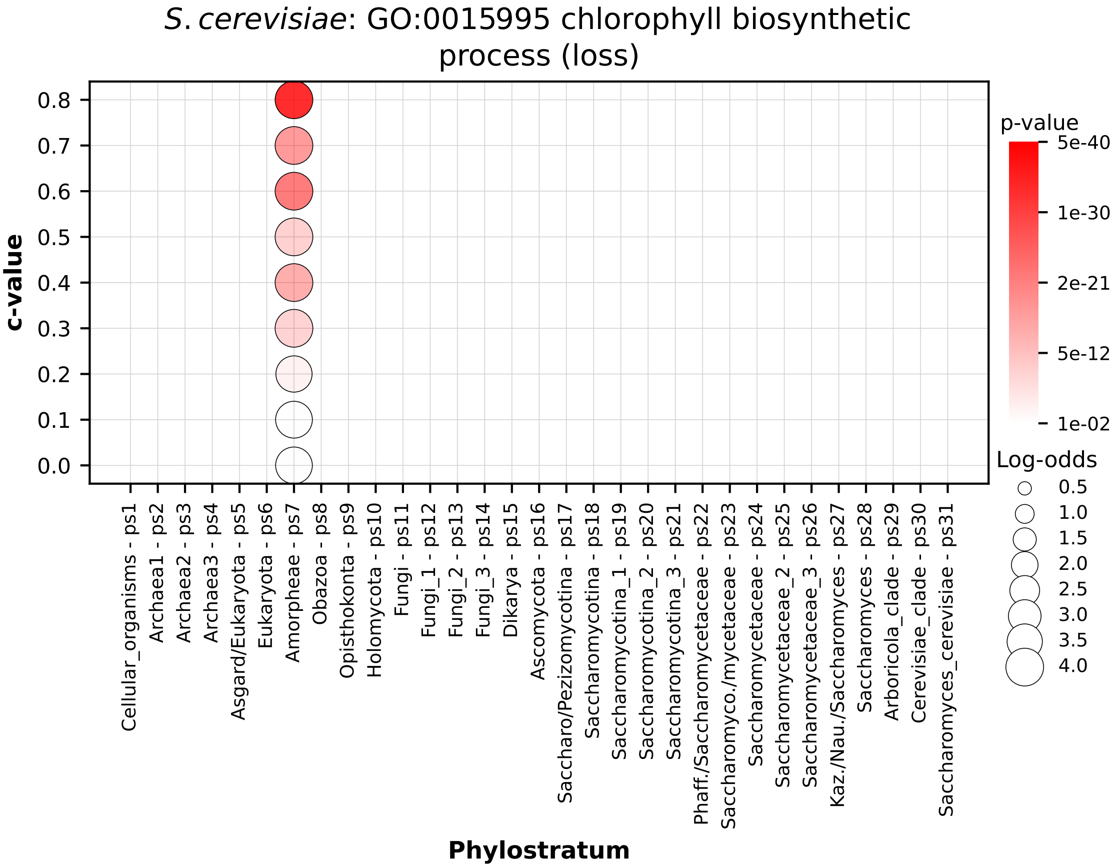
S. cerevisiae: GO:0015995 chlorophyll biosynthetic process (loss)
c-value
0.8
0.7
0.6
0.5
0.4
0.3
0.2
0.1
0.0
p-value
5e-40
1e-30
2e-21
5e-12
1e-02
Log-odds
0.5
1.0
1.5
2.0
2.5
3.0
3.5
4.0
Cellular_organisms - ps1
Archaea1 - ps2
Archaea2 - ps3
Archaea3 - ps4
Asgard/Eukaryota - ps5
Eukaryota - ps6
Amorpheae - ps7
Obazoa - ps8
Opisthokonta - ps9
Holomycota - ps10
Fungi - ps11
Fungi_1 - ps12
Fungi_2 - ps13
Fungi_3 - ps14
Dikarya - ps15
Ascomycota - ps16
Saccharo/Pezizomycotina - ps17
Saccharomycotina - ps18
Saccharomycotina_1 - ps19
Saccharomycotina_2 - ps20
Saccharomycotina_3 - ps21
Phaff./Saccharomycetaceae - ps22
Saccharomyco./mycetaceae - ps23
Saccharomycetaceae - ps24
Saccharomycetaceae_2 - ps25
Saccharomycetaceae_3 - ps26
Kaz./Nau./Saccharomyces - ps27
Saccharomyces - ps28
Arboricola_clade - ps29
Cerevisiae_clade - ps30
Saccharomyces_cerevisiae - ps31
Phylostratum

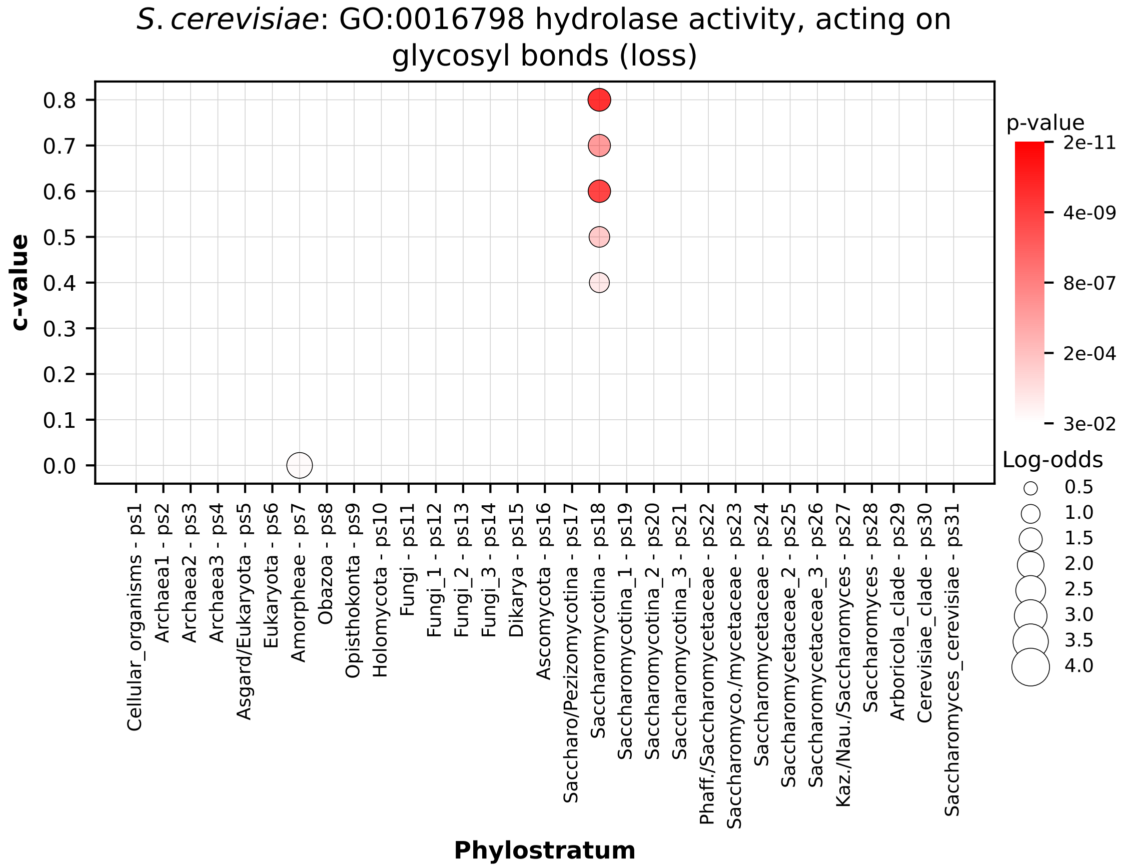
S. cerevisiae: GO:0016798 hydrolase activity, acting on glycosyl bonds (loss)
c-value
0.8
0.7
0.6
0.5
0.4
0.3
0.2
0.1
0.0
p-value
2e-11
4e-09
8e-07
2e-04
3e-02
Log-odds
0.5
1.0
1.5
2.0
2.5
3.0
3.5
4.0
Cellular_organisms - ps1
Archaea1 - ps2
Archaea2 - ps3
Archaea3 - ps4
Asgard/Eukaryota - ps5
Eukaryota - ps6
Amorpheae - ps7
Obazoa - ps8
Opisthokonta - ps9
Holomycota - ps10
Fungi - ps11
Fungi_1 - ps12
Fungi_2 - ps13
Fungi_3 - ps14
Dikarya - ps15
Ascomycota - ps16
Saccharo/Pezizomycotina - ps17
Saccharomycotina - ps18
Saccharomycotina_1 - ps19
Saccharomycotina_2 - ps20
Saccharomycotina_3 - ps21
Phaff./Saccharomycetaceae - ps22
Saccharomyco./mycetaceae - ps23
Saccharomycetaceae - ps24
Saccharomycetaceae_2 - ps25
Saccharomycetaceae_3 - ps26
Kaz./Nau./Saccharomyces - ps27
Saccharomyces - ps28
Arboricola_clade - ps29
Cerevisiae_clade - ps30
Saccharomyces_cerevisiae - ps31
Phylostratum

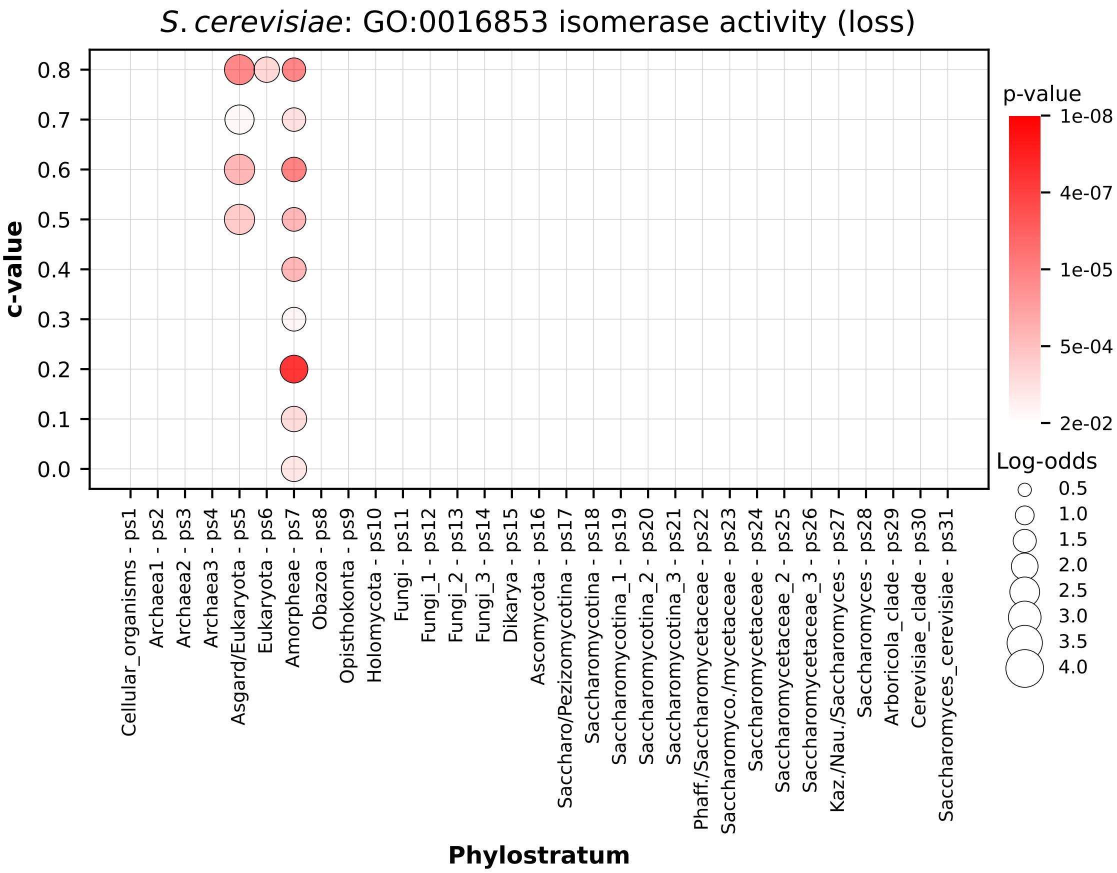

S. cerevisiae: GO:0016853 isomerase activity (loss)
c-value
0.8
0.7
0.6
0.5
0.4
0.3
0.2
0.1
0.0
p-value
1e-08
4e-07
1e-05
5e-04
2e-02
Log-odds
0.5
1.0
1.5
2.0
2.5
3.0
3.5
4.0
Cellular_organisms - ps1
Archaea1 - ps2
Archaea2 - ps3
Archaea3 - ps4
Asgard/Eukaryota - ps5
Eukaryota - ps6
Amorpheae - ps7
Obazoa - ps8
Opisthokonta - ps9
Holomycota - ps10
Fungi - ps11
Fungi_1 - ps12
Fungi_2 - ps13
Fungi_3 - ps14
Dikarya - ps15
Ascomycota - ps16
Saccharo/Pezizomycotina - ps17
Saccharomycotina - ps18
Saccharomycotina_1 - ps19
Saccharomycotina_2 - ps20
Saccharomycotina_3 - ps21
Phaff./Saccharomycetaceae - ps22
Saccharomyco./mycetaceae - ps23
Saccharomycetaceae - ps24
Saccharomycetaceae_2 - ps25
Saccharomycetaceae_3 - ps26
Kaz./Nau./Saccharomyces - ps27
Saccharomyces - ps28
Arboricola_clade - ps29
Cerevisiae_clade - ps30
Saccharomyces_cerevisiae - ps31
Phylostratum

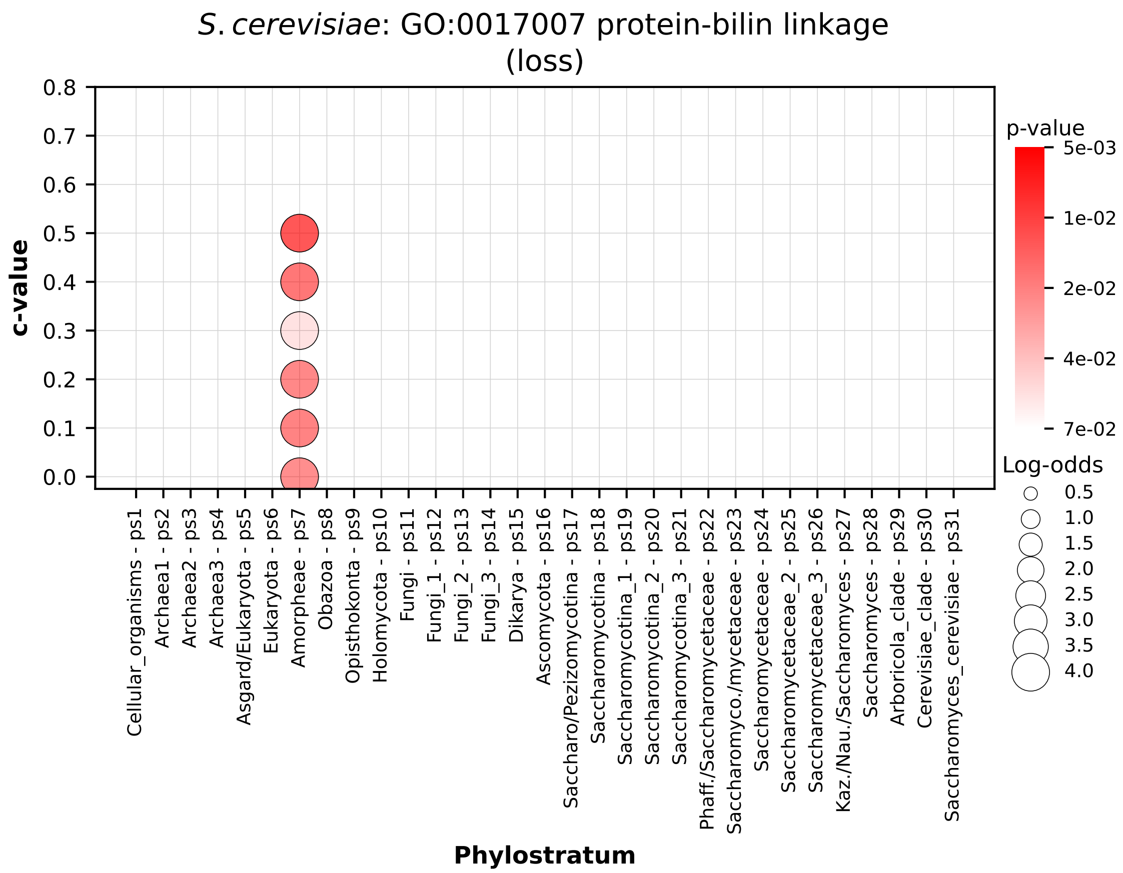

S. cerevisiae: GO:0017007 protein-bilin linkage
(loss)
c-value
0.8
0.7
0.6
0.5
0.4
0.3
0.2
0.1
0.0
p-value
5e-03
1e-02
2e-02
4e-02
7e-02
Log-odds
0.5
1.0
1.5
2.0
2.5
3.0
3.5
4.0
Cellular_organisms - ps1
Archaea1 - ps2
Archaea2 - ps3
Archaea3 - ps4
Asgard/Eukaryota - ps5
Eukaryota - ps6
Amorpheae - ps7
Obazoa - ps8
Opisthokonta - ps9
Holomycota - ps10
Fungi - ps11
Fungi_1 - ps12
Fungi_2 - ps13
Fungi_3 - ps14
Dikarya - ps15
Ascomycota - ps16
Saccharo/Pezizomycotina - ps17
Saccharomycotina - ps18
Saccharomycotina_1 - ps19
Saccharomycotina_2 - ps20
Saccharomycotina_3 - ps21
Phaff./Saccharomycetaceae - ps22
Saccharomyco./mycetaceae - ps23
Saccharomycetaceae - ps24
Saccharomycetaceae_2 - ps25
Saccharomycetaceae_3 - ps26
Kaz./Nau./Saccharomyces - ps27
Saccharomyces - ps28
Arboricola_clade - ps29
Cerevisiae_clade - ps30
Saccharomyces_cerevisiae - ps31
Phylostratum

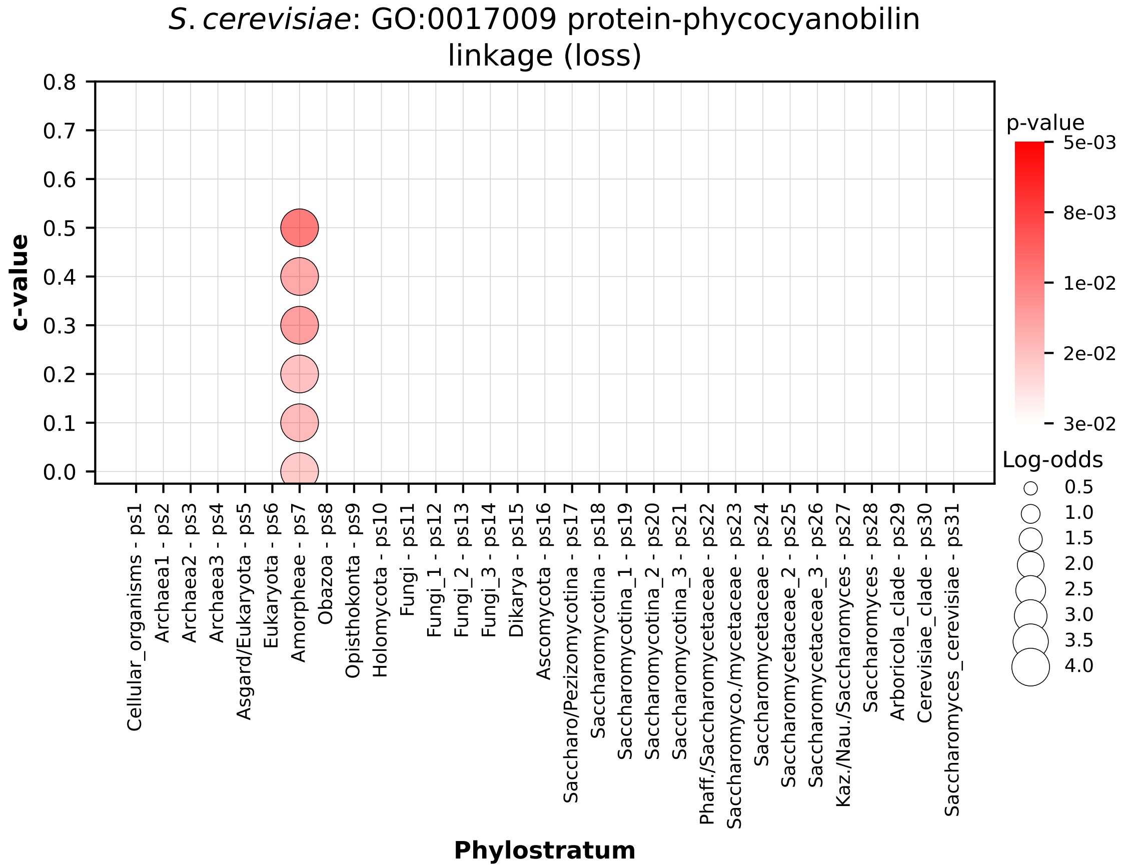
S. cerevisiae: GO:0017009 protein-phycocyanobilin linkage (loss)
c-value
0.8
0.7
0.6
0.5
0.4
0.3
0.2
0.1
0.0
p-value
5e-03
8e-03
1e-02
2e-02
3e-02
Log-odds
0.5
1.0
1.5
2.0
2.5
3.0
3.5
4.0
Cellular_organisms - ps1
Archaea1 - ps2
Archaea2 - ps3
Archaea3 - ps4
Asgard/Eukaryota - ps5
Eukaryota - ps6
Amorpheae - ps7
Obazoa - ps8
Opisthokonta - ps9
Holomycota - ps10
Fungi - ps11
Fungi_1 - ps12
Fungi_2 - ps13
Fungi_3 - ps14
Dikarya - ps15
Ascomycota - ps16
Saccharo/Pezizomycotina - ps17
Saccharomycotina - ps18
Saccharomycotina_1 - ps19
Saccharomycotina_2 - ps20
Saccharomycotina_3 - ps21
Phaff./Saccharomycetaceae - ps22
Saccharomyco./mycetaceae - ps23
Saccharomycetaceae - ps24
Saccharomycetaceae_2 - ps25
Saccharomycetaceae_3 - ps26
Kaz./Nau./Saccharomyces - ps27
Saccharomyces - ps28
Arboricola_clade - ps29
Cerevisiae_clade - ps30
Saccharomyces_cerevisiae - ps31
Phylostratum

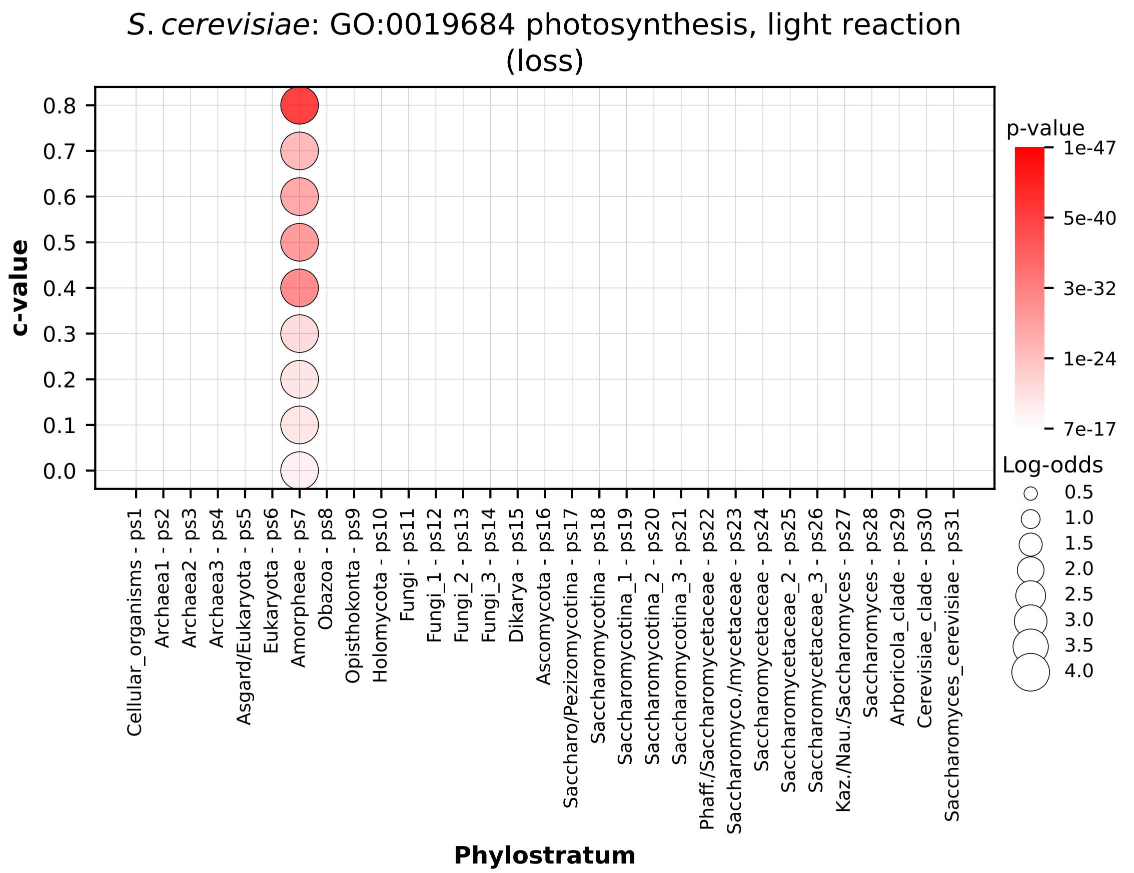

S. cerevisiae: GO:0019684 photosynthesis, light reaction
(loss)
c-value
0.8
0.7
0.6
0.5
0.4
0.3
0.2
0.1
0.0
p-value
1e-47
5e-40
3e-32
1e-24
7e-17
Log-odds
0.5
1.0
1.5
2.0
2.5
3.0
3.5
4.0
Cellular_organisms - ps1
Archaea1 - ps2
Archaea2 - ps3
Archaea3 - ps4
Asgard/Eukaryota - ps5
Eukaryota - ps6
Amorpheae - ps7
Obazoa - ps8
Opisthokonta - ps9
Holomycota - ps10
Fungi - ps11
Fungi_1 - ps12
Fungi_2 - ps13
Fungi_3 - ps14
Dikarya - ps15
Ascomycota - ps16
Saccharo/Pezizomycotina - ps17
Saccharomycotina - ps18
Saccharomycotina_1 - ps19
Saccharomycotina_2 - ps20
Saccharomycotina_3 - ps21
Phaff./Saccharomycetaceae - ps22
Saccharomyco./mycetaceae - ps23
Saccharomycetaceae - ps24
Saccharomycetaceae_2 - ps25
Saccharomycetaceae_3 - ps26
Kaz./Nau./Saccharomyces - ps27
Saccharomyces - ps28
Arboricola_clade - ps29
Cerevisiae_clade - ps30
Saccharomyces_cerevisiae - ps31
Phylostratum

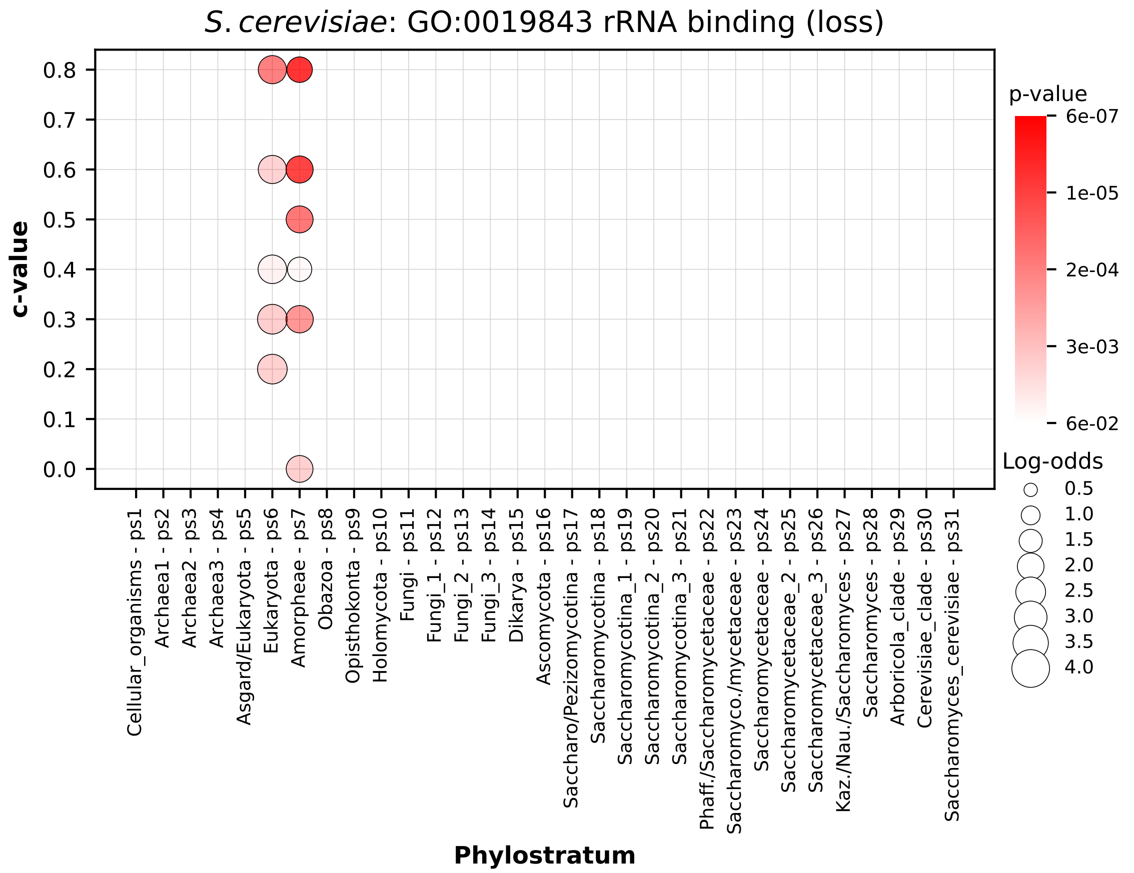
S. cerevisiae: GO:0019843 rRNA binding (loss)
c-value
0.0
0.1
0.2
0.3
0.4
0.5
0.6
0.7
0.8
Phylostratum
Cellular_organisms - ps1
Archaea1 - ps2
Archaea2 - ps3
Archaea3 - ps4
Asgard/Eukaryota - ps5
Eukaryota - ps6
Amorpheae - ps7
Obazoa - ps8
Opisthokonta - ps9
Holomycota - ps10
Fungi - ps11
Fungi_1 - ps12
Fungi_2 - ps13
Fungi_3 - ps14
Dikarya - ps15
Ascomycota - ps16
Saccharo/Pezizomycotina - ps17
Saccharomycotina - ps18
Saccharomycotina_1 - ps19
Saccharomycotina_2 - ps20
Saccharomycotina_3 - ps21
Phaff./Saccharomycetaceae - ps22
Saccharomyco./mycetaceae - ps23
Saccharomycetaceae - ps24
Saccharomycetaceae_2 - ps25
Saccharomycetaceae_3 - ps26
Kaz./Nau./Saccharomyces - ps27
Saccharomyces - ps28
Arboricola_clade - ps29
Cerevisiae_clade - ps30
Saccharomyces_cerevisiae - ps31
p-value
6e-07
1e-05
2e-04
3e-03
6e-02
Log-odds
0.5
1.0
1.5
2.0
2.5
3.0
3.5
4.0

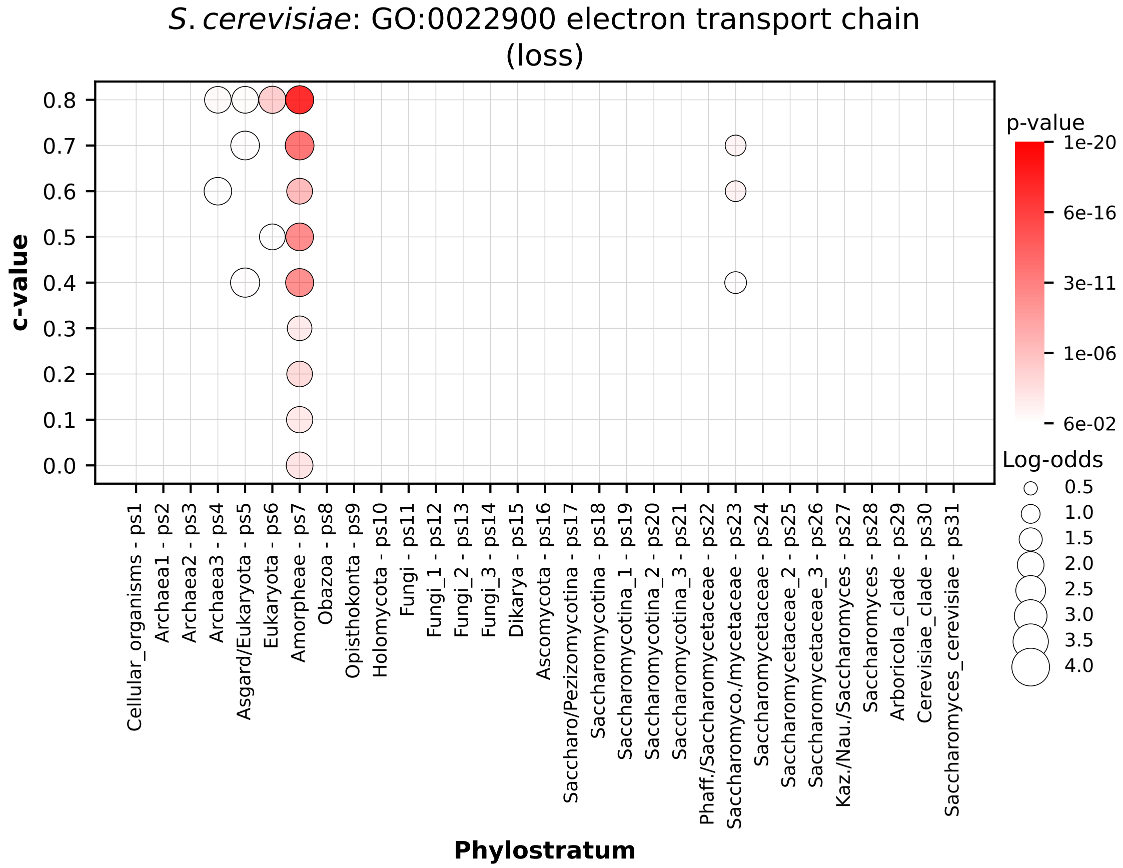

S. cerevisiae: GO:0022900 electron transport chain (loss)
c-value
0.8
0.7
0.6
0.5
0.4
0.3
0.2
0.1
0.0
Phylostratum
Cellular_organisms - ps1
Archaea1 - ps2
Archaea2 - ps3
Archaea3 - ps4
Asgard/Eukaryota - ps5
Eukaryota - ps6
Amorpheae - ps7
Obazoa - ps8
Opisthokonta - ps9
Holomycota - ps10
Fungi - ps11
Fungi_1 - ps12
Fungi_2 - ps13
Fungi_3 - ps14
Dikarya - ps15
Ascomycota - ps16
Saccharo/Pezizomycotina - ps17
Saccharomycotina - ps18
Saccharomycotina_1 - ps19
Saccharomycotina_2 - ps20
Saccharomycotina_3 - ps21
Phaff./Saccharomycetaceae - ps22
Saccharomyco./mycetaceae - ps23
Saccharomycetaceae - ps24
Saccharomycetaceae_2 - ps25
Saccharomycetaceae_3 - ps26
Kaz./Nau./Saccharomyces - ps27
Saccharomyces - ps28
Arboricola_clade - ps29
Cerevisiae_clade - ps30
Saccharomyces_cerevisiae - ps31
p-value
1e-20
6e-16
3e-11
1e-06
6e-02
Log-odds
0.5
1.0
1.5
2.0
2.5
3.0
3.5
4.0

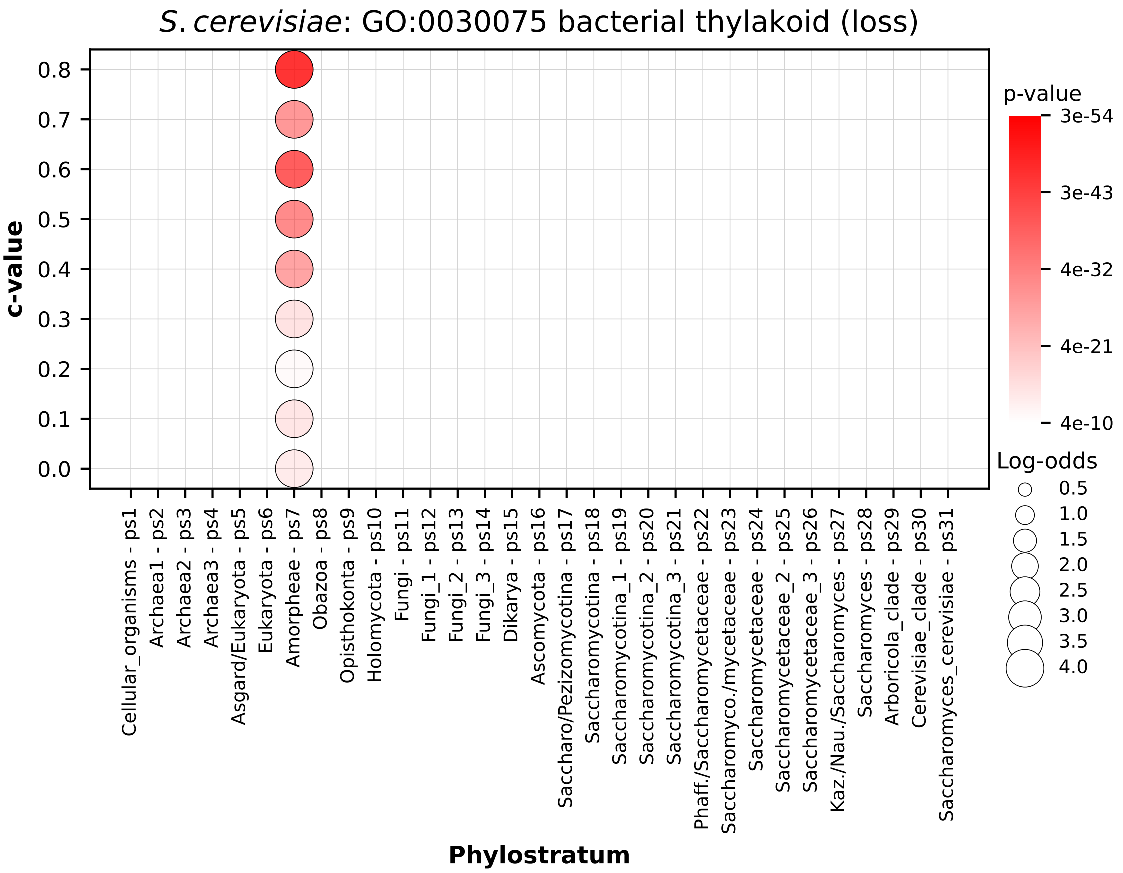

S. cerevisiae: GO:0030075 bacterial thylakoid (loss)
c-value
0.8
0.7
0.6
0.5
0.4
0.3
0.2
0.1
0.0
Phylostratum
Cellular_organisms - ps1
Archaea1 - ps2
Archaea2 - ps3
Archaea3 - ps4
Asgard/Eukaryota - ps5
Eukaryota - ps6
Amorpheae - ps7
Obazoa - ps8
Opisthokonta - ps9
Holomycota - ps10
Fungi - ps11
Fungi_1 - ps12
Fungi_2 - ps13
Fungi_3 - ps14
Dikarya - ps15
Ascomycota - ps16
Saccharo/Pezizomycotina - ps17
Saccharomycotina - ps18
Saccharomycotina_1 - ps19
Saccharomycotina_2 - ps20
Saccharomycotina_3 - ps21
Phaff./Saccharomycetaceae - ps22
Saccharomyco./mycetaceae - ps23
Saccharomycetaceae - ps24
Saccharomycetaceae_2 - ps25
Saccharomycetaceae_3 - ps26
Kaz./Nau./Saccharomyces - ps27
Saccharomyces - ps28
Arboricola_clade - ps29
Cerevisiae_clade - ps30
Saccharomyces_cerevisiae - ps31
p-value
3e-54
3e-43
4e-32
4e-21
4e-10
Log-odds
0.5
1.0
1.5
2.0
2.5
3.0
3.5
4.0

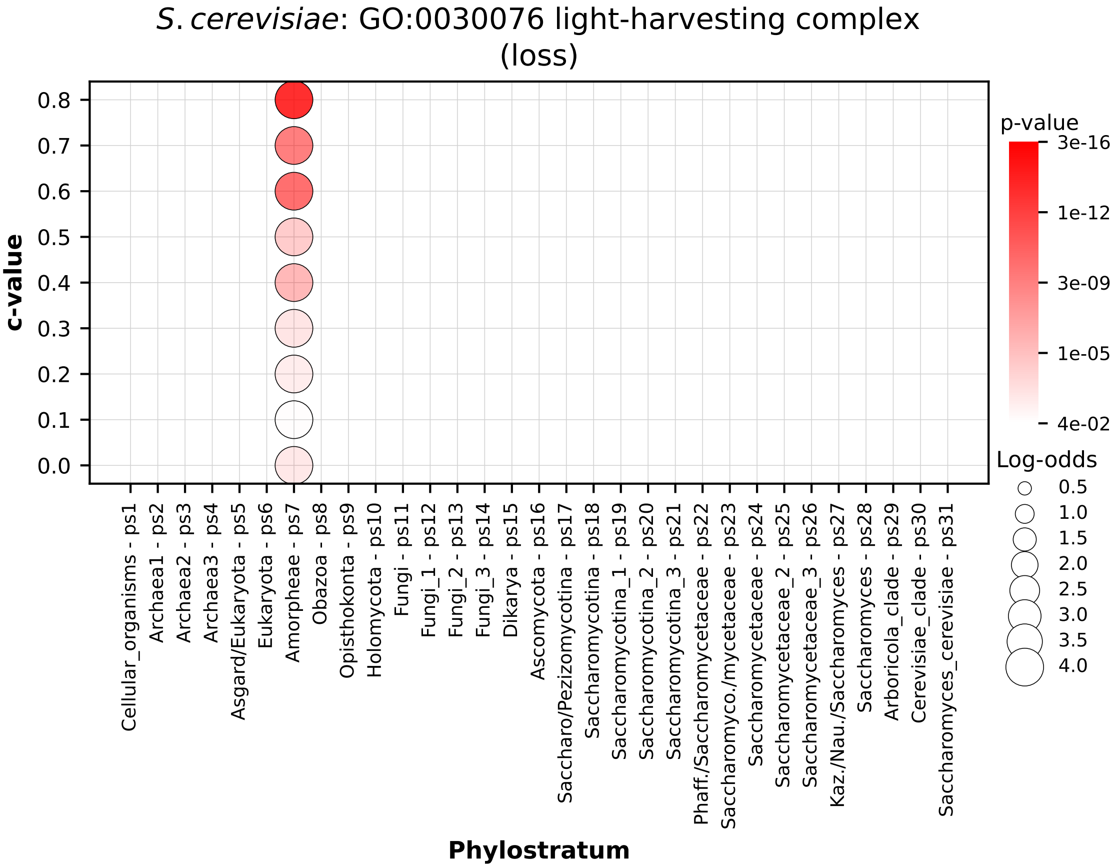
S. cerevisiae: GO:0030076 light-harvesting complex
(loss)
c-value
0.8
0.7
0.6
0.5
0.4
0.3
0.2
0.1
0.0
p-value
3e-16
1e-12
3e-09
1e-05
4e-02
Log-odds
0.5
1.0
1.5
2.0
2.5
3.0
3.5
4.0
Cellular_organisms - ps1
Archaea1 - ps2
Archaea2 - ps3
Archaea3 - ps4
Asgard/Eukaryota - ps5
Eukaryota - ps6
Amorpheae - ps7
Obazoa - ps8
Opisthokonta - ps9
Holomycota - ps10
Fungi - ps11
Fungi_1 - ps12
Fungi_2 - ps13
Fungi_3 - ps14
Dikarya - ps15
Ascomycota - ps16
Saccharo/Pezizomycotina - ps17
Saccharomycotina - ps18
Saccharomycotina_1 - ps19
Saccharomycotina_2 - ps20
Saccharomycotina_3 - ps21
Phaff./Saccharomycetaceae - ps22
Saccharomyco./mycetaceae - ps23
Saccharomycetaceae - ps24
Saccharomycetaceae_2 - ps25
Saccharomycetaceae_3 - ps26
Kaz./Nau./Saccharomyces - ps27
Saccharomyces - ps28
Arboricola_clade - ps29
Cerevisiae_clade - ps30
Saccharomyces_cerevisiae - ps31
Phylostratum

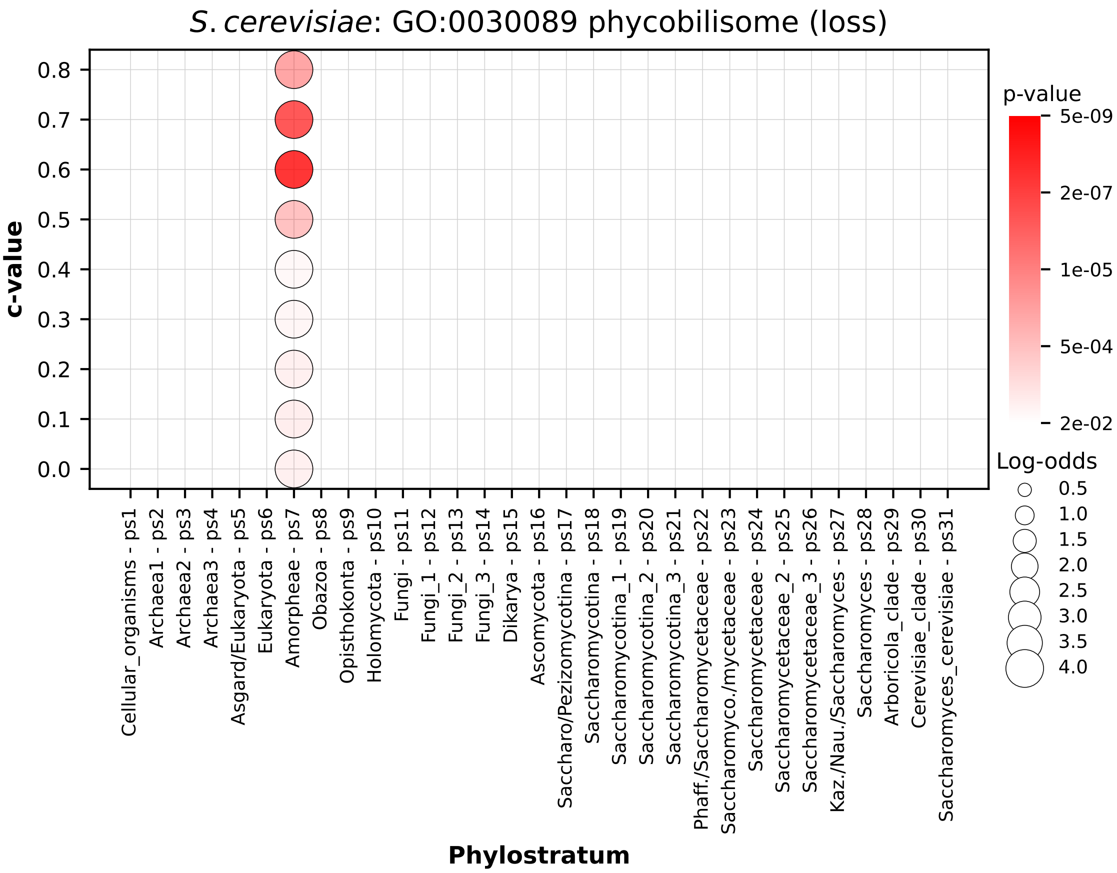

S. cerevisiae: GO:0030089 phycobilisome (loss)
c-value
0.8
0.7
0.6
0.5
0.4
0.3
0.2
0.1
0.0
p-value
5e-09
2e-07
1e-05
5e-04
2e-02
Log-odds
0.5
1.0
1.5
2.0
2.5
3.0
3.5
4.0
Cellular_organisms - ps1
Archaea1 - ps2
Archaea2 - ps3
Archaea3 - ps4
Asgard/Eukaryota - ps5
Eukaryota - ps6
Amorpheae - ps7
Obazoa - ps8
Opisthokonta - ps9
Holomycota - ps10
Fungi - ps11
Fungi_1 - ps12
Fungi_2 - ps13
Fungi_3 - ps14
Dikarya - ps15
Ascomycota - ps16
Saccharo/Pezizomycotina - ps17
Saccharomycotina - ps18
Saccharomycotina_1 - ps19
Saccharomycotina_2 - ps20
Saccharomycotina_3 - ps21
Phaff./Saccharomycetaceae - ps22
Saccharomyco./mycetaceae - ps23
Saccharomycetaceae - ps24
Saccharomycetaceae_2 - ps25
Saccharomycetaceae_3 - ps26
Kaz./Nau./Saccharomyces - ps27
Saccharomyces - ps28
Arboricola_clade - ps29
Cerevisiae_clade - ps30
Saccharomyces_cerevisiae - ps31
Phylostratum

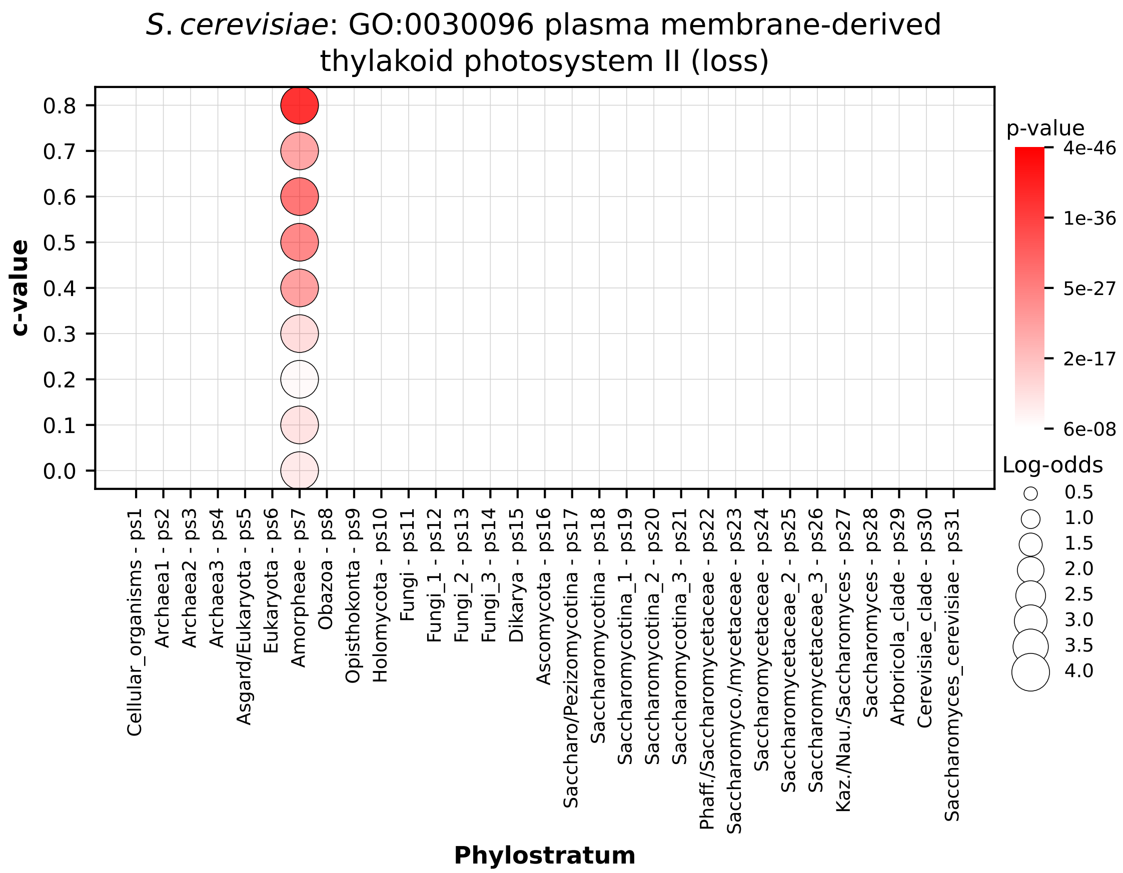
S. cerevisiae: GO:0030096 plasma membrane-derived thylakoid photosystem II (loss)
c-value
0.8
0.7
0.6
0.5
0.4
0.3
0.2
0.1
0.0
p-value
4e-46
1e-36
5e-27
2e-17
6e-08
Log-odds
0.5
1.0
1.5
2.0
2.5
3.0
3.5
4.0
Cellular_organisms - ps1
Archaea1 - ps2
Archaea2 - ps3
Archaea3 - ps4
Asgard/Eukaryota - ps5
Eukaryota - ps6
Amorpheae - ps7
Obazoa - ps8
Opisthokonta - ps9
Holomycota - ps10
Fungi - ps11
Fungi_1 - ps12
Fungi_2 - ps13
Fungi_3 - ps14
Dikarya - ps15
Ascomycota - ps16
Saccharo/Pezizomycotina - ps17
Saccharomycotina - ps18
Saccharomycotina_1 - ps19
Saccharomycotina_2 - ps20
Saccharomycotina_3 - ps21
Phaff./Saccharomycetaceae - ps22
Saccharomyco./mycetaceae - ps23
Saccharomycetaceae - ps24
Saccharomycetaceae_2 - ps25
Saccharomycetaceae_3 - ps26
Kaz./Nau./Saccharomyces - ps27
Saccharomyces - ps28
Arboricola_clade - ps29
Cerevisiae_clade - ps30
Saccharomyces_cerevisiae - ps31
Phylostratum

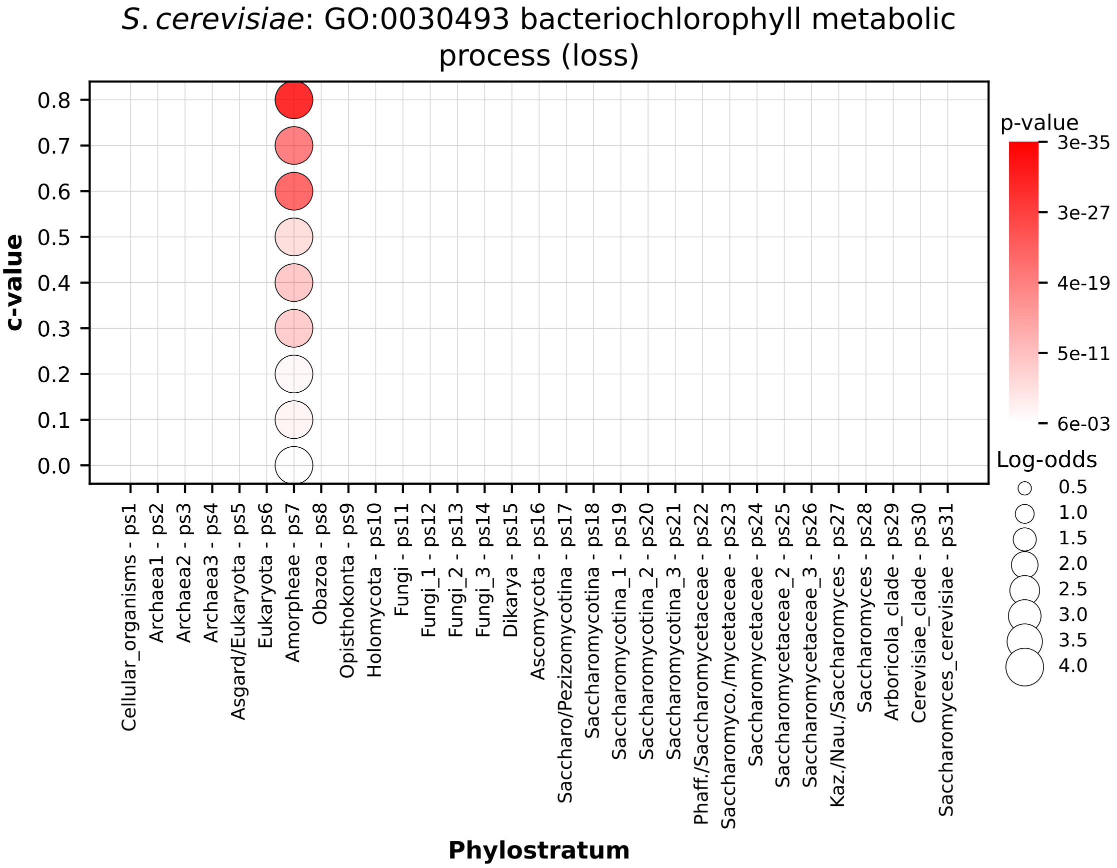

S. cerevisiae: GO:0030493 bacteriochlorophyll metabolic process (loss)
c-value
0.8
0.7
0.6
0.5
0.4
0.3
0.2
0.1
0.0
Phylostratum
Cellular_organisms - ps1
Archaea1 - ps2
Archaea2 - ps3
Archaea3 - ps4
Asgard/Eukaryota - ps5
Eukaryota - ps6
Amorpheae - ps7
Obazoa - ps8
Opisthokonta - ps9
Holomycota - ps10
Fungi - ps11
Fungi_1 - ps12
Fungi_2 - ps13
Fungi_3 - ps14
Dikarya - ps15
Ascomycota - ps16
Saccharo/Pezizomycotina - ps17
Saccharomycotina - ps18
Saccharomycotina_1 - ps19
Saccharomycotina_2 - ps20
Saccharomycotina_3 - ps21
Phaff./Saccharomycetaceae - ps22
Saccharomyco./mycetaceae - ps23
Saccharomycetaceae - ps24
Saccharomycetaceae_2 - ps25
Saccharomycetaceae_3 - ps26
Kaz./Nau./Saccharomyces - ps27
Saccharomyces - ps28
Arboricola_clade - ps29
Cerevisiae_clade - ps30
Saccharomyces_cerevisiae - ps31
p-value
3e-35
3e-27
4e-19
5e-11
6e-03
Log-odds
0.5
1.0
1.5
2.0
2.5
3.0
3.5
4.0

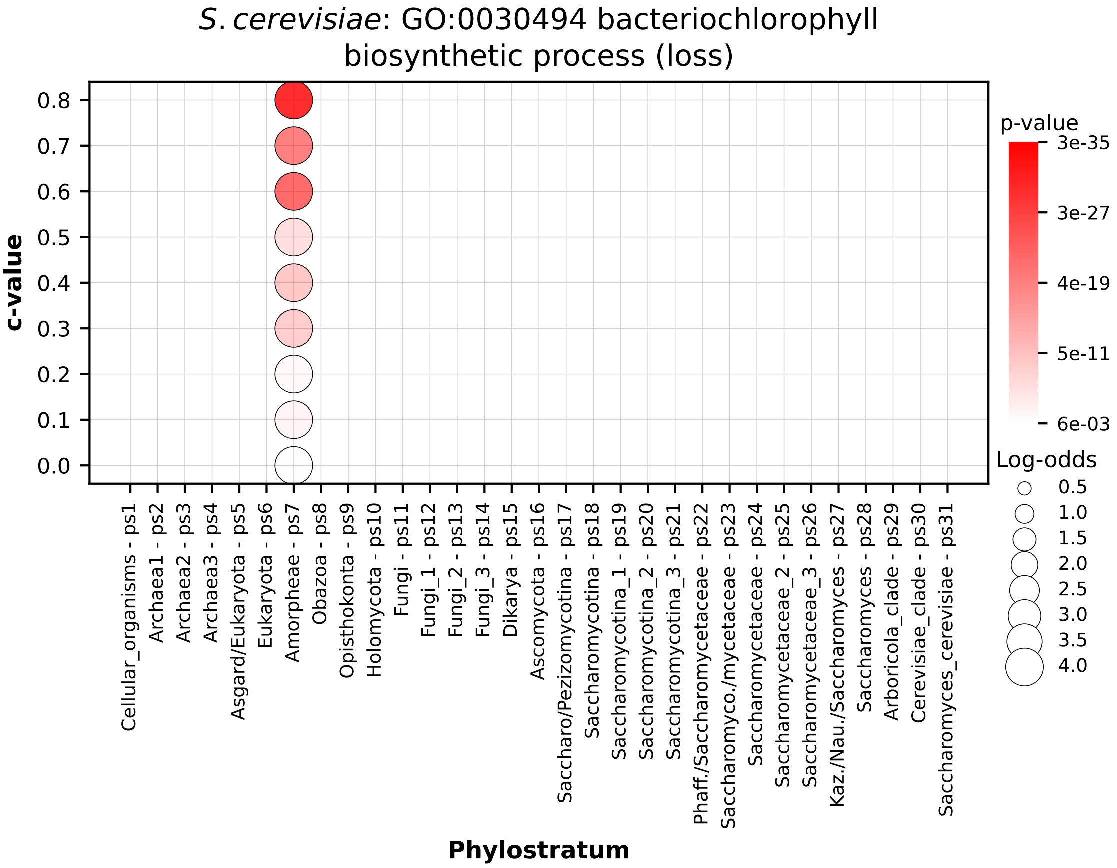

S. cerevisiae: GO:0030494 bacteriochlorophyll biosynthetic process (loss)
c-value
0.8
0.7
0.6
0.5
0.4
0.3
0.2
0.1
0.0
p-value
3e-35
3e-27
4e-19
5e-11
6e-03
Log-odds
0.5
1.0
1.5
2.0
2.5
3.0
3.5
4.0
Cellular_organisms - ps1
Archaea1 - ps2
Archaea2 - ps3
Archaea3 - ps4
Asgard/Eukaryota - ps5
Eukaryota - ps6
Amorpheae - ps7
Obazoa - ps8
Opisthokonta - ps9
Holomycota - ps10
Fungi - ps11
Fungi_1 - ps12
Fungi_2 - ps13
Fungi_3 - ps14
Dikarya - ps15
Ascomycota - ps16
Saccharo/Pezizomycotina - ps17
Saccharomycotina - ps18
Saccharomycotina_1 - ps19
Saccharomycotina_2 - ps20
Saccharomycotina_3 - ps21
Phaff./Saccharomycetaceae - ps22
Saccharomyco./mycetaceae - ps23
Saccharomycetaceae - ps24
Saccharomycetaceae_2 - ps25
Saccharomycetaceae_3 - ps26
Kaz./Nau./Saccharomyces - ps27
Saccharomyces - ps28
Arboricola_clade - ps29
Cerevisiae_clade - ps30
Saccharomyces_cerevisiae - ps31
Phylostratum

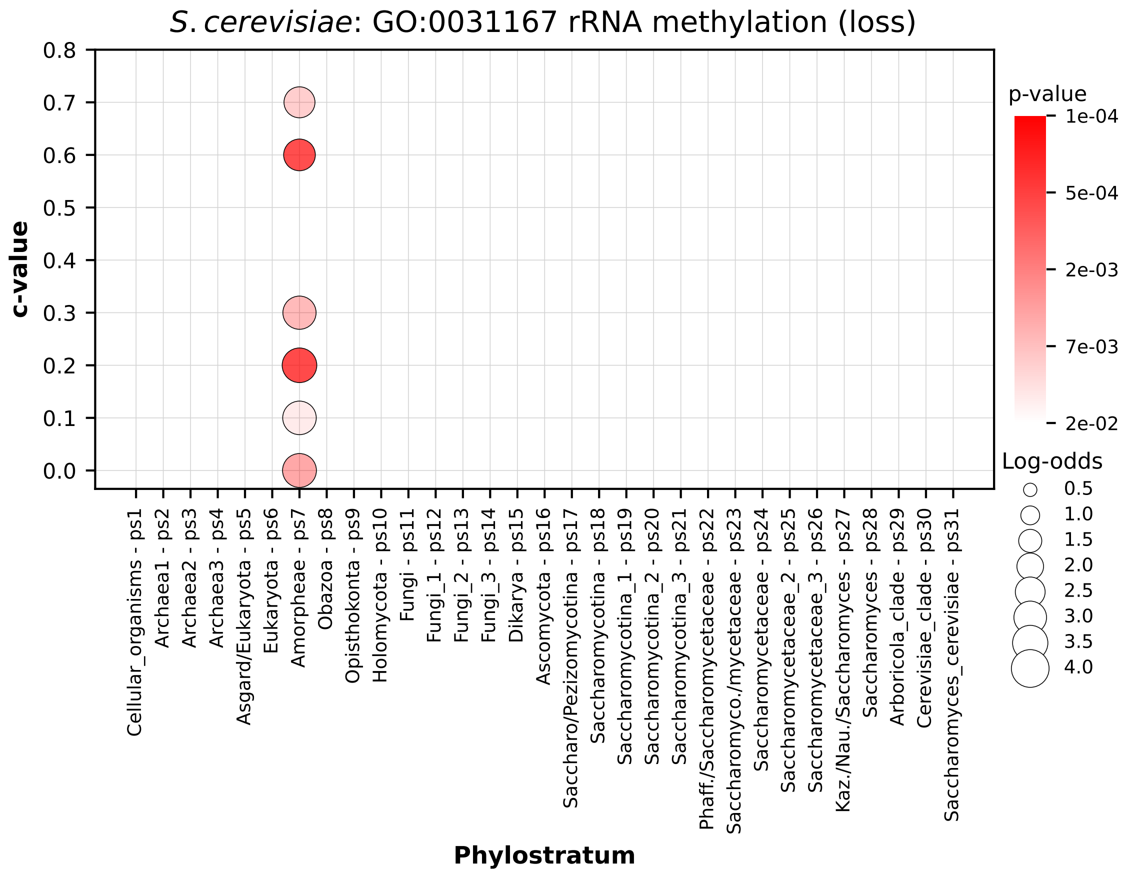

S. cerevisiae: GO:0031167 rRNA methylation (loss)
c-value
0.8
0.7
0.6
0.5
0.4
0.3
0.2
0.1
0.0
p-value
1e-04
5e-04
2e-03
7e-03
2e-02
Log-odds
0.5
1.0
1.5
2.0
2.5
3.0
3.5
4.0
Cellular_organisms - ps1
Archaea1 - ps2
Archaea2 - ps3
Archaea3 - ps4
Asgard/Eukaryota - ps5
Eukaryota - ps6
Amorpheae - ps7
Obazoa - ps8
Opisthokonta - ps9
Holomycota - ps10
Fungi - ps11
Fungi_1 - ps12
Fungi_2 - ps13
Fungi_3 - ps14
Dikarya - ps15
Ascomycota - ps16
Saccharo/Pezizomycotina - ps17
Saccharomycotina - ps18
Saccharomycotina_1 - ps19
Saccharomycotina_2 - ps20
Saccharomycotina_3 - ps21
Phaff./Saccharomycetaceae - ps22
Saccharomyco./mycetaceae - ps23
Saccharomycetaceae - ps24
Saccharomycetaceae_2 - ps25
Saccharomycetaceae_3 - ps26
Kaz./Nau./Saccharomyces - ps27
Saccharomyces - ps28
Arboricola_clade - ps29
Cerevisiae_clade - ps30
Saccharomyces_cerevisiae - ps31
Phylostratum

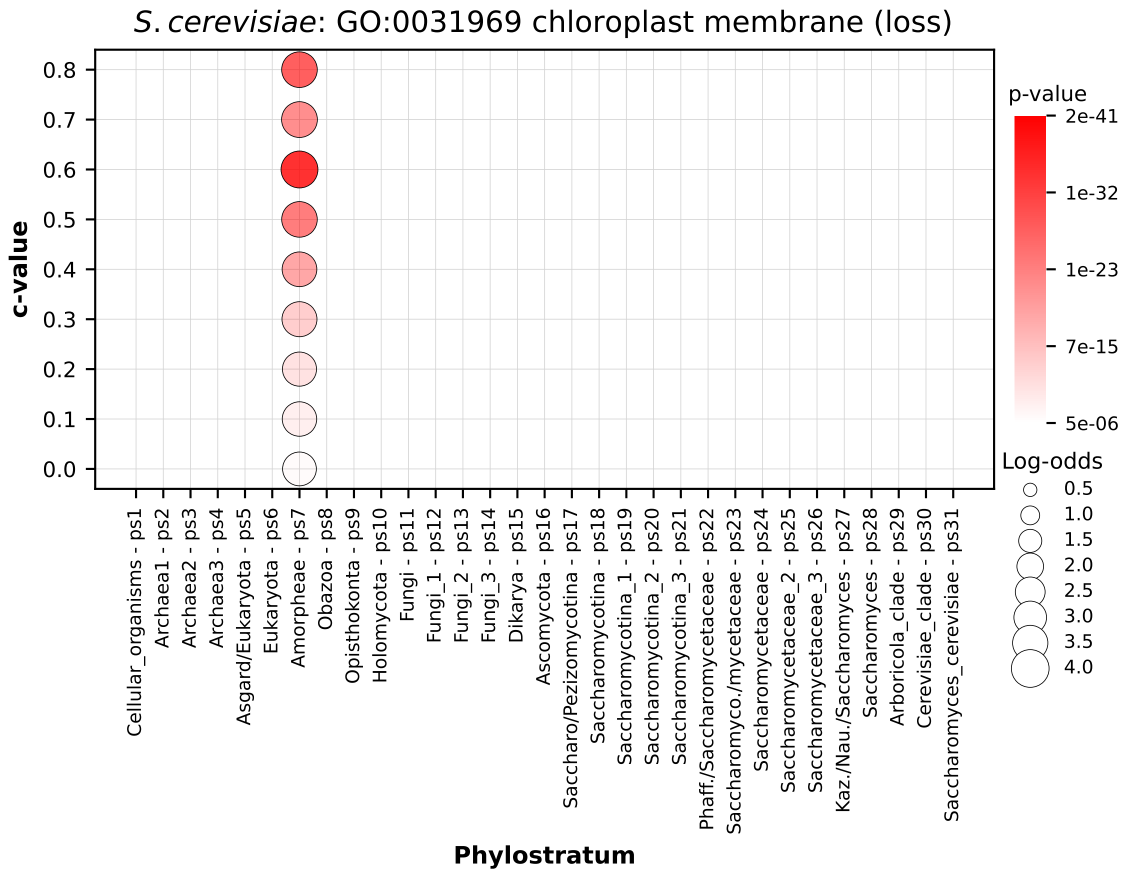

S. cerevisiae: GO:0031969 chloroplast membrane (loss)
c-value
0.0
0.1
0.2
0.3
0.4
0.5
0.6
0.7
0.8
Phylostratum
Cellular_organisms - ps1
Archaea1 - ps2
Archaea2 - ps3
Archaea3 - ps4
Asgard/Eukaryota - ps5
Eukaryota - ps6
Amorpheae - ps7
Obazoa - ps8
Opisthokonta - ps9
Holomycota - ps10
Fungi - ps11
Fungi_1 - ps12
Fungi_2 - ps13
Fungi_3 - ps14
Dikarya - ps15
Ascomycota - ps16
Saccharo/Pezizomycotina - ps17
Saccharomycotina - ps18
Saccharomycotina_1 - ps19
Saccharomycotina_2 - ps20
Saccharomycotina_3 - ps21
Phaff./Saccharomycetaceae - ps22
Saccharomyco./mycetaceae - ps23
Saccharomycetaceae - ps24
Saccharomycetaceae_2 - ps25
Saccharomycetaceae_3 - ps26
Kaz./Nau./Saccharomyces - ps27
Saccharomyces - ps28
Arboricola_clade - ps29
Cerevisiae_clade - ps30
Saccharomyces_cerevisiae - ps31
p-value
2e-41
1e-32
1e-23
7e-15
5e-06
Log-odds
0.5
1.0
1.5
2.0
2.5
3.0
3.5
4.0

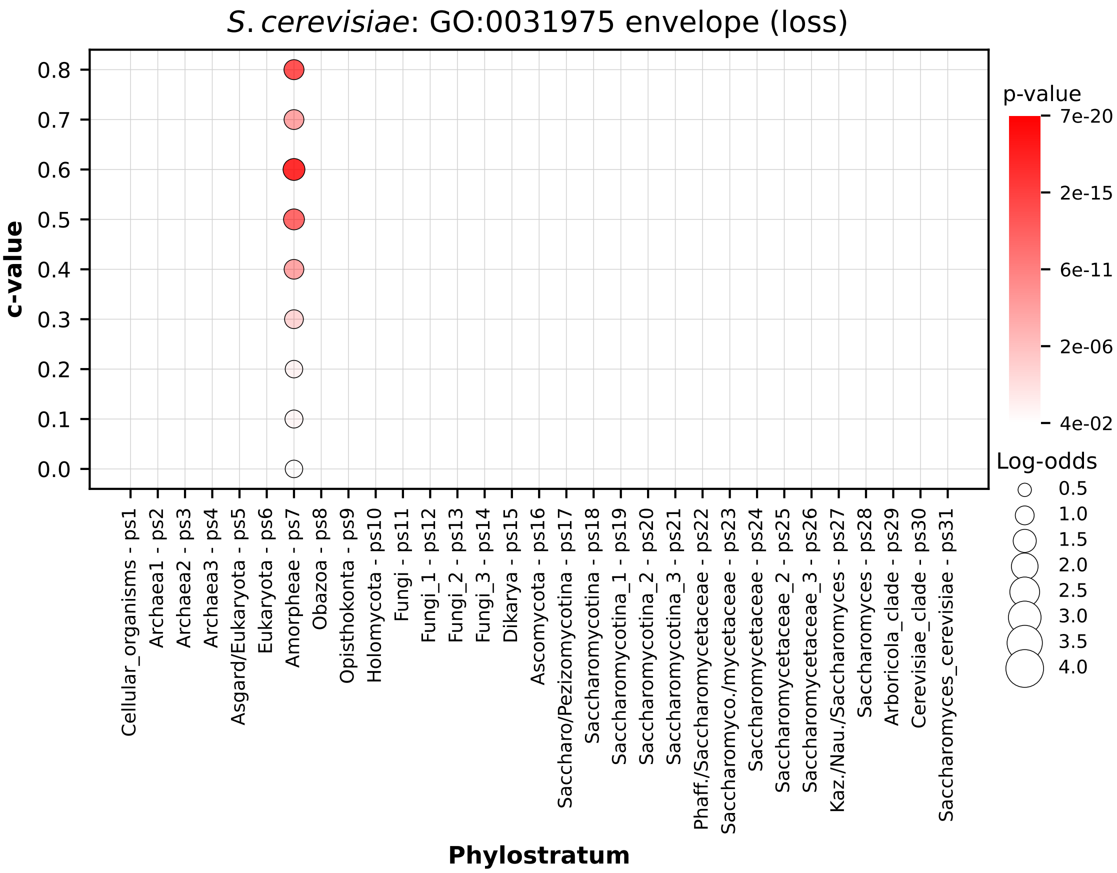

S. cerevisiae: GO:0031975 envelope (loss)
c-value
0.0
0.1
0.2
0.3
0.4
0.5
0.6
0.7
0.8
p-value
7e-20
2e-15
6e-11
2e-06
4e-02
Log-odds
0.5
1.0
1.5
2.0
2.5
3.0
3.5
4.0
Cellular_organisms - ps1
Archaea1 - ps2
Archaea2 - ps3
Archaea3 - ps4
Asgard/Eukaryota - ps5
Eukaryota - ps6
Amorpheae - ps7
Obazoa - ps8
Opisthokonta - ps9
Holomycota - ps10
Fungi - ps11
Fungi_1 - ps12
Fungi_2 - ps13
Fungi_3 - ps14
Dikarya - ps15
Ascomycota - ps16
Saccharo/Pezizomycotina - ps17
Saccharomycotina - ps18
Saccharomycotina_1 - ps19
Saccharomycotina_2 - ps20
Saccharomycotina_3 - ps21
Phaff./Saccharomycetaceae - ps22
Saccharomyco./mycetaceae - ps23
Saccharomycetaceae - ps24
Saccharomycetaceae_2 - ps25
Saccharomycetaceae_3 - ps26
Kaz./Nau./Saccharomyces - ps27
Saccharomyces - ps28
Arboricola_clade - ps29
Cerevisiae_clade - ps30
Saccharomyces_cerevisiae - ps31
Phylostratum

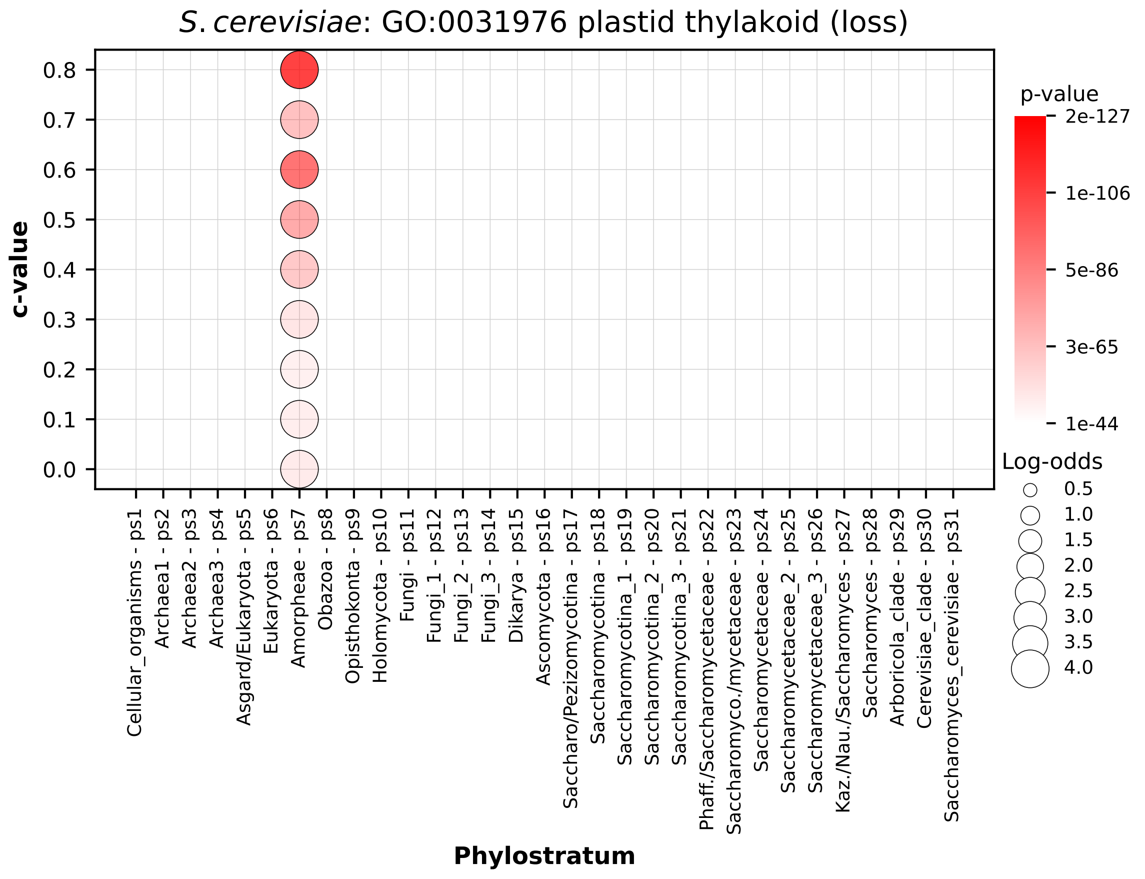

S. cerevisiae: GO:0031976 plastid thylakoid (loss)
c-value
0.8
0.7
0.6
0.5
0.4
0.3
0.2
0.1
0.0
p-value
2e-127
1e-106
5e-86
3e-65
1e-44
Log-odds
0.5
1.0
1.5
2.0
2.5
3.0
3.5
4.0
Cellular_organisms - ps1
Archaea1 - ps2
Archaea2 - ps3
Archaea3 - ps4
Asgard/Eukaryota - ps5
Eukaryota - ps6
Amorpheae - ps7
Obazoa - ps8
Opisthokonta - ps9
Holomycota - ps10
Fungi - ps11
Fungi_1 - ps12
Fungi_2 - ps13
Fungi_3 - ps14
Dikarya - ps15
Ascomycota - ps16
Saccharo/Pezizomycotina - ps17
Saccharomycotina - ps18
Saccharomycotina_1 - ps19
Saccharomycotina_2 - ps20
Saccharomycotina_3 - ps21
Phaff./Saccharomycetaceae - ps22
Saccharomyco./mycetaceae - ps23
Saccharomycetaceae - ps24
Saccharomycetaceae_2 - ps25
Saccharomycetaceae_3 - ps26
Kaz./Nau./Saccharomyces - ps27
Saccharomyces - ps28
Arboricola_clade - ps29
Cerevisiae_clade - ps30
Saccharomyces_cerevisiae - ps31
Phylostratum

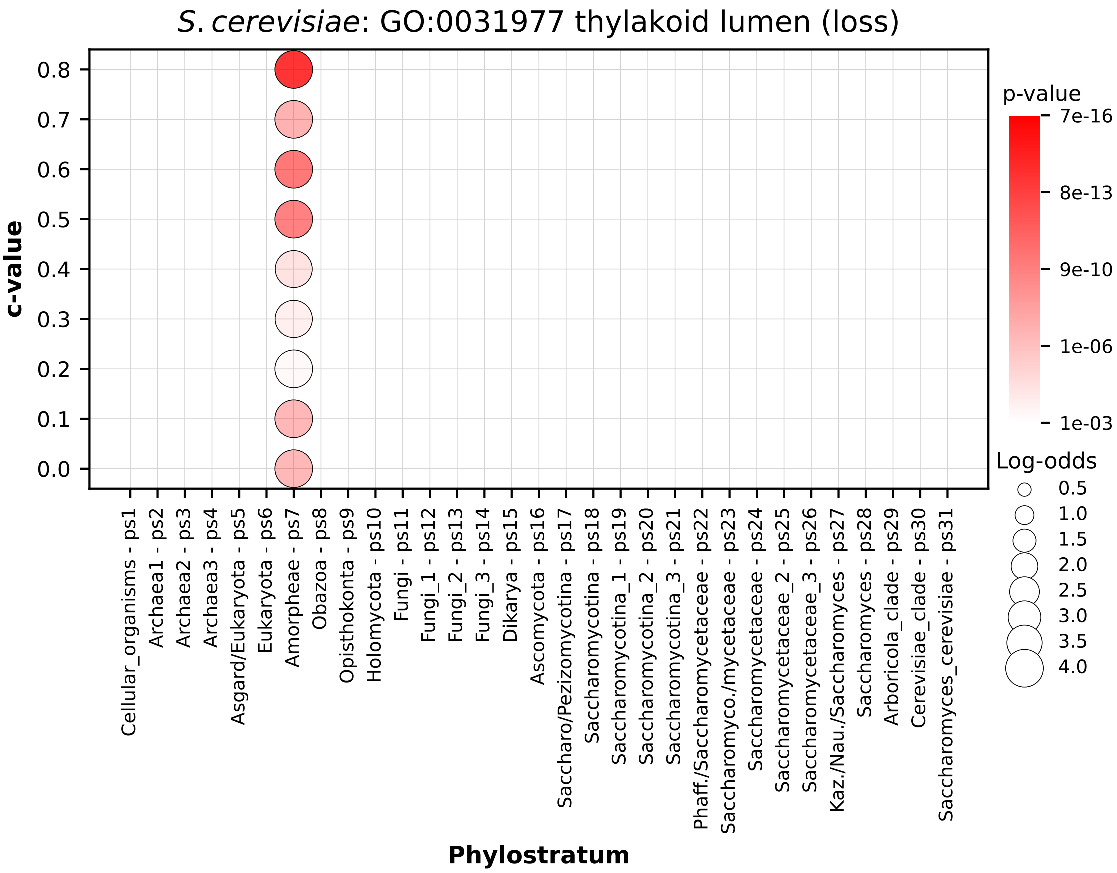

S. cerevisiae: GO:0031977 thylakoid lumen (loss)
c-value
0.0
0.1
0.2
0.3
0.4
0.5
0.6
0.7
0.8
p-value
7e-16
8e-13
9e-10
1e-06
1e-03
Log-odds
0.5
1.0
1.5
2.0
2.5
3.0
3.5
4.0
Cellular_organisms - ps1
Archaea1 - ps2
Archaea2 - ps3
Archaea3 - ps4
Asgard/Eukaryota - ps5
Eukaryota - ps6
Amorpheae - ps7
Obazoa - ps8
Opisthokonta - ps9
Holomycota - ps10
Fungi - ps11
Fungi_1 - ps12
Fungi_2 - ps13
Fungi_3 - ps14
Dikarya - ps15
Ascomycota - ps16
Saccharo/Pezizomycotina - ps17
Saccharomycotina - ps18
Saccharomycotina_1 - ps19
Saccharomycotina_2 - ps20
Saccharomycotina_3 - ps21
Phaff./Saccharomycetaceae - ps22
Saccharomyco./mycetaceae - ps23
Saccharomycetaceae - ps24
Saccharomycetaceae_2 - ps25
Saccharomycetaceae_3 - ps26
Kaz./Nau./Saccharomyces - ps27
Saccharomyces - ps28
Arboricola_clade - ps29
Cerevisiae_clade - ps30
Saccharomyces_cerevisiae - ps31
Phylostratum

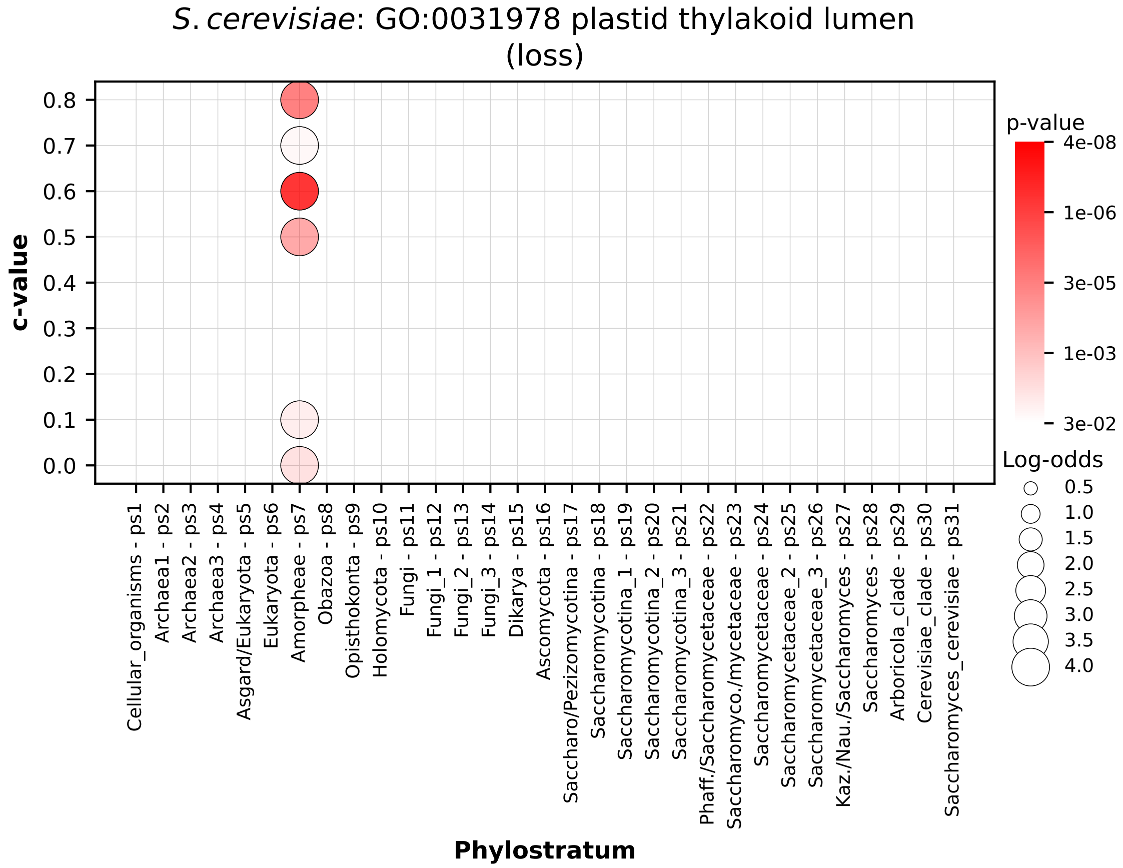

S. cerevisiae: GO:0031978 plastid thylakoid lumen
(loss)
c-value
0.8
0.7
0.6
0.5
0.4
0.3
0.2
0.1
0.0
p-value
4e-08
1e-06
3e-05
1e-03
3e-02
Log-odds
0.5
1.0
1.5
2.0
2.5
3.0
3.5
4.0
Cellular_organisms - ps1
Archaea1 - ps2
Archaea2 - ps3
Archaea3 - ps4
Asgard/Eukaryota - ps5
Eukaryota - ps6
Amorpheae - ps7
Obazoa - ps8
Opisthokonta - ps9
Holomycota - ps10
Fungi - ps11
Fungi_1 - ps12
Fungi_2 - ps13
Fungi_3 - ps14
Dikarya - ps15
Ascomycota - ps16
Saccharo/Pezizomycotina - ps17
Saccharomycotina - ps18
Saccharomycotina_1 - ps19
Saccharomycotina_2 - ps20
Saccharomycotina_3 - ps21
Phaff./Saccharomycetaceae - ps22
Saccharomyco./mycetaceae - ps23
Saccharomycetaceae - ps24
Saccharomycetaceae_2 - ps25
Saccharomycetaceae_3 - ps26
Kaz./Nau./Saccharomyces - ps27
Saccharomyces - ps28
Arboricola_clade - ps29
Cerevisiae_clade - ps30
Saccharomyces_cerevisiae - ps31
Phylostratum

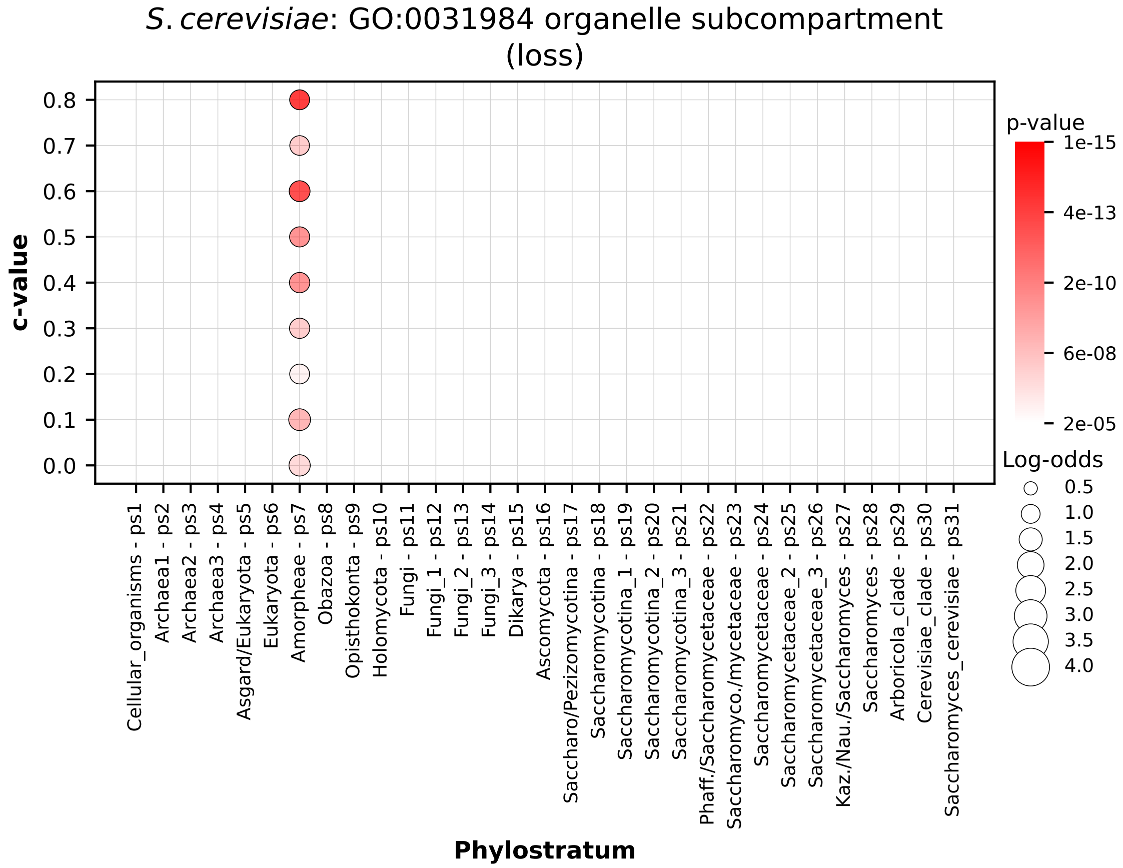

S. cerevisiae: GO:0031984 organelle subcompartment
(loss)
c-value
0.8
0.7
0.6
0.5
0.4
0.3
0.2
0.1
0.0
p-value
1e-15
4e-13
2e-10
6e-08
2e-05
Log-odds
0.5
1.0
1.5
2.0
2.5
3.0
3.5
4.0
Cellular_organisms - ps1
Archaea1 - ps2
Archaea2 - ps3
Archaea3 - ps4
Asgard/Eukaryota - ps5
Eukaryota - ps6
Amorpheae - ps7
Obazoa - ps8
Opisthokonta - ps9
Holomycota - ps10
Fungi - ps11
Fungi_1 - ps12
Fungi_2 - ps13
Fungi_3 - ps14
Dikarya - ps15
Ascomycota - ps16
Saccharo/Pezizomycotina - ps17
Saccharomycotina - ps18
Saccharomycotina_1 - ps19
Saccharomycotina_2 - ps20
Saccharomycotina_3 - ps21
Phaff./Saccharomycetaceae - ps22
Saccharomyco./mycetaceae - ps23
Saccharomycetaceae - ps24
Saccharomycetaceae_2 - ps25
Saccharomycetaceae_3 - ps26
Kaz./Nau./Saccharomyces - ps27
Saccharomyces - ps28
Arboricola_clade - ps29
Cerevisiae_clade - ps30
Saccharomyces_cerevisiae - ps31
Phylostratum

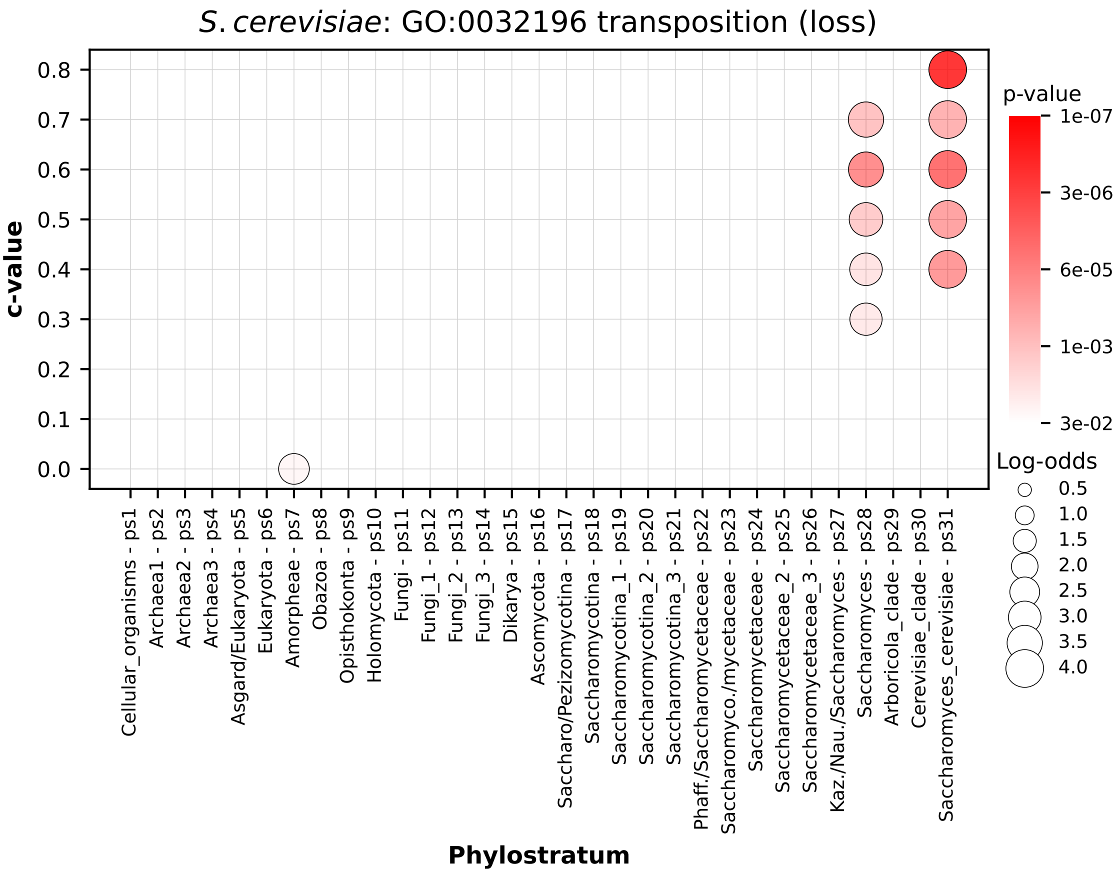

S. cerevisiae: GO:0032196 transposition (loss)
c-value
0.8
0.7
0.6
0.5
0.4
0.3
0.2
0.1
0.0
p-value
1e-07
3e-06
6e-05
1e-03
3e-02
Log-odds
0.5
1.0
1.5
2.0
2.5
3.0
3.5
4.0
Cellular_organisms - ps1
Archaea1 - ps2
Archaea2 - ps3
Archaea3 - ps4
Asgard/Eukaryota - ps5
Eukaryota - ps6
Amorpheae - ps7
Obazoa - ps8
Opisthokonta - ps9
Holomycota - ps10
Fungi - ps11
Fungi_1 - ps12
Fungi_2 - ps13
Fungi_3 - ps14
Dikarya - ps15
Ascomycota - ps16
Saccharo/Pezizomycotina - ps17
Saccharomycotina - ps18
Saccharomycotina_1 - ps19
Saccharomycotina_2 - ps20
Saccharomycotina_3 - ps21
Phaff./Saccharomycetaceae - ps22
Saccharomyco./mycetaceae - ps23
Saccharomycetaceae - ps24
Saccharomycetaceae_2 - ps25
Saccharomycetaceae_3 - ps26
Kaz./Nau./Saccharomyces - ps27
Saccharomyces - ps28
Arboricola_clade - ps29
Cerevisiae_clade - ps30
Saccharomyces_cerevisiae - ps31
Phylostratum

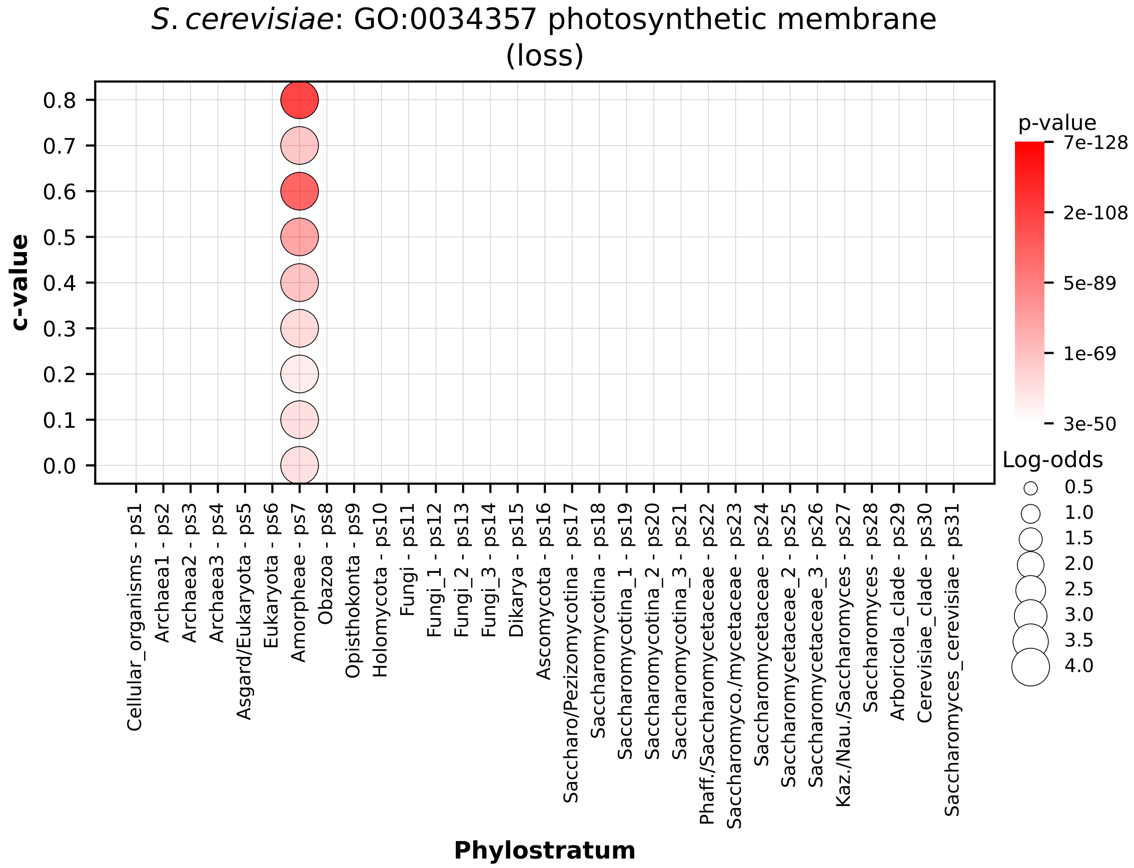
S. cerevisiae: GO:0034357 photosynthetic membrane
(loss)
c-value
0.8
0.7
0.6
0.5
0.4
0.3
0.2
0.1
0.0
p-value
7e-128
2e-108
5e-89
1e-69
3e-50
Log-odds
0.5
1.0
1.5
2.0
2.5
3.0
3.5
4.0
Cellular_organisms - ps1
Archaea1 - ps2
Archaea2 - ps3
Archaea3 - ps4
Asgard/Eukaryota - ps5
Eukaryota - ps6
Amorpheae - ps7
Obazoa - ps8
Opisthokonta - ps9
Holomycota - ps10
Fungi - ps11
Fungi_1 - ps12
Fungi_2 - ps13
Fungi_3 - ps14
Dikarya - ps15
Ascomycota - ps16
Saccharo/Pezizomycotina - ps17
Saccharomycotina - ps18
Saccharomycotina_1 - ps19
Saccharomycotina_2 - ps20
Saccharomycotina_3 - ps21
Phaff./Saccharomycetaceae - ps22
Saccharomyco./mycetaceae - ps23
Saccharomycetaceae - ps24
Saccharomycetaceae_2 - ps25
Saccharomycetaceae_3 - ps26
Kaz./Nau./Saccharomyces - ps27
Saccharomyces - ps28
Arboricola_clade - ps29
Cerevisiae_clade - ps30
Saccharomyces_cerevisiae - ps31
Phylostratum

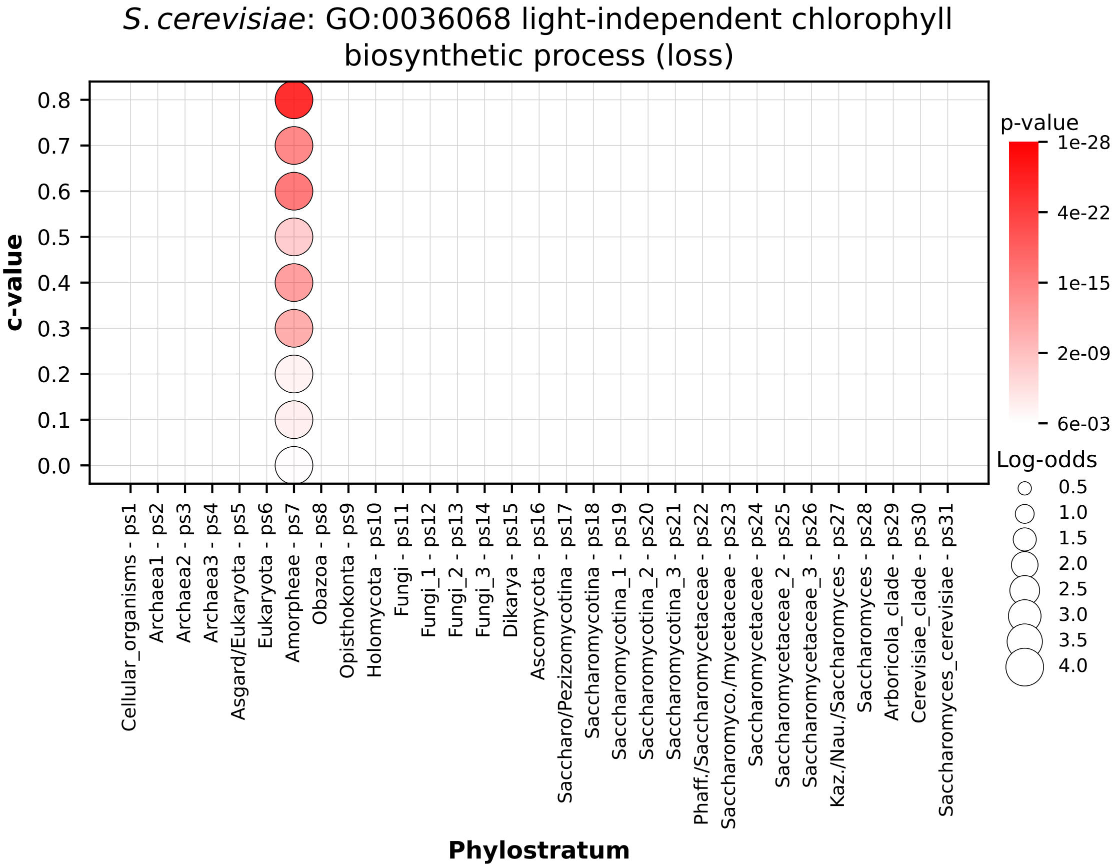
S. cerevisiae: GO:0036068 light-independent chlorophyll biosynthetic process (loss)
c-value
0.0
0.1
0.2
0.3
0.4
0.5
0.6
0.7
0.8
Phylostratum
Cellular_organisms - ps1
Archaea1 - ps2
Archaea2 - ps3
Archaea3 - ps4
Asgard/Eukaryota - ps5
Eukaryota - ps6
Amorpheae - ps7
Obazoa - ps8
Opisthokonta - ps9
Holomycota - ps10
Fungi - ps11
Fungi_1 - ps12
Fungi_2 - ps13
Fungi_3 - ps14
Dikarya - ps15
Ascomycota - ps16
Saccharo/Pezizomycotina - ps17
Saccharomycotina - ps18
Saccharomycotina_1 - ps19
Saccharomycotina_2 - ps20
Saccharomycotina_3 - ps21
Phaff./Saccharomycetaceae - ps22
Saccharomyco./mycetaceae - ps23
Saccharomycetaceae - ps24
Saccharomycetaceae_2 - ps25
Saccharomycetaceae_3 - ps26
Kaz./Nau./Saccharomyces - ps27
Saccharomyces - ps28
Arboricola_clade - ps29
Cerevisiae_clade - ps30
Saccharomyces_cerevisiae - ps31
p-value
1e-28
4e-22
1e-15
2e-09
6e-03
Log-odds
0.5
1.0
1.5
2.0
2.5
3.0
3.5
4.0

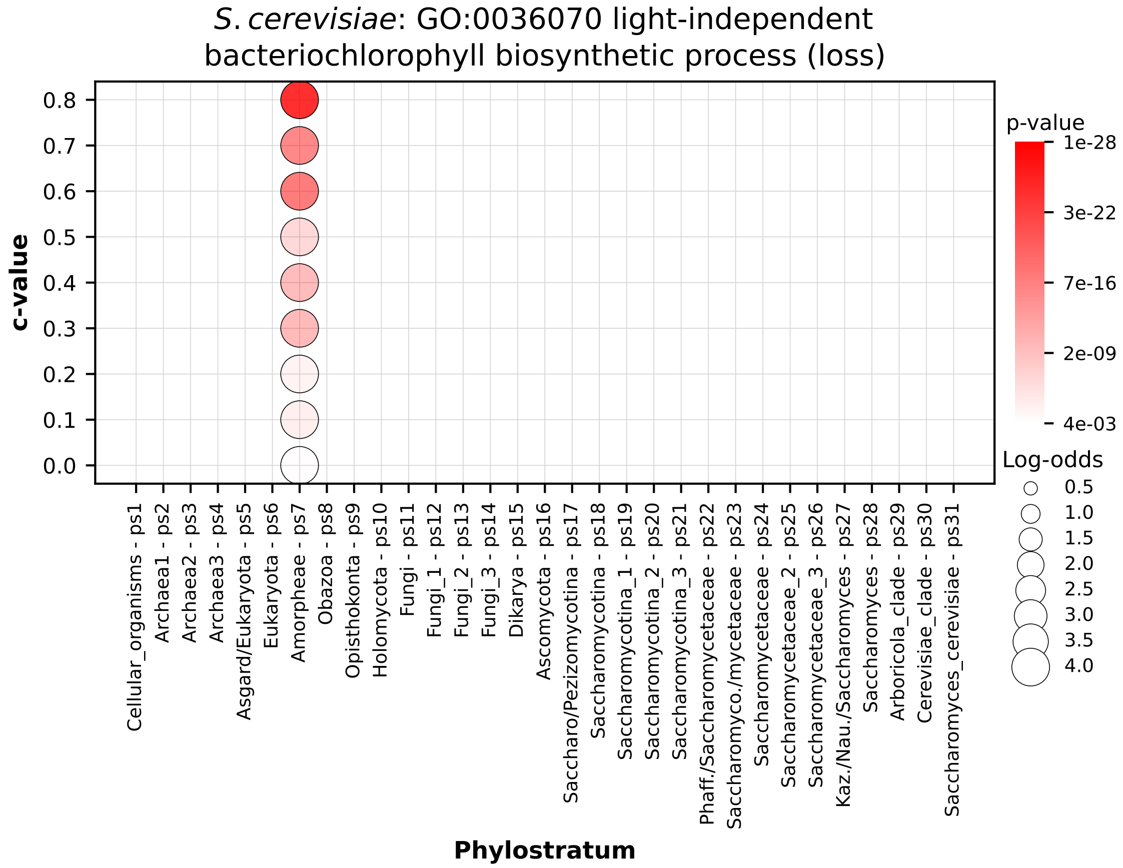
S. cerevisiae: GO:0036070 light-independent bacteriochlorophyll biosynthetic process (loss)
c-value
0.8
0.7
0.6
0.5
0.4
0.3
0.2
0.1
0.0
p-value
1e-28
3e-22
7e-16
2e-09
4e-03
Log-odds
0.5
1.0
1.5
2.0
2.5
3.0
3.5
4.0
Cellular_organisms - ps1
Archaea1 - ps2
Archaea2 - ps3
Archaea3 - ps4
Asgard/Eukaryota - ps5
Eukaryota - ps6
Amorpheae - ps7
Obazoa - ps8
Opisthokonta - ps9
Holomycota - ps10
Fungi - ps11
Fungi_1 - ps12
Fungi_2 - ps13
Fungi_3 - ps14
Dikarya - ps15
Ascomycota - ps16
Saccharo/Pezizomycotina - ps17
Saccharomycotina - ps18
Saccharomycotina_1 - ps19
Saccharomycotina_2 - ps20
Saccharomycotina_3 - ps21
Phaff./Saccharomycetaceae - ps22
Saccharomyco./mycetaceae - ps23
Saccharomycetaceae - ps24
Saccharomycetaceae_2 - ps25
Saccharomycetaceae_3 - ps26
Kaz./Nau./Saccharomyces - ps27
Saccharomyces - ps28
Arboricola_clade - ps29
Cerevisiae_clade - ps30
Saccharomyces_cerevisiae - ps31
Phylostratum

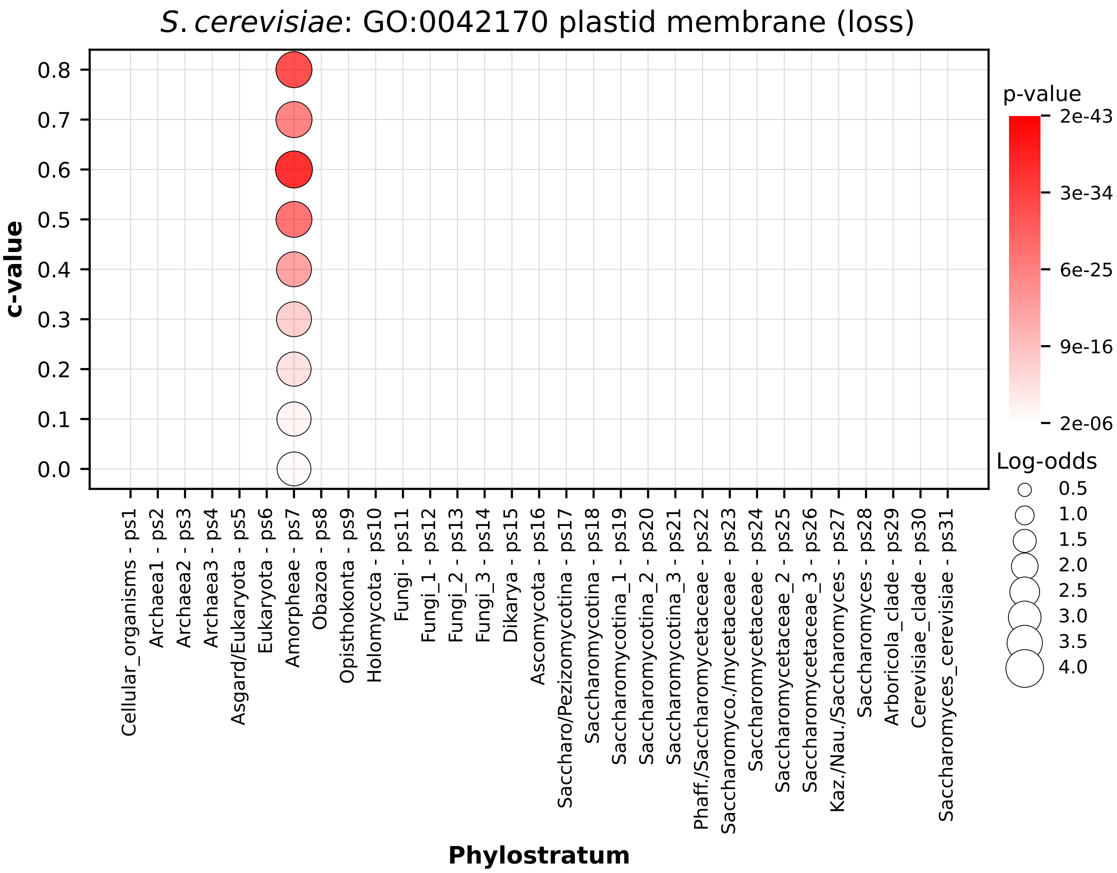
S. cerevisiae: GO:0042170 plastid membrane (loss)
c-value
0.8
0.7
0.6
0.5
0.4
0.3
0.2
0.1
0.0
p-value
2e-43
3e-34
6e-25
9e-16
2e-06
Log-odds
0.5
1.0
1.5
2.0
2.5
3.0
3.5
4.0
Cellular_organisms - ps1
Archaea1 - ps2
Archaea2 - ps3
Archaea3 - ps4
Asgard/Eukaryota - ps5
Eukaryota - ps6
Amorpheae - ps7
Obazoa - ps8
Opisthokonta - ps9
Holomycota - ps10
Fungi - ps11
Fungi_1 - ps12
Fungi_2 - ps13
Fungi_3 - ps14
Dikarya - ps15
Ascomycota - ps16
Saccharo/Pezizomycotina - ps17
Saccharomycotina - ps18
Saccharomycotina_1 - ps19
Saccharomycotina_2 - ps20
Saccharomycotina_3 - ps21
Phaff./Saccharomycetaceae - ps22
Saccharomyco./mycetaceae - ps23
Saccharomycetaceae - ps24
Saccharomycetaceae_2 - ps25
Saccharomycetaceae_3 - ps26
Kaz./Nau./Saccharomyces - ps27
Saccharomyces - ps28
Arboricola_clade - ps29
Cerevisiae_clade - ps30
Saccharomyces_cerevisiae - ps31
Phylostratum

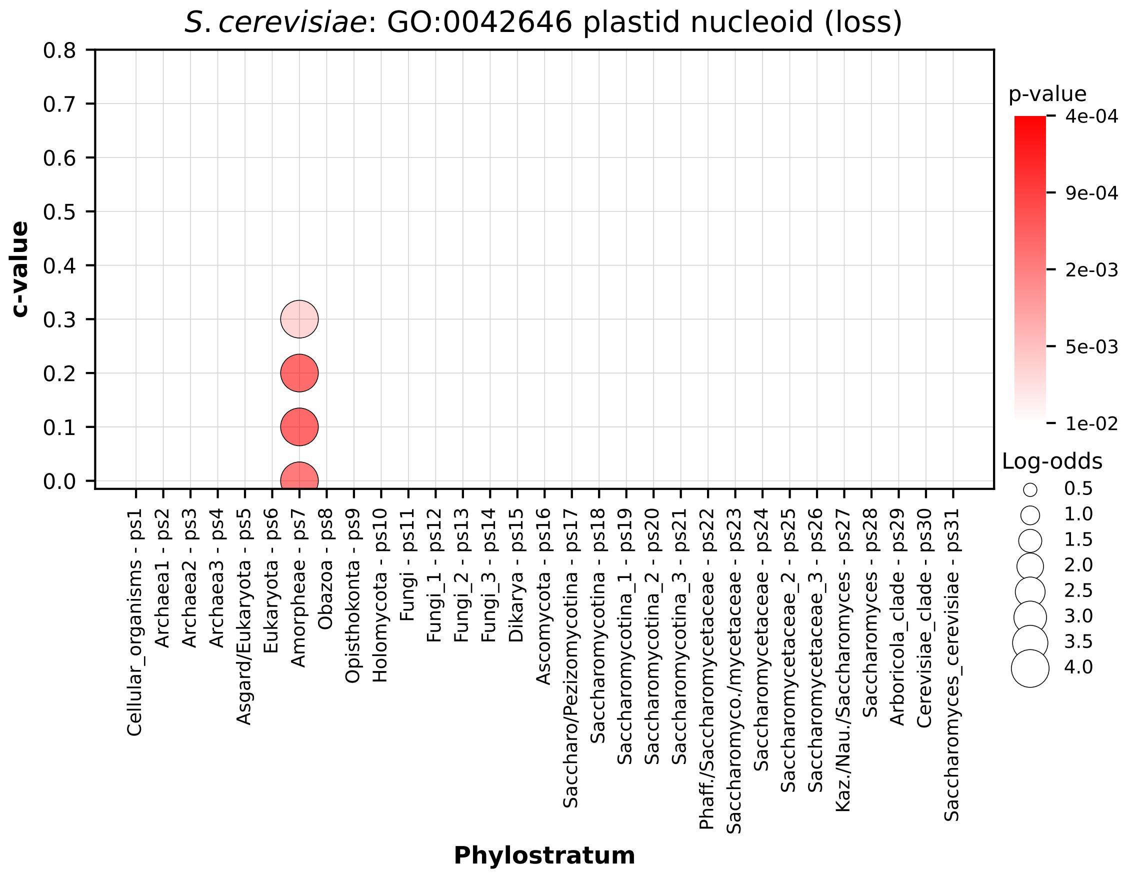

S. cerevisiae: GO:0042646 plastid nucleoid (loss)
c-value
0.0
0.1
0.2
0.3
0.4
0.5
0.6
0.7
0.8
Phylostratum
Cellular_organisms - ps1
Archaea1 - ps2
Archaea2 - ps3
Archaea3 - ps4
Asgard/Eukaryota - ps5
Eukaryota - ps6
Amorpheae - ps7
Obazoa - ps8
Opisthokonta - ps9
Holomycota - ps10
Fungi - ps11
Fungi_1 - ps12
Fungi_2 - ps13
Fungi_3 - ps14
Dikarya - ps15
Ascomycota - ps16
Saccharo/Pezizomycotina - ps17
Saccharomycotina - ps18
Saccharomycotina_1 - ps19
Saccharomycotina_2 - ps20
Saccharomycotina_3 - ps21
Phaff./Saccharomycetaceae - ps22
Saccharomyco./mycetaceae - ps23
Saccharomycetaceae - ps24
Saccharomycetaceae_2 - ps25
Saccharomycetaceae_3 - ps26
Kaz./Nau./Saccharomyces - ps27
Saccharomyces - ps28
Arboricola_clade - ps29
Cerevisiae_clade - ps30
Saccharomyces_cerevisiae - ps31
p-value
4e-04
9e-04
2e-03
5e-03
1e-02
Log-odds
0.5
1.0
1.5
2.0
2.5
3.0
3.5
4.0

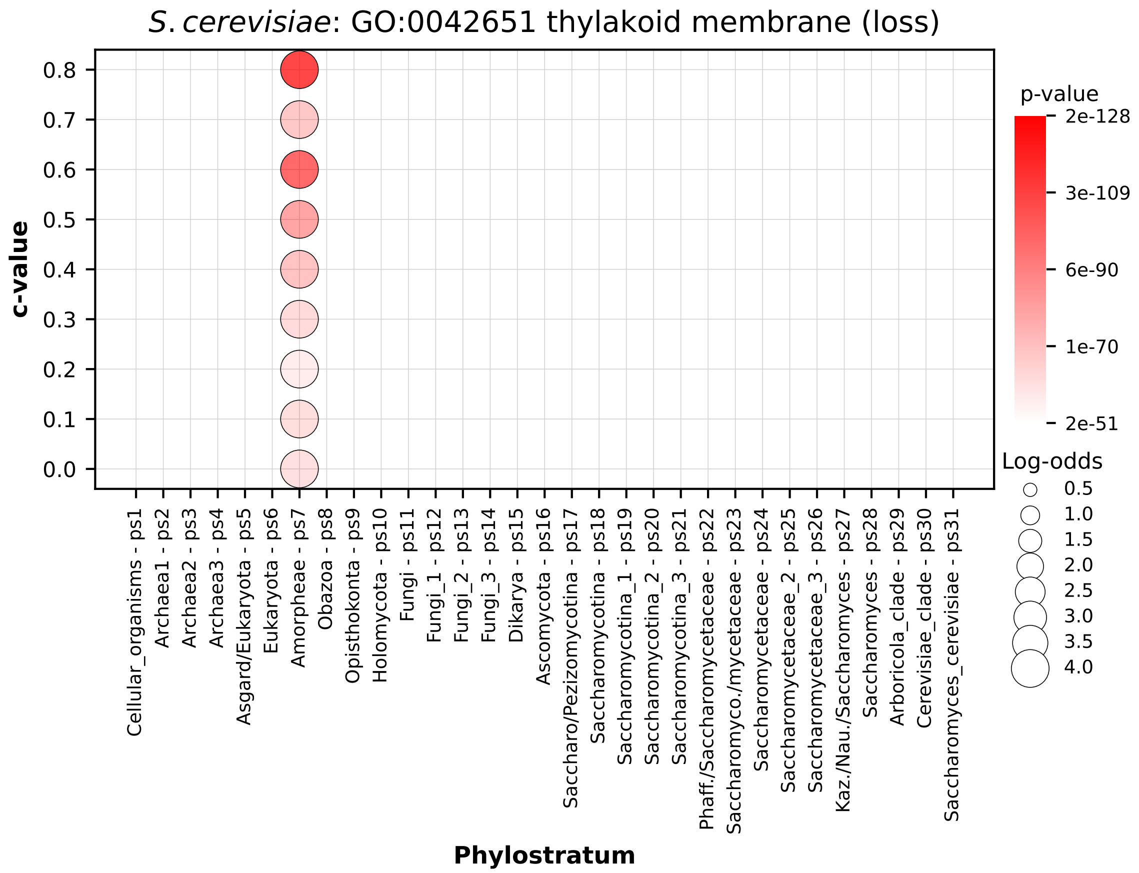

S. cerevisiae: GO:0042651 thylakoid membrane (loss)
c-value
0.8
0.7
0.6
0.5
0.4
0.3
0.2
0.1
0.0
p-value
2e-128
3e-109
6e-90
1e-70
2e-51
Log-odds
0.5
1.0
1.5
2.0
2.5
3.0
3.5
4.0
Cellular_organisms - ps1
Archaea1 - ps2
Archaea2 - ps3
Archaea3 - ps4
Asgard/Eukaryota - ps5
Eukaryota - ps6
Amorpheae - ps7
Obazoa - ps8
Opisthokonta - ps9
Holomycota - ps10
Fungi - ps11
Fungi_1 - ps12
Fungi_2 - ps13
Fungi_3 - ps14
Dikarya - ps15
Ascomycota - ps16
Saccharo/Pezizomycotina - ps17
Saccharomycotina - ps18
Saccharomycotina_1 - ps19
Saccharomycotina_2 - ps20
Saccharomycotina_3 - ps21
Phaff./Saccharomycetaceae - ps22
Saccharomyco./mycetaceae - ps23
Saccharomycetaceae - ps24
Saccharomycetaceae_2 - ps25
Saccharomycetaceae_3 - ps26
Kaz./Nau./Saccharomyces - ps27
Saccharomyces - ps28
Arboricola_clade - ps29
Cerevisiae_clade - ps30
Saccharomyces_cerevisiae - ps31
Phylostratum

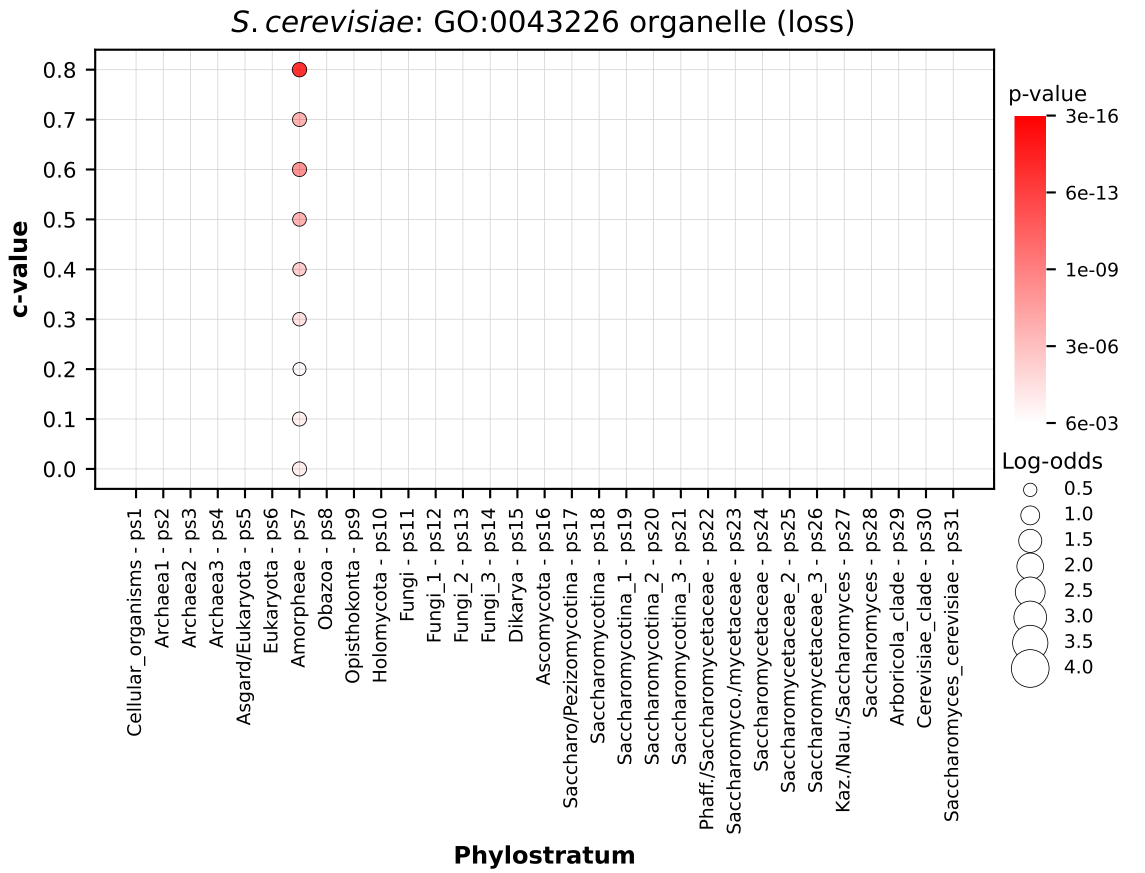
S. cerevisiae: GO:0043226 organelle (loss)
c-value
0.8
0.7
0.6
0.5
0.4
0.3
0.2
0.1
0.0
p-value
3e-16
6e-13
1e-09
3e-06
6e-03
Log-odds
0.5
1.0
1.5
2.0
2.5
3.0
3.5
4.0
Cellular_organisms - ps1
Archaea1 - ps2
Archaea2 - ps3
Archaea3 - ps4
Asgard/Eukaryota - ps5
Eukaryota - ps6
Amorpheae - ps7
Obazoa - ps8
Opisthokonta - ps9
Holomycota - ps10
Fungi - ps11
Fungi_1 - ps12
Fungi_2 - ps13
Fungi_3 - ps14
Dikarya - ps15
Ascomycota - ps16
Saccharo/Pezizomycotina - ps17
Saccharomycotina - ps18
Saccharomycotina_1 - ps19
Saccharomycotina_2 - ps20
Saccharomycotina_3 - ps21
Phaff./Saccharomycetaceae - ps22
Saccharomyco./mycetaceae - ps23
Saccharomycetaceae - ps24
Saccharomycetaceae_2 - ps25
Saccharomycetaceae_3 - ps26
Kaz./Nau./Saccharomyces - ps27
Saccharomyces - ps28
Arboricola_clade - ps29
Cerevisiae_clade - ps30
Saccharomyces_cerevisiae - ps31
Phylostratum

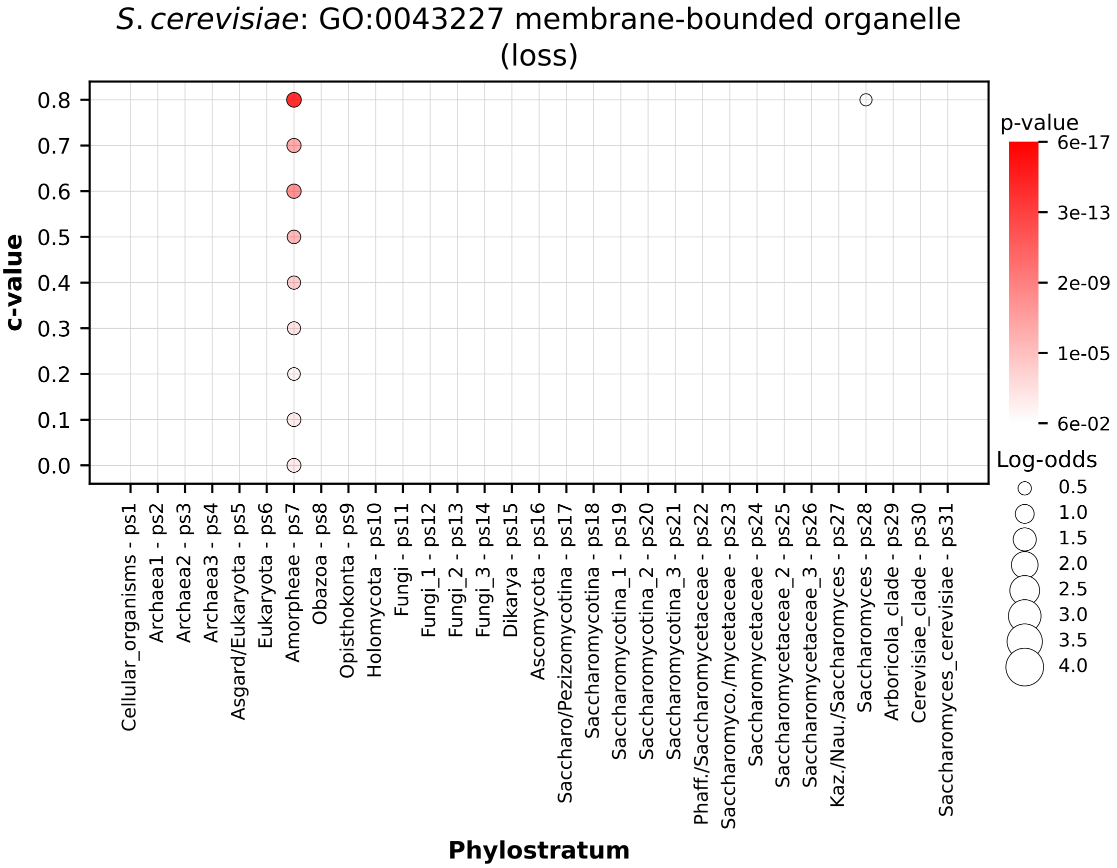

S. cerevisiae: GO:0043227 membrane-bounded organelle (loss)
c-value
0.8
0.7
0.6
0.5
0.4
0.3
0.2
0.1
0.0
Phylostratum
Cellular_organisms - ps1
Archaea1 - ps2
Archaea2 - ps3
Archaea3 - ps4
Asgard/Eukaryota - ps5
Eukaryota - ps6
Amorpheae - ps7
Obazoa - ps8
Opisthokonta - ps9
Holomycota - ps10
Fungi - ps11
Fungi_1 - ps12
Fungi_2 - ps13
Fungi_3 - ps14
Dikarya - ps15
Ascomycota - ps16
Saccharo/Pezizomycotina - ps17
Saccharomycotina - ps18
Saccharomycotina_1 - ps19
Saccharomycotina_2 - ps20
Saccharomycotina_3 - ps21
Phaff./Saccharomycetaceae - ps22
Saccharomyco./mycetaceae - ps23
Saccharomycetaceae - ps24
Saccharomycetaceae_2 - ps25
Saccharomycetaceae_3 - ps26
Kaz./Nau./Saccharomyces - ps27
Saccharomyces - ps28
Arboricola_clade - ps29
Cerevisiae_clade - ps30
Saccharomyces_cerevisiae - ps31
p-value
6e-17
3e-13
2e-09
1e-05
6e-02
Log-odds
0.5
1.0
1.5
2.0
2.5
3.0
3.5
4.0

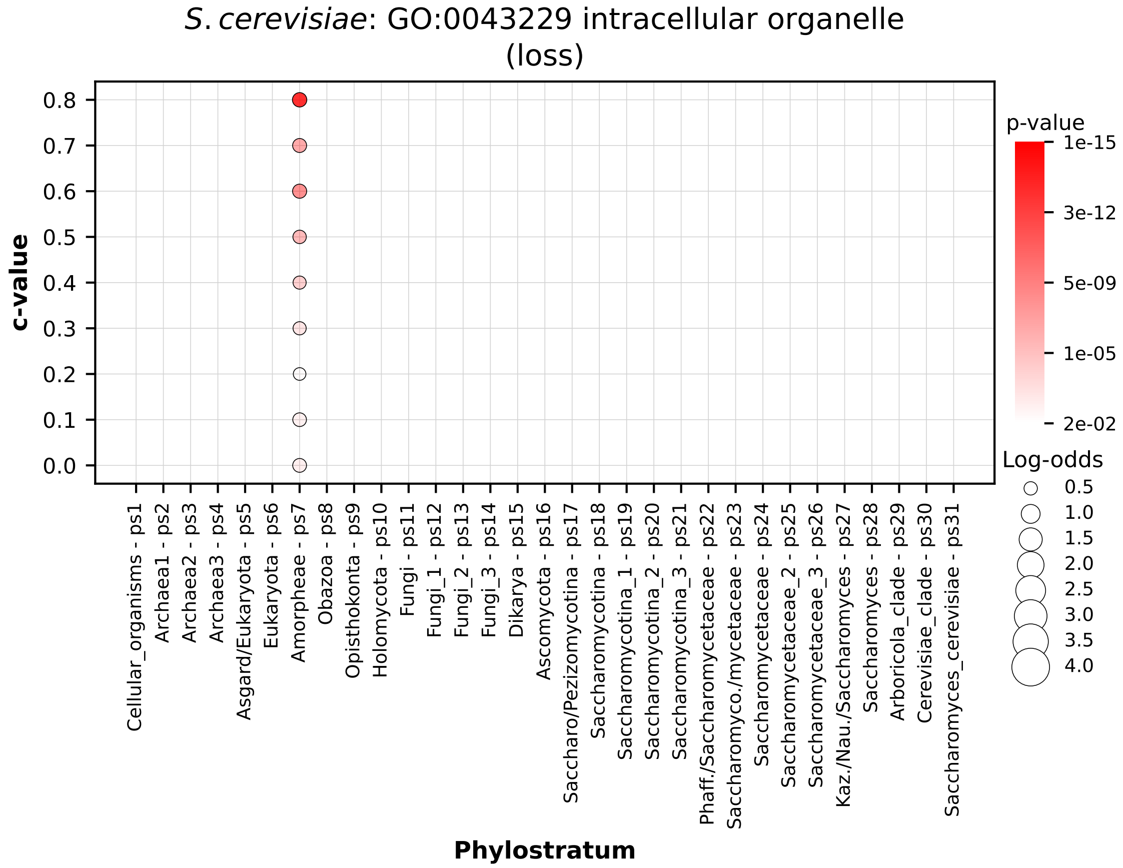
S. cerevisiae: GO:0043229 intracellular organelle
(loss)
c-value
0.8
0.7
0.6
0.5
0.4
0.3
0.2
0.1
0.0
p-value
1e-15
3e-12
5e-09
1e-05
2e-02
Log-odds
0.5
1.0
1.5
2.0
2.5
3.0
3.5
4.0
Cellular_organisms - ps1
Archaea1 - ps2
Archaea2 - ps3
Archaea3 - ps4
Asgard/Eukaryota - ps5
Eukaryota - ps6
Amorpheae - ps7
Obazoa - ps8
Opisthokonta - ps9
Holomycota - ps10
Fungi - ps11
Fungi_1 - ps12
Fungi_2 - ps13
Fungi_3 - ps14
Dikarya - ps15
Ascomycota - ps16
Saccharo/Pezizomycotina - ps17
Saccharomycotina - ps18
Saccharomycotina_1 - ps19
Saccharomycotina_2 - ps20
Saccharomycotina_3 - ps21
Phaff./Saccharomycetaceae - ps22
Saccharomyco./mycetaceae - ps23
Saccharomycetaceae - ps24
Saccharomycetaceae_2 - ps25
Saccharomycetaceae_3 - ps26
Kaz./Nau./Saccharomyces - ps27
Saccharomyces - ps28
Arboricola_clade - ps29
Cerevisiae_clade - ps30
Saccharomyces_cerevisiae - ps31
Phylostratum

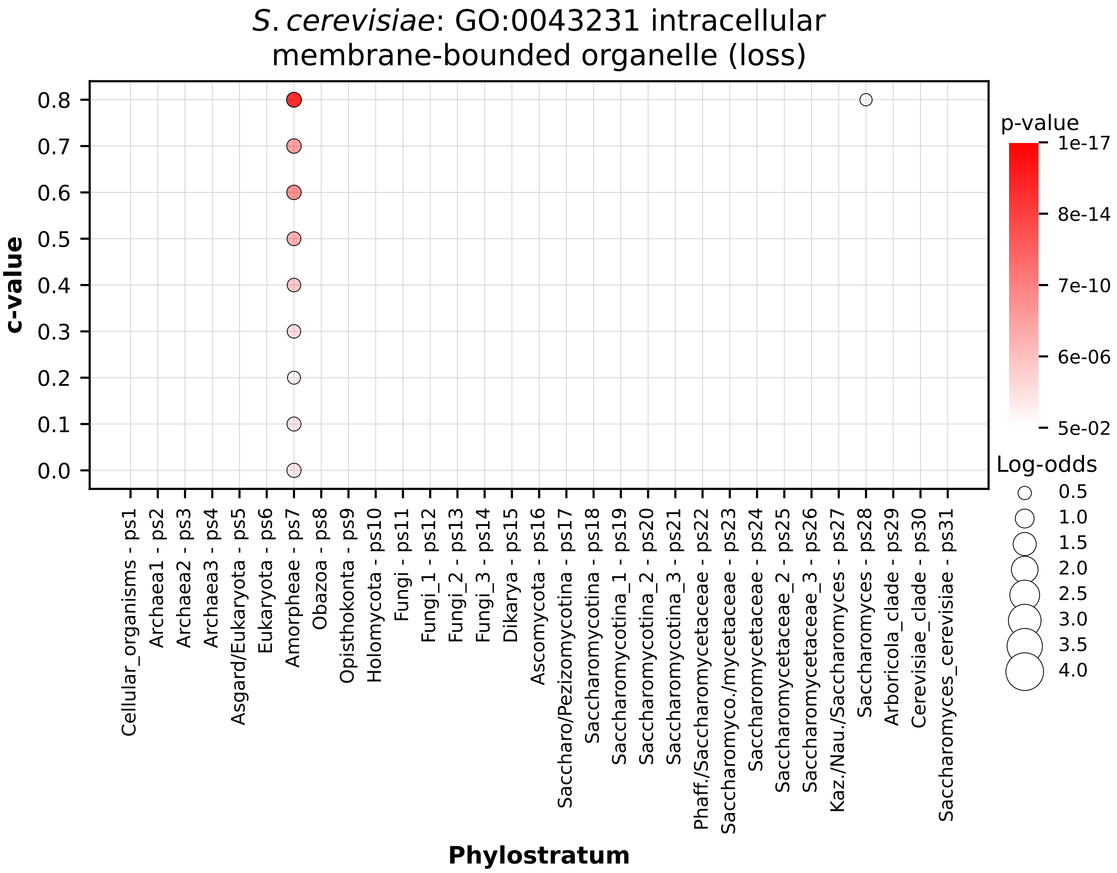

S. cerevisiae: GO:0043231 intracellular membrane-bounded organelle (loss)
c-value
0.8
0.7
0.6
0.5
0.4
0.3
0.2
0.1
0.0
p-value
1e-17
8e-14
7e-10
6e-06
5e-02
Log-odds
0.5
1.0
1.5
2.0
2.5
3.0
3.5
4.0
Cellular_organisms - ps1
Archaea1 - ps2
Archaea2 - ps3
Archaea3 - ps4
Asgard/Eukaryota - ps5
Eukaryota - ps6
Amorpheae - ps7
Obazoa - ps8
Opisthokonta - ps9
Holomycota - ps10
Fungi - ps11
Fungi_1 - ps12
Fungi_2 - ps13
Fungi_3 - ps14
Dikarya - ps15
Ascomycota - ps16
Saccharo/Pezizomycotina - ps17
Saccharomycotina - ps18
Saccharomycotina_1 - ps19
Saccharomycotina_2 - ps20
Saccharomycotina_3 - ps21
Phaff./Saccharomycetaceae - ps22
Saccharomyco./mycetaceae - ps23
Saccharomycetaceae - ps24
Saccharomycetaceae_2 - ps25
Saccharomycetaceae_3 - ps26
Kaz./Nau./Saccharomyces - ps27
Saccharomyces - ps28
Arboricola_clade - ps29
Cerevisiae_clade - ps30
Saccharomyces_cerevisiae - ps31
Phylostratum

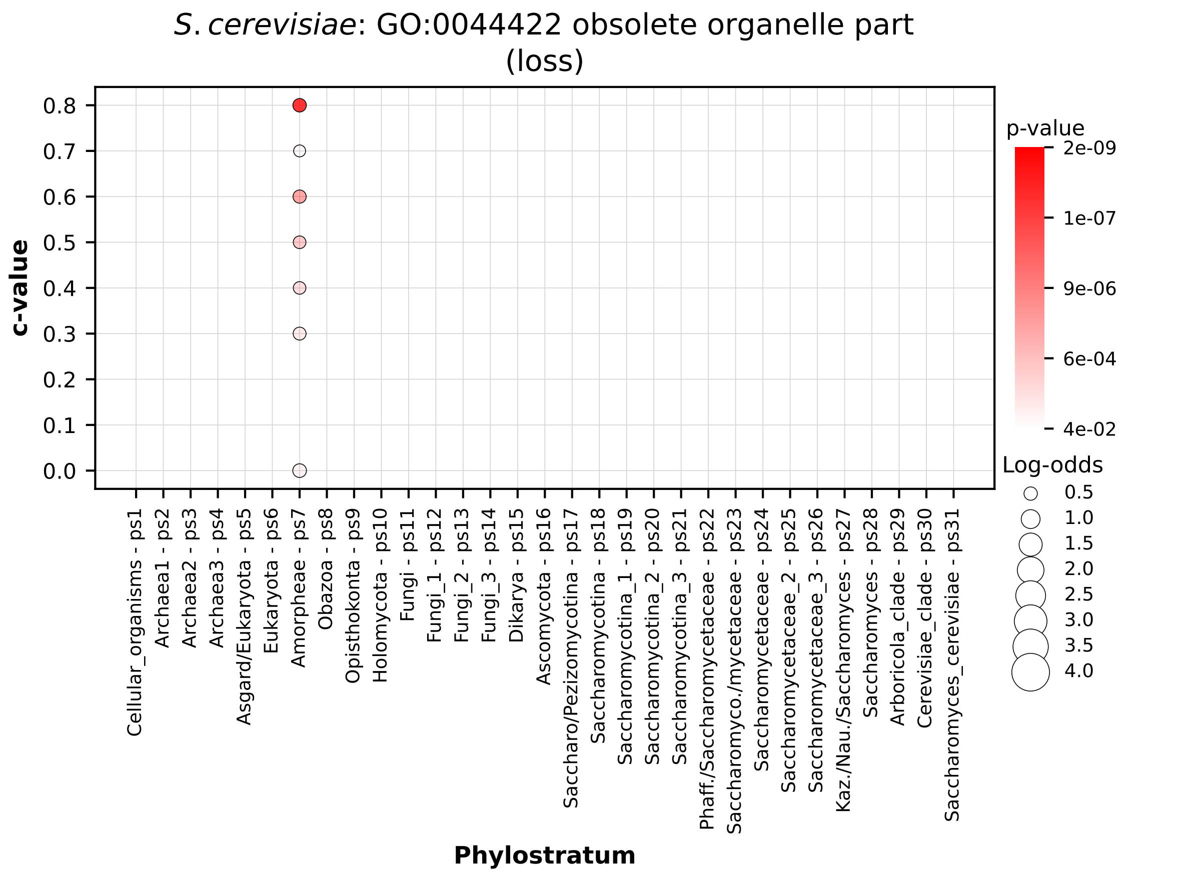
S. cerevisiae: GO:0044422 obsolete organelle part
(loss)
c-value
0.0
0.1
0.2
0.3
0.4
0.5
0.6
0.7
0.8
p-value
2e-09
1e-07
9e-06
6e-04
4e-02
Log-odds
0.5
1.0
1.5
2.0
2.5
3.0
3.5
4.0
Cellular_organisms - ps1
Archaea1 - ps2
Archaea2 - ps3
Archaea3 - ps4
Asgard/Eukaryota - ps5
Eukaryota - ps6
Amorpheae - ps7
Obazoa - ps8
Opisthokonta - ps9
Holomycota - ps10
Fungi - ps11
Fungi_1 - ps12
Fungi_2 - ps13
Fungi_3 - ps14
Dikarya - ps15
Ascomycota - ps16
Saccharo/Pezizomycotina - ps17
Saccharomycotina - ps18
Saccharomycotina_1 - ps19
Saccharomycotina_2 - ps20
Saccharomycotina_3 - ps21
Phaff./Saccharomycetaceae - ps22
Saccharomyco./mycetaceae - ps23
Saccharomycetaceae - ps24
Saccharomycetaceae_2 - ps25
Saccharomycetaceae_3 - ps26
Kaz./Nau./Saccharomyces - ps27
Saccharomyces - ps28
Arboricola_clade - ps29
Cerevisiae_clade - ps30
Saccharomyces_cerevisiae - ps31
Phylostratum

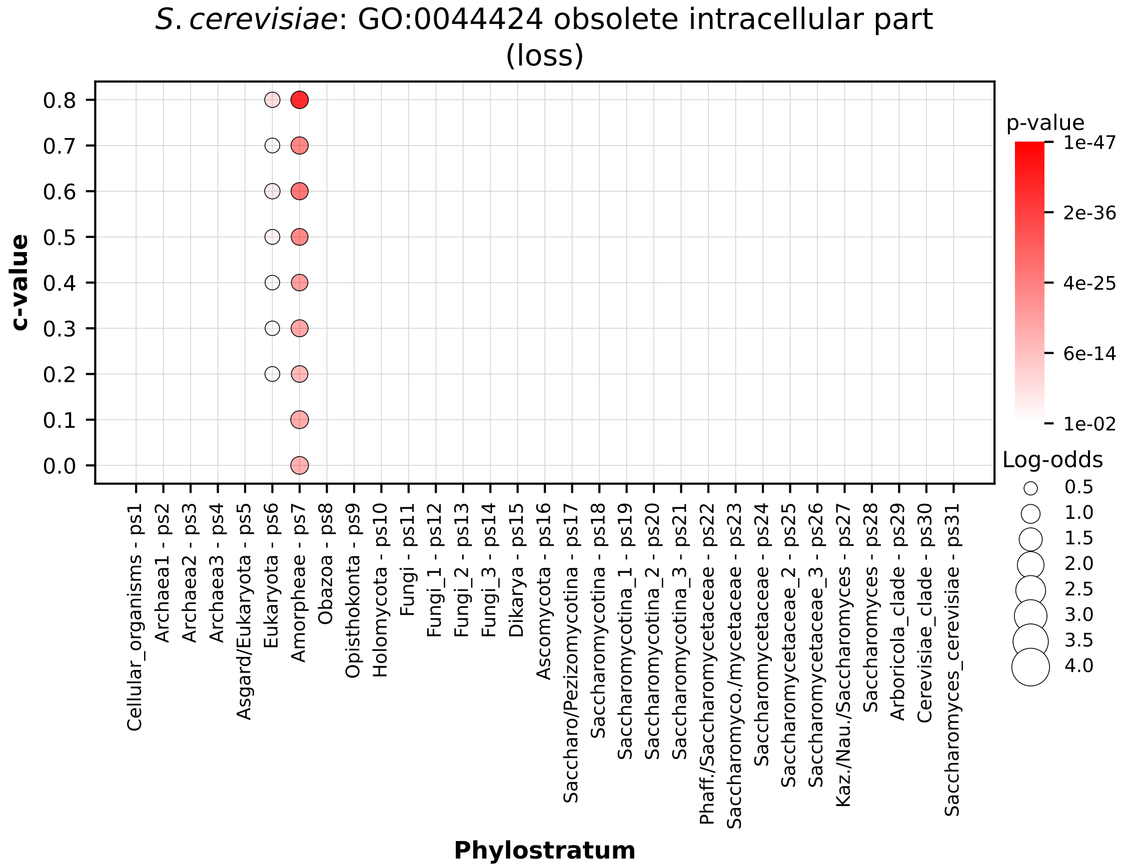

S. cerevisiae: GO:0044424 obsolete intracellular part
(loss)
c-value
0.8
0.7
0.6
0.5
0.4
0.3
0.2
0.1
0.0
p-value
1e-47
2e-36
4e-25
6e-14
1e-02
Log-odds
0.5
1.0
1.5
2.0
2.5
3.0
3.5
4.0
Cellular_organisms - ps1
Archaea1 - ps2
Archaea2 - ps3
Archaea3 - ps4
Asgard/Eukaryota - ps5
Eukaryota - ps6
Amorpheae - ps7
Obazoa - ps8
Opisthokonta - ps9
Holomycota - ps10
Fungi - ps11
Fungi_1 - ps12
Fungi_2 - ps13
Fungi_3 - ps14
Dikarya - ps15
Ascomycota - ps16
Saccharo/Pezizomycotina - ps17
Saccharomycotina - ps18
Saccharomycotina_1 - ps19
Saccharomycotina_2 - ps20
Saccharomycotina_3 - ps21
Phaff./Saccharomycetaceae - ps22
Saccharomyco./mycetaceae - ps23
Saccharomycetaceae - ps24
Saccharomycetaceae_2 - ps25
Saccharomycetaceae_3 - ps26
Kaz./Nau./Saccharomyces - ps27
Saccharomyces - ps28
Arboricola_clade - ps29
Cerevisiae_clade - ps30
Saccharomyces_cerevisiae - ps31
Phylostratum

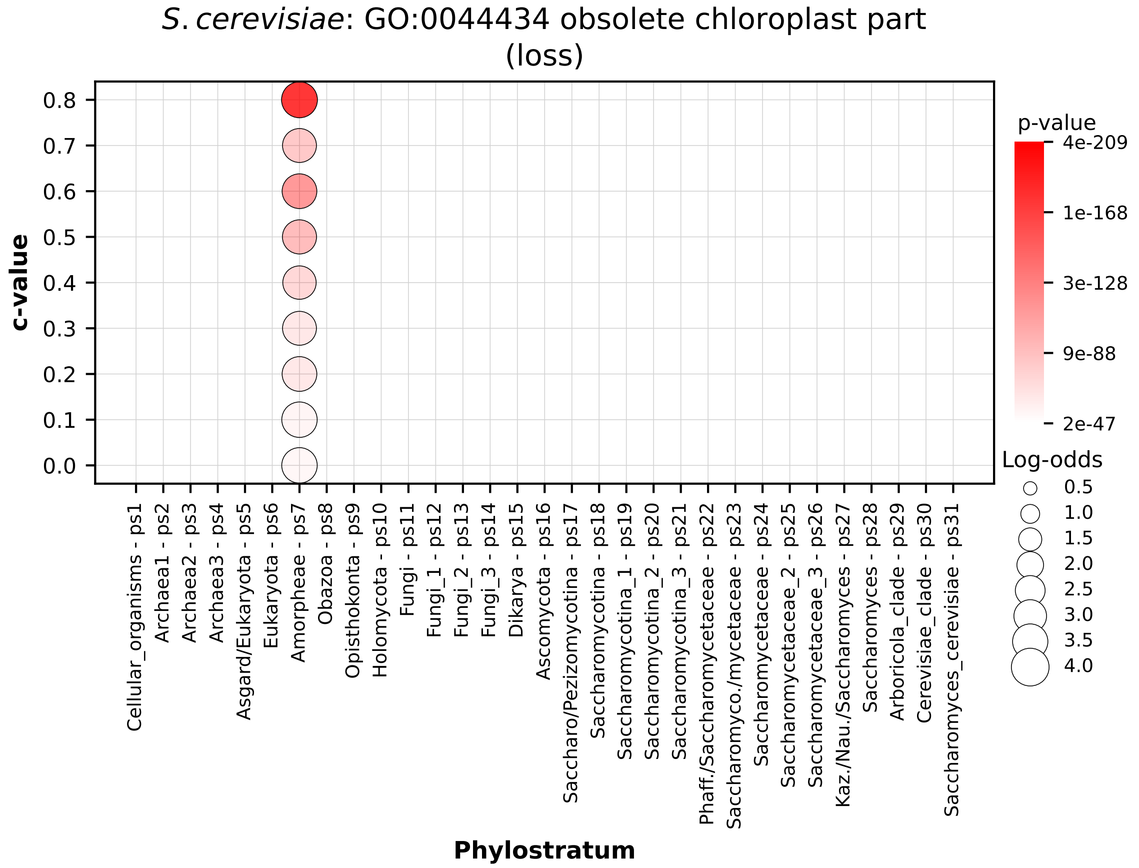
S. cerevisiae: GO:0044434 obsolete chloroplast part
(loss)
c-value
0.8
0.7
0.6
0.5
0.4
0.3
0.2
0.1
0.0
p-value
4e-209
1e-168
3e-128
9e-88
2e-47
Log-odds
0.5
1.0
1.5
2.0
2.5
3.0
3.5
4.0
Cellular_organisms - ps1
Archaea1 - ps2
Archaea2 - ps3
Archaea3 - ps4
Asgard/Eukaryota - ps5
Eukaryota - ps6
Amorpheae - ps7
Obazoa - ps8
Opisthokonta - ps9
Holomycota - ps10
Fungi - ps11
Fungi_1 - ps12
Fungi_2 - ps13
Fungi_3 - ps14
Dikarya - ps15
Ascomycota - ps16
Saccharo/Pezizomycotina - ps17
Saccharomycotina - ps18
Saccharomycotina_1 - ps19
Saccharomycotina_2 - ps20
Saccharomycotina_3 - ps21
Phaff./Saccharomycetaceae - ps22
Saccharomyco./mycetaceae - ps23
Saccharomycetaceae - ps24
Saccharomycetaceae_2 - ps25
Saccharomycetaceae_3 - ps26
Kaz./Nau./Saccharomyces - ps27
Saccharomyces - ps28
Arboricola_clade - ps29
Cerevisiae_clade - ps30
Saccharomyces_cerevisiae - ps31
Phylostratum

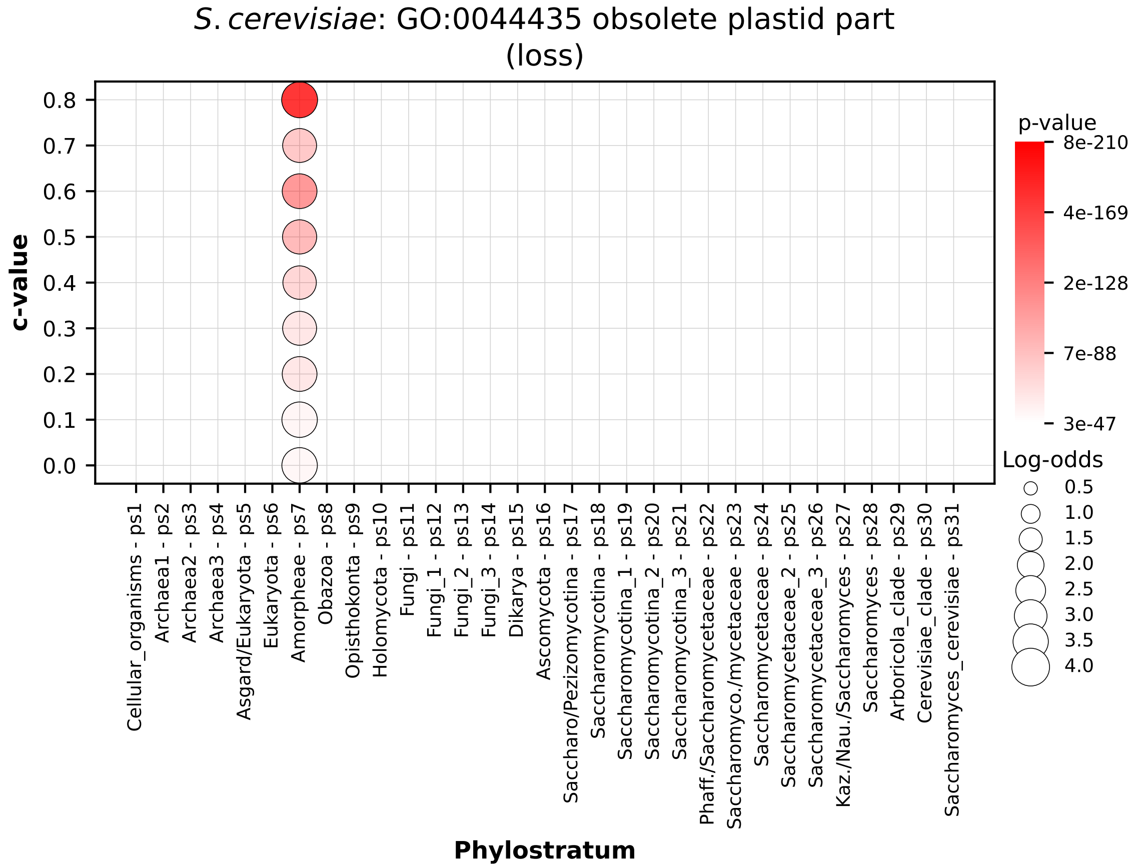

S. cerevisiae: GO:0044435 obsolete plastid part
(loss)
c-value
0.8
0.7
0.6
0.5
0.4
0.3
0.2
0.1
0.0
p-value
8e-210
4e-169
2e-128
7e-88
3e-47
Log-odds
0.5
1.0
1.5
2.0
2.5
3.0
3.5
4.0
Cellular_organisms - ps1
Archaea1 - ps2
Archaea2 - ps3
Archaea3 - ps4
Asgard/Eukaryota - ps5
Eukaryota - ps6
Amorpheae - ps7
Obazoa - ps8
Opisthokonta - ps9
Holomycota - ps10
Fungi - ps11
Fungi_1 - ps12
Fungi_2 - ps13
Fungi_3 - ps14
Dikarya - ps15
Ascomycota - ps16
Saccharo/Pezizomycotina - ps17
Saccharomycotina - ps18
Saccharomycotina_1 - ps19
Saccharomycotina_2 - ps20
Saccharomycotina_3 - ps21
Phaff./Saccharomycetaceae - ps22
Saccharomyco./mycetaceae - ps23
Saccharomycetaceae - ps24
Saccharomycetaceae_2 - ps25
Saccharomycetaceae_3 - ps26
Kaz./Nau./Saccharomyces - ps27
Saccharomyces - ps28
Arboricola_clade - ps29
Cerevisiae_clade - ps30
Saccharomyces_cerevisiae - ps31
Phylostratum

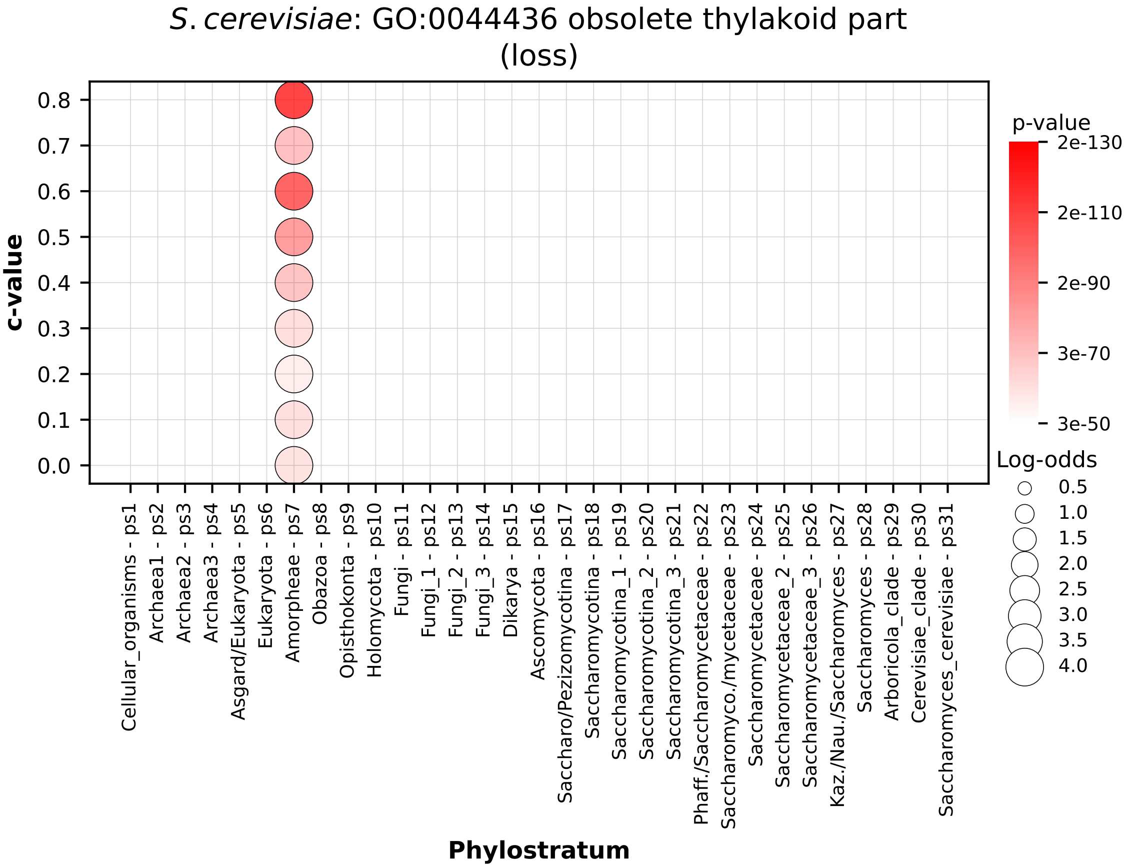
S. cerevisiae: GO:0044436 obsolete thylakoid part
(loss)
c-value
0.8
0.7
0.6
0.5
0.4
0.3
0.2
0.1
0.0
p-value
2e-130
2e-110
2e-90
3e-70
3e-50
Log-odds
0.5
1.0
1.5
2.0
2.5
3.0
3.5
4.0
Cellular_organisms - ps1
Archaea1 - ps2
Archaea2 - ps3
Archaea3 - ps4
Asgard/Eukaryota - ps5
Eukaryota - ps6
Amorpheae - ps7
Obazoa - ps8
Opisthokonta - ps9
Holomycota - ps10
Fungi - ps11
Fungi_1 - ps12
Fungi_2 - ps13
Fungi_3 - ps14
Dikarya - ps15
Ascomycota - ps16
Saccharo/Pezizomycotina - ps17
Saccharomycotina - ps18
Saccharomycotina_1 - ps19
Saccharomycotina_2 - ps20
Saccharomycotina_3 - ps21
Phaff./Saccharomycetaceae - ps22
Saccharomyco./mycetaceae - ps23
Saccharomycetaceae - ps24
Saccharomycetaceae_2 - ps25
Saccharomycetaceae_3 - ps26
Kaz./Nau./Saccharomyces - ps27
Saccharomyces - ps28
Arboricola_clade - ps29
Cerevisiae_clade - ps30
Saccharomyces_cerevisiae - ps31
Phylostratum

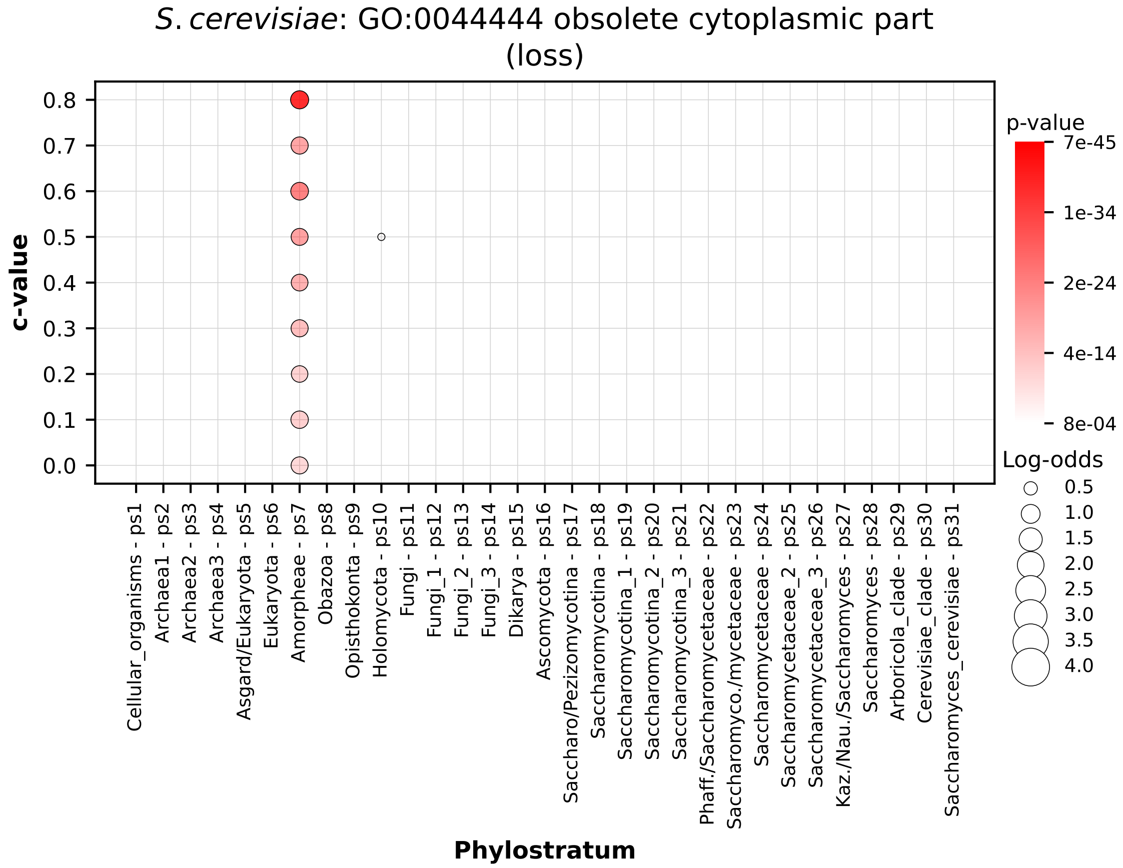

S. cerevisiae: GO:0044444 obsolete cytoplasmic part
(loss)
c-value
0.8
0.7
0.6
0.5
0.4
0.3
0.2
0.1
0.0
Phylostratum
Cellular_organisms - ps1
Archaea1 - ps2
Archaea2 - ps3
Archaea3 - ps4
Asgard/Eukaryota - ps5
Eukaryota - ps6
Amorpheae - ps7
Obazoa - ps8
Opisthokonta - ps9
Holomycota - ps10
Fungi - ps11
Fungi_1 - ps12
Fungi_2 - ps13
Fungi_3 - ps14
Dikarya - ps15
Ascomycota - ps16
Saccharo/Pezizomycotina - ps17
Saccharomycotina - ps18
Saccharomycotina_1 - ps19
Saccharomycotina_2 - ps20
Saccharomycotina_3 - ps21
Phaff./Saccharomycetaceae - ps22
Saccharomyco./mycetaceae - ps23
Saccharomycetaceae - ps24
Saccharomycetaceae_2 - ps25
Saccharomycetaceae_3 - ps26
Kaz./Nau./Saccharomyces - ps27
Saccharomyces - ps28
Arboricola_clade - ps29
Cerevisiae_clade - ps30
Saccharomyces_cerevisiae - ps31
p-value
7e-45
1e-34
2e-24
4e-14
8e-04
Log-odds
0.5
1.0
1.5
2.0
2.5
3.0
3.5
4.0

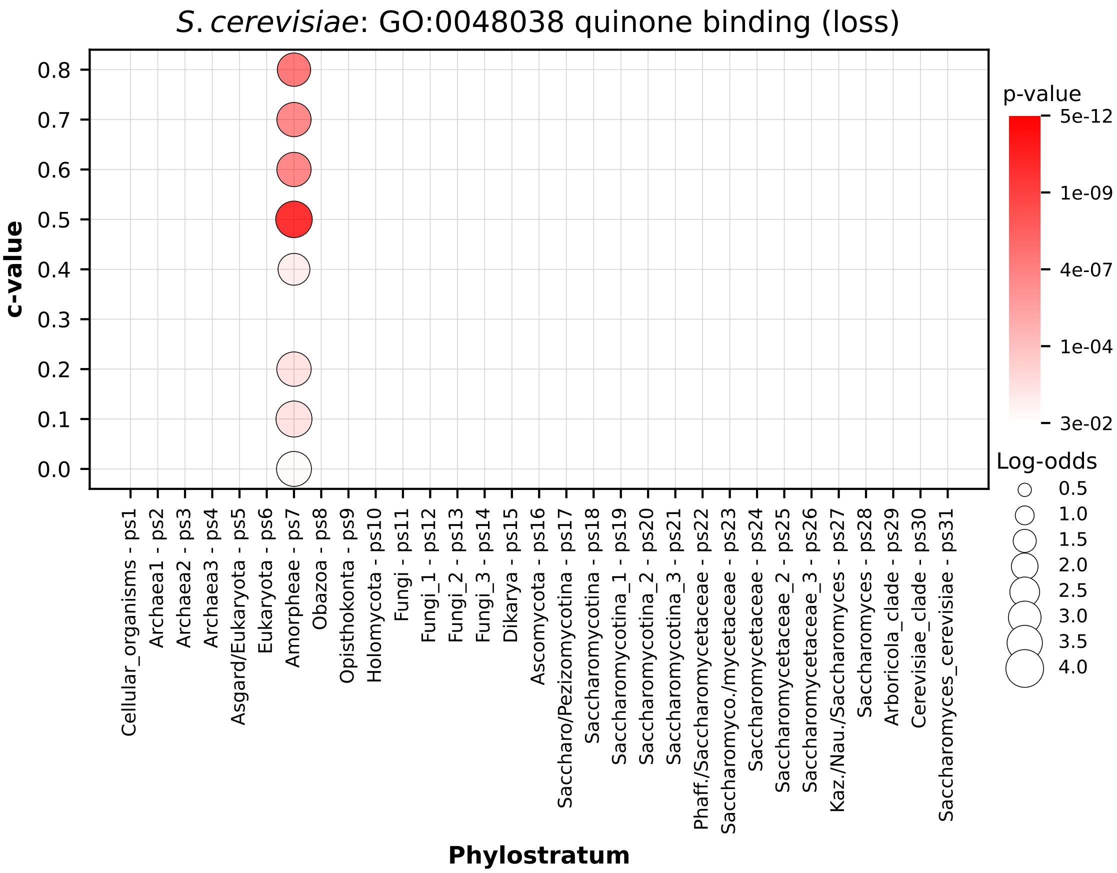

S. cerevisiae: GO:0048038 quinone binding (loss)
c-value
0.0
0.1
0.2
0.3
0.4
0.5
0.6
0.7
0.8
Phylostratum
Cellular_organisms - ps1
Archaea1 - ps2
Archaea2 - ps3
Archaea3 - ps4
Asgard/Eukaryota - ps5
Eukaryota - ps6
Amorpheae - ps7
Obazoa - ps8
Opisthokonta - ps9
Holomycota - ps10
Fungi - ps11
Fungi_1 - ps12
Fungi_2 - ps13
Fungi_3 - ps14
Dikarya - ps15
Ascomycota - ps16
Saccharo/Pezizomycotina - ps17
Saccharomycotina - ps18
Saccharomycotina_1 - ps19
Saccharomycotina_2 - ps20
Saccharomycotina_3 - ps21
Phaff./Saccharomycetaceae - ps22
Saccharomyco./mycetaceae - ps23
Saccharomycetaceae - ps24
Saccharomycetaceae_2 - ps25
Saccharomycetaceae_3 - ps26
Kaz./Nau./Saccharomyces - ps27
Saccharomyces - ps28
Arboricola_clade - ps29
Cerevisiae_clade - ps30
Saccharomyces_cerevisiae - ps31
p-value
5e-12
1e-09
4e-07
1e-04
3e-02
Log-odds
0.5
1.0
1.5
2.0
2.5
3.0
3.5
4.0

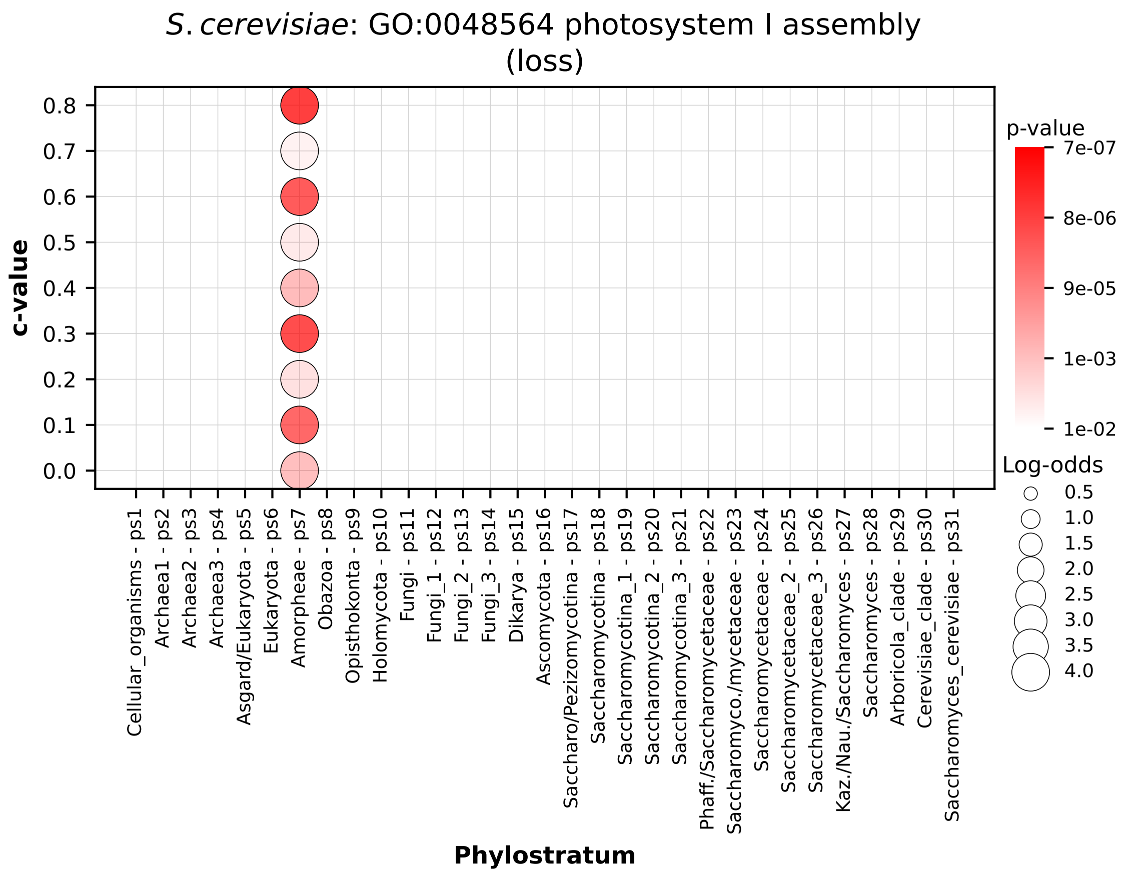

S. cerevisiae: GO:0048564 photosystem I assembly
(loss)
c-value
0.8
0.7
0.6
0.5
0.4
0.3
0.2
0.1
0.0
p-value
7e-07
8e-06
9e-05
1e-03
1e-02
Log-odds
0.5
1.0
1.5
2.0
2.5
3.0
3.5
4.0
Cellular_organisms - ps1
Archaea1 - ps2
Archaea2 - ps3
Archaea3 - ps4
Asgard/Eukaryota - ps5
Eukaryota - ps6
Amorpheae - ps7
Obazoa - ps8
Opisthokonta - ps9
Holomycota - ps10
Fungi - ps11
Fungi_1 - ps12
Fungi_2 - ps13
Fungi_3 - ps14
Dikarya - ps15
Ascomycota - ps16
Saccharo/Pezizomycotina - ps17
Saccharomycotina - ps18
Saccharomycotina_1 - ps19
Saccharomycotina_2 - ps20
Saccharomycotina_3 - ps21
Phaff./Saccharomycetaceae - ps22
Saccharomyco./mycetaceae - ps23
Saccharomycetaceae - ps24
Saccharomycetaceae_2 - ps25
Saccharomycetaceae_3 - ps26
Kaz./Nau./Saccharomyces - ps27
Saccharomyces - ps28
Arboricola_clade - ps29
Cerevisiae_clade - ps30
Saccharomyces_cerevisiae - ps31
Phylostratum

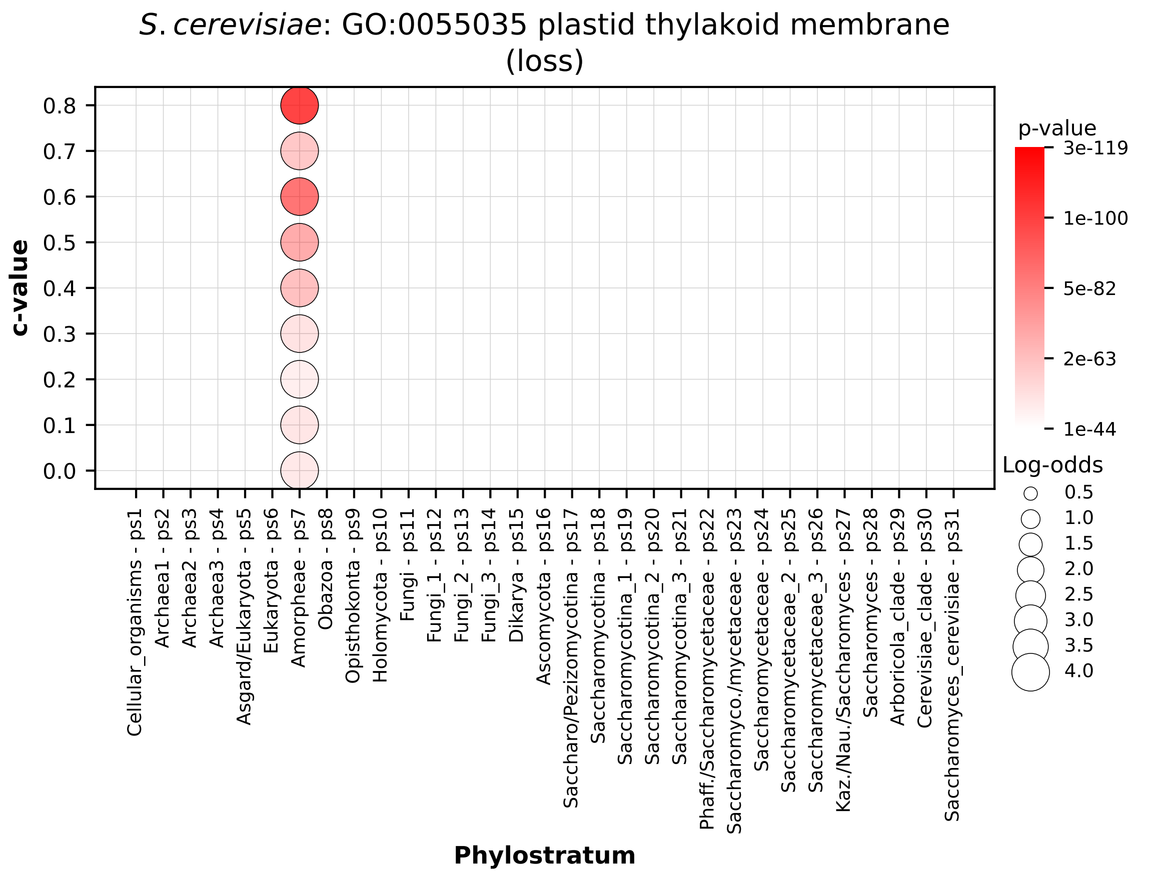

S. cerevisiae: GO:0055035 plastid thylakoid membrane (loss)
c-value
0.8
0.7
0.6
0.5
0.4
0.3
0.2
0.1
0.0
p-value
3e-119
1e-100
5e-82
2e-63
1e-44
Log-odds
0.5
1.0
1.5
2.0
2.5
3.0
3.5
4.0
Cellular_organisms - ps1
Archaea1 - ps2
Archaea2 - ps3
Archaea3 - ps4
Asgard/Eukaryota - ps5
Eukaryota - ps6
Amorpheae - ps7
Obazoa - ps8
Opisthokonta - ps9
Holomycota - ps10
Fungi - ps11
Fungi_1 - ps12
Fungi_2 - ps13
Fungi_3 - ps14
Dikarya - ps15
Ascomycota - ps16
Saccharo/Pezizomycotina - ps17
Saccharomycotina - ps18
Saccharomycotina_1 - ps19
Saccharomycotina_2 - ps20
Saccharomycotina_3 - ps21
Phaff./Saccharomycetaceae - ps22
Saccharomyco./mycetaceae - ps23
Saccharomycetaceae - ps24
Saccharomycetaceae_2 - ps25
Saccharomycetaceae_3 - ps26
Kaz./Nau./Saccharomyces - ps27
Saccharomyces - ps28
Arboricola_clade - ps29
Cerevisiae_clade - ps30
Saccharomyces_cerevisiae - ps31
Phylostratum

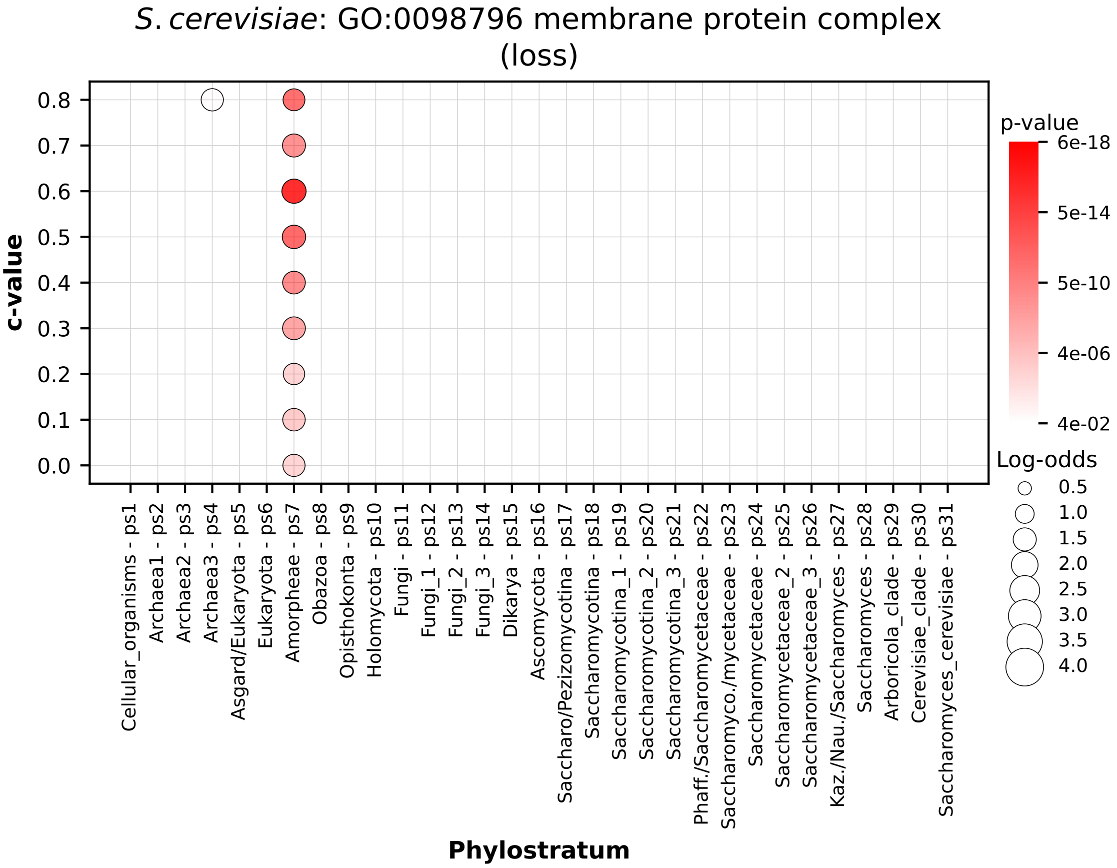

S. cerevisiae: GO:0098796 membrane protein complex
(loss)
c-value
0.8
0.7
0.6
0.5
0.4
0.3
0.2
0.1
0.0
p-value
6e-18
5e-14
5e-10
4e-06
4e-02
Log-odds
0.5
1.0
1.5
2.0
2.5
3.0
3.5
4.0
Cellular_organisms - ps1
Archaea1 - ps2
Archaea2 - ps3
Archaea3 - ps4
Asgard/Eukaryota - ps5
Eukaryota - ps6
Amorpheae - ps7
Obazoa - ps8
Opisthokonta - ps9
Holomycota - ps10
Fungi - ps11
Fungi_1 - ps12
Fungi_2 - ps13
Fungi_3 - ps14
Dikarya - ps15
Ascomycota - ps16
Saccharo/Pezizomycotina - ps17
Saccharomycotina - ps18
Saccharomycotina_1 - ps19
Saccharomycotina_2 - ps20
Saccharomycotina_3 - ps21
Phaff./Saccharomycetaceae - ps22
Saccharomyco./mycetaceae - ps23
Saccharomycetaceae - ps24
Saccharomycetaceae_2 - ps25
Saccharomycetaceae_3 - ps26
Kaz./Nau./Saccharomyces - ps27
Saccharomyces - ps28
Arboricola_clade - ps29
Cerevisiae_clade - ps30
Saccharomyces_cerevisiae - ps31
Phylostratum

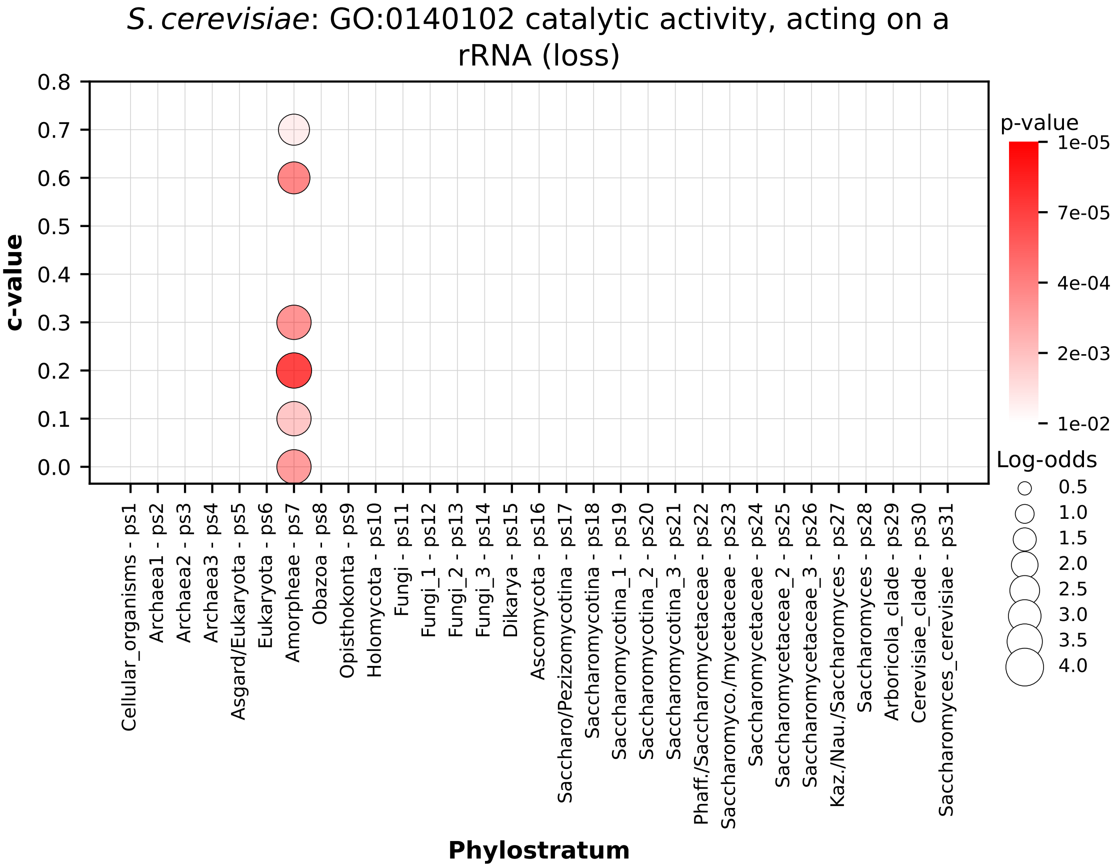
S. cerevisiae: GO:0140102 catalytic activity, acting on a rRNA (loss)
c-value
0.8
0.7
0.6
0.5
0.4
0.3
0.2
0.1
0.0
p-value
1e-05
7e-05
4e-04
2e-03
1e-02
Log-odds
0.5
1.0
1.5
2.0
2.5
3.0
3.5
4.0
Cellular_organisms - ps1
Archaea1 - ps2
Archaea2 - ps3
Archaea3 - ps4
Asgard/Eukaryota - ps5
Eukaryota - ps6
Amorpheae - ps7
Obazoa - ps8
Opisthokonta - ps9
Holomycota - ps10
Fungi - ps11
Fungi_1 - ps12
Fungi_2 - ps13
Fungi_3 - ps14
Dikarya - ps15
Ascomycota - ps16
Saccharo/Pezizomycotina - ps17
Saccharomycotina - ps18
Saccharomycotina_1 - ps19
Saccharomycotina_2 - ps20
Saccharomycotina_3 - ps21
Phaff./Saccharomycetaceae - ps22
Saccharomyco./mycetaceae - ps23
Saccharomycetaceae - ps24
Saccharomycetaceae_2 - ps25
Saccharomycetaceae_3 - ps26
Kaz./Nau./Saccharomyces - ps27
Saccharomyces - ps28
Arboricola_clade - ps29
Cerevisiae_clade - ps30
Saccharomyces_cerevisiae - ps31
Phylostratum

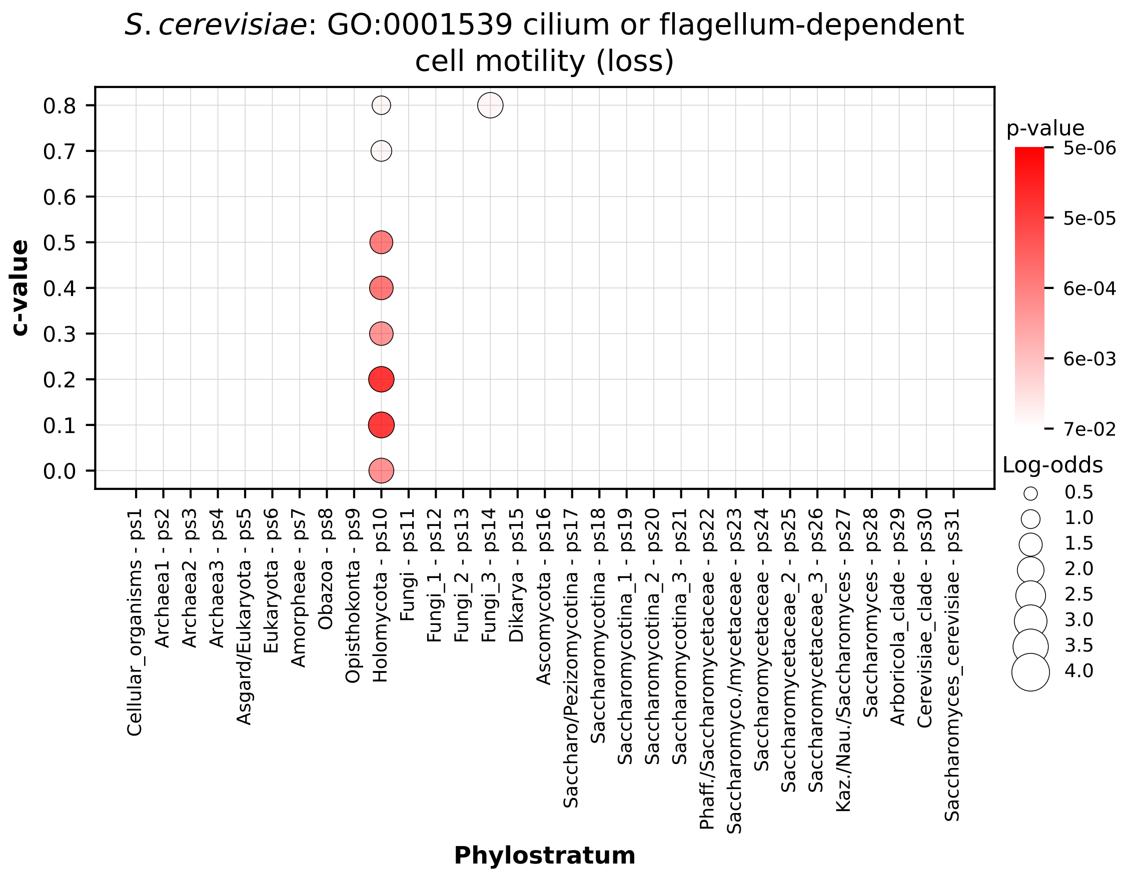
S. cerevisiae: GO:0001539 cilium or flagellum-dependent cell motility (loss)
c-value
0.8
0.7
0.6
0.5
0.4
0.3
0.2
0.1
0.0
p-value
5e-06
5e-05
6e-04
6e-03
7e-02
Log-odds
0.5
1.0
1.5
2.0
2.5
3.0
3.5
4.0
Cellular_organisms - ps1
Archaea1 - ps2
Archaea2 - ps3
Archaea3 - ps4
Asgard/Eukaryota - ps5
Eukaryota - ps6
Amorpheae - ps7
Obazoa - ps8
Opisthokonta - ps9
Holomycota - ps10
Fungi - ps11
Fungi_1 - ps12
Fungi_2 - ps13
Fungi_3 - ps14
Dikarya - ps15
Ascomycota - ps16
Saccharo/Pezizomycotina - ps17
Saccharomycotina - ps18
Saccharomycotina_1 - ps19
Saccharomycotina_2 - ps20
Saccharomycotina_3 - ps21
Phaff./Saccharomycetaceae - ps22
Saccharomyco./mycetaceae - ps23
Saccharomycetaceae - ps24
Saccharomycetaceae_2 - ps25
Saccharomycetaceae_3 - ps26
Kaz./Nau./Saccharomyces - ps27
Saccharomyces - ps28
Arboricola_clade - ps29
Cerevisiae_clade - ps30
Saccharomyces_cerevisiae - ps31
Phylostratum

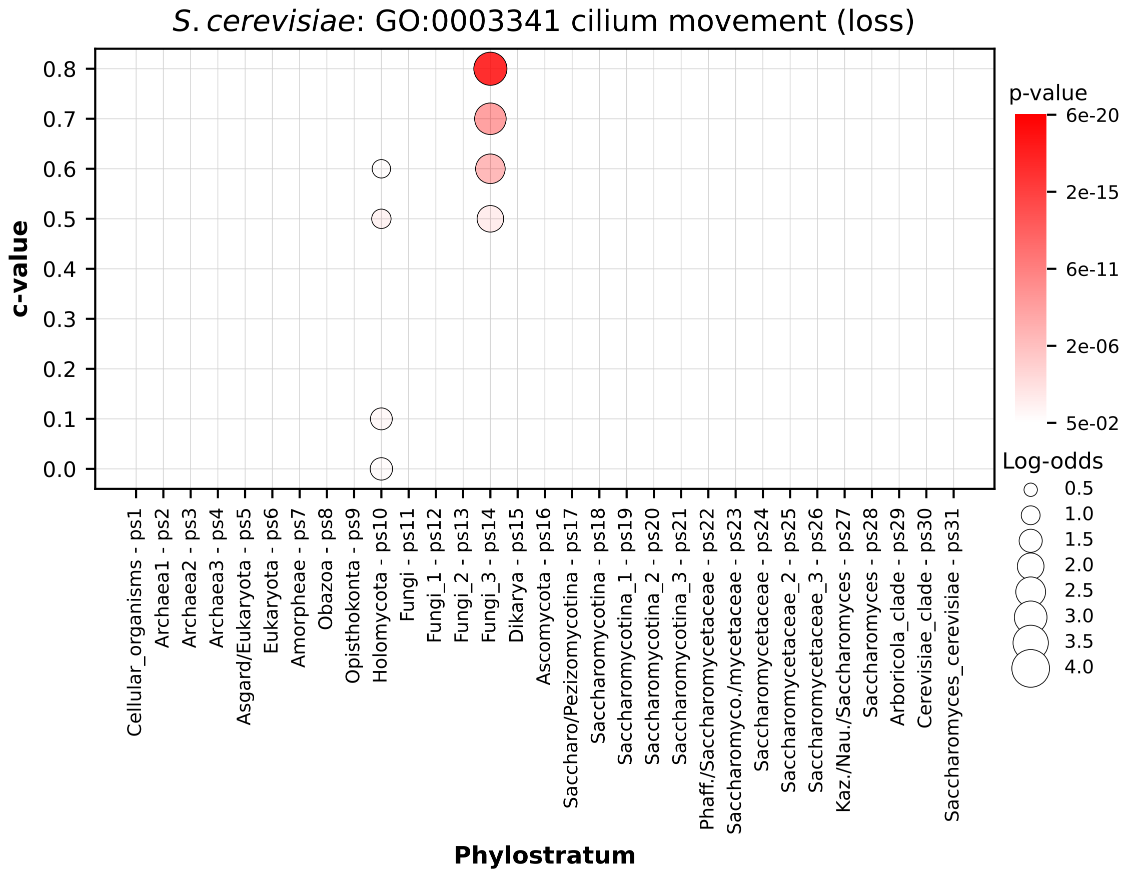

S. cerevisiae: GO:0003341 cilium movement (loss)
c-value
0.8
0.7
0.6
0.5
0.4
0.3
0.2
0.1
0.0
p-value
6e-20
2e-15
6e-11
2e-06
5e-02
Log-odds
0.5
1.0
1.5
2.0
2.5
3.0
3.5
4.0
Cellular_organisms - ps1
Archaea1 - ps2
Archaea2 - ps3
Archaea3 - ps4
Asgard/Eukaryota - ps5
Eukaryota - ps6
Amorpheae - ps7
Obazoa - ps8
Opisthokonta - ps9
Holomycota - ps10
Fungi - ps11
Fungi_1 - ps12
Fungi_2 - ps13
Fungi_3 - ps14
Dikarya - ps15
Ascomycota - ps16
Saccharo/Pezizomycotina - ps17
Saccharomycotina - ps18
Saccharomycotina_1 - ps19
Saccharomycotina_2 - ps20
Saccharomycotina_3 - ps21
Phaff./Saccharomycetaceae - ps22
Saccharomyco./mycetaceae - ps23
Saccharomycetaceae - ps24
Saccharomycetaceae_2 - ps25
Saccharomycetaceae_3 - ps26
Kaz./Nau./Saccharomyces - ps27
Saccharomyces - ps28
Arboricola_clade - ps29
Cerevisiae_clade - ps30
Saccharomyces_cerevisiae - ps31
Phylostratum

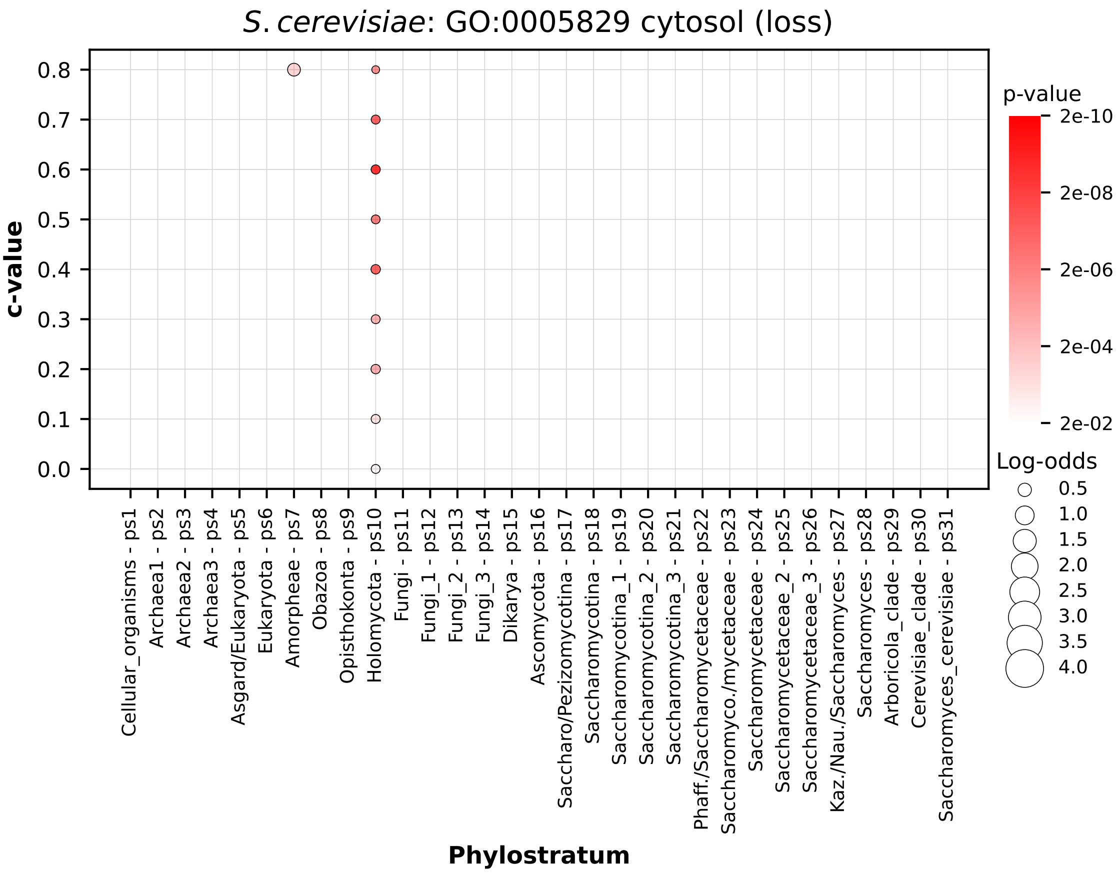

$S. cerevisiae$: GO:0005829 cytosol (loss)
c-value
0.0
0.1
0.2
0.3
0.4
0.5
0.6
0.7
0.8
Phylostratum
Cellular_organisms - ps1
Archaea1 - ps2
Archaea2 - ps3
Archaea3 - ps4
Asgard/Eukaryota - ps5
Eukaryota - ps6
Amorpheae - ps7
Obazoa - ps8
Opisthokonta - ps9
Holomycota - ps10
Fungi - ps11
Fungi_1 - ps12
Fungi_2 - ps13
Fungi_3 - ps14
Dikarya - ps15
Ascomycota - ps16
Saccharo/Pezizomycotina - ps17
Saccharomycotina - ps18
Saccharomycotina_1 - ps19
Saccharomycotina_2 - ps20
Saccharomycotina_3 - ps21
Phaff./Saccharomycetaceae - ps22
Saccharomyco./mycetaceae - ps23
Saccharomycetaceae - ps24
Saccharomycetaceae_2 - ps25
Saccharomycetaceae_3 - ps26
Kaz./Nau./Saccharomyces - ps27
Saccharomyces - ps28
Arboricola_clade - ps29
Cerevisiae_clade - ps30
Saccharomyces_cerevisiae - ps31
p-value
2e-10
2e-08
2e-06
2e-04
2e-02
Log-odds
0.5
1.0
1.5
2.0
2.5
3.0
3.5
4.0

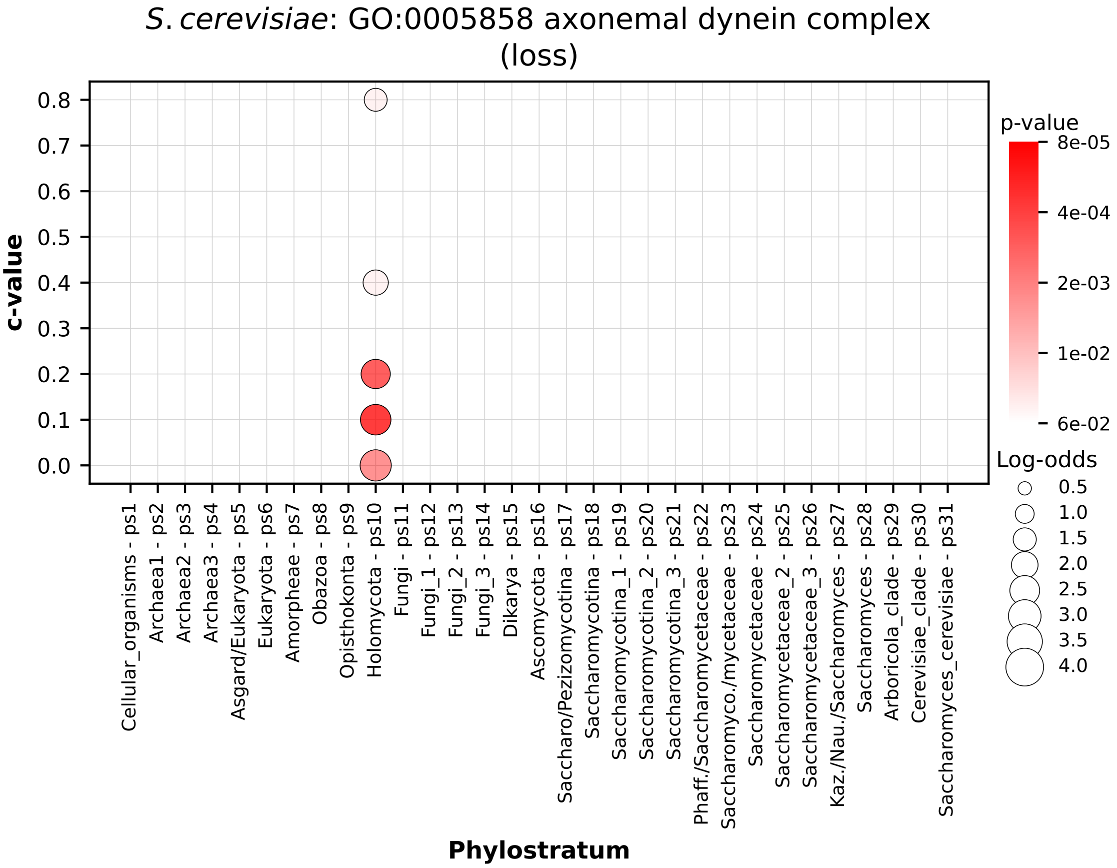

S. cerevisiae: GO:0005858 axonemal dynein complex
(loss)
c-value
0.8
0.7
0.6
0.5
0.4
0.3
0.2
0.1
0.0
p-value
8e-05
4e-04
2e-03
1e-02
6e-02
Log-odds
0.5
1.0
1.5
2.0
2.5
3.0
3.5
4.0
Cellular_organisms - ps1
Archaea1 - ps2
Archaea2 - ps3
Archaea3 - ps4
Asgard/Eukaryota - ps5
Eukaryota - ps6
Amorpheae - ps7
Obazoa - ps8
Opisthokonta - ps9
Holomycota - ps10
Fungi - ps11
Fungi_1 - ps12
Fungi_2 - ps13
Fungi_3 - ps14
Dikarya - ps15
Ascomycota - ps16
Saccharo/Pezizomycotina - ps17
Saccharomycotina - ps18
Saccharomycotina_1 - ps19
Saccharomycotina_2 - ps20
Saccharomycotina_3 - ps21
Phaff./Saccharomycetaceae - ps22
Saccharomyco./mycetaceae - ps23
Saccharomycetaceae - ps24
Saccharomycetaceae_2 - ps25
Saccharomycetaceae_3 - ps26
Kaz./Nau./Saccharomyces - ps27
Saccharomyces - ps28
Arboricola_clade - ps29
Cerevisiae_clade - ps30
Saccharomyces_cerevisiae - ps31
Phylostratum

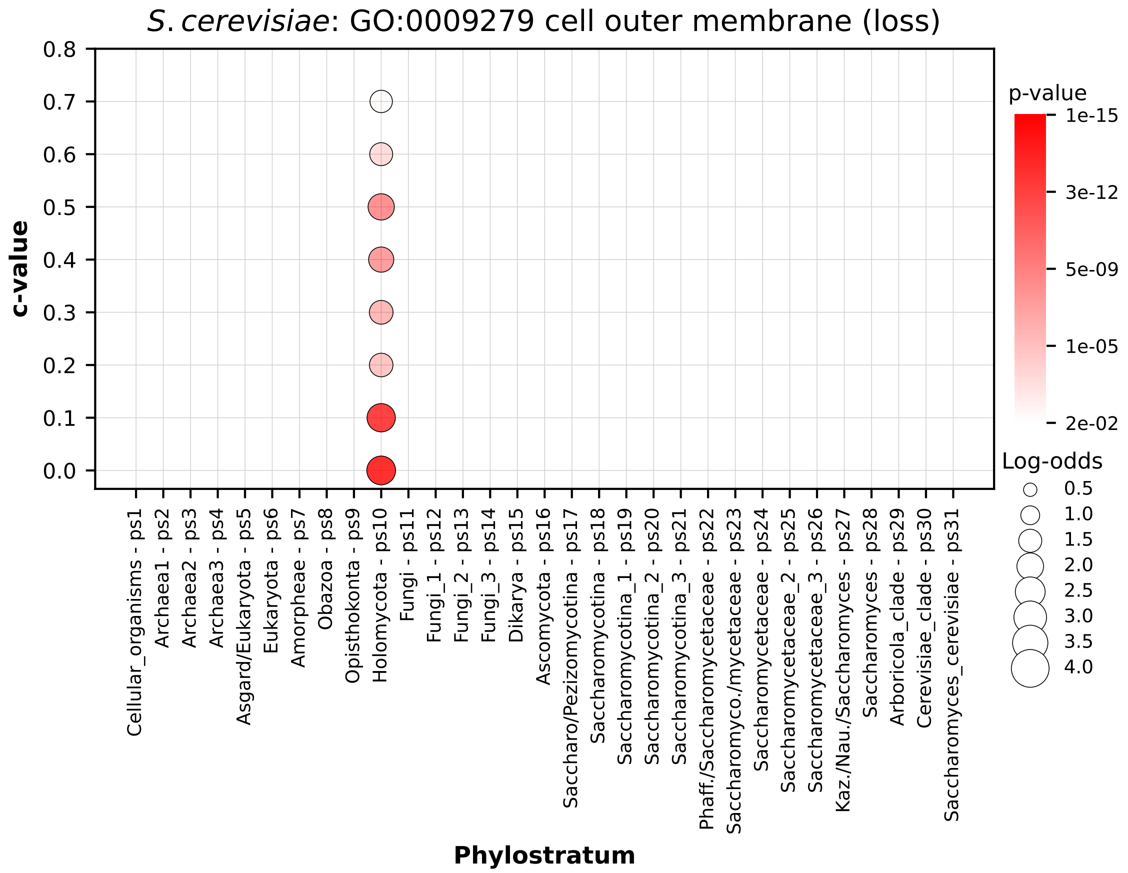
S. cerevisiae: GO:0009279 cell outer membrane (loss)
c-value
0.8
0.7
0.6
0.5
0.4
0.3
0.2
0.1
0.0
Phylostratum
Cellular_organisms - ps1
Archaea1 - ps2
Archaea2 - ps3
Archaea3 - ps4
Asgard/Eukaryota - ps5
Eukaryota - ps6
Amorpheae - ps7
Obazoa - ps8
Opisthokonta - ps9
Holomycota - ps10
Fungi - ps11
Fungi_1 - ps12
Fungi_2 - ps13
Fungi_3 - ps14
Dikarya - ps15
Ascomycota - ps16
Saccharo/Pezizomycotina - ps17
Saccharomycotina - ps18
Saccharomycotina_1 - ps19
Saccharomycotina_2 - ps20
Saccharomycotina_3 - ps21
Phaff./Saccharomycetaceae - ps22
Saccharomyco./mycetaceae - ps23
Saccharomycetaceae - ps24
Saccharomycetaceae_2 - ps25
Saccharomycetaceae_3 - ps26
Kaz./Nau./Saccharomyces - ps27
Saccharomyces - ps28
Arboricola_clade - ps29
Cerevisiae_clade - ps30
Saccharomyces_cerevisiae - ps31
p-value
1e-15
3e-12
5e-09
1e-05
2e-02
Log-odds
0.5
1.0
1.5
2.0
2.5
3.0
3.5
4.0

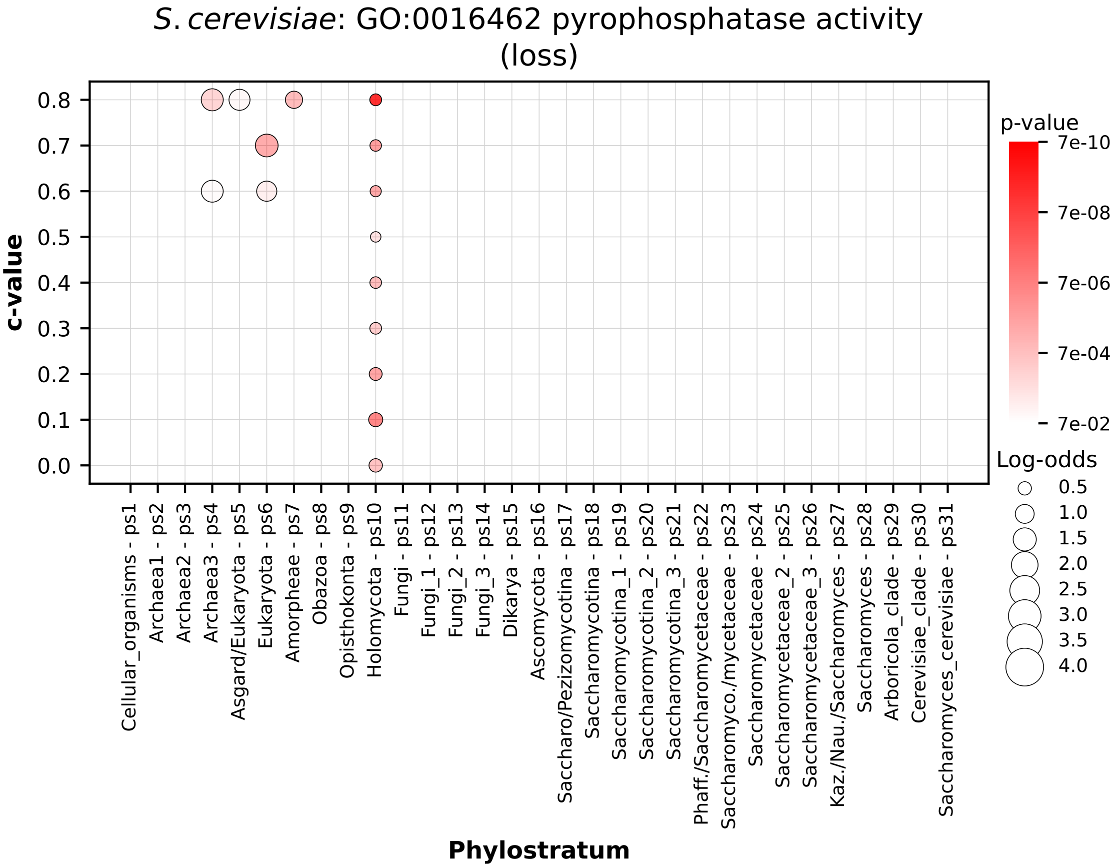
S. cerevisiae: GO:0016462 pyrophosphatase activity
(loss)
c-value
0.8
0.7
0.6
0.5
0.4
0.3
0.2
0.1
0.0
p-value
7e-10
7e-08
7e-06
7e-04
7e-02
Log-odds
0.5
1.0
1.5
2.0
2.5
3.0
3.5
4.0
Cellular_organisms - ps1
Archaea1 - ps2
Archaea2 - ps3
Archaea3 - ps4
Asgard/Eukaryota - ps5
Eukaryota - ps6
Amorpheae - ps7
Obazoa - ps8
Opisthokonta - ps9
Holomycota - ps10
Fungi - ps11
Fungi_1 - ps12
Fungi_2 - ps13
Fungi_3 - ps14
Dikarya - ps15
Ascomycota - ps16
Saccharo/Pezizomycotina - ps17
Saccharomycotina - ps18
Saccharomycotina_1 - ps19
Saccharomycotina_2 - ps20
Saccharomycotina_3 - ps21
Phaff./Saccharomycetaceae - ps22
Saccharomyco./mycetaceae - ps23
Saccharomycetaceae - ps24
Saccharomycetaceae_2 - ps25
Saccharomycetaceae_3 - ps26
Kaz./Nau./Saccharomyces - ps27
Saccharomyces - ps28
Arboricola_clade - ps29
Cerevisiae_clade - ps30
Saccharomyces_cerevisiae - ps31
Phylostratum

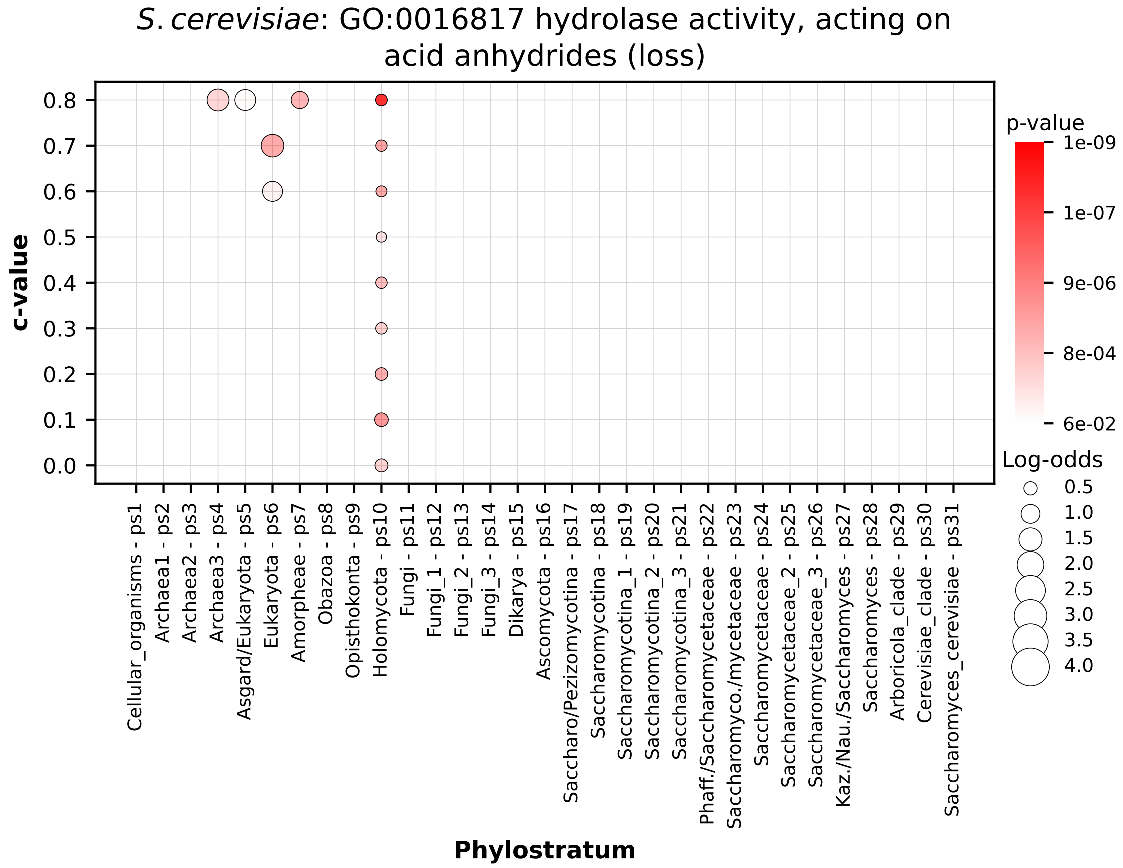
S. cerevisiae: GO:0016817 hydrolase activity, acting on acid anhydrides (loss)
c-value
0.8
0.7
0.6
0.5
0.4
0.3
0.2
0.1
0.0
Phylostratum
Cellular_organisms - ps1
Archaea1 - ps2
Archaea2 - ps3
Archaea3 - ps4
Asgard/Eukaryota - ps5
Eukaryota - ps6
Amorpheae - ps7
Obazoa - ps8
Opisthokonta - ps9
Holomycota - ps10
Fungi - ps11
Fungi_1 - ps12
Fungi_2 - ps13
Fungi_3 - ps14
Dikarya - ps15
Ascomycota - ps16
Saccharo/Pezizomycotina - ps17
Saccharomycotina - ps18
Saccharomycotina_1 - ps19
Saccharomycotina_2 - ps20
Saccharomycotina_3 - ps21
Phaff./Saccharomycetaceae - ps22
Saccharomyco./mycetaceae - ps23
Saccharomycetaceae - ps24
Saccharomycetaceae_2 - ps25
Saccharomycetaceae_3 - ps26
Kaz./Nau./Saccharomyces - ps27
Saccharomyces - ps28
Arboricola_clade - ps29
Cerevisiae_clade - ps30
Saccharomyces_cerevisiae - ps31
p-value
1e-09
1e-07
9e-06
8e-04
6e-02
Log-odds
0.5
1.0
1.5
2.0
2.5
3.0
3.5
4.0

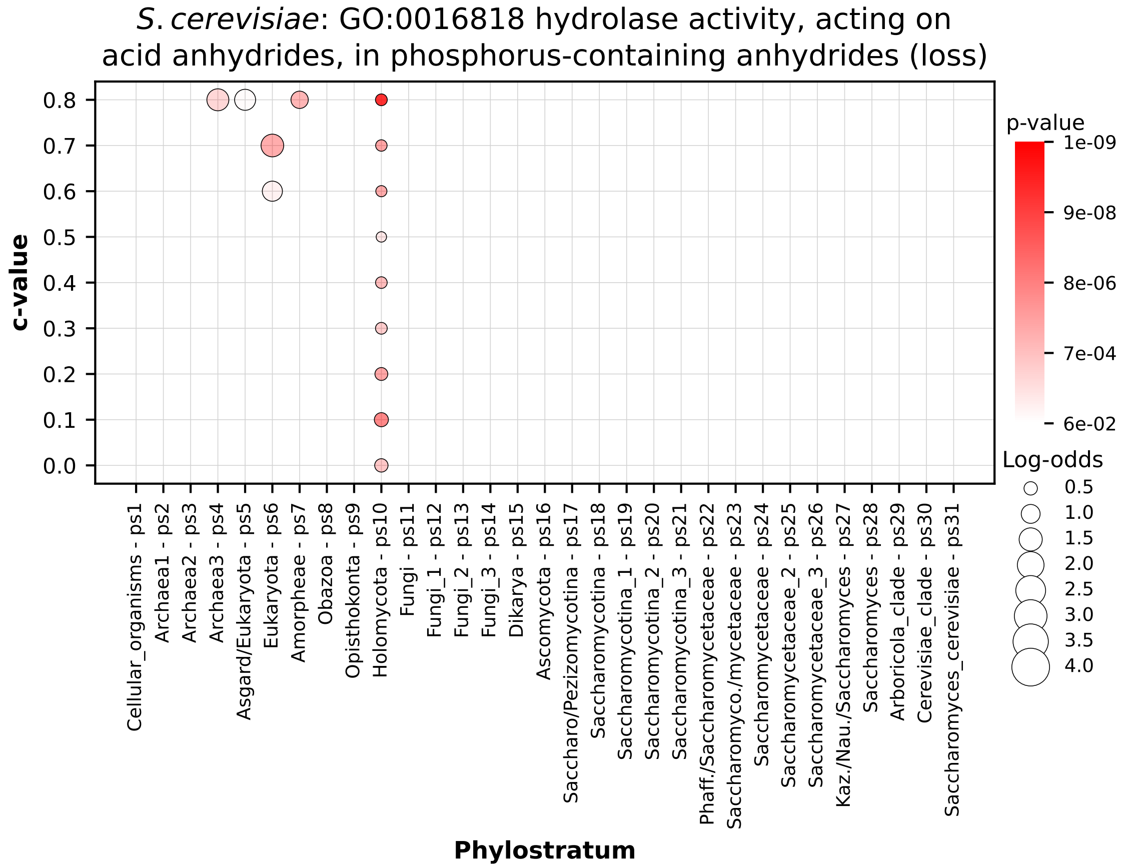

S. cerevisiae: GO:0016818 hydrolase activity, acting on acid anhydrides, in phosphorus-containing anhydrides (loss)
c-value
0.8
0.7
0.6
0.5
0.4
0.3
0.2
0.1
0.0
p-value
1e-09
9e-08
8e-06
7e-04
6e-02
Log-odds
0.5
1.0
1.5
2.0
2.5
3.0
3.5
4.0
Cellular_organisms - ps1
Archaea1 - ps2
Archaea2 - ps3
Archaea3 - ps4
Asgard/Eukaryota - ps5
Eukaryota - ps6
Amorpheae - ps7
Obazoa - ps8
Opisthokonta - ps9
Holomycota - ps10
Fungi - ps11
Fungi_1 - ps12
Fungi_2 - ps13
Fungi_3 - ps14
Dikarya - ps15
Ascomycota - ps16
Saccharo/Pezizomycotina - ps17
Saccharomycotina - ps18
Saccharomycotina_1 - ps19
Saccharomycotina_2 - ps20
Saccharomycotina_3 - ps21
Phaff./Saccharomycetaceae - ps22
Saccharomyco./mycetaceae - ps23
Saccharomycetaceae - ps24
Saccharomycetaceae_2 - ps25
Saccharomycetaceae_3 - ps26
Kaz./Nau./Saccharomyces - ps27
Saccharomyces - ps28
Arboricola_clade - ps29
Cerevisiae_clade - ps30
Saccharomyces_cerevisiae - ps31
Phylostratum

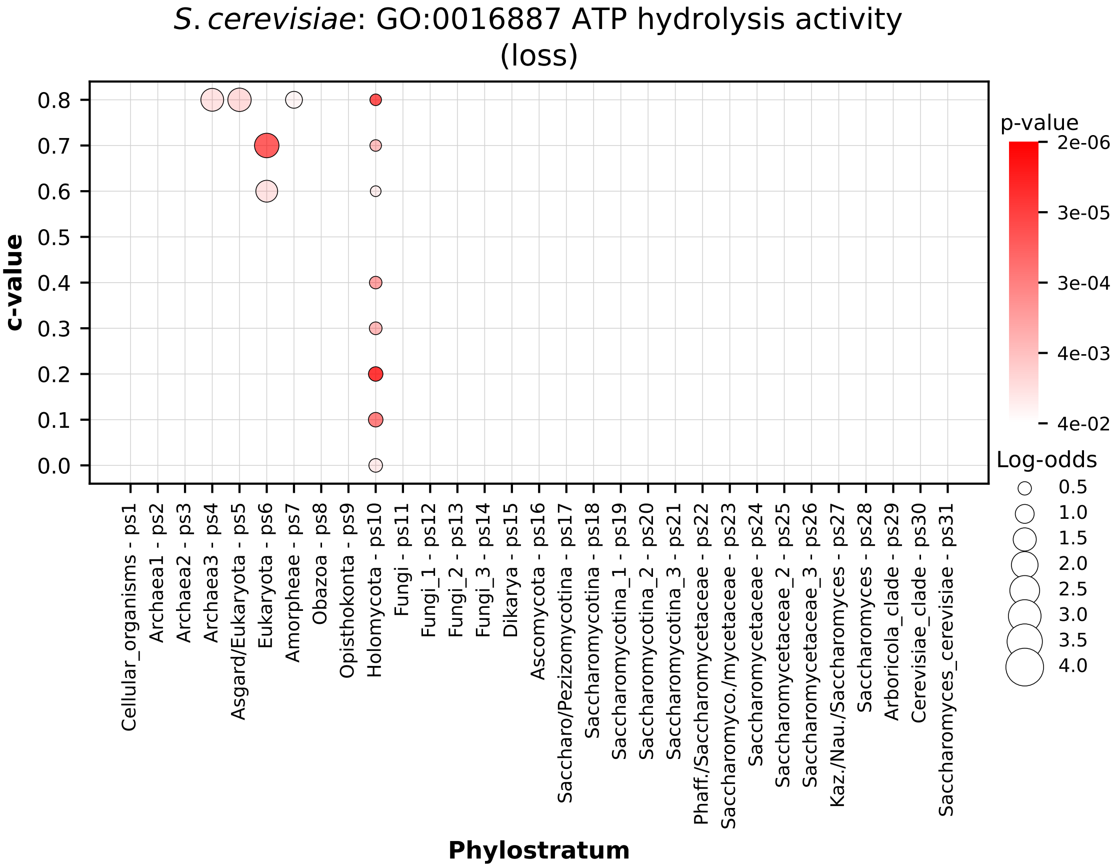

S. cerevisiae: GO:0016887 ATP hydrolysis activity (loss)
c-value
0.8
0.7
0.6
0.5
0.4
0.3
0.2
0.1
0.0
Phylostratum
Cellular_organisms - ps1
Archaea1 - ps2
Archaea2 - ps3
Archaea3 - ps4
Asgard/Eukaryota - ps5
Eukaryota - ps6
Amorpheae - ps7
Obazoa - ps8
Opisthokonta - ps9
Holomycota - ps10
Fungi - ps11
Fungi_1 - ps12
Fungi_2 - ps13
Fungi_3 - ps14
Dikarya - ps15
Ascomycota - ps16
Saccharo/Pezizomycotina - ps17
Saccharomycotina - ps18
Saccharomycotina_1 - ps19
Saccharomycotina_2 - ps20
Saccharomycotina_3 - ps21
Phaff./Saccharomycetaceae - ps22
Saccharomyco./mycetaceae - ps23
Saccharomycetaceae - ps24
Saccharomycetaceae_2 - ps25
Saccharomycetaceae_3 - ps26
Kaz./Nau./Saccharomyces - ps27
Saccharomyces - ps28
Arboricola_clade - ps29
Cerevisiae_clade - ps30
Saccharomyces_cerevisiae - ps31
p-value
2e-06
3e-05
3e-04
4e-03
4e-02
Log-odds
0.5
1.0
1.5
2.0
2.5
3.0
3.5
4.0

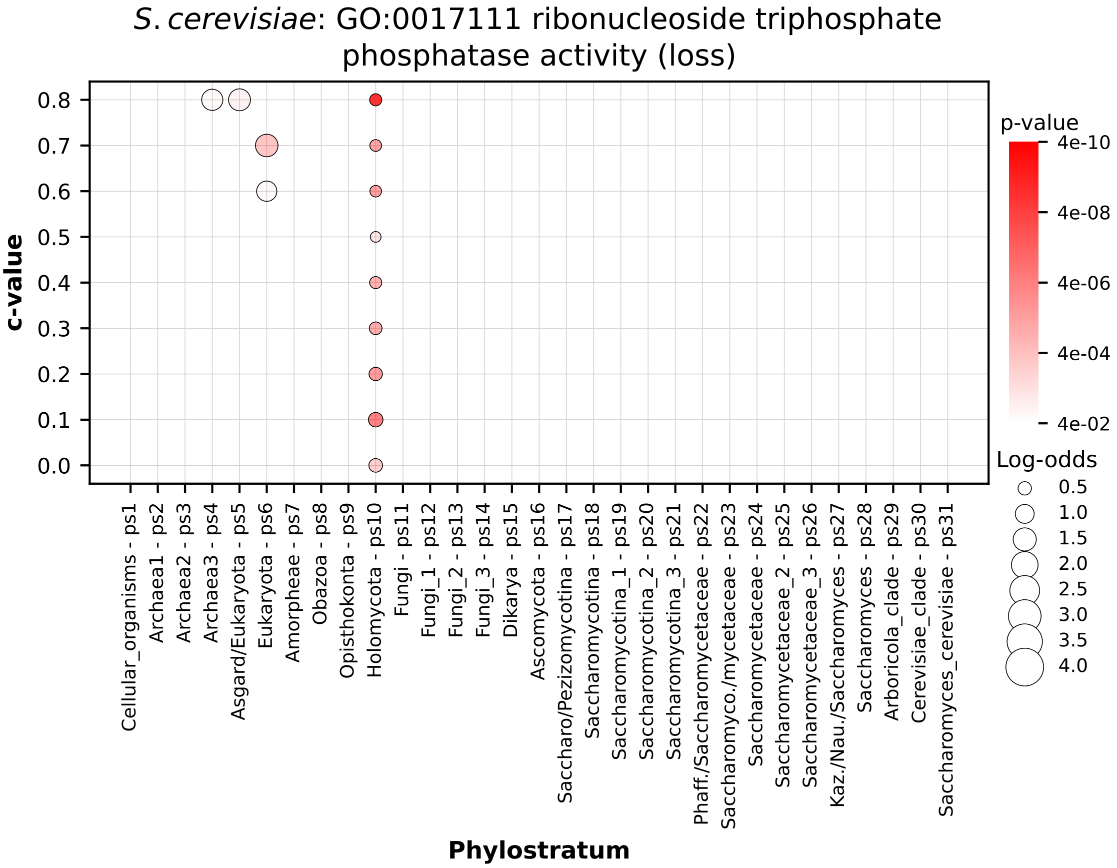
S. cerevisiae: GO:0017111 ribonucleoside triphosphate phosphatase activity (loss)
c-value
0.8
0.7
0.6
0.5
0.4
0.3
0.2
0.1
0.0
p-value
4e-10
4e-08
4e-06
4e-04
4e-02
Log-odds
0.5
1.0
1.5
2.0
2.5
3.0
3.5
4.0
Cellular_organisms - ps1
Archaea1 - ps2
Archaea2 - ps3
Archaea3 - ps4
Asgard/Eukaryota - ps5
Eukaryota - ps6
Amorpheae - ps7
Obazoa - ps8
Opisthokonta - ps9
Holomycota - ps10
Fungi - ps11
Fungi_1 - ps12
Fungi_2 - ps13
Fungi_3 - ps14
Dikarya - ps15
Ascomycota - ps16
Saccharo/Pezizomycotina - ps17
Saccharomycotina - ps18
Saccharomycotina_1 - ps19
Saccharomycotina_2 - ps20
Saccharomycotina_3 - ps21
Phaff./Saccharomycetaceae - ps22
Saccharomyco./mycetaceae - ps23
Saccharomycetaceae - ps24
Saccharomycetaceae_2 - ps25
Saccharomycetaceae_3 - ps26
Kaz./Nau./Saccharomyces - ps27
Saccharomyces - ps28
Arboricola_clade - ps29
Cerevisiae_clade - ps30
Saccharomyces_cerevisiae - ps31
Phylostratum

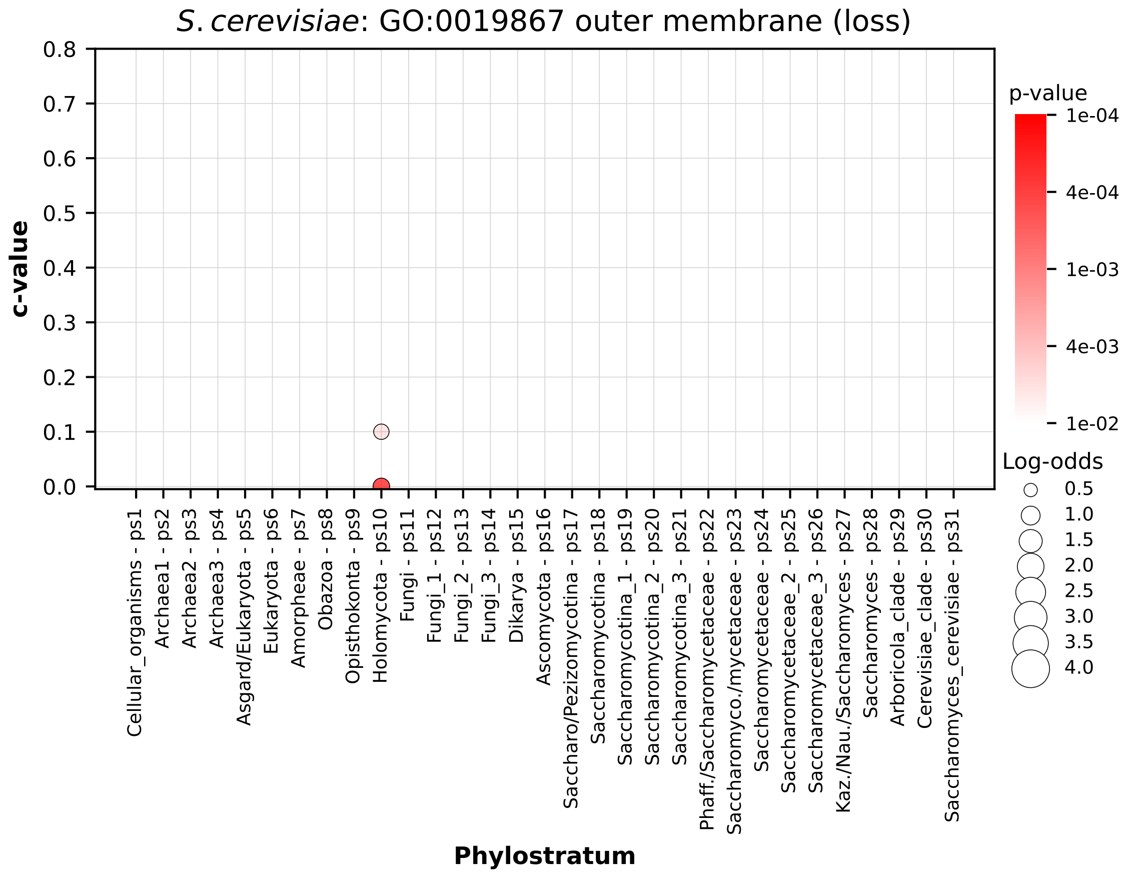
S. cerevisiae: GO:0019867 outer membrane (loss)
c-value
0.8
0.7
0.6
0.5
0.4
0.3
0.2
0.1
0.0
p-value
1e-04
4e-04
1e-03
4e-03
1e-02
Log-odds
0.5
1.0
1.5
2.0
2.5
3.0
3.5
4.0
Cellular_organisms - ps1
Archaea1 - ps2
Archaea2 - ps3
Archaea3 - ps4
Asgard/Eukaryota - ps5
Eukaryota - ps6
Amorpheae - ps7
Obazoa - ps8
Opisthokonta - ps9
Holomycota - ps10
Fungi - ps11
Fungi_1 - ps12
Fungi_2 - ps13
Fungi_3 - ps14
Dikarya - ps15
Ascomycota - ps16
Saccharo/Pezizomycotina - ps17
Saccharomycotina - ps18
Saccharomycotina_1 - ps19
Saccharomycotina_2 - ps20
Saccharomycotina_3 - ps21
Phaff./Saccharomycetaceae - ps22
Saccharomyco./mycetaceae - ps23
Saccharomycetaceae - ps24
Saccharomycetaceae_2 - ps25
Saccharomycetaceae_3 - ps26
Kaz./Nau./Saccharomyces - ps27
Saccharomyces - ps28
Arboricola_clade - ps29
Cerevisiae_clade - ps30
Saccharomyces_cerevisiae - ps31
Phylostratum

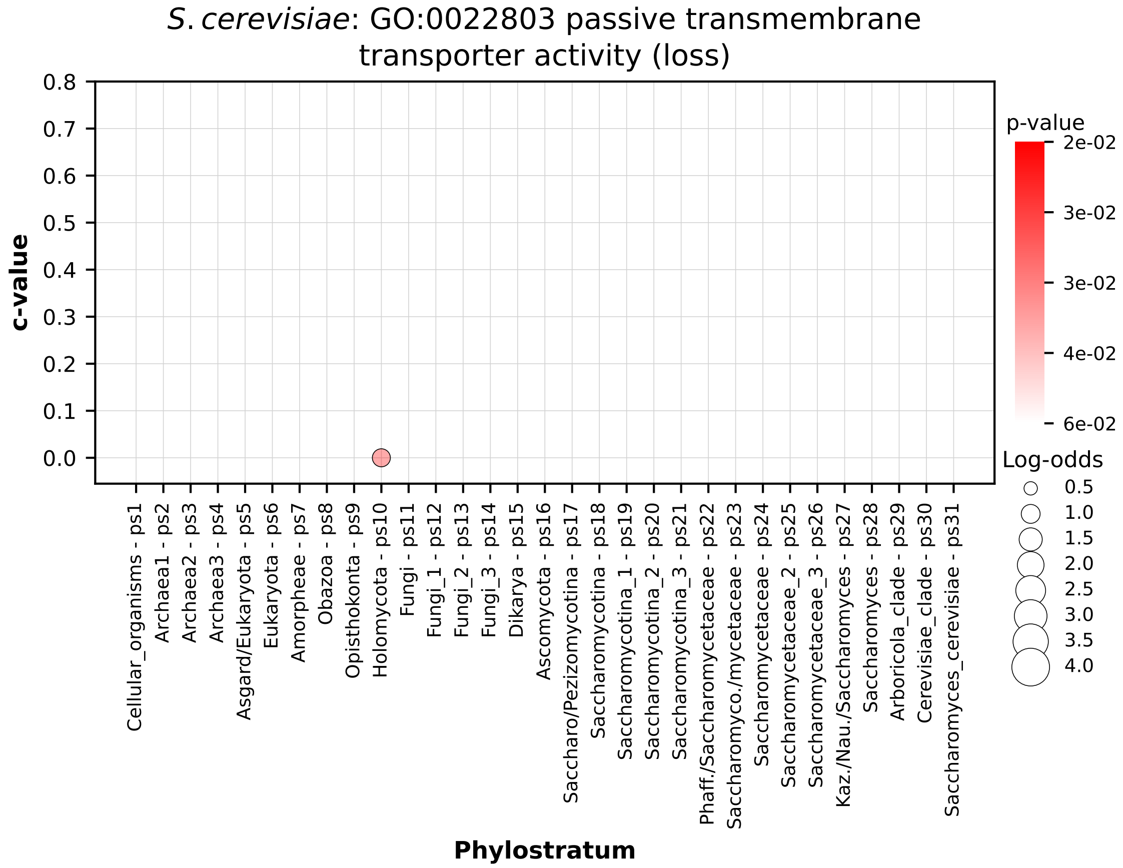
S. cerevisiae: GO:0022803 passive transmembrane transporter activity (loss)
c-value
0.8
0.7
0.6
0.5
0.4
0.3
0.2
0.1
0.0
p-value
2e-02
3e-02
3e-02
4e-02
6e-02
Log-odds
0.5
1.0
1.5
2.0
2.5
3.0
3.5
4.0
Cellular_organisms - ps1
Archaea1 - ps2
Archaea2 - ps3
Archaea3 - ps4
Asgard/Eukaryota - ps5
Eukaryota - ps6
Amorpheae - ps7
Obazoa - ps8
Opisthokonta - ps9
Holomycota - ps10
Fungi - ps11
Fungi_1 - ps12
Fungi_2 - ps13
Fungi_3 - ps14
Dikarya - ps15
Ascomycota - ps16
Saccharo/Pezizomycotina - ps17
Saccharomycotina - ps18
Saccharomycotina_1 - ps19
Saccharomycotina_2 - ps20
Saccharomycotina_3 - ps21
Phaff./Saccharomycetaceae - ps22
Saccharomyco./mycetaceae - ps23
Saccharomycetaceae - ps24
Saccharomycetaceae_2 - ps25
Saccharomycetaceae_3 - ps26
Kaz./Nau./Saccharomyces - ps27
Saccharomyces - ps28
Arboricola_clade - ps29
Cerevisiae_clade - ps30
Saccharomyces_cerevisiae - ps31
Phylostratum

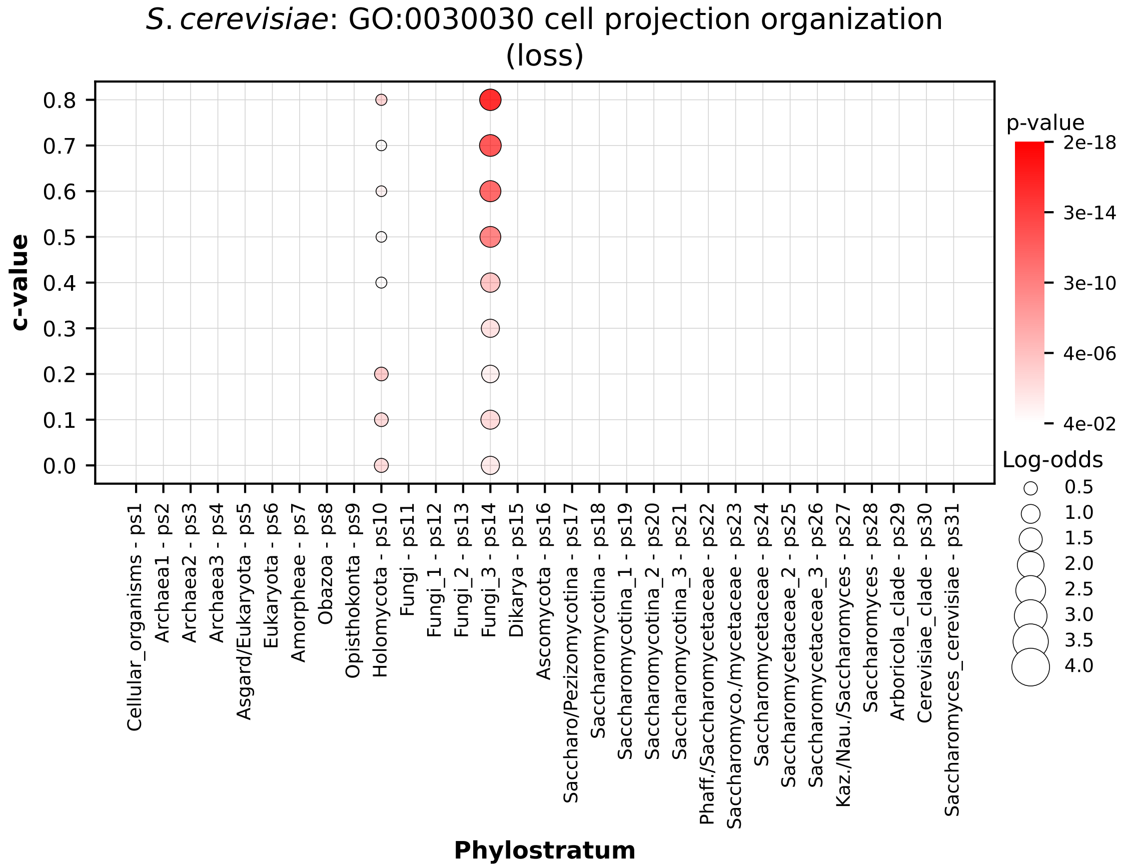
S. cerevisiae: GO:0030030 cell projection organization
(loss)
c-value
0.8
0.7
0.6
0.5
0.4
0.3
0.2
0.1
0.0
p-value
2e-18
3e-14
3e-10
4e-06
4e-02
Log-odds
0.5
1.0
1.5
2.0
2.5
3.0
3.5
4.0
Cellular_organisms - ps1
Archaea1 - ps2
Archaea2 - ps3
Archaea3 - ps4
Asgard/Eukaryota - ps5
Eukaryota - ps6
Amorpheae - ps7
Obazoa - ps8
Opisthokonta - ps9
Holomycota - ps10
Fungi - ps11
Fungi_1 - ps12
Fungi_2 - ps13
Fungi_3 - ps14
Dikarya - ps15
Ascomycota - ps16
Saccharo/Pezizomycotina - ps17
Saccharomycotina - ps18
Saccharomycotina_1 - ps19
Saccharomycotina_2 - ps20
Saccharomycotina_3 - ps21
Phaff./Saccharomycetaceae - ps22
Saccharomyco./mycetaceae - ps23
Saccharomycetaceae - ps24
Saccharomycetaceae_2 - ps25
Saccharomycetaceae_3 - ps26
Kaz./Nau./Saccharomyces - ps27
Saccharomyces - ps28
Arboricola_clade - ps29
Cerevisiae_clade - ps30
Saccharomyces_cerevisiae - ps31
Phylostratum

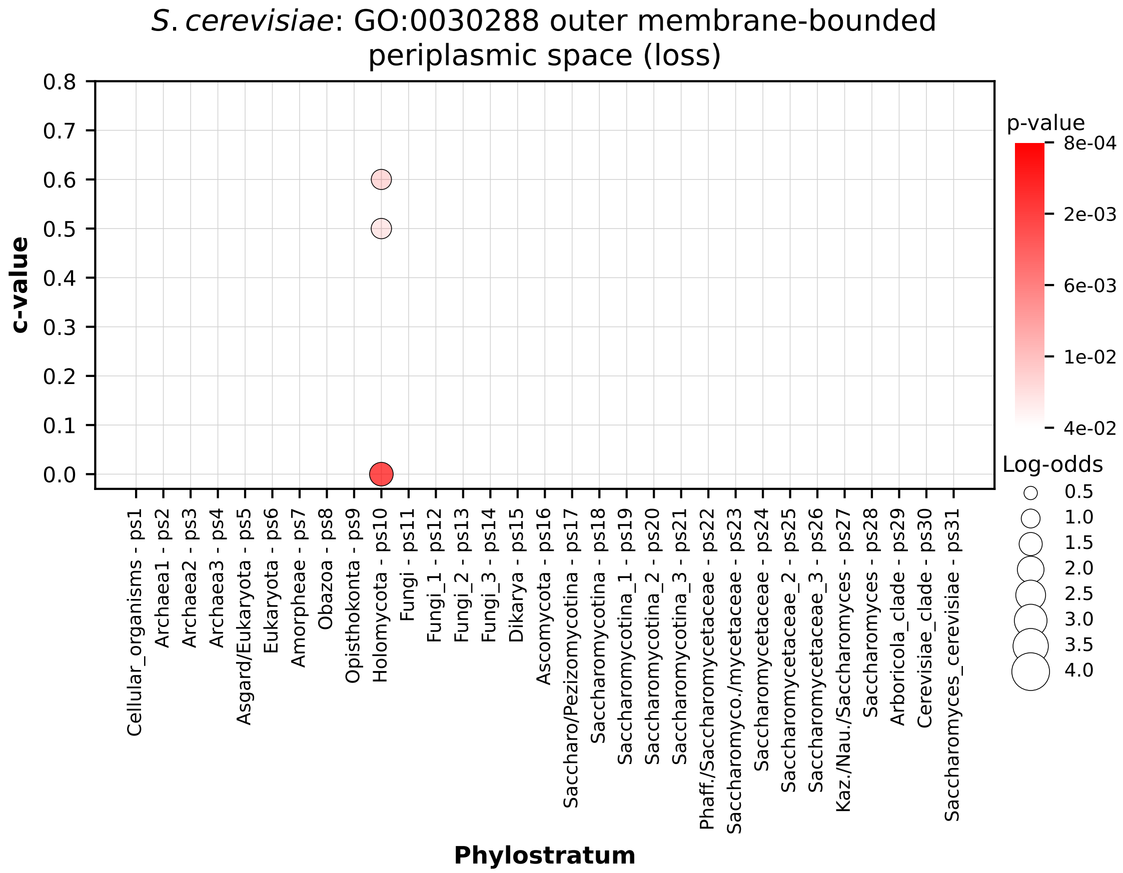
S. cerevisiae: GO:0030288 outer membrane-bounded periplasmic space (loss)
c-value
0.8
0.7
0.6
0.5
0.4
0.3
0.2
0.1
0.0
Phylostratum
Cellular_organisms - ps1
Archaea1 - ps2
Archaea2 - ps3
Archaea3 - ps4
Asgard/Eukaryota - ps5
Eukaryota - ps6
Amorpheae - ps7
Obazoa - ps8
Opisthokonta - ps9
Holomycota - ps10
Fungi - ps11
Fungi_1 - ps12
Fungi_2 - ps13
Fungi_3 - ps14
Dikarya - ps15
Ascomycota - ps16
Saccharo/Pezizomycotina - ps17
Saccharomycotina - ps18
Saccharomycotina_1 - ps19
Saccharomycotina_2 - ps20
Saccharomycotina_3 - ps21
Phaff./Saccharomycetaceae - ps22
Saccharomyco./mycetaceae - ps23
Saccharomycetaceae - ps24
Saccharomycetaceae_2 - ps25
Saccharomycetaceae_3 - ps26
Kaz./Nau./Saccharomyces - ps27
Saccharomyces - ps28
Arboricola_clade - ps29
Cerevisiae_clade - ps30
Saccharomyces_cerevisiae - ps31
p-value
8e-04
2e-03
6e-03
1e-02
4e-02
Log-odds
0.5
1.0
1.5
2.0
2.5
3.0
3.5
4.0

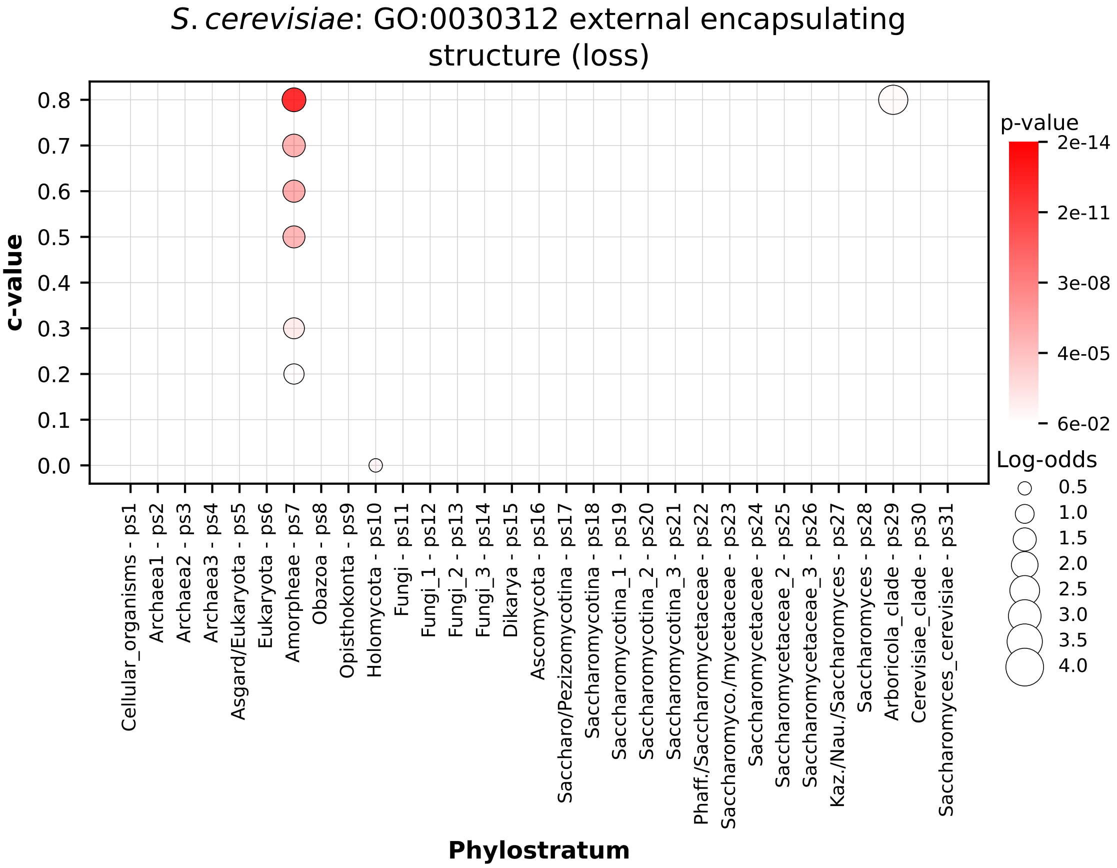

S. cerevisiae: GO:0030312 external encapsulating structure (loss)
c-value
0.8
0.7
0.6
0.5
0.4
0.3
0.2
0.1
0.0
p-value
2e-14
2e-11
3e-08
4e-05
6e-02
Log-odds
0.5
1.0
1.5
2.0
2.5
3.0
3.5
4.0
Cellular_organisms - ps1
Archaea1 - ps2
Archaea2 - ps3
Archaea3 - ps4
Asgard/Eukaryota - ps5
Eukaryota - ps6
Amorpheae - ps7
Obazoa - ps8
Opisthokonta - ps9
Holomycota - ps10
Fungi - ps11
Fungi_1 - ps12
Fungi_2 - ps13
Fungi_3 - ps14
Dikarya - ps15
Ascomycota - ps16
Saccharo/Pezizomycotina - ps17
Saccharomycotina - ps18
Saccharomycotina_1 - ps19
Saccharomycotina_2 - ps20
Saccharomycotina_3 - ps21
Phaff./Saccharomycetaceae - ps22
Saccharomyco./mycetaceae - ps23
Saccharomycetaceae - ps24
Saccharomycetaceae_2 - ps25
Saccharomycetaceae_3 - ps26
Kaz./Nau./Saccharomyces - ps27
Saccharomyces - ps28
Arboricola_clade - ps29
Cerevisiae_clade - ps30
Saccharomyces_cerevisiae - ps31
Phylostratum

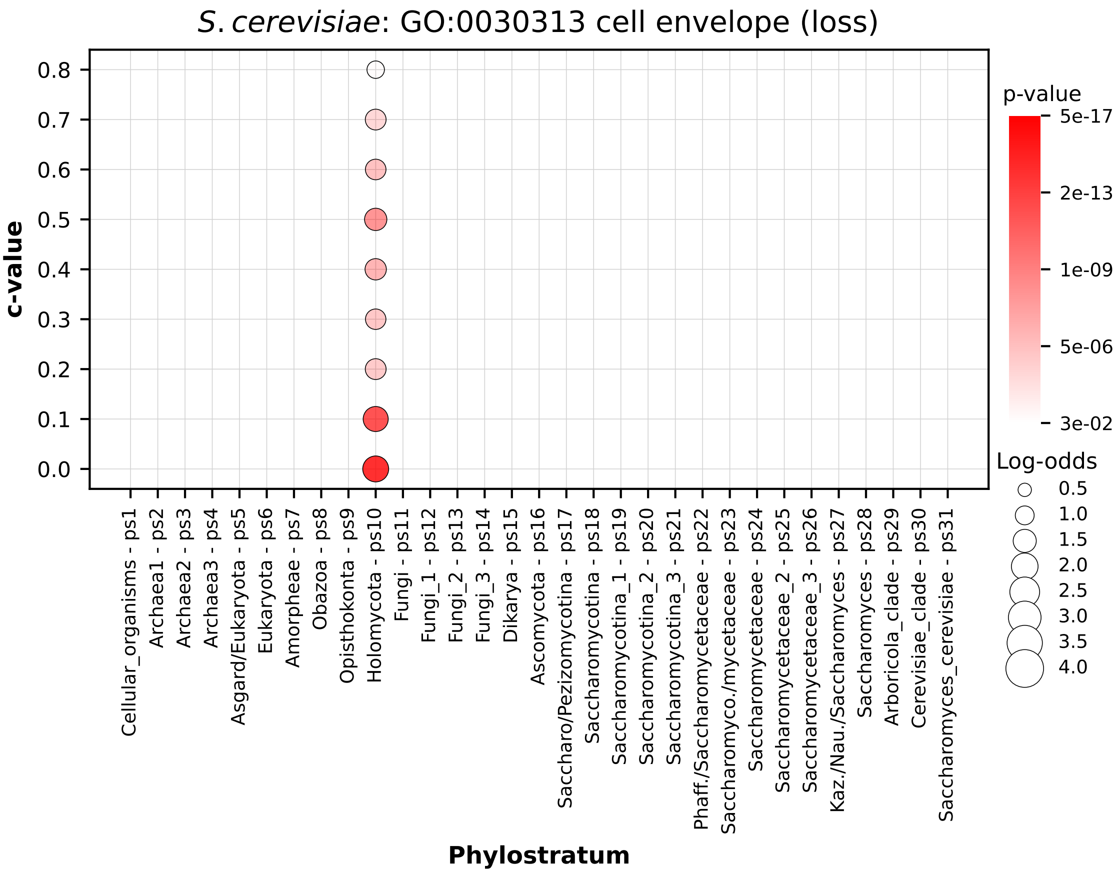

S. cerevisiae: GO:0030313 cell envelope (loss)
c-value
0.8
0.7
0.6
0.5
0.4
0.3
0.2
0.1
0.0
p-value
5e-17
2e-13
1e-09
5e-06
3e-02
Log-odds
0.5
1.0
1.5
2.0
2.5
3.0
3.5
4.0
Cellular_organisms - ps1
Archaea1 - ps2
Archaea2 - ps3
Archaea3 - ps4
Asgard/Eukaryota - ps5
Eukaryota - ps6
Amorpheae - ps7
Obazoa - ps8
Opisthokonta - ps9
Holomycota - ps10
Fungi - ps11
Fungi_1 - ps12
Fungi_2 - ps13
Fungi_3 - ps14
Dikarya - ps15
Ascomycota - ps16
Saccharo/Pezizomycotina - ps17
Saccharomycotina - ps18
Saccharomycotina_1 - ps19
Saccharomycotina_2 - ps20
Saccharomycotina_3 - ps21
Phaff./Saccharomycetaceae - ps22
Saccharomyco./mycetaceae - ps23
Saccharomycetaceae - ps24
Saccharomycetaceae_2 - ps25
Saccharomycetaceae_3 - ps26
Kaz./Nau./Saccharomyces - ps27
Saccharomyces - ps28
Arboricola_clade - ps29
Cerevisiae_clade - ps30
Saccharomyces_cerevisiae - ps31
Phylostratum

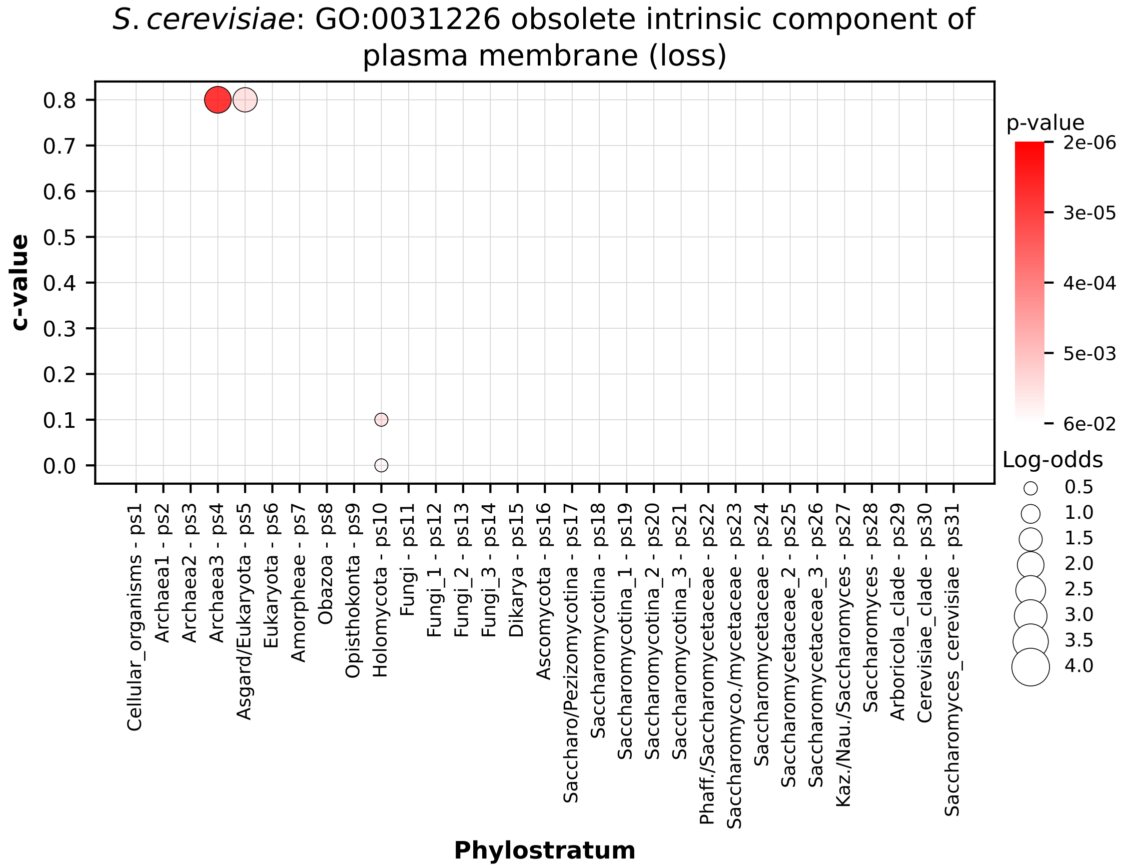

S. cerevisiae: GO:0031226 obsolete intrinsic component of plasma membrane (loss)
c-value
0.8
0.7
0.6
0.5
0.4
0.3
0.2
0.1
0.0
p-value
2e-06
3e-05
4e-04
5e-03
6e-02
Log-odds
0.5
1.0
1.5
2.0
2.5
3.0
3.5
4.0
Cellular_organisms - ps1
Archaea1 - ps2
Archaea2 - ps3
Archaea3 - ps4
Asgard/Eukaryota - ps5
Eukaryota - ps6
Amorpheae - ps7
Obazoa - ps8
Opisthokonta - ps9
Holomycota - ps10
Fungi - ps11
Fungi_1 - ps12
Fungi_2 - ps13
Fungi_3 - ps14
Dikarya - ps15
Ascomycota - ps16
Saccharo/Pezizomycotina - ps17
Saccharomycotina - ps18
Saccharomycotina_1 - ps19
Saccharomycotina_2 - ps20
Saccharomycotina_3 - ps21
Phaff./Saccharomycetaceae - ps22
Saccharomyco./mycetaceae - ps23
Saccharomycetaceae - ps24
Saccharomycetaceae_2 - ps25
Saccharomycetaceae_3 - ps26
Kaz./Nau./Saccharomyces - ps27
Saccharomyces - ps28
Arboricola_clade - ps29
Cerevisiae_clade - ps30
Saccharomyces_cerevisiae - ps31
Phylostratum

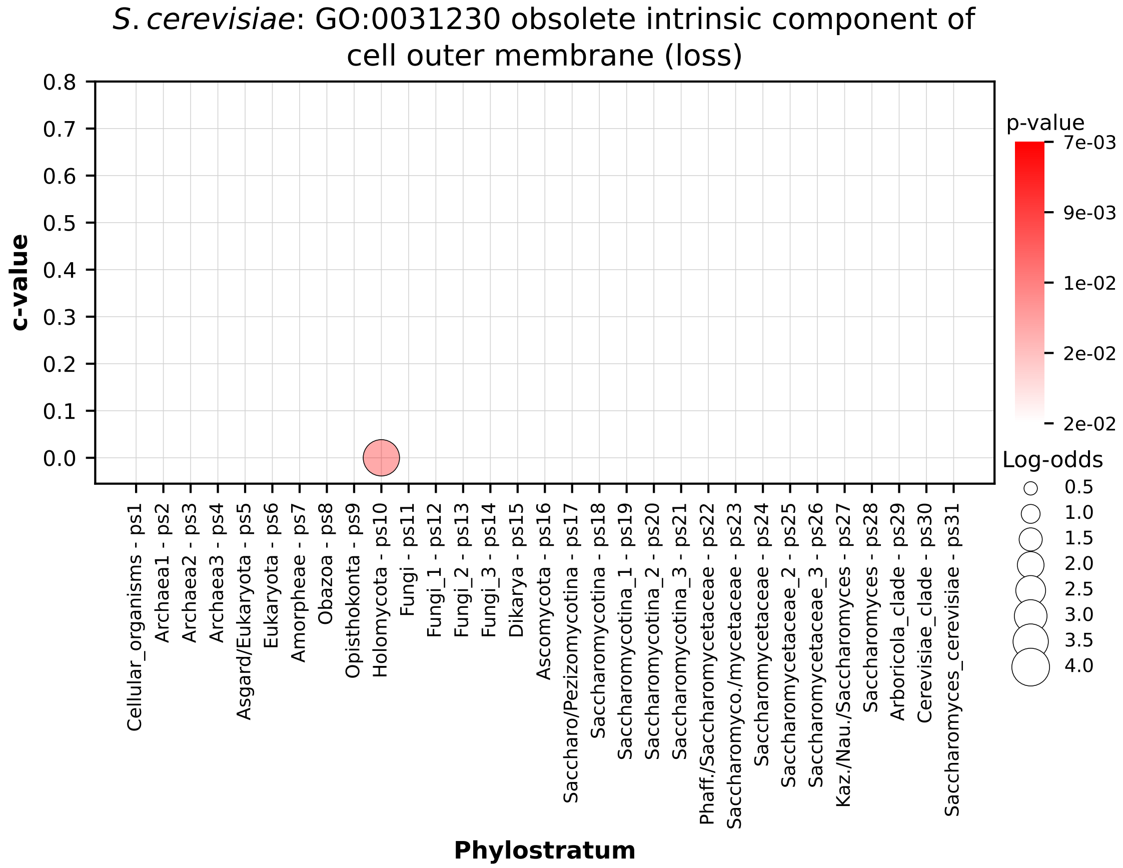

S. cerevisiae: GO:0031230 obsolete intrinsic component of cell outer membrane (loss)
c-value
0.8
0.7
0.6
0.5
0.4
0.3
0.2
0.1
0.0
p-value
7e-03
9e-03
1e-02
2e-02
2e-02
Log-odds
0.5
1.0
1.5
2.0
2.5
3.0
3.5
4.0
Cellular_organisms - ps1
Archaea1 - ps2
Archaea2 - ps3
Archaea3 - ps4
Asgard/Eukaryota - ps5
Eukaryota - ps6
Amorpheae - ps7
Obazoa - ps8
Opisthokonta - ps9
Holomycota - ps10
Fungi - ps11
Fungi_1 - ps12
Fungi_2 - ps13
Fungi_3 - ps14
Dikarya - ps15
Ascomycota - ps16
Saccharo/Pezizomycotina - ps17
Saccharomycotina - ps18
Saccharomycotina_1 - ps19
Saccharomycotina_2 - ps20
Saccharomycotina_3 - ps21
Phaff./Saccharomycetaceae - ps22
Saccharomyco./mycetaceae - ps23
Saccharomycetaceae - ps24
Saccharomycetaceae_2 - ps25
Saccharomycetaceae_3 - ps26
Kaz./Nau./Saccharomyces - ps27
Saccharomyces - ps28
Arboricola_clade - ps29
Cerevisiae_clade - ps30
Saccharomyces_cerevisiae - ps31
Phylostratum

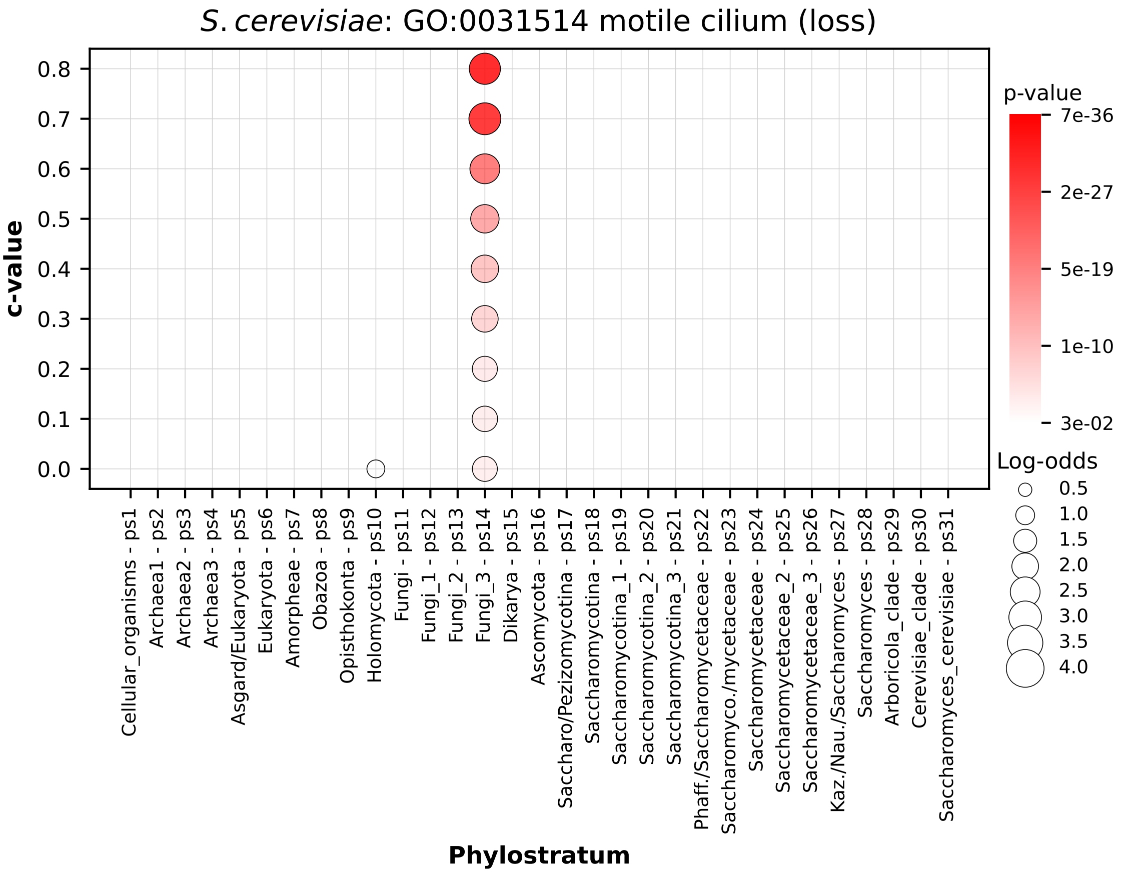

S. cerevisiae: GO:0031514 motile cilium (loss)
c-value
0.8
0.7
0.6
0.5
0.4
0.3
0.2
0.1
0.0
p-value
7e-36
2e-27
5e-19
1e-10
3e-02
Log-odds
0.5
1.0
1.5
2.0
2.5
3.0
3.5
4.0
Cellular_organisms - ps1
Archaea1 - ps2
Archaea2 - ps3
Archaea3 - ps4
Asgard/Eukaryota - ps5
Eukaryota - ps6
Amorpheae - ps7
Obazoa - ps8
Opisthokonta - ps9
Holomycota - ps10
Fungi - ps11
Fungi_1 - ps12
Fungi_2 - ps13
Fungi_3 - ps14
Dikarya - ps15
Ascomycota - ps16
Saccharo/Pezizomycotina - ps17
Saccharomycotina - ps18
Saccharomycotina_1 - ps19
Saccharomycotina_2 - ps20
Saccharomycotina_3 - ps21
Phaff./Saccharomycetaceae - ps22
Saccharomyco./mycetaceae - ps23
Saccharomycetaceae - ps24
Saccharomycetaceae_2 - ps25
Saccharomycetaceae_3 - ps26
Kaz./Nau./Saccharomyces - ps27
Saccharomyces - ps28
Arboricola_clade - ps29
Cerevisiae_clade - ps30
Saccharomyces_cerevisiae - ps31
Phylostratum

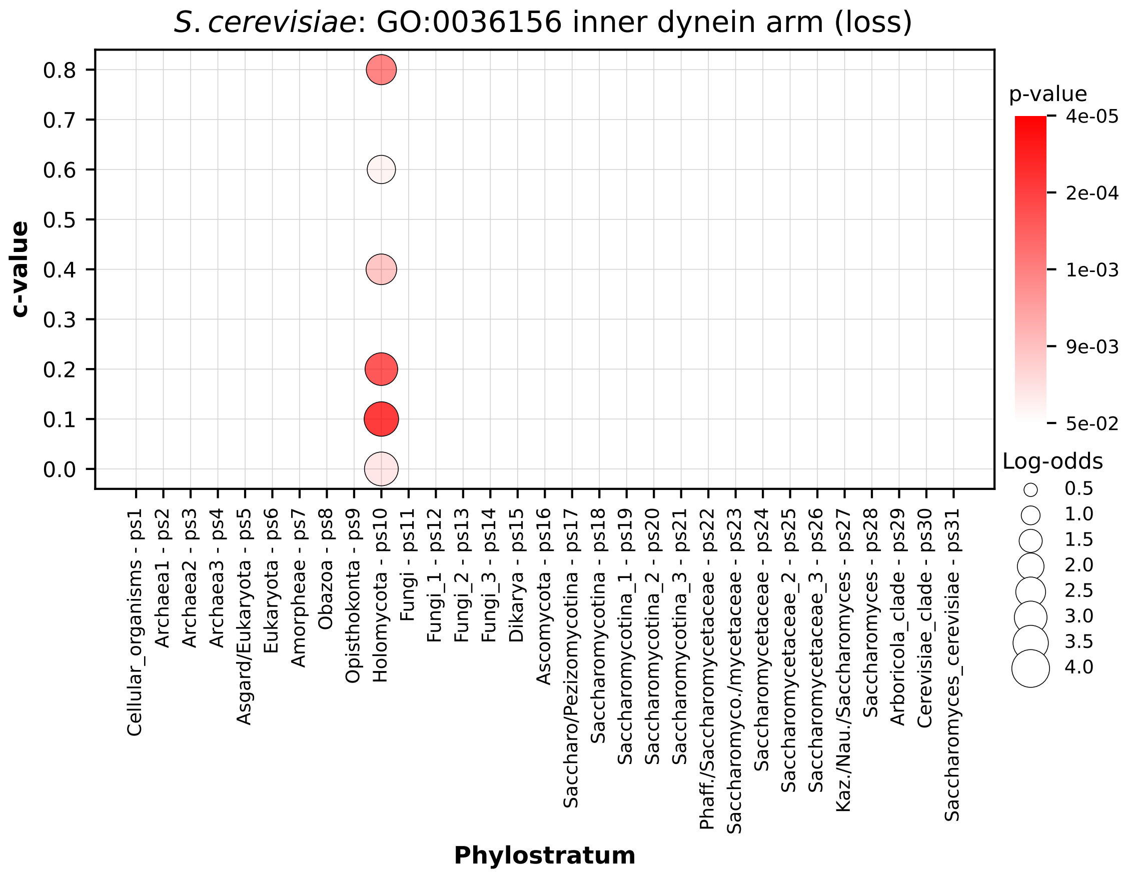

S. cerevisiae: GO:0036156 inner dynein arm (loss)
c-value
0.8
0.7
0.6
0.5
0.4
0.3
0.2
0.1
0.0
p-value
4e-05
2e-04
1e-03
9e-03
5e-02
Log-odds
0.5
1.0
1.5
2.0
2.5
3.0
3.5
4.0
Cellular_organisms - ps1
Archaea1 - ps2
Archaea2 - ps3
Archaea3 - ps4
Asgard/Eukaryota - ps5
Eukaryota - ps6
Amorpheae - ps7
Obazoa - ps8
Opisthokonta - ps9
Holomycota - ps10
Fungi - ps11
Fungi_1 - ps12
Fungi_2 - ps13
Fungi_3 - ps14
Dikarya - ps15
Ascomycota - ps16
Saccharo/Pezizomycotina - ps17
Saccharomycotina - ps18
Saccharomycotina_1 - ps19
Saccharomycotina_2 - ps20
Saccharomycotina_3 - ps21
Phaff./Saccharomycetaceae - ps22
Saccharomyco./mycetaceae - ps23
Saccharomycetaceae - ps24
Saccharomycetaceae_2 - ps25
Saccharomycetaceae_3 - ps26
Kaz./Nau./Saccharomyces - ps27
Saccharomyces - ps28
Arboricola_clade - ps29
Cerevisiae_clade - ps30
Saccharomyces_cerevisiae - ps31
Phylostratum

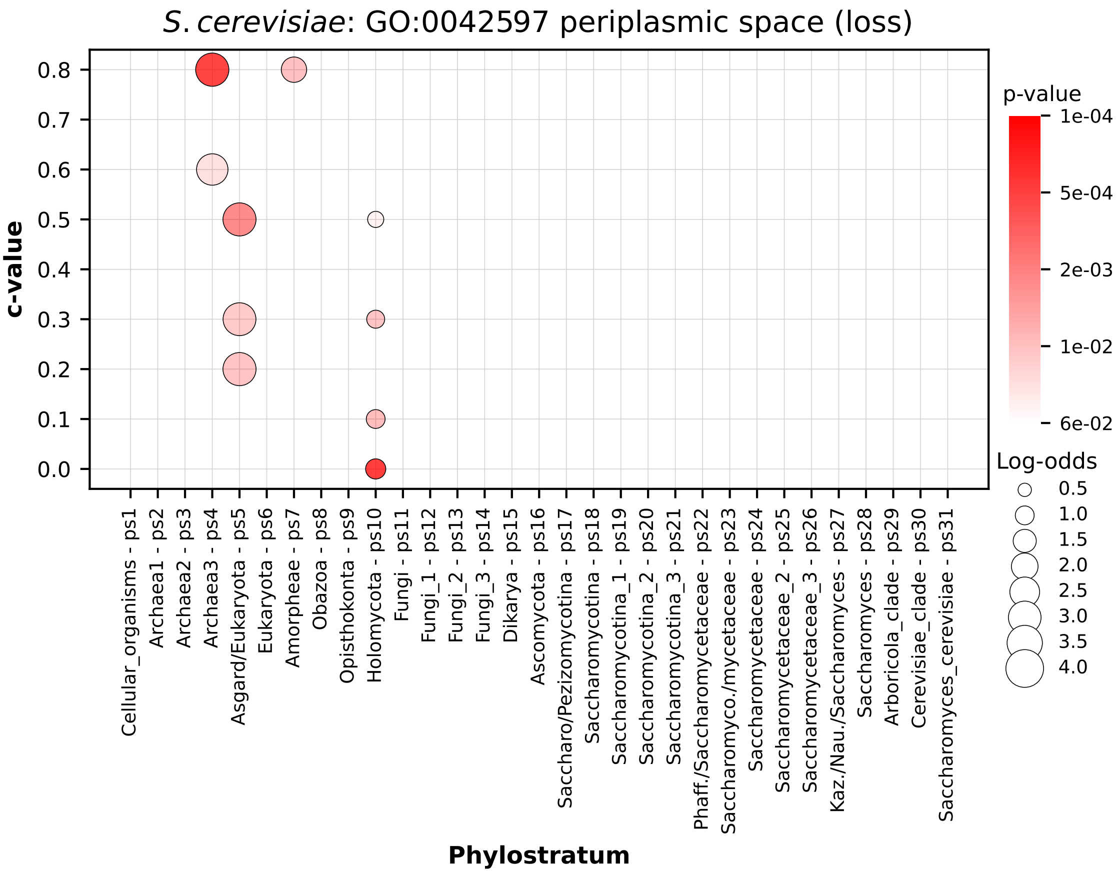

S. cerevisiae: GO:0042597 periplasmic space (loss)
c-value
0.0
0.1
0.2
0.3
0.4
0.5
0.6
0.7
0.8
Phylostratum
Cellular_organisms - ps1
Archaea1 - ps2
Archaea2 - ps3
Archaea3 - ps4
Asgard/Eukaryota - ps5
Eukaryota - ps6
Amorpheae - ps7
Obazoa - ps8
Opisthokonta - ps9
Holomycota - ps10
Fungi - ps11
Fungi_1 - ps12
Fungi_2 - ps13
Fungi_3 - ps14
Dikarya - ps15
Ascomycota - ps16
Saccharo/Pezizomycotina - ps17
Saccharomycotina - ps18
Saccharomycotina_1 - ps19
Saccharomycotina_2 - ps20
Saccharomycotina_3 - ps21
Phaff./Saccharomycetaceae - ps22
Saccharomyco./mycetaceae - ps23
Saccharomycetaceae - ps24
Saccharomycetaceae_2 - ps25
Saccharomycetaceae_3 - ps26
Kaz./Nau./Saccharomyces - ps27
Saccharomyces - ps28
Arboricola_clade - ps29
Cerevisiae_clade - ps30
Saccharomyces_cerevisiae - ps31
p-value
1e-04
5e-04
2e-03
1e-02
6e-02
Log-odds
0.5
1.0
1.5
2.0
2.5
3.0
3.5
4.0

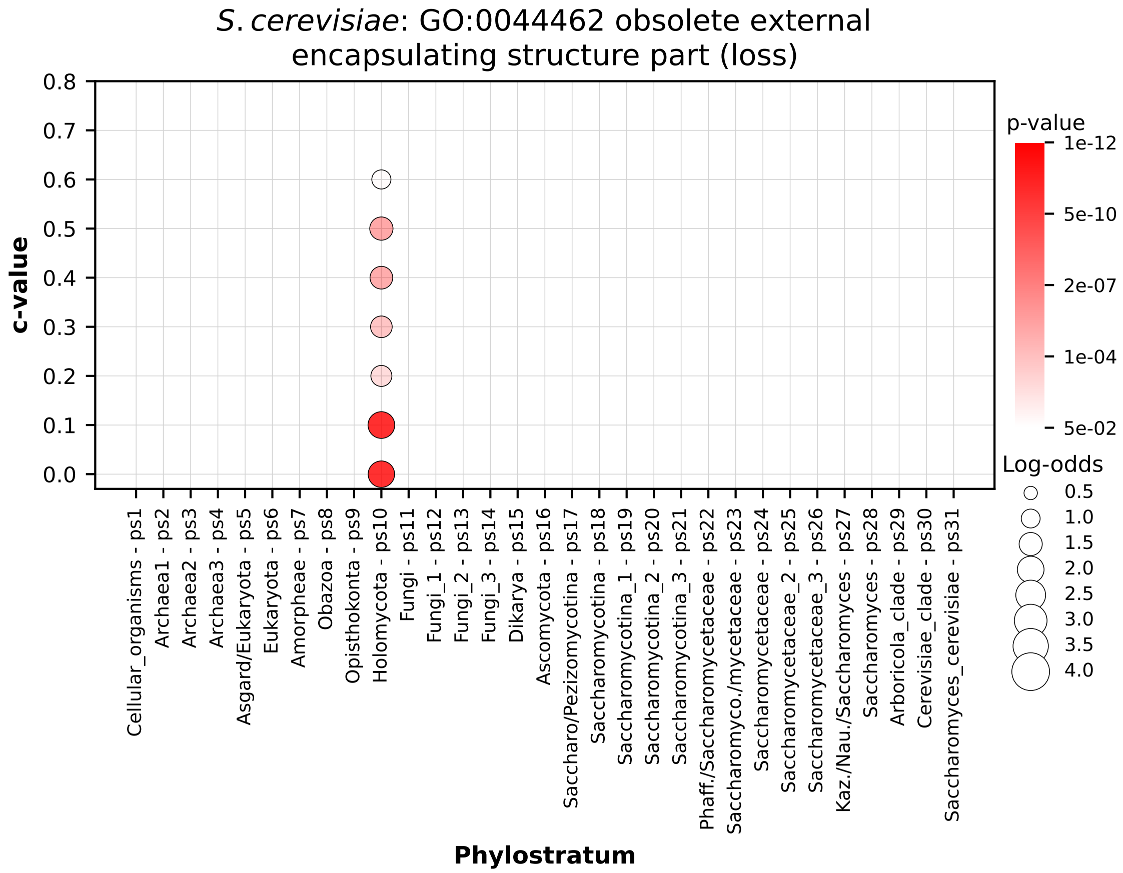

S. cerevisiae: GO:0044462 obsolete external encapsulating structure part (loss)
c-value
0.8
0.7
0.6
0.5
0.4
0.3
0.2
0.1
0.0
Phylostratum
Cellular_organisms - ps1
Archaea1 - ps2
Archaea2 - ps3
Archaea3 - ps4
Asgard/Eukaryota - ps5
Eukaryota - ps6
Amorpheae - ps7
Obazoa - ps8
Opisthokonta - ps9
Holomycota - ps10
Fungi - ps11
Fungi_1 - ps12
Fungi_2 - ps13
Fungi_3 - ps14
Dikarya - ps15
Ascomycota - ps16
Saccharo/Pezizomycotina - ps17
Saccharomycotina - ps18
Saccharomycotina_1 - ps19
Saccharomycotina_2 - ps20
Saccharomycotina_3 - ps21
Phaff./Saccharomycetaceae - ps22
Saccharomyco./mycetaceae - ps23
Saccharomycetaceae - ps24
Saccharomycetaceae_2 - ps25
Saccharomycetaceae_3 - ps26
Kaz./Nau./Saccharomyces - ps27
Saccharomyces - ps28
Arboricola_clade - ps29
Cerevisiae_clade - ps30
Saccharomyces_cerevisiae - ps31
p-value
1e-12
5e-10
2e-07
1e-04
5e-02
Log-odds
0.5
1.0
1.5
2.0
2.5
3.0
3.5
4.0

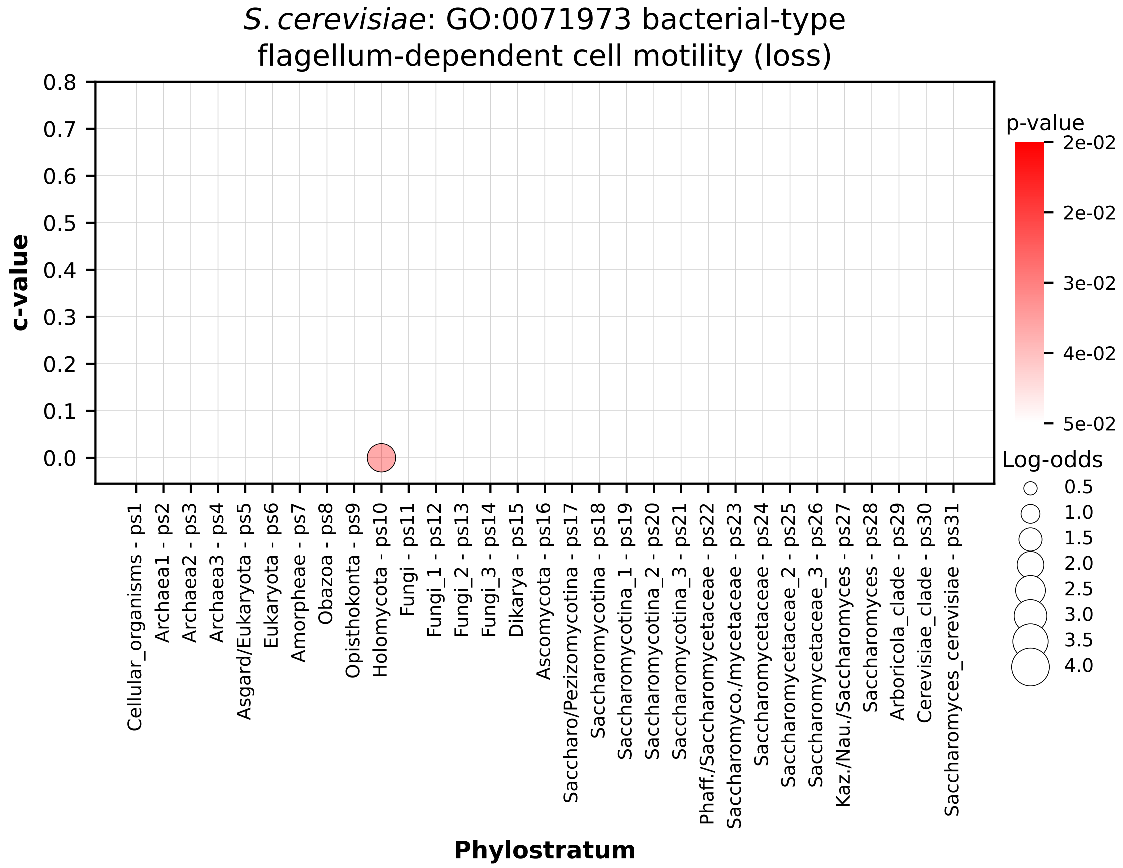

S. cerevisiae: GO:0071973 bacterial-type flagellum-dependent cell motility (loss)
c-value
0.8
0.7
0.6
0.5
0.4
0.3
0.2
0.1
0.0
p-value
2e-02
2e-02
3e-02
4e-02
5e-02
Log-odds
0.5
1.0
1.5
2.0
2.5
3.0
3.5
4.0
Cellular_organisms - ps1
Archaea1 - ps2
Archaea2 - ps3
Archaea3 - ps4
Asgard/Eukaryota - ps5
Eukaryota - ps6
Amorpheae - ps7
Obazoa - ps8
Opisthokonta - ps9
Holomycota - ps10
Fungi - ps11
Fungi_1 - ps12
Fungi_2 - ps13
Fungi_3 - ps14
Dikarya - ps15
Ascomycota - ps16
Saccharo/Pezizomycotina - ps17
Saccharomycotina - ps18
Saccharomycotina_1 - ps19
Saccharomycotina_2 - ps20
Saccharomycotina_3 - ps21
Phaff./Saccharomycetaceae - ps22
Saccharomyco./mycetaceae - ps23
Saccharomycetaceae - ps24
Saccharomycetaceae_2 - ps25
Saccharomycetaceae_3 - ps26
Kaz./Nau./Saccharomyces - ps27
Saccharomyces - ps28
Arboricola_clade - ps29
Cerevisiae_clade - ps30
Saccharomyces_cerevisiae - ps31
Phylostratum

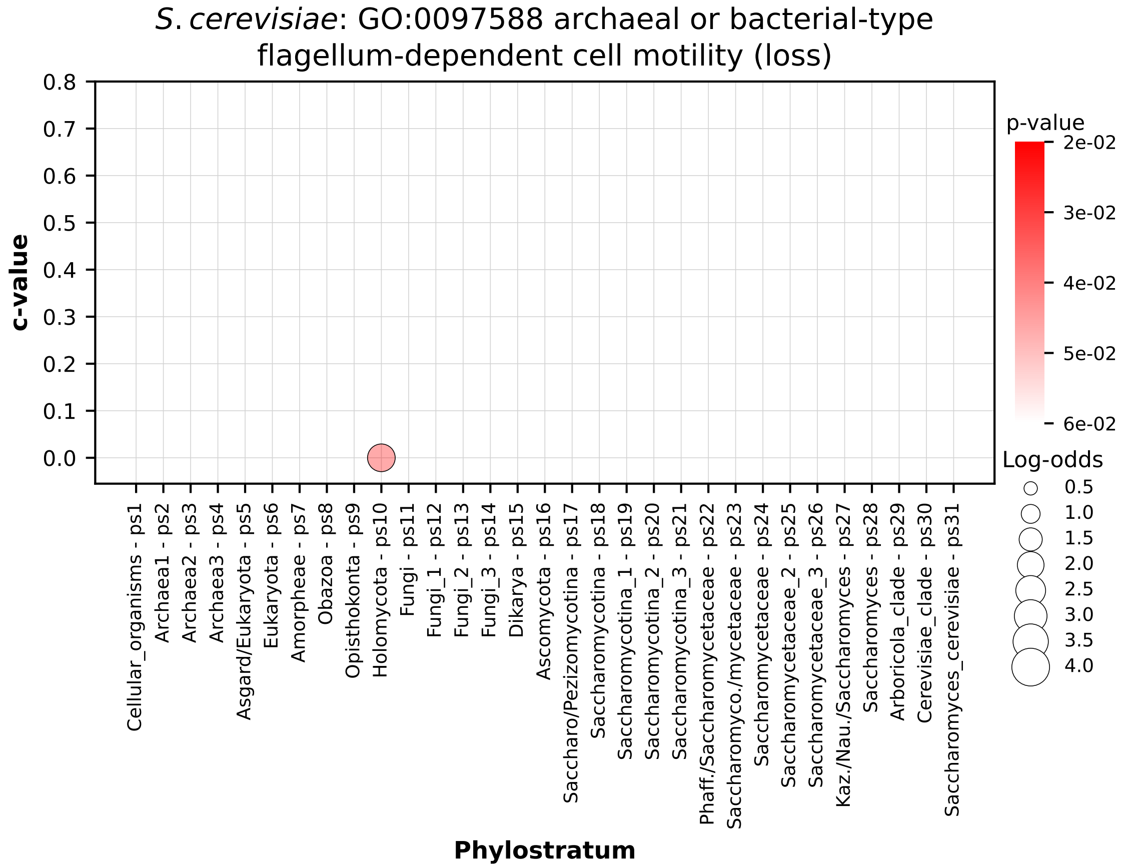

S. cerevisiae: GO:0097588 archaeal or bacterial-type flagellum-dependent cell motility (loss)
c-value
0.8
0.7
0.6
0.5
0.4
0.3
0.2
0.1
0.0
Phylostratum
Cellular_organisms - ps1
Archaea1 - ps2
Archaea2 - ps3
Archaea3 - ps4
Asgard/Eukaryota - ps5
Eukaryota - ps6
Amorpheae - ps7
Obazoa - ps8
Opisthokonta - ps9
Holomycota - ps10
Fungi - ps11
Fungi_1 - ps12
Fungi_2 - ps13
Fungi_3 - ps14
Dikarya - ps15
Ascomycota - ps16
Saccharo/Pezizomycotina - ps17
Saccharomycotina - ps18
Saccharomycotina_1 - ps19
Saccharomycotina_2 - ps20
Saccharomycotina_3 - ps21
Phaff./Saccharomycetaceae - ps22
Saccharomyco./mycetaceae - ps23
Saccharomycetaceae - ps24
Saccharomycetaceae_2 - ps25
Saccharomycetaceae_3 - ps26
Kaz./Nau./Saccharomyces - ps27
Saccharomyces - ps28
Arboricola_clade - ps29
Cerevisiae_clade - ps30
Saccharomyces_cerevisiae - ps31
p-value
2e-02
3e-02
4e-02
5e-02
6e-02
Log-odds
0.5
1.0
1.5
2.0
2.5
3.0
3.5
4.0

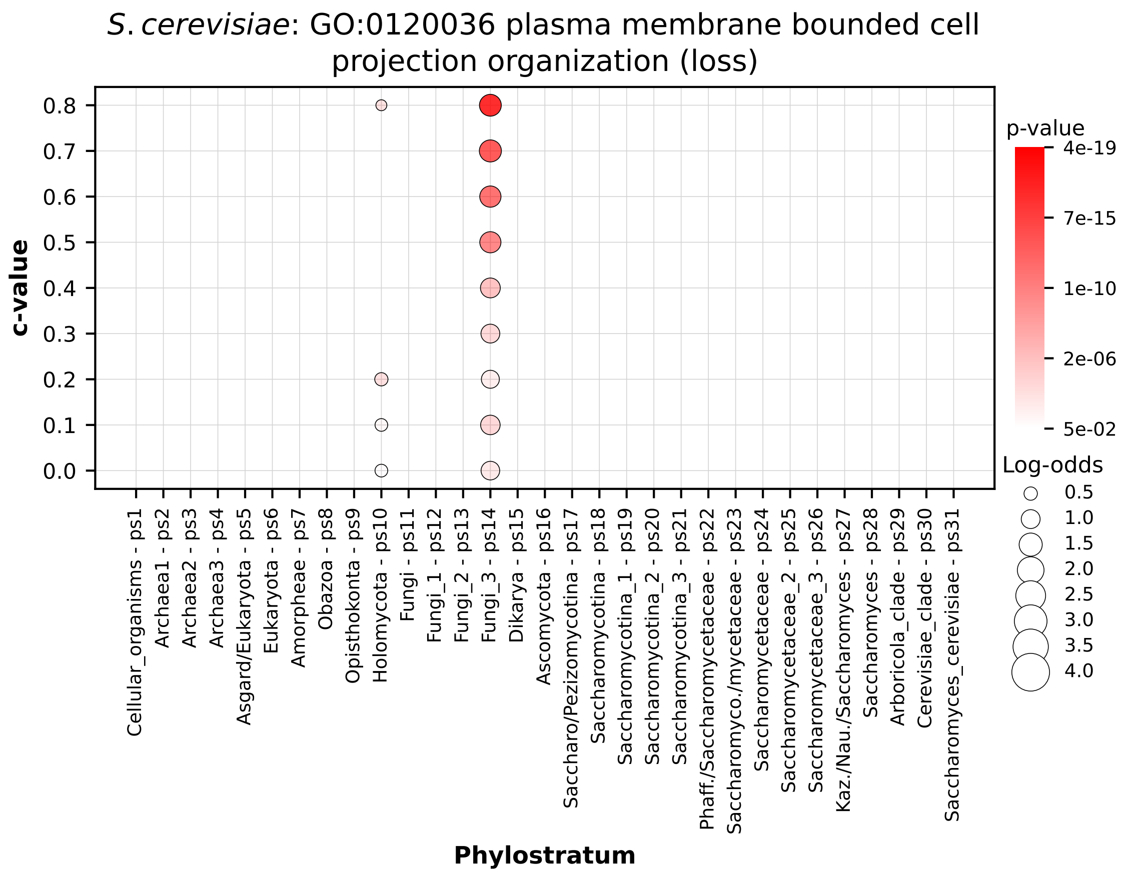

S. cerevisiae: GO:0120036 plasma membrane bounded cell projection organization (loss)
c-value
0.8
0.7
0.6
0.5
0.4
0.3
0.2
0.1
0.0
p-value
4e-19
7e-15
1e-10
2e-06
5e-02
Log-odds
0.5
1.0
1.5
2.0
2.5
3.0
3.5
4.0
Cellular_organisms - ps1
Archaea1 - ps2
Archaea2 - ps3
Archaea3 - ps4
Asgard/Eukaryota - ps5
Eukaryota - ps6
Amorpheae - ps7
Obazoa - ps8
Opisthokonta - ps9
Holomycota - ps10
Fungi - ps11
Fungi_1 - ps12
Fungi_2 - ps13
Fungi_3 - ps14
Dikarya - ps15
Ascomycota - ps16
Saccharo/Pezizomycotina - ps17
Saccharomycotina - ps18
Saccharomycotina_1 - ps19
Saccharomycotina_2 - ps20
Saccharomycotina_3 - ps21
Phaff./Saccharomycetaceae - ps22
Saccharomyco./mycetaceae - ps23
Saccharomycetaceae - ps24
Saccharomycetaceae_2 - ps25
Saccharomycetaceae_3 - ps26
Kaz./Nau./Saccharomyces - ps27
Saccharomyces - ps28
Arboricola_clade - ps29
Cerevisiae_clade - ps30
Saccharomyces_cerevisiae - ps31
Phylostratum

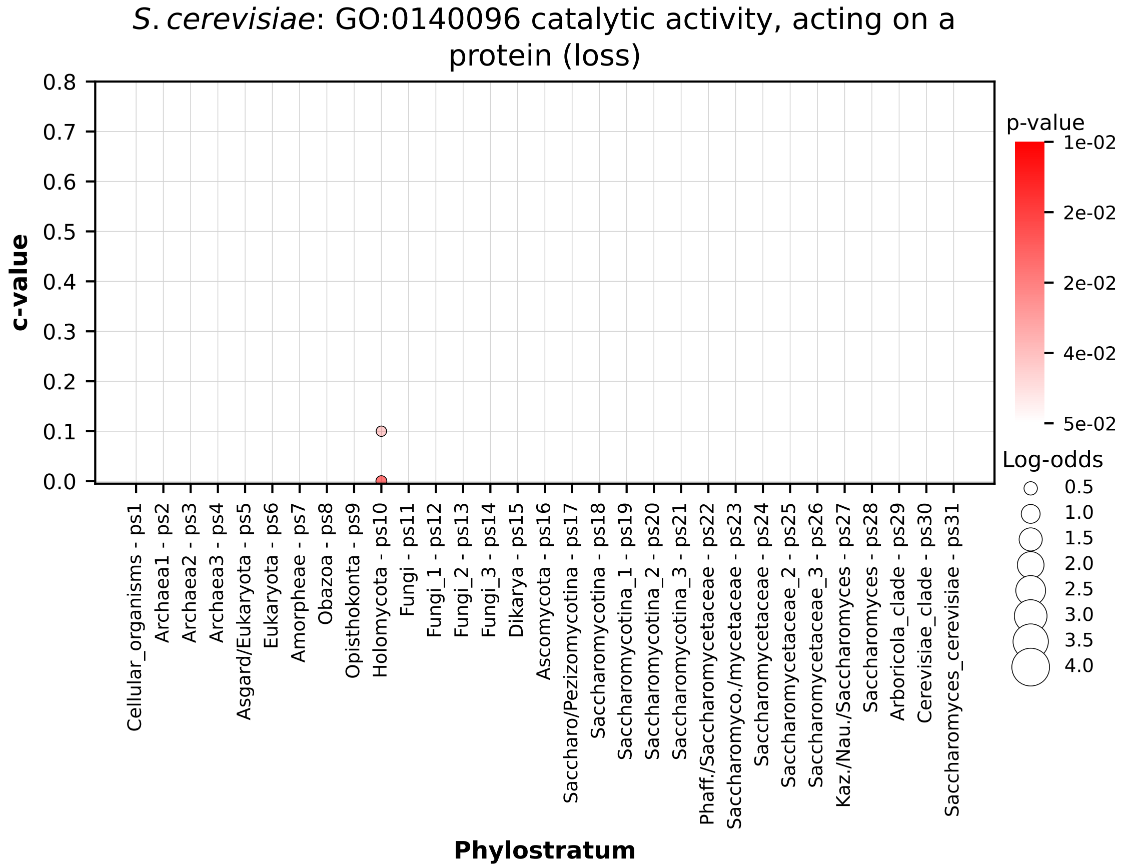
S. cerevisiae: GO:0140096 catalytic activity, acting on a protein (loss)
c-value
0.8
0.7
0.6
0.5
0.4
0.3
0.2
0.1
0.0
Phylostratum
Cellular_organisms - ps1
Archaea1 - ps2
Archaea2 - ps3
Archaea3 - ps4
Asgard/Eukaryota - ps5
Eukaryota - ps6
Amorpheae - ps7
Obazoa - ps8
Opisthokonta - ps9
Holomycota - ps10
Fungi - ps11
Fungi_1 - ps12
Fungi_2 - ps13
Fungi_3 - ps14
Dikarya - ps15
Ascomycota - ps16
Saccharo/Pezizomycotina - ps17
Saccharomycotina - ps18
Saccharomycotina_1 - ps19
Saccharomycotina_2 - ps20
Saccharomycotina_3 - ps21
Phaff./Saccharomycetaceae - ps22
Saccharomyco./mycetaceae - ps23
Saccharomycetaceae - ps24
Saccharomycetaceae_2 - ps25
Saccharomycetaceae_3 - ps26
Kaz./Nau./Saccharomyces - ps27
Saccharomyces - ps28
Arboricola_clade - ps29
Cerevisiae_clade - ps30
Saccharomyces_cerevisiae - ps31
p-value
1e-02
2e-02
2e-02
4e-02
5e-02
Log-odds
0.5
1.0
1.5
2.0
2.5
3.0
3.5
4.0

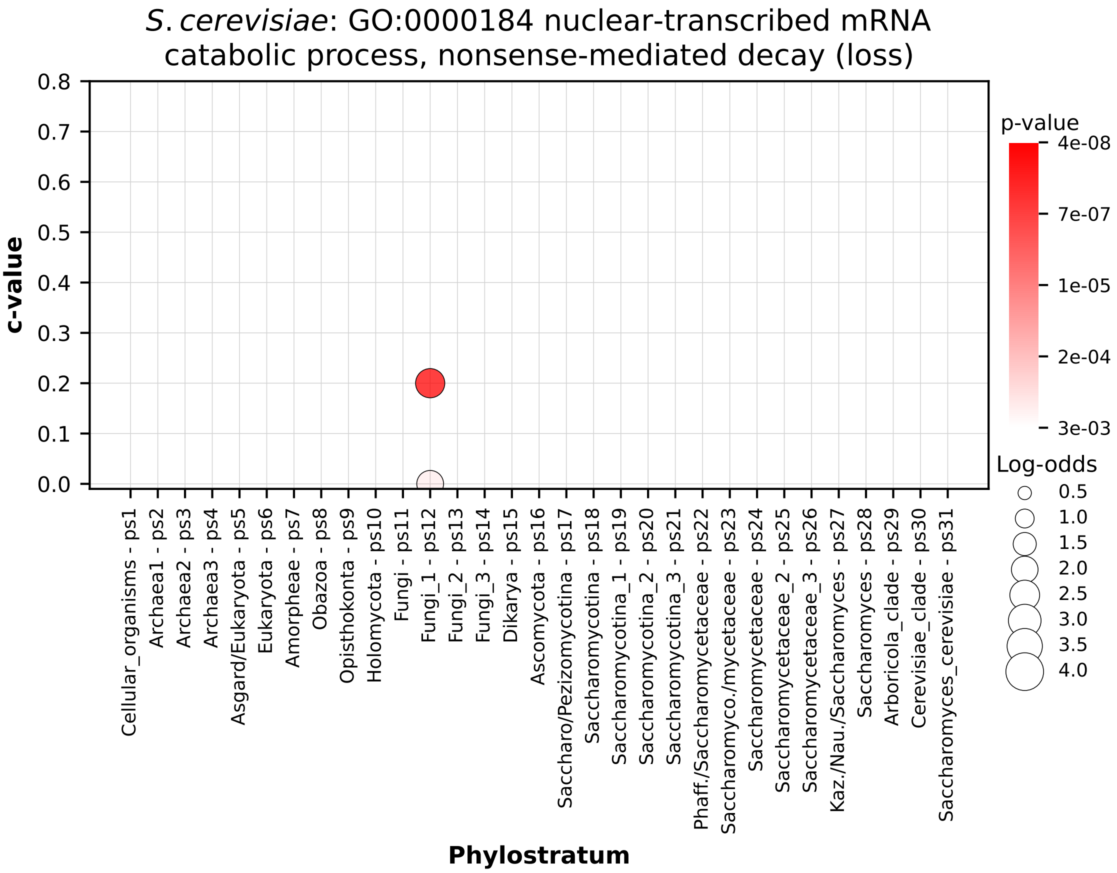

S. cerevisiae: GO:0000184 nuclear-transcribed mRNA catabolic process, nonsense-mediated decay (loss)
c-value
0.8
0.7
0.6
0.5
0.4
0.3
0.2
0.1
0.0
Phylostratum
Cellular_organisms - ps1
Archaea1 - ps2
Archaea2 - ps3
Archaea3 - ps4
Asgard/Eukaryota - ps5
Eukaryota - ps6
Amorpheae - ps7
Obazoa - ps8
Opisthokonta - ps9
Holomycota - ps10
Fungi - ps11
Fungi_1 - ps12
Fungi_2 - ps13
Fungi_3 - ps14
Dikarya - ps15
Ascomycota - ps16
Saccharo/Pezizomycotina - ps17
Saccharomycotina - ps18
Saccharomycotina_1 - ps19
Saccharomycotina_2 - ps20
Saccharomycotina_3 - ps21
Phaff./Saccharomycetaceae - ps22
Saccharomyco./mycetaceae - ps23
Saccharomycetaceae - ps24
Saccharomycetaceae_2 - ps25
Saccharomycetaceae_3 - ps26
Kaz./Nau./Saccharomyces - ps27
Saccharomyces - ps28
Arboricola_clade - ps29
Cerevisiae_clade - ps30
Saccharomyces_cerevisiae - ps31
p-value
4e-08
7e-07
1e-05
2e-04
3e-03
Log-odds
0.5
1.0
1.5
2.0
2.5
3.0
3.5
4.0

S. cerevisiae: GO:0000956 nuclear-transcribed mRNA catabolic process (loss)

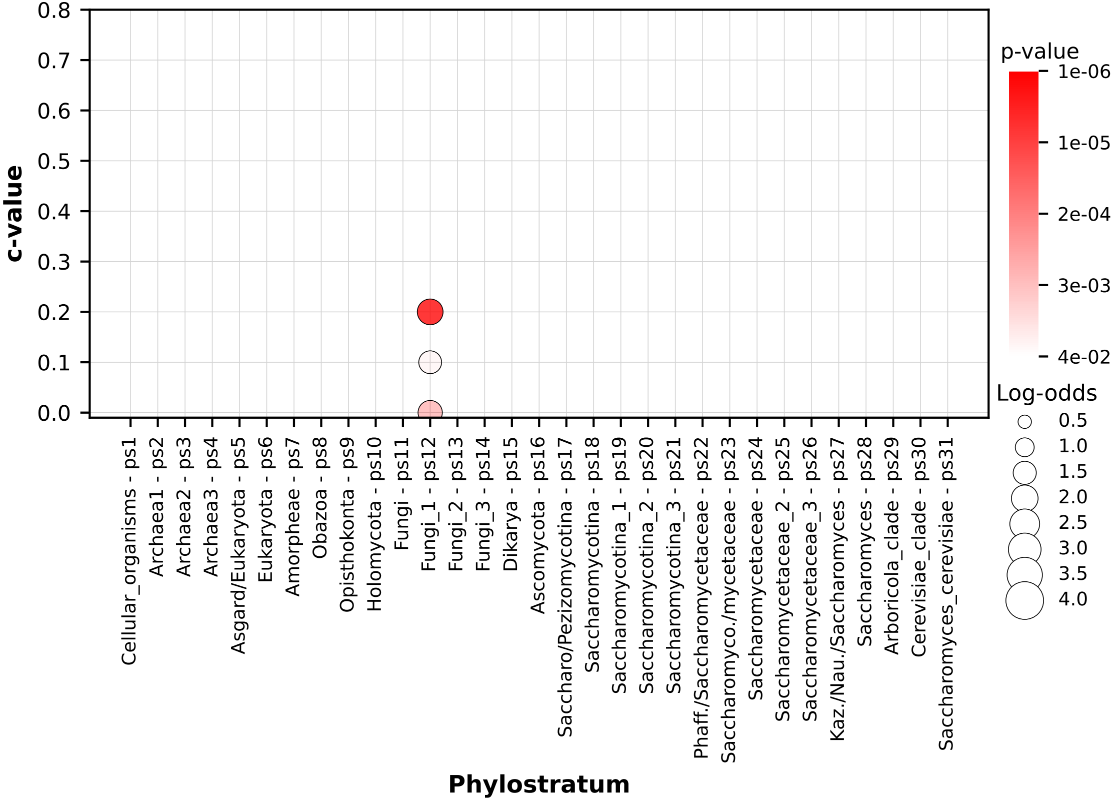

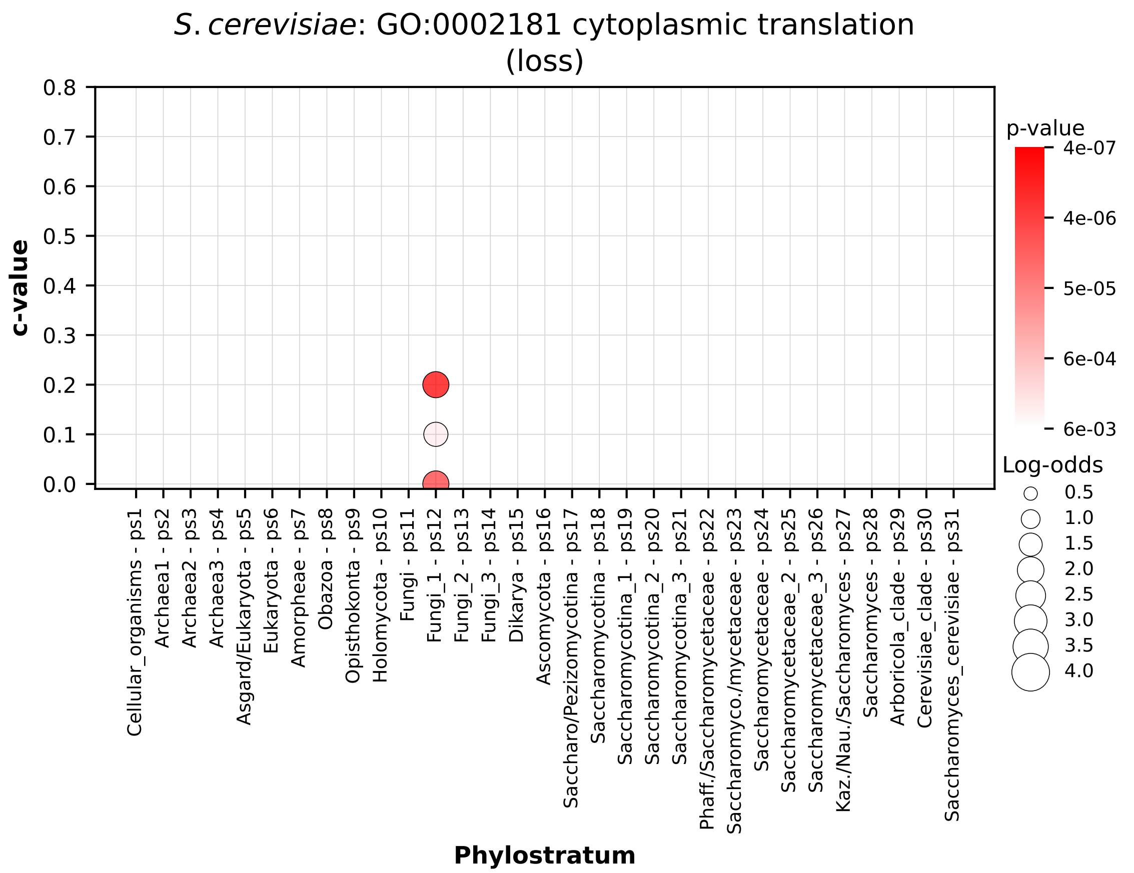
S. cerevisiae: GO:0002181 cytoplasmic translation
(loss)
c-value
0.8
0.7
0.6
0.5
0.4
0.3
0.2
0.1
0.0
Phylostratum
Cellular_organisms - ps1
Archaea1 - ps2
Archaea2 - ps3
Archaea3 - ps4
Asgard/Eukaryota - ps5
Eukaryota - ps6
Amorpheae - ps7
Obazoa - ps8
Opisthokonta - ps9
Holomycota - ps10
Fungi - ps11
Fungi_1 - ps12
Fungi_2 - ps13
Fungi_3 - ps14
Dikarya - ps15
Ascomycota - ps16
Saccharo/Pezizomycotina - ps17
Saccharomycotina - ps18
Saccharomycotina_1 - ps19
Saccharomycotina_2 - ps20
Saccharomycotina_3 - ps21
Phaff./Saccharomycetaceae - ps22
Saccharomyco./mycetaceae - ps23
Saccharomycetaceae - ps24
Saccharomycetaceae_2 - ps25
Saccharomycetaceae_3 - ps26
Kaz./Nau./Saccharomyces - ps27
Saccharomyces - ps28
Arboricola_clade - ps29
Cerevisiae_clade - ps30
Saccharomyces_cerevisiae - ps31
p-value
4e-07
4e-06
5e-05
6e-04
6e-03
Log-odds
0.5
1.0
1.5
2.0
2.5
3.0
3.5
4.0

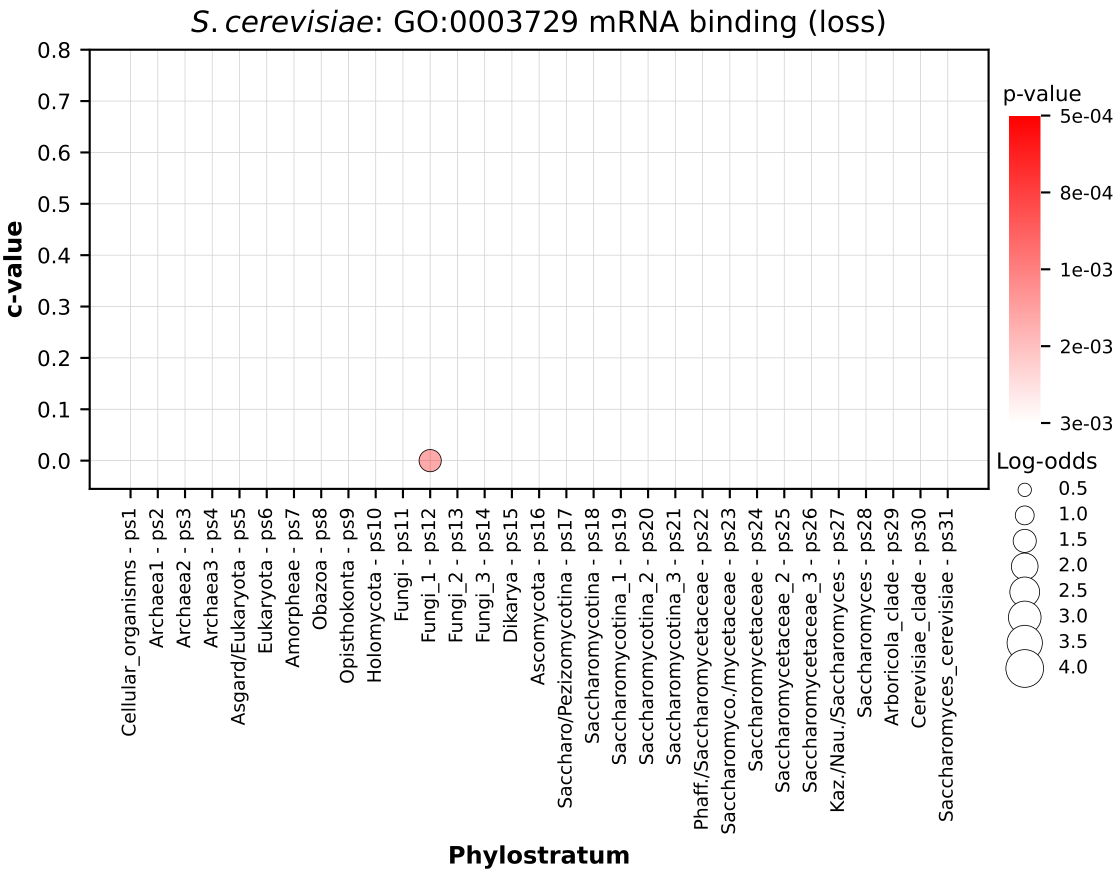

S. cerevisiae: GO:0003729 mRNA binding (loss)
c-value
0.8
0.7
0.6
0.5
0.4
0.3
0.2
0.1
0.0
Phylostratum
Cellular_organisms - ps1
Archaea1 - ps2
Archaea2 - ps3
Archaea3 - ps4
Asgard/Eukaryota - ps5
Eukaryota - ps6
Amorpheae - ps7
Obazoa - ps8
Opisthokonta - ps9
Holomycota - ps10
Fungi - ps11
Fungi_1 - ps12
Fungi_2 - ps13
Fungi_3 - ps14
Dikarya - ps15
Ascomycota - ps16
Saccharo/Pezizomycotina - ps17
Saccharomycotina - ps18
Saccharomycotina_1 - ps19
Saccharomycotina_2 - ps20
Saccharomycotina_3 - ps21
Phaff./Saccharomycetaceae - ps22
Saccharomyco./mycetaceae - ps23
Saccharomycetaceae - ps24
Saccharomycetaceae_2 - ps25
Saccharomycetaceae_3 - ps26
Kaz./Nau./Saccharomyces - ps27
Saccharomyces - ps28
Arboricola_clade - ps29
Cerevisiae_clade - ps30
Saccharomyces_cerevisiae - ps31
p-value
5e-04
8e-04
1e-03
2e-03
3e-03
Log-odds
0.5
1.0
1.5
2.0
2.5
3.0
3.5
4.0

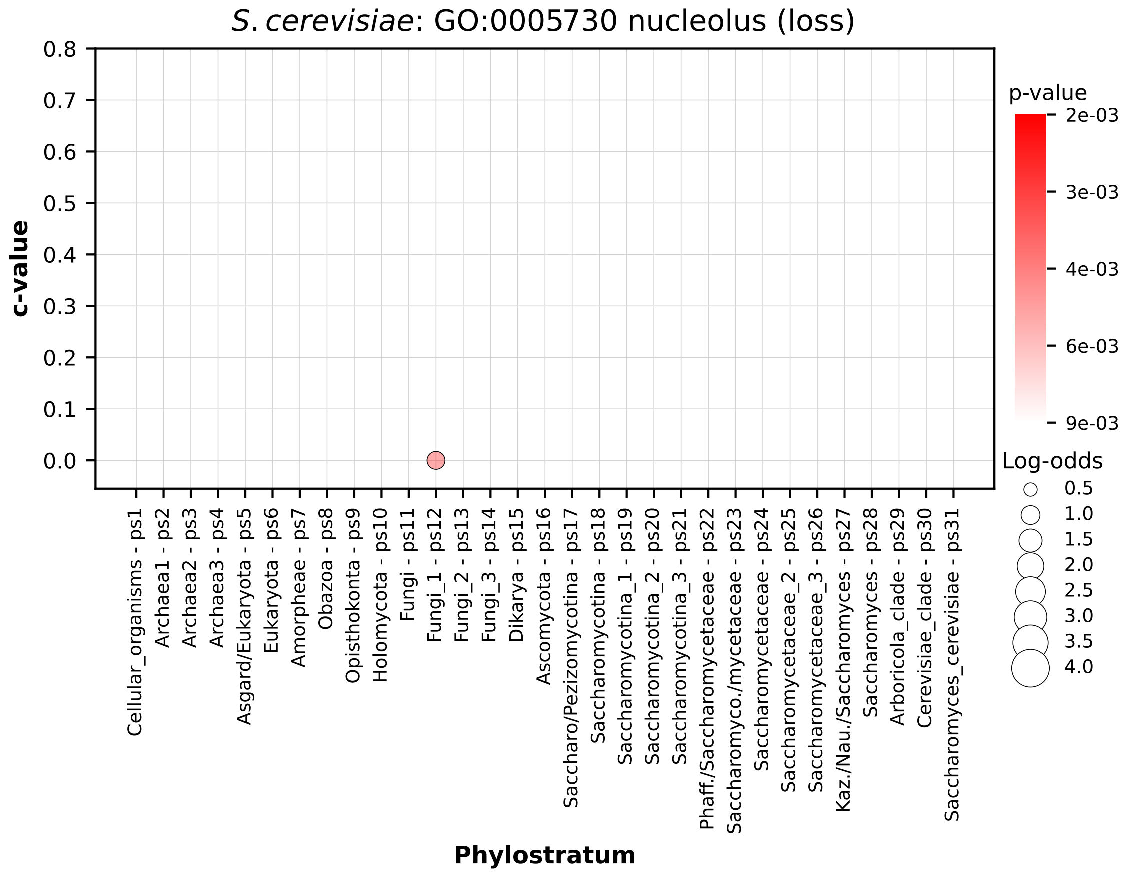

S. cerevisiae: GO:0005730 nucleolus (loss)
c-value
0.8
0.7
0.6
0.5
0.4
0.3
0.2
0.1
0.0
Phylostratum
Cellular_organisms - ps1
Archaea1 - ps2
Archaea2 - ps3
Archaea3 - ps4
Asgard/Eukaryota - ps5
Eukaryota - ps6
Amorpheae - ps7
Obazoa - ps8
Opisthokonta - ps9
Holomycota - ps10
Fungi - ps11
Fungi_1 - ps12
Fungi_2 - ps13
Fungi_3 - ps14
Dikarya - ps15
Ascomycota - ps16
Saccharo/Pezizomycotina - ps17
Saccharomycotina - ps18
Saccharomycotina_1 - ps19
Saccharomycotina_2 - ps20
Saccharomycotina_3 - ps21
Phaff./Saccharomycetaceae - ps22
Saccharomyco./mycetaceae - ps23
Saccharomycetaceae - ps24
Saccharomycetaceae_2 - ps25
Saccharomycetaceae_3 - ps26
Kaz./Nau./Saccharomyces - ps27
Saccharomyces - ps28
Arboricola_clade - ps29
Cerevisiae_clade - ps30
Saccharomyces_cerevisiae - ps31
p-value
2e-03
3e-03
4e-03
6e-03
9e-03
Log-odds
0.5
1.0
1.5
2.0
2.5
3.0
3.5
4.0

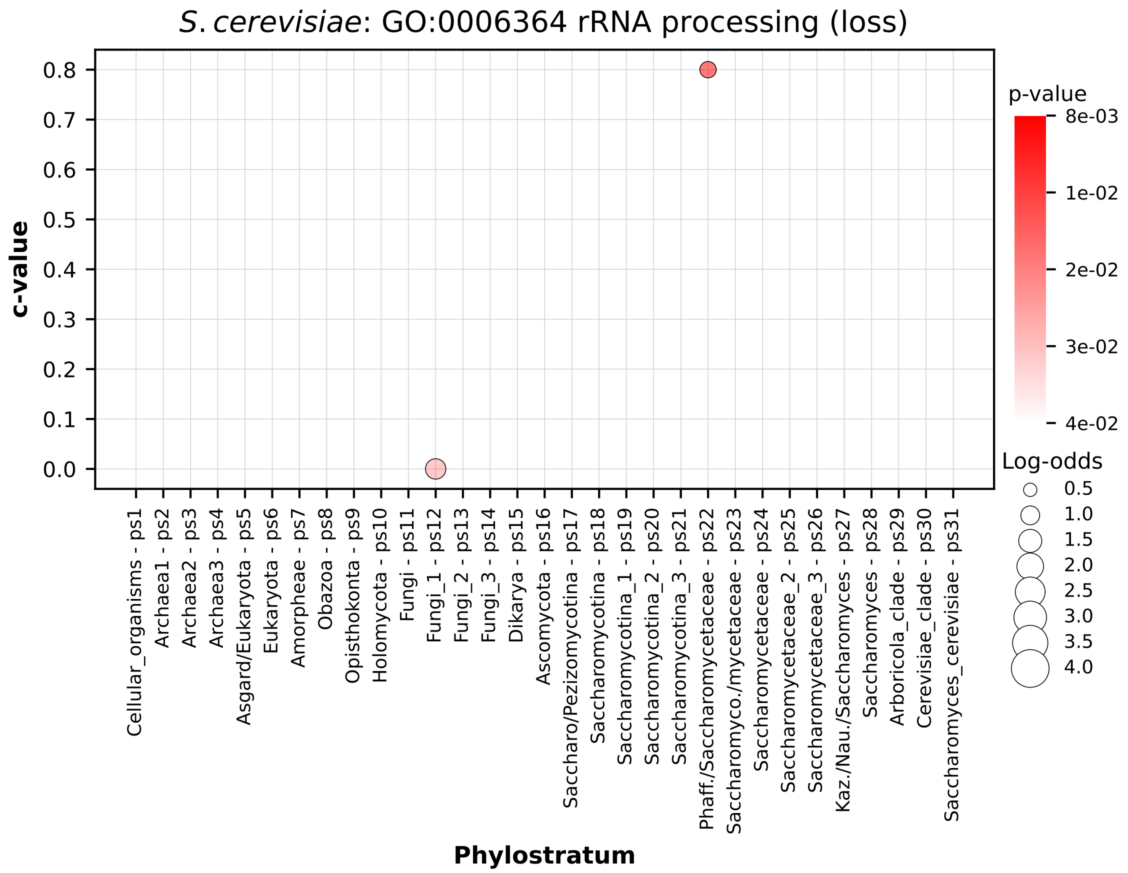
S. cerevisiae: GO:0006364 rRNA processing (loss)
c-value
0.0
0.1
0.2
0.3
0.4
0.5
0.6
0.7
0.8
p-value
8e-03
1e-02
2e-02
3e-02
4e-02
Log-odds
0.5
1.0
1.5
2.0
2.5
3.0
3.5
4.0
Cellular_organisms - ps1
Archaea1 - ps2
Archaea2 - ps3
Archaea3 - ps4
Asgard/Eukaryota - ps5
Eukaryota - ps6
Amorpheae - ps7
Obazoa - ps8
Opisthokonta - ps9
Holomycota - ps10
Fungi - ps11
Fungi_1 - ps12
Fungi_2 - ps13
Fungi_3 - ps14
Dikarya - ps15
Ascomycota - ps16
Saccharo/Pezizomycotina - ps17
Saccharomycotina - ps18
Saccharomycotina_1 - ps19
Saccharomycotina_2 - ps20
Saccharomycotina_3 - ps21
Phaff./Saccharomycetaceae - ps22
Saccharomyco./mycetaceae - ps23
Saccharomycetaceae - ps24
Saccharomycetaceae_2 - ps25
Saccharomycetaceae_3 - ps26
Kaz./Nau./Saccharomyces - ps27
Saccharomyces - ps28
Arboricola_clade - ps29
Cerevisiae_clade - ps30
Saccharomyces_cerevisiae - ps31
Phylostratum

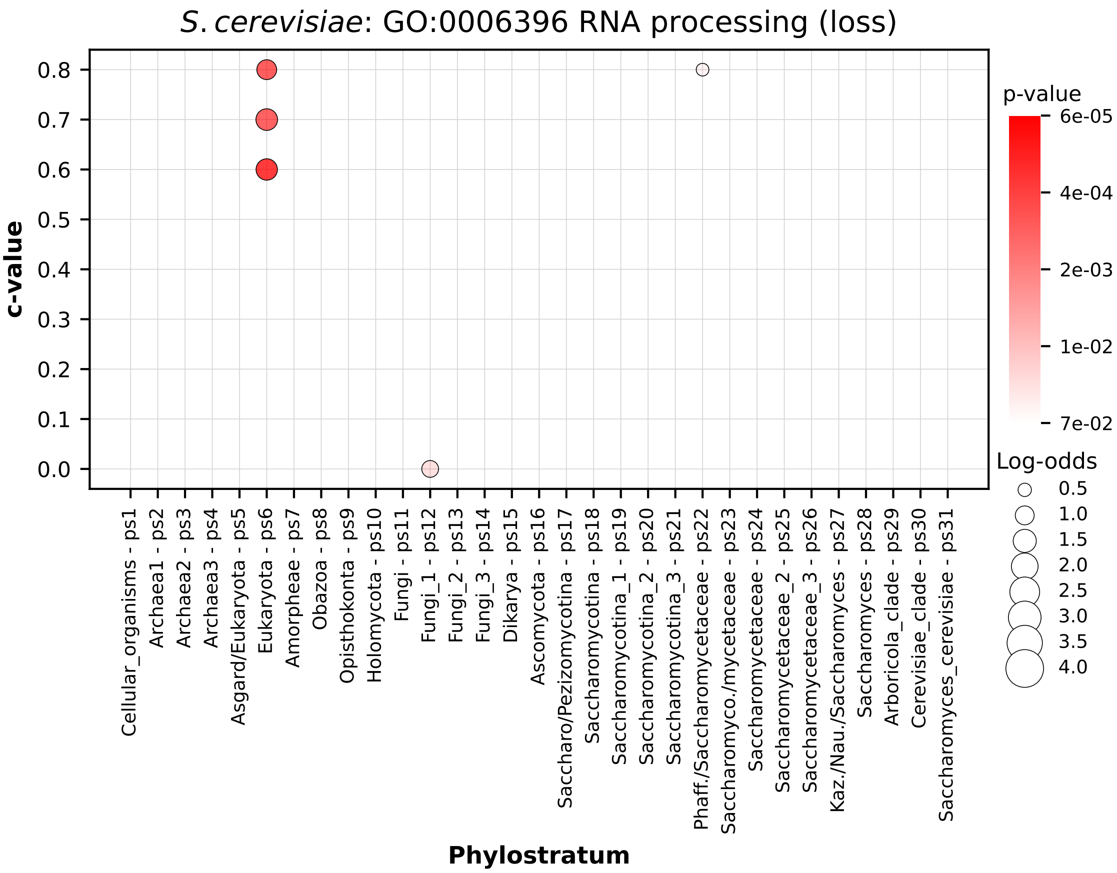
S. cerevisiae: GO:0006396 RNA processing (loss)
c-value
0.8
0.7
0.6
0.5
0.4
0.3
0.2
0.1
0.0
p-value
6e-05
4e-04
2e-03
1e-02
7e-02
Log-odds
0.5
1.0
1.5
2.0
2.5
3.0
3.5
4.0
Cellular_organisms - ps1
Archaea1 - ps2
Archaea2 - ps3
Archaea3 - ps4
Asgard/Eukaryota - ps5
Eukaryota - ps6
Amorpheae - ps7
Obazoa - ps8
Opisthokonta - ps9
Holomycota - ps10
Fungi - ps11
Fungi_1 - ps12
Fungi_2 - ps13
Fungi_3 - ps14
Dikarya - ps15
Ascomycota - ps16
Saccharo/Pezizomycotina - ps17
Saccharomycotina - ps18
Saccharomycotina_1 - ps19
Saccharomycotina_2 - ps20
Saccharomycotina_3 - ps21
Phaff./Saccharomycetaceae - ps22
Saccharomyco./mycetaceae - ps23
Saccharomycetaceae - ps24
Saccharomycetaceae_2 - ps25
Saccharomycetaceae_3 - ps26
Kaz./Nau./Saccharomyces - ps27
Saccharomyces - ps28
Arboricola_clade - ps29
Cerevisiae_clade - ps30
Saccharomyces_cerevisiae - ps31
Phylostratum

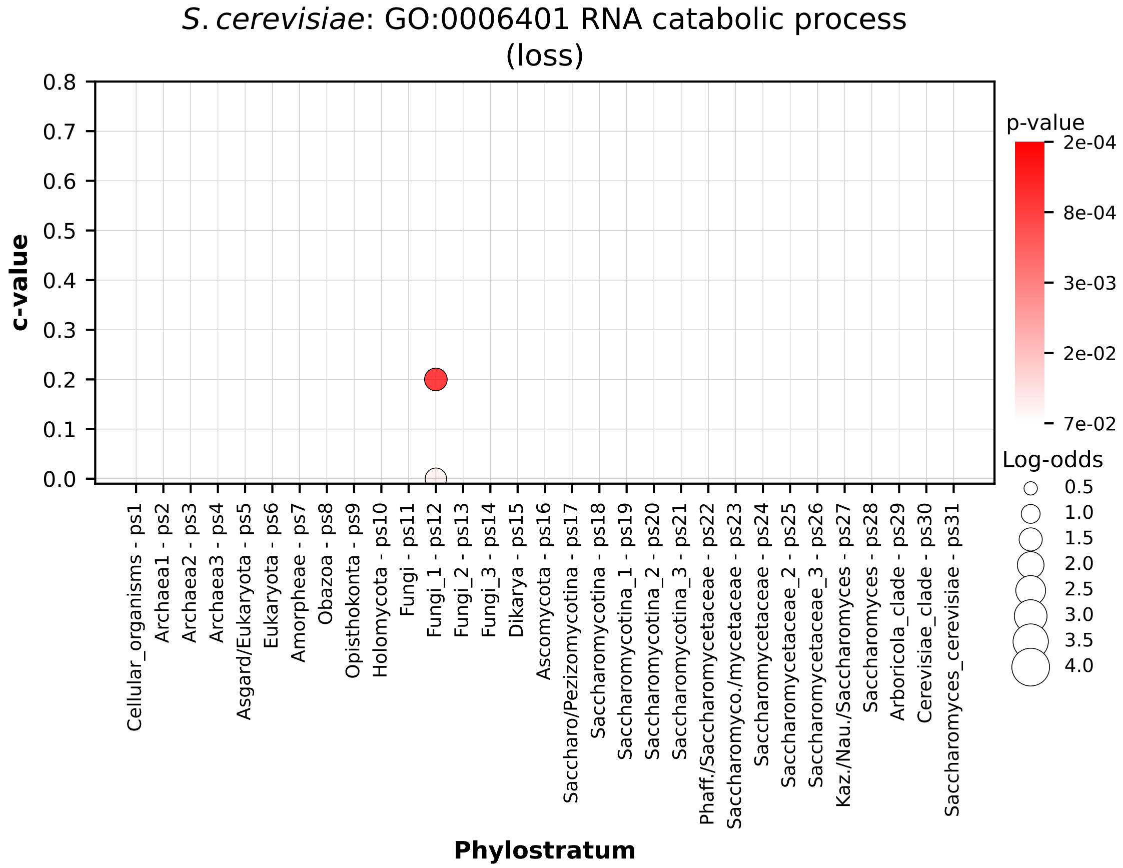

S. cerevisiae: GO:0006401 RNA catabolic process
(loss)
c-value
0.0
0.1
0.2
0.3
0.4
0.5
0.6
0.7
0.8
Phylostratum
Cellular_organisms - ps1
Archaea1 - ps2
Archaea2 - ps3
Archaea3 - ps4
Asgard/Eukaryota - ps5
Eukaryota - ps6
Amorpheae - ps7
Obazoa - ps8
Opisthokonta - ps9
Holomycota - ps10
Fungi - ps11
Fungi_1 - ps12
Fungi_2 - ps13
Fungi_3 - ps14
Dikarya - ps15
Ascomycota - ps16
Saccharo/Pezizomycotina - ps17
Saccharomycotina - ps18
Saccharomycotina_1 - ps19
Saccharomycotina_2 - ps20
Saccharomycotina_3 - ps21
Phaff./Saccharomycetaceae - ps22
Saccharomyco./mycetaceae - ps23
Saccharomycetaceae - ps24
Saccharomycetaceae_2 - ps25
Saccharomycetaceae_3 - ps26
Kaz./Nau./Saccharomyces - ps27
Saccharomyces - ps28
Arboricola_clade - ps29
Cerevisiae_clade - ps30
Saccharomyces_cerevisiae - ps31
p-value
2e-04
8e-04
3e-03
2e-02
7e-02
Log-odds
0.5
1.0
1.5
2.0
2.5
3.0
3.5
4.0

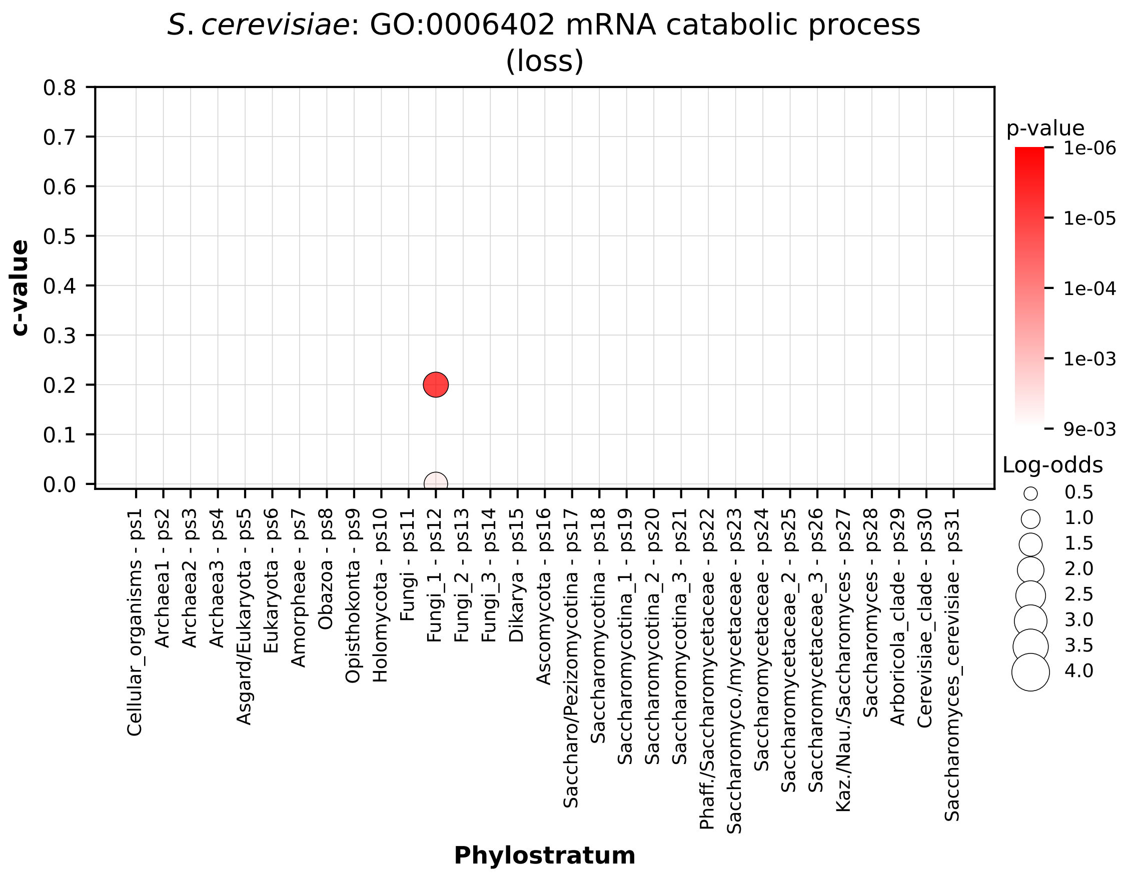

S. cerevisiae: GO:0006402 mRNA catabolic process
(loss)
c-value
0.0
0.1
0.2
0.3
0.4
0.5
0.6
0.7
0.8
Phylostratum
Cellular_organisms - ps1
Archaea1 - ps2
Archaea2 - ps3
Archaea3 - ps4
Asgard/Eukaryota - ps5
Eukaryota - ps6
Amorpheae - ps7
Obazoa - ps8
Opisthokonta - ps9
Holomycota - ps10
Fungi - ps11
Fungi_1 - ps12
Fungi_2 - ps13
Fungi_3 - ps14
Dikarya - ps15
Ascomycota - ps16
Saccharo/Pezizomycotina - ps17
Saccharomycotina - ps18
Saccharomycotina_1 - ps19
Saccharomycotina_2 - ps20
Saccharomycotina_3 - ps21
Phaff./Saccharomycetaceae - ps22
Saccharomyco./mycetaceae - ps23
Saccharomycetaceae - ps24
Saccharomycetaceae_2 - ps25
Saccharomycetaceae_3 - ps26
Kaz./Nau./Saccharomyces - ps27
Saccharomyces - ps28
Arboricola_clade - ps29
Cerevisiae_clade - ps30
Saccharomyces_cerevisiae - ps31
p-value
1e-06
1e-05
1e-04
1e-03
9e-03
Log-odds
0.5
1.0
1.5
2.0
2.5
3.0
3.5
4.0

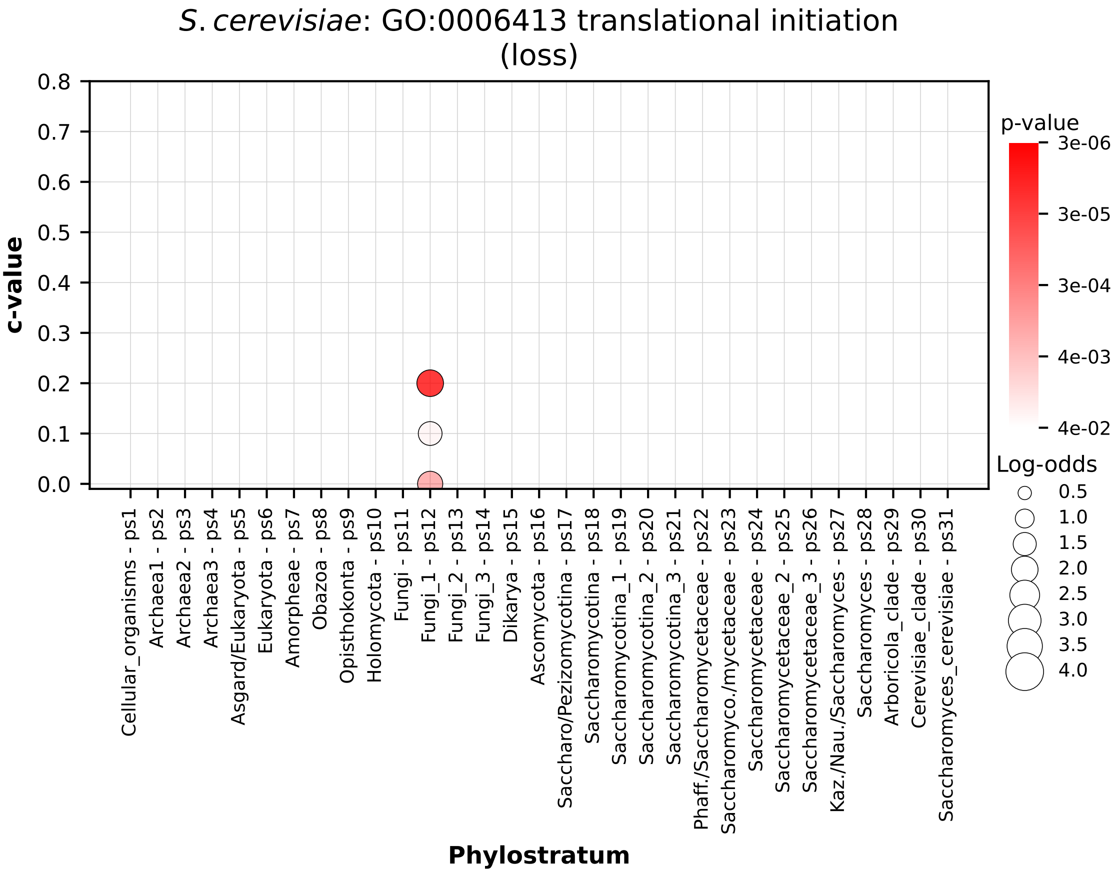
S. cerevisiae: GO:0006413 translational initiation
(loss)
c-value
0.8
0.7
0.6
0.5
0.4
0.3
0.2
0.1
0.0
Phylostratum
Cellular_organisms - ps1
Archaea1 - ps2
Archaea2 - ps3
Archaea3 - ps4
Asgard/Eukaryota - ps5
Eukaryota - ps6
Amorpheae - ps7
Obazoa - ps8
Opisthokonta - ps9
Holomycota - ps10
Fungi - ps11
Fungi_1 - ps12
Fungi_2 - ps13
Fungi_3 - ps14
Dikarya - ps15
Ascomycota - ps16
Saccharo/Pezizomycotina - ps17
Saccharomycotina - ps18
Saccharomycotina_1 - ps19
Saccharomycotina_2 - ps20
Saccharomycotina_3 - ps21
Phaff./Saccharomycetaceae - ps22
Saccharomyco./mycetaceae - ps23
Saccharomycetaceae - ps24
Saccharomycetaceae_2 - ps25
Saccharomycetaceae_3 - ps26
Kaz./Nau./Saccharomyces - ps27
Saccharomyces - ps28
Arboricola_clade - ps29
Cerevisiae_clade - ps30
Saccharomyces_cerevisiae - ps31
p-value
3e-06
3e-05
3e-04
4e-03
4e-02
Log-odds
0.5
1.0
1.5
2.0
2.5
3.0
3.5
4.0

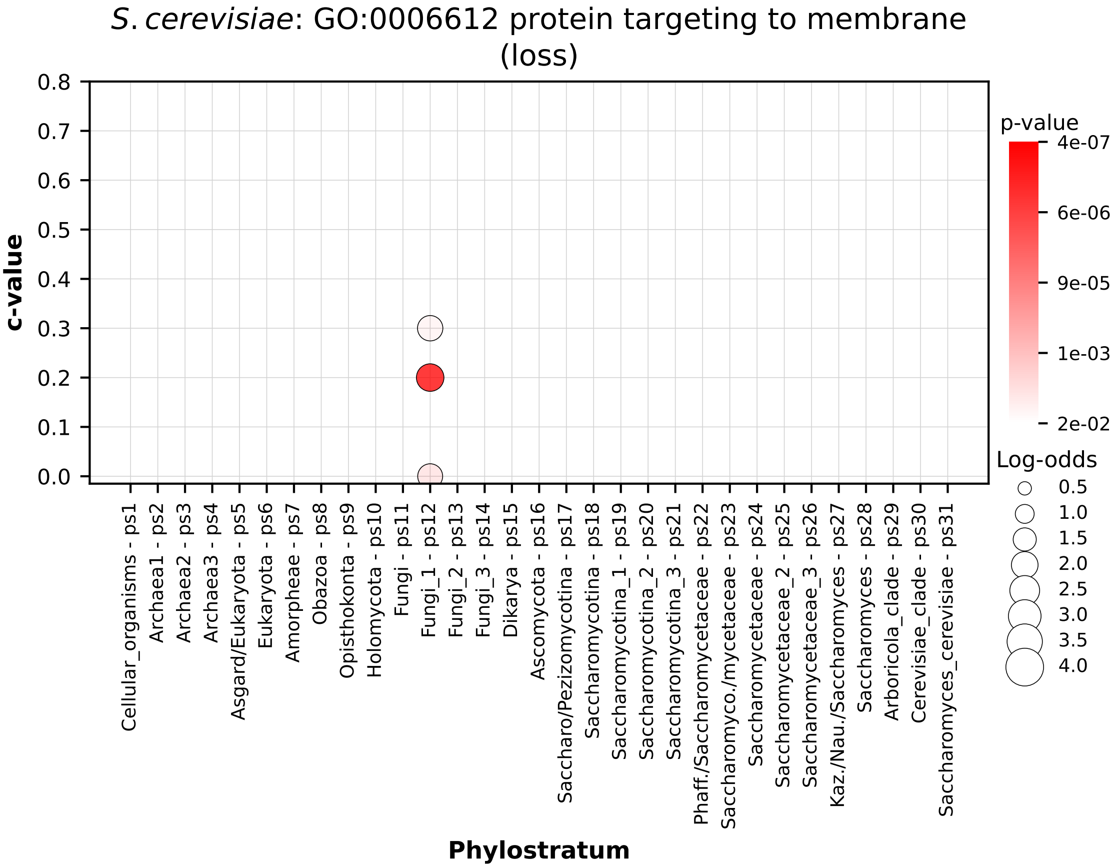
S. cerevisiae: GO:0006612 protein targeting to membrane
(loss)
c-value
0.8
0.7
0.6
0.5
0.4
0.3
0.2
0.1
0.0
Phylostratum
Cellular_organisms - ps1
Archaea1 - ps2
Archaea2 - ps3
Archaea3 - ps4
Asgard/Eukaryota - ps5
Eukaryota - ps6
Amorpheae - ps7
Obazoa - ps8
Opisthokonta - ps9
Holomycota - ps10
Fungi - ps11
Fungi_1 - ps12
Fungi_2 - ps13
Fungi_3 - ps14
Dikarya - ps15
Ascomycota - ps16
Saccharo/Pezizomycotina - ps17
Saccharomycotina - ps18
Saccharomycotina_1 - ps19
Saccharomycotina_2 - ps20
Saccharomycotina_3 - ps21
Phaff./Saccharomycetaceae - ps22
Saccharomyco./mycetaceae - ps23
Saccharomycetaceae - ps24
Saccharomycetaceae_2 - ps25
Saccharomycetaceae_3 - ps26
Kaz./Nau./Saccharomyces - ps27
Saccharomyces - ps28
Arboricola_clade - ps29
Cerevisiae_clade - ps30
Saccharomyces_cerevisiae - ps31
p-value
4e-07
6e-06
9e-05
1e-03
2e-02
Log-odds
0.5
1.0
1.5
2.0
2.5
3.0
3.5
4.0

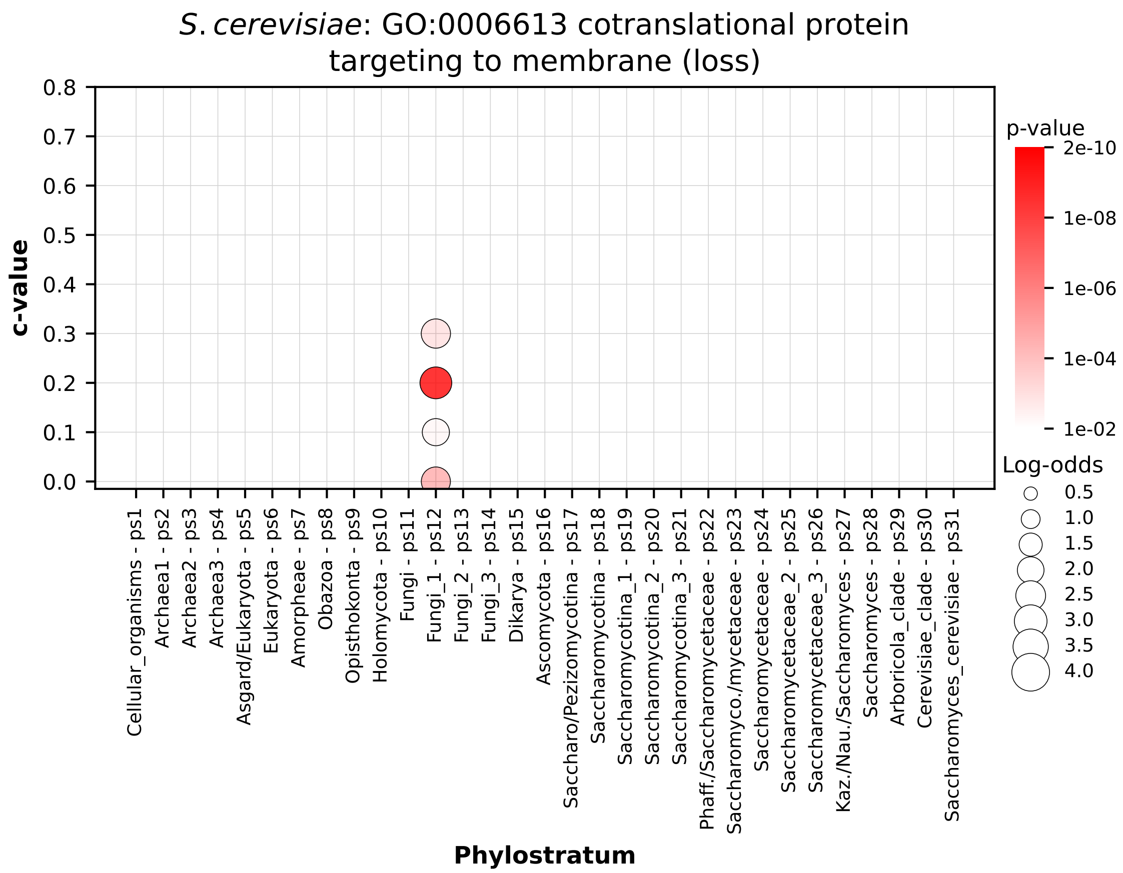

S. cerevisiae: GO:0006613 cotranslational protein targeting to membrane (loss)
c-value
0.8
0.7
0.6
0.5
0.4
0.3
0.2
0.1
0.0
p-value
2e-10
1e-08
1e-06
1e-04
1e-02
Log-odds
0.5
1.0
1.5
2.0
2.5
3.0
3.5
4.0
Cellular_organisms - ps1
Archaea1 - ps2
Archaea2 - ps3
Archaea3 - ps4
Asgard/Eukaryota - ps5
Eukaryota - ps6
Amorpheae - ps7
Obazoa - ps8
Opisthokonta - ps9
Holomycota - ps10
Fungi - ps11
Fungi_1 - ps12
Fungi_2 - ps13
Fungi_3 - ps14
Dikarya - ps15
Ascomycota - ps16
Saccharo/Pezizomycotina - ps17
Saccharomycotina - ps18
Saccharomycotina_1 - ps19
Saccharomycotina_2 - ps20
Saccharomycotina_3 - ps21
Phaff./Saccharomycetaceae - ps22
Saccharomyco./mycetaceae - ps23
Saccharomycetaceae - ps24
Saccharomycetaceae_2 - ps25
Saccharomycetaceae_3 - ps26
Kaz./Nau./Saccharomyces - ps27
Saccharomyces - ps28
Arboricola_clade - ps29
Cerevisiae_clade - ps30
Saccharomyces_cerevisiae - ps31
Phylostratum

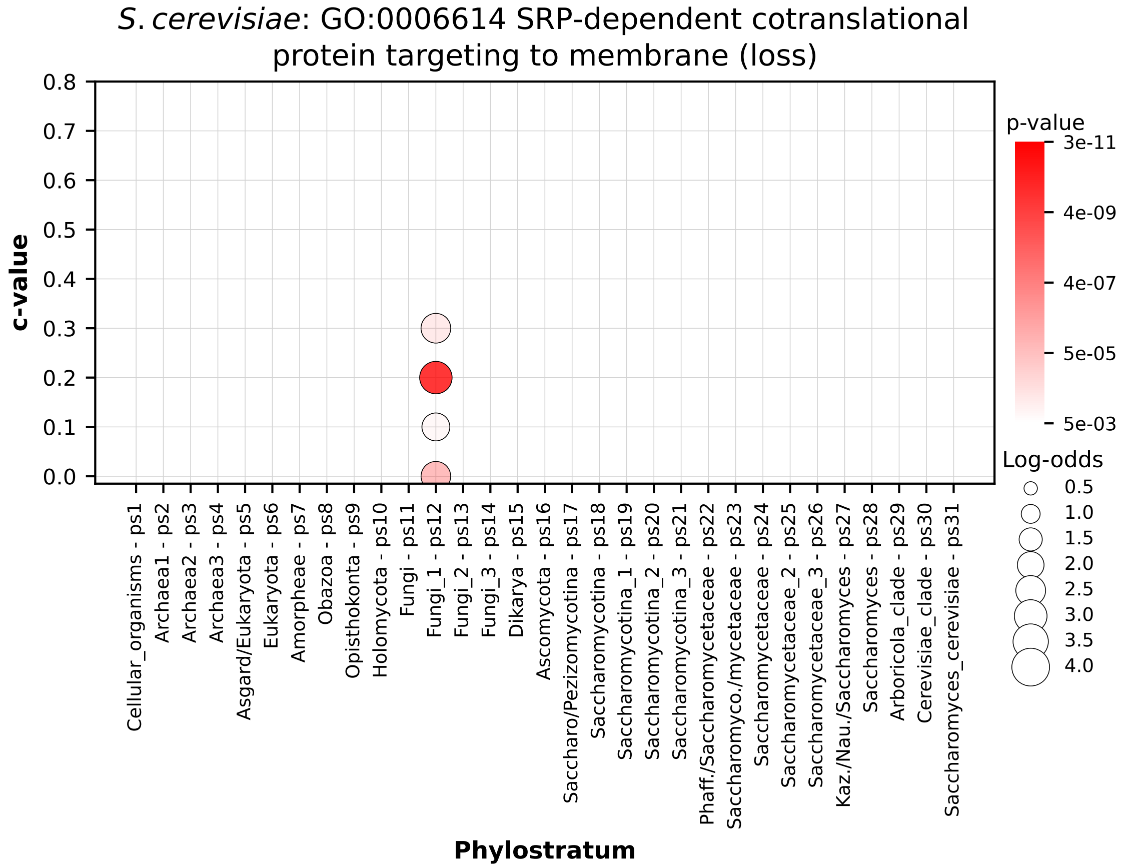

S. cerevisiae: GO:0006614 SRP-dependent cotranslational protein targeting to membrane (loss)
c-value
0.8
0.7
0.6
0.5
0.4
0.3
0.2
0.1
0.0
p-value
3e-11
4e-09
4e-07
5e-05
5e-03
Log-odds
0.5
1.0
1.5
2.0
2.5
3.0
3.5
4.0
Cellular_organisms - ps1
Archaea1 - ps2
Archaea2 - ps3
Archaea3 - ps4
Asgard/Eukaryota - ps5
Eukaryota - ps6
Amorpheae - ps7
Obazoa - ps8
Opisthokonta - ps9
Holomycota - ps10
Fungi - ps11
Fungi_1 - ps12
Fungi_2 - ps13
Fungi_3 - ps14
Dikarya - ps15
Ascomycota - ps16
Saccharo/Pezizomycotina - ps17
Saccharomycotina - ps18
Saccharomycotina_1 - ps19
Saccharomycotina_2 - ps20
Saccharomycotina_3 - ps21
Phaff./Saccharomycetaceae - ps22
Saccharomyco./mycetaceae - ps23
Saccharomycetaceae - ps24
Saccharomycetaceae_2 - ps25
Saccharomycetaceae_3 - ps26
Kaz./Nau./Saccharomyces - ps27
Saccharomyces - ps28
Arboricola_clade - ps29
Cerevisiae_clade - ps30
Saccharomyces_cerevisiae - ps31
Phylostratum

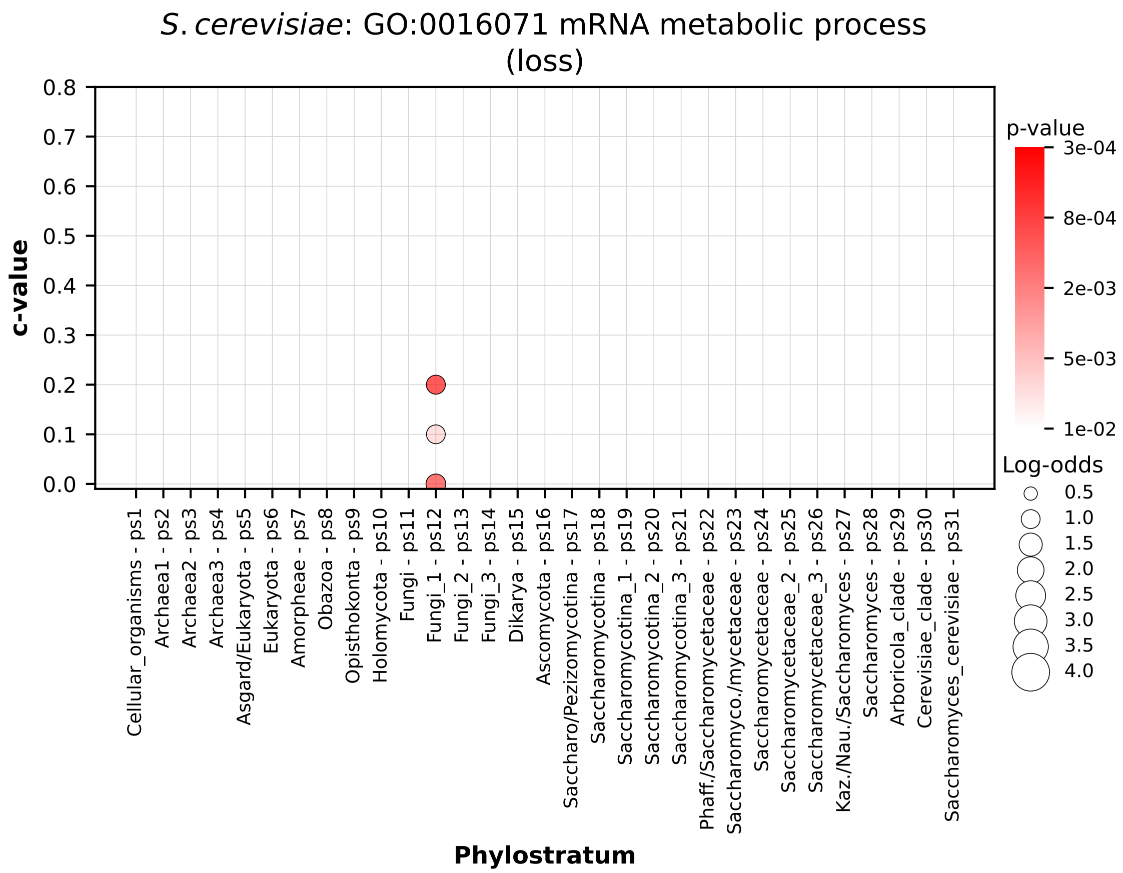
S. cerevisiae: GO:0016071 mRNA metabolic process
(loss)
c-value
0.8
0.7
0.6
0.5
0.4
0.3
0.2
0.1
0.0
Phylostratum
Cellular_organisms - ps1
Archaea1 - ps2
Archaea2 - ps3
Archaea3 - ps4
Asgard/Eukaryota - ps5
Eukaryota - ps6
Amorpheae - ps7
Obazoa - ps8
Opisthokonta - ps9
Holomycota - ps10
Fungi - ps11
Fungi_1 - ps12
Fungi_2 - ps13
Fungi_3 - ps14
Dikarya - ps15
Ascomycota - ps16
Saccharo/Pezizomycotina - ps17
Saccharomycotina - ps18
Saccharomycotina_1 - ps19
Saccharomycotina_2 - ps20
Saccharomycotina_3 - ps21
Phaff./Saccharomycetaceae - ps22
Saccharomyco./mycetaceae - ps23
Saccharomycetaceae - ps24
Saccharomycetaceae_2 - ps25
Saccharomycetaceae_3 - ps26
Kaz./Nau./Saccharomyces - ps27
Saccharomyces - ps28
Arboricola_clade - ps29
Cerevisiae_clade - ps30
Saccharomyces_cerevisiae - ps31
p-value
3e-04
8e-04
2e-03
5e-03
1e-02
Log-odds
0.5
1.0
1.5
2.0
2.5
3.0
3.5
4.0

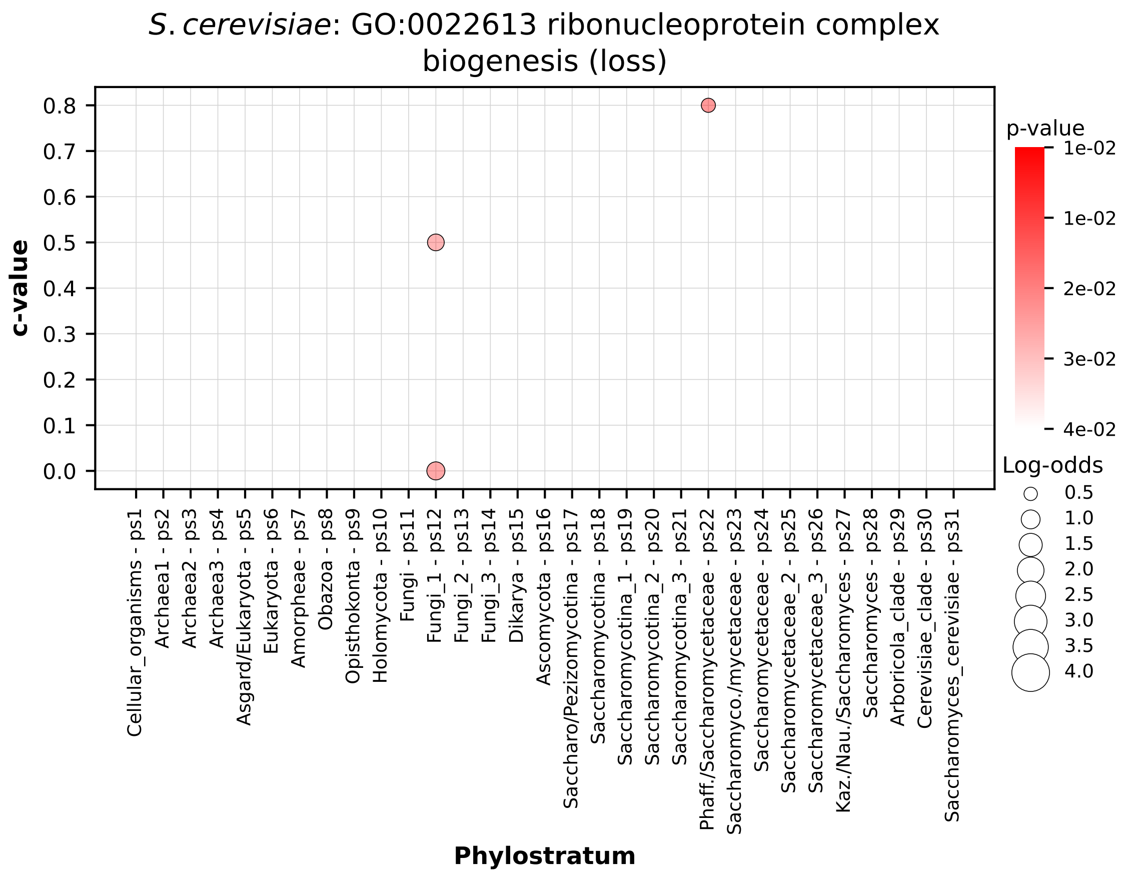

S. cerevisiae: GO:0022613 ribonucleoprotein complex biogenesis (loss)
c-value
0.8
0.7
0.6
0.5
0.4
0.3
0.2
0.1
0.0
p-value
1e-02
1e-02
2e-02
3e-02
4e-02
Log-odds
0.5
1.0
1.5
2.0
2.5
3.0
3.5
4.0
Cellular_organisms - ps1
Archaea1 - ps2
Archaea2 - ps3
Archaea3 - ps4
Asgard/Eukaryota - ps5
Eukaryota - ps6
Amorpheae - ps7
Obazoa - ps8
Opisthokonta - ps9
Holomycota - ps10
Fungi - ps11
Fungi_1 - ps12
Fungi_2 - ps13
Fungi_3 - ps14
Dikarya - ps15
Ascomycota - ps16
Saccharo/Pezizomycotina - ps17
Saccharomycotina - ps18
Saccharomycotina_1 - ps19
Saccharomycotina_2 - ps20
Saccharomycotina_3 - ps21
Phaff./Saccharomycetaceae - ps22
Saccharomyco./mycetaceae - ps23
Saccharomycetaceae - ps24
Saccharomycetaceae_2 - ps25
Saccharomycetaceae_3 - ps26
Kaz./Nau./Saccharomyces - ps27
Saccharomyces - ps28
Arboricola_clade - ps29
Cerevisiae_clade - ps30
Saccharomyces_cerevisiae - ps31
Phylostratum

S. cerevisiae: GO:0042254 ribosome biogenesis (loss)

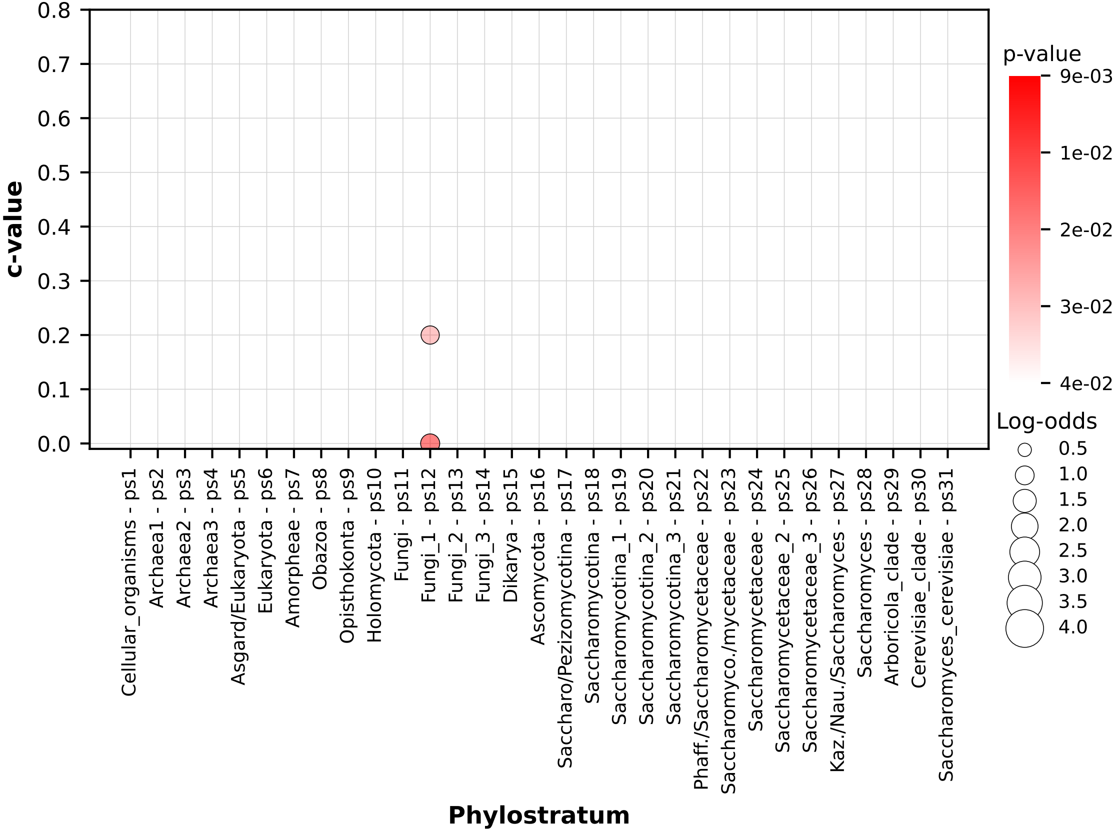

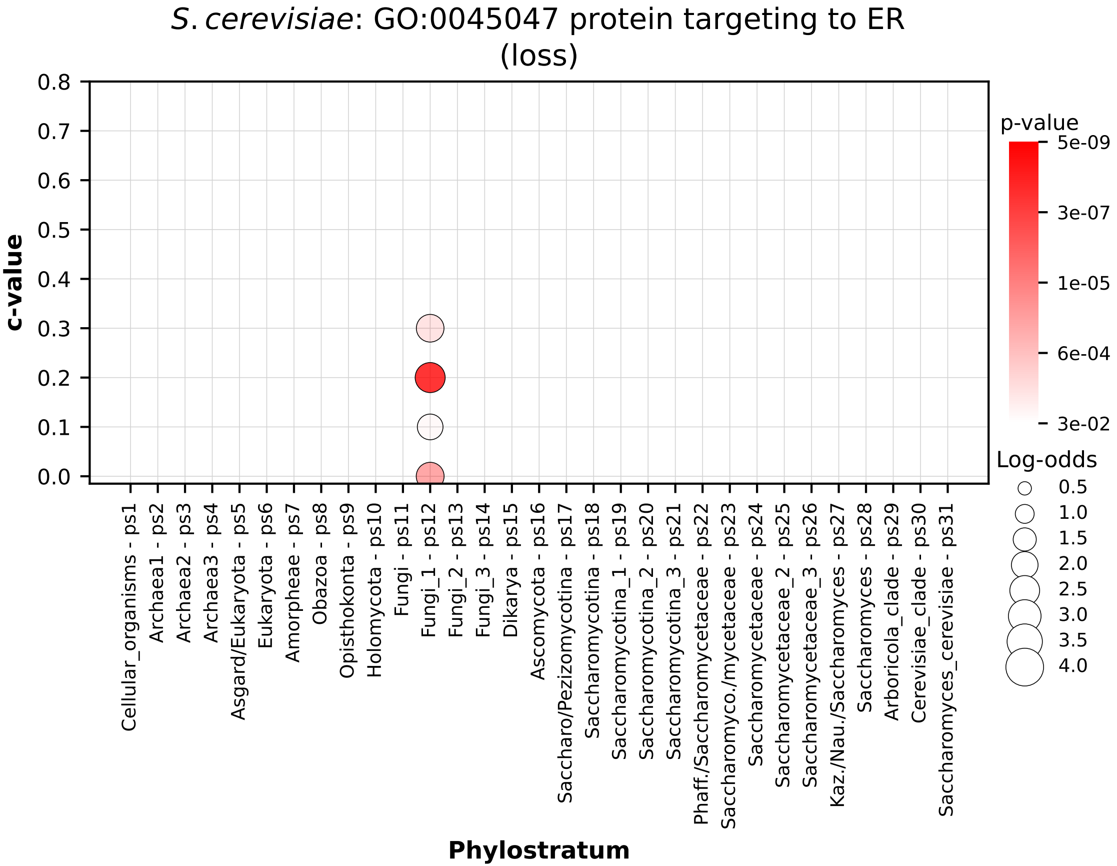

S. cerevisiae: GO:0045047 protein targeting to ER
(loss)
c-value
0.8
0.7
0.6
0.5
0.4
0.3
0.2
0.1
0.0
p-value
5e-09
3e-07
1e-05
6e-04
3e-02
Log-odds
0.5
1.0
1.5
2.0
2.5
3.0
3.5
4.0
Cellular_organisms - ps1
Archaea1 - ps2
Archaea2 - ps3
Archaea3 - ps4
Asgard/Eukaryota - ps5
Eukaryota - ps6
Amorpheae - ps7
Obazoa - ps8
Opisthokonta - ps9
Holomycota - ps10
Fungi - ps11
Fungi_1 - ps12
Fungi_2 - ps13
Fungi_3 - ps14
Dikarya - ps15
Ascomycota - ps16
Saccharo/Pezizomycotina - ps17
Saccharomycotina - ps18
Saccharomycotina_1 - ps19
Saccharomycotina_2 - ps20
Saccharomycotina_3 - ps21
Phaff./Saccharomycetaceae - ps22
Saccharomyco./mycetaceae - ps23
Saccharomycetaceae - ps24
Saccharomycetaceae_2 - ps25
Saccharomycetaceae_3 - ps26
Kaz./Nau./Saccharomyces - ps27
Saccharomyces - ps28
Arboricola_clade - ps29
Cerevisiae_clade - ps30
Saccharomyces_cerevisiae - ps31
Phylostratum

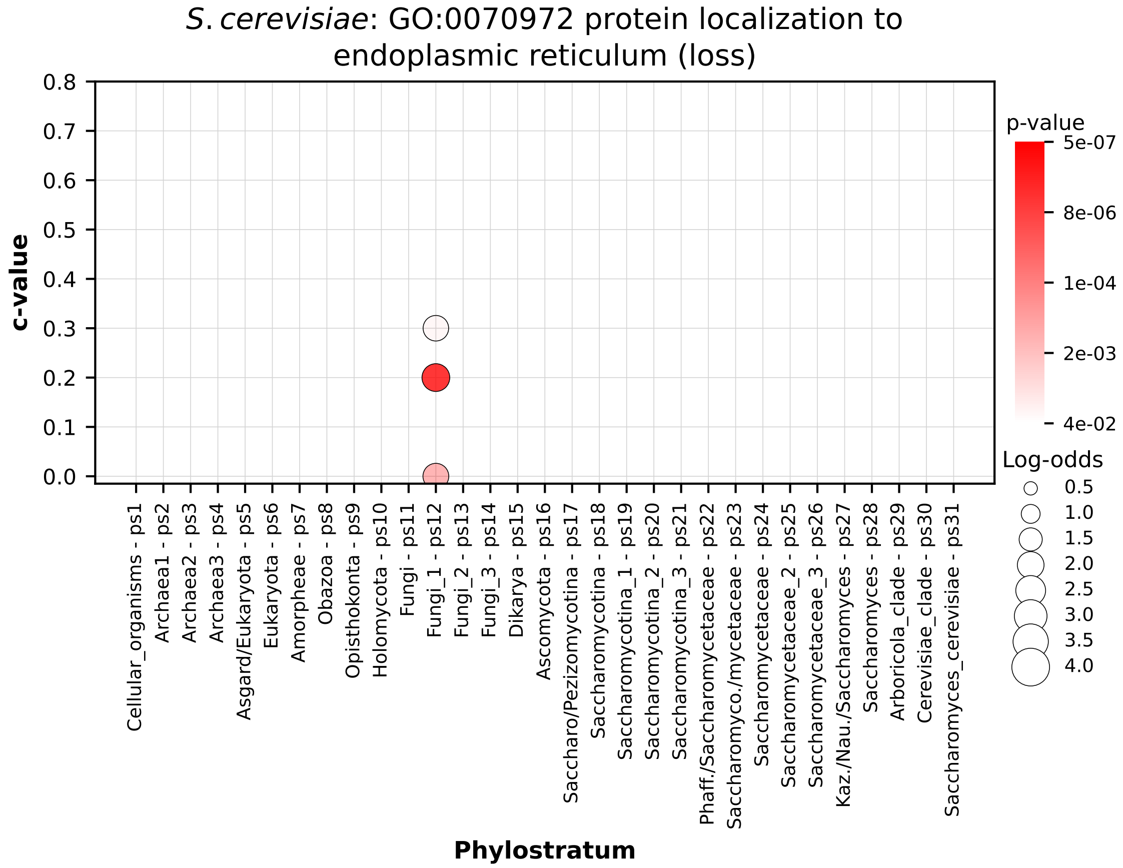

$S. cerevisiae$: GO:0070972 protein localization to endoplasmic reticulum (loss)
c-value
0.8
0.7
0.6
0.5
0.4
0.3
0.2
0.1
0.0
p-value
5e-07
8e-06
1e-04
2e-03
4e-02
Log-odds
0.5
1.0
1.5
2.0
2.5
3.0
3.5
4.0
Cellular_organisms - ps1
Archaea1 - ps2
Archaea2 - ps3
Archaea3 - ps4
Asgard/Eukaryota - ps5
Eukaryota - ps6
Amorpheae - ps7
Obazoa - ps8
Opisthokonta - ps9
Holomycota - ps10
Fungi - ps11
Fungi_1 - ps12
Fungi_2 - ps13
Fungi_3 - ps14
Dikarya - ps15
Ascomycota - ps16
Saccharo/Pezizomycotina - ps17
Saccharomycotina - ps18
Saccharomycotina_1 - ps19
Saccharomycotina_2 - ps20
Saccharomycotina_3 - ps21
Phaff./Saccharomycetaceae - ps22
Saccharomyco./mycetaceae - ps23
Saccharomycetaceae - ps24
Saccharomycetaceae_2 - ps25
Saccharomycetaceae_3 - ps26
Kaz./Nau./Saccharomyces - ps27
Saccharomyces - ps28
Arboricola_clade - ps29
Cerevisiae_clade - ps30
Saccharomyces_cerevisiae - ps31
Phylostratum

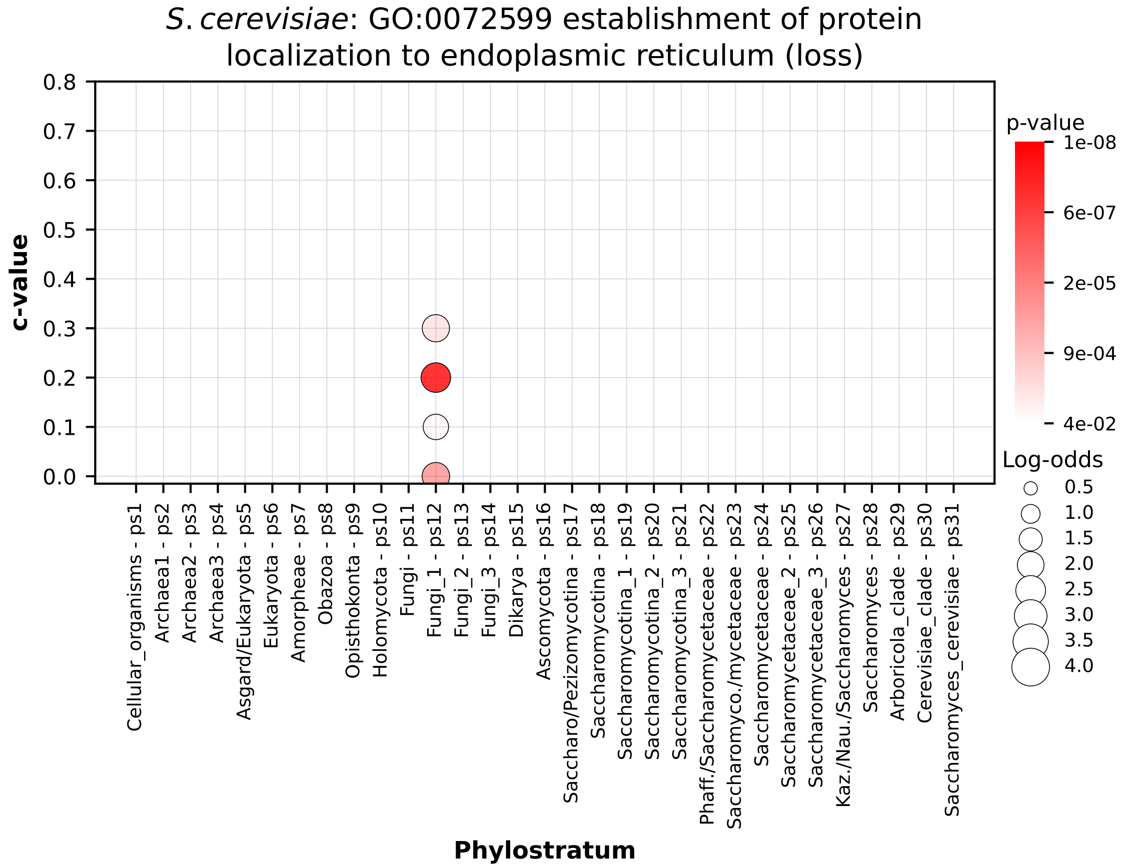

S. cerevisiae: GO:0072599 establishment of protein localization to endoplasmic reticulum (loss)
c-value
0.8
0.7
0.6
0.5
0.4
0.3
0.2
0.1
0.0
p-value
1e-08
6e-07
2e-05
9e-04
4e-02
Log-odds
0.5
1.0
1.5
2.0
2.5
3.0
3.5
4.0
Cellular_organisms - ps1
Archaea1 - ps2
Archaea2 - ps3
Archaea3 - ps4
Asgard/Eukaryota - ps5
Eukaryota - ps6
Amorpheae - ps7
Obazoa - ps8
Opisthokonta - ps9
Holomycota - ps10
Fungi - ps11
Fungi_1 - ps12
Fungi_2 - ps13
Fungi_3 - ps14
Dikarya - ps15
Ascomycota - ps16
Saccharo/Pezizomycotina - ps17
Saccharomycotina - ps18
Saccharomycotina_1 - ps19
Saccharomycotina_2 - ps20
Saccharomycotina_3 - ps21
Phaff./Saccharomycetaceae - ps22
Saccharomyco./mycetaceae - ps23
Saccharomycetaceae - ps24
Saccharomycetaceae_2 - ps25
Saccharomycetaceae_3 - ps26
Kaz./Nau./Saccharomyces - ps27
Saccharomyces - ps28
Arboricola_clade - ps29
Cerevisiae_clade - ps30
Saccharomyces_cerevisiae - ps31
Phylostratum

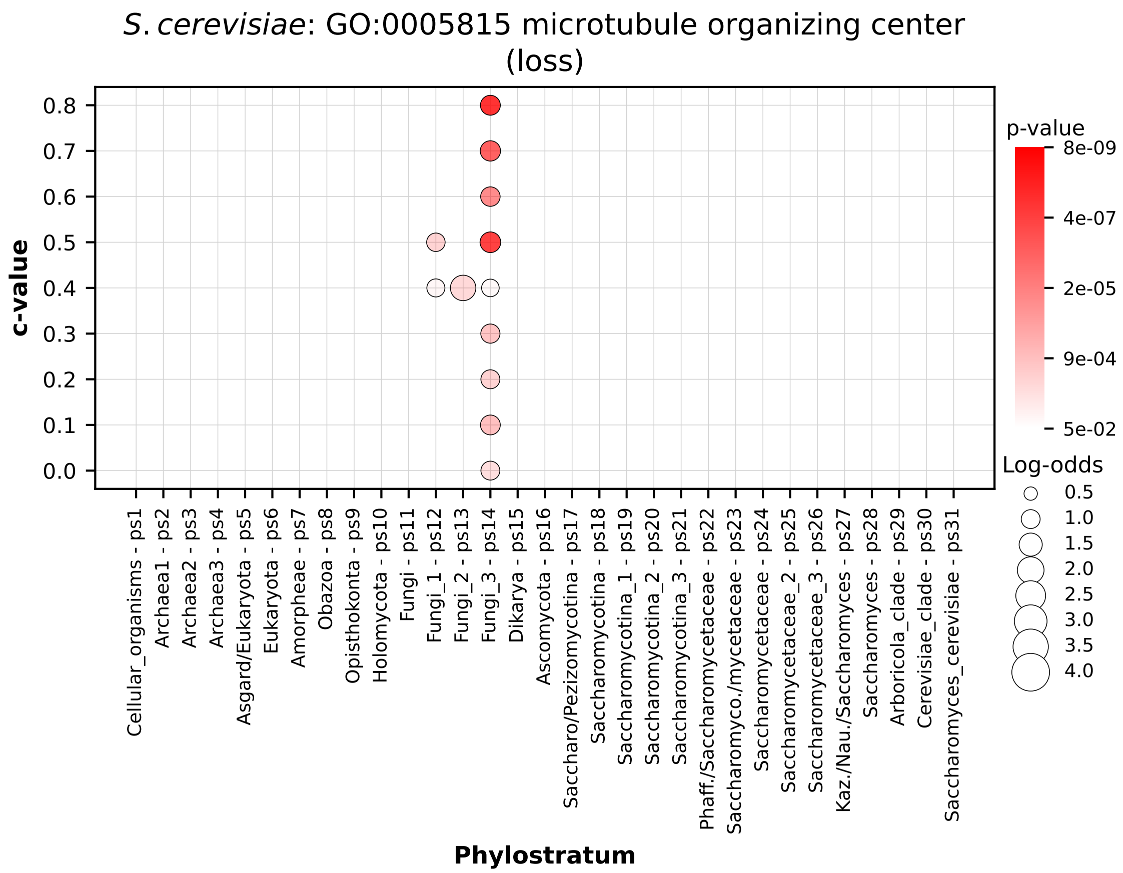

S. cerevisiae: GO:0005815 microtubule organizing center
(loss)
c-value
0.8
0.7
0.6
0.5
0.4
0.3
0.2
0.1
0.0
p-value
8e-09
4e-07
2e-05
9e-04
5e-02
Log-odds
0.5
1.0
1.5
2.0
2.5
3.0
3.5
4.0
Cellular_organisms - ps1
Archaea1 - ps2
Archaea2 - ps3
Archaea3 - ps4
Asgard/Eukaryota - ps5
Eukaryota - ps6
Amorpheae - ps7
Obazoa - ps8
Opisthokonta - ps9
Holomycota - ps10
Fungi - ps11
Fungi_1 - ps12
Fungi_2 - ps13
Fungi_3 - ps14
Dikarya - ps15
Ascomycota - ps16
Saccharo/Pezizomycotina - ps17
Saccharomycotina - ps18
Saccharomycotina_1 - ps19
Saccharomycotina_2 - ps20
Saccharomycotina_3 - ps21
Phaff./Saccharomycetaceae - ps22
Saccharomyco./mycetaceae - ps23
Saccharomycetaceae - ps24
Saccharomycetaceae_2 - ps25
Saccharomycetaceae_3 - ps26
Kaz./Nau./Saccharomyces - ps27
Saccharomyces - ps28
Arboricola_clade - ps29
Cerevisiae_clade - ps30
Saccharomyces_cerevisiae - ps31
Phylostratum

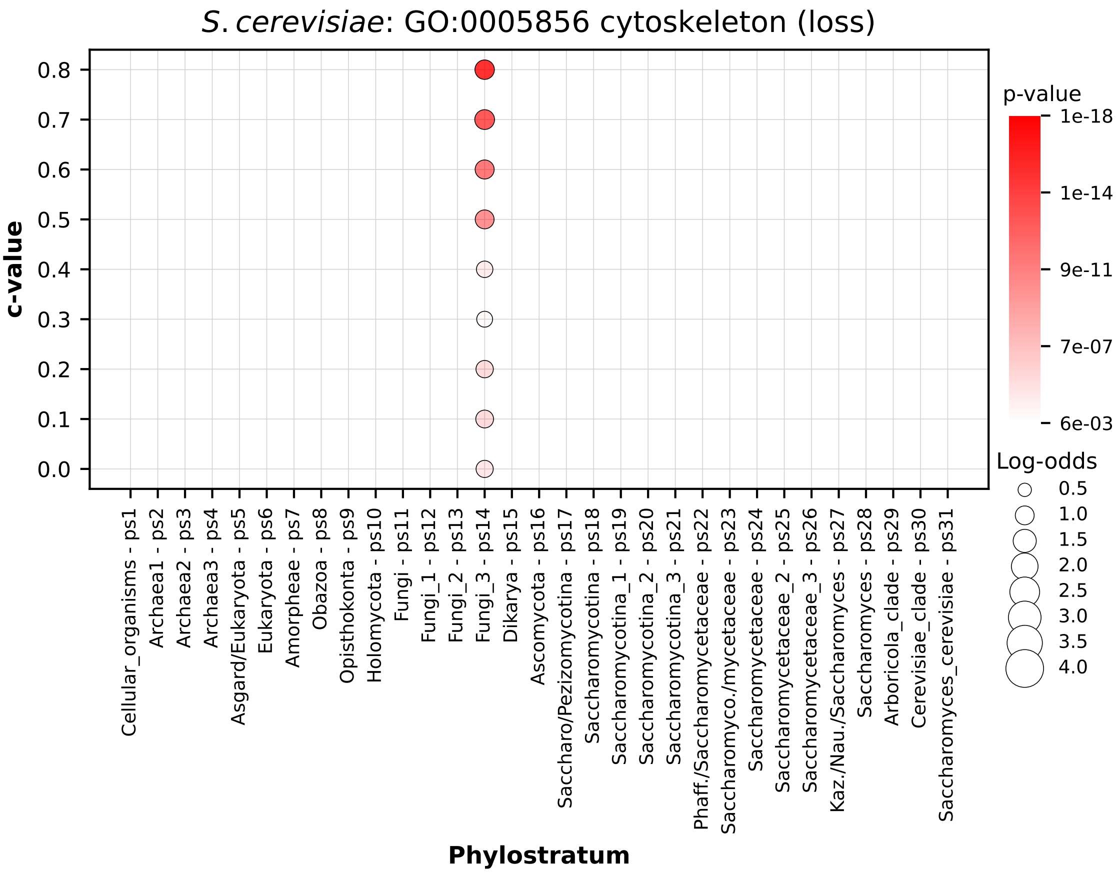
S. cerevisiae: GO:0005856 cytoskeleton (loss)
c-value
0.0
0.1
0.2
0.3
0.4
0.5
0.6
0.7
0.8
p-value
1e-18
1e-14
9e-11
7e-07
6e-03
Log-odds
0.5
1.0
1.5
2.0
2.5
3.0
3.5
4.0
Cellular_organisms - ps1
Archaea1 - ps2
Archaea2 - ps3
Archaea3 - ps4
Asgard/Eukaryota - ps5
Eukaryota - ps6
Amorpheae - ps7
Obazoa - ps8
Opisthokonta - ps9
Holomycota - ps10
Fungi - ps11
Fungi_1 - ps12
Fungi_2 - ps13
Fungi_3 - ps14
Dikarya - ps15
Ascomycota - ps16
Saccharo/Pezizomycotina - ps17
Saccharomycotina - ps18
Saccharomycotina_1 - ps19
Saccharomycotina_2 - ps20
Saccharomycotina_3 - ps21
Phaff./Saccharomycetaceae - ps22
Saccharomyco./mycetaceae - ps23
Saccharomycetaceae - ps24
Saccharomycetaceae_2 - ps25
Saccharomycetaceae_3 - ps26
Kaz./Nau./Saccharomyces - ps27
Saccharomyces - ps28
Arboricola_clade - ps29
Cerevisiae_clade - ps30
Saccharomyces_cerevisiae - ps31
Phylostratum

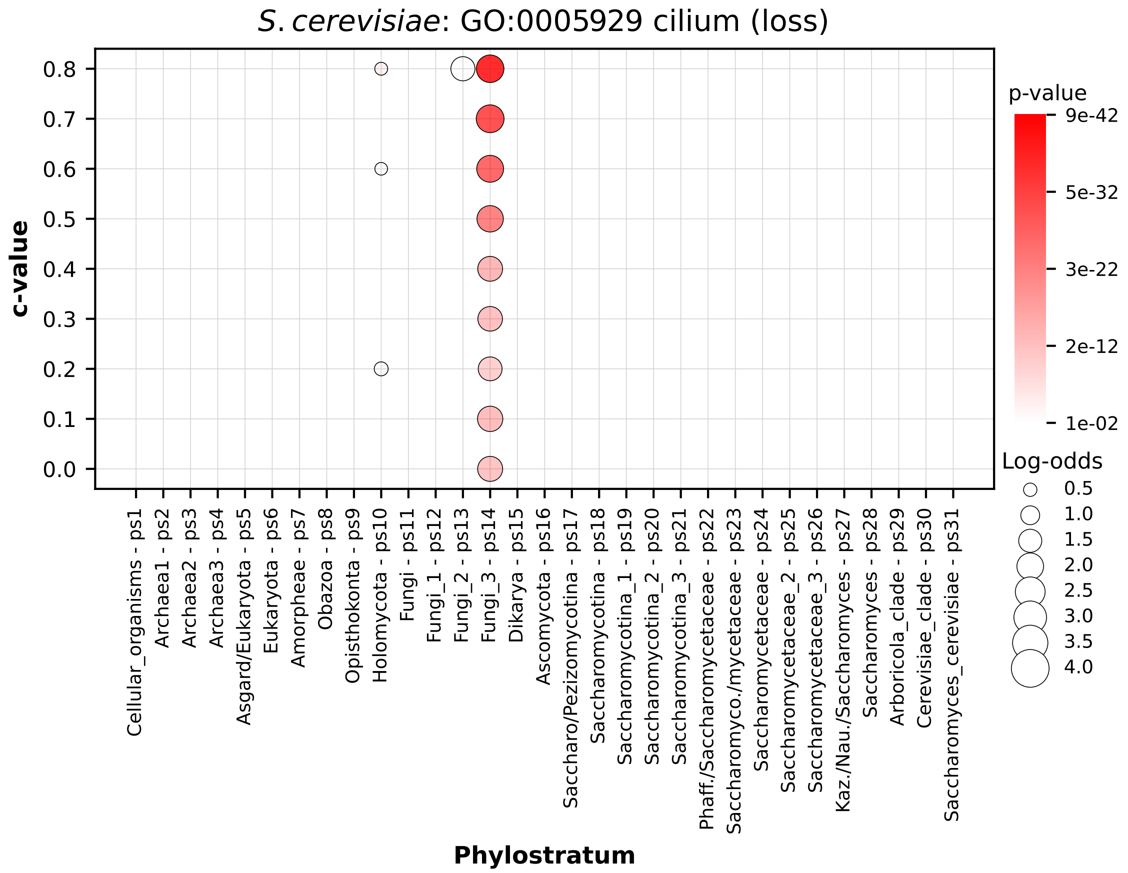
S. cerevisiae: GO:0005929 cilium (loss)
c-value
0.8
0.7
0.6
0.5
0.4
0.3
0.2
0.1
0.0
p-value
9e-42
5e-32
3e-22
2e-12
1e-02
Log-odds
0.5
1.0
1.5
2.0
2.5
3.0
3.5
4.0
Cellular_organisms - ps1
Archaea1 - ps2
Archaea2 - ps3
Archaea3 - ps4
Asgard/Eukaryota - ps5
Eukaryota - ps6
Amorpheae - ps7
Obazoa - ps8
Opisthokonta - ps9
Holomycota - ps10
Fungi - ps11
Fungi_1 - ps12
Fungi_2 - ps13
Fungi_3 - ps14
Dikarya - ps15
Ascomycota - ps16
Saccharo/Pezizomycotina - ps17
Saccharomycotina - ps18
Saccharomycotina_1 - ps19
Saccharomycotina_2 - ps20
Saccharomycotina_3 - ps21
Phaff./Saccharomycetaceae - ps22
Saccharomyco./mycetaceae - ps23
Saccharomycetaceae - ps24
Saccharomycetaceae_2 - ps25
Saccharomycetaceae_3 - ps26
Kaz./Nau./Saccharomyces - ps27
Saccharomyces - ps28
Arboricola_clade - ps29
Cerevisiae_clade - ps30
Saccharomyces_cerevisiae - ps31
Phylostratum

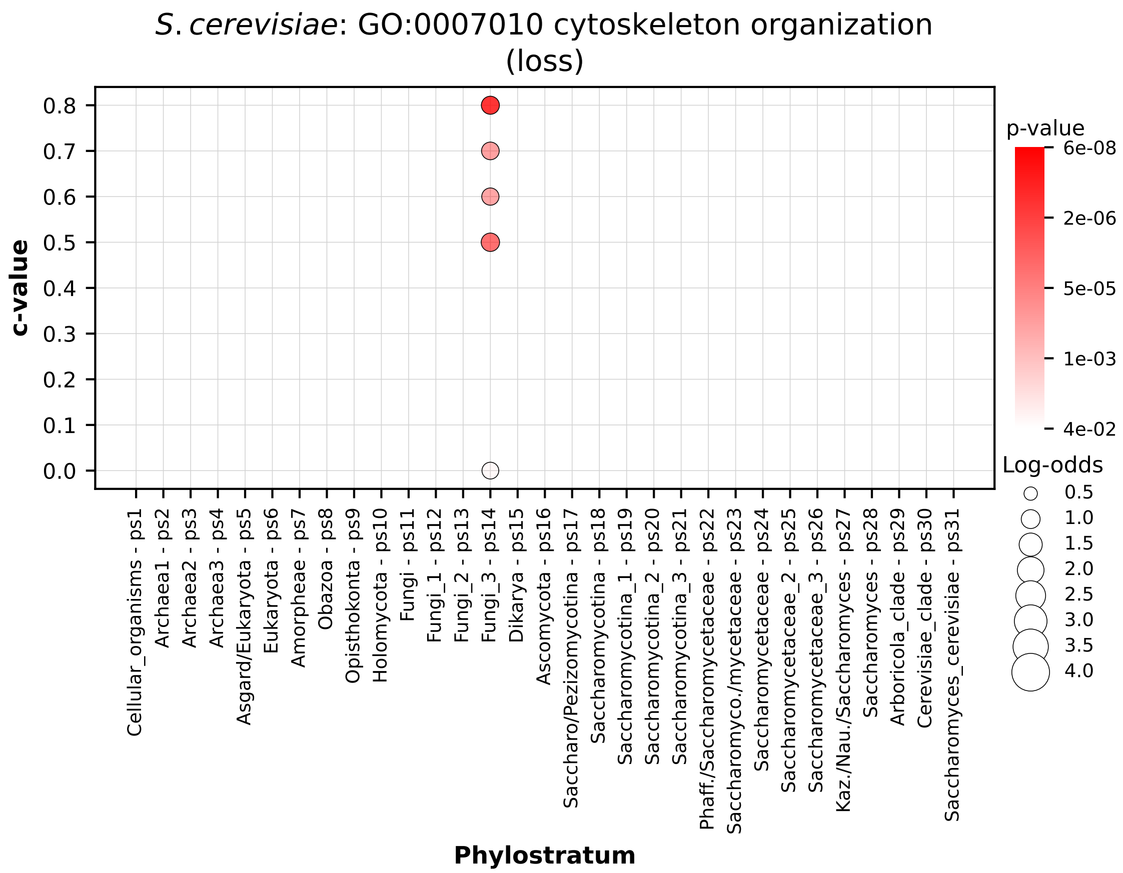

S. cerevisiae: GO:0007010 cytoskeleton organization
(loss)
c-value
0.0
0.1
0.2
0.3
0.4
0.5
0.6
0.7
0.8
Phylostratum
Cellular_organisms - ps1
Archaea1 - ps2
Archaea2 - ps3
Archaea3 - ps4
Asgard/Eukaryota - ps5
Eukaryota - ps6
Amorpheae - ps7
Obazoa - ps8
Opisthokonta - ps9
Holomycota - ps10
Fungi - ps11
Fungi_1 - ps12
Fungi_2 - ps13
Fungi_3 - ps14
Dikarya - ps15
Ascomycota - ps16
Saccharo/Pezizomycotina - ps17
Saccharomycotina - ps18
Saccharomycotina_1 - ps19
Saccharomycotina_2 - ps20
Saccharomycotina_3 - ps21
Phaff./Saccharomycetaceae - ps22
Saccharomyco./mycetaceae - ps23
Saccharomycetaceae - ps24
Saccharomycetaceae_2 - ps25
Saccharomycetaceae_3 - ps26
Kaz./Nau./Saccharomyces - ps27
Saccharomyces - ps28
Arboricola_clade - ps29
Cerevisiae_clade - ps30
Saccharomyces_cerevisiae - ps31
p-value
6e-08
2e-06
5e-05
1e-03
4e-02
Log-odds
0.5
1.0
1.5
2.0
2.5
3.0
3.5
4.0

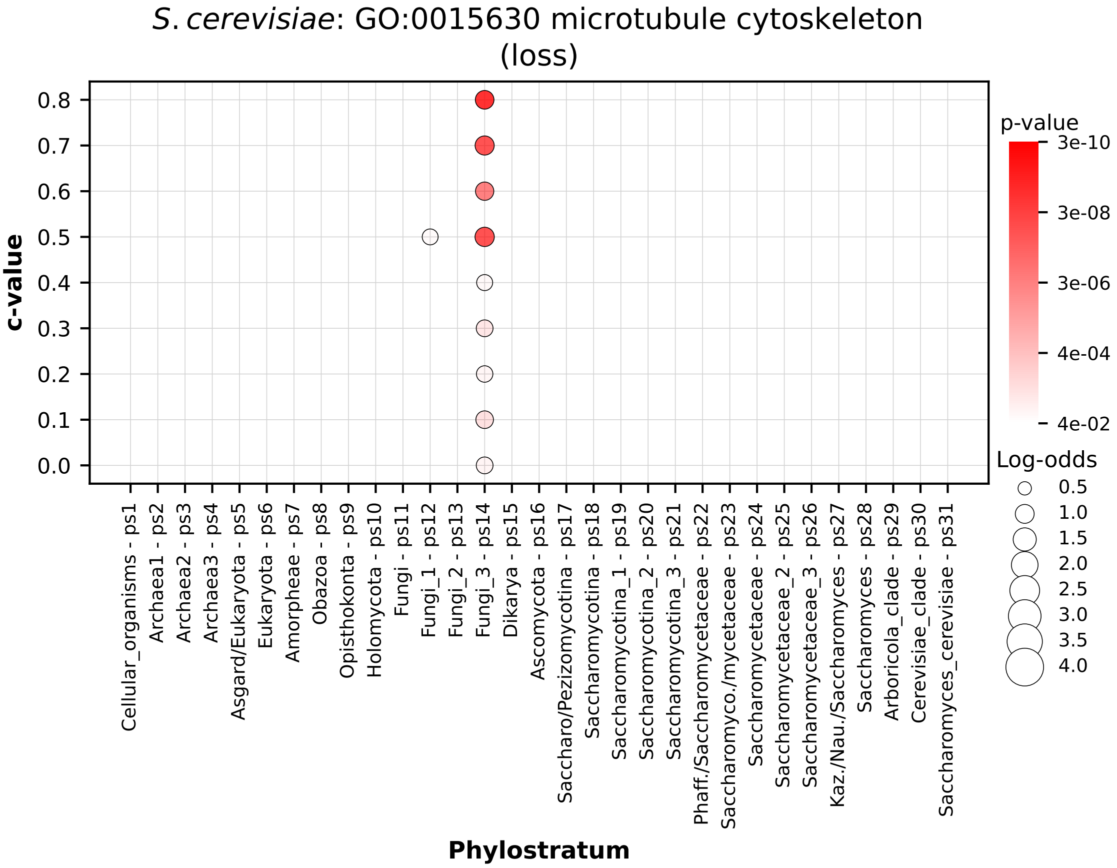

S. cerevisiae: GO:0015630 microtubule cytoskeleton (loss)
c-value
0.8
0.7
0.6
0.5
0.4
0.3
0.2
0.1
0.0
Phylostratum
Cellular_organisms - ps1
Archaea1 - ps2
Archaea2 - ps3
Archaea3 - ps4
Asgard/Eukaryota - ps5
Eukaryota - ps6
Amorpheae - ps7
Obazoa - ps8
Opisthokonta - ps9
Holomycota - ps10
Fungi - ps11
Fungi_1 - ps12
Fungi_2 - ps13
Fungi_3 - ps14
Dikarya - ps15
Ascomycota - ps16
Saccharo/Pezizomycotina - ps17
Saccharomycotina - ps18
Saccharomycotina_1 - ps19
Saccharomycotina_2 - ps20
Saccharomycotina_3 - ps21
Phaff./Saccharomycetaceae - ps22
Saccharomyco./mycetaceae - ps23
Saccharomycetaceae - ps24
Saccharomycetaceae_2 - ps25
Saccharomycetaceae_3 - ps26
Kaz./Nau./Saccharomyces - ps27
Saccharomyces - ps28
Arboricola_clade - ps29
Cerevisiae_clade - ps30
Saccharomyces_cerevisiae - ps31
p-value
3e-10
3e-08
3e-06
4e-04
4e-02
Log-odds
0.5
1.0
1.5
2.0
2.5
3.0
3.5
4.0

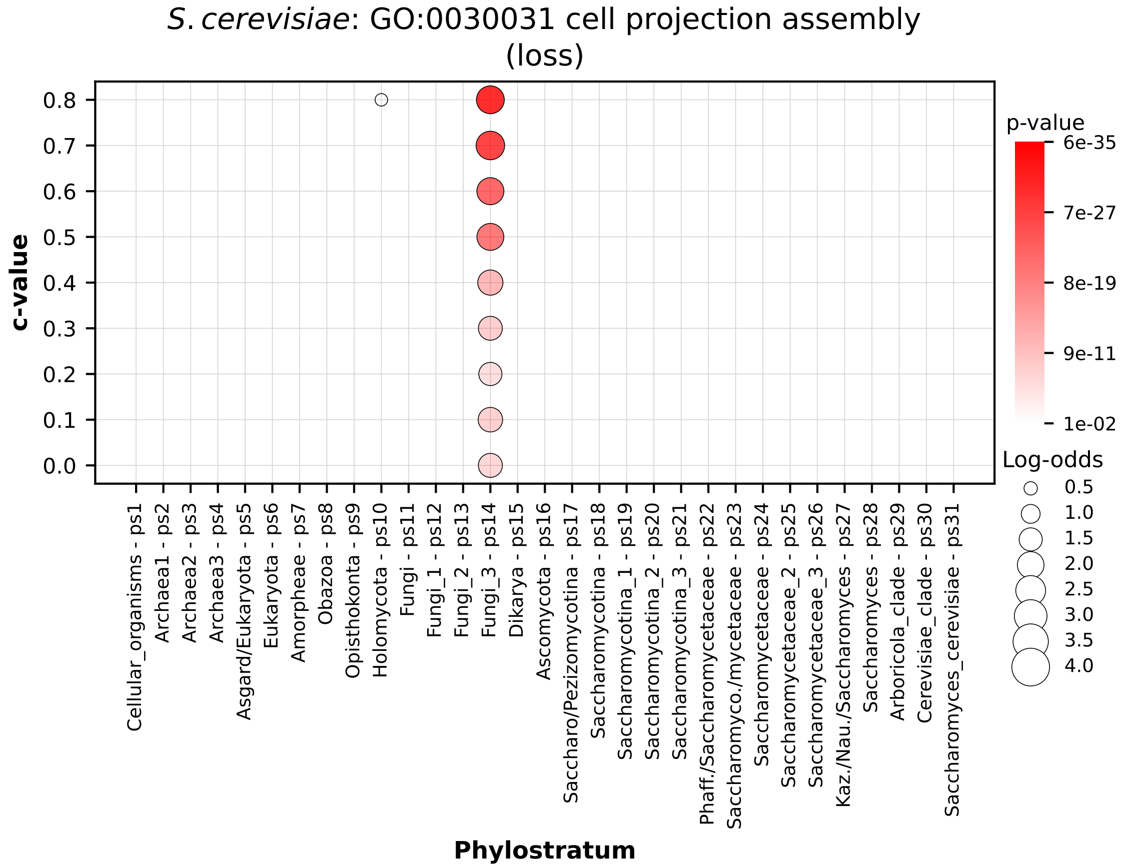
S. cerevisiae: GO:0030031 cell projection assembly
(loss)
c-value
0.8
0.7
0.6
0.5
0.4
0.3
0.2
0.1
0.0
p-value
6e-35
7e-27
8e-19
9e-11
1e-02
Log-odds
0.5
1.0
1.5
2.0
2.5
3.0
3.5
4.0
Cellular_organisms - ps1
Archaea1 - ps2
Archaea2 - ps3
Archaea3 - ps4
Asgard/Eukaryota - ps5
Eukaryota - ps6
Amorpheae - ps7
Obazoa - ps8
Opisthokonta - ps9
Holomycota - ps10
Fungi - ps11
Fungi_1 - ps12
Fungi_2 - ps13
Fungi_3 - ps14
Dikarya - ps15
Ascomycota - ps16
Saccharo/Pezizomycotina - ps17
Saccharomycotina - ps18
Saccharomycotina_1 - ps19
Saccharomycotina_2 - ps20
Saccharomycotina_3 - ps21
Phaff./Saccharomycetaceae - ps22
Saccharomyco./mycetaceae - ps23
Saccharomycetaceae - ps24
Saccharomycetaceae_2 - ps25
Saccharomycetaceae_3 - ps26
Kaz./Nau./Saccharomyces - ps27
Saccharomyces - ps28
Arboricola_clade - ps29
Cerevisiae_clade - ps30
Saccharomyces_cerevisiae - ps31
Phylostratum

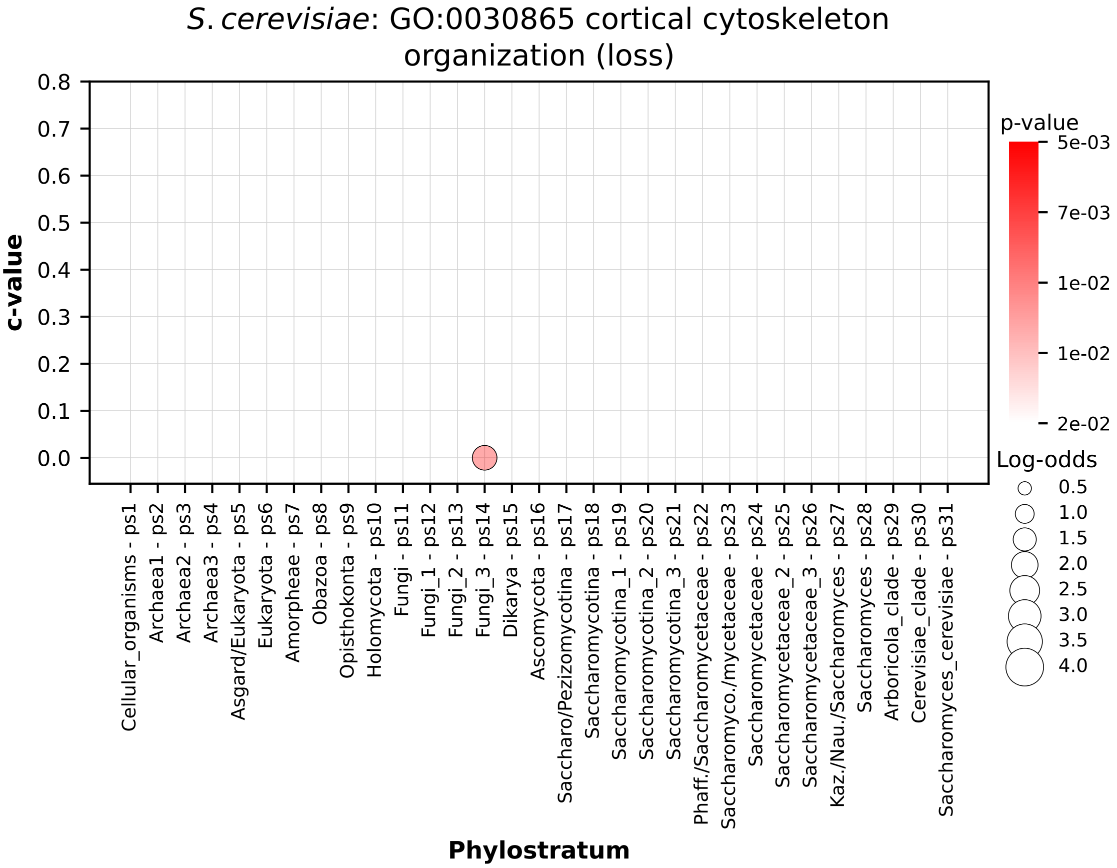

S. cerevisiae: GO:0030865 cortical cytoskeleton organization (loss)
c-value
0.8
0.7
0.6
0.5
0.4
0.3
0.2
0.1
0.0
p-value
5e-03
7e-03
1e-02
1e-02
2e-02
Log-odds
0.5
1.0
1.5
2.0
2.5
3.0
3.5
4.0
Cellular_organisms - ps1
Archaea1 - ps2
Archaea2 - ps3
Archaea3 - ps4
Asgard/Eukaryota - ps5
Eukaryota - ps6
Amorpheae - ps7
Obazoa - ps8
Opisthokonta - ps9
Holomycota - ps10
Fungi - ps11
Fungi_1 - ps12
Fungi_2 - ps13
Fungi_3 - ps14
Dikarya - ps15
Ascomycota - ps16
Saccharo/Pezizomycotina - ps17
Saccharomycotina - ps18
Saccharomycotina_1 - ps19
Saccharomycotina_2 - ps20
Saccharomycotina_3 - ps21
Phaff./Saccharomycetaceae - ps22
Saccharomyco./mycetaceae - ps23
Saccharomycetaceae - ps24
Saccharomycetaceae_2 - ps25
Saccharomycetaceae_3 - ps26
Kaz./Nau./Saccharomyces - ps27
Saccharomyces - ps28
Arboricola_clade - ps29
Cerevisiae_clade - ps30
Saccharomyces_cerevisiae - ps31
Phylostratum

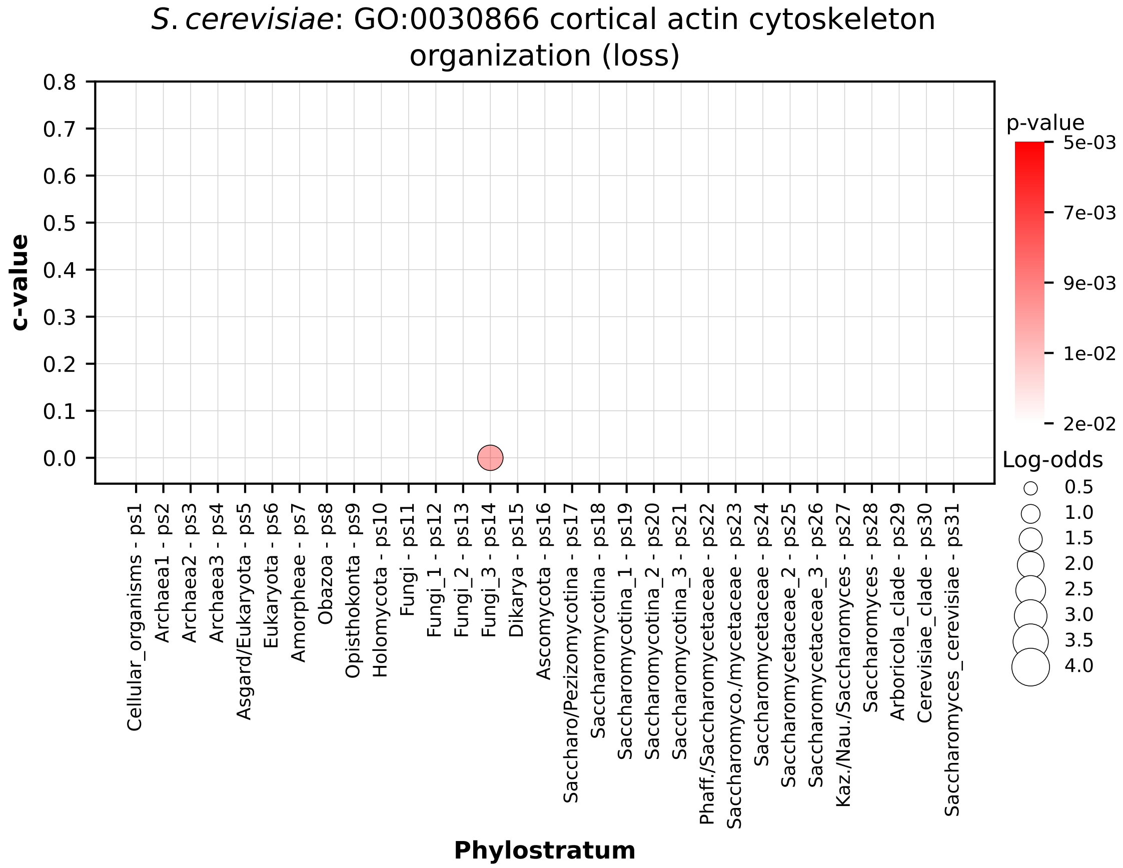
S. cerevisiae: GO:0030866 cortical actin cytoskeleton organization (loss)
c-value
0.8
0.7
0.6
0.5
0.4
0.3
0.2
0.1
0.0
p-value
5e-03
7e-03
9e-03
1e-02
2e-02
Log-odds
0.5
1.0
1.5
2.0
2.5
3.0
3.5
4.0
Cellular_organisms - ps1
Archaea1 - ps2
Archaea2 - ps3
Archaea3 - ps4
Asgard/Eukaryota - ps5
Eukaryota - ps6
Amorpheae - ps7
Obazoa - ps8
Opisthokonta - ps9
Holomycota - ps10
Fungi - ps11
Fungi_1 - ps12
Fungi_2 - ps13
Fungi_3 - ps14
Dikarya - ps15
Ascomycota - ps16
Saccharo/Pezizomycotina - ps17
Saccharomycotina - ps18
Saccharomycotina_1 - ps19
Saccharomycotina_2 - ps20
Saccharomycotina_3 - ps21
Phaff./Saccharomycetaceae - ps22
Saccharomyco./mycetaceae - ps23
Saccharomycetaceae - ps24
Saccharomycetaceae_2 - ps25
Saccharomycetaceae_3 - ps26
Kaz./Nau./Saccharomyces - ps27
Saccharomyces - ps28
Arboricola_clade - ps29
Cerevisiae_clade - ps30
Saccharomyces_cerevisiae - ps31
Phylostratum

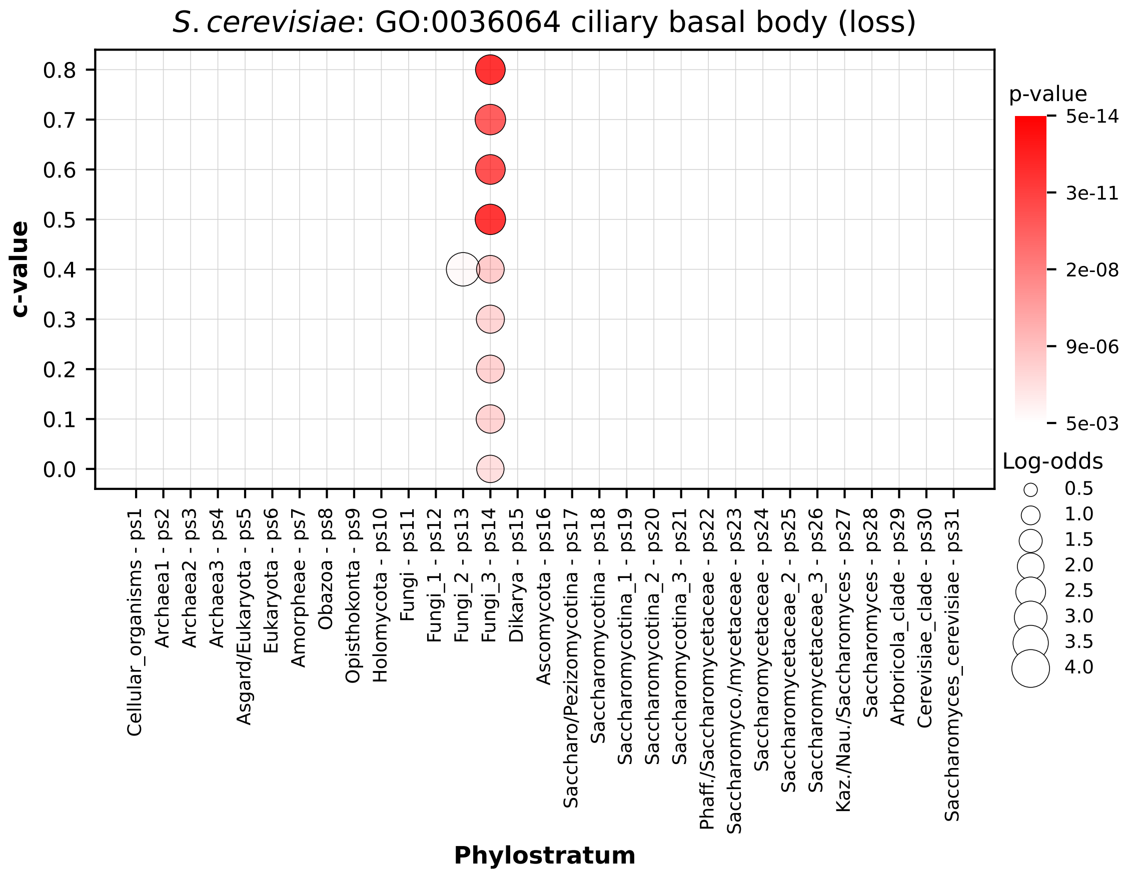

S. cerevisiae: GO:0036064 ciliary basal body (loss)
c-value
0.8
0.7
0.6
0.5
0.4
0.3
0.2
0.1
0.0
p-value
5e-14
3e-11
2e-08
9e-06
5e-03
Log-odds
0.5
1.0
1.5
2.0
2.5
3.0
3.5
4.0
Cellular_organisms - ps1
Archaea1 - ps2
Archaea2 - ps3
Archaea3 - ps4
Asgard/Eukaryota - ps5
Eukaryota - ps6
Amorpheae - ps7
Obazoa - ps8
Opisthokonta - ps9
Holomycota - ps10
Fungi - ps11
Fungi_1 - ps12
Fungi_2 - ps13
Fungi_3 - ps14
Dikarya - ps15
Ascomycota - ps16
Saccharo/Pezizomycotina - ps17
Saccharomycotina - ps18
Saccharomycotina_1 - ps19
Saccharomycotina_2 - ps20
Saccharomycotina_3 - ps21
Phaff./Saccharomycetaceae - ps22
Saccharomyco./mycetaceae - ps23
Saccharomycetaceae - ps24
Saccharomycetaceae_2 - ps25
Saccharomycetaceae_3 - ps26
Kaz./Nau./Saccharomyces - ps27
Saccharomyces - ps28
Arboricola_clade - ps29
Cerevisiae_clade - ps30
Saccharomyces_cerevisiae - ps31
Phylostratum

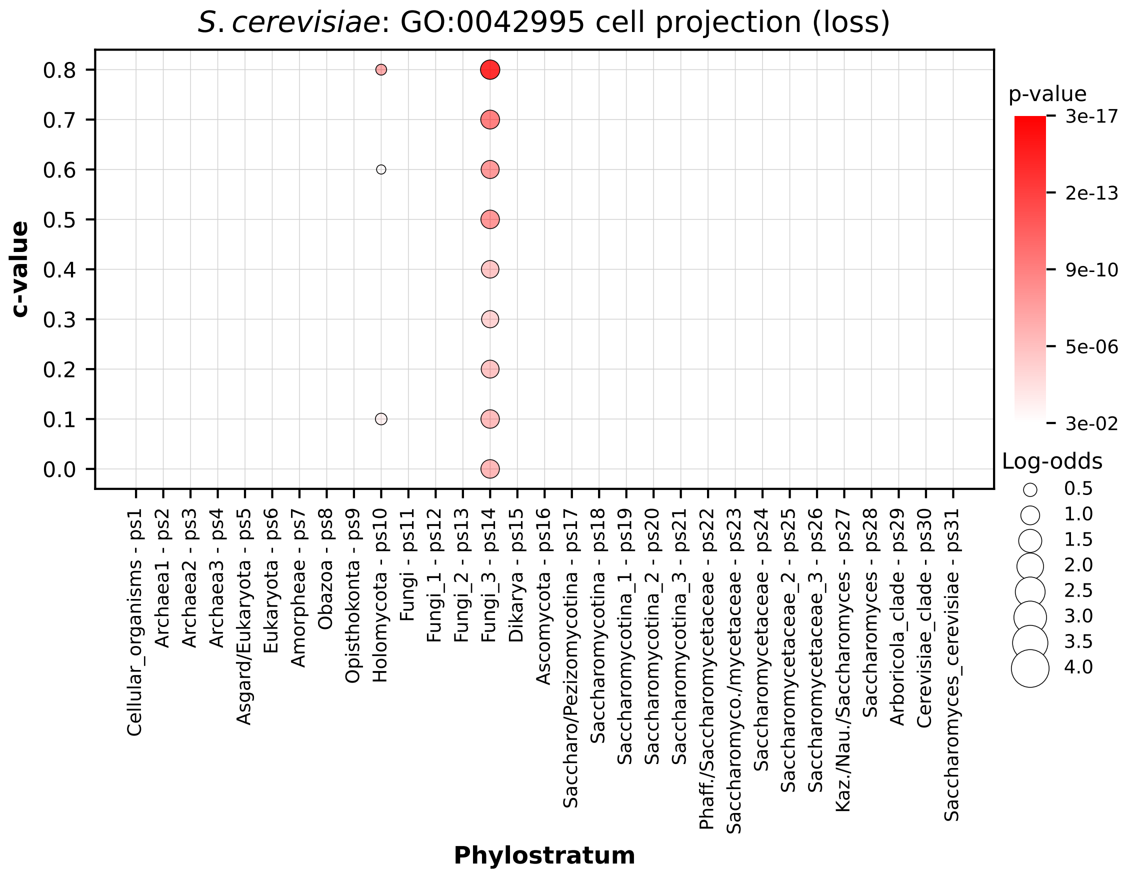
S. cerevisiae: GO:0042995 cell projection (loss)
c-value
0.8
0.7
0.6
0.5
0.4
0.3
0.2
0.1
0.0
Phylostratum
Cellular_organisms - ps1
Archaea1 - ps2
Archaea2 - ps3
Archaea3 - ps4
Asgard/Eukaryota - ps5
Eukaryota - ps6
Amorpheae - ps7
Obazoa - ps8
Opisthokonta - ps9
Holomycota - ps10
Fungi - ps11
Fungi_1 - ps12
Fungi_2 - ps13
Fungi_3 - ps14
Dikarya - ps15
Ascomycota - ps16
Saccharo/Pezizomycotina - ps17
Saccharomycotina - ps18
Saccharomycotina_1 - ps19
Saccharomycotina_2 - ps20
Saccharomycotina_3 - ps21
Phaff./Saccharomycetaceae - ps22
Saccharomyco./mycetaceae - ps23
Saccharomycetaceae - ps24
Saccharomycetaceae_2 - ps25
Saccharomycetaceae_3 - ps26
Kaz./Nau./Saccharomyces - ps27
Saccharomyces - ps28
Arboricola_clade - ps29
Cerevisiae_clade - ps30
Saccharomyces_cerevisiae - ps31
p-value
3e-17
2e-13
9e-10
5e-06
3e-02
Log-odds
0.5
1.0
1.5
2.0
2.5
3.0
3.5
4.0

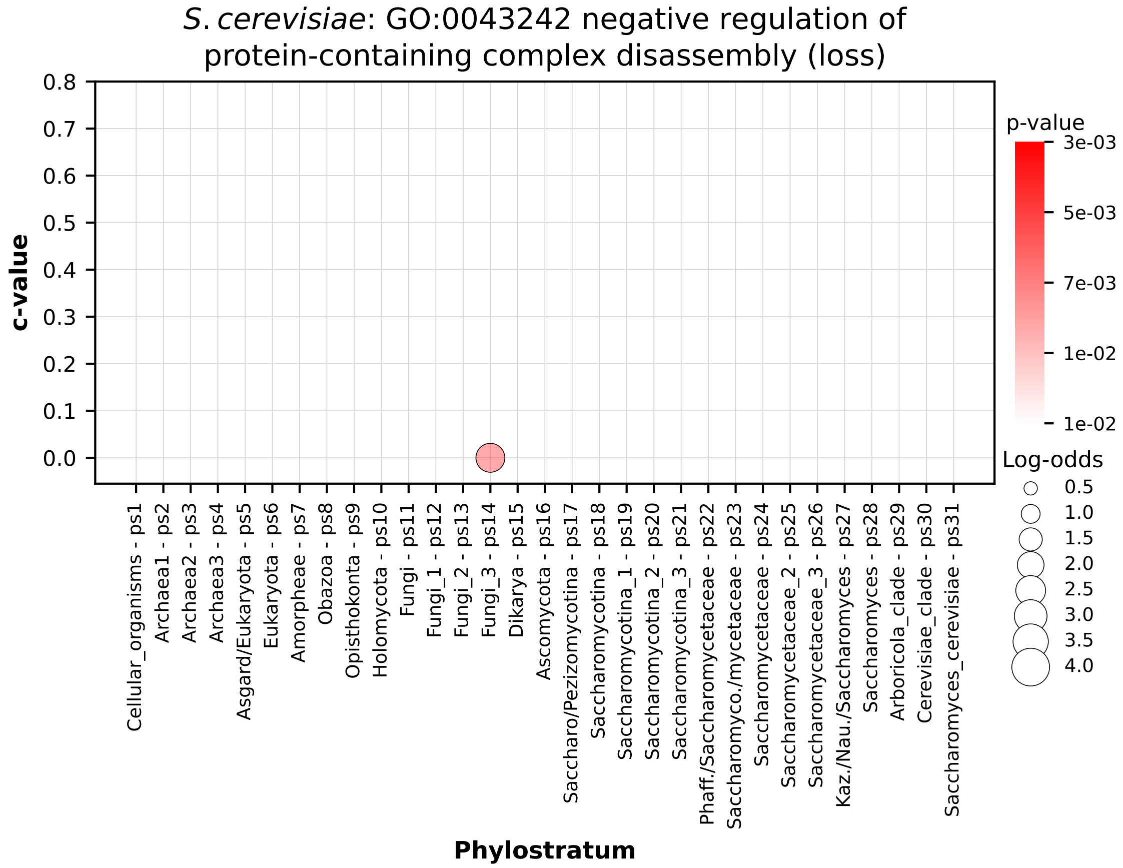
S. cerevisiae: GO:0043242 negative regulation of protein-containing complex disassembly (loss)
c-value
0.8
0.7
0.6
0.5
0.4
0.3
0.2
0.1
0.0
Phylostratum
Cellular_organisms - ps1
Archaea1 - ps2
Archaea2 - ps3
Archaea3 - ps4
Asgard/Eukaryota - ps5
Eukaryota - ps6
Amorpheae - ps7
Obazoa - ps8
Opisthokonta - ps9
Holomycota - ps10
Fungi - ps11
Fungi_1 - ps12
Fungi_2 - ps13
Fungi_3 - ps14
Dikarya - ps15
Ascomycota - ps16
Saccharo/Pezizomycotina - ps17
Saccharomycotina - ps18
Saccharomycotina_1 - ps19
Saccharomycotina_2 - ps20
Saccharomycotina_3 - ps21
Phaff./Saccharomycetaceae - ps22
Saccharomyco./mycetaceae - ps23
Saccharomycetaceae - ps24
Saccharomycetaceae_2 - ps25
Saccharomycetaceae_3 - ps26
Kaz./Nau./Saccharomyces - ps27
Saccharomyces - ps28
Arboricola_clade - ps29
Cerevisiae_clade - ps30
Saccharomyces_cerevisiae - ps31
p-value
3e-03
5e-03
7e-03
1e-02
1e-02
Log-odds
0.5
1.0
1.5
2.0
2.5
3.0
3.5
4.0

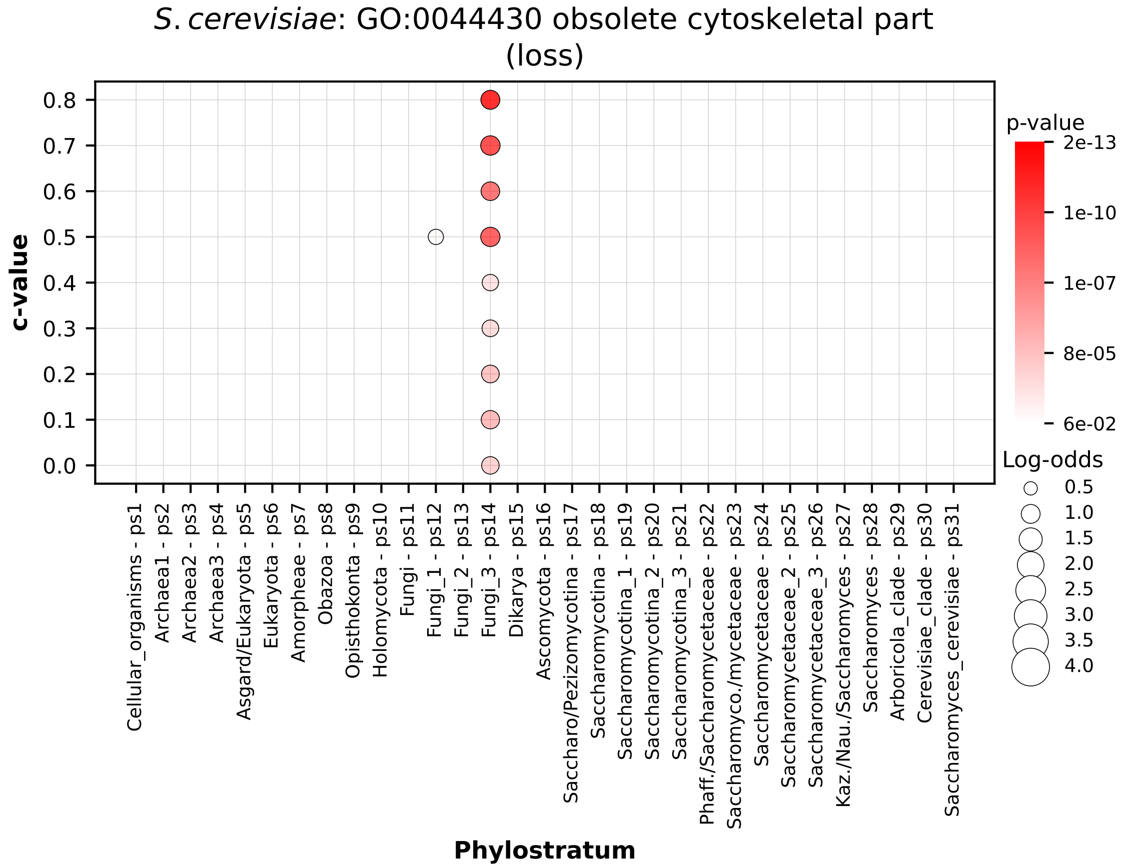

S. cerevisiae: GO:0044430 obsolete cytoskeletal part (loss)
c-value
0.8
0.7
0.6
0.5
0.4
0.3
0.2
0.1
0.0
Phylostratum
Cellular_organisms - ps1
Archaea1 - ps2
Archaea2 - ps3
Archaea3 - ps4
Asgard/Eukaryota - ps5
Eukaryota - ps6
Amorpheae - ps7
Obazoa - ps8
Opisthokonta - ps9
Holomycota - ps10
Fungi - ps11
Fungi_1 - ps12
Fungi_2 - ps13
Fungi_3 - ps14
Dikarya - ps15
Ascomycota - ps16
Saccharo/Pezizomycotina - ps17
Saccharomycotina - ps18
Saccharomycotina_1 - ps19
Saccharomycotina_2 - ps20
Saccharomycotina_3 - ps21
Phaff./Saccharomycetaceae - ps22
Saccharomyco./mycetaceae - ps23
Saccharomycetaceae - ps24
Saccharomycetaceae_2 - ps25
Saccharomycetaceae_3 - ps26
Kaz./Nau./Saccharomyces - ps27
Saccharomyces - ps28
Arboricola_clade - ps29
Cerevisiae_clade - ps30
Saccharomyces_cerevisiae - ps31
p-value
2e-13
1e-10
1e-07
8e-05
6e-02
Log-odds
0.5
1.0
1.5
2.0
2.5
3.0
3.5
4.0

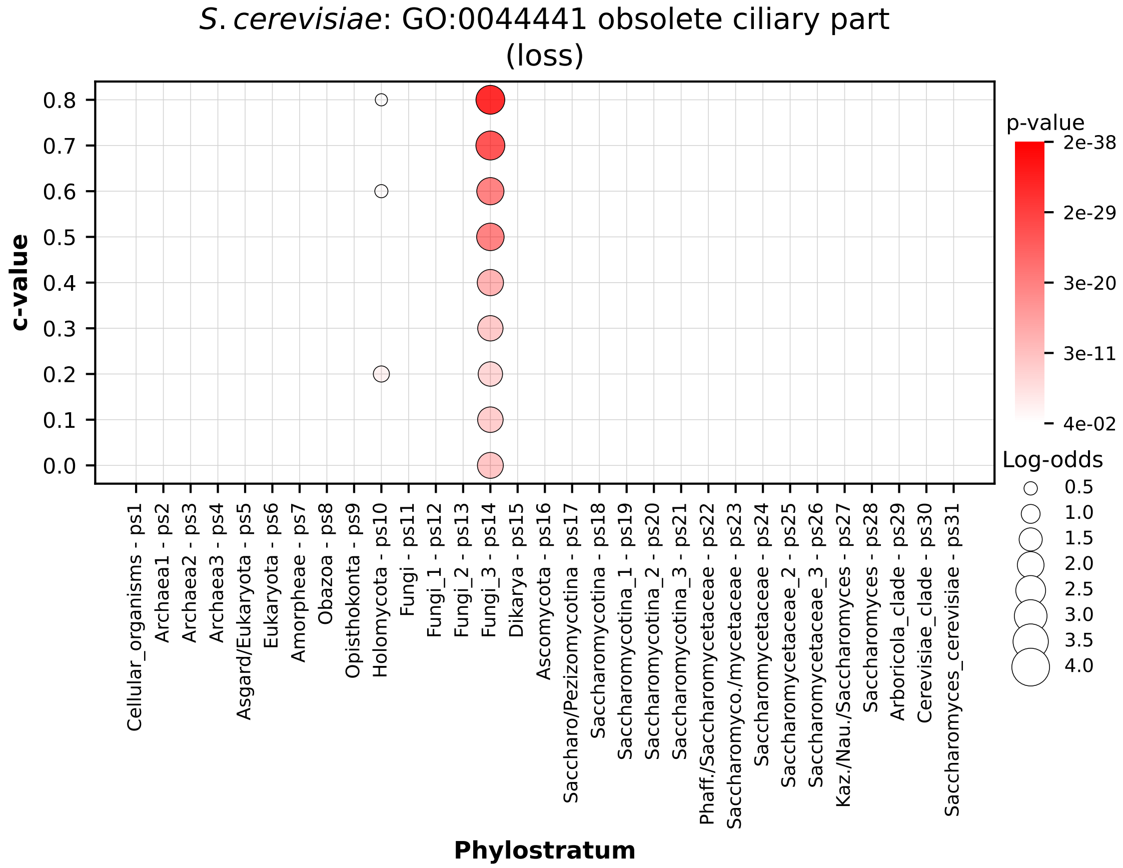

S. cerevisiae: GO:0044441 obsolete ciliary part
(loss)
c-value
0.8
0.7
0.6
0.5
0.4
0.3
0.2
0.1
0.0
p-value
2e-38
2e-29
3e-20
3e-11
4e-02
Log-odds
0.5
1.0
1.5
2.0
2.5
3.0
3.5
4.0
Cellular_organisms - ps1
Archaea1 - ps2
Archaea2 - ps3
Archaea3 - ps4
Asgard/Eukaryota - ps5
Eukaryota - ps6
Amorpheae - ps7
Obazoa - ps8
Opisthokonta - ps9
Holomycota - ps10
Fungi - ps11
Fungi_1 - ps12
Fungi_2 - ps13
Fungi_3 - ps14
Dikarya - ps15
Ascomycota - ps16
Saccharo/Pezizomycotina - ps17
Saccharomycotina - ps18
Saccharomycotina_1 - ps19
Saccharomycotina_2 - ps20
Saccharomycotina_3 - ps21
Phaff./Saccharomycetaceae - ps22
Saccharomyco./mycetaceae - ps23
Saccharomycetaceae - ps24
Saccharomycetaceae_2 - ps25
Saccharomycetaceae_3 - ps26
Kaz./Nau./Saccharomyces - ps27
Saccharomyces - ps28
Arboricola_clade - ps29
Cerevisiae_clade - ps30
Saccharomyces_cerevisiae - ps31
Phylostratum

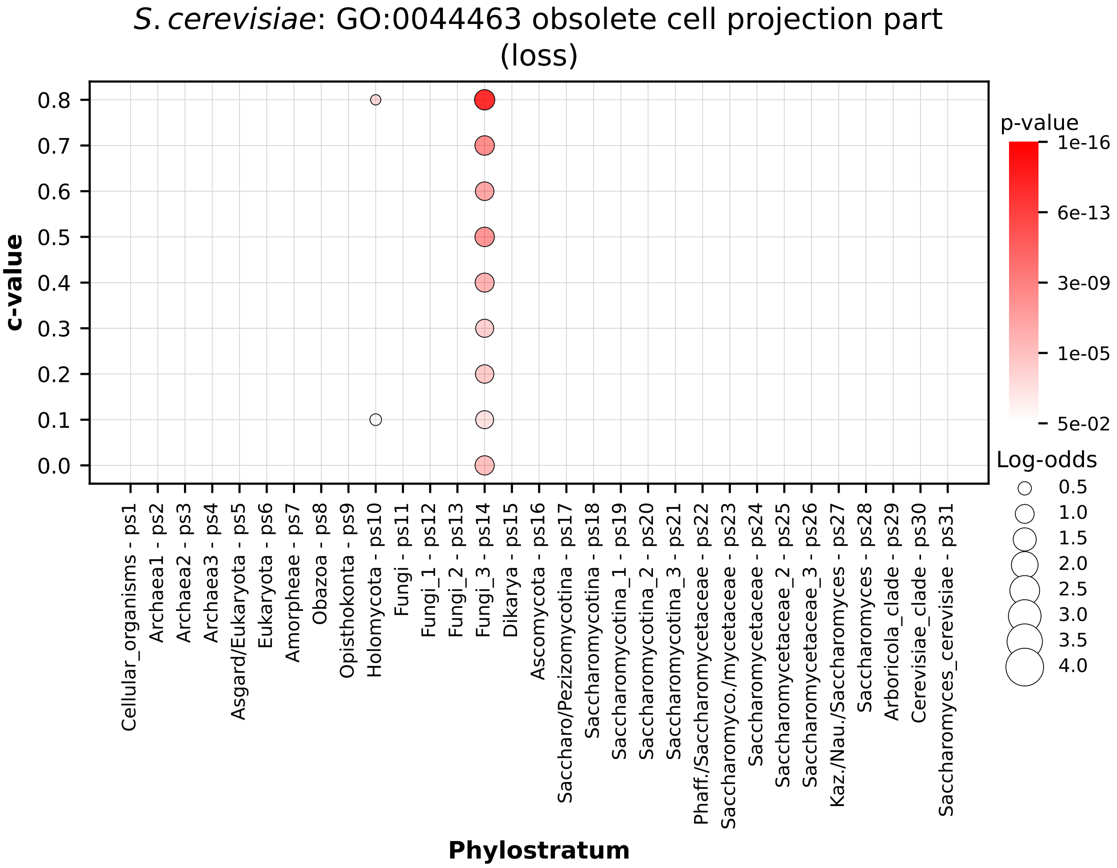

S. cerevisiae: GO:0044463 obsolete cell projection part (loss)
c-value
0.8
0.7
0.6
0.5
0.4
0.3
0.2
0.1
0.0
p-value
1e-16
6e-13
3e-09
1e-05
5e-02
Log-odds
0.5
1.0
1.5
2.0
2.5
3.0
3.5
4.0
Cellular_organisms - ps1
Archaea1 - ps2
Archaea2 - ps3
Archaea3 - ps4
Asgard/Eukaryota - ps5
Eukaryota - ps6
Amorpheae - ps7
Obazoa - ps8
Opisthokonta - ps9
Holomycota - ps10
Fungi - ps11
Fungi_1 - ps12
Fungi_2 - ps13
Fungi_3 - ps14
Dikarya - ps15
Ascomycota - ps16
Saccharo/Pezizomycotina - ps17
Saccharomycotina - ps18
Saccharomycotina_1 - ps19
Saccharomycotina_2 - ps20
Saccharomycotina_3 - ps21
Phaff./Saccharomycetaceae - ps22
Saccharomyco./mycetaceae - ps23
Saccharomycetaceae - ps24
Saccharomycetaceae_2 - ps25
Saccharomycetaceae_3 - ps26
Kaz./Nau./Saccharomyces - ps27
Saccharomyces - ps28
Arboricola_clade - ps29
Cerevisiae_clade - ps30
Saccharomyces_cerevisiae - ps31
Phylostratum

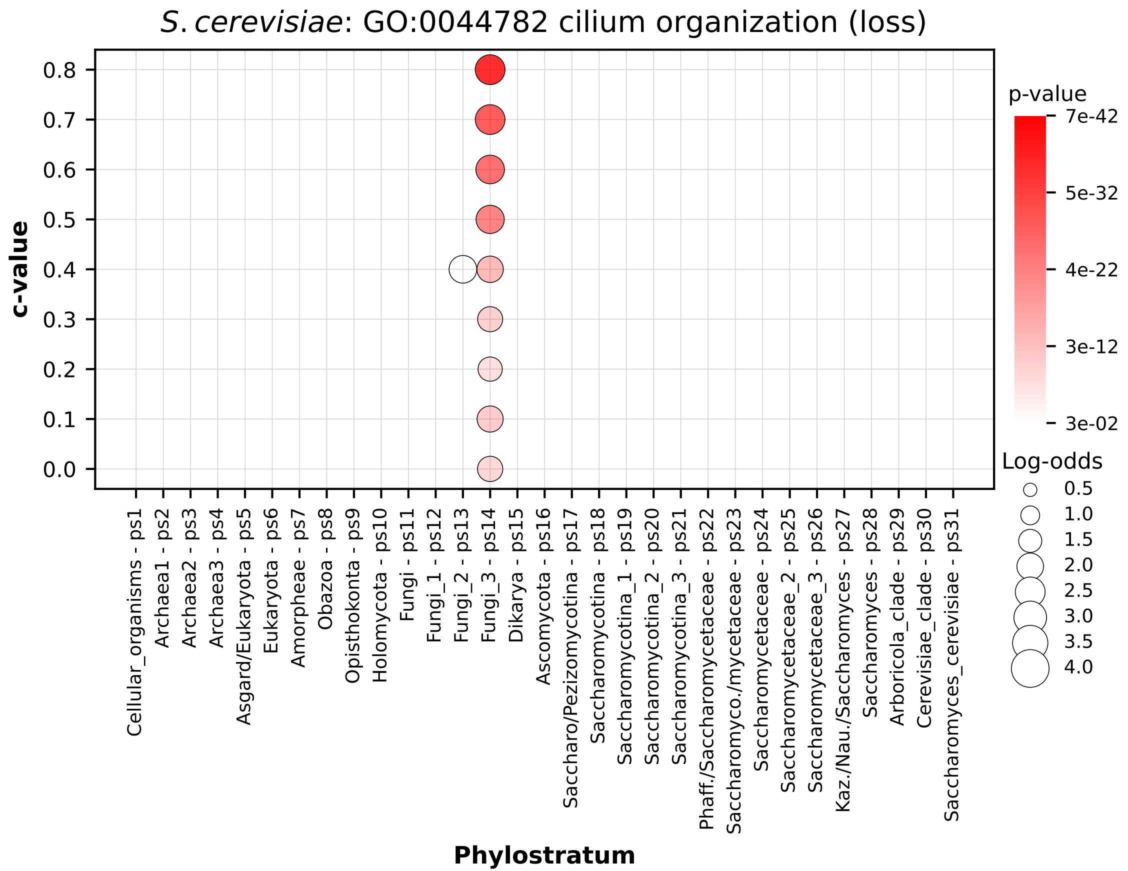

S. cerevisiae: GO:0044782 cilium organization (loss)
c-value
0.0
0.1
0.2
0.3
0.4
0.5
0.6
0.7
0.8
Phylostratum
Cellular_organisms - ps1
Archaea1 - ps2
Archaea2 - ps3
Archaea3 - ps4
Asgard/Eukaryota - ps5
Eukaryota - ps6
Amorpheae - ps7
Obazoa - ps8
Opisthokonta - ps9
Holomycota - ps10
Fungi - ps11
Fungi_1 - ps12
Fungi_2 - ps13
Fungi_3 - ps14
Dikarya - ps15
Ascomycota - ps16
Saccharo/Pezizomycotina - ps17
Saccharomycotina - ps18
Saccharomycotina_1 - ps19
Saccharomycotina_2 - ps20
Saccharomycotina_3 - ps21
Phaff./Saccharomycetaceae - ps22
Saccharomyco./mycetaceae - ps23
Saccharomycetaceae - ps24
Saccharomycetaceae_2 - ps25
Saccharomycetaceae_3 - ps26
Kaz./Nau./Saccharomyces - ps27
Saccharomyces - ps28
Arboricola_clade - ps29
Cerevisiae_clade - ps30
Saccharomyces_cerevisiae - ps31
p-value
7e-42
5e-32
4e-22
3e-12
3e-02
Log-odds
0.5
1.0
1.5
2.0
2.5
3.0
3.5
4.0

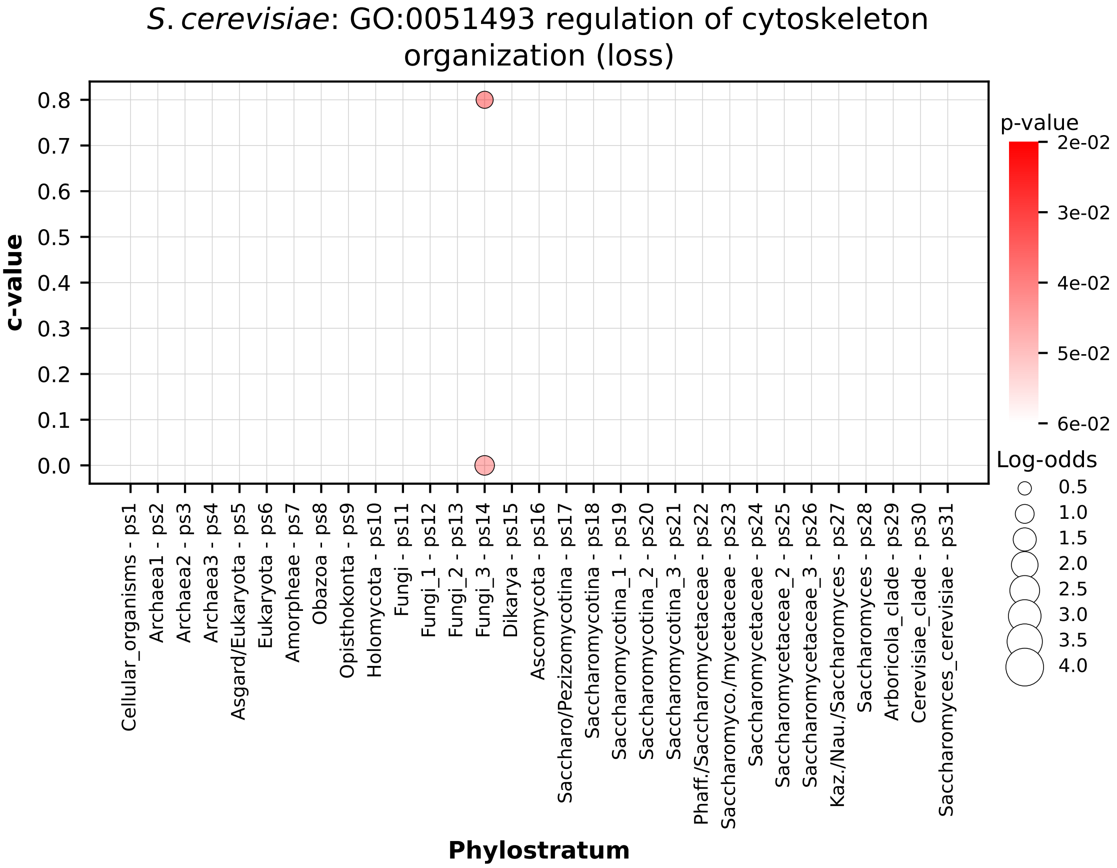

S. cerevisiae: GO:0051493 regulation of cytoskeleton organization (loss)
c-value
0.0
0.1
0.2
0.3
0.4
0.5
0.6
0.7
0.8
p-value
2e-02
3e-02
4e-02
5e-02
6e-02
Log-odds
0.5
1.0
1.5
2.0
2.5
3.0
3.5
4.0
Cellular_organisms - ps1
Archaea1 - ps2
Archaea2 - ps3
Archaea3 - ps4
Asgard/Eukaryota - ps5
Eukaryota - ps6
Amorpheae - ps7
Obazoa - ps8
Opisthokonta - ps9
Holomycota - ps10
Fungi - ps11
Fungi_1 - ps12
Fungi_2 - ps13
Fungi_3 - ps14
Dikarya - ps15
Ascomycota - ps16
Saccharo/Pezizomycotina - ps17
Saccharomycotina - ps18
Saccharomycotina_1 - ps19
Saccharomycotina_2 - ps20
Saccharomycotina_3 - ps21
Phaff./Saccharomycetaceae - ps22
Saccharomyco./mycetaceae - ps23
Saccharomycetaceae - ps24
Saccharomycetaceae_2 - ps25
Saccharomycetaceae_3 - ps26
Kaz./Nau./Saccharomyces - ps27
Saccharomyces - ps28
Arboricola_clade - ps29
Cerevisiae_clade - ps30
Saccharomyces_cerevisiae - ps31
Phylostratum

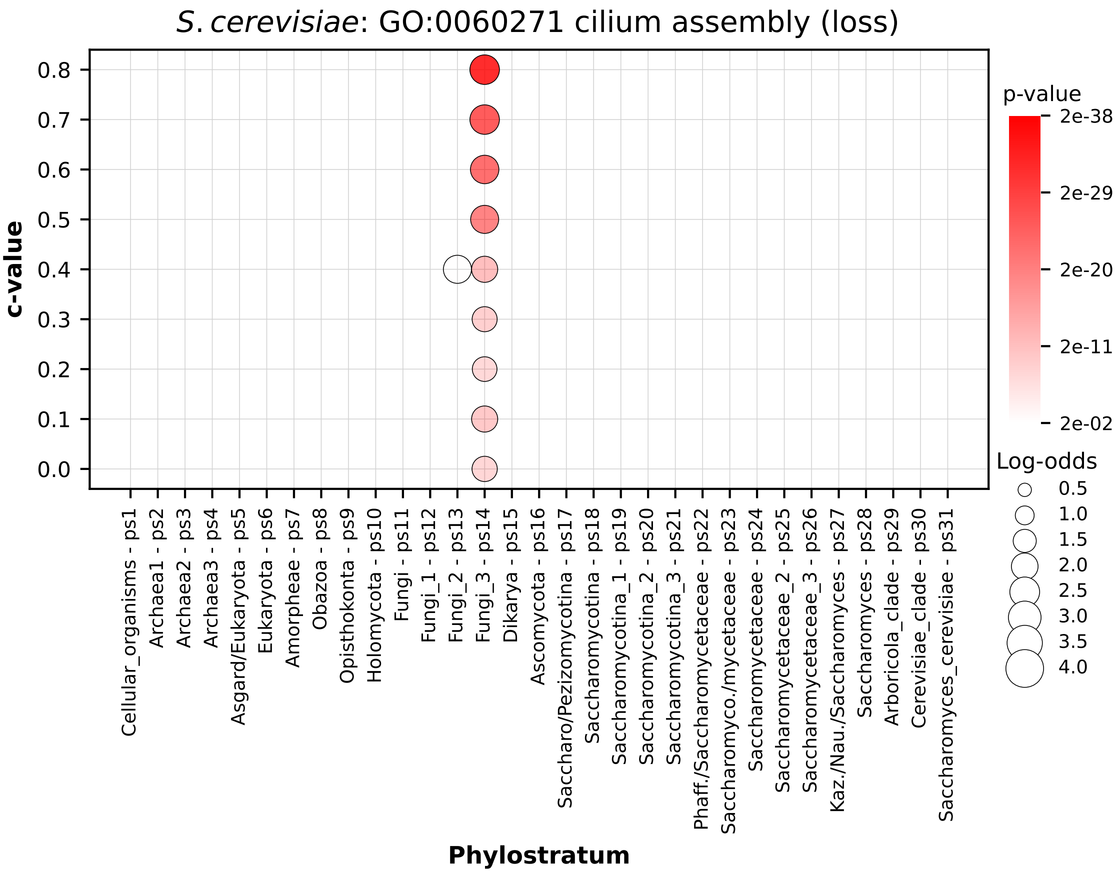

S. cerevisiae: GO:0060271 cilium assembly (loss)
c-value
0.0
0.1
0.2
0.3
0.4
0.5
0.6
0.7
0.8
Phylostratum
Cellular_organisms - ps1
Archaea1 - ps2
Archaea2 - ps3
Archaea3 - ps4
Asgard/Eukaryota - ps5
Eukaryota - ps6
Amorpheae - ps7
Obazoa - ps8
Opisthokonta - ps9
Holomycota - ps10
Fungi - ps11
Fungi_1 - ps12
Fungi_2 - ps13
Fungi_3 - ps14
Dikarya - ps15
Ascomycota - ps16
Saccharo/Pezizomycotina - ps17
Saccharomycotina - ps18
Saccharomycotina_1 - ps19
Saccharomycotina_2 - ps20
Saccharomycotina_3 - ps21
Phaff./Saccharomycetaceae - ps22
Saccharomyco./mycetaceae - ps23
Saccharomycetaceae - ps24
Saccharomycetaceae_2 - ps25
Saccharomycetaceae_3 - ps26
Kaz./Nau./Saccharomyces - ps27
Saccharomyces - ps28
Arboricola_clade - ps29
Cerevisiae_clade - ps30
Saccharomyces_cerevisiae - ps31
p-value
2e-38
2e-29
2e-20
2e-11
2e-02
Log-odds
0.5
1.0
1.5
2.0
2.5
3.0
3.5
4.0

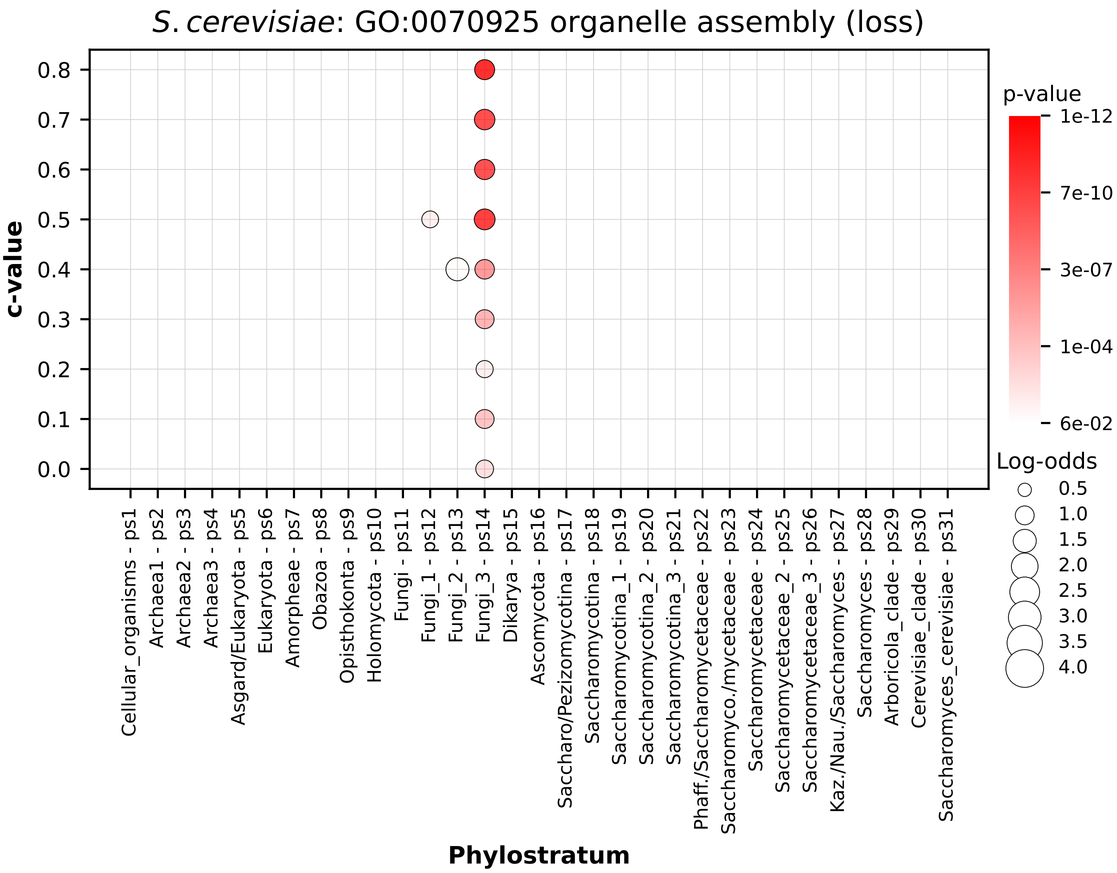

S. cerevisiae: GO:0070925 organelle assembly (loss)
c-value
0.8
0.7
0.6
0.5
0.4
0.3
0.2
0.1
0.0
p-value
1e-12
7e-10
3e-07
1e-04
6e-02
Log-odds
0.5
1.0
1.5
2.0
2.5
3.0
3.5
4.0
Cellular_organisms - ps1
Archaea1 - ps2
Archaea2 - ps3
Archaea3 - ps4
Asgard/Eukaryota - ps5
Eukaryota - ps6
Amorpheae - ps7
Obazoa - ps8
Opisthokonta - ps9
Holomycota - ps10
Fungi - ps11
Fungi_1 - ps12
Fungi_2 - ps13
Fungi_3 - ps14
Dikarya - ps15
Ascomycota - ps16
Saccharo/Pezizomycotina - ps17
Saccharomycotina - ps18
Saccharomycotina_1 - ps19
Saccharomycotina_2 - ps20
Saccharomycotina_3 - ps21
Phaff./Saccharomycetaceae - ps22
Saccharomyco./mycetaceae - ps23
Saccharomycetaceae - ps24
Saccharomycetaceae_2 - ps25
Saccharomycetaceae_3 - ps26
Kaz./Nau./Saccharomyces - ps27
Saccharomyces - ps28
Arboricola_clade - ps29
Cerevisiae_clade - ps30
Saccharomyces_cerevisiae - ps31
Phylostratum

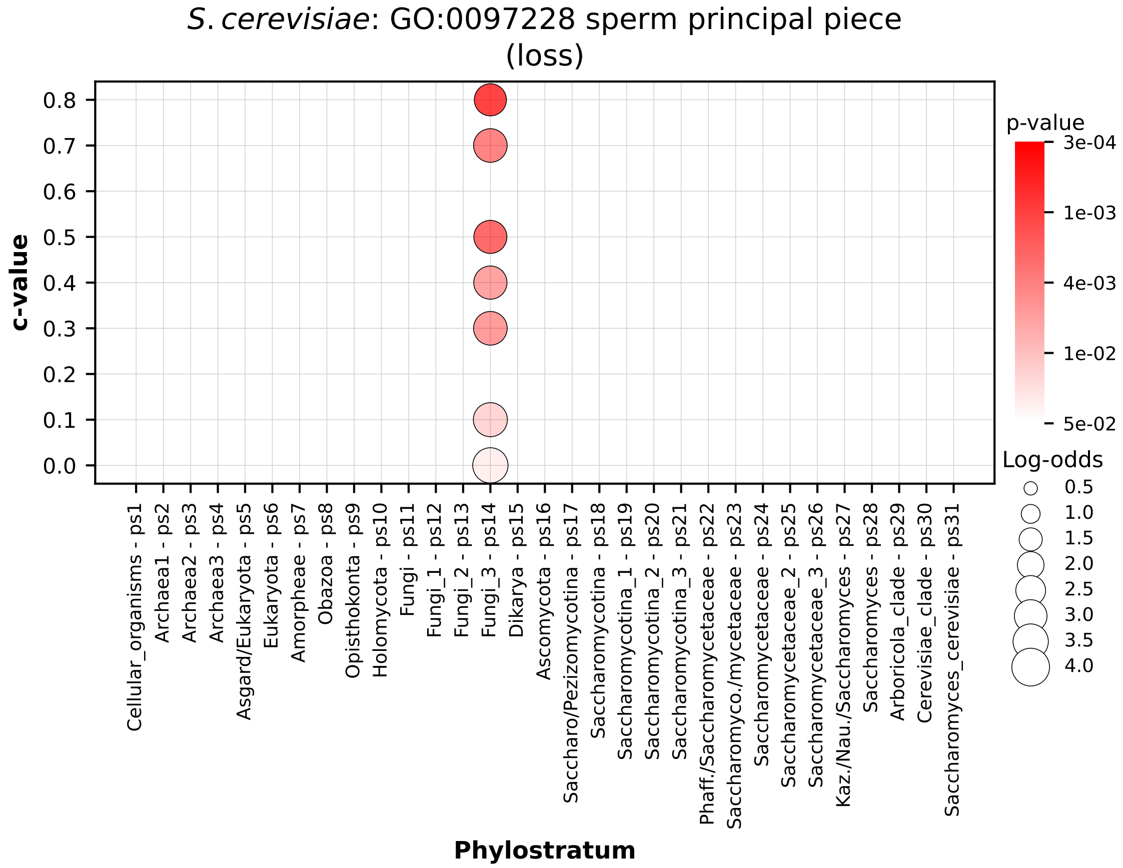

S. cerevisiae: GO:0097228 sperm principal piece
(loss)
c-value
0.0
0.1
0.2
0.3
0.4
0.5
0.6
0.7
0.8
Phylostratum
Cellular_organisms - ps1
Archaea1 - ps2
Archaea2 - ps3
Archaea3 - ps4
Asgard/Eukaryota - ps5
Eukaryota - ps6
Amorpheae - ps7
Obazoa - ps8
Opisthokonta - ps9
Holomycota - ps10
Fungi - ps11
Fungi_1 - ps12
Fungi_2 - ps13
Fungi_3 - ps14
Dikarya - ps15
Ascomycota - ps16
Saccharo/Pezizomycotina - ps17
Saccharomycotina - ps18
Saccharomycotina_1 - ps19
Saccharomycotina_2 - ps20
Saccharomycotina_3 - ps21
Phaff./Saccharomycetaceae - ps22
Saccharomyco./mycetaceae - ps23
Saccharomycetaceae - ps24
Saccharomycetaceae_2 - ps25
Saccharomycetaceae_3 - ps26
Kaz./Nau./Saccharomyces - ps27
Saccharomyces - ps28
Arboricola_clade - ps29
Cerevisiae_clade - ps30
Saccharomyces_cerevisiae - ps31
p-value
3e-04
1e-03
4e-03
1e-02
5e-02
Log-odds
0.5
1.0
1.5
2.0
2.5
3.0
3.5
4.0

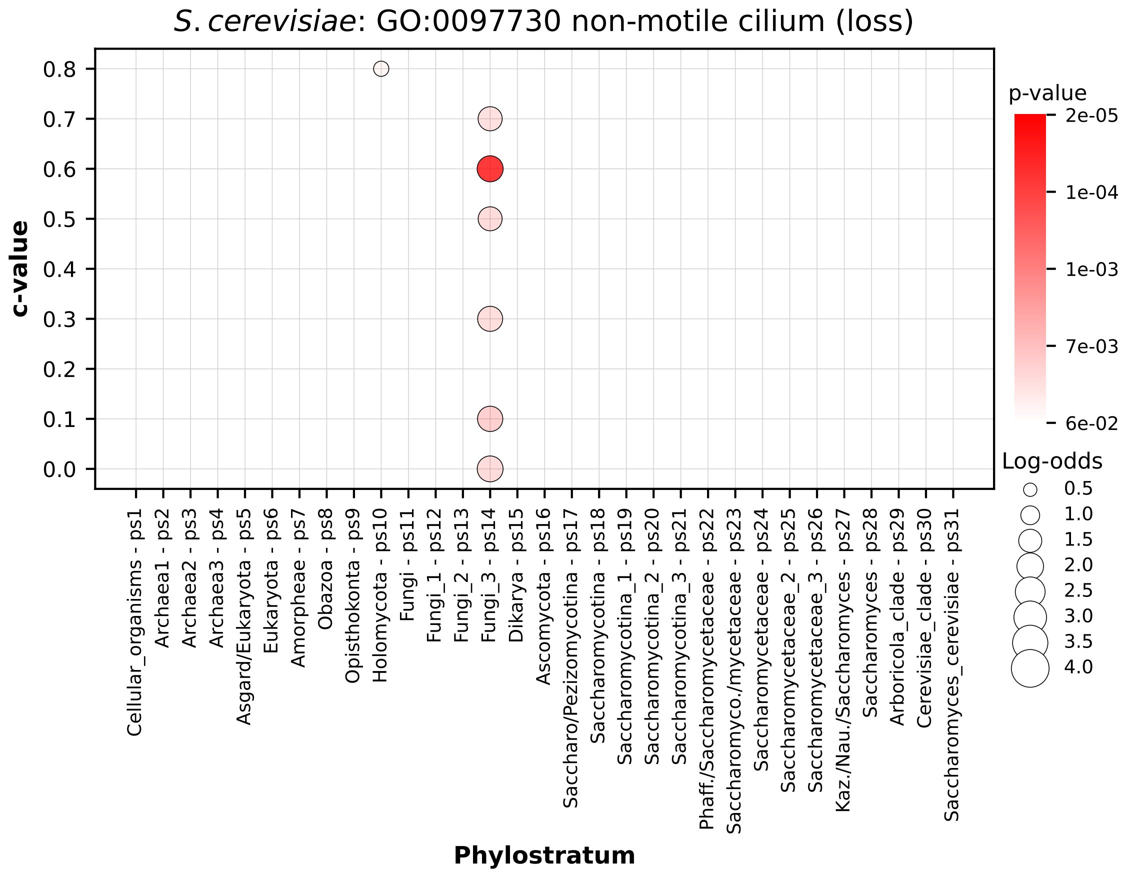

S. cerevisiae: GO:0097730 non-motile cilium (loss)
c-value
0.0
0.1
0.2
0.3
0.4
0.5
0.6
0.7
0.8
p-value
2e-05
1e-04
1e-03
7e-03
6e-02
Log-odds
0.5
1.0
1.5
2.0
2.5
3.0
3.5
4.0
Cellular_organisms - ps1
Archaea1 - ps2
Archaea2 - ps3
Archaea3 - ps4
Asgard/Eukaryota - ps5
Eukaryota - ps6
Amorpheae - ps7
Obazoa - ps8
Opisthokonta - ps9
Holomycota - ps10
Fungi - ps11
Fungi_1 - ps12
Fungi_2 - ps13
Fungi_3 - ps14
Dikarya - ps15
Ascomycota - ps16
Saccharo/Pezizomycotina - ps17
Saccharomycotina - ps18
Saccharomycotina_1 - ps19
Saccharomycotina_2 - ps20
Saccharomycotina_3 - ps21
Phaff./Saccharomycetaceae - ps22
Saccharomyco./mycetaceae - ps23
Saccharomycetaceae - ps24
Saccharomycetaceae_2 - ps25
Saccharomycetaceae_3 - ps26
Kaz./Nau./Saccharomyces - ps27
Saccharomyces - ps28
Arboricola_clade - ps29
Cerevisiae_clade - ps30
Saccharomyces_cerevisiae - ps31
Phylostratum

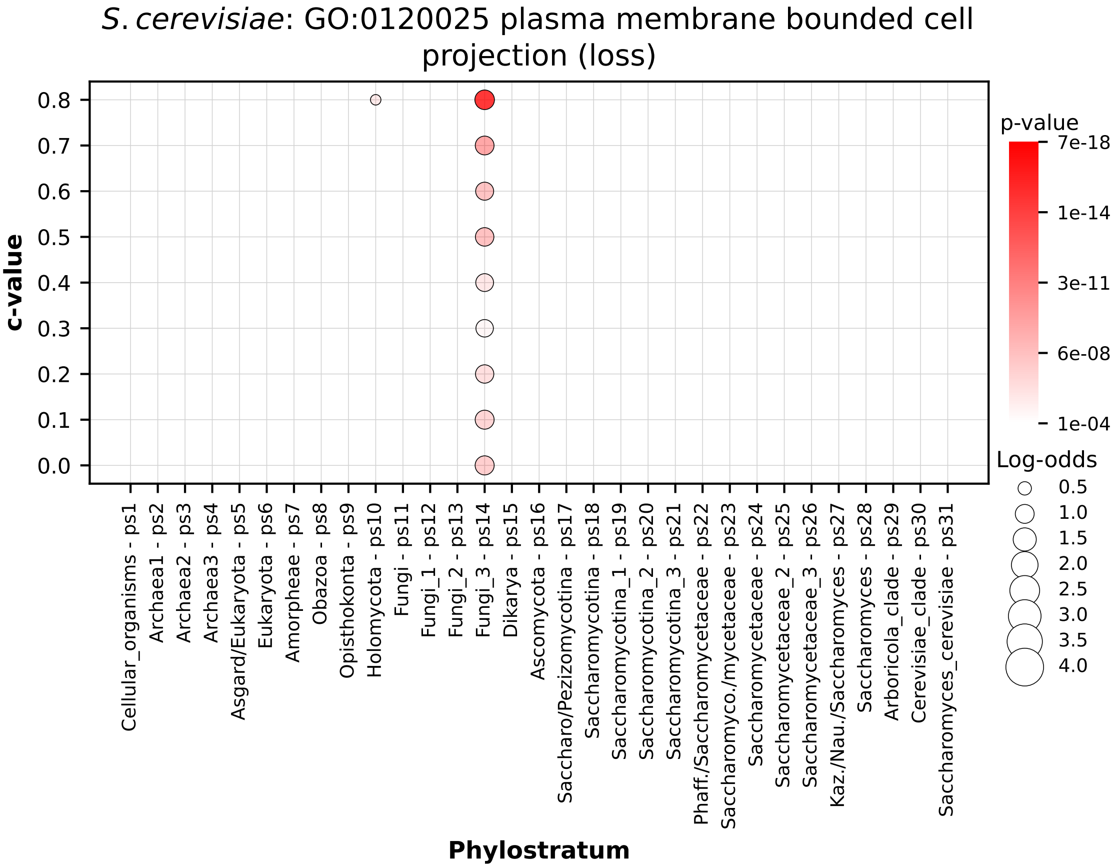

S. cerevisiae: GO:0120025 plasma membrane bounded cell projection (loss)
c-value
0.8
0.7
0.6
0.5
0.4
0.3
0.2
0.1
0.0
p-value
7e-18
1e-14
3e-11
6e-08
1e-04
Log-odds
0.5
1.0
1.5
2.0
2.5
3.0
3.5
4.0
Cellular_organisms - ps1
Archaea1 - ps2
Archaea2 - ps3
Archaea3 - ps4
Asgard/Eukaryota - ps5
Eukaryota - ps6
Amorpheae - ps7
Obazoa - ps8
Opisthokonta - ps9
Holomycota - ps10
Fungi - ps11
Fungi_1 - ps12
Fungi_2 - ps13
Fungi_3 - ps14
Dikarya - ps15
Ascomycota - ps16
Saccharo/Pezizomycotina - ps17
Saccharomycotina - ps18
Saccharomycotina_1 - ps19
Saccharomycotina_2 - ps20
Saccharomycotina_3 - ps21
Phaff./Saccharomycetaceae - ps22
Saccharomyco./mycetaceae - ps23
Saccharomycetaceae - ps24
Saccharomycetaceae_2 - ps25
Saccharomycetaceae_3 - ps26
Kaz./Nau./Saccharomyces - ps27
Saccharomyces - ps28
Arboricola_clade - ps29
Cerevisiae_clade - ps30
Saccharomyces_cerevisiae - ps31
Phylostratum

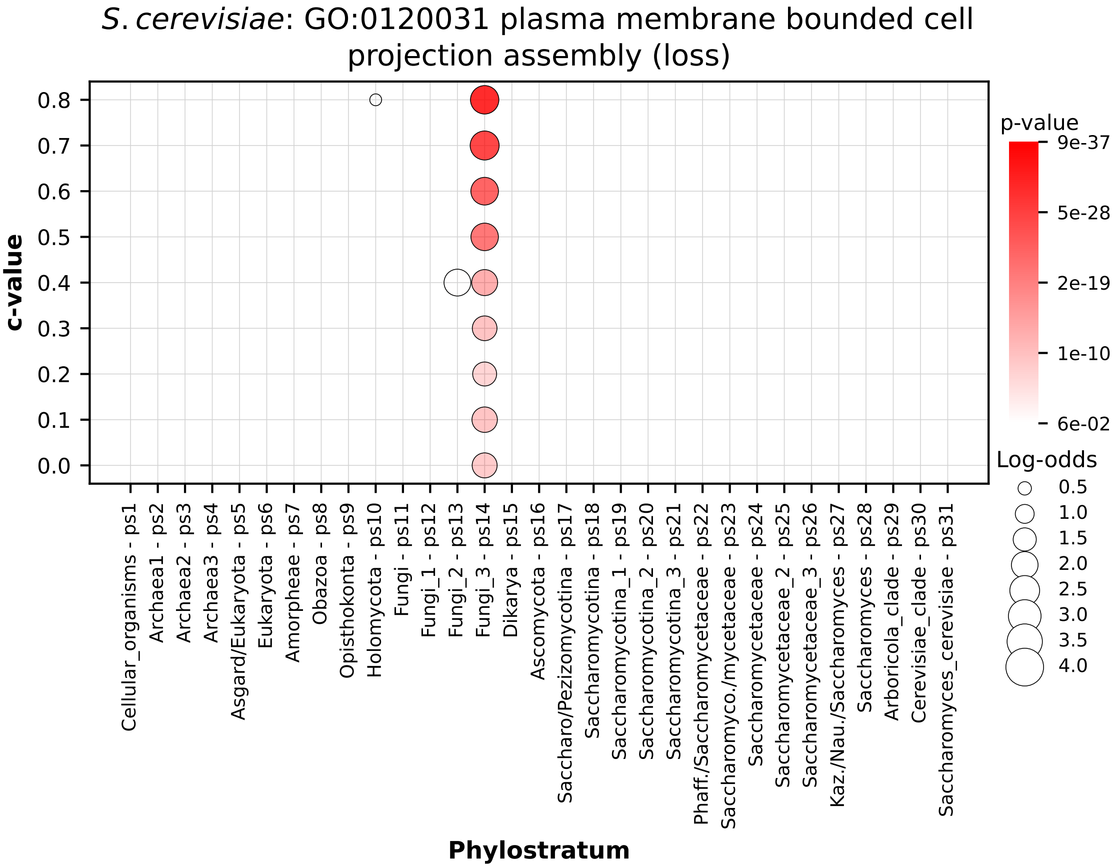
S. cerevisiae: GO:0120031 plasma membrane bounded cell projection assembly (loss)
c-value
0.8
0.7
0.6
0.5
0.4
0.3
0.2
0.1
0.0
p-value
9e-37
5e-28
2e-19
1e-10
6e-02
Log-odds
0.5
1.0
1.5
2.0
2.5
3.0
3.5
4.0
Cellular_organisms - ps1
Archaea1 - ps2
Archaea2 - ps3
Archaea3 - ps4
Asgard/Eukaryota - ps5
Eukaryota - ps6
Amorpheae - ps7
Obazoa - ps8
Opisthokonta - ps9
Holomycota - ps10
Fungi - ps11
Fungi_1 - ps12
Fungi_2 - ps13
Fungi_3 - ps14
Dikarya - ps15
Ascomycota - ps16
Saccharo/Pezizomycotina - ps17
Saccharomycotina - ps18
Saccharomycotina_1 - ps19
Saccharomycotina_2 - ps20
Saccharomycotina_3 - ps21
Phaff./Saccharomycetaceae - ps22
Saccharomyco./mycetaceae - ps23
Saccharomycetaceae - ps24
Saccharomycetaceae_2 - ps25
Saccharomycetaceae_3 - ps26
Kaz./Nau./Saccharomyces - ps27
Saccharomyces - ps28
Arboricola_clade - ps29
Cerevisiae_clade - ps30
Saccharomyces_cerevisiae - ps31
Phylostratum

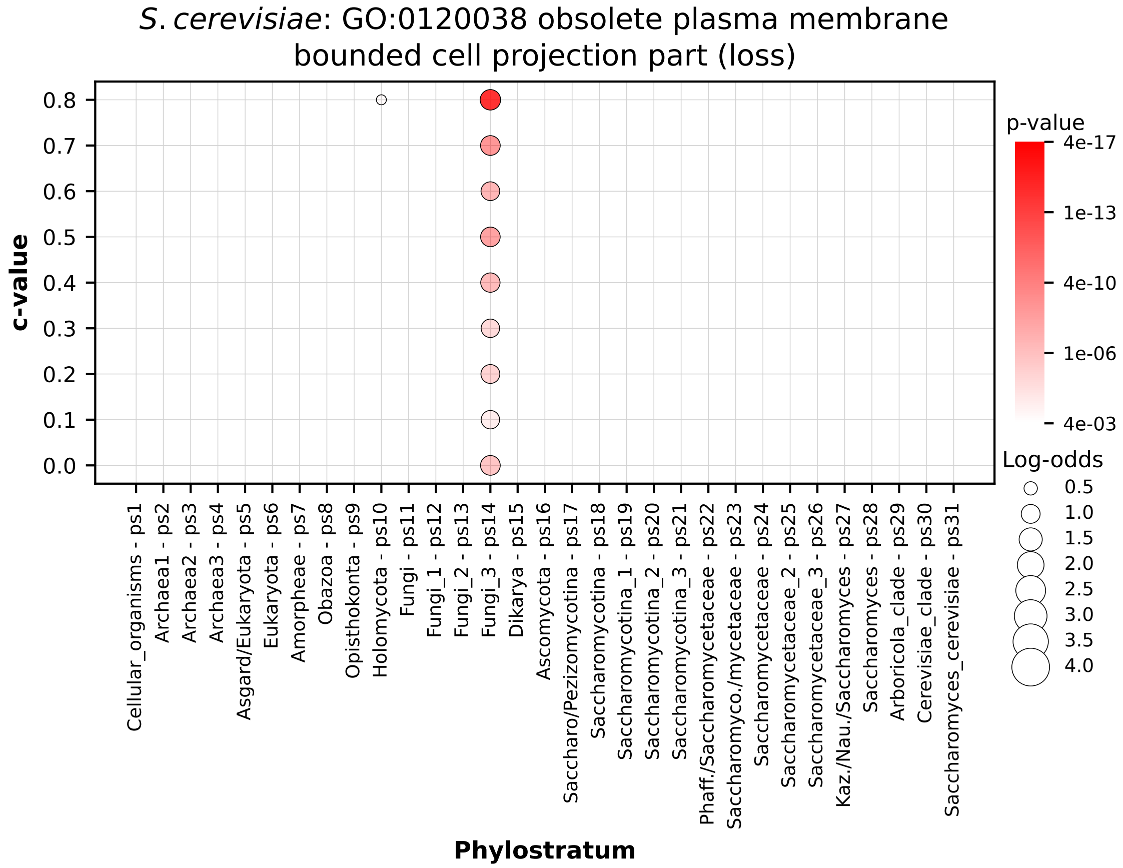

S. cerevisiae: GO:0120038 obsolete plasma membrane bounded cell projection part (loss)
c-value
0.8
0.7
0.6
0.5
0.4
0.3
0.2
0.1
0.0
Phylostratum
Cellular_organisms - ps1
Archaea1 - ps2
Archaea2 - ps3
Archaea3 - ps4
Asgard/Eukaryota - ps5
Eukaryota - ps6
Amorpheae - ps7
Obazoa - ps8
Opisthokonta - ps9
Holomycota - ps10
Fungi - ps11
Fungi_1 - ps12
Fungi_2 - ps13
Fungi_3 - ps14
Dikarya - ps15
Ascomycota - ps16
Saccharo/Pezizomycotina - ps17
Saccharomycotina - ps18
Saccharomycotina_1 - ps19
Saccharomycotina_2 - ps20
Saccharomycotina_3 - ps21
Phaff./Saccharomycetaceae - ps22
Saccharomyco./mycetaceae - ps23
Saccharomycetaceae - ps24
Saccharomycetaceae_2 - ps25
Saccharomycetaceae_3 - ps26
Kaz./Nau./Saccharomyces - ps27
Saccharomyces - ps28
Arboricola_clade - ps29
Cerevisiae_clade - ps30
Saccharomyces_cerevisiae - ps31
p-value
4e-17
1e-13
4e-10
1e-06
4e-03
Log-odds
0.5
1.0
1.5
2.0
2.5
3.0
3.5
4.0

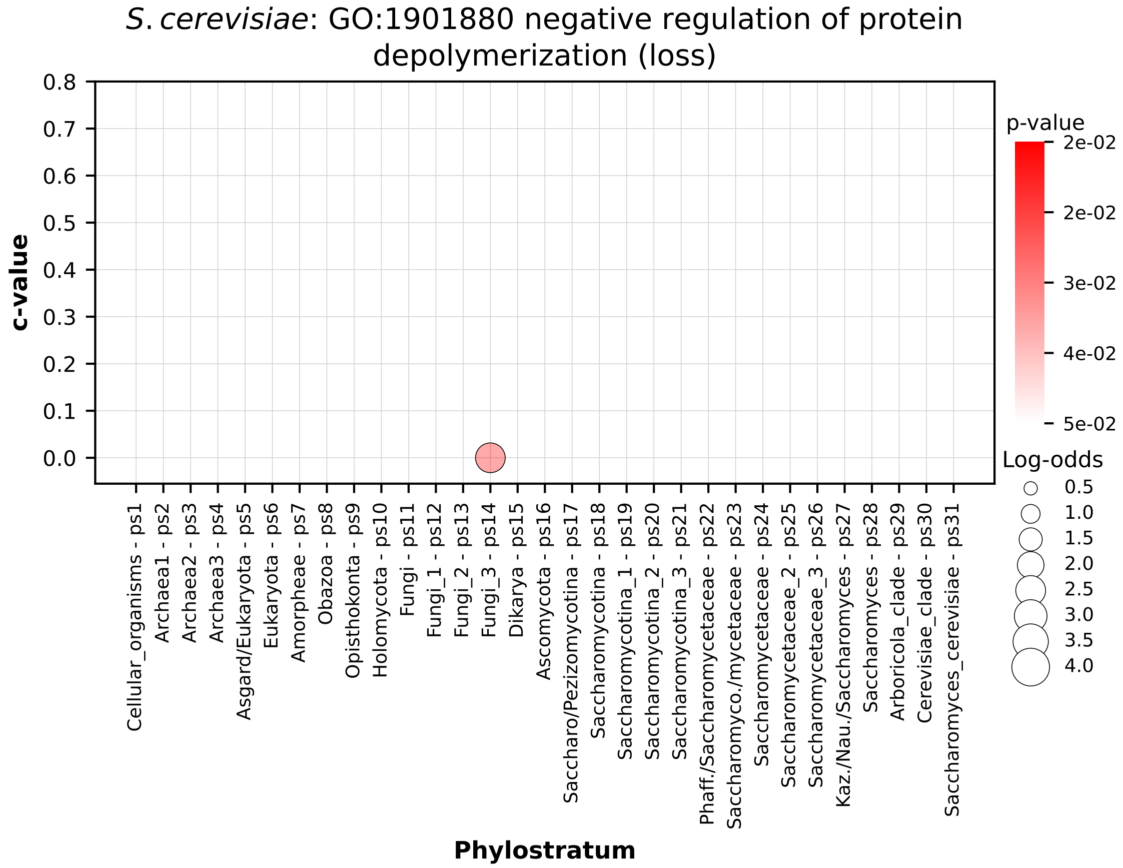

S. cerevisiae: GO:1901880 negative regulation of protein depolymerization (loss)
c-value
0.8
0.7
0.6
0.5
0.4
0.3
0.2
0.1
0.0
Phylostratum
Cellular_organisms - ps1
Archaea1 - ps2
Archaea2 - ps3
Archaea3 - ps4
Asgard/Eukaryota - ps5
Eukaryota - ps6
Amorpheae - ps7
Obazoa - ps8
Opisthokonta - ps9
Holomycota - ps10
Fungi - ps11
Fungi_1 - ps12
Fungi_2 - ps13
Fungi_3 - ps14
Dikarya - ps15
Ascomycota - ps16
Saccharo/Pezizomycotina - ps17
Saccharomycotina - ps18
Saccharomycotina_1 - ps19
Saccharomycotina_2 - ps20
Saccharomycotina_3 - ps21
Phaff./Saccharomycetaceae - ps22
Saccharomyco./mycetaceae - ps23
Saccharomycetaceae - ps24
Saccharomycetaceae_2 - ps25
Saccharomycetaceae_3 - ps26
Kaz./Nau./Saccharomyces - ps27
Saccharomyces - ps28
Arboricola_clade - ps29
Cerevisiae_clade - ps30
Saccharomyces_cerevisiae - ps31
p-value
2e-02
2e-02
3e-02
4e-02
5e-02
Log-odds
0.5
1.0
1.5
2.0
2.5
3.0
3.5
4.0

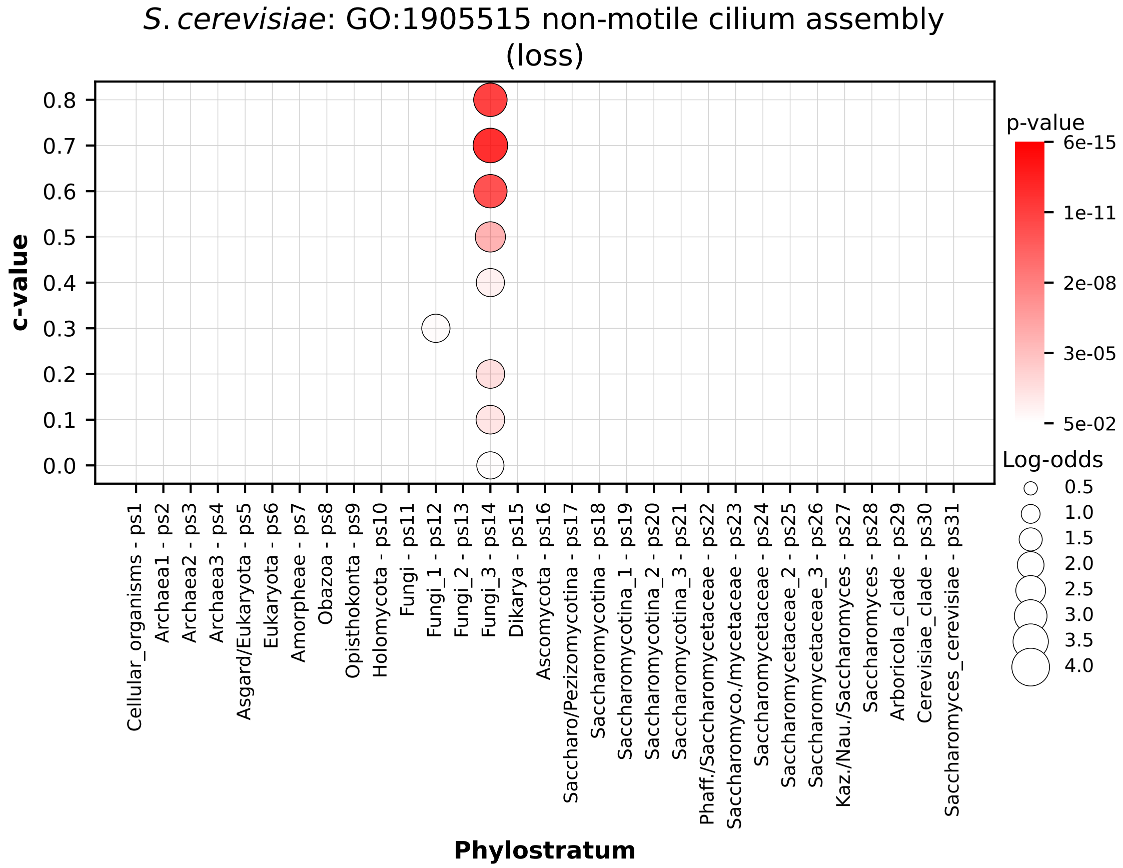

S. cerevisiae: GO:1905515 non-motile cilium assembly
(loss)
c-value
0.8
0.7
0.6
0.5
0.4
0.3
0.2
0.1
0.0
p-value
6e-15
1e-11
2e-08
3e-05
5e-02
Log-odds
0.5
1.0
1.5
2.0
2.5
3.0
3.5
4.0
Cellular_organisms - ps1
Archaea1 - ps2
Archaea2 - ps3
Archaea3 - ps4
Asgard/Eukaryota - ps5
Eukaryota - ps6
Amorpheae - ps7
Obazoa - ps8
Opisthokonta - ps9
Holomycota - ps10
Fungi - ps11
Fungi_1 - ps12
Fungi_2 - ps13
Fungi_3 - ps14
Dikarya - ps15
Ascomycota - ps16
Saccharo/Pezizomycotina - ps17
Saccharomycotina - ps18
Saccharomycotina_1 - ps19
Saccharomycotina_2 - ps20
Saccharomycotina_3 - ps21
Phaff./Saccharomycetaceae - ps22
Saccharomyco./mycetaceae - ps23
Saccharomycetaceae - ps24
Saccharomycetaceae_2 - ps25
Saccharomycetaceae_3 - ps26
Kaz./Nau./Saccharomyces - ps27
Saccharomyces - ps28
Arboricola_clade - ps29
Cerevisiae_clade - ps30
Saccharomyces_cerevisiae - ps31
Phylostratum

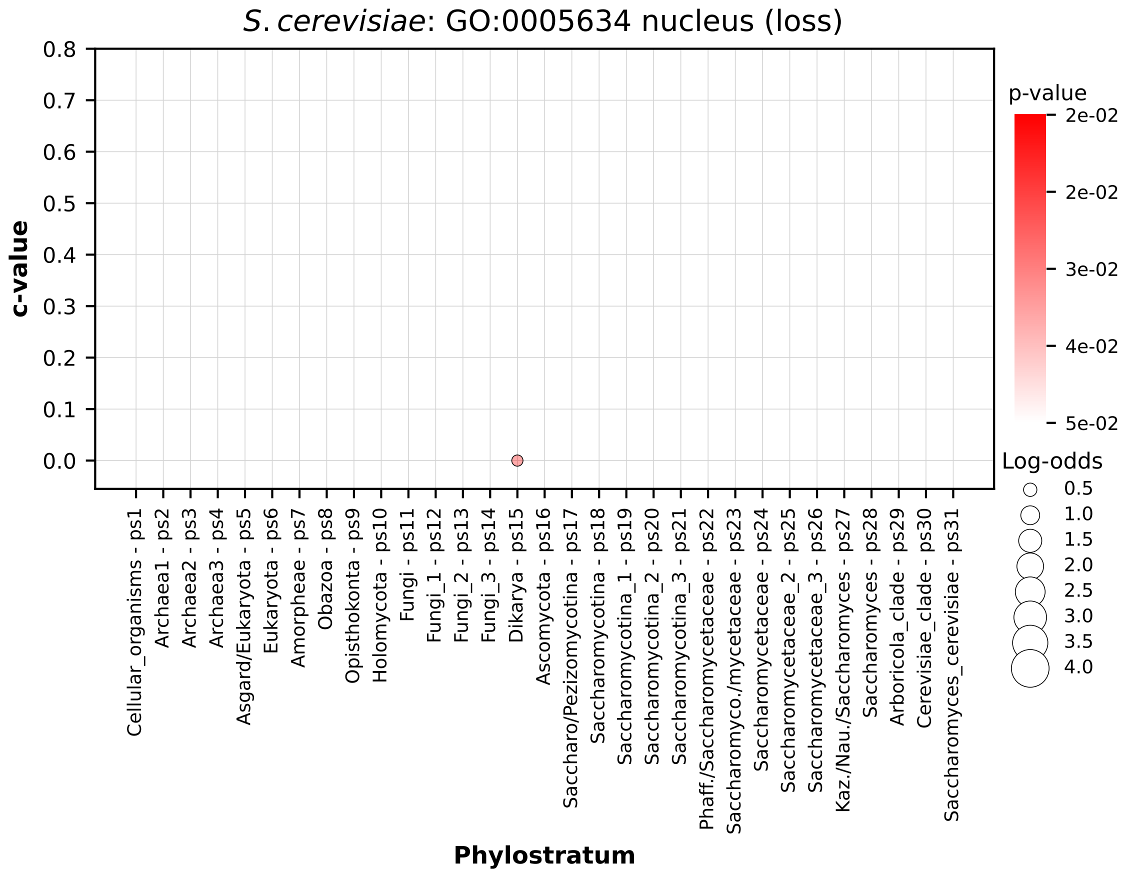

S. cerevisiae: GO:0005634 nucleus (loss)
c-value
0.8
0.7
0.6
0.5
0.4
0.3
0.2
0.1
0.0
p-value
2e-02
2e-02
3e-02
4e-02
5e-02
Log-odds
0.5
1.0
1.5
2.0
2.5
3.0
3.5
4.0
Cellular_organisms - ps1
Archaea1 - ps2
Archaea2 - ps3
Archaea3 - ps4
Asgard/Eukaryota - ps5
Eukaryota - ps6
Amorpheae - ps7
Obazoa - ps8
Opisthokonta - ps9
Holomycota - ps10
Fungi - ps11
Fungi_1 - ps12
Fungi_2 - ps13
Fungi_3 - ps14
Dikarya - ps15
Ascomycota - ps16
Saccharo/Pezizomycotina - ps17
Saccharomycotina - ps18
Saccharomycotina_1 - ps19
Saccharomycotina_2 - ps20
Saccharomycotina_3 - ps21
Phaff./Saccharomycetaceae - ps22
Saccharomyco./mycetaceae - ps23
Saccharomycetaceae - ps24
Saccharomycetaceae_2 - ps25
Saccharomycetaceae_3 - ps26
Kaz./Nau./Saccharomyces - ps27
Saccharomyces - ps28
Arboricola_clade - ps29
Cerevisiae_clade - ps30
Saccharomyces_cerevisiae - ps31
Phylostratum

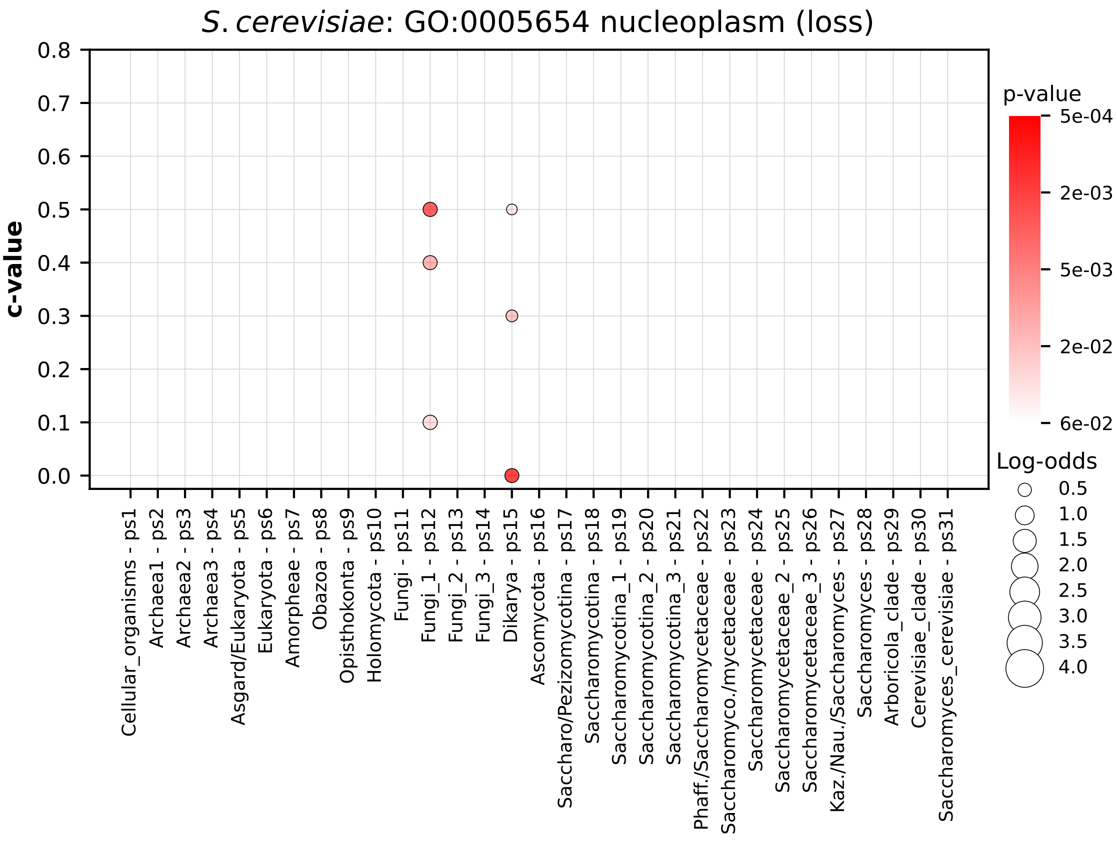
S. cerevisiae: GO:0005654 nucleoplasm (loss)
c-value
0.8
0.7
0.6
0.5
0.4
0.3
0.2
0.1
0.0
Phylostratum
Cellular_organisms - ps1
Archaea1 - ps2
Archaea2 - ps3
Archaea3 - ps4
Asgard/Eukaryota - ps5
Eukaryota - ps6
Amorpheae - ps7
Obazoa - ps8
Opisthokonta - ps9
Holomycota - ps10
Fungi - ps11
Fungi_1 - ps12
Fungi_2 - ps13
Fungi_3 - ps14
Dikarya - ps15
Ascomycota - ps16
Saccharo/Pezizomycotina - ps17
Saccharomycotina - ps18
Saccharomycotina_1 - ps19
Saccharomycotina_2 - ps20
Saccharomycotina_3 - ps21
Phaff./Saccharomycetaceae - ps22
Saccharomyco./mycetaceae - ps23
Saccharomycetaceae - ps24
Saccharomycetaceae_2 - ps25
Saccharomycetaceae_3 - ps26
Kaz./Nau./Saccharomyces - ps27
Saccharomyces - ps28
Arboricola_clade - ps29
Cerevisiae_clade - ps30
Saccharomyces_cerevisiae - ps31
p-value
5e-04
2e-03
5e-03
2e-02
6e-02
Log-odds
0.5
1.0
1.5
2.0
2.5
3.0
3.5
4.0

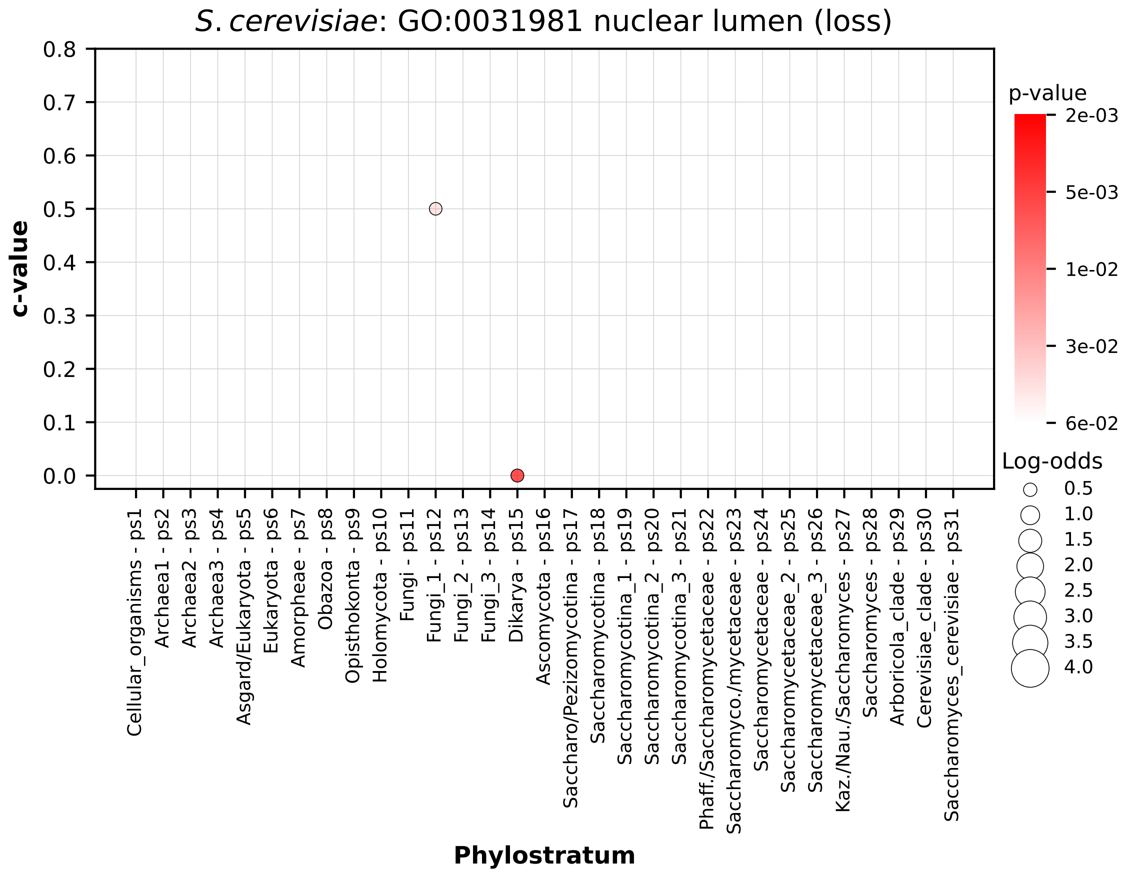

S. cerevisiae: GO:0031981 nuclear lumen (loss)
c-value
0.8
0.7
0.6
0.5
0.4
0.3
0.2
0.1
0.0
p-value
2e-03
5e-03
1e-02
3e-02
6e-02
Log-odds
0.5
1.0
1.5
2.0
2.5
3.0
3.5
4.0
Cellular_organisms - ps1
Archaea1 - ps2
Archaea2 - ps3
Archaea3 - ps4
Asgard/Eukaryota - ps5
Eukaryota - ps6
Amorpheae - ps7
Obazoa - ps8
Opisthokonta - ps9
Holomycota - ps10
Fungi - ps11
Fungi_1 - ps12
Fungi_2 - ps13
Fungi_3 - ps14
Dikarya - ps15
Ascomycota - ps16
Saccharo/Pezizomycotina - ps17
Saccharomycotina - ps18
Saccharomycotina_1 - ps19
Saccharomycotina_2 - ps20
Saccharomycotina_3 - ps21
Phaff./Saccharomycetaceae - ps22
Saccharomyco./mycetaceae - ps23
Saccharomycetaceae - ps24
Saccharomycetaceae_2 - ps25
Saccharomycetaceae_3 - ps26
Kaz./Nau./Saccharomyces - ps27
Saccharomyces - ps28
Arboricola_clade - ps29
Cerevisiae_clade - ps30
Saccharomyces_cerevisiae - ps31
Phylostratum

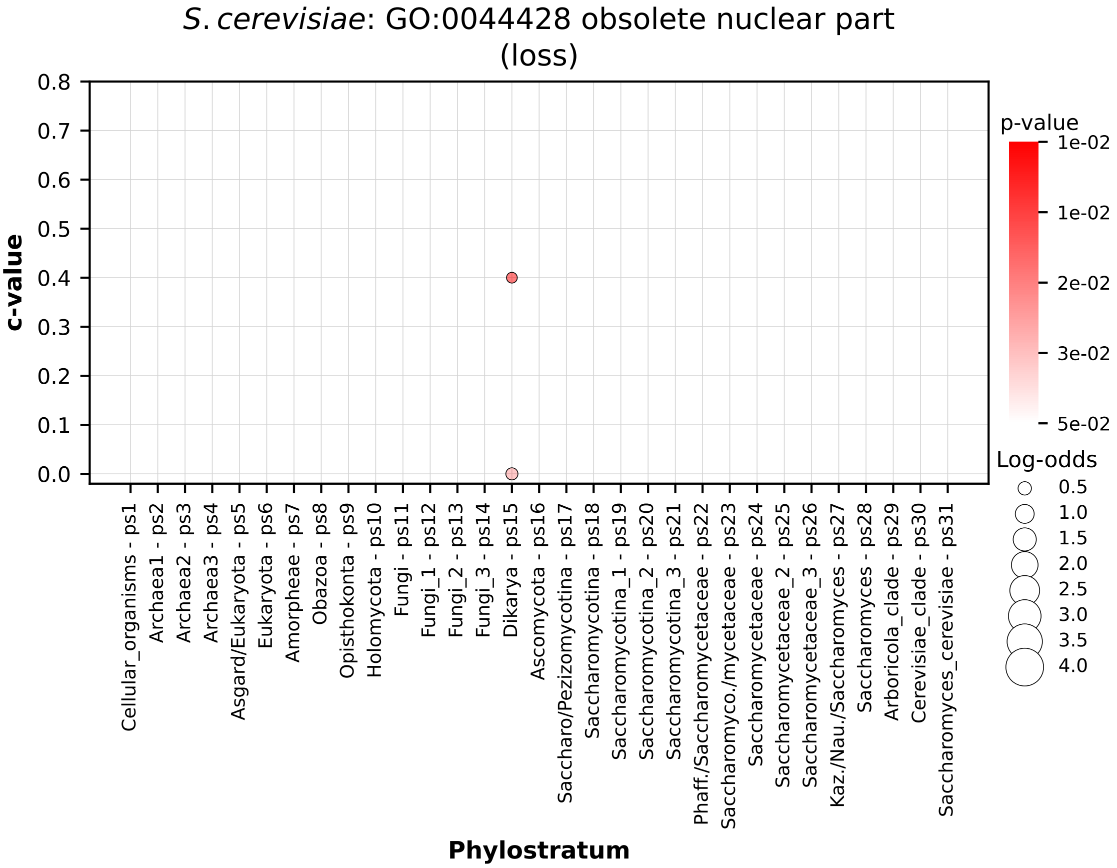

S. cerevisiae: GO:0044428 obsolete nuclear part
(loss)
c-value
0.8
0.7
0.6
0.5
0.4
0.3
0.2
0.1
0.0
Phylostratum
Cellular_organisms - ps1
Archaea1 - ps2
Archaea2 - ps3
Archaea3 - ps4
Asgard/Eukaryota - ps5
Eukaryota - ps6
Amorpheae - ps7
Obazoa - ps8
Opisthokonta - ps9
Holomycota - ps10
Fungi - ps11
Fungi_1 - ps12
Fungi_2 - ps13
Fungi_3 - ps14
Dikarya - ps15
Ascomycota - ps16
Saccharo/Pezizomycotina - ps17
Saccharomycotina - ps18
Saccharomycotina_1 - ps19
Saccharomycotina_2 - ps20
Saccharomycotina_3 - ps21
Phaff./Saccharomycetaceae - ps22
Saccharomyco./mycetaceae - ps23
Saccharomycetaceae - ps24
Saccharomycetaceae_2 - ps25
Saccharomycetaceae_3 - ps26
Kaz./Nau./Saccharomyces - ps27
Saccharomyces - ps28
Arboricola_clade - ps29
Cerevisiae_clade - ps30
Saccharomyces_cerevisiae - ps31
p-value
1e-02
1e-02
2e-02
3e-02
5e-02
Log-odds
0.5
1.0
1.5
2.0
2.5
3.0
3.5
4.0

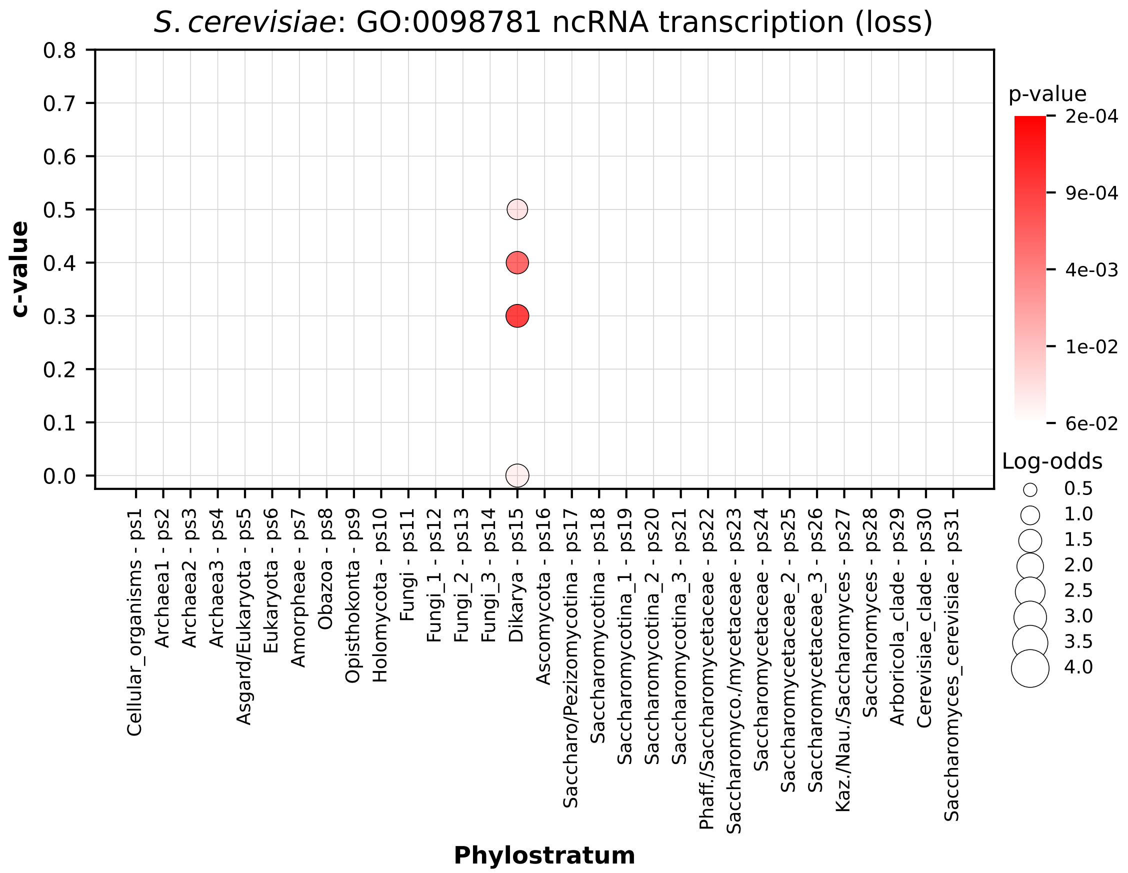

S. cerevisiae: GO:0098781 ncRNA transcription (loss)
c-value
0.8
0.7
0.6
0.5
0.4
0.3
0.2
0.1
0.0
p-value
2e-04
9e-04
4e-03
1e-02
6e-02
Log-odds
0.5
1.0
1.5
2.0
2.5
3.0
3.5
4.0
Cellular_organisms - ps1
Archaea1 - ps2
Archaea2 - ps3
Archaea3 - ps4
Asgard/Eukaryota - ps5
Eukaryota - ps6
Amorpheae - ps7
Obazoa - ps8
Opisthokonta - ps9
Holomycota - ps10
Fungi - ps11
Fungi_1 - ps12
Fungi_2 - ps13
Fungi_3 - ps14
Dikarya - ps15
Ascomycota - ps16
Saccharo/Pezizomycotina - ps17
Saccharomycotina - ps18
Saccharomycotina_1 - ps19
Saccharomycotina_2 - ps20
Saccharomycotina_3 - ps21
Phaff./Saccharomycetaceae - ps22
Saccharomyco./mycetaceae - ps23
Saccharomycetaceae - ps24
Saccharomycetaceae_2 - ps25
Saccharomycetaceae_3 - ps26
Kaz./Nau./Saccharomyces - ps27
Saccharomyces - ps28
Arboricola_clade - ps29
Cerevisiae_clade - ps30
Saccharomyces_cerevisiae - ps31
Phylostratum

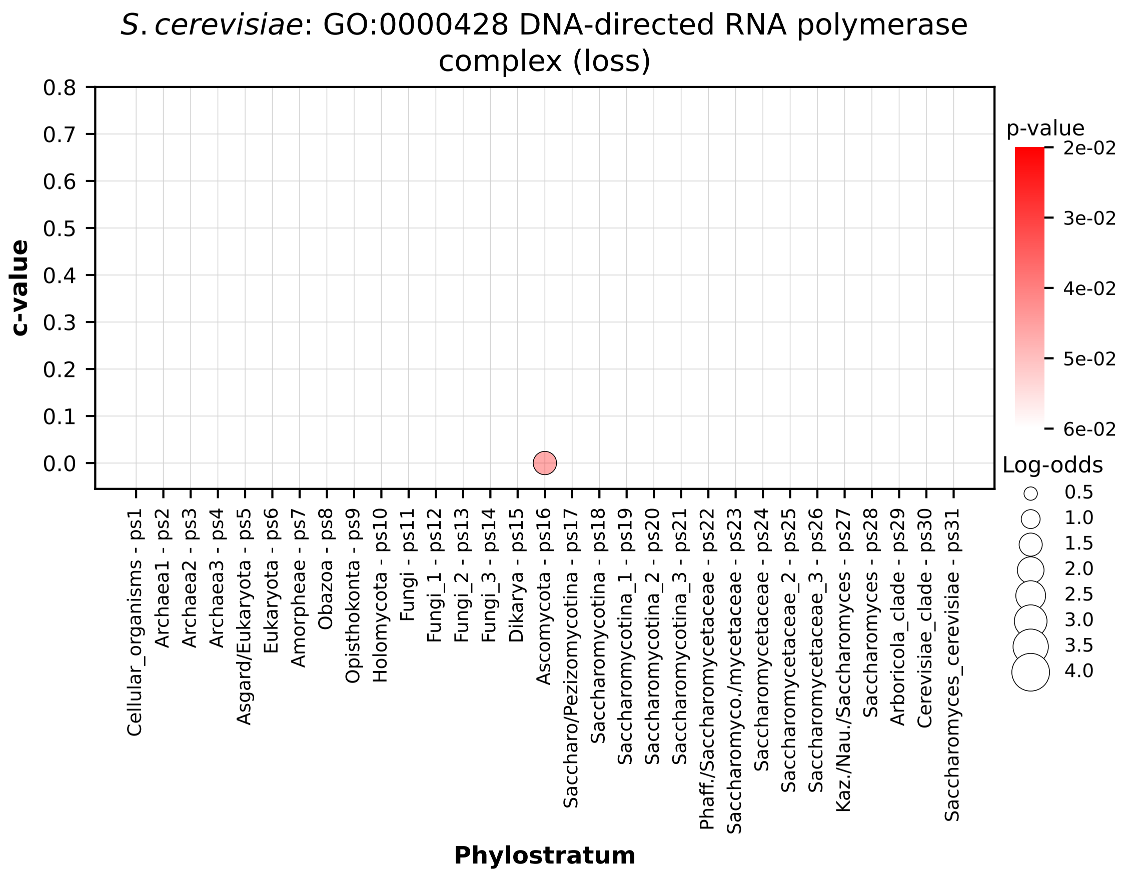

S. cerevisiae: GO:0000428 DNA-directed RNA polymerase complex (loss)
c-value
0.8
0.7
0.6
0.5
0.4
0.3
0.2
0.1
0.0
p-value
2e-02
3e-02
4e-02
5e-02
6e-02
Log-odds
0.5
1.0
1.5
2.0
2.5
3.0
3.5
4.0
Cellular_organisms - ps1
Archaea1 - ps2
Archaea2 - ps3
Archaea3 - ps4
Asgard/Eukaryota - ps5
Eukaryota - ps6
Amorpheae - ps7
Obazoa - ps8
Opisthokonta - ps9
Holomycota - ps10
Fungi - ps11
Fungi_1 - ps12
Fungi_2 - ps13
Fungi_3 - ps14
Dikarya - ps15
Ascomycota - ps16
Saccharo/Pezizomycotina - ps17
Saccharomycotina - ps18
Saccharomycotina_1 - ps19
Saccharomycotina_2 - ps20
Saccharomycotina_3 - ps21
Phaff./Saccharomycetaceae - ps22
Saccharomyco./mycetaceae - ps23
Saccharomycetaceae - ps24
Saccharomycetaceae_2 - ps25
Saccharomycetaceae_3 - ps26
Kaz./Nau./Saccharomyces - ps27
Saccharomyces - ps28
Arboricola_clade - ps29
Cerevisiae_clade - ps30
Saccharomyces_cerevisiae - ps31
Phylostratum

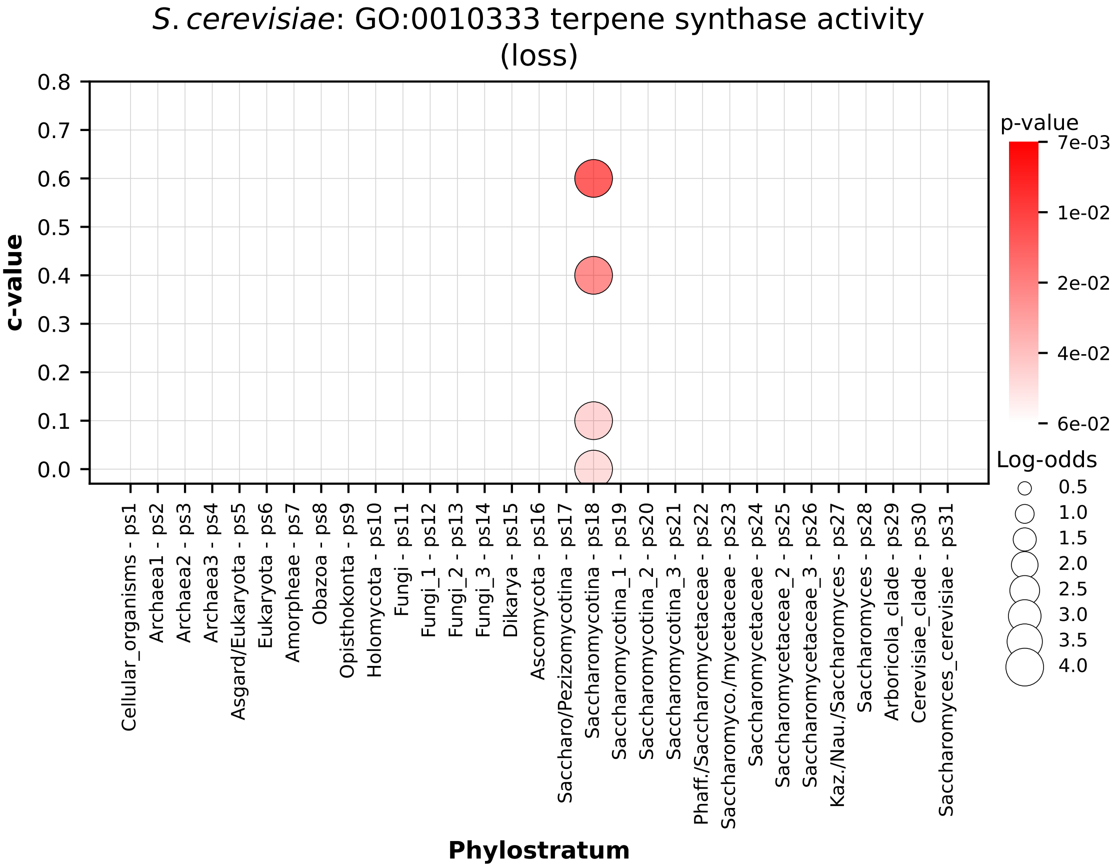

S. cerevisiae: GO:0010333 terpene synthase activity
(loss)
c-value
0.8
0.7
0.6
0.5
0.4
0.3
0.2
0.1
0.0
p-value
7e-03
1e-02
2e-02
4e-02
6e-02
Log-odds
0.5
1.0
1.5
2.0
2.5
3.0
3.5
4.0
Cellular_organisms - ps1
Archaea1 - ps2
Archaea2 - ps3
Archaea3 - ps4
Asgard/Eukaryota - ps5
Eukaryota - ps6
Amorpheae - ps7
Obazoa - ps8
Opisthokonta - ps9
Holomycota - ps10
Fungi - ps11
Fungi_1 - ps12
Fungi_2 - ps13
Fungi_3 - ps14
Dikarya - ps15
Ascomycota - ps16
Saccharo/Pezizomycotina - ps17
Saccharomycotina - ps18
Saccharomycotina_1 - ps19
Saccharomycotina_2 - ps20
Saccharomycotina_3 - ps21
Phaff./Saccharomycetaceae - ps22
Saccharomyco./mycetaceae - ps23
Saccharomycetaceae - ps24
Saccharomycetaceae_2 - ps25
Saccharomycetaceae_3 - ps26
Kaz./Nau./Saccharomyces - ps27
Saccharomyces - ps28
Arboricola_clade - ps29
Cerevisiae_clade - ps30
Saccharomyces_cerevisiae - ps31
Phylostratum

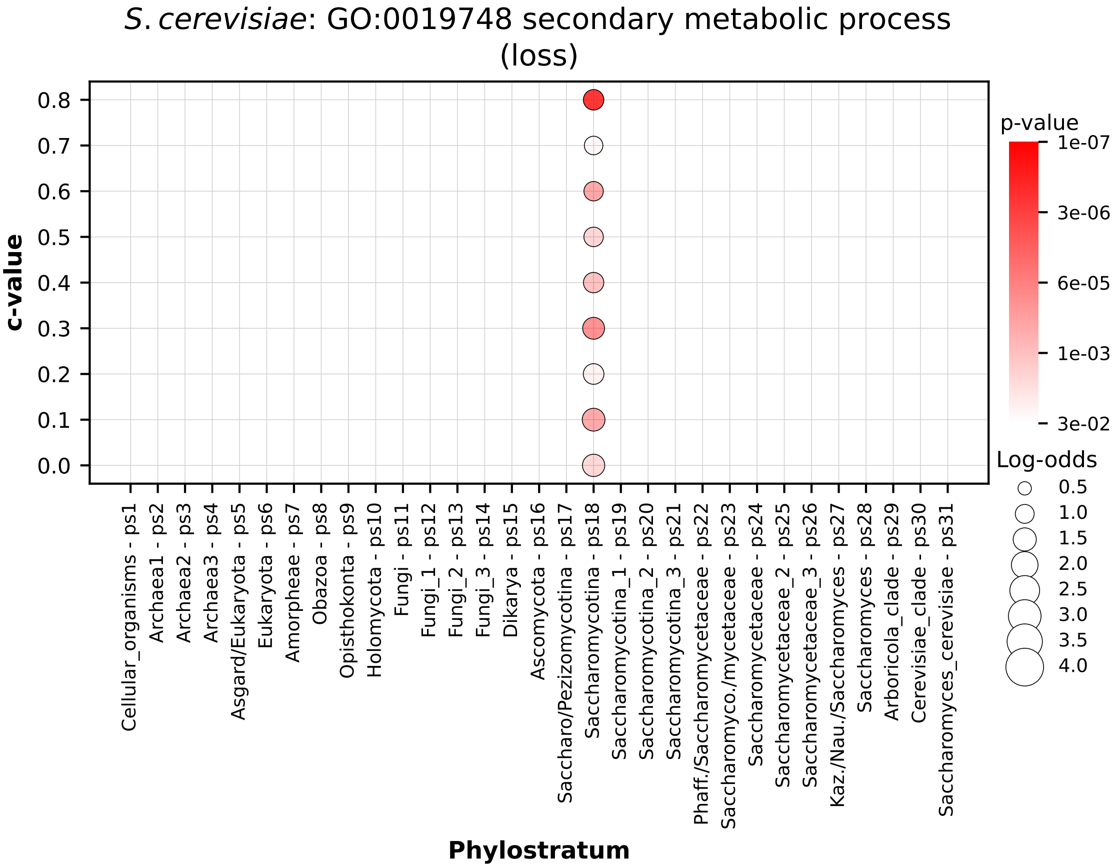

S. cerevisiae: GO:0019748 secondary metabolic process
(loss)
c-value
0.8
0.7
0.6
0.5
0.4
0.3
0.2
0.1
0.0
Phylostratum
Cellular_organisms - ps1
Archaea1 - ps2
Archaea2 - ps3
Archaea3 - ps4
Asgard/Eukaryota - ps5
Eukaryota - ps6
Amorpheae - ps7
Obazoa - ps8
Opisthokonta - ps9
Holomycota - ps10
Fungi - ps11
Fungi_1 - ps12
Fungi_2 - ps13
Fungi_3 - ps14
Dikarya - ps15
Ascomycota - ps16
Saccharo/Pezizomycotina - ps17
Saccharomycotina - ps18
Saccharomycotina_1 - ps19
Saccharomycotina_2 - ps20
Saccharomycotina_3 - ps21
Phaff./Saccharomycetaceae - ps22
Saccharomyco./mycetaceae - ps23
Saccharomycetaceae - ps24
Saccharomycetaceae_2 - ps25
Saccharomycetaceae_3 - ps26
Kaz./Nau./Saccharomyces - ps27
Saccharomyces - ps28
Arboricola_clade - ps29
Cerevisiae_clade - ps30
Saccharomyces_cerevisiae - ps31
p-value
1e-07
3e-06
6e-05
1e-03
3e-02
Log-odds
0.5
1.0
1.5
2.0
2.5
3.0
3.5
4.0

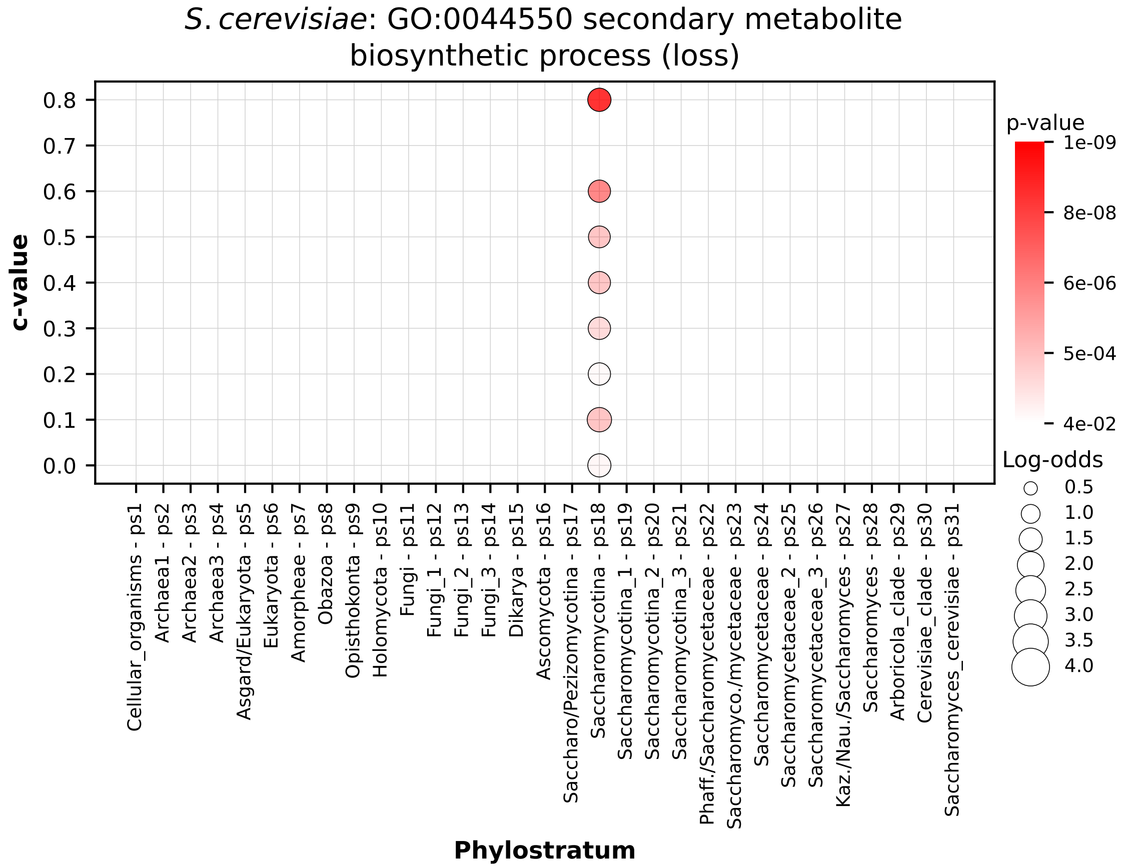
S. cerevisiae: GO:0044550 secondary metabolite biosynthetic process (loss)
c-value
0.8
0.7
0.6
0.5
0.4
0.3
0.2
0.1
0.0
p-value
1e-09
8e-08
6e-06
5e-04
4e-02
Log-odds
0.5
1.0
1.5
2.0
2.5
3.0
3.5
4.0
Cellular_organisms - ps1
Archaea1 - ps2
Archaea2 - ps3
Archaea3 - ps4
Asgard/Eukaryota - ps5
Eukaryota - ps6
Amorpheae - ps7
Obazoa - ps8
Opisthokonta - ps9
Holomycota - ps10
Fungi - ps11
Fungi_1 - ps12
Fungi_2 - ps13
Fungi_3 - ps14
Dikarya - ps15
Ascomycota - ps16
Saccharo/Pezizomycotina - ps17
Saccharomycotina - ps18
Saccharomycotina_1 - ps19
Saccharomycotina_2 - ps20
Saccharomycotina_3 - ps21
Phaff./Saccharomycetaceae - ps22
Saccharomyco./mycetaceae - ps23
Saccharomycetaceae - ps24
Saccharomycetaceae_2 - ps25
Saccharomycetaceae_3 - ps26
Kaz./Nau./Saccharomyces - ps27
Saccharomyces - ps28
Arboricola_clade - ps29
Cerevisiae_clade - ps30
Saccharomyces_cerevisiae - ps31
Phylostratum

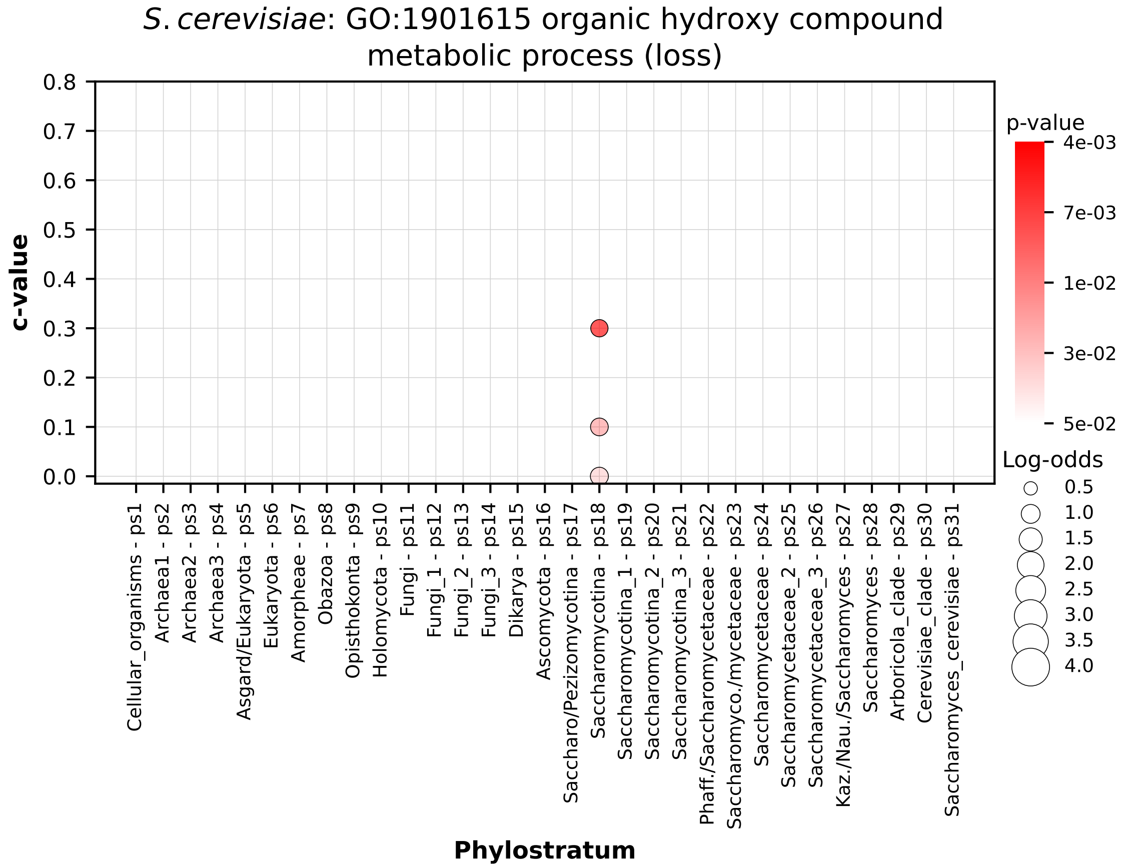

S. cerevisiae: GO:1901615 organic hydroxy compound metabolic process (loss)
c-value
0.8
0.7
0.6
0.5
0.4
0.3
0.2
0.1
0.0
Phylostratum
Cellular_organisms - ps1
Archaea1 - ps2
Archaea2 - ps3
Archaea3 - ps4
Asgard/Eukaryota - ps5
Eukaryota - ps6
Amorpheae - ps7
Obazoa - ps8
Opisthokonta - ps9
Holomycota - ps10
Fungi - ps11
Fungi_1 - ps12
Fungi_2 - ps13
Fungi_3 - ps14
Dikarya - ps15
Ascomycota - ps16
Saccharo/Pezizomycotina - ps17
Saccharomycotina - ps18
Saccharomycotina_1 - ps19
Saccharomycotina_2 - ps20
Saccharomycotina_3 - ps21
Phaff./Saccharomycetaceae - ps22
Saccharomyco./mycetaceae - ps23
Saccharomycetaceae - ps24
Saccharomycetaceae_2 - ps25
Saccharomycetaceae_3 - ps26
Kaz./Nau./Saccharomyces - ps27
Saccharomyces - ps28
Arboricola_clade - ps29
Cerevisiae_clade - ps30
Saccharomyces_cerevisiae - ps31
p-value
4e-03
7e-03
1e-02
3e-02
5e-02
Log-odds
0.5
1.0
1.5
2.0
2.5
3.0
3.5
4.0

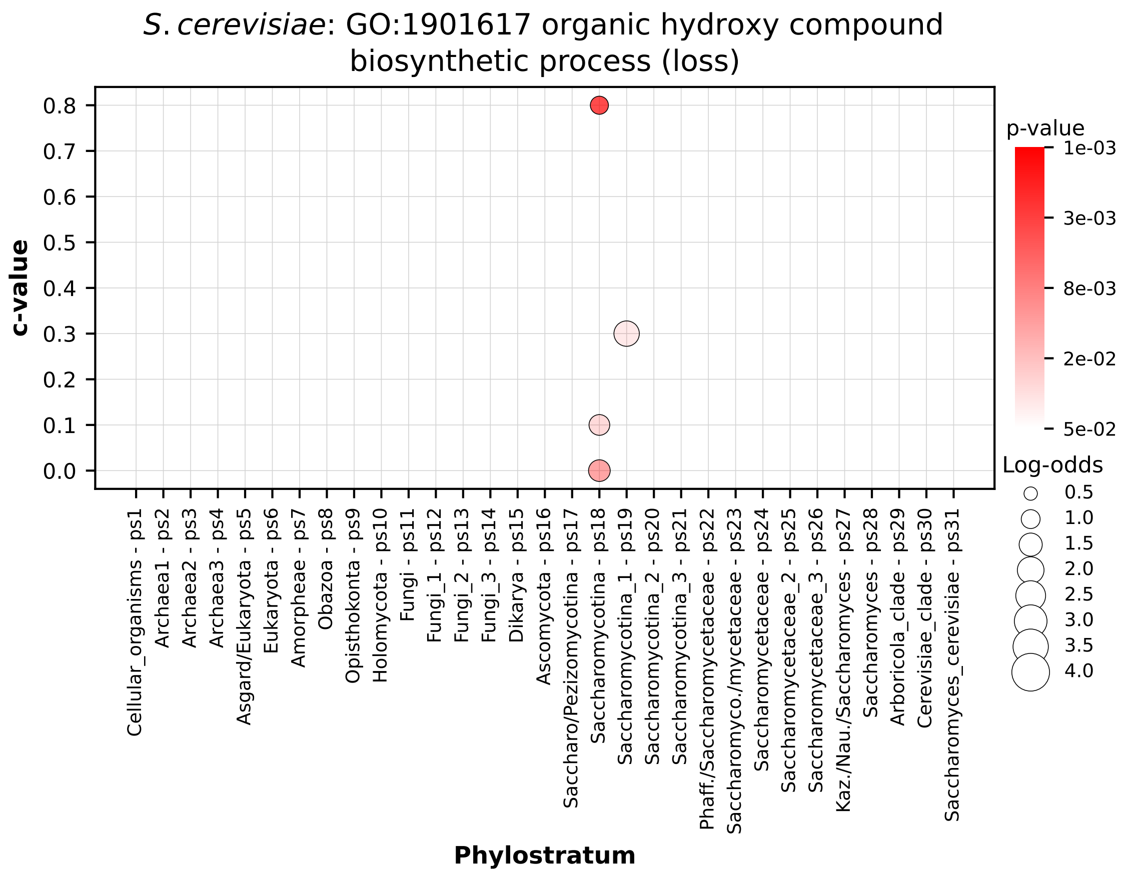
S. cerevisiae: GO:1901617 organic hydroxy compound biosynthetic process (loss)
c-value
0.8
0.7
0.6
0.5
0.4
0.3
0.2
0.1
0.0
p-value
1e-03
3e-03
8e-03
2e-02
5e-02
Log-odds
0.5
1.0
1.5
2.0
2.5
3.0
3.5
4.0
Cellular_organisms - ps1
Archaea1 - ps2
Archaea2 - ps3
Archaea3 - ps4
Asgard/Eukaryota - ps5
Eukaryota - ps6
Amorpheae - ps7
Obazoa - ps8
Opisthokonta - ps9
Holomycota - ps10
Fungi - ps11
Fungi_1 - ps12
Fungi_2 - ps13
Fungi_3 - ps14
Dikarya - ps15
Ascomycota - ps16
Saccharo/Pezizomycotina - ps17
Saccharomycotina - ps18
Saccharomycotina_1 - ps19
Saccharomycotina_2 - ps20
Saccharomycotina_3 - ps21
Phaff./Saccharomycetaceae - ps22
Saccharomyco./mycetaceae - ps23
Saccharomycetaceae - ps24
Saccharomycetaceae_2 - ps25
Saccharomycetaceae_3 - ps26
Kaz./Nau./Saccharomyces - ps27
Saccharomyces - ps28
Arboricola_clade - ps29
Cerevisiae_clade - ps30
Saccharomyces_cerevisiae - ps31
Phylostratum

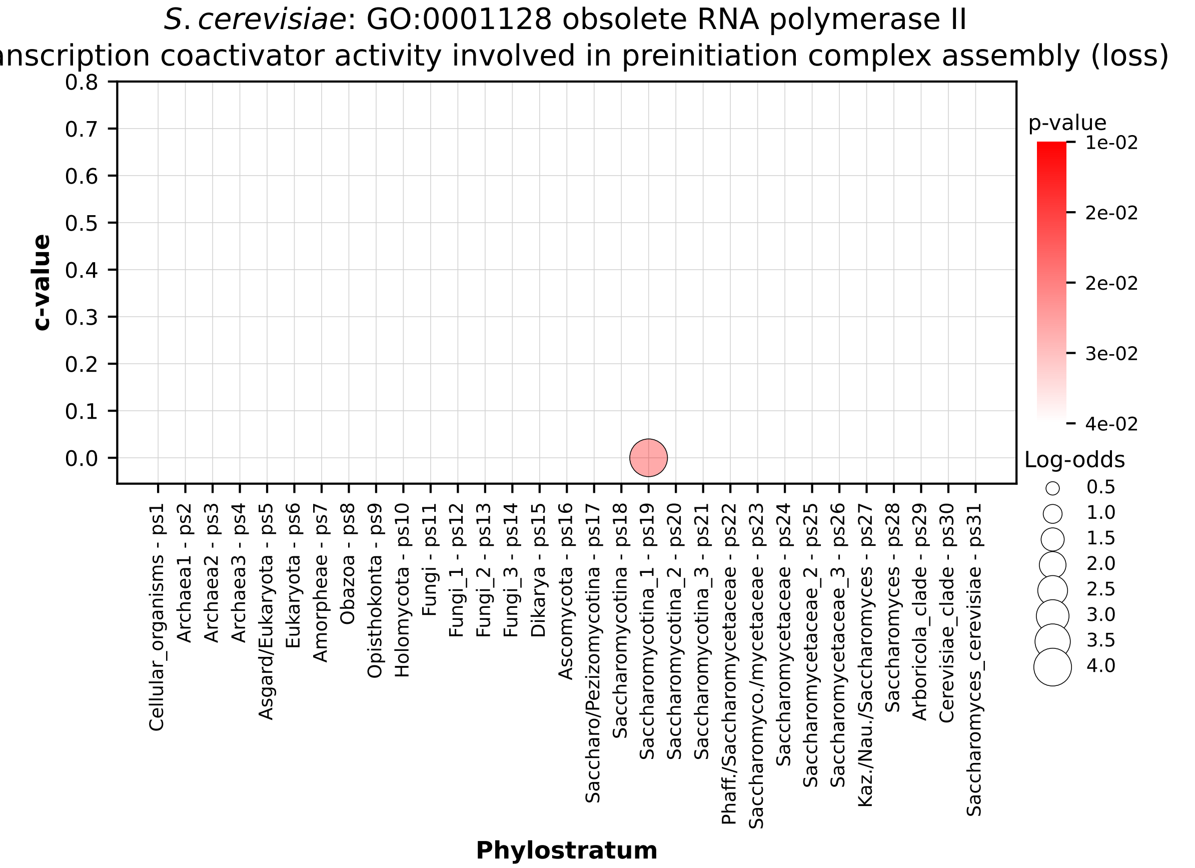

S. cerevisiae: GO:0001128 obsolete RNA polymerase II
anscription coactivator activity involved in preinitiation complex assembly (loss)
c-value
0.8
0.7
0.6
0.5
0.4
0.3
0.2
0.1
0.0
p-value
1e-02
2e-02
2e-02
3e-02
4e-02
Log-odds
0.5
1.0
1.5
2.0
2.5
3.0
3.5
4.0
Cellular_organisms - ps1
Archaea1 - ps2
Archaea2 - ps3
Archaea3 - ps4
Asgard/Eukaryota - ps5
Eukaryota - ps6
Amorpheae - ps7
Obazoa - ps8
Opisthokonta - ps9
Holomycota - ps10
Fungi - ps11
Fungi_1 - ps12
Fungi_2 - ps13
Fungi_3 - ps14
Dikarya - ps15
Ascomycota - ps16
Saccharo/Pezizomycotina - ps17
Saccharomycotina - ps18
Saccharomycotina_1 - ps19
Saccharomycotina_2 - ps20
Saccharomycotina_3 - ps21
Phaff./Saccharomycetaceae - ps22
Saccharomyco./mycetaceae - ps23
Saccharomycetaceae - ps24
Saccharomycetaceae_2 - ps25
Saccharomycetaceae_3 - ps26
Kaz./Nau./Saccharomyces - ps27
Saccharomyces - ps28
Arboricola_clade - ps29
Cerevisiae_clade - ps30
Saccharomyces_cerevisiae - ps31
Phylostratum

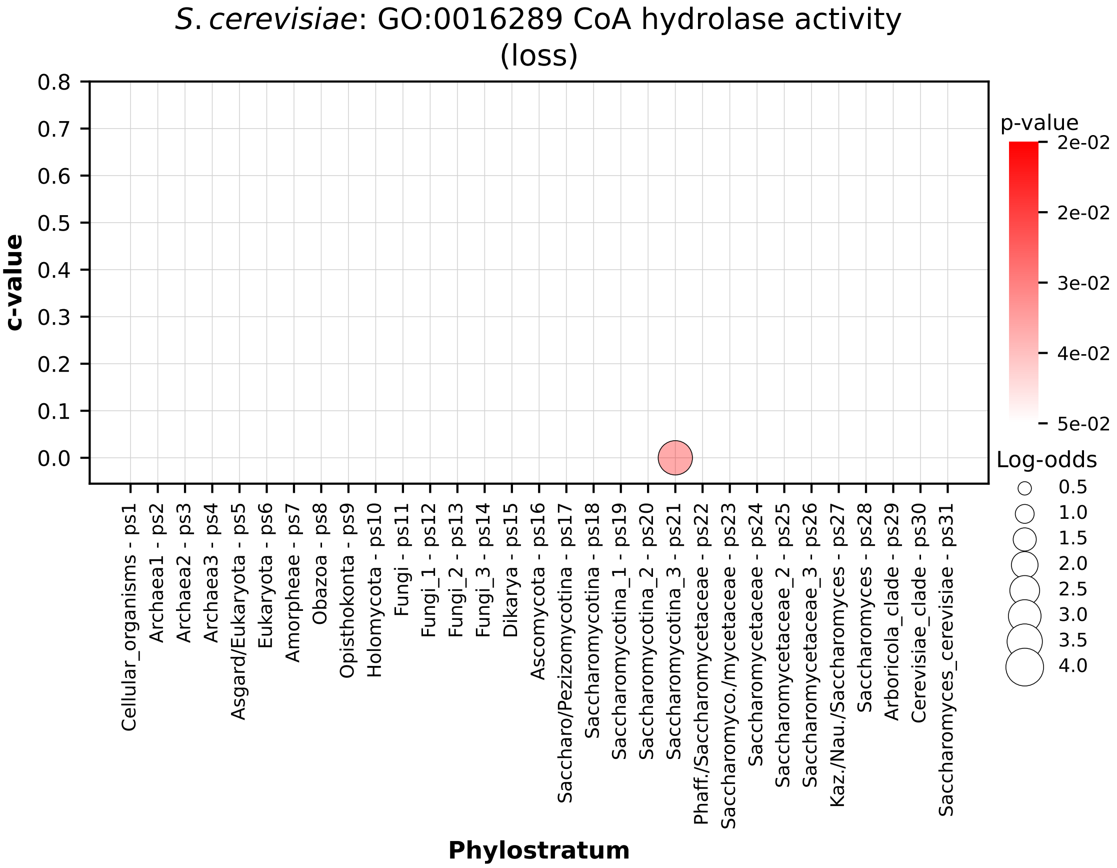

S. cerevisiae: GO:0016289 CoA hydrolase activity (loss)
c-value
0.8
0.7
0.6
0.5
0.4
0.3
0.2
0.1
0.0
p-value
2e-02
2e-02
3e-02
4e-02
5e-02
Log-odds
0.5
1.0
1.5
2.0
2.5
3.0
3.5
4.0
Cellular_organisms - ps1
Archaea1 - ps2
Archaea2 - ps3
Archaea3 - ps4
Asgard/Eukaryota - ps5
Eukaryota - ps6
Amorpheae - ps7
Obazoa - ps8
Opisthokonta - ps9
Holomycota - ps10
Fungi - ps11
Fungi_1 - ps12
Fungi_2 - ps13
Fungi_3 - ps14
Dikarya - ps15
Ascomycota - ps16
Saccharo/Pezizomycotina - ps17
Saccharomycotina - ps18
Saccharomycotina_1 - ps19
Saccharomycotina_2 - ps20
Saccharomycotina_3 - ps21
Phaff./Saccharomycetaceae - ps22
Saccharomyco./mycetaceae - ps23
Saccharomycetaceae - ps24
Saccharomycetaceae_2 - ps25
Saccharomycetaceae_3 - ps26
Kaz./Nau./Saccharomyces - ps27
Saccharomyces - ps28
Arboricola_clade - ps29
Cerevisiae_clade - ps30
Saccharomyces_cerevisiae - ps31
Phylostratum

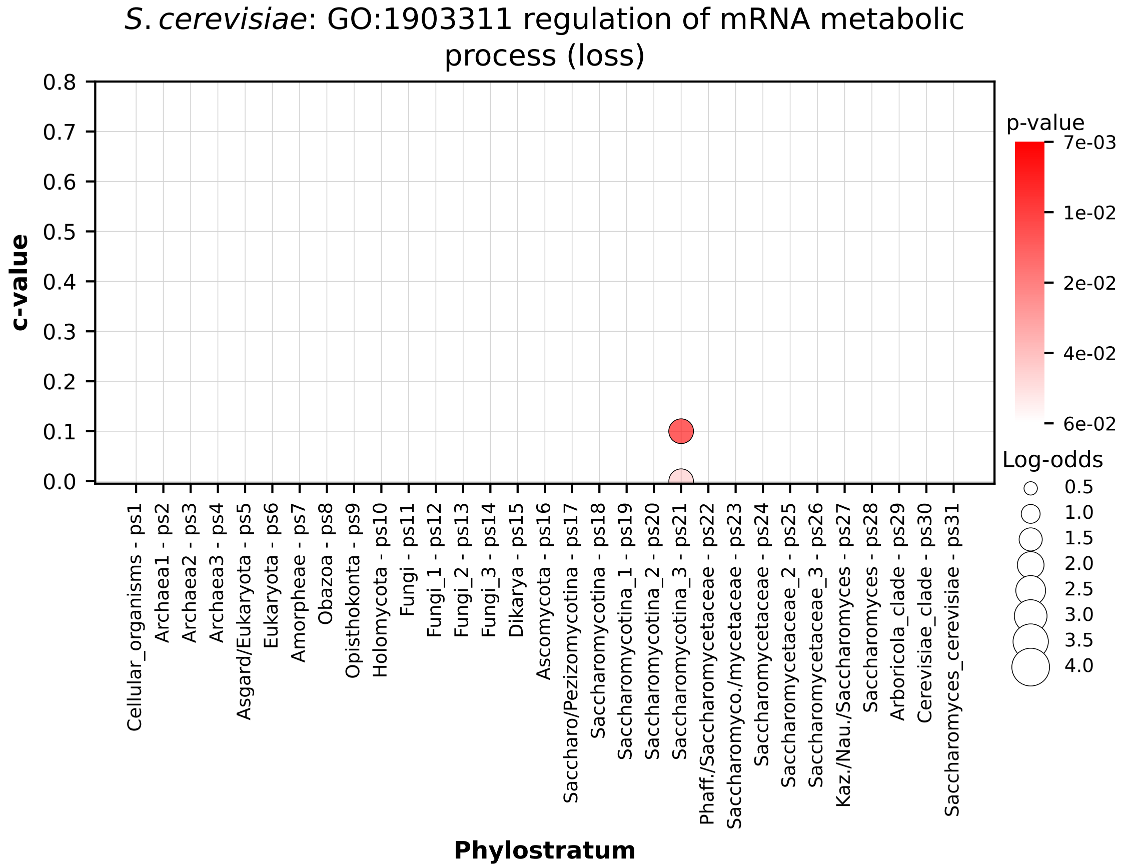
S. cerevisiae: GO:1903311 regulation of mRNA metabolic process (loss)
c-value
0.8
0.7
0.6
0.5
0.4
0.3
0.2
0.1
0.0
Phylostratum
Cellular_organisms - ps1
Archaea1 - ps2
Archaea2 - ps3
Archaea3 - ps4
Asgard/Eukaryota - ps5
Eukaryota - ps6
Amorpheae - ps7
Obazoa - ps8
Opisthokonta - ps9
Holomycota - ps10
Fungi - ps11
Fungi_1 - ps12
Fungi_2 - ps13
Fungi_3 - ps14
Dikarya - ps15
Ascomycota - ps16
Saccharo/Pezizomycotina - ps17
Saccharomycotina - ps18
Saccharomycotina_1 - ps19
Saccharomycotina_2 - ps20
Saccharomycotina_3 - ps21
Phaff./Saccharomycetaceae - ps22
Saccharomyco./mycetaceae - ps23
Saccharomycetaceae - ps24
Saccharomycetaceae_2 - ps25
Saccharomycetaceae_3 - ps26
Kaz./Nau./Saccharomyces - ps27
Saccharomyces - ps28
Arboricola_clade - ps29
Cerevisiae_clade - ps30
Saccharomyces_cerevisiae - ps31
p-value
7e-03
1e-02
2e-02
4e-02
6e-02
Log-odds
0.5
1.0
1.5
2.0
2.5
3.0
3.5
4.0

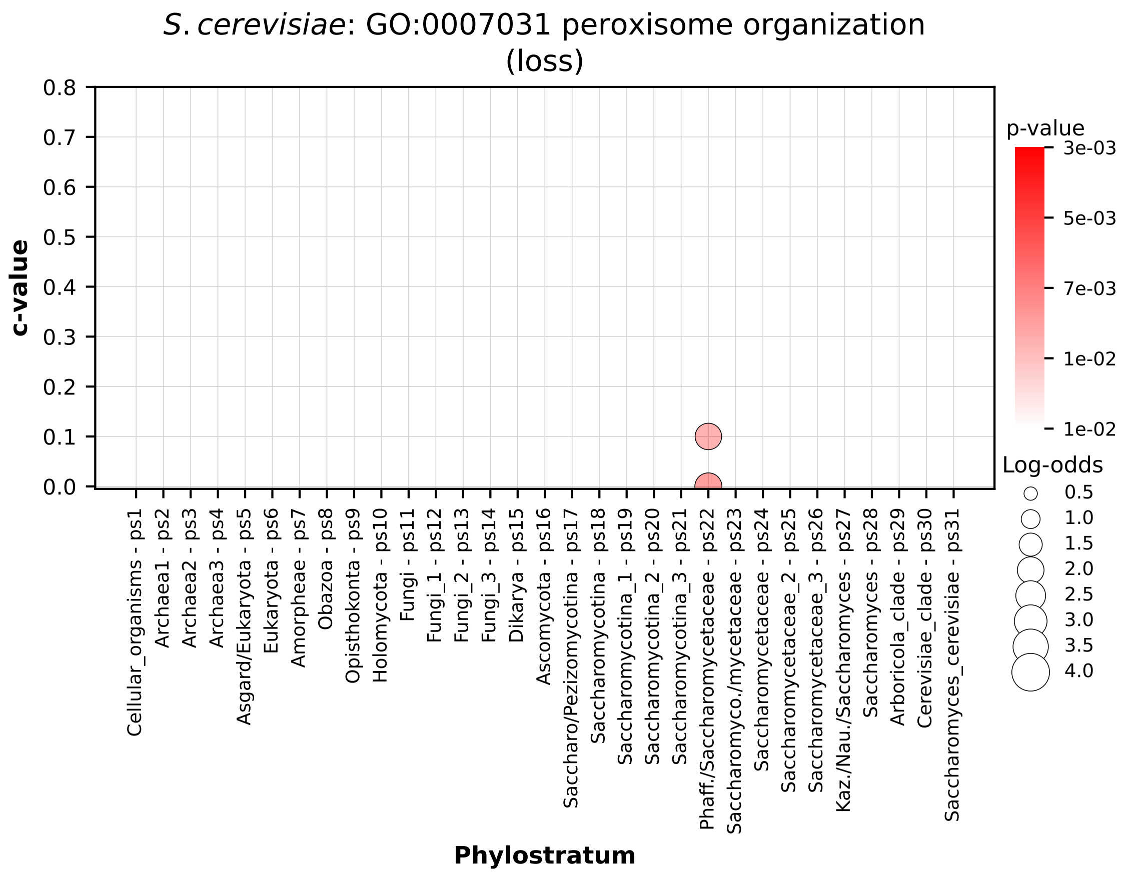

S. cerevisiae: GO:0007031 peroxisome organization
(loss)
c-value
0.0
0.1
0.2
0.3
0.4
0.5
0.6
0.7
0.8
Phylostratum
Cellular_organisms - ps1
Archaea1 - ps2
Archaea2 - ps3
Archaea3 - ps4
Asgard/Eukaryota - ps5
Eukaryota - ps6
Amorpheae - ps7
Obazoa - ps8
Opisthokonta - ps9
Holomycota - ps10
Fungi - ps11
Fungi_1 - ps12
Fungi_2 - ps13
Fungi_3 - ps14
Dikarya - ps15
Ascomycota - ps16
Saccharo/Pezizomycotina - ps17
Saccharomycotina - ps18
Saccharomycotina_1 - ps19
Saccharomycotina_2 - ps20
Saccharomycotina_3 - ps21
Phaff./Saccharomycetaceae - ps22
Saccharomyco./mycetaceae - ps23
Saccharomycetaceae - ps24
Saccharomycetaceae_2 - ps25
Saccharomycetaceae_3 - ps26
Kaz./Nau./Saccharomyces - ps27
Saccharomyces - ps28
Arboricola_clade - ps29
Cerevisiae_clade - ps30
Saccharomyces_cerevisiae - ps31
p-value
3e-03
5e-03
7e-03
1e-02
1e-02
Log-odds
0.5
1.0
1.5
2.0
2.5
3.0
3.5
4.0

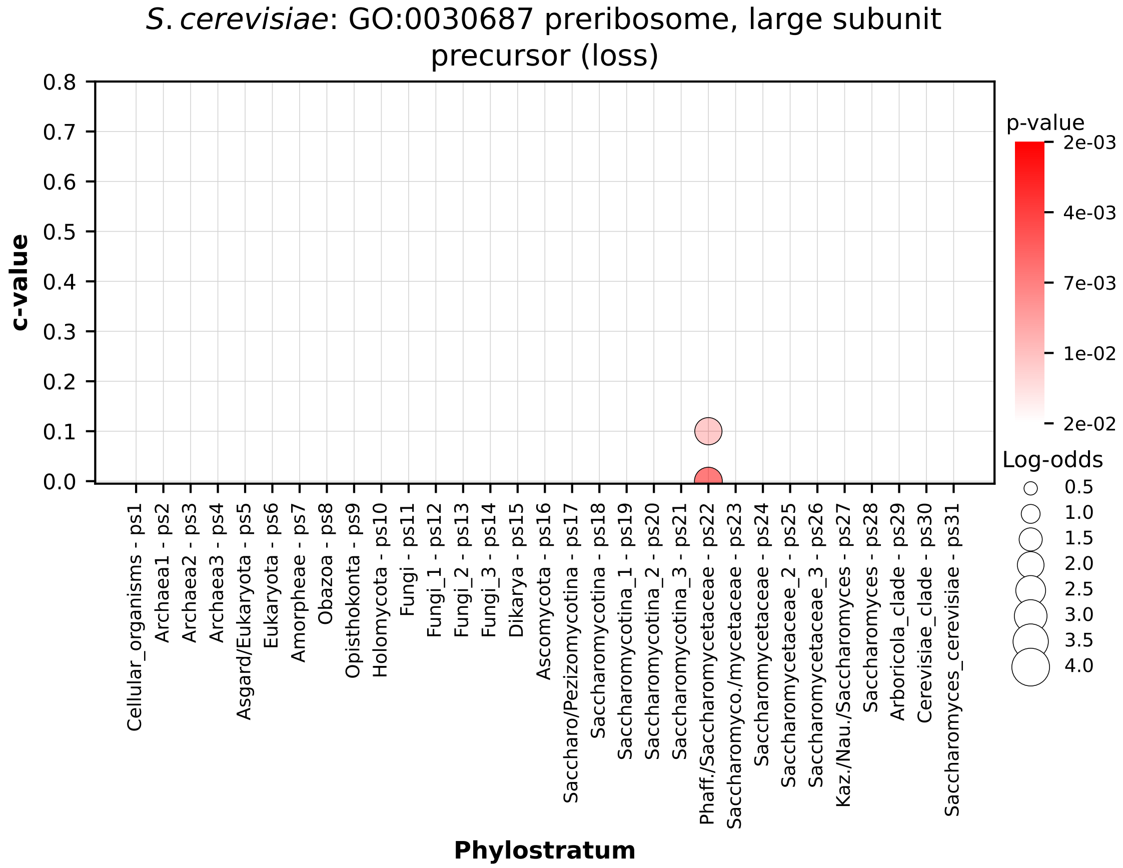

S. cerevisiae: GO:0030687 preribosome, large subunit precursor (loss)
c-value
0.8
0.7
0.6
0.5
0.4
0.3
0.2
0.1
0.0
p-value
2e-03
4e-03
7e-03
1e-02
2e-02
Log-odds
0.5
1.0
1.5
2.0
2.5
3.0
3.5
4.0
Cellular_organisms - ps1
Archaea1 - ps2
Archaea2 - ps3
Archaea3 - ps4
Asgard/Eukaryota - ps5
Eukaryota - ps6
Amorpheae - ps7
Obazoa - ps8
Opisthokonta - ps9
Holomycota - ps10
Fungi - ps11
Fungi_1 - ps12
Fungi_2 - ps13
Fungi_3 - ps14
Dikarya - ps15
Ascomycota - ps16
Saccharo/Pezizomycotina - ps17
Saccharomycotina - ps18
Saccharomycotina_1 - ps19
Saccharomycotina_2 - ps20
Saccharomycotina_3 - ps21
Phaff./Saccharomycetaceae - ps22
Saccharomyco./mycetaceae - ps23
Saccharomycetaceae - ps24
Saccharomycetaceae_2 - ps25
Saccharomycetaceae_3 - ps26
Kaz./Nau./Saccharomyces - ps27
Saccharomyces - ps28
Arboricola_clade - ps29
Cerevisiae_clade - ps30
Saccharomyces_cerevisiae - ps31
Phylostratum

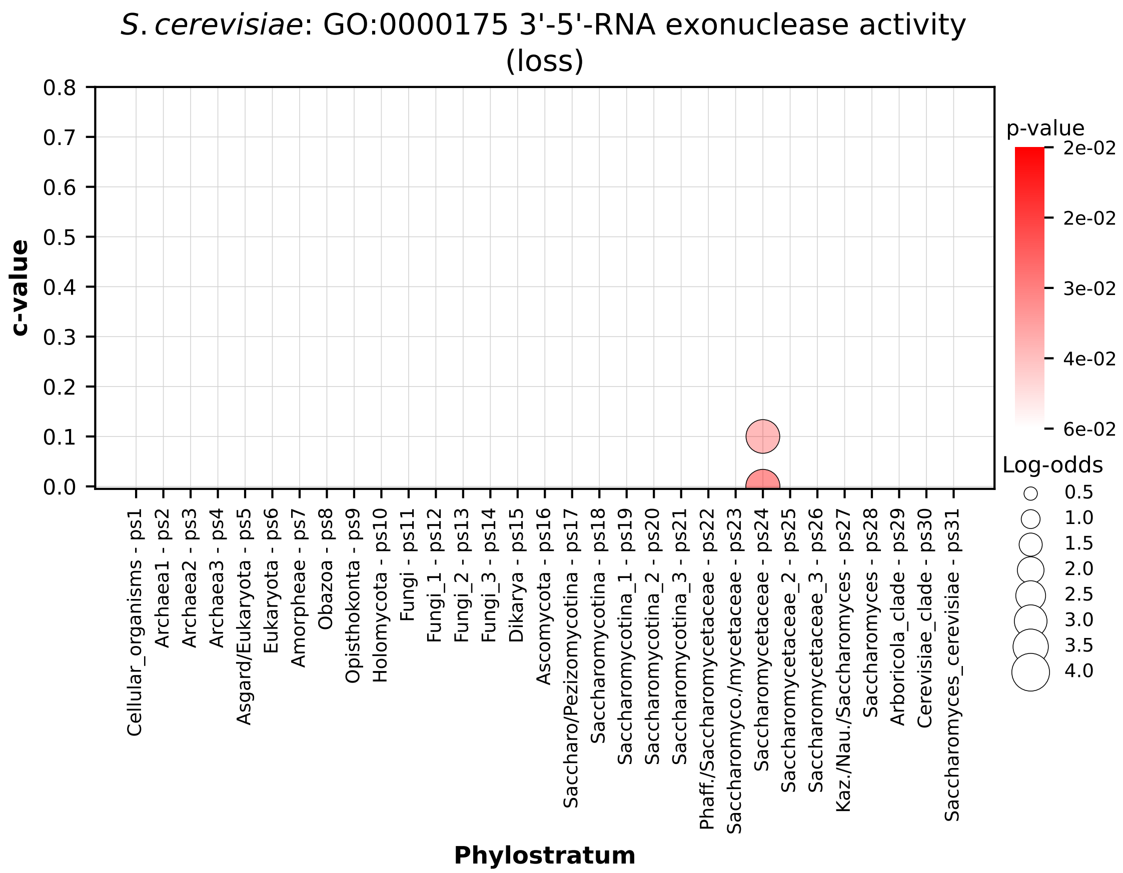

S. cerevisiae: GO:0000175 3'-5'-RNA exonuclease activity
(loss)
c-value
0.0
0.1
0.2
0.3
0.4
0.5
0.6
0.7
0.8
p-value
2e-02
2e-02
3e-02
4e-02
6e-02
Log-odds
0.5
1.0
1.5
2.0
2.5
3.0
3.5
4.0
Cellular_organisms - ps1
Archaea1 - ps2
Archaea2 - ps3
Archaea3 - ps4
Asgard/Eukaryota - ps5
Eukaryota - ps6
Amorpheae - ps7
Obazoa - ps8
Opisthokonta - ps9
Holomycota - ps10
Fungi - ps11
Fungi_1 - ps12
Fungi_2 - ps13
Fungi_3 - ps14
Dikarya - ps15
Ascomycota - ps16
Saccharo/Pezizomycotina - ps17
Saccharomycotina - ps18
Saccharomycotina_1 - ps19
Saccharomycotina_2 - ps20
Saccharomycotina_3 - ps21
Phaff./Saccharomycetaceae - ps22
Saccharomyco./mycetaceae - ps23
Saccharomycetaceae - ps24
Saccharomycetaceae_2 - ps25
Saccharomycetaceae_3 - ps26
Kaz./Nau./Saccharomyces - ps27
Saccharomyces - ps28
Arboricola_clade - ps29
Cerevisiae_clade - ps30
Saccharomyces_cerevisiae - ps31
Phylostratum

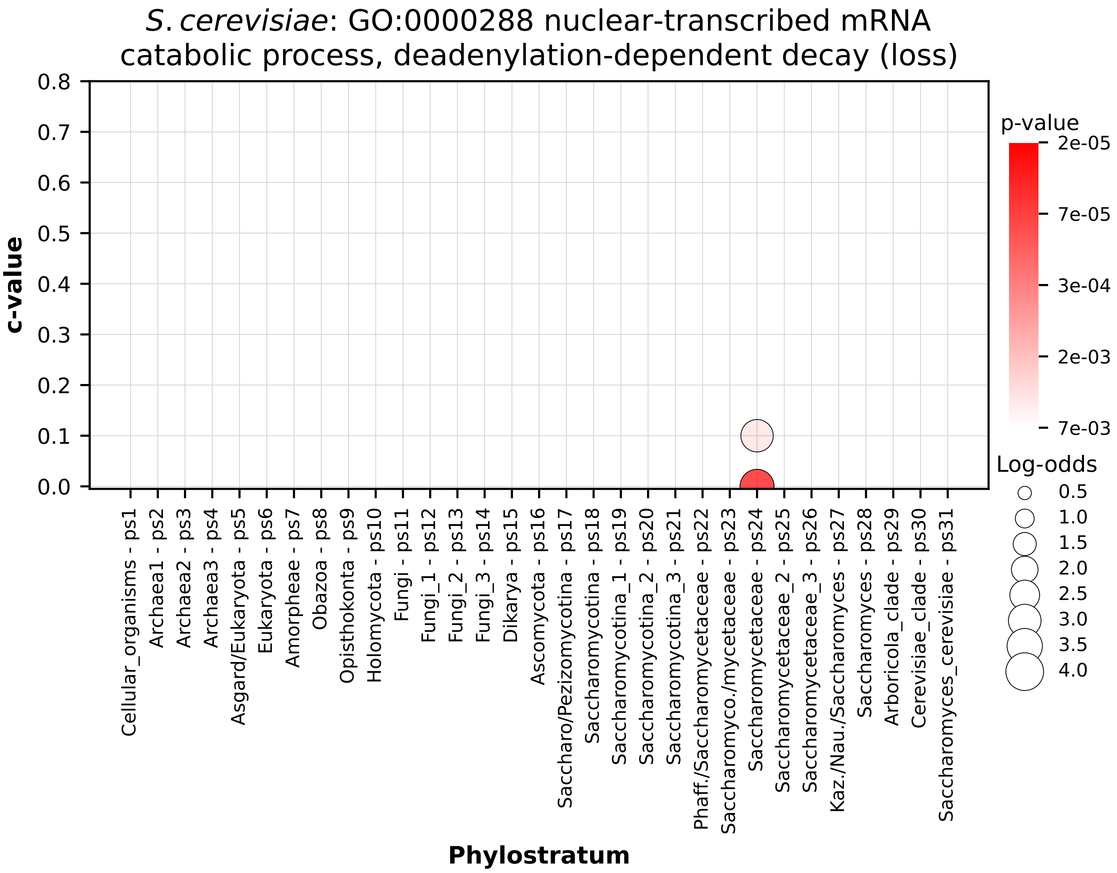

S. cerevisiae: GO:0000288 nuclear-transcribed mRNA
catabolic process, deadenylation-dependent decay (loss)
c-value
0.8
0.7
0.6
0.5
0.4
0.3
0.2
0.1
0.0
Phylostratum
Cellular_organisms - ps1
Archaea1 - ps2
Archaea2 - ps3
Archaea3 - ps4
Asgard/Eukaryota - ps5
Eukaryota - ps6
Amorpheae - ps7
Obazoa - ps8
Opisthokonta - ps9
Holomycota - ps10
Fungi - ps11
Fungi_1 - ps12
Fungi_2 - ps13
Fungi_3 - ps14
Dikarya - ps15
Ascomycota - ps16
Saccharo/Pezizomycotina - ps17
Saccharomycotina - ps18
Saccharomycotina_1 - ps19
Saccharomycotina_2 - ps20
Saccharomycotina_3 - ps21
Phaff./Saccharomycetaceae - ps22
Saccharomyco./mycetaceae - ps23
Saccharomycetaceae - ps24
Saccharomycetaceae_2 - ps25
Saccharomycetaceae_3 - ps26
Kaz./Nau./Saccharomyces - ps27
Saccharomyces - ps28
Arboricola_clade - ps29
Cerevisiae_clade - ps30
Saccharomyces_cerevisiae - ps31
p-value
2e-05
7e-05
3e-04
2e-03
7e-03
Log-odds
0.5
1.0
1.5
2.0
2.5
3.0
3.5
4.0

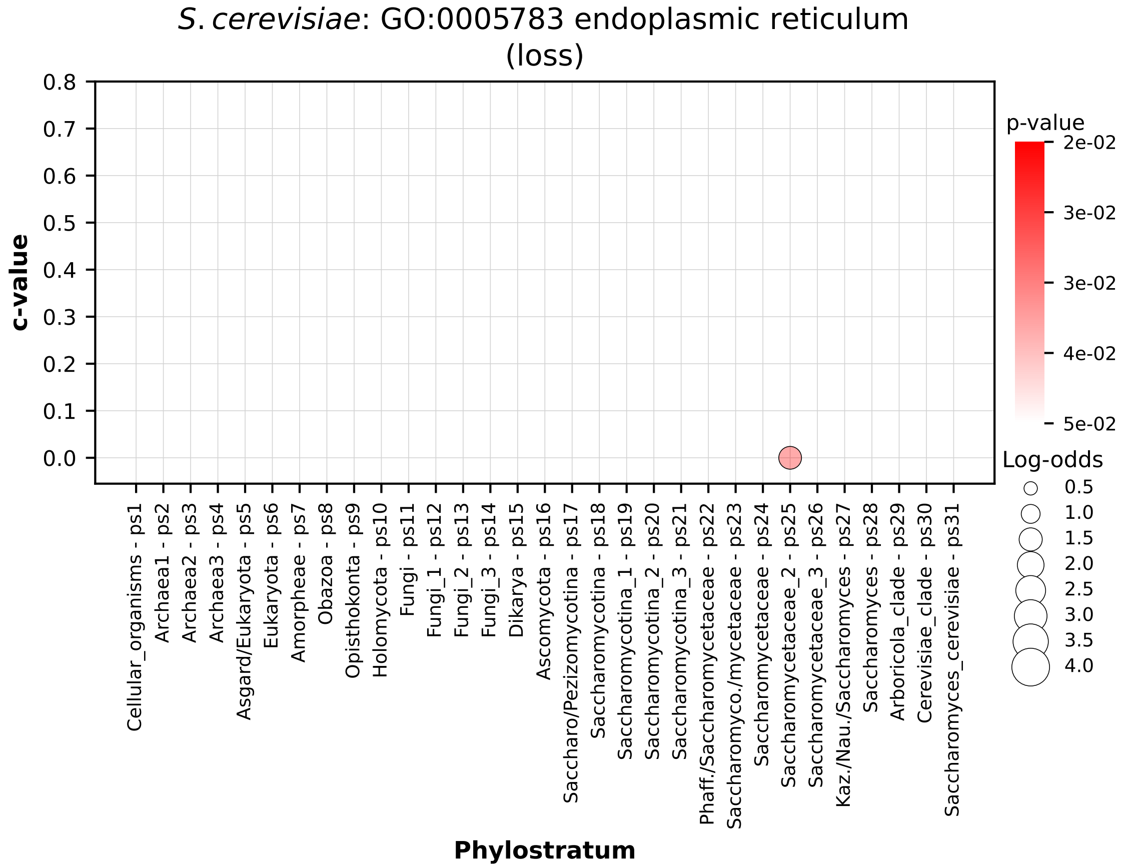

S. cerevisiae: GO:0005783 endoplasmic reticulum
(loss)
c-value
0.8
0.7
0.6
0.5
0.4
0.3
0.2
0.1
0.0
p-value
2e-02
3e-02
3e-02
4e-02
5e-02
Log-odds
0.5
1.0
1.5
2.0
2.5
3.0
3.5
4.0
Cellular_organisms - ps1
Archaea1 - ps2
Archaea2 - ps3
Archaea3 - ps4
Asgard/Eukaryota - ps5
Eukaryota - ps6
Amorpheae - ps7
Obazoa - ps8
Opisthokonta - ps9
Holomycota - ps10
Fungi - ps11
Fungi_1 - ps12
Fungi_2 - ps13
Fungi_3 - ps14
Dikarya - ps15
Ascomycota - ps16
Saccharo/Pezizomycotina - ps17
Saccharomycotina - ps18
Saccharomycotina_1 - ps19
Saccharomycotina_2 - ps20
Saccharomycotina_3 - ps21
Phaff./Saccharomycetaceae - ps22
Saccharomyco./mycetaceae - ps23
Saccharomycetaceae - ps24
Saccharomycetaceae_2 - ps25
Saccharomycetaceae_3 - ps26
Kaz./Nau./Saccharomyces - ps27
Saccharomyces - ps28
Arboricola_clade - ps29
Cerevisiae_clade - ps30
Saccharomyces_cerevisiae - ps31
Phylostratum

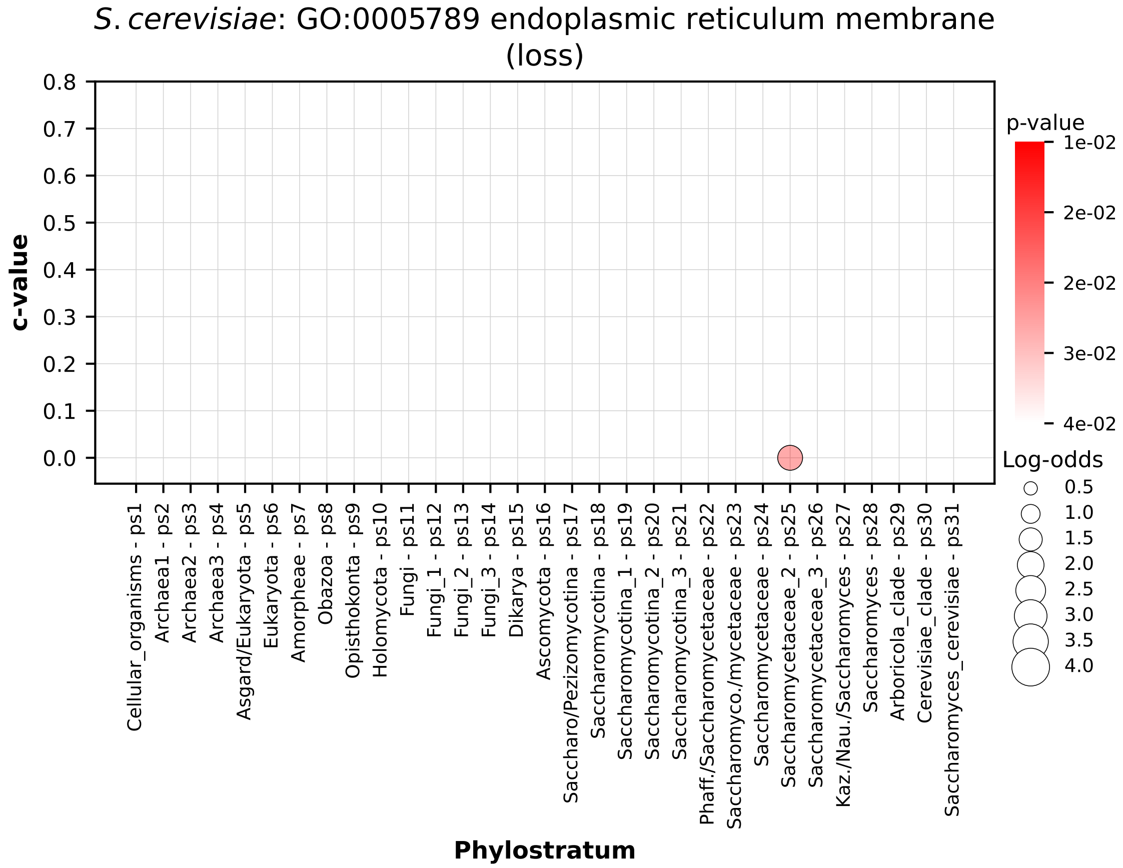

S. cerevisiae: GO:0005789 endoplasmic reticulum membrane (loss)
c-value
0.8
0.7
0.6
0.5
0.4
0.3
0.2
0.1
0.0
p-value
1e-02
2e-02
2e-02
3e-02
4e-02
Log-odds
0.5
1.0
1.5
2.0
2.5
3.0
3.5
4.0
Cellular_organisms - ps1
Archaea1 - ps2
Archaea2 - ps3
Archaea3 - ps4
Asgard/Eukaryota - ps5
Eukaryota - ps6
Amorpheae - ps7
Obazoa - ps8
Opisthokonta - ps9
Holomycota - ps10
Fungi - ps11
Fungi_1 - ps12
Fungi_2 - ps13
Fungi_3 - ps14
Dikarya - ps15
Ascomycota - ps16
Saccharo/Pezizomycotina - ps17
Saccharomycotina - ps18
Saccharomycotina_1 - ps19
Saccharomycotina_2 - ps20
Saccharomycotina_3 - ps21
Phaff./Saccharomycetaceae - ps22
Saccharomyco./mycetaceae - ps23
Saccharomycetaceae - ps24
Saccharomycetaceae_2 - ps25
Saccharomycetaceae_3 - ps26
Kaz./Nau./Saccharomyces - ps27
Saccharomyces - ps28
Arboricola_clade - ps29
Cerevisiae_clade - ps30
Saccharomyces_cerevisiae - ps31
Phylostratum

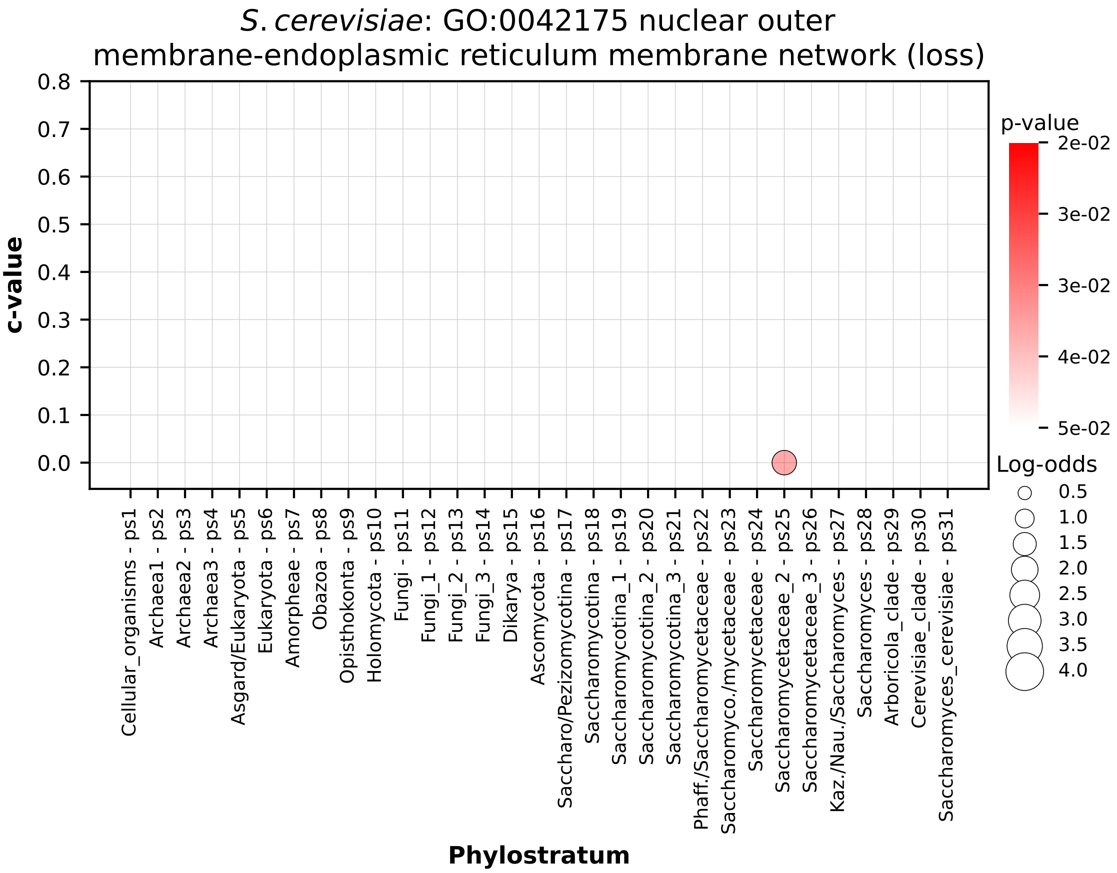
S. cerevisiae: GO:0042175 nuclear outer membrane-endoplasmic reticulum membrane network (loss)
c-value
0.8
0.7
0.6
0.5
0.4
0.3
0.2
0.1
0.0
Phylostratum
Cellular_organisms - ps1
Archaea1 - ps2
Archaea2 - ps3
Archaea3 - ps4
Asgard/Eukaryota - ps5
Eukaryota - ps6
Amorpheae - ps7
Obazoa - ps8
Opisthokonta - ps9
Holomycota - ps10
Fungi - ps11
Fungi_1 - ps12
Fungi_2 - ps13
Fungi_3 - ps14
Dikarya - ps15
Ascomycota - ps16
Saccharo/Pezizomycotina - ps17
Saccharomycotina - ps18
Saccharomycotina_1 - ps19
Saccharomycotina_2 - ps20
Saccharomycotina_3 - ps21
Phaff./Saccharomycetaceae - ps22
Saccharomyco./mycetaceae - ps23
Saccharomycetaceae - ps24
Saccharomycetaceae_2 - ps25
Saccharomycetaceae_3 - ps26
Kaz./Nau./Saccharomyces - ps27
Saccharomyces - ps28
Arboricola_clade - ps29
Cerevisiae_clade - ps30
Saccharomyces_cerevisiae - ps31
p-value
2e-02
3e-02
3e-02
4e-02
5e-02
Log-odds
0.5
1.0
1.5
2.0
2.5
3.0
3.5
4.0

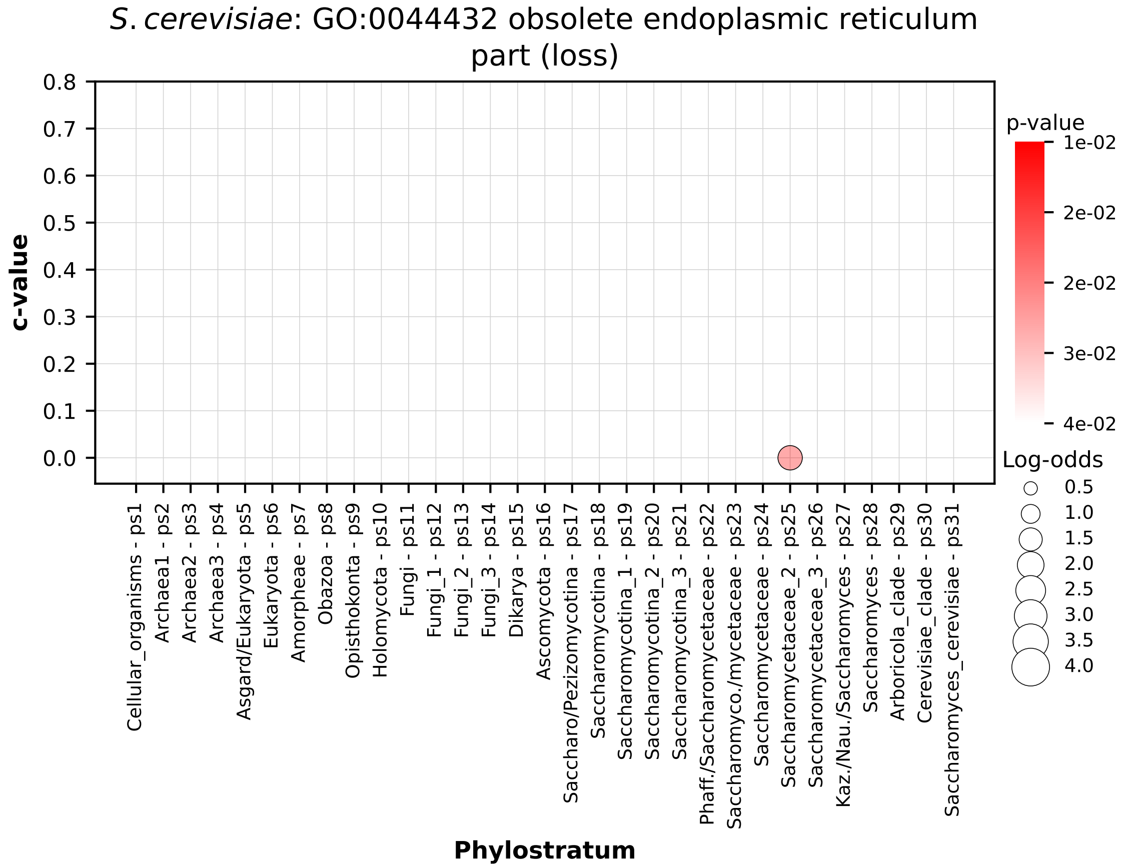

S. cerevisiae: GO:0044432 obsolete endoplasmic reticulum part (loss)
c-value
0.8
0.7
0.6
0.5
0.4
0.3
0.2
0.1
0.0
p-value
1e-02
2e-02
2e-02
3e-02
4e-02
Log-odds
0.5
1.0
1.5
2.0
2.5
3.0
3.5
4.0
Cellular_organisms - ps1
Archaea1 - ps2
Archaea2 - ps3
Archaea3 - ps4
Asgard/Eukaryota - ps5
Eukaryota - ps6
Amorpheae - ps7
Obazoa - ps8
Opisthokonta - ps9
Holomycota - ps10
Fungi - ps11
Fungi_1 - ps12
Fungi_2 - ps13
Fungi_3 - ps14
Dikarya - ps15
Ascomycota - ps16
Saccharo/Pezizomycotina - ps17
Saccharomycotina - ps18
Saccharomycotina_1 - ps19
Saccharomycotina_2 - ps20
Saccharomycotina_3 - ps21
Phaff./Saccharomycetaceae - ps22
Saccharomyco./mycetaceae - ps23
Saccharomycetaceae - ps24
Saccharomycetaceae_2 - ps25
Saccharomycetaceae_3 - ps26
Kaz./Nau./Saccharomyces - ps27
Saccharomyces - ps28
Arboricola_clade - ps29
Cerevisiae_clade - ps30
Saccharomyces_cerevisiae - ps31
Phylostratum

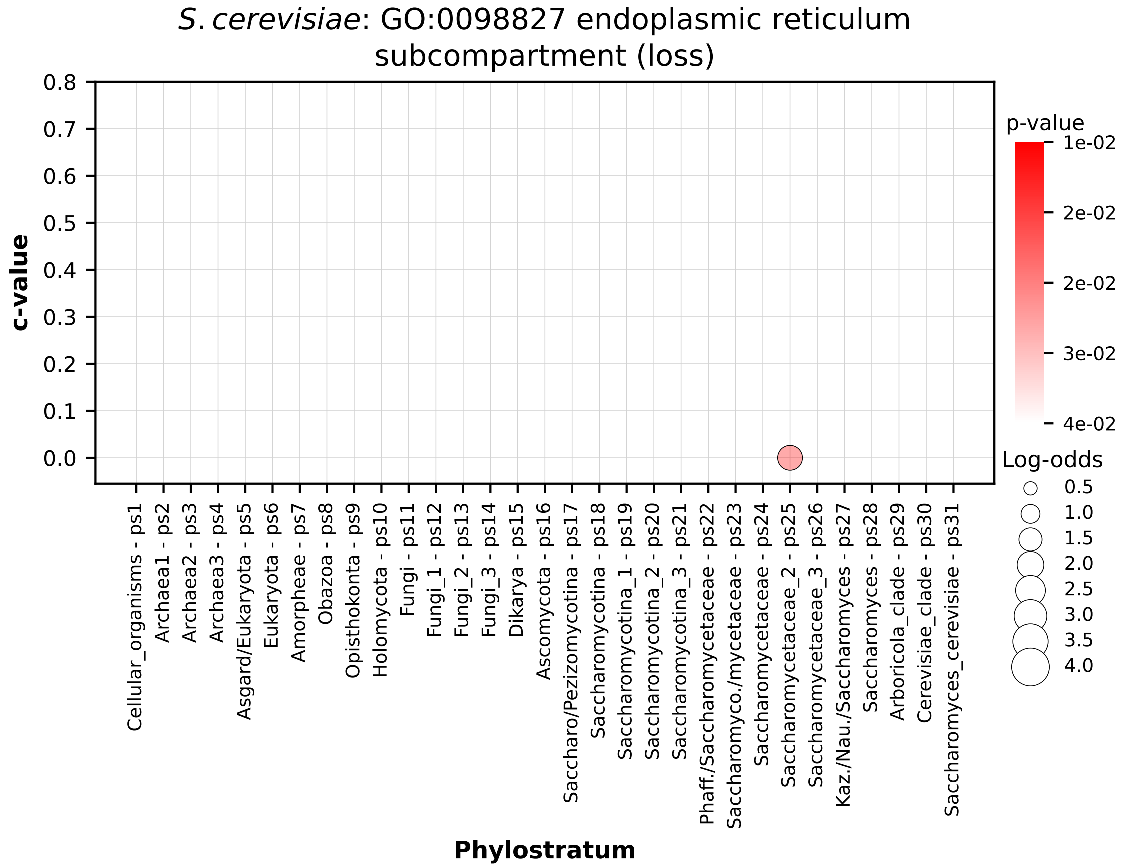
S. cerevisiae: GO:0098827 endoplasmic reticulum subcompartment (loss)
c-value
0.8
0.7
0.6
0.5
0.4
0.3
0.2
0.1
0.0
Phylostratum
Cellular_organisms - ps1
Archaea1 - ps2
Archaea2 - ps3
Archaea3 - ps4
Asgard/Eukaryota - ps5
Eukaryota - ps6
Amorpheae - ps7
Obazoa - ps8
Opisthokonta - ps9
Holomycota - ps10
Fungi - ps11
Fungi_1 - ps12
Fungi_2 - ps13
Fungi_3 - ps14
Dikarya - ps15
Ascomycota - ps16
Saccharo/Pezizomycotina - ps17
Saccharomycotina - ps18
Saccharomycotina_1 - ps19
Saccharomycotina_2 - ps20
Saccharomycotina_3 - ps21
Phaff./Saccharomycetaceae - ps22
Saccharomyco./mycetaceae - ps23
Saccharomycetaceae - ps24
Saccharomycetaceae_2 - ps25
Saccharomycetaceae_3 - ps26
Kaz./Nau./Saccharomyces - ps27
Saccharomyces - ps28
Arboricola_clade - ps29
Cerevisiae_clade - ps30
Saccharomyces_cerevisiae - ps31
p-value
1e-02
2e-02
2e-02
3e-02
4e-02
Log-odds
0.5
1.0
1.5
2.0
2.5
3.0
3.5
4.0

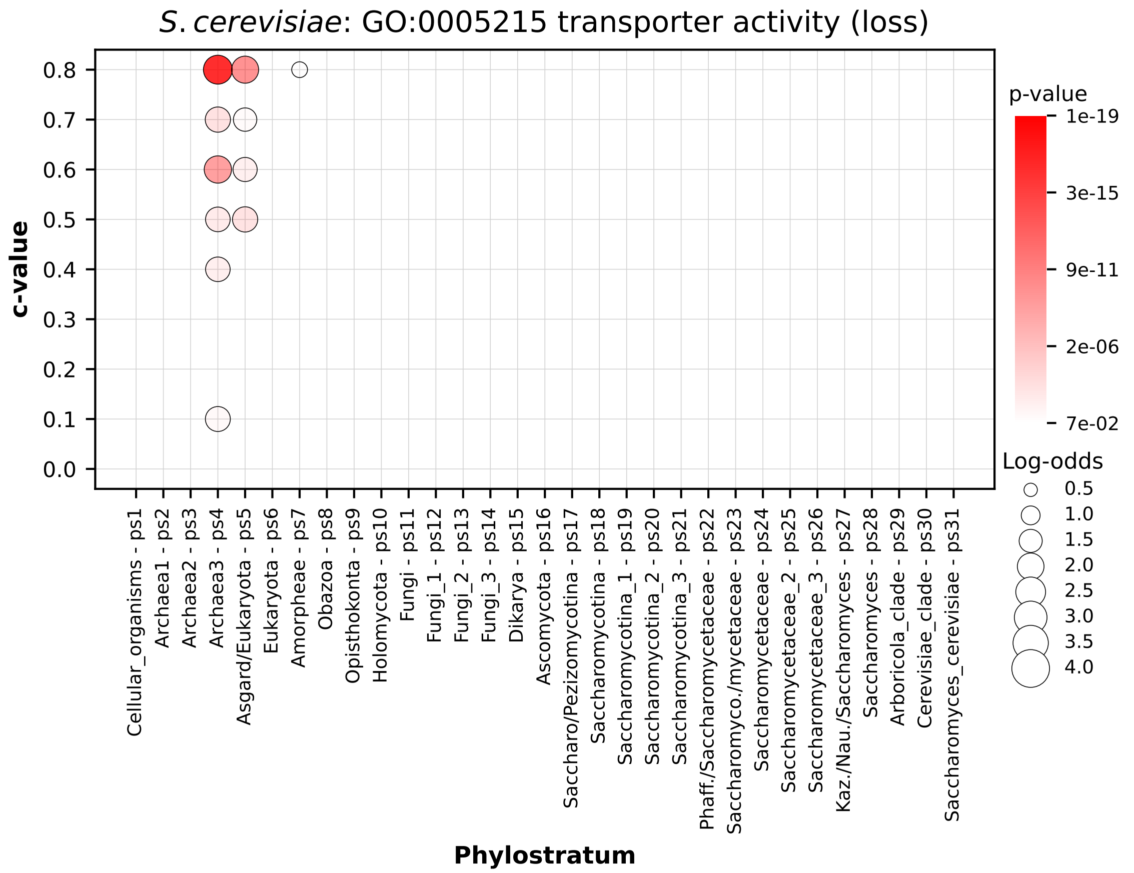

S. cerevisiae: GO:0005215 transporter activity (loss)
c-value
0.0
0.1
0.2
0.3
0.4
0.5
0.6
0.7
0.8
Phylostratum
Cellular_organisms - ps1
Archaea1 - ps2
Archaea2 - ps3
Archaea3 - ps4
Asgard/Eukaryota - ps5
Eukaryota - ps6
Amorpheae - ps7
Obazoa - ps8
Opisthokonta - ps9
Holomycota - ps10
Fungi - ps11
Fungi_1 - ps12
Fungi_2 - ps13
Fungi_3 - ps14
Dikarya - ps15
Ascomycota - ps16
Saccharo/Pezizomycotina - ps17
Saccharomycotina - ps18
Saccharomycotina_1 - ps19
Saccharomycotina_2 - ps20
Saccharomycotina_3 - ps21
Phaff./Saccharomycetaceae - ps22
Saccharomyco./mycetaceae - ps23
Saccharomycetaceae - ps24
Saccharomycetaceae_2 - ps25
Saccharomycetaceae_3 - ps26
Kaz./Nau./Saccharomyces - ps27
Saccharomyces - ps28
Arboricola_clade - ps29
Cerevisiae_clade - ps30
Saccharomyces_cerevisiae - ps31
p-value
1e-19
3e-15
9e-11
2e-06
7e-02
Log-odds
0.5
1.0
1.5
2.0
2.5
3.0
3.5
4.0

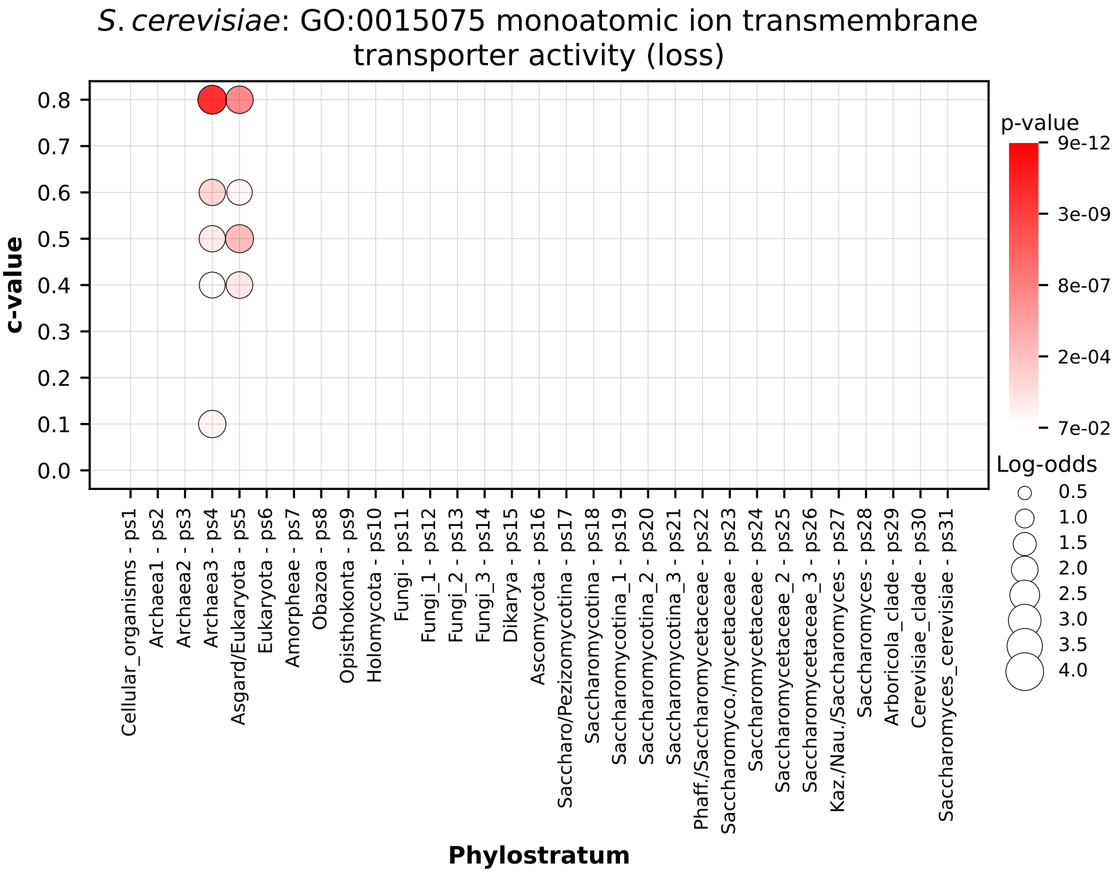
S. cerevisiae: GO:0015075 monoatomic ion transmembrane transporter activity (loss)
c-value
0.0
0.1
0.2
0.3
0.4
0.5
0.6
0.7
0.8
Phylostratum
Cellular_organisms - ps1
Archaea1 - ps2
Archaea2 - ps3
Archaea3 - ps4
Asgard/Eukaryota - ps5
Eukaryota - ps6
Amorpheae - ps7
Obazoa - ps8
Opisthokonta - ps9
Holomycota - ps10
Fungi - ps11
Fungi_1 - ps12
Fungi_2 - ps13
Fungi_3 - ps14
Dikarya - ps15
Ascomycota - ps16
Saccharo/Pezizomycotina - ps17
Saccharomycotina - ps18
Saccharomycotina_1 - ps19
Saccharomycotina_2 - ps20
Saccharomycotina_3 - ps21
Phaff./Saccharomycetaceae - ps22
Saccharomyco./mycetaceae - ps23
Saccharomycetaceae - ps24
Saccharomycetaceae_2 - ps25
Saccharomycetaceae_3 - ps26
Kaz./Nau./Saccharomyces - ps27
Saccharomyces - ps28
Arboricola_clade - ps29
Cerevisiae_clade - ps30
Saccharomyces_cerevisiae - ps31
p-value
9e-12
3e-09
8e-07
2e-04
7e-02
Log-odds
0.5
1.0
1.5
2.0
2.5
3.0
3.5
4.0

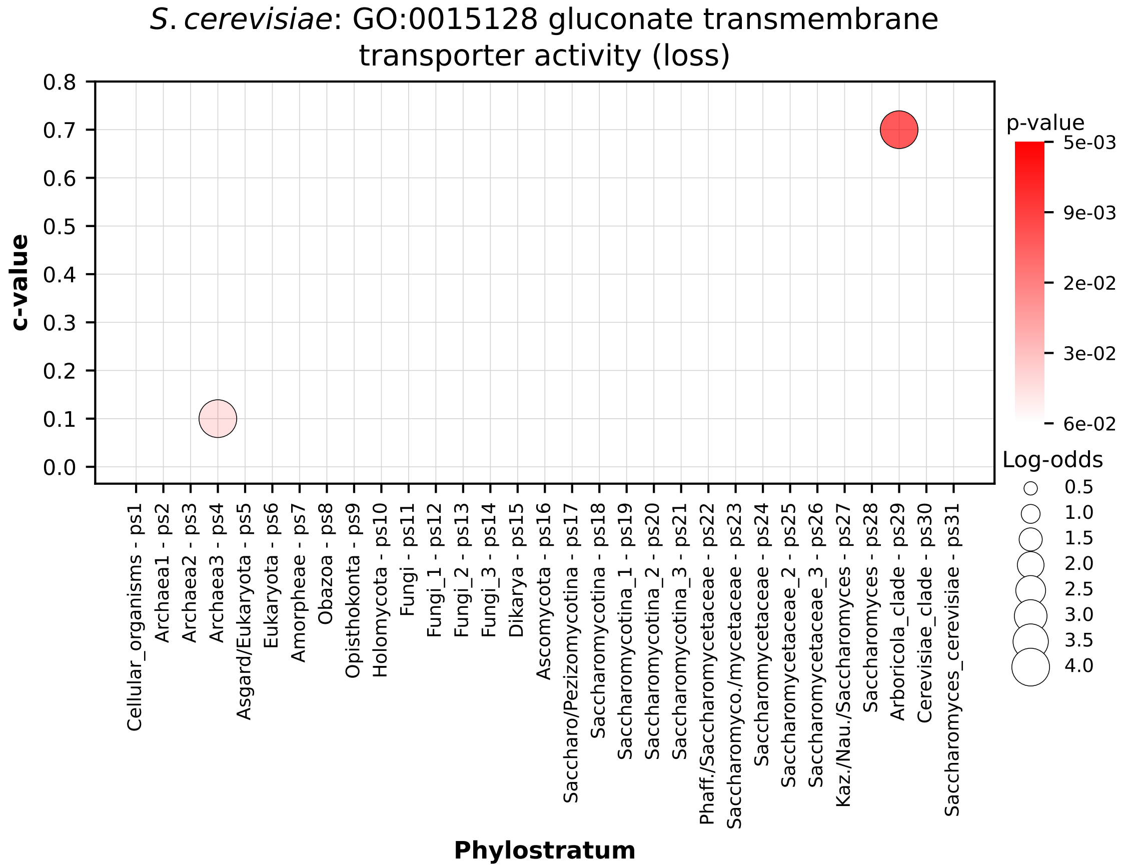
S. cerevisiae: GO:0015128 gluconate transmembrane transporter activity (loss)
c-value
0.8
0.7
0.6
0.5
0.4
0.3
0.2
0.1
0.0
Phylostratum
Cellular_organisms - ps1
Archaea1 - ps2
Archaea2 - ps3
Archaea3 - ps4
Asgard/Eukaryota - ps5
Eukaryota - ps6
Amorpheae - ps7
Obazoa - ps8
Opisthokonta - ps9
Holomycota - ps10
Fungi - ps11
Fungi_1 - ps12
Fungi_2 - ps13
Fungi_3 - ps14
Dikarya - ps15
Ascomycota - ps16
Saccharo/Pezizomycotina - ps17
Saccharomycotina - ps18
Saccharomycotina_1 - ps19
Saccharomycotina_2 - ps20
Saccharomycotina_3 - ps21
Phaff./Saccharomycetaceae - ps22
Saccharomyco./mycetaceae - ps23
Saccharomycetaceae - ps24
Saccharomycetaceae_2 - ps25
Saccharomycetaceae_3 - ps26
Kaz./Nau./Saccharomyces - ps27
Saccharomyces - ps28
Arboricola_clade - ps29
Cerevisiae_clade - ps30
Saccharomyces_cerevisiae - ps31
p-value
5e-03
9e-03
2e-02
3e-02
6e-02
Log-odds
0.5
1.0
1.5
2.0
2.5
3.0
3.5
4.0

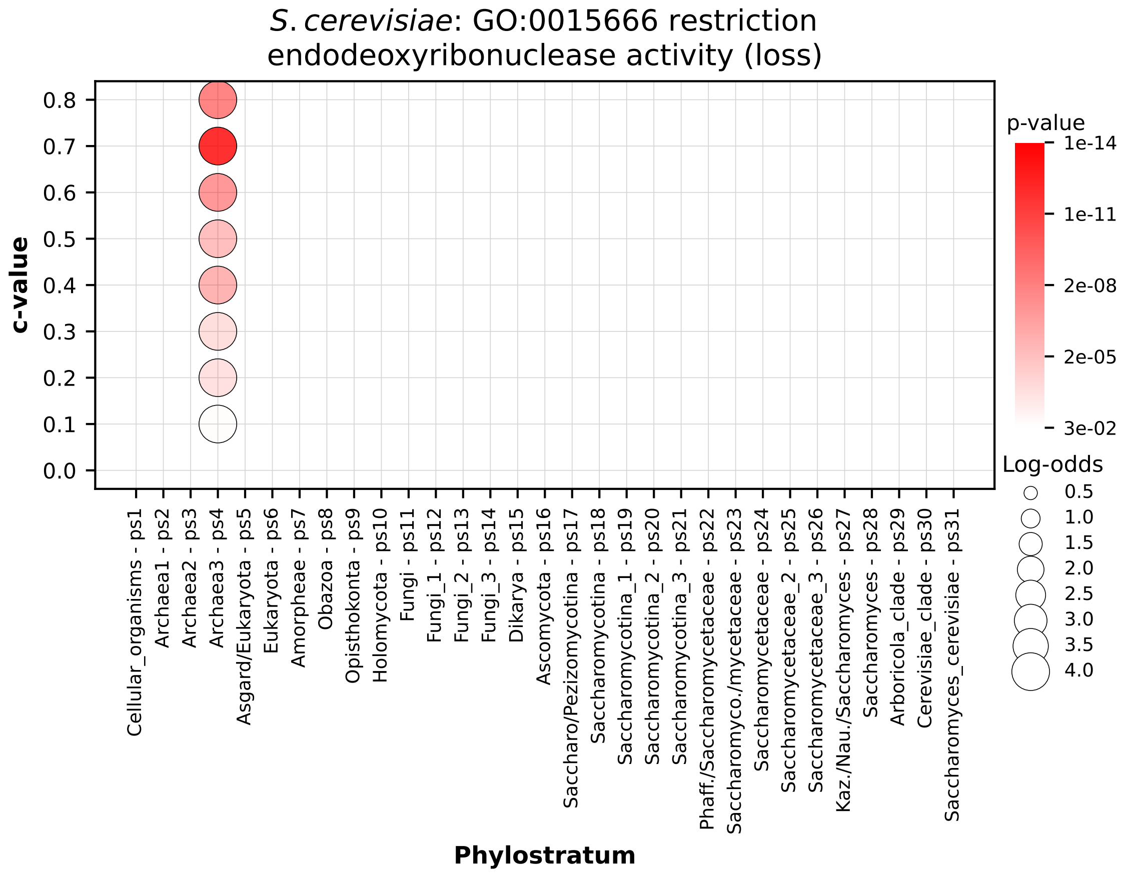

S. cerevisiae: GO:0015666 restriction endodeoxyribonuclease activity (loss)
c-value
0.0
0.1
0.2
0.3
0.4
0.5
0.6
0.7
0.8
p-value
1e-14
1e-11
2e-08
2e-05
3e-02
Log-odds
0.5
1.0
1.5
2.0
2.5
3.0
3.5
4.0
Cellular_organisms - ps1
Archaea1 - ps2
Archaea2 - ps3
Archaea3 - ps4
Asgard/Eukaryota - ps5
Eukaryota - ps6
Amorpheae - ps7
Obazoa - ps8
Opisthokonta - ps9
Holomycota - ps10
Fungi - ps11
Fungi_1 - ps12
Fungi_2 - ps13
Fungi_3 - ps14
Dikarya - ps15
Ascomycota - ps16
Saccharo/Pezizomycotina - ps17
Saccharomycotina - ps18
Saccharomycotina_1 - ps19
Saccharomycotina_2 - ps20
Saccharomycotina_3 - ps21
Phaff./Saccharomycetaceae - ps22
Saccharomyco./mycetaceae - ps23
Saccharomycetaceae - ps24
Saccharomycetaceae_2 - ps25
Saccharomycetaceae_3 - ps26
Kaz./Nau./Saccharomyces - ps27
Saccharomyces - ps28
Arboricola_clade - ps29
Cerevisiae_clade - ps30
Saccharomyces_cerevisiae - ps31
Phylostratum

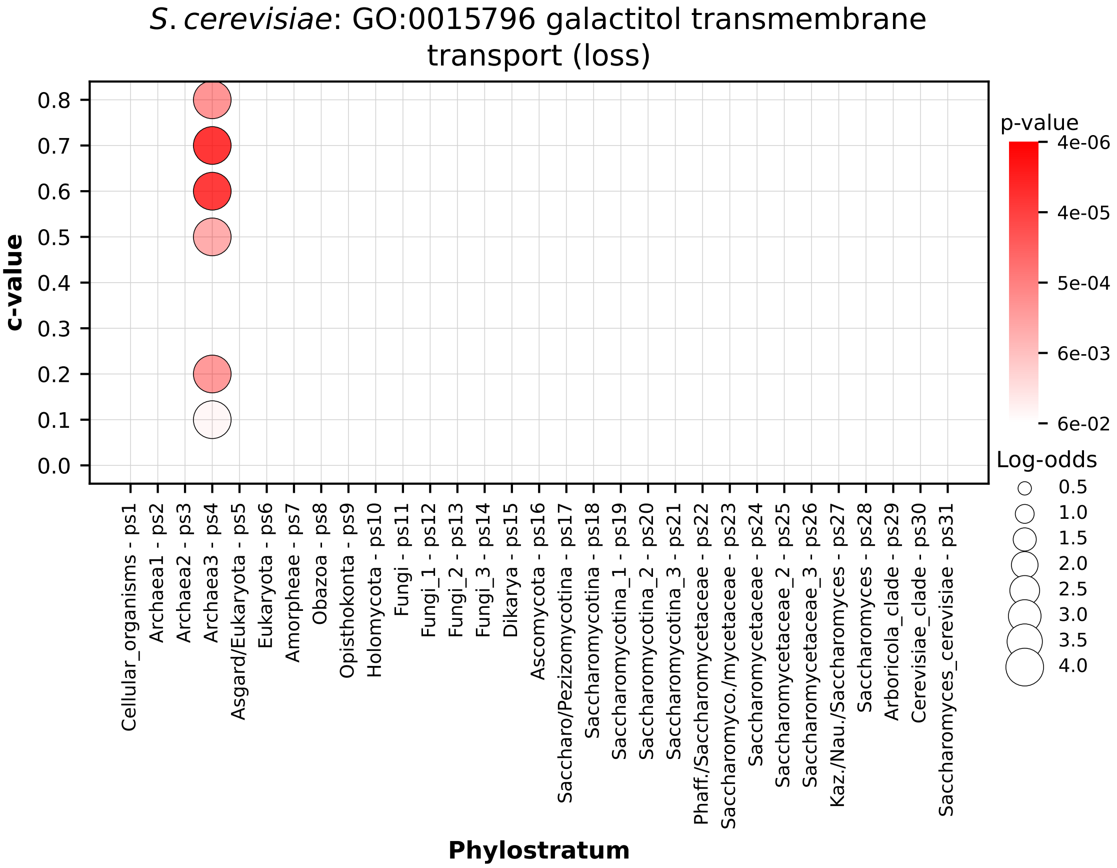
S. cerevisiae: GO:0015796 galactitol transmembrane transport (loss)
c-value
0.8
0.7
0.6
0.5
0.4
0.3
0.2
0.1
0.0
p-value
4e-06
4e-05
5e-04
6e-03
6e-02
Log-odds
0.5
1.0
1.5
2.0
2.5
3.0
3.5
4.0
Cellular_organisms - ps1
Archaea1 - ps2
Archaea2 - ps3
Archaea3 - ps4
Asgard/Eukaryota - ps5
Eukaryota - ps6
Amorpheae - ps7
Obazoa - ps8
Opisthokonta - ps9
Holomycota - ps10
Fungi - ps11
Fungi_1 - ps12
Fungi_2 - ps13
Fungi_3 - ps14
Dikarya - ps15
Ascomycota - ps16
Saccharo/Pezizomycotina - ps17
Saccharomycotina - ps18
Saccharomycotina_1 - ps19
Saccharomycotina_2 - ps20
Saccharomycotina_3 - ps21
Phaff./Saccharomycetaceae - ps22
Saccharomyco./mycetaceae - ps23
Saccharomycetaceae - ps24
Saccharomycetaceae_2 - ps25
Saccharomycetaceae_3 - ps26
Kaz./Nau./Saccharomyces - ps27
Saccharomyces - ps28
Arboricola_clade - ps29
Cerevisiae_clade - ps30
Saccharomyces_cerevisiae - ps31
Phylostratum

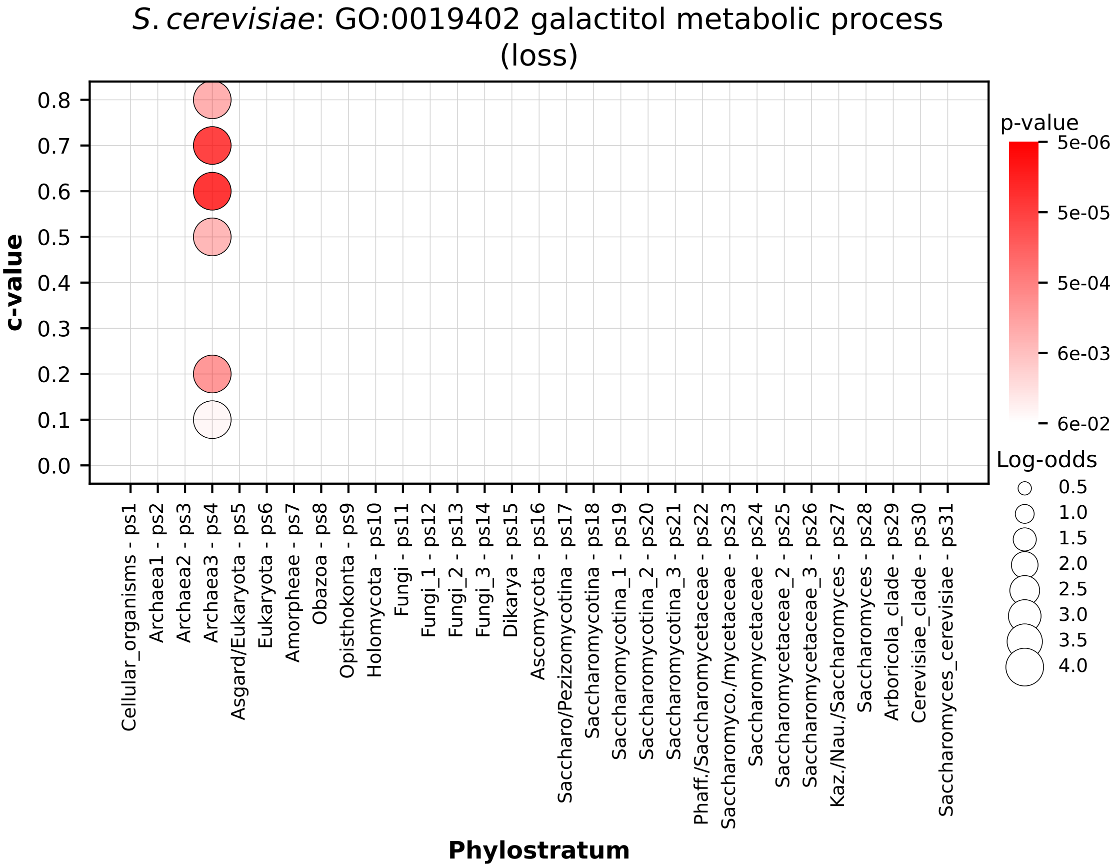
S. cerevisiae: GO:0019402 galactitol metabolic process
(loss)
c-value
0.0
0.1
0.2
0.3
0.4
0.5
0.6
0.7
0.8
Phylostratum
Cellular_organisms - ps1
Archaea1 - ps2
Archaea2 - ps3
Archaea3 - ps4
Asgard/Eukaryota - ps5
Eukaryota - ps6
Amorpheae - ps7
Obazoa - ps8
Opisthokonta - ps9
Holomycota - ps10
Fungi - ps11
Fungi_1 - ps12
Fungi_2 - ps13
Fungi_3 - ps14
Dikarya - ps15
Ascomycota - ps16
Saccharo/Pezizomycotina - ps17
Saccharomycotina - ps18
Saccharomycotina_1 - ps19
Saccharomycotina_2 - ps20
Saccharomycotina_3 - ps21
Phaff./Saccharomycetaceae - ps22
Saccharomyco./mycetaceae - ps23
Saccharomycetaceae - ps24
Saccharomycetaceae_2 - ps25
Saccharomycetaceae_3 - ps26
Kaz./Nau./Saccharomyces - ps27
Saccharomyces - ps28
Arboricola_clade - ps29
Cerevisiae_clade - ps30
Saccharomyces_cerevisiae - ps31
p-value
5e-06
5e-05
5e-04
6e-03
6e-02
Log-odds
0.5
1.0
1.5
2.0
2.5
3.0
3.5
4.0

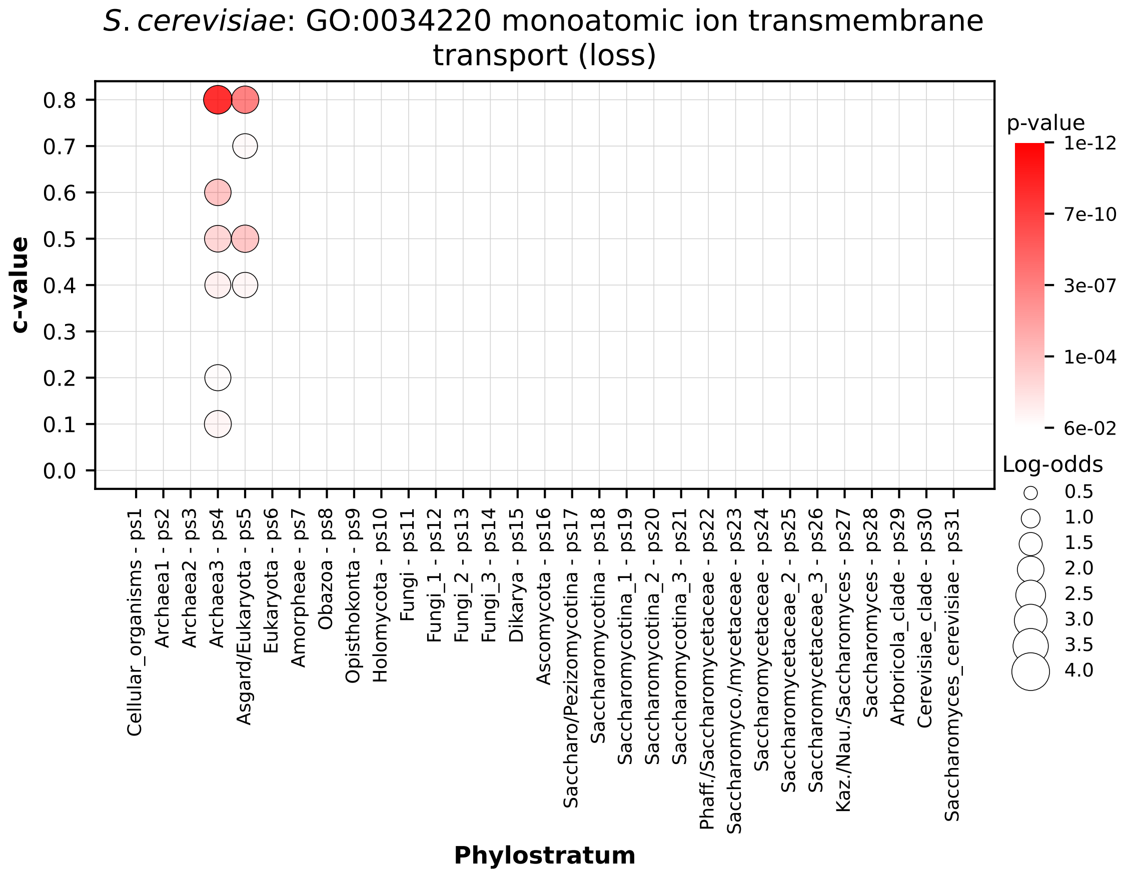

S. cerevisiae: GO:0034220 monoatomic ion transmembrane transport (loss)
c-value
0.0
0.1
0.2
0.3
0.4
0.5
0.6
0.7
0.8
p-value
1e-12
7e-10
3e-07
1e-04
6e-02
Log-odds
0.5
1.0
1.5
2.0
2.5
3.0
3.5
4.0
Cellular_organisms - ps1
Archaea1 - ps2
Archaea2 - ps3
Archaea3 - ps4
Asgard/Eukaryota - ps5
Eukaryota - ps6
Amorpheae - ps7
Obazoa - ps8
Opisthokonta - ps9
Holomycota - ps10
Fungi - ps11
Fungi_1 - ps12
Fungi_2 - ps13
Fungi_3 - ps14
Dikarya - ps15
Ascomycota - ps16
Saccharo/Pezizomycotina - ps17
Saccharomycotina - ps18
Saccharomycotina_1 - ps19
Saccharomycotina_2 - ps20
Saccharomycotina_3 - ps21
Phaff./Saccharomycetaceae - ps22
Saccharomyco./mycetaceae - ps23
Saccharomycetaceae - ps24
Saccharomycetaceae_2 - ps25
Saccharomycetaceae_3 - ps26
Kaz./Nau./Saccharomyces - ps27
Saccharomyces - ps28
Arboricola_clade - ps29
Cerevisiae_clade - ps30
Saccharomyces_cerevisiae - ps31
Phylostratum

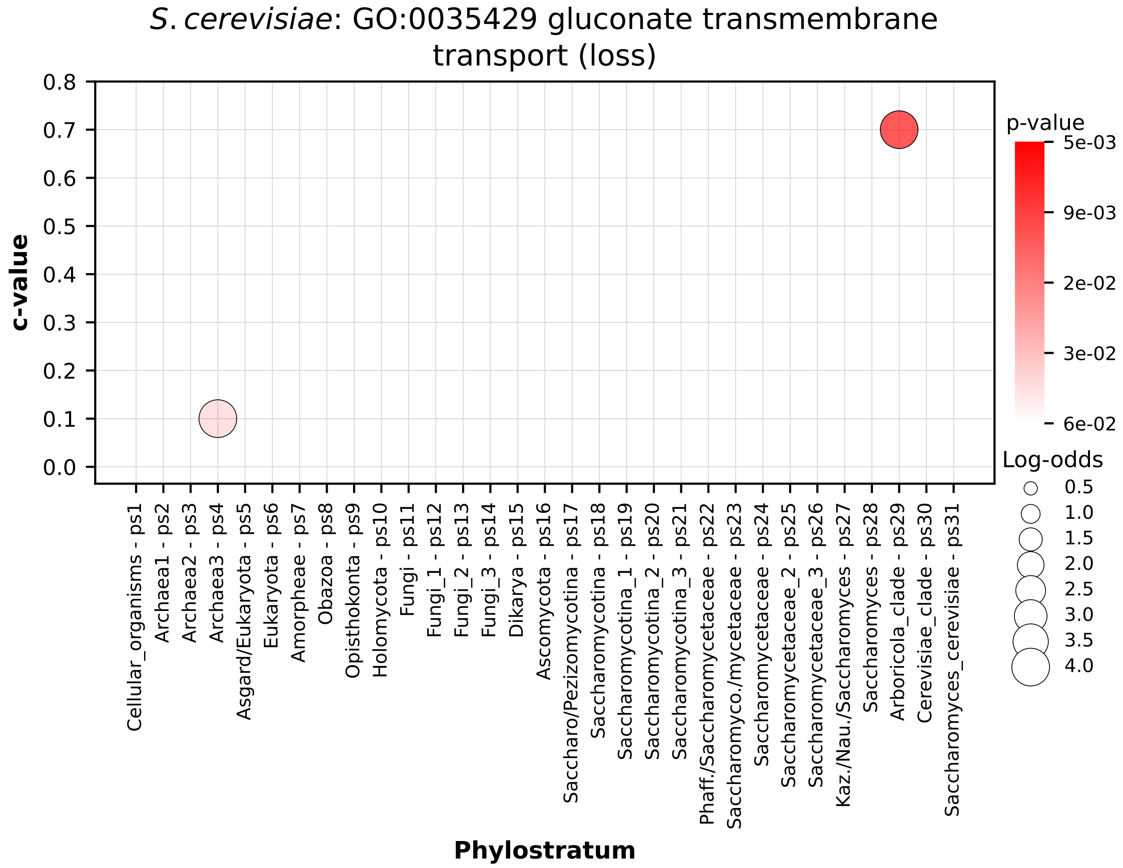
S. cerevisiae: GO:0035429 gluconate transmembrane transport (loss)
c-value
0.8
0.7
0.6
0.5
0.4
0.3
0.2
0.1
0.0
p-value
5e-03
9e-03
2e-02
3e-02
6e-02
Log-odds
0.5
1.0
1.5
2.0
2.5
3.0
3.5
4.0
Cellular_organisms - ps1
Archaea1 - ps2
Archaea2 - ps3
Archaea3 - ps4
Asgard/Eukaryota - ps5
Eukaryota - ps6
Amorpheae - ps7
Obazoa - ps8
Opisthokonta - ps9
Holomycota - ps10
Fungi - ps11
Fungi_1 - ps12
Fungi_2 - ps13
Fungi_3 - ps14
Dikarya - ps15
Ascomycota - ps16
Saccharo/Pezizomycotina - ps17
Saccharomycotina - ps18
Saccharomycotina_1 - ps19
Saccharomycotina_2 - ps20
Saccharomycotina_3 - ps21
Phaff./Saccharomycetaceae - ps22
Saccharomyco./mycetaceae - ps23
Saccharomycetaceae - ps24
Saccharomycetaceae_2 - ps25
Saccharomycetaceae_3 - ps26
Kaz./Nau./Saccharomyces - ps27
Saccharomyces - ps28
Arboricola_clade - ps29
Cerevisiae_clade - ps30
Saccharomyces_cerevisiae - ps31
Phylostratum

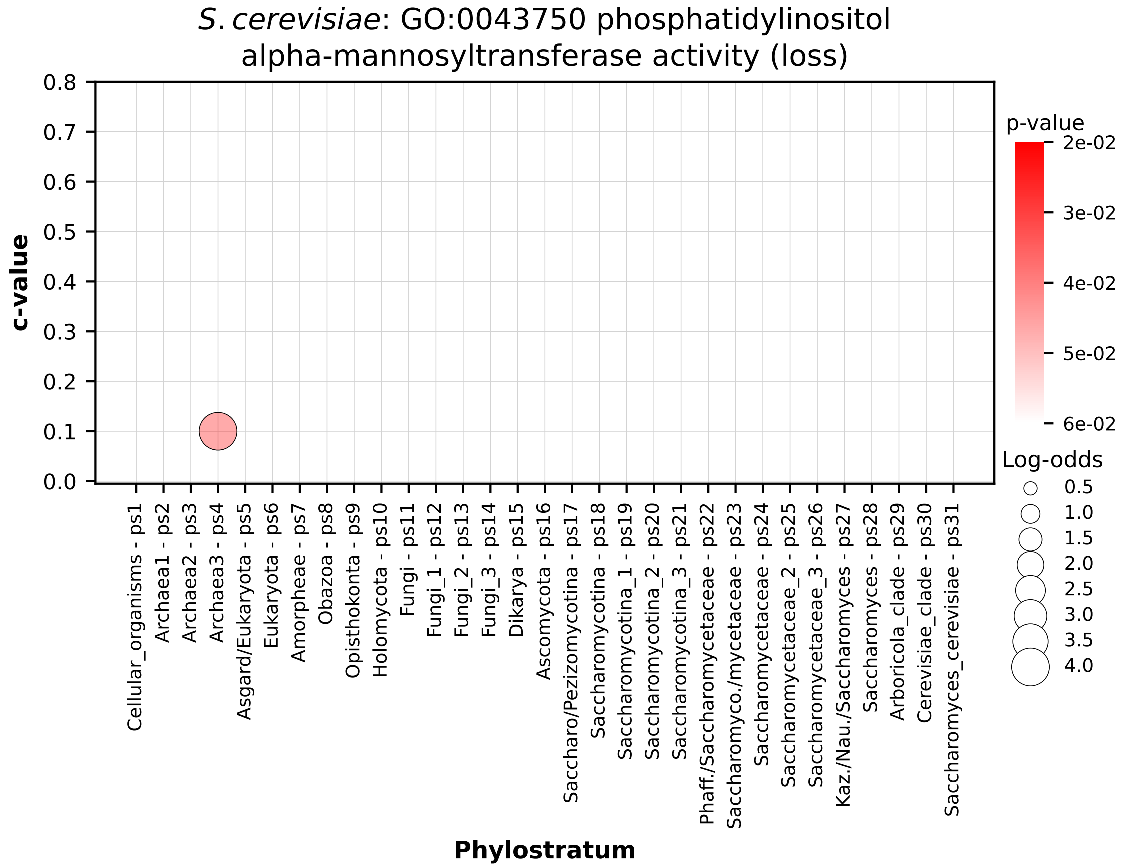

S. cerevisiae: GO:0043750 phosphatidylinositol alpha-mannosyltransferase activity (loss)
c-value
0.8
0.7
0.6
0.5
0.4
0.3
0.2
0.1
0.0
Phylostratum
Cellular_organisms - ps1
Archaea1 - ps2
Archaea2 - ps3
Archaea3 - ps4
Asgard/Eukaryota - ps5
Eukaryota - ps6
Amorpheae - ps7
Obazoa - ps8
Opisthokonta - ps9
Holomycota - ps10
Fungi - ps11
Fungi_1 - ps12
Fungi_2 - ps13
Fungi_3 - ps14
Dikarya - ps15
Ascomycota - ps16
Saccharo/Pezizomycotina - ps17
Saccharomycotina - ps18
Saccharomycotina_1 - ps19
Saccharomycotina_2 - ps20
Saccharomycotina_3 - ps21
Phaff./Saccharomycetaceae - ps22
Saccharomyco./mycetaceae - ps23
Saccharomycetaceae - ps24
Saccharomycetaceae_2 - ps25
Saccharomycetaceae_3 - ps26
Kaz./Nau./Saccharomyces - ps27
Saccharomyces - ps28
Arboricola_clade - ps29
Cerevisiae_clade - ps30
Saccharomyces_cerevisiae - ps31
p-value
2e-02
3e-02
4e-02
5e-02
6e-02
Log-odds
0.5
1.0
1.5
2.0
2.5
3.0
3.5
4.0

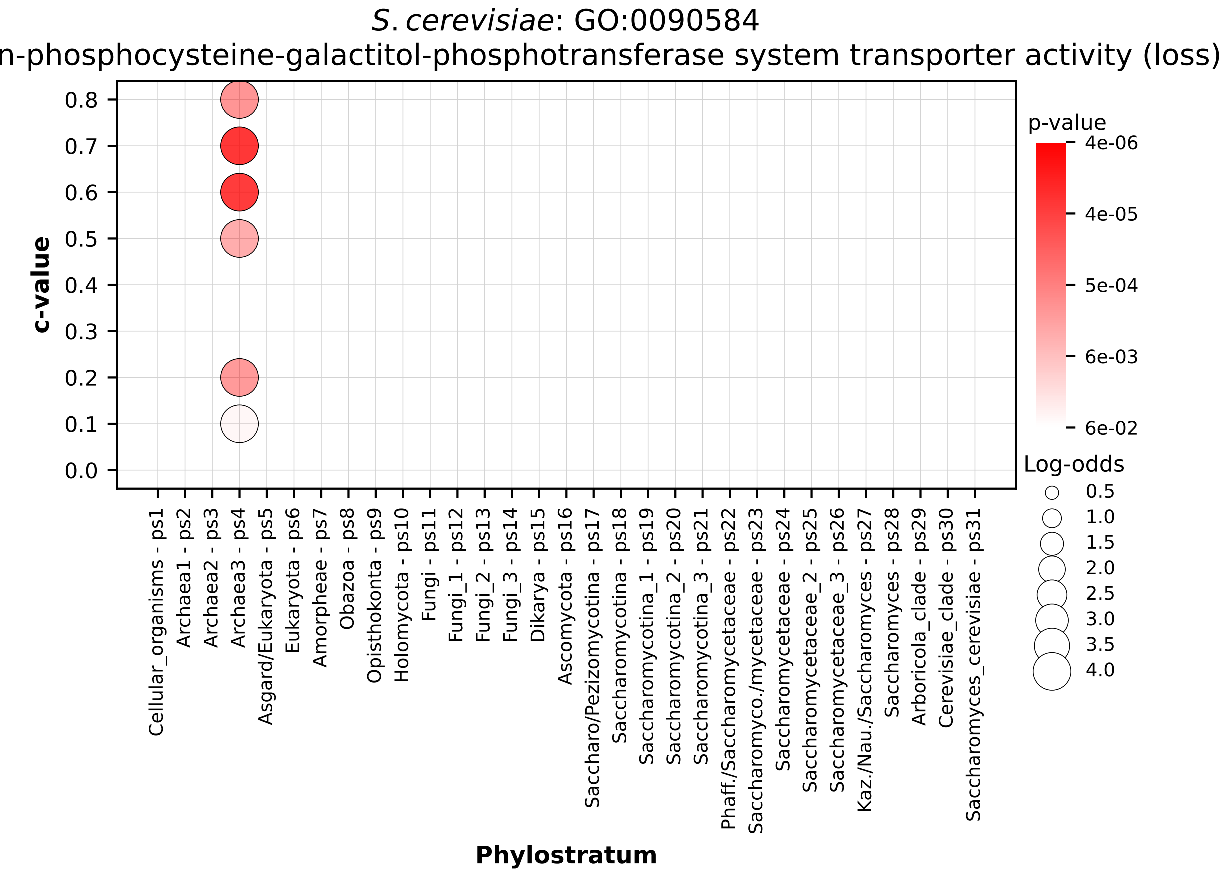

S. cerevisiae: GO:0090584
n-phosphocysteine-galactitol-phosphotransferase system transporter activity (loss)
c-value
0.8
0.7
0.6
0.5
0.4
0.3
0.2
0.1
0.0
p-value
4e-06
4e-05
5e-04
6e-03
6e-02
Log-odds
0.5
1.0
1.5
2.0
2.5
3.0
3.5
4.0
Cellular_organisms - ps1
Archaea1 - ps2
Archaea2 - ps3
Archaea3 - ps4
Asgard/Eukaryota - ps5
Eukaryota - ps6
Amorpheae - ps7
Obazoa - ps8
Opisthokonta - ps9
Holomycota - ps10
Fungi - ps11
Fungi_1 - ps12
Fungi_2 - ps13
Fungi_3 - ps14
Dikarya - ps15
Ascomycota - ps16
Saccharo/Pezizomycotina - ps17
Saccharomycotina - ps18
Saccharomycotina_1 - ps19
Saccharomycotina_2 - ps20
Saccharomycotina_3 - ps21
Phaff./Saccharomycetaceae - ps22
Saccharomyco./mycetaceae - ps23
Saccharomycetaceae - ps24
Saccharomycetaceae_2 - ps25
Saccharomycetaceae_3 - ps26
Kaz./Nau./Saccharomyces - ps27
Saccharomyces - ps28
Arboricola_clade - ps29
Cerevisiae_clade - ps30
Saccharomyces_cerevisiae - ps31
Phylostratum

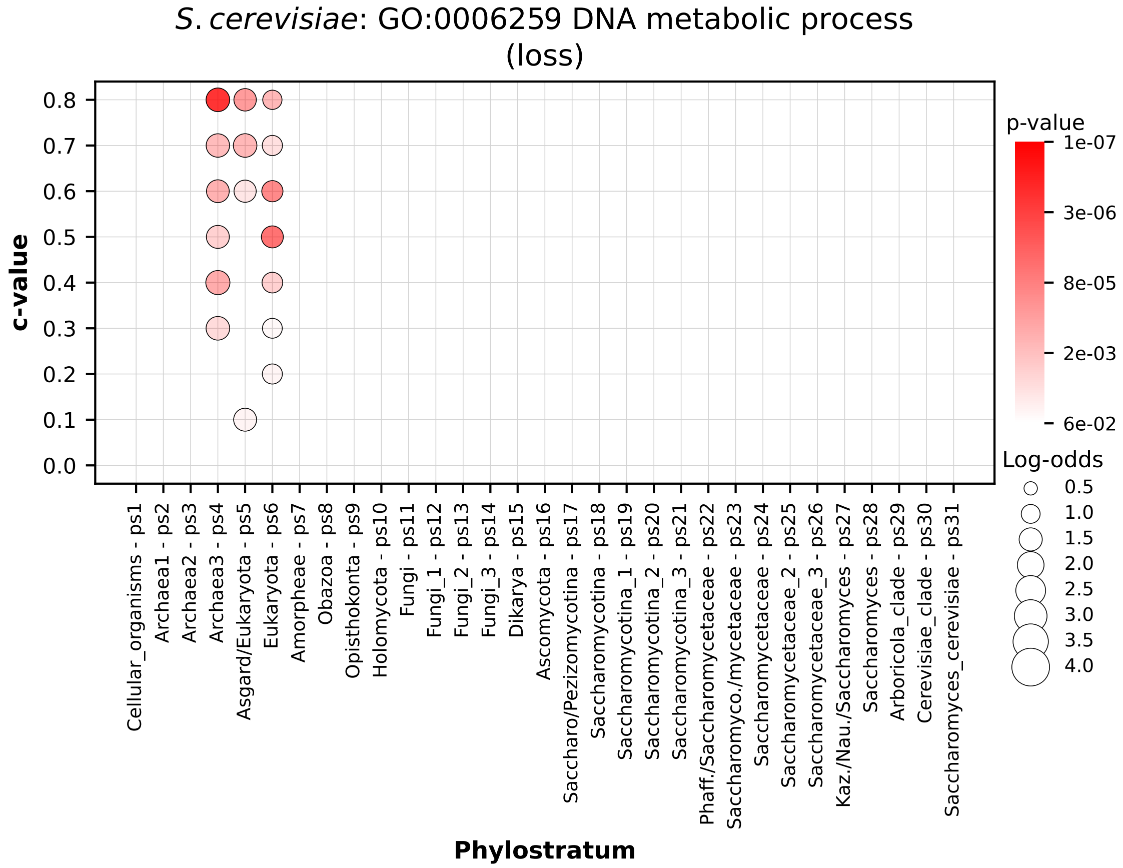

S. cerevisiae: GO:0006259 DNA metabolic process (loss)
c-value
0.8
0.7
0.6
0.5
0.4
0.3
0.2
0.1
0.0
p-value
1e-07
3e-06
8e-05
2e-03
6e-02
Log-odds
0.5
1.0
1.5
2.0
2.5
3.0
3.5
4.0
Cellular_organisms - ps1
Archaea1 - ps2
Archaea2 - ps3
Archaea3 - ps4
Asgard/Eukaryota - ps5
Eukaryota - ps6
Amorpheae - ps7
Obazoa - ps8
Opisthokonta - ps9
Holomycota - ps10
Fungi - ps11
Fungi_1 - ps12
Fungi_2 - ps13
Fungi_3 - ps14
Dikarya - ps15
Ascomycota - ps16
Saccharo/Pezizomycotina - ps17
Saccharomycotina - ps18
Saccharomycotina_1 - ps19
Saccharomycotina_2 - ps20
Saccharomycotina_3 - ps21
Phaff./Saccharomycetaceae - ps22
Saccharomyco./mycetaceae - ps23
Saccharomycetaceae - ps24
Saccharomycetaceae_2 - ps25
Saccharomycetaceae_3 - ps26
Kaz./Nau./Saccharomyces - ps27
Saccharomyces - ps28
Arboricola_clade - ps29
Cerevisiae_clade - ps30
Saccharomyces_cerevisiae - ps31
Phylostratum

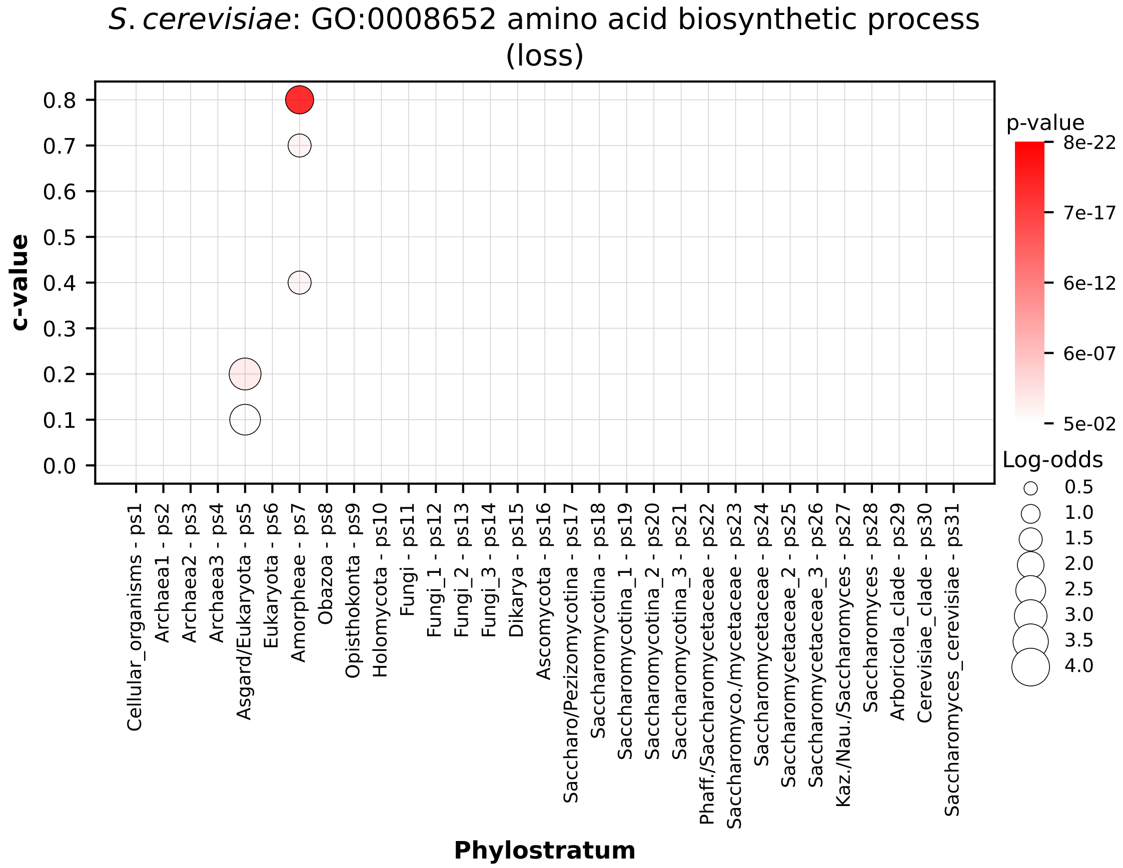

S. cerevisiae: GO:0008652 amino acid biosynthetic process (loss)
c-value
0.0
0.1
0.2
0.3
0.4
0.5
0.6
0.7
0.8
Phylostratum
Cellular_organisms - ps1
Archaea1 - ps2
Archaea2 - ps3
Archaea3 - ps4
Asgard/Eukaryota - ps5
Eukaryota - ps6
Amorpheae - ps7
Obazoa - ps8
Opisthokonta - ps9
Holomycota - ps10
Fungi - ps11
Fungi_1 - ps12
Fungi_2 - ps13
Fungi_3 - ps14
Dikarya - ps15
Ascomycota - ps16
Saccharo/Pezizomycotina - ps17
Saccharomycotina - ps18
Saccharomycotina_1 - ps19
Saccharomycotina_2 - ps20
Saccharomycotina_3 - ps21
Phaff./Saccharomycetaceae - ps22
Saccharomyco./mycetaceae - ps23
Saccharomycetaceae - ps24
Saccharomycetaceae_2 - ps25
Saccharomycetaceae_3 - ps26
Kaz./Nau./Saccharomyces - ps27
Saccharomyces - ps28
Arboricola_clade - ps29
Cerevisiae_clade - ps30
Saccharomyces_cerevisiae - ps31
p-value
8e-22
7e-17
6e-12
6e-07
5e-02
Log-odds
0.5
1.0
1.5
2.0
2.5
3.0
3.5
4.0

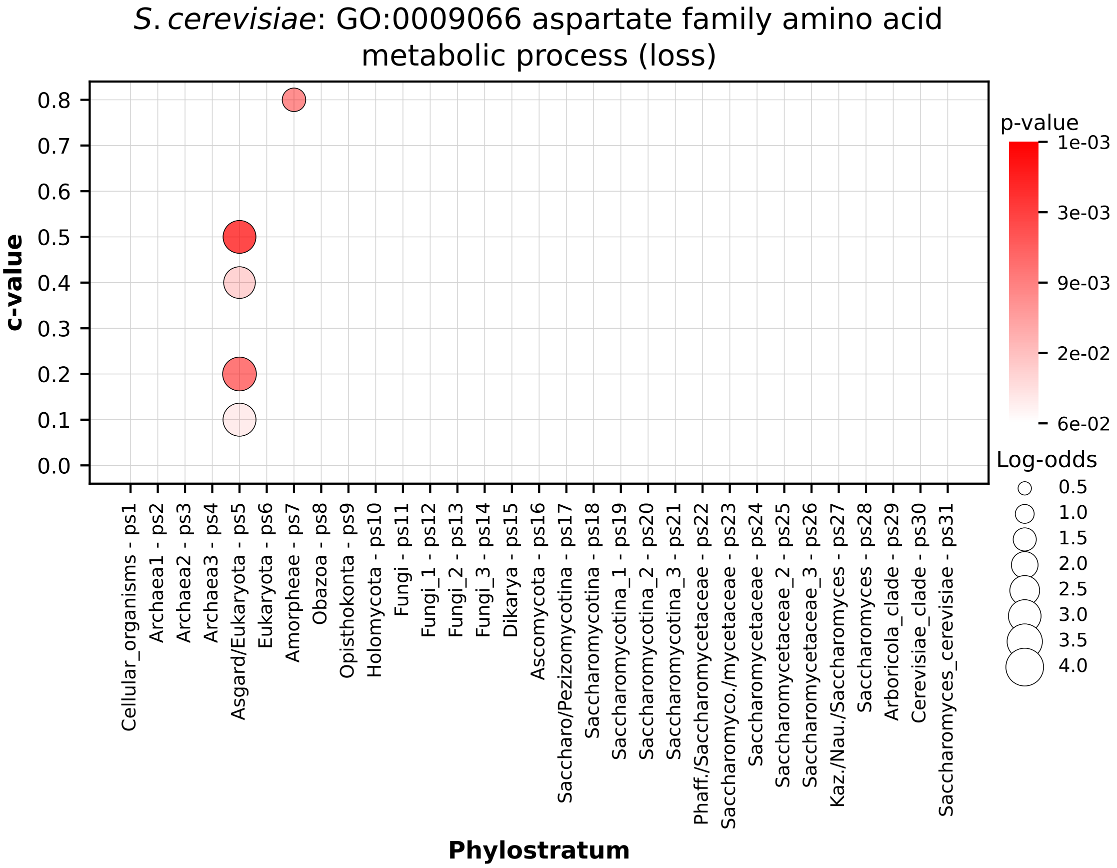

S. cerevisiae: GO:0009066 aspartate family amino acid metabolic process (loss)
c-value
0.0
0.1
0.2
0.3
0.4
0.5
0.6
0.7
0.8
p-value
1e-03
3e-03
9e-03
2e-02
6e-02
Log-odds
0.5
1.0
1.5
2.0
2.5
3.0
3.5
4.0
Cellular_organisms - ps1
Archaea1 - ps2
Archaea2 - ps3
Archaea3 - ps4
Asgard/Eukaryota - ps5
Eukaryota - ps6
Amorpheae - ps7
Obazoa - ps8
Opisthokonta - ps9
Holomycota - ps10
Fungi - ps11
Fungi_1 - ps12
Fungi_2 - ps13
Fungi_3 - ps14
Dikarya - ps15
Ascomycota - ps16
Saccharo/Pezizomycotina - ps17
Saccharomycotina - ps18
Saccharomycotina_1 - ps19
Saccharomycotina_2 - ps20
Saccharomycotina_3 - ps21
Phaff./Saccharomycetaceae - ps22
Saccharomyco./mycetaceae - ps23
Saccharomycetaceae - ps24
Saccharomycetaceae_2 - ps25
Saccharomycetaceae_3 - ps26
Kaz./Nau./Saccharomyces - ps27
Saccharomyces - ps28
Arboricola_clade - ps29
Cerevisiae_clade - ps30
Saccharomyces_cerevisiae - ps31
Phylostratum

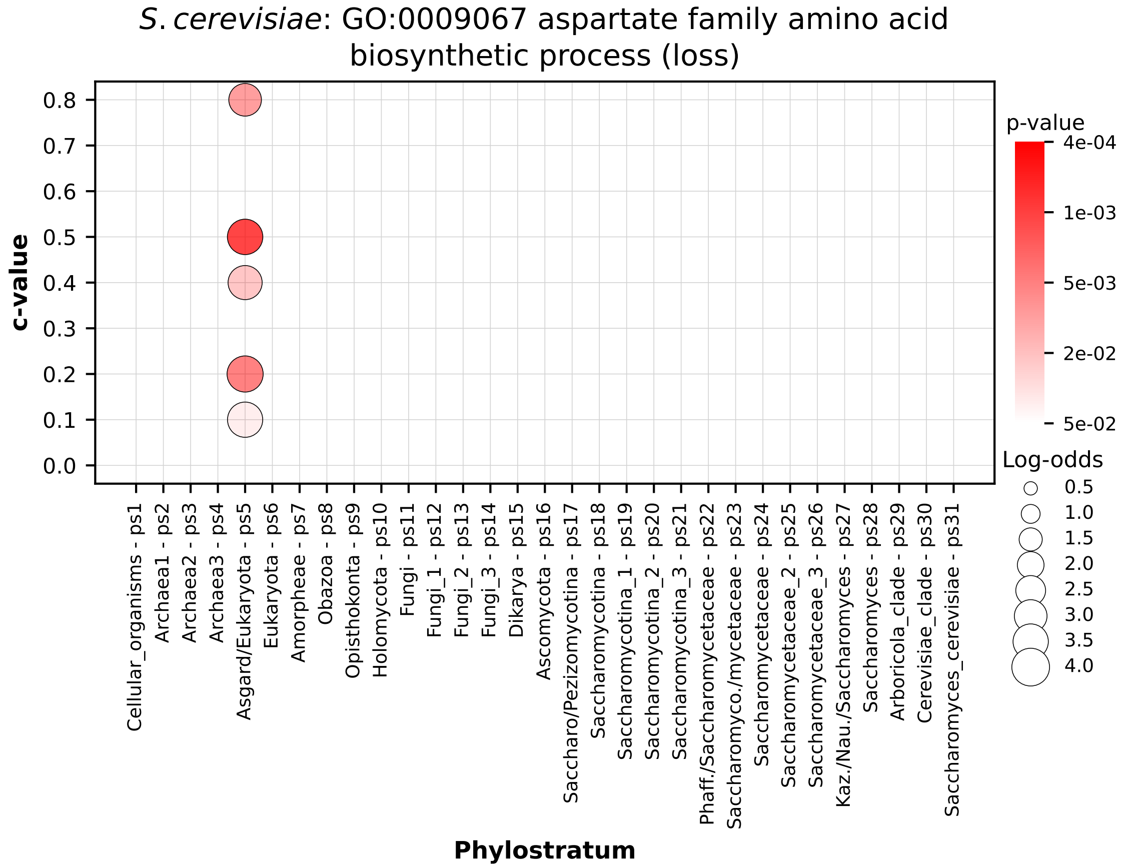

S. cerevisiae: GO:0009067 aspartate family amino acid biosynthetic process (loss)
c-value
0.8
0.7
0.6
0.5
0.4
0.3
0.2
0.1
0.0
p-value
4e-04
1e-03
5e-03
2e-02
5e-02
Log-odds
0.5
1.0
1.5
2.0
2.5
3.0
3.5
4.0
Cellular_organisms - ps1
Archaea1 - ps2
Archaea2 - ps3
Archaea3 - ps4
Asgard/Eukaryota - ps5
Eukaryota - ps6
Amorpheae - ps7
Obazoa - ps8
Opisthokonta - ps9
Holomycota - ps10
Fungi - ps11
Fungi_1 - ps12
Fungi_2 - ps13
Fungi_3 - ps14
Dikarya - ps15
Ascomycota - ps16
Saccharo/Pezizomycotina - ps17
Saccharomycotina - ps18
Saccharomycotina_1 - ps19
Saccharomycotina_2 - ps20
Saccharomycotina_3 - ps21
Phaff./Saccharomycetaceae - ps22
Saccharomyco./mycetaceae - ps23
Saccharomycetaceae - ps24
Saccharomycetaceae_2 - ps25
Saccharomycetaceae_3 - ps26
Kaz./Nau./Saccharomyces - ps27
Saccharomyces - ps28
Arboricola_clade - ps29
Cerevisiae_clade - ps30
Saccharomyces_cerevisiae - ps31
Phylostratum

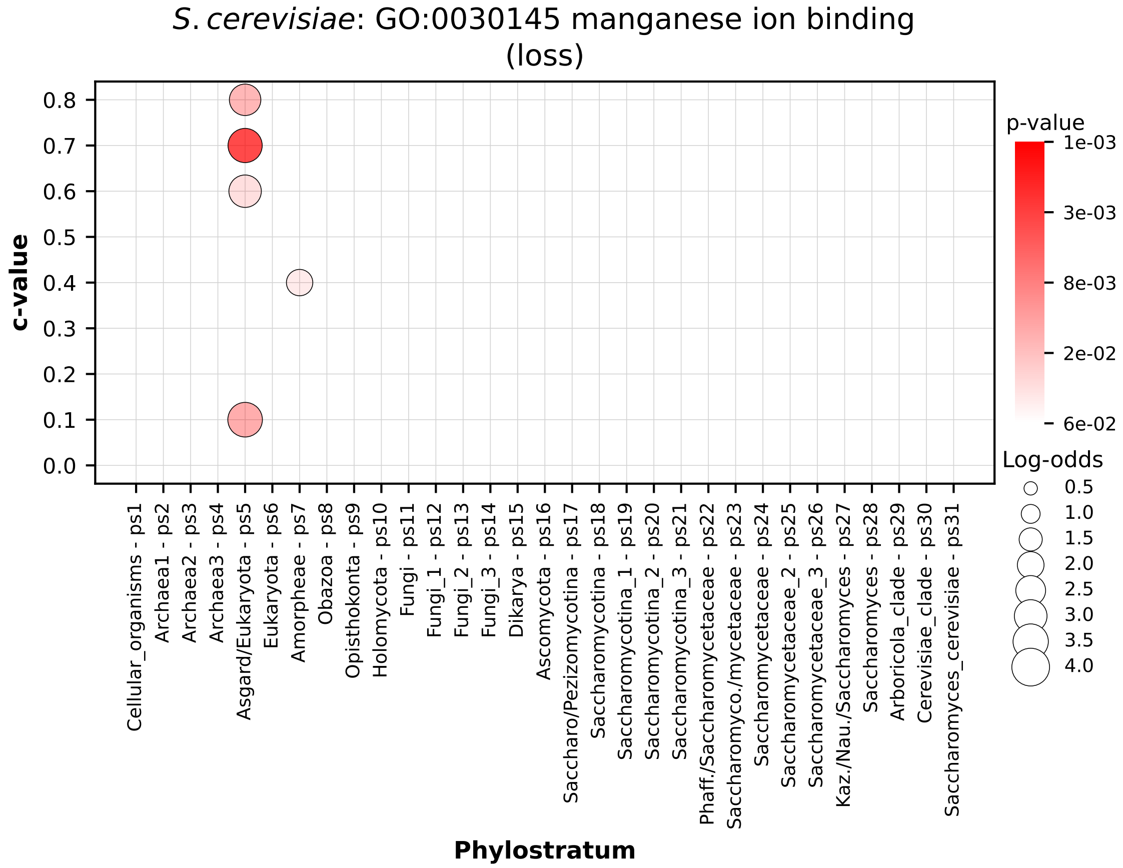

S. cerevisiae: GO:0030145 manganese ion binding
(loss)
c-value
0.8
0.7
0.6
0.5
0.4
0.3
0.2
0.1
0.0
p-value
1e-03
3e-03
8e-03
2e-02
6e-02
Log-odds
0.5
1.0
1.5
2.0
2.5
3.0
3.5
4.0
Cellular_organisms - ps1
Archaea1 - ps2
Archaea2 - ps3
Archaea3 - ps4
Asgard/Eukaryota - ps5
Eukaryota - ps6
Amorpheae - ps7
Obazoa - ps8
Opisthokonta - ps9
Holomycota - ps10
Fungi - ps11
Fungi_1 - ps12
Fungi_2 - ps13
Fungi_3 - ps14
Dikarya - ps15
Ascomycota - ps16
Saccharo/Pezizomycotina - ps17
Saccharomycotina - ps18
Saccharomycotina_1 - ps19
Saccharomycotina_2 - ps20
Saccharomycotina_3 - ps21
Phaff./Saccharomycetaceae - ps22
Saccharomyco./mycetaceae - ps23
Saccharomycetaceae - ps24
Saccharomycetaceae_2 - ps25
Saccharomycetaceae_3 - ps26
Kaz./Nau./Saccharomyces - ps27
Saccharomyces - ps28
Arboricola_clade - ps29
Cerevisiae_clade - ps30
Saccharomyces_cerevisiae - ps31
Phylostratum

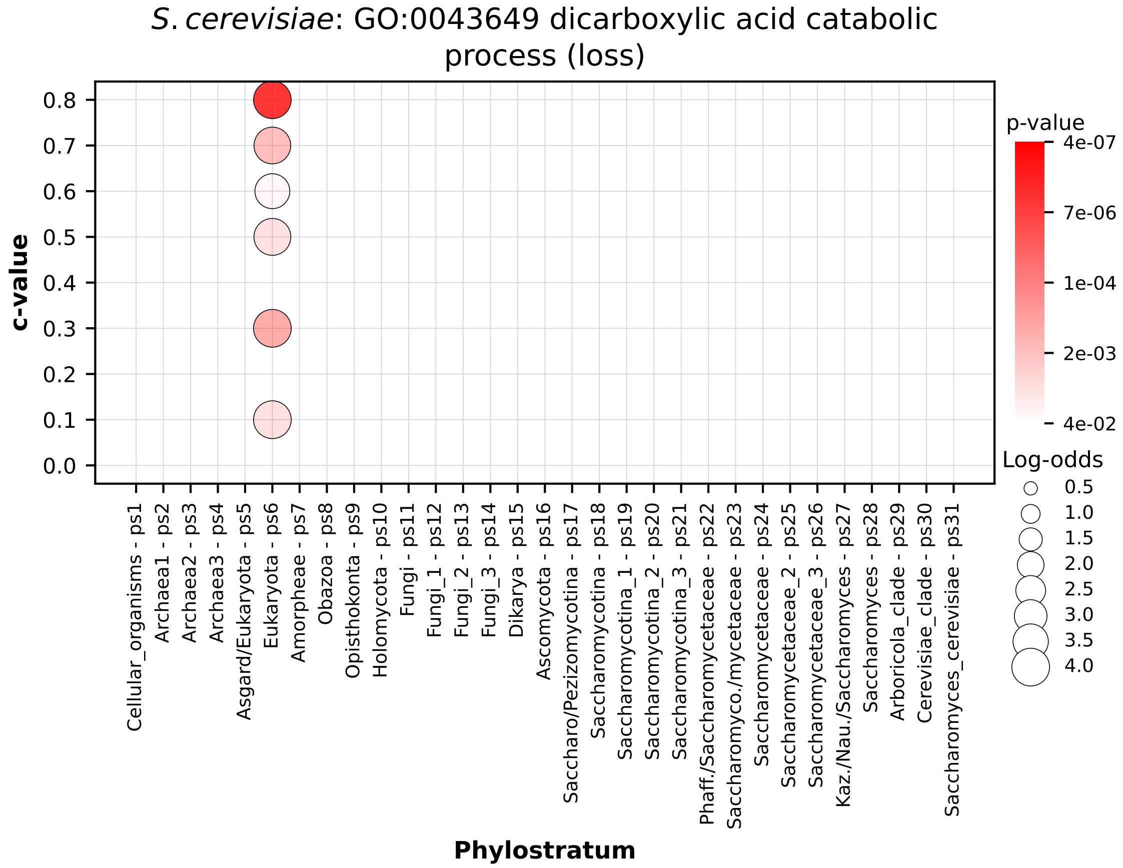

S. cerevisiae: GO:0043649 dicarboxylic acid catabolic process (loss)
c-value
0.0
0.1
0.2
0.3
0.4
0.5
0.6
0.7
0.8
p-value
4e-07
7e-06
1e-04
2e-03
4e-02
Log-odds
0.5
1.0
1.5
2.0
2.5
3.0
3.5
4.0
Cellular_organisms - ps1
Archaea1 - ps2
Archaea2 - ps3
Archaea3 - ps4
Asgard/Eukaryota - ps5
Eukaryota - ps6
Amorpheae - ps7
Obazoa - ps8
Opisthokonta - ps9
Holomycota - ps10
Fungi - ps11
Fungi_1 - ps12
Fungi_2 - ps13
Fungi_3 - ps14
Dikarya - ps15
Ascomycota - ps16
Saccharo/Pezizomycotina - ps17
Saccharomycotina - ps18
Saccharomycotina_1 - ps19
Saccharomycotina_2 - ps20
Saccharomycotina_3 - ps21
Phaff./Saccharomycetaceae - ps22
Saccharomyco./mycetaceae - ps23
Saccharomycetaceae - ps24
Saccharomycetaceae_2 - ps25
Saccharomycetaceae_3 - ps26
Kaz./Nau./Saccharomyces - ps27
Saccharomyces - ps28
Arboricola_clade - ps29
Cerevisiae_clade - ps30
Saccharomyces_cerevisiae - ps31
Phylostratum

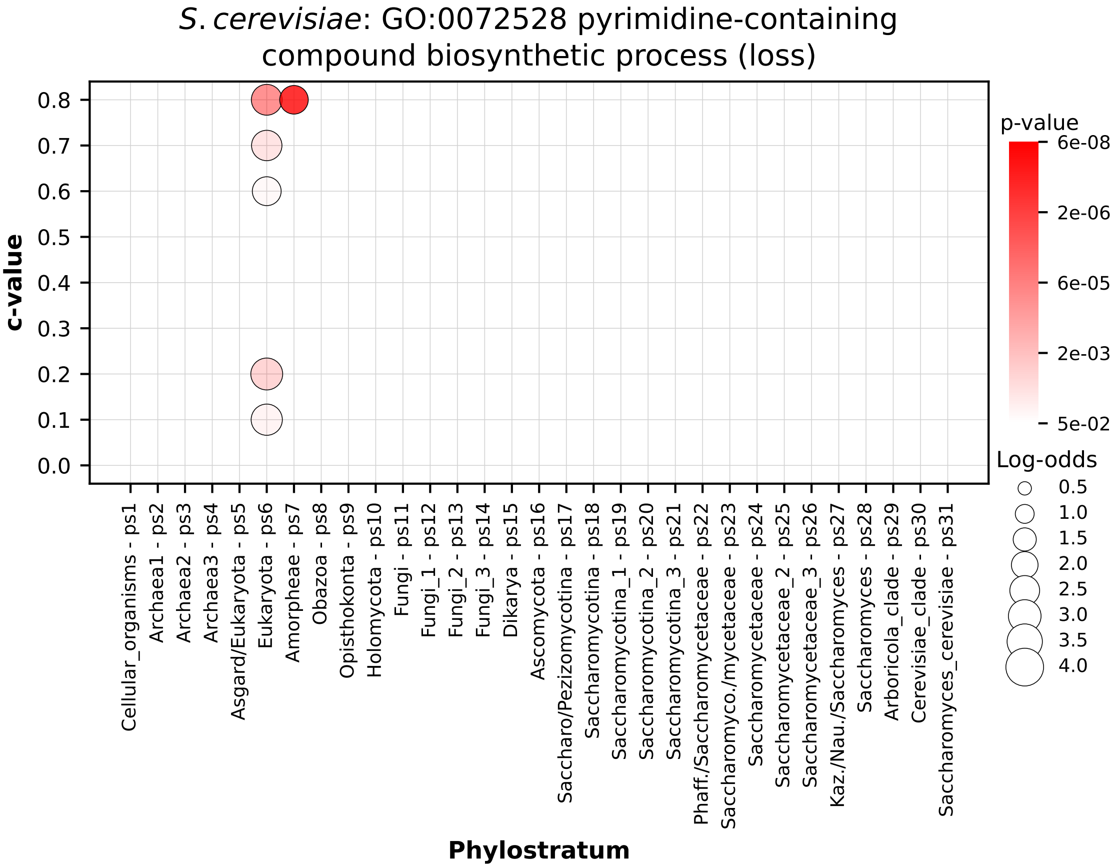

S. cerevisiae: GO:0072528 pyrimidine-containing compound biosynthetic process (loss)
c-value
0.8
0.7
0.6
0.5
0.4
0.3
0.2
0.1
0.0
p-value
6e-08
2e-06
6e-05
2e-03
5e-02
Log-odds
0.5
1.0
1.5
2.0
2.5
3.0
3.5
4.0
Cellular_organisms - ps1
Archaea1 - ps2
Archaea2 - ps3
Archaea3 - ps4
Asgard/Eukaryota - ps5
Eukaryota - ps6
Amorpheae - ps7
Obazoa - ps8
Opisthokonta - ps9
Holomycota - ps10
Fungi - ps11
Fungi_1 - ps12
Fungi_2 - ps13
Fungi_3 - ps14
Dikarya - ps15
Ascomycota - ps16
Saccharo/Pezizomycotina - ps17
Saccharomycotina - ps18
Saccharomycotina_1 - ps19
Saccharomycotina_2 - ps20
Saccharomycotina_3 - ps21
Phaff./Saccharomycetaceae - ps22
Saccharomyco./mycetaceae - ps23
Saccharomycetaceae - ps24
Saccharomycetaceae_2 - ps25
Saccharomycetaceae_3 - ps26
Kaz./Nau./Saccharomyces - ps27
Saccharomyces - ps28
Arboricola_clade - ps29
Cerevisiae_clade - ps30
Saccharomyces_cerevisiae - ps31
Phylostratum

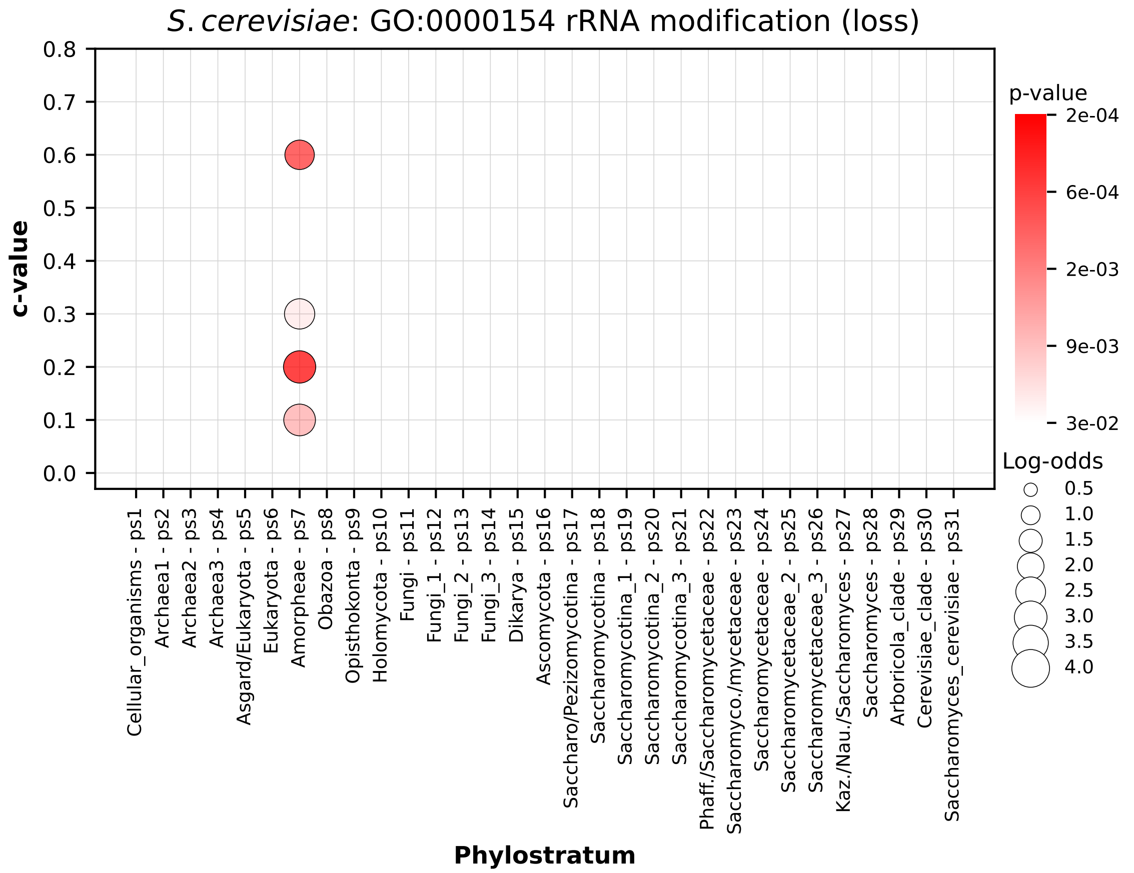
S. cerevisiae: GO:0000154 rRNA modification (loss)
c-value
0.8
0.7
0.6
0.5
0.4
0.3
0.2
0.1
0.0
p-value
2e-04
6e-04
2e-03
9e-03
3e-02
Log-odds
0.5
1.0
1.5
2.0
2.5
3.0
3.5
4.0
Cellular_organisms - ps1
Archaea1 - ps2
Archaea2 - ps3
Archaea3 - ps4
Asgard/Eukaryota - ps5
Eukaryota - ps6
Amorpheae - ps7
Obazoa - ps8
Opisthokonta - ps9
Holomycota - ps10
Fungi - ps11
Fungi_1 - ps12
Fungi_2 - ps13
Fungi_3 - ps14
Dikarya - ps15
Ascomycota - ps16
Saccharo/Pezizomycotina - ps17
Saccharomycotina - ps18
Saccharomycotina_1 - ps19
Saccharomycotina_2 - ps20
Saccharomycotina_3 - ps21
Phaff./Saccharomycetaceae - ps22
Saccharomyco./mycetaceae - ps23
Saccharomycetaceae - ps24
Saccharomycetaceae_2 - ps25
Saccharomycetaceae_3 - ps26
Kaz./Nau./Saccharomyces - ps27
Saccharomyces - ps28
Arboricola_clade - ps29
Cerevisiae_clade - ps30
Saccharomyces_cerevisiae - ps31
Phylostratum

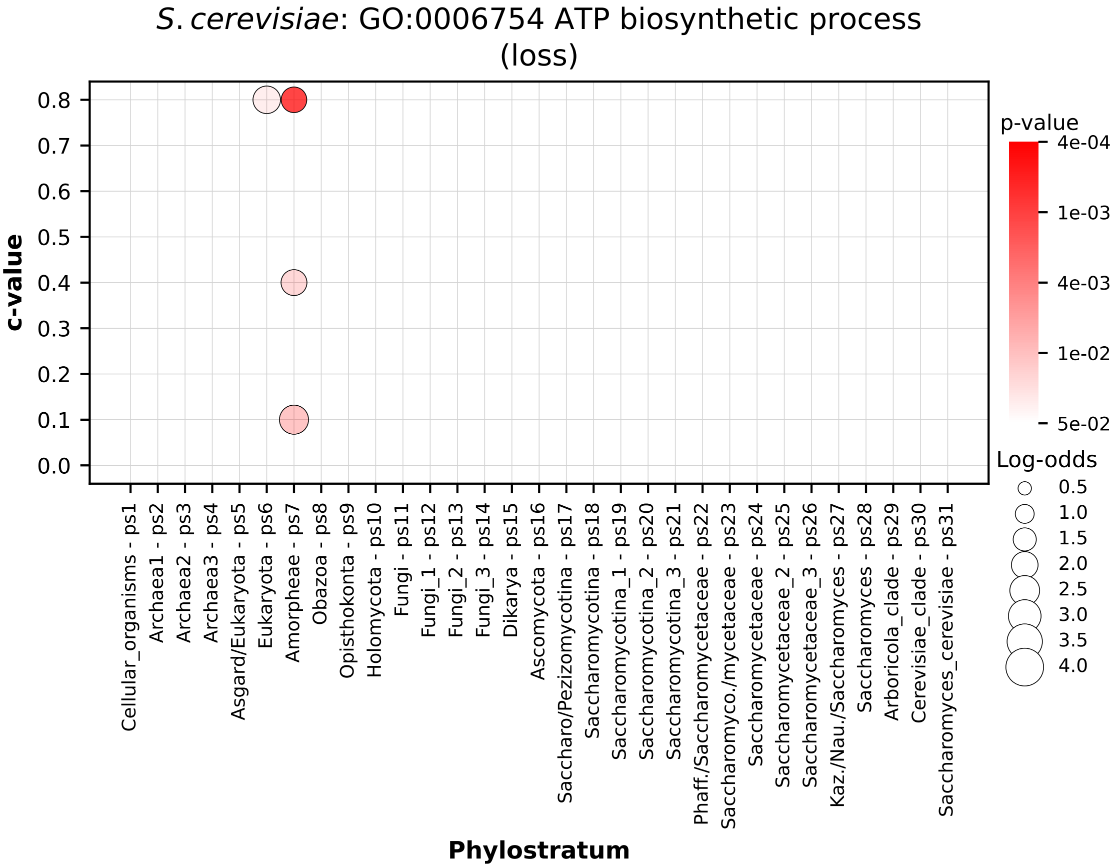
S. cerevisiae: GO:0006754 ATP biosynthetic process
(loss)
c-value
0.8
0.7
0.6
0.5
0.4
0.3
0.2
0.1
0.0
p-value
4e-04
1e-03
4e-03
1e-02
5e-02
Log-odds
0.5
1.0
1.5
2.0
2.5
3.0
3.5
4.0
Cellular_organisms - ps1
Archaea1 - ps2
Archaea2 - ps3
Archaea3 - ps4
Asgard/Eukaryota - ps5
Eukaryota - ps6
Amorpheae - ps7
Obazoa - ps8
Opisthokonta - ps9
Holomycota - ps10
Fungi - ps11
Fungi_1 - ps12
Fungi_2 - ps13
Fungi_3 - ps14
Dikarya - ps15
Ascomycota - ps16
Saccharo/Pezizomycotina - ps17
Saccharomycotina - ps18
Saccharomycotina_1 - ps19
Saccharomycotina_2 - ps20
Saccharomycotina_3 - ps21
Phaff./Saccharomycetaceae - ps22
Saccharomyco./mycetaceae - ps23
Saccharomycetaceae - ps24
Saccharomycetaceae_2 - ps25
Saccharomycetaceae_3 - ps26
Kaz./Nau./Saccharomyces - ps27
Saccharomyces - ps28
Arboricola_clade - ps29
Cerevisiae_clade - ps30
Saccharomyces_cerevisiae - ps31
Phylostratum

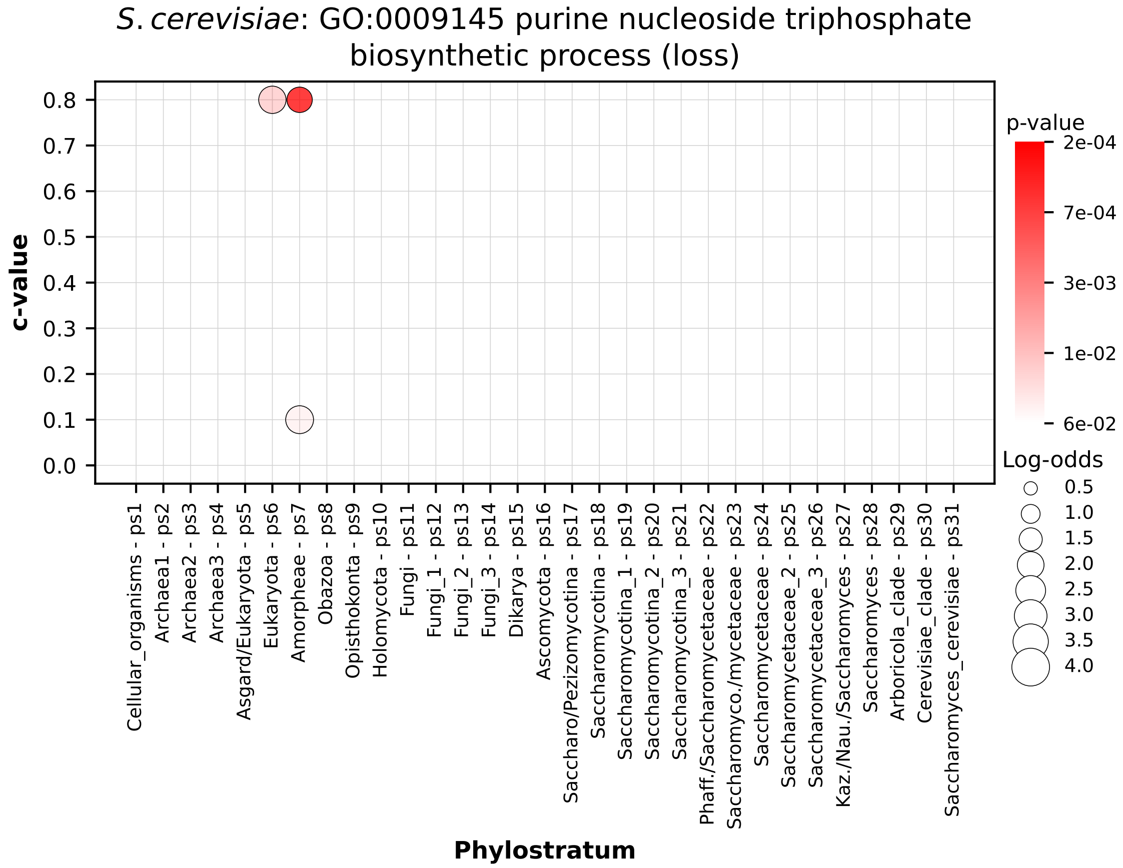

S. cerevisiae: GO:0009145 purine nucleoside triphosphate biosynthetic process (loss)
c-value
0.8
0.7
0.6
0.5
0.4
0.3
0.2
0.1
0.0
Phylostratum
Cellular_organisms - ps1
Archaea1 - ps2
Archaea2 - ps3
Archaea3 - ps4
Asgard/Eukaryota - ps5
Eukaryota - ps6
Amorpheae - ps7
Obazoa - ps8
Opisthokonta - ps9
Holomycota - ps10
Fungi - ps11
Fungi_1 - ps12
Fungi_2 - ps13
Fungi_3 - ps14
Dikarya - ps15
Ascomycota - ps16
Saccharo/Pezizomycotina - ps17
Saccharomycotina - ps18
Saccharomycotina_1 - ps19
Saccharomycotina_2 - ps20
Saccharomycotina_3 - ps21
Phaff./Saccharomycetaceae - ps22
Saccharomyco./mycetaceae - ps23
Saccharomycetaceae - ps24
Saccharomycetaceae_2 - ps25
Saccharomycetaceae_3 - ps26
Kaz./Nau./Saccharomyces - ps27
Saccharomyces - ps28
Arboricola_clade - ps29
Cerevisiae_clade - ps30
Saccharomyces_cerevisiae - ps31
p-value
2e-04
7e-04
3e-03
1e-02
6e-02
Log-odds
0.5
1.0
1.5
2.0
2.5
3.0
3.5
4.0

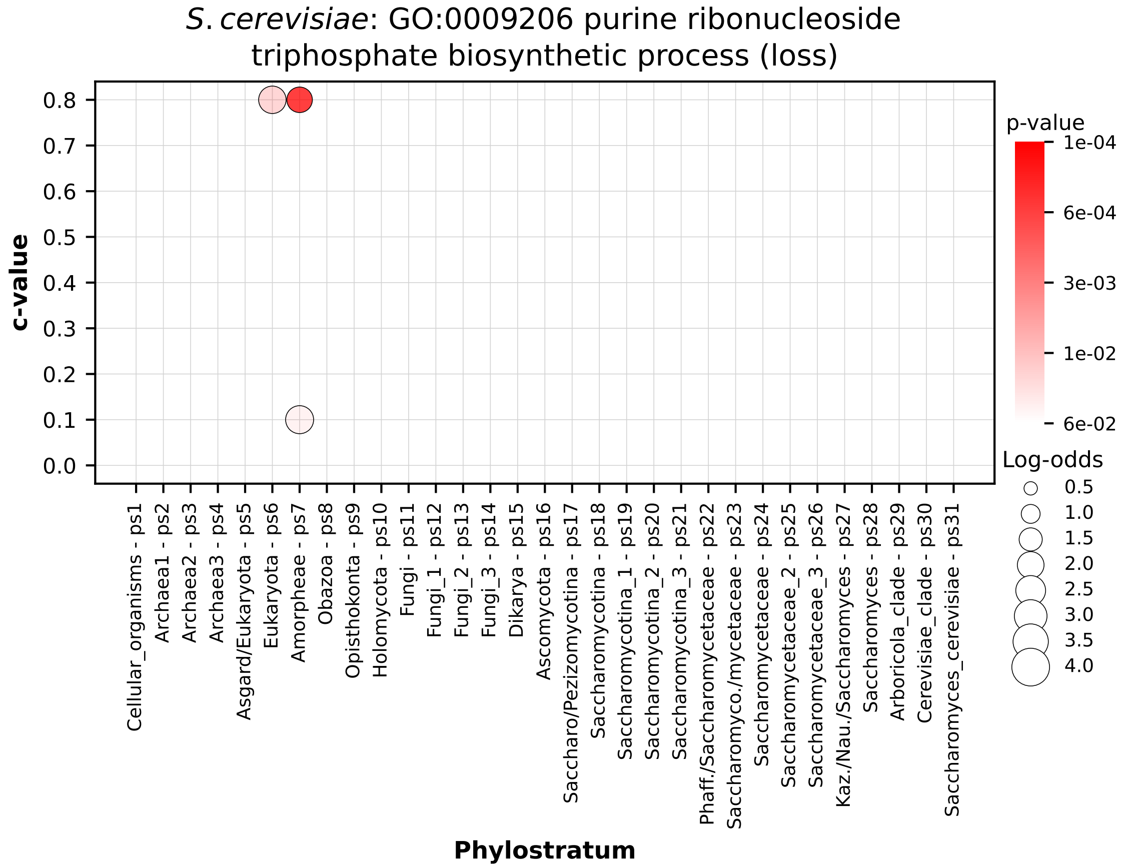

S. cerevisiae: GO:0009206 purine ribonucleoside triphosphate biosynthetic process (loss)
c-value
0.8
0.7
0.6
0.5
0.4
0.3
0.2
0.1
0.0
p-value
1e-04
6e-04
3e-03
1e-02
6e-02
Log-odds
0.5
1.0
1.5
2.0
2.5
3.0
3.5
4.0
Cellular_organisms - ps1
Archaea1 - ps2
Archaea2 - ps3
Archaea3 - ps4
Asgard/Eukaryota - ps5
Eukaryota - ps6
Amorpheae - ps7
Obazoa - ps8
Opisthokonta - ps9
Holomycota - ps10
Fungi - ps11
Fungi_1 - ps12
Fungi_2 - ps13
Fungi_3 - ps14
Dikarya - ps15
Ascomycota - ps16
Saccharo/Pezizomycotina - ps17
Saccharomycotina - ps18
Saccharomycotina_1 - ps19
Saccharomycotina_2 - ps20
Saccharomycotina_3 - ps21
Phaff./Saccharomycetaceae - ps22
Saccharomyco./mycetaceae - ps23
Saccharomycetaceae - ps24
Saccharomycetaceae_2 - ps25
Saccharomycetaceae_3 - ps26
Kaz./Nau./Saccharomyces - ps27
Saccharomyces - ps28
Arboricola_clade - ps29
Cerevisiae_clade - ps30
Saccharomyces_cerevisiae - ps31
Phylostratum

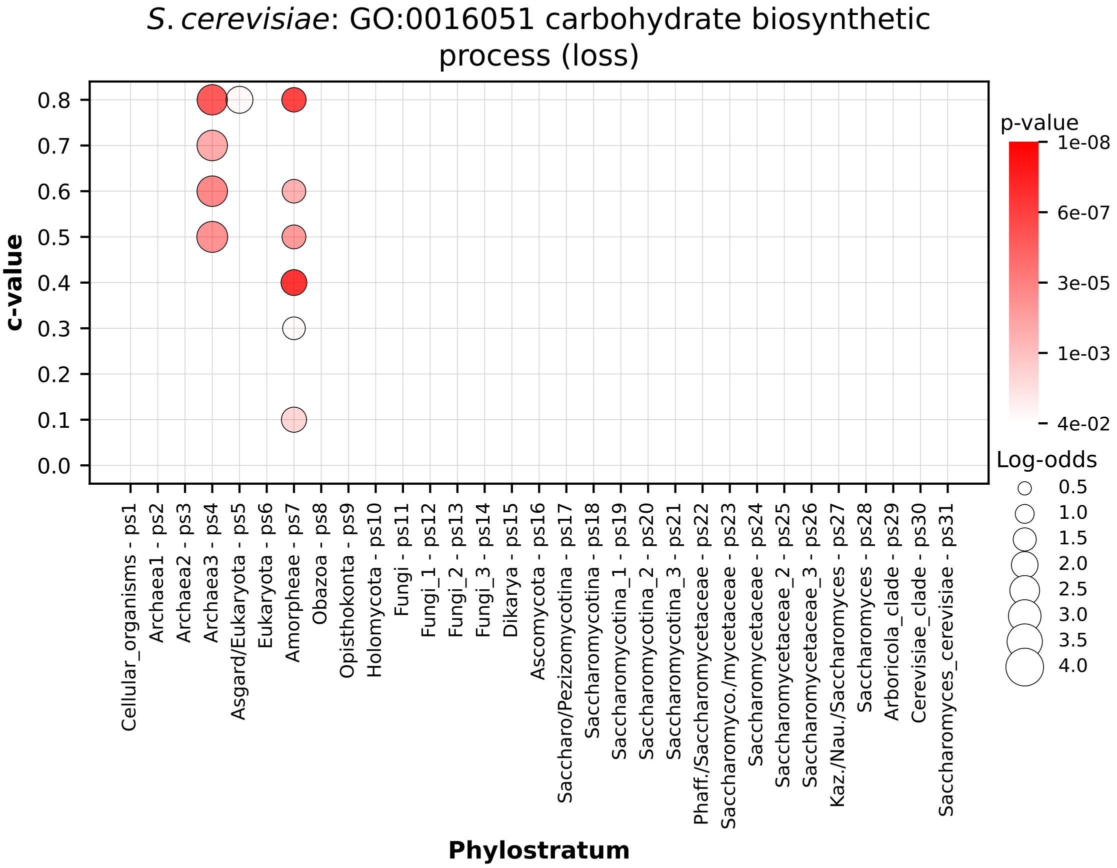
S. cerevisiae: GO:0016051 carbohydrate biosynthetic process (loss)
c-value
0.0
0.1
0.2
0.3
0.4
0.5
0.6
0.7
0.8
p-value
1e-08
6e-07
3e-05
1e-03
4e-02
Log-odds
0.5
1.0
1.5
2.0
2.5
3.0
3.5
4.0
Cellular_organisms - ps1
Archaea1 - ps2
Archaea2 - ps3
Archaea3 - ps4
Asgard/Eukaryota - ps5
Eukaryota - ps6
Amorpheae - ps7
Obazoa - ps8
Opisthokonta - ps9
Holomycota - ps10
Fungi - ps11
Fungi_1 - ps12
Fungi_2 - ps13
Fungi_3 - ps14
Dikarya - ps15
Ascomycota - ps16
Saccharo/Pezizomycotina - ps17
Saccharomycotina - ps18
Saccharomycotina_1 - ps19
Saccharomycotina_2 - ps20
Saccharomycotina_3 - ps21
Phaff./Saccharomycetaceae - ps22
Saccharomyco./mycetaceae - ps23
Saccharomycetaceae - ps24
Saccharomycetaceae_2 - ps25
Saccharomycetaceae_3 - ps26
Kaz./Nau./Saccharomyces - ps27
Saccharomyces - ps28
Arboricola_clade - ps29
Cerevisiae_clade - ps30
Saccharomyces_cerevisiae - ps31
Phylostratum

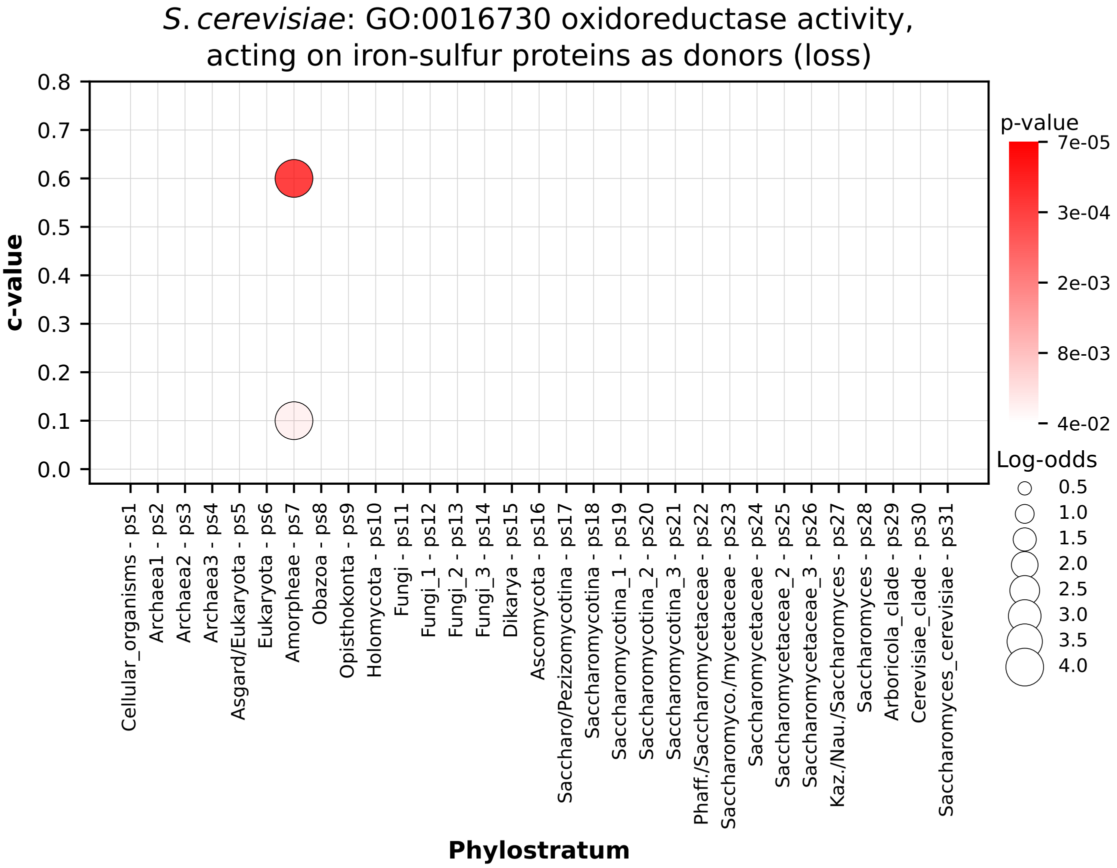
S. cerevisiae: GO:0016730 oxidoreductase activity, acting on iron-sulfur proteins as donors (loss)
c-value
0.8
0.7
0.6
0.5
0.4
0.3
0.2
0.1
0.0
p-value
7e-05
3e-04
2e-03
8e-03
4e-02
Log-odds
0.5
1.0
1.5
2.0
2.5
3.0
3.5
4.0
Cellular_organisms - ps1
Archaea1 - ps2
Archaea2 - ps3
Archaea3 - ps4
Asgard/Eukaryota - ps5
Eukaryota - ps6
Amorpheae - ps7
Obazoa - ps8
Opisthokonta - ps9
Holomycota - ps10
Fungi - ps11
Fungi_1 - ps12
Fungi_2 - ps13
Fungi_3 - ps14
Dikarya - ps15
Ascomycota - ps16
Saccharo/Pezizomycotina - ps17
Saccharomycotina - ps18
Saccharomycotina_1 - ps19
Saccharomycotina_2 - ps20
Saccharomycotina_3 - ps21
Phaff./Saccharomycetaceae - ps22
Saccharomyco./mycetaceae - ps23
Saccharomycetaceae - ps24
Saccharomycetaceae_2 - ps25
Saccharomycetaceae_3 - ps26
Kaz./Nau./Saccharomyces - ps27
Saccharomyces - ps28
Arboricola_clade - ps29
Cerevisiae_clade - ps30
Saccharomyces_cerevisiae - ps31
Phylostratum

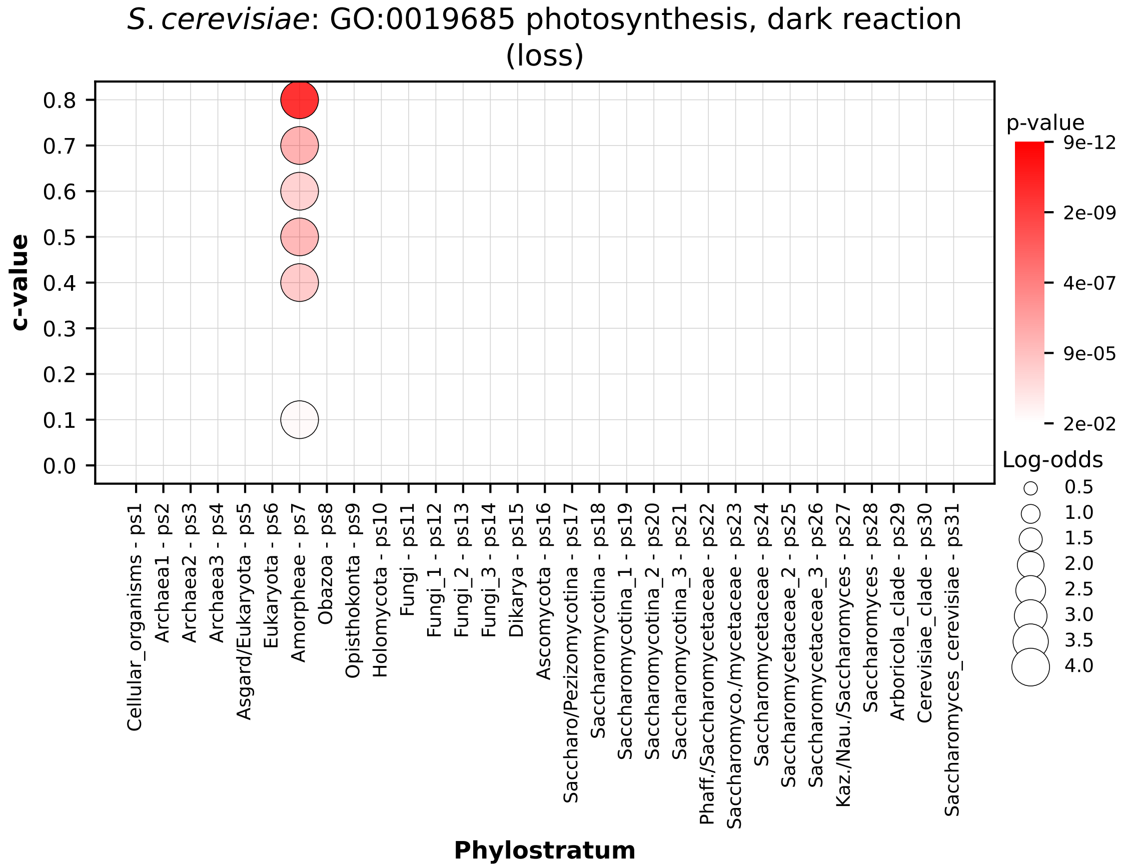
S. cerevisiae: GO:0019685 photosynthesis, dark reaction (loss)
c-value
0.8
0.7
0.6
0.5
0.4
0.3
0.2
0.1
0.0
p-value
9e-12
2e-09
4e-07
9e-05
2e-02
Log-odds
0.5
1.0
1.5
2.0
2.5
3.0
3.5
4.0
Cellular_organisms - ps1
Archaea1 - ps2
Archaea2 - ps3
Archaea3 - ps4
Asgard/Eukaryota - ps5
Eukaryota - ps6
Amorpheae - ps7
Obazoa - ps8
Opisthokonta - ps9
Holomycota - ps10
Fungi - ps11
Fungi_1 - ps12
Fungi_2 - ps13
Fungi_3 - ps14
Dikarya - ps15
Ascomycota - ps16
Saccharo/Pezizomycotina - ps17
Saccharomycotina - ps18
Saccharomycotina_1 - ps19
Saccharomycotina_2 - ps20
Saccharomycotina_3 - ps21
Phaff./Saccharomycetaceae - ps22
Saccharomyco./mycetaceae - ps23
Saccharomycetaceae - ps24
Saccharomycetaceae_2 - ps25
Saccharomycetaceae_3 - ps26
Kaz./Nau./Saccharomyces - ps27
Saccharomyces - ps28
Arboricola_clade - ps29
Cerevisiae_clade - ps30
Saccharomyces_cerevisiae - ps31
Phylostratum

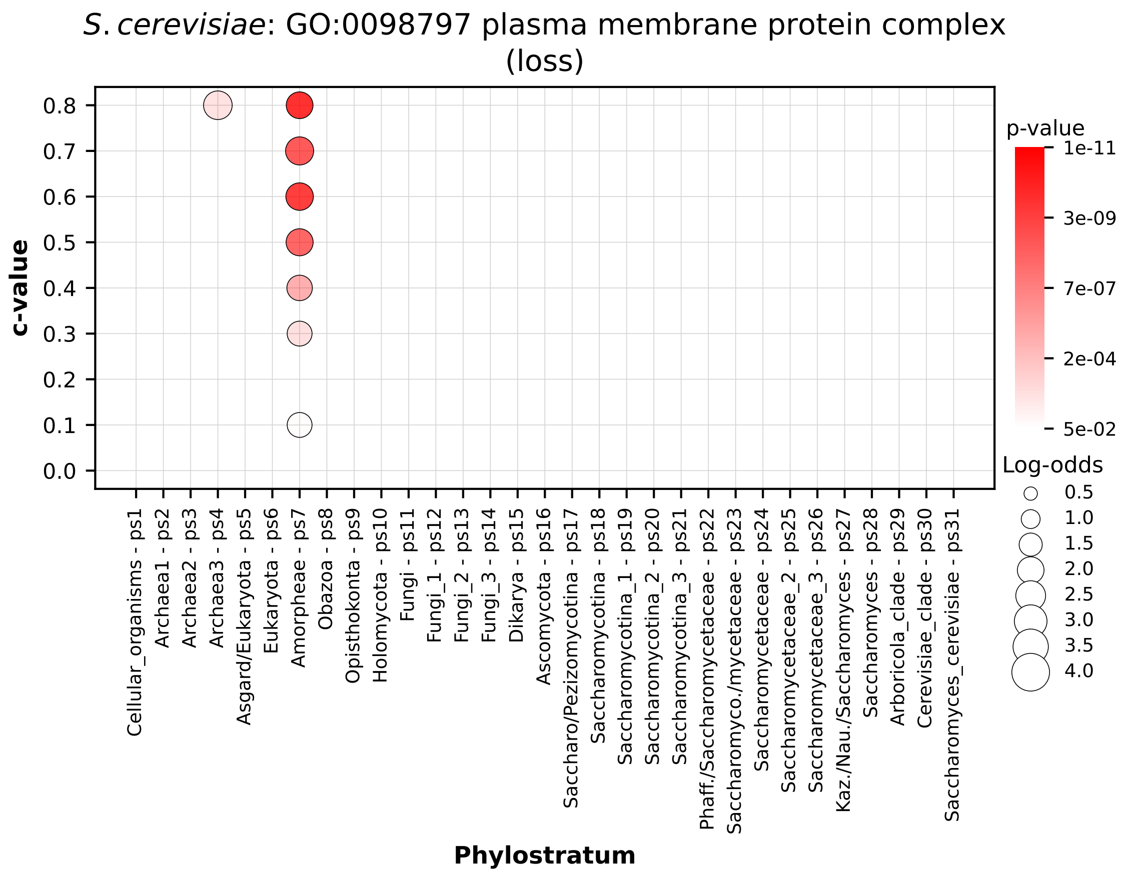
S. cerevisiae: GO:0098797 plasma membrane protein complex (loss)
c-value
Phylostratum
p-value
1e-11
3e-09
7e-07
2e-04
5e-02
Log-odds
0.5
1.0
1.5
2.0
2.5
3.0
3.5
4.0
Cellular_organisms - ps1
Archaea1 - ps2
Archaea2 - ps3
Archaea3 - ps4
Asgard/Eukaryota - ps5
Eukaryota - ps6
Amorpheae - ps7
Obazoa - ps8
Opisthokonta - ps9
Holomycota - ps10
Fungi - ps11
Fungi_1 - ps12
Fungi_2 - ps13
Fungi_3 - ps14
Dikarya - ps15
Ascomycota - ps16
Saccharo/Pezizomycotina - ps17
Saccharomycotina - ps18
Saccharomycotina_1 - ps19
Saccharomycotina_2 - ps20
Saccharomycotina_3 - ps21
Phaff./Saccharomycetaceae - ps22
Saccharomyco./mycetaceae - ps23
Saccharomycetaceae - ps24
Saccharomycetaceae_2 - ps25
Saccharomycetaceae_3 - ps26
Kaz./Nau./Saccharomyces - ps27
Saccharomyces - ps28
Arboricola_clade - ps29
Cerevisiae_clade - ps30
Saccharomyces_cerevisiae - ps31

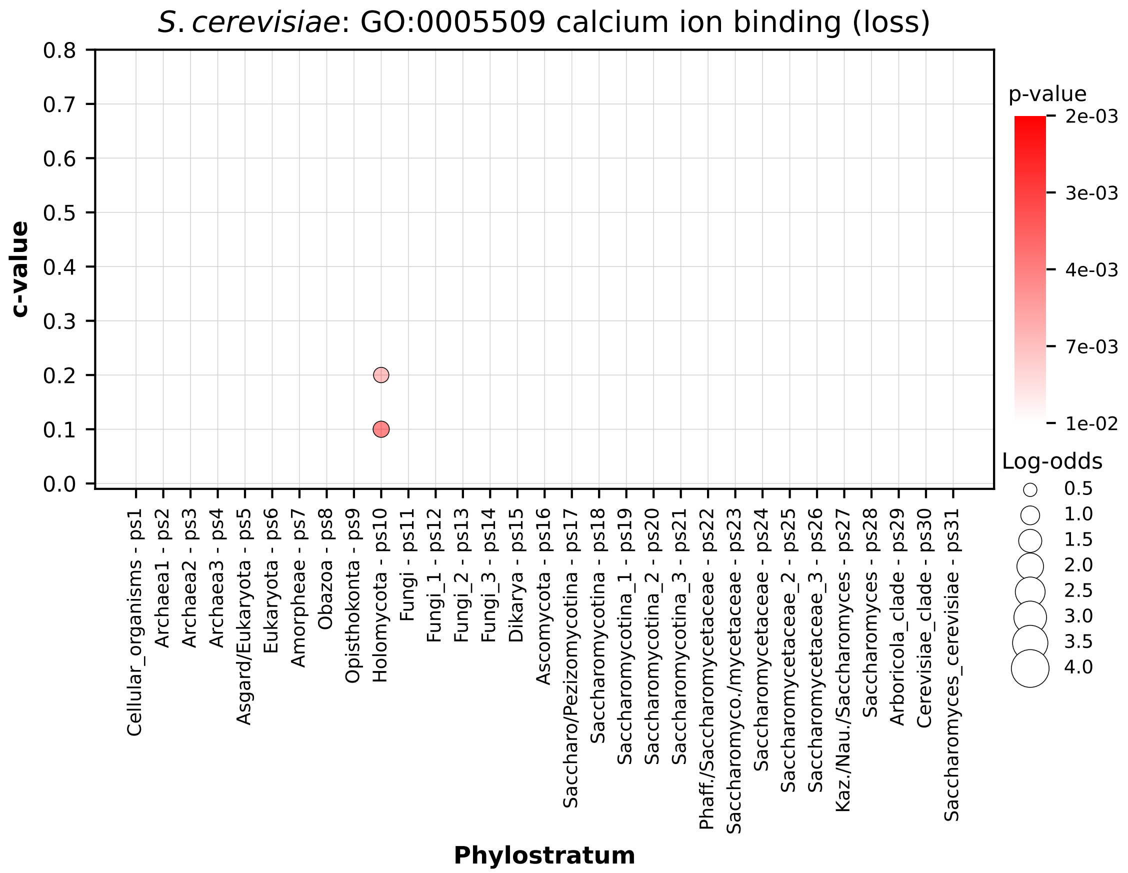
S. cerevisiae: GO:0005509 calcium ion binding (loss)
c-value
0.8
0.7
0.6
0.5
0.4
0.3
0.2
0.1
0.0
Phylostratum
Cellular_organisms - ps1
Archaea1 - ps2
Archaea2 - ps3
Archaea3 - ps4
Asgard/Eukaryota - ps5
Eukaryota - ps6
Amorpheae - ps7
Obazoa - ps8
Opisthokonta - ps9
Holomycota - ps10
Fungi - ps11
Fungi_1 - ps12
Fungi_2 - ps13
Fungi_3 - ps14
Dikarya - ps15
Ascomycota - ps16
Saccharo/Pezizomycotina - ps17
Saccharomycotina - ps18
Saccharomycotina_1 - ps19
Saccharomycotina_2 - ps20
Saccharomycotina_3 - ps21
Phaff./Saccharomycetaceae - ps22
Saccharomyco./mycetaceae - ps23
Saccharomycetaceae - ps24
Saccharomycetaceae_2 - ps25
Saccharomycetaceae_3 - ps26
Kaz./Nau./Saccharomyces - ps27
Saccharomyces - ps28
Arboricola_clade - ps29
Cerevisiae_clade - ps30
Saccharomyces_cerevisiae - ps31
p-value
2e-03
3e-03
4e-03
7e-03
1e-02
Log-odds
0.5
1.0
1.5
2.0
2.5
3.0
3.5
4.0

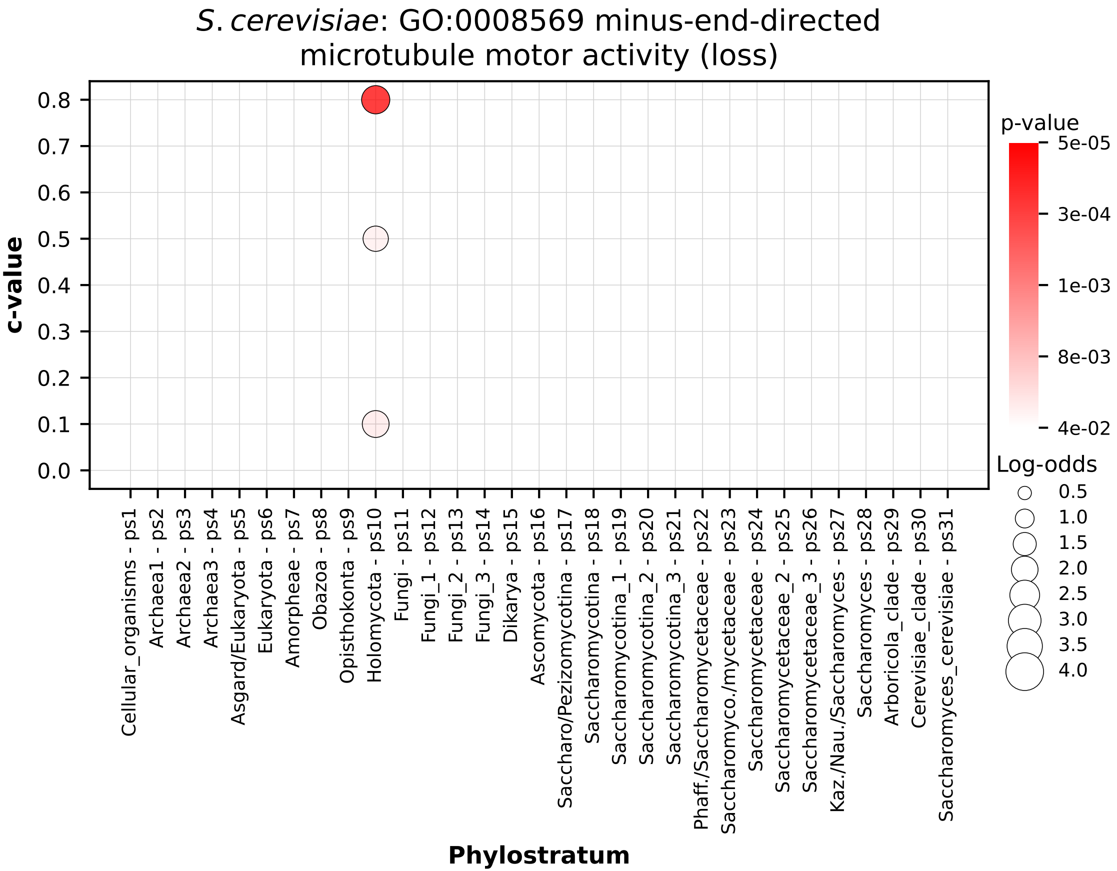
S. cerevisiae: GO:0008569 minus-end-directed microtubule motor activity (loss)
c-value
0.0
0.1
0.2
0.3
0.4
0.5
0.6
0.7
0.8
Phylostratum
Cellular_organisms - ps1
Archaea1 - ps2
Archaea2 - ps3
Archaea3 - ps4
Asgard/Eukaryota - ps5
Eukaryota - ps6
Amorpheae - ps7
Obazoa - ps8
Opisthokonta - ps9
Holomycota - ps10
Fungi - ps11
Fungi_1 - ps12
Fungi_2 - ps13
Fungi_3 - ps14
Dikarya - ps15
Ascomycota - ps16
Saccharo/Pezizomycotina - ps17
Saccharomycotina - ps18
Saccharomycotina_1 - ps19
Saccharomycotina_2 - ps20
Saccharomycotina_3 - ps21
Phaff./Saccharomycetaceae - ps22
Saccharomyco./mycetaceae - ps23
Saccharomycetaceae - ps24
Saccharomycetaceae_2 - ps25
Saccharomycetaceae_3 - ps26
Kaz./Nau./Saccharomyces - ps27
Saccharomyces - ps28
Arboricola_clade - ps29
Cerevisiae_clade - ps30
Saccharomyces_cerevisiae - ps31
p-value
5e-05
3e-04
1e-03
8e-03
4e-02
Log-odds
0.5
1.0
1.5
2.0
2.5
3.0
3.5
4.0

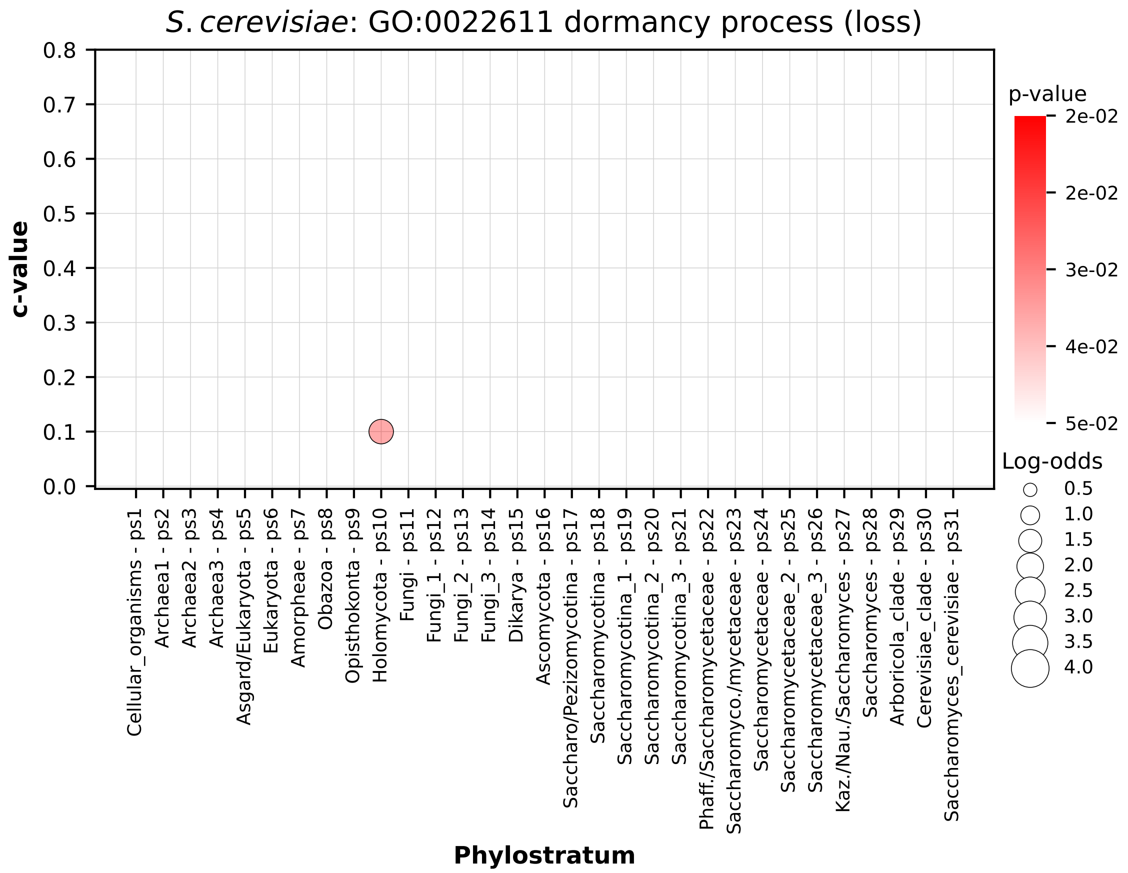

S. cerevisiae: GO:0022611 dormancy process (loss)
c-value
0.0
0.1
0.2
0.3
0.4
0.5
0.6
0.7
0.8
Phylostratum
Cellular_organisms - ps1
Archaea1 - ps2
Archaea2 - ps3
Archaea3 - ps4
Asgard/Eukaryota - ps5
Eukaryota - ps6
Amorpheae - ps7
Obazoa - ps8
Opisthokonta - ps9
Holomycota - ps10
Fungi - ps11
Fungi_1 - ps12
Fungi_2 - ps13
Fungi_3 - ps14
Dikarya - ps15
Ascomycota - ps16
Saccharo/Pezizomycotina - ps17
Saccharomycotina - ps18
Saccharomycotina_1 - ps19
Saccharomycotina_2 - ps20
Saccharomycotina_3 - ps21
Phaff./Saccharomycetaceae - ps22
Saccharomyco./mycetaceae - ps23
Saccharomycetaceae - ps24
Saccharomycetaceae_2 - ps25
Saccharomycetaceae_3 - ps26
Kaz./Nau./Saccharomyces - ps27
Saccharomyces - ps28
Arboricola_clade - ps29
Cerevisiae_clade - ps30
Saccharomyces_cerevisiae - ps31
p-value
2e-02
2e-02
3e-02
4e-02
5e-02
Log-odds
0.5
1.0
1.5
2.0
2.5
3.0
3.5
4.0

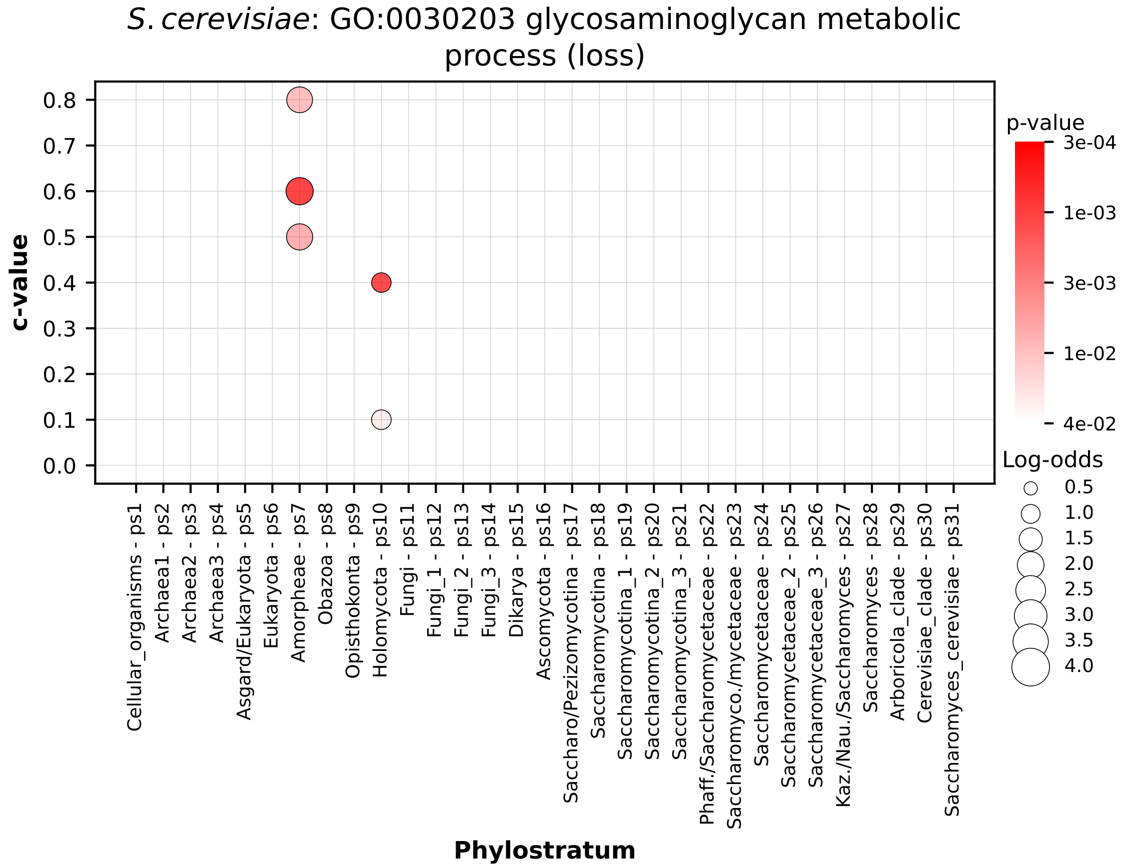

S. cerevisiae: GO:0030203 glycosaminoglycan metabolic process (loss)
c-value
0.8
0.7
0.6
0.5
0.4
0.3
0.2
0.1
0.0
Phylostratum
Cellular_organisms - ps1
Archaea1 - ps2
Archaea2 - ps3
Archaea3 - ps4
Asgard/Eukaryota - ps5
Eukaryota - ps6
Amorpheae - ps7
Obazoa - ps8
Opisthokonta - ps9
Holomycota - ps10
Fungi - ps11
Fungi_1 - ps12
Fungi_2 - ps13
Fungi_3 - ps14
Dikarya - ps15
Ascomycota - ps16
Saccharo/Pezizomycotina - ps17
Saccharomycotina - ps18
Saccharomycotina_1 - ps19
Saccharomycotina_2 - ps20
Saccharomycotina_3 - ps21
Phaff./Saccharomycetaceae - ps22
Saccharomyco./mycetaceae - ps23
Saccharomycetaceae - ps24
Saccharomycetaceae_2 - ps25
Saccharomycetaceae_3 - ps26
Kaz./Nau./Saccharomyces - ps27
Saccharomyces - ps28
Arboricola_clade - ps29
Cerevisiae_clade - ps30
Saccharomyces_cerevisiae - ps31
p-value
3e-04
1e-03
3e-03
1e-02
4e-02
Log-odds
0.5
1.0
1.5
2.0
2.5
3.0
3.5
4.0

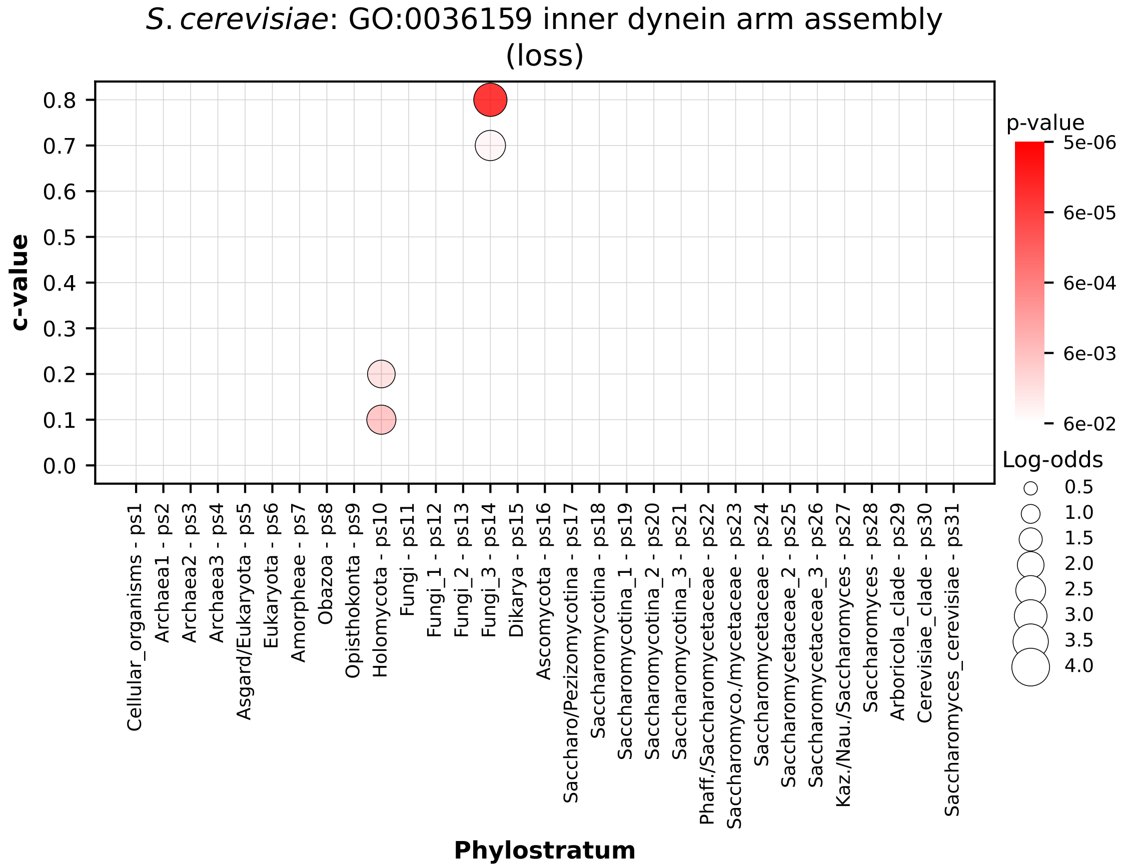

S. cerevisiae: GO:0036159 inner dynein arm assembly
(loss)
c-value
0.8
0.7
0.6
0.5
0.4
0.3
0.2
0.1
0.0
p-value
5e-06
6e-05
6e-04
6e-03
6e-02
Log-odds
0.5
1.0
1.5
2.0
2.5
3.0
3.5
4.0
Cellular_organisms - ps1
Archaea1 - ps2
Archaea2 - ps3
Archaea3 - ps4
Asgard/Eukaryota - ps5
Eukaryota - ps6
Amorpheae - ps7
Obazoa - ps8
Opisthokonta - ps9
Holomycota - ps10
Fungi - ps11
Fungi_1 - ps12
Fungi_2 - ps13
Fungi_3 - ps14
Dikarya - ps15
Ascomycota - ps16
Saccharo/Pezizomycotina - ps17
Saccharomycotina - ps18
Saccharomycotina_1 - ps19
Saccharomycotina_2 - ps20
Saccharomycotina_3 - ps21
Phaff./Saccharomycetaceae - ps22
Saccharomyco./mycetaceae - ps23
Saccharomycetaceae - ps24
Saccharomycetaceae_2 - ps25
Saccharomycetaceae_3 - ps26
Kaz./Nau./Saccharomyces - ps27
Saccharomyces - ps28
Arboricola_clade - ps29
Cerevisiae_clade - ps30
Saccharomyces_cerevisiae - ps31
Phylostratum

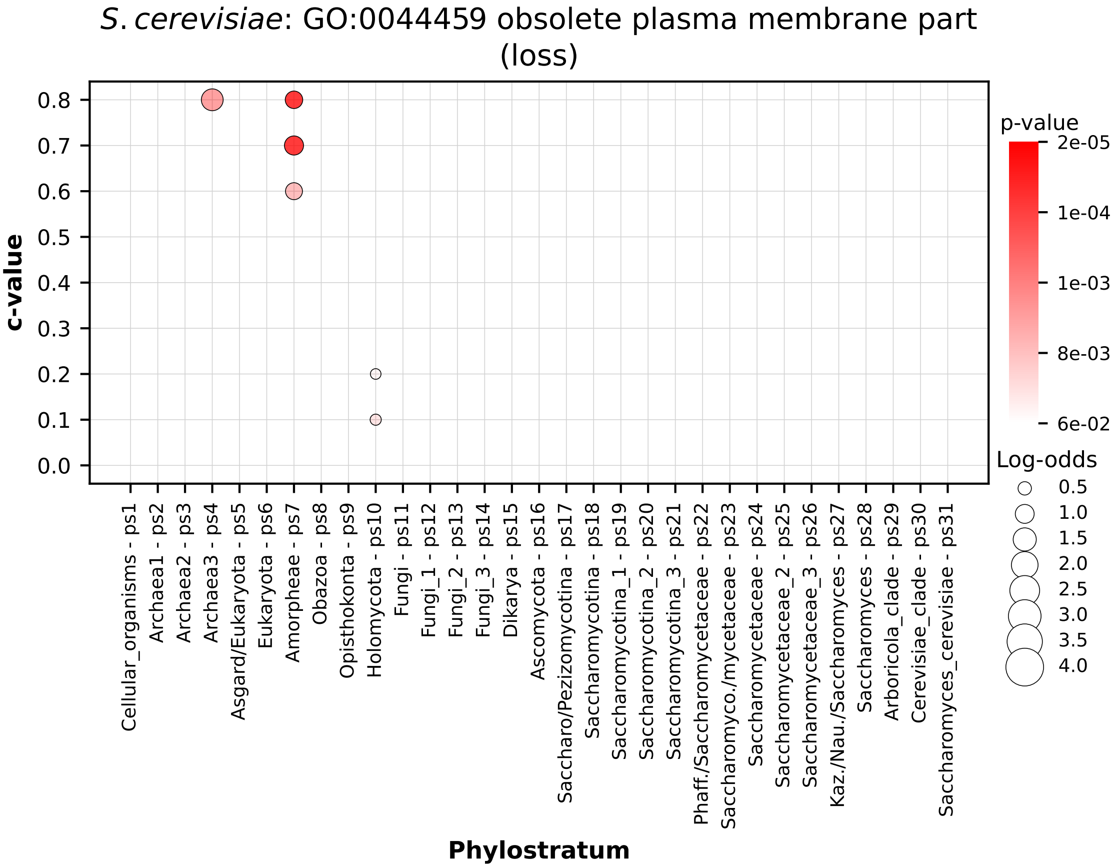

S. cerevisiae: GO:0044459 obsolete plasma membrane part
(loss)
c-value
0.8
0.7
0.6
0.5
0.4
0.3
0.2
0.1
0.0
p-value
2e-05
1e-04
1e-03
8e-03
6e-02
Log-odds
0.5
1.0
1.5
2.0
2.5
3.0
3.5
4.0
Cellular_organisms - ps1
Archaea1 - ps2
Archaea2 - ps3
Archaea3 - ps4
Asgard/Eukaryota - ps5
Eukaryota - ps6
Amorpheae - ps7
Obazoa - ps8
Opisthokonta - ps9
Holomycota - ps10
Fungi - ps11
Fungi_1 - ps12
Fungi_2 - ps13
Fungi_3 - ps14
Dikarya - ps15
Ascomycota - ps16
Saccharo/Pezizomycotina - ps17
Saccharomycotina - ps18
Saccharomycotina_1 - ps19
Saccharomycotina_2 - ps20
Saccharomycotina_3 - ps21
Phaff./Saccharomycetaceae - ps22
Saccharomyco./mycetaceae - ps23
Saccharomycetaceae - ps24
Saccharomycetaceae_2 - ps25
Saccharomycetaceae_3 - ps26
Kaz./Nau./Saccharomyces - ps27
Saccharomyces - ps28
Arboricola_clade - ps29
Cerevisiae_clade - ps30
Saccharomyces_cerevisiae - ps31
Phylostratum

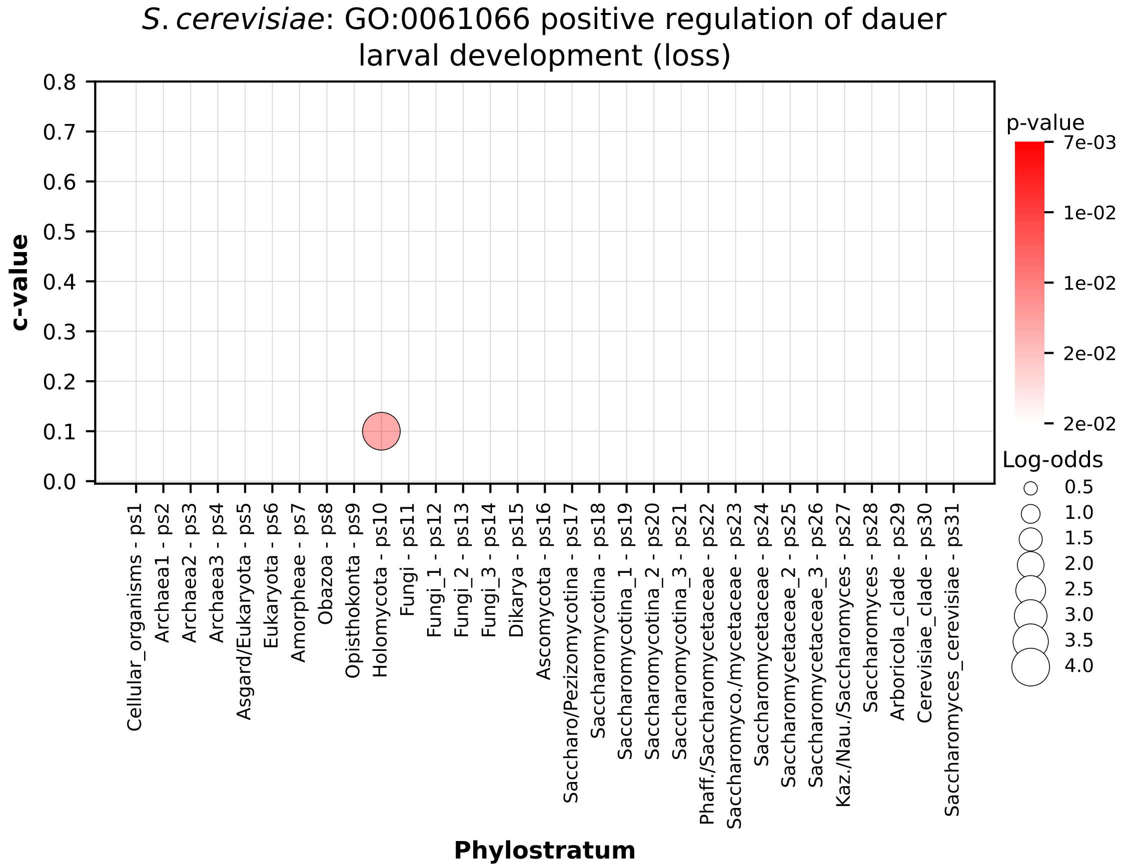

S. cerevisiae: GO:0061066 positive regulation of dauer larval development (loss)
c-value
0.8
0.7
0.6
0.5
0.4
0.3
0.2
0.1
0.0
p-value
7e-03
1e-02
1e-02
2e-02
2e-02
Log-odds
0.5
1.0
1.5
2.0
2.5
3.0
3.5
4.0
Cellular_organisms - ps1
Archaea1 - ps2
Archaea2 - ps3
Archaea3 - ps4
Asgard/Eukaryota - ps5
Eukaryota - ps6
Amorpheae - ps7
Obazoa - ps8
Opisthokonta - ps9
Holomycota - ps10
Fungi - ps11
Fungi_1 - ps12
Fungi_2 - ps13
Fungi_3 - ps14
Dikarya - ps15
Ascomycota - ps16
Saccharo/Pezizomycotina - ps17
Saccharomycotina - ps18
Saccharomycotina_1 - ps19
Saccharomycotina_2 - ps20
Saccharomycotina_3 - ps21
Phaff./Saccharomycetaceae - ps22
Saccharomyco./mycetaceae - ps23
Saccharomycetaceae - ps24
Saccharomycetaceae_2 - ps25
Saccharomycetaceae_3 - ps26
Kaz./Nau./Saccharomyces - ps27
Saccharomyces - ps28
Arboricola_clade - ps29
Cerevisiae_clade - ps30
Saccharomyces_cerevisiae - ps31
Phylostratum

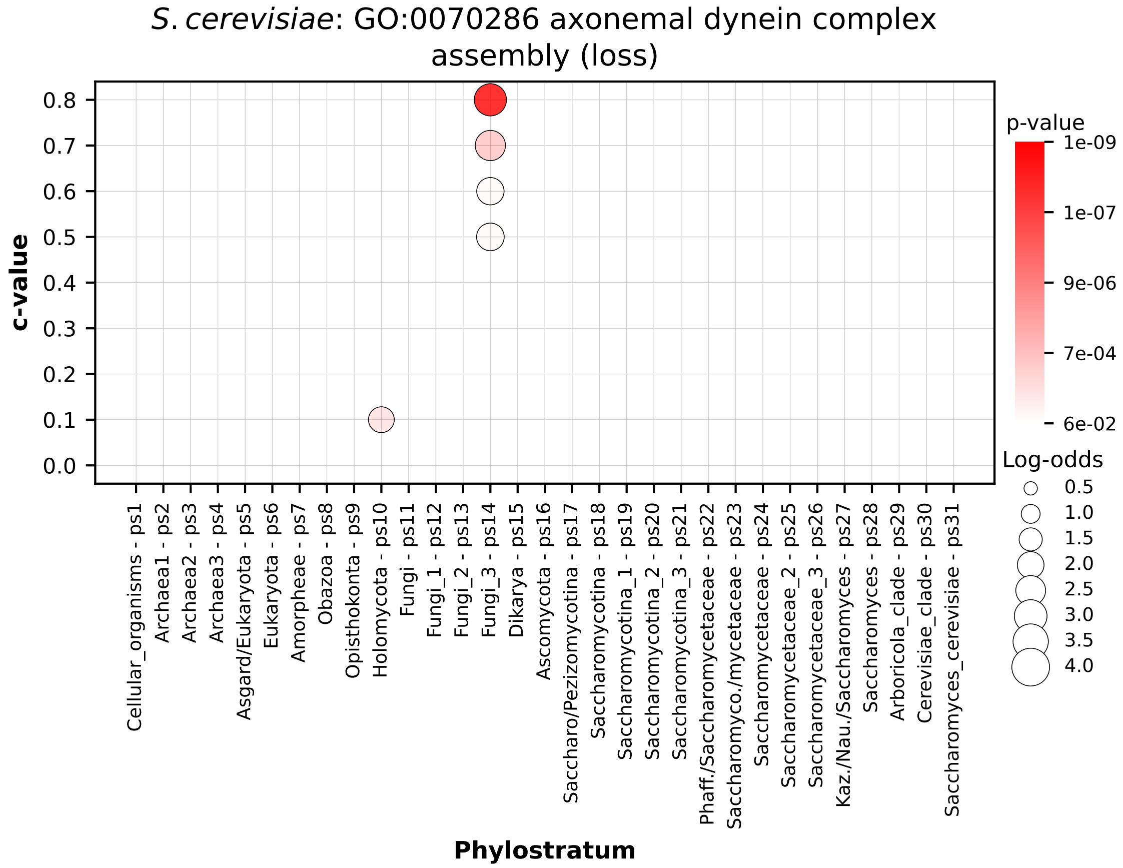
S. cerevisiae: GO:0070286 axonemal dynein complex assembly (loss)
c-value
0.8
0.7
0.6
0.5
0.4
0.3
0.2
0.1
0.0
p-value
1e-09
1e-07
9e-06
7e-04
6e-02
Log-odds
0.5
1.0
1.5
2.0
2.5
3.0
3.5
4.0
Cellular_organisms - ps1
Archaea1 - ps2
Archaea2 - ps3
Archaea3 - ps4
Asgard/Eukaryota - ps5
Eukaryota - ps6
Amorpheae - ps7
Obazoa - ps8
Opisthokonta - ps9
Holomycota - ps10
Fungi - ps11
Fungi_1 - ps12
Fungi_2 - ps13
Fungi_3 - ps14
Dikarya - ps15
Ascomycota - ps16
Saccharo/Pezizomycotina - ps17
Saccharomycotina - ps18
Saccharomycotina_1 - ps19
Saccharomycotina_2 - ps20
Saccharomycotina_3 - ps21
Phaff./Saccharomycetaceae - ps22
Saccharomyco./mycetaceae - ps23
Saccharomycetaceae - ps24
Saccharomycetaceae_2 - ps25
Saccharomycetaceae_3 - ps26
Kaz./Nau./Saccharomyces - ps27
Saccharomyces - ps28
Arboricola_clade - ps29
Cerevisiae_clade - ps30
Saccharomyces_cerevisiae - ps31
Phylostratum

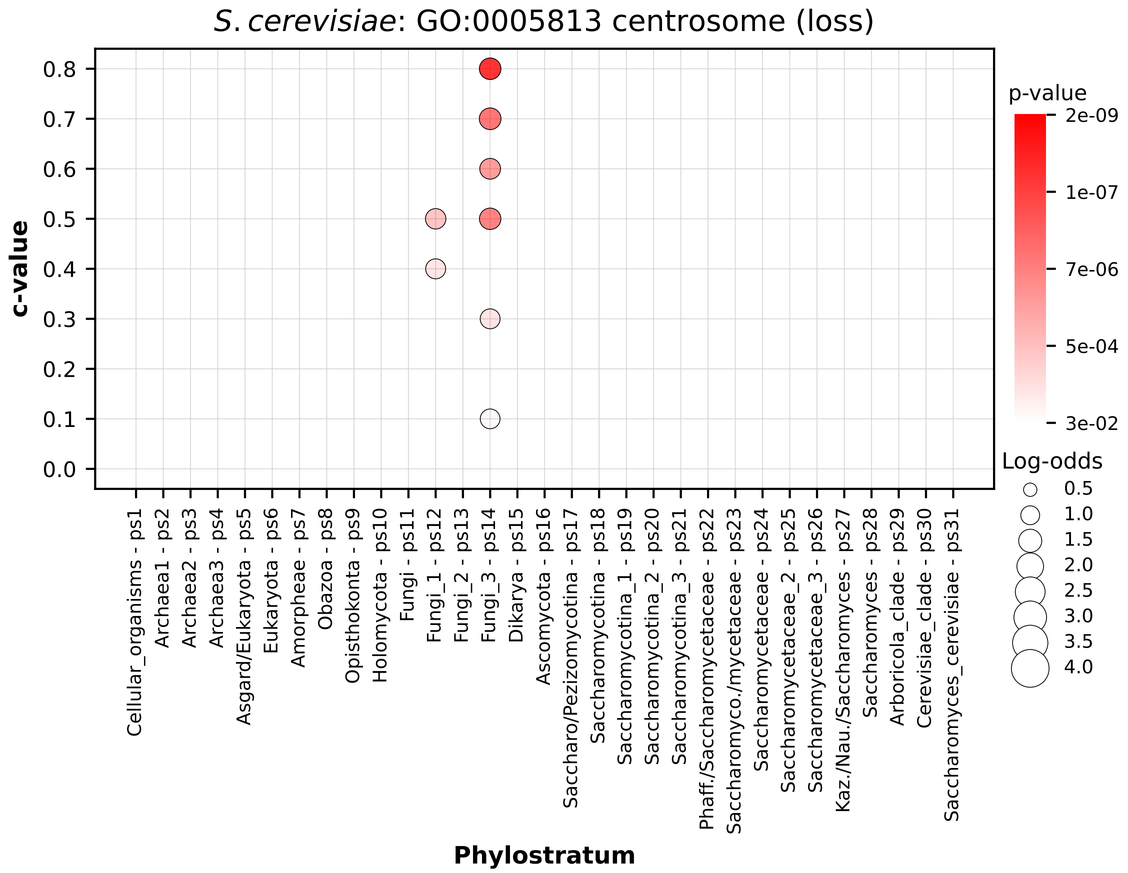

S. cerevisiae: GO:0005813 centrosome (loss)
c-value
0.0
0.1
0.2
0.3
0.4
0.5
0.6
0.7
0.8
Phylostratum
Cellular_organisms - ps1
Archaea1 - ps2
Archaea2 - ps3
Archaea3 - ps4
Asgard/Eukaryota - ps5
Eukaryota - ps6
Amorpheae - ps7
Obazoa - ps8
Opisthokonta - ps9
Holomycota - ps10
Fungi - ps11
Fungi_1 - ps12
Fungi_2 - ps13
Fungi_3 - ps14
Dikarya - ps15
Ascomycota - ps16
Saccharo/Pezizomycotina - ps17
Saccharomycotina - ps18
Saccharomycotina_1 - ps19
Saccharomycotina_2 - ps20
Saccharomycotina_3 - ps21
Phaff./Saccharomycetaceae - ps22
Saccharomyco./mycetaceae - ps23
Saccharomycetaceae - ps24
Saccharomycetaceae_2 - ps25
Saccharomycetaceae_3 - ps26
Kaz./Nau./Saccharomyces - ps27
Saccharomyces - ps28
Arboricola_clade - ps29
Cerevisiae_clade - ps30
Saccharomyces_cerevisiae - ps31
p-value
2e-09
1e-07
7e-06
5e-04
3e-02
Log-odds
0.5
1.0
1.5
2.0
2.5
3.0
3.5
4.0

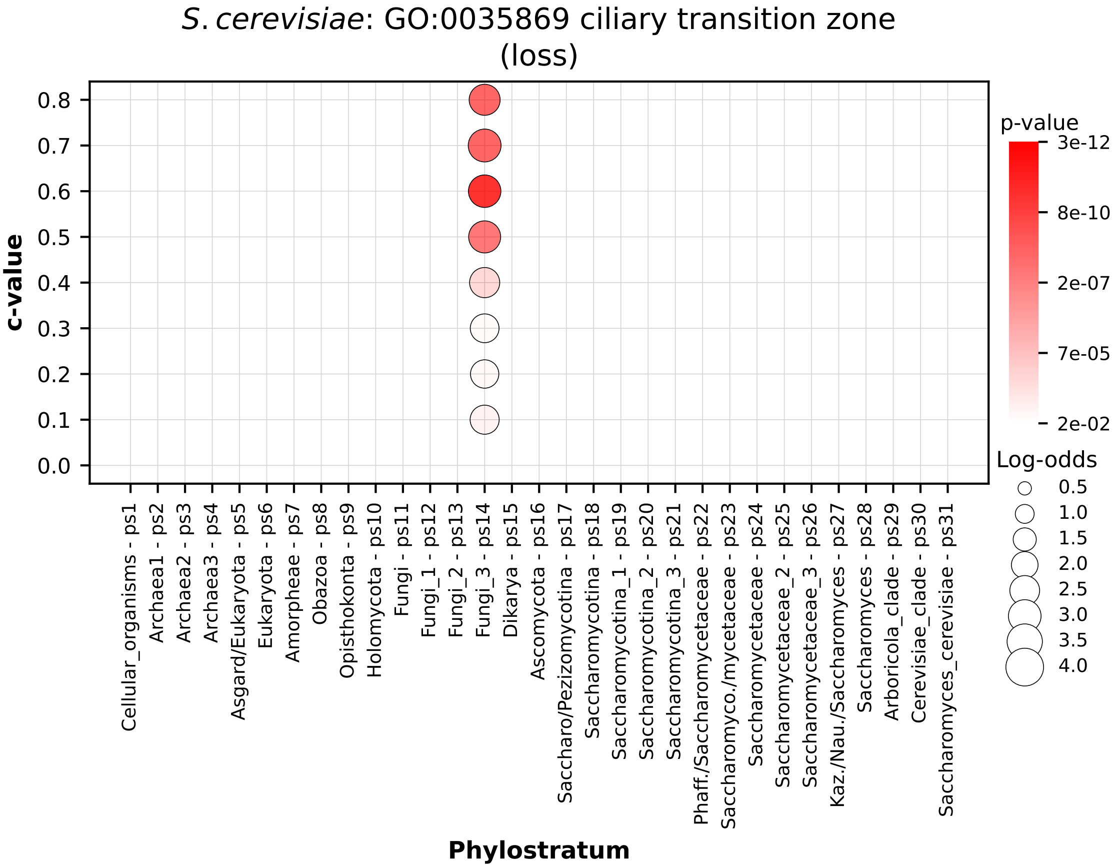
S. cerevisiae: GO:0035869 ciliary transition zone
(loss)
c-value
0.8
0.7
0.6
0.5
0.4
0.3
0.2
0.1
0.0
p-value
3e-12
8e-10
2e-07
7e-05
2e-02
Log-odds
0.5
1.0
1.5
2.0
2.5
3.0
3.5
4.0
Cellular_organisms - ps1
Archaea1 - ps2
Archaea2 - ps3
Archaea3 - ps4
Asgard/Eukaryota - ps5
Eukaryota - ps6
Amorpheae - ps7
Obazoa - ps8
Opisthokonta - ps9
Holomycota - ps10
Fungi - ps11
Fungi_1 - ps12
Fungi_2 - ps13
Fungi_3 - ps14
Dikarya - ps15
Ascomycota - ps16
Saccharo/Pezizomycotina - ps17
Saccharomycotina - ps18
Saccharomycotina_1 - ps19
Saccharomycotina_2 - ps20
Saccharomycotina_3 - ps21
Phaff./Saccharomycetaceae - ps22
Saccharomyco./mycetaceae - ps23
Saccharomycetaceae - ps24
Saccharomycetaceae_2 - ps25
Saccharomycetaceae_3 - ps26
Kaz./Nau./Saccharomyces - ps27
Saccharomyces - ps28
Arboricola_clade - ps29
Cerevisiae_clade - ps30
Saccharomyces_cerevisiae - ps31
Phylostratum

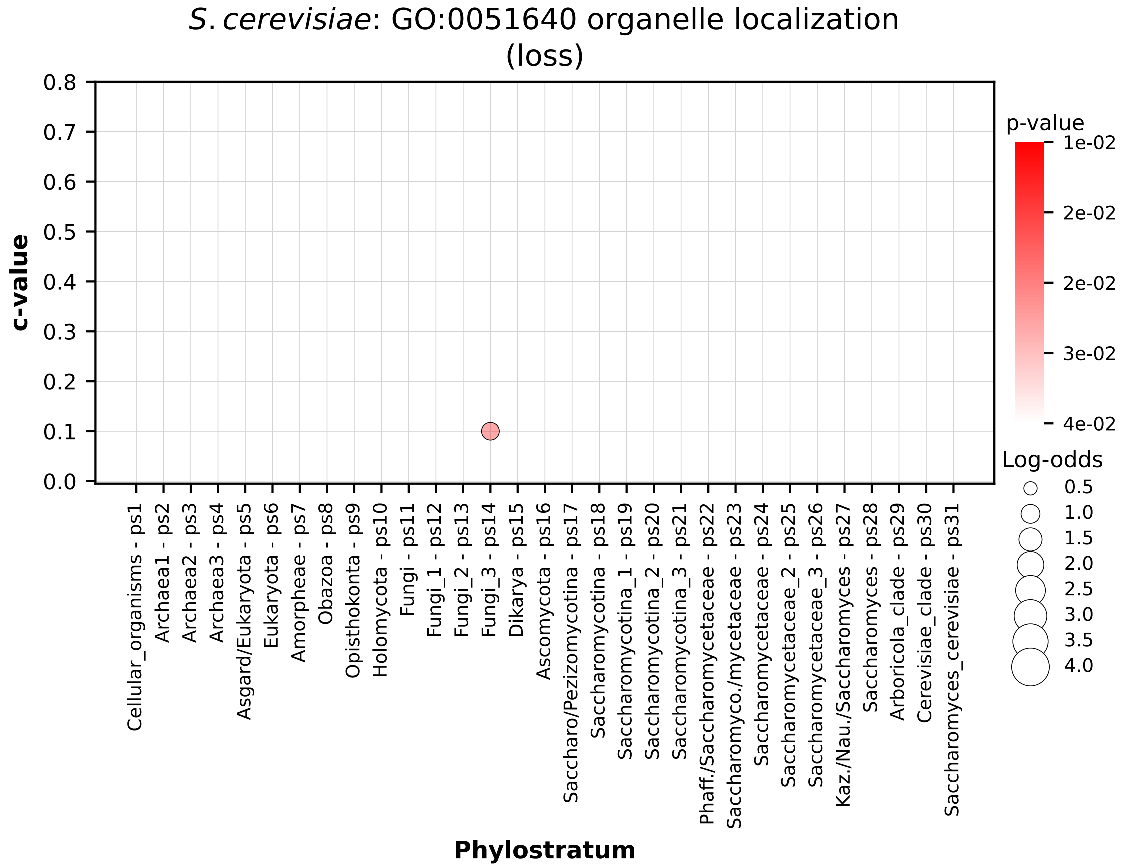

S. cerevisiae: GO:0051640 organelle localization
(loss)
c-value
0.0
0.1
0.2
0.3
0.4
0.5
0.6
0.7
0.8
Phylostratum
Cellular_organisms - ps1
Archaea1 - ps2
Archaea2 - ps3
Archaea3 - ps4
Asgard/Eukaryota - ps5
Eukaryota - ps6
Amorpheae - ps7
Obazoa - ps8
Opisthokonta - ps9
Holomycota - ps10
Fungi - ps11
Fungi_1 - ps12
Fungi_2 - ps13
Fungi_3 - ps14
Dikarya - ps15
Ascomycota - ps16
Saccharo/Pezizomycotina - ps17
Saccharomycotina - ps18
Saccharomycotina_1 - ps19
Saccharomycotina_2 - ps20
Saccharomycotina_3 - ps21
Phaff./Saccharomycetaceae - ps22
Saccharomyco./mycetaceae - ps23
Saccharomycetaceae - ps24
Saccharomycetaceae_2 - ps25
Saccharomycetaceae_3 - ps26
Kaz./Nau./Saccharomyces - ps27
Saccharomyces - ps28
Arboricola_clade - ps29
Cerevisiae_clade - ps30
Saccharomyces_cerevisiae - ps31
p-value
1e-02
2e-02
2e-02
3e-02
4e-02
Log-odds
0.5
1.0
1.5
2.0
2.5
3.0
3.5
4.0

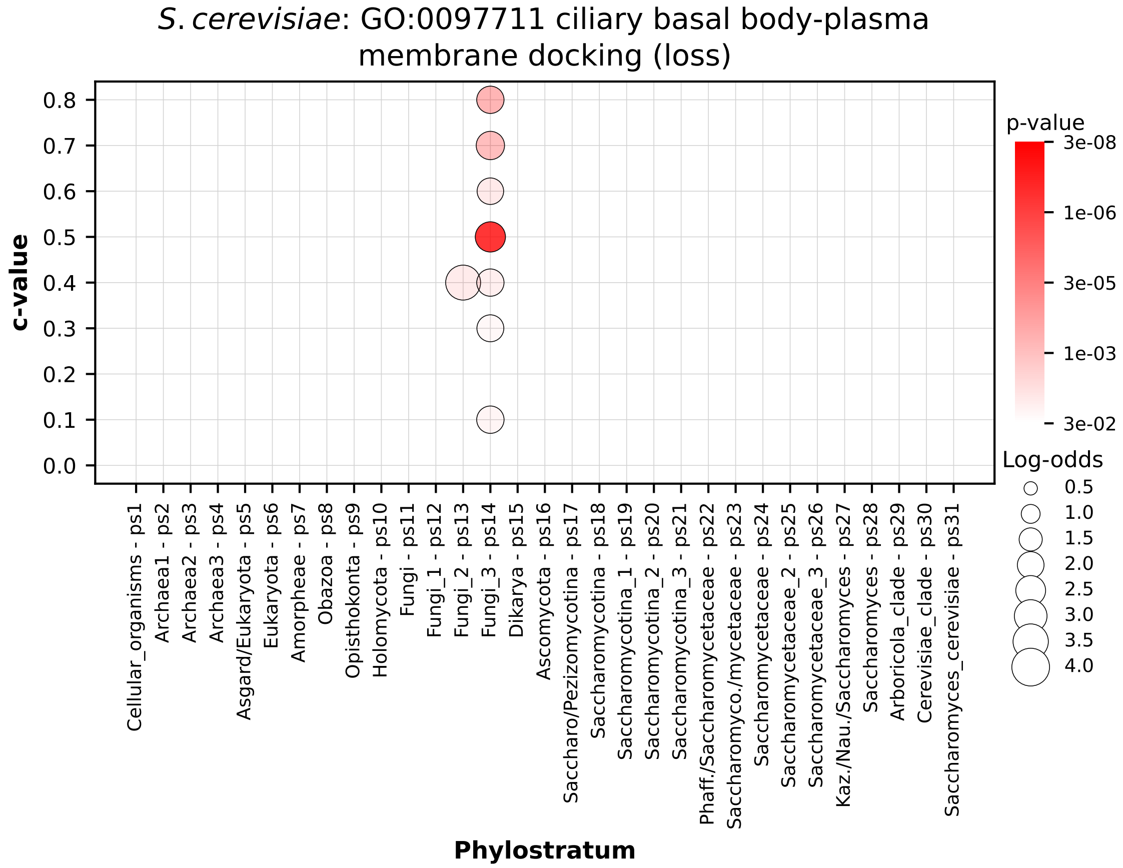

S. cerevisiae: GO:0097711 ciliary basal body-plasma membrane docking (loss)
c-value
0.0
0.1
0.2
0.3
0.4
0.5
0.6
0.7
0.8
p-value
3e-08
1e-06
3e-05
1e-03
3e-02
Log-odds
0.5
1.0
1.5
2.0
2.5
3.0
3.5
4.0
Cellular_organisms - ps1
Archaea1 - ps2
Archaea2 - ps3
Archaea3 - ps4
Asgard/Eukaryota - ps5
Eukaryota - ps6
Amorpheae - ps7
Obazoa - ps8
Opisthokonta - ps9
Holomycota - ps10
Fungi - ps11
Fungi_1 - ps12
Fungi_2 - ps13
Fungi_3 - ps14
Dikarya - ps15
Ascomycota - ps16
Saccharo/Pezizomycotina - ps17
Saccharomycotina - ps18
Saccharomycotina_1 - ps19
Saccharomycotina_2 - ps20
Saccharomycotina_3 - ps21
Phaff./Saccharomycetaceae - ps22
Saccharomyco./mycetaceae - ps23
Saccharomycetaceae - ps24
Saccharomycetaceae_2 - ps25
Saccharomycetaceae_3 - ps26
Kaz./Nau./Saccharomyces - ps27
Saccharomyces - ps28
Arboricola_clade - ps29
Cerevisiae_clade - ps30
Saccharomyces_cerevisiae - ps31
Phylostratum

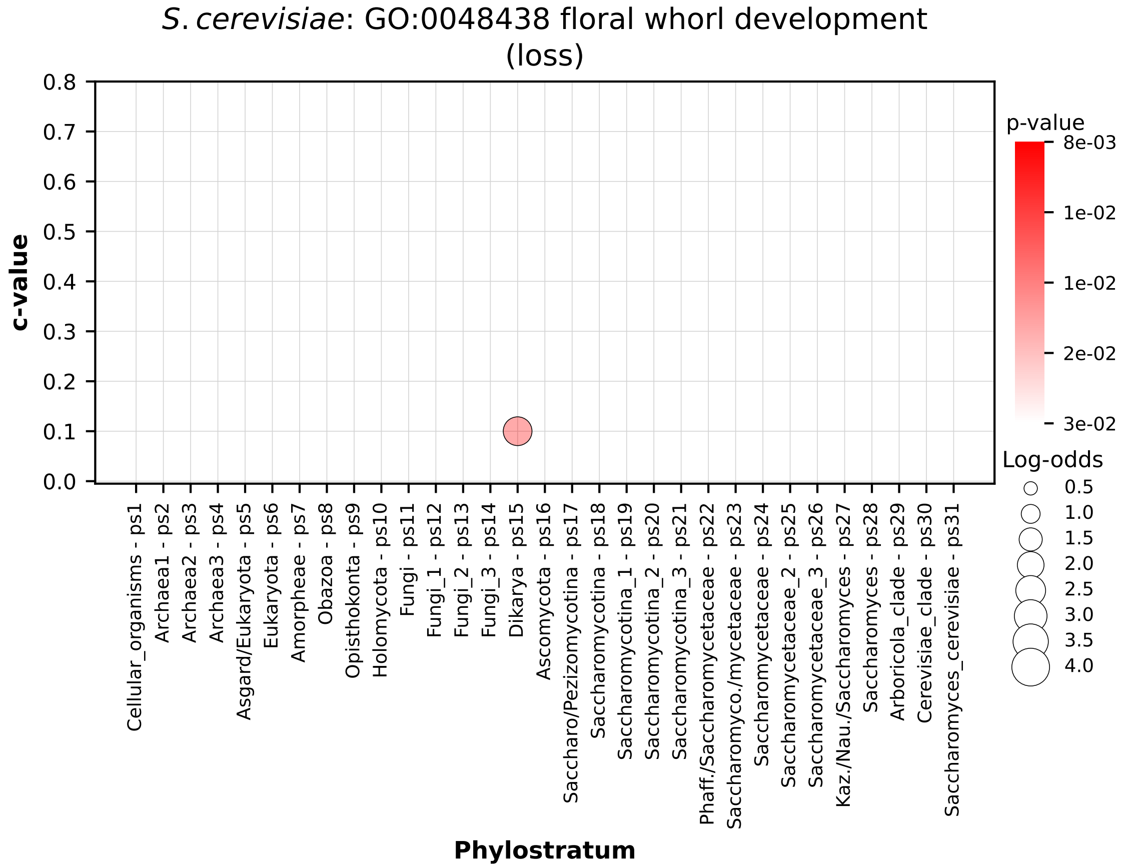

S. cerevisiae: GO:0048438 floral whorl development
(loss)
c-value
0.0
0.1
0.2
0.3
0.4
0.5
0.6
0.7
0.8
Phylostratum
Cellular_organisms - ps1
Archaea1 - ps2
Archaea2 - ps3
Archaea3 - ps4
Asgard/Eukaryota - ps5
Eukaryota - ps6
Amorpheae - ps7
Obazoa - ps8
Opisthokonta - ps9
Holomycota - ps10
Fungi - ps11
Fungi_1 - ps12
Fungi_2 - ps13
Fungi_3 - ps14
Dikarya - ps15
Ascomycota - ps16
Saccharo/Pezizomycotina - ps17
Saccharomycotina - ps18
Saccharomycotina_1 - ps19
Saccharomycotina_2 - ps20
Saccharomycotina_3 - ps21
Phaff./Saccharomycetaceae - ps22
Saccharomyco./mycetaceae - ps23
Saccharomycetaceae - ps24
Saccharomycetaceae_2 - ps25
Saccharomycetaceae_3 - ps26
Kaz./Nau./Saccharomyces - ps27
Saccharomyces - ps28
Arboricola_clade - ps29
Cerevisiae_clade - ps30
Saccharomyces_cerevisiae - ps31
p-value
8e-03
1e-02
1e-02
2e-02
3e-02
Log-odds
0.5
1.0
1.5
2.0
2.5
3.0
3.5
4.0

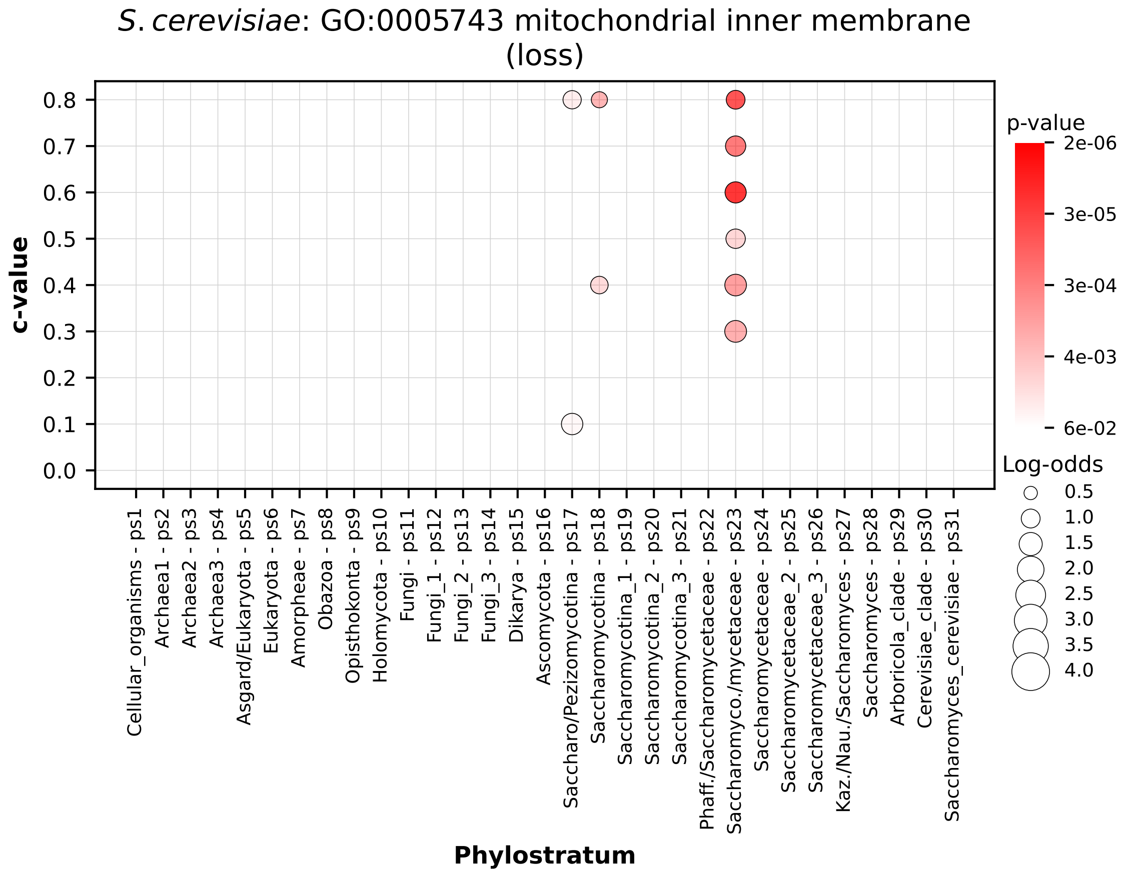

S. cerevisiae: GO:0005743 mitochondrial inner membrane
(loss)
c-value
0.8
0.7
0.6
0.5
0.4
0.3
0.2
0.1
0.0
p-value
2e-06
3e-05
3e-04
4e-03
6e-02
Log-odds
0.5
1.0
1.5
2.0
2.5
3.0
3.5
4.0
Cellular_organisms - ps1
Archaea1 - ps2
Archaea2 - ps3
Archaea3 - ps4
Asgard/Eukaryota - ps5
Eukaryota - ps6
Amorpheae - ps7
Obazoa - ps8
Opisthokonta - ps9
Holomycota - ps10
Fungi - ps11
Fungi_1 - ps12
Fungi_2 - ps13
Fungi_3 - ps14
Dikarya - ps15
Ascomycota - ps16
Saccharo/Pezizomycotina - ps17
Saccharomycotina - ps18
Saccharomycotina_1 - ps19
Saccharomycotina_2 - ps20
Saccharomycotina_3 - ps21
Phaff./Saccharomycetaceae - ps22
Saccharomyco./mycetaceae - ps23
Saccharomycetaceae - ps24
Saccharomycetaceae_2 - ps25
Saccharomycetaceae_3 - ps26
Kaz./Nau./Saccharomyces - ps27
Saccharomyces - ps28
Arboricola_clade - ps29
Cerevisiae_clade - ps30
Saccharomyces_cerevisiae - ps31
Phylostratum

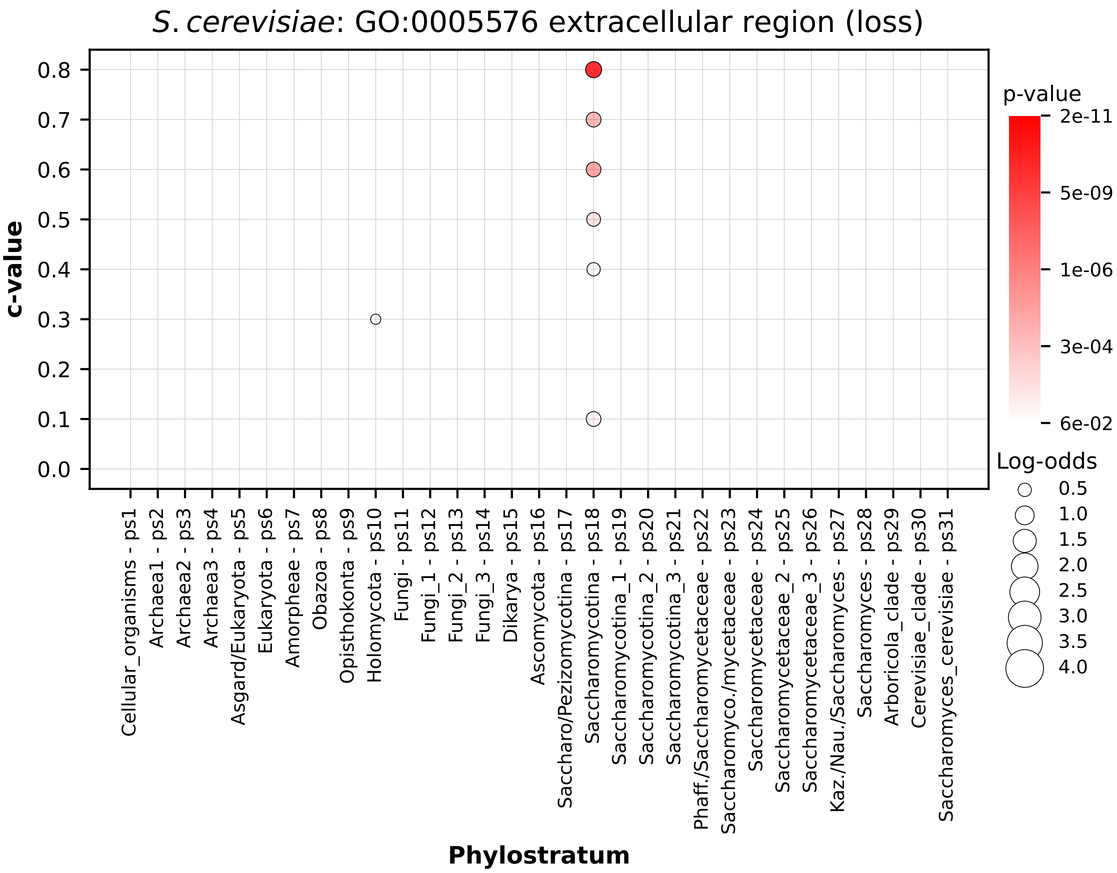

S. cerevisiae: GO:0005576 extracellular region (loss)
c-value
0.0
0.1
0.2
0.3
0.4
0.5
0.6
0.7
0.8
p-value
2e-11
5e-09
1e-06
3e-04
6e-02
Log-odds
0.5
1.0
1.5
2.0
2.5
3.0
3.5
4.0
Cellular_organisms - ps1
Archaea1 - ps2
Archaea2 - ps3
Archaea3 - ps4
Asgard/Eukaryota - ps5
Eukaryota - ps6
Amorpheae - ps7
Obazoa - ps8
Opisthokonta - ps9
Holomycota - ps10
Fungi - ps11
Fungi_1 - ps12
Fungi_2 - ps13
Fungi_3 - ps14
Dikarya - ps15
Ascomycota - ps16
Saccharo/Pezizomycotina - ps17
Saccharomycotina - ps18
Saccharomycotina_1 - ps19
Saccharomycotina_2 - ps20
Saccharomycotina_3 - ps21
Phaff./Saccharomycetaceae - ps22
Saccharomyco./mycetaceae - ps23
Saccharomycetaceae - ps24
Saccharomycetaceae_2 - ps25
Saccharomycetaceae_3 - ps26
Kaz./Nau./Saccharomyces - ps27
Saccharomyces - ps28
Arboricola_clade - ps29
Cerevisiae_clade - ps30
Saccharomyces_cerevisiae - ps31
Phylostratum

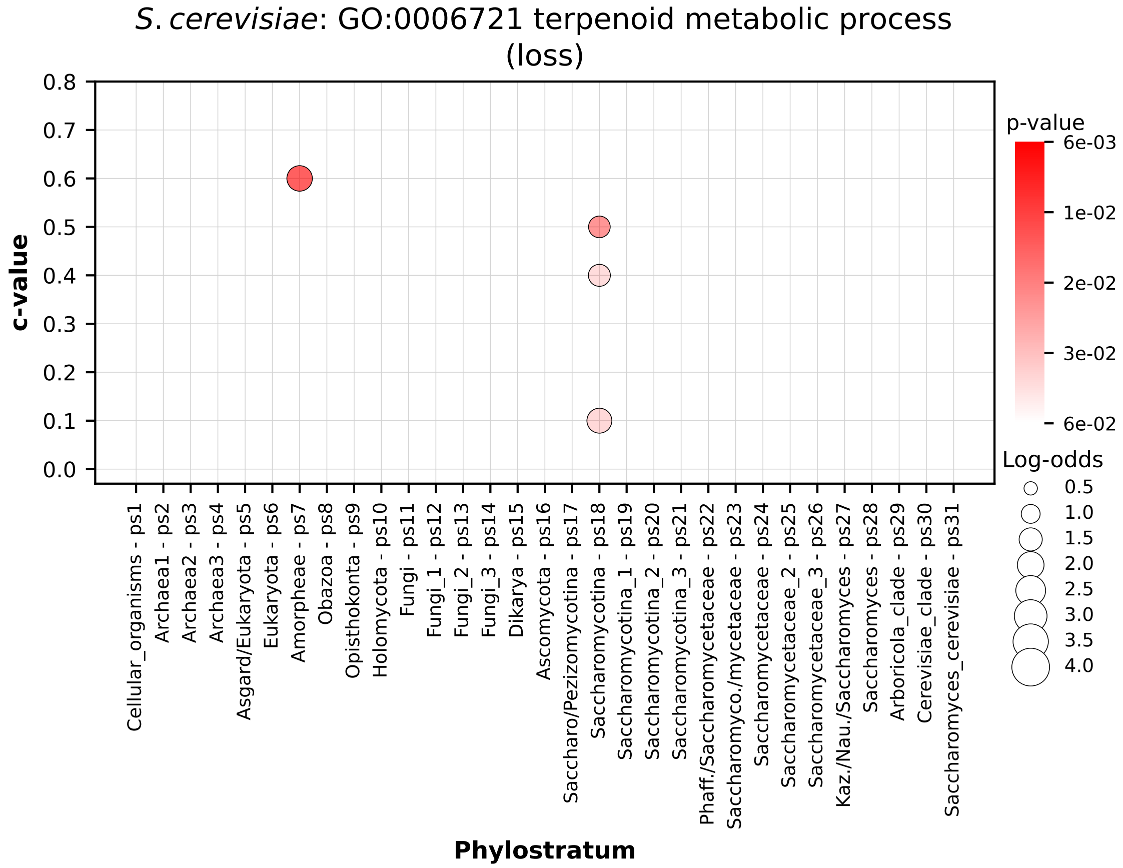

S. cerevisiae: GO:0006721 terpenoid metabolic process
(loss)
c-value
0.8
0.7
0.6
0.5
0.4
0.3
0.2
0.1
0.0
p-value
6e-03
1e-02
2e-02
3e-02
6e-02
Log-odds
0.5
1.0
1.5
2.0
2.5
3.0
3.5
4.0
Cellular_organisms - ps1
Archaea1 - ps2
Archaea2 - ps3
Archaea3 - ps4
Asgard/Eukaryota - ps5
Eukaryota - ps6
Amorpheae - ps7
Obazoa - ps8
Opisthokonta - ps9
Holomycota - ps10
Fungi - ps11
Fungi_1 - ps12
Fungi_2 - ps13
Fungi_3 - ps14
Dikarya - ps15
Ascomycota - ps16
Saccharo/Pezizomycotina - ps17
Saccharomycotina - ps18
Saccharomycotina_1 - ps19
Saccharomycotina_2 - ps20
Saccharomycotina_3 - ps21
Phaff./Saccharomycetaceae - ps22
Saccharomyco./mycetaceae - ps23
Saccharomycetaceae - ps24
Saccharomycetaceae_2 - ps25
Saccharomycetaceae_3 - ps26
Kaz./Nau./Saccharomyces - ps27
Saccharomyces - ps28
Arboricola_clade - ps29
Cerevisiae_clade - ps30
Saccharomyces_cerevisiae - ps31
Phylostratum

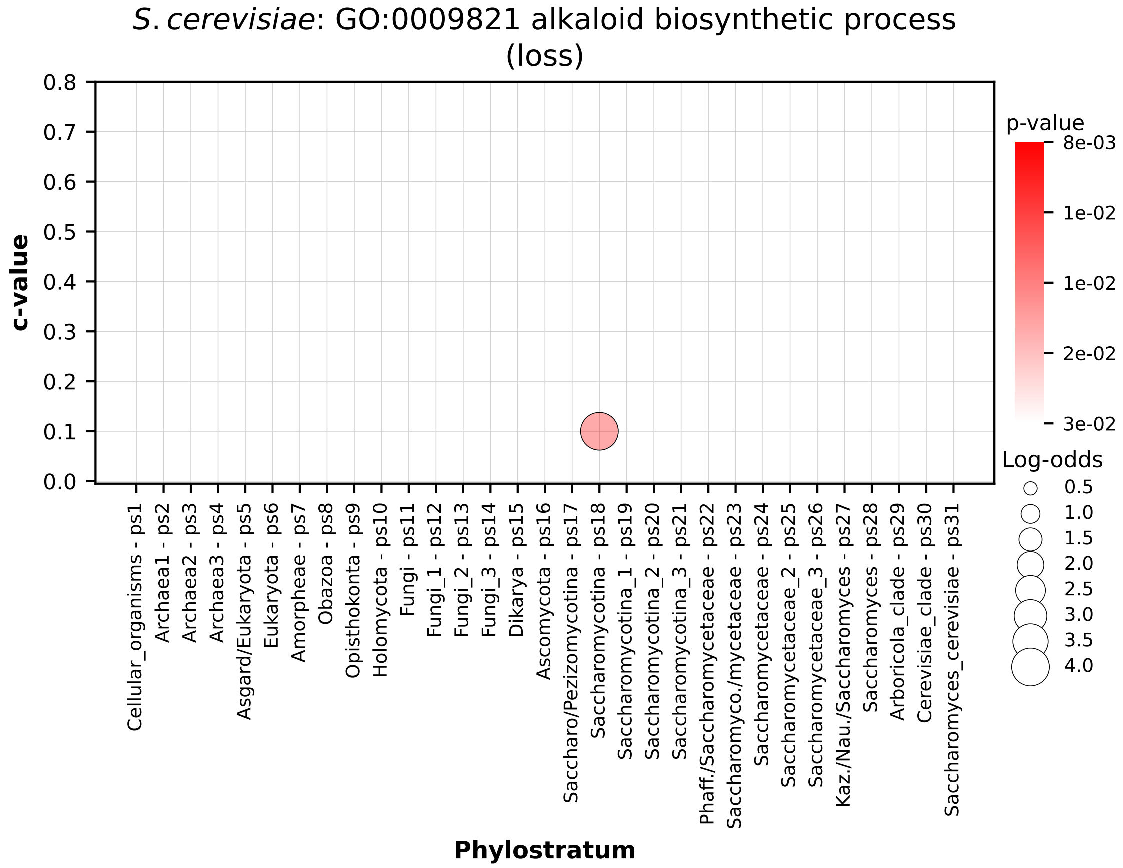

S. cerevisiae: GO:0009821 alkaloid biosynthetic process (loss)
c-value
0.0
0.1
0.2
0.3
0.4
0.5
0.6
0.7
0.8
Phylostratum
Cellular_organisms - ps1
Archaea1 - ps2
Archaea2 - ps3
Archaea3 - ps4
Asgard/Eukaryota - ps5
Eukaryota - ps6
Amorpheae - ps7
Obazoa - ps8
Opisthokonta - ps9
Holomycota - ps10
Fungi - ps11
Fungi_1 - ps12
Fungi_2 - ps13
Fungi_3 - ps14
Dikarya - ps15
Ascomycota - ps16
Saccharo/Pezizomycotina - ps17
Saccharomycotina - ps18
Saccharomycotina_1 - ps19
Saccharomycotina_2 - ps20
Saccharomycotina_3 - ps21
Phaff./Saccharomycetaceae - ps22
Saccharomyco./mycetaceae - ps23
Saccharomycetaceae - ps24
Saccharomycetaceae_2 - ps25
Saccharomycetaceae_3 - ps26
Kaz./Nau./Saccharomyces - ps27
Saccharomyces - ps28
Arboricola_clade - ps29
Cerevisiae_clade - ps30
Saccharomyces_cerevisiae - ps31
p-value
8e-03
1e-02
1e-02
2e-02
3e-02
Log-odds
0.5
1.0
1.5
2.0
2.5
3.0
3.5
4.0

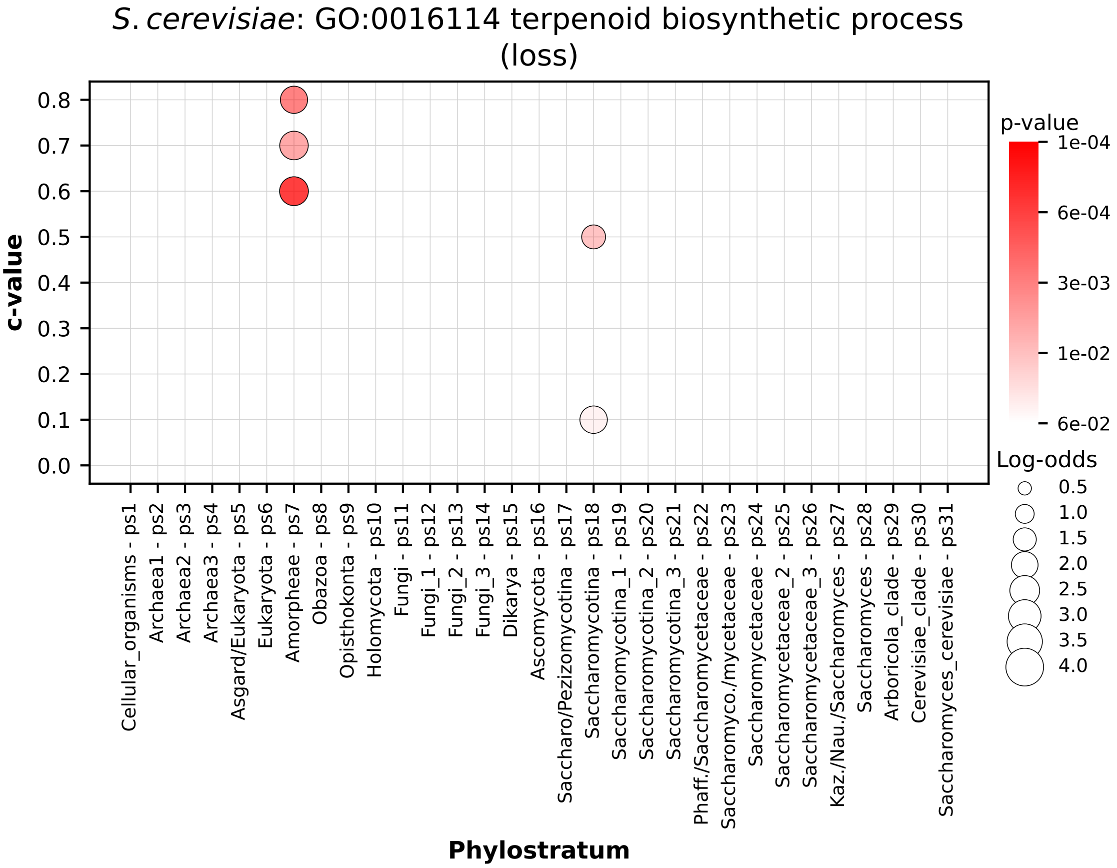

S. cerevisiae: GO:0016114 terpenoid biosynthetic process
(loss)
c-value
0.0
0.1
0.2
0.3
0.4
0.5
0.6
0.7
0.8
p-value
1e-04
6e-04
3e-03
1e-02
6e-02
Log-odds
0.5
1.0
1.5
2.0
2.5
3.0
3.5
4.0
Cellular_organisms - ps1
Archaea1 - ps2
Archaea2 - ps3
Archaea3 - ps4
Asgard/Eukaryota - ps5
Eukaryota - ps6
Amorpheae - ps7
Obazoa - ps8
Opisthokonta - ps9
Holomycota - ps10
Fungi - ps11
Fungi_1 - ps12
Fungi_2 - ps13
Fungi_3 - ps14
Dikarya - ps15
Ascomycota - ps16
Saccharo/Pezizomycotina - ps17
Saccharomycotina - ps18
Saccharomycotina_1 - ps19
Saccharomycotina_2 - ps20
Saccharomycotina_3 - ps21
Phaff./Saccharomycetaceae - ps22
Saccharomyco./mycetaceae - ps23
Saccharomycetaceae - ps24
Saccharomycetaceae_2 - ps25
Saccharomycetaceae_3 - ps26
Kaz./Nau./Saccharomyces - ps27
Saccharomyces - ps28
Arboricola_clade - ps29
Cerevisiae_clade - ps30
Saccharomyces_cerevisiae - ps31
Phylostratum

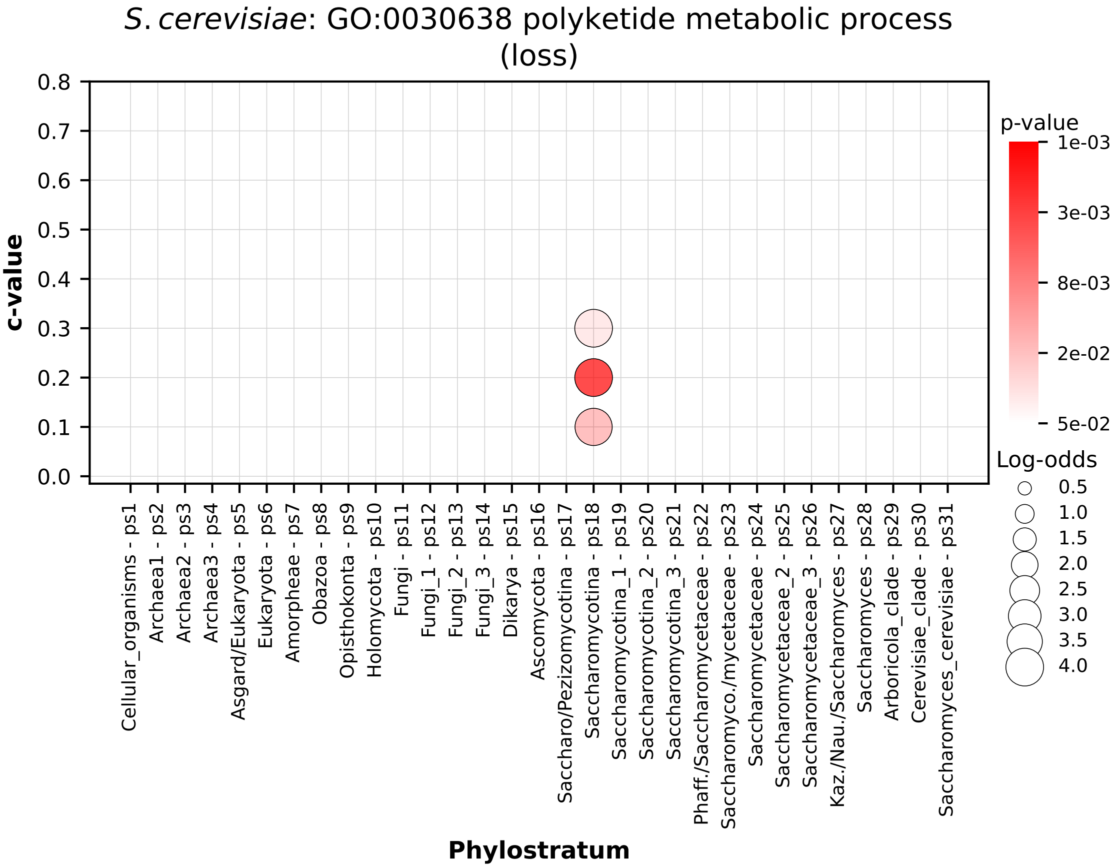

S. cerevisiae: GO:0030638 polyketide metabolic process (loss)
c-value
0.0
0.1
0.2
0.3
0.4
0.5
0.6
0.7
0.8
Phylostratum
Cellular_organisms - ps1
Archaea1 - ps2
Archaea2 - ps3
Archaea3 - ps4
Asgard/Eukaryota - ps5
Eukaryota - ps6
Amorpheae - ps7
Obazoa - ps8
Opisthokonta - ps9
Holomycota - ps10
Fungi - ps11
Fungi_1 - ps12
Fungi_2 - ps13
Fungi_3 - ps14
Dikarya - ps15
Ascomycota - ps16
Saccharo/Pezizomycotina - ps17
Saccharomycotina - ps18
Saccharomycotina_1 - ps19
Saccharomycotina_2 - ps20
Saccharomycotina_3 - ps21
Phaff./Saccharomycetaceae - ps22
Saccharomyco./mycetaceae - ps23
Saccharomycetaceae - ps24
Saccharomycetaceae_2 - ps25
Saccharomycetaceae_3 - ps26
Kaz./Nau./Saccharomyces - ps27
Saccharomyces - ps28
Arboricola_clade - ps29
Cerevisiae_clade - ps30
Saccharomyces_cerevisiae - ps31
p-value
1e-03
3e-03
8e-03
2e-02
5e-02
Log-odds
0.5
1.0
1.5
2.0
2.5
3.0
3.5
4.0

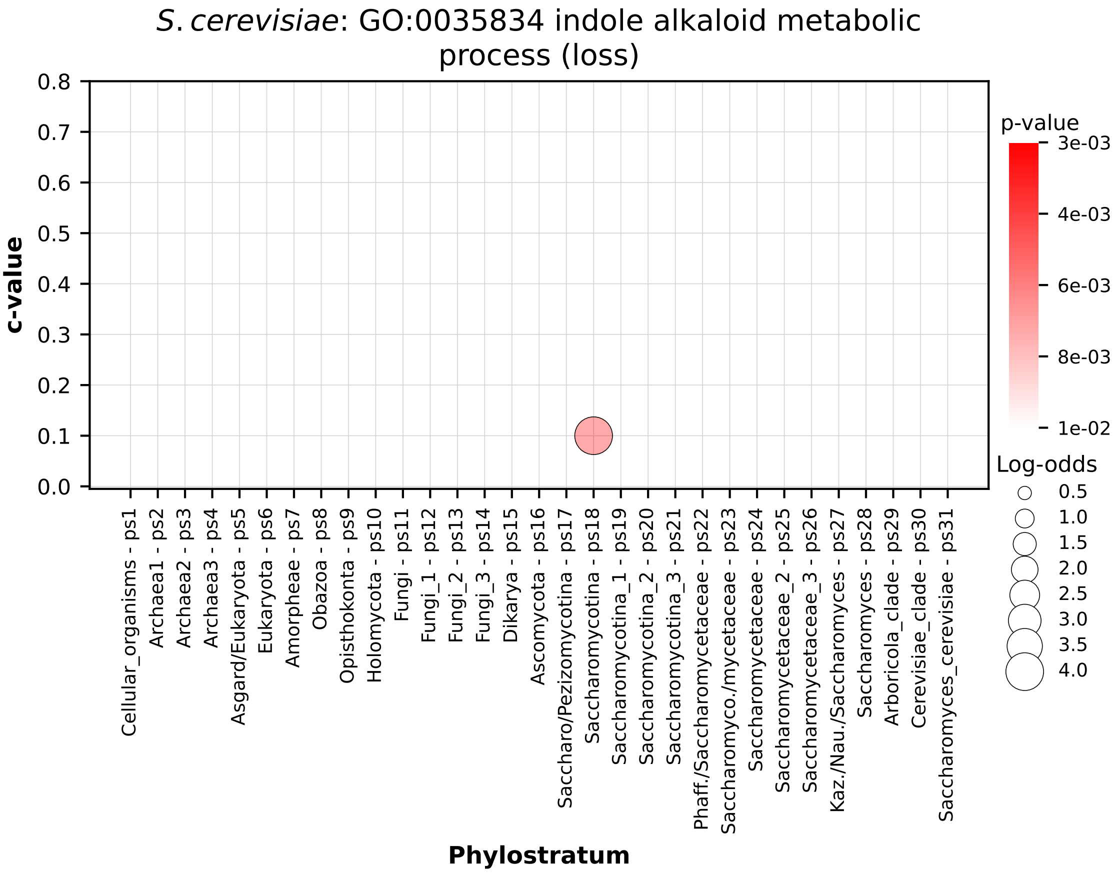

S. cerevisiae: GO:0035834 indole alkaloid metabolic process (loss)
c-value
0.8
0.7
0.6
0.5
0.4
0.3
0.2
0.1
0.0
p-value
3e-03
4e-03
6e-03
8e-03
1e-02
Log-odds
0.5
1.0
1.5
2.0
2.5
3.0
3.5
4.0
Cellular_organisms - ps1
Archaea1 - ps2
Archaea2 - ps3
Archaea3 - ps4
Asgard/Eukaryota - ps5
Eukaryota - ps6
Amorpheae - ps7
Obazoa - ps8
Opisthokonta - ps9
Holomycota - ps10
Fungi - ps11
Fungi_1 - ps12
Fungi_2 - ps13
Fungi_3 - ps14
Dikarya - ps15
Ascomycota - ps16
Saccharo/Pezizomycotina - ps17
Saccharomycotina - ps18
Saccharomycotina_1 - ps19
Saccharomycotina_2 - ps20
Saccharomycotina_3 - ps21
Phaff./Saccharomycetaceae - ps22
Saccharomyco./mycetaceae - ps23
Saccharomycetaceae - ps24
Saccharomycetaceae_2 - ps25
Saccharomycetaceae_3 - ps26
Kaz./Nau./Saccharomyces - ps27
Saccharomyces - ps28
Arboricola_clade - ps29
Cerevisiae_clade - ps30
Saccharomyces_cerevisiae - ps31
Phylostratum

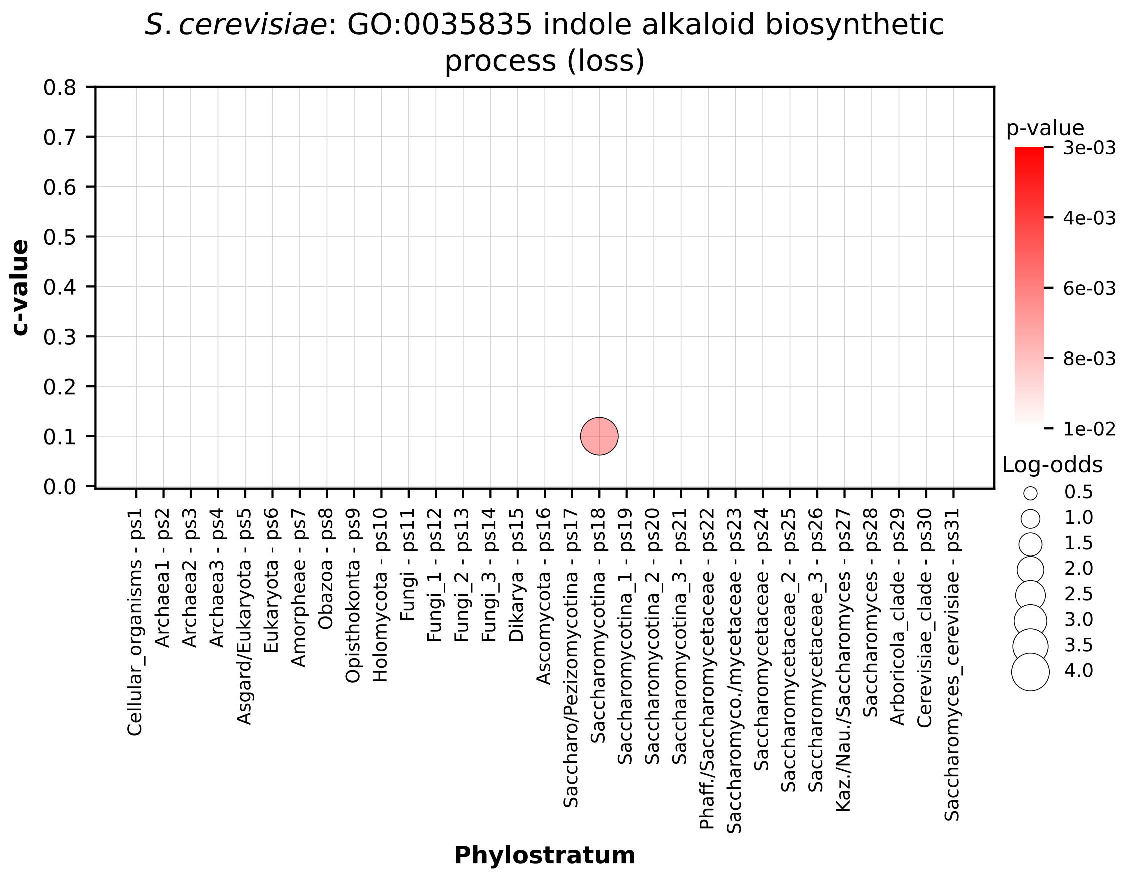

S. cerevisiae: GO:0035835 indole alkaloid biosynthetic process (loss)
c-value
0.0
0.1
0.2
0.3
0.4
0.5
0.6
0.7
0.8
Phylostratum
Cellular_organisms - ps1
Archaea1 - ps2
Archaea2 - ps3
Archaea3 - ps4
Asgard/Eukaryota - ps5
Eukaryota - ps6
Amorpheae - ps7
Obazoa - ps8
Opisthokonta - ps9
Holomycota - ps10
Fungi - ps11
Fungi_1 - ps12
Fungi_2 - ps13
Fungi_3 - ps14
Dikarya - ps15
Ascomycota - ps16
Saccharo/Pezizomycotina - ps17
Saccharomycotina - ps18
Saccharomycotina_1 - ps19
Saccharomycotina_2 - ps20
Saccharomycotina_3 - ps21
Phaff./Saccharomycetaceae - ps22
Saccharomyco./mycetaceae - ps23
Saccharomycetaceae - ps24
Saccharomycetaceae_2 - ps25
Saccharomycetaceae_3 - ps26
Kaz./Nau./Saccharomyces - ps27
Saccharomyces - ps28
Arboricola_clade - ps29
Cerevisiae_clade - ps30
Saccharomyces_cerevisiae - ps31
p-value
3e-03
4e-03
6e-03
8e-03
1e-02
Log-odds
0.5
1.0
1.5
2.0
2.5
3.0
3.5
4.0

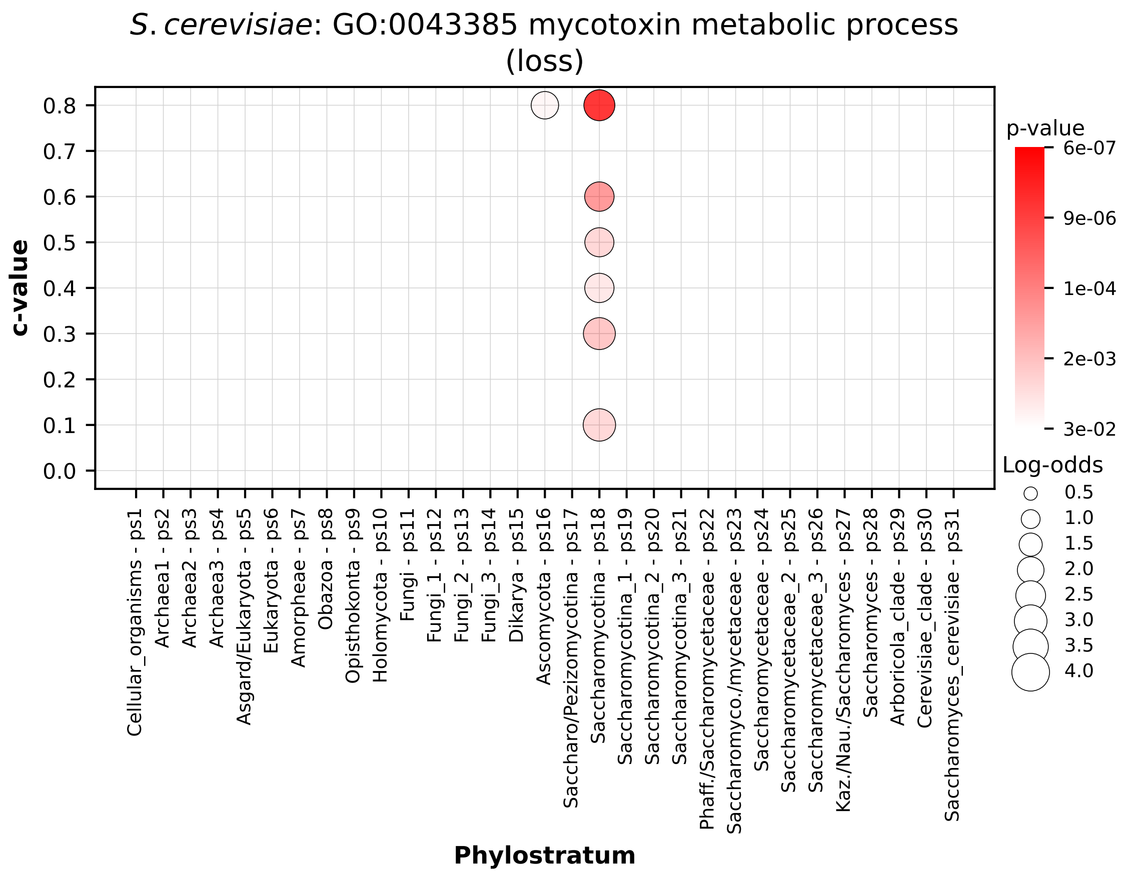

S. cerevisiae: GO:0043385 mycotoxin metabolic process
(loss)
c-value
0.0
0.1
0.2
0.3
0.4
0.5
0.6
0.7
0.8
p-value
6e-07
9e-06
1e-04
2e-03
3e-02
Log-odds
0.5
1.0
1.5
2.0
2.5
3.0
3.5
4.0
Cellular_organisms - ps1
Archaea1 - ps2
Archaea2 - ps3
Archaea3 - ps4
Asgard/Eukaryota - ps5
Eukaryota - ps6
Amorpheae - ps7
Obazoa - ps8
Opisthokonta - ps9
Holomycota - ps10
Fungi - ps11
Fungi_1 - ps12
Fungi_2 - ps13
Fungi_3 - ps14
Dikarya - ps15
Ascomycota - ps16
Saccharo/Pezizomycotina - ps17
Saccharomycotina - ps18
Saccharomycotina_1 - ps19
Saccharomycotina_2 - ps20
Saccharomycotina_3 - ps21
Phaff./Saccharomycetaceae - ps22
Saccharomyco./mycetaceae - ps23
Saccharomycetaceae - ps24
Saccharomycetaceae_2 - ps25
Saccharomycetaceae_3 - ps26
Kaz./Nau./Saccharomyces - ps27
Saccharomyces - ps28
Arboricola_clade - ps29
Cerevisiae_clade - ps30
Saccharomyces_cerevisiae - ps31
Phylostratum

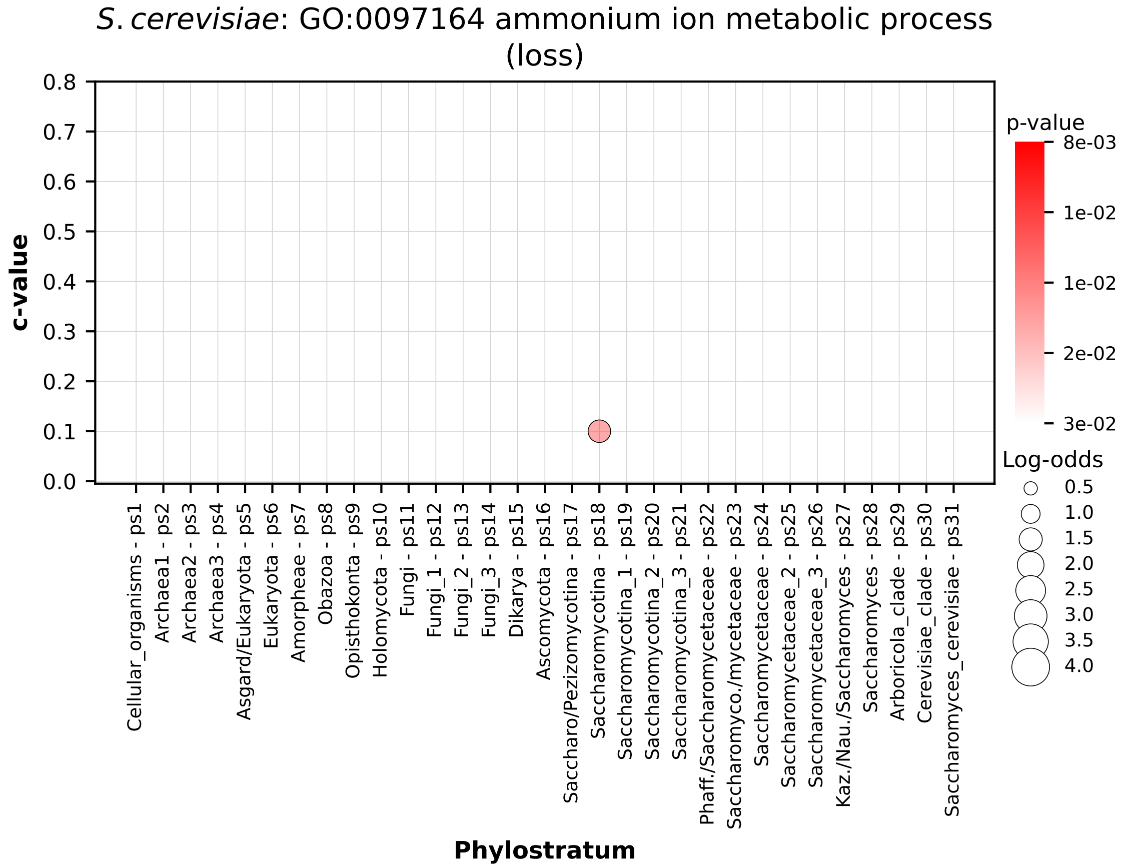

S. cerevisiae: GO:0097164 ammonium ion metabolic process (loss)
c-value
0.8
0.7
0.6
0.5
0.4
0.3
0.2
0.1
0.0
p-value
8e-03
1e-02
1e-02
2e-02
3e-02
Log-odds
0.5
1.0
1.5
2.0
2.5
3.0
3.5
4.0
Cellular_organisms - ps1
Archaea1 - ps2
Archaea2 - ps3
Archaea3 - ps4
Asgard/Eukaryota - ps5
Eukaryota - ps6
Amorpheae - ps7
Obazoa - ps8
Opisthokonta - ps9
Holomycota - ps10
Fungi - ps11
Fungi_1 - ps12
Fungi_2 - ps13
Fungi_3 - ps14
Dikarya - ps15
Ascomycota - ps16
Saccharo/Pezizomycotina - ps17
Saccharomycotina - ps18
Saccharomycotina_1 - ps19
Saccharomycotina_2 - ps20
Saccharomycotina_3 - ps21
Phaff./Saccharomycetaceae - ps22
Saccharomyco./mycetaceae - ps23
Saccharomycetaceae - ps24
Saccharomycetaceae_2 - ps25
Saccharomycetaceae_3 - ps26
Kaz./Nau./Saccharomyces - ps27
Saccharomyces - ps28
Arboricola_clade - ps29
Cerevisiae_clade - ps30
Saccharomyces_cerevisiae - ps31
Phylostratum

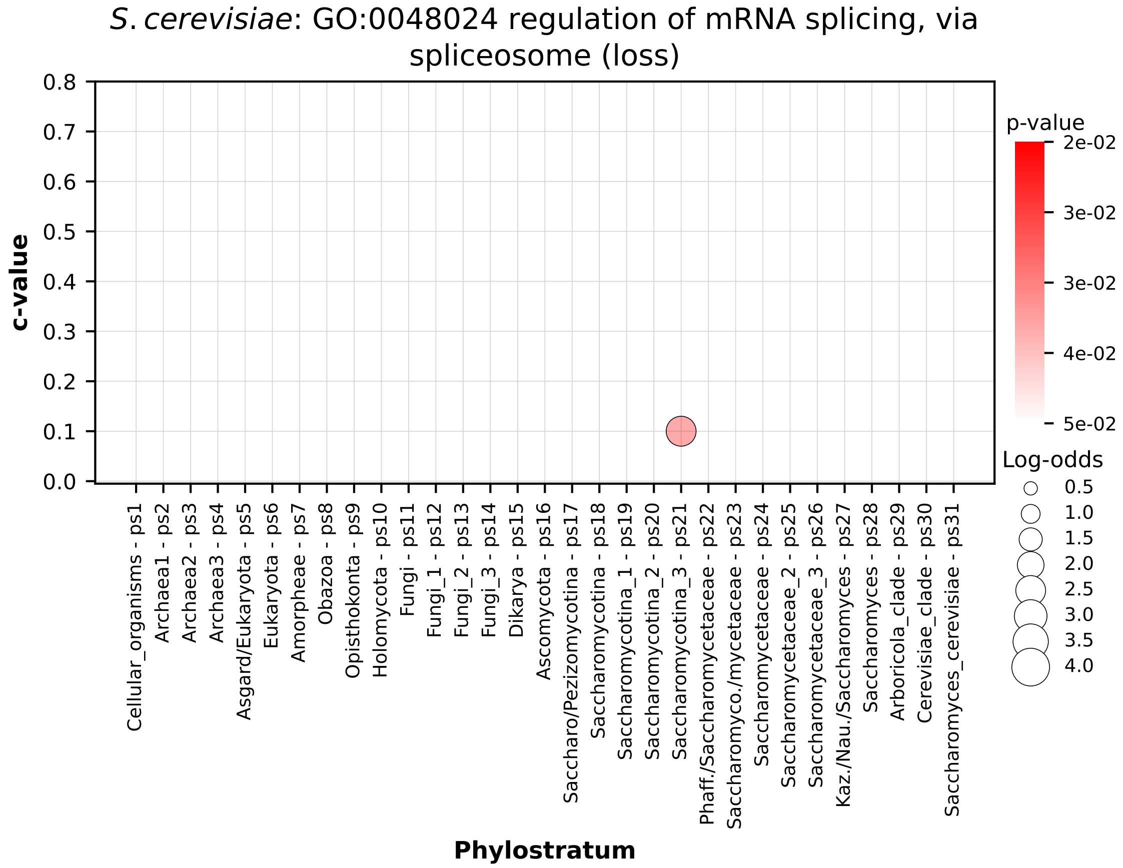

S. cerevisiae: GO:0048024 regulation of mRNA splicing, via spliceosome (loss)
c-value
0.0
0.1
0.2
0.3
0.4
0.5
0.6
0.7
0.8
p-value
2e-02
3e-02
3e-02
4e-02
5e-02
Log-odds
0.5
1.0
1.5
2.0
2.5
3.0
3.5
4.0
Cellular_organisms - ps1
Archaea1 - ps2
Archaea2 - ps3
Archaea3 - ps4
Asgard/Eukaryota - ps5
Eukaryota - ps6
Amorpheae - ps7
Obazoa - ps8
Opisthokonta - ps9
Holomycota - ps10
Fungi - ps11
Fungi_1 - ps12
Fungi_2 - ps13
Fungi_3 - ps14
Dikarya - ps15
Ascomycota - ps16
Saccharo/Pezizomycotina - ps17
Saccharomycotina - ps18
Saccharomycotina_1 - ps19
Saccharomycotina_2 - ps20
Saccharomycotina_3 - ps21
Phaff./Saccharomycetaceae - ps22
Saccharomyco./mycetaceae - ps23
Saccharomycetaceae - ps24
Saccharomycetaceae_2 - ps25
Saccharomycetaceae_3 - ps26
Kaz./Nau./Saccharomyces - ps27
Saccharomyces - ps28
Arboricola_clade - ps29
Cerevisiae_clade - ps30
Saccharomyces_cerevisiae - ps31
Phylostratum

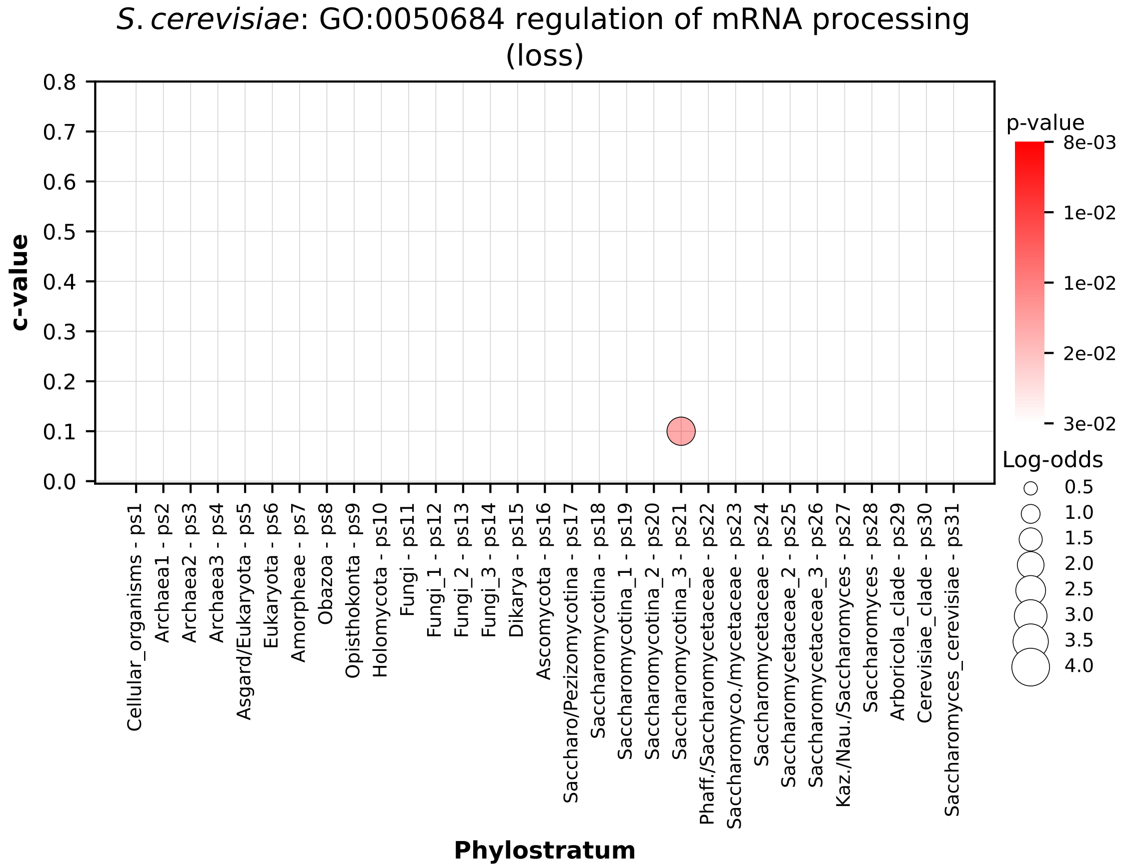
S. cerevisiae: GO:0050684 regulation of mRNA processing (loss)
c-value
0.8
0.7
0.6
0.5
0.4
0.3
0.2
0.1
0.0
Phylostratum
Cellular_organisms - ps1
Archaea1 - ps2
Archaea2 - ps3
Archaea3 - ps4
Asgard/Eukaryota - ps5
Eukaryota - ps6
Amorpheae - ps7
Obazoa - ps8
Opisthokonta - ps9
Holomycota - ps10
Fungi - ps11
Fungi_1 - ps12
Fungi_2 - ps13
Fungi_3 - ps14
Dikarya - ps15
Ascomycota - ps16
Saccharo/Pezizomycotina - ps17
Saccharomycotina - ps18
Saccharomycotina_1 - ps19
Saccharomycotina_2 - ps20
Saccharomycotina_3 - ps21
Phaff./Saccharomycetaceae - ps22
Saccharomyco./mycetaceae - ps23
Saccharomycetaceae - ps24
Saccharomycetaceae_2 - ps25
Saccharomycetaceae_3 - ps26
Kaz./Nau./Saccharomyces - ps27
Saccharomyces - ps28
Arboricola_clade - ps29
Cerevisiae_clade - ps30
Saccharomyces_cerevisiae - ps31
p-value
8e-03
1e-02
1e-02
2e-02
3e-02
Log-odds
0.5
1.0
1.5
2.0
2.5
3.0
3.5
4.0

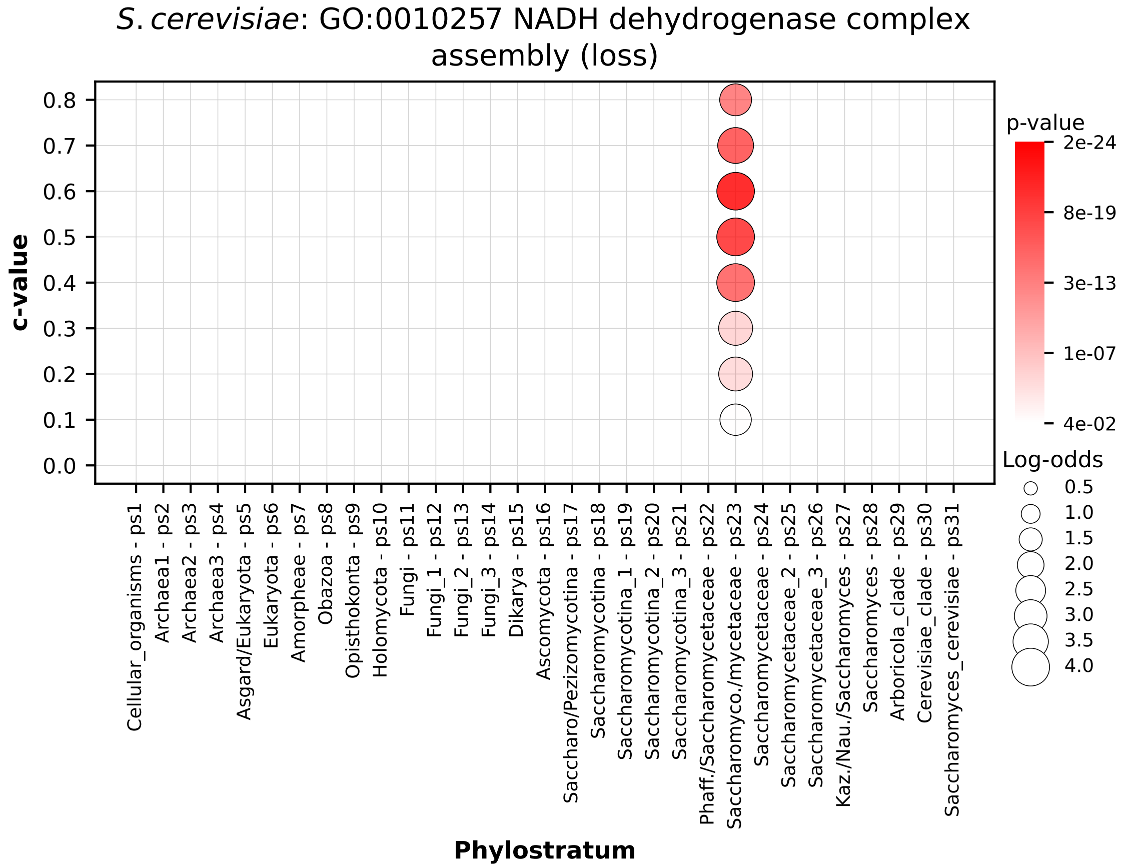
S. cerevisiae: GO:0010257 NADH dehydrogenase complex assembly (loss)
c-value
0.0
0.1
0.2
0.3
0.4
0.5
0.6
0.7
0.8
p-value
2e-24
8e-19
3e-13
1e-07
4e-02
Log-odds
0.5
1.0
1.5
2.0
2.5
3.0
3.5
4.0
Cellular_organisms - ps1
Archaea1 - ps2
Archaea2 - ps3
Archaea3 - ps4
Asgard/Eukaryota - ps5
Eukaryota - ps6
Amorpheae - ps7
Obazoa - ps8
Opisthokonta - ps9
Holomycota - ps10
Fungi - ps11
Fungi_1 - ps12
Fungi_2 - ps13
Fungi_3 - ps14
Dikarya - ps15
Ascomycota - ps16
Saccharo/Pezizomycotina - ps17
Saccharomycotina - ps18
Saccharomycotina_1 - ps19
Saccharomycotina_2 - ps20
Saccharomycotina_3 - ps21
Phaff./Saccharomycetaceae - ps22
Saccharomyco./mycetaceae - ps23
Saccharomycetaceae - ps24
Saccharomycetaceae_2 - ps25
Saccharomycetaceae_3 - ps26
Kaz./Nau./Saccharomyces - ps27
Saccharomyces - ps28
Arboricola_clade - ps29
Cerevisiae_clade - ps30
Saccharomyces_cerevisiae - ps31
Phylostratum

S. cerevisiae: GO:0032981 mitochondrial respiratory chain complex I assembly (loss)

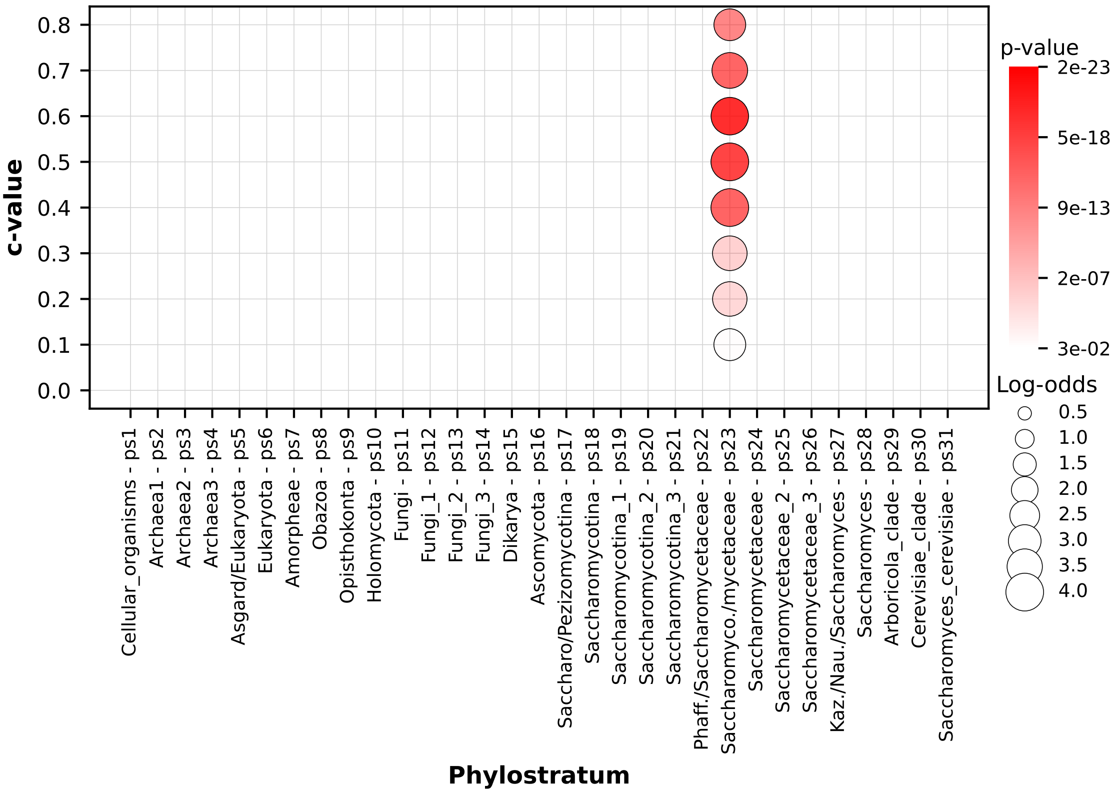

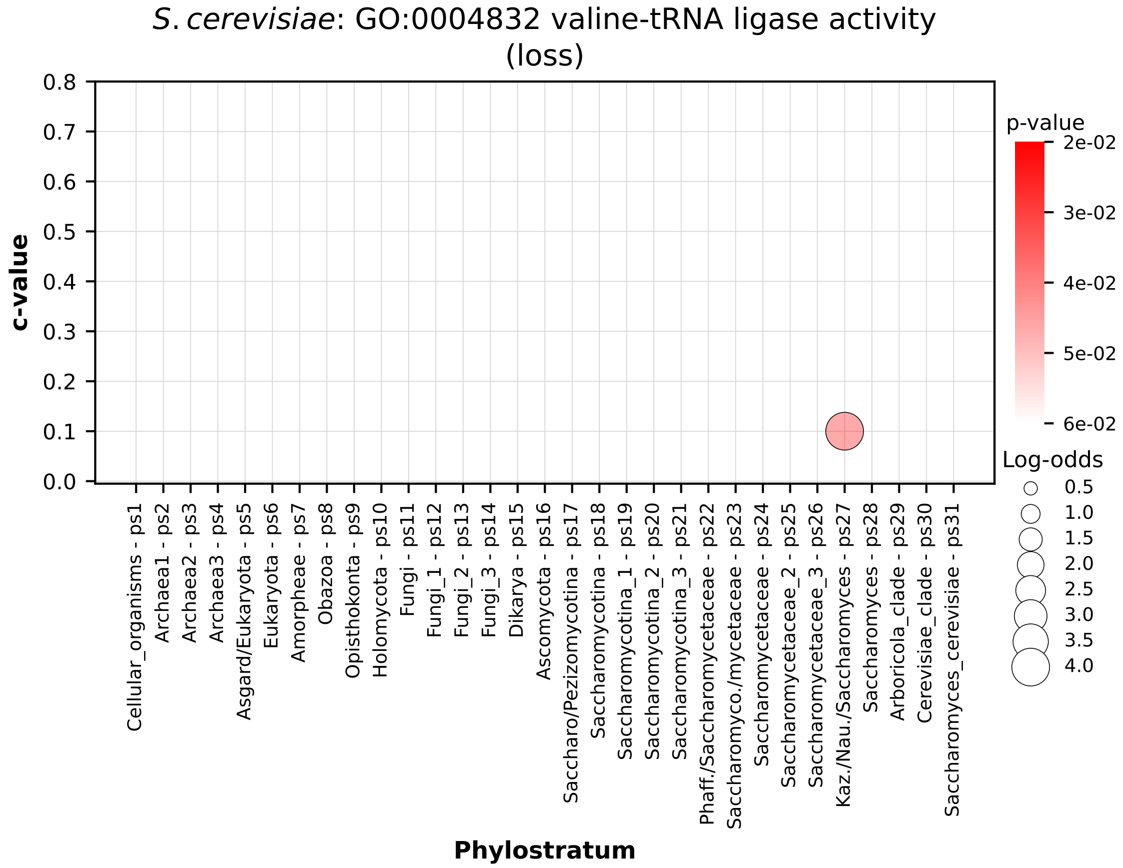

S. cerevisiae: GO:0004832 valine-tRNA ligase activity
(loss)
c-value
0.8
0.7
0.6
0.5
0.4
0.3
0.2
0.1
0.0
p-value
2e-02
3e-02
4e-02
5e-02
6e-02
Log-odds
0.5
1.0
1.5
2.0
2.5
3.0
3.5
4.0
Cellular_organisms - ps1
Archaea1 - ps2
Archaea2 - ps3
Archaea3 - ps4
Asgard/Eukaryota - ps5
Eukaryota - ps6
Amorpheae - ps7
Obazoa - ps8
Opisthokonta - ps9
Holomycota - ps10
Fungi - ps11
Fungi_1 - ps12
Fungi_2 - ps13
Fungi_3 - ps14
Dikarya - ps15
Ascomycota - ps16
Saccharo/Pezizomycotina - ps17
Saccharomycotina - ps18
Saccharomycotina_1 - ps19
Saccharomycotina_2 - ps20
Saccharomycotina_3 - ps21
Phaff./Saccharomycetaceae - ps22
Saccharomyco./mycetaceae - ps23
Saccharomycetaceae - ps24
Saccharomycetaceae_2 - ps25
Saccharomycetaceae_3 - ps26
Kaz./Nau./Saccharomyces - ps27
Saccharomyces - ps28
Arboricola_clade - ps29
Cerevisiae_clade - ps30
Saccharomyces_cerevisiae - ps31
Phylostratum

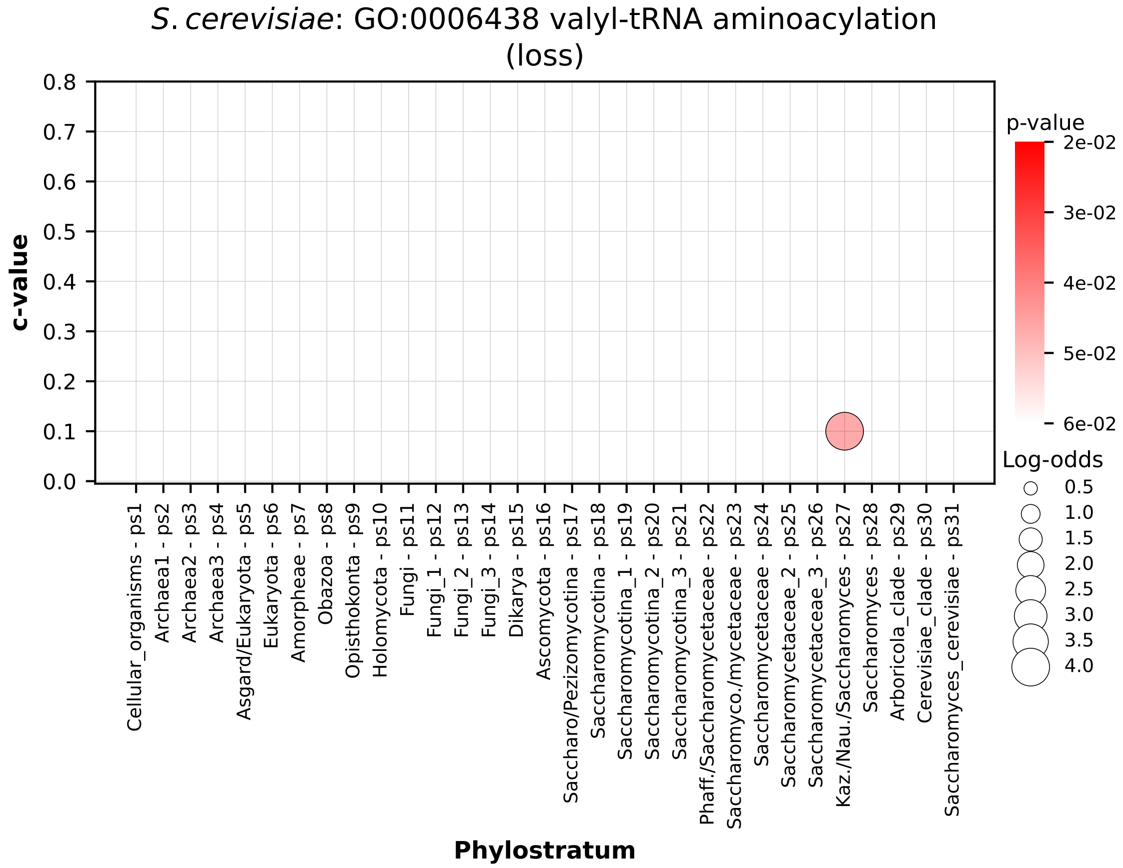
S. cerevisiae: GO:0006438 valyl-tRNA aminoacylation
(loss)
c-value
0.8
0.7
0.6
0.5
0.4
0.3
0.2
0.1
0.0
p-value
2e-02
3e-02
4e-02
5e-02
6e-02
Log-odds
0.5
1.0
1.5
2.0
2.5
3.0
3.5
4.0
Cellular_organisms - ps1
Archaea1 - ps2
Archaea2 - ps3
Archaea3 - ps4
Asgard/Eukaryota - ps5
Eukaryota - ps6
Amorpheae - ps7
Obazoa - ps8
Opisthokonta - ps9
Holomycota - ps10
Fungi - ps11
Fungi_1 - ps12
Fungi_2 - ps13
Fungi_3 - ps14
Dikarya - ps15
Ascomycota - ps16
Saccharo/Pezizomycotina - ps17
Saccharomycotina - ps18
Saccharomycotina_1 - ps19
Saccharomycotina_2 - ps20
Saccharomycotina_3 - ps21
Phaff./Saccharomycetaceae - ps22
Saccharomyco./mycetaceae - ps23
Saccharomycetaceae - ps24
Saccharomycetaceae_2 - ps25
Saccharomycetaceae_3 - ps26
Kaz./Nau./Saccharomyces - ps27
Saccharomyces - ps28
Arboricola_clade - ps29
Cerevisiae_clade - ps30
Saccharomyces_cerevisiae - ps31
Phylostratum

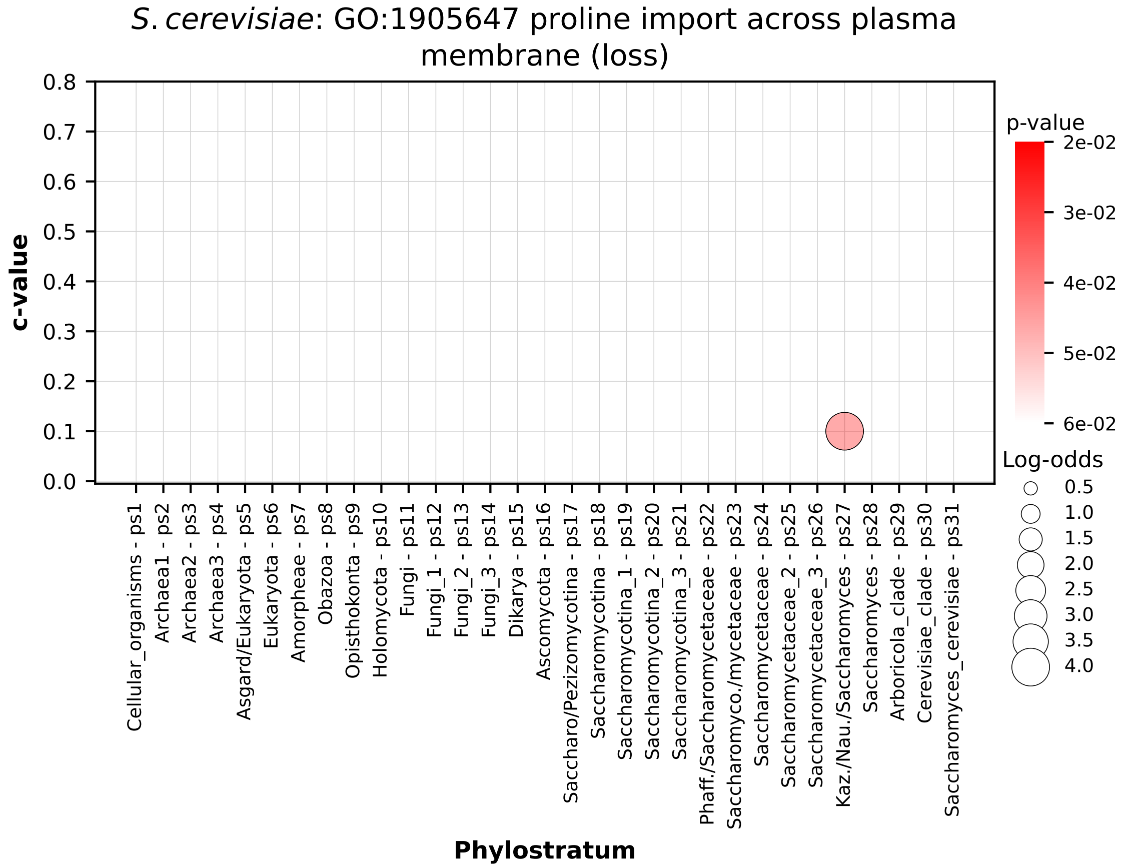
S. cerevisiae: GO:1905647 proline import across plasma membrane (loss)
c-value
0.8
0.7
0.6
0.5
0.4
0.3
0.2
0.1
0.0
p-value
2e-02
3e-02
4e-02
5e-02
6e-02
Log-odds
0.5
1.0
1.5
2.0
2.5
3.0
3.5
4.0
Cellular_organisms - ps1
Archaea1 - ps2
Archaea2 - ps3
Archaea3 - ps4
Asgard/Eukaryota - ps5
Eukaryota - ps6
Amorpheae - ps7
Obazoa - ps8
Opisthokonta - ps9
Holomycota - ps10
Fungi - ps11
Fungi_1 - ps12
Fungi_2 - ps13
Fungi_3 - ps14
Dikarya - ps15
Ascomycota - ps16
Saccharo/Pezizomycotina - ps17
Saccharomycotina - ps18
Saccharomycotina_1 - ps19
Saccharomycotina_2 - ps20
Saccharomycotina_3 - ps21
Phaff./Saccharomycetaceae - ps22
Saccharomyco./mycetaceae - ps23
Saccharomycetaceae - ps24
Saccharomycetaceae_2 - ps25
Saccharomycetaceae_3 - ps26
Kaz./Nau./Saccharomyces - ps27
Saccharomyces - ps28
Arboricola_clade - ps29
Cerevisiae_clade - ps30
Saccharomyces_cerevisiae - ps31
Phylostratum

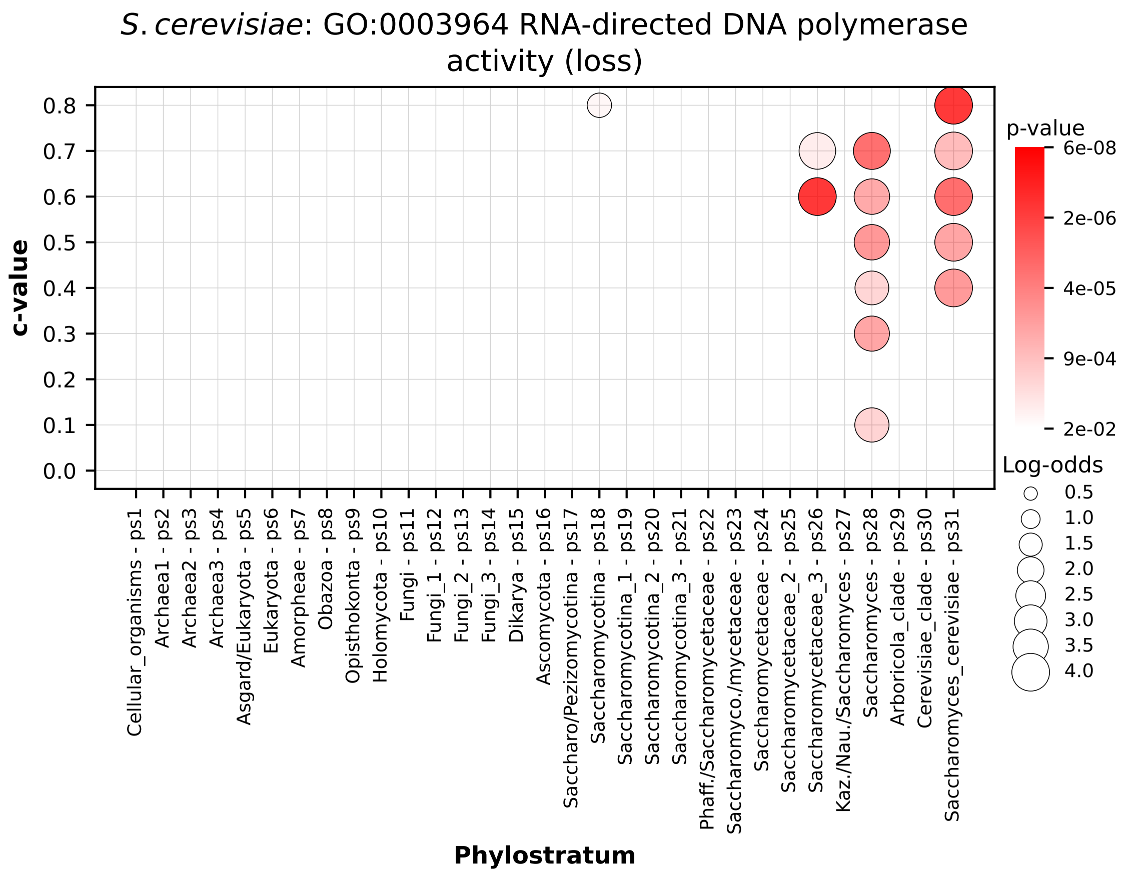
S. cerevisiae: GO:0003964 RNA-directed DNA polymerase activity (loss)
c-value
0.8
0.7
0.6
0.5
0.4
0.3
0.2
0.1
0.0
p-value
6e-08
2e-06
4e-05
9e-04
2e-02
Log-odds
0.5
1.0
1.5
2.0
2.5
3.0
3.5
4.0
Cellular_organisms - ps1
Archaea1 - ps2
Archaea2 - ps3
Archaea3 - ps4
Asgard/Eukaryota - ps5
Eukaryota - ps6
Amorpheae - ps7
Obazoa - ps8
Opisthokonta - ps9
Holomycota - ps10
Fungi - ps11
Fungi_1 - ps12
Fungi_2 - ps13
Fungi_3 - ps14
Dikarya - ps15
Ascomycota - ps16
Saccharo/Pezizomycotina - ps17
Saccharomycotina - ps18
Saccharomycotina_1 - ps19
Saccharomycotina_2 - ps20
Saccharomycotina_3 - ps21
Phaff./Saccharomycetaceae - ps22
Saccharomyco./mycetaceae - ps23
Saccharomycetaceae - ps24
Saccharomycetaceae_2 - ps25
Saccharomycetaceae_3 - ps26
Kaz./Nau./Saccharomyces - ps27
Saccharomyces - ps28
Arboricola_clade - ps29
Cerevisiae_clade - ps30
Saccharomyces_cerevisiae - ps31
Phylostratum

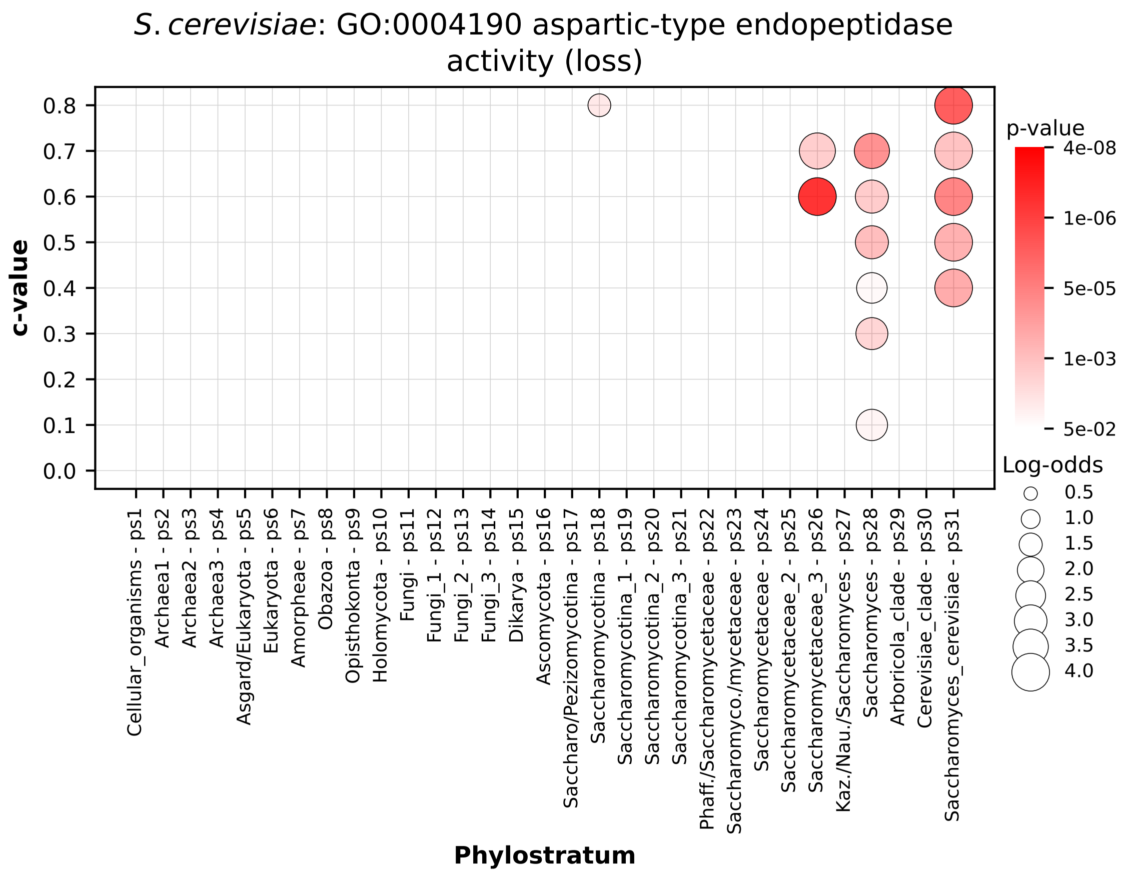
S. cerevisiae: GO:0004190 aspartic-type endopeptidase activity (loss)
c-value
0.8
0.7
0.6
0.5
0.4
0.3
0.2
0.1
0.0
p-value
4e-08
1e-06
5e-05
1e-03
5e-02
Log-odds
0.5
1.0
1.5
2.0
2.5
3.0
3.5
4.0
Cellular_organisms - ps1
Archaea1 - ps2
Archaea2 - ps3
Archaea3 - ps4
Asgard/Eukaryota - ps5
Eukaryota - ps6
Amorpheae - ps7
Obazoa - ps8
Opisthokonta - ps9
Holomycota - ps10
Fungi - ps11
Fungi_1 - ps12
Fungi_2 - ps13
Fungi_3 - ps14
Dikarya - ps15
Ascomycota - ps16
Saccharo/Pezizomycotina - ps17
Saccharomycotina - ps18
Saccharomycotina_1 - ps19
Saccharomycotina_2 - ps20
Saccharomycotina_3 - ps21
Phaff./Saccharomycetaceae - ps22
Saccharomyco./mycetaceae - ps23
Saccharomycetaceae - ps24
Saccharomycetaceae_2 - ps25
Saccharomycetaceae_3 - ps26
Kaz./Nau./Saccharomyces - ps27
Saccharomyces - ps28
Arboricola_clade - ps29
Cerevisiae_clade - ps30
Saccharomyces_cerevisiae - ps31
Phylostratum

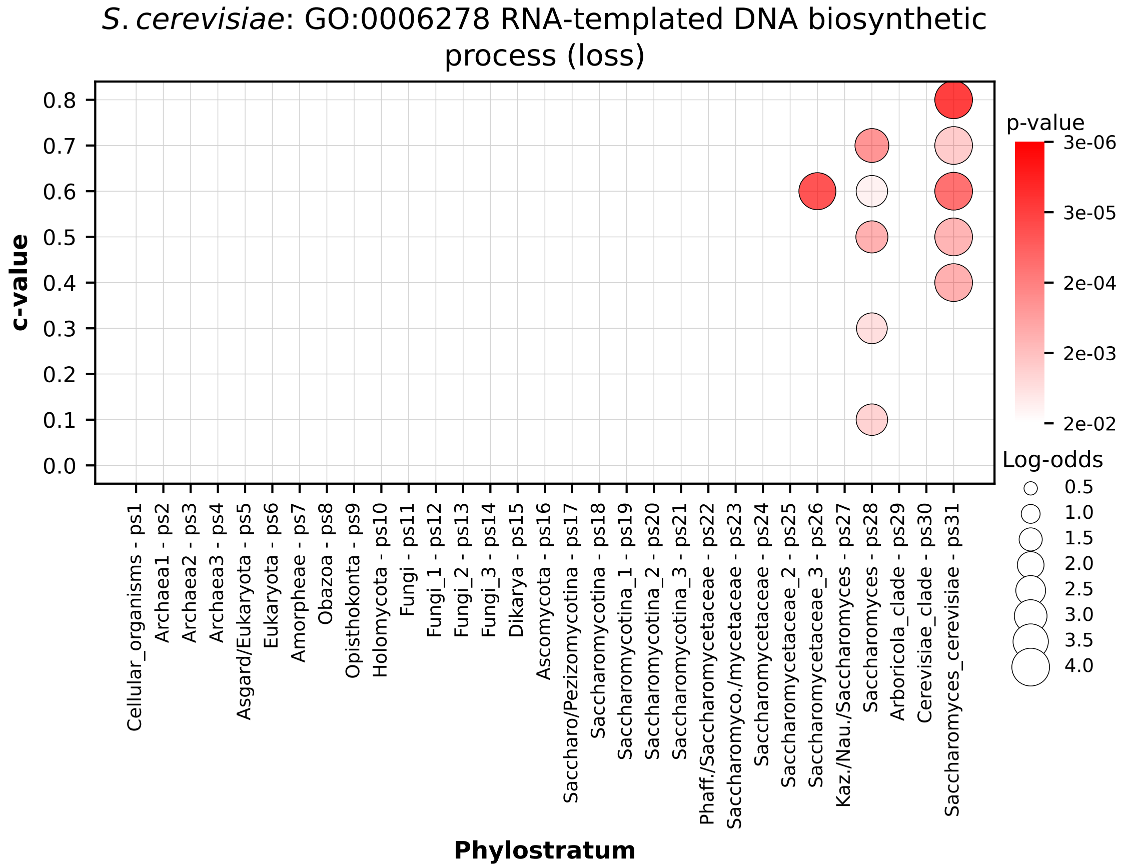

S. cerevisiae: GO:0006278 RNA-templated DNA biosynthetic process (loss)
c-value
0.0
0.1
0.2
0.3
0.4
0.5
0.6
0.7
0.8
Phylostratum
Cellular_organisms - ps1
Archaea1 - ps2
Archaea2 - ps3
Archaea3 - ps4
Asgard/Eukaryota - ps5
Eukaryota - ps6
Amorpheae - ps7
Obazoa - ps8
Opisthokonta - ps9
Holomycota - ps10
Fungi - ps11
Fungi_1 - ps12
Fungi_2 - ps13
Fungi_3 - ps14
Dikarya - ps15
Ascomycota - ps16
Saccharo/Pezizomycotina - ps17
Saccharomycotina - ps18
Saccharomycotina_1 - ps19
Saccharomycotina_2 - ps20
Saccharomycotina_3 - ps21
Phaff./Saccharomycetaceae - ps22
Saccharomyco./mycetaceae - ps23
Saccharomycetaceae - ps24
Saccharomycetaceae_2 - ps25
Saccharomycetaceae_3 - ps26
Kaz./Nau./Saccharomyces - ps27
Saccharomyces - ps28
Arboricola_clade - ps29
Cerevisiae_clade - ps30
Saccharomyces_cerevisiae - ps31
p-value
3e-06
3e-05
2e-04
2e-03
2e-02
Log-odds
0.5
1.0
1.5
2.0
2.5
3.0
3.5
4.0

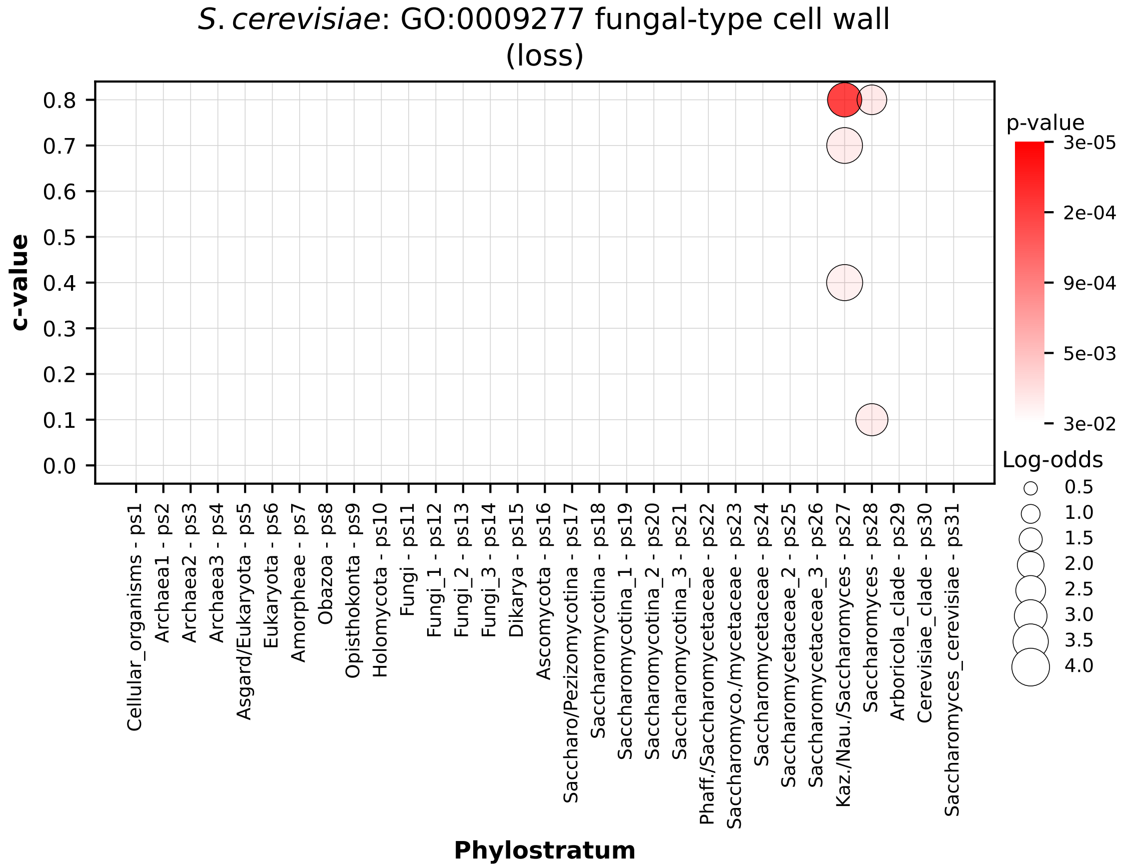

S. cerevisiae: GO:0009277 fungal-type cell wall
(loss)
c-value
0.0
0.1
0.2
0.3
0.4
0.5
0.6
0.7
0.8
Phylostratum
Cellular_organisms - ps1
Archaea1 - ps2
Archaea2 - ps3
Archaea3 - ps4
Asgard/Eukaryota - ps5
Eukaryota - ps6
Amorpheae - ps7
Obazoa - ps8
Opisthokonta - ps9
Holomycota - ps10
Fungi - ps11
Fungi_1 - ps12
Fungi_2 - ps13
Fungi_3 - ps14
Dikarya - ps15
Ascomycota - ps16
Saccharo/Pezizomycotina - ps17
Saccharomycotina - ps18
Saccharomycotina_1 - ps19
Saccharomycotina_2 - ps20
Saccharomycotina_3 - ps21
Phaff./Saccharomycetaceae - ps22
Saccharomyco./mycetaceae - ps23
Saccharomycetaceae - ps24
Saccharomycetaceae_2 - ps25
Saccharomycetaceae_3 - ps26
Kaz./Nau./Saccharomyces - ps27
Saccharomyces - ps28
Arboricola_clade - ps29
Cerevisiae_clade - ps30
Saccharomyces_cerevisiae - ps31
p-value
3e-05
2e-04
9e-04
5e-03
3e-02
Log-odds
0.5
1.0
1.5
2.0
2.5
3.0
3.5
4.0

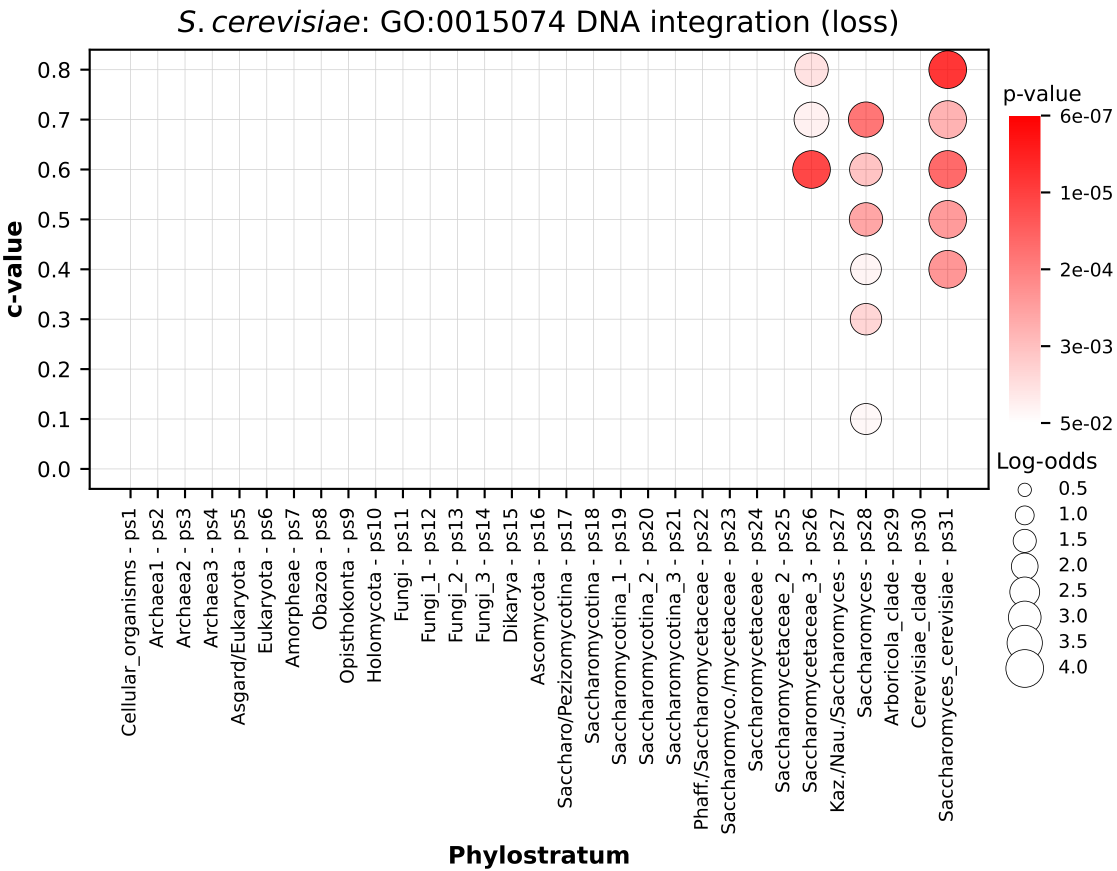

S. cerevisiae: GO:0015074 DNA integration (loss)
c-value
0.8
0.7
0.6
0.5
0.4
0.3
0.2
0.1
0.0
Phylostratum
Cellular_organisms - ps1
Archaea1 - ps2
Archaea2 - ps3
Archaea3 - ps4
Asgard/Eukaryota - ps5
Eukaryota - ps6
Amorpheae - ps7
Obazoa - ps8
Opisthokonta - ps9
Holomycota - ps10
Fungi - ps11
Fungi_1 - ps12
Fungi_2 - ps13
Fungi_3 - ps14
Dikarya - ps15
Ascomycota - ps16
Saccharo/Pezizomycotina - ps17
Saccharomycotina - ps18
Saccharomycotina_1 - ps19
Saccharomycotina_2 - ps20
Saccharomycotina_3 - ps21
Phaff./Saccharomycetaceae - ps22
Saccharomyco./mycetaceae - ps23
Saccharomycetaceae - ps24
Saccharomycetaceae_2 - ps25
Saccharomycetaceae_3 - ps26
Kaz./Nau./Saccharomyces - ps27
Saccharomyces - ps28
Arboricola_clade - ps29
Cerevisiae_clade - ps30
Saccharomyces_cerevisiae - ps31
p-value
6e-07
1e-05
2e-04
3e-03
5e-02
Log-odds
0.5
1.0
1.5
2.0
2.5
3.0
3.5
4.0

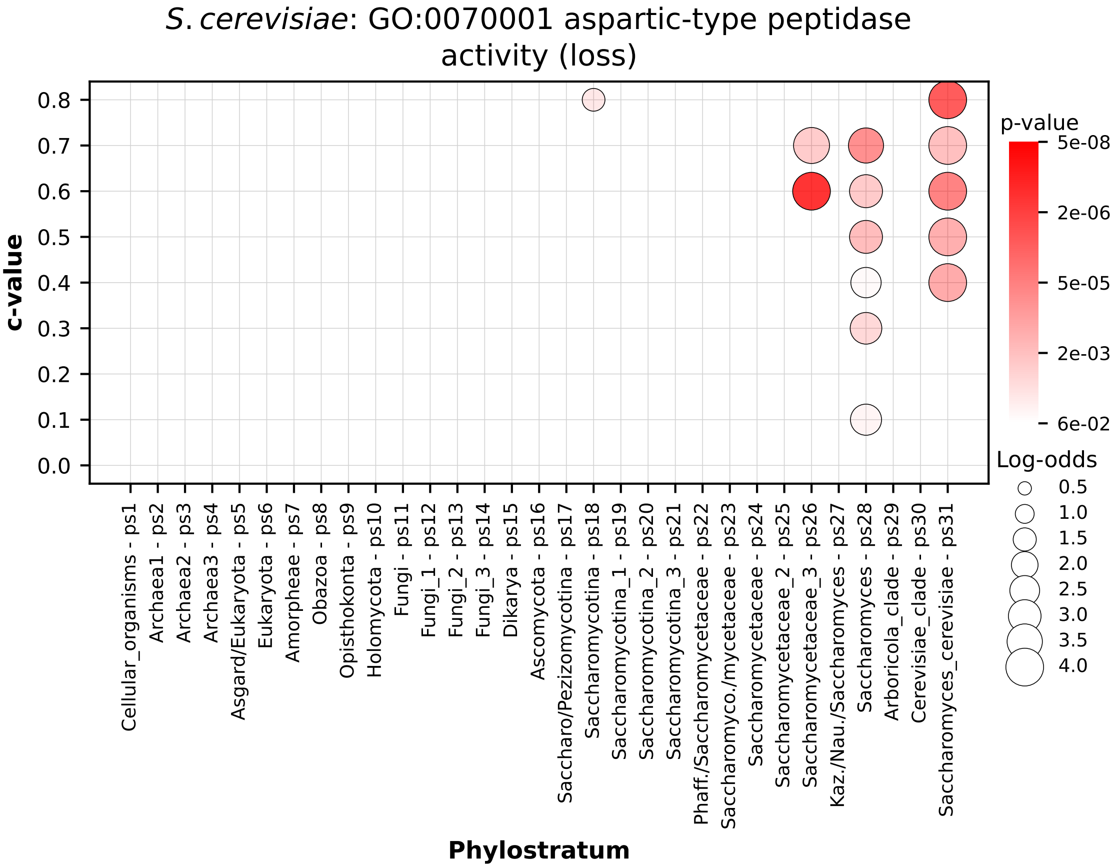

S. cerevisiae: GO:0070001 aspartic-type peptidase activity (loss)
c-value
0.8
0.7
0.6
0.5
0.4
0.3
0.2
0.1
0.0
Phylostratum
Cellular_organisms - ps1
Archaea1 - ps2
Archaea2 - ps3
Archaea3 - ps4
Asgard/Eukaryota - ps5
Eukaryota - ps6
Amorpheae - ps7
Obazoa - ps8
Opisthokonta - ps9
Holomycota - ps10
Fungi - ps11
Fungi_1 - ps12
Fungi_2 - ps13
Fungi_3 - ps14
Dikarya - ps15
Ascomycota - ps16
Saccharo/Pezizomycotina - ps17
Saccharomycotina - ps18
Saccharomycotina_1 - ps19
Saccharomycotina_2 - ps20
Saccharomycotina_3 - ps21
Phaff./Saccharomycetaceae - ps22
Saccharomyco./mycetaceae - ps23
Saccharomycetaceae - ps24
Saccharomycetaceae_2 - ps25
Saccharomycetaceae_3 - ps26
Kaz./Nau./Saccharomyces - ps27
Saccharomyces - ps28
Arboricola_clade - ps29
Cerevisiae_clade - ps30
Saccharomyces_cerevisiae - ps31
p-value
5e-08
2e-06
5e-05
2e-03
6e-02
Log-odds
0.5
1.0
1.5
2.0
2.5
3.0
3.5
4.0

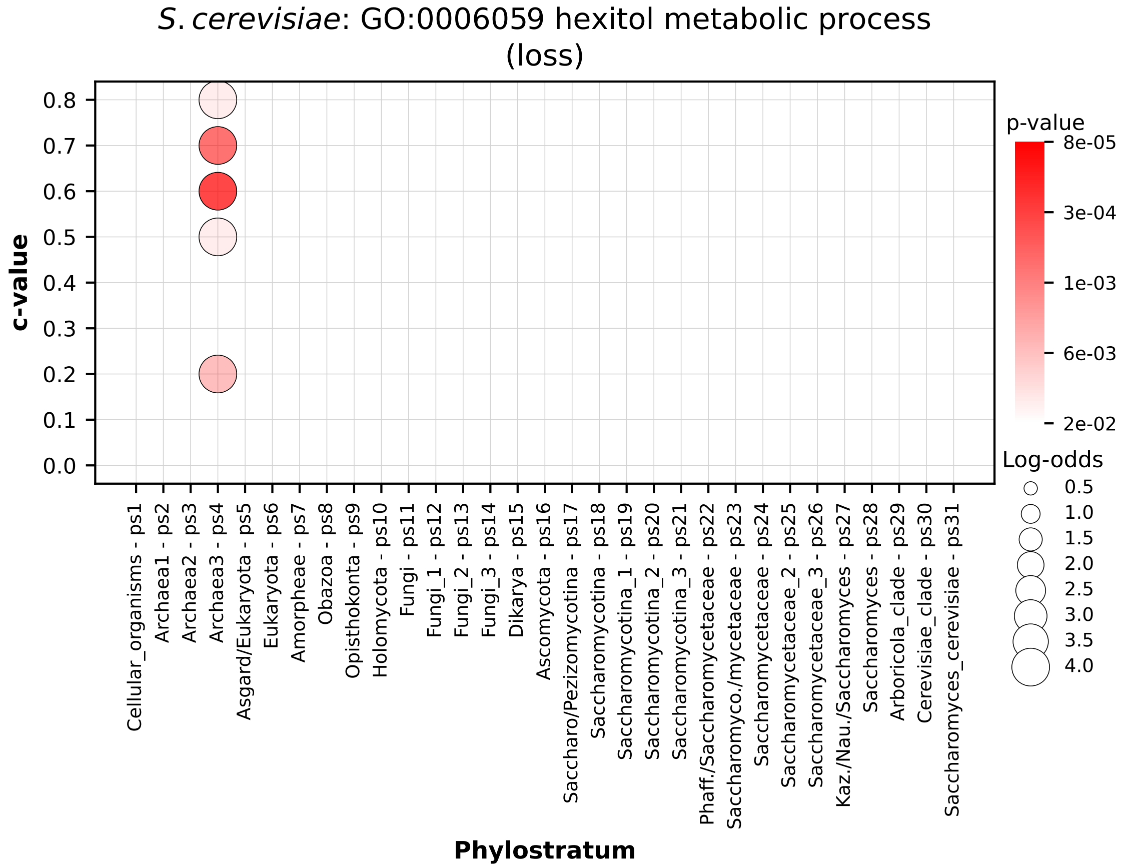

S. cerevisiae: GO:0006059 hexitol metabolic process
(loss)
c-value
0.8
0.7
0.6
0.5
0.4
0.3
0.2
0.1
0.0
p-value
8e-05
3e-04
1e-03
6e-03
2e-02
Log-odds
0.5
1.0
1.5
2.0
2.5
3.0
3.5
4.0
Cellular_organisms - ps1
Archaea1 - ps2
Archaea2 - ps3
Archaea3 - ps4
Asgard/Eukaryota - ps5
Eukaryota - ps6
Amorpheae - ps7
Obazoa - ps8
Opisthokonta - ps9
Holomycota - ps10
Fungi - ps11
Fungi_1 - ps12
Fungi_2 - ps13
Fungi_3 - ps14
Dikarya - ps15
Ascomycota - ps16
Saccharo/Pezizomycotina - ps17
Saccharomycotina - ps18
Saccharomycotina_1 - ps19
Saccharomycotina_2 - ps20
Saccharomycotina_3 - ps21
Phaff./Saccharomycetaceae - ps22
Saccharomyco./mycetaceae - ps23
Saccharomycetaceae - ps24
Saccharomycetaceae_2 - ps25
Saccharomycetaceae_3 - ps26
Kaz./Nau./Saccharomyces - ps27
Saccharomyces - ps28
Arboricola_clade - ps29
Cerevisiae_clade - ps30
Saccharomyces_cerevisiae - ps31
Phylostratum

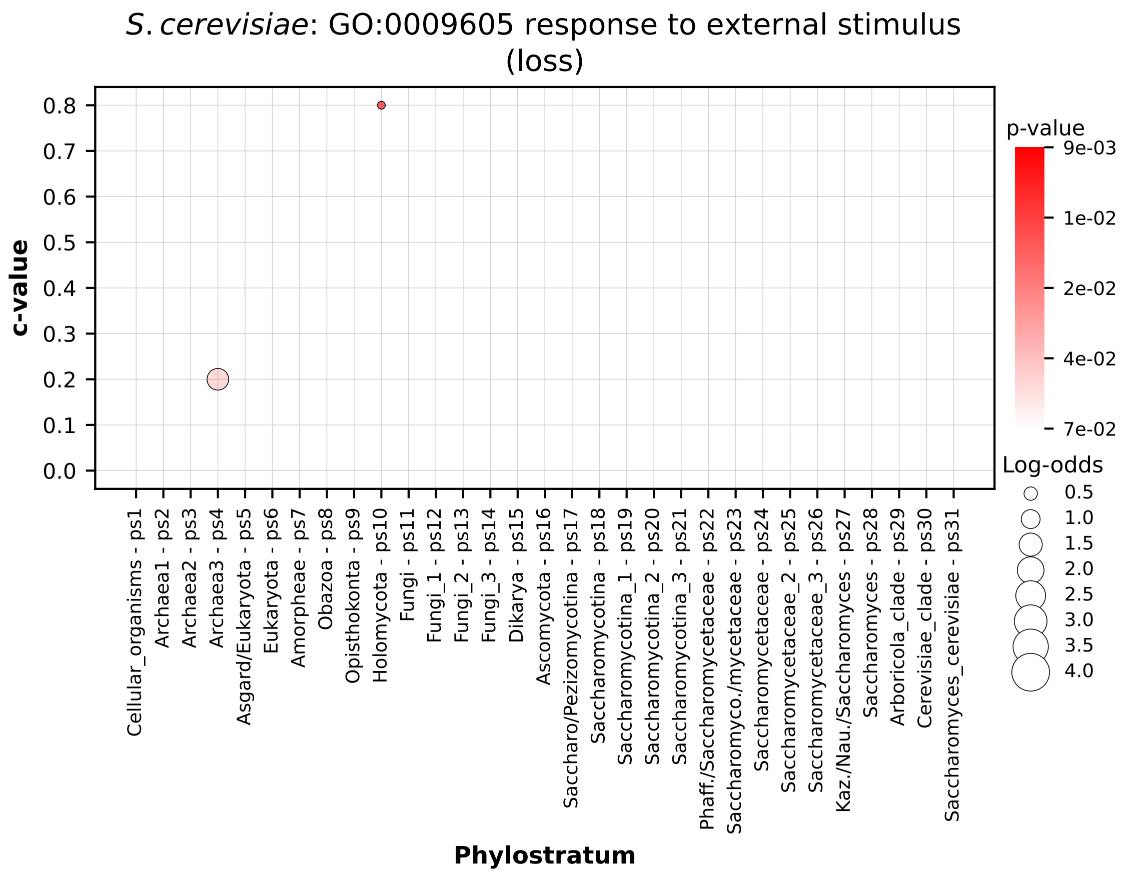

S. cerevisiae: GO:0009605 response to external stimulus
(loss)
c-value
0.0
0.1
0.2
0.3
0.4
0.5
0.6
0.7
0.8
Phylostratum
Cellular_organisms - ps1
Archaea1 - ps2
Archaea2 - ps3
Archaea3 - ps4
Asgard/Eukaryota - ps5
Eukaryota - ps6
Amorpheae - ps7
Obazoa - ps8
Opisthokonta - ps9
Holomycota - ps10
Fungi - ps11
Fungi_1 - ps12
Fungi_2 - ps13
Fungi_3 - ps14
Dikarya - ps15
Ascomycota - ps16
Saccharo/Pezizomycotina - ps17
Saccharomycotina - ps18
Saccharomycotina_1 - ps19
Saccharomycotina_2 - ps20
Saccharomycotina_3 - ps21
Phaff./Saccharomycetaceae - ps22
Saccharomyco./mycetaceae - ps23
Saccharomycetaceae - ps24
Saccharomycetaceae_2 - ps25
Saccharomycetaceae_3 - ps26
Kaz./Nau./Saccharomyces - ps27
Saccharomyces - ps28
Arboricola_clade - ps29
Cerevisiae_clade - ps30
Saccharomyces_cerevisiae - ps31
p-value
9e-03
1e-02
2e-02
4e-02
7e-02
Log-odds
0.5
1.0
1.5
2.0
2.5
3.0
3.5
4.0

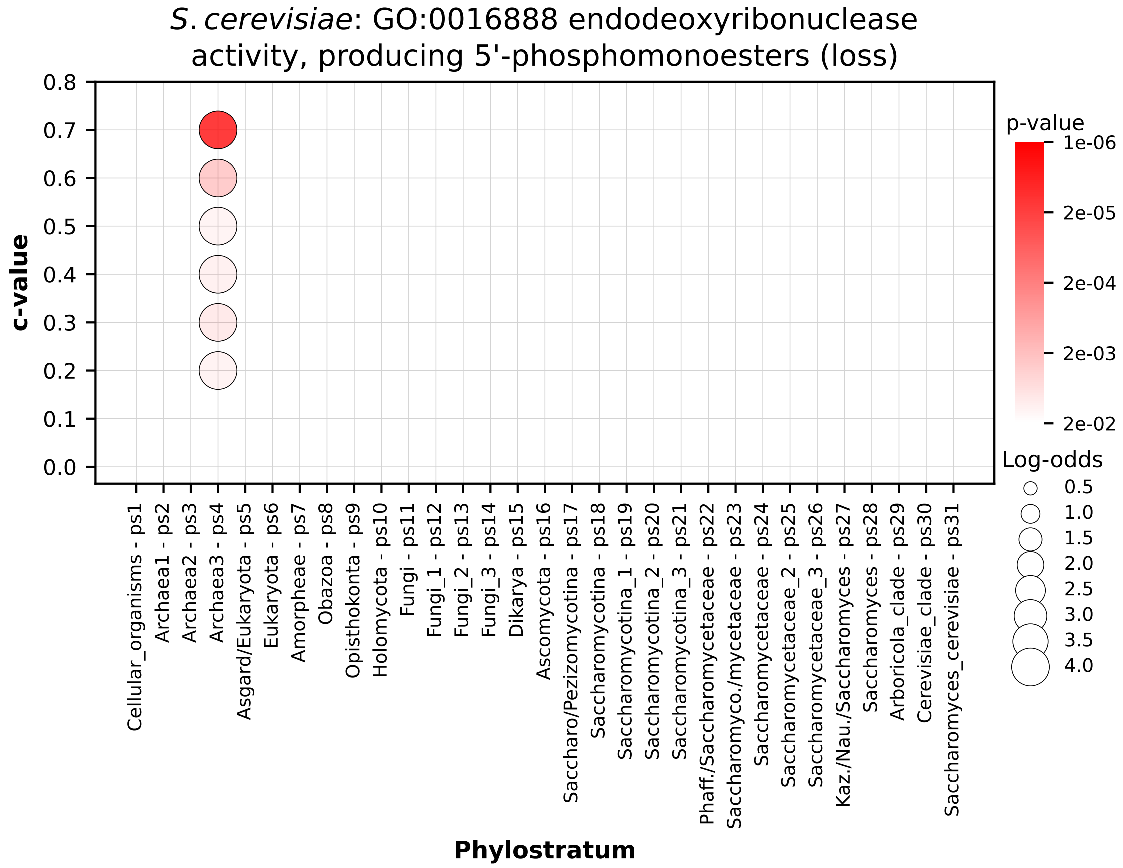

S. cerevisiae: GO:0016888 endodeoxyribonuclease activity, producing 5'-phosphomonoesters (loss)
c-value
0.0
0.1
0.2
0.3
0.4
0.5
0.6
0.7
0.8
p-value
1e-06
2e-05
2e-04
2e-03
2e-02
Log-odds
0.5
1.0
1.5
2.0
2.5
3.0
3.5
4.0
Cellular_organisms - ps1
Archaea1 - ps2
Archaea2 - ps3
Archaea3 - ps4
Asgard/Eukaryota - ps5
Eukaryota - ps6
Amorpheae - ps7
Obazoa - ps8
Opisthokonta - ps9
Holomycota - ps10
Fungi - ps11
Fungi_1 - ps12
Fungi_2 - ps13
Fungi_3 - ps14
Dikarya - ps15
Ascomycota - ps16
Saccharo/Pezizomycotina - ps17
Saccharomycotina - ps18
Saccharomycotina_1 - ps19
Saccharomycotina_2 - ps20
Saccharomycotina_3 - ps21
Phaff./Saccharomycetaceae - ps22
Saccharomyco./mycetaceae - ps23
Saccharomycetaceae - ps24
Saccharomycetaceae_2 - ps25
Saccharomycetaceae_3 - ps26
Kaz./Nau./Saccharomyces - ps27
Saccharomyces - ps28
Arboricola_clade - ps29
Cerevisiae_clade - ps30
Saccharomyces_cerevisiae - ps31
Phylostratum

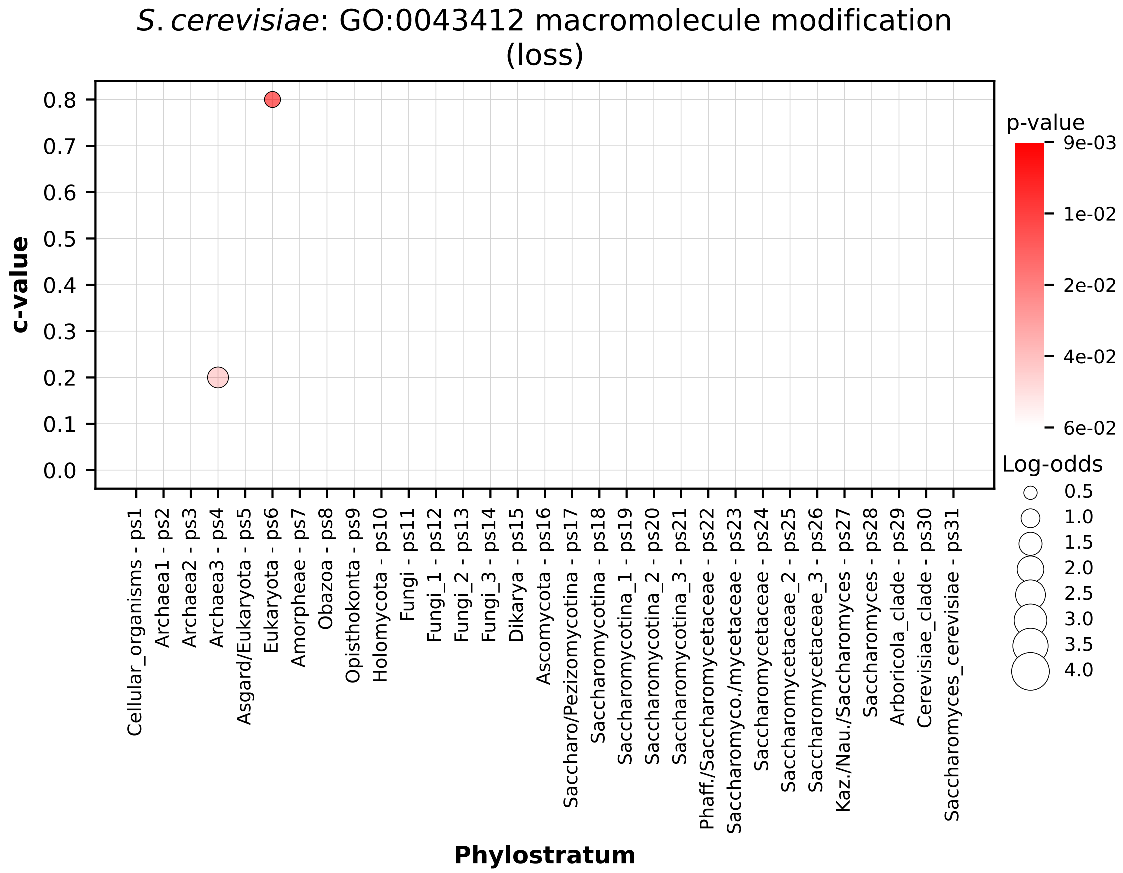

S. cerevisiae: GO:0043412 macromolecule modification
(loss)
c-value
0.0
0.1
0.2
0.3
0.4
0.5
0.6
0.7
0.8
p-value
9e-03
1e-02
2e-02
4e-02
6e-02
Log-odds
0.5
1.0
1.5
2.0
2.5
3.0
3.5
4.0
Cellular_organisms - ps1
Archaea1 - ps2
Archaea2 - ps3
Archaea3 - ps4
Asgard/Eukaryota - ps5
Eukaryota - ps6
Amorpheae - ps7
Obazoa - ps8
Opisthokonta - ps9
Holomycota - ps10
Fungi - ps11
Fungi_1 - ps12
Fungi_2 - ps13
Fungi_3 - ps14
Dikarya - ps15
Ascomycota - ps16
Saccharo/Pezizomycotina - ps17
Saccharomycotina - ps18
Saccharomycotina_1 - ps19
Saccharomycotina_2 - ps20
Saccharomycotina_3 - ps21
Phaff./Saccharomycetaceae - ps22
Saccharomyco./mycetaceae - ps23
Saccharomycetaceae - ps24
Saccharomycetaceae_2 - ps25
Saccharomycetaceae_3 - ps26
Kaz./Nau./Saccharomyces - ps27
Saccharomyces - ps28
Arboricola_clade - ps29
Cerevisiae_clade - ps30
Saccharomyces_cerevisiae - ps31
Phylostratum

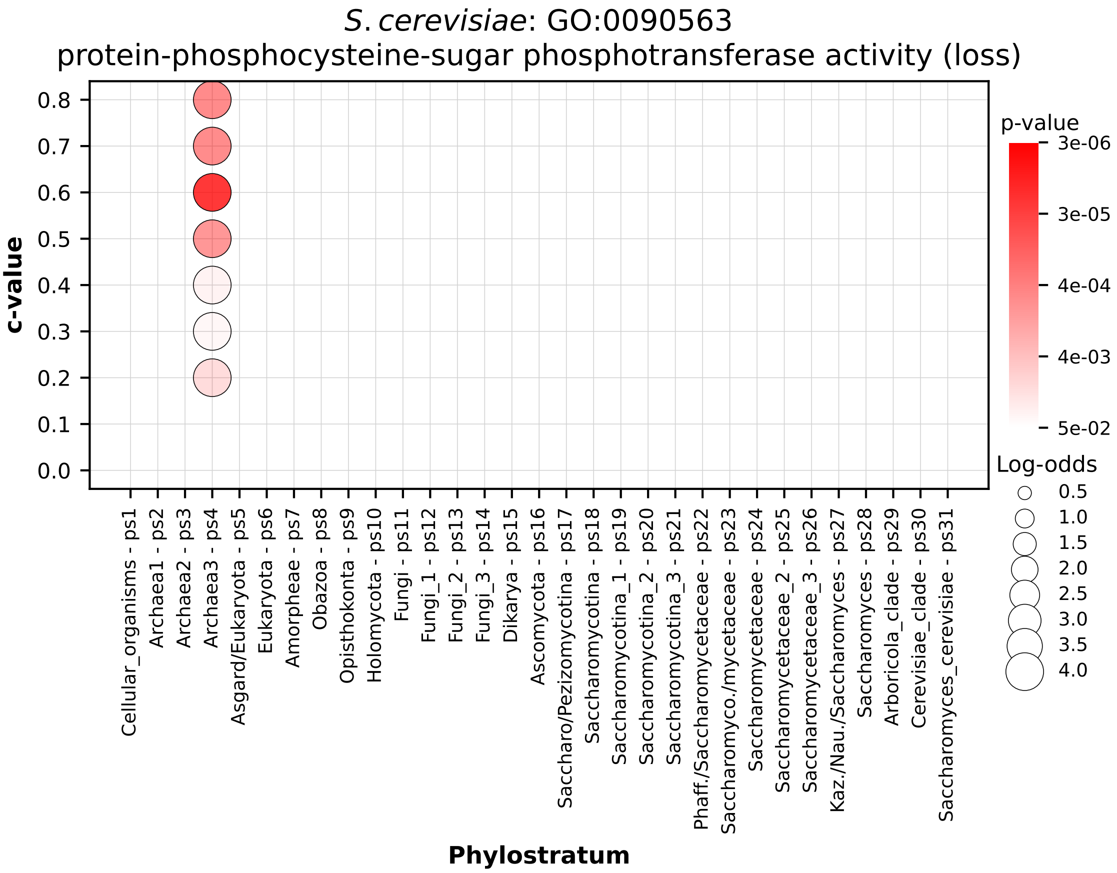

S. cerevisiae: GO:0090563
protein-phosphocysteine-sugar phosphotransferase activity (loss)
c-value
0.8
0.7
0.6
0.5
0.4
0.3
0.2
0.1
0.0
p-value
3e-06
3e-05
4e-04
4e-03
5e-02
Log-odds
0.5
1.0
1.5
2.0
2.5
3.0
3.5
4.0
Cellular_organisms - ps1
Archaea1 - ps2
Archaea2 - ps3
Archaea3 - ps4
Asgard/Eukaryota - ps5
Eukaryota - ps6
Amorpheae - ps7
Obazoa - ps8
Opisthokonta - ps9
Holomycota - ps10
Fungi - ps11
Fungi_1 - ps12
Fungi_2 - ps13
Fungi_3 - ps14
Dikarya - ps15
Ascomycota - ps16
Saccharo/Pezizomycotina - ps17
Saccharomycotina - ps18
Saccharomycotina_1 - ps19
Saccharomycotina_2 - ps20
Saccharomycotina_3 - ps21
Phaff./Saccharomycetaceae - ps22
Saccharomyco./mycetaceae - ps23
Saccharomycetaceae - ps24
Saccharomycetaceae_2 - ps25
Saccharomycetaceae_3 - ps26
Kaz./Nau./Saccharomyces - ps27
Saccharomyces - ps28
Arboricola_clade - ps29
Cerevisiae_clade - ps30
Saccharomyces_cerevisiae - ps31
Phylostratum

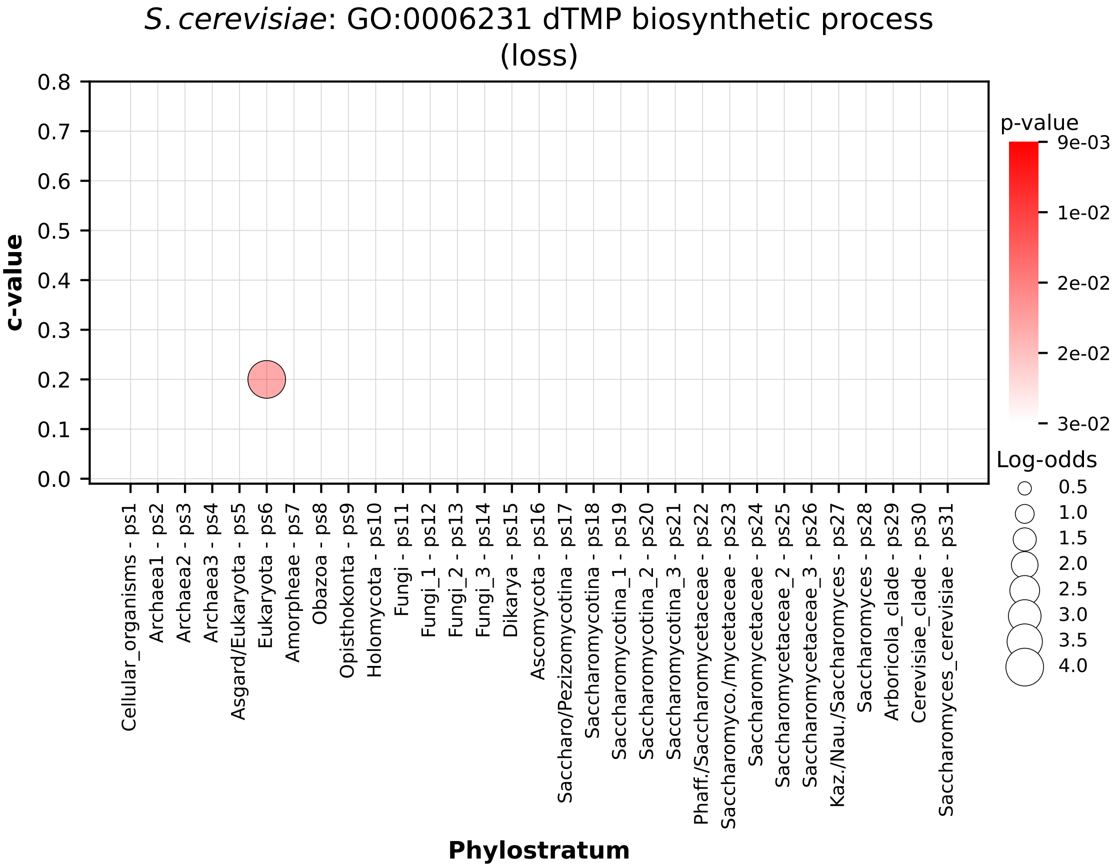

S. cerevisiae: GO:0006231 dTMP biosynthetic process
(loss)
c-value
0.8
0.7
0.6
0.5
0.4
0.3
0.2
0.1
0.0
p-value
9e-03
1e-02
2e-02
2e-02
3e-02
Log-odds
0.5
1.0
1.5
2.0
2.5
3.0
3.5
4.0
Cellular_organisms - ps1
Archaea1 - ps2
Archaea2 - ps3
Archaea3 - ps4
Asgard/Eukaryota - ps5
Eukaryota - ps6
Amorpheae - ps7
Obazoa - ps8
Opisthokonta - ps9
Holomycota - ps10
Fungi - ps11
Fungi_1 - ps12
Fungi_2 - ps13
Fungi_3 - ps14
Dikarya - ps15
Ascomycota - ps16
Saccharo/Pezizomycotina - ps17
Saccharomycotina - ps18
Saccharomycotina_1 - ps19
Saccharomycotina_2 - ps20
Saccharomycotina_3 - ps21
Phaff./Saccharomycetaceae - ps22
Saccharomyco./mycetaceae - ps23
Saccharomycetaceae - ps24
Saccharomycetaceae_2 - ps25
Saccharomycetaceae_3 - ps26
Kaz./Nau./Saccharomyces - ps27
Saccharomyces - ps28
Arboricola_clade - ps29
Cerevisiae_clade - ps30
Saccharomyces_cerevisiae - ps31
Phylostratum

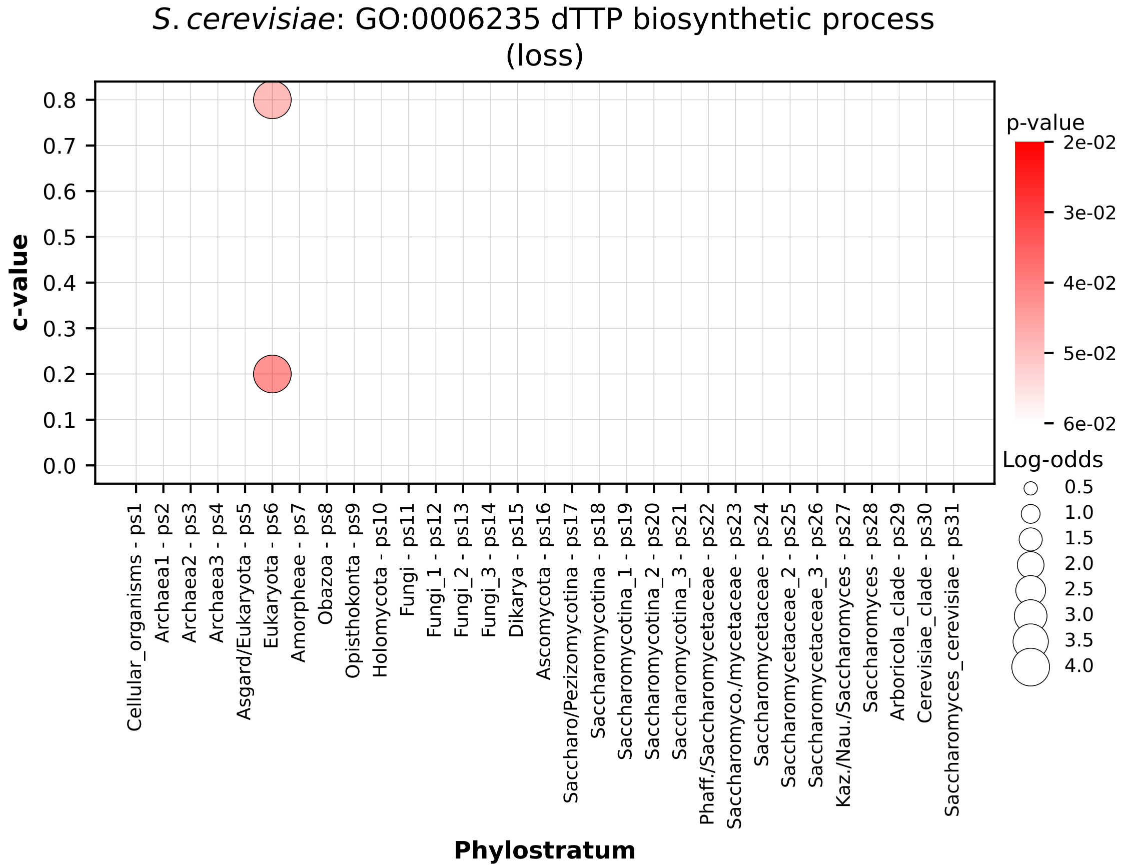

S. cerevisiae: GO:0006235 dTTP biosynthetic process (loss)
c-value
0.8
0.7
0.6
0.5
0.4
0.3
0.2
0.1
0.0
p-value
2e-02
3e-02
4e-02
5e-02
6e-02
Log-odds
0.5
1.0
1.5
2.0
2.5
3.0
3.5
4.0
Cellular_organisms - ps1
Archaea1 - ps2
Archaea2 - ps3
Archaea3 - ps4
Asgard/Eukaryota - ps5
Eukaryota - ps6
Amorpheae - ps7
Obazoa - ps8
Opisthokonta - ps9
Holomycota - ps10
Fungi - ps11
Fungi_1 - ps12
Fungi_2 - ps13
Fungi_3 - ps14
Dikarya - ps15
Ascomycota - ps16
Saccharo/Pezizomycotina - ps17
Saccharomycotina - ps18
Saccharomycotina_1 - ps19
Saccharomycotina_2 - ps20
Saccharomycotina_3 - ps21
Phaff./Saccharomycetaceae - ps22
Saccharomyco./mycetaceae - ps23
Saccharomycetaceae - ps24
Saccharomycetaceae_2 - ps25
Saccharomycetaceae_3 - ps26
Kaz./Nau./Saccharomyces - ps27
Saccharomyces - ps28
Arboricola_clade - ps29
Cerevisiae_clade - ps30
Saccharomyces_cerevisiae - ps31
Phylostratum

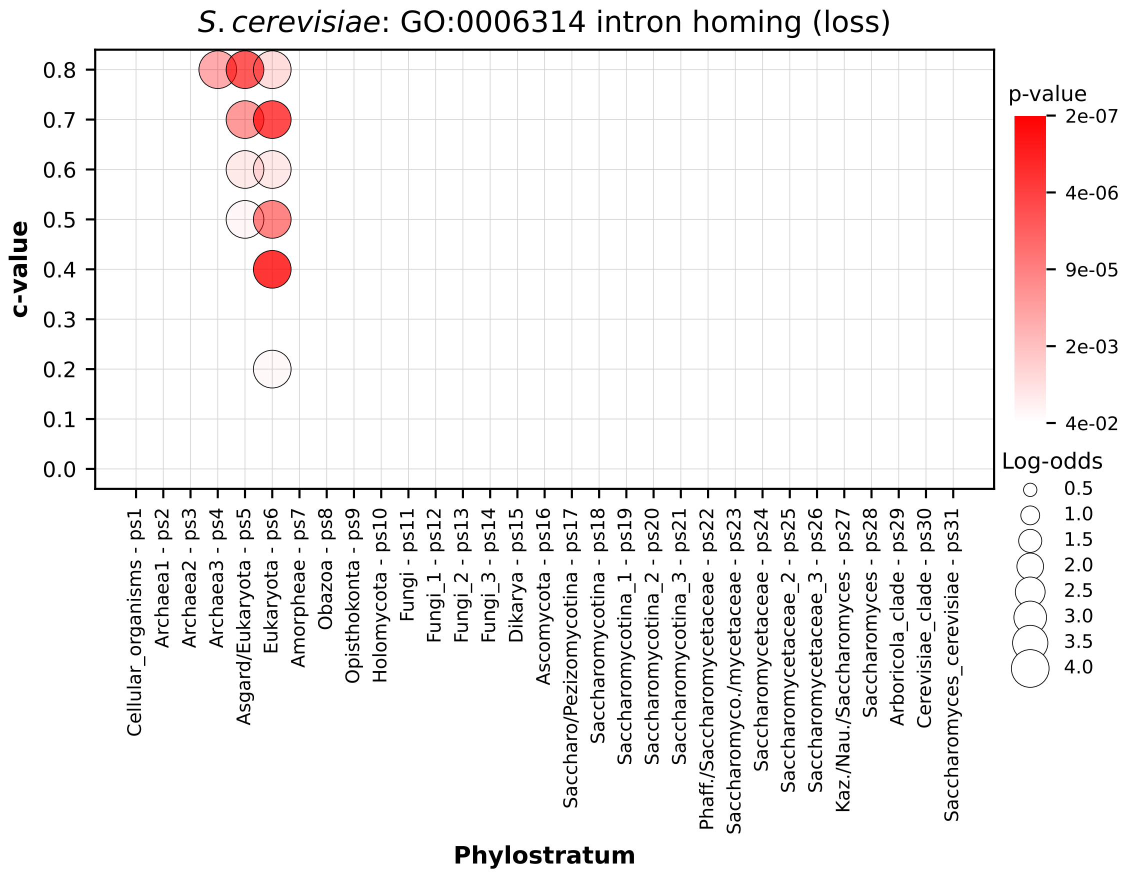
S. cerevisiae: GO:0006314 intron homing (loss)
c-value
0.0
0.1
0.2
0.3
0.4
0.5
0.6
0.7
0.8
Phylostratum
Cellular_organisms - ps1
Archaea1 - ps2
Archaea2 - ps3
Archaea3 - ps4
Asgard/Eukaryota - ps5
Eukaryota - ps6
Amorpheae - ps7
Obazoa - ps8
Opisthokonta - ps9
Holomycota - ps10
Fungi - ps11
Fungi_1 - ps12
Fungi_2 - ps13
Fungi_3 - ps14
Dikarya - ps15
Ascomycota - ps16
Saccharo/Pezizomycotina - ps17
Saccharomycotina - ps18
Saccharomycotina_1 - ps19
Saccharomycotina_2 - ps20
Saccharomycotina_3 - ps21
Phaff./Saccharomycetaceae - ps22
Saccharomyco./mycetaceae - ps23
Saccharomycetaceae - ps24
Saccharomycetaceae_2 - ps25
Saccharomycetaceae_3 - ps26
Kaz./Nau./Saccharomyces - ps27
Saccharomyces - ps28
Arboricola_clade - ps29
Cerevisiae_clade - ps30
Saccharomyces_cerevisiae - ps31
p-value
2e-07
4e-06
9e-05
2e-03
4e-02
Log-odds
0.5
1.0
1.5
2.0
2.5
3.0
3.5
4.0

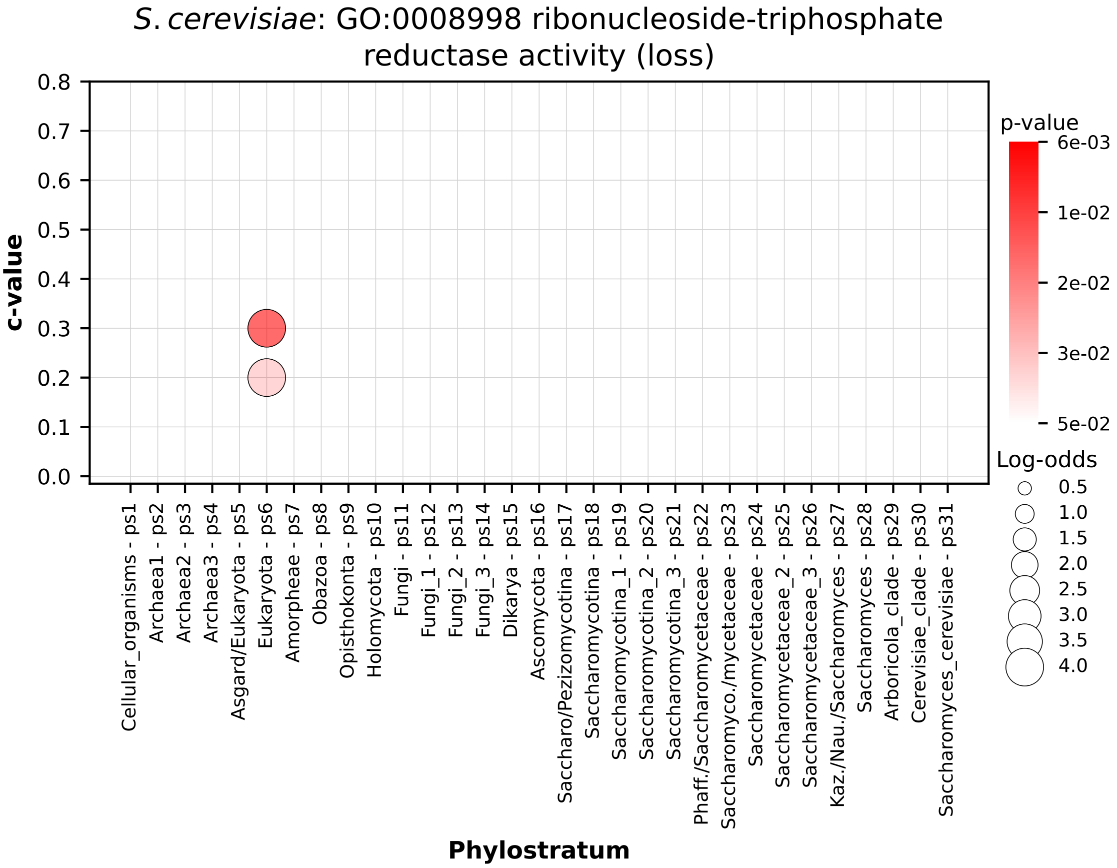

$S. cerevisiae$: GO:0008998 ribonucleoside-triphosphate reductase activity (loss)
c-value
0.8
0.7
0.6
0.5
0.4
0.3
0.2
0.1
0.0
p-value
6e-03
1e-02
2e-02
3e-02
5e-02
Log-odds
0.5
1.0
1.5
2.0
2.5
3.0
3.5
4.0
Cellular_organisms - ps1
Archaea1 - ps2
Archaea2 - ps3
Archaea3 - ps4
Asgard/Eukaryota - ps5
Eukaryota - ps6
Amorpheae - ps7
Obazoa - ps8
Opisthokonta - ps9
Holomycota - ps10
Fungi - ps11
Fungi_1 - ps12
Fungi_2 - ps13
Fungi_3 - ps14
Dikarya - ps15
Ascomycota - ps16
Saccharo/Pezizomycotina - ps17
Saccharomycotina - ps18
Saccharomycotina_1 - ps19
Saccharomycotina_2 - ps20
Saccharomycotina_3 - ps21
Phaff./Saccharomycetaceae - ps22
Saccharomyco./mycetaceae - ps23
Saccharomycetaceae - ps24
Saccharomycetaceae_2 - ps25
Saccharomycetaceae_3 - ps26
Kaz./Nau./Saccharomyces - ps27
Saccharomyces - ps28
Arboricola_clade - ps29
Cerevisiae_clade - ps30
Saccharomyces_cerevisiae - ps31
Phylostratum

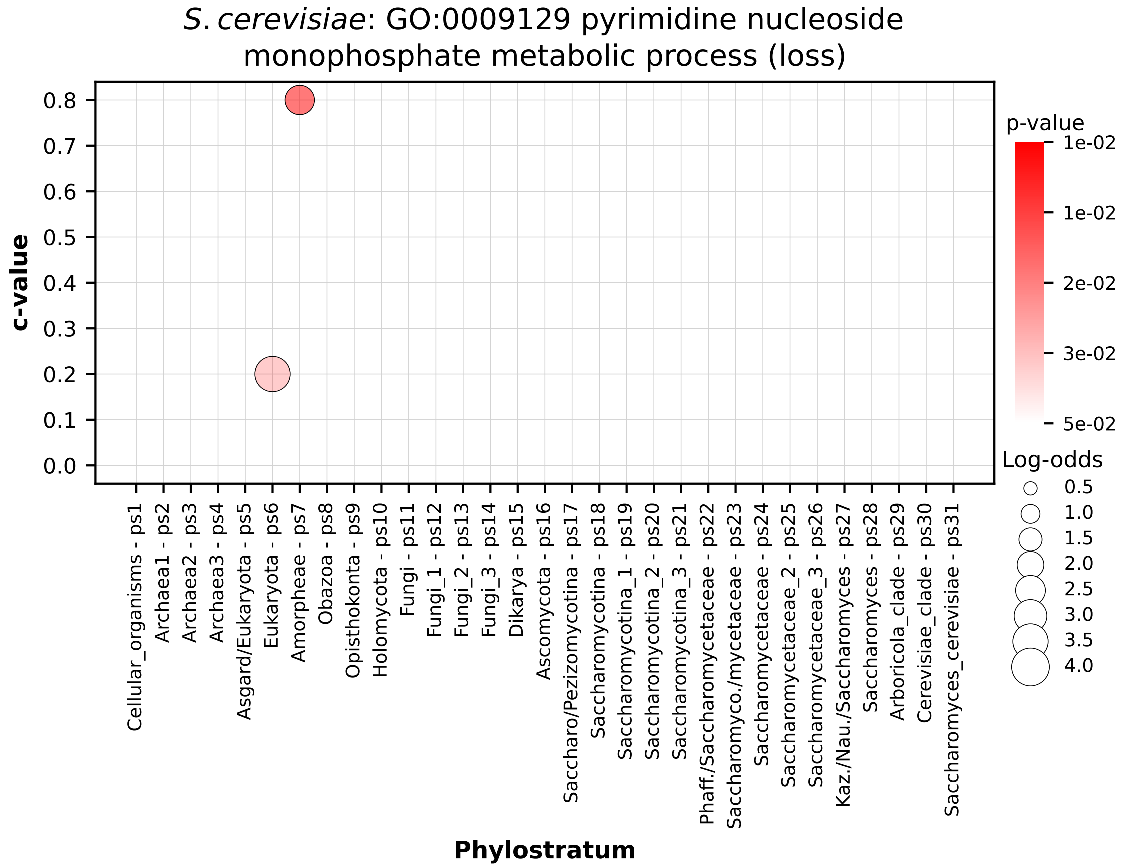
S. cerevisiae: GO:0009129 pyrimidine nucleoside monophosphate metabolic process (loss)
c-value
0.0
0.1
0.2
0.3
0.4
0.5
0.6
0.7
0.8
Phylostratum
Cellular_organisms - ps1
Archaea1 - ps2
Archaea2 - ps3
Archaea3 - ps4
Asgard/Eukaryota - ps5
Eukaryota - ps6
Amorpheae - ps7
Obazoa - ps8
Opisthokonta - ps9
Holomycota - ps10
Fungi - ps11
Fungi_1 - ps12
Fungi_2 - ps13
Fungi_3 - ps14
Dikarya - ps15
Ascomycota - ps16
Saccharo/Pezizomycotina - ps17
Saccharomycotina - ps18
Saccharomycotina_1 - ps19
Saccharomycotina_2 - ps20
Saccharomycotina_3 - ps21
Phaff./Saccharomycetaceae - ps22
Saccharomyco./mycetaceae - ps23
Saccharomycetaceae - ps24
Saccharomycetaceae_2 - ps25
Saccharomycetaceae_3 - ps26
Kaz./Nau./Saccharomyces - ps27
Saccharomyces - ps28
Arboricola_clade - ps29
Cerevisiae_clade - ps30
Saccharomyces_cerevisiae - ps31
p-value
1e-02
1e-02
2e-02
3e-02
5e-02
Log-odds
0.5
1.0
1.5
2.0
2.5
3.0
3.5
4.0

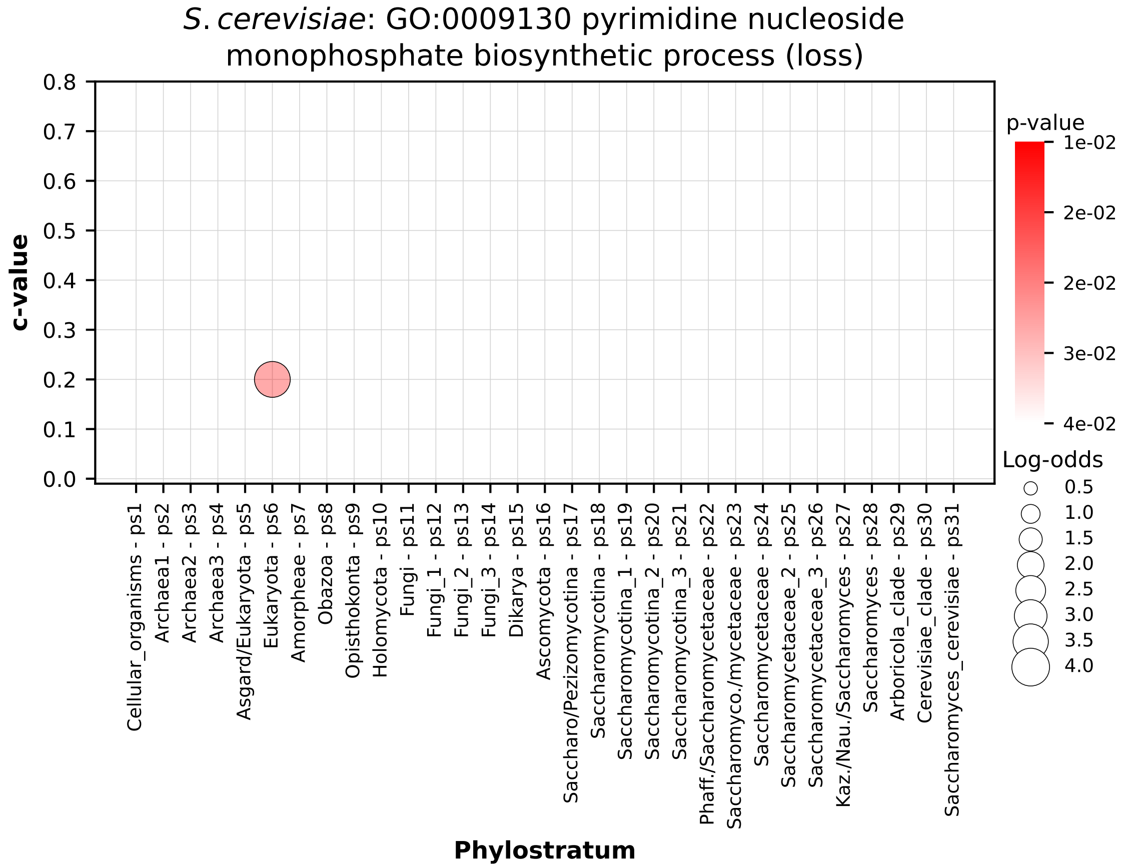

S. cerevisiae: GO:0009130 pyrimidine nucleoside monophosphate biosynthetic process (loss)
c-value
0.8
0.7
0.6
0.5
0.4
0.3
0.2
0.1
0.0
Phylostratum
Cellular_organisms - ps1
Archaea1 - ps2
Archaea2 - ps3
Archaea3 - ps4
Asgard/Eukaryota - ps5
Eukaryota - ps6
Amorpheae - ps7
Obazoa - ps8
Opisthokonta - ps9
Holomycota - ps10
Fungi - ps11
Fungi_1 - ps12
Fungi_2 - ps13
Fungi_3 - ps14
Dikarya - ps15
Ascomycota - ps16
Saccharo/Pezizomycotina - ps17
Saccharomycotina - ps18
Saccharomycotina_1 - ps19
Saccharomycotina_2 - ps20
Saccharomycotina_3 - ps21
Phaff./Saccharomycetaceae - ps22
Saccharomyco./mycetaceae - ps23
Saccharomycetaceae - ps24
Saccharomycetaceae_2 - ps25
Saccharomycetaceae_3 - ps26
Kaz./Nau./Saccharomyces - ps27
Saccharomyces - ps28
Arboricola_clade - ps29
Cerevisiae_clade - ps30
Saccharomyces_cerevisiae - ps31
p-value
1e-02
2e-02
2e-02
3e-02
4e-02
Log-odds
0.5
1.0
1.5
2.0
2.5
3.0
3.5
4.0

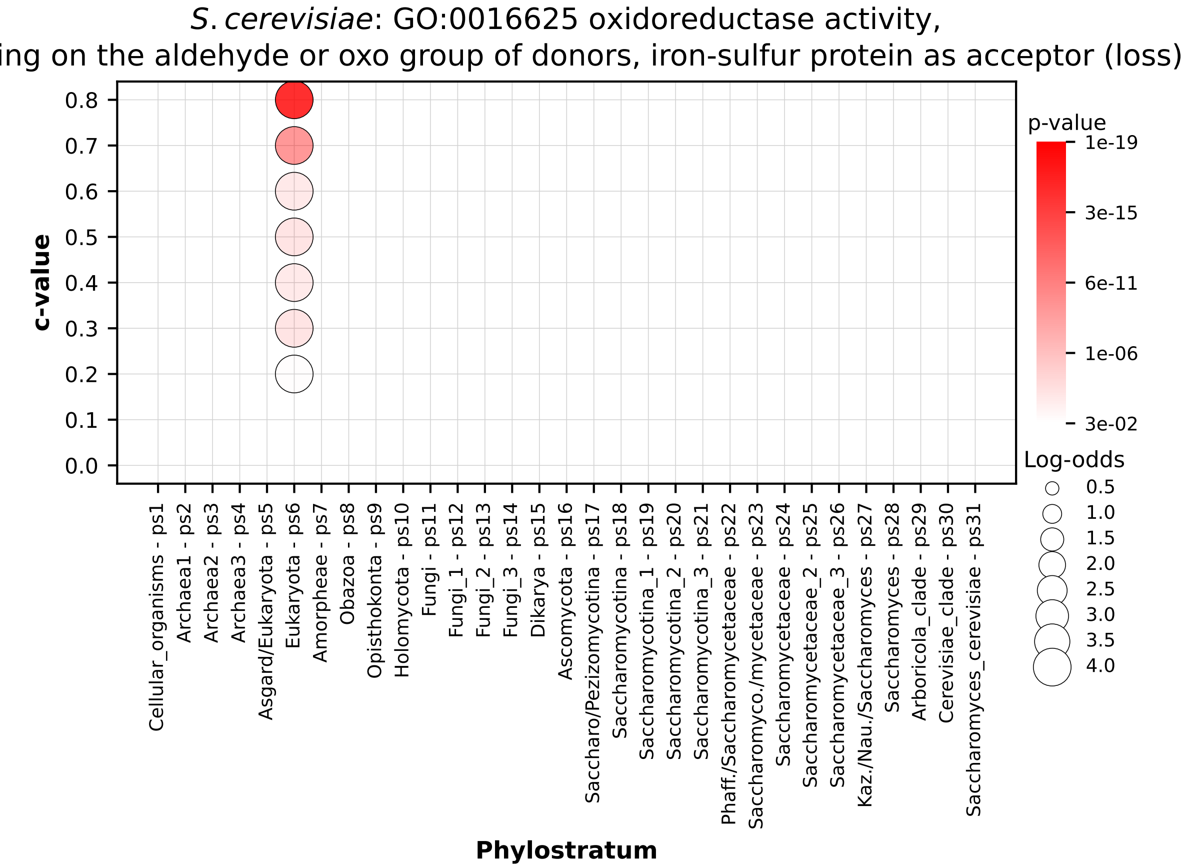

S. cerevisiae: GO:0016625 oxidoreductase activity,
ing on the aldehyde or oxo group of donors, iron-sulfur protein as acceptor (loss)
c-value
0.8
0.7
0.6
0.5
0.4
0.3
0.2
0.1
0.0
p-value
1e-19
3e-15
6e-11
1e-06
3e-02
Log-odds
0.5
1.0
1.5
2.0
2.5
3.0
3.5
4.0
Cellular_organisms - ps1
Archaea1 - ps2
Archaea2 - ps3
Archaea3 - ps4
Asgard/Eukaryota - ps5
Eukaryota - ps6
Amorpheae - ps7
Obazoa - ps8
Opisthokonta - ps9
Holomycota - ps10
Fungi - ps11
Fungi_1 - ps12
Fungi_2 - ps13
Fungi_3 - ps14
Dikarya - ps15
Ascomycota - ps16
Saccharo/Pezizomycotina - ps17
Saccharomycotina - ps18
Saccharomycotina_1 - ps19
Saccharomycotina_2 - ps20
Saccharomycotina_3 - ps21
Phaff./Saccharomycetaceae - ps22
Saccharomyco./mycetaceae - ps23
Saccharomycetaceae - ps24
Saccharomycetaceae_2 - ps25
Saccharomycetaceae_3 - ps26
Kaz./Nau./Saccharomyces - ps27
Saccharomyces - ps28
Arboricola_clade - ps29
Cerevisiae_clade - ps30
Saccharomyces_cerevisiae - ps31
Phylostratum

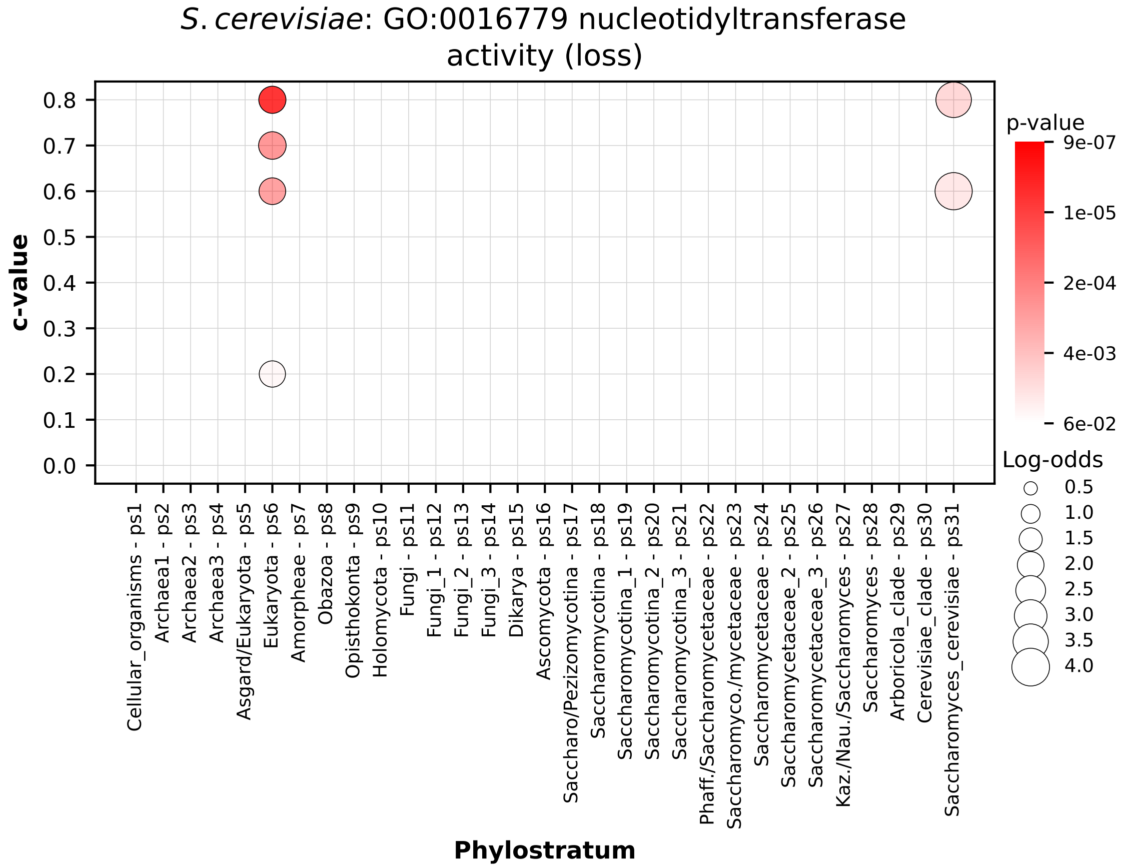

S. cerevisiae: GO:0016779 nucleotidyltransferase activity (loss)
c-value
0.8
0.7
0.6
0.5
0.4
0.3
0.2
0.1
0.0
p-value
9e-07
1e-05
2e-04
4e-03
6e-02
Log-odds
0.5
1.0
1.5
2.0
2.5
3.0
3.5
4.0
Cellular_organisms - ps1
Archaea1 - ps2
Archaea2 - ps3
Archaea3 - ps4
Asgard/Eukaryota - ps5
Eukaryota - ps6
Amorpheae - ps7
Obazoa - ps8
Opisthokonta - ps9
Holomycota - ps10
Fungi - ps11
Fungi_1 - ps12
Fungi_2 - ps13
Fungi_3 - ps14
Dikarya - ps15
Ascomycota - ps16
Saccharo/Pezizomycotina - ps17
Saccharomycotina - ps18
Saccharomycotina_1 - ps19
Saccharomycotina_2 - ps20
Saccharomycotina_3 - ps21
Phaff./Saccharomycetaceae - ps22
Saccharomyco./mycetaceae - ps23
Saccharomycetaceae - ps24
Saccharomycetaceae_2 - ps25
Saccharomycetaceae_3 - ps26
Kaz./Nau./Saccharomyces - ps27
Saccharomyces - ps28
Arboricola_clade - ps29
Cerevisiae_clade - ps30
Saccharomyces_cerevisiae - ps31
Phylostratum

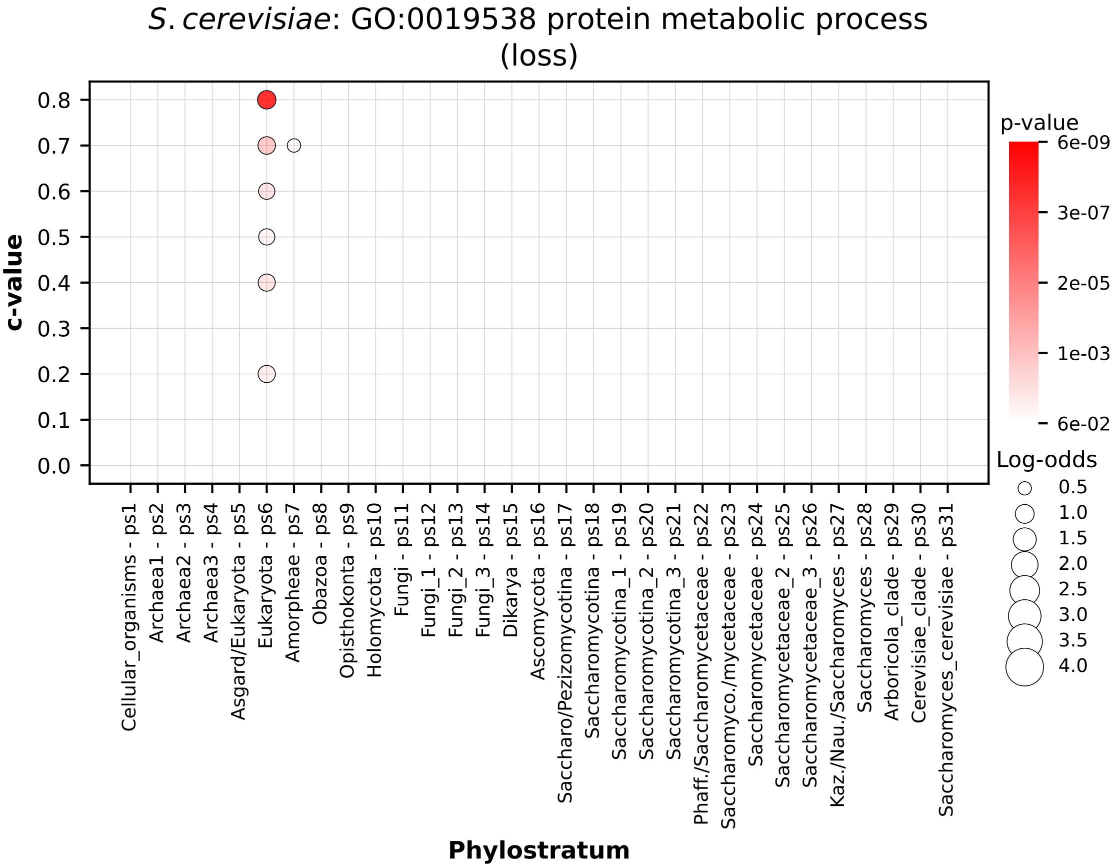
S. cerevisiae: GO:0019538 protein metabolic process
(loss)
c-value
0.0
0.1
0.2
0.3
0.4
0.5
0.6
0.7
0.8
p-value
6e-09
3e-07
2e-05
1e-03
6e-02
Log-odds
0.5
1.0
1.5
2.0
2.5
3.0
3.5
4.0
Cellular_organisms - ps1
Archaea1 - ps2
Archaea2 - ps3
Archaea3 - ps4
Asgard/Eukaryota - ps5
Eukaryota - ps6
Amorpheae - ps7
Obazoa - ps8
Opisthokonta - ps9
Holomycota - ps10
Fungi - ps11
Fungi_1 - ps12
Fungi_2 - ps13
Fungi_3 - ps14
Dikarya - ps15
Ascomycota - ps16
Saccharo/Pezizomycotina - ps17
Saccharomycotina - ps18
Saccharomycotina_1 - ps19
Saccharomycotina_2 - ps20
Saccharomycotina_3 - ps21
Phaff./Saccharomycetaceae - ps22
Saccharomyco./mycetaceae - ps23
Saccharomycetaceae - ps24
Saccharomycetaceae_2 - ps25
Saccharomycetaceae_3 - ps26
Kaz./Nau./Saccharomyces - ps27
Saccharomyces - ps28
Arboricola_clade - ps29
Cerevisiae_clade - ps30
Saccharomyces_cerevisiae - ps31
Phylostratum

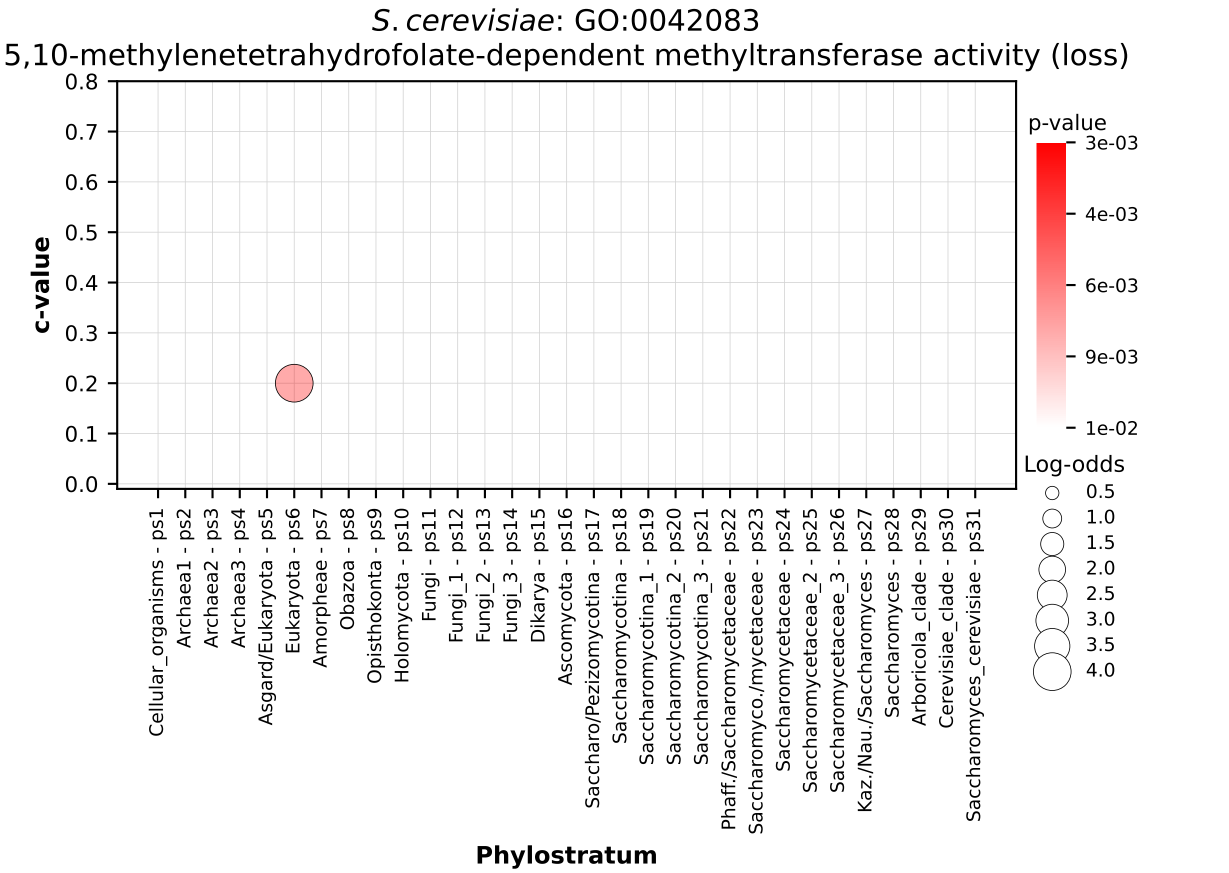

S. cerevisiae: GO:0042083
5,10-methylenetetrahydrofolate-dependent methyltransferase activity (loss)
c-value
0.8
0.7
0.6
0.5
0.4
0.3
0.2
0.1
0.0
Phylostratum
Cellular_organisms - ps1
Archaea1 - ps2
Archaea2 - ps3
Archaea3 - ps4
Asgard/Eukaryota - ps5
Eukaryota - ps6
Amorpheae - ps7
Obazoa - ps8
Opisthokonta - ps9
Holomycota - ps10
Fungi - ps11
Fungi_1 - ps12
Fungi_2 - ps13
Fungi_3 - ps14
Dikarya - ps15
Ascomycota - ps16
Saccharo/Pezizomycotina - ps17
Saccharomycotina - ps18
Saccharomycotina_1 - ps19
Saccharomycotina_2 - ps20
Saccharomycotina_3 - ps21
Phaff./Saccharomycetaceae - ps22
Saccharomyco./mycetaceae - ps23
Saccharomycetaceae - ps24
Saccharomycetaceae_2 - ps25
Saccharomycetaceae_3 - ps26
Kaz./Nau./Saccharomyces - ps27
Saccharomyces - ps28
Arboricola_clade - ps29
Cerevisiae_clade - ps30
Saccharomyces_cerevisiae - ps31
p-value
3e-03
4e-03
6e-03
9e-03
1e-02
Log-odds
0.5
1.0
1.5
2.0
2.5
3.0
3.5
4.0

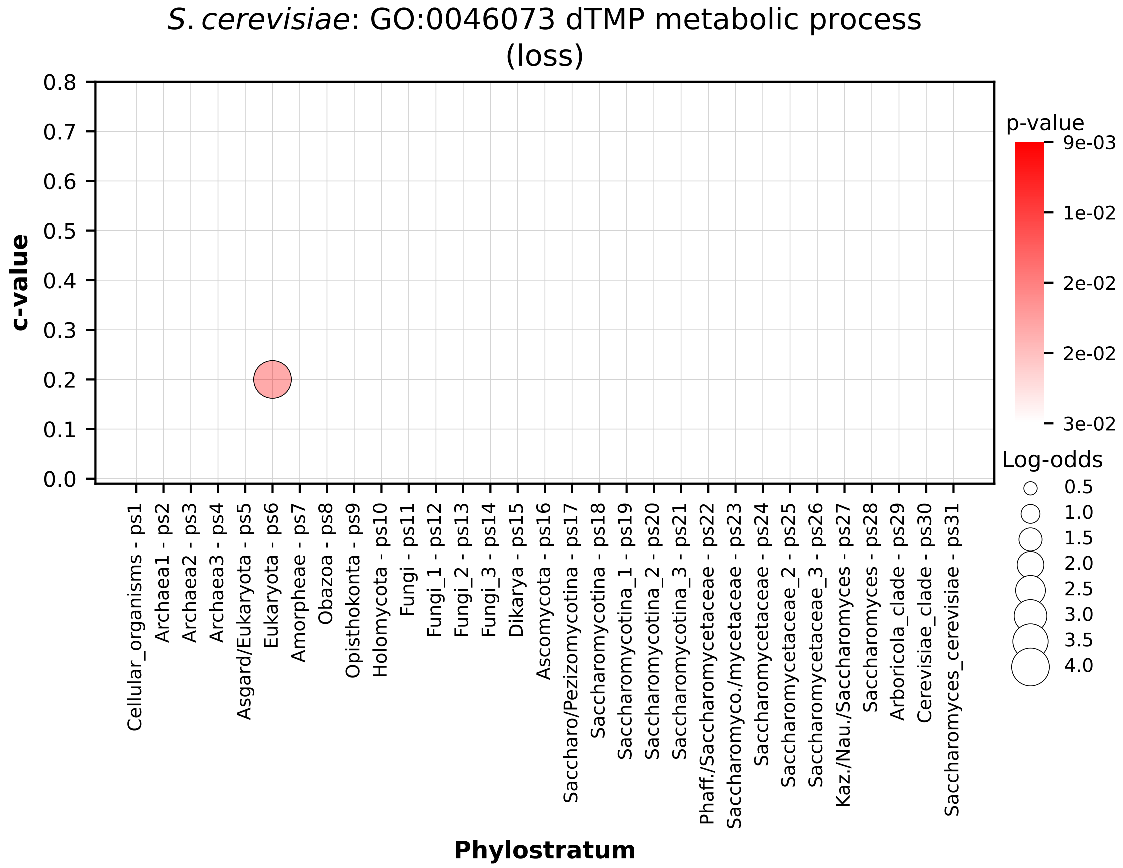

S. cerevisiae: GO:0046073 dTMP metabolic process
(loss)
c-value
0.8
0.7
0.6
0.5
0.4
0.3
0.2
0.1
0.0
p-value
9e-03
1e-02
2e-02
2e-02
3e-02
Log-odds
0.5
1.0
1.5
2.0
2.5
3.0
3.5
4.0
Cellular_organisms - ps1
Archaea1 - ps2
Archaea2 - ps3
Archaea3 - ps4
Asgard/Eukaryota - ps5
Eukaryota - ps6
Amorpheae - ps7
Obazoa - ps8
Opisthokonta - ps9
Holomycota - ps10
Fungi - ps11
Fungi_1 - ps12
Fungi_2 - ps13
Fungi_3 - ps14
Dikarya - ps15
Ascomycota - ps16
Saccharo/Pezizomycotina - ps17
Saccharomycotina - ps18
Saccharomycotina_1 - ps19
Saccharomycotina_2 - ps20
Saccharomycotina_3 - ps21
Phaff./Saccharomycetaceae - ps22
Saccharomyco./mycetaceae - ps23
Saccharomycetaceae - ps24
Saccharomycetaceae_2 - ps25
Saccharomycetaceae_3 - ps26
Kaz./Nau./Saccharomyces - ps27
Saccharomyces - ps28
Arboricola_clade - ps29
Cerevisiae_clade - ps30
Saccharomyces_cerevisiae - ps31
Phylostratum

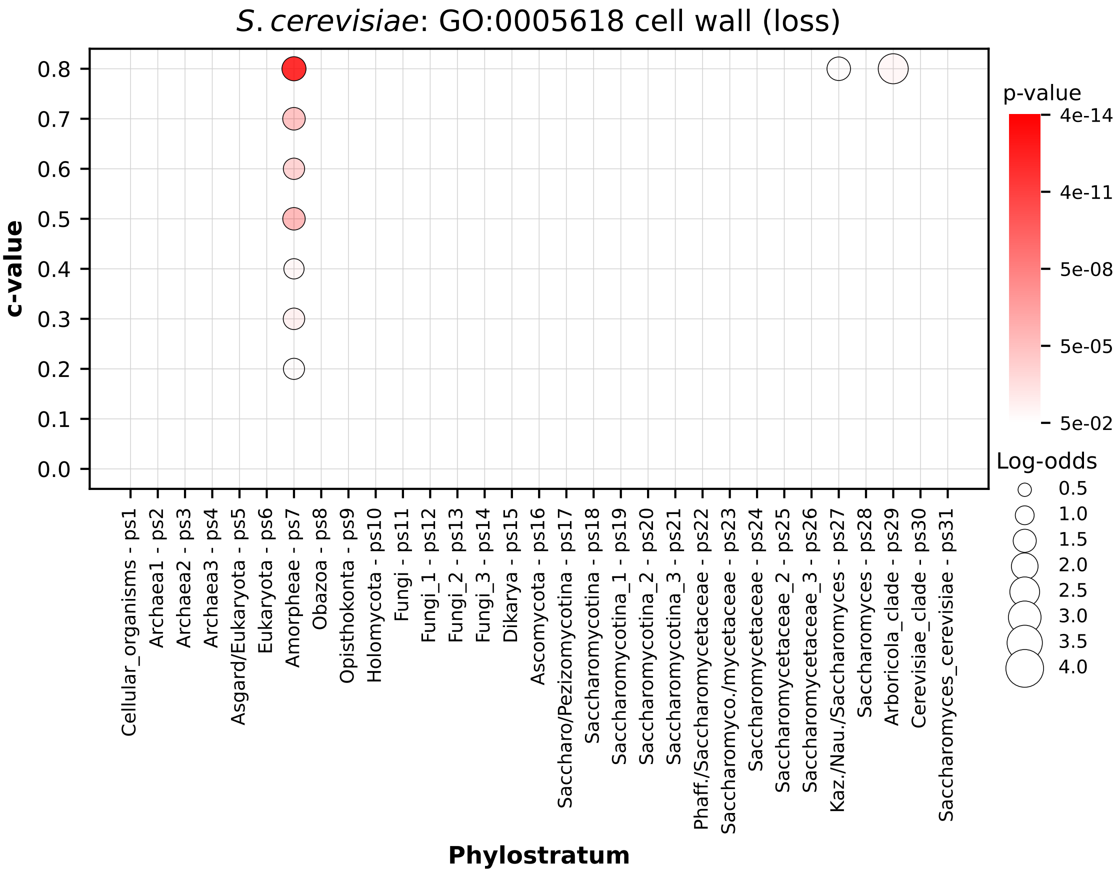

S. cerevisiae: GO:0005618 cell wall (loss)
c-value
0.0
0.1
0.2
0.3
0.4
0.5
0.6
0.7
0.8
p-value
4e-14
4e-11
5e-08
5e-05
5e-02
Log-odds
0.5
1.0
1.5
2.0
2.5
3.0
3.5
4.0
Cellular_organisms - ps1
Archaea1 - ps2
Archaea2 - ps3
Archaea3 - ps4
Asgard/Eukaryota - ps5
Eukaryota - ps6
Amorpheae - ps7
Obazoa - ps8
Opisthokonta - ps9
Holomycota - ps10
Fungi - ps11
Fungi_1 - ps12
Fungi_2 - ps13
Fungi_3 - ps14
Dikarya - ps15
Ascomycota - ps16
Saccharo/Pezizomycotina - ps17
Saccharomycotina - ps18
Saccharomycotina_1 - ps19
Saccharomycotina_2 - ps20
Saccharomycotina_3 - ps21
Phaff./Saccharomycetaceae - ps22
Saccharomyco./mycetaceae - ps23
Saccharomycetaceae - ps24
Saccharomycetaceae_2 - ps25
Saccharomycetaceae_3 - ps26
Kaz./Nau./Saccharomyces - ps27
Saccharomyces - ps28
Arboricola_clade - ps29
Cerevisiae_clade - ps30
Saccharomyces_cerevisiae - ps31
Phylostratum

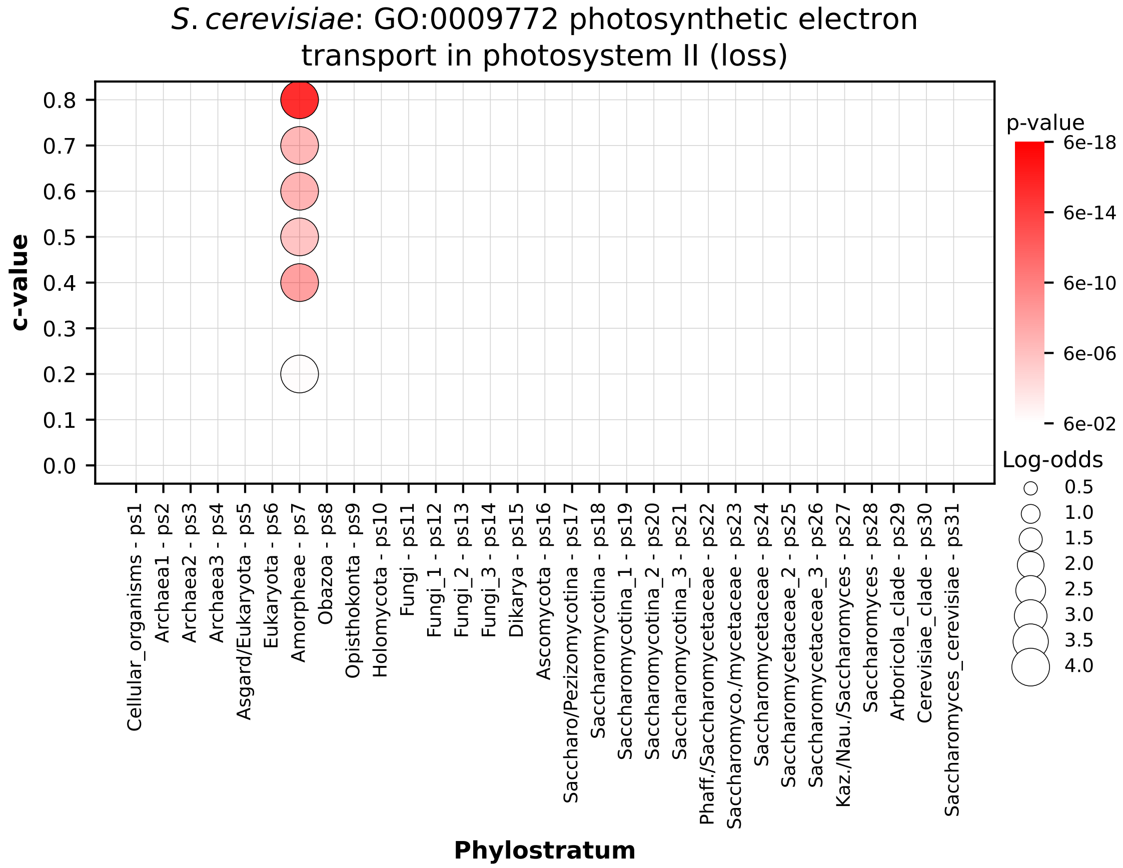

S. cerevisiae: GO:0009772 photosynthetic electron transport in photosystem II (loss)
c-value
0.0
0.1
0.2
0.3
0.4
0.5
0.6
0.7
0.8
p-value
6e-18
6e-14
6e-10
6e-06
6e-02
Log-odds
0.5
1.0
1.5
2.0
2.5
3.0
3.5
4.0
Cellular_organisms - ps1
Archaea1 - ps2
Archaea2 - ps3
Archaea3 - ps4
Asgard/Eukaryota - ps5
Eukaryota - ps6
Amorpheae - ps7
Obazoa - ps8
Opisthokonta - ps9
Holomycota - ps10
Fungi - ps11
Fungi_1 - ps12
Fungi_2 - ps13
Fungi_3 - ps14
Dikarya - ps15
Ascomycota - ps16
Saccharo/Pezizomycotina - ps17
Saccharomycotina - ps18
Saccharomycotina_1 - ps19
Saccharomycotina_2 - ps20
Saccharomycotina_3 - ps21
Phaff./Saccharomycetaceae - ps22
Saccharomyco./mycetaceae - ps23
Saccharomycetaceae - ps24
Saccharomycetaceae_2 - ps25
Saccharomycetaceae_3 - ps26
Kaz./Nau./Saccharomyces - ps27
Saccharomyces - ps28
Arboricola_clade - ps29
Cerevisiae_clade - ps30
Saccharomyces_cerevisiae - ps31
Phylostratum

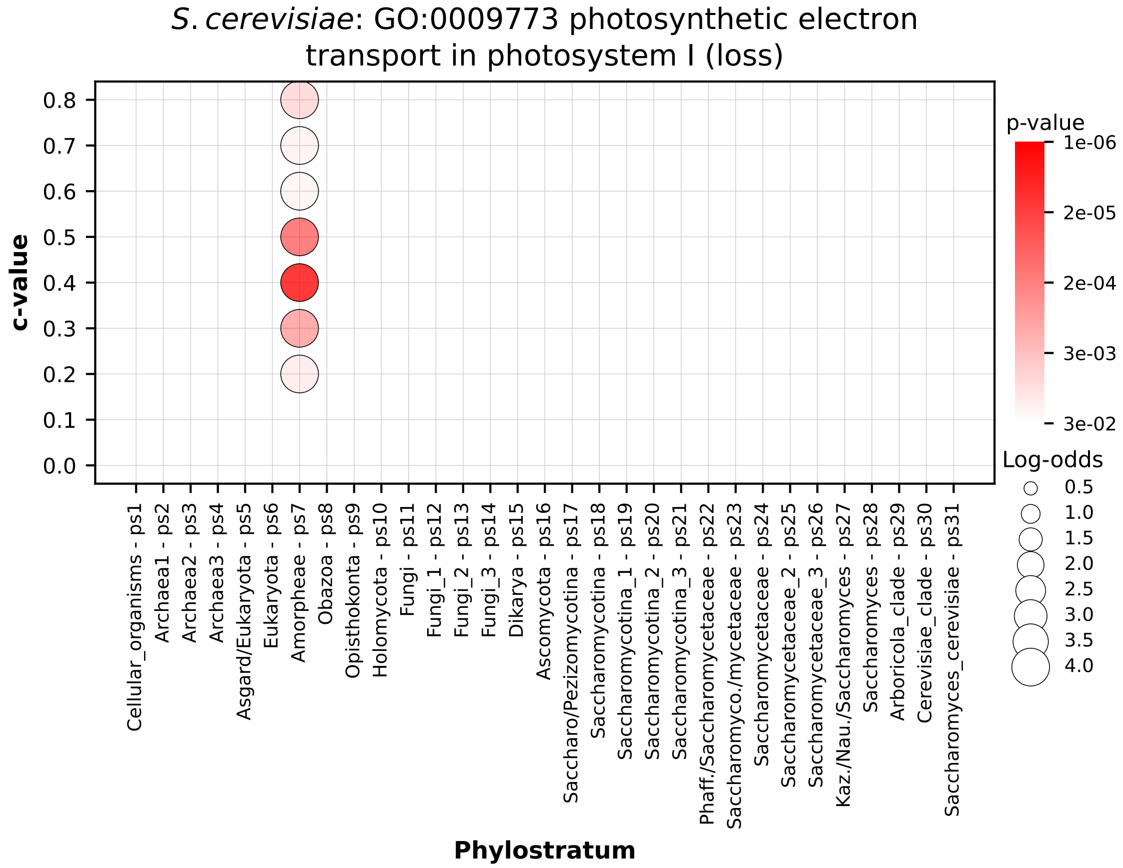

S. cerevisiae: GO:0009773 photosynthetic electron transport in photosystem I (loss)
c-value
0.8
0.7
0.6
0.5
0.4
0.3
0.2
0.1
0.0
p-value
1e-06
2e-05
2e-04
3e-03
3e-02
Log-odds
0.5
1.0
1.5
2.0
2.5
3.0
3.5
4.0
Cellular_organisms - ps1
Archaea1 - ps2
Archaea2 - ps3
Archaea3 - ps4
Asgard/Eukaryota - ps5
Eukaryota - ps6
Amorpheae - ps7
Obazoa - ps8
Opisthokonta - ps9
Holomycota - ps10
Fungi - ps11
Fungi_1 - ps12
Fungi_2 - ps13
Fungi_3 - ps14
Dikarya - ps15
Ascomycota - ps16
Saccharo/Pezizomycotina - ps17
Saccharomycotina - ps18
Saccharomycotina_1 - ps19
Saccharomycotina_2 - ps20
Saccharomycotina_3 - ps21
Phaff./Saccharomycetaceae - ps22
Saccharomyco./mycetaceae - ps23
Saccharomycetaceae - ps24
Saccharomycetaceae_2 - ps25
Saccharomycetaceae_3 - ps26
Kaz./Nau./Saccharomyces - ps27
Saccharomyces - ps28
Arboricola_clade - ps29
Cerevisiae_clade - ps30
Saccharomyces_cerevisiae - ps31
Phylostratum

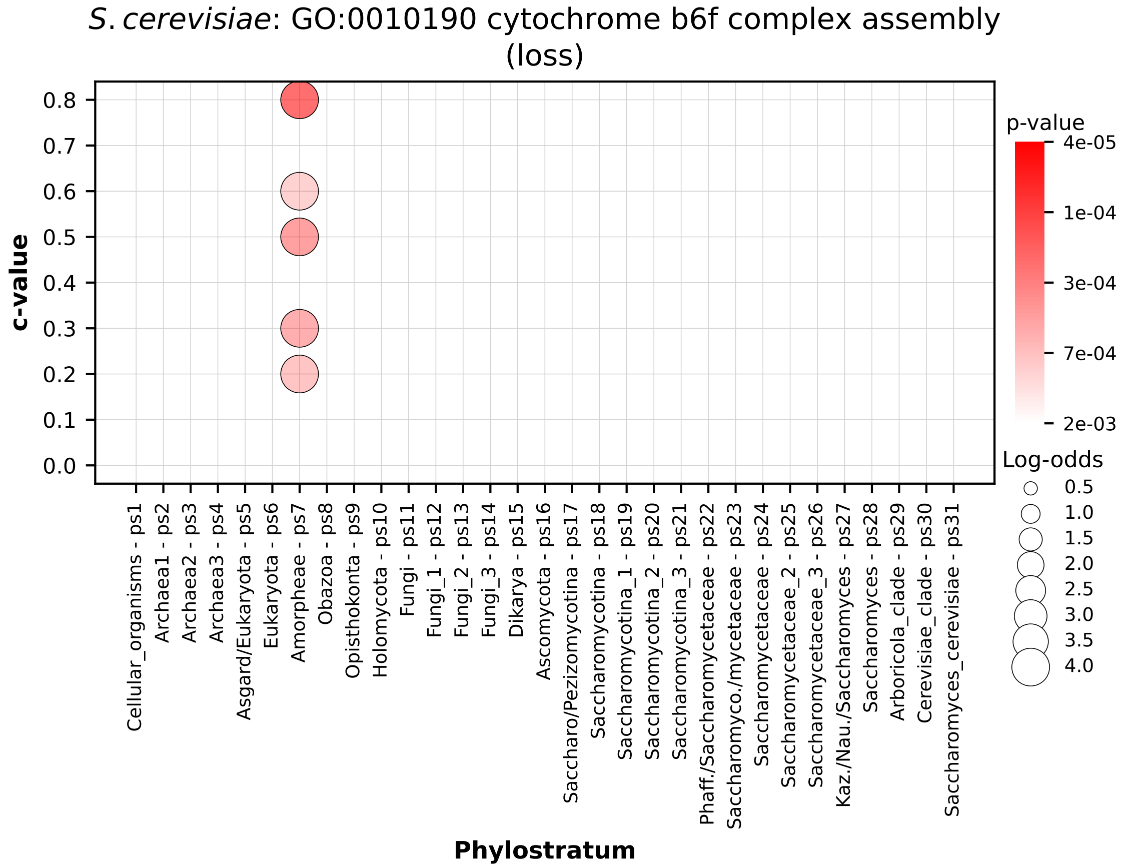

S. cerevisiae: GO:0010190 cytochrome b6f complex assembly
(loss)
c-value
0.0
0.1
0.2
0.3
0.4
0.5
0.6
0.7
0.8
p-value
4e-05
1e-04
3e-04
7e-04
2e-03
Log-odds
0.5
1.0
1.5
2.0
2.5
3.0
3.5
4.0
Cellular_organisms - ps1
Archaea1 - ps2
Archaea2 - ps3
Archaea3 - ps4
Asgard/Eukaryota - ps5
Eukaryota - ps6
Amorpheae - ps7
Obazoa - ps8
Opisthokonta - ps9
Holomycota - ps10
Fungi - ps11
Fungi_1 - ps12
Fungi_2 - ps13
Fungi_3 - ps14
Dikarya - ps15
Ascomycota - ps16
Saccharo/Pezizomycotina - ps17
Saccharomycotina - ps18
Saccharomycotina_1 - ps19
Saccharomycotina_2 - ps20
Saccharomycotina_3 - ps21
Phaff./Saccharomycetaceae - ps22
Saccharomyco./mycetaceae - ps23
Saccharomycetaceae - ps24
Saccharomycetaceae_2 - ps25
Saccharomycetaceae_3 - ps26
Kaz./Nau./Saccharomyces - ps27
Saccharomyces - ps28
Arboricola_clade - ps29
Cerevisiae_clade - ps30
Saccharomyces_cerevisiae - ps31
Phylostratum

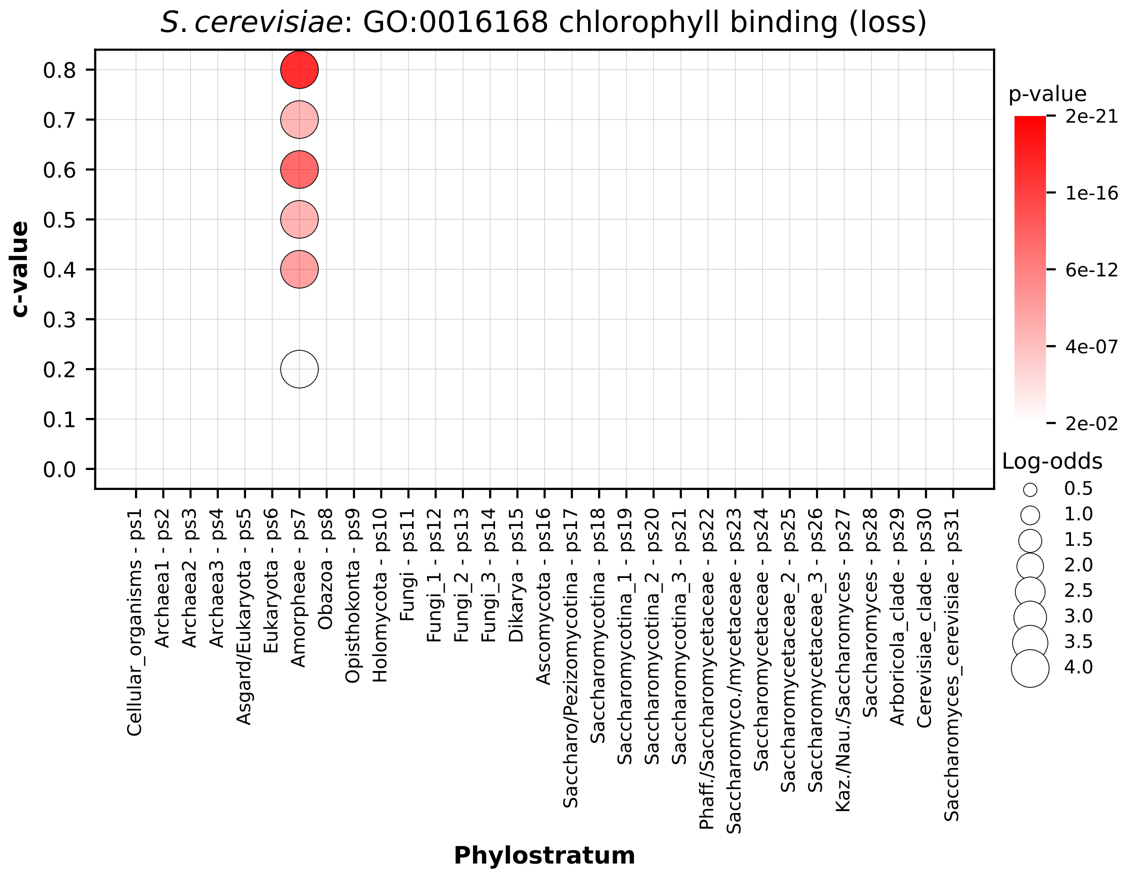

S. cerevisiae: GO:0016168 chlorophyll binding (loss)
c-value
0.8
0.7
0.6
0.5
0.4
0.3
0.2
0.1
0.0
Phylostratum
Cellular_organisms - ps1
Archaea1 - ps2
Archaea2 - ps3
Archaea3 - ps4
Asgard/Eukaryota - ps5
Eukaryota - ps6
Amorpheae - ps7
Obazoa - ps8
Opisthokonta - ps9
Holomycota - ps10
Fungi - ps11
Fungi_1 - ps12
Fungi_2 - ps13
Fungi_3 - ps14
Dikarya - ps15
Ascomycota - ps16
Saccharo/Pezizomycotina - ps17
Saccharomycotina - ps18
Saccharomycotina_1 - ps19
Saccharomycotina_2 - ps20
Saccharomycotina_3 - ps21
Phaff./Saccharomycetaceae - ps22
Saccharomyco./mycetaceae - ps23
Saccharomycetaceae - ps24
Saccharomycetaceae_2 - ps25
Saccharomycetaceae_3 - ps26
Kaz./Nau./Saccharomyces - ps27
Saccharomyces - ps28
Arboricola_clade - ps29
Cerevisiae_clade - ps30
Saccharomyces_cerevisiae - ps31
p-value
2e-21
1e-16
6e-12
4e-07
2e-02
Log-odds
0.5
1.0
1.5
2.0
2.5
3.0
3.5
4.0

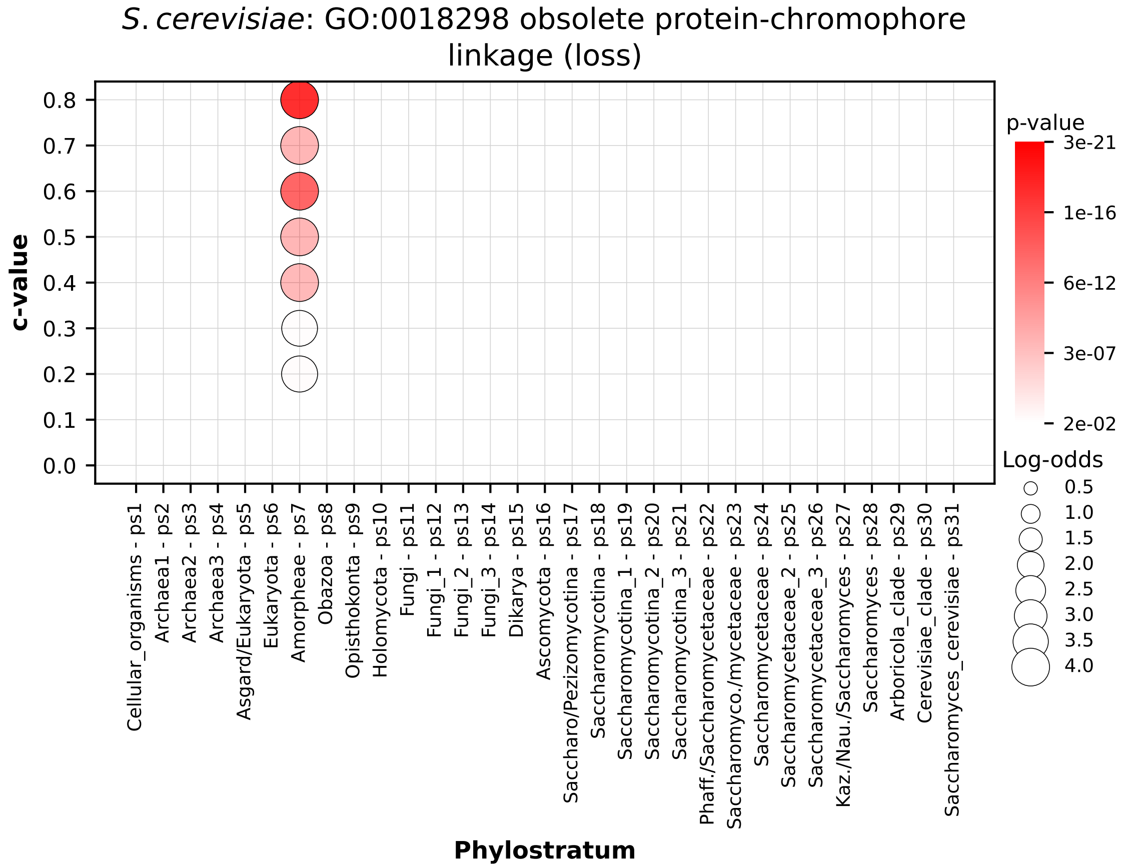
S. cerevisiae: GO:0018298 obsolete protein-chromophore linkage (loss)
c-value
0.8
0.7
0.6
0.5
0.4
0.3
0.2
0.1
0.0
p-value
3e-21
1e-16
6e-12
3e-07
2e-02
Log-odds
0.5
1.0
1.5
2.0
2.5
3.0
3.5
4.0
Cellular_organisms - ps1
Archaea1 - ps2
Archaea2 - ps3
Archaea3 - ps4
Asgard/Eukaryota - ps5
Eukaryota - ps6
Amorpheae - ps7
Obazoa - ps8
Opisthokonta - ps9
Holomycota - ps10
Fungi - ps11
Fungi_1 - ps12
Fungi_2 - ps13
Fungi_3 - ps14
Dikarya - ps15
Ascomycota - ps16
Saccharo/Pezizomycotina - ps17
Saccharomycotina - ps18
Saccharomycotina_1 - ps19
Saccharomycotina_2 - ps20
Saccharomycotina_3 - ps21
Phaff./Saccharomycetaceae - ps22
Saccharomyco./mycetaceae - ps23
Saccharomycetaceae - ps24
Saccharomycetaceae_2 - ps25
Saccharomycetaceae_3 - ps26
Kaz./Nau./Saccharomyces - ps27
Saccharomyces - ps28
Arboricola_clade - ps29
Cerevisiae_clade - ps30
Saccharomyces_cerevisiae - ps31
Phylostratum

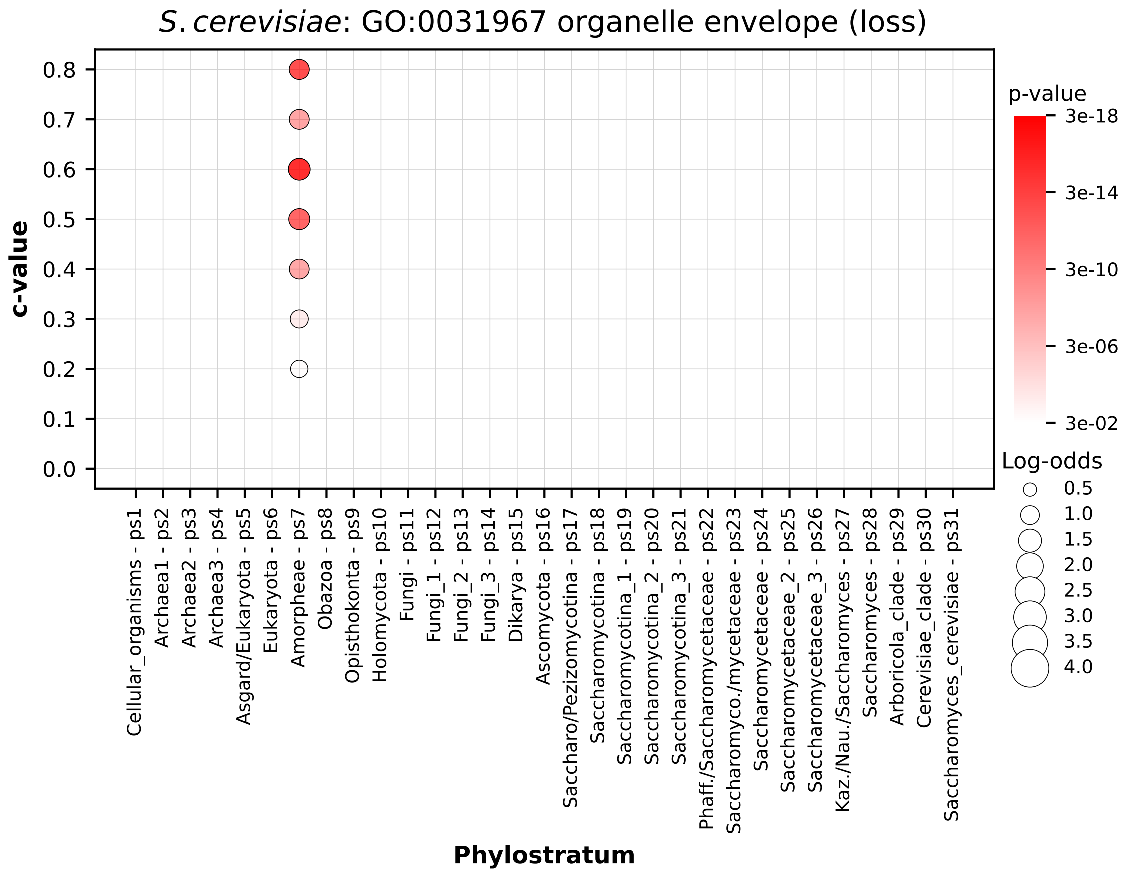

S. cerevisiae: GO:0031967 organelle envelope (loss)
c-value
0.0
0.1
0.2
0.3
0.4
0.5
0.6
0.7
0.8
Phylostratum
Cellular_organisms - ps1
Archaea1 - ps2
Archaea2 - ps3
Archaea3 - ps4
Asgard/Eukaryota - ps5
Eukaryota - ps6
Amorpheae - ps7
Obazoa - ps8
Opisthokonta - ps9
Holomycota - ps10
Fungi - ps11
Fungi_1 - ps12
Fungi_2 - ps13
Fungi_3 - ps14
Dikarya - ps15
Ascomycota - ps16
Saccharo/Pezizomycotina - ps17
Saccharomycotina - ps18
Saccharomycotina_1 - ps19
Saccharomycotina_2 - ps20
Saccharomycotina_3 - ps21
Phaff./Saccharomycetaceae - ps22
Saccharomyco./mycetaceae - ps23
Saccharomycetaceae - ps24
Saccharomycetaceae_2 - ps25
Saccharomycetaceae_3 - ps26
Kaz./Nau./Saccharomyces - ps27
Saccharomyces - ps28
Arboricola_clade - ps29
Cerevisiae_clade - ps30
Saccharomyces_cerevisiae - ps31
p-value
3e-18
3e-14
3e-10
3e-06
3e-02
Log-odds
0.5
1.0
1.5
2.0
2.5
3.0
3.5
4.0

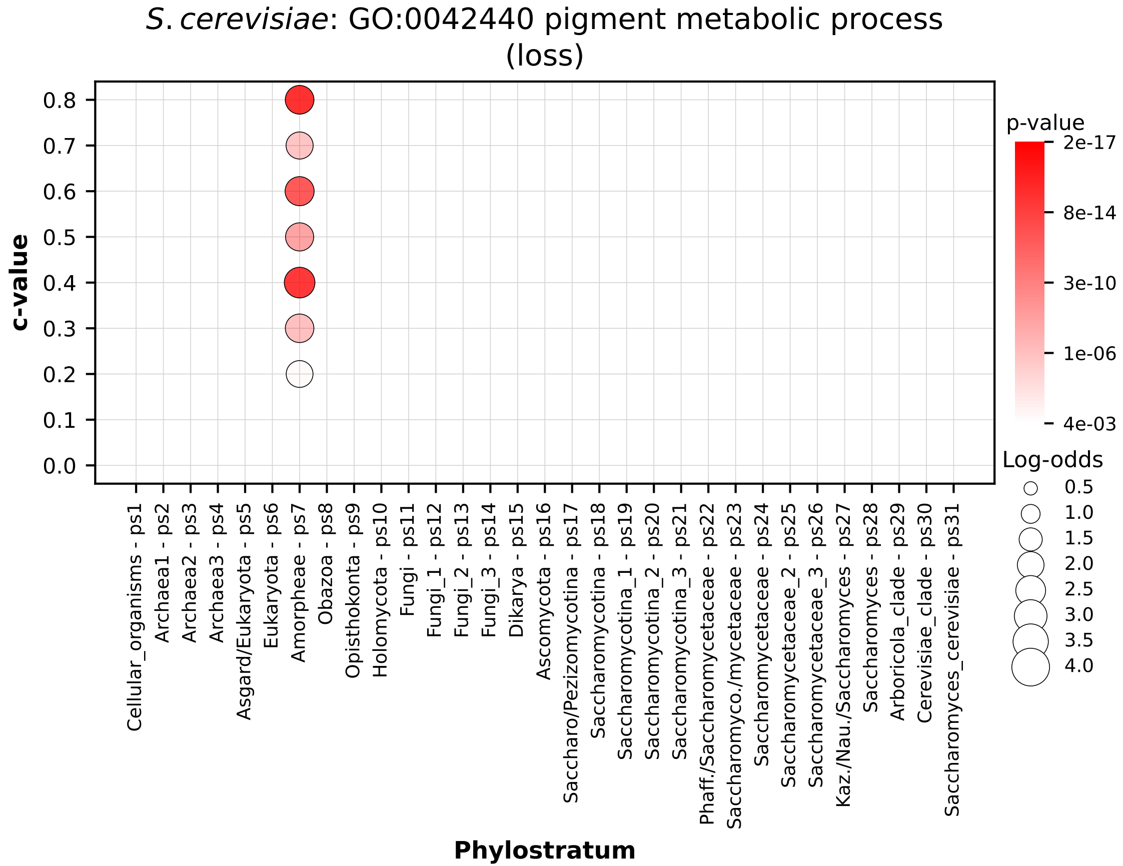

S. cerevisiae: GO:0042440 pigment metabolic process (loss)
c-value
0.8
0.7
0.6
0.5
0.4
0.3
0.2
0.1
0.0
p-value
2e-17
8e-14
3e-10
1e-06
4e-03
Log-odds
0.5
1.0
1.5
2.0
2.5
3.0
3.5
4.0
Cellular_organisms - ps1
Archaea1 - ps2
Archaea2 - ps3
Archaea3 - ps4
Asgard/Eukaryota - ps5
Eukaryota - ps6
Amorpheae - ps7
Obazoa - ps8
Opisthokonta - ps9
Holomycota - ps10
Fungi - ps11
Fungi_1 - ps12
Fungi_2 - ps13
Fungi_3 - ps14
Dikarya - ps15
Ascomycota - ps16
Saccharo/Pezizomycotina - ps17
Saccharomycotina - ps18
Saccharomycotina_1 - ps19
Saccharomycotina_2 - ps20
Saccharomycotina_3 - ps21
Phaff./Saccharomycetaceae - ps22
Saccharomyco./mycetaceae - ps23
Saccharomycetaceae - ps24
Saccharomycetaceae_2 - ps25
Saccharomycetaceae_3 - ps26
Kaz./Nau./Saccharomyces - ps27
Saccharomyces - ps28
Arboricola_clade - ps29
Cerevisiae_clade - ps30
Saccharomyces_cerevisiae - ps31
Phylostratum

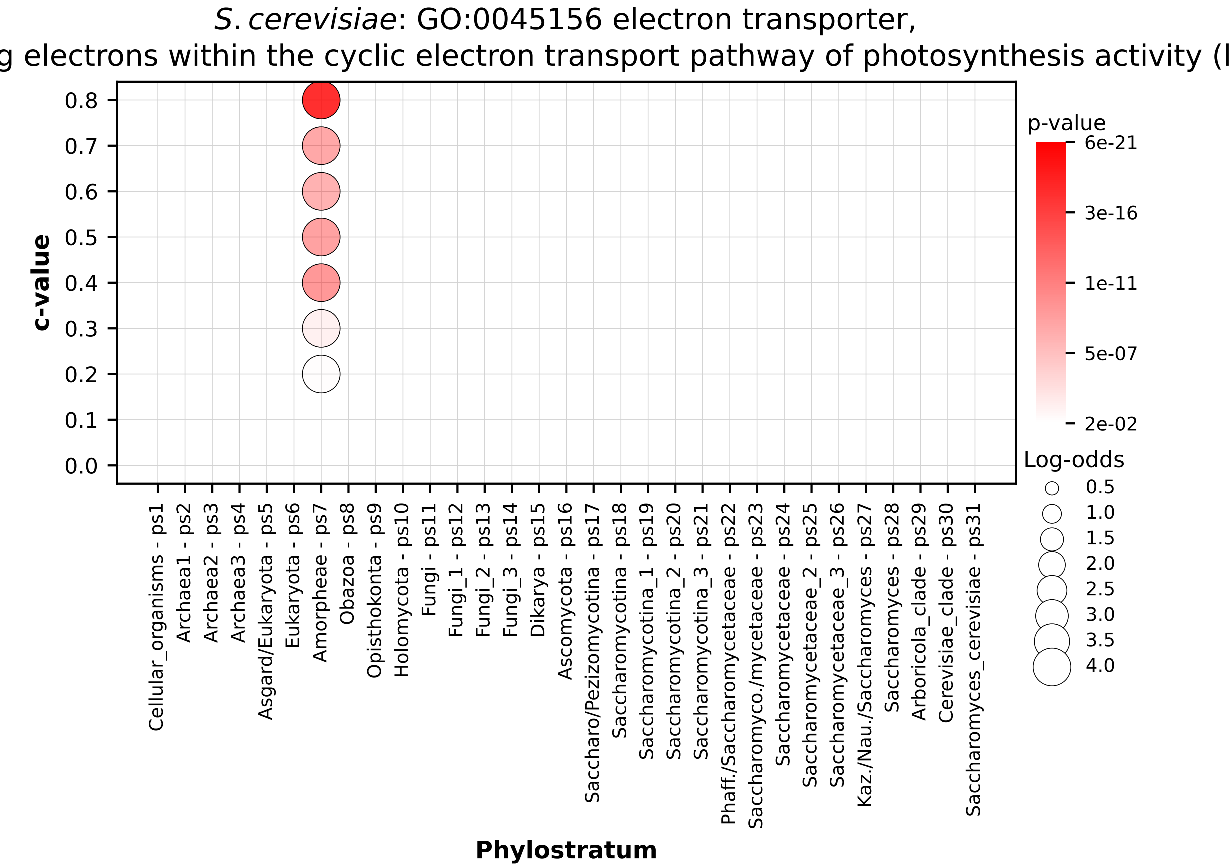
S. cerevisiae: GO:0045156 electron transporter,
g electrons within the cyclic electron transport pathway of photosynthesis activity (I
c-value
0.8
0.7
0.6
0.5
0.4
0.3
0.2
0.1
0.0
p-value
6e-21
3e-16
1e-11
5e-07
2e-02
Log-odds
0.5
1.0
1.5
2.0
2.5
3.0
3.5
4.0
Cellular_organisms - ps1
Archaea1 - ps2
Archaea2 - ps3
Archaea3 - ps4
Asgard/Eukaryota - ps5
Eukaryota - ps6
Amorpheae - ps7
Obazoa - ps8
Opisthokonta - ps9
Holomycota - ps10
Fungi - ps11
Fungi_1 - ps12
Fungi_2 - ps13
Fungi_3 - ps14
Dikarya - ps15
Ascomycota - ps16
Saccharo/Pezizomycotina - ps17
Saccharomycotina - ps18
Saccharomycotina_1 - ps19
Saccharomycotina_2 - ps20
Saccharomycotina_3 - ps21
Phaff./Saccharomycetaceae - ps22
Saccharomyco./mycetaceae - ps23
Saccharomycetaceae - ps24
Saccharomycetaceae_2 - ps25
Saccharomycetaceae_3 - ps26
Kaz./Nau./Saccharomyces - ps27
Saccharomyces - ps28
Arboricola_clade - ps29
Cerevisiae_clade - ps30
Saccharomyces_cerevisiae - ps31
Phylostratum

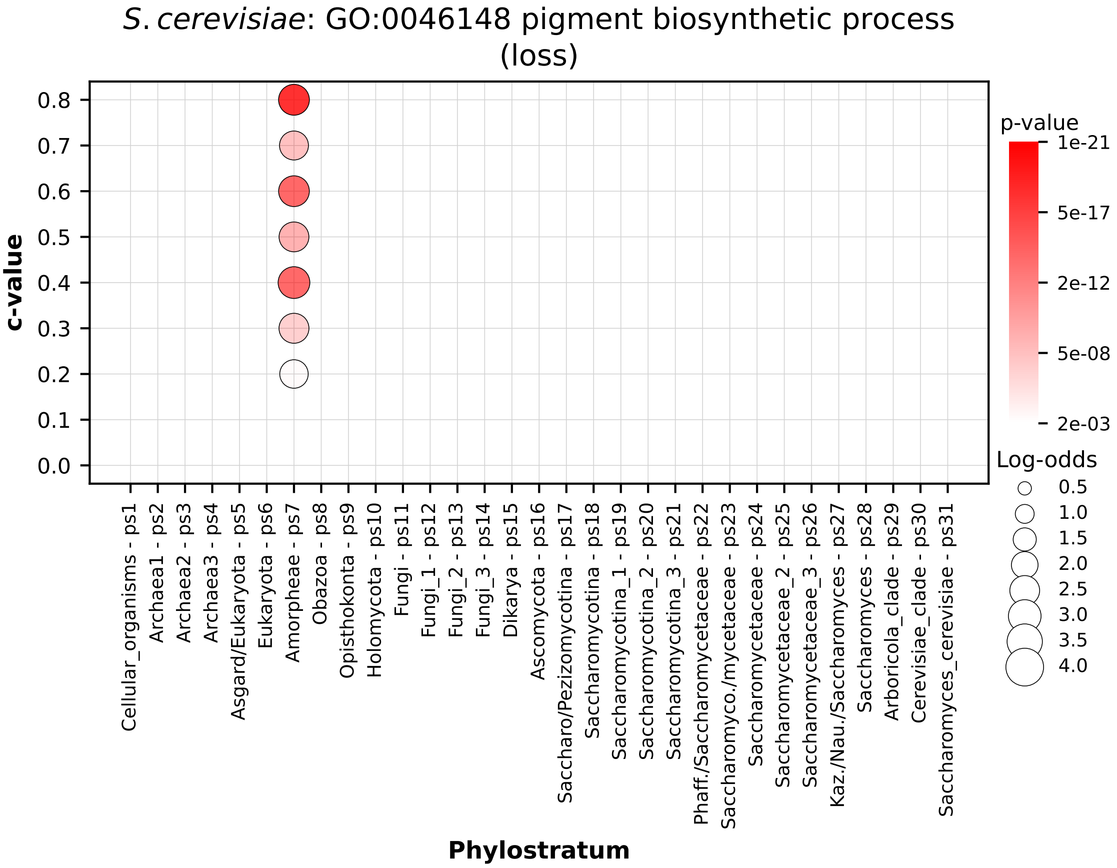

S. cerevisiae: GO:0046148 pigment biosynthetic process
(loss)
c-value
0.0
0.1
0.2
0.3
0.4
0.5
0.6
0.7
0.8
Phylostratum
Cellular_organisms - ps1
Archaea1 - ps2
Archaea2 - ps3
Archaea3 - ps4
Asgard/Eukaryota - ps5
Eukaryota - ps6
Amorpheae - ps7
Obazoa - ps8
Opisthokonta - ps9
Holomycota - ps10
Fungi - ps11
Fungi_1 - ps12
Fungi_2 - ps13
Fungi_3 - ps14
Dikarya - ps15
Ascomycota - ps16
Saccharo/Pezizomycotina - ps17
Saccharomycotina - ps18
Saccharomycotina_1 - ps19
Saccharomycotina_2 - ps20
Saccharomycotina_3 - ps21
Phaff./Saccharomycetaceae - ps22
Saccharomyco./mycetaceae - ps23
Saccharomycetaceae - ps24
Saccharomycetaceae_2 - ps25
Saccharomycetaceae_3 - ps26
Kaz./Nau./Saccharomyces - ps27
Saccharomyces - ps28
Arboricola_clade - ps29
Cerevisiae_clade - ps30
Saccharomyces_cerevisiae - ps31
p-value
1e-21
5e-17
2e-12
5e-08
2e-03
Log-odds
0.5
1.0
1.5
2.0
2.5
3.0
3.5
4.0

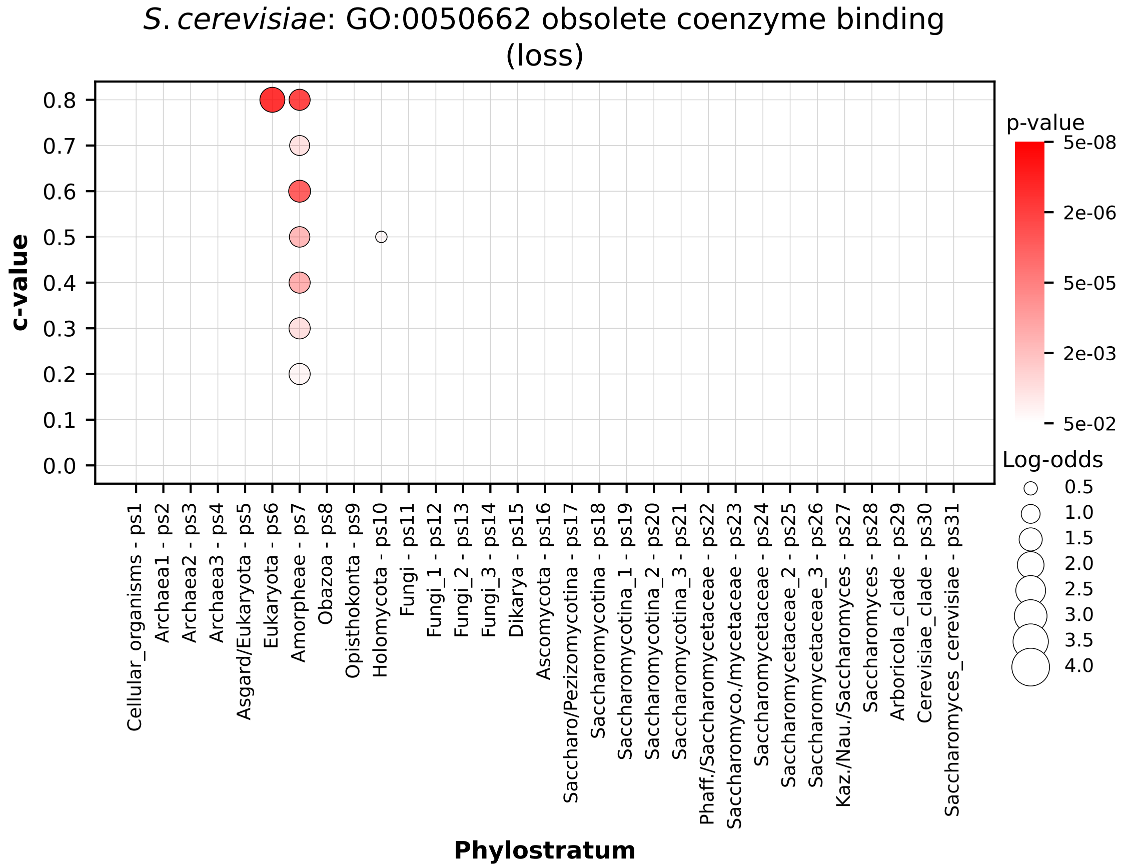
S. cerevisiae: GO:0050662 obsolete coenzyme binding
(loss)
c-value
0.8
0.7
0.6
0.5
0.4
0.3
0.2
0.1
0.0
p-value
5e-08
2e-06
5e-05
2e-03
5e-02
Log-odds
0.5
1.0
1.5
2.0
2.5
3.0
3.5
4.0
Cellular_organisms - ps1
Archaea1 - ps2
Archaea2 - ps3
Archaea3 - ps4
Asgard/Eukaryota - ps5
Eukaryota - ps6
Amorpheae - ps7
Obazoa - ps8
Opisthokonta - ps9
Holomycota - ps10
Fungi - ps11
Fungi_1 - ps12
Fungi_2 - ps13
Fungi_3 - ps14
Dikarya - ps15
Ascomycota - ps16
Saccharo/Pezizomycotina - ps17
Saccharomycotina - ps18
Saccharomycotina_1 - ps19
Saccharomycotina_2 - ps20
Saccharomycotina_3 - ps21
Phaff./Saccharomycetaceae - ps22
Saccharomyco./mycetaceae - ps23
Saccharomycetaceae - ps24
Saccharomycetaceae_2 - ps25
Saccharomycetaceae_3 - ps26
Kaz./Nau./Saccharomyces - ps27
Saccharomyces - ps28
Arboricola_clade - ps29
Cerevisiae_clade - ps30
Saccharomyces_cerevisiae - ps31
Phylostratum

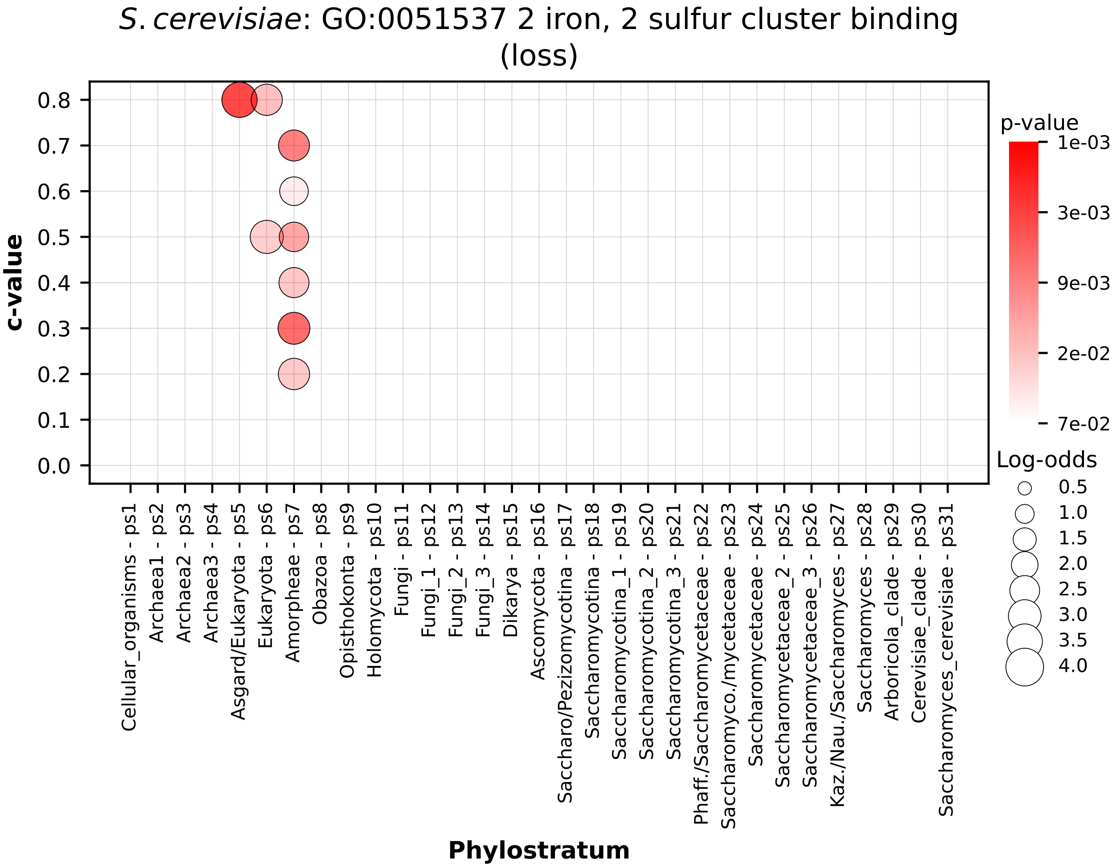

S. cerevisiae: GO:0051537 2 iron, 2 sulfur cluster binding
(loss)
c-value
0.8
0.7
0.6
0.5
0.4
0.3
0.2
0.1
0.0
p-value
1e-03
3e-03
9e-03
2e-02
7e-02
Log-odds
0.5
1.0
1.5
2.0
2.5
3.0
3.5
4.0
Cellular_organisms - ps1
Archaea1 - ps2
Archaea2 - ps3
Archaea3 - ps4
Asgard/Eukaryota - ps5
Eukaryota - ps6
Amorpheae - ps7
Obazoa - ps8
Opisthokonta - ps9
Holomycota - ps10
Fungi - ps11
Fungi_1 - ps12
Fungi_2 - ps13
Fungi_3 - ps14
Dikarya - ps15
Ascomycota - ps16
Saccharo/Pezizomycotina - ps17
Saccharomycotina - ps18
Saccharomycotina_1 - ps19
Saccharomycotina_2 - ps20
Saccharomycotina_3 - ps21
Phaff./Saccharomycetaceae - ps22
Saccharomyco./mycetaceae - ps23
Saccharomycetaceae - ps24
Saccharomycetaceae_2 - ps25
Saccharomycetaceae_3 - ps26
Kaz./Nau./Saccharomyces - ps27
Saccharomyces - ps28
Arboricola_clade - ps29
Cerevisiae_clade - ps30
Saccharomyces_cerevisiae - ps31
Phylostratum

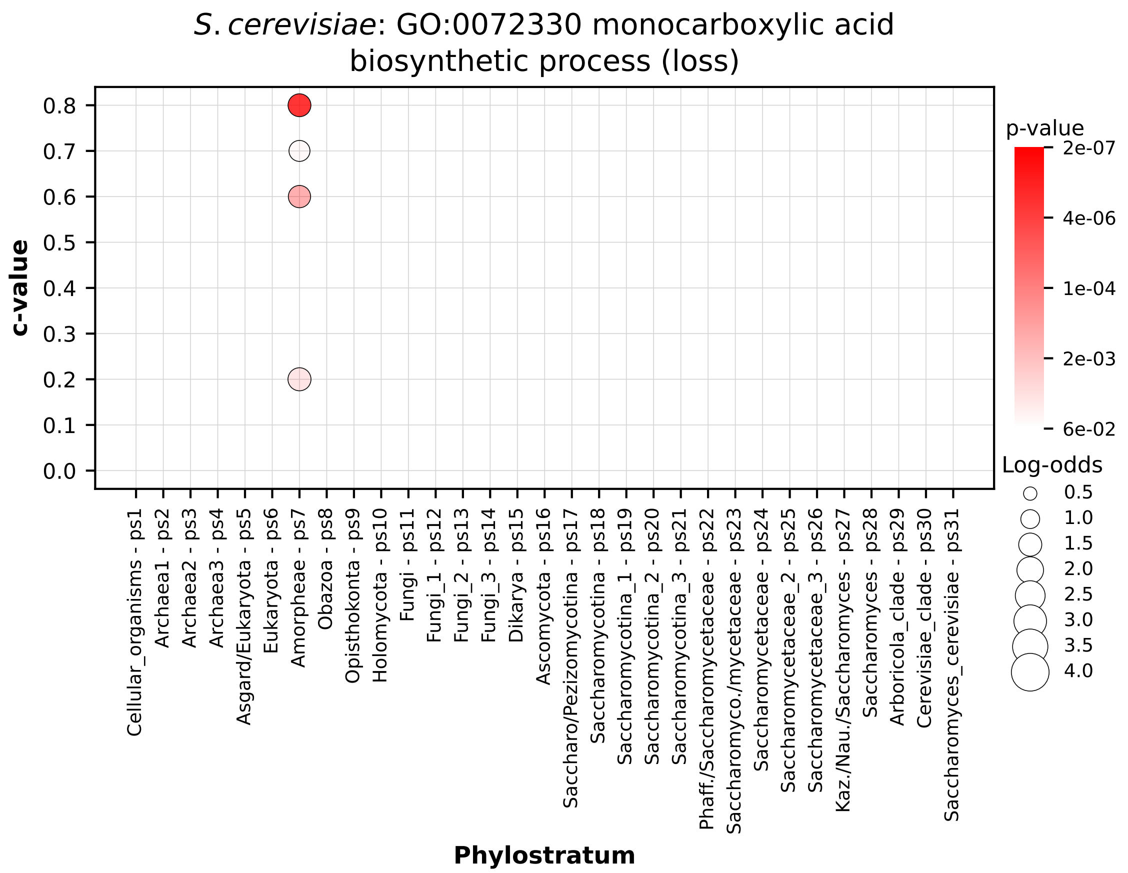
S. cerevisiae: GO:0072330 monocarboxylic acid biosynthetic process (loss)
c-value
0.8
0.7
0.6
0.5
0.4
0.3
0.2
0.1
0.0
p-value
2e-07
4e-06
1e-04
2e-03
6e-02
Log-odds
0.5
1.0
1.5
2.0
2.5
3.0
3.5
4.0
Cellular_organisms - ps1
Archaea1 - ps2
Archaea2 - ps3
Archaea3 - ps4
Asgard/Eukaryota - ps5
Eukaryota - ps6
Amorpheae - ps7
Obazoa - ps8
Opisthokonta - ps9
Holomycota - ps10
Fungi - ps11
Fungi_1 - ps12
Fungi_2 - ps13
Fungi_3 - ps14
Dikarya - ps15
Ascomycota - ps16
Saccharo/Pezizomycotina - ps17
Saccharomycotina - ps18
Saccharomycotina_1 - ps19
Saccharomycotina_2 - ps20
Saccharomycotina_3 - ps21
Phaff./Saccharomycetaceae - ps22
Saccharomyco./mycetaceae - ps23
Saccharomycetaceae - ps24
Saccharomycetaceae_2 - ps25
Saccharomycetaceae_3 - ps26
Kaz./Nau./Saccharomyces - ps27
Saccharomyces - ps28
Arboricola_clade - ps29
Cerevisiae_clade - ps30
Saccharomyces_cerevisiae - ps31
Phylostratum

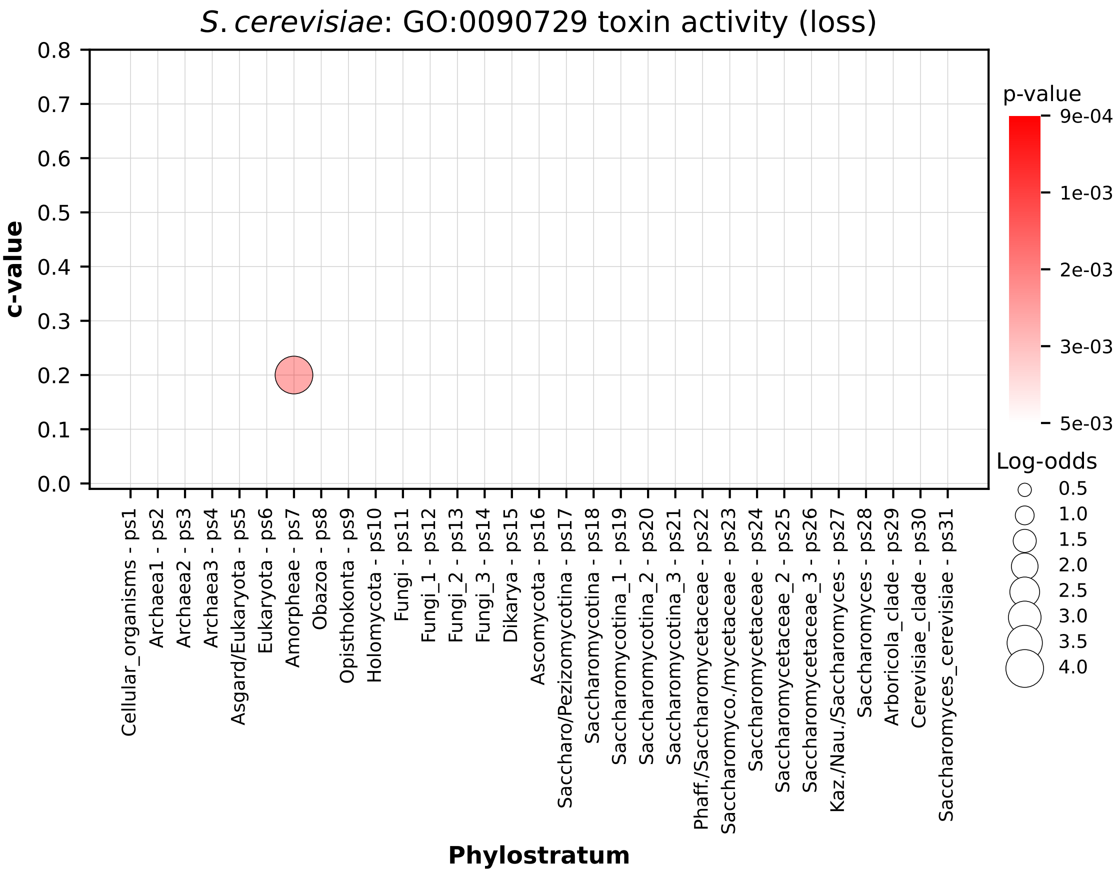

S. cerevisiae: GO:0090729 toxin activity (loss)
c-value
0.8
0.7
0.6
0.5
0.4
0.3
0.2
0.1
0.0
Phylostratum
Cellular_organisms - ps1
Archaea1 - ps2
Archaea2 - ps3
Archaea3 - ps4
Asgard/Eukaryota - ps5
Eukaryota - ps6
Amorpheae - ps7
Obazoa - ps8
Opisthokonta - ps9
Holomycota - ps10
Fungi - ps11
Fungi_1 - ps12
Fungi_2 - ps13
Fungi_3 - ps14
Dikarya - ps15
Ascomycota - ps16
Saccharo/Pezizomycotina - ps17
Saccharomycotina - ps18
Saccharomycotina_1 - ps19
Saccharomycotina_2 - ps20
Saccharomycotina_3 - ps21
Phaff./Saccharomycetaceae - ps22
Saccharomyco./mycetaceae - ps23
Saccharomycetaceae - ps24
Saccharomycetaceae_2 - ps25
Saccharomycetaceae_3 - ps26
Kaz./Nau./Saccharomyces - ps27
Saccharomyces - ps28
Arboricola_clade - ps29
Cerevisiae_clade - ps30
Saccharomyces_cerevisiae - ps31
p-value
9e-04
1e-03
2e-03
3e-03
5e-03
Log-odds
0.5
1.0
1.5
2.0
2.5
3.0
3.5
4.0

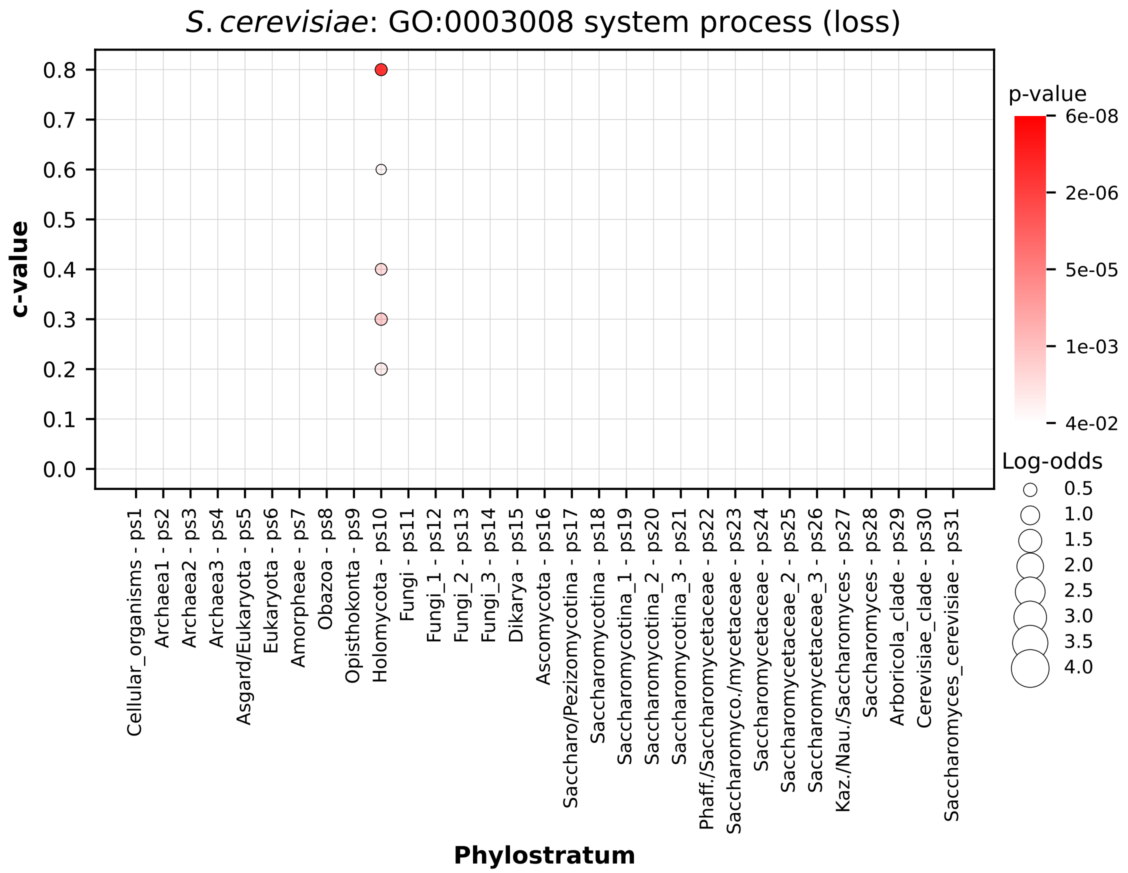

S. cerevisiae: GO:0003008 system process (loss)
c-value
0.0
0.1
0.2
0.3
0.4
0.5
0.6
0.7
0.8
p-value
6e-08
2e-06
5e-05
1e-03
4e-02
Log-odds
0.5
1.0
1.5
2.0
2.5
3.0
3.5
4.0
Cellular_organisms - ps1
Archaea1 - ps2
Archaea2 - ps3
Archaea3 - ps4
Asgard/Eukaryota - ps5
Eukaryota - ps6
Amorpheae - ps7
Obazoa - ps8
Opisthokonta - ps9
Holomycota - ps10
Fungi - ps11
Fungi_1 - ps12
Fungi_2 - ps13
Fungi_3 - ps14
Dikarya - ps15
Ascomycota - ps16
Saccharo/Pezizomycotina - ps17
Saccharomycotina - ps18
Saccharomycotina_1 - ps19
Saccharomycotina_2 - ps20
Saccharomycotina_3 - ps21
Phaff./Saccharomycetaceae - ps22
Saccharomyco./mycetaceae - ps23
Saccharomycetaceae - ps24
Saccharomycetaceae_2 - ps25
Saccharomycetaceae_3 - ps26
Kaz./Nau./Saccharomyces - ps27
Saccharomyces - ps28
Arboricola_clade - ps29
Cerevisiae_clade - ps30
Saccharomyces_cerevisiae - ps31
Phylostratum

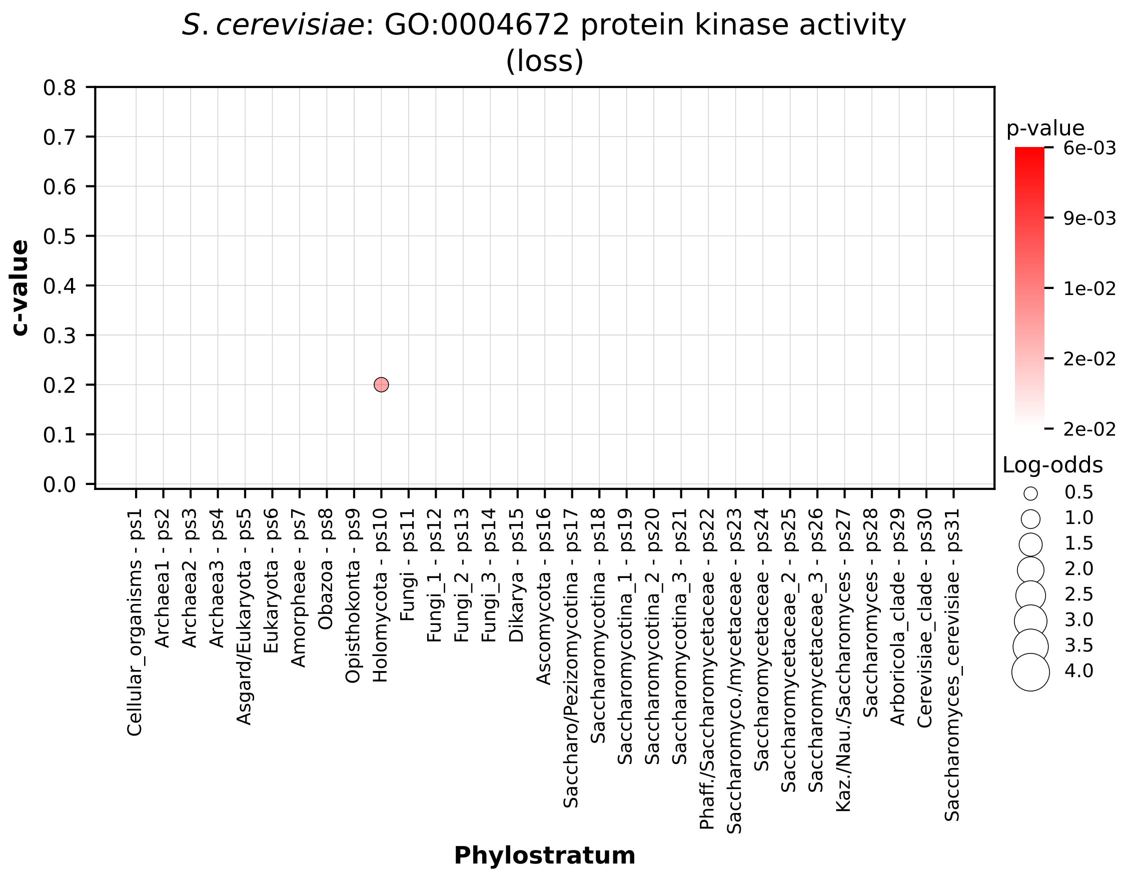
S. cerevisiae: GO:0004672 protein kinase activity (loss)
c-value
0.0
0.1
0.2
0.3
0.4
0.5
0.6
0.7
0.8
Phylostratum
Cellular_organisms - ps1
Archaea1 - ps2
Archaea2 - ps3
Archaea3 - ps4
Asgard/Eukaryota - ps5
Eukaryota - ps6
Amorpheae - ps7
Obazoa - ps8
Opisthokonta - ps9
Holomycota - ps10
Fungi - ps11
Fungi_1 - ps12
Fungi_2 - ps13
Fungi_3 - ps14
Dikarya - ps15
Ascomycota - ps16
Saccharo/Pezizomycotina - ps17
Saccharomycotina - ps18
Saccharomycotina_1 - ps19
Saccharomycotina_2 - ps20
Saccharomycotina_3 - ps21
Phaff./Saccharomycetaceae - ps22
Saccharomyco./mycetaceae - ps23
Saccharomycetaceae - ps24
Saccharomycetaceae_2 - ps25
Saccharomycetaceae_3 - ps26
Kaz./Nau./Saccharomyces - ps27
Saccharomyces - ps28
Arboricola_clade - ps29
Cerevisiae_clade - ps30
Saccharomyces_cerevisiae - ps31
p-value
6e-03
9e-03
1e-02
2e-02
2e-02
Log-odds
0.5
1.0
1.5
2.0
2.5
3.0
3.5
4.0

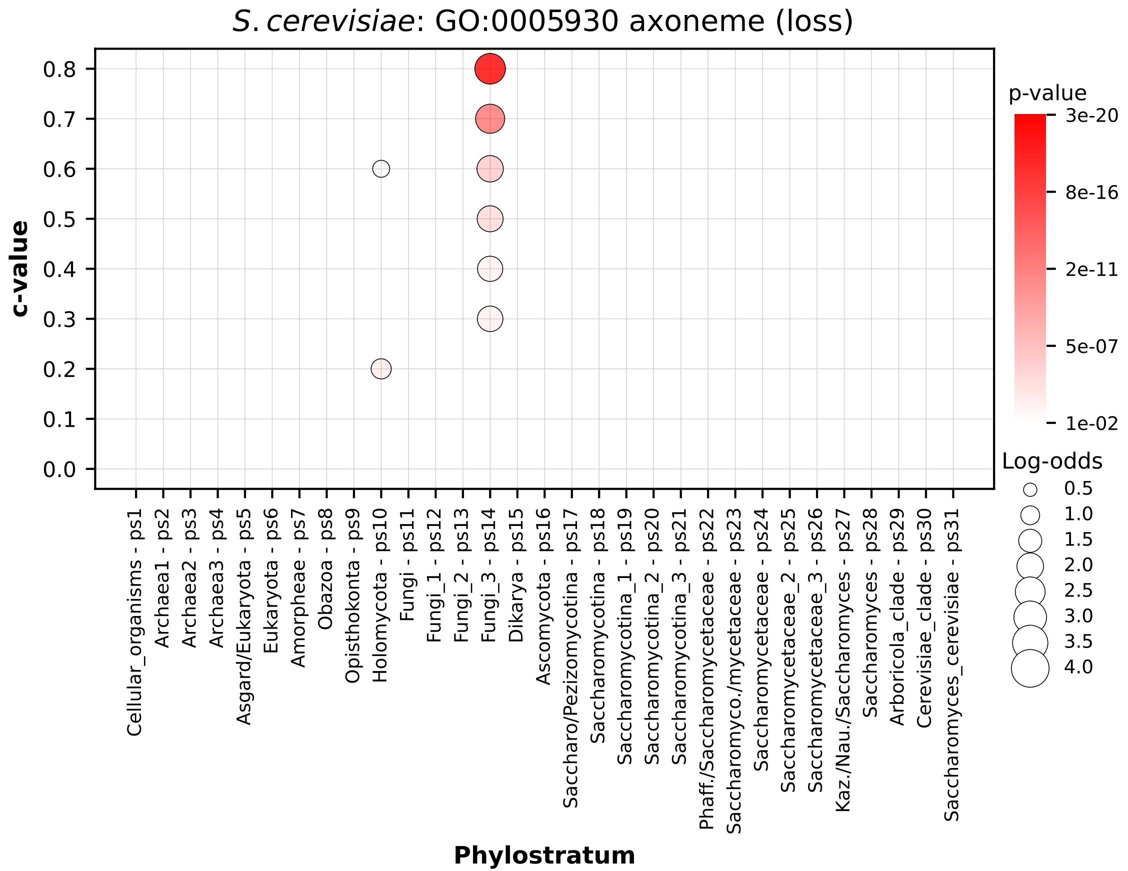

S. cerevisiae: GO:0005930 axoneme (loss)
c-value
0.8
0.7
0.6
0.5
0.4
0.3
0.2
0.1
0.0
Phylostratum
Cellular_organisms - ps1
Archaea1 - ps2
Archaea2 - ps3
Archaea3 - ps4
Asgard/Eukaryota - ps5
Eukaryota - ps6
Amorpheae - ps7
Obazoa - ps8
Opisthokonta - ps9
Holomycota - ps10
Fungi - ps11
Fungi_1 - ps12
Fungi_2 - ps13
Fungi_3 - ps14
Dikarya - ps15
Ascomycota - ps16
Saccharo/Pezizomycotina - ps17
Saccharomycotina - ps18
Saccharomycotina_1 - ps19
Saccharomycotina_2 - ps20
Saccharomycotina_3 - ps21
Phaff./Saccharomycetaceae - ps22
Saccharomyco./mycetaceae - ps23
Saccharomycetaceae - ps24
Saccharomycetaceae_2 - ps25
Saccharomycetaceae_3 - ps26
Kaz./Nau./Saccharomyces - ps27
Saccharomyces - ps28
Arboricola_clade - ps29
Cerevisiae_clade - ps30
Saccharomyces_cerevisiae - ps31
p-value
3e-20
8e-16
2e-11
5e-07
1e-02
Log-odds
0.5
1.0
1.5
2.0
2.5
3.0
3.5
4.0

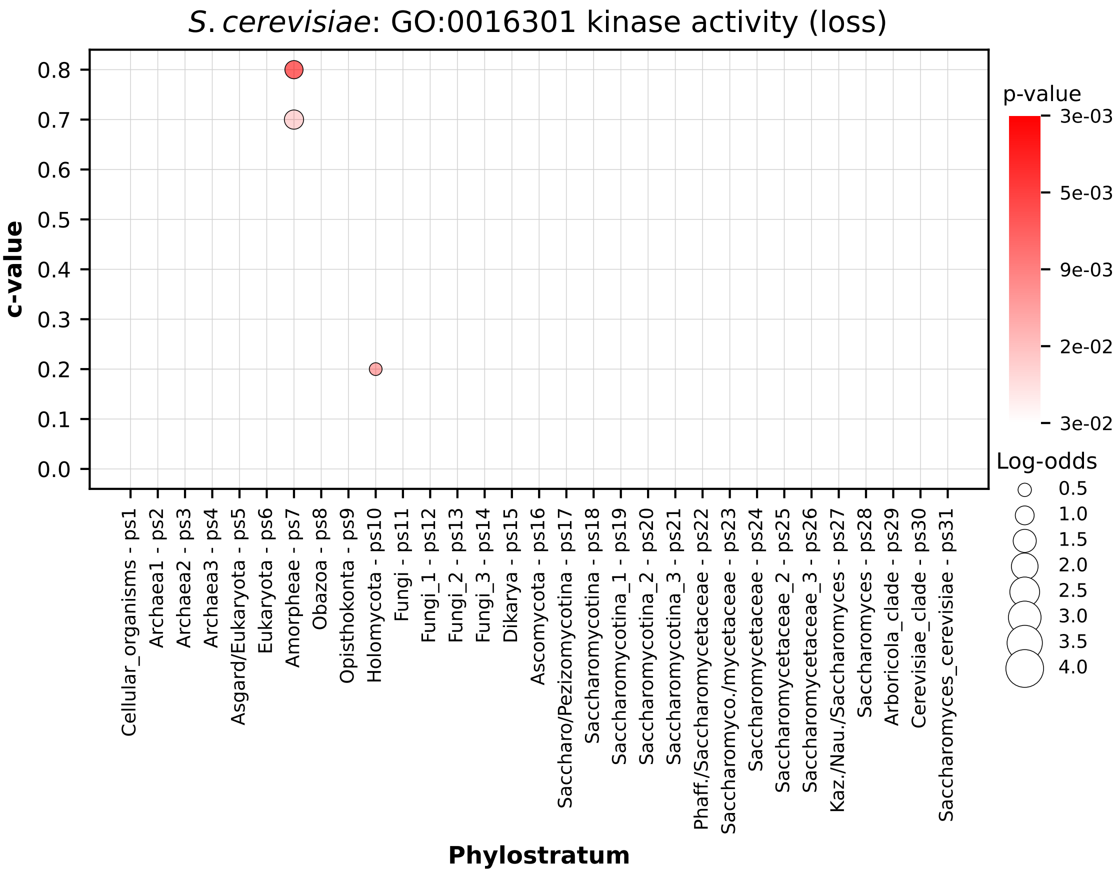

S. cerevisiae: GO:0016301 kinase activity (loss)
c-value
0.0
0.1
0.2
0.3
0.4
0.5
0.6
0.7
0.8
p-value
3e-03
5e-03
9e-03
2e-02
3e-02
Log-odds
0.5
1.0
1.5
2.0
2.5
3.0
3.5
4.0
Cellular_organisms - ps1
Archaea1 - ps2
Archaea2 - ps3
Archaea3 - ps4
Asgard/Eukaryota - ps5
Eukaryota - ps6
Amorpheae - ps7
Obazoa - ps8
Opisthokonta - ps9
Holomycota - ps10
Fungi - ps11
Fungi_1 - ps12
Fungi_2 - ps13
Fungi_3 - ps14
Dikarya - ps15
Ascomycota - ps16
Saccharo/Pezizomycotina - ps17
Saccharomycotina - ps18
Saccharomycotina_1 - ps19
Saccharomycotina_2 - ps20
Saccharomycotina_3 - ps21
Phaff./Saccharomycetaceae - ps22
Saccharomyco./mycetaceae - ps23
Saccharomycetaceae - ps24
Saccharomycetaceae_2 - ps25
Saccharomycetaceae_3 - ps26
Kaz./Nau./Saccharomyces - ps27
Saccharomyces - ps28
Arboricola_clade - ps29
Cerevisiae_clade - ps30
Saccharomyces_cerevisiae - ps31
Phylostratum

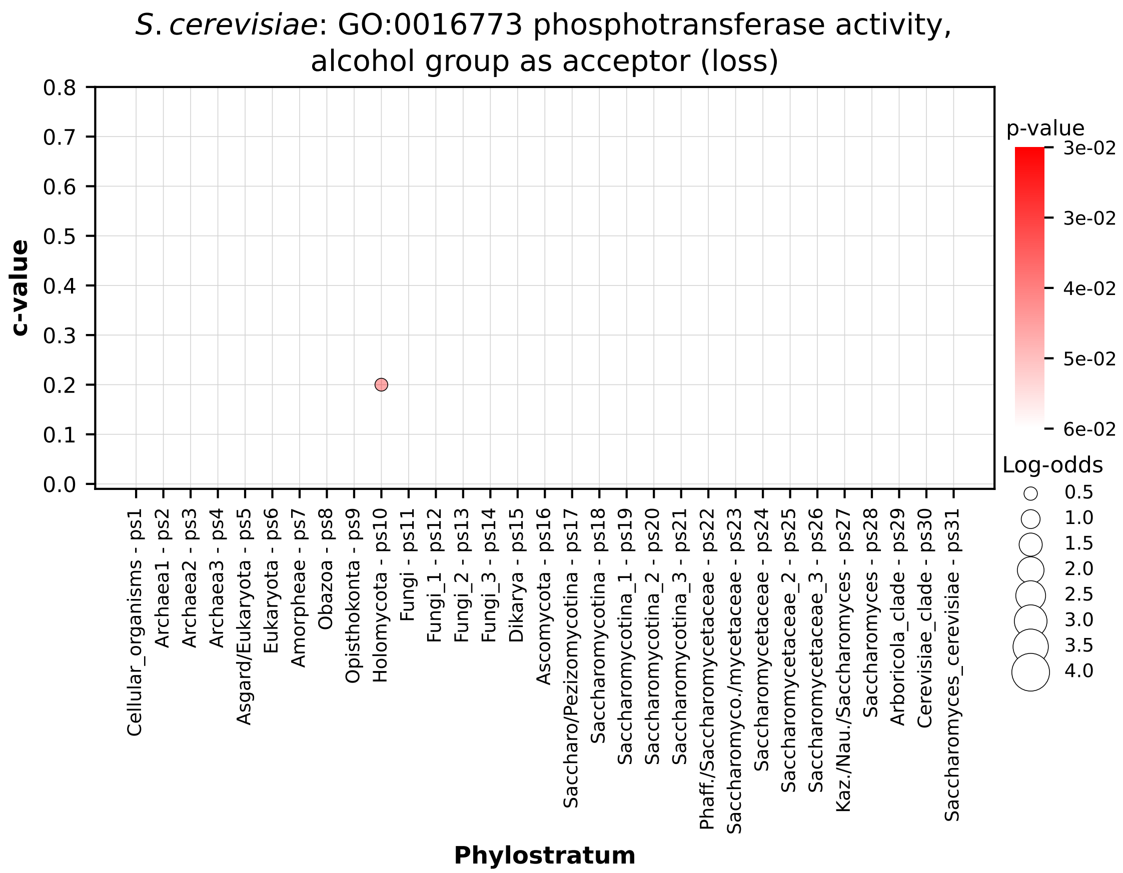

S. cerevisiae: GO:0016773 phosphotransferase activity, alcohol group as acceptor (loss)
c-value
0.8
0.7
0.6
0.5
0.4
0.3
0.2
0.1
0.0
p-value
3e-02
3e-02
4e-02
5e-02
6e-02
Log-odds
0.5
1.0
1.5
2.0
2.5
3.0
3.5
4.0
Cellular_organisms - ps1
Archaea1 - ps2
Archaea2 - ps3
Archaea3 - ps4
Asgard/Eukaryota - ps5
Eukaryota - ps6
Amorpheae - ps7
Obazoa - ps8
Opisthokonta - ps9
Holomycota - ps10
Fungi - ps11
Fungi_1 - ps12
Fungi_2 - ps13
Fungi_3 - ps14
Dikarya - ps15
Ascomycota - ps16
Saccharo/Pezizomycotina - ps17
Saccharomycotina - ps18
Saccharomycotina_1 - ps19
Saccharomycotina_2 - ps20
Saccharomycotina_3 - ps21
Phaff./Saccharomycetaceae - ps22
Saccharomyco./mycetaceae - ps23
Saccharomycetaceae - ps24
Saccharomycetaceae_2 - ps25
Saccharomycetaceae_3 - ps26
Kaz./Nau./Saccharomyces - ps27
Saccharomyces - ps28
Arboricola_clade - ps29
Cerevisiae_clade - ps30
Saccharomyces_cerevisiae - ps31
Phylostratum

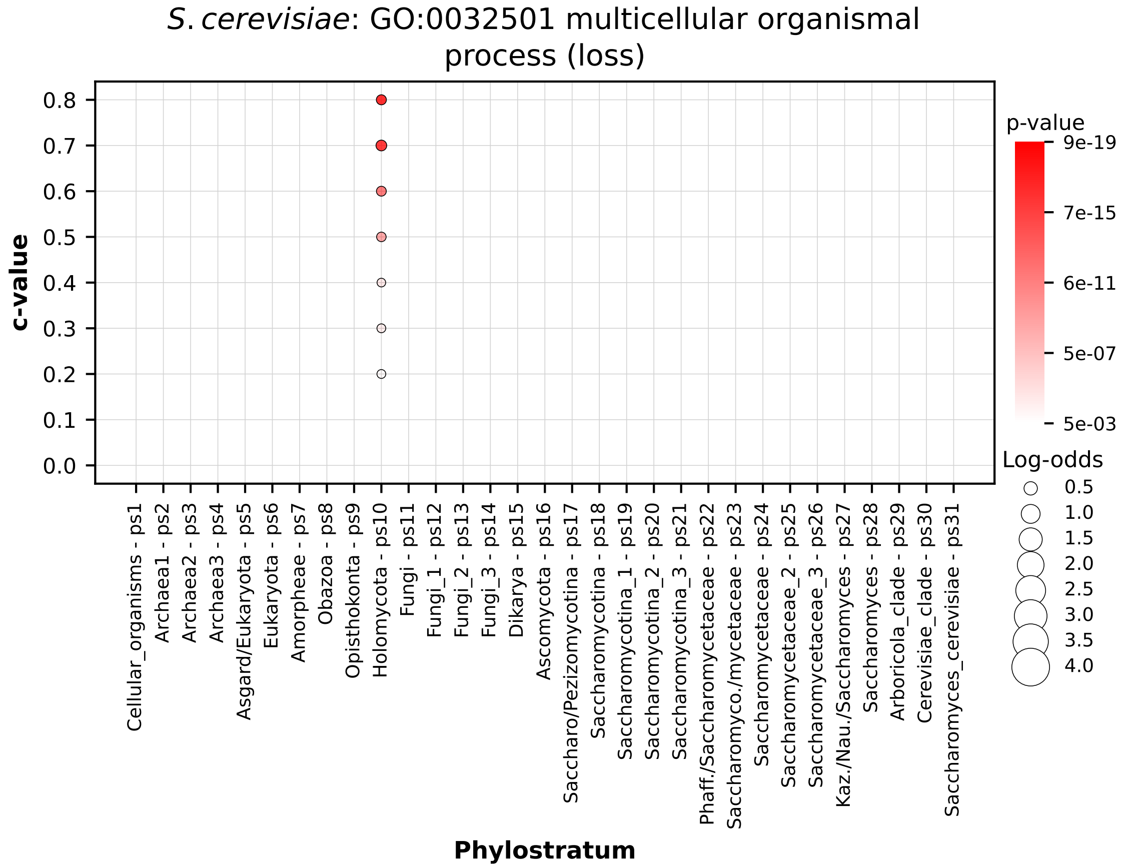
S. cerevisiae: GO:0032501 multicellular organismal process (loss)
c-value
0.0
0.1
0.2
0.3
0.4
0.5
0.6
0.7
0.8
Phylostratum
Cellular_organisms - ps1
Archaea1 - ps2
Archaea2 - ps3
Archaea3 - ps4
Asgard/Eukaryota - ps5
Eukaryota - ps6
Amorpheae - ps7
Obazoa - ps8
Opisthokonta - ps9
Holomycota - ps10
Fungi - ps11
Fungi_1 - ps12
Fungi_2 - ps13
Fungi_3 - ps14
Dikarya - ps15
Ascomycota - ps16
Saccharo/Pezizomycotina - ps17
Saccharomycotina - ps18
Saccharomycotina_1 - ps19
Saccharomycotina_2 - ps20
Saccharomycotina_3 - ps21
Phaff./Saccharomycetaceae - ps22
Saccharomyco./mycetaceae - ps23
Saccharomycetaceae - ps24
Saccharomycetaceae_2 - ps25
Saccharomycetaceae_3 - ps26
Kaz./Nau./Saccharomyces - ps27
Saccharomyces - ps28
Arboricola_clade - ps29
Cerevisiae_clade - ps30
Saccharomyces_cerevisiae - ps31
p-value
9e-19
7e-15
6e-11
5e-07
5e-03
Log-odds
0.5
1.0
1.5
2.0
2.5
3.0
3.5
4.0

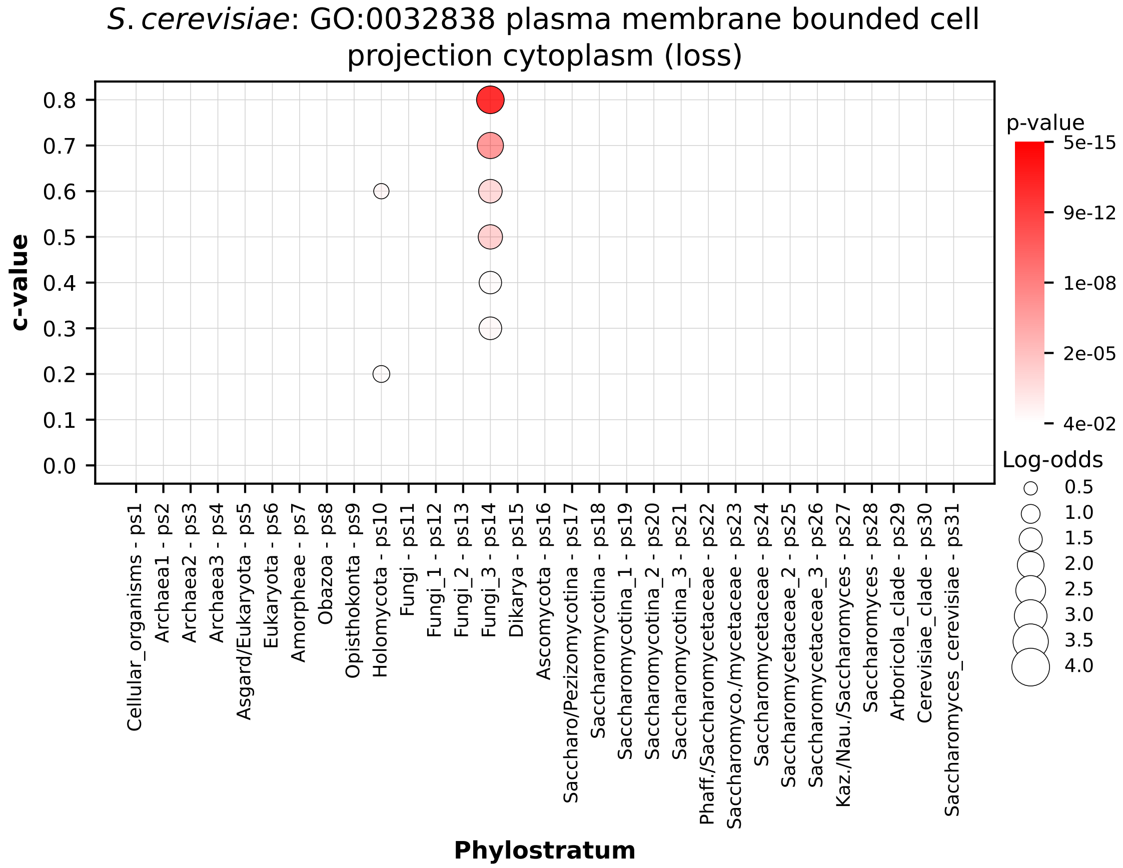

S. cerevisiae: GO:0032838 plasma membrane bounded cell projection cytoplasm (loss)
c-value
0.8
0.7
0.6
0.5
0.4
0.3
0.2
0.1
0.0
Phylostratum
Cellular_organisms - ps1
Archaea1 - ps2
Archaea2 - ps3
Archaea3 - ps4
Asgard/Eukaryota - ps5
Eukaryota - ps6
Amorpheae - ps7
Obazoa - ps8
Opisthokonta - ps9
Holomycota - ps10
Fungi - ps11
Fungi_1 - ps12
Fungi_2 - ps13
Fungi_3 - ps14
Dikarya - ps15
Ascomycota - ps16
Saccharo/Pezizomycotina - ps17
Saccharomycotina - ps18
Saccharomycotina_1 - ps19
Saccharomycotina_2 - ps20
Saccharomycotina_3 - ps21
Phaff./Saccharomycetaceae - ps22
Saccharomyco./mycetaceae - ps23
Saccharomycetaceae - ps24
Saccharomycetaceae_2 - ps25
Saccharomycetaceae_3 - ps26
Kaz./Nau./Saccharomyces - ps27
Saccharomyces - ps28
Arboricola_clade - ps29
Cerevisiae_clade - ps30
Saccharomyces_cerevisiae - ps31
p-value
5e-15
9e-12
1e-08
2e-05
4e-02
Log-odds
0.5
1.0
1.5
2.0
2.5
3.0
3.5
4.0

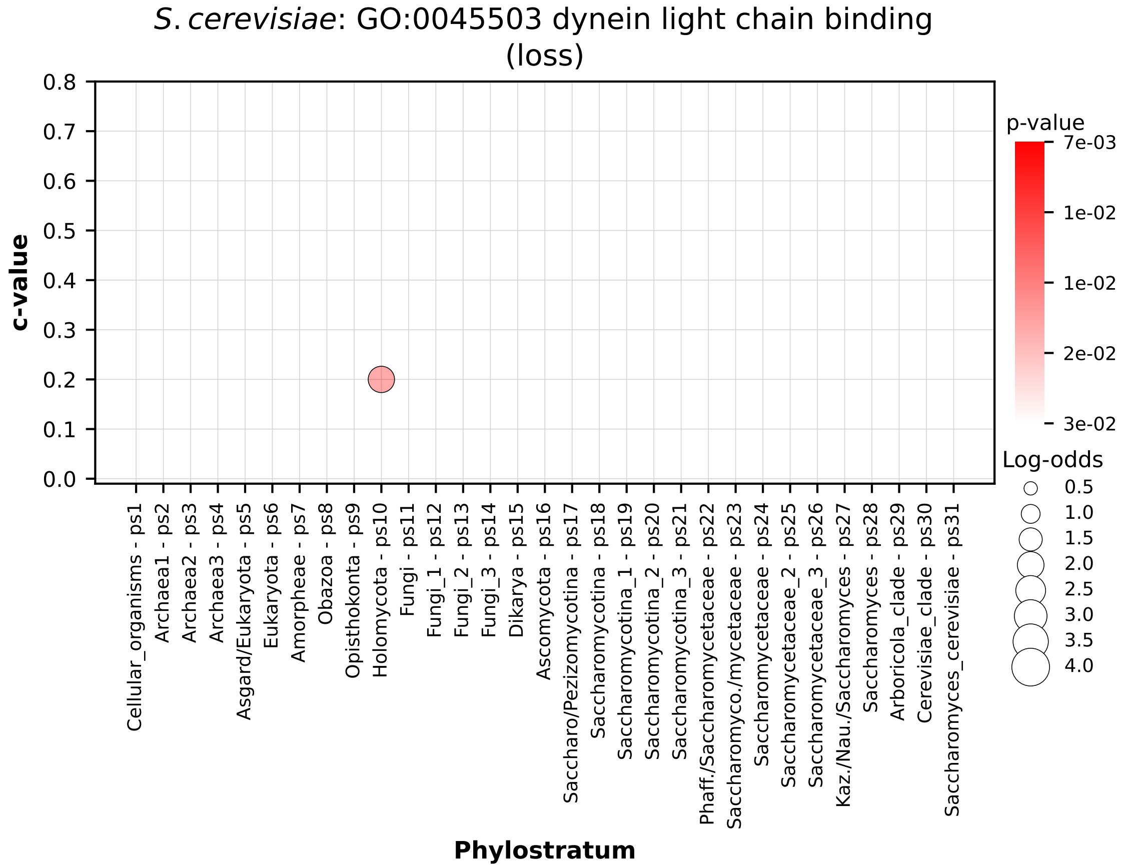

S. cerevisiae: GO:0045503 dynein light chain binding (loss)
c-value
0.0
0.1
0.2
0.3
0.4
0.5
0.6
0.7
0.8
p-value
7e-03
1e-02
1e-02
2e-02
3e-02
Log-odds
0.5
1.0
1.5
2.0
2.5
3.0
3.5
4.0
Cellular_organisms - ps1
Archaea1 - ps2
Archaea2 - ps3
Archaea3 - ps4
Asgard/Eukaryota - ps5
Eukaryota - ps6
Amorpheae - ps7
Obazoa - ps8
Opisthokonta - ps9
Holomycota - ps10
Fungi - ps11
Fungi_1 - ps12
Fungi_2 - ps13
Fungi_3 - ps14
Dikarya - ps15
Ascomycota - ps16
Saccharo/Pezizomycotina - ps17
Saccharomycotina - ps18
Saccharomycotina_1 - ps19
Saccharomycotina_2 - ps20
Saccharomycotina_3 - ps21
Phaff./Saccharomycetaceae - ps22
Saccharomyco./mycetaceae - ps23
Saccharomycetaceae - ps24
Saccharomycetaceae_2 - ps25
Saccharomycetaceae_3 - ps26
Kaz./Nau./Saccharomyces - ps27
Saccharomyces - ps28
Arboricola_clade - ps29
Cerevisiae_clade - ps30
Saccharomyces_cerevisiae - ps31
Phylostratum

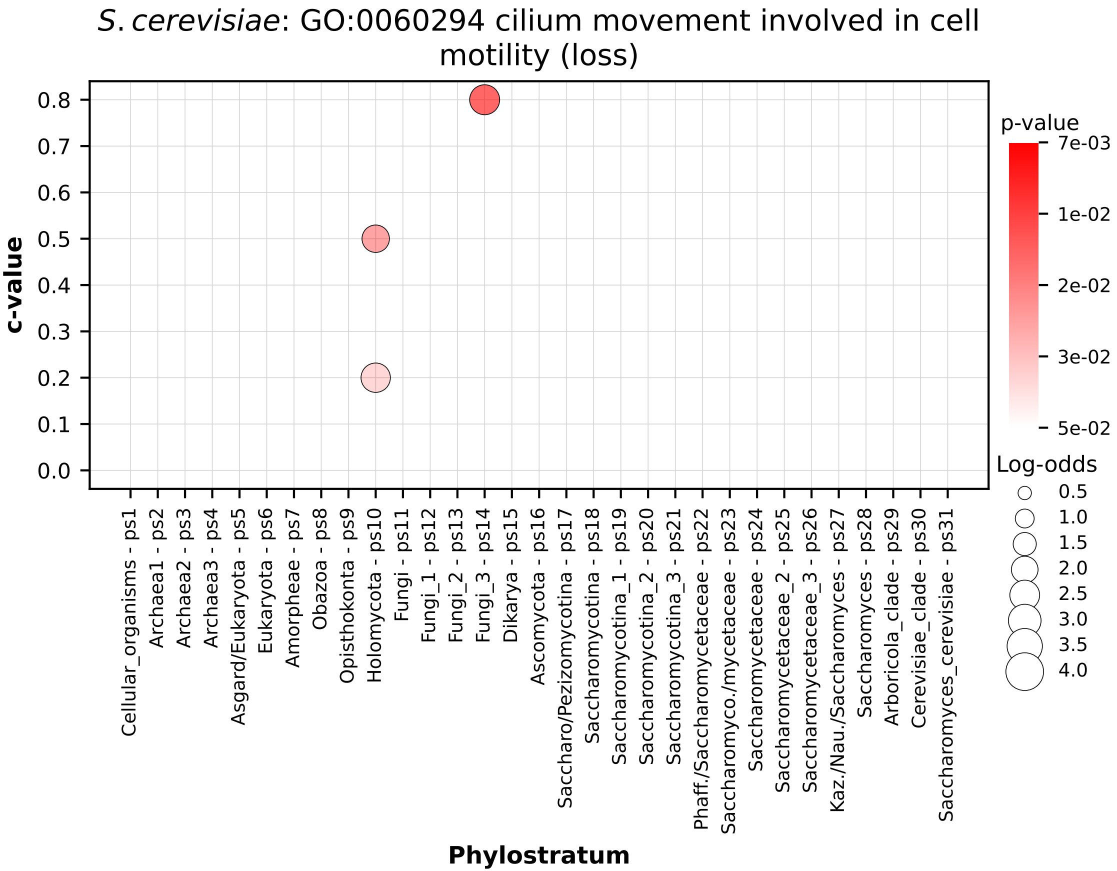
S. cerevisiae: GO:0060294 cilium movement involved in cell motility (loss)
c-value
0.8
0.7
0.6
0.5
0.4
0.3
0.2
0.1
0.0
Phylostratum
Cellular_organisms - ps1
Archaea1 - ps2
Archaea2 - ps3
Archaea3 - ps4
Asgard/Eukaryota - ps5
Eukaryota - ps6
Amorpheae - ps7
Obazoa - ps8
Opisthokonta - ps9
Holomycota - ps10
Fungi - ps11
Fungi_1 - ps12
Fungi_2 - ps13
Fungi_3 - ps14
Dikarya - ps15
Ascomycota - ps16
Saccharo/Pezizomycotina - ps17
Saccharomycotina - ps18
Saccharomycotina_1 - ps19
Saccharomycotina_2 - ps20
Saccharomycotina_3 - ps21
Phaff./Saccharomycetaceae - ps22
Saccharomyco./mycetaceae - ps23
Saccharomycetaceae - ps24
Saccharomycetaceae_2 - ps25
Saccharomycetaceae_3 - ps26
Kaz./Nau./Saccharomyces - ps27
Saccharomyces - ps28
Arboricola_clade - ps29
Cerevisiae_clade - ps30
Saccharomyces_cerevisiae - ps31
p-value
7e-03
1e-02
2e-02
3e-02
5e-02
Log-odds
0.5
1.0
1.5
2.0
2.5
3.0
3.5
4.0

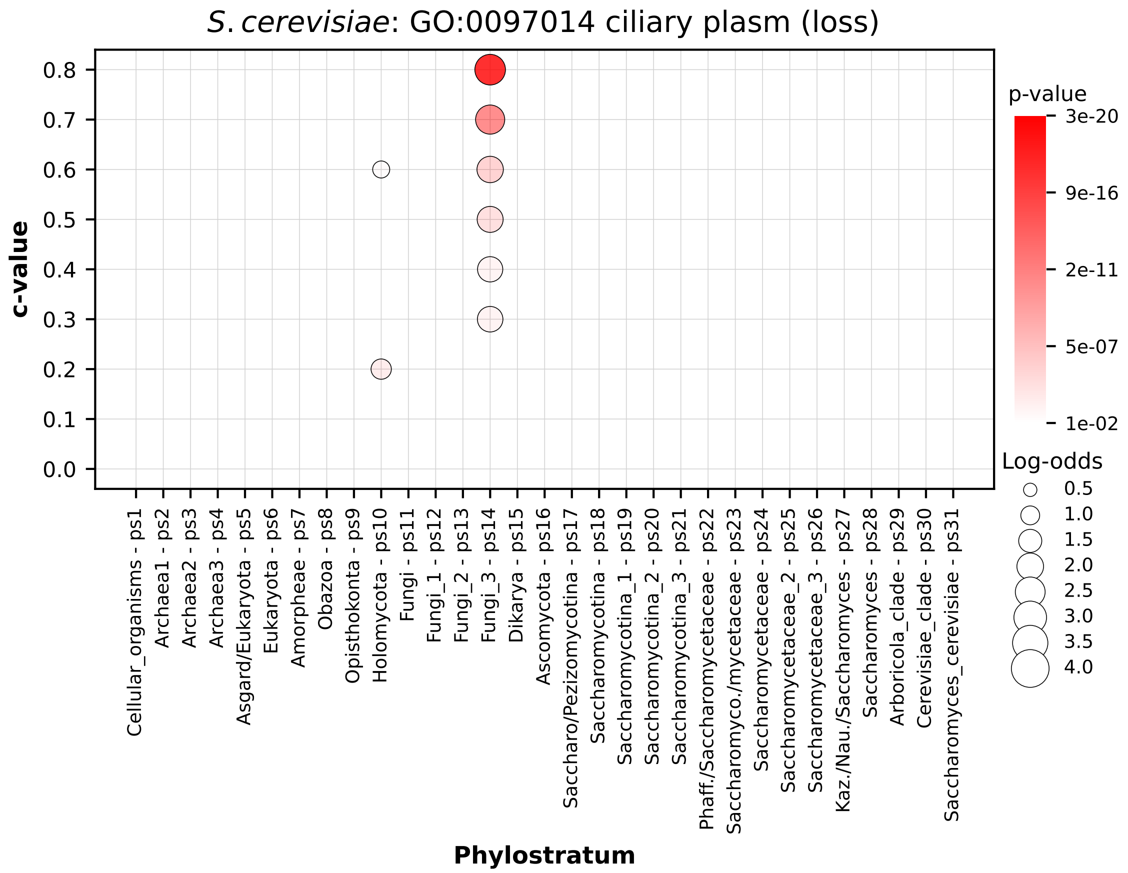

S. cerevisiae: GO:0097014 ciliary plasm (loss)
c-value
0.8
0.7
0.6
0.5
0.4
0.3
0.2
0.1
0.0
p-value
3e-20
9e-16
2e-11
5e-07
1e-02
Log-odds
0.5
1.0
1.5
2.0
2.5
3.0
3.5
4.0
Cellular_organisms - ps1
Archaea1 - ps2
Archaea2 - ps3
Archaea3 - ps4
Asgard/Eukaryota - ps5
Eukaryota - ps6
Amorpheae - ps7
Obazoa - ps8
Opisthokonta - ps9
Holomycota - ps10
Fungi - ps11
Fungi_1 - ps12
Fungi_2 - ps13
Fungi_3 - ps14
Dikarya - ps15
Ascomycota - ps16
Saccharo/Pezizomycotina - ps17
Saccharomycotina - ps18
Saccharomycotina_1 - ps19
Saccharomycotina_2 - ps20
Saccharomycotina_3 - ps21
Phaff./Saccharomycetaceae - ps22
Saccharomyco./mycetaceae - ps23
Saccharomycetaceae - ps24
Saccharomycetaceae_2 - ps25
Saccharomycetaceae_3 - ps26
Kaz./Nau./Saccharomyces - ps27
Saccharomyces - ps28
Arboricola_clade - ps29
Cerevisiae_clade - ps30
Saccharomyces_cerevisiae - ps31
Phylostratum

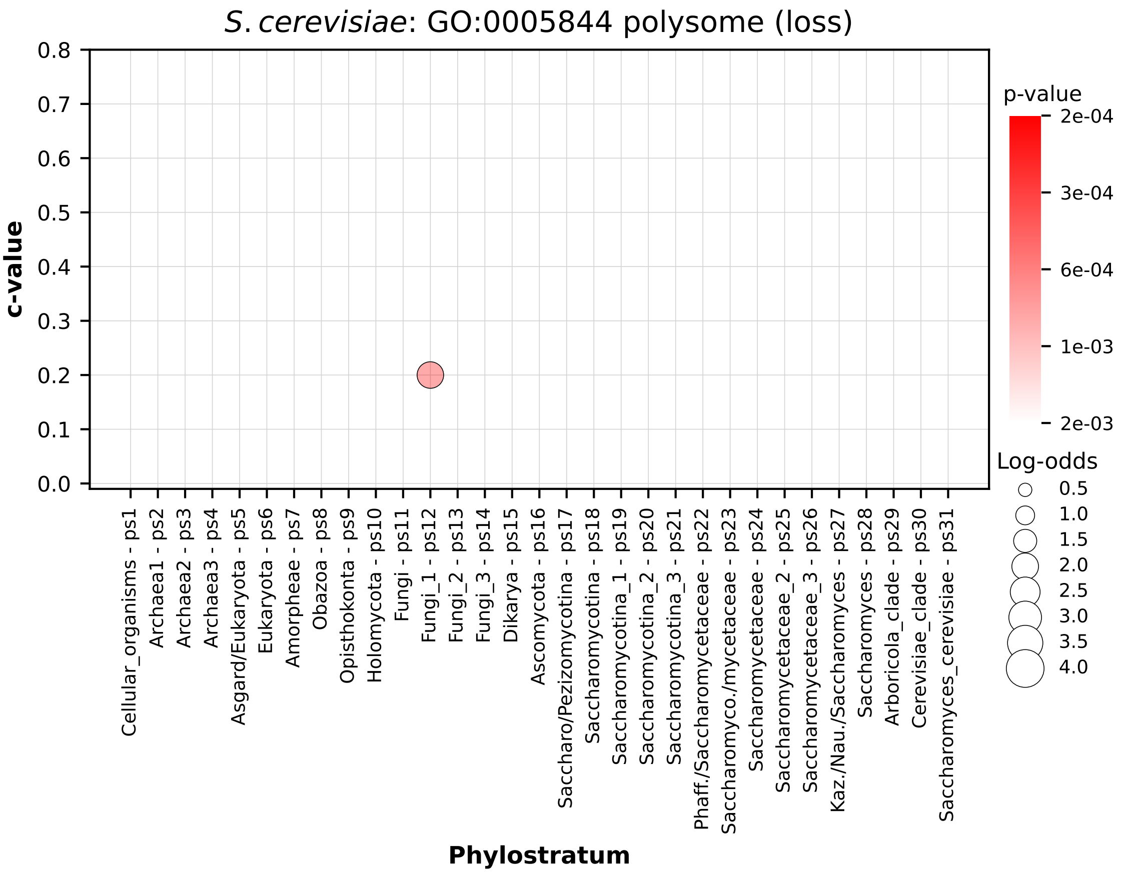

S. cerevisiae: GO:0005844 polysome (loss)
c-value
0.0
0.1
0.2
0.3
0.4
0.5
0.6
0.7
0.8
Phylostratum
Cellular_organisms - ps1
Archaea1 - ps2
Archaea2 - ps3
Archaea3 - ps4
Asgard/Eukaryota - ps5
Eukaryota - ps6
Amorpheae - ps7
Obazoa - ps8
Opisthokonta - ps9
Holomycota - ps10
Fungi - ps11
Fungi_1 - ps12
Fungi_2 - ps13
Fungi_3 - ps14
Dikarya - ps15
Ascomycota - ps16
Saccharo/Pezizomycotina - ps17
Saccharomycotina - ps18
Saccharomycotina_1 - ps19
Saccharomycotina_2 - ps20
Saccharomycotina_3 - ps21
Phaff./Saccharomycetaceae - ps22
Saccharomyco./mycetaceae - ps23
Saccharomycetaceae - ps24
Saccharomycetaceae_2 - ps25
Saccharomycetaceae_3 - ps26
Kaz./Nau./Saccharomyces - ps27
Saccharomyces - ps28
Arboricola_clade - ps29
Cerevisiae_clade - ps30
Saccharomyces_cerevisiae - ps31
p-value
2e-04
3e-04
6e-04
1e-03
2e-03
Log-odds
0.5
1.0
1.5
2.0
2.5
3.0
3.5
4.0

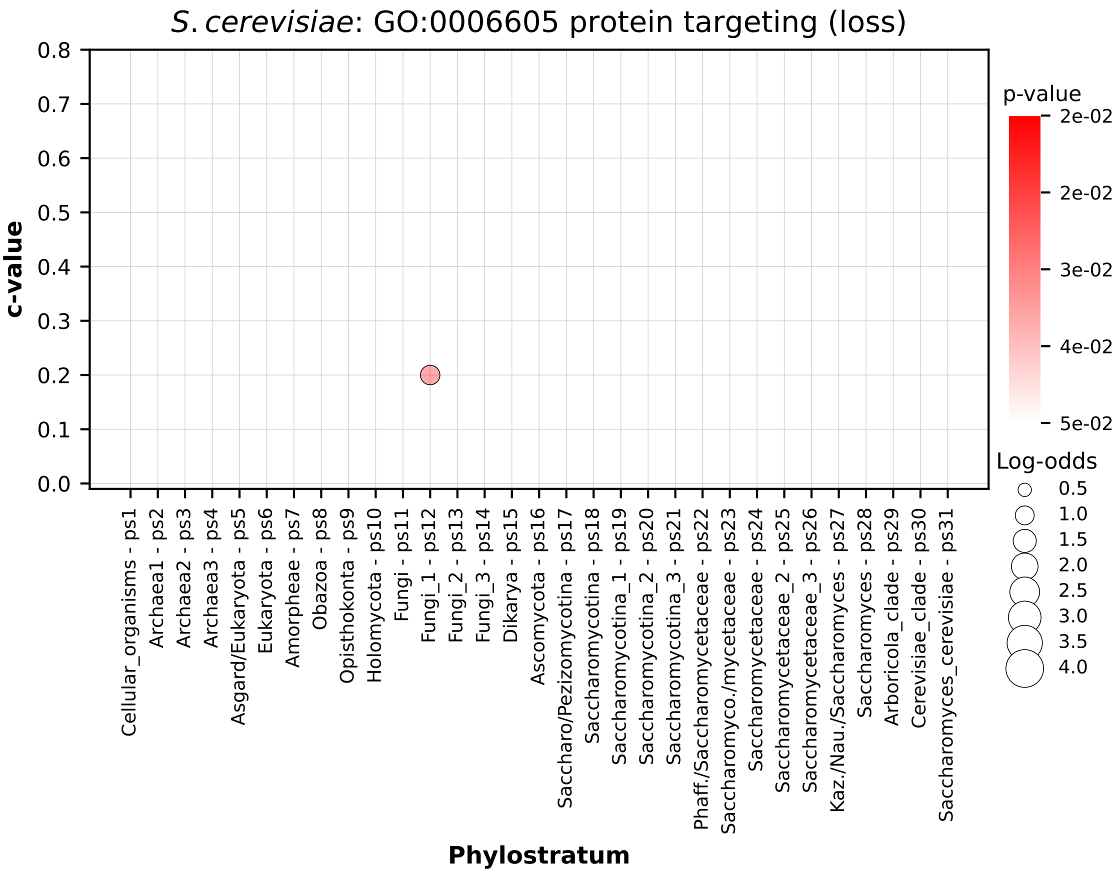

S. cerevisiae: GO:0006605 protein targeting (loss)
c-value
0.8
0.7
0.6
0.5
0.4
0.3
0.2
0.1
0.0
p-value
2e-02
2e-02
3e-02
4e-02
5e-02
Log-odds
0.5
1.0
1.5
2.0
2.5
3.0
3.5
4.0
Cellular_organisms - ps1
Archaea1 - ps2
Archaea2 - ps3
Archaea3 - ps4
Asgard/Eukaryota - ps5
Eukaryota - ps6
Amorpheae - ps7
Obazoa - ps8
Opisthokonta - ps9
Holomycota - ps10
Fungi - ps11
Fungi_1 - ps12
Fungi_2 - ps13
Fungi_3 - ps14
Dikarya - ps15
Ascomycota - ps16
Saccharo/Pezizomycotina - ps17
Saccharomycotina - ps18
Saccharomycotina_1 - ps19
Saccharomycotina_2 - ps20
Saccharomycotina_3 - ps21
Phaff./Saccharomycetaceae - ps22
Saccharomyco./mycetaceae - ps23
Saccharomycetaceae - ps24
Saccharomycetaceae_2 - ps25
Saccharomycetaceae_3 - ps26
Kaz./Nau./Saccharomyces - ps27
Saccharomyces - ps28
Arboricola_clade - ps29
Cerevisiae_clade - ps30
Saccharomyces_cerevisiae - ps31
Phylostratum

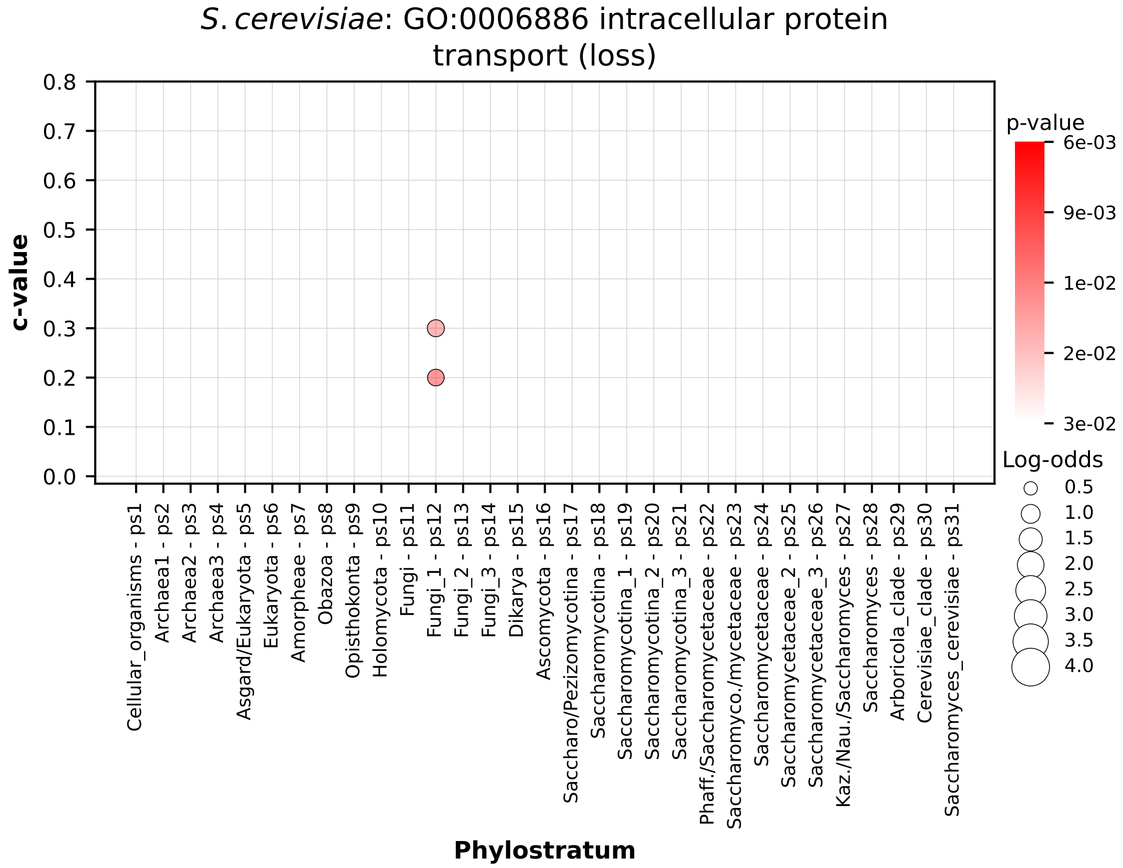

S. cerevisiae: GO:0006886 intracellular protein transport (loss)
c-value
0.8
0.7
0.6
0.5
0.4
0.3
0.2
0.1
0.0
p-value
6e-03
9e-03
1e-02
2e-02
3e-02
Log-odds
0.5
1.0
1.5
2.0
2.5
3.0
3.5
4.0
Cellular_organisms - ps1
Archaea1 - ps2
Archaea2 - ps3
Archaea3 - ps4
Asgard/Eukaryota - ps5
Eukaryota - ps6
Amorpheae - ps7
Obazoa - ps8
Opisthokonta - ps9
Holomycota - ps10
Fungi - ps11
Fungi_1 - ps12
Fungi_2 - ps13
Fungi_3 - ps14
Dikarya - ps15
Ascomycota - ps16
Saccharo/Pezizomycotina - ps17
Saccharomycotina - ps18
Saccharomycotina_1 - ps19
Saccharomycotina_2 - ps20
Saccharomycotina_3 - ps21
Phaff./Saccharomycetaceae - ps22
Saccharomyco./mycetaceae - ps23
Saccharomycetaceae - ps24
Saccharomycetaceae_2 - ps25
Saccharomycetaceae_3 - ps26
Kaz./Nau./Saccharomyces - ps27
Saccharomyces - ps28
Arboricola_clade - ps29
Cerevisiae_clade - ps30
Saccharomyces_cerevisiae - ps31
Phylostratum

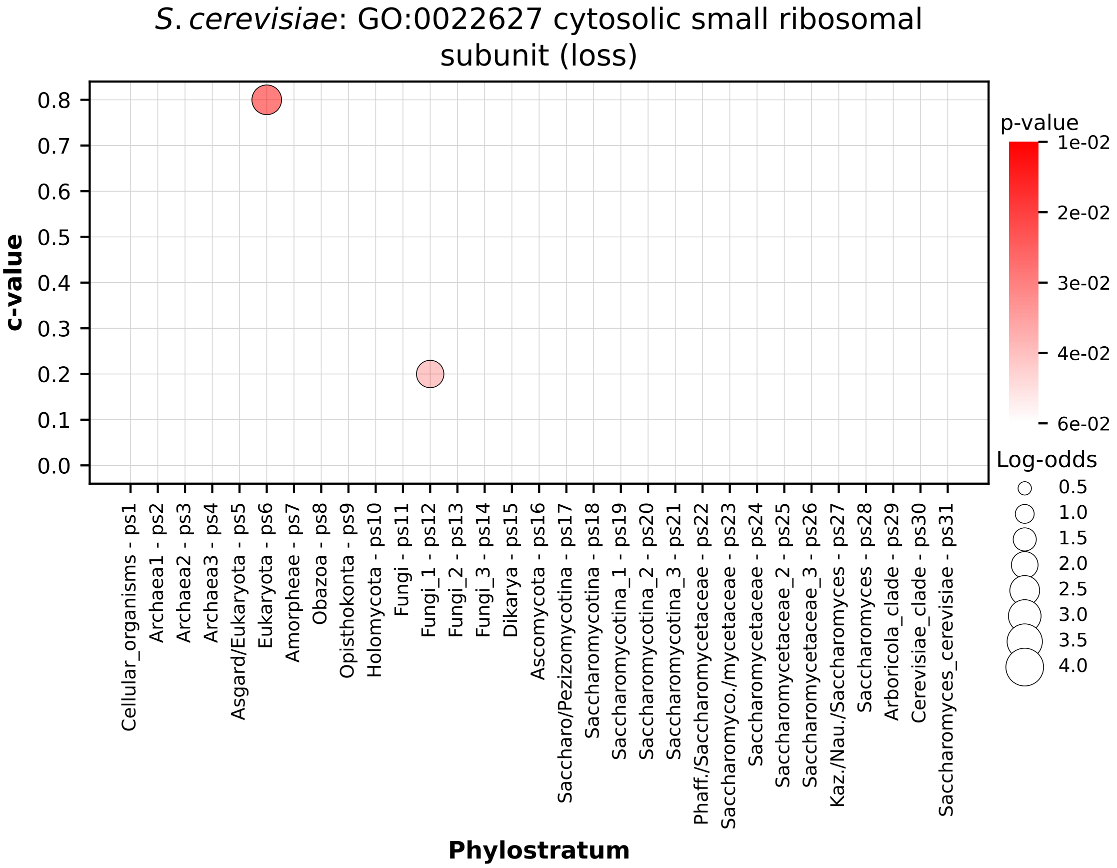
S. cerevisiae: GO:0022627 cytosolic small ribosomal subunit (loss)
c-value
0.8
0.7
0.6
0.5
0.4
0.3
0.2
0.1
0.0
Phylostratum
Cellular_organisms - ps1
Archaea1 - ps2
Archaea2 - ps3
Archaea3 - ps4
Asgard/Eukaryota - ps5
Eukaryota - ps6
Amorpheae - ps7
Obazoa - ps8
Opisthokonta - ps9
Holomycota - ps10
Fungi - ps11
Fungi_1 - ps12
Fungi_2 - ps13
Fungi_3 - ps14
Dikarya - ps15
Ascomycota - ps16
Saccharo/Pezizomycotina - ps17
Saccharomycotina - ps18
Saccharomycotina_1 - ps19
Saccharomycotina_2 - ps20
Saccharomycotina_3 - ps21
Phaff./Saccharomycetaceae - ps22
Saccharomyco./mycetaceae - ps23
Saccharomycetaceae - ps24
Saccharomycetaceae_2 - ps25
Saccharomycetaceae_3 - ps26
Kaz./Nau./Saccharomyces - ps27
Saccharomyces - ps28
Arboricola_clade - ps29
Cerevisiae_clade - ps30
Saccharomyces_cerevisiae - ps31
p-value
1e-02
2e-02
3e-02
4e-02
6e-02
Log-odds
0.5
1.0
1.5
2.0
2.5
3.0
3.5
4.0

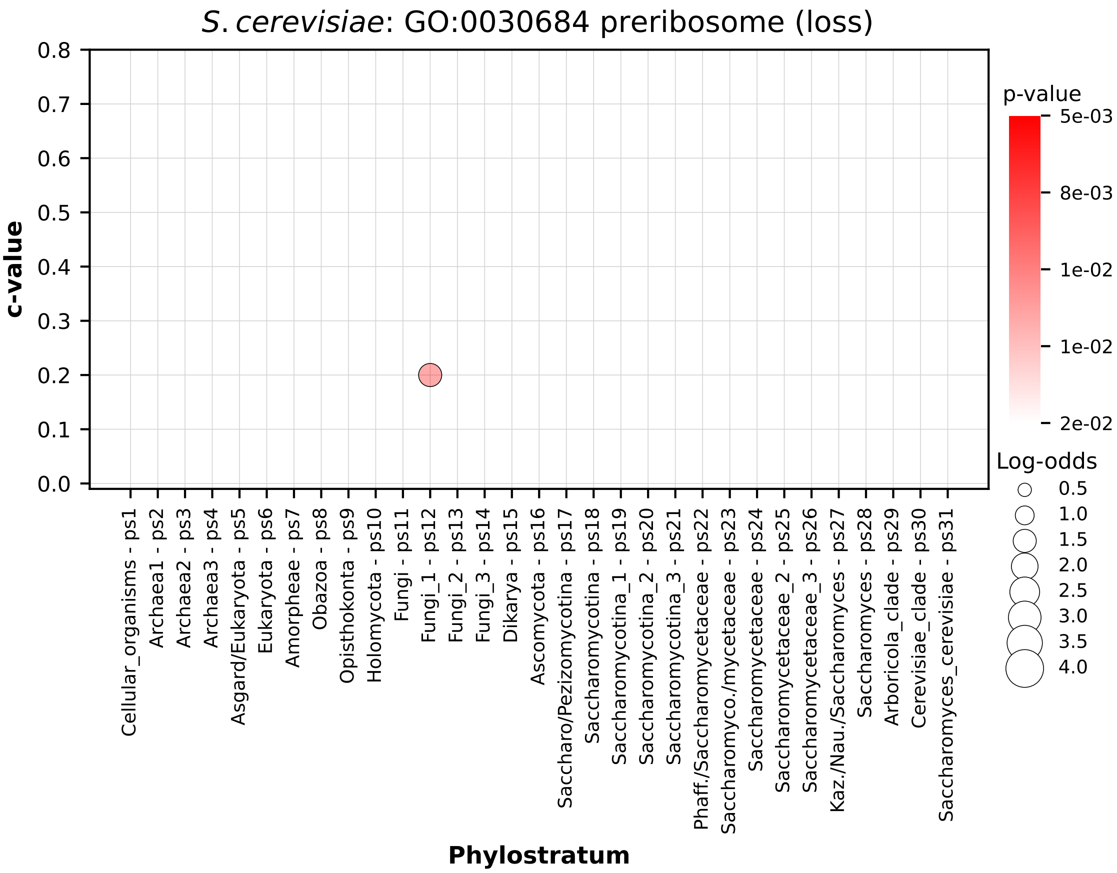
S. cerevisiae: GO:0030684 preribosome (loss)
c-value
0.0
0.1
0.2
0.3
0.4
0.5
0.6
0.7
0.8
p-value
5e-03
8e-03
1e-02
1e-02
2e-02
Log-odds
0.5
1.0
1.5
2.0
2.5
3.0
3.5
4.0
Cellular_organisms - ps1
Archaea1 - ps2
Archaea2 - ps3
Archaea3 - ps4
Asgard/Eukaryota - ps5
Eukaryota - ps6
Amorpheae - ps7
Obazoa - ps8
Opisthokonta - ps9
Holomycota - ps10
Fungi - ps11
Fungi_1 - ps12
Fungi_2 - ps13
Fungi_3 - ps14
Dikarya - ps15
Ascomycota - ps16
Saccharo/Pezizomycotina - ps17
Saccharomycotina - ps18
Saccharomycotina_1 - ps19
Saccharomycotina_2 - ps20
Saccharomycotina_3 - ps21
Phaff./Saccharomycetaceae - ps22
Saccharomyco./mycetaceae - ps23
Saccharomycetaceae - ps24
Saccharomycetaceae_2 - ps25
Saccharomycetaceae_3 - ps26
Kaz./Nau./Saccharomyces - ps27
Saccharomyces - ps28
Arboricola_clade - ps29
Cerevisiae_clade - ps30
Saccharomyces_cerevisiae - ps31
Phylostratum

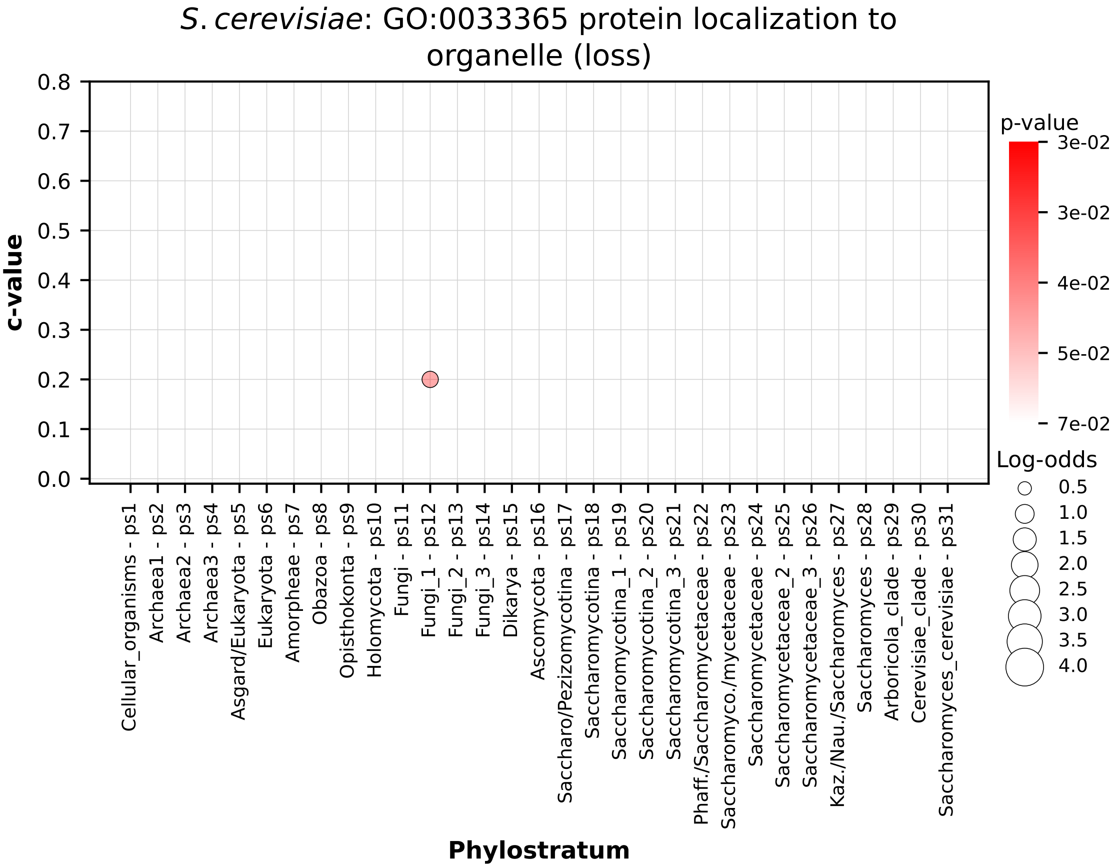

S. cerevisiae: GO:0033365 protein localization to organelle (loss)
c-value
0.0
0.1
0.2
0.3
0.4
0.5
0.6
0.7
0.8
p-value
3e-02
3e-02
4e-02
5e-02
7e-02
Log-odds
0.5
1.0
1.5
2.0
2.5
3.0
3.5
4.0
Cellular_organisms - ps1
Archaea1 - ps2
Archaea2 - ps3
Archaea3 - ps4
Asgard/Eukaryota - ps5
Eukaryota - ps6
Amorpheae - ps7
Obazoa - ps8
Opisthokonta - ps9
Holomycota - ps10
Fungi - ps11
Fungi_1 - ps12
Fungi_2 - ps13
Fungi_3 - ps14
Dikarya - ps15
Ascomycota - ps16
Saccharo/Pezizomycotina - ps17
Saccharomycotina - ps18
Saccharomycotina_1 - ps19
Saccharomycotina_2 - ps20
Saccharomycotina_3 - ps21
Phaff./Saccharomycetaceae - ps22
Saccharomyco./mycetaceae - ps23
Saccharomycetaceae - ps24
Saccharomycetaceae_2 - ps25
Saccharomycetaceae_3 - ps26
Kaz./Nau./Saccharomyces - ps27
Saccharomyces - ps28
Arboricola_clade - ps29
Cerevisiae_clade - ps30
Saccharomyces_cerevisiae - ps31
Phylostratum

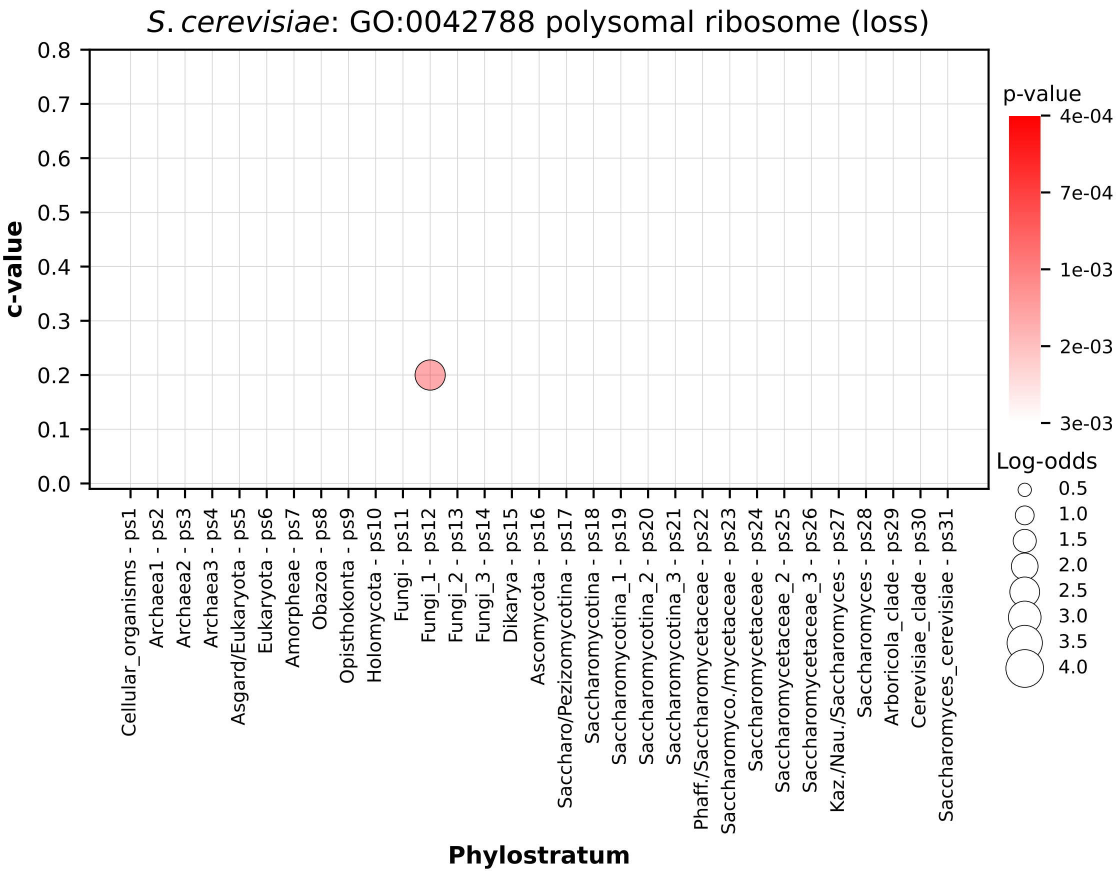
S. cerevisiae: GO:0042788 polysomal ribosome (loss)
c-value
0.8
0.7
0.6
0.5
0.4
0.3
0.2
0.1
0.0
p-value
4e-04
7e-04
1e-03
2e-03
3e-03
Log-odds
0.5
1.0
1.5
2.0
2.5
3.0
3.5
4.0
Cellular_organisms - ps1
Archaea1 - ps2
Archaea2 - ps3
Archaea3 - ps4
Asgard/Eukaryota - ps5
Eukaryota - ps6
Amorpheae - ps7
Obazoa - ps8
Opisthokonta - ps9
Holomycota - ps10
Fungi - ps11
Fungi_1 - ps12
Fungi_2 - ps13
Fungi_3 - ps14
Dikarya - ps15
Ascomycota - ps16
Saccharo/Pezizomycotina - ps17
Saccharomycotina - ps18
Saccharomycotina_1 - ps19
Saccharomycotina_2 - ps20
Saccharomycotina_3 - ps21
Phaff./Saccharomycetaceae - ps22
Saccharomyco./mycetaceae - ps23
Saccharomycetaceae - ps24
Saccharomycetaceae_2 - ps25
Saccharomycetaceae_3 - ps26
Kaz./Nau./Saccharomyces - ps27
Saccharomyces - ps28
Arboricola_clade - ps29
Cerevisiae_clade - ps30
Saccharomyces_cerevisiae - ps31
Phylostratum

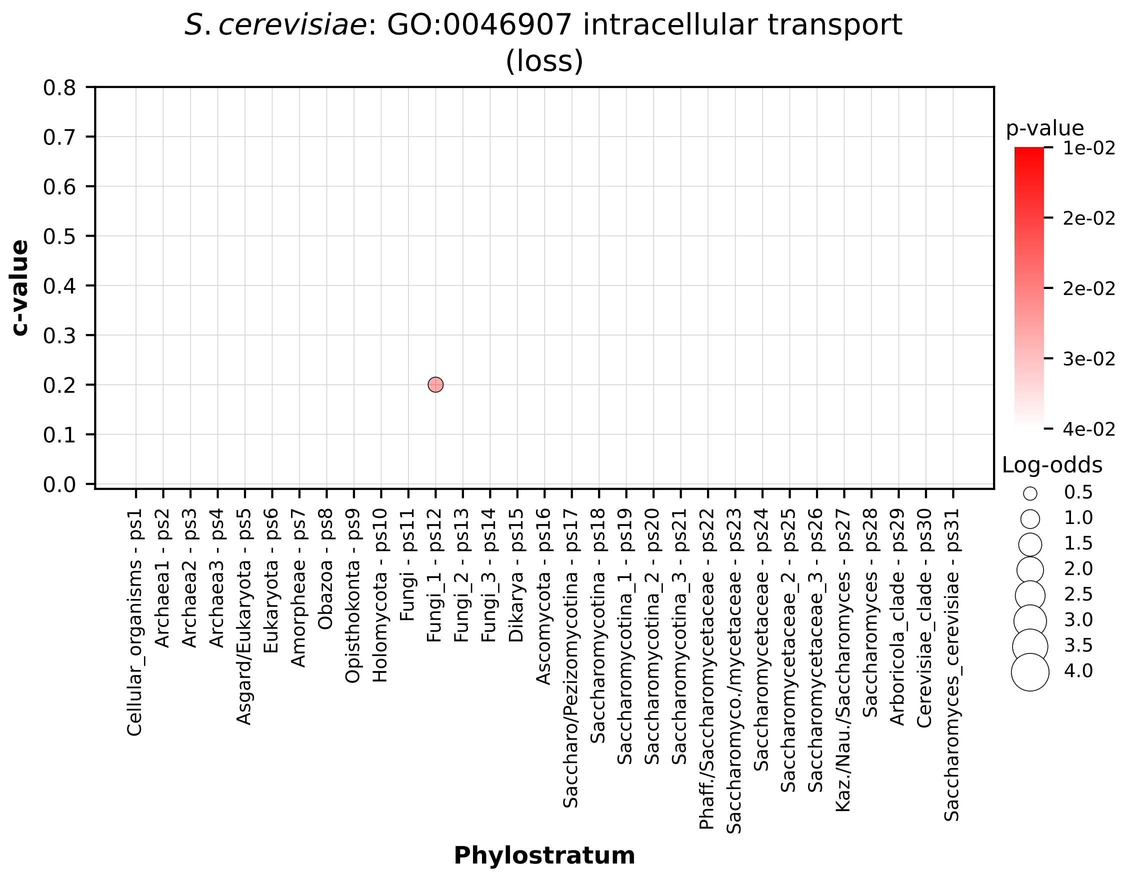
S. cerevisiae: GO:0046907 intracellular transport
(loss)
c-value
0.0
0.1
0.2
0.3
0.4
0.5
0.6
0.7
0.8
p-value
1e-02
2e-02
2e-02
3e-02
4e-02
Log-odds
0.5
1.0
1.5
2.0
2.5
3.0
3.5
4.0
Cellular_organisms - ps1
Archaea1 - ps2
Archaea2 - ps3
Archaea3 - ps4
Asgard/Eukaryota - ps5
Eukaryota - ps6
Amorpheae - ps7
Obazoa - ps8
Opisthokonta - ps9
Holomycota - ps10
Fungi - ps11
Fungi_1 - ps12
Fungi_2 - ps13
Fungi_3 - ps14
Dikarya - ps15
Ascomycota - ps16
Saccharo/Pezizomycotina - ps17
Saccharomycotina - ps18
Saccharomycotina_1 - ps19
Saccharomycotina_2 - ps20
Saccharomycotina_3 - ps21
Phaff./Saccharomycetaceae - ps22
Saccharomyco./mycetaceae - ps23
Saccharomycetaceae - ps24
Saccharomycetaceae_2 - ps25
Saccharomycetaceae_3 - ps26
Kaz./Nau./Saccharomyces - ps27
Saccharomyces - ps28
Arboricola_clade - ps29
Cerevisiae_clade - ps30
Saccharomyces_cerevisiae - ps31
Phylostratum

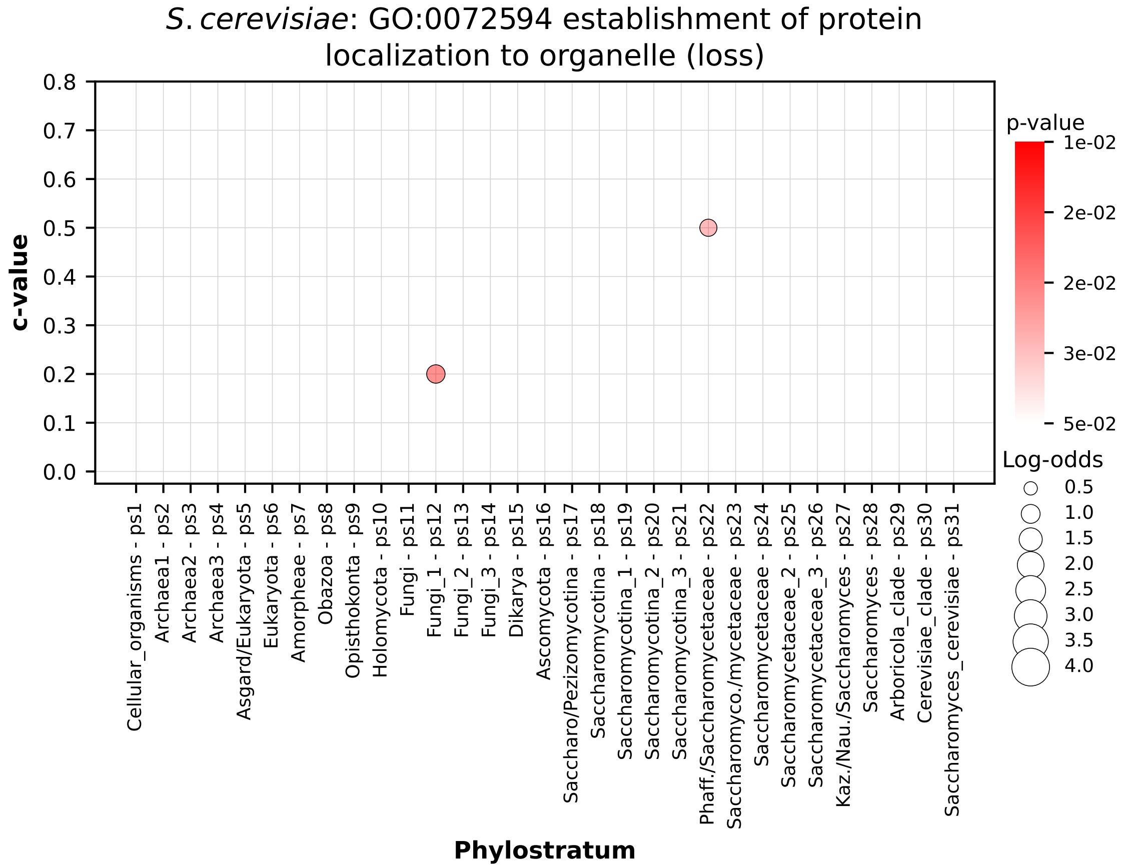

S. cerevisiae: GO:0072594 establishment of protein localization to organelle (loss)
c-value
0.0
0.1
0.2
0.3
0.4
0.5
0.6
0.7
0.8
p-value
1e-02
2e-02
2e-02
3e-02
5e-02
Log-odds
0.5
1.0
1.5
2.0
2.5
3.0
3.5
4.0
Cellular_organisms - ps1
Archaea1 - ps2
Archaea2 - ps3
Archaea3 - ps4
Asgard/Eukaryota - ps5
Eukaryota - ps6
Amorpheae - ps7
Obazoa - ps8
Opisthokonta - ps9
Holomycota - ps10
Fungi - ps11
Fungi_1 - ps12
Fungi_2 - ps13
Fungi_3 - ps14
Dikarya - ps15
Ascomycota - ps16
Saccharo/Pezizomycotina - ps17
Saccharomycotina - ps18
Saccharomycotina_1 - ps19
Saccharomycotina_2 - ps20
Saccharomycotina_3 - ps21
Phaff./Saccharomycetaceae - ps22
Saccharomyco./mycetaceae - ps23
Saccharomycetaceae - ps24
Saccharomycetaceae_2 - ps25
Saccharomycetaceae_3 - ps26
Kaz./Nau./Saccharomyces - ps27
Saccharomyces - ps28
Arboricola_clade - ps29
Cerevisiae_clade - ps30
Saccharomyces_cerevisiae - ps31
Phylostratum

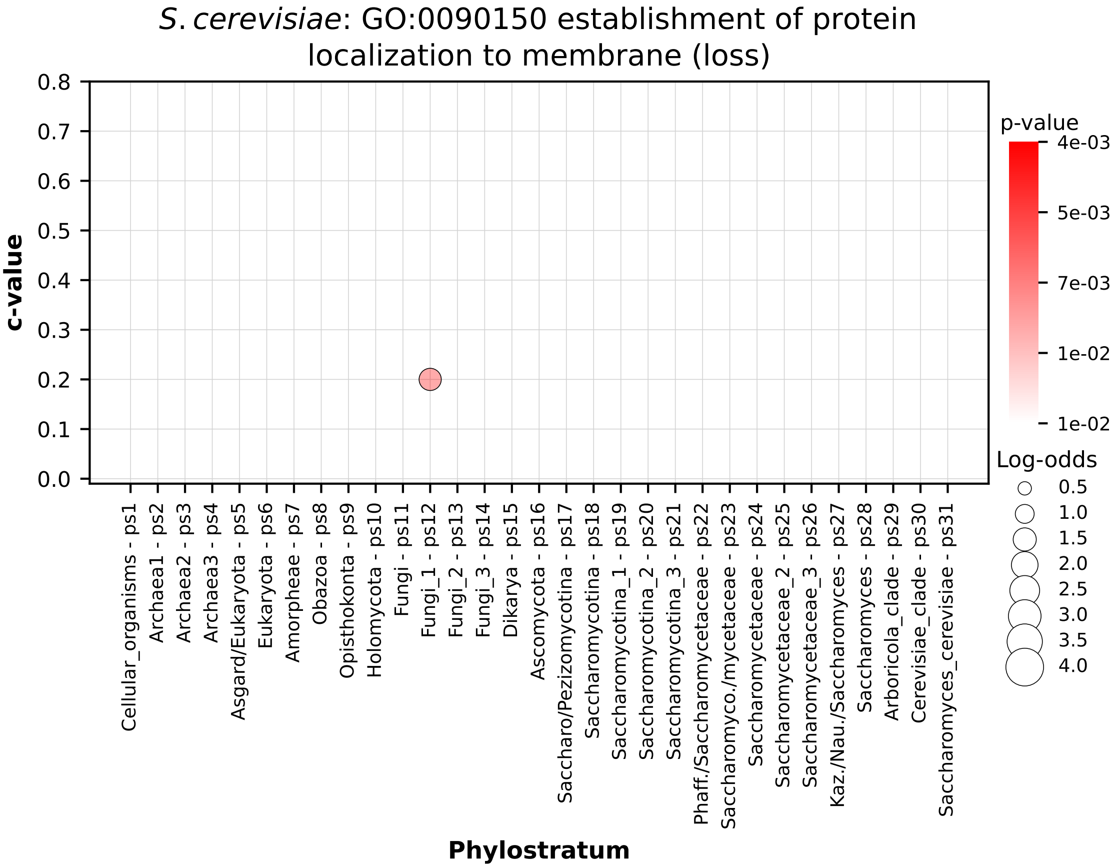

S. cerevisiae: GO:0090150 establishment of protein localization to membrane (loss)
c-value
0.8
0.7
0.6
0.5
0.4
0.3
0.2
0.1
0.0
p-value
4e-03
5e-03
7e-03
1e-02
1e-02
Log-odds
0.5
1.0
1.5
2.0
2.5
3.0
3.5
4.0
Cellular_organisms - ps1
Archaea1 - ps2
Archaea2 - ps3
Archaea3 - ps4
Asgard/Eukaryota - ps5
Eukaryota - ps6
Amorpheae - ps7
Obazoa - ps8
Opisthokonta - ps9
Holomycota - ps10
Fungi - ps11
Fungi_1 - ps12
Fungi_2 - ps13
Fungi_3 - ps14
Dikarya - ps15
Ascomycota - ps16
Saccharo/Pezizomycotina - ps17
Saccharomycotina - ps18
Saccharomycotina_1 - ps19
Saccharomycotina_2 - ps20
Saccharomycotina_3 - ps21
Phaff./Saccharomycetaceae - ps22
Saccharomyco./mycetaceae - ps23
Saccharomycetaceae - ps24
Saccharomycetaceae_2 - ps25
Saccharomycetaceae_3 - ps26
Kaz./Nau./Saccharomyces - ps27
Saccharomyces - ps28
Arboricola_clade - ps29
Cerevisiae_clade - ps30
Saccharomyces_cerevisiae - ps31
Phylostratum

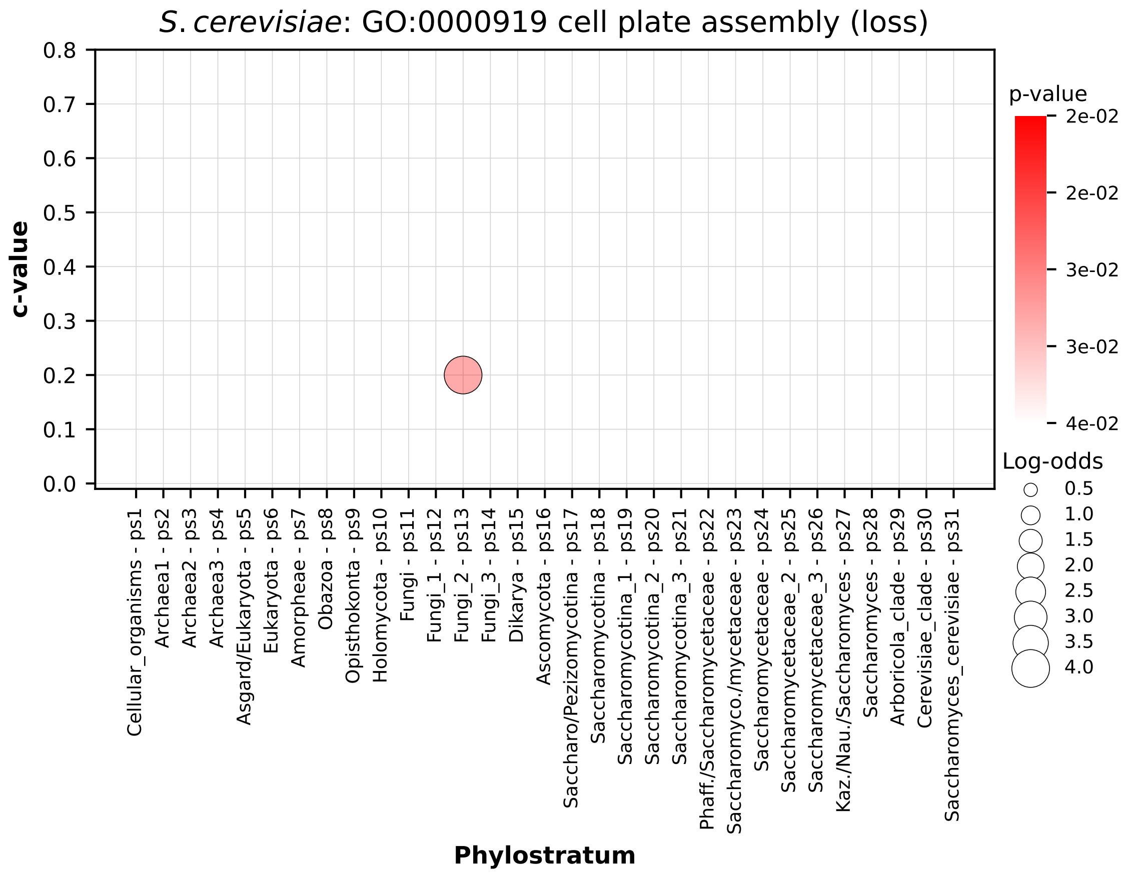

S. cerevisiae: GO:0000919 cell plate assembly (loss)
c-value
0.8
0.7
0.6
0.5
0.4
0.3
0.2
0.1
0.0
p-value
2e-02
2e-02
3e-02
3e-02
4e-02
Log-odds
0.5
1.0
1.5
2.0
2.5
3.0
3.5
4.0
Cellular_organisms - ps1
Archaea1 - ps2
Archaea2 - ps3
Archaea3 - ps4
Asgard/Eukaryota - ps5
Eukaryota - ps6
Amorpheae - ps7
Obazoa - ps8
Opisthokonta - ps9
Holomycota - ps10
Fungi - ps11
Fungi_1 - ps12
Fungi_2 - ps13
Fungi_3 - ps14
Dikarya - ps15
Ascomycota - ps16
Saccharo/Pezizomycotina - ps17
Saccharomycotina - ps18
Saccharomycotina_1 - ps19
Saccharomycotina_2 - ps20
Saccharomycotina_3 - ps21
Phaff./Saccharomycetaceae - ps22
Saccharomyco./mycetaceae - ps23
Saccharomycetaceae - ps24
Saccharomycetaceae_2 - ps25
Saccharomycetaceae_3 - ps26
Kaz./Nau./Saccharomyces - ps27
Saccharomyces - ps28
Arboricola_clade - ps29
Cerevisiae_clade - ps30
Saccharomyces_cerevisiae - ps31
Phylostratum

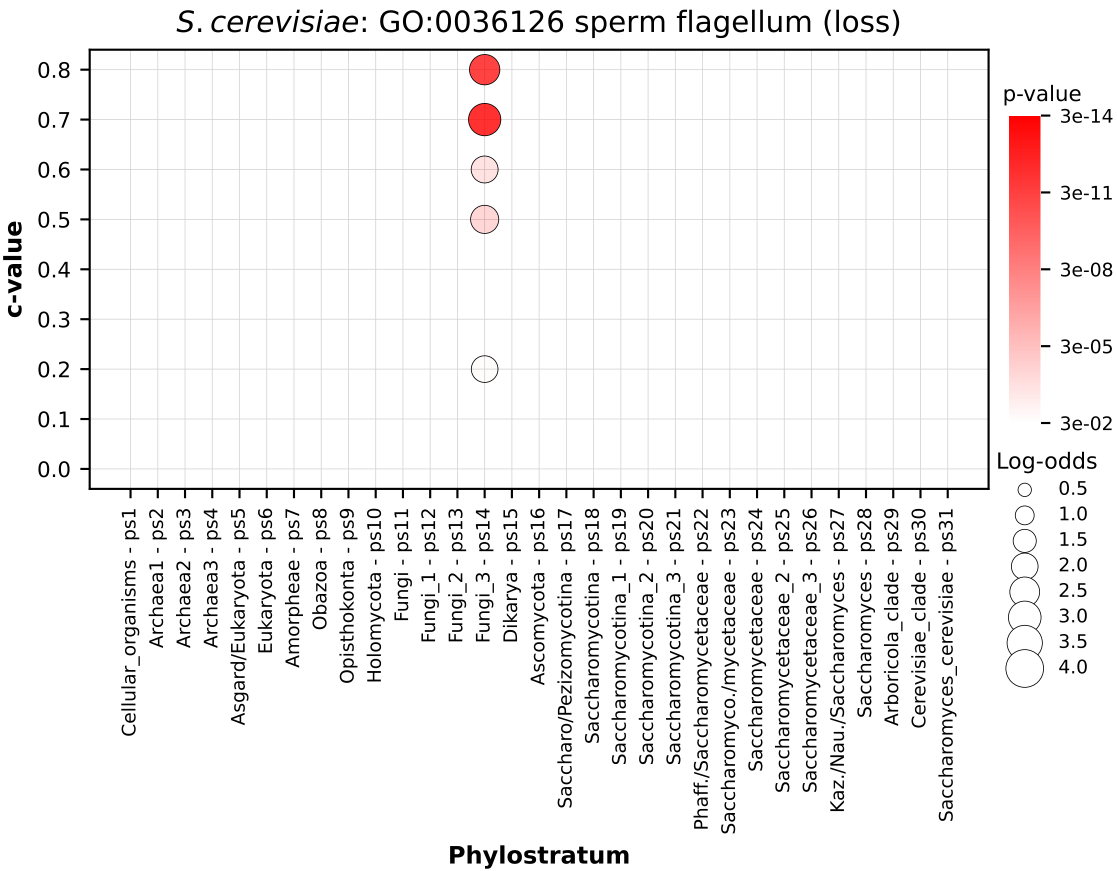

S. cerevisiae: GO:0036126 sperm flagellum (loss)
c-value
0.8
0.7
0.6
0.5
0.4
0.3
0.2
0.1
0.0
Phylostratum
Cellular_organisms - ps1
Archaea1 - ps2
Archaea2 - ps3
Archaea3 - ps4
Asgard/Eukaryota - ps5
Eukaryota - ps6
Amorpheae - ps7
Obazoa - ps8
Opisthokonta - ps9
Holomycota - ps10
Fungi - ps11
Fungi_1 - ps12
Fungi_2 - ps13
Fungi_3 - ps14
Dikarya - ps15
Ascomycota - ps16
Saccharo/Pezizomycotina - ps17
Saccharomycotina - ps18
Saccharomycotina_1 - ps19
Saccharomycotina_2 - ps20
Saccharomycotina_3 - ps21
Phaff./Saccharomycetaceae - ps22
Saccharomyco./mycetaceae - ps23
Saccharomycetaceae - ps24
Saccharomycetaceae_2 - ps25
Saccharomycetaceae_3 - ps26
Kaz./Nau./Saccharomyces - ps27
Saccharomyces - ps28
Arboricola_clade - ps29
Cerevisiae_clade - ps30
Saccharomyces_cerevisiae - ps31
p-value
3e-14
3e-11
3e-08
3e-05
3e-02
Log-odds
0.5
1.0
1.5
2.0
2.5
3.0
3.5
4.0

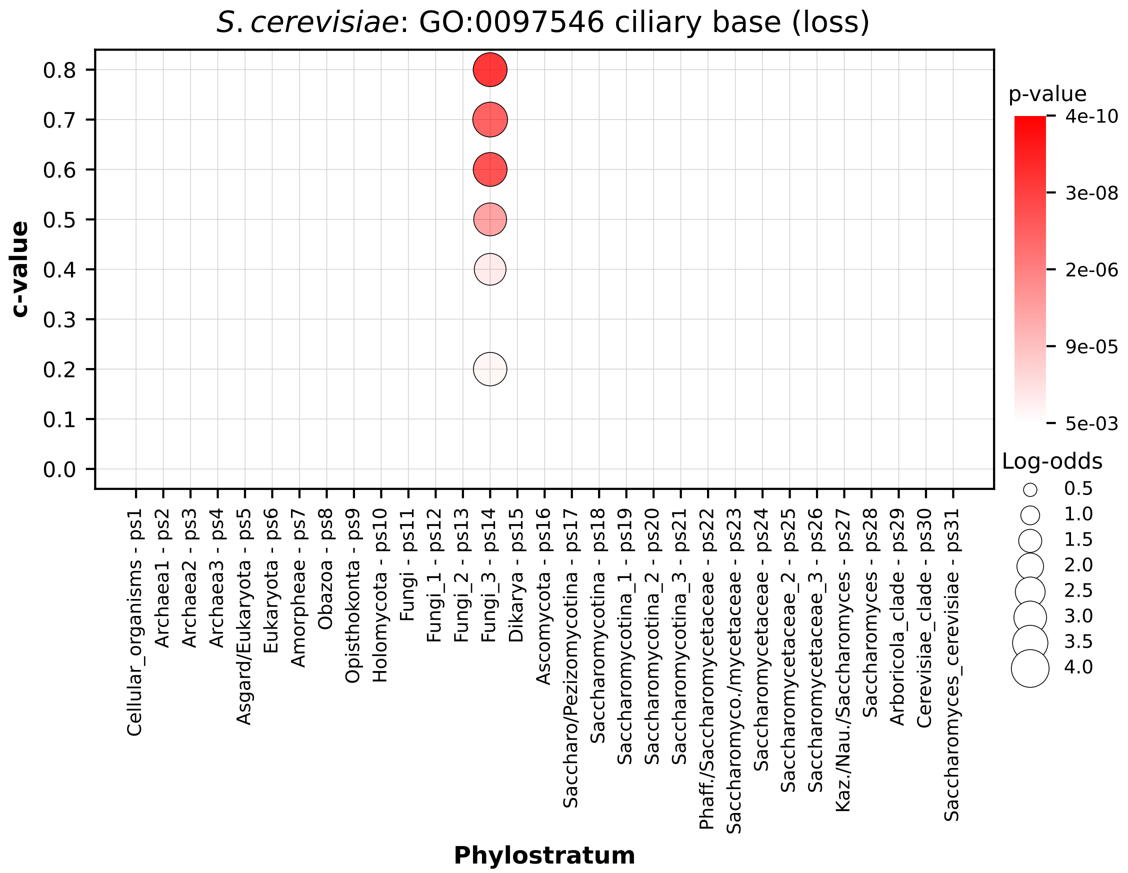

S. cerevisiae: GO:0097546 ciliary base (loss)
c-value
0.0
0.1
0.2
0.3
0.4
0.5
0.6
0.7
0.8
Phylostratum
Cellular_organisms - ps1
Archaea1 - ps2
Archaea2 - ps3
Archaea3 - ps4
Asgard/Eukaryota - ps5
Eukaryota - ps6
Amorpheae - ps7
Obazoa - ps8
Opisthokonta - ps9
Holomycota - ps10
Fungi - ps11
Fungi_1 - ps12
Fungi_2 - ps13
Fungi_3 - ps14
Dikarya - ps15
Ascomycota - ps16
Saccharo/Pezizomycotina - ps17
Saccharomycotina - ps18
Saccharomycotina_1 - ps19
Saccharomycotina_2 - ps20
Saccharomycotina_3 - ps21
Phaff./Saccharomycetaceae - ps22
Saccharomyco./mycetaceae - ps23
Saccharomycetaceae - ps24
Saccharomycetaceae_2 - ps25
Saccharomycetaceae_3 - ps26
Kaz./Nau./Saccharomyces - ps27
Saccharomyces - ps28
Arboricola_clade - ps29
Cerevisiae_clade - ps30
Saccharomyces_cerevisiae - ps31
p-value
4e-10
3e-08
2e-06
9e-05
5e-03
Log-odds
0.5
1.0
1.5
2.0
2.5
3.0
3.5
4.0

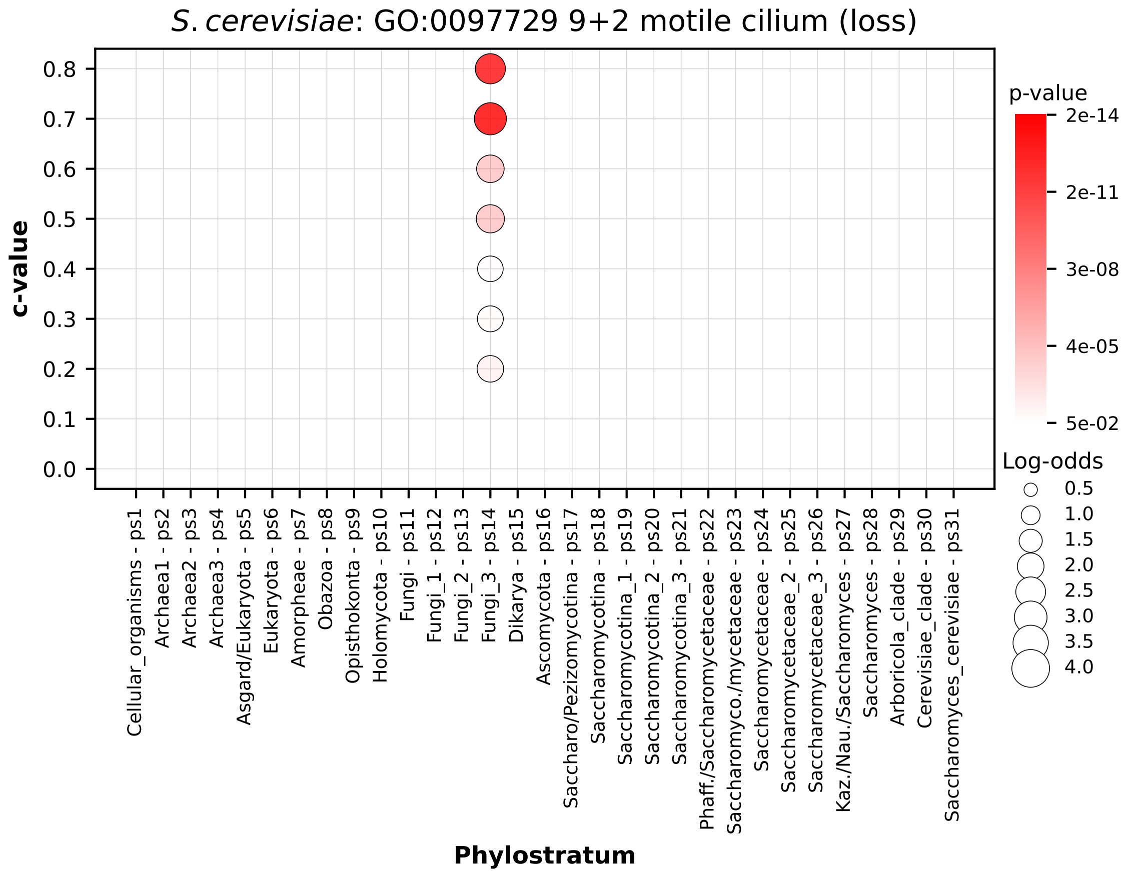
S. cerevisiae: GO:0097729 9+2 motile cilium (loss)
c-value
0.0
0.1
0.2
0.3
0.4
0.5
0.6
0.7
0.8
p-value
2e-14
2e-11
3e-08
4e-05
5e-02
Log-odds
0.5
1.0
1.5
2.0
2.5
3.0
3.5
4.0
Cellular_organisms - ps1
Archaea1 - ps2
Archaea2 - ps3
Archaea3 - ps4
Asgard/Eukaryota - ps5
Eukaryota - ps6
Amorpheae - ps7
Obazoa - ps8
Opisthokonta - ps9
Holomycota - ps10
Fungi - ps11
Fungi_1 - ps12
Fungi_2 - ps13
Fungi_3 - ps14
Dikarya - ps15
Ascomycota - ps16
Saccharo/Pezizomycotina - ps17
Saccharomycotina - ps18
Saccharomycotina_1 - ps19
Saccharomycotina_2 - ps20
Saccharomycotina_3 - ps21
Phaff./Saccharomycetaceae - ps22
Saccharomyco./mycetaceae - ps23
Saccharomycetaceae - ps24
Saccharomycetaceae_2 - ps25
Saccharomycetaceae_3 - ps26
Kaz./Nau./Saccharomyces - ps27
Saccharomyces - ps28
Arboricola_clade - ps29
Cerevisiae_clade - ps30
Saccharomyces_cerevisiae - ps31
Phylostratum

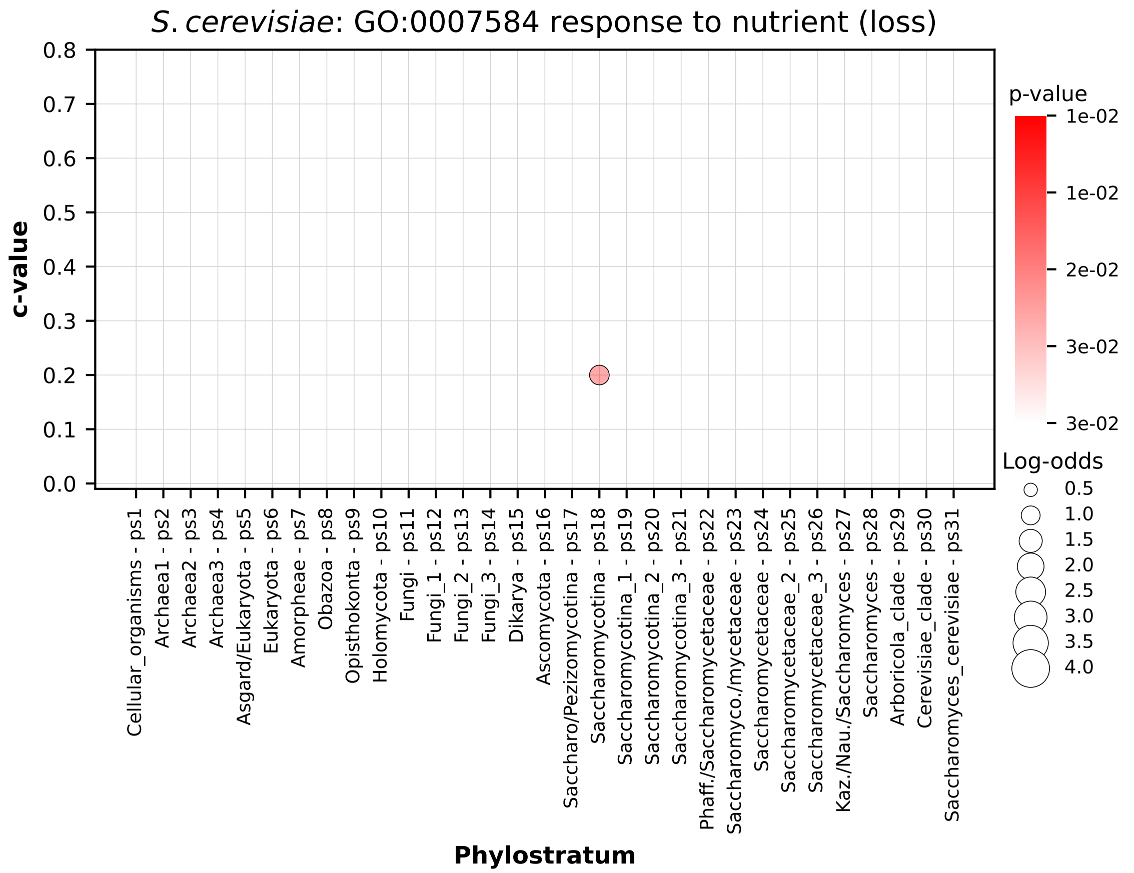

S. cerevisiae: GO:0007584 response to nutrient (loss)
c-value
0.8
0.7
0.6
0.5
0.4
0.3
0.2
0.1
0.0
p-value
1e-02
1e-02
2e-02
3e-02
3e-02
Log-odds
0.5
1.0
1.5
2.0
2.5
3.0
3.5
4.0
Cellular_organisms - ps1
Archaea1 - ps2
Archaea2 - ps3
Archaea3 - ps4
Asgard/Eukaryota - ps5
Eukaryota - ps6
Amorpheae - ps7
Obazoa - ps8
Opisthokonta - ps9
Holomycota - ps10
Fungi - ps11
Fungi_1 - ps12
Fungi_2 - ps13
Fungi_3 - ps14
Dikarya - ps15
Ascomycota - ps16
Saccharo/Pezizomycotina - ps17
Saccharomycotina - ps18
Saccharomycotina_1 - ps19
Saccharomycotina_2 - ps20
Saccharomycotina_3 - ps21
Phaff./Saccharomycetaceae - ps22
Saccharomyco./mycetaceae - ps23
Saccharomycetaceae - ps24
Saccharomycetaceae_2 - ps25
Saccharomycetaceae_3 - ps26
Kaz./Nau./Saccharomyces - ps27
Saccharomyces - ps28
Arboricola_clade - ps29
Cerevisiae_clade - ps30
Saccharomyces_cerevisiae - ps31
Phylostratum

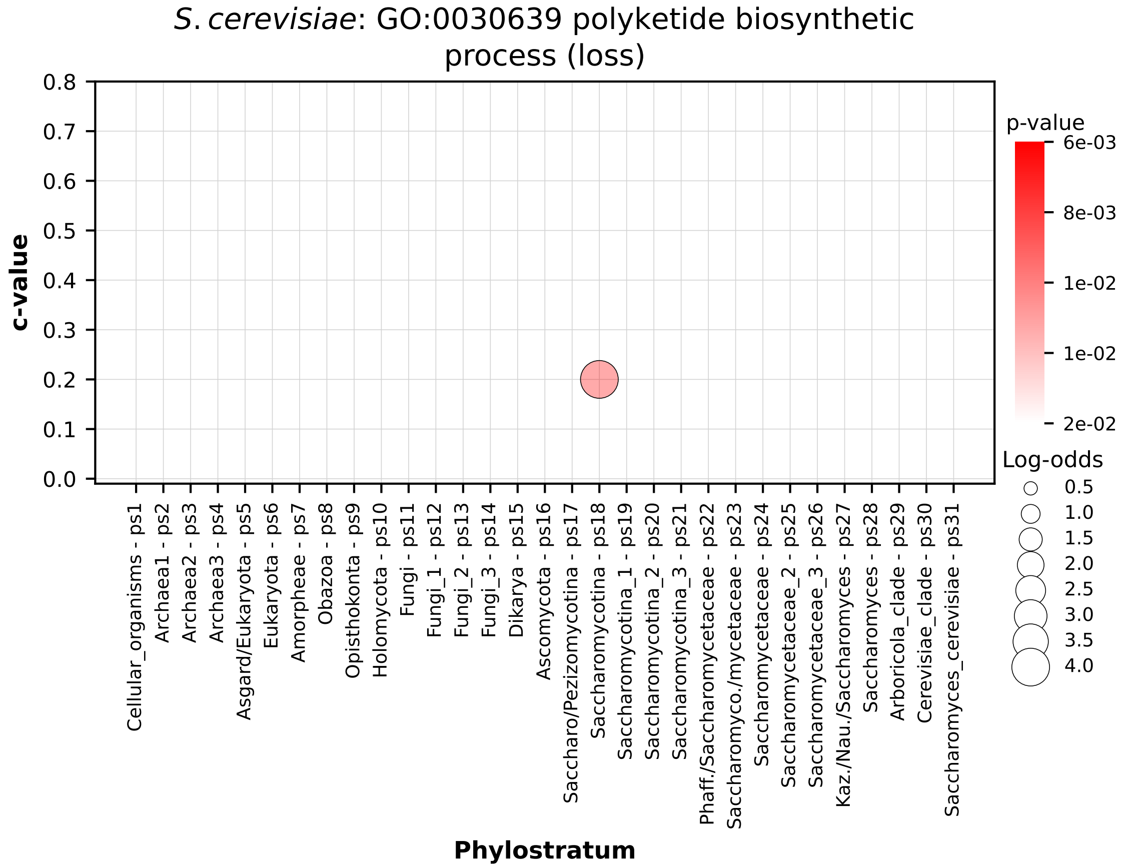

S. cerevisiae: GO:0030639 polyketide biosynthetic process (loss)
c-value
0.8
0.7
0.6
0.5
0.4
0.3
0.2
0.1
0.0
Phylostratum
Cellular_organisms - ps1
Archaea1 - ps2
Archaea2 - ps3
Archaea3 - ps4
Asgard/Eukaryota - ps5
Eukaryota - ps6
Amorpheae - ps7
Obazoa - ps8
Opisthokonta - ps9
Holomycota - ps10
Fungi - ps11
Fungi_1 - ps12
Fungi_2 - ps13
Fungi_3 - ps14
Dikarya - ps15
Ascomycota - ps16
Saccharo/Pezizomycotina - ps17
Saccharomycotina - ps18
Saccharomycotina_1 - ps19
Saccharomycotina_2 - ps20
Saccharomycotina_3 - ps21
Phaff./Saccharomycetaceae - ps22
Saccharomyco./mycetaceae - ps23
Saccharomycetaceae - ps24
Saccharomycetaceae_2 - ps25
Saccharomycetaceae_3 - ps26
Kaz./Nau./Saccharomyces - ps27
Saccharomyces - ps28
Arboricola_clade - ps29
Cerevisiae_clade - ps30
Saccharomyces_cerevisiae - ps31
p-value
6e-03
8e-03
1e-02
1e-02
2e-02
Log-odds
0.5
1.0
1.5
2.0
2.5
3.0
3.5
4.0

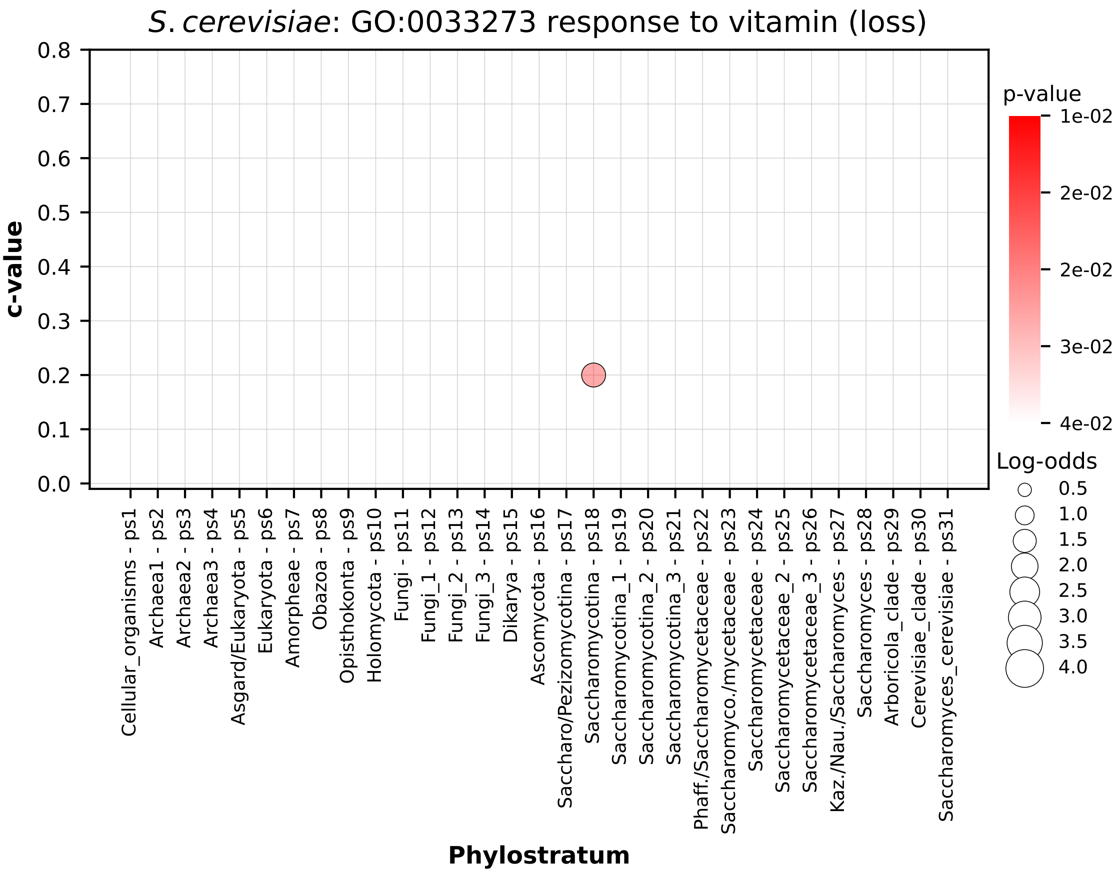
S. cerevisiae: GO:0033273 response to vitamin (loss)
c-value
0.0
0.1
0.2
0.3
0.4
0.5
0.6
0.7
0.8
Phylostratum
Cellular_organisms - ps1
Archaea1 - ps2
Archaea2 - ps3
Archaea3 - ps4
Asgard/Eukaryota - ps5
Eukaryota - ps6
Amorpheae - ps7
Obazoa - ps8
Opisthokonta - ps9
Holomycota - ps10
Fungi - ps11
Fungi_1 - ps12
Fungi_2 - ps13
Fungi_3 - ps14
Dikarya - ps15
Ascomycota - ps16
Saccharo/Pezizomycotina - ps17
Saccharomycotina - ps18
Saccharomycotina_1 - ps19
Saccharomycotina_2 - ps20
Saccharomycotina_3 - ps21
Phaff./Saccharomycetaceae - ps22
Saccharomyco./mycetaceae - ps23
Saccharomycetaceae - ps24
Saccharomycetaceae_2 - ps25
Saccharomycetaceae_3 - ps26
Kaz./Nau./Saccharomyces - ps27
Saccharomyces - ps28
Arboricola_clade - ps29
Cerevisiae_clade - ps30
Saccharomyces_cerevisiae - ps31
p-value
1e-02
2e-02
2e-02
3e-02
4e-02
Log-odds
0.5
1.0
1.5
2.0
2.5
3.0
3.5
4.0

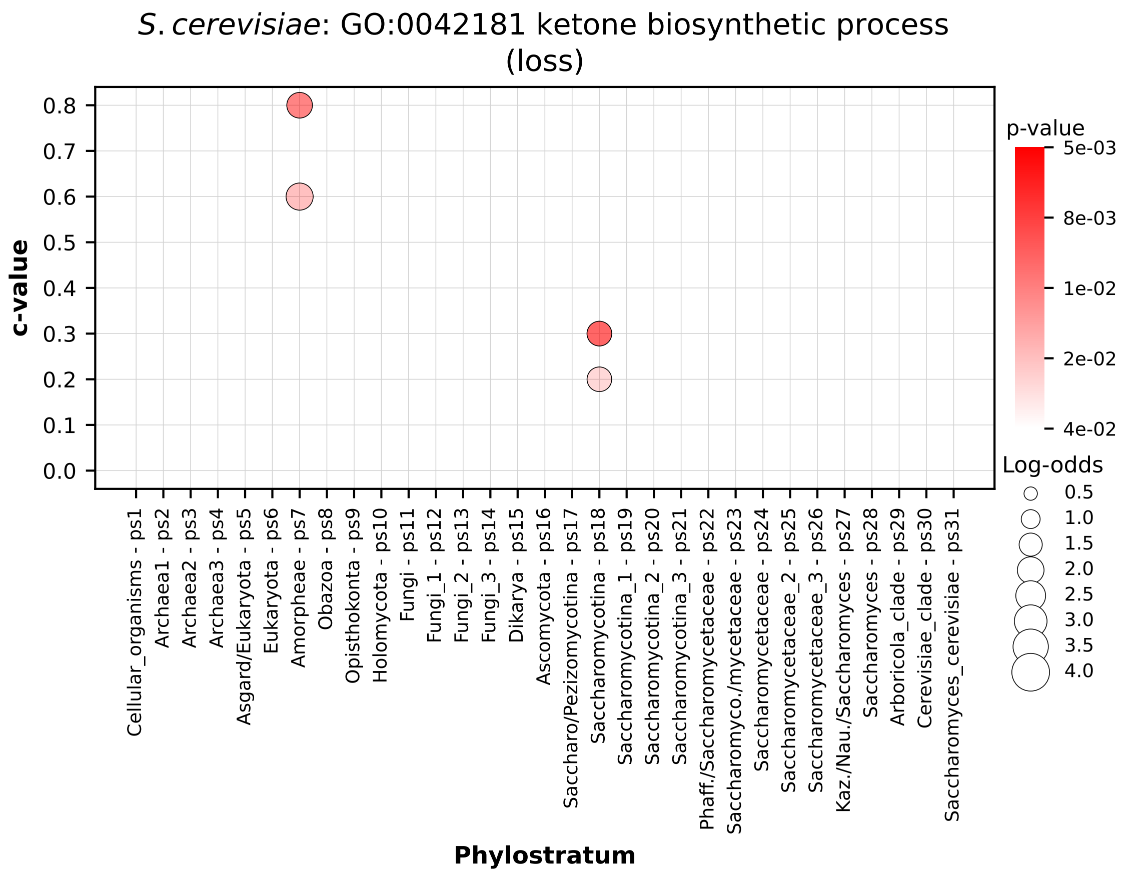
S. cerevisiae: GO:0042181 ketone biosynthetic process
(loss)
c-value
0.8
0.7
0.6
0.5
0.4
0.3
0.2
0.1
0.0
Phylostratum
Cellular_organisms - ps1
Archaea1 - ps2
Archaea2 - ps3
Archaea3 - ps4
Asgard/Eukaryota - ps5
Eukaryota - ps6
Amorpheae - ps7
Obazoa - ps8
Opisthokonta - ps9
Holomycota - ps10
Fungi - ps11
Fungi_1 - ps12
Fungi_2 - ps13
Fungi_3 - ps14
Dikarya - ps15
Ascomycota - ps16
Saccharo/Pezizomycotina - ps17
Saccharomycotina - ps18
Saccharomycotina_1 - ps19
Saccharomycotina_2 - ps20
Saccharomycotina_3 - ps21
Phaff./Saccharomycetaceae - ps22
Saccharomyco./mycetaceae - ps23
Saccharomycetaceae - ps24
Saccharomycetaceae_2 - ps25
Saccharomycetaceae_3 - ps26
Kaz./Nau./Saccharomyces - ps27
Saccharomyces - ps28
Arboricola_clade - ps29
Cerevisiae_clade - ps30
Saccharomyces_cerevisiae - ps31
p-value
5e-03
8e-03
1e-02
2e-02
4e-02
Log-odds
0.5
1.0
1.5
2.0
2.5
3.0
3.5
4.0

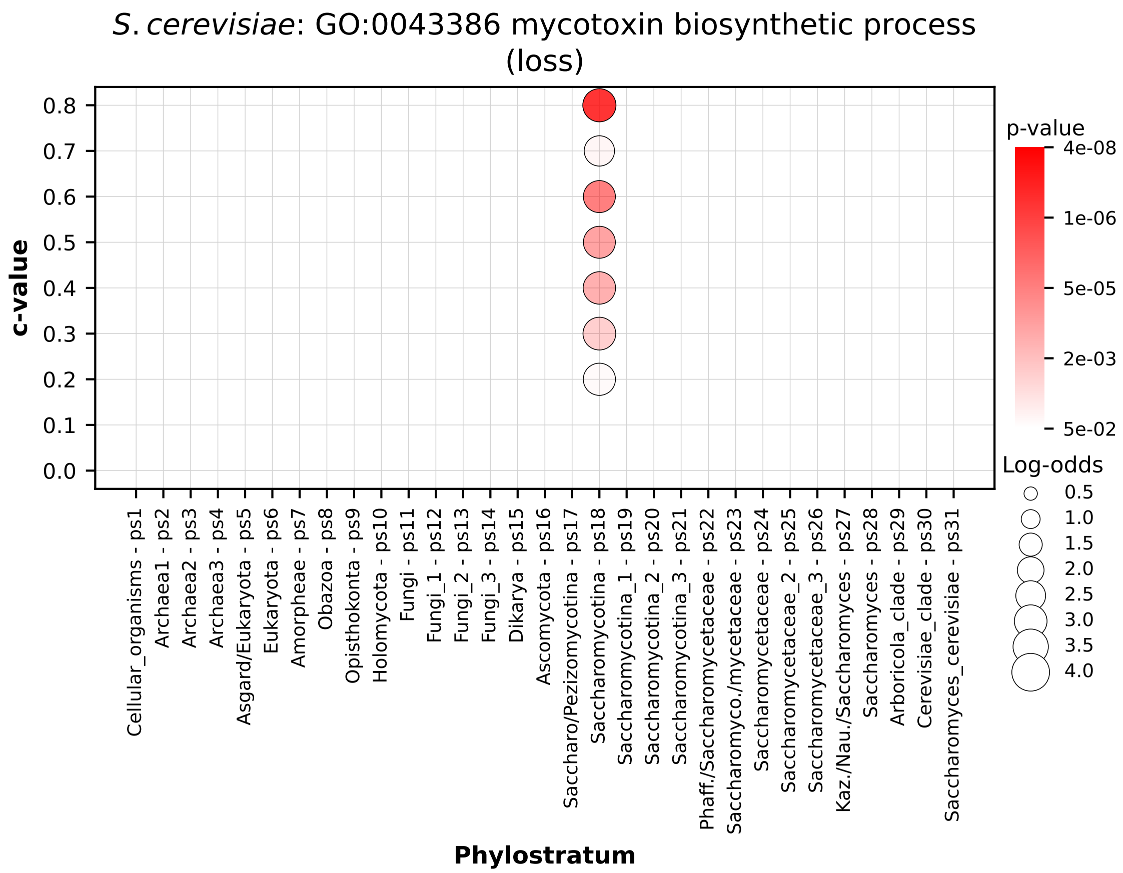

S. cerevisiae: GO:0043386 mycotoxin biosynthetic process
(loss)
c-value
0.8
0.7
0.6
0.5
0.4
0.3
0.2
0.1
0.0
p-value
4e-08
1e-06
5e-05
2e-03
5e-02
Log-odds
0.5
1.0
1.5
2.0
2.5
3.0
3.5
4.0
Cellular_organisms - ps1
Archaea1 - ps2
Archaea2 - ps3
Archaea3 - ps4
Asgard/Eukaryota - ps5
Eukaryota - ps6
Amorpheae - ps7
Obazoa - ps8
Opisthokonta - ps9
Holomycota - ps10
Fungi - ps11
Fungi_1 - ps12
Fungi_2 - ps13
Fungi_3 - ps14
Dikarya - ps15
Ascomycota - ps16
Saccharo/Pezizomycotina - ps17
Saccharomycotina - ps18
Saccharomycotina_1 - ps19
Saccharomycotina_2 - ps20
Saccharomycotina_3 - ps21
Phaff./Saccharomycetaceae - ps22
Saccharomyco./mycetaceae - ps23
Saccharomycetaceae - ps24
Saccharomycetaceae_2 - ps25
Saccharomycetaceae_3 - ps26
Kaz./Nau./Saccharomyces - ps27
Saccharomyces - ps28
Arboricola_clade - ps29
Cerevisiae_clade - ps30
Saccharomyces_cerevisiae - ps31
Phylostratum

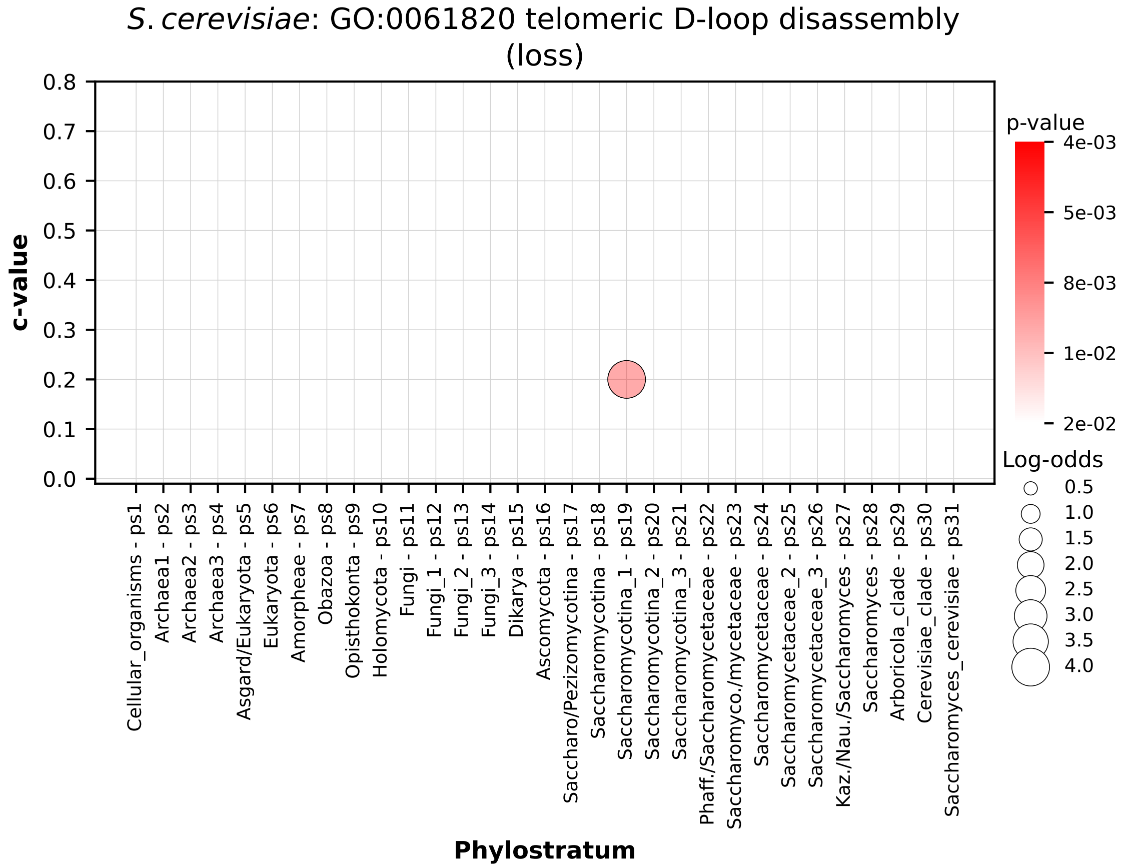

S. cerevisiae: GO:0061820 telomeric D-loop disassembly
(loss)
c-value
0.8
0.7
0.6
0.5
0.4
0.3
0.2
0.1
0.0
Phylostratum
Cellular_organisms - ps1
Archaea1 - ps2
Archaea2 - ps3
Archaea3 - ps4
Asgard/Eukaryota - ps5
Eukaryota - ps6
Amorpheae - ps7
Obazoa - ps8
Opisthokonta - ps9
Holomycota - ps10
Fungi - ps11
Fungi_1 - ps12
Fungi_2 - ps13
Fungi_3 - ps14
Dikarya - ps15
Ascomycota - ps16
Saccharo/Pezizomycotina - ps17
Saccharomycotina - ps18
Saccharomycotina_1 - ps19
Saccharomycotina_2 - ps20
Saccharomycotina_3 - ps21
Phaff./Saccharomycetaceae - ps22
Saccharomyco./mycetaceae - ps23
Saccharomycetaceae - ps24
Saccharomycetaceae_2 - ps25
Saccharomycetaceae_3 - ps26
Kaz./Nau./Saccharomyces - ps27
Saccharomyces - ps28
Arboricola_clade - ps29
Cerevisiae_clade - ps30
Saccharomyces_cerevisiae - ps31
p-value
4e-03
5e-03
8e-03
1e-02
2e-02
Log-odds
0.5
1.0
1.5
2.0
2.5
3.0
3.5
4.0

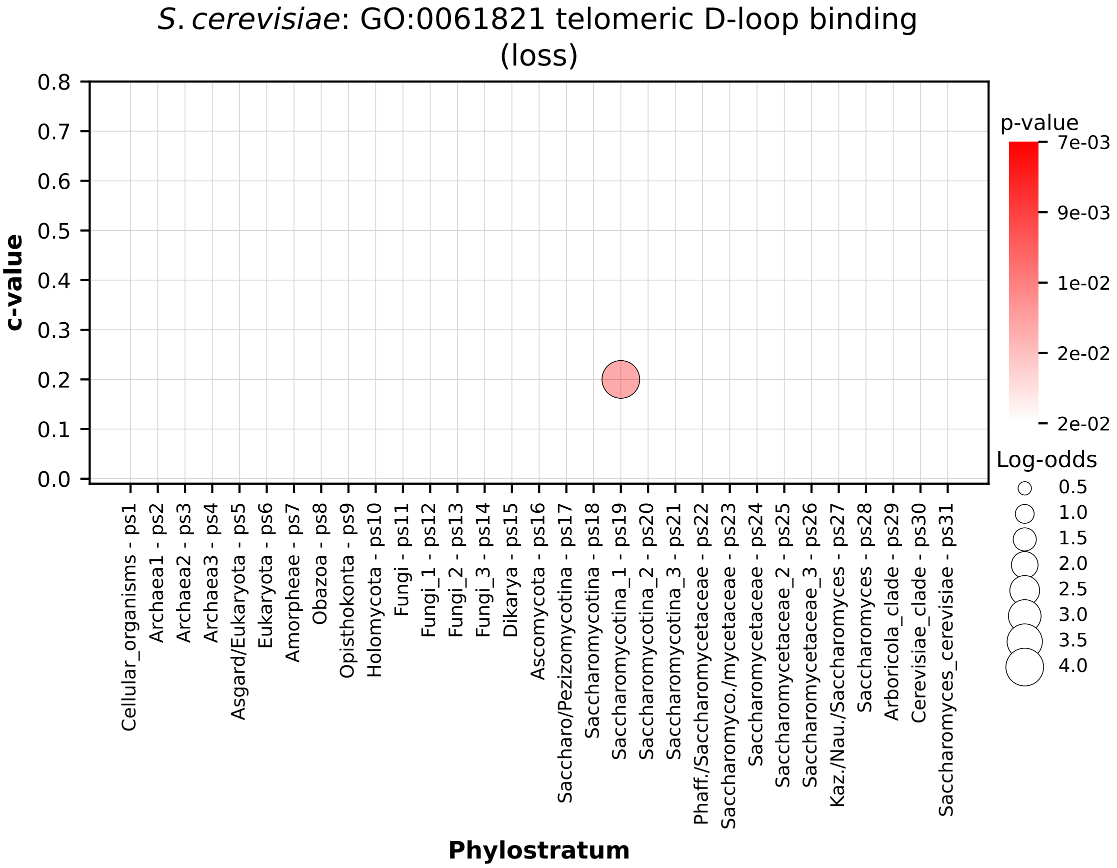

S. cerevisiae: GO:0061821 telomeric D-loop binding
(loss)
c-value
0.0
0.1
0.2
0.3
0.4
0.5
0.6
0.7
0.8
Phylostratum
Cellular_organisms - ps1
Archaea1 - ps2
Archaea2 - ps3
Archaea3 - ps4
Asgard/Eukaryota - ps5
Eukaryota - ps6
Amorpheae - ps7
Obazoa - ps8
Opisthokonta - ps9
Holomycota - ps10
Fungi - ps11
Fungi_1 - ps12
Fungi_2 - ps13
Fungi_3 - ps14
Dikarya - ps15
Ascomycota - ps16
Saccharo/Pezizomycotina - ps17
Saccharomycotina - ps18
Saccharomycotina_1 - ps19
Saccharomycotina_2 - ps20
Saccharomycotina_3 - ps21
Phaff./Saccharomycetaceae - ps22
Saccharomyco./mycetaceae - ps23
Saccharomycetaceae - ps24
Saccharomycetaceae_2 - ps25
Saccharomycetaceae_3 - ps26
Kaz./Nau./Saccharomyces - ps27
Saccharomyces - ps28
Arboricola_clade - ps29
Cerevisiae_clade - ps30
Saccharomyces_cerevisiae - ps31
p-value
7e-03
9e-03
1e-02
2e-02
2e-02
Log-odds
0.5
1.0
1.5
2.0
2.5
3.0
3.5
4.0

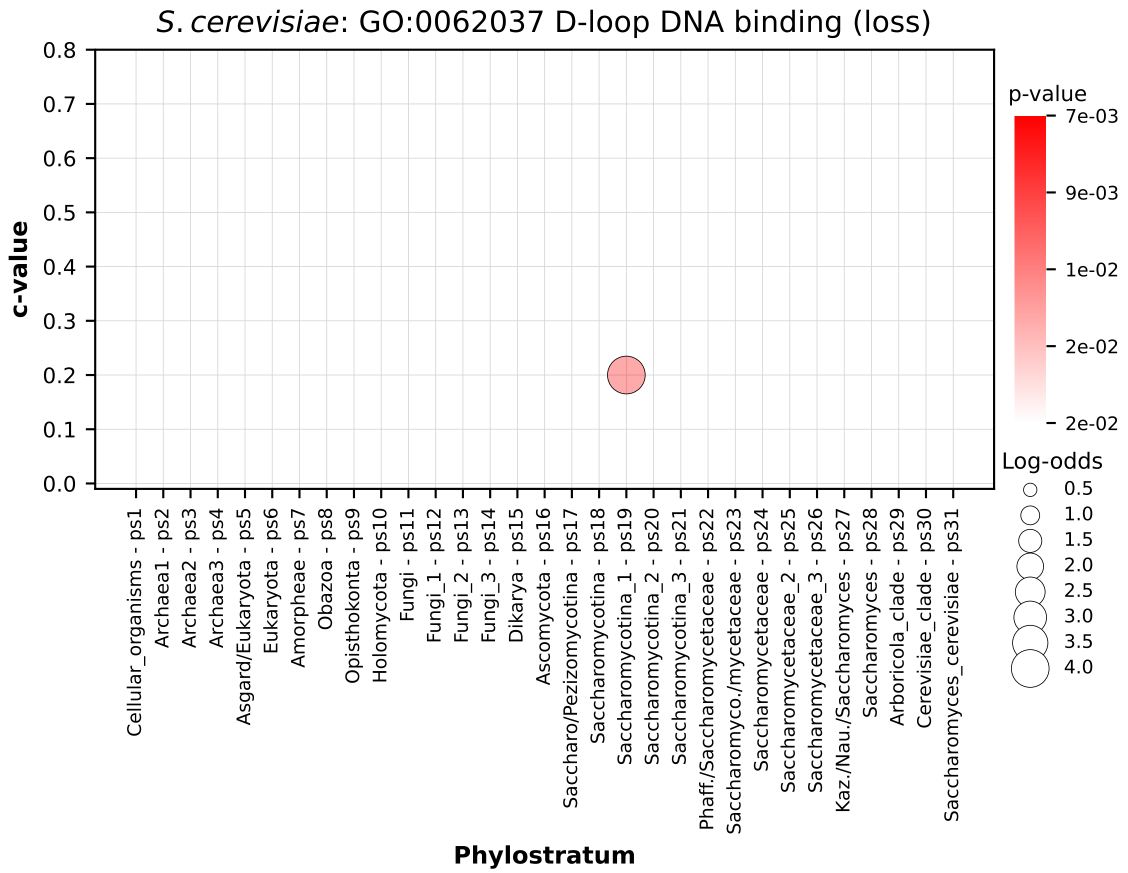

S. cerevisiae: GO:0062037 D-loop DNA binding (loss)
c-value
0.8
0.7
0.6
0.5
0.4
0.3
0.2
0.1
0.0
Phylostratum
Cellular_organisms - ps1
Archaea1 - ps2
Archaea2 - ps3
Archaea3 - ps4
Asgard/Eukaryota - ps5
Eukaryota - ps6
Amorpheae - ps7
Obazoa - ps8
Opisthokonta - ps9
Holomycota - ps10
Fungi - ps11
Fungi_1 - ps12
Fungi_2 - ps13
Fungi_3 - ps14
Dikarya - ps15
Ascomycota - ps16
Saccharo/Pezizomycotina - ps17
Saccharomycotina - ps18
Saccharomycotina_1 - ps19
Saccharomycotina_2 - ps20
Saccharomycotina_3 - ps21
Phaff./Saccharomycetaceae - ps22
Saccharomyco./mycetaceae - ps23
Saccharomycetaceae - ps24
Saccharomycetaceae_2 - ps25
Saccharomycetaceae_3 - ps26
Kaz./Nau./Saccharomyces - ps27
Saccharomyces - ps28
Arboricola_clade - ps29
Cerevisiae_clade - ps30
Saccharomyces_cerevisiae - ps31
p-value
7e-03
9e-03
1e-02
2e-02
2e-02
Log-odds
0.5
1.0
1.5
2.0
2.5
3.0
3.5
4.0

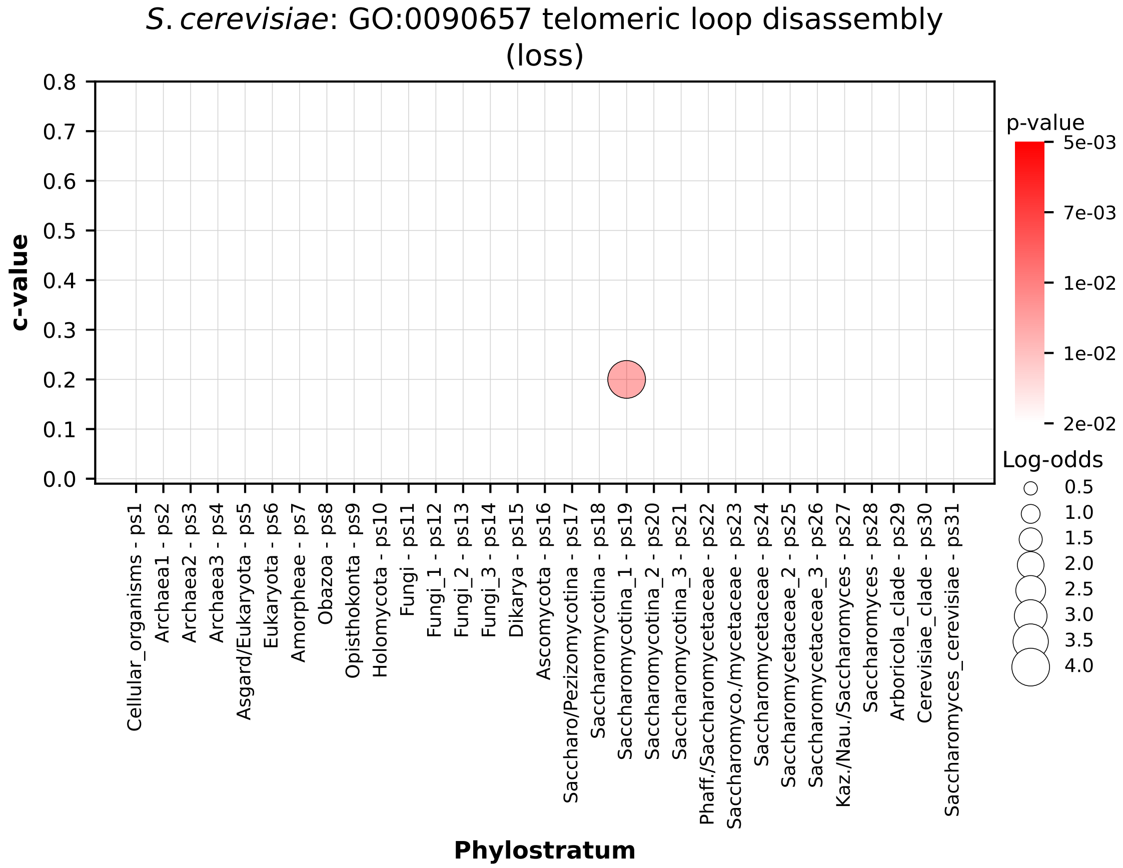
S. cerevisiae: GO:0090657 telomeric loop disassembly
(loss)
c-value
0.8
0.7
0.6
0.5
0.4
0.3
0.2
0.1
0.0
p-value
5e-03
7e-03
1e-02
1e-02
2e-02
Log-odds
0.5
1.0
1.5
2.0
2.5
3.0
3.5
4.0
Cellular_organisms - ps1
Archaea1 - ps2
Archaea2 - ps3
Archaea3 - ps4
Asgard/Eukaryota - ps5
Eukaryota - ps6
Amorpheae - ps7
Obazoa - ps8
Opisthokonta - ps9
Holomycota - ps10
Fungi - ps11
Fungi_1 - ps12
Fungi_2 - ps13
Fungi_3 - ps14
Dikarya - ps15
Ascomycota - ps16
Saccharo/Pezizomycotina - ps17
Saccharomycotina - ps18
Saccharomycotina_1 - ps19
Saccharomycotina_2 - ps20
Saccharomycotina_3 - ps21
Phaff./Saccharomycetaceae - ps22
Saccharomyco./mycetaceae - ps23
Saccharomycetaceae - ps24
Saccharomycetaceae_2 - ps25
Saccharomycetaceae_3 - ps26
Kaz./Nau./Saccharomyces - ps27
Saccharomyces - ps28
Arboricola_clade - ps29
Cerevisiae_clade - ps30
Saccharomyces_cerevisiae - ps31
Phylostratum

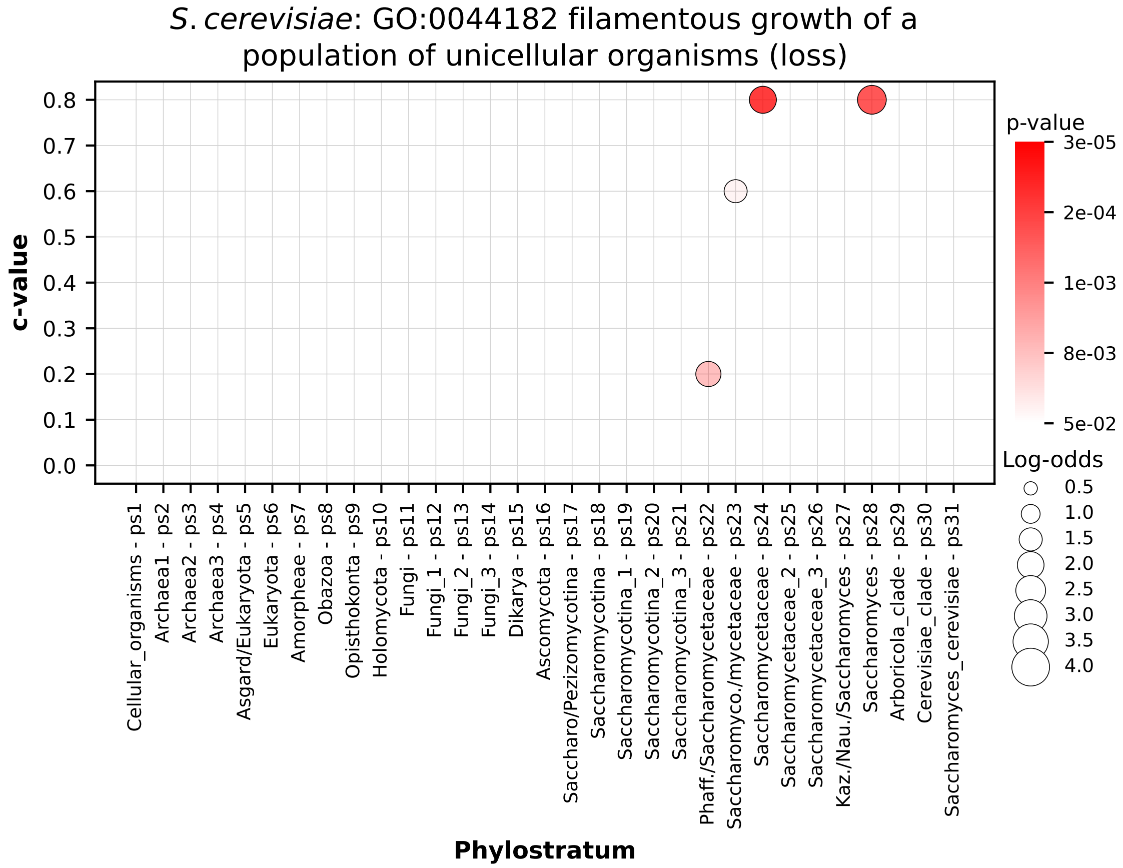

S. cerevisiae: GO:0044182 filamentous growth of a population of unicellular organisms (loss)
c-value
0.0
0.1
0.2
0.3
0.4
0.5
0.6
0.7
0.8
Phylostratum
Cellular_organisms - ps1
Archaea1 - ps2
Archaea2 - ps3
Archaea3 - ps4
Asgard/Eukaryota - ps5
Eukaryota - ps6
Amorpheae - ps7
Obazoa - ps8
Opisthokonta - ps9
Holomycota - ps10
Fungi - ps11
Fungi_1 - ps12
Fungi_2 - ps13
Fungi_3 - ps14
Dikarya - ps15
Ascomycota - ps16
Saccharo/Pezizomycotina - ps17
Saccharomycotina - ps18
Saccharomycotina_1 - ps19
Saccharomycotina_2 - ps20
Saccharomycotina_3 - ps21
Phaff./Saccharomycetaceae - ps22
Saccharomyco./mycetaceae - ps23
Saccharomycetaceae - ps24
Saccharomycetaceae_2 - ps25
Saccharomycetaceae_3 - ps26
Kaz./Nau./Saccharomyces - ps27
Saccharomyces - ps28
Arboricola_clade - ps29
Cerevisiae_clade - ps30
Saccharomyces_cerevisiae - ps31
p-value
3e-05
2e-04
1e-03
8e-03
5e-02
Log-odds
0.5
1.0
1.5
2.0
2.5
3.0
3.5
4.0

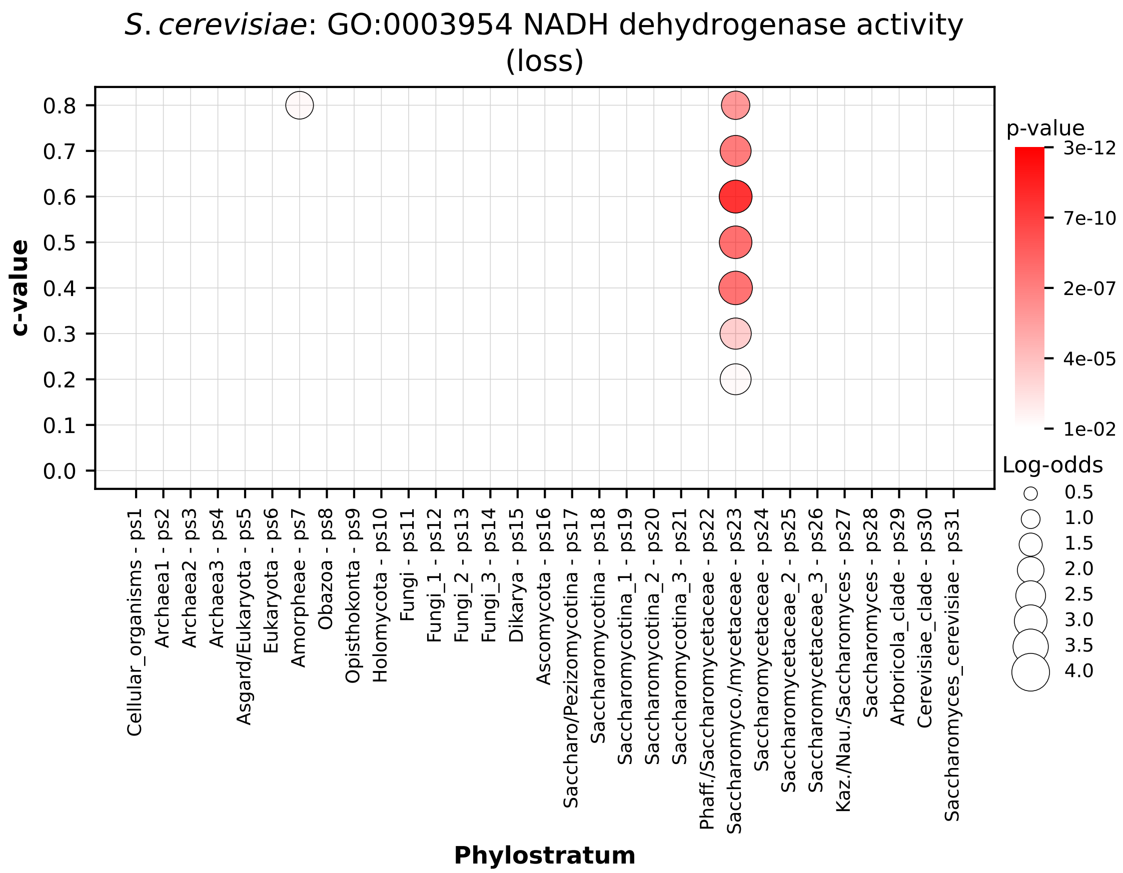
S. cerevisiae: GO:0003954 NADH dehydrogenase activity
(loss)
c-value
0.0
0.1
0.2
0.3
0.4
0.5
0.6
0.7
0.8
p-value
3e-12
7e-10
2e-07
4e-05
1e-02
Log-odds
0.5
1.0
1.5
2.0
2.5
3.0
3.5
4.0
Cellular_organisms - ps1
Archaea1 - ps2
Archaea2 - ps3
Archaea3 - ps4
Asgard/Eukaryota - ps5
Eukaryota - ps6
Amorpheae - ps7
Obazoa - ps8
Opisthokonta - ps9
Holomycota - ps10
Fungi - ps11
Fungi_1 - ps12
Fungi_2 - ps13
Fungi_3 - ps14
Dikarya - ps15
Ascomycota - ps16
Saccharo/Pezizomycotina - ps17
Saccharomycotina - ps18
Saccharomycotina_1 - ps19
Saccharomycotina_2 - ps20
Saccharomycotina_3 - ps21
Phaff./Saccharomycetaceae - ps22
Saccharomyco./mycetaceae - ps23
Saccharomycetaceae - ps24
Saccharomycetaceae_2 - ps25
Saccharomycetaceae_3 - ps26
Kaz./Nau./Saccharomyces - ps27
Saccharomyces - ps28
Arboricola_clade - ps29
Cerevisiae_clade - ps30
Saccharomyces_cerevisiae - ps31
Phylostratum

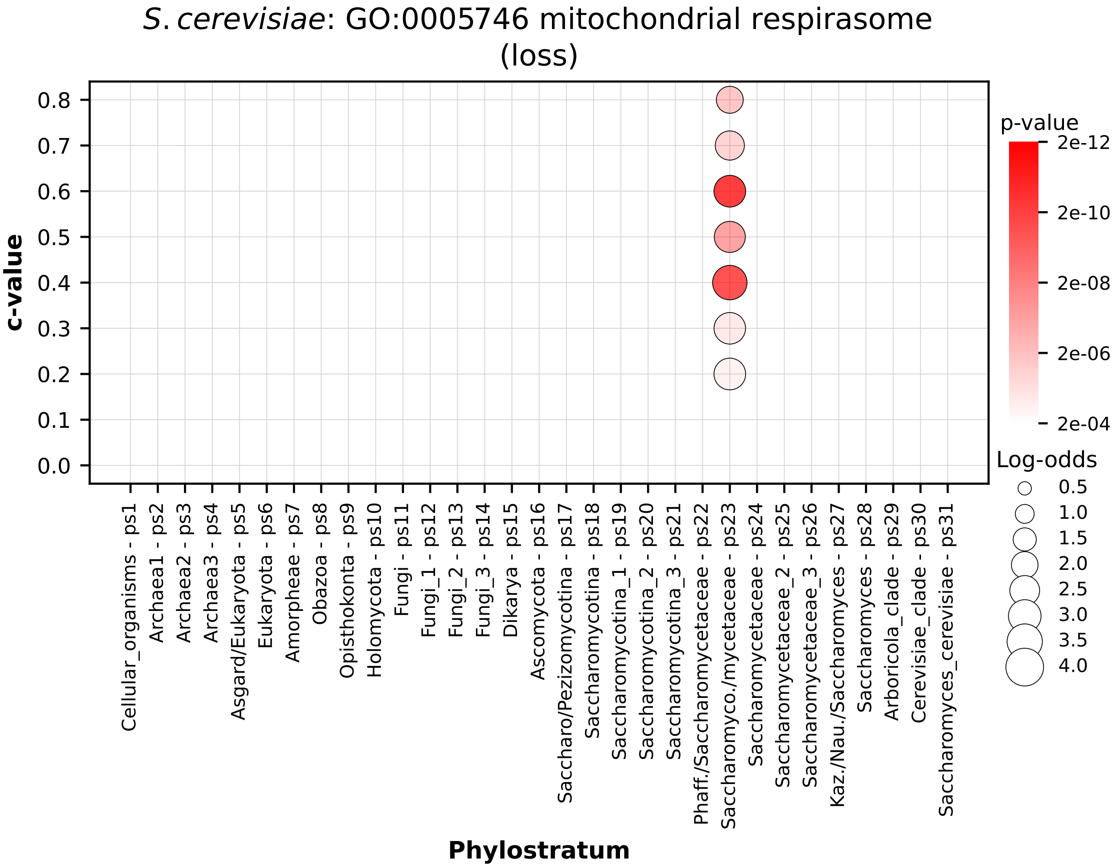

S. cerevisiae: GO:0005746 mitochondrial respirasome
(loss)
c-value
0.0
0.1
0.2
0.3
0.4
0.5
0.6
0.7
0.8
Phylostratum
Cellular_organisms - ps1
Archaea1 - ps2
Archaea2 - ps3
Archaea3 - ps4
Asgard/Eukaryota - ps5
Eukaryota - ps6
Amorpheae - ps7
Obazoa - ps8
Opisthokonta - ps9
Holomycota - ps10
Fungi - ps11
Fungi_1 - ps12
Fungi_2 - ps13
Fungi_3 - ps14
Dikarya - ps15
Ascomycota - ps16
Saccharo/Pezizomycotina - ps17
Saccharomycotina - ps18
Saccharomycotina_1 - ps19
Saccharomycotina_2 - ps20
Saccharomycotina_3 - ps21
Phaff./Saccharomycetaceae - ps22
Saccharomyco./mycetaceae - ps23
Saccharomycetaceae - ps24
Saccharomycetaceae_2 - ps25
Saccharomycetaceae_3 - ps26
Kaz./Nau./Saccharomyces - ps27
Saccharomyces - ps28
Arboricola_clade - ps29
Cerevisiae_clade - ps30
Saccharomyces_cerevisiae - ps31
p-value
2e-12
2e-10
2e-08
2e-06
2e-04
Log-odds
0.5
1.0
1.5
2.0
2.5
3.0
3.5
4.0

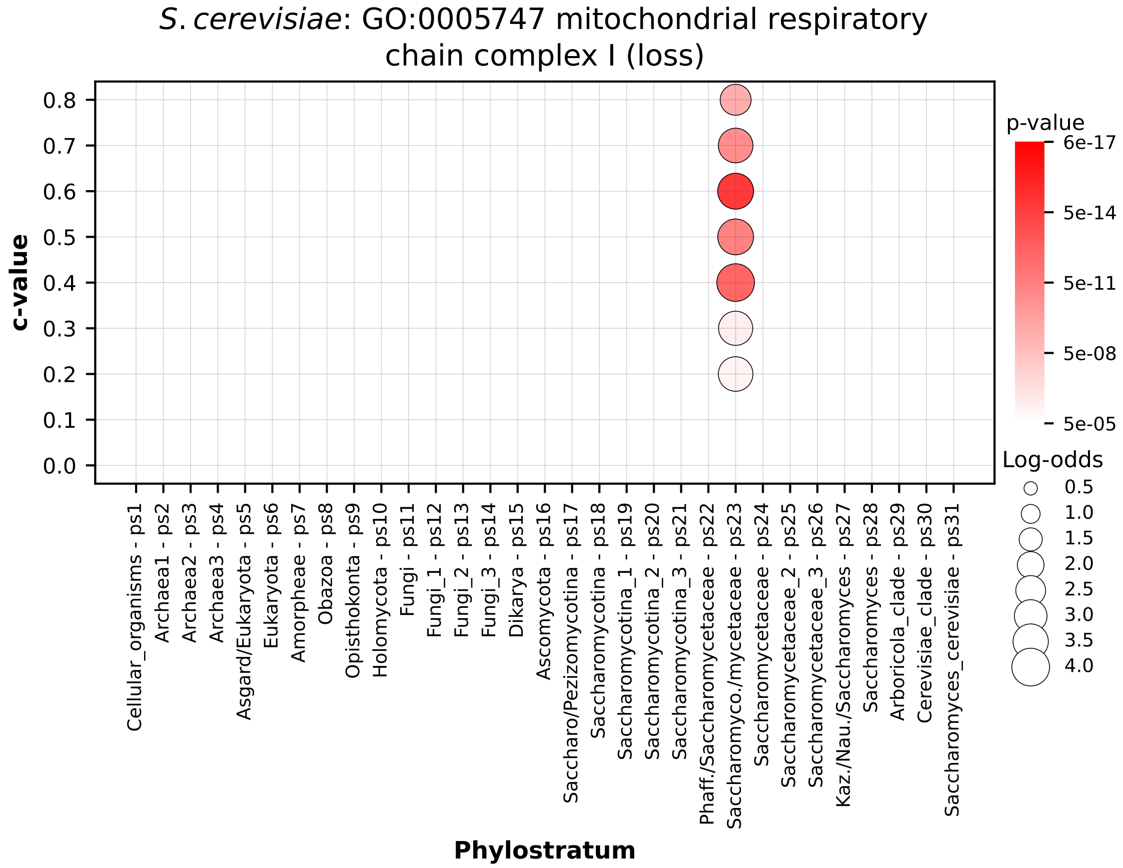

S. cerevisiae: GO:0005747 mitochondrial respiratory chain complex I (loss)
c-value
0.0
0.1
0.2
0.3
0.4
0.5
0.6
0.7
0.8
p-value
6e-17
5e-14
5e-11
5e-08
5e-05
Log-odds
0.5
1.0
1.5
2.0
2.5
3.0
3.5
4.0
Cellular_organisms - ps1
Archaea1 - ps2
Archaea2 - ps3
Archaea3 - ps4
Asgard/Eukaryota - ps5
Eukaryota - ps6
Amorpheae - ps7
Obazoa - ps8
Opisthokonta - ps9
Holomycota - ps10
Fungi - ps11
Fungi_1 - ps12
Fungi_2 - ps13
Fungi_3 - ps14
Dikarya - ps15
Ascomycota - ps16
Saccharo/Pezizomycotina - ps17
Saccharomycotina - ps18
Saccharomycotina_1 - ps19
Saccharomycotina_2 - ps20
Saccharomycotina_3 - ps21
Phaff./Saccharomycetaceae - ps22
Saccharomyco./mycetaceae - ps23
Saccharomycetaceae - ps24
Saccharomycetaceae_2 - ps25
Saccharomycetaceae_3 - ps26
Kaz./Nau./Saccharomyces - ps27
Saccharomyces - ps28
Arboricola_clade - ps29
Cerevisiae_clade - ps30
Saccharomyces_cerevisiae - ps31
Phylostratum

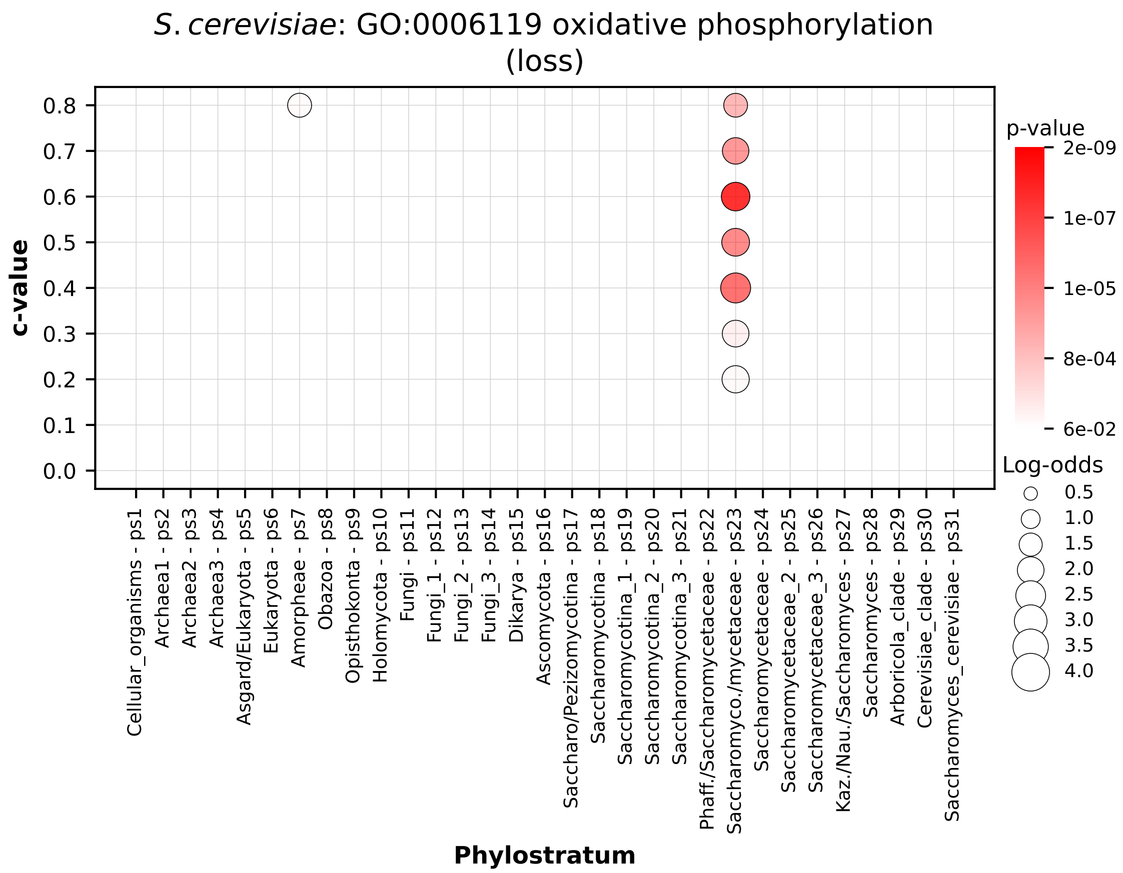

S. cerevisiae: GO:0006119 oxidative phosphorylation
(loss)
c-value
0.0
0.1
0.2
0.3
0.4
0.5
0.6
0.7
0.8
Phylostratum
Cellular_organisms - ps1
Archaea1 - ps2
Archaea2 - ps3
Archaea3 - ps4
Asgard/Eukaryota - ps5
Eukaryota - ps6
Amorpheae - ps7
Obazoa - ps8
Opisthokonta - ps9
Holomycota - ps10
Fungi - ps11
Fungi_1 - ps12
Fungi_2 - ps13
Fungi_3 - ps14
Dikarya - ps15
Ascomycota - ps16
Saccharo/Pezizomycotina - ps17
Saccharomycotina - ps18
Saccharomycotina_1 - ps19
Saccharomycotina_2 - ps20
Saccharomycotina_3 - ps21
Phaff./Saccharomycetaceae - ps22
Saccharomyco./mycetaceae - ps23
Saccharomycetaceae - ps24
Saccharomycetaceae_2 - ps25
Saccharomycetaceae_3 - ps26
Kaz./Nau./Saccharomyces - ps27
Saccharomyces - ps28
Arboricola_clade - ps29
Cerevisiae_clade - ps30
Saccharomyces_cerevisiae - ps31
p-value
2e-09
1e-07
1e-05
8e-04
6e-02
Log-odds
0.5
1.0
1.5
2.0
2.5
3.0
3.5
4.0

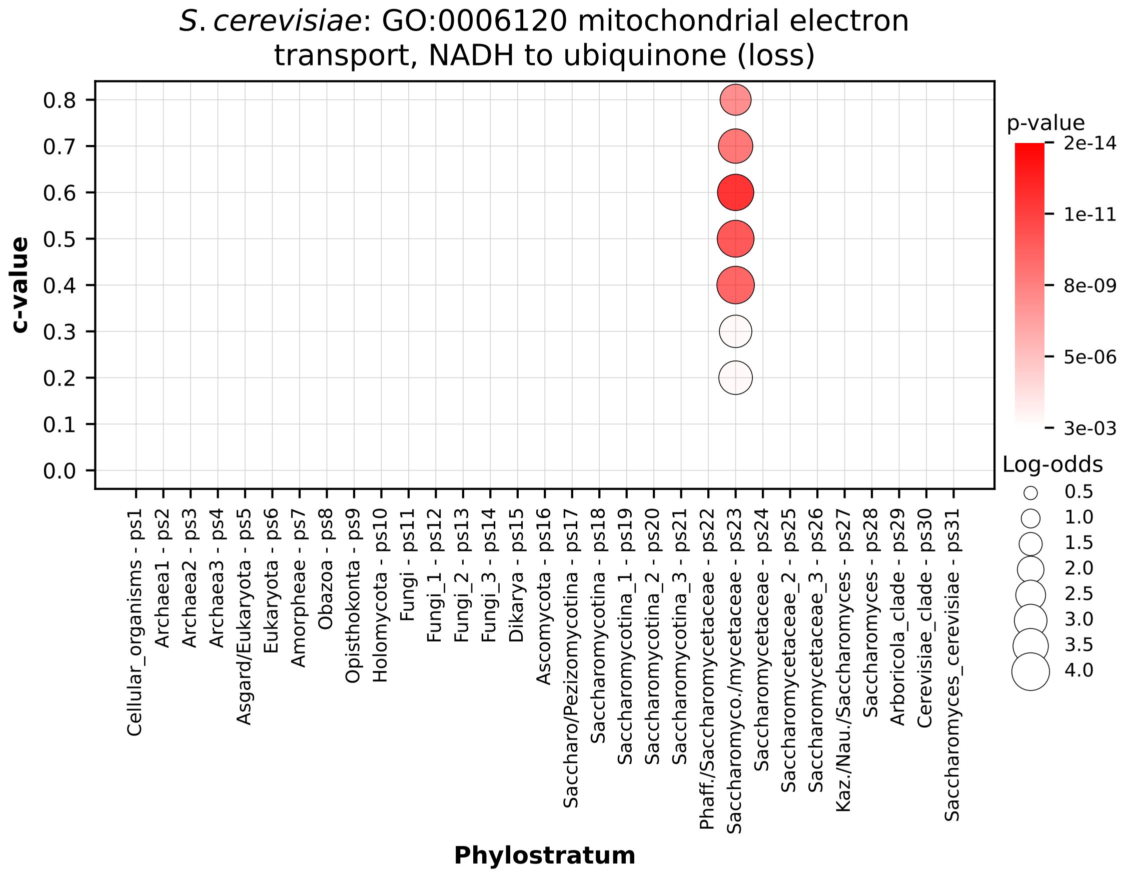

S. cerevisiae: GO:0006120 mitochondrial electron transport, NADH to ubiquinone (loss)
c-value
0.8
0.7
0.6
0.5
0.4
0.3
0.2
0.1
0.0
p-value
2e-14
1e-11
8e-09
5e-06
3e-03
Log-odds
0.5
1.0
1.5
2.0
2.5
3.0
3.5
4.0
Cellular_organisms - ps1
Archaea1 - ps2
Archaea2 - ps3
Archaea3 - ps4
Asgard/Eukaryota - ps5
Eukaryota - ps6
Amorpheae - ps7
Obazoa - ps8
Opisthokonta - ps9
Holomycota - ps10
Fungi - ps11
Fungi_1 - ps12
Fungi_2 - ps13
Fungi_3 - ps14
Dikarya - ps15
Ascomycota - ps16
Saccharo/Pezizomycotina - ps17
Saccharomycotina - ps18
Saccharomycotina_1 - ps19
Saccharomycotina_2 - ps20
Saccharomycotina_3 - ps21
Phaff./Saccharomycetaceae - ps22
Saccharomyco./mycetaceae - ps23
Saccharomycetaceae - ps24
Saccharomycetaceae_2 - ps25
Saccharomycetaceae_3 - ps26
Kaz./Nau./Saccharomyces - ps27
Saccharomyces - ps28
Arboricola_clade - ps29
Cerevisiae_clade - ps30
Saccharomyces_cerevisiae - ps31
Phylostratum

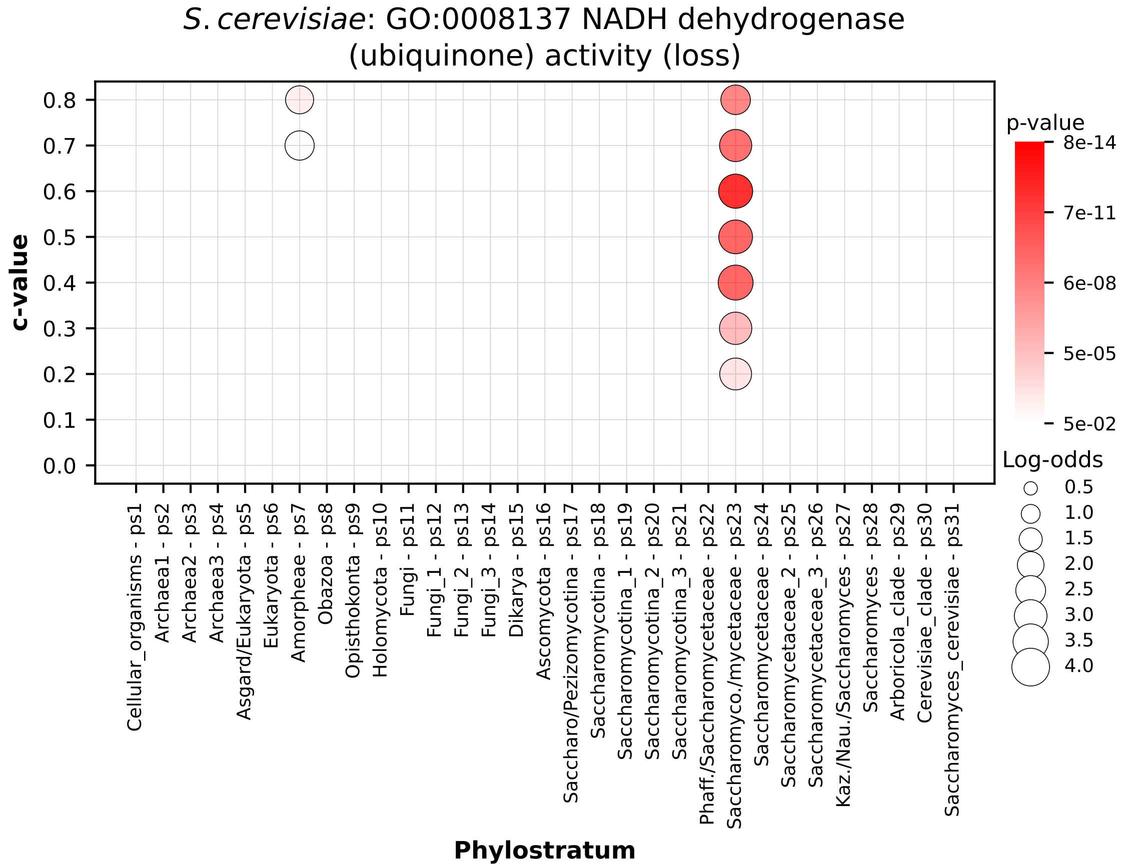

S. cerevisiae: GO:0008137 NADH dehydrogenase (ubiquinone) activity (loss)
c-value
0.0
0.1
0.2
0.3
0.4
0.5
0.6
0.7
0.8
p-value
8e-14
7e-11
6e-08
5e-05
5e-02
Log-odds
0.5
1.0
1.5
2.0
2.5
3.0
3.5
4.0
Cellular_organisms - ps1
Archaea1 - ps2
Archaea2 - ps3
Archaea3 - ps4
Asgard/Eukaryota - ps5
Eukaryota - ps6
Amorpheae - ps7
Obazoa - ps8
Opisthokonta - ps9
Holomycota - ps10
Fungi - ps11
Fungi_1 - ps12
Fungi_2 - ps13
Fungi_3 - ps14
Dikarya - ps15
Ascomycota - ps16
Saccharo/Pezizomycotina - ps17
Saccharomycotina - ps18
Saccharomycotina_1 - ps19
Saccharomycotina_2 - ps20
Saccharomycotina_3 - ps21
Phaff./Saccharomycetaceae - ps22
Saccharomyco./mycetaceae - ps23
Saccharomycetaceae - ps24
Saccharomycetaceae_2 - ps25
Saccharomycetaceae_3 - ps26
Kaz./Nau./Saccharomyces - ps27
Saccharomyces - ps28
Arboricola_clade - ps29
Cerevisiae_clade - ps30
Saccharomyces_cerevisiae - ps31
Phylostratum

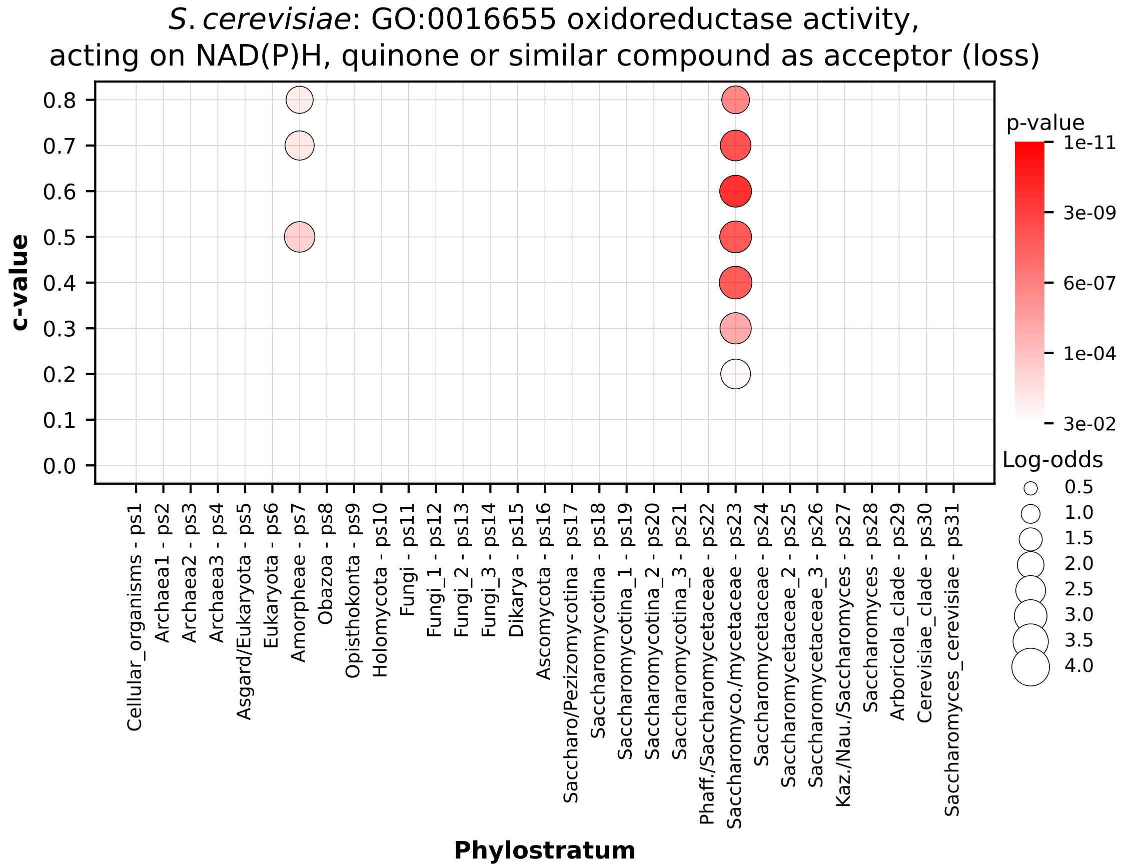
S. cerevisiae: GO:0016655 oxidoreductase activity,
acting on NAD(P)H, quinone or similar compound as acceptor (loss)
c-value
0.8
0.7
0.6
0.5
0.4
0.3
0.2
0.1
0.0
p-value
1e-11
3e-09
6e-07
1e-04
3e-02
Log-odds
0.5
1.0
1.5
2.0
2.5
3.0
3.5
4.0
Cellular_organisms - ps1
Archaea1 - ps2
Archaea2 - ps3
Archaea3 - ps4
Asgard/Eukaryota - ps5
Eukaryota - ps6
Amorpheae - ps7
Obazoa - ps8
Opisthokonta - ps9
Holomycota - ps10
Fungi - ps11
Fungi_1 - ps12
Fungi_2 - ps13
Fungi_3 - ps14
Dikarya - ps15
Ascomycota - ps16
Saccharo/Pezizomycotina - ps17
Saccharomycotina - ps18
Saccharomycotina_1 - ps19
Saccharomycotina_2 - ps20
Saccharomycotina_3 - ps21
Phaff./Saccharomycetaceae - ps22
Saccharomyco./mycetaceae - ps23
Saccharomycetaceae - ps24
Saccharomycetaceae_2 - ps25
Saccharomycetaceae_3 - ps26
Kaz./Nau./Saccharomyces - ps27
Saccharomyces - ps28
Arboricola_clade - ps29
Cerevisiae_clade - ps30
Saccharomyces_cerevisiae - ps31
Phylostratum

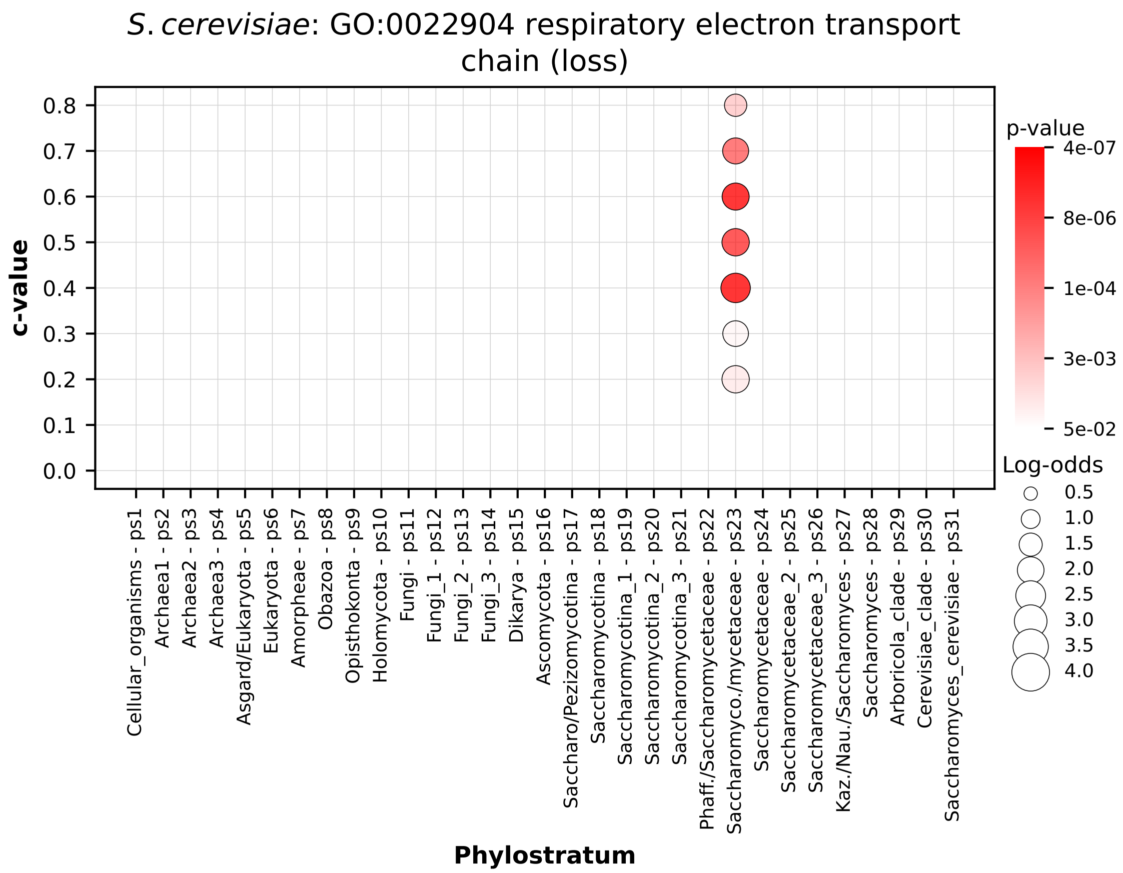
S. cerevisiae: GO:0022904 respiratory electron transport chain (loss)
c-value
0.8
0.7
0.6
0.5
0.4
0.3
0.2
0.1
0.0
Phylostratum
Cellular_organisms - ps1
Archaea1 - ps2
Archaea2 - ps3
Archaea3 - ps4
Asgard/Eukaryota - ps5
Eukaryota - ps6
Amorpheae - ps7
Obazoa - ps8
Opisthokonta - ps9
Holomycota - ps10
Fungi - ps11
Fungi_1 - ps12
Fungi_2 - ps13
Fungi_3 - ps14
Dikarya - ps15
Ascomycota - ps16
Saccharo/Pezizomycotina - ps17
Saccharomycotina - ps18
Saccharomycotina_1 - ps19
Saccharomycotina_2 - ps20
Saccharomycotina_3 - ps21
Phaff./Saccharomycetaceae - ps22
Saccharomyco./mycetaceae - ps23
Saccharomycetaceae - ps24
Saccharomycetaceae_2 - ps25
Saccharomycetaceae_3 - ps26
Kaz./Nau./Saccharomyces - ps27
Saccharomyces - ps28
Arboricola_clade - ps29
Cerevisiae_clade - ps30
Saccharomyces_cerevisiae - ps31
p-value
4e-07
8e-06
1e-04
3e-03
5e-02
Log-odds
0.5
1.0
1.5
2.0
2.5
3.0
3.5
4.0

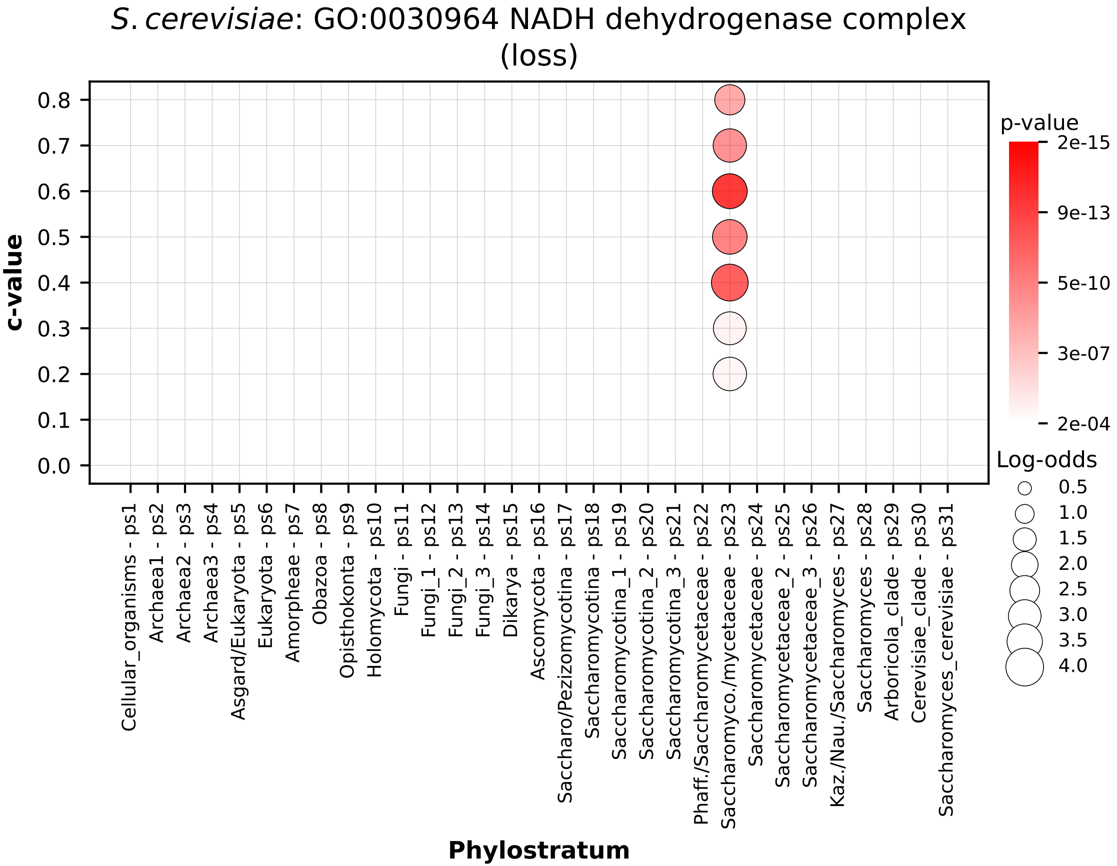

S. cerevisiae: GO:0030964 NADH dehydrogenase complex
(loss)
c-value
0.8
0.7
0.6
0.5
0.4
0.3
0.2
0.1
0.0
p-value
2e-15
9e-13
5e-10
3e-07
2e-04
Log-odds
0.5
1.0
1.5
2.0
2.5
3.0
3.5
4.0
Cellular_organisms - ps1
Archaea1 - ps2
Archaea2 - ps3
Archaea3 - ps4
Asgard/Eukaryota - ps5
Eukaryota - ps6
Amorpheae - ps7
Obazoa - ps8
Opisthokonta - ps9
Holomycota - ps10
Fungi - ps11
Fungi_1 - ps12
Fungi_2 - ps13
Fungi_3 - ps14
Dikarya - ps15
Ascomycota - ps16
Saccharo/Pezizomycotina - ps17
Saccharomycotina - ps18
Saccharomycotina_1 - ps19
Saccharomycotina_2 - ps20
Saccharomycotina_3 - ps21
Phaff./Saccharomycetaceae - ps22
Saccharomyco./mycetaceae - ps23
Saccharomycetaceae - ps24
Saccharomycetaceae_2 - ps25
Saccharomycetaceae_3 - ps26
Kaz./Nau./Saccharomyces - ps27
Saccharomyces - ps28
Arboricola_clade - ps29
Cerevisiae_clade - ps30
Saccharomyces_cerevisiae - ps31
Phylostratum

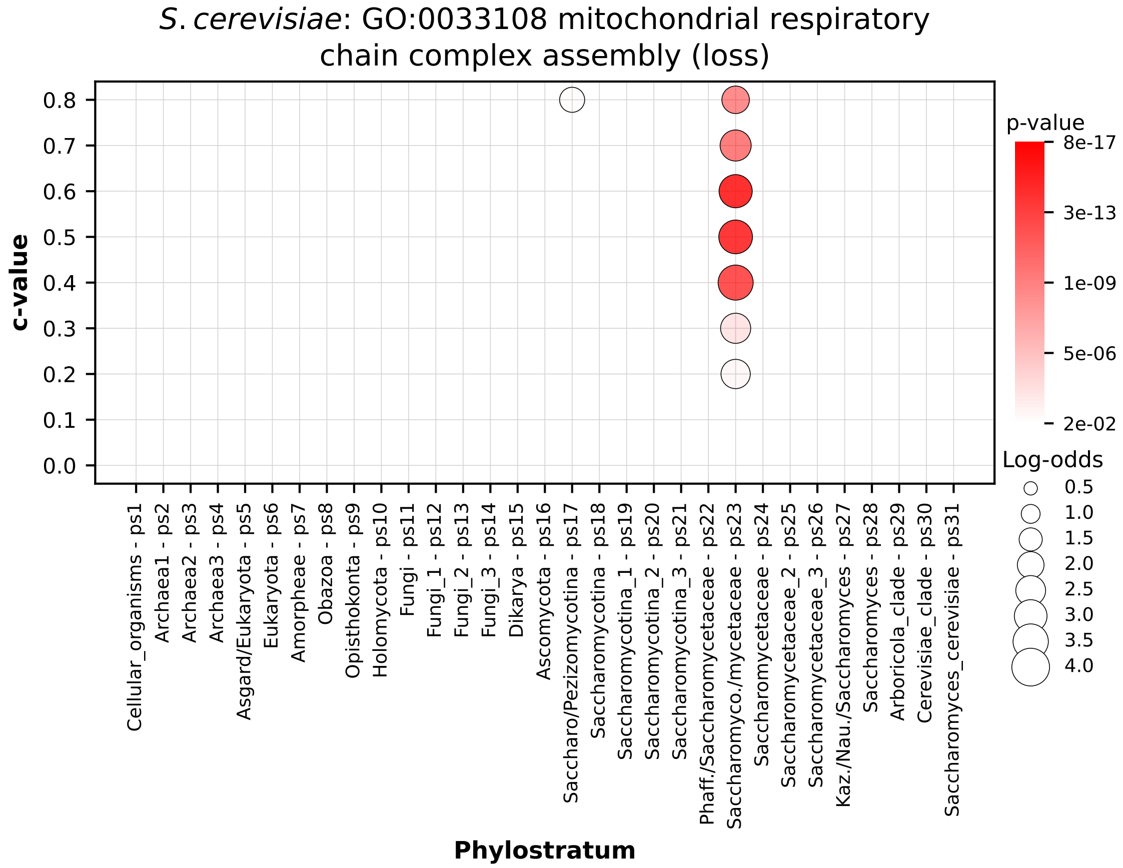

S. cerevisiae: GO:0033108 mitochondrial respiratory chain complex assembly (loss)
c-value
0.8
0.7
0.6
0.5
0.4
0.3
0.2
0.1
0.0
p-value
8e-17
3e-13
1e-09
5e-06
2e-02
Log-odds
0.5
1.0
1.5
2.0
2.5
3.0
3.5
4.0
Cellular_organisms - ps1
Archaea1 - ps2
Archaea2 - ps3
Archaea3 - ps4
Asgard/Eukaryota - ps5
Eukaryota - ps6
Amorpheae - ps7
Obazoa - ps8
Opisthokonta - ps9
Holomycota - ps10
Fungi - ps11
Fungi_1 - ps12
Fungi_2 - ps13
Fungi_3 - ps14
Dikarya - ps15
Ascomycota - ps16
Saccharo/Pezizomycotina - ps17
Saccharomycotina - ps18
Saccharomycotina_1 - ps19
Saccharomycotina_2 - ps20
Saccharomycotina_3 - ps21
Phaff./Saccharomycetaceae - ps22
Saccharomyco./mycetaceae - ps23
Saccharomycetaceae - ps24
Saccharomycetaceae_2 - ps25
Saccharomycetaceae_3 - ps26
Kaz./Nau./Saccharomyces - ps27
Saccharomyces - ps28
Arboricola_clade - ps29
Cerevisiae_clade - ps30
Saccharomyces_cerevisiae - ps31
Phylostratum

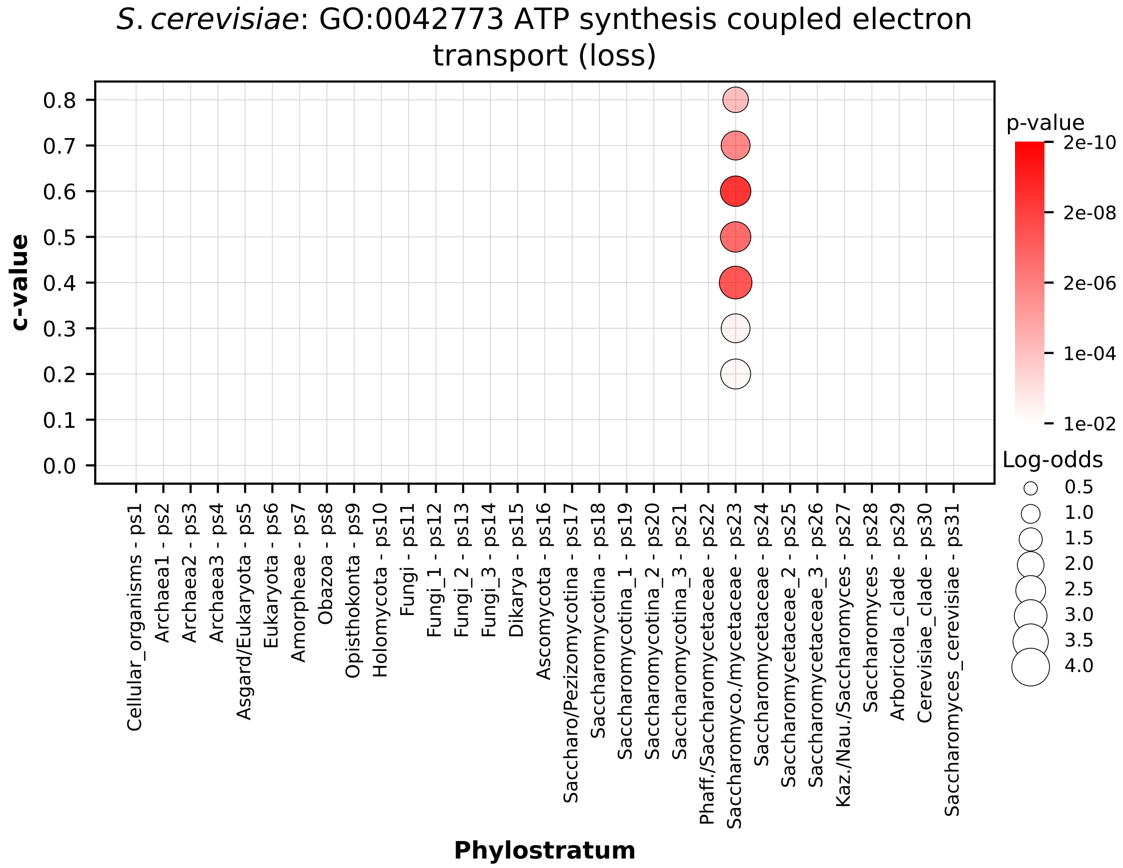

S. cerevisiae: GO:0042773 ATP synthesis coupled electron transport (loss)
c-value
0.0
0.1
0.2
0.3
0.4
0.5
0.6
0.7
0.8
p-value
2e-10
2e-08
2e-06
1e-04
1e-02
Log-odds
0.5
1.0
1.5
2.0
2.5
3.0
3.5
4.0
Cellular_organisms - ps1
Archaea1 - ps2
Archaea2 - ps3
Archaea3 - ps4
Asgard/Eukaryota - ps5
Eukaryota - ps6
Amorpheae - ps7
Obazoa - ps8
Opisthokonta - ps9
Holomycota - ps10
Fungi - ps11
Fungi_1 - ps12
Fungi_2 - ps13
Fungi_3 - ps14
Dikarya - ps15
Ascomycota - ps16
Saccharo/Pezizomycotina - ps17
Saccharomycotina - ps18
Saccharomycotina_1 - ps19
Saccharomycotina_2 - ps20
Saccharomycotina_3 - ps21
Phaff./Saccharomycetaceae - ps22
Saccharomyco./mycetaceae - ps23
Saccharomycetaceae - ps24
Saccharomycetaceae_2 - ps25
Saccharomycetaceae_3 - ps26
Kaz./Nau./Saccharomyces - ps27
Saccharomyces - ps28
Arboricola_clade - ps29
Cerevisiae_clade - ps30
Saccharomyces_cerevisiae - ps31
Phylostratum

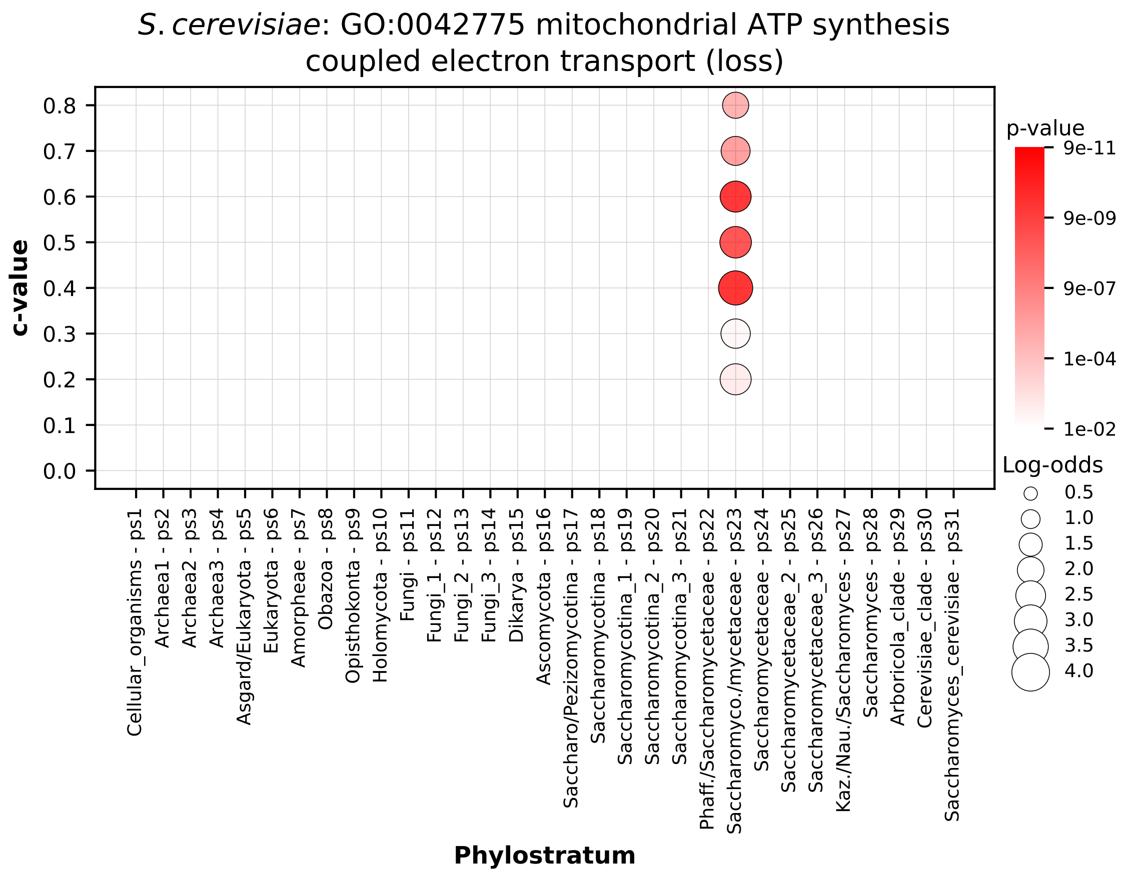

S. cerevisiae: GO:0042775 mitochondrial ATP synthesis coupled electron transport (loss)
c-value
0.8
0.7
0.6
0.5
0.4
0.3
0.2
0.1
0.0
p-value
9e-11
9e-09
9e-07
1e-04
1e-02
Log-odds
0.5
1.0
1.5
2.0
2.5
3.0
3.5
4.0
Cellular_organisms - ps1
Archaea1 - ps2
Archaea2 - ps3
Archaea3 - ps4
Asgard/Eukaryota - ps5
Eukaryota - ps6
Amorpheae - ps7
Obazoa - ps8
Opisthokonta - ps9
Holomycota - ps10
Fungi - ps11
Fungi_1 - ps12
Fungi_2 - ps13
Fungi_3 - ps14
Dikarya - ps15
Ascomycota - ps16
Saccharo/Pezizomycotina - ps17
Saccharomycotina - ps18
Saccharomycotina_1 - ps19
Saccharomycotina_2 - ps20
Saccharomycotina_3 - ps21
Phaff./Saccharomycetaceae - ps22
Saccharomyco./mycetaceae - ps23
Saccharomycetaceae - ps24
Saccharomycetaceae_2 - ps25
Saccharomycetaceae_3 - ps26
Kaz./Nau./Saccharomyces - ps27
Saccharomyces - ps28
Arboricola_clade - ps29
Cerevisiae_clade - ps30
Saccharomyces_cerevisiae - ps31
Phylostratum

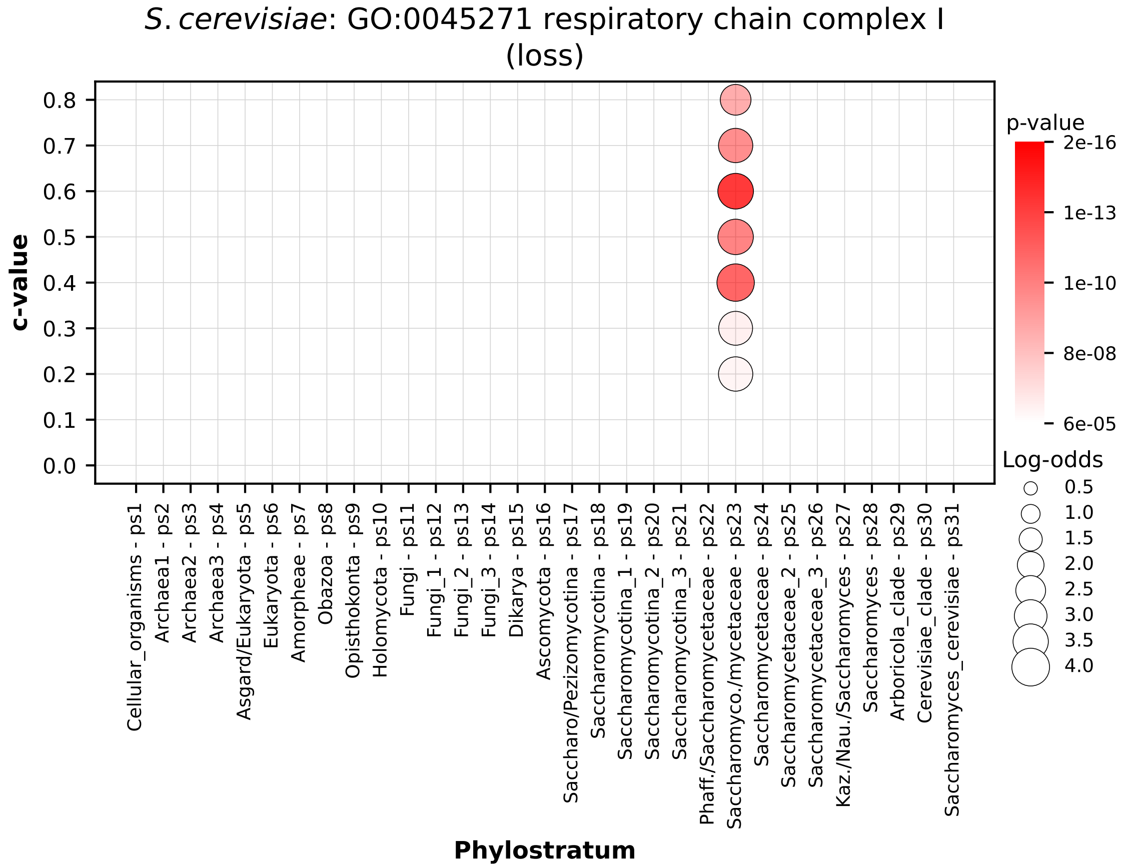

S. cerevisiae: GO:0045271 respiratory chain complex I
(loss)
c-value
0.8
0.7
0.6
0.5
0.4
0.3
0.2
0.1
0.0
p-value
2e-16
1e-13
1e-10
8e-08
6e-05
Log-odds
0.5
1.0
1.5
2.0
2.5
3.0
3.5
4.0
Cellular_organisms - ps1
Archaea1 - ps2
Archaea2 - ps3
Archaea3 - ps4
Asgard/Eukaryota - ps5
Eukaryota - ps6
Amorpheae - ps7
Obazoa - ps8
Opisthokonta - ps9
Holomycota - ps10
Fungi - ps11
Fungi_1 - ps12
Fungi_2 - ps13
Fungi_3 - ps14
Dikarya - ps15
Ascomycota - ps16
Saccharo/Pezizomycotina - ps17
Saccharomycotina - ps18
Saccharomycotina_1 - ps19
Saccharomycotina_2 - ps20
Saccharomycotina_3 - ps21
Phaff./Saccharomycetaceae - ps22
Saccharomyco./mycetaceae - ps23
Saccharomycetaceae - ps24
Saccharomycetaceae_2 - ps25
Saccharomycetaceae_3 - ps26
Kaz./Nau./Saccharomyces - ps27
Saccharomyces - ps28
Arboricola_clade - ps29
Cerevisiae_clade - ps30
Saccharomyces_cerevisiae - ps31
Phylostratum

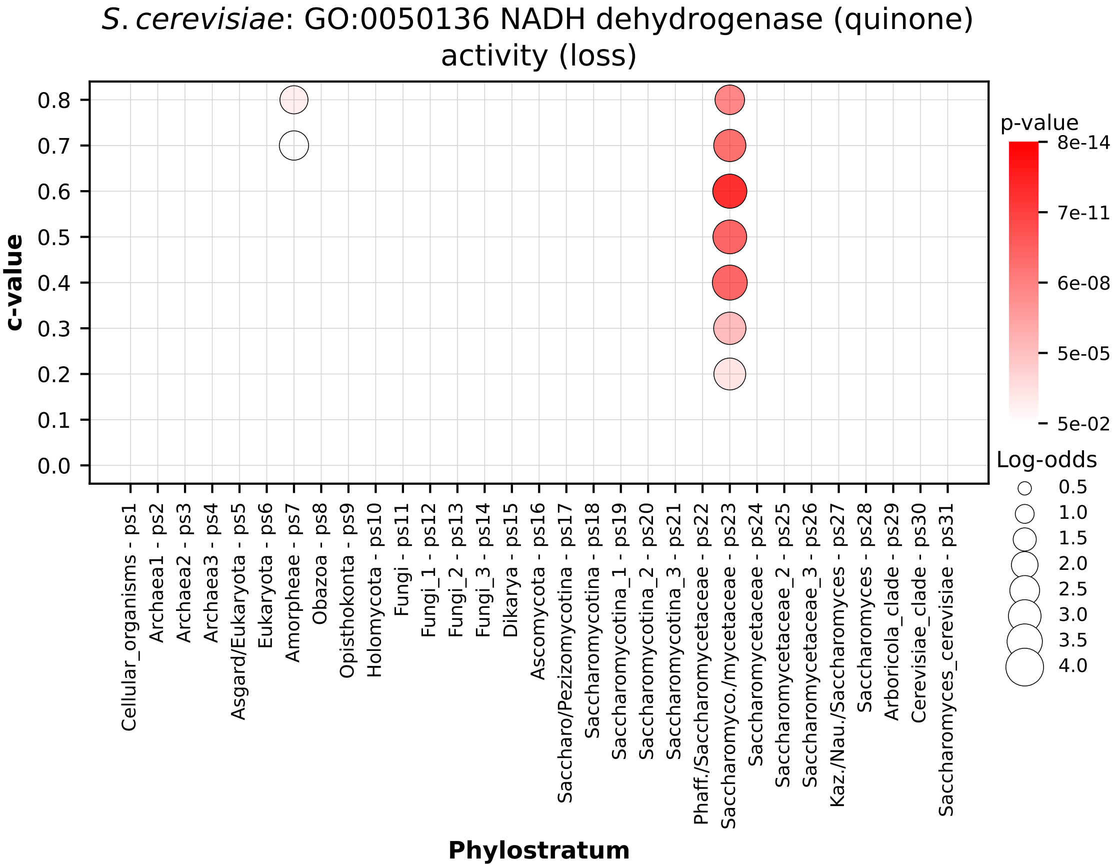

S. cerevisiae: GO:0050136 NADH dehydrogenase (quinone) activity (loss)
c-value
0.8
0.7
0.6
0.5
0.4
0.3
0.2
0.1
0.0
p-value
8e-14
7e-11
6e-08
5e-05
5e-02
Log-odds
0.5
1.0
1.5
2.0
2.5
3.0
3.5
4.0
Cellular_organisms - ps1
Archaea1 - ps2
Archaea2 - ps3
Archaea3 - ps4
Asgard/Eukaryota - ps5
Eukaryota - ps6
Amorpheae - ps7
Obazoa - ps8
Opisthokonta - ps9
Holomycota - ps10
Fungi - ps11
Fungi_1 - ps12
Fungi_2 - ps13
Fungi_3 - ps14
Dikarya - ps15
Ascomycota - ps16
Saccharo/Pezizomycotina - ps17
Saccharomycotina - ps18
Saccharomycotina_1 - ps19
Saccharomycotina_2 - ps20
Saccharomycotina_3 - ps21
Phaff./Saccharomycetaceae - ps22
Saccharomyco./mycetaceae - ps23
Saccharomycetaceae - ps24
Saccharomycetaceae_2 - ps25
Saccharomycetaceae_3 - ps26
Kaz./Nau./Saccharomyces - ps27
Saccharomyces - ps28
Arboricola_clade - ps29
Cerevisiae_clade - ps30
Saccharomyces_cerevisiae - ps31
Phylostratum

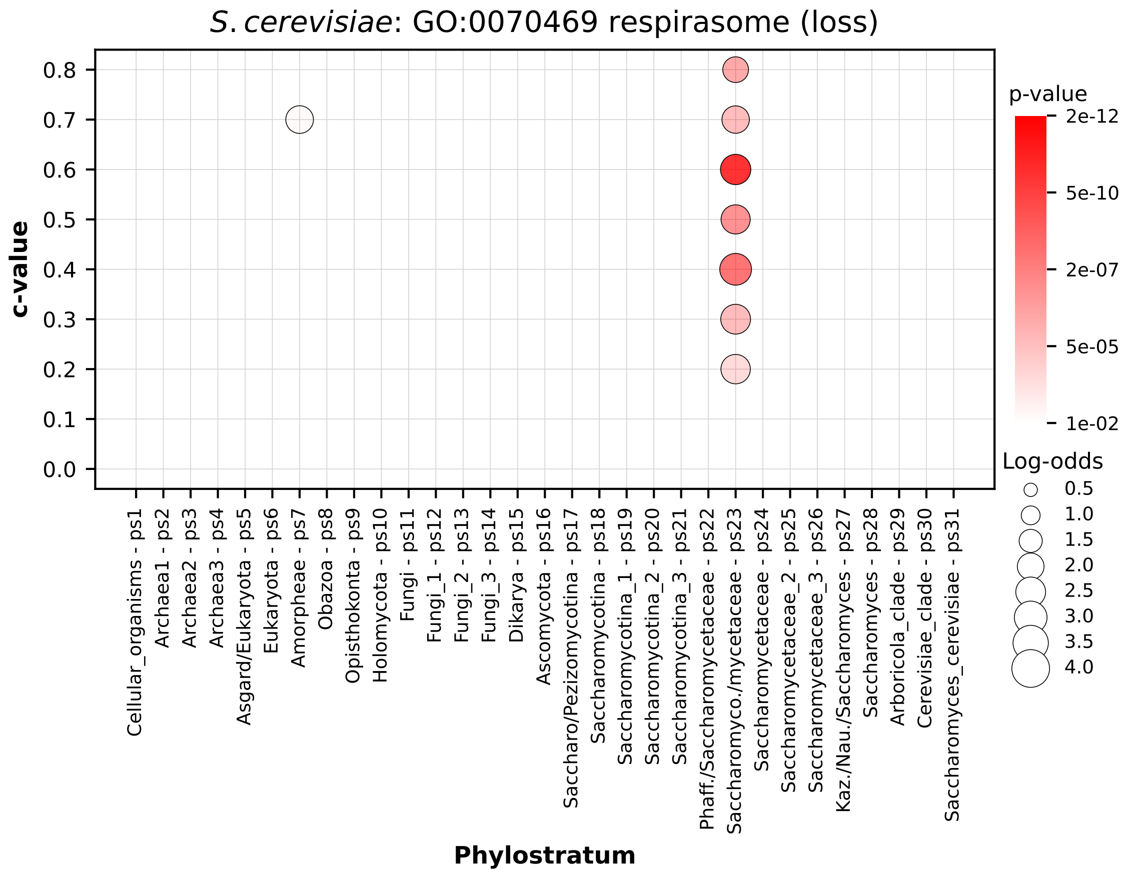

S. cerevisiae: GO:0070469 respirasome (loss)
c-value
0.8
0.7
0.6
0.5
0.4
0.3
0.2
0.1
0.0
p-value
2e-12
5e-10
2e-07
5e-05
1e-02
Log-odds
0.5
1.0
1.5
2.0
2.5
3.0
3.5
4.0
Cellular_organisms - ps1
Archaea1 - ps2
Archaea2 - ps3
Archaea3 - ps4
Asgard/Eukaryota - ps5
Eukaryota - ps6
Amorpheae - ps7
Obazoa - ps8
Opisthokonta - ps9
Holomycota - ps10
Fungi - ps11
Fungi_1 - ps12
Fungi_2 - ps13
Fungi_3 - ps14
Dikarya - ps15
Ascomycota - ps16
Saccharo/Pezizomycotina - ps17
Saccharomycotina - ps18
Saccharomycotina_1 - ps19
Saccharomycotina_2 - ps20
Saccharomycotina_3 - ps21
Phaff./Saccharomycetaceae - ps22
Saccharomyco./mycetaceae - ps23
Saccharomycetaceae - ps24
Saccharomycetaceae_2 - ps25
Saccharomycetaceae_3 - ps26
Kaz./Nau./Saccharomyces - ps27
Saccharomyces - ps28
Arboricola_clade - ps29
Cerevisiae_clade - ps30
Saccharomyces_cerevisiae - ps31
Phylostratum

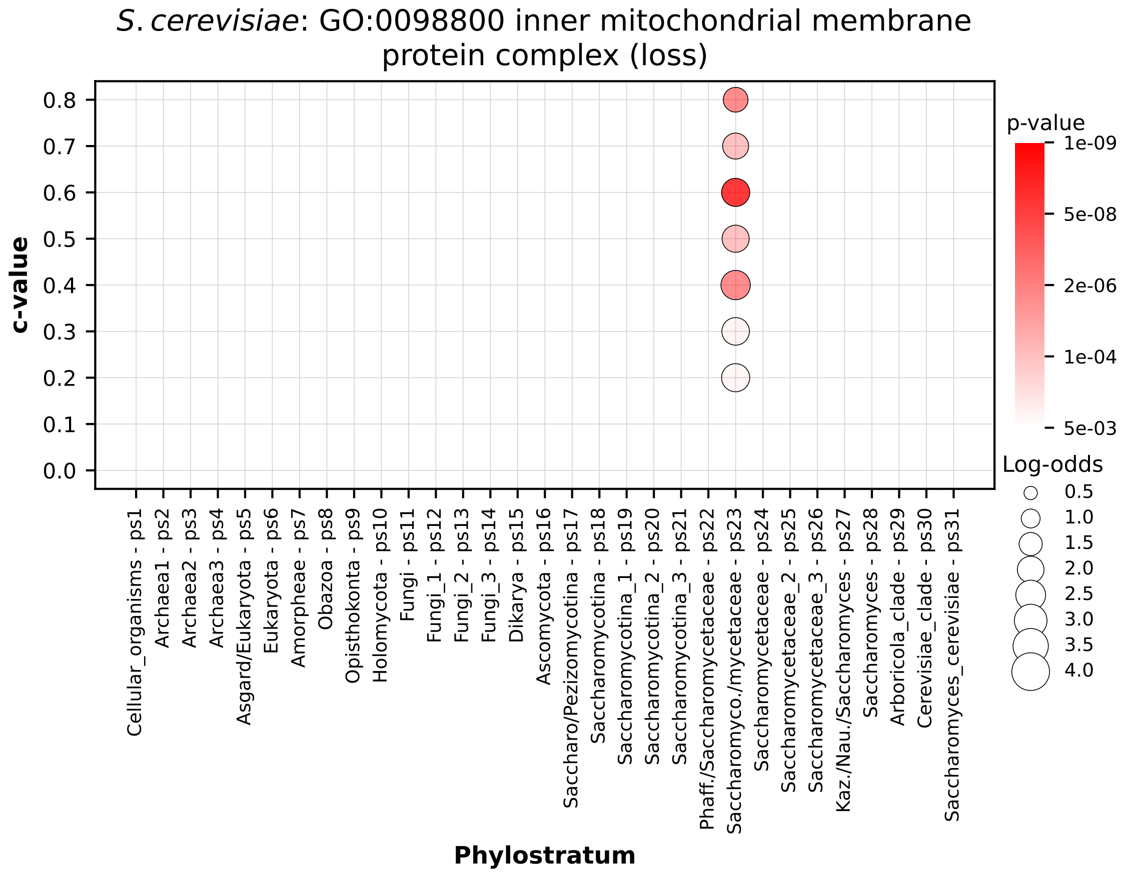

S. cerevisiae: GO:0098800 inner mitochondrial membrane protein complex (loss)
c-value
0.8
0.7
0.6
0.5
0.4
0.3
0.2
0.1
0.0
p-value
1e-09
5e-08
2e-06
1e-04
5e-03
Log-odds
0.5
1.0
1.5
2.0
2.5
3.0
3.5
4.0
Cellular_organisms - ps1
Archaea1 - ps2
Archaea2 - ps3
Archaea3 - ps4
Asgard/Eukaryota - ps5
Eukaryota - ps6
Amorpheae - ps7
Obazoa - ps8
Opisthokonta - ps9
Holomycota - ps10
Fungi - ps11
Fungi_1 - ps12
Fungi_2 - ps13
Fungi_3 - ps14
Dikarya - ps15
Ascomycota - ps16
Saccharo/Pezizomycotina - ps17
Saccharomycotina - ps18
Saccharomycotina_1 - ps19
Saccharomycotina_2 - ps20
Saccharomycotina_3 - ps21
Phaff./Saccharomycetaceae - ps22
Saccharomyco./mycetaceae - ps23
Saccharomycetaceae - ps24
Saccharomycetaceae_2 - ps25
Saccharomycetaceae_3 - ps26
Kaz./Nau./Saccharomyces - ps27
Saccharomyces - ps28
Arboricola_clade - ps29
Cerevisiae_clade - ps30
Saccharomyces_cerevisiae - ps31
Phylostratum

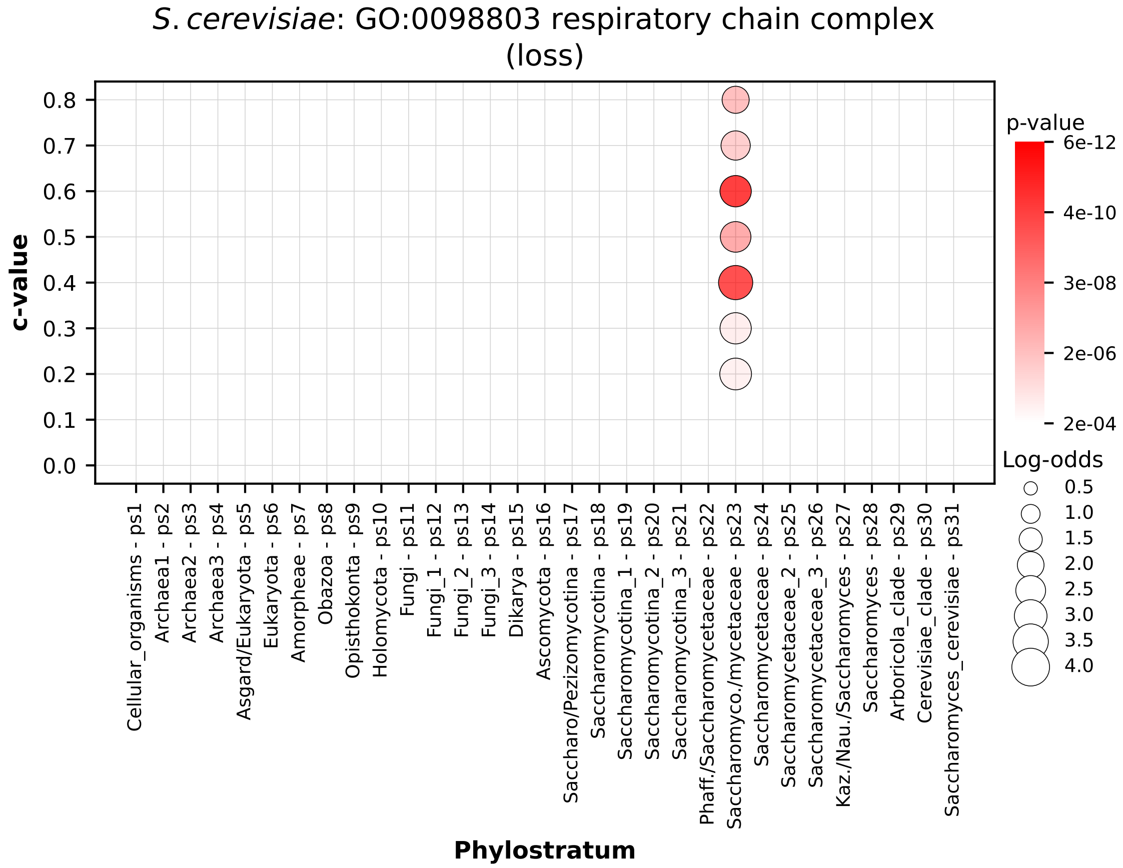

S. cerevisiae: GO:0098803 respiratory chain complex
(loss)
c-value
0.8
0.7
0.6
0.5
0.4
0.3
0.2
0.1
0.0
p-value
6e-12
4e-10
3e-08
2e-06
2e-04
Log-odds
0.5
1.0
1.5
2.0
2.5
3.0
3.5
4.0
Cellular_organisms - ps1
Archaea1 - ps2
Archaea2 - ps3
Archaea3 - ps4
Asgard/Eukaryota - ps5
Eukaryota - ps6
Amorpheae - ps7
Obazoa - ps8
Opisthokonta - ps9
Holomycota - ps10
Fungi - ps11
Fungi_1 - ps12
Fungi_2 - ps13
Fungi_3 - ps14
Dikarya - ps15
Ascomycota - ps16
Saccharo/Pezizomycotina - ps17
Saccharomycotina - ps18
Saccharomycotina_1 - ps19
Saccharomycotina_2 - ps20
Saccharomycotina_3 - ps21
Phaff./Saccharomycetaceae - ps22
Saccharomyco./mycetaceae - ps23
Saccharomycetaceae - ps24
Saccharomycetaceae_2 - ps25
Saccharomycetaceae_3 - ps26
Kaz./Nau./Saccharomyces - ps27
Saccharomyces - ps28
Arboricola_clade - ps29
Cerevisiae_clade - ps30
Saccharomyces_cerevisiae - ps31
Phylostratum

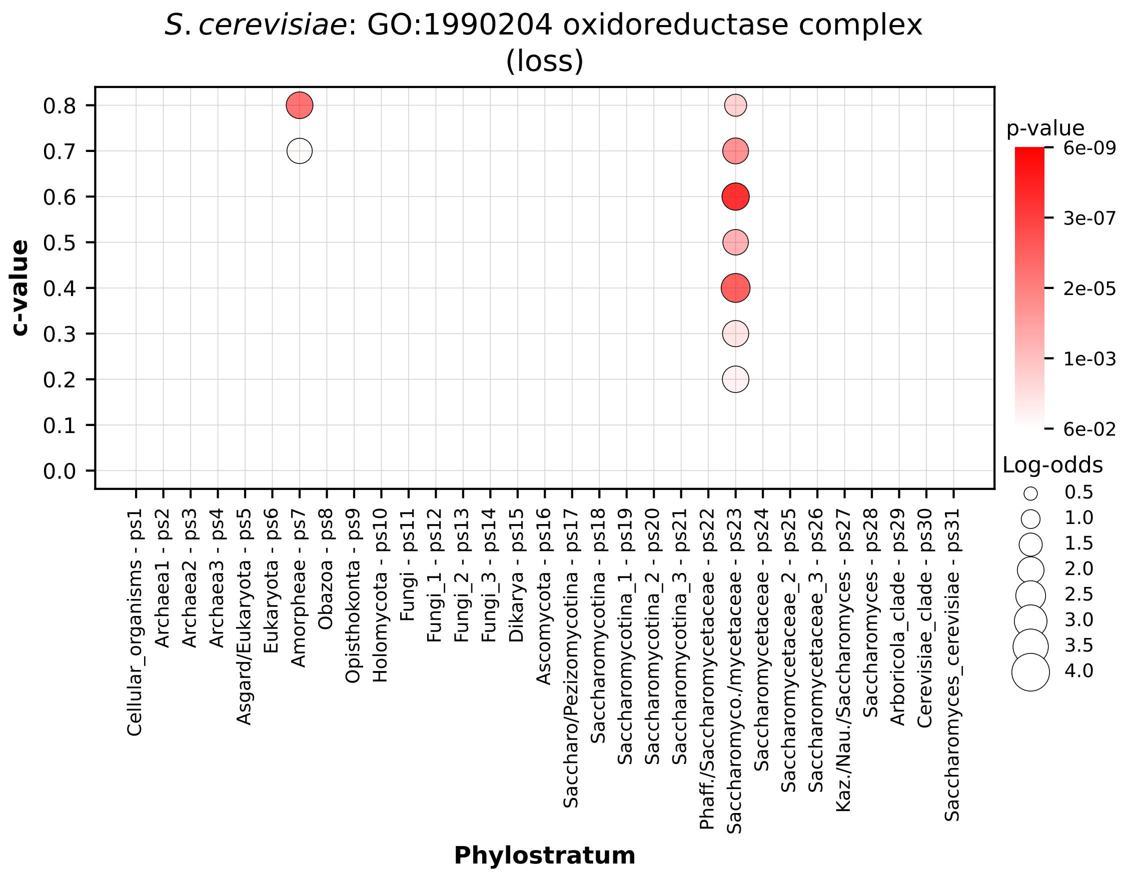
S. cerevisiae: GO:1990204 oxidoreductase complex
(loss)
c-value
0.8
0.7
0.6
0.5
0.4
0.3
0.2
0.1
0.0
p-value
6e-09
3e-07
2e-05
1e-03
6e-02
Log-odds
0.5
1.0
1.5
2.0
2.5
3.0
3.5
4.0
Cellular_organisms - ps1
Archaea1 - ps2
Archaea2 - ps3
Archaea3 - ps4
Asgard/Eukaryota - ps5
Eukaryota - ps6
Amorpheae - ps7
Obazoa - ps8
Opisthokonta - ps9
Holomycota - ps10
Fungi - ps11
Fungi_1 - ps12
Fungi_2 - ps13
Fungi_3 - ps14
Dikarya - ps15
Ascomycota - ps16
Saccharo/Pezizomycotina - ps17
Saccharomycotina - ps18
Saccharomycotina_1 - ps19
Saccharomycotina_2 - ps20
Saccharomycotina_3 - ps21
Phaff./Saccharomycetaceae - ps22
Saccharomyco./mycetaceae - ps23
Saccharomycetaceae - ps24
Saccharomycetaceae_2 - ps25
Saccharomycetaceae_3 - ps26
Kaz./Nau./Saccharomyces - ps27
Saccharomyces - ps28
Arboricola_clade - ps29
Cerevisiae_clade - ps30
Saccharomyces_cerevisiae - ps31
Phylostratum

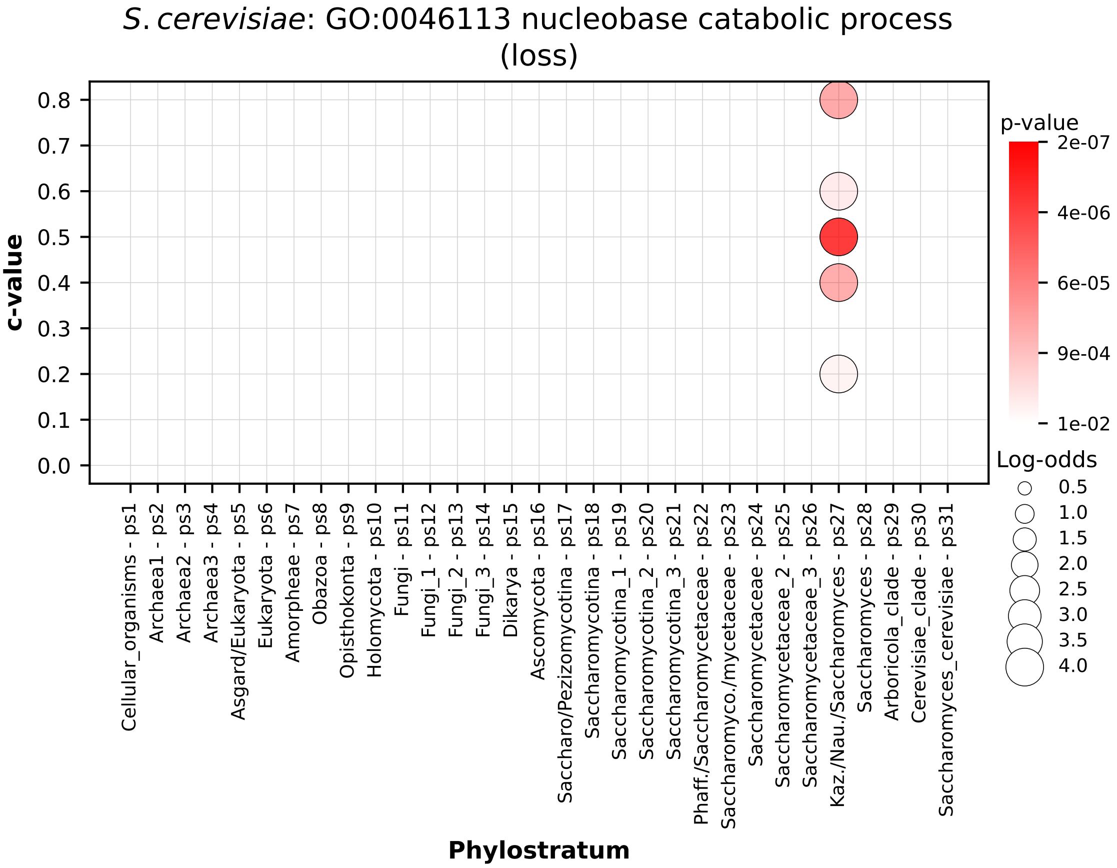

S. cerevisiae: GO:0046113 nucleobase catabolic process
(loss)
c-value
0.0
0.1
0.2
0.3
0.4
0.5
0.6
0.7
0.8
Phylostratum
Cellular_organisms - ps1
Archaea1 - ps2
Archaea2 - ps3
Archaea3 - ps4
Asgard/Eukaryota - ps5
Eukaryota - ps6
Amorpheae - ps7
Obazoa - ps8
Opisthokonta - ps9
Holomycota - ps10
Fungi - ps11
Fungi_1 - ps12
Fungi_2 - ps13
Fungi_3 - ps14
Dikarya - ps15
Ascomycota - ps16
Saccharo/Pezizomycotina - ps17
Saccharomycotina - ps18
Saccharomycotina_1 - ps19
Saccharomycotina_2 - ps20
Saccharomycotina_3 - ps21
Phaff./Saccharomycetaceae - ps22
Saccharomyco./mycetaceae - ps23
Saccharomycetaceae - ps24
Saccharomycetaceae_2 - ps25
Saccharomycetaceae_3 - ps26
Kaz./Nau./Saccharomyces - ps27
Saccharomyces - ps28
Arboricola_clade - ps29
Cerevisiae_clade - ps30
Saccharomyces_cerevisiae - ps31
p-value
2e-07
4e-06
6e-05
9e-04
1e-02
Log-odds
0.5
1.0
1.5
2.0
2.5
3.0
3.5
4.0

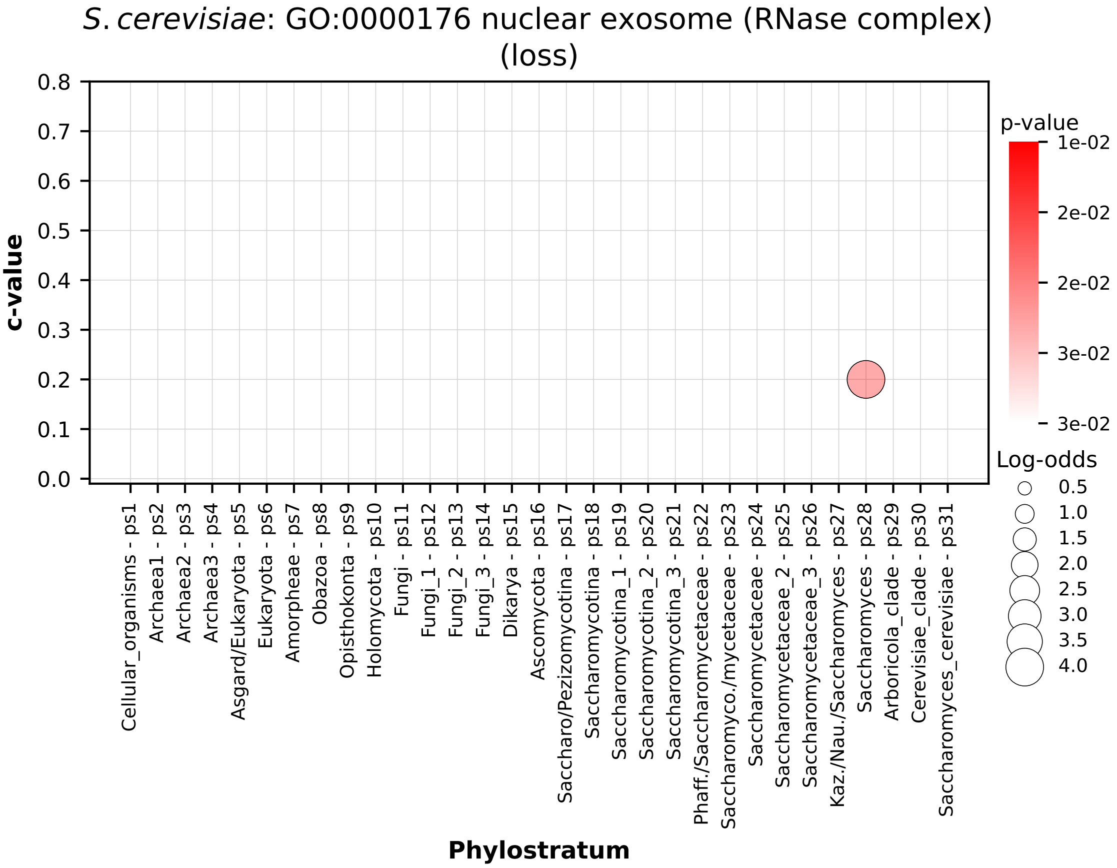

S. cerevisiae: GO:0000176 nuclear exosome (RNase complex)
(loss)
c-value
0.8
0.7
0.6
0.5
0.4
0.3
0.2
0.1
0.0
p-value
1e-02
2e-02
2e-02
3e-02
3e-02
Log-odds
0.5
1.0
1.5
2.0
2.5
3.0
3.5
4.0
Cellular_organisms - ps1
Archaea1 - ps2
Archaea2 - ps3
Archaea3 - ps4
Asgard/Eukaryota - ps5
Eukaryota - ps6
Amorpheae - ps7
Obazoa - ps8
Opisthokonta - ps9
Holomycota - ps10
Fungi - ps11
Fungi_1 - ps12
Fungi_2 - ps13
Fungi_3 - ps14
Dikarya - ps15
Ascomycota - ps16
Saccharo/Pezizomycotina - ps17
Saccharomycotina - ps18
Saccharomycotina_1 - ps19
Saccharomycotina_2 - ps20
Saccharomycotina_3 - ps21
Phaff./Saccharomycetaceae - ps22
Saccharomyco./mycetaceae - ps23
Saccharomycetaceae - ps24
Saccharomycetaceae_2 - ps25
Saccharomycetaceae_3 - ps26
Kaz./Nau./Saccharomyces - ps27
Saccharomyces - ps28
Arboricola_clade - ps29
Cerevisiae_clade - ps30
Saccharomyces_cerevisiae - ps31
Phylostratum

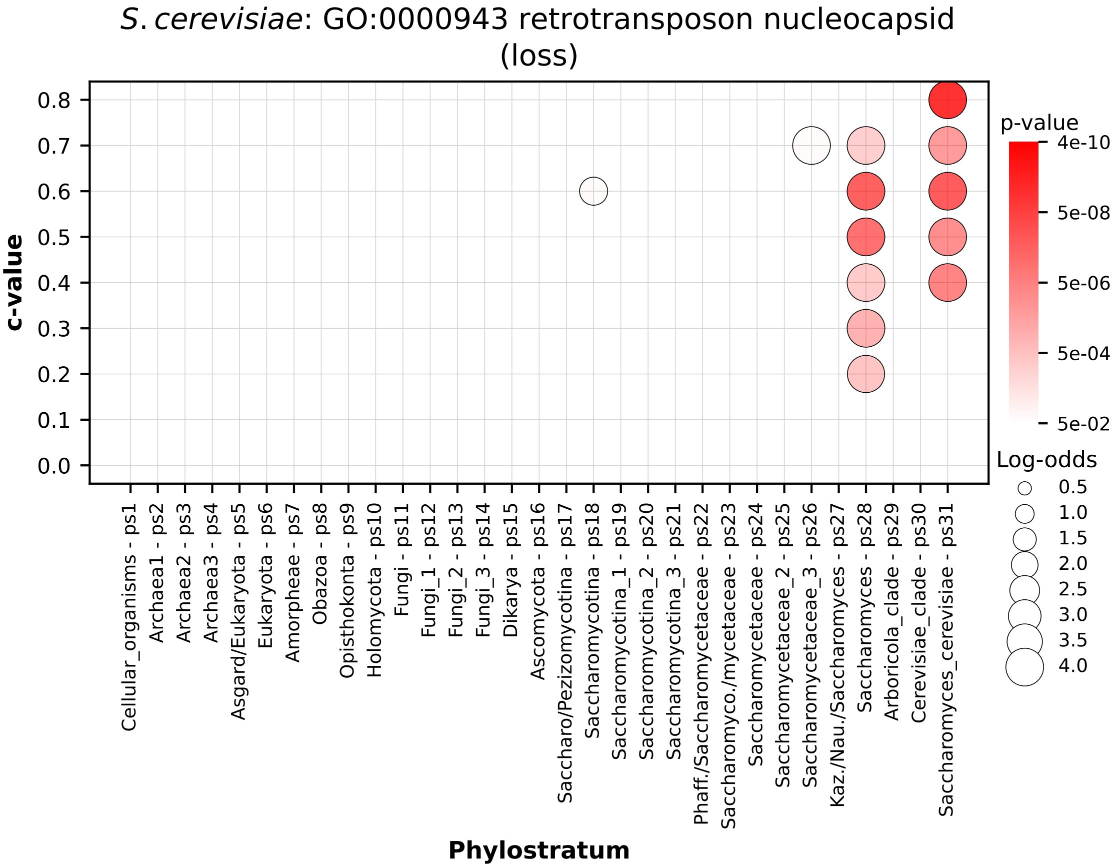
S. cerevisiae: GO:0000943 retrotransposon nucleocapsid (loss)
c-value
0.8
0.7
0.6
0.5
0.4
0.3
0.2
0.1
0.0
p-value
4e-10
5e-08
5e-06
5e-04
5e-02
Log-odds
0.5
1.0
1.5
2.0
2.5
3.0
3.5
4.0
Cellular_organisms - ps1
Archaea1 - ps2
Archaea2 - ps3
Archaea3 - ps4
Asgard/Eukaryota - ps5
Eukaryota - ps6
Amorpheae - ps7
Obazoa - ps8
Opisthokonta - ps9
Holomycota - ps10
Fungi - ps11
Fungi_1 - ps12
Fungi_2 - ps13
Fungi_3 - ps14
Dikarya - ps15
Ascomycota - ps16
Saccharo/Pezizomycotina - ps17
Saccharomycotina - ps18
Saccharomycotina_1 - ps19
Saccharomycotina_2 - ps20
Saccharomycotina_3 - ps21
Phaff./Saccharomycetaceae - ps22
Saccharomyco./mycetaceae - ps23
Saccharomycetaceae - ps24
Saccharomycetaceae_2 - ps25
Saccharomycetaceae_3 - ps26
Kaz./Nau./Saccharomyces - ps27
Saccharomyces - ps28
Arboricola_clade - ps29
Cerevisiae_clade - ps30
Saccharomyces_cerevisiae - ps31
Phylostratum

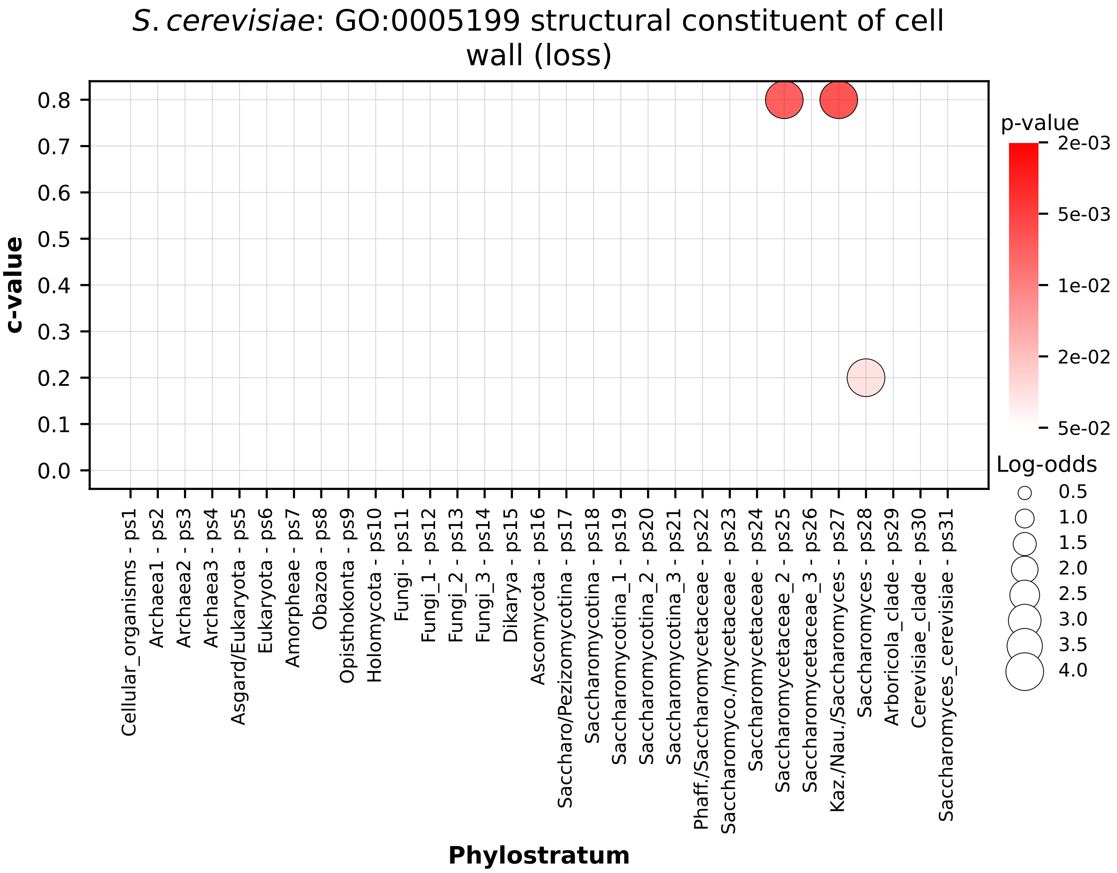

S. cerevisiae: GO:0005199 structural constituent of cell wall (loss)
c-value
0.0
0.1
0.2
0.3
0.4
0.5
0.6
0.7
0.8
p-value
2e-03
5e-03
1e-02
2e-02
5e-02
Log-odds
0.5
1.0
1.5
2.0
2.5
3.0
3.5
4.0
Cellular_organisms - ps1
Archaea1 - ps2
Archaea2 - ps3
Archaea3 - ps4
Asgard/Eukaryota - ps5
Eukaryota - ps6
Amorpheae - ps7
Obazoa - ps8
Opisthokonta - ps9
Holomycota - ps10
Fungi - ps11
Fungi_1 - ps12
Fungi_2 - ps13
Fungi_3 - ps14
Dikarya - ps15
Ascomycota - ps16
Saccharo/Pezizomycotina - ps17
Saccharomycotina - ps18
Saccharomycotina_1 - ps19
Saccharomycotina_2 - ps20
Saccharomycotina_3 - ps21
Phaff./Saccharomycetaceae - ps22
Saccharomyco./mycetaceae - ps23
Saccharomycetaceae - ps24
Saccharomycetaceae_2 - ps25
Saccharomycetaceae_3 - ps26
Kaz./Nau./Saccharomyces - ps27
Saccharomyces - ps28
Arboricola_clade - ps29
Cerevisiae_clade - ps30
Saccharomyces_cerevisiae - ps31
Phylostratum

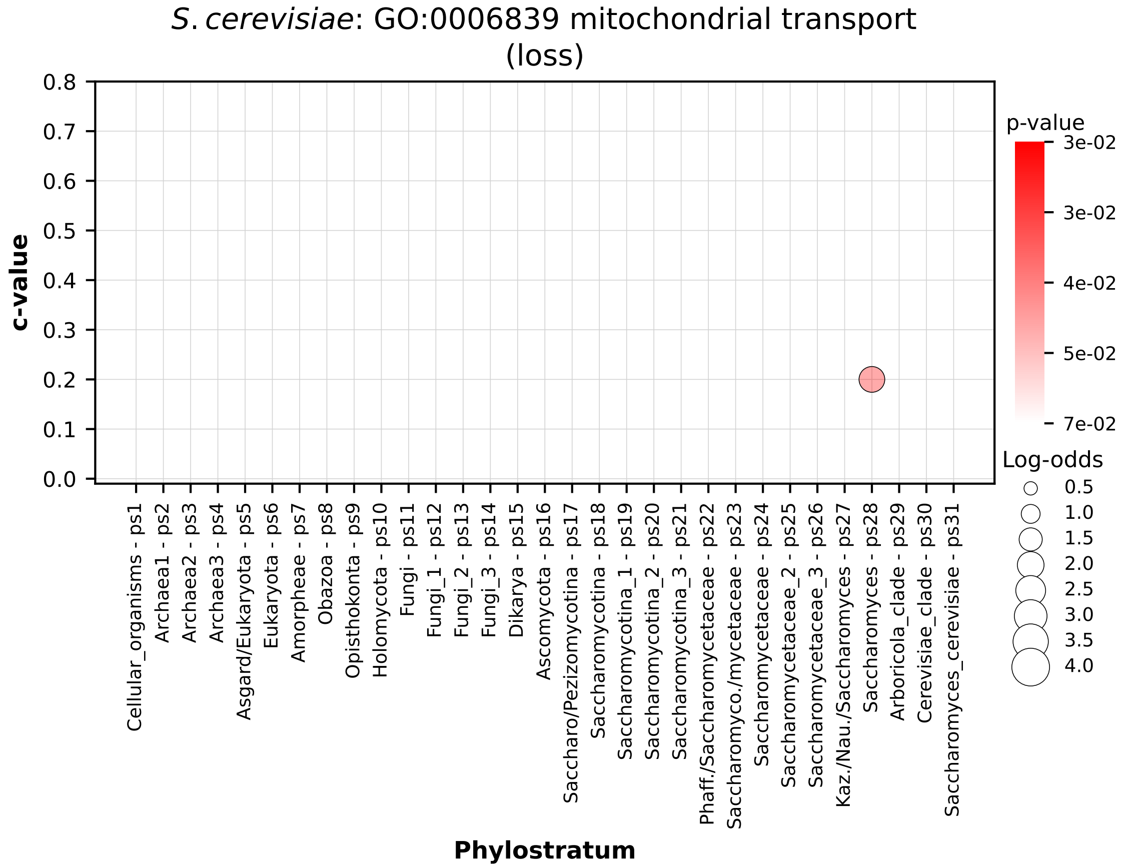

S. cerevisiae: GO:0006839 mitochondrial transport
(loss)
c-value
0.8
0.7
0.6
0.5
0.4
0.3
0.2
0.1
0.0
Phylostratum
Cellular_organisms - ps1
Archaea1 - ps2
Archaea2 - ps3
Archaea3 - ps4
Asgard/Eukaryota - ps5
Eukaryota - ps6
Amorpheae - ps7
Obazoa - ps8
Opisthokonta - ps9
Holomycota - ps10
Fungi - ps11
Fungi_1 - ps12
Fungi_2 - ps13
Fungi_3 - ps14
Dikarya - ps15
Ascomycota - ps16
Saccharo/Pezizomycotina - ps17
Saccharomycotina - ps18
Saccharomycotina_1 - ps19
Saccharomycotina_2 - ps20
Saccharomycotina_3 - ps21
Phaff./Saccharomycetaceae - ps22
Saccharomyco./mycetaceae - ps23
Saccharomycetaceae - ps24
Saccharomycetaceae_2 - ps25
Saccharomycetaceae_3 - ps26
Kaz./Nau./Saccharomyces - ps27
Saccharomyces - ps28
Arboricola_clade - ps29
Cerevisiae_clade - ps30
Saccharomyces_cerevisiae - ps31
p-value
3e-02
3e-02
4e-02
5e-02
7e-02
Log-odds
0.5
1.0
1.5
2.0
2.5
3.0
3.5
4.0

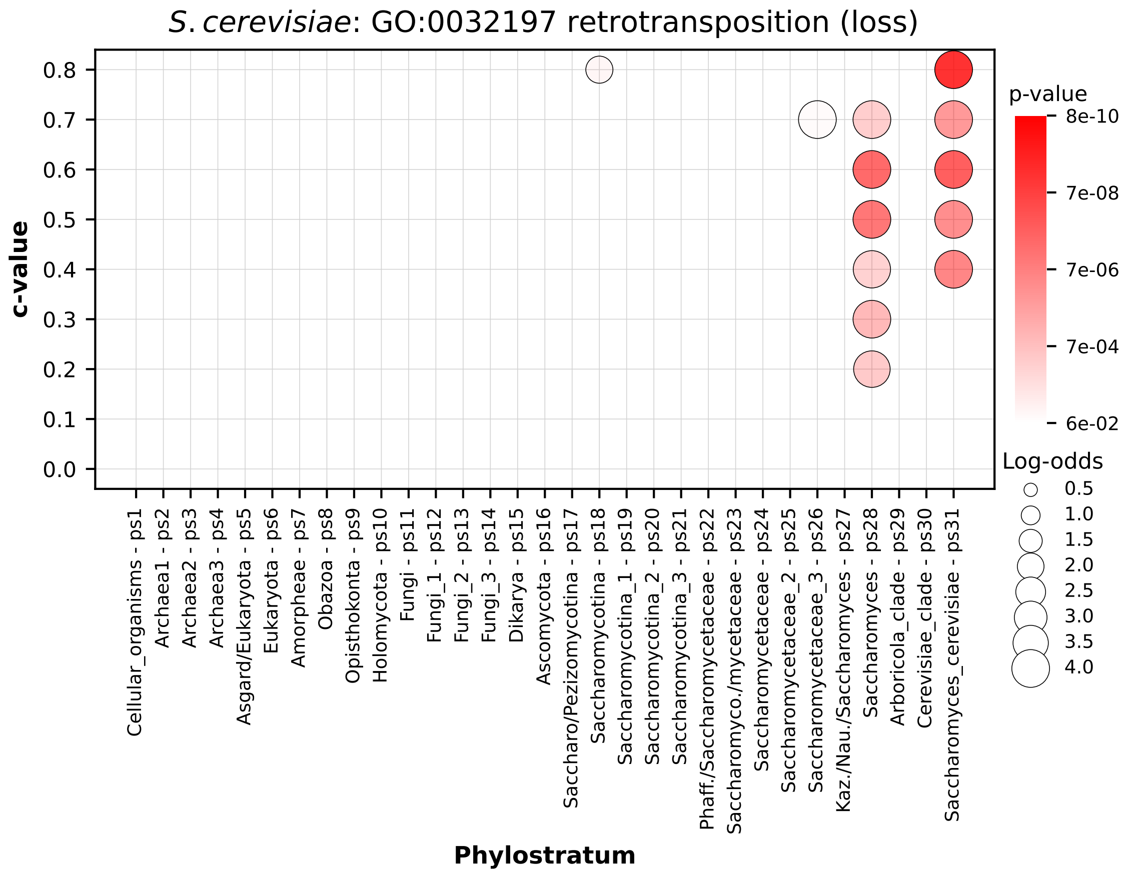
S. cerevisiae: GO:0032197 retrotransposition (loss)
c-value
0.8
0.7
0.6
0.5
0.4
0.3
0.2
0.1
0.0
Phylostratum
Cellular_organisms - ps1
Archaea1 - ps2
Archaea2 - ps3
Archaea3 - ps4
Asgard/Eukaryota - ps5
Eukaryota - ps6
Amorpheae - ps7
Obazoa - ps8
Opisthokonta - ps9
Holomycota - ps10
Fungi - ps11
Fungi_1 - ps12
Fungi_2 - ps13
Fungi_3 - ps14
Dikarya - ps15
Ascomycota - ps16
Saccharo/Pezizomycotina - ps17
Saccharomycotina - ps18
Saccharomycotina_1 - ps19
Saccharomycotina_2 - ps20
Saccharomycotina_3 - ps21
Phaff./Saccharomycetaceae - ps22
Saccharomyco./mycetaceae - ps23
Saccharomycetaceae - ps24
Saccharomycetaceae_2 - ps25
Saccharomycetaceae_3 - ps26
Kaz./Nau./Saccharomyces - ps27
Saccharomyces - ps28
Arboricola_clade - ps29
Cerevisiae_clade - ps30
Saccharomyces_cerevisiae - ps31
p-value
8e-10
7e-08
7e-06
7e-04
6e-02
Log-odds
0.5
1.0
1.5
2.0
2.5
3.0
3.5
4.0

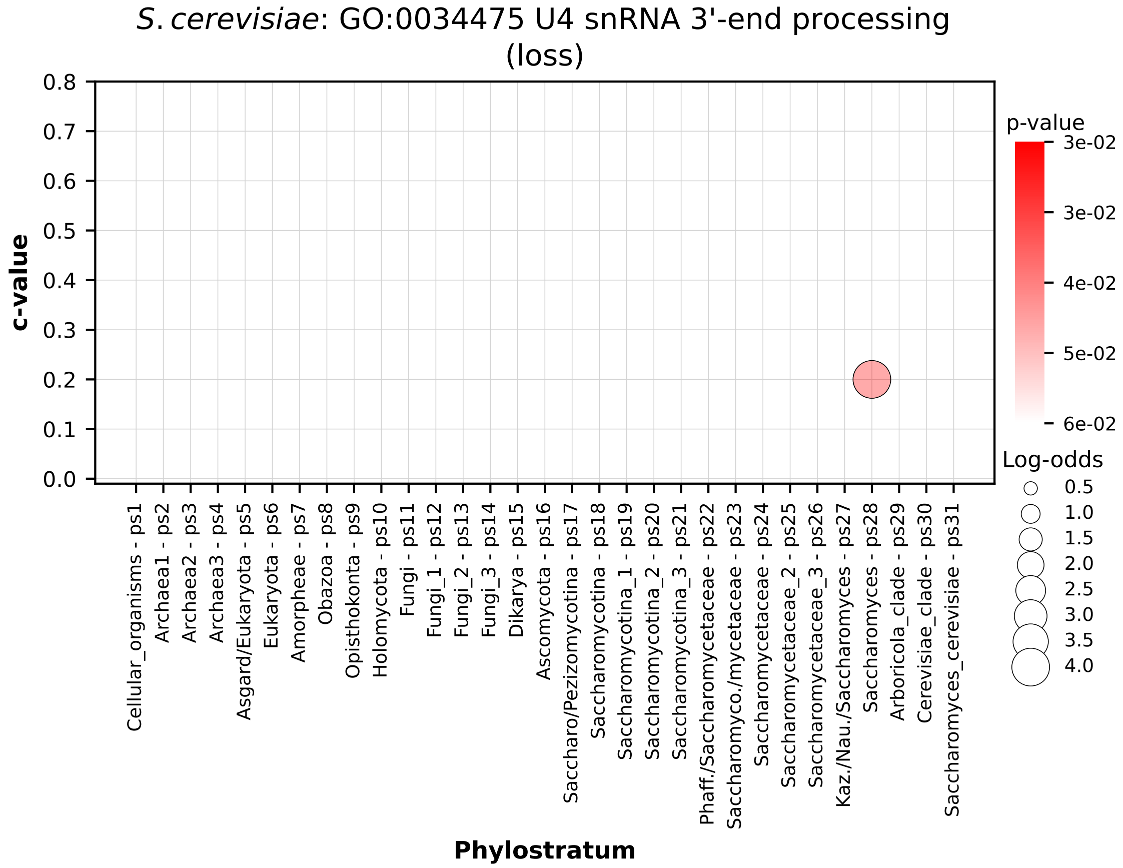
S. cerevisiae: GO:0034475 U4 snRNA 3'-end processing
(loss)
c-value
0.0
0.1
0.2
0.3
0.4
0.5
0.6
0.7
0.8
p-value
3e-02
3e-02
4e-02
5e-02
6e-02
Log-odds
0.5
1.0
1.5
2.0
2.5
3.0
3.5
4.0
Cellular_organisms - ps1
Archaea1 - ps2
Archaea2 - ps3
Archaea3 - ps4
Asgard/Eukaryota - ps5
Eukaryota - ps6
Amorpheae - ps7
Obazoa - ps8
Opisthokonta - ps9
Holomycota - ps10
Fungi - ps11
Fungi_1 - ps12
Fungi_2 - ps13
Fungi_3 - ps14
Dikarya - ps15
Ascomycota - ps16
Saccharo/Pezizomycotina - ps17
Saccharomycotina - ps18
Saccharomycotina_1 - ps19
Saccharomycotina_2 - ps20
Saccharomycotina_3 - ps21
Phaff./Saccharomycetaceae - ps22
Saccharomyco./mycetaceae - ps23
Saccharomycetaceae - ps24
Saccharomycetaceae_2 - ps25
Saccharomycetaceae_3 - ps26
Kaz./Nau./Saccharomyces - ps27
Saccharomyces - ps28
Arboricola_clade - ps29
Cerevisiae_clade - ps30
Saccharomyces_cerevisiae - ps31
Phylostratum

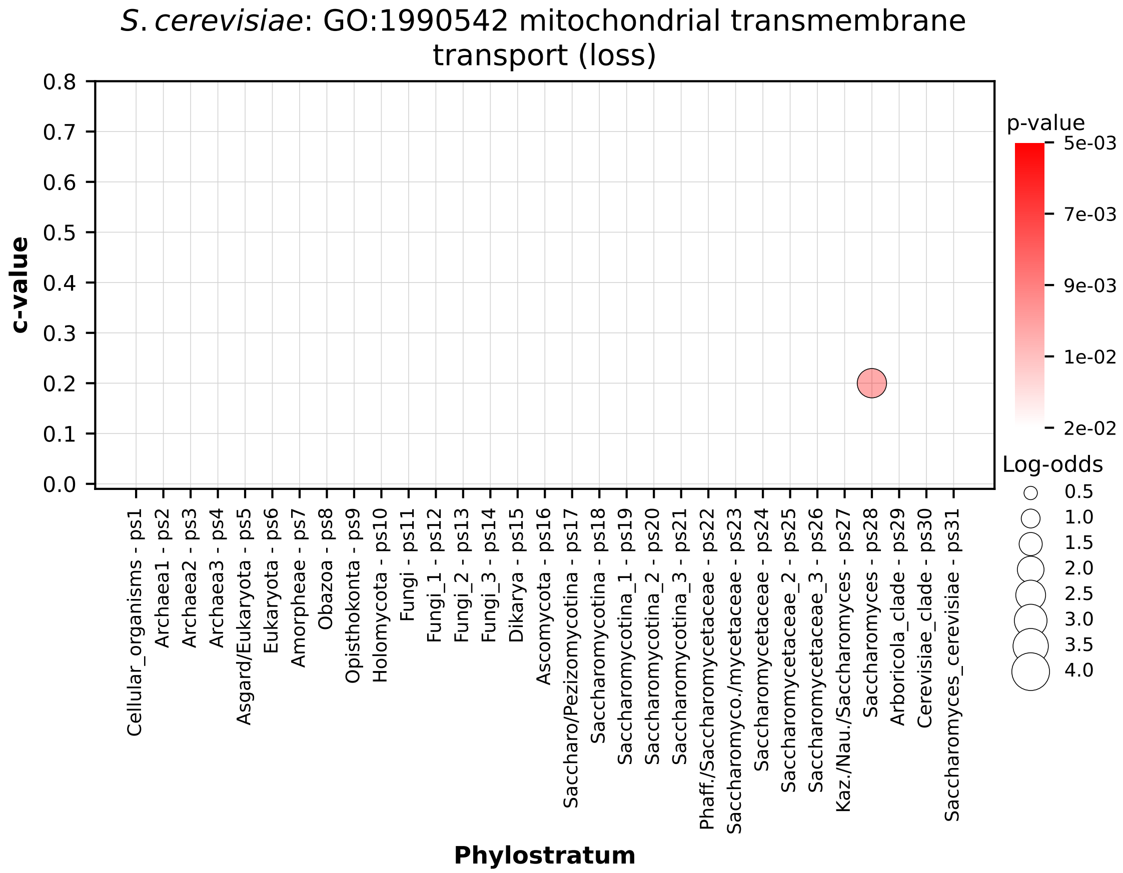

S. cerevisiae: GO:1990542 mitochondrial transmembrane transport (loss)
c-value
0.0
0.1
0.2
0.3
0.4
0.5
0.6
0.7
0.8
Phylostratum
Cellular_organisms - ps1
Archaea1 - ps2
Archaea2 - ps3
Archaea3 - ps4
Asgard/Eukaryota - ps5
Eukaryota - ps6
Amorpheae - ps7
Obazoa - ps8
Opisthokonta - ps9
Holomycota - ps10
Fungi - ps11
Fungi_1 - ps12
Fungi_2 - ps13
Fungi_3 - ps14
Dikarya - ps15
Ascomycota - ps16
Saccharo/Pezizomycotina - ps17
Saccharomycotina - ps18
Saccharomycotina_1 - ps19
Saccharomycotina_2 - ps20
Saccharomycotina_3 - ps21
Phaff./Saccharomycetaceae - ps22
Saccharomyco./mycetaceae - ps23
Saccharomycetaceae - ps24
Saccharomycetaceae_2 - ps25
Saccharomycetaceae_3 - ps26
Kaz./Nau./Saccharomyces - ps27
Saccharomyces - ps28
Arboricola_clade - ps29
Cerevisiae_clade - ps30
Saccharomyces_cerevisiae - ps31
p-value
5e-03
7e-03
9e-03
1e-02
2e-02
Log-odds
0.5
1.0
1.5
2.0
2.5
3.0
3.5
4.0

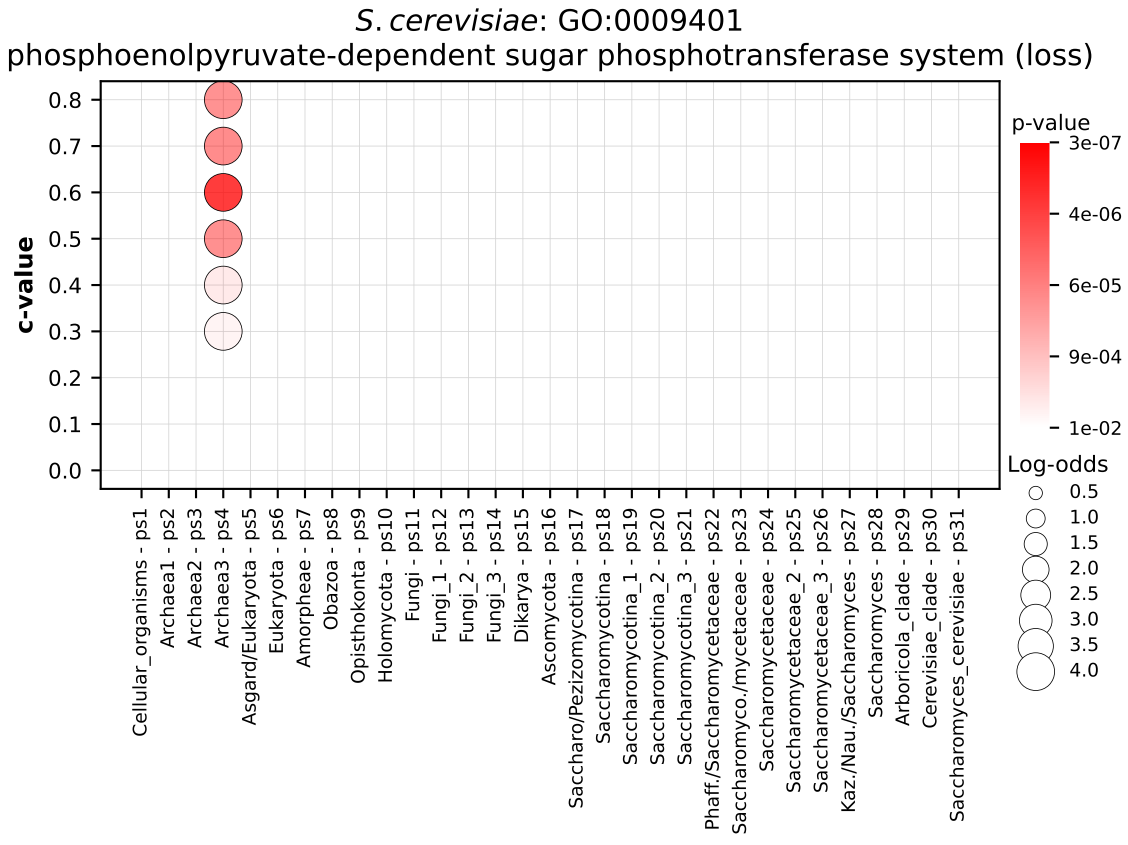

S. cerevisiae: GO:0009401
phosphoenolpyruvate-dependent sugar phosphotransferase system (loss)
c-value
0.8
0.7
0.6
0.5
0.4
0.3
0.2
0.1
0.0
Phylostratum
Cellular_organisms - ps1
Archaea1 - ps2
Archaea2 - ps3
Archaea3 - ps4
Asgard/Eukaryota - ps5
Eukaryota - ps6
Amorpheae - ps7
Obazoa - ps8
Opisthokonta - ps9
Holomycota - ps10
Fungi - ps11
Fungi_1 - ps12
Fungi_2 - ps13
Fungi_3 - ps14
Dikarya - ps15
Ascomycota - ps16
Saccharo/Pezizomycotina - ps17
Saccharomycotina - ps18
Saccharomycotina_1 - ps19
Saccharomycotina_2 - ps20
Saccharomycotina_3 - ps21
Phaff./Saccharomycetaceae - ps22
Saccharomyco./mycetaceae - ps23
Saccharomycetaceae - ps24
Saccharomycetaceae_2 - ps25
Saccharomycetaceae_3 - ps26
Kaz./Nau./Saccharomyces - ps27
Saccharomyces - ps28
Arboricola_clade - ps29
Cerevisiae_clade - ps30
Saccharomyces_cerevisiae - ps31
p-value
3e-07
4e-06
6e-05
9e-04
1e-02
Log-odds
0.5
1.0
1.5
2.0
2.5
3.0
3.5
4.0

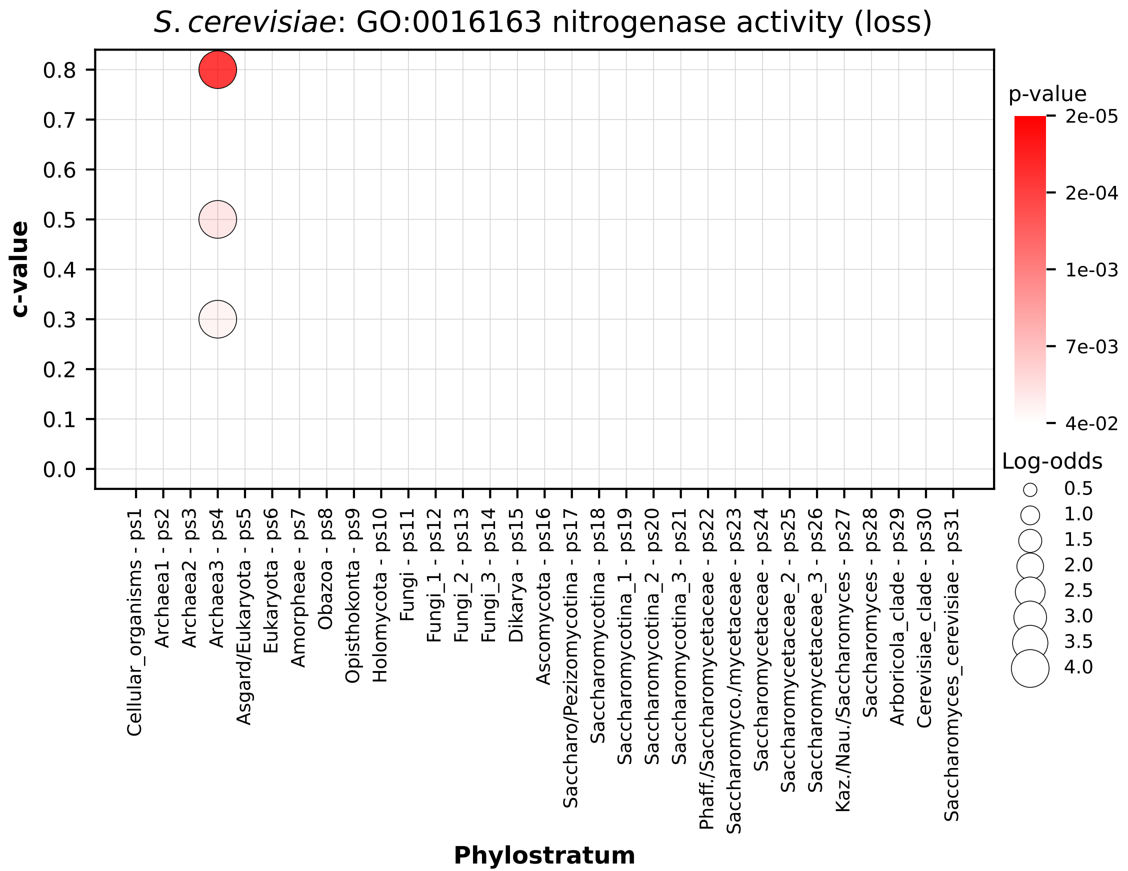
S. cerevisiae: GO:0016163 nitrogenase activity (loss)
c-value
0.8
0.7
0.6
0.5
0.4
0.3
0.2
0.1
0.0
p-value
2e-05
2e-04
1e-03
7e-03
4e-02
Log-odds
0.5
1.0
1.5
2.0
2.5
3.0
3.5
4.0
Cellular_organisms - ps1
Archaea1 - ps2
Archaea2 - ps3
Archaea3 - ps4
Asgard/Eukaryota - ps5
Eukaryota - ps6
Amorpheae - ps7
Obazoa - ps8
Opisthokonta - ps9
Holomycota - ps10
Fungi - ps11
Fungi_1 - ps12
Fungi_2 - ps13
Fungi_3 - ps14
Dikarya - ps15
Ascomycota - ps16
Saccharo/Pezizomycotina - ps17
Saccharomycotina - ps18
Saccharomycotina_1 - ps19
Saccharomycotina_2 - ps20
Saccharomycotina_3 - ps21
Phaff./Saccharomycetaceae - ps22
Saccharomyco./mycetaceae - ps23
Saccharomycetaceae - ps24
Saccharomycetaceae_2 - ps25
Saccharomycetaceae_3 - ps26
Kaz./Nau./Saccharomyces - ps27
Saccharomyces - ps28
Arboricola_clade - ps29
Cerevisiae_clade - ps30
Saccharomyces_cerevisiae - ps31
Phylostratum

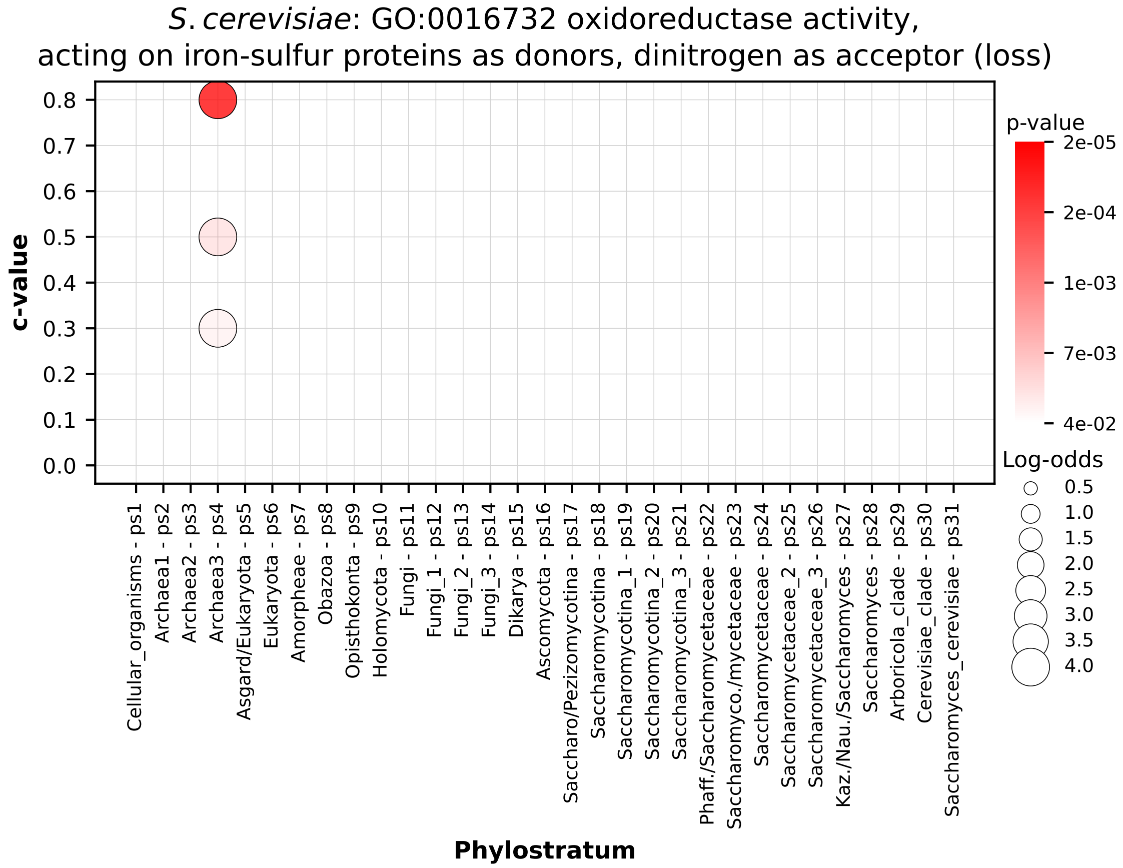

S. cerevisiae: GO:0016732 oxidoreductase activity,
acting on iron-sulfur proteins as donors, dinitrogen as acceptor (loss)
c-value
0.8
0.7
0.6
0.5
0.4
0.3
0.2
0.1
0.0
p-value
2e-05
2e-04
1e-03
7e-03
4e-02
Log-odds
0.5
1.0
1.5
2.0
2.5
3.0
3.5
4.0
Cellular_organisms - ps1
Archaea1 - ps2
Archaea2 - ps3
Archaea3 - ps4
Asgard/Eukaryota - ps5
Eukaryota - ps6
Amorpheae - ps7
Obazoa - ps8
Opisthokonta - ps9
Holomycota - ps10
Fungi - ps11
Fungi_1 - ps12
Fungi_2 - ps13
Fungi_3 - ps14
Dikarya - ps15
Ascomycota - ps16
Saccharo/Pezizomycotina - ps17
Saccharomycotina - ps18
Saccharomycotina_1 - ps19
Saccharomycotina_2 - ps20
Saccharomycotina_3 - ps21
Phaff./Saccharomycetaceae - ps22
Saccharomyco./mycetaceae - ps23
Saccharomycetaceae - ps24
Saccharomycetaceae_2 - ps25
Saccharomycetaceae_3 - ps26
Kaz./Nau./Saccharomyces - ps27
Saccharomyces - ps28
Arboricola_clade - ps29
Cerevisiae_clade - ps30
Saccharomyces_cerevisiae - ps31
Phylostratum

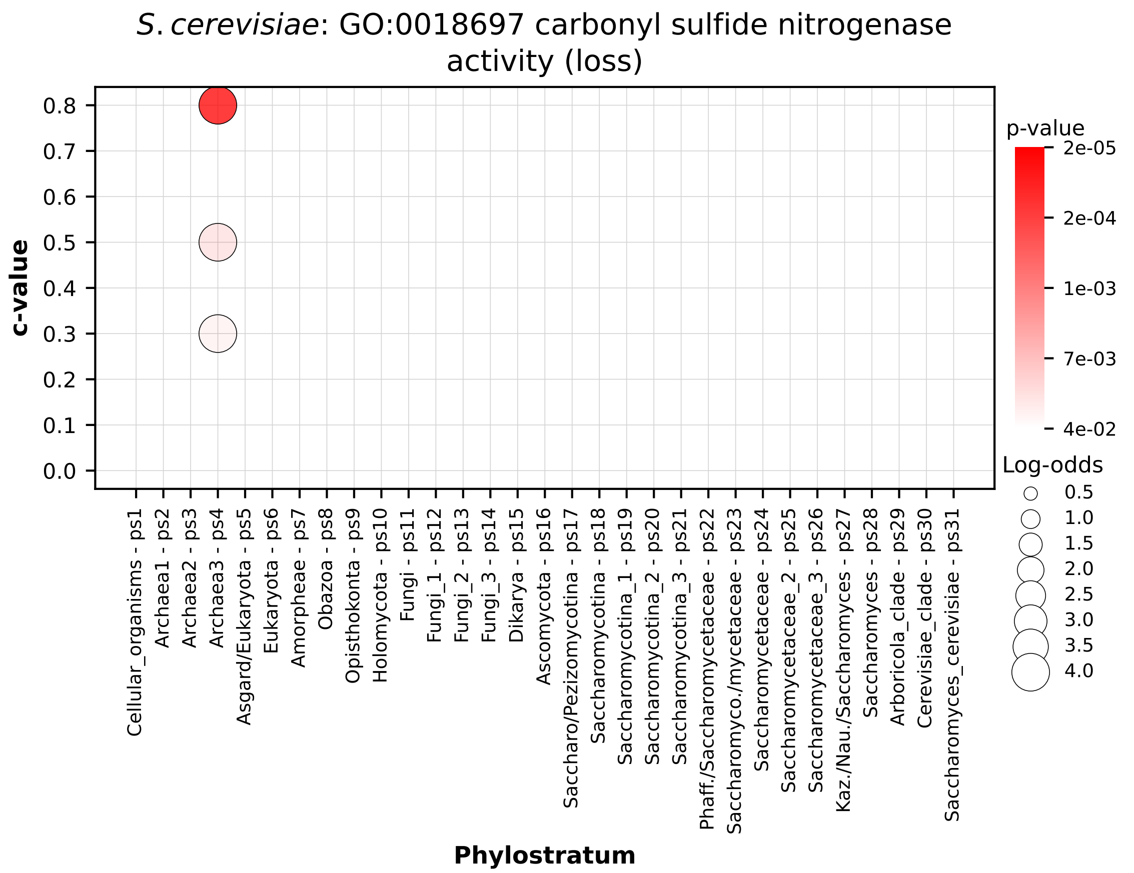

S. cerevisiae: GO:0018697 carbonyl sulfide nitrogenase activity (loss)
c-value
0.0
0.1
0.2
0.3
0.4
0.5
0.6
0.7
0.8
Phylostratum
Cellular_organisms - ps1
Archaea1 - ps2
Archaea2 - ps3
Archaea3 - ps4
Asgard/Eukaryota - ps5
Eukaryota - ps6
Amorpheae - ps7
Obazoa - ps8
Opisthokonta - ps9
Holomycota - ps10
Fungi - ps11
Fungi_1 - ps12
Fungi_2 - ps13
Fungi_3 - ps14
Dikarya - ps15
Ascomycota - ps16
Saccharo/Pezizomycotina - ps17
Saccharomycotina - ps18
Saccharomycotina_1 - ps19
Saccharomycotina_2 - ps20
Saccharomycotina_3 - ps21
Phaff./Saccharomycetaceae - ps22
Saccharomyco./mycetaceae - ps23
Saccharomycetaceae - ps24
Saccharomycetaceae_2 - ps25
Saccharomycetaceae_3 - ps26
Kaz./Nau./Saccharomyces - ps27
Saccharomyces - ps28
Arboricola_clade - ps29
Cerevisiae_clade - ps30
Saccharomyces_cerevisiae - ps31
p-value
2e-05
2e-04
1e-03
7e-03
4e-02
Log-odds
0.5
1.0
1.5
2.0
2.5
3.0
3.5
4.0

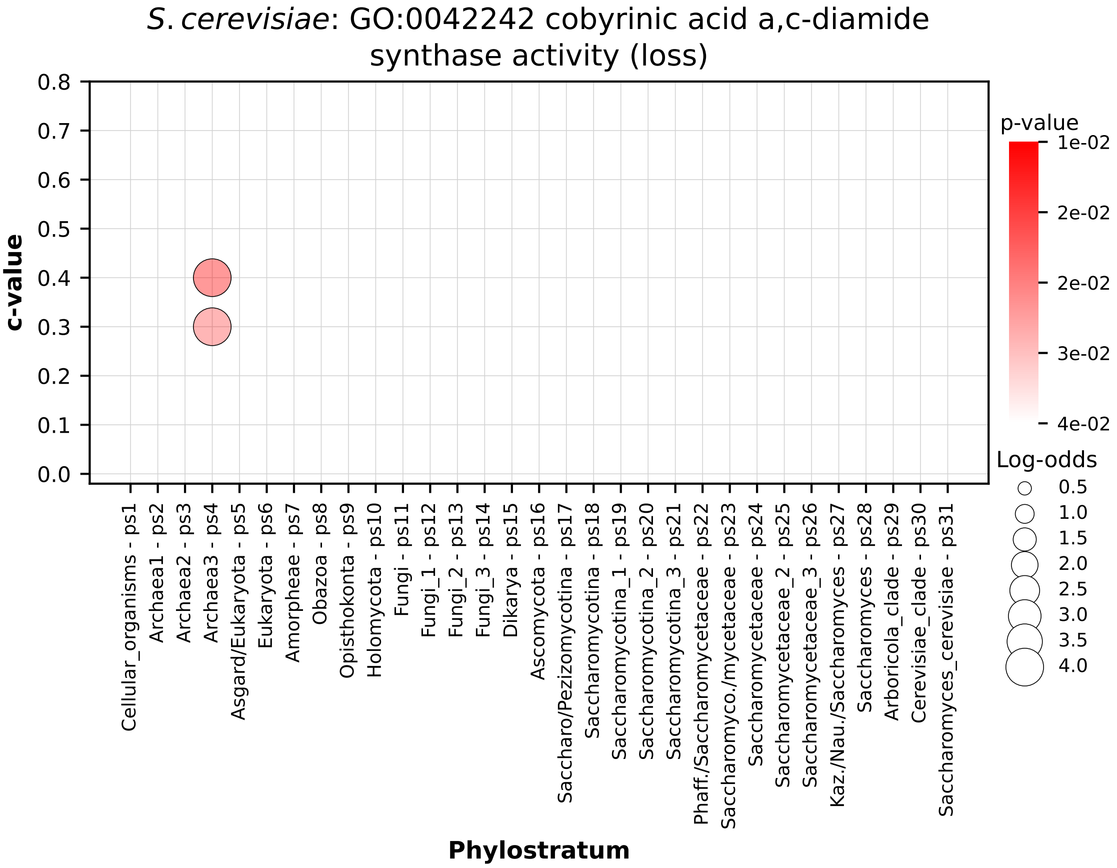

S. cerevisiae: GO:0042242 cobyrinic acid a,c-diamide synthase activity (loss)
c-value
0.8
0.7
0.6
0.5
0.4
0.3
0.2
0.1
0.0
p-value
1e-02
2e-02
2e-02
3e-02
4e-02
Log-odds
0.5
1.0
1.5
2.0
2.5
3.0
3.5
4.0
Cellular_organisms - ps1
Archaea1 - ps2
Archaea2 - ps3
Archaea3 - ps4
Asgard/Eukaryota - ps5
Eukaryota - ps6
Amorpheae - ps7
Obazoa - ps8
Opisthokonta - ps9
Holomycota - ps10
Fungi - ps11
Fungi_1 - ps12
Fungi_2 - ps13
Fungi_3 - ps14
Dikarya - ps15
Ascomycota - ps16
Saccharo/Pezizomycotina - ps17
Saccharomycotina - ps18
Saccharomycotina_1 - ps19
Saccharomycotina_2 - ps20
Saccharomycotina_3 - ps21
Phaff./Saccharomycetaceae - ps22
Saccharomyco./mycetaceae - ps23
Saccharomycetaceae - ps24
Saccharomycetaceae_2 - ps25
Saccharomycetaceae_3 - ps26
Kaz./Nau./Saccharomyces - ps27
Saccharomyces - ps28
Arboricola_clade - ps29
Cerevisiae_clade - ps30
Saccharomyces_cerevisiae - ps31
Phylostratum

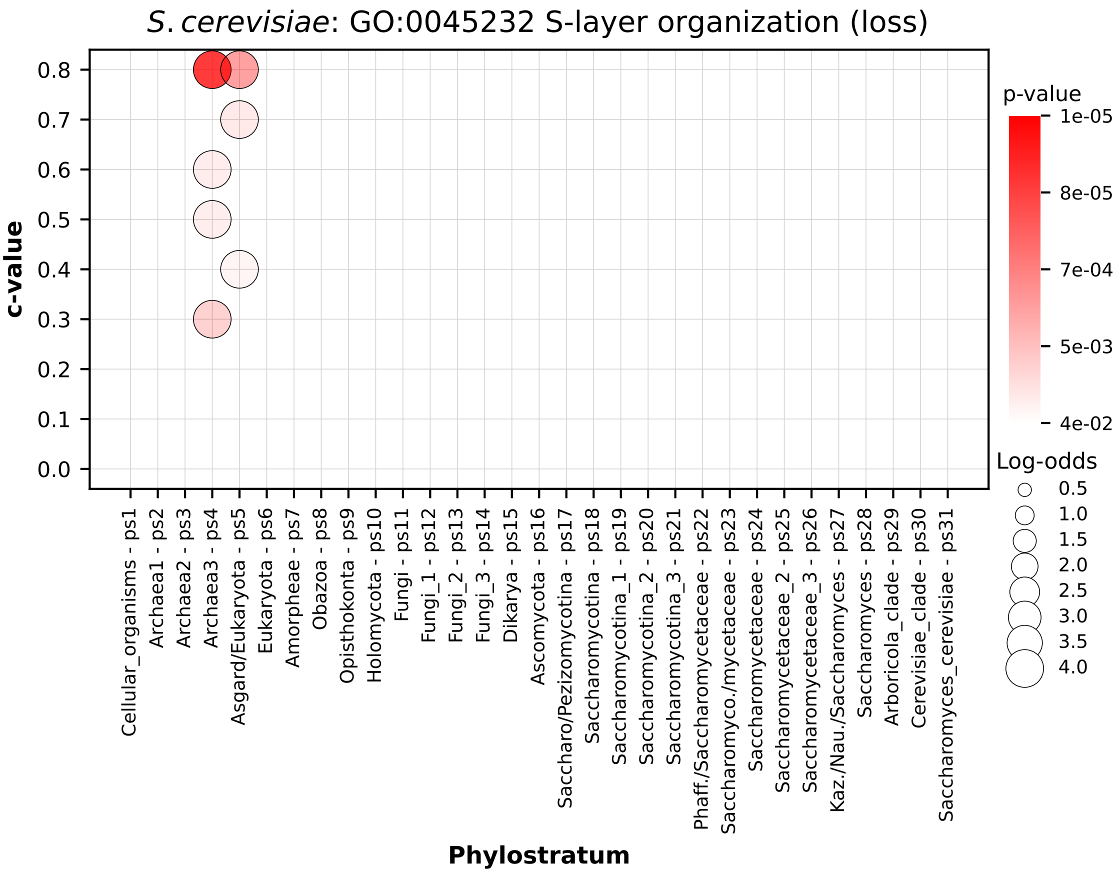

S. cerevisiae: GO:0045232 S-layer organization (loss)
c-value
0.0
0.1
0.2
0.3
0.4
0.5
0.6
0.7
0.8
Phylostratum
Cellular_organisms - ps1
Archaea1 - ps2
Archaea2 - ps3
Archaea3 - ps4
Asgard/Eukaryota - ps5
Eukaryota - ps6
Amorpheae - ps7
Obazoa - ps8
Opisthokonta - ps9
Holomycota - ps10
Fungi - ps11
Fungi_1 - ps12
Fungi_2 - ps13
Fungi_3 - ps14
Dikarya - ps15
Ascomycota - ps16
Saccharo/Pezizomycotina - ps17
Saccharomycotina - ps18
Saccharomycotina_1 - ps19
Saccharomycotina_2 - ps20
Saccharomycotina_3 - ps21
Phaff./Saccharomycetaceae - ps22
Saccharomyco./mycetaceae - ps23
Saccharomycetaceae - ps24
Saccharomycetaceae_2 - ps25
Saccharomycetaceae_3 - ps26
Kaz./Nau./Saccharomyces - ps27
Saccharomyces - ps28
Arboricola_clade - ps29
Cerevisiae_clade - ps30
Saccharomyces_cerevisiae - ps31
p-value
1e-05
8e-05
7e-04
5e-03
4e-02
Log-odds
0.5
1.0
1.5
2.0
2.5
3.0
3.5
4.0

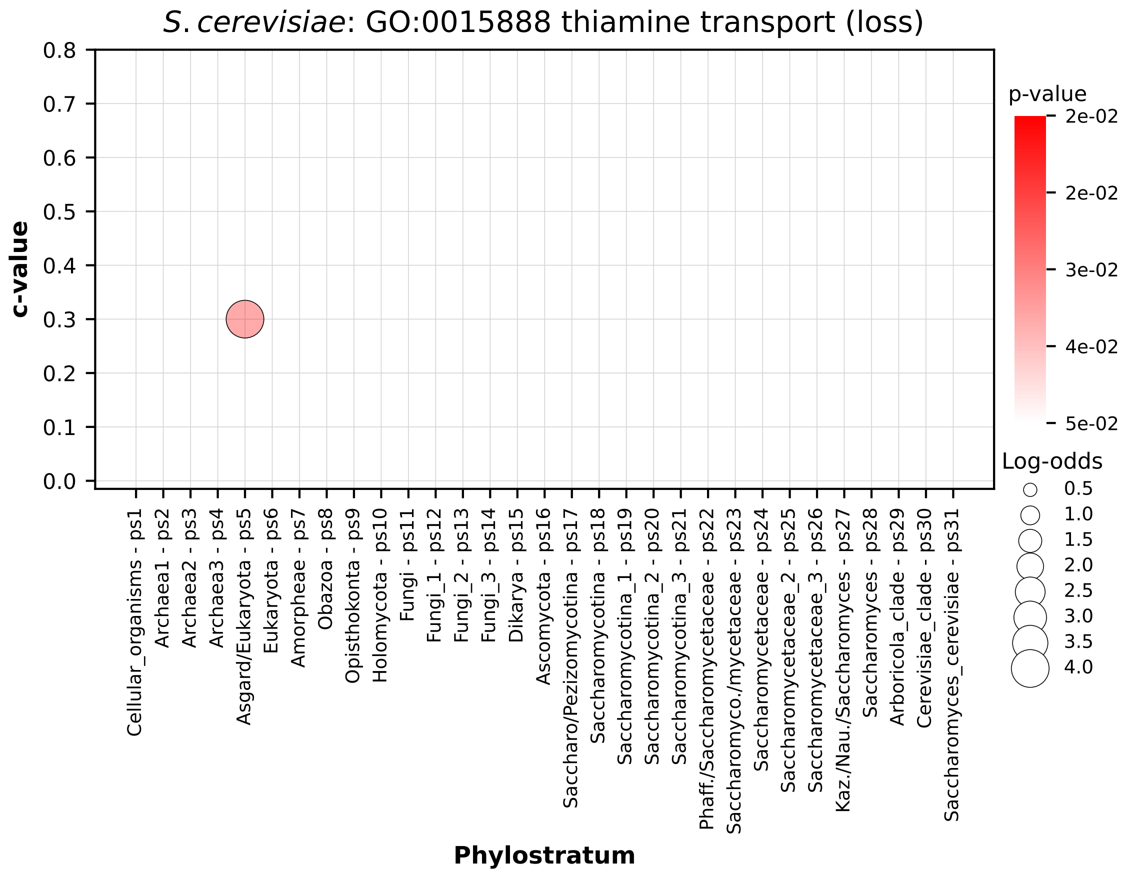
S. cerevisiae: GO:0015888 thiamine transport (loss)
c-value
0.8
0.7
0.6
0.5
0.4
0.3
0.2
0.1
0.0
Phylostratum
Cellular_organisms - ps1
Archaea1 - ps2
Archaea2 - ps3
Archaea3 - ps4
Asgard/Eukaryota - ps5
Eukaryota - ps6
Amorpheae - ps7
Obazoa - ps8
Opisthokonta - ps9
Holomycota - ps10
Fungi - ps11
Fungi_1 - ps12
Fungi_2 - ps13
Fungi_3 - ps14
Dikarya - ps15
Ascomycota - ps16
Saccharo/Pezizomycotina - ps17
Saccharomycotina - ps18
Saccharomycotina_1 - ps19
Saccharomycotina_2 - ps20
Saccharomycotina_3 - ps21
Phaff./Saccharomycetaceae - ps22
Saccharomyco./mycetaceae - ps23
Saccharomycetaceae - ps24
Saccharomycetaceae_2 - ps25
Saccharomycetaceae_3 - ps26
Kaz./Nau./Saccharomyces - ps27
Saccharomyces - ps28
Arboricola_clade - ps29
Cerevisiae_clade - ps30
Saccharomyces_cerevisiae - ps31
p-value
2e-02
2e-02
3e-02
4e-02
5e-02
Log-odds
0.5
1.0
1.5
2.0
2.5
3.0
3.5
4.0

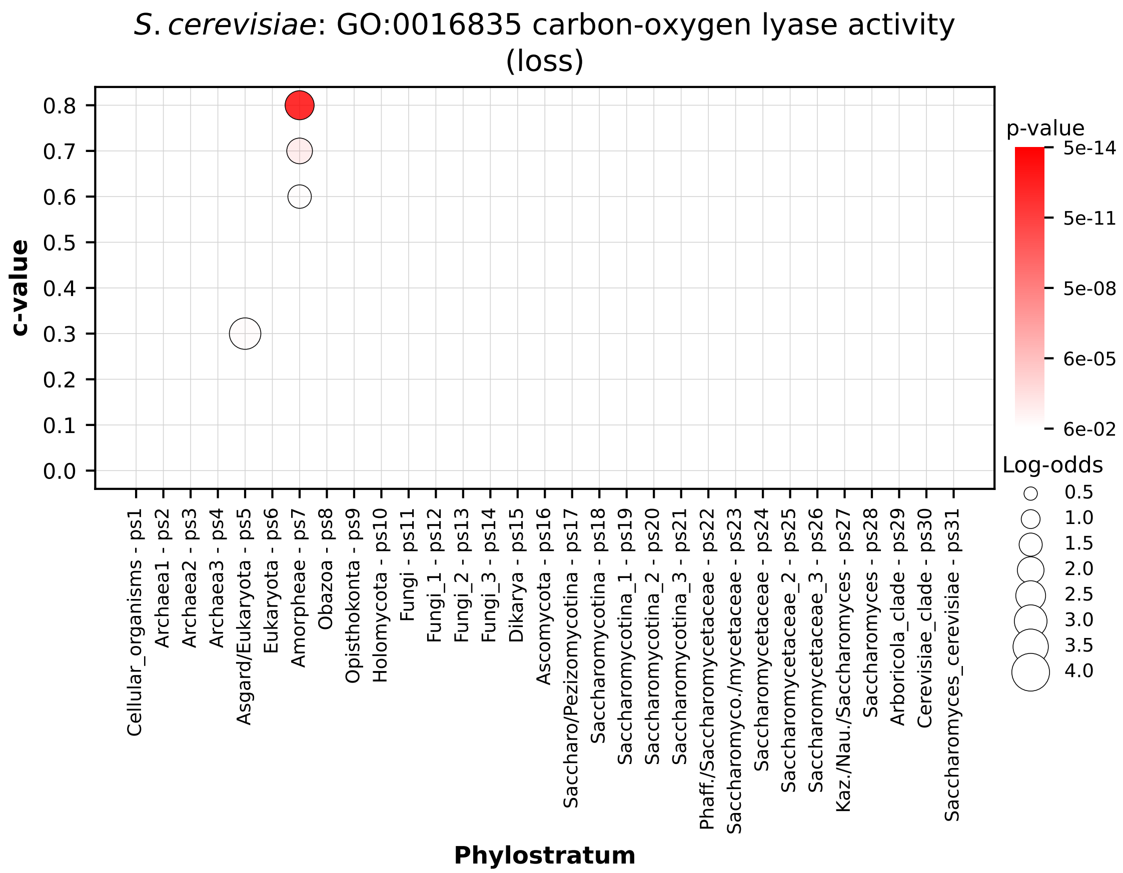

S. cerevisiae: GO:0016835 carbon-oxygen lyase activity (loss)
c-value
0.8
0.7
0.6
0.5
0.4
0.3
0.2
0.1
0.0
Phylostratum
Cellular_organisms - ps1
Archaea1 - ps2
Archaea2 - ps3
Archaea3 - ps4
Asgard/Eukaryota - ps5
Eukaryota - ps6
Amorpheae - ps7
Obazoa - ps8
Opisthokonta - ps9
Holomycota - ps10
Fungi - ps11
Fungi_1 - ps12
Fungi_2 - ps13
Fungi_3 - ps14
Dikarya - ps15
Ascomycota - ps16
Saccharo/Pezizomycotina - ps17
Saccharomycotina - ps18
Saccharomycotina_1 - ps19
Saccharomycotina_2 - ps20
Saccharomycotina_3 - ps21
Phaff./Saccharomycetaceae - ps22
Saccharomyco./mycetaceae - ps23
Saccharomycetaceae - ps24
Saccharomycetaceae_2 - ps25
Saccharomycetaceae_3 - ps26
Kaz./Nau./Saccharomyces - ps27
Saccharomyces - ps28
Arboricola_clade - ps29
Cerevisiae_clade - ps30
Saccharomyces_cerevisiae - ps31
p-value
5e-14
5e-11
5e-08
6e-05
6e-02
Log-odds
0.5
1.0
1.5
2.0
2.5
3.0
3.5
4.0

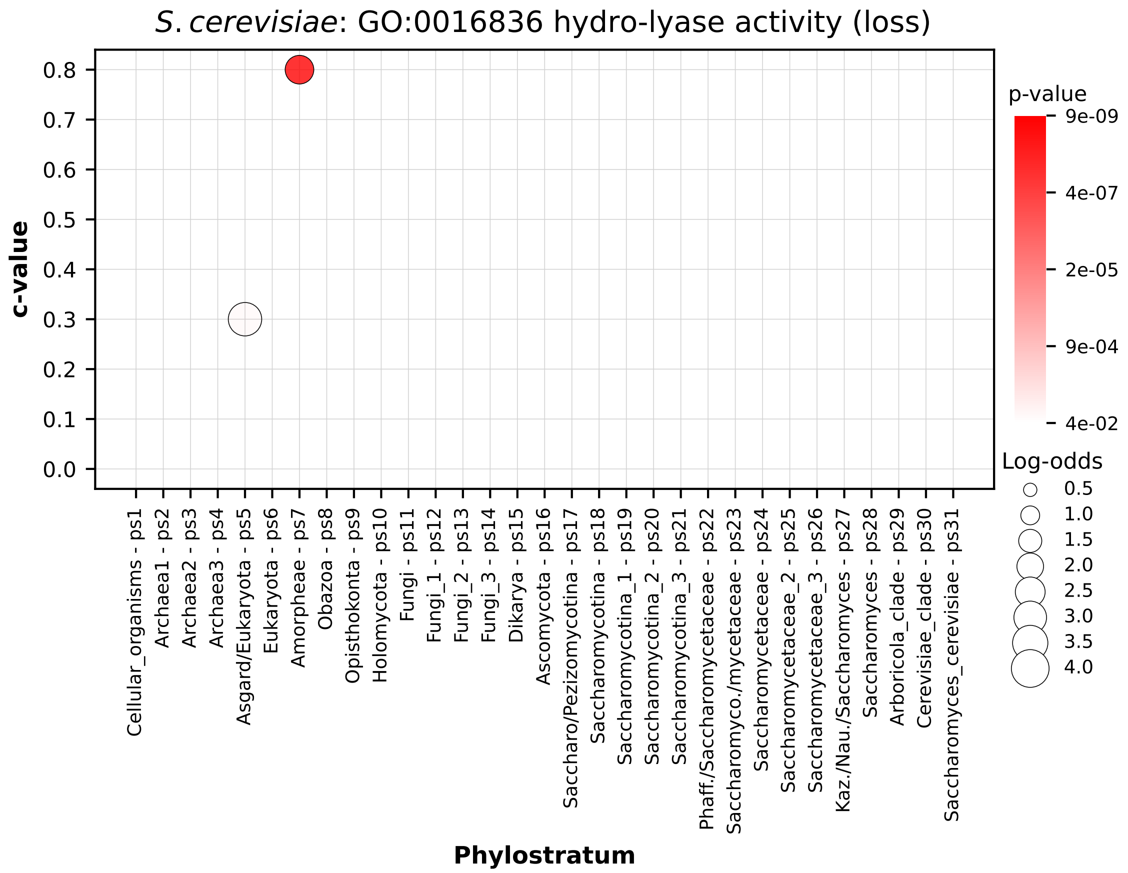
S. cerevisiae: GO:0016836 hydro-lyase activity (loss)
c-value
0.0
0.1
0.2
0.3
0.4
0.5
0.6
0.7
0.8
p-value
9e-09
4e-07
2e-05
9e-04
4e-02
Log-odds
0.5
1.0
1.5
2.0
2.5
3.0
3.5
4.0
Cellular_organisms - ps1
Archaea1 - ps2
Archaea2 - ps3
Archaea3 - ps4
Asgard/Eukaryota - ps5
Eukaryota - ps6
Amorpheae - ps7
Obazoa - ps8
Opisthokonta - ps9
Holomycota - ps10
Fungi - ps11
Fungi_1 - ps12
Fungi_2 - ps13
Fungi_3 - ps14
Dikarya - ps15
Ascomycota - ps16
Saccharo/Pezizomycotina - ps17
Saccharomycotina - ps18
Saccharomycotina_1 - ps19
Saccharomycotina_2 - ps20
Saccharomycotina_3 - ps21
Phaff./Saccharomycetaceae - ps22
Saccharomyco./mycetaceae - ps23
Saccharomycetaceae - ps24
Saccharomycetaceae_2 - ps25
Saccharomycetaceae_3 - ps26
Kaz./Nau./Saccharomyces - ps27
Saccharomyces - ps28
Arboricola_clade - ps29
Cerevisiae_clade - ps30
Saccharomyces_cerevisiae - ps31
Phylostratum

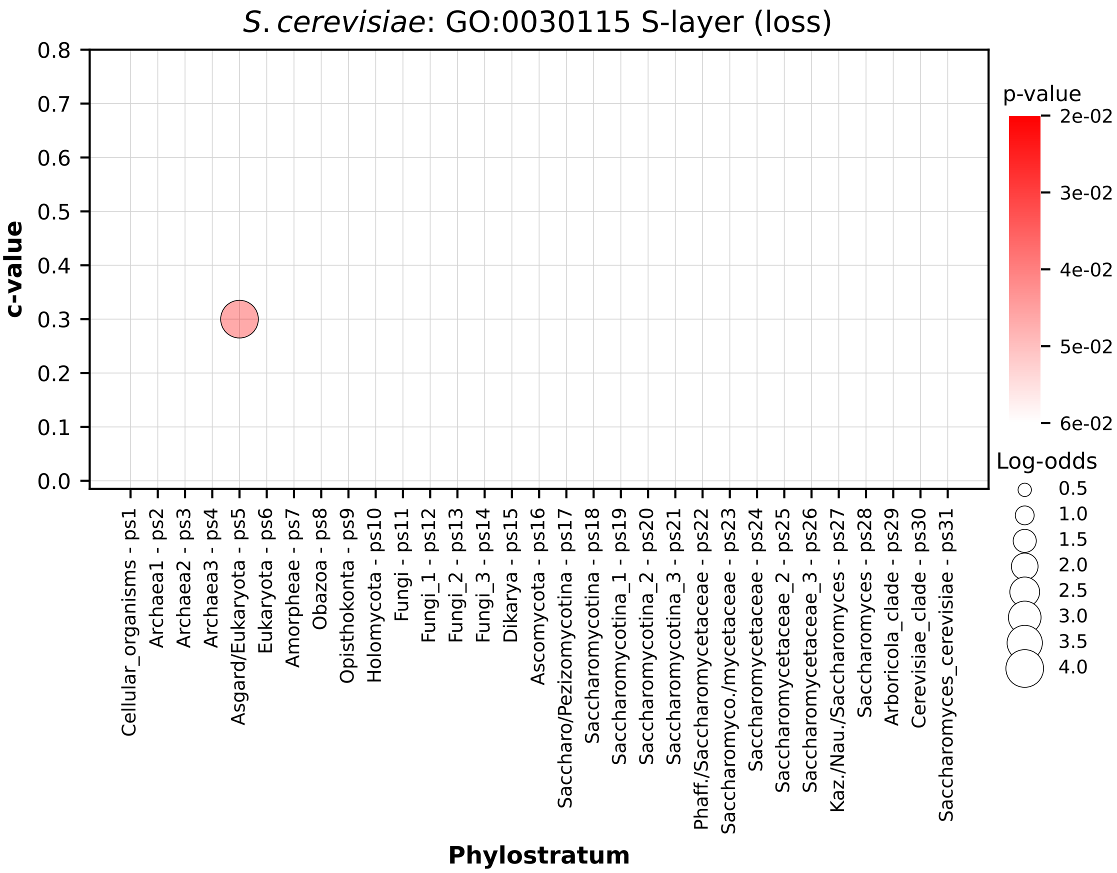

S. cerevisiae: GO:0030115 S-layer (loss)
c-value
0.8
0.7
0.6
0.5
0.4
0.3
0.2
0.1
0.0
p-value
2e-02
3e-02
4e-02
5e-02
6e-02
Log-odds
0.5
1.0
1.5
2.0
2.5
3.0
3.5
4.0
Cellular_organisms - ps1
Archaea1 - ps2
Archaea2 - ps3
Archaea3 - ps4
Asgard/Eukaryota - ps5
Eukaryota - ps6
Amorpheae - ps7
Obazoa - ps8
Opisthokonta - ps9
Holomycota - ps10
Fungi - ps11
Fungi_1 - ps12
Fungi_2 - ps13
Fungi_3 - ps14
Dikarya - ps15
Ascomycota - ps16
Saccharo/Pezizomycotina - ps17
Saccharomycotina - ps18
Saccharomycotina_1 - ps19
Saccharomycotina_2 - ps20
Saccharomycotina_3 - ps21
Phaff./Saccharomycetaceae - ps22
Saccharomyco./mycetaceae - ps23
Saccharomycetaceae - ps24
Saccharomycetaceae_2 - ps25
Saccharomycetaceae_3 - ps26
Kaz./Nau./Saccharomyces - ps27
Saccharomyces - ps28
Arboricola_clade - ps29
Cerevisiae_clade - ps30
Saccharomyces_cerevisiae - ps31
Phylostratum

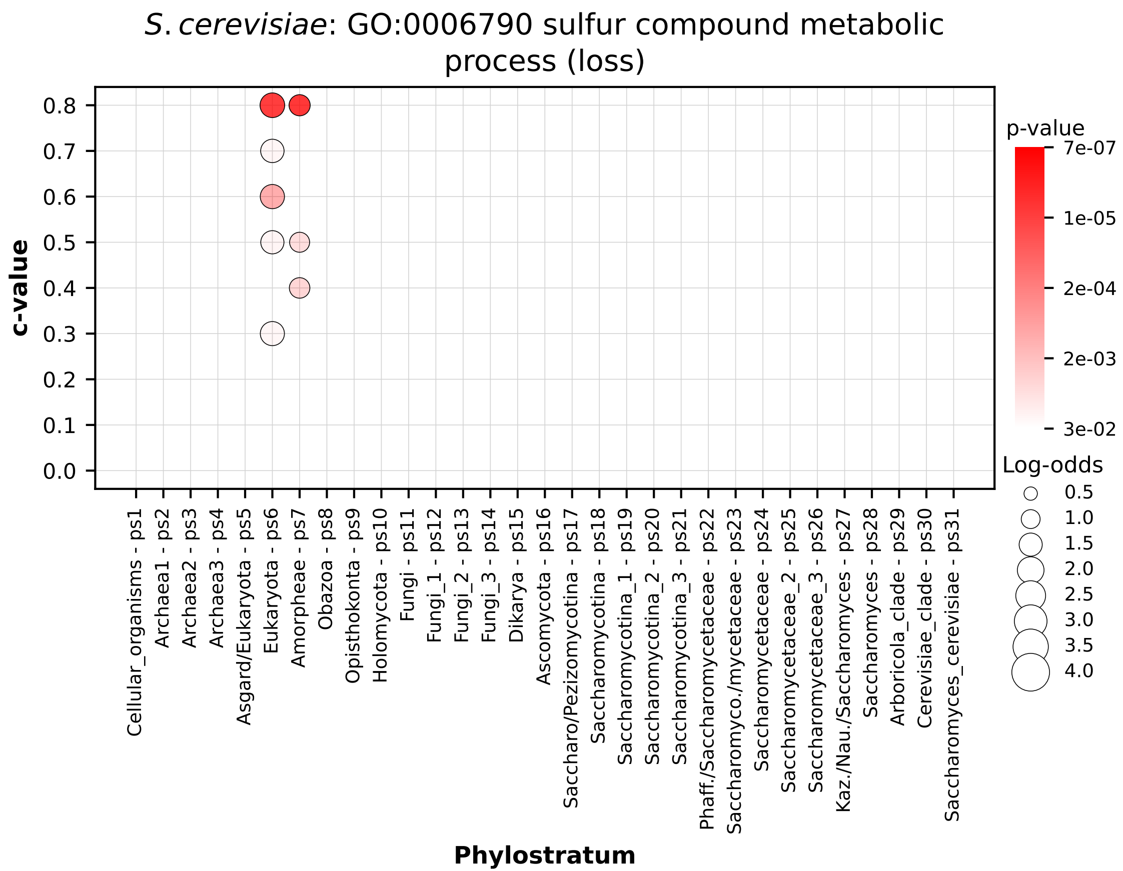
S. cerevisiae: GO:0006790 sulfur compound metabolic process (loss)
c-value
0.8
0.7
0.6
0.5
0.4
0.3
0.2
0.1
0.0
p-value
7e-07
1e-05
2e-04
2e-03
3e-02
Log-odds
0.5
1.0
1.5
2.0
2.5
3.0
3.5
4.0
Cellular_organisms - ps1
Archaea1 - ps2
Archaea2 - ps3
Archaea3 - ps4
Asgard/Eukaryota - ps5
Eukaryota - ps6
Amorpheae - ps7
Obazoa - ps8
Opisthokonta - ps9
Holomycota - ps10
Fungi - ps11
Fungi_1 - ps12
Fungi_2 - ps13
Fungi_3 - ps14
Dikarya - ps15
Ascomycota - ps16
Saccharo/Pezizomycotina - ps17
Saccharomycotina - ps18
Saccharomycotina_1 - ps19
Saccharomycotina_2 - ps20
Saccharomycotina_3 - ps21
Phaff./Saccharomycetaceae - ps22
Saccharomyco./mycetaceae - ps23
Saccharomycetaceae - ps24
Saccharomycetaceae_2 - ps25
Saccharomycetaceae_3 - ps26
Kaz./Nau./Saccharomyces - ps27
Saccharomyces - ps28
Arboricola_clade - ps29
Cerevisiae_clade - ps30
Saccharomyces_cerevisiae - ps31
Phylostratum

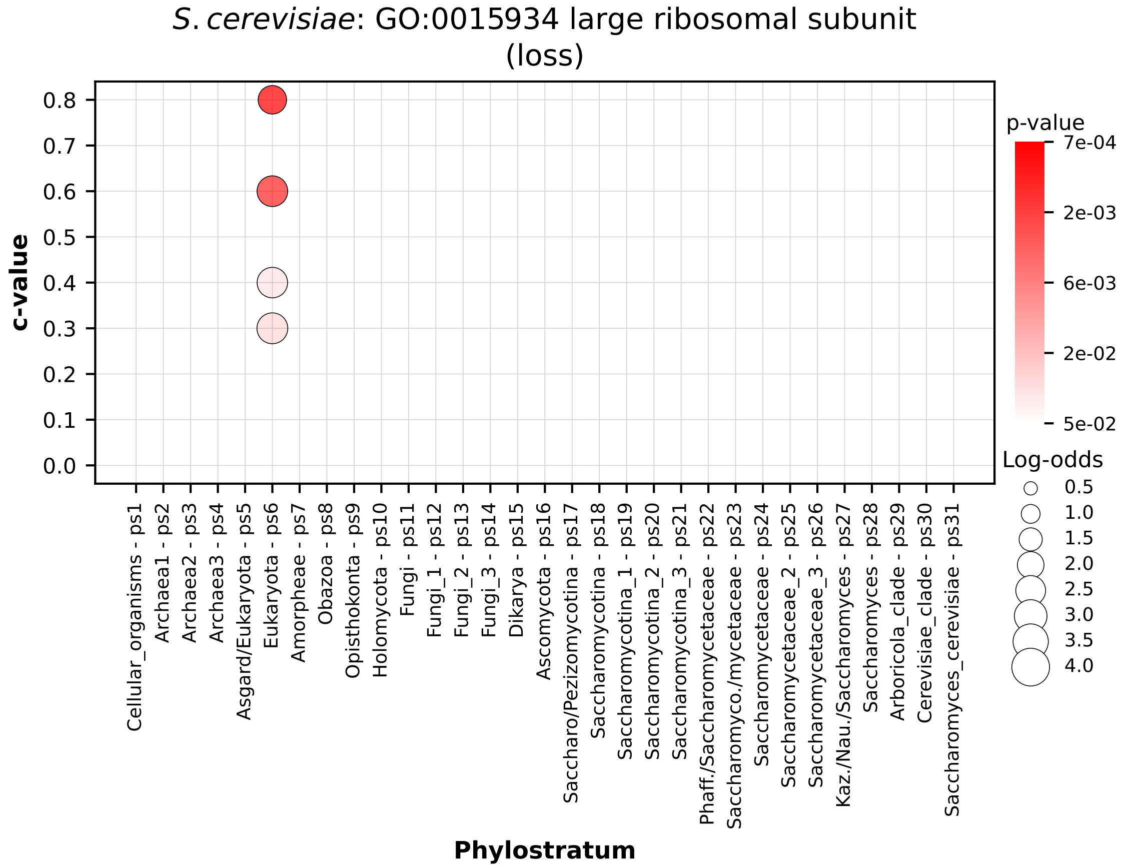
S. cerevisiae: GO:0015934 large ribosomal subunit
(loss)
c-value
0.8
0.7
0.6
0.5
0.4
0.3
0.2
0.1
0.0
p-value
7e-04
2e-03
6e-03
2e-02
5e-02
Log-odds
0.5
1.0
1.5
2.0
2.5
3.0
3.5
4.0
Cellular_organisms - ps1
Archaea1 - ps2
Archaea2 - ps3
Archaea3 - ps4
Asgard/Eukaryota - ps5
Eukaryota - ps6
Amorpheae - ps7
Obazoa - ps8
Opisthokonta - ps9
Holomycota - ps10
Fungi - ps11
Fungi_1 - ps12
Fungi_2 - ps13
Fungi_3 - ps14
Dikarya - ps15
Ascomycota - ps16
Saccharo/Pezizomycotina - ps17
Saccharomycotina - ps18
Saccharomycotina_1 - ps19
Saccharomycotina_2 - ps20
Saccharomycotina_3 - ps21
Phaff./Saccharomycetaceae - ps22
Saccharomyco./mycetaceae - ps23
Saccharomycetaceae - ps24
Saccharomycetaceae_2 - ps25
Saccharomycetaceae_3 - ps26
Kaz./Nau./Saccharomyces - ps27
Saccharomyces - ps28
Arboricola_clade - ps29
Cerevisiae_clade - ps30
Saccharomyces_cerevisiae - ps31
Phylostratum

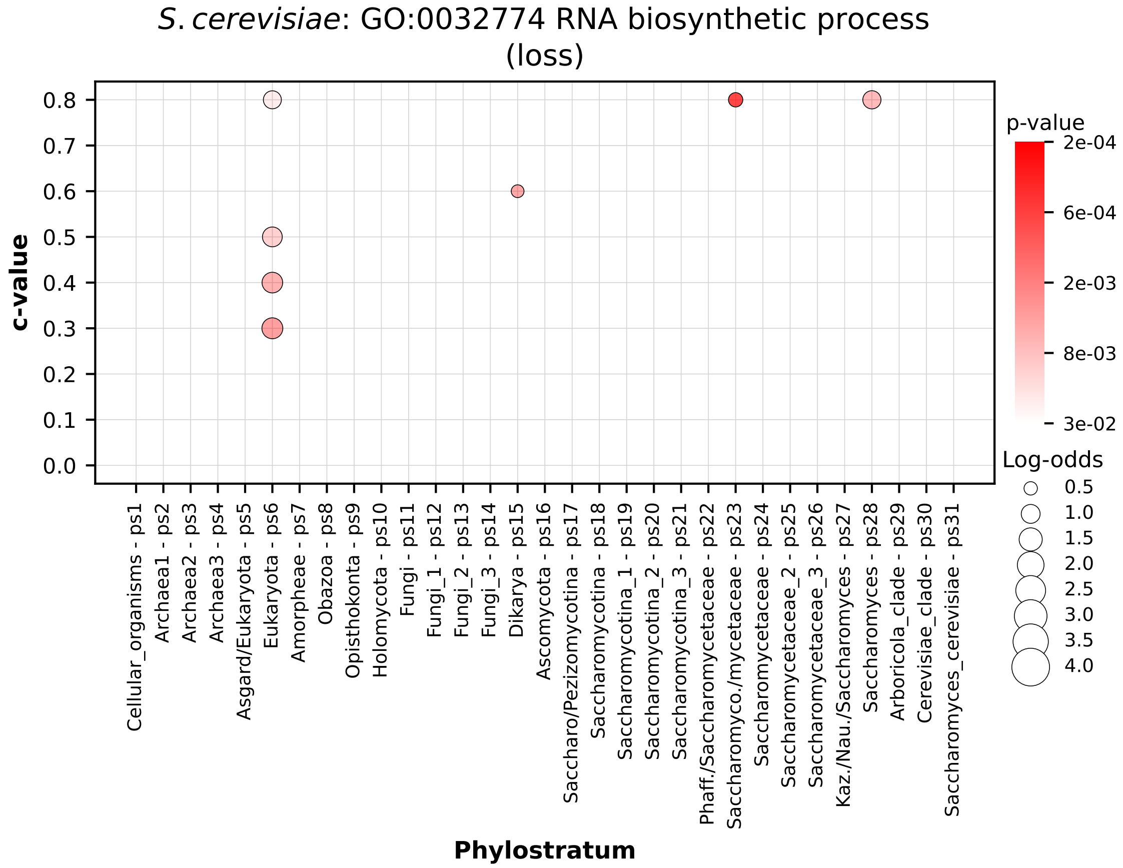

S. cerevisiae: GO:0032774 RNA biosynthetic process
(loss)
c-value
0.8
0.7
0.6
0.5
0.4
0.3
0.2
0.1
0.0
Phylostratum
Cellular_organisms - ps1
Archaea1 - ps2
Archaea2 - ps3
Archaea3 - ps4
Asgard/Eukaryota - ps5
Eukaryota - ps6
Amorpheae - ps7
Obazoa - ps8
Opisthokonta - ps9
Holomycota - ps10
Fungi - ps11
Fungi_1 - ps12
Fungi_2 - ps13
Fungi_3 - ps14
Dikarya - ps15
Ascomycota - ps16
Saccharo/Pezizomycotina - ps17
Saccharomycotina - ps18
Saccharomycotina_1 - ps19
Saccharomycotina_2 - ps20
Saccharomycotina_3 - ps21
Phaff./Saccharomycetaceae - ps22
Saccharomyco./mycetaceae - ps23
Saccharomycetaceae - ps24
Saccharomycetaceae_2 - ps25
Saccharomycetaceae_3 - ps26
Kaz./Nau./Saccharomyces - ps27
Saccharomyces - ps28
Arboricola_clade - ps29
Cerevisiae_clade - ps30
Saccharomyces_cerevisiae - ps31
p-value
2e-04
6e-04
2e-03
8e-03
3e-02
Log-odds
0.5
1.0
1.5
2.0
2.5
3.0
3.5
4.0

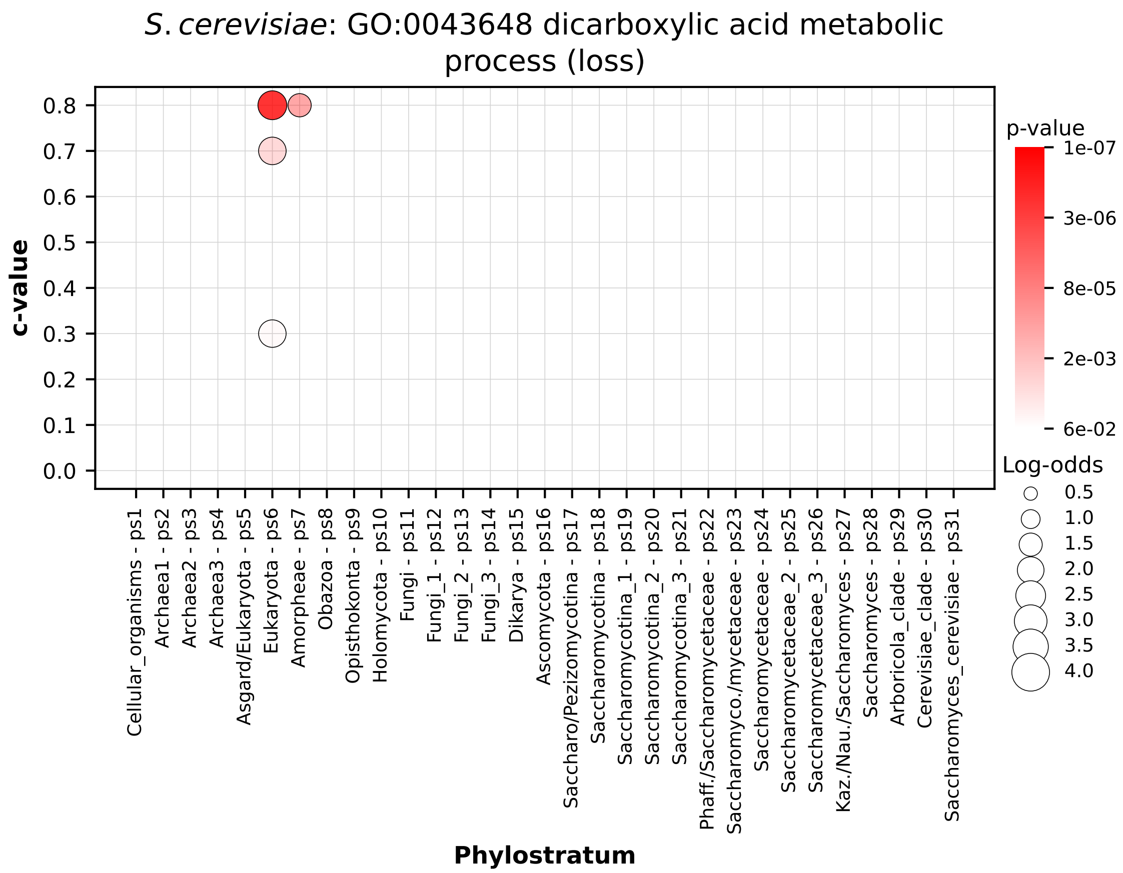

S. cerevisiae: GO:0043648 dicarboxylic acid metabolic process (loss)
c-value
0.0
0.1
0.2
0.3
0.4
0.5
0.6
0.7
0.8
p-value
1e-07
3e-06
8e-05
2e-03
6e-02
Log-odds
0.5
1.0
1.5
2.0
2.5
3.0
3.5
4.0
Cellular_organisms - ps1
Archaea1 - ps2
Archaea2 - ps3
Archaea3 - ps4
Asgard/Eukaryota - ps5
Eukaryota - ps6
Amorpheae - ps7
Obazoa - ps8
Opisthokonta - ps9
Holomycota - ps10
Fungi - ps11
Fungi_1 - ps12
Fungi_2 - ps13
Fungi_3 - ps14
Dikarya - ps15
Ascomycota - ps16
Saccharo/Pezizomycotina - ps17
Saccharomycotina - ps18
Saccharomycotina_1 - ps19
Saccharomycotina_2 - ps20
Saccharomycotina_3 - ps21
Phaff./Saccharomycetaceae - ps22
Saccharomyco./mycetaceae - ps23
Saccharomycetaceae - ps24
Saccharomycetaceae_2 - ps25
Saccharomycetaceae_3 - ps26
Kaz./Nau./Saccharomyces - ps27
Saccharomyces - ps28
Arboricola_clade - ps29
Cerevisiae_clade - ps30
Saccharomyces_cerevisiae - ps31
Phylostratum

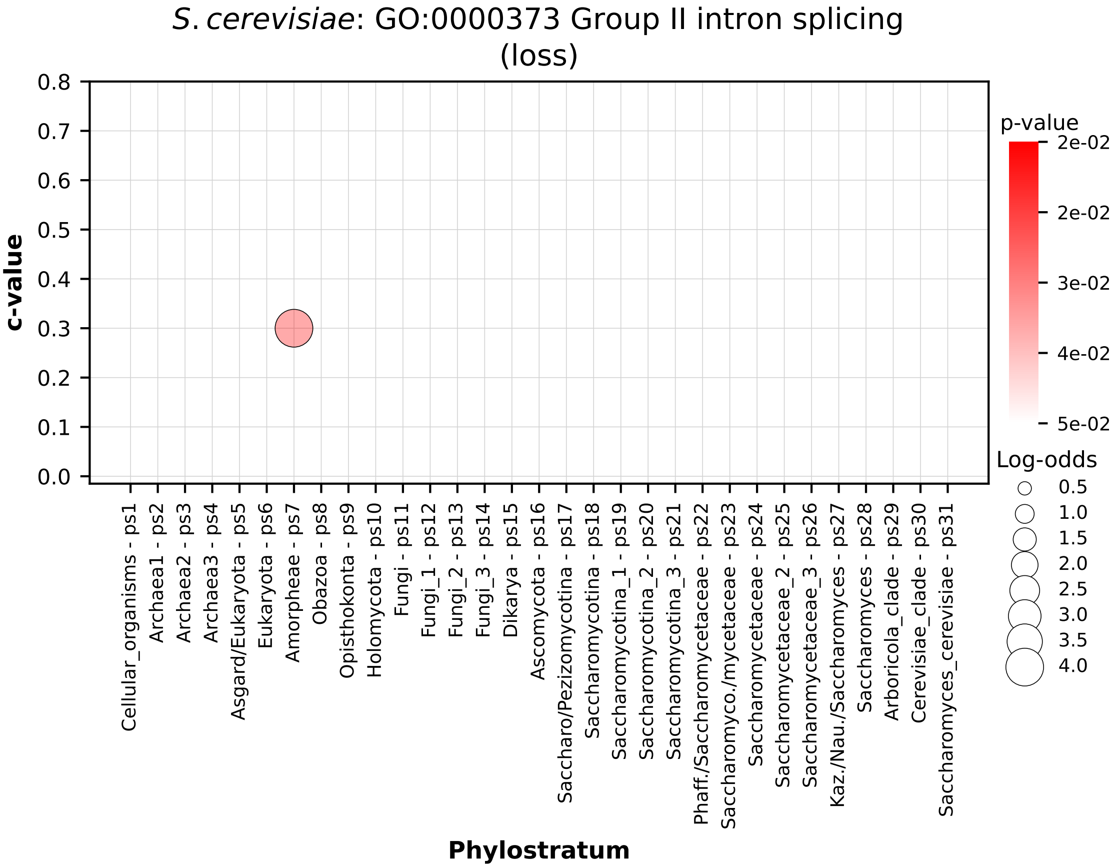
S. cerevisiae: GO:0000373 Group II intron splicing
(loss)
c-value
0.8
0.7
0.6
0.5
0.4
0.3
0.2
0.1
0.0
p-value
2e-02
2e-02
3e-02
4e-02
5e-02
Log-odds
0.5
1.0
1.5
2.0
2.5
3.0
3.5
4.0
Cellular_organisms - ps1
Archaea1 - ps2
Archaea2 - ps3
Archaea3 - ps4
Asgard/Eukaryota - ps5
Eukaryota - ps6
Amorpheae - ps7
Obazoa - ps8
Opisthokonta - ps9
Holomycota - ps10
Fungi - ps11
Fungi_1 - ps12
Fungi_2 - ps13
Fungi_3 - ps14
Dikarya - ps15
Ascomycota - ps16
Saccharo/Pezizomycotina - ps17
Saccharomycotina - ps18
Saccharomycotina_1 - ps19
Saccharomycotina_2 - ps20
Saccharomycotina_3 - ps21
Phaff./Saccharomycetaceae - ps22
Saccharomyco./mycetaceae - ps23
Saccharomycetaceae - ps24
Saccharomycetaceae_2 - ps25
Saccharomycetaceae_3 - ps26
Kaz./Nau./Saccharomyces - ps27
Saccharomyces - ps28
Arboricola_clade - ps29
Cerevisiae_clade - ps30
Saccharomyces_cerevisiae - ps31
Phylostratum

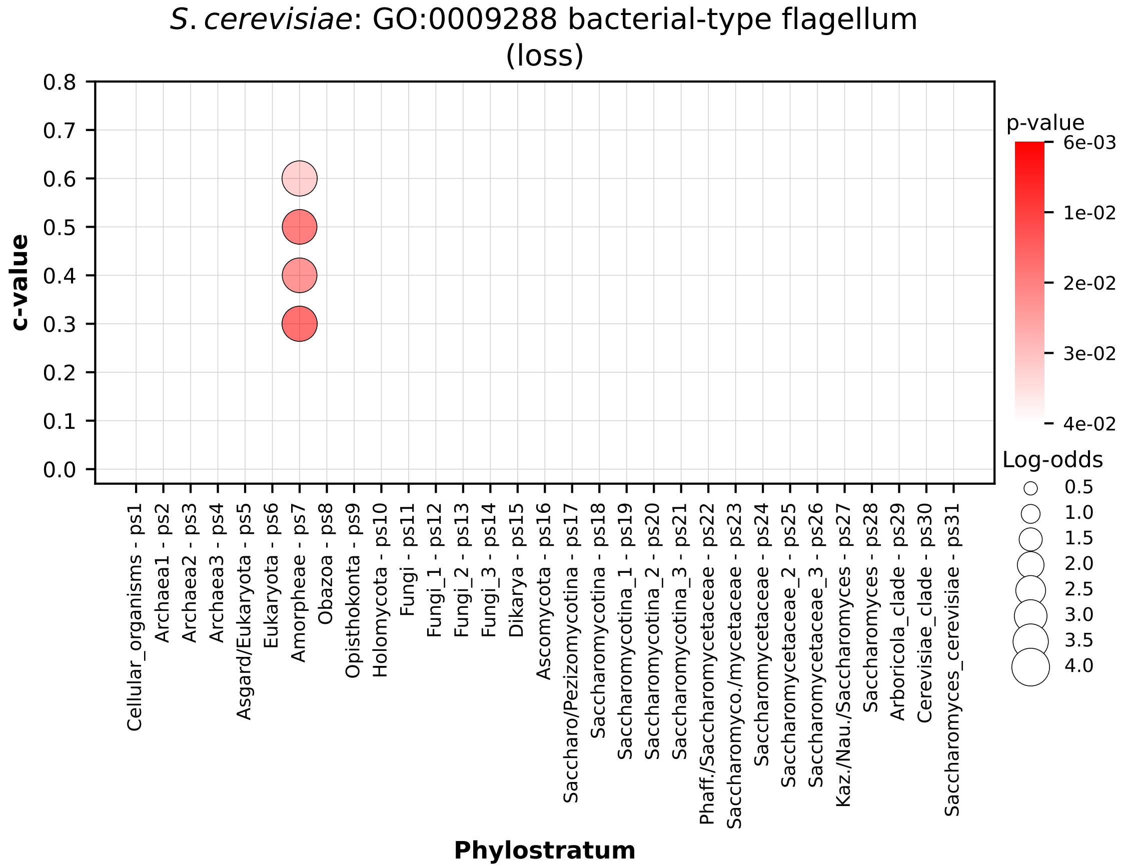
S. cerevisiae: GO:0009288 bacterial-type flagellum
(loss)
c-value
0.8
0.7
0.6
0.5
0.4
0.3
0.2
0.1
0.0
p-value
6e-03
1e-02
2e-02
3e-02
4e-02
Log-odds
0.5
1.0
1.5
2.0
2.5
3.0
3.5
4.0
Cellular_organisms - ps1
Archaea1 - ps2
Archaea2 - ps3
Archaea3 - ps4
Asgard/Eukaryota - ps5
Eukaryota - ps6
Amorpheae - ps7
Obazoa - ps8
Opisthokonta - ps9
Holomycota - ps10
Fungi - ps11
Fungi_1 - ps12
Fungi_2 - ps13
Fungi_3 - ps14
Dikarya - ps15
Ascomycota - ps16
Saccharo/Pezizomycotina - ps17
Saccharomycotina - ps18
Saccharomycotina_1 - ps19
Saccharomycotina_2 - ps20
Saccharomycotina_3 - ps21
Phaff./Saccharomycetaceae - ps22
Saccharomyco./mycetaceae - ps23
Saccharomycetaceae - ps24
Saccharomycetaceae_2 - ps25
Saccharomycetaceae_3 - ps26
Kaz./Nau./Saccharomyces - ps27
Saccharomyces - ps28
Arboricola_clade - ps29
Cerevisiae_clade - ps30
Saccharomyces_cerevisiae - ps31
Phylostratum

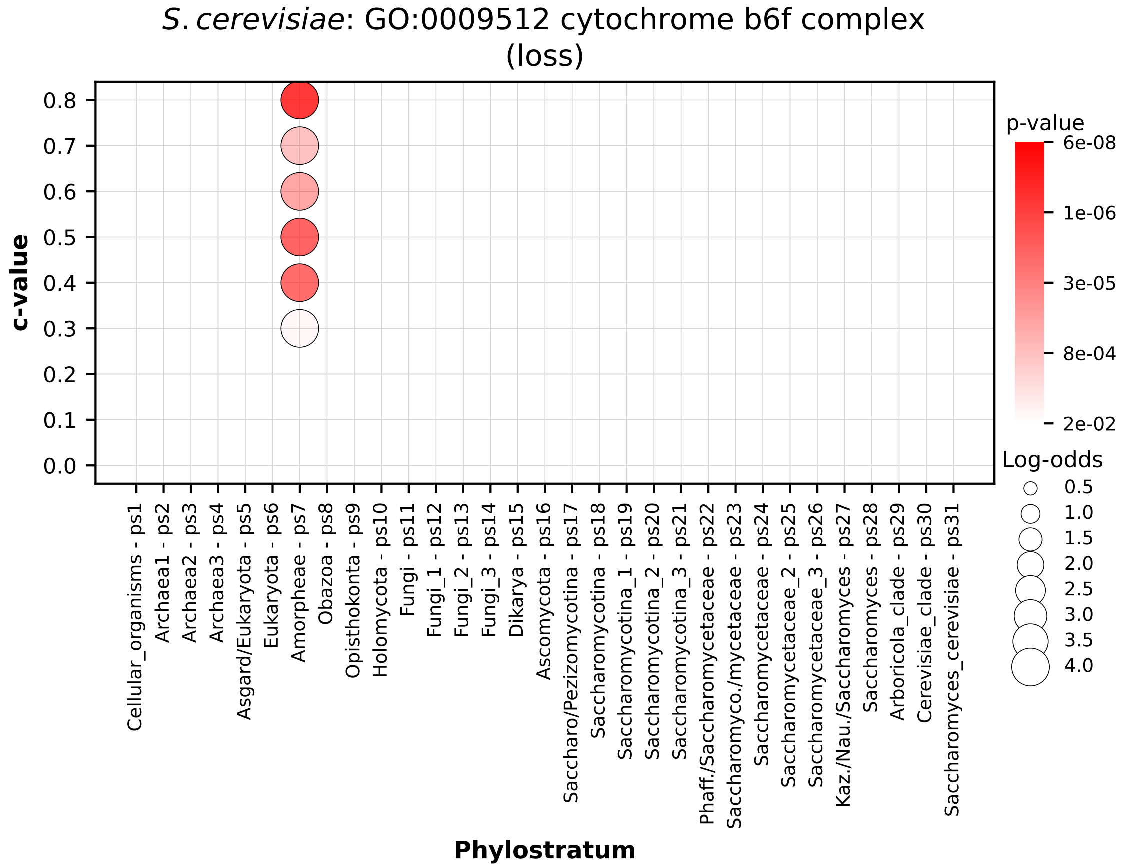
S. cerevisiae: GO:0009512 cytochrome b6f complex
(loss)
c-value
0.8
0.7
0.6
0.5
0.4
0.3
0.2
0.1
0.0
p-value
6e-08
1e-06
3e-05
8e-04
2e-02
Log-odds
0.5
1.0
1.5
2.0
2.5
3.0
3.5
4.0
Cellular_organisms - ps1
Archaea1 - ps2
Archaea2 - ps3
Archaea3 - ps4
Asgard/Eukaryota - ps5
Eukaryota - ps6
Amorpheae - ps7
Obazoa - ps8
Opisthokonta - ps9
Holomycota - ps10
Fungi - ps11
Fungi_1 - ps12
Fungi_2 - ps13
Fungi_3 - ps14
Dikarya - ps15
Ascomycota - ps16
Saccharo/Pezizomycotina - ps17
Saccharomycotina - ps18
Saccharomycotina_1 - ps19
Saccharomycotina_2 - ps20
Saccharomycotina_3 - ps21
Phaff./Saccharomycetaceae - ps22
Saccharomyco./mycetaceae - ps23
Saccharomycetaceae - ps24
Saccharomycetaceae_2 - ps25
Saccharomycetaceae_3 - ps26
Kaz./Nau./Saccharomyces - ps27
Saccharomyces - ps28
Arboricola_clade - ps29
Cerevisiae_clade - ps30
Saccharomyces_cerevisiae - ps31
Phylostratum

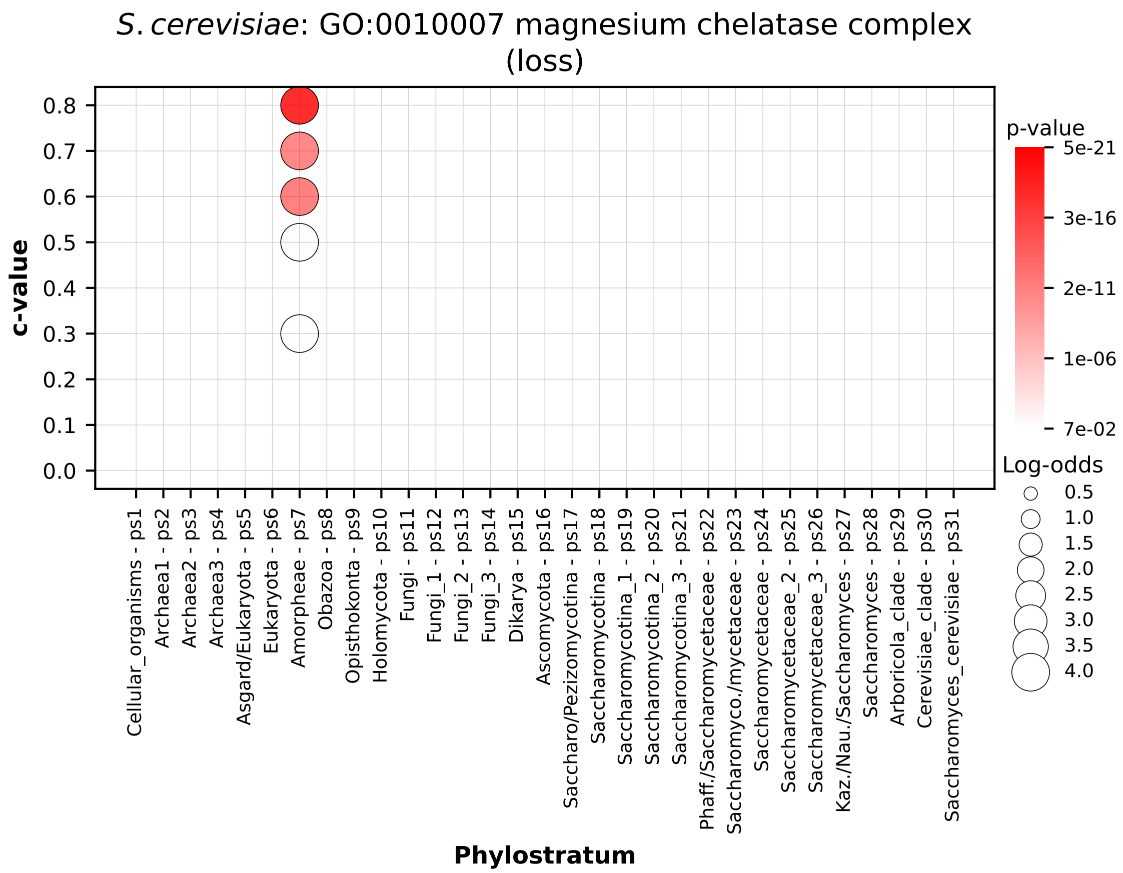

S. cerevisiae: GO:0010007 magnesium chelatase complex
(loss)
c-value
0.8
0.7
0.6
0.5
0.4
0.3
0.2
0.1
0.0
p-value
5e-21
3e-16
2e-11
1e-06
7e-02
Log-odds
0.5
1.0
1.5
2.0
2.5
3.0
3.5
4.0
Cellular_organisms - ps1
Archaea1 - ps2
Archaea2 - ps3
Archaea3 - ps4
Asgard/Eukaryota - ps5
Eukaryota - ps6
Amorpheae - ps7
Obazoa - ps8
Opisthokonta - ps9
Holomycota - ps10
Fungi - ps11
Fungi_1 - ps12
Fungi_2 - ps13
Fungi_3 - ps14
Dikarya - ps15
Ascomycota - ps16
Saccharo/Pezizomycotina - ps17
Saccharomycotina - ps18
Saccharomycotina_1 - ps19
Saccharomycotina_2 - ps20
Saccharomycotina_3 - ps21
Phaff./Saccharomycetaceae - ps22
Saccharomyco./mycetaceae - ps23
Saccharomycetaceae - ps24
Saccharomycetaceae_2 - ps25
Saccharomycetaceae_3 - ps26
Kaz./Nau./Saccharomyces - ps27
Saccharomyces - ps28
Arboricola_clade - ps29
Cerevisiae_clade - ps30
Saccharomyces_cerevisiae - ps31
Phylostratum

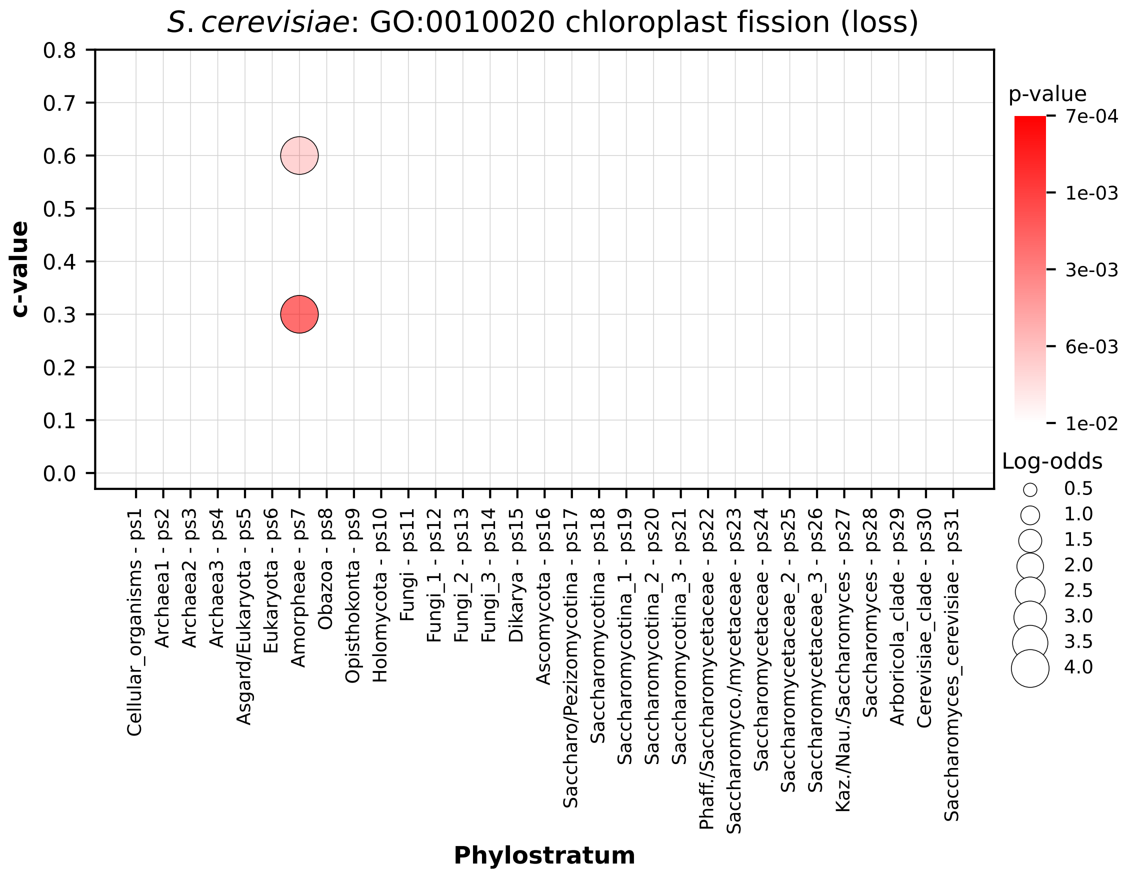
S. cerevisiae: GO:0010020 chloroplast fission (loss)
c-value
0.8
0.7
0.6
0.5
0.4
0.3
0.2
0.1
0.0
p-value
7e-04
1e-03
3e-03
6e-03
1e-02
Log-odds
0.5
1.0
1.5
2.0
2.5
3.0
3.5
4.0
Cellular_organisms - ps1
Archaea1 - ps2
Archaea2 - ps3
Archaea3 - ps4
Asgard/Eukaryota - ps5
Eukaryota - ps6
Amorpheae - ps7
Obazoa - ps8
Opisthokonta - ps9
Holomycota - ps10
Fungi - ps11
Fungi_1 - ps12
Fungi_2 - ps13
Fungi_3 - ps14
Dikarya - ps15
Ascomycota - ps16
Saccharo/Pezizomycotina - ps17
Saccharomycotina - ps18
Saccharomycotina_1 - ps19
Saccharomycotina_2 - ps20
Saccharomycotina_3 - ps21
Phaff./Saccharomycetaceae - ps22
Saccharomyco./mycetaceae - ps23
Saccharomycetaceae - ps24
Saccharomycetaceae_2 - ps25
Saccharomycetaceae_3 - ps26
Kaz./Nau./Saccharomyces - ps27
Saccharomyces - ps28
Arboricola_clade - ps29
Cerevisiae_clade - ps30
Saccharomyces_cerevisiae - ps31
Phylostratum

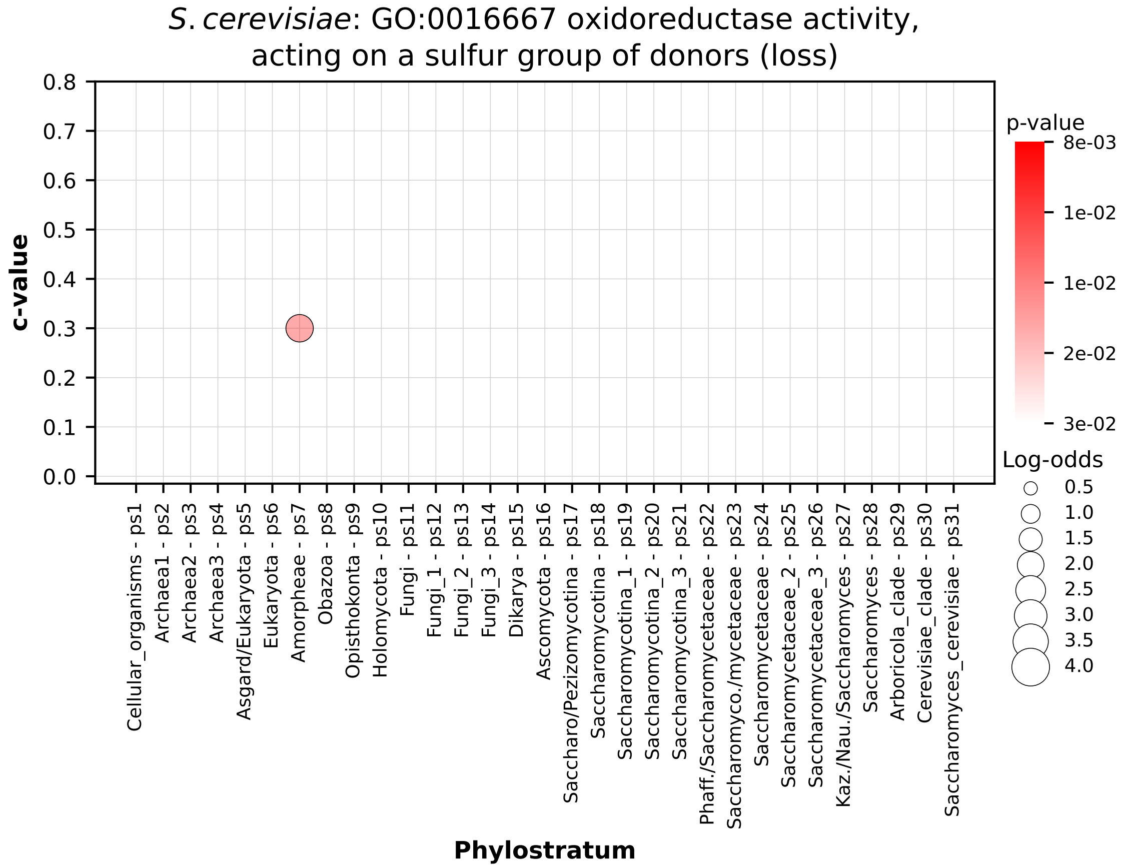

S. cerevisiae: GO:0016667 oxidoreductase activity, acting on a sulfur group of donors (loss)
c-value
0.8
0.7
0.6
0.5
0.4
0.3
0.2
0.1
0.0
p-value
8e-03
1e-02
1e-02
2e-02
3e-02
Log-odds
0.5
1.0
1.5
2.0
2.5
3.0
3.5
4.0
Cellular_organisms - ps1
Archaea1 - ps2
Archaea2 - ps3
Archaea3 - ps4
Asgard/Eukaryota - ps5
Eukaryota - ps6
Amorpheae - ps7
Obazoa - ps8
Opisthokonta - ps9
Holomycota - ps10
Fungi - ps11
Fungi_1 - ps12
Fungi_2 - ps13
Fungi_3 - ps14
Dikarya - ps15
Ascomycota - ps16
Saccharo/Pezizomycotina - ps17
Saccharomycotina - ps18
Saccharomycotina_1 - ps19
Saccharomycotina_2 - ps20
Saccharomycotina_3 - ps21
Phaff./Saccharomycetaceae - ps22
Saccharomyco./mycetaceae - ps23
Saccharomycetaceae - ps24
Saccharomycetaceae_2 - ps25
Saccharomycetaceae_3 - ps26
Kaz./Nau./Saccharomyces - ps27
Saccharomyces - ps28
Arboricola_clade - ps29
Cerevisiae_clade - ps30
Saccharomyces_cerevisiae - ps31
Phylostratum

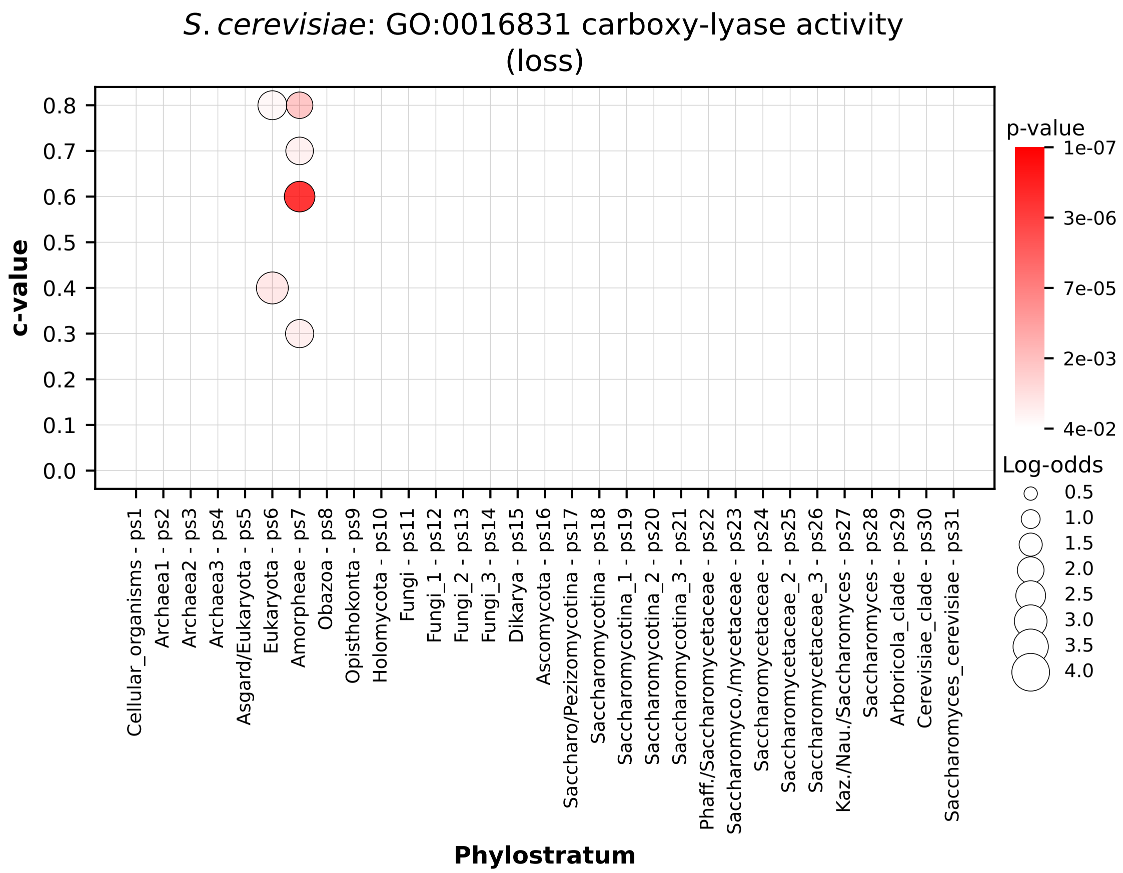
S. cerevisiae: GO:0016831 carboxy-lyase activity
(loss)
c-value
0.8
0.7
0.6
0.5
0.4
0.3
0.2
0.1
0.0
Phylostratum
Cellular_organisms - ps1
Archaea1 - ps2
Archaea2 - ps3
Archaea3 - ps4
Asgard/Eukaryota - ps5
Eukaryota - ps6
Amorpheae - ps7
Obazoa - ps8
Opisthokonta - ps9
Holomycota - ps10
Fungi - ps11
Fungi_1 - ps12
Fungi_2 - ps13
Fungi_3 - ps14
Dikarya - ps15
Ascomycota - ps16
Saccharo/Pezizomycotina - ps17
Saccharomycotina - ps18
Saccharomycotina_1 - ps19
Saccharomycotina_2 - ps20
Saccharomycotina_3 - ps21
Phaff./Saccharomycetaceae - ps22
Saccharomyco./mycetaceae - ps23
Saccharomycetaceae - ps24
Saccharomycetaceae_2 - ps25
Saccharomycetaceae_3 - ps26
Kaz./Nau./Saccharomyces - ps27
Saccharomyces - ps28
Arboricola_clade - ps29
Cerevisiae_clade - ps30
Saccharomyces_cerevisiae - ps31
p-value
1e-07
3e-06
7e-05
2e-03
4e-02
Log-odds
0.5
1.0
1.5
2.0
2.5
3.0
3.5
4.0

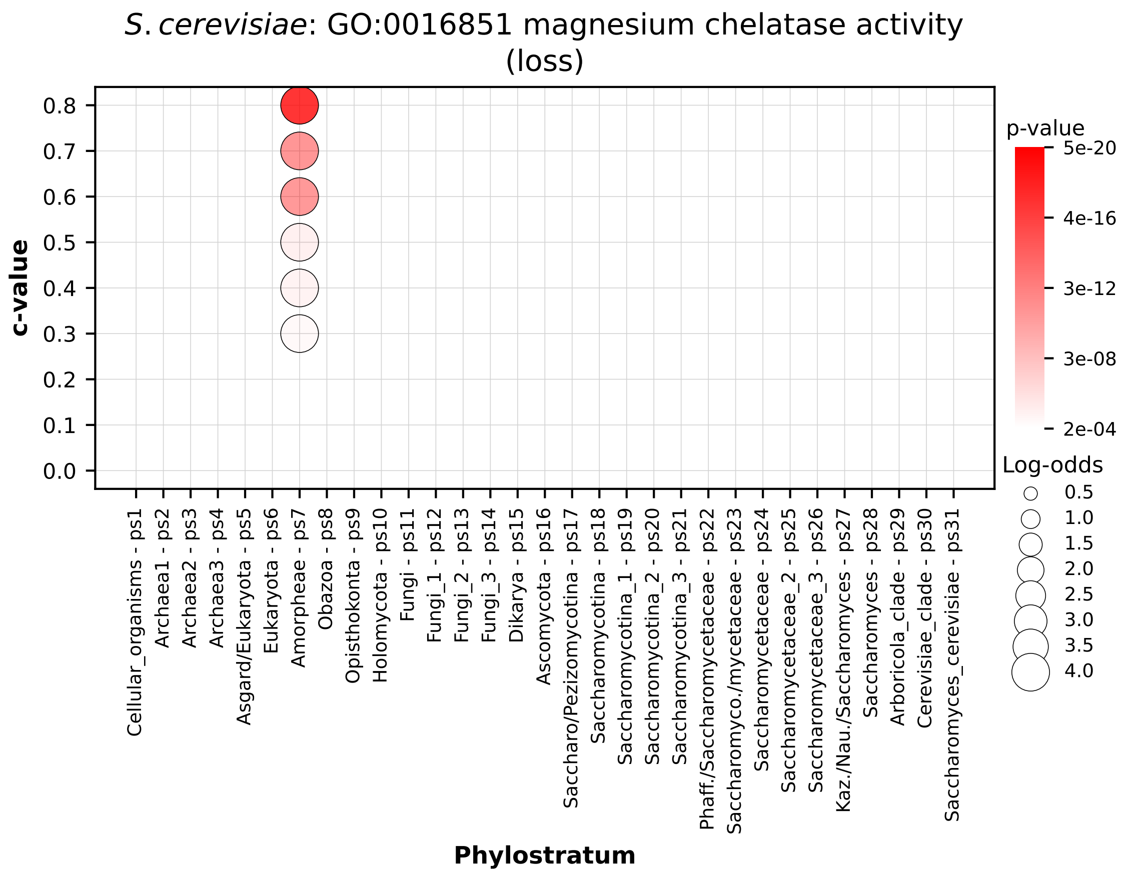

S. cerevisiae: GO:0016851 magnesium chelatase activity
(loss)
c-value
0.0
0.1
0.2
0.3
0.4
0.5
0.6
0.7
0.8
p-value
5e-20
4e-16
3e-12
3e-08
2e-04
Log-odds
0.5
1.0
1.5
2.0
2.5
3.0
3.5
4.0
Cellular_organisms - ps1
Archaea1 - ps2
Archaea2 - ps3
Archaea3 - ps4
Asgard/Eukaryota - ps5
Eukaryota - ps6
Amorpheae - ps7
Obazoa - ps8
Opisthokonta - ps9
Holomycota - ps10
Fungi - ps11
Fungi_1 - ps12
Fungi_2 - ps13
Fungi_3 - ps14
Dikarya - ps15
Ascomycota - ps16
Saccharo/Pezizomycotina - ps17
Saccharomycotina - ps18
Saccharomycotina_1 - ps19
Saccharomycotina_2 - ps20
Saccharomycotina_3 - ps21
Phaff./Saccharomycetaceae - ps22
Saccharomyco./mycetaceae - ps23
Saccharomycetaceae - ps24
Saccharomycetaceae_2 - ps25
Saccharomycetaceae_3 - ps26
Kaz./Nau./Saccharomyces - ps27
Saccharomyces - ps28
Arboricola_clade - ps29
Cerevisiae_clade - ps30
Saccharomyces_cerevisiae - ps31
Phylostratum

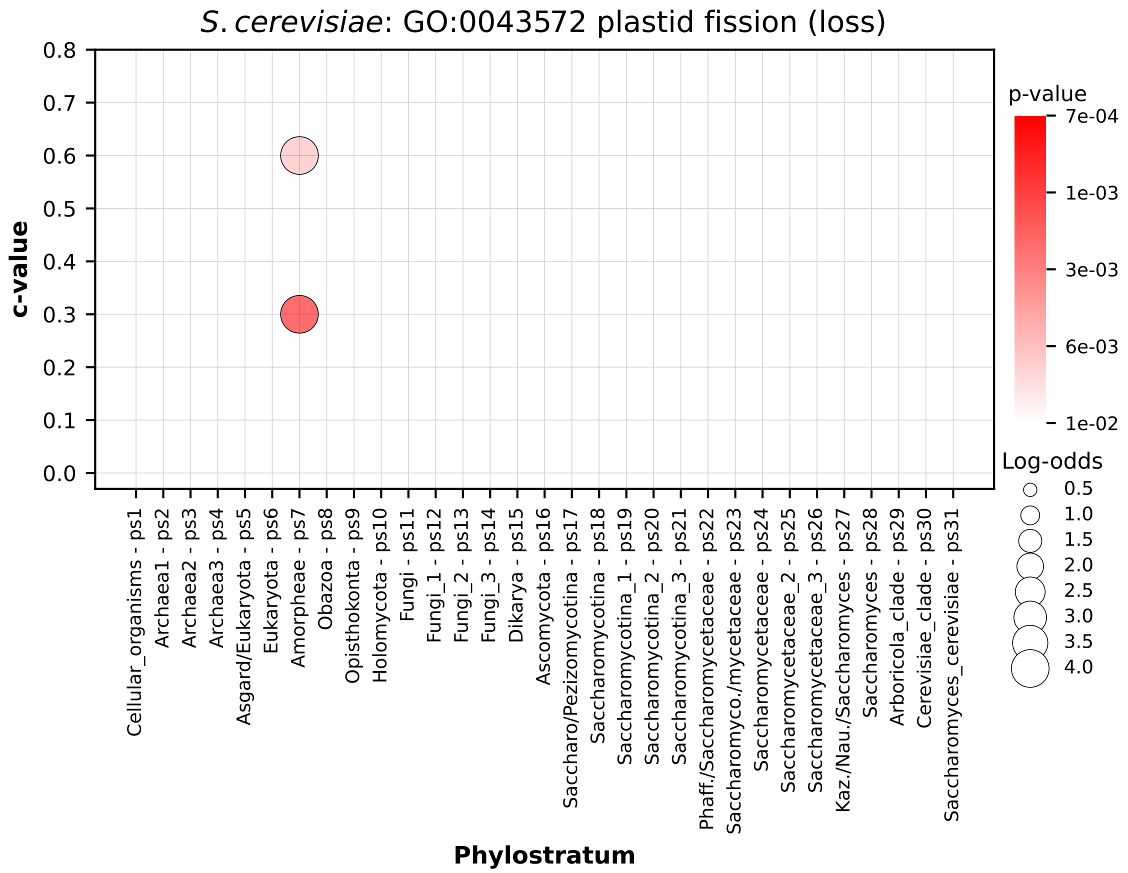

S. cerevisiae: GO:0043572 plastid fission (loss)
c-value
0.8
0.7
0.6
0.5
0.4
0.3
0.2
0.1
0.0
p-value
7e-04
1e-03
3e-03
6e-03
1e-02
Log-odds
0.5
1.0
1.5
2.0
2.5
3.0
3.5
4.0
Cellular_organisms - ps1
Archaea1 - ps2
Archaea2 - ps3
Archaea3 - ps4
Asgard/Eukaryota - ps5
Eukaryota - ps6
Amorpheae - ps7
Obazoa - ps8
Opisthokonta - ps9
Holomycota - ps10
Fungi - ps11
Fungi_1 - ps12
Fungi_2 - ps13
Fungi_3 - ps14
Dikarya - ps15
Ascomycota - ps16
Saccharo/Pezizomycotina - ps17
Saccharomycotina - ps18
Saccharomycotina_1 - ps19
Saccharomycotina_2 - ps20
Saccharomycotina_3 - ps21
Phaff./Saccharomycetaceae - ps22
Saccharomyco./mycetaceae - ps23
Saccharomycetaceae - ps24
Saccharomycetaceae_2 - ps25
Saccharomycetaceae_3 - ps26
Kaz./Nau./Saccharomyces - ps27
Saccharomyces - ps28
Arboricola_clade - ps29
Cerevisiae_clade - ps30
Saccharomyces_cerevisiae - ps31
Phylostratum

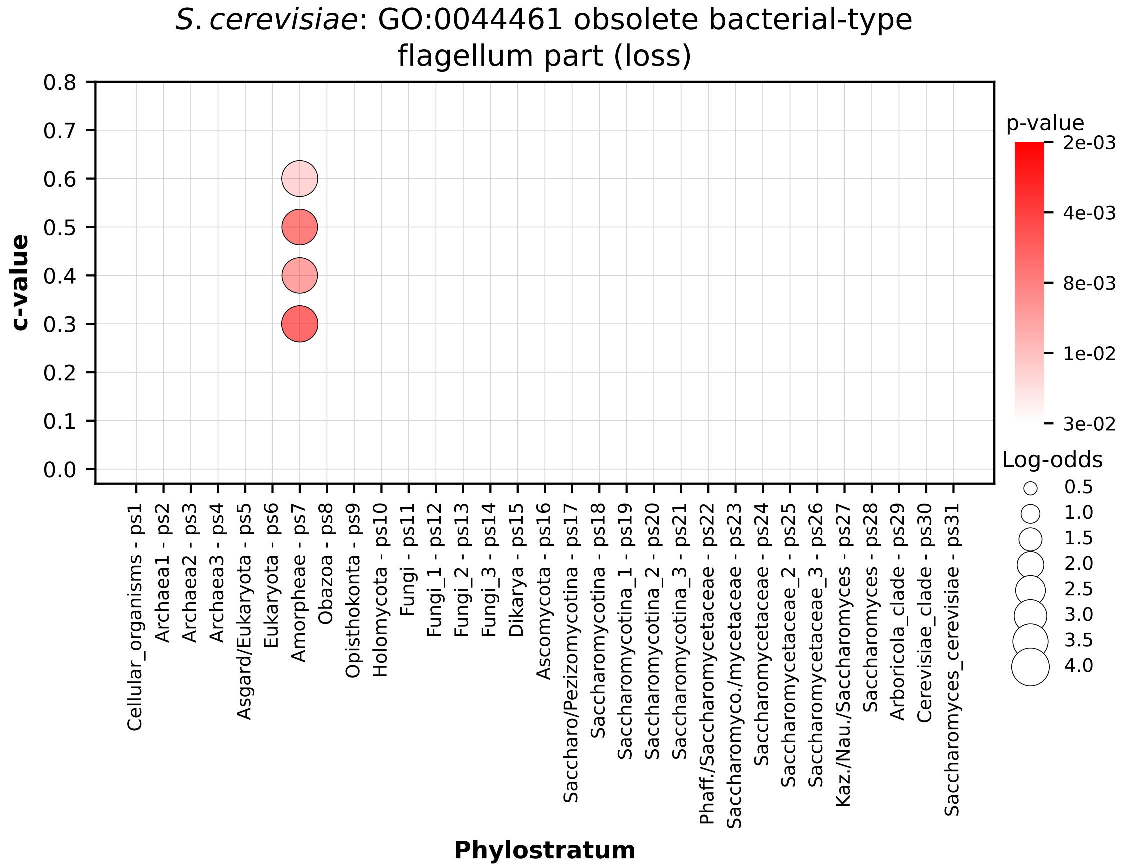
S. cerevisiae: GO:0044461 obsolete bacterial-type flagellum part (loss)
c-value
0.8
0.7
0.6
0.5
0.4
0.3
0.2
0.1
0.0
p-value
2e-03
4e-03
8e-03
1e-02
3e-02
Log-odds
0.5
1.0
1.5
2.0
2.5
3.0
3.5
4.0
Cellular_organisms - ps1
Archaea1 - ps2
Archaea2 - ps3
Archaea3 - ps4
Asgard/Eukaryota - ps5
Eukaryota - ps6
Amorpheae - ps7
Obazoa - ps8
Opisthokonta - ps9
Holomycota - ps10
Fungi - ps11
Fungi_1 - ps12
Fungi_2 - ps13
Fungi_3 - ps14
Dikarya - ps15
Ascomycota - ps16
Saccharo/Pezizomycotina - ps17
Saccharomycotina - ps18
Saccharomycotina_1 - ps19
Saccharomycotina_2 - ps20
Saccharomycotina_3 - ps21
Phaff./Saccharomycetaceae - ps22
Saccharomyco./mycetaceae - ps23
Saccharomycetaceae - ps24
Saccharomycetaceae_2 - ps25
Saccharomycetaceae_3 - ps26
Kaz./Nau./Saccharomyces - ps27
Saccharomyces - ps28
Arboricola_clade - ps29
Cerevisiae_clade - ps30
Saccharomyces_cerevisiae - ps31
Phylostratum

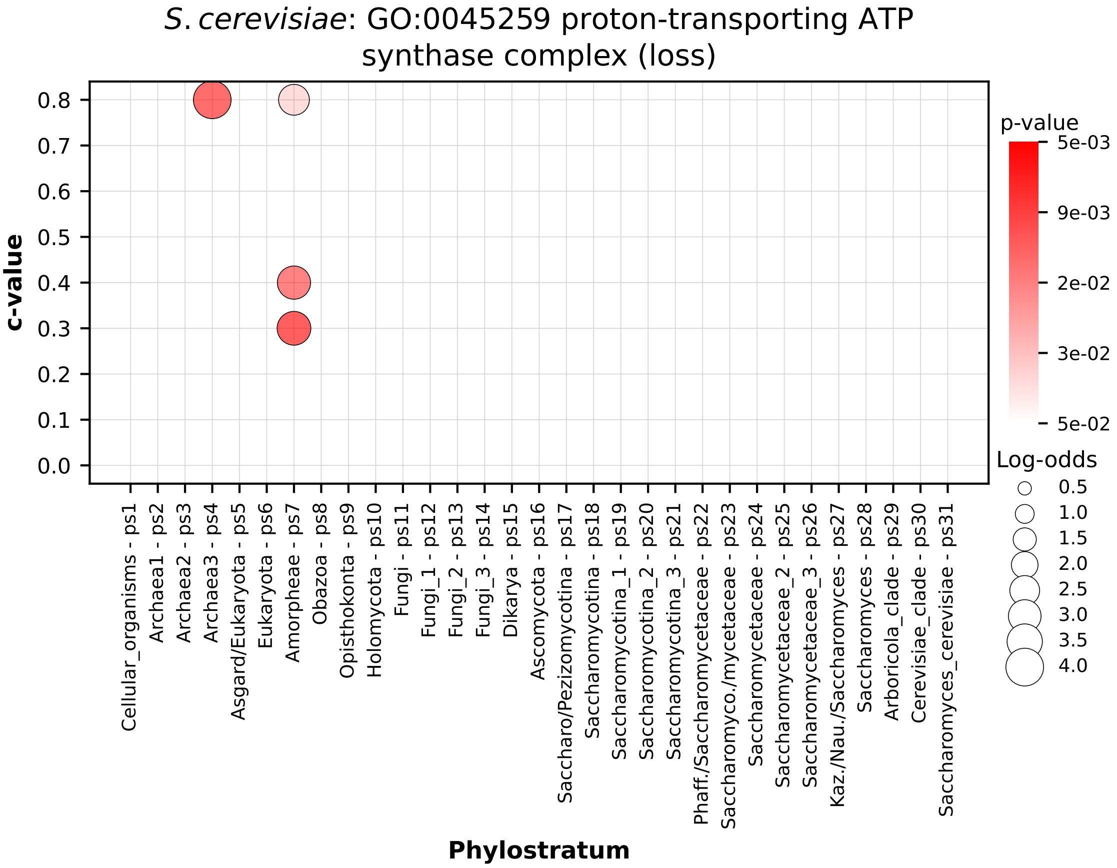
S. cerevisiae: GO:0045259 proton-transporting ATP synthase complex (loss)
c-value
0.0
0.1
0.2
0.3
0.4
0.5
0.6
0.7
0.8
p-value
5e-03
9e-03
2e-02
3e-02
5e-02
Log-odds
0.5
1.0
1.5
2.0
2.5
3.0
3.5
4.0
Cellular_organisms - ps1
Archaea1 - ps2
Archaea2 - ps3
Archaea3 - ps4
Asgard/Eukaryota - ps5
Eukaryota - ps6
Amorpheae - ps7
Obazoa - ps8
Opisthokonta - ps9
Holomycota - ps10
Fungi - ps11
Fungi_1 - ps12
Fungi_2 - ps13
Fungi_3 - ps14
Dikarya - ps15
Ascomycota - ps16
Saccharo/Pezizomycotina - ps17
Saccharomycotina - ps18
Saccharomycotina_1 - ps19
Saccharomycotina_2 - ps20
Saccharomycotina_3 - ps21
Phaff./Saccharomycetaceae - ps22
Saccharomyco./mycetaceae - ps23
Saccharomycetaceae - ps24
Saccharomycetaceae_2 - ps25
Saccharomycetaceae_3 - ps26
Kaz./Nau./Saccharomyces - ps27
Saccharomyces - ps28
Arboricola_clade - ps29
Cerevisiae_clade - ps30
Saccharomyces_cerevisiae - ps31
Phylostratum

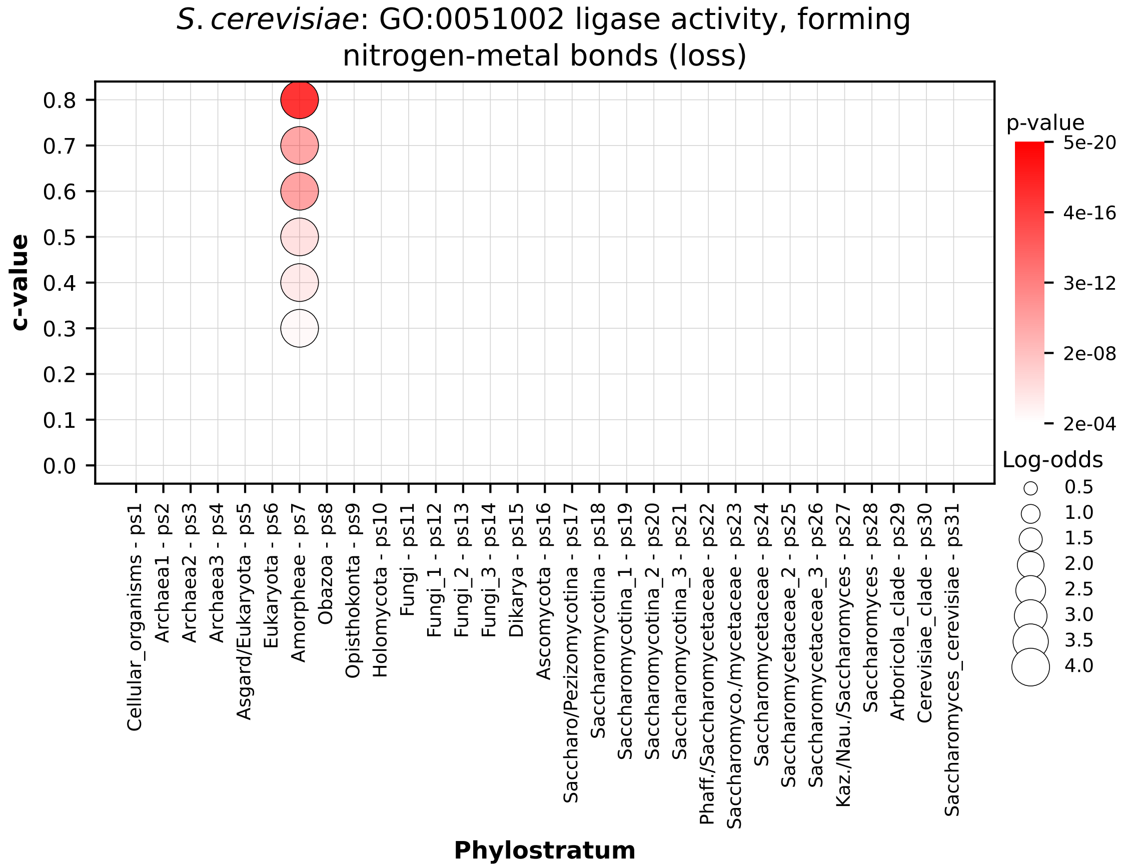

S. cerevisiae: GO:0051002 ligase activity, forming nitrogen-metal bonds (loss)
c-value
0.0
0.1
0.2
0.3
0.4
0.5
0.6
0.7
0.8
Phylostratum
Cellular_organisms - ps1
Archaea1 - ps2
Archaea2 - ps3
Archaea3 - ps4
Asgard/Eukaryota - ps5
Eukaryota - ps6
Amorpheae - ps7
Obazoa - ps8
Opisthokonta - ps9
Holomycota - ps10
Fungi - ps11
Fungi_1 - ps12
Fungi_2 - ps13
Fungi_3 - ps14
Dikarya - ps15
Ascomycota - ps16
Saccharo/Pezizomycotina - ps17
Saccharomycotina - ps18
Saccharomycotina_1 - ps19
Saccharomycotina_2 - ps20
Saccharomycotina_3 - ps21
Phaff./Saccharomycetaceae - ps22
Saccharomyco./mycetaceae - ps23
Saccharomycetaceae - ps24
Saccharomycetaceae_2 - ps25
Saccharomycetaceae_3 - ps26
Kaz./Nau./Saccharomyces - ps27
Saccharomyces - ps28
Arboricola_clade - ps29
Cerevisiae_clade - ps30
Saccharomyces_cerevisiae - ps31
p-value
5e-20
4e-16
3e-12
2e-08
2e-04
Log-odds
0.5
1.0
1.5
2.0
2.5
3.0
3.5
4.0

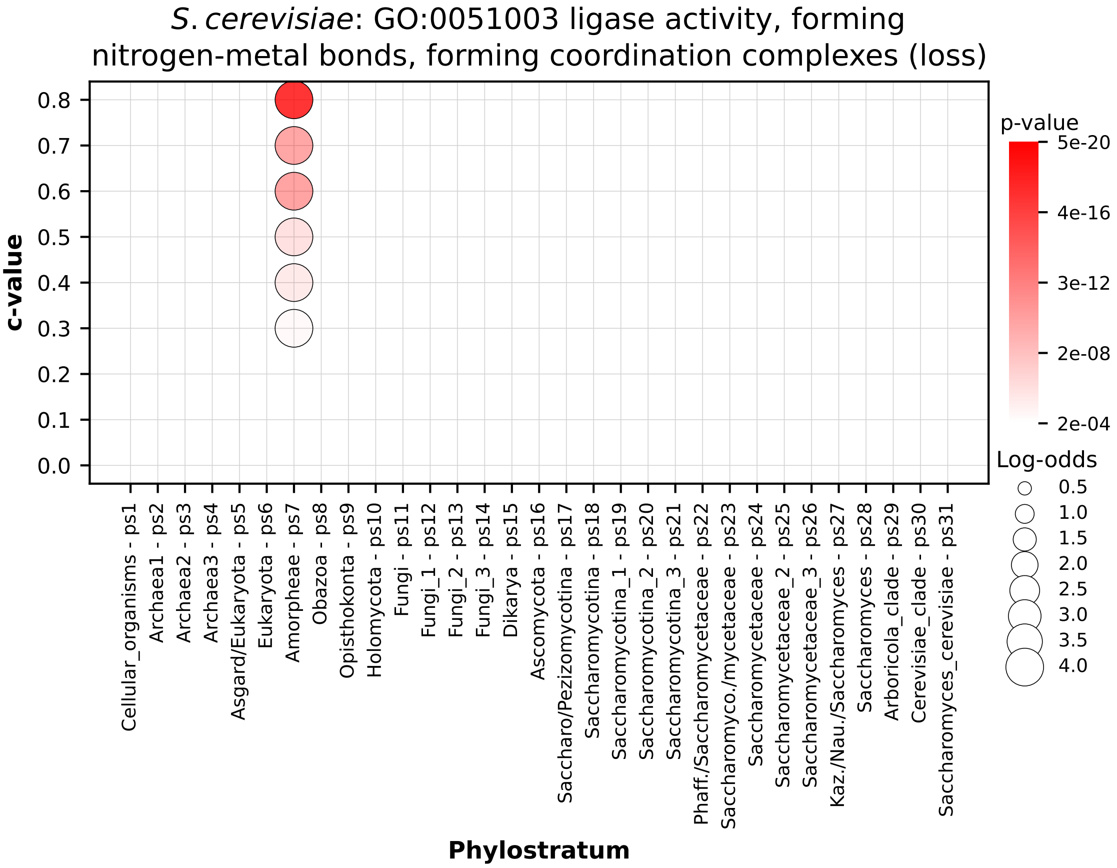
S. cerevisiae: GO:0051003 ligase activity, forming nitrogen-metal bonds, forming coordination complexes (loss)
c-value
0.0
0.1
0.2
0.3
0.4
0.5
0.6
0.7
0.8
p-value
5e-20
4e-16
3e-12
2e-08
2e-04
Log-odds
0.5
1.0
1.5
2.0
2.5
3.0
3.5
4.0
Cellular_organisms - ps1
Archaea1 - ps2
Archaea2 - ps3
Archaea3 - ps4
Asgard/Eukaryota - ps5
Eukaryota - ps6
Amorpheae - ps7
Obazoa - ps8
Opisthokonta - ps9
Holomycota - ps10
Fungi - ps11
Fungi_1 - ps12
Fungi_2 - ps13
Fungi_3 - ps14
Dikarya - ps15
Ascomycota - ps16
Saccharo/Pezizomycotina - ps17
Saccharomycotina - ps18
Saccharomycotina_1 - ps19
Saccharomycotina_2 - ps20
Saccharomycotina_3 - ps21
Phaff./Saccharomycetaceae - ps22
Saccharomyco./mycetaceae - ps23
Saccharomycetaceae - ps24
Saccharomycetaceae_2 - ps25
Saccharomycetaceae_3 - ps26
Kaz./Nau./Saccharomyces - ps27
Saccharomyces - ps28
Arboricola_clade - ps29
Cerevisiae_clade - ps30
Saccharomyces_cerevisiae - ps31
Phylostratum

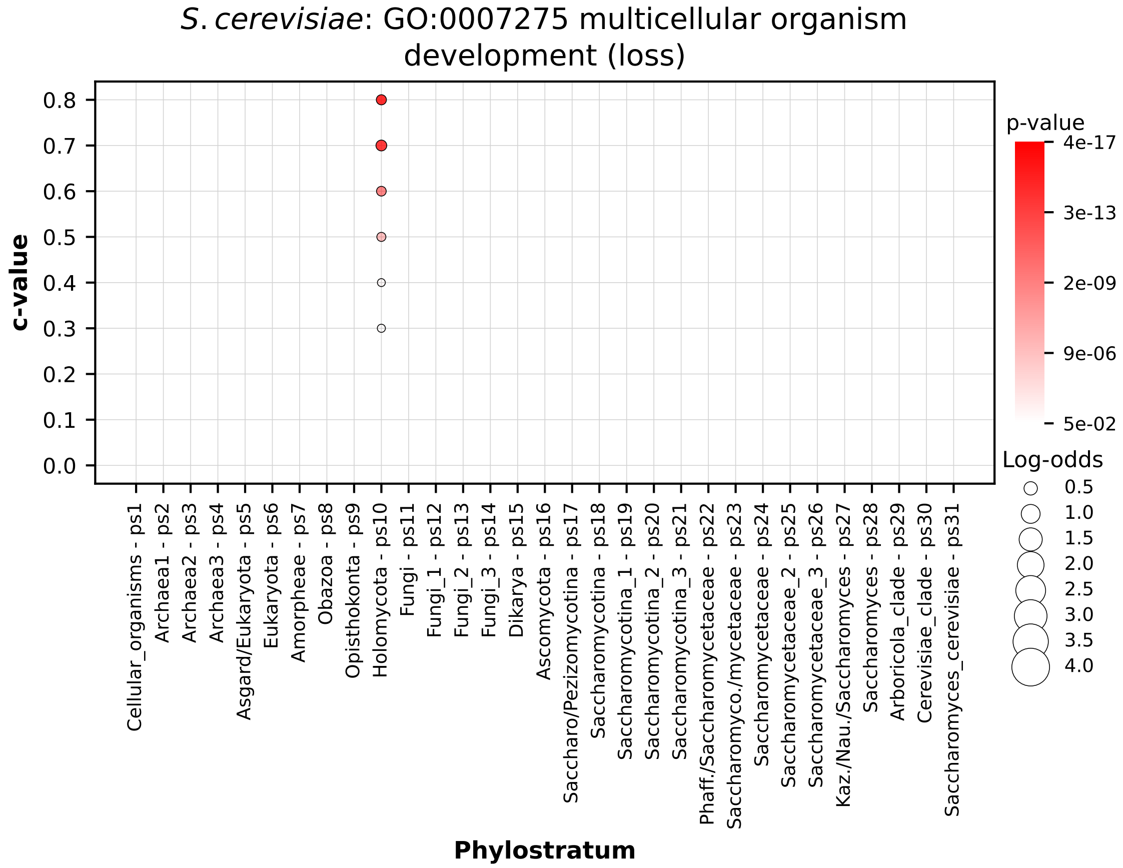

S. cerevisiae: GO:0007275 multicellular organism development (loss)
c-value
0.8
0.7
0.6
0.5
0.4
0.3
0.2
0.1
0.0
p-value
4e-17
3e-13
2e-09
9e-06
5e-02
Log-odds
0.5
1.0
1.5
2.0
2.5
3.0
3.5
4.0
Cellular_organisms - ps1
Archaea1 - ps2
Archaea2 - ps3
Archaea3 - ps4
Asgard/Eukaryota - ps5
Eukaryota - ps6
Amorpheae - ps7
Obazoa - ps8
Opisthokonta - ps9
Holomycota - ps10
Fungi - ps11
Fungi_1 - ps12
Fungi_2 - ps13
Fungi_3 - ps14
Dikarya - ps15
Ascomycota - ps16
Saccharo/Pezizomycotina - ps17
Saccharomycotina - ps18
Saccharomycotina_1 - ps19
Saccharomycotina_2 - ps20
Saccharomycotina_3 - ps21
Phaff./Saccharomycetaceae - ps22
Saccharomyco./mycetaceae - ps23
Saccharomycetaceae - ps24
Saccharomycetaceae_2 - ps25
Saccharomycetaceae_3 - ps26
Kaz./Nau./Saccharomyces - ps27
Saccharomyces - ps28
Arboricola_clade - ps29
Cerevisiae_clade - ps30
Saccharomyces_cerevisiae - ps31
Phylostratum

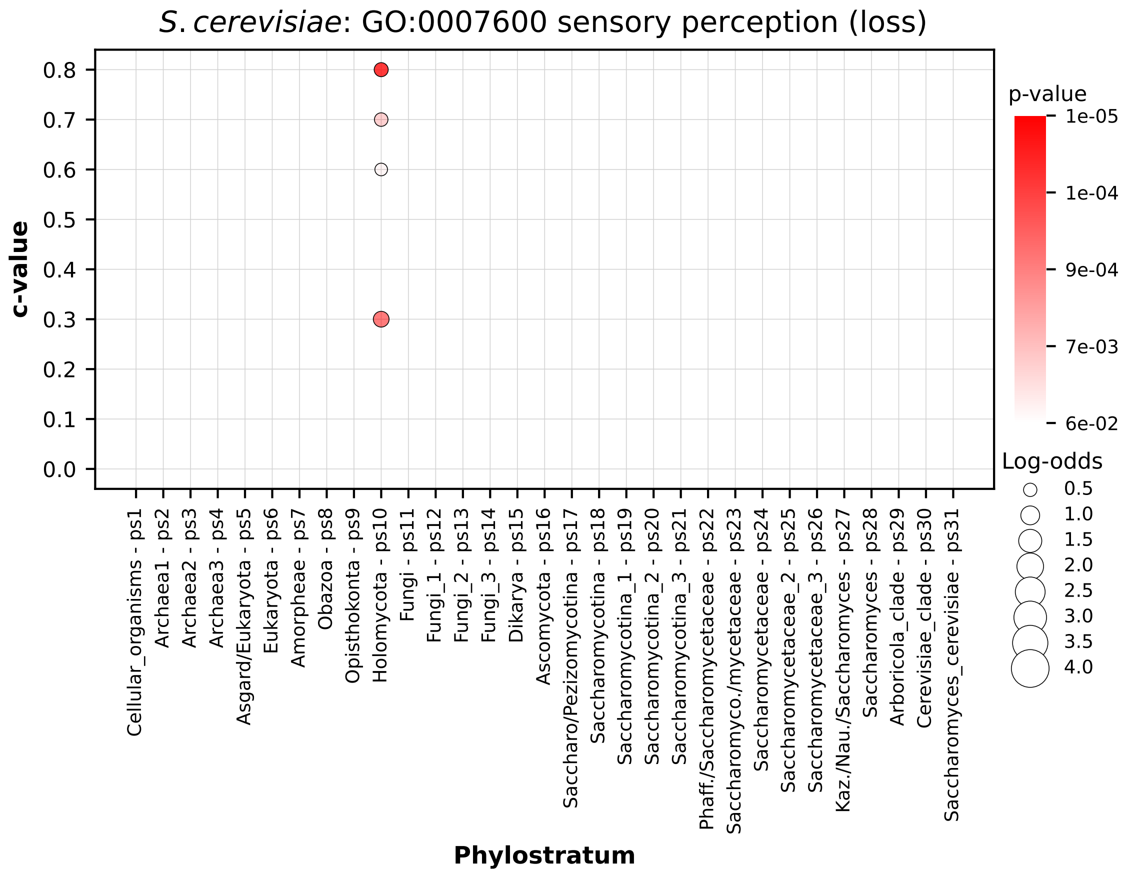
S. cerevisiae: GO:0007600 sensory perception (loss)
c-value
0.8
0.7
0.6
0.5
0.4
0.3
0.2
0.1
0.0
Phylostratum
Cellular_organisms - ps1
Archaea1 - ps2
Archaea2 - ps3
Archaea3 - ps4
Asgard/Eukaryota - ps5
Eukaryota - ps6
Amorpheae - ps7
Obazoa - ps8
Opisthokonta - ps9
Holomycota - ps10
Fungi - ps11
Fungi_1 - ps12
Fungi_2 - ps13
Fungi_3 - ps14
Dikarya - ps15
Ascomycota - ps16
Saccharo/Pezizomycotina - ps17
Saccharomycotina - ps18
Saccharomycotina_1 - ps19
Saccharomycotina_2 - ps20
Saccharomycotina_3 - ps21
Phaff./Saccharomycetaceae - ps22
Saccharomyco./mycetaceae - ps23
Saccharomycetaceae - ps24
Saccharomycetaceae_2 - ps25
Saccharomycetaceae_3 - ps26
Kaz./Nau./Saccharomyces - ps27
Saccharomyces - ps28
Arboricola_clade - ps29
Cerevisiae_clade - ps30
Saccharomyces_cerevisiae - ps31
p-value
1e-05
1e-04
9e-04
7e-03
6e-02
Log-odds
0.5
1.0
1.5
2.0
2.5
3.0
3.5
4.0

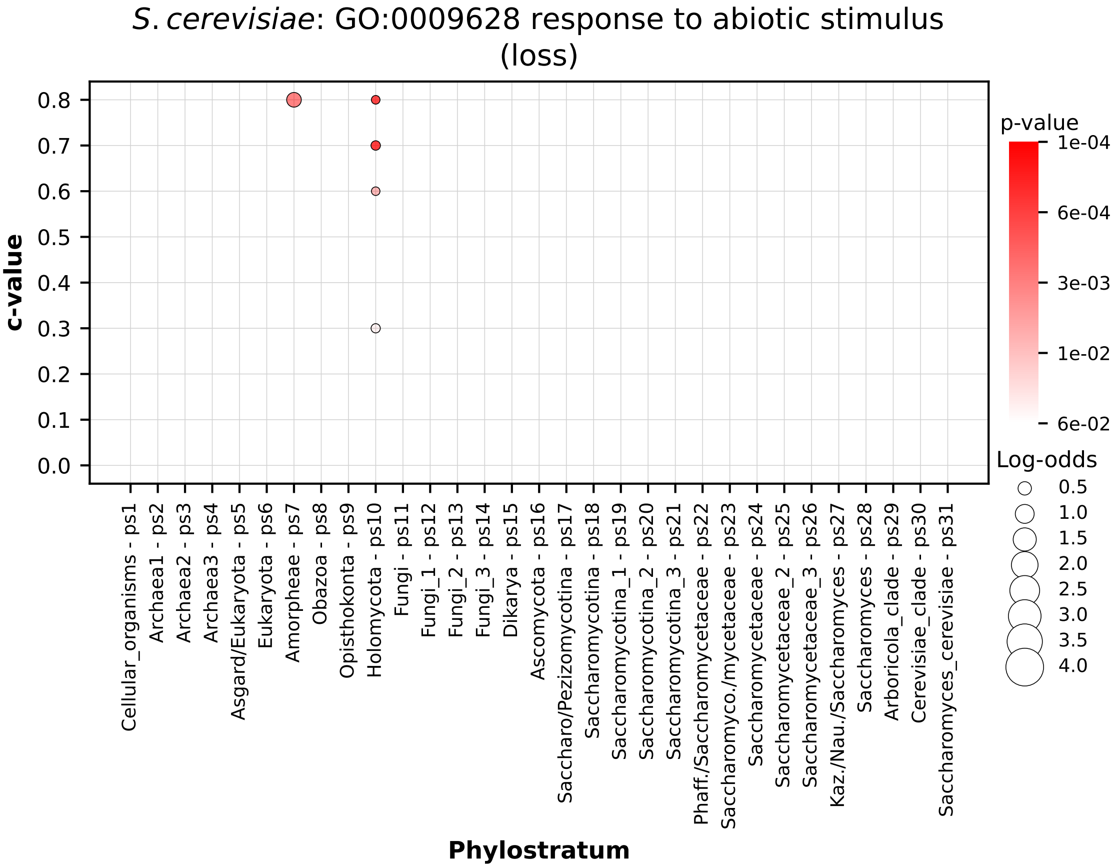
S. cerevisiae: GO:0009628 response to abiotic stimulus
(loss)
c-value
0.0
0.1
0.2
0.3
0.4
0.5
0.6
0.7
0.8
p-value
1e-04
6e-04
3e-03
1e-02
6e-02
Log-odds
0.5
1.0
1.5
2.0
2.5
3.0
3.5
4.0
Cellular_organisms - ps1
Archaea1 - ps2
Archaea2 - ps3
Archaea3 - ps4
Asgard/Eukaryota - ps5
Eukaryota - ps6
Amorpheae - ps7
Obazoa - ps8
Opisthokonta - ps9
Holomycota - ps10
Fungi - ps11
Fungi_1 - ps12
Fungi_2 - ps13
Fungi_3 - ps14
Dikarya - ps15
Ascomycota - ps16
Saccharo/Pezizomycotina - ps17
Saccharomycotina - ps18
Saccharomycotina_1 - ps19
Saccharomycotina_2 - ps20
Saccharomycotina_3 - ps21
Phaff./Saccharomycetaceae - ps22
Saccharomyco./mycetaceae - ps23
Saccharomycetaceae - ps24
Saccharomycetaceae_2 - ps25
Saccharomycetaceae_3 - ps26
Kaz./Nau./Saccharomyces - ps27
Saccharomyces - ps28
Arboricola_clade - ps29
Cerevisiae_clade - ps30
Saccharomyces_cerevisiae - ps31
Phylostratum

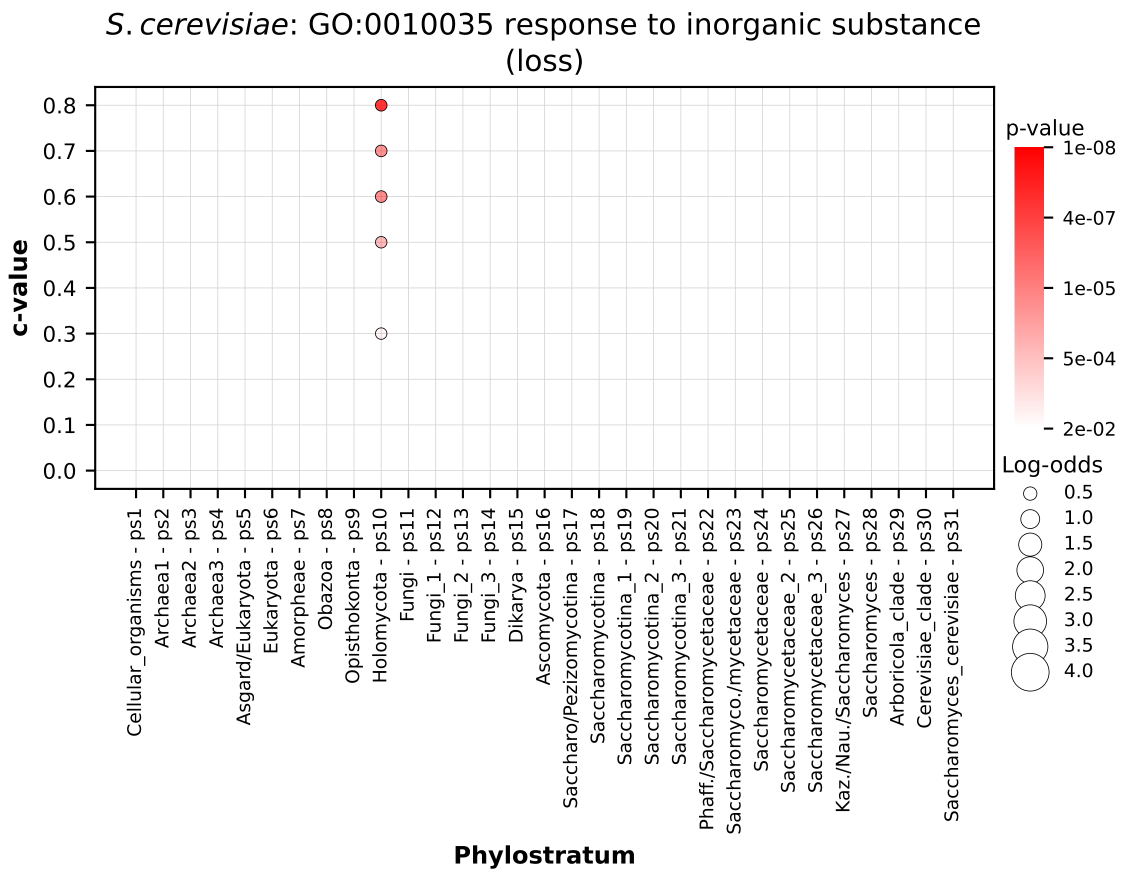

S. cerevisiae: GO:0010035 response to inorganic substance
(loss)
c-value
0.8
0.7
0.6
0.5
0.4
0.3
0.2
0.1
0.0
p-value
1e-08
4e-07
1e-05
5e-04
2e-02
Log-odds
0.5
1.0
1.5
2.0
2.5
3.0
3.5
4.0
Cellular_organisms - ps1
Archaea1 - ps2
Archaea2 - ps3
Archaea3 - ps4
Asgard/Eukaryota - ps5
Eukaryota - ps6
Amorpheae - ps7
Obazoa - ps8
Opisthokonta - ps9
Holomycota - ps10
Fungi - ps11
Fungi_1 - ps12
Fungi_2 - ps13
Fungi_3 - ps14
Dikarya - ps15
Ascomycota - ps16
Saccharo/Pezizomycotina - ps17
Saccharomycotina - ps18
Saccharomycotina_1 - ps19
Saccharomycotina_2 - ps20
Saccharomycotina_3 - ps21
Phaff./Saccharomycetaceae - ps22
Saccharomyco./mycetaceae - ps23
Saccharomycetaceae - ps24
Saccharomycetaceae_2 - ps25
Saccharomycetaceae_3 - ps26
Kaz./Nau./Saccharomyces - ps27
Saccharomyces - ps28
Arboricola_clade - ps29
Cerevisiae_clade - ps30
Saccharomyces_cerevisiae - ps31
Phylostratum

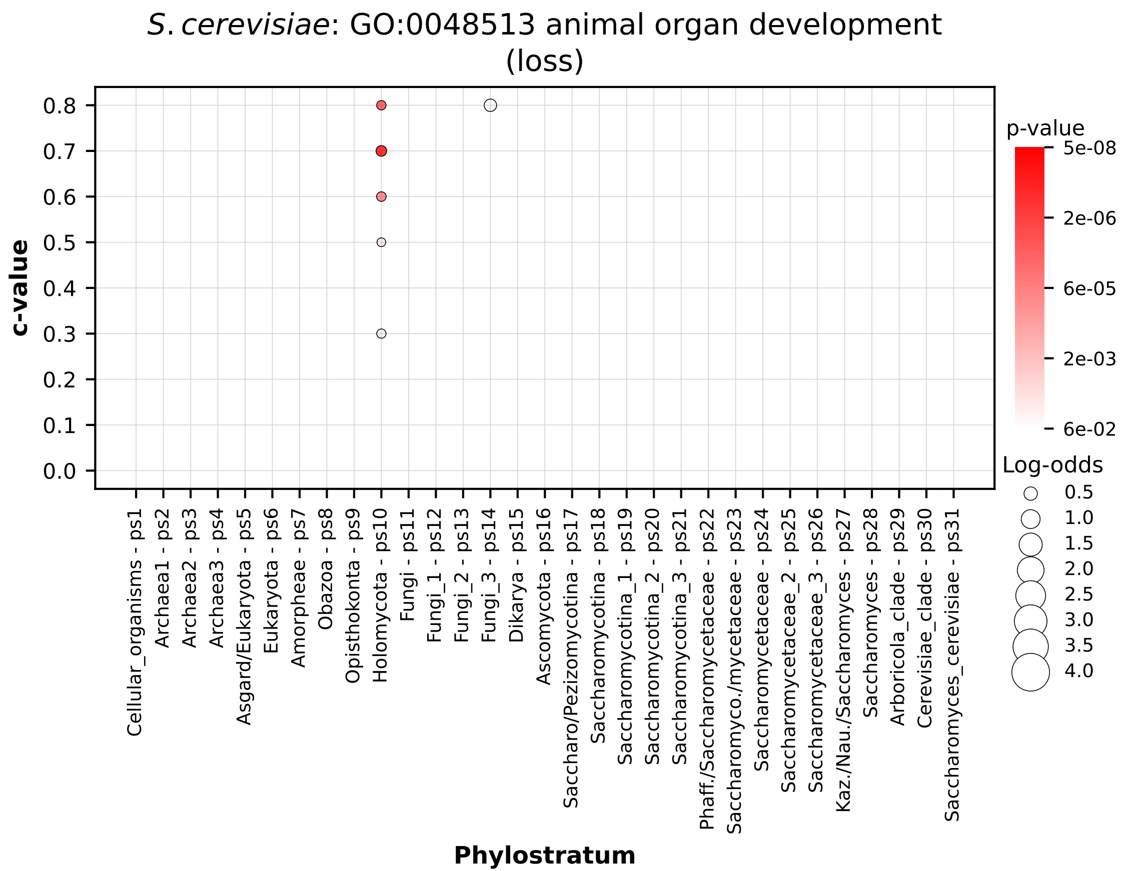

S. cerevisiae: GO:0048513 animal organ development
(loss)
c-value
0.0
0.1
0.2
0.3
0.4
0.5
0.6
0.7
0.8
p-value
5e-08
2e-06
6e-05
2e-03
6e-02
Log-odds
0.5
1.0
1.5
2.0
2.5
3.0
3.5
4.0
Cellular_organisms - ps1
Archaea1 - ps2
Archaea2 - ps3
Archaea3 - ps4
Asgard/Eukaryota - ps5
Eukaryota - ps6
Amorpheae - ps7
Obazoa - ps8
Opisthokonta - ps9
Holomycota - ps10
Fungi - ps11
Fungi_1 - ps12
Fungi_2 - ps13
Fungi_3 - ps14
Dikarya - ps15
Ascomycota - ps16
Saccharo/Pezizomycotina - ps17
Saccharomycotina - ps18
Saccharomycotina_1 - ps19
Saccharomycotina_2 - ps20
Saccharomycotina_3 - ps21
Phaff./Saccharomycetaceae - ps22
Saccharomyco./mycetaceae - ps23
Saccharomycetaceae - ps24
Saccharomycetaceae_2 - ps25
Saccharomycetaceae_3 - ps26
Kaz./Nau./Saccharomyces - ps27
Saccharomyces - ps28
Arboricola_clade - ps29
Cerevisiae_clade - ps30
Saccharomyces_cerevisiae - ps31
Phylostratum

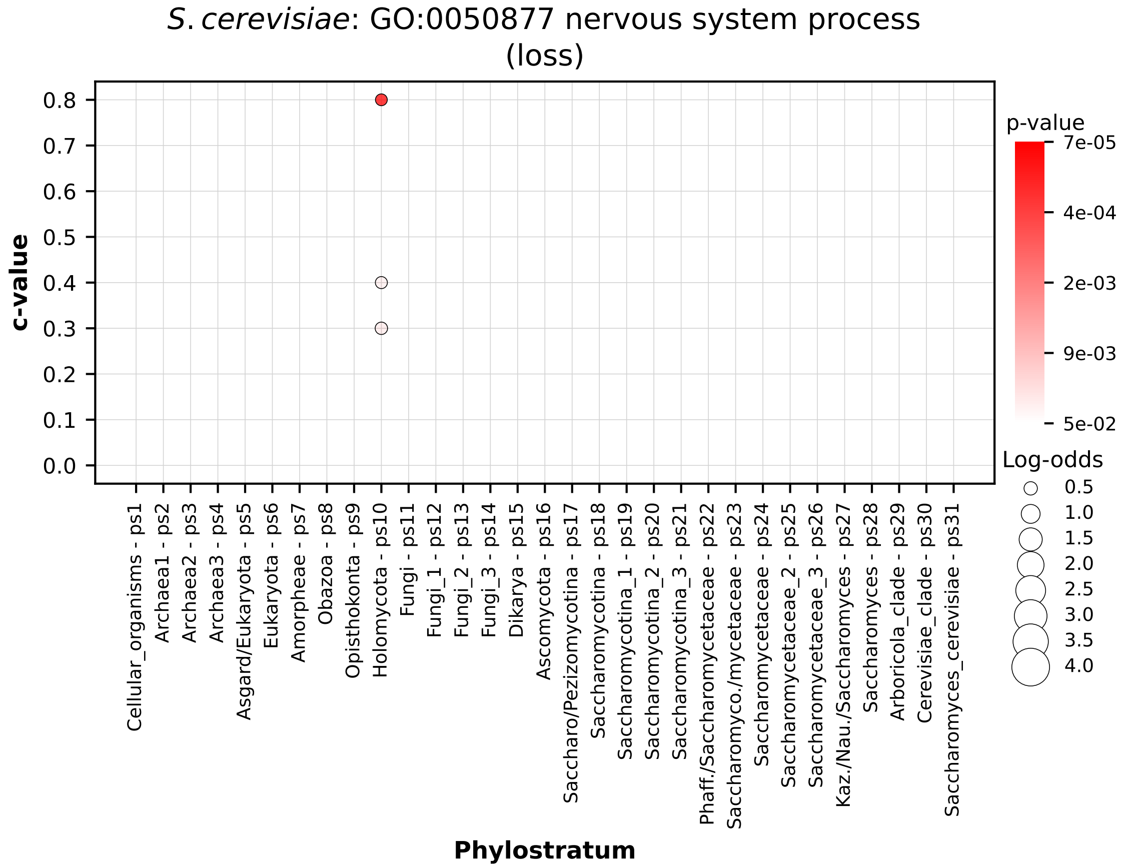

S. cerevisiae: GO:0050877 nervous system process
(loss)
c-value
0.0
0.1
0.2
0.3
0.4
0.5
0.6
0.7
0.8
Phylostratum
Cellular_organisms - ps1
Archaea1 - ps2
Archaea2 - ps3
Archaea3 - ps4
Asgard/Eukaryota - ps5
Eukaryota - ps6
Amorpheae - ps7
Obazoa - ps8
Opisthokonta - ps9
Holomycota - ps10
Fungi - ps11
Fungi_1 - ps12
Fungi_2 - ps13
Fungi_3 - ps14
Dikarya - ps15
Ascomycota - ps16
Saccharo/Pezizomycotina - ps17
Saccharomycotina - ps18
Saccharomycotina_1 - ps19
Saccharomycotina_2 - ps20
Saccharomycotina_3 - ps21
Phaff./Saccharomycetaceae - ps22
Saccharomyco./mycetaceae - ps23
Saccharomycetaceae - ps24
Saccharomycetaceae_2 - ps25
Saccharomycetaceae_3 - ps26
Kaz./Nau./Saccharomyces - ps27
Saccharomyces - ps28
Arboricola_clade - ps29
Cerevisiae_clade - ps30
Saccharomyces_cerevisiae - ps31
p-value
7e-05
4e-04
2e-03
9e-03
5e-02
Log-odds
0.5
1.0
1.5
2.0
2.5
3.0
3.5
4.0

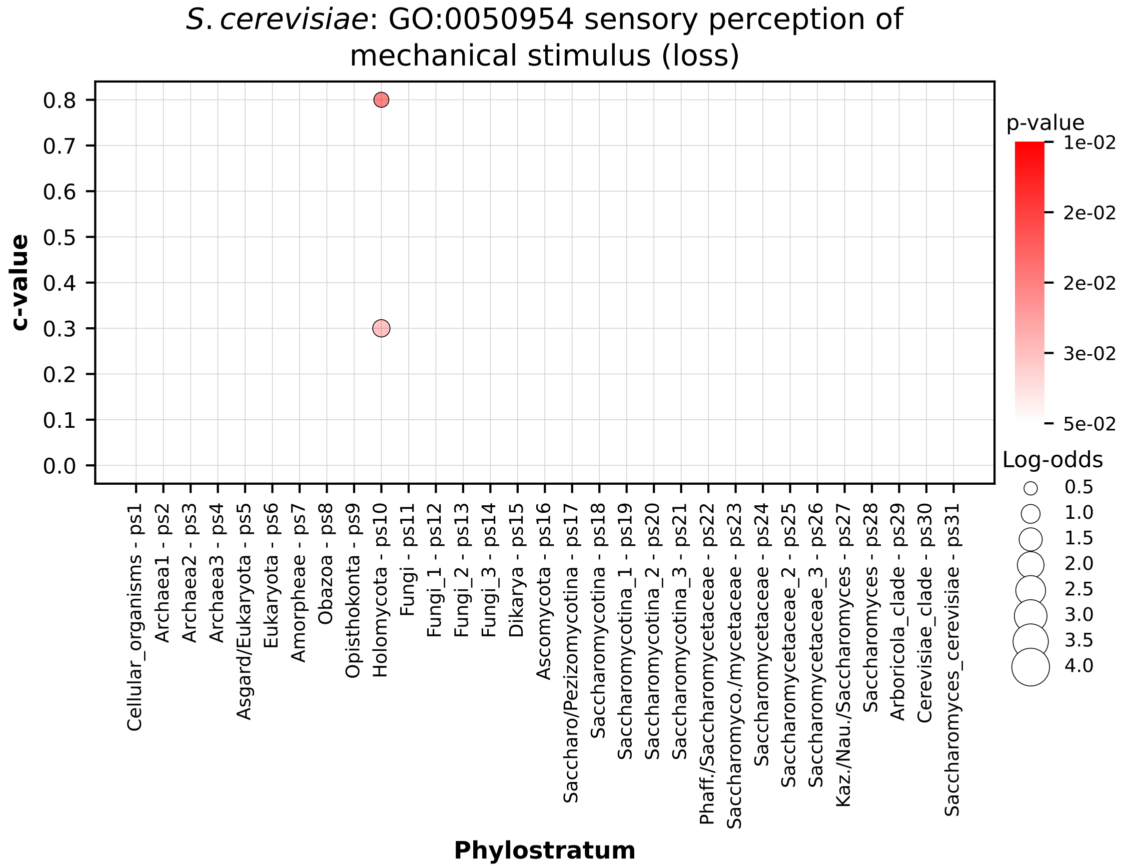

S. cerevisiae: GO:0050954 sensory perception of mechanical stimulus (loss)
c-value
0.0
0.1
0.2
0.3
0.4
0.5
0.6
0.7
0.8
Phylostratum
Cellular_organisms - ps1
Archaea1 - ps2
Archaea2 - ps3
Archaea3 - ps4
Asgard/Eukaryota - ps5
Eukaryota - ps6
Amorpheae - ps7
Obazoa - ps8
Opisthokonta - ps9
Holomycota - ps10
Fungi - ps11
Fungi_1 - ps12
Fungi_2 - ps13
Fungi_3 - ps14
Dikarya - ps15
Ascomycota - ps16
Saccharo/Pezizomycotina - ps17
Saccharomycotina - ps18
Saccharomycotina_1 - ps19
Saccharomycotina_2 - ps20
Saccharomycotina_3 - ps21
Phaff./Saccharomycetaceae - ps22
Saccharomyco./mycetaceae - ps23
Saccharomycetaceae - ps24
Saccharomycetaceae_2 - ps25
Saccharomycetaceae_3 - ps26
Kaz./Nau./Saccharomyces - ps27
Saccharomyces - ps28
Arboricola_clade - ps29
Cerevisiae_clade - ps30
Saccharomyces_cerevisiae - ps31
p-value
1e-02
2e-02
2e-02
3e-02
5e-02
Log-odds
0.5
1.0
1.5
2.0
2.5
3.0
3.5
4.0

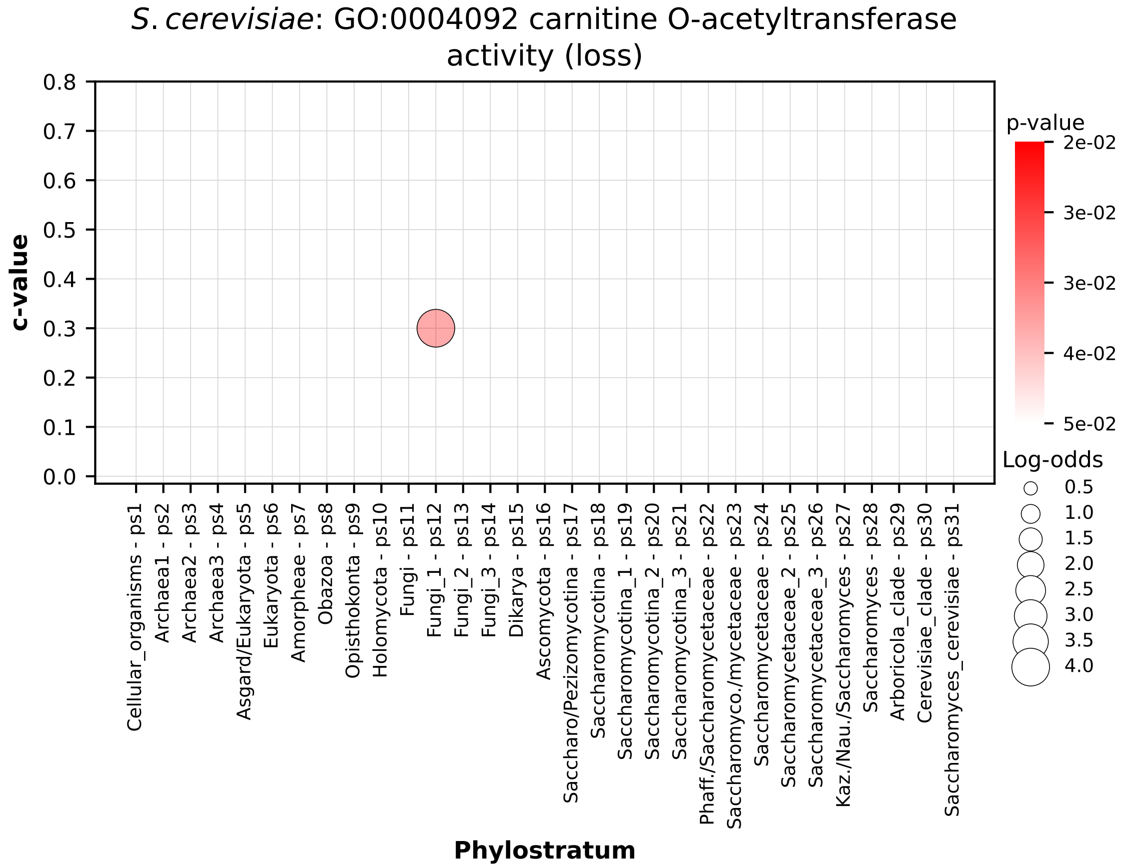
S. cerevisiae: GO:0004092 carnitine O-acetyltransferase activity (loss)
c-value
0.8
0.7
0.6
0.5
0.4
0.3
0.2
0.1
0.0
p-value
2e-02
3e-02
3e-02
4e-02
5e-02
Log-odds
0.5
1.0
1.5
2.0
2.5
3.0
3.5
4.0
Cellular_organisms - ps1
Archaea1 - ps2
Archaea2 - ps3
Archaea3 - ps4
Asgard/Eukaryota - ps5
Eukaryota - ps6
Amorpheae - ps7
Obazoa - ps8
Opisthokonta - ps9
Holomycota - ps10
Fungi - ps11
Fungi_1 - ps12
Fungi_2 - ps13
Fungi_3 - ps14
Dikarya - ps15
Ascomycota - ps16
Saccharo/Pezizomycotina - ps17
Saccharomycotina - ps18
Saccharomycotina_1 - ps19
Saccharomycotina_2 - ps20
Saccharomycotina_3 - ps21
Phaff./Saccharomycetaceae - ps22
Saccharomyco./mycetaceae - ps23
Saccharomycetaceae - ps24
Saccharomycetaceae_2 - ps25
Saccharomycetaceae_3 - ps26
Kaz./Nau./Saccharomyces - ps27
Saccharomyces - ps28
Arboricola_clade - ps29
Cerevisiae_clade - ps30
Saccharomyces_cerevisiae - ps31
Phylostratum

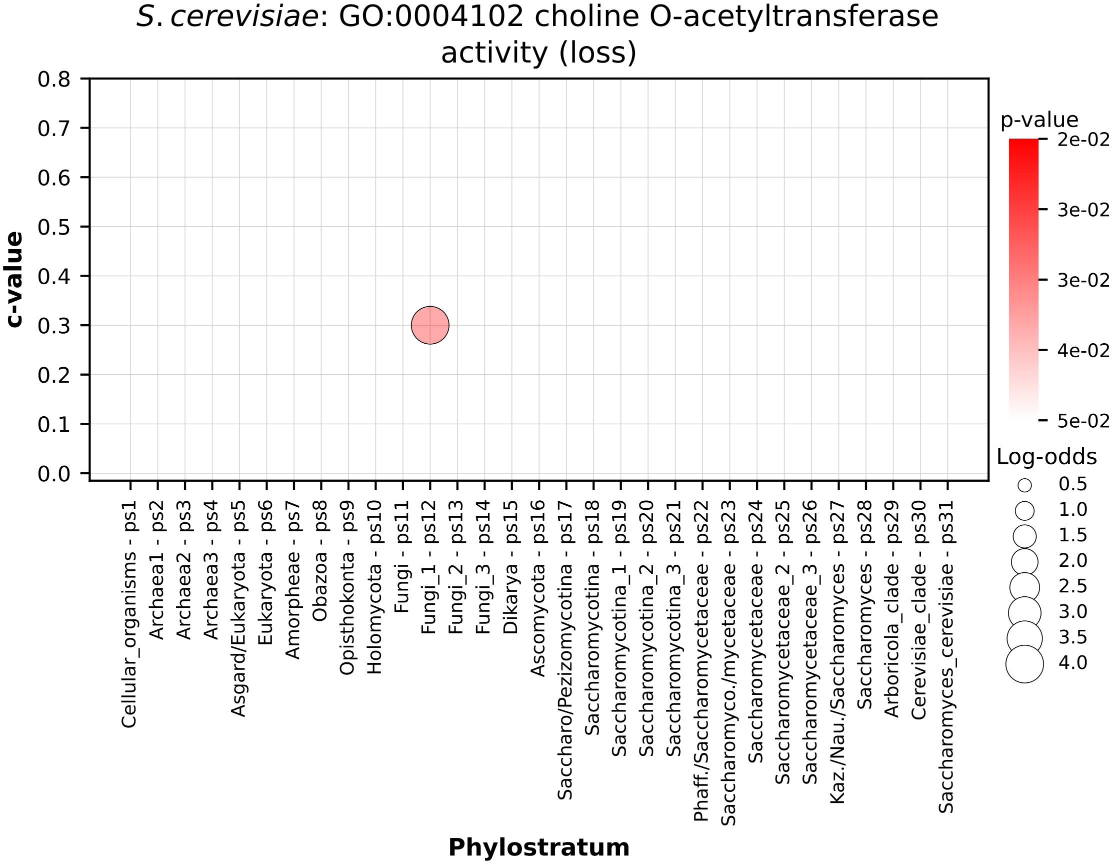

S. cerevisiae: GO:0004102 choline O-acetyltransferase activity (loss)
c-value
0.8
0.7
0.6
0.5
0.4
0.3
0.2
0.1
0.0
p-value
2e-02
3e-02
3e-02
4e-02
5e-02
Log-odds
0.5
1.0
1.5
2.0
2.5
3.0
3.5
4.0
Cellular_organisms - ps1
Archaea1 - ps2
Archaea2 - ps3
Archaea3 - ps4
Asgard/Eukaryota - ps5
Eukaryota - ps6
Amorpheae - ps7
Obazoa - ps8
Opisthokonta - ps9
Holomycota - ps10
Fungi - ps11
Fungi_1 - ps12
Fungi_2 - ps13
Fungi_3 - ps14
Dikarya - ps15
Ascomycota - ps16
Saccharo/Pezizomycotina - ps17
Saccharomycotina - ps18
Saccharomycotina_1 - ps19
Saccharomycotina_2 - ps20
Saccharomycotina_3 - ps21
Phaff./Saccharomycetaceae - ps22
Saccharomyco./mycetaceae - ps23
Saccharomycetaceae - ps24
Saccharomycetaceae_2 - ps25
Saccharomycetaceae_3 - ps26
Kaz./Nau./Saccharomyces - ps27
Saccharomyces - ps28
Arboricola_clade - ps29
Cerevisiae_clade - ps30
Saccharomyces_cerevisiae - ps31
Phylostratum

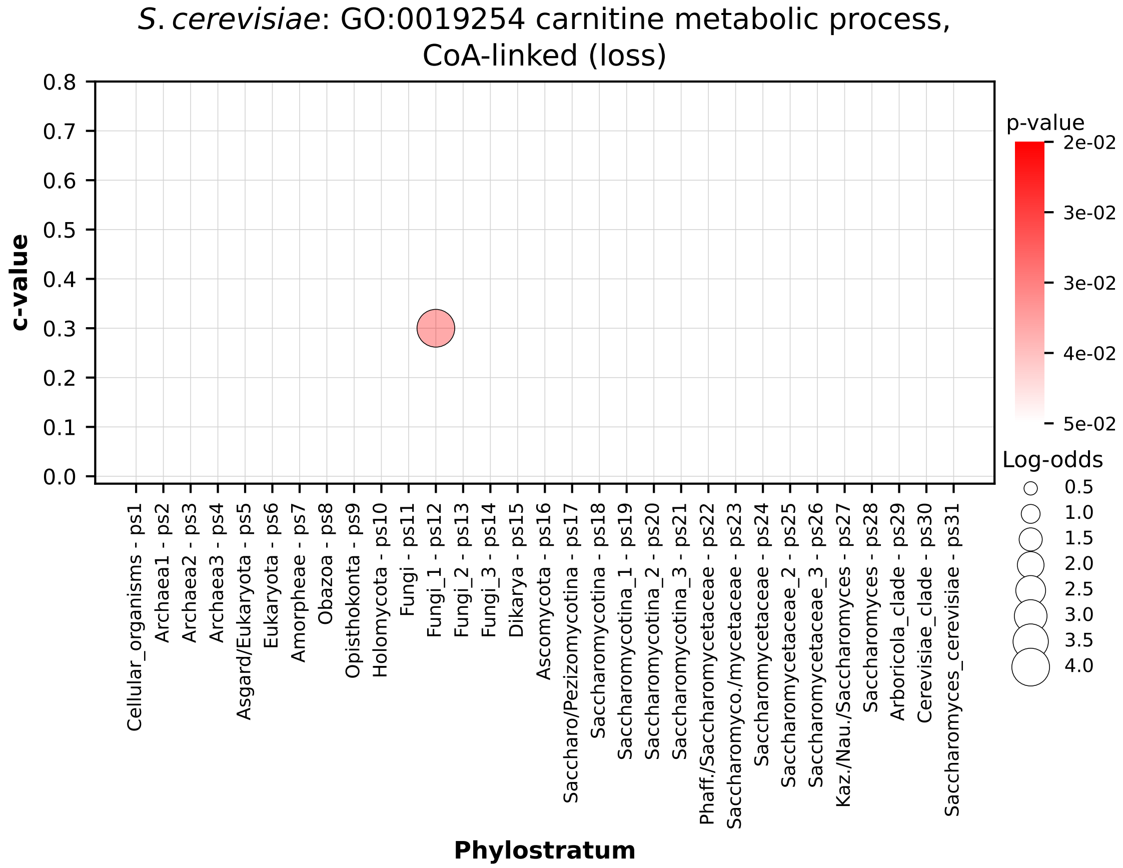

S. cerevisiae: GO:0019254 carnitine metabolic process, CoA-linked (loss)
c-value
0.8
0.7
0.6
0.5
0.4
0.3
0.2
0.1
0.0
p-value
2e-02
3e-02
3e-02
4e-02
5e-02
Log-odds
0.5
1.0
1.5
2.0
2.5
3.0
3.5
4.0
Cellular_organisms - ps1
Archaea1 - ps2
Archaea2 - ps3
Archaea3 - ps4
Asgard/Eukaryota - ps5
Eukaryota - ps6
Amorpheae - ps7
Obazoa - ps8
Opisthokonta - ps9
Holomycota - ps10
Fungi - ps11
Fungi_1 - ps12
Fungi_2 - ps13
Fungi_3 - ps14
Dikarya - ps15
Ascomycota - ps16
Saccharo/Pezizomycotina - ps17
Saccharomycotina - ps18
Saccharomycotina_1 - ps19
Saccharomycotina_2 - ps20
Saccharomycotina_3 - ps21
Phaff./Saccharomycetaceae - ps22
Saccharomyco./mycetaceae - ps23
Saccharomycetaceae - ps24
Saccharomycetaceae_2 - ps25
Saccharomycetaceae_3 - ps26
Kaz./Nau./Saccharomyces - ps27
Saccharomyces - ps28
Arboricola_clade - ps29
Cerevisiae_clade - ps30
Saccharomyces_cerevisiae - ps31
Phylostratum

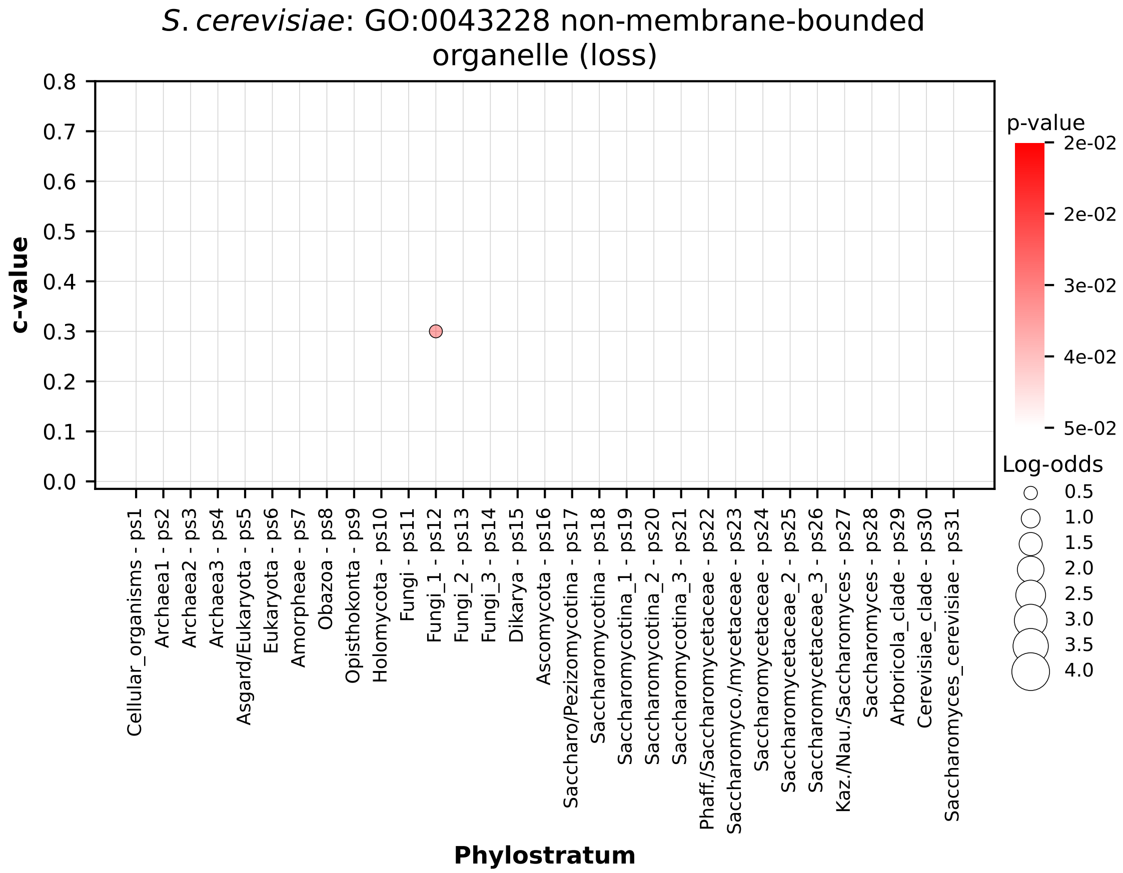

S. cerevisiae: GO:0043228 non-membrane-bounded organelle (loss)
c-value
0.0
0.1
0.2
0.3
0.4
0.5
0.6
0.7
0.8
Phylostratum
Cellular_organisms - ps1
Archaea1 - ps2
Archaea2 - ps3
Archaea3 - ps4
Asgard/Eukaryota - ps5
Eukaryota - ps6
Amorpheae - ps7
Obazoa - ps8
Opisthokonta - ps9
Holomycota - ps10
Fungi - ps11
Fungi_1 - ps12
Fungi_2 - ps13
Fungi_3 - ps14
Dikarya - ps15
Ascomycota - ps16
Saccharo/Pezizomycotina - ps17
Saccharomycotina - ps18
Saccharomycotina_1 - ps19
Saccharomycotina_2 - ps20
Saccharomycotina_3 - ps21
Phaff./Saccharomycetaceae - ps22
Saccharomyco./mycetaceae - ps23
Saccharomycetaceae - ps24
Saccharomycetaceae_2 - ps25
Saccharomycetaceae_3 - ps26
Kaz./Nau./Saccharomyces - ps27
Saccharomyces - ps28
Arboricola_clade - ps29
Cerevisiae_clade - ps30
Saccharomyces_cerevisiae - ps31
p-value
2e-02
2e-02
3e-02
4e-02
5e-02
Log-odds
0.5
1.0
1.5
2.0
2.5
3.0
3.5
4.0

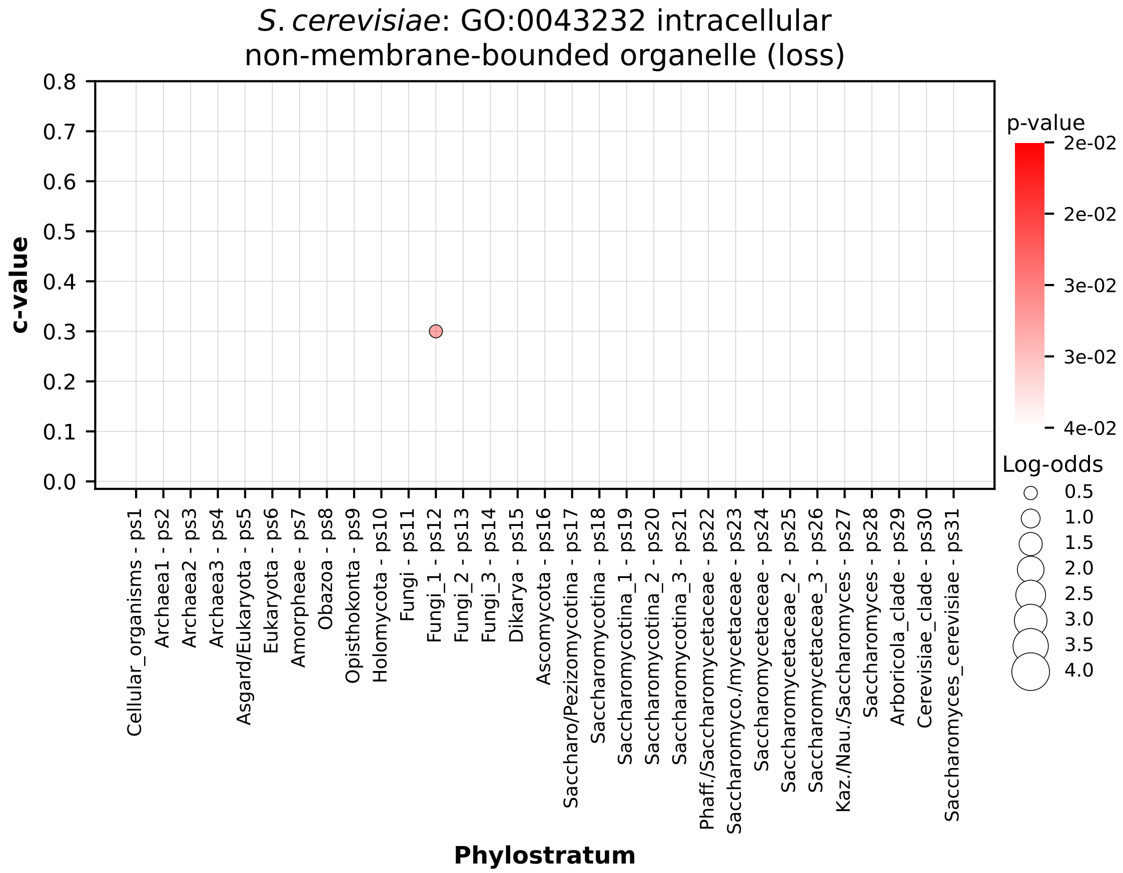
S. cerevisiae: GO:0043232 intracellular non-membrane-bounded organelle (loss)
c-value
Phylostratum
p-value
2e-02
2e-02
3e-02
3e-02
4e-02
Log-odds
0.5
1.0
1.5
2.0
2.5
3.0
3.5
4.0
Cellular_organisms - ps1
Archaea1 - ps2
Archaea2 - ps3
Archaea3 - ps4
Asgard/Eukaryota - ps5
Eukaryota - ps6
Amorpheae - ps7
Obazoa - ps8
Opisthokonta - ps9
Holomycota - ps10
Fungi - ps11
Fungi_1 - ps12
Fungi_2 - ps13
Fungi_3 - ps14
Dikarya - ps15
Ascomycota - ps16
Saccharo/Pezizomycotina - ps17
Saccharomycotina - ps18
Saccharomycotina_1 - ps19
Saccharomycotina_2 - ps20
Saccharomycotina_3 - ps21
Phaff./Saccharomycetaceae - ps22
Saccharomyco./mycetaceae - ps23
Saccharomycetaceae - ps24
Saccharomycetaceae_2 - ps25
Saccharomycetaceae_3 - ps26
Kaz./Nau./Saccharomyces - ps27
Saccharomyces - ps28
Arboricola_clade - ps29
Cerevisiae_clade - ps30
Saccharomyces_cerevisiae - ps31

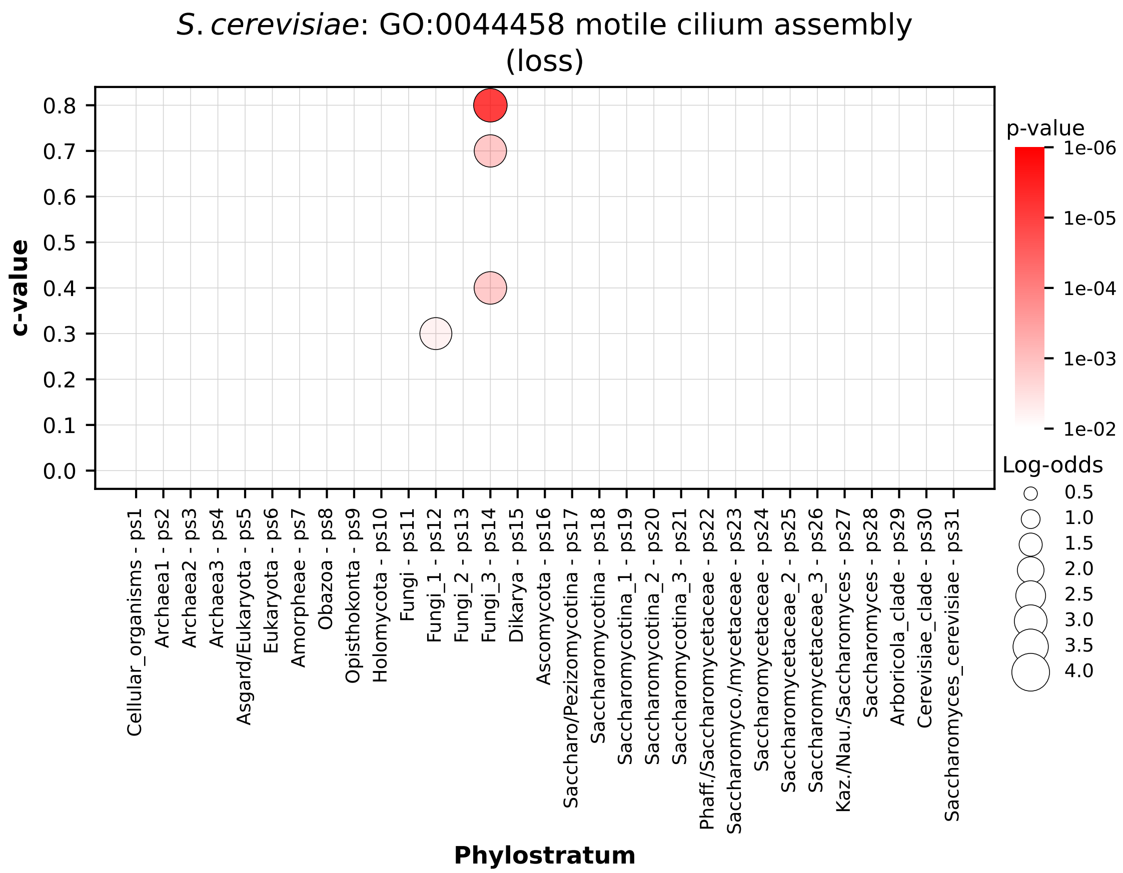

S. cerevisiae: GO:0044458 motile cilium assembly
(loss)
c-value
0.0
0.1
0.2
0.3
0.4
0.5
0.6
0.7
0.8
Phylostratum
Cellular_organisms - ps1
Archaea1 - ps2
Archaea2 - ps3
Archaea3 - ps4
Asgard/Eukaryota - ps5
Eukaryota - ps6
Amorpheae - ps7
Obazoa - ps8
Opisthokonta - ps9
Holomycota - ps10
Fungi - ps11
Fungi_1 - ps12
Fungi_2 - ps13
Fungi_3 - ps14
Dikarya - ps15
Ascomycota - ps16
Saccharo/Pezizomycotina - ps17
Saccharomycotina - ps18
Saccharomycotina_1 - ps19
Saccharomycotina_2 - ps20
Saccharomycotina_3 - ps21
Phaff./Saccharomycetaceae - ps22
Saccharomyco./mycetaceae - ps23
Saccharomycetaceae - ps24
Saccharomycetaceae_2 - ps25
Saccharomycetaceae_3 - ps26
Kaz./Nau./Saccharomyces - ps27
Saccharomyces - ps28
Arboricola_clade - ps29
Cerevisiae_clade - ps30
Saccharomyces_cerevisiae - ps31
p-value
1e-06
1e-05
1e-04
1e-03
1e-02
Log-odds
0.5
1.0
1.5
2.0
2.5
3.0
3.5
4.0

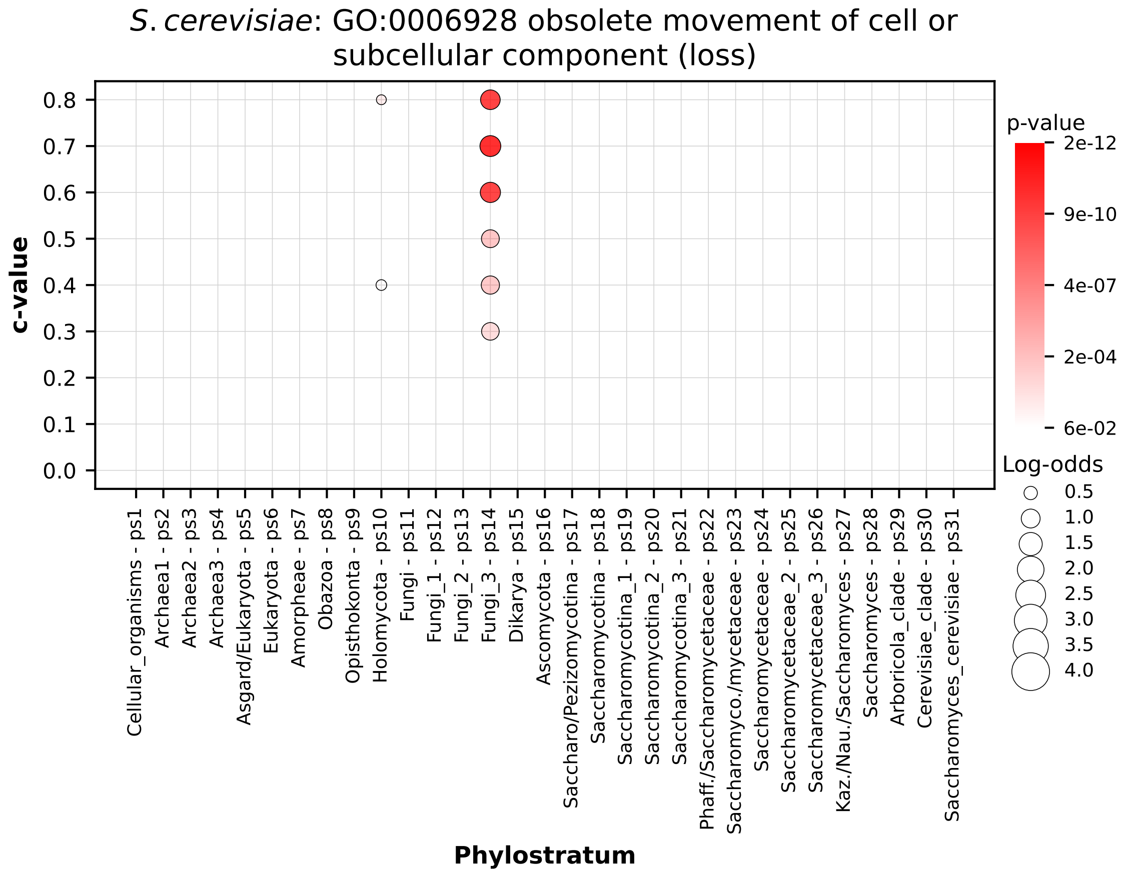
S. cerevisiae: GO:0006928 obsolete movement of cell or subcellular component (loss)
c-value
0.0
0.1
0.2
0.3
0.4
0.5
0.6
0.7
0.8
Phylostratum
Cellular_organisms - ps1
Archaea1 - ps2
Archaea2 - ps3
Archaea3 - ps4
Asgard/Eukaryota - ps5
Eukaryota - ps6
Amorpheae - ps7
Obazoa - ps8
Opisthokonta - ps9
Holomycota - ps10
Fungi - ps11
Fungi_1 - ps12
Fungi_2 - ps13
Fungi_3 - ps14
Dikarya - ps15
Ascomycota - ps16
Saccharo/Pezizomycotina - ps17
Saccharomycotina - ps18
Saccharomycotina_1 - ps19
Saccharomycotina_2 - ps20
Saccharomycotina_3 - ps21
Phaff./Saccharomycetaceae - ps22
Saccharomyco./mycetaceae - ps23
Saccharomycetaceae - ps24
Saccharomycetaceae_2 - ps25
Saccharomycetaceae_3 - ps26
Kaz./Nau./Saccharomyces - ps27
Saccharomyces - ps28
Arboricola_clade - ps29
Cerevisiae_clade - ps30
Saccharomyces_cerevisiae - ps31
p-value
2e-12
9e-10
4e-07
2e-04
6e-02
Log-odds
0.5
1.0
1.5
2.0
2.5
3.0
3.5
4.0

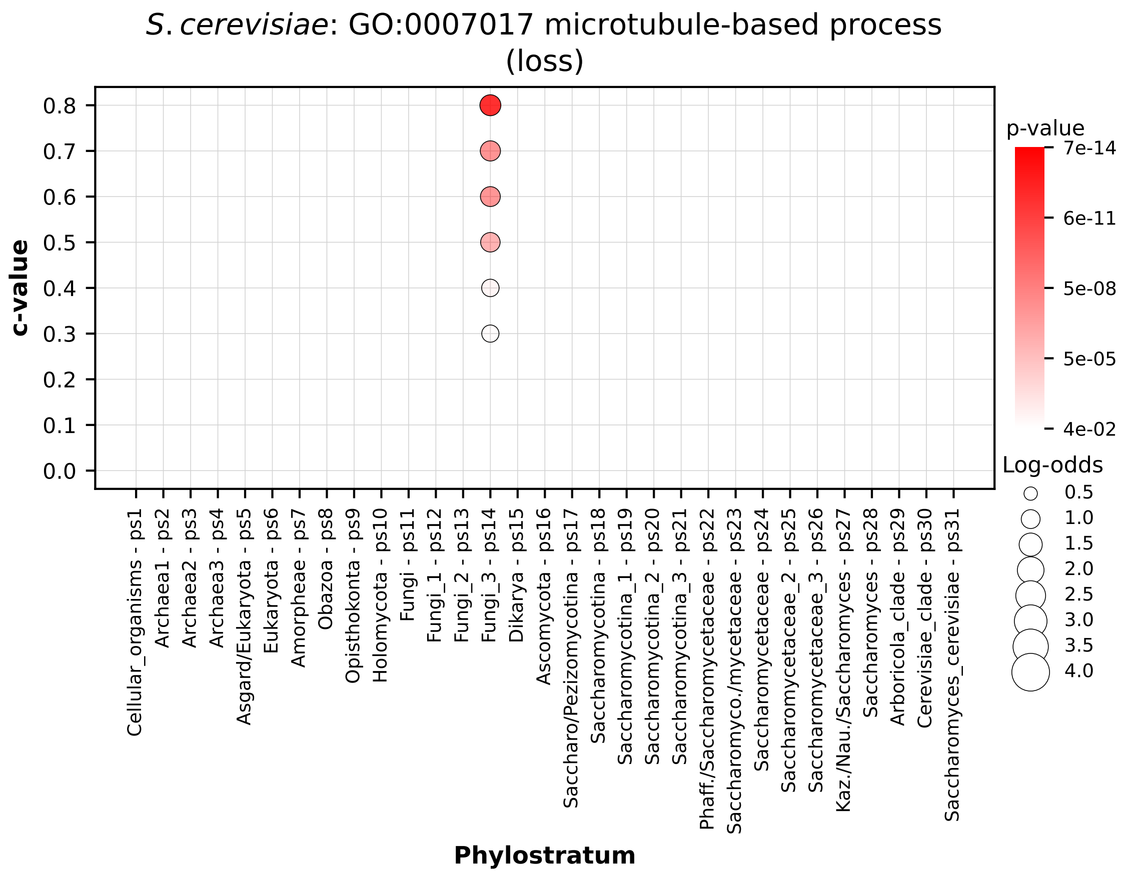

S. cerevisiae: GO:0007017 microtubule-based process
(loss)
c-value
0.0
0.1
0.2
0.3
0.4
0.5
0.6
0.7
0.8
p-value
7e-14
6e-11
5e-08
5e-05
4e-02
Log-odds
0.5
1.0
1.5
2.0
2.5
3.0
3.5
4.0
Cellular_organisms - ps1
Archaea1 - ps2
Archaea2 - ps3
Archaea3 - ps4
Asgard/Eukaryota - ps5
Eukaryota - ps6
Amorpheae - ps7
Obazoa - ps8
Opisthokonta - ps9
Holomycota - ps10
Fungi - ps11
Fungi_1 - ps12
Fungi_2 - ps13
Fungi_3 - ps14
Dikarya - ps15
Ascomycota - ps16
Saccharo/Pezizomycotina - ps17
Saccharomycotina - ps18
Saccharomycotina_1 - ps19
Saccharomycotina_2 - ps20
Saccharomycotina_3 - ps21
Phaff./Saccharomycetaceae - ps22
Saccharomyco./mycetaceae - ps23
Saccharomycetaceae - ps24
Saccharomycetaceae_2 - ps25
Saccharomycetaceae_3 - ps26
Kaz./Nau./Saccharomyces - ps27
Saccharomyces - ps28
Arboricola_clade - ps29
Cerevisiae_clade - ps30
Saccharomyces_cerevisiae - ps31
Phylostratum

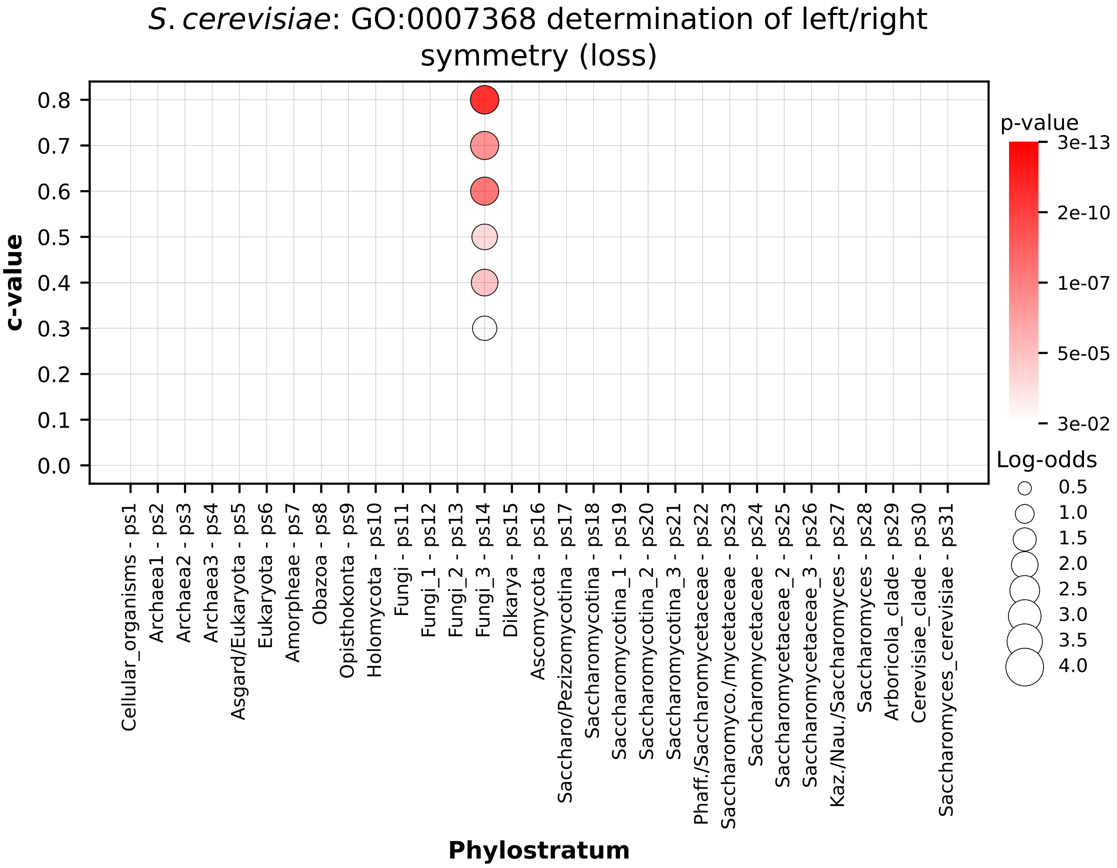
S. cerevisiae: GO:0007368 determination of left/right symmetry (loss)
c-value
0.8
0.7
0.6
0.5
0.4
0.3
0.2
0.1
0.0
p-value
3e-13
2e-10
1e-07
5e-05
3e-02
Log-odds
0.5
1.0
1.5
2.0
2.5
3.0
3.5
4.0
Cellular_organisms - ps1
Archaea1 - ps2
Archaea2 - ps3
Archaea3 - ps4
Asgard/Eukaryota - ps5
Eukaryota - ps6
Amorpheae - ps7
Obazoa - ps8
Opisthokonta - ps9
Holomycota - ps10
Fungi - ps11
Fungi_1 - ps12
Fungi_2 - ps13
Fungi_3 - ps14
Dikarya - ps15
Ascomycota - ps16
Saccharo/Pezizomycotina - ps17
Saccharomycotina - ps18
Saccharomycotina_1 - ps19
Saccharomycotina_2 - ps20
Saccharomycotina_3 - ps21
Phaff./Saccharomycetaceae - ps22
Saccharomyco./mycetaceae - ps23
Saccharomycetaceae - ps24
Saccharomycetaceae_2 - ps25
Saccharomycetaceae_3 - ps26
Kaz./Nau./Saccharomyces - ps27
Saccharomyces - ps28
Arboricola_clade - ps29
Cerevisiae_clade - ps30
Saccharomyces_cerevisiae - ps31
Phylostratum

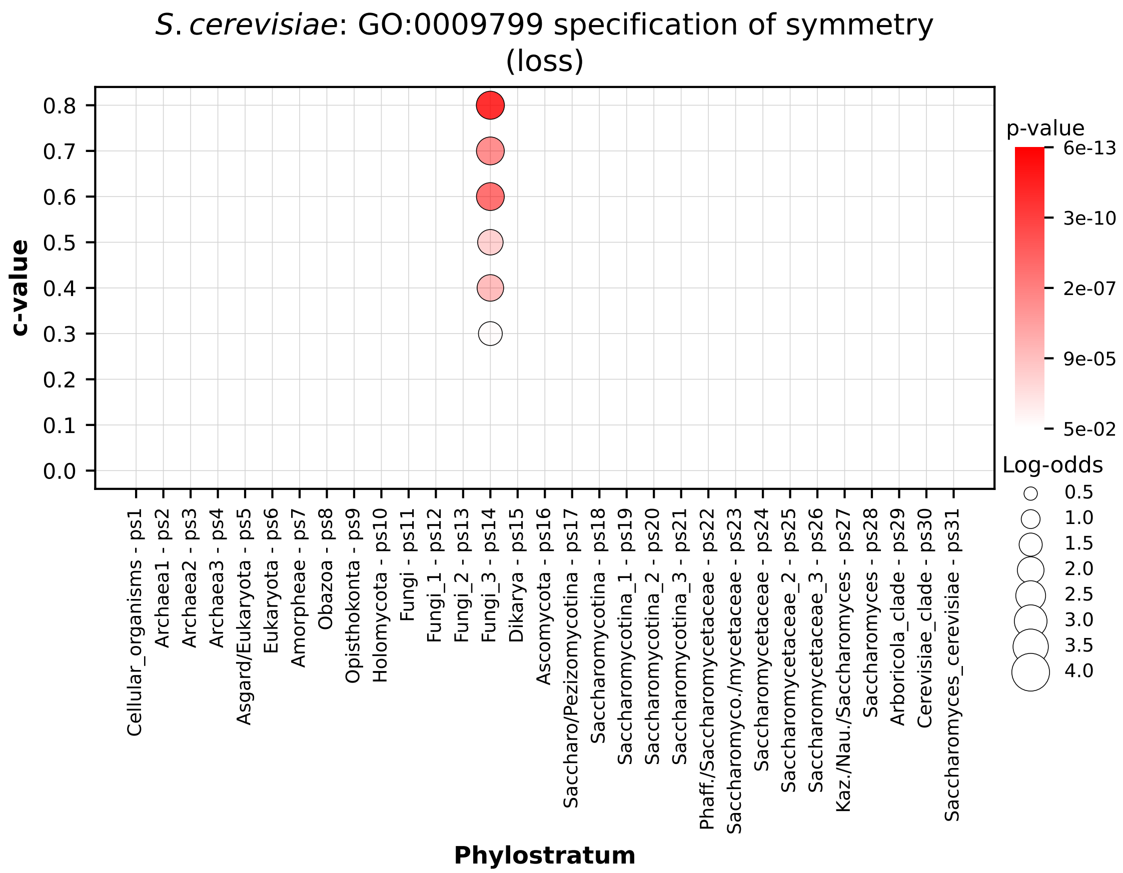

S. cerevisiae: GO:0009799 specification of symmetry
(loss)
c-value
0.8
0.7
0.6
0.5
0.4
0.3
0.2
0.1
0.0
p-value
6e-13
3e-10
2e-07
9e-05
5e-02
Log-odds
0.5
1.0
1.5
2.0
2.5
3.0
3.5
4.0
Cellular_organisms - ps1
Archaea1 - ps2
Archaea2 - ps3
Archaea3 - ps4
Asgard/Eukaryota - ps5
Eukaryota - ps6
Amorpheae - ps7
Obazoa - ps8
Opisthokonta - ps9
Holomycota - ps10
Fungi - ps11
Fungi_1 - ps12
Fungi_2 - ps13
Fungi_3 - ps14
Dikarya - ps15
Ascomycota - ps16
Saccharo/Pezizomycotina - ps17
Saccharomycotina - ps18
Saccharomycotina_1 - ps19
Saccharomycotina_2 - ps20
Saccharomycotina_3 - ps21
Phaff./Saccharomycetaceae - ps22
Saccharomyco./mycetaceae - ps23
Saccharomycetaceae - ps24
Saccharomycetaceae_2 - ps25
Saccharomycetaceae_3 - ps26
Kaz./Nau./Saccharomyces - ps27
Saccharomyces - ps28
Arboricola_clade - ps29
Cerevisiae_clade - ps30
Saccharomyces_cerevisiae - ps31
Phylostratum

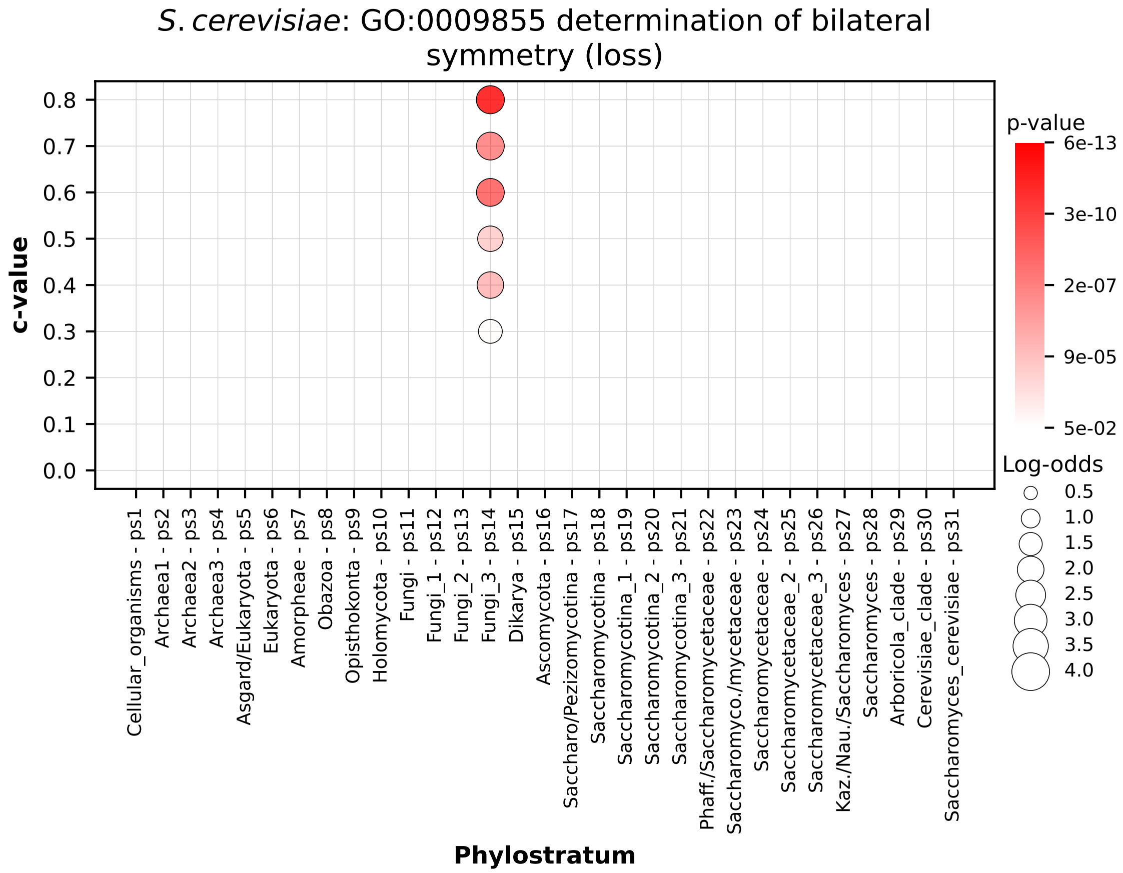
S. cerevisiae: GO:0009855 determination of bilateral symmetry (loss)
c-value
0.0
0.1
0.2
0.3
0.4
0.5
0.6
0.7
0.8
p-value
6e-13
3e-10
2e-07
9e-05
5e-02
Log-odds
0.5
1.0
1.5
2.0
2.5
3.0
3.5
4.0
Cellular_organisms - ps1
Archaea1 - ps2
Archaea2 - ps3
Archaea3 - ps4
Asgard/Eukaryota - ps5
Eukaryota - ps6
Amorpheae - ps7
Obazoa - ps8
Opisthokonta - ps9
Holomycota - ps10
Fungi - ps11
Fungi_1 - ps12
Fungi_2 - ps13
Fungi_3 - ps14
Dikarya - ps15
Ascomycota - ps16
Saccharo/Pezizomycotina - ps17
Saccharomycotina - ps18
Saccharomycotina_1 - ps19
Saccharomycotina_2 - ps20
Saccharomycotina_3 - ps21
Phaff./Saccharomycetaceae - ps22
Saccharomyco./mycetaceae - ps23
Saccharomycetaceae - ps24
Saccharomycetaceae_2 - ps25
Saccharomycetaceae_3 - ps26
Kaz./Nau./Saccharomyces - ps27
Saccharomyces - ps28
Arboricola_clade - ps29
Cerevisiae_clade - ps30
Saccharomyces_cerevisiae - ps31
Phylostratum

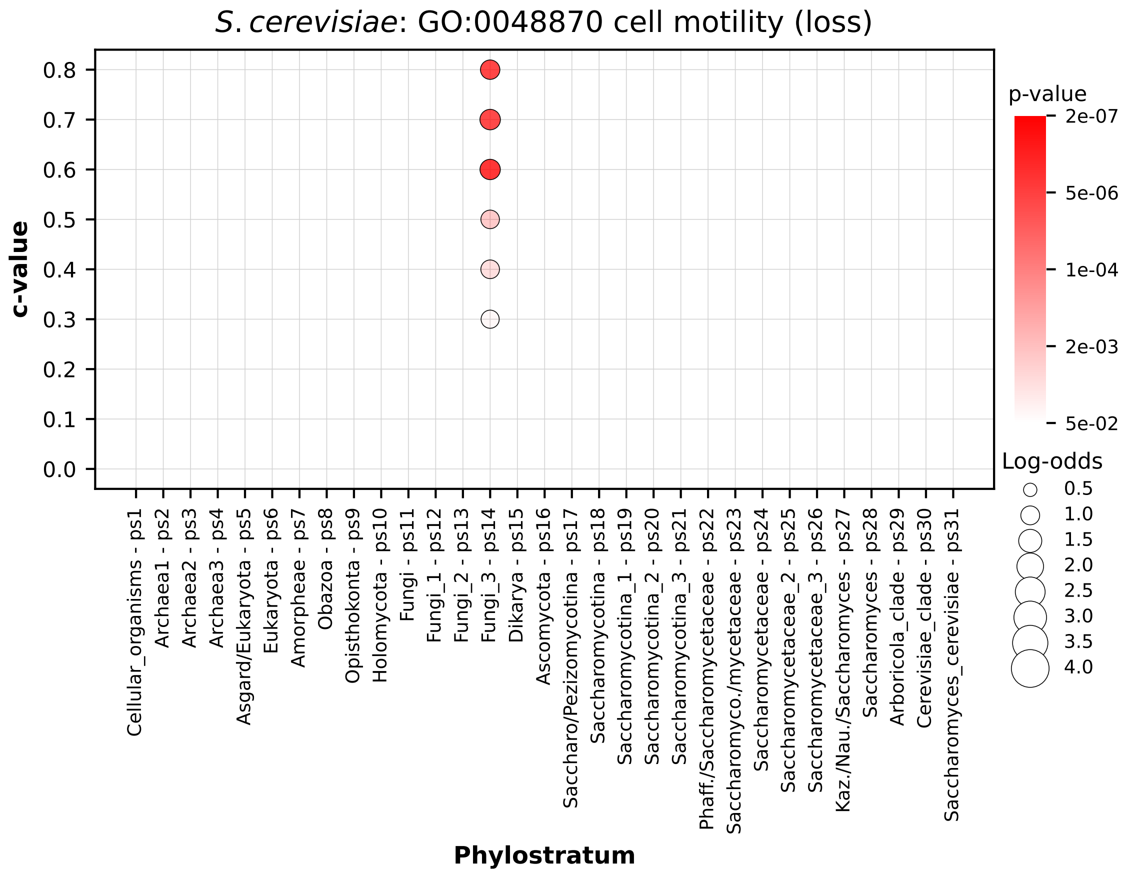

S. cerevisiae: GO:0048870 cell motility (loss)
c-value
0.8
0.7
0.6
0.5
0.4
0.3
0.2
0.1
0.0
p-value
2e-07
5e-06
1e-04
2e-03
5e-02
Log-odds
0.5
1.0
1.5
2.0
2.5
3.0
3.5
4.0
Cellular_organisms - ps1
Archaea1 - ps2
Archaea2 - ps3
Archaea3 - ps4
Asgard/Eukaryota - ps5
Eukaryota - ps6
Amorpheae - ps7
Obazoa - ps8
Opisthokonta - ps9
Holomycota - ps10
Fungi - ps11
Fungi_1 - ps12
Fungi_2 - ps13
Fungi_3 - ps14
Dikarya - ps15
Ascomycota - ps16
Saccharo/Pezizomycotina - ps17
Saccharomycotina - ps18
Saccharomycotina_1 - ps19
Saccharomycotina_2 - ps20
Saccharomycotina_3 - ps21
Phaff./Saccharomycetaceae - ps22
Saccharomyco./mycetaceae - ps23
Saccharomycetaceae - ps24
Saccharomycetaceae_2 - ps25
Saccharomycetaceae_3 - ps26
Kaz./Nau./Saccharomyces - ps27
Saccharomyces - ps28
Arboricola_clade - ps29
Cerevisiae_clade - ps30
Saccharomyces_cerevisiae - ps31
Phylostratum

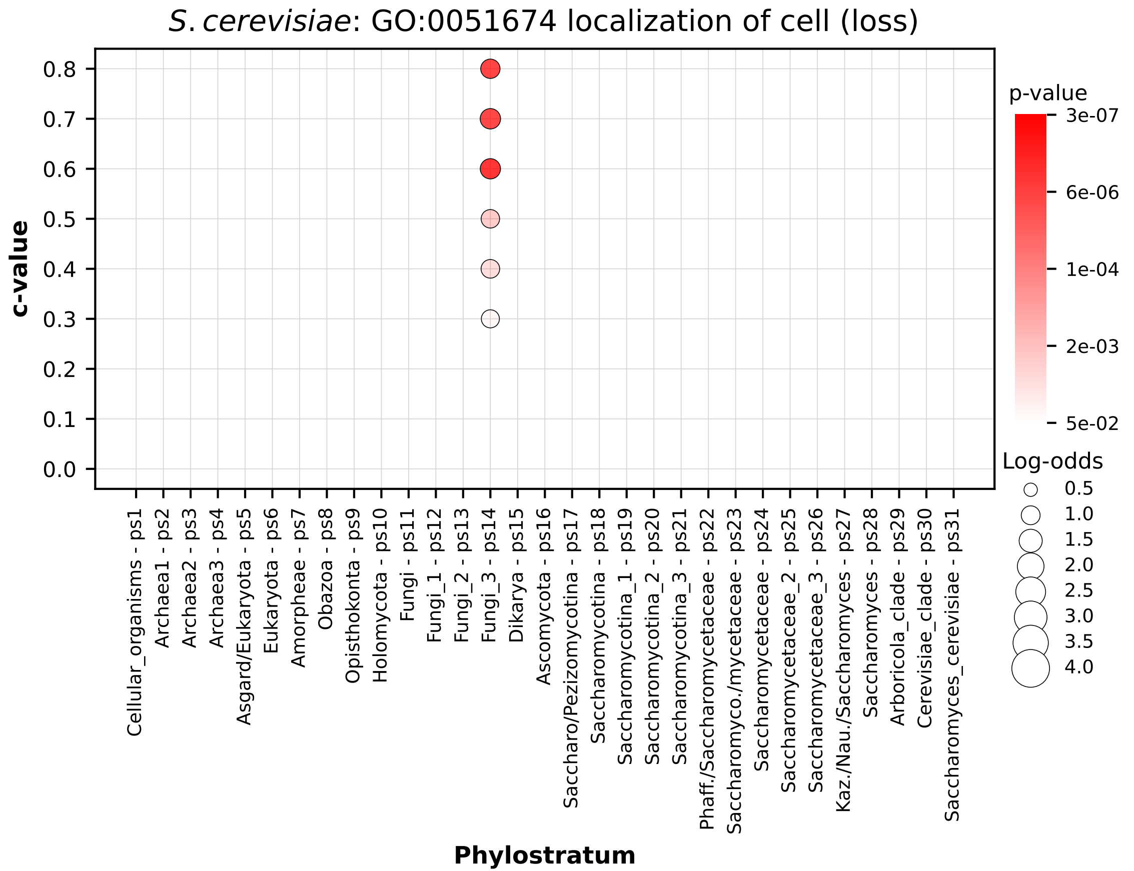

S. cerevisiae: GO:0051674 localization of cell (loss)
c-value
0.0
0.1
0.2
0.3
0.4
0.5
0.6
0.7
0.8
Phylostratum
Cellular_organisms - ps1
Archaea1 - ps2
Archaea2 - ps3
Archaea3 - ps4
Asgard/Eukaryota - ps5
Eukaryota - ps6
Amorpheae - ps7
Obazoa - ps8
Opisthokonta - ps9
Holomycota - ps10
Fungi - ps11
Fungi_1 - ps12
Fungi_2 - ps13
Fungi_3 - ps14
Dikarya - ps15
Ascomycota - ps16
Saccharo/Pezizomycotina - ps17
Saccharomycotina - ps18
Saccharomycotina_1 - ps19
Saccharomycotina_2 - ps20
Saccharomycotina_3 - ps21
Phaff./Saccharomycetaceae - ps22
Saccharomyco./mycetaceae - ps23
Saccharomycetaceae - ps24
Saccharomycetaceae_2 - ps25
Saccharomycetaceae_3 - ps26
Kaz./Nau./Saccharomyces - ps27
Saccharomyces - ps28
Arboricola_clade - ps29
Cerevisiae_clade - ps30
Saccharomyces_cerevisiae - ps31
p-value
3e-07
6e-06
1e-04
2e-03
5e-02
Log-odds
0.5
1.0
1.5
2.0
2.5
3.0
3.5
4.0

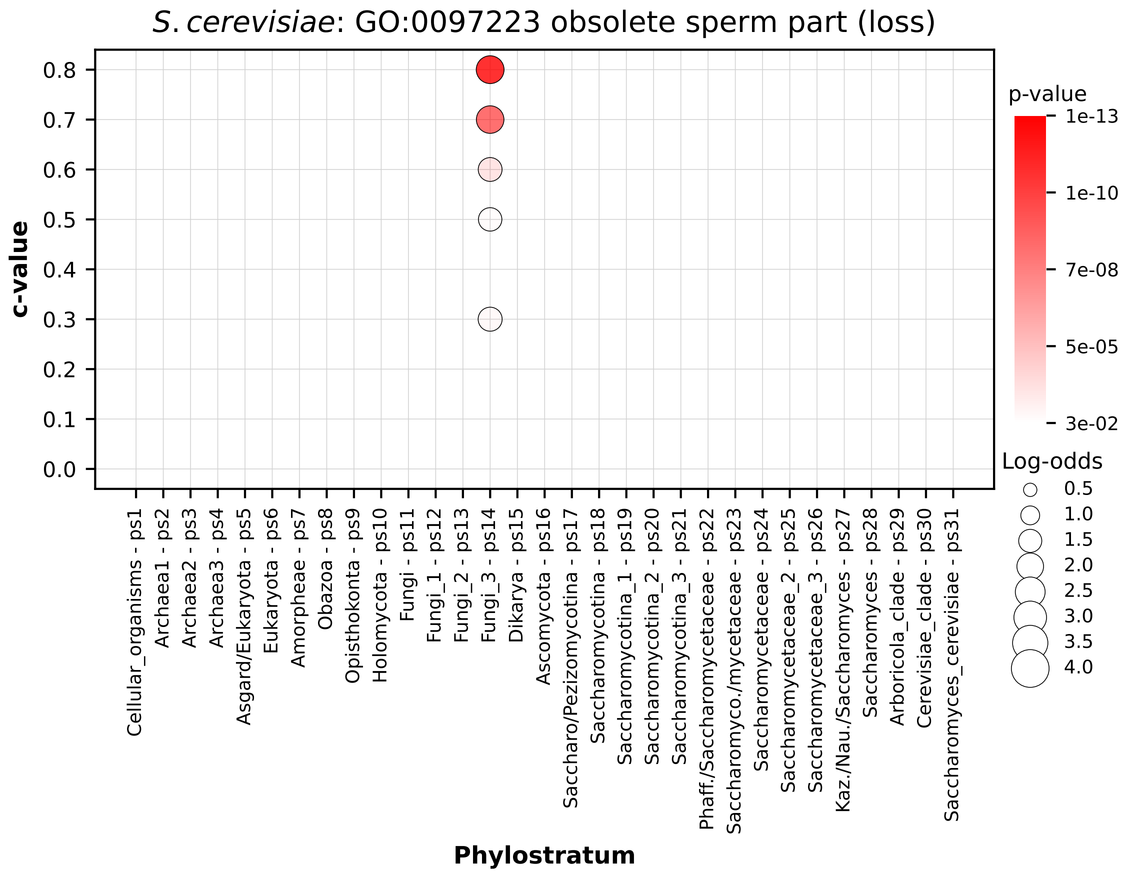
S. cerevisiae: GO:0097223 obsolete sperm part (loss)
c-value
0.0
0.1
0.2
0.3
0.4
0.5
0.6
0.7
0.8
p-value
1e-13
1e-10
7e-08
5e-05
3e-02
Log-odds
0.5
1.0
1.5
2.0
2.5
3.0
3.5
4.0
Cellular_organisms - ps1
Archaea1 - ps2
Archaea2 - ps3
Archaea3 - ps4
Asgard/Eukaryota - ps5
Eukaryota - ps6
Amorpheae - ps7
Obazoa - ps8
Opisthokonta - ps9
Holomycota - ps10
Fungi - ps11
Fungi_1 - ps12
Fungi_2 - ps13
Fungi_3 - ps14
Dikarya - ps15
Ascomycota - ps16
Saccharo/Pezizomycotina - ps17
Saccharomycotina - ps18
Saccharomycotina_1 - ps19
Saccharomycotina_2 - ps20
Saccharomycotina_3 - ps21
Phaff./Saccharomycetaceae - ps22
Saccharomyco./mycetaceae - ps23
Saccharomycetaceae - ps24
Saccharomycetaceae_2 - ps25
Saccharomycetaceae_3 - ps26
Kaz./Nau./Saccharomyces - ps27
Saccharomyces - ps28
Arboricola_clade - ps29
Cerevisiae_clade - ps30
Saccharomyces_cerevisiae - ps31
Phylostratum

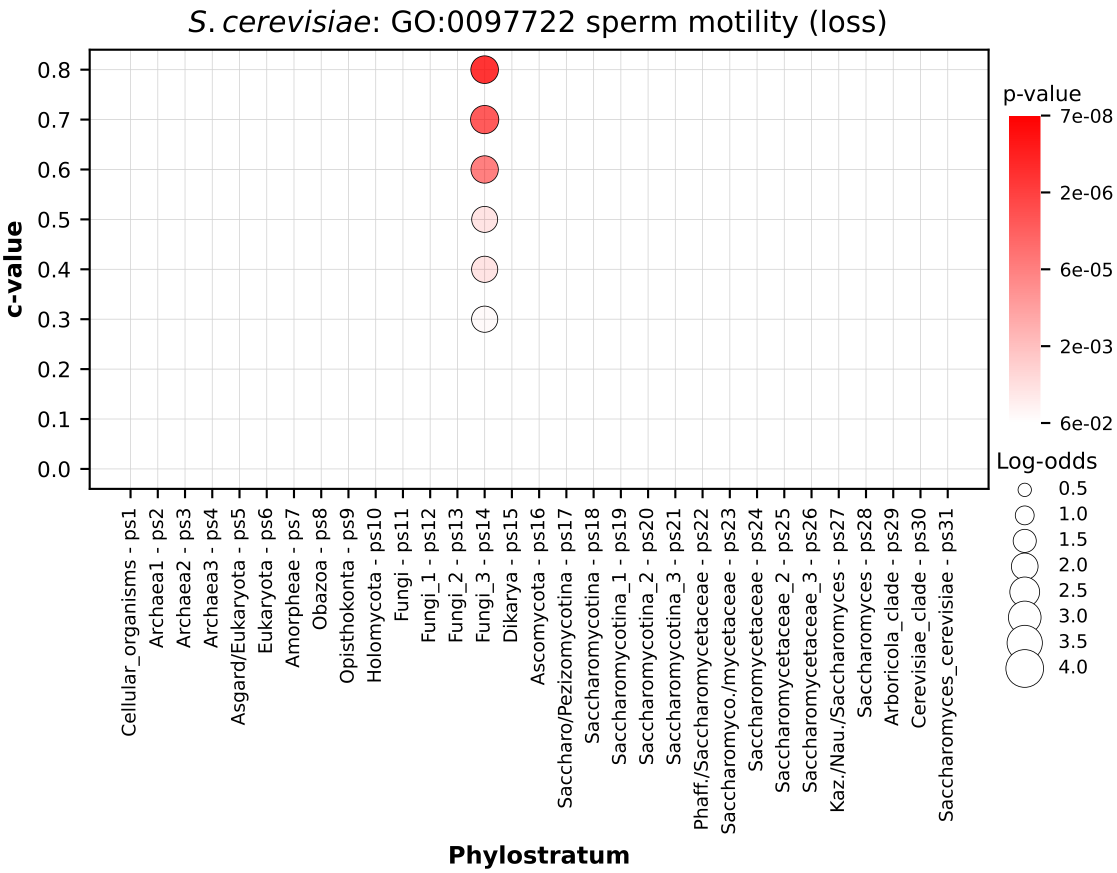

S. cerevisiae: GO:0097722 sperm motility (loss)
c-value
0.8
0.7
0.6
0.5
0.4
0.3
0.2
0.1
0.0
p-value
7e-08
2e-06
6e-05
2e-03
6e-02
Log-odds
0.5
1.0
1.5
2.0
2.5
3.0
3.5
4.0
Cellular_organisms - ps1
Archaea1 - ps2
Archaea2 - ps3
Archaea3 - ps4
Asgard/Eukaryota - ps5
Eukaryota - ps6
Amorpheae - ps7
Obazoa - ps8
Opisthokonta - ps9
Holomycota - ps10
Fungi - ps11
Fungi_1 - ps12
Fungi_2 - ps13
Fungi_3 - ps14
Dikarya - ps15
Ascomycota - ps16
Saccharo/Pezizomycotina - ps17
Saccharomycotina - ps18
Saccharomycotina_1 - ps19
Saccharomycotina_2 - ps20
Saccharomycotina_3 - ps21
Phaff./Saccharomycetaceae - ps22
Saccharomyco./mycetaceae - ps23
Saccharomycetaceae - ps24
Saccharomycetaceae_2 - ps25
Saccharomycetaceae_3 - ps26
Kaz./Nau./Saccharomyces - ps27
Saccharomyces - ps28
Arboricola_clade - ps29
Cerevisiae_clade - ps30
Saccharomyces_cerevisiae - ps31
Phylostratum

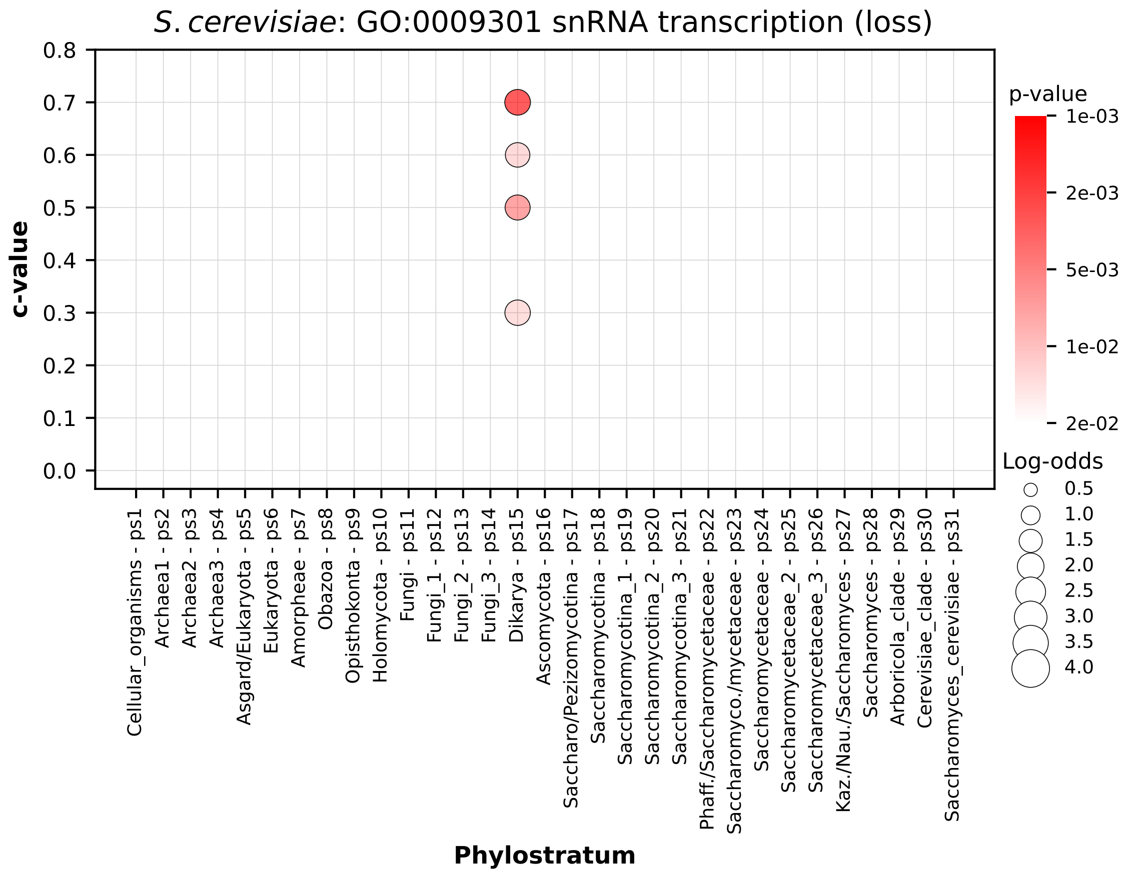
S. cerevisiae: GO:0009301 snRNA transcription (loss)
c-value
0.8
0.7
0.6
0.5
0.4
0.3
0.2
0.1
0.0
p-value
1e-03
2e-03
5e-03
1e-02
2e-02
Log-odds
0.5
1.0
1.5
2.0
2.5
3.0
3.5
4.0
Cellular_organisms - ps1
Archaea1 - ps2
Archaea2 - ps3
Archaea3 - ps4
Asgard/Eukaryota - ps5
Eukaryota - ps6
Amorpheae - ps7
Obazoa - ps8
Opisthokonta - ps9
Holomycota - ps10
Fungi - ps11
Fungi_1 - ps12
Fungi_2 - ps13
Fungi_3 - ps14
Dikarya - ps15
Ascomycota - ps16
Saccharo/Pezizomycotina - ps17
Saccharomycotina - ps18
Saccharomycotina_1 - ps19
Saccharomycotina_2 - ps20
Saccharomycotina_3 - ps21
Phaff./Saccharomycetaceae - ps22
Saccharomyco./mycetaceae - ps23
Saccharomycetaceae - ps24
Saccharomycetaceae_2 - ps25
Saccharomycetaceae_3 - ps26
Kaz./Nau./Saccharomyces - ps27
Saccharomyces - ps28
Arboricola_clade - ps29
Cerevisiae_clade - ps30
Saccharomyces_cerevisiae - ps31
Phylostratum

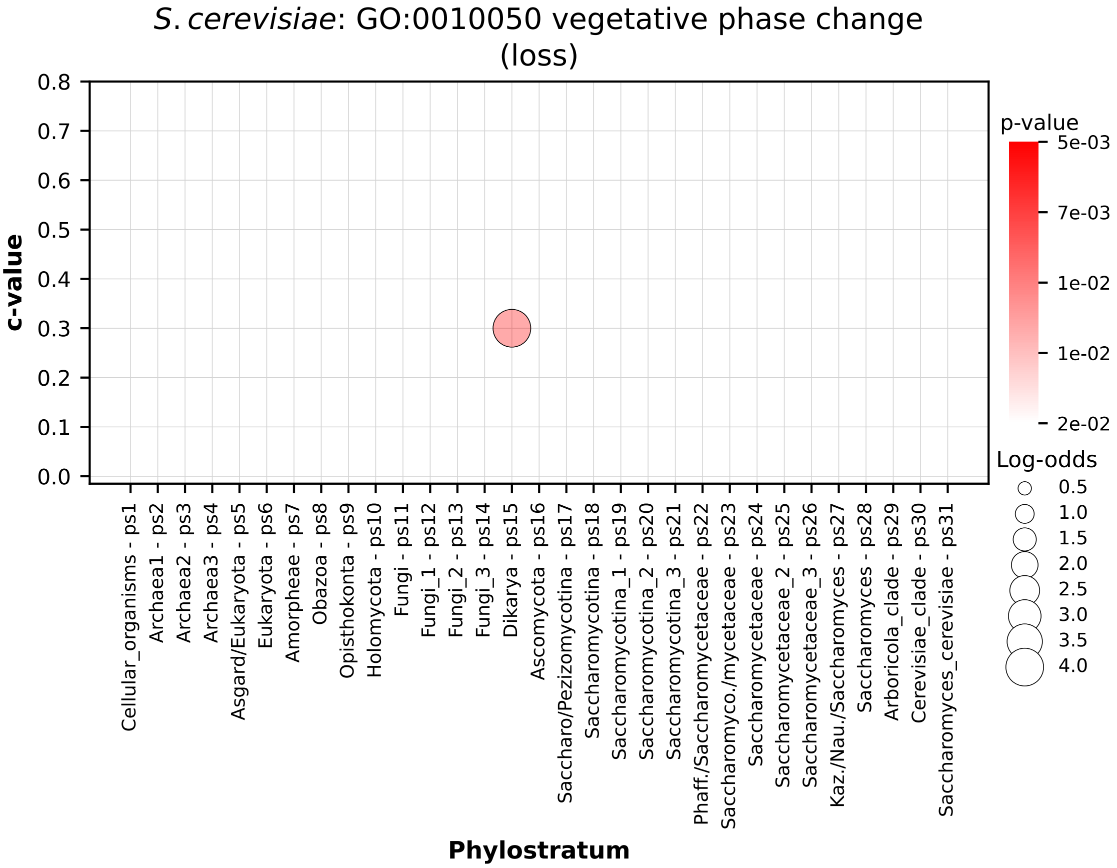

S. cerevisiae: GO:0010050 vegetative phase change
(loss)
c-value
0.8
0.7
0.6
0.5
0.4
0.3
0.2
0.1
0.0
p-value
5e-03
7e-03
1e-02
1e-02
2e-02
Log-odds
0.5
1.0
1.5
2.0
2.5
3.0
3.5
4.0
Cellular_organisms - ps1
Archaea1 - ps2
Archaea2 - ps3
Archaea3 - ps4
Asgard/Eukaryota - ps5
Eukaryota - ps6
Amorpheae - ps7
Obazoa - ps8
Opisthokonta - ps9
Holomycota - ps10
Fungi - ps11
Fungi_1 - ps12
Fungi_2 - ps13
Fungi_3 - ps14
Dikarya - ps15
Ascomycota - ps16
Saccharo/Pezizomycotina - ps17
Saccharomycotina - ps18
Saccharomycotina_1 - ps19
Saccharomycotina_2 - ps20
Saccharomycotina_3 - ps21
Phaff./Saccharomycetaceae - ps22
Saccharomyco./mycetaceae - ps23
Saccharomycetaceae - ps24
Saccharomycetaceae_2 - ps25
Saccharomycetaceae_3 - ps26
Kaz./Nau./Saccharomyces - ps27
Saccharomyces - ps28
Arboricola_clade - ps29
Cerevisiae_clade - ps30
Saccharomyces_cerevisiae - ps31
Phylostratum

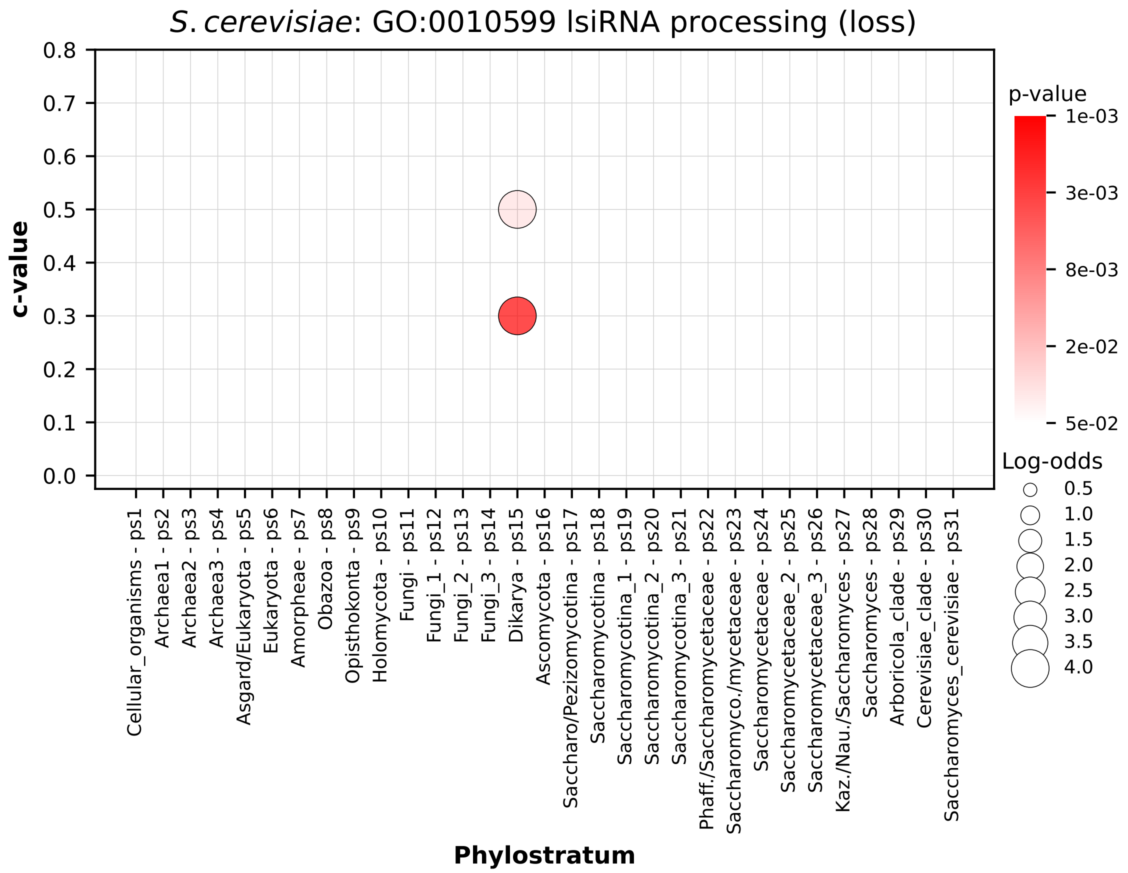
S. cerevisiae: GO:0010599 lsiRNA processing (loss)
c-value
0.0
0.1
0.2
0.3
0.4
0.5
0.6
0.7
0.8
p-value
1e-03
3e-03
8e-03
2e-02
5e-02
Log-odds
0.5
1.0
1.5
2.0
2.5
3.0
3.5
4.0
Cellular_organisms - ps1
Archaea1 - ps2
Archaea2 - ps3
Archaea3 - ps4
Asgard/Eukaryota - ps5
Eukaryota - ps6
Amorpheae - ps7
Obazoa - ps8
Opisthokonta - ps9
Holomycota - ps10
Fungi - ps11
Fungi_1 - ps12
Fungi_2 - ps13
Fungi_3 - ps14
Dikarya - ps15
Ascomycota - ps16
Saccharo/Pezizomycotina - ps17
Saccharomycotina - ps18
Saccharomycotina_1 - ps19
Saccharomycotina_2 - ps20
Saccharomycotina_3 - ps21
Phaff./Saccharomycetaceae - ps22
Saccharomyco./mycetaceae - ps23
Saccharomycetaceae - ps24
Saccharomycetaceae_2 - ps25
Saccharomycetaceae_3 - ps26
Kaz./Nau./Saccharomyces - ps27
Saccharomyces - ps28
Arboricola_clade - ps29
Cerevisiae_clade - ps30
Saccharomyces_cerevisiae - ps31
Phylostratum

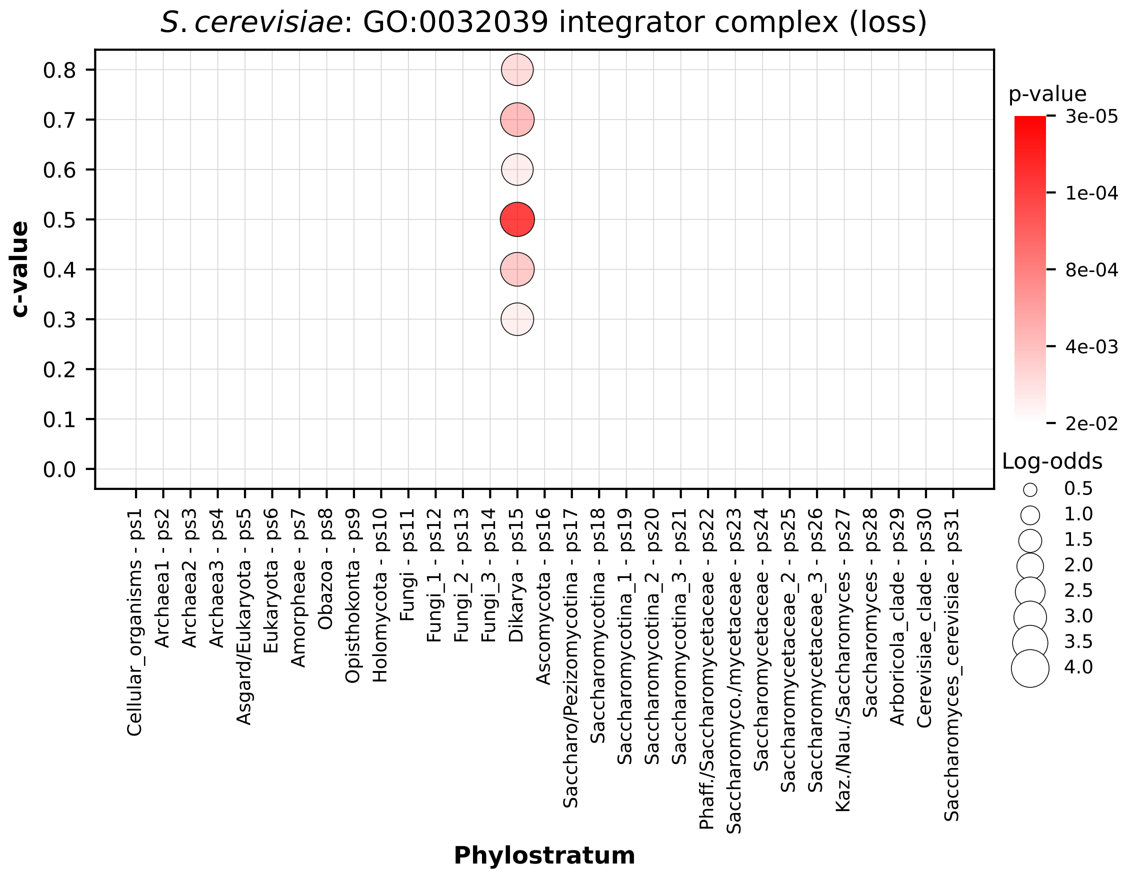
S. cerevisiae: GO:0032039 integrator complex (loss)
c-value
0.8
0.7
0.6
0.5
0.4
0.3
0.2
0.1
0.0
p-value
3e-05
1e-04
8e-04
4e-03
2e-02
Log-odds
0.5
1.0
1.5
2.0
2.5
3.0
3.5
4.0
Cellular_organisms - ps1
Archaea1 - ps2
Archaea2 - ps3
Archaea3 - ps4
Asgard/Eukaryota - ps5
Eukaryota - ps6
Amorpheae - ps7
Obazoa - ps8
Opisthokonta - ps9
Holomycota - ps10
Fungi - ps11
Fungi_1 - ps12
Fungi_2 - ps13
Fungi_3 - ps14
Dikarya - ps15
Ascomycota - ps16
Saccharo/Pezizomycotina - ps17
Saccharomycotina - ps18
Saccharomycotina_1 - ps19
Saccharomycotina_2 - ps20
Saccharomycotina_3 - ps21
Phaff./Saccharomycetaceae - ps22
Saccharomyco./mycetaceae - ps23
Saccharomycetaceae - ps24
Saccharomycetaceae_2 - ps25
Saccharomycetaceae_3 - ps26
Kaz./Nau./Saccharomyces - ps27
Saccharomyces - ps28
Arboricola_clade - ps29
Cerevisiae_clade - ps30
Saccharomyces_cerevisiae - ps31
Phylostratum

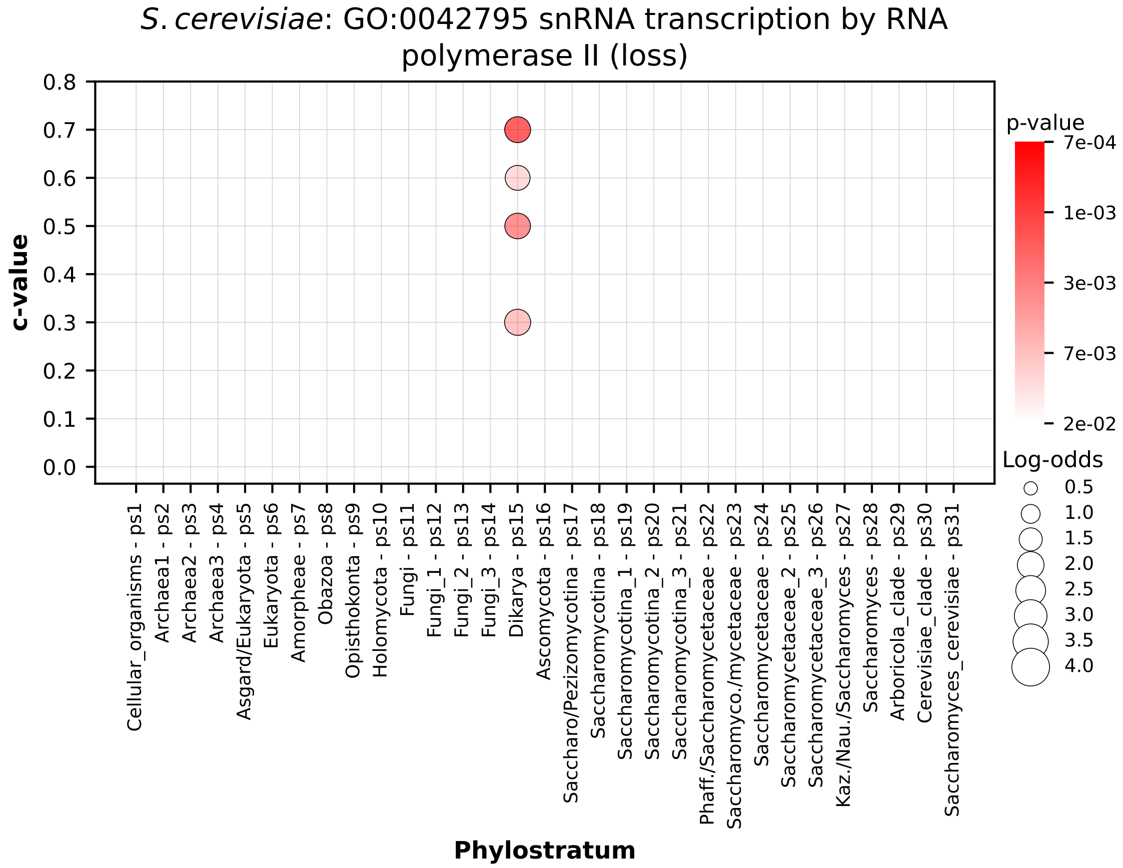

S. cerevisiae: GO:0042795 snRNA transcription by RNA polymerase II (loss)
c-value
0.8
0.7
0.6
0.5
0.4
0.3
0.2
0.1
0.0
Phylostratum
Cellular_organisms - ps1
Archaea1 - ps2
Archaea2 - ps3
Archaea3 - ps4
Asgard/Eukaryota - ps5
Eukaryota - ps6
Amorpheae - ps7
Obazoa - ps8
Opisthokonta - ps9
Holomycota - ps10
Fungi - ps11
Fungi_1 - ps12
Fungi_2 - ps13
Fungi_3 - ps14
Dikarya - ps15
Ascomycota - ps16
Saccharo/Pezizomycotina - ps17
Saccharomycotina - ps18
Saccharomycotina_1 - ps19
Saccharomycotina_2 - ps20
Saccharomycotina_3 - ps21
Phaff./Saccharomycetaceae - ps22
Saccharomyco./mycetaceae - ps23
Saccharomycetaceae - ps24
Saccharomycetaceae_2 - ps25
Saccharomycetaceae_3 - ps26
Kaz./Nau./Saccharomyces - ps27
Saccharomyces - ps28
Arboricola_clade - ps29
Cerevisiae_clade - ps30
Saccharomyces_cerevisiae - ps31
p-value
7e-04
1e-03
3e-03
7e-03
2e-02
Log-odds
0.5
1.0
1.5
2.0
2.5
3.0
3.5
4.0

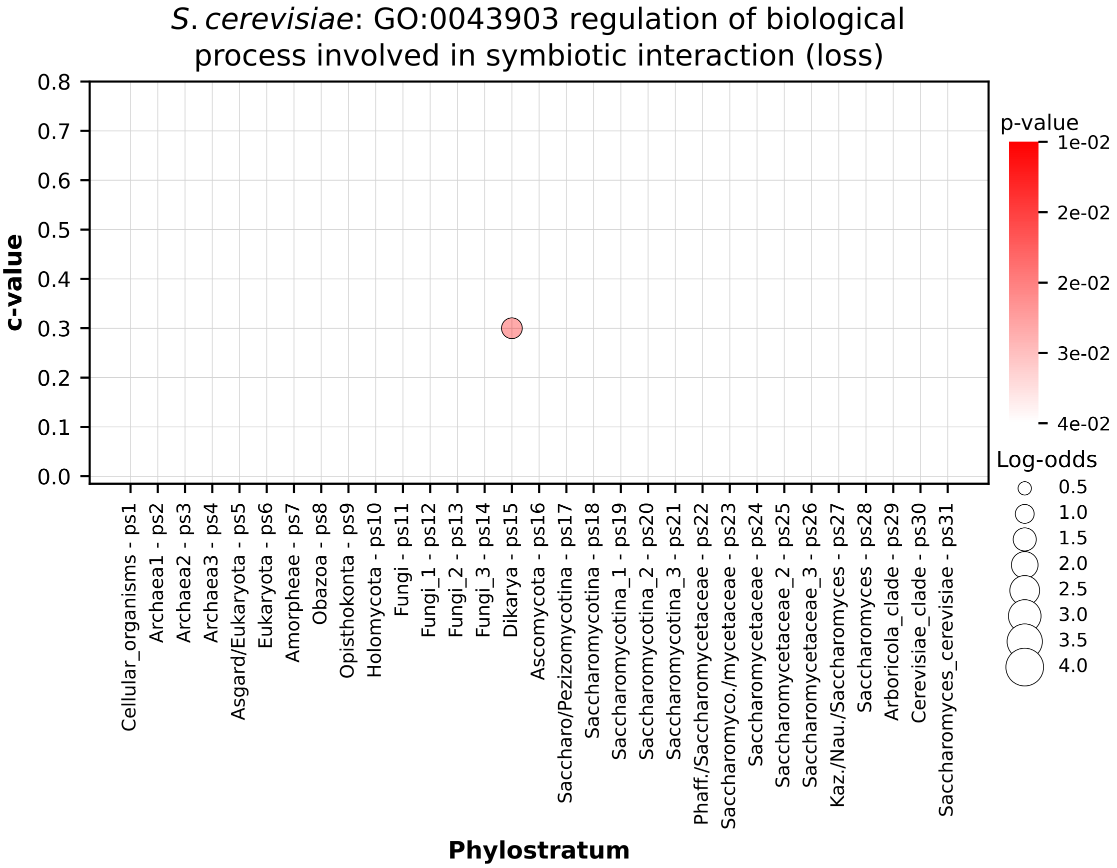
S. cerevisiae: GO:0043903 regulation of biological process involved in symbiotic interaction (loss)
c-value
0.8
0.7
0.6
0.5
0.4
0.3
0.2
0.1
0.0
p-value
1e-02
2e-02
2e-02
3e-02
4e-02
Log-odds
0.5
1.0
1.5
2.0
2.5
3.0
3.5
4.0
Cellular_organisms - ps1
Archaea1 - ps2
Archaea2 - ps3
Archaea3 - ps4
Asgard/Eukaryota - ps5
Eukaryota - ps6
Amorpheae - ps7
Obazoa - ps8
Opisthokonta - ps9
Holomycota - ps10
Fungi - ps11
Fungi_1 - ps12
Fungi_2 - ps13
Fungi_3 - ps14
Dikarya - ps15
Ascomycota - ps16
Saccharo/Pezizomycotina - ps17
Saccharomycotina - ps18
Saccharomycotina_1 - ps19
Saccharomycotina_2 - ps20
Saccharomycotina_3 - ps21
Phaff./Saccharomycetaceae - ps22
Saccharomyco./mycetaceae - ps23
Saccharomycetaceae - ps24
Saccharomycetaceae_2 - ps25
Saccharomycetaceae_3 - ps26
Kaz./Nau./Saccharomyces - ps27
Saccharomyces - ps28
Arboricola_clade - ps29
Cerevisiae_clade - ps30
Saccharomyces_cerevisiae - ps31
Phylostratum

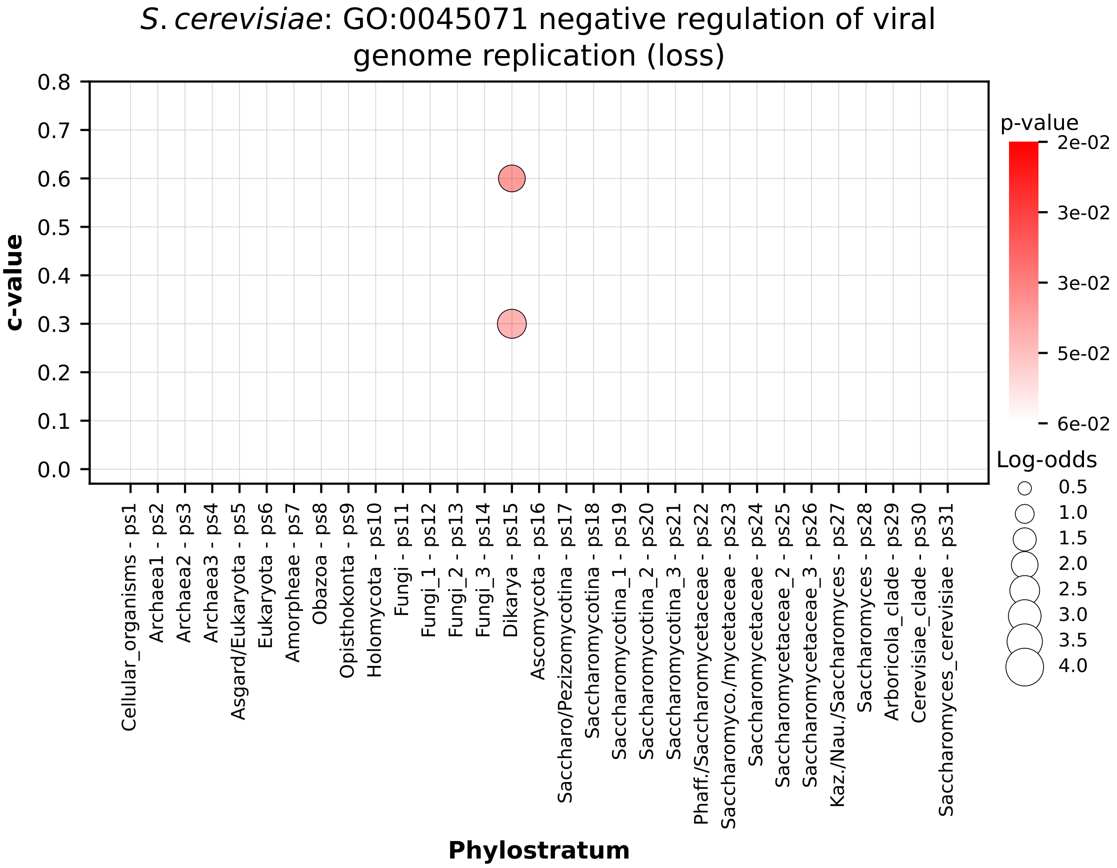

S. cerevisiae: GO:0045071 negative regulation of viral genome replication (loss)
c-value
0.0
0.1
0.2
0.3
0.4
0.5
0.6
0.7
0.8
p-value
2e-02
3e-02
3e-02
5e-02
6e-02
Log-odds
0.5
1.0
1.5
2.0
2.5
3.0
3.5
4.0
Cellular_organisms - ps1
Archaea1 - ps2
Archaea2 - ps3
Archaea3 - ps4
Asgard/Eukaryota - ps5
Eukaryota - ps6
Amorpheae - ps7
Obazoa - ps8
Opisthokonta - ps9
Holomycota - ps10
Fungi - ps11
Fungi_1 - ps12
Fungi_2 - ps13
Fungi_3 - ps14
Dikarya - ps15
Ascomycota - ps16
Saccharo/Pezizomycotina - ps17
Saccharomycotina - ps18
Saccharomycotina_1 - ps19
Saccharomycotina_2 - ps20
Saccharomycotina_3 - ps21
Phaff./Saccharomycetaceae - ps22
Saccharomyco./mycetaceae - ps23
Saccharomycetaceae - ps24
Saccharomycetaceae_2 - ps25
Saccharomycetaceae_3 - ps26
Kaz./Nau./Saccharomyces - ps27
Saccharomyces - ps28
Arboricola_clade - ps29
Cerevisiae_clade - ps30
Saccharomyces_cerevisiae - ps31
Phylostratum

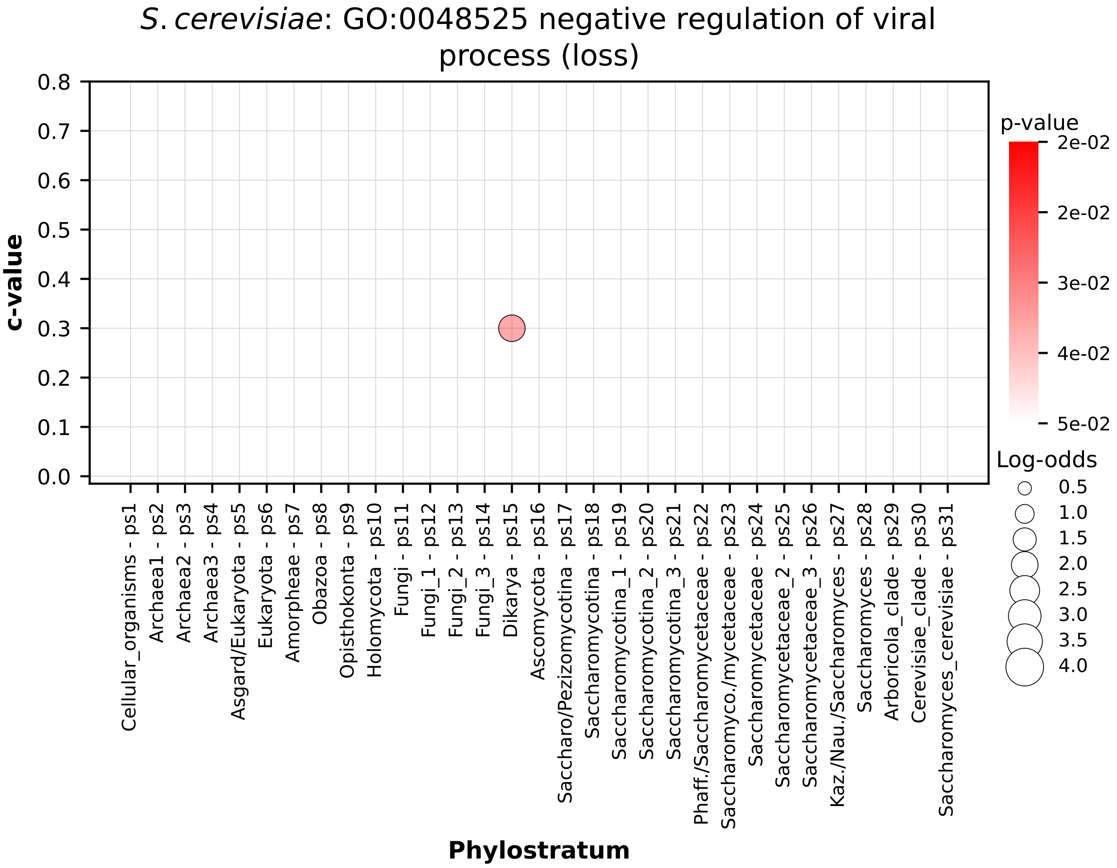
S. cerevisiae: GO:0048525 negative regulation of viral process (loss)
c-value
0.8
0.7
0.6
0.5
0.4
0.3
0.2
0.1
0.0
p-value
2e-02
2e-02
3e-02
4e-02
5e-02
Log-odds
0.5
1.0
1.5
2.0
2.5
3.0
3.5
4.0
Cellular_organisms - ps1
Archaea1 - ps2
Archaea2 - ps3
Archaea3 - ps4
Asgard/Eukaryota - ps5
Eukaryota - ps6
Amorpheae - ps7
Obazoa - ps8
Opisthokonta - ps9
Holomycota - ps10
Fungi - ps11
Fungi_1 - ps12
Fungi_2 - ps13
Fungi_3 - ps14
Dikarya - ps15
Ascomycota - ps16
Saccharo/Pezizomycotina - ps17
Saccharomycotina - ps18
Saccharomycotina_1 - ps19
Saccharomycotina_2 - ps20
Saccharomycotina_3 - ps21
Phaff./Saccharomycetaceae - ps22
Saccharomyco./mycetaceae - ps23
Saccharomycetaceae - ps24
Saccharomycetaceae_2 - ps25
Saccharomycetaceae_3 - ps26
Kaz./Nau./Saccharomyces - ps27
Saccharomyces - ps28
Arboricola_clade - ps29
Cerevisiae_clade - ps30
Saccharomyces_cerevisiae - ps31
Phylostratum

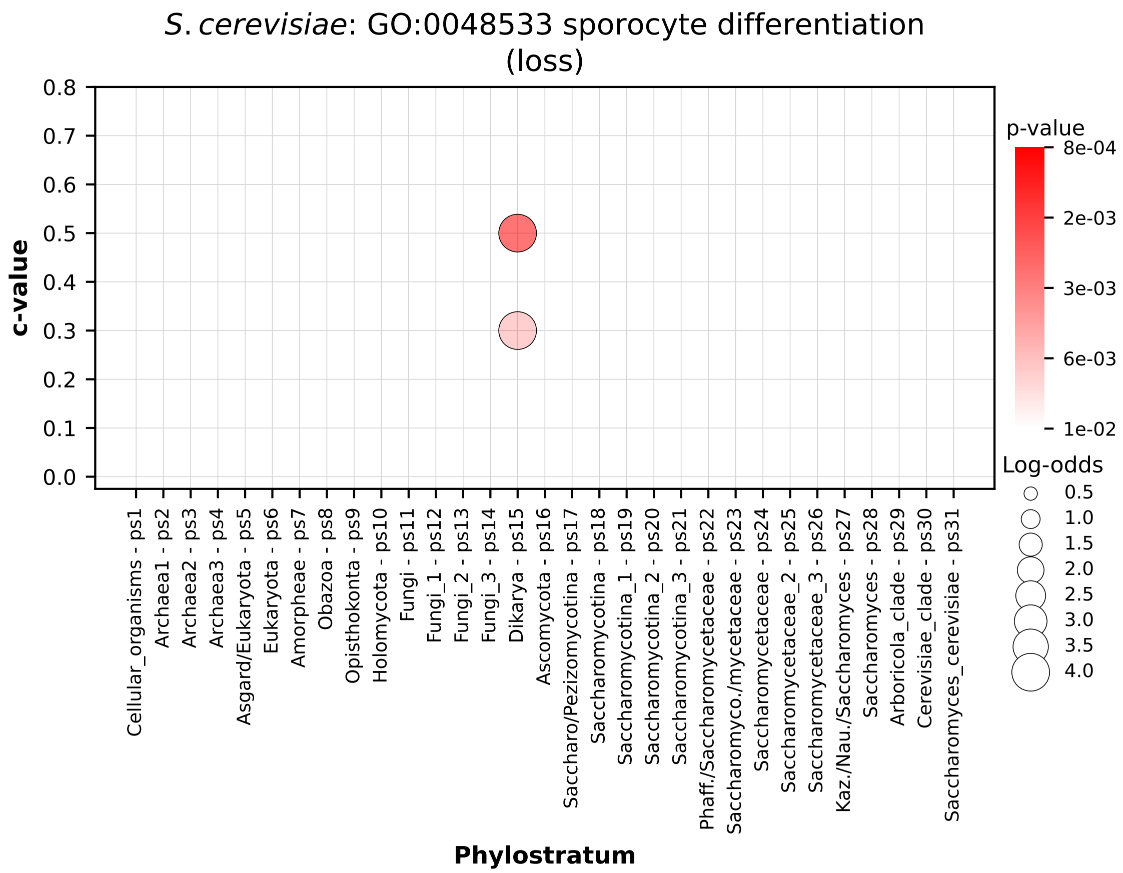

S. cerevisiae: GO:0048533 sporocyte differentiation
(loss)
c-value
0.8
0.7
0.6
0.5
0.4
0.3
0.2
0.1
0.0
p-value
8e-04
2e-03
3e-03
6e-03
1e-02
Log-odds
0.5
1.0
1.5
2.0
2.5
3.0
3.5
4.0
Cellular_organisms - ps1
Archaea1 - ps2
Archaea2 - ps3
Archaea3 - ps4
Asgard/Eukaryota - ps5
Eukaryota - ps6
Amorpheae - ps7
Obazoa - ps8
Opisthokonta - ps9
Holomycota - ps10
Fungi - ps11
Fungi_1 - ps12
Fungi_2 - ps13
Fungi_3 - ps14
Dikarya - ps15
Ascomycota - ps16
Saccharo/Pezizomycotina - ps17
Saccharomycotina - ps18
Saccharomycotina_1 - ps19
Saccharomycotina_2 - ps20
Saccharomycotina_3 - ps21
Phaff./Saccharomycetaceae - ps22
Saccharomyco./mycetaceae - ps23
Saccharomycetaceae - ps24
Saccharomycetaceae_2 - ps25
Saccharomycetaceae_3 - ps26
Kaz./Nau./Saccharomyces - ps27
Saccharomyces - ps28
Arboricola_clade - ps29
Cerevisiae_clade - ps30
Saccharomyces_cerevisiae - ps31
Phylostratum

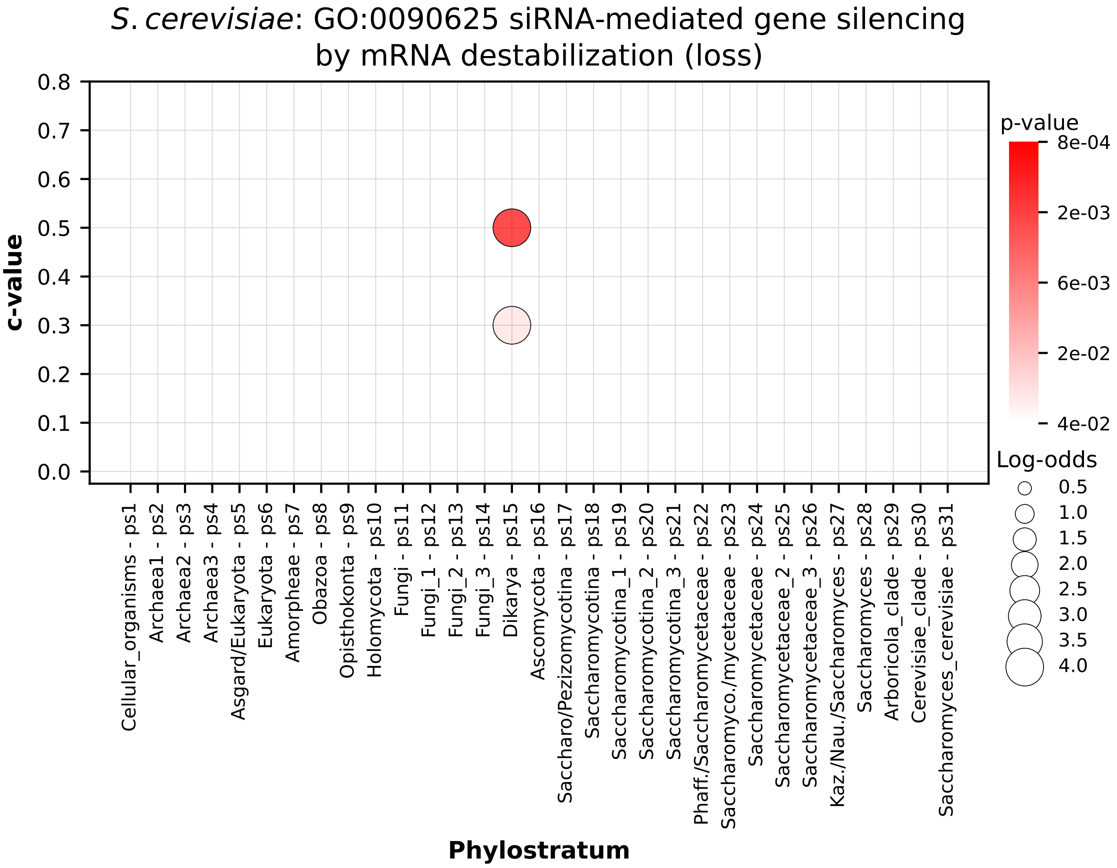

S. cerevisiae: GO:0090625 siRNA-mediated gene silencing by mRNA destabilization (loss)
c-value
0.8
0.7
0.6
0.5
0.4
0.3
0.2
0.1
0.0
p-value
8e-04
2e-03
6e-03
2e-02
4e-02
Log-odds
0.5
1.0
1.5
2.0
2.5
3.0
3.5
4.0
Cellular_organisms - ps1
Archaea1 - ps2
Archaea2 - ps3
Archaea3 - ps4
Asgard/Eukaryota - ps5
Eukaryota - ps6
Amorpheae - ps7
Obazoa - ps8
Opisthokonta - ps9
Holomycota - ps10
Fungi - ps11
Fungi_1 - ps12
Fungi_2 - ps13
Fungi_3 - ps14
Dikarya - ps15
Ascomycota - ps16
Saccharo/Pezizomycotina - ps17
Saccharomycotina - ps18
Saccharomycotina_1 - ps19
Saccharomycotina_2 - ps20
Saccharomycotina_3 - ps21
Phaff./Saccharomycetaceae - ps22
Saccharomyco./mycetaceae - ps23
Saccharomycetaceae - ps24
Saccharomycetaceae_2 - ps25
Saccharomycetaceae_3 - ps26
Kaz./Nau./Saccharomyces - ps27
Saccharomyces - ps28
Arboricola_clade - ps29
Cerevisiae_clade - ps30
Saccharomyces_cerevisiae - ps31
Phylostratum

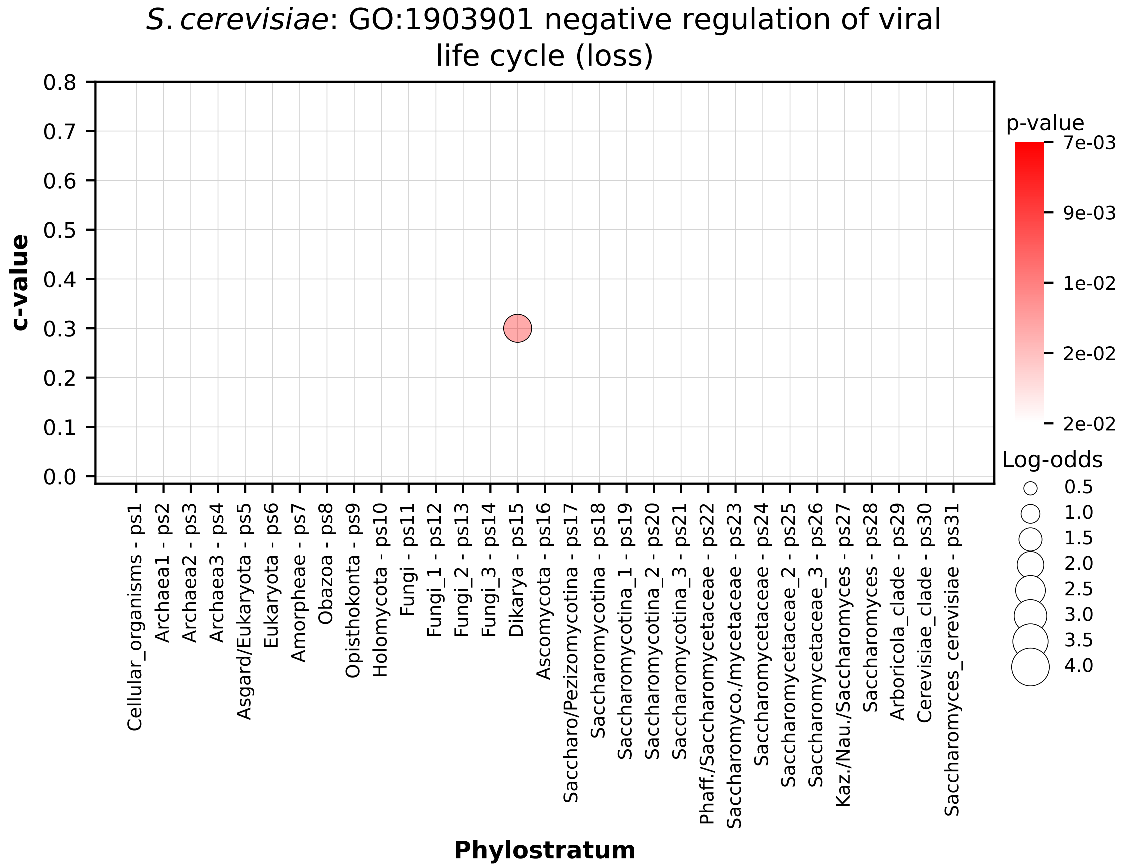
S. cerevisiae: GO:1903901 negative regulation of viral life cycle (loss)
c-value
0.8
0.7
0.6
0.5
0.4
0.3
0.2
0.1
0.0
p-value
7e-03
9e-03
1e-02
2e-02
2e-02
Log-odds
0.5
1.0
1.5
2.0
2.5
3.0
3.5
4.0
Cellular_organisms - ps1
Archaea1 - ps2
Archaea2 - ps3
Archaea3 - ps4
Asgard/Eukaryota - ps5
Eukaryota - ps6
Amorpheae - ps7
Obazoa - ps8
Opisthokonta - ps9
Holomycota - ps10
Fungi - ps11
Fungi_1 - ps12
Fungi_2 - ps13
Fungi_3 - ps14
Dikarya - ps15
Ascomycota - ps16
Saccharo/Pezizomycotina - ps17
Saccharomycotina - ps18
Saccharomycotina_1 - ps19
Saccharomycotina_2 - ps20
Saccharomycotina_3 - ps21
Phaff./Saccharomycetaceae - ps22
Saccharomyco./mycetaceae - ps23
Saccharomycetaceae - ps24
Saccharomycetaceae_2 - ps25
Saccharomycetaceae_3 - ps26
Kaz./Nau./Saccharomyces - ps27
Saccharomyces - ps28
Arboricola_clade - ps29
Cerevisiae_clade - ps30
Saccharomyces_cerevisiae - ps31
Phylostratum

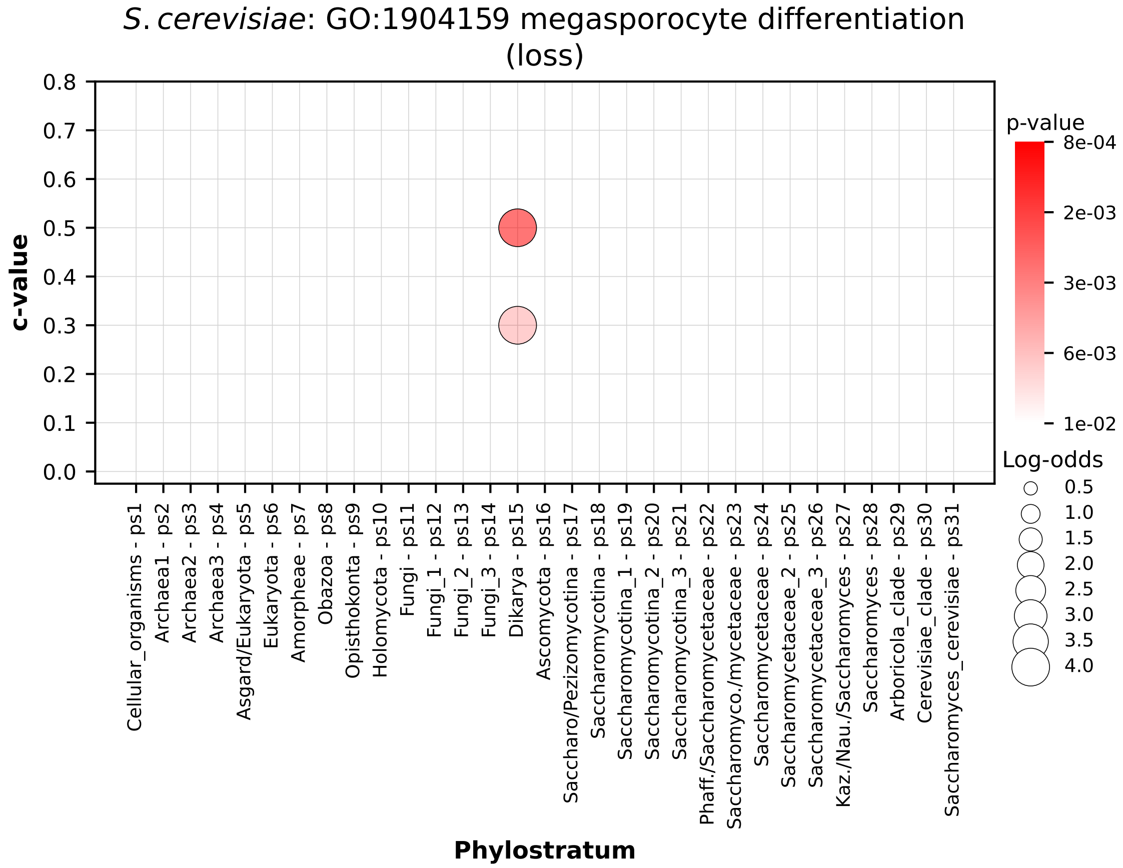
S. cerevisiae: GO:1904159 megasporocyte differentiation
(loss)
c-value
0.8
0.7
0.6
0.5
0.4
0.3
0.2
0.1
0.0
p-value
8e-04
2e-03
3e-03
6e-03
1e-02
Log-odds
0.5
1.0
1.5
2.0
2.5
3.0
3.5
4.0
Cellular_organisms - ps1
Archaea1 - ps2
Archaea2 - ps3
Archaea3 - ps4
Asgard/Eukaryota - ps5
Eukaryota - ps6
Amorpheae - ps7
Obazoa - ps8
Opisthokonta - ps9
Holomycota - ps10
Fungi - ps11
Fungi_1 - ps12
Fungi_2 - ps13
Fungi_3 - ps14
Dikarya - ps15
Ascomycota - ps16
Saccharo/Pezizomycotina - ps17
Saccharomycotina - ps18
Saccharomycotina_1 - ps19
Saccharomycotina_2 - ps20
Saccharomycotina_3 - ps21
Phaff./Saccharomycetaceae - ps22
Saccharomyco./mycetaceae - ps23
Saccharomycetaceae - ps24
Saccharomycetaceae_2 - ps25
Saccharomycetaceae_3 - ps26
Kaz./Nau./Saccharomyces - ps27
Saccharomyces - ps28
Arboricola_clade - ps29
Cerevisiae_clade - ps30
Saccharomyces_cerevisiae - ps31
Phylostratum

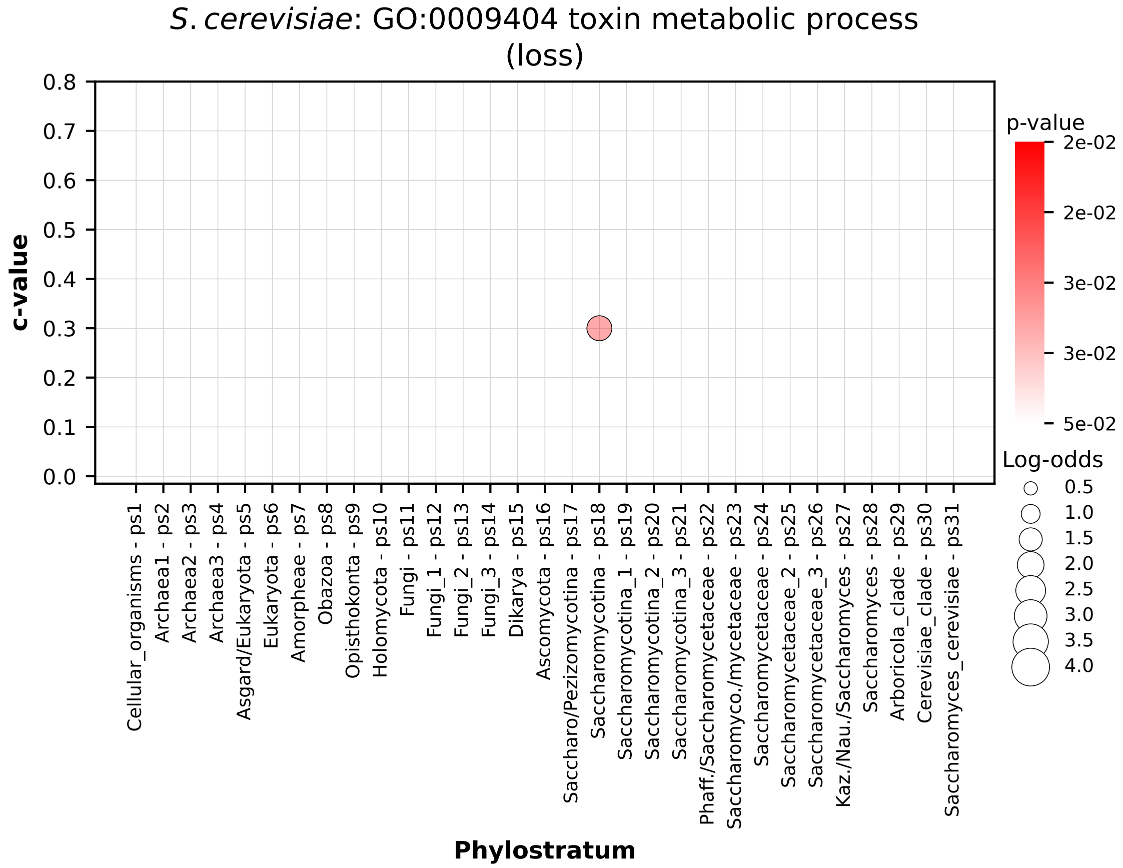

S. cerevisiae: GO:0009404 toxin metabolic process (loss)
c-value
0.8
0.7
0.6
0.5
0.4
0.3
0.2
0.1
0.0
Phylostratum
Cellular_organisms - ps1
Archaea1 - ps2
Archaea2 - ps3
Archaea3 - ps4
Asgard/Eukaryota - ps5
Eukaryota - ps6
Amorpheae - ps7
Obazoa - ps8
Opisthokonta - ps9
Holomycota - ps10
Fungi - ps11
Fungi_1 - ps12
Fungi_2 - ps13
Fungi_3 - ps14
Dikarya - ps15
Ascomycota - ps16
Saccharo/Pezizomycotina - ps17
Saccharomycotina - ps18
Saccharomycotina_1 - ps19
Saccharomycotina_2 - ps20
Saccharomycotina_3 - ps21
Phaff./Saccharomycetaceae - ps22
Saccharomyco./mycetaceae - ps23
Saccharomycetaceae - ps24
Saccharomycetaceae_2 - ps25
Saccharomycetaceae_3 - ps26
Kaz./Nau./Saccharomyces - ps27
Saccharomyces - ps28
Arboricola_clade - ps29
Cerevisiae_clade - ps30
Saccharomyces_cerevisiae - ps31
p-value
2e-02
2e-02
3e-02
3e-02
5e-02
Log-odds
0.5
1.0
1.5
2.0
2.5
3.0
3.5
4.0

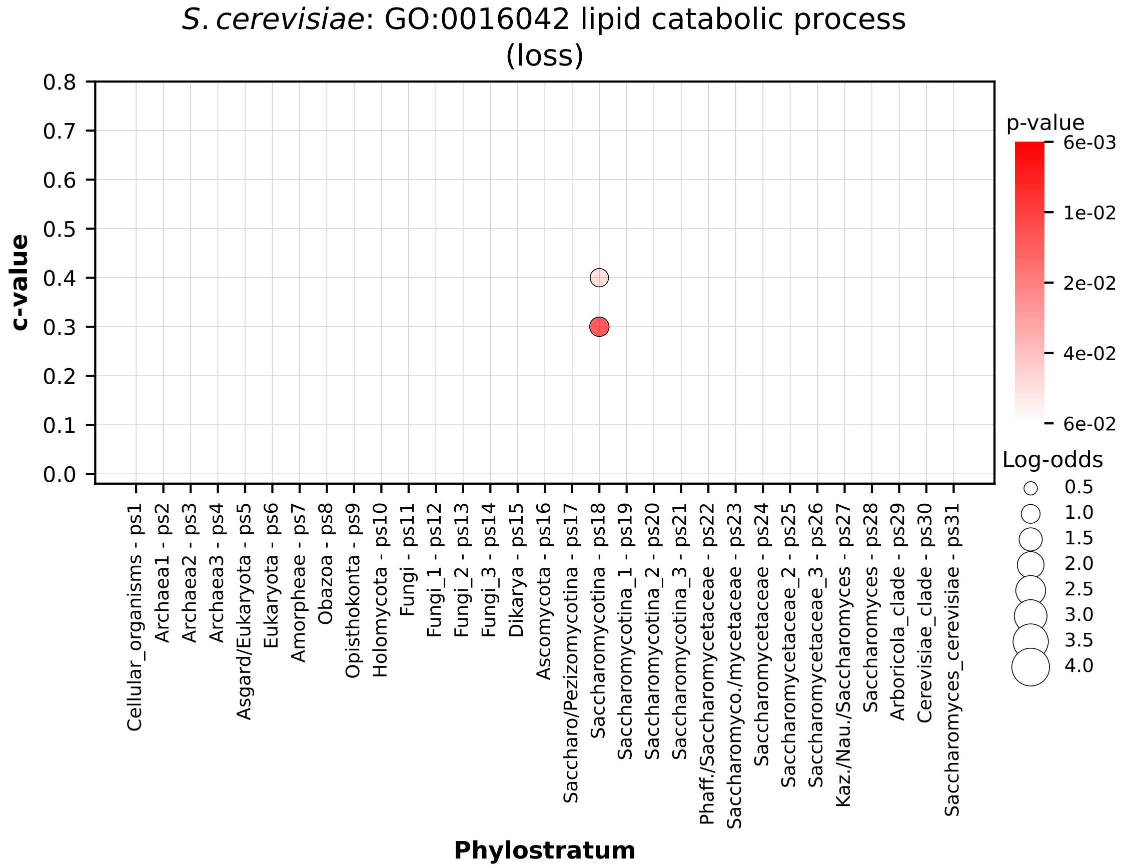

S. cerevisiae: GO:0016042 lipid catabolic process
(loss)
c-value
0.8
0.7
0.6
0.5
0.4
0.3
0.2
0.1
0.0
p-value
6e-03
1e-02
2e-02
4e-02
6e-02
Log-odds
0.5
1.0
1.5
2.0
2.5
3.0
3.5
4.0
Cellular_organisms - ps1
Archaea1 - ps2
Archaea2 - ps3
Archaea3 - ps4
Asgard/Eukaryota - ps5
Eukaryota - ps6
Amorpheae - ps7
Obazoa - ps8
Opisthokonta - ps9
Holomycota - ps10
Fungi - ps11
Fungi_1 - ps12
Fungi_2 - ps13
Fungi_3 - ps14
Dikarya - ps15
Ascomycota - ps16
Saccharo/Pezizomycotina - ps17
Saccharomycotina - ps18
Saccharomycotina_1 - ps19
Saccharomycotina_2 - ps20
Saccharomycotina_3 - ps21
Phaff./Saccharomycetaceae - ps22
Saccharomyco./mycetaceae - ps23
Saccharomycetaceae - ps24
Saccharomycetaceae_2 - ps25
Saccharomycetaceae_3 - ps26
Kaz./Nau./Saccharomyces - ps27
Saccharomyces - ps28
Arboricola_clade - ps29
Cerevisiae_clade - ps30
Saccharomyces_cerevisiae - ps31
Phylostratum

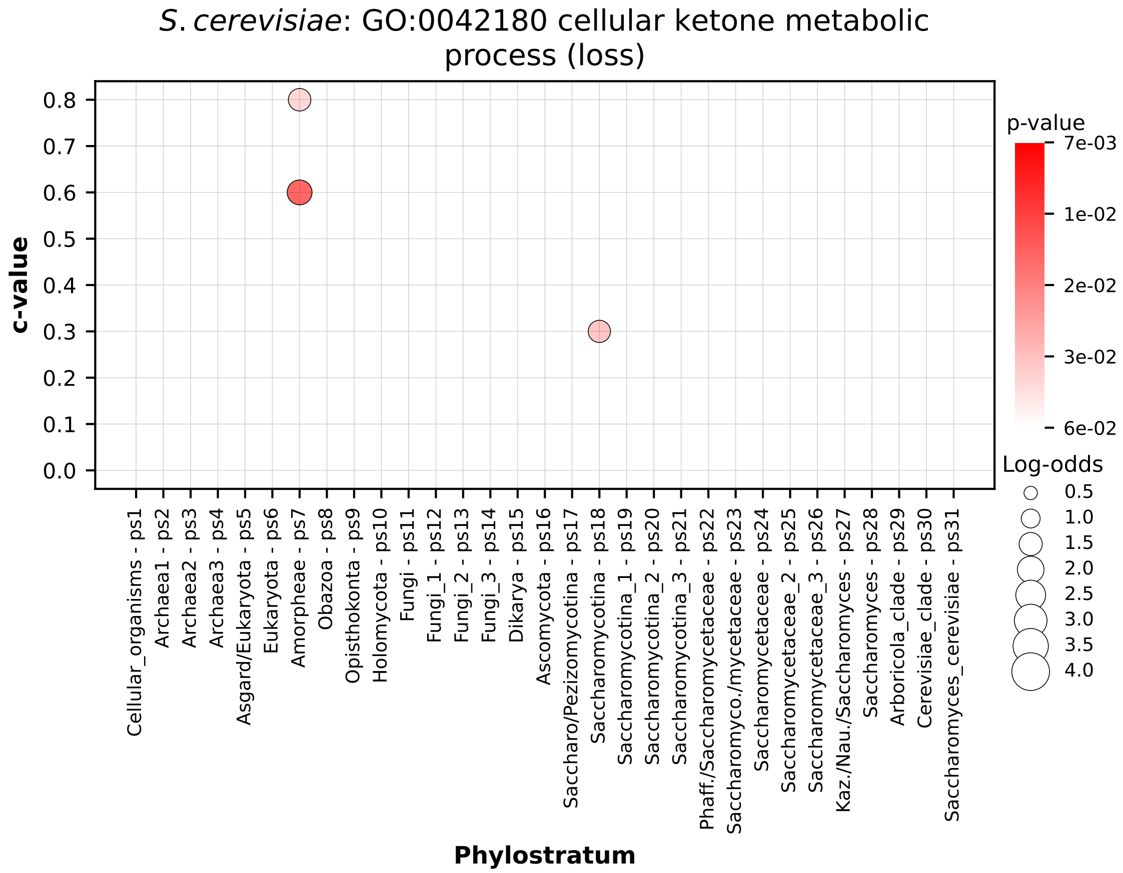

S. cerevisiae: GO:0042180 cellular ketone metabolic process (loss)
c-value
0.8
0.7
0.6
0.5
0.4
0.3
0.2
0.1
0.0
p-value
7e-03
1e-02
2e-02
3e-02
6e-02
Log-odds
0.5
1.0
1.5
2.0
2.5
3.0
3.5
4.0
Cellular_organisms - ps1
Archaea1 - ps2
Archaea2 - ps3
Archaea3 - ps4
Asgard/Eukaryota - ps5
Eukaryota - ps6
Amorpheae - ps7
Obazoa - ps8
Opisthokonta - ps9
Holomycota - ps10
Fungi - ps11
Fungi_1 - ps12
Fungi_2 - ps13
Fungi_3 - ps14
Dikarya - ps15
Ascomycota - ps16
Saccharo/Pezizomycotina - ps17
Saccharomycotina - ps18
Saccharomycotina_1 - ps19
Saccharomycotina_2 - ps20
Saccharomycotina_3 - ps21
Phaff./Saccharomycetaceae - ps22
Saccharomyco./mycetaceae - ps23
Saccharomycetaceae - ps24
Saccharomycetaceae_2 - ps25
Saccharomycetaceae_3 - ps26
Kaz./Nau./Saccharomyces - ps27
Saccharomyces - ps28
Arboricola_clade - ps29
Cerevisiae_clade - ps30
Saccharomyces_cerevisiae - ps31
Phylostratum

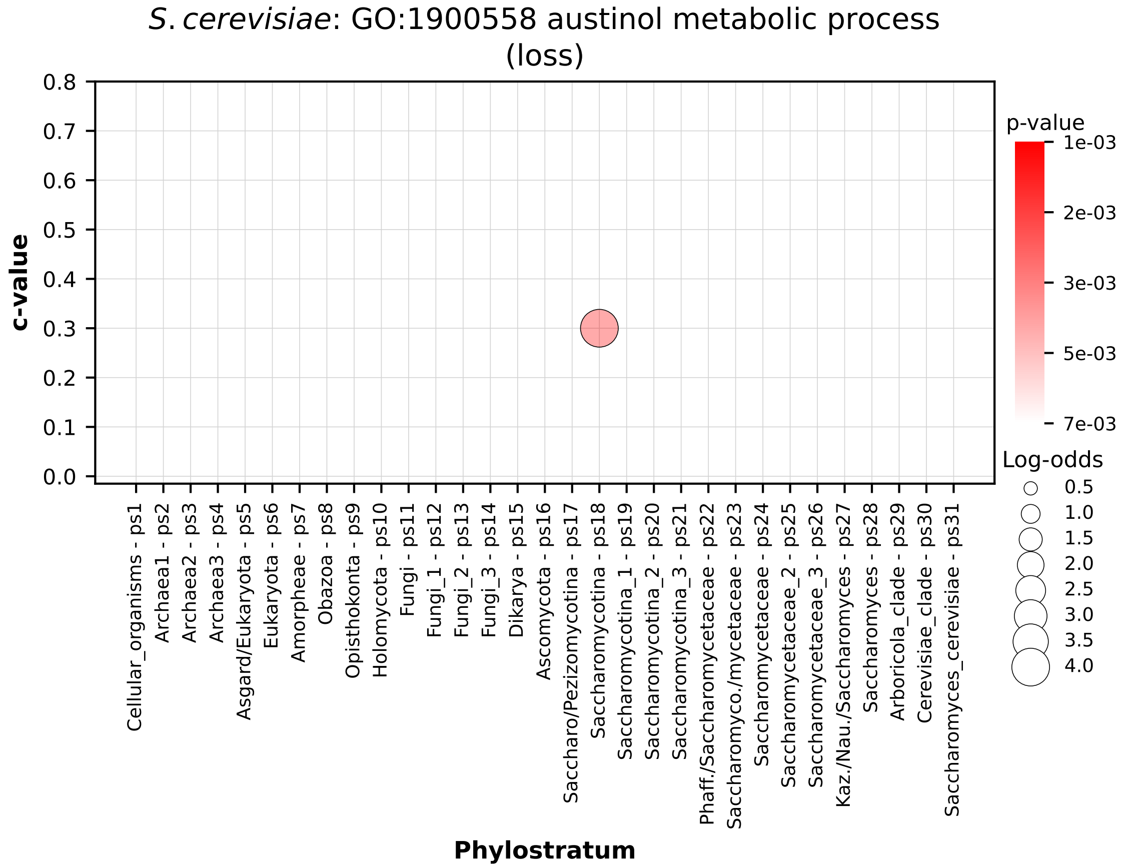

S. cerevisiae: GO:1900558 austinol metabolic process (loss)
c-value
0.0
0.1
0.2
0.3
0.4
0.5
0.6
0.7
0.8
Phylostratum
Cellular_organisms - ps1
Archaea1 - ps2
Archaea2 - ps3
Archaea3 - ps4
Asgard/Eukaryota - ps5
Eukaryota - ps6
Amorpheae - ps7
Obazoa - ps8
Opisthokonta - ps9
Holomycota - ps10
Fungi - ps11
Fungi_1 - ps12
Fungi_2 - ps13
Fungi_3 - ps14
Dikarya - ps15
Ascomycota - ps16
Saccharo/Pezizomycotina - ps17
Saccharomycotina - ps18
Saccharomycotina_1 - ps19
Saccharomycotina_2 - ps20
Saccharomycotina_3 - ps21
Phaff./Saccharomycetaceae - ps22
Saccharomyco./mycetaceae - ps23
Saccharomycetaceae - ps24
Saccharomycetaceae_2 - ps25
Saccharomycetaceae_3 - ps26
Kaz./Nau./Saccharomyces - ps27
Saccharomyces - ps28
Arboricola_clade - ps29
Cerevisiae_clade - ps30
Saccharomyces_cerevisiae - ps31
p-value
1e-03
2e-03
3e-03
5e-03
7e-03
Log-odds
0.5
1.0
1.5
2.0
2.5
3.0
3.5
4.0

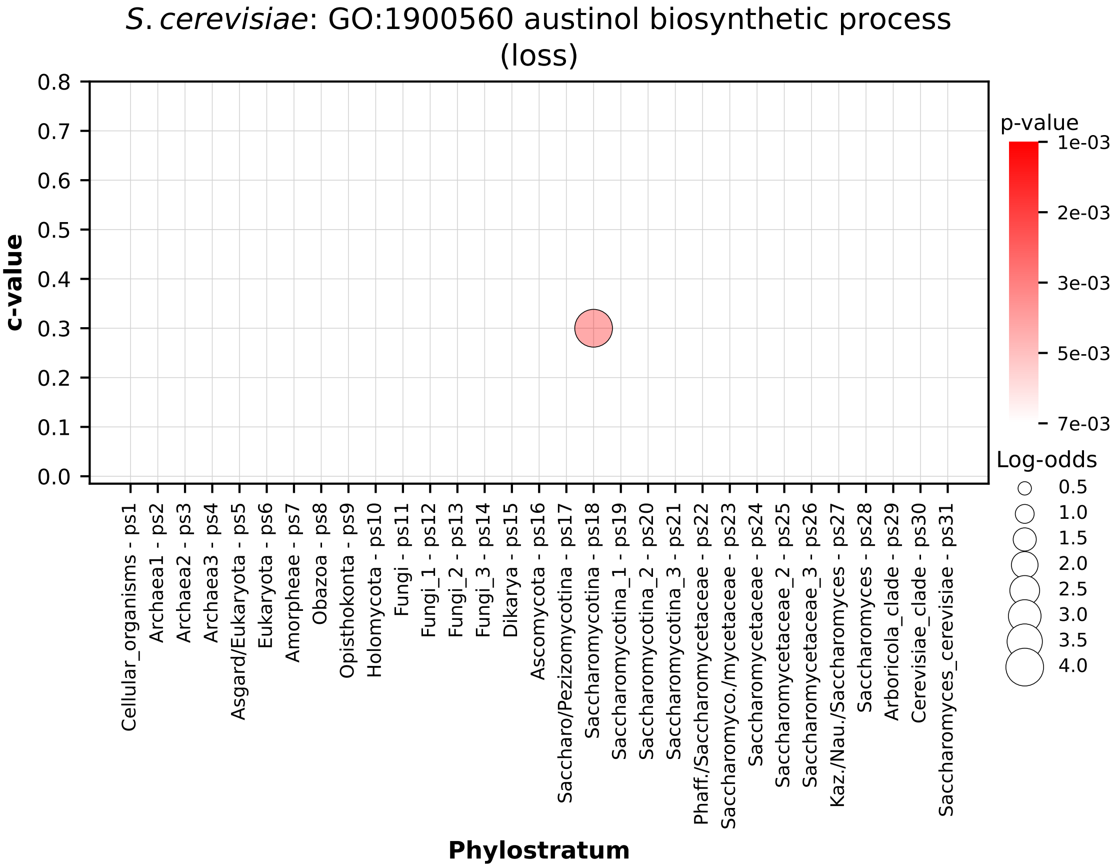

S. cerevisiae: GO:1900560 austinol biosynthetic process
(loss)
c-value
0.8
0.7
0.6
0.5
0.4
0.3
0.2
0.1
0.0
p-value
1e-03
2e-03
3e-03
5e-03
7e-03
Log-odds
0.5
1.0
1.5
2.0
2.5
3.0
3.5
4.0
Cellular_organisms - ps1
Archaea1 - ps2
Archaea2 - ps3
Archaea3 - ps4
Asgard/Eukaryota - ps5
Eukaryota - ps6
Amorpheae - ps7
Obazoa - ps8
Opisthokonta - ps9
Holomycota - ps10
Fungi - ps11
Fungi_1 - ps12
Fungi_2 - ps13
Fungi_3 - ps14
Dikarya - ps15
Ascomycota - ps16
Saccharo/Pezizomycotina - ps17
Saccharomycotina - ps18
Saccharomycotina_1 - ps19
Saccharomycotina_2 - ps20
Saccharomycotina_3 - ps21
Phaff./Saccharomycetaceae - ps22
Saccharomyco./mycetaceae - ps23
Saccharomycetaceae - ps24
Saccharomycetaceae_2 - ps25
Saccharomycetaceae_3 - ps26
Kaz./Nau./Saccharomyces - ps27
Saccharomyces - ps28
Arboricola_clade - ps29
Cerevisiae_clade - ps30
Saccharomyces_cerevisiae - ps31
Phylostratum

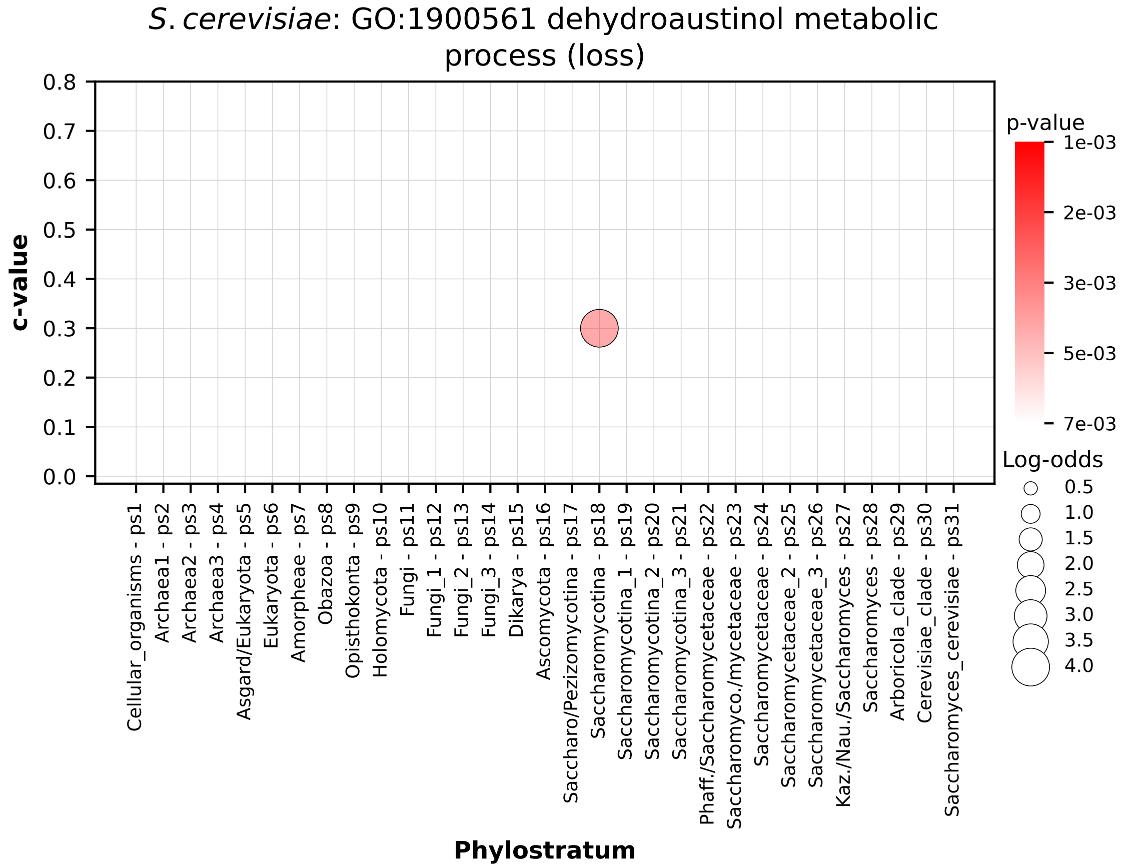
S. cerevisiae: GO:1900561 dehydroaustinol metabolic process (loss)
c-value
0.8
0.7
0.6
0.5
0.4
0.3
0.2
0.1
0.0
Cellular_organisms - ps1
Archaea1 - ps2
Archaea2 - ps3
Archaea3 - ps4
Asgard/Eukaryota - ps5
Eukaryota - ps6
Amorpheae - ps7
Obazoa - ps8
Opisthokonta - ps9
Holomycota - ps10
Fungi - ps11
Fungi_1 - ps12
Fungi_2 - ps13
Fungi_3 - ps14
Dikarya - ps15
Ascomycota - ps16
Saccharo/Pezizomycotina - ps17
Saccharomycotina - ps18
Saccharomycotina_1 - ps19
Saccharomycotina_2 - ps20
Saccharomycotina_3 - ps21
Phaff./Saccharomycetaceae - ps22
Saccharomyco./mycetaceae - ps23
Saccharomycetaceae - ps24
Saccharomycetaceae_2 - ps25
Saccharomycetaceae_3 - ps26
Kaz./Nau./Saccharomyces - ps27
Saccharomyces - ps28
Arboricola_clade - ps29
Cerevisiae_clade - ps30
Saccharomyces_cerevisiae - ps31
Phylostratum
p-value
1e-03
2e-03
3e-03
5e-03
7e-03
Log-odds
0.5
1.0
1.5
2.0
2.5
3.0
3.5
4.0

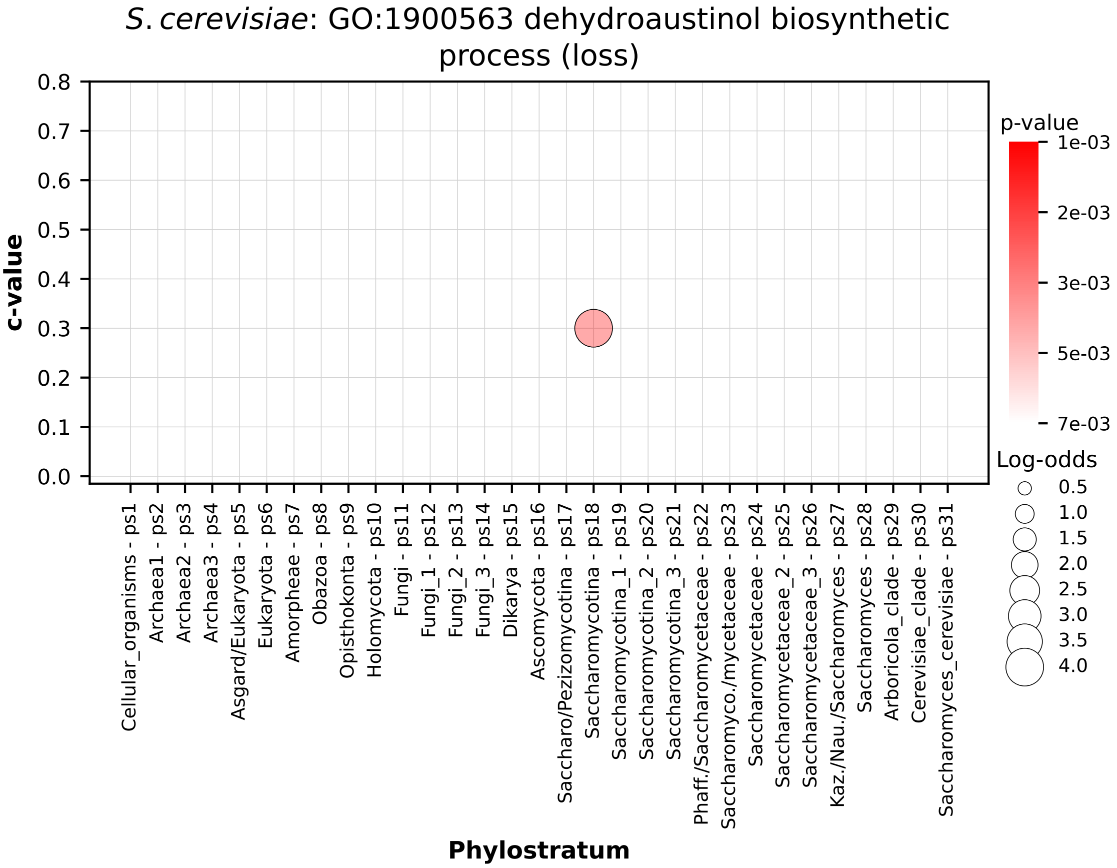

S. cerevisiae: GO:1900563 dehydroaustinol biosynthetic process (loss)
c-value
0.8
0.7
0.6
0.5
0.4
0.3
0.2
0.1
0.0
Phylostratum
Cellular_organisms - ps1
Archaea1 - ps2
Archaea2 - ps3
Archaea3 - ps4
Asgard/Eukaryota - ps5
Eukaryota - ps6
Amorpheae - ps7
Obazoa - ps8
Opisthokonta - ps9
Holomycota - ps10
Fungi - ps11
Fungi_1 - ps12
Fungi_2 - ps13
Fungi_3 - ps14
Dikarya - ps15
Ascomycota - ps16
Saccharo/Pezizomycotina - ps17
Saccharomycotina - ps18
Saccharomycotina_1 - ps19
Saccharomycotina_2 - ps20
Saccharomycotina_3 - ps21
Phaff./Saccharomycetaceae - ps22
Saccharomyco./mycetaceae - ps23
Saccharomycetaceae - ps24
Saccharomycetaceae_2 - ps25
Saccharomycetaceae_3 - ps26
Kaz./Nau./Saccharomyces - ps27
Saccharomyces - ps28
Arboricola_clade - ps29
Cerevisiae_clade - ps30
Saccharomyces_cerevisiae - ps31
p-value
1e-03
2e-03
3e-03
5e-03
7e-03
Log-odds
0.5
1.0
1.5
2.0
2.5
3.0
3.5
4.0

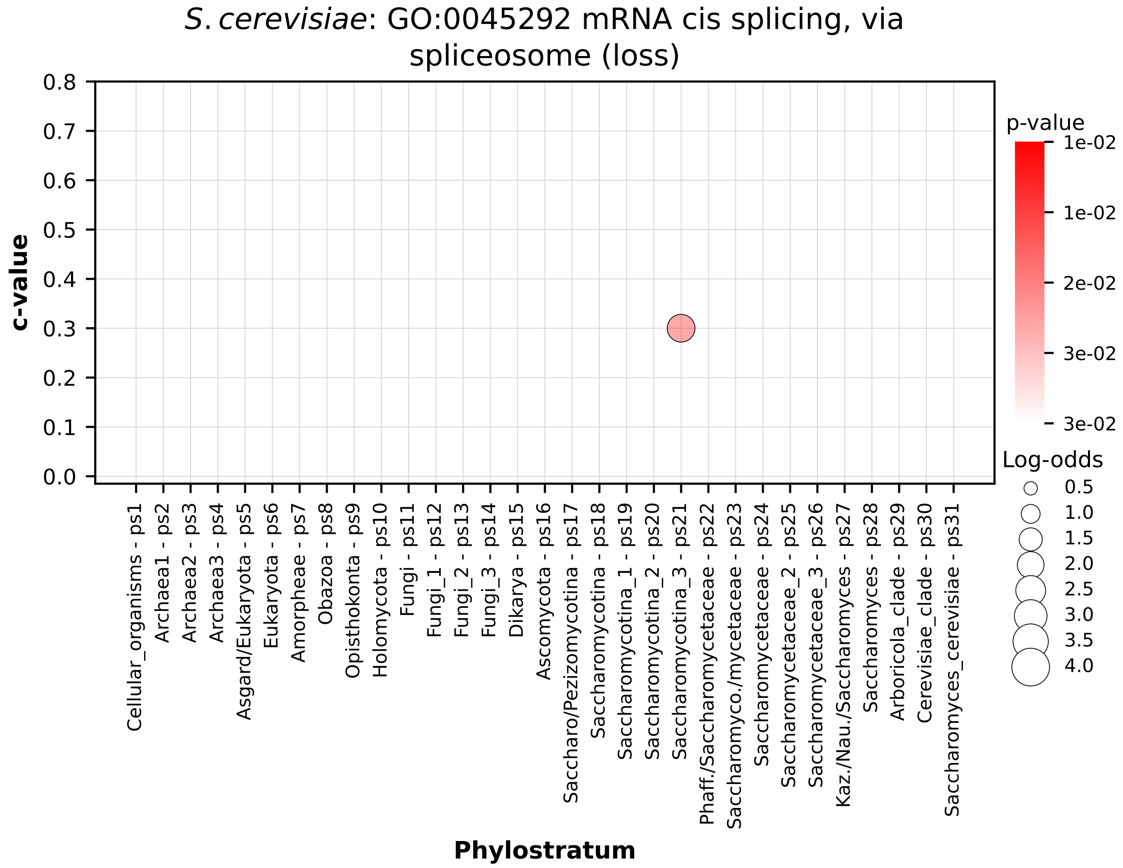

S. cerevisiae: GO:0045292 mRNA cis splicing, via spliceosome (loss)
c-value
0.8
0.7
0.6
0.5
0.4
0.3
0.2
0.1
0.0
Phylostratum
Cellular_organisms - ps1
Archaea1 - ps2
Archaea2 - ps3
Archaea3 - ps4
Asgard/Eukaryota - ps5
Eukaryota - ps6
Amorpheae - ps7
Obazoa - ps8
Opisthokonta - ps9
Holomycota - ps10
Fungi - ps11
Fungi_1 - ps12
Fungi_2 - ps13
Fungi_3 - ps14
Dikarya - ps15
Ascomycota - ps16
Saccharo/Pezizomycotina - ps17
Saccharomycotina - ps18
Saccharomycotina_1 - ps19
Saccharomycotina_2 - ps20
Saccharomycotina_3 - ps21
Phaff./Saccharomycetaceae - ps22
Saccharomyco./mycetaceae - ps23
Saccharomycetaceae - ps24
Saccharomycetaceae_2 - ps25
Saccharomycetaceae_3 - ps26
Kaz./Nau./Saccharomyces - ps27
Saccharomyces - ps28
Arboricola_clade - ps29
Cerevisiae_clade - ps30
Saccharomyces_cerevisiae - ps31
p-value
1e-02
1e-02
2e-02
3e-02
3e-02
Log-odds
0.5
1.0
1.5
2.0
2.5
3.0
3.5
4.0

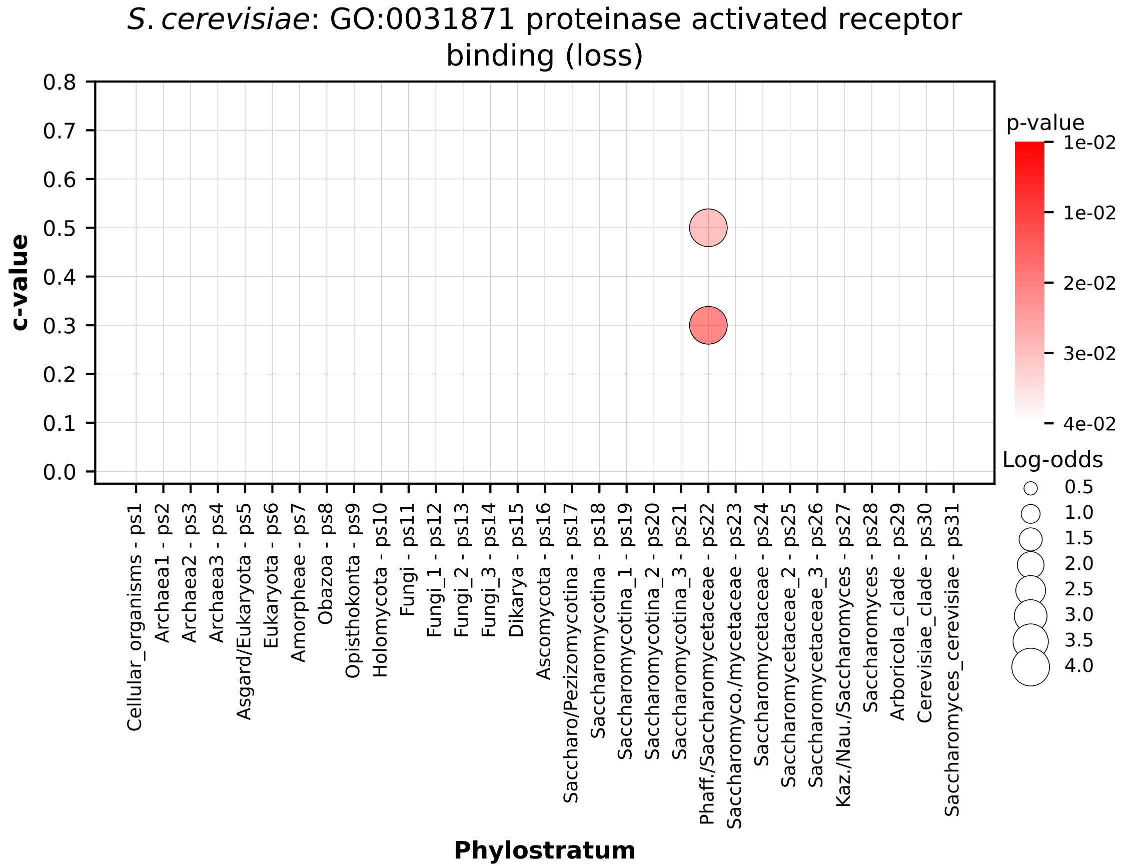
S. cerevisiae: GO:0031871 proteinase activated receptor binding (loss)
c-value
0.8
0.7
0.6
0.5
0.4
0.3
0.2
0.1
0.0
Phylostratum
Cellular_organisms - ps1
Archaea1 - ps2
Archaea2 - ps3
Archaea3 - ps4
Asgard/Eukaryota - ps5
Eukaryota - ps6
Amorpheae - ps7
Obazoa - ps8
Opisthokonta - ps9
Holomycota - ps10
Fungi - ps11
Fungi_1 - ps12
Fungi_2 - ps13
Fungi_3 - ps14
Dikarya - ps15
Ascomycota - ps16
Saccharo/Pezizomycotina - ps17
Saccharomycotina - ps18
Saccharomycotina_1 - ps19
Saccharomycotina_2 - ps20
Saccharomycotina_3 - ps21
Phaff./Saccharomycetaceae - ps22
Saccharomyco./mycetaceae - ps23
Saccharomycetaceae - ps24
Saccharomycetaceae_2 - ps25
Saccharomycetaceae_3 - ps26
Kaz./Nau./Saccharomyces - ps27
Saccharomyces - ps28
Arboricola_clade - ps29
Cerevisiae_clade - ps30
Saccharomyces_cerevisiae - ps31
p-value
1e-02
1e-02
2e-02
3e-02
4e-02
Log-odds
0.5
1.0
1.5
2.0
2.5
3.0
3.5
4.0

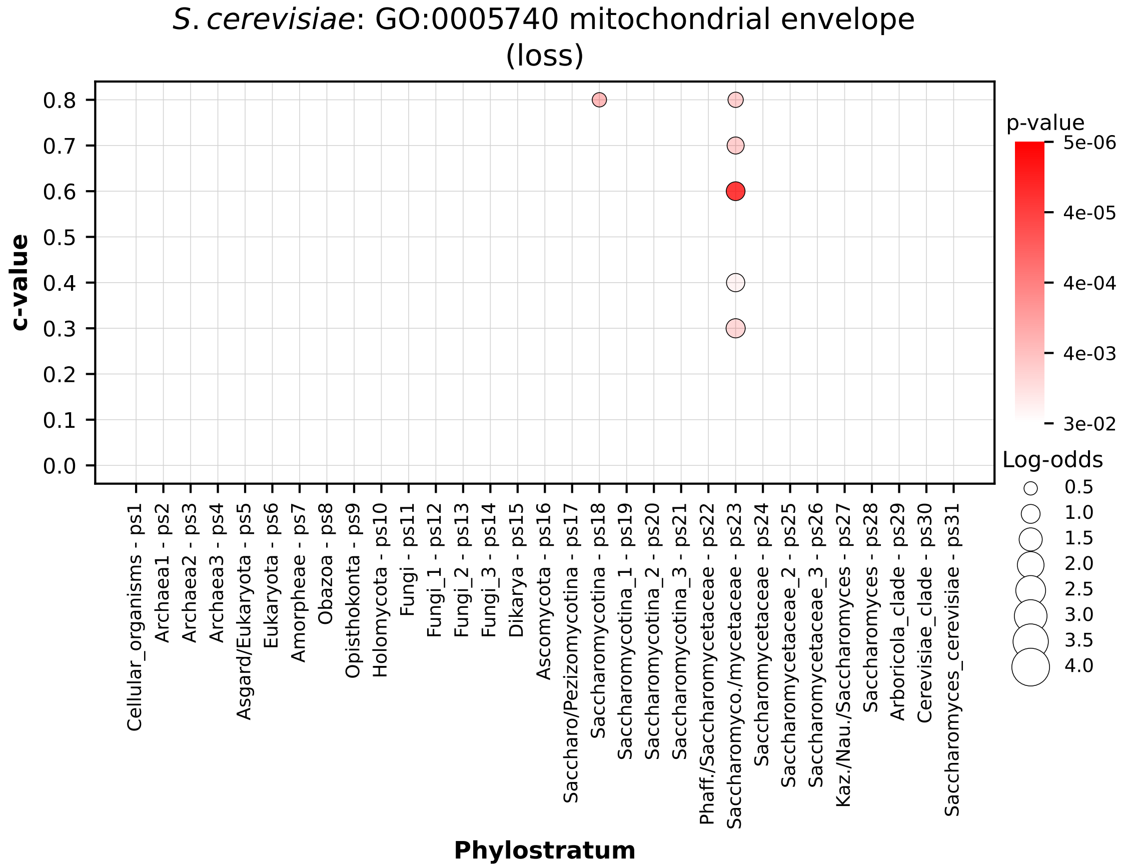
S. cerevisiae: GO:0005740 mitochondrial envelope
(loss)
c-value
0.8
0.7
0.6
0.5
0.4
0.3
0.2
0.1
0.0
p-value
5e-06
4e-05
4e-04
4e-03
3e-02
Log-odds
0.5
1.0
1.5
2.0
2.5
3.0
3.5
4.0
Cellular_organisms - ps1
Archaea1 - ps2
Archaea2 - ps3
Archaea3 - ps4
Asgard/Eukaryota - ps5
Eukaryota - ps6
Amorpheae - ps7
Obazoa - ps8
Opisthokonta - ps9
Holomycota - ps10
Fungi - ps11
Fungi_1 - ps12
Fungi_2 - ps13
Fungi_3 - ps14
Dikarya - ps15
Ascomycota - ps16
Saccharo/Pezizomycotina - ps17
Saccharomycotina - ps18
Saccharomycotina_1 - ps19
Saccharomycotina_2 - ps20
Saccharomycotina_3 - ps21
Phaff./Saccharomycetaceae - ps22
Saccharomyco./mycetaceae - ps23
Saccharomycetaceae - ps24
Saccharomycetaceae_2 - ps25
Saccharomycetaceae_3 - ps26
Kaz./Nau./Saccharomyces - ps27
Saccharomyces - ps28
Arboricola_clade - ps29
Cerevisiae_clade - ps30
Saccharomyces_cerevisiae - ps31
Phylostratum

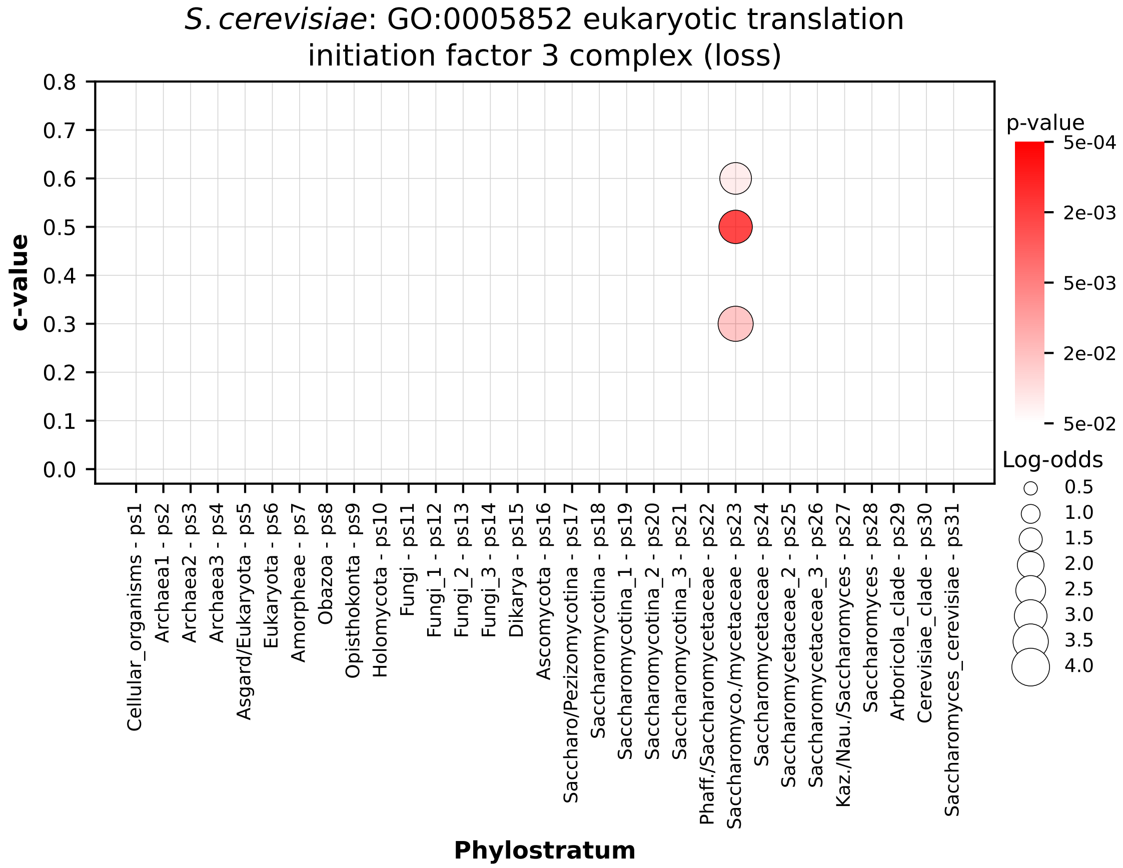

S. cerevisiae: GO:0005852 eukaryotic translation initiation factor 3 complex (loss)
c-value
0.8
0.7
0.6
0.5
0.4
0.3
0.2
0.1
0.0
p-value
5e-04
2e-03
5e-03
2e-02
5e-02
Log-odds
0.5
1.0
1.5
2.0
2.5
3.0
3.5
4.0
Cellular_organisms - ps1
Archaea1 - ps2
Archaea2 - ps3
Archaea3 - ps4
Asgard/Eukaryota - ps5
Eukaryota - ps6
Amorpheae - ps7
Obazoa - ps8
Opisthokonta - ps9
Holomycota - ps10
Fungi - ps11
Fungi_1 - ps12
Fungi_2 - ps13
Fungi_3 - ps14
Dikarya - ps15
Ascomycota - ps16
Saccharo/Pezizomycotina - ps17
Saccharomycotina - ps18
Saccharomycotina_1 - ps19
Saccharomycotina_2 - ps20
Saccharomycotina_3 - ps21
Phaff./Saccharomycetaceae - ps22
Saccharomyco./mycetaceae - ps23
Saccharomycetaceae - ps24
Saccharomycetaceae_2 - ps25
Saccharomycetaceae_3 - ps26
Kaz./Nau./Saccharomyces - ps27
Saccharomyces - ps28
Arboricola_clade - ps29
Cerevisiae_clade - ps30
Saccharomyces_cerevisiae - ps31
Phylostratum

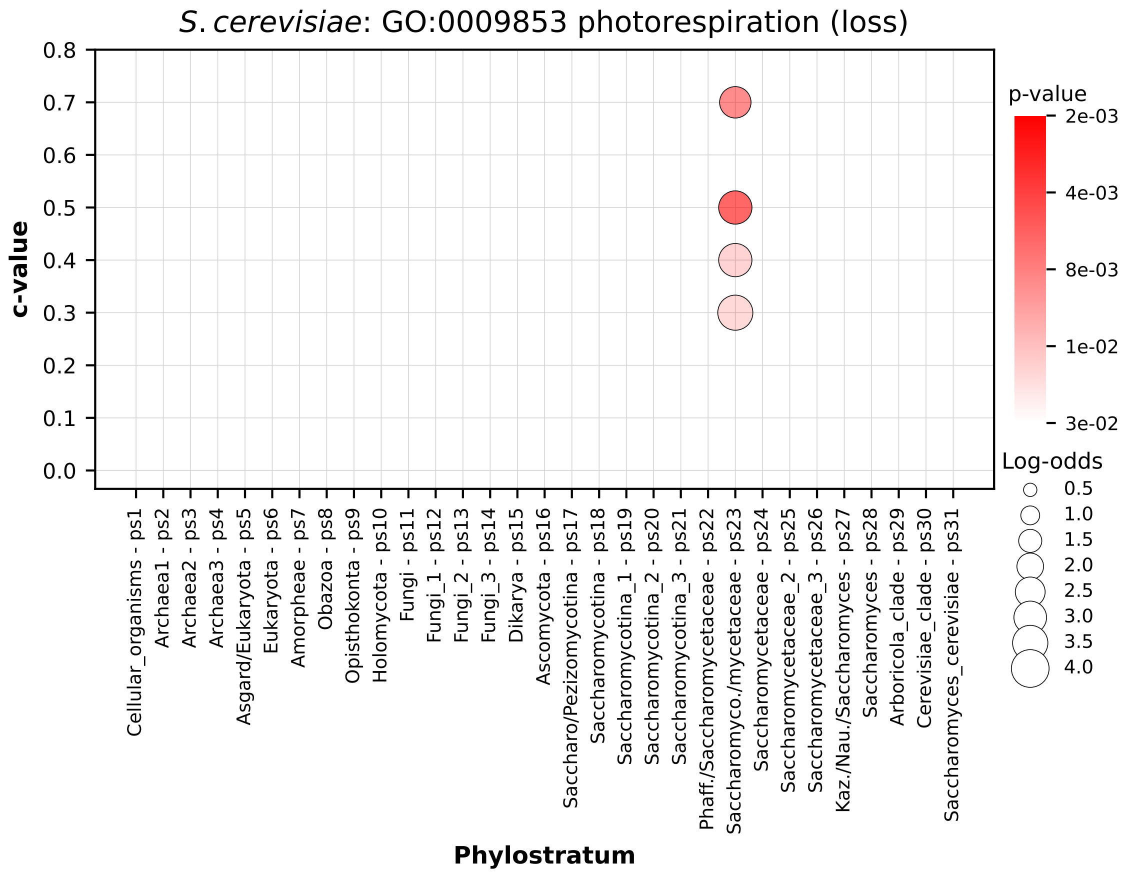

S. cerevisiae: GO:0009853 photorespiration (loss)
c-value
0.0
0.1
0.2
0.3
0.4
0.5
0.6
0.7
0.8
Phylostratum
Cellular_organisms - ps1
Archaea1 - ps2
Archaea2 - ps3
Archaea3 - ps4
Asgard/Eukaryota - ps5
Eukaryota - ps6
Amorpheae - ps7
Obazoa - ps8
Opisthokonta - ps9
Holomycota - ps10
Fungi - ps11
Fungi_1 - ps12
Fungi_2 - ps13
Fungi_3 - ps14
Dikarya - ps15
Ascomycota - ps16
Saccharo/Pezizomycotina - ps17
Saccharomycotina - ps18
Saccharomycotina_1 - ps19
Saccharomycotina_2 - ps20
Saccharomycotina_3 - ps21
Phaff./Saccharomycetaceae - ps22
Saccharomyco./mycetaceae - ps23
Saccharomycetaceae - ps24
Saccharomycetaceae_2 - ps25
Saccharomycetaceae_3 - ps26
Kaz./Nau./Saccharomyces - ps27
Saccharomyces - ps28
Arboricola_clade - ps29
Cerevisiae_clade - ps30
Saccharomyces_cerevisiae - ps31
p-value
2e-03
4e-03
8e-03
1e-02
3e-02
Log-odds
0.5
1.0
1.5
2.0
2.5
3.0
3.5
4.0

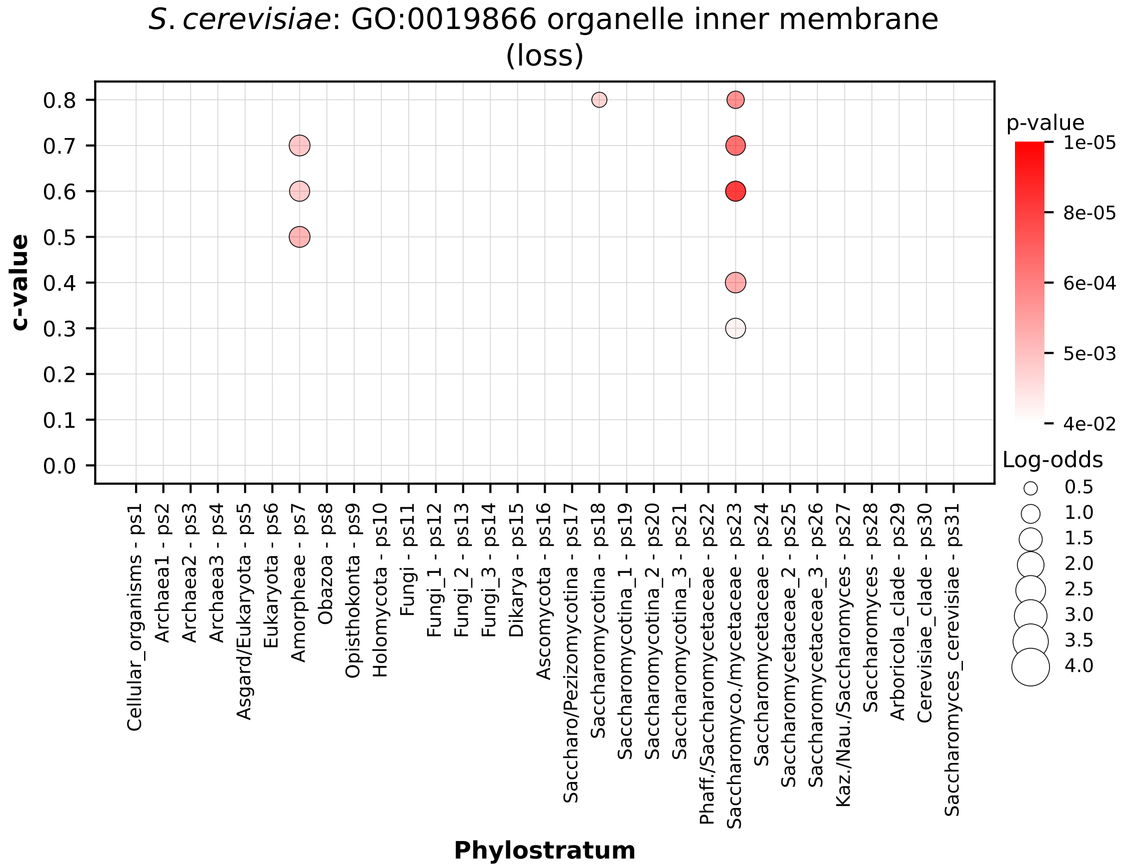

S. cerevisiae: GO:0019866 organelle inner membrane
(loss)
c-value
0.8
0.7
0.6
0.5
0.4
0.3
0.2
0.1
0.0
p-value
1e-05
8e-05
6e-04
5e-03
4e-02
Log-odds
0.5
1.0
1.5
2.0
2.5
3.0
3.5
4.0
Cellular_organisms - ps1
Archaea1 - ps2
Archaea2 - ps3
Archaea3 - ps4
Asgard/Eukaryota - ps5
Eukaryota - ps6
Amorpheae - ps7
Obazoa - ps8
Opisthokonta - ps9
Holomycota - ps10
Fungi - ps11
Fungi_1 - ps12
Fungi_2 - ps13
Fungi_3 - ps14
Dikarya - ps15
Ascomycota - ps16
Saccharo/Pezizomycotina - ps17
Saccharomycotina - ps18
Saccharomycotina_1 - ps19
Saccharomycotina_2 - ps20
Saccharomycotina_3 - ps21
Phaff./Saccharomycetaceae - ps22
Saccharomyco./mycetaceae - ps23
Saccharomycetaceae - ps24
Saccharomycetaceae_2 - ps25
Saccharomycetaceae_3 - ps26
Kaz./Nau./Saccharomyces - ps27
Saccharomyces - ps28
Arboricola_clade - ps29
Cerevisiae_clade - ps30
Saccharomyces_cerevisiae - ps31
Phylostratum

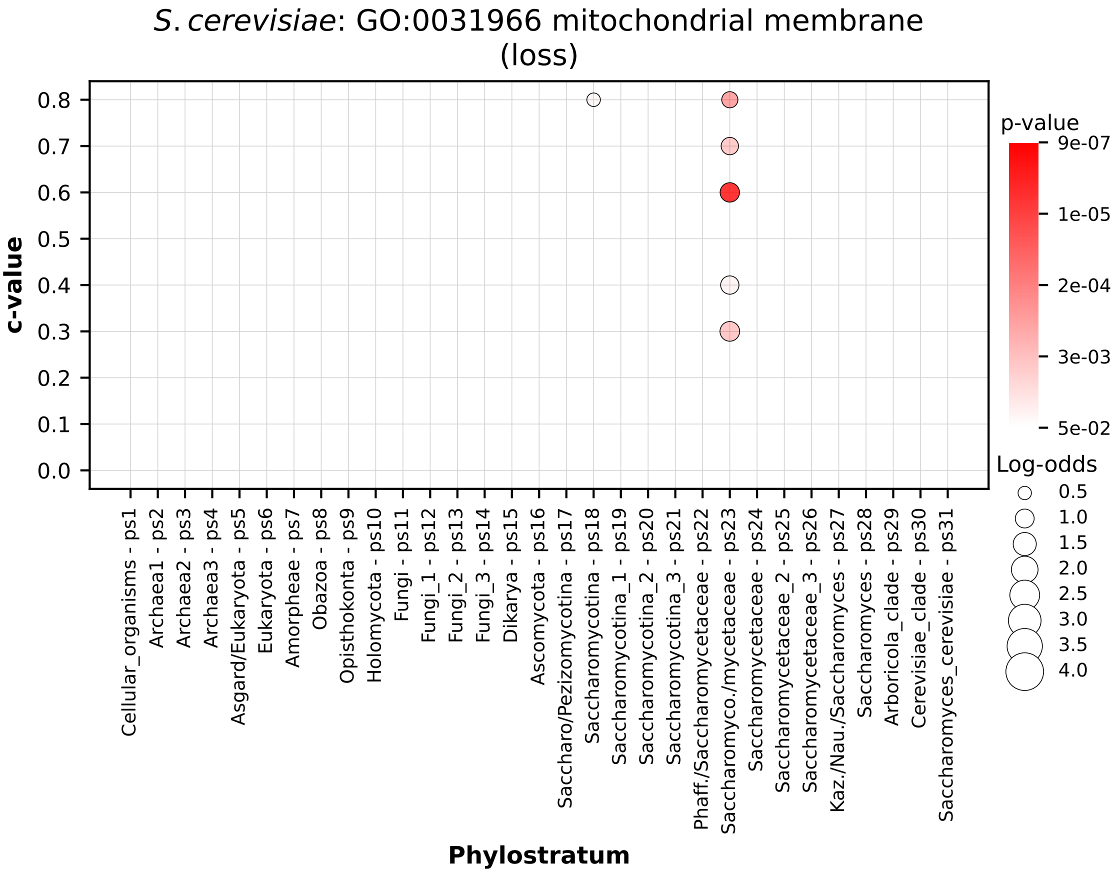

S. cerevisiae: GO:0031966 mitochondrial membrane
(loss)
c-value
0.8
0.7
0.6
0.5
0.4
0.3
0.2
0.1
0.0
p-value
9e-07
1e-05
2e-04
3e-03
5e-02
Log-odds
0.5
1.0
1.5
2.0
2.5
3.0
3.5
4.0
Cellular_organisms - ps1
Archaea1 - ps2
Archaea2 - ps3
Archaea3 - ps4
Asgard/Eukaryota - ps5
Eukaryota - ps6
Amorpheae - ps7
Obazoa - ps8
Opisthokonta - ps9
Holomycota - ps10
Fungi - ps11
Fungi_1 - ps12
Fungi_2 - ps13
Fungi_3 - ps14
Dikarya - ps15
Ascomycota - ps16
Saccharo/Pezizomycotina - ps17
Saccharomycotina - ps18
Saccharomycotina_1 - ps19
Saccharomycotina_2 - ps20
Saccharomycotina_3 - ps21
Phaff./Saccharomycetaceae - ps22
Saccharomyco./mycetaceae - ps23
Saccharomycetaceae - ps24
Saccharomycetaceae_2 - ps25
Saccharomycetaceae_3 - ps26
Kaz./Nau./Saccharomyces - ps27
Saccharomyces - ps28
Arboricola_clade - ps29
Cerevisiae_clade - ps30
Saccharomyces_cerevisiae - ps31
Phylostratum

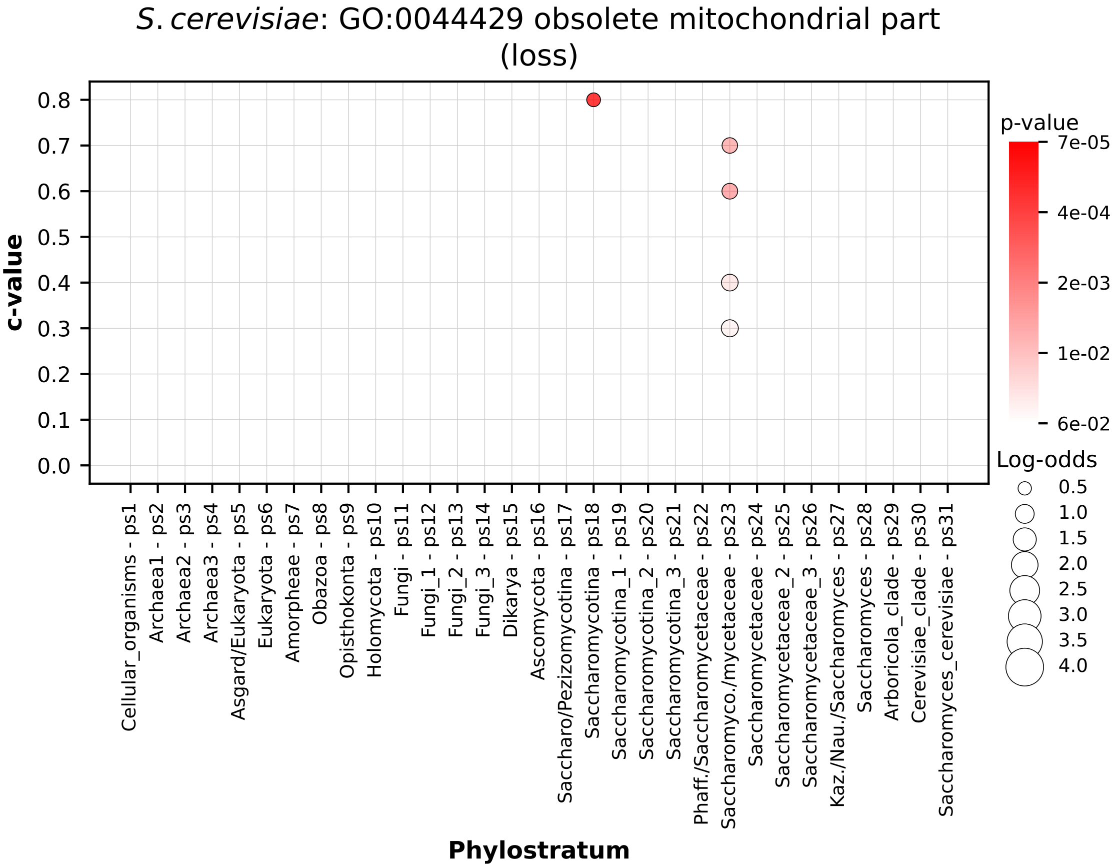

S. cerevisiae: GO:0044429 obsolete mitochondrial part
(loss)
c-value
0.0
0.1
0.2
0.3
0.4
0.5
0.6
0.7
0.8
p-value
7e-05
4e-04
2e-03
1e-02
6e-02
Log-odds
0.5
1.0
1.5
2.0
2.5
3.0
3.5
4.0
Cellular_organisms - ps1
Archaea1 - ps2
Archaea2 - ps3
Archaea3 - ps4
Asgard/Eukaryota - ps5
Eukaryota - ps6
Amorpheae - ps7
Obazoa - ps8
Opisthokonta - ps9
Holomycota - ps10
Fungi - ps11
Fungi_1 - ps12
Fungi_2 - ps13
Fungi_3 - ps14
Dikarya - ps15
Ascomycota - ps16
Saccharo/Pezizomycotina - ps17
Saccharomycotina - ps18
Saccharomycotina_1 - ps19
Saccharomycotina_2 - ps20
Saccharomycotina_3 - ps21
Phaff./Saccharomycetaceae - ps22
Saccharomyco./mycetaceae - ps23
Saccharomycetaceae - ps24
Saccharomycetaceae_2 - ps25
Saccharomycetaceae_3 - ps26
Kaz./Nau./Saccharomyces - ps27
Saccharomyces - ps28
Arboricola_clade - ps29
Cerevisiae_clade - ps30
Saccharomyces_cerevisiae - ps31
Phylostratum

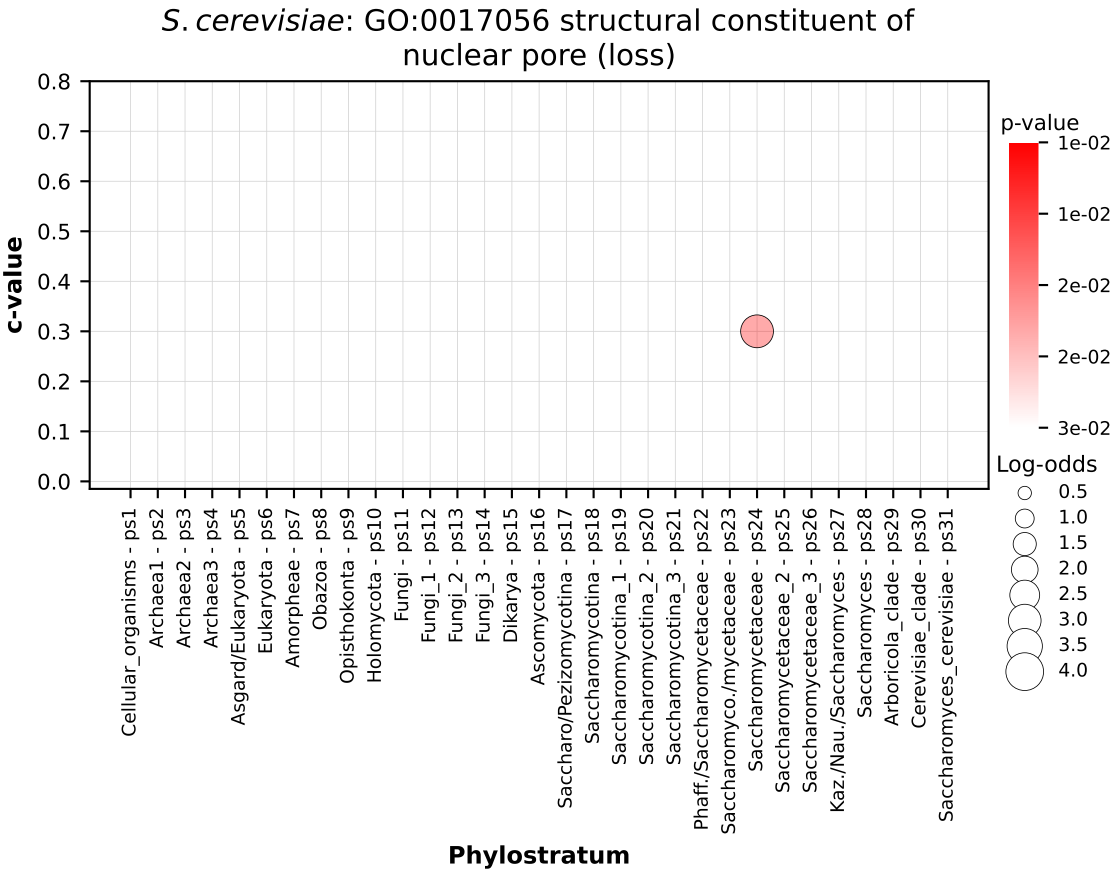

S. cerevisiae: GO:0017056 structural constituent of nuclear pore (loss)
c-value
0.8
0.7
0.6
0.5
0.4
0.3
0.2
0.1
0.0
p-value
1e-02
1e-02
2e-02
2e-02
3e-02
Log-odds
0.5
1.0
1.5
2.0
2.5
3.0
3.5
4.0
Cellular_organisms - ps1
Archaea1 - ps2
Archaea2 - ps3
Archaea3 - ps4
Asgard/Eukaryota - ps5
Eukaryota - ps6
Amorpheae - ps7
Obazoa - ps8
Opisthokonta - ps9
Holomycota - ps10
Fungi - ps11
Fungi_1 - ps12
Fungi_2 - ps13
Fungi_3 - ps14
Dikarya - ps15
Ascomycota - ps16
Saccharo/Pezizomycotina - ps17
Saccharomycotina - ps18
Saccharomycotina_1 - ps19
Saccharomycotina_2 - ps20
Saccharomycotina_3 - ps21
Phaff./Saccharomycetaceae - ps22
Saccharomyco./mycetaceae - ps23
Saccharomycetaceae - ps24
Saccharomycetaceae_2 - ps25
Saccharomycetaceae_3 - ps26
Kaz./Nau./Saccharomyces - ps27
Saccharomyces - ps28
Arboricola_clade - ps29
Cerevisiae_clade - ps30
Saccharomyces_cerevisiae - ps31
Phylostratum

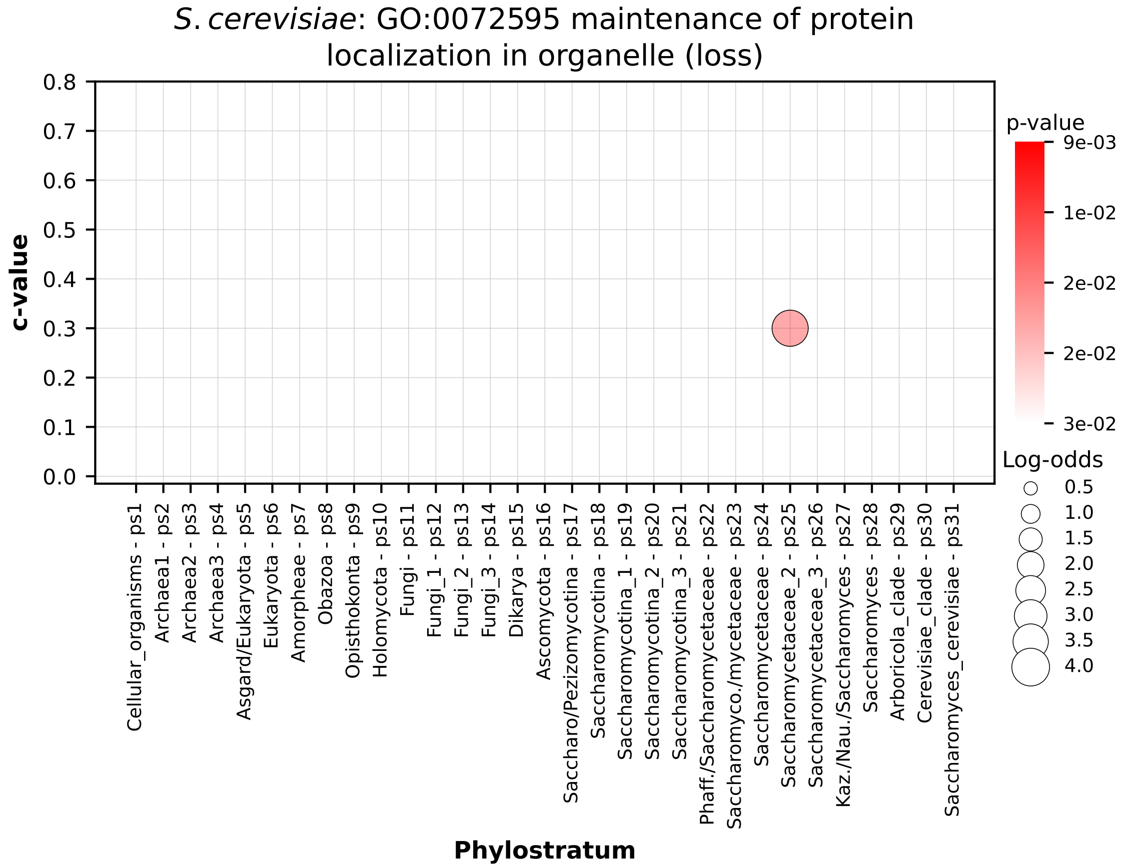

S. cerevisiae: GO:0072595 maintenance of protein localization in organelle (loss)
c-value
0.8
0.7
0.6
0.5
0.4
0.3
0.2
0.1
0.0
Phylostratum
Cellular_organisms - ps1
Archaea1 - ps2
Archaea2 - ps3
Archaea3 - ps4
Asgard/Eukaryota - ps5
Eukaryota - ps6
Amorpheae - ps7
Obazoa - ps8
Opisthokonta - ps9
Holomycota - ps10
Fungi - ps11
Fungi_1 - ps12
Fungi_2 - ps13
Fungi_3 - ps14
Dikarya - ps15
Ascomycota - ps16
Saccharo/Pezizomycotina - ps17
Saccharomycotina - ps18
Saccharomycotina_1 - ps19
Saccharomycotina_2 - ps20
Saccharomycotina_3 - ps21
Phaff./Saccharomycetaceae - ps22
Saccharomyco./mycetaceae - ps23
Saccharomycetaceae - ps24
Saccharomycetaceae_2 - ps25
Saccharomycetaceae_3 - ps26
Kaz./Nau./Saccharomyces - ps27
Saccharomyces - ps28
Arboricola_clade - ps29
Cerevisiae_clade - ps30
Saccharomyces_cerevisiae - ps31
p-value
9e-03
1e-02
2e-02
2e-02
3e-02
Log-odds
0.5
1.0
1.5
2.0
2.5
3.0
3.5
4.0

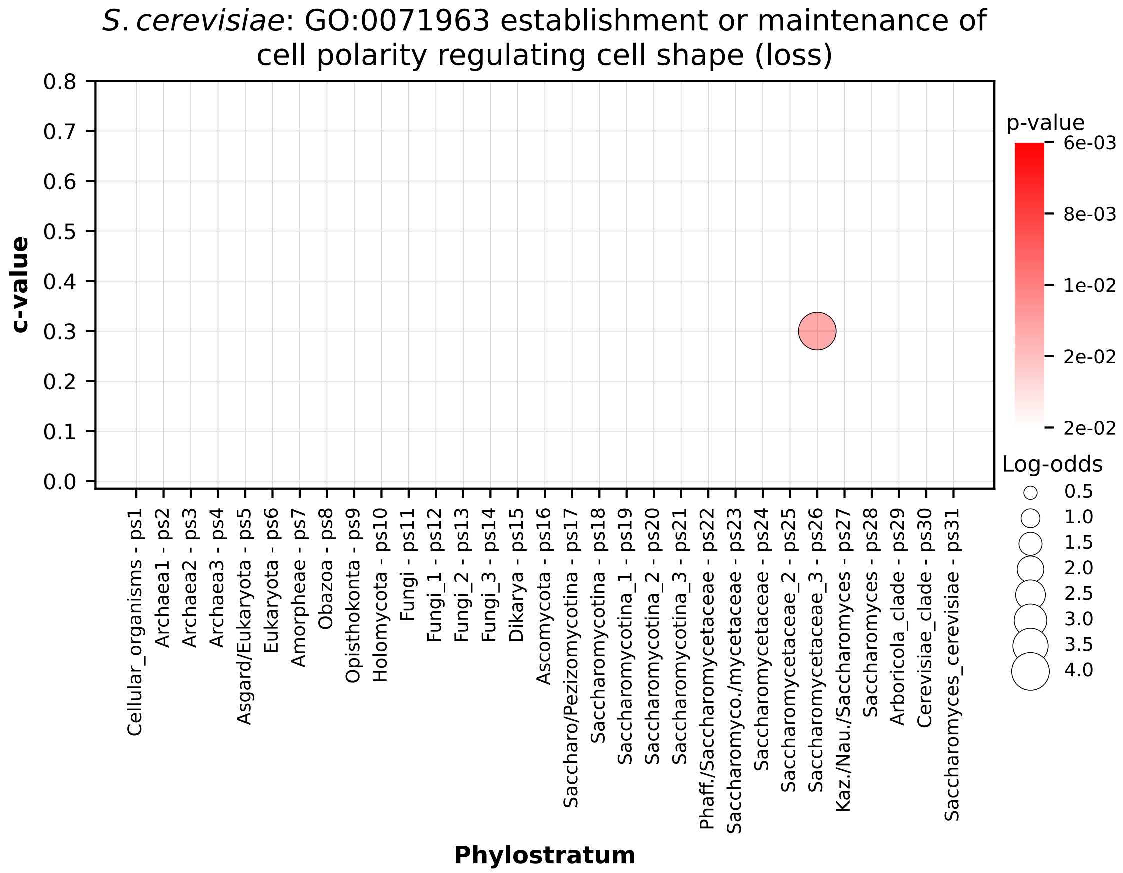

S. cerevisiae: GO:0071963 establishment or maintenance of cell polarity regulating cell shape (loss)
c-value
0.8
0.7
0.6
0.5
0.4
0.3
0.2
0.1
0.0
Phylostratum
Cellular_organisms - ps1
Archaea1 - ps2
Archaea2 - ps3
Archaea3 - ps4
Asgard/Eukaryota - ps5
Eukaryota - ps6
Amorpheae - ps7
Obazoa - ps8
Opisthokonta - ps9
Holomycota - ps10
Fungi - ps11
Fungi_1 - ps12
Fungi_2 - ps13
Fungi_3 - ps14
Dikarya - ps15
Ascomycota - ps16
Saccharo/Pezizomycotina - ps17
Saccharomycotina - ps18
Saccharomycotina_1 - ps19
Saccharomycotina_2 - ps20
Saccharomycotina_3 - ps21
Phaff./Saccharomycetaceae - ps22
Saccharomyco./mycetaceae - ps23
Saccharomycetaceae - ps24
Saccharomycetaceae_2 - ps25
Saccharomycetaceae_3 - ps26
Kaz./Nau./Saccharomyces - ps27
Saccharomyces - ps28
Arboricola_clade - ps29
Cerevisiae_clade - ps30
Saccharomyces_cerevisiae - ps31
p-value
6e-03
8e-03
1e-02
2e-02
2e-02
Log-odds
0.5
1.0
1.5
2.0
2.5
3.0
3.5
4.0

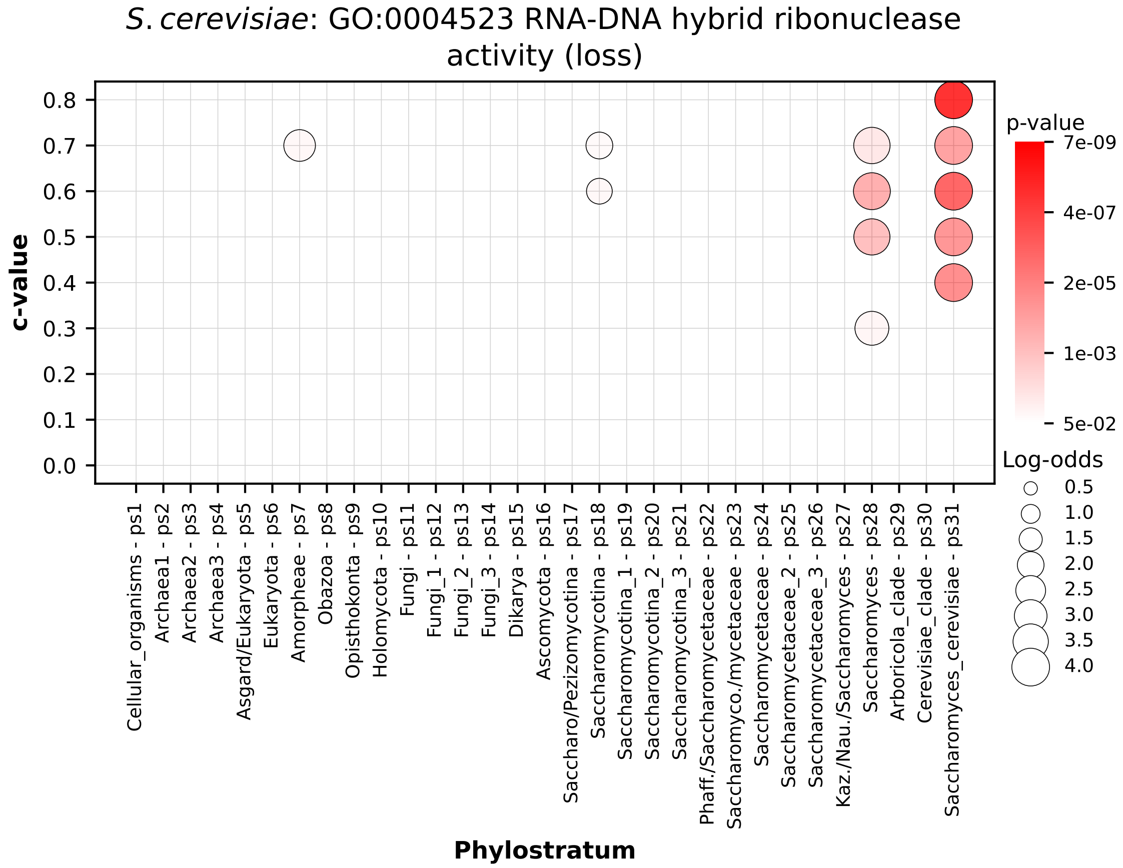

S. cerevisiae: GO:0004523 RNA-DNA hybrid ribonuclease activity (loss)
c-value
0.0
0.1
0.2
0.3
0.4
0.5
0.6
0.7
0.8
Phylostratum
Cellular_organisms - ps1
Archaea1 - ps2
Archaea2 - ps3
Archaea3 - ps4
Asgard/Eukaryota - ps5
Eukaryota - ps6
Amorpheae - ps7
Obazoa - ps8
Opisthokonta - ps9
Holomycota - ps10
Fungi - ps11
Fungi_1 - ps12
Fungi_2 - ps13
Fungi_3 - ps14
Dikarya - ps15
Ascomycota - ps16
Saccharo/Pezizomycotina - ps17
Saccharomycotina - ps18
Saccharomycotina_1 - ps19
Saccharomycotina_2 - ps20
Saccharomycotina_3 - ps21
Phaff./Saccharomycetaceae - ps22
Saccharomyco./mycetaceae - ps23
Saccharomycetaceae - ps24
Saccharomycetaceae_2 - ps25
Saccharomycetaceae_3 - ps26
Kaz./Nau./Saccharomyces - ps27
Saccharomyces - ps28
Arboricola_clade - ps29
Cerevisiae_clade - ps30
Saccharomyces_cerevisiae - ps31
p-value
7e-09
4e-07
2e-05
1e-03
5e-02
Log-odds
0.5
1.0
1.5
2.0
2.5
3.0
3.5
4.0

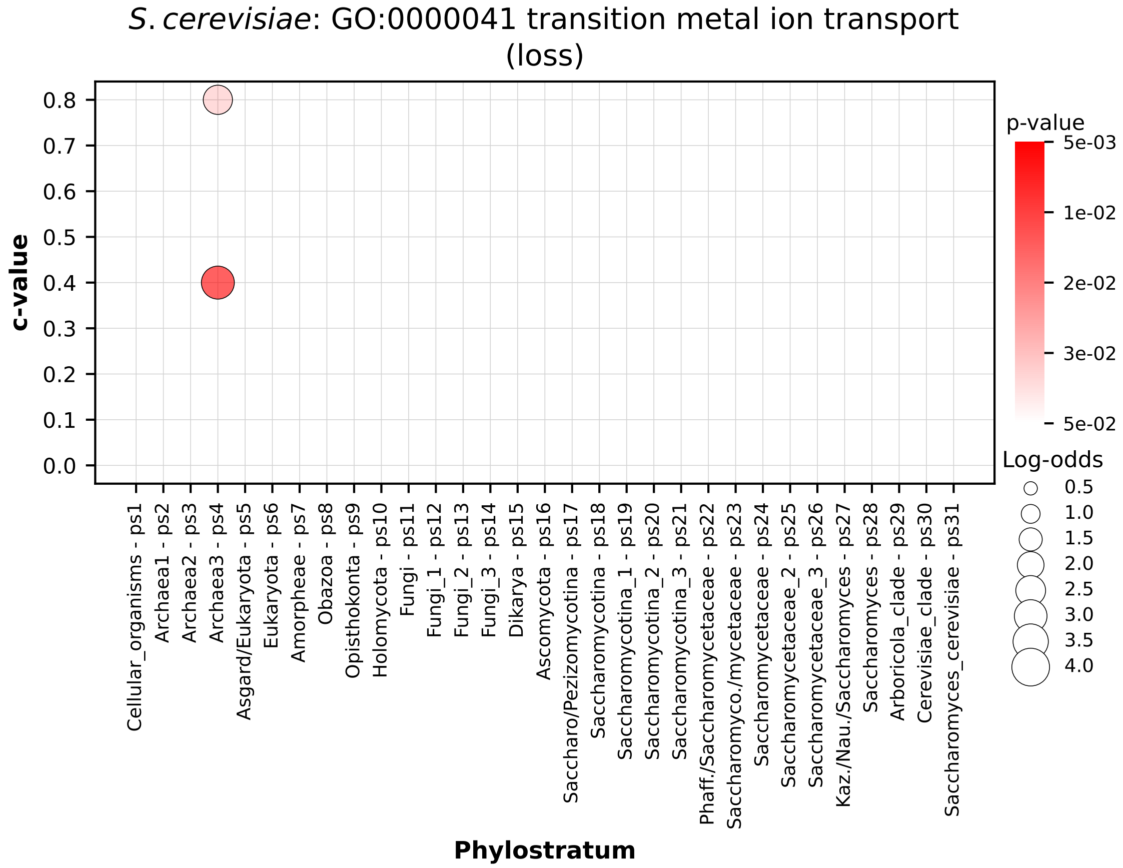

S. cerevisiae: GO:0000041 transition metal ion transport
(loss)
c-value
0.8
0.7
0.6
0.5
0.4
0.3
0.2
0.1
0.0
p-value
5e-03
1e-02
2e-02
3e-02
5e-02
Log-odds
0.5
1.0
1.5
2.0
2.5
3.0
3.5
4.0
Cellular_organisms - ps1
Archaea1 - ps2
Archaea2 - ps3
Archaea3 - ps4
Asgard/Eukaryota - ps5
Eukaryota - ps6
Amorpheae - ps7
Obazoa - ps8
Opisthokonta - ps9
Holomycota - ps10
Fungi - ps11
Fungi_1 - ps12
Fungi_2 - ps13
Fungi_3 - ps14
Dikarya - ps15
Ascomycota - ps16
Saccharo/Pezizomycotina - ps17
Saccharomycotina - ps18
Saccharomycotina_1 - ps19
Saccharomycotina_2 - ps20
Saccharomycotina_3 - ps21
Phaff./Saccharomycetaceae - ps22
Saccharomyco./mycetaceae - ps23
Saccharomycetaceae - ps24
Saccharomycetaceae_2 - ps25
Saccharomycetaceae_3 - ps26
Kaz./Nau./Saccharomyces - ps27
Saccharomyces - ps28
Arboricola_clade - ps29
Cerevisiae_clade - ps30
Saccharomyces_cerevisiae - ps31
Phylostratum

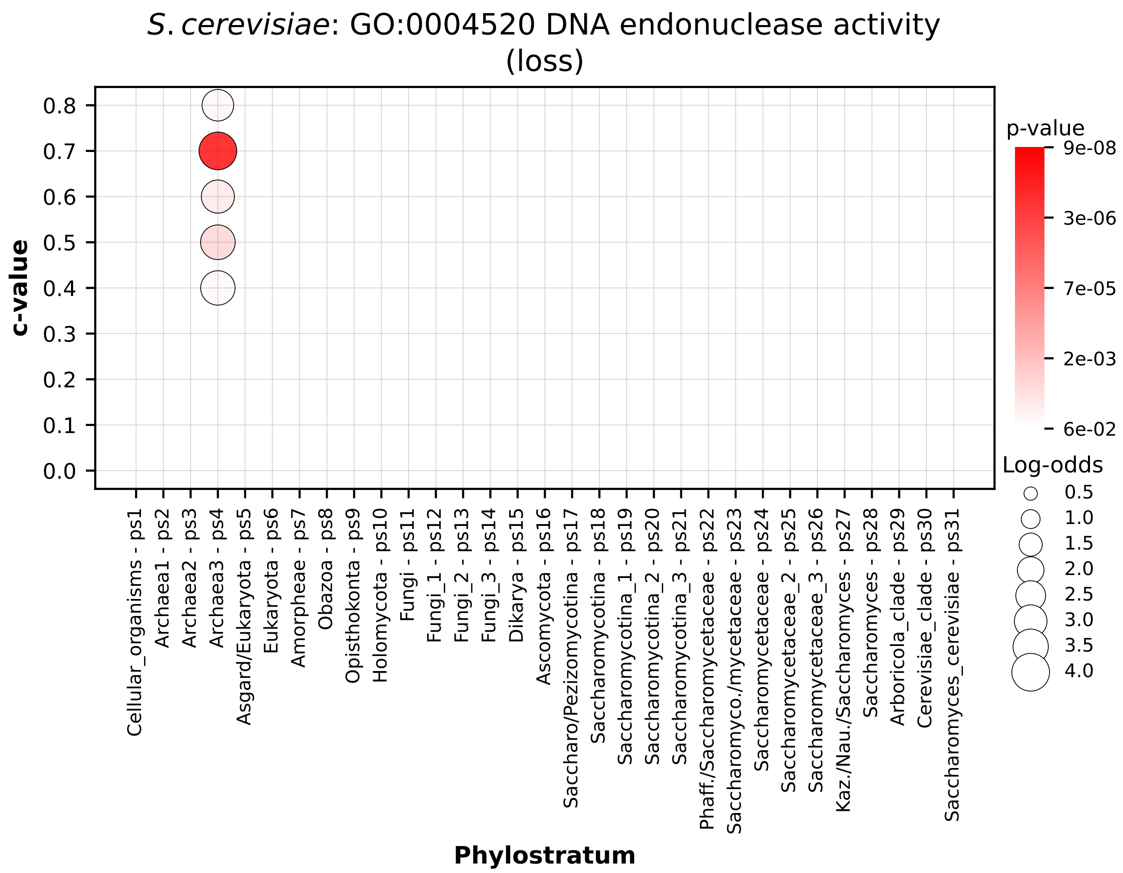

S. cerevisiae: GO:0004520 DNA endonuclease activity
(loss)
c-value
0.8
0.7
0.6
0.5
0.4
0.3
0.2
0.1
0.0
p-value
9e-08
3e-06
7e-05
2e-03
6e-02
Log-odds
0.5
1.0
1.5
2.0
2.5
3.0
3.5
4.0
Cellular_organisms - ps1
Archaea1 - ps2
Archaea2 - ps3
Archaea3 - ps4
Asgard/Eukaryota - ps5
Eukaryota - ps6
Amorpheae - ps7
Obazoa - ps8
Opisthokonta - ps9
Holomycota - ps10
Fungi - ps11
Fungi_1 - ps12
Fungi_2 - ps13
Fungi_3 - ps14
Dikarya - ps15
Ascomycota - ps16
Saccharo/Pezizomycotina - ps17
Saccharomycotina - ps18
Saccharomycotina_1 - ps19
Saccharomycotina_2 - ps20
Saccharomycotina_3 - ps21
Phaff./Saccharomycetaceae - ps22
Saccharomyco./mycetaceae - ps23
Saccharomycetaceae - ps24
Saccharomycetaceae_2 - ps25
Saccharomycetaceae_3 - ps26
Kaz./Nau./Saccharomyces - ps27
Saccharomyces - ps28
Arboricola_clade - ps29
Cerevisiae_clade - ps30
Saccharomyces_cerevisiae - ps31
Phylostratum

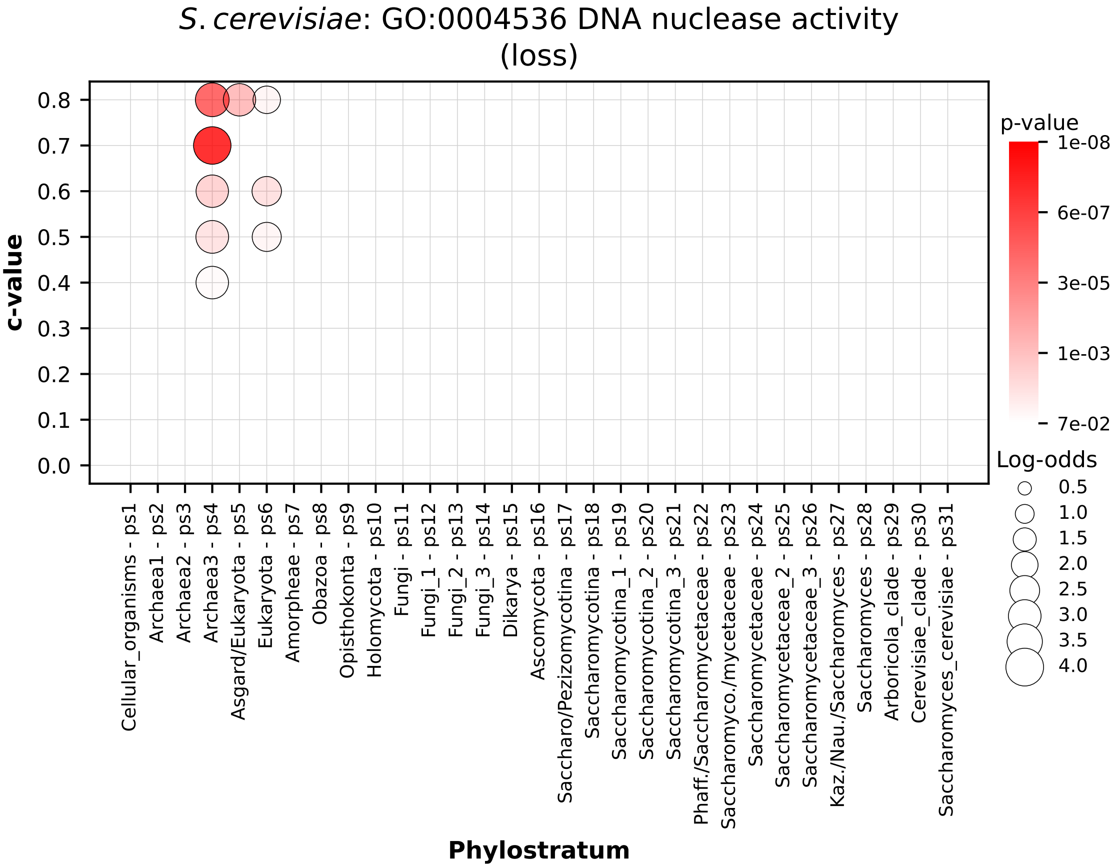
S. cerevisiae: GO:0004536 DNA nuclease activity
(loss)
c-value
0.8
0.7
0.6
0.5
0.4
0.3
0.2
0.1
0.0
Phylostratum
Cellular_organisms - ps1
Archaea1 - ps2
Archaea2 - ps3
Archaea3 - ps4
Asgard/Eukaryota - ps5
Eukaryota - ps6
Amorpheae - ps7
Obazoa - ps8
Opisthokonta - ps9
Holomycota - ps10
Fungi - ps11
Fungi_1 - ps12
Fungi_2 - ps13
Fungi_3 - ps14
Dikarya - ps15
Ascomycota - ps16
Saccharo/Pezizomycotina - ps17
Saccharomycotina - ps18
Saccharomycotina_1 - ps19
Saccharomycotina_2 - ps20
Saccharomycotina_3 - ps21
Phaff./Saccharomycetaceae - ps22
Saccharomyco./mycetaceae - ps23
Saccharomycetaceae - ps24
Saccharomycetaceae_2 - ps25
Saccharomycetaceae_3 - ps26
Kaz./Nau./Saccharomyces - ps27
Saccharomyces - ps28
Arboricola_clade - ps29
Cerevisiae_clade - ps30
Saccharomyces_cerevisiae - ps31
p-value
1e-08
6e-07
3e-05
1e-03
7e-02
Log-odds
0.5
1.0
1.5
2.0
2.5
3.0
3.5
4.0

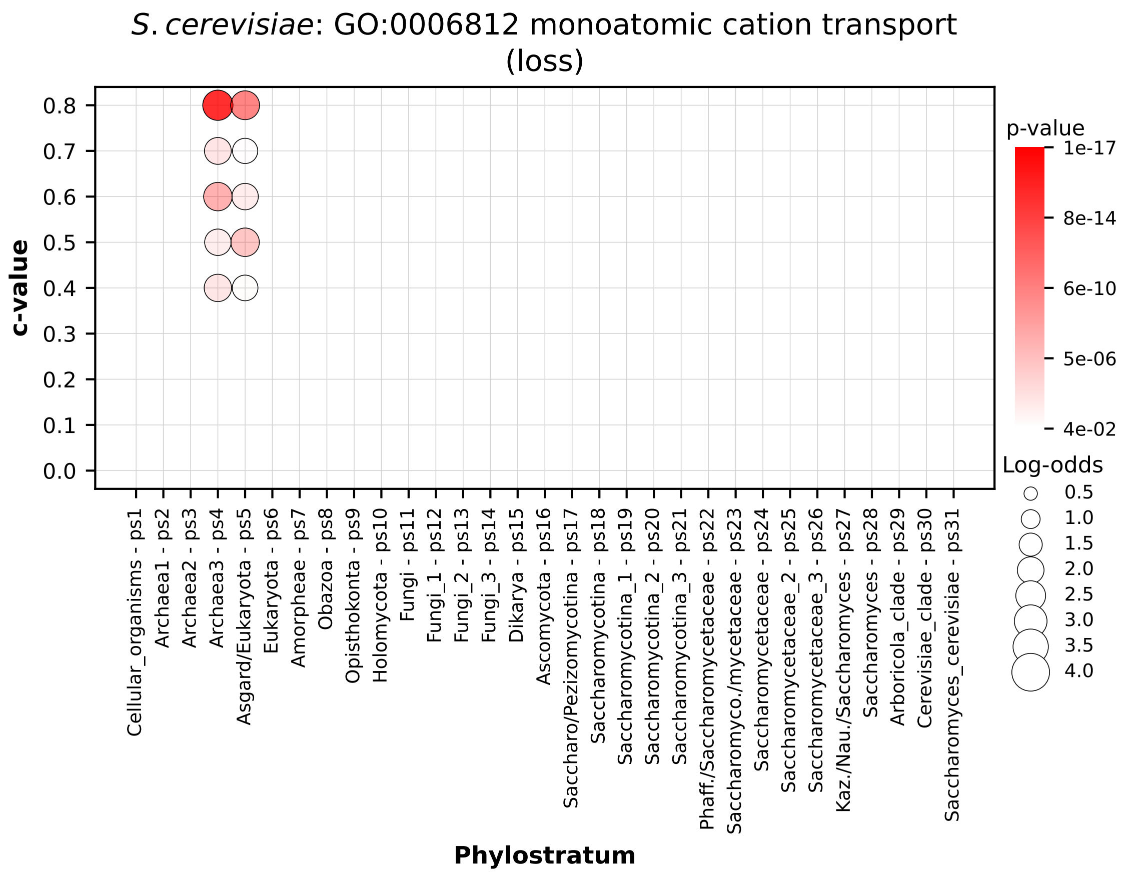
S. cerevisiae: GO:0006812 monoatomic cation transport
(loss)
c-value
0.8
0.7
0.6
0.5
0.4
0.3
0.2
0.1
0.0
p-value
1e-17
8e-14
6e-10
5e-06
4e-02
Log-odds
0.5
1.0
1.5
2.0
2.5
3.0
3.5
4.0
Cellular_organisms - ps1
Archaea1 - ps2
Archaea2 - ps3
Archaea3 - ps4
Asgard/Eukaryota - ps5
Eukaryota - ps6
Amorpheae - ps7
Obazoa - ps8
Opisthokonta - ps9
Holomycota - ps10
Fungi - ps11
Fungi_1 - ps12
Fungi_2 - ps13
Fungi_3 - ps14
Dikarya - ps15
Ascomycota - ps16
Saccharo/Pezizomycotina - ps17
Saccharomycotina - ps18
Saccharomycotina_1 - ps19
Saccharomycotina_2 - ps20
Saccharomycotina_3 - ps21
Phaff./Saccharomycetaceae - ps22
Saccharomyco./mycetaceae - ps23
Saccharomycetaceae - ps24
Saccharomycetaceae_2 - ps25
Saccharomycetaceae_3 - ps26
Kaz./Nau./Saccharomyces - ps27
Saccharomyces - ps28
Arboricola_clade - ps29
Cerevisiae_clade - ps30
Saccharomyces_cerevisiae - ps31
Phylostratum

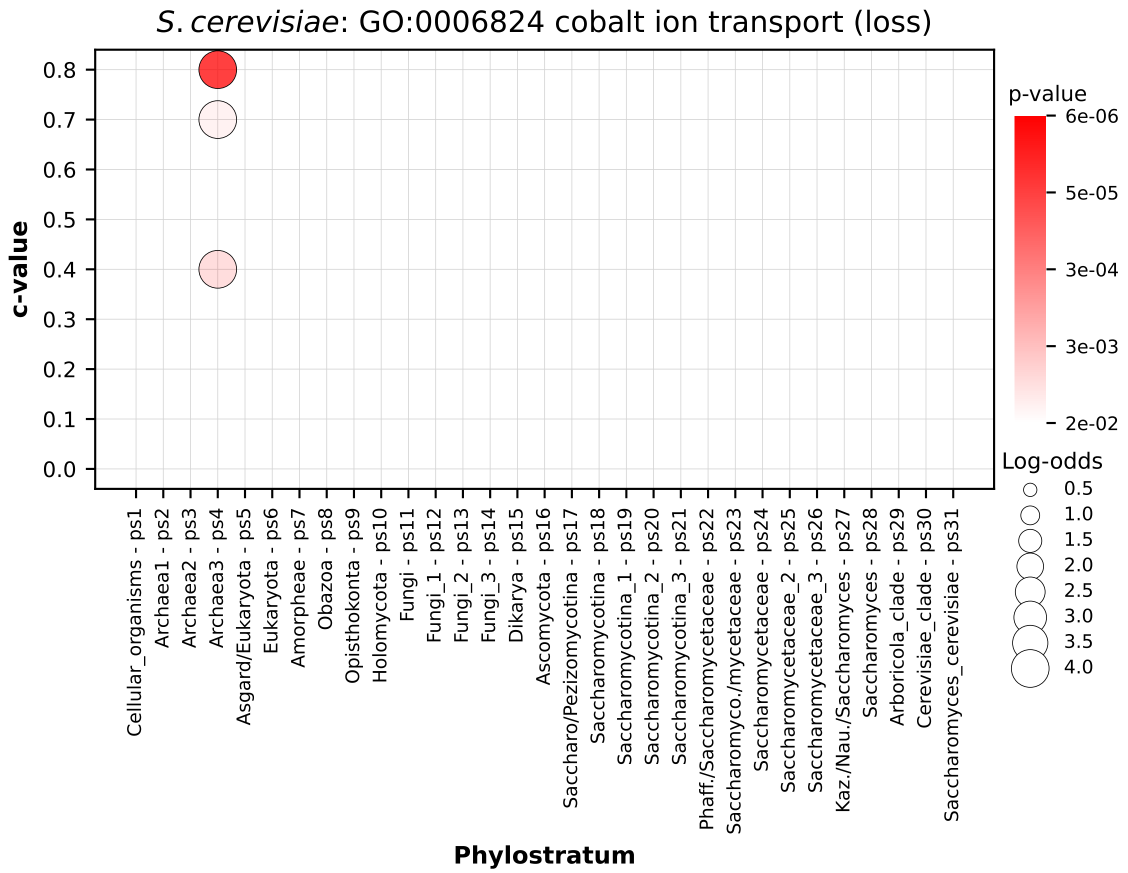

S. cerevisiae: GO:0006824 cobalt ion transport (loss)
c-value
0.8
0.7
0.6
0.5
0.4
0.3
0.2
0.1
0.0
p-value
6e-06
5e-05
3e-04
3e-03
2e-02
Log-odds
0.5
1.0
1.5
2.0
2.5
3.0
3.5
4.0
Cellular_organisms - ps1
Archaea1 - ps2
Archaea2 - ps3
Archaea3 - ps4
Asgard/Eukaryota - ps5
Eukaryota - ps6
Amorpheae - ps7
Obazoa - ps8
Opisthokonta - ps9
Holomycota - ps10
Fungi - ps11
Fungi_1 - ps12
Fungi_2 - ps13
Fungi_3 - ps14
Dikarya - ps15
Ascomycota - ps16
Saccharo/Pezizomycotina - ps17
Saccharomycotina - ps18
Saccharomycotina_1 - ps19
Saccharomycotina_2 - ps20
Saccharomycotina_3 - ps21
Phaff./Saccharomycetaceae - ps22
Saccharomyco./mycetaceae - ps23
Saccharomycetaceae - ps24
Saccharomycetaceae_2 - ps25
Saccharomycetaceae_3 - ps26
Kaz./Nau./Saccharomyces - ps27
Saccharomyces - ps28
Arboricola_clade - ps29
Cerevisiae_clade - ps30
Saccharomyces_cerevisiae - ps31
Phylostratum

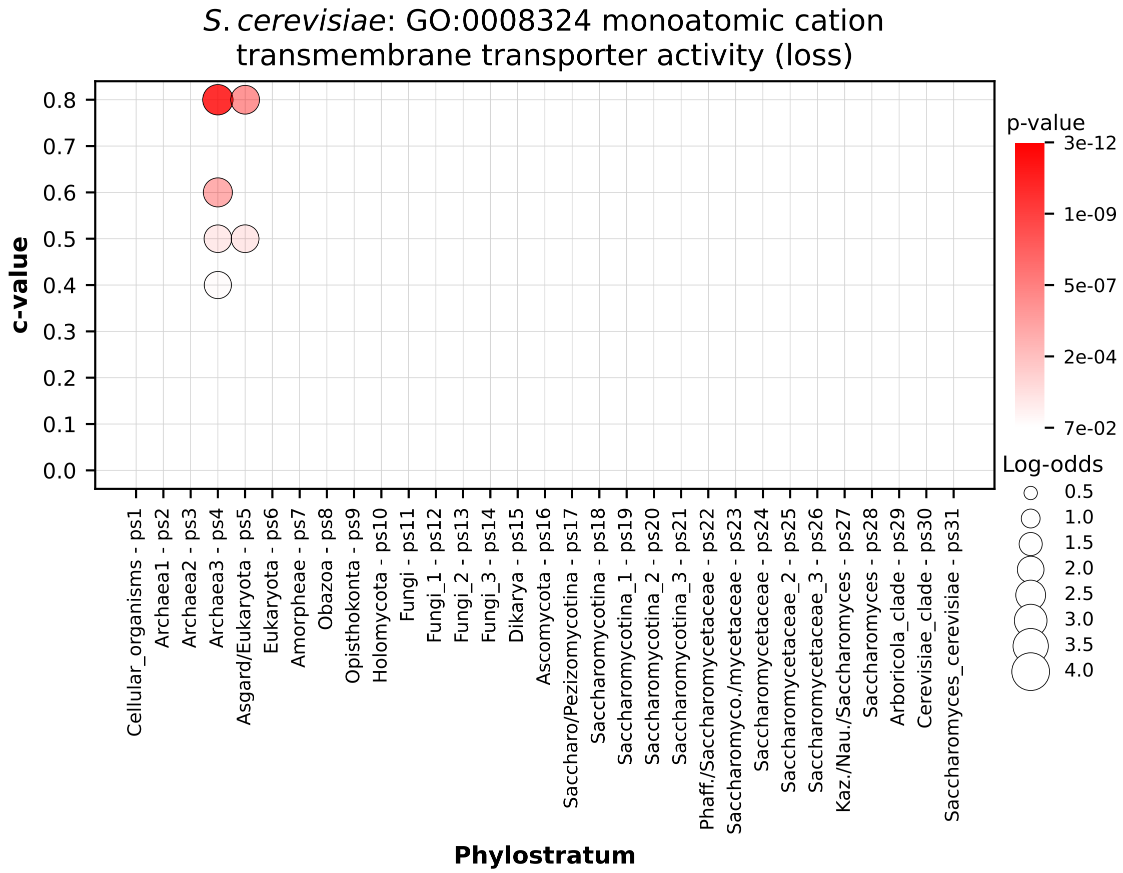

S. cerevisiae: GO:0008324 monoatomic cation transmembrane transporter activity (loss)
c-value
0.8
0.7
0.6
0.5
0.4
0.3
0.2
0.1
0.0
p-value
3e-12
1e-09
5e-07
2e-04
7e-02
Log-odds
0.5
1.0
1.5
2.0
2.5
3.0
3.5
4.0
Cellular_organisms - ps1
Archaea1 - ps2
Archaea2 - ps3
Archaea3 - ps4
Asgard/Eukaryota - ps5
Eukaryota - ps6
Amorpheae - ps7
Obazoa - ps8
Opisthokonta - ps9
Holomycota - ps10
Fungi - ps11
Fungi_1 - ps12
Fungi_2 - ps13
Fungi_3 - ps14
Dikarya - ps15
Ascomycota - ps16
Saccharo/Pezizomycotina - ps17
Saccharomycotina - ps18
Saccharomycotina_1 - ps19
Saccharomycotina_2 - ps20
Saccharomycotina_3 - ps21
Phaff./Saccharomycetaceae - ps22
Saccharomyco./mycetaceae - ps23
Saccharomycetaceae - ps24
Saccharomycetaceae_2 - ps25
Saccharomycetaceae_3 - ps26
Kaz./Nau./Saccharomyces - ps27
Saccharomyces - ps28
Arboricola_clade - ps29
Cerevisiae_clade - ps30
Saccharomyces_cerevisiae - ps31
Phylostratum

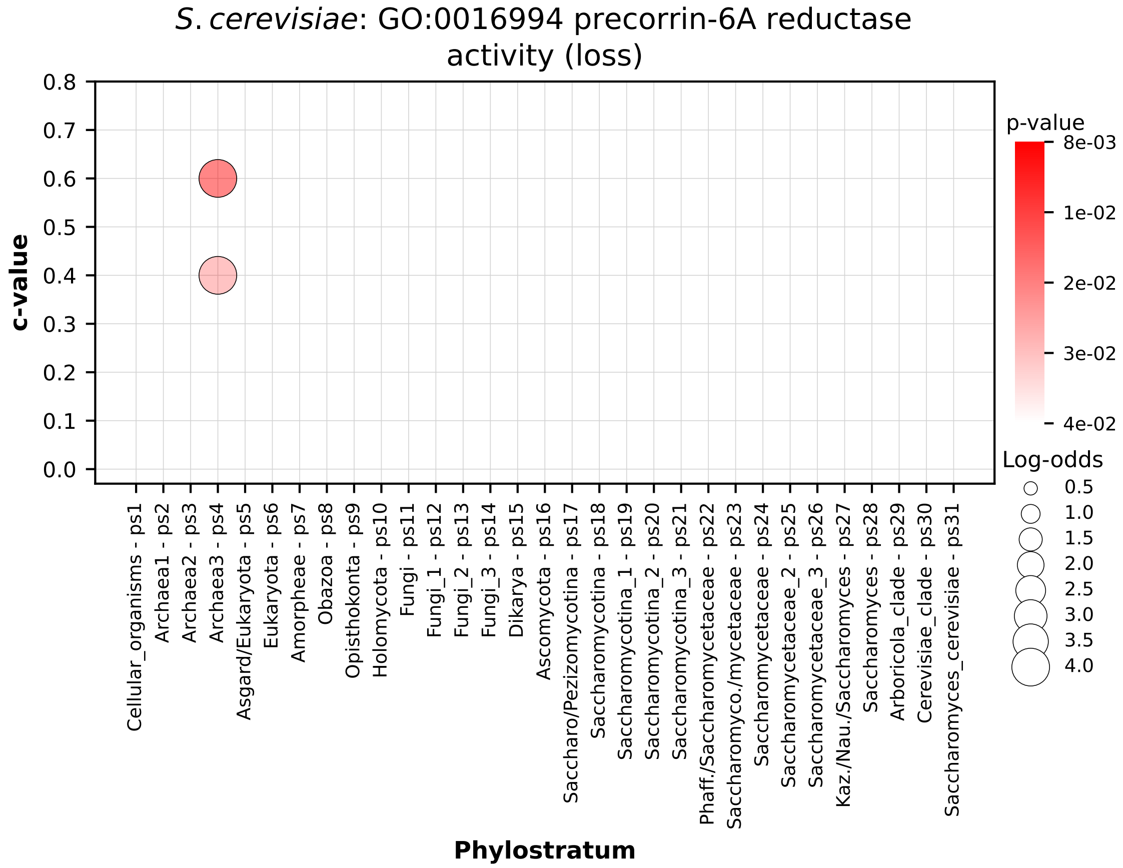

S. cerevisiae: GO:0016994 precorrin-6A reductase activity (loss)
c-value
0.8
0.7
0.6
0.5
0.4
0.3
0.2
0.1
0.0
p-value
8e-03
1e-02
2e-02
3e-02
4e-02
Log-odds
0.5
1.0
1.5
2.0
2.5
3.0
3.5
4.0
Cellular_organisms - ps1
Archaea1 - ps2
Archaea2 - ps3
Archaea3 - ps4
Asgard/Eukaryota - ps5
Eukaryota - ps6
Amorpheae - ps7
Obazoa - ps8
Opisthokonta - ps9
Holomycota - ps10
Fungi - ps11
Fungi_1 - ps12
Fungi_2 - ps13
Fungi_3 - ps14
Dikarya - ps15
Ascomycota - ps16
Saccharo/Pezizomycotina - ps17
Saccharomycotina - ps18
Saccharomycotina_1 - ps19
Saccharomycotina_2 - ps20
Saccharomycotina_3 - ps21
Phaff./Saccharomycetaceae - ps22
Saccharomyco./mycetaceae - ps23
Saccharomycetaceae - ps24
Saccharomycetaceae_2 - ps25
Saccharomycetaceae_3 - ps26
Kaz./Nau./Saccharomyces - ps27
Saccharomyces - ps28
Arboricola_clade - ps29
Cerevisiae_clade - ps30
Saccharomyces_cerevisiae - ps31
Phylostratum

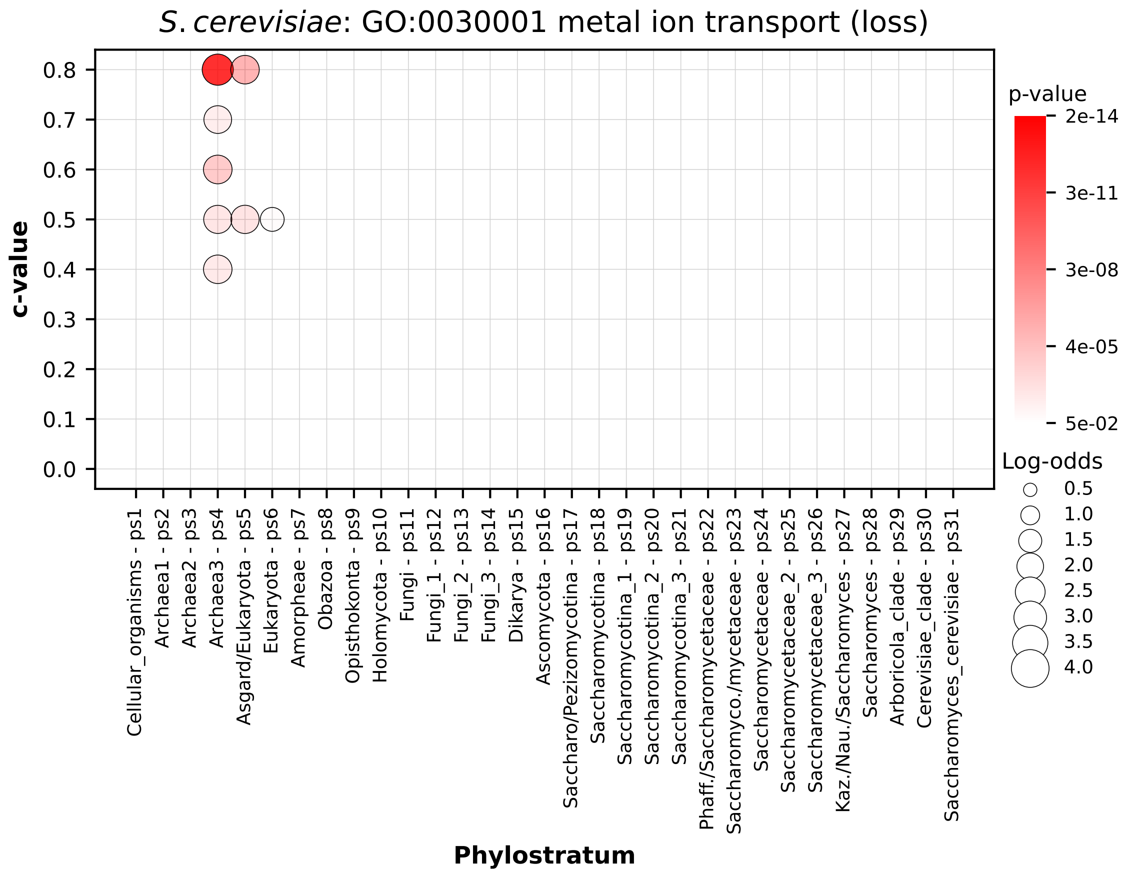

S. cerevisiae: GO:0030001 metal ion transport (loss)
c-value
0.0
0.1
0.2
0.3
0.4
0.5
0.6
0.7
0.8
Phylostratum
Cellular_organisms - ps1
Archaea1 - ps2
Archaea2 - ps3
Archaea3 - ps4
Asgard/Eukaryota - ps5
Eukaryota - ps6
Amorpheae - ps7
Obazoa - ps8
Opisthokonta - ps9
Holomycota - ps10
Fungi - ps11
Fungi_1 - ps12
Fungi_2 - ps13
Fungi_3 - ps14
Dikarya - ps15
Ascomycota - ps16
Saccharo/Pezizomycotina - ps17
Saccharomycotina - ps18
Saccharomycotina_1 - ps19
Saccharomycotina_2 - ps20
Saccharomycotina_3 - ps21
Phaff./Saccharomycetaceae - ps22
Saccharomyco./mycetaceae - ps23
Saccharomycetaceae - ps24
Saccharomycetaceae_2 - ps25
Saccharomycetaceae_3 - ps26
Kaz./Nau./Saccharomyces - ps27
Saccharomyces - ps28
Arboricola_clade - ps29
Cerevisiae_clade - ps30
Saccharomyces_cerevisiae - ps31
p-value
2e-14
3e-11
3e-08
4e-05
5e-02
Log-odds
0.5
1.0
1.5
2.0
2.5
3.0
3.5
4.0

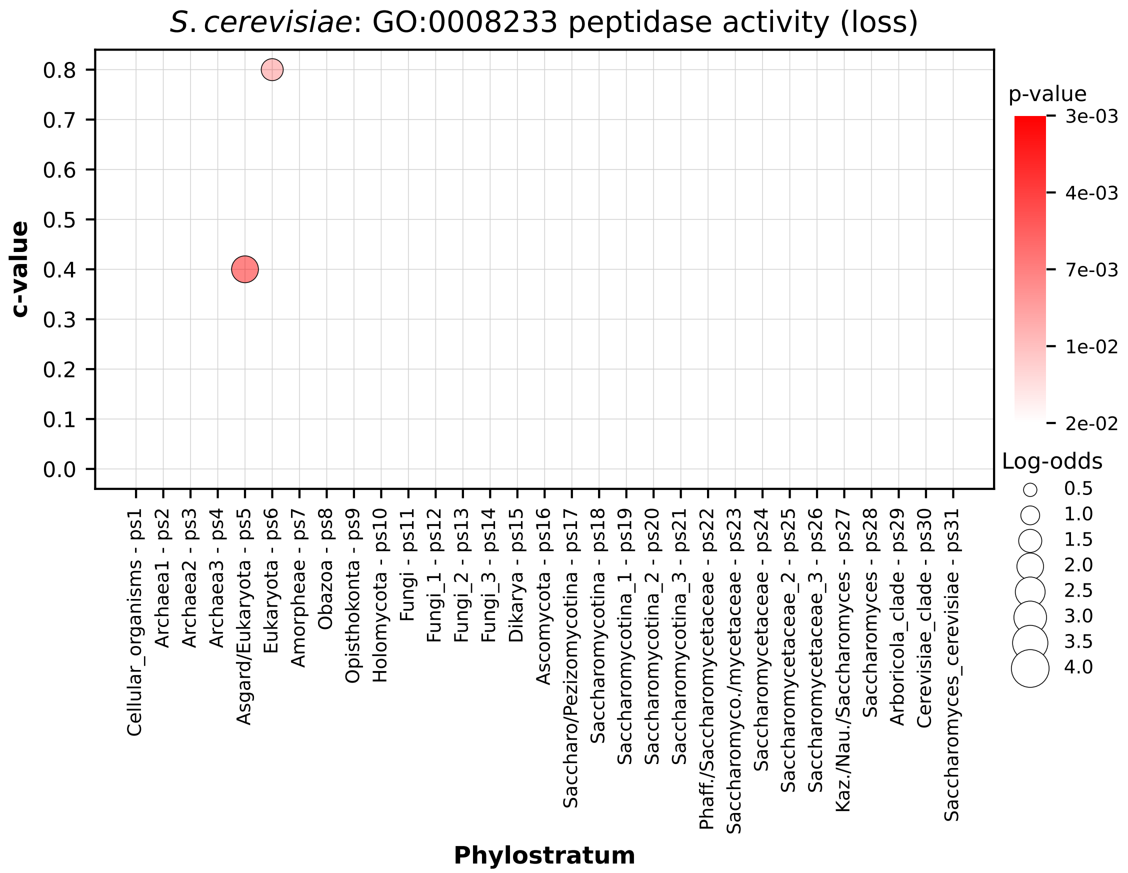

S. cerevisiae: GO:0008233 peptidase activity (loss)
c-value
0.0
0.1
0.2
0.3
0.4
0.5
0.6
0.7
0.8
Phylostratum
Cellular_organisms - ps1
Archaea1 - ps2
Archaea2 - ps3
Archaea3 - ps4
Asgard/Eukaryota - ps5
Eukaryota - ps6
Amorpheae - ps7
Obazoa - ps8
Opisthokonta - ps9
Holomycota - ps10
Fungi - ps11
Fungi_1 - ps12
Fungi_2 - ps13
Fungi_3 - ps14
Dikarya - ps15
Ascomycota - ps16
Saccharo/Pezizomycotina - ps17
Saccharomycotina - ps18
Saccharomycotina_1 - ps19
Saccharomycotina_2 - ps20
Saccharomycotina_3 - ps21
Phaff./Saccharomycetaceae - ps22
Saccharomyco./mycetaceae - ps23
Saccharomycetaceae - ps24
Saccharomycetaceae_2 - ps25
Saccharomycetaceae_3 - ps26
Kaz./Nau./Saccharomyces - ps27
Saccharomyces - ps28
Arboricola_clade - ps29
Cerevisiae_clade - ps30
Saccharomyces_cerevisiae - ps31
p-value
3e-03
4e-03
7e-03
1e-02
2e-02
Log-odds
0.5
1.0
1.5
2.0
2.5
3.0
3.5
4.0

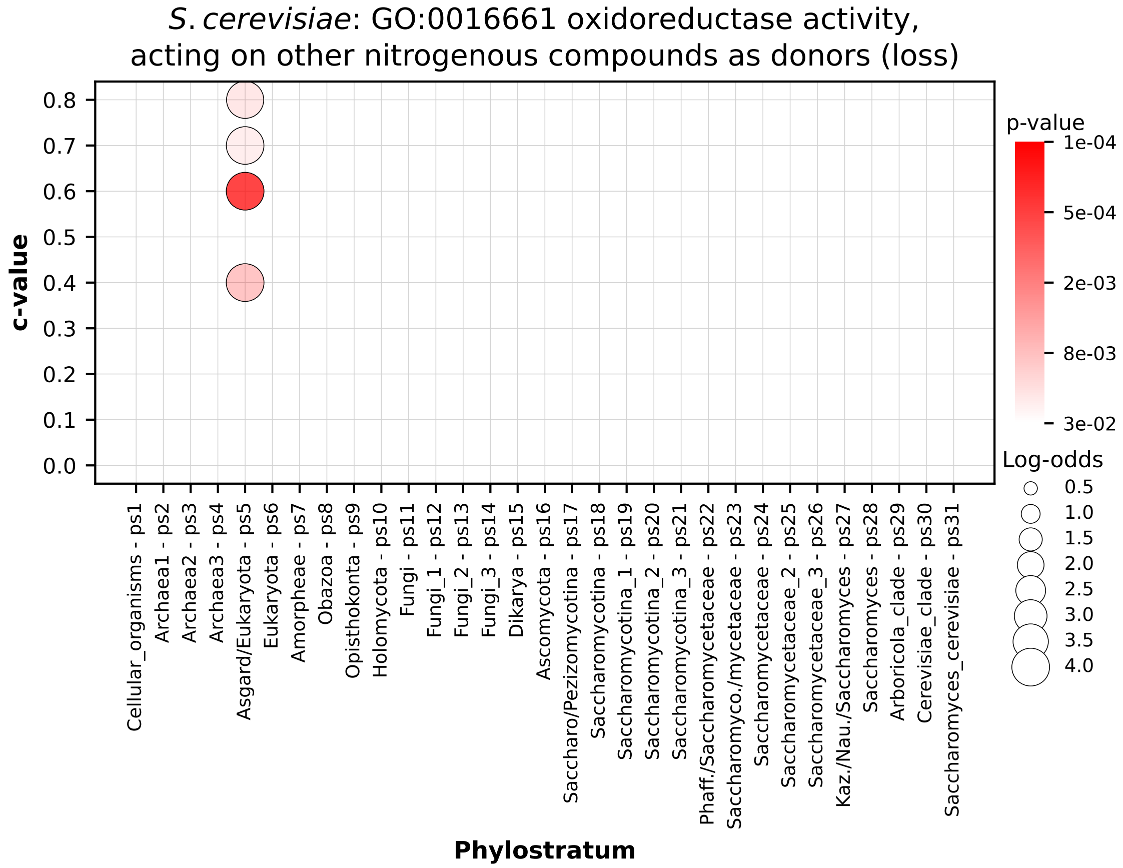
S. cerevisiae: GO:0016661 oxidoreductase activity,
acting on other nitrogenous compounds as donors (loss)
c-value
0.0
0.1
0.2
0.3
0.4
0.5
0.6
0.7
0.8
Phylostratum
Cellular_organisms - ps1
Archaea1 - ps2
Archaea2 - ps3
Archaea3 - ps4
Asgard/Eukaryota - ps5
Eukaryota - ps6
Amorpheae - ps7
Obazoa - ps8
Opisthokonta - ps9
Holomycota - ps10
Fungi - ps11
Fungi_1 - ps12
Fungi_2 - ps13
Fungi_3 - ps14
Dikarya - ps15
Ascomycota - ps16
Saccharo/Pezizomycotina - ps17
Saccharomycotina - ps18
Saccharomycotina_1 - ps19
Saccharomycotina_2 - ps20
Saccharomycotina_3 - ps21
Phaff./Saccharomycetaceae - ps22
Saccharomyco./mycetaceae - ps23
Saccharomycetaceae - ps24
Saccharomycetaceae_2 - ps25
Saccharomycetaceae_3 - ps26
Kaz./Nau./Saccharomyces - ps27
Saccharomyces - ps28
Arboricola_clade - ps29
Cerevisiae_clade - ps30
Saccharomyces_cerevisiae - ps31
p-value
1e-04
5e-04
2e-03
8e-03
3e-02
Log-odds
0.5
1.0
1.5
2.0
2.5
3.0
3.5
4.0

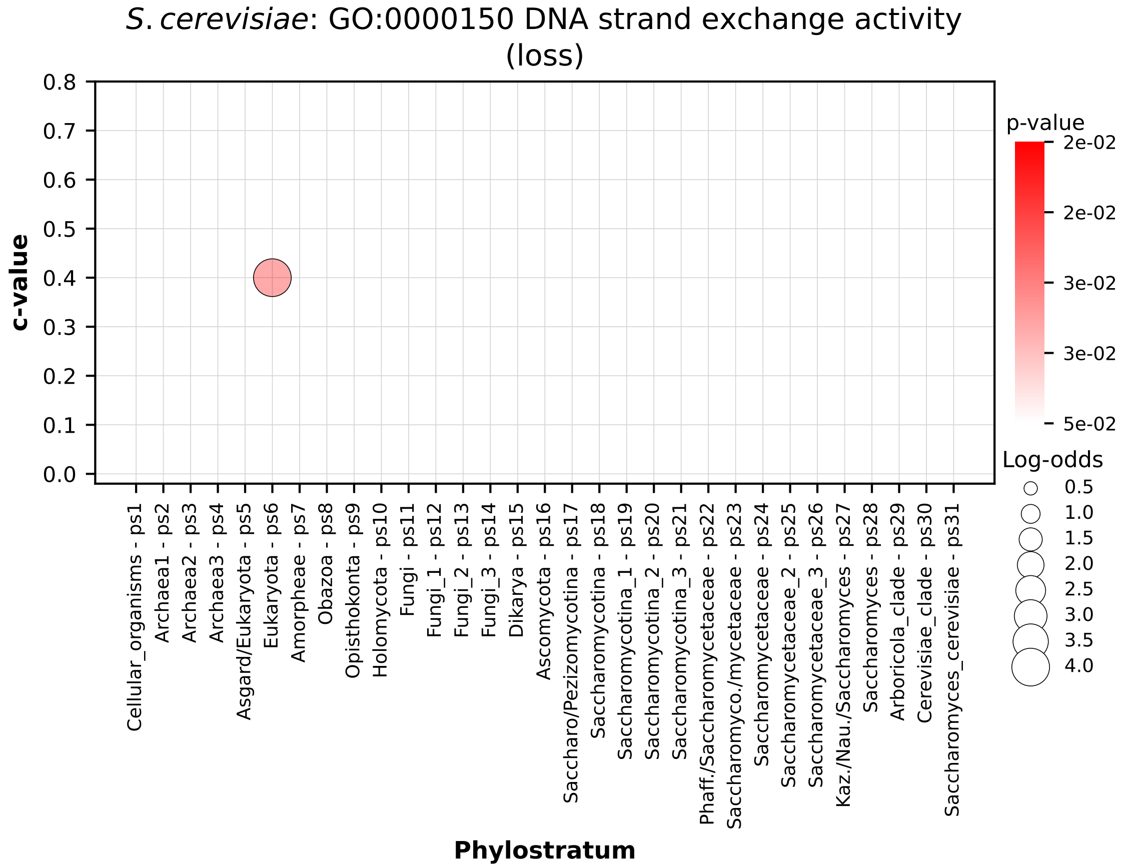

S. cerevisiae: GO:0000150 DNA strand exchange activity
(loss)
c-value
0.8
0.7
0.6
0.5
0.4
0.3
0.2
0.1
0.0
p-value
2e-02
2e-02
3e-02
3e-02
5e-02
Log-odds
0.5
1.0
1.5
2.0
2.5
3.0
3.5
4.0
Cellular_organisms - ps1
Archaea1 - ps2
Archaea2 - ps3
Archaea3 - ps4
Asgard/Eukaryota - ps5
Eukaryota - ps6
Amorpheae - ps7
Obazoa - ps8
Opisthokonta - ps9
Holomycota - ps10
Fungi - ps11
Fungi_1 - ps12
Fungi_2 - ps13
Fungi_3 - ps14
Dikarya - ps15
Ascomycota - ps16
Saccharo/Pezizomycotina - ps17
Saccharomycotina - ps18
Saccharomycotina_1 - ps19
Saccharomycotina_2 - ps20
Saccharomycotina_3 - ps21
Phaff./Saccharomycetaceae - ps22
Saccharomyco./mycetaceae - ps23
Saccharomycetaceae - ps24
Saccharomycetaceae_2 - ps25
Saccharomycetaceae_3 - ps26
Kaz./Nau./Saccharomyces - ps27
Saccharomyces - ps28
Arboricola_clade - ps29
Cerevisiae_clade - ps30
Saccharomyces_cerevisiae - ps31
Phylostratum

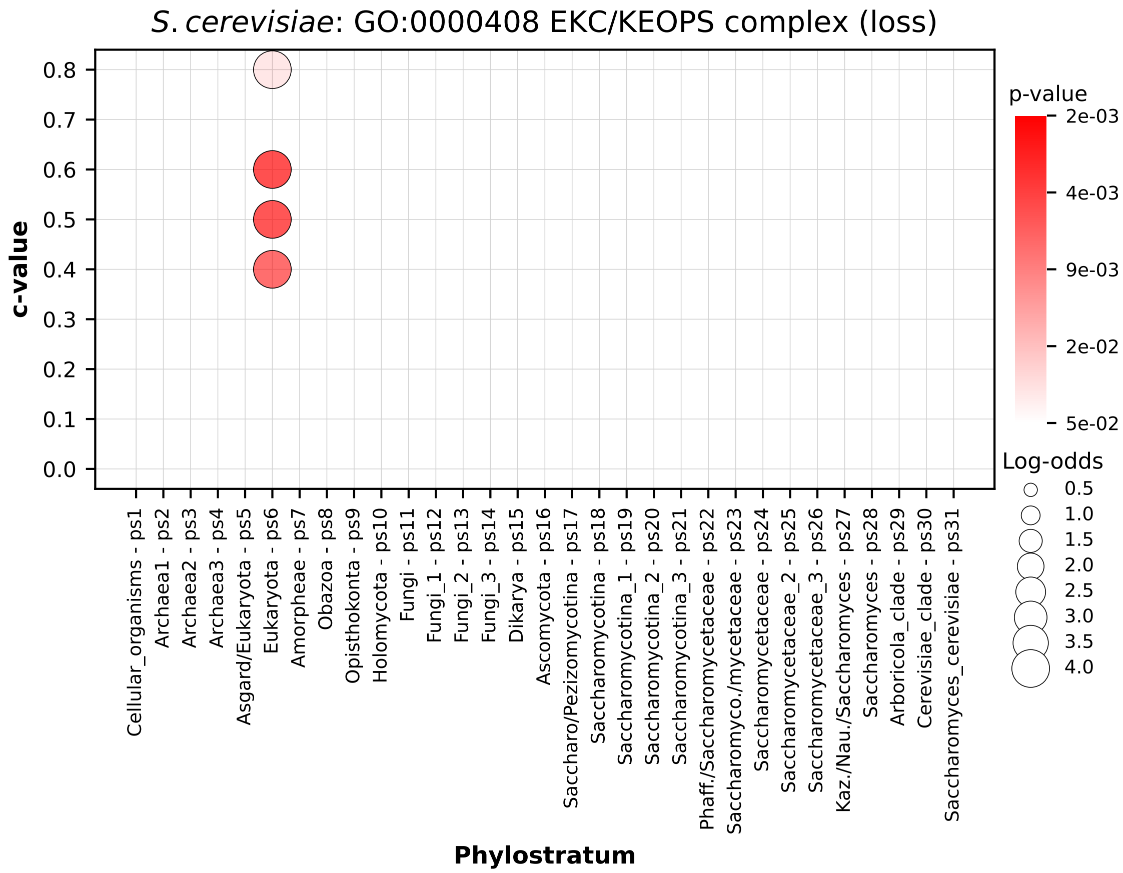

S. cerevisiae: GO:0000408 EKC/KEOPS complex (loss)
c-value
0.8
0.7
0.6
0.5
0.4
0.3
0.2
0.1
0.0
p-value
2e-03
4e-03
9e-03
2e-02
5e-02
Log-odds
0.5
1.0
1.5
2.0
2.5
3.0
3.5
4.0
Cellular_organisms - ps1
Archaea1 - ps2
Archaea2 - ps3
Archaea3 - ps4
Asgard/Eukaryota - ps5
Eukaryota - ps6
Amorpheae - ps7
Obazoa - ps8
Opisthokonta - ps9
Holomycota - ps10
Fungi - ps11
Fungi_1 - ps12
Fungi_2 - ps13
Fungi_3 - ps14
Dikarya - ps15
Ascomycota - ps16
Saccharo/Pezizomycotina - ps17
Saccharomycotina - ps18
Saccharomycotina_1 - ps19
Saccharomycotina_2 - ps20
Saccharomycotina_3 - ps21
Phaff./Saccharomycetaceae - ps22
Saccharomyco./mycetaceae - ps23
Saccharomycetaceae - ps24
Saccharomycetaceae_2 - ps25
Saccharomycetaceae_3 - ps26
Kaz./Nau./Saccharomyces - ps27
Saccharomyces - ps28
Arboricola_clade - ps29
Cerevisiae_clade - ps30
Saccharomyces_cerevisiae - ps31
Phylostratum

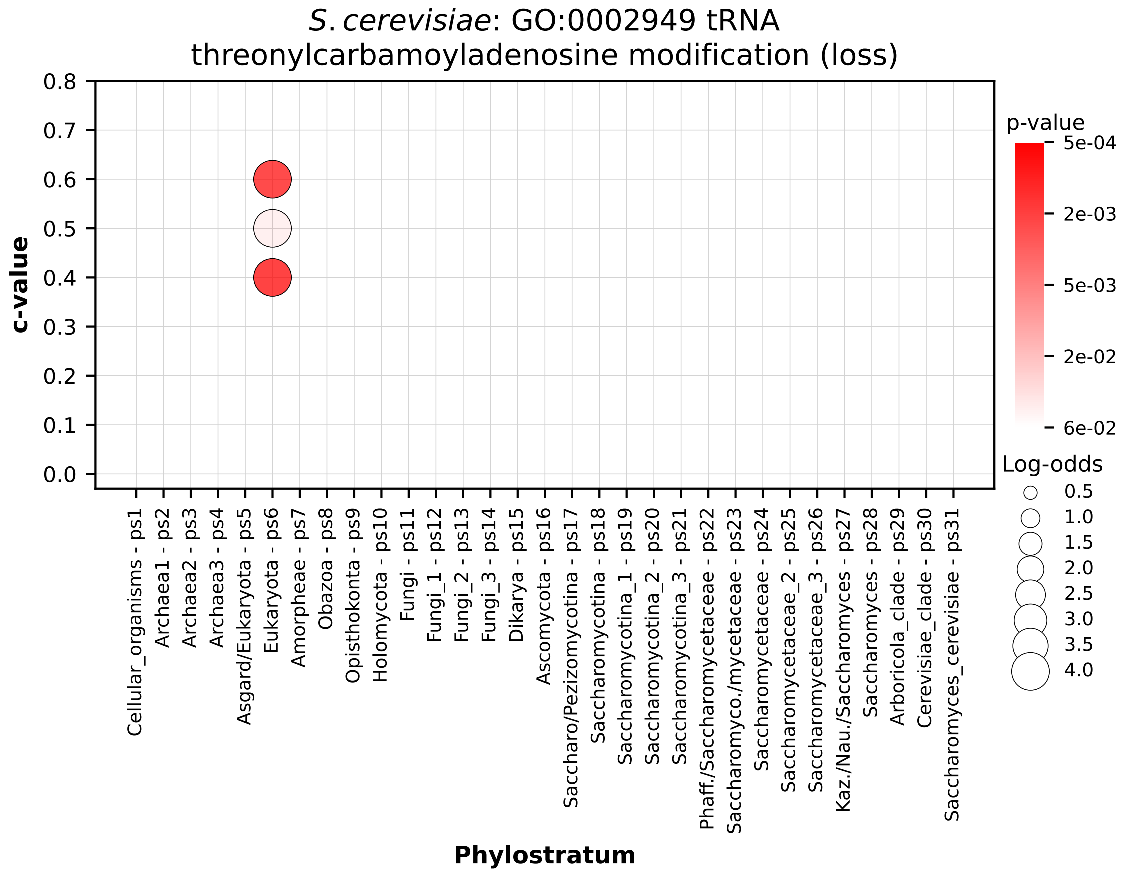

S. cerevisiae: GO:0002949 tRNA threonylcarbamoyladenosine modification (loss)
c-value
0.8
0.7
0.6
0.5
0.4
0.3
0.2
0.1
0.0
p-value
5e-04
2e-03
5e-03
2e-02
6e-02
Log-odds
0.5
1.0
1.5
2.0
2.5
3.0
3.5
4.0
Cellular_organisms - ps1
Archaea1 - ps2
Archaea2 - ps3
Archaea3 - ps4
Asgard/Eukaryota - ps5
Eukaryota - ps6
Amorpheae - ps7
Obazoa - ps8
Opisthokonta - ps9
Holomycota - ps10
Fungi - ps11
Fungi_1 - ps12
Fungi_2 - ps13
Fungi_3 - ps14
Dikarya - ps15
Ascomycota - ps16
Saccharo/Pezizomycotina - ps17
Saccharomycotina - ps18
Saccharomycotina_1 - ps19
Saccharomycotina_2 - ps20
Saccharomycotina_3 - ps21
Phaff./Saccharomycetaceae - ps22
Saccharomyco./mycetaceae - ps23
Saccharomycetaceae - ps24
Saccharomycetaceae_2 - ps25
Saccharomycetaceae_3 - ps26
Kaz./Nau./Saccharomyces - ps27
Saccharomyces - ps28
Arboricola_clade - ps29
Cerevisiae_clade - ps30
Saccharomyces_cerevisiae - ps31
Phylostratum

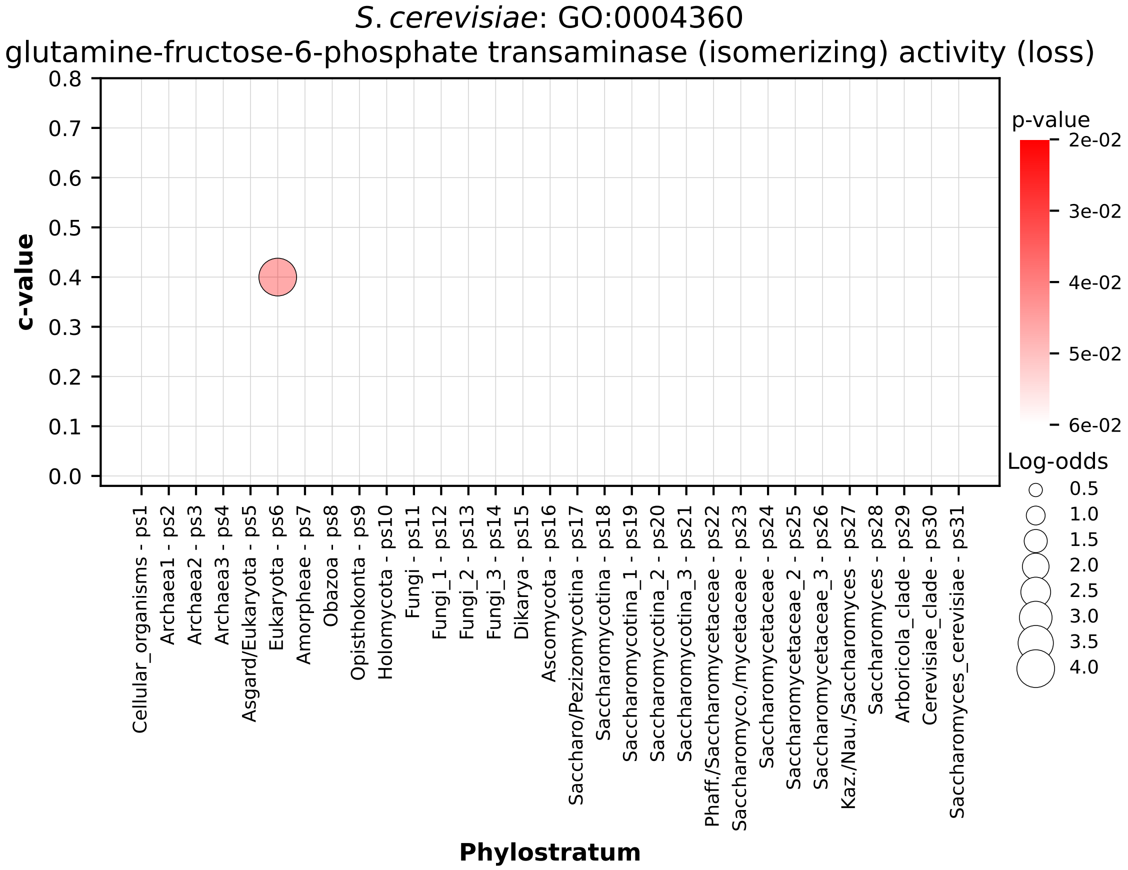

S. cerevisiae: GO:0004360
glutamine-fructose-6-phosphate transaminase (isomerizing) activity (loss)
c-value
0.8
0.7
0.6
0.5
0.4
0.3
0.2
0.1
0.0
p-value
2e-02
3e-02
4e-02
5e-02
6e-02
Log-odds
0.5
1.0
1.5
2.0
2.5
3.0
3.5
4.0
Cellular_organisms - ps1
Archaea1 - ps2
Archaea2 - ps3
Archaea3 - ps4
Asgard/Eukaryota - ps5
Eukaryota - ps6
Amorpheae - ps7
Obazoa - ps8
Opisthokonta - ps9
Holomycota - ps10
Fungi - ps11
Fungi_1 - ps12
Fungi_2 - ps13
Fungi_3 - ps14
Dikarya - ps15
Ascomycota - ps16
Saccharo/Pezizomycotina - ps17
Saccharomycotina - ps18
Saccharomycotina_1 - ps19
Saccharomycotina_2 - ps20
Saccharomycotina_3 - ps21
Phaff./Saccharomycetaceae - ps22
Saccharomyco./mycetaceae - ps23
Saccharomycetaceae - ps24
Saccharomycetaceae_2 - ps25
Saccharomycetaceae_3 - ps26
Kaz./Nau./Saccharomyces - ps27
Saccharomyces - ps28
Arboricola_clade - ps29
Cerevisiae_clade - ps30
Saccharomyces_cerevisiae - ps31
Phylostratum

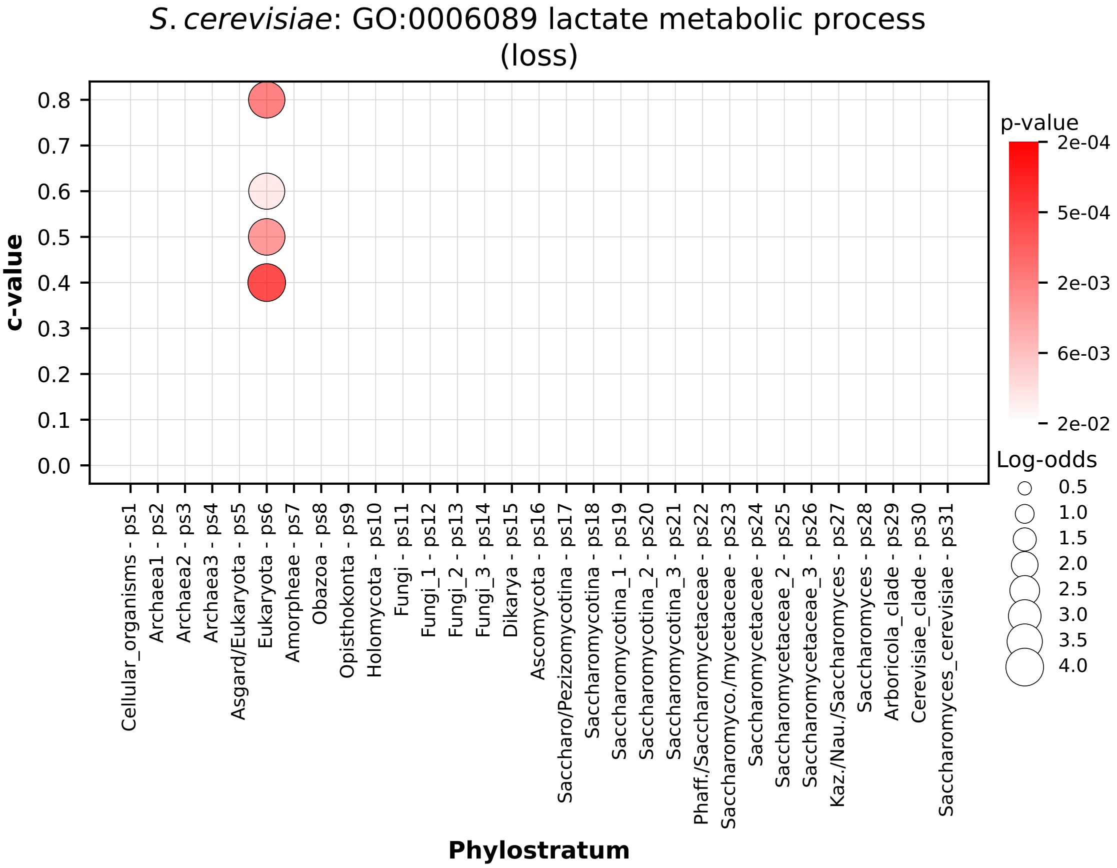
S. cerevisiae: GO:0006089 lactate metabolic process
(loss)
c-value
0.8
0.7
0.6
0.5
0.4
0.3
0.2
0.1
0.0
p-value
2e-04
5e-04
2e-03
6e-03
2e-02
Log-odds
0.5
1.0
1.5
2.0
2.5
3.0
3.5
4.0
Cellular_organisms - ps1
Archaea1 - ps2
Archaea2 - ps3
Archaea3 - ps4
Asgard/Eukaryota - ps5
Eukaryota - ps6
Amorpheae - ps7
Obazoa - ps8
Opisthokonta - ps9
Holomycota - ps10
Fungi - ps11
Fungi_1 - ps12
Fungi_2 - ps13
Fungi_3 - ps14
Dikarya - ps15
Ascomycota - ps16
Saccharo/Pezizomycotina - ps17
Saccharomycotina - ps18
Saccharomycotina_1 - ps19
Saccharomycotina_2 - ps20
Saccharomycotina_3 - ps21
Phaff./Saccharomycetaceae - ps22
Saccharomyco./mycetaceae - ps23
Saccharomycetaceae - ps24
Saccharomycetaceae_2 - ps25
Saccharomycetaceae_3 - ps26
Kaz./Nau./Saccharomyces - ps27
Saccharomyces - ps28
Arboricola_clade - ps29
Cerevisiae_clade - ps30
Saccharomyces_cerevisiae - ps31
Phylostratum

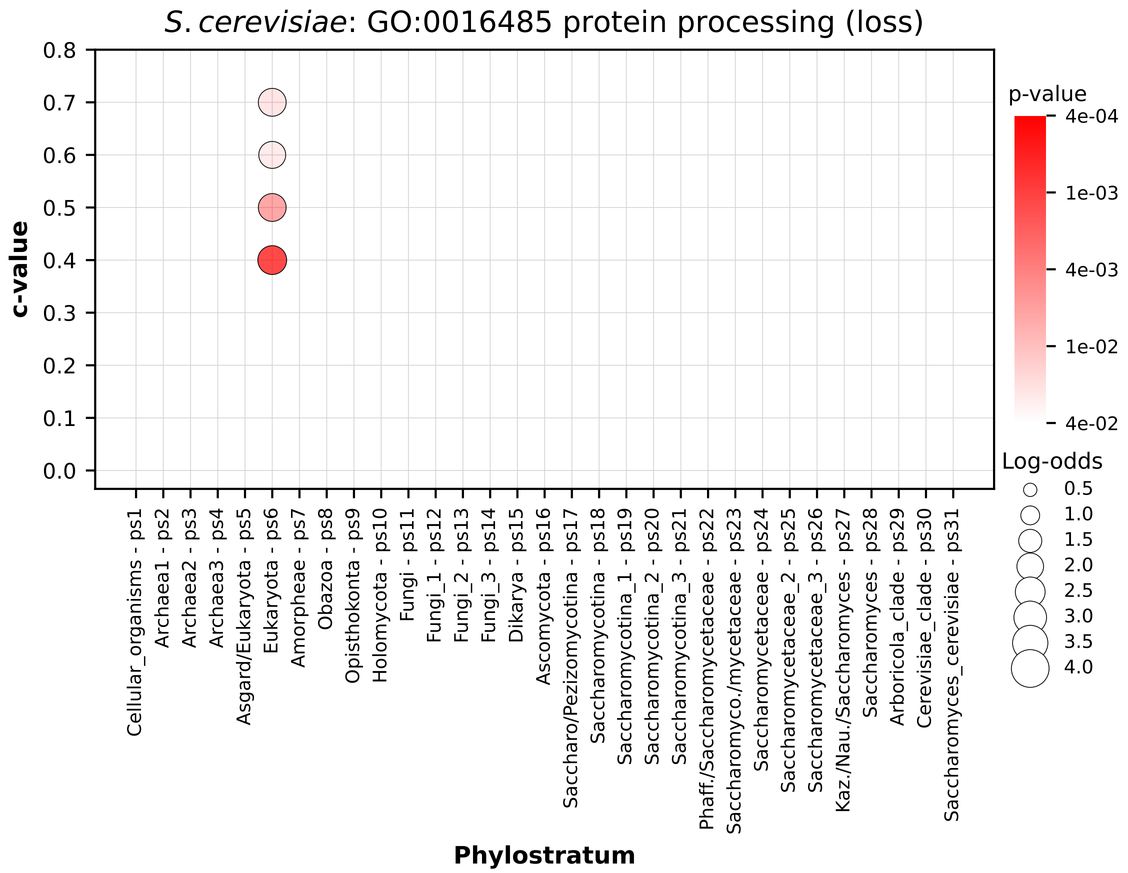

S. cerevisiae: GO:0016485 protein processing (loss)
c-value
0.8
0.7
0.6
0.5
0.4
0.3
0.2
0.1
0.0
p-value
4e-04
1e-03
4e-03
1e-02
4e-02
Log-odds
0.5
1.0
1.5
2.0
2.5
3.0
3.5
4.0
Cellular_organisms - ps1
Archaea1 - ps2
Archaea2 - ps3
Archaea3 - ps4
Asgard/Eukaryota - ps5
Eukaryota - ps6
Amorpheae - ps7
Obazoa - ps8
Opisthokonta - ps9
Holomycota - ps10
Fungi - ps11
Fungi_1 - ps12
Fungi_2 - ps13
Fungi_3 - ps14
Dikarya - ps15
Ascomycota - ps16
Saccharo/Pezizomycotina - ps17
Saccharomycotina - ps18
Saccharomycotina_1 - ps19
Saccharomycotina_2 - ps20
Saccharomycotina_3 - ps21
Phaff./Saccharomycetaceae - ps22
Saccharomyco./mycetaceae - ps23
Saccharomycetaceae - ps24
Saccharomycetaceae_2 - ps25
Saccharomycetaceae_3 - ps26
Kaz./Nau./Saccharomyces - ps27
Saccharomyces - ps28
Arboricola_clade - ps29
Cerevisiae_clade - ps30
Saccharomyces_cerevisiae - ps31
Phylostratum

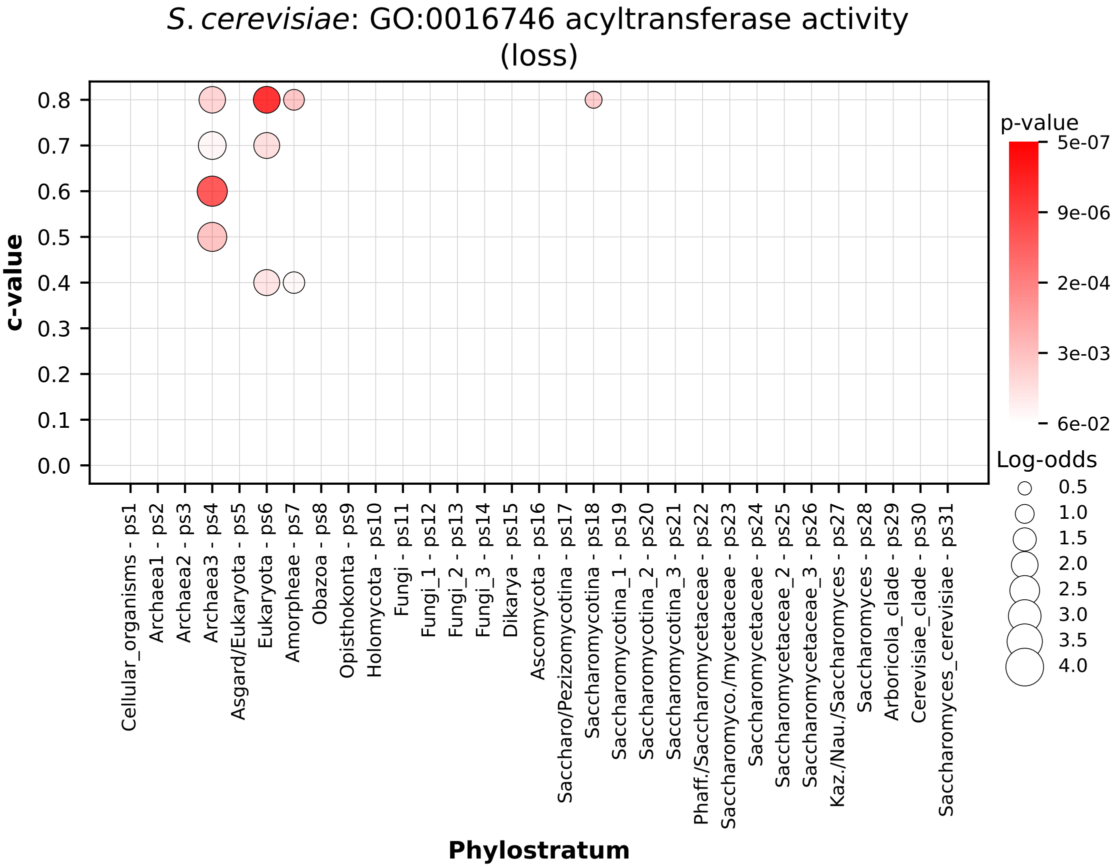
S. cerevisiae: GO:0016746 acyltransferase activity
(loss)
c-value
0.0
0.1
0.2
0.3
0.4
0.5
0.6
0.7
0.8
p-value
5e-07
9e-06
2e-04
3e-03
6e-02
Log-odds
0.5
1.0
1.5
2.0
2.5
3.0
3.5
4.0
Cellular_organisms - ps1
Archaea1 - ps2
Archaea2 - ps3
Archaea3 - ps4
Asgard/Eukaryota - ps5
Eukaryota - ps6
Amorpheae - ps7
Obazoa - ps8
Opisthokonta - ps9
Holomycota - ps10
Fungi - ps11
Fungi_1 - ps12
Fungi_2 - ps13
Fungi_3 - ps14
Dikarya - ps15
Ascomycota - ps16
Saccharo/Pezizomycotina - ps17
Saccharomycotina - ps18
Saccharomycotina_1 - ps19
Saccharomycotina_2 - ps20
Saccharomycotina_3 - ps21
Phaff./Saccharomycetaceae - ps22
Saccharomyco./mycetaceae - ps23
Saccharomycetaceae - ps24
Saccharomycetaceae_2 - ps25
Saccharomycetaceae_3 - ps26
Kaz./Nau./Saccharomyces - ps27
Saccharomyces - ps28
Arboricola_clade - ps29
Cerevisiae_clade - ps30
Saccharomyces_cerevisiae - ps31
Phylostratum

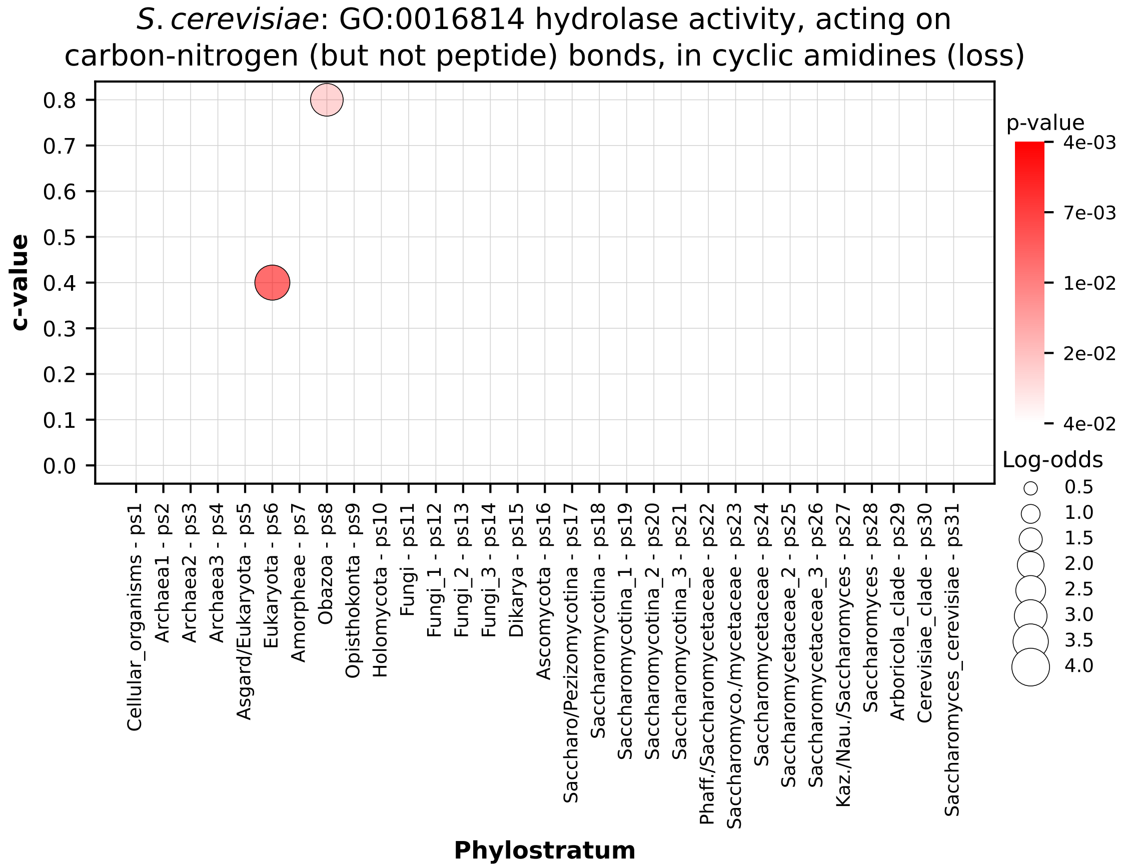

S. cerevisiae: GO:0016814 hydrolase activity, acting on carbon-nitrogen (but not peptide) bonds, in cyclic amidines (loss)
c-value
0.8
0.7
0.6
0.5
0.4
0.3
0.2
0.1
0.0
p-value
4e-03
7e-03
1e-02
2e-02
4e-02
Log-odds
0.5
1.0
1.5
2.0
2.5
3.0
3.5
4.0
Cellular_organisms - ps1
Archaea1 - ps2
Archaea2 - ps3
Archaea3 - ps4
Asgard/Eukaryota - ps5
Eukaryota - ps6
Amorpheae - ps7
Obazoa - ps8
Opisthokonta - ps9
Holomycota - ps10
Fungi - ps11
Fungi_1 - ps12
Fungi_2 - ps13
Fungi_3 - ps14
Dikarya - ps15
Ascomycota - ps16
Saccharo/Pezizomycotina - ps17
Saccharomycotina - ps18
Saccharomycotina_1 - ps19
Saccharomycotina_2 - ps20
Saccharomycotina_3 - ps21
Phaff./Saccharomycetaceae - ps22
Saccharomyco./mycetaceae - ps23
Saccharomycetaceae - ps24
Saccharomycetaceae_2 - ps25
Saccharomycetaceae_3 - ps26
Kaz./Nau./Saccharomyces - ps27
Saccharomyces - ps28
Arboricola_clade - ps29
Cerevisiae_clade - ps30
Saccharomyces_cerevisiae - ps31
Phylostratum

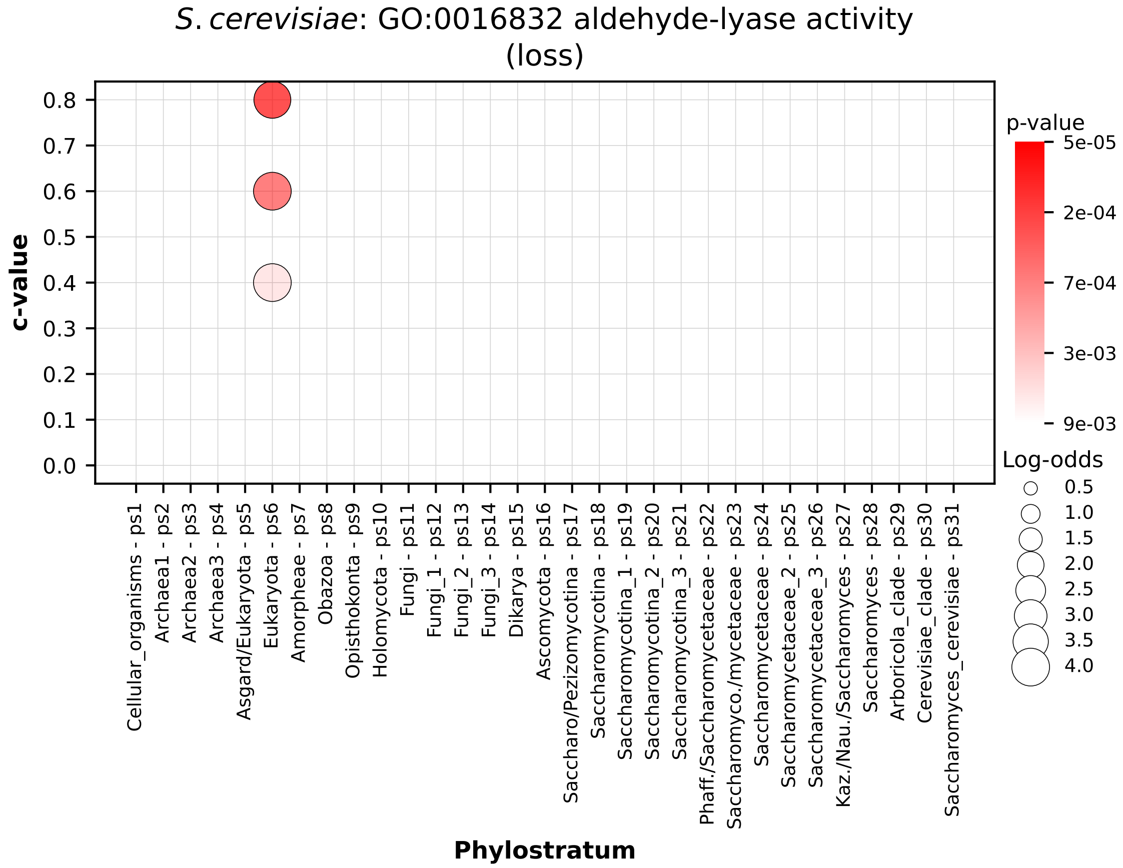
S. cerevisiae: GO:0016832 aldehyde-lyase activity
(loss)
c-value
0.8
0.7
0.6
0.5
0.4
0.3
0.2
0.1
0.0
p-value
5e-05
2e-04
7e-04
3e-03
9e-03
Log-odds
0.5
1.0
1.5
2.0
2.5
3.0
3.5
4.0
Cellular_organisms - ps1
Archaea1 - ps2
Archaea2 - ps3
Archaea3 - ps4
Asgard/Eukaryota - ps5
Eukaryota - ps6
Amorpheae - ps7
Obazoa - ps8
Opisthokonta - ps9
Holomycota - ps10
Fungi - ps11
Fungi_1 - ps12
Fungi_2 - ps13
Fungi_3 - ps14
Dikarya - ps15
Ascomycota - ps16
Saccharo/Pezizomycotina - ps17
Saccharomycotina - ps18
Saccharomycotina_1 - ps19
Saccharomycotina_2 - ps20
Saccharomycotina_3 - ps21
Phaff./Saccharomycetaceae - ps22
Saccharomyco./mycetaceae - ps23
Saccharomycetaceae - ps24
Saccharomycetaceae_2 - ps25
Saccharomycetaceae_3 - ps26
Kaz./Nau./Saccharomyces - ps27
Saccharomyces - ps28
Arboricola_clade - ps29
Cerevisiae_clade - ps30
Saccharomyces_cerevisiae - ps31
Phylostratum

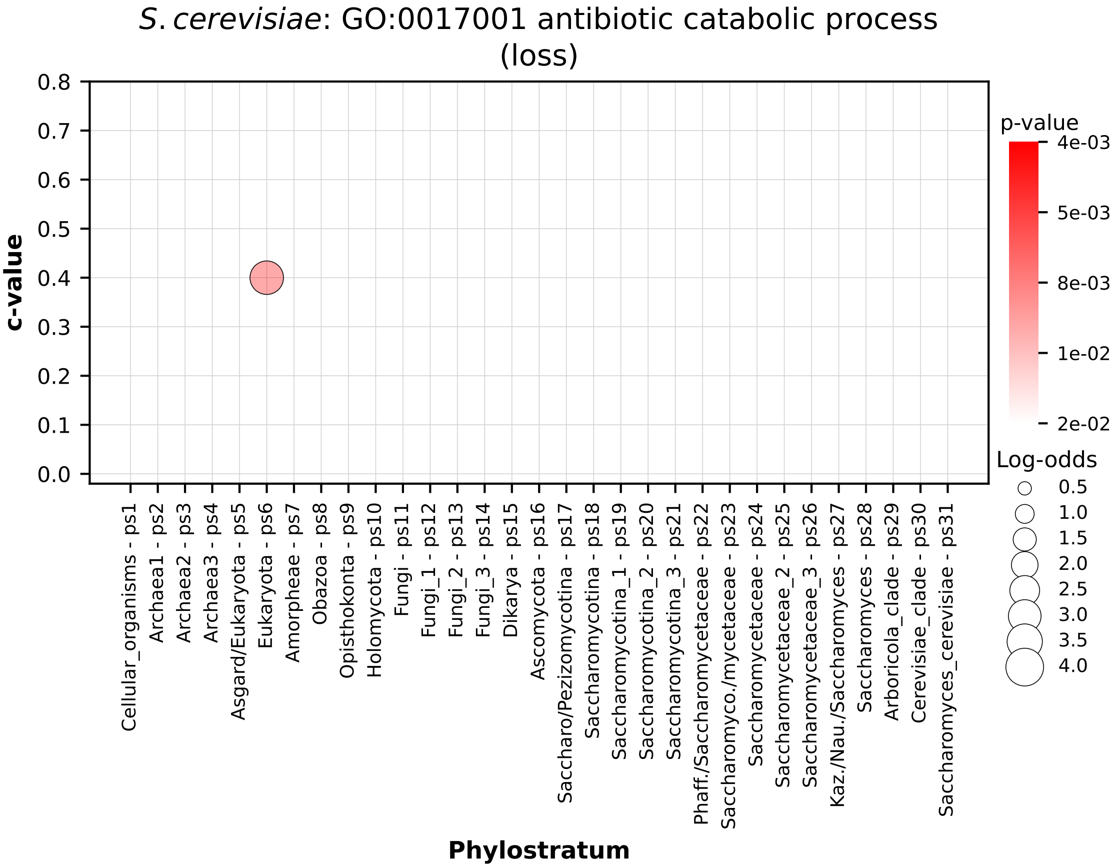
S. cerevisiae: GO:0017001 antibiotic catabolic process
(loss)
c-value
0.8
0.7
0.6
0.5
0.4
0.3
0.2
0.1
0.0
Phylostratum
Cellular_organisms - ps1
Archaea1 - ps2
Archaea2 - ps3
Archaea3 - ps4
Asgard/Eukaryota - ps5
Eukaryota - ps6
Amorpheae - ps7
Obazoa - ps8
Opisthokonta - ps9
Holomycota - ps10
Fungi - ps11
Fungi_1 - ps12
Fungi_2 - ps13
Fungi_3 - ps14
Dikarya - ps15
Ascomycota - ps16
Saccharo/Pezizomycotina - ps17
Saccharomycotina - ps18
Saccharomycotina_1 - ps19
Saccharomycotina_2 - ps20
Saccharomycotina_3 - ps21
Phaff./Saccharomycetaceae - ps22
Saccharomyco./mycetaceae - ps23
Saccharomycetaceae - ps24
Saccharomycetaceae_2 - ps25
Saccharomycetaceae_3 - ps26
Kaz./Nau./Saccharomyces - ps27
Saccharomyces - ps28
Arboricola_clade - ps29
Cerevisiae_clade - ps30
Saccharomyces_cerevisiae - ps31
p-value
4e-03
5e-03
8e-03
1e-02
2e-02
Log-odds
0.5
1.0
1.5
2.0
2.5
3.0
3.5
4.0

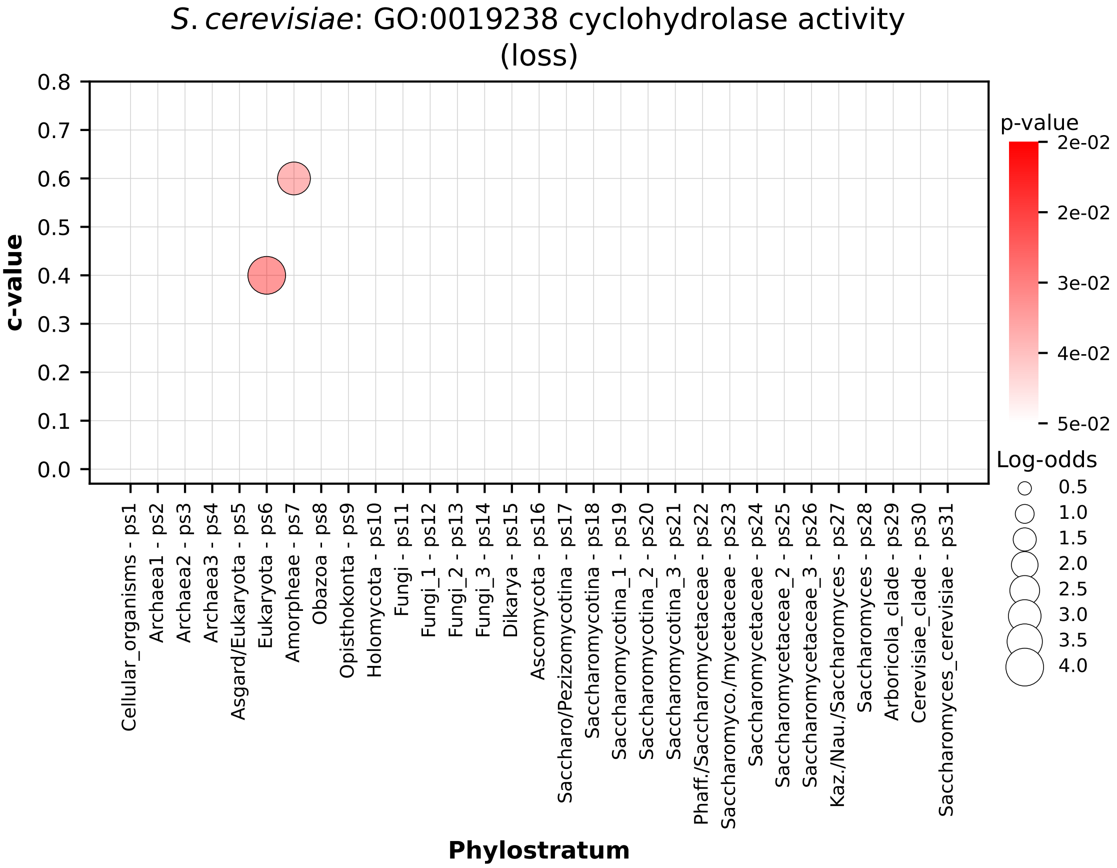
S. cerevisiae: GO:0019238 cyclohydrolase activity
(loss)
c-value
0.8
0.7
0.6
0.5
0.4
0.3
0.2
0.1
0.0
p-value
2e-02
2e-02
3e-02
4e-02
5e-02
Log-odds
0.5
1.0
1.5
2.0
2.5
3.0
3.5
4.0
Cellular_organisms - ps1
Archaea1 - ps2
Archaea2 - ps3
Archaea3 - ps4
Asgard/Eukaryota - ps5
Eukaryota - ps6
Amorpheae - ps7
Obazoa - ps8
Opisthokonta - ps9
Holomycota - ps10
Fungi - ps11
Fungi_1 - ps12
Fungi_2 - ps13
Fungi_3 - ps14
Dikarya - ps15
Ascomycota - ps16
Saccharo/Pezizomycotina - ps17
Saccharomycotina - ps18
Saccharomycotina_1 - ps19
Saccharomycotina_2 - ps20
Saccharomycotina_3 - ps21
Phaff./Saccharomycetaceae - ps22
Saccharomyco./mycetaceae - ps23
Saccharomycetaceae - ps24
Saccharomycetaceae_2 - ps25
Saccharomycetaceae_3 - ps26
Kaz./Nau./Saccharomyces - ps27
Saccharomyces - ps28
Arboricola_clade - ps29
Cerevisiae_clade - ps30
Saccharomyces_cerevisiae - ps31
Phylostratum

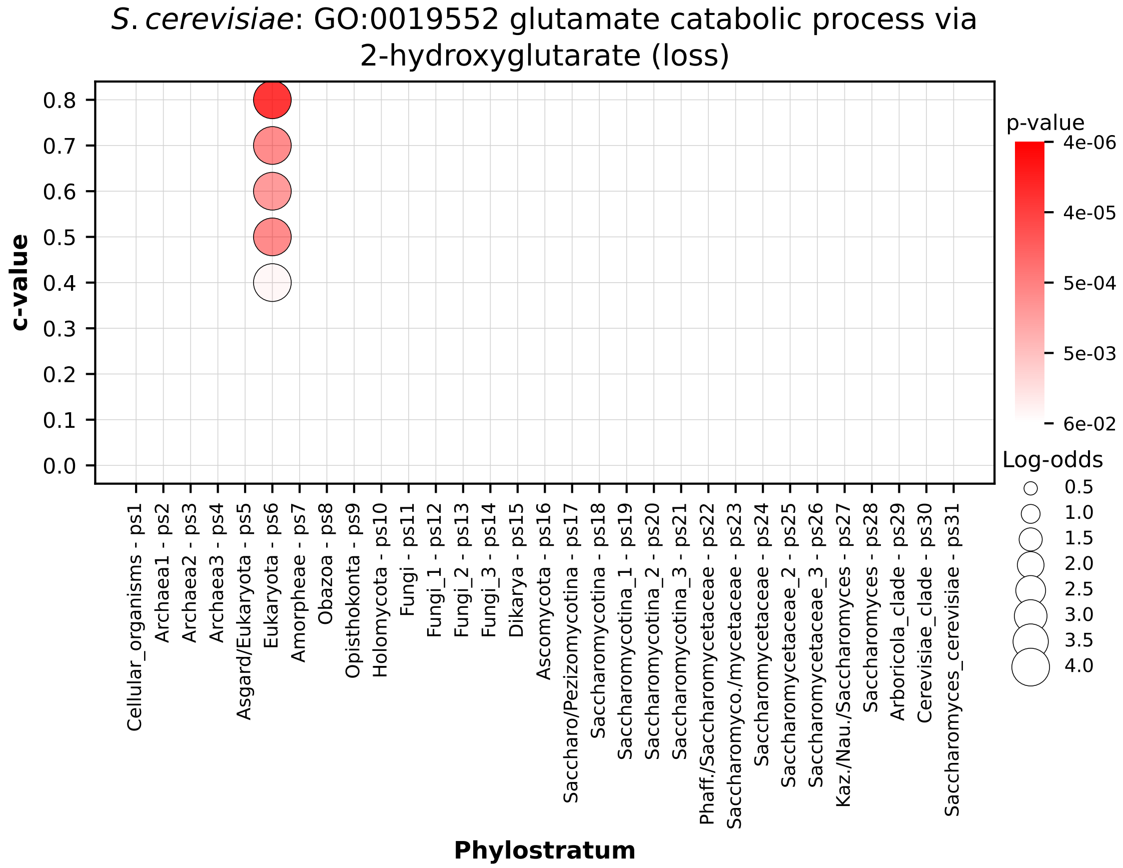
S. cerevisiae: GO:0019552 glutamate catabolic process via 2-hydroxyglutarate (loss)
c-value
0.8
0.7
0.6
0.5
0.4
0.3
0.2
0.1
0.0
p-value
4e-06
4e-05
5e-04
5e-03
6e-02
Log-odds
0.5
1.0
1.5
2.0
2.5
3.0
3.5
4.0
Cellular_organisms - ps1
Archaea1 - ps2
Archaea2 - ps3
Archaea3 - ps4
Asgard/Eukaryota - ps5
Eukaryota - ps6
Amorpheae - ps7
Obazoa - ps8
Opisthokonta - ps9
Holomycota - ps10
Fungi - ps11
Fungi_1 - ps12
Fungi_2 - ps13
Fungi_3 - ps14
Dikarya - ps15
Ascomycota - ps16
Saccharo/Pezizomycotina - ps17
Saccharomycotina - ps18
Saccharomycotina_1 - ps19
Saccharomycotina_2 - ps20
Saccharomycotina_3 - ps21
Phaff./Saccharomycetaceae - ps22
Saccharomyco./mycetaceae - ps23
Saccharomycetaceae - ps24
Saccharomycetaceae_2 - ps25
Saccharomycetaceae_3 - ps26
Kaz./Nau./Saccharomyces - ps27
Saccharomyces - ps28
Arboricola_clade - ps29
Cerevisiae_clade - ps30
Saccharomyces_cerevisiae - ps31
Phylostratum

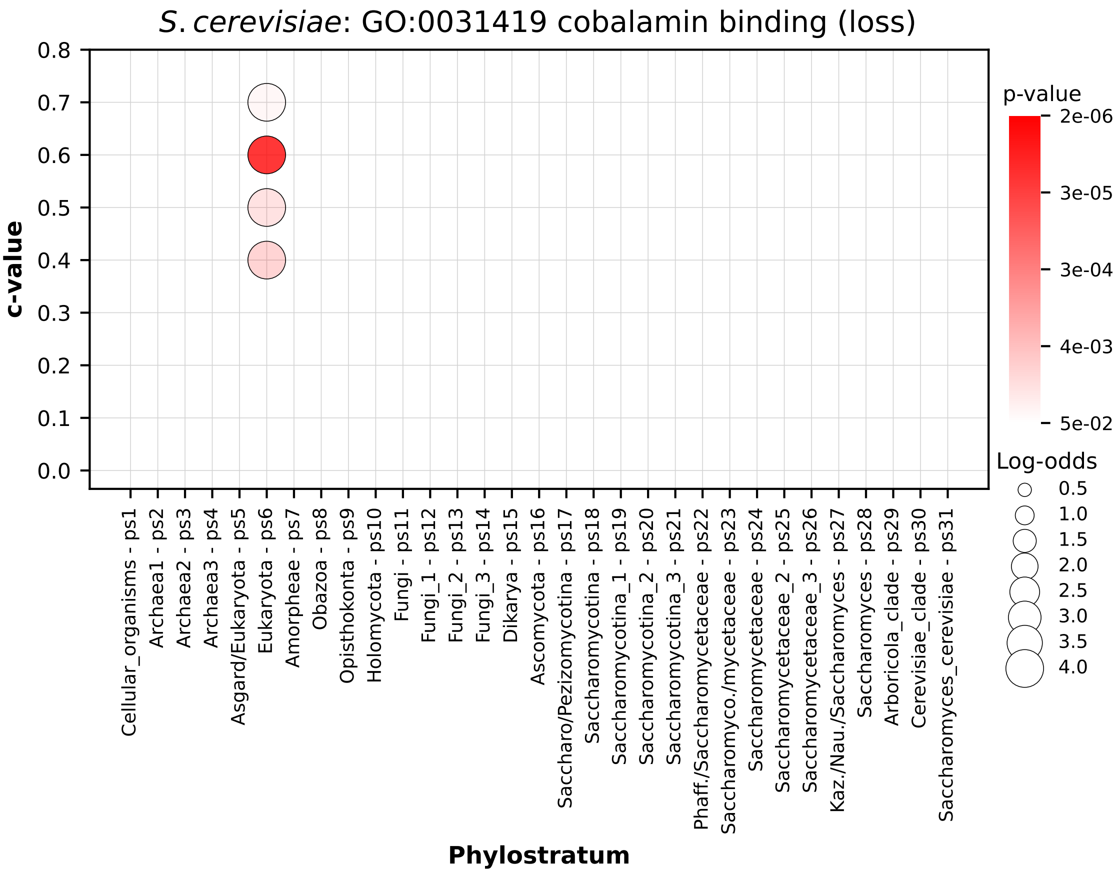
S. cerevisiae: GO:0031419 cobalamin binding (loss)
c-value
0.8
0.7
0.6
0.5
0.4
0.3
0.2
0.1
0.0
p-value
2e-06
3e-05
3e-04
4e-03
5e-02
Log-odds
0.5
1.0
1.5
2.0
2.5
3.0
3.5
4.0
Cellular_organisms - ps1
Archaea1 - ps2
Archaea2 - ps3
Archaea3 - ps4
Asgard/Eukaryota - ps5
Eukaryota - ps6
Amorpheae - ps7
Obazoa - ps8
Opisthokonta - ps9
Holomycota - ps10
Fungi - ps11
Fungi_1 - ps12
Fungi_2 - ps13
Fungi_3 - ps14
Dikarya - ps15
Ascomycota - ps16
Saccharo/Pezizomycotina - ps17
Saccharomycotina - ps18
Saccharomycotina_1 - ps19
Saccharomycotina_2 - ps20
Saccharomycotina_3 - ps21
Phaff./Saccharomycetaceae - ps22
Saccharomyco./mycetaceae - ps23
Saccharomycetaceae - ps24
Saccharomycetaceae_2 - ps25
Saccharomycetaceae_3 - ps26
Kaz./Nau./Saccharomyces - ps27
Saccharomyces - ps28
Arboricola_clade - ps29
Cerevisiae_clade - ps30
Saccharomyces_cerevisiae - ps31
Phylostratum

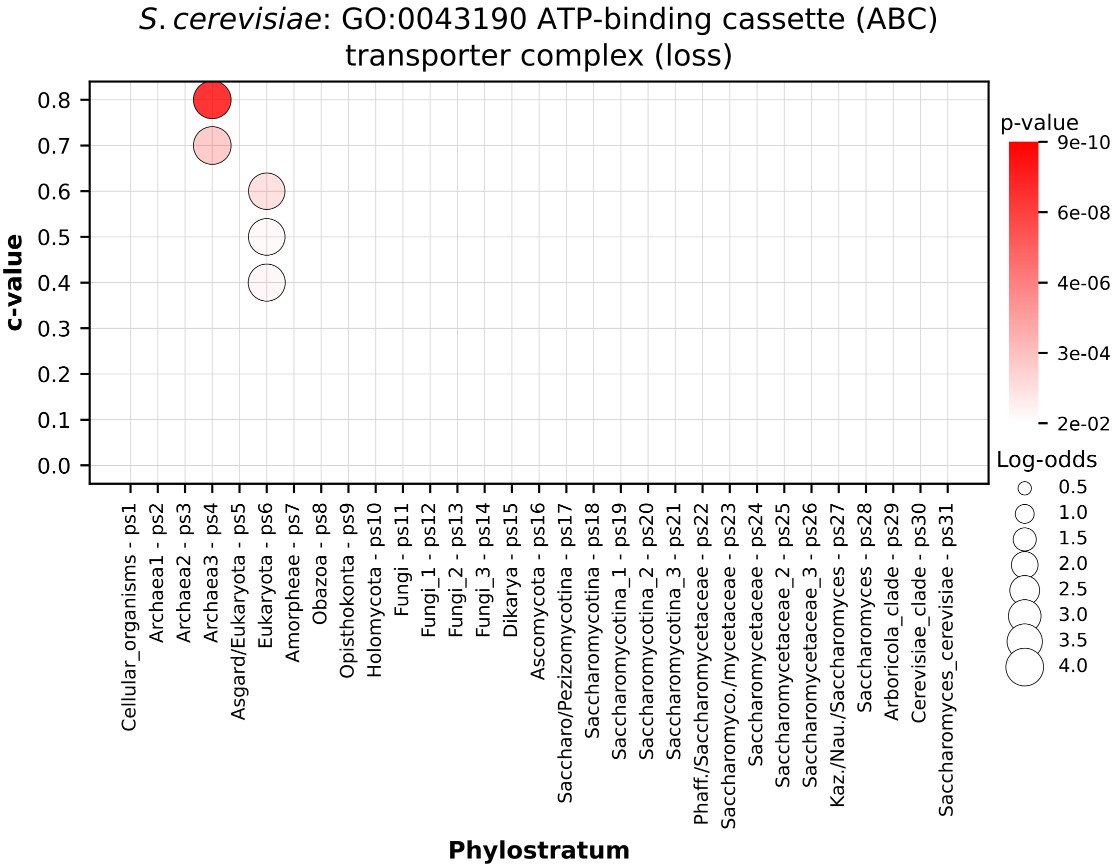

S. cerevisiae: GO:0043190 ATP-binding cassette (ABC) transporter complex (loss)
c-value
0.8
0.7
0.6
0.5
0.4
0.3
0.2
0.1
0.0
p-value
9e-10
6e-08
4e-06
3e-04
2e-02
Log-odds
0.5
1.0
1.5
2.0
2.5
3.0
3.5
4.0
Cellular_organisms - ps1
Archaea1 - ps2
Archaea2 - ps3
Archaea3 - ps4
Asgard/Eukaryota - ps5
Eukaryota - ps6
Amorpheae - ps7
Obazoa - ps8
Opisthokonta - ps9
Holomycota - ps10
Fungi - ps11
Fungi_1 - ps12
Fungi_2 - ps13
Fungi_3 - ps14
Dikarya - ps15
Ascomycota - ps16
Saccharo/Pezizomycotina - ps17
Saccharomycotina - ps18
Saccharomycotina_1 - ps19
Saccharomycotina_2 - ps20
Saccharomycotina_3 - ps21
Phaff./Saccharomycetaceae - ps22
Saccharomyco./mycetaceae - ps23
Saccharomycetaceae - ps24
Saccharomycetaceae_2 - ps25
Saccharomycetaceae_3 - ps26
Kaz./Nau./Saccharomyces - ps27
Saccharomyces - ps28
Arboricola_clade - ps29
Cerevisiae_clade - ps30
Saccharomyces_cerevisiae - ps31
Phylostratum

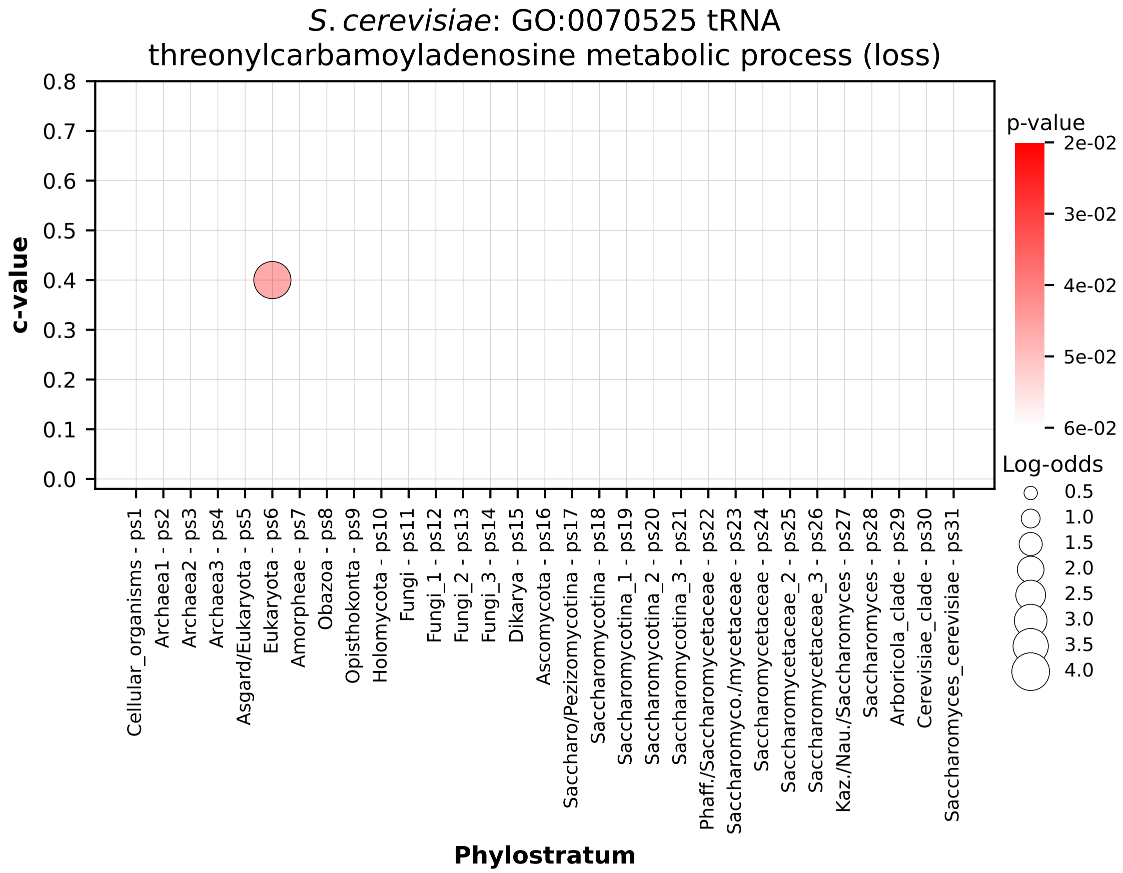

S. cerevisiae: GO:0070525 tRNA threonylcarbamoyladenosine metabolic process (loss)
c-value
0.8
0.7
0.6
0.5
0.4
0.3
0.2
0.1
0.0
Phylostratum
Cellular_organisms - ps1
Archaea1 - ps2
Archaea2 - ps3
Archaea3 - ps4
Asgard/Eukaryota - ps5
Eukaryota - ps6
Amorpheae - ps7
Obazoa - ps8
Opisthokonta - ps9
Holomycota - ps10
Fungi - ps11
Fungi_1 - ps12
Fungi_2 - ps13
Fungi_3 - ps14
Dikarya - ps15
Ascomycota - ps16
Saccharo/Pezizomycotina - ps17
Saccharomycotina - ps18
Saccharomycotina_1 - ps19
Saccharomycotina_2 - ps20
Saccharomycotina_3 - ps21
Phaff./Saccharomycetaceae - ps22
Saccharomyco./mycetaceae - ps23
Saccharomycetaceae - ps24
Saccharomycetaceae_2 - ps25
Saccharomycetaceae_3 - ps26
Kaz./Nau./Saccharomyces - ps27
Saccharomyces - ps28
Arboricola_clade - ps29
Cerevisiae_clade - ps30
Saccharomyces_cerevisiae - ps31
p-value
2e-02
3e-02
4e-02
5e-02
6e-02
Log-odds
0.5
1.0
1.5
2.0
2.5
3.0
3.5
4.0

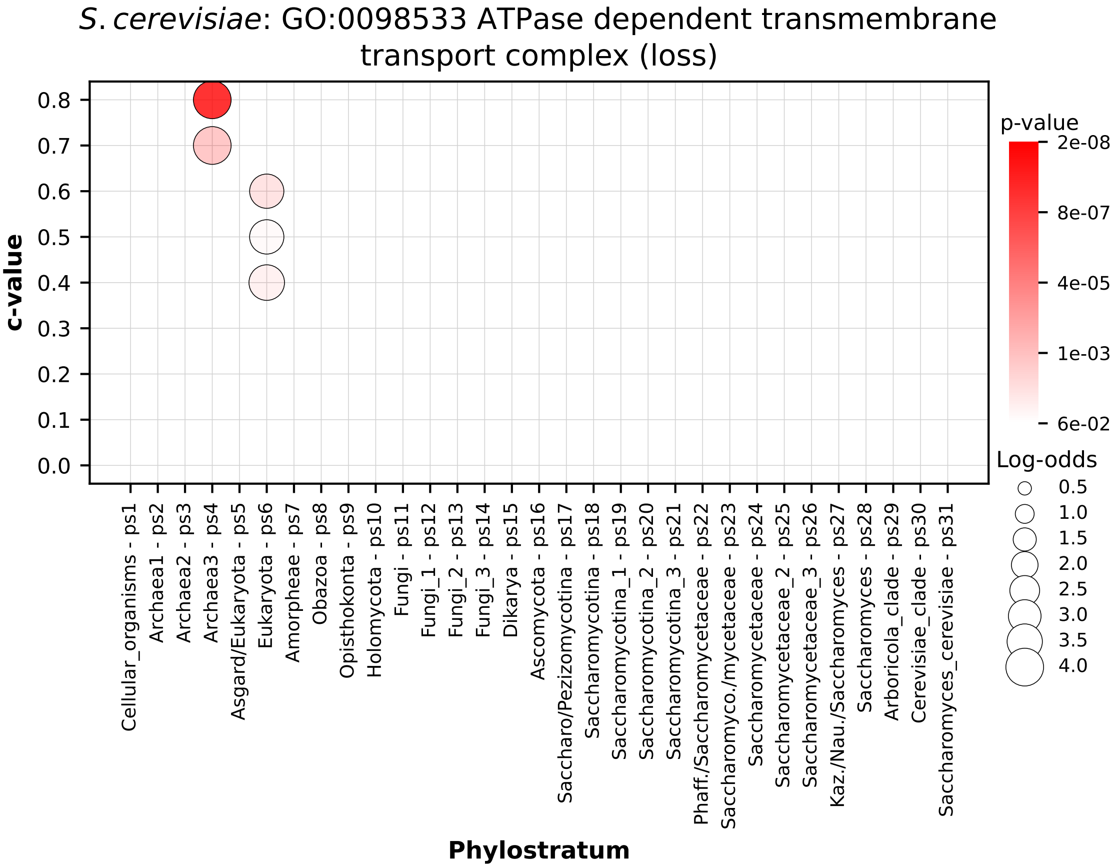
S. cerevisiae: GO:0098533 ATPase dependent transmembrane transport complex (loss)
c-value
0.0
0.1
0.2
0.3
0.4
0.5
0.6
0.7
0.8
p-value
2e-08
8e-07
4e-05
1e-03
6e-02
Log-odds
0.5
1.0
1.5
2.0
2.5
3.0
3.5
4.0
Cellular_organisms - ps1
Archaea1 - ps2
Archaea2 - ps3
Archaea3 - ps4
Asgard/Eukaryota - ps5
Eukaryota - ps6
Amorpheae - ps7
Obazoa - ps8
Opisthokonta - ps9
Holomycota - ps10
Fungi - ps11
Fungi_1 - ps12
Fungi_2 - ps13
Fungi_3 - ps14
Dikarya - ps15
Ascomycota - ps16
Saccharo/Pezizomycotina - ps17
Saccharomycotina - ps18
Saccharomycotina_1 - ps19
Saccharomycotina_2 - ps20
Saccharomycotina_3 - ps21
Phaff./Saccharomycetaceae - ps22
Saccharomyco./mycetaceae - ps23
Saccharomycetaceae - ps24
Saccharomycetaceae_2 - ps25
Saccharomycetaceae_3 - ps26
Kaz./Nau./Saccharomyces - ps27
Saccharomyces - ps28
Arboricola_clade - ps29
Cerevisiae_clade - ps30
Saccharomyces_cerevisiae - ps31
Phylostratum

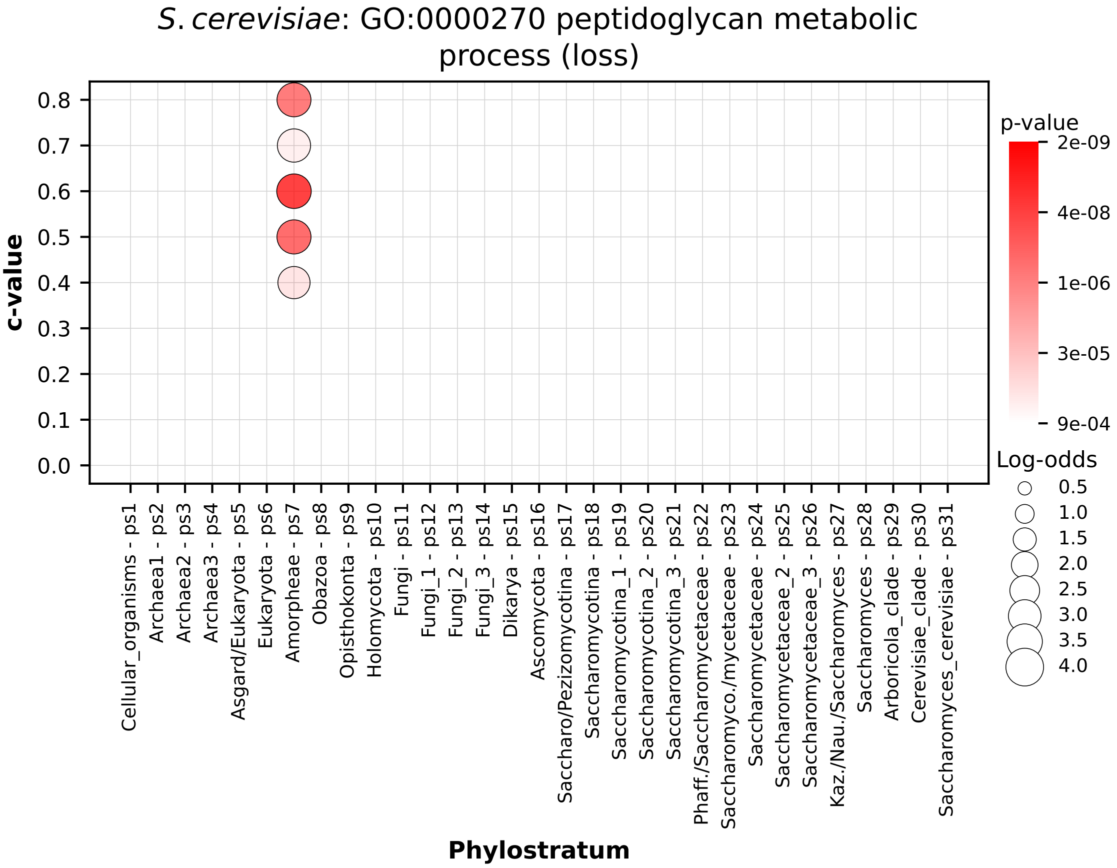
S. cerevisiae: GO:0000270 peptidoglycan metabolic process (loss)
c-value
0.8
0.7
0.6
0.5
0.4
0.3
0.2
0.1
0.0
p-value
2e-09
4e-08
1e-06
3e-05
9e-04
Log-odds
0.5
1.0
1.5
2.0
2.5
3.0
3.5
4.0
Cellular_organisms - ps1
Archaea1 - ps2
Archaea2 - ps3
Archaea3 - ps4
Asgard/Eukaryota - ps5
Eukaryota - ps6
Amorpheae - ps7
Obazoa - ps8
Opisthokonta - ps9
Holomycota - ps10
Fungi - ps11
Fungi_1 - ps12
Fungi_2 - ps13
Fungi_3 - ps14
Dikarya - ps15
Ascomycota - ps16
Saccharo/Pezizomycotina - ps17
Saccharomycotina - ps18
Saccharomycotina_1 - ps19
Saccharomycotina_2 - ps20
Saccharomycotina_3 - ps21
Phaff./Saccharomycetaceae - ps22
Saccharomyco./mycetaceae - ps23
Saccharomycetaceae - ps24
Saccharomycetaceae_2 - ps25
Saccharomycetaceae_3 - ps26
Kaz./Nau./Saccharomyces - ps27
Saccharomyces - ps28
Arboricola_clade - ps29
Cerevisiae_clade - ps30
Saccharomyces_cerevisiae - ps31
Phylostratum

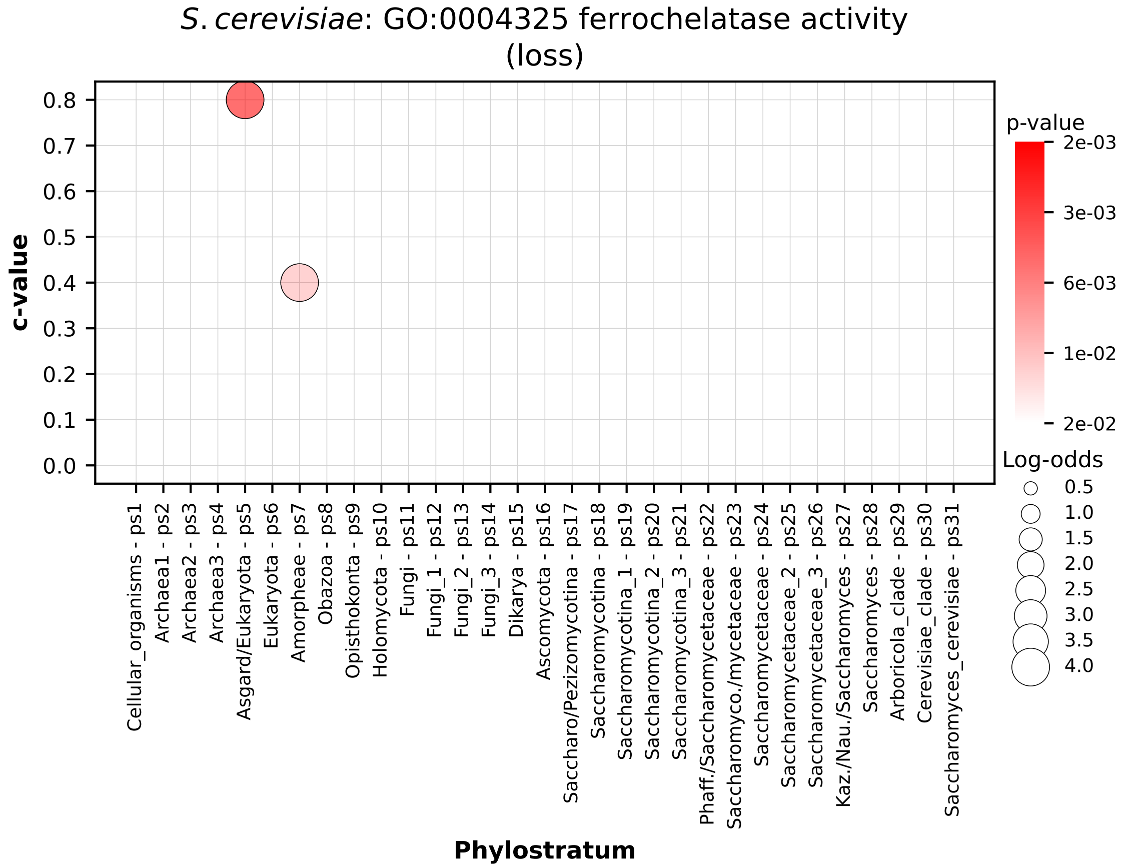

S. cerevisiae: GO:0004325 ferrochelatase activity
(loss)
c-value
0.0
0.1
0.2
0.3
0.4
0.5
0.6
0.7
0.8
Phylostratum
Cellular_organisms - ps1
Archaea1 - ps2
Archaea2 - ps3
Archaea3 - ps4
Asgard/Eukaryota - ps5
Eukaryota - ps6
Amorpheae - ps7
Obazoa - ps8
Opisthokonta - ps9
Holomycota - ps10
Fungi - ps11
Fungi_1 - ps12
Fungi_2 - ps13
Fungi_3 - ps14
Dikarya - ps15
Ascomycota - ps16
Saccharo/Pezizomycotina - ps17
Saccharomycotina - ps18
Saccharomycotina_1 - ps19
Saccharomycotina_2 - ps20
Saccharomycotina_3 - ps21
Phaff./Saccharomycetaceae - ps22
Saccharomyco./mycetaceae - ps23
Saccharomycetaceae - ps24
Saccharomycetaceae_2 - ps25
Saccharomycetaceae_3 - ps26
Kaz./Nau./Saccharomyces - ps27
Saccharomyces - ps28
Arboricola_clade - ps29
Cerevisiae_clade - ps30
Saccharomyces_cerevisiae - ps31
p-value
2e-03
3e-03
6e-03
1e-02
2e-02
Log-odds
0.5
1.0
1.5
2.0
2.5
3.0
3.5
4.0

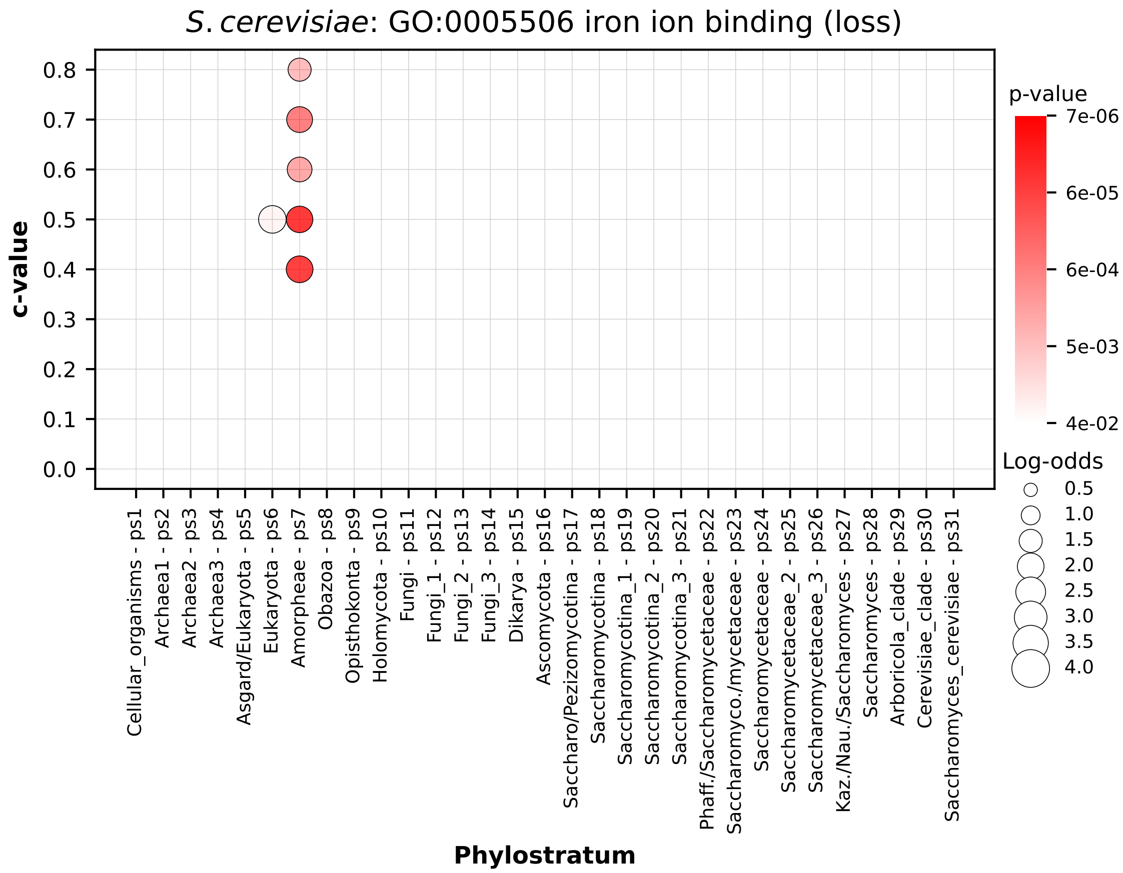

S. cerevisiae: GO:0005506 iron ion binding (loss)
c-value
0.0
0.1
0.2
0.3
0.4
0.5
0.6
0.7
0.8
p-value
7e-06
6e-05
6e-04
5e-03
4e-02
Log-odds
0.5
1.0
1.5
2.0
2.5
3.0
3.5
4.0
Cellular_organisms - ps1
Archaea1 - ps2
Archaea2 - ps3
Archaea3 - ps4
Asgard/Eukaryota - ps5
Eukaryota - ps6
Amorpheae - ps7
Obazoa - ps8
Opisthokonta - ps9
Holomycota - ps10
Fungi - ps11
Fungi_1 - ps12
Fungi_2 - ps13
Fungi_3 - ps14
Dikarya - ps15
Ascomycota - ps16
Saccharo/Pezizomycotina - ps17
Saccharomycotina - ps18
Saccharomycotina_1 - ps19
Saccharomycotina_2 - ps20
Saccharomycotina_3 - ps21
Phaff./Saccharomycetaceae - ps22
Saccharomyco./mycetaceae - ps23
Saccharomycetaceae - ps24
Saccharomycetaceae_2 - ps25
Saccharomycetaceae_3 - ps26
Kaz./Nau./Saccharomyces - ps27
Saccharomyces - ps28
Arboricola_clade - ps29
Cerevisiae_clade - ps30
Saccharomyces_cerevisiae - ps31
Phylostratum

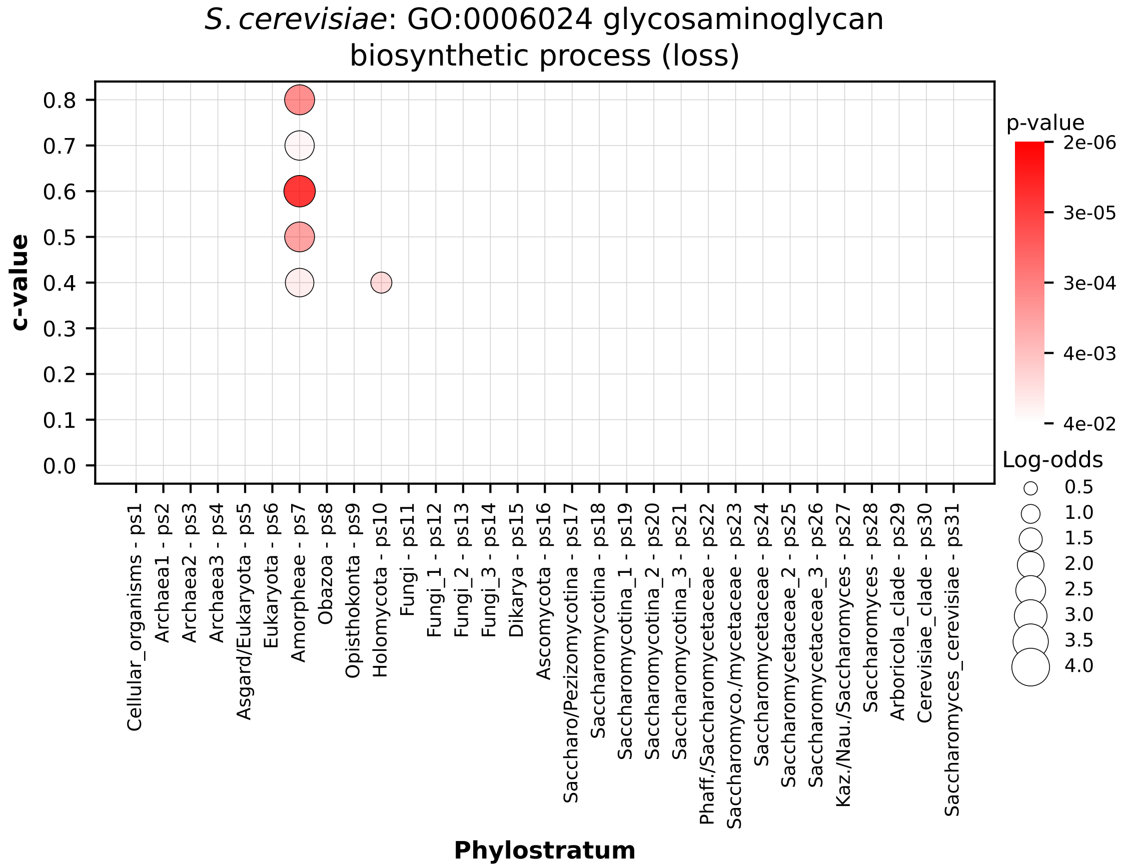

S. cerevisiae: GO:0006024 glycosaminoglycan biosynthetic process (loss)
c-value
0.8
0.7
0.6
0.5
0.4
0.3
0.2
0.1
0.0
Phylostratum
Cellular_organisms - ps1
Archaea1 - ps2
Archaea2 - ps3
Archaea3 - ps4
Asgard/Eukaryota - ps5
Eukaryota - ps6
Amorpheae - ps7
Obazoa - ps8
Opisthokonta - ps9
Holomycota - ps10
Fungi - ps11
Fungi_1 - ps12
Fungi_2 - ps13
Fungi_3 - ps14
Dikarya - ps15
Ascomycota - ps16
Saccharo/Pezizomycotina - ps17
Saccharomycotina - ps18
Saccharomycotina_1 - ps19
Saccharomycotina_2 - ps20
Saccharomycotina_3 - ps21
Phaff./Saccharomycetaceae - ps22
Saccharomyco./mycetaceae - ps23
Saccharomycetaceae - ps24
Saccharomycetaceae_2 - ps25
Saccharomycetaceae_3 - ps26
Kaz./Nau./Saccharomyces - ps27
Saccharomyces - ps28
Arboricola_clade - ps29
Cerevisiae_clade - ps30
Saccharomyces_cerevisiae - ps31
p-value
2e-06
3e-05
3e-04
4e-03
4e-02
Log-odds
0.5
1.0
1.5
2.0
2.5
3.0
3.5
4.0

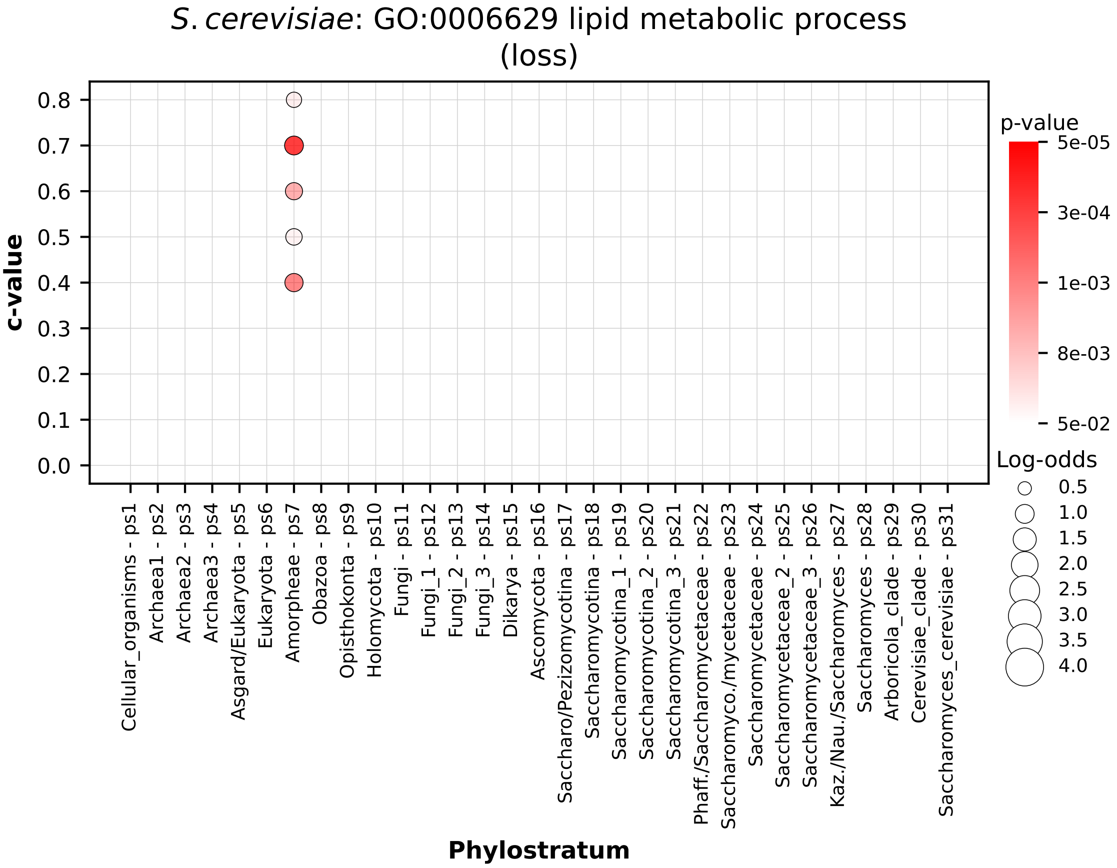

S. cerevisiae: GO:0006629 lipid metabolic process
(loss)
c-value
0.0
0.1
0.2
0.3
0.4
0.5
0.6
0.7
0.8
p-value
5e-05
3e-04
1e-03
8e-03
5e-02
Log-odds
0.5
1.0
1.5
2.0
2.5
3.0
3.5
4.0
Cellular_organisms - ps1
Archaea1 - ps2
Archaea2 - ps3
Archaea3 - ps4
Asgard/Eukaryota - ps5
Eukaryota - ps6
Amorpheae - ps7
Obazoa - ps8
Opisthokonta - ps9
Holomycota - ps10
Fungi - ps11
Fungi_1 - ps12
Fungi_2 - ps13
Fungi_3 - ps14
Dikarya - ps15
Ascomycota - ps16
Saccharo/Pezizomycotina - ps17
Saccharomycotina - ps18
Saccharomycotina_1 - ps19
Saccharomycotina_2 - ps20
Saccharomycotina_3 - ps21
Phaff./Saccharomycetaceae - ps22
Saccharomyco./mycetaceae - ps23
Saccharomycetaceae - ps24
Saccharomycetaceae_2 - ps25
Saccharomycetaceae_3 - ps26
Kaz./Nau./Saccharomyces - ps27
Saccharomyces - ps28
Arboricola_clade - ps29
Cerevisiae_clade - ps30
Saccharomyces_cerevisiae - ps31
Phylostratum

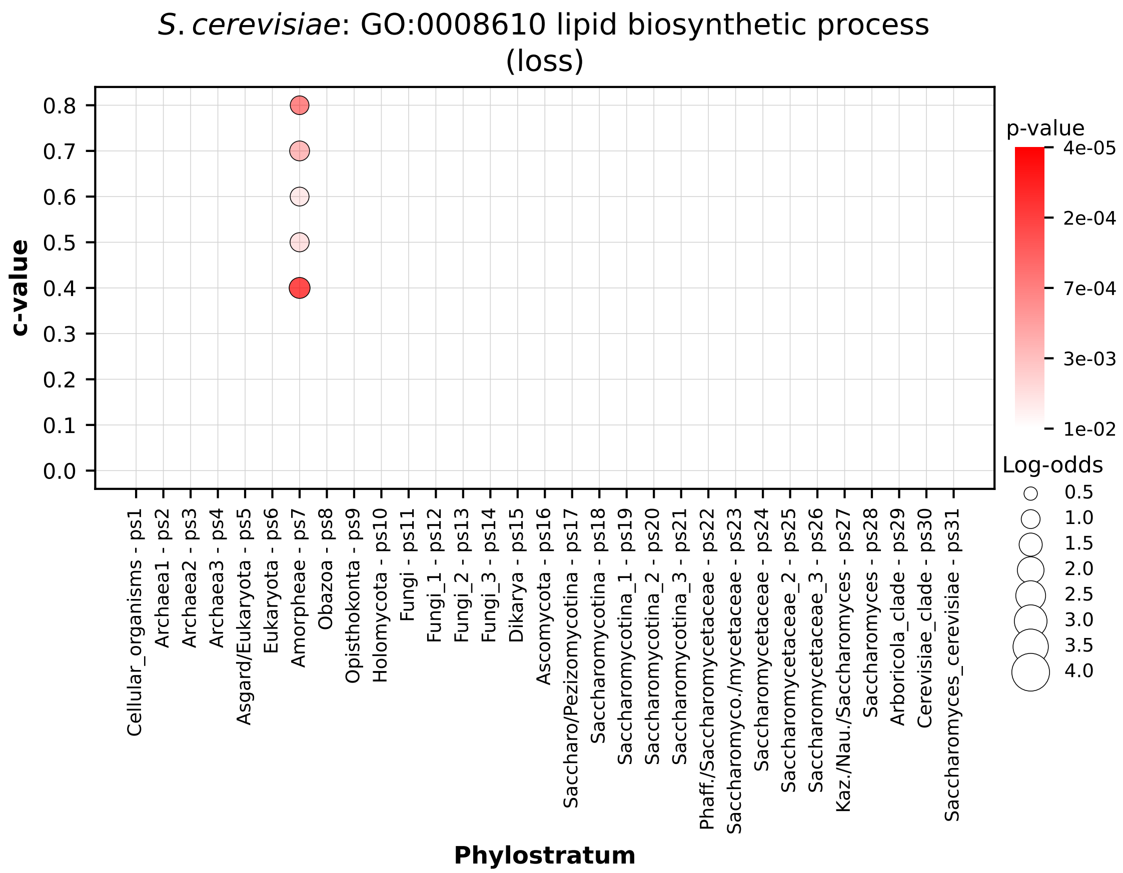

S. cerevisiae: GO:0008610 lipid biosynthetic process
(loss)
c-value
0.8
0.7
0.6
0.5
0.4
0.3
0.2
0.1
0.0
p-value
4e-05
2e-04
7e-04
3e-03
1e-02
Log-odds
0.5
1.0
1.5
2.0
2.5
3.0
3.5
4.0
Cellular_organisms - ps1
Archaea1 - ps2
Archaea2 - ps3
Archaea3 - ps4
Asgard/Eukaryota - ps5
Eukaryota - ps6
Amorpheae - ps7
Obazoa - ps8
Opisthokonta - ps9
Holomycota - ps10
Fungi - ps11
Fungi_1 - ps12
Fungi_2 - ps13
Fungi_3 - ps14
Dikarya - ps15
Ascomycota - ps16
Saccharo/Pezizomycotina - ps17
Saccharomycotina - ps18
Saccharomycotina_1 - ps19
Saccharomycotina_2 - ps20
Saccharomycotina_3 - ps21
Phaff./Saccharomycetaceae - ps22
Saccharomyco./mycetaceae - ps23
Saccharomycetaceae - ps24
Saccharomycetaceae_2 - ps25
Saccharomycetaceae_3 - ps26
Kaz./Nau./Saccharomyces - ps27
Saccharomyces - ps28
Arboricola_clade - ps29
Cerevisiae_clade - ps30
Saccharomyces_cerevisiae - ps31
Phylostratum

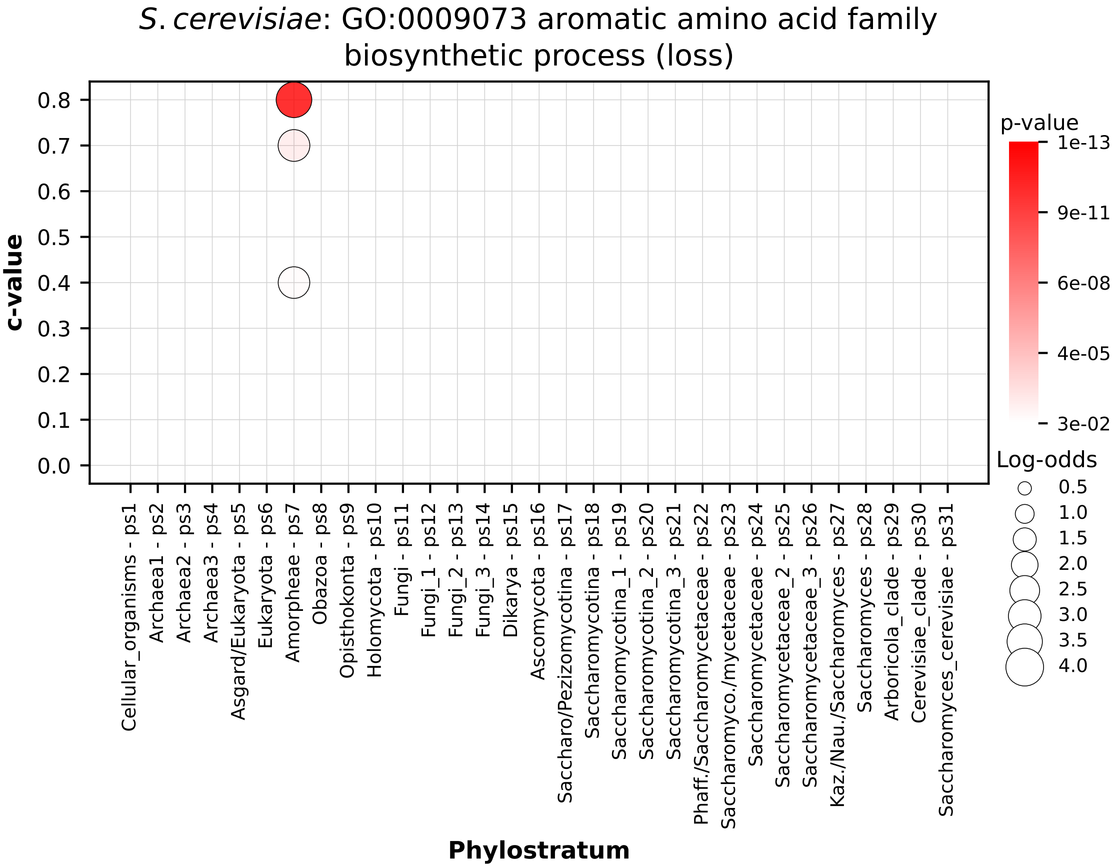

S. cerevisiae: GO:0009073 aromatic amino acid family biosynthetic process (loss)
c-value
0.0
0.1
0.2
0.3
0.4
0.5
0.6
0.7
0.8
Phylostratum
Cellular_organisms - ps1
Archaea1 - ps2
Archaea2 - ps3
Archaea3 - ps4
Asgard/Eukaryota - ps5
Eukaryota - ps6
Amorpheae - ps7
Obazoa - ps8
Opisthokonta - ps9
Holomycota - ps10
Fungi - ps11
Fungi_1 - ps12
Fungi_2 - ps13
Fungi_3 - ps14
Dikarya - ps15
Ascomycota - ps16
Saccharo/Pezizomycotina - ps17
Saccharomycotina - ps18
Saccharomycotina_1 - ps19
Saccharomycotina_2 - ps20
Saccharomycotina_3 - ps21
Phaff./Saccharomycetaceae - ps22
Saccharomyco./mycetaceae - ps23
Saccharomycetaceae - ps24
Saccharomycetaceae_2 - ps25
Saccharomycetaceae_3 - ps26
Kaz./Nau./Saccharomyces - ps27
Saccharomyces - ps28
Arboricola_clade - ps29
Cerevisiae_clade - ps30
Saccharomyces_cerevisiae - ps31
p-value
1e-13
9e-11
6e-08
4e-05
3e-02
Log-odds
0.5
1.0
1.5
2.0
2.5
3.0
3.5
4.0

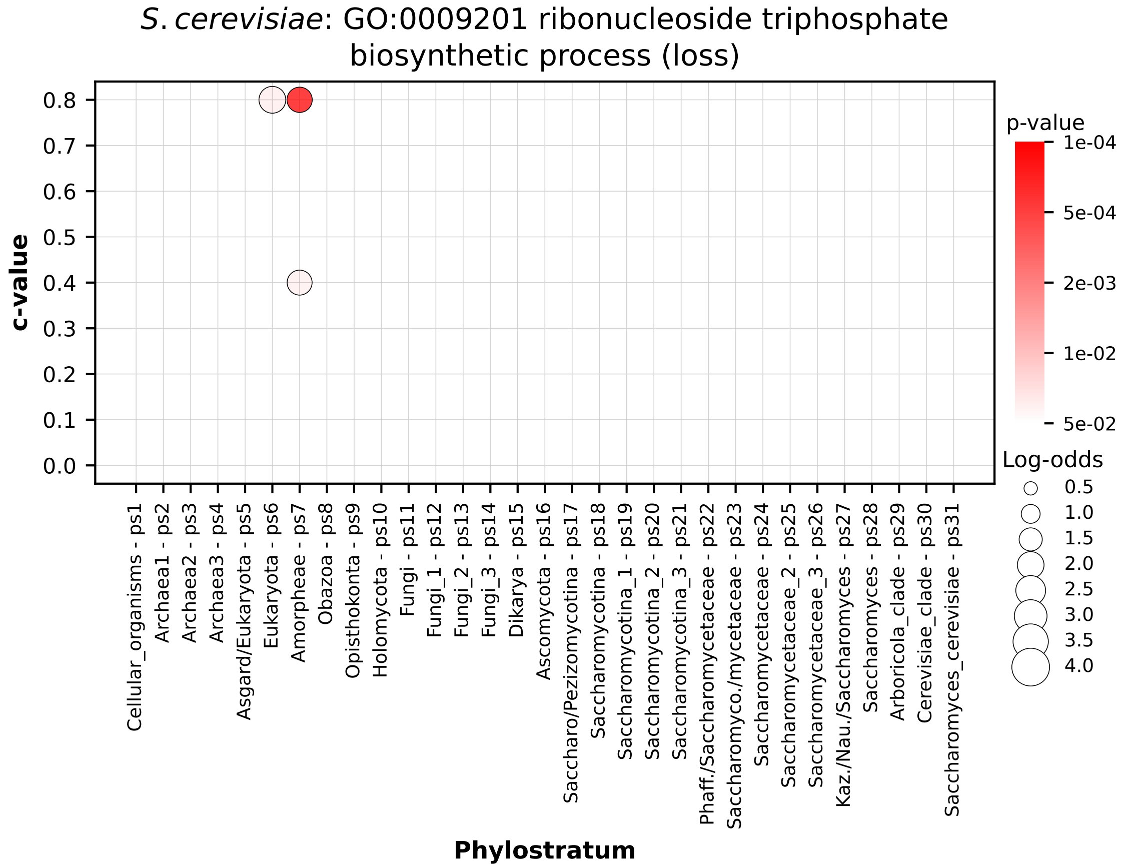
S. cerevisiae: GO:0009201 ribonucleoside triphosphate biosynthetic process (loss)
c-value
0.8
0.7
0.6
0.5
0.4
0.3
0.2
0.1
0.0
p-value
1e-04
5e-04
2e-03
1e-02
5e-02
Log-odds
0.5
1.0
1.5
2.0
2.5
3.0
3.5
4.0
Cellular_organisms - ps1
Archaea1 - ps2
Archaea2 - ps3
Archaea3 - ps4
Asgard/Eukaryota - ps5
Eukaryota - ps6
Amorpheae - ps7
Obazoa - ps8
Opisthokonta - ps9
Holomycota - ps10
Fungi - ps11
Fungi_1 - ps12
Fungi_2 - ps13
Fungi_3 - ps14
Dikarya - ps15
Ascomycota - ps16
Saccharo/Pezizomycotina - ps17
Saccharomycotina - ps18
Saccharomycotina_1 - ps19
Saccharomycotina_2 - ps20
Saccharomycotina_3 - ps21
Phaff./Saccharomycetaceae - ps22
Saccharomyco./mycetaceae - ps23
Saccharomycetaceae - ps24
Saccharomycetaceae_2 - ps25
Saccharomycetaceae_3 - ps26
Kaz./Nau./Saccharomyces - ps27
Saccharomyces - ps28
Arboricola_clade - ps29
Cerevisiae_clade - ps30
Saccharomyces_cerevisiae - ps31
Phylostratum

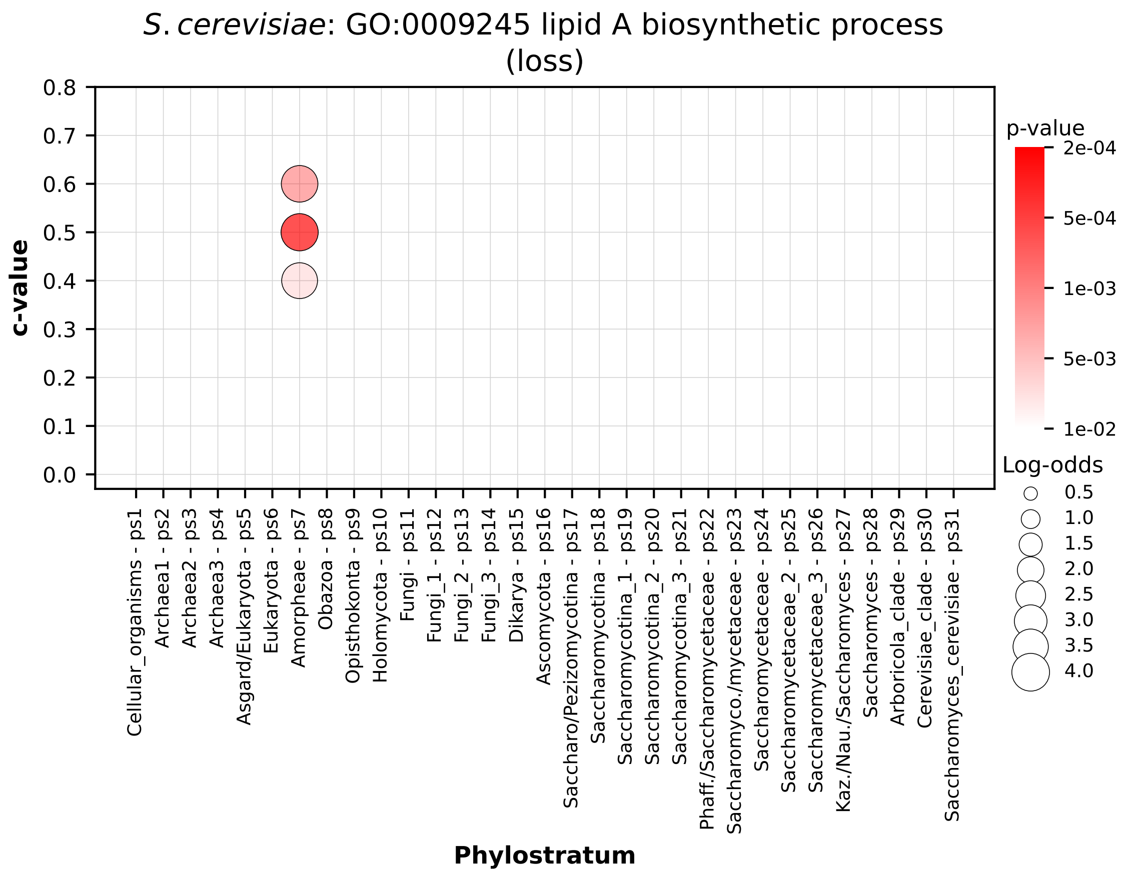

S. cerevisiae: GO:0009245 lipid A biosynthetic process (loss)
c-value
0.0
0.1
0.2
0.3
0.4
0.5
0.6
0.7
0.8
p-value
2e-04
5e-04
1e-03
5e-03
1e-02
Log-odds
0.5
1.0
1.5
2.0
2.5
3.0
3.5
4.0
Cellular_organisms - ps1
Archaea1 - ps2
Archaea2 - ps3
Archaea3 - ps4
Asgard/Eukaryota - ps5
Eukaryota - ps6
Amorpheae - ps7
Obazoa - ps8
Opisthokonta - ps9
Holomycota - ps10
Fungi - ps11
Fungi_1 - ps12
Fungi_2 - ps13
Fungi_3 - ps14
Dikarya - ps15
Ascomycota - ps16
Saccharo/Pezizomycotina - ps17
Saccharomycotina - ps18
Saccharomycotina_1 - ps19
Saccharomycotina_2 - ps20
Saccharomycotina_3 - ps21
Phaff./Saccharomycetaceae - ps22
Saccharomyco./mycetaceae - ps23
Saccharomycetaceae - ps24
Saccharomycetaceae_2 - ps25
Saccharomycetaceae_3 - ps26
Kaz./Nau./Saccharomyces - ps27
Saccharomyces - ps28
Arboricola_clade - ps29
Cerevisiae_clade - ps30
Saccharomyces_cerevisiae - ps31
Phylostratum

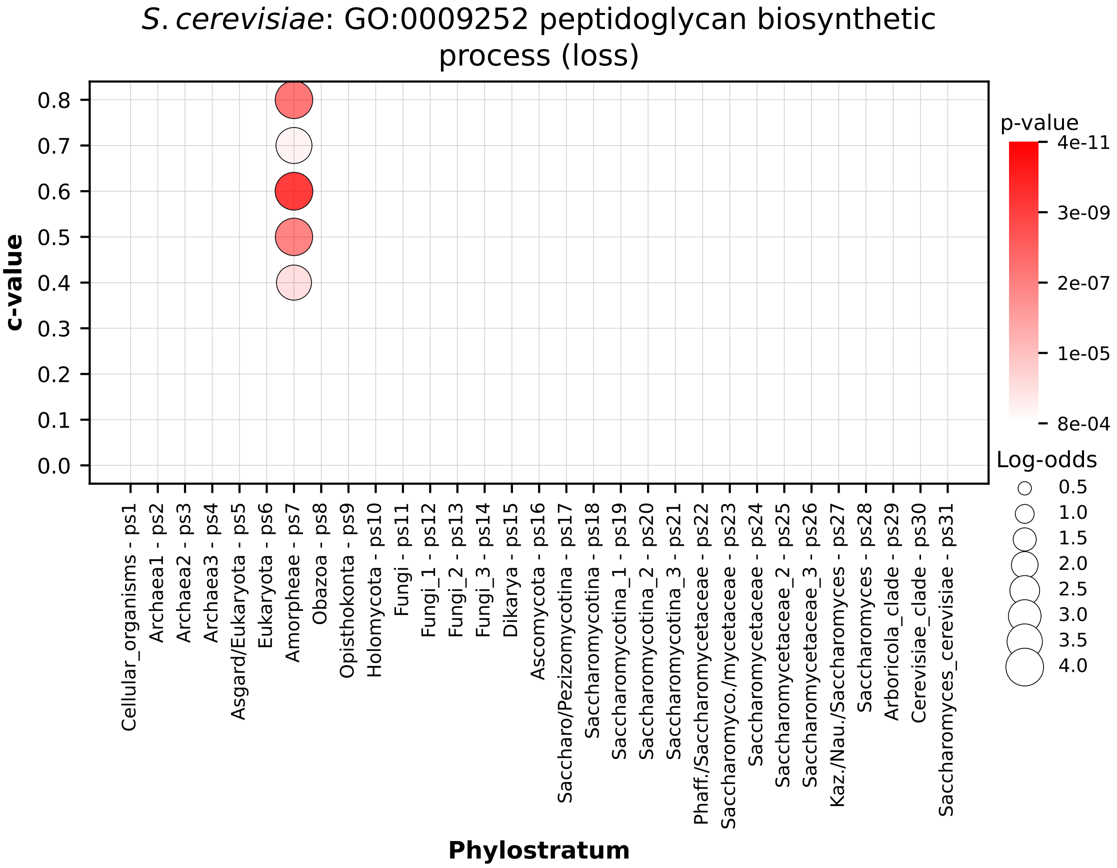

S. cerevisiae: GO:0009252 peptidoglycan biosynthetic process (loss)
c-value
0.0
0.1
0.2
0.3
0.4
0.5
0.6
0.7
0.8
Phylostratum
Cellular_organisms - ps1
Archaea1 - ps2
Archaea2 - ps3
Archaea3 - ps4
Asgard/Eukaryota - ps5
Eukaryota - ps6
Amorpheae - ps7
Obazoa - ps8
Opisthokonta - ps9
Holomycota - ps10
Fungi - ps11
Fungi_1 - ps12
Fungi_2 - ps13
Fungi_3 - ps14
Dikarya - ps15
Ascomycota - ps16
Saccharo/Pezizomycotina - ps17
Saccharomycotina - ps18
Saccharomycotina_1 - ps19
Saccharomycotina_2 - ps20
Saccharomycotina_3 - ps21
Phaff./Saccharomycetaceae - ps22
Saccharomyco./mycetaceae - ps23
Saccharomycetaceae - ps24
Saccharomycetaceae_2 - ps25
Saccharomycetaceae_3 - ps26
Kaz./Nau./Saccharomyces - ps27
Saccharomyces - ps28
Arboricola_clade - ps29
Cerevisiae_clade - ps30
Saccharomyces_cerevisiae - ps31
p-value
4e-11
3e-09
2e-07
1e-05
8e-04
Log-odds
0.5
1.0
1.5
2.0
2.5
3.0
3.5
4.0

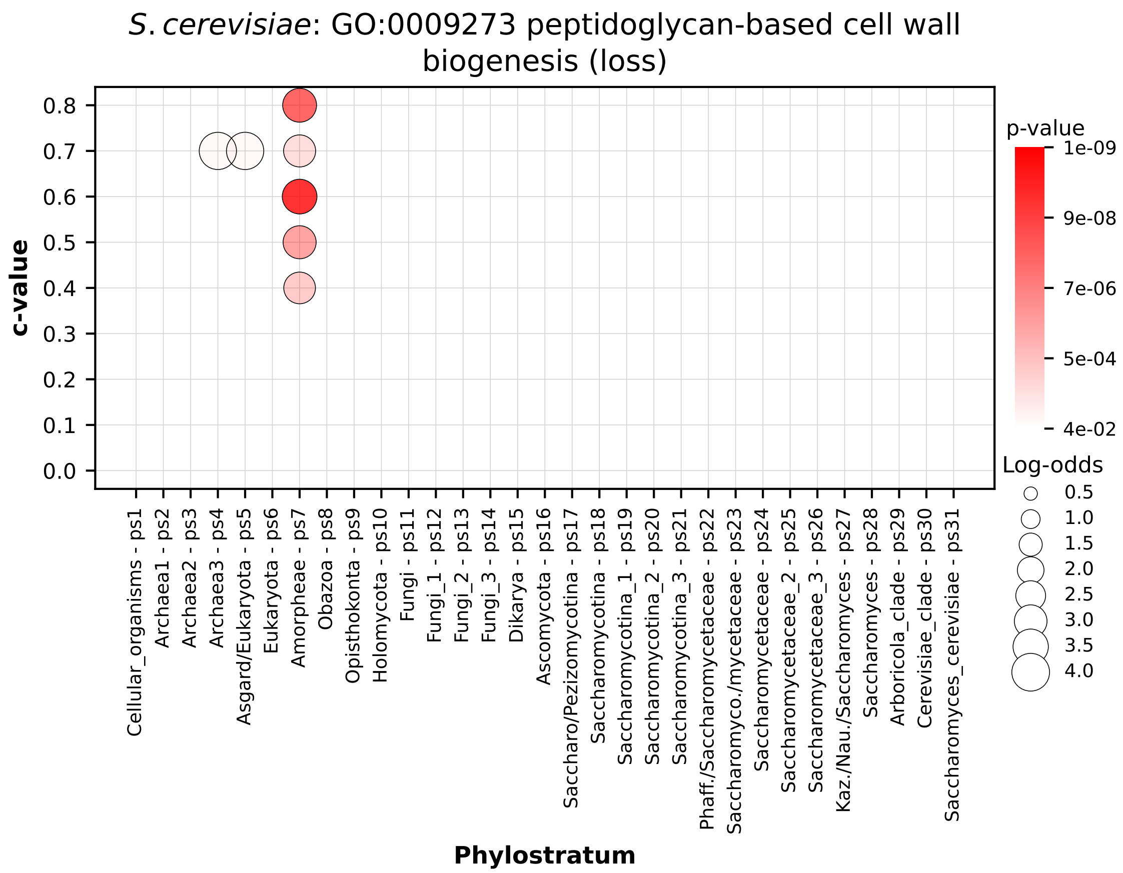

S. cerevisiae: GO:0009273 peptidoglycan-based cell wall biogenesis (loss)
c-value
0.8
0.7
0.6
0.5
0.4
0.3
0.2
0.1
0.0
p-value
1e-09
9e-08
7e-06
5e-04
4e-02
Log-odds
0.5
1.0
1.5
2.0
2.5
3.0
3.5
4.0
Cellular_organisms - ps1
Archaea1 - ps2
Archaea2 - ps3
Archaea3 - ps4
Asgard/Eukaryota - ps5
Eukaryota - ps6
Amorpheae - ps7
Obazoa - ps8
Opisthokonta - ps9
Holomycota - ps10
Fungi - ps11
Fungi_1 - ps12
Fungi_2 - ps13
Fungi_3 - ps14
Dikarya - ps15
Ascomycota - ps16
Saccharo/Pezizomycotina - ps17
Saccharomycotina - ps18
Saccharomycotina_1 - ps19
Saccharomycotina_2 - ps20
Saccharomycotina_3 - ps21
Phaff./Saccharomycetaceae - ps22
Saccharomyco./mycetaceae - ps23
Saccharomycetaceae - ps24
Saccharomycetaceae_2 - ps25
Saccharomycetaceae_3 - ps26
Kaz./Nau./Saccharomyces - ps27
Saccharomyces - ps28
Arboricola_clade - ps29
Cerevisiae_clade - ps30
Saccharomyces_cerevisiae - ps31
Phylostratum

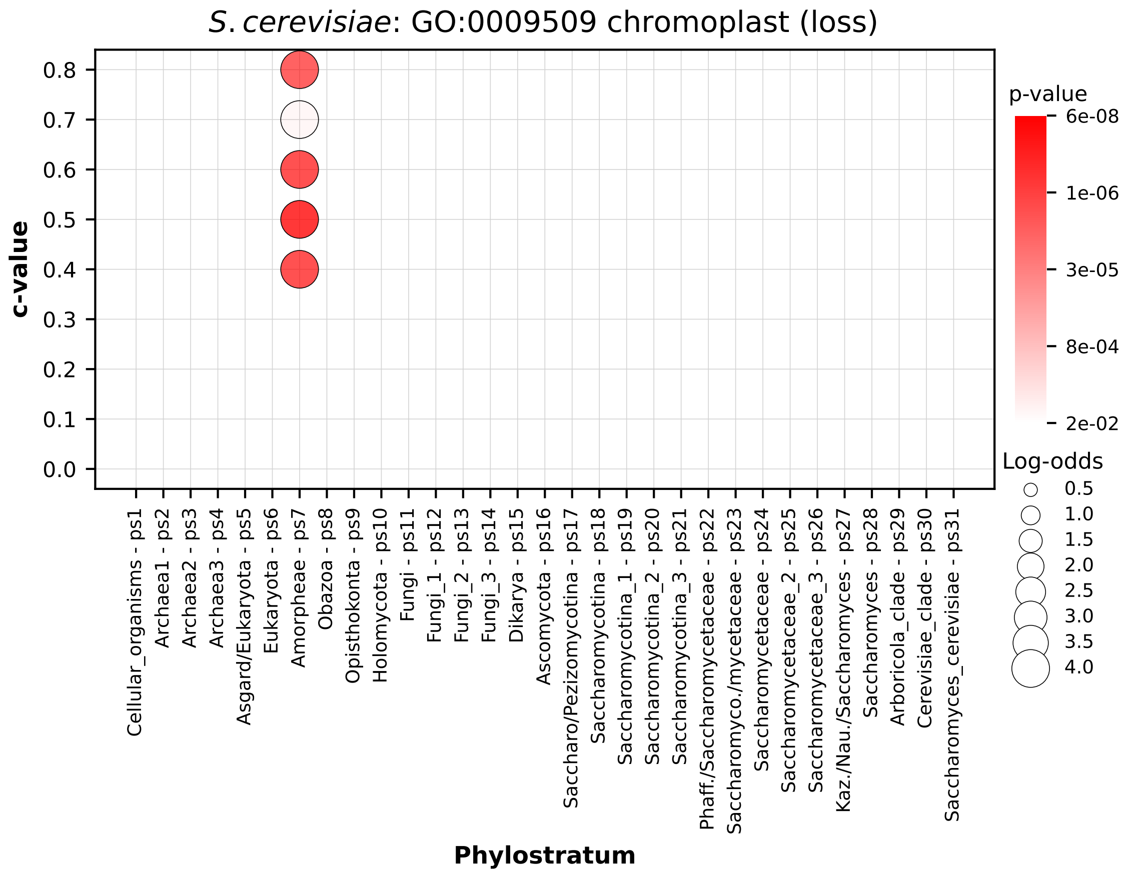

S. cerevisiae: GO:0009509 chromoplast (loss)
c-value
0.8
0.7
0.6
0.5
0.4
0.3
0.2
0.1
0.0
p-value
6e-08
1e-06
3e-05
8e-04
2e-02
Log-odds
0.5
1.0
1.5
2.0
2.5
3.0
3.5
4.0
Cellular_organisms - ps1
Archaea1 - ps2
Archaea2 - ps3
Archaea3 - ps4
Asgard/Eukaryota - ps5
Eukaryota - ps6
Amorpheae - ps7
Obazoa - ps8
Opisthokonta - ps9
Holomycota - ps10
Fungi - ps11
Fungi_1 - ps12
Fungi_2 - ps13
Fungi_3 - ps14
Dikarya - ps15
Ascomycota - ps16
Saccharo/Pezizomycotina - ps17
Saccharomycotina - ps18
Saccharomycotina_1 - ps19
Saccharomycotina_2 - ps20
Saccharomycotina_3 - ps21
Phaff./Saccharomycetaceae - ps22
Saccharomyco./mycetaceae - ps23
Saccharomycetaceae - ps24
Saccharomycetaceae_2 - ps25
Saccharomycetaceae_3 - ps26
Kaz./Nau./Saccharomyces - ps27
Saccharomyces - ps28
Arboricola_clade - ps29
Cerevisiae_clade - ps30
Saccharomyces_cerevisiae - ps31
Phylostratum

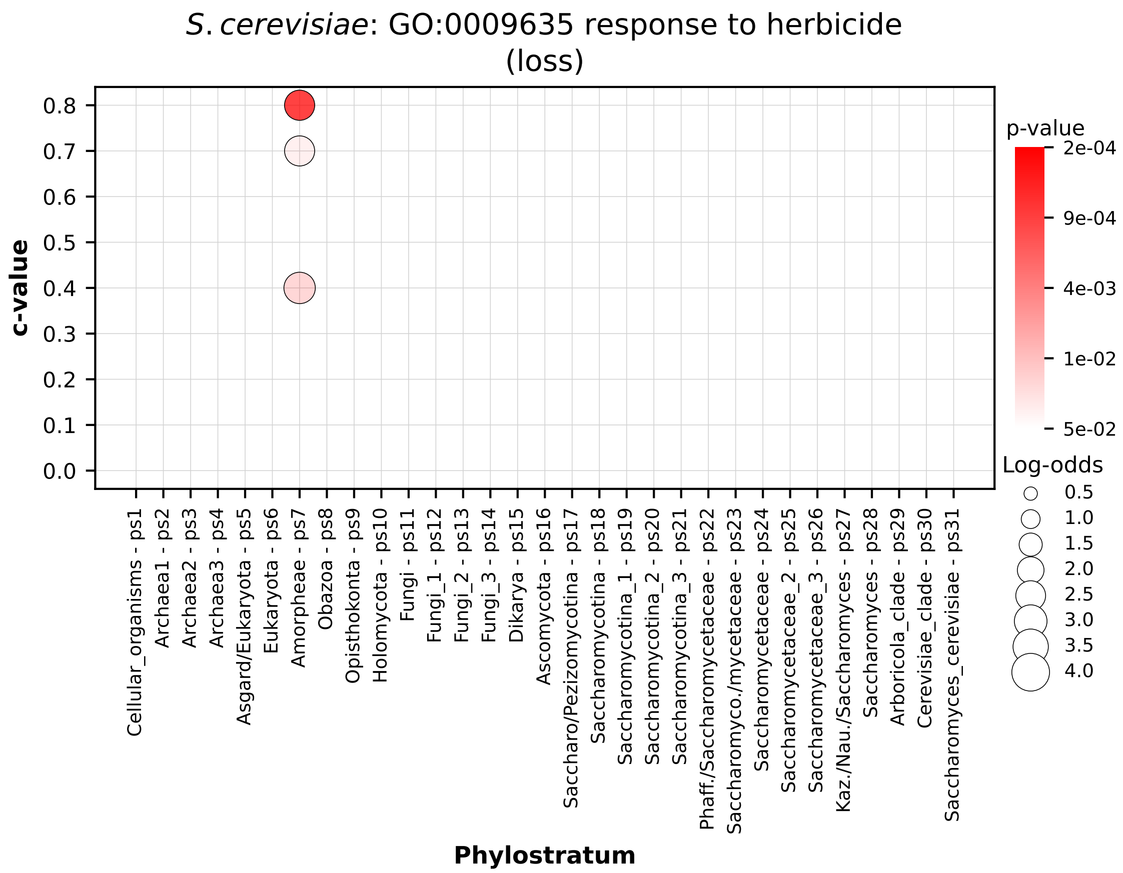

S. cerevisiae: GO:0009635 response to herbicide
(loss)
c-value
0.8
0.7
0.6
0.5
0.4
0.3
0.2
0.1
0.0
p-value
2e-04
9e-04
4e-03
1e-02
5e-02
Log-odds
0.5
1.0
1.5
2.0
2.5
3.0
3.5
4.0
Cellular_organisms - ps1
Archaea1 - ps2
Archaea2 - ps3
Archaea3 - ps4
Asgard/Eukaryota - ps5
Eukaryota - ps6
Amorpheae - ps7
Obazoa - ps8
Opisthokonta - ps9
Holomycota - ps10
Fungi - ps11
Fungi_1 - ps12
Fungi_2 - ps13
Fungi_3 - ps14
Dikarya - ps15
Ascomycota - ps16
Saccharo/Pezizomycotina - ps17
Saccharomycotina - ps18
Saccharomycotina_1 - ps19
Saccharomycotina_2 - ps20
Saccharomycotina_3 - ps21
Phaff./Saccharomycetaceae - ps22
Saccharomyco./mycetaceae - ps23
Saccharomycetaceae - ps24
Saccharomycetaceae_2 - ps25
Saccharomycetaceae_3 - ps26
Kaz./Nau./Saccharomyces - ps27
Saccharomyces - ps28
Arboricola_clade - ps29
Cerevisiae_clade - ps30
Saccharomyces_cerevisiae - ps31
Phylostratum

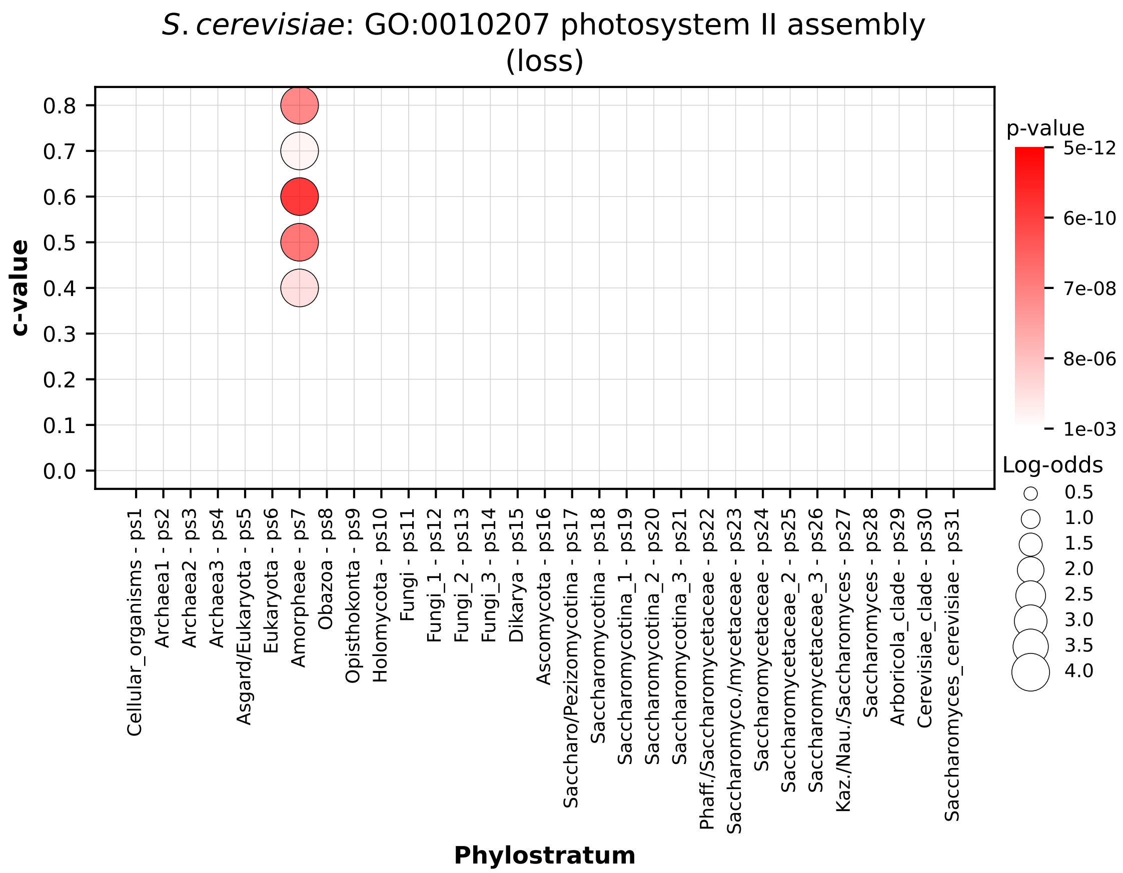

S. cerevisiae: GO:0010207 photosystem II assembly
(loss)
c-value
0.0
0.1
0.2
0.3
0.4
0.5
0.6
0.7
0.8
p-value
5e-12
6e-10
7e-08
8e-06
1e-03
Log-odds
0.5
1.0
1.5
2.0
2.5
3.0
3.5
4.0
Cellular_organisms - ps1
Archaea1 - ps2
Archaea2 - ps3
Archaea3 - ps4
Asgard/Eukaryota - ps5
Eukaryota - ps6
Amorpheae - ps7
Obazoa - ps8
Opisthokonta - ps9
Holomycota - ps10
Fungi - ps11
Fungi_1 - ps12
Fungi_2 - ps13
Fungi_3 - ps14
Dikarya - ps15
Ascomycota - ps16
Saccharo/Pezizomycotina - ps17
Saccharomycotina - ps18
Saccharomycotina_1 - ps19
Saccharomycotina_2 - ps20
Saccharomycotina_3 - ps21
Phaff./Saccharomycetaceae - ps22
Saccharomyco./mycetaceae - ps23
Saccharomycetaceae - ps24
Saccharomycetaceae_2 - ps25
Saccharomycetaceae_3 - ps26
Kaz./Nau./Saccharomyces - ps27
Saccharomyces - ps28
Arboricola_clade - ps29
Cerevisiae_clade - ps30
Saccharomyces_cerevisiae - ps31
Phylostratum

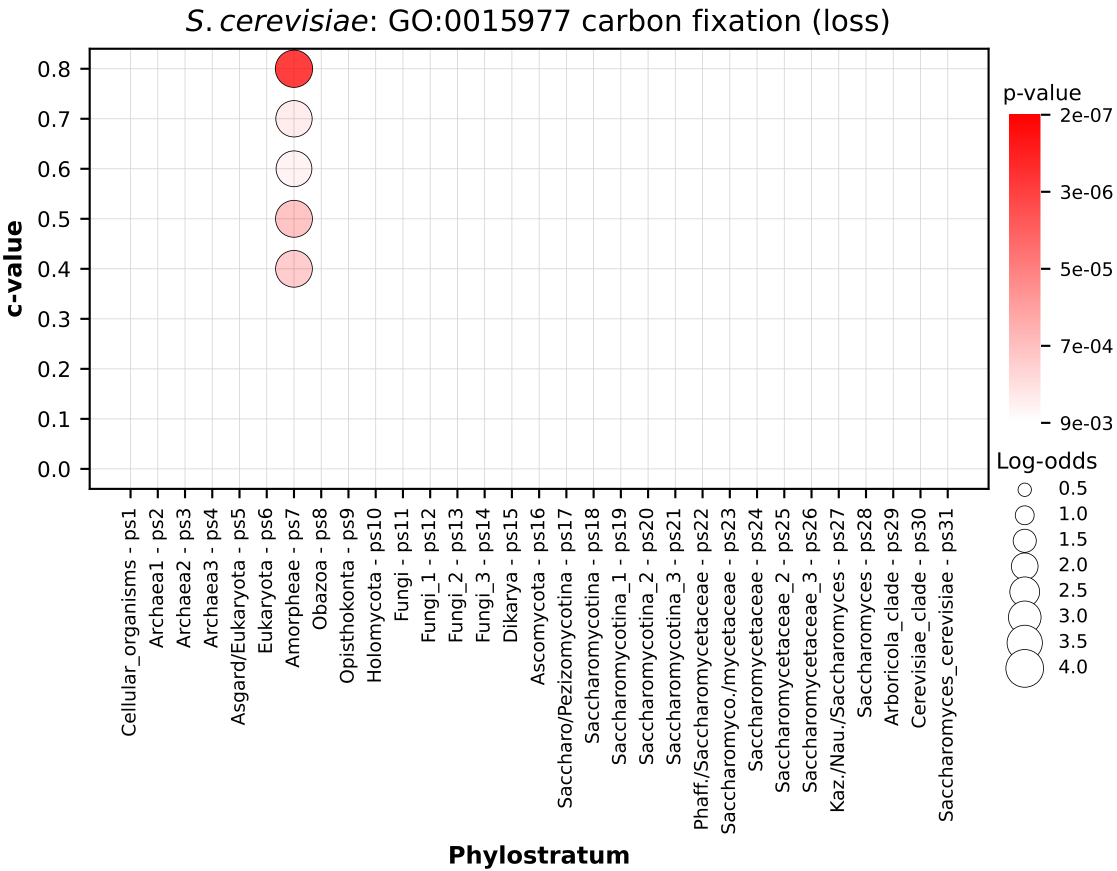
S. cerevisiae: GO:0015977 carbon fixation (loss)
c-value
0.8
0.7
0.6
0.5
0.4
0.3
0.2
0.1
0.0
p-value
2e-07
3e-06
5e-05
7e-04
9e-03
Log-odds
0.5
1.0
1.5
2.0
2.5
3.0
3.5
4.0
Cellular_organisms - ps1
Archaea1 - ps2
Archaea2 - ps3
Archaea3 - ps4
Asgard/Eukaryota - ps5
Eukaryota - ps6
Amorpheae - ps7
Obazoa - ps8
Opisthokonta - ps9
Holomycota - ps10
Fungi - ps11
Fungi_1 - ps12
Fungi_2 - ps13
Fungi_3 - ps14
Dikarya - ps15
Ascomycota - ps16
Saccharo/Pezizomycotina - ps17
Saccharomycotina - ps18
Saccharomycotina_1 - ps19
Saccharomycotina_2 - ps20
Saccharomycotina_3 - ps21
Phaff./Saccharomycetaceae - ps22
Saccharomyco./mycetaceae - ps23
Saccharomycetaceae - ps24
Saccharomycetaceae_2 - ps25
Saccharomycetaceae_3 - ps26
Kaz./Nau./Saccharomyces - ps27
Saccharomyces - ps28
Arboricola_clade - ps29
Cerevisiae_clade - ps30
Saccharomyces_cerevisiae - ps31
Phylostratum

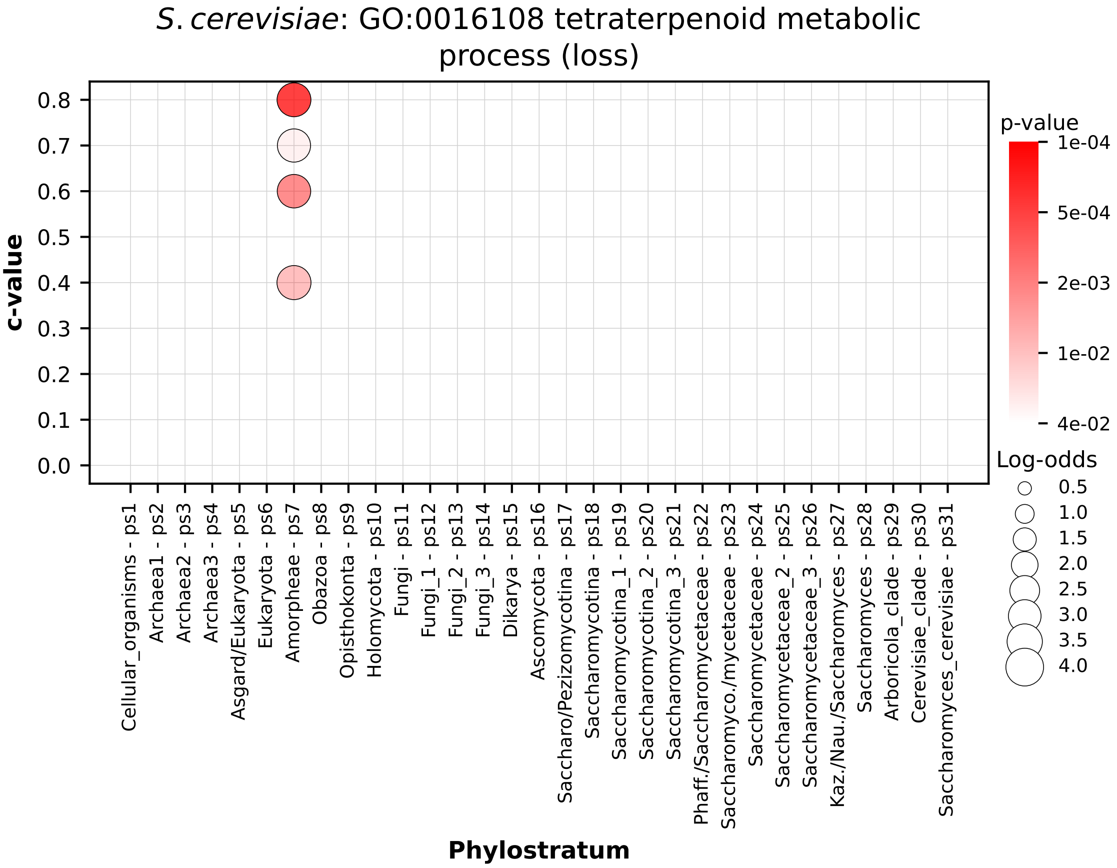
S. cerevisiae: GO:0016108 tetraterpenoid metabolic process (loss)
c-value
0.0
0.1
0.2
0.3
0.4
0.5
0.6
0.7
0.8
p-value
1e-04
5e-04
2e-03
1e-02
4e-02
Log-odds
0.5
1.0
1.5
2.0
2.5
3.0
3.5
4.0
Cellular_organisms - ps1
Archaea1 - ps2
Archaea2 - ps3
Archaea3 - ps4
Asgard/Eukaryota - ps5
Eukaryota - ps6
Amorpheae - ps7
Obazoa - ps8
Opisthokonta - ps9
Holomycota - ps10
Fungi - ps11
Fungi_1 - ps12
Fungi_2 - ps13
Fungi_3 - ps14
Dikarya - ps15
Ascomycota - ps16
Saccharo/Pezizomycotina - ps17
Saccharomycotina - ps18
Saccharomycotina_1 - ps19
Saccharomycotina_2 - ps20
Saccharomycotina_3 - ps21
Phaff./Saccharomycetaceae - ps22
Saccharomyco./mycetaceae - ps23
Saccharomycetaceae - ps24
Saccharomycetaceae_2 - ps25
Saccharomycetaceae_3 - ps26
Kaz./Nau./Saccharomyces - ps27
Saccharomyces - ps28
Arboricola_clade - ps29
Cerevisiae_clade - ps30
Saccharomyces_cerevisiae - ps31
Phylostratum

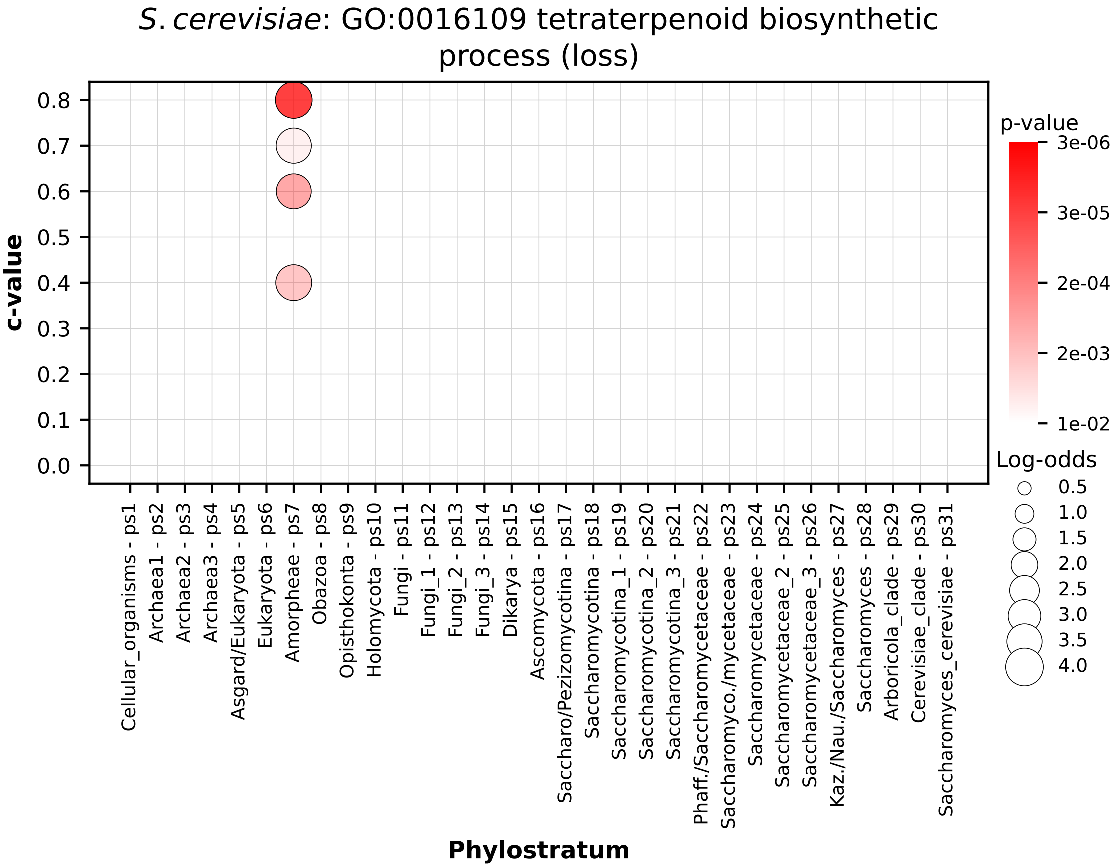
S. cerevisiae: GO:0016109 tetraterpenoid biosynthetic process (loss)
c-value
0.0
0.1
0.2
0.3
0.4
0.5
0.6
0.7
0.8
Phylostratum
Cellular_organisms - ps1
Archaea1 - ps2
Archaea2 - ps3
Archaea3 - ps4
Asgard/Eukaryota - ps5
Eukaryota - ps6
Amorpheae - ps7
Obazoa - ps8
Opisthokonta - ps9
Holomycota - ps10
Fungi - ps11
Fungi_1 - ps12
Fungi_2 - ps13
Fungi_3 - ps14
Dikarya - ps15
Ascomycota - ps16
Saccharo/Pezizomycotina - ps17
Saccharomycotina - ps18
Saccharomycotina_1 - ps19
Saccharomycotina_2 - ps20
Saccharomycotina_3 - ps21
Phaff./Saccharomycetaceae - ps22
Saccharomyco./mycetaceae - ps23
Saccharomycetaceae - ps24
Saccharomycetaceae_2 - ps25
Saccharomycetaceae_3 - ps26
Kaz./Nau./Saccharomyces - ps27
Saccharomyces - ps28
Arboricola_clade - ps29
Cerevisiae_clade - ps30
Saccharomyces_cerevisiae - ps31
p-value
3e-06
3e-05
2e-04
2e-03
1e-02
Log-odds
0.5
1.0
1.5
2.0
2.5
3.0
3.5
4.0

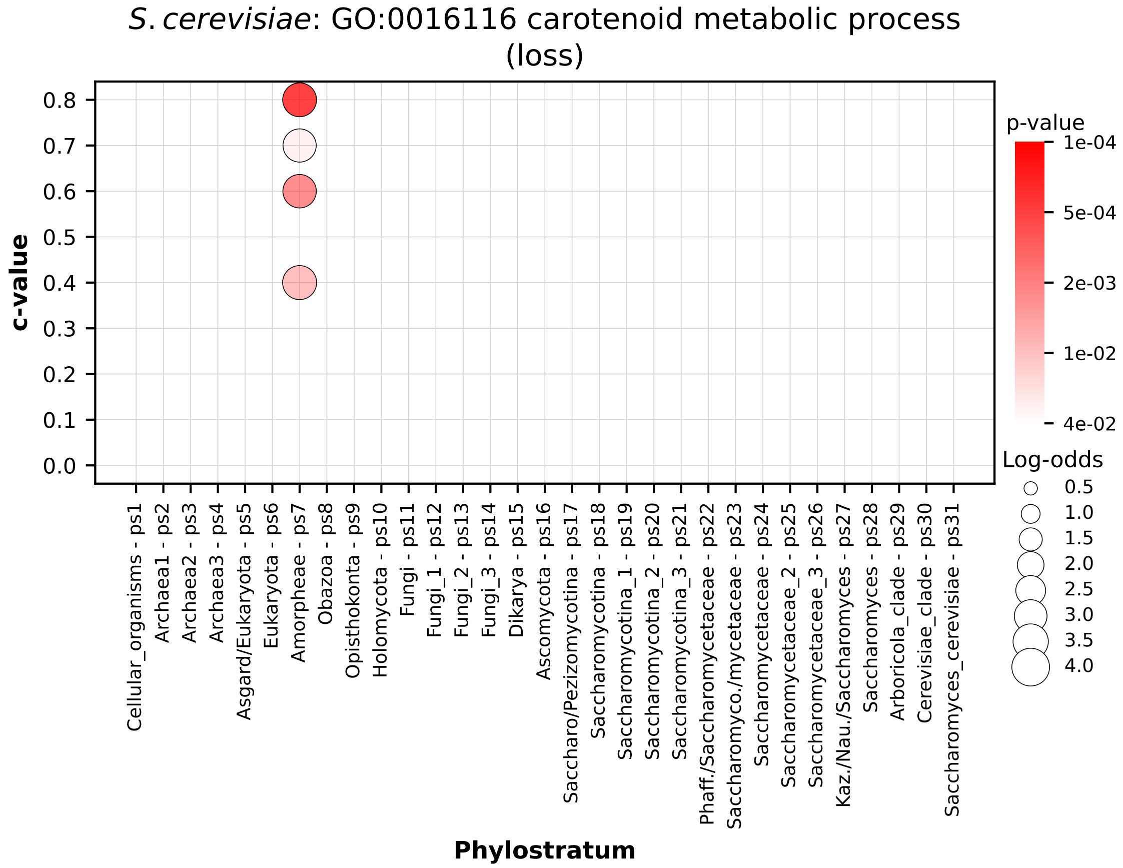

S. cerevisiae: GO:0016116 carotenoid metabolic process
(loss)
c-value
0.0
0.1
0.2
0.3
0.4
0.5
0.6
0.7
0.8
p-value
1e-04
5e-04
2e-03
1e-02
4e-02
Log-odds
0.5
1.0
1.5
2.0
2.5
3.0
3.5
4.0
Cellular_organisms - ps1
Archaea1 - ps2
Archaea2 - ps3
Archaea3 - ps4
Asgard/Eukaryota - ps5
Eukaryota - ps6
Amorpheae - ps7
Obazoa - ps8
Opisthokonta - ps9
Holomycota - ps10
Fungi - ps11
Fungi_1 - ps12
Fungi_2 - ps13
Fungi_3 - ps14
Dikarya - ps15
Ascomycota - ps16
Saccharo/Pezizomycotina - ps17
Saccharomycotina - ps18
Saccharomycotina_1 - ps19
Saccharomycotina_2 - ps20
Saccharomycotina_3 - ps21
Phaff./Saccharomycetaceae - ps22
Saccharomyco./mycetaceae - ps23
Saccharomycetaceae - ps24
Saccharomycetaceae_2 - ps25
Saccharomycetaceae_3 - ps26
Kaz./Nau./Saccharomyces - ps27
Saccharomyces - ps28
Arboricola_clade - ps29
Cerevisiae_clade - ps30
Saccharomyces_cerevisiae - ps31
Phylostratum

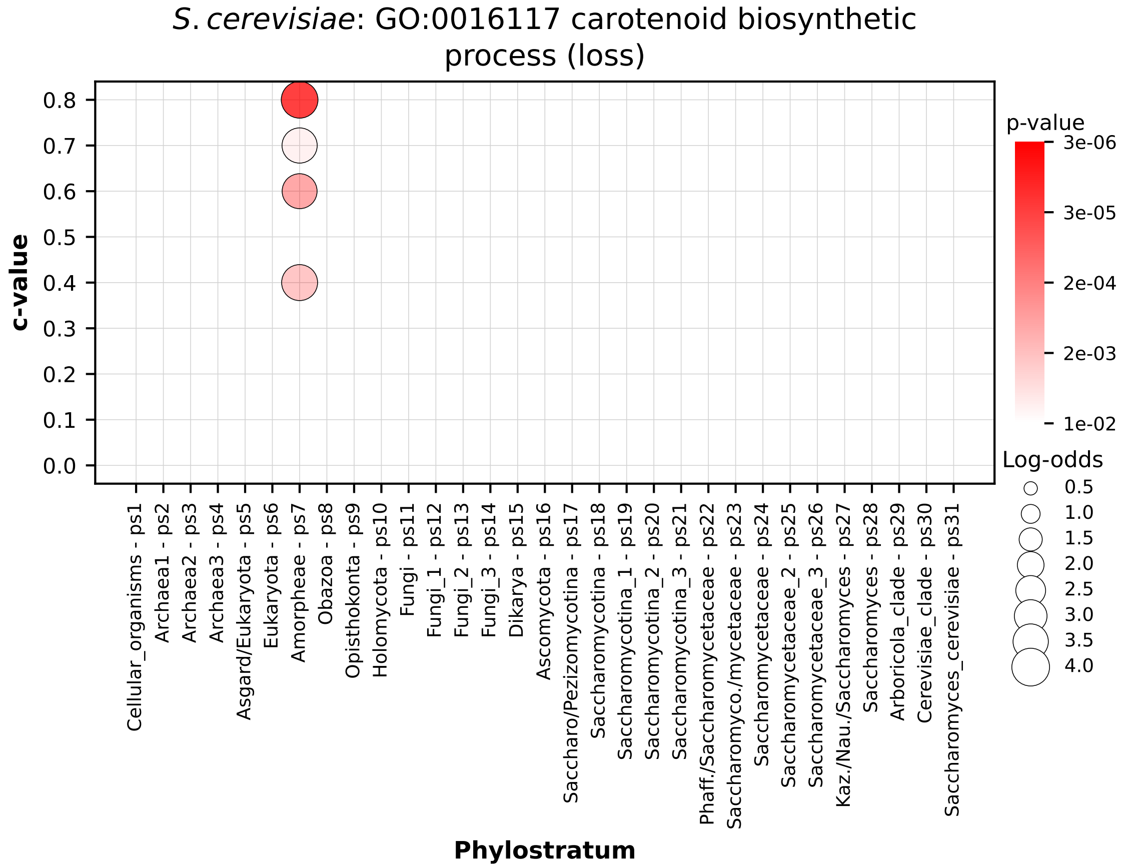

S. cerevisiae: GO:0016117 carotenoid biosynthetic process (loss)
c-value
0.0
0.1
0.2
0.3
0.4
0.5
0.6
0.7
0.8
Phylostratum
Cellular_organisms - ps1
Archaea1 - ps2
Archaea2 - ps3
Archaea3 - ps4
Asgard/Eukaryota - ps5
Eukaryota - ps6
Amorpheae - ps7
Obazoa - ps8
Opisthokonta - ps9
Holomycota - ps10
Fungi - ps11
Fungi_1 - ps12
Fungi_2 - ps13
Fungi_3 - ps14
Dikarya - ps15
Ascomycota - ps16
Saccharo/Pezizomycotina - ps17
Saccharomycotina - ps18
Saccharomycotina_1 - ps19
Saccharomycotina_2 - ps20
Saccharomycotina_3 - ps21
Phaff./Saccharomycetaceae - ps22
Saccharomyco./mycetaceae - ps23
Saccharomycetaceae - ps24
Saccharomycetaceae_2 - ps25
Saccharomycetaceae_3 - ps26
Kaz./Nau./Saccharomyces - ps27
Saccharomyces - ps28
Arboricola_clade - ps29
Cerevisiae_clade - ps30
Saccharomyces_cerevisiae - ps31
p-value
3e-06
3e-05
2e-04
2e-03
1e-02
Log-odds
0.5
1.0
1.5
2.0
2.5
3.0
3.5
4.0

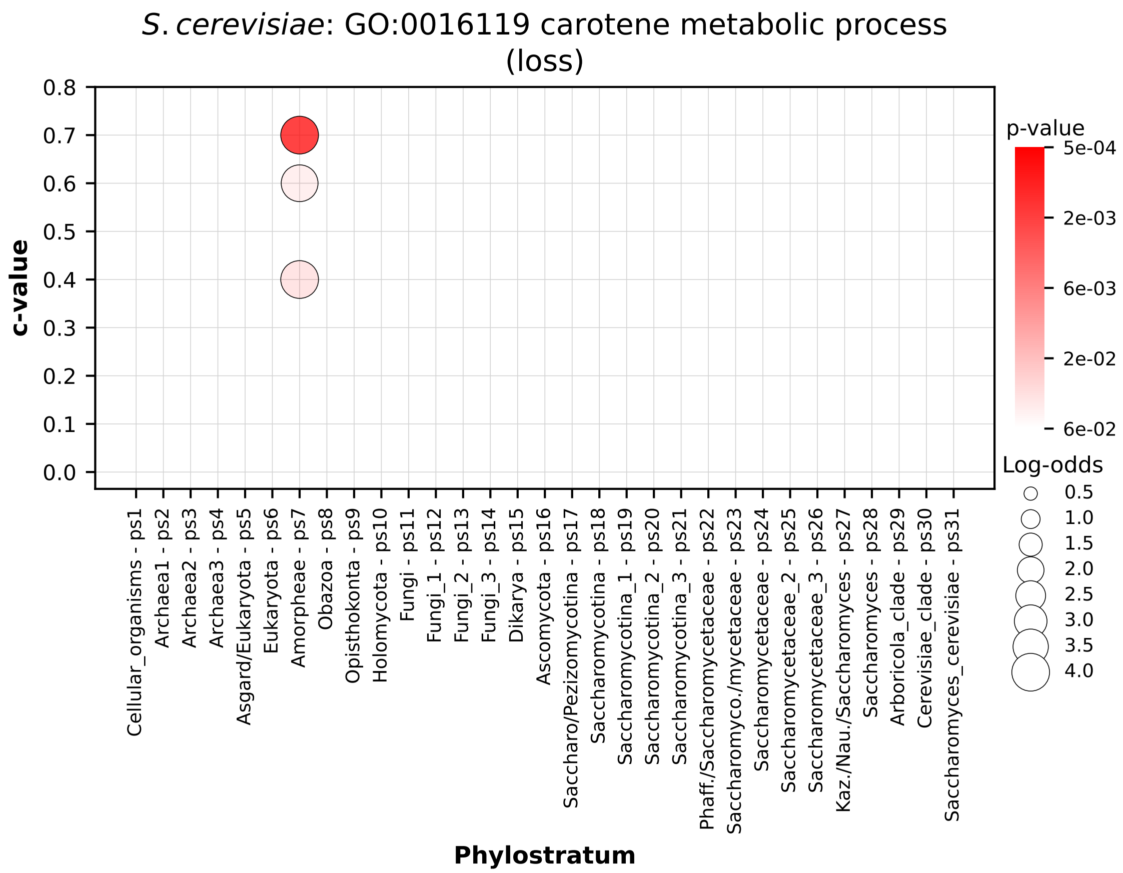
S. cerevisiae: GO:0016119 carotene metabolic process
(loss)
c-value
0.8
0.7
0.6
0.5
0.4
0.3
0.2
0.1
0.0
Phylostratum
Cellular_organisms - ps1
Archaea1 - ps2
Archaea2 - ps3
Archaea3 - ps4
Asgard/Eukaryota - ps5
Eukaryota - ps6
Amorpheae - ps7
Obazoa - ps8
Opisthokonta - ps9
Holomycota - ps10
Fungi - ps11
Fungi_1 - ps12
Fungi_2 - ps13
Fungi_3 - ps14
Dikarya - ps15
Ascomycota - ps16
Saccharo/Pezizomycotina - ps17
Saccharomycotina - ps18
Saccharomycotina_1 - ps19
Saccharomycotina_2 - ps20
Saccharomycotina_3 - ps21
Phaff./Saccharomycetaceae - ps22
Saccharomyco./mycetaceae - ps23
Saccharomycetaceae - ps24
Saccharomycetaceae_2 - ps25
Saccharomycetaceae_3 - ps26
Kaz./Nau./Saccharomyces - ps27
Saccharomyces - ps28
Arboricola_clade - ps29
Cerevisiae_clade - ps30
Saccharomyces_cerevisiae - ps31
p-value
5e-04
2e-03
6e-03
2e-02
6e-02
Log-odds
0.5
1.0
1.5
2.0
2.5
3.0
3.5
4.0

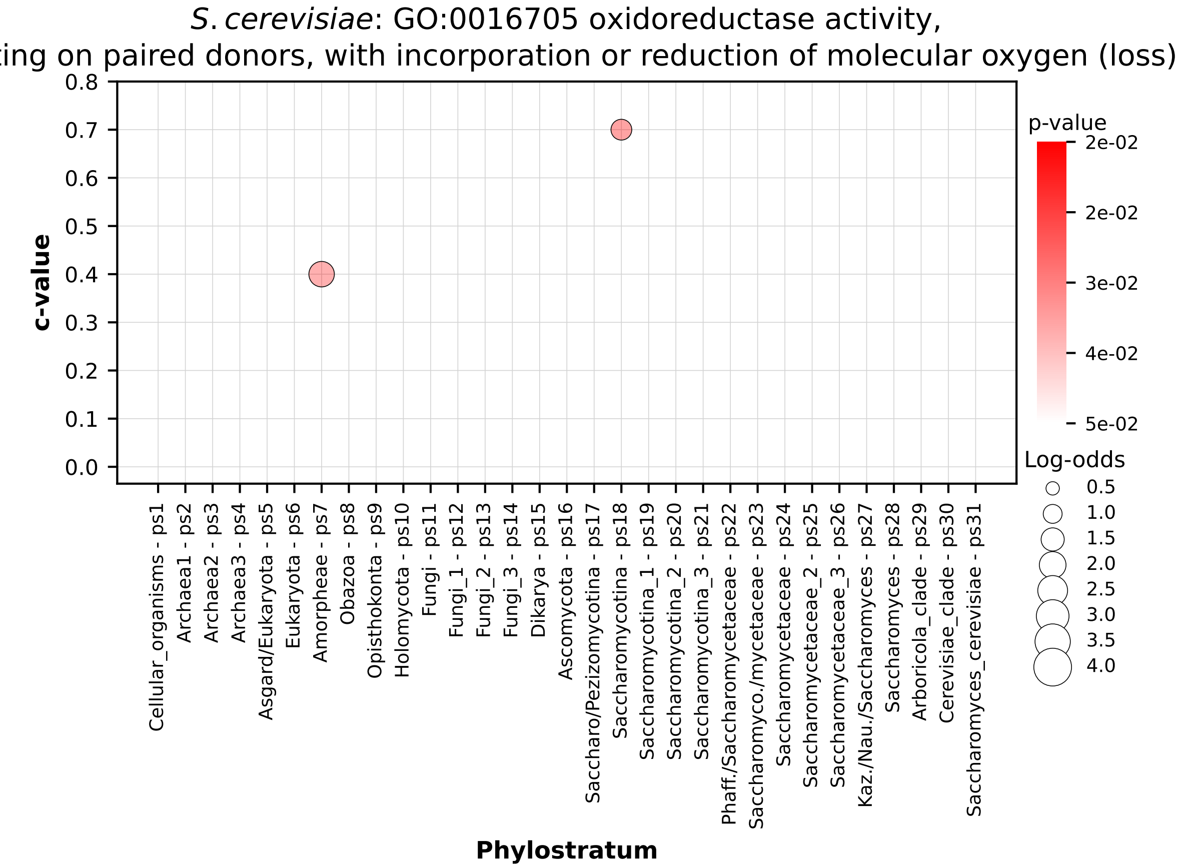
S. cerevisiae: GO:0016705 oxidoreductase activity,
ting on paired donors, with incorporation or reduction of molecular oxygen (loss)
c-value
0.8
0.7
0.6
0.5
0.4
0.3
0.2
0.1
0.0
p-value
2e-02
2e-02
3e-02
4e-02
5e-02
Log-odds
0.5
1.0
1.5
2.0
2.5
3.0
3.5
4.0
Cellular_organisms - ps1
Archaea1 - ps2
Archaea2 - ps3
Archaea3 - ps4
Asgard/Eukaryota - ps5
Eukaryota - ps6
Amorpheae - ps7
Obazoa - ps8
Opisthokonta - ps9
Holomycota - ps10
Fungi - ps11
Fungi_1 - ps12
Fungi_2 - ps13
Fungi_3 - ps14
Dikarya - ps15
Ascomycota - ps16
Saccharo/Pezizomycotina - ps17
Saccharomycotina - ps18
Saccharomycotina_1 - ps19
Saccharomycotina_2 - ps20
Saccharomycotina_3 - ps21
Phaff./Saccharomycetaceae - ps22
Saccharomyco./mycetaceae - ps23
Saccharomycetaceae - ps24
Saccharomycetaceae_2 - ps25
Saccharomycetaceae_3 - ps26
Kaz./Nau./Saccharomyces - ps27
Saccharomyces - ps28
Arboricola_clade - ps29
Cerevisiae_clade - ps30
Saccharomyces_cerevisiae - ps31
Phylostratum

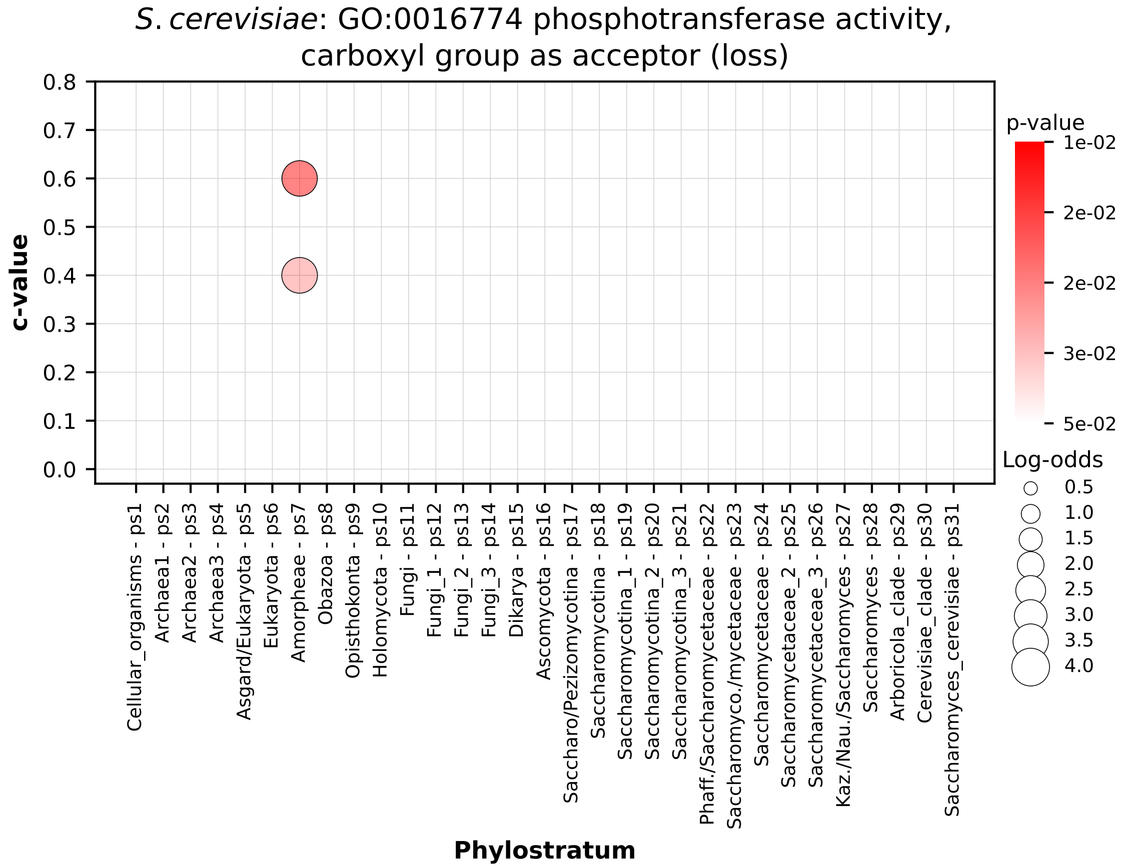

S. cerevisiae: GO:0016774 phosphotransferase activity, carboxyl group as acceptor (loss)
c-value
0.8
0.7
0.6
0.5
0.4
0.3
0.2
0.1
0.0
p-value
1e-02
2e-02
2e-02
3e-02
5e-02
Log-odds
0.5
1.0
1.5
2.0
2.5
3.0
3.5
4.0
Cellular_organisms - ps1
Archaea1 - ps2
Archaea2 - ps3
Archaea3 - ps4
Asgard/Eukaryota - ps5
Eukaryota - ps6
Amorpheae - ps7
Obazoa - ps8
Opisthokonta - ps9
Holomycota - ps10
Fungi - ps11
Fungi_1 - ps12
Fungi_2 - ps13
Fungi_3 - ps14
Dikarya - ps15
Ascomycota - ps16
Saccharo/Pezizomycotina - ps17
Saccharomycotina - ps18
Saccharomycotina_1 - ps19
Saccharomycotina_2 - ps20
Saccharomycotina_3 - ps21
Phaff./Saccharomycetaceae - ps22
Saccharomyco./mycetaceae - ps23
Saccharomycetaceae - ps24
Saccharomycetaceae_2 - ps25
Saccharomycetaceae_3 - ps26
Kaz./Nau./Saccharomyces - ps27
Saccharomyces - ps28
Arboricola_clade - ps29
Cerevisiae_clade - ps30
Saccharomyces_cerevisiae - ps31
Phylostratum

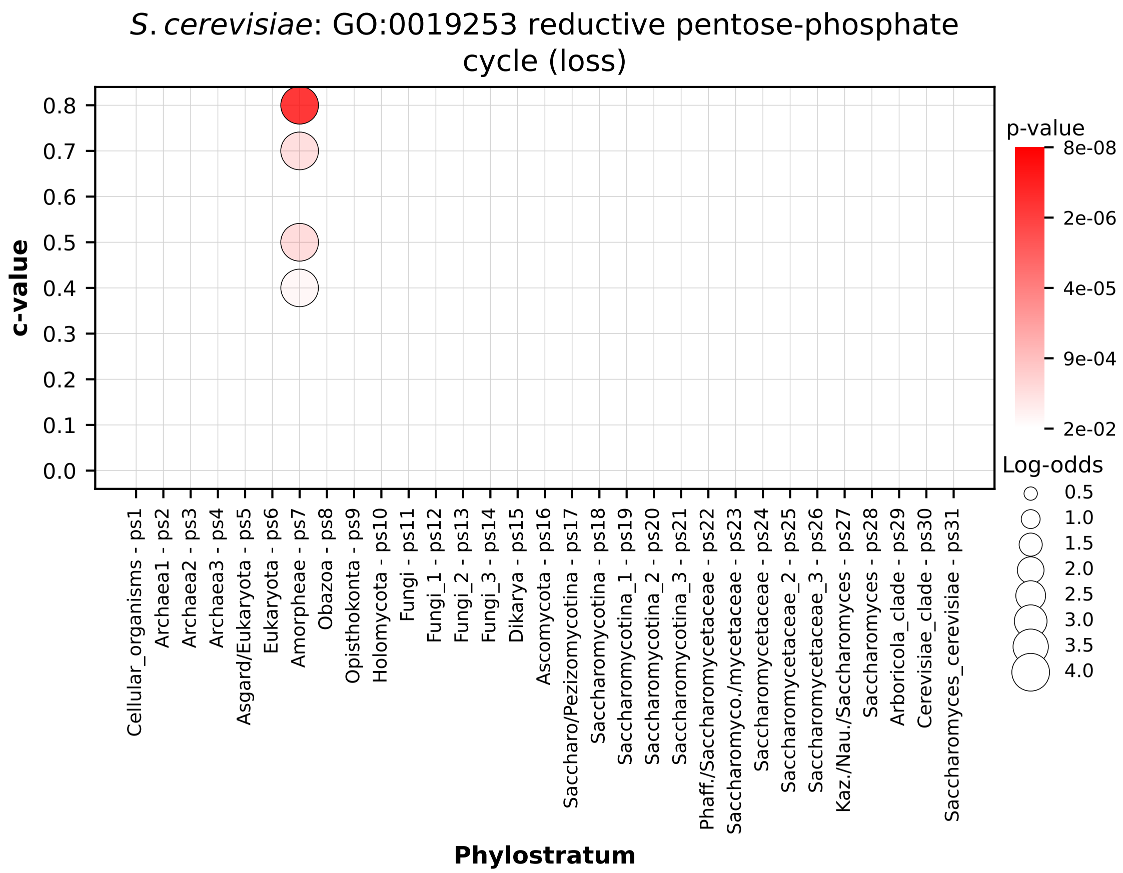

S. cerevisiae: GO:0019253 reductive pentose-phosphate cycle (loss)
c-value
0.8
0.7
0.6
0.5
0.4
0.3
0.2
0.1
0.0
p-value
8e-08
2e-06
4e-05
9e-04
2e-02
Log-odds
0.5
1.0
1.5
2.0
2.5
3.0
3.5
4.0
Cellular_organisms - ps1
Archaea1 - ps2
Archaea2 - ps3
Archaea3 - ps4
Asgard/Eukaryota - ps5
Eukaryota - ps6
Amorpheae - ps7
Obazoa - ps8
Opisthokonta - ps9
Holomycota - ps10
Fungi - ps11
Fungi_1 - ps12
Fungi_2 - ps13
Fungi_3 - ps14
Dikarya - ps15
Ascomycota - ps16
Saccharo/Pezizomycotina - ps17
Saccharomycotina - ps18
Saccharomycotina_1 - ps19
Saccharomycotina_2 - ps20
Saccharomycotina_3 - ps21
Phaff./Saccharomycetaceae - ps22
Saccharomyco./mycetaceae - ps23
Saccharomycetaceae - ps24
Saccharomycetaceae_2 - ps25
Saccharomycetaceae_3 - ps26
Kaz./Nau./Saccharomyces - ps27
Saccharomyces - ps28
Arboricola_clade - ps29
Cerevisiae_clade - ps30
Saccharomyces_cerevisiae - ps31
Phylostratum

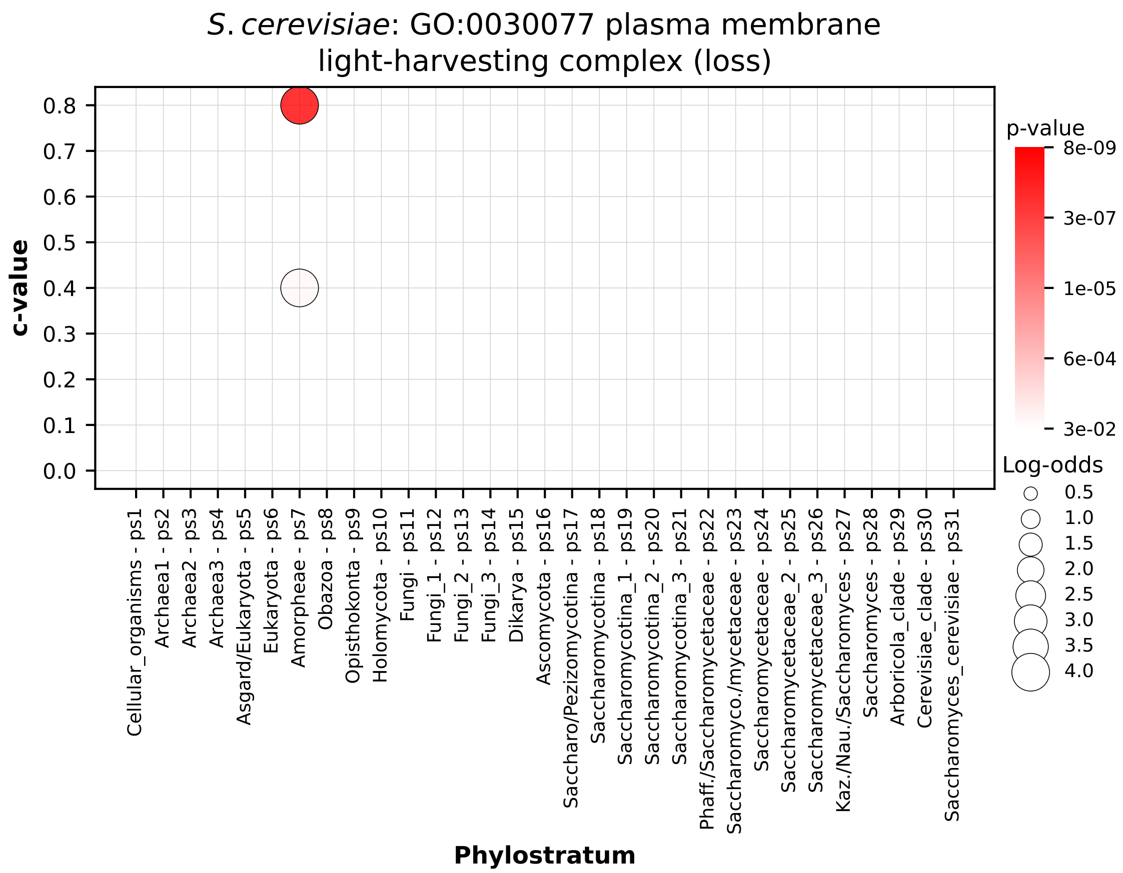

S. cerevisiae: GO:0030077 plasma membrane light-harvesting complex (loss)
c-value
0.0
0.1
0.2
0.3
0.4
0.5
0.6
0.7
0.8
Phylostratum
Cellular_organisms - ps1
Archaea1 - ps2
Archaea2 - ps3
Archaea3 - ps4
Asgard/Eukaryota - ps5
Eukaryota - ps6
Amorpheae - ps7
Obazoa - ps8
Opisthokonta - ps9
Holomycota - ps10
Fungi - ps11
Fungi_1 - ps12
Fungi_2 - ps13
Fungi_3 - ps14
Dikarya - ps15
Ascomycota - ps16
Saccharo/Pezizomycotina - ps17
Saccharomycotina - ps18
Saccharomycotina_1 - ps19
Saccharomycotina_2 - ps20
Saccharomycotina_3 - ps21
Phaff./Saccharomycetaceae - ps22
Saccharomyco./mycetaceae - ps23
Saccharomycetaceae - ps24
Saccharomycetaceae_2 - ps25
Saccharomycetaceae_3 - ps26
Kaz./Nau./Saccharomyces - ps27
Saccharomyces - ps28
Arboricola_clade - ps29
Cerevisiae_clade - ps30
Saccharomyces_cerevisiae - ps31
p-value
8e-09
3e-07
1e-05
6e-04
3e-02
Log-odds
0.5
1.0
1.5
2.0
2.5
3.0
3.5
4.0

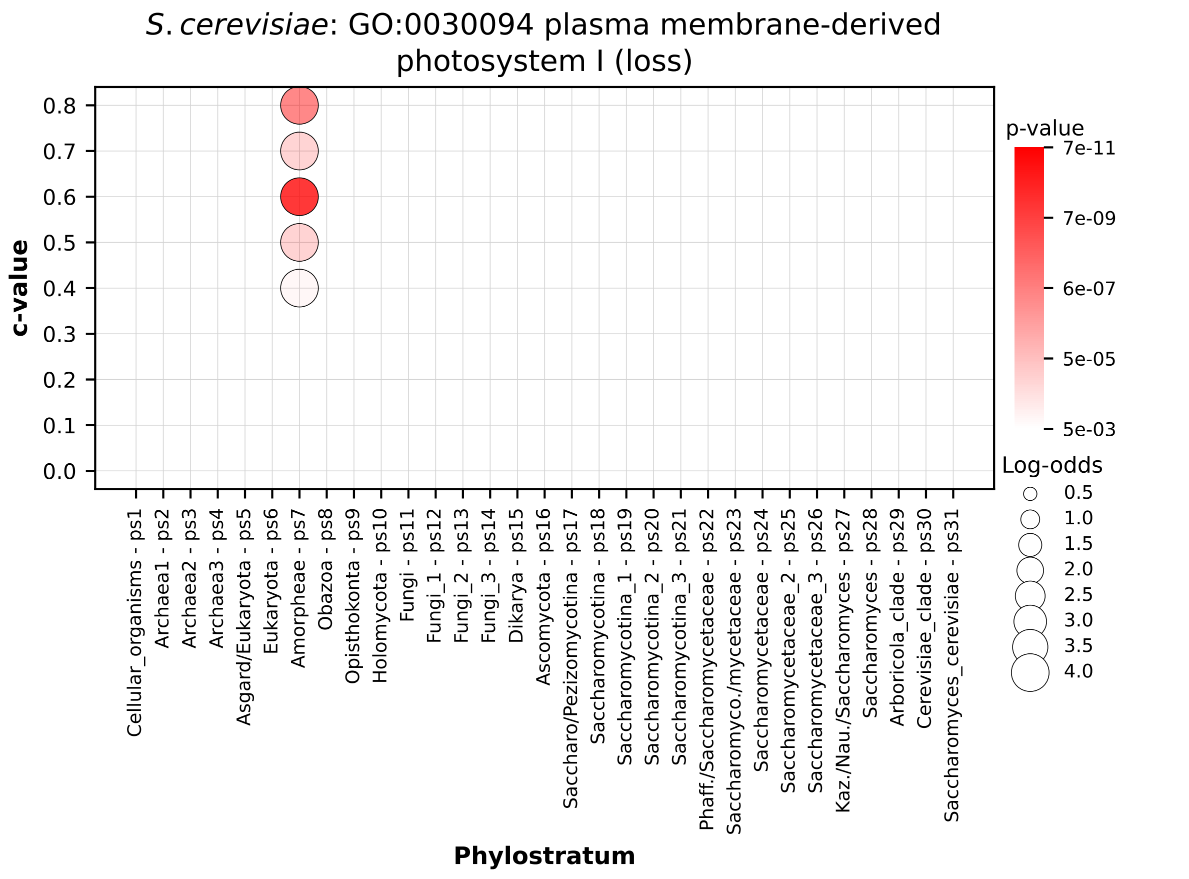

S. cerevisiae: GO:0030094 plasma membrane-derived photosystem I (loss)
c-value
0.0
0.1
0.2
0.3
0.4
0.5
0.6
0.7
0.8
p-value
7e-11
7e-09
6e-07
5e-05
5e-03
Log-odds
0.5
1.0
1.5
2.0
2.5
3.0
3.5
4.0
Cellular_organisms - ps1
Archaea1 - ps2
Archaea2 - ps3
Archaea3 - ps4
Asgard/Eukaryota - ps5
Eukaryota - ps6
Amorpheae - ps7
Obazoa - ps8
Opisthokonta - ps9
Holomycota - ps10
Fungi - ps11
Fungi_1 - ps12
Fungi_2 - ps13
Fungi_3 - ps14
Dikarya - ps15
Ascomycota - ps16
Saccharo/Pezizomycotina - ps17
Saccharomycotina - ps18
Saccharomycotina_1 - ps19
Saccharomycotina_2 - ps20
Saccharomycotina_3 - ps21
Phaff./Saccharomycetaceae - ps22
Saccharomyco./mycetaceae - ps23
Saccharomycetaceae - ps24
Saccharomycetaceae_2 - ps25
Saccharomycetaceae_3 - ps26
Kaz./Nau./Saccharomyces - ps27
Saccharomyces - ps28
Arboricola_clade - ps29
Cerevisiae_clade - ps30
Saccharomyces_cerevisiae - ps31
Phylostratum

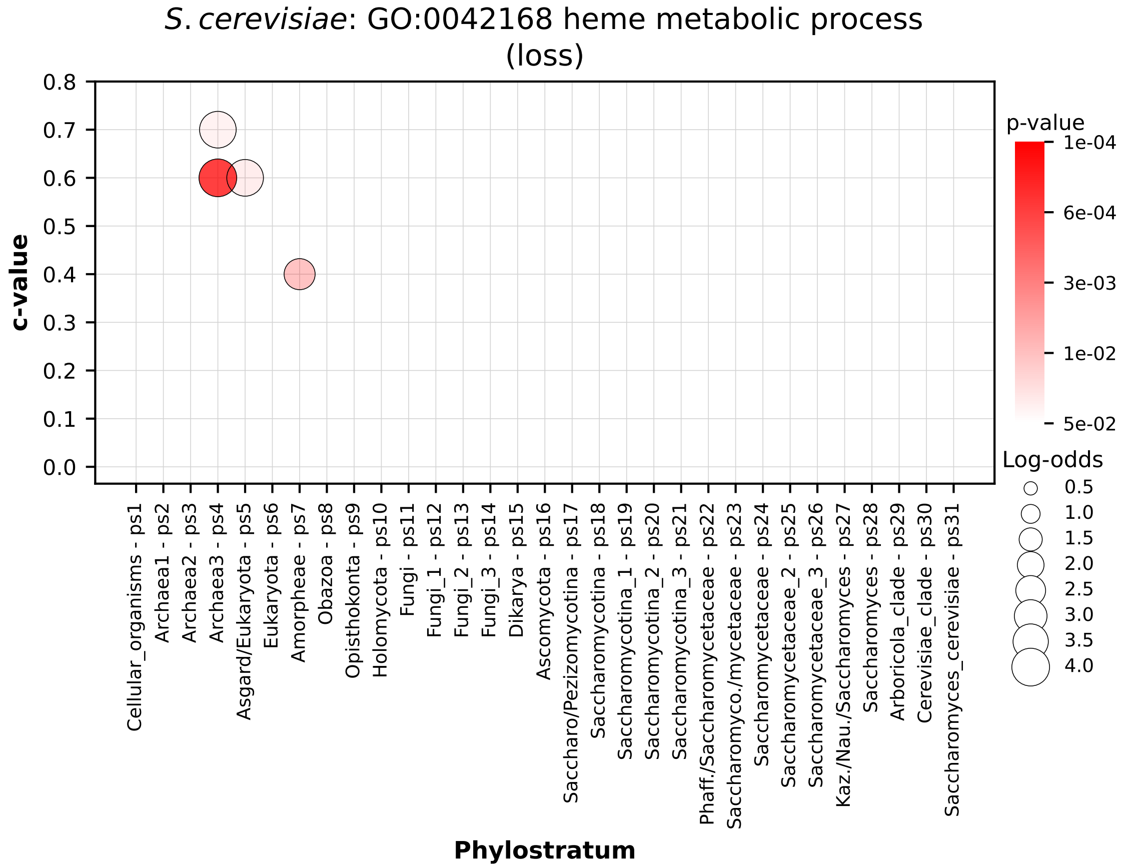

S. cerevisiae: GO:0042168 heme metabolic process
(loss)
c-value
0.8
0.7
0.6
0.5
0.4
0.3
0.2
0.1
0.0
Phylostratum
Cellular_organisms - ps1
Archaea1 - ps2
Archaea2 - ps3
Archaea3 - ps4
Asgard/Eukaryota - ps5
Eukaryota - ps6
Amorpheae - ps7
Obazoa - ps8
Opisthokonta - ps9
Holomycota - ps10
Fungi - ps11
Fungi_1 - ps12
Fungi_2 - ps13
Fungi_3 - ps14
Dikarya - ps15
Ascomycota - ps16
Saccharo/Pezizomycotina - ps17
Saccharomycotina - ps18
Saccharomycotina_1 - ps19
Saccharomycotina_2 - ps20
Saccharomycotina_3 - ps21
Phaff./Saccharomycetaceae - ps22
Saccharomyco./mycetaceae - ps23
Saccharomycetaceae - ps24
Saccharomycetaceae_2 - ps25
Saccharomycetaceae_3 - ps26
Kaz./Nau./Saccharomyces - ps27
Saccharomyces - ps28
Arboricola_clade - ps29
Cerevisiae_clade - ps30
Saccharomyces_cerevisiae - ps31
p-value
1e-04
6e-04
3e-03
1e-02
5e-02
Log-odds
0.5
1.0
1.5
2.0
2.5
3.0
3.5
4.0

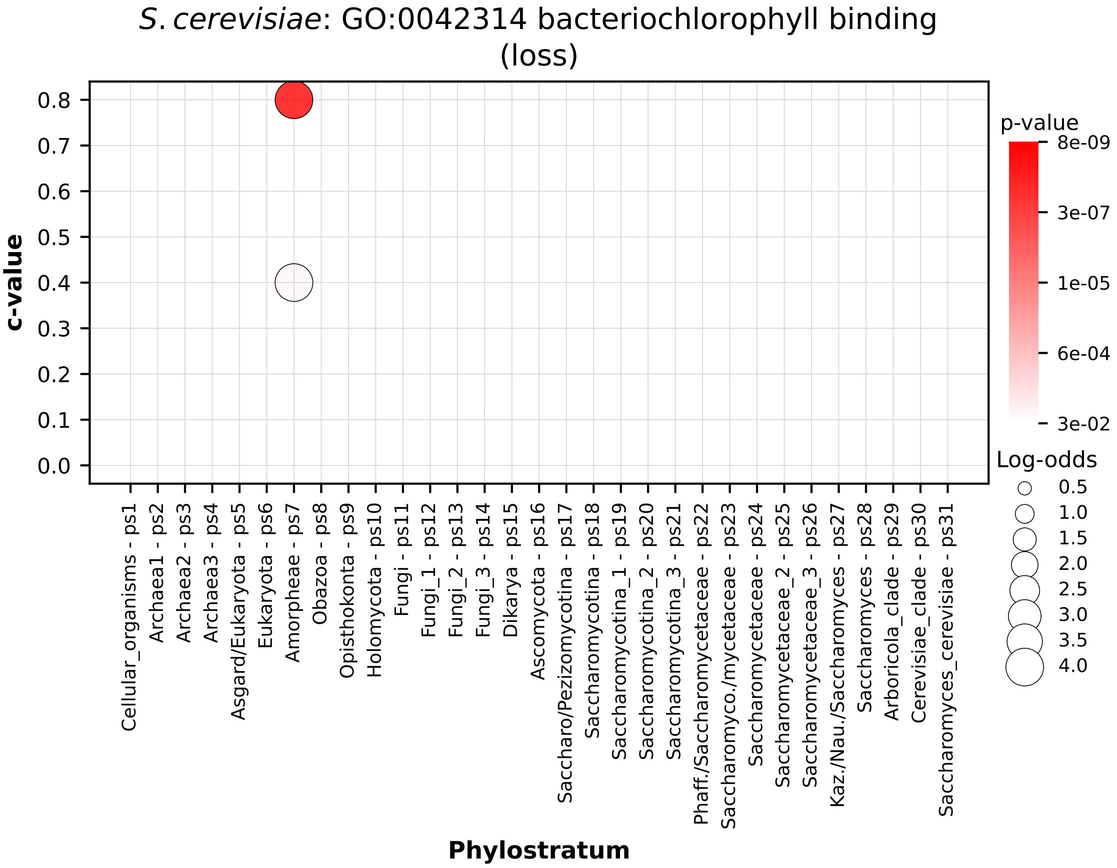

$S. cerevisiae$: GO:0042314 bacteriochlorophyll binding
(loss)
c-value
0.8
0.7
0.6
0.5
0.4
0.3
0.2
0.1
0.0
p-value
8e-09
3e-07
1e-05
6e-04
3e-02
Log-odds
0.5
1.0
1.5
2.0
2.5
3.0
3.5
4.0
Cellular_organisms - ps1
Archaea1 - ps2
Archaea2 - ps3
Archaea3 - ps4
Asgard/Eukaryota - ps5
Eukaryota - ps6
Amorpheae - ps7
Obazoa - ps8
Opisthokonta - ps9
Holomycota - ps10
Fungi - ps11
Fungi_1 - ps12
Fungi_2 - ps13
Fungi_3 - ps14
Dikarya - ps15
Ascomycota - ps16
Saccharo/Pezizomycotina - ps17
Saccharomycotina - ps18
Saccharomycotina_1 - ps19
Saccharomycotina_2 - ps20
Saccharomycotina_3 - ps21
Phaff./Saccharomycetaceae - ps22
Saccharomyco./mycetaceae - ps23
Saccharomycetaceae - ps24
Saccharomycetaceae_2 - ps25
Saccharomycetaceae_3 - ps26
Kaz./Nau./Saccharomyces - ps27
Saccharomyces - ps28
Arboricola_clade - ps29
Cerevisiae_clade - ps30
Saccharomyces_cerevisiae - ps31
Phylostratum

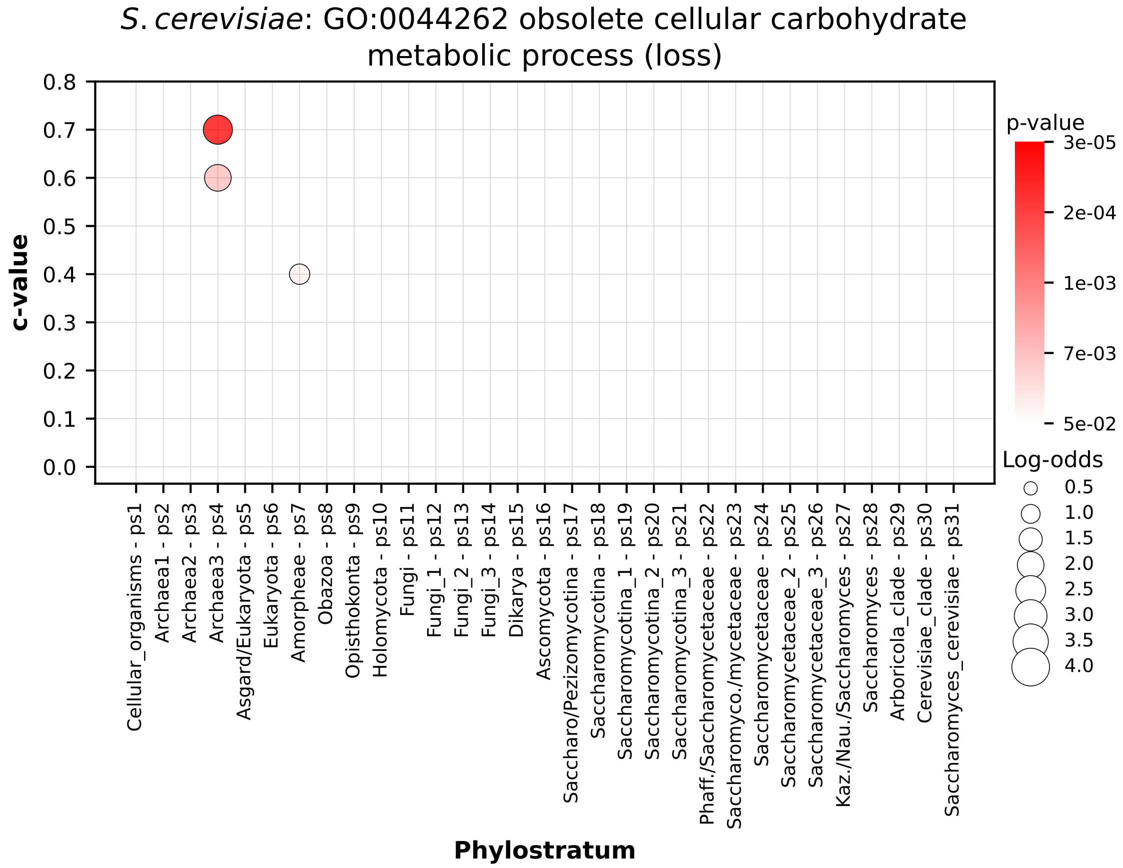

S. cerevisiae: GO:0044262 obsolete cellular carbohydrate metabolic process (loss)
c-value
0.0
0.1
0.2
0.3
0.4
0.5
0.6
0.7
0.8
Phylostratum
Cellular_organisms - ps1
Archaea1 - ps2
Archaea2 - ps3
Archaea3 - ps4
Asgard/Eukaryota - ps5
Eukaryota - ps6
Amorpheae - ps7
Obazoa - ps8
Opisthokonta - ps9
Holomycota - ps10
Fungi - ps11
Fungi_1 - ps12
Fungi_2 - ps13
Fungi_3 - ps14
Dikarya - ps15
Ascomycota - ps16
Saccharo/Pezizomycotina - ps17
Saccharomycotina - ps18
Saccharomycotina_1 - ps19
Saccharomycotina_2 - ps20
Saccharomycotina_3 - ps21
Phaff./Saccharomycetaceae - ps22
Saccharomyco./mycetaceae - ps23
Saccharomycetaceae - ps24
Saccharomycetaceae_2 - ps25
Saccharomycetaceae_3 - ps26
Kaz./Nau./Saccharomyces - ps27
Saccharomyces - ps28
Arboricola_clade - ps29
Cerevisiae_clade - ps30
Saccharomyces_cerevisiae - ps31
p-value
3e-05
2e-04
1e-03
7e-03
5e-02
Log-odds
0.5
1.0
1.5
2.0
2.5
3.0
3.5
4.0

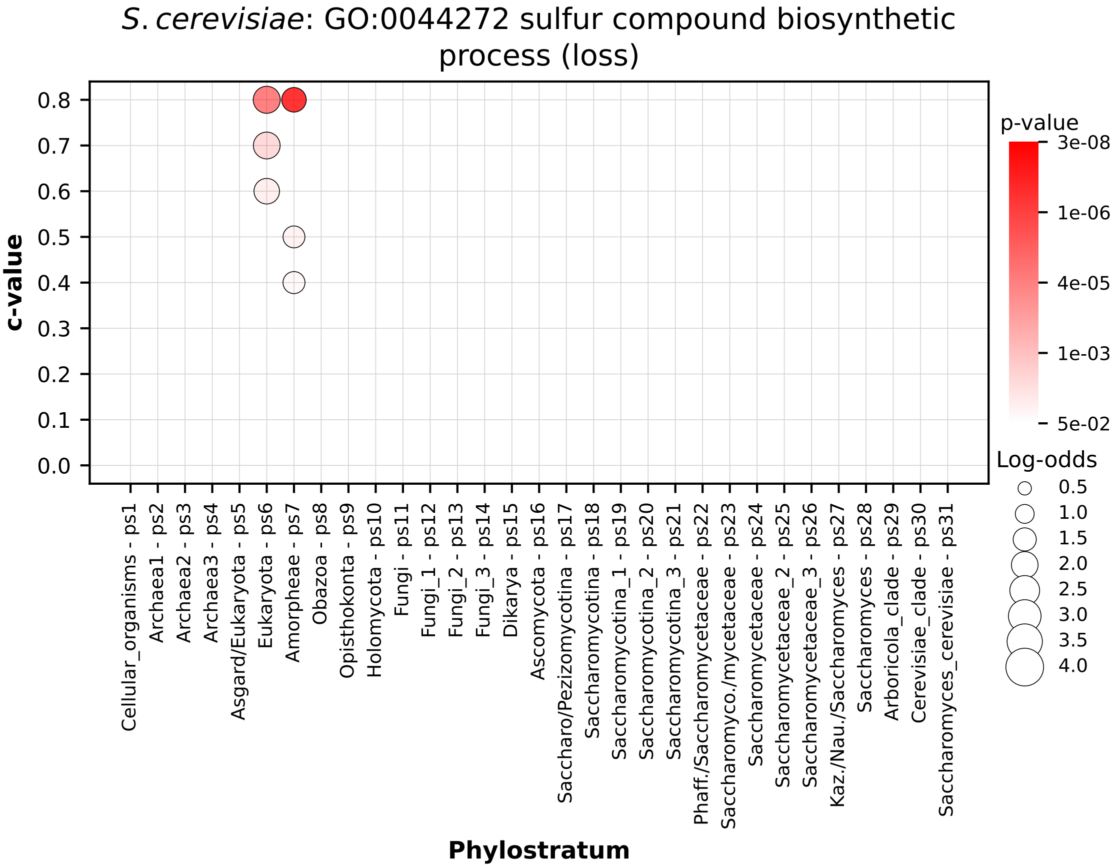

S. cerevisiae: GO:0044272 sulfur compound biosynthetic process (loss)
c-value
0.8
0.7
0.6
0.5
0.4
0.3
0.2
0.1
0.0
p-value
3e-08
1e-06
4e-05
1e-03
5e-02
Log-odds
0.5
1.0
1.5
2.0
2.5
3.0
3.5
4.0
Cellular_organisms - ps1
Archaea1 - ps2
Archaea2 - ps3
Archaea3 - ps4
Asgard/Eukaryota - ps5
Eukaryota - ps6
Amorpheae - ps7
Obazoa - ps8
Opisthokonta - ps9
Holomycota - ps10
Fungi - ps11
Fungi_1 - ps12
Fungi_2 - ps13
Fungi_3 - ps14
Dikarya - ps15
Ascomycota - ps16
Saccharo/Pezizomycotina - ps17
Saccharomycotina - ps18
Saccharomycotina_1 - ps19
Saccharomycotina_2 - ps20
Saccharomycotina_3 - ps21
Phaff./Saccharomycetaceae - ps22
Saccharomyco./mycetaceae - ps23
Saccharomycetaceae - ps24
Saccharomycetaceae_2 - ps25
Saccharomycetaceae_3 - ps26
Kaz./Nau./Saccharomyces - ps27
Saccharomyces - ps28
Arboricola_clade - ps29
Cerevisiae_clade - ps30
Saccharomyces_cerevisiae - ps31
Phylostratum

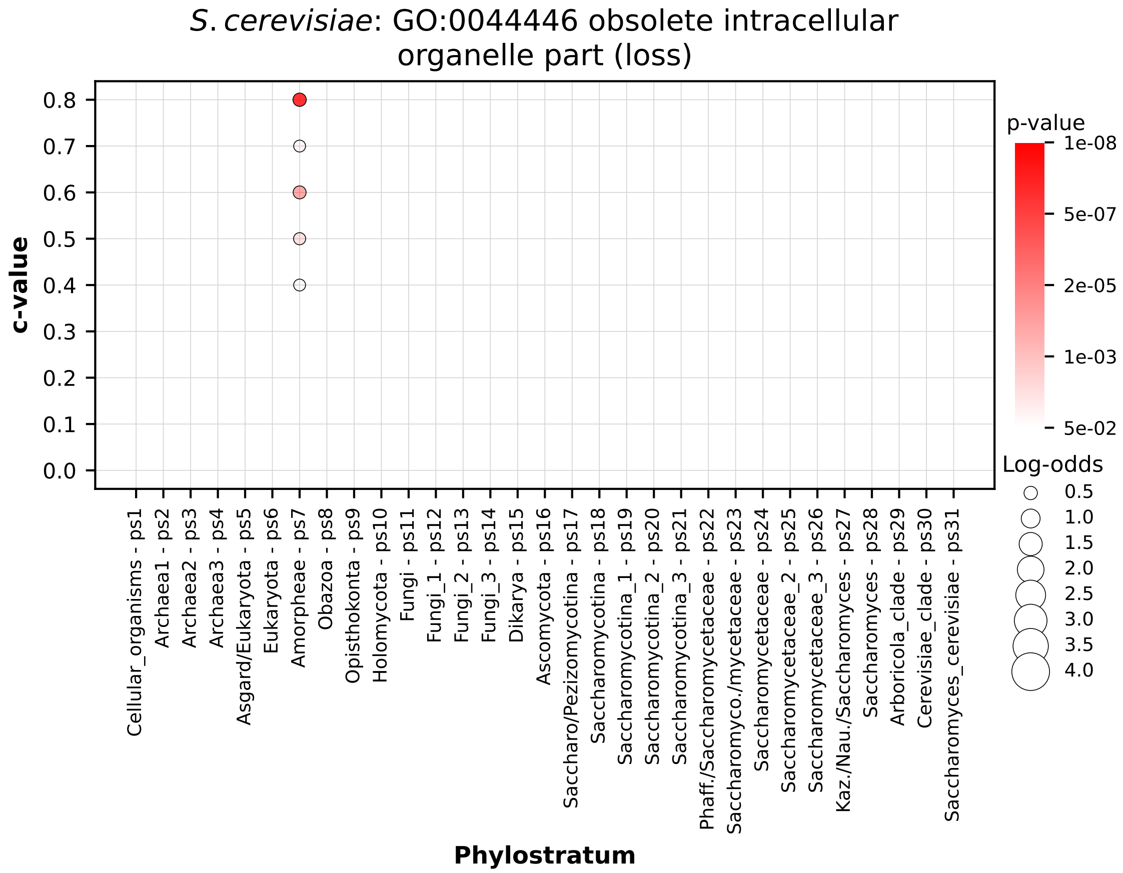

S. cerevisiae: GO:0044446 obsolete intracellular organelle part (loss)
c-value
0.0
0.1
0.2
0.3
0.4
0.5
0.6
0.7
0.8
Phylostratum
Cellular_organisms - ps1
Archaea1 - ps2
Archaea2 - ps3
Archaea3 - ps4
Asgard/Eukaryota - ps5
Eukaryota - ps6
Amorpheae - ps7
Obazoa - ps8
Opisthokonta - ps9
Holomycota - ps10
Fungi - ps11
Fungi_1 - ps12
Fungi_2 - ps13
Fungi_3 - ps14
Dikarya - ps15
Ascomycota - ps16
Saccharo/Pezizomycotina - ps17
Saccharomycotina - ps18
Saccharomycotina_1 - ps19
Saccharomycotina_2 - ps20
Saccharomycotina_3 - ps21
Phaff./Saccharomycetaceae - ps22
Saccharomyco./mycetaceae - ps23
Saccharomycetaceae - ps24
Saccharomycetaceae_2 - ps25
Saccharomycetaceae_3 - ps26
Kaz./Nau./Saccharomyces - ps27
Saccharomyces - ps28
Arboricola_clade - ps29
Cerevisiae_clade - ps30
Saccharomyces_cerevisiae - ps31
p-value
1e-08
5e-07
2e-05
1e-03
5e-02
Log-odds
0.5
1.0
1.5
2.0
2.5
3.0
3.5
4.0

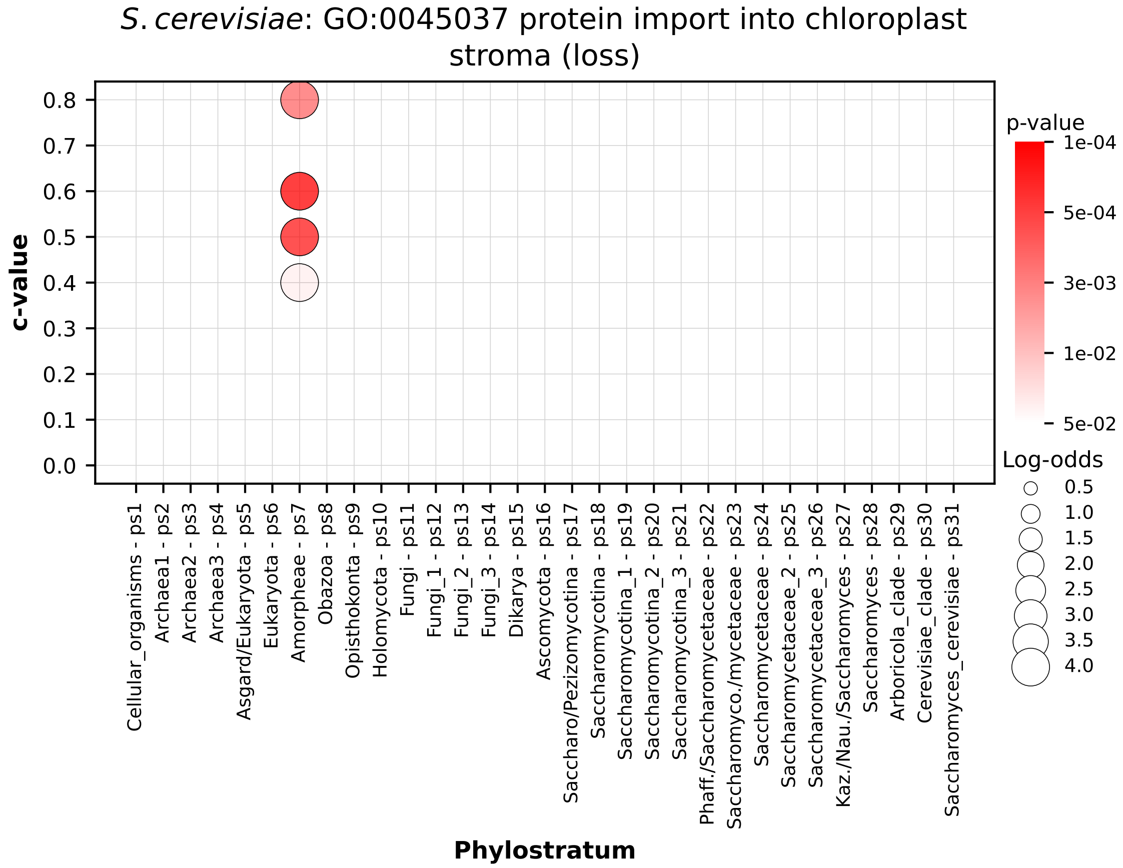

S. cerevisiae: GO:0045037 protein import into chloroplast stroma (loss)
c-value
0.0
0.1
0.2
0.3
0.4
0.5
0.6
0.7
0.8
Phylostratum
Cellular_organisms - ps1
Archaea1 - ps2
Archaea2 - ps3
Archaea3 - ps4
Asgard/Eukaryota - ps5
Eukaryota - ps6
Amorpheae - ps7
Obazoa - ps8
Opisthokonta - ps9
Holomycota - ps10
Fungi - ps11
Fungi_1 - ps12
Fungi_2 - ps13
Fungi_3 - ps14
Dikarya - ps15
Ascomycota - ps16
Saccharo/Pezizomycotina - ps17
Saccharomycotina - ps18
Saccharomycotina_1 - ps19
Saccharomycotina_2 - ps20
Saccharomycotina_3 - ps21
Phaff./Saccharomycetaceae - ps22
Saccharomyco./mycetaceae - ps23
Saccharomycetaceae - ps24
Saccharomycetaceae_2 - ps25
Saccharomycetaceae_3 - ps26
Kaz./Nau./Saccharomyces - ps27
Saccharomyces - ps28
Arboricola_clade - ps29
Cerevisiae_clade - ps30
Saccharomyces_cerevisiae - ps31
p-value
1e-04
5e-04
3e-03
1e-02
5e-02
Log-odds
0.5
1.0
1.5
2.0
2.5
3.0
3.5
4.0

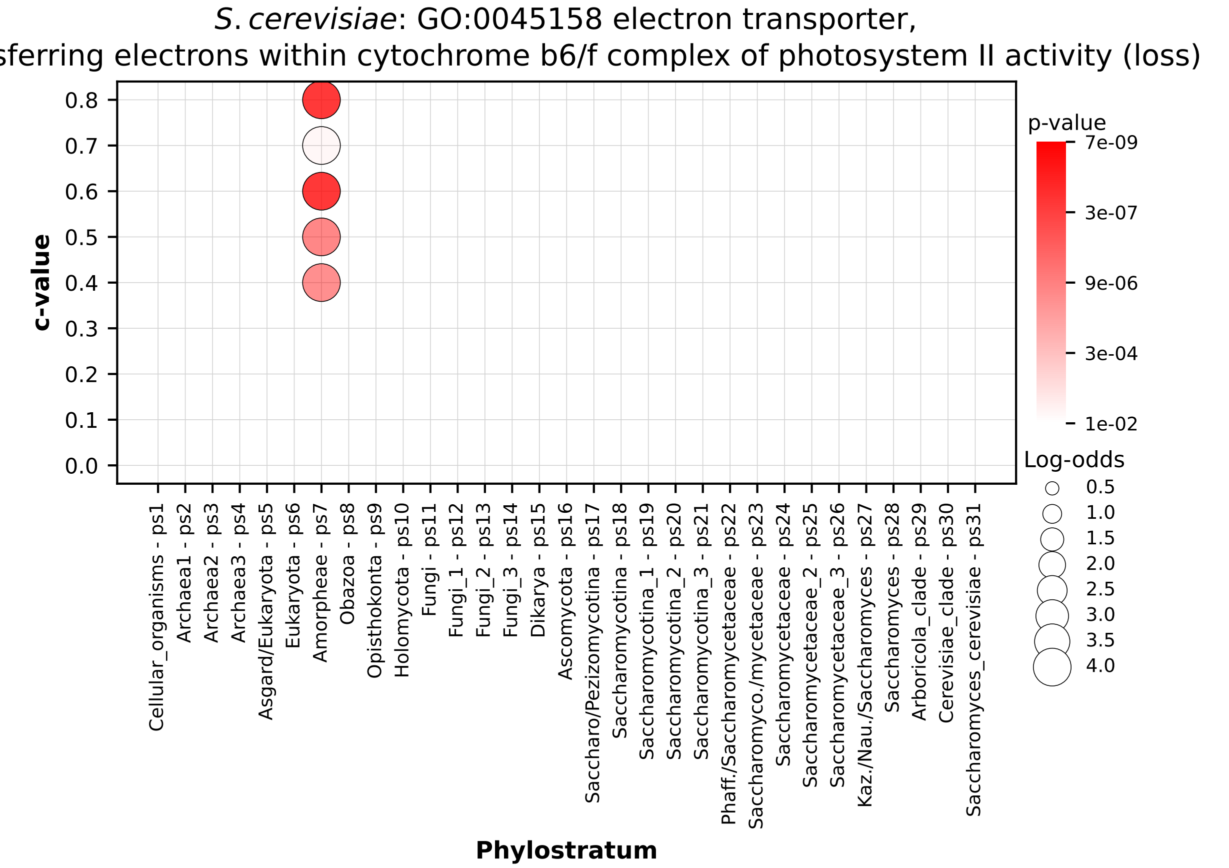
S. cerevisiae: GO:0045158 electron transporter,
sferring electrons within cytochrome b6/f complex of photosystem II activity (loss)
c-value
0.8
0.7
0.6
0.5
0.4
0.3
0.2
0.1
0.0
p-value
7e-09
3e-07
9e-06
3e-04
1e-02
Log-odds
0.5
1.0
1.5
2.0
2.5
3.0
3.5
4.0
Cellular_organisms - ps1
Archaea1 - ps2
Archaea2 - ps3
Archaea3 - ps4
Asgard/Eukaryota - ps5
Eukaryota - ps6
Amorpheae - ps7
Obazoa - ps8
Opisthokonta - ps9
Holomycota - ps10
Fungi - ps11
Fungi_1 - ps12
Fungi_2 - ps13
Fungi_3 - ps14
Dikarya - ps15
Ascomycota - ps16
Saccharo/Pezizomycotina - ps17
Saccharomycotina - ps18
Saccharomycotina_1 - ps19
Saccharomycotina_2 - ps20
Saccharomycotina_3 - ps21
Phaff./Saccharomycetaceae - ps22
Saccharomyco./mycetaceae - ps23
Saccharomycetaceae - ps24
Saccharomycetaceae_2 - ps25
Saccharomycetaceae_3 - ps26
Kaz./Nau./Saccharomyces - ps27
Saccharomyces - ps28
Arboricola_clade - ps29
Cerevisiae_clade - ps30
Saccharomyces_cerevisiae - ps31
Phylostratum

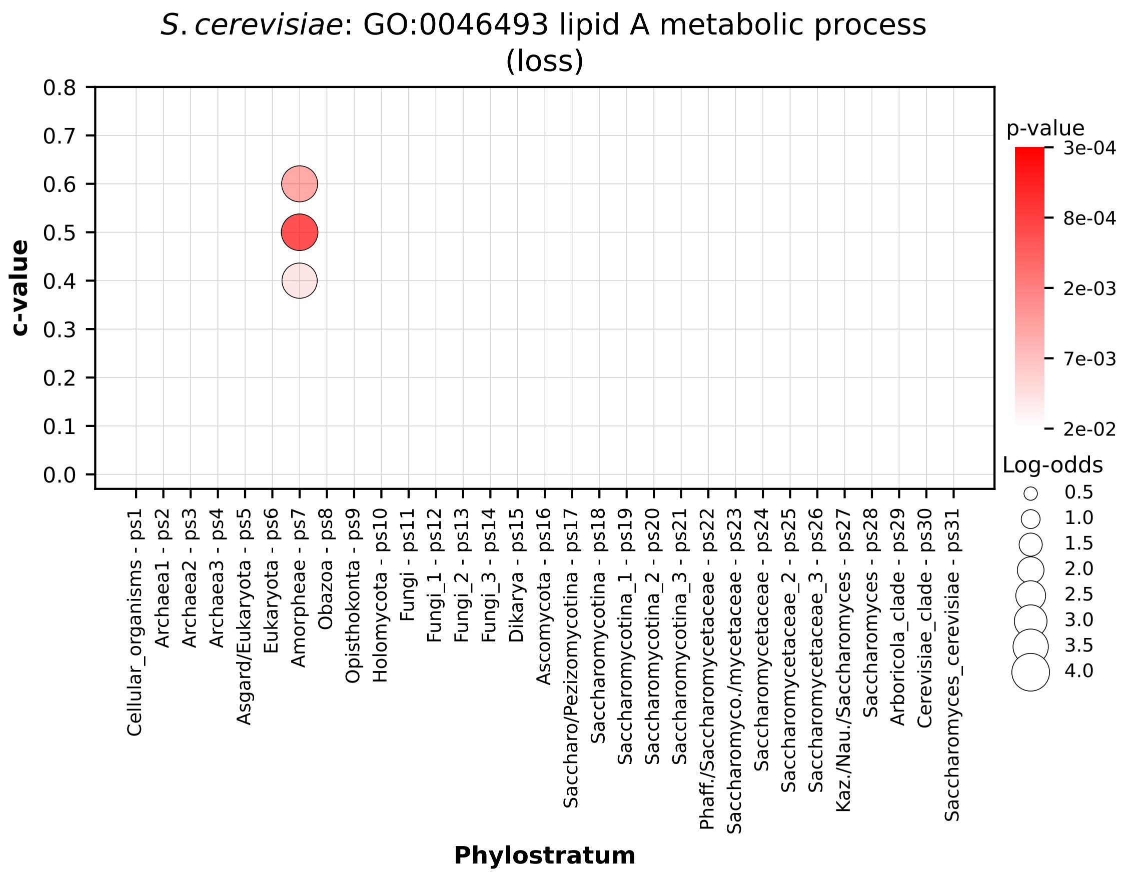

S. cerevisiae: GO:0046493 lipid A metabolic process
(loss)
c-value
0.8
0.7
0.6
0.5
0.4
0.3
0.2
0.1
0.0
p-value
3e-04
8e-04
2e-03
7e-03
2e-02
Log-odds
0.5
1.0
1.5
2.0
2.5
3.0
3.5
4.0
Cellular_organisms - ps1
Archaea1 - ps2
Archaea2 - ps3
Archaea3 - ps4
Asgard/Eukaryota - ps5
Eukaryota - ps6
Amorpheae - ps7
Obazoa - ps8
Opisthokonta - ps9
Holomycota - ps10
Fungi - ps11
Fungi_1 - ps12
Fungi_2 - ps13
Fungi_3 - ps14
Dikarya - ps15
Ascomycota - ps16
Saccharo/Pezizomycotina - ps17
Saccharomycotina - ps18
Saccharomycotina_1 - ps19
Saccharomycotina_2 - ps20
Saccharomycotina_3 - ps21
Phaff./Saccharomycetaceae - ps22
Saccharomyco./mycetaceae - ps23
Saccharomycetaceae - ps24
Saccharomycetaceae_2 - ps25
Saccharomycetaceae_3 - ps26
Kaz./Nau./Saccharomyces - ps27
Saccharomyces - ps28
Arboricola_clade - ps29
Cerevisiae_clade - ps30
Saccharomyces_cerevisiae - ps31
Phylostratum

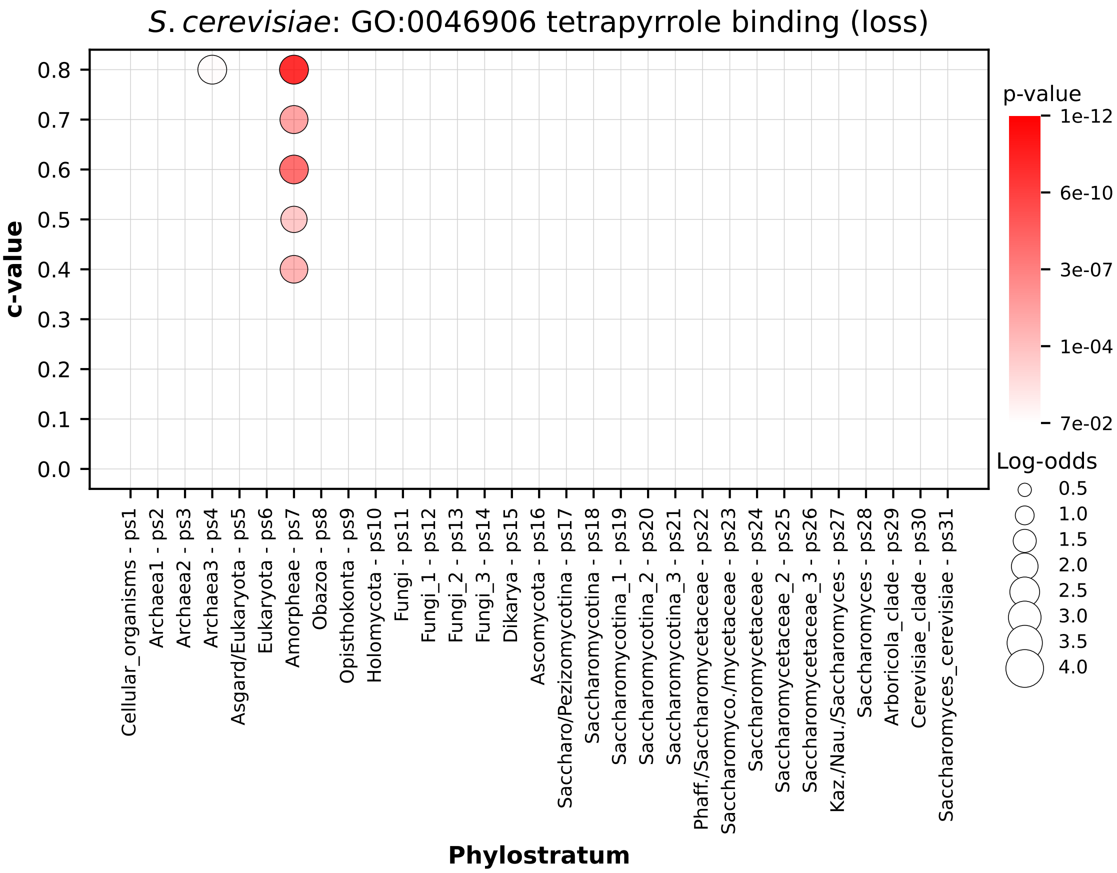
S. cerevisiae: GO:0046906 tetrapyrrole binding (loss)
c-value
0.0
0.1
0.2
0.3
0.4
0.5
0.6
0.7
0.8
p-value
1e-12
6e-10
3e-07
1e-04
7e-02
Log-odds
0.5
1.0
1.5
2.0
2.5
3.0
3.5
4.0
Cellular_organisms - ps1
Archaea1 - ps2
Archaea2 - ps3
Archaea3 - ps4
Asgard/Eukaryota - ps5
Eukaryota - ps6
Amorpheae - ps7
Obazoa - ps8
Opisthokonta - ps9
Holomycota - ps10
Fungi - ps11
Fungi_1 - ps12
Fungi_2 - ps13
Fungi_3 - ps14
Dikarya - ps15
Ascomycota - ps16
Saccharo/Pezizomycotina - ps17
Saccharomycotina - ps18
Saccharomycotina_1 - ps19
Saccharomycotina_2 - ps20
Saccharomycotina_3 - ps21
Phaff./Saccharomycetaceae - ps22
Saccharomyco./mycetaceae - ps23
Saccharomycetaceae - ps24
Saccharomycetaceae_2 - ps25
Saccharomycetaceae_3 - ps26
Kaz./Nau./Saccharomyces - ps27
Saccharomyces - ps28
Arboricola_clade - ps29
Cerevisiae_clade - ps30
Saccharomyces_cerevisiae - ps31
Phylostratum

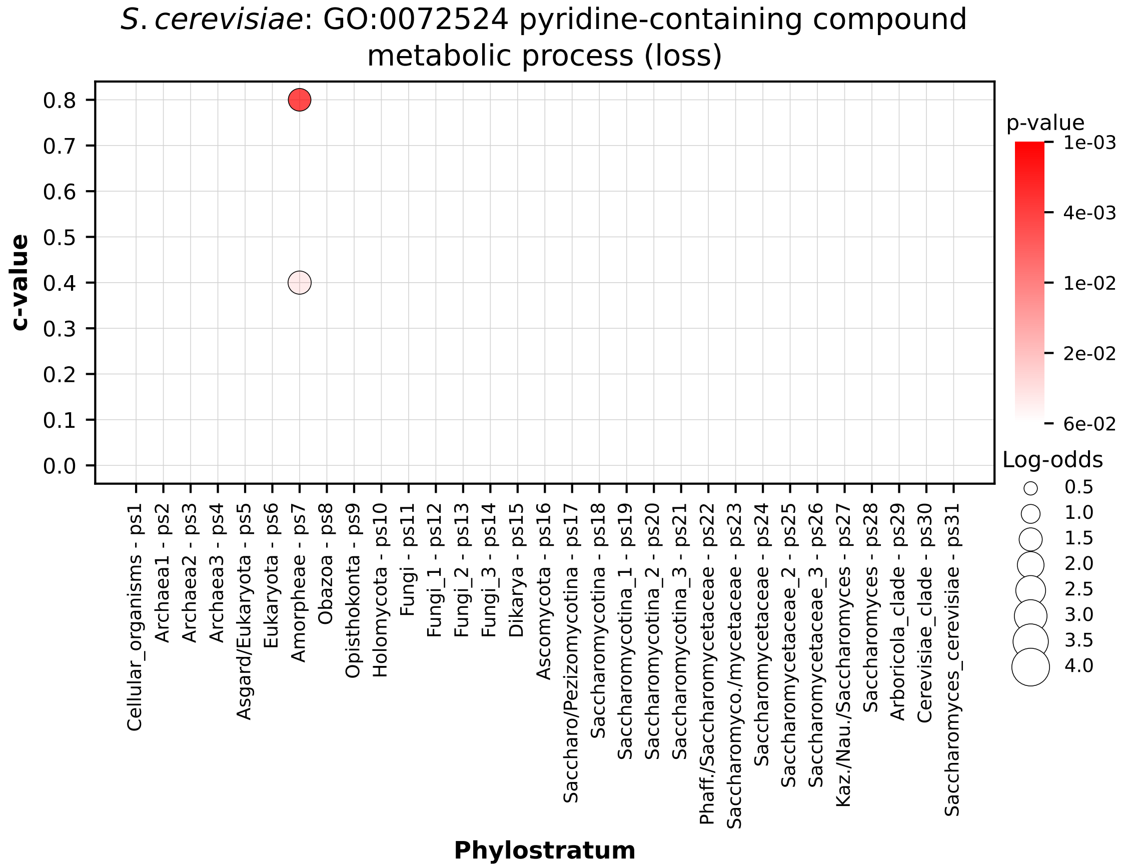
S. cerevisiae: GO:0072524 pyridine-containing compound metabolic process (loss)
c-value
0.0
0.1
0.2
0.3
0.4
0.5
0.6
0.7
0.8
Phylostratum
Cellular_organisms - ps1
Archaea1 - ps2
Archaea2 - ps3
Archaea3 - ps4
Asgard/Eukaryota - ps5
Eukaryota - ps6
Amorpheae - ps7
Obazoa - ps8
Opisthokonta - ps9
Holomycota - ps10
Fungi - ps11
Fungi_1 - ps12
Fungi_2 - ps13
Fungi_3 - ps14
Dikarya - ps15
Ascomycota - ps16
Saccharo/Pezizomycotina - ps17
Saccharomycotina - ps18
Saccharomycotina_1 - ps19
Saccharomycotina_2 - ps20
Saccharomycotina_3 - ps21
Phaff./Saccharomycetaceae - ps22
Saccharomyco./mycetaceae - ps23
Saccharomycetaceae - ps24
Saccharomycetaceae_2 - ps25
Saccharomycetaceae_3 - ps26
Kaz./Nau./Saccharomyces - ps27
Saccharomyces - ps28
Arboricola_clade - ps29
Cerevisiae_clade - ps30
Saccharomyces_cerevisiae - ps31
p-value
1e-03
4e-03
1e-02
2e-02
6e-02
Log-odds
0.5
1.0
1.5
2.0
2.5
3.0
3.5
4.0

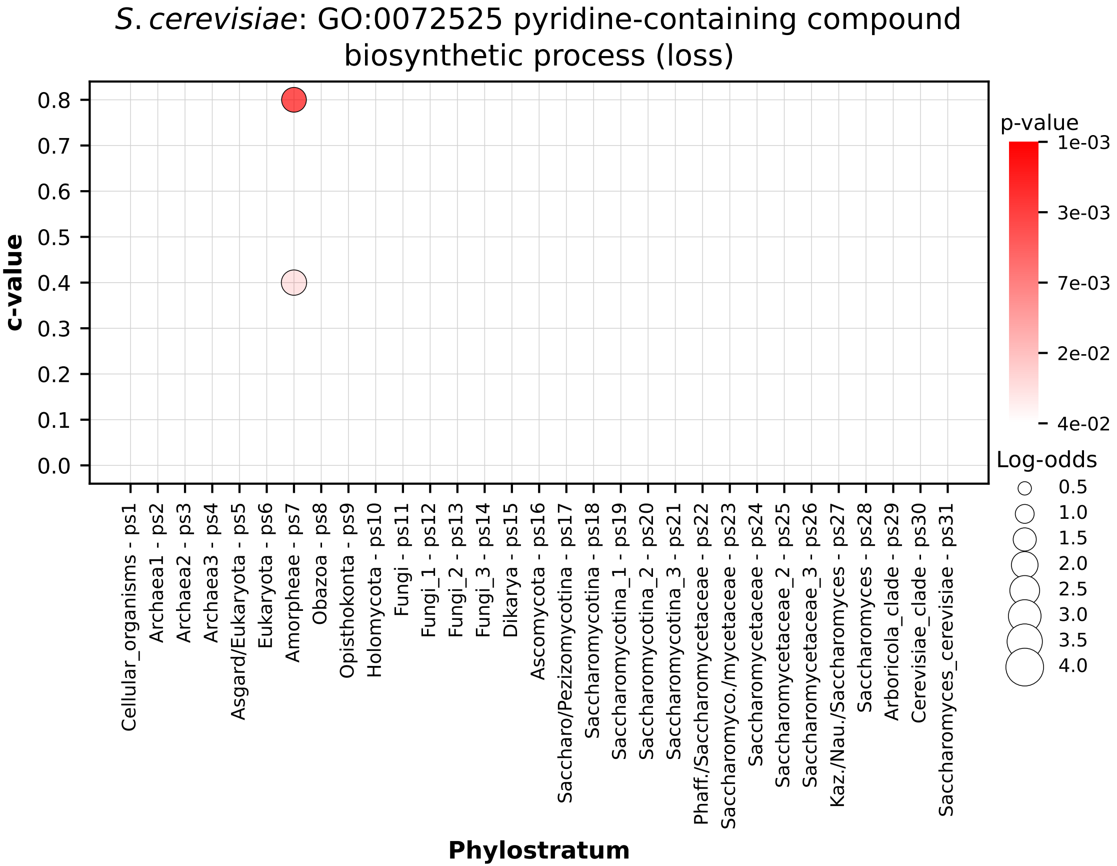

S. cerevisiae: GO:0072525 pyridine-containing compound biosynthetic process (loss)
c-value
0.0
0.1
0.2
0.3
0.4
0.5
0.6
0.7
0.8
Phylostratum
Cellular_organisms - ps1
Archaea1 - ps2
Archaea2 - ps3
Archaea3 - ps4
Asgard/Eukaryota - ps5
Eukaryota - ps6
Amorpheae - ps7
Obazoa - ps8
Opisthokonta - ps9
Holomycota - ps10
Fungi - ps11
Fungi_1 - ps12
Fungi_2 - ps13
Fungi_3 - ps14
Dikarya - ps15
Ascomycota - ps16
Saccharo/Pezizomycotina - ps17
Saccharomycotina - ps18
Saccharomycotina_1 - ps19
Saccharomycotina_2 - ps20
Saccharomycotina_3 - ps21
Phaff./Saccharomycetaceae - ps22
Saccharomyco./mycetaceae - ps23
Saccharomycetaceae - ps24
Saccharomycetaceae_2 - ps25
Saccharomycetaceae_3 - ps26
Kaz./Nau./Saccharomyces - ps27
Saccharomyces - ps28
Arboricola_clade - ps29
Cerevisiae_clade - ps30
Saccharomyces_cerevisiae - ps31
p-value
1e-03
3e-03
7e-03
2e-02
4e-02
Log-odds
0.5
1.0
1.5
2.0
2.5
3.0
3.5
4.0

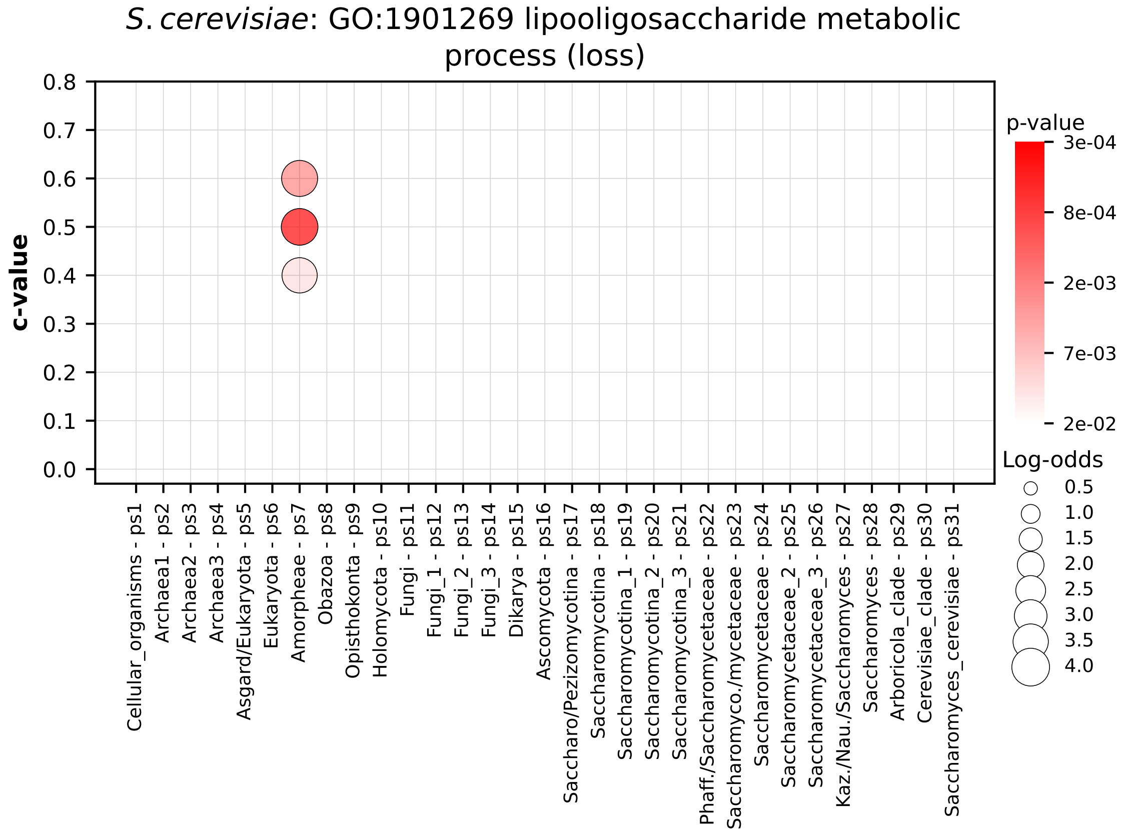

S. cerevisiae: GO:1901269 lipooligosaccharide metabolic process (loss)
c-value
0.8
0.7
0.6
0.5
0.4
0.3
0.2
0.1
0.0
p-value
3e-04
8e-04
2e-03
7e-03
2e-02
Log-odds
0.5
1.0
1.5
2.0
2.5
3.0
3.5
4.0
Cellular_organisms - ps1
Archaea1 - ps2
Archaea2 - ps3
Archaea3 - ps4
Asgard/Eukaryota - ps5
Eukaryota - ps6
Amorpheae - ps7
Obazoa - ps8
Opisthokonta - ps9
Holomycota - ps10
Fungi - ps11
Fungi_1 - ps12
Fungi_2 - ps13
Fungi_3 - ps14
Dikarya - ps15
Ascomycota - ps16
Saccharo/Pezizomycotina - ps17
Saccharomycotina - ps18
Saccharomycotina_1 - ps19
Saccharomycotina_2 - ps20
Saccharomycotina_3 - ps21
Phaff./Saccharomycetaceae - ps22
Saccharomyco./mycetaceae - ps23
Saccharomycetaceae - ps24
Saccharomycetaceae_2 - ps25
Saccharomycetaceae_3 - ps26
Kaz./Nau./Saccharomyces - ps27
Saccharomyces - ps28
Arboricola_clade - ps29
Cerevisiae_clade - ps30
Saccharomyces_cerevisiae - ps31
Phylostratum

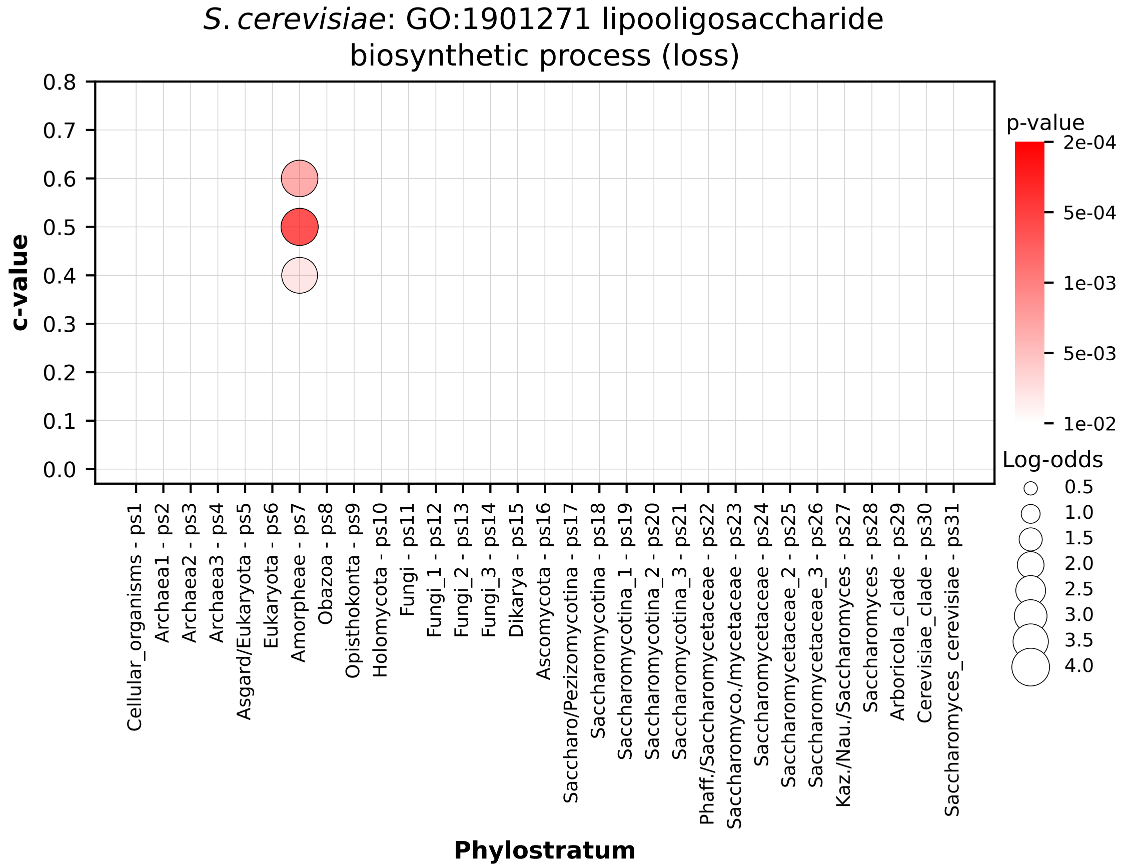
S. cerevisiae: GO:1901271 lipooligosaccharide biosynthetic process (loss)
c-value
0.8
0.7
0.6
0.5
0.4
0.3
0.2
0.1
0.0
Phylostratum
Cellular_organisms - ps1
Archaea1 - ps2
Archaea2 - ps3
Archaea3 - ps4
Asgard/Eukaryota - ps5
Eukaryota - ps6
Amorpheae - ps7
Obazoa - ps8
Opisthokonta - ps9
Holomycota - ps10
Fungi - ps11
Fungi_1 - ps12
Fungi_2 - ps13
Fungi_3 - ps14
Dikarya - ps15
Ascomycota - ps16
Saccharo/Pezizomycotina - ps17
Saccharomycotina - ps18
Saccharomycotina_1 - ps19
Saccharomycotina_2 - ps20
Saccharomycotina_3 - ps21
Phaff./Saccharomycetaceae - ps22
Saccharomyco./mycetaceae - ps23
Saccharomycetaceae - ps24
Saccharomycetaceae_2 - ps25
Saccharomycetaceae_3 - ps26
Kaz./Nau./Saccharomyces - ps27
Saccharomyces - ps28
Arboricola_clade - ps29
Cerevisiae_clade - ps30
Saccharomyces_cerevisiae - ps31
p-value
2e-04
5e-04
1e-03
5e-03
1e-02
Log-odds
0.5
1.0
1.5
2.0
2.5
3.0
3.5
4.0

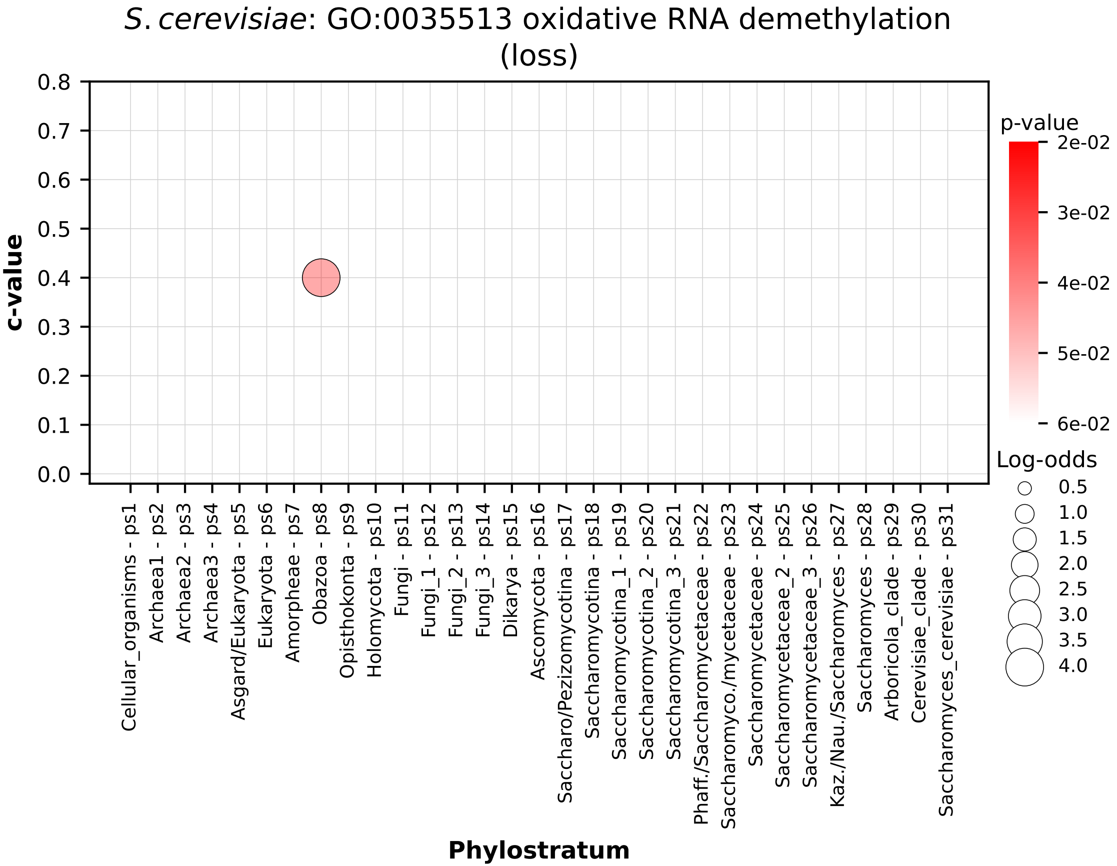

S. cerevisiae: GO:0035513 oxidative RNA demethylation (loss)
c-value
0.0
0.1
0.2
0.3
0.4
0.5
0.6
0.7
0.8
Phylostratum
Cellular_organisms - ps1
Archaea1 - ps2
Archaea2 - ps3
Archaea3 - ps4
Asgard/Eukaryota - ps5
Eukaryota - ps6
Amorpheae - ps7
Obazoa - ps8
Opisthokonta - ps9
Holomycota - ps10
Fungi - ps11
Fungi_1 - ps12
Fungi_2 - ps13
Fungi_3 - ps14
Dikarya - ps15
Ascomycota - ps16
Saccharo/Pezizomycotina - ps17
Saccharomycotina - ps18
Saccharomycotina_1 - ps19
Saccharomycotina_2 - ps20
Saccharomycotina_3 - ps21
Phaff./Saccharomycetaceae - ps22
Saccharomyco./mycetaceae - ps23
Saccharomycetaceae - ps24
Saccharomycetaceae_2 - ps25
Saccharomycetaceae_3 - ps26
Kaz./Nau./Saccharomyces - ps27
Saccharomyces - ps28
Arboricola_clade - ps29
Cerevisiae_clade - ps30
Saccharomyces_cerevisiae - ps31
p-value
2e-02
3e-02
4e-02
5e-02
6e-02
Log-odds
0.5
1.0
1.5
2.0
2.5
3.0
3.5
4.0

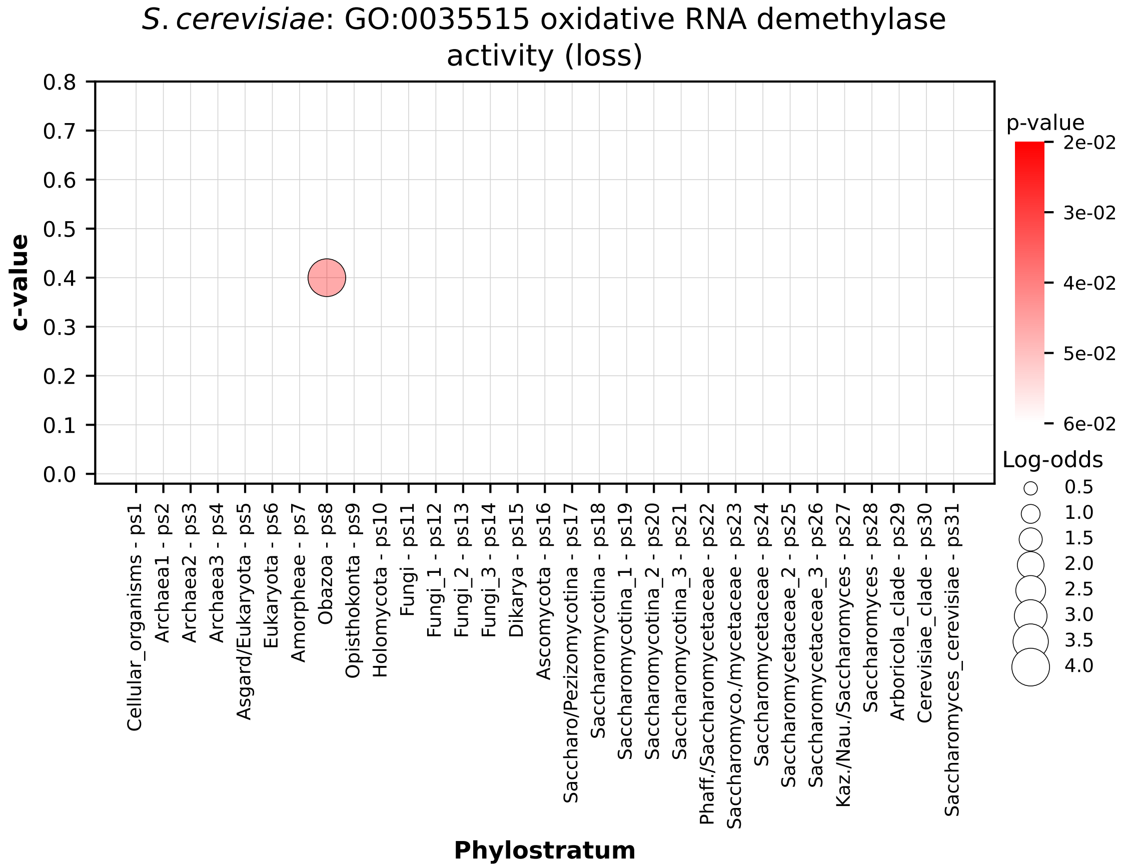
S. cerevisiae: GO:0035515 oxidative RNA demethylase activity (loss)
c-value
0.8
0.7
0.6
0.5
0.4
0.3
0.2
0.1
0.0
Phylostratum
Cellular_organisms - ps1
Archaea1 - ps2
Archaea2 - ps3
Archaea3 - ps4
Asgard/Eukaryota - ps5
Eukaryota - ps6
Amorpheae - ps7
Obazoa - ps8
Opisthokonta - ps9
Holomycota - ps10
Fungi - ps11
Fungi_1 - ps12
Fungi_2 - ps13
Fungi_3 - ps14
Dikarya - ps15
Ascomycota - ps16
Saccharo/Pezizomycotina - ps17
Saccharomycotina - ps18
Saccharomycotina_1 - ps19
Saccharomycotina_2 - ps20
Saccharomycotina_3 - ps21
Phaff./Saccharomycetaceae - ps22
Saccharomyco./mycetaceae - ps23
Saccharomycetaceae - ps24
Saccharomycetaceae_2 - ps25
Saccharomycetaceae_3 - ps26
Kaz./Nau./Saccharomyces - ps27
Saccharomyces - ps28
Arboricola_clade - ps29
Cerevisiae_clade - ps30
Saccharomyces_cerevisiae - ps31
p-value
2e-02
3e-02
4e-02
5e-02
6e-02
Log-odds
0.5
1.0
1.5
2.0
2.5
3.0
3.5
4.0

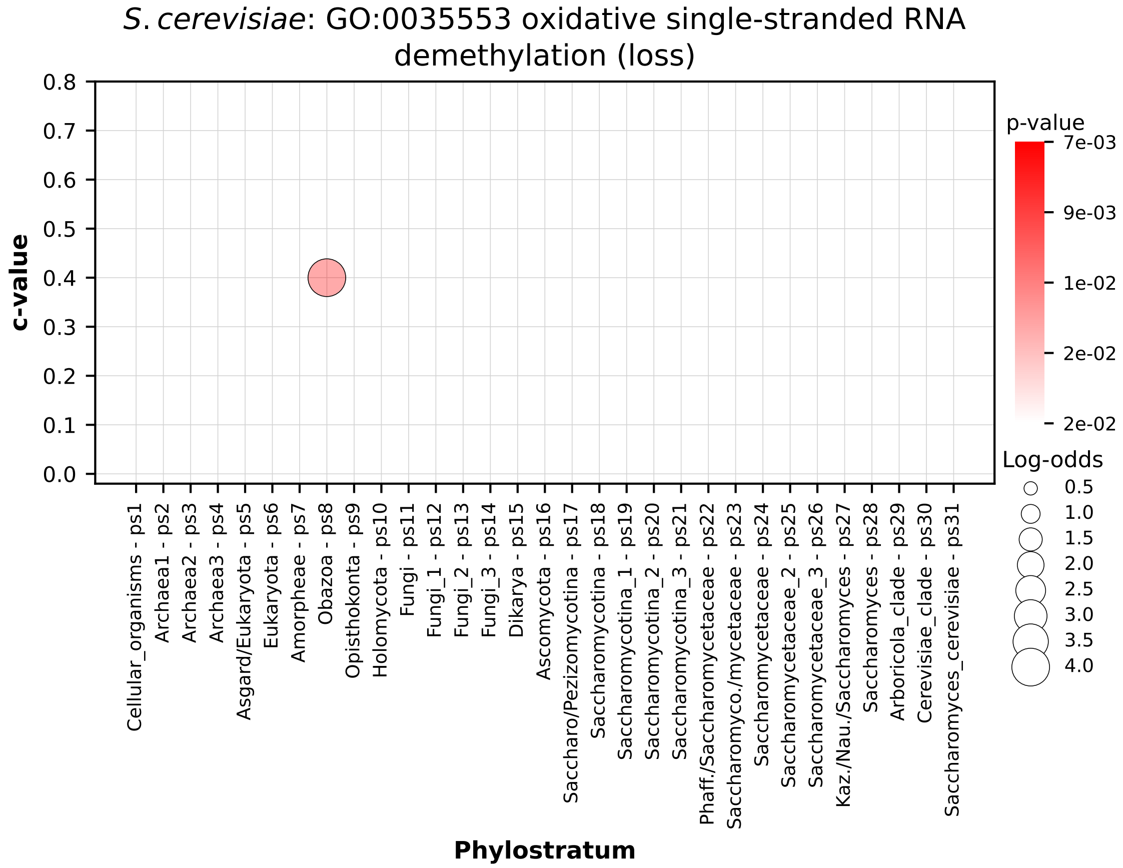

S. cerevisiae: GO:0035553 oxidative single-stranded RNA demethylation (loss)
c-value
0.8
0.7
0.6
0.5
0.4
0.3
0.2
0.1
0.0
Phylostratum
Cellular_organisms - ps1
Archaea1 - ps2
Archaea2 - ps3
Archaea3 - ps4
Asgard/Eukaryota - ps5
Eukaryota - ps6
Amorpheae - ps7
Obazoa - ps8
Opisthokonta - ps9
Holomycota - ps10
Fungi - ps11
Fungi_1 - ps12
Fungi_2 - ps13
Fungi_3 - ps14
Dikarya - ps15
Ascomycota - ps16
Saccharo/Pezizomycotina - ps17
Saccharomycotina - ps18
Saccharomycotina_1 - ps19
Saccharomycotina_2 - ps20
Saccharomycotina_3 - ps21
Phaff./Saccharomycetaceae - ps22
Saccharomyco./mycetaceae - ps23
Saccharomycetaceae - ps24
Saccharomycetaceae_2 - ps25
Saccharomycetaceae_3 - ps26
Kaz./Nau./Saccharomyces - ps27
Saccharomyces - ps28
Arboricola_clade - ps29
Cerevisiae_clade - ps30
Saccharomyces_cerevisiae - ps31
p-value
7e-03
9e-03
1e-02
2e-02
2e-02
Log-odds
0.5
1.0
1.5
2.0
2.5
3.0
3.5
4.0

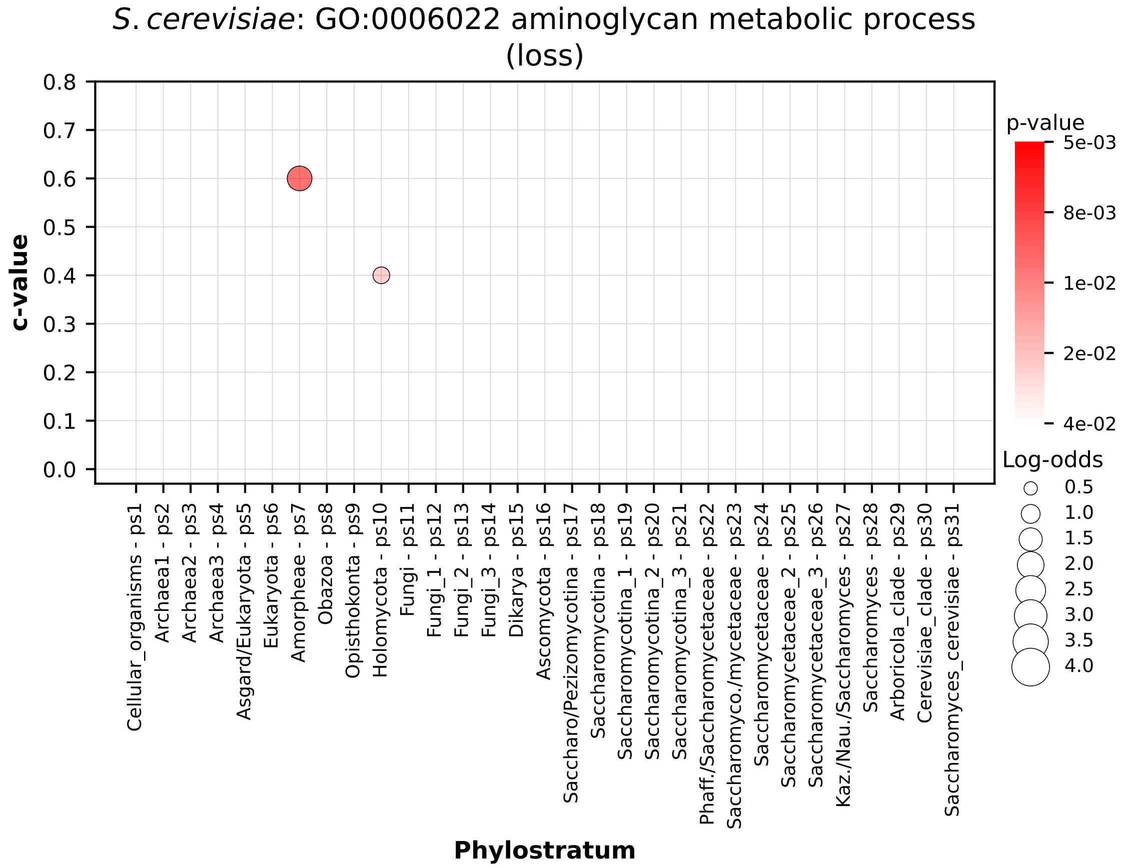

S. cerevisiae: GO:0006022 aminoglycan metabolic process
(loss)
c-value
0.8
0.7
0.6
0.5
0.4
0.3
0.2
0.1
0.0
p-value
5e-03
8e-03
1e-02
2e-02
4e-02
Log-odds
0.5
1.0
1.5
2.0
2.5
3.0
3.5
4.0
Cellular_organisms - ps1
Archaea1 - ps2
Archaea2 - ps3
Archaea3 - ps4
Asgard/Eukaryota - ps5
Eukaryota - ps6
Amorpheae - ps7
Obazoa - ps8
Opisthokonta - ps9
Holomycota - ps10
Fungi - ps11
Fungi_1 - ps12
Fungi_2 - ps13
Fungi_3 - ps14
Dikarya - ps15
Ascomycota - ps16
Saccharo/Pezizomycotina - ps17
Saccharomycotina - ps18
Saccharomycotina_1 - ps19
Saccharomycotina_2 - ps20
Saccharomycotina_3 - ps21
Phaff./Saccharomycetaceae - ps22
Saccharomyco./mycetaceae - ps23
Saccharomycetaceae - ps24
Saccharomycetaceae_2 - ps25
Saccharomycetaceae_3 - ps26
Kaz./Nau./Saccharomyces - ps27
Saccharomyces - ps28
Arboricola_clade - ps29
Cerevisiae_clade - ps30
Saccharomyces_cerevisiae - ps31
Phylostratum

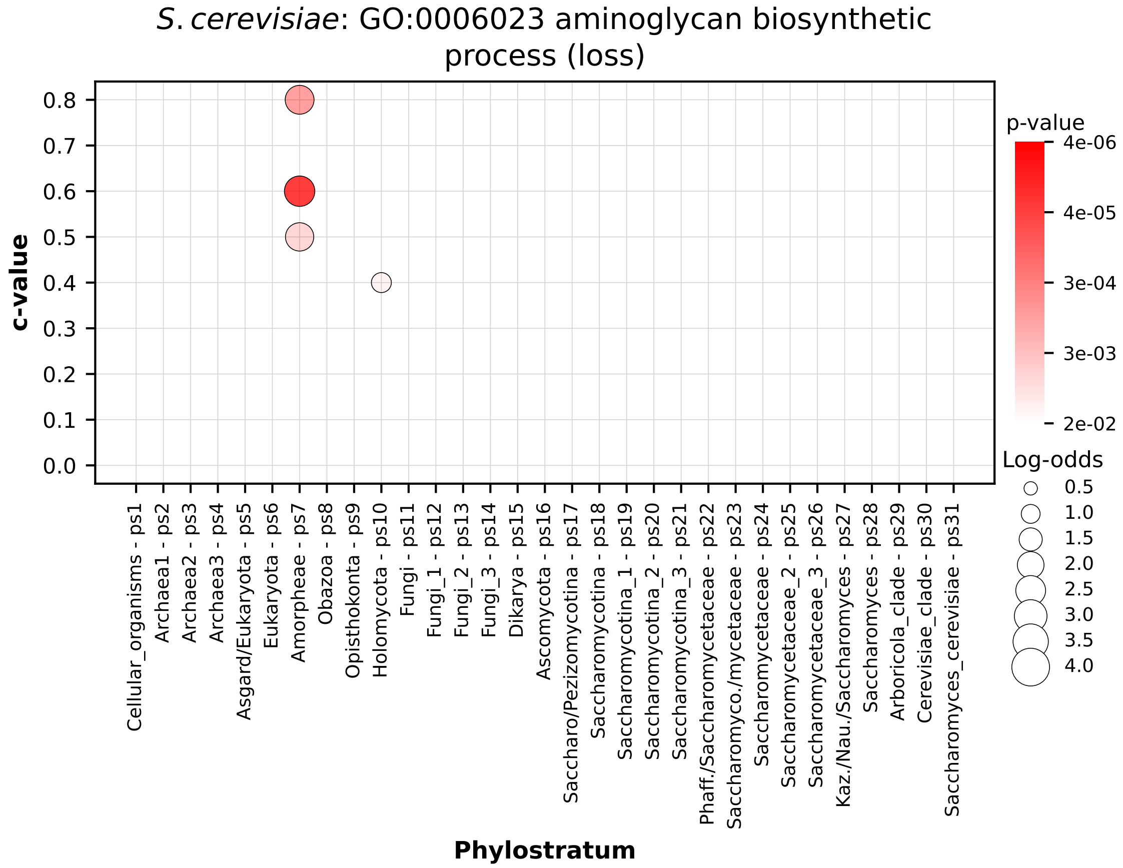

S. cerevisiae: GO:0006023 aminoglycan biosynthetic process (loss)
c-value
0.8
0.7
0.6
0.5
0.4
0.3
0.2
0.1
0.0
Phylostratum
Cellular_organisms - ps1
Archaea1 - ps2
Archaea2 - ps3
Archaea3 - ps4
Asgard/Eukaryota - ps5
Eukaryota - ps6
Amorpheae - ps7
Obazoa - ps8
Opisthokonta - ps9
Holomycota - ps10
Fungi - ps11
Fungi_1 - ps12
Fungi_2 - ps13
Fungi_3 - ps14
Dikarya - ps15
Ascomycota - ps16
Saccharo/Pezizomycotina - ps17
Saccharomycotina - ps18
Saccharomycotina_1 - ps19
Saccharomycotina_2 - ps20
Saccharomycotina_3 - ps21
Phaff./Saccharomycetaceae - ps22
Saccharomyco./mycetaceae - ps23
Saccharomycetaceae - ps24
Saccharomycetaceae_2 - ps25
Saccharomycetaceae_3 - ps26
Kaz./Nau./Saccharomyces - ps27
Saccharomyces - ps28
Arboricola_clade - ps29
Cerevisiae_clade - ps30
Saccharomyces_cerevisiae - ps31
p-value
4e-06
4e-05
3e-04
3e-03
2e-02
Log-odds
0.5
1.0
1.5
2.0
2.5
3.0
3.5
4.0

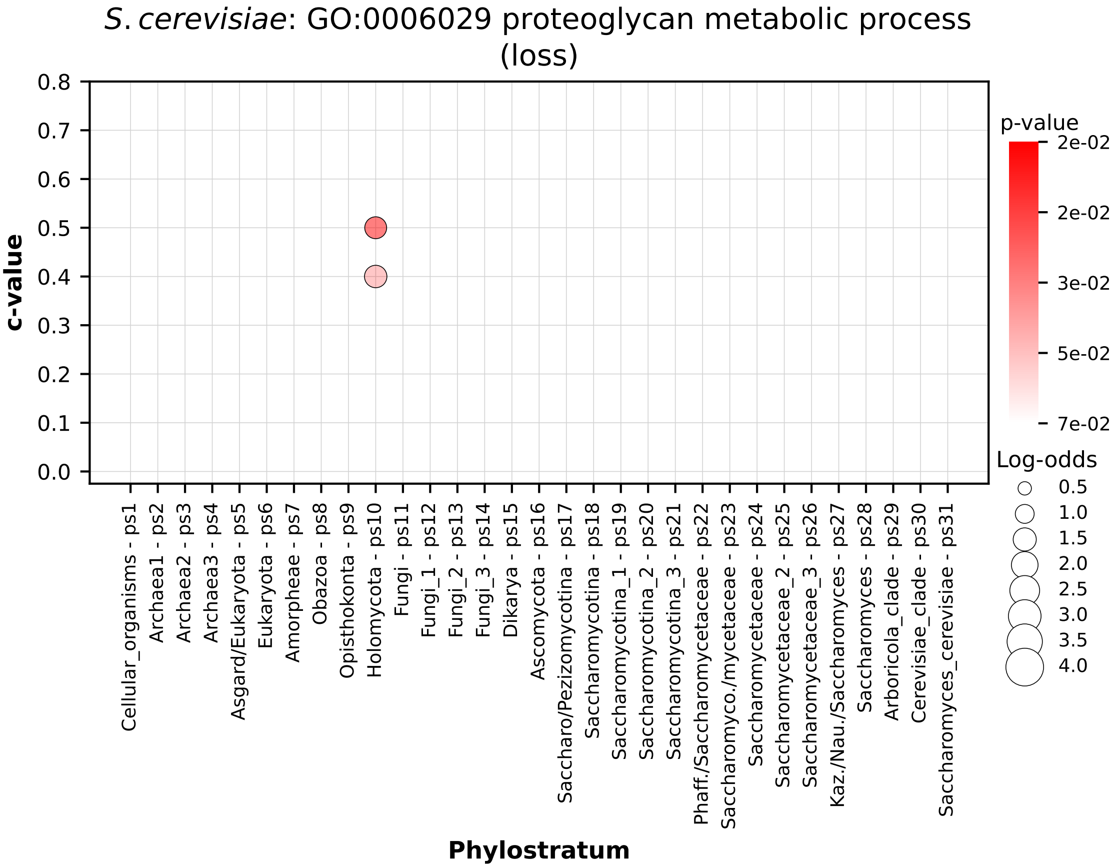
S. cerevisiae: GO:0006029 proteoglycan metabolic process
(loss)
c-value
0.8
0.7
0.6
0.5
0.4
0.3
0.2
0.1
0.0
p-value
2e-02
2e-02
3e-02
5e-02
7e-02
Log-odds
0.5
1.0
1.5
2.0
2.5
3.0
3.5
4.0
Cellular_organisms - ps1
Archaea1 - ps2
Archaea2 - ps3
Archaea3 - ps4
Asgard/Eukaryota - ps5
Eukaryota - ps6
Amorpheae - ps7
Obazoa - ps8
Opisthokonta - ps9
Holomycota - ps10
Fungi - ps11
Fungi_1 - ps12
Fungi_2 - ps13
Fungi_3 - ps14
Dikarya - ps15
Ascomycota - ps16
Saccharo/Pezizomycotina - ps17
Saccharomycotina - ps18
Saccharomycotina_1 - ps19
Saccharomycotina_2 - ps20
Saccharomycotina_3 - ps21
Phaff./Saccharomycetaceae - ps22
Saccharomyco./mycetaceae - ps23
Saccharomycetaceae - ps24
Saccharomycetaceae_2 - ps25
Saccharomycetaceae_3 - ps26
Kaz./Nau./Saccharomyces - ps27
Saccharomyces - ps28
Arboricola_clade - ps29
Cerevisiae_clade - ps30
Saccharomyces_cerevisiae - ps31
Phylostratum

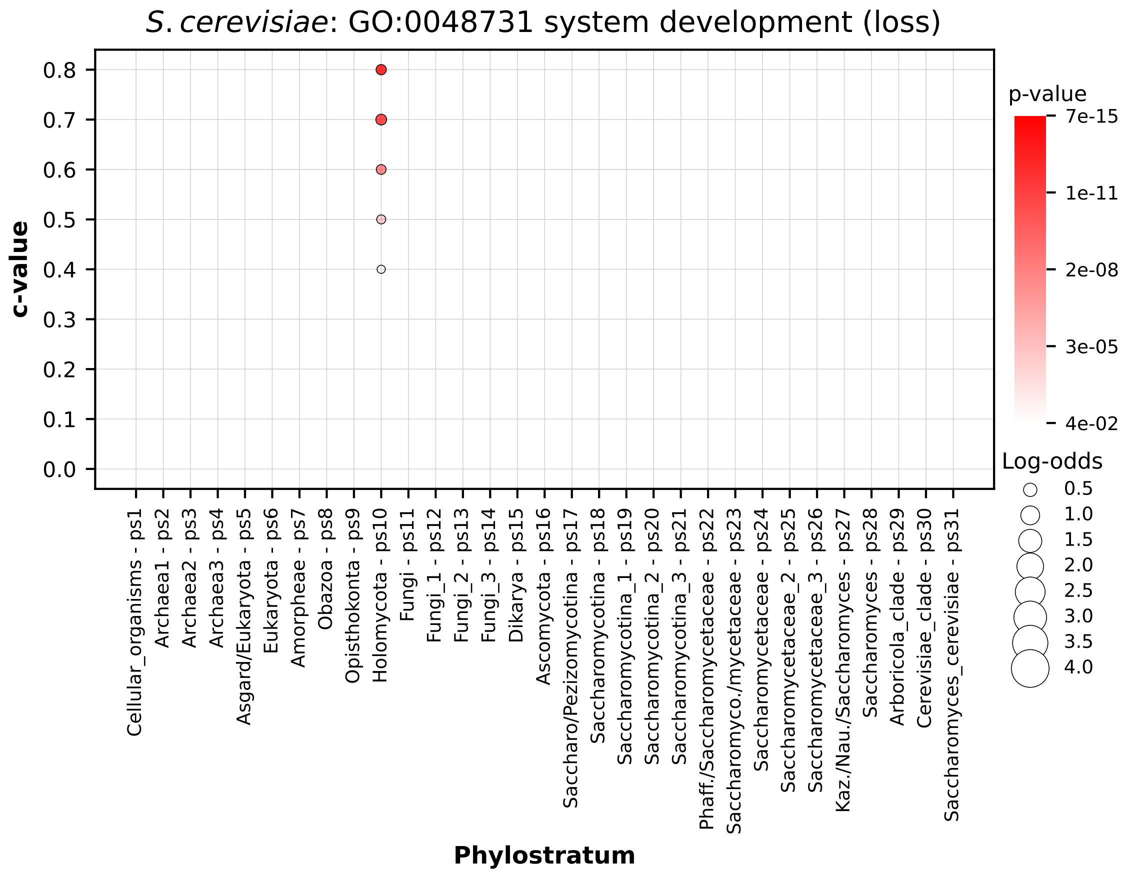
S. cerevisiae: GO:0048731 system development (loss)
c-value
0.8
0.7
0.6
0.5
0.4
0.3
0.2
0.1
0.0
Phylostratum
Cellular_organisms - ps1
Archaea1 - ps2
Archaea2 - ps3
Archaea3 - ps4
Asgard/Eukaryota - ps5
Eukaryota - ps6
Amorpheae - ps7
Obazoa - ps8
Opisthokonta - ps9
Holomycota - ps10
Fungi - ps11
Fungi_1 - ps12
Fungi_2 - ps13
Fungi_3 - ps14
Dikarya - ps15
Ascomycota - ps16
Saccharo/Pezizomycotina - ps17
Saccharomycotina - ps18
Saccharomycotina_1 - ps19
Saccharomycotina_2 - ps20
Saccharomycotina_3 - ps21
Phaff./Saccharomycetaceae - ps22
Saccharomyco./mycetaceae - ps23
Saccharomycetaceae - ps24
Saccharomycetaceae_2 - ps25
Saccharomycetaceae_3 - ps26
Kaz./Nau./Saccharomyces - ps27
Saccharomyces - ps28
Arboricola_clade - ps29
Cerevisiae_clade - ps30
Saccharomyces_cerevisiae - ps31
p-value
7e-15
1e-11
2e-08
3e-05
4e-02
Log-odds
0.5
1.0
1.5
2.0
2.5
3.0
3.5
4.0

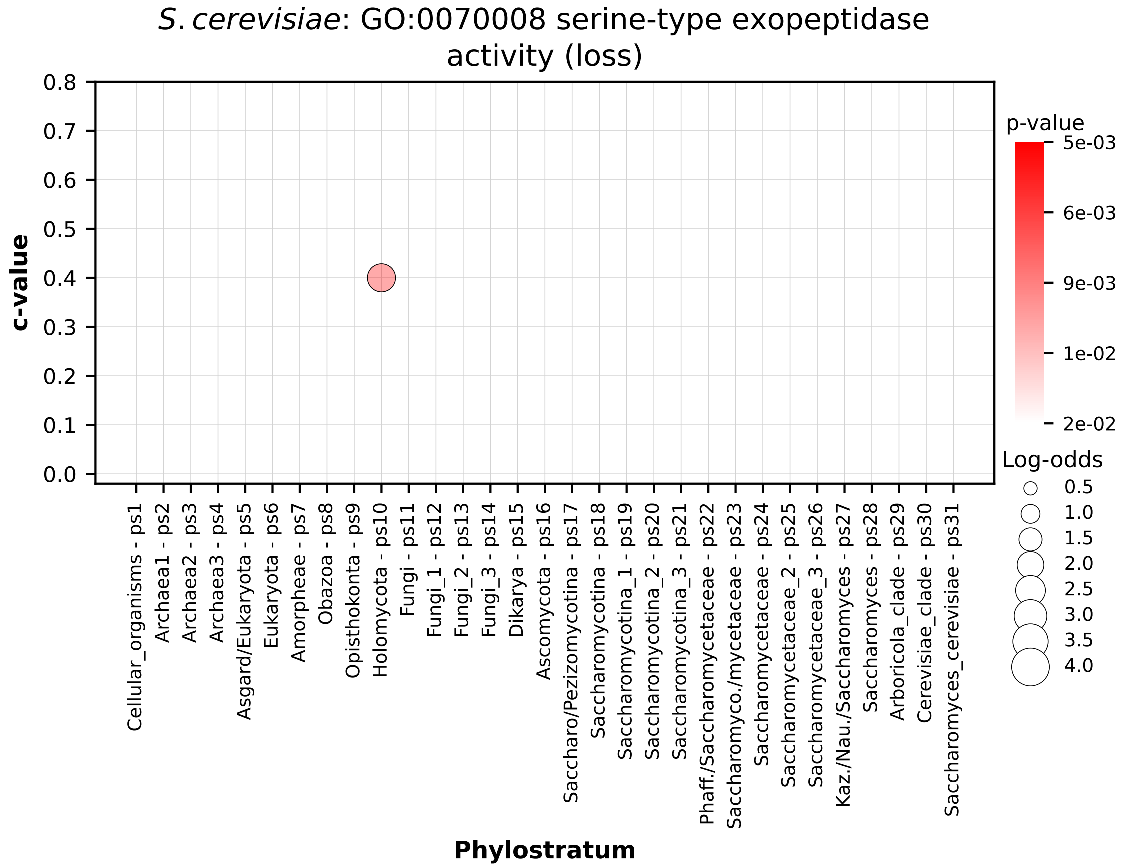

S. cerevisiae: GO:0070008 serine-type exopeptidase activity (loss)
c-value
0.8
0.7
0.6
0.5
0.4
0.3
0.2
0.1
0.0
Phylostratum
Cellular_organisms - ps1
Archaea1 - ps2
Archaea2 - ps3
Archaea3 - ps4
Asgard/Eukaryota - ps5
Eukaryota - ps6
Amorpheae - ps7
Obazoa - ps8
Opisthokonta - ps9
Holomycota - ps10
Fungi - ps11
Fungi_1 - ps12
Fungi_2 - ps13
Fungi_3 - ps14
Dikarya - ps15
Ascomycota - ps16
Saccharo/Pezizomycotina - ps17
Saccharomycotina - ps18
Saccharomycotina_1 - ps19
Saccharomycotina_2 - ps20
Saccharomycotina_3 - ps21
Phaff./Saccharomycetaceae - ps22
Saccharomyco./mycetaceae - ps23
Saccharomycetaceae - ps24
Saccharomycetaceae_2 - ps25
Saccharomycetaceae_3 - ps26
Kaz./Nau./Saccharomyces - ps27
Saccharomyces - ps28
Arboricola_clade - ps29
Cerevisiae_clade - ps30
Saccharomyces_cerevisiae - ps31
p-value
5e-03
6e-03
9e-03
1e-02
2e-02
Log-odds
0.5
1.0
1.5
2.0
2.5
3.0
3.5
4.0

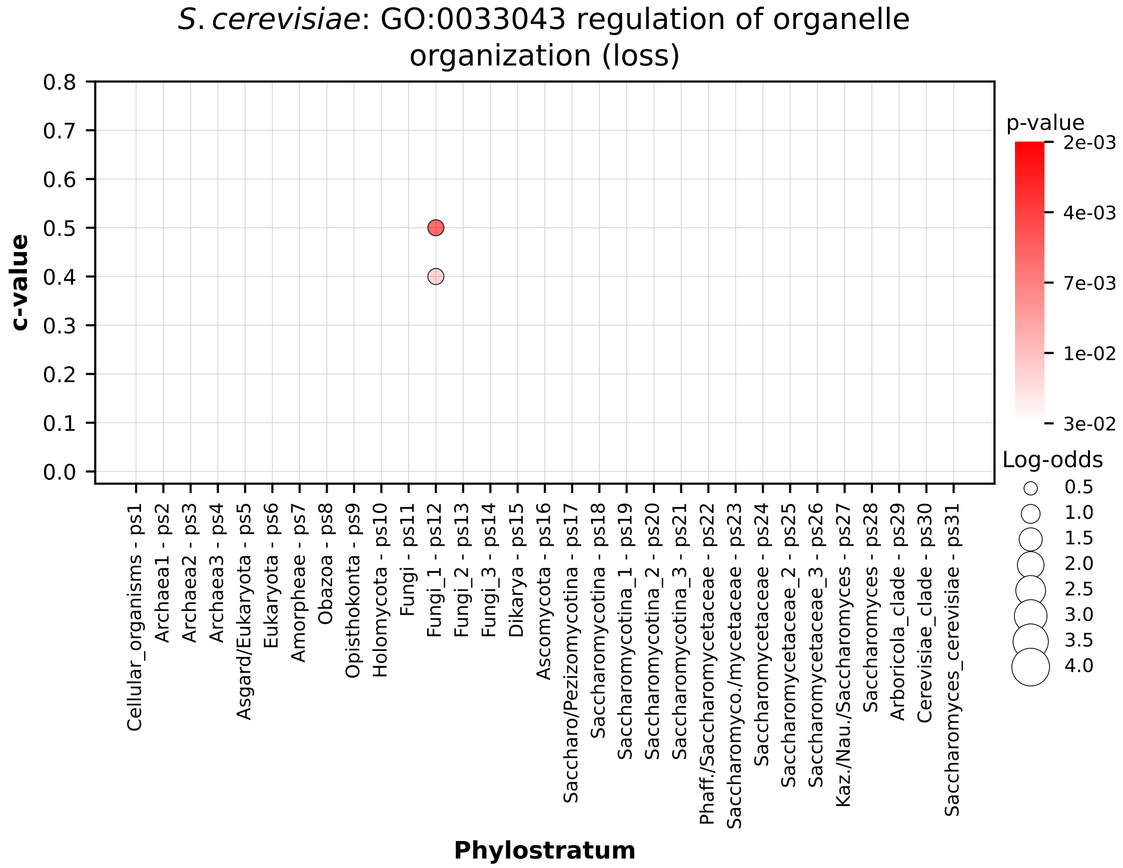

S. cerevisiae: GO:0033043 regulation of organelle organization (loss)
c-value
0.0
0.1
0.2
0.3
0.4
0.5
0.6
0.7
0.8
Phylostratum
Cellular_organisms - ps1
Archaea1 - ps2
Archaea2 - ps3
Archaea3 - ps4
Asgard/Eukaryota - ps5
Eukaryota - ps6
Amorpheae - ps7
Obazoa - ps8
Opisthokonta - ps9
Holomycota - ps10
Fungi - ps11
Fungi_1 - ps12
Fungi_2 - ps13
Fungi_3 - ps14
Dikarya - ps15
Ascomycota - ps16
Saccharo/Pezizomycotina - ps17
Saccharomycotina - ps18
Saccharomycotina_1 - ps19
Saccharomycotina_2 - ps20
Saccharomycotina_3 - ps21
Phaff./Saccharomycetaceae - ps22
Saccharomyco./mycetaceae - ps23
Saccharomycetaceae - ps24
Saccharomycetaceae_2 - ps25
Saccharomycetaceae_3 - ps26
Kaz./Nau./Saccharomyces - ps27
Saccharomyces - ps28
Arboricola_clade - ps29
Cerevisiae_clade - ps30
Saccharomyces_cerevisiae - ps31
p-value
2e-03
4e-03
7e-03
1e-02
3e-02
Log-odds
0.5
1.0
1.5
2.0
2.5
3.0
3.5
4.0

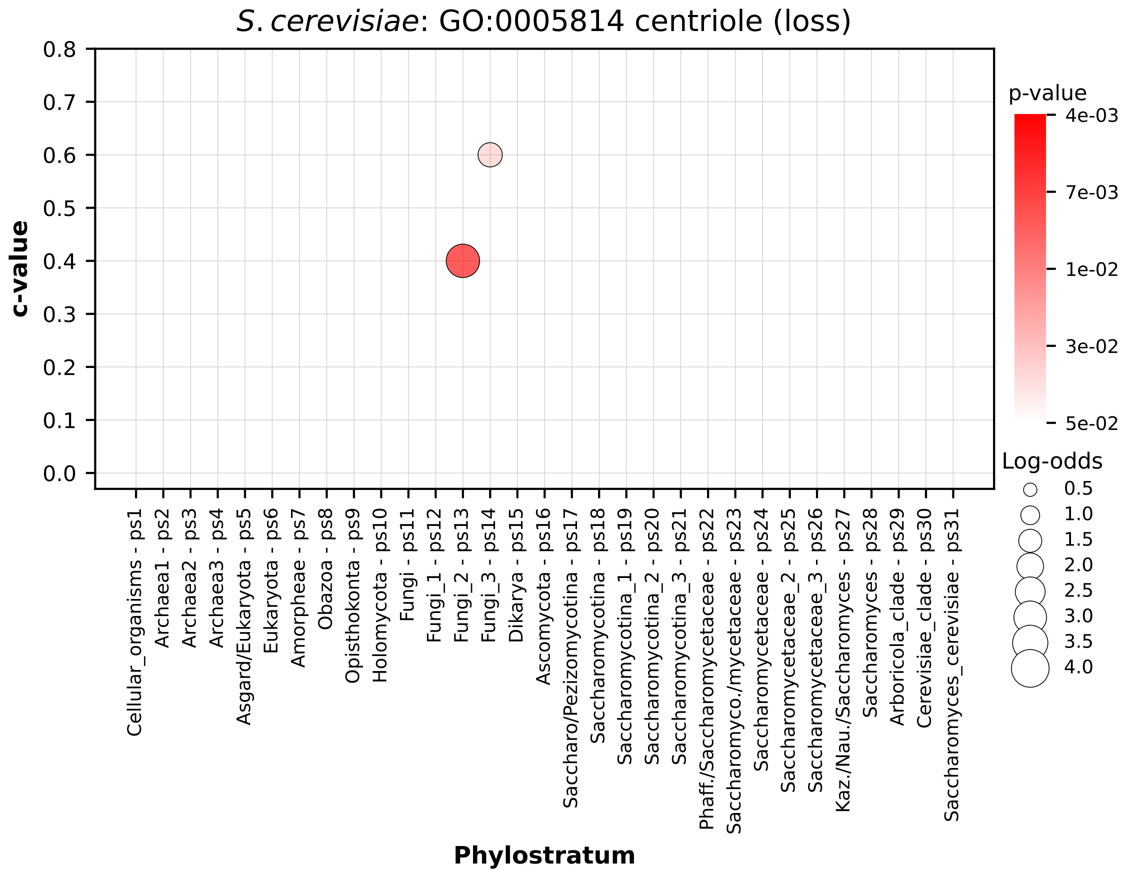
S. cerevisiae: GO:0005814 centriole (loss)
c-value
0.8
0.7
0.6
0.5
0.4
0.3
0.2
0.1
0.0
p-value
4e-03
7e-03
1e-02
3e-02
5e-02
Log-odds
0.5
1.0
1.5
2.0
2.5
3.0
3.5
4.0
Cellular_organisms - ps1
Archaea1 - ps2
Archaea2 - ps3
Archaea3 - ps4
Asgard/Eukaryota - ps5
Eukaryota - ps6
Amorpheae - ps7
Obazoa - ps8
Opisthokonta - ps9
Holomycota - ps10
Fungi - ps11
Fungi_1 - ps12
Fungi_2 - ps13
Fungi_3 - ps14
Dikarya - ps15
Ascomycota - ps16
Saccharo/Pezizomycotina - ps17
Saccharomycotina - ps18
Saccharomycotina_1 - ps19
Saccharomycotina_2 - ps20
Saccharomycotina_3 - ps21
Phaff./Saccharomycetaceae - ps22
Saccharomyco./mycetaceae - ps23
Saccharomycetaceae - ps24
Saccharomycetaceae_2 - ps25
Saccharomycetaceae_3 - ps26
Kaz./Nau./Saccharomyces - ps27
Saccharomyces - ps28
Arboricola_clade - ps29
Cerevisiae_clade - ps30
Saccharomyces_cerevisiae - ps31
Phylostratum

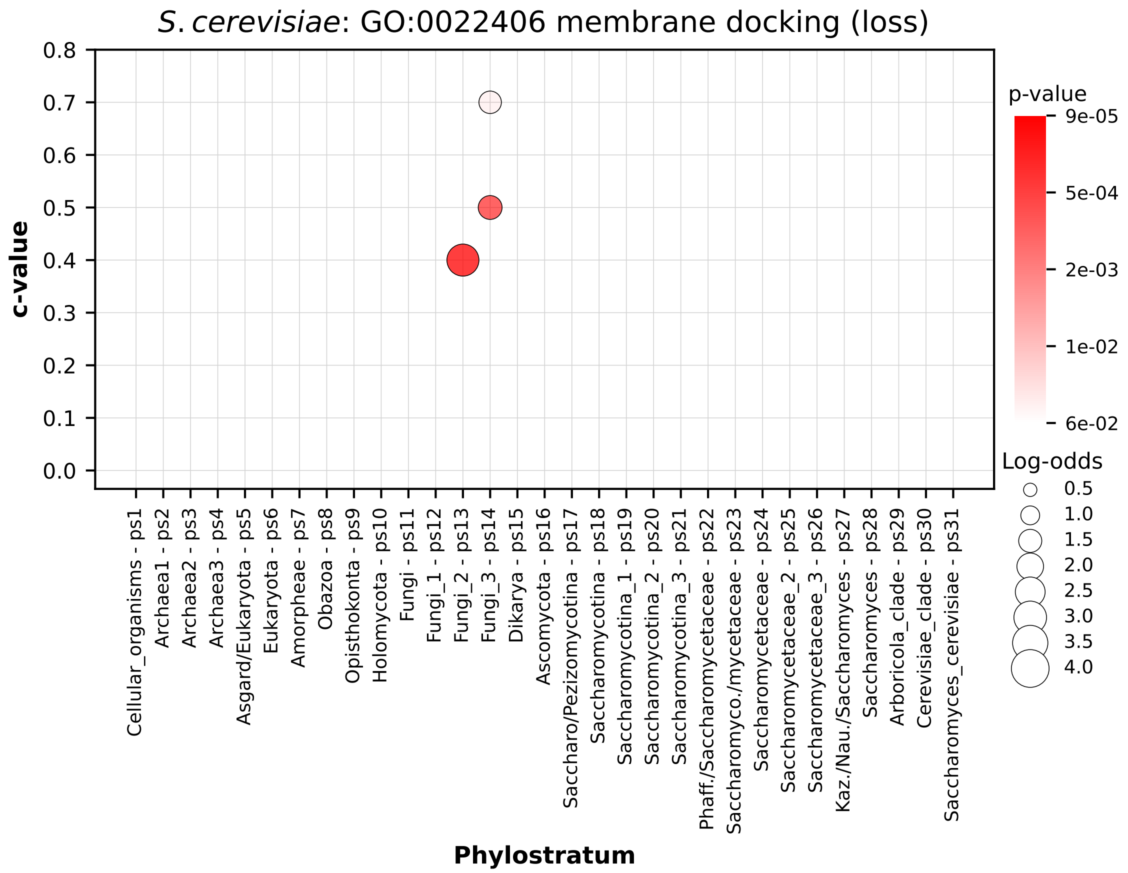

S. cerevisiae: GO:0022406 membrane docking (loss)
c-value
0.8
0.7
0.6
0.5
0.4
0.3
0.2
0.1
0.0
p-value
9e-05
5e-04
2e-03
1e-02
6e-02
Log-odds
0.5
1.0
1.5
2.0
2.5
3.0
3.5
4.0
Cellular_organisms - ps1
Archaea1 - ps2
Archaea2 - ps3
Archaea3 - ps4
Asgard/Eukaryota - ps5
Eukaryota - ps6
Amorpheae - ps7
Obazoa - ps8
Opisthokonta - ps9
Holomycota - ps10
Fungi - ps11
Fungi_1 - ps12
Fungi_2 - ps13
Fungi_3 - ps14
Dikarya - ps15
Ascomycota - ps16
Saccharo/Pezizomycotina - ps17
Saccharomycotina - ps18
Saccharomycotina_1 - ps19
Saccharomycotina_2 - ps20
Saccharomycotina_3 - ps21
Phaff./Saccharomycetaceae - ps22
Saccharomyco./mycetaceae - ps23
Saccharomycetaceae - ps24
Saccharomycetaceae_2 - ps25
Saccharomycetaceae_3 - ps26
Kaz./Nau./Saccharomyces - ps27
Saccharomyces - ps28
Arboricola_clade - ps29
Cerevisiae_clade - ps30
Saccharomyces_cerevisiae - ps31
Phylostratum

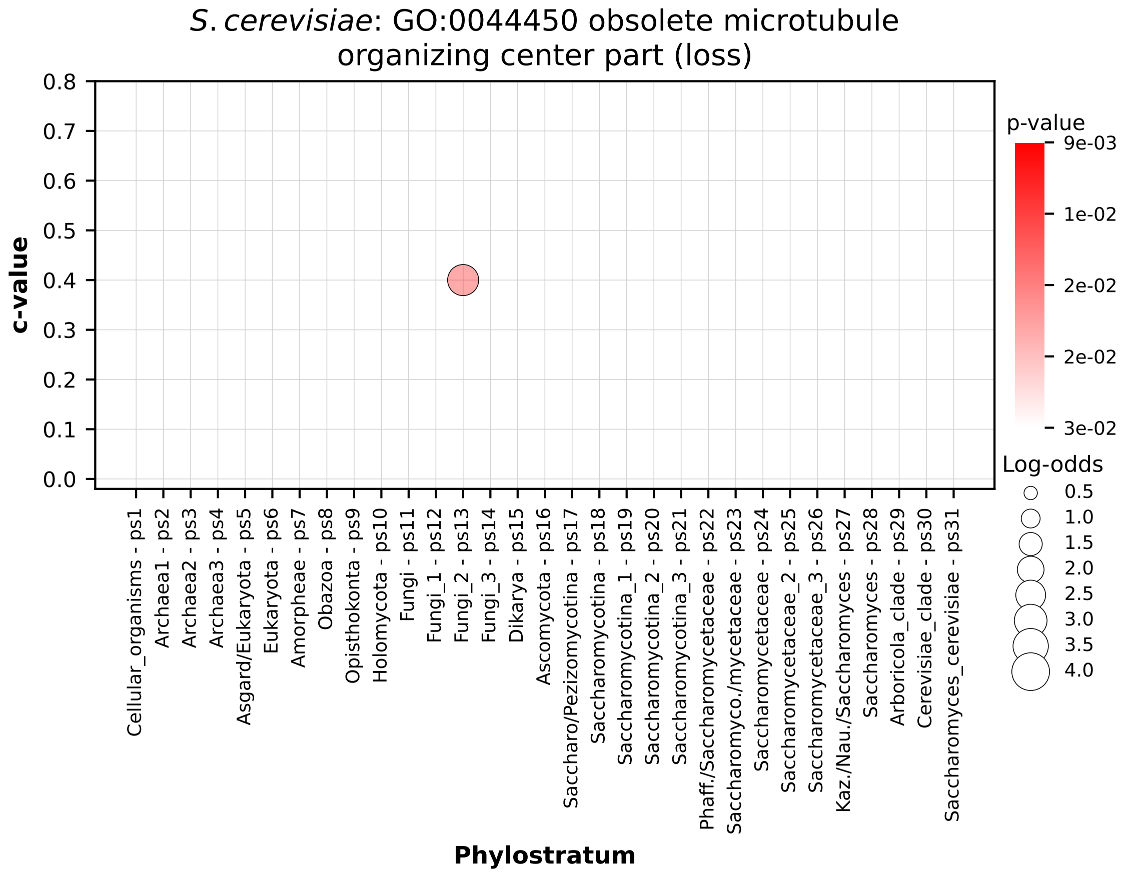
S. cerevisiae: GO:0044450 obsolete microtubule organizing center part (loss)
c-value
0.8
0.7
0.6
0.5
0.4
0.3
0.2
0.1
0.0
p-value
9e-03
1e-02
2e-02
2e-02
3e-02
Log-odds
0.5
1.0
1.5
2.0
2.5
3.0
3.5
4.0
Cellular_organisms - ps1
Archaea1 - ps2
Archaea2 - ps3
Archaea3 - ps4
Asgard/Eukaryota - ps5
Eukaryota - ps6
Amorpheae - ps7
Obazoa - ps8
Opisthokonta - ps9
Holomycota - ps10
Fungi - ps11
Fungi_1 - ps12
Fungi_2 - ps13
Fungi_3 - ps14
Dikarya - ps15
Ascomycota - ps16
Saccharo/Pezizomycotina - ps17
Saccharomycotina - ps18
Saccharomycotina_1 - ps19
Saccharomycotina_2 - ps20
Saccharomycotina_3 - ps21
Phaff./Saccharomycetaceae - ps22
Saccharomyco./mycetaceae - ps23
Saccharomycetaceae - ps24
Saccharomycetaceae_2 - ps25
Saccharomycetaceae_3 - ps26
Kaz./Nau./Saccharomyces - ps27
Saccharomyces - ps28
Arboricola_clade - ps29
Cerevisiae_clade - ps30
Saccharomyces_cerevisiae - ps31
Phylostratum

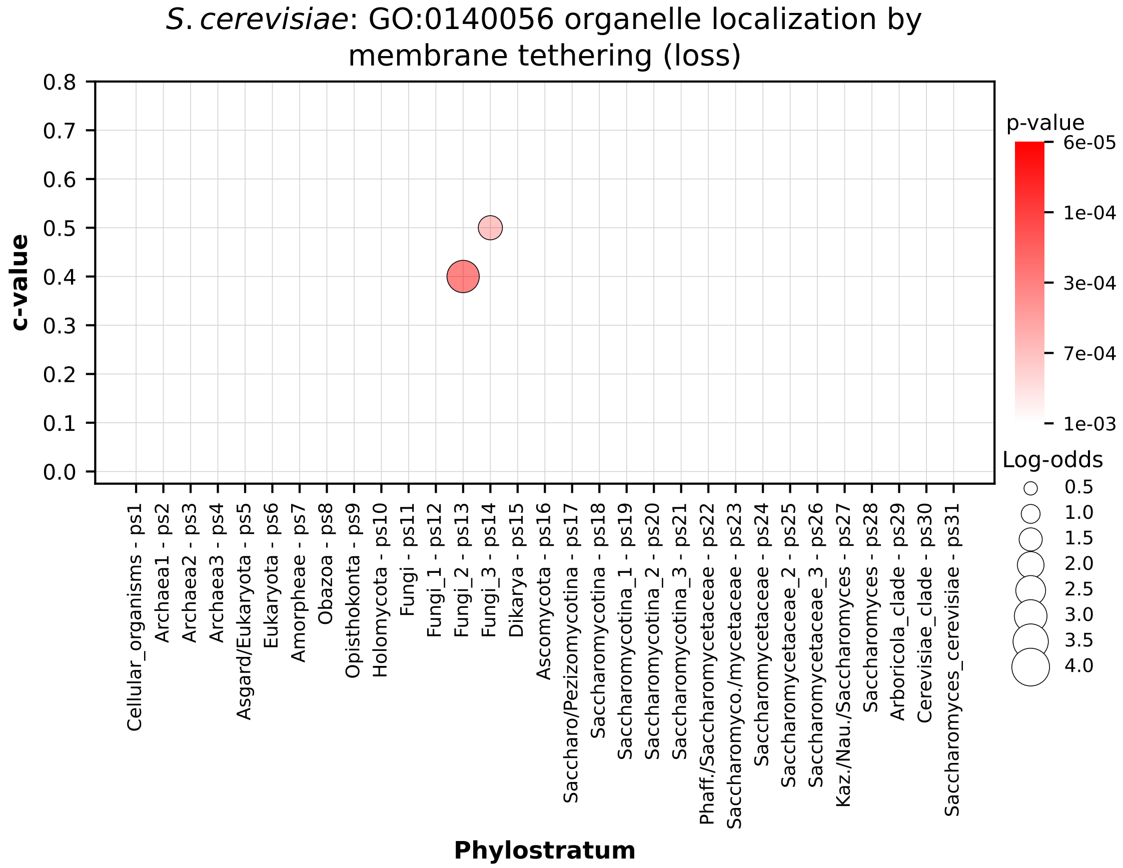

S. cerevisiae: GO:0140056 organelle localization by membrane tethering (loss)
c-value
0.0
0.1
0.2
0.3
0.4
0.5
0.6
0.7
0.8
Phylostratum
Cellular_organisms - ps1
Archaea1 - ps2
Archaea2 - ps3
Archaea3 - ps4
Asgard/Eukaryota - ps5
Eukaryota - ps6
Amorpheae - ps7
Obazoa - ps8
Opisthokonta - ps9
Holomycota - ps10
Fungi - ps11
Fungi_1 - ps12
Fungi_2 - ps13
Fungi_3 - ps14
Dikarya - ps15
Ascomycota - ps16
Saccharo/Pezizomycotina - ps17
Saccharomycotina - ps18
Saccharomycotina_1 - ps19
Saccharomycotina_2 - ps20
Saccharomycotina_3 - ps21
Phaff./Saccharomycetaceae - ps22
Saccharomyco./mycetaceae - ps23
Saccharomycetaceae - ps24
Saccharomycetaceae_2 - ps25
Saccharomycetaceae_3 - ps26
Kaz./Nau./Saccharomyces - ps27
Saccharomyces - ps28
Arboricola_clade - ps29
Cerevisiae_clade - ps30
Saccharomyces_cerevisiae - ps31
p-value
6e-05
1e-04
3e-04
7e-04
1e-03
Log-odds
0.5
1.0
1.5
2.0
2.5
3.0
3.5
4.0

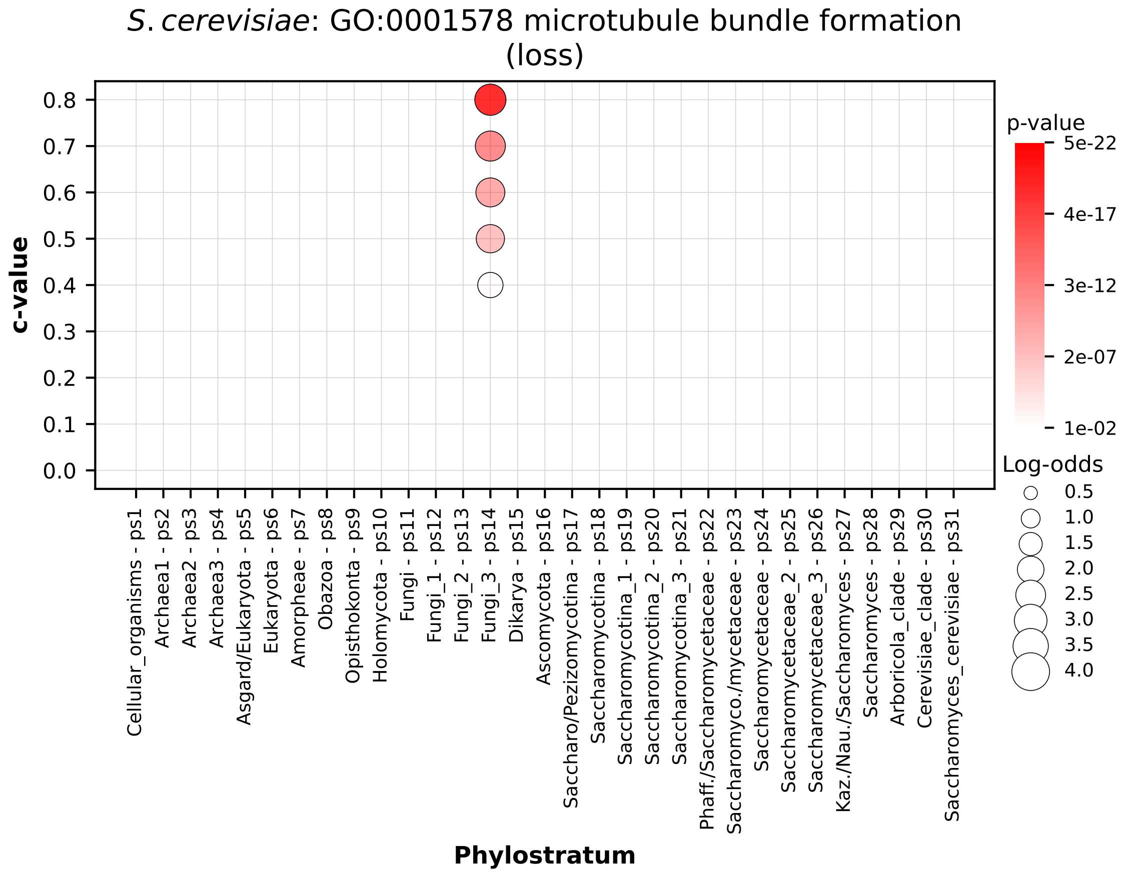

S. cerevisiae: GO:0001578 microtubule bundle formation (loss)
c-value
0.8
0.7
0.6
0.5
0.4
0.3
0.2
0.1
0.0
p-value
5e-22
4e-17
3e-12
2e-07
1e-02
Log-odds
0.5
1.0
1.5
2.0
2.5
3.0
3.5
4.0
Cellular_organisms - ps1
Archaea1 - ps2
Archaea2 - ps3
Archaea3 - ps4
Asgard/Eukaryota - ps5
Eukaryota - ps6
Amorpheae - ps7
Obazoa - ps8
Opisthokonta - ps9
Holomycota - ps10
Fungi - ps11
Fungi_1 - ps12
Fungi_2 - ps13
Fungi_3 - ps14
Dikarya - ps15
Ascomycota - ps16
Saccharo/Pezizomycotina - ps17
Saccharomycotina - ps18
Saccharomycotina_1 - ps19
Saccharomycotina_2 - ps20
Saccharomycotina_3 - ps21
Phaff./Saccharomycetaceae - ps22
Saccharomyco./mycetaceae - ps23
Saccharomycetaceae - ps24
Saccharomycetaceae_2 - ps25
Saccharomycetaceae_3 - ps26
Kaz./Nau./Saccharomyces - ps27
Saccharomyces - ps28
Arboricola_clade - ps29
Cerevisiae_clade - ps30
Saccharomyces_cerevisiae - ps31
Phylostratum

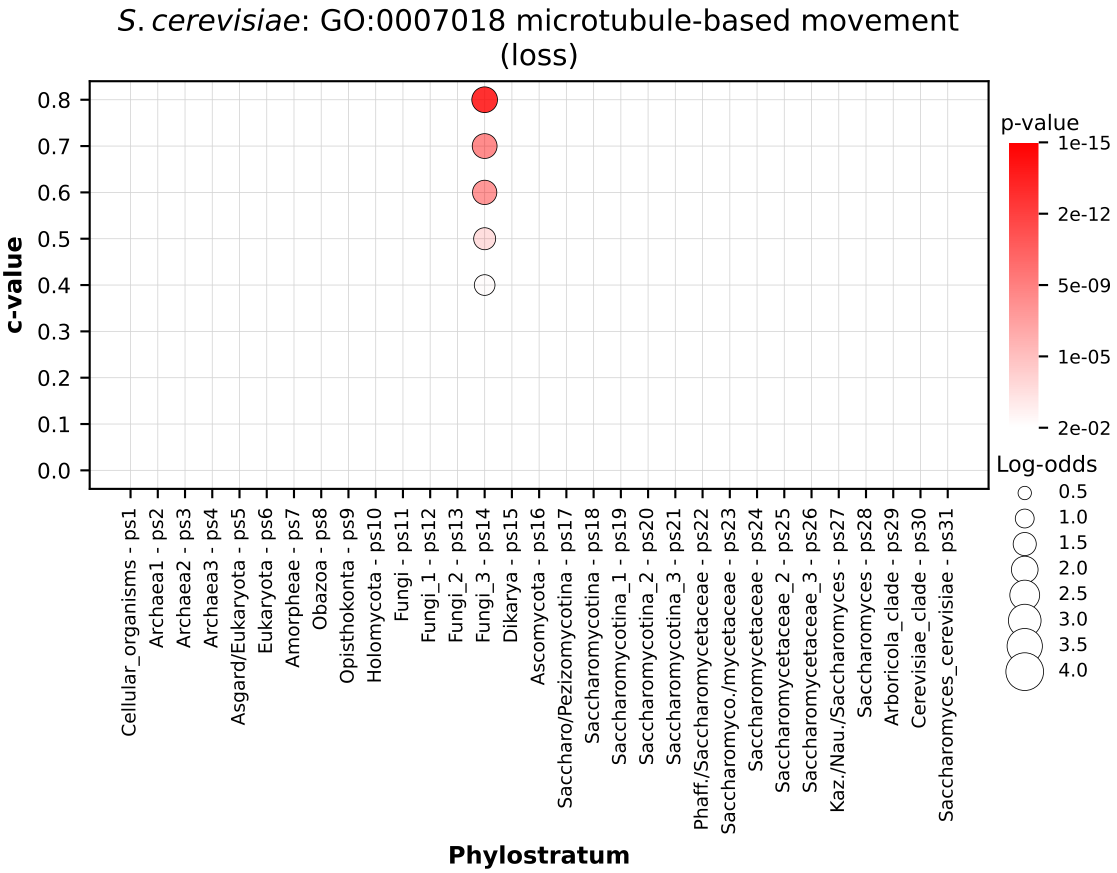
S. cerevisiae: GO:0007018 microtubule-based movement
(loss)
c-value
0.0
0.1
0.2
0.3
0.4
0.5
0.6
0.7
0.8
p-value
1e-15
2e-12
5e-09
1e-05
2e-02
Log-odds
0.5
1.0
1.5
2.0
2.5
3.0
3.5
4.0
Cellular_organisms - ps1
Archaea1 - ps2
Archaea2 - ps3
Archaea3 - ps4
Asgard/Eukaryota - ps5
Eukaryota - ps6
Amorpheae - ps7
Obazoa - ps8
Opisthokonta - ps9
Holomycota - ps10
Fungi - ps11
Fungi_1 - ps12
Fungi_2 - ps13
Fungi_3 - ps14
Dikarya - ps15
Ascomycota - ps16
Saccharo/Pezizomycotina - ps17
Saccharomycotina - ps18
Saccharomycotina_1 - ps19
Saccharomycotina_2 - ps20
Saccharomycotina_3 - ps21
Phaff./Saccharomycetaceae - ps22
Saccharomyco./mycetaceae - ps23
Saccharomycetaceae - ps24
Saccharomycetaceae_2 - ps25
Saccharomycetaceae_3 - ps26
Kaz./Nau./Saccharomyces - ps27
Saccharomyces - ps28
Arboricola_clade - ps29
Cerevisiae_clade - ps30
Saccharomyces_cerevisiae - ps31
Phylostratum

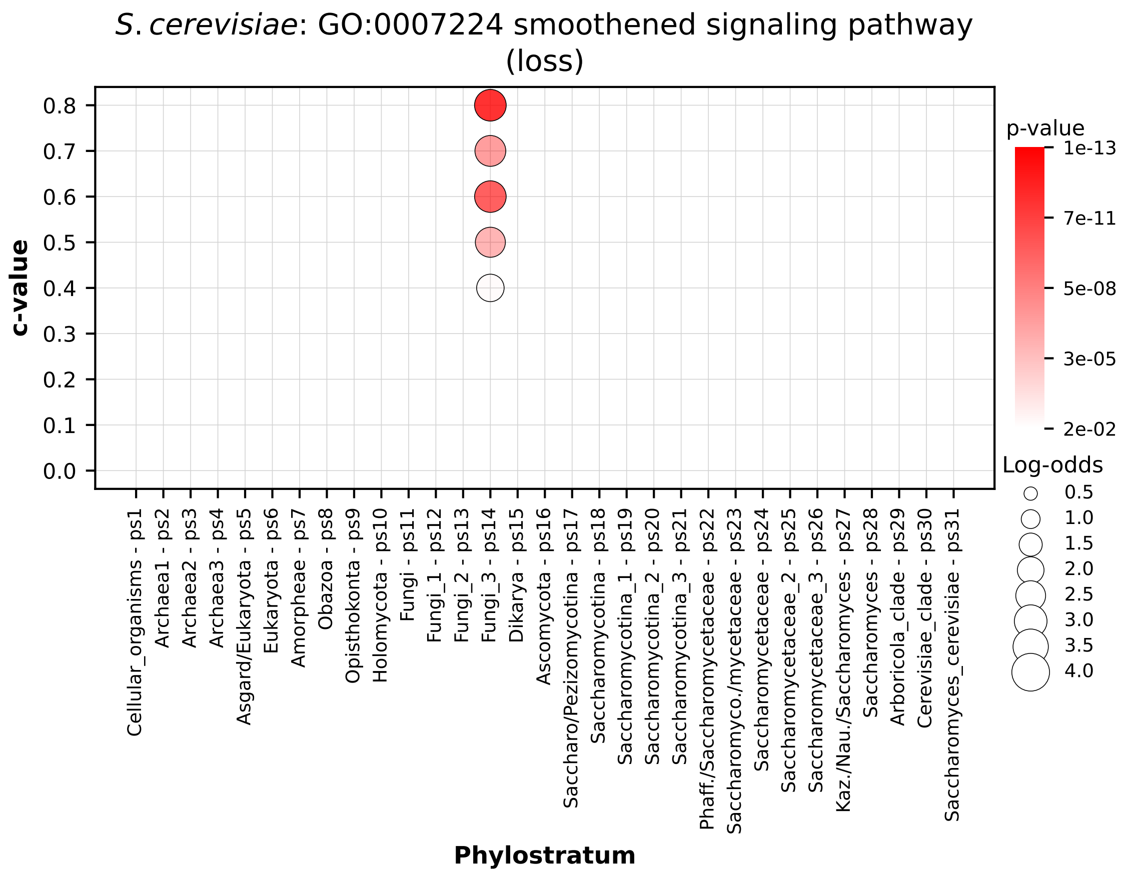

S. cerevisiae: GO:0007224 smoothened signaling pathway (loss)
c-value
0.0
0.1
0.2
0.3
0.4
0.5
0.6
0.7
0.8
p-value
1e-13
7e-11
5e-08
3e-05
2e-02
Log-odds
0.5
1.0
1.5
2.0
2.5
3.0
3.5
4.0
Cellular_organisms - ps1
Archaea1 - ps2
Archaea2 - ps3
Archaea3 - ps4
Asgard/Eukaryota - ps5
Eukaryota - ps6
Amorpheae - ps7
Obazoa - ps8
Opisthokonta - ps9
Holomycota - ps10
Fungi - ps11
Fungi_1 - ps12
Fungi_2 - ps13
Fungi_3 - ps14
Dikarya - ps15
Ascomycota - ps16
Saccharo/Pezizomycotina - ps17
Saccharomycotina - ps18
Saccharomycotina_1 - ps19
Saccharomycotina_2 - ps20
Saccharomycotina_3 - ps21
Phaff./Saccharomycetaceae - ps22
Saccharomyco./mycetaceae - ps23
Saccharomycetaceae - ps24
Saccharomycetaceae_2 - ps25
Saccharomycetaceae_3 - ps26
Kaz./Nau./Saccharomyces - ps27
Saccharomyces - ps28
Arboricola_clade - ps29
Cerevisiae_clade - ps30
Saccharomyces_cerevisiae - ps31
Phylostratum

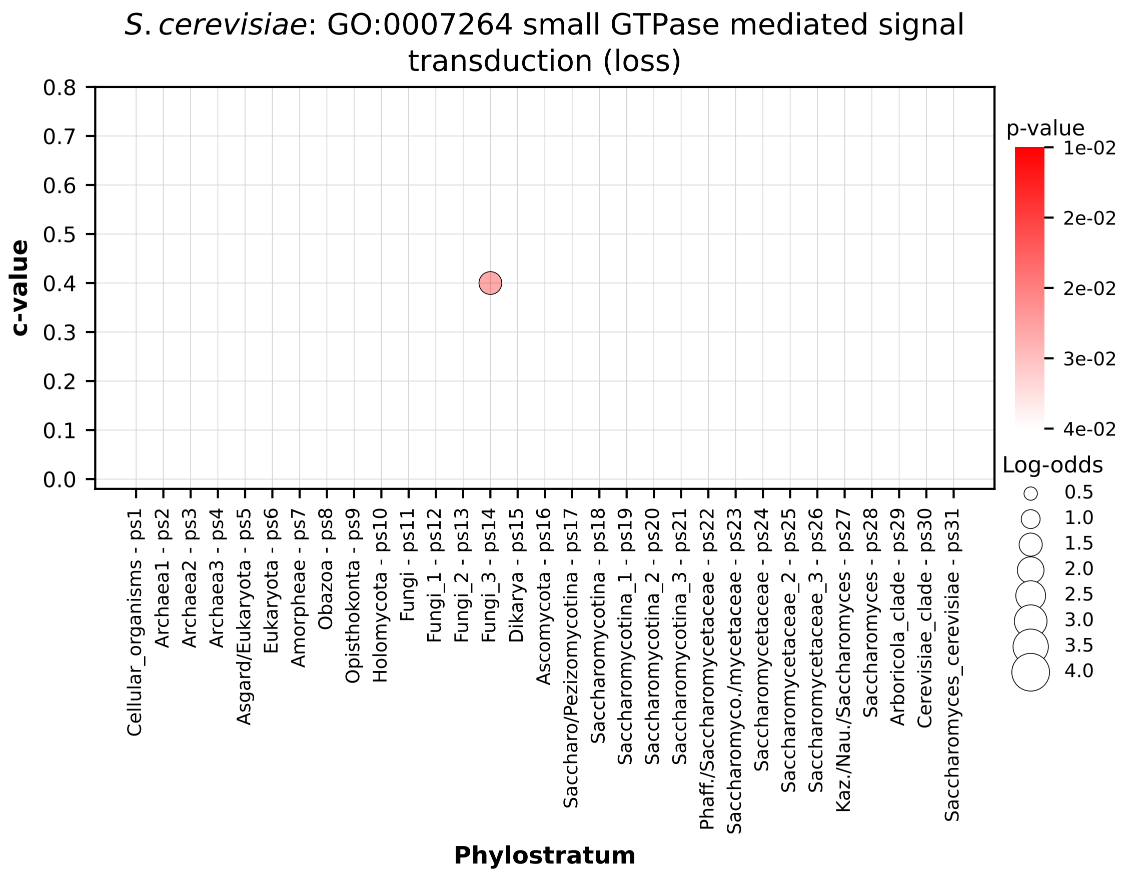

S. cerevisiae: GO:0007264 small GTPase mediated signal transduction (loss)
c-value
0.8
0.7
0.6
0.5
0.4
0.3
0.2
0.1
0.0
p-value
1e-02
2e-02
2e-02
3e-02
4e-02
Log-odds
0.5
1.0
1.5
2.0
2.5
3.0
3.5
4.0
Cellular_organisms - ps1
Archaea1 - ps2
Archaea2 - ps3
Archaea3 - ps4
Asgard/Eukaryota - ps5
Eukaryota - ps6
Amorpheae - ps7
Obazoa - ps8
Opisthokonta - ps9
Holomycota - ps10
Fungi - ps11
Fungi_1 - ps12
Fungi_2 - ps13
Fungi_3 - ps14
Dikarya - ps15
Ascomycota - ps16
Saccharo/Pezizomycotina - ps17
Saccharomycotina - ps18
Saccharomycotina_1 - ps19
Saccharomycotina_2 - ps20
Saccharomycotina_3 - ps21
Phaff./Saccharomycetaceae - ps22
Saccharomyco./mycetaceae - ps23
Saccharomycetaceae - ps24
Saccharomycetaceae_2 - ps25
Saccharomycetaceae_3 - ps26
Kaz./Nau./Saccharomyces - ps27
Saccharomyces - ps28
Arboricola_clade - ps29
Cerevisiae_clade - ps30
Saccharomyces_cerevisiae - ps31
Phylostratum

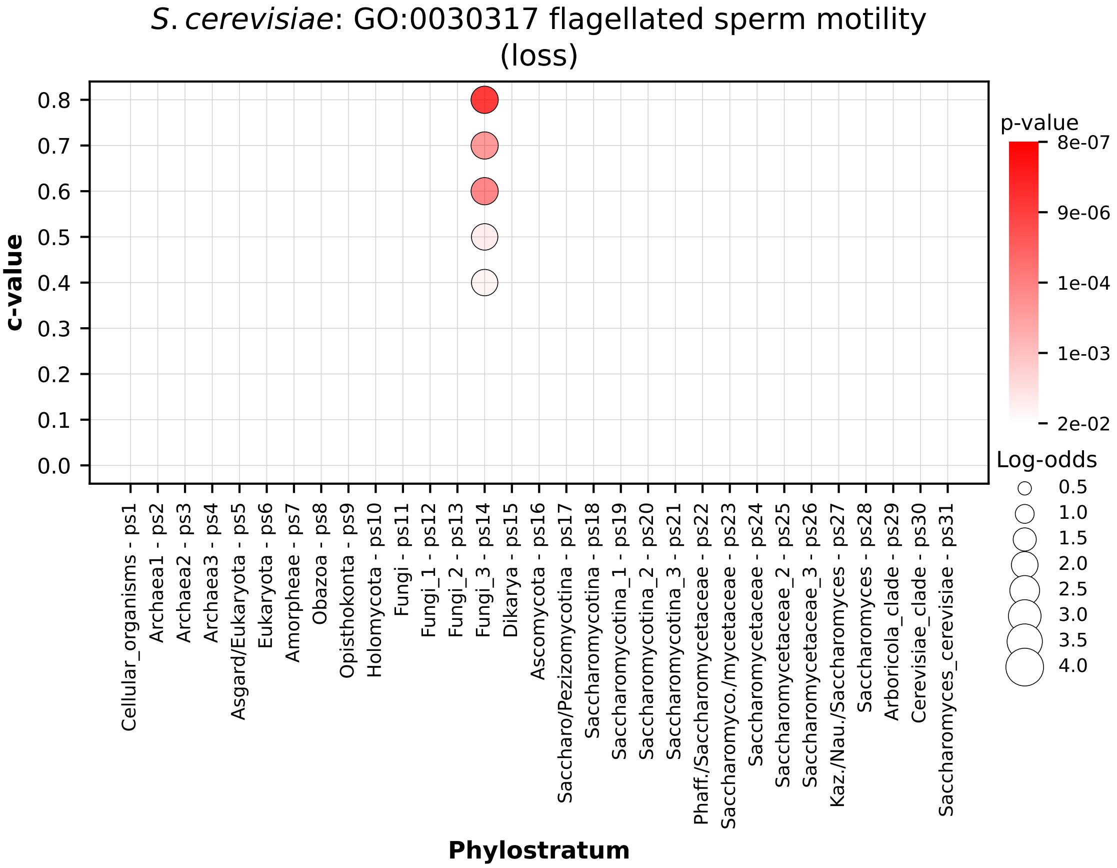

S. cerevisiae: GO:0030317 flagellated sperm motility
(loss)
c-value
0.8
0.7
0.6
0.5
0.4
0.3
0.2
0.1
0.0
p-value
8e-07
9e-06
1e-04
1e-03
2e-02
Log-odds
0.5
1.0
1.5
2.0
2.5
3.0
3.5
4.0
Cellular_organisms - ps1
Archaea1 - ps2
Archaea2 - ps3
Archaea3 - ps4
Asgard/Eukaryota - ps5
Eukaryota - ps6
Amorpheae - ps7
Obazoa - ps8
Opisthokonta - ps9
Holomycota - ps10
Fungi - ps11
Fungi_1 - ps12
Fungi_2 - ps13
Fungi_3 - ps14
Dikarya - ps15
Ascomycota - ps16
Saccharo/Pezizomycotina - ps17
Saccharomycotina - ps18
Saccharomycotina_1 - ps19
Saccharomycotina_2 - ps20
Saccharomycotina_3 - ps21
Phaff./Saccharomycetaceae - ps22
Saccharomyco./mycetaceae - ps23
Saccharomycetaceae - ps24
Saccharomycetaceae_2 - ps25
Saccharomycetaceae_3 - ps26
Kaz./Nau./Saccharomyces - ps27
Saccharomyces - ps28
Arboricola_clade - ps29
Cerevisiae_clade - ps30
Saccharomyces_cerevisiae - ps31
Phylostratum

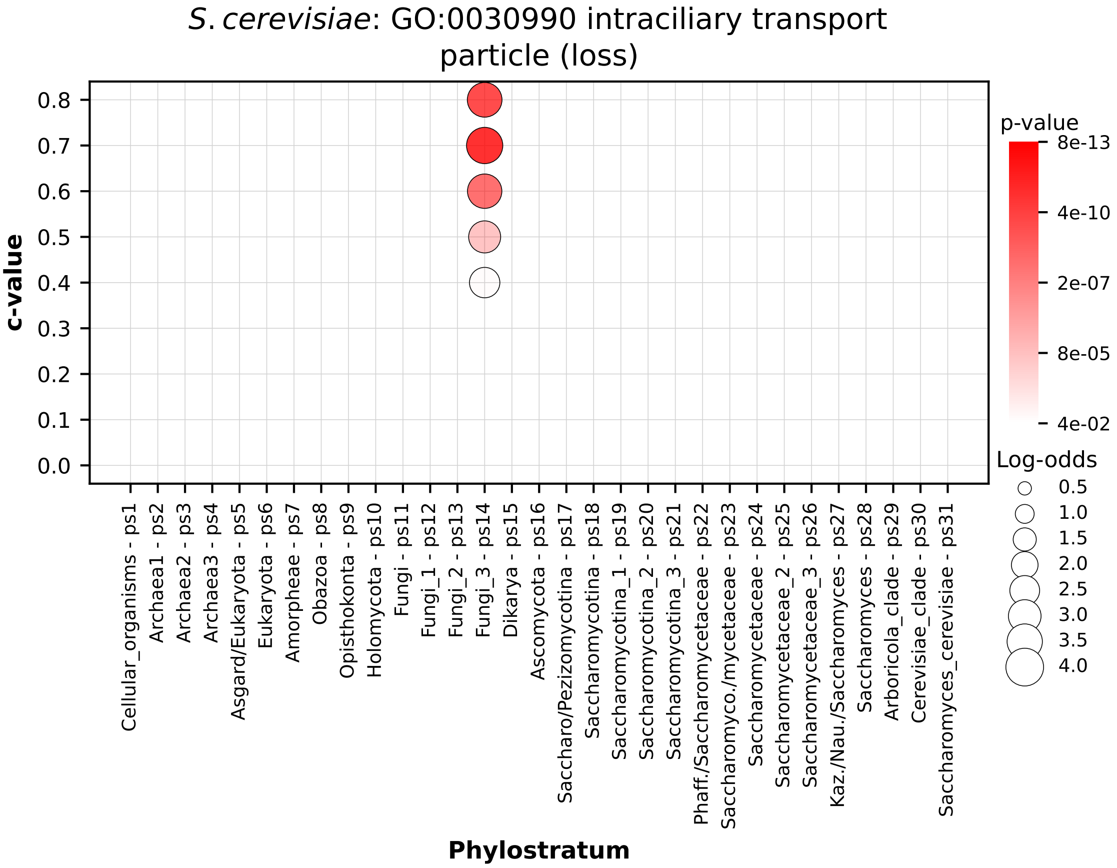

S. cerevisiae: GO:0030990 intraciliary transport particle (loss)
c-value
0.8
0.7
0.6
0.5
0.4
0.3
0.2
0.1
0.0
p-value
8e-13
4e-10
2e-07
8e-05
4e-02
Log-odds
0.5
1.0
1.5
2.0
2.5
3.0
3.5
4.0
Cellular_organisms - ps1
Archaea1 - ps2
Archaea2 - ps3
Archaea3 - ps4
Asgard/Eukaryota - ps5
Eukaryota - ps6
Amorpheae - ps7
Obazoa - ps8
Opisthokonta - ps9
Holomycota - ps10
Fungi - ps11
Fungi_1 - ps12
Fungi_2 - ps13
Fungi_3 - ps14
Dikarya - ps15
Ascomycota - ps16
Saccharo/Pezizomycotina - ps17
Saccharomycotina - ps18
Saccharomycotina_1 - ps19
Saccharomycotina_2 - ps20
Saccharomycotina_3 - ps21
Phaff./Saccharomycetaceae - ps22
Saccharomyco./mycetaceae - ps23
Saccharomycetaceae - ps24
Saccharomycetaceae_2 - ps25
Saccharomycetaceae_3 - ps26
Kaz./Nau./Saccharomyces - ps27
Saccharomyces - ps28
Arboricola_clade - ps29
Cerevisiae_clade - ps30
Saccharomyces_cerevisiae - ps31
Phylostratum

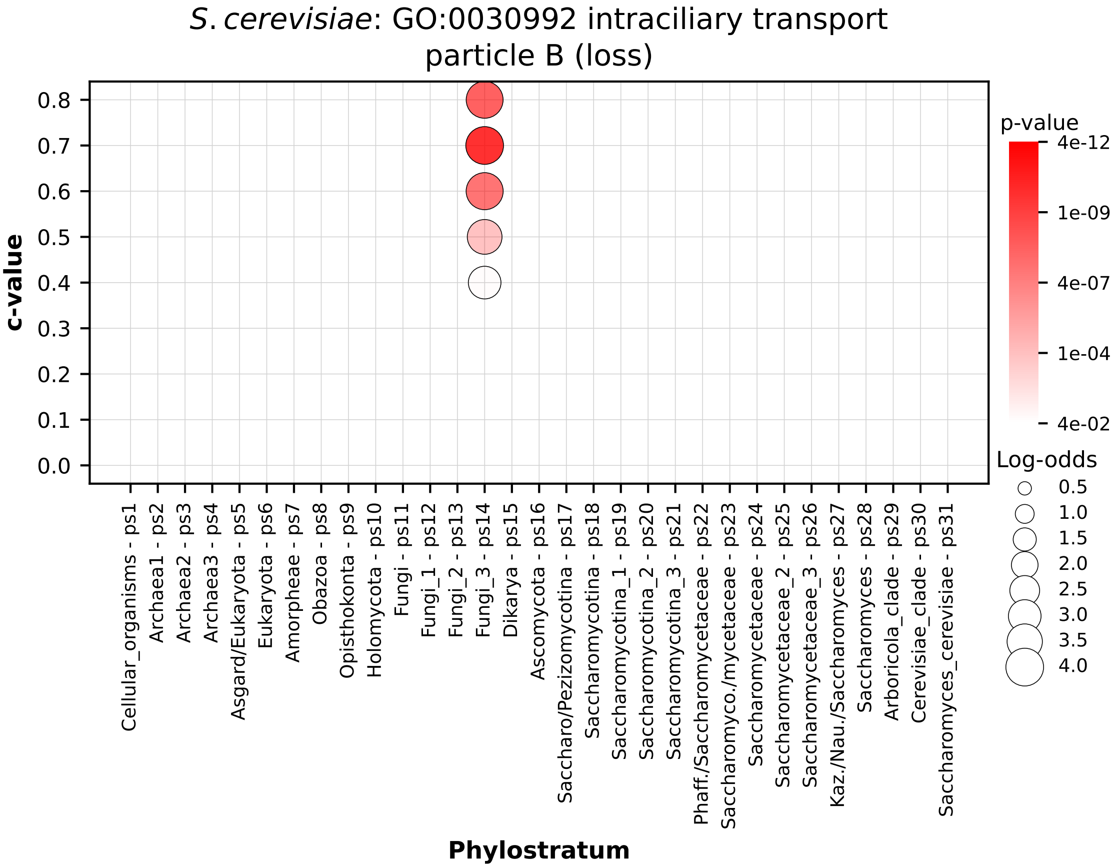

S. cerevisiae: GO:0030992 intraciliary transport particle B (loss)
c-value
0.0
0.1
0.2
0.3
0.4
0.5
0.6
0.7
0.8
p-value
4e-12
1e-09
4e-07
1e-04
4e-02
Log-odds
0.5
1.0
1.5
2.0
2.5
3.0
3.5
4.0
Cellular_organisms - ps1
Archaea1 - ps2
Archaea2 - ps3
Archaea3 - ps4
Asgard/Eukaryota - ps5
Eukaryota - ps6
Amorpheae - ps7
Obazoa - ps8
Opisthokonta - ps9
Holomycota - ps10
Fungi - ps11
Fungi_1 - ps12
Fungi_2 - ps13
Fungi_3 - ps14
Dikarya - ps15
Ascomycota - ps16
Saccharo/Pezizomycotina - ps17
Saccharomycotina - ps18
Saccharomycotina_1 - ps19
Saccharomycotina_2 - ps20
Saccharomycotina_3 - ps21
Phaff./Saccharomycetaceae - ps22
Saccharomyco./mycetaceae - ps23
Saccharomycetaceae - ps24
Saccharomycetaceae_2 - ps25
Saccharomycetaceae_3 - ps26
Kaz./Nau./Saccharomyces - ps27
Saccharomyces - ps28
Arboricola_clade - ps29
Cerevisiae_clade - ps30
Saccharomyces_cerevisiae - ps31
Phylostratum

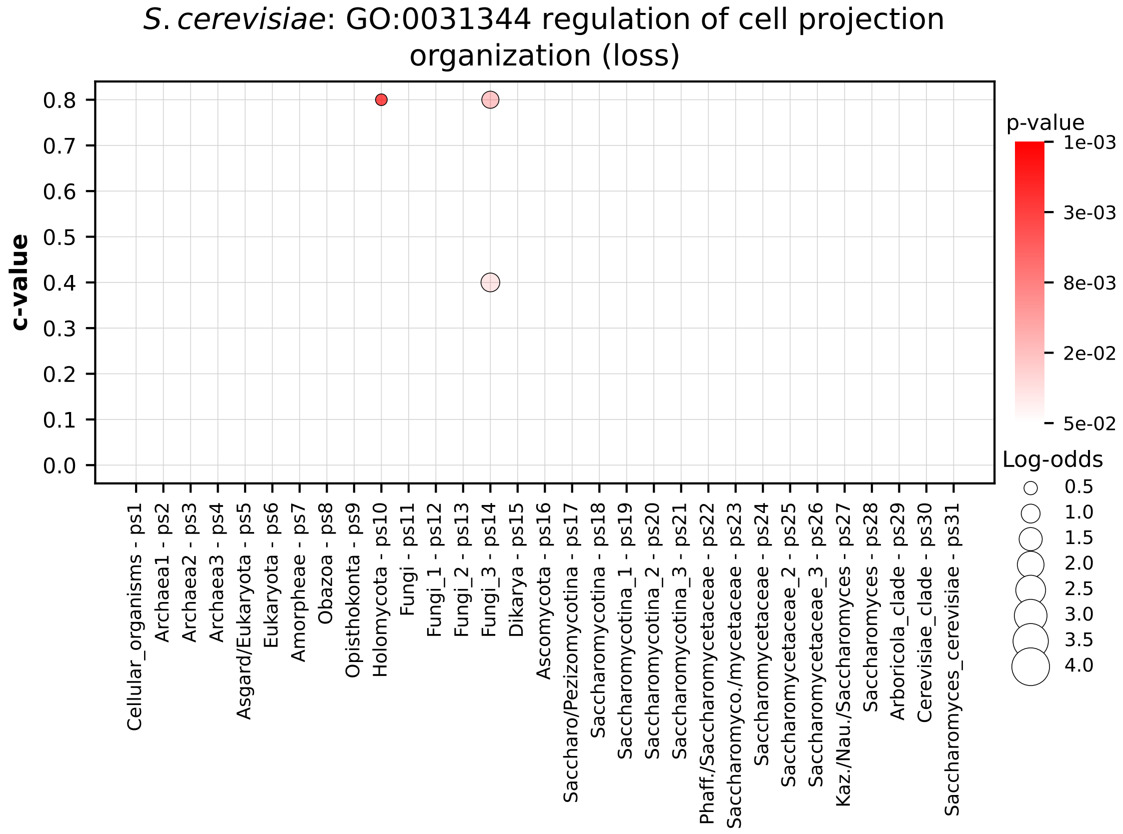

S. cerevisiae: GO:0031344 regulation of cell projection organization (loss)
c-value
0.8
0.7
0.6
0.5
0.4
0.3
0.2
0.1
0.0
Phylostratum
Cellular_organisms - ps1
Archaea1 - ps2
Archaea2 - ps3
Archaea3 - ps4
Asgard/Eukaryota - ps5
Eukaryota - ps6
Amorpheae - ps7
Obazoa - ps8
Opisthokonta - ps9
Holomycota - ps10
Fungi - ps11
Fungi_1 - ps12
Fungi_2 - ps13
Fungi_3 - ps14
Dikarya - ps15
Ascomycota - ps16
Saccharo/Pezizomycotina - ps17
Saccharomycotina - ps18
Saccharomycotina_1 - ps19
Saccharomycotina_2 - ps20
Saccharomycotina_3 - ps21
Phaff./Saccharomycetaceae - ps22
Saccharomyco./mycetaceae - ps23
Saccharomycetaceae - ps24
Saccharomycetaceae_2 - ps25
Saccharomycetaceae_3 - ps26
Kaz./Nau./Saccharomyces - ps27
Saccharomyces - ps28
Arboricola_clade - ps29
Cerevisiae_clade - ps30
Saccharomyces_cerevisiae - ps31
p-value
1e-03
3e-03
8e-03
2e-02
5e-02
Log-odds
0.5
1.0
1.5
2.0
2.5
3.0
3.5
4.0

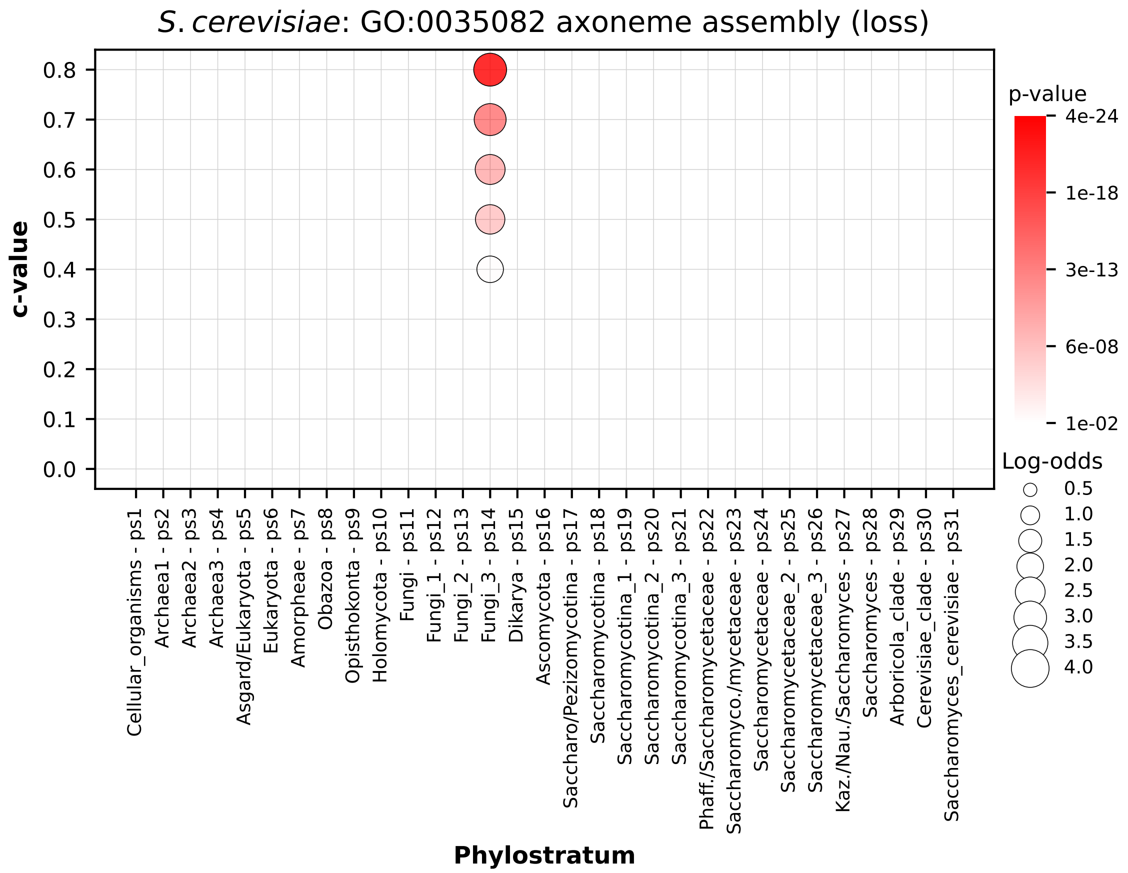

S. cerevisiae: GO:0035082 axoneme assembly (loss)
c-value
0.8
0.7
0.6
0.5
0.4
0.3
0.2
0.1
0.0
p-value
4e-24
1e-18
3e-13
6e-08
1e-02
Log-odds
0.5
1.0
1.5
2.0
2.5
3.0
3.5
4.0
Cellular_organisms - ps1
Archaea1 - ps2
Archaea2 - ps3
Archaea3 - ps4
Asgard/Eukaryota - ps5
Eukaryota - ps6
Amorpheae - ps7
Obazoa - ps8
Opisthokonta - ps9
Holomycota - ps10
Fungi - ps11
Fungi_1 - ps12
Fungi_2 - ps13
Fungi_3 - ps14
Dikarya - ps15
Ascomycota - ps16
Saccharo/Pezizomycotina - ps17
Saccharomycotina - ps18
Saccharomycotina_1 - ps19
Saccharomycotina_2 - ps20
Saccharomycotina_3 - ps21
Phaff./Saccharomycetaceae - ps22
Saccharomyco./mycetaceae - ps23
Saccharomycetaceae - ps24
Saccharomycetaceae_2 - ps25
Saccharomycetaceae_3 - ps26
Kaz./Nau./Saccharomyces - ps27
Saccharomyces - ps28
Arboricola_clade - ps29
Cerevisiae_clade - ps30
Saccharomyces_cerevisiae - ps31
Phylostratum

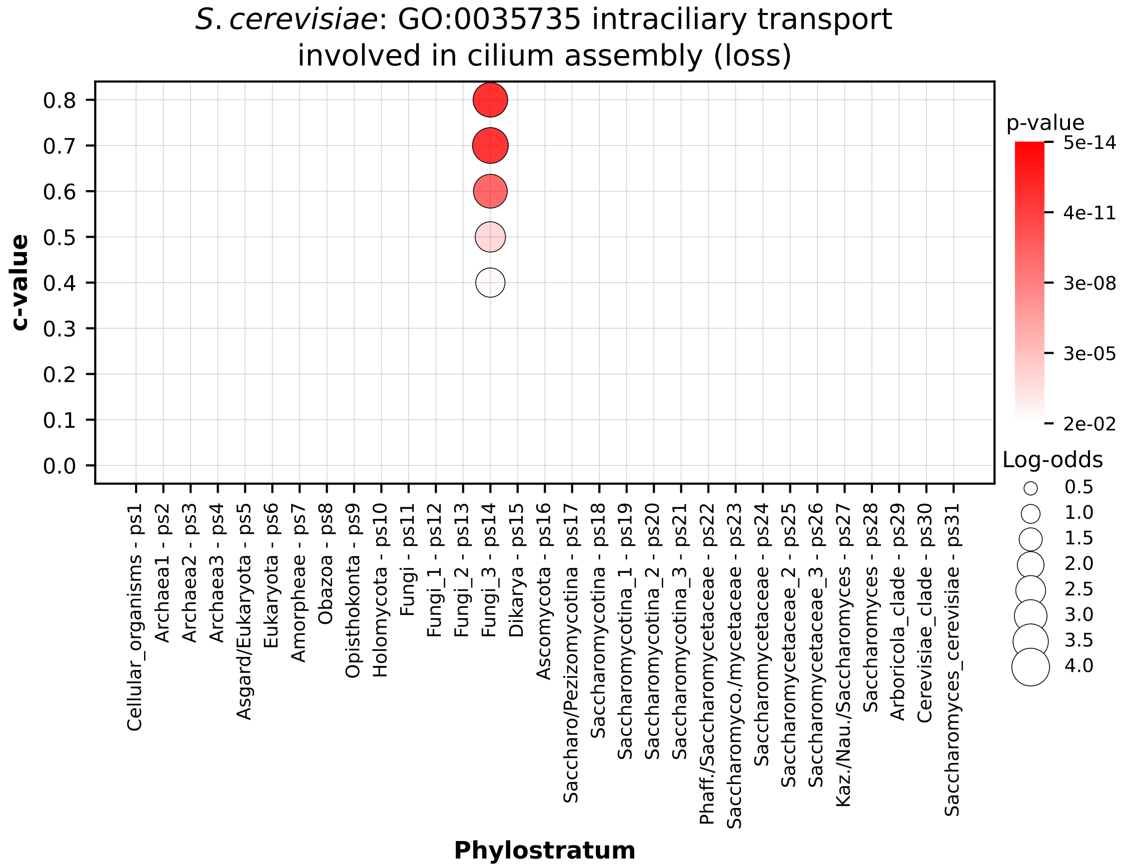

S. cerevisiae: GO:0035735 intraciliary transport involved in cilium assembly (loss)
c-value
0.8
0.7
0.6
0.5
0.4
0.3
0.2
0.1
0.0
p-value
5e-14
4e-11
3e-08
3e-05
2e-02
Log-odds
0.5
1.0
1.5
2.0
2.5
3.0
3.5
4.0
Cellular_organisms - ps1
Archaea1 - ps2
Archaea2 - ps3
Archaea3 - ps4
Asgard/Eukaryota - ps5
Eukaryota - ps6
Amorpheae - ps7
Obazoa - ps8
Opisthokonta - ps9
Holomycota - ps10
Fungi - ps11
Fungi_1 - ps12
Fungi_2 - ps13
Fungi_3 - ps14
Dikarya - ps15
Ascomycota - ps16
Saccharo/Pezizomycotina - ps17
Saccharomycotina - ps18
Saccharomycotina_1 - ps19
Saccharomycotina_2 - ps20
Saccharomycotina_3 - ps21
Phaff./Saccharomycetaceae - ps22
Saccharomyco./mycetaceae - ps23
Saccharomycetaceae - ps24
Saccharomycetaceae_2 - ps25
Saccharomycetaceae_3 - ps26
Kaz./Nau./Saccharomyces - ps27
Saccharomyces - ps28
Arboricola_clade - ps29
Cerevisiae_clade - ps30
Saccharomyces_cerevisiae - ps31
Phylostratum

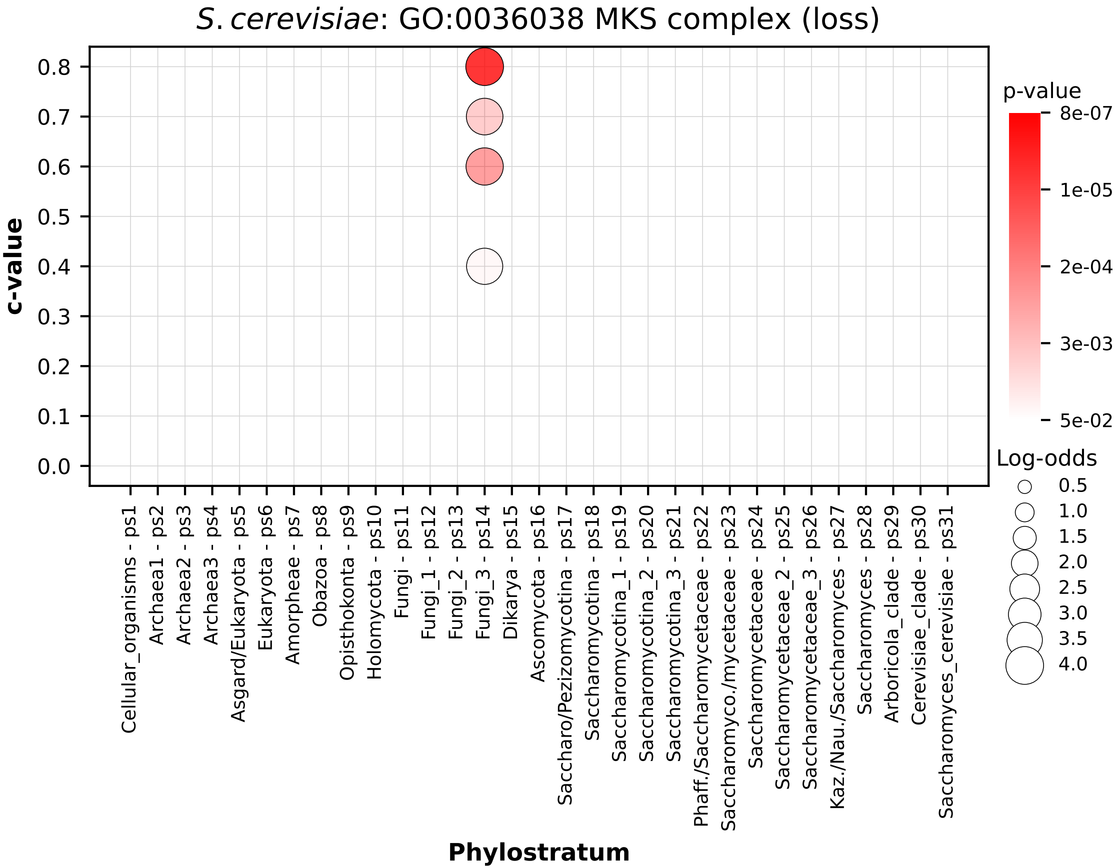

S. cerevisiae: GO:0036038 MKS complex (loss)
c-value
0.0
0.1
0.2
0.3
0.4
0.5
0.6
0.7
0.8
p-value
8e-07
1e-05
2e-04
3e-03
5e-02
Log-odds
0.5
1.0
1.5
2.0
2.5
3.0
3.5
4.0
Cellular_organisms - ps1
Archaea1 - ps2
Archaea2 - ps3
Archaea3 - ps4
Asgard/Eukaryota - ps5
Eukaryota - ps6
Amorpheae - ps7
Obazoa - ps8
Opisthokonta - ps9
Holomycota - ps10
Fungi - ps11
Fungi_1 - ps12
Fungi_2 - ps13
Fungi_3 - ps14
Dikarya - ps15
Ascomycota - ps16
Saccharo/Pezizomycotina - ps17
Saccharomycotina - ps18
Saccharomycotina_1 - ps19
Saccharomycotina_2 - ps20
Saccharomycotina_3 - ps21
Phaff./Saccharomycetaceae - ps22
Saccharomyco./mycetaceae - ps23
Saccharomycetaceae - ps24
Saccharomycetaceae_2 - ps25
Saccharomycetaceae_3 - ps26
Kaz./Nau./Saccharomyces - ps27
Saccharomyces - ps28
Arboricola_clade - ps29
Cerevisiae_clade - ps30
Saccharomyces_cerevisiae - ps31
Phylostratum

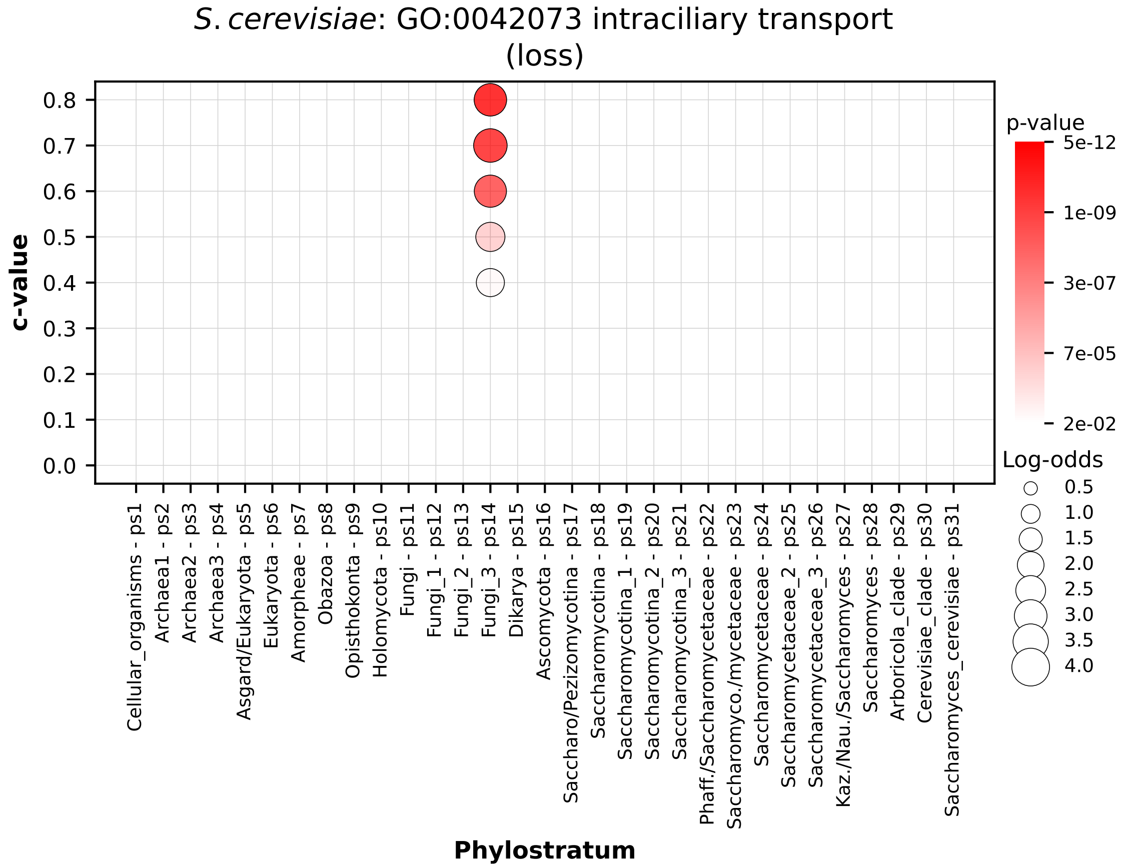

S. cerevisiae: GO:0042073 intraciliary transport
(loss)
c-value
0.0
0.1
0.2
0.3
0.4
0.5
0.6
0.7
0.8
p-value
5e-12
1e-09
3e-07
7e-05
2e-02
Log-odds
0.5
1.0
1.5
2.0
2.5
3.0
3.5
4.0
Cellular_organisms - ps1
Archaea1 - ps2
Archaea2 - ps3
Archaea3 - ps4
Asgard/Eukaryota - ps5
Eukaryota - ps6
Amorpheae - ps7
Obazoa - ps8
Opisthokonta - ps9
Holomycota - ps10
Fungi - ps11
Fungi_1 - ps12
Fungi_2 - ps13
Fungi_3 - ps14
Dikarya - ps15
Ascomycota - ps16
Saccharo/Pezizomycotina - ps17
Saccharomycotina - ps18
Saccharomycotina_1 - ps19
Saccharomycotina_2 - ps20
Saccharomycotina_3 - ps21
Phaff./Saccharomycetaceae - ps22
Saccharomyco./mycetaceae - ps23
Saccharomycetaceae - ps24
Saccharomycetaceae_2 - ps25
Saccharomycetaceae_3 - ps26
Kaz./Nau./Saccharomyces - ps27
Saccharomyces - ps28
Arboricola_clade - ps29
Cerevisiae_clade - ps30
Saccharomyces_cerevisiae - ps31
Phylostratum

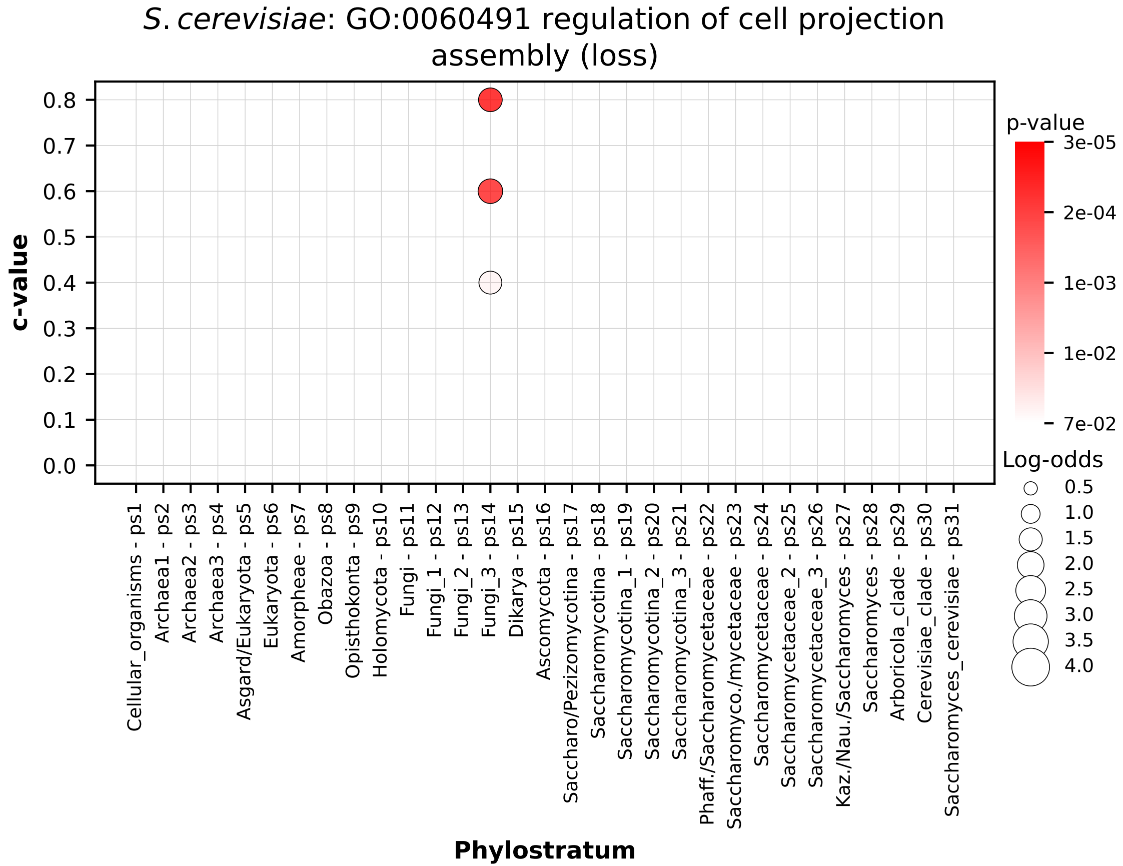

S. cerevisiae: GO:0060491 regulation of cell projection assembly (loss)
c-value
0.0
0.1
0.2
0.3
0.4
0.5
0.6
0.7
0.8
Phylostratum
Cellular_organisms - ps1
Archaea1 - ps2
Archaea2 - ps3
Archaea3 - ps4
Asgard/Eukaryota - ps5
Eukaryota - ps6
Amorpheae - ps7
Obazoa - ps8
Opisthokonta - ps9
Holomycota - ps10
Fungi - ps11
Fungi_1 - ps12
Fungi_2 - ps13
Fungi_3 - ps14
Dikarya - ps15
Ascomycota - ps16
Saccharo/Pezizomycotina - ps17
Saccharomycotina - ps18
Saccharomycotina_1 - ps19
Saccharomycotina_2 - ps20
Saccharomycotina_3 - ps21
Phaff./Saccharomycetaceae - ps22
Saccharomyco./mycetaceae - ps23
Saccharomycetaceae - ps24
Saccharomycetaceae_2 - ps25
Saccharomycetaceae_3 - ps26
Kaz./Nau./Saccharomyces - ps27
Saccharomyces - ps28
Arboricola_clade - ps29
Cerevisiae_clade - ps30
Saccharomyces_cerevisiae - ps31
p-value
3e-05
2e-04
1e-03
1e-02
7e-02
Log-odds
0.5
1.0
1.5
2.0
2.5
3.0
3.5
4.0

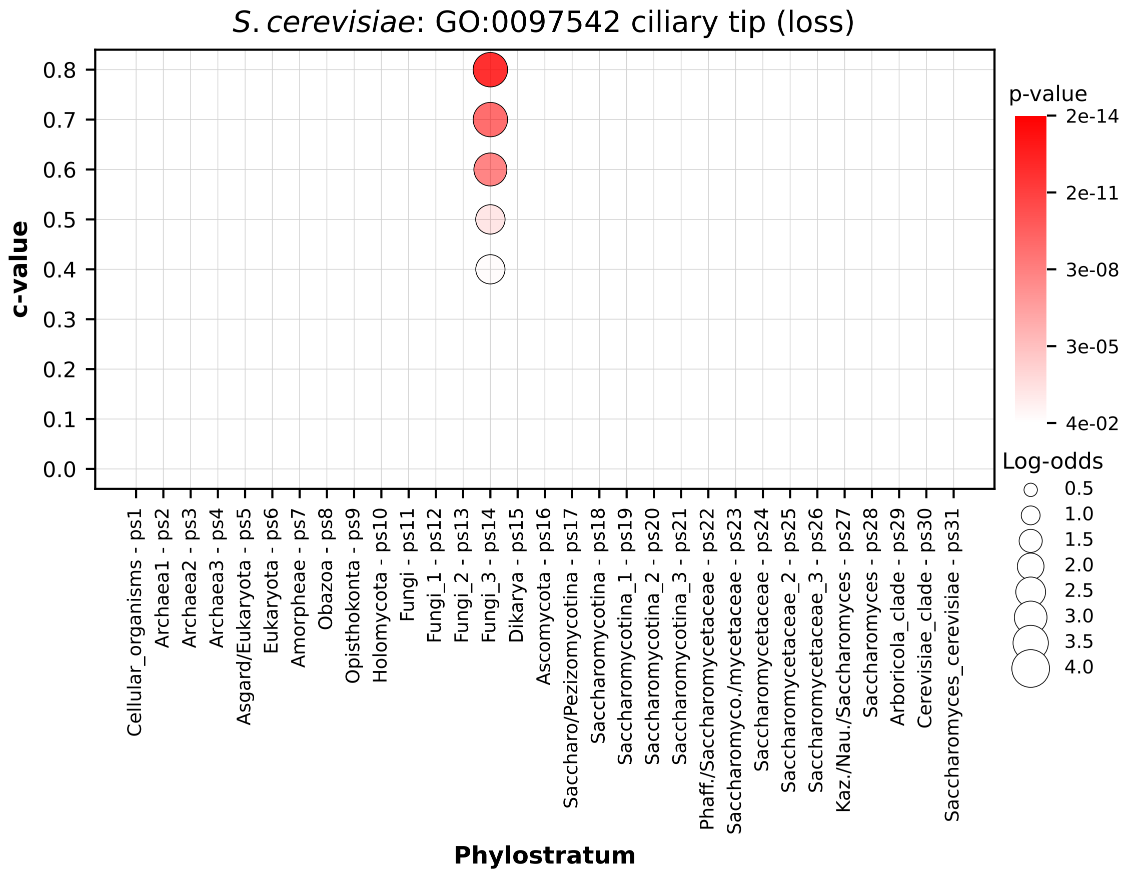

S. cerevisiae: GO:0097542 ciliary tip (loss)
c-value
0.0
0.1
0.2
0.3
0.4
0.5
0.6
0.7
0.8
p-value
2e-14
2e-11
3e-08
3e-05
4e-02
Log-odds
0.5
1.0
1.5
2.0
2.5
3.0
3.5
4.0
Cellular_organisms - ps1
Archaea1 - ps2
Archaea2 - ps3
Archaea3 - ps4
Asgard/Eukaryota - ps5
Eukaryota - ps6
Amorpheae - ps7
Obazoa - ps8
Opisthokonta - ps9
Holomycota - ps10
Fungi - ps11
Fungi_1 - ps12
Fungi_2 - ps13
Fungi_3 - ps14
Dikarya - ps15
Ascomycota - ps16
Saccharo/Pezizomycotina - ps17
Saccharomycotina - ps18
Saccharomycotina_1 - ps19
Saccharomycotina_2 - ps20
Saccharomycotina_3 - ps21
Phaff./Saccharomycetaceae - ps22
Saccharomyco./mycetaceae - ps23
Saccharomycetaceae - ps24
Saccharomycetaceae_2 - ps25
Saccharomycetaceae_3 - ps26
Kaz./Nau./Saccharomyces - ps27
Saccharomyces - ps28
Arboricola_clade - ps29
Cerevisiae_clade - ps30
Saccharomyces_cerevisiae - ps31
Phylostratum

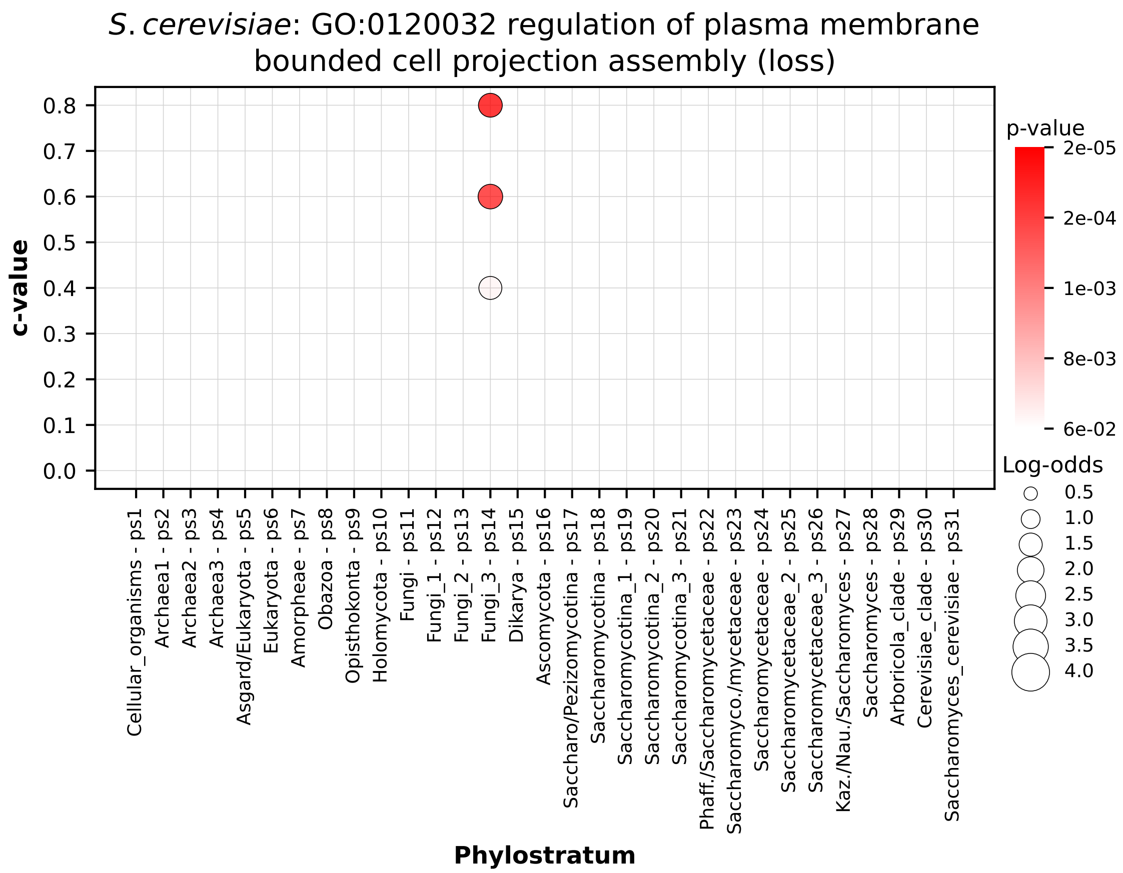
S. cerevisiae: GO:0120032 regulation of plasma membrane bounded cell projection assembly (loss)
c-value
0.8
0.7
0.6
0.5
0.4
0.3
0.2
0.1
0.0
p-value
2e-05
2e-04
1e-03
8e-03
6e-02
Log-odds
0.5
1.0
1.5
2.0
2.5
3.0
3.5
4.0
Cellular_organisms - ps1
Archaea1 - ps2
Archaea2 - ps3
Archaea3 - ps4
Asgard/Eukaryota - ps5
Eukaryota - ps6
Amorpheae - ps7
Obazoa - ps8
Opisthokonta - ps9
Holomycota - ps10
Fungi - ps11
Fungi_1 - ps12
Fungi_2 - ps13
Fungi_3 - ps14
Dikarya - ps15
Ascomycota - ps16
Saccharo/Pezizomycotina - ps17
Saccharomycotina - ps18
Saccharomycotina_1 - ps19
Saccharomycotina_2 - ps20
Saccharomycotina_3 - ps21
Phaff./Saccharomycetaceae - ps22
Saccharomyco./mycetaceae - ps23
Saccharomycetaceae - ps24
Saccharomycetaceae_2 - ps25
Saccharomycetaceae_3 - ps26
Kaz./Nau./Saccharomyces - ps27
Saccharomyces - ps28
Arboricola_clade - ps29
Cerevisiae_clade - ps30
Saccharomyces_cerevisiae - ps31
Phylostratum

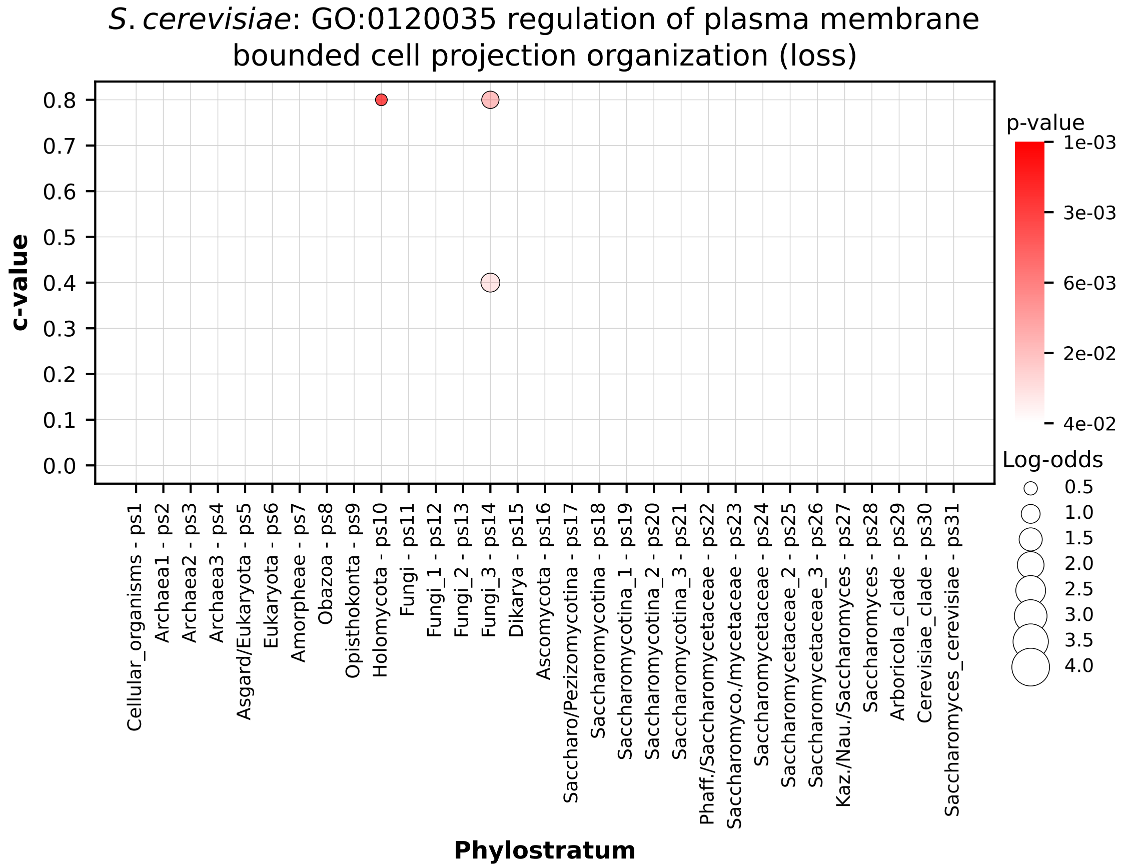

S. cerevisiae: GO:0120035 regulation of plasma membrane bounded cell projection organization (loss)
c-value
0.8
0.7
0.6
0.5
0.4
0.3
0.2
0.1
0.0
p-value
1e-03
3e-03
6e-03
2e-02
4e-02
Log-odds
0.5
1.0
1.5
2.0
2.5
3.0
3.5
4.0
Cellular_organisms - ps1
Archaea1 - ps2
Archaea2 - ps3
Archaea3 - ps4
Asgard/Eukaryota - ps5
Eukaryota - ps6
Amorpheae - ps7
Obazoa - ps8
Opisthokonta - ps9
Holomycota - ps10
Fungi - ps11
Fungi_1 - ps12
Fungi_2 - ps13
Fungi_3 - ps14
Dikarya - ps15
Ascomycota - ps16
Saccharo/Pezizomycotina - ps17
Saccharomycotina - ps18
Saccharomycotina_1 - ps19
Saccharomycotina_2 - ps20
Saccharomycotina_3 - ps21
Phaff./Saccharomycetaceae - ps22
Saccharomyco./mycetaceae - ps23
Saccharomycetaceae - ps24
Saccharomycetaceae_2 - ps25
Saccharomycetaceae_3 - ps26
Kaz./Nau./Saccharomyces - ps27
Saccharomyces - ps28
Arboricola_clade - ps29
Cerevisiae_clade - ps30
Saccharomyces_cerevisiae - ps31
Phylostratum

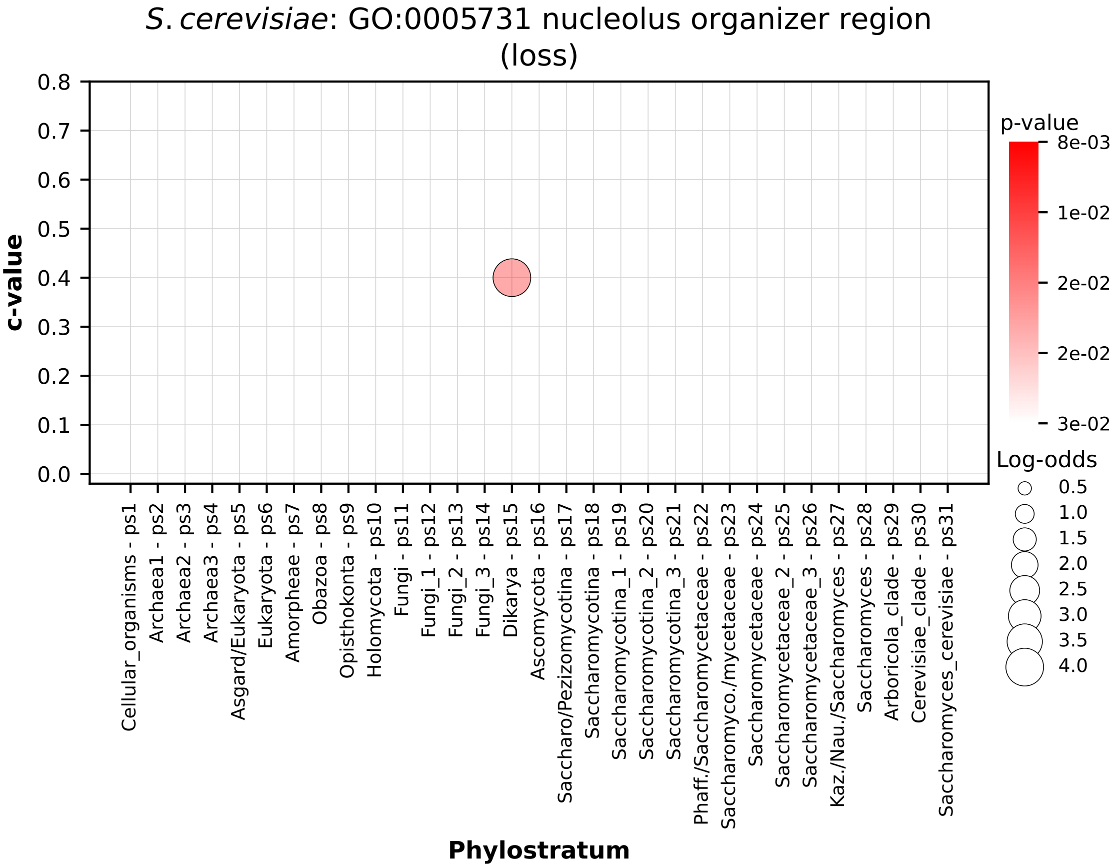

S. cerevisiae: GO:0005731 nucleolus organizer region
(loss)
c-value
0.8
0.7
0.6
0.5
0.4
0.3
0.2
0.1
0.0
p-value
8e-03
1e-02
2e-02
2e-02
3e-02
Log-odds
0.5
1.0
1.5
2.0
2.5
3.0
3.5
4.0
Cellular_organisms - ps1
Archaea1 - ps2
Archaea2 - ps3
Archaea3 - ps4
Asgard/Eukaryota - ps5
Eukaryota - ps6
Amorpheae - ps7
Obazoa - ps8
Opisthokonta - ps9
Holomycota - ps10
Fungi - ps11
Fungi_1 - ps12
Fungi_2 - ps13
Fungi_3 - ps14
Dikarya - ps15
Ascomycota - ps16
Saccharo/Pezizomycotina - ps17
Saccharomycotina - ps18
Saccharomycotina_1 - ps19
Saccharomycotina_2 - ps20
Saccharomycotina_3 - ps21
Phaff./Saccharomycetaceae - ps22
Saccharomyco./mycetaceae - ps23
Saccharomycetaceae - ps24
Saccharomycetaceae_2 - ps25
Saccharomycetaceae_3 - ps26
Kaz./Nau./Saccharomyces - ps27
Saccharomyces - ps28
Arboricola_clade - ps29
Cerevisiae_clade - ps30
Saccharomyces_cerevisiae - ps31
Phylostratum

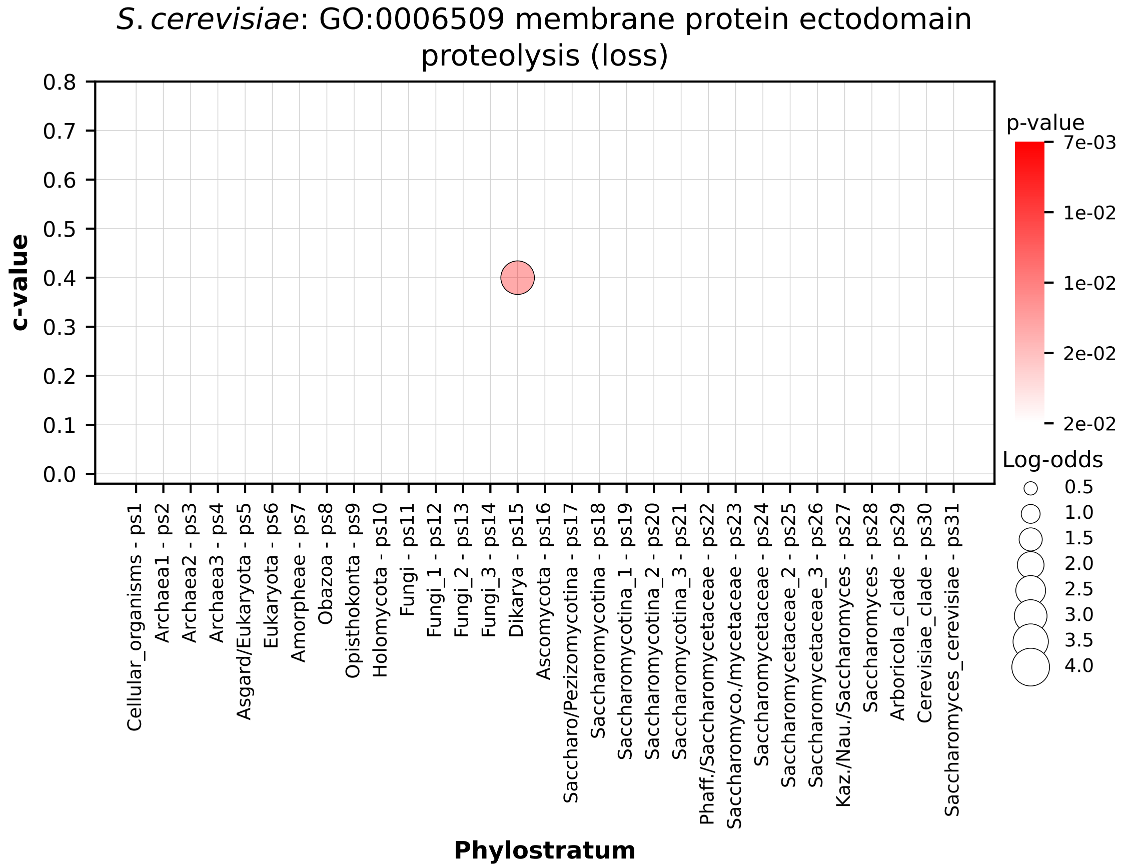
S. cerevisiae: GO:0006509 membrane protein ectodomain proteolysis (loss)
c-value
0.8
0.7
0.6
0.5
0.4
0.3
0.2
0.1
0.0
p-value
7e-03
1e-02
1e-02
2e-02
2e-02
Log-odds
0.5
1.0
1.5
2.0
2.5
3.0
3.5
4.0
Cellular_organisms - ps1
Archaea1 - ps2
Archaea2 - ps3
Archaea3 - ps4
Asgard/Eukaryota - ps5
Eukaryota - ps6
Amorpheae - ps7
Obazoa - ps8
Opisthokonta - ps9
Holomycota - ps10
Fungi - ps11
Fungi_1 - ps12
Fungi_2 - ps13
Fungi_3 - ps14
Dikarya - ps15
Ascomycota - ps16
Saccharo/Pezizomycotina - ps17
Saccharomycotina - ps18
Saccharomycotina_1 - ps19
Saccharomycotina_2 - ps20
Saccharomycotina_3 - ps21
Phaff./Saccharomycetaceae - ps22
Saccharomyco./mycetaceae - ps23
Saccharomycetaceae - ps24
Saccharomycetaceae_2 - ps25
Saccharomycetaceae_3 - ps26
Kaz./Nau./Saccharomyces - ps27
Saccharomyces - ps28
Arboricola_clade - ps29
Cerevisiae_clade - ps30
Saccharomyces_cerevisiae - ps31
Phylostratum

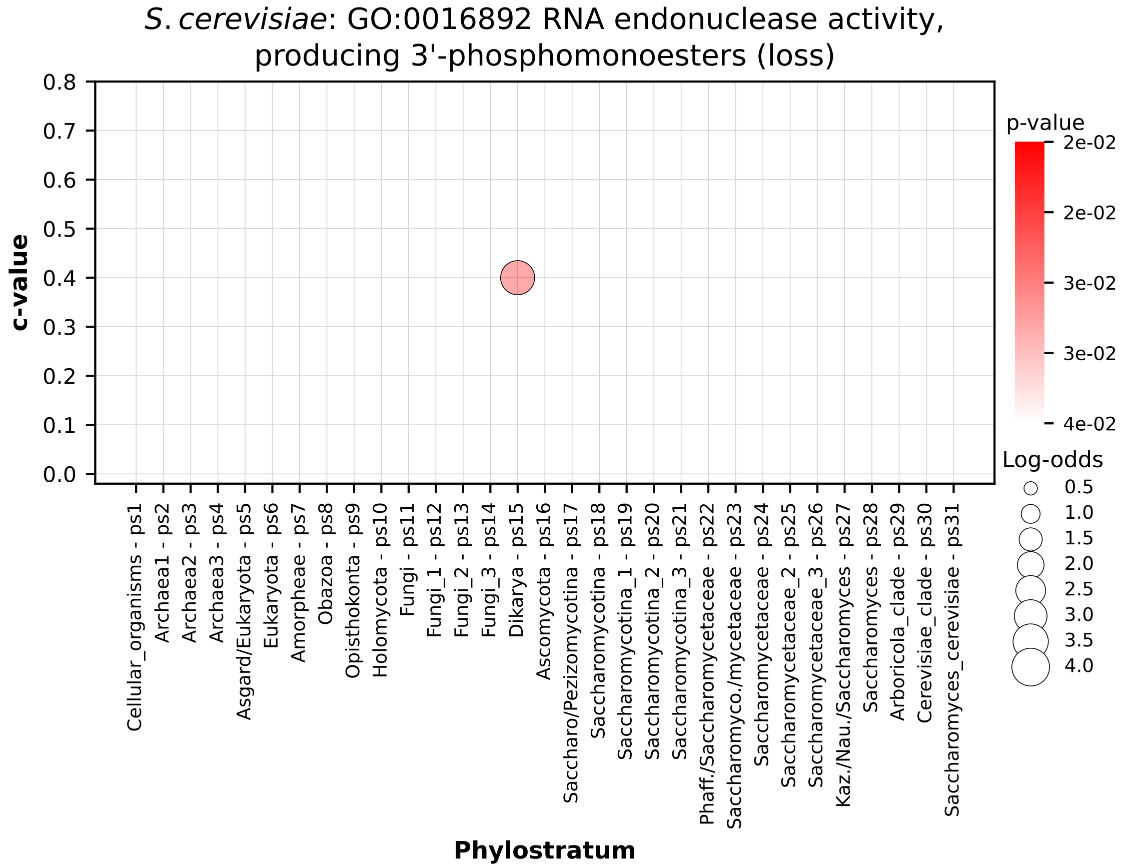

S. cerevisiae: GO:0016892 RNA endonuclease activity, producing 3'-phosphomonoesters (loss)
c-value
0.8
0.7
0.6
0.5
0.4
0.3
0.2
0.1
0.0
Phylostratum
Cellular_organisms - ps1
Archaea1 - ps2
Archaea2 - ps3
Archaea3 - ps4
Asgard/Eukaryota - ps5
Eukaryota - ps6
Amorpheae - ps7
Obazoa - ps8
Opisthokonta - ps9
Holomycota - ps10
Fungi - ps11
Fungi_1 - ps12
Fungi_2 - ps13
Fungi_3 - ps14
Dikarya - ps15
Ascomycota - ps16
Saccharo/Pezizomycotina - ps17
Saccharomycotina - ps18
Saccharomycotina_1 - ps19
Saccharomycotina_2 - ps20
Saccharomycotina_3 - ps21
Phaff./Saccharomycetaceae - ps22
Saccharomyco./mycetaceae - ps23
Saccharomycetaceae - ps24
Saccharomycetaceae_2 - ps25
Saccharomycetaceae_3 - ps26
Kaz./Nau./Saccharomyces - ps27
Saccharomyces - ps28
Arboricola_clade - ps29
Cerevisiae_clade - ps30
Saccharomyces_cerevisiae - ps31
p-value
2e-02
2e-02
3e-02
3e-02
4e-02
Log-odds
0.5
1.0
1.5
2.0
2.5
3.0
3.5
4.0

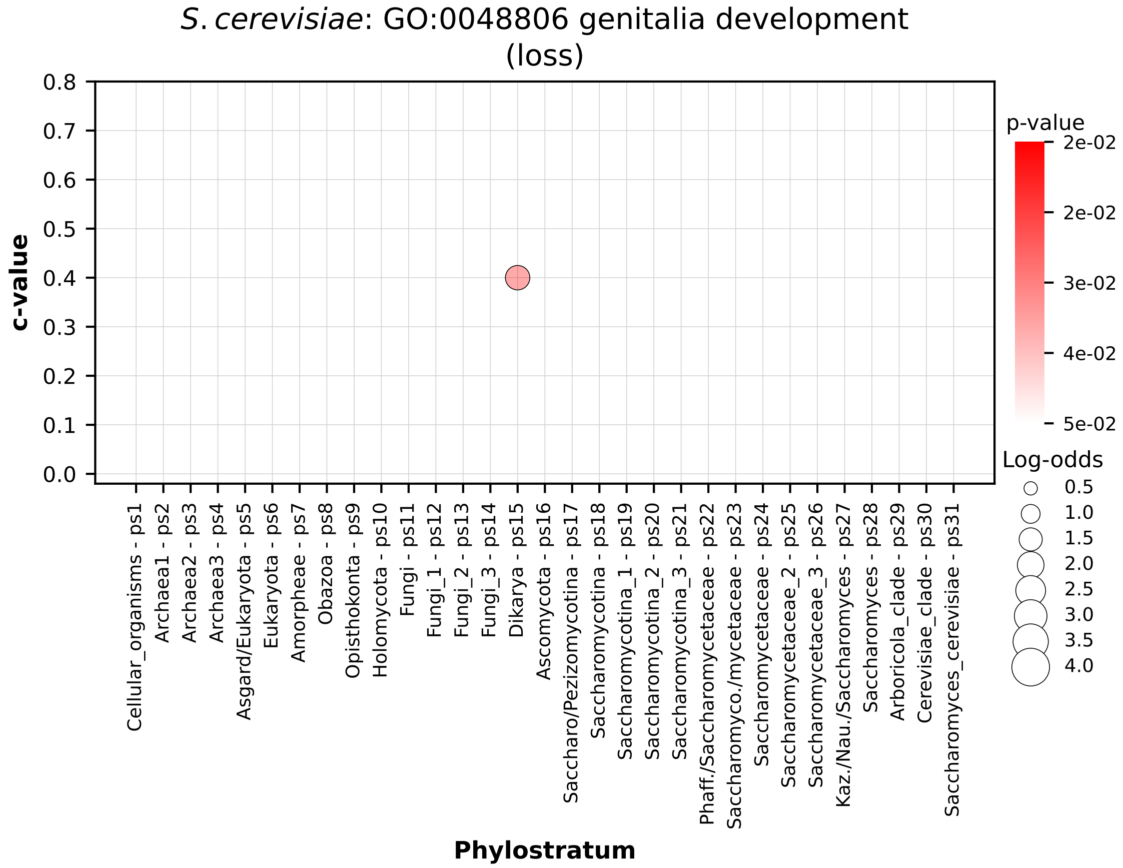

S. cerevisiae: GO:0048806 genitalia development
(loss)
c-value
0.8
0.7
0.6
0.5
0.4
0.3
0.2
0.1
0.0
p-value
2e-02
2e-02
3e-02
4e-02
5e-02
Log-odds
0.5
1.0
1.5
2.0
2.5
3.0
3.5
4.0
Cellular_organisms - ps1
Archaea1 - ps2
Archaea2 - ps3
Archaea3 - ps4
Asgard/Eukaryota - ps5
Eukaryota - ps6
Amorpheae - ps7
Obazoa - ps8
Opisthokonta - ps9
Holomycota - ps10
Fungi - ps11
Fungi_1 - ps12
Fungi_2 - ps13
Fungi_3 - ps14
Dikarya - ps15
Ascomycota - ps16
Saccharo/Pezizomycotina - ps17
Saccharomycotina - ps18
Saccharomycotina_1 - ps19
Saccharomycotina_2 - ps20
Saccharomycotina_3 - ps21
Phaff./Saccharomycetaceae - ps22
Saccharomyco./mycetaceae - ps23
Saccharomycetaceae - ps24
Saccharomycetaceae_2 - ps25
Saccharomycetaceae_3 - ps26
Kaz./Nau./Saccharomyces - ps27
Saccharomyces - ps28
Arboricola_clade - ps29
Cerevisiae_clade - ps30
Saccharomyces_cerevisiae - ps31
Phylostratum

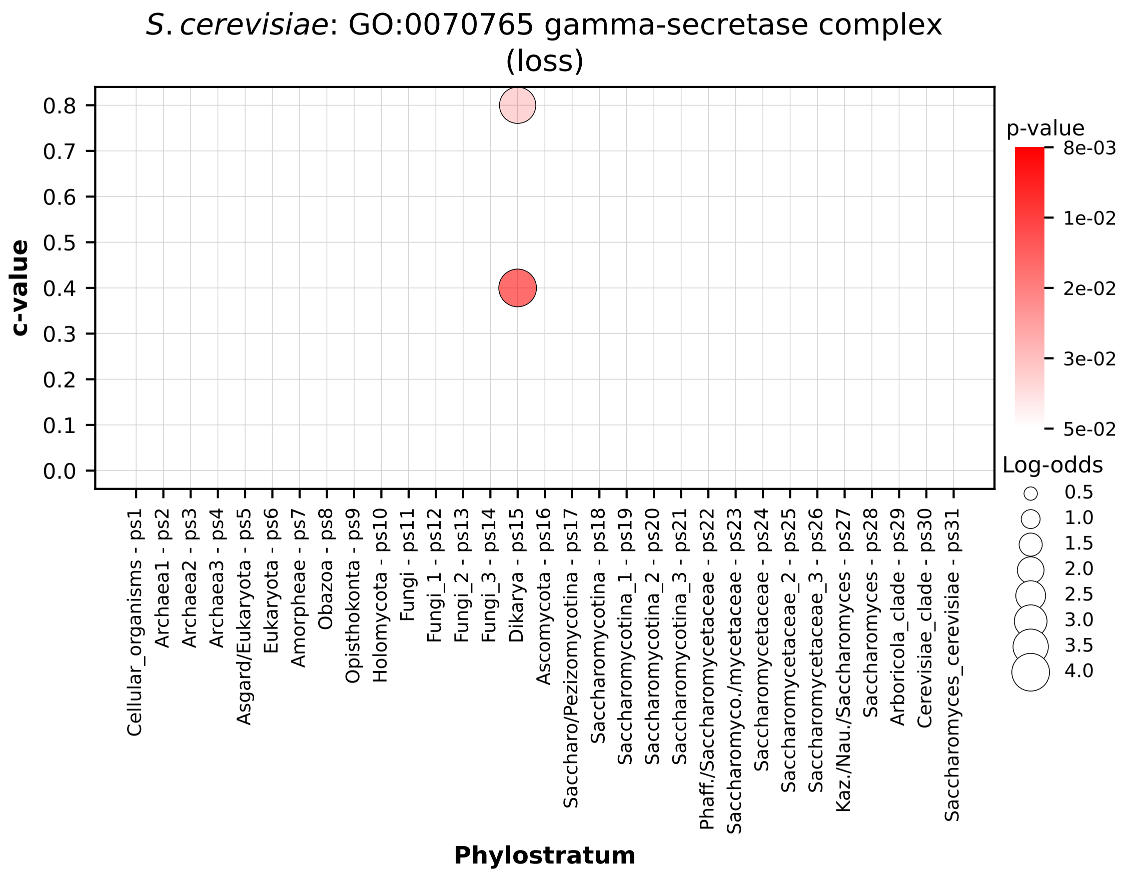
S. cerevisiae: GO:0070765 gamma-secretase complex
(loss)
c-value
0.0
0.1
0.2
0.3
0.4
0.5
0.6
0.7
0.8
p-value
8e-03
1e-02
2e-02
3e-02
5e-02
Log-odds
0.5
1.0
1.5
2.0
2.5
3.0
3.5
4.0
Cellular_organisms - ps1
Archaea1 - ps2
Archaea2 - ps3
Archaea3 - ps4
Asgard/Eukaryota - ps5
Eukaryota - ps6
Amorpheae - ps7
Obazoa - ps8
Opisthokonta - ps9
Holomycota - ps10
Fungi - ps11
Fungi_1 - ps12
Fungi_2 - ps13
Fungi_3 - ps14
Dikarya - ps15
Ascomycota - ps16
Saccharo/Pezizomycotina - ps17
Saccharomycotina - ps18
Saccharomycotina_1 - ps19
Saccharomycotina_2 - ps20
Saccharomycotina_3 - ps21
Phaff./Saccharomycetaceae - ps22
Saccharomyco./mycetaceae - ps23
Saccharomycetaceae - ps24
Saccharomycetaceae_2 - ps25
Saccharomycetaceae_3 - ps26
Kaz./Nau./Saccharomyces - ps27
Saccharomyces - ps28
Arboricola_clade - ps29
Cerevisiae_clade - ps30
Saccharomyces_cerevisiae - ps31
Phylostratum

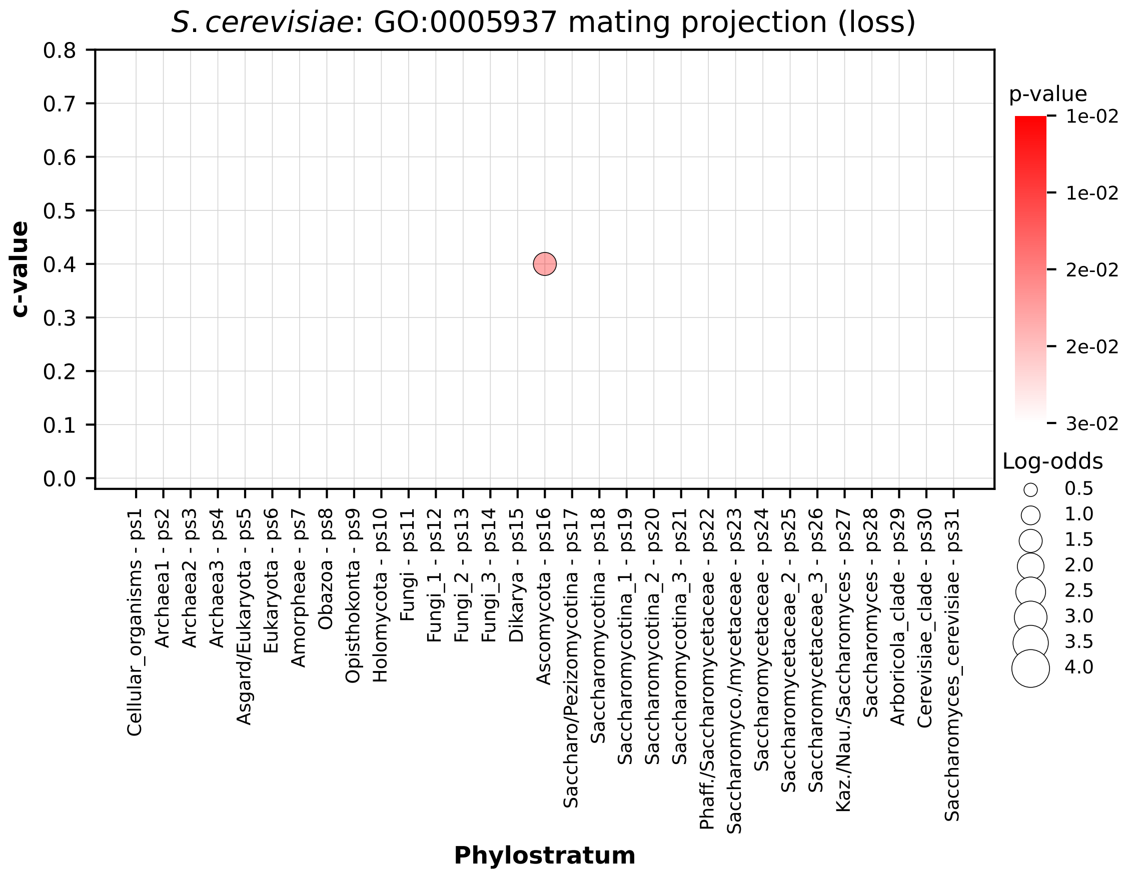

S. cerevisiae: GO:0005937 mating projection (loss)
c-value
0.8
0.7
0.6
0.5
0.4
0.3
0.2
0.1
0.0
Phylostratum
Cellular_organisms - ps1
Archaea1 - ps2
Archaea2 - ps3
Archaea3 - ps4
Asgard/Eukaryota - ps5
Eukaryota - ps6
Amorpheae - ps7
Obazoa - ps8
Opisthokonta - ps9
Holomycota - ps10
Fungi - ps11
Fungi_1 - ps12
Fungi_2 - ps13
Fungi_3 - ps14
Dikarya - ps15
Ascomycota - ps16
Saccharo/Pezizomycotina - ps17
Saccharomycotina - ps18
Saccharomycotina_1 - ps19
Saccharomycotina_2 - ps20
Saccharomycotina_3 - ps21
Phaff./Saccharomycetaceae - ps22
Saccharomyco./mycetaceae - ps23
Saccharomycetaceae - ps24
Saccharomycetaceae_2 - ps25
Saccharomycetaceae_3 - ps26
Kaz./Nau./Saccharomyces - ps27
Saccharomyces - ps28
Arboricola_clade - ps29
Cerevisiae_clade - ps30
Saccharomyces_cerevisiae - ps31
p-value
1e-02
1e-02
2e-02
2e-02
3e-02
Log-odds
0.5
1.0
1.5
2.0
2.5
3.0
3.5
4.0

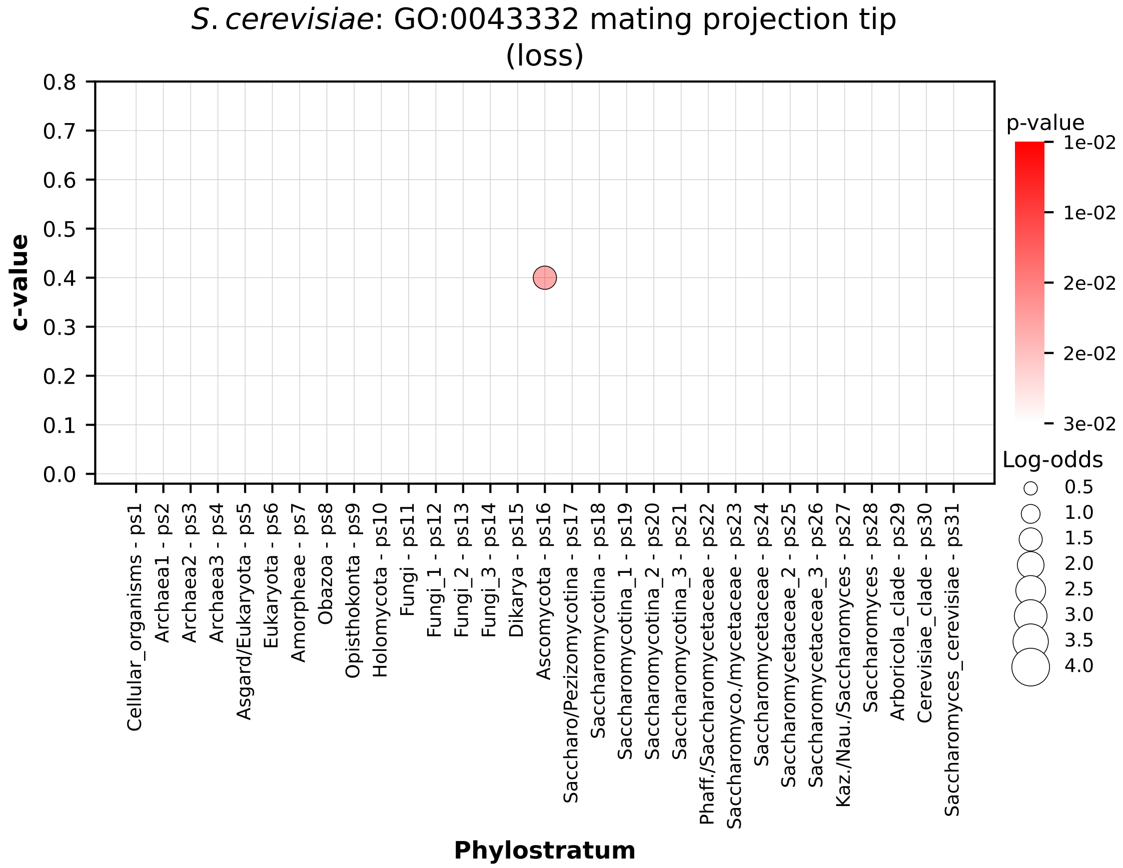

S. cerevisiae: GO:0043332 mating projection tip
(loss)
c-value
0.8
0.7
0.6
0.5
0.4
0.3
0.2
0.1
0.0
p-value
1e-02
1e-02
2e-02
2e-02
3e-02
Log-odds
0.5
1.0
1.5
2.0
2.5
3.0
3.5
4.0
Cellular_organisms - ps1
Archaea1 - ps2
Archaea2 - ps3
Archaea3 - ps4
Asgard/Eukaryota - ps5
Eukaryota - ps6
Amorpheae - ps7
Obazoa - ps8
Opisthokonta - ps9
Holomycota - ps10
Fungi - ps11
Fungi_1 - ps12
Fungi_2 - ps13
Fungi_3 - ps14
Dikarya - ps15
Ascomycota - ps16
Saccharo/Pezizomycotina - ps17
Saccharomycotina - ps18
Saccharomycotina_1 - ps19
Saccharomycotina_2 - ps20
Saccharomycotina_3 - ps21
Phaff./Saccharomycetaceae - ps22
Saccharomyco./mycetaceae - ps23
Saccharomycetaceae - ps24
Saccharomycetaceae_2 - ps25
Saccharomycetaceae_3 - ps26
Kaz./Nau./Saccharomyces - ps27
Saccharomyces - ps28
Arboricola_clade - ps29
Cerevisiae_clade - ps30
Saccharomyces_cerevisiae - ps31
Phylostratum

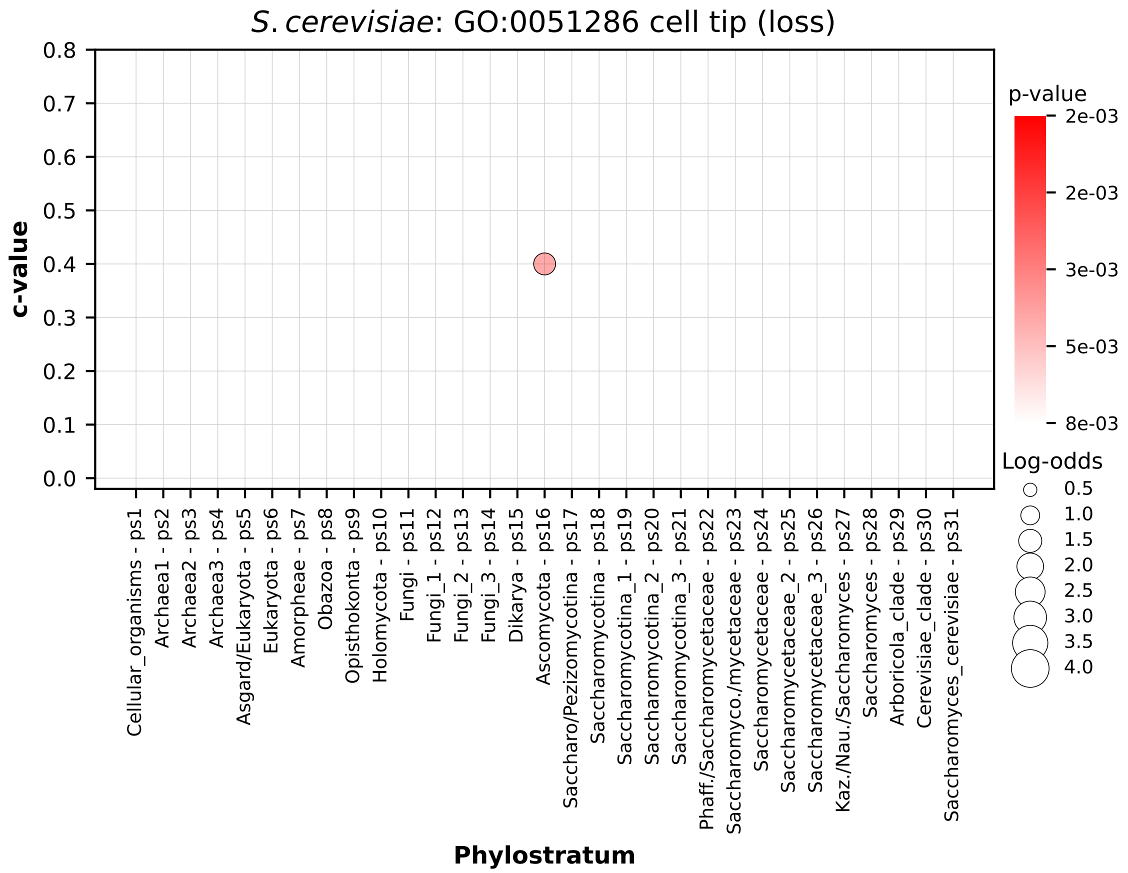
S. cerevisiae: GO:0051286 cell tip (loss)
c-value
0.8
0.7
0.6
0.5
0.4
0.3
0.2
0.1
0.0
p-value
2e-03
2e-03
3e-03
5e-03
8e-03
Log-odds
0.5
1.0
1.5
2.0
2.5
3.0
3.5
4.0
Cellular_organisms - ps1
Archaea1 - ps2
Archaea2 - ps3
Archaea3 - ps4
Asgard/Eukaryota - ps5
Eukaryota - ps6
Amorpheae - ps7
Obazoa - ps8
Opisthokonta - ps9
Holomycota - ps10
Fungi - ps11
Fungi_1 - ps12
Fungi_2 - ps13
Fungi_3 - ps14
Dikarya - ps15
Ascomycota - ps16
Saccharo/Pezizomycotina - ps17
Saccharomycotina - ps18
Saccharomycotina_1 - ps19
Saccharomycotina_2 - ps20
Saccharomycotina_3 - ps21
Phaff./Saccharomycetaceae - ps22
Saccharomyco./mycetaceae - ps23
Saccharomycetaceae - ps24
Saccharomycetaceae_2 - ps25
Saccharomycetaceae_3 - ps26
Kaz./Nau./Saccharomyces - ps27
Saccharomyces - ps28
Arboricola_clade - ps29
Cerevisiae_clade - ps30
Saccharomyces_cerevisiae - ps31
Phylostratum

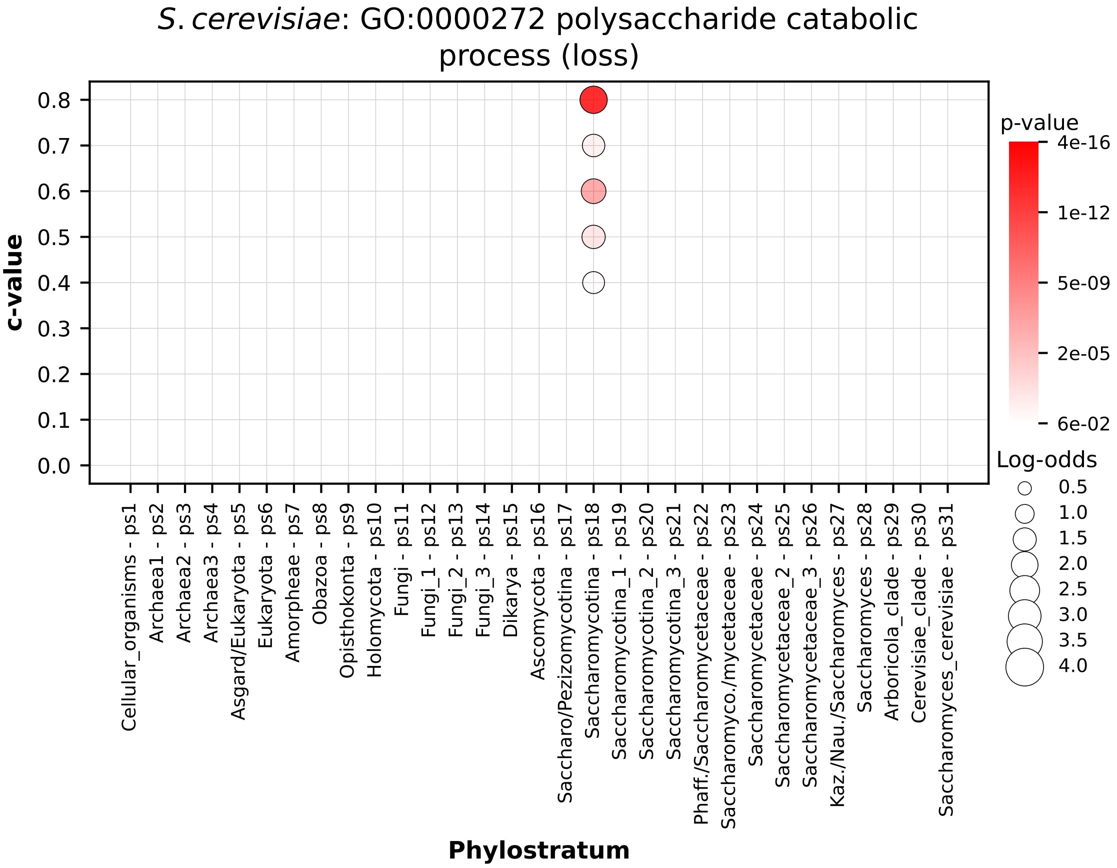
S. cerevisiae: GO:0000272 polysaccharide catabolic process (loss)
c-value
0.0
0.1
0.2
0.3
0.4
0.5
0.6
0.7
0.8
p-value
4e-16
1e-12
5e-09
2e-05
6e-02
Log-odds
0.5
1.0
1.5
2.0
2.5
3.0
3.5
4.0
Cellular_organisms - ps1
Archaea1 - ps2
Archaea2 - ps3
Archaea3 - ps4
Asgard/Eukaryota - ps5
Eukaryota - ps6
Amorpheae - ps7
Obazoa - ps8
Opisthokonta - ps9
Holomycota - ps10
Fungi - ps11
Fungi_1 - ps12
Fungi_2 - ps13
Fungi_3 - ps14
Dikarya - ps15
Ascomycota - ps16
Saccharo/Pezizomycotina - ps17
Saccharomycotina - ps18
Saccharomycotina_1 - ps19
Saccharomycotina_2 - ps20
Saccharomycotina_3 - ps21
Phaff./Saccharomycetaceae - ps22
Saccharomyco./mycetaceae - ps23
Saccharomycetaceae - ps24
Saccharomycetaceae_2 - ps25
Saccharomycetaceae_3 - ps26
Kaz./Nau./Saccharomyces - ps27
Saccharomyces - ps28
Arboricola_clade - ps29
Cerevisiae_clade - ps30
Saccharomyces_cerevisiae - ps31
Phylostratum

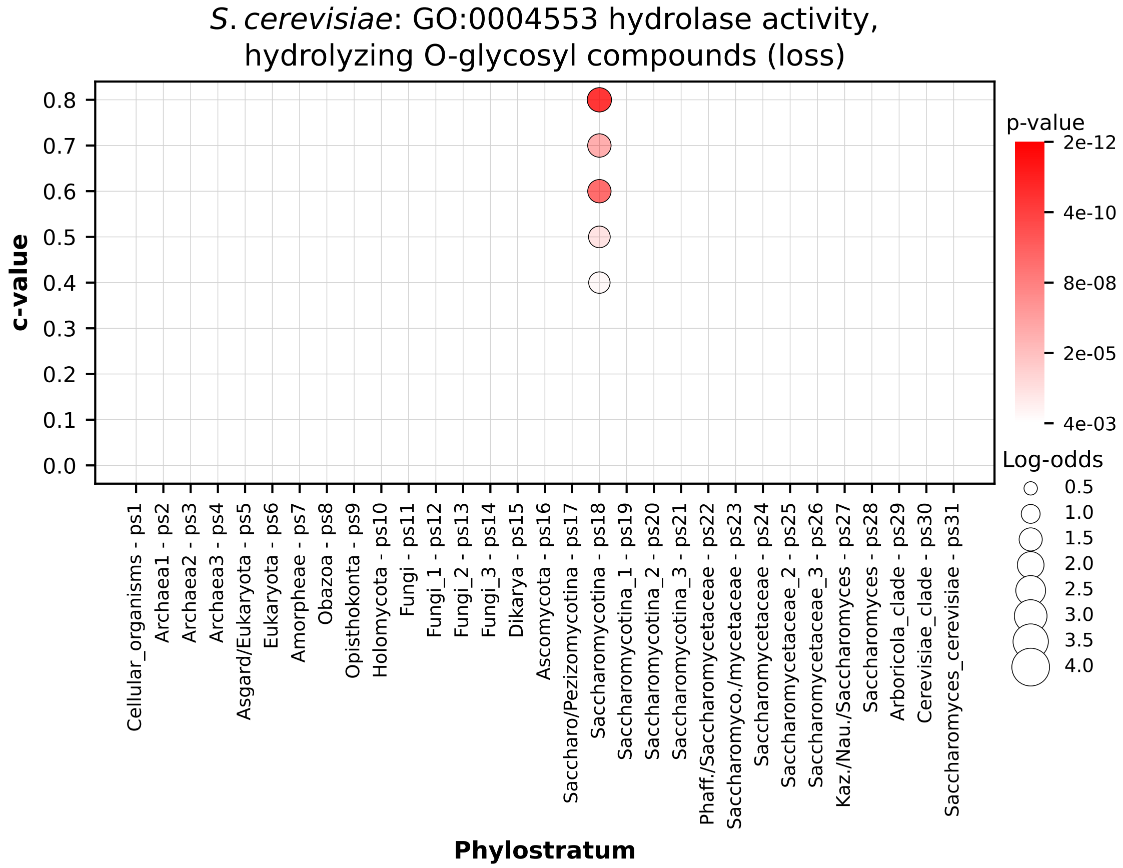

S. cerevisiae: GO:0004553 hydrolase activity, hydrolyzing O-glycosyl compounds (loss)
c-value
0.8
0.7
0.6
0.5
0.4
0.3
0.2
0.1
0.0
p-value
2e-12
4e-10
8e-08
2e-05
4e-03
Log-odds
0.5
1.0
1.5
2.0
2.5
3.0
3.5
4.0
Cellular_organisms - ps1
Archaea1 - ps2
Archaea2 - ps3
Archaea3 - ps4
Asgard/Eukaryota - ps5
Eukaryota - ps6
Amorpheae - ps7
Obazoa - ps8
Opisthokonta - ps9
Holomycota - ps10
Fungi - ps11
Fungi_1 - ps12
Fungi_2 - ps13
Fungi_3 - ps14
Dikarya - ps15
Ascomycota - ps16
Saccharo/Pezizomycotina - ps17
Saccharomycotina - ps18
Saccharomycotina_1 - ps19
Saccharomycotina_2 - ps20
Saccharomycotina_3 - ps21
Phaff./Saccharomycetaceae - ps22
Saccharomyco./mycetaceae - ps23
Saccharomycetaceae - ps24
Saccharomycetaceae_2 - ps25
Saccharomycetaceae_3 - ps26
Kaz./Nau./Saccharomyces - ps27
Saccharomyces - ps28
Arboricola_clade - ps29
Cerevisiae_clade - ps30
Saccharomyces_cerevisiae - ps31
Phylostratum

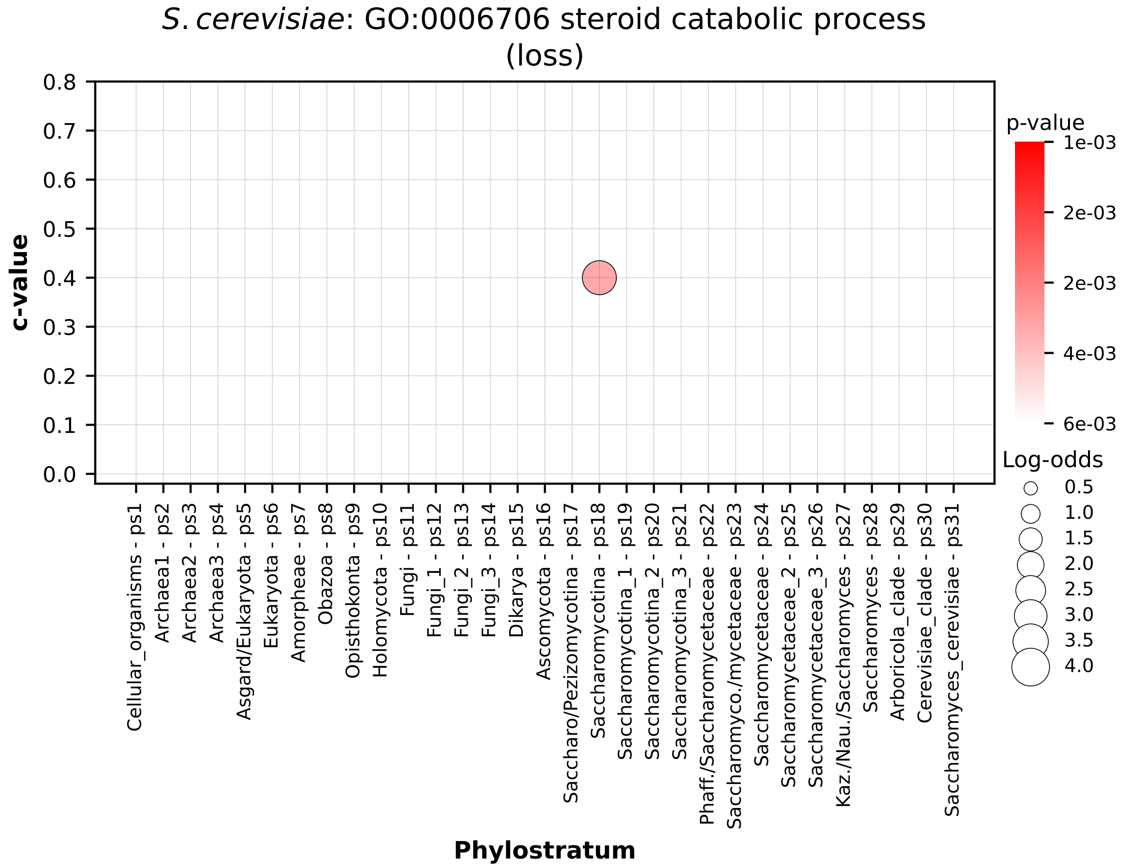

S. cerevisiae: GO:0006706 steroid catabolic process
(loss)
c-value
0.8
0.7
0.6
0.5
0.4
0.3
0.2
0.1
0.0
p-value
1e-03
2e-03
2e-03
4e-03
6e-03
Log-odds
0.5
1.0
1.5
2.0
2.5
3.0
3.5
4.0
Cellular_organisms - ps1
Archaea1 - ps2
Archaea2 - ps3
Archaea3 - ps4
Asgard/Eukaryota - ps5
Eukaryota - ps6
Amorpheae - ps7
Obazoa - ps8
Opisthokonta - ps9
Holomycota - ps10
Fungi - ps11
Fungi_1 - ps12
Fungi_2 - ps13
Fungi_3 - ps14
Dikarya - ps15
Ascomycota - ps16
Saccharo/Pezizomycotina - ps17
Saccharomycotina - ps18
Saccharomycotina_1 - ps19
Saccharomycotina_2 - ps20
Saccharomycotina_3 - ps21
Phaff./Saccharomycetaceae - ps22
Saccharomyco./mycetaceae - ps23
Saccharomycetaceae - ps24
Saccharomycetaceae_2 - ps25
Saccharomycetaceae_3 - ps26
Kaz./Nau./Saccharomyces - ps27
Saccharomyces - ps28
Arboricola_clade - ps29
Cerevisiae_clade - ps30
Saccharomyces_cerevisiae - ps31
Phylostratum

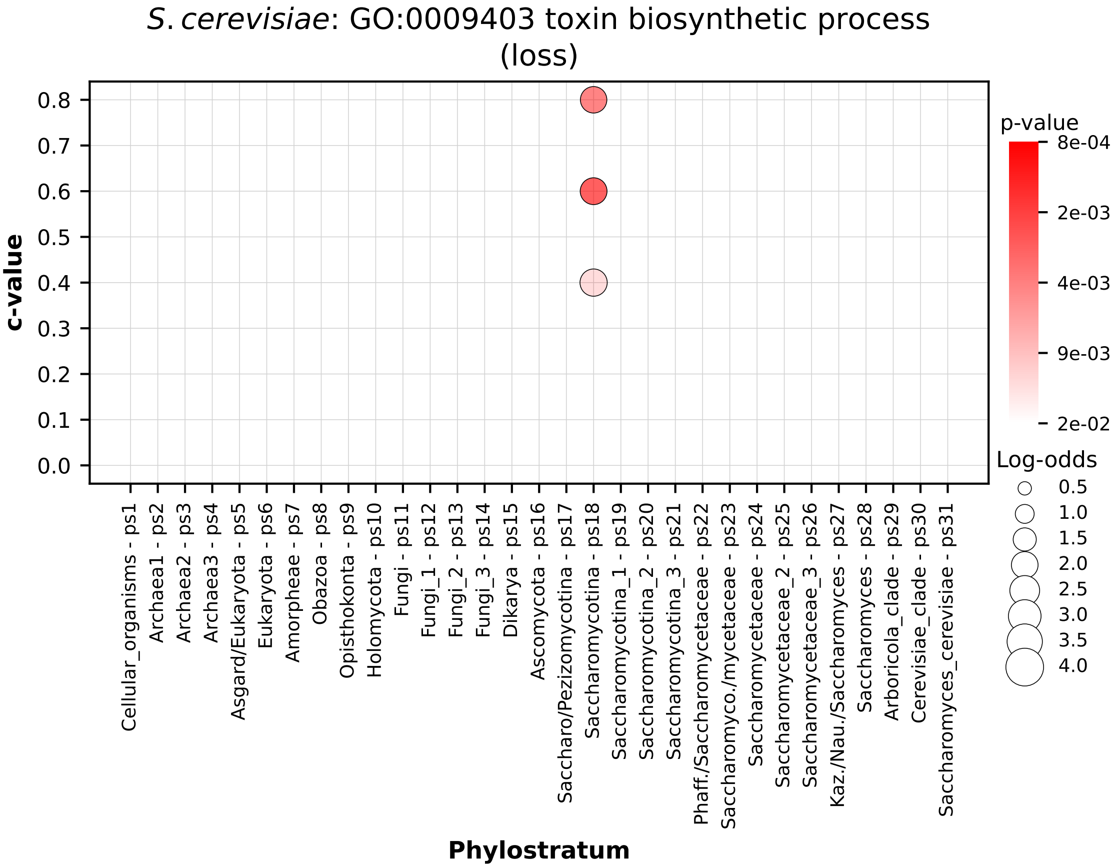
S. cerevisiae: GO:0009403 toxin biosynthetic process
(loss)
c-value
0.8
0.7
0.6
0.5
0.4
0.3
0.2
0.1
0.0
p-value
8e-04
2e-03
4e-03
9e-03
2e-02
Log-odds
0.5
1.0
1.5
2.0
2.5
3.0
3.5
4.0
Cellular_organisms - ps1
Archaea1 - ps2
Archaea2 - ps3
Archaea3 - ps4
Asgard/Eukaryota - ps5
Eukaryota - ps6
Amorpheae - ps7
Obazoa - ps8
Opisthokonta - ps9
Holomycota - ps10
Fungi - ps11
Fungi_1 - ps12
Fungi_2 - ps13
Fungi_3 - ps14
Dikarya - ps15
Ascomycota - ps16
Saccharo/Pezizomycotina - ps17
Saccharomycotina - ps18
Saccharomycotina_1 - ps19
Saccharomycotina_2 - ps20
Saccharomycotina_3 - ps21
Phaff./Saccharomycetaceae - ps22
Saccharomyco./mycetaceae - ps23
Saccharomycetaceae - ps24
Saccharomycetaceae_2 - ps25
Saccharomycetaceae_3 - ps26
Kaz./Nau./Saccharomyces - ps27
Saccharomyces - ps28
Arboricola_clade - ps29
Cerevisiae_clade - ps30
Saccharomyces_cerevisiae - ps31
Phylostratum

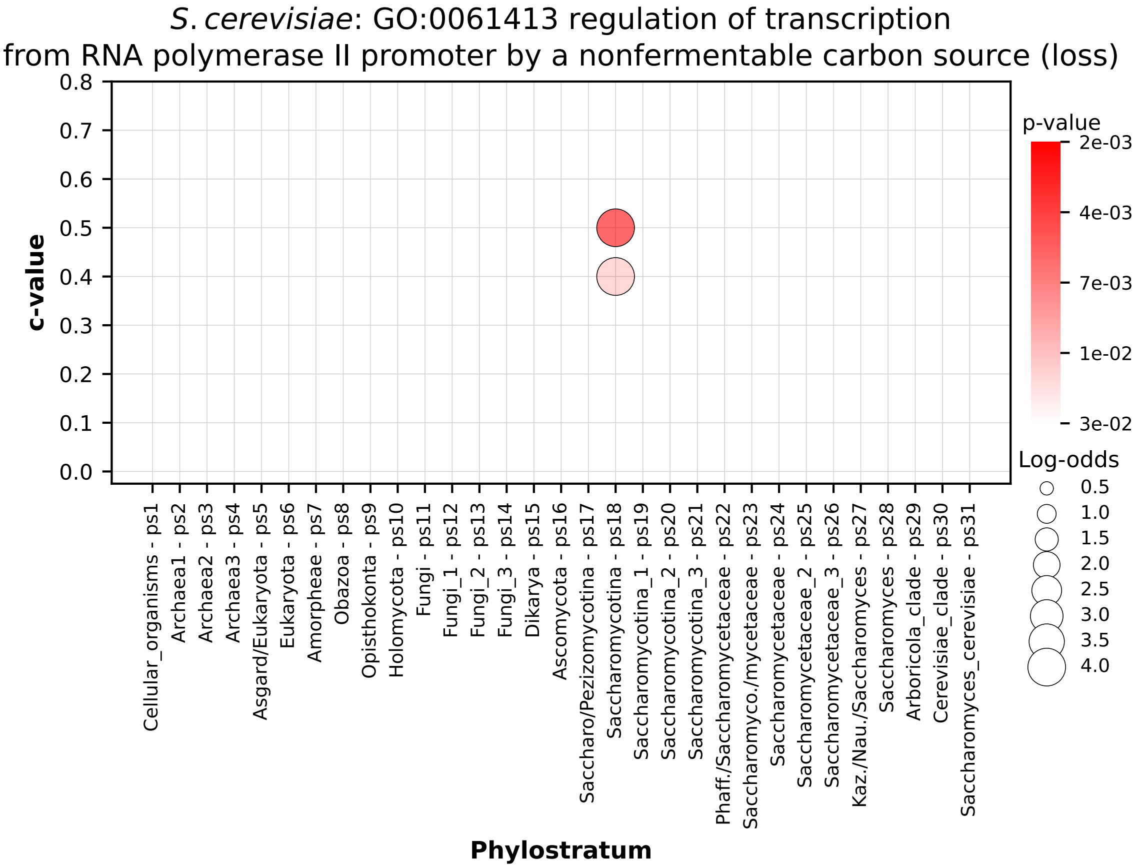

S. cerevisiae: GO:0061413 regulation of transcription
from RNA polymerase II promoter by a nonfermentable carbon source (loss)
c-value
0.8
0.7
0.6
0.5
0.4
0.3
0.2
0.1
0.0
Phylostratum
Cellular_organisms - ps1
Archaea1 - ps2
Archaea2 - ps3
Archaea3 - ps4
Asgard/Eukaryota - ps5
Eukaryota - ps6
Amorpheae - ps7
Obazoa - ps8
Opisthokonta - ps9
Holomycota - ps10
Fungi - ps11
Fungi_1 - ps12
Fungi_2 - ps13
Fungi_3 - ps14
Dikarya - ps15
Ascomycota - ps16
Saccharo/Pezizomycotina - ps17
Saccharomycotina - ps18
Saccharomycotina_1 - ps19
Saccharomycotina_2 - ps20
Saccharomycotina_3 - ps21
Phaff./Saccharomycetaceae - ps22
Saccharomyco./mycetaceae - ps23
Saccharomycetaceae - ps24
Saccharomycetaceae_2 - ps25
Saccharomycetaceae_3 - ps26
Kaz./Nau./Saccharomyces - ps27
Saccharomyces - ps28
Arboricola_clade - ps29
Cerevisiae_clade - ps30
Saccharomyces_cerevisiae - ps31
p-value
2e-03
4e-03
7e-03
1e-02
3e-02
Log-odds
0.5
1.0
1.5
2.0
2.5
3.0
3.5
4.0

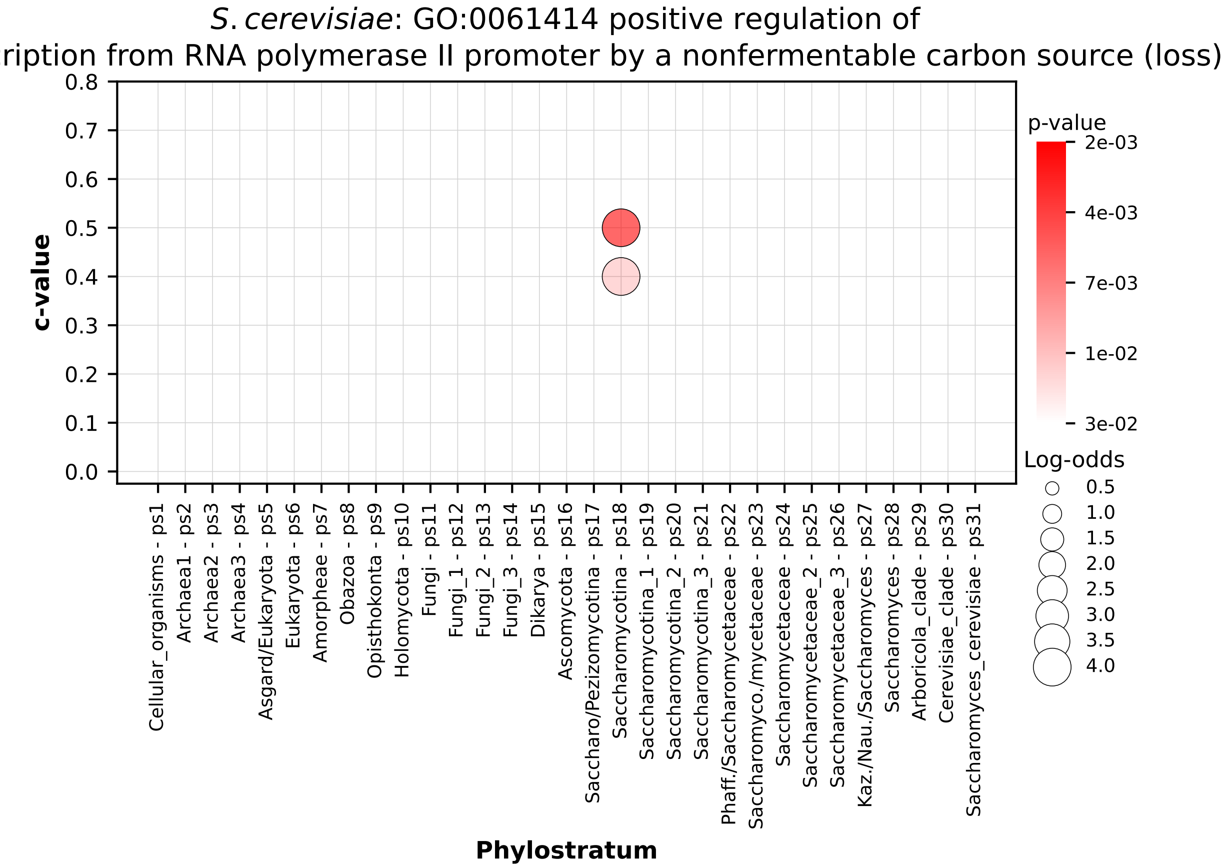

S. cerevisiae: GO:0061414 positive regulation of
ription from RNA polymerase II promoter by a nonfermentable carbon source (loss)
c-value
0.8
0.7
0.6
0.5
0.4
0.3
0.2
0.1
0.0
p-value
2e-03
4e-03
7e-03
1e-02
3e-02
Log-odds
0.5
1.0
1.5
2.0
2.5
3.0
3.5
4.0
Cellular_organisms - ps1
Archaea1 - ps2
Archaea2 - ps3
Archaea3 - ps4
Asgard/Eukaryota - ps5
Eukaryota - ps6
Amorpheae - ps7
Obazoa - ps8
Opisthokonta - ps9
Holomycota - ps10
Fungi - ps11
Fungi_1 - ps12
Fungi_2 - ps13
Fungi_3 - ps14
Dikarya - ps15
Ascomycota - ps16
Saccharo/Pezizomycotina - ps17
Saccharomycotina - ps18
Saccharomycotina_1 - ps19
Saccharomycotina_2 - ps20
Saccharomycotina_3 - ps21
Phaff./Saccharomycetaceae - ps22
Saccharomyco./mycetaceae - ps23
Saccharomycetaceae - ps24
Saccharomycetaceae_2 - ps25
Saccharomycetaceae_3 - ps26
Kaz./Nau./Saccharomyces - ps27
Saccharomyces - ps28
Arboricola_clade - ps29
Cerevisiae_clade - ps30
Saccharomyces_cerevisiae - ps31
Phylostratum

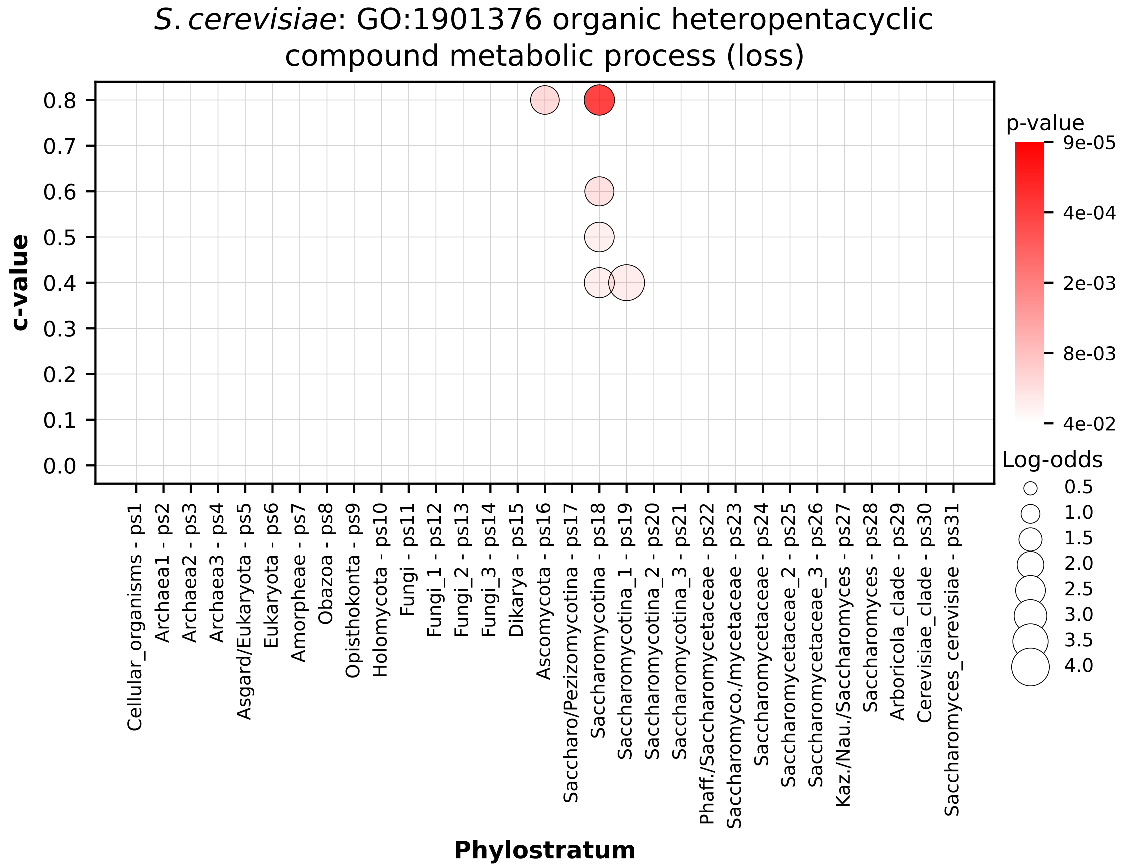
S. cerevisiae: GO:1901376 organic heteropentacyclic compound metabolic process (loss)
c-value
0.0
0.1
0.2
0.3
0.4
0.5
0.6
0.7
0.8
p-value
9e-05
4e-04
2e-03
8e-03
4e-02
Log-odds
0.5
1.0
1.5
2.0
2.5
3.0
3.5
4.0
Cellular_organisms - ps1
Archaea1 - ps2
Archaea2 - ps3
Archaea3 - ps4
Asgard/Eukaryota - ps5
Eukaryota - ps6
Amorpheae - ps7
Obazoa - ps8
Opisthokonta - ps9
Holomycota - ps10
Fungi - ps11
Fungi_1 - ps12
Fungi_2 - ps13
Fungi_3 - ps14
Dikarya - ps15
Ascomycota - ps16
Saccharo/Pezizomycotina - ps17
Saccharomycotina - ps18
Saccharomycotina_1 - ps19
Saccharomycotina_2 - ps20
Saccharomycotina_3 - ps21
Phaff./Saccharomycetaceae - ps22
Saccharomyco./mycetaceae - ps23
Saccharomycetaceae - ps24
Saccharomycetaceae_2 - ps25
Saccharomycetaceae_3 - ps26
Kaz./Nau./Saccharomyces - ps27
Saccharomyces - ps28
Arboricola_clade - ps29
Cerevisiae_clade - ps30
Saccharomyces_cerevisiae - ps31
Phylostratum

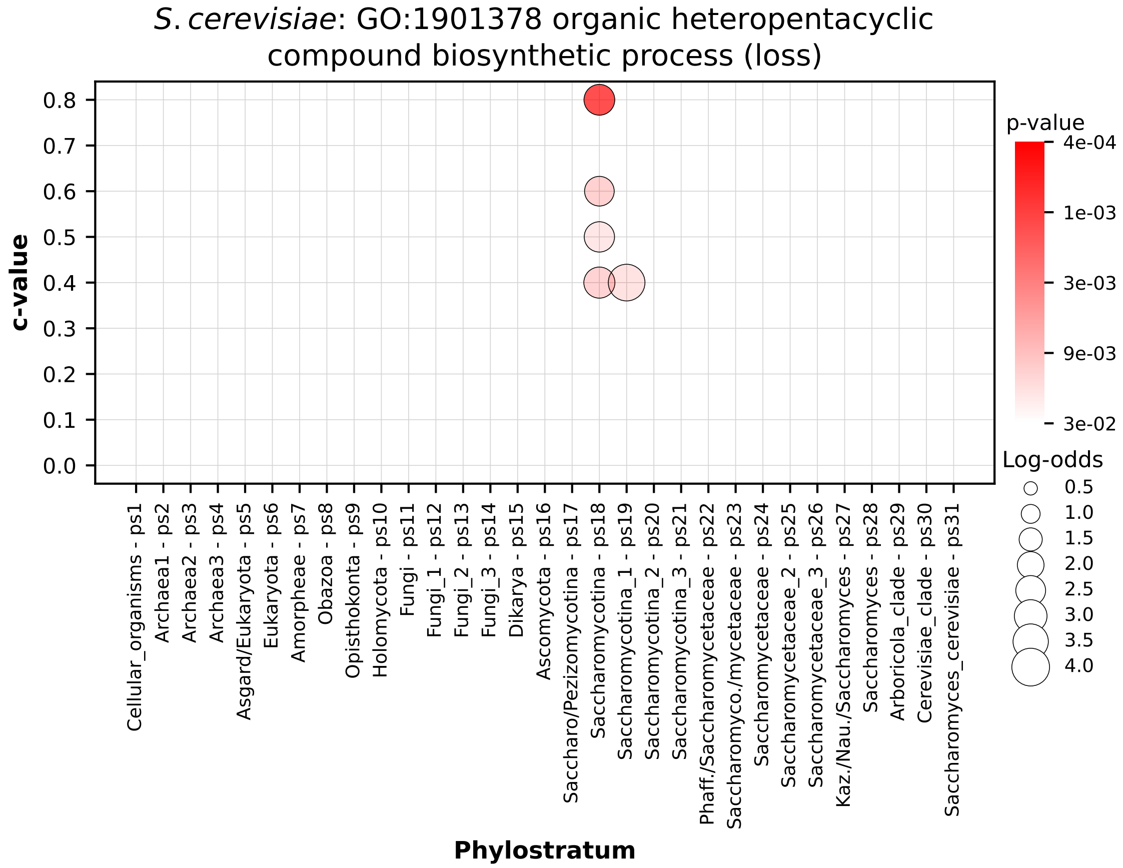

S. cerevisiae: GO:1901378 organic heteropentacyclic compound biosynthetic process (loss)
c-value
0.0
0.1
0.2
0.3
0.4
0.5
0.6
0.7
0.8
p-value
4e-04
1e-03
3e-03
9e-03
3e-02
Log-odds
0.5
1.0
1.5
2.0
2.5
3.0
3.5
4.0
Cellular_organisms - ps1
Archaea1 - ps2
Archaea2 - ps3
Archaea3 - ps4
Asgard/Eukaryota - ps5
Eukaryota - ps6
Amorpheae - ps7
Obazoa - ps8
Opisthokonta - ps9
Holomycota - ps10
Fungi - ps11
Fungi_1 - ps12
Fungi_2 - ps13
Fungi_3 - ps14
Dikarya - ps15
Ascomycota - ps16
Saccharo/Pezizomycotina - ps17
Saccharomycotina - ps18
Saccharomycotina_1 - ps19
Saccharomycotina_2 - ps20
Saccharomycotina_3 - ps21
Phaff./Saccharomycetaceae - ps22
Saccharomyco./mycetaceae - ps23
Saccharomycetaceae - ps24
Saccharomycetaceae_2 - ps25
Saccharomycetaceae_3 - ps26
Kaz./Nau./Saccharomyces - ps27
Saccharomyces - ps28
Arboricola_clade - ps29
Cerevisiae_clade - ps30
Saccharomyces_cerevisiae - ps31
Phylostratum

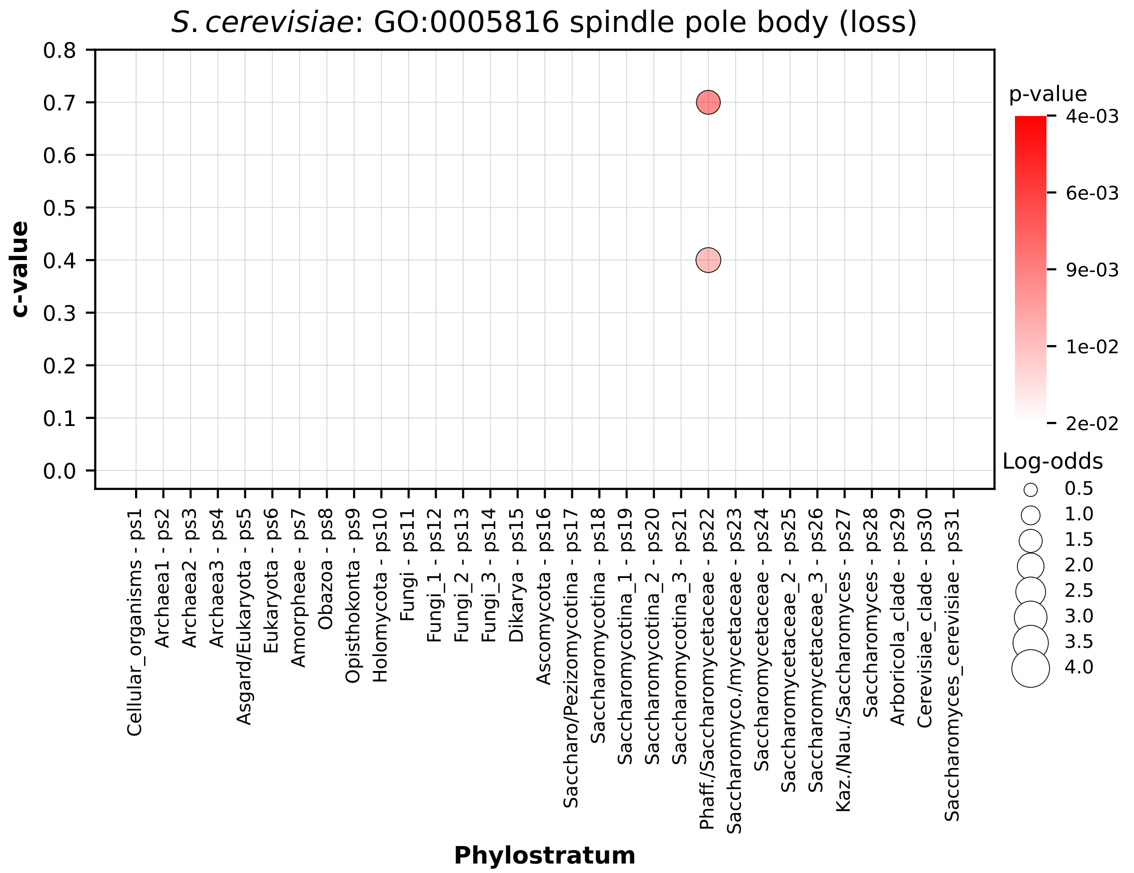

S. cerevisiae: GO:0005816 spindle pole body (loss)
c-value
0.8
0.7
0.6
0.5
0.4
0.3
0.2
0.1
0.0
p-value
4e-03
6e-03
9e-03
1e-02
2e-02
Log-odds
0.5
1.0
1.5
2.0
2.5
3.0
3.5
4.0
Cellular_organisms - ps1
Archaea1 - ps2
Archaea2 - ps3
Archaea3 - ps4
Asgard/Eukaryota - ps5
Eukaryota - ps6
Amorpheae - ps7
Obazoa - ps8
Opisthokonta - ps9
Holomycota - ps10
Fungi - ps11
Fungi_1 - ps12
Fungi_2 - ps13
Fungi_3 - ps14
Dikarya - ps15
Ascomycota - ps16
Saccharo/Pezizomycotina - ps17
Saccharomycotina - ps18
Saccharomycotina_1 - ps19
Saccharomycotina_2 - ps20
Saccharomycotina_3 - ps21
Phaff./Saccharomycetaceae - ps22
Saccharomyco./mycetaceae - ps23
Saccharomycetaceae - ps24
Saccharomycetaceae_2 - ps25
Saccharomycetaceae_3 - ps26
Kaz./Nau./Saccharomyces - ps27
Saccharomyces - ps28
Arboricola_clade - ps29
Cerevisiae_clade - ps30
Saccharomyces_cerevisiae - ps31
Phylostratum

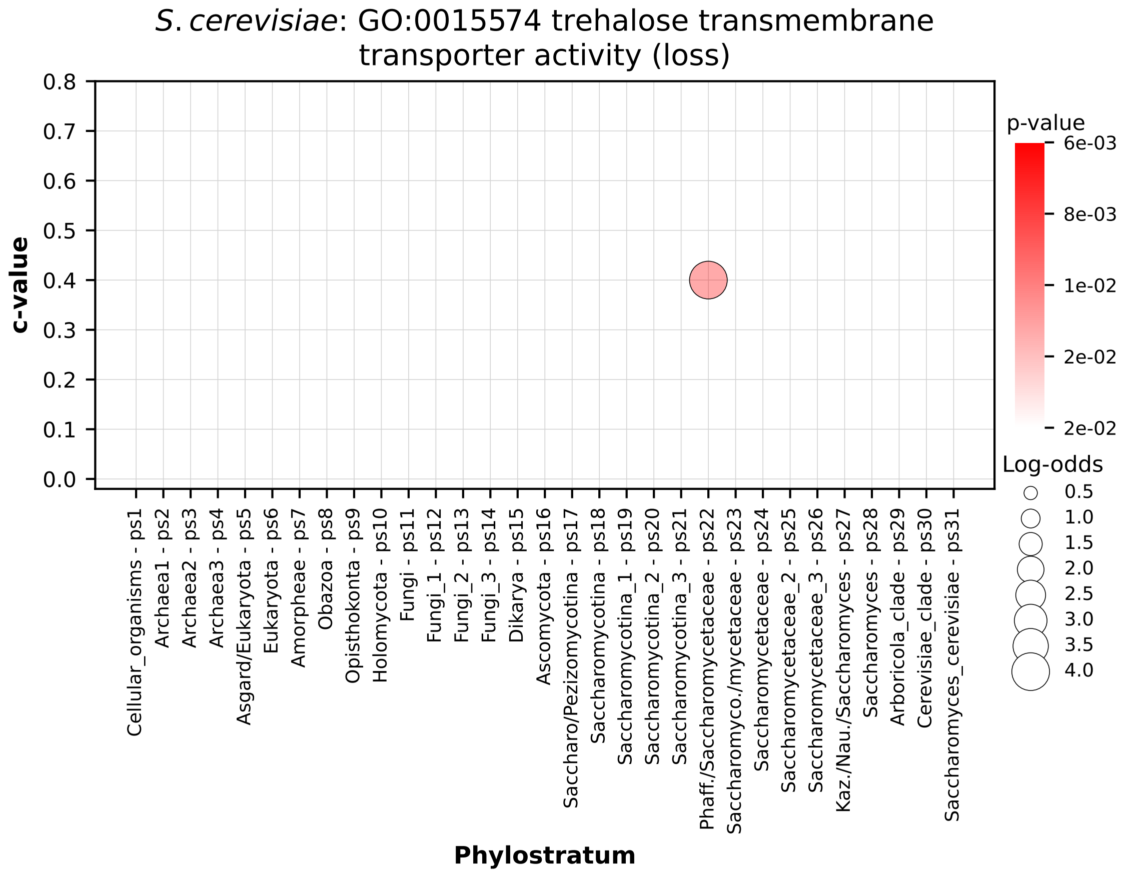

S. cerevisiae: GO:0015574 trehalose transmembrane transporter activity (loss)
c-value
0.8
0.7
0.6
0.5
0.4
0.3
0.2
0.1
0.0
p-value
6e-03
8e-03
1e-02
2e-02
2e-02
Log-odds
0.5
1.0
1.5
2.0
2.5
3.0
3.5
4.0
Cellular_organisms - ps1
Archaea1 - ps2
Archaea2 - ps3
Archaea3 - ps4
Asgard/Eukaryota - ps5
Eukaryota - ps6
Amorpheae - ps7
Obazoa - ps8
Opisthokonta - ps9
Holomycota - ps10
Fungi - ps11
Fungi_1 - ps12
Fungi_2 - ps13
Fungi_3 - ps14
Dikarya - ps15
Ascomycota - ps16
Saccharo/Pezizomycotina - ps17
Saccharomycotina - ps18
Saccharomycotina_1 - ps19
Saccharomycotina_2 - ps20
Saccharomycotina_3 - ps21
Phaff./Saccharomycetaceae - ps22
Saccharomyco./mycetaceae - ps23
Saccharomycetaceae - ps24
Saccharomycetaceae_2 - ps25
Saccharomycetaceae_3 - ps26
Kaz./Nau./Saccharomyces - ps27
Saccharomyces - ps28
Arboricola_clade - ps29
Cerevisiae_clade - ps30
Saccharomyces_cerevisiae - ps31
Phylostratum

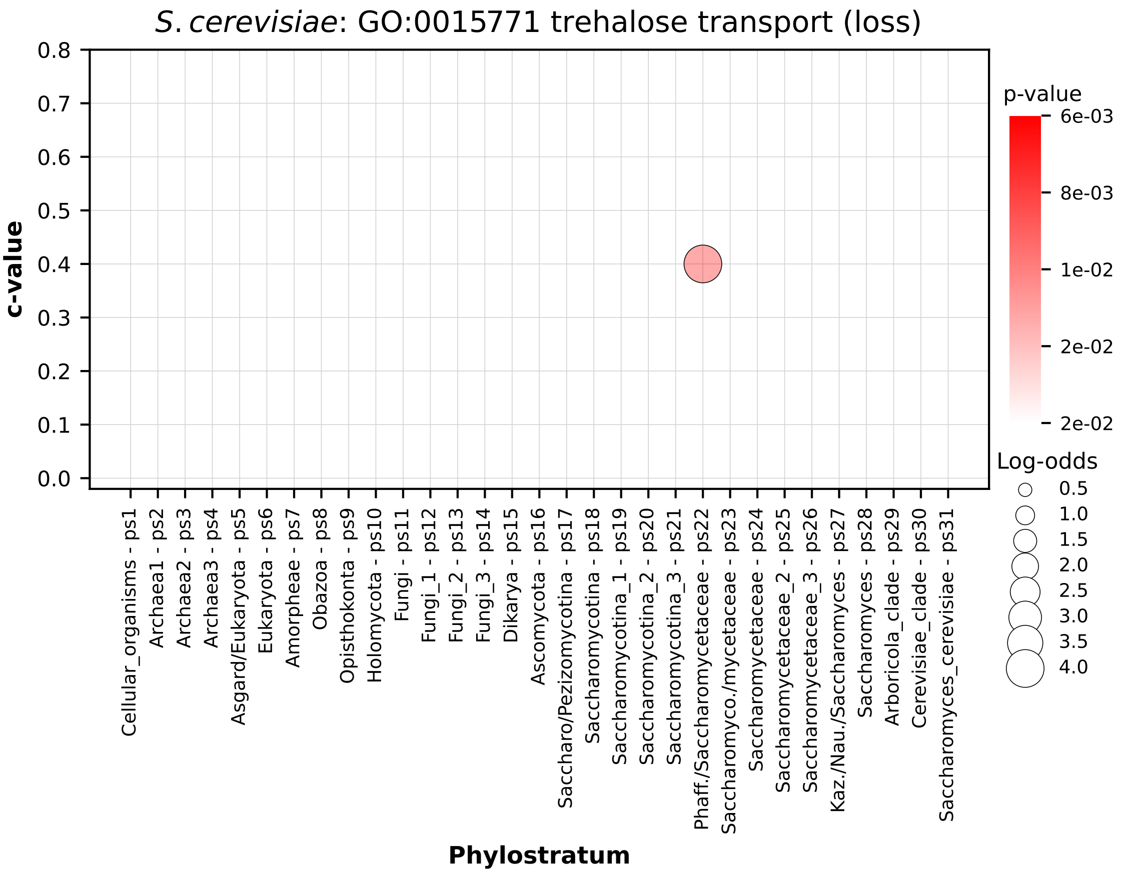

S. cerevisiae: GO:0015771 trehalose transport (loss)
c-value
0.8
0.7
0.6
0.5
0.4
0.3
0.2
0.1
0.0
Phylostratum
Cellular_organisms - ps1
Archaea1 - ps2
Archaea2 - ps3
Archaea3 - ps4
Asgard/Eukaryota - ps5
Eukaryota - ps6
Amorpheae - ps7
Obazoa - ps8
Opisthokonta - ps9
Holomycota - ps10
Fungi - ps11
Fungi_1 - ps12
Fungi_2 - ps13
Fungi_3 - ps14
Dikarya - ps15
Ascomycota - ps16
Saccharo/Pezizomycotina - ps17
Saccharomycotina - ps18
Saccharomycotina_1 - ps19
Saccharomycotina_2 - ps20
Saccharomycotina_3 - ps21
Phaff./Saccharomycetaceae - ps22
Saccharomyco./mycetaceae - ps23
Saccharomycetaceae - ps24
Saccharomycetaceae_2 - ps25
Saccharomycetaceae_3 - ps26
Kaz./Nau./Saccharomyces - ps27
Saccharomyces - ps28
Arboricola_clade - ps29
Cerevisiae_clade - ps30
Saccharomyces_cerevisiae - ps31
p-value
6e-03
8e-03
1e-02
2e-02
2e-02
Log-odds
0.5
1.0
1.5
2.0
2.5
3.0
3.5
4.0

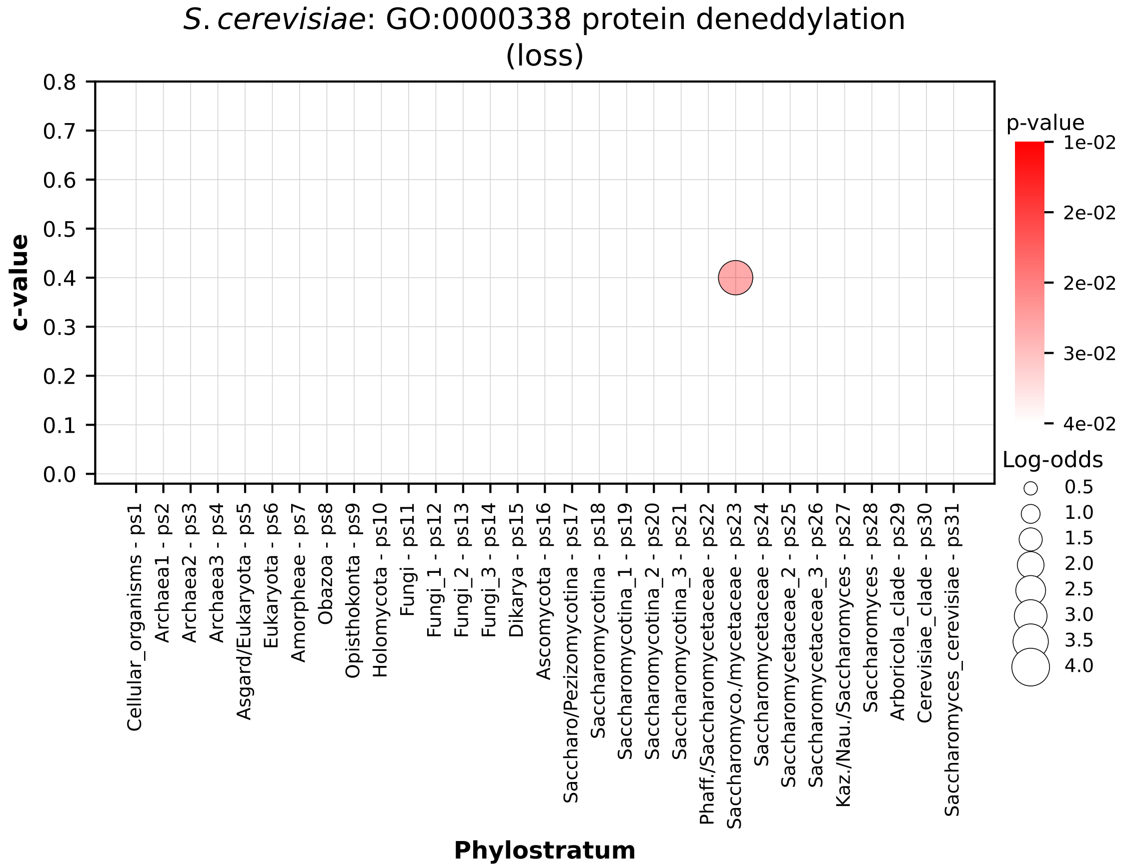

S. cerevisiae: GO:0000338 protein deneddylation
(loss)
c-value
0.8
0.7
0.6
0.5
0.4
0.3
0.2
0.1
0.0
p-value
1e-02
2e-02
2e-02
3e-02
4e-02
Log-odds
0.5
1.0
1.5
2.0
2.5
3.0
3.5
4.0
Cellular_organisms - ps1
Archaea1 - ps2
Archaea2 - ps3
Archaea3 - ps4
Asgard/Eukaryota - ps5
Eukaryota - ps6
Amorpheae - ps7
Obazoa - ps8
Opisthokonta - ps9
Holomycota - ps10
Fungi - ps11
Fungi_1 - ps12
Fungi_2 - ps13
Fungi_3 - ps14
Dikarya - ps15
Ascomycota - ps16
Saccharo/Pezizomycotina - ps17
Saccharomycotina - ps18
Saccharomycotina_1 - ps19
Saccharomycotina_2 - ps20
Saccharomycotina_3 - ps21
Phaff./Saccharomycetaceae - ps22
Saccharomyco./mycetaceae - ps23
Saccharomycetaceae - ps24
Saccharomycetaceae_2 - ps25
Saccharomycetaceae_3 - ps26
Kaz./Nau./Saccharomyces - ps27
Saccharomyces - ps28
Arboricola_clade - ps29
Cerevisiae_clade - ps30
Saccharomyces_cerevisiae - ps31
Phylostratum

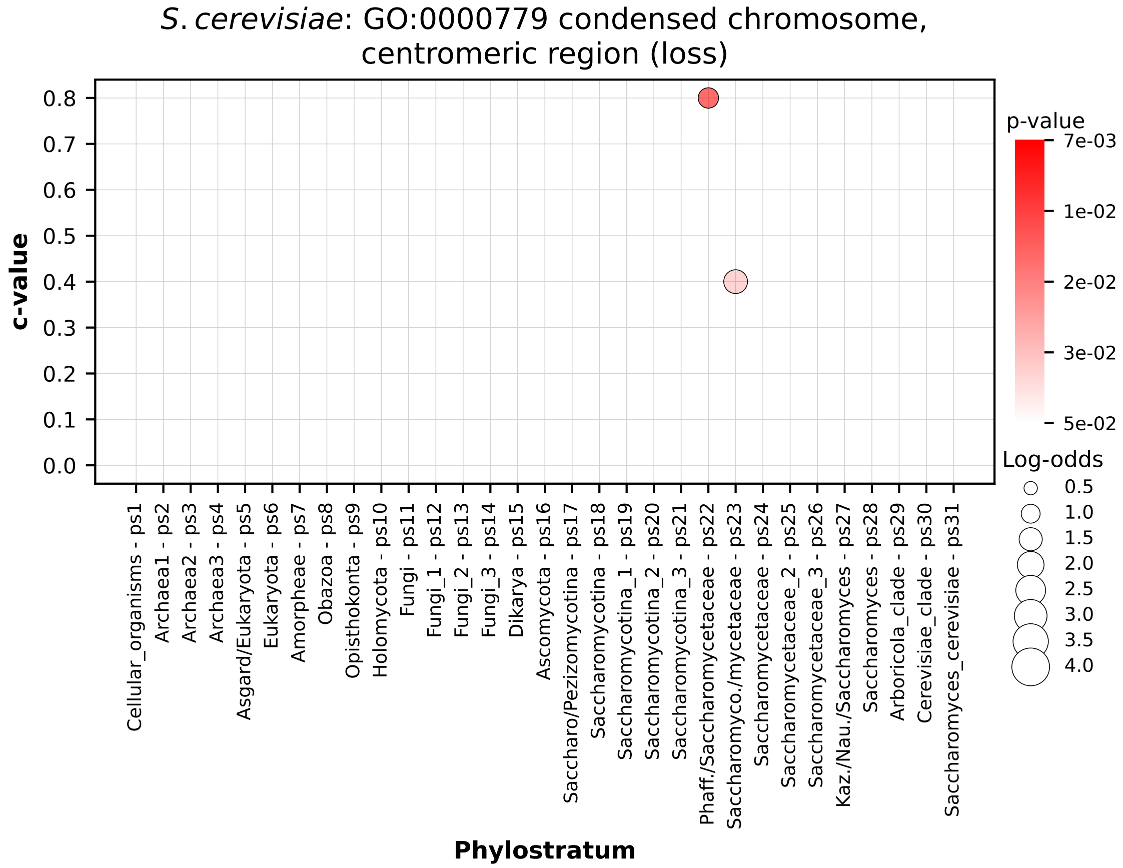

S. cerevisiae: GO:0000779 condensed chromosome, centromeric region (loss)
c-value
0.8
0.7
0.6
0.5
0.4
0.3
0.2
0.1
0.0
p-value
7e-03
1e-02
2e-02
3e-02
5e-02
Log-odds
0.5
1.0
1.5
2.0
2.5
3.0
3.5
4.0
Cellular_organisms - ps1
Archaea1 - ps2
Archaea2 - ps3
Archaea3 - ps4
Asgard/Eukaryota - ps5
Eukaryota - ps6
Amorpheae - ps7
Obazoa - ps8
Opisthokonta - ps9
Holomycota - ps10
Fungi - ps11
Fungi_1 - ps12
Fungi_2 - ps13
Fungi_3 - ps14
Dikarya - ps15
Ascomycota - ps16
Saccharo/Pezizomycotina - ps17
Saccharomycotina - ps18
Saccharomycotina_1 - ps19
Saccharomycotina_2 - ps20
Saccharomycotina_3 - ps21
Phaff./Saccharomycetaceae - ps22
Saccharomyco./mycetaceae - ps23
Saccharomycetaceae - ps24
Saccharomycetaceae_2 - ps25
Saccharomycetaceae_3 - ps26
Kaz./Nau./Saccharomyces - ps27
Saccharomyces - ps28
Arboricola_clade - ps29
Cerevisiae_clade - ps30
Saccharomyces_cerevisiae - ps31
Phylostratum

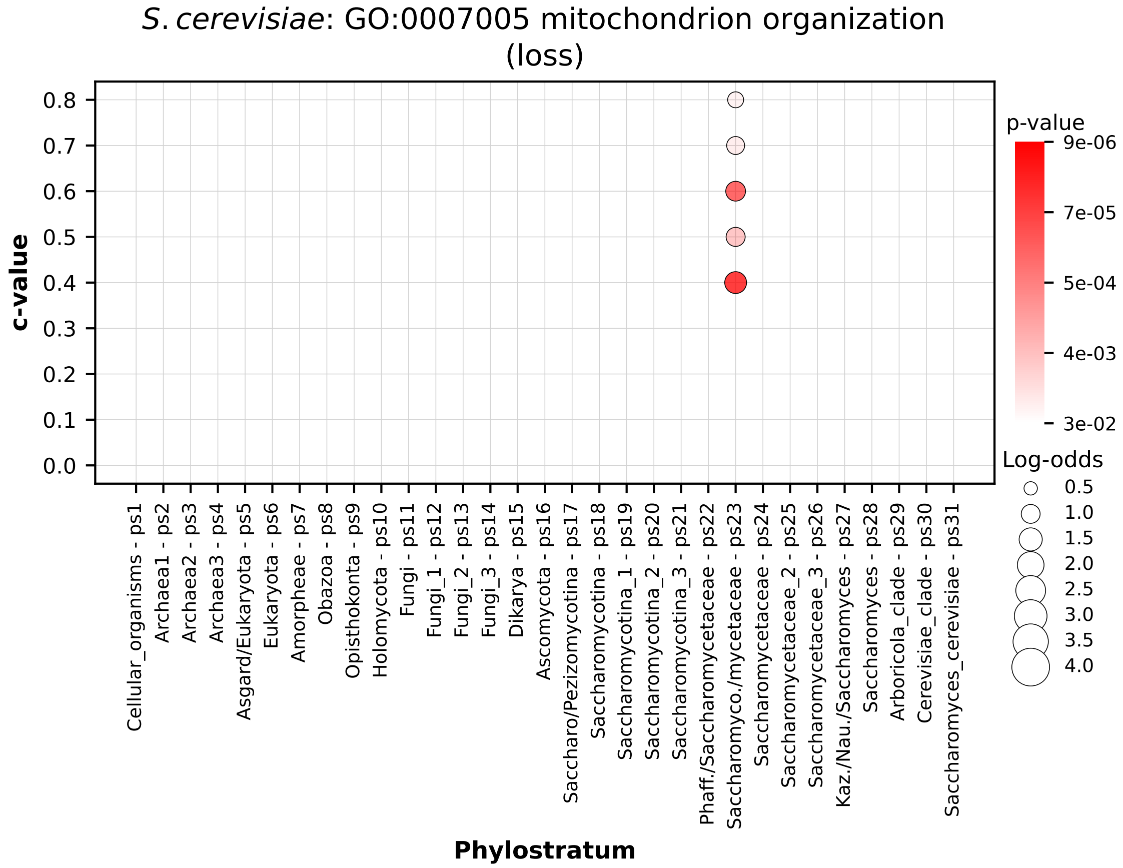

S. cerevisiae: GO:0007005 mitochondrion organization
(loss)
c-value
0.8
0.7
0.6
0.5
0.4
0.3
0.2
0.1
0.0
p-value
9e-06
7e-05
5e-04
4e-03
3e-02
Log-odds
0.5
1.0
1.5
2.0
2.5
3.0
3.5
4.0
Cellular_organisms - ps1
Archaea1 - ps2
Archaea2 - ps3
Archaea3 - ps4
Asgard/Eukaryota - ps5
Eukaryota - ps6
Amorpheae - ps7
Obazoa - ps8
Opisthokonta - ps9
Holomycota - ps10
Fungi - ps11
Fungi_1 - ps12
Fungi_2 - ps13
Fungi_3 - ps14
Dikarya - ps15
Ascomycota - ps16
Saccharo/Pezizomycotina - ps17
Saccharomycotina - ps18
Saccharomycotina_1 - ps19
Saccharomycotina_2 - ps20
Saccharomycotina_3 - ps21
Phaff./Saccharomycetaceae - ps22
Saccharomyco./mycetaceae - ps23
Saccharomycetaceae - ps24
Saccharomycetaceae_2 - ps25
Saccharomycetaceae_3 - ps26
Kaz./Nau./Saccharomyces - ps27
Saccharomyces - ps28
Arboricola_clade - ps29
Cerevisiae_clade - ps30
Saccharomyces_cerevisiae - ps31
Phylostratum

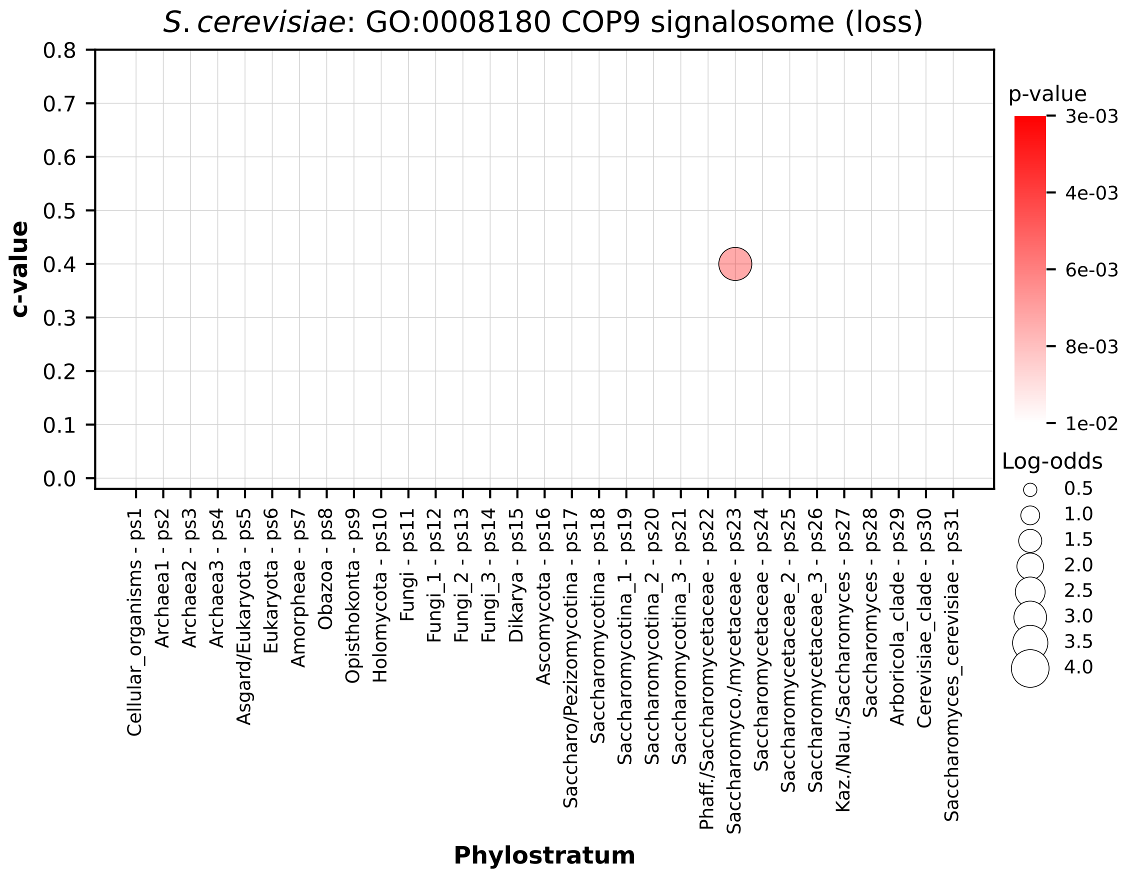

S. cerevisiae: GO:0008180 COP9 signalosome (loss)
c-value
0.8
0.7
0.6
0.5
0.4
0.3
0.2
0.1
0.0
Phylostratum
Cellular_organisms - ps1
Archaea1 - ps2
Archaea2 - ps3
Archaea3 - ps4
Asgard/Eukaryota - ps5
Eukaryota - ps6
Amorpheae - ps7
Obazoa - ps8
Opisthokonta - ps9
Holomycota - ps10
Fungi - ps11
Fungi_1 - ps12
Fungi_2 - ps13
Fungi_3 - ps14
Dikarya - ps15
Ascomycota - ps16
Saccharo/Pezizomycotina - ps17
Saccharomycotina - ps18
Saccharomycotina_1 - ps19
Saccharomycotina_2 - ps20
Saccharomycotina_3 - ps21
Phaff./Saccharomycetaceae - ps22
Saccharomyco./mycetaceae - ps23
Saccharomycetaceae - ps24
Saccharomycetaceae_2 - ps25
Saccharomycetaceae_3 - ps26
Kaz./Nau./Saccharomyces - ps27
Saccharomyces - ps28
Arboricola_clade - ps29
Cerevisiae_clade - ps30
Saccharomyces_cerevisiae - ps31
p-value
3e-03
4e-03
6e-03
8e-03
1e-02
Log-odds
0.5
1.0
1.5
2.0
2.5
3.0
3.5
4.0

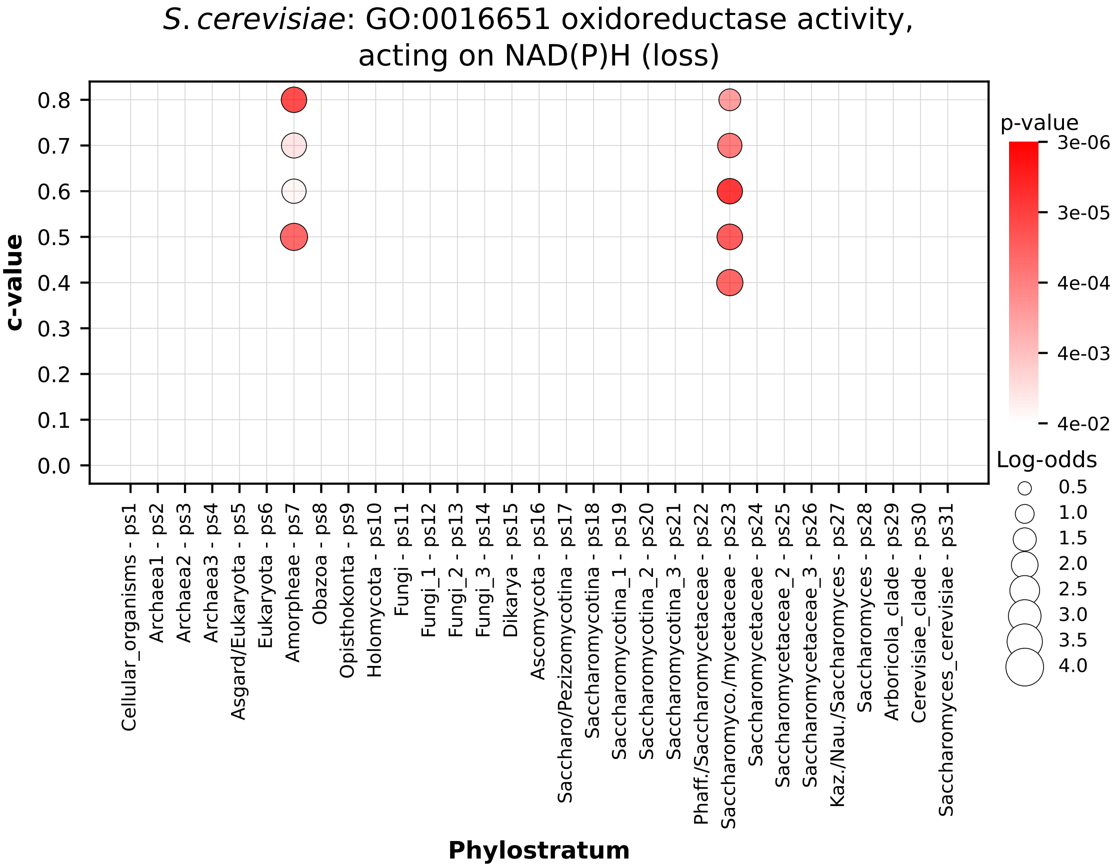

S. cerevisiae: GO:0016651 oxidoreductase activity, acting on NAD(P)H (loss)
c-value
0.8
0.7
0.6
0.5
0.4
0.3
0.2
0.1
0.0
p-value
3e-06
3e-05
4e-04
4e-03
4e-02
Log-odds
0.5
1.0
1.5
2.0
2.5
3.0
3.5
4.0
Cellular_organisms - ps1
Archaea1 - ps2
Archaea2 - ps3
Archaea3 - ps4
Asgard/Eukaryota - ps5
Eukaryota - ps6
Amorpheae - ps7
Obazoa - ps8
Opisthokonta - ps9
Holomycota - ps10
Fungi - ps11
Fungi_1 - ps12
Fungi_2 - ps13
Fungi_3 - ps14
Dikarya - ps15
Ascomycota - ps16
Saccharo/Pezizomycotina - ps17
Saccharomycotina - ps18
Saccharomycotina_1 - ps19
Saccharomycotina_2 - ps20
Saccharomycotina_3 - ps21
Phaff./Saccharomycetaceae - ps22
Saccharomyco./mycetaceae - ps23
Saccharomycetaceae - ps24
Saccharomycetaceae_2 - ps25
Saccharomycetaceae_3 - ps26
Kaz./Nau./Saccharomyces - ps27
Saccharomyces - ps28
Arboricola_clade - ps29
Cerevisiae_clade - ps30
Saccharomyces_cerevisiae - ps31
Phylostratum

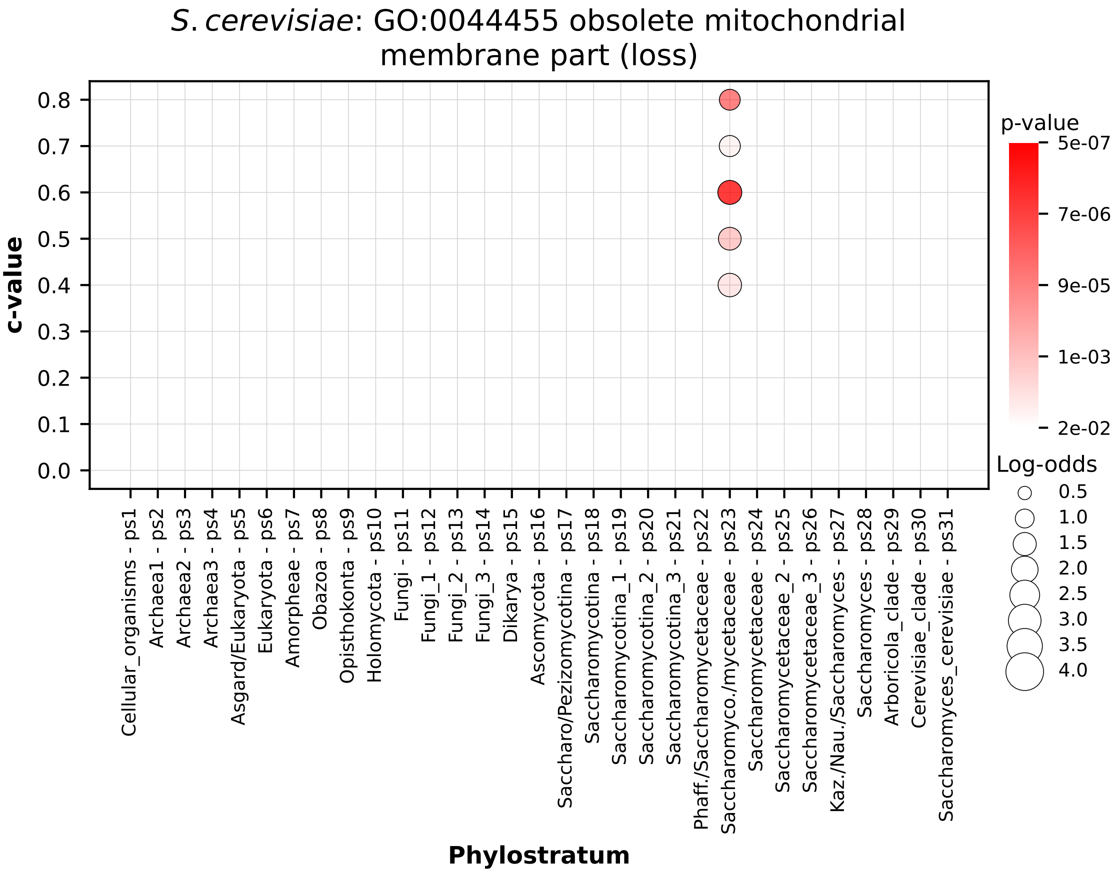

S. cerevisiae: GO:0044455 obsolete mitochondrial membrane part (loss)
c-value
0.8
0.7
0.6
0.5
0.4
0.3
0.2
0.1
0.0
p-value
5e-07
7e-06
9e-05
1e-03
2e-02
Log-odds
0.5
1.0
1.5
2.0
2.5
3.0
3.5
4.0
Cellular_organisms - ps1
Archaea1 - ps2
Archaea2 - ps3
Archaea3 - ps4
Asgard/Eukaryota - ps5
Eukaryota - ps6
Amorpheae - ps7
Obazoa - ps8
Opisthokonta - ps9
Holomycota - ps10
Fungi - ps11
Fungi_1 - ps12
Fungi_2 - ps13
Fungi_3 - ps14
Dikarya - ps15
Ascomycota - ps16
Saccharo/Pezizomycotina - ps17
Saccharomycotina - ps18
Saccharomycotina_1 - ps19
Saccharomycotina_2 - ps20
Saccharomycotina_3 - ps21
Phaff./Saccharomycetaceae - ps22
Saccharomyco./mycetaceae - ps23
Saccharomycetaceae - ps24
Saccharomycetaceae_2 - ps25
Saccharomycetaceae_3 - ps26
Kaz./Nau./Saccharomyces - ps27
Saccharomyces - ps28
Arboricola_clade - ps29
Cerevisiae_clade - ps30
Saccharomyces_cerevisiae - ps31
Phylostratum

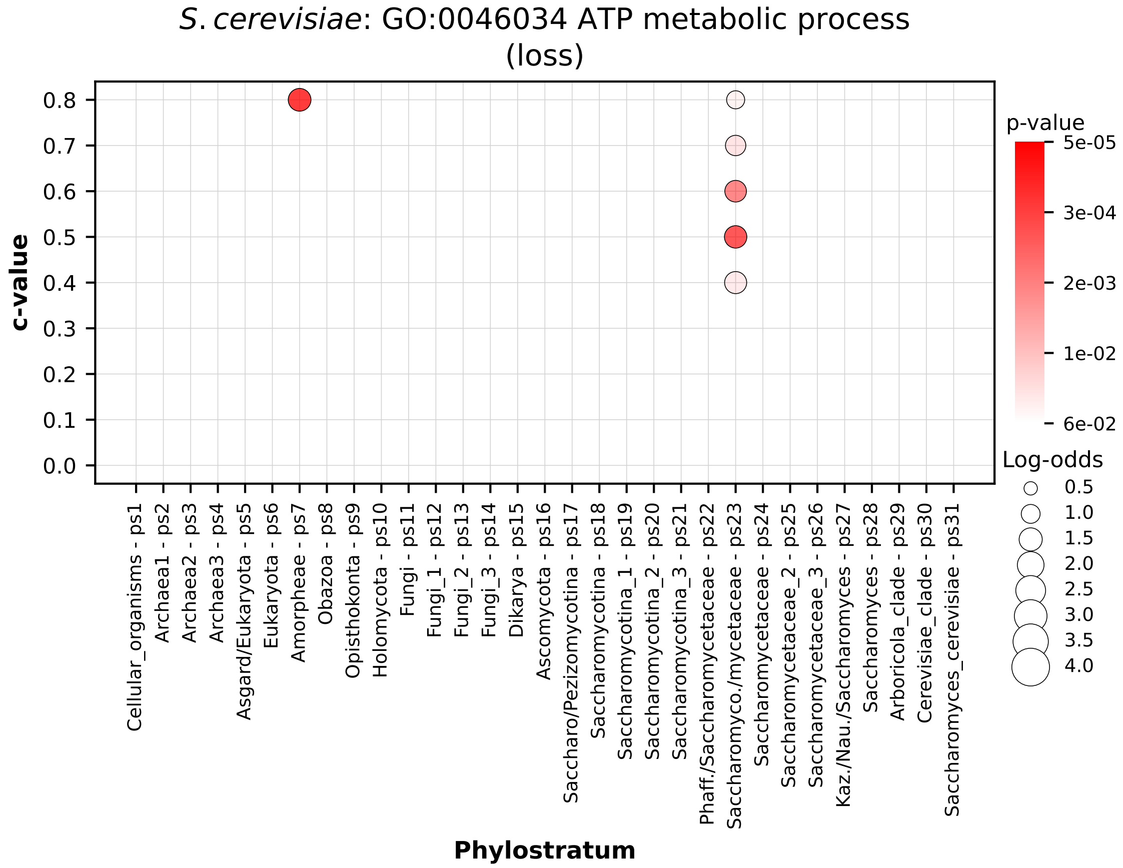

S. cerevisiae: GO:0046034 ATP metabolic process
(loss)
c-value
0.8
0.7
0.6
0.5
0.4
0.3
0.2
0.1
0.0
p-value
5e-05
3e-04
2e-03
1e-02
6e-02
Log-odds
0.5
1.0
1.5
2.0
2.5
3.0
3.5
4.0
Cellular_organisms - ps1
Archaea1 - ps2
Archaea2 - ps3
Archaea3 - ps4
Asgard/Eukaryota - ps5
Eukaryota - ps6
Amorpheae - ps7
Obazoa - ps8
Opisthokonta - ps9
Holomycota - ps10
Fungi - ps11
Fungi_1 - ps12
Fungi_2 - ps13
Fungi_3 - ps14
Dikarya - ps15
Ascomycota - ps16
Saccharo/Pezizomycotina - ps17
Saccharomycotina - ps18
Saccharomycotina_1 - ps19
Saccharomycotina_2 - ps20
Saccharomycotina_3 - ps21
Phaff./Saccharomycetaceae - ps22
Saccharomyco./mycetaceae - ps23
Saccharomycetaceae - ps24
Saccharomycetaceae_2 - ps25
Saccharomycetaceae_3 - ps26
Kaz./Nau./Saccharomyces - ps27
Saccharomyces - ps28
Arboricola_clade - ps29
Cerevisiae_clade - ps30
Saccharomyces_cerevisiae - ps31
Phylostratum

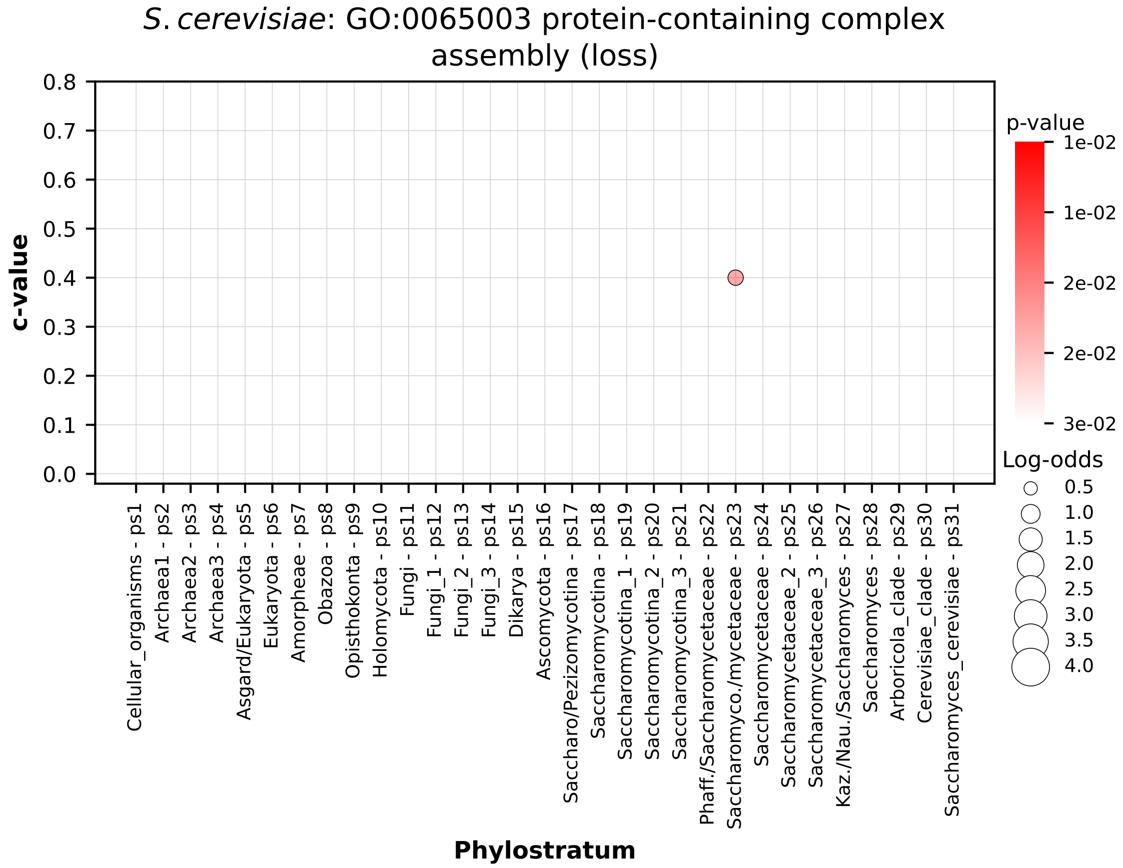

S. cerevisiae: GO:0065003 protein-containing complex assembly (loss)
c-value
0.8
0.7
0.6
0.5
0.4
0.3
0.2
0.1
0.0
p-value
1e-02
1e-02
2e-02
2e-02
3e-02
Log-odds
0.5
1.0
1.5
2.0
2.5
3.0
3.5
4.0
Cellular_organisms - ps1
Archaea1 - ps2
Archaea2 - ps3
Archaea3 - ps4
Asgard/Eukaryota - ps5
Eukaryota - ps6
Amorpheae - ps7
Obazoa - ps8
Opisthokonta - ps9
Holomycota - ps10
Fungi - ps11
Fungi_1 - ps12
Fungi_2 - ps13
Fungi_3 - ps14
Dikarya - ps15
Ascomycota - ps16
Saccharo/Pezizomycotina - ps17
Saccharomycotina - ps18
Saccharomycotina_1 - ps19
Saccharomycotina_2 - ps20
Saccharomycotina_3 - ps21
Phaff./Saccharomycetaceae - ps22
Saccharomyco./mycetaceae - ps23
Saccharomycetaceae - ps24
Saccharomycetaceae_2 - ps25
Saccharomycetaceae_3 - ps26
Kaz./Nau./Saccharomyces - ps27
Saccharomyces - ps28
Arboricola_clade - ps29
Cerevisiae_clade - ps30
Saccharomyces_cerevisiae - ps31
Phylostratum

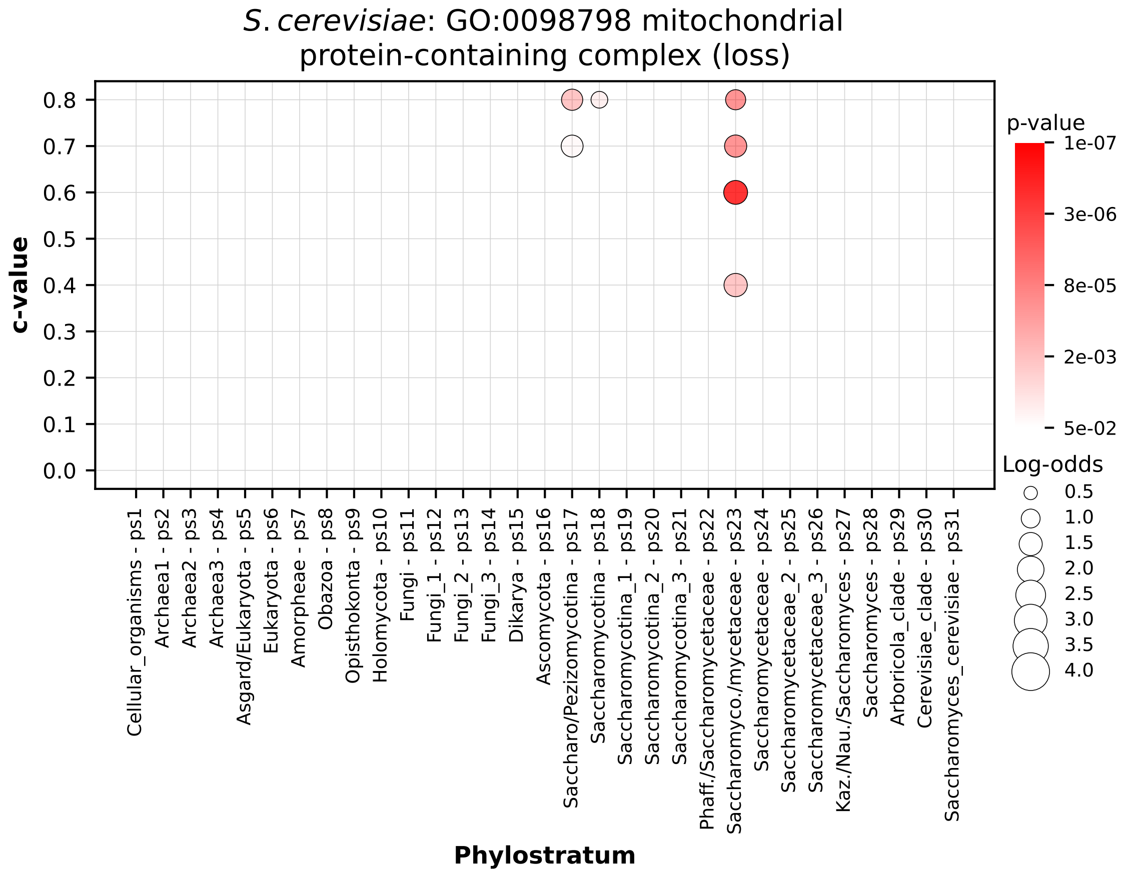

S. cerevisiae: GO:0098798 mitochondrial protein-containing complex (loss)
c-value
0.8
0.7
0.6
0.5
0.4
0.3
0.2
0.1
0.0
Phylostratum
Cellular_organisms - ps1
Archaea1 - ps2
Archaea2 - ps3
Archaea3 - ps4
Asgard/Eukaryota - ps5
Eukaryota - ps6
Amorpheae - ps7
Obazoa - ps8
Opisthokonta - ps9
Holomycota - ps10
Fungi - ps11
Fungi_1 - ps12
Fungi_2 - ps13
Fungi_3 - ps14
Dikarya - ps15
Ascomycota - ps16
Saccharo/Pezizomycotina - ps17
Saccharomycotina - ps18
Saccharomycotina_1 - ps19
Saccharomycotina_2 - ps20
Saccharomycotina_3 - ps21
Phaff./Saccharomycetaceae - ps22
Saccharomyco./mycetaceae - ps23
Saccharomycetaceae - ps24
Saccharomycetaceae_2 - ps25
Saccharomycetaceae_3 - ps26
Kaz./Nau./Saccharomyces - ps27
Saccharomyces - ps28
Arboricola_clade - ps29
Cerevisiae_clade - ps30
Saccharomyces_cerevisiae - ps31
p-value
1e-07
3e-06
8e-05
2e-03
5e-02
Log-odds
0.5
1.0
1.5
2.0
2.5
3.0
3.5
4.0

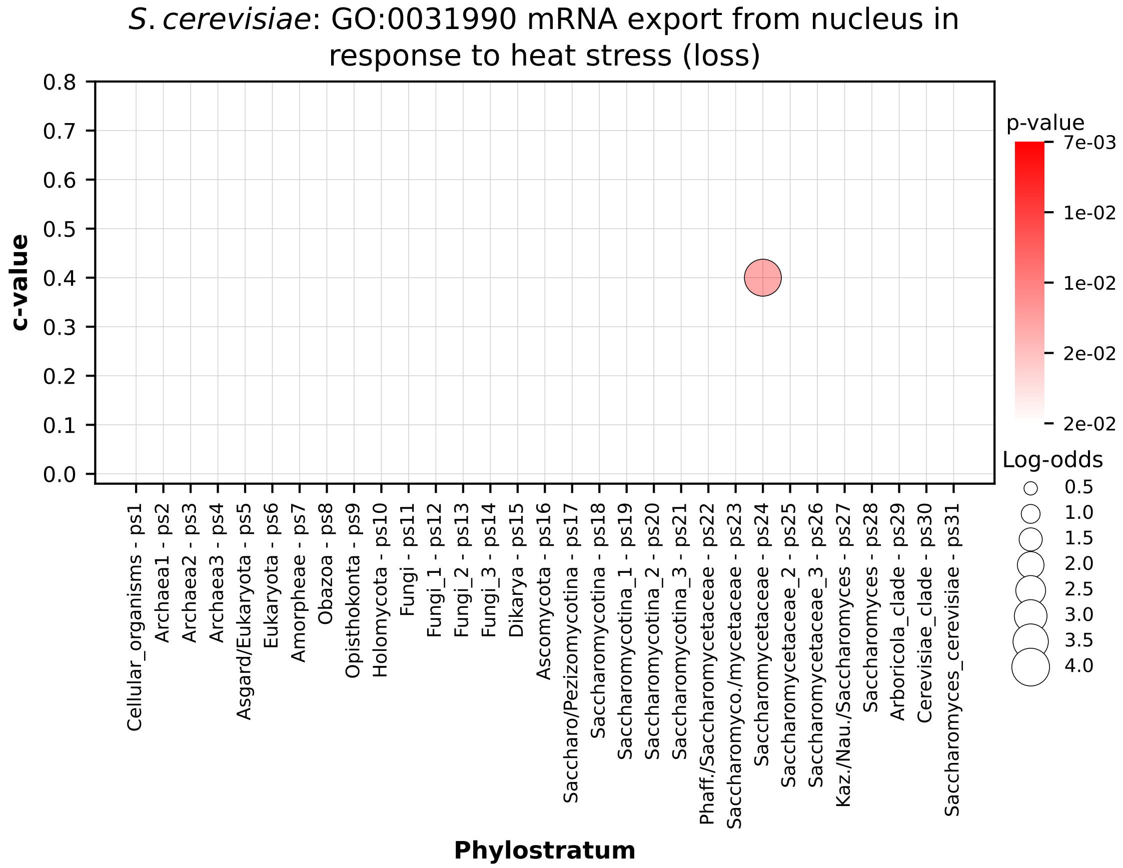

S. cerevisiae: GO:0031990 mRNA export from nucleus in response to heat stress (loss)
c-value
0.8
0.7
0.6
0.5
0.4
0.3
0.2
0.1
0.0
p-value
7e-03
1e-02
1e-02
2e-02
2e-02
Log-odds
0.5
1.0
1.5
2.0
2.5
3.0
3.5
4.0
Cellular_organisms - ps1
Archaea1 - ps2
Archaea2 - ps3
Archaea3 - ps4
Asgard/Eukaryota - ps5
Eukaryota - ps6
Amorpheae - ps7
Obazoa - ps8
Opisthokonta - ps9
Holomycota - ps10
Fungi - ps11
Fungi_1 - ps12
Fungi_2 - ps13
Fungi_3 - ps14
Dikarya - ps15
Ascomycota - ps16
Saccharo/Pezizomycotina - ps17
Saccharomycotina - ps18
Saccharomycotina_1 - ps19
Saccharomycotina_2 - ps20
Saccharomycotina_3 - ps21
Phaff./Saccharomycetaceae - ps22
Saccharomyco./mycetaceae - ps23
Saccharomycetaceae - ps24
Saccharomycetaceae_2 - ps25
Saccharomycetaceae_3 - ps26
Kaz./Nau./Saccharomyces - ps27
Saccharomyces - ps28
Arboricola_clade - ps29
Cerevisiae_clade - ps30
Saccharomyces_cerevisiae - ps31
Phylostratum

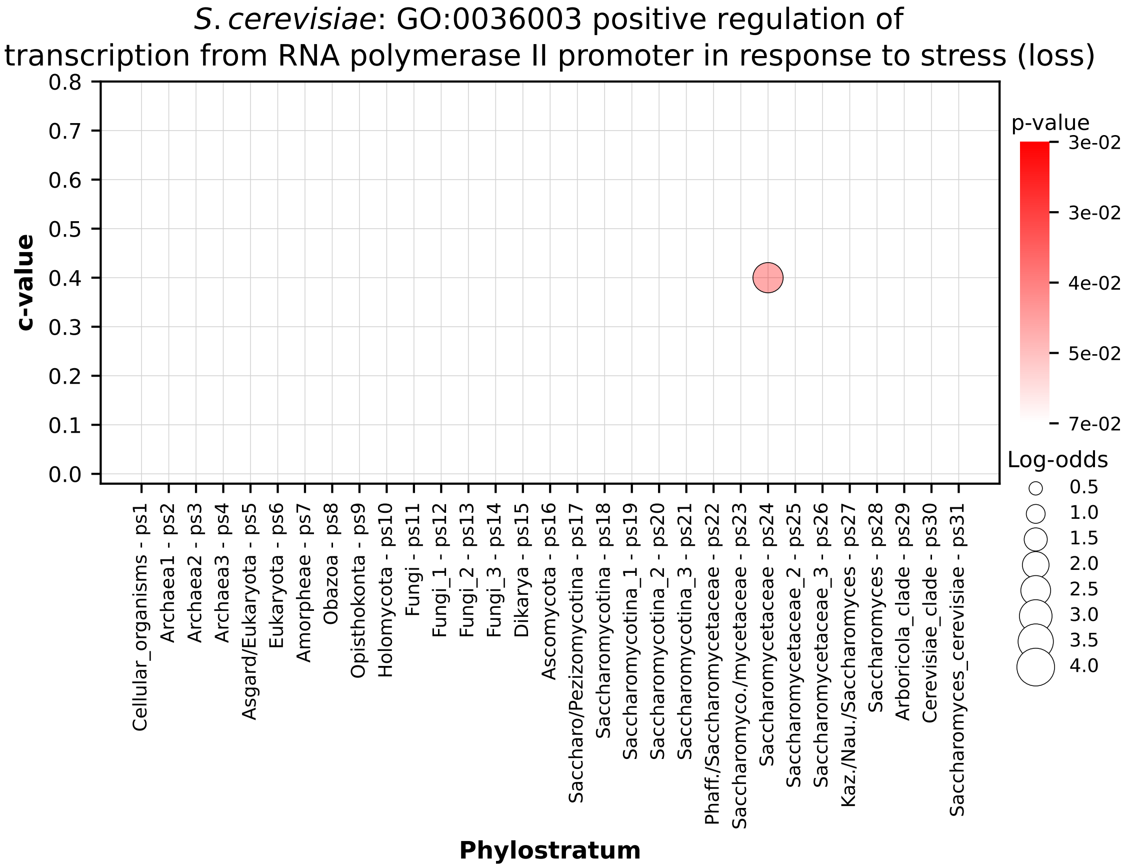

S. cerevisiae: GO:0036003 positive regulation of transcription from RNA polymerase II promoter in response to stress (loss)
c-value
0.0
0.1
0.2
0.3
0.4
0.5
0.6
0.7
0.8
Phylostratum
Cellular_organisms - ps1
Archaea1 - ps2
Archaea2 - ps3
Archaea3 - ps4
Asgard/Eukaryota - ps5
Eukaryota - ps6
Amorpheae - ps7
Obazoa - ps8
Opisthokonta - ps9
Holomycota - ps10
Fungi - ps11
Fungi_1 - ps12
Fungi_2 - ps13
Fungi_3 - ps14
Dikarya - ps15
Ascomycota - ps16
Saccharo/Pezizomycotina - ps17
Saccharomycotina - ps18
Saccharomycotina_1 - ps19
Saccharomycotina_2 - ps20
Saccharomycotina_3 - ps21
Phaff./Saccharomycetaceae - ps22
Saccharomyco./mycetaceae - ps23
Saccharomycetaceae - ps24
Saccharomycetaceae_2 - ps25
Saccharomycetaceae_3 - ps26
Kaz./Nau./Saccharomyces - ps27
Saccharomyces - ps28
Arboricola_clade - ps29
Cerevisiae_clade - ps30
Saccharomyces_cerevisiae - ps31
p-value
3e-02
3e-02
4e-02
5e-02
7e-02
Log-odds
0.5
1.0
1.5
2.0
2.5
3.0
3.5
4.0

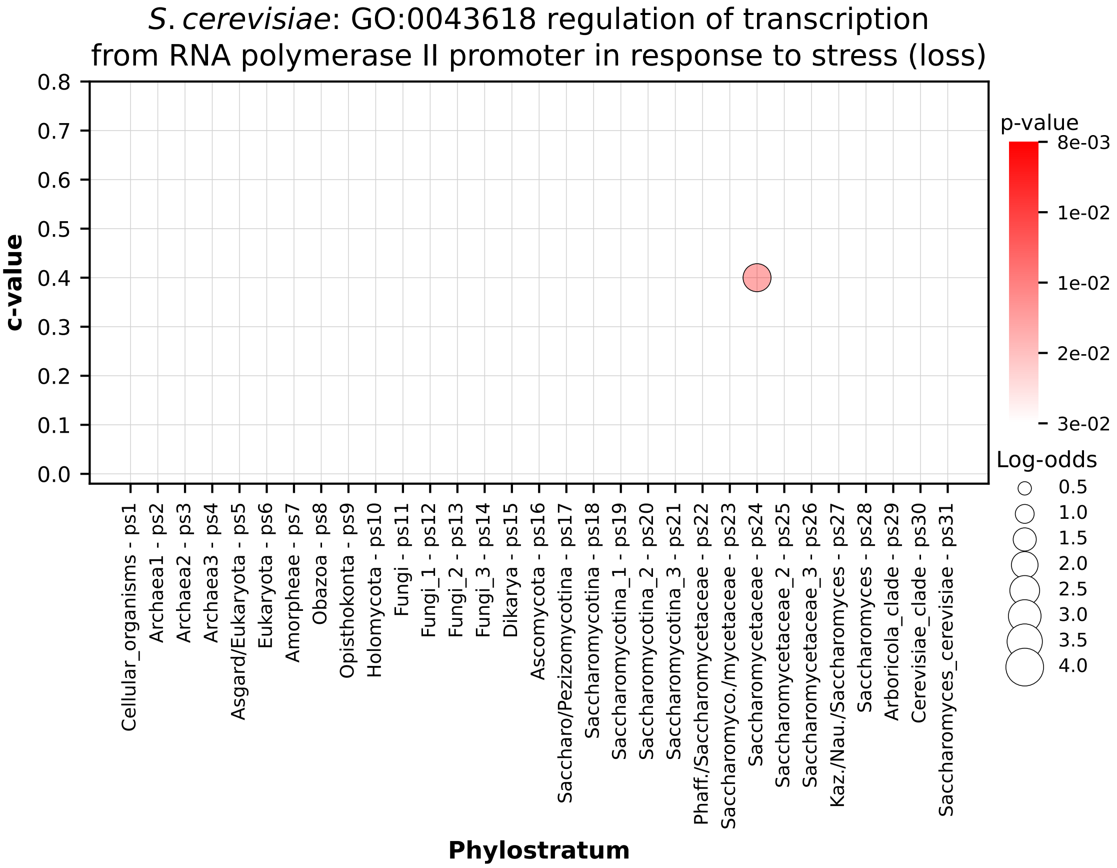
S. cerevisiae: GO:0043618 regulation of transcription from RNA polymerase II promoter in response to stress (loss)
c-value
0.8
0.7
0.6
0.5
0.4
0.3
0.2
0.1
0.0
p-value
8e-03
1e-02
1e-02
2e-02
3e-02
Log-odds
0.5
1.0
1.5
2.0
2.5
3.0
3.5
4.0
Cellular_organisms - ps1
Archaea1 - ps2
Archaea2 - ps3
Archaea3 - ps4
Asgard/Eukaryota - ps5
Eukaryota - ps6
Amorpheae - ps7
Obazoa - ps8
Opisthokonta - ps9
Holomycota - ps10
Fungi - ps11
Fungi_1 - ps12
Fungi_2 - ps13
Fungi_3 - ps14
Dikarya - ps15
Ascomycota - ps16
Saccharo/Pezizomycotina - ps17
Saccharomycotina - ps18
Saccharomycotina_1 - ps19
Saccharomycotina_2 - ps20
Saccharomycotina_3 - ps21
Phaff./Saccharomycetaceae - ps22
Saccharomyco./mycetaceae - ps23
Saccharomycetaceae - ps24
Saccharomycetaceae_2 - ps25
Saccharomycetaceae_3 - ps26
Kaz./Nau./Saccharomyces - ps27
Saccharomyces - ps28
Arboricola_clade - ps29
Cerevisiae_clade - ps30
Saccharomyces_cerevisiae - ps31
Phylostratum

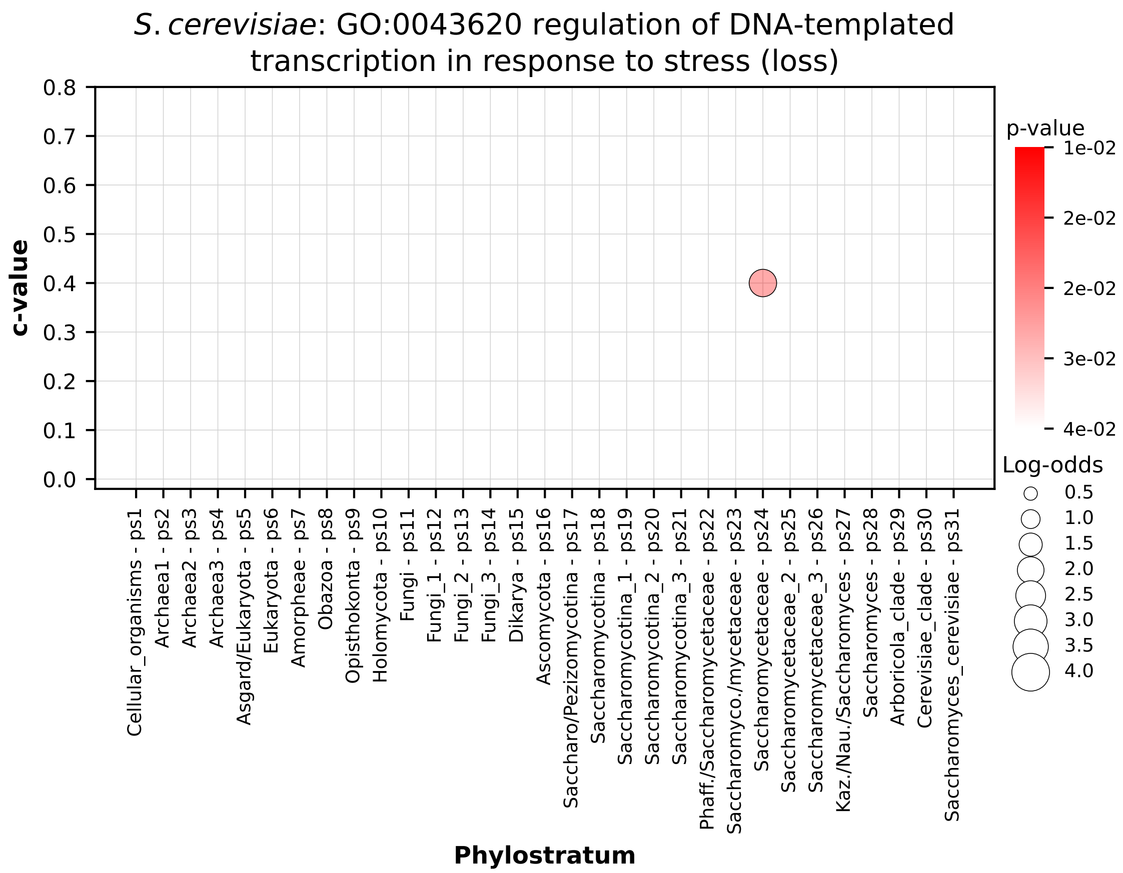
S. cerevisiae: GO:0043620 regulation of DNA-templated transcription in response to stress (loss)
c-value
0.8
0.7
0.6
0.5
0.4
0.3
0.2
0.1
0.0
p-value
1e-02
2e-02
2e-02
3e-02
4e-02
Log-odds
0.5
1.0
1.5
2.0
2.5
3.0
3.5
4.0
Cellular_organisms - ps1
Archaea1 - ps2
Archaea2 - ps3
Archaea3 - ps4
Asgard/Eukaryota - ps5
Eukaryota - ps6
Amorpheae - ps7
Obazoa - ps8
Opisthokonta - ps9
Holomycota - ps10
Fungi - ps11
Fungi_1 - ps12
Fungi_2 - ps13
Fungi_3 - ps14
Dikarya - ps15
Ascomycota - ps16
Saccharo/Pezizomycotina - ps17
Saccharomycotina - ps18
Saccharomycotina_1 - ps19
Saccharomycotina_2 - ps20
Saccharomycotina_3 - ps21
Phaff./Saccharomycetaceae - ps22
Saccharomyco./mycetaceae - ps23
Saccharomycetaceae - ps24
Saccharomycetaceae_2 - ps25
Saccharomycetaceae_3 - ps26
Kaz./Nau./Saccharomyces - ps27
Saccharomyces - ps28
Arboricola_clade - ps29
Cerevisiae_clade - ps30
Saccharomyces_cerevisiae - ps31
Phylostratum

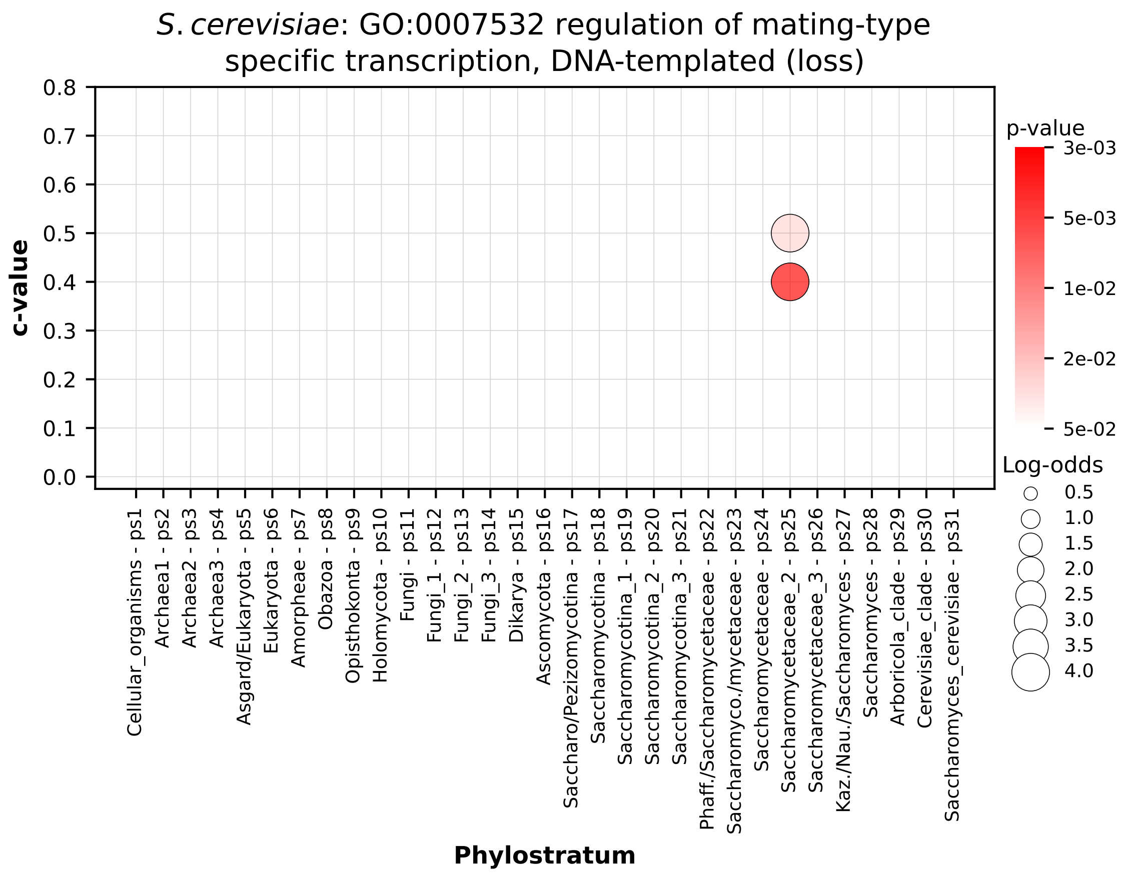

S. cerevisiae: GO:0007532 regulation of mating-type specific transcription, DNA-templated (loss)
c-value
0.8
0.7
0.6
0.5
0.4
0.3
0.2
0.1
0.0
p-value
3e-03
5e-03
1e-02
2e-02
5e-02
Log-odds
0.5
1.0
1.5
2.0
2.5
3.0
3.5
4.0
Cellular_organisms - ps1
Archaea1 - ps2
Archaea2 - ps3
Archaea3 - ps4
Asgard/Eukaryota - ps5
Eukaryota - ps6
Amorpheae - ps7
Obazoa - ps8
Opisthokonta - ps9
Holomycota - ps10
Fungi - ps11
Fungi_1 - ps12
Fungi_2 - ps13
Fungi_3 - ps14
Dikarya - ps15
Ascomycota - ps16
Saccharo/Pezizomycotina - ps17
Saccharomycotina - ps18
Saccharomycotina_1 - ps19
Saccharomycotina_2 - ps20
Saccharomycotina_3 - ps21
Phaff./Saccharomycetaceae - ps22
Saccharomyco./mycetaceae - ps23
Saccharomycetaceae - ps24
Saccharomycetaceae_2 - ps25
Saccharomycetaceae_3 - ps26
Kaz./Nau./Saccharomyces - ps27
Saccharomyces - ps28
Arboricola_clade - ps29
Cerevisiae_clade - ps30
Saccharomyces_cerevisiae - ps31
Phylostratum

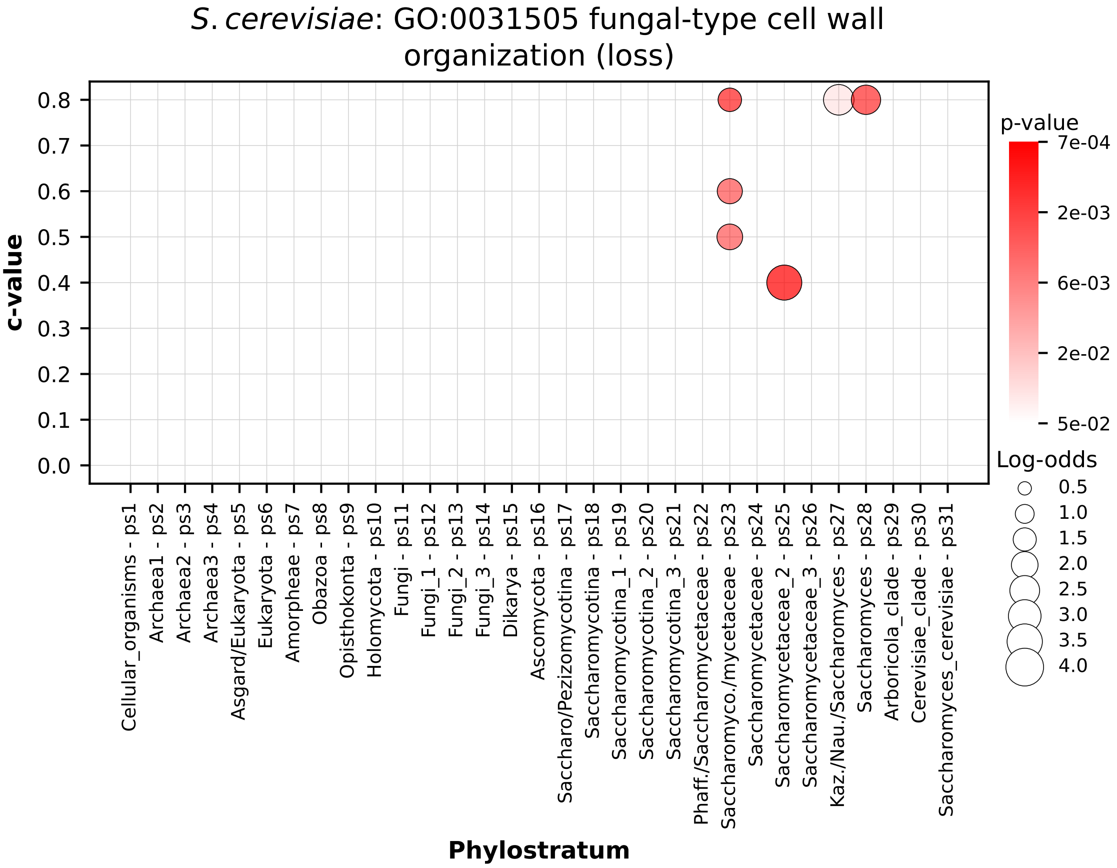
S. cerevisiae: GO:0031505 fungal-type cell wall organization (loss)
c-value
0.8
0.7
0.6
0.5
0.4
0.3
0.2
0.1
0.0
p-value
7e-04
2e-03
6e-03
2e-02
5e-02
Log-odds
0.5
1.0
1.5
2.0
2.5
3.0
3.5
4.0
Cellular_organisms - ps1
Archaea1 - ps2
Archaea2 - ps3
Archaea3 - ps4
Asgard/Eukaryota - ps5
Eukaryota - ps6
Amorpheae - ps7
Obazoa - ps8
Opisthokonta - ps9
Holomycota - ps10
Fungi - ps11
Fungi_1 - ps12
Fungi_2 - ps13
Fungi_3 - ps14
Dikarya - ps15
Ascomycota - ps16
Saccharo/Pezizomycotina - ps17
Saccharomycotina - ps18
Saccharomycotina_1 - ps19
Saccharomycotina_2 - ps20
Saccharomycotina_3 - ps21
Phaff./Saccharomycetaceae - ps22
Saccharomyco./mycetaceae - ps23
Saccharomycetaceae - ps24
Saccharomycetaceae_2 - ps25
Saccharomycetaceae_3 - ps26
Kaz./Nau./Saccharomyces - ps27
Saccharomyces - ps28
Arboricola_clade - ps29
Cerevisiae_clade - ps30
Saccharomyces_cerevisiae - ps31
Phylostratum

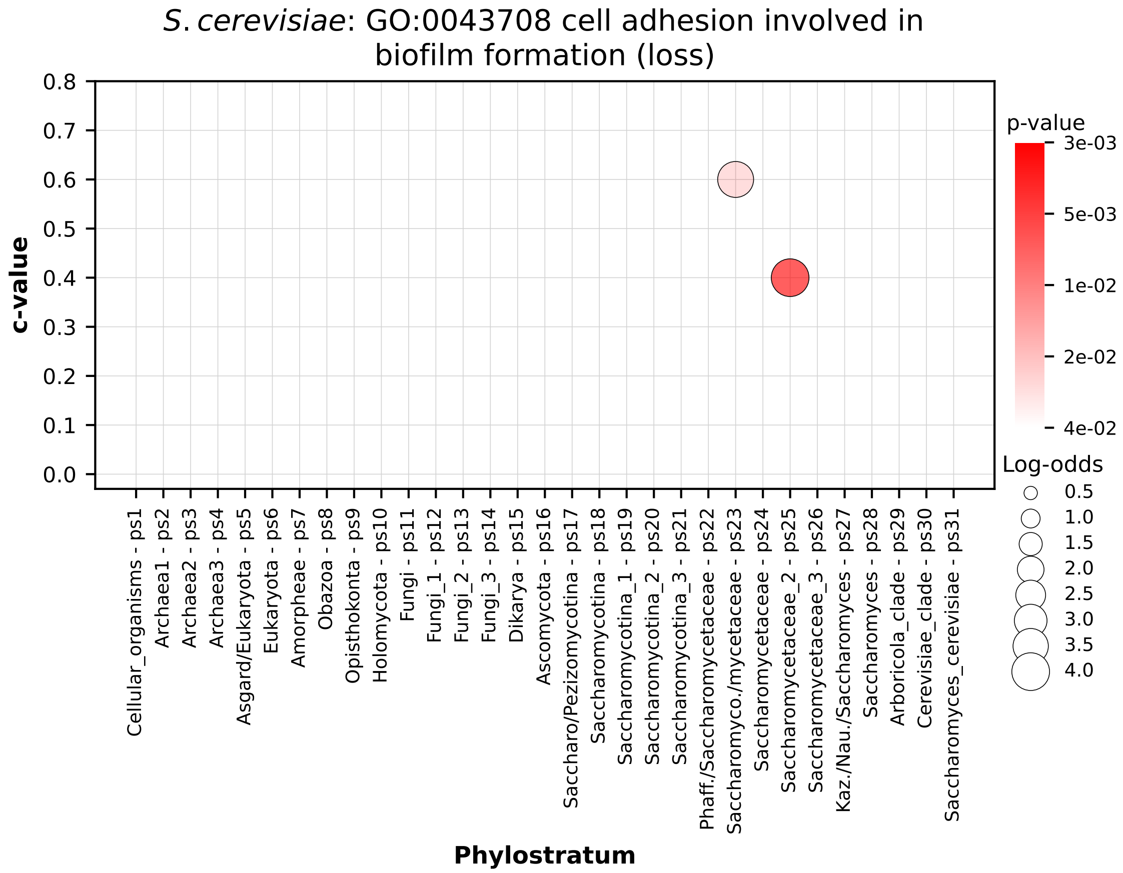

S. cerevisiae: GO:0043708 cell adhesion involved in biofilm formation (loss)
c-value
0.8
0.7
0.6
0.5
0.4
0.3
0.2
0.1
0.0
Phylostratum
Cellular_organisms - ps1
Archaea1 - ps2
Archaea2 - ps3
Archaea3 - ps4
Asgard/Eukaryota - ps5
Eukaryota - ps6
Amorpheae - ps7
Obazoa - ps8
Opisthokonta - ps9
Holomycota - ps10
Fungi - ps11
Fungi_1 - ps12
Fungi_2 - ps13
Fungi_3 - ps14
Dikarya - ps15
Ascomycota - ps16
Saccharo/Pezizomycotina - ps17
Saccharomycotina - ps18
Saccharomycotina_1 - ps19
Saccharomycotina_2 - ps20
Saccharomycotina_3 - ps21
Phaff./Saccharomycetaceae - ps22
Saccharomyco./mycetaceae - ps23
Saccharomycetaceae - ps24
Saccharomycetaceae_2 - ps25
Saccharomycetaceae_3 - ps26
Kaz./Nau./Saccharomyces - ps27
Saccharomyces - ps28
Arboricola_clade - ps29
Cerevisiae_clade - ps30
Saccharomyces_cerevisiae - ps31
p-value
3e-03
5e-03
1e-02
2e-02
4e-02
Log-odds
0.5
1.0
1.5
2.0
2.5
3.0
3.5
4.0

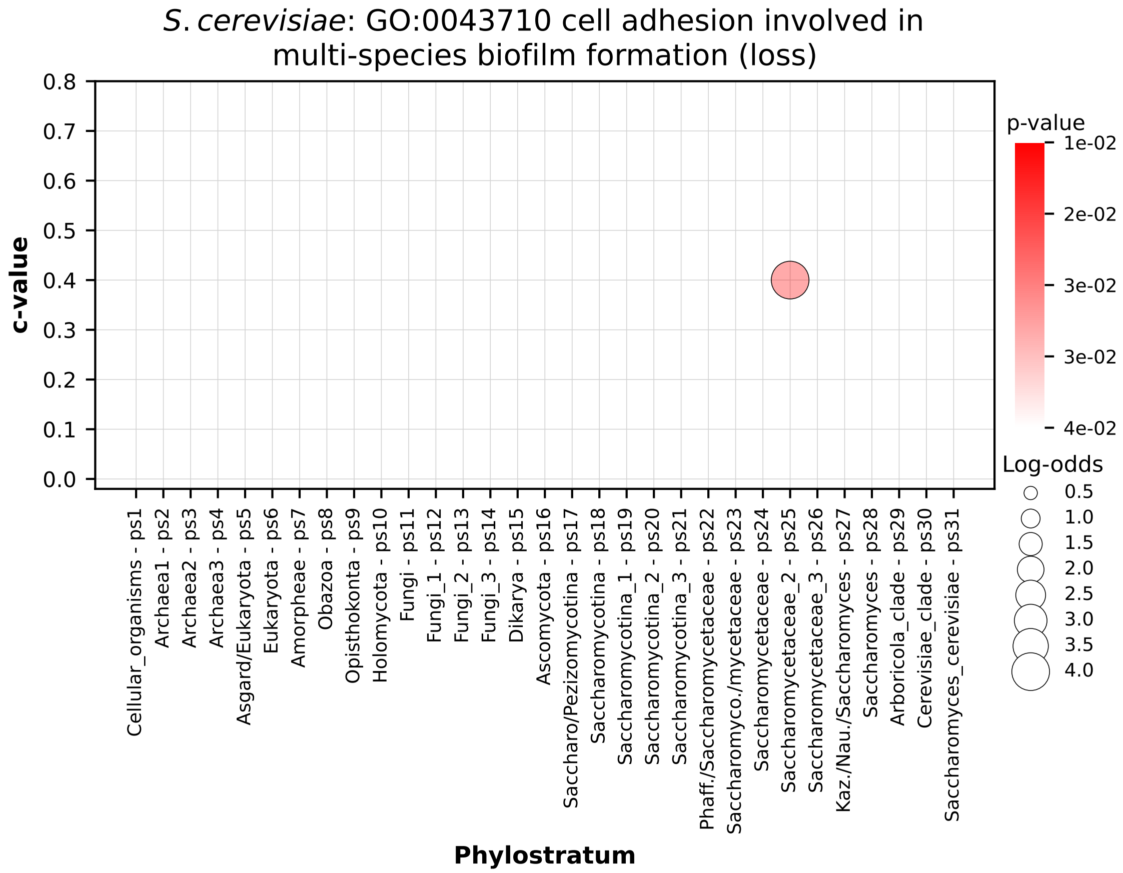

S. cerevisiae: GO:0043710 cell adhesion involved in multi-species biofilm formation (loss)
c-value
0.8
0.7
0.6
0.5
0.4
0.3
0.2
0.1
0.0
Phylostratum
Cellular_organisms - ps1
Archaea1 - ps2
Archaea2 - ps3
Archaea3 - ps4
Asgard/Eukaryota - ps5
Eukaryota - ps6
Amorpheae - ps7
Obazoa - ps8
Opisthokonta - ps9
Holomycota - ps10
Fungi - ps11
Fungi_1 - ps12
Fungi_2 - ps13
Fungi_3 - ps14
Dikarya - ps15
Ascomycota - ps16
Saccharo/Pezizomycotina - ps17
Saccharomycotina - ps18
Saccharomycotina_1 - ps19
Saccharomycotina_2 - ps20
Saccharomycotina_3 - ps21
Phaff./Saccharomycetaceae - ps22
Saccharomyco./mycetaceae - ps23
Saccharomycetaceae - ps24
Saccharomycetaceae_2 - ps25
Saccharomycetaceae_3 - ps26
Kaz./Nau./Saccharomyces - ps27
Saccharomyces - ps28
Arboricola_clade - ps29
Cerevisiae_clade - ps30
Saccharomyces_cerevisiae - ps31
p-value
1e-02
2e-02
3e-02
3e-02
4e-02
Log-odds
0.5
1.0
1.5
2.0
2.5
3.0
3.5
4.0

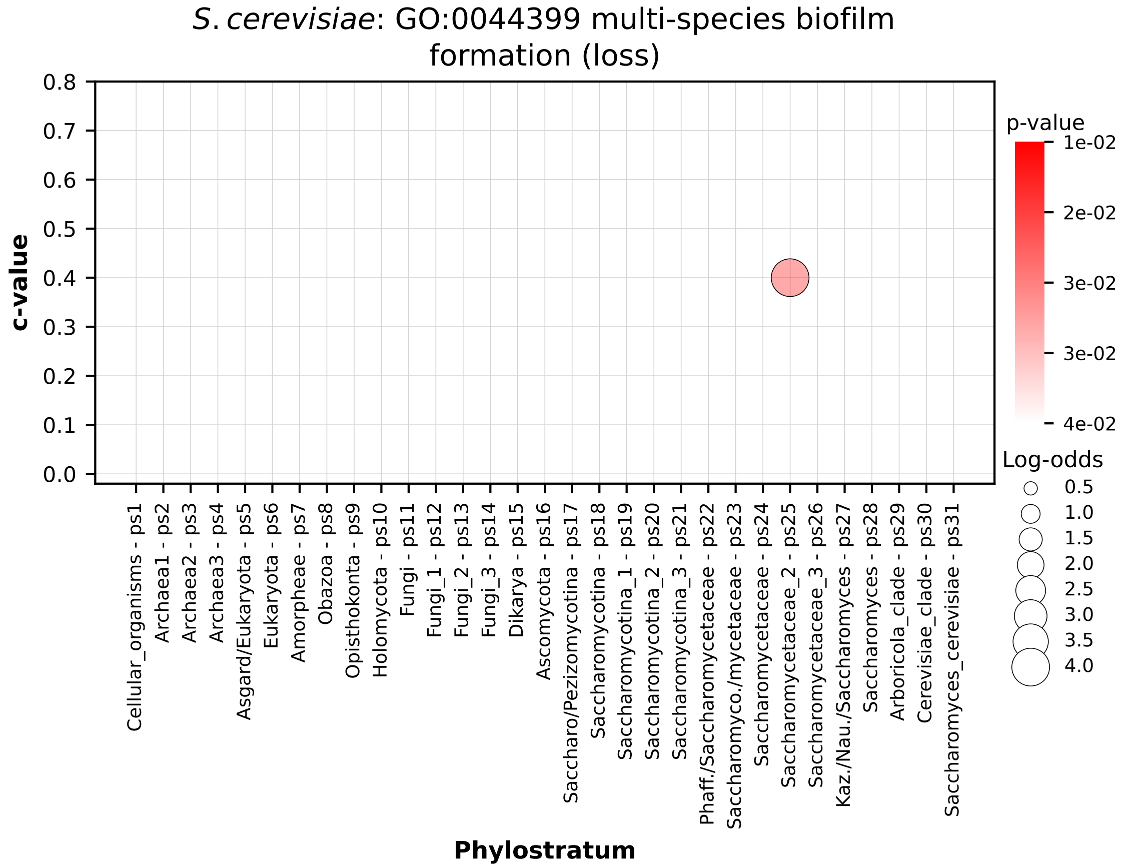

S. cerevisiae: GO:0044399 multi-species biofilm formation (loss)
c-value
0.0
0.1
0.2
0.3
0.4
0.5
0.6
0.7
0.8
Phylostratum
Cellular_organisms - ps1
Archaea1 - ps2
Archaea2 - ps3
Archaea3 - ps4
Asgard/Eukaryota - ps5
Eukaryota - ps6
Amorpheae - ps7
Obazoa - ps8
Opisthokonta - ps9
Holomycota - ps10
Fungi - ps11
Fungi_1 - ps12
Fungi_2 - ps13
Fungi_3 - ps14
Dikarya - ps15
Ascomycota - ps16
Saccharo/Pezizomycotina - ps17
Saccharomycotina - ps18
Saccharomycotina_1 - ps19
Saccharomycotina_2 - ps20
Saccharomycotina_3 - ps21
Phaff./Saccharomycetaceae - ps22
Saccharomyco./mycetaceae - ps23
Saccharomycetaceae - ps24
Saccharomycetaceae_2 - ps25
Saccharomycetaceae_3 - ps26
Kaz./Nau./Saccharomyces - ps27
Saccharomyces - ps28
Arboricola_clade - ps29
Cerevisiae_clade - ps30
Saccharomyces_cerevisiae - ps31
p-value
1e-02
2e-02
3e-02
3e-02
4e-02
Log-odds
0.5
1.0
1.5
2.0
2.5
3.0
3.5
4.0

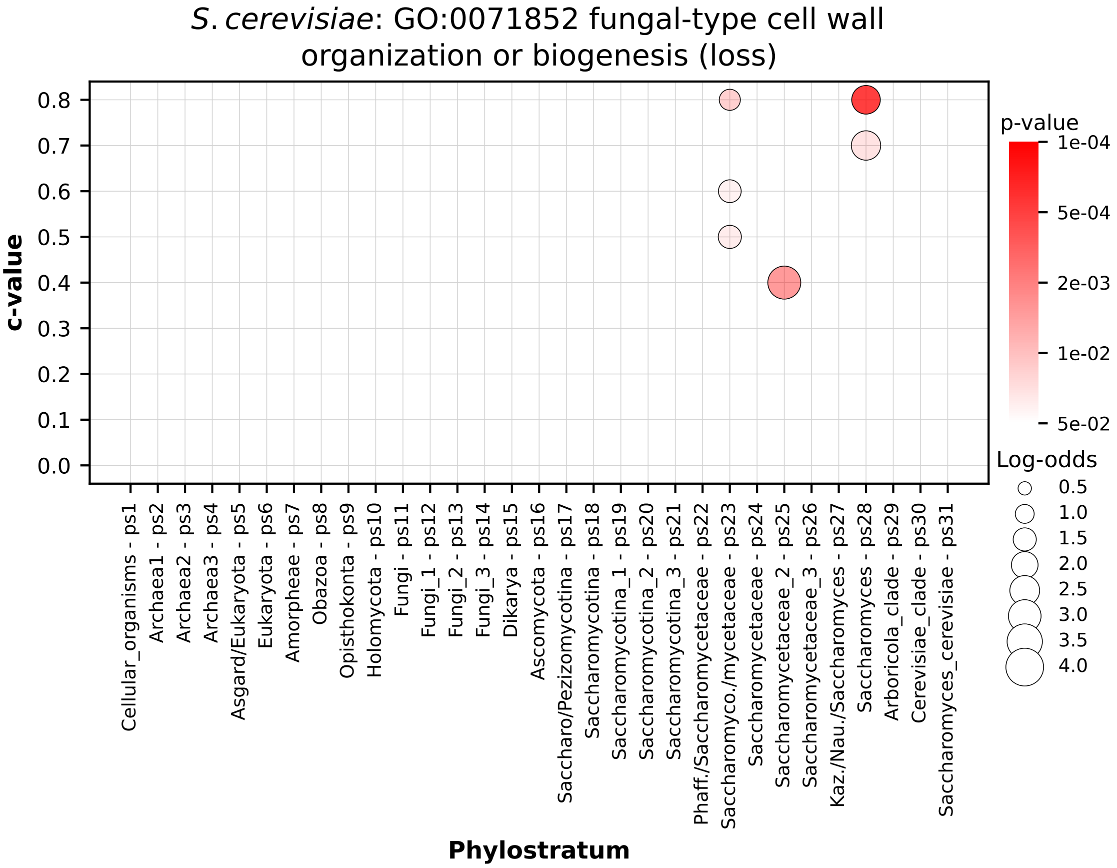

S. cerevisiae: GO:0071852 fungal-type cell wall organization or biogenesis (loss)
c-value
0.0
0.1
0.2
0.3
0.4
0.5
0.6
0.7
0.8
p-value
1e-04
5e-04
2e-03
1e-02
5e-02
Log-odds
0.5
1.0
1.5
2.0
2.5
3.0
3.5
4.0
Cellular_organisms - ps1
Archaea1 - ps2
Archaea2 - ps3
Archaea3 - ps4
Asgard/Eukaryota - ps5
Eukaryota - ps6
Amorpheae - ps7
Obazoa - ps8
Opisthokonta - ps9
Holomycota - ps10
Fungi - ps11
Fungi_1 - ps12
Fungi_2 - ps13
Fungi_3 - ps14
Dikarya - ps15
Ascomycota - ps16
Saccharo/Pezizomycotina - ps17
Saccharomycotina - ps18
Saccharomycotina_1 - ps19
Saccharomycotina_2 - ps20
Saccharomycotina_3 - ps21
Phaff./Saccharomycetaceae - ps22
Saccharomyco./mycetaceae - ps23
Saccharomycetaceae - ps24
Saccharomycetaceae_2 - ps25
Saccharomycetaceae_3 - ps26
Kaz./Nau./Saccharomyces - ps27
Saccharomyces - ps28
Arboricola_clade - ps29
Cerevisiae_clade - ps30
Saccharomyces_cerevisiae - ps31
Phylostratum

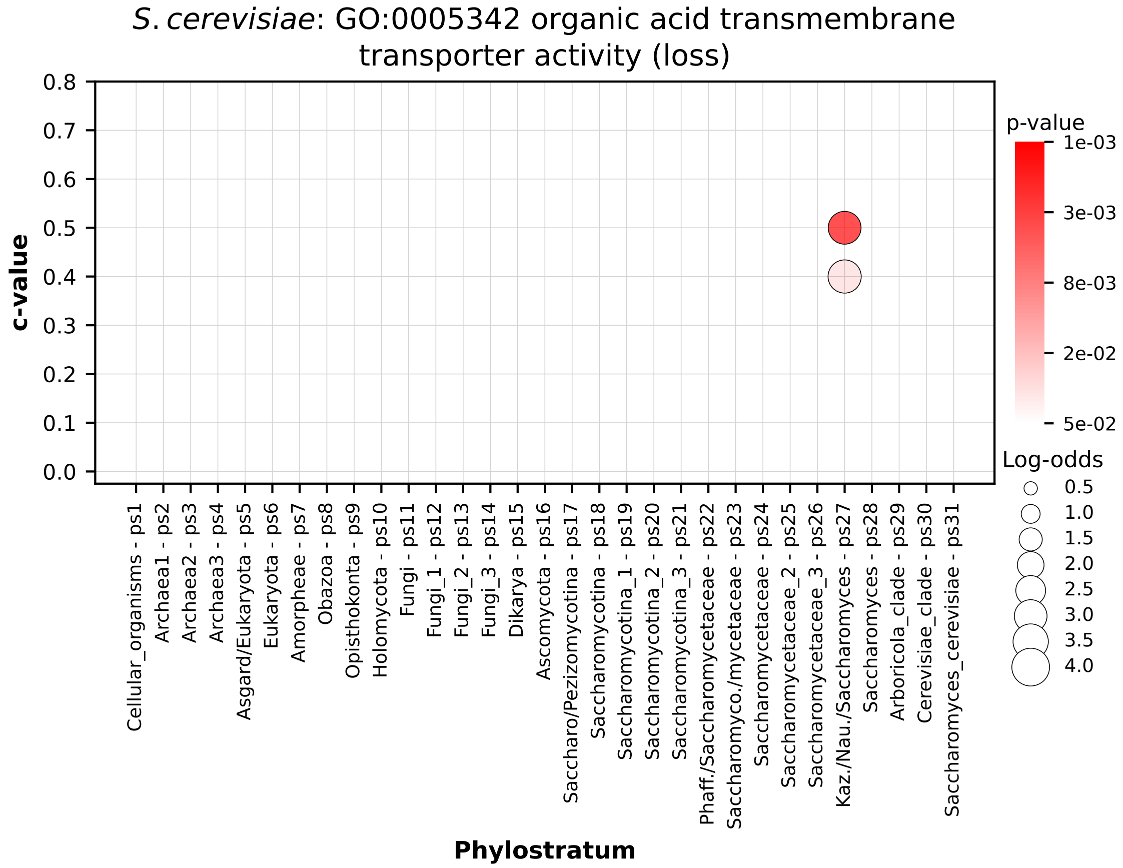

S. cerevisiae: GO:0005342 organic acid transmembrane transporter activity (loss)
c-value
0.8
0.7
0.6
0.5
0.4
0.3
0.2
0.1
0.0
Phylostratum
Cellular_organisms - ps1
Archaea1 - ps2
Archaea2 - ps3
Archaea3 - ps4
Asgard/Eukaryota - ps5
Eukaryota - ps6
Amorpheae - ps7
Obazoa - ps8
Opisthokonta - ps9
Holomycota - ps10
Fungi - ps11
Fungi_1 - ps12
Fungi_2 - ps13
Fungi_3 - ps14
Dikarya - ps15
Ascomycota - ps16
Saccharo/Pezizomycotina - ps17
Saccharomycotina - ps18
Saccharomycotina_1 - ps19
Saccharomycotina_2 - ps20
Saccharomycotina_3 - ps21
Phaff./Saccharomycetaceae - ps22
Saccharomyco./mycetaceae - ps23
Saccharomycetaceae - ps24
Saccharomycetaceae_2 - ps25
Saccharomycetaceae_3 - ps26
Kaz./Nau./Saccharomyces - ps27
Saccharomyces - ps28
Arboricola_clade - ps29
Cerevisiae_clade - ps30
Saccharomyces_cerevisiae - ps31
p-value
1e-03
3e-03
8e-03
2e-02
5e-02
Log-odds
0.5
1.0
1.5
2.0
2.5
3.0
3.5
4.0

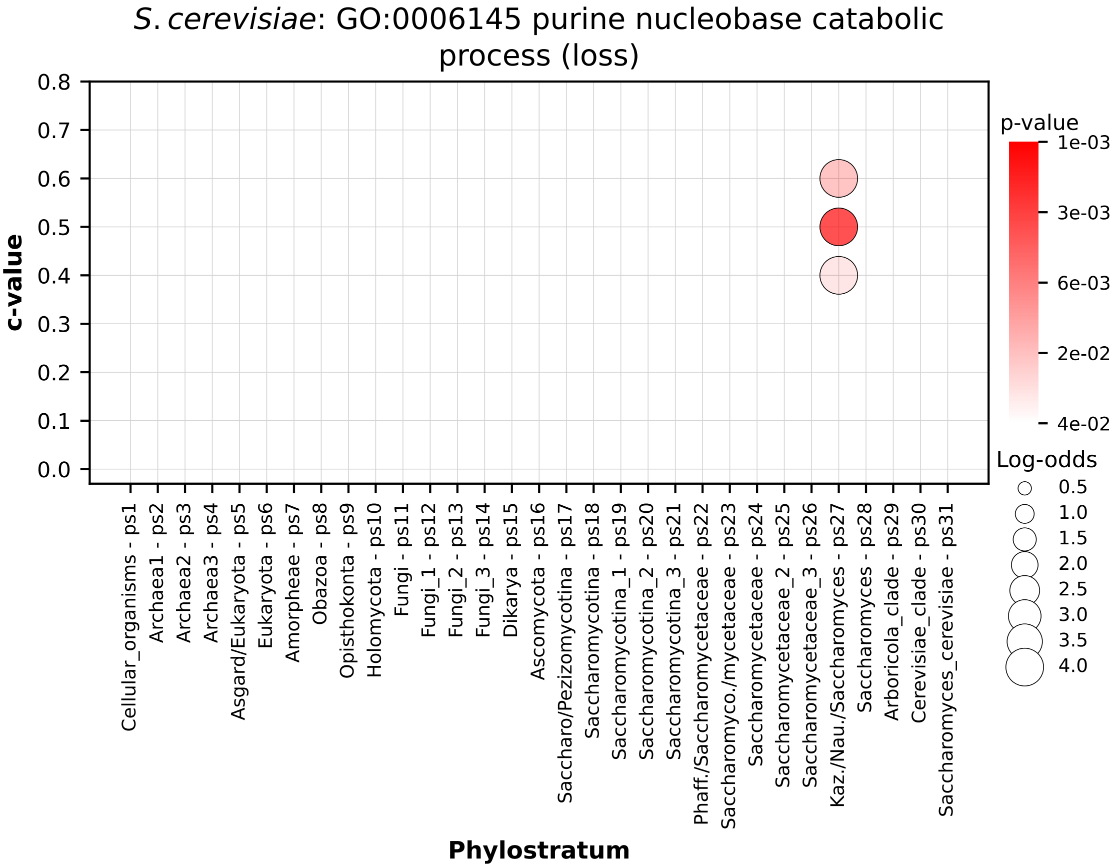

S. cerevisiae: GO:0006145 purine nucleobase catabolic process (loss)
c-value
0.8
0.7
0.6
0.5
0.4
0.3
0.2
0.1
0.0
p-value
1e-03
3e-03
6e-03
2e-02
4e-02
Log-odds
0.5
1.0
1.5
2.0
2.5
3.0
3.5
4.0
Cellular_organisms - ps1
Archaea1 - ps2
Archaea2 - ps3
Archaea3 - ps4
Asgard/Eukaryota - ps5
Eukaryota - ps6
Amorpheae - ps7
Obazoa - ps8
Opisthokonta - ps9
Holomycota - ps10
Fungi - ps11
Fungi_1 - ps12
Fungi_2 - ps13
Fungi_3 - ps14
Dikarya - ps15
Ascomycota - ps16
Saccharo/Pezizomycotina - ps17
Saccharomycotina - ps18
Saccharomycotina_1 - ps19
Saccharomycotina_2 - ps20
Saccharomycotina_3 - ps21
Phaff./Saccharomycetaceae - ps22
Saccharomyco./mycetaceae - ps23
Saccharomycetaceae - ps24
Saccharomycetaceae_2 - ps25
Saccharomycetaceae_3 - ps26
Kaz./Nau./Saccharomyces - ps27
Saccharomyces - ps28
Arboricola_clade - ps29
Cerevisiae_clade - ps30
Saccharomyces_cerevisiae - ps31
Phylostratum

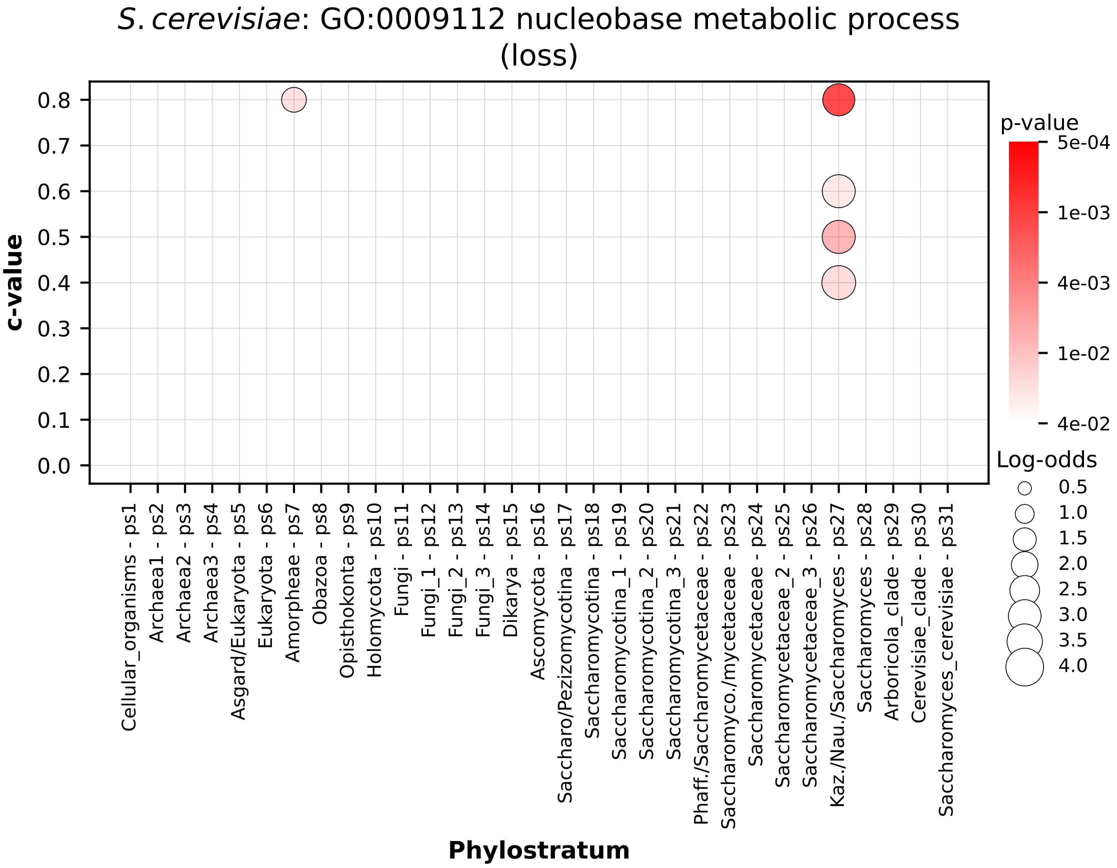

S. cerevisiae: GO:0009112 nucleobase metabolic process
(loss)
c-value
0.0
0.1
0.2
0.3
0.4
0.5
0.6
0.7
0.8
p-value
5e-04
1e-03
4e-03
1e-02
4e-02
Log-odds
0.5
1.0
1.5
2.0
2.5
3.0
3.5
4.0
Cellular_organisms - ps1
Archaea1 - ps2
Archaea2 - ps3
Archaea3 - ps4
Asgard/Eukaryota - ps5
Eukaryota - ps6
Amorpheae - ps7
Obazoa - ps8
Opisthokonta - ps9
Holomycota - ps10
Fungi - ps11
Fungi_1 - ps12
Fungi_2 - ps13
Fungi_3 - ps14
Dikarya - ps15
Ascomycota - ps16
Saccharo/Pezizomycotina - ps17
Saccharomycotina - ps18
Saccharomycotina_1 - ps19
Saccharomycotina_2 - ps20
Saccharomycotina_3 - ps21
Phaff./Saccharomycetaceae - ps22
Saccharomyco./mycetaceae - ps23
Saccharomycetaceae - ps24
Saccharomycetaceae_2 - ps25
Saccharomycetaceae_3 - ps26
Kaz./Nau./Saccharomyces - ps27
Saccharomyces - ps28
Arboricola_clade - ps29
Cerevisiae_clade - ps30
Saccharomyces_cerevisiae - ps31
Phylostratum

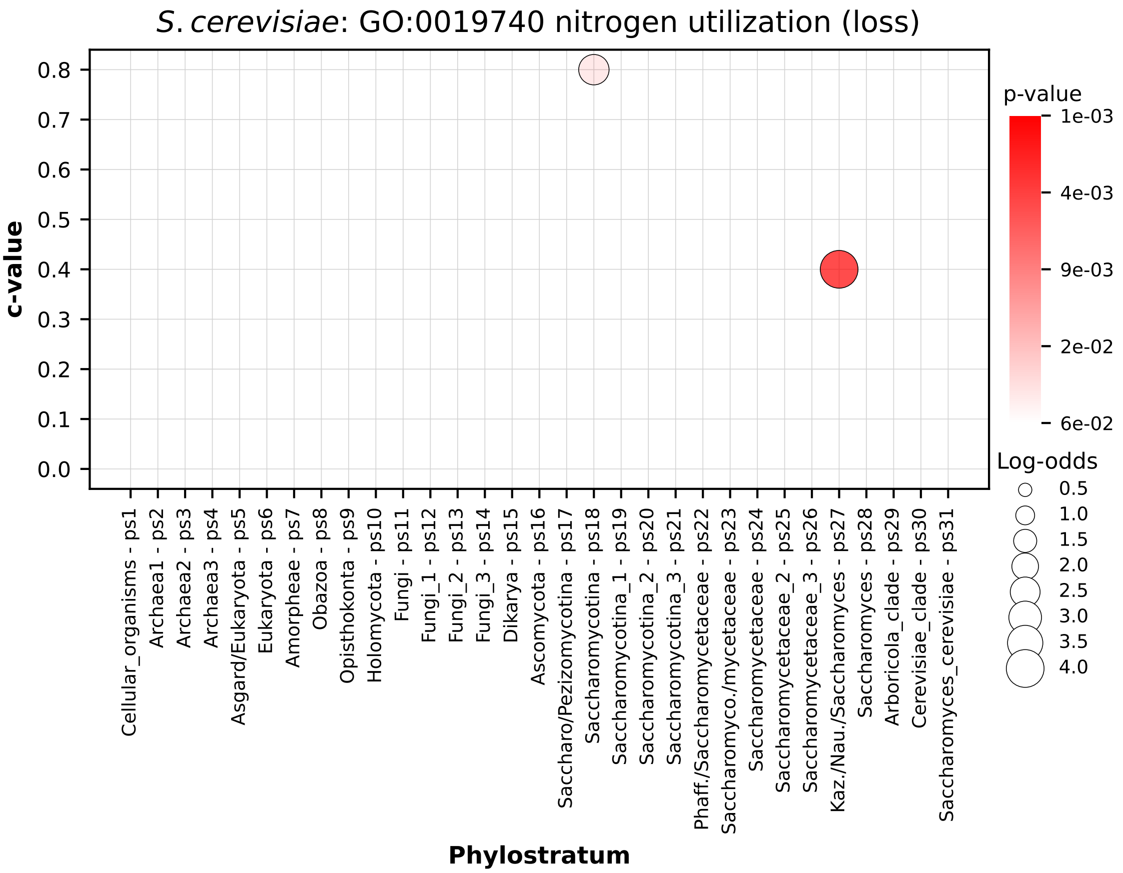

S. cerevisiae: GO:0019740 nitrogen utilization (loss)
c-value
0.0
0.1
0.2
0.3
0.4
0.5
0.6
0.7
0.8
Phylostratum
Cellular_organisms - ps1
Archaea1 - ps2
Archaea2 - ps3
Archaea3 - ps4
Asgard/Eukaryota - ps5
Eukaryota - ps6
Amorpheae - ps7
Obazoa - ps8
Opisthokonta - ps9
Holomycota - ps10
Fungi - ps11
Fungi_1 - ps12
Fungi_2 - ps13
Fungi_3 - ps14
Dikarya - ps15
Ascomycota - ps16
Saccharo/Pezizomycotina - ps17
Saccharomycotina - ps18
Saccharomycotina_1 - ps19
Saccharomycotina_2 - ps20
Saccharomycotina_3 - ps21
Phaff./Saccharomycetaceae - ps22
Saccharomyco./mycetaceae - ps23
Saccharomycetaceae - ps24
Saccharomycetaceae_2 - ps25
Saccharomycetaceae_3 - ps26
Kaz./Nau./Saccharomyces - ps27
Saccharomyces - ps28
Arboricola_clade - ps29
Cerevisiae_clade - ps30
Saccharomyces_cerevisiae - ps31
p-value
1e-03
4e-03
9e-03
2e-02
6e-02
Log-odds
0.5
1.0
1.5
2.0
2.5
3.0
3.5
4.0

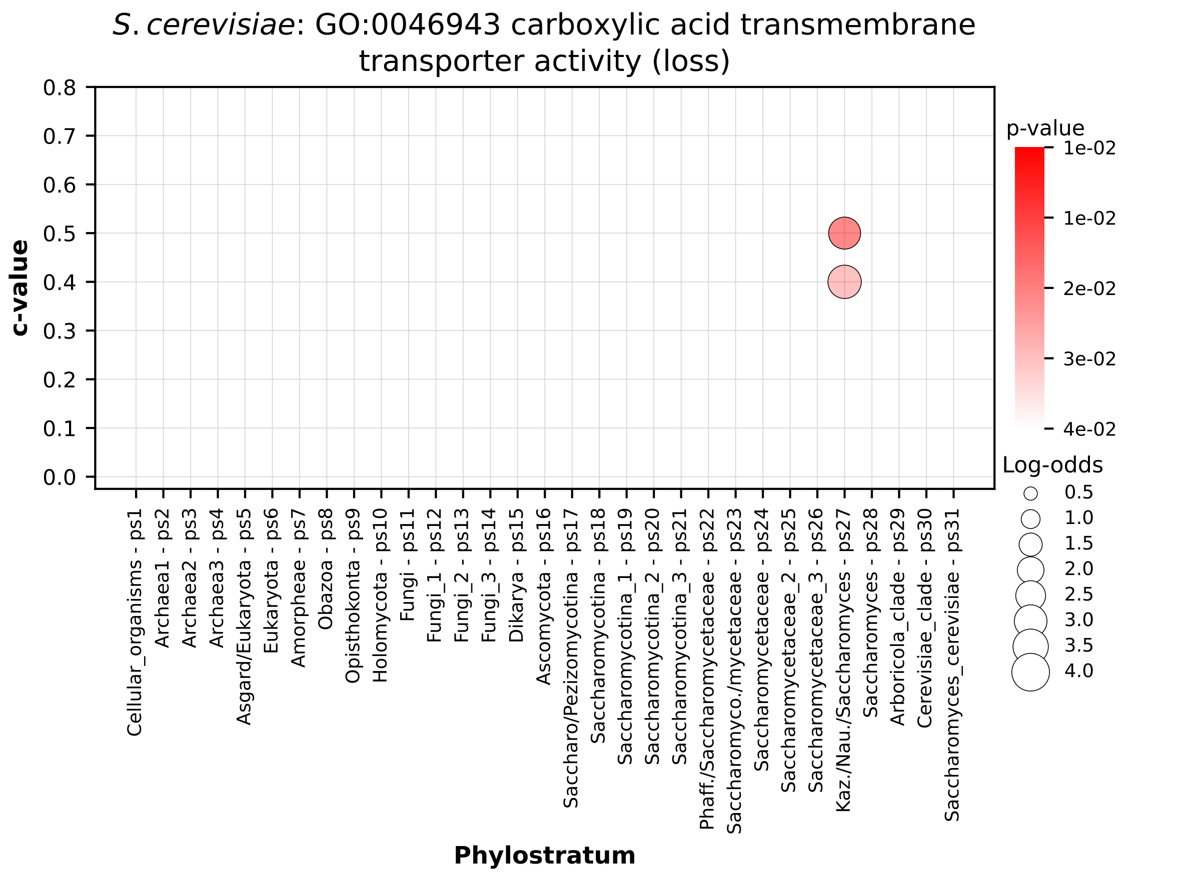
S. cerevisiae: GO:0046943 carboxylic acid transmembrane transporter activity (loss)
c-value
0.8
0.7
0.6
0.5
0.4
0.3
0.2
0.1
0.0
Phylostratum
Cellular_organisms - ps1
Archaea1 - ps2
Archaea2 - ps3
Archaea3 - ps4
Asgard/Eukaryota - ps5
Eukaryota - ps6
Amorpheae - ps7
Obazoa - ps8
Opisthokonta - ps9
Holomycota - ps10
Fungi - ps11
Fungi_1 - ps12
Fungi_2 - ps13
Fungi_3 - ps14
Dikarya - ps15
Ascomycota - ps16
Saccharo/Pezizomycotina - ps17
Saccharomycotina - ps18
Saccharomycotina_1 - ps19
Saccharomycotina_2 - ps20
Saccharomycotina_3 - ps21
Phaff./Saccharomycetaceae - ps22
Saccharomyco./mycetaceae - ps23
Saccharomycetaceae - ps24
Saccharomycetaceae_2 - ps25
Saccharomycetaceae_3 - ps26
Kaz./Nau./Saccharomyces - ps27
Saccharomyces - ps28
Arboricola_clade - ps29
Cerevisiae_clade - ps30
Saccharomyces_cerevisiae - ps31
p-value
1e-02
1e-02
2e-02
3e-02
4e-02
Log-odds
0.5
1.0
1.5
2.0
2.5
3.0
3.5
4.0

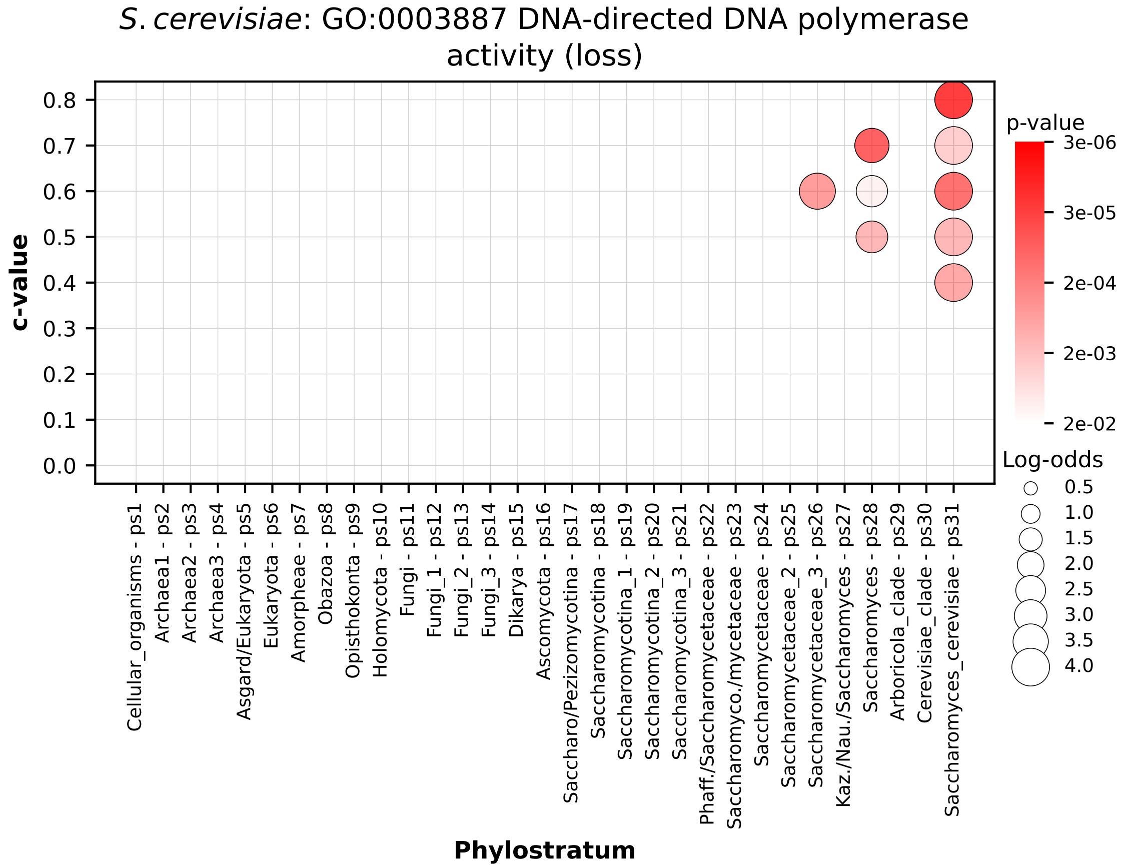

S. cerevisiae: GO:0003887 DNA-directed DNA polymerase activity (loss)
c-value
0.8
0.7
0.6
0.5
0.4
0.3
0.2
0.1
0.0
p-value
3e-06
3e-05
2e-04
2e-03
2e-02
Log-odds
0.5
1.0
1.5
2.0
2.5
3.0
3.5
4.0
Cellular_organisms - ps1
Archaea1 - ps2
Archaea2 - ps3
Archaea3 - ps4
Asgard/Eukaryota - ps5
Eukaryota - ps6
Amorpheae - ps7
Obazoa - ps8
Opisthokonta - ps9
Holomycota - ps10
Fungi - ps11
Fungi_1 - ps12
Fungi_2 - ps13
Fungi_3 - ps14
Dikarya - ps15
Ascomycota - ps16
Saccharo/Pezizomycotina - ps17
Saccharomycotina - ps18
Saccharomycotina_1 - ps19
Saccharomycotina_2 - ps20
Saccharomycotina_3 - ps21
Phaff./Saccharomycetaceae - ps22
Saccharomyco./mycetaceae - ps23
Saccharomycetaceae - ps24
Saccharomycetaceae_2 - ps25
Saccharomycetaceae_3 - ps26
Kaz./Nau./Saccharomyces - ps27
Saccharomyces - ps28
Arboricola_clade - ps29
Cerevisiae_clade - ps30
Saccharomyces_cerevisiae - ps31
Phylostratum

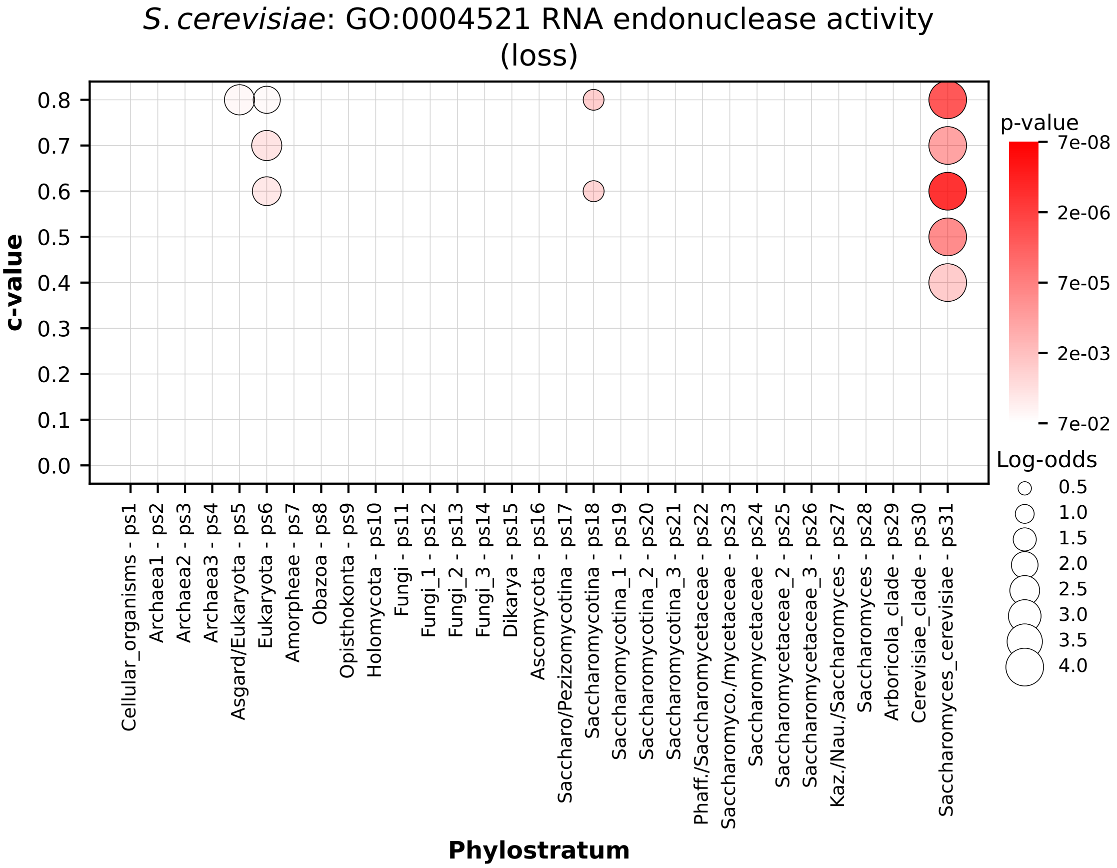

$S. cerevisiae$: GO:0004521 RNA endonuclease activity
(loss)
c-value
0.0
0.1
0.2
0.3
0.4
0.5
0.6
0.7
0.8
Phylostratum
Cellular_organisms - ps1
Archaea1 - ps2
Archaea2 - ps3
Archaea3 - ps4
Asgard/Eukaryota - ps5
Eukaryota - ps6
Amorpheae - ps7
Obazoa - ps8
Opisthokonta - ps9
Holomycota - ps10
Fungi - ps11
Fungi_1 - ps12
Fungi_2 - ps13
Fungi_3 - ps14
Dikarya - ps15
Ascomycota - ps16
Saccharo/Pezizomycotina - ps17
Saccharomycotina - ps18
Saccharomycotina_1 - ps19
Saccharomycotina_2 - ps20
Saccharomycotina_3 - ps21
Phaff./Saccharomycetaceae - ps22
Saccharomyco./mycetaceae - ps23
Saccharomycetaceae - ps24
Saccharomycetaceae_2 - ps25
Saccharomycetaceae_3 - ps26
Kaz./Nau./Saccharomyces - ps27
Saccharomyces - ps28
Arboricola_clade - ps29
Cerevisiae_clade - ps30
Saccharomyces_cerevisiae - ps31
p-value
7e-08
2e-06
7e-05
2e-03
7e-02
Log-odds
0.5
1.0
1.5
2.0
2.5
3.0
3.5
4.0

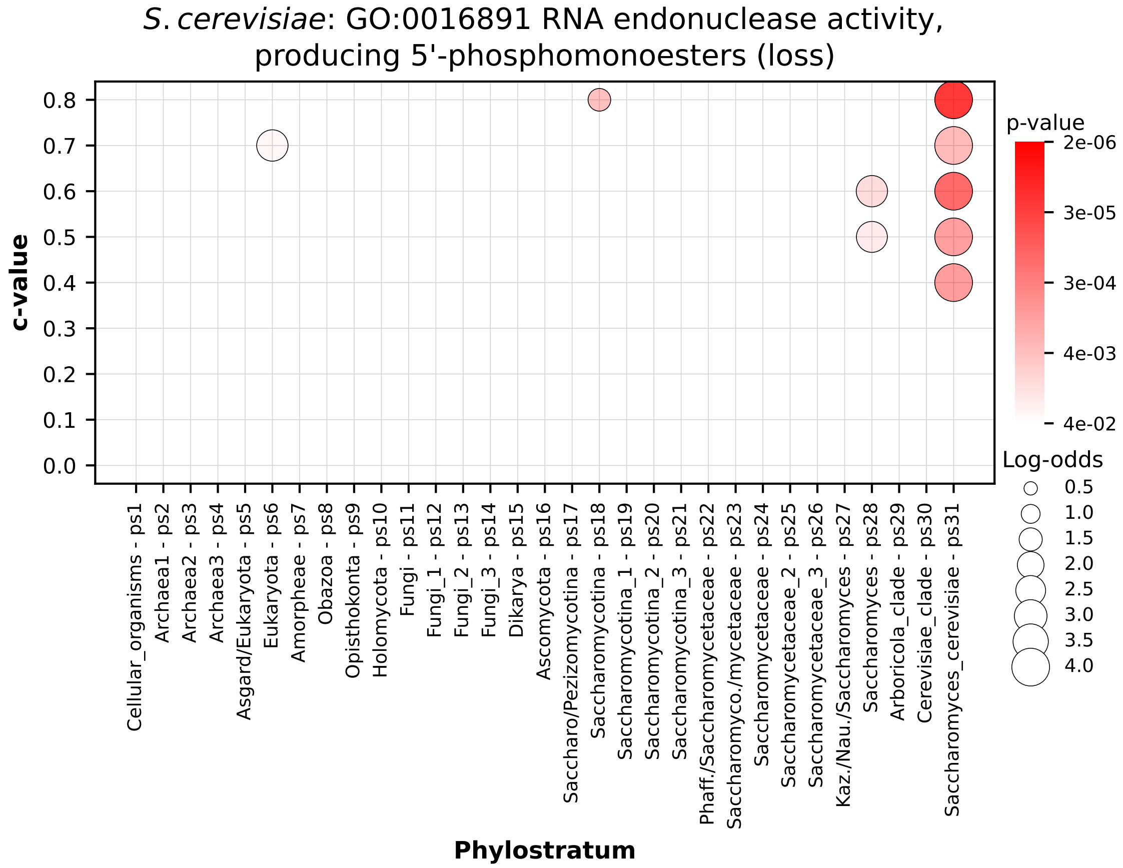

S. cerevisiae: GO:0016891 RNA endonuclease activity, producing 5'-phosphomonoesters (loss)
c-value
0.8
0.7
0.6
0.5
0.4
0.3
0.2
0.1
0.0
p-value
2e-06
3e-05
3e-04
4e-03
4e-02
Log-odds
0.5
1.0
1.5
2.0
2.5
3.0
3.5
4.0
Cellular_organisms - ps1
Archaea1 - ps2
Archaea2 - ps3
Archaea3 - ps4
Asgard/Eukaryota - ps5
Eukaryota - ps6
Amorpheae - ps7
Obazoa - ps8
Opisthokonta - ps9
Holomycota - ps10
Fungi - ps11
Fungi_1 - ps12
Fungi_2 - ps13
Fungi_3 - ps14
Dikarya - ps15
Ascomycota - ps16
Saccharo/Pezizomycotina - ps17
Saccharomycotina - ps18
Saccharomycotina_1 - ps19
Saccharomycotina_2 - ps20
Saccharomycotina_3 - ps21
Phaff./Saccharomycetaceae - ps22
Saccharomyco./mycetaceae - ps23
Saccharomycetaceae - ps24
Saccharomycetaceae_2 - ps25
Saccharomycetaceae_3 - ps26
Kaz./Nau./Saccharomyces - ps27
Saccharomyces - ps28
Arboricola_clade - ps29
Cerevisiae_clade - ps30
Saccharomyces_cerevisiae - ps31
Phylostratum

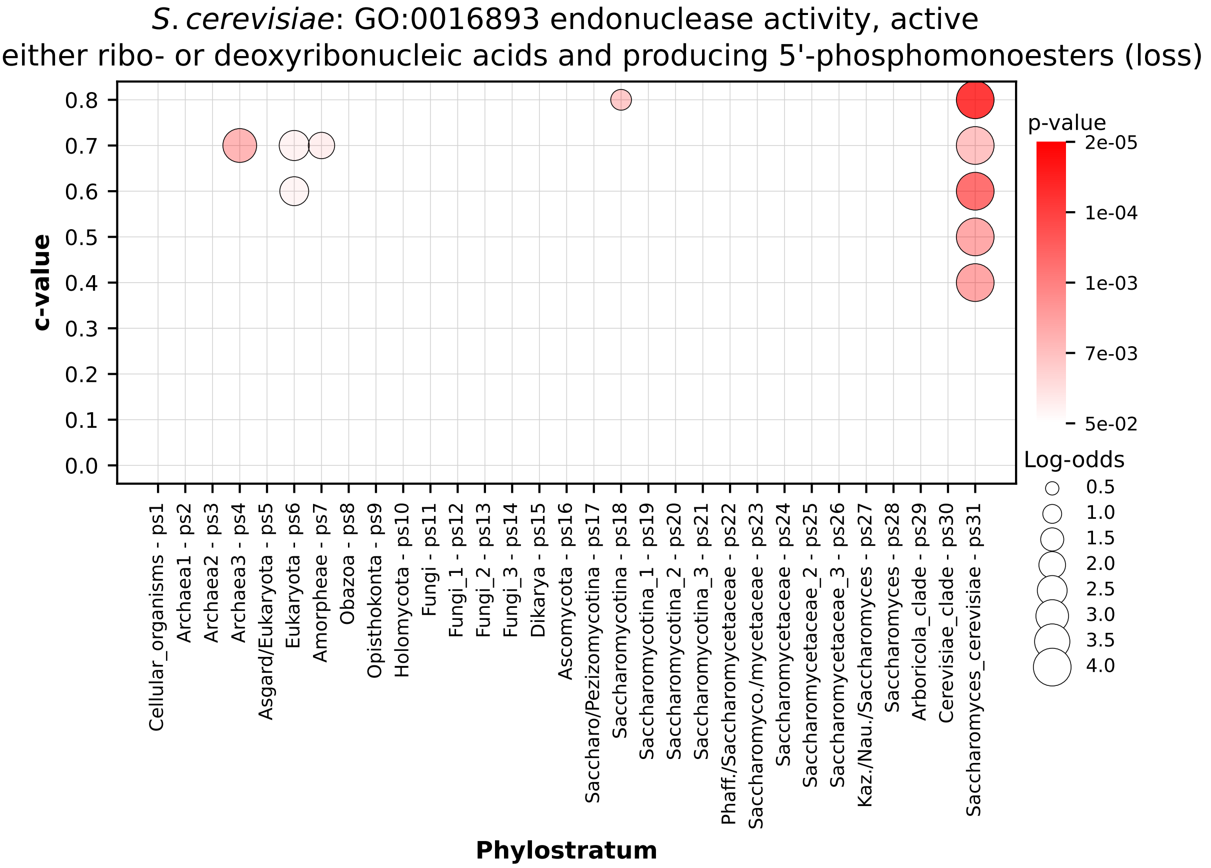

S. cerevisiae: GO:0016893 endonuclease activity, active
either ribo- or deoxyribonucleic acids and producing 5'-phosphomonoesters (loss)
c-value
0.8
0.7
0.6
0.5
0.4
0.3
0.2
0.1
0.0
p-value
2e-05
1e-04
1e-03
7e-03
5e-02
Log-odds
0.5
1.0
1.5
2.0
2.5
3.0
3.5
4.0
Cellular_organisms - ps1
Archaea1 - ps2
Archaea2 - ps3
Archaea3 - ps4
Asgard/Eukaryota - ps5
Eukaryota - ps6
Amorpheae - ps7
Obazoa - ps8
Opisthokonta - ps9
Holomycota - ps10
Fungi - ps11
Fungi_1 - ps12
Fungi_2 - ps13
Fungi_3 - ps14
Dikarya - ps15
Ascomycota - ps16
Saccharo/Pezizomycotina - ps17
Saccharomycotina - ps18
Saccharomycotina_1 - ps19
Saccharomycotina_2 - ps20
Saccharomycotina_3 - ps21
Phaff./Saccharomycetaceae - ps22
Saccharomyco./mycetaceae - ps23
Saccharomycetaceae - ps24
Saccharomycetaceae_2 - ps25
Saccharomycetaceae_3 - ps26
Kaz./Nau./Saccharomyces - ps27
Saccharomyces - ps28
Arboricola_clade - ps29
Cerevisiae_clade - ps30
Saccharomyces_cerevisiae - ps31
Phylostratum

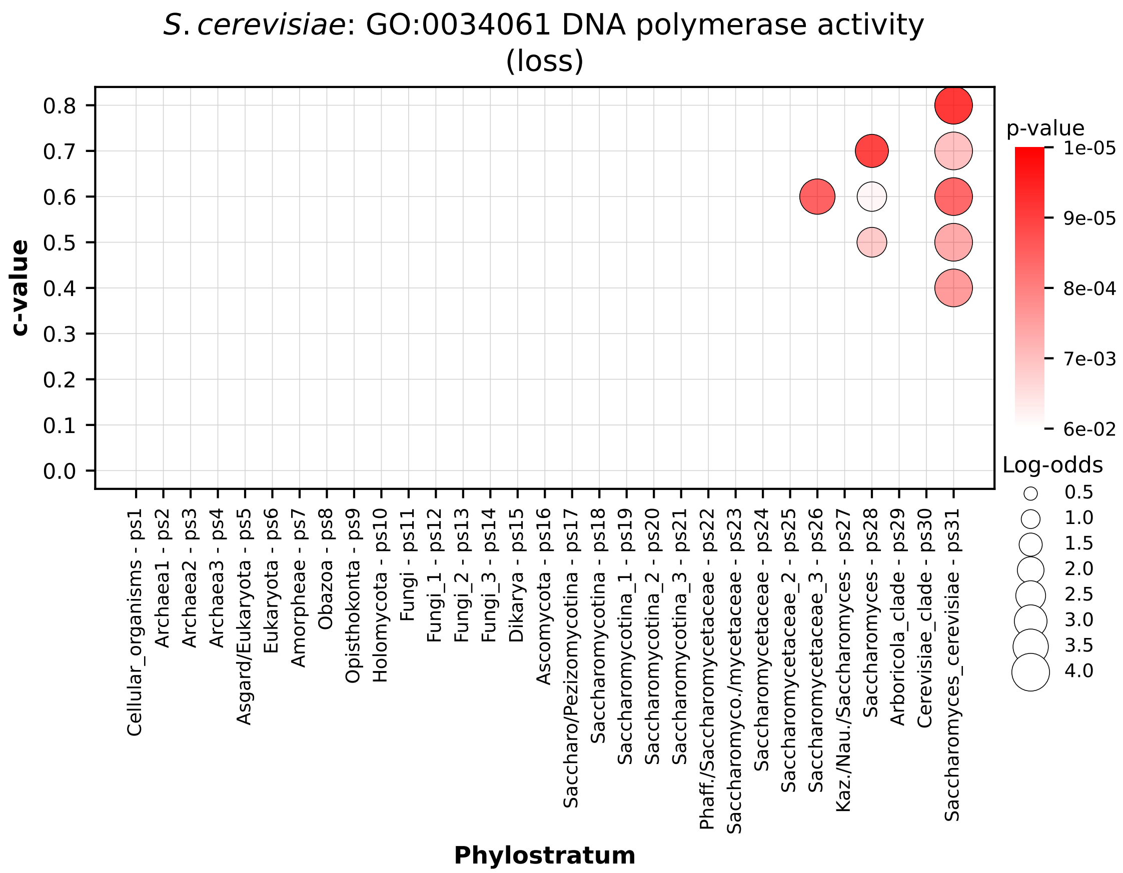

S. cerevisiae: GO:0034061 DNA polymerase activity
(loss)
c-value
0.8
0.7
0.6
0.5
0.4
0.3
0.2
0.1
0.0
p-value
1e-05
9e-05
8e-04
7e-03
6e-02
Log-odds
0.5
1.0
1.5
2.0
2.5
3.0
3.5
4.0
Cellular_organisms - ps1
Archaea1 - ps2
Archaea2 - ps3
Archaea3 - ps4
Asgard/Eukaryota - ps5
Eukaryota - ps6
Amorpheae - ps7
Obazoa - ps8
Opisthokonta - ps9
Holomycota - ps10
Fungi - ps11
Fungi_1 - ps12
Fungi_2 - ps13
Fungi_3 - ps14
Dikarya - ps15
Ascomycota - ps16
Saccharo/Pezizomycotina - ps17
Saccharomycotina - ps18
Saccharomycotina_1 - ps19
Saccharomycotina_2 - ps20
Saccharomycotina_3 - ps21
Phaff./Saccharomycetaceae - ps22
Saccharomyco./mycetaceae - ps23
Saccharomycetaceae - ps24
Saccharomycetaceae_2 - ps25
Saccharomycetaceae_3 - ps26
Kaz./Nau./Saccharomyces - ps27
Saccharomyces - ps28
Arboricola_clade - ps29
Cerevisiae_clade - ps30
Saccharomyces_cerevisiae - ps31
Phylostratum

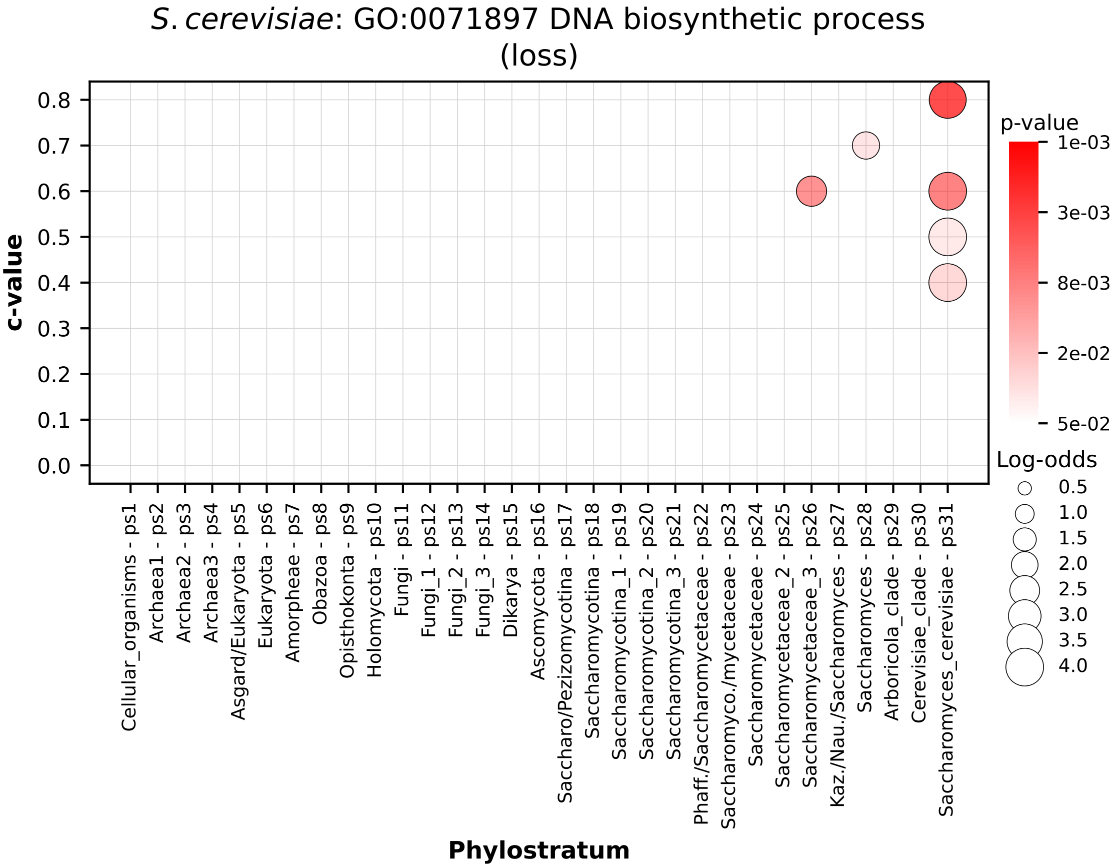
S. cerevisiae: GO:0071897 DNA biosynthetic process
(loss)
c-value
0.8
0.7
0.6
0.5
0.4
0.3
0.2
0.1
0.0
p-value
1e-03
3e-03
8e-03
2e-02
5e-02
Log-odds
0.5
1.0
1.5
2.0
2.5
3.0
3.5
4.0
Cellular_organisms - ps1
Archaea1 - ps2
Archaea2 - ps3
Archaea3 - ps4
Asgard/Eukaryota - ps5
Eukaryota - ps6
Amorpheae - ps7
Obazoa - ps8
Opisthokonta - ps9
Holomycota - ps10
Fungi - ps11
Fungi_1 - ps12
Fungi_2 - ps13
Fungi_3 - ps14
Dikarya - ps15
Ascomycota - ps16
Saccharo/Pezizomycotina - ps17
Saccharomycotina - ps18
Saccharomycotina_1 - ps19
Saccharomycotina_2 - ps20
Saccharomycotina_3 - ps21
Phaff./Saccharomycetaceae - ps22
Saccharomyco./mycetaceae - ps23
Saccharomycetaceae - ps24
Saccharomycetaceae_2 - ps25
Saccharomycetaceae_3 - ps26
Kaz./Nau./Saccharomyces - ps27
Saccharomyces - ps28
Arboricola_clade - ps29
Cerevisiae_clade - ps30
Saccharomyces_cerevisiae - ps31
Phylostratum

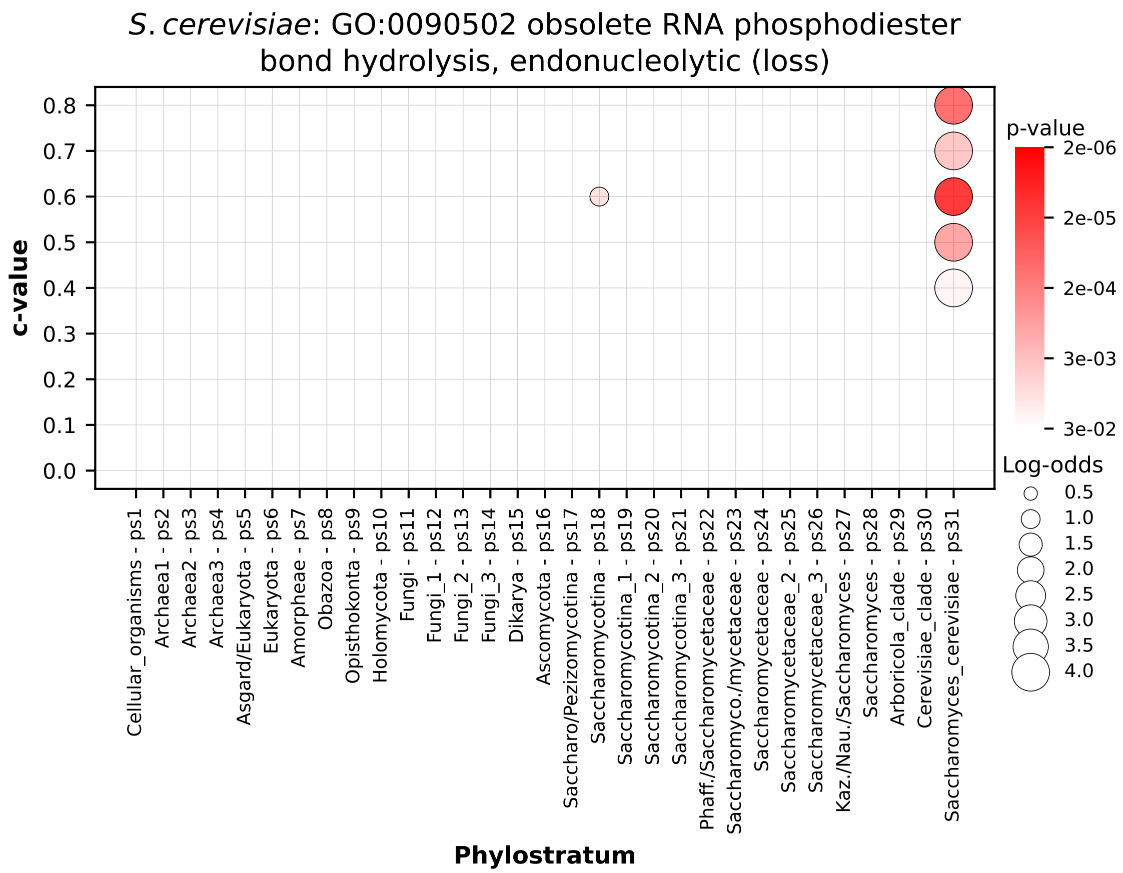

S. cerevisiae: GO:0090502 obsolete RNA phosphodiester bond hydrolysis, endonucleolytic (loss)
c-value
Phylostratum
p-value
2e-06
2e-05
2e-04
3e-03
3e-02
Log-odds
0.5
1.0
1.5
2.0
2.5
3.0
3.5
4.0
Cellular_organisms - ps1
Archaea1 - ps2
Archaea2 - ps3
Archaea3 - ps4
Asgard/Eukaryota - ps5
Eukaryota - ps6
Amorpheae - ps7
Obazoa - ps8
Opisthokonta - ps9
Holomycota - ps10
Fungi - ps11
Fungi_1 - ps12
Fungi_2 - ps13
Fungi_3 - ps14
Dikarya - ps15
Ascomycota - ps16
Saccharo/Pezizomycotina - ps17
Saccharomycotina - ps18
Saccharomycotina_1 - ps19
Saccharomycotina_2 - ps20
Saccharomycotina_3 - ps21
Phaff./Saccharomycetaceae - ps22
Saccharomyco./mycetaceae - ps23
Saccharomycetaceae - ps24
Saccharomycetaceae_2 - ps25
Saccharomycetaceae_3 - ps26
Kaz./Nau./Saccharomyces - ps27
Saccharomyces - ps28
Arboricola_clade - ps29
Cerevisiae_clade - ps30
Saccharomyces_cerevisiae - ps31

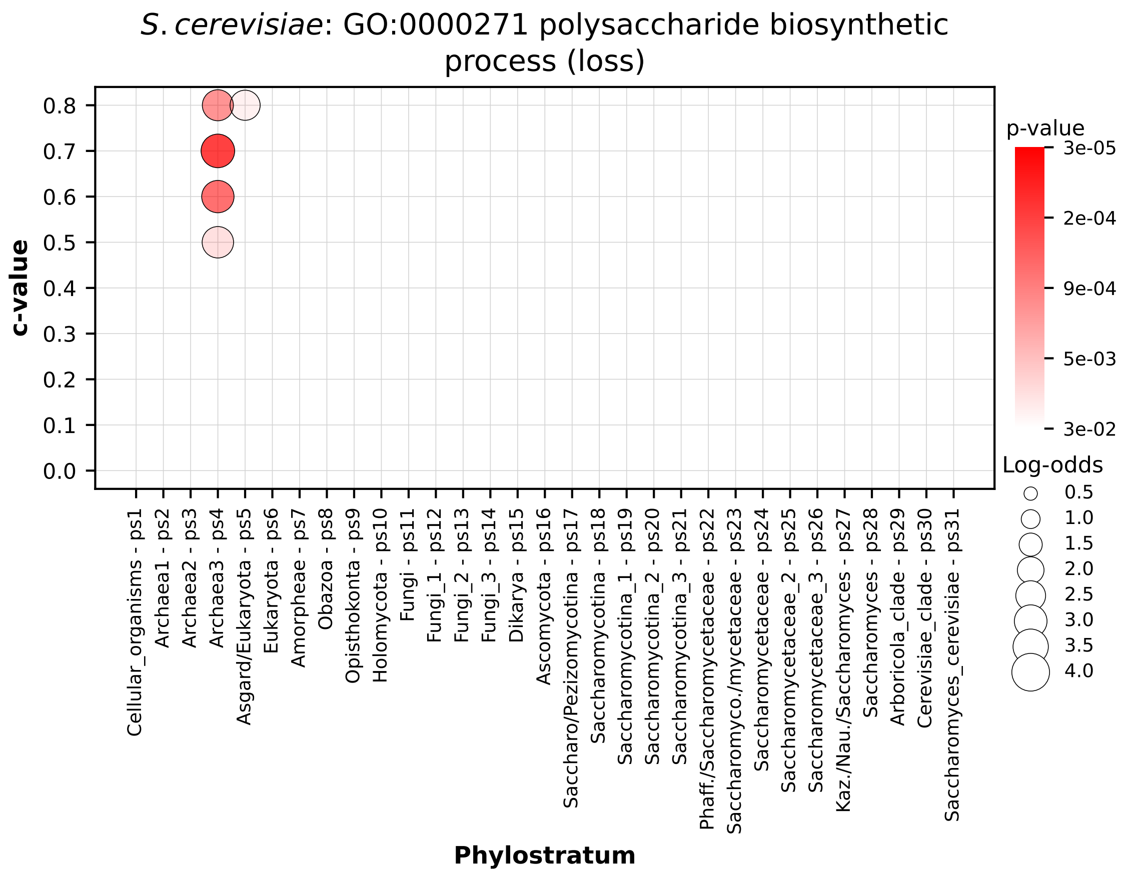

S. cerevisiae: GO:0000271 polysaccharide biosynthetic process (loss)
c-value
0.0
0.1
0.2
0.3
0.4
0.5
0.6
0.7
0.8
p-value
3e-05
2e-04
9e-04
5e-03
3e-02
Log-odds
0.5
1.0
1.5
2.0
2.5
3.0
3.5
4.0
Cellular_organisms - ps1
Archaea1 - ps2
Archaea2 - ps3
Archaea3 - ps4
Asgard/Eukaryota - ps5
Eukaryota - ps6
Amorpheae - ps7
Obazoa - ps8
Opisthokonta - ps9
Holomycota - ps10
Fungi - ps11
Fungi_1 - ps12
Fungi_2 - ps13
Fungi_3 - ps14
Dikarya - ps15
Ascomycota - ps16
Saccharo/Pezizomycotina - ps17
Saccharomycotina - ps18
Saccharomycotina_1 - ps19
Saccharomycotina_2 - ps20
Saccharomycotina_3 - ps21
Phaff./Saccharomycetaceae - ps22
Saccharomyco./mycetaceae - ps23
Saccharomycetaceae - ps24
Saccharomycetaceae_2 - ps25
Saccharomycetaceae_3 - ps26
Kaz./Nau./Saccharomyces - ps27
Saccharomyces - ps28
Arboricola_clade - ps29
Cerevisiae_clade - ps30
Saccharomyces_cerevisiae - ps31
Phylostratum

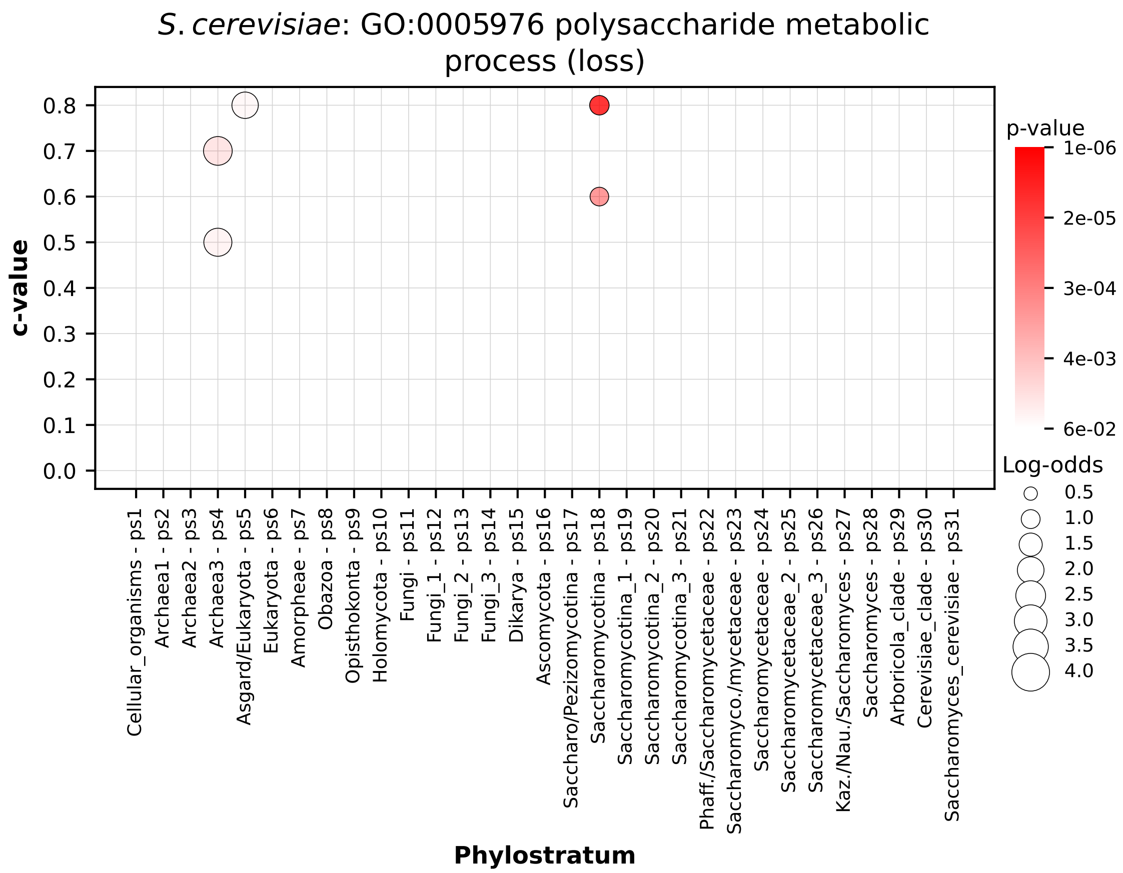

S. cerevisiae: GO:0005976 polysaccharide metabolic process (loss)
c-value
0.8
0.7
0.6
0.5
0.4
0.3
0.2
0.1
0.0
Phylostratum
Cellular_organisms - ps1
Archaea1 - ps2
Archaea2 - ps3
Archaea3 - ps4
Asgard/Eukaryota - ps5
Eukaryota - ps6
Amorpheae - ps7
Obazoa - ps8
Opisthokonta - ps9
Holomycota - ps10
Fungi - ps11
Fungi_1 - ps12
Fungi_2 - ps13
Fungi_3 - ps14
Dikarya - ps15
Ascomycota - ps16
Saccharo/Pezizomycotina - ps17
Saccharomycotina - ps18
Saccharomycotina_1 - ps19
Saccharomycotina_2 - ps20
Saccharomycotina_3 - ps21
Phaff./Saccharomycetaceae - ps22
Saccharomyco./mycetaceae - ps23
Saccharomycetaceae - ps24
Saccharomycetaceae_2 - ps25
Saccharomycetaceae_3 - ps26
Kaz./Nau./Saccharomyces - ps27
Saccharomyces - ps28
Arboricola_clade - ps29
Cerevisiae_clade - ps30
Saccharomyces_cerevisiae - ps31
p-value
1e-06
2e-05
3e-04
4e-03
6e-02
Log-odds
0.5
1.0
1.5
2.0
2.5
3.0
3.5
4.0

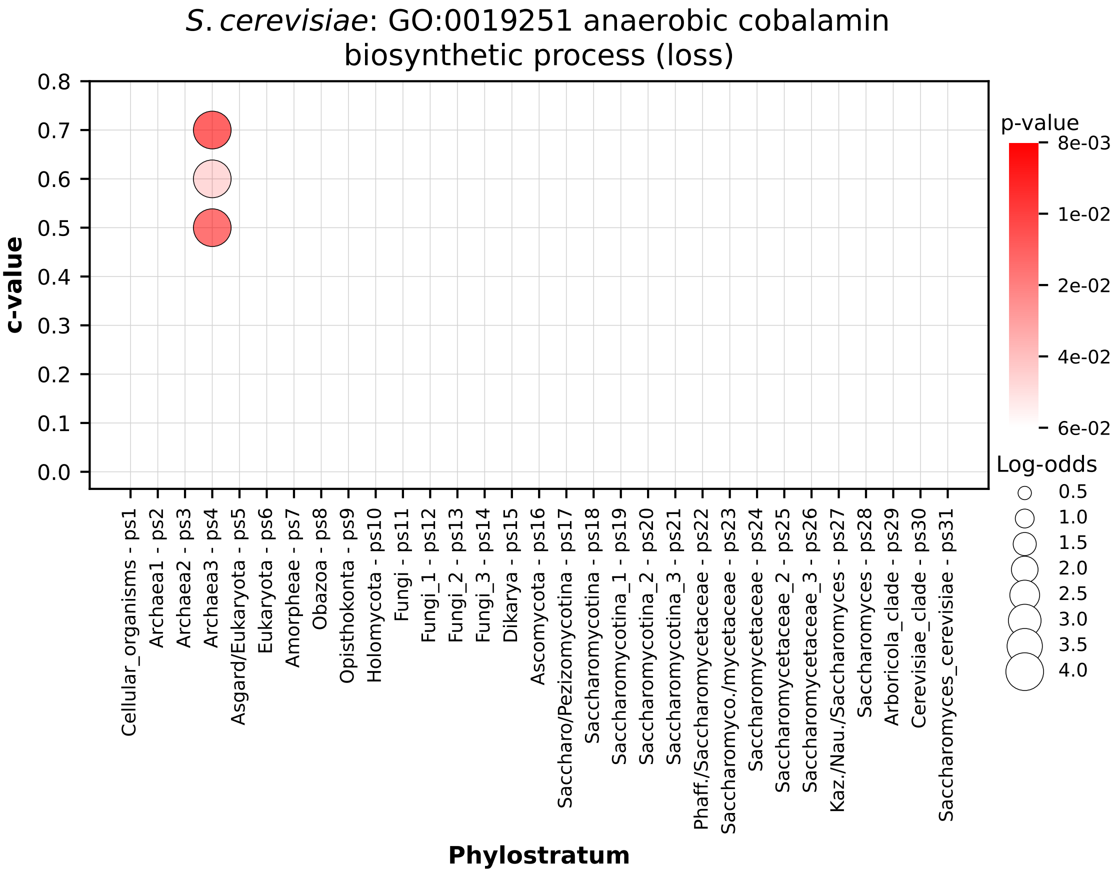

S. cerevisiae: GO:0019251 anaerobic cobalamin biosynthetic process (loss)
c-value
0.8
0.7
0.6
0.5
0.4
0.3
0.2
0.1
0.0
p-value
8e-03
1e-02
2e-02
4e-02
6e-02
Log-odds
0.5
1.0
1.5
2.0
2.5
3.0
3.5
4.0
Cellular_organisms - ps1
Archaea1 - ps2
Archaea2 - ps3
Archaea3 - ps4
Asgard/Eukaryota - ps5
Eukaryota - ps6
Amorpheae - ps7
Obazoa - ps8
Opisthokonta - ps9
Holomycota - ps10
Fungi - ps11
Fungi_1 - ps12
Fungi_2 - ps13
Fungi_3 - ps14
Dikarya - ps15
Ascomycota - ps16
Saccharo/Pezizomycotina - ps17
Saccharomycotina - ps18
Saccharomycotina_1 - ps19
Saccharomycotina_2 - ps20
Saccharomycotina_3 - ps21
Phaff./Saccharomycetaceae - ps22
Saccharomyco./mycetaceae - ps23
Saccharomycetaceae - ps24
Saccharomycetaceae_2 - ps25
Saccharomycetaceae_3 - ps26
Kaz./Nau./Saccharomyces - ps27
Saccharomyces - ps28
Arboricola_clade - ps29
Cerevisiae_clade - ps30
Saccharomyces_cerevisiae - ps31
Phylostratum

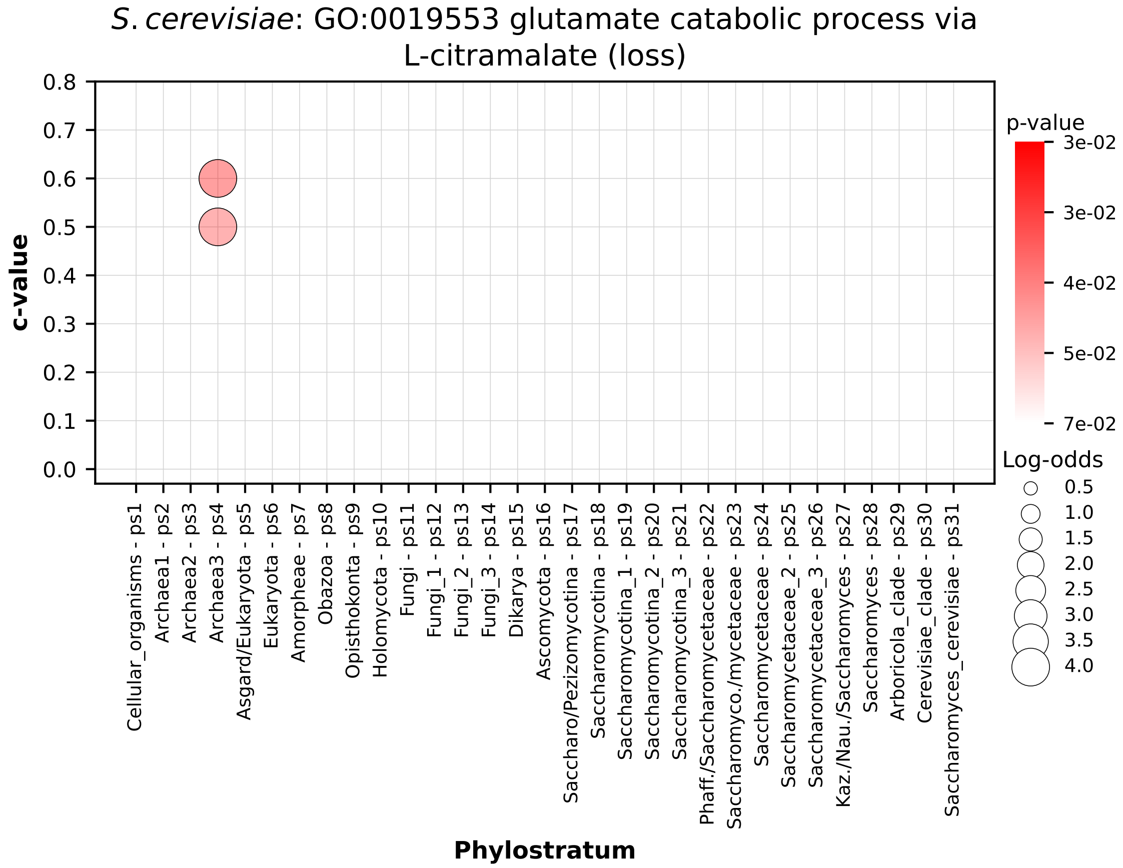

S. cerevisiae: GO:0019553 glutamate catabolic process via L-citramalate (loss)
c-value
0.8
0.7
0.6
0.5
0.4
0.3
0.2
0.1
0.0
p-value
3e-02
3e-02
4e-02
5e-02
7e-02
Log-odds
0.5
1.0
1.5
2.0
2.5
3.0
3.5
4.0
Cellular_organisms - ps1
Archaea1 - ps2
Archaea2 - ps3
Archaea3 - ps4
Asgard/Eukaryota - ps5
Eukaryota - ps6
Amorpheae - ps7
Obazoa - ps8
Opisthokonta - ps9
Holomycota - ps10
Fungi - ps11
Fungi_1 - ps12
Fungi_2 - ps13
Fungi_3 - ps14
Dikarya - ps15
Ascomycota - ps16
Saccharo/Pezizomycotina - ps17
Saccharomycotina - ps18
Saccharomycotina_1 - ps19
Saccharomycotina_2 - ps20
Saccharomycotina_3 - ps21
Phaff./Saccharomycetaceae - ps22
Saccharomyco./mycetaceae - ps23
Saccharomycetaceae - ps24
Saccharomycetaceae_2 - ps25
Saccharomycetaceae_3 - ps26
Kaz./Nau./Saccharomyces - ps27
Saccharomyces - ps28
Arboricola_clade - ps29
Cerevisiae_clade - ps30
Saccharomyces_cerevisiae - ps31
Phylostratum

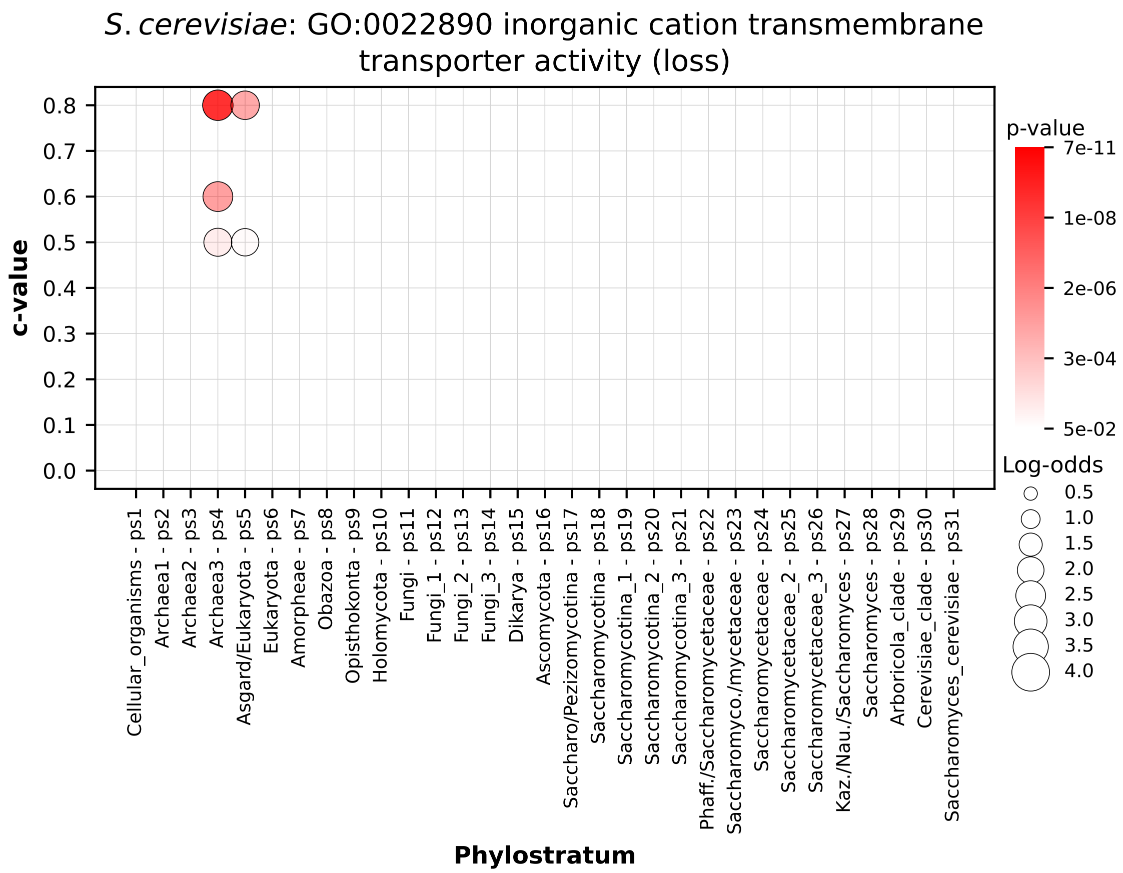
S. cerevisiae: GO:0022890 inorganic cation transmembrane transporter activity (loss)
c-value
0.8
0.7
0.6
0.5
0.4
0.3
0.2
0.1
0.0
p-value
7e-11
1e-08
2e-06
3e-04
5e-02
Log-odds
0.5
1.0
1.5
2.0
2.5
3.0
3.5
4.0
Cellular_organisms - ps1
Archaea1 - ps2
Archaea2 - ps3
Archaea3 - ps4
Asgard/Eukaryota - ps5
Eukaryota - ps6
Amorpheae - ps7
Obazoa - ps8
Opisthokonta - ps9
Holomycota - ps10
Fungi - ps11
Fungi_1 - ps12
Fungi_2 - ps13
Fungi_3 - ps14
Dikarya - ps15
Ascomycota - ps16
Saccharo/Pezizomycotina - ps17
Saccharomycotina - ps18
Saccharomycotina_1 - ps19
Saccharomycotina_2 - ps20
Saccharomycotina_3 - ps21
Phaff./Saccharomycetaceae - ps22
Saccharomyco./mycetaceae - ps23
Saccharomycetaceae - ps24
Saccharomycetaceae_2 - ps25
Saccharomycetaceae_3 - ps26
Kaz./Nau./Saccharomyces - ps27
Saccharomyces - ps28
Arboricola_clade - ps29
Cerevisiae_clade - ps30
Saccharomyces_cerevisiae - ps31
Phylostratum

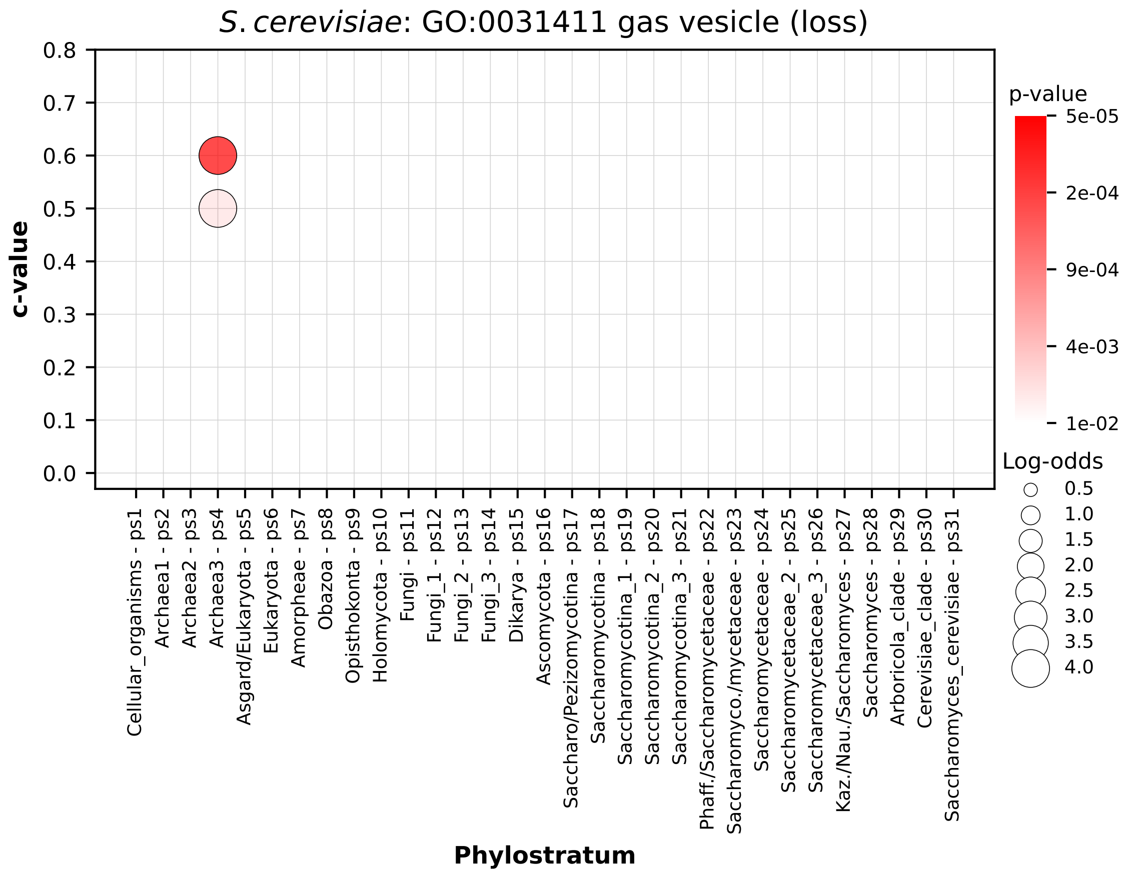
S. cerevisiae: GO:0031411 gas vesicle (loss)
c-value
0.0
0.1
0.2
0.3
0.4
0.5
0.6
0.7
0.8
Phylostratum
Cellular_organisms - ps1
Archaea1 - ps2
Archaea2 - ps3
Archaea3 - ps4
Asgard/Eukaryota - ps5
Eukaryota - ps6
Amorpheae - ps7
Obazoa - ps8
Opisthokonta - ps9
Holomycota - ps10
Fungi - ps11
Fungi_1 - ps12
Fungi_2 - ps13
Fungi_3 - ps14
Dikarya - ps15
Ascomycota - ps16
Saccharo/Pezizomycotina - ps17
Saccharomycotina - ps18
Saccharomycotina_1 - ps19
Saccharomycotina_2 - ps20
Saccharomycotina_3 - ps21
Phaff./Saccharomycetaceae - ps22
Saccharomyco./mycetaceae - ps23
Saccharomycetaceae - ps24
Saccharomycetaceae_2 - ps25
Saccharomycetaceae_3 - ps26
Kaz./Nau./Saccharomyces - ps27
Saccharomyces - ps28
Arboricola_clade - ps29
Cerevisiae_clade - ps30
Saccharomyces_cerevisiae - ps31
p-value
5e-05
2e-04
9e-04
4e-03
1e-02
Log-odds
0.5
1.0
1.5
2.0
2.5
3.0
3.5
4.0

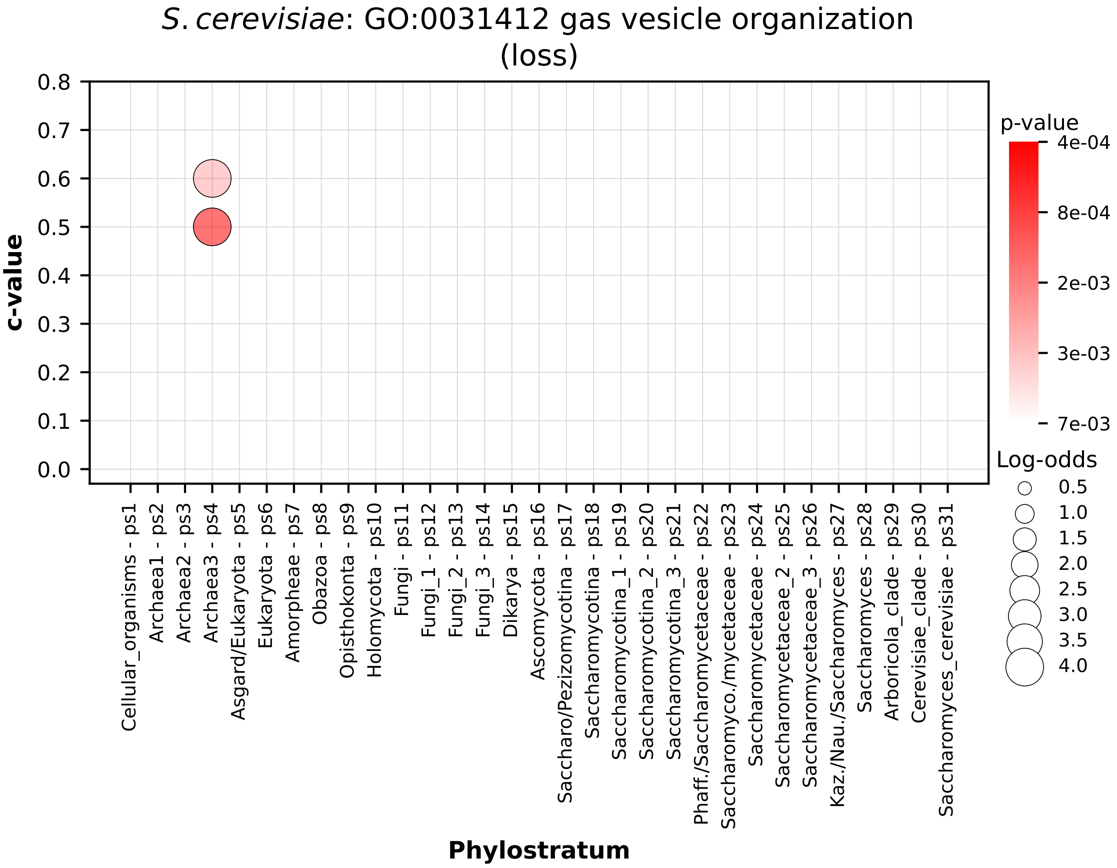

S. cerevisiae: GO:0031412 gas vesicle organization
(loss)
c-value
0.8
0.7
0.6
0.5
0.4
0.3
0.2
0.1
0.0
p-value
4e-04
8e-04
2e-03
3e-03
7e-03
Log-odds
0.5
1.0
1.5
2.0
2.5
3.0
3.5
4.0
Cellular_organisms - ps1
Archaea1 - ps2
Archaea2 - ps3
Archaea3 - ps4
Asgard/Eukaryota - ps5
Eukaryota - ps6
Amorpheae - ps7
Obazoa - ps8
Opisthokonta - ps9
Holomycota - ps10
Fungi - ps11
Fungi_1 - ps12
Fungi_2 - ps13
Fungi_3 - ps14
Dikarya - ps15
Ascomycota - ps16
Saccharo/Pezizomycotina - ps17
Saccharomycotina - ps18
Saccharomycotina_1 - ps19
Saccharomycotina_2 - ps20
Saccharomycotina_3 - ps21
Phaff./Saccharomycetaceae - ps22
Saccharomyco./mycetaceae - ps23
Saccharomycetaceae - ps24
Saccharomycetaceae_2 - ps25
Saccharomycetaceae_3 - ps26
Kaz./Nau./Saccharomyces - ps27
Saccharomyces - ps28
Arboricola_clade - ps29
Cerevisiae_clade - ps30
Saccharomyces_cerevisiae - ps31
Phylostratum

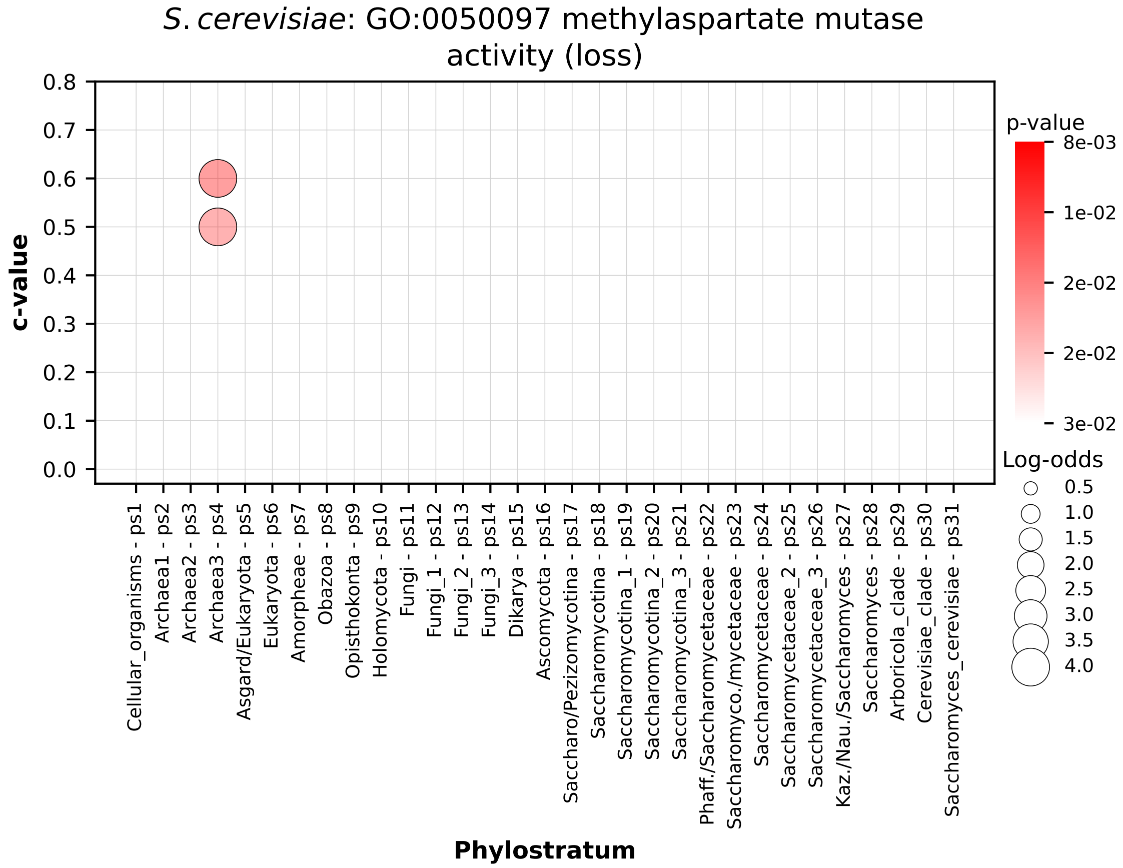
S. cerevisiae: GO:0050097 methylaspartate mutase activity (loss)
c-value
0.8
0.7
0.6
0.5
0.4
0.3
0.2
0.1
0.0
p-value
8e-03
1e-02
2e-02
2e-02
3e-02
Log-odds
0.5
1.0
1.5
2.0
2.5
3.0
3.5
4.0
Cellular_organisms - ps1
Archaea1 - ps2
Archaea2 - ps3
Archaea3 - ps4
Asgard/Eukaryota - ps5
Eukaryota - ps6
Amorpheae - ps7
Obazoa - ps8
Opisthokonta - ps9
Holomycota - ps10
Fungi - ps11
Fungi_1 - ps12
Fungi_2 - ps13
Fungi_3 - ps14
Dikarya - ps15
Ascomycota - ps16
Saccharo/Pezizomycotina - ps17
Saccharomycotina - ps18
Saccharomycotina_1 - ps19
Saccharomycotina_2 - ps20
Saccharomycotina_3 - ps21
Phaff./Saccharomycetaceae - ps22
Saccharomyco./mycetaceae - ps23
Saccharomycetaceae - ps24
Saccharomycetaceae_2 - ps25
Saccharomycetaceae_3 - ps26
Kaz./Nau./Saccharomyces - ps27
Saccharomyces - ps28
Arboricola_clade - ps29
Cerevisiae_clade - ps30
Saccharomyces_cerevisiae - ps31
Phylostratum

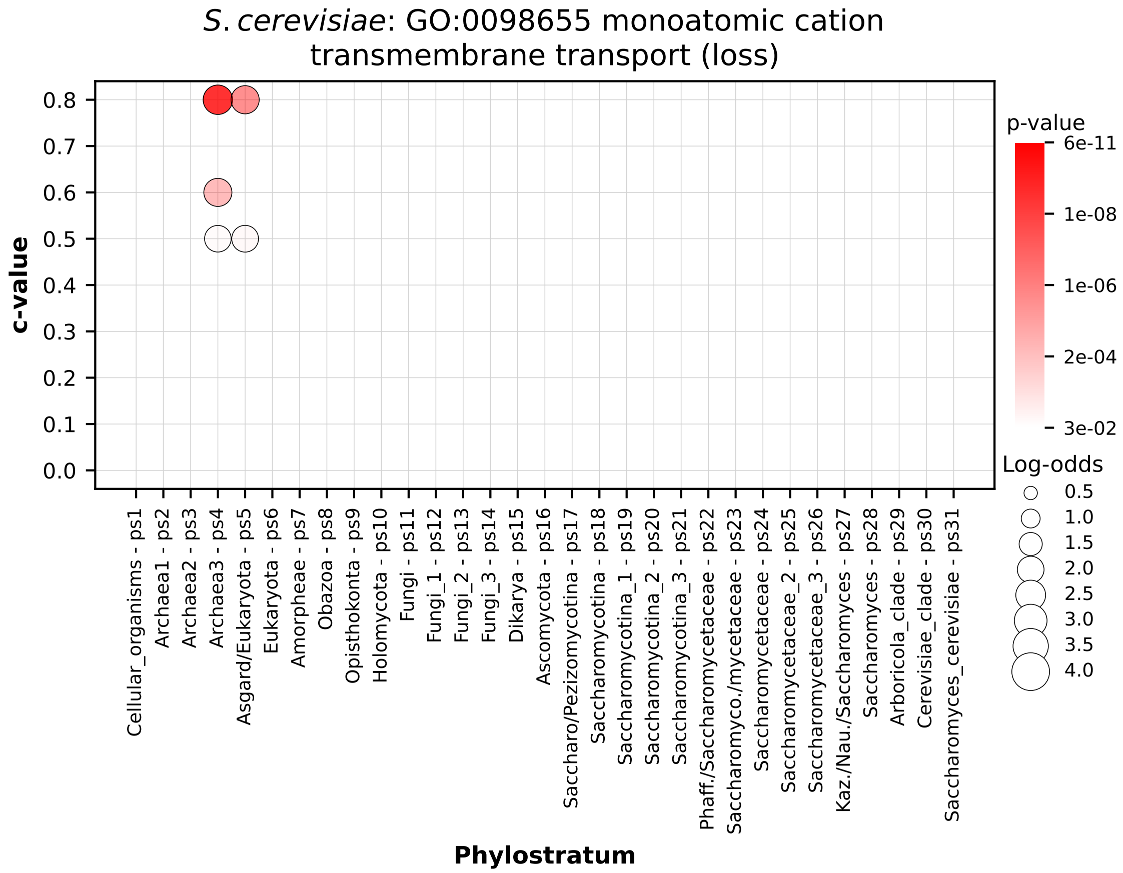

S. cerevisiae: GO:0098655 monoatomic cation transmembrane transport (loss)
c-value
0.0
0.1
0.2
0.3
0.4
0.5
0.6
0.7
0.8
Phylostratum
Cellular_organisms - ps1
Archaea1 - ps2
Archaea2 - ps3
Archaea3 - ps4
Asgard/Eukaryota - ps5
Eukaryota - ps6
Amorpheae - ps7
Obazoa - ps8
Opisthokonta - ps9
Holomycota - ps10
Fungi - ps11
Fungi_1 - ps12
Fungi_2 - ps13
Fungi_3 - ps14
Dikarya - ps15
Ascomycota - ps16
Saccharo/Pezizomycotina - ps17
Saccharomycotina - ps18
Saccharomycotina_1 - ps19
Saccharomycotina_2 - ps20
Saccharomycotina_3 - ps21
Phaff./Saccharomycetaceae - ps22
Saccharomyco./mycetaceae - ps23
Saccharomycetaceae - ps24
Saccharomycetaceae_2 - ps25
Saccharomycetaceae_3 - ps26
Kaz./Nau./Saccharomyces - ps27
Saccharomyces - ps28
Arboricola_clade - ps29
Cerevisiae_clade - ps30
Saccharomyces_cerevisiae - ps31
p-value
6e-11
1e-08
1e-06
2e-04
3e-02
Log-odds
0.5
1.0
1.5
2.0
2.5
3.0
3.5
4.0

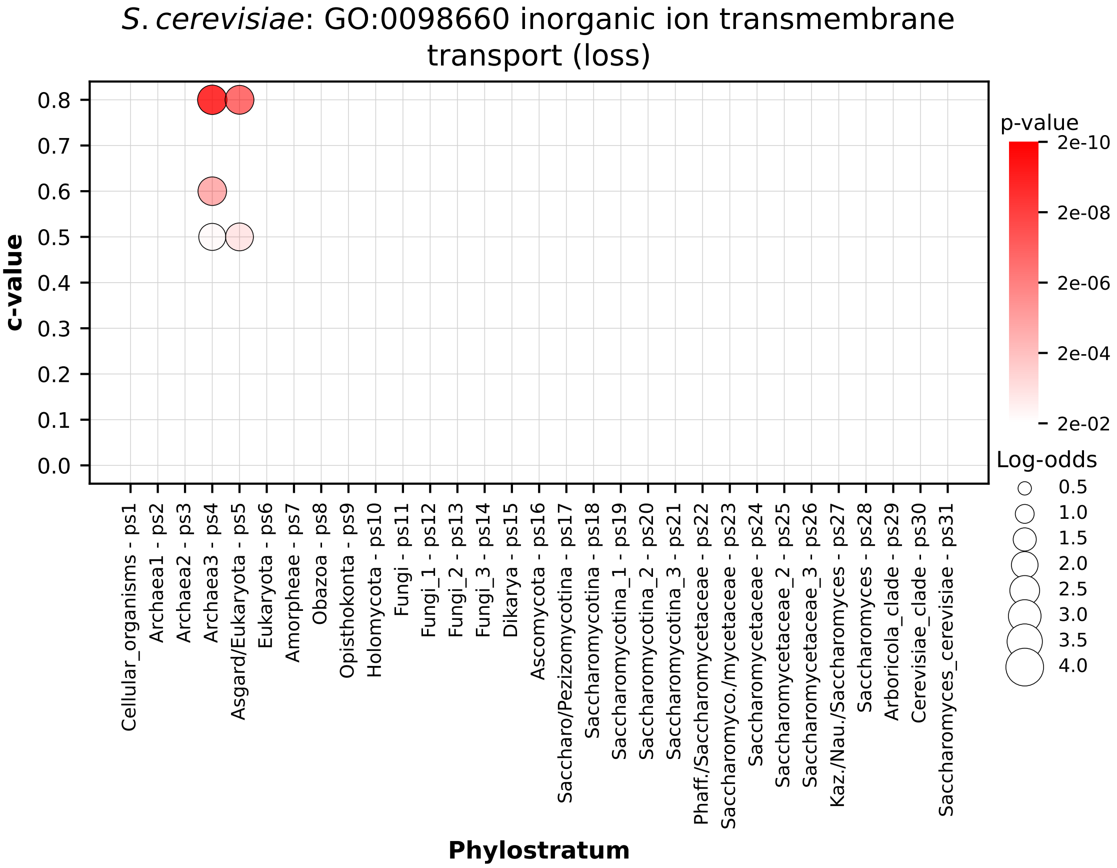
S. cerevisiae: GO:0098660 inorganic ion transmembrane transport (loss)
c-value
0.8
0.7
0.6
0.5
0.4
0.3
0.2
0.1
0.0
p-value
2e-10
2e-08
2e-06
2e-04
2e-02
Log-odds
0.5
1.0
1.5
2.0
2.5
3.0
3.5
4.0
Cellular_organisms - ps1
Archaea1 - ps2
Archaea2 - ps3
Archaea3 - ps4
Asgard/Eukaryota - ps5
Eukaryota - ps6
Amorpheae - ps7
Obazoa - ps8
Opisthokonta - ps9
Holomycota - ps10
Fungi - ps11
Fungi_1 - ps12
Fungi_2 - ps13
Fungi_3 - ps14
Dikarya - ps15
Ascomycota - ps16
Saccharo/Pezizomycotina - ps17
Saccharomycotina - ps18
Saccharomycotina_1 - ps19
Saccharomycotina_2 - ps20
Saccharomycotina_3 - ps21
Phaff./Saccharomycetaceae - ps22
Saccharomyco./mycetaceae - ps23
Saccharomycetaceae - ps24
Saccharomycetaceae_2 - ps25
Saccharomycetaceae_3 - ps26
Kaz./Nau./Saccharomyces - ps27
Saccharomyces - ps28
Arboricola_clade - ps29
Cerevisiae_clade - ps30
Saccharomyces_cerevisiae - ps31
Phylostratum

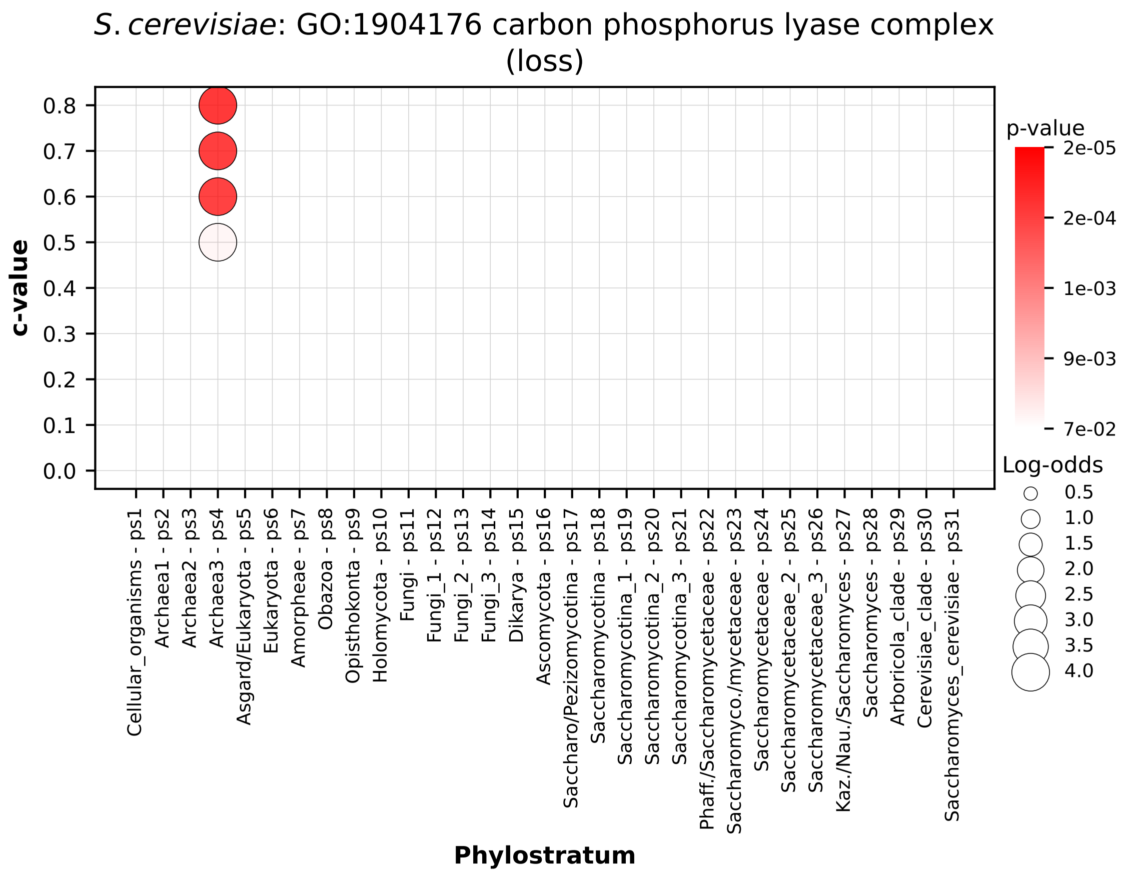
S. cerevisiae: GO:1904176 carbon phosphorus lyase complex (loss)
c-value
0.8
0.7
0.6
0.5
0.4
0.3
0.2
0.1
0.0
p-value
2e-05
2e-04
1e-03
9e-03
7e-02
Log-odds
0.5
1.0
1.5
2.0
2.5
3.0
3.5
4.0
Cellular_organisms - ps1
Archaea1 - ps2
Archaea2 - ps3
Archaea3 - ps4
Asgard/Eukaryota - ps5
Eukaryota - ps6
Amorpheae - ps7
Obazoa - ps8
Opisthokonta - ps9
Holomycota - ps10
Fungi - ps11
Fungi_1 - ps12
Fungi_2 - ps13
Fungi_3 - ps14
Dikarya - ps15
Ascomycota - ps16
Saccharo/Pezizomycotina - ps17
Saccharomycotina - ps18
Saccharomycotina_1 - ps19
Saccharomycotina_2 - ps20
Saccharomycotina_3 - ps21
Phaff./Saccharomycetaceae - ps22
Saccharomyco./mycetaceae - ps23
Saccharomycetaceae - ps24
Saccharomycetaceae_2 - ps25
Saccharomycetaceae_3 - ps26
Kaz./Nau./Saccharomyces - ps27
Saccharomyces - ps28
Arboricola_clade - ps29
Cerevisiae_clade - ps30
Saccharomyces_cerevisiae - ps31
Phylostratum

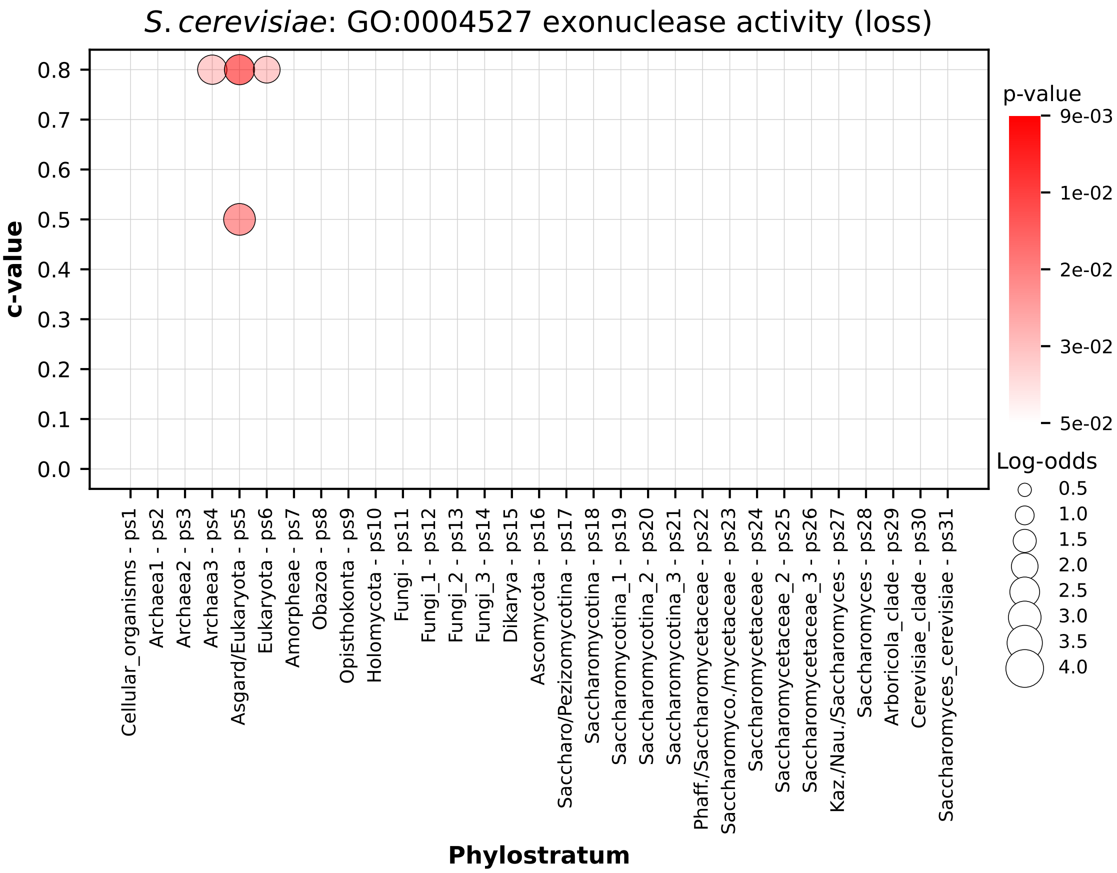

S. cerevisiae: GO:0004527 exonuclease activity (loss)
c-value
0.8
0.7
0.6
0.5
0.4
0.3
0.2
0.1
0.0
p-value
9e-03
1e-02
2e-02
3e-02
5e-02
Log-odds
0.5
1.0
1.5
2.0
2.5
3.0
3.5
4.0
Cellular_organisms - ps1
Archaea1 - ps2
Archaea2 - ps3
Archaea3 - ps4
Asgard/Eukaryota - ps5
Eukaryota - ps6
Amorpheae - ps7
Obazoa - ps8
Opisthokonta - ps9
Holomycota - ps10
Fungi - ps11
Fungi_1 - ps12
Fungi_2 - ps13
Fungi_3 - ps14
Dikarya - ps15
Ascomycota - ps16
Saccharo/Pezizomycotina - ps17
Saccharomycotina - ps18
Saccharomycotina_1 - ps19
Saccharomycotina_2 - ps20
Saccharomycotina_3 - ps21
Phaff./Saccharomycetaceae - ps22
Saccharomyco./mycetaceae - ps23
Saccharomycetaceae - ps24
Saccharomycetaceae_2 - ps25
Saccharomycetaceae_3 - ps26
Kaz./Nau./Saccharomyces - ps27
Saccharomyces - ps28
Arboricola_clade - ps29
Cerevisiae_clade - ps30
Saccharomyces_cerevisiae - ps31
Phylostratum

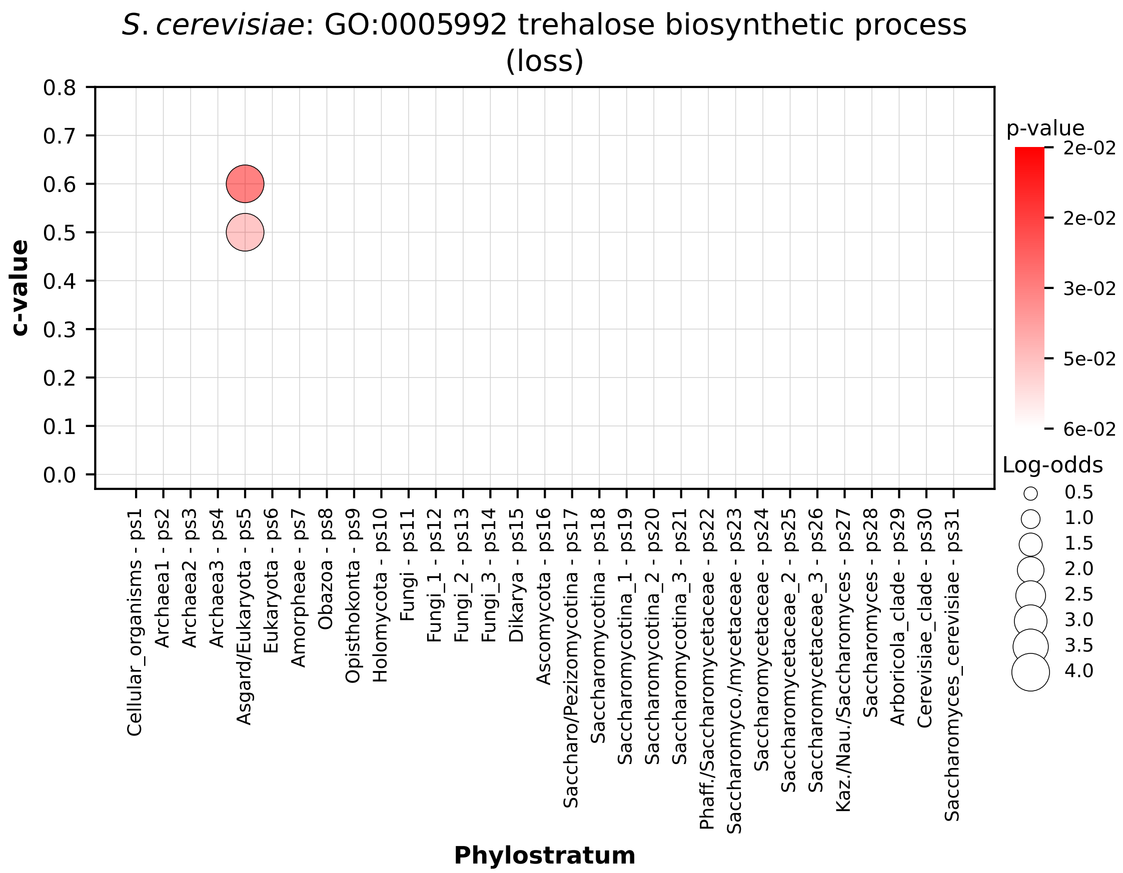
S. cerevisiae: GO:0005992 trehalose biosynthetic process
(loss)
c-value
0.8
0.7
0.6
0.5
0.4
0.3
0.2
0.1
0.0
p-value
2e-02
2e-02
3e-02
5e-02
6e-02
Log-odds
0.5
1.0
1.5
2.0
2.5
3.0
3.5
4.0
Cellular_organisms - ps1
Archaea1 - ps2
Archaea2 - ps3
Archaea3 - ps4
Asgard/Eukaryota - ps5
Eukaryota - ps6
Amorpheae - ps7
Obazoa - ps8
Opisthokonta - ps9
Holomycota - ps10
Fungi - ps11
Fungi_1 - ps12
Fungi_2 - ps13
Fungi_3 - ps14
Dikarya - ps15
Ascomycota - ps16
Saccharo/Pezizomycotina - ps17
Saccharomycotina - ps18
Saccharomycotina_1 - ps19
Saccharomycotina_2 - ps20
Saccharomycotina_3 - ps21
Phaff./Saccharomycetaceae - ps22
Saccharomyco./mycetaceae - ps23
Saccharomycetaceae - ps24
Saccharomycetaceae_2 - ps25
Saccharomycetaceae_3 - ps26
Kaz./Nau./Saccharomyces - ps27
Saccharomyces - ps28
Arboricola_clade - ps29
Cerevisiae_clade - ps30
Saccharomyces_cerevisiae - ps31
Phylostratum

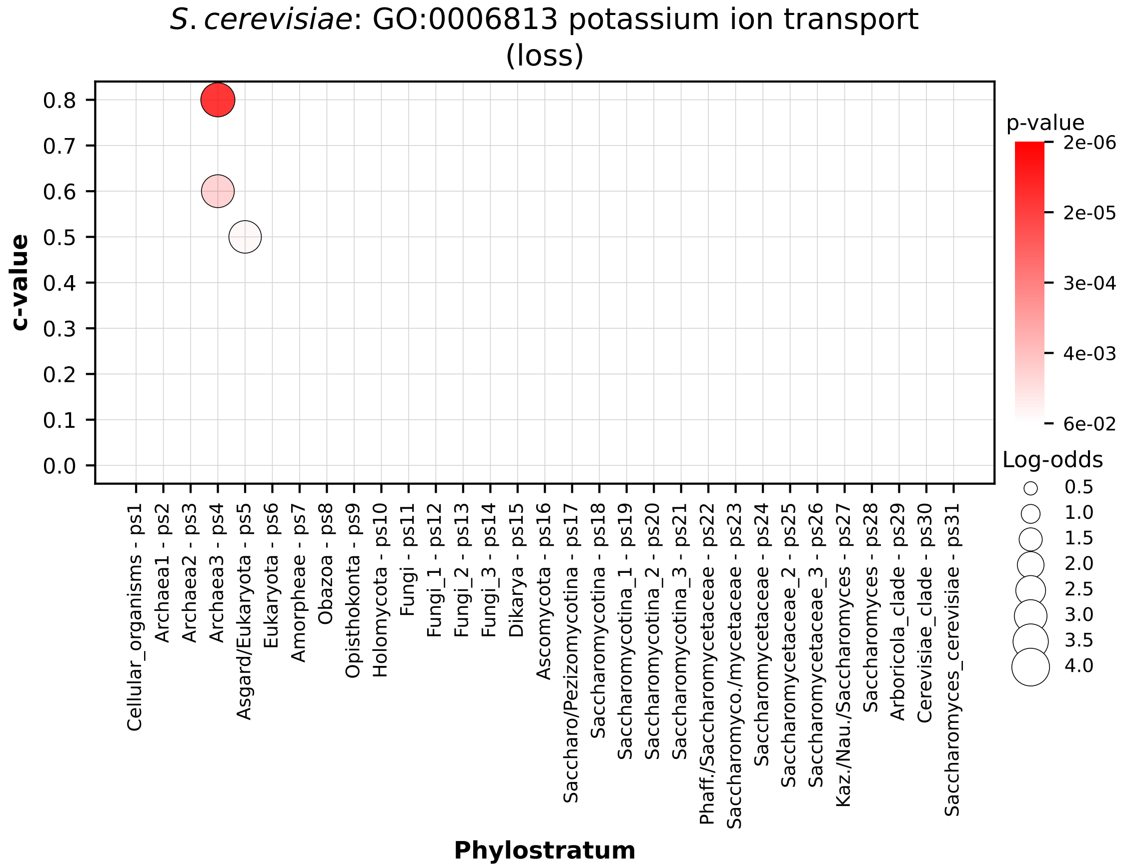
S. cerevisiae: GO:0006813 potassium ion transport
(loss)
c-value
0.8
0.7
0.6
0.5
0.4
0.3
0.2
0.1
0.0
Phylostratum
Cellular_organisms - ps1
Archaea1 - ps2
Archaea2 - ps3
Archaea3 - ps4
Asgard/Eukaryota - ps5
Eukaryota - ps6
Amorpheae - ps7
Obazoa - ps8
Opisthokonta - ps9
Holomycota - ps10
Fungi - ps11
Fungi_1 - ps12
Fungi_2 - ps13
Fungi_3 - ps14
Dikarya - ps15
Ascomycota - ps16
Saccharo/Pezizomycotina - ps17
Saccharomycotina - ps18
Saccharomycotina_1 - ps19
Saccharomycotina_2 - ps20
Saccharomycotina_3 - ps21
Phaff./Saccharomycetaceae - ps22
Saccharomyco./mycetaceae - ps23
Saccharomycetaceae - ps24
Saccharomycetaceae_2 - ps25
Saccharomycetaceae_3 - ps26
Kaz./Nau./Saccharomyces - ps27
Saccharomyces - ps28
Arboricola_clade - ps29
Cerevisiae_clade - ps30
Saccharomyces_cerevisiae - ps31
p-value
2e-06
2e-05
3e-04
4e-03
6e-02
Log-odds
0.5
1.0
1.5
2.0
2.5
3.0
3.5
4.0

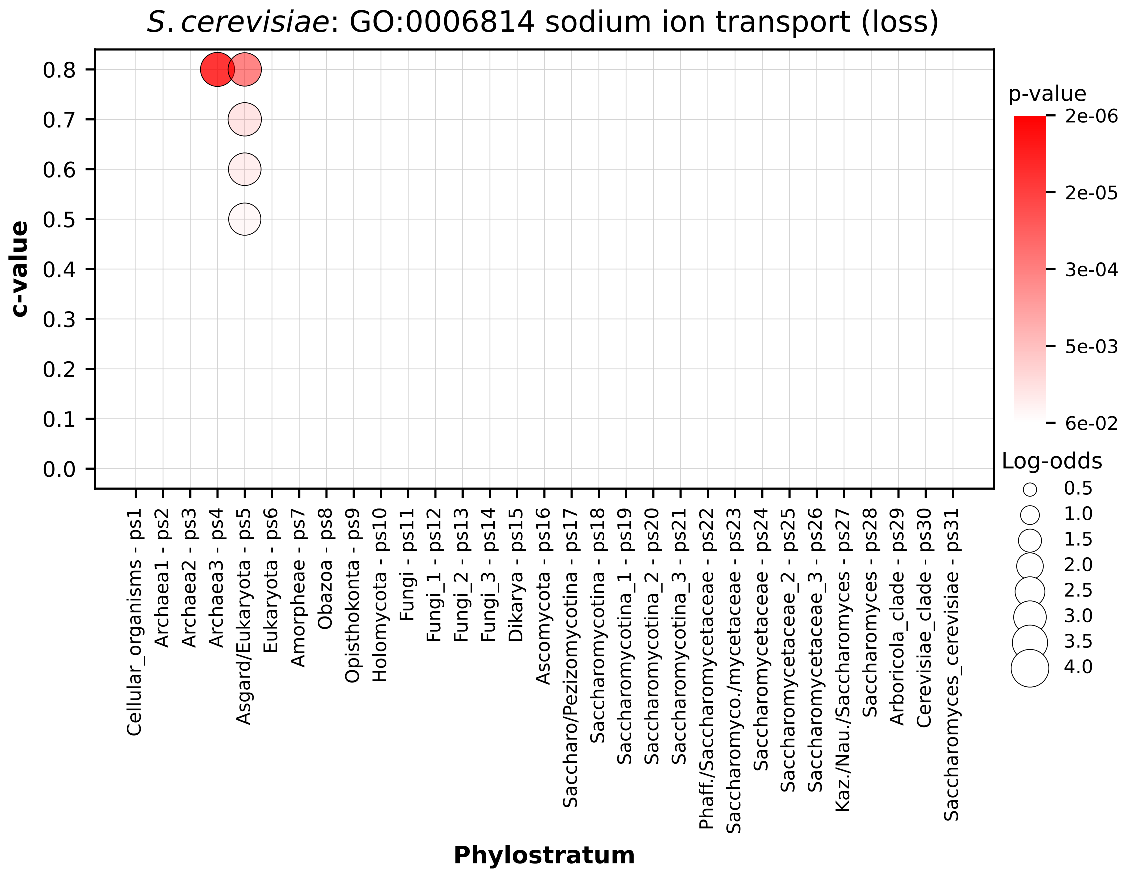
S. cerevisiae: GO:0006814 sodium ion transport (loss)
c-value
0.0
0.1
0.2
0.3
0.4
0.5
0.6
0.7
0.8
Phylostratum
Cellular_organisms - ps1
Archaea1 - ps2
Archaea2 - ps3
Archaea3 - ps4
Asgard/Eukaryota - ps5
Eukaryota - ps6
Amorpheae - ps7
Obazoa - ps8
Opisthokonta - ps9
Holomycota - ps10
Fungi - ps11
Fungi_1 - ps12
Fungi_2 - ps13
Fungi_3 - ps14
Dikarya - ps15
Ascomycota - ps16
Saccharo/Pezizomycotina - ps17
Saccharomycotina - ps18
Saccharomycotina_1 - ps19
Saccharomycotina_2 - ps20
Saccharomycotina_3 - ps21
Phaff./Saccharomycetaceae - ps22
Saccharomyco./mycetaceae - ps23
Saccharomycetaceae - ps24
Saccharomycetaceae_2 - ps25
Saccharomycetaceae_3 - ps26
Kaz./Nau./Saccharomyces - ps27
Saccharomyces - ps28
Arboricola_clade - ps29
Cerevisiae_clade - ps30
Saccharomyces_cerevisiae - ps31
p-value
2e-06
2e-05
3e-04
5e-03
6e-02
Log-odds
0.5
1.0
1.5
2.0
2.5
3.0
3.5
4.0

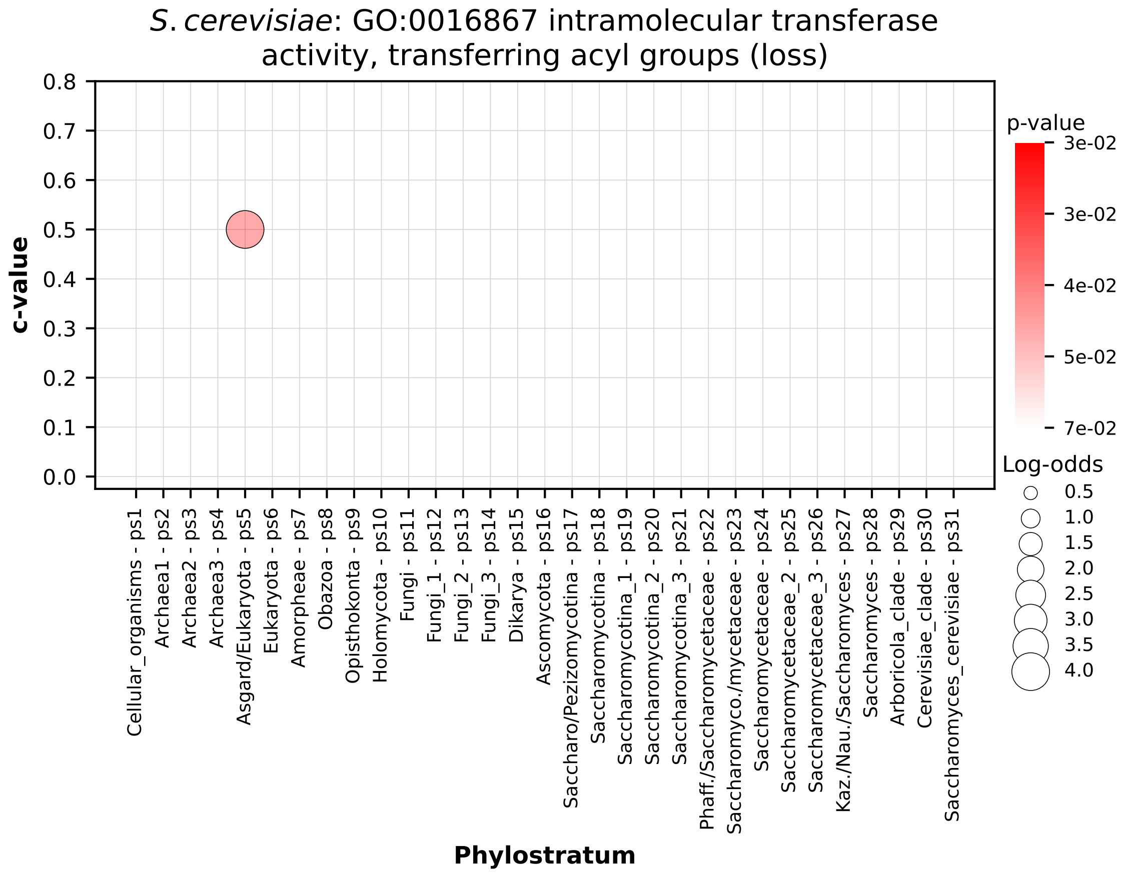
S. cerevisiae: GO:0016867 intramolecular transferase activity, transferring acyl groups (loss)
c-value
0.0
0.1
0.2
0.3
0.4
0.5
0.6
0.7
0.8
p-value
3e-02
3e-02
4e-02
5e-02
7e-02
Log-odds
0.5
1.0
1.5
2.0
2.5
3.0
3.5
4.0
Cellular_organisms - ps1
Archaea1 - ps2
Archaea2 - ps3
Archaea3 - ps4
Asgard/Eukaryota - ps5
Eukaryota - ps6
Amorpheae - ps7
Obazoa - ps8
Opisthokonta - ps9
Holomycota - ps10
Fungi - ps11
Fungi_1 - ps12
Fungi_2 - ps13
Fungi_3 - ps14
Dikarya - ps15
Ascomycota - ps16
Saccharo/Pezizomycotina - ps17
Saccharomycotina - ps18
Saccharomycotina_1 - ps19
Saccharomycotina_2 - ps20
Saccharomycotina_3 - ps21
Phaff./Saccharomycetaceae - ps22
Saccharomyco./mycetaceae - ps23
Saccharomycetaceae - ps24
Saccharomycetaceae_2 - ps25
Saccharomycetaceae_3 - ps26
Kaz./Nau./Saccharomyces - ps27
Saccharomyces - ps28
Arboricola_clade - ps29
Cerevisiae_clade - ps30
Saccharomyces_cerevisiae - ps31
Phylostratum

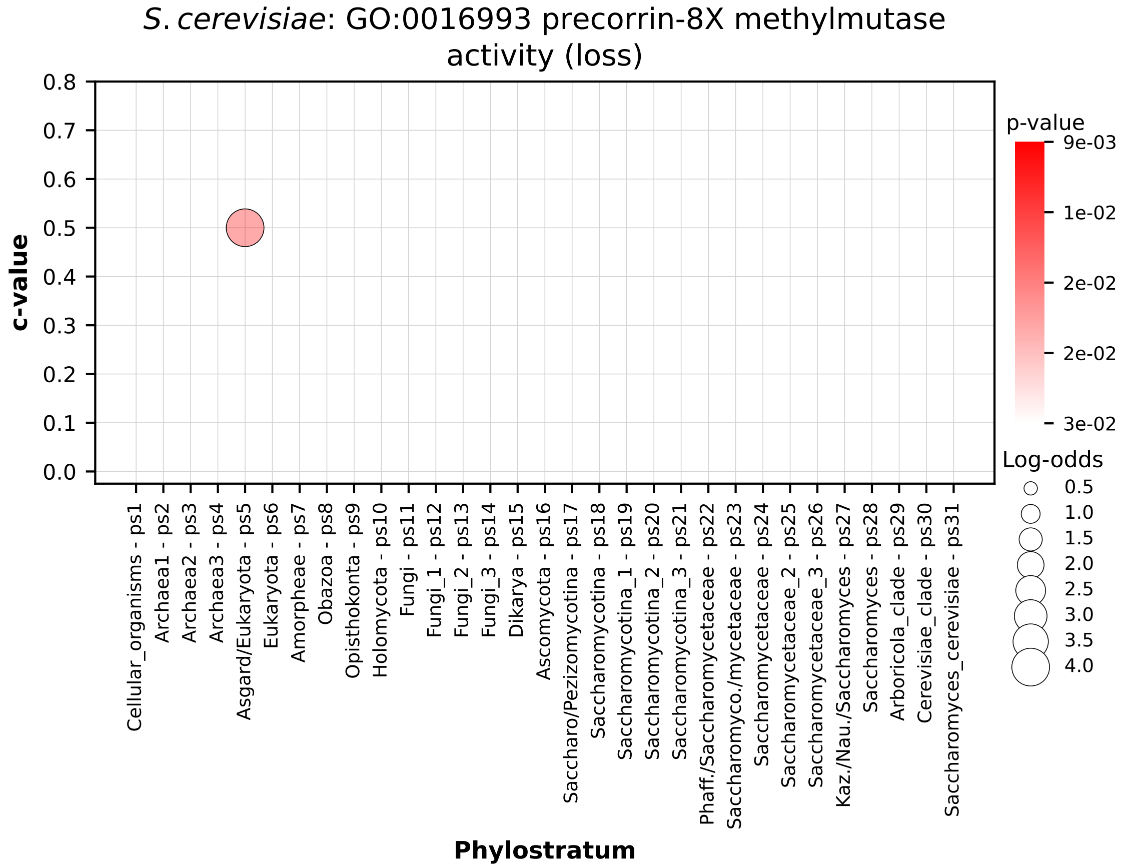
S. cerevisiae: GO:0016993 precorrin-8X methylmutase activity (loss)
c-value
0.8
0.7
0.6
0.5
0.4
0.3
0.2
0.1
0.0
p-value
9e-03
1e-02
2e-02
2e-02
3e-02
Log-odds
0.5
1.0
1.5
2.0
2.5
3.0
3.5
4.0
Cellular_organisms - ps1
Archaea1 - ps2
Archaea2 - ps3
Archaea3 - ps4
Asgard/Eukaryota - ps5
Eukaryota - ps6
Amorpheae - ps7
Obazoa - ps8
Opisthokonta - ps9
Holomycota - ps10
Fungi - ps11
Fungi_1 - ps12
Fungi_2 - ps13
Fungi_3 - ps14
Dikarya - ps15
Ascomycota - ps16
Saccharo/Pezizomycotina - ps17
Saccharomycotina - ps18
Saccharomycotina_1 - ps19
Saccharomycotina_2 - ps20
Saccharomycotina_3 - ps21
Phaff./Saccharomycetaceae - ps22
Saccharomyco./mycetaceae - ps23
Saccharomycetaceae - ps24
Saccharomycetaceae_2 - ps25
Saccharomycetaceae_3 - ps26
Kaz./Nau./Saccharomyces - ps27
Saccharomyces - ps28
Arboricola_clade - ps29
Cerevisiae_clade - ps30
Saccharomyces_cerevisiae - ps31
Phylostratum

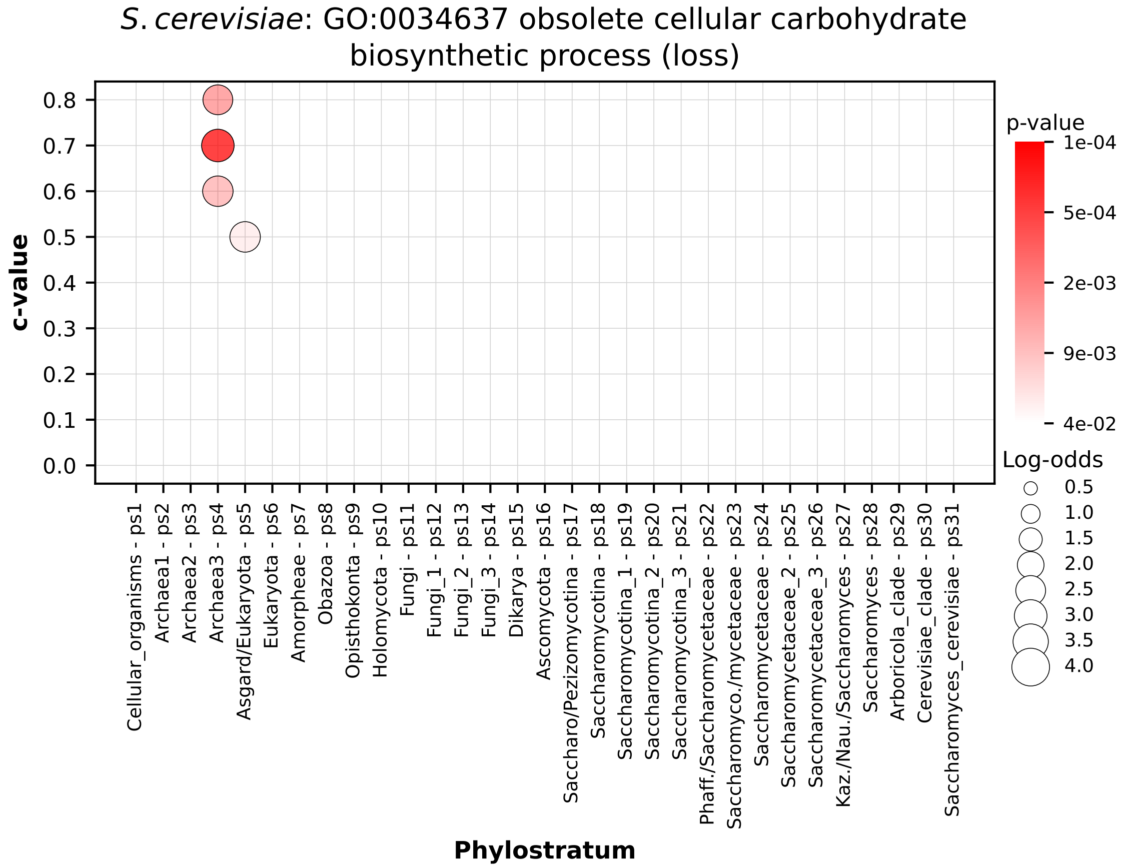

$S. cerevisiae$: GO:0034637 obsolete cellular carbohydrate biosynthetic process (loss)
c-value
0.8
0.7
0.6
0.5
0.4
0.3
0.2
0.1
0.0
p-value
1e-04
5e-04
2e-03
9e-03
4e-02
Log-odds
0.5
1.0
1.5
2.0
2.5
3.0
3.5
4.0
Cellular_organisms - ps1
Archaea1 - ps2
Archaea2 - ps3
Archaea3 - ps4
Asgard/Eukaryota - ps5
Eukaryota - ps6
Amorpheae - ps7
Obazoa - ps8
Opisthokonta - ps9
Holomycota - ps10
Fungi - ps11
Fungi_1 - ps12
Fungi_2 - ps13
Fungi_3 - ps14
Dikarya - ps15
Ascomycota - ps16
Saccharo/Pezizomycotina - ps17
Saccharomycotina - ps18
Saccharomycotina_1 - ps19
Saccharomycotina_2 - ps20
Saccharomycotina_3 - ps21
Phaff./Saccharomycetaceae - ps22
Saccharomyco./mycetaceae - ps23
Saccharomycetaceae - ps24
Saccharomycetaceae_2 - ps25
Saccharomycetaceae_3 - ps26
Kaz./Nau./Saccharomyces - ps27
Saccharomyces - ps28
Arboricola_clade - ps29
Cerevisiae_clade - ps30
Saccharomyces_cerevisiae - ps31
Phylostratum

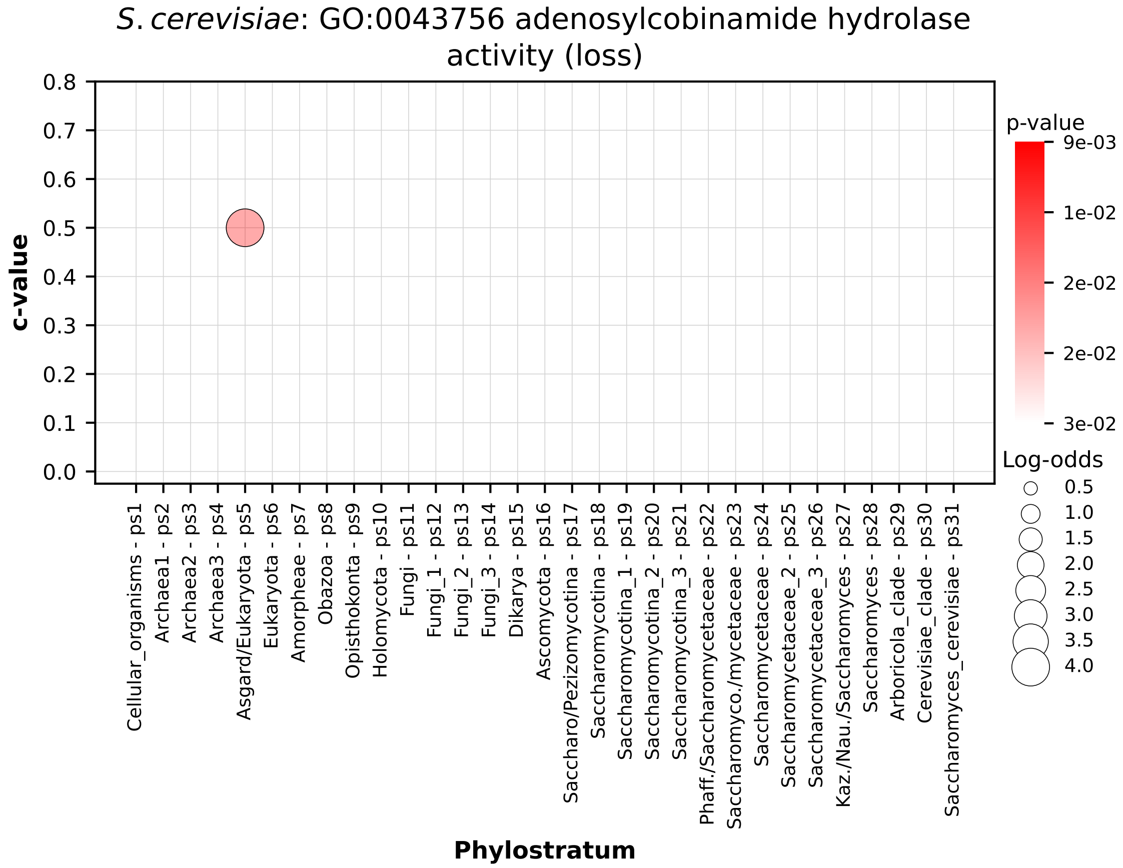

S. cerevisiae: GO:0043756 adenosylcobinamide hydrolase activity (loss)
c-value
0.8
0.7
0.6
0.5
0.4
0.3
0.2
0.1
0.0
Phylostratum
Cellular_organisms - ps1
Archaea1 - ps2
Archaea2 - ps3
Archaea3 - ps4
Asgard/Eukaryota - ps5
Eukaryota - ps6
Amorpheae - ps7
Obazoa - ps8
Opisthokonta - ps9
Holomycota - ps10
Fungi - ps11
Fungi_1 - ps12
Fungi_2 - ps13
Fungi_3 - ps14
Dikarya - ps15
Ascomycota - ps16
Saccharo/Pezizomycotina - ps17
Saccharomycotina - ps18
Saccharomycotina_1 - ps19
Saccharomycotina_2 - ps20
Saccharomycotina_3 - ps21
Phaff./Saccharomycetaceae - ps22
Saccharomyco./mycetaceae - ps23
Saccharomycetaceae - ps24
Saccharomycetaceae_2 - ps25
Saccharomycetaceae_3 - ps26
Kaz./Nau./Saccharomyces - ps27
Saccharomyces - ps28
Arboricola_clade - ps29
Cerevisiae_clade - ps30
Saccharomyces_cerevisiae - ps31
p-value
9e-03
1e-02
2e-02
2e-02
3e-02
Log-odds
0.5
1.0
1.5
2.0
2.5
3.0
3.5
4.0

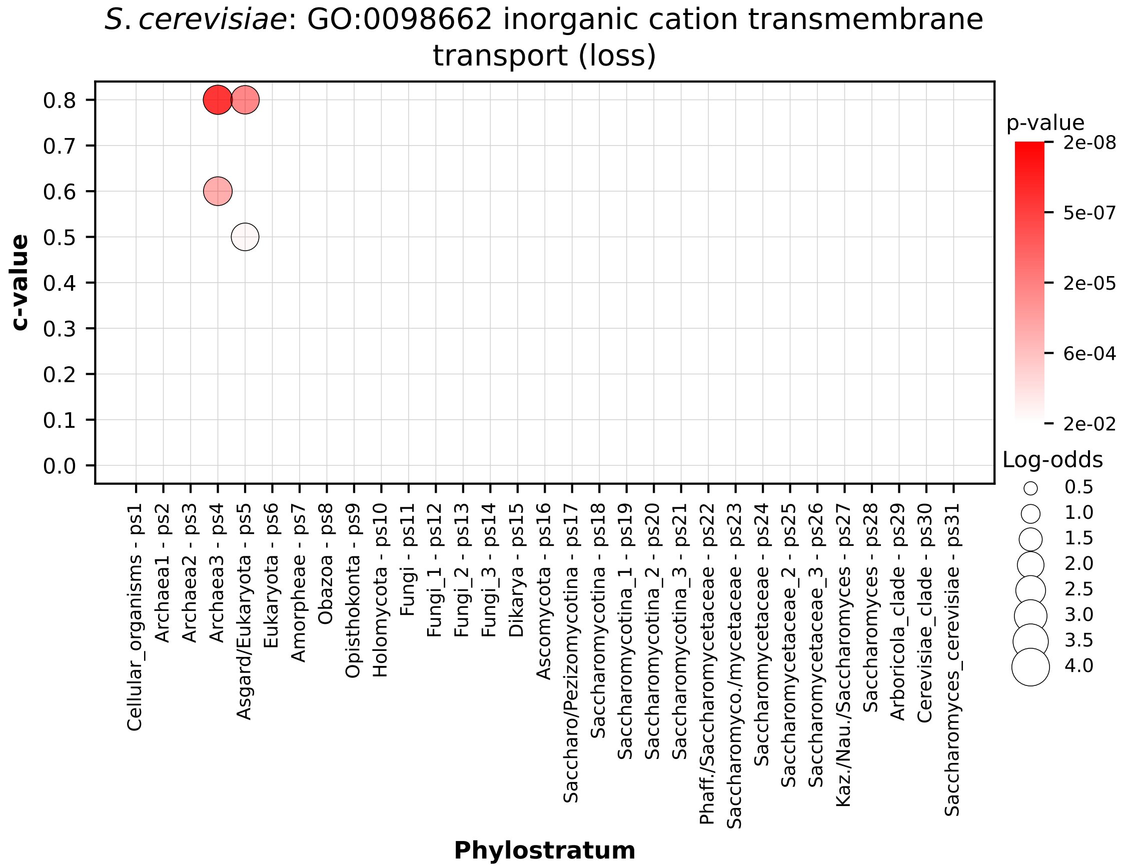

S. cerevisiae: GO:0098662 inorganic cation transmembrane transport (loss)
c-value
0.0
0.1
0.2
0.3
0.4
0.5
0.6
0.7
0.8
p-value
2e-08
5e-07
2e-05
6e-04
2e-02
Log-odds
0.5
1.0
1.5
2.0
2.5
3.0
3.5
4.0
Cellular_organisms - ps1
Archaea1 - ps2
Archaea2 - ps3
Archaea3 - ps4
Asgard/Eukaryota - ps5
Eukaryota - ps6
Amorpheae - ps7
Obazoa - ps8
Opisthokonta - ps9
Holomycota - ps10
Fungi - ps11
Fungi_1 - ps12
Fungi_2 - ps13
Fungi_3 - ps14
Dikarya - ps15
Ascomycota - ps16
Saccharo/Pezizomycotina - ps17
Saccharomycotina - ps18
Saccharomycotina_1 - ps19
Saccharomycotina_2 - ps20
Saccharomycotina_3 - ps21
Phaff./Saccharomycetaceae - ps22
Saccharomyco./mycetaceae - ps23
Saccharomycetaceae - ps24
Saccharomycetaceae_2 - ps25
Saccharomycetaceae_3 - ps26
Kaz./Nau./Saccharomyces - ps27
Saccharomyces - ps28
Arboricola_clade - ps29
Cerevisiae_clade - ps30
Saccharomyces_cerevisiae - ps31
Phylostratum

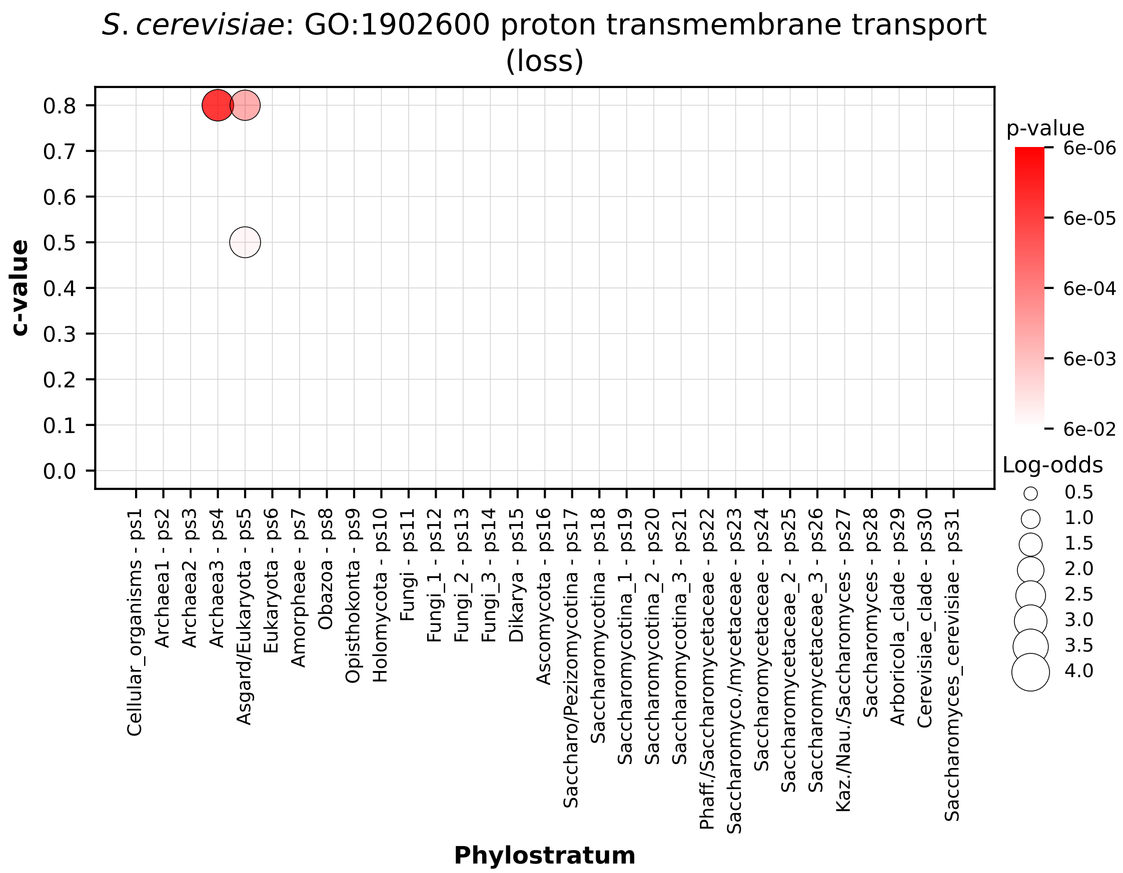

S. cerevisiae: GO:1902600 proton transmembrane transport
(loss)
c-value
0.8
0.7
0.6
0.5
0.4
0.3
0.2
0.1
0.0
Phylostratum
Cellular_organisms - ps1
Archaea1 - ps2
Archaea2 - ps3
Archaea3 - ps4
Asgard/Eukaryota - ps5
Eukaryota - ps6
Amorpheae - ps7
Obazoa - ps8
Opisthokonta - ps9
Holomycota - ps10
Fungi - ps11
Fungi_1 - ps12
Fungi_2 - ps13
Fungi_3 - ps14
Dikarya - ps15
Ascomycota - ps16
Saccharo/Pezizomycotina - ps17
Saccharomycotina - ps18
Saccharomycotina_1 - ps19
Saccharomycotina_2 - ps20
Saccharomycotina_3 - ps21
Phaff./Saccharomycetaceae - ps22
Saccharomyco./mycetaceae - ps23
Saccharomycetaceae - ps24
Saccharomycetaceae_2 - ps25
Saccharomycetaceae_3 - ps26
Kaz./Nau./Saccharomyces - ps27
Saccharomyces - ps28
Arboricola_clade - ps29
Cerevisiae_clade - ps30
Saccharomyces_cerevisiae - ps31
p-value
6e-06
6e-05
6e-04
6e-03
6e-02
Log-odds
0.5
1.0
1.5
2.0
2.5
3.0
3.5
4.0

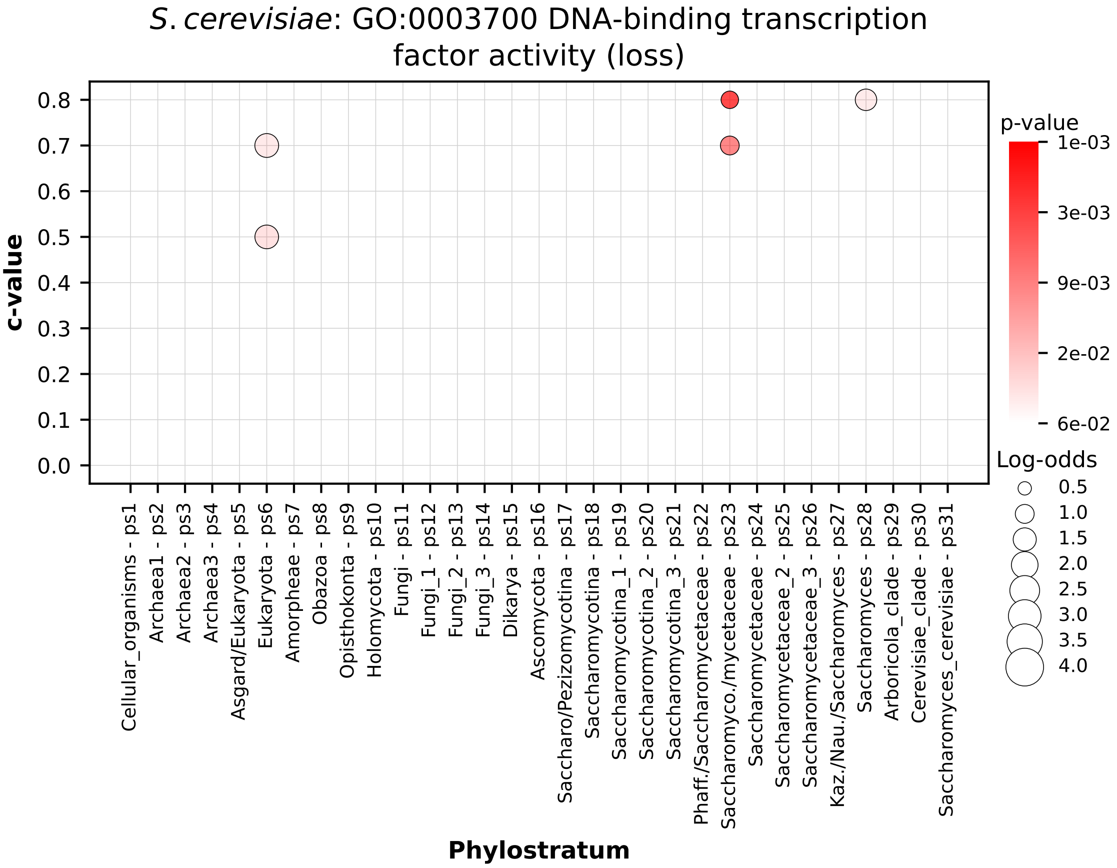

S. cerevisiae: GO:0003700 DNA-binding transcription factor activity (loss)
c-value
0.8
0.7
0.6
0.5
0.4
0.3
0.2
0.1
0.0
Phylostratum
Cellular_organisms - ps1
Archaea1 - ps2
Archaea2 - ps3
Archaea3 - ps4
Asgard/Eukaryota - ps5
Eukaryota - ps6
Amorpheae - ps7
Obazoa - ps8
Opisthokonta - ps9
Holomycota - ps10
Fungi - ps11
Fungi_1 - ps12
Fungi_2 - ps13
Fungi_3 - ps14
Dikarya - ps15
Ascomycota - ps16
Saccharo/Pezizomycotina - ps17
Saccharomycotina - ps18
Saccharomycotina_1 - ps19
Saccharomycotina_2 - ps20
Saccharomycotina_3 - ps21
Phaff./Saccharomycetaceae - ps22
Saccharomyco./mycetaceae - ps23
Saccharomycetaceae - ps24
Saccharomycetaceae_2 - ps25
Saccharomycetaceae_3 - ps26
Kaz./Nau./Saccharomyces - ps27
Saccharomyces - ps28
Arboricola_clade - ps29
Cerevisiae_clade - ps30
Saccharomyces_cerevisiae - ps31
p-value
1e-03
3e-03
9e-03
2e-02
6e-02
Log-odds
0.5
1.0
1.5
2.0
2.5
3.0
3.5
4.0

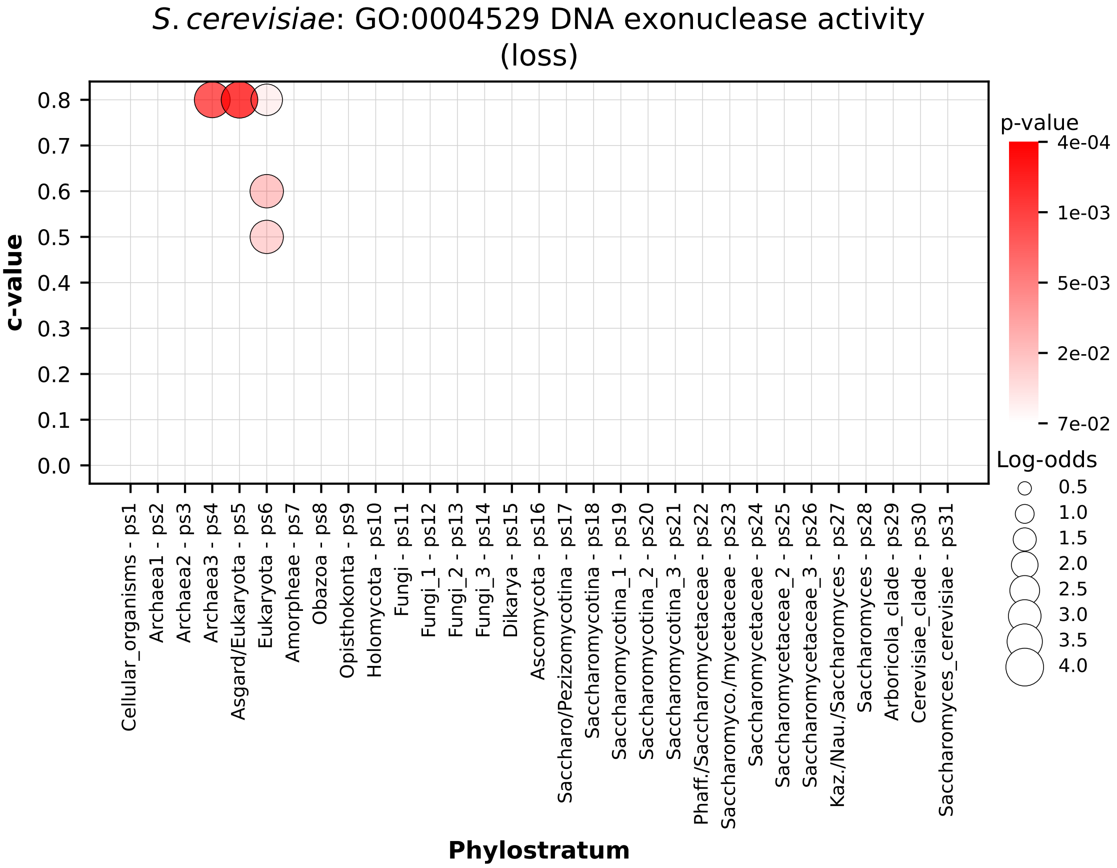
S. cerevisiae: GO:0004529 DNA exonuclease activity (loss)
c-value
0.8
0.7
0.6
0.5
0.4
0.3
0.2
0.1
0.0
p-value
4e-04
1e-03
5e-03
2e-02
7e-02
Log-odds
0.5
1.0
1.5
2.0
2.5
3.0
3.5
4.0
Cellular_organisms - ps1
Archaea1 - ps2
Archaea2 - ps3
Archaea3 - ps4
Asgard/Eukaryota - ps5
Eukaryota - ps6
Amorpheae - ps7
Obazoa - ps8
Opisthokonta - ps9
Holomycota - ps10
Fungi - ps11
Fungi_1 - ps12
Fungi_2 - ps13
Fungi_3 - ps14
Dikarya - ps15
Ascomycota - ps16
Saccharo/Pezizomycotina - ps17
Saccharomycotina - ps18
Saccharomycotina_1 - ps19
Saccharomycotina_2 - ps20
Saccharomycotina_3 - ps21
Phaff./Saccharomycetaceae - ps22
Saccharomyco./mycetaceae - ps23
Saccharomycetaceae - ps24
Saccharomycetaceae_2 - ps25
Saccharomycetaceae_3 - ps26
Kaz./Nau./Saccharomyces - ps27
Saccharomyces - ps28
Arboricola_clade - ps29
Cerevisiae_clade - ps30
Saccharomyces_cerevisiae - ps31
Phylostratum

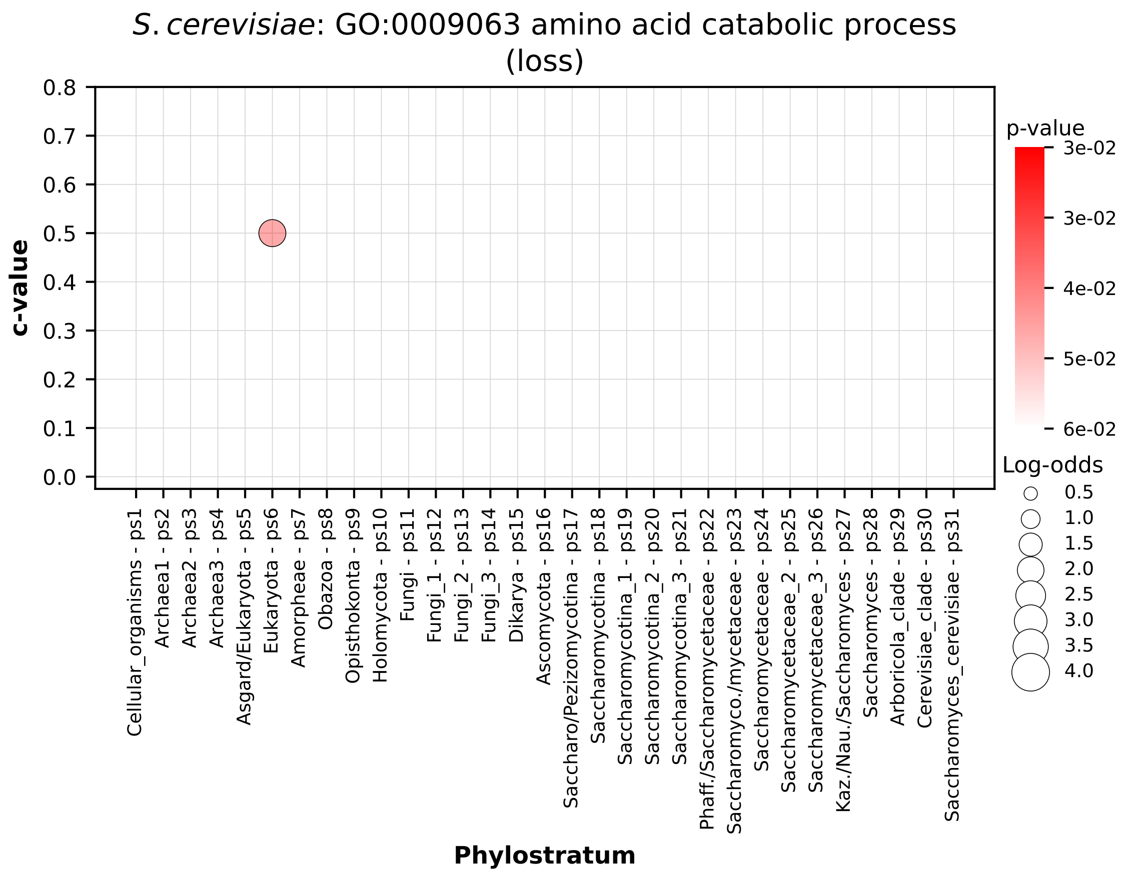
S. cerevisiae: GO:0009063 amino acid catabolic process
(loss)
c-value
0.8
0.7
0.6
0.5
0.4
0.3
0.2
0.1
0.0
p-value
3e-02
3e-02
4e-02
5e-02
6e-02
Log-odds
0.5
1.0
1.5
2.0
2.5
3.0
3.5
4.0
Cellular_organisms - ps1
Archaea1 - ps2
Archaea2 - ps3
Archaea3 - ps4
Asgard/Eukaryota - ps5
Eukaryota - ps6
Amorpheae - ps7
Obazoa - ps8
Opisthokonta - ps9
Holomycota - ps10
Fungi - ps11
Fungi_1 - ps12
Fungi_2 - ps13
Fungi_3 - ps14
Dikarya - ps15
Ascomycota - ps16
Saccharo/Pezizomycotina - ps17
Saccharomycotina - ps18
Saccharomycotina_1 - ps19
Saccharomycotina_2 - ps20
Saccharomycotina_3 - ps21
Phaff./Saccharomycetaceae - ps22
Saccharomyco./mycetaceae - ps23
Saccharomycetaceae - ps24
Saccharomycetaceae_2 - ps25
Saccharomycetaceae_3 - ps26
Kaz./Nau./Saccharomyces - ps27
Saccharomyces - ps28
Arboricola_clade - ps29
Cerevisiae_clade - ps30
Saccharomyces_cerevisiae - ps31
Phylostratum

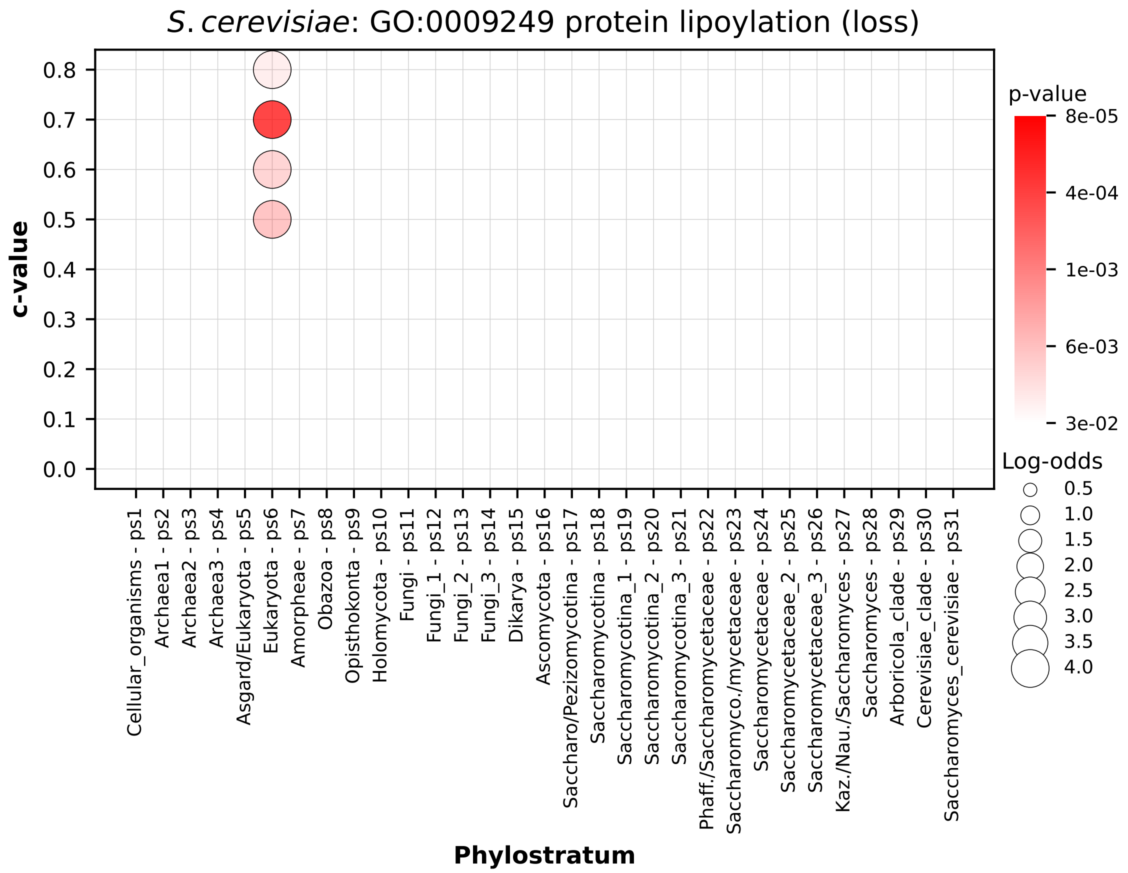

S. cerevisiae: GO:0009249 protein lipoylation (loss)
c-value
0.0
0.1
0.2
0.3
0.4
0.5
0.6
0.7
0.8
Phylostratum
Cellular_organisms - ps1
Archaea1 - ps2
Archaea2 - ps3
Archaea3 - ps4
Asgard/Eukaryota - ps5
Eukaryota - ps6
Amorpheae - ps7
Obazoa - ps8
Opisthokonta - ps9
Holomycota - ps10
Fungi - ps11
Fungi_1 - ps12
Fungi_2 - ps13
Fungi_3 - ps14
Dikarya - ps15
Ascomycota - ps16
Saccharo/Pezizomycotina - ps17
Saccharomycotina - ps18
Saccharomycotina_1 - ps19
Saccharomycotina_2 - ps20
Saccharomycotina_3 - ps21
Phaff./Saccharomycetaceae - ps22
Saccharomyco./mycetaceae - ps23
Saccharomycetaceae - ps24
Saccharomycetaceae_2 - ps25
Saccharomycetaceae_3 - ps26
Kaz./Nau./Saccharomyces - ps27
Saccharomyces - ps28
Arboricola_clade - ps29
Cerevisiae_clade - ps30
Saccharomyces_cerevisiae - ps31
p-value
8e-05
4e-04
1e-03
6e-03
3e-02
Log-odds
0.5
1.0
1.5
2.0
2.5
3.0
3.5
4.0

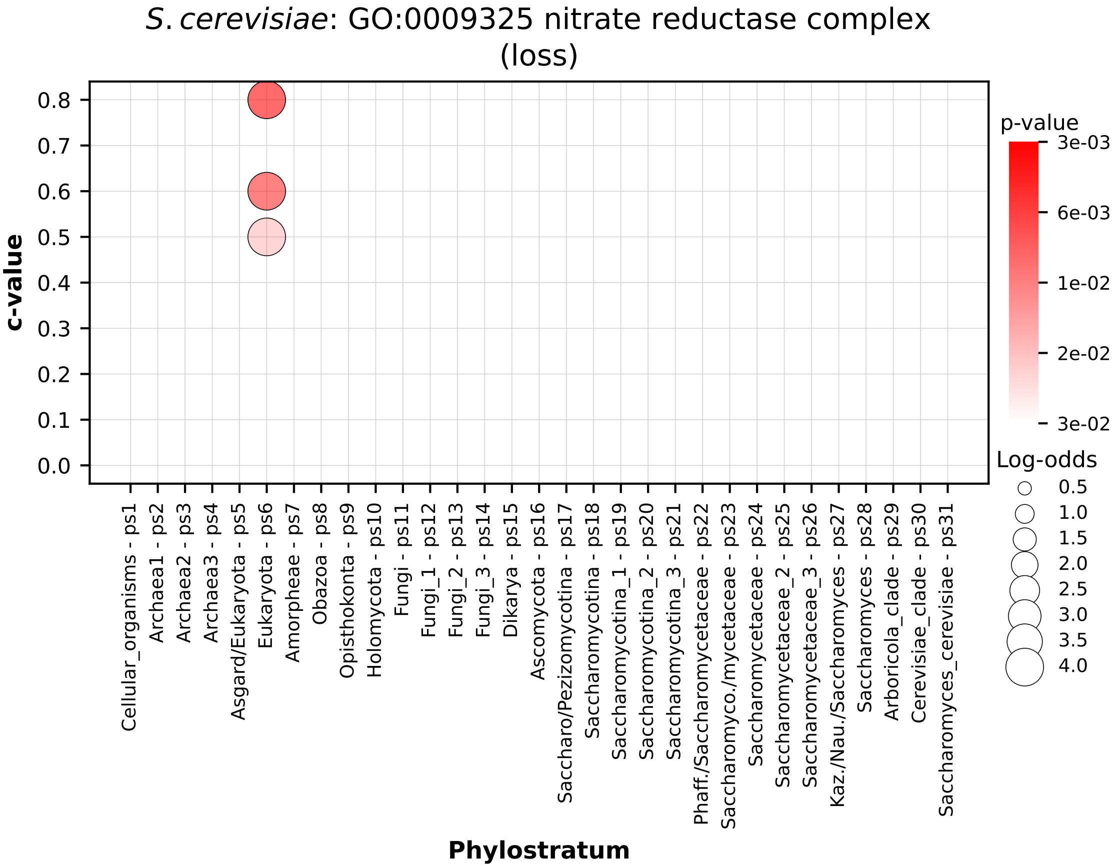

S. cerevisiae: GO:0009325 nitrate reductase complex
(loss)
c-value
0.0
0.1
0.2
0.3
0.4
0.5
0.6
0.7
0.8
Phylostratum
Cellular_organisms - ps1
Archaea1 - ps2
Archaea2 - ps3
Archaea3 - ps4
Asgard/Eukaryota - ps5
Eukaryota - ps6
Amorpheae - ps7
Obazoa - ps8
Opisthokonta - ps9
Holomycota - ps10
Fungi - ps11
Fungi_1 - ps12
Fungi_2 - ps13
Fungi_3 - ps14
Dikarya - ps15
Ascomycota - ps16
Saccharo/Pezizomycotina - ps17
Saccharomycotina - ps18
Saccharomycotina_1 - ps19
Saccharomycotina_2 - ps20
Saccharomycotina_3 - ps21
Phaff./Saccharomycetaceae - ps22
Saccharomyco./mycetaceae - ps23
Saccharomycetaceae - ps24
Saccharomycetaceae_2 - ps25
Saccharomycetaceae_3 - ps26
Kaz./Nau./Saccharomyces - ps27
Saccharomyces - ps28
Arboricola_clade - ps29
Cerevisiae_clade - ps30
Saccharomyces_cerevisiae - ps31
p-value
3e-03
6e-03
1e-02
2e-02
3e-02
Log-odds
0.5
1.0
1.5
2.0
2.5
3.0
3.5
4.0

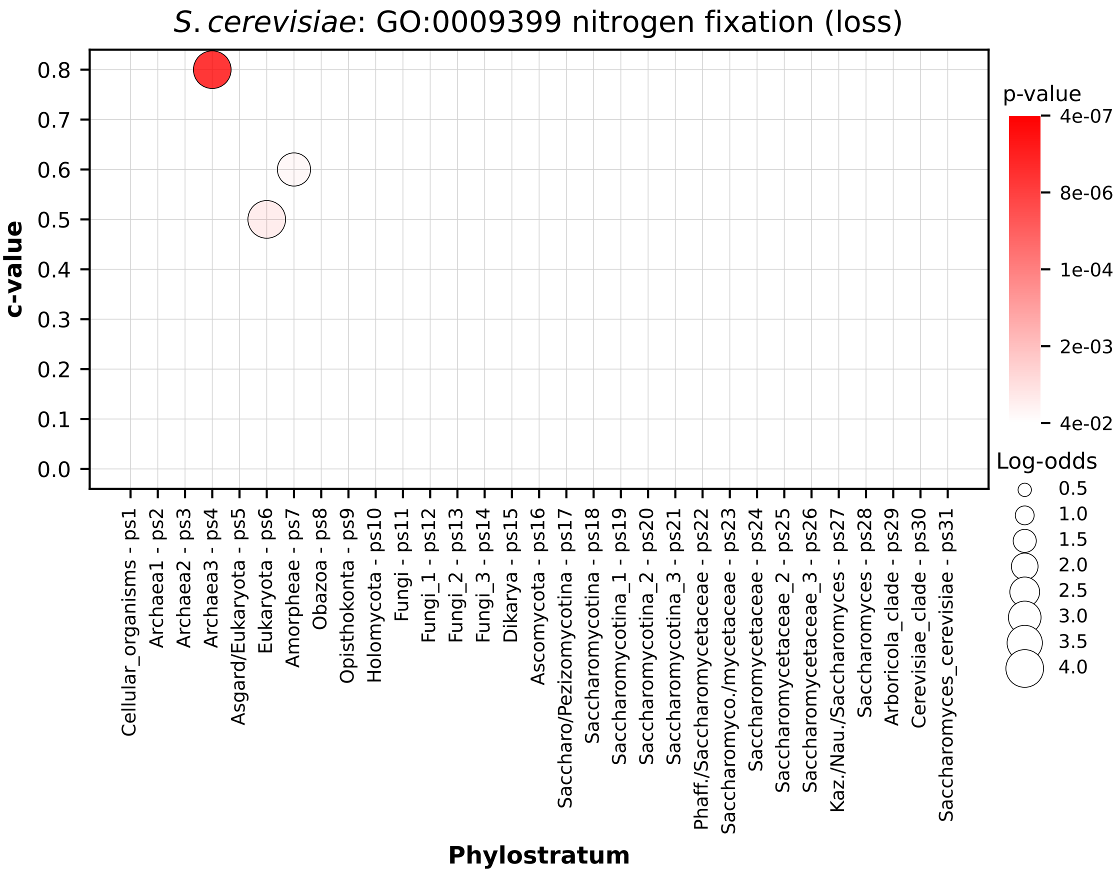
S. cerevisiae: GO:0009399 nitrogen fixation (loss)
c-value
0.0
0.1
0.2
0.3
0.4
0.5
0.6
0.7
0.8
Phylostratum
Cellular_organisms - ps1
Archaea1 - ps2
Archaea2 - ps3
Archaea3 - ps4
Asgard/Eukaryota - ps5
Eukaryota - ps6
Amorpheae - ps7
Obazoa - ps8
Opisthokonta - ps9
Holomycota - ps10
Fungi - ps11
Fungi_1 - ps12
Fungi_2 - ps13
Fungi_3 - ps14
Dikarya - ps15
Ascomycota - ps16
Saccharo/Pezizomycotina - ps17
Saccharomycotina - ps18
Saccharomycotina_1 - ps19
Saccharomycotina_2 - ps20
Saccharomycotina_3 - ps21
Phaff./Saccharomycetaceae - ps22
Saccharomyco./mycetaceae - ps23
Saccharomycetaceae - ps24
Saccharomycetaceae_2 - ps25
Saccharomycetaceae_3 - ps26
Kaz./Nau./Saccharomyces - ps27
Saccharomyces - ps28
Arboricola_clade - ps29
Cerevisiae_clade - ps30
Saccharomyces_cerevisiae - ps31
p-value
4e-07
8e-06
1e-04
2e-03
4e-02
Log-odds
0.5
1.0
1.5
2.0
2.5
3.0
3.5
4.0

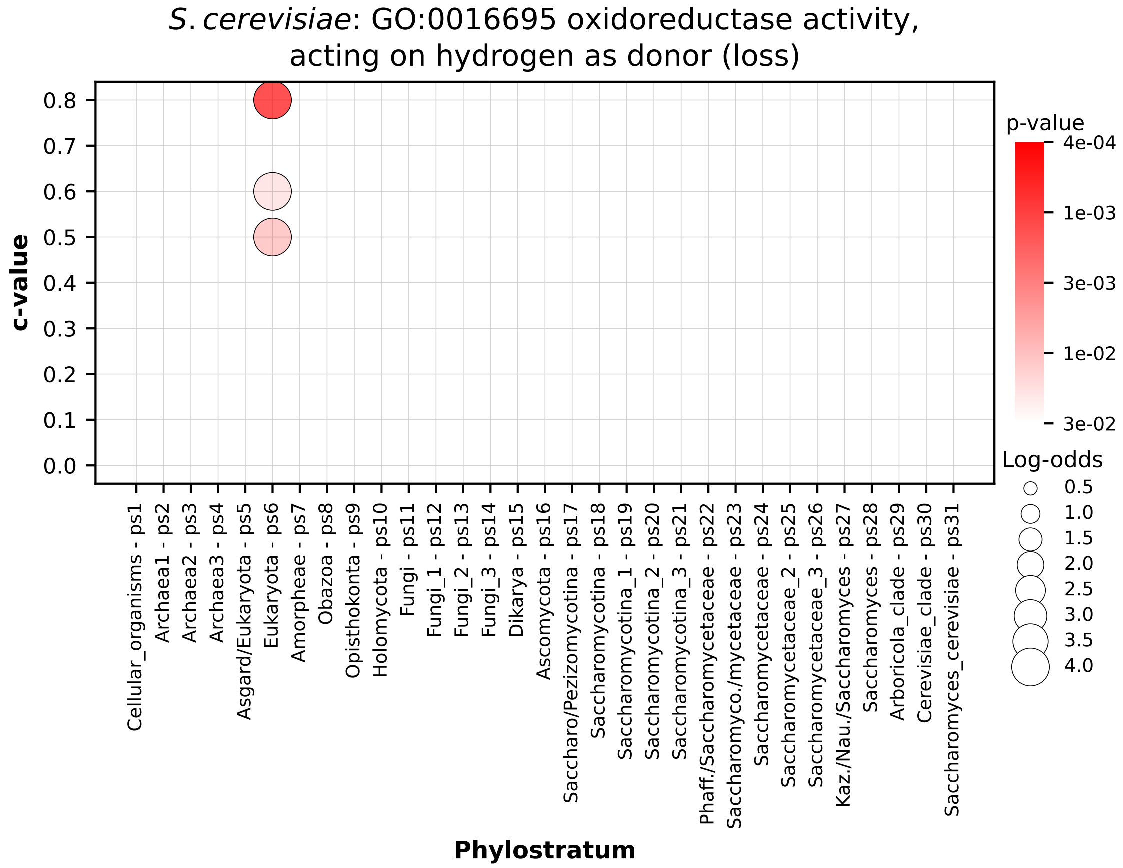
S. cerevisiae: GO:0016695 oxidoreductase activity, acting on hydrogen as donor (loss)
c-value
0.8
0.7
0.6
0.5
0.4
0.3
0.2
0.1
0.0
p-value
4e-04
1e-03
3e-03
1e-02
3e-02
Log-odds
0.5
1.0
1.5
2.0
2.5
3.0
3.5
4.0
Cellular_organisms - ps1
Archaea1 - ps2
Archaea2 - ps3
Archaea3 - ps4
Asgard/Eukaryota - ps5
Eukaryota - ps6
Amorpheae - ps7
Obazoa - ps8
Opisthokonta - ps9
Holomycota - ps10
Fungi - ps11
Fungi_1 - ps12
Fungi_2 - ps13
Fungi_3 - ps14
Dikarya - ps15
Ascomycota - ps16
Saccharo/Pezizomycotina - ps17
Saccharomycotina - ps18
Saccharomycotina_1 - ps19
Saccharomycotina_2 - ps20
Saccharomycotina_3 - ps21
Phaff./Saccharomycetaceae - ps22
Saccharomyco./mycetaceae - ps23
Saccharomycetaceae - ps24
Saccharomycetaceae_2 - ps25
Saccharomycetaceae_3 - ps26
Kaz./Nau./Saccharomyces - ps27
Saccharomyces - ps28
Arboricola_clade - ps29
Cerevisiae_clade - ps30
Saccharomyces_cerevisiae - ps31
Phylostratum

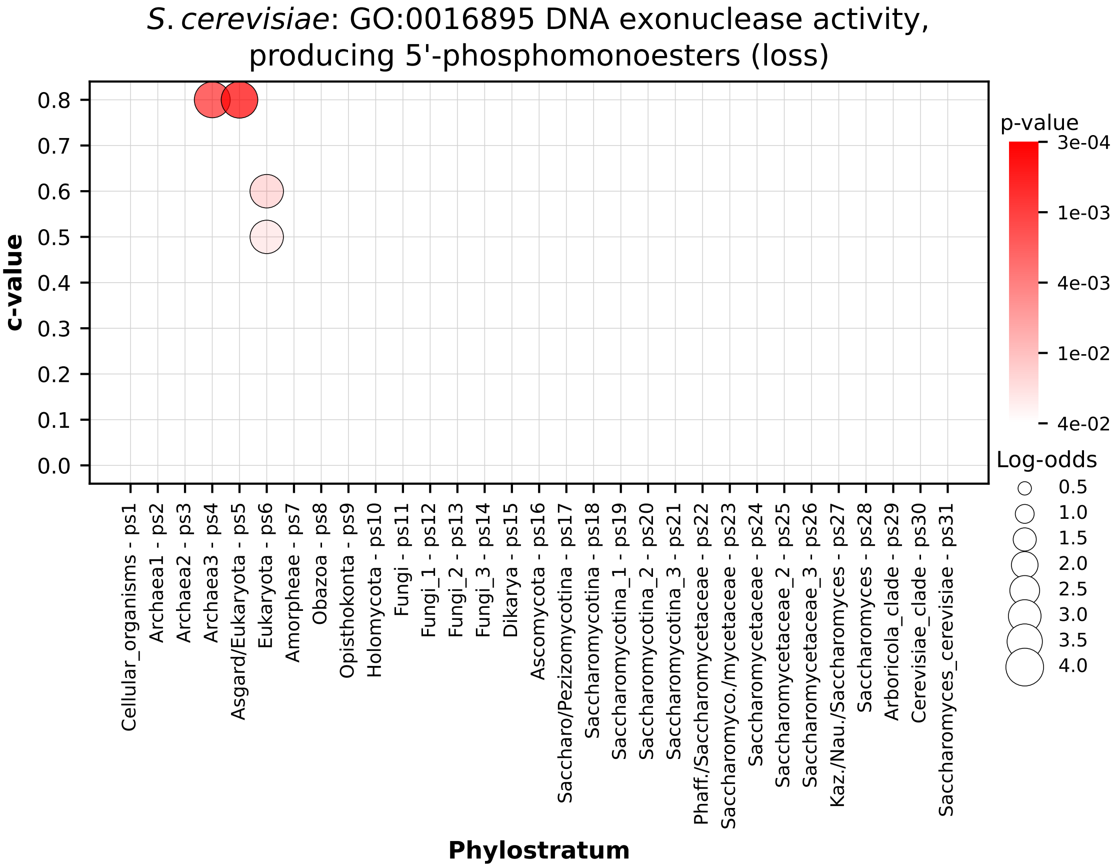
S. cerevisiae: GO:0016895 DNA exonuclease activity, producing 5'-phosphomonoesters (loss)
c-value
0.0
0.1
0.2
0.3
0.4
0.5
0.6
0.7
0.8
Phylostratum
Cellular_organisms - ps1
Archaea1 - ps2
Archaea2 - ps3
Archaea3 - ps4
Asgard/Eukaryota - ps5
Eukaryota - ps6
Amorpheae - ps7
Obazoa - ps8
Opisthokonta - ps9
Holomycota - ps10
Fungi - ps11
Fungi_1 - ps12
Fungi_2 - ps13
Fungi_3 - ps14
Dikarya - ps15
Ascomycota - ps16
Saccharo/Pezizomycotina - ps17
Saccharomycotina - ps18
Saccharomycotina_1 - ps19
Saccharomycotina_2 - ps20
Saccharomycotina_3 - ps21
Phaff./Saccharomycetaceae - ps22
Saccharomyco./mycetaceae - ps23
Saccharomycetaceae - ps24
Saccharomycetaceae_2 - ps25
Saccharomycetaceae_3 - ps26
Kaz./Nau./Saccharomyces - ps27
Saccharomyces - ps28
Arboricola_clade - ps29
Cerevisiae_clade - ps30
Saccharomyces_cerevisiae - ps31
p-value
3e-04
1e-03
4e-03
1e-02
4e-02
Log-odds
0.5
1.0
1.5
2.0
2.5
3.0
3.5
4.0

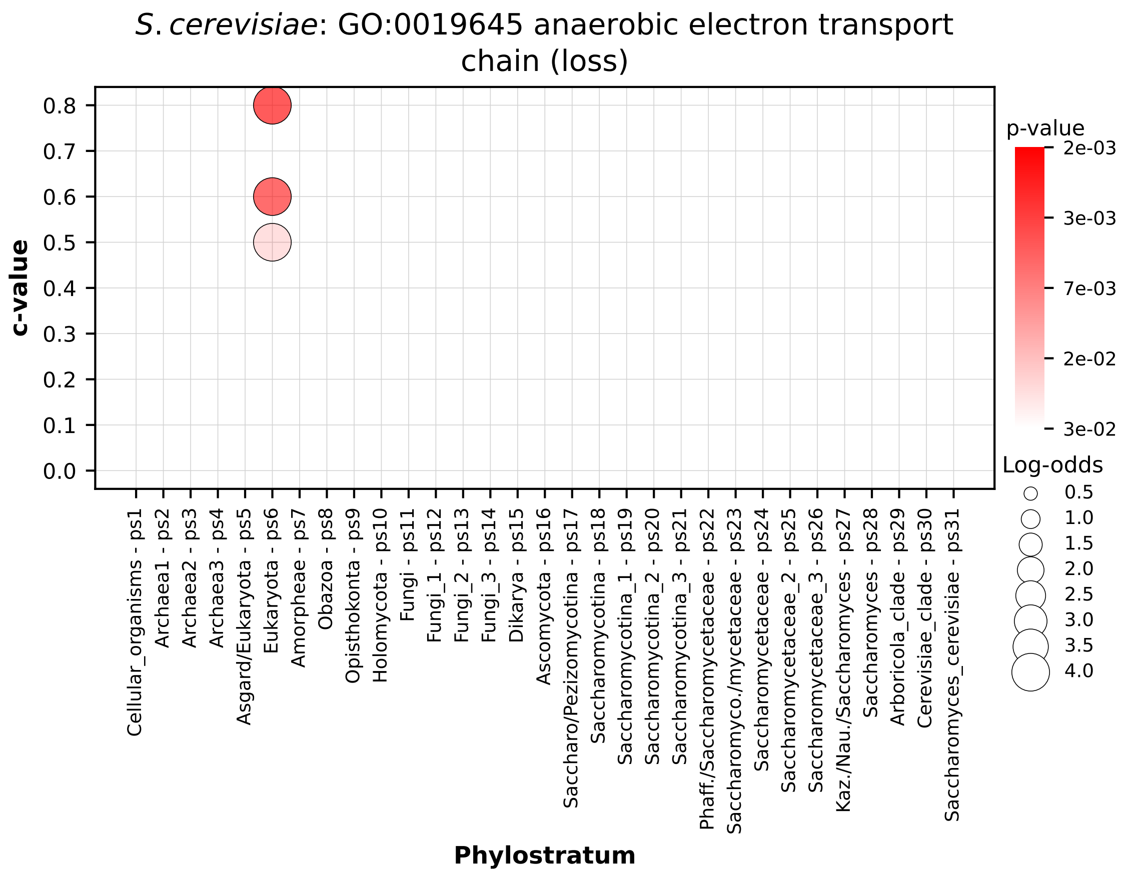

S. cerevisiae: GO:0019645 anaerobic electron transport chain (loss)
c-value
0.8
0.7
0.6
0.5
0.4
0.3
0.2
0.1
0.0
p-value
2e-03
3e-03
7e-03
2e-02
3e-02
Log-odds
0.5
1.0
1.5
2.0
2.5
3.0
3.5
4.0
Cellular_organisms - ps1
Archaea1 - ps2
Archaea2 - ps3
Archaea3 - ps4
Asgard/Eukaryota - ps5
Eukaryota - ps6
Amorpheae - ps7
Obazoa - ps8
Opisthokonta - ps9
Holomycota - ps10
Fungi - ps11
Fungi_1 - ps12
Fungi_2 - ps13
Fungi_3 - ps14
Dikarya - ps15
Ascomycota - ps16
Saccharo/Pezizomycotina - ps17
Saccharomycotina - ps18
Saccharomycotina_1 - ps19
Saccharomycotina_2 - ps20
Saccharomycotina_3 - ps21
Phaff./Saccharomycetaceae - ps22
Saccharomyco./mycetaceae - ps23
Saccharomycetaceae - ps24
Saccharomycetaceae_2 - ps25
Saccharomycetaceae_3 - ps26
Kaz./Nau./Saccharomyces - ps27
Saccharomyces - ps28
Arboricola_clade - ps29
Cerevisiae_clade - ps30
Saccharomyces_cerevisiae - ps31
Phylostratum

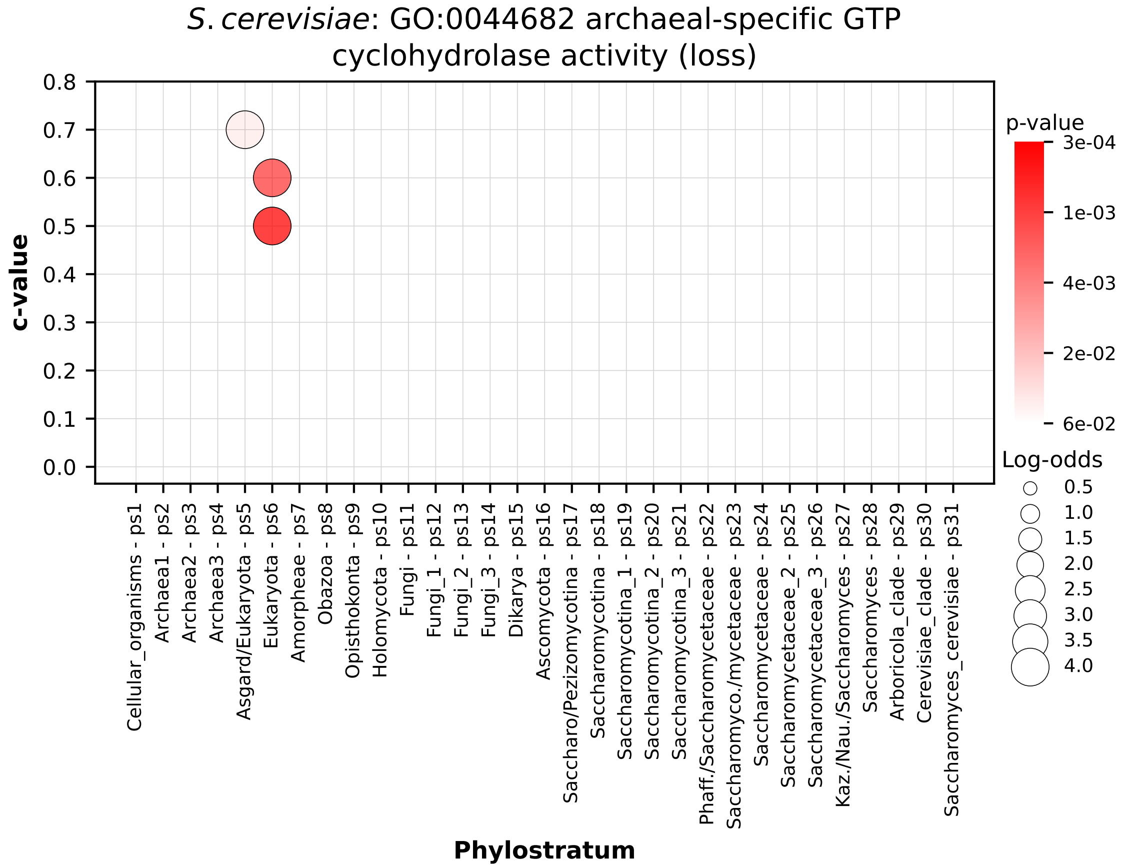
S. cerevisiae: GO:0044682 archaeal-specific GTP cyclohydrolase activity (loss)
c-value
0.8
0.7
0.6
0.5
0.4
0.3
0.2
0.1
0.0
p-value
3e-04
1e-03
4e-03
2e-02
6e-02
Log-odds
0.5
1.0
1.5
2.0
2.5
3.0
3.5
4.0
Cellular_organisms - ps1
Archaea1 - ps2
Archaea2 - ps3
Archaea3 - ps4
Asgard/Eukaryota - ps5
Eukaryota - ps6
Amorpheae - ps7
Obazoa - ps8
Opisthokonta - ps9
Holomycota - ps10
Fungi - ps11
Fungi_1 - ps12
Fungi_2 - ps13
Fungi_3 - ps14
Dikarya - ps15
Ascomycota - ps16
Saccharo/Pezizomycotina - ps17
Saccharomycotina - ps18
Saccharomycotina_1 - ps19
Saccharomycotina_2 - ps20
Saccharomycotina_3 - ps21
Phaff./Saccharomycetaceae - ps22
Saccharomyco./mycetaceae - ps23
Saccharomycetaceae - ps24
Saccharomycetaceae_2 - ps25
Saccharomycetaceae_3 - ps26
Kaz./Nau./Saccharomyces - ps27
Saccharomyces - ps28
Arboricola_clade - ps29
Cerevisiae_clade - ps30
Saccharomyces_cerevisiae - ps31
Phylostratum

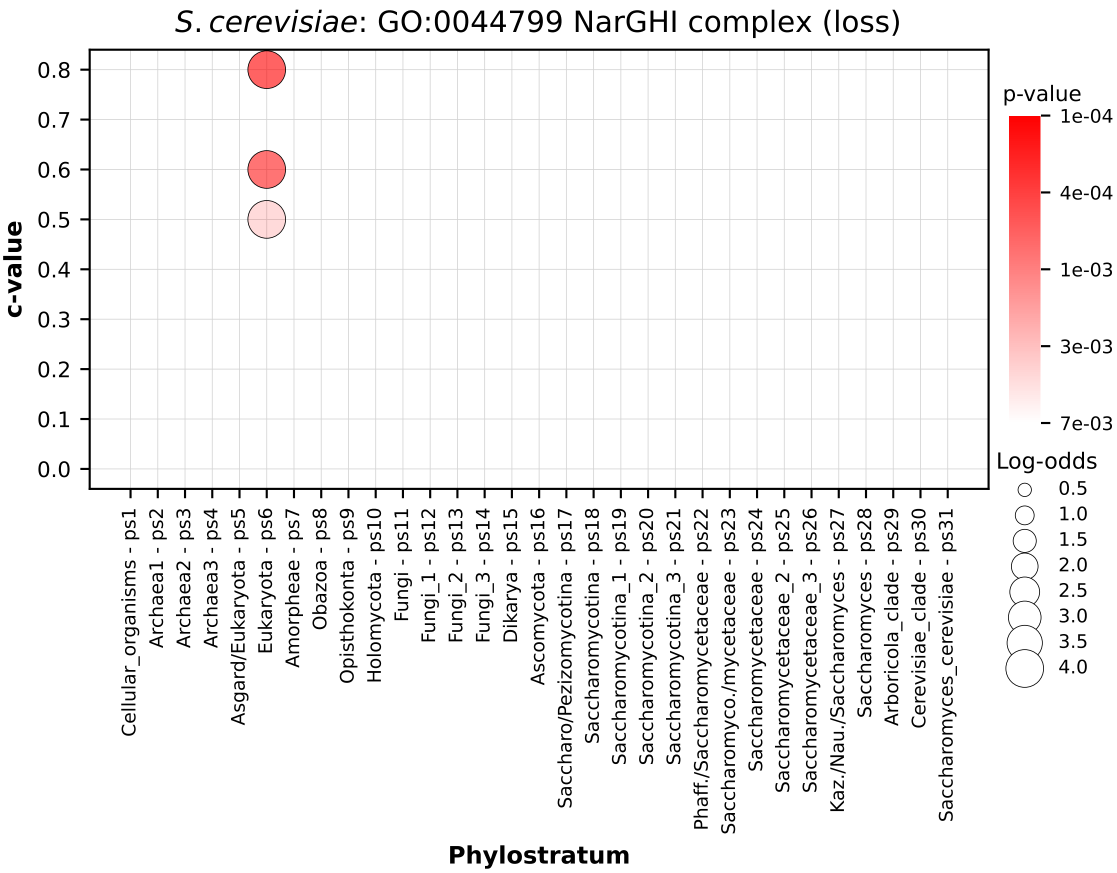

S. cerevisiae: GO:0044799 NarGHI complex (loss)
c-value
0.0
0.1
0.2
0.3
0.4
0.5
0.6
0.7
0.8
Phylostratum
Cellular_organisms - ps1
Archaea1 - ps2
Archaea2 - ps3
Archaea3 - ps4
Asgard/Eukaryota - ps5
Eukaryota - ps6
Amorpheae - ps7
Obazoa - ps8
Opisthokonta - ps9
Holomycota - ps10
Fungi - ps11
Fungi_1 - ps12
Fungi_2 - ps13
Fungi_3 - ps14
Dikarya - ps15
Ascomycota - ps16
Saccharo/Pezizomycotina - ps17
Saccharomycotina - ps18
Saccharomycotina_1 - ps19
Saccharomycotina_2 - ps20
Saccharomycotina_3 - ps21
Phaff./Saccharomycetaceae - ps22
Saccharomyco./mycetaceae - ps23
Saccharomycetaceae - ps24
Saccharomycetaceae_2 - ps25
Saccharomycetaceae_3 - ps26
Kaz./Nau./Saccharomyces - ps27
Saccharomyces - ps28
Arboricola_clade - ps29
Cerevisiae_clade - ps30
Saccharomyces_cerevisiae - ps31
p-value
1e-04
4e-04
1e-03
3e-03
7e-03
Log-odds
0.5
1.0
1.5
2.0
2.5
3.0
3.5
4.0

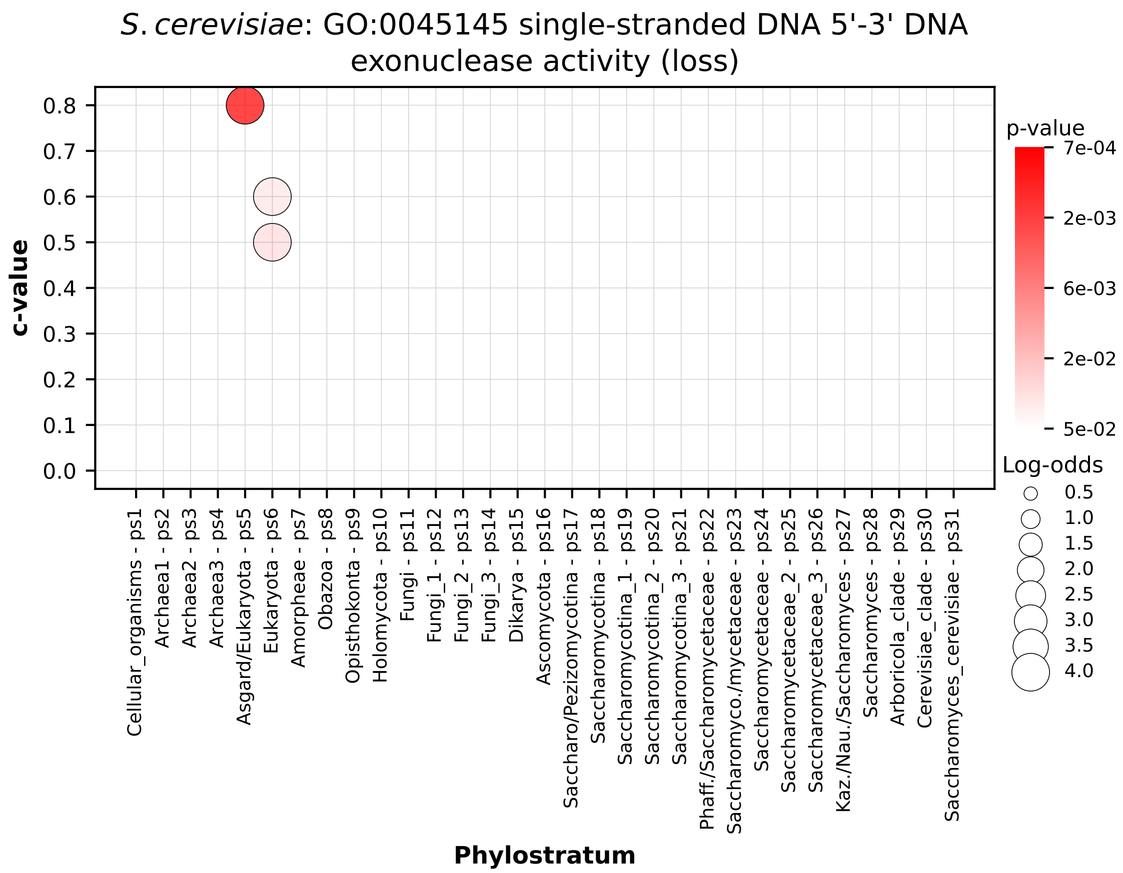

S. cerevisiae: GO:0045145 single-stranded DNA 5'-3' DNA exonuclease activity (loss)
c-value
0.0
0.1
0.2
0.3
0.4
0.5
0.6
0.7
0.8
p-value
7e-04
2e-03
6e-03
2e-02
5e-02
Log-odds
0.5
1.0
1.5
2.0
2.5
3.0
3.5
4.0
Cellular_organisms - ps1
Archaea1 - ps2
Archaea2 - ps3
Archaea3 - ps4
Asgard/Eukaryota - ps5
Eukaryota - ps6
Amorpheae - ps7
Obazoa - ps8
Opisthokonta - ps9
Holomycota - ps10
Fungi - ps11
Fungi_1 - ps12
Fungi_2 - ps13
Fungi_3 - ps14
Dikarya - ps15
Ascomycota - ps16
Saccharo/Pezizomycotina - ps17
Saccharomycotina - ps18
Saccharomycotina_1 - ps19
Saccharomycotina_2 - ps20
Saccharomycotina_3 - ps21
Phaff./Saccharomycetaceae - ps22
Saccharomyco./mycetaceae - ps23
Saccharomycetaceae - ps24
Saccharomycetaceae_2 - ps25
Saccharomycetaceae_3 - ps26
Kaz./Nau./Saccharomyces - ps27
Saccharomyces - ps28
Arboricola_clade - ps29
Cerevisiae_clade - ps30
Saccharomyces_cerevisiae - ps31
Phylostratum

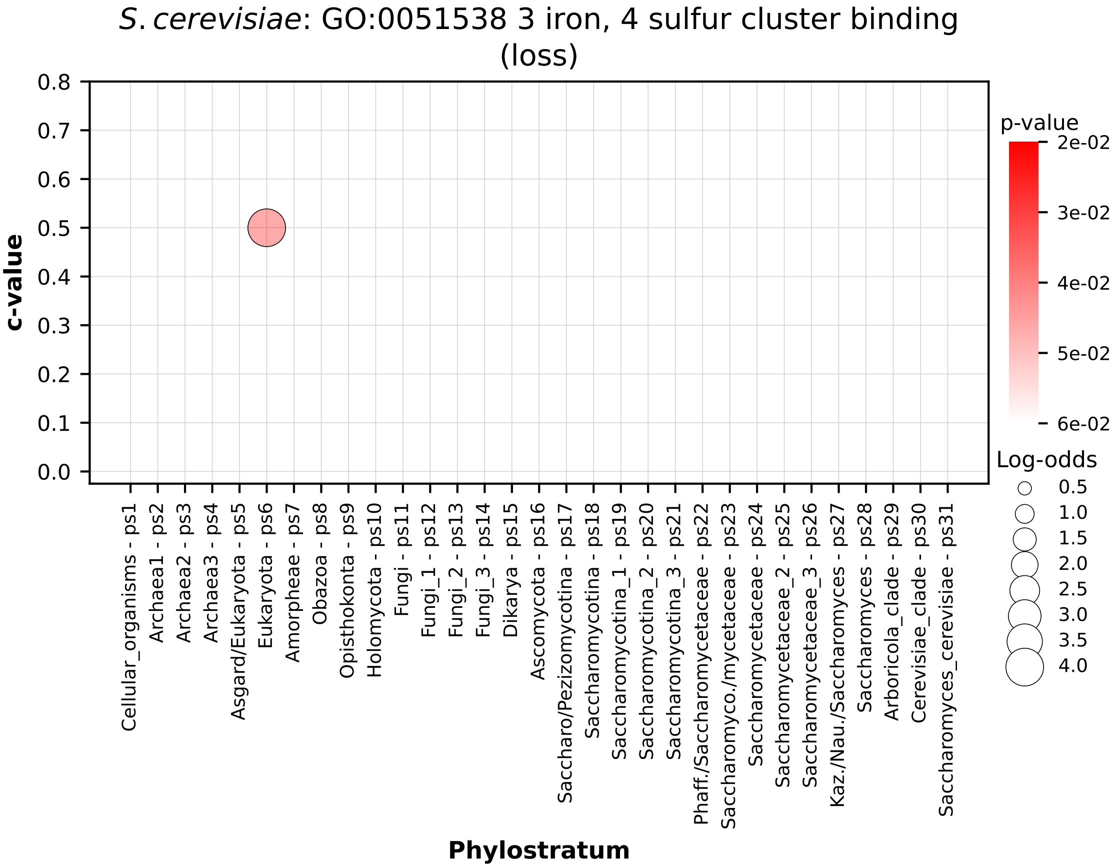

S. cerevisiae: GO:0051538 3 iron, 4 sulfur cluster binding (loss)
c-value
0.8
0.7
0.6
0.5
0.4
0.3
0.2
0.1
0.0
p-value
2e-02
3e-02
4e-02
5e-02
6e-02
Log-odds
0.5
1.0
1.5
2.0
2.5
3.0
3.5
4.0
Cellular_organisms - ps1
Archaea1 - ps2
Archaea2 - ps3
Archaea3 - ps4
Asgard/Eukaryota - ps5
Eukaryota - ps6
Amorpheae - ps7
Obazoa - ps8
Opisthokonta - ps9
Holomycota - ps10
Fungi - ps11
Fungi_1 - ps12
Fungi_2 - ps13
Fungi_3 - ps14
Dikarya - ps15
Ascomycota - ps16
Saccharo/Pezizomycotina - ps17
Saccharomycotina - ps18
Saccharomycotina_1 - ps19
Saccharomycotina_2 - ps20
Saccharomycotina_3 - ps21
Phaff./Saccharomycetaceae - ps22
Saccharomyco./mycetaceae - ps23
Saccharomycetaceae - ps24
Saccharomycetaceae_2 - ps25
Saccharomycetaceae_3 - ps26
Kaz./Nau./Saccharomyces - ps27
Saccharomyces - ps28
Arboricola_clade - ps29
Cerevisiae_clade - ps30
Saccharomyces_cerevisiae - ps31
Phylostratum

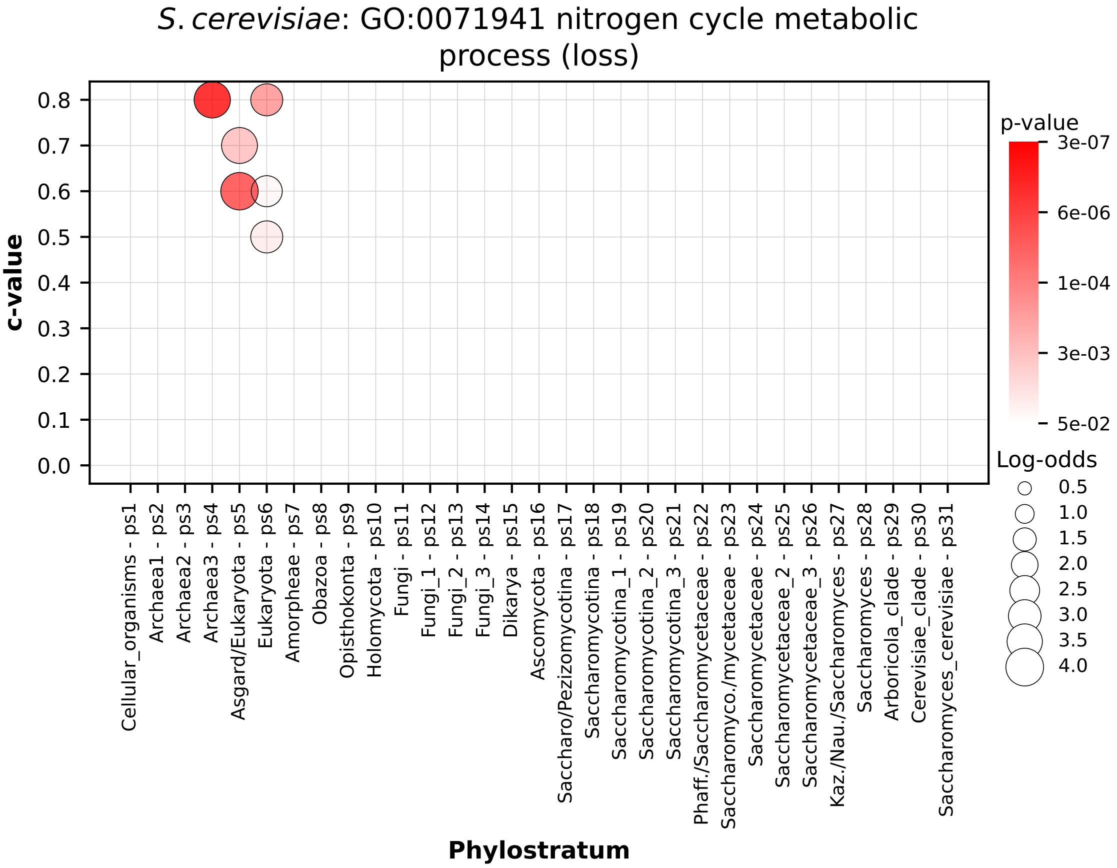

$S. cerevisiae$: GO:0071941 nitrogen cycle metabolic process (loss)
c-value
0.8
0.7
0.6
0.5
0.4
0.3
0.2
0.1
0.0
Phylostratum
Cellular_organisms - ps1
Archaea1 - ps2
Archaea2 - ps3
Archaea3 - ps4
Asgard/Eukaryota - ps5
Eukaryota - ps6
Amorpheae - ps7
Obazoa - ps8
Opisthokonta - ps9
Holomycota - ps10
Fungi - ps11
Fungi_1 - ps12
Fungi_2 - ps13
Fungi_3 - ps14
Dikarya - ps15
Ascomycota - ps16
Saccharo/Pezizomycotina - ps17
Saccharomycotina - ps18
Saccharomycotina_1 - ps19
Saccharomycotina_2 - ps20
Saccharomycotina_3 - ps21
Phaff./Saccharomycetaceae - ps22
Saccharomyco./mycetaceae - ps23
Saccharomycetaceae - ps24
Saccharomycetaceae_2 - ps25
Saccharomycetaceae_3 - ps26
Kaz./Nau./Saccharomyces - ps27
Saccharomyces - ps28
Arboricola_clade - ps29
Cerevisiae_clade - ps30
Saccharomyces_cerevisiae - ps31
p-value
3e-07
6e-06
1e-04
3e-03
5e-02
Log-odds
0.5
1.0
1.5
2.0
2.5
3.0
3.5
4.0

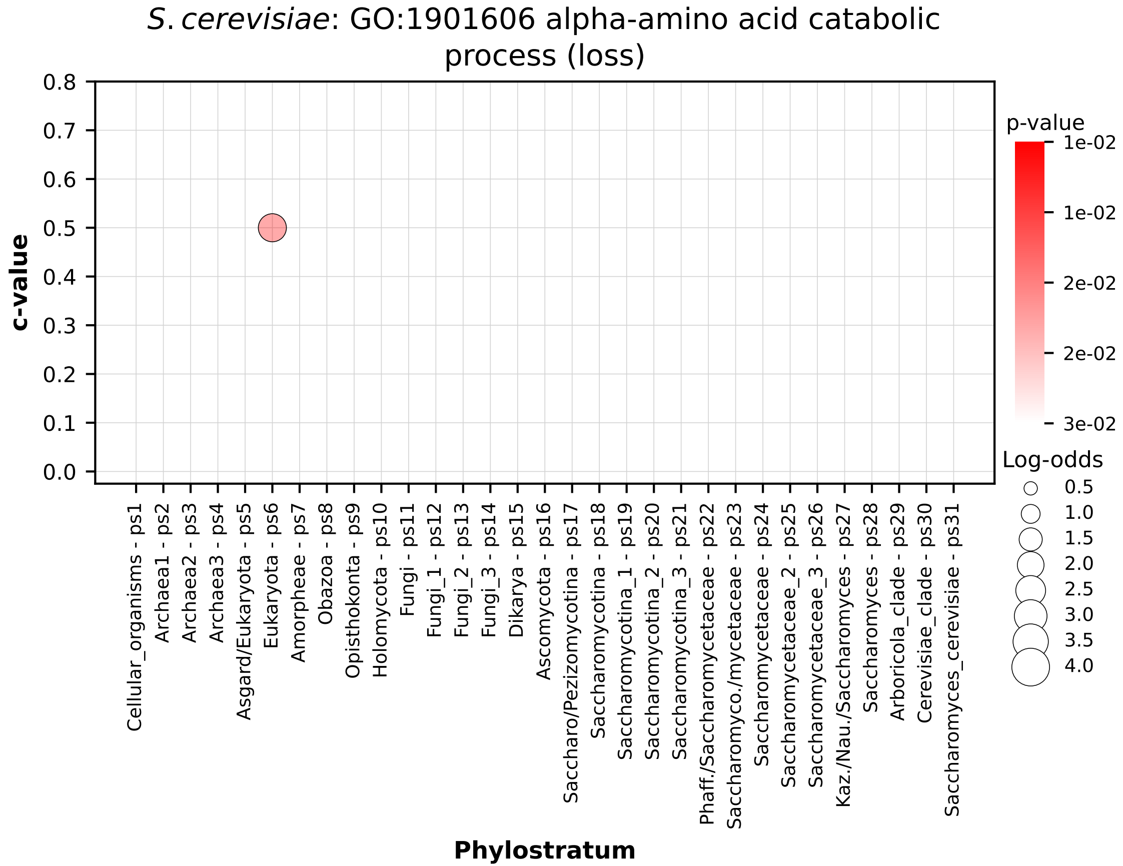

S. cerevisiae: GO:1901606 alpha-amino acid catabolic process (loss)
c-value
0.8
0.7
0.6
0.5
0.4
0.3
0.2
0.1
0.0
p-value
1e-02
1e-02
2e-02
2e-02
3e-02
Log-odds
0.5
1.0
1.5
2.0
2.5
3.0
3.5
4.0
Cellular_organisms - ps1
Archaea1 - ps2
Archaea2 - ps3
Archaea3 - ps4
Asgard/Eukaryota - ps5
Eukaryota - ps6
Amorpheae - ps7
Obazoa - ps8
Opisthokonta - ps9
Holomycota - ps10
Fungi - ps11
Fungi_1 - ps12
Fungi_2 - ps13
Fungi_3 - ps14
Dikarya - ps15
Ascomycota - ps16
Saccharo/Pezizomycotina - ps17
Saccharomycotina - ps18
Saccharomycotina_1 - ps19
Saccharomycotina_2 - ps20
Saccharomycotina_3 - ps21
Phaff./Saccharomycetaceae - ps22
Saccharomyco./mycetaceae - ps23
Saccharomycetaceae - ps24
Saccharomycetaceae_2 - ps25
Saccharomycetaceae_3 - ps26
Kaz./Nau./Saccharomyces - ps27
Saccharomyces - ps28
Arboricola_clade - ps29
Cerevisiae_clade - ps30
Saccharomyces_cerevisiae - ps31
Phylostratum

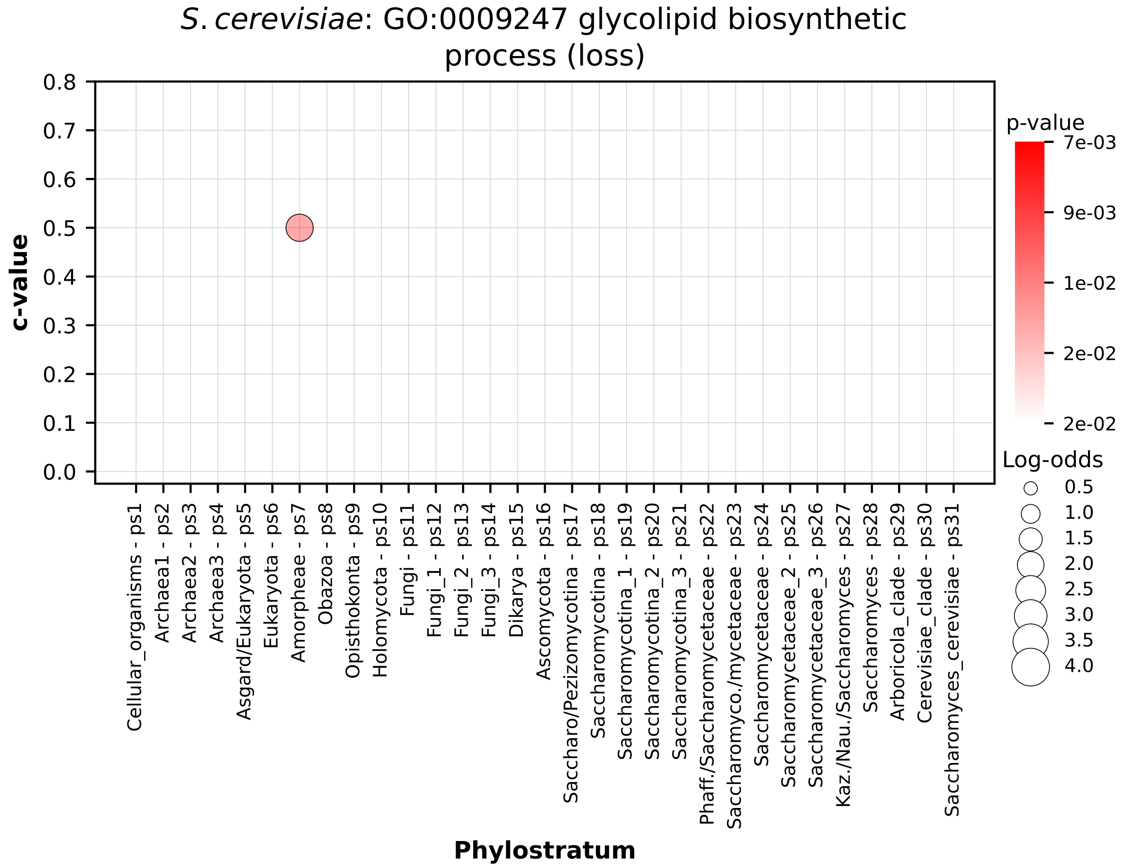

S. cerevisiae: GO:0009247 glycolipid biosynthetic process (loss)
c-value
0.8
0.7
0.6
0.5
0.4
0.3
0.2
0.1
0.0
Phylostratum
Cellular_organisms - ps1
Archaea1 - ps2
Archaea2 - ps3
Archaea3 - ps4
Asgard/Eukaryota - ps5
Eukaryota - ps6
Amorpheae - ps7
Obazoa - ps8
Opisthokonta - ps9
Holomycota - ps10
Fungi - ps11
Fungi_1 - ps12
Fungi_2 - ps13
Fungi_3 - ps14
Dikarya - ps15
Ascomycota - ps16
Saccharo/Pezizomycotina - ps17
Saccharomycotina - ps18
Saccharomycotina_1 - ps19
Saccharomycotina_2 - ps20
Saccharomycotina_3 - ps21
Phaff./Saccharomycetaceae - ps22
Saccharomyco./mycetaceae - ps23
Saccharomycetaceae - ps24
Saccharomycetaceae_2 - ps25
Saccharomycetaceae_3 - ps26
Kaz./Nau./Saccharomyces - ps27
Saccharomyces - ps28
Arboricola_clade - ps29
Cerevisiae_clade - ps30
Saccharomyces_cerevisiae - ps31
p-value
7e-03
9e-03
1e-02
2e-02
2e-02
Log-odds
0.5
1.0
1.5
2.0
2.5
3.0
3.5
4.0

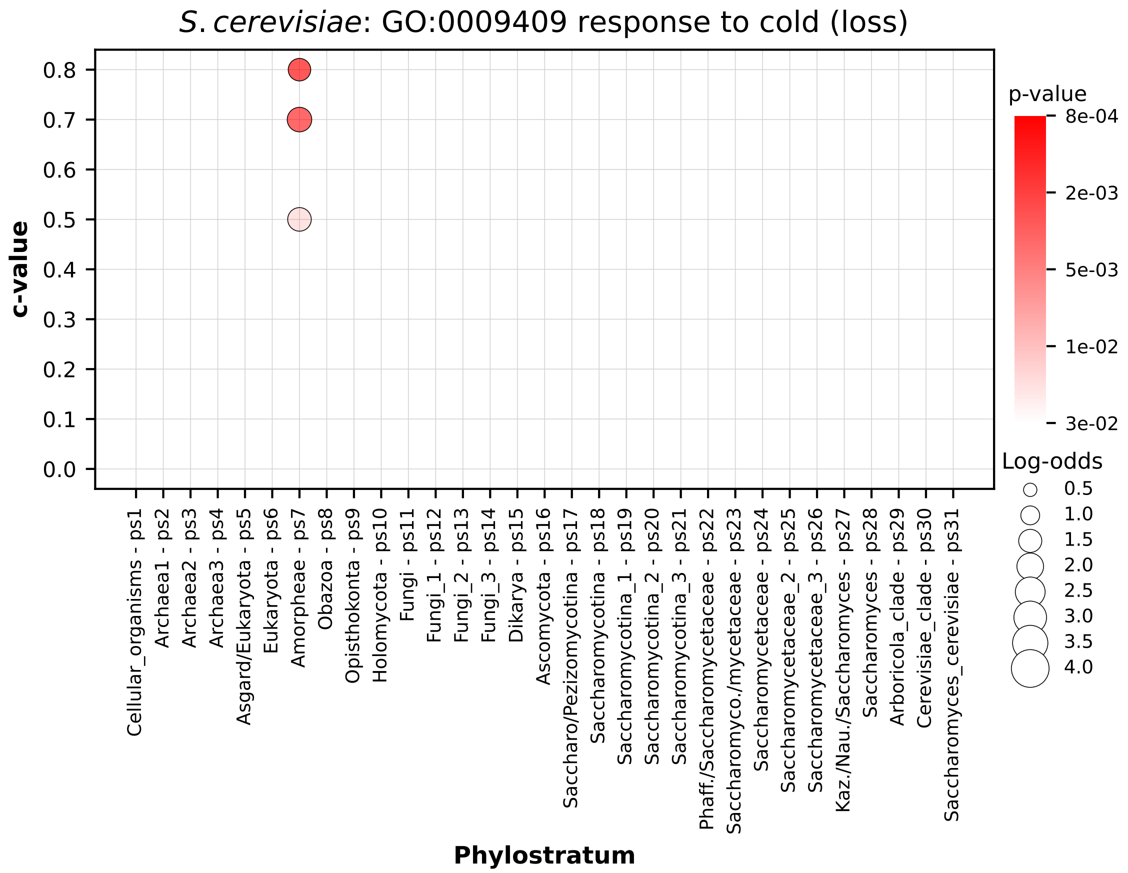

S. cerevisiae: GO:0009409 response to cold (loss)
c-value
0.8
0.7
0.6
0.5
0.4
0.3
0.2
0.1
0.0
p-value
8e-04
2e-03
5e-03
1e-02
3e-02
Log-odds
0.5
1.0
1.5
2.0
2.5
3.0
3.5
4.0
Cellular_organisms - ps1
Archaea1 - ps2
Archaea2 - ps3
Archaea3 - ps4
Asgard/Eukaryota - ps5
Eukaryota - ps6
Amorpheae - ps7
Obazoa - ps8
Opisthokonta - ps9
Holomycota - ps10
Fungi - ps11
Fungi_1 - ps12
Fungi_2 - ps13
Fungi_3 - ps14
Dikarya - ps15
Ascomycota - ps16
Saccharo/Pezizomycotina - ps17
Saccharomycotina - ps18
Saccharomycotina_1 - ps19
Saccharomycotina_2 - ps20
Saccharomycotina_3 - ps21
Phaff./Saccharomycetaceae - ps22
Saccharomyco./mycetaceae - ps23
Saccharomycetaceae - ps24
Saccharomycetaceae_2 - ps25
Saccharomycetaceae_3 - ps26
Kaz./Nau./Saccharomyces - ps27
Saccharomyces - ps28
Arboricola_clade - ps29
Cerevisiae_clade - ps30
Saccharomyces_cerevisiae - ps31
Phylostratum

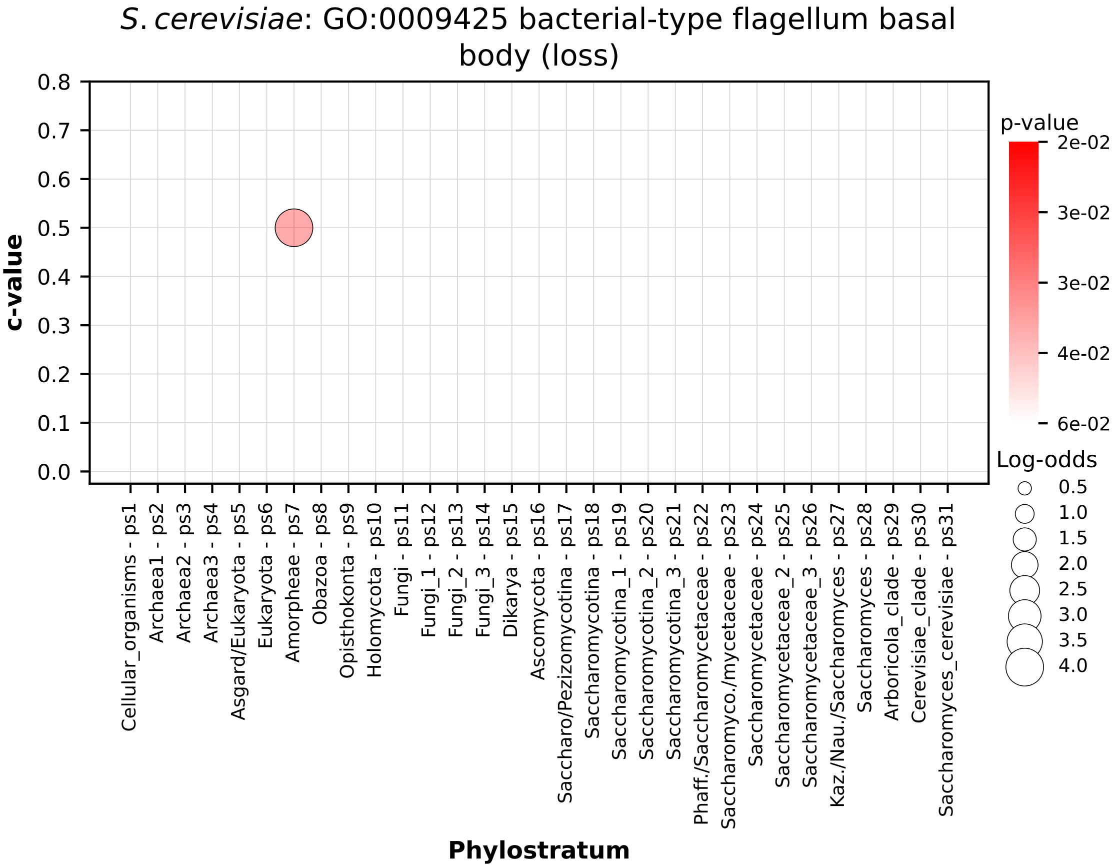

S. cerevisiae: GO:0009425 bacterial-type flagellum basal body (loss)
c-value
0.8
0.7
0.6
0.5
0.4
0.3
0.2
0.1
0.0
p-value
2e-02
3e-02
3e-02
4e-02
6e-02
Log-odds
0.5
1.0
1.5
2.0
2.5
3.0
3.5
4.0
Cellular_organisms - ps1
Archaea1 - ps2
Archaea2 - ps3
Archaea3 - ps4
Asgard/Eukaryota - ps5
Eukaryota - ps6
Amorpheae - ps7
Obazoa - ps8
Opisthokonta - ps9
Holomycota - ps10
Fungi - ps11
Fungi_1 - ps12
Fungi_2 - ps13
Fungi_3 - ps14
Dikarya - ps15
Ascomycota - ps16
Saccharo/Pezizomycotina - ps17
Saccharomycotina - ps18
Saccharomycotina_1 - ps19
Saccharomycotina_2 - ps20
Saccharomycotina_3 - ps21
Phaff./Saccharomycetaceae - ps22
Saccharomyco./mycetaceae - ps23
Saccharomycetaceae - ps24
Saccharomycetaceae_2 - ps25
Saccharomycetaceae_3 - ps26
Kaz./Nau./Saccharomyces - ps27
Saccharomyces - ps28
Arboricola_clade - ps29
Cerevisiae_clade - ps30
Saccharomyces_cerevisiae - ps31
Phylostratum

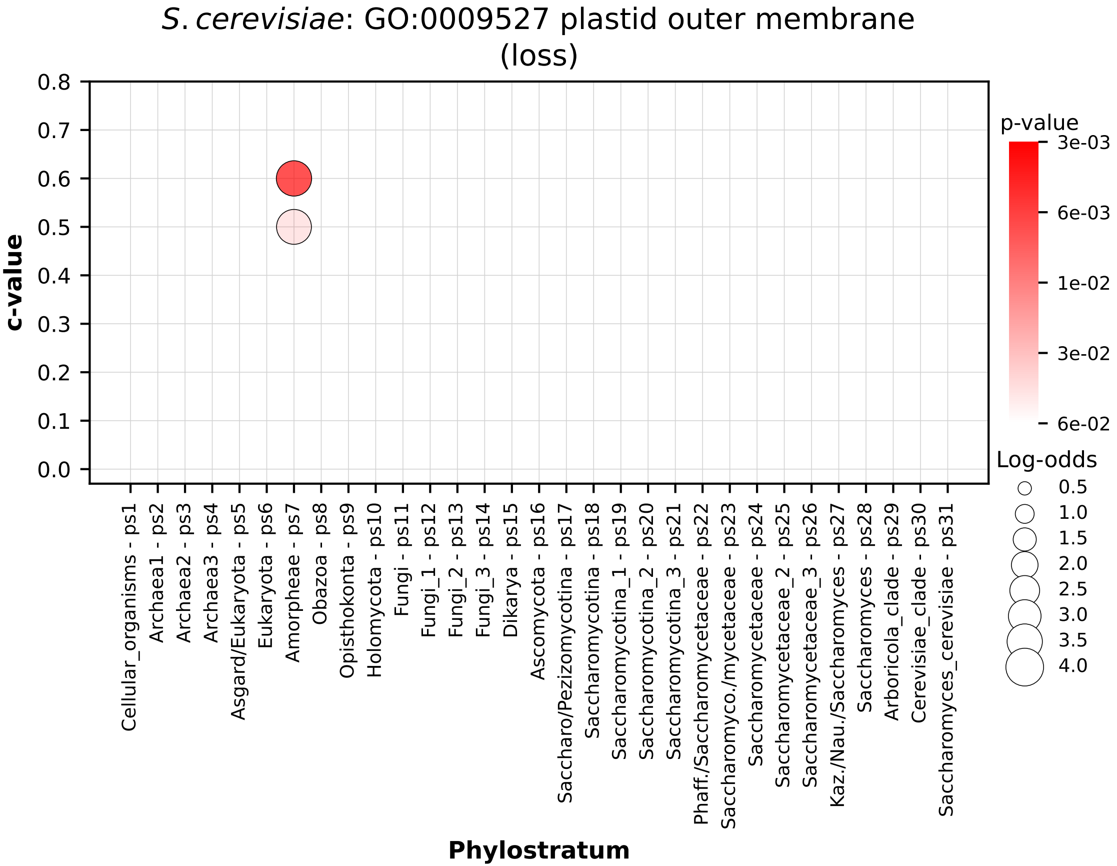

S. cerevisiae: GO:0009527 plastid outer membrane
(loss)
c-value
0.0
0.1
0.2
0.3
0.4
0.5
0.6
0.7
0.8
p-value
3e-03
6e-03
1e-02
3e-02
6e-02
Log-odds
0.5
1.0
1.5
2.0
2.5
3.0
3.5
4.0
Cellular_organisms - ps1
Archaea1 - ps2
Archaea2 - ps3
Archaea3 - ps4
Asgard/Eukaryota - ps5
Eukaryota - ps6
Amorpheae - ps7
Obazoa - ps8
Opisthokonta - ps9
Holomycota - ps10
Fungi - ps11
Fungi_1 - ps12
Fungi_2 - ps13
Fungi_3 - ps14
Dikarya - ps15
Ascomycota - ps16
Saccharo/Pezizomycotina - ps17
Saccharomycotina - ps18
Saccharomycotina_1 - ps19
Saccharomycotina_2 - ps20
Saccharomycotina_3 - ps21
Phaff./Saccharomycetaceae - ps22
Saccharomyco./mycetaceae - ps23
Saccharomycetaceae - ps24
Saccharomycetaceae_2 - ps25
Saccharomycetaceae_3 - ps26
Kaz./Nau./Saccharomyces - ps27
Saccharomyces - ps28
Arboricola_clade - ps29
Cerevisiae_clade - ps30
Saccharomyces_cerevisiae - ps31
Phylostratum

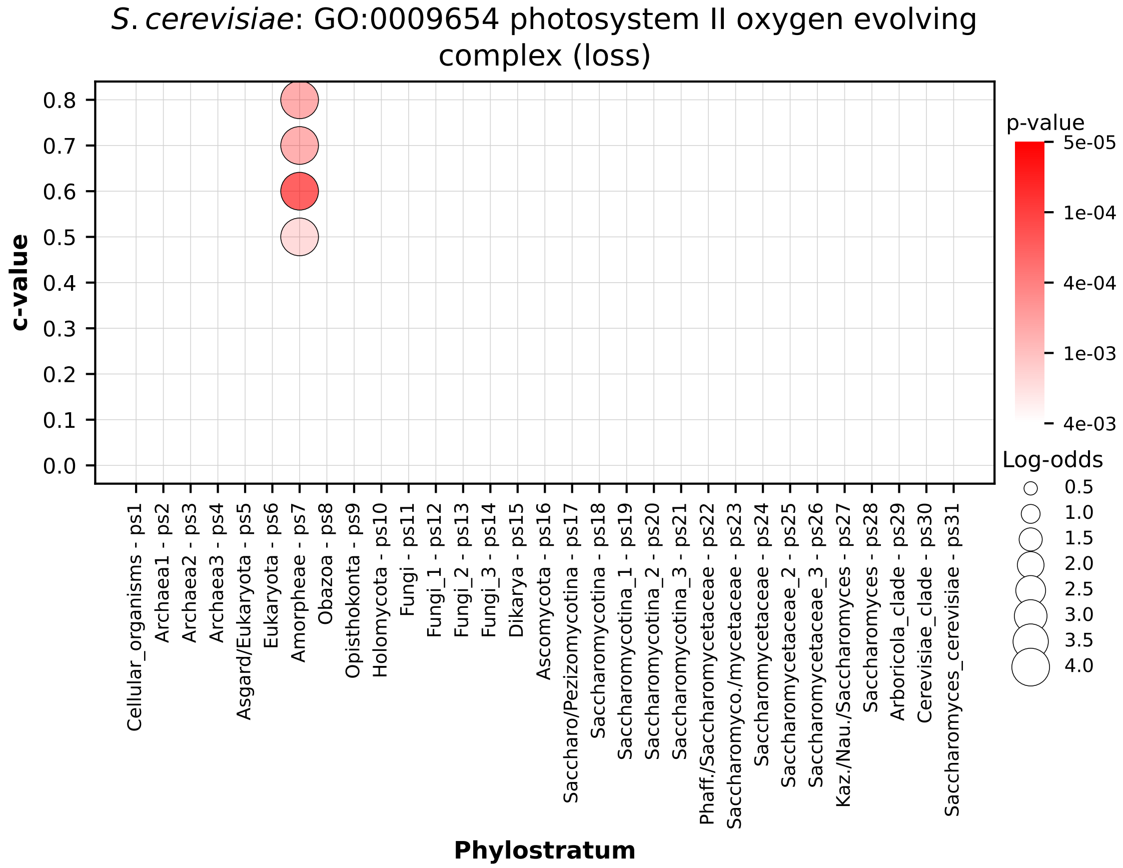

S. cerevisiae: GO:0009654 photosystem II oxygen evolving complex (loss)
c-value
0.0
0.1
0.2
0.3
0.4
0.5
0.6
0.7
0.8
Phylostratum
Cellular_organisms - ps1
Archaea1 - ps2
Archaea2 - ps3
Archaea3 - ps4
Asgard/Eukaryota - ps5
Eukaryota - ps6
Amorpheae - ps7
Obazoa - ps8
Opisthokonta - ps9
Holomycota - ps10
Fungi - ps11
Fungi_1 - ps12
Fungi_2 - ps13
Fungi_3 - ps14
Dikarya - ps15
Ascomycota - ps16
Saccharo/Pezizomycotina - ps17
Saccharomycotina - ps18
Saccharomycotina_1 - ps19
Saccharomycotina_2 - ps20
Saccharomycotina_3 - ps21
Phaff./Saccharomycetaceae - ps22
Saccharomyco./mycetaceae - ps23
Saccharomycetaceae - ps24
Saccharomycetaceae_2 - ps25
Saccharomycetaceae_3 - ps26
Kaz./Nau./Saccharomyces - ps27
Saccharomyces - ps28
Arboricola_clade - ps29
Cerevisiae_clade - ps30
Saccharomyces_cerevisiae - ps31
p-value
5e-05
1e-04
4e-04
1e-03
4e-03
Log-odds
0.5
1.0
1.5
2.0
2.5
3.0
3.5
4.0

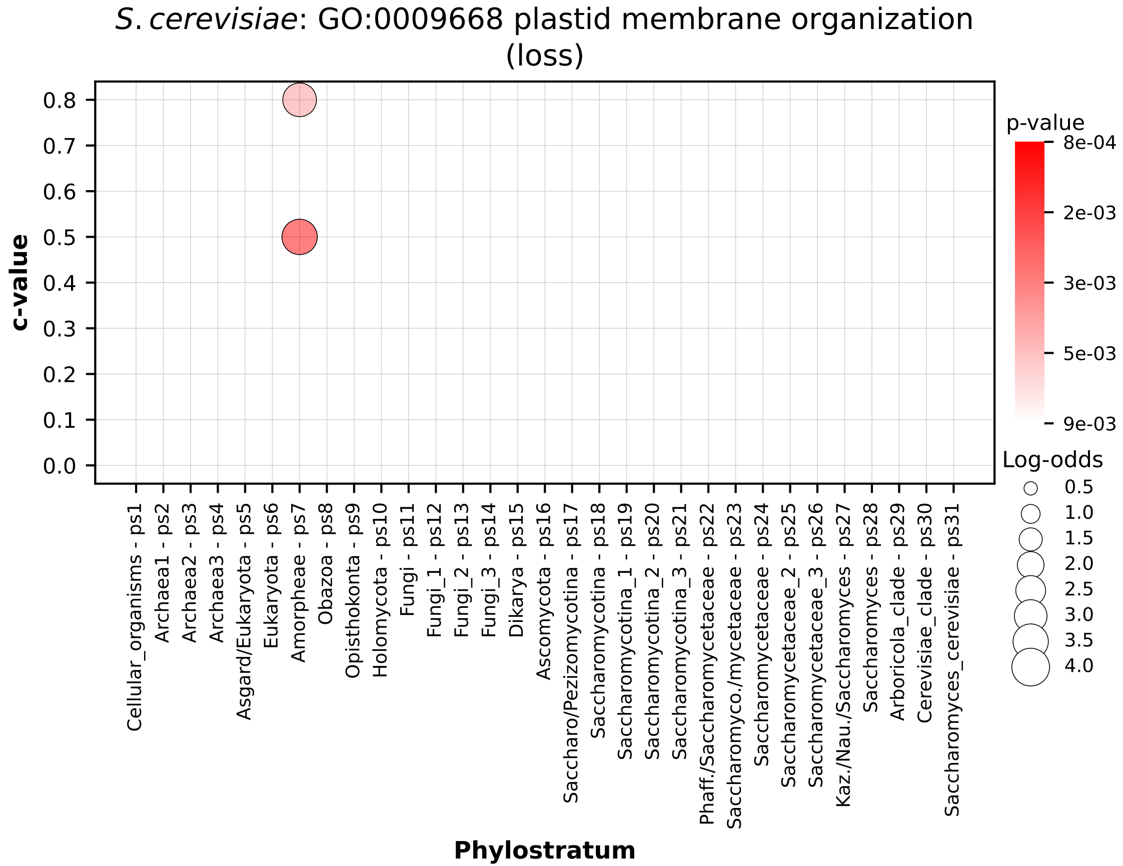
S. cerevisiae: GO:0009668 plastid membrane organization
(loss)
c-value
0.0
0.1
0.2
0.3
0.4
0.5
0.6
0.7
0.8
p-value
8e-04
2e-03
3e-03
5e-03
9e-03
Log-odds
0.5
1.0
1.5
2.0
2.5
3.0
3.5
4.0
Cellular_organisms - ps1
Archaea1 - ps2
Archaea2 - ps3
Archaea3 - ps4
Asgard/Eukaryota - ps5
Eukaryota - ps6
Amorpheae - ps7
Obazoa - ps8
Opisthokonta - ps9
Holomycota - ps10
Fungi - ps11
Fungi_1 - ps12
Fungi_2 - ps13
Fungi_3 - ps14
Dikarya - ps15
Ascomycota - ps16
Saccharo/Pezizomycotina - ps17
Saccharomycotina - ps18
Saccharomycotina_1 - ps19
Saccharomycotina_2 - ps20
Saccharomycotina_3 - ps21
Phaff./Saccharomycetaceae - ps22
Saccharomyco./mycetaceae - ps23
Saccharomycetaceae - ps24
Saccharomycetaceae_2 - ps25
Saccharomycetaceae_3 - ps26
Kaz./Nau./Saccharomyces - ps27
Saccharomyces - ps28
Arboricola_clade - ps29
Cerevisiae_clade - ps30
Saccharomyces_cerevisiae - ps31
Phylostratum

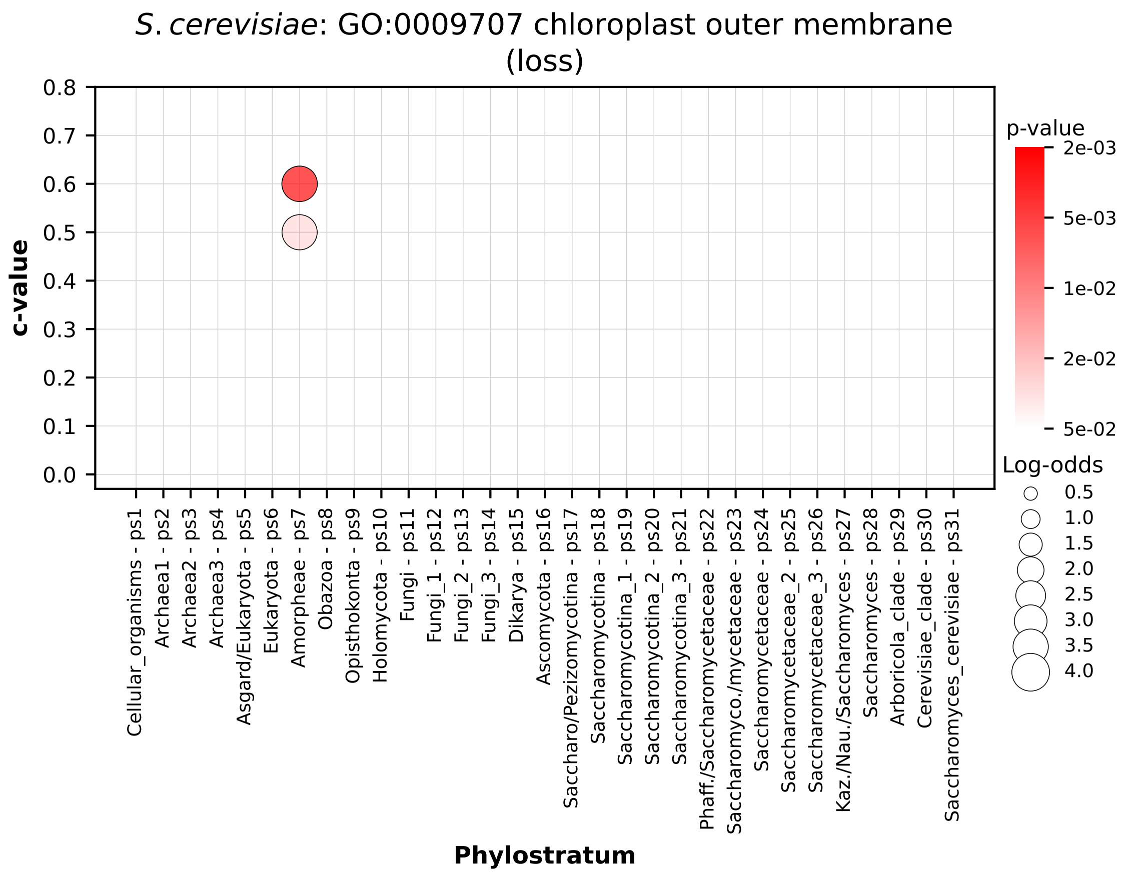

S. cerevisiae: GO:0009707 chloroplast outer membrane
(loss)
c-value
0.8
0.7
0.6
0.5
0.4
0.3
0.2
0.1
0.0
Phylostratum
Cellular_organisms - ps1
Archaea1 - ps2
Archaea2 - ps3
Archaea3 - ps4
Asgard/Eukaryota - ps5
Eukaryota - ps6
Amorpheae - ps7
Obazoa - ps8
Opisthokonta - ps9
Holomycota - ps10
Fungi - ps11
Fungi_1 - ps12
Fungi_2 - ps13
Fungi_3 - ps14
Dikarya - ps15
Ascomycota - ps16
Saccharo/Pezizomycotina - ps17
Saccharomycotina - ps18
Saccharomycotina_1 - ps19
Saccharomycotina_2 - ps20
Saccharomycotina_3 - ps21
Phaff./Saccharomycetaceae - ps22
Saccharomyco./mycetaceae - ps23
Saccharomycetaceae - ps24
Saccharomycetaceae_2 - ps25
Saccharomycetaceae_3 - ps26
Kaz./Nau./Saccharomyces - ps27
Saccharomyces - ps28
Arboricola_clade - ps29
Cerevisiae_clade - ps30
Saccharomyces_cerevisiae - ps31
p-value
2e-03
5e-03
1e-02
2e-02
5e-02
Log-odds
0.5
1.0
1.5
2.0
2.5
3.0
3.5
4.0

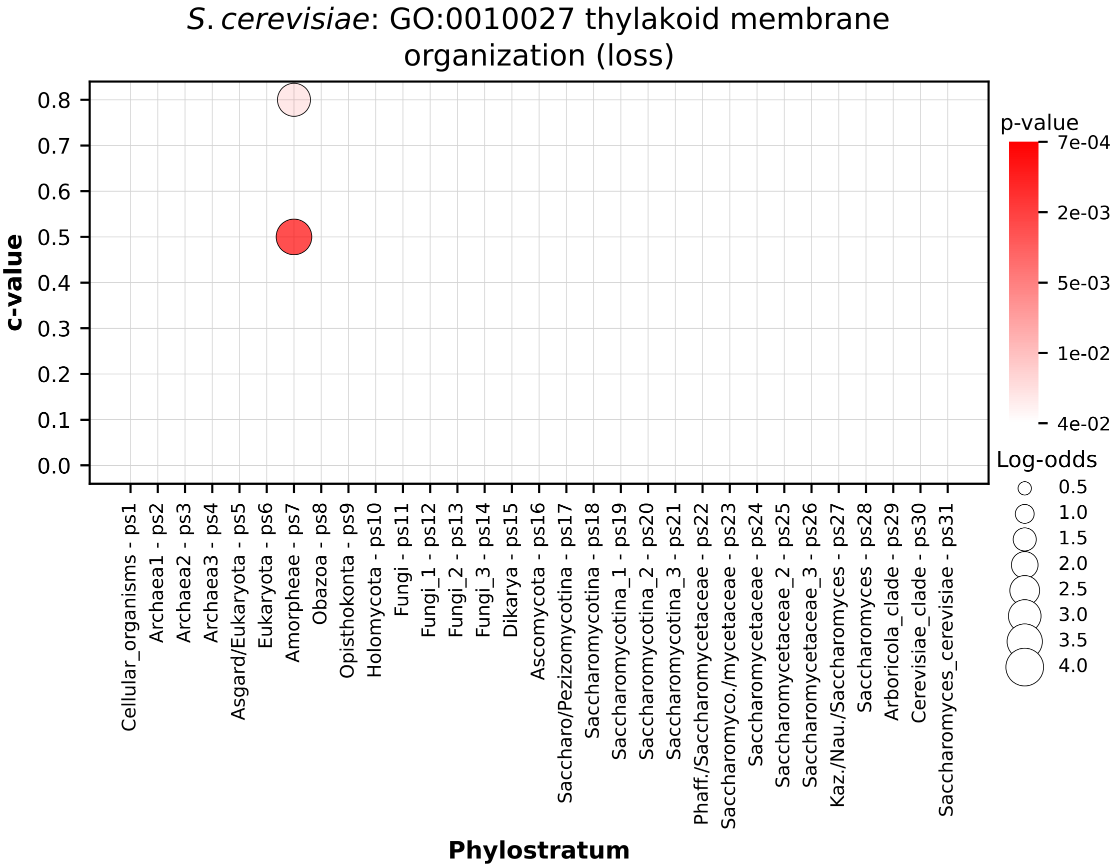

S. cerevisiae: GO:0010027 thylakoid membrane organization (loss)
c-value
0.0
0.1
0.2
0.3
0.4
0.5
0.6
0.7
0.8
p-value
7e-04
2e-03
5e-03
1e-02
4e-02
Log-odds
0.5
1.0
1.5
2.0
2.5
3.0
3.5
4.0
Cellular_organisms - ps1
Archaea1 - ps2
Archaea2 - ps3
Archaea3 - ps4
Asgard/Eukaryota - ps5
Eukaryota - ps6
Amorpheae - ps7
Obazoa - ps8
Opisthokonta - ps9
Holomycota - ps10
Fungi - ps11
Fungi_1 - ps12
Fungi_2 - ps13
Fungi_3 - ps14
Dikarya - ps15
Ascomycota - ps16
Saccharo/Pezizomycotina - ps17
Saccharomycotina - ps18
Saccharomycotina_1 - ps19
Saccharomycotina_2 - ps20
Saccharomycotina_3 - ps21
Phaff./Saccharomycetaceae - ps22
Saccharomyco./mycetaceae - ps23
Saccharomycetaceae - ps24
Saccharomycetaceae_2 - ps25
Saccharomycetaceae_3 - ps26
Kaz./Nau./Saccharomyces - ps27
Saccharomyces - ps28
Arboricola_clade - ps29
Cerevisiae_clade - ps30
Saccharomyces_cerevisiae - ps31
Phylostratum

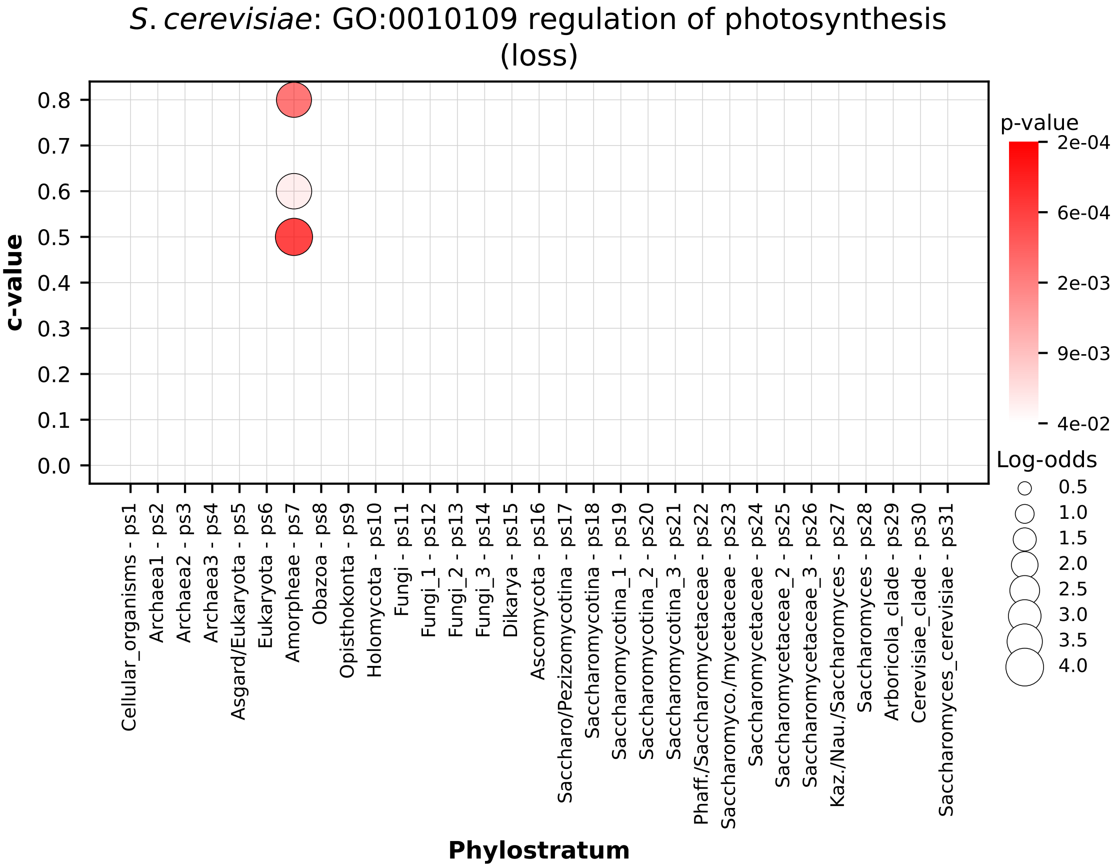
S. cerevisiae: GO:0010109 regulation of photosynthesis (loss)
c-value
0.8
0.7
0.6
0.5
0.4
0.3
0.2
0.1
0.0
p-value
2e-04
6e-04
2e-03
9e-03
4e-02
Log-odds
0.5
1.0
1.5
2.0
2.5
3.0
3.5
4.0
Cellular_organisms - ps1
Archaea1 - ps2
Archaea2 - ps3
Archaea3 - ps4
Asgard/Eukaryota - ps5
Eukaryota - ps6
Amorpheae - ps7
Obazoa - ps8
Opisthokonta - ps9
Holomycota - ps10
Fungi - ps11
Fungi_1 - ps12
Fungi_2 - ps13
Fungi_3 - ps14
Dikarya - ps15
Ascomycota - ps16
Saccharo/Pezizomycotina - ps17
Saccharomycotina - ps18
Saccharomycotina_1 - ps19
Saccharomycotina_2 - ps20
Saccharomycotina_3 - ps21
Phaff./Saccharomycetaceae - ps22
Saccharomyco./mycetaceae - ps23
Saccharomycetaceae - ps24
Saccharomycetaceae_2 - ps25
Saccharomycetaceae_3 - ps26
Kaz./Nau./Saccharomyces - ps27
Saccharomyces - ps28
Arboricola_clade - ps29
Cerevisiae_clade - ps30
Saccharomyces_cerevisiae - ps31
Phylostratum

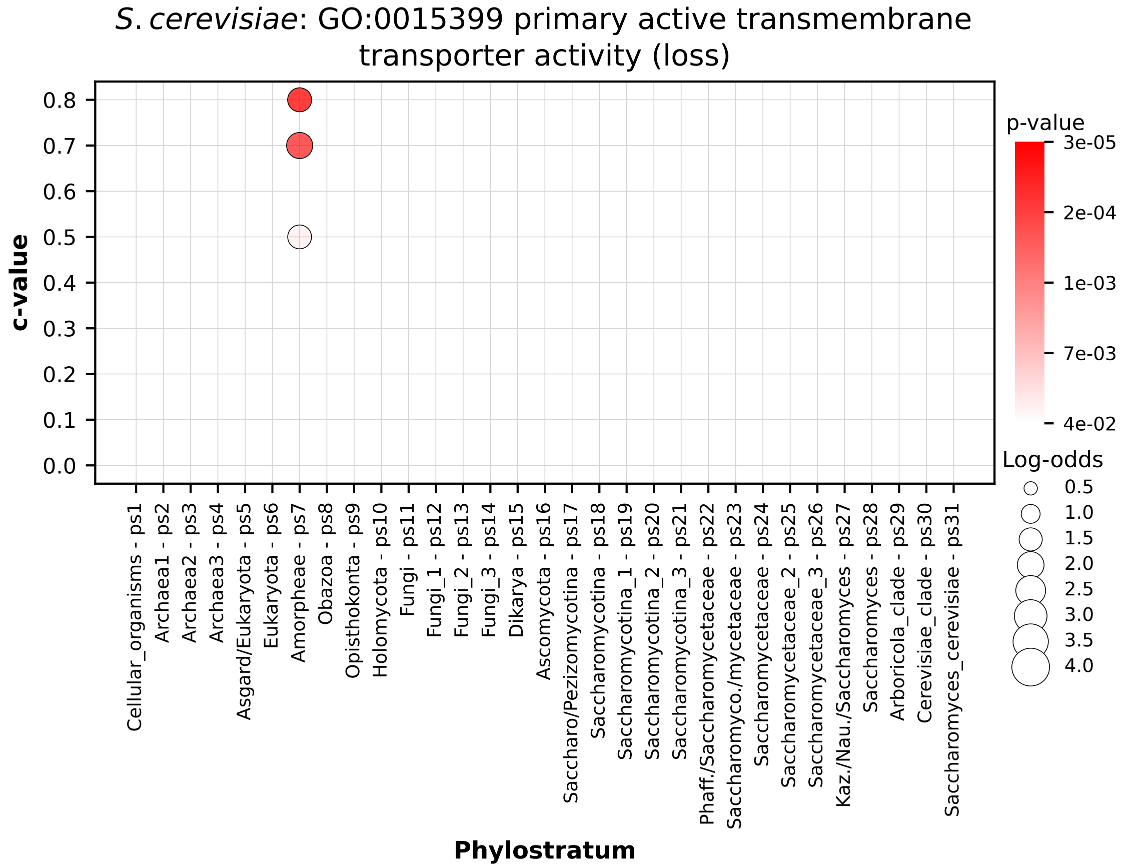

S. cerevisiae: GO:0015399 primary active transmembrane transporter activity (loss)
c-value
0.8
0.7
0.6
0.5
0.4
0.3
0.2
0.1
0.0
p-value
3e-05
2e-04
1e-03
7e-03
4e-02
Log-odds
0.5
1.0
1.5
2.0
2.5
3.0
3.5
4.0
Cellular_organisms - ps1
Archaea1 - ps2
Archaea2 - ps3
Archaea3 - ps4
Asgard/Eukaryota - ps5
Eukaryota - ps6
Amorpheae - ps7
Obazoa - ps8
Opisthokonta - ps9
Holomycota - ps10
Fungi - ps11
Fungi_1 - ps12
Fungi_2 - ps13
Fungi_3 - ps14
Dikarya - ps15
Ascomycota - ps16
Saccharo/Pezizomycotina - ps17
Saccharomycotina - ps18
Saccharomycotina_1 - ps19
Saccharomycotina_2 - ps20
Saccharomycotina_3 - ps21
Phaff./Saccharomycetaceae - ps22
Saccharomyco./mycetaceae - ps23
Saccharomycetaceae - ps24
Saccharomycetaceae_2 - ps25
Saccharomycetaceae_3 - ps26
Kaz./Nau./Saccharomyces - ps27
Saccharomyces - ps28
Arboricola_clade - ps29
Cerevisiae_clade - ps30
Saccharomyces_cerevisiae - ps31
Phylostratum

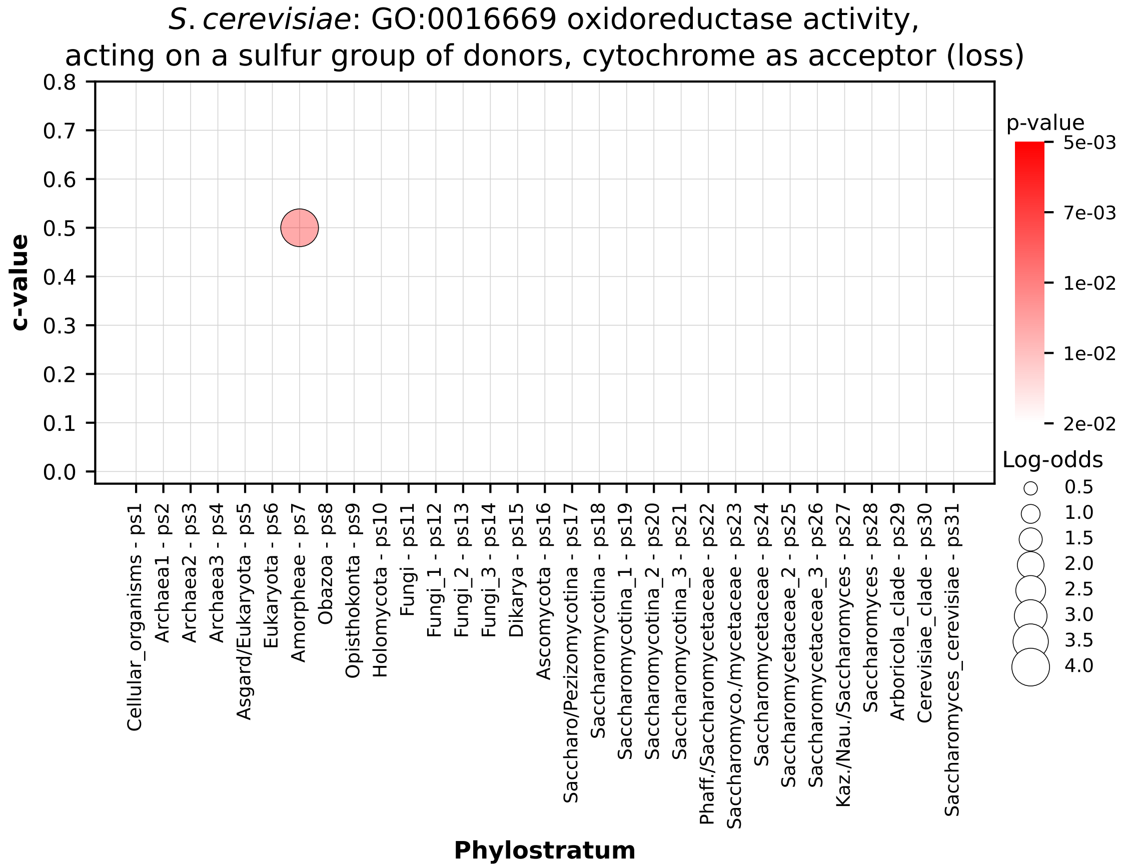

S. cerevisiae: GO:0016669 oxidoreductase activity,
acting on a sulfur group of donors, cytochrome as acceptor (loss)
c-value
0.8
0.7
0.6
0.5
0.4
0.3
0.2
0.1
0.0
p-value
5e-03
7e-03
1e-02
1e-02
2e-02
Log-odds
0.5
1.0
1.5
2.0
2.5
3.0
3.5
4.0
Cellular_organisms - ps1
Archaea1 - ps2
Archaea2 - ps3
Archaea3 - ps4
Asgard/Eukaryota - ps5
Eukaryota - ps6
Amorpheae - ps7
Obazoa - ps8
Opisthokonta - ps9
Holomycota - ps10
Fungi - ps11
Fungi_1 - ps12
Fungi_2 - ps13
Fungi_3 - ps14
Dikarya - ps15
Ascomycota - ps16
Saccharo/Pezizomycotina - ps17
Saccharomycotina - ps18
Saccharomycotina_1 - ps19
Saccharomycotina_2 - ps20
Saccharomycotina_3 - ps21
Phaff./Saccharomycetaceae - ps22
Saccharomyco./mycetaceae - ps23
Saccharomycetaceae - ps24
Saccharomycetaceae_2 - ps25
Saccharomycetaceae_3 - ps26
Kaz./Nau./Saccharomyces - ps27
Saccharomyces - ps28
Arboricola_clade - ps29
Cerevisiae_clade - ps30
Saccharomyces_cerevisiae - ps31
Phylostratum

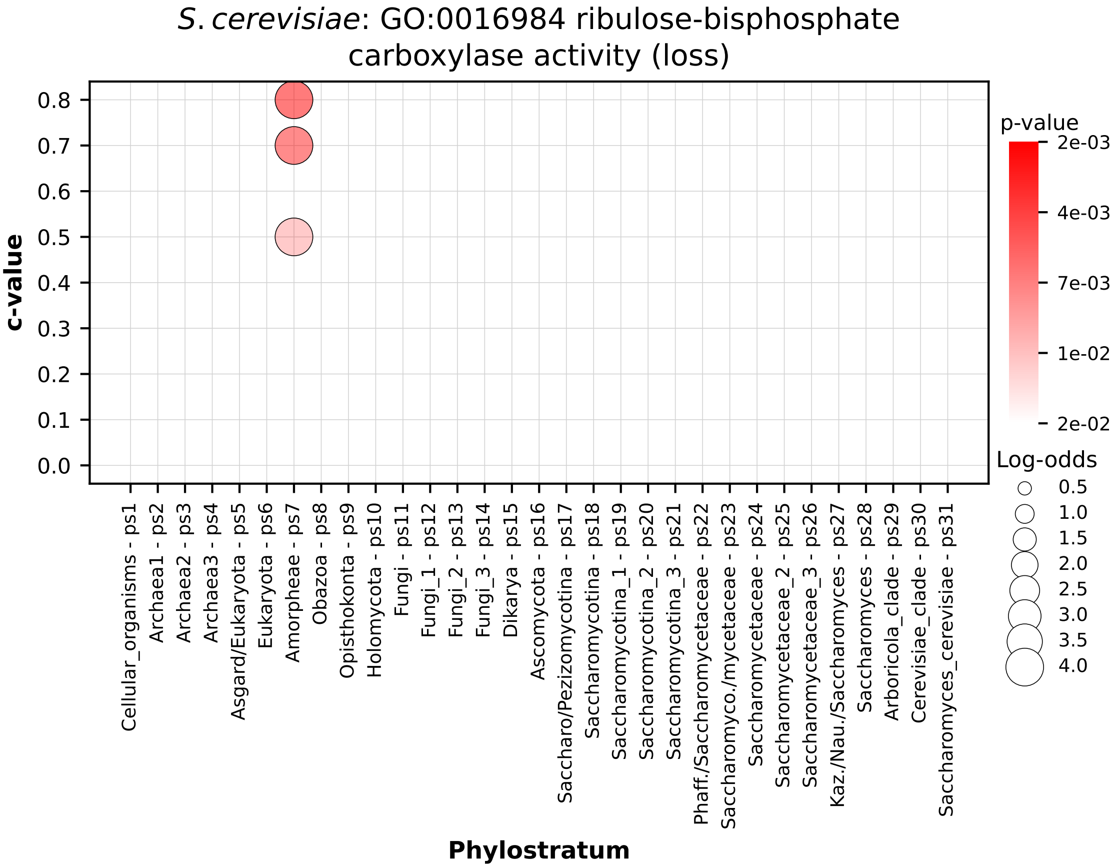
S. cerevisiae: GO:0016984 ribulose-bisphosphate carboxylase activity (loss)
c-value
0.0
0.1
0.2
0.3
0.4
0.5
0.6
0.7
0.8
Phylostratum
Cellular_organisms - ps1
Archaea1 - ps2
Archaea2 - ps3
Archaea3 - ps4
Asgard/Eukaryota - ps5
Eukaryota - ps6
Amorpheae - ps7
Obazoa - ps8
Opisthokonta - ps9
Holomycota - ps10
Fungi - ps11
Fungi_1 - ps12
Fungi_2 - ps13
Fungi_3 - ps14
Dikarya - ps15
Ascomycota - ps16
Saccharo/Pezizomycotina - ps17
Saccharomycotina - ps18
Saccharomycotina_1 - ps19
Saccharomycotina_2 - ps20
Saccharomycotina_3 - ps21
Phaff./Saccharomycetaceae - ps22
Saccharomyco./mycetaceae - ps23
Saccharomycetaceae - ps24
Saccharomycetaceae_2 - ps25
Saccharomycetaceae_3 - ps26
Kaz./Nau./Saccharomyces - ps27
Saccharomyces - ps28
Arboricola_clade - ps29
Cerevisiae_clade - ps30
Saccharomyces_cerevisiae - ps31
p-value
2e-03
4e-03
7e-03
1e-02
2e-02
Log-odds
0.5
1.0
1.5
2.0
2.5
3.0
3.5
4.0

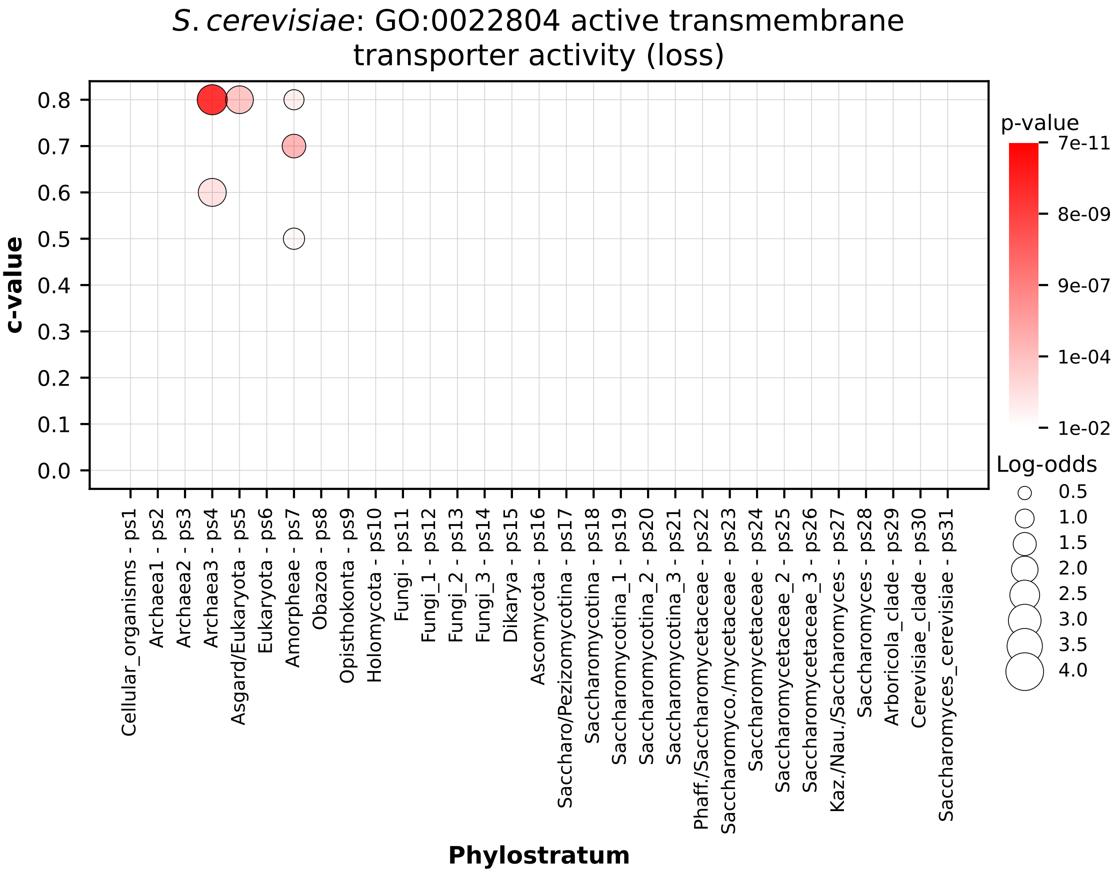

S. cerevisiae: GO:0022804 active transmembrane transporter activity (loss)
c-value
0.8
0.7
0.6
0.5
0.4
0.3
0.2
0.1
0.0
p-value
7e-11
8e-09
9e-07
1e-04
1e-02
Log-odds
0.5
1.0
1.5
2.0
2.5
3.0
3.5
4.0
Cellular_organisms - ps1
Archaea1 - ps2
Archaea2 - ps3
Archaea3 - ps4
Asgard/Eukaryota - ps5
Eukaryota - ps6
Amorpheae - ps7
Obazoa - ps8
Opisthokonta - ps9
Holomycota - ps10
Fungi - ps11
Fungi_1 - ps12
Fungi_2 - ps13
Fungi_3 - ps14
Dikarya - ps15
Ascomycota - ps16
Saccharo/Pezizomycotina - ps17
Saccharomycotina - ps18
Saccharomycotina_1 - ps19
Saccharomycotina_2 - ps20
Saccharomycotina_3 - ps21
Phaff./Saccharomycetaceae - ps22
Saccharomyco./mycetaceae - ps23
Saccharomycetaceae - ps24
Saccharomycetaceae_2 - ps25
Saccharomycetaceae_3 - ps26
Kaz./Nau./Saccharomyces - ps27
Saccharomyces - ps28
Arboricola_clade - ps29
Cerevisiae_clade - ps30
Saccharomyces_cerevisiae - ps31
Phylostratum

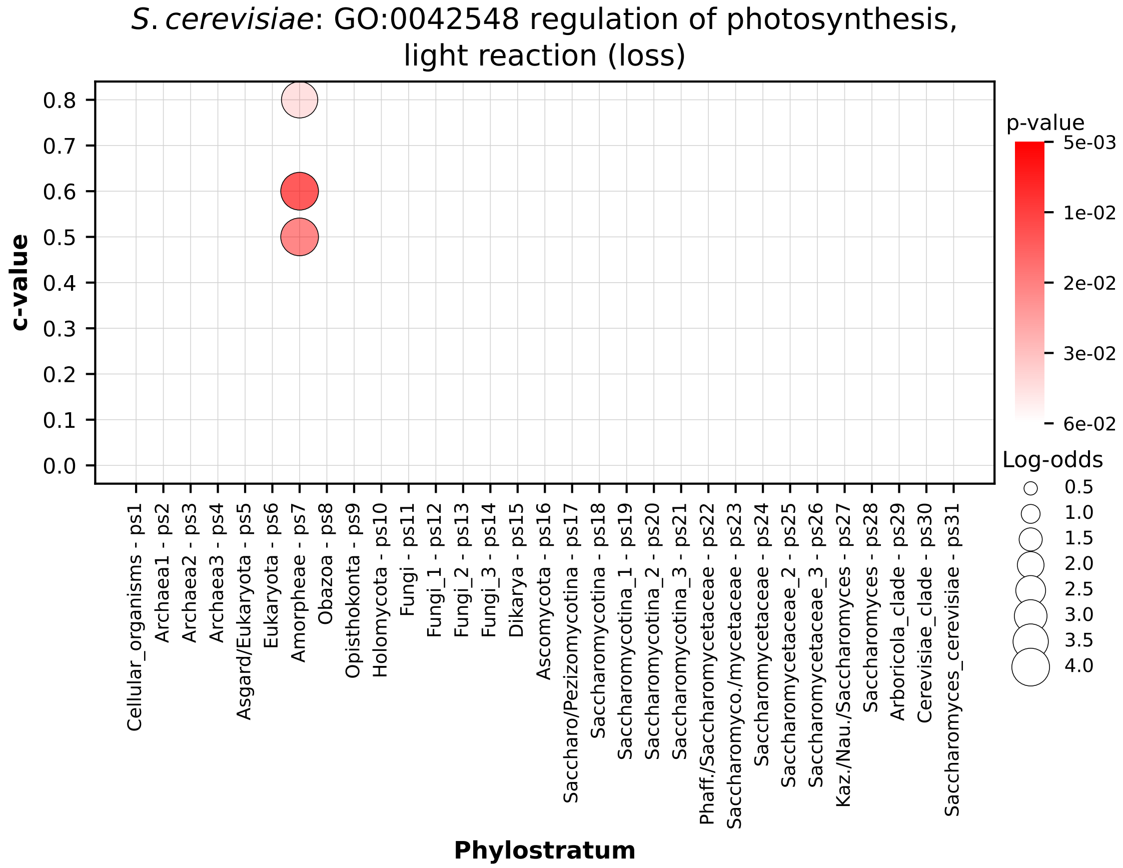
S. cerevisiae: GO:0042548 regulation of photosynthesis, light reaction (loss)
c-value
0.0
0.1
0.2
0.3
0.4
0.5
0.6
0.7
0.8
p-value
5e-03
1e-02
2e-02
3e-02
6e-02
Log-odds
0.5
1.0
1.5
2.0
2.5
3.0
3.5
4.0
Cellular_organisms - ps1
Archaea1 - ps2
Archaea2 - ps3
Archaea3 - ps4
Asgard/Eukaryota - ps5
Eukaryota - ps6
Amorpheae - ps7
Obazoa - ps8
Opisthokonta - ps9
Holomycota - ps10
Fungi - ps11
Fungi_1 - ps12
Fungi_2 - ps13
Fungi_3 - ps14
Dikarya - ps15
Ascomycota - ps16
Saccharo/Pezizomycotina - ps17
Saccharomycotina - ps18
Saccharomycotina_1 - ps19
Saccharomycotina_2 - ps20
Saccharomycotina_3 - ps21
Phaff./Saccharomycetaceae - ps22
Saccharomyco./mycetaceae - ps23
Saccharomycetaceae - ps24
Saccharomycetaceae_2 - ps25
Saccharomycetaceae_3 - ps26
Kaz./Nau./Saccharomyces - ps27
Saccharomyces - ps28
Arboricola_clade - ps29
Cerevisiae_clade - ps30
Saccharomyces_cerevisiae - ps31
Phylostratum

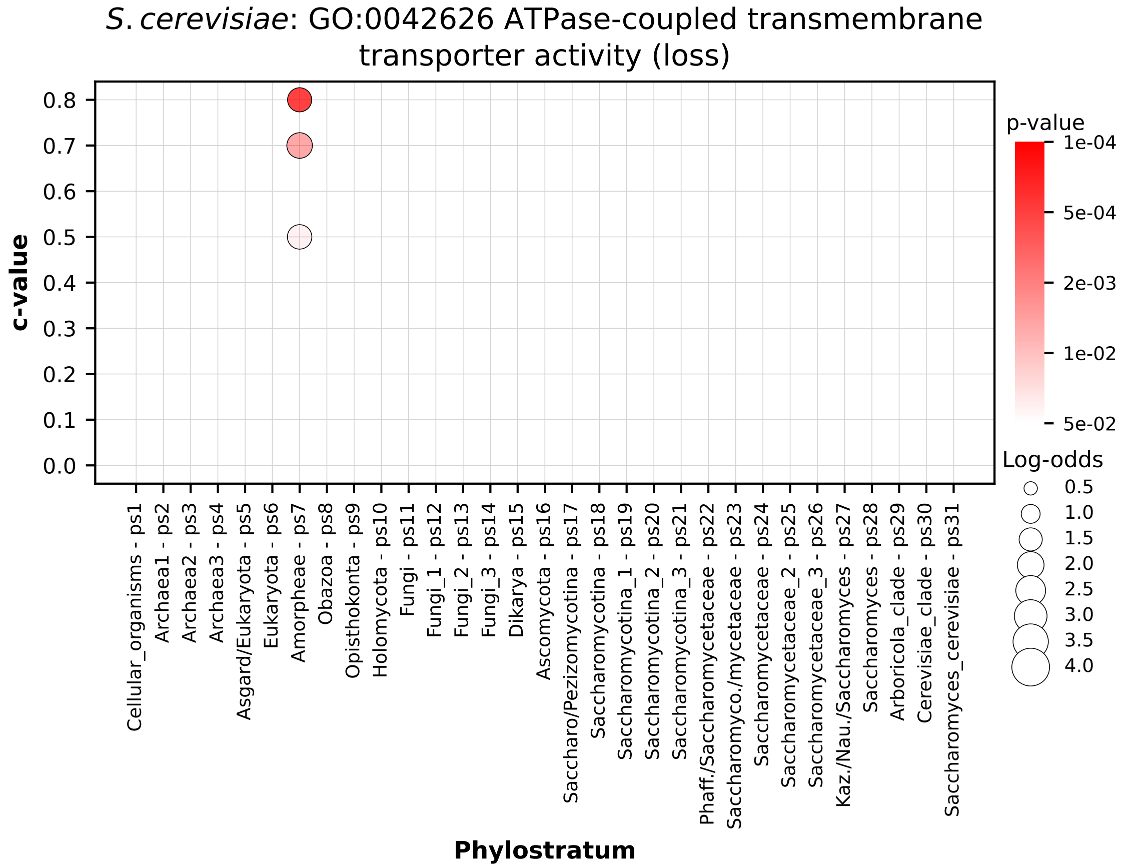

S. cerevisiae: GO:0042626 ATPase-coupled transmembrane transporter activity (loss)
c-value
0.0
0.1
0.2
0.3
0.4
0.5
0.6
0.7
0.8
p-value
1e-04
5e-04
2e-03
1e-02
5e-02
Log-odds
0.5
1.0
1.5
2.0
2.5
3.0
3.5
4.0
Cellular_organisms - ps1
Archaea1 - ps2
Archaea2 - ps3
Archaea3 - ps4
Asgard/Eukaryota - ps5
Eukaryota - ps6
Amorpheae - ps7
Obazoa - ps8
Opisthokonta - ps9
Holomycota - ps10
Fungi - ps11
Fungi_1 - ps12
Fungi_2 - ps13
Fungi_3 - ps14
Dikarya - ps15
Ascomycota - ps16
Saccharo/Pezizomycotina - ps17
Saccharomycotina - ps18
Saccharomycotina_1 - ps19
Saccharomycotina_2 - ps20
Saccharomycotina_3 - ps21
Phaff./Saccharomycetaceae - ps22
Saccharomyco./mycetaceae - ps23
Saccharomycetaceae - ps24
Saccharomycetaceae_2 - ps25
Saccharomycetaceae_3 - ps26
Kaz./Nau./Saccharomyces - ps27
Saccharomyces - ps28
Arboricola_clade - ps29
Cerevisiae_clade - ps30
Saccharomyces_cerevisiae - ps31
Phylostratum

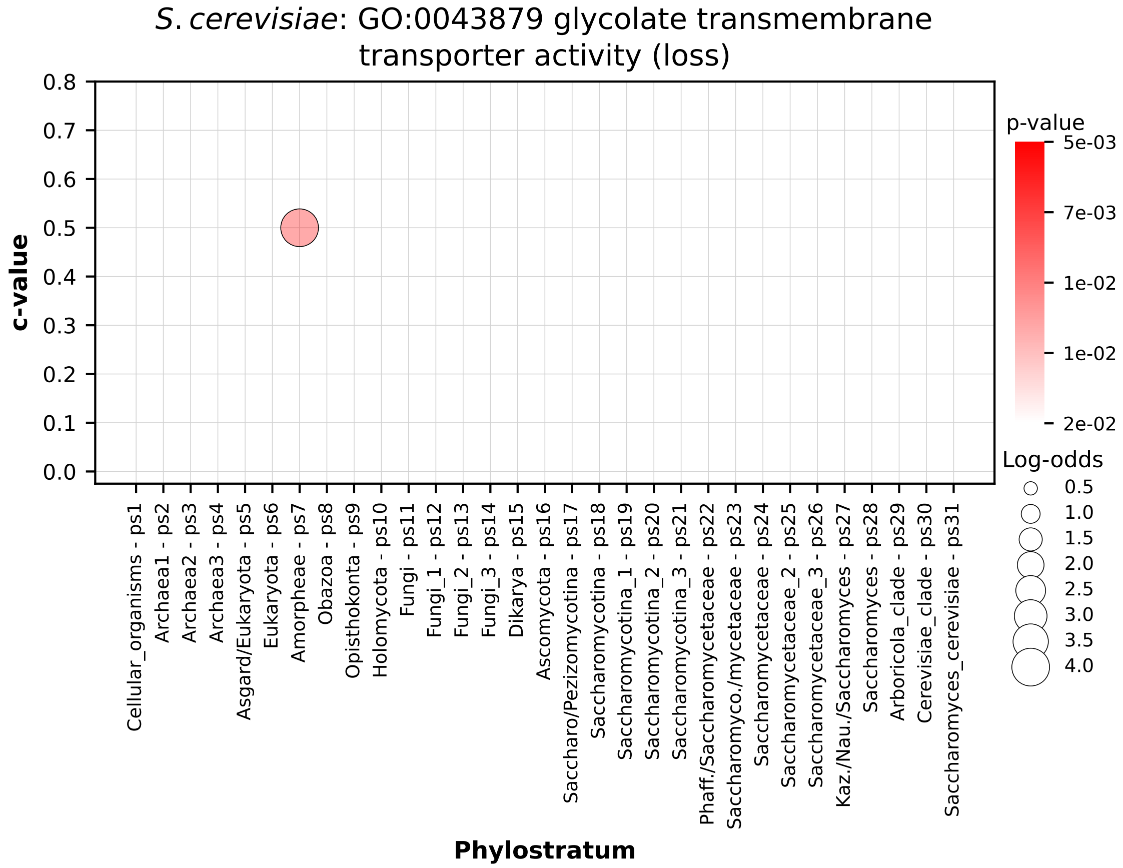

S. cerevisiae: GO:0043879 glycolate transmembrane transporter activity (loss)
c-value
0.8
0.7
0.6
0.5
0.4
0.3
0.2
0.1
0.0
Phylostratum
Cellular_organisms - ps1
Archaea1 - ps2
Archaea2 - ps3
Archaea3 - ps4
Asgard/Eukaryota - ps5
Eukaryota - ps6
Amorpheae - ps7
Obazoa - ps8
Opisthokonta - ps9
Holomycota - ps10
Fungi - ps11
Fungi_1 - ps12
Fungi_2 - ps13
Fungi_3 - ps14
Dikarya - ps15
Ascomycota - ps16
Saccharo/Pezizomycotina - ps17
Saccharomycotina - ps18
Saccharomycotina_1 - ps19
Saccharomycotina_2 - ps20
Saccharomycotina_3 - ps21
Phaff./Saccharomycetaceae - ps22
Saccharomyco./mycetaceae - ps23
Saccharomycetaceae - ps24
Saccharomycetaceae_2 - ps25
Saccharomycetaceae_3 - ps26
Kaz./Nau./Saccharomyces - ps27
Saccharomyces - ps28
Arboricola_clade - ps29
Cerevisiae_clade - ps30
Saccharomyces_cerevisiae - ps31
p-value
5e-03
7e-03
1e-02
1e-02
2e-02
Log-odds
0.5
1.0
1.5
2.0
2.5
3.0
3.5
4.0

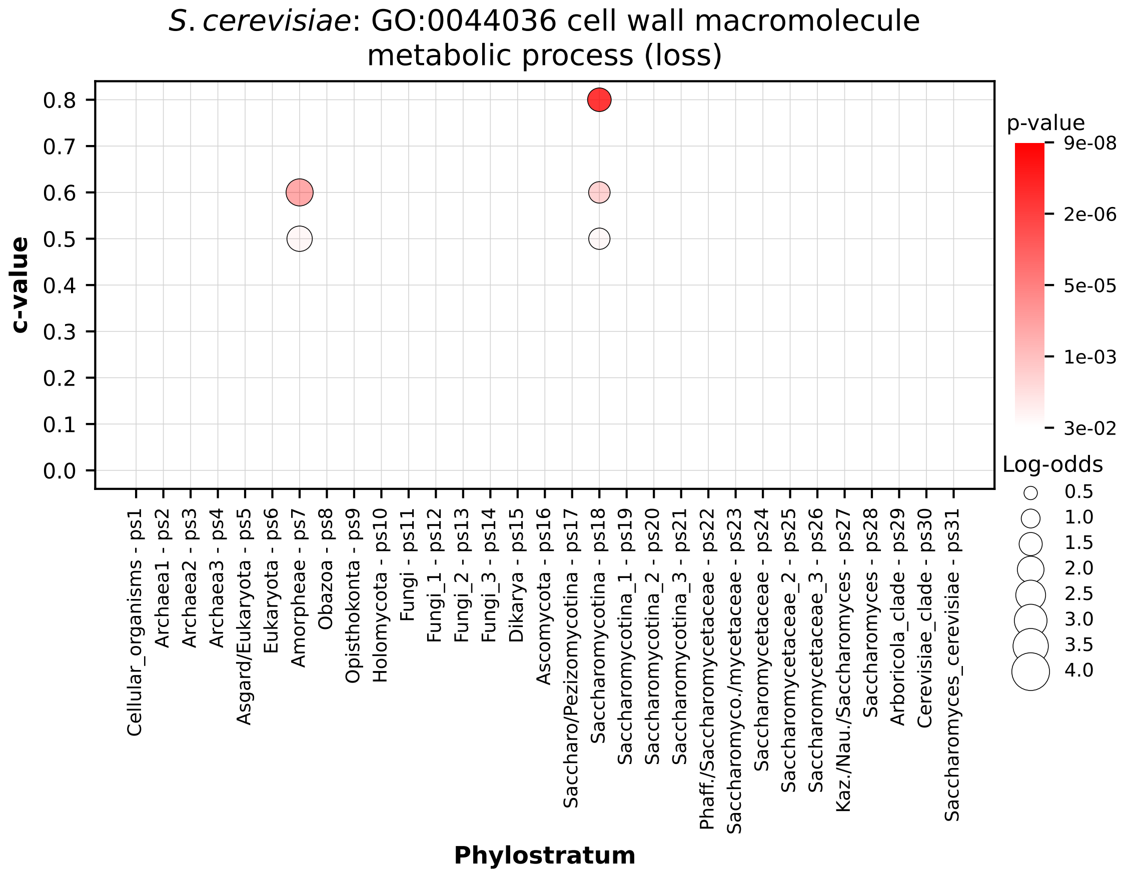

S. cerevisiae: GO:0044036 cell wall macromolecule metabolic process (loss)
c-value
0.8
0.7
0.6
0.5
0.4
0.3
0.2
0.1
0.0
p-value
9e-08
2e-06
5e-05
1e-03
3e-02
Log-odds
0.5
1.0
1.5
2.0
2.5
3.0
3.5
4.0
Cellular_organisms - ps1
Archaea1 - ps2
Archaea2 - ps3
Archaea3 - ps4
Asgard/Eukaryota - ps5
Eukaryota - ps6
Amorpheae - ps7
Obazoa - ps8
Opisthokonta - ps9
Holomycota - ps10
Fungi - ps11
Fungi_1 - ps12
Fungi_2 - ps13
Fungi_3 - ps14
Dikarya - ps15
Ascomycota - ps16
Saccharo/Pezizomycotina - ps17
Saccharomycotina - ps18
Saccharomycotina_1 - ps19
Saccharomycotina_2 - ps20
Saccharomycotina_3 - ps21
Phaff./Saccharomycetaceae - ps22
Saccharomyco./mycetaceae - ps23
Saccharomycetaceae - ps24
Saccharomycetaceae_2 - ps25
Saccharomycetaceae_3 - ps26
Kaz./Nau./Saccharomyces - ps27
Saccharomyces - ps28
Arboricola_clade - ps29
Cerevisiae_clade - ps30
Saccharomyces_cerevisiae - ps31
Phylostratum

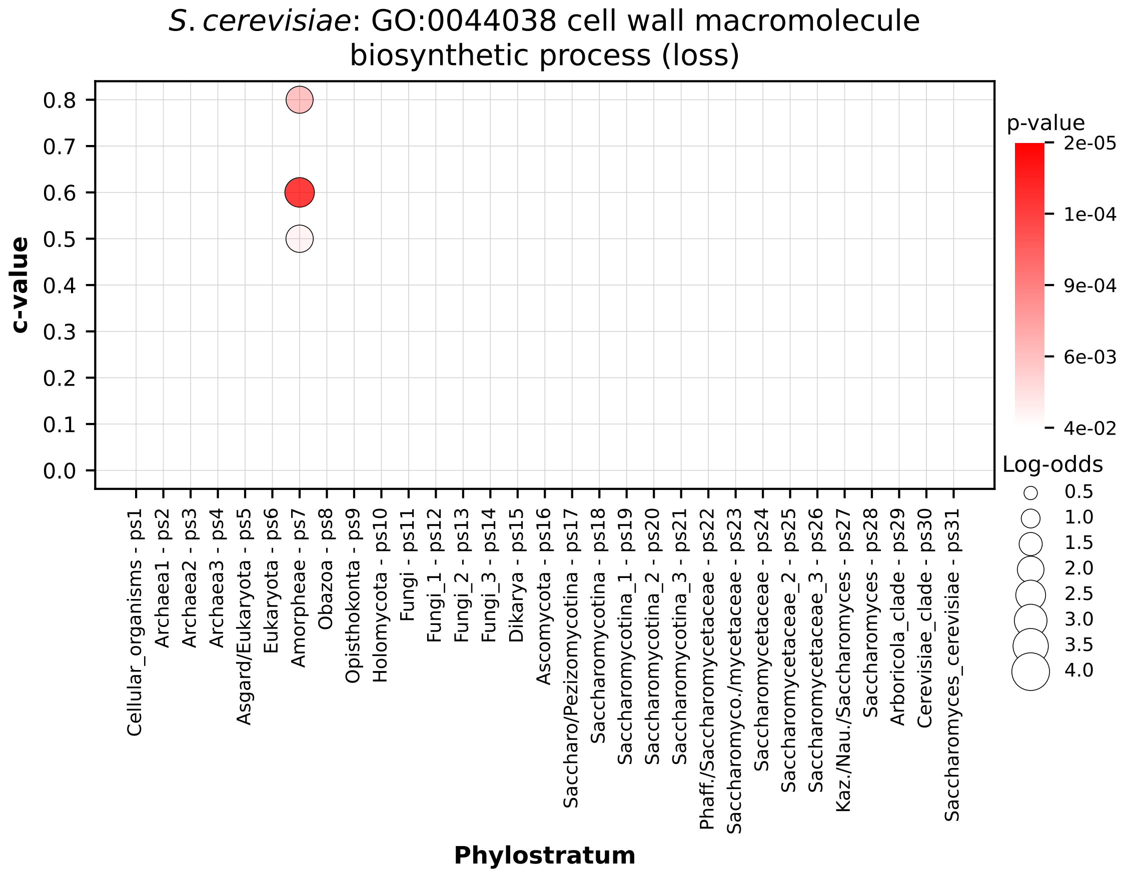

S. cerevisiae: GO:0044038 cell wall macromolecule biosynthetic process (loss)
c-value
0.8
0.7
0.6
0.5
0.4
0.3
0.2
0.1
0.0
p-value
2e-05
1e-04
9e-04
6e-03
4e-02
Log-odds
0.5
1.0
1.5
2.0
2.5
3.0
3.5
4.0
Cellular_organisms - ps1
Archaea1 - ps2
Archaea2 - ps3
Archaea3 - ps4
Asgard/Eukaryota - ps5
Eukaryota - ps6
Amorpheae - ps7
Obazoa - ps8
Opisthokonta - ps9
Holomycota - ps10
Fungi - ps11
Fungi_1 - ps12
Fungi_2 - ps13
Fungi_3 - ps14
Dikarya - ps15
Ascomycota - ps16
Saccharo/Pezizomycotina - ps17
Saccharomycotina - ps18
Saccharomycotina_1 - ps19
Saccharomycotina_2 - ps20
Saccharomycotina_3 - ps21
Phaff./Saccharomycetaceae - ps22
Saccharomyco./mycetaceae - ps23
Saccharomycetaceae - ps24
Saccharomycetaceae_2 - ps25
Saccharomycetaceae_3 - ps26
Kaz./Nau./Saccharomyces - ps27
Saccharomyces - ps28
Arboricola_clade - ps29
Cerevisiae_clade - ps30
Saccharomyces_cerevisiae - ps31
Phylostratum

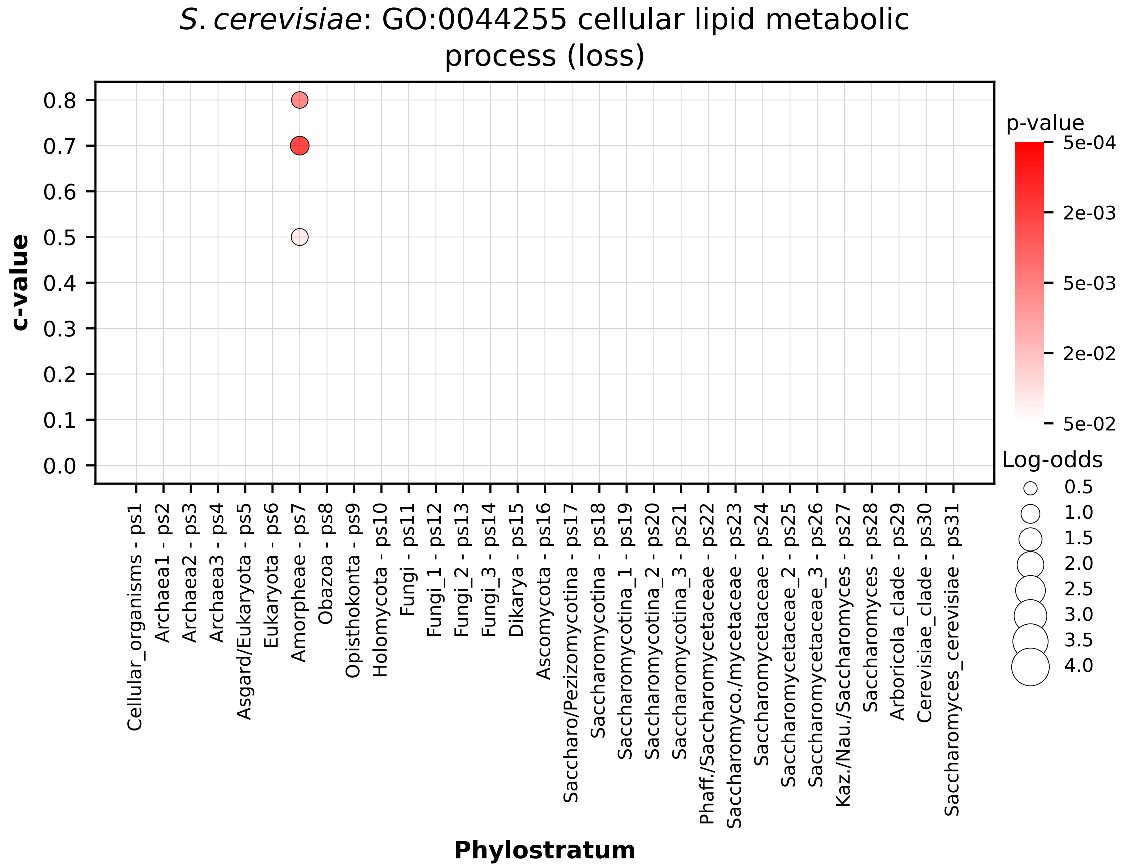
S. cerevisiae: GO:0044255 cellular lipid metabolic process (loss)
c-value
0.0
0.1
0.2
0.3
0.4
0.5
0.6
0.7
0.8
p-value
5e-04
2e-03
5e-03
2e-02
5e-02
Log-odds
0.5
1.0
1.5
2.0
2.5
3.0
3.5
4.0
Cellular_organisms - ps1
Archaea1 - ps2
Archaea2 - ps3
Archaea3 - ps4
Asgard/Eukaryota - ps5
Eukaryota - ps6
Amorpheae - ps7
Obazoa - ps8
Opisthokonta - ps9
Holomycota - ps10
Fungi - ps11
Fungi_1 - ps12
Fungi_2 - ps13
Fungi_3 - ps14
Dikarya - ps15
Ascomycota - ps16
Saccharo/Pezizomycotina - ps17
Saccharomycotina - ps18
Saccharomycotina_1 - ps19
Saccharomycotina_2 - ps20
Saccharomycotina_3 - ps21
Phaff./Saccharomycetaceae - ps22
Saccharomyco./mycetaceae - ps23
Saccharomycetaceae - ps24
Saccharomycetaceae_2 - ps25
Saccharomycetaceae_3 - ps26
Kaz./Nau./Saccharomyces - ps27
Saccharomyces - ps28
Arboricola_clade - ps29
Cerevisiae_clade - ps30
Saccharomyces_cerevisiae - ps31
Phylostratum

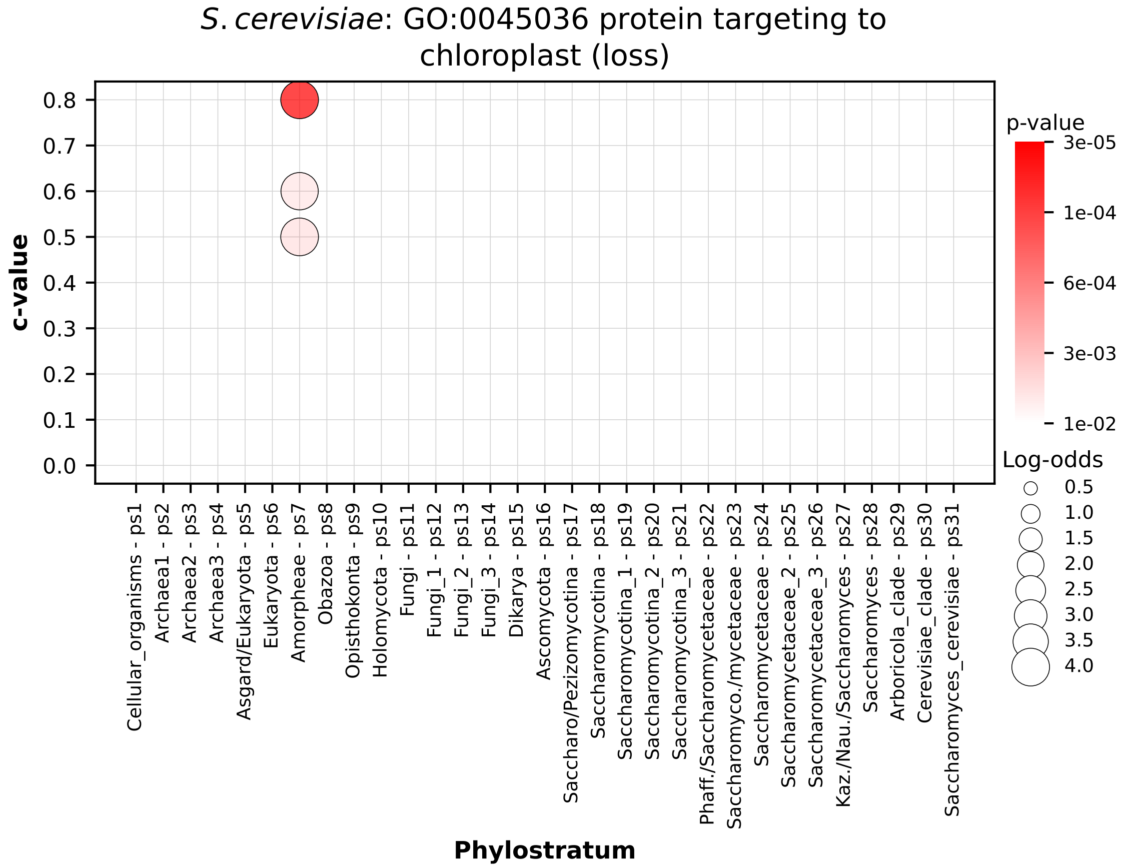

S. cerevisiae: GO:0045036 protein targeting to chloroplast (loss)
c-value
0.0
0.1
0.2
0.3
0.4
0.5
0.6
0.7
0.8
Phylostratum
Cellular_organisms - ps1
Archaea1 - ps2
Archaea2 - ps3
Archaea3 - ps4
Asgard/Eukaryota - ps5
Eukaryota - ps6
Amorpheae - ps7
Obazoa - ps8
Opisthokonta - ps9
Holomycota - ps10
Fungi - ps11
Fungi_1 - ps12
Fungi_2 - ps13
Fungi_3 - ps14
Dikarya - ps15
Ascomycota - ps16
Saccharo/Pezizomycotina - ps17
Saccharomycotina - ps18
Saccharomycotina_1 - ps19
Saccharomycotina_2 - ps20
Saccharomycotina_3 - ps21
Phaff./Saccharomycetaceae - ps22
Saccharomyco./mycetaceae - ps23
Saccharomycetaceae - ps24
Saccharomycetaceae_2 - ps25
Saccharomycetaceae_3 - ps26
Kaz./Nau./Saccharomyces - ps27
Saccharomyces - ps28
Arboricola_clade - ps29
Cerevisiae_clade - ps30
Saccharomyces_cerevisiae - ps31
p-value
3e-05
1e-04
6e-04
3e-03
1e-02
Log-odds
0.5
1.0
1.5
2.0
2.5
3.0
3.5
4.0

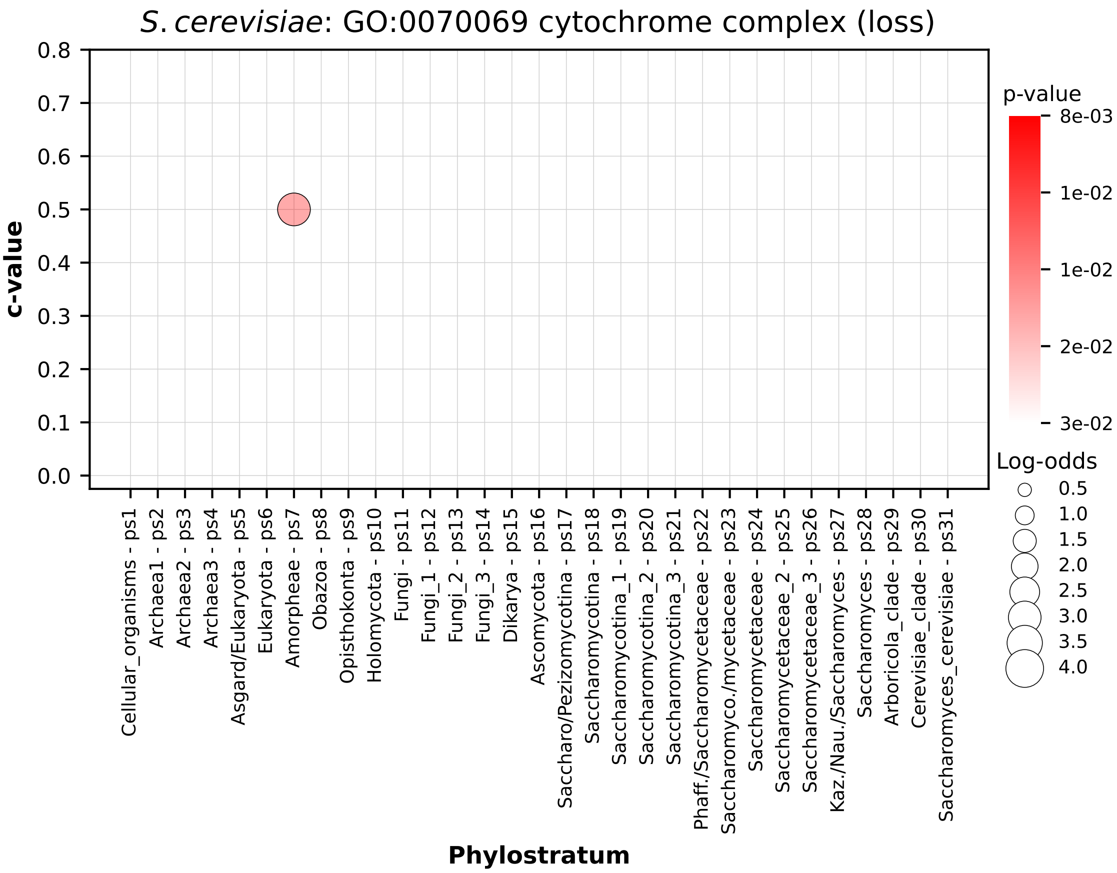

S. cerevisiae: GO:0070069 cytochrome complex (loss)
c-value
0.0
0.1
0.2
0.3
0.4
0.5
0.6
0.7
0.8
Phylostratum
Cellular_organisms - ps1
Archaea1 - ps2
Archaea2 - ps3
Archaea3 - ps4
Asgard/Eukaryota - ps5
Eukaryota - ps6
Amorpheae - ps7
Obazoa - ps8
Opisthokonta - ps9
Holomycota - ps10
Fungi - ps11
Fungi_1 - ps12
Fungi_2 - ps13
Fungi_3 - ps14
Dikarya - ps15
Ascomycota - ps16
Saccharo/Pezizomycotina - ps17
Saccharomycotina - ps18
Saccharomycotina_1 - ps19
Saccharomycotina_2 - ps20
Saccharomycotina_3 - ps21
Phaff./Saccharomycetaceae - ps22
Saccharomyco./mycetaceae - ps23
Saccharomycetaceae - ps24
Saccharomycetaceae_2 - ps25
Saccharomycetaceae_3 - ps26
Kaz./Nau./Saccharomyces - ps27
Saccharomyces - ps28
Arboricola_clade - ps29
Cerevisiae_clade - ps30
Saccharomyces_cerevisiae - ps31
p-value
8e-03
1e-02
1e-02
2e-02
3e-02
Log-odds
0.5
1.0
1.5
2.0
2.5
3.0
3.5
4.0

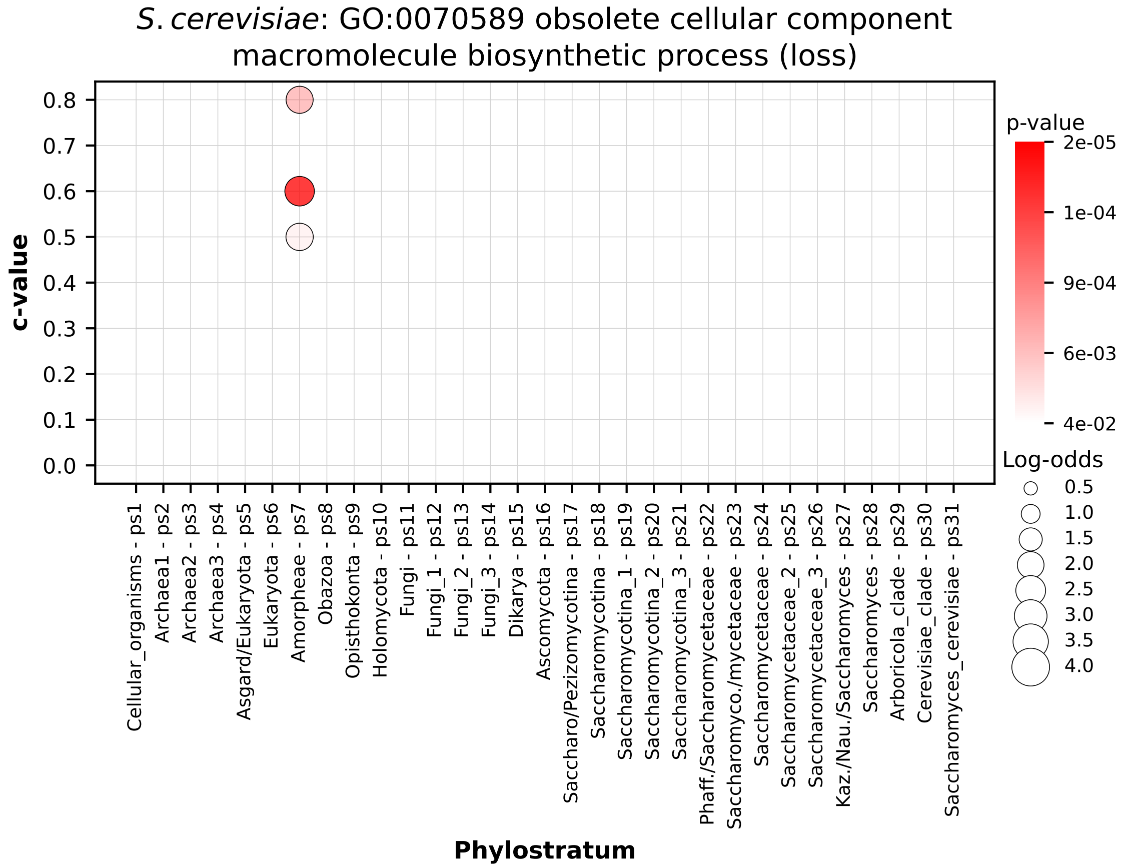
S. cerevisiae: GO:0070589 obsolete cellular component macromolecule biosynthetic process (loss)
c-value
0.8
0.7
0.6
0.5
0.4
0.3
0.2
0.1
0.0
p-value
2e-05
1e-04
9e-04
6e-03
4e-02
Log-odds
0.5
1.0
1.5
2.0
2.5
3.0
3.5
4.0
Cellular_organisms - ps1
Archaea1 - ps2
Archaea2 - ps3
Archaea3 - ps4
Asgard/Eukaryota - ps5
Eukaryota - ps6
Amorpheae - ps7
Obazoa - ps8
Opisthokonta - ps9
Holomycota - ps10
Fungi - ps11
Fungi_1 - ps12
Fungi_2 - ps13
Fungi_3 - ps14
Dikarya - ps15
Ascomycota - ps16
Saccharo/Pezizomycotina - ps17
Saccharomycotina - ps18
Saccharomycotina_1 - ps19
Saccharomycotina_2 - ps20
Saccharomycotina_3 - ps21
Phaff./Saccharomycetaceae - ps22
Saccharomyco./mycetaceae - ps23
Saccharomycetaceae - ps24
Saccharomycetaceae_2 - ps25
Saccharomycetaceae_3 - ps26
Kaz./Nau./Saccharomyces - ps27
Saccharomyces - ps28
Arboricola_clade - ps29
Cerevisiae_clade - ps30
Saccharomyces_cerevisiae - ps31
Phylostratum

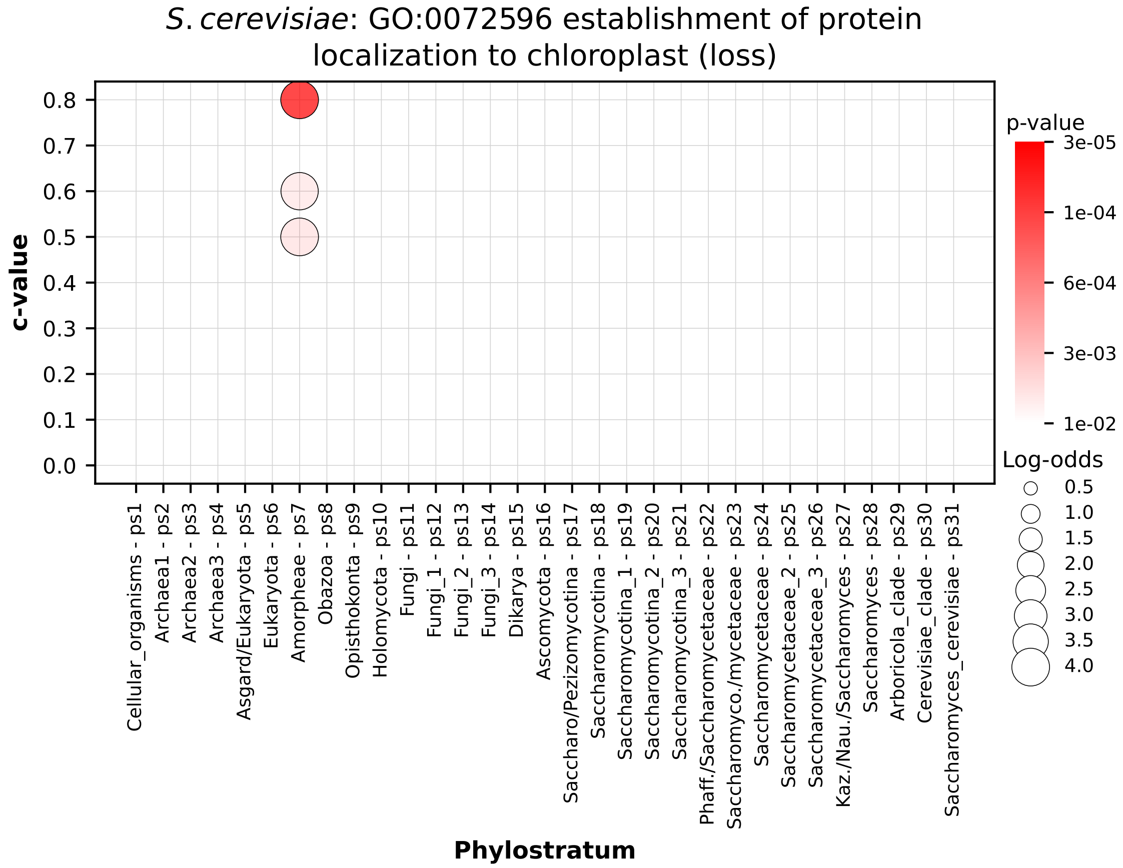

S. cerevisiae: GO:0072596 establishment of protein localization to chloroplast (loss)
c-value
0.8
0.7
0.6
0.5
0.4
0.3
0.2
0.1
0.0
Phylostratum
Cellular_organisms - ps1
Archaea1 - ps2
Archaea2 - ps3
Archaea3 - ps4
Asgard/Eukaryota - ps5
Eukaryota - ps6
Amorpheae - ps7
Obazoa - ps8
Opisthokonta - ps9
Holomycota - ps10
Fungi - ps11
Fungi_1 - ps12
Fungi_2 - ps13
Fungi_3 - ps14
Dikarya - ps15
Ascomycota - ps16
Saccharo/Pezizomycotina - ps17
Saccharomycotina - ps18
Saccharomycotina_1 - ps19
Saccharomycotina_2 - ps20
Saccharomycotina_3 - ps21
Phaff./Saccharomycetaceae - ps22
Saccharomyco./mycetaceae - ps23
Saccharomycetaceae - ps24
Saccharomycetaceae_2 - ps25
Saccharomycetaceae_3 - ps26
Kaz./Nau./Saccharomyces - ps27
Saccharomyces - ps28
Arboricola_clade - ps29
Cerevisiae_clade - ps30
Saccharomyces_cerevisiae - ps31
p-value
3e-05
1e-04
6e-04
3e-03
1e-02
Log-odds
0.5
1.0
1.5
2.0
2.5
3.0
3.5
4.0

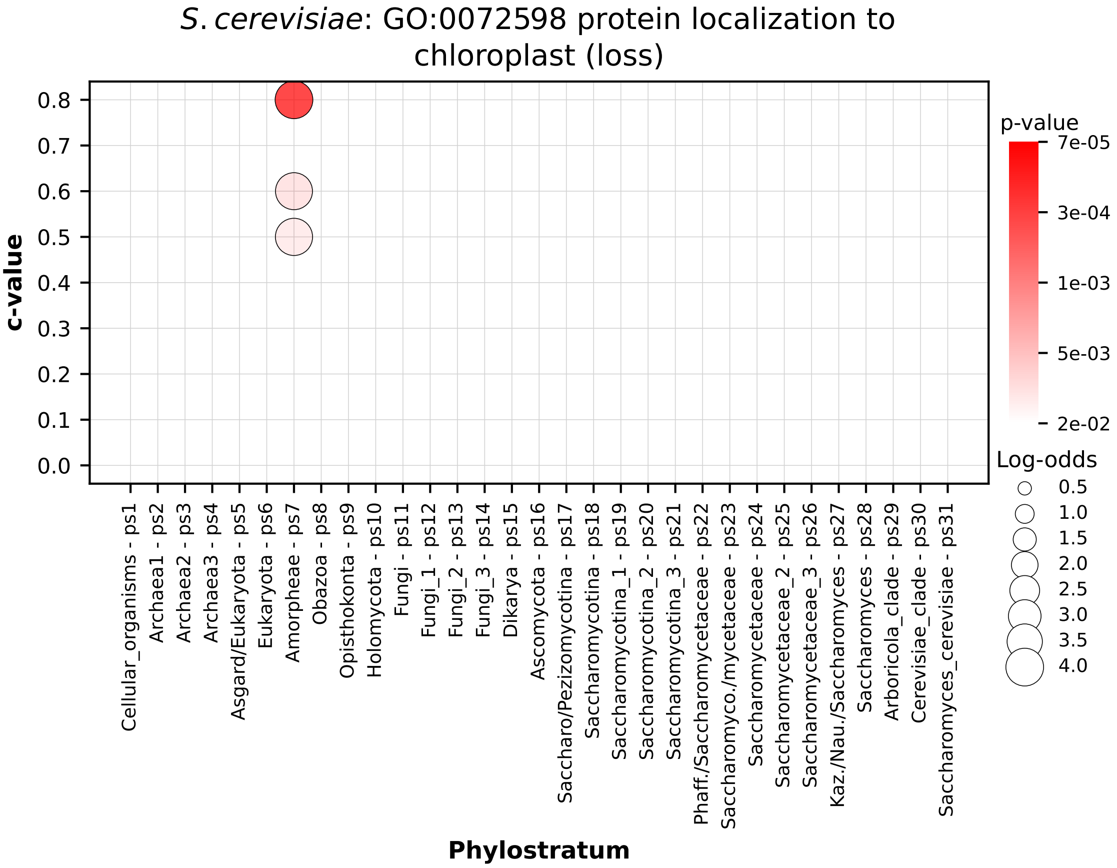

S. cerevisiae: GO:0072598 protein localization to chloroplast (loss)
c-value
0.8
0.7
0.6
0.5
0.4
0.3
0.2
0.1
0.0
Phylostratum
Cellular_organisms - ps1
Archaea1 - ps2
Archaea2 - ps3
Archaea3 - ps4
Asgard/Eukaryota - ps5
Eukaryota - ps6
Amorpheae - ps7
Obazoa - ps8
Opisthokonta - ps9
Holomycota - ps10
Fungi - ps11
Fungi_1 - ps12
Fungi_2 - ps13
Fungi_3 - ps14
Dikarya - ps15
Ascomycota - ps16
Saccharo/Pezizomycotina - ps17
Saccharomycotina - ps18
Saccharomycotina_1 - ps19
Saccharomycotina_2 - ps20
Saccharomycotina_3 - ps21
Phaff./Saccharomycetaceae - ps22
Saccharomyco./mycetaceae - ps23
Saccharomycetaceae - ps24
Saccharomycetaceae_2 - ps25
Saccharomycetaceae_3 - ps26
Kaz./Nau./Saccharomyces - ps27
Saccharomyces - ps28
Arboricola_clade - ps29
Cerevisiae_clade - ps30
Saccharomyces_cerevisiae - ps31
p-value
7e-05
3e-04
1e-03
5e-03
2e-02
Log-odds
0.5
1.0
1.5
2.0
2.5
3.0
3.5
4.0

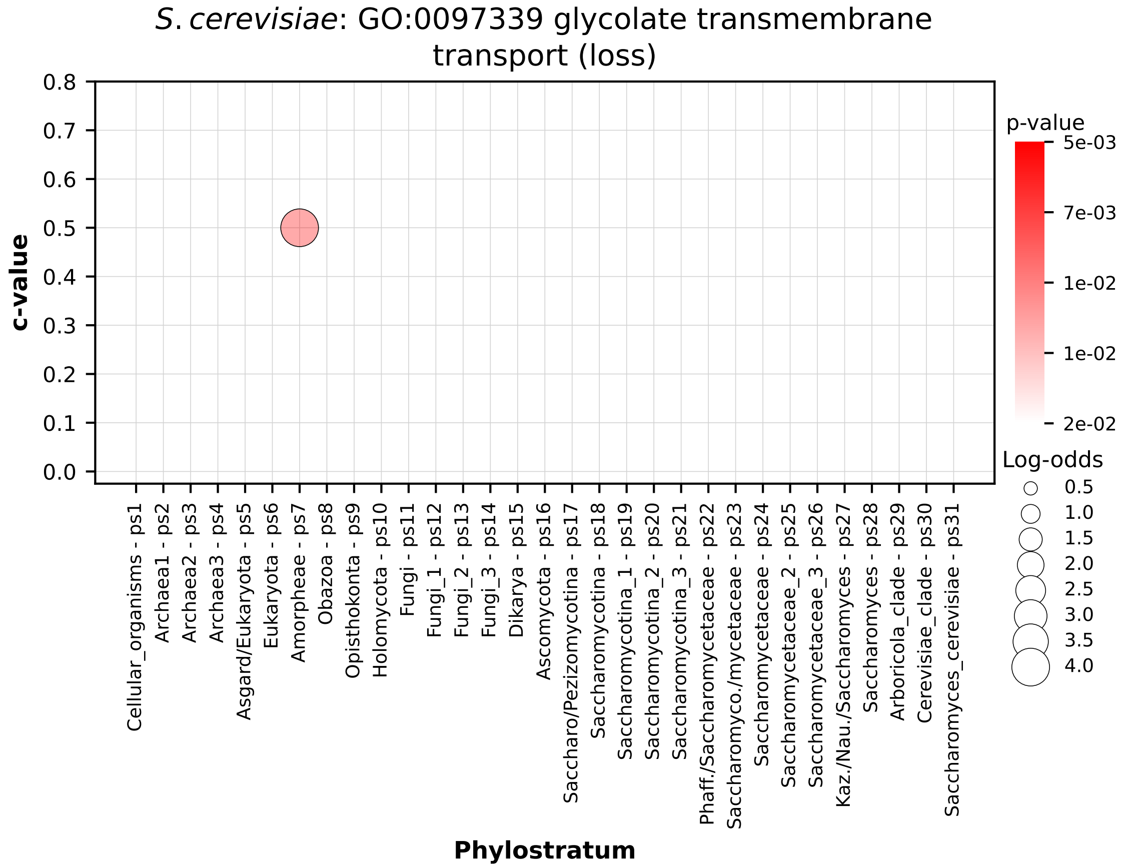

S. cerevisiae: GO:0097339 glycolate transmembrane transport (loss)
c-value
0.8
0.7
0.6
0.5
0.4
0.3
0.2
0.1
0.0
Phylostratum
Cellular_organisms - ps1
Archaea1 - ps2
Archaea2 - ps3
Archaea3 - ps4
Asgard/Eukaryota - ps5
Eukaryota - ps6
Amorpheae - ps7
Obazoa - ps8
Opisthokonta - ps9
Holomycota - ps10
Fungi - ps11
Fungi_1 - ps12
Fungi_2 - ps13
Fungi_3 - ps14
Dikarya - ps15
Ascomycota - ps16
Saccharo/Pezizomycotina - ps17
Saccharomycotina - ps18
Saccharomycotina_1 - ps19
Saccharomycotina_2 - ps20
Saccharomycotina_3 - ps21
Phaff./Saccharomycetaceae - ps22
Saccharomyco./mycetaceae - ps23
Saccharomycetaceae - ps24
Saccharomycetaceae_2 - ps25
Saccharomycetaceae_3 - ps26
Kaz./Nau./Saccharomyces - ps27
Saccharomyces - ps28
Arboricola_clade - ps29
Cerevisiae_clade - ps30
Saccharomyces_cerevisiae - ps31
p-value
5e-03
7e-03
1e-02
1e-02
2e-02
Log-odds
0.5
1.0
1.5
2.0
2.5
3.0
3.5
4.0

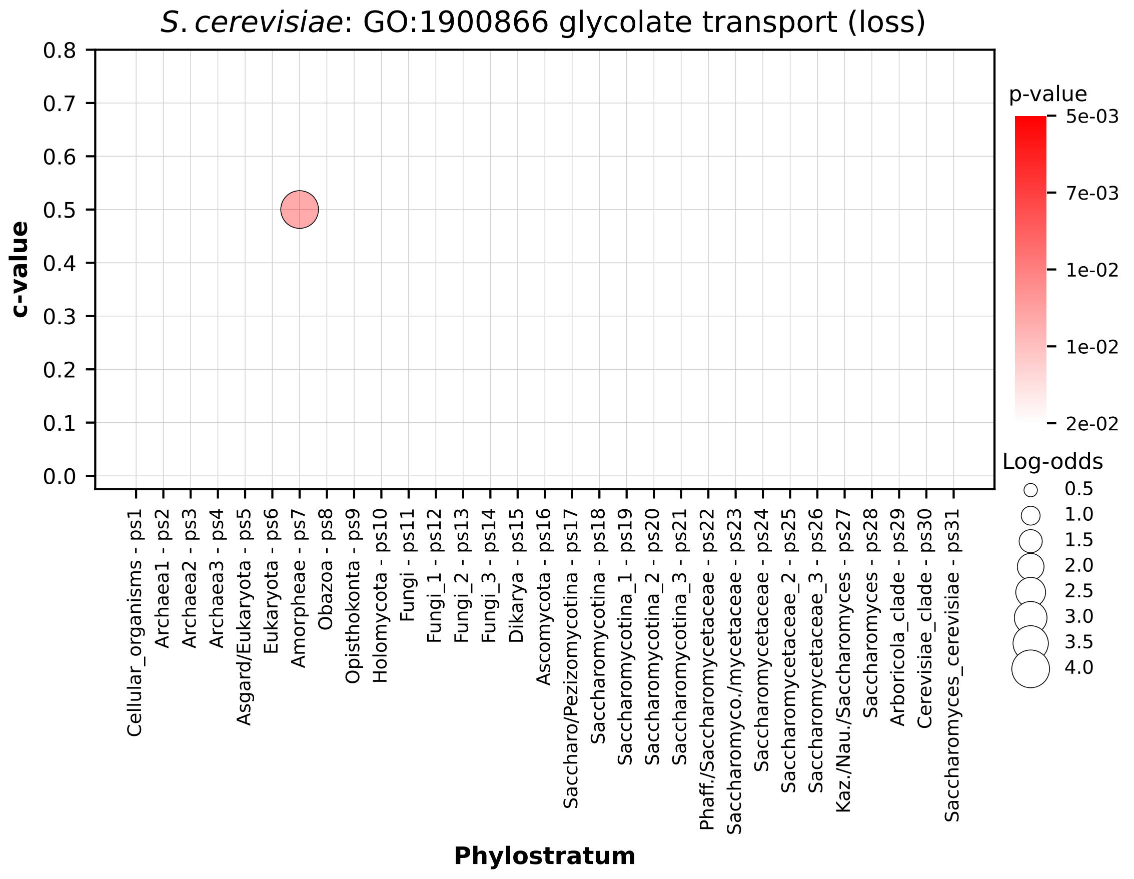

S. cerevisiae: GO:1900866 glycolate transport (loss)
c-value
0.8
0.7
0.6
0.5
0.4
0.3
0.2
0.1
0.0
p-value
5e-03
7e-03
1e-02
1e-02
2e-02
Log-odds
0.5
1.0
1.5
2.0
2.5
3.0
3.5
4.0
Cellular_organisms - ps1
Archaea1 - ps2
Archaea2 - ps3
Archaea3 - ps4
Asgard/Eukaryota - ps5
Eukaryota - ps6
Amorpheae - ps7
Obazoa - ps8
Opisthokonta - ps9
Holomycota - ps10
Fungi - ps11
Fungi_1 - ps12
Fungi_2 - ps13
Fungi_3 - ps14
Dikarya - ps15
Ascomycota - ps16
Saccharo/Pezizomycotina - ps17
Saccharomycotina - ps18
Saccharomycotina_1 - ps19
Saccharomycotina_2 - ps20
Saccharomycotina_3 - ps21
Phaff./Saccharomycetaceae - ps22
Saccharomyco./mycetaceae - ps23
Saccharomycetaceae - ps24
Saccharomycetaceae_2 - ps25
Saccharomycetaceae_3 - ps26
Kaz./Nau./Saccharomyces - ps27
Saccharomyces - ps28
Arboricola_clade - ps29
Cerevisiae_clade - ps30
Saccharomyces_cerevisiae - ps31
Phylostratum

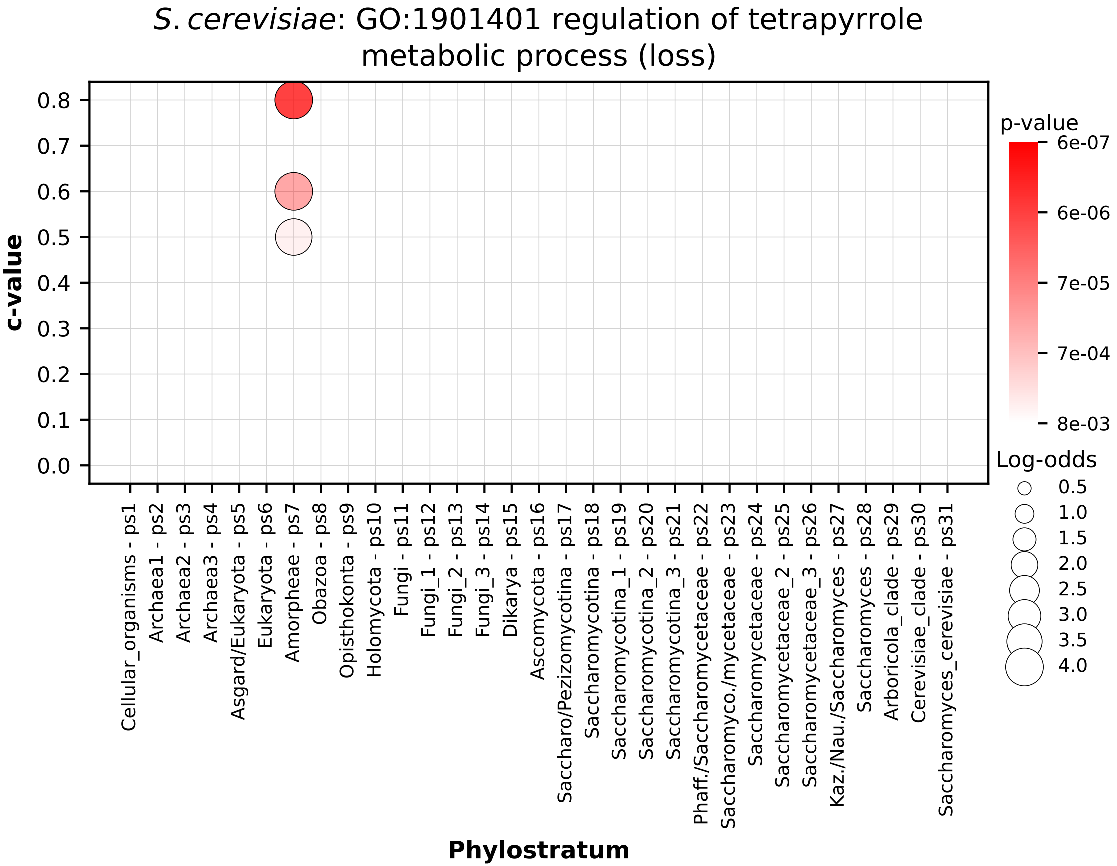

S. cerevisiae: GO:1901401 regulation of tetrapyrrole metabolic process (loss)
c-value
0.0
0.1
0.2
0.3
0.4
0.5
0.6
0.7
0.8
p-value
6e-07
6e-06
7e-05
7e-04
8e-03
Log-odds
0.5
1.0
1.5
2.0
2.5
3.0
3.5
4.0
Cellular_organisms - ps1
Archaea1 - ps2
Archaea2 - ps3
Archaea3 - ps4
Asgard/Eukaryota - ps5
Eukaryota - ps6
Amorpheae - ps7
Obazoa - ps8
Opisthokonta - ps9
Holomycota - ps10
Fungi - ps11
Fungi_1 - ps12
Fungi_2 - ps13
Fungi_3 - ps14
Dikarya - ps15
Ascomycota - ps16
Saccharo/Pezizomycotina - ps17
Saccharomycotina - ps18
Saccharomycotina_1 - ps19
Saccharomycotina_2 - ps20
Saccharomycotina_3 - ps21
Phaff./Saccharomycetaceae - ps22
Saccharomyco./mycetaceae - ps23
Saccharomycetaceae - ps24
Saccharomycetaceae_2 - ps25
Saccharomycetaceae_3 - ps26
Kaz./Nau./Saccharomyces - ps27
Saccharomyces - ps28
Arboricola_clade - ps29
Cerevisiae_clade - ps30
Saccharomyces_cerevisiae - ps31
Phylostratum

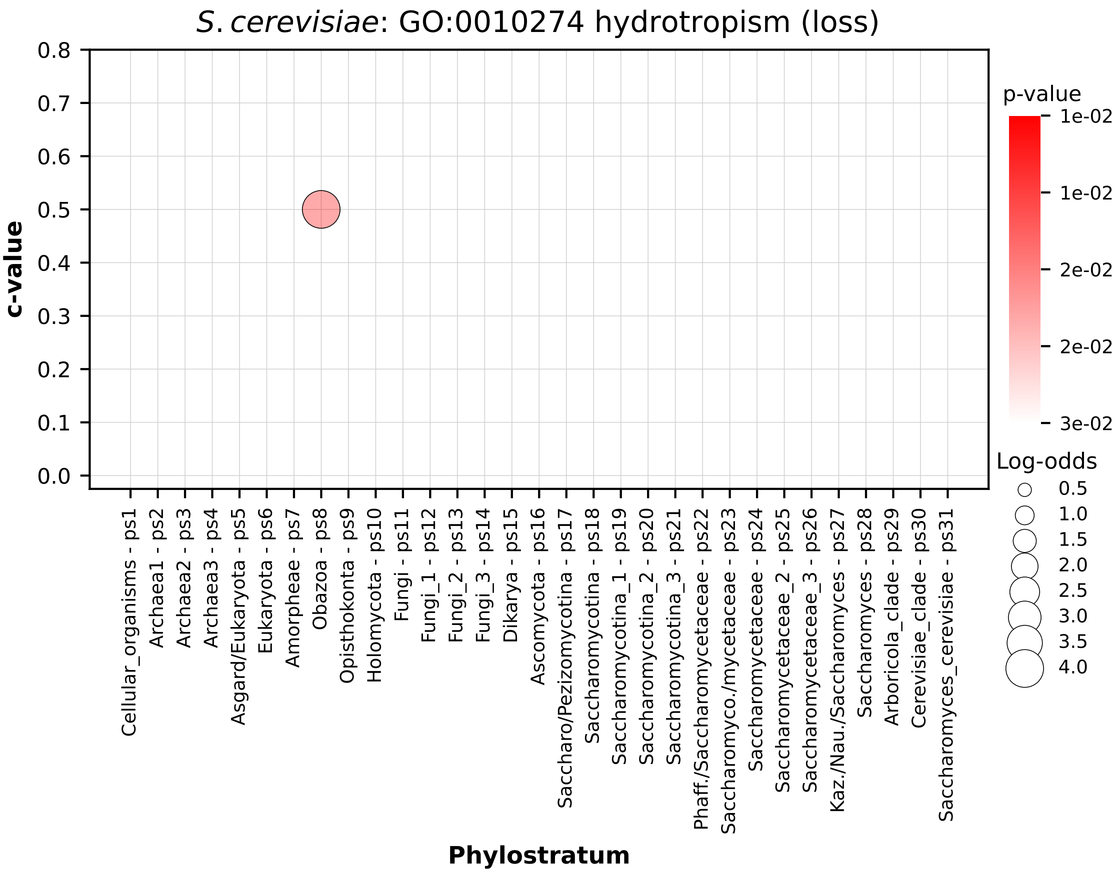

S. cerevisiae: GO:0010274 hydrotropism (loss)
c-value
0.8
0.7
0.6
0.5
0.4
0.3
0.2
0.1
0.0
Phylostratum
Cellular_organisms - ps1
Archaea1 - ps2
Archaea2 - ps3
Archaea3 - ps4
Asgard/Eukaryota - ps5
Eukaryota - ps6
Amorpheae - ps7
Obazoa - ps8
Opisthokonta - ps9
Holomycota - ps10
Fungi - ps11
Fungi_1 - ps12
Fungi_2 - ps13
Fungi_3 - ps14
Dikarya - ps15
Ascomycota - ps16
Saccharo/Pezizomycotina - ps17
Saccharomycotina - ps18
Saccharomycotina_1 - ps19
Saccharomycotina_2 - ps20
Saccharomycotina_3 - ps21
Phaff./Saccharomycetaceae - ps22
Saccharomyco./mycetaceae - ps23
Saccharomycetaceae - ps24
Saccharomycetaceae_2 - ps25
Saccharomycetaceae_3 - ps26
Kaz./Nau./Saccharomyces - ps27
Saccharomyces - ps28
Arboricola_clade - ps29
Cerevisiae_clade - ps30
Saccharomyces_cerevisiae - ps31
p-value
1e-02
1e-02
2e-02
2e-02
3e-02
Log-odds
0.5
1.0
1.5
2.0
2.5
3.0
3.5
4.0

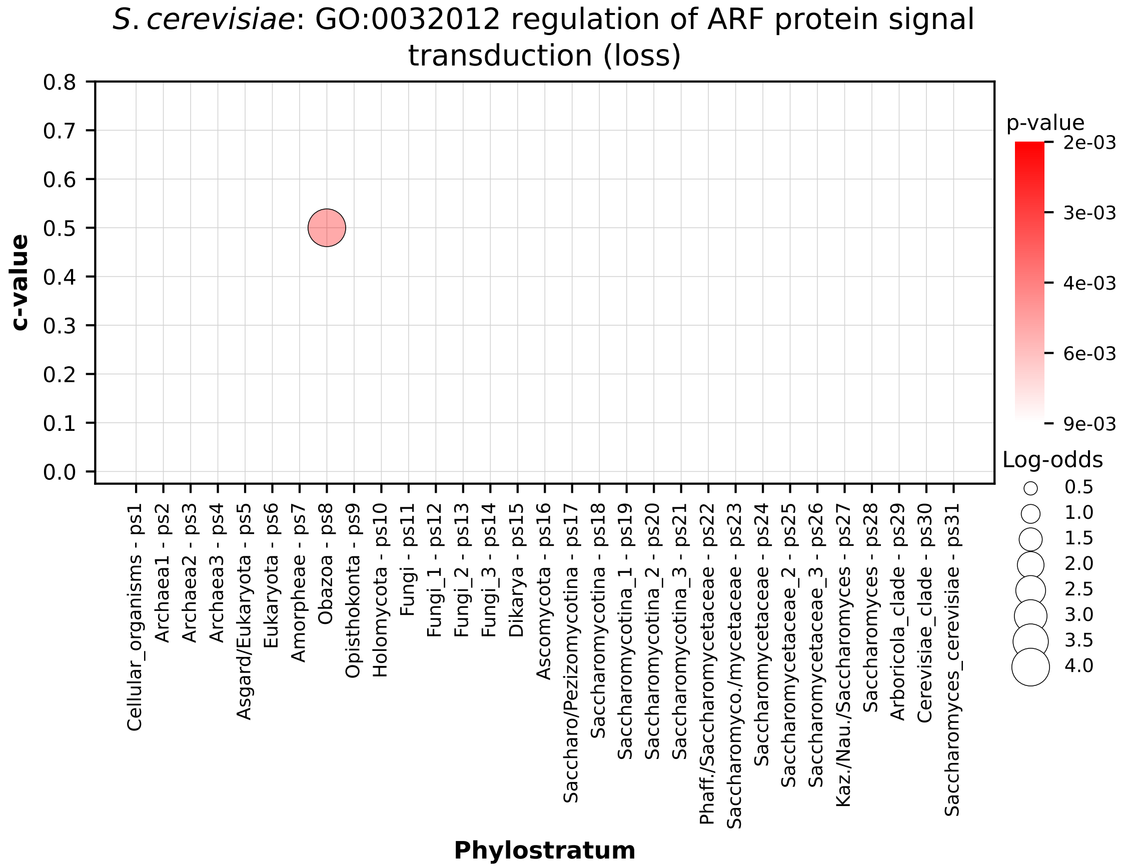

S. cerevisiae: GO:0032012 regulation of ARF protein signal transduction (loss)
c-value
0.8
0.7
0.6
0.5
0.4
0.3
0.2
0.1
0.0
p-value
2e-03
3e-03
4e-03
6e-03
9e-03
Log-odds
0.5
1.0
1.5
2.0
2.5
3.0
3.5
4.0
Cellular_organisms - ps1
Archaea1 - ps2
Archaea2 - ps3
Archaea3 - ps4
Asgard/Eukaryota - ps5
Eukaryota - ps6
Amorpheae - ps7
Obazoa - ps8
Opisthokonta - ps9
Holomycota - ps10
Fungi - ps11
Fungi_1 - ps12
Fungi_2 - ps13
Fungi_3 - ps14
Dikarya - ps15
Ascomycota - ps16
Saccharo/Pezizomycotina - ps17
Saccharomycotina - ps18
Saccharomycotina_1 - ps19
Saccharomycotina_2 - ps20
Saccharomycotina_3 - ps21
Phaff./Saccharomycetaceae - ps22
Saccharomyco./mycetaceae - ps23
Saccharomycetaceae - ps24
Saccharomycetaceae_2 - ps25
Saccharomycetaceae_3 - ps26
Kaz./Nau./Saccharomyces - ps27
Saccharomyces - ps28
Arboricola_clade - ps29
Cerevisiae_clade - ps30
Saccharomyces_cerevisiae - ps31
Phylostratum

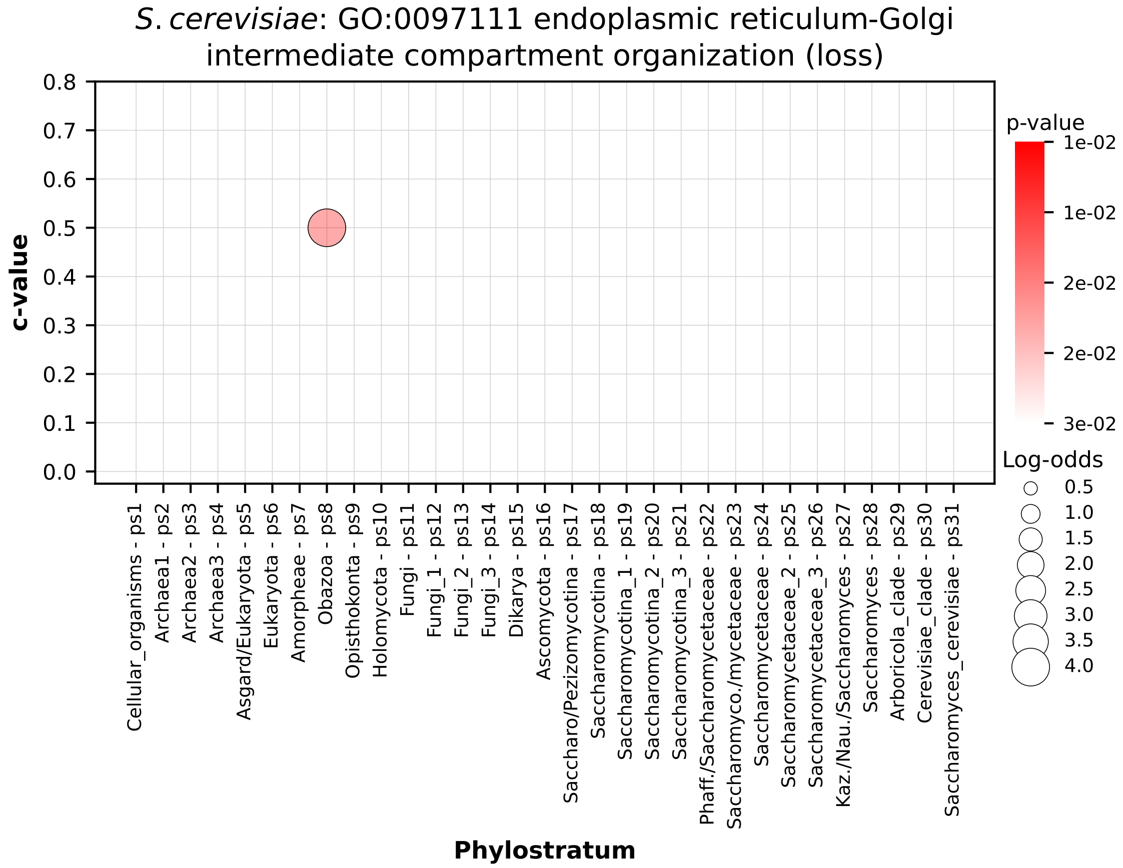

S. cerevisiae: GO:0097111 endoplasmic reticulum-Golgi intermediate compartment organization (loss)
c-value
0.8
0.7
0.6
0.5
0.4
0.3
0.2
0.1
0.0
Phylostratum
Cellular_organisms - ps1
Archaea1 - ps2
Archaea2 - ps3
Archaea3 - ps4
Asgard/Eukaryota - ps5
Eukaryota - ps6
Amorpheae - ps7
Obazoa - ps8
Opisthokonta - ps9
Holomycota - ps10
Fungi - ps11
Fungi_1 - ps12
Fungi_2 - ps13
Fungi_3 - ps14
Dikarya - ps15
Ascomycota - ps16
Saccharo/Pezizomycotina - ps17
Saccharomycotina - ps18
Saccharomycotina_1 - ps19
Saccharomycotina_2 - ps20
Saccharomycotina_3 - ps21
Phaff./Saccharomycetaceae - ps22
Saccharomyco./mycetaceae - ps23
Saccharomycetaceae - ps24
Saccharomycetaceae_2 - ps25
Saccharomycetaceae_3 - ps26
Kaz./Nau./Saccharomyces - ps27
Saccharomyces - ps28
Arboricola_clade - ps29
Cerevisiae_clade - ps30
Saccharomyces_cerevisiae - ps31
p-value
1e-02
1e-02
2e-02
2e-02
3e-02
Log-odds
0.5
1.0
1.5
2.0
2.5
3.0
3.5
4.0

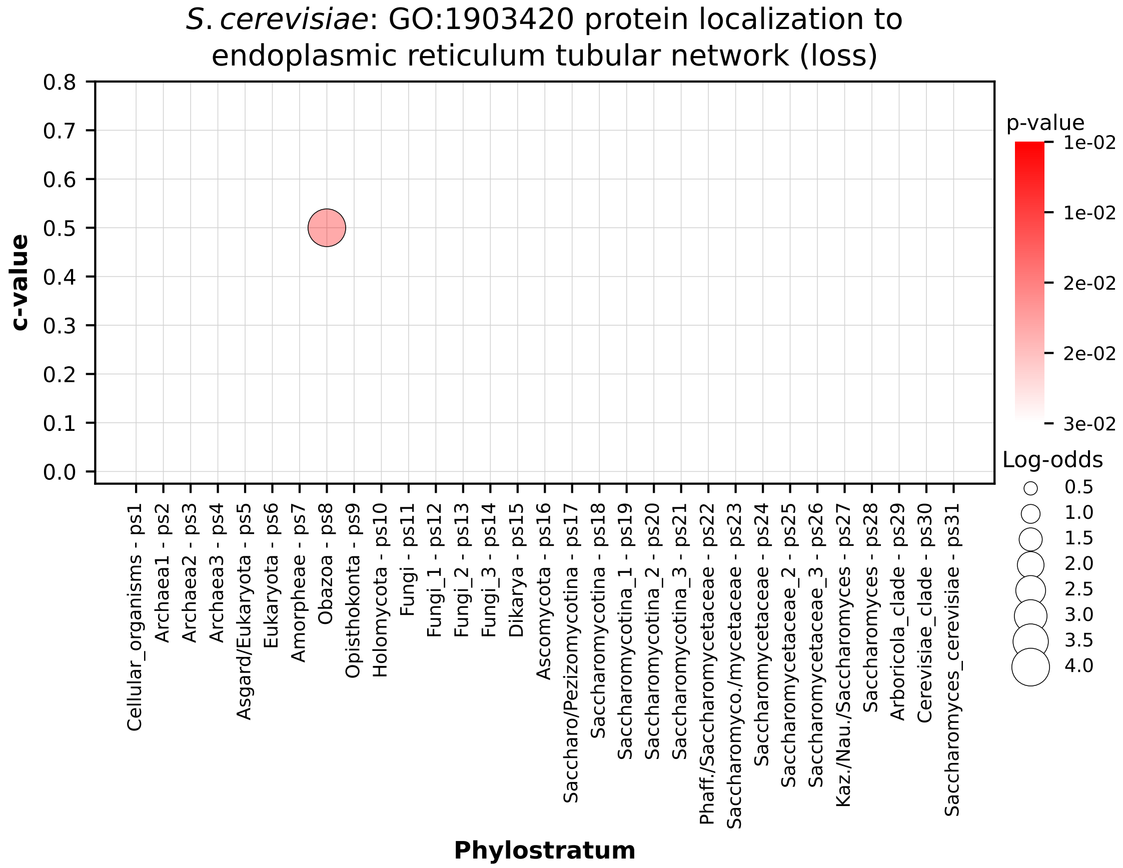

S. cerevisiae: GO:1903420 protein localization to endoplasmic reticulum tubular network (loss)
c-value
0.0
0.1
0.2
0.3
0.4
0.5
0.6
0.7
0.8
Phylostratum
Cellular_organisms - ps1
Archaea1 - ps2
Archaea2 - ps3
Archaea3 - ps4
Asgard/Eukaryota - ps5
Eukaryota - ps6
Amorpheae - ps7
Obazoa - ps8
Opisthokonta - ps9
Holomycota - ps10
Fungi - ps11
Fungi_1 - ps12
Fungi_2 - ps13
Fungi_3 - ps14
Dikarya - ps15
Ascomycota - ps16
Saccharo/Pezizomycotina - ps17
Saccharomycotina - ps18
Saccharomycotina_1 - ps19
Saccharomycotina_2 - ps20
Saccharomycotina_3 - ps21
Phaff./Saccharomycetaceae - ps22
Saccharomyco./mycetaceae - ps23
Saccharomycetaceae - ps24
Saccharomycetaceae_2 - ps25
Saccharomycetaceae_3 - ps26
Kaz./Nau./Saccharomyces - ps27
Saccharomyces - ps28
Arboricola_clade - ps29
Cerevisiae_clade - ps30
Saccharomyces_cerevisiae - ps31
p-value
1e-02
1e-02
2e-02
2e-02
3e-02
Log-odds
0.5
1.0
1.5
2.0
2.5
3.0
3.5
4.0

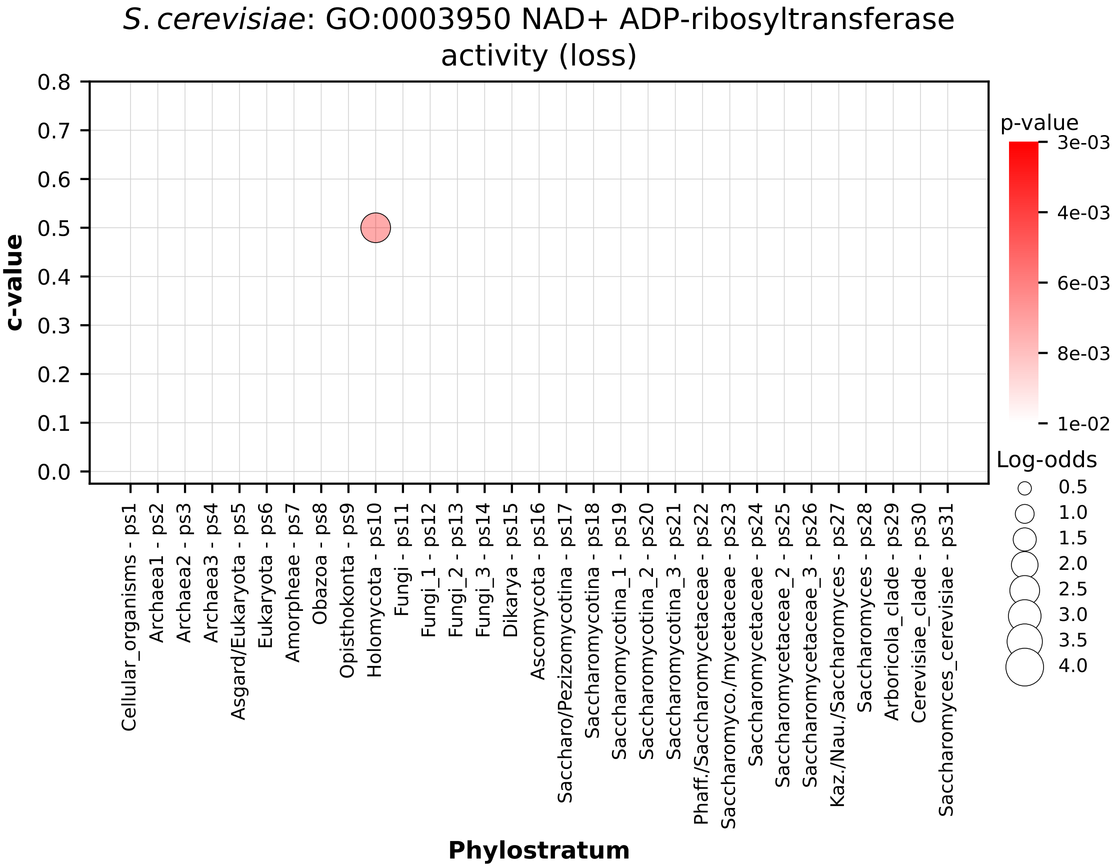

S. cerevisiae: GO:0003950 NAD+ ADP-ribosyltransferase activity (loss)
c-value
0.8
0.7
0.6
0.5
0.4
0.3
0.2
0.1
0.0
p-value
3e-03
4e-03
6e-03
8e-03
1e-02
Log-odds
0.5
1.0
1.5
2.0
2.5
3.0
3.5
4.0
Cellular_organisms - ps1
Archaea1 - ps2
Archaea2 - ps3
Archaea3 - ps4
Asgard/Eukaryota - ps5
Eukaryota - ps6
Amorpheae - ps7
Obazoa - ps8
Opisthokonta - ps9
Holomycota - ps10
Fungi - ps11
Fungi_1 - ps12
Fungi_2 - ps13
Fungi_3 - ps14
Dikarya - ps15
Ascomycota - ps16
Saccharo/Pezizomycotina - ps17
Saccharomycotina - ps18
Saccharomycotina_1 - ps19
Saccharomycotina_2 - ps20
Saccharomycotina_3 - ps21
Phaff./Saccharomycetaceae - ps22
Saccharomyco./mycetaceae - ps23
Saccharomycetaceae - ps24
Saccharomycetaceae_2 - ps25
Saccharomycetaceae_3 - ps26
Kaz./Nau./Saccharomyces - ps27
Saccharomyces - ps28
Arboricola_clade - ps29
Cerevisiae_clade - ps30
Saccharomyces_cerevisiae - ps31
Phylostratum

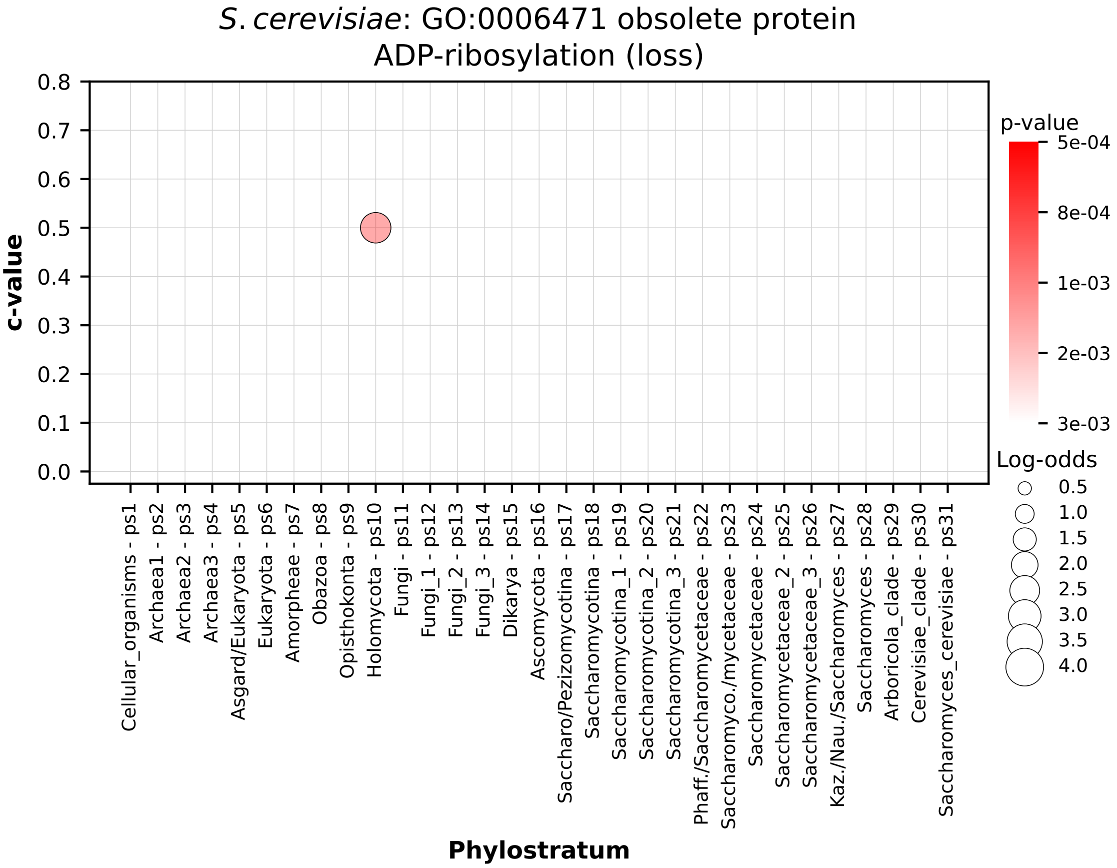

S. cerevisiae: GO:0006471 obsolete protein ADP-ribosylation (loss)
c-value
0.8
0.7
0.6
0.5
0.4
0.3
0.2
0.1
0.0
Phylostratum
Cellular_organisms - ps1
Archaea1 - ps2
Archaea2 - ps3
Archaea3 - ps4
Asgard/Eukaryota - ps5
Eukaryota - ps6
Amorpheae - ps7
Obazoa - ps8
Opisthokonta - ps9
Holomycota - ps10
Fungi - ps11
Fungi_1 - ps12
Fungi_2 - ps13
Fungi_3 - ps14
Dikarya - ps15
Ascomycota - ps16
Saccharo/Pezizomycotina - ps17
Saccharomycotina - ps18
Saccharomycotina_1 - ps19
Saccharomycotina_2 - ps20
Saccharomycotina_3 - ps21
Phaff./Saccharomycetaceae - ps22
Saccharomyco./mycetaceae - ps23
Saccharomycetaceae - ps24
Saccharomycetaceae_2 - ps25
Saccharomycetaceae_3 - ps26
Kaz./Nau./Saccharomyces - ps27
Saccharomyces - ps28
Arboricola_clade - ps29
Cerevisiae_clade - ps30
Saccharomyces_cerevisiae - ps31
p-value
5e-04
8e-04
1e-03
2e-03
3e-03
Log-odds
0.5
1.0
1.5
2.0
2.5
3.0
3.5
4.0

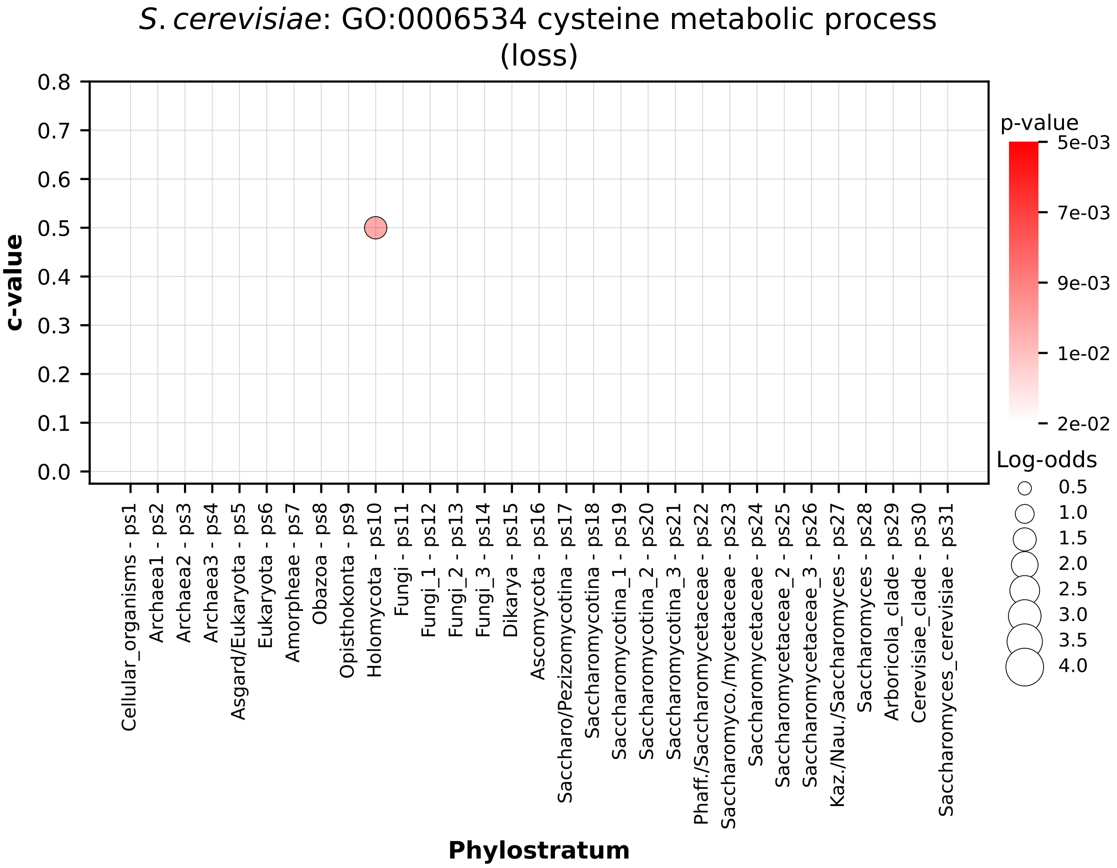

S. cerevisiae: GO:0006534 cysteine metabolic process
(loss)
c-value
0.8
0.7
0.6
0.5
0.4
0.3
0.2
0.1
0.0
Phylostratum
Cellular_organisms - ps1
Archaea1 - ps2
Archaea2 - ps3
Archaea3 - ps4
Asgard/Eukaryota - ps5
Eukaryota - ps6
Amorpheae - ps7
Obazoa - ps8
Opisthokonta - ps9
Holomycota - ps10
Fungi - ps11
Fungi_1 - ps12
Fungi_2 - ps13
Fungi_3 - ps14
Dikarya - ps15
Ascomycota - ps16
Saccharo/Pezizomycotina - ps17
Saccharomycotina - ps18
Saccharomycotina_1 - ps19
Saccharomycotina_2 - ps20
Saccharomycotina_3 - ps21
Phaff./Saccharomycetaceae - ps22
Saccharomyco./mycetaceae - ps23
Saccharomycetaceae - ps24
Saccharomycetaceae_2 - ps25
Saccharomycetaceae_3 - ps26
Kaz./Nau./Saccharomyces - ps27
Saccharomyces - ps28
Arboricola_clade - ps29
Cerevisiae_clade - ps30
Saccharomyces_cerevisiae - ps31
p-value
5e-03
7e-03
9e-03
1e-02
2e-02
Log-odds
0.5
1.0
1.5
2.0
2.5
3.0
3.5
4.0

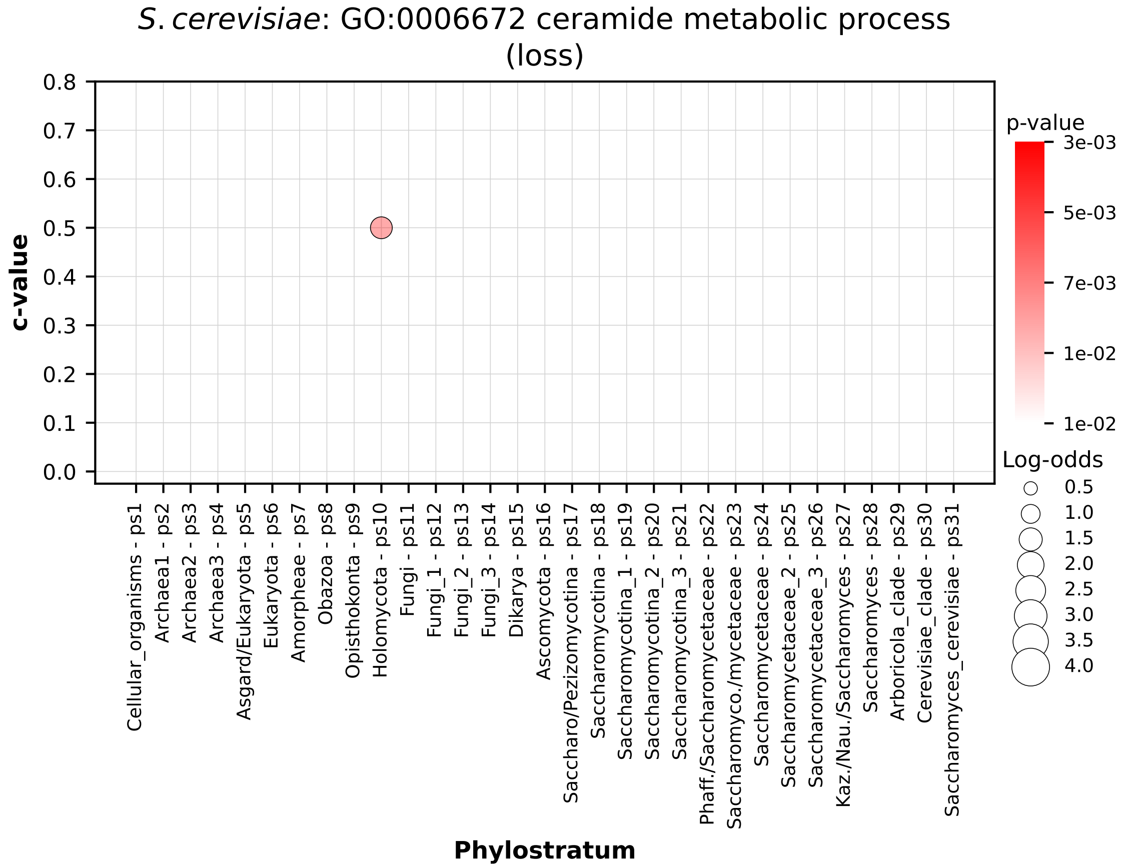

S. cerevisiae: GO:0006672 ceramide metabolic process (loss)
c-value
0.8
0.7
0.6
0.5
0.4
0.3
0.2
0.1
0.0
p-value
3e-03
5e-03
7e-03
1e-02
1e-02
Log-odds
0.5
1.0
1.5
2.0
2.5
3.0
3.5
4.0
Cellular_organisms - ps1
Archaea1 - ps2
Archaea2 - ps3
Archaea3 - ps4
Asgard/Eukaryota - ps5
Eukaryota - ps6
Amorpheae - ps7
Obazoa - ps8
Opisthokonta - ps9
Holomycota - ps10
Fungi - ps11
Fungi_1 - ps12
Fungi_2 - ps13
Fungi_3 - ps14
Dikarya - ps15
Ascomycota - ps16
Saccharo/Pezizomycotina - ps17
Saccharomycotina - ps18
Saccharomycotina_1 - ps19
Saccharomycotina_2 - ps20
Saccharomycotina_3 - ps21
Phaff./Saccharomycetaceae - ps22
Saccharomyco./mycetaceae - ps23
Saccharomycetaceae - ps24
Saccharomycetaceae_2 - ps25
Saccharomycetaceae_3 - ps26
Kaz./Nau./Saccharomyces - ps27
Saccharomyces - ps28
Arboricola_clade - ps29
Cerevisiae_clade - ps30
Saccharomyces_cerevisiae - ps31
Phylostratum

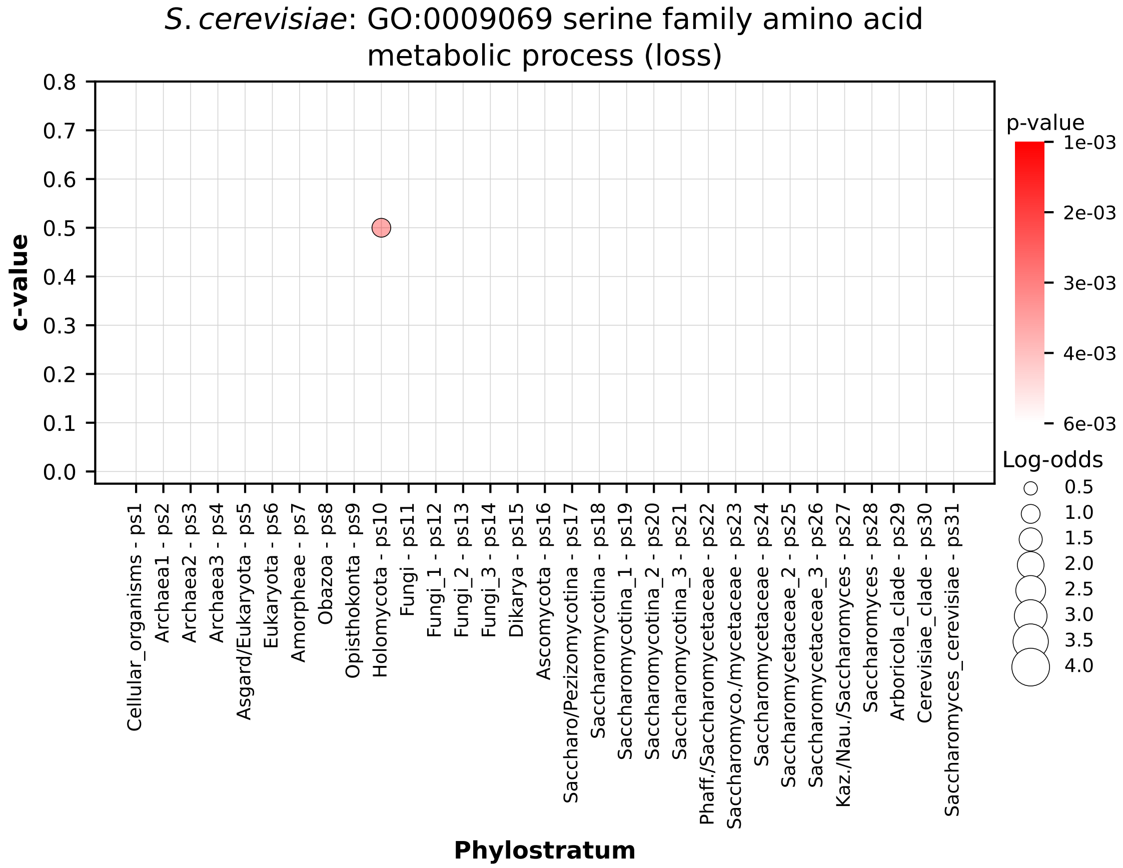

S. cerevisiae: GO:0009069 serine family amino acid metabolic process (loss)
c-value
0.0
0.1
0.2
0.3
0.4
0.5
0.6
0.7
0.8
p-value
1e-03
2e-03
3e-03
4e-03
6e-03
Log-odds
0.5
1.0
1.5
2.0
2.5
3.0
3.5
4.0
Cellular_organisms - ps1
Archaea1 - ps2
Archaea2 - ps3
Archaea3 - ps4
Asgard/Eukaryota - ps5
Eukaryota - ps6
Amorpheae - ps7
Obazoa - ps8
Opisthokonta - ps9
Holomycota - ps10
Fungi - ps11
Fungi_1 - ps12
Fungi_2 - ps13
Fungi_3 - ps14
Dikarya - ps15
Ascomycota - ps16
Saccharo/Pezizomycotina - ps17
Saccharomycotina - ps18
Saccharomycotina_1 - ps19
Saccharomycotina_2 - ps20
Saccharomycotina_3 - ps21
Phaff./Saccharomycetaceae - ps22
Saccharomyco./mycetaceae - ps23
Saccharomycetaceae - ps24
Saccharomycetaceae_2 - ps25
Saccharomycetaceae_3 - ps26
Kaz./Nau./Saccharomyces - ps27
Saccharomyces - ps28
Arboricola_clade - ps29
Cerevisiae_clade - ps30
Saccharomyces_cerevisiae - ps31
Phylostratum

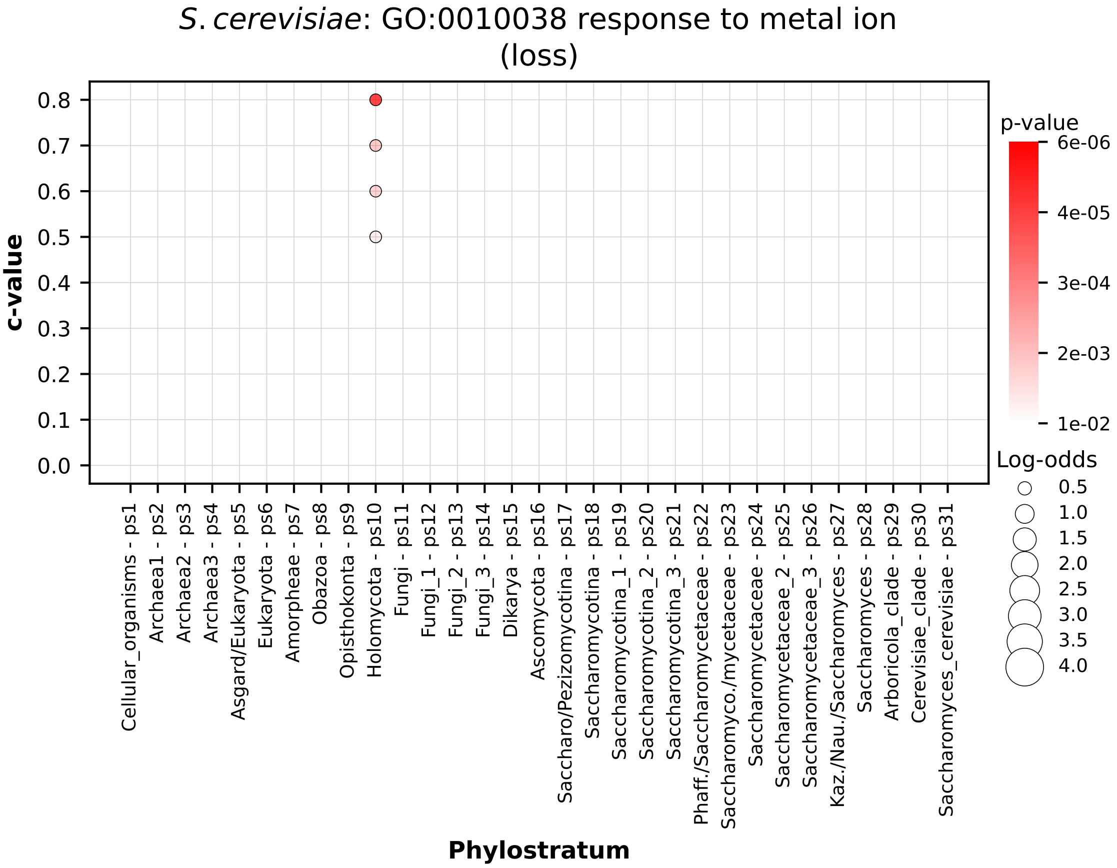
S. cerevisiae: GO:0010038 response to metal ion
(loss)
c-value
0.8
0.7
0.6
0.5
0.4
0.3
0.2
0.1
0.0
p-value
6e-06
4e-05
3e-04
2e-03
1e-02
Log-odds
0.5
1.0
1.5
2.0
2.5
3.0
3.5
4.0
Cellular_organisms - ps1
Archaea1 - ps2
Archaea2 - ps3
Archaea3 - ps4
Asgard/Eukaryota - ps5
Eukaryota - ps6
Amorpheae - ps7
Obazoa - ps8
Opisthokonta - ps9
Holomycota - ps10
Fungi - ps11
Fungi_1 - ps12
Fungi_2 - ps13
Fungi_3 - ps14
Dikarya - ps15
Ascomycota - ps16
Saccharo/Pezizomycotina - ps17
Saccharomycotina - ps18
Saccharomycotina_1 - ps19
Saccharomycotina_2 - ps20
Saccharomycotina_3 - ps21
Phaff./Saccharomycetaceae - ps22
Saccharomyco./mycetaceae - ps23
Saccharomycetaceae - ps24
Saccharomycetaceae_2 - ps25
Saccharomycetaceae_3 - ps26
Kaz./Nau./Saccharomyces - ps27
Saccharomyces - ps28
Arboricola_clade - ps29
Cerevisiae_clade - ps30
Saccharomyces_cerevisiae - ps31
Phylostratum

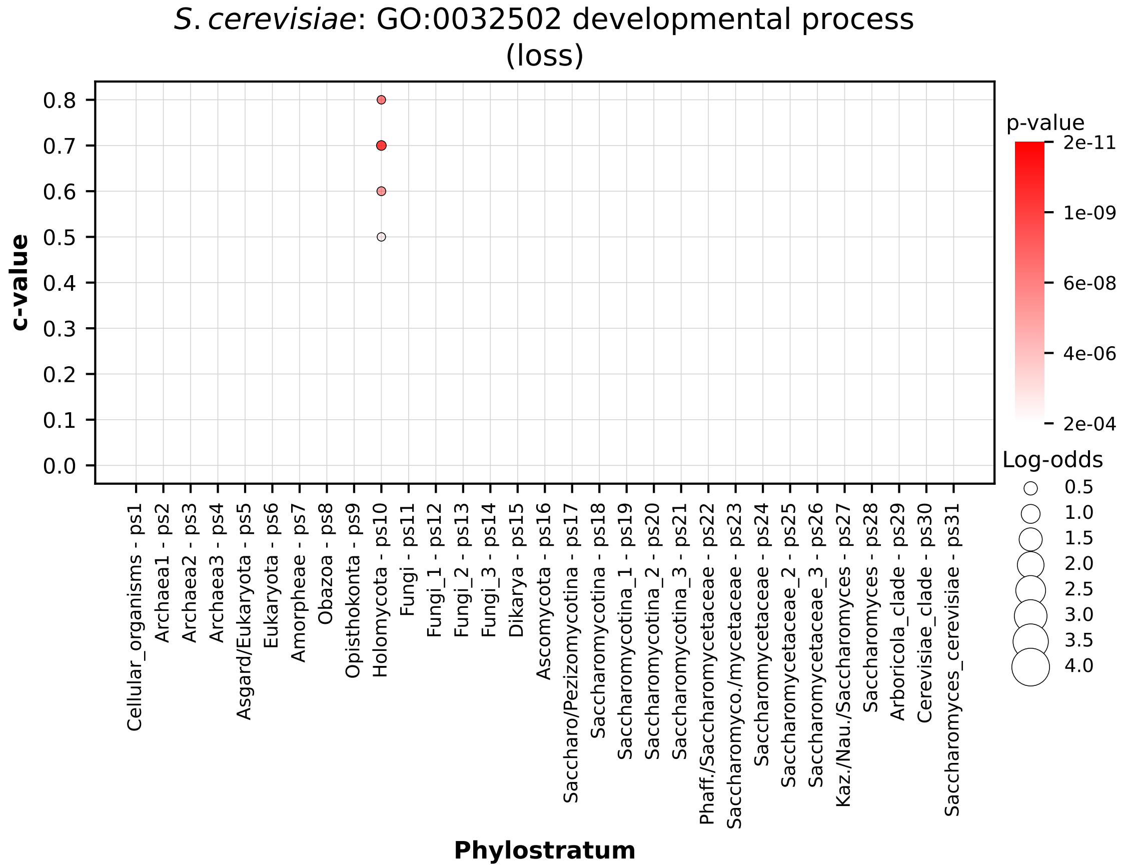

S. cerevisiae: GO:0032502 developmental process
(loss)
c-value
0.0
0.1
0.2
0.3
0.4
0.5
0.6
0.7
0.8
Phylostratum
Cellular_organisms - ps1
Archaea1 - ps2
Archaea2 - ps3
Archaea3 - ps4
Asgard/Eukaryota - ps5
Eukaryota - ps6
Amorpheae - ps7
Obazoa - ps8
Opisthokonta - ps9
Holomycota - ps10
Fungi - ps11
Fungi_1 - ps12
Fungi_2 - ps13
Fungi_3 - ps14
Dikarya - ps15
Ascomycota - ps16
Saccharo/Pezizomycotina - ps17
Saccharomycotina - ps18
Saccharomycotina_1 - ps19
Saccharomycotina_2 - ps20
Saccharomycotina_3 - ps21
Phaff./Saccharomycetaceae - ps22
Saccharomyco./mycetaceae - ps23
Saccharomycetaceae - ps24
Saccharomycetaceae_2 - ps25
Saccharomycetaceae_3 - ps26
Kaz./Nau./Saccharomyces - ps27
Saccharomyces - ps28
Arboricola_clade - ps29
Cerevisiae_clade - ps30
Saccharomyces_cerevisiae - ps31
p-value
2e-11
1e-09
6e-08
4e-06
2e-04
Log-odds
0.5
1.0
1.5
2.0
2.5
3.0
3.5
4.0

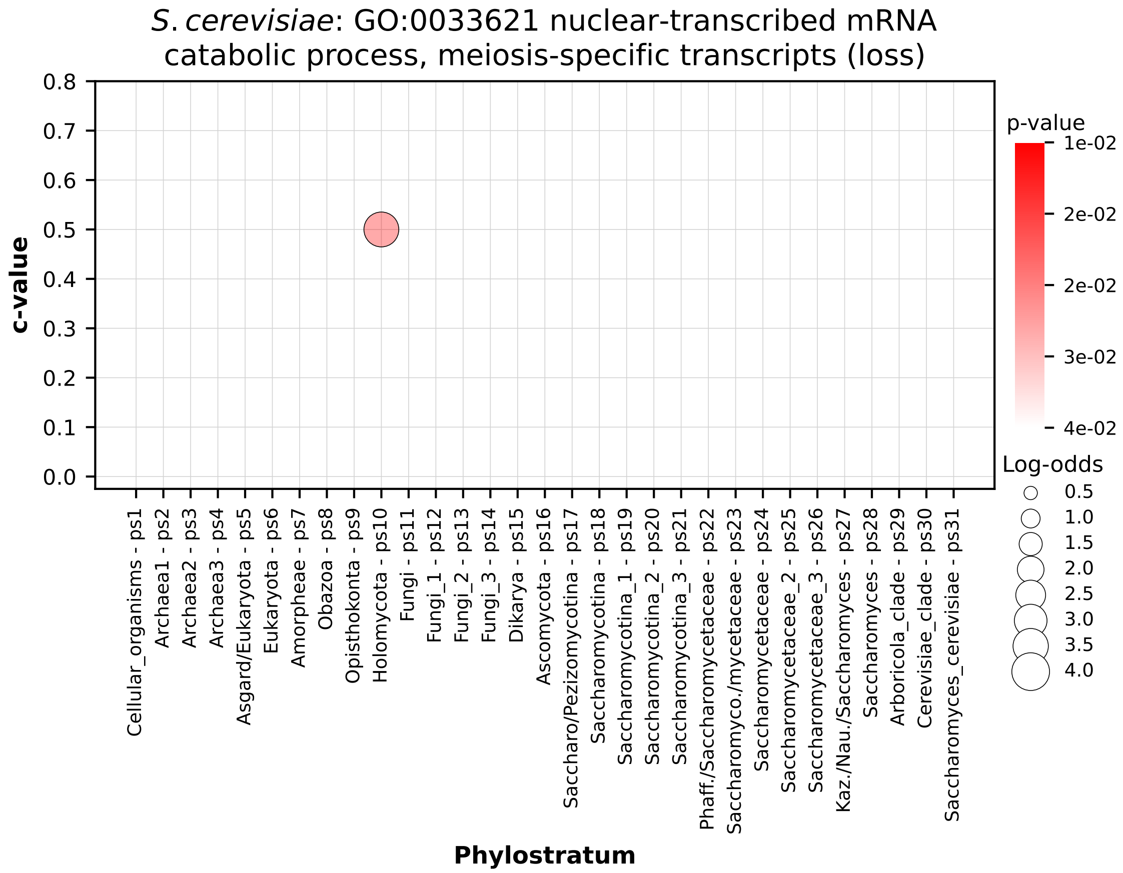
S. cerevisiae: GO:0033621 nuclear-transcribed mRNA catabolic process, meiosis-specific transcripts (loss)
c-value
0.8
0.7
0.6
0.5
0.4
0.3
0.2
0.1
0.0
p-value
1e-02
2e-02
2e-02
3e-02
4e-02
Log-odds
0.5
1.0
1.5
2.0
2.5
3.0
3.5
4.0
Cellular_organisms - ps1
Archaea1 - ps2
Archaea2 - ps3
Archaea3 - ps4
Asgard/Eukaryota - ps5
Eukaryota - ps6
Amorpheae - ps7
Obazoa - ps8
Opisthokonta - ps9
Holomycota - ps10
Fungi - ps11
Fungi_1 - ps12
Fungi_2 - ps13
Fungi_3 - ps14
Dikarya - ps15
Ascomycota - ps16
Saccharo/Pezizomycotina - ps17
Saccharomycotina - ps18
Saccharomycotina_1 - ps19
Saccharomycotina_2 - ps20
Saccharomycotina_3 - ps21
Phaff./Saccharomycetaceae - ps22
Saccharomyco./mycetaceae - ps23
Saccharomycetaceae - ps24
Saccharomycetaceae_2 - ps25
Saccharomycetaceae_3 - ps26
Kaz./Nau./Saccharomyces - ps27
Saccharomyces - ps28
Arboricola_clade - ps29
Cerevisiae_clade - ps30
Saccharomyces_cerevisiae - ps31
Phylostratum

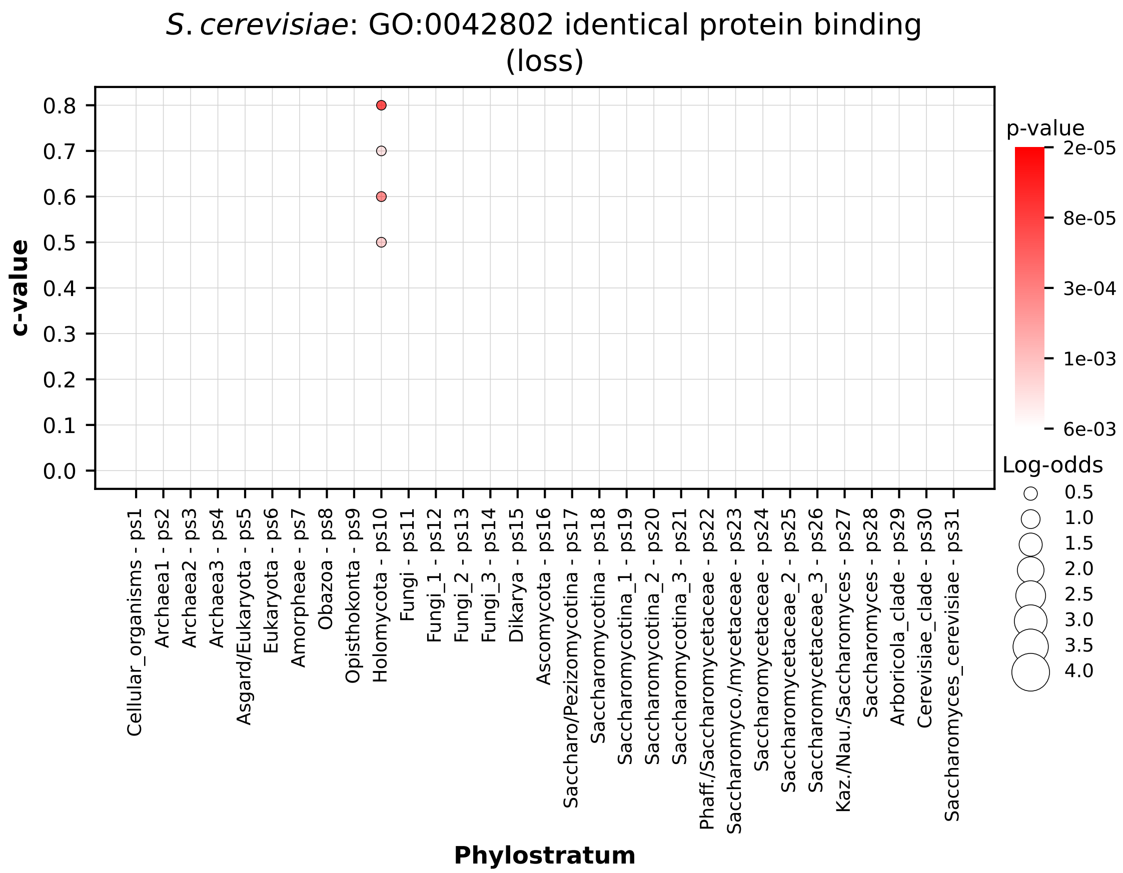

S. cerevisiae: GO:0042802 identical protein binding
(loss)
c-value
0.8
0.7
0.6
0.5
0.4
0.3
0.2
0.1
0.0
p-value
2e-05
8e-05
3e-04
1e-03
6e-03
Log-odds
0.5
1.0
1.5
2.0
2.5
3.0
3.5
4.0
Cellular_organisms - ps1
Archaea1 - ps2
Archaea2 - ps3
Archaea3 - ps4
Asgard/Eukaryota - ps5
Eukaryota - ps6
Amorpheae - ps7
Obazoa - ps8
Opisthokonta - ps9
Holomycota - ps10
Fungi - ps11
Fungi_1 - ps12
Fungi_2 - ps13
Fungi_3 - ps14
Dikarya - ps15
Ascomycota - ps16
Saccharo/Pezizomycotina - ps17
Saccharomycotina - ps18
Saccharomycotina_1 - ps19
Saccharomycotina_2 - ps20
Saccharomycotina_3 - ps21
Phaff./Saccharomycetaceae - ps22
Saccharomyco./mycetaceae - ps23
Saccharomycetaceae - ps24
Saccharomycetaceae_2 - ps25
Saccharomycetaceae_3 - ps26
Kaz./Nau./Saccharomyces - ps27
Saccharomyces - ps28
Arboricola_clade - ps29
Cerevisiae_clade - ps30
Saccharomyces_cerevisiae - ps31
Phylostratum

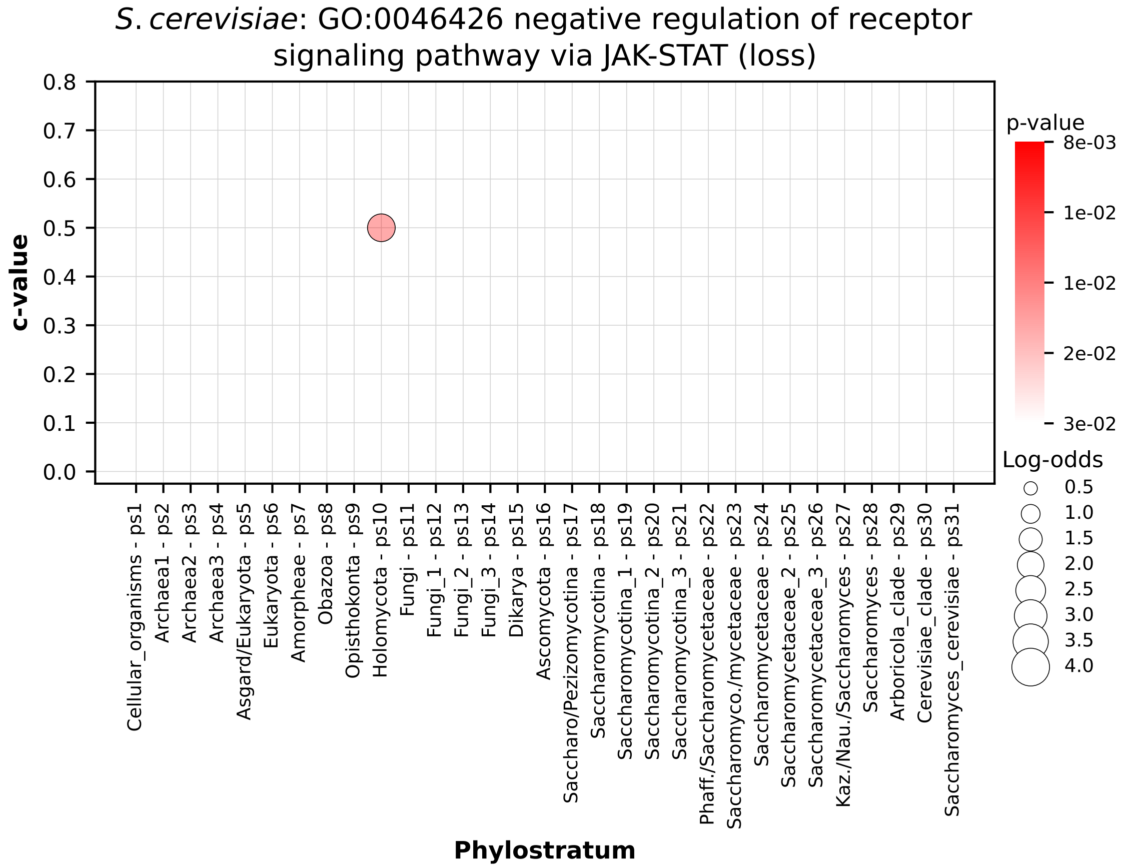

S. cerevisiae: GO:0046426 negative regulation of receptor signaling pathway via JAK-STAT (loss)
c-value
0.8
0.7
0.6
0.5
0.4
0.3
0.2
0.1
0.0
p-value
8e-03
1e-02
1e-02
2e-02
3e-02
Log-odds
0.5
1.0
1.5
2.0
2.5
3.0
3.5
4.0
Cellular_organisms - ps1
Archaea1 - ps2
Archaea2 - ps3
Archaea3 - ps4
Asgard/Eukaryota - ps5
Eukaryota - ps6
Amorpheae - ps7
Obazoa - ps8
Opisthokonta - ps9
Holomycota - ps10
Fungi - ps11
Fungi_1 - ps12
Fungi_2 - ps13
Fungi_3 - ps14
Dikarya - ps15
Ascomycota - ps16
Saccharo/Pezizomycotina - ps17
Saccharomycotina - ps18
Saccharomycotina_1 - ps19
Saccharomycotina_2 - ps20
Saccharomycotina_3 - ps21
Phaff./Saccharomycetaceae - ps22
Saccharomyco./mycetaceae - ps23
Saccharomycetaceae - ps24
Saccharomycetaceae_2 - ps25
Saccharomycetaceae_3 - ps26
Kaz./Nau./Saccharomyces - ps27
Saccharomyces - ps28
Arboricola_clade - ps29
Cerevisiae_clade - ps30
Saccharomyces_cerevisiae - ps31
Phylostratum

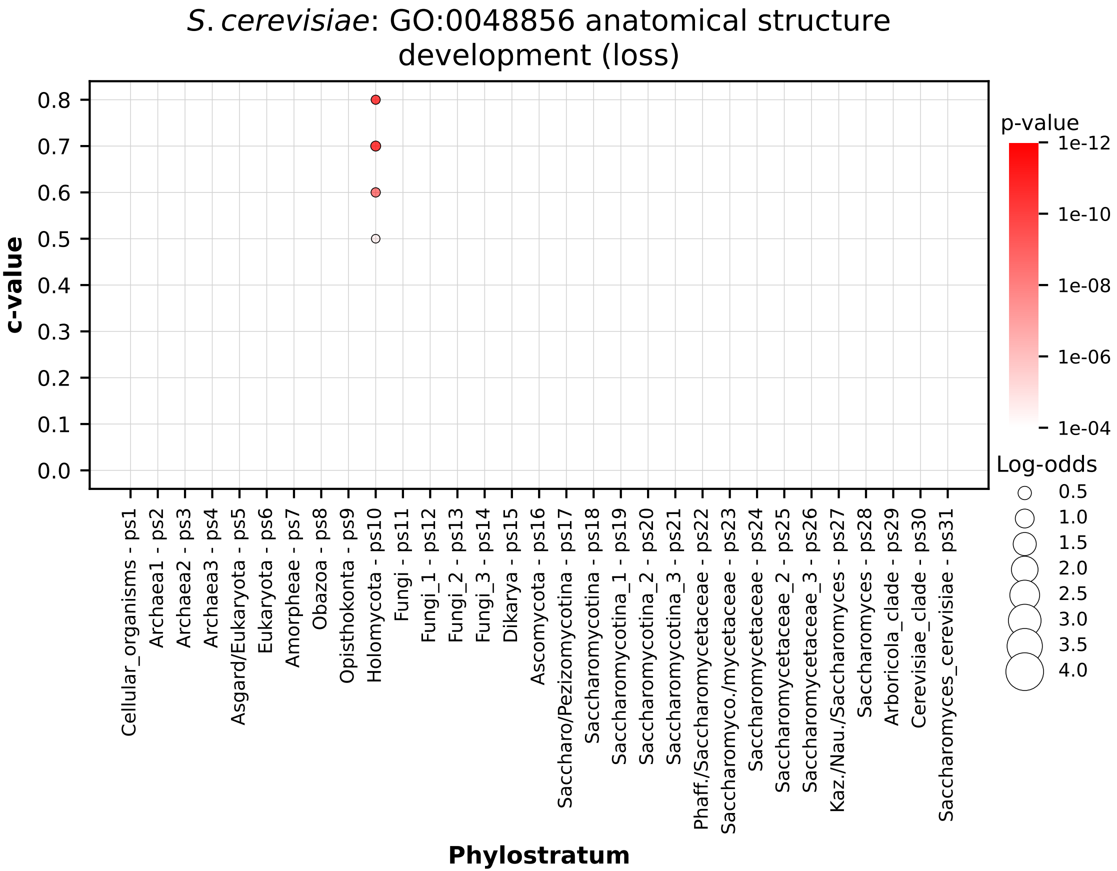

S. cerevisiae: GO:0048856 anatomical structure development (loss)
c-value
0.0
0.1
0.2
0.3
0.4
0.5
0.6
0.7
0.8
Phylostratum
Cellular_organisms - ps1
Archaea1 - ps2
Archaea2 - ps3
Archaea3 - ps4
Asgard/Eukaryota - ps5
Eukaryota - ps6
Amorpheae - ps7
Obazoa - ps8
Opisthokonta - ps9
Holomycota - ps10
Fungi - ps11
Fungi_1 - ps12
Fungi_2 - ps13
Fungi_3 - ps14
Dikarya - ps15
Ascomycota - ps16
Saccharo/Pezizomycotina - ps17
Saccharomycotina - ps18
Saccharomycotina_1 - ps19
Saccharomycotina_2 - ps20
Saccharomycotina_3 - ps21
Phaff./Saccharomycetaceae - ps22
Saccharomyco./mycetaceae - ps23
Saccharomycetaceae - ps24
Saccharomycetaceae_2 - ps25
Saccharomycetaceae_3 - ps26
Kaz./Nau./Saccharomyces - ps27
Saccharomyces - ps28
Arboricola_clade - ps29
Cerevisiae_clade - ps30
Saccharomyces_cerevisiae - ps31
p-value
1e-12
1e-10
1e-08
1e-06
1e-04
Log-odds
0.5
1.0
1.5
2.0
2.5
3.0
3.5
4.0

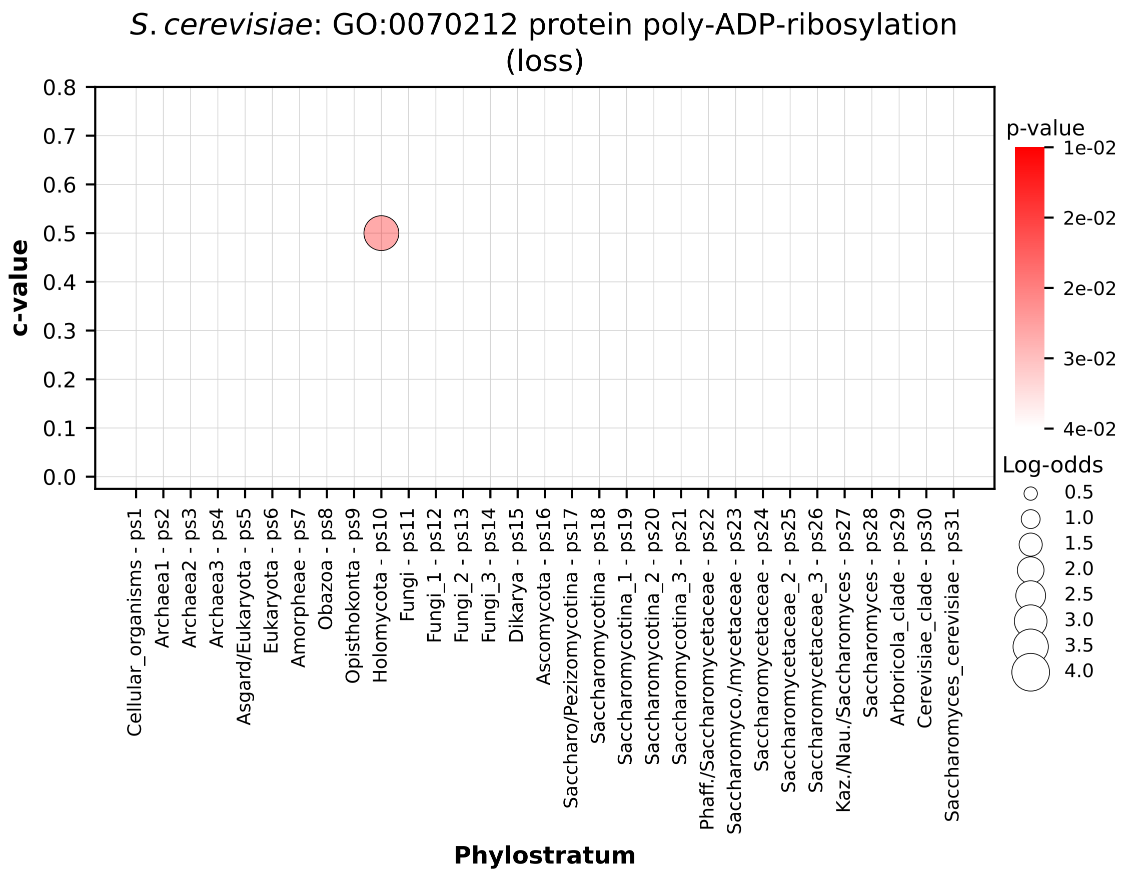

S. cerevisiae: GO:0070212 protein poly-ADP-ribosylation (loss)
c-value
0.8
0.7
0.6
0.5
0.4
0.3
0.2
0.1
0.0
Phylostratum
Cellular_organisms - ps1
Archaea1 - ps2
Archaea2 - ps3
Archaea3 - ps4
Asgard/Eukaryota - ps5
Eukaryota - ps6
Amorpheae - ps7
Obazoa - ps8
Opisthokonta - ps9
Holomycota - ps10
Fungi - ps11
Fungi_1 - ps12
Fungi_2 - ps13
Fungi_3 - ps14
Dikarya - ps15
Ascomycota - ps16
Saccharo/Pezizomycotina - ps17
Saccharomycotina - ps18
Saccharomycotina_1 - ps19
Saccharomycotina_2 - ps20
Saccharomycotina_3 - ps21
Phaff./Saccharomycetaceae - ps22
Saccharomyco./mycetaceae - ps23
Saccharomycetaceae - ps24
Saccharomycetaceae_2 - ps25
Saccharomycetaceae_3 - ps26
Kaz./Nau./Saccharomyces - ps27
Saccharomyces - ps28
Arboricola_clade - ps29
Cerevisiae_clade - ps30
Saccharomyces_cerevisiae - ps31
p-value
1e-02
2e-02
2e-02
3e-02
4e-02
Log-odds
0.5
1.0
1.5
2.0
2.5
3.0
3.5
4.0

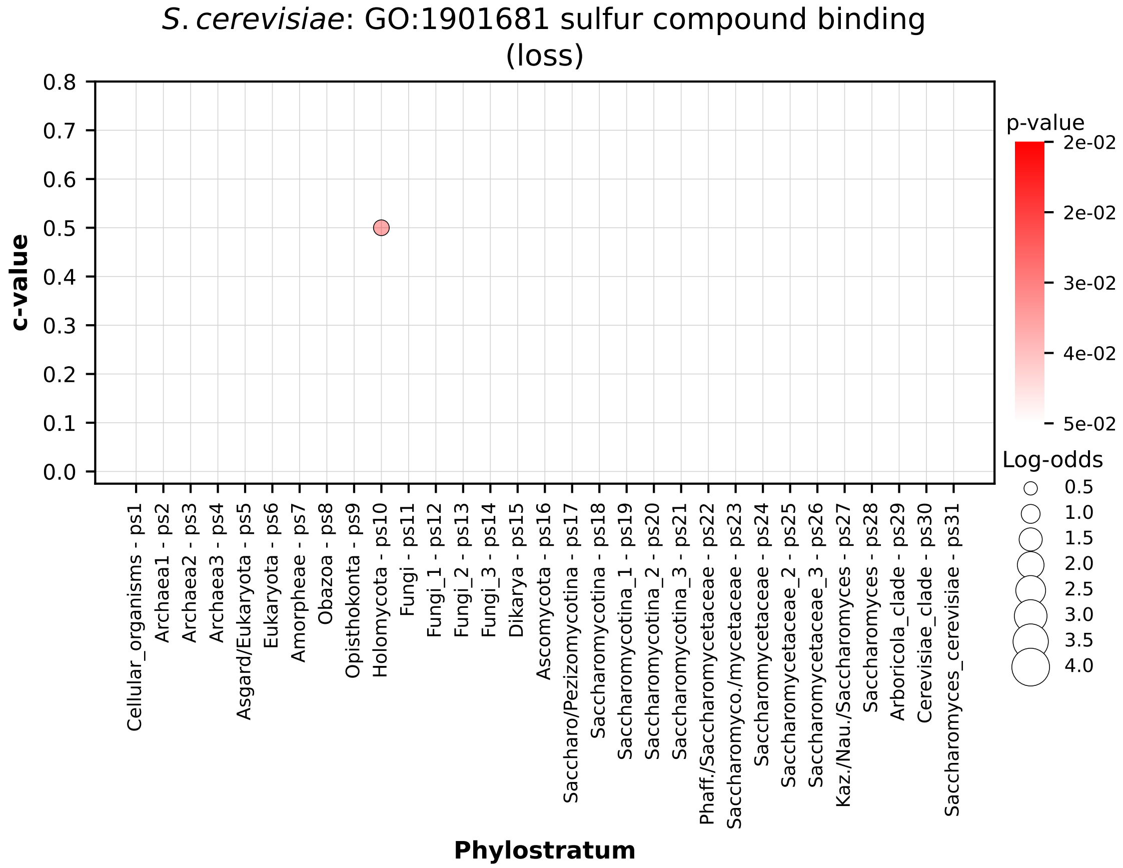
S. cerevisiae: GO:1901681 sulfur compound binding
(loss)
c-value
0.8
0.7
0.6
0.5
0.4
0.3
0.2
0.1
0.0
Phylostratum
Cellular_organisms - ps1
Archaea1 - ps2
Archaea2 - ps3
Archaea3 - ps4
Asgard/Eukaryota - ps5
Eukaryota - ps6
Amorpheae - ps7
Obazoa - ps8
Opisthokonta - ps9
Holomycota - ps10
Fungi - ps11
Fungi_1 - ps12
Fungi_2 - ps13
Fungi_3 - ps14
Dikarya - ps15
Ascomycota - ps16
Saccharo/Pezizomycotina - ps17
Saccharomycotina - ps18
Saccharomycotina_1 - ps19
Saccharomycotina_2 - ps20
Saccharomycotina_3 - ps21
Phaff./Saccharomycetaceae - ps22
Saccharomyco./mycetaceae - ps23
Saccharomycetaceae - ps24
Saccharomycetaceae_2 - ps25
Saccharomycetaceae_3 - ps26
Kaz./Nau./Saccharomyces - ps27
Saccharomyces - ps28
Arboricola_clade - ps29
Cerevisiae_clade - ps30
Saccharomyces_cerevisiae - ps31
p-value
2e-02
2e-02
3e-02
4e-02
5e-02
Log-odds
0.5
1.0
1.5
2.0
2.5
3.0
3.5
4.0

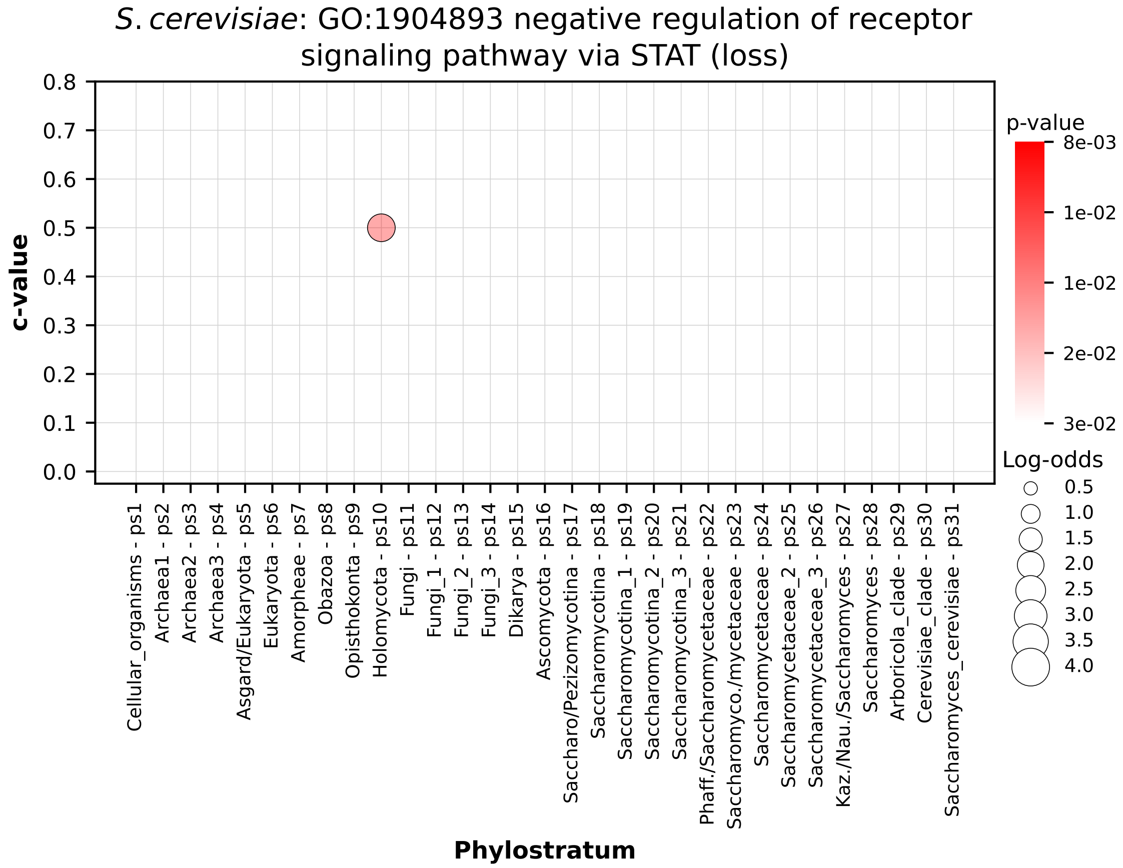

S. cerevisiae: GO:1904893 negative regulation of receptor signaling pathway via STAT (loss)
c-value
0.8
0.7
0.6
0.5
0.4
0.3
0.2
0.1
0.0
p-value
8e-03
1e-02
1e-02
2e-02
3e-02
Log-odds
0.5
1.0
1.5
2.0
2.5
3.0
3.5
4.0
Cellular_organisms - ps1
Archaea1 - ps2
Archaea2 - ps3
Archaea3 - ps4
Asgard/Eukaryota - ps5
Eukaryota - ps6
Amorpheae - ps7
Obazoa - ps8
Opisthokonta - ps9
Holomycota - ps10
Fungi - ps11
Fungi_1 - ps12
Fungi_2 - ps13
Fungi_3 - ps14
Dikarya - ps15
Ascomycota - ps16
Saccharo/Pezizomycotina - ps17
Saccharomycotina - ps18
Saccharomycotina_1 - ps19
Saccharomycotina_2 - ps20
Saccharomycotina_3 - ps21
Phaff./Saccharomycetaceae - ps22
Saccharomyco./mycetaceae - ps23
Saccharomycetaceae - ps24
Saccharomycetaceae_2 - ps25
Saccharomycetaceae_3 - ps26
Kaz./Nau./Saccharomyces - ps27
Saccharomyces - ps28
Arboricola_clade - ps29
Cerevisiae_clade - ps30
Saccharomyces_cerevisiae - ps31
Phylostratum

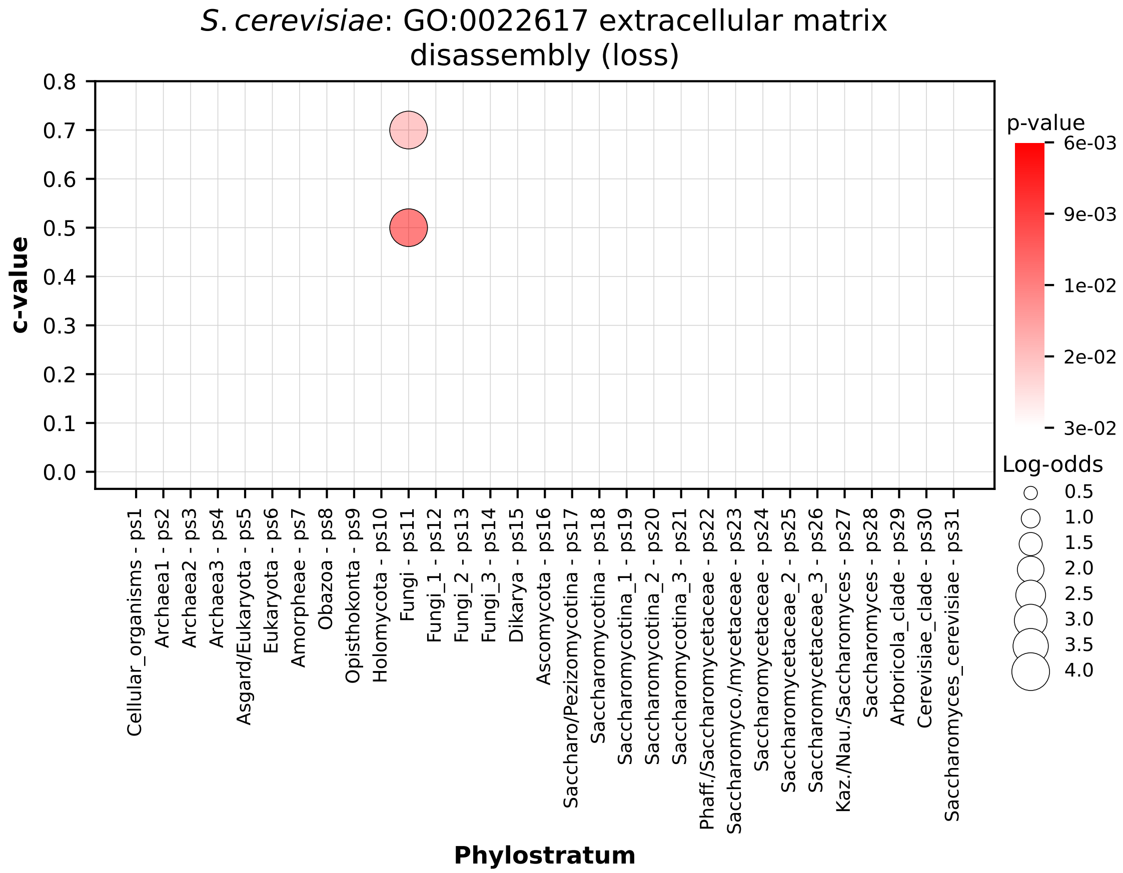

S. cerevisiae: GO:0022617 extracellular matrix disassembly (loss)
c-value
0.8
0.7
0.6
0.5
0.4
0.3
0.2
0.1
0.0
Phylostratum
Cellular_organisms - ps1
Archaea1 - ps2
Archaea2 - ps3
Archaea3 - ps4
Asgard/Eukaryota - ps5
Eukaryota - ps6
Amorpheae - ps7
Obazoa - ps8
Opisthokonta - ps9
Holomycota - ps10
Fungi - ps11
Fungi_1 - ps12
Fungi_2 - ps13
Fungi_3 - ps14
Dikarya - ps15
Ascomycota - ps16
Saccharo/Pezizomycotina - ps17
Saccharomycotina - ps18
Saccharomycotina_1 - ps19
Saccharomycotina_2 - ps20
Saccharomycotina_3 - ps21
Phaff./Saccharomycetaceae - ps22
Saccharomyco./mycetaceae - ps23
Saccharomycetaceae - ps24
Saccharomycetaceae_2 - ps25
Saccharomycetaceae_3 - ps26
Kaz./Nau./Saccharomyces - ps27
Saccharomyces - ps28
Arboricola_clade - ps29
Cerevisiae_clade - ps30
Saccharomyces_cerevisiae - ps31
p-value
6e-03
9e-03
1e-02
2e-02
3e-02
Log-odds
0.5
1.0
1.5
2.0
2.5
3.0
3.5
4.0

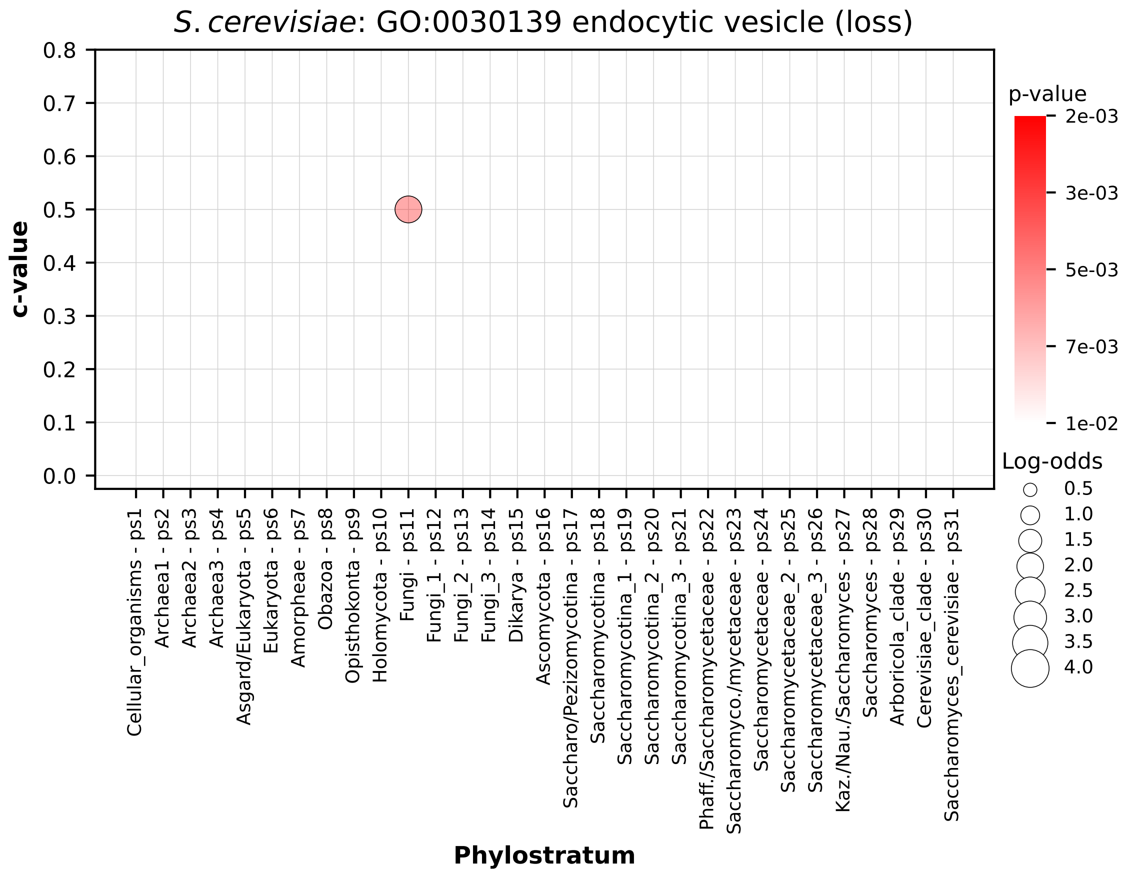

S. cerevisiae: GO:0030139 endocytic vesicle (loss)
c-value
0.8
0.7
0.6
0.5
0.4
0.3
0.2
0.1
0.0
p-value
2e-03
3e-03
5e-03
7e-03
1e-02
Log-odds
0.5
1.0
1.5
2.0
2.5
3.0
3.5
4.0
Cellular_organisms - ps1
Archaea1 - ps2
Archaea2 - ps3
Archaea3 - ps4
Asgard/Eukaryota - ps5
Eukaryota - ps6
Amorpheae - ps7
Obazoa - ps8
Opisthokonta - ps9
Holomycota - ps10
Fungi - ps11
Fungi_1 - ps12
Fungi_2 - ps13
Fungi_3 - ps14
Dikarya - ps15
Ascomycota - ps16
Saccharo/Pezizomycotina - ps17
Saccharomycotina - ps18
Saccharomycotina_1 - ps19
Saccharomycotina_2 - ps20
Saccharomycotina_3 - ps21
Phaff./Saccharomycetaceae - ps22
Saccharomyco./mycetaceae - ps23
Saccharomycetaceae - ps24
Saccharomycetaceae_2 - ps25
Saccharomycetaceae_3 - ps26
Kaz./Nau./Saccharomyces - ps27
Saccharomyces - ps28
Arboricola_clade - ps29
Cerevisiae_clade - ps30
Saccharomyces_cerevisiae - ps31
Phylostratum

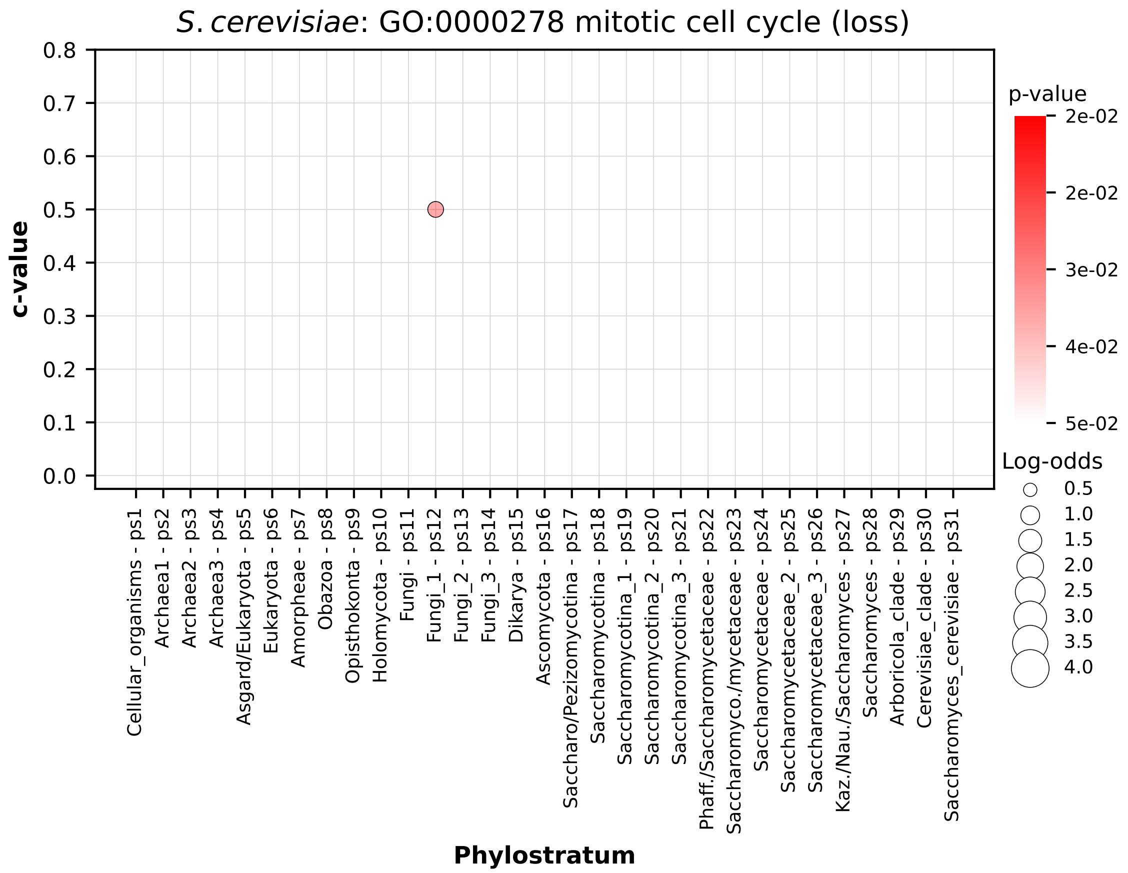
S. cerevisiae: GO:0000278 mitotic cell cycle (loss)
c-value
0.8
0.7
0.6
0.5
0.4
0.3
0.2
0.1
0.0
p-value
2e-02
2e-02
3e-02
4e-02
5e-02
Log-odds
0.5
1.0
1.5
2.0
2.5
3.0
3.5
4.0
Cellular_organisms - ps1
Archaea1 - ps2
Archaea2 - ps3
Archaea3 - ps4
Asgard/Eukaryota - ps5
Eukaryota - ps6
Amorpheae - ps7
Obazoa - ps8
Opisthokonta - ps9
Holomycota - ps10
Fungi - ps11
Fungi_1 - ps12
Fungi_2 - ps13
Fungi_3 - ps14
Dikarya - ps15
Ascomycota - ps16
Saccharo/Pezizomycotina - ps17
Saccharomycotina - ps18
Saccharomycotina_1 - ps19
Saccharomycotina_2 - ps20
Saccharomycotina_3 - ps21
Phaff./Saccharomycetaceae - ps22
Saccharomyco./mycetaceae - ps23
Saccharomycetaceae - ps24
Saccharomycetaceae_2 - ps25
Saccharomycetaceae_3 - ps26
Kaz./Nau./Saccharomyces - ps27
Saccharomyces - ps28
Arboricola_clade - ps29
Cerevisiae_clade - ps30
Saccharomyces_cerevisiae - ps31
Phylostratum

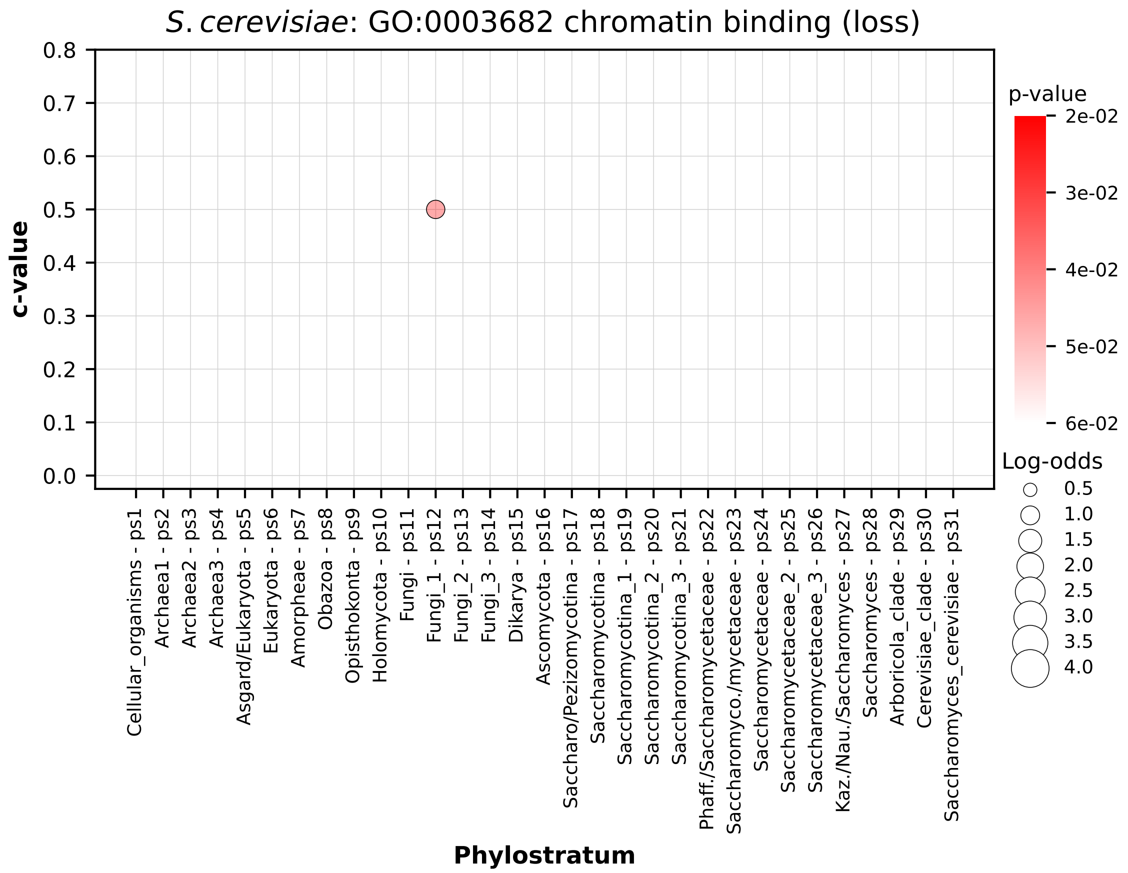
S. cerevisiae: GO:0003682 chromatin binding (loss)
c-value
0.8
0.7
0.6
0.5
0.4
0.3
0.2
0.1
0.0
p-value
2e-02
3e-02
4e-02
5e-02
6e-02
Log-odds
0.5
1.0
1.5
2.0
2.5
3.0
3.5
4.0
Cellular_organisms - ps1
Archaea1 - ps2
Archaea2 - ps3
Archaea3 - ps4
Asgard/Eukaryota - ps5
Eukaryota - ps6
Amorpheae - ps7
Obazoa - ps8
Opisthokonta - ps9
Holomycota - ps10
Fungi - ps11
Fungi_1 - ps12
Fungi_2 - ps13
Fungi_3 - ps14
Dikarya - ps15
Ascomycota - ps16
Saccharo/Pezizomycotina - ps17
Saccharomycotina - ps18
Saccharomycotina_1 - ps19
Saccharomycotina_2 - ps20
Saccharomycotina_3 - ps21
Phaff./Saccharomycetaceae - ps22
Saccharomyco./mycetaceae - ps23
Saccharomycetaceae - ps24
Saccharomycetaceae_2 - ps25
Saccharomycetaceae_3 - ps26
Kaz./Nau./Saccharomyces - ps27
Saccharomyces - ps28
Arboricola_clade - ps29
Cerevisiae_clade - ps30
Saccharomyces_cerevisiae - ps31
Phylostratum

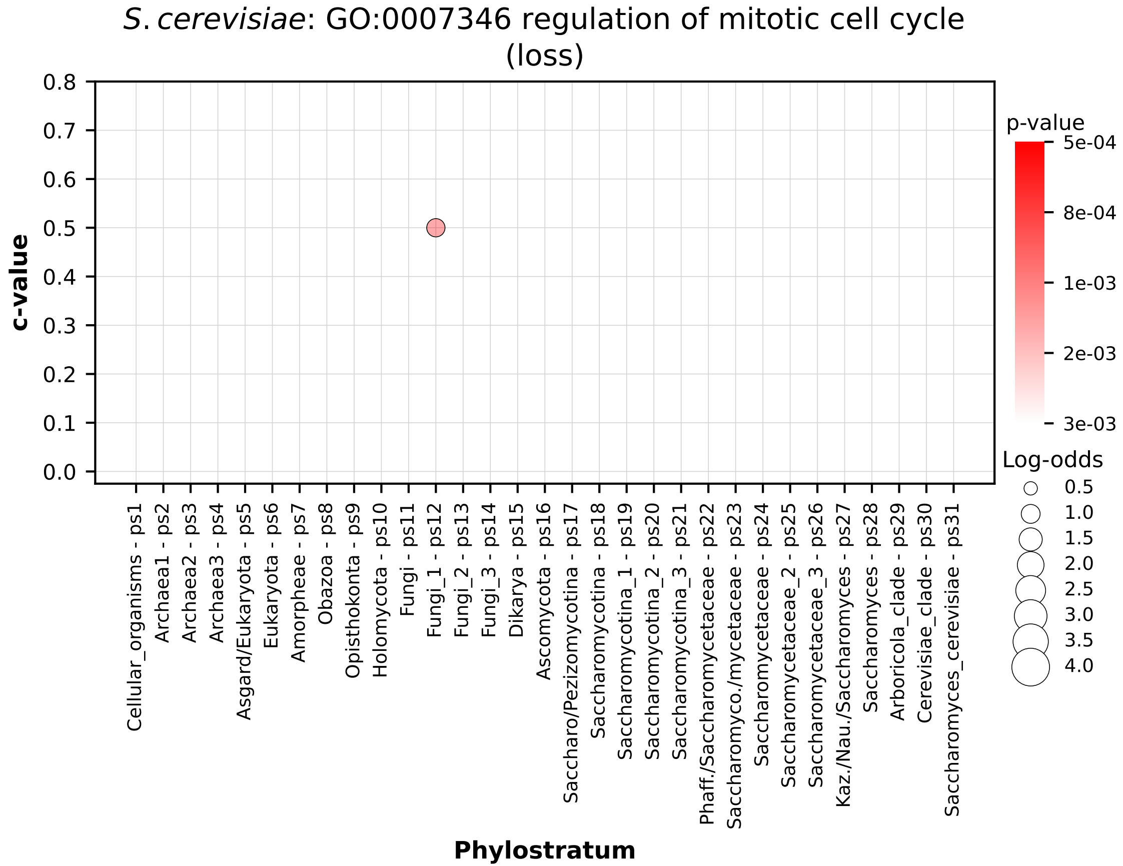
S. cerevisiae: GO:0007346 regulation of mitotic cell cycle
(loss)
c-value
0.8
0.7
0.6
0.5
0.4
0.3
0.2
0.1
0.0
p-value
5e-04
8e-04
1e-03
2e-03
3e-03
Log-odds
0.5
1.0
1.5
2.0
2.5
3.0
3.5
4.0
Cellular_organisms - ps1
Archaea1 - ps2
Archaea2 - ps3
Archaea3 - ps4
Asgard/Eukaryota - ps5
Eukaryota - ps6
Amorpheae - ps7
Obazoa - ps8
Opisthokonta - ps9
Holomycota - ps10
Fungi - ps11
Fungi_1 - ps12
Fungi_2 - ps13
Fungi_3 - ps14
Dikarya - ps15
Ascomycota - ps16
Saccharo/Pezizomycotina - ps17
Saccharomycotina - ps18
Saccharomycotina_1 - ps19
Saccharomycotina_2 - ps20
Saccharomycotina_3 - ps21
Phaff./Saccharomycetaceae - ps22
Saccharomyco./mycetaceae - ps23
Saccharomycetaceae - ps24
Saccharomycetaceae_2 - ps25
Saccharomycetaceae_3 - ps26
Kaz./Nau./Saccharomyces - ps27
Saccharomyces - ps28
Arboricola_clade - ps29
Cerevisiae_clade - ps30
Saccharomyces_cerevisiae - ps31
Phylostratum

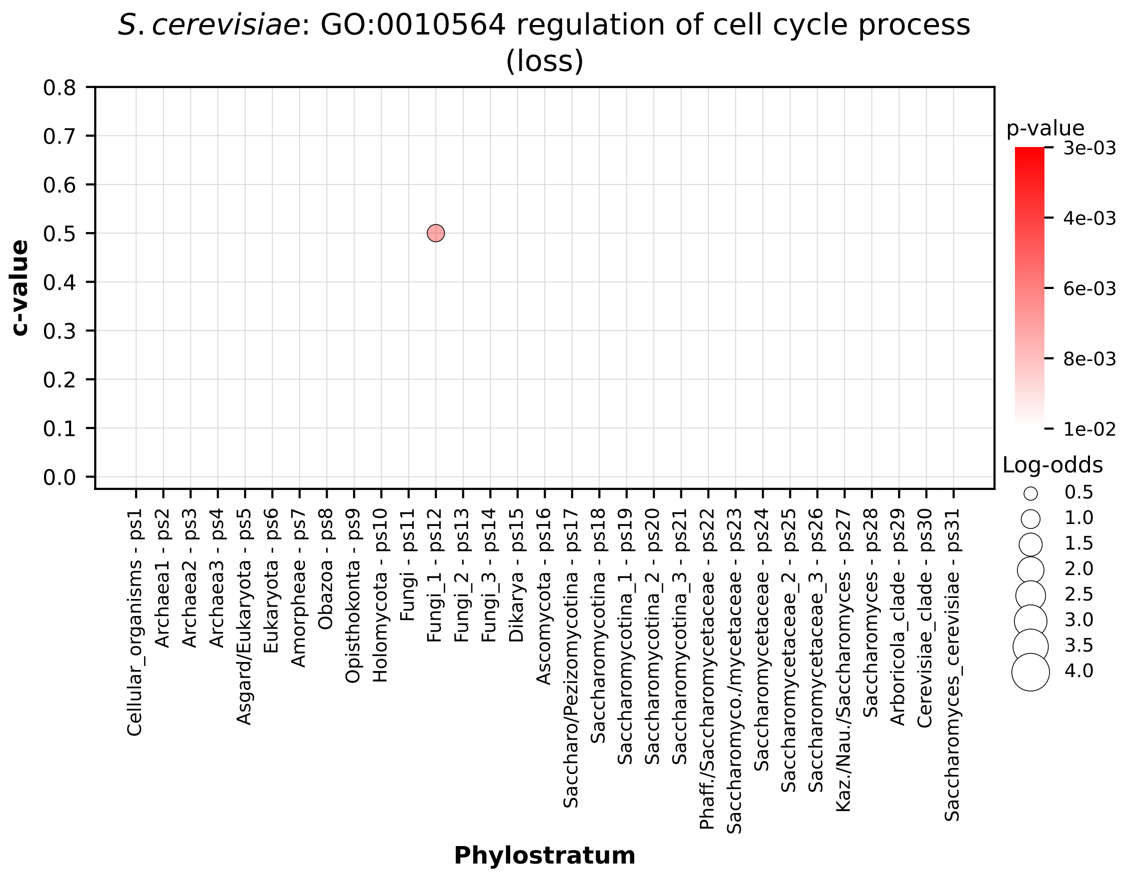

S. cerevisiae: GO:0010564 regulation of cell cycle process
(loss)
c-value
0.8
0.7
0.6
0.5
0.4
0.3
0.2
0.1
0.0
Phylostratum
Cellular_organisms - ps1
Archaea1 - ps2
Archaea2 - ps3
Archaea3 - ps4
Asgard/Eukaryota - ps5
Eukaryota - ps6
Amorpheae - ps7
Obazoa - ps8
Opisthokonta - ps9
Holomycota - ps10
Fungi - ps11
Fungi_1 - ps12
Fungi_2 - ps13
Fungi_3 - ps14
Dikarya - ps15
Ascomycota - ps16
Saccharo/Pezizomycotina - ps17
Saccharomycotina - ps18
Saccharomycotina_1 - ps19
Saccharomycotina_2 - ps20
Saccharomycotina_3 - ps21
Phaff./Saccharomycetaceae - ps22
Saccharomyco./mycetaceae - ps23
Saccharomycetaceae - ps24
Saccharomycetaceae_2 - ps25
Saccharomycetaceae_3 - ps26
Kaz./Nau./Saccharomyces - ps27
Saccharomyces - ps28
Arboricola_clade - ps29
Cerevisiae_clade - ps30
Saccharomyces_cerevisiae - ps31
p-value
3e-03
4e-03
6e-03
8e-03
1e-02
Log-odds
0.5
1.0
1.5
2.0
2.5
3.0
3.5
4.0

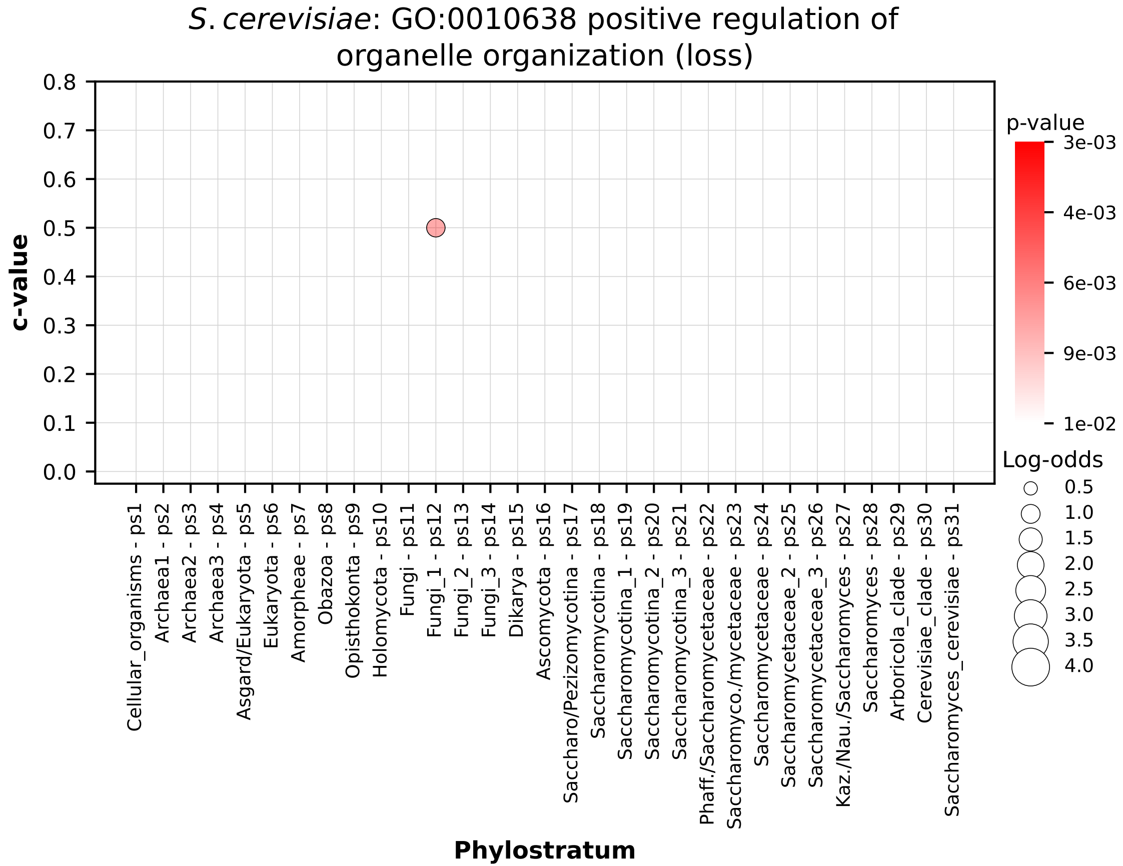
S. cerevisiae: GO:0010638 positive regulation of organelle organization (loss)
c-value
0.8
0.7
0.6
0.5
0.4
0.3
0.2
0.1
0.0
Phylostratum
Cellular_organisms - ps1
Archaea1 - ps2
Archaea2 - ps3
Archaea3 - ps4
Asgard/Eukaryota - ps5
Eukaryota - ps6
Amorpheae - ps7
Obazoa - ps8
Opisthokonta - ps9
Holomycota - ps10
Fungi - ps11
Fungi_1 - ps12
Fungi_2 - ps13
Fungi_3 - ps14
Dikarya - ps15
Ascomycota - ps16
Saccharo/Pezizomycotina - ps17
Saccharomycotina - ps18
Saccharomycotina_1 - ps19
Saccharomycotina_2 - ps20
Saccharomycotina_3 - ps21
Phaff./Saccharomycetaceae - ps22
Saccharomyco./mycetaceae - ps23
Saccharomycetaceae - ps24
Saccharomycetaceae_2 - ps25
Saccharomycetaceae_3 - ps26
Kaz./Nau./Saccharomyces - ps27
Saccharomyces - ps28
Arboricola_clade - ps29
Cerevisiae_clade - ps30
Saccharomyces_cerevisiae - ps31
p-value
3e-03
4e-03
6e-03
9e-03
1e-02
Log-odds
0.5
1.0
1.5
2.0
2.5
3.0
3.5
4.0

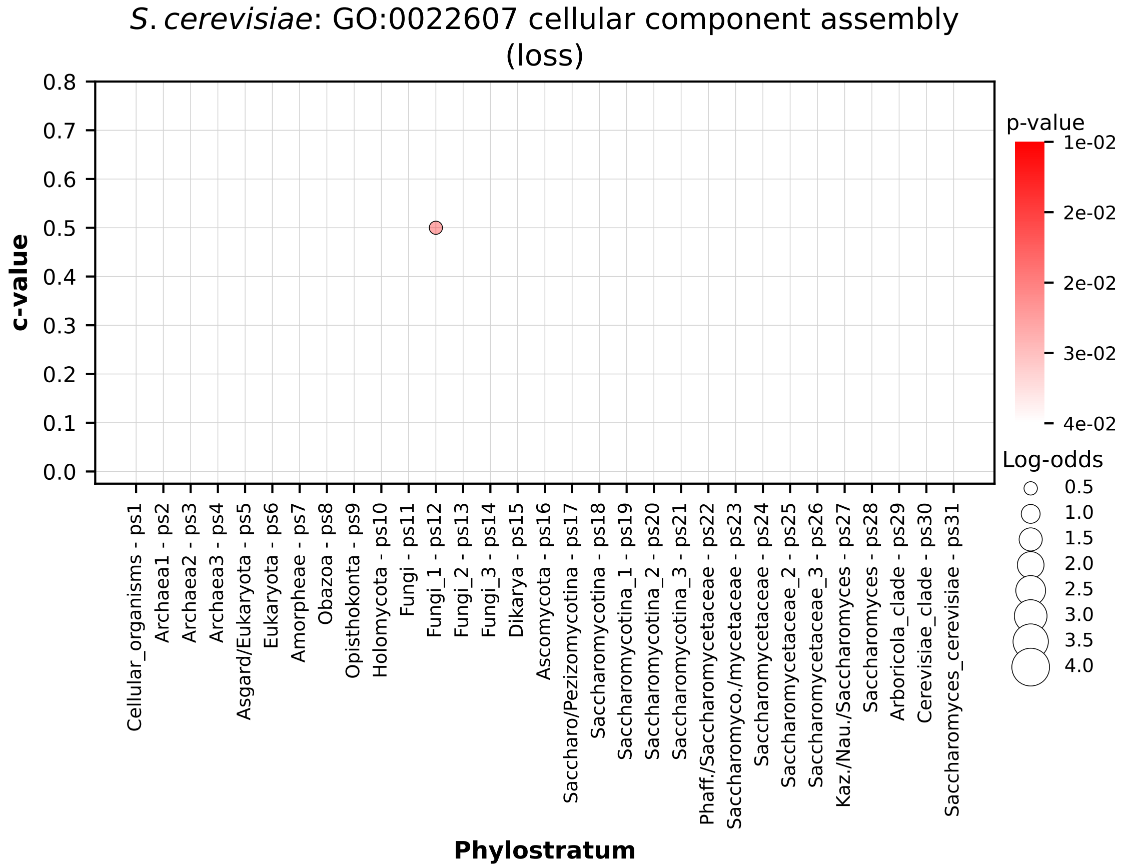
S. cerevisiae: GO:0022607 cellular component assembly
(loss)
c-value
0.0
0.1
0.2
0.3
0.4
0.5
0.6
0.7
0.8
Phylostratum
Cellular_organisms - ps1
Archaea1 - ps2
Archaea2 - ps3
Archaea3 - ps4
Asgard/Eukaryota - ps5
Eukaryota - ps6
Amorpheae - ps7
Obazoa - ps8
Opisthokonta - ps9
Holomycota - ps10
Fungi - ps11
Fungi_1 - ps12
Fungi_2 - ps13
Fungi_3 - ps14
Dikarya - ps15
Ascomycota - ps16
Saccharo/Pezizomycotina - ps17
Saccharomycotina - ps18
Saccharomycotina_1 - ps19
Saccharomycotina_2 - ps20
Saccharomycotina_3 - ps21
Phaff./Saccharomycetaceae - ps22
Saccharomyco./mycetaceae - ps23
Saccharomycetaceae - ps24
Saccharomycetaceae_2 - ps25
Saccharomycetaceae_3 - ps26
Kaz./Nau./Saccharomyces - ps27
Saccharomyces - ps28
Arboricola_clade - ps29
Cerevisiae_clade - ps30
Saccharomyces_cerevisiae - ps31
p-value
1e-02
2e-02
2e-02
3e-02
4e-02
Log-odds
0.5
1.0
1.5
2.0
2.5
3.0
3.5
4.0

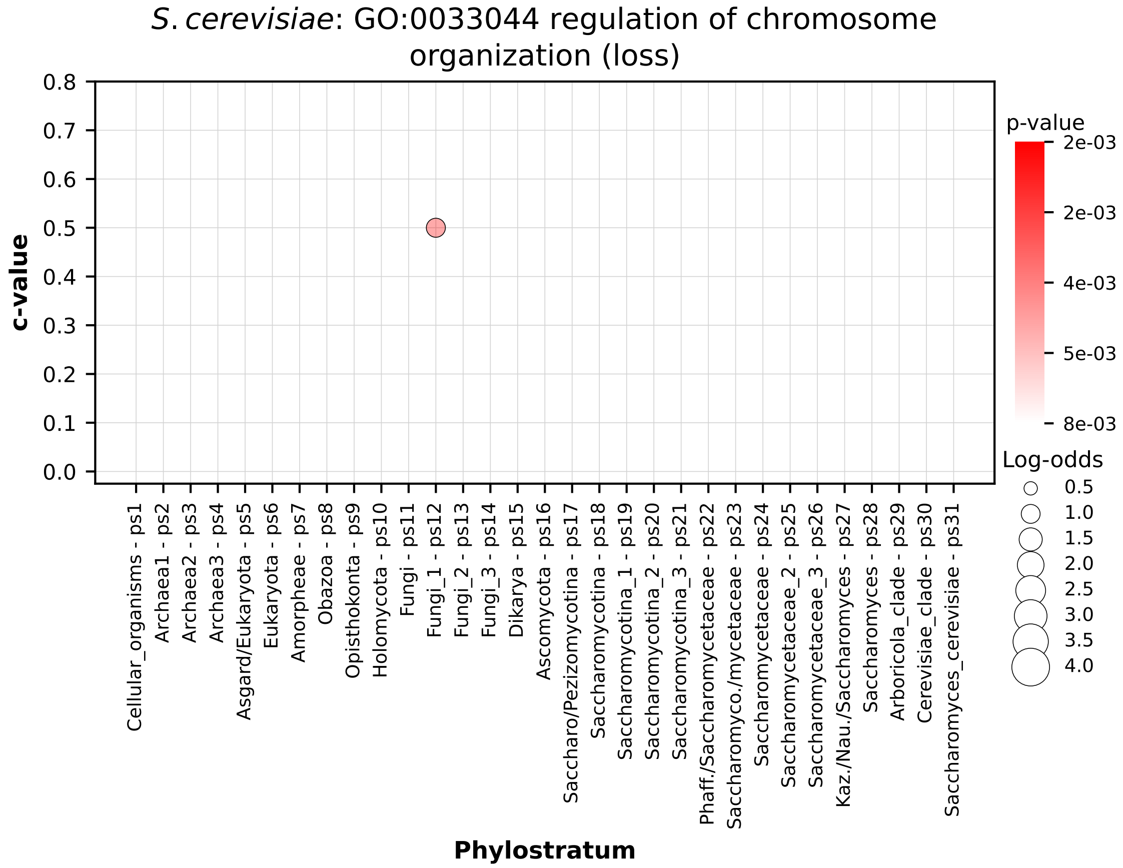

S. cerevisiae: GO:0033044 regulation of chromosome organization (loss)
c-value
0.8
0.7
0.6
0.5
0.4
0.3
0.2
0.1
0.0
p-value
2e-03
2e-03
4e-03
5e-03
8e-03
Log-odds
0.5
1.0
1.5
2.0
2.5
3.0
3.5
4.0
Cellular_organisms - ps1
Archaea1 - ps2
Archaea2 - ps3
Archaea3 - ps4
Asgard/Eukaryota - ps5
Eukaryota - ps6
Amorpheae - ps7
Obazoa - ps8
Opisthokonta - ps9
Holomycota - ps10
Fungi - ps11
Fungi_1 - ps12
Fungi_2 - ps13
Fungi_3 - ps14
Dikarya - ps15
Ascomycota - ps16
Saccharo/Pezizomycotina - ps17
Saccharomycotina - ps18
Saccharomycotina_1 - ps19
Saccharomycotina_2 - ps20
Saccharomycotina_3 - ps21
Phaff./Saccharomycetaceae - ps22
Saccharomyco./mycetaceae - ps23
Saccharomycetaceae - ps24
Saccharomycetaceae_2 - ps25
Saccharomycetaceae_3 - ps26
Kaz./Nau./Saccharomyces - ps27
Saccharomyces - ps28
Arboricola_clade - ps29
Cerevisiae_clade - ps30
Saccharomyces_cerevisiae - ps31
Phylostratum

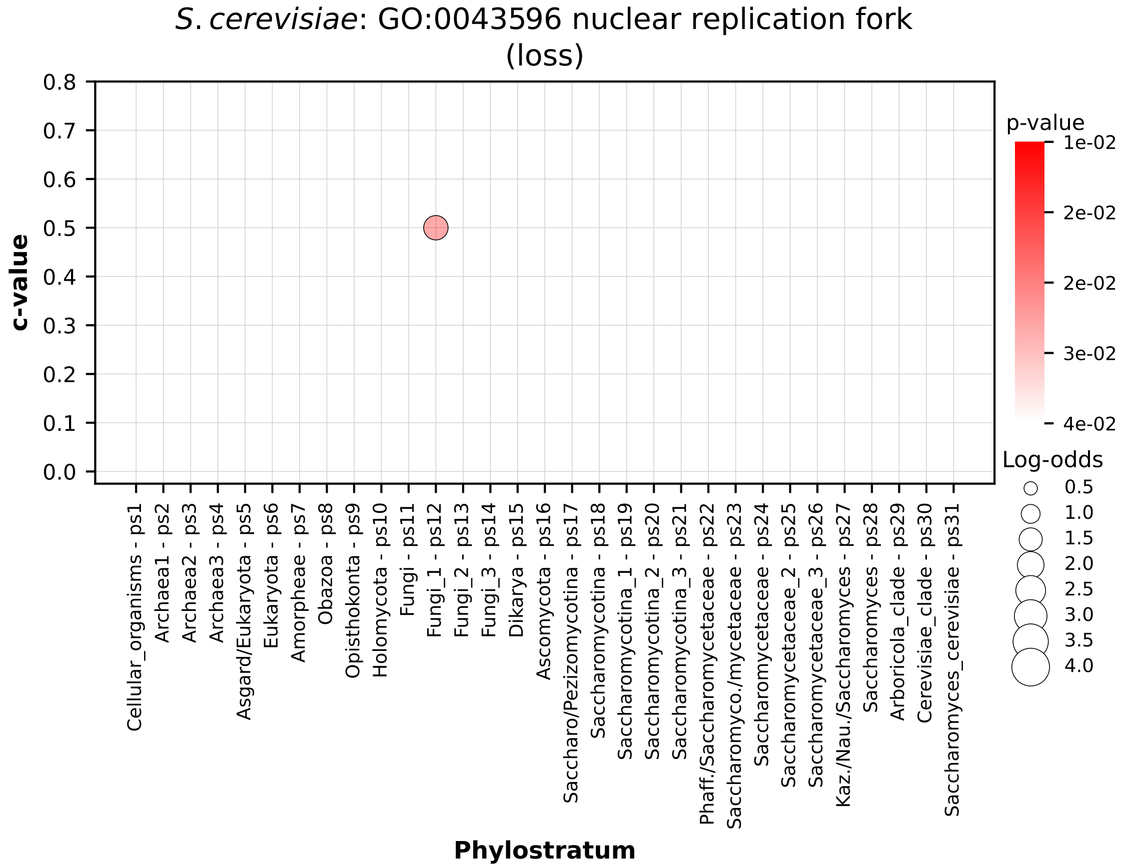
S. cerevisiae: GO:0043596 nuclear replication fork
(loss)
c-value
0.8
0.7
0.6
0.5
0.4
0.3
0.2
0.1
0.0
p-value
1e-02
2e-02
2e-02
3e-02
4e-02
Log-odds
0.5
1.0
1.5
2.0
2.5
3.0
3.5
4.0
Cellular_organisms - ps1
Archaea1 - ps2
Archaea2 - ps3
Archaea3 - ps4
Asgard/Eukaryota - ps5
Eukaryota - ps6
Amorpheae - ps7
Obazoa - ps8
Opisthokonta - ps9
Holomycota - ps10
Fungi - ps11
Fungi_1 - ps12
Fungi_2 - ps13
Fungi_3 - ps14
Dikarya - ps15
Ascomycota - ps16
Saccharo/Pezizomycotina - ps17
Saccharomycotina - ps18
Saccharomycotina_1 - ps19
Saccharomycotina_2 - ps20
Saccharomycotina_3 - ps21
Phaff./Saccharomycetaceae - ps22
Saccharomyco./mycetaceae - ps23
Saccharomycetaceae - ps24
Saccharomycetaceae_2 - ps25
Saccharomycetaceae_3 - ps26
Kaz./Nau./Saccharomyces - ps27
Saccharomyces - ps28
Arboricola_clade - ps29
Cerevisiae_clade - ps30
Saccharomyces_cerevisiae - ps31
Phylostratum

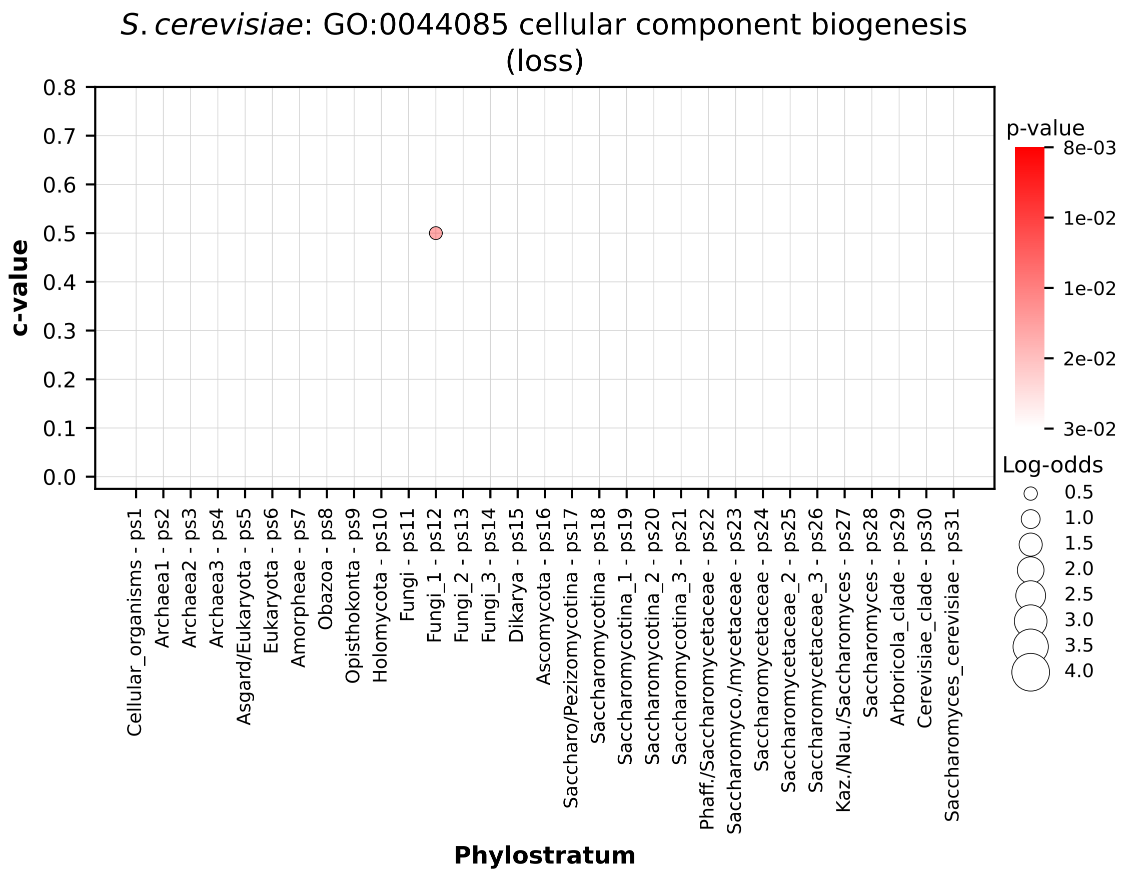
S. cerevisiae: GO:0044085 cellular component biogenesis
(loss)
c-value
0.8
0.7
0.6
0.5
0.4
0.3
0.2
0.1
0.0
Phylostratum
Cellular_organisms - ps1
Archaea1 - ps2
Archaea2 - ps3
Archaea3 - ps4
Asgard/Eukaryota - ps5
Eukaryota - ps6
Amorpheae - ps7
Obazoa - ps8
Opisthokonta - ps9
Holomycota - ps10
Fungi - ps11
Fungi_1 - ps12
Fungi_2 - ps13
Fungi_3 - ps14
Dikarya - ps15
Ascomycota - ps16
Saccharo/Pezizomycotina - ps17
Saccharomycotina - ps18
Saccharomycotina_1 - ps19
Saccharomycotina_2 - ps20
Saccharomycotina_3 - ps21
Phaff./Saccharomycetaceae - ps22
Saccharomyco./mycetaceae - ps23
Saccharomycetaceae - ps24
Saccharomycetaceae_2 - ps25
Saccharomycetaceae_3 - ps26
Kaz./Nau./Saccharomyces - ps27
Saccharomyces - ps28
Arboricola_clade - ps29
Cerevisiae_clade - ps30
Saccharomyces_cerevisiae - ps31
p-value
8e-03
1e-02
1e-02
2e-02
3e-02
Log-odds
0.5
1.0
1.5
2.0
2.5
3.0
3.5
4.0

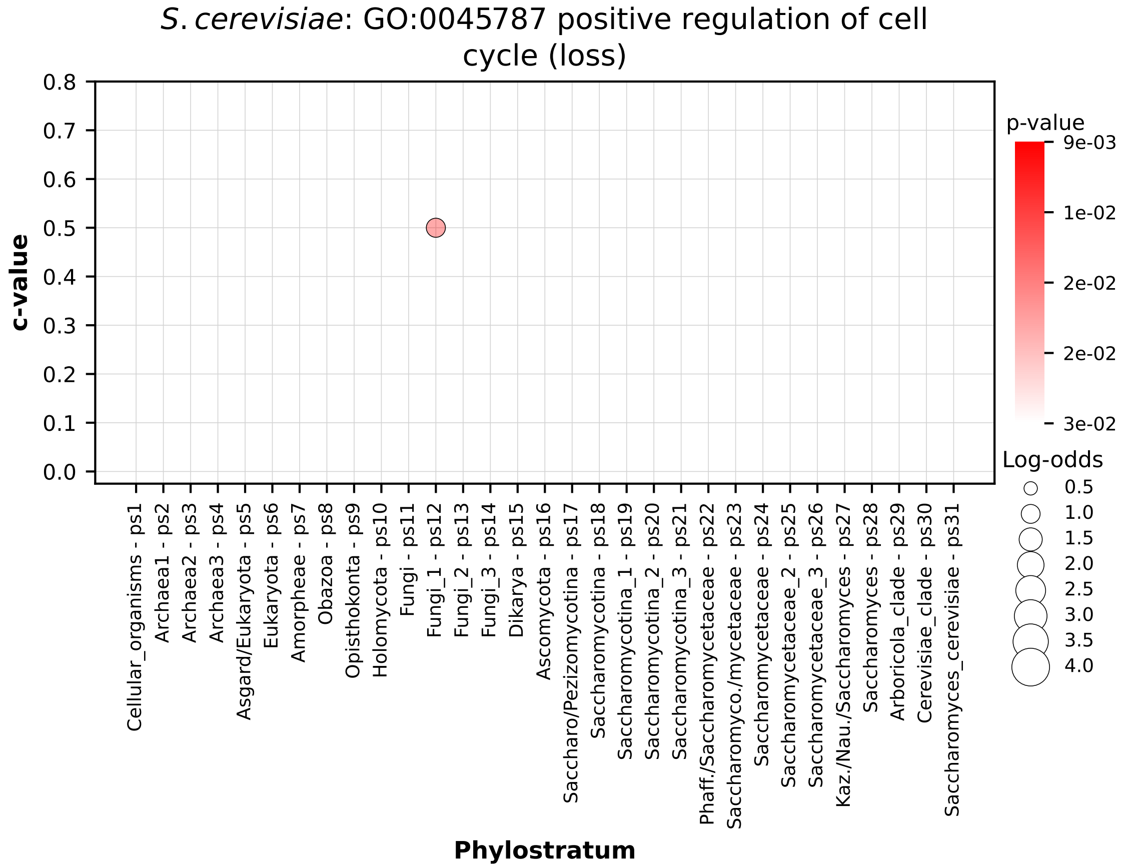

S. cerevisiae: GO:0045787 positive regulation of cell cycle (loss)
c-value
0.8
0.7
0.6
0.5
0.4
0.3
0.2
0.1
0.0
p-value
9e-03
1e-02
2e-02
2e-02
3e-02
Log-odds
0.5
1.0
1.5
2.0
2.5
3.0
3.5
4.0
Cellular_organisms - ps1
Archaea1 - ps2
Archaea2 - ps3
Archaea3 - ps4
Asgard/Eukaryota - ps5
Eukaryota - ps6
Amorpheae - ps7
Obazoa - ps8
Opisthokonta - ps9
Holomycota - ps10
Fungi - ps11
Fungi_1 - ps12
Fungi_2 - ps13
Fungi_3 - ps14
Dikarya - ps15
Ascomycota - ps16
Saccharo/Pezizomycotina - ps17
Saccharomycotina - ps18
Saccharomycotina_1 - ps19
Saccharomycotina_2 - ps20
Saccharomycotina_3 - ps21
Phaff./Saccharomycetaceae - ps22
Saccharomyco./mycetaceae - ps23
Saccharomycetaceae - ps24
Saccharomycetaceae_2 - ps25
Saccharomycetaceae_3 - ps26
Kaz./Nau./Saccharomyces - ps27
Saccharomyces - ps28
Arboricola_clade - ps29
Cerevisiae_clade - ps30
Saccharomyces_cerevisiae - ps31
Phylostratum

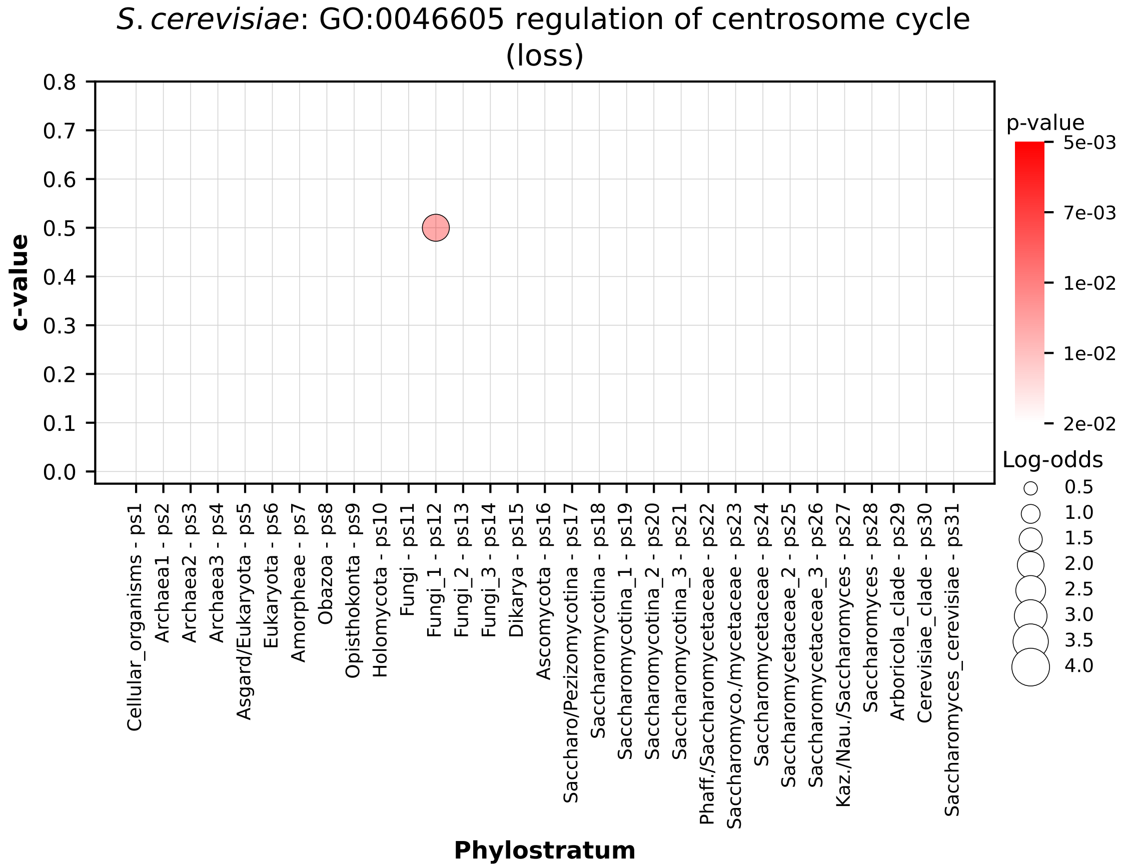

S. cerevisiae: GO:0046605 regulation of centrosome cycle (loss)
c-value
0.8
0.7
0.6
0.5
0.4
0.3
0.2
0.1
0.0
Phylostratum
Cellular_organisms - ps1
Archaea1 - ps2
Archaea2 - ps3
Archaea3 - ps4
Asgard/Eukaryota - ps5
Eukaryota - ps6
Amorpheae - ps7
Obazoa - ps8
Opisthokonta - ps9
Holomycota - ps10
Fungi - ps11
Fungi_1 - ps12
Fungi_2 - ps13
Fungi_3 - ps14
Dikarya - ps15
Ascomycota - ps16
Saccharo/Pezizomycotina - ps17
Saccharomycotina - ps18
Saccharomycotina_1 - ps19
Saccharomycotina_2 - ps20
Saccharomycotina_3 - ps21
Phaff./Saccharomycetaceae - ps22
Saccharomyco./mycetaceae - ps23
Saccharomycetaceae - ps24
Saccharomycetaceae_2 - ps25
Saccharomycetaceae_3 - ps26
Kaz./Nau./Saccharomyces - ps27
Saccharomyces - ps28
Arboricola_clade - ps29
Cerevisiae_clade - ps30
Saccharomyces_cerevisiae - ps31
p-value
5e-03
7e-03
1e-02
1e-02
2e-02
Log-odds
0.5
1.0
1.5
2.0
2.5
3.0
3.5
4.0

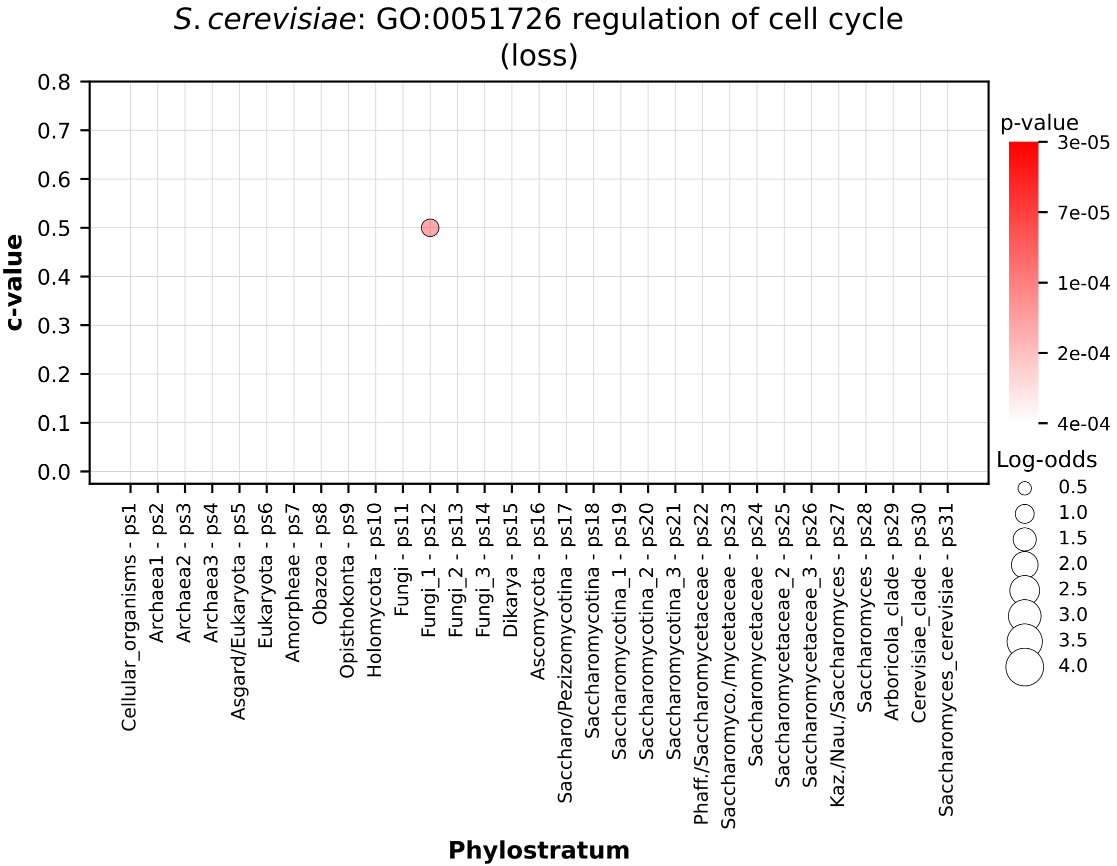
S. cerevisiae: GO:0051726 regulation of cell cycle
(loss)
c-value
0.8
0.7
0.6
0.5
0.4
0.3
0.2
0.1
0.0
p-value
3e-05
7e-05
1e-04
2e-04
4e-04
Log-odds
0.5
1.0
1.5
2.0
2.5
3.0
3.5
4.0
Cellular_organisms - ps1
Archaea1 - ps2
Archaea2 - ps3
Archaea3 - ps4
Asgard/Eukaryota - ps5
Eukaryota - ps6
Amorpheae - ps7
Obazoa - ps8
Opisthokonta - ps9
Holomycota - ps10
Fungi - ps11
Fungi_1 - ps12
Fungi_2 - ps13
Fungi_3 - ps14
Dikarya - ps15
Ascomycota - ps16
Saccharo/Pezizomycotina - ps17
Saccharomycotina - ps18
Saccharomycotina_1 - ps19
Saccharomycotina_2 - ps20
Saccharomycotina_3 - ps21
Phaff./Saccharomycetaceae - ps22
Saccharomyco./mycetaceae - ps23
Saccharomycetaceae - ps24
Saccharomycetaceae_2 - ps25
Saccharomycetaceae_3 - ps26
Kaz./Nau./Saccharomyces - ps27
Saccharomyces - ps28
Arboricola_clade - ps29
Cerevisiae_clade - ps30
Saccharomyces_cerevisiae - ps31
Phylostratum

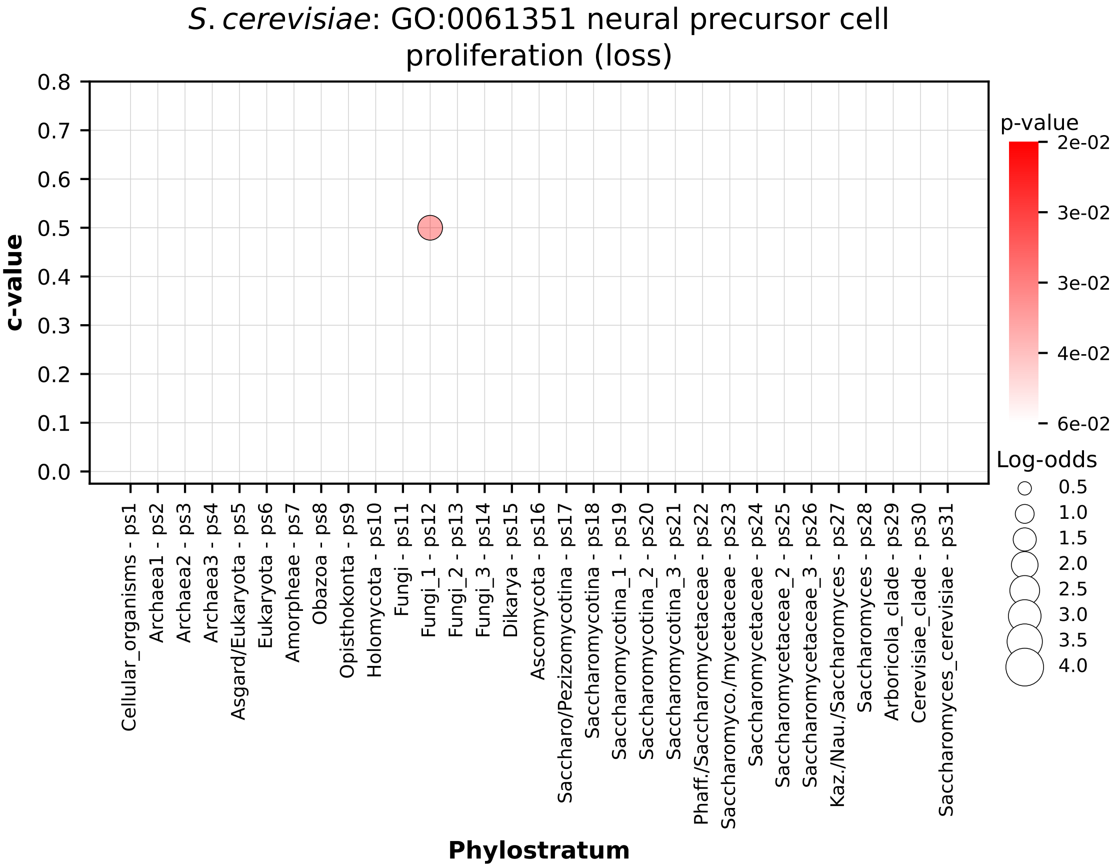

S. cerevisiae: GO:0061351 neural precursor cell proliferation (loss)
c-value
0.8
0.7
0.6
0.5
0.4
0.3
0.2
0.1
0.0
p-value
2e-02
3e-02
3e-02
4e-02
6e-02
Log-odds
0.5
1.0
1.5
2.0
2.5
3.0
3.5
4.0
Cellular_organisms - ps1
Archaea1 - ps2
Archaea2 - ps3
Archaea3 - ps4
Asgard/Eukaryota - ps5
Eukaryota - ps6
Amorpheae - ps7
Obazoa - ps8
Opisthokonta - ps9
Holomycota - ps10
Fungi - ps11
Fungi_1 - ps12
Fungi_2 - ps13
Fungi_3 - ps14
Dikarya - ps15
Ascomycota - ps16
Saccharo/Pezizomycotina - ps17
Saccharomycotina - ps18
Saccharomycotina_1 - ps19
Saccharomycotina_2 - ps20
Saccharomycotina_3 - ps21
Phaff./Saccharomycetaceae - ps22
Saccharomyco./mycetaceae - ps23
Saccharomycetaceae - ps24
Saccharomycetaceae_2 - ps25
Saccharomycetaceae_3 - ps26
Kaz./Nau./Saccharomyces - ps27
Saccharomyces - ps28
Arboricola_clade - ps29
Cerevisiae_clade - ps30
Saccharomyces_cerevisiae - ps31
Phylostratum

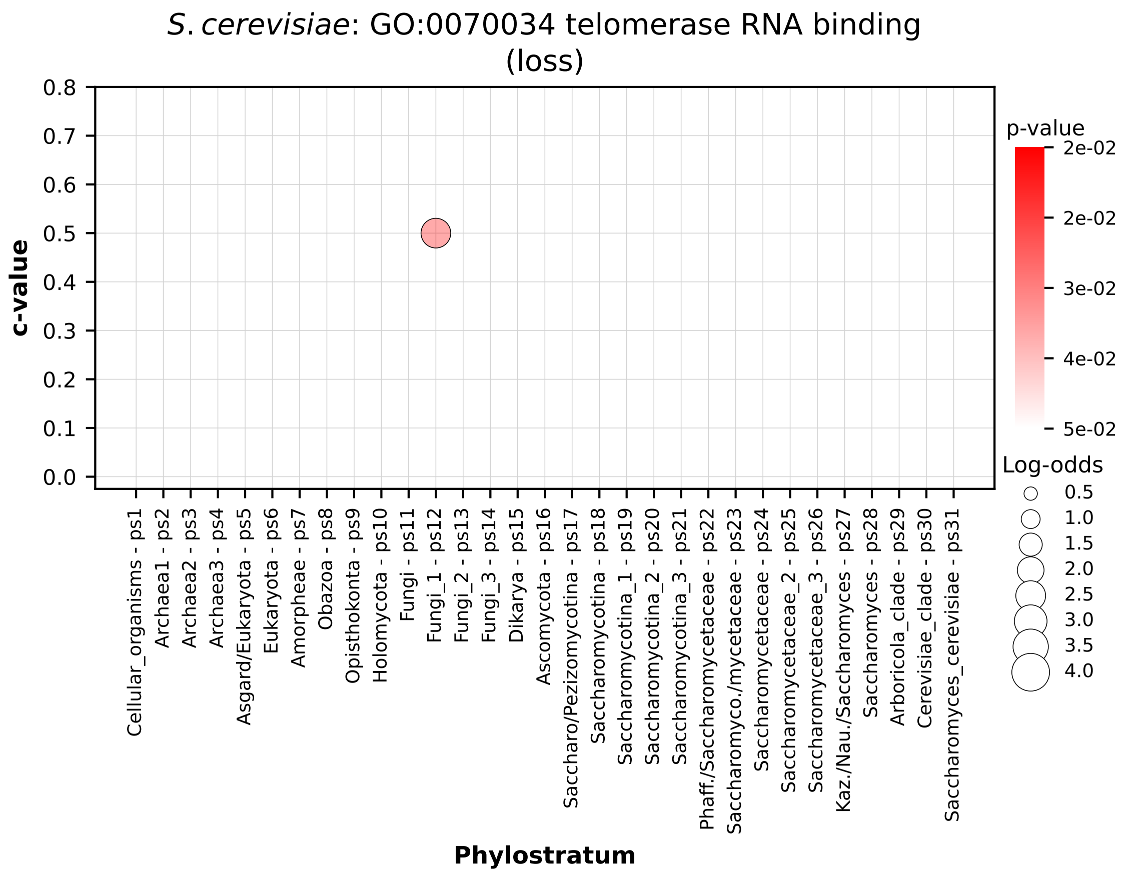
S. cerevisiae: GO:0070034 telomerase RNA binding
(loss)
c-value
0.8
0.7
0.6
0.5
0.4
0.3
0.2
0.1
0.0
Phylostratum
Cellular_organisms - ps1
Archaea1 - ps2
Archaea2 - ps3
Archaea3 - ps4
Asgard/Eukaryota - ps5
Eukaryota - ps6
Amorpheae - ps7
Obazoa - ps8
Opisthokonta - ps9
Holomycota - ps10
Fungi - ps11
Fungi_1 - ps12
Fungi_2 - ps13
Fungi_3 - ps14
Dikarya - ps15
Ascomycota - ps16
Saccharo/Pezizomycotina - ps17
Saccharomycotina - ps18
Saccharomycotina_1 - ps19
Saccharomycotina_2 - ps20
Saccharomycotina_3 - ps21
Phaff./Saccharomycetaceae - ps22
Saccharomyco./mycetaceae - ps23
Saccharomycetaceae - ps24
Saccharomycetaceae_2 - ps25
Saccharomycetaceae_3 - ps26
Kaz./Nau./Saccharomyces - ps27
Saccharomyces - ps28
Arboricola_clade - ps29
Cerevisiae_clade - ps30
Saccharomyces_cerevisiae - ps31
p-value
2e-02
2e-02
3e-02
4e-02
5e-02
Log-odds
0.5
1.0
1.5
2.0
2.5
3.0
3.5
4.0

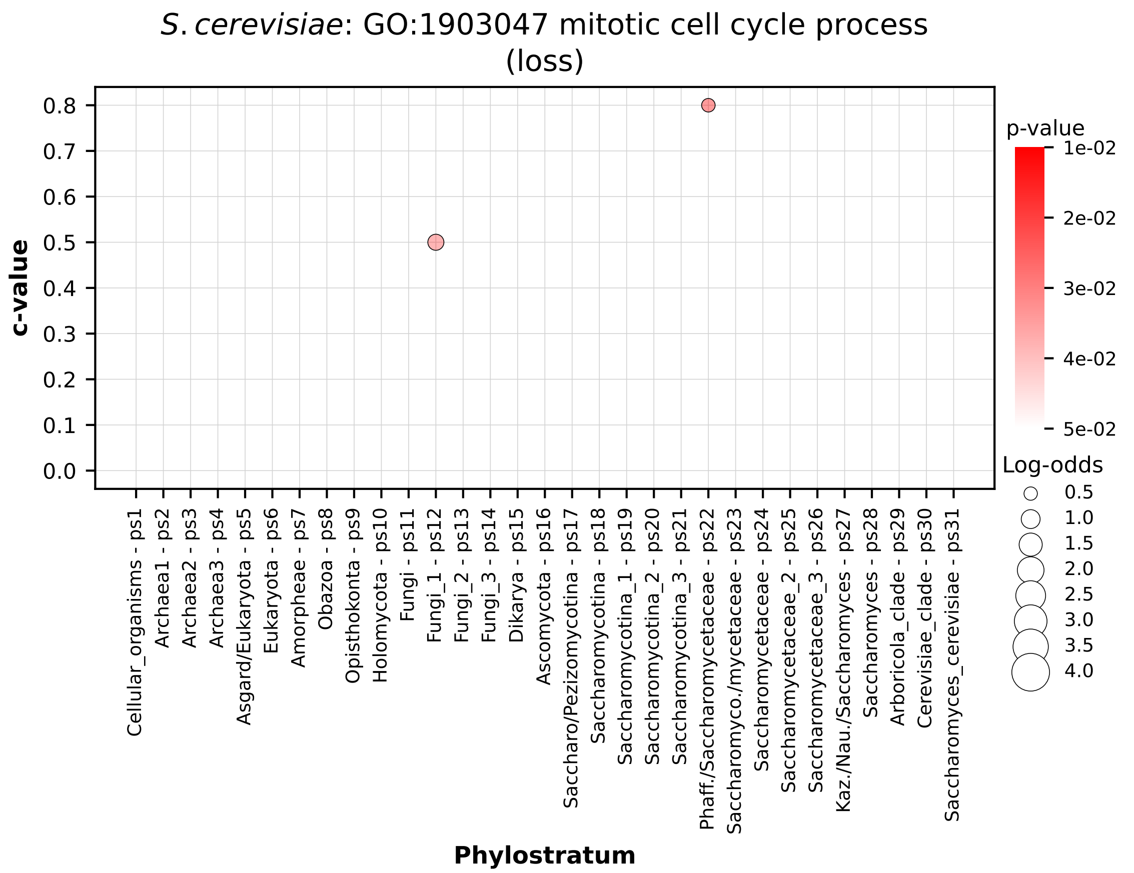

S. cerevisiae: GO:1903047 mitotic cell cycle process
(loss)
c-value
0.0
0.1
0.2
0.3
0.4
0.5
0.6
0.7
0.8
p-value
1e-02
2e-02
3e-02
4e-02
5e-02
Log-odds
0.5
1.0
1.5
2.0
2.5
3.0
3.5
4.0
Cellular_organisms - ps1
Archaea1 - ps2
Archaea2 - ps3
Archaea3 - ps4
Asgard/Eukaryota - ps5
Eukaryota - ps6
Amorpheae - ps7
Obazoa - ps8
Opisthokonta - ps9
Holomycota - ps10
Fungi - ps11
Fungi_1 - ps12
Fungi_2 - ps13
Fungi_3 - ps14
Dikarya - ps15
Ascomycota - ps16
Saccharo/Pezizomycotina - ps17
Saccharomycotina - ps18
Saccharomycotina_1 - ps19
Saccharomycotina_2 - ps20
Saccharomycotina_3 - ps21
Phaff./Saccharomycetaceae - ps22
Saccharomyco./mycetaceae - ps23
Saccharomycetaceae - ps24
Saccharomycetaceae_2 - ps25
Saccharomycetaceae_3 - ps26
Kaz./Nau./Saccharomyces - ps27
Saccharomyces - ps28
Arboricola_clade - ps29
Cerevisiae_clade - ps30
Saccharomyces_cerevisiae - ps31
Phylostratum

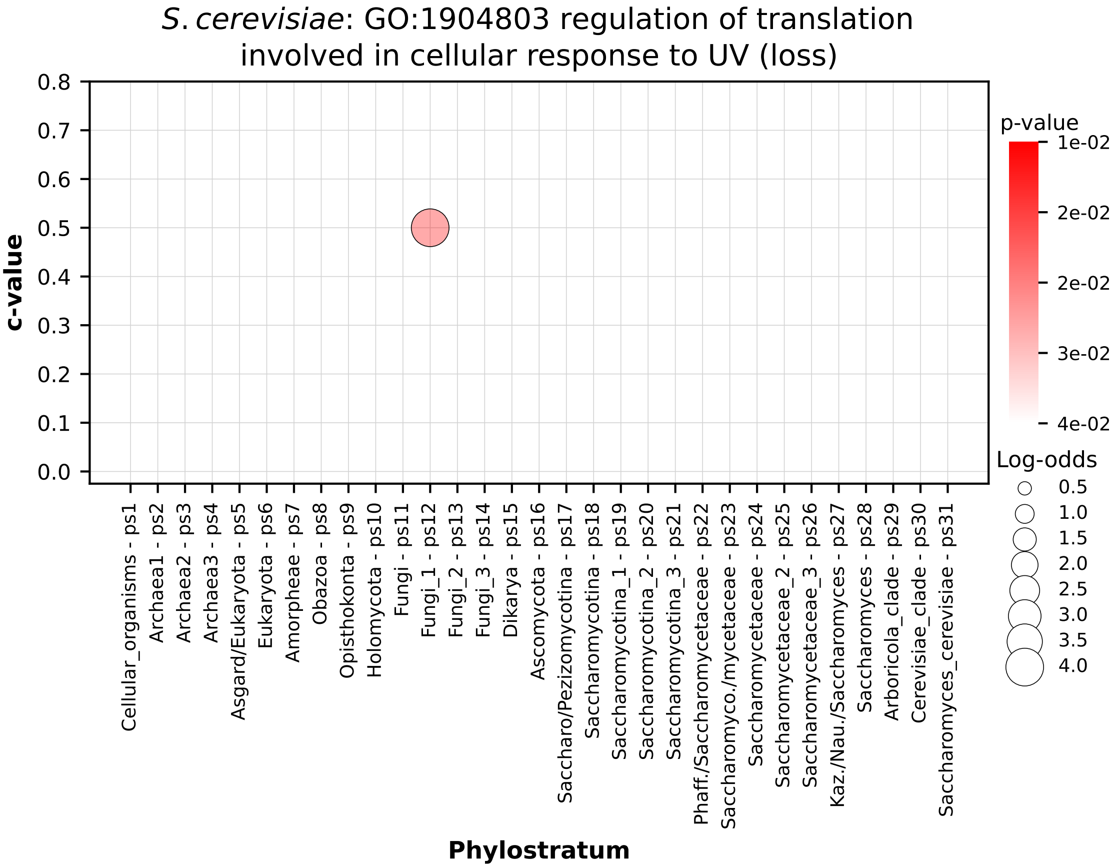
S. cerevisiae: GO:1904803 regulation of translation involved in cellular response to UV (loss)
c-value
0.8
0.7
0.6
0.5
0.4
0.3
0.2
0.1
0.0
Phylostratum
Cellular_organisms - ps1
Archaea1 - ps2
Archaea2 - ps3
Archaea3 - ps4
Asgard/Eukaryota - ps5
Eukaryota - ps6
Amorpheae - ps7
Obazoa - ps8
Opisthokonta - ps9
Holomycota - ps10
Fungi - ps11
Fungi_1 - ps12
Fungi_2 - ps13
Fungi_3 - ps14
Dikarya - ps15
Ascomycota - ps16
Saccharo/Pezizomycotina - ps17
Saccharomycotina - ps18
Saccharomycotina_1 - ps19
Saccharomycotina_2 - ps20
Saccharomycotina_3 - ps21
Phaff./Saccharomycetaceae - ps22
Saccharomyco./mycetaceae - ps23
Saccharomycetaceae - ps24
Saccharomycetaceae_2 - ps25
Saccharomycetaceae_3 - ps26
Kaz./Nau./Saccharomyces - ps27
Saccharomyces - ps28
Arboricola_clade - ps29
Cerevisiae_clade - ps30
Saccharomyces_cerevisiae - ps31
p-value
1e-02
2e-02
2e-02
3e-02
4e-02
Log-odds
0.5
1.0
1.5
2.0
2.5
3.0
3.5
4.0

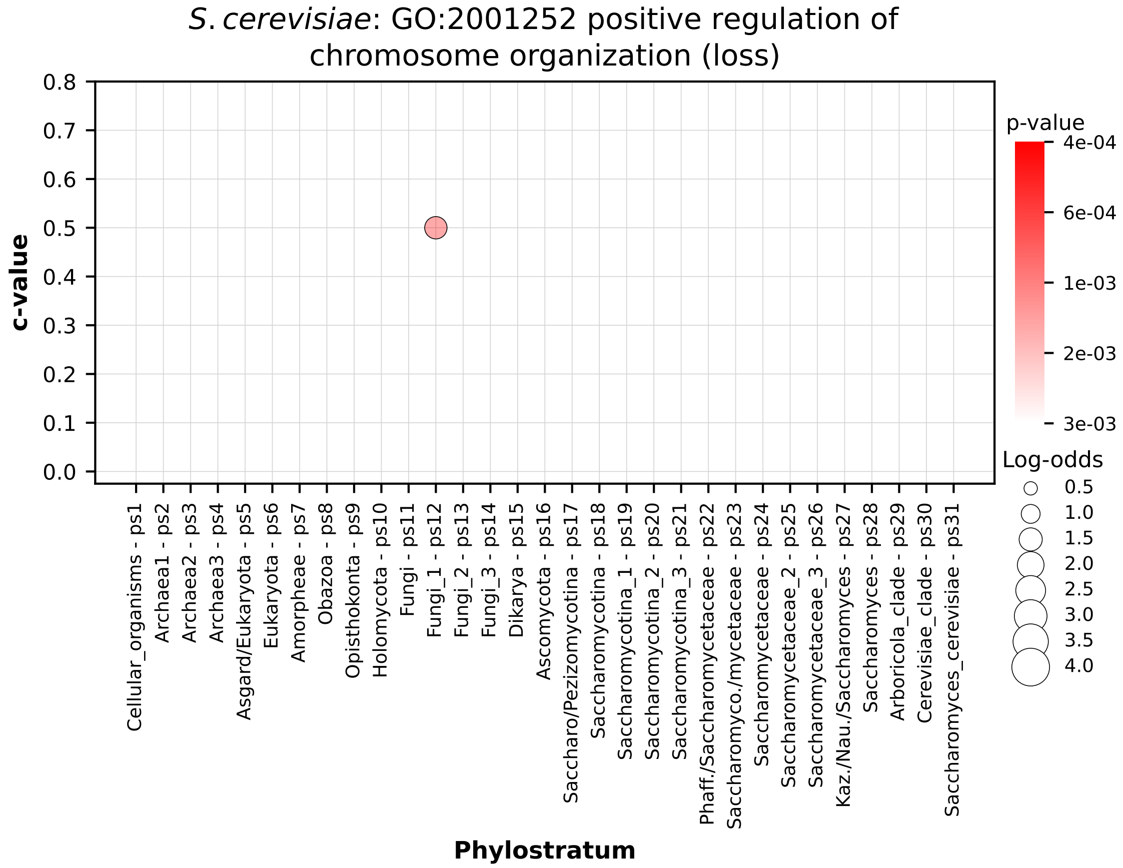

S. cerevisiae: GO:2001252 positive regulation of chromosome organization (loss)
c-value
0.8
0.7
0.6
0.5
0.4
0.3
0.2
0.1
0.0
Phylostratum
Cellular_organisms - ps1
Archaea1 - ps2
Archaea2 - ps3
Archaea3 - ps4
Asgard/Eukaryota - ps5
Eukaryota - ps6
Amorpheae - ps7
Obazoa - ps8
Opisthokonta - ps9
Holomycota - ps10
Fungi - ps11
Fungi_1 - ps12
Fungi_2 - ps13
Fungi_3 - ps14
Dikarya - ps15
Ascomycota - ps16
Saccharo/Pezizomycotina - ps17
Saccharomycotina - ps18
Saccharomycotina_1 - ps19
Saccharomycotina_2 - ps20
Saccharomycotina_3 - ps21
Phaff./Saccharomycetaceae - ps22
Saccharomyco./mycetaceae - ps23
Saccharomycetaceae - ps24
Saccharomycetaceae_2 - ps25
Saccharomycetaceae_3 - ps26
Kaz./Nau./Saccharomyces - ps27
Saccharomyces - ps28
Arboricola_clade - ps29
Cerevisiae_clade - ps30
Saccharomyces_cerevisiae - ps31
p-value
4e-04
6e-04
1e-03
2e-03
3e-03
Log-odds
0.5
1.0
1.5
2.0
2.5
3.0
3.5
4.0

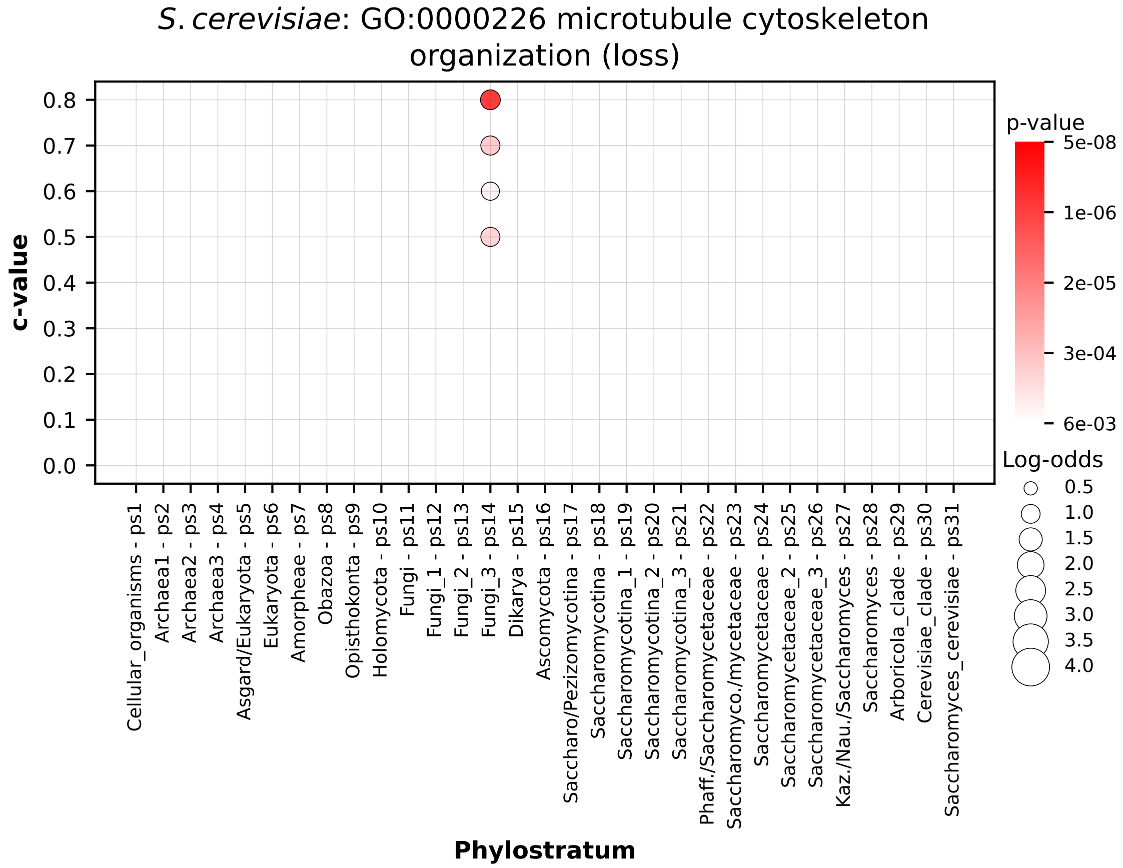

S. cerevisiae: GO:0000226 microtubule cytoskeleton organization (loss)
c-value
0.0
0.1
0.2
0.3
0.4
0.5
0.6
0.7
0.8
p-value
5e-08
1e-06
2e-05
3e-04
6e-03
Log-odds
0.5
1.0
1.5
2.0
2.5
3.0
3.5
4.0
Cellular_organisms - ps1
Archaea1 - ps2
Archaea2 - ps3
Archaea3 - ps4
Asgard/Eukaryota - ps5
Eukaryota - ps6
Amorpheae - ps7
Obazoa - ps8
Opisthokonta - ps9
Holomycota - ps10
Fungi - ps11
Fungi_1 - ps12
Fungi_2 - ps13
Fungi_3 - ps14
Dikarya - ps15
Ascomycota - ps16
Saccharo/Pezizomycotina - ps17
Saccharomycotina - ps18
Saccharomycotina_1 - ps19
Saccharomycotina_2 - ps20
Saccharomycotina_3 - ps21
Phaff./Saccharomycetaceae - ps22
Saccharomyco./mycetaceae - ps23
Saccharomycetaceae - ps24
Saccharomycetaceae_2 - ps25
Saccharomycetaceae_3 - ps26
Kaz./Nau./Saccharomyces - ps27
Saccharomyces - ps28
Arboricola_clade - ps29
Cerevisiae_clade - ps30
Saccharomyces_cerevisiae - ps31
Phylostratum

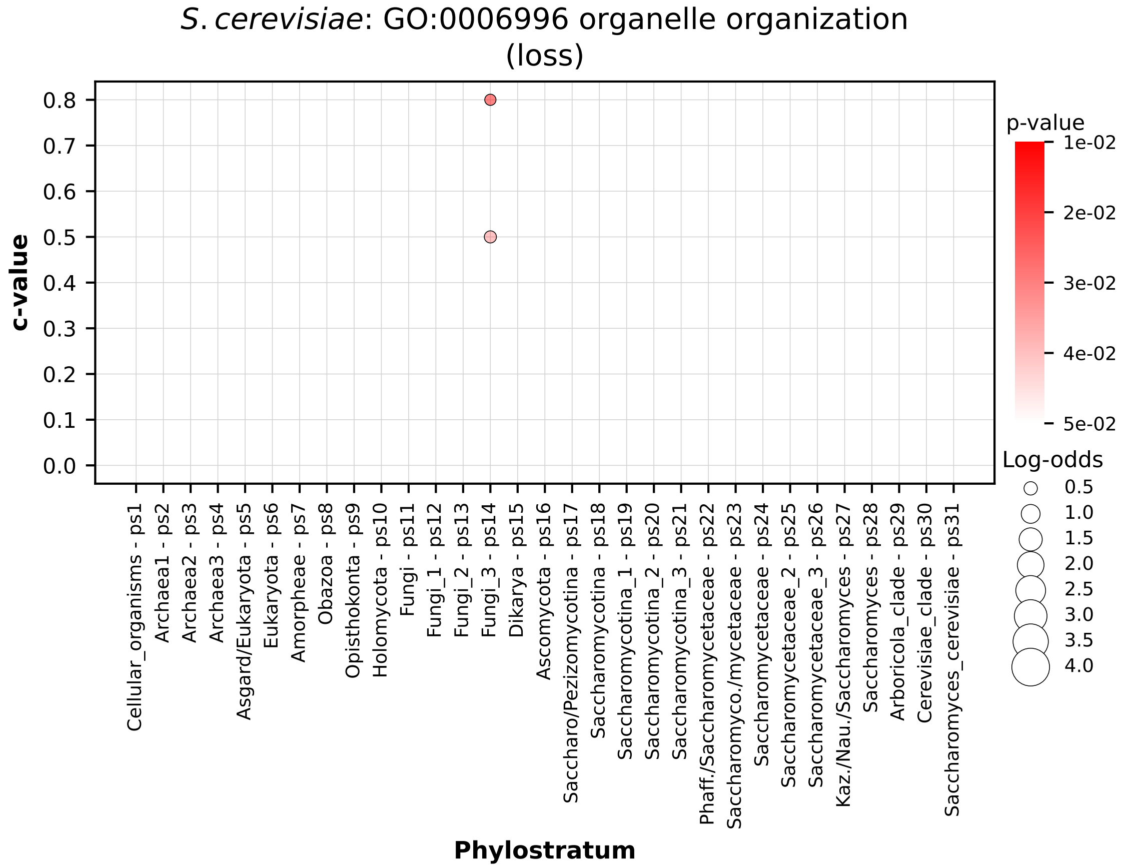

S. cerevisiae: GO:0006996 organelle organization
(loss)
c-value
0.0
0.1
0.2
0.3
0.4
0.5
0.6
0.7
0.8
p-value
1e-02
2e-02
3e-02
4e-02
5e-02
Log-odds
0.5
1.0
1.5
2.0
2.5
3.0
3.5
4.0
Cellular_organisms - ps1
Archaea1 - ps2
Archaea2 - ps3
Archaea3 - ps4
Asgard/Eukaryota - ps5
Eukaryota - ps6
Amorpheae - ps7
Obazoa - ps8
Opisthokonta - ps9
Holomycota - ps10
Fungi - ps11
Fungi_1 - ps12
Fungi_2 - ps13
Fungi_3 - ps14
Dikarya - ps15
Ascomycota - ps16
Saccharo/Pezizomycotina - ps17
Saccharomycotina - ps18
Saccharomycotina_1 - ps19
Saccharomycotina_2 - ps20
Saccharomycotina_3 - ps21
Phaff./Saccharomycetaceae - ps22
Saccharomyco./mycetaceae - ps23
Saccharomycetaceae - ps24
Saccharomycetaceae_2 - ps25
Saccharomycetaceae_3 - ps26
Kaz./Nau./Saccharomyces - ps27
Saccharomyces - ps28
Arboricola_clade - ps29
Cerevisiae_clade - ps30
Saccharomyces_cerevisiae - ps31
Phylostratum

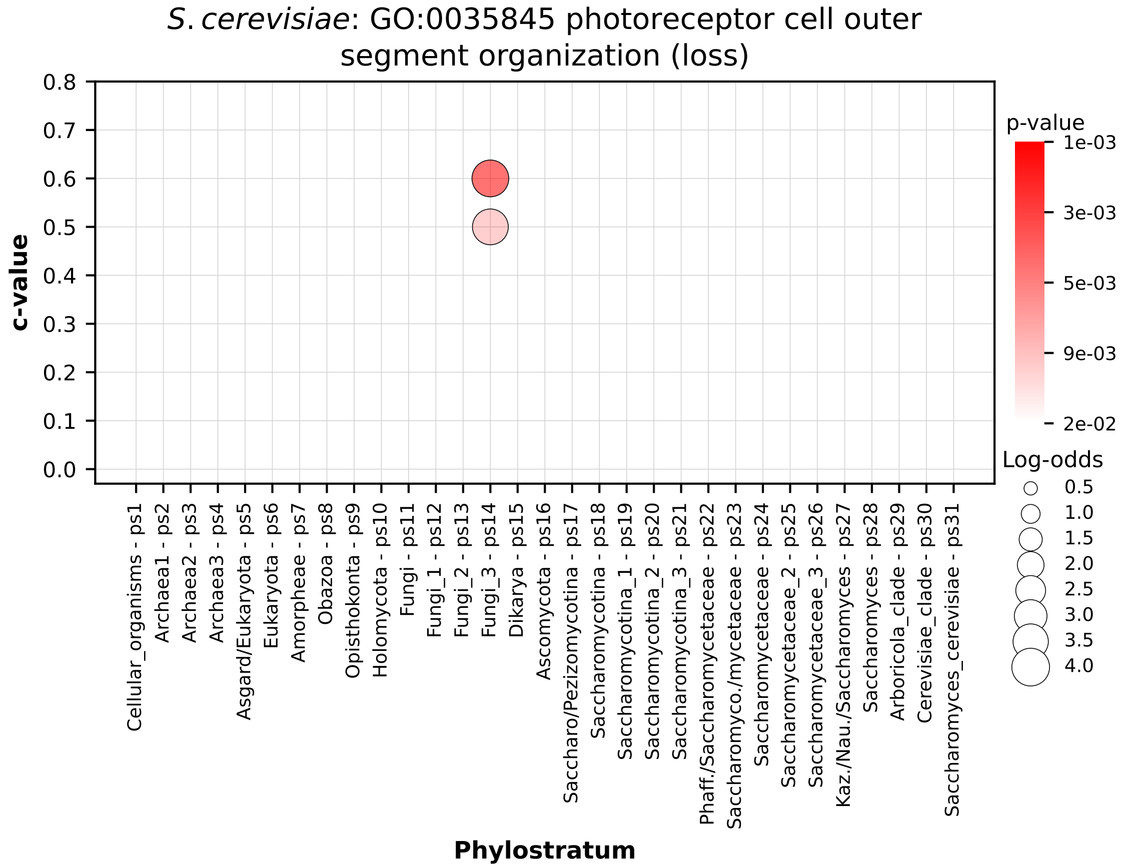

S. cerevisiae: GO:0035845 photoreceptor cell outer segment organization (loss)
c-value
0.8
0.7
0.6
0.5
0.4
0.3
0.2
0.1
0.0
Phylostratum
Cellular_organisms - ps1
Archaea1 - ps2
Archaea2 - ps3
Archaea3 - ps4
Asgard/Eukaryota - ps5
Eukaryota - ps6
Amorpheae - ps7
Obazoa - ps8
Opisthokonta - ps9
Holomycota - ps10
Fungi - ps11
Fungi_1 - ps12
Fungi_2 - ps13
Fungi_3 - ps14
Dikarya - ps15
Ascomycota - ps16
Saccharo/Pezizomycotina - ps17
Saccharomycotina - ps18
Saccharomycotina_1 - ps19
Saccharomycotina_2 - ps20
Saccharomycotina_3 - ps21
Phaff./Saccharomycetaceae - ps22
Saccharomyco./mycetaceae - ps23
Saccharomycetaceae - ps24
Saccharomycetaceae_2 - ps25
Saccharomycetaceae_3 - ps26
Kaz./Nau./Saccharomyces - ps27
Saccharomyces - ps28
Arboricola_clade - ps29
Cerevisiae_clade - ps30
Saccharomyces_cerevisiae - ps31
p-value
1e-03
3e-03
5e-03
9e-03
2e-02
Log-odds
0.5
1.0
1.5
2.0
2.5
3.0
3.5
4.0

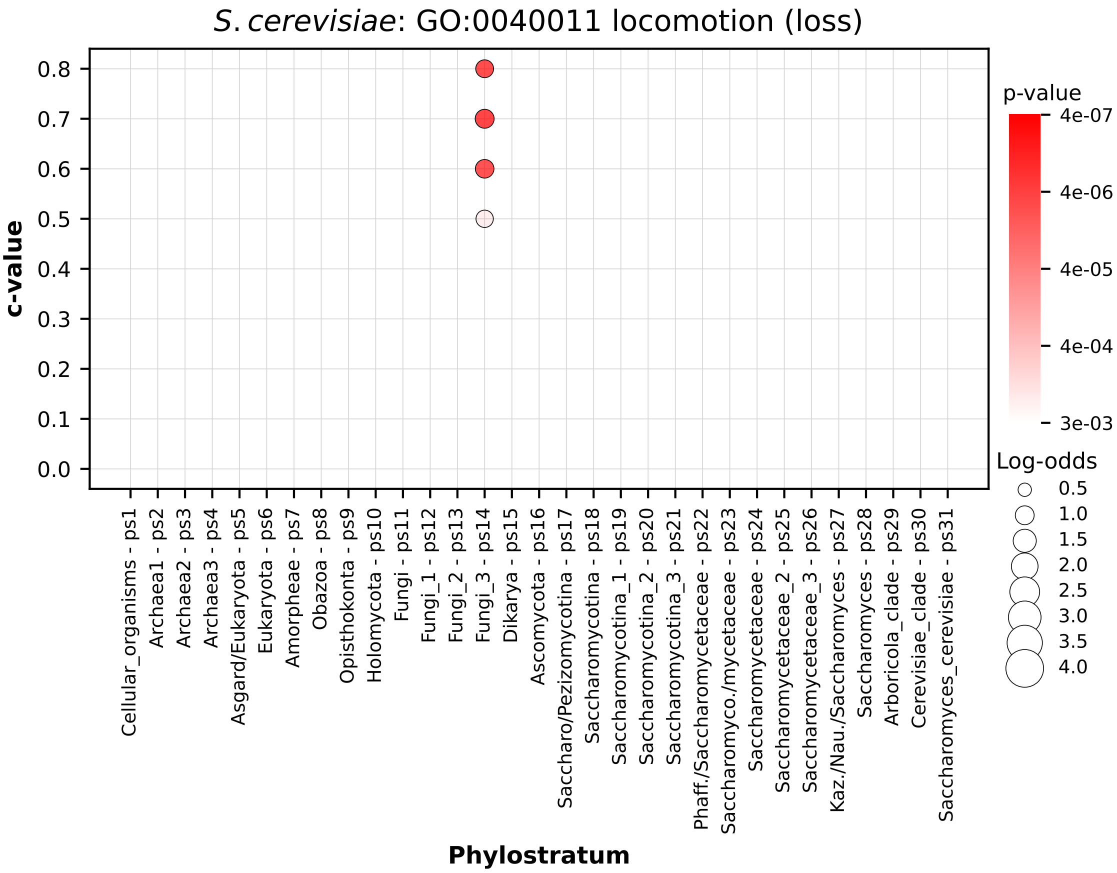
S. cerevisiae: GO:0040011 locomotion (loss)
c-value
0.8
0.7
0.6
0.5
0.4
0.3
0.2
0.1
0.0
p-value
4e-07
4e-06
4e-05
4e-04
3e-03
Log-odds
0.5
1.0
1.5
2.0
2.5
3.0
3.5
4.0
Cellular_organisms - ps1
Archaea1 - ps2
Archaea2 - ps3
Archaea3 - ps4
Asgard/Eukaryota - ps5
Eukaryota - ps6
Amorpheae - ps7
Obazoa - ps8
Opisthokonta - ps9
Holomycota - ps10
Fungi - ps11
Fungi_1 - ps12
Fungi_2 - ps13
Fungi_3 - ps14
Dikarya - ps15
Ascomycota - ps16
Saccharo/Pezizomycotina - ps17
Saccharomycotina - ps18
Saccharomycotina_1 - ps19
Saccharomycotina_2 - ps20
Saccharomycotina_3 - ps21
Phaff./Saccharomycetaceae - ps22
Saccharomyco./mycetaceae - ps23
Saccharomycetaceae - ps24
Saccharomycetaceae_2 - ps25
Saccharomycetaceae_3 - ps26
Kaz./Nau./Saccharomyces - ps27
Saccharomyces - ps28
Arboricola_clade - ps29
Cerevisiae_clade - ps30
Saccharomyces_cerevisiae - ps31
Phylostratum

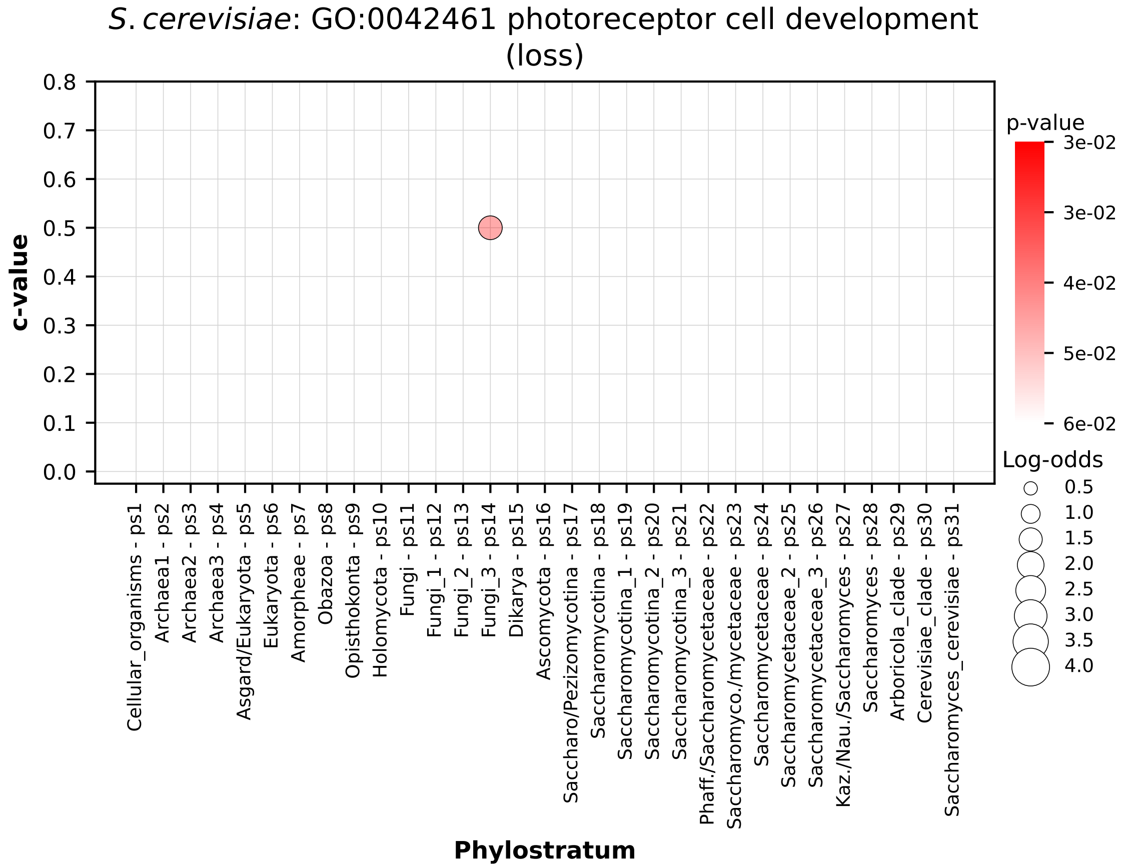

S. cerevisiae: GO:0042461 photoreceptor cell development (loss)
c-value
0.8
0.7
0.6
0.5
0.4
0.3
0.2
0.1
0.0
p-value
3e-02
3e-02
4e-02
5e-02
6e-02
Log-odds
0.5
1.0
1.5
2.0
2.5
3.0
3.5
4.0
Cellular_organisms - ps1
Archaea1 - ps2
Archaea2 - ps3
Archaea3 - ps4
Asgard/Eukaryota - ps5
Eukaryota - ps6
Amorpheae - ps7
Obazoa - ps8
Opisthokonta - ps9
Holomycota - ps10
Fungi - ps11
Fungi_1 - ps12
Fungi_2 - ps13
Fungi_3 - ps14
Dikarya - ps15
Ascomycota - ps16
Saccharo/Pezizomycotina - ps17
Saccharomycotina - ps18
Saccharomycotina_1 - ps19
Saccharomycotina_2 - ps20
Saccharomycotina_3 - ps21
Phaff./Saccharomycetaceae - ps22
Saccharomyco./mycetaceae - ps23
Saccharomycetaceae - ps24
Saccharomycetaceae_2 - ps25
Saccharomycetaceae_3 - ps26
Kaz./Nau./Saccharomyces - ps27
Saccharomyces - ps28
Arboricola_clade - ps29
Cerevisiae_clade - ps30
Saccharomyces_cerevisiae - ps31
Phylostratum

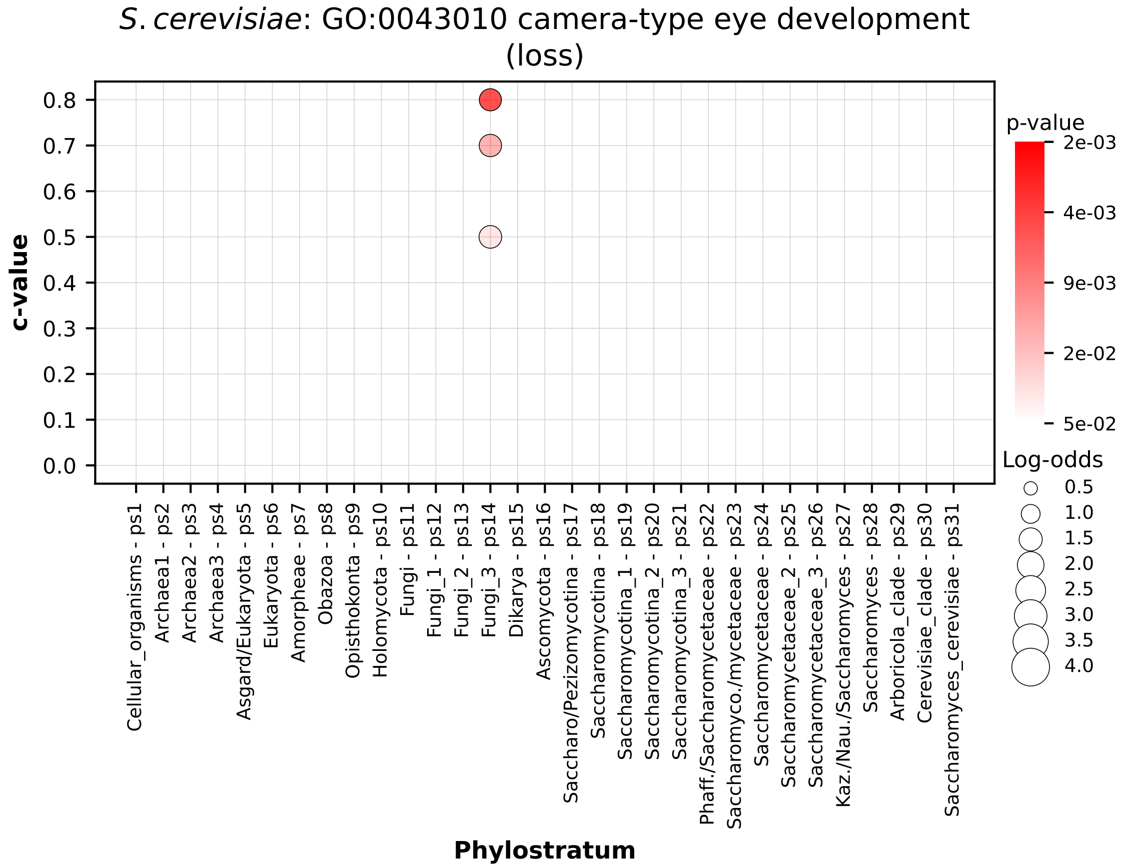
S. cerevisiae: GO:0043010 camera-type eye development (loss)
c-value
0.8
0.7
0.6
0.5
0.4
0.3
0.2
0.1
0.0
p-value
2e-03
4e-03
9e-03
2e-02
5e-02
Log-odds
0.5
1.0
1.5
2.0
2.5
3.0
3.5
4.0
Cellular_organisms - ps1
Archaea1 - ps2
Archaea2 - ps3
Archaea3 - ps4
Asgard/Eukaryota - ps5
Eukaryota - ps6
Amorpheae - ps7
Obazoa - ps8
Opisthokonta - ps9
Holomycota - ps10
Fungi - ps11
Fungi_1 - ps12
Fungi_2 - ps13
Fungi_3 - ps14
Dikarya - ps15
Ascomycota - ps16
Saccharo/Pezizomycotina - ps17
Saccharomycotina - ps18
Saccharomycotina_1 - ps19
Saccharomycotina_2 - ps20
Saccharomycotina_3 - ps21
Phaff./Saccharomycetaceae - ps22
Saccharomyco./mycetaceae - ps23
Saccharomycetaceae - ps24
Saccharomycetaceae_2 - ps25
Saccharomycetaceae_3 - ps26
Kaz./Nau./Saccharomyces - ps27
Saccharomyces - ps28
Arboricola_clade - ps29
Cerevisiae_clade - ps30
Saccharomyces_cerevisiae - ps31
Phylostratum

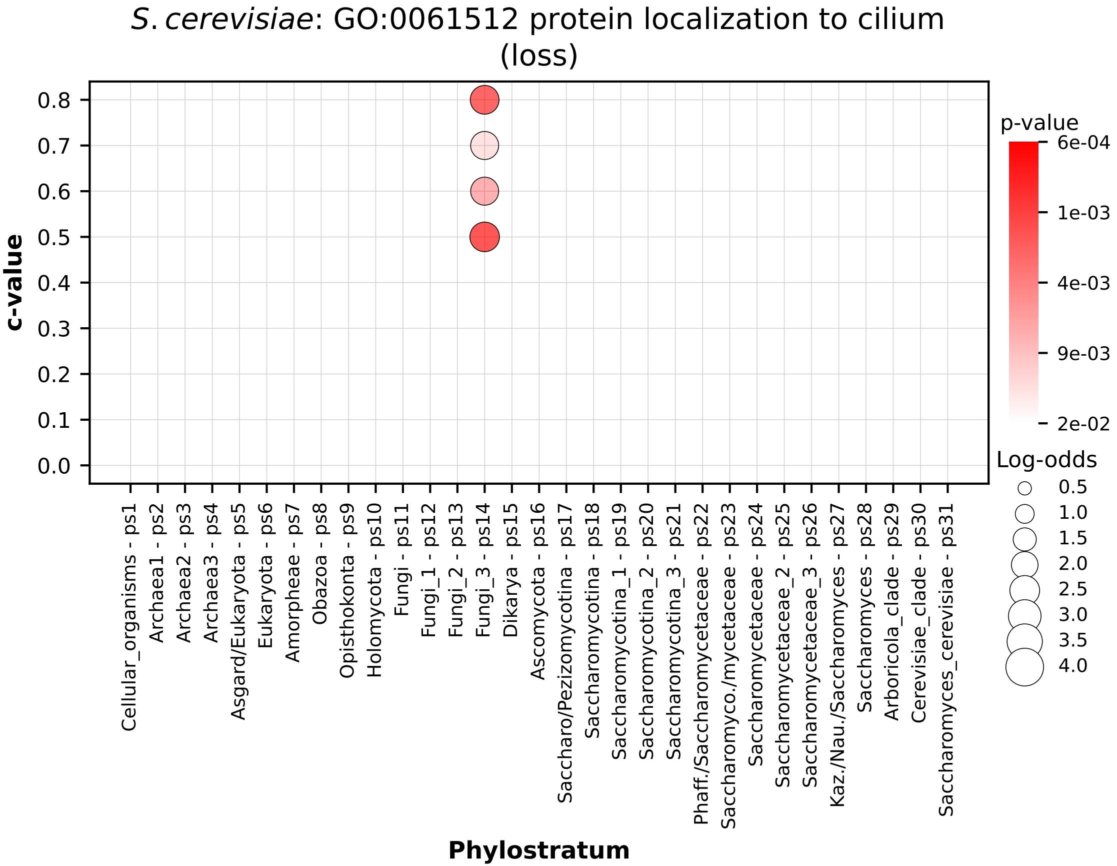
S. cerevisiae: GO:0061512 protein localization to cilium
(loss)
c-value
0.8
0.7
0.6
0.5
0.4
0.3
0.2
0.1
0.0
p-value
6e-04
1e-03
4e-03
9e-03
2e-02
Log-odds
0.5
1.0
1.5
2.0
2.5
3.0
3.5
4.0
Cellular_organisms - ps1
Archaea1 - ps2
Archaea2 - ps3
Archaea3 - ps4
Asgard/Eukaryota - ps5
Eukaryota - ps6
Amorpheae - ps7
Obazoa - ps8
Opisthokonta - ps9
Holomycota - ps10
Fungi - ps11
Fungi_1 - ps12
Fungi_2 - ps13
Fungi_3 - ps14
Dikarya - ps15
Ascomycota - ps16
Saccharo/Pezizomycotina - ps17
Saccharomycotina - ps18
Saccharomycotina_1 - ps19
Saccharomycotina_2 - ps20
Saccharomycotina_3 - ps21
Phaff./Saccharomycetaceae - ps22
Saccharomyco./mycetaceae - ps23
Saccharomycetaceae - ps24
Saccharomycetaceae_2 - ps25
Saccharomycetaceae_3 - ps26
Kaz./Nau./Saccharomyces - ps27
Saccharomyces - ps28
Arboricola_clade - ps29
Cerevisiae_clade - ps30
Saccharomyces_cerevisiae - ps31
Phylostratum

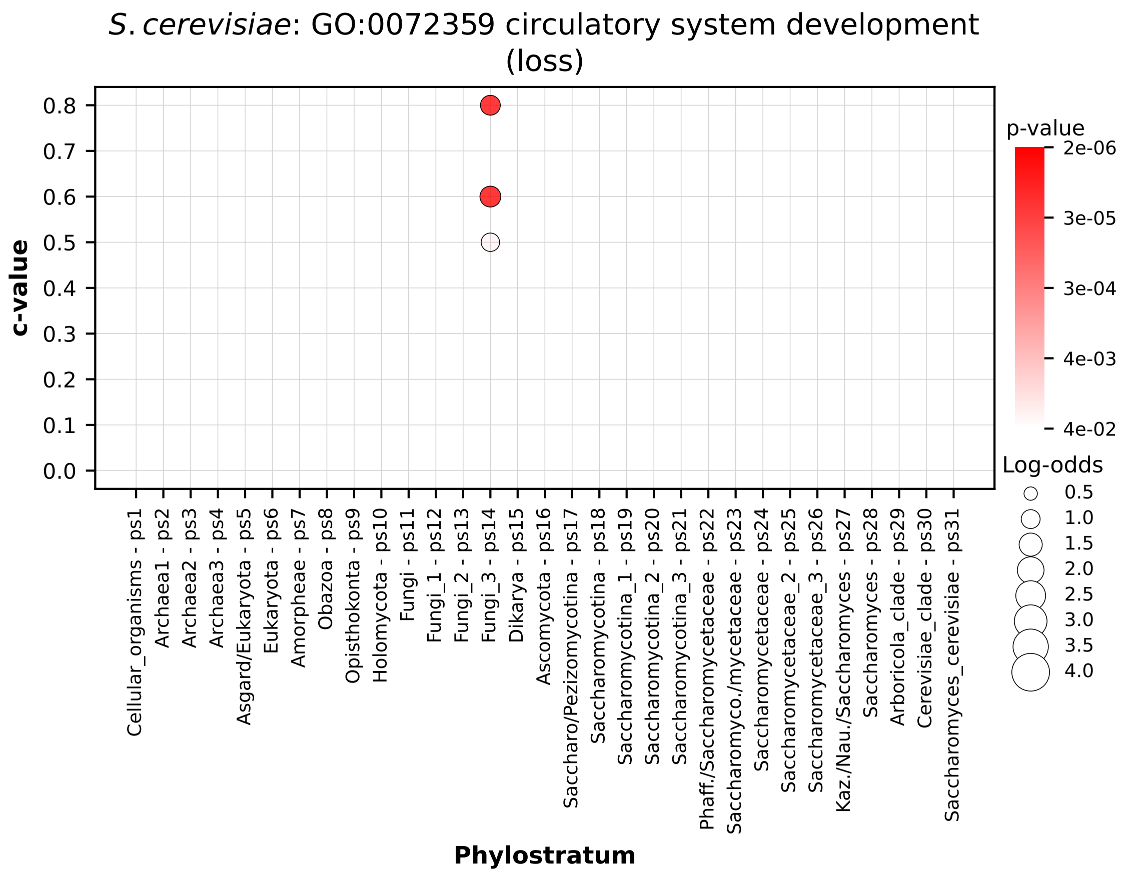
S. cerevisiae: GO:0072359 circulatory system development
(loss)
c-value
0.8
0.7
0.6
0.5
0.4
0.3
0.2
0.1
0.0
p-value
2e-06
3e-05
3e-04
4e-03
4e-02
Log-odds
0.5
1.0
1.5
2.0
2.5
3.0
3.5
4.0
Cellular_organisms - ps1
Archaea1 - ps2
Archaea2 - ps3
Archaea3 - ps4
Asgard/Eukaryota - ps5
Eukaryota - ps6
Amorpheae - ps7
Obazoa - ps8
Opisthokonta - ps9
Holomycota - ps10
Fungi - ps11
Fungi_1 - ps12
Fungi_2 - ps13
Fungi_3 - ps14
Dikarya - ps15
Ascomycota - ps16
Saccharo/Pezizomycotina - ps17
Saccharomycotina - ps18
Saccharomycotina_1 - ps19
Saccharomycotina_2 - ps20
Saccharomycotina_3 - ps21
Phaff./Saccharomycetaceae - ps22
Saccharomyco./mycetaceae - ps23
Saccharomycetaceae - ps24
Saccharomycetaceae_2 - ps25
Saccharomycetaceae_3 - ps26
Kaz./Nau./Saccharomyces - ps27
Saccharomyces - ps28
Arboricola_clade - ps29
Cerevisiae_clade - ps30
Saccharomyces_cerevisiae - ps31
Phylostratum

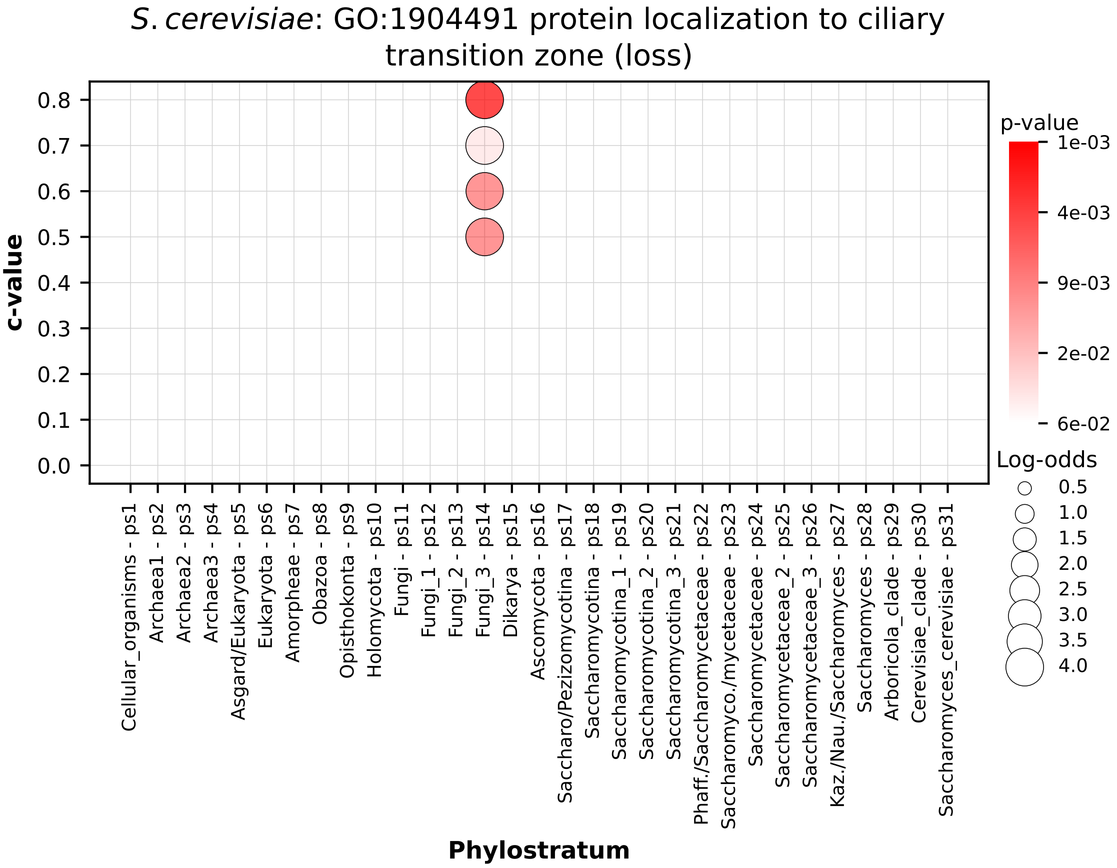

S. cerevisiae: GO:1904491 protein localization to ciliary transition zone (loss)
c-value
0.8
0.7
0.6
0.5
0.4
0.3
0.2
0.1
0.0
p-value
1e-03
4e-03
9e-03
2e-02
6e-02
Log-odds
0.5
1.0
1.5
2.0
2.5
3.0
3.5
4.0
Cellular_organisms - ps1
Archaea1 - ps2
Archaea2 - ps3
Archaea3 - ps4
Asgard/Eukaryota - ps5
Eukaryota - ps6
Amorpheae - ps7
Obazoa - ps8
Opisthokonta - ps9
Holomycota - ps10
Fungi - ps11
Fungi_1 - ps12
Fungi_2 - ps13
Fungi_3 - ps14
Dikarya - ps15
Ascomycota - ps16
Saccharo/Pezizomycotina - ps17
Saccharomycotina - ps18
Saccharomycotina_1 - ps19
Saccharomycotina_2 - ps20
Saccharomycotina_3 - ps21
Phaff./Saccharomycetaceae - ps22
Saccharomyco./mycetaceae - ps23
Saccharomycetaceae - ps24
Saccharomycetaceae_2 - ps25
Saccharomycetaceae_3 - ps26
Kaz./Nau./Saccharomyces - ps27
Saccharomyces - ps28
Arboricola_clade - ps29
Cerevisiae_clade - ps30
Saccharomyces_cerevisiae - ps31
Phylostratum

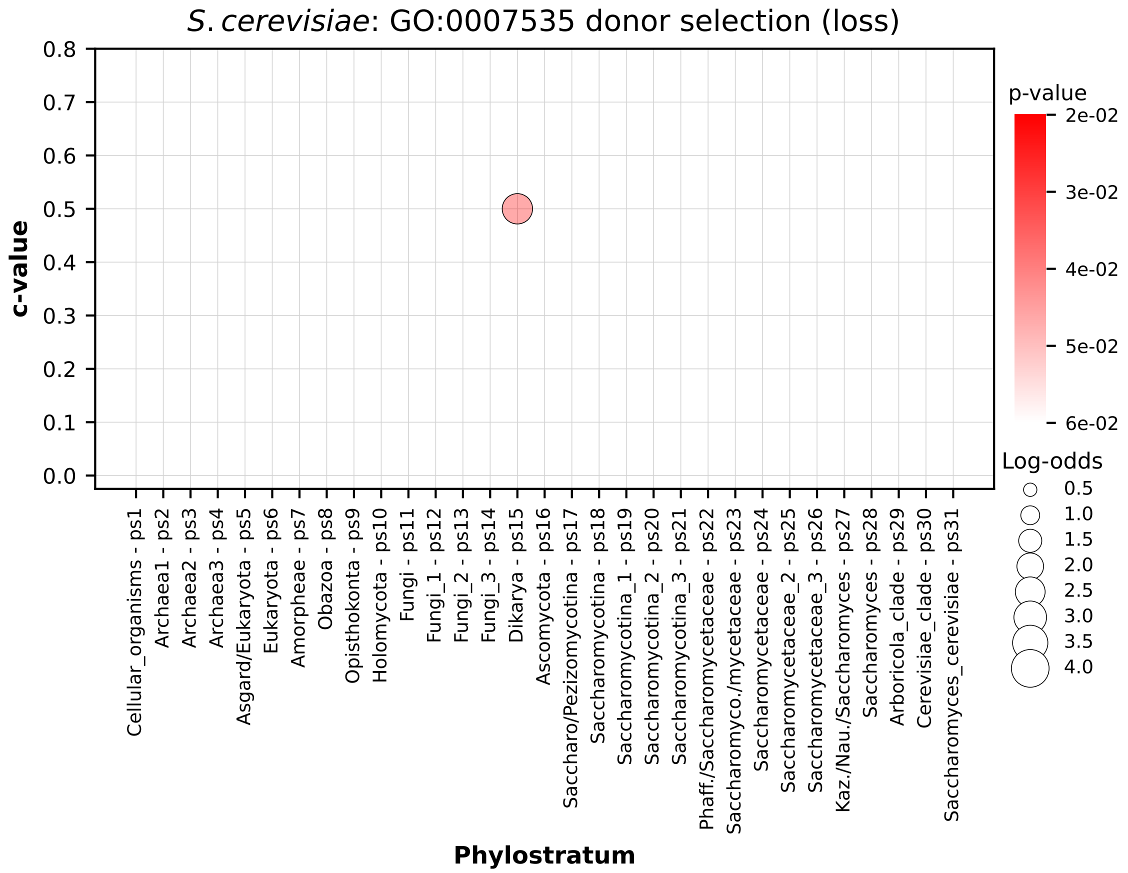
S. cerevisiae: GO:0007535 donor selection (loss)
c-value
0.8
0.7
0.6
0.5
0.4
0.3
0.2
0.1
0.0
p-value
2e-02
3e-02
4e-02
5e-02
6e-02
Log-odds
0.5
1.0
1.5
2.0
2.5
3.0
3.5
4.0
Cellular_organisms - ps1
Archaea1 - ps2
Archaea2 - ps3
Archaea3 - ps4
Asgard/Eukaryota - ps5
Eukaryota - ps6
Amorpheae - ps7
Obazoa - ps8
Opisthokonta - ps9
Holomycota - ps10
Fungi - ps11
Fungi_1 - ps12
Fungi_2 - ps13
Fungi_3 - ps14
Dikarya - ps15
Ascomycota - ps16
Saccharo/Pezizomycotina - ps17
Saccharomycotina - ps18
Saccharomycotina_1 - ps19
Saccharomycotina_2 - ps20
Saccharomycotina_3 - ps21
Phaff./Saccharomycetaceae - ps22
Saccharomyco./mycetaceae - ps23
Saccharomycetaceae - ps24
Saccharomycetaceae_2 - ps25
Saccharomycetaceae_3 - ps26
Kaz./Nau./Saccharomyces - ps27
Saccharomyces - ps28
Arboricola_clade - ps29
Cerevisiae_clade - ps30
Saccharomyces_cerevisiae - ps31
Phylostratum

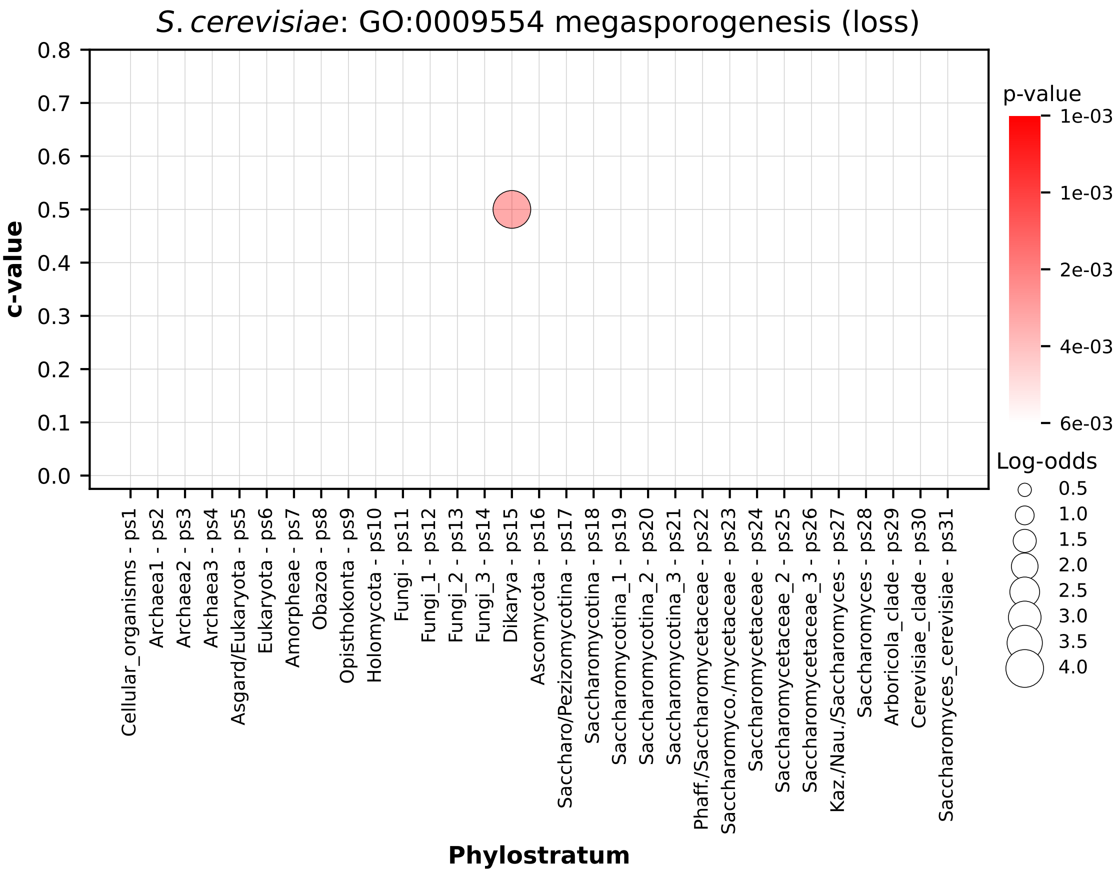
S. cerevisiae: GO:0009554 megasporogenesis (loss)
c-value
0.8
0.7
0.6
0.5
0.4
0.3
0.2
0.1
0.0
Phylostratum
Cellular_organisms - ps1
Archaea1 - ps2
Archaea2 - ps3
Archaea3 - ps4
Asgard/Eukaryota - ps5
Eukaryota - ps6
Amorpheae - ps7
Obazoa - ps8
Opisthokonta - ps9
Holomycota - ps10
Fungi - ps11
Fungi_1 - ps12
Fungi_2 - ps13
Fungi_3 - ps14
Dikarya - ps15
Ascomycota - ps16
Saccharo/Pezizomycotina - ps17
Saccharomycotina - ps18
Saccharomycotina_1 - ps19
Saccharomycotina_2 - ps20
Saccharomycotina_3 - ps21
Phaff./Saccharomycetaceae - ps22
Saccharomyco./mycetaceae - ps23
Saccharomycetaceae - ps24
Saccharomycetaceae_2 - ps25
Saccharomycetaceae_3 - ps26
Kaz./Nau./Saccharomyces - ps27
Saccharomyces - ps28
Arboricola_clade - ps29
Cerevisiae_clade - ps30
Saccharomyces_cerevisiae - ps31
p-value
1e-03
1e-03
2e-03
4e-03
6e-03
Log-odds
0.5
1.0
1.5
2.0
2.5
3.0
3.5
4.0

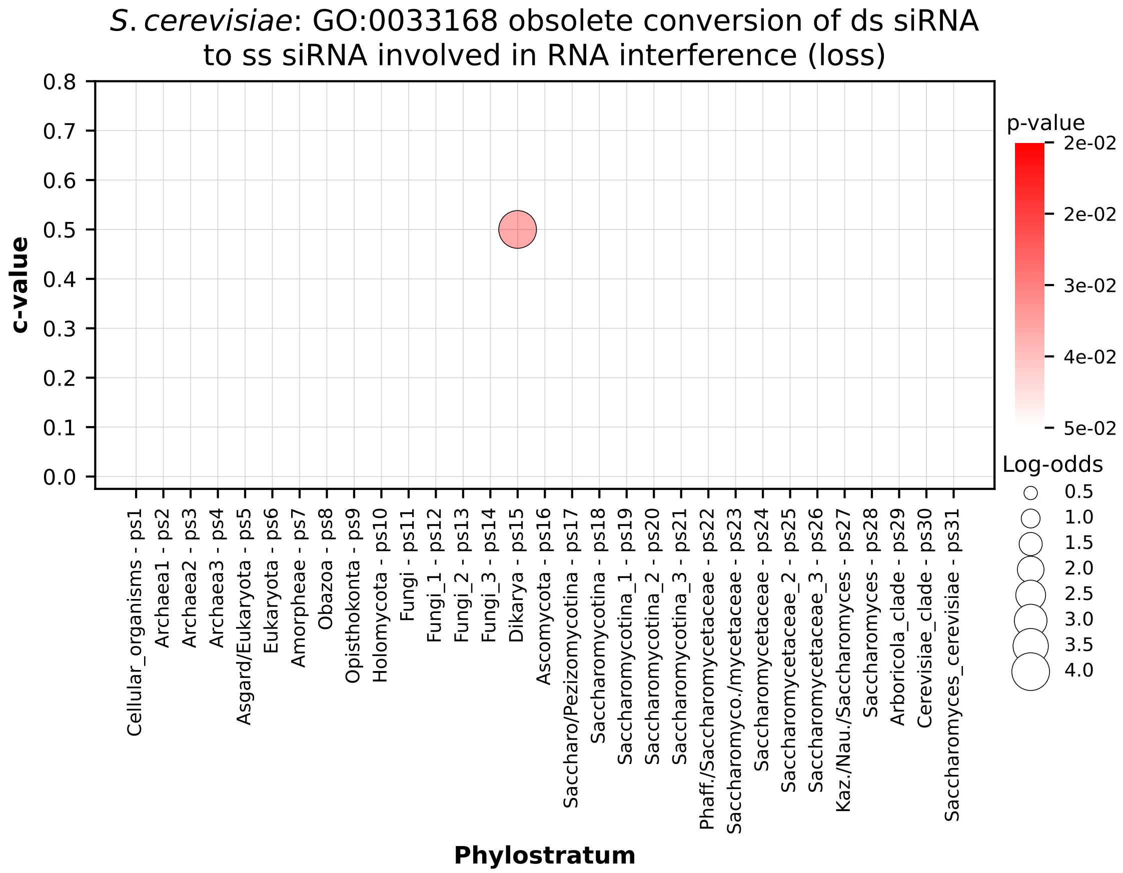
S. cerevisiae: GO:0033168 obsolete conversion of ds siRNA to ss siRNA involved in RNA interference (loss)
c-value
0.0
0.1
0.2
0.3
0.4
0.5
0.6
0.7
0.8
Phylostratum
Cellular_organisms - ps1
Archaea1 - ps2
Archaea2 - ps3
Archaea3 - ps4
Asgard/Eukaryota - ps5
Eukaryota - ps6
Amorpheae - ps7
Obazoa - ps8
Opisthokonta - ps9
Holomycota - ps10
Fungi - ps11
Fungi_1 - ps12
Fungi_2 - ps13
Fungi_3 - ps14
Dikarya - ps15
Ascomycota - ps16
Saccharo/Pezizomycotina - ps17
Saccharomycotina - ps18
Saccharomycotina_1 - ps19
Saccharomycotina_2 - ps20
Saccharomycotina_3 - ps21
Phaff./Saccharomycetaceae - ps22
Saccharomyco./mycetaceae - ps23
Saccharomycetaceae - ps24
Saccharomycetaceae_2 - ps25
Saccharomycetaceae_3 - ps26
Kaz./Nau./Saccharomyces - ps27
Saccharomyces - ps28
Arboricola_clade - ps29
Cerevisiae_clade - ps30
Saccharomyces_cerevisiae - ps31
p-value
2e-02
2e-02
3e-02
4e-02
5e-02
Log-odds
0.5
1.0
1.5
2.0
2.5
3.0
3.5
4.0

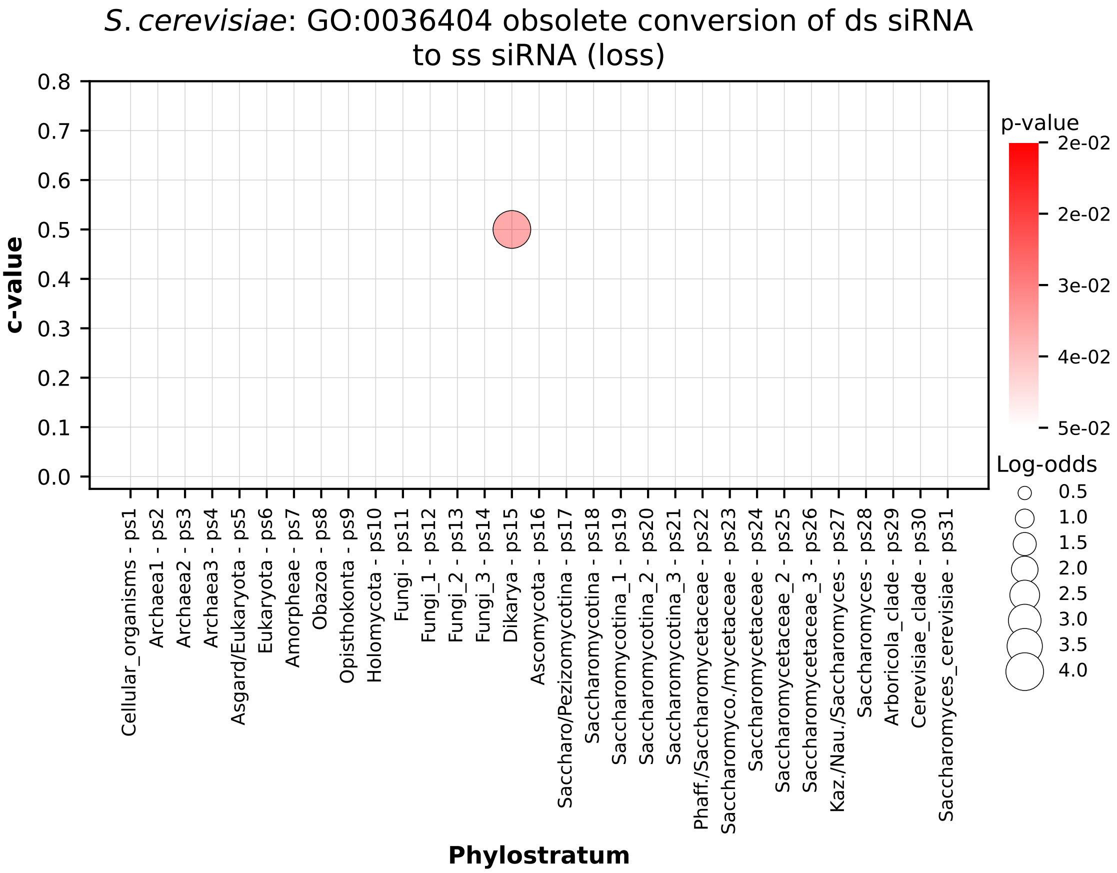
S. cerevisiae: GO:0036404 obsolete conversion of ds siRNA to ss siRNA (loss)
c-value
0.8
0.7
0.6
0.5
0.4
0.3
0.2
0.1
0.0
p-value
2e-02
2e-02
3e-02
4e-02
5e-02
Log-odds
0.5
1.0
1.5
2.0
2.5
3.0
3.5
4.0
Cellular_organisms - ps1
Archaea1 - ps2
Archaea2 - ps3
Archaea3 - ps4
Asgard/Eukaryota - ps5
Eukaryota - ps6
Amorpheae - ps7
Obazoa - ps8
Opisthokonta - ps9
Holomycota - ps10
Fungi - ps11
Fungi_1 - ps12
Fungi_2 - ps13
Fungi_3 - ps14
Dikarya - ps15
Ascomycota - ps16
Saccharo/Pezizomycotina - ps17
Saccharomycotina - ps18
Saccharomycotina_1 - ps19
Saccharomycotina_2 - ps20
Saccharomycotina_3 - ps21
Phaff./Saccharomycetaceae - ps22
Saccharomyco./mycetaceae - ps23
Saccharomycetaceae - ps24
Saccharomycetaceae_2 - ps25
Saccharomycetaceae_3 - ps26
Kaz./Nau./Saccharomyces - ps27
Saccharomyces - ps28
Arboricola_clade - ps29
Cerevisiae_clade - ps30
Saccharomyces_cerevisiae - ps31
Phylostratum

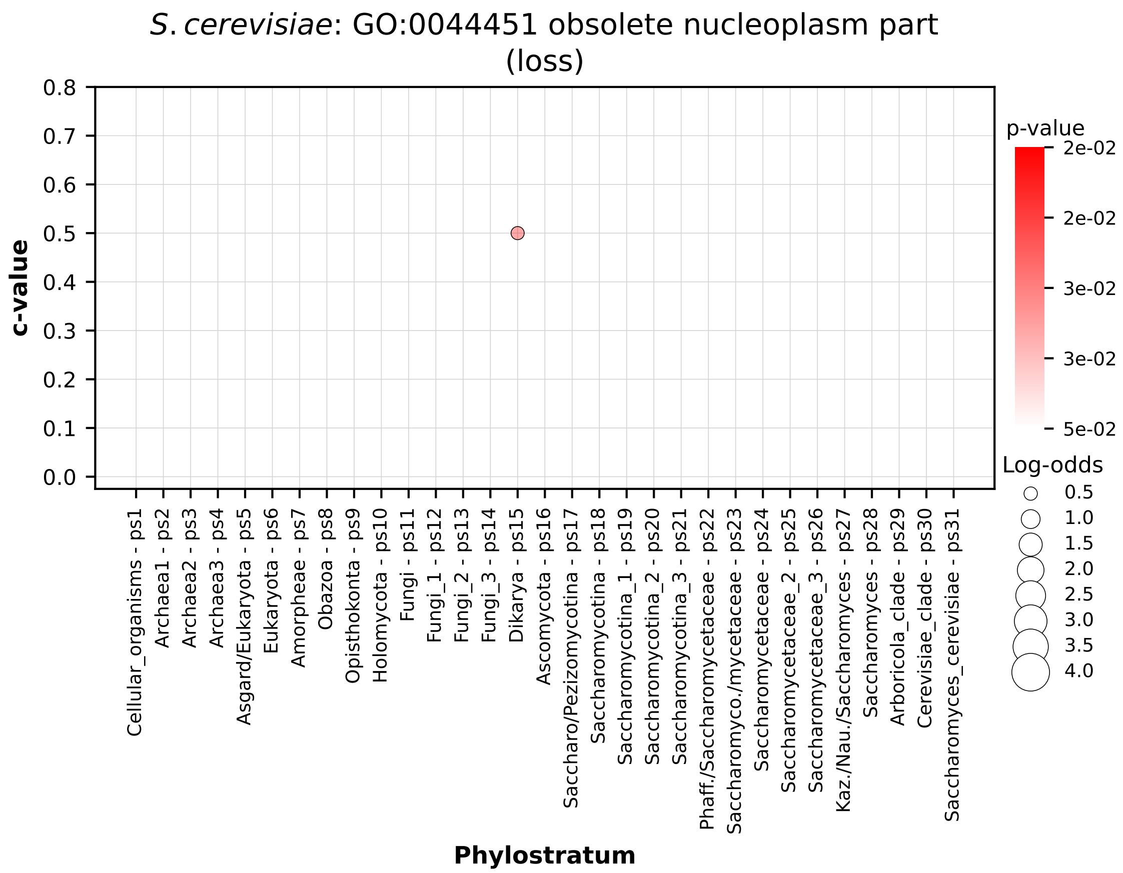

S. cerevisiae: GO:0044451 obsolete nucleoplasm part
(loss)
c-value
0.8
0.7
0.6
0.5
0.4
0.3
0.2
0.1
0.0
p-value
2e-02
2e-02
3e-02
3e-02
5e-02
Log-odds
0.5
1.0
1.5
2.0
2.5
3.0
3.5
4.0
Cellular_organisms - ps1
Archaea1 - ps2
Archaea2 - ps3
Archaea3 - ps4
Asgard/Eukaryota - ps5
Eukaryota - ps6
Amorpheae - ps7
Obazoa - ps8
Opisthokonta - ps9
Holomycota - ps10
Fungi - ps11
Fungi_1 - ps12
Fungi_2 - ps13
Fungi_3 - ps14
Dikarya - ps15
Ascomycota - ps16
Saccharo/Pezizomycotina - ps17
Saccharomycotina - ps18
Saccharomycotina_1 - ps19
Saccharomycotina_2 - ps20
Saccharomycotina_3 - ps21
Phaff./Saccharomycetaceae - ps22
Saccharomyco./mycetaceae - ps23
Saccharomycetaceae - ps24
Saccharomycetaceae_2 - ps25
Saccharomycetaceae_3 - ps26
Kaz./Nau./Saccharomyces - ps27
Saccharomyces - ps28
Arboricola_clade - ps29
Cerevisiae_clade - ps30
Saccharomyces_cerevisiae - ps31
Phylostratum

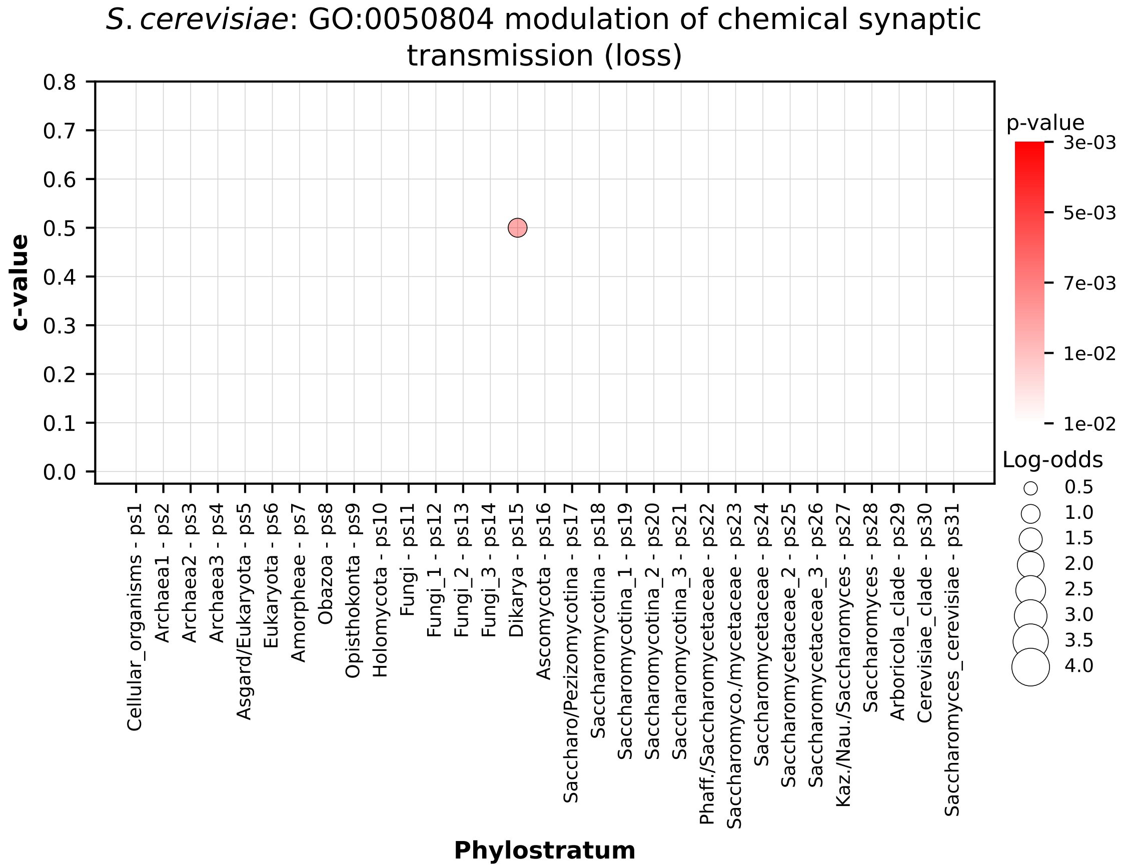

S. cerevisiae: GO:0050804 modulation of chemical synaptic transmission (loss)
c-value
0.8
0.7
0.6
0.5
0.4
0.3
0.2
0.1
0.0
Phylostratum
Cellular_organisms - ps1
Archaea1 - ps2
Archaea2 - ps3
Archaea3 - ps4
Asgard/Eukaryota - ps5
Eukaryota - ps6
Amorpheae - ps7
Obazoa - ps8
Opisthokonta - ps9
Holomycota - ps10
Fungi - ps11
Fungi_1 - ps12
Fungi_2 - ps13
Fungi_3 - ps14
Dikarya - ps15
Ascomycota - ps16
Saccharo/Pezizomycotina - ps17
Saccharomycotina - ps18
Saccharomycotina_1 - ps19
Saccharomycotina_2 - ps20
Saccharomycotina_3 - ps21
Phaff./Saccharomycetaceae - ps22
Saccharomyco./mycetaceae - ps23
Saccharomycetaceae - ps24
Saccharomycetaceae_2 - ps25
Saccharomycetaceae_3 - ps26
Kaz./Nau./Saccharomyces - ps27
Saccharomyces - ps28
Arboricola_clade - ps29
Cerevisiae_clade - ps30
Saccharomyces_cerevisiae - ps31
p-value
3e-03
5e-03
7e-03
1e-02
1e-02
Log-odds
0.5
1.0
1.5
2.0
2.5
3.0
3.5
4.0

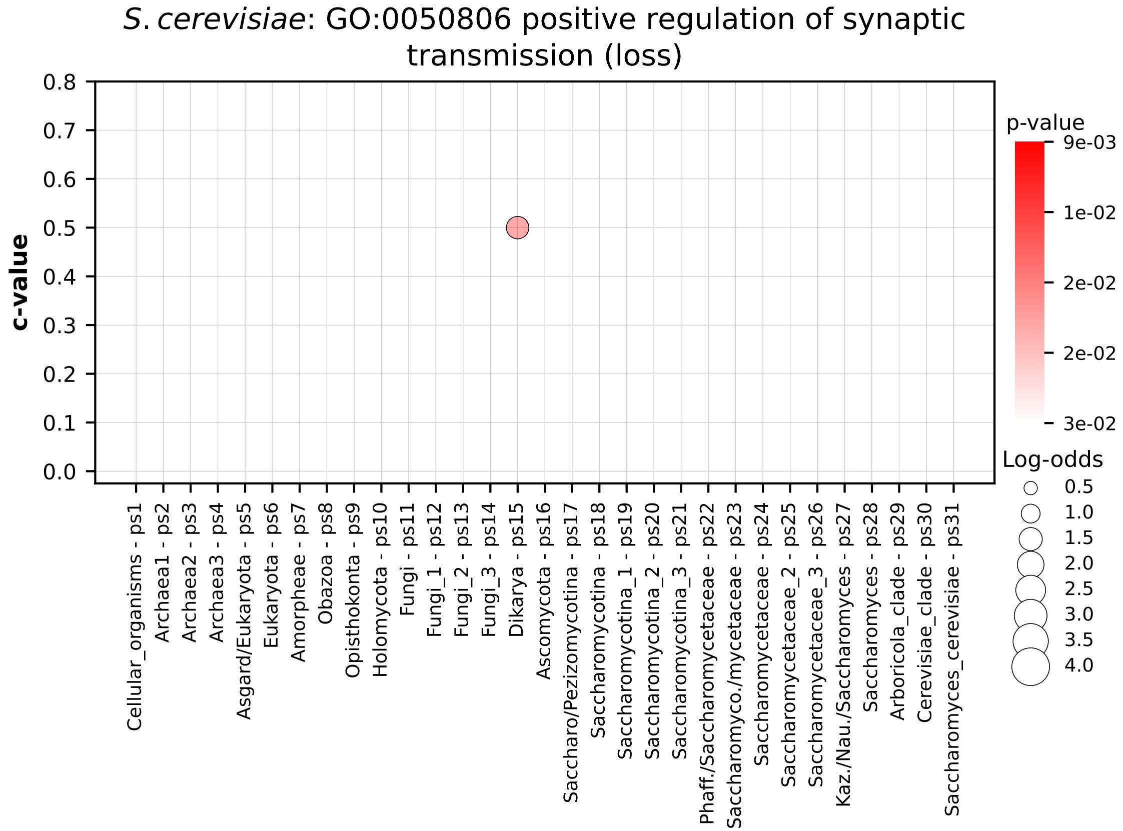

S. cerevisiae: GO:0050806 positive regulation of synaptic transmission (loss)
c-value
0.8
0.7
0.6
0.5
0.4
0.3
0.2
0.1
0.0
Phylostratum
Cellular_organisms - ps1
Archaea1 - ps2
Archaea2 - ps3
Archaea3 - ps4
Asgard/Eukaryota - ps5
Eukaryota - ps6
Amorpheae - ps7
Obazoa - ps8
Opisthokonta - ps9
Holomycota - ps10
Fungi - ps11
Fungi_1 - ps12
Fungi_2 - ps13
Fungi_3 - ps14
Dikarya - ps15
Ascomycota - ps16
Saccharo/Pezizomycotina - ps17
Saccharomycotina - ps18
Saccharomycotina_1 - ps19
Saccharomycotina_2 - ps20
Saccharomycotina_3 - ps21
Phaff./Saccharomycetaceae - ps22
Saccharomyco./mycetaceae - ps23
Saccharomycetaceae - ps24
Saccharomycetaceae_2 - ps25
Saccharomycetaceae_3 - ps26
Kaz./Nau./Saccharomyces - ps27
Saccharomyces - ps28
Arboricola_clade - ps29
Cerevisiae_clade - ps30
Saccharomyces_cerevisiae - ps31
p-value
9e-03
1e-02
2e-02
2e-02
3e-02
Log-odds
0.5
1.0
1.5
2.0
2.5
3.0
3.5
4.0

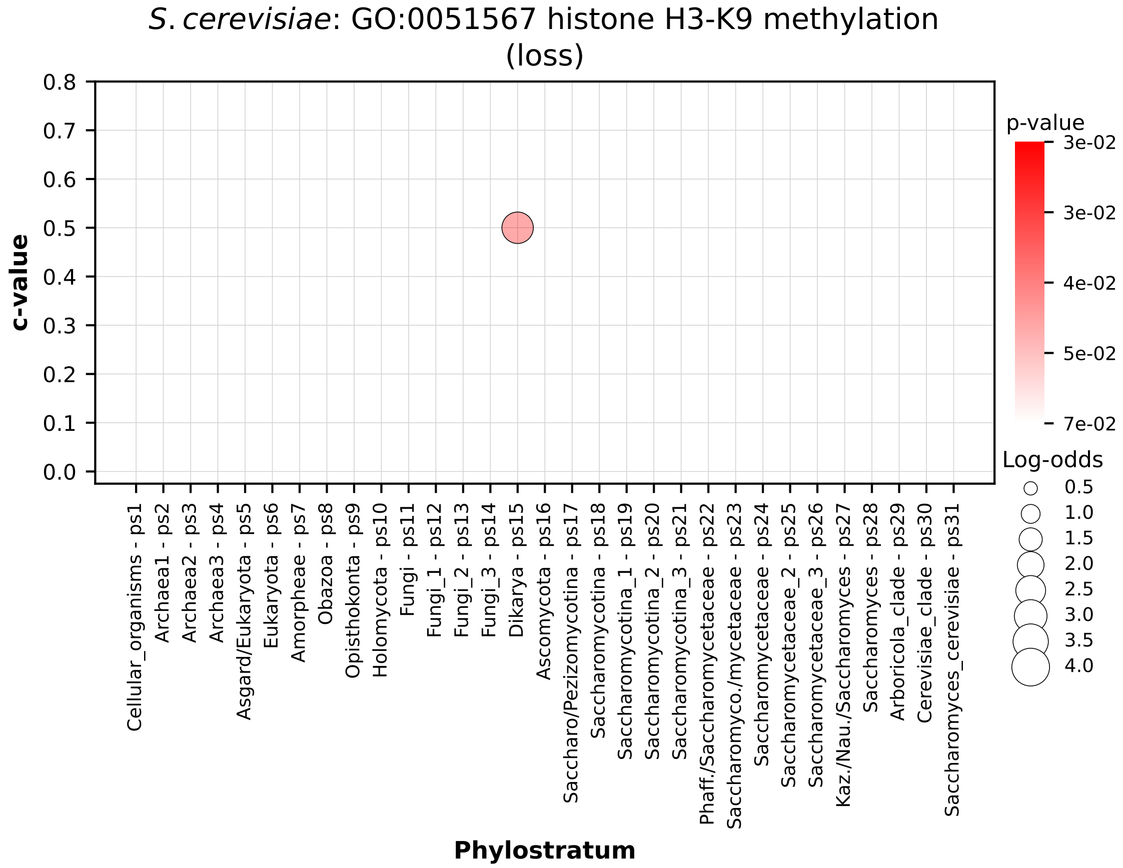
S. cerevisiae: GO:0051567 histone H3-K9 methylation
(loss)
c-value
0.8
0.7
0.6
0.5
0.4
0.3
0.2
0.1
0.0
p-value
3e-02
3e-02
4e-02
5e-02
7e-02
Log-odds
0.5
1.0
1.5
2.0
2.5
3.0
3.5
4.0
Cellular_organisms - ps1
Archaea1 - ps2
Archaea2 - ps3
Archaea3 - ps4
Asgard/Eukaryota - ps5
Eukaryota - ps6
Amorpheae - ps7
Obazoa - ps8
Opisthokonta - ps9
Holomycota - ps10
Fungi - ps11
Fungi_1 - ps12
Fungi_2 - ps13
Fungi_3 - ps14
Dikarya - ps15
Ascomycota - ps16
Saccharo/Pezizomycotina - ps17
Saccharomycotina - ps18
Saccharomycotina_1 - ps19
Saccharomycotina_2 - ps20
Saccharomycotina_3 - ps21
Phaff./Saccharomycetaceae - ps22
Saccharomyco./mycetaceae - ps23
Saccharomycetaceae - ps24
Saccharomycetaceae_2 - ps25
Saccharomycetaceae_3 - ps26
Kaz./Nau./Saccharomyces - ps27
Saccharomyces - ps28
Arboricola_clade - ps29
Cerevisiae_clade - ps30
Saccharomyces_cerevisiae - ps31
Phylostratum

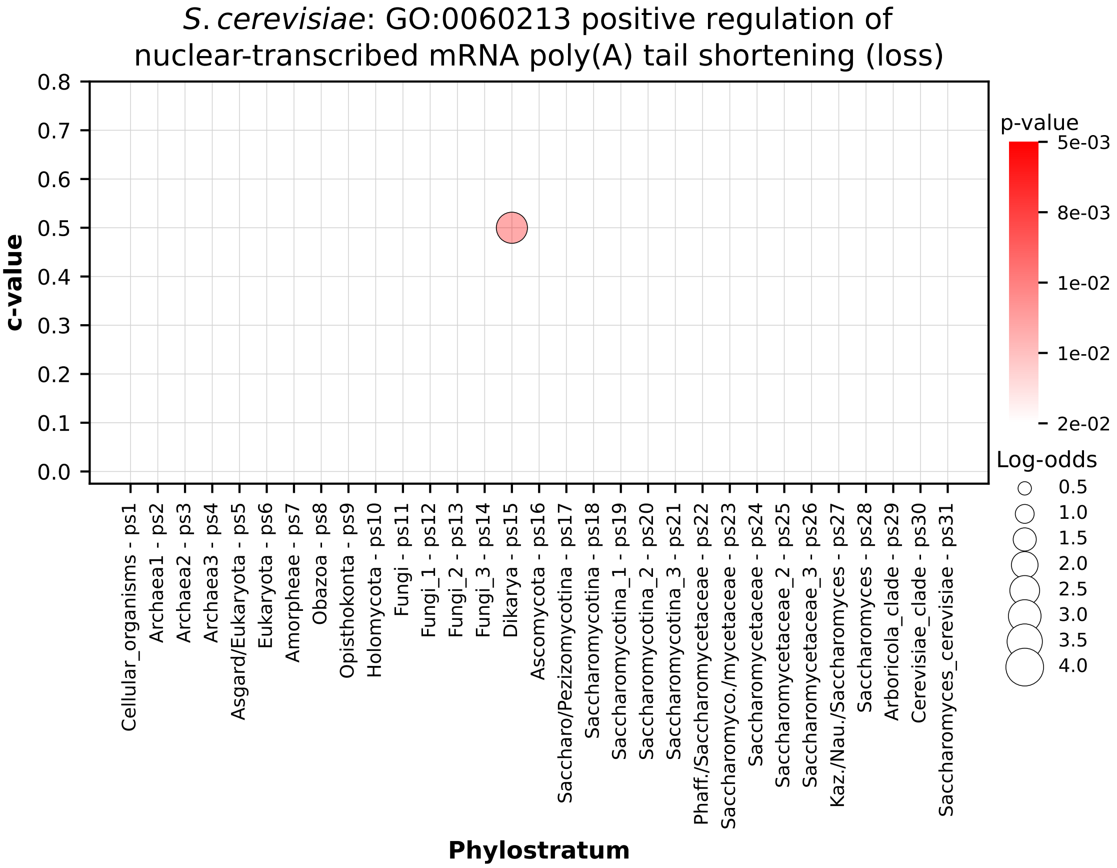

S. cerevisiae: GO:0060213 positive regulation of nuclear-transcribed mRNA poly(A) tail shortening (loss)
c-value
0.8
0.7
0.6
0.5
0.4
0.3
0.2
0.1
0.0
p-value
5e-03
8e-03
1e-02
1e-02
2e-02
Log-odds
0.5
1.0
1.5
2.0
2.5
3.0
3.5
4.0
Cellular_organisms - ps1
Archaea1 - ps2
Archaea2 - ps3
Archaea3 - ps4
Asgard/Eukaryota - ps5
Eukaryota - ps6
Amorpheae - ps7
Obazoa - ps8
Opisthokonta - ps9
Holomycota - ps10
Fungi - ps11
Fungi_1 - ps12
Fungi_2 - ps13
Fungi_3 - ps14
Dikarya - ps15
Ascomycota - ps16
Saccharo/Pezizomycotina - ps17
Saccharomycotina - ps18
Saccharomycotina_1 - ps19
Saccharomycotina_2 - ps20
Saccharomycotina_3 - ps21
Phaff./Saccharomycetaceae - ps22
Saccharomyco./mycetaceae - ps23
Saccharomycetaceae - ps24
Saccharomycetaceae_2 - ps25
Saccharomycetaceae_3 - ps26
Kaz./Nau./Saccharomyces - ps27
Saccharomyces - ps28
Arboricola_clade - ps29
Cerevisiae_clade - ps30
Saccharomyces_cerevisiae - ps31
Phylostratum

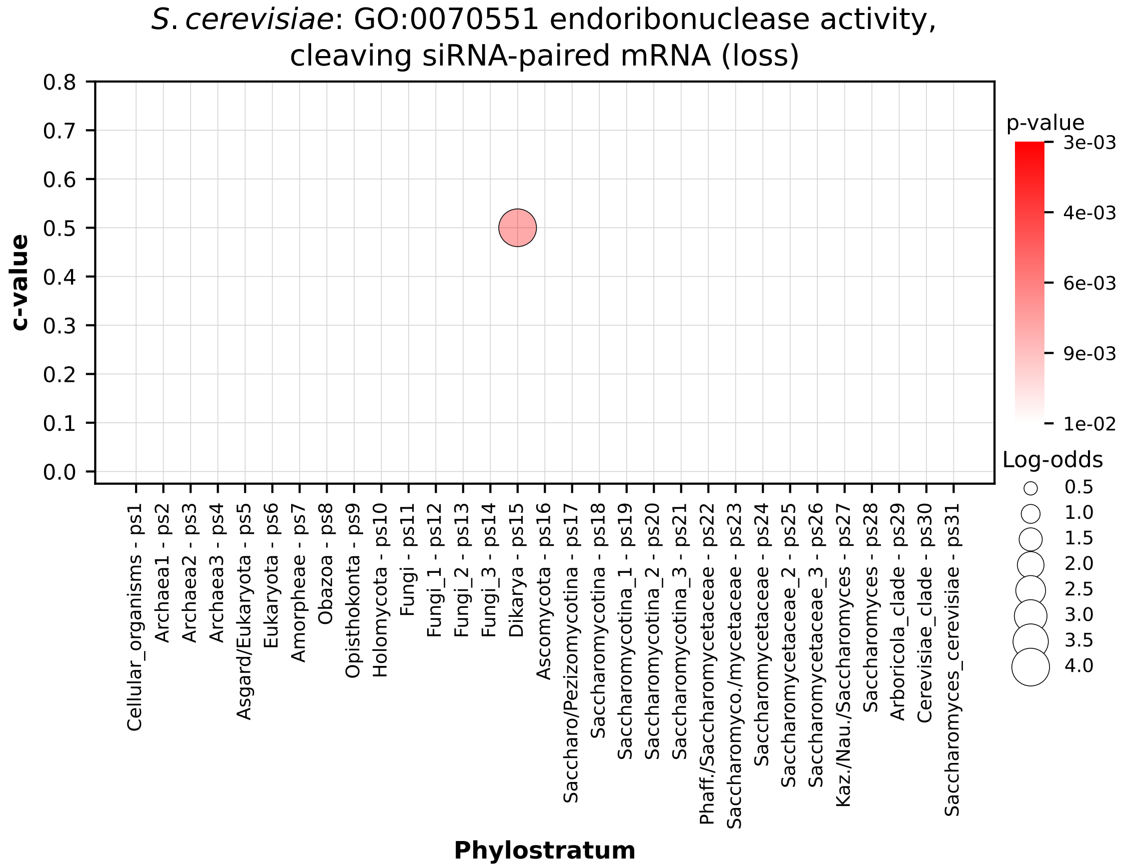

S. cerevisiae: GO:0070551 endoribonuclease activity, cleaving siRNA-paired mRNA (loss)
c-value
0.0
0.1
0.2
0.3
0.4
0.5
0.6
0.7
0.8
Phylostratum
Cellular_organisms - ps1
Archaea1 - ps2
Archaea2 - ps3
Archaea3 - ps4
Asgard/Eukaryota - ps5
Eukaryota - ps6
Amorpheae - ps7
Obazoa - ps8
Opisthokonta - ps9
Holomycota - ps10
Fungi - ps11
Fungi_1 - ps12
Fungi_2 - ps13
Fungi_3 - ps14
Dikarya - ps15
Ascomycota - ps16
Saccharo/Pezizomycotina - ps17
Saccharomycotina - ps18
Saccharomycotina_1 - ps19
Saccharomycotina_2 - ps20
Saccharomycotina_3 - ps21
Phaff./Saccharomycetaceae - ps22
Saccharomyco./mycetaceae - ps23
Saccharomycetaceae - ps24
Saccharomycetaceae_2 - ps25
Saccharomycetaceae_3 - ps26
Kaz./Nau./Saccharomyces - ps27
Saccharomyces - ps28
Arboricola_clade - ps29
Cerevisiae_clade - ps30
Saccharomyces_cerevisiae - ps31
p-value
3e-03
4e-03
6e-03
9e-03
1e-02
Log-odds
0.5
1.0
1.5
2.0
2.5
3.0
3.5
4.0

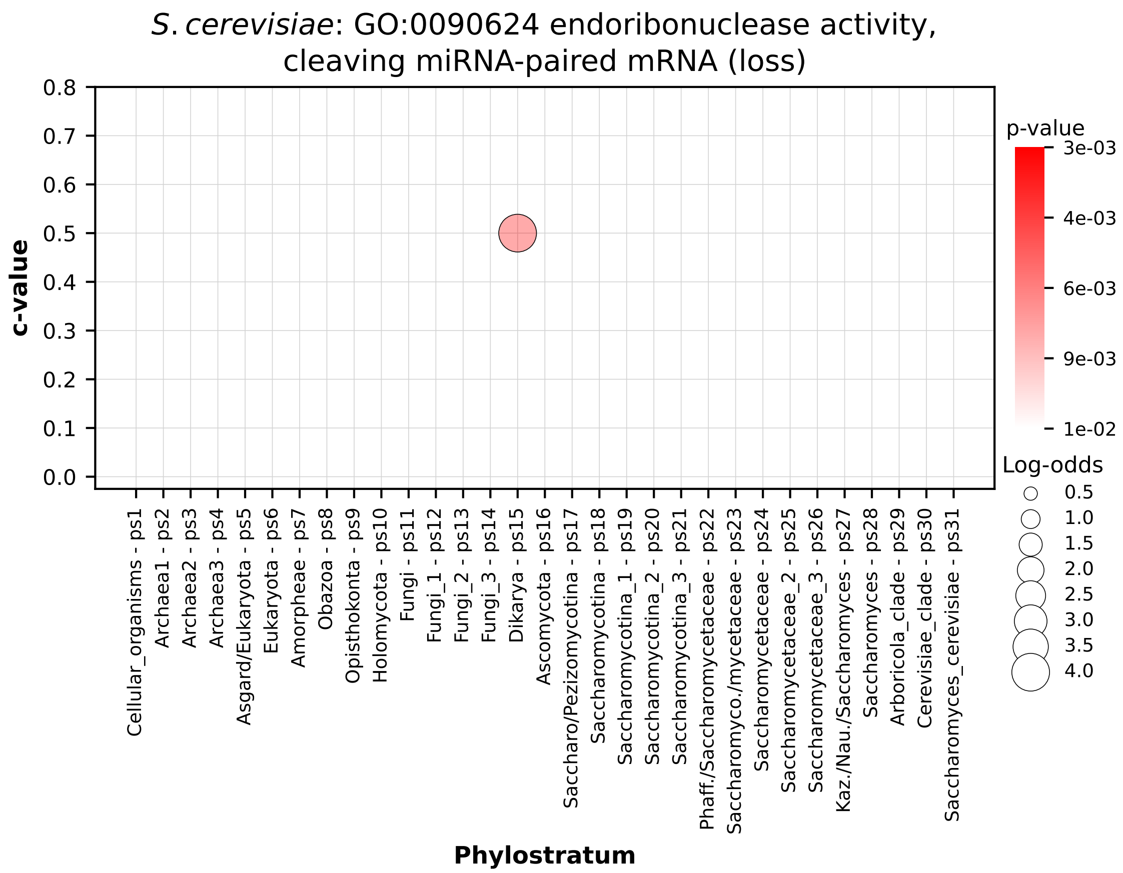

S. cerevisiae: GO:0090624 endoribonuclease activity, cleaving miRNA-paired mRNA (loss)
c-value
0.8
0.7
0.6
0.5
0.4
0.3
0.2
0.1
0.0
Phylostratum
Cellular_organisms - ps1
Archaea1 - ps2
Archaea2 - ps3
Archaea3 - ps4
Asgard/Eukaryota - ps5
Eukaryota - ps6
Amorpheae - ps7
Obazoa - ps8
Opisthokonta - ps9
Holomycota - ps10
Fungi - ps11
Fungi_1 - ps12
Fungi_2 - ps13
Fungi_3 - ps14
Dikarya - ps15
Ascomycota - ps16
Saccharo/Pezizomycotina - ps17
Saccharomycotina - ps18
Saccharomycotina_1 - ps19
Saccharomycotina_2 - ps20
Saccharomycotina_3 - ps21
Phaff./Saccharomycetaceae - ps22
Saccharomyco./mycetaceae - ps23
Saccharomycetaceae - ps24
Saccharomycetaceae_2 - ps25
Saccharomycetaceae_3 - ps26
Kaz./Nau./Saccharomyces - ps27
Saccharomyces - ps28
Arboricola_clade - ps29
Cerevisiae_clade - ps30
Saccharomyces_cerevisiae - ps31
p-value
3e-03
4e-03
6e-03
9e-03
1e-02
Log-odds
0.5
1.0
1.5
2.0
2.5
3.0
3.5
4.0

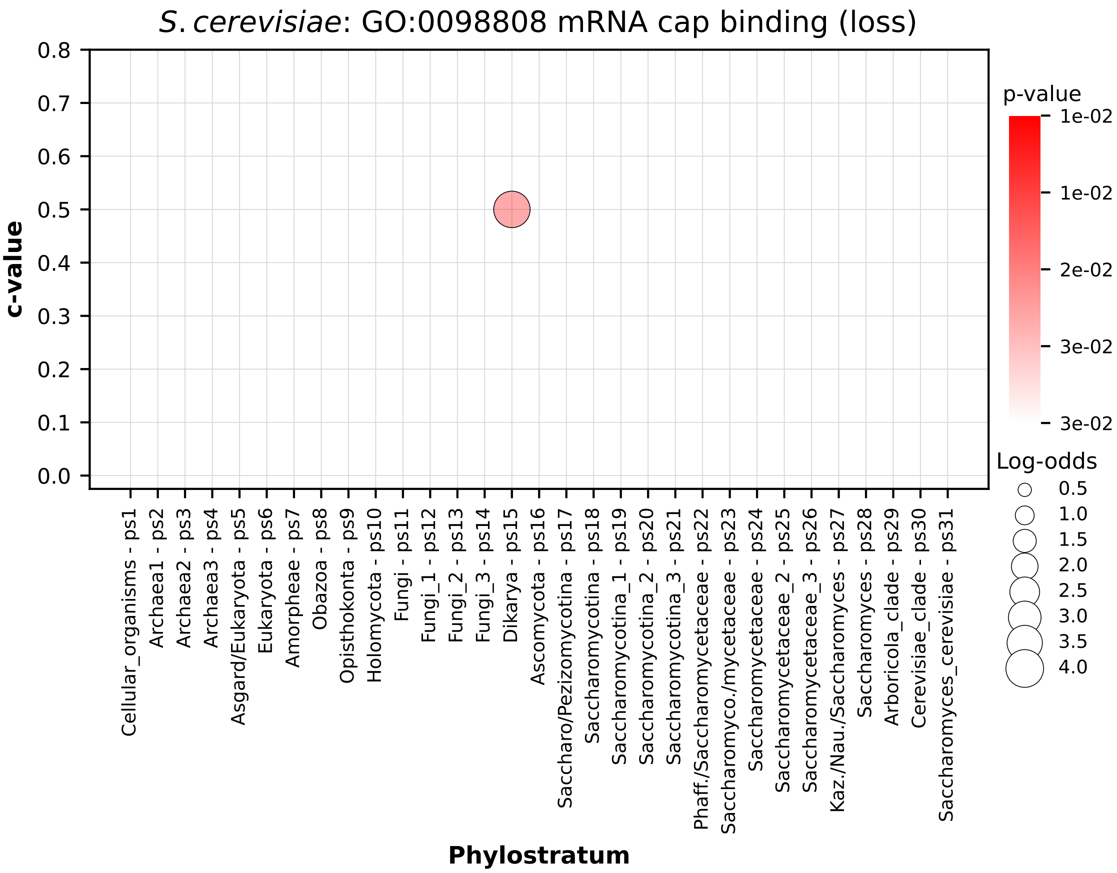
S. cerevisiae: GO:0098808 mRNA cap binding (loss)
c-value
0.8
0.7
0.6
0.5
0.4
0.3
0.2
0.1
0.0
p-value
1e-02
1e-02
2e-02
3e-02
3e-02
Log-odds
0.5
1.0
1.5
2.0
2.5
3.0
3.5
4.0
Cellular_organisms - ps1
Archaea1 - ps2
Archaea2 - ps3
Archaea3 - ps4
Asgard/Eukaryota - ps5
Eukaryota - ps6
Amorpheae - ps7
Obazoa - ps8
Opisthokonta - ps9
Holomycota - ps10
Fungi - ps11
Fungi_1 - ps12
Fungi_2 - ps13
Fungi_3 - ps14
Dikarya - ps15
Ascomycota - ps16
Saccharo/Pezizomycotina - ps17
Saccharomycotina - ps18
Saccharomycotina_1 - ps19
Saccharomycotina_2 - ps20
Saccharomycotina_3 - ps21
Phaff./Saccharomycetaceae - ps22
Saccharomyco./mycetaceae - ps23
Saccharomycetaceae - ps24
Saccharomycetaceae_2 - ps25
Saccharomycetaceae_3 - ps26
Kaz./Nau./Saccharomyces - ps27
Saccharomyces - ps28
Arboricola_clade - ps29
Cerevisiae_clade - ps30
Saccharomyces_cerevisiae - ps31
Phylostratum

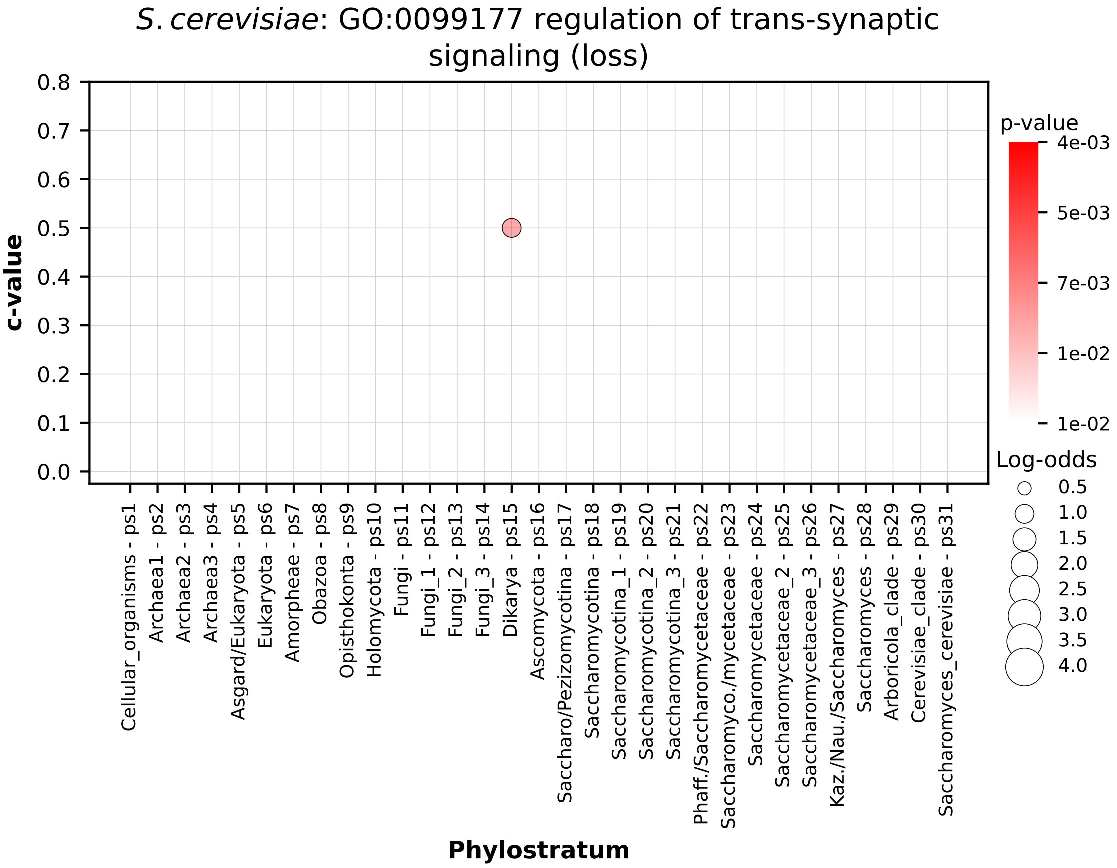
S. cerevisiae: GO:0099177 regulation of trans-synaptic signaling (loss)
c-value
0.0
0.1
0.2
0.3
0.4
0.5
0.6
0.7
0.8
Phylostratum
Cellular_organisms - ps1
Archaea1 - ps2
Archaea2 - ps3
Archaea3 - ps4
Asgard/Eukaryota - ps5
Eukaryota - ps6
Amorpheae - ps7
Obazoa - ps8
Opisthokonta - ps9
Holomycota - ps10
Fungi - ps11
Fungi_1 - ps12
Fungi_2 - ps13
Fungi_3 - ps14
Dikarya - ps15
Ascomycota - ps16
Saccharo/Pezizomycotina - ps17
Saccharomycotina - ps18
Saccharomycotina_1 - ps19
Saccharomycotina_2 - ps20
Saccharomycotina_3 - ps21
Phaff./Saccharomycetaceae - ps22
Saccharomyco./mycetaceae - ps23
Saccharomycetaceae - ps24
Saccharomycetaceae_2 - ps25
Saccharomycetaceae_3 - ps26
Kaz./Nau./Saccharomyces - ps27
Saccharomyces - ps28
Arboricola_clade - ps29
Cerevisiae_clade - ps30
Saccharomyces_cerevisiae - ps31
p-value
4e-03
5e-03
7e-03
1e-02
1e-02
Log-odds
0.5
1.0
1.5
2.0
2.5
3.0
3.5
4.0

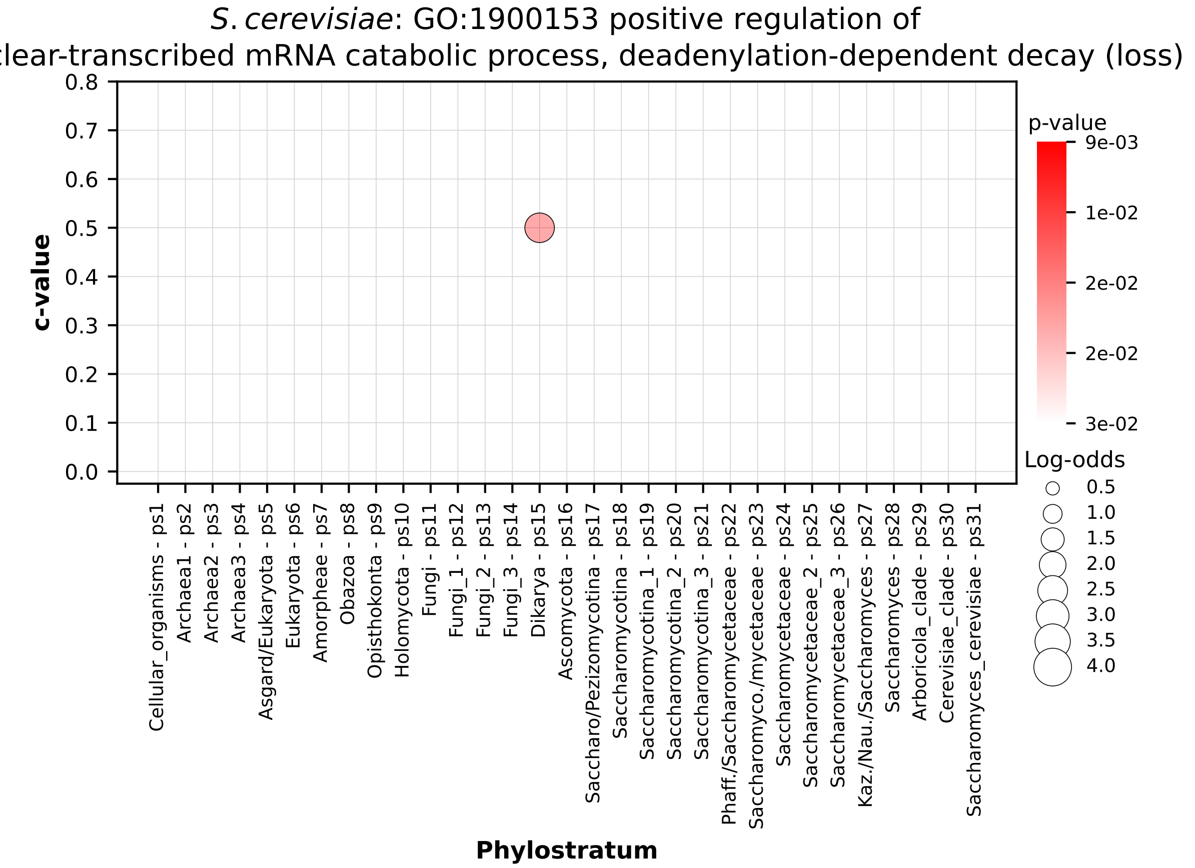

S. cerevisiae: GO:1900153 positive regulation of
clear-transcribed mRNA catabolic process, deadenylation-dependent decay (loss)
c-value
0.0
0.1
0.2
0.3
0.4
0.5
0.6
0.7
0.8
p-value
9e-03
1e-02
2e-02
2e-02
3e-02
Log-odds
0.5
1.0
1.5
2.0
2.5
3.0
3.5
4.0
Cellular_organisms - ps1
Archaea1 - ps2
Archaea2 - ps3
Archaea3 - ps4
Asgard/Eukaryota - ps5
Eukaryota - ps6
Amorpheae - ps7
Obazoa - ps8
Opisthokonta - ps9
Holomycota - ps10
Fungi - ps11
Fungi_1 - ps12
Fungi_2 - ps13
Fungi_3 - ps14
Dikarya - ps15
Ascomycota - ps16
Saccharo/Pezizomycotina - ps17
Saccharomycotina - ps18
Saccharomycotina_1 - ps19
Saccharomycotina_2 - ps20
Saccharomycotina_3 - ps21
Phaff./Saccharomycetaceae - ps22
Saccharomyco./mycetaceae - ps23
Saccharomycetaceae - ps24
Saccharomycetaceae_2 - ps25
Saccharomycetaceae_3 - ps26
Kaz./Nau./Saccharomyces - ps27
Saccharomyces - ps28
Arboricola_clade - ps29
Cerevisiae_clade - ps30
Saccharomyces_cerevisiae - ps31
Phylostratum

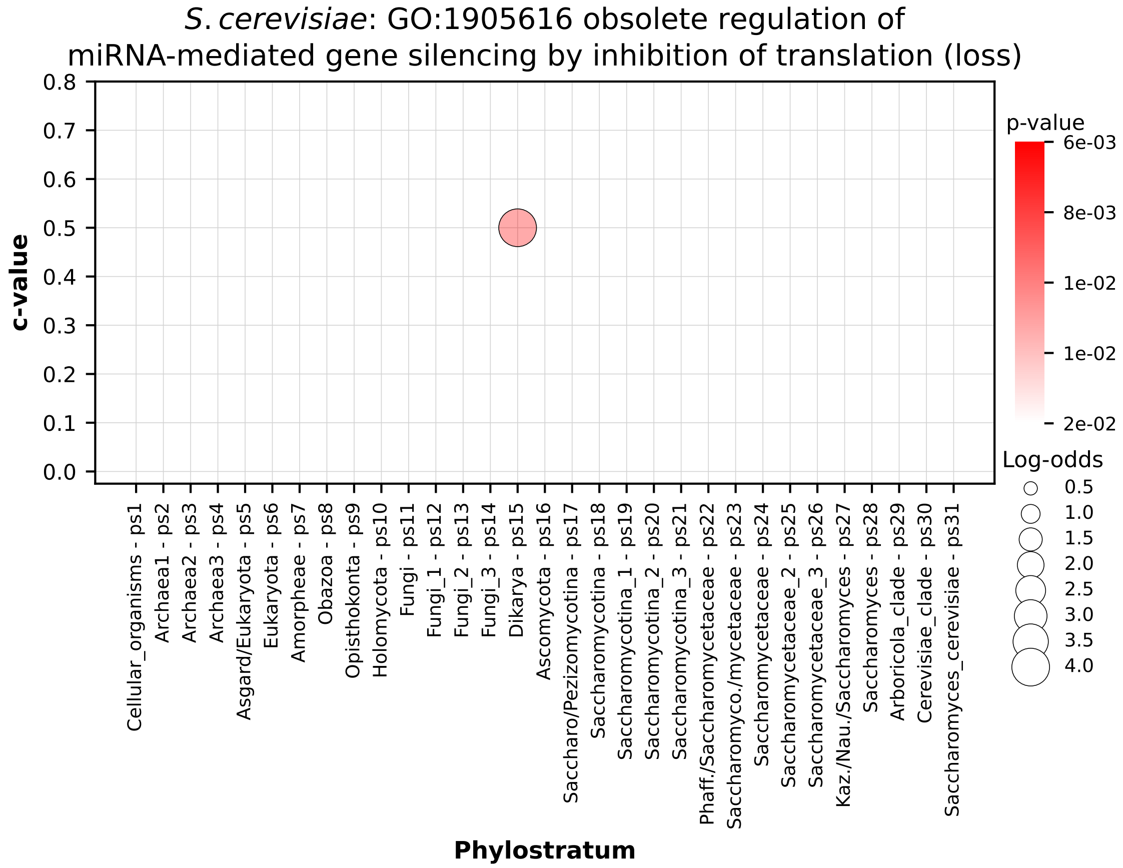

S. cerevisiae: GO:1905616 obsolete regulation of miRNA-mediated gene silencing by inhibition of translation (loss)
c-value
0.8
0.7
0.6
0.5
0.4
0.3
0.2
0.1
0.0
p-value
6e-03
8e-03
1e-02
1e-02
2e-02
Log-odds
0.5
1.0
1.5
2.0
2.5
3.0
3.5
4.0
Cellular_organisms - ps1
Archaea1 - ps2
Archaea2 - ps3
Archaea3 - ps4
Asgard/Eukaryota - ps5
Eukaryota - ps6
Amorpheae - ps7
Obazoa - ps8
Opisthokonta - ps9
Holomycota - ps10
Fungi - ps11
Fungi_1 - ps12
Fungi_2 - ps13
Fungi_3 - ps14
Dikarya - ps15
Ascomycota - ps16
Saccharo/Pezizomycotina - ps17
Saccharomycotina - ps18
Saccharomycotina_1 - ps19
Saccharomycotina_2 - ps20
Saccharomycotina_3 - ps21
Phaff./Saccharomycetaceae - ps22
Saccharomyco./mycetaceae - ps23
Saccharomycetaceae - ps24
Saccharomycetaceae_2 - ps25
Saccharomycetaceae_3 - ps26
Kaz./Nau./Saccharomyces - ps27
Saccharomyces - ps28
Arboricola_clade - ps29
Cerevisiae_clade - ps30
Saccharomyces_cerevisiae - ps31
Phylostratum

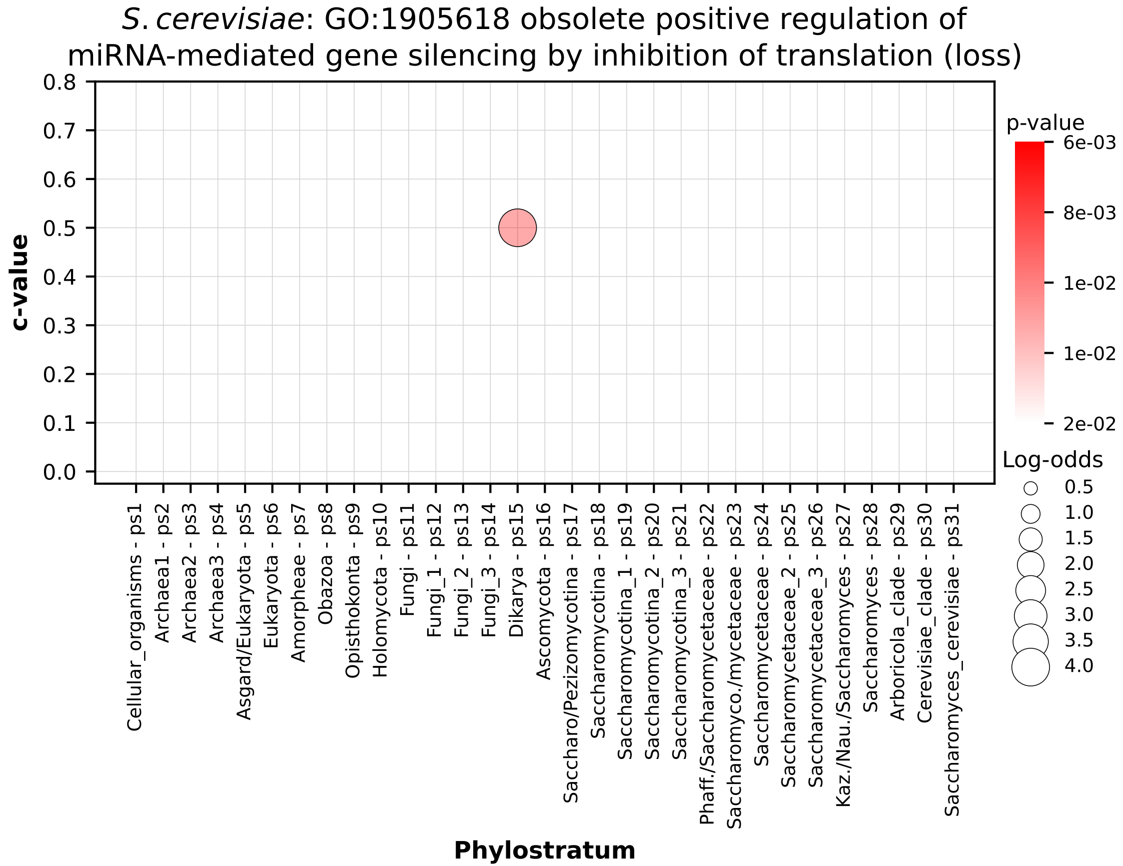

S. cerevisiae: GO:1905618 obsolete positive regulation of miRNA-mediated gene silencing by inhibition of translation (loss)
c-value
0.8
0.7
0.6
0.5
0.4
0.3
0.2
0.1
0.0
Phylostratum
Cellular_organisms - ps1
Archaea1 - ps2
Archaea2 - ps3
Archaea3 - ps4
Asgard/Eukaryota - ps5
Eukaryota - ps6
Amorpheae - ps7
Obazoa - ps8
Opisthokonta - ps9
Holomycota - ps10
Fungi - ps11
Fungi_1 - ps12
Fungi_2 - ps13
Fungi_3 - ps14
Dikarya - ps15
Ascomycota - ps16
Saccharo/Pezizomycotina - ps17
Saccharomycotina - ps18
Saccharomycotina_1 - ps19
Saccharomycotina_2 - ps20
Saccharomycotina_3 - ps21
Phaff./Saccharomycetaceae - ps22
Saccharomyco./mycetaceae - ps23
Saccharomycetaceae - ps24
Saccharomycetaceae_2 - ps25
Saccharomycetaceae_3 - ps26
Kaz./Nau./Saccharomyces - ps27
Saccharomyces - ps28
Arboricola_clade - ps29
Cerevisiae_clade - ps30
Saccharomyces_cerevisiae - ps31
p-value
6e-03
8e-03
1e-02
1e-02
2e-02
Log-odds
0.5
1.0
1.5
2.0
2.5
3.0
3.5
4.0

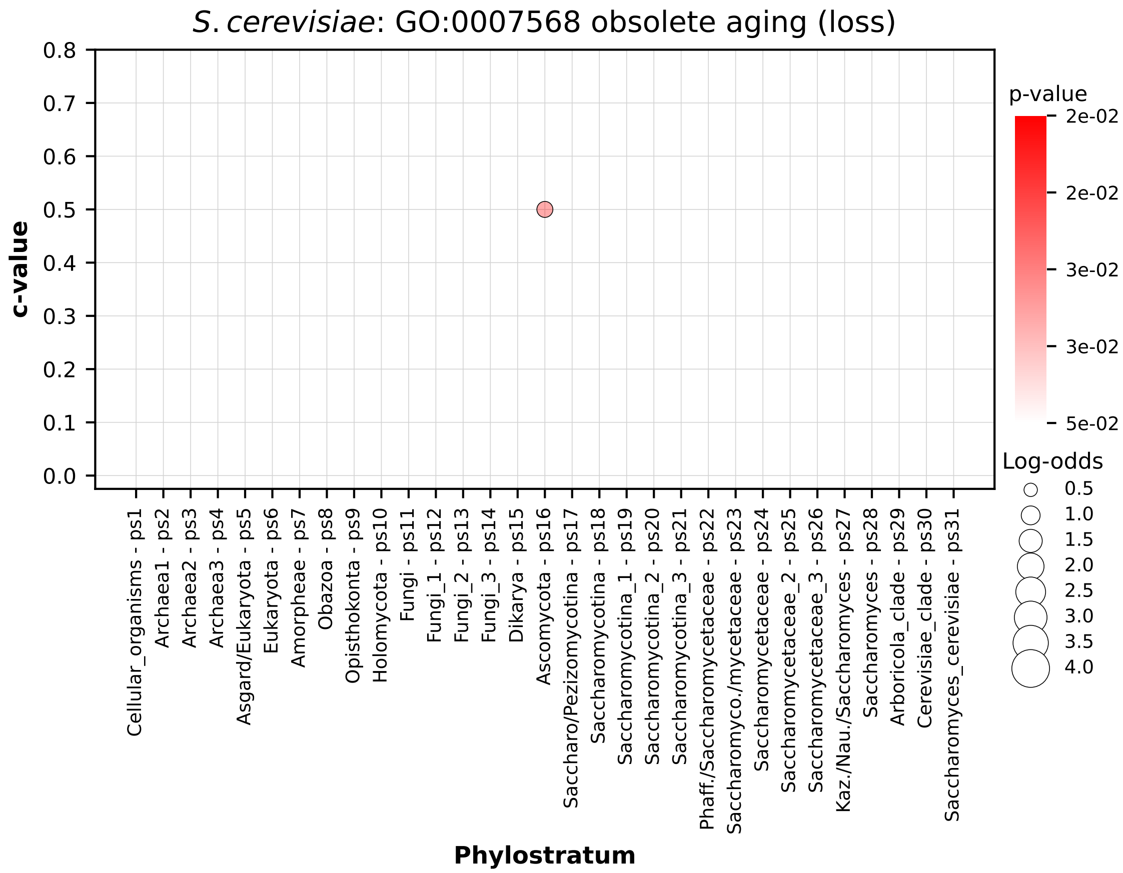
S. cerevisiae: GO:0007568 obsolete aging (loss)
c-value
0.8
0.7
0.6
0.5
0.4
0.3
0.2
0.1
0.0
p-value
2e-02
2e-02
3e-02
3e-02
5e-02
Log-odds
0.5
1.0
1.5
2.0
2.5
3.0
3.5
4.0
Cellular_organisms - ps1
Archaea1 - ps2
Archaea2 - ps3
Archaea3 - ps4
Asgard/Eukaryota - ps5
Eukaryota - ps6
Amorpheae - ps7
Obazoa - ps8
Opisthokonta - ps9
Holomycota - ps10
Fungi - ps11
Fungi_1 - ps12
Fungi_2 - ps13
Fungi_3 - ps14
Dikarya - ps15
Ascomycota - ps16
Saccharo/Pezizomycotina - ps17
Saccharomycotina - ps18
Saccharomycotina_1 - ps19
Saccharomycotina_2 - ps20
Saccharomycotina_3 - ps21
Phaff./Saccharomycetaceae - ps22
Saccharomyco./mycetaceae - ps23
Saccharomycetaceae - ps24
Saccharomycetaceae_2 - ps25
Saccharomycetaceae_3 - ps26
Kaz./Nau./Saccharomyces - ps27
Saccharomyces - ps28
Arboricola_clade - ps29
Cerevisiae_clade - ps30
Saccharomyces_cerevisiae - ps31
Phylostratum

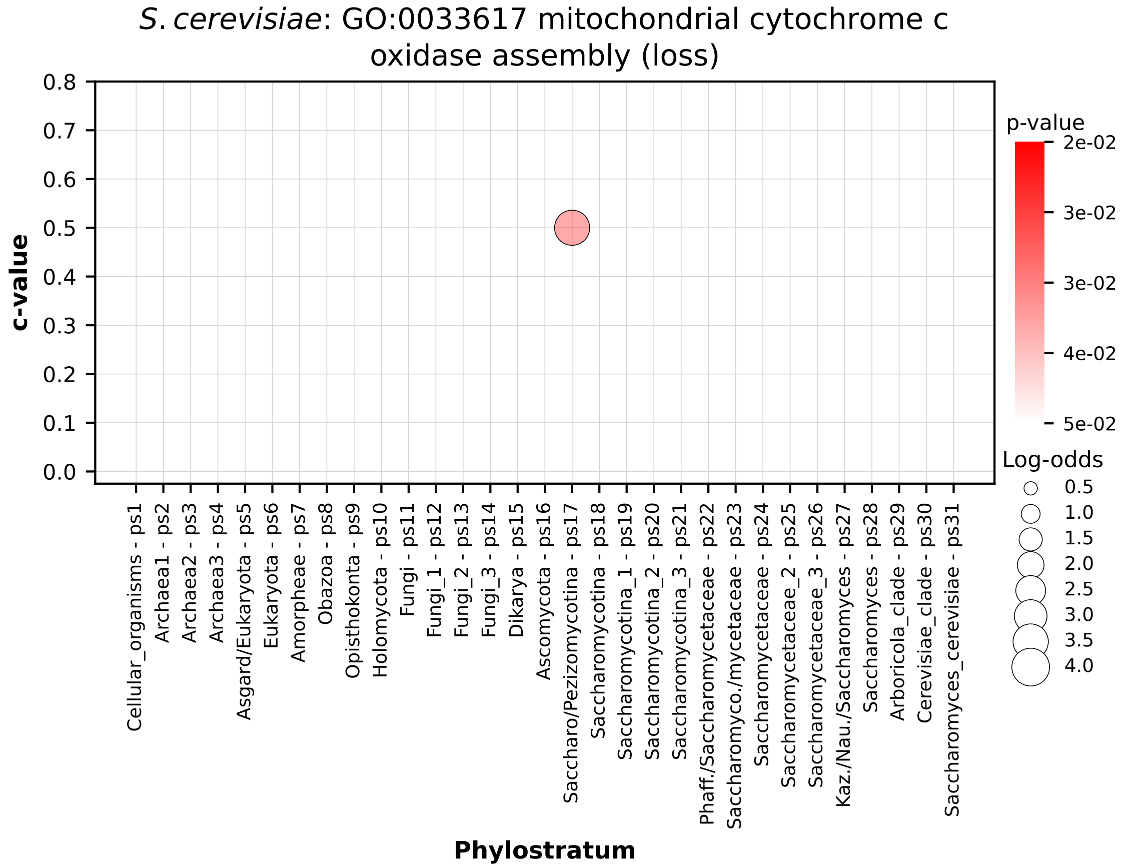
S. cerevisiae: GO:0033617 mitochondrial cytochrome c oxidase assembly (loss)
c-value
0.0
0.1
0.2
0.3
0.4
0.5
0.6
0.7
0.8
p-value
2e-02
3e-02
3e-02
4e-02
5e-02
Log-odds
0.5
1.0
1.5
2.0
2.5
3.0
3.5
4.0
Cellular_organisms - ps1
Archaea1 - ps2
Archaea2 - ps3
Archaea3 - ps4
Asgard/Eukaryota - ps5
Eukaryota - ps6
Amorpheae - ps7
Obazoa - ps8
Opisthokonta - ps9
Holomycota - ps10
Fungi - ps11
Fungi_1 - ps12
Fungi_2 - ps13
Fungi_3 - ps14
Dikarya - ps15
Ascomycota - ps16
Saccharo/Pezizomycotina - ps17
Saccharomycotina - ps18
Saccharomycotina_1 - ps19
Saccharomycotina_2 - ps20
Saccharomycotina_3 - ps21
Phaff./Saccharomycetaceae - ps22
Saccharomyco./mycetaceae - ps23
Saccharomycetaceae - ps24
Saccharomycetaceae_2 - ps25
Saccharomycetaceae_3 - ps26
Kaz./Nau./Saccharomyces - ps27
Saccharomyces - ps28
Arboricola_clade - ps29
Cerevisiae_clade - ps30
Saccharomyces_cerevisiae - ps31
Phylostratum

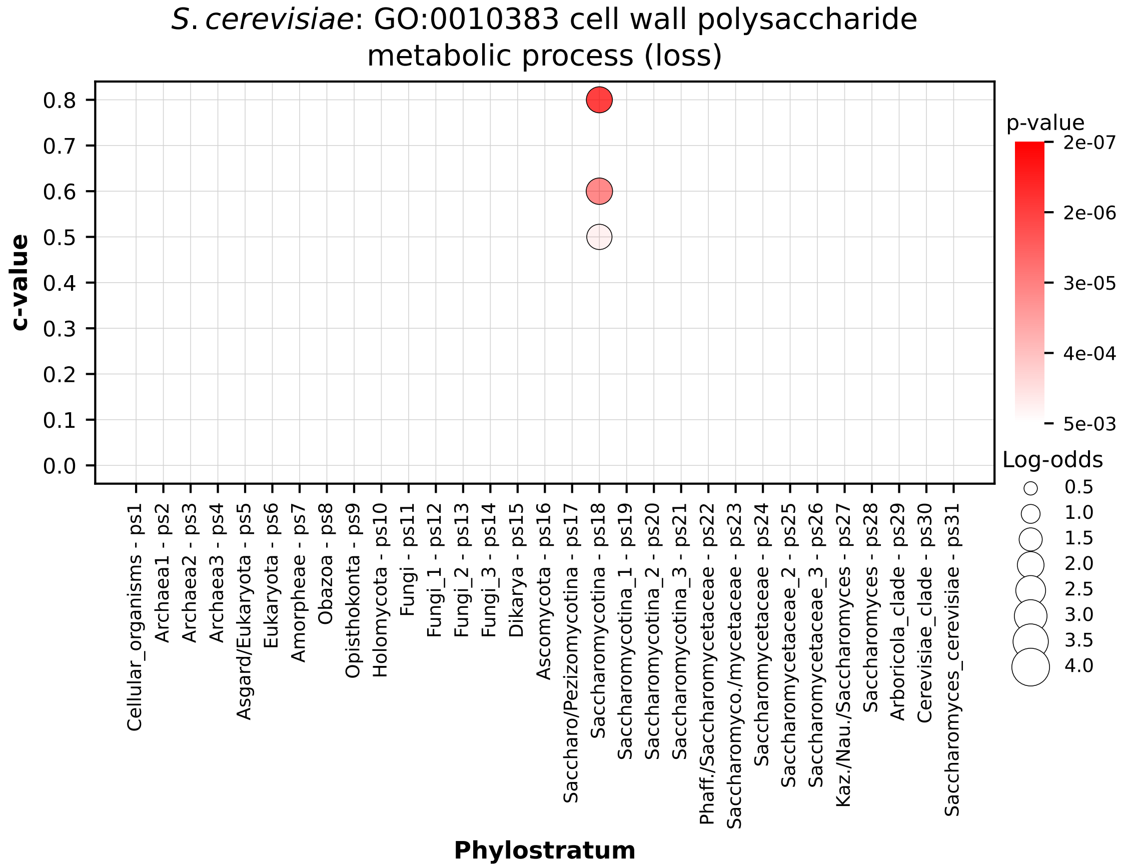

S. cerevisiae: GO:0010383 cell wall polysaccharide metabolic process (loss)
c-value
0.0
0.1
0.2
0.3
0.4
0.5
0.6
0.7
0.8
p-value
2e-07
2e-06
3e-05
4e-04
5e-03
Log-odds
0.5
1.0
1.5
2.0
2.5
3.0
3.5
4.0
Cellular_organisms - ps1
Archaea1 - ps2
Archaea2 - ps3
Archaea3 - ps4
Asgard/Eukaryota - ps5
Eukaryota - ps6
Amorpheae - ps7
Obazoa - ps8
Opisthokonta - ps9
Holomycota - ps10
Fungi - ps11
Fungi_1 - ps12
Fungi_2 - ps13
Fungi_3 - ps14
Dikarya - ps15
Ascomycota - ps16
Saccharo/Pezizomycotina - ps17
Saccharomycotina - ps18
Saccharomycotina_1 - ps19
Saccharomycotina_2 - ps20
Saccharomycotina_3 - ps21
Phaff./Saccharomycetaceae - ps22
Saccharomyco./mycetaceae - ps23
Saccharomycetaceae - ps24
Saccharomycetaceae_2 - ps25
Saccharomycetaceae_3 - ps26
Kaz./Nau./Saccharomyces - ps27
Saccharomyces - ps28
Arboricola_clade - ps29
Cerevisiae_clade - ps30
Saccharomyces_cerevisiae - ps31
Phylostratum

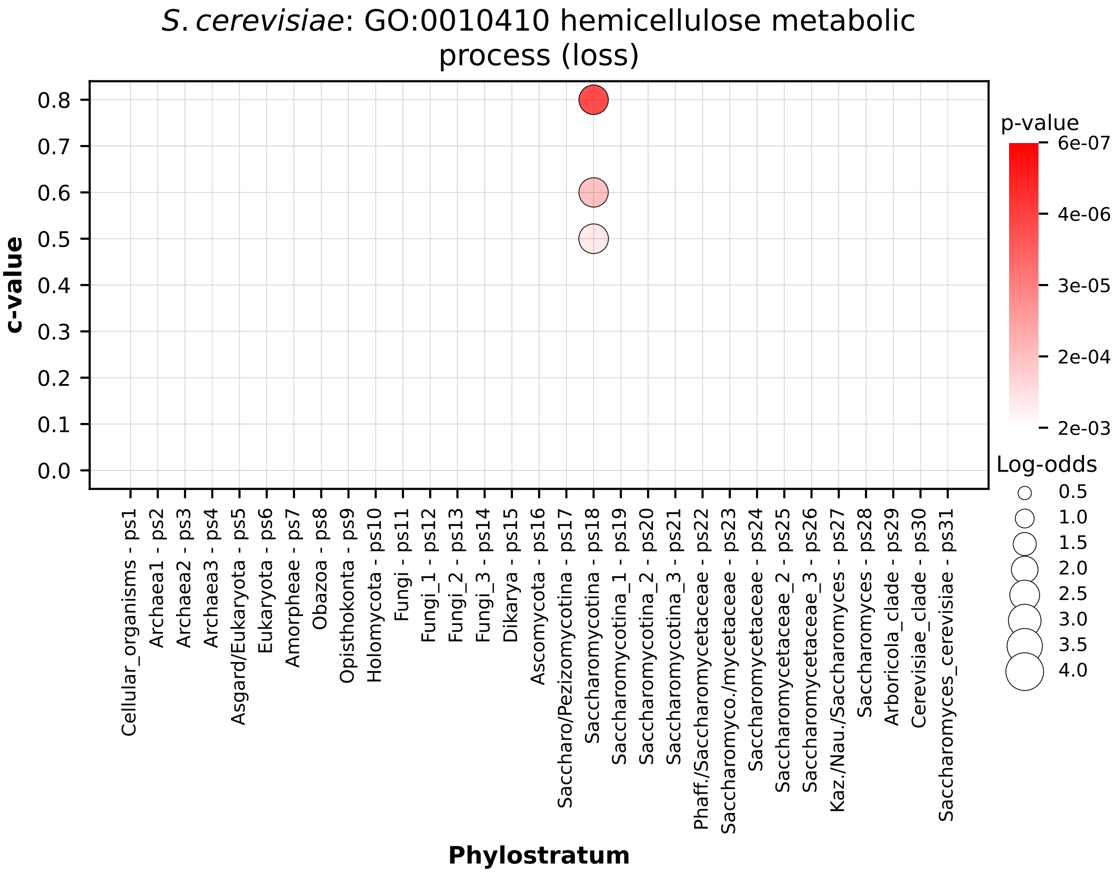
S. cerevisiae: GO:0010410 hemicellulose metabolic process (loss)
c-value
0.0
0.1
0.2
0.3
0.4
0.5
0.6
0.7
0.8
p-value
6e-07
4e-06
3e-05
2e-04
2e-03
Log-odds
0.5
1.0
1.5
2.0
2.5
3.0
3.5
4.0
Cellular_organisms - ps1
Archaea1 - ps2
Archaea2 - ps3
Archaea3 - ps4
Asgard/Eukaryota - ps5
Eukaryota - ps6
Amorpheae - ps7
Obazoa - ps8
Opisthokonta - ps9
Holomycota - ps10
Fungi - ps11
Fungi_1 - ps12
Fungi_2 - ps13
Fungi_3 - ps14
Dikarya - ps15
Ascomycota - ps16
Saccharo/Pezizomycotina - ps17
Saccharomycotina - ps18
Saccharomycotina_1 - ps19
Saccharomycotina_2 - ps20
Saccharomycotina_3 - ps21
Phaff./Saccharomycetaceae - ps22
Saccharomyco./mycetaceae - ps23
Saccharomycetaceae - ps24
Saccharomycetaceae_2 - ps25
Saccharomycetaceae_3 - ps26
Kaz./Nau./Saccharomyces - ps27
Saccharomyces - ps28
Arboricola_clade - ps29
Cerevisiae_clade - ps30
Saccharomyces_cerevisiae - ps31
Phylostratum

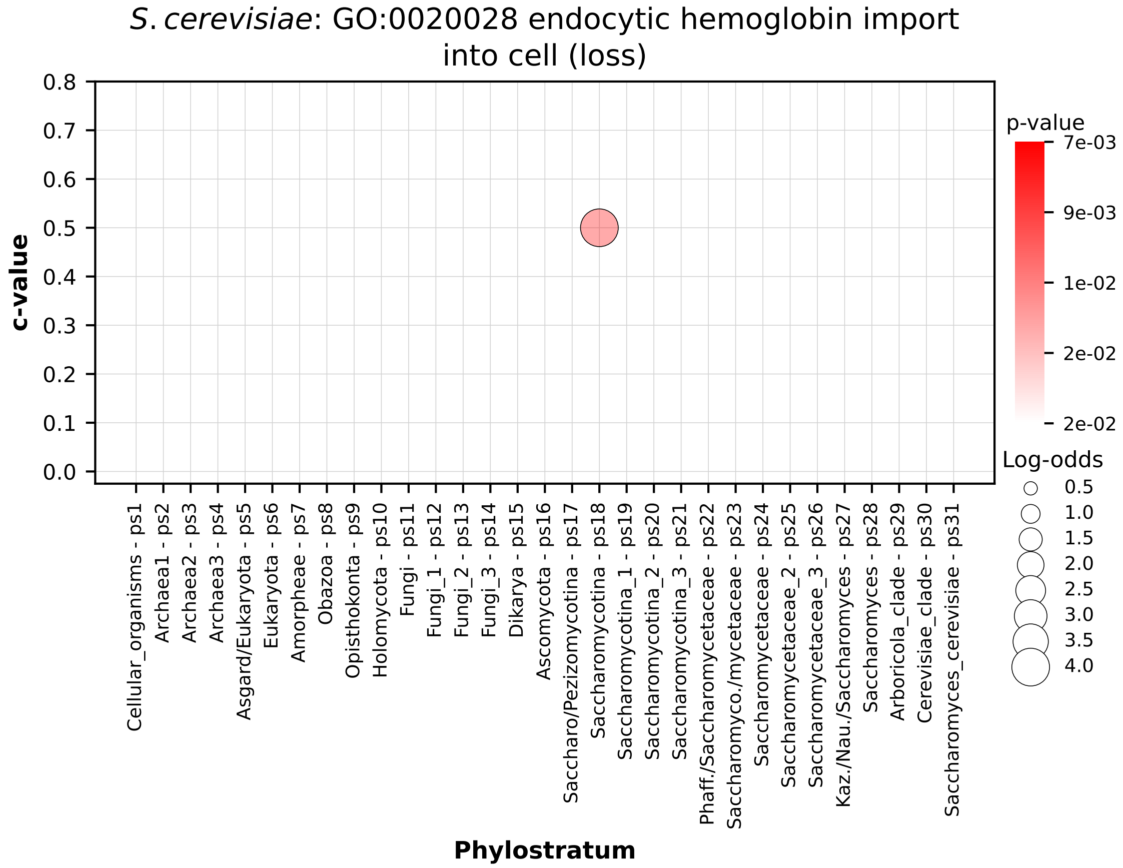

S. cerevisiae: GO:0020028 endocytic hemoglobin import into cell (loss)
c-value
0.0
0.1
0.2
0.3
0.4
0.5
0.6
0.7
0.8
Phylostratum
Cellular_organisms - ps1
Archaea1 - ps2
Archaea2 - ps3
Archaea3 - ps4
Asgard/Eukaryota - ps5
Eukaryota - ps6
Amorpheae - ps7
Obazoa - ps8
Opisthokonta - ps9
Holomycota - ps10
Fungi - ps11
Fungi_1 - ps12
Fungi_2 - ps13
Fungi_3 - ps14
Dikarya - ps15
Ascomycota - ps16
Saccharo/Pezizomycotina - ps17
Saccharomycotina - ps18
Saccharomycotina_1 - ps19
Saccharomycotina_2 - ps20
Saccharomycotina_3 - ps21
Phaff./Saccharomycetaceae - ps22
Saccharomyco./mycetaceae - ps23
Saccharomycetaceae - ps24
Saccharomycetaceae_2 - ps25
Saccharomycetaceae_3 - ps26
Kaz./Nau./Saccharomyces - ps27
Saccharomyces - ps28
Arboricola_clade - ps29
Cerevisiae_clade - ps30
Saccharomyces_cerevisiae - ps31
p-value
7e-03
9e-03
1e-02
2e-02
2e-02
Log-odds
0.5
1.0
1.5
2.0
2.5
3.0
3.5
4.0

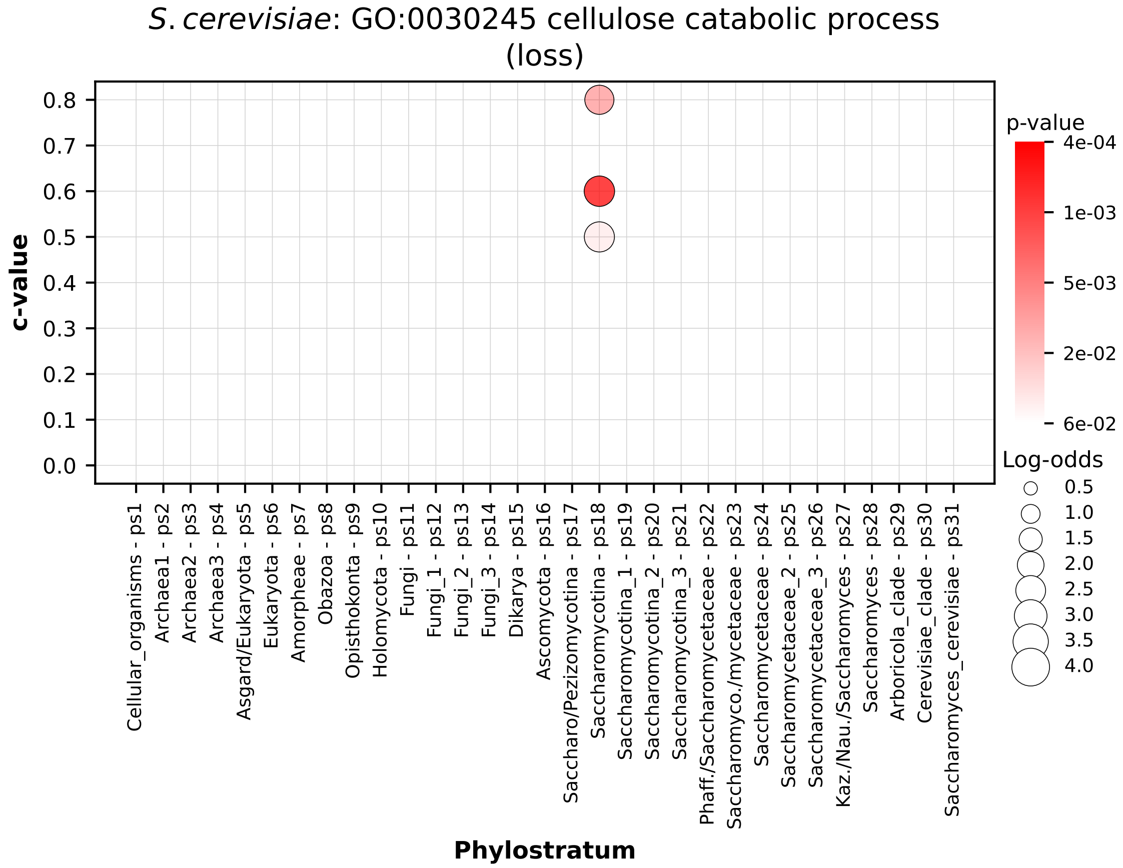

S. cerevisiae: GO:0030245 cellulose catabolic process (loss)
c-value
0.8
0.7
0.6
0.5
0.4
0.3
0.2
0.1
0.0
p-value
4e-04
1e-03
5e-03
2e-02
6e-02
Log-odds
0.5
1.0
1.5
2.0
2.5
3.0
3.5
4.0
Cellular_organisms - ps1
Archaea1 - ps2
Archaea2 - ps3
Archaea3 - ps4
Asgard/Eukaryota - ps5
Eukaryota - ps6
Amorpheae - ps7
Obazoa - ps8
Opisthokonta - ps9
Holomycota - ps10
Fungi - ps11
Fungi_1 - ps12
Fungi_2 - ps13
Fungi_3 - ps14
Dikarya - ps15
Ascomycota - ps16
Saccharo/Pezizomycotina - ps17
Saccharomycotina - ps18
Saccharomycotina_1 - ps19
Saccharomycotina_2 - ps20
Saccharomycotina_3 - ps21
Phaff./Saccharomycetaceae - ps22
Saccharomyco./mycetaceae - ps23
Saccharomycetaceae - ps24
Saccharomycetaceae_2 - ps25
Saccharomycetaceae_3 - ps26
Kaz./Nau./Saccharomyces - ps27
Saccharomyces - ps28
Arboricola_clade - ps29
Cerevisiae_clade - ps30
Saccharomyces_cerevisiae - ps31
Phylostratum

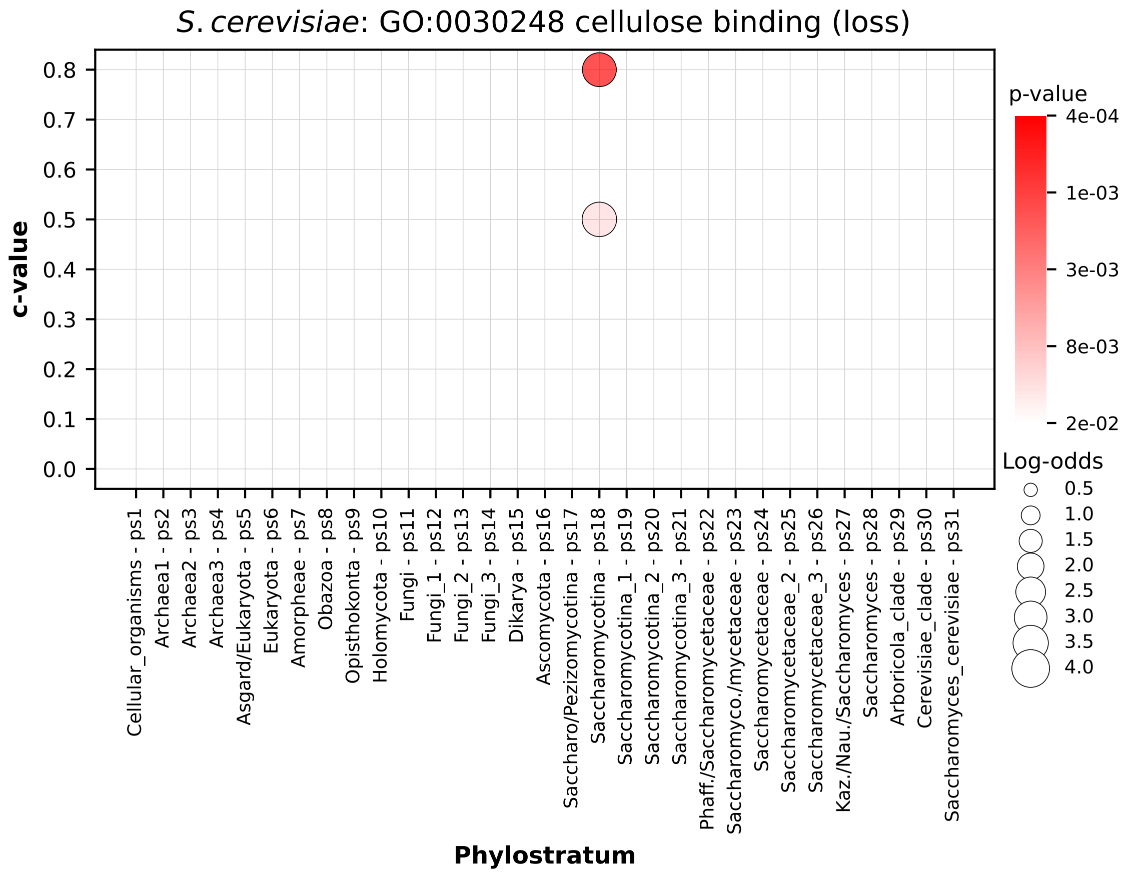
S. cerevisiae: GO:0030248 cellulose binding (loss)
c-value
0.8
0.7
0.6
0.5
0.4
0.3
0.2
0.1
0.0
p-value
4e-04
1e-03
3e-03
8e-03
2e-02
Log-odds
0.5
1.0
1.5
2.0
2.5
3.0
3.5
4.0
Cellular_organisms - ps1
Archaea1 - ps2
Archaea2 - ps3
Archaea3 - ps4
Asgard/Eukaryota - ps5
Eukaryota - ps6
Amorpheae - ps7
Obazoa - ps8
Opisthokonta - ps9
Holomycota - ps10
Fungi - ps11
Fungi_1 - ps12
Fungi_2 - ps13
Fungi_3 - ps14
Dikarya - ps15
Ascomycota - ps16
Saccharo/Pezizomycotina - ps17
Saccharomycotina - ps18
Saccharomycotina_1 - ps19
Saccharomycotina_2 - ps20
Saccharomycotina_3 - ps21
Phaff./Saccharomycetaceae - ps22
Saccharomyco./mycetaceae - ps23
Saccharomycetaceae - ps24
Saccharomycetaceae_2 - ps25
Saccharomycetaceae_3 - ps26
Kaz./Nau./Saccharomyces - ps27
Saccharomyces - ps28
Arboricola_clade - ps29
Cerevisiae_clade - ps30
Saccharomyces_cerevisiae - ps31
Phylostratum

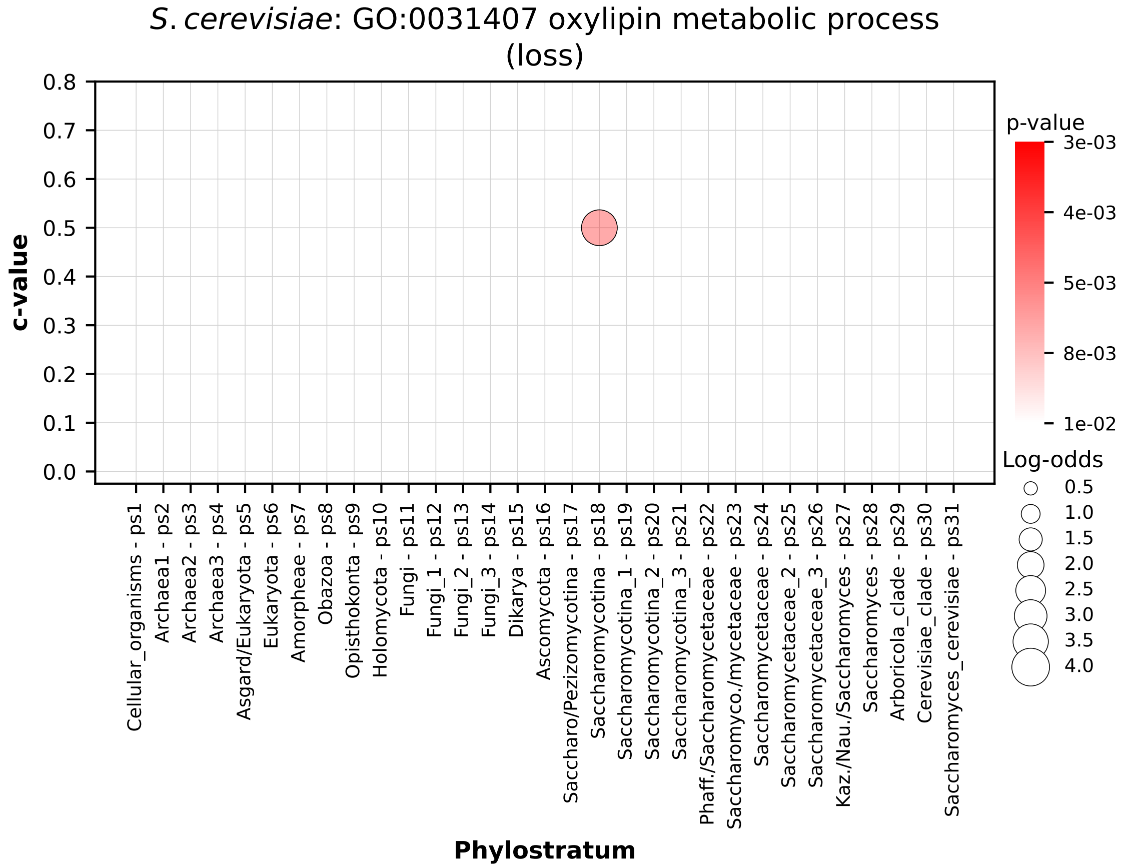

S. cerevisiae: GO:0031407 oxylipin metabolic process
(loss)
c-value
0.8
0.7
0.6
0.5
0.4
0.3
0.2
0.1
0.0
p-value
3e-03
4e-03
5e-03
8e-03
1e-02
Log-odds
0.5
1.0
1.5
2.0
2.5
3.0
3.5
4.0
Cellular_organisms - ps1
Archaea1 - ps2
Archaea2 - ps3
Archaea3 - ps4
Asgard/Eukaryota - ps5
Eukaryota - ps6
Amorpheae - ps7
Obazoa - ps8
Opisthokonta - ps9
Holomycota - ps10
Fungi - ps11
Fungi_1 - ps12
Fungi_2 - ps13
Fungi_3 - ps14
Dikarya - ps15
Ascomycota - ps16
Saccharo/Pezizomycotina - ps17
Saccharomycotina - ps18
Saccharomycotina_1 - ps19
Saccharomycotina_2 - ps20
Saccharomycotina_3 - ps21
Phaff./Saccharomycetaceae - ps22
Saccharomyco./mycetaceae - ps23
Saccharomycetaceae - ps24
Saccharomycetaceae_2 - ps25
Saccharomycetaceae_3 - ps26
Kaz./Nau./Saccharomyces - ps27
Saccharomyces - ps28
Arboricola_clade - ps29
Cerevisiae_clade - ps30
Saccharomyces_cerevisiae - ps31
Phylostratum

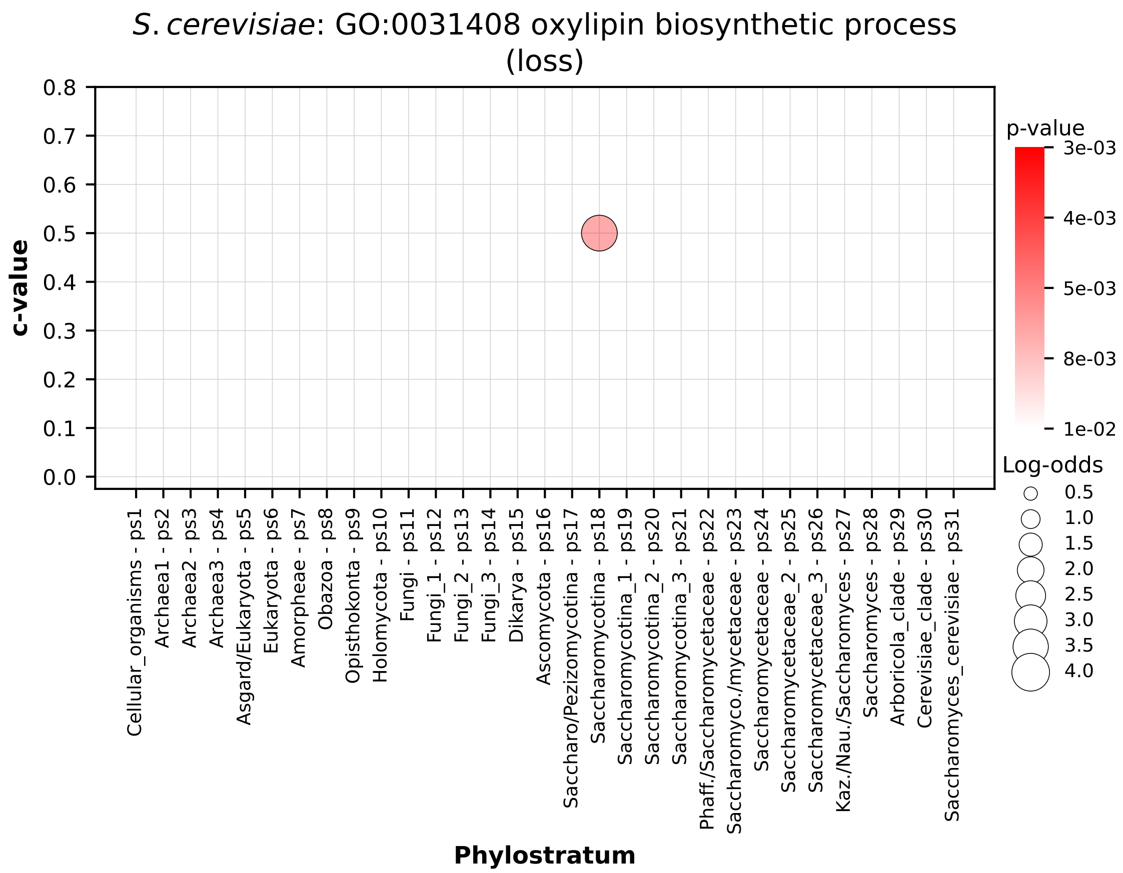

S. cerevisiae: GO:0031408 oxylipin biosynthetic process
(loss)
c-value
0.8
0.7
0.6
0.5
0.4
0.3
0.2
0.1
0.0
Phylostratum
Cellular_organisms - ps1
Archaea1 - ps2
Archaea2 - ps3
Archaea3 - ps4
Asgard/Eukaryota - ps5
Eukaryota - ps6
Amorpheae - ps7
Obazoa - ps8
Opisthokonta - ps9
Holomycota - ps10
Fungi - ps11
Fungi_1 - ps12
Fungi_2 - ps13
Fungi_3 - ps14
Dikarya - ps15
Ascomycota - ps16
Saccharo/Pezizomycotina - ps17
Saccharomycotina - ps18
Saccharomycotina_1 - ps19
Saccharomycotina_2 - ps20
Saccharomycotina_3 - ps21
Phaff./Saccharomycetaceae - ps22
Saccharomyco./mycetaceae - ps23
Saccharomycetaceae - ps24
Saccharomycetaceae_2 - ps25
Saccharomycetaceae_3 - ps26
Kaz./Nau./Saccharomyces - ps27
Saccharomyces - ps28
Arboricola_clade - ps29
Cerevisiae_clade - ps30
Saccharomyces_cerevisiae - ps31
p-value
3e-03
4e-03
5e-03
8e-03
1e-02
Log-odds
0.5
1.0
1.5
2.0
2.5
3.0
3.5
4.0

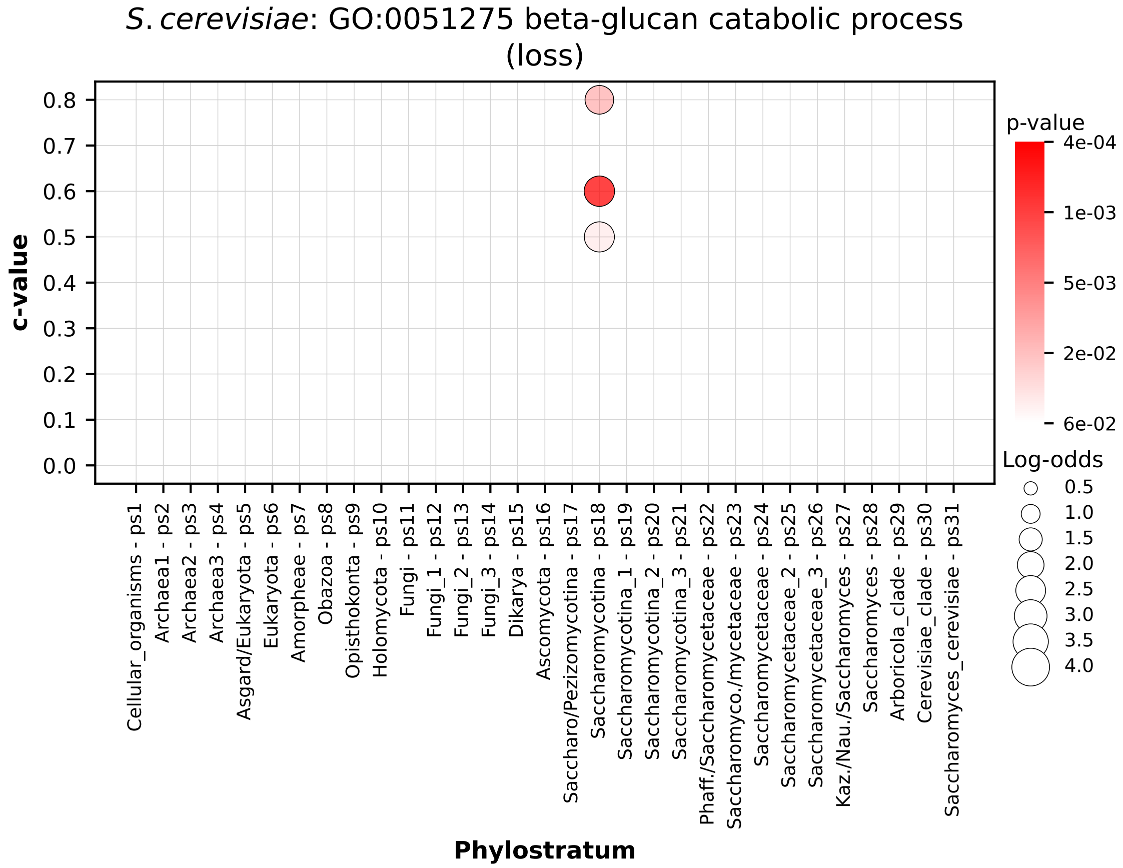

S. cerevisiae: GO:0051275 beta-glucan catabolic process
(loss)
c-value
0.8
0.7
0.6
0.5
0.4
0.3
0.2
0.1
0.0
Phylostratum
Cellular_organisms - ps1
Archaea1 - ps2
Archaea2 - ps3
Archaea3 - ps4
Asgard/Eukaryota - ps5
Eukaryota - ps6
Amorpheae - ps7
Obazoa - ps8
Opisthokonta - ps9
Holomycota - ps10
Fungi - ps11
Fungi_1 - ps12
Fungi_2 - ps13
Fungi_3 - ps14
Dikarya - ps15
Ascomycota - ps16
Saccharo/Pezizomycotina - ps17
Saccharomycotina - ps18
Saccharomycotina_1 - ps19
Saccharomycotina_2 - ps20
Saccharomycotina_3 - ps21
Phaff./Saccharomycetaceae - ps22
Saccharomyco./mycetaceae - ps23
Saccharomycetaceae - ps24
Saccharomycetaceae_2 - ps25
Saccharomycetaceae_3 - ps26
Kaz./Nau./Saccharomyces - ps27
Saccharomyces - ps28
Arboricola_clade - ps29
Cerevisiae_clade - ps30
Saccharomyces_cerevisiae - ps31
p-value
4e-04
1e-03
5e-03
2e-02
6e-02
Log-odds
0.5
1.0
1.5
2.0
2.5
3.0
3.5
4.0

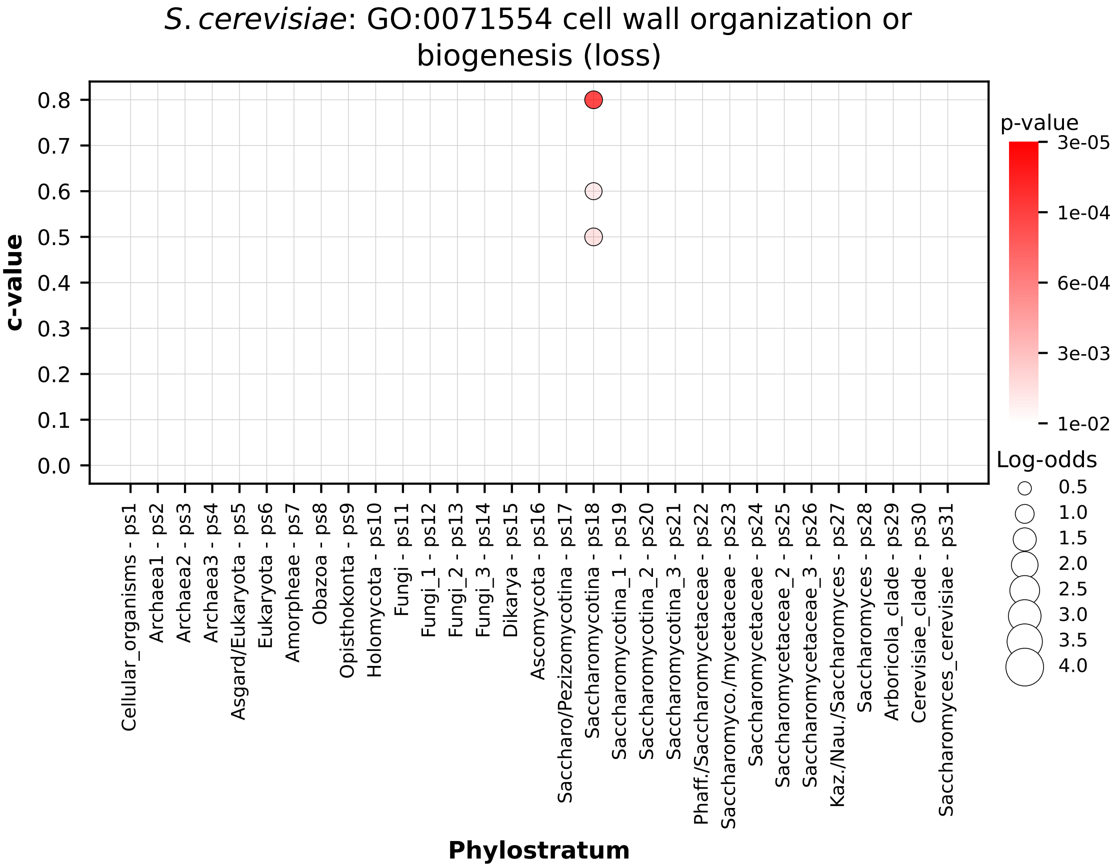
S. cerevisiae: GO:0071554 cell wall organization or biogenesis (loss)
c-value
0.0
0.1
0.2
0.3
0.4
0.5
0.6
0.7
0.8
p-value
3e-05
1e-04
6e-04
3e-03
1e-02
Log-odds
0.5
1.0
1.5
2.0
2.5
3.0
3.5
4.0
Cellular_organisms - ps1
Archaea1 - ps2
Archaea2 - ps3
Archaea3 - ps4
Asgard/Eukaryota - ps5
Eukaryota - ps6
Amorpheae - ps7
Obazoa - ps8
Opisthokonta - ps9
Holomycota - ps10
Fungi - ps11
Fungi_1 - ps12
Fungi_2 - ps13
Fungi_3 - ps14
Dikarya - ps15
Ascomycota - ps16
Saccharo/Pezizomycotina - ps17
Saccharomycotina - ps18
Saccharomycotina_1 - ps19
Saccharomycotina_2 - ps20
Saccharomycotina_3 - ps21
Phaff./Saccharomycetaceae - ps22
Saccharomyco./mycetaceae - ps23
Saccharomycetaceae - ps24
Saccharomycetaceae_2 - ps25
Saccharomycetaceae_3 - ps26
Kaz./Nau./Saccharomyces - ps27
Saccharomyces - ps28
Arboricola_clade - ps29
Cerevisiae_clade - ps30
Saccharomyces_cerevisiae - ps31
Phylostratum

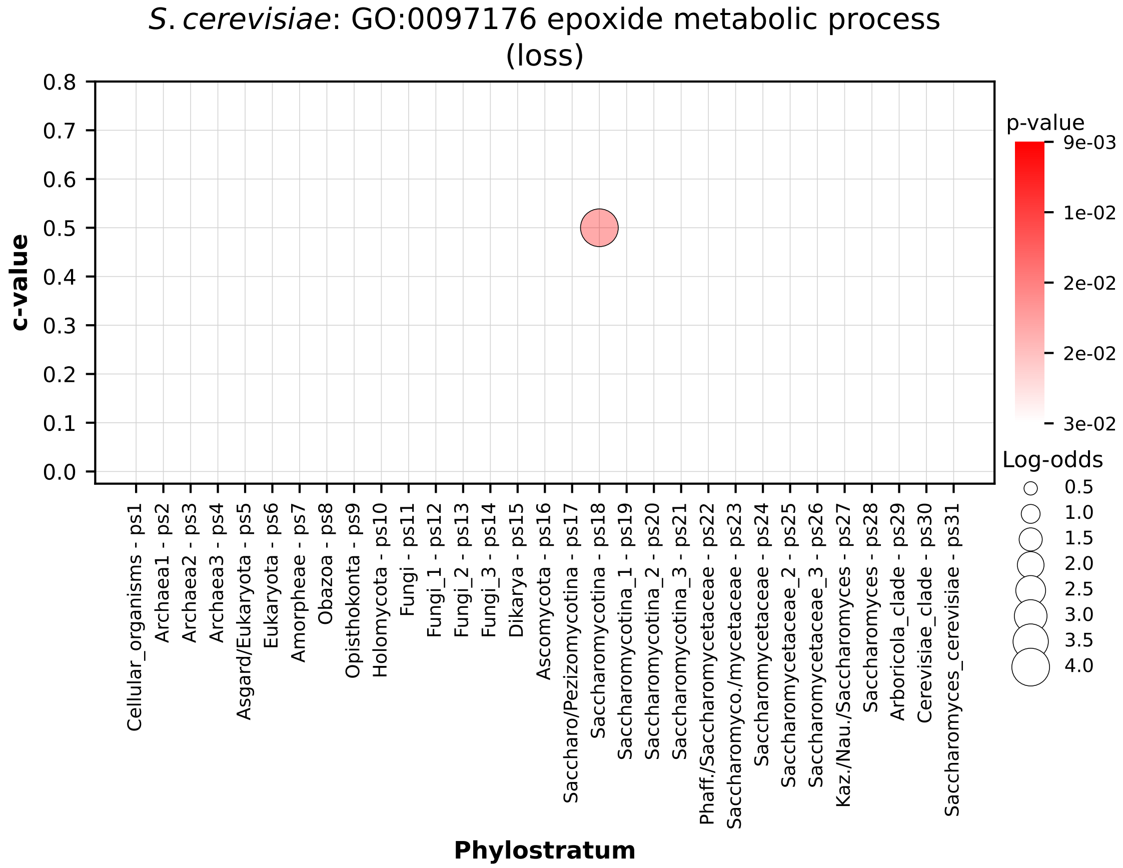
S. cerevisiae: GO:0097176 epoxide metabolic process
(loss)
c-value
0.8
0.7
0.6
0.5
0.4
0.3
0.2
0.1
0.0
p-value
9e-03
1e-02
2e-02
2e-02
3e-02
Log-odds
0.5
1.0
1.5
2.0
2.5
3.0
3.5
4.0
Cellular_organisms - ps1
Archaea1 - ps2
Archaea2 - ps3
Archaea3 - ps4
Asgard/Eukaryota - ps5
Eukaryota - ps6
Amorpheae - ps7
Obazoa - ps8
Opisthokonta - ps9
Holomycota - ps10
Fungi - ps11
Fungi_1 - ps12
Fungi_2 - ps13
Fungi_3 - ps14
Dikarya - ps15
Ascomycota - ps16
Saccharo/Pezizomycotina - ps17
Saccharomycotina - ps18
Saccharomycotina_1 - ps19
Saccharomycotina_2 - ps20
Saccharomycotina_3 - ps21
Phaff./Saccharomycetaceae - ps22
Saccharomyco./mycetaceae - ps23
Saccharomycetaceae - ps24
Saccharomycetaceae_2 - ps25
Saccharomycetaceae_3 - ps26
Kaz./Nau./Saccharomyces - ps27
Saccharomyces - ps28
Arboricola_clade - ps29
Cerevisiae_clade - ps30
Saccharomyces_cerevisiae - ps31
Phylostratum

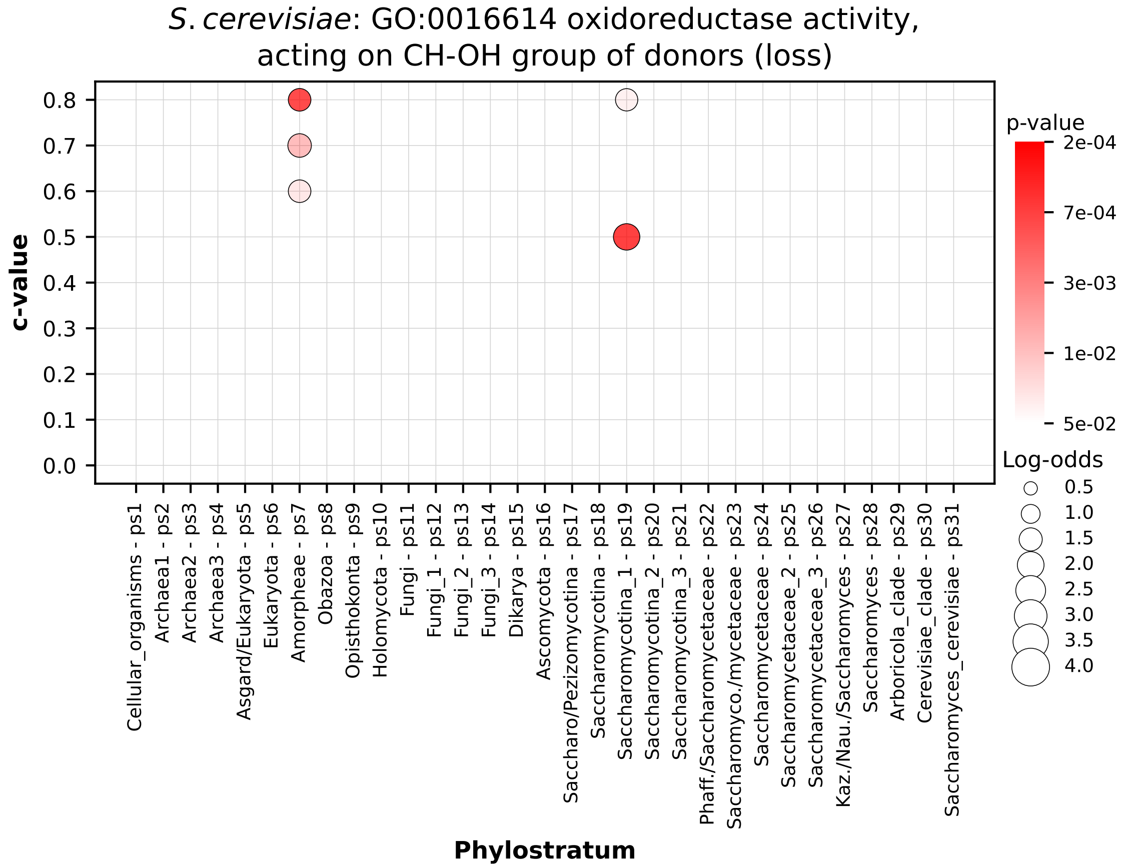

S. cerevisiae: GO:0016614 oxidoreductase activity, acting on CH-OH group of donors (loss)
c-value
0.0
0.1
0.2
0.3
0.4
0.5
0.6
0.7
0.8
p-value
2e-04
7e-04
3e-03
1e-02
5e-02
Log-odds
0.5
1.0
1.5
2.0
2.5
3.0
3.5
4.0
Cellular_organisms - ps1
Archaea1 - ps2
Archaea2 - ps3
Archaea3 - ps4
Asgard/Eukaryota - ps5
Eukaryota - ps6
Amorpheae - ps7
Obazoa - ps8
Opisthokonta - ps9
Holomycota - ps10
Fungi - ps11
Fungi_1 - ps12
Fungi_2 - ps13
Fungi_3 - ps14
Dikarya - ps15
Ascomycota - ps16
Saccharo/Pezizomycotina - ps17
Saccharomycotina - ps18
Saccharomycotina_1 - ps19
Saccharomycotina_2 - ps20
Saccharomycotina_3 - ps21
Phaff./Saccharomycetaceae - ps22
Saccharomyco./mycetaceae - ps23
Saccharomycetaceae - ps24
Saccharomycetaceae_2 - ps25
Saccharomycetaceae_3 - ps26
Kaz./Nau./Saccharomyces - ps27
Saccharomyces - ps28
Arboricola_clade - ps29
Cerevisiae_clade - ps30
Saccharomyces_cerevisiae - ps31
Phylostratum

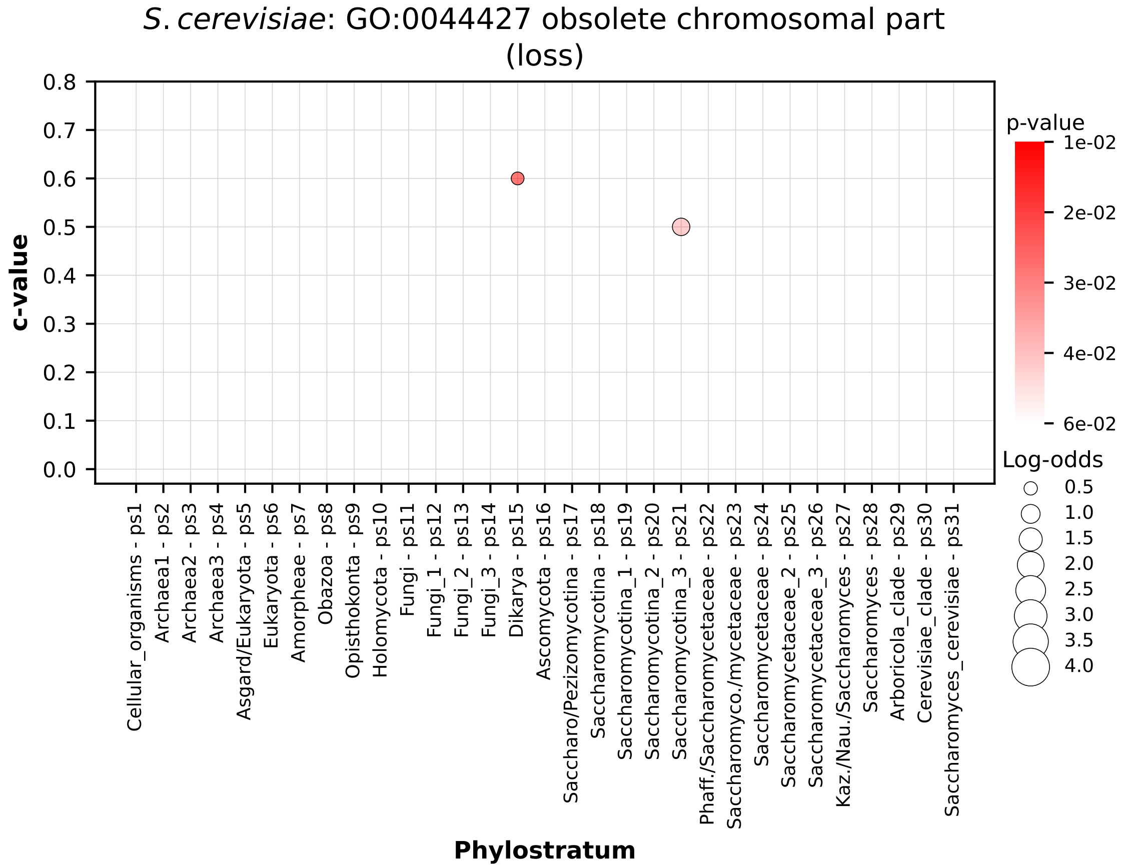

S. cerevisiae: GO:0044427 obsolete chromosomal part
(loss)
c-value
0.8
0.7
0.6
0.5
0.4
0.3
0.2
0.1
0.0
Phylostratum
Cellular_organisms - ps1
Archaea1 - ps2
Archaea2 - ps3
Archaea3 - ps4
Asgard/Eukaryota - ps5
Eukaryota - ps6
Amorpheae - ps7
Obazoa - ps8
Opisthokonta - ps9
Holomycota - ps10
Fungi - ps11
Fungi_1 - ps12
Fungi_2 - ps13
Fungi_3 - ps14
Dikarya - ps15
Ascomycota - ps16
Saccharo/Pezizomycotina - ps17
Saccharomycotina - ps18
Saccharomycotina_1 - ps19
Saccharomycotina_2 - ps20
Saccharomycotina_3 - ps21
Phaff./Saccharomycetaceae - ps22
Saccharomyco./mycetaceae - ps23
Saccharomycetaceae - ps24
Saccharomycetaceae_2 - ps25
Saccharomycetaceae_3 - ps26
Kaz./Nau./Saccharomyces - ps27
Saccharomyces - ps28
Arboricola_clade - ps29
Cerevisiae_clade - ps30
Saccharomyces_cerevisiae - ps31
p-value
1e-02
2e-02
3e-02
4e-02
6e-02
Log-odds
0.5
1.0
1.5
2.0
2.5
3.0
3.5
4.0

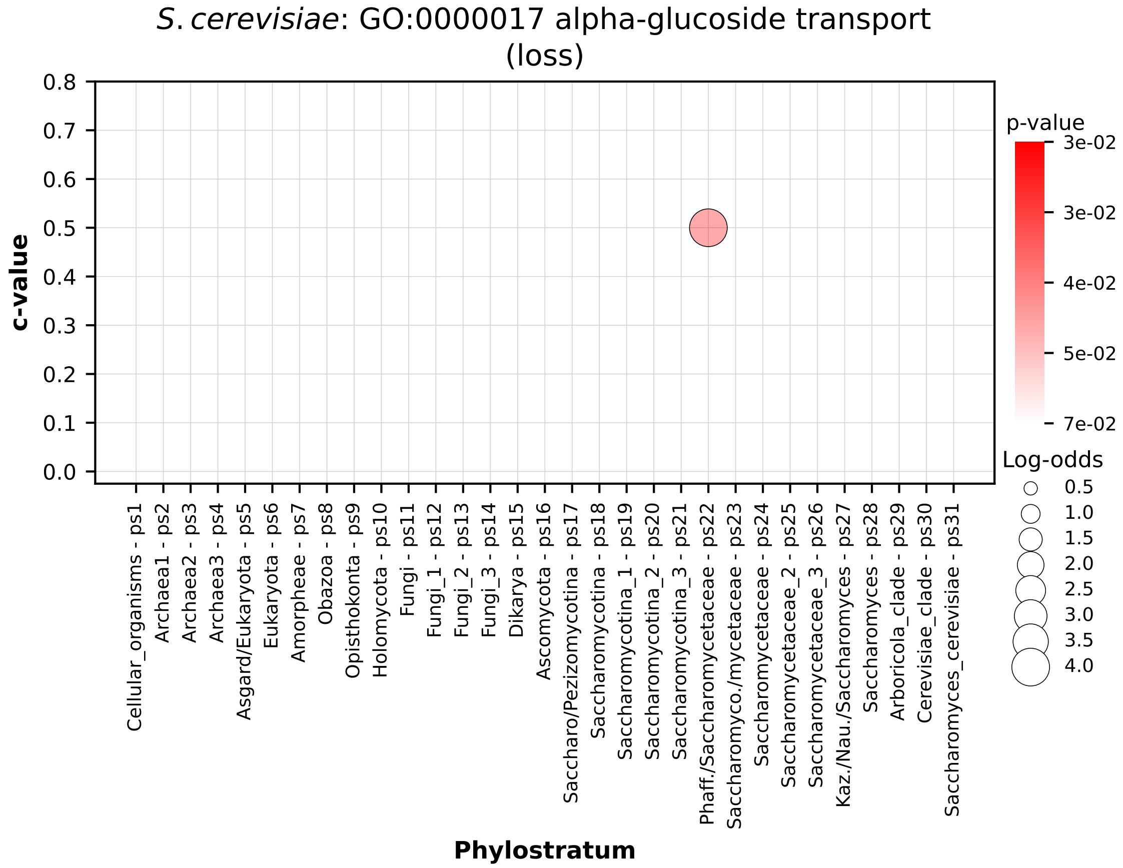

S. cerevisiae: GO:0000017 alpha-glucoside transport
(loss)
c-value
0.8
0.7
0.6
0.5
0.4
0.3
0.2
0.1
0.0
p-value
3e-02
3e-02
4e-02
5e-02
7e-02
Log-odds
0.5
1.0
1.5
2.0
2.5
3.0
3.5
4.0
Cellular_organisms - ps1
Archaea1 - ps2
Archaea2 - ps3
Archaea3 - ps4
Asgard/Eukaryota - ps5
Eukaryota - ps6
Amorpheae - ps7
Obazoa - ps8
Opisthokonta - ps9
Holomycota - ps10
Fungi - ps11
Fungi_1 - ps12
Fungi_2 - ps13
Fungi_3 - ps14
Dikarya - ps15
Ascomycota - ps16
Saccharo/Pezizomycotina - ps17
Saccharomycotina - ps18
Saccharomycotina_1 - ps19
Saccharomycotina_2 - ps20
Saccharomycotina_3 - ps21
Phaff./Saccharomycetaceae - ps22
Saccharomyco./mycetaceae - ps23
Saccharomycetaceae - ps24
Saccharomycetaceae_2 - ps25
Saccharomycetaceae_3 - ps26
Kaz./Nau./Saccharomyces - ps27
Saccharomyces - ps28
Arboricola_clade - ps29
Cerevisiae_clade - ps30
Saccharomyces_cerevisiae - ps31
Phylostratum

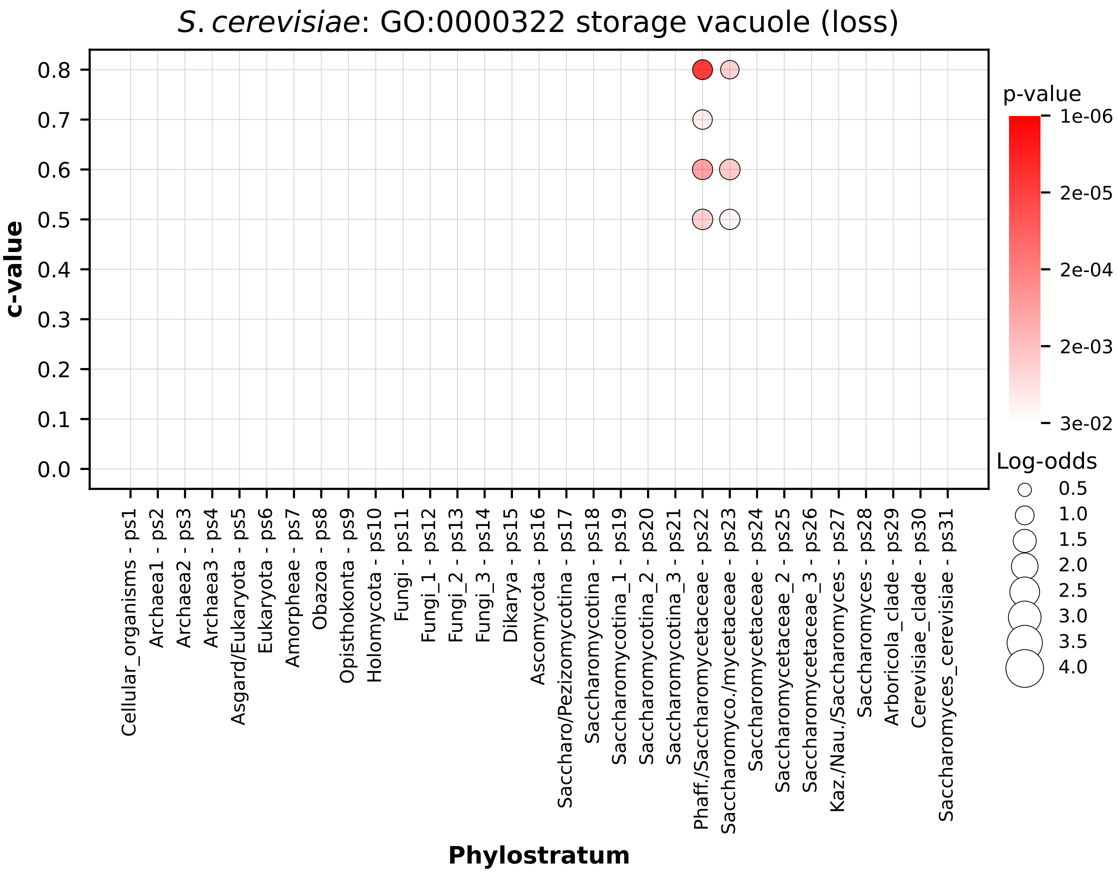

S. cerevisiae: GO:0000322 storage vacuole (loss)
c-value
0.8
0.7
0.6
0.5
0.4
0.3
0.2
0.1
0.0
p-value
1e-06
2e-05
2e-04
2e-03
3e-02
Log-odds
0.5
1.0
1.5
2.0
2.5
3.0
3.5
4.0
Cellular_organisms - ps1
Archaea1 - ps2
Archaea2 - ps3
Archaea3 - ps4
Asgard/Eukaryota - ps5
Eukaryota - ps6
Amorpheae - ps7
Obazoa - ps8
Opisthokonta - ps9
Holomycota - ps10
Fungi - ps11
Fungi_1 - ps12
Fungi_2 - ps13
Fungi_3 - ps14
Dikarya - ps15
Ascomycota - ps16
Saccharo/Pezizomycotina - ps17
Saccharomycotina - ps18
Saccharomycotina_1 - ps19
Saccharomycotina_2 - ps20
Saccharomycotina_3 - ps21
Phaff./Saccharomycetaceae - ps22
Saccharomyco./mycetaceae - ps23
Saccharomycetaceae - ps24
Saccharomycetaceae_2 - ps25
Saccharomycetaceae_3 - ps26
Kaz./Nau./Saccharomyces - ps27
Saccharomyces - ps28
Arboricola_clade - ps29
Cerevisiae_clade - ps30
Saccharomyces_cerevisiae - ps31
Phylostratum

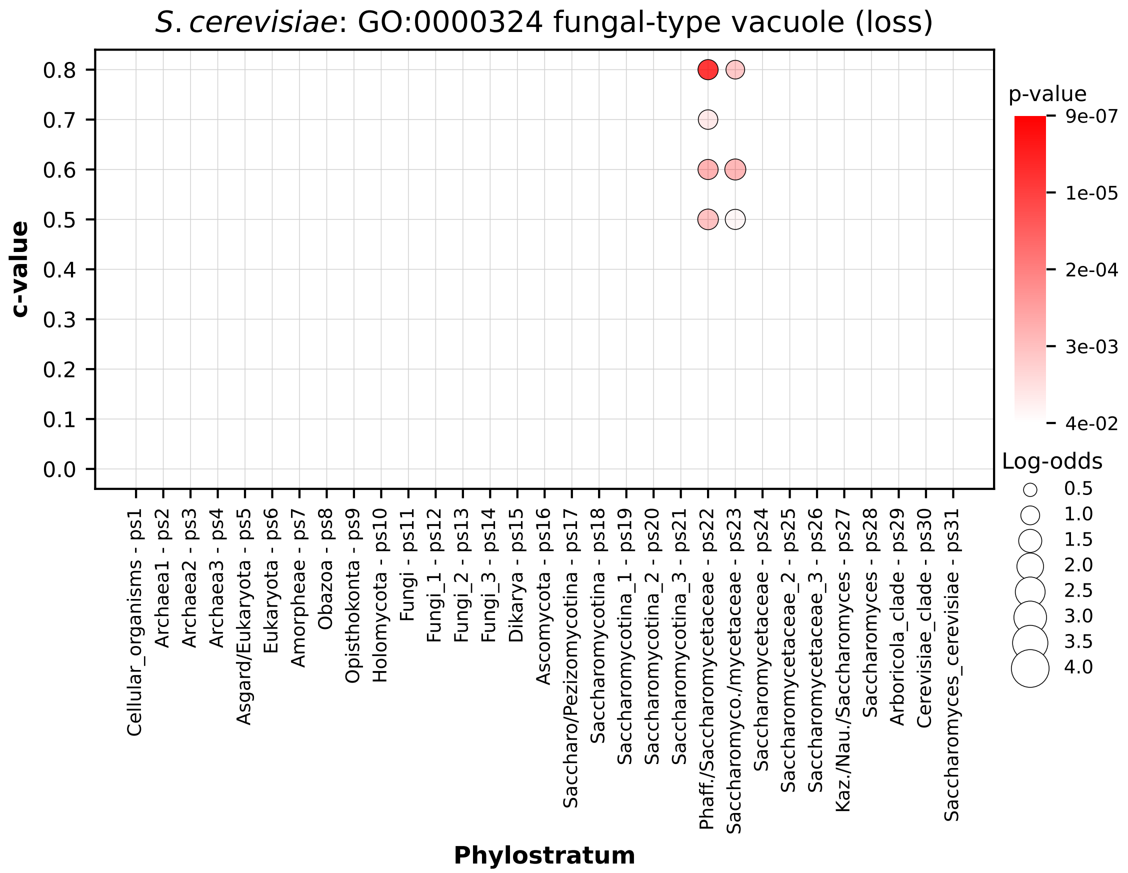
S. cerevisiae: GO:0000324 fungal-type vacuole (loss)
c-value
0.0
0.1
0.2
0.3
0.4
0.5
0.6
0.7
0.8
Phylostratum
Cellular_organisms - ps1
Archaea1 - ps2
Archaea2 - ps3
Archaea3 - ps4
Asgard/Eukaryota - ps5
Eukaryota - ps6
Amorpheae - ps7
Obazoa - ps8
Opisthokonta - ps9
Holomycota - ps10
Fungi - ps11
Fungi_1 - ps12
Fungi_2 - ps13
Fungi_3 - ps14
Dikarya - ps15
Ascomycota - ps16
Saccharo/Pezizomycotina - ps17
Saccharomycotina - ps18
Saccharomycotina_1 - ps19
Saccharomycotina_2 - ps20
Saccharomycotina_3 - ps21
Phaff./Saccharomycetaceae - ps22
Saccharomyco./mycetaceae - ps23
Saccharomycetaceae - ps24
Saccharomycetaceae_2 - ps25
Saccharomycetaceae_3 - ps26
Kaz./Nau./Saccharomyces - ps27
Saccharomyces - ps28
Arboricola_clade - ps29
Cerevisiae_clade - ps30
Saccharomyces_cerevisiae - ps31
p-value
9e-07
1e-05
2e-04
3e-03
4e-02
Log-odds
0.5
1.0
1.5
2.0
2.5
3.0
3.5
4.0

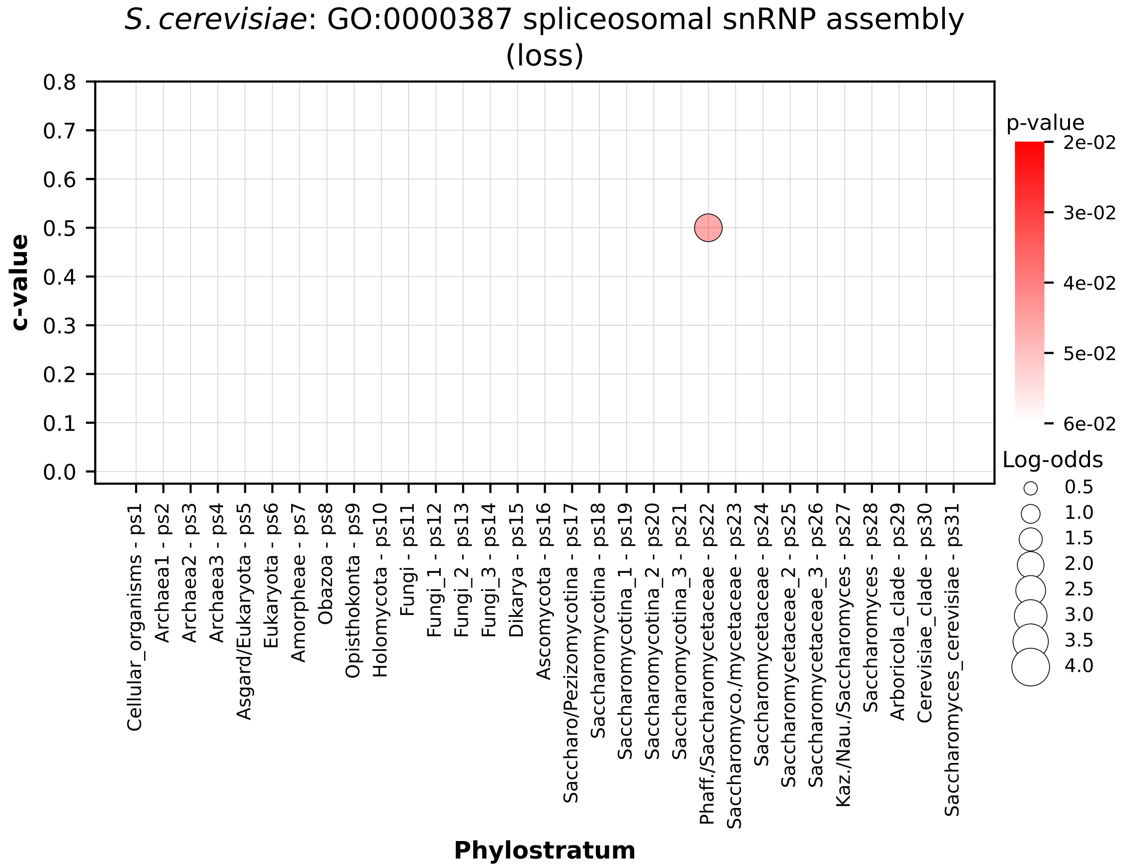

S. cerevisiae: GO:0000387 spliceosomal snRNP assembly (loss)
c-value
0.8
0.7
0.6
0.5
0.4
0.3
0.2
0.1
0.0
p-value
2e-02
3e-02
4e-02
5e-02
6e-02
Log-odds
0.5
1.0
1.5
2.0
2.5
3.0
3.5
4.0
Cellular_organisms - ps1
Archaea1 - ps2
Archaea2 - ps3
Archaea3 - ps4
Asgard/Eukaryota - ps5
Eukaryota - ps6
Amorpheae - ps7
Obazoa - ps8
Opisthokonta - ps9
Holomycota - ps10
Fungi - ps11
Fungi_1 - ps12
Fungi_2 - ps13
Fungi_3 - ps14
Dikarya - ps15
Ascomycota - ps16
Saccharo/Pezizomycotina - ps17
Saccharomycotina - ps18
Saccharomycotina_1 - ps19
Saccharomycotina_2 - ps20
Saccharomycotina_3 - ps21
Phaff./Saccharomycetaceae - ps22
Saccharomyco./mycetaceae - ps23
Saccharomycetaceae - ps24
Saccharomycetaceae_2 - ps25
Saccharomycetaceae_3 - ps26
Kaz./Nau./Saccharomyces - ps27
Saccharomyces - ps28
Arboricola_clade - ps29
Cerevisiae_clade - ps30
Saccharomyces_cerevisiae - ps31
Phylostratum

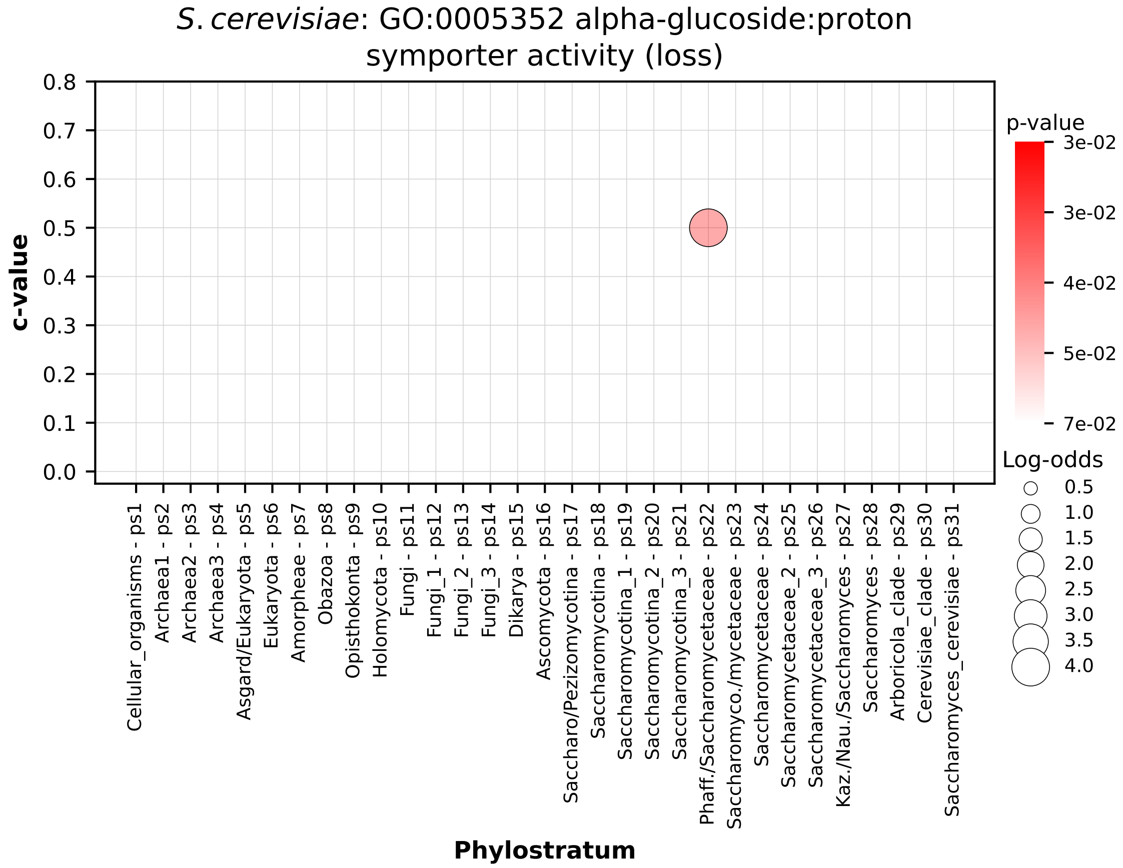

S. cerevisiae: GO:0005352 alpha-glucoside:proton symporter activity (loss)
c-value
0.0
0.1
0.2
0.3
0.4
0.5
0.6
0.7
0.8
p-value
3e-02
3e-02
4e-02
5e-02
7e-02
Log-odds
0.5
1.0
1.5
2.0
2.5
3.0
3.5
4.0
Cellular_organisms - ps1
Archaea1 - ps2
Archaea2 - ps3
Archaea3 - ps4
Asgard/Eukaryota - ps5
Eukaryota - ps6
Amorpheae - ps7
Obazoa - ps8
Opisthokonta - ps9
Holomycota - ps10
Fungi - ps11
Fungi_1 - ps12
Fungi_2 - ps13
Fungi_3 - ps14
Dikarya - ps15
Ascomycota - ps16
Saccharo/Pezizomycotina - ps17
Saccharomycotina - ps18
Saccharomycotina_1 - ps19
Saccharomycotina_2 - ps20
Saccharomycotina_3 - ps21
Phaff./Saccharomycetaceae - ps22
Saccharomyco./mycetaceae - ps23
Saccharomycetaceae - ps24
Saccharomycetaceae_2 - ps25
Saccharomycetaceae_3 - ps26
Kaz./Nau./Saccharomyces - ps27
Saccharomyces - ps28
Arboricola_clade - ps29
Cerevisiae_clade - ps30
Saccharomyces_cerevisiae - ps31
Phylostratum

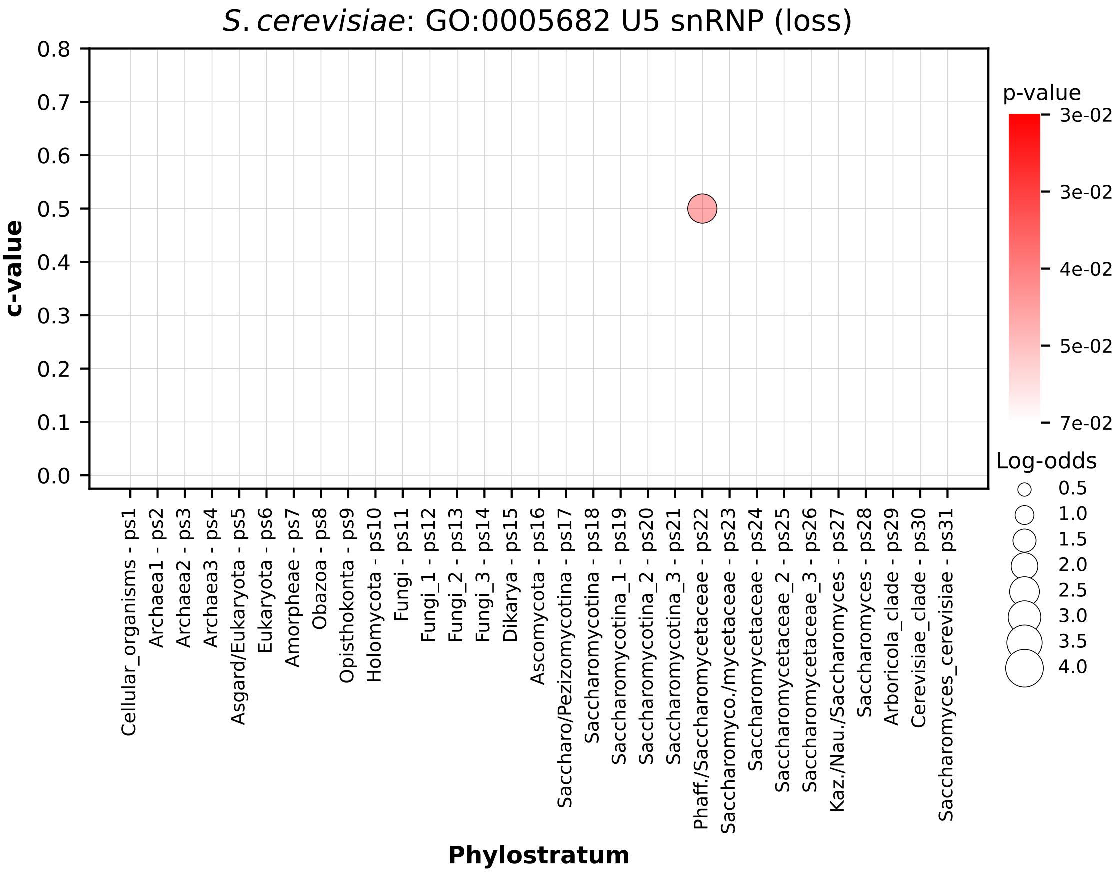

S. cerevisiae: GO:0005682 U5 snRNP (loss)
c-value
0.8
0.7
0.6
0.5
0.4
0.3
0.2
0.1
0.0
p-value
3e-02
3e-02
4e-02
5e-02
7e-02
Log-odds
0.5
1.0
1.5
2.0
2.5
3.0
3.5
4.0
Cellular_organisms - ps1
Archaea1 - ps2
Archaea2 - ps3
Archaea3 - ps4
Asgard/Eukaryota - ps5
Eukaryota - ps6
Amorpheae - ps7
Obazoa - ps8
Opisthokonta - ps9
Holomycota - ps10
Fungi - ps11
Fungi_1 - ps12
Fungi_2 - ps13
Fungi_3 - ps14
Dikarya - ps15
Ascomycota - ps16
Saccharo/Pezizomycotina - ps17
Saccharomycotina - ps18
Saccharomycotina_1 - ps19
Saccharomycotina_2 - ps20
Saccharomycotina_3 - ps21
Phaff./Saccharomycetaceae - ps22
Saccharomyco./mycetaceae - ps23
Saccharomycetaceae - ps24
Saccharomycetaceae_2 - ps25
Saccharomycetaceae_3 - ps26
Kaz./Nau./Saccharomyces - ps27
Saccharomyces - ps28
Arboricola_clade - ps29
Cerevisiae_clade - ps30
Saccharomyces_cerevisiae - ps31
Phylostratum

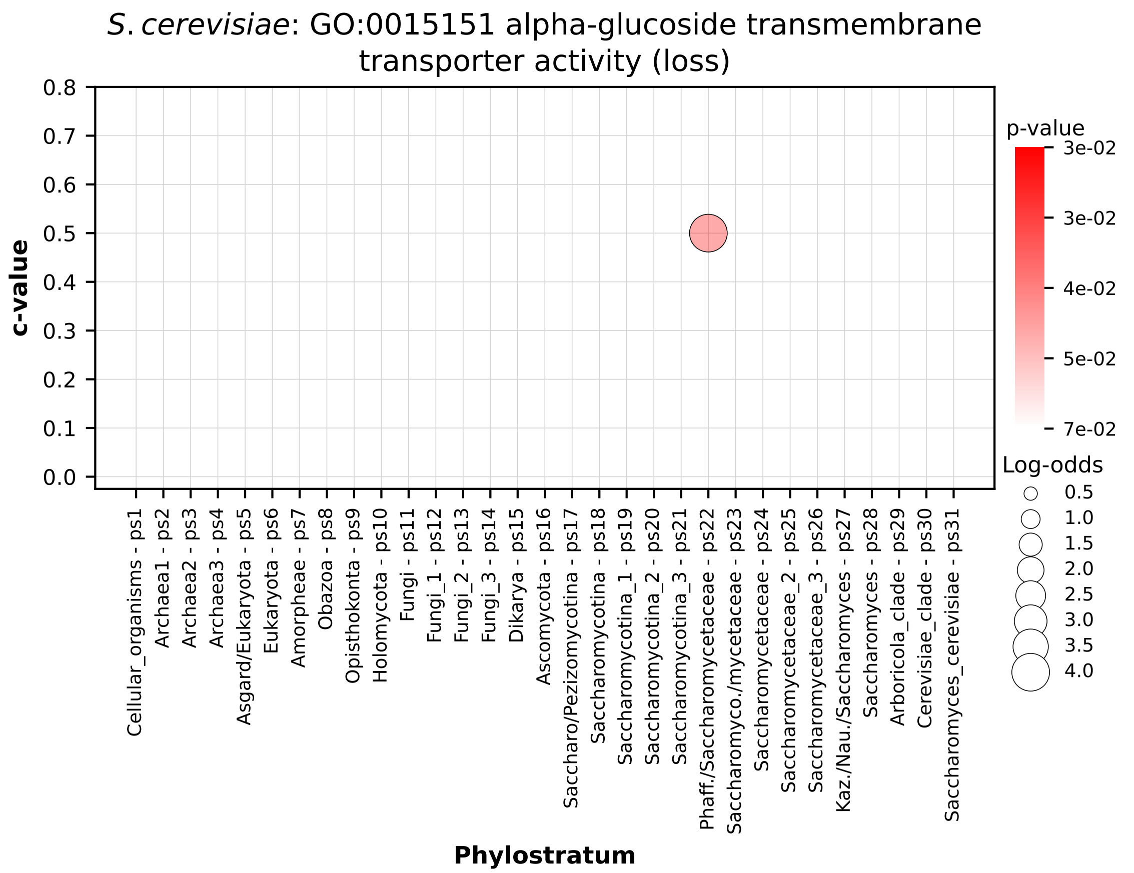

S. cerevisiae: GO:0015151 alpha-glucoside transmembrane transporter activity (loss)
c-value
0.8
0.7
0.6
0.5
0.4
0.3
0.2
0.1
0.0
Phylostratum
Cellular_organisms - ps1
Archaea1 - ps2
Archaea2 - ps3
Archaea3 - ps4
Asgard/Eukaryota - ps5
Eukaryota - ps6
Amorpheae - ps7
Obazoa - ps8
Opisthokonta - ps9
Holomycota - ps10
Fungi - ps11
Fungi_1 - ps12
Fungi_2 - ps13
Fungi_3 - ps14
Dikarya - ps15
Ascomycota - ps16
Saccharo/Pezizomycotina - ps17
Saccharomycotina - ps18
Saccharomycotina_1 - ps19
Saccharomycotina_2 - ps20
Saccharomycotina_3 - ps21
Phaff./Saccharomycetaceae - ps22
Saccharomyco./mycetaceae - ps23
Saccharomycetaceae - ps24
Saccharomycetaceae_2 - ps25
Saccharomycetaceae_3 - ps26
Kaz./Nau./Saccharomyces - ps27
Saccharomyces - ps28
Arboricola_clade - ps29
Cerevisiae_clade - ps30
Saccharomyces_cerevisiae - ps31
p-value
3e-02
3e-02
4e-02
5e-02
7e-02
Log-odds
0.5
1.0
1.5
2.0
2.5
3.0
3.5
4.0

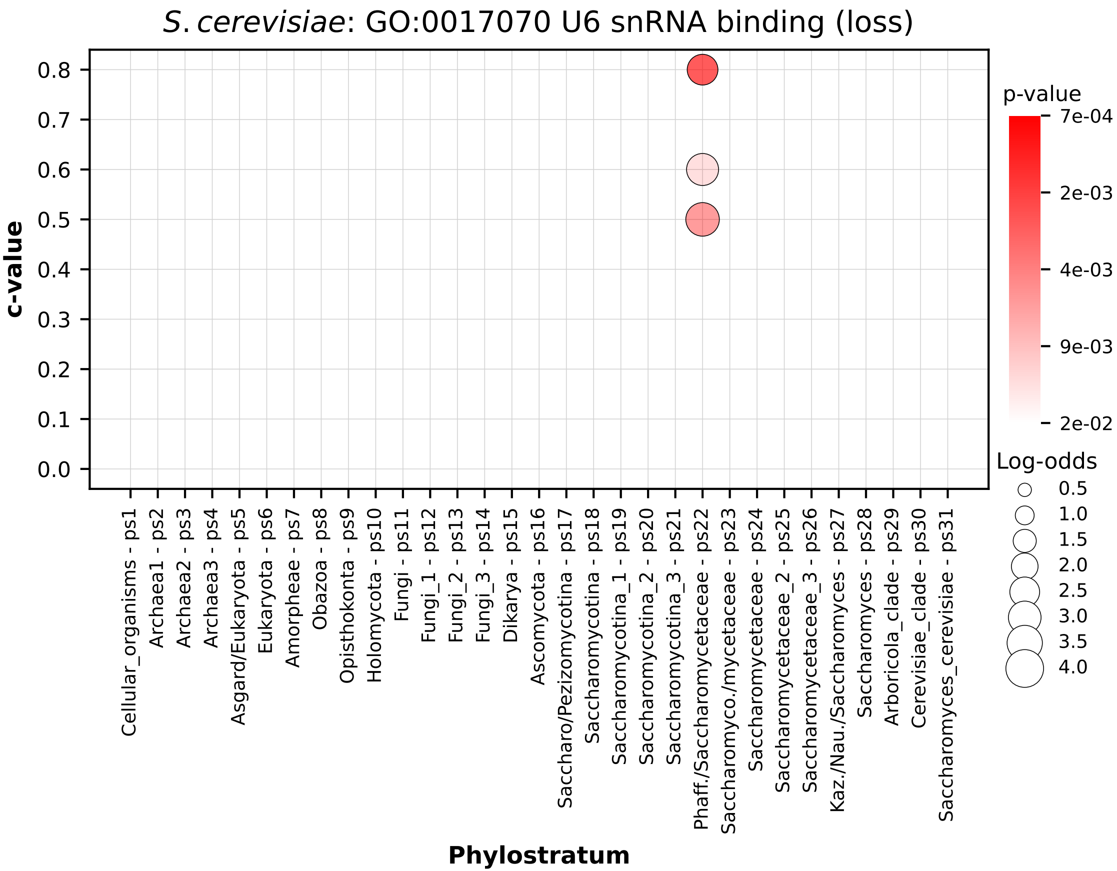

S. cerevisiae: GO:0017070 U6 snRNA binding (loss)
c-value
0.8
0.7
0.6
0.5
0.4
0.3
0.2
0.1
0.0
p-value
7e-04
2e-03
4e-03
9e-03
2e-02
Log-odds
0.5
1.0
1.5
2.0
2.5
3.0
3.5
4.0
Cellular_organisms - ps1
Archaea1 - ps2
Archaea2 - ps3
Archaea3 - ps4
Asgard/Eukaryota - ps5
Eukaryota - ps6
Amorpheae - ps7
Obazoa - ps8
Opisthokonta - ps9
Holomycota - ps10
Fungi - ps11
Fungi_1 - ps12
Fungi_2 - ps13
Fungi_3 - ps14
Dikarya - ps15
Ascomycota - ps16
Saccharo/Pezizomycotina - ps17
Saccharomycotina - ps18
Saccharomycotina_1 - ps19
Saccharomycotina_2 - ps20
Saccharomycotina_3 - ps21
Phaff./Saccharomycetaceae - ps22
Saccharomyco./mycetaceae - ps23
Saccharomycetaceae - ps24
Saccharomycetaceae_2 - ps25
Saccharomycetaceae_3 - ps26
Kaz./Nau./Saccharomyces - ps27
Saccharomyces - ps28
Arboricola_clade - ps29
Cerevisiae_clade - ps30
Saccharomyces_cerevisiae - ps31
Phylostratum

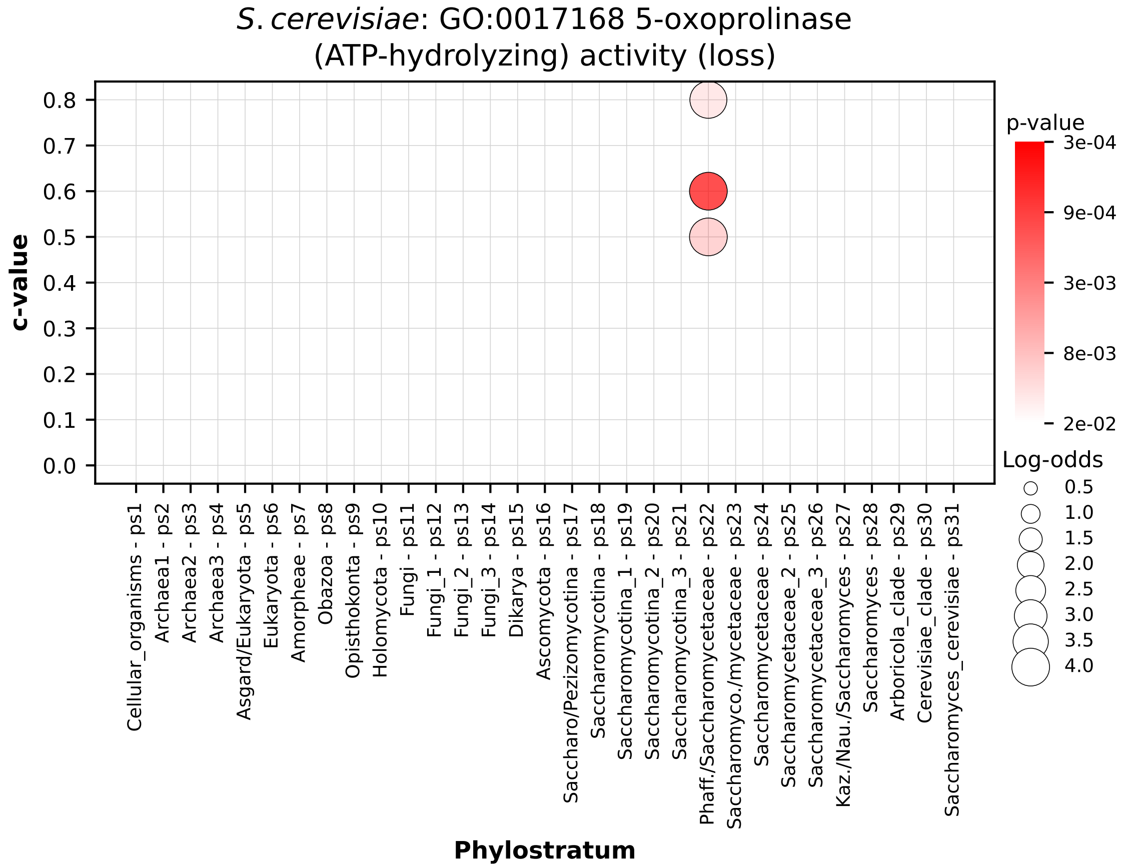

S. cerevisiae: GO:0017168 5-oxoprolinase (ATP-hydrolyzing) activity (loss)
c-value
0.0
0.1
0.2
0.3
0.4
0.5
0.6
0.7
0.8
p-value
3e-04
9e-04
3e-03
8e-03
2e-02
Log-odds
0.5
1.0
1.5
2.0
2.5
3.0
3.5
4.0
Cellular_organisms - ps1
Archaea1 - ps2
Archaea2 - ps3
Archaea3 - ps4
Asgard/Eukaryota - ps5
Eukaryota - ps6
Amorpheae - ps7
Obazoa - ps8
Opisthokonta - ps9
Holomycota - ps10
Fungi - ps11
Fungi_1 - ps12
Fungi_2 - ps13
Fungi_3 - ps14
Dikarya - ps15
Ascomycota - ps16
Saccharo/Pezizomycotina - ps17
Saccharomycotina - ps18
Saccharomycotina_1 - ps19
Saccharomycotina_2 - ps20
Saccharomycotina_3 - ps21
Phaff./Saccharomycetaceae - ps22
Saccharomyco./mycetaceae - ps23
Saccharomycetaceae - ps24
Saccharomycetaceae_2 - ps25
Saccharomycetaceae_3 - ps26
Kaz./Nau./Saccharomyces - ps27
Saccharomyces - ps28
Arboricola_clade - ps29
Cerevisiae_clade - ps30
Saccharomyces_cerevisiae - ps31
Phylostratum

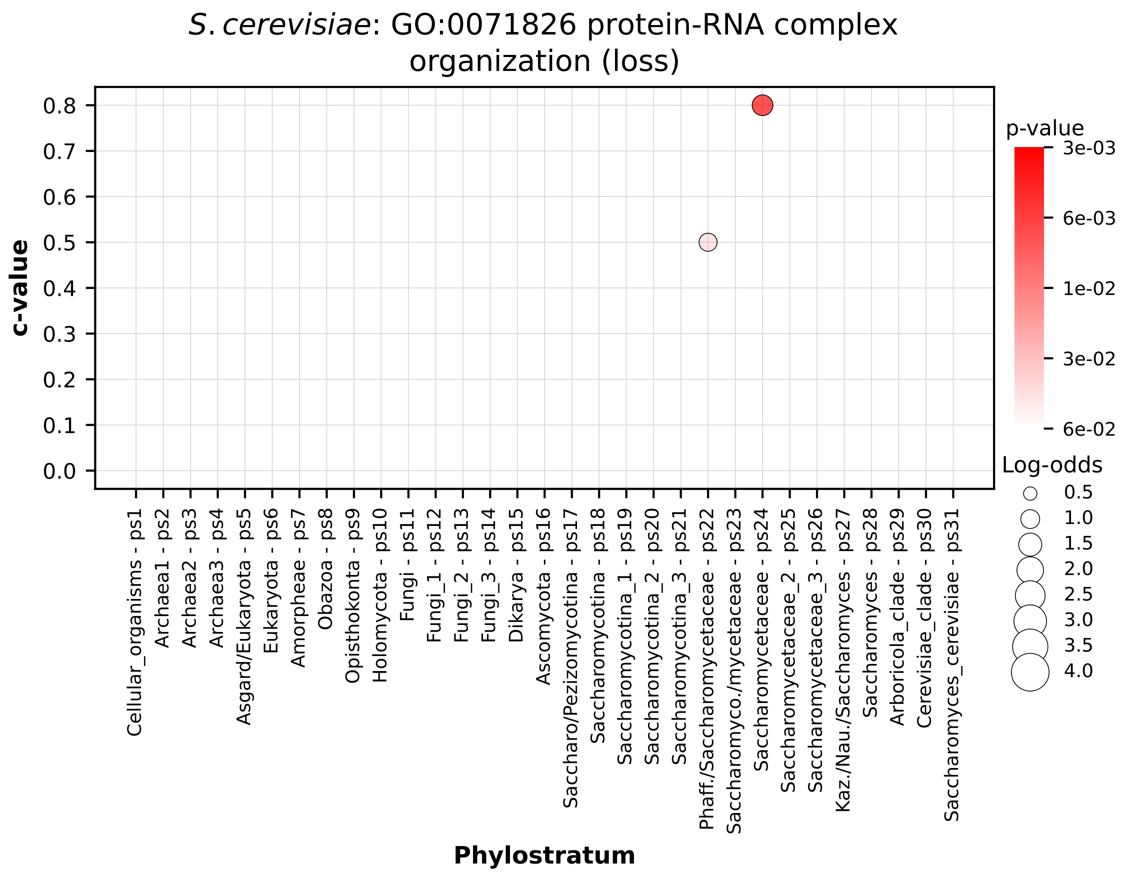
S. cerevisiae: GO:0071826 protein-RNA complex organization (loss)
c-value
0.8
0.7
0.6
0.5
0.4
0.3
0.2
0.1
0.0
p-value
3e-03
6e-03
1e-02
3e-02
6e-02
Log-odds
0.5
1.0
1.5
2.0
2.5
3.0
3.5
4.0
Cellular_organisms - ps1
Archaea1 - ps2
Archaea2 - ps3
Archaea3 - ps4
Asgard/Eukaryota - ps5
Eukaryota - ps6
Amorpheae - ps7
Obazoa - ps8
Opisthokonta - ps9
Holomycota - ps10
Fungi - ps11
Fungi_1 - ps12
Fungi_2 - ps13
Fungi_3 - ps14
Dikarya - ps15
Ascomycota - ps16
Saccharo/Pezizomycotina - ps17
Saccharomycotina - ps18
Saccharomycotina_1 - ps19
Saccharomycotina_2 - ps20
Saccharomycotina_3 - ps21
Phaff./Saccharomycetaceae - ps22
Saccharomyco./mycetaceae - ps23
Saccharomycetaceae - ps24
Saccharomycetaceae_2 - ps25
Saccharomycetaceae_3 - ps26
Kaz./Nau./Saccharomyces - ps27
Saccharomyces - ps28
Arboricola_clade - ps29
Cerevisiae_clade - ps30
Saccharomyces_cerevisiae - ps31
Phylostratum

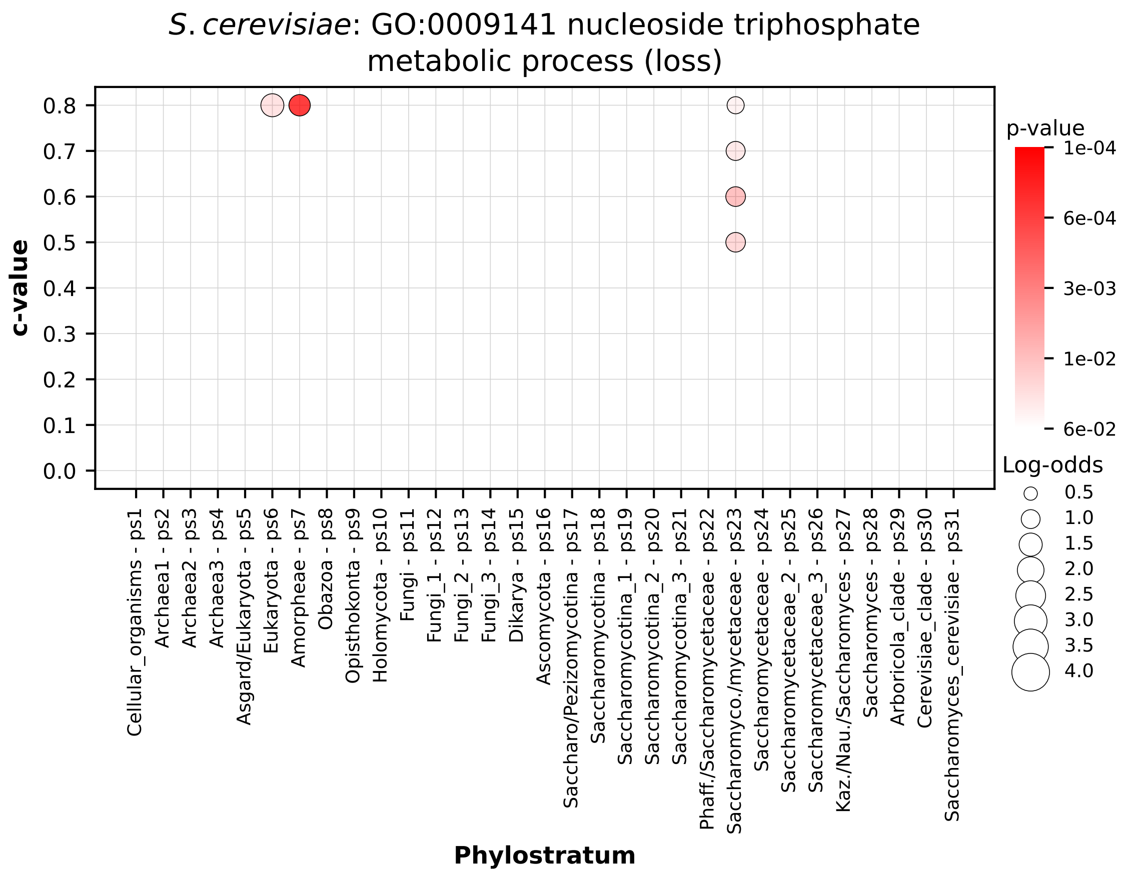

$S. cerevisiae$: GO:0009141 nucleoside triphosphate metabolic process (loss)
c-value
0.8
0.7
0.6
0.5
0.4
0.3
0.2
0.1
0.0
p-value
1e-04
6e-04
3e-03
1e-02
6e-02
Log-odds
0.5
1.0
1.5
2.0
2.5
3.0
3.5
4.0
Cellular_organisms - ps1
Archaea1 - ps2
Archaea2 - ps3
Archaea3 - ps4
Asgard/Eukaryota - ps5
Eukaryota - ps6
Amorpheae - ps7
Obazoa - ps8
Opisthokonta - ps9
Holomycota - ps10
Fungi - ps11
Fungi_1 - ps12
Fungi_2 - ps13
Fungi_3 - ps14
Dikarya - ps15
Ascomycota - ps16
Saccharo/Pezizomycotina - ps17
Saccharomycotina - ps18
Saccharomycotina_1 - ps19
Saccharomycotina_2 - ps20
Saccharomycotina_3 - ps21
Phaff./Saccharomycetaceae - ps22
Saccharomyco./mycetaceae - ps23
Saccharomycetaceae - ps24
Saccharomycetaceae_2 - ps25
Saccharomycetaceae_3 - ps26
Kaz./Nau./Saccharomyces - ps27
Saccharomyces - ps28
Arboricola_clade - ps29
Cerevisiae_clade - ps30
Saccharomyces_cerevisiae - ps31
Phylostratum

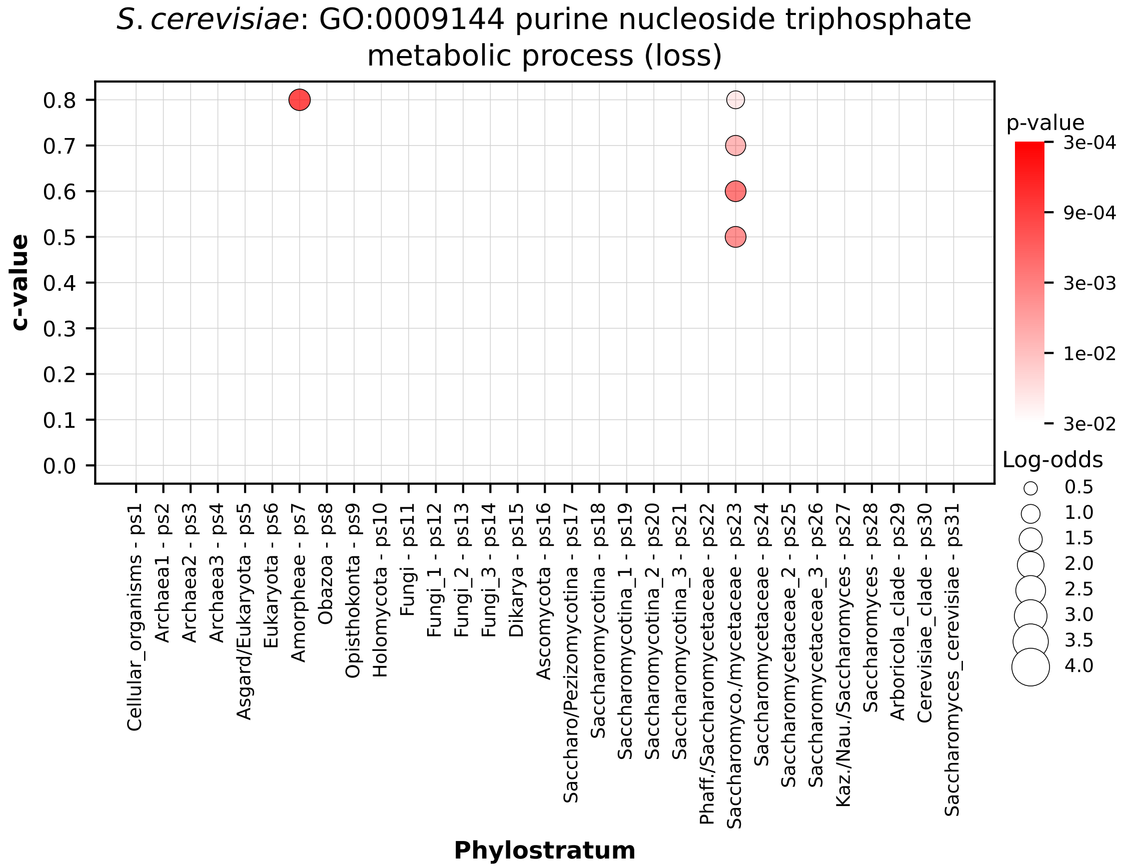

S. cerevisiae: GO:0009144 purine nucleoside triphosphate metabolic process (loss)
c-value
0.8
0.7
0.6
0.5
0.4
0.3
0.2
0.1
0.0
p-value
3e-04
9e-04
3e-03
1e-02
3e-02
Log-odds
0.5
1.0
1.5
2.0
2.5
3.0
3.5
4.0
Cellular_organisms - ps1
Archaea1 - ps2
Archaea2 - ps3
Archaea3 - ps4
Asgard/Eukaryota - ps5
Eukaryota - ps6
Amorpheae - ps7
Obazoa - ps8
Opisthokonta - ps9
Holomycota - ps10
Fungi - ps11
Fungi_1 - ps12
Fungi_2 - ps13
Fungi_3 - ps14
Dikarya - ps15
Ascomycota - ps16
Saccharo/Pezizomycotina - ps17
Saccharomycotina - ps18
Saccharomycotina_1 - ps19
Saccharomycotina_2 - ps20
Saccharomycotina_3 - ps21
Phaff./Saccharomycetaceae - ps22
Saccharomyco./mycetaceae - ps23
Saccharomycetaceae - ps24
Saccharomycetaceae_2 - ps25
Saccharomycetaceae_3 - ps26
Kaz./Nau./Saccharomyces - ps27
Saccharomyces - ps28
Arboricola_clade - ps29
Cerevisiae_clade - ps30
Saccharomyces_cerevisiae - ps31
Phylostratum

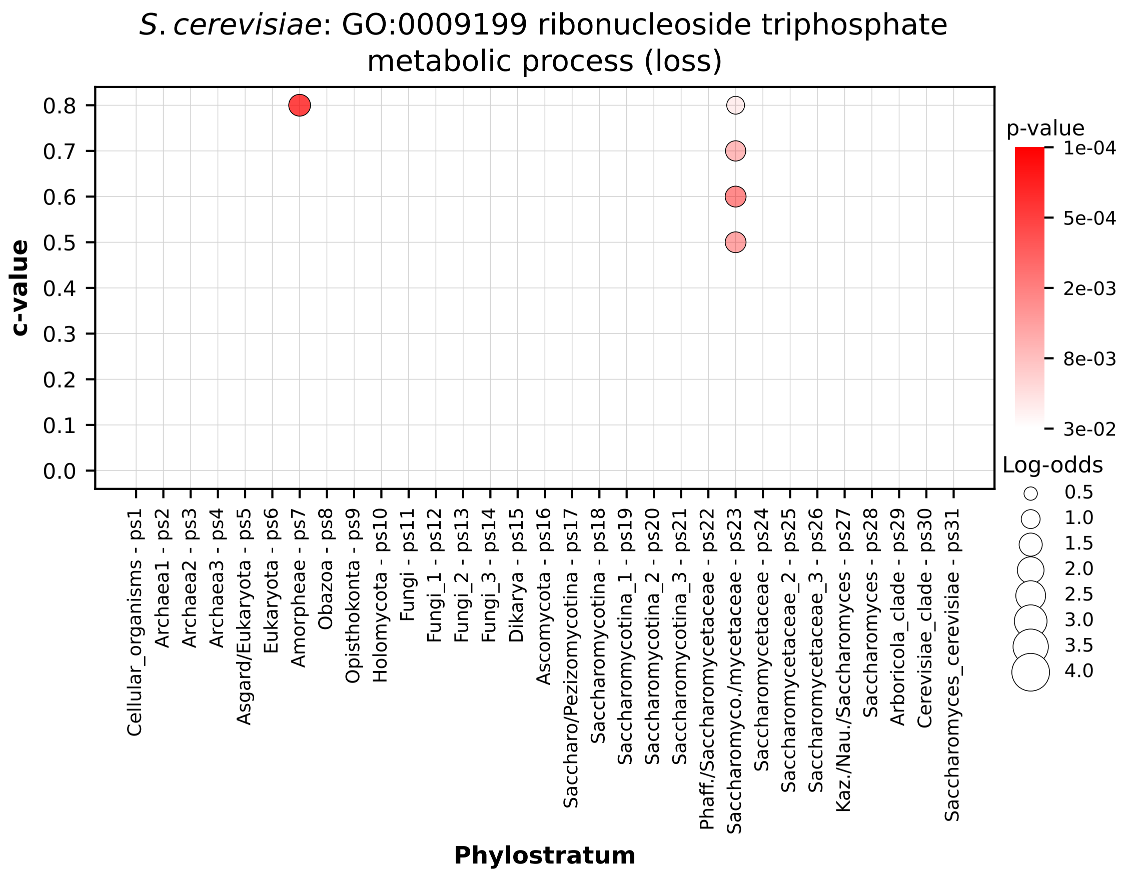

S. cerevisiae: GO:0009199 ribonucleoside triphosphate metabolic process (loss)
c-value
0.0
0.1
0.2
0.3
0.4
0.5
0.6
0.7
0.8
Phylostratum
Cellular_organisms - ps1
Archaea1 - ps2
Archaea2 - ps3
Archaea3 - ps4
Asgard/Eukaryota - ps5
Eukaryota - ps6
Amorpheae - ps7
Obazoa - ps8
Opisthokonta - ps9
Holomycota - ps10
Fungi - ps11
Fungi_1 - ps12
Fungi_2 - ps13
Fungi_3 - ps14
Dikarya - ps15
Ascomycota - ps16
Saccharo/Pezizomycotina - ps17
Saccharomycotina - ps18
Saccharomycotina_1 - ps19
Saccharomycotina_2 - ps20
Saccharomycotina_3 - ps21
Phaff./Saccharomycetaceae - ps22
Saccharomyco./mycetaceae - ps23
Saccharomycetaceae - ps24
Saccharomycetaceae_2 - ps25
Saccharomycetaceae_3 - ps26
Kaz./Nau./Saccharomyces - ps27
Saccharomyces - ps28
Arboricola_clade - ps29
Cerevisiae_clade - ps30
Saccharomyces_cerevisiae - ps31
p-value
1e-04
5e-04
2e-03
8e-03
3e-02
Log-odds
0.5
1.0
1.5
2.0
2.5
3.0
3.5
4.0

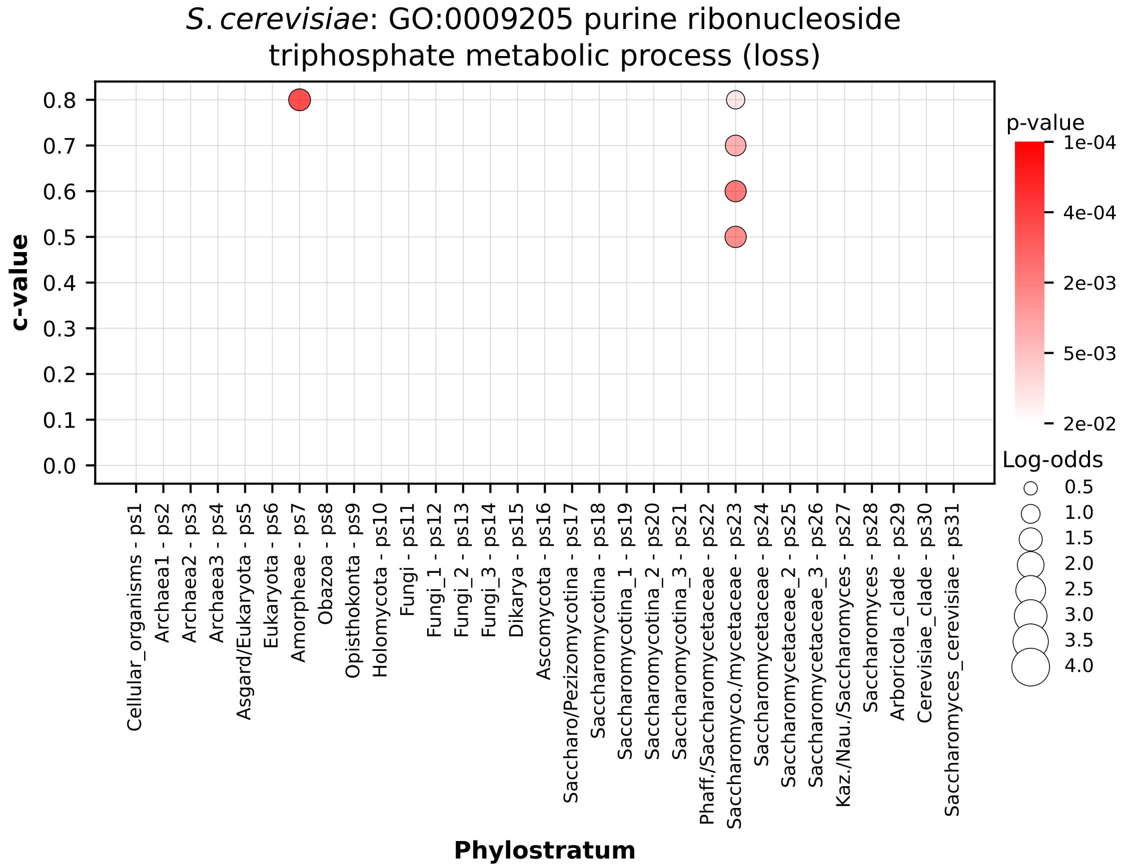
S. cerevisiae: GO:0009205 purine ribonucleoside triphosphate metabolic process (loss)
c-value
0.8
0.7
0.6
0.5
0.4
0.3
0.2
0.1
0.0
p-value
1e-04
4e-04
2e-03
5e-03
2e-02
Log-odds
0.5
1.0
1.5
2.0
2.5
3.0
3.5
4.0
Cellular_organisms - ps1
Archaea1 - ps2
Archaea2 - ps3
Archaea3 - ps4
Asgard/Eukaryota - ps5
Eukaryota - ps6
Amorpheae - ps7
Obazoa - ps8
Opisthokonta - ps9
Holomycota - ps10
Fungi - ps11
Fungi_1 - ps12
Fungi_2 - ps13
Fungi_3 - ps14
Dikarya - ps15
Ascomycota - ps16
Saccharo/Pezizomycotina - ps17
Saccharomycotina - ps18
Saccharomycotina_1 - ps19
Saccharomycotina_2 - ps20
Saccharomycotina_3 - ps21
Phaff./Saccharomycetaceae - ps22
Saccharomyco./mycetaceae - ps23
Saccharomycetaceae - ps24
Saccharomycetaceae_2 - ps25
Saccharomycetaceae_3 - ps26
Kaz./Nau./Saccharomyces - ps27
Saccharomyces - ps28
Arboricola_clade - ps29
Cerevisiae_clade - ps30
Saccharomyces_cerevisiae - ps31
Phylostratum

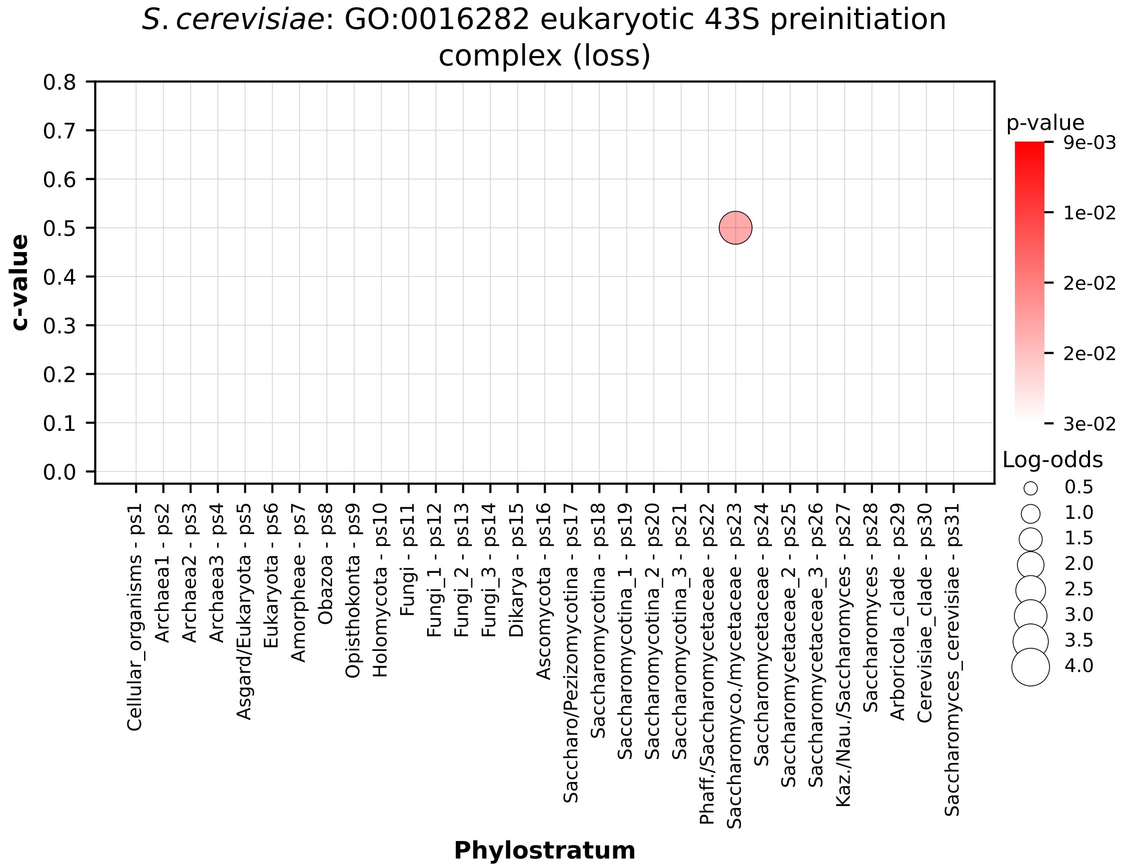

S. cerevisiae: GO:0016282 eukaryotic 43S preinitiation complex (loss)
c-value
0.8
0.7
0.6
0.5
0.4
0.3
0.2
0.1
0.0
Cellular_organisms - ps1
Archaea1 - ps2
Archaea2 - ps3
Archaea3 - ps4
Asgard/Eukaryota - ps5
Eukaryota - ps6
Amorpheae - ps7
Obazoa - ps8
Opisthokonta - ps9
Holomycota - ps10
Fungi - ps11
Fungi_1 - ps12
Fungi_2 - ps13
Fungi_3 - ps14
Dikarya - ps15
Ascomycota - ps16
Saccharo/Pezizomycotina - ps17
Saccharomycotina - ps18
Saccharomycotina_1 - ps19
Saccharomycotina_2 - ps20
Saccharomycotina_3 - ps21
Phaff./Saccharomycetaceae - ps22
Saccharomyco./mycetaceae - ps23
Saccharomycetaceae - ps24
Saccharomycetaceae_2 - ps25
Saccharomycetaceae_3 - ps26
Kaz./Nau./Saccharomyces - ps27
Saccharomyces - ps28
Arboricola_clade - ps29
Cerevisiae_clade - ps30
Saccharomyces_cerevisiae - ps31
Phylostratum
p-value
9e-03
1e-02
2e-02
2e-02
3e-02
Log-odds
0.5
1.0
1.5
2.0
2.5
3.0
3.5
4.0

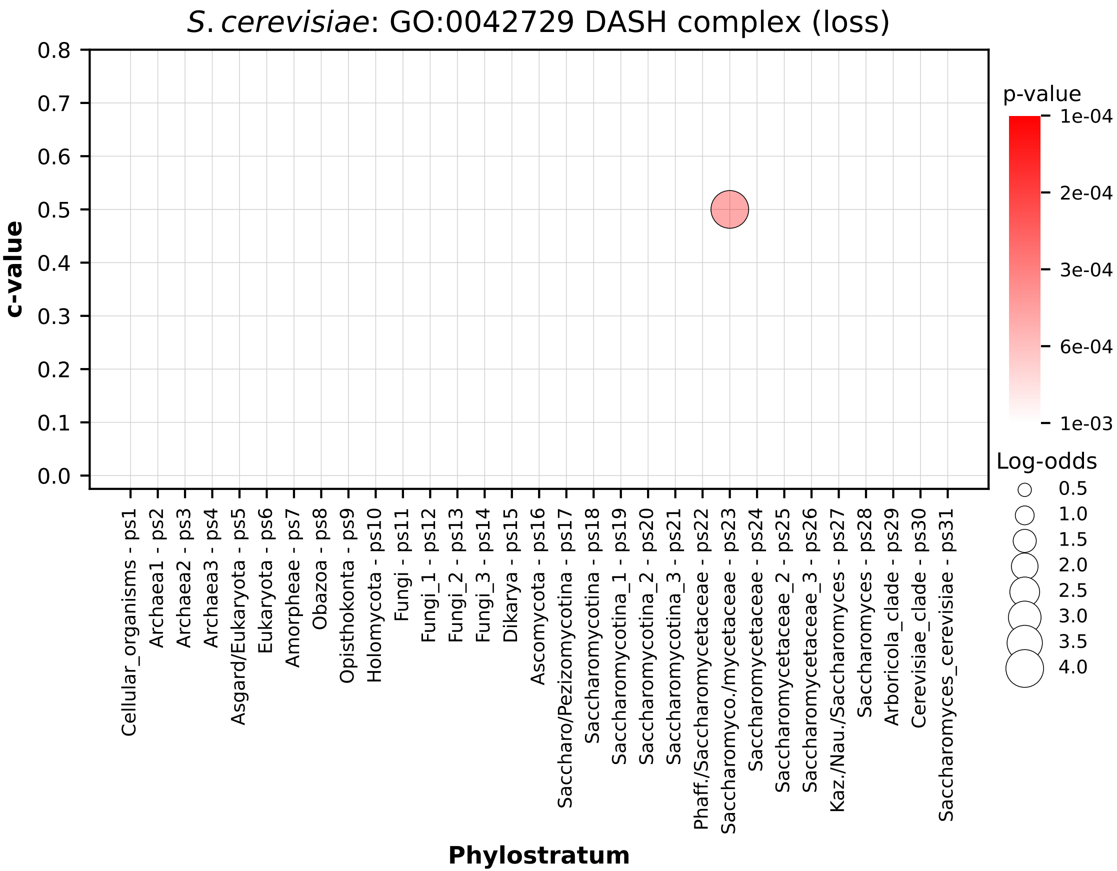

$S. cerevisiae$: GO:0042729 DASH complex (loss)
c-value
0.0
0.1
0.2
0.3
0.4
0.5
0.6
0.7
0.8
p-value
1e-04
2e-04
3e-04
6e-04
1e-03
Log-odds
0.5
1.0
1.5
2.0
2.5
3.0
3.5
4.0
Cellular_organisms - ps1
Archaea1 - ps2
Archaea2 - ps3
Archaea3 - ps4
Asgard/Eukaryota - ps5
Eukaryota - ps6
Amorpheae - ps7
Obazoa - ps8
Opisthokonta - ps9
Holomycota - ps10
Fungi - ps11
Fungi_1 - ps12
Fungi_2 - ps13
Fungi_3 - ps14
Dikarya - ps15
Ascomycota - ps16
Saccharo/Pezizomycotina - ps17
Saccharomycotina - ps18
Saccharomycotina_1 - ps19
Saccharomycotina_2 - ps20
Saccharomycotina_3 - ps21
Phaff./Saccharomycetaceae - ps22
Saccharomyco./mycetaceae - ps23
Saccharomycetaceae - ps24
Saccharomycetaceae_2 - ps25
Saccharomycetaceae_3 - ps26
Kaz./Nau./Saccharomyces - ps27
Saccharomyces - ps28
Arboricola_clade - ps29
Cerevisiae_clade - ps30
Saccharomyces_cerevisiae - ps31
Phylostratum

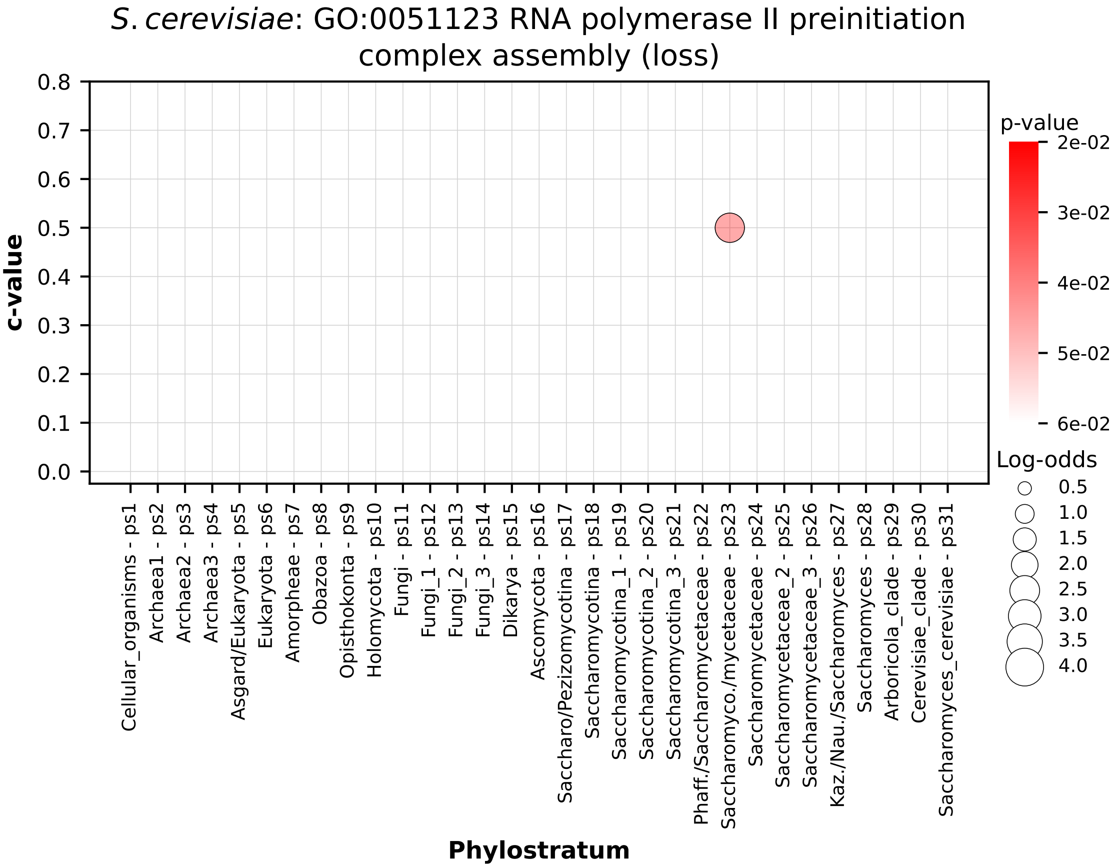
S. cerevisiae: GO:0051123 RNA polymerase II preinitiation complex assembly (loss)
c-value
0.8
0.7
0.6
0.5
0.4
0.3
0.2
0.1
0.0
p-value
2e-02
3e-02
4e-02
5e-02
6e-02
Log-odds
0.5
1.0
1.5
2.0
2.5
3.0
3.5
4.0
Cellular_organisms - ps1
Archaea1 - ps2
Archaea2 - ps3
Archaea3 - ps4
Asgard/Eukaryota - ps5
Eukaryota - ps6
Amorpheae - ps7
Obazoa - ps8
Opisthokonta - ps9
Holomycota - ps10
Fungi - ps11
Fungi_1 - ps12
Fungi_2 - ps13
Fungi_3 - ps14
Dikarya - ps15
Ascomycota - ps16
Saccharo/Pezizomycotina - ps17
Saccharomycotina - ps18
Saccharomycotina_1 - ps19
Saccharomycotina_2 - ps20
Saccharomycotina_3 - ps21
Phaff./Saccharomycetaceae - ps22
Saccharomyco./mycetaceae - ps23
Saccharomycetaceae - ps24
Saccharomycetaceae_2 - ps25
Saccharomycetaceae_3 - ps26
Kaz./Nau./Saccharomyces - ps27
Saccharomyces - ps28
Arboricola_clade - ps29
Cerevisiae_clade - ps30
Saccharomyces_cerevisiae - ps31
Phylostratum

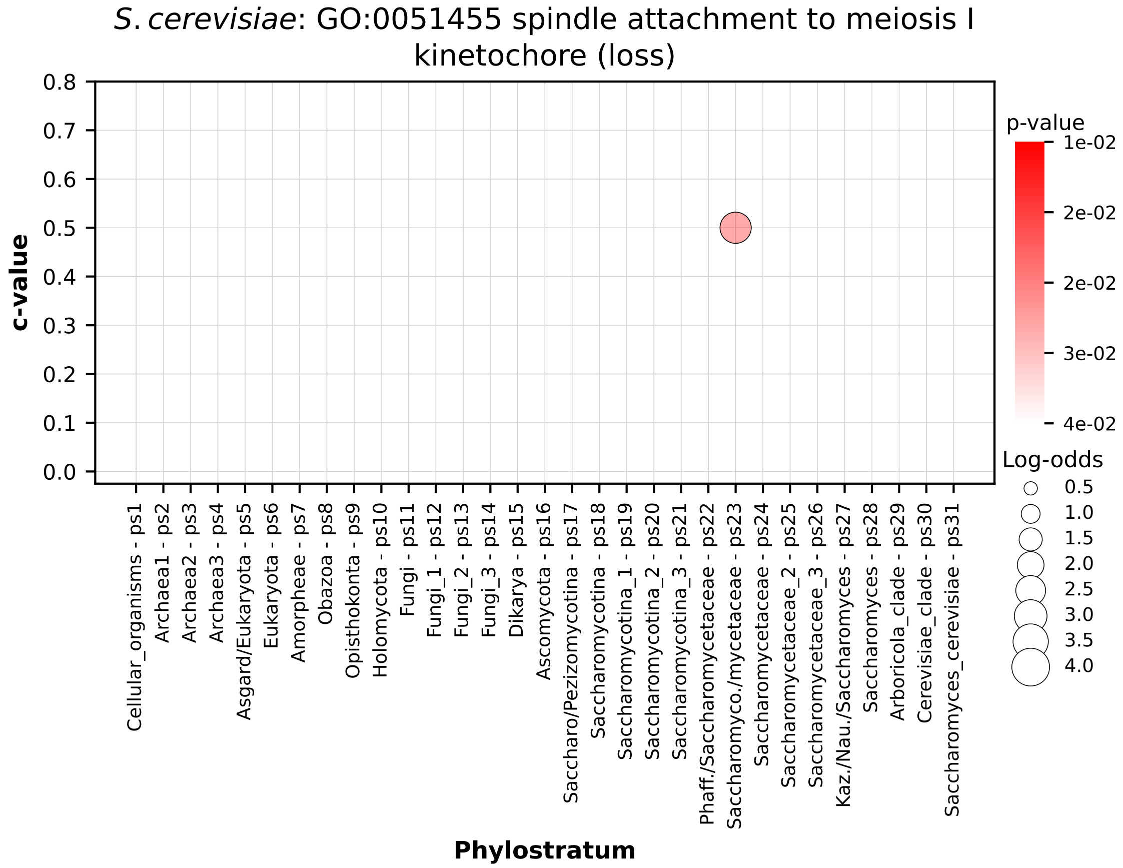

S. cerevisiae: GO:0051455 spindle attachment to meiosis I kinetochore (loss)
c-value
0.8
0.7
0.6
0.5
0.4
0.3
0.2
0.1
0.0
Phylostratum
Cellular_organisms - ps1
Archaea1 - ps2
Archaea2 - ps3
Archaea3 - ps4
Asgard/Eukaryota - ps5
Eukaryota - ps6
Amorpheae - ps7
Obazoa - ps8
Opisthokonta - ps9
Holomycota - ps10
Fungi - ps11
Fungi_1 - ps12
Fungi_2 - ps13
Fungi_3 - ps14
Dikarya - ps15
Ascomycota - ps16
Saccharo/Pezizomycotina - ps17
Saccharomycotina - ps18
Saccharomycotina_1 - ps19
Saccharomycotina_2 - ps20
Saccharomycotina_3 - ps21
Phaff./Saccharomycetaceae - ps22
Saccharomyco./mycetaceae - ps23
Saccharomycetaceae - ps24
Saccharomycetaceae_2 - ps25
Saccharomycetaceae_3 - ps26
Kaz./Nau./Saccharomyces - ps27
Saccharomyces - ps28
Arboricola_clade - ps29
Cerevisiae_clade - ps30
Saccharomyces_cerevisiae - ps31
p-value
1e-02
2e-02
2e-02
3e-02
4e-02
Log-odds
0.5
1.0
1.5
2.0
2.5
3.0
3.5
4.0

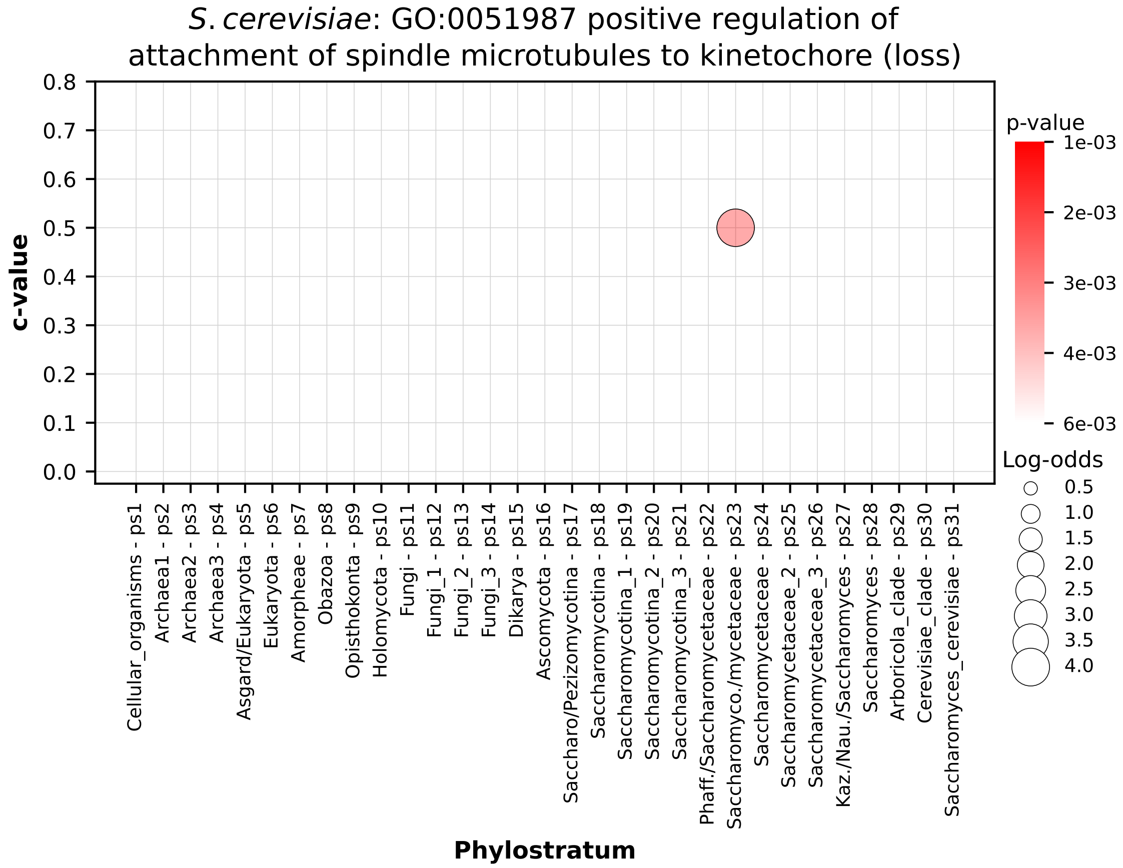
S. cerevisiae: GO:0051987 positive regulation of attachment of spindle microtubules to kinetochore (loss)
c-value
0.8
0.7
0.6
0.5
0.4
0.3
0.2
0.1
0.0
p-value
1e-03
2e-03
3e-03
4e-03
6e-03
Log-odds
0.5
1.0
1.5
2.0
2.5
3.0
3.5
4.0
Cellular_organisms - ps1
Archaea1 - ps2
Archaea2 - ps3
Archaea3 - ps4
Asgard/Eukaryota - ps5
Eukaryota - ps6
Amorpheae - ps7
Obazoa - ps8
Opisthokonta - ps9
Holomycota - ps10
Fungi - ps11
Fungi_1 - ps12
Fungi_2 - ps13
Fungi_3 - ps14
Dikarya - ps15
Ascomycota - ps16
Saccharo/Pezizomycotina - ps17
Saccharomycotina - ps18
Saccharomycotina_1 - ps19
Saccharomycotina_2 - ps20
Saccharomycotina_3 - ps21
Phaff./Saccharomycetaceae - ps22
Saccharomyco./mycetaceae - ps23
Saccharomycetaceae - ps24
Saccharomycetaceae_2 - ps25
Saccharomycetaceae_3 - ps26
Kaz./Nau./Saccharomyces - ps27
Saccharomyces - ps28
Arboricola_clade - ps29
Cerevisiae_clade - ps30
Saccharomyces_cerevisiae - ps31
Phylostratum

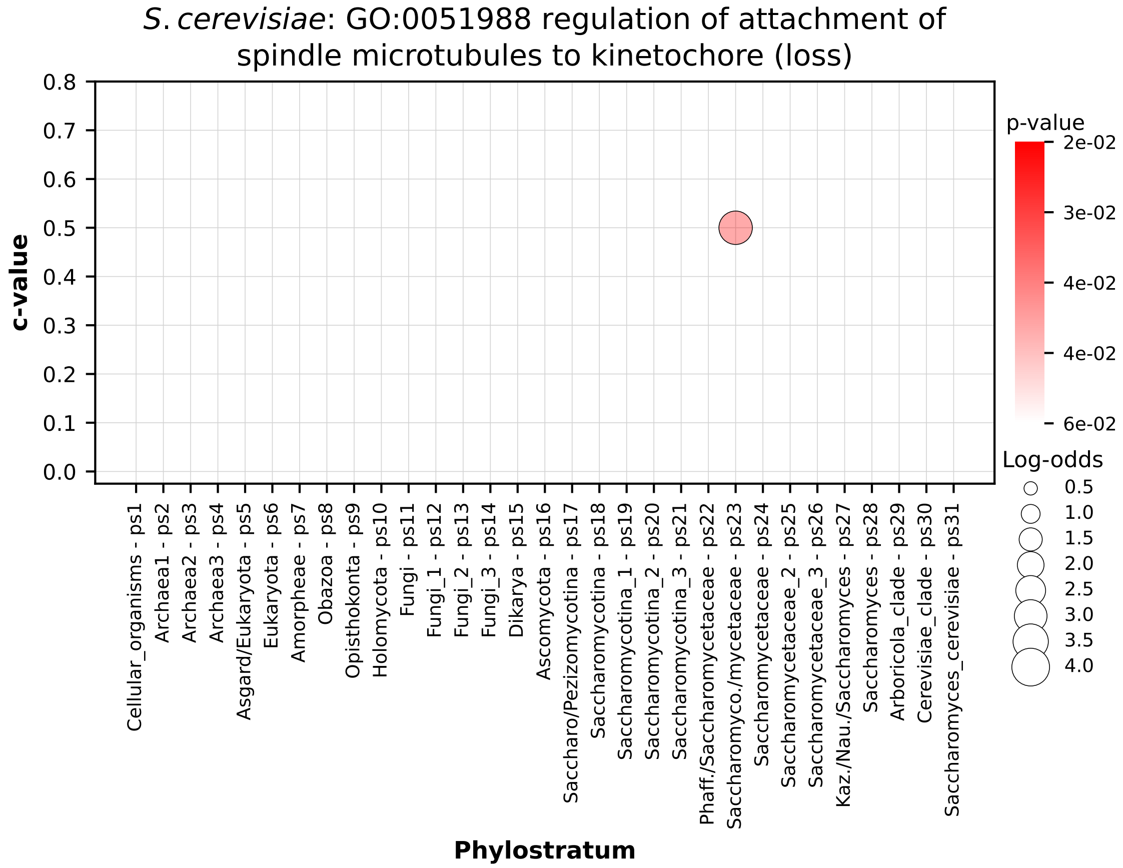

S. cerevisiae: GO:0051988 regulation of attachment of spindle microtubules to kinetochore (loss)
c-value
0.8
0.7
0.6
0.5
0.4
0.3
0.2
0.1
0.0
p-value
2e-02
3e-02
4e-02
4e-02
6e-02
Log-odds
0.5
1.0
1.5
2.0
2.5
3.0
3.5
4.0
Cellular_organisms - ps1
Archaea1 - ps2
Archaea2 - ps3
Archaea3 - ps4
Asgard/Eukaryota - ps5
Eukaryota - ps6
Amorpheae - ps7
Obazoa - ps8
Opisthokonta - ps9
Holomycota - ps10
Fungi - ps11
Fungi_1 - ps12
Fungi_2 - ps13
Fungi_3 - ps14
Dikarya - ps15
Ascomycota - ps16
Saccharo/Pezizomycotina - ps17
Saccharomycotina - ps18
Saccharomycotina_1 - ps19
Saccharomycotina_2 - ps20
Saccharomycotina_3 - ps21
Phaff./Saccharomycetaceae - ps22
Saccharomyco./mycetaceae - ps23
Saccharomycetaceae - ps24
Saccharomycetaceae_2 - ps25
Saccharomycetaceae_3 - ps26
Kaz./Nau./Saccharomyces - ps27
Saccharomyces - ps28
Arboricola_clade - ps29
Cerevisiae_clade - ps30
Saccharomyces_cerevisiae - ps31
Phylostratum

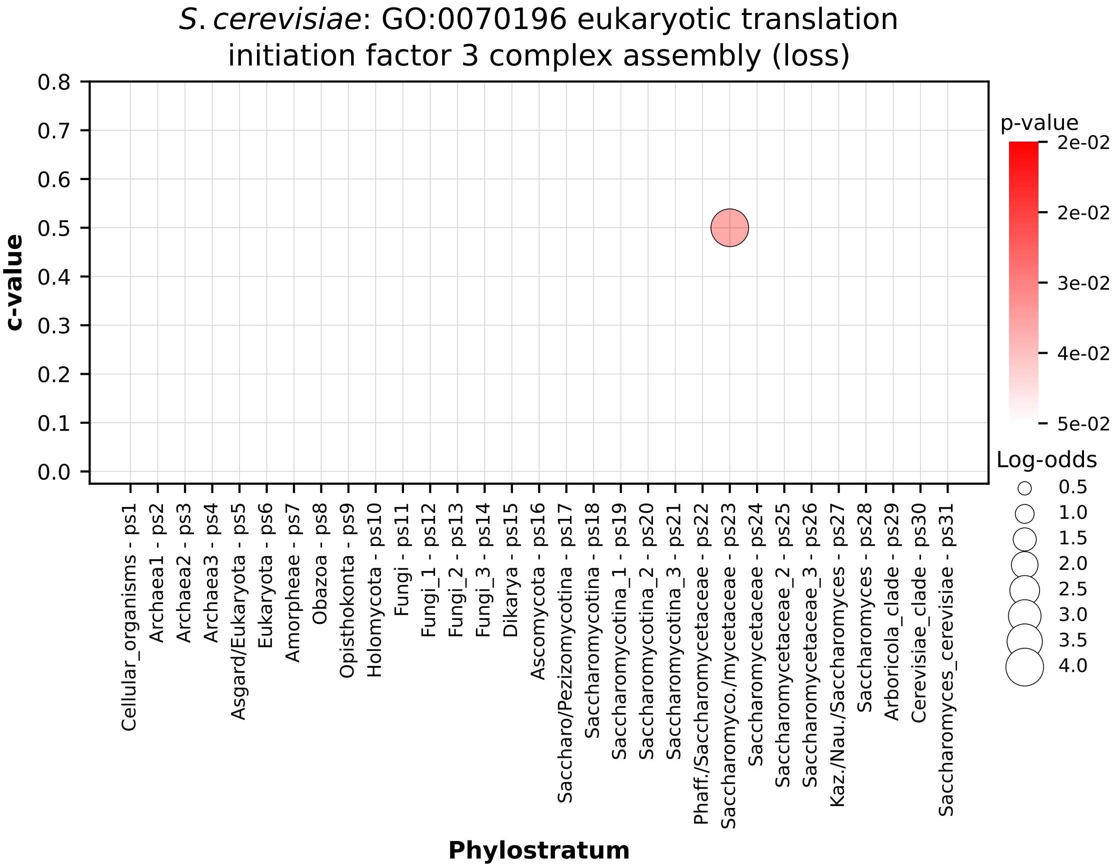
S. cerevisiae: GO:0070196 eukaryotic translation initiation factor 3 complex assembly (loss)
c-value
0.8
0.7
0.6
0.5
0.4
0.3
0.2
0.1
0.0
Phylostratum
Cellular_organisms - ps1
Archaea1 - ps2
Archaea2 - ps3
Archaea3 - ps4
Asgard/Eukaryota - ps5
Eukaryota - ps6
Amorpheae - ps7
Obazoa - ps8
Opisthokonta - ps9
Holomycota - ps10
Fungi - ps11
Fungi_1 - ps12
Fungi_2 - ps13
Fungi_3 - ps14
Dikarya - ps15
Ascomycota - ps16
Saccharo/Pezizomycotina - ps17
Saccharomycotina - ps18
Saccharomycotina_1 - ps19
Saccharomycotina_2 - ps20
Saccharomycotina_3 - ps21
Phaff./Saccharomycetaceae - ps22
Saccharomyco./mycetaceae - ps23
Saccharomycetaceae - ps24
Saccharomycetaceae_2 - ps25
Saccharomycetaceae_3 - ps26
Kaz./Nau./Saccharomyces - ps27
Saccharomyces - ps28
Arboricola_clade - ps29
Cerevisiae_clade - ps30
Saccharomyces_cerevisiae - ps31
p-value
2e-02
2e-02
3e-02
4e-02
5e-02
Log-odds
0.5
1.0
1.5
2.0
2.5
3.0
3.5
4.0

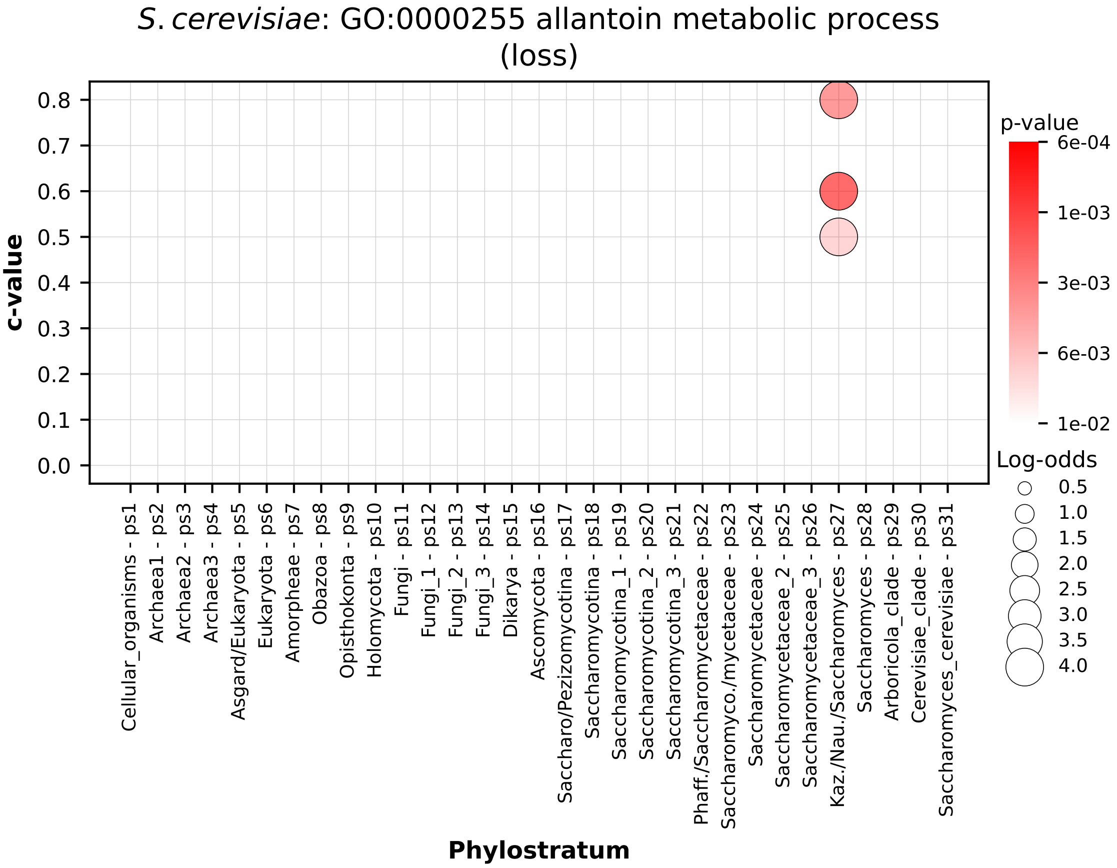

S. cerevisiae: GO:0000255 allantoin metabolic process
(loss)
c-value
0.8
0.7
0.6
0.5
0.4
0.3
0.2
0.1
0.0
p-value
6e-04
1e-03
3e-03
6e-03
1e-02
Log-odds
0.5
1.0
1.5
2.0
2.5
3.0
3.5
4.0
Cellular_organisms - ps1
Archaea1 - ps2
Archaea2 - ps3
Archaea3 - ps4
Asgard/Eukaryota - ps5
Eukaryota - ps6
Amorpheae - ps7
Obazoa - ps8
Opisthokonta - ps9
Holomycota - ps10
Fungi - ps11
Fungi_1 - ps12
Fungi_2 - ps13
Fungi_3 - ps14
Dikarya - ps15
Ascomycota - ps16
Saccharo/Pezizomycotina - ps17
Saccharomycotina - ps18
Saccharomycotina_1 - ps19
Saccharomycotina_2 - ps20
Saccharomycotina_3 - ps21
Phaff./Saccharomycetaceae - ps22
Saccharomyco./mycetaceae - ps23
Saccharomycetaceae - ps24
Saccharomycetaceae_2 - ps25
Saccharomycetaceae_3 - ps26
Kaz./Nau./Saccharomyces - ps27
Saccharomyces - ps28
Arboricola_clade - ps29
Cerevisiae_clade - ps30
Saccharomyces_cerevisiae - ps31
Phylostratum

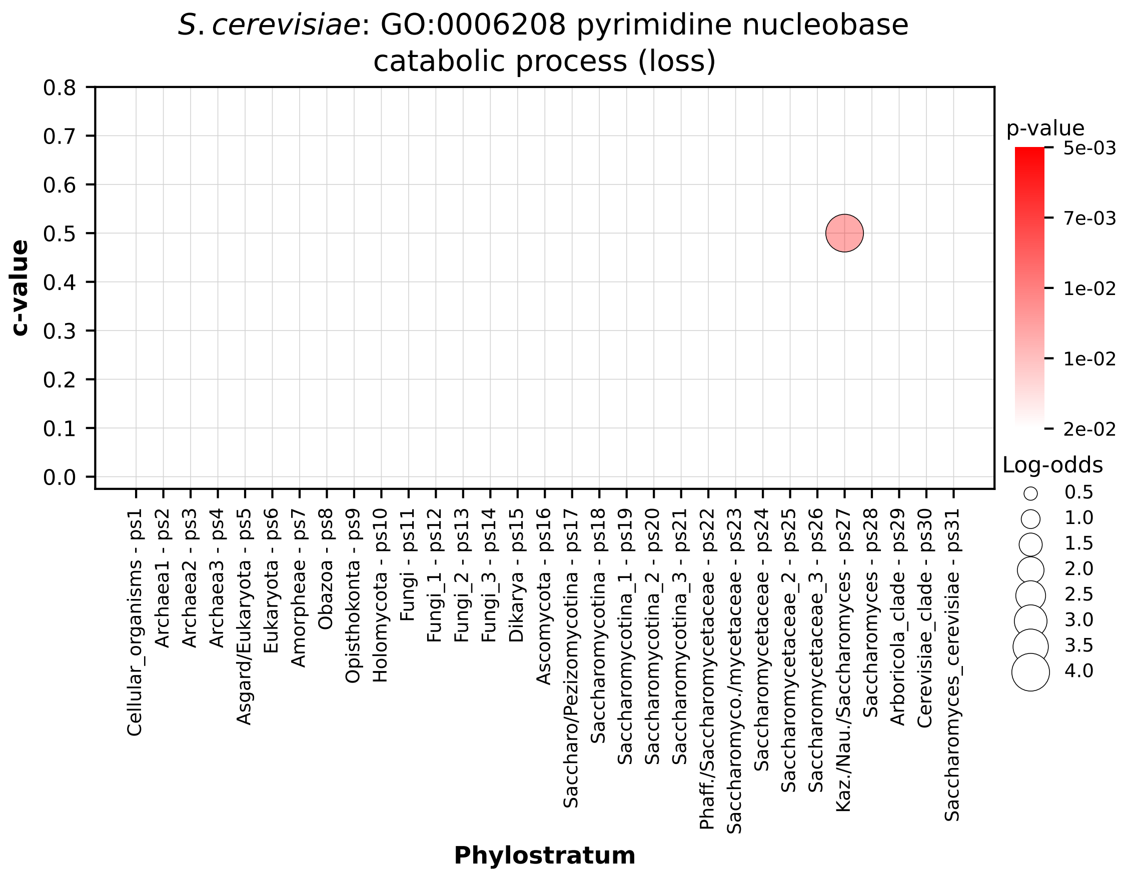

S. cerevisiae: GO:0006208 pyrimidine nucleobase catabolic process (loss)
c-value
0.0
0.1
0.2
0.3
0.4
0.5
0.6
0.7
0.8
Phylostratum
Cellular_organisms - ps1
Archaea1 - ps2
Archaea2 - ps3
Archaea3 - ps4
Asgard/Eukaryota - ps5
Eukaryota - ps6
Amorpheae - ps7
Obazoa - ps8
Opisthokonta - ps9
Holomycota - ps10
Fungi - ps11
Fungi_1 - ps12
Fungi_2 - ps13
Fungi_3 - ps14
Dikarya - ps15
Ascomycota - ps16
Saccharo/Pezizomycotina - ps17
Saccharomycotina - ps18
Saccharomycotina_1 - ps19
Saccharomycotina_2 - ps20
Saccharomycotina_3 - ps21
Phaff./Saccharomycetaceae - ps22
Saccharomyco./mycetaceae - ps23
Saccharomycetaceae - ps24
Saccharomycetaceae_2 - ps25
Saccharomycetaceae_3 - ps26
Kaz./Nau./Saccharomyces - ps27
Saccharomyces - ps28
Arboricola_clade - ps29
Cerevisiae_clade - ps30
Saccharomyces_cerevisiae - ps31
p-value
5e-03
7e-03
1e-02
1e-02
2e-02
Log-odds
0.5
1.0
1.5
2.0
2.5
3.0
3.5
4.0

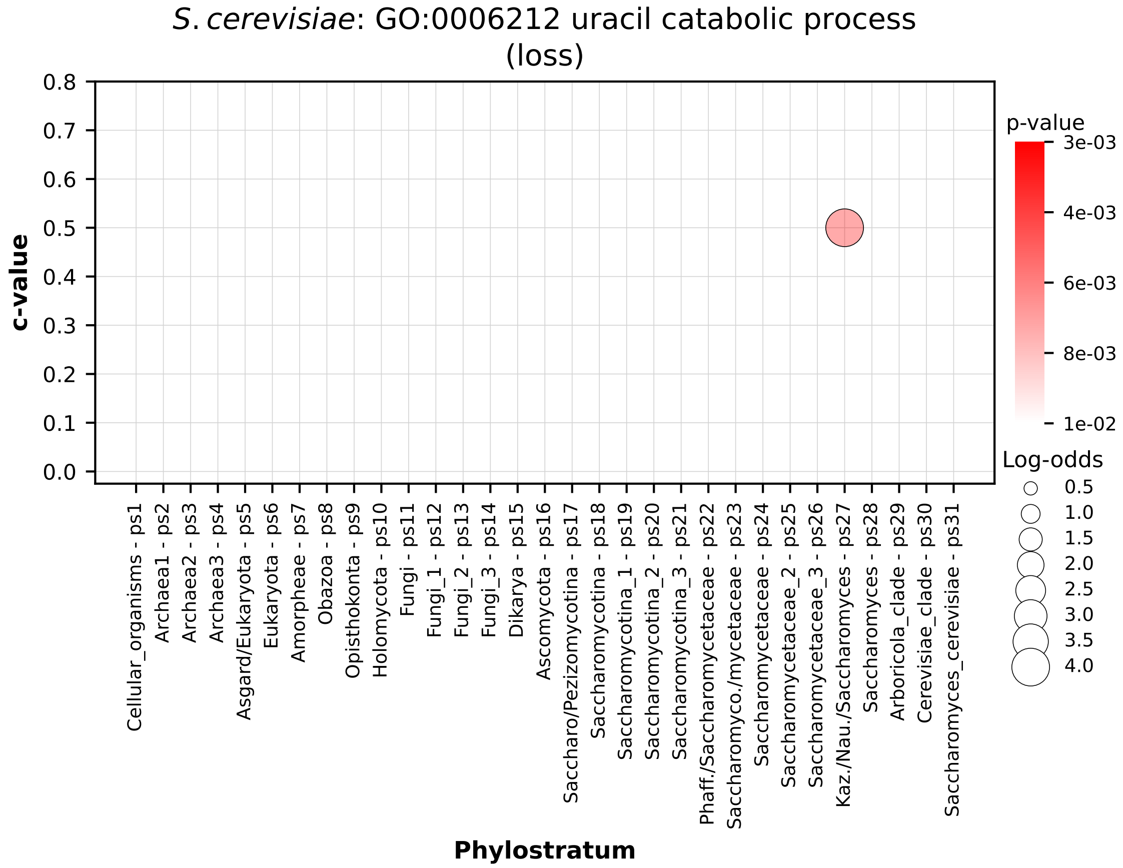

S. cerevisiae: GO:0006212 uracil catabolic process
(loss)
c-value
0.8
0.7
0.6
0.5
0.4
0.3
0.2
0.1
0.0
Phylostratum
Cellular_organisms - ps1
Archaea1 - ps2
Archaea2 - ps3
Archaea3 - ps4
Asgard/Eukaryota - ps5
Eukaryota - ps6
Amorpheae - ps7
Obazoa - ps8
Opisthokonta - ps9
Holomycota - ps10
Fungi - ps11
Fungi_1 - ps12
Fungi_2 - ps13
Fungi_3 - ps14
Dikarya - ps15
Ascomycota - ps16
Saccharo/Pezizomycotina - ps17
Saccharomycotina - ps18
Saccharomycotina_1 - ps19
Saccharomycotina_2 - ps20
Saccharomycotina_3 - ps21
Phaff./Saccharomycetaceae - ps22
Saccharomyco./mycetaceae - ps23
Saccharomycetaceae - ps24
Saccharomycetaceae_2 - ps25
Saccharomycetaceae_3 - ps26
Kaz./Nau./Saccharomyces - ps27
Saccharomyces - ps28
Arboricola_clade - ps29
Cerevisiae_clade - ps30
Saccharomyces_cerevisiae - ps31
p-value
3e-03
4e-03
6e-03
8e-03
1e-02
Log-odds
0.5
1.0
1.5
2.0
2.5
3.0
3.5
4.0

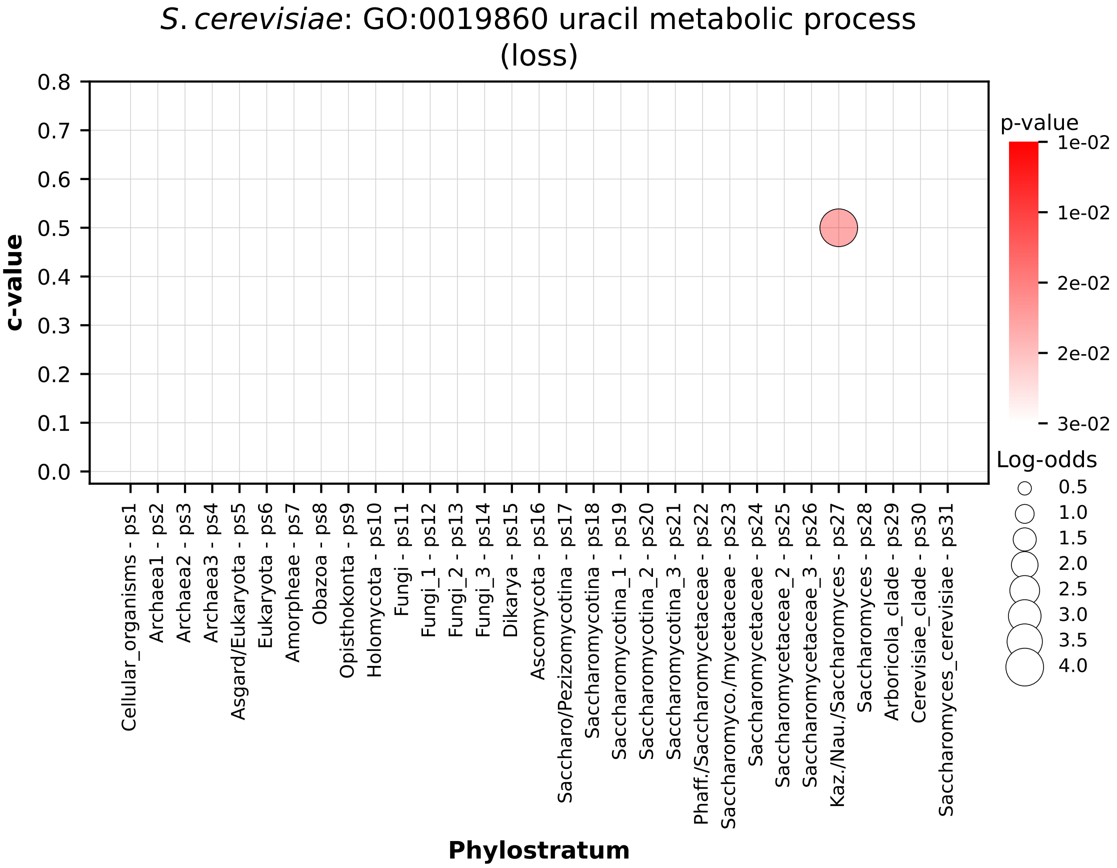

S. cerevisiae: GO:0019860 uracil metabolic process
(loss)
c-value
0.8
0.7
0.6
0.5
0.4
0.3
0.2
0.1
0.0
Phylostratum
Cellular_organisms - ps1
Archaea1 - ps2
Archaea2 - ps3
Archaea3 - ps4
Asgard/Eukaryota - ps5
Eukaryota - ps6
Amorpheae - ps7
Obazoa - ps8
Opisthokonta - ps9
Holomycota - ps10
Fungi - ps11
Fungi_1 - ps12
Fungi_2 - ps13
Fungi_3 - ps14
Dikarya - ps15
Ascomycota - ps16
Saccharo/Pezizomycotina - ps17
Saccharomycotina - ps18
Saccharomycotina_1 - ps19
Saccharomycotina_2 - ps20
Saccharomycotina_3 - ps21
Phaff./Saccharomycetaceae - ps22
Saccharomyco./mycetaceae - ps23
Saccharomycetaceae - ps24
Saccharomycetaceae_2 - ps25
Saccharomycetaceae_3 - ps26
Kaz./Nau./Saccharomyces - ps27
Saccharomyces - ps28
Arboricola_clade - ps29
Cerevisiae_clade - ps30
Saccharomyces_cerevisiae - ps31
p-value
1e-02
1e-02
2e-02
2e-02
3e-02
Log-odds
0.5
1.0
1.5
2.0
2.5
3.0
3.5
4.0

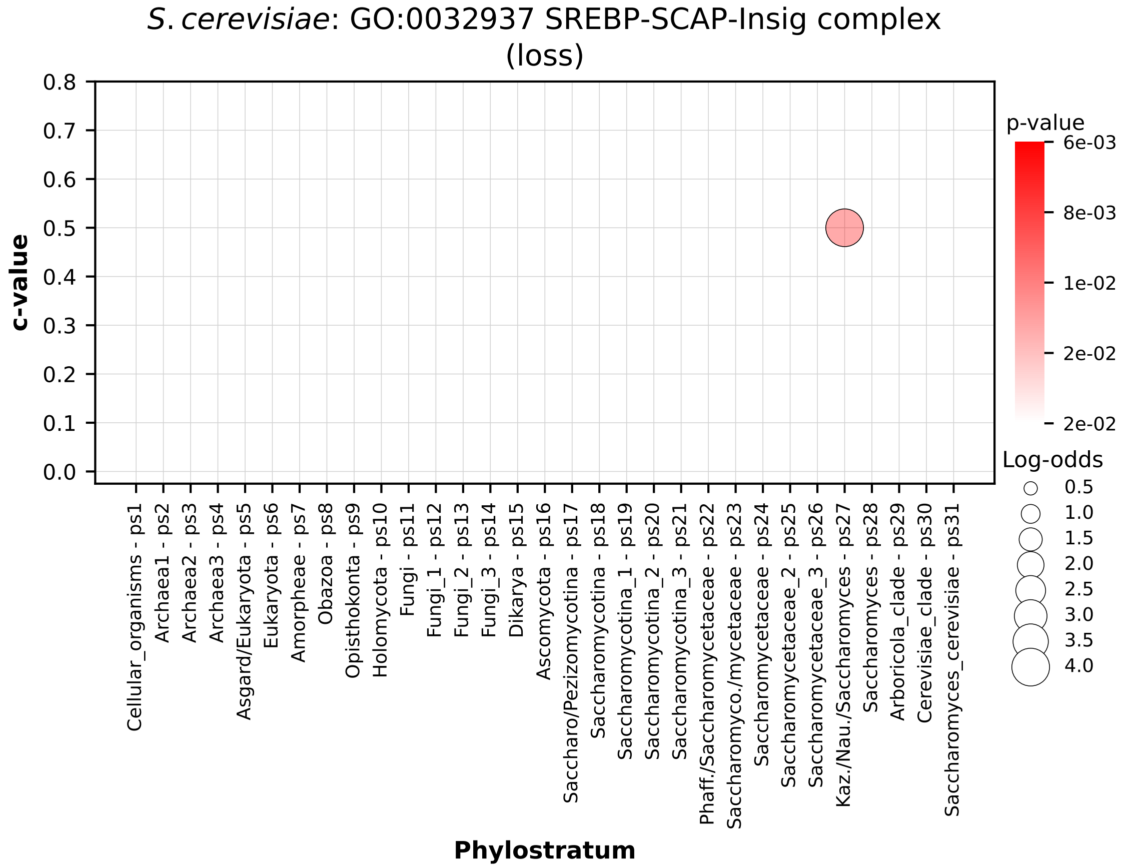
S. cerevisiae: GO:0032937 SREBP-SCAP-Insig complex
(loss)
c-value
0.8
0.7
0.6
0.5
0.4
0.3
0.2
0.1
0.0
p-value
6e-03
8e-03
1e-02
2e-02
2e-02
Log-odds
0.5
1.0
1.5
2.0
2.5
3.0
3.5
4.0
Cellular_organisms - ps1
Archaea1 - ps2
Archaea2 - ps3
Archaea3 - ps4
Asgard/Eukaryota - ps5
Eukaryota - ps6
Amorpheae - ps7
Obazoa - ps8
Opisthokonta - ps9
Holomycota - ps10
Fungi - ps11
Fungi_1 - ps12
Fungi_2 - ps13
Fungi_3 - ps14
Dikarya - ps15
Ascomycota - ps16
Saccharo/Pezizomycotina - ps17
Saccharomycotina - ps18
Saccharomycotina_1 - ps19
Saccharomycotina_2 - ps20
Saccharomycotina_3 - ps21
Phaff./Saccharomycetaceae - ps22
Saccharomyco./mycetaceae - ps23
Saccharomycetaceae - ps24
Saccharomycetaceae_2 - ps25
Saccharomycetaceae_3 - ps26
Kaz./Nau./Saccharomyces - ps27
Saccharomyces - ps28
Arboricola_clade - ps29
Cerevisiae_clade - ps30
Saccharomyces_cerevisiae - ps31
Phylostratum

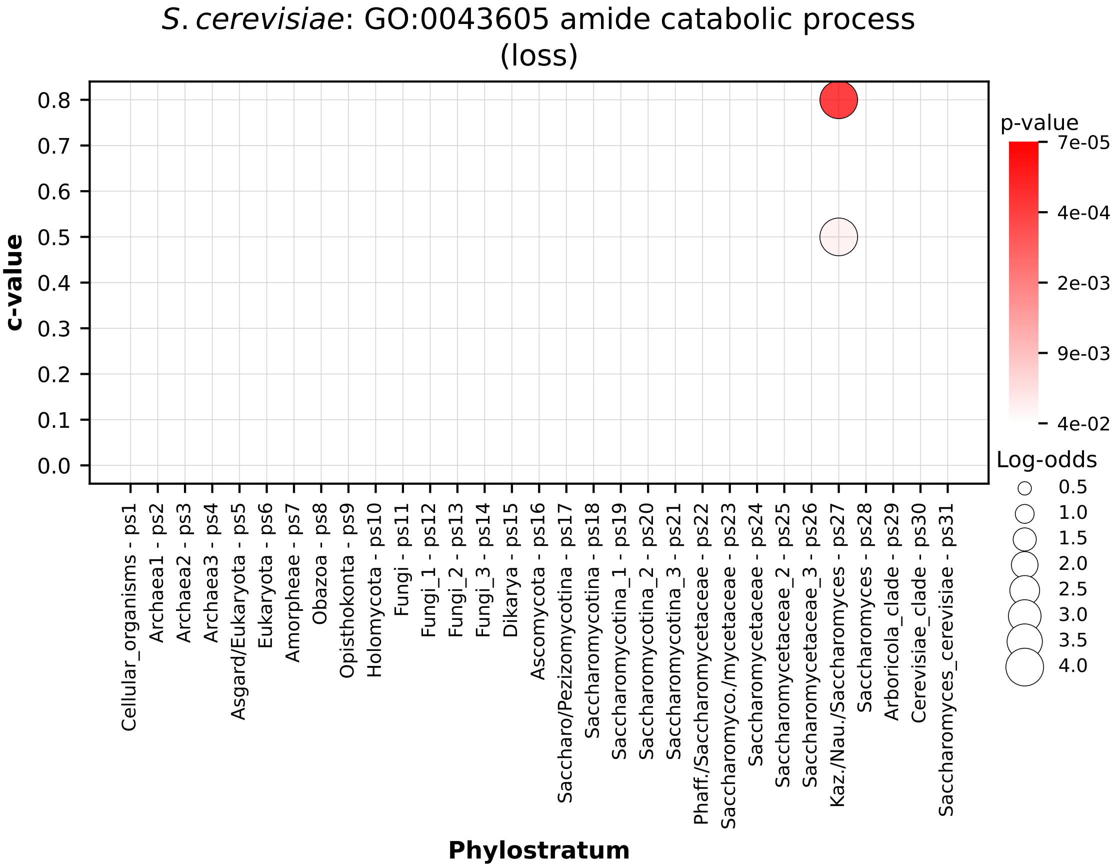

S. cerevisiae: GO:0043605 amide catabolic process
(loss)
c-value
0.0
0.1
0.2
0.3
0.4
0.5
0.6
0.7
0.8
Phylostratum
Cellular_organisms - ps1
Archaea1 - ps2
Archaea2 - ps3
Archaea3 - ps4
Asgard/Eukaryota - ps5
Eukaryota - ps6
Amorpheae - ps7
Obazoa - ps8
Opisthokonta - ps9
Holomycota - ps10
Fungi - ps11
Fungi_1 - ps12
Fungi_2 - ps13
Fungi_3 - ps14
Dikarya - ps15
Ascomycota - ps16
Saccharo/Pezizomycotina - ps17
Saccharomycotina - ps18
Saccharomycotina_1 - ps19
Saccharomycotina_2 - ps20
Saccharomycotina_3 - ps21
Phaff./Saccharomycetaceae - ps22
Saccharomyco./mycetaceae - ps23
Saccharomycetaceae - ps24
Saccharomycetaceae_2 - ps25
Saccharomycetaceae_3 - ps26
Kaz./Nau./Saccharomyces - ps27
Saccharomyces - ps28
Arboricola_clade - ps29
Cerevisiae_clade - ps30
Saccharomyces_cerevisiae - ps31
p-value
7e-05
4e-04
2e-03
9e-03
4e-02
Log-odds
0.5
1.0
1.5
2.0
2.5
3.0
3.5
4.0

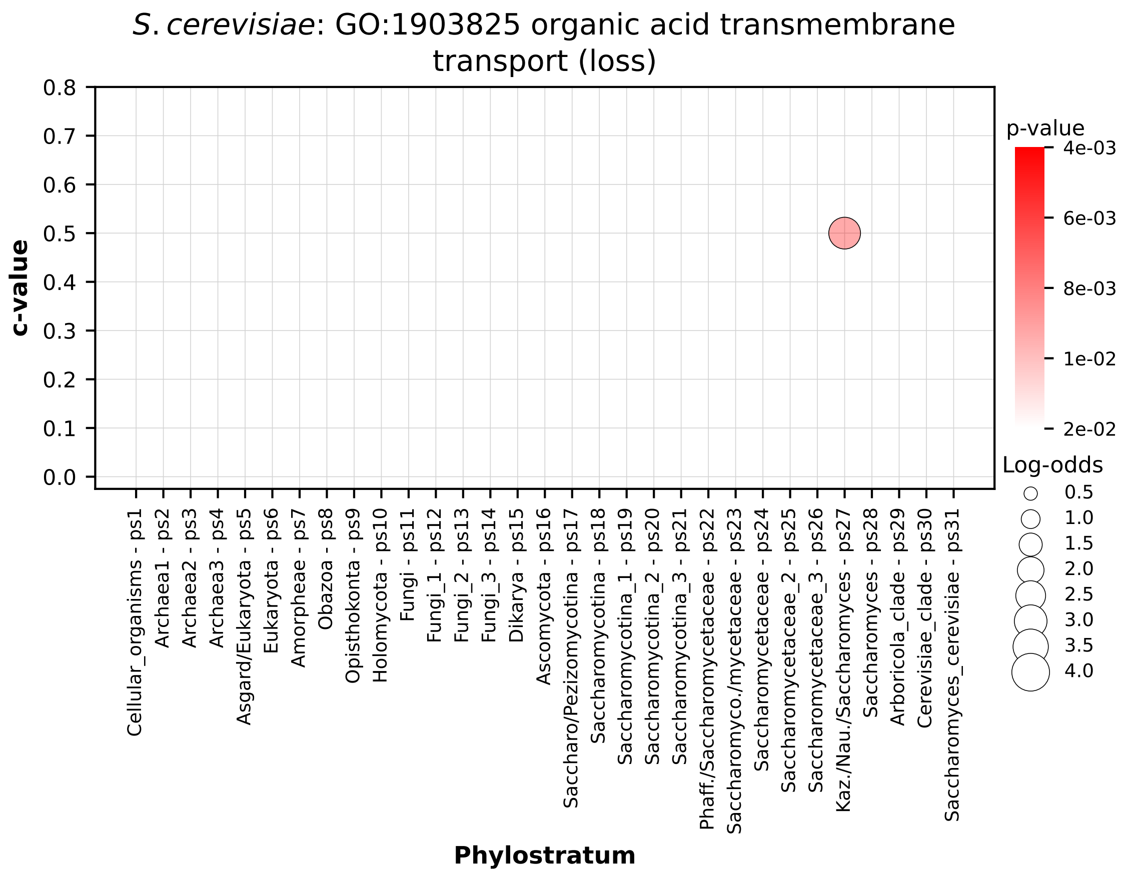

S. cerevisiae: GO:1903825 organic acid transmembrane transport (loss)
c-value
0.0
0.1
0.2
0.3
0.4
0.5
0.6
0.7
0.8
Phylostratum
Cellular_organisms - ps1
Archaea1 - ps2
Archaea2 - ps3
Archaea3 - ps4
Asgard/Eukaryota - ps5
Eukaryota - ps6
Amorpheae - ps7
Obazoa - ps8
Opisthokonta - ps9
Holomycota - ps10
Fungi - ps11
Fungi_1 - ps12
Fungi_2 - ps13
Fungi_3 - ps14
Dikarya - ps15
Ascomycota - ps16
Saccharo/Pezizomycotina - ps17
Saccharomycotina - ps18
Saccharomycotina_1 - ps19
Saccharomycotina_2 - ps20
Saccharomycotina_3 - ps21
Phaff./Saccharomycetaceae - ps22
Saccharomyco./mycetaceae - ps23
Saccharomycetaceae - ps24
Saccharomycetaceae_2 - ps25
Saccharomycetaceae_3 - ps26
Kaz./Nau./Saccharomyces - ps27
Saccharomyces - ps28
Arboricola_clade - ps29
Cerevisiae_clade - ps30
Saccharomyces_cerevisiae - ps31
p-value
4e-03
6e-03
8e-03
1e-02
2e-02
Log-odds
0.5
1.0
1.5
2.0
2.5
3.0
3.5
4.0

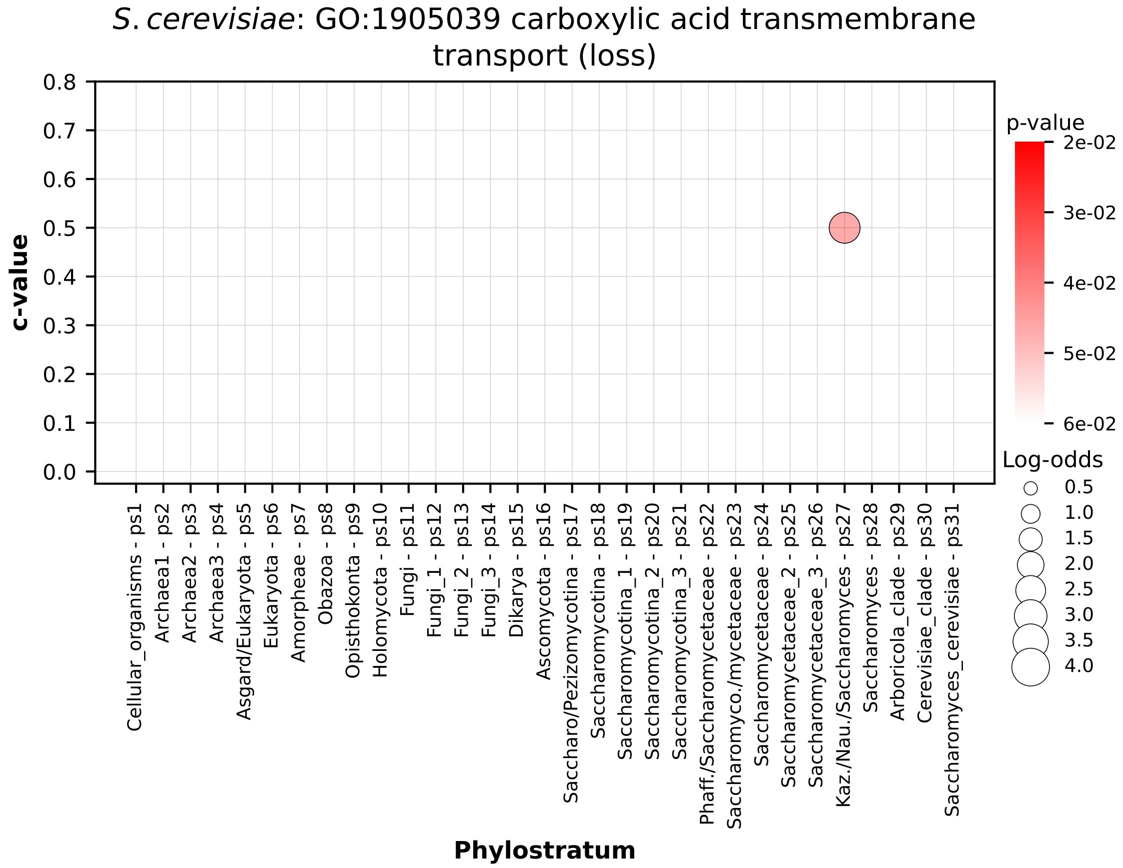

S. cerevisiae: GO:1905039 carboxylic acid transmembrane transport (loss)
c-value
0.8
0.7
0.6
0.5
0.4
0.3
0.2
0.1
0.0
Phylostratum
Cellular_organisms - ps1
Archaea1 - ps2
Archaea2 - ps3
Archaea3 - ps4
Asgard/Eukaryota - ps5
Eukaryota - ps6
Amorpheae - ps7
Obazoa - ps8
Opisthokonta - ps9
Holomycota - ps10
Fungi - ps11
Fungi_1 - ps12
Fungi_2 - ps13
Fungi_3 - ps14
Dikarya - ps15
Ascomycota - ps16
Saccharo/Pezizomycotina - ps17
Saccharomycotina - ps18
Saccharomycotina_1 - ps19
Saccharomycotina_2 - ps20
Saccharomycotina_3 - ps21
Phaff./Saccharomycetaceae - ps22
Saccharomyco./mycetaceae - ps23
Saccharomycetaceae - ps24
Saccharomycetaceae_2 - ps25
Saccharomycetaceae_3 - ps26
Kaz./Nau./Saccharomyces - ps27
Saccharomyces - ps28
Arboricola_clade - ps29
Cerevisiae_clade - ps30
Saccharomyces_cerevisiae - ps31
p-value
2e-02
3e-02
4e-02
5e-02
6e-02
Log-odds
0.5
1.0
1.5
2.0
2.5
3.0
3.5
4.0

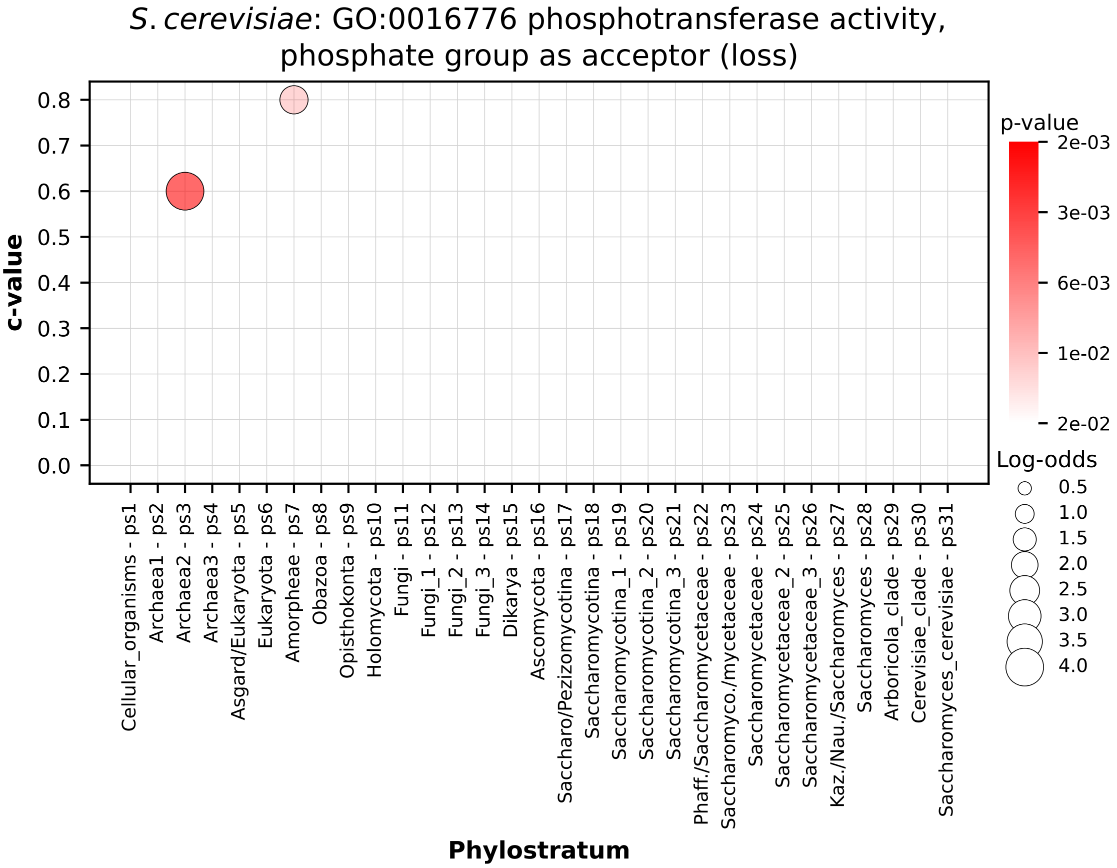

S. cerevisiae: GO:0016776 phosphotransferase activity, phosphate group as acceptor (loss)
c-value
0.8
0.7
0.6
0.5
0.4
0.3
0.2
0.1
0.0
Cellular_organisms - ps1
Archaea1 - ps2
Archaea2 - ps3
Archaea3 - ps4
Asgard/Eukaryota - ps5
Eukaryota - ps6
Amorpheae - ps7
Obazoa - ps8
Opisthokonta - ps9
Holomycota - ps10
Fungi - ps11
Fungi_1 - ps12
Fungi_2 - ps13
Fungi_3 - ps14
Dikarya - ps15
Ascomycota - ps16
Saccharo/Pezizomycotina - ps17
Saccharomycotina - ps18
Saccharomycotina_1 - ps19
Saccharomycotina_2 - ps20
Saccharomycotina_3 - ps21
Phaff./Saccharomycetaceae - ps22
Saccharomyco./mycetaceae - ps23
Saccharomycetaceae - ps24
Saccharomycetaceae_2 - ps25
Saccharomycetaceae_3 - ps26
Kaz./Nau./Saccharomyces - ps27
Saccharomyces - ps28
Arboricola_clade - ps29
Cerevisiae_clade - ps30
Saccharomyces_cerevisiae - ps31
Phylostratum
p-value
2e-03
3e-03
6e-03
1e-02
2e-02
Log-odds
0.5
1.0
1.5
2.0
2.5
3.0
3.5
4.0

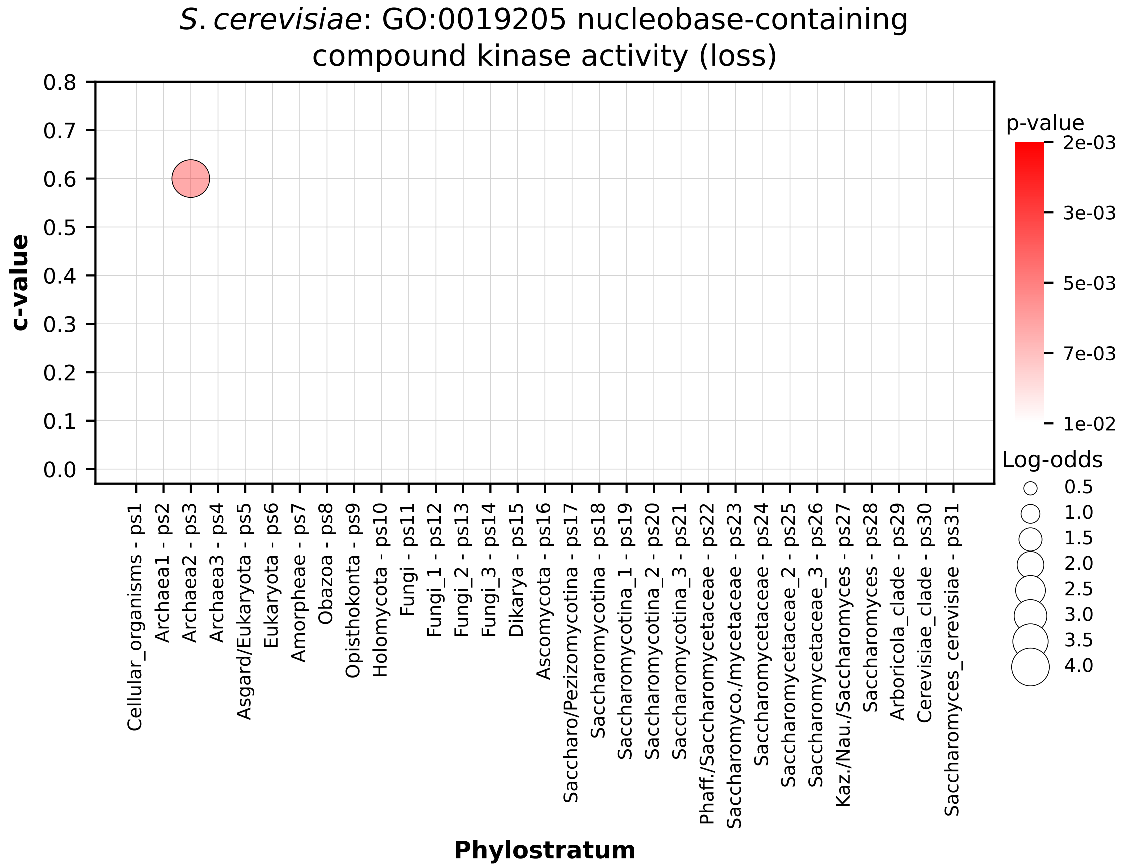

S. cerevisiae: GO:0019205 nucleobase-containing compound kinase activity (loss)
c-value
0.8
0.7
0.6
0.5
0.4
0.3
0.2
0.1
0.0
Phylostratum
Cellular_organisms - ps1
Archaea1 - ps2
Archaea2 - ps3
Archaea3 - ps4
Asgard/Eukaryota - ps5
Eukaryota - ps6
Amorpheae - ps7
Obazoa - ps8
Opisthokonta - ps9
Holomycota - ps10
Fungi - ps11
Fungi_1 - ps12
Fungi_2 - ps13
Fungi_3 - ps14
Dikarya - ps15
Ascomycota - ps16
Saccharo/Pezizomycotina - ps17
Saccharomycotina - ps18
Saccharomycotina_1 - ps19
Saccharomycotina_2 - ps20
Saccharomycotina_3 - ps21
Phaff./Saccharomycetaceae - ps22
Saccharomyco./mycetaceae - ps23
Saccharomycetaceae - ps24
Saccharomycetaceae_2 - ps25
Saccharomycetaceae_3 - ps26
Kaz./Nau./Saccharomyces - ps27
Saccharomyces - ps28
Arboricola_clade - ps29
Cerevisiae_clade - ps30
Saccharomyces_cerevisiae - ps31
p-value
2e-03
3e-03
5e-03
7e-03
1e-02
Log-odds
0.5
1.0
1.5
2.0
2.5
3.0
3.5
4.0

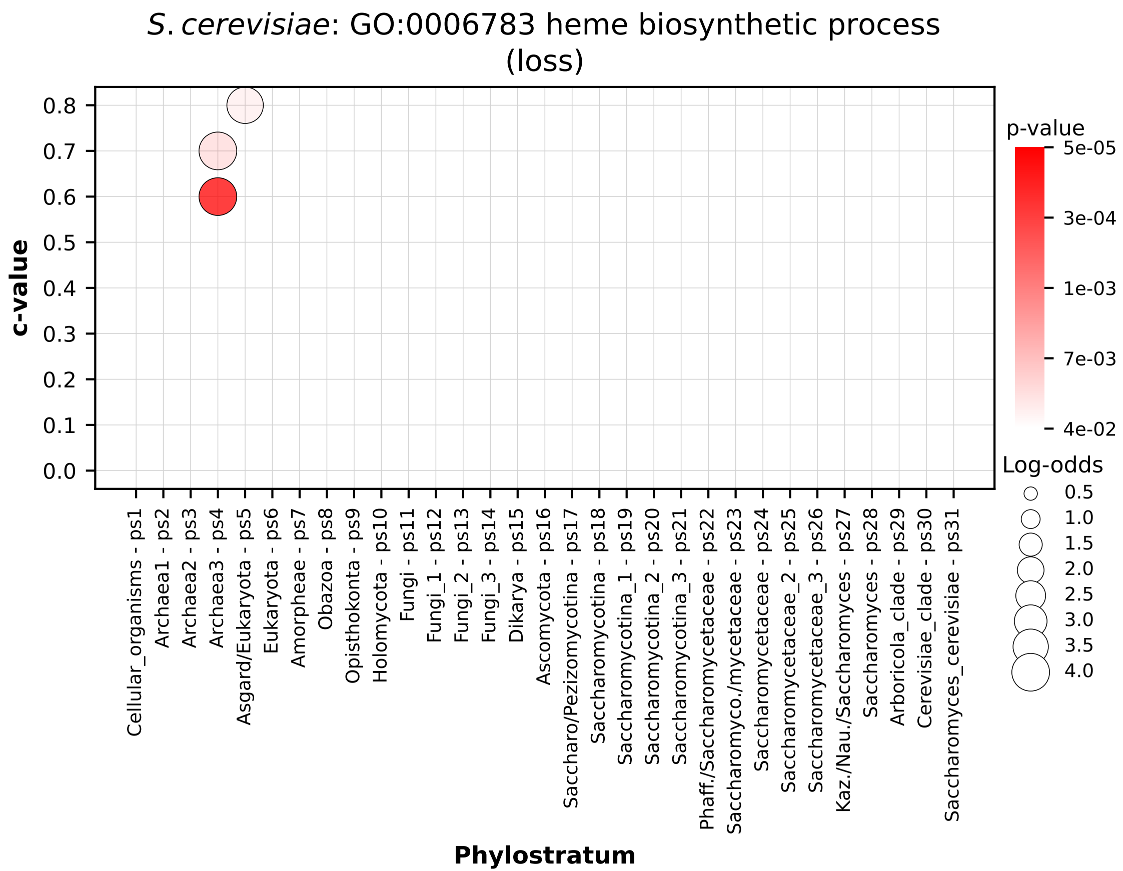

S. cerevisiae: GO:0006783 heme biosynthetic process
(loss)
c-value
0.8
0.7
0.6
0.5
0.4
0.3
0.2
0.1
0.0
Phylostratum
Cellular_organisms - ps1
Archaea1 - ps2
Archaea2 - ps3
Archaea3 - ps4
Asgard/Eukaryota - ps5
Eukaryota - ps6
Amorpheae - ps7
Obazoa - ps8
Opisthokonta - ps9
Holomycota - ps10
Fungi - ps11
Fungi_1 - ps12
Fungi_2 - ps13
Fungi_3 - ps14
Dikarya - ps15
Ascomycota - ps16
Saccharo/Pezizomycotina - ps17
Saccharomycotina - ps18
Saccharomycotina_1 - ps19
Saccharomycotina_2 - ps20
Saccharomycotina_3 - ps21
Phaff./Saccharomycetaceae - ps22
Saccharomyco./mycetaceae - ps23
Saccharomycetaceae - ps24
Saccharomycetaceae_2 - ps25
Saccharomycetaceae_3 - ps26
Kaz./Nau./Saccharomyces - ps27
Saccharomyces - ps28
Arboricola_clade - ps29
Cerevisiae_clade - ps30
Saccharomyces_cerevisiae - ps31
p-value
5e-05
3e-04
1e-03
7e-03
4e-02
Log-odds
0.5
1.0
1.5
2.0
2.5
3.0
3.5
4.0

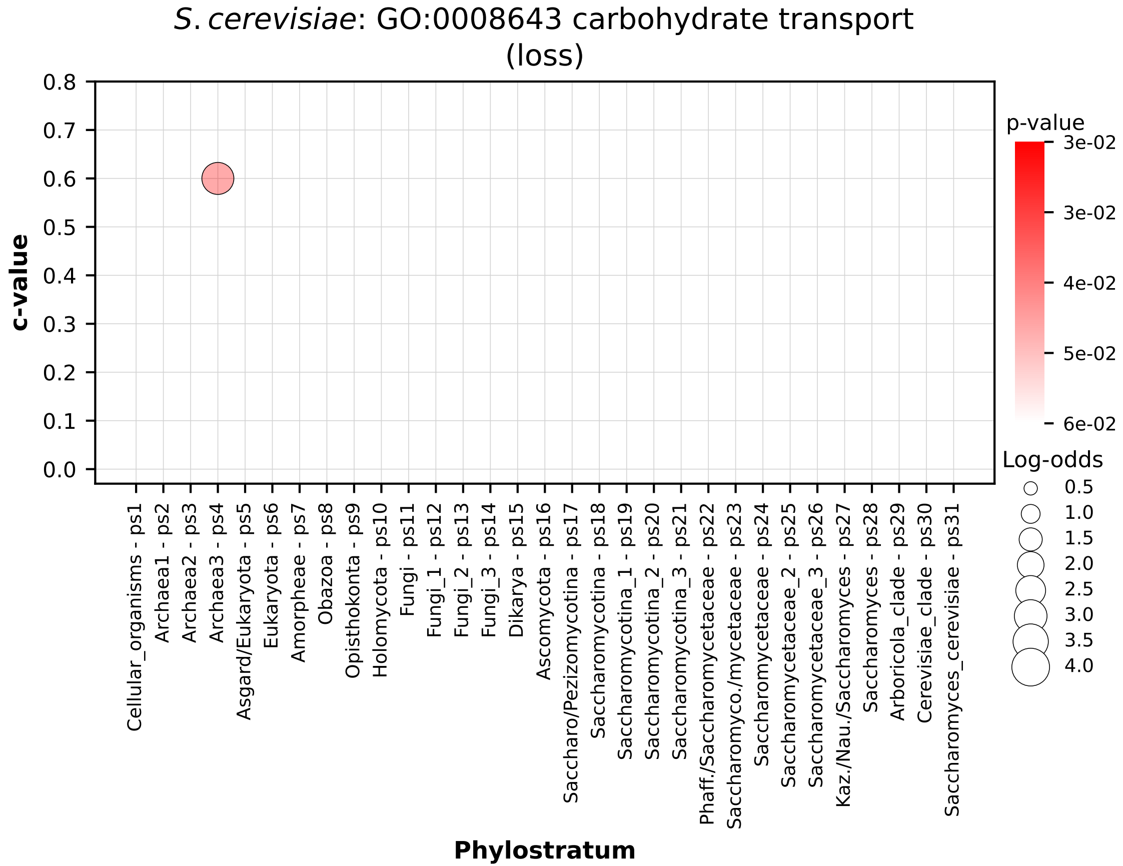
S. cerevisiae: GO:0008643 carbohydrate transport
(loss)
c-value
0.8
0.7
0.6
0.5
0.4
0.3
0.2
0.1
0.0
Phylostratum
Cellular_organisms - ps1
Archaea1 - ps2
Archaea2 - ps3
Archaea3 - ps4
Asgard/Eukaryota - ps5
Eukaryota - ps6
Amorpheae - ps7
Obazoa - ps8
Opisthokonta - ps9
Holomycota - ps10
Fungi - ps11
Fungi_1 - ps12
Fungi_2 - ps13
Fungi_3 - ps14
Dikarya - ps15
Ascomycota - ps16
Saccharo/Pezizomycotina - ps17
Saccharomycotina - ps18
Saccharomycotina_1 - ps19
Saccharomycotina_2 - ps20
Saccharomycotina_3 - ps21
Phaff./Saccharomycetaceae - ps22
Saccharomyco./mycetaceae - ps23
Saccharomycetaceae - ps24
Saccharomycetaceae_2 - ps25
Saccharomycetaceae_3 - ps26
Kaz./Nau./Saccharomyces - ps27
Saccharomyces - ps28
Arboricola_clade - ps29
Cerevisiae_clade - ps30
Saccharomyces_cerevisiae - ps31
p-value
3e-02
3e-02
4e-02
5e-02
6e-02
Log-odds
0.5
1.0
1.5
2.0
2.5
3.0
3.5
4.0

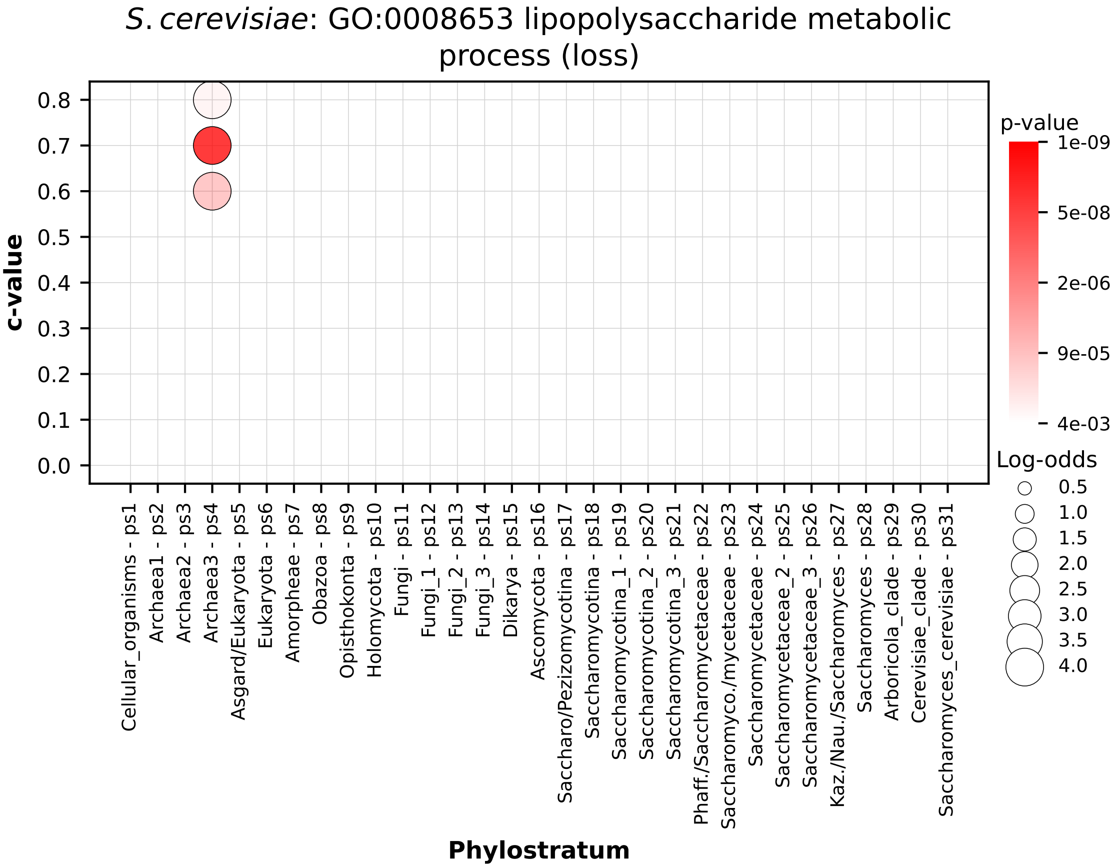
S. cerevisiae: GO:0008653 lipopolysaccharide metabolic process (loss)
c-value
0.8
0.7
0.6
0.5
0.4
0.3
0.2
0.1
0.0
p-value
1e-09
5e-08
2e-06
9e-05
4e-03
Log-odds
0.5
1.0
1.5
2.0
2.5
3.0
3.5
4.0
Cellular_organisms - ps1
Archaea1 - ps2
Archaea2 - ps3
Archaea3 - ps4
Asgard/Eukaryota - ps5
Eukaryota - ps6
Amorpheae - ps7
Obazoa - ps8
Opisthokonta - ps9
Holomycota - ps10
Fungi - ps11
Fungi_1 - ps12
Fungi_2 - ps13
Fungi_3 - ps14
Dikarya - ps15
Ascomycota - ps16
Saccharo/Pezizomycotina - ps17
Saccharomycotina - ps18
Saccharomycotina_1 - ps19
Saccharomycotina_2 - ps20
Saccharomycotina_3 - ps21
Phaff./Saccharomycetaceae - ps22
Saccharomyco./mycetaceae - ps23
Saccharomycetaceae - ps24
Saccharomycetaceae_2 - ps25
Saccharomycetaceae_3 - ps26
Kaz./Nau./Saccharomyces - ps27
Saccharomyces - ps28
Arboricola_clade - ps29
Cerevisiae_clade - ps30
Saccharomyces_cerevisiae - ps31
Phylostratum

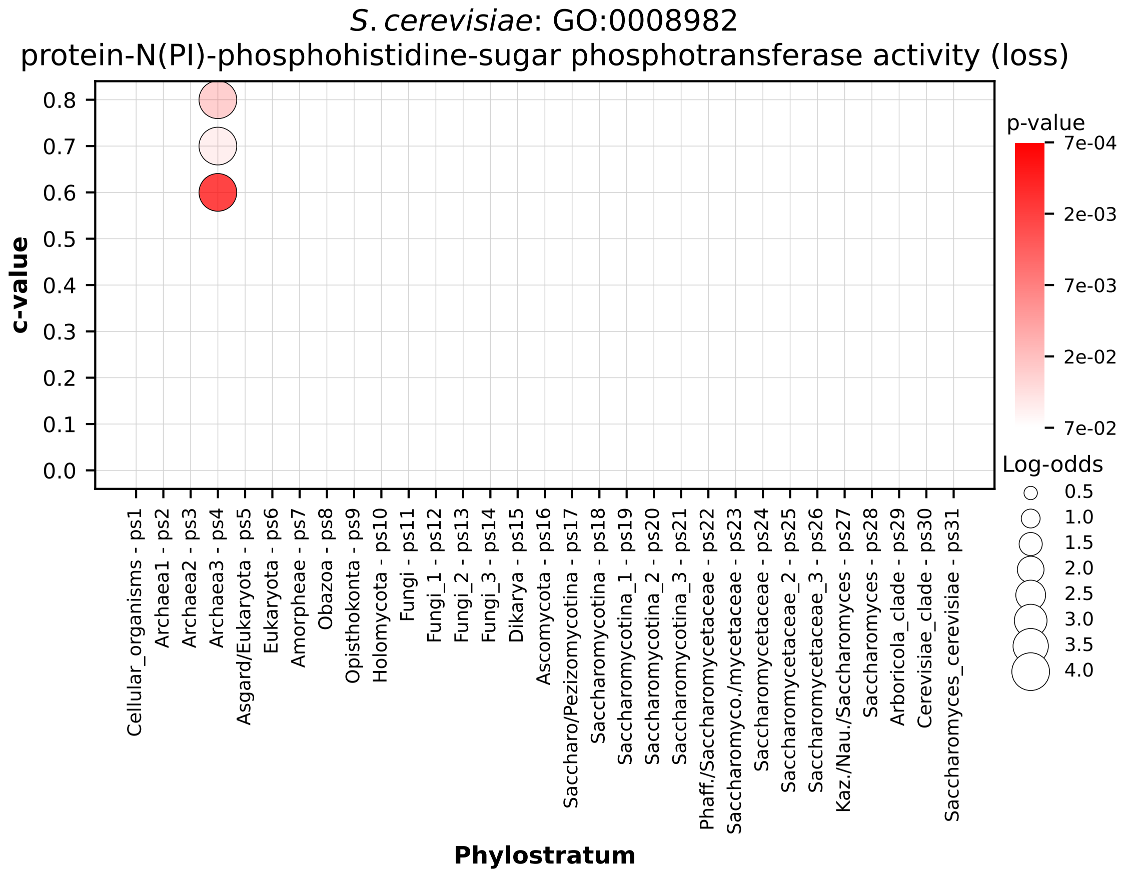

S. cerevisiae: GO:0008982
protein-N(PI)-phosphohistidine-sugar phosphotransferase activity (loss)
c-value
0.0
0.1
0.2
0.3
0.4
0.5
0.6
0.7
0.8
p-value
7e-04
2e-03
7e-03
2e-02
7e-02
Log-odds
0.5
1.0
1.5
2.0
2.5
3.0
3.5
4.0
Cellular_organisms - ps1
Archaea1 - ps2
Archaea2 - ps3
Archaea3 - ps4
Asgard/Eukaryota - ps5
Eukaryota - ps6
Amorpheae - ps7
Obazoa - ps8
Opisthokonta - ps9
Holomycota - ps10
Fungi - ps11
Fungi_1 - ps12
Fungi_2 - ps13
Fungi_3 - ps14
Dikarya - ps15
Ascomycota - ps16
Saccharo/Pezizomycotina - ps17
Saccharomycotina - ps18
Saccharomycotina_1 - ps19
Saccharomycotina_2 - ps20
Saccharomycotina_3 - ps21
Phaff./Saccharomycetaceae - ps22
Saccharomyco./mycetaceae - ps23
Saccharomycetaceae - ps24
Saccharomycetaceae_2 - ps25
Saccharomycetaceae_3 - ps26
Kaz./Nau./Saccharomyces - ps27
Saccharomyces - ps28
Arboricola_clade - ps29
Cerevisiae_clade - ps30
Saccharomyces_cerevisiae - ps31
Phylostratum

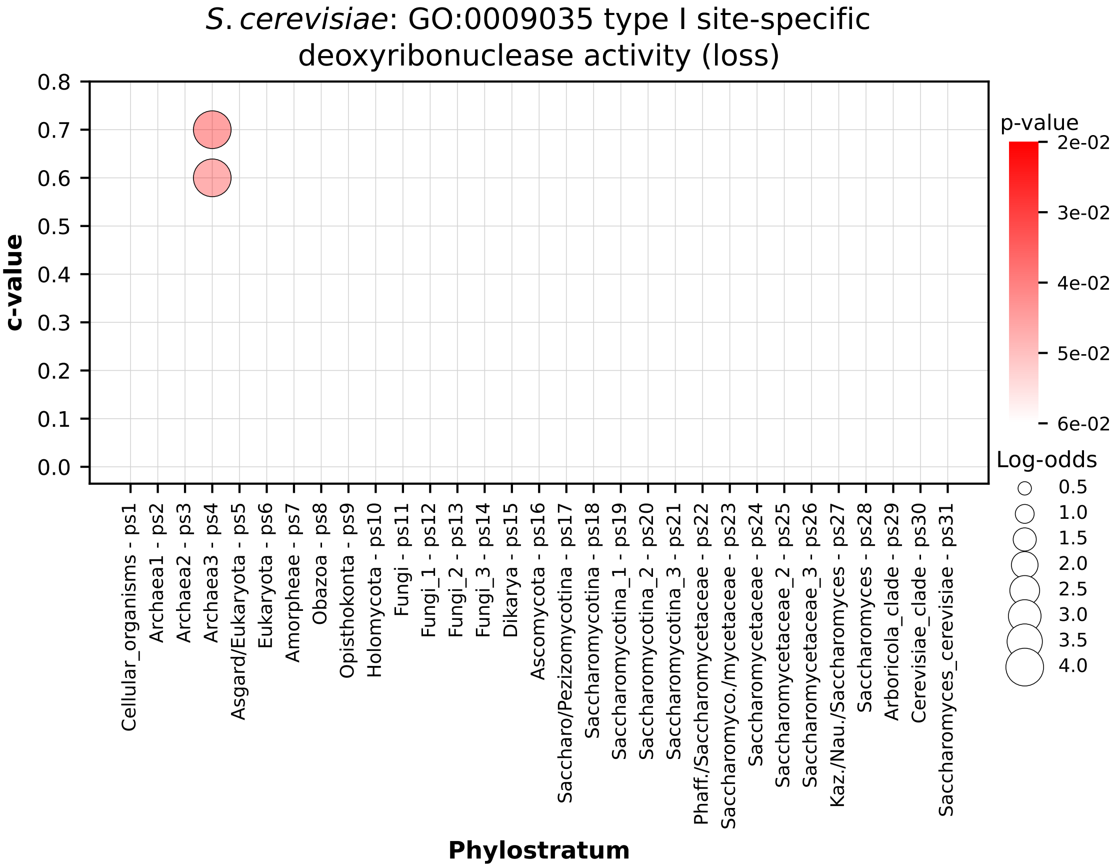

S. cerevisiae: GO:0009035 type I site-specific deoxyribonuclease activity (loss)
c-value
0.8
0.7
0.6
0.5
0.4
0.3
0.2
0.1
0.0
p-value
2e-02
3e-02
4e-02
5e-02
6e-02
Log-odds
0.5
1.0
1.5
2.0
2.5
3.0
3.5
4.0
Cellular_organisms - ps1
Archaea1 - ps2
Archaea2 - ps3
Archaea3 - ps4
Asgard/Eukaryota - ps5
Eukaryota - ps6
Amorpheae - ps7
Obazoa - ps8
Opisthokonta - ps9
Holomycota - ps10
Fungi - ps11
Fungi_1 - ps12
Fungi_2 - ps13
Fungi_3 - ps14
Dikarya - ps15
Ascomycota - ps16
Saccharo/Pezizomycotina - ps17
Saccharomycotina - ps18
Saccharomycotina_1 - ps19
Saccharomycotina_2 - ps20
Saccharomycotina_3 - ps21
Phaff./Saccharomycetaceae - ps22
Saccharomyco./mycetaceae - ps23
Saccharomycetaceae - ps24
Saccharomycetaceae_2 - ps25
Saccharomycetaceae_3 - ps26
Kaz./Nau./Saccharomyces - ps27
Saccharomyces - ps28
Arboricola_clade - ps29
Cerevisiae_clade - ps30
Saccharomyces_cerevisiae - ps31
Phylostratum

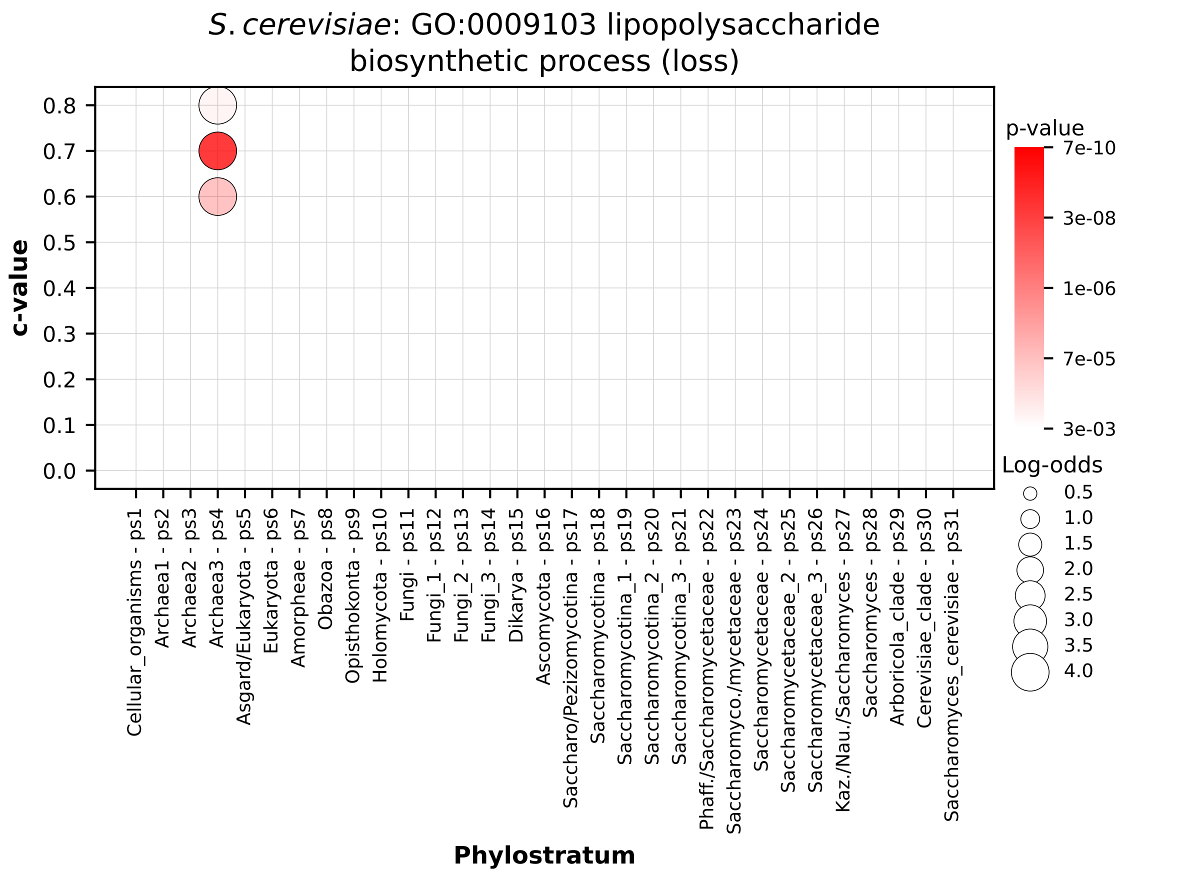

$S. cerevisiae$: GO:0009103 lipopolysaccharide biosynthetic process (loss)
c-value
0.0
0.1
0.2
0.3
0.4
0.5
0.6
0.7
0.8
p-value
7e-10
3e-08
1e-06
7e-05
3e-03
Log-odds
0.5
1.0
1.5
2.0
2.5
3.0
3.5
4.0
Cellular_organisms - ps1
Archaea1 - ps2
Archaea2 - ps3
Archaea3 - ps4
Asgard/Eukaryota - ps5
Eukaryota - ps6
Amorpheae - ps7
Obazoa - ps8
Opisthokonta - ps9
Holomycota - ps10
Fungi - ps11
Fungi_1 - ps12
Fungi_2 - ps13
Fungi_3 - ps14
Dikarya - ps15
Ascomycota - ps16
Saccharo/Pezizomycotina - ps17
Saccharomycotina - ps18
Saccharomycotina_1 - ps19
Saccharomycotina_2 - ps20
Saccharomycotina_3 - ps21
Phaff./Saccharomycetaceae - ps22
Saccharomyco./mycetaceae - ps23
Saccharomycetaceae - ps24
Saccharomycetaceae_2 - ps25
Saccharomycetaceae_3 - ps26
Kaz./Nau./Saccharomyces - ps27
Saccharomyces - ps28
Arboricola_clade - ps29
Cerevisiae_clade - ps30
Saccharomyces_cerevisiae - ps31
Phylostratum

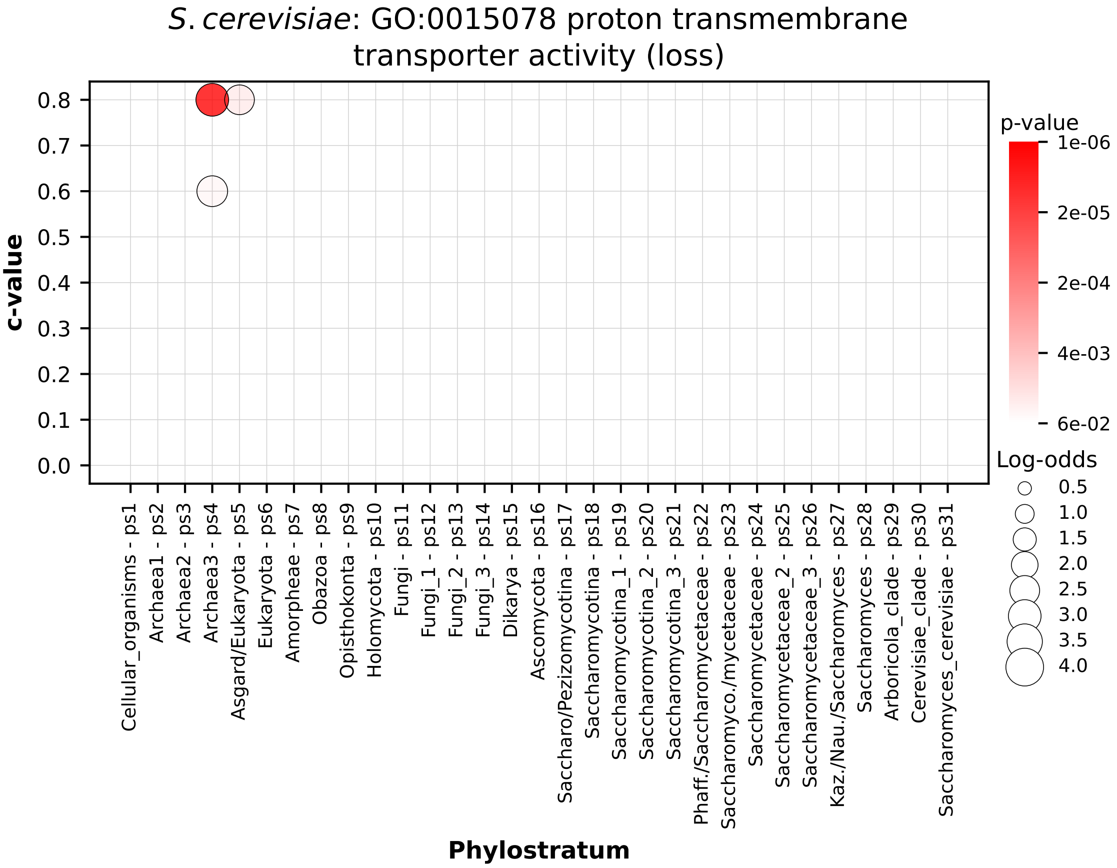
S. cerevisiae: GO:0015078 proton transmembrane transporter activity (loss)
c-value
0.8
0.7
0.6
0.5
0.4
0.3
0.2
0.1
0.0
Phylostratum
Cellular_organisms - ps1
Archaea1 - ps2
Archaea2 - ps3
Archaea3 - ps4
Asgard/Eukaryota - ps5
Eukaryota - ps6
Amorpheae - ps7
Obazoa - ps8
Opisthokonta - ps9
Holomycota - ps10
Fungi - ps11
Fungi_1 - ps12
Fungi_2 - ps13
Fungi_3 - ps14
Dikarya - ps15
Ascomycota - ps16
Saccharo/Pezizomycotina - ps17
Saccharomycotina - ps18
Saccharomycotina_1 - ps19
Saccharomycotina_2 - ps20
Saccharomycotina_3 - ps21
Phaff./Saccharomycetaceae - ps22
Saccharomyco./mycetaceae - ps23
Saccharomycetaceae - ps24
Saccharomycetaceae_2 - ps25
Saccharomycetaceae_3 - ps26
Kaz./Nau./Saccharomyces - ps27
Saccharomyces - ps28
Arboricola_clade - ps29
Cerevisiae_clade - ps30
Saccharomyces_cerevisiae - ps31
p-value
1e-06
2e-05
2e-04
4e-03
6e-02
Log-odds
0.5
1.0
1.5
2.0
2.5
3.0
3.5
4.0

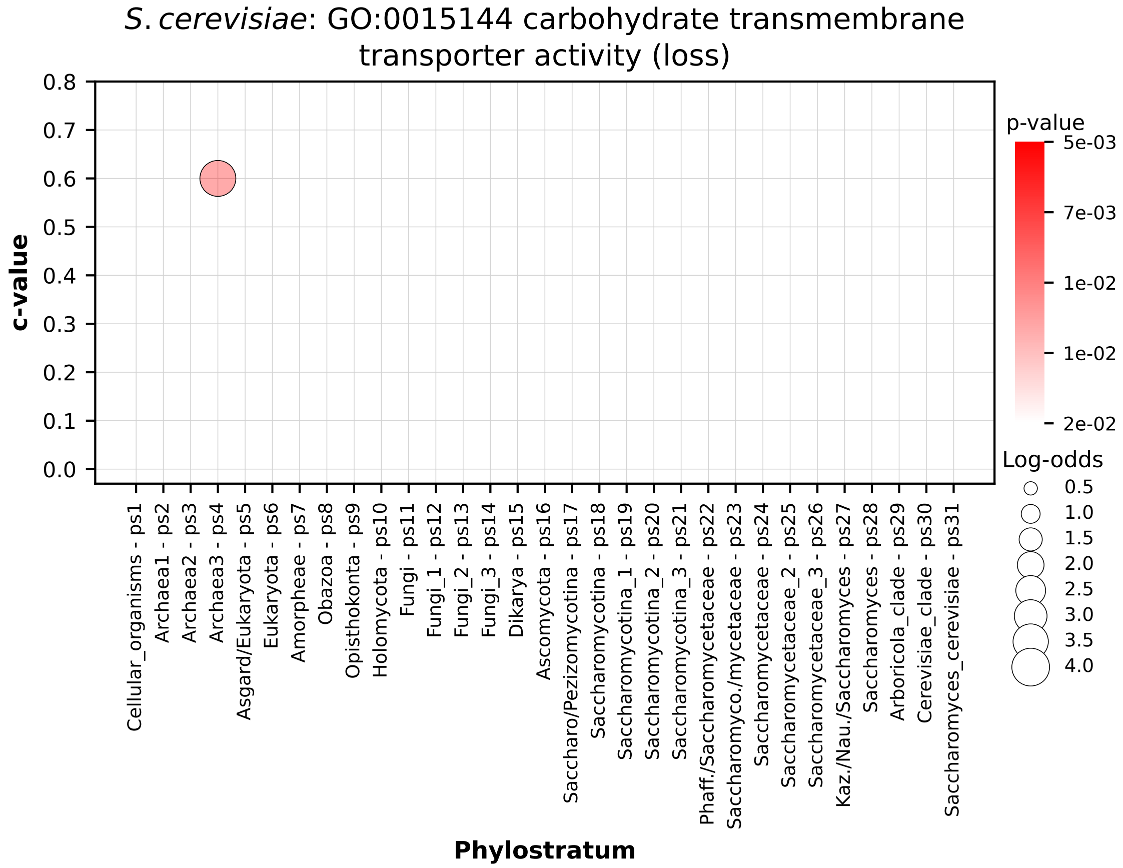
S. cerevisiae: GO:0015144 carbohydrate transmembrane transporter activity (loss)
c-value
0.8
0.7
0.6
0.5
0.4
0.3
0.2
0.1
0.0
Phylostratum
Cellular_organisms - ps1
Archaea1 - ps2
Archaea2 - ps3
Archaea3 - ps4
Asgard/Eukaryota - ps5
Eukaryota - ps6
Amorpheae - ps7
Obazoa - ps8
Opisthokonta - ps9
Holomycota - ps10
Fungi - ps11
Fungi_1 - ps12
Fungi_2 - ps13
Fungi_3 - ps14
Dikarya - ps15
Ascomycota - ps16
Saccharo/Pezizomycotina - ps17
Saccharomycotina - ps18
Saccharomycotina_1 - ps19
Saccharomycotina_2 - ps20
Saccharomycotina_3 - ps21
Phaff./Saccharomycetaceae - ps22
Saccharomyco./mycetaceae - ps23
Saccharomycetaceae - ps24
Saccharomycetaceae_2 - ps25
Saccharomycetaceae_3 - ps26
Kaz./Nau./Saccharomyces - ps27
Saccharomyces - ps28
Arboricola_clade - ps29
Cerevisiae_clade - ps30
Saccharomyces_cerevisiae - ps31
p-value
5e-03
7e-03
1e-02
1e-02
2e-02
Log-odds
0.5
1.0
1.5
2.0
2.5
3.0
3.5
4.0

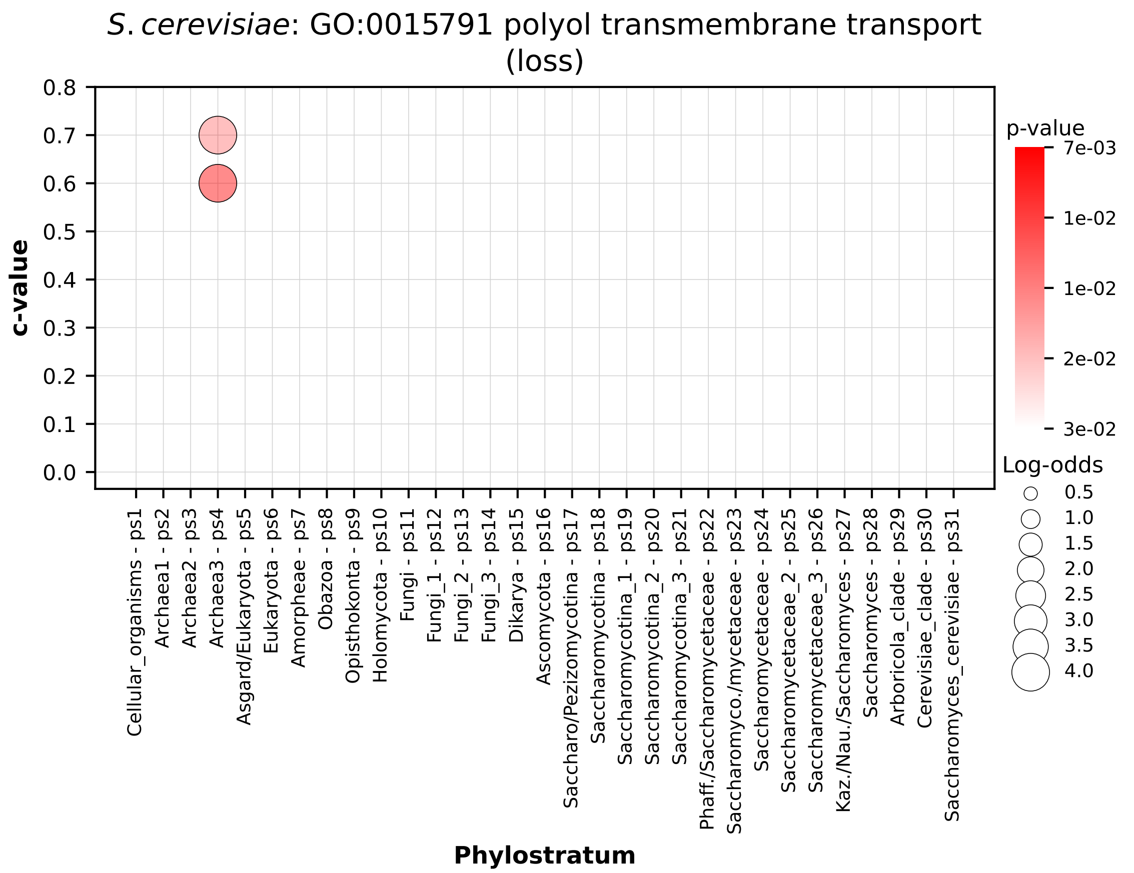
S. cerevisiae: GO:0015791 polyol transmembrane transport
(loss)
c-value
0.8
0.7
0.6
0.5
0.4
0.3
0.2
0.1
0.0
p-value
7e-03
1e-02
1e-02
2e-02
3e-02
Log-odds
0.5
1.0
1.5
2.0
2.5
3.0
3.5
4.0
Cellular_organisms - ps1
Archaea1 - ps2
Archaea2 - ps3
Archaea3 - ps4
Asgard/Eukaryota - ps5
Eukaryota - ps6
Amorpheae - ps7
Obazoa - ps8
Opisthokonta - ps9
Holomycota - ps10
Fungi - ps11
Fungi_1 - ps12
Fungi_2 - ps13
Fungi_3 - ps14
Dikarya - ps15
Ascomycota - ps16
Saccharo/Pezizomycotina - ps17
Saccharomycotina - ps18
Saccharomycotina_1 - ps19
Saccharomycotina_2 - ps20
Saccharomycotina_3 - ps21
Phaff./Saccharomycetaceae - ps22
Saccharomyco./mycetaceae - ps23
Saccharomycetaceae - ps24
Saccharomycetaceae_2 - ps25
Saccharomycetaceae_3 - ps26
Kaz./Nau./Saccharomyces - ps27
Saccharomyces - ps28
Arboricola_clade - ps29
Cerevisiae_clade - ps30
Saccharomyces_cerevisiae - ps31
Phylostratum

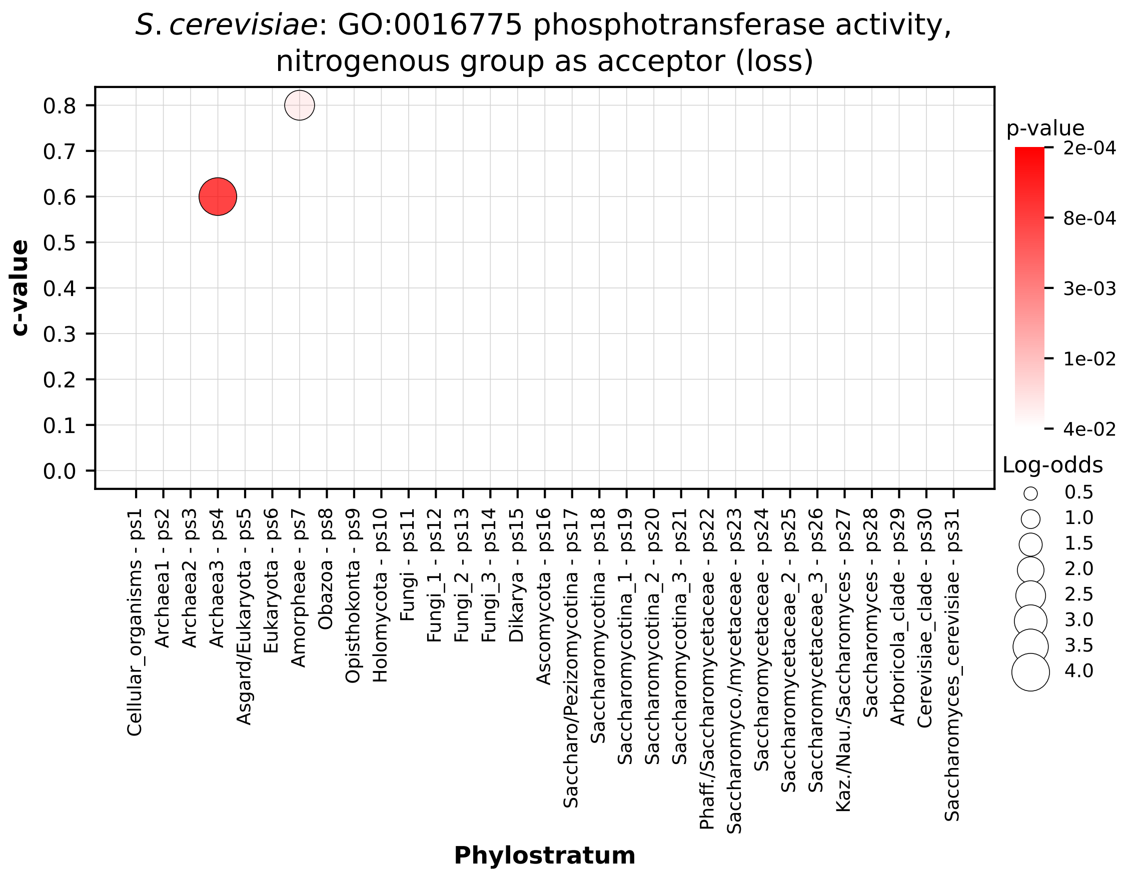

S. cerevisiae: GO:0016775 phosphotransferase activity, nitrogenous group as acceptor (loss)
c-value
0.0
0.1
0.2
0.3
0.4
0.5
0.6
0.7
0.8
Phylostratum
Cellular_organisms - ps1
Archaea1 - ps2
Archaea2 - ps3
Archaea3 - ps4
Asgard/Eukaryota - ps5
Eukaryota - ps6
Amorpheae - ps7
Obazoa - ps8
Opisthokonta - ps9
Holomycota - ps10
Fungi - ps11
Fungi_1 - ps12
Fungi_2 - ps13
Fungi_3 - ps14
Dikarya - ps15
Ascomycota - ps16
Saccharo/Pezizomycotina - ps17
Saccharomycotina - ps18
Saccharomycotina_1 - ps19
Saccharomycotina_2 - ps20
Saccharomycotina_3 - ps21
Phaff./Saccharomycetaceae - ps22
Saccharomyco./mycetaceae - ps23
Saccharomycetaceae - ps24
Saccharomycetaceae_2 - ps25
Saccharomycetaceae_3 - ps26
Kaz./Nau./Saccharomyces - ps27
Saccharomyces - ps28
Arboricola_clade - ps29
Cerevisiae_clade - ps30
Saccharomyces_cerevisiae - ps31
p-value
2e-04
8e-04
3e-03
1e-02
4e-02
Log-odds
0.5
1.0
1.5
2.0
2.5
3.0
3.5
4.0

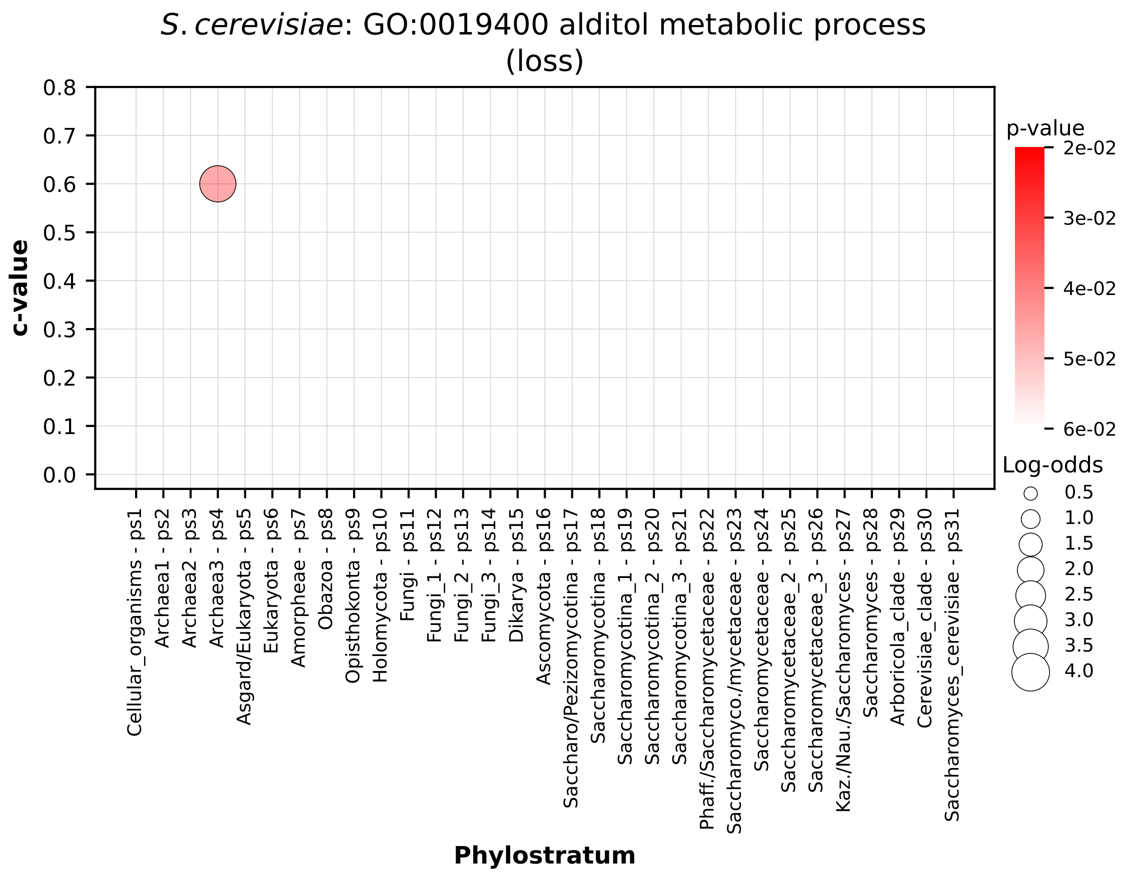

S. cerevisiae: GO:0019400 alditol metabolic process
(loss)
c-value
0.8
0.7
0.6
0.5
0.4
0.3
0.2
0.1
0.0
Phylostratum
Cellular_organisms - ps1
Archaea1 - ps2
Archaea2 - ps3
Archaea3 - ps4
Asgard/Eukaryota - ps5
Eukaryota - ps6
Amorpheae - ps7
Obazoa - ps8
Opisthokonta - ps9
Holomycota - ps10
Fungi - ps11
Fungi_1 - ps12
Fungi_2 - ps13
Fungi_3 - ps14
Dikarya - ps15
Ascomycota - ps16
Saccharo/Pezizomycotina - ps17
Saccharomycotina - ps18
Saccharomycotina_1 - ps19
Saccharomycotina_2 - ps20
Saccharomycotina_3 - ps21
Phaff./Saccharomycetaceae - ps22
Saccharomyco./mycetaceae - ps23
Saccharomycetaceae - ps24
Saccharomycetaceae_2 - ps25
Saccharomycetaceae_3 - ps26
Kaz./Nau./Saccharomyces - ps27
Saccharomyces - ps28
Arboricola_clade - ps29
Cerevisiae_clade - ps30
Saccharomyces_cerevisiae - ps31
p-value
2e-02
3e-02
4e-02
5e-02
6e-02
Log-odds
0.5
1.0
1.5
2.0
2.5
3.0
3.5
4.0

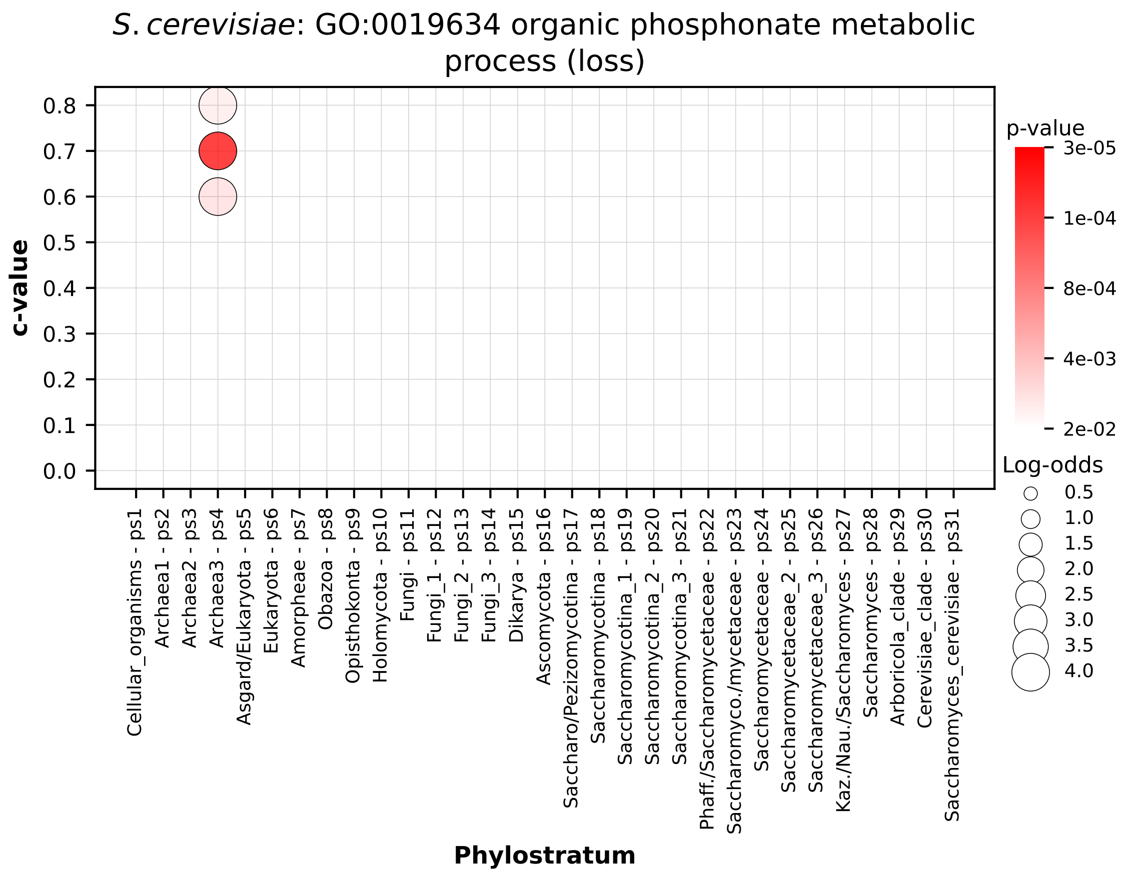

S. cerevisiae: GO:0019634 organic phosphonate metabolic process (loss)
c-value
0.8
0.7
0.6
0.5
0.4
0.3
0.2
0.1
0.0
Phylostratum
Cellular_organisms - ps1
Archaea1 - ps2
Archaea2 - ps3
Archaea3 - ps4
Asgard/Eukaryota - ps5
Eukaryota - ps6
Amorpheae - ps7
Obazoa - ps8
Opisthokonta - ps9
Holomycota - ps10
Fungi - ps11
Fungi_1 - ps12
Fungi_2 - ps13
Fungi_3 - ps14
Dikarya - ps15
Ascomycota - ps16
Saccharo/Pezizomycotina - ps17
Saccharomycotina - ps18
Saccharomycotina_1 - ps19
Saccharomycotina_2 - ps20
Saccharomycotina_3 - ps21
Phaff./Saccharomycetaceae - ps22
Saccharomyco./mycetaceae - ps23
Saccharomycetaceae - ps24
Saccharomycetaceae_2 - ps25
Saccharomycetaceae_3 - ps26
Kaz./Nau./Saccharomyces - ps27
Saccharomyces - ps28
Arboricola_clade - ps29
Cerevisiae_clade - ps30
Saccharomyces_cerevisiae - ps31
p-value
3e-05
1e-04
8e-04
4e-03
2e-02
Log-odds
0.5
1.0
1.5
2.0
2.5
3.0
3.5
4.0

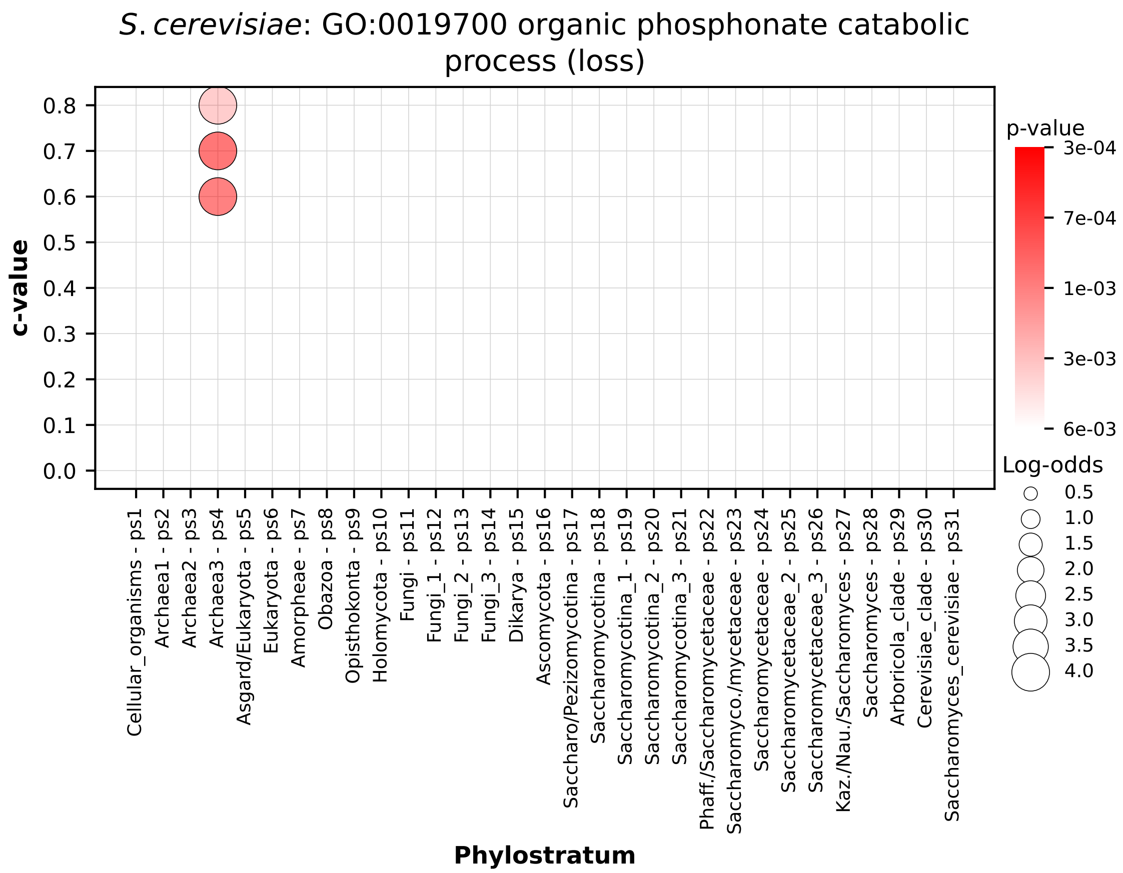

S. cerevisiae: GO:0019700 organic phosphonate catabolic process (loss)
c-value
0.8
0.7
0.6
0.5
0.4
0.3
0.2
0.1
0.0
Phylostratum
Cellular_organisms - ps1
Archaea1 - ps2
Archaea2 - ps3
Archaea3 - ps4
Asgard/Eukaryota - ps5
Eukaryota - ps6
Amorpheae - ps7
Obazoa - ps8
Opisthokonta - ps9
Holomycota - ps10
Fungi - ps11
Fungi_1 - ps12
Fungi_2 - ps13
Fungi_3 - ps14
Dikarya - ps15
Ascomycota - ps16
Saccharo/Pezizomycotina - ps17
Saccharomycotina - ps18
Saccharomycotina_1 - ps19
Saccharomycotina_2 - ps20
Saccharomycotina_3 - ps21
Phaff./Saccharomycetaceae - ps22
Saccharomyco./mycetaceae - ps23
Saccharomycetaceae - ps24
Saccharomycetaceae_2 - ps25
Saccharomycetaceae_3 - ps26
Kaz./Nau./Saccharomyces - ps27
Saccharomyces - ps28
Arboricola_clade - ps29
Cerevisiae_clade - ps30
Saccharomyces_cerevisiae - ps31
p-value
3e-04
7e-04
1e-03
3e-03
6e-03
Log-odds
0.5
1.0
1.5
2.0
2.5
3.0
3.5
4.0

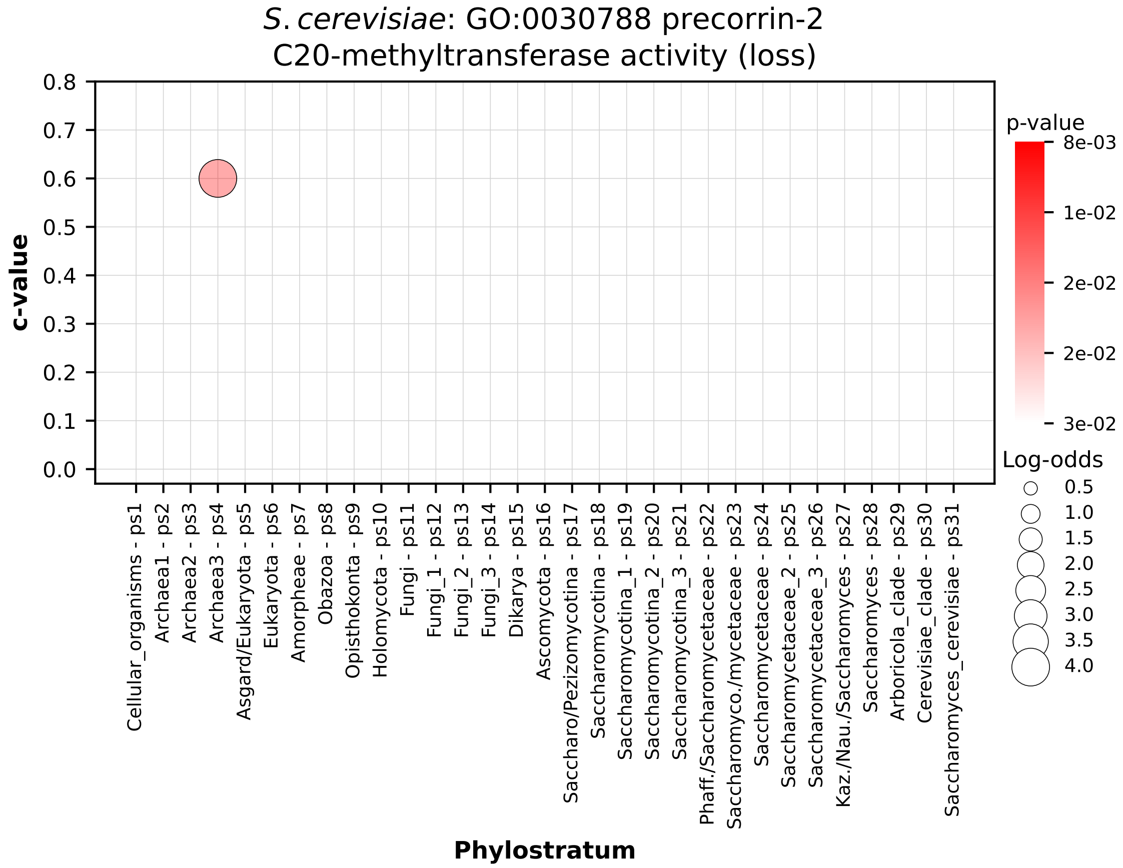

S. cerevisiae: GO:0030788 precorrin-2 C20-methyltransferase activity (loss)
c-value
0.8
0.7
0.6
0.5
0.4
0.3
0.2
0.1
0.0
Phylostratum
Cellular_organisms - ps1
Archaea1 - ps2
Archaea2 - ps3
Archaea3 - ps4
Asgard/Eukaryota - ps5
Eukaryota - ps6
Amorpheae - ps7
Obazoa - ps8
Opisthokonta - ps9
Holomycota - ps10
Fungi - ps11
Fungi_1 - ps12
Fungi_2 - ps13
Fungi_3 - ps14
Dikarya - ps15
Ascomycota - ps16
Saccharo/Pezizomycotina - ps17
Saccharomycotina - ps18
Saccharomycotina_1 - ps19
Saccharomycotina_2 - ps20
Saccharomycotina_3 - ps21
Phaff./Saccharomycetaceae - ps22
Saccharomyco./mycetaceae - ps23
Saccharomycetaceae - ps24
Saccharomycetaceae_2 - ps25
Saccharomycetaceae_3 - ps26
Kaz./Nau./Saccharomyces - ps27
Saccharomyces - ps28
Arboricola_clade - ps29
Cerevisiae_clade - ps30
Saccharomyces_cerevisiae - ps31
p-value
8e-03
1e-02
2e-02
2e-02
3e-02
Log-odds
0.5
1.0
1.5
2.0
2.5
3.0
3.5
4.0

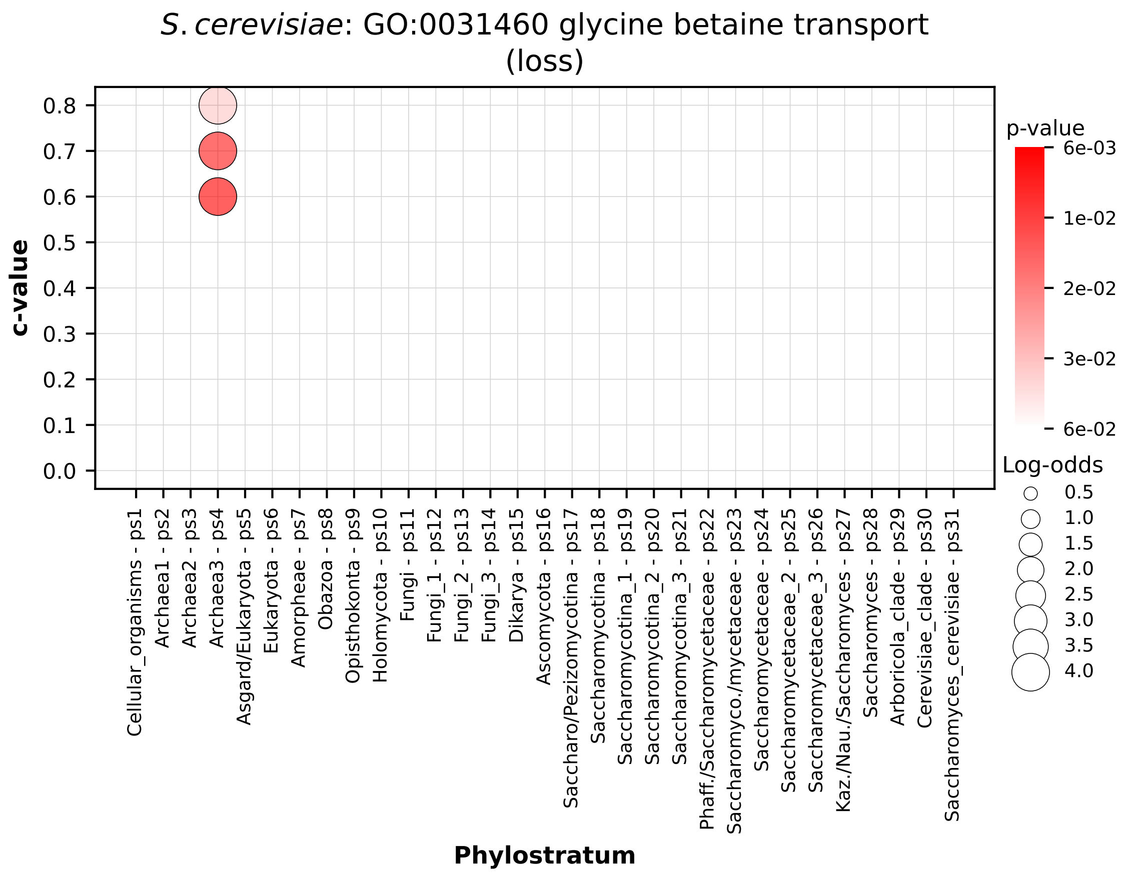

S. cerevisiae: GO:0031460 glycine betaine transport
(loss)
c-value
0.8
0.7
0.6
0.5
0.4
0.3
0.2
0.1
0.0
p-value
6e-03
1e-02
2e-02
3e-02
6e-02
Log-odds
0.5
1.0
1.5
2.0
2.5
3.0
3.5
4.0
Cellular_organisms - ps1
Archaea1 - ps2
Archaea2 - ps3
Archaea3 - ps4
Asgard/Eukaryota - ps5
Eukaryota - ps6
Amorpheae - ps7
Obazoa - ps8
Opisthokonta - ps9
Holomycota - ps10
Fungi - ps11
Fungi_1 - ps12
Fungi_2 - ps13
Fungi_3 - ps14
Dikarya - ps15
Ascomycota - ps16
Saccharo/Pezizomycotina - ps17
Saccharomycotina - ps18
Saccharomycotina_1 - ps19
Saccharomycotina_2 - ps20
Saccharomycotina_3 - ps21
Phaff./Saccharomycetaceae - ps22
Saccharomyco./mycetaceae - ps23
Saccharomycetaceae - ps24
Saccharomycetaceae_2 - ps25
Saccharomycetaceae_3 - ps26
Kaz./Nau./Saccharomyces - ps27
Saccharomyces - ps28
Arboricola_clade - ps29
Cerevisiae_clade - ps30
Saccharomyces_cerevisiae - ps31
Phylostratum

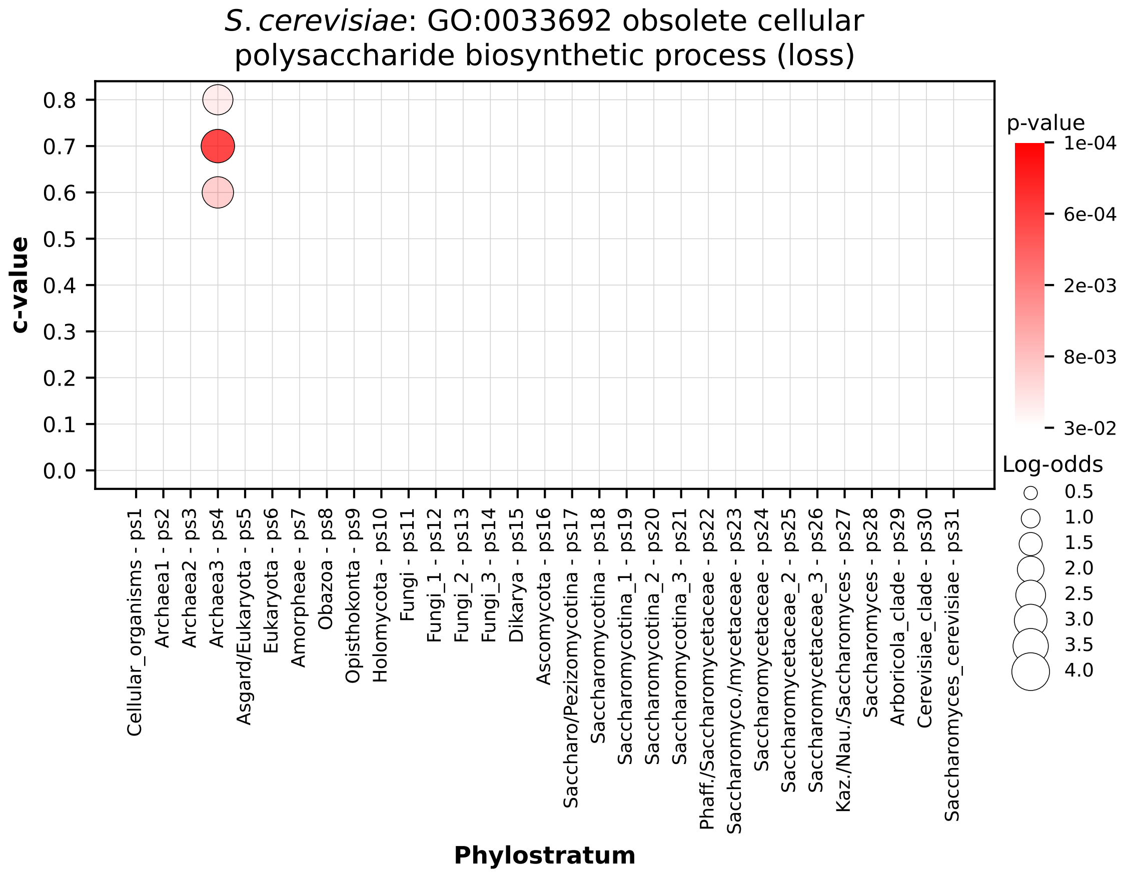

S. cerevisiae: GO:0033692 obsolete cellular polysaccharide biosynthetic process (loss)
c-value
0.0
0.1
0.2
0.3
0.4
0.5
0.6
0.7
0.8
p-value
1e-04
6e-04
2e-03
8e-03
3e-02
Log-odds
0.5
1.0
1.5
2.0
2.5
3.0
3.5
4.0
Cellular_organisms - ps1
Archaea1 - ps2
Archaea2 - ps3
Archaea3 - ps4
Asgard/Eukaryota - ps5
Eukaryota - ps6
Amorpheae - ps7
Obazoa - ps8
Opisthokonta - ps9
Holomycota - ps10
Fungi - ps11
Fungi_1 - ps12
Fungi_2 - ps13
Fungi_3 - ps14
Dikarya - ps15
Ascomycota - ps16
Saccharo/Pezizomycotina - ps17
Saccharomycotina - ps18
Saccharomycotina_1 - ps19
Saccharomycotina_2 - ps20
Saccharomycotina_3 - ps21
Phaff./Saccharomycetaceae - ps22
Saccharomyco./mycetaceae - ps23
Saccharomycetaceae - ps24
Saccharomycetaceae_2 - ps25
Saccharomycetaceae_3 - ps26
Kaz./Nau./Saccharomyces - ps27
Saccharomyces - ps28
Arboricola_clade - ps29
Cerevisiae_clade - ps30
Saccharomyces_cerevisiae - ps31
Phylostratum

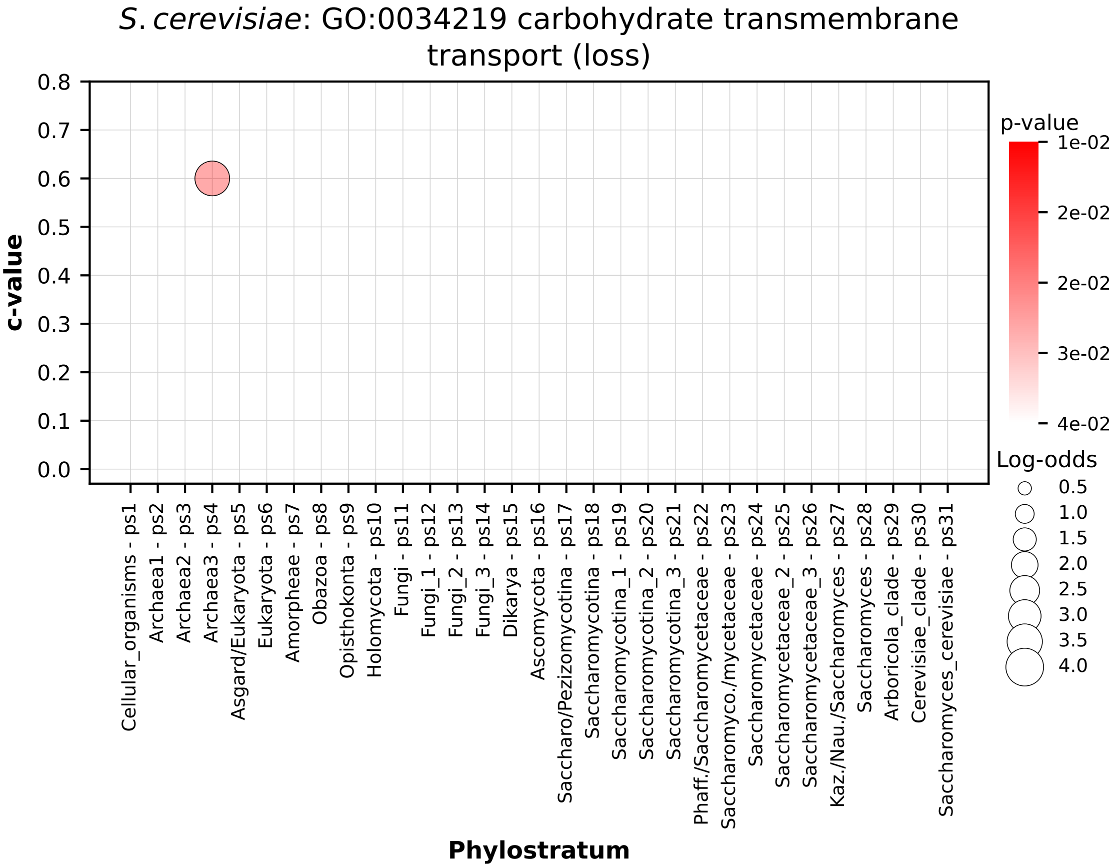
S. cerevisiae: GO:0034219 carbohydrate transmembrane transport (loss)
c-value
0.8
0.7
0.6
0.5
0.4
0.3
0.2
0.1
0.0
Phylostratum
Cellular_organisms - ps1
Archaea1 - ps2
Archaea2 - ps3
Archaea3 - ps4
Asgard/Eukaryota - ps5
Eukaryota - ps6
Amorpheae - ps7
Obazoa - ps8
Opisthokonta - ps9
Holomycota - ps10
Fungi - ps11
Fungi_1 - ps12
Fungi_2 - ps13
Fungi_3 - ps14
Dikarya - ps15
Ascomycota - ps16
Saccharo/Pezizomycotina - ps17
Saccharomycotina - ps18
Saccharomycotina_1 - ps19
Saccharomycotina_2 - ps20
Saccharomycotina_3 - ps21
Phaff./Saccharomycetaceae - ps22
Saccharomyco./mycetaceae - ps23
Saccharomycetaceae - ps24
Saccharomycetaceae_2 - ps25
Saccharomycetaceae_3 - ps26
Kaz./Nau./Saccharomyces - ps27
Saccharomyces - ps28
Arboricola_clade - ps29
Cerevisiae_clade - ps30
Saccharomyces_cerevisiae - ps31
p-value
1e-02
2e-02
2e-02
3e-02
4e-02
Log-odds
0.5
1.0
1.5
2.0
2.5
3.0
3.5
4.0

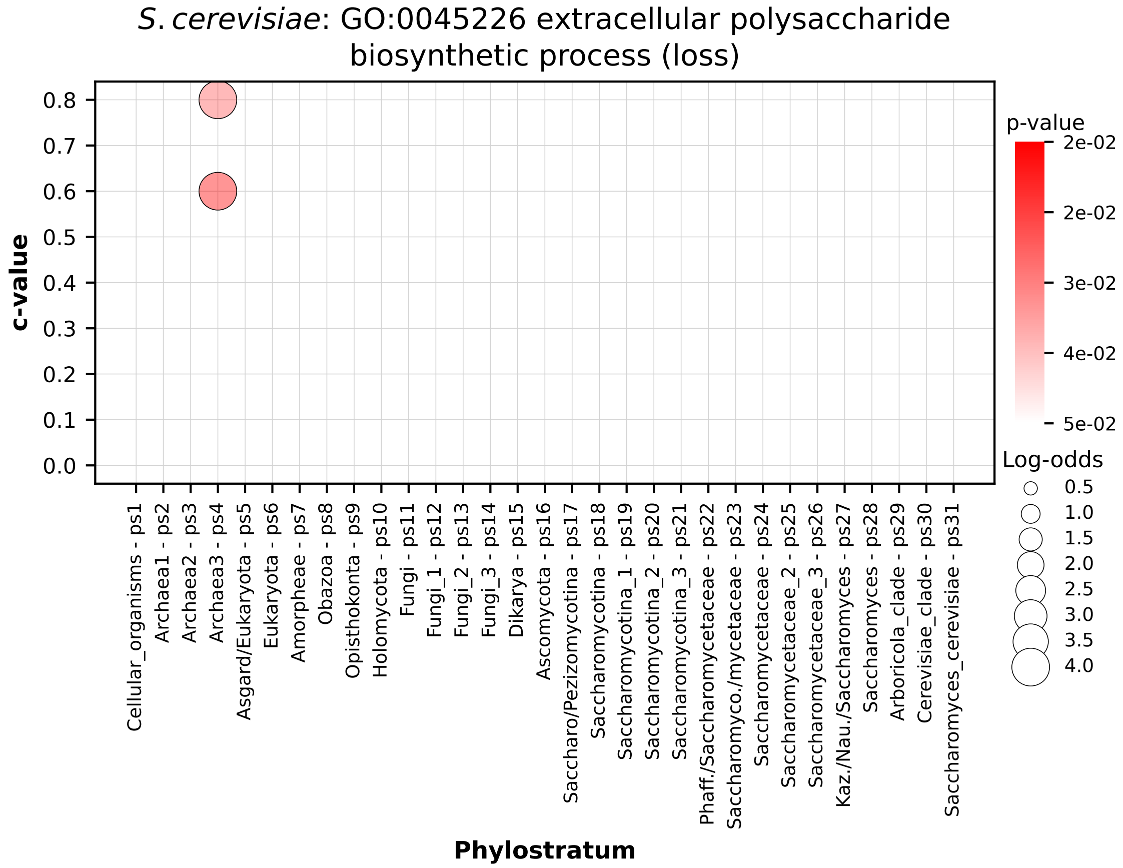

S. cerevisiae: GO:0045226 extracellular polysaccharide biosynthetic process (loss)
c-value
0.0
0.1
0.2
0.3
0.4
0.5
0.6
0.7
0.8
Phylostratum
Cellular_organisms - ps1
Archaea1 - ps2
Archaea2 - ps3
Archaea3 - ps4
Asgard/Eukaryota - ps5
Eukaryota - ps6
Amorpheae - ps7
Obazoa - ps8
Opisthokonta - ps9
Holomycota - ps10
Fungi - ps11
Fungi_1 - ps12
Fungi_2 - ps13
Fungi_3 - ps14
Dikarya - ps15
Ascomycota - ps16
Saccharo/Pezizomycotina - ps17
Saccharomycotina - ps18
Saccharomycotina_1 - ps19
Saccharomycotina_2 - ps20
Saccharomycotina_3 - ps21
Phaff./Saccharomycetaceae - ps22
Saccharomyco./mycetaceae - ps23
Saccharomycetaceae - ps24
Saccharomycetaceae_2 - ps25
Saccharomycetaceae_3 - ps26
Kaz./Nau./Saccharomyces - ps27
Saccharomyces - ps28
Arboricola_clade - ps29
Cerevisiae_clade - ps30
Saccharomyces_cerevisiae - ps31
p-value
2e-02
2e-02
3e-02
4e-02
5e-02
Log-odds
0.5
1.0
1.5
2.0
2.5
3.0
3.5
4.0

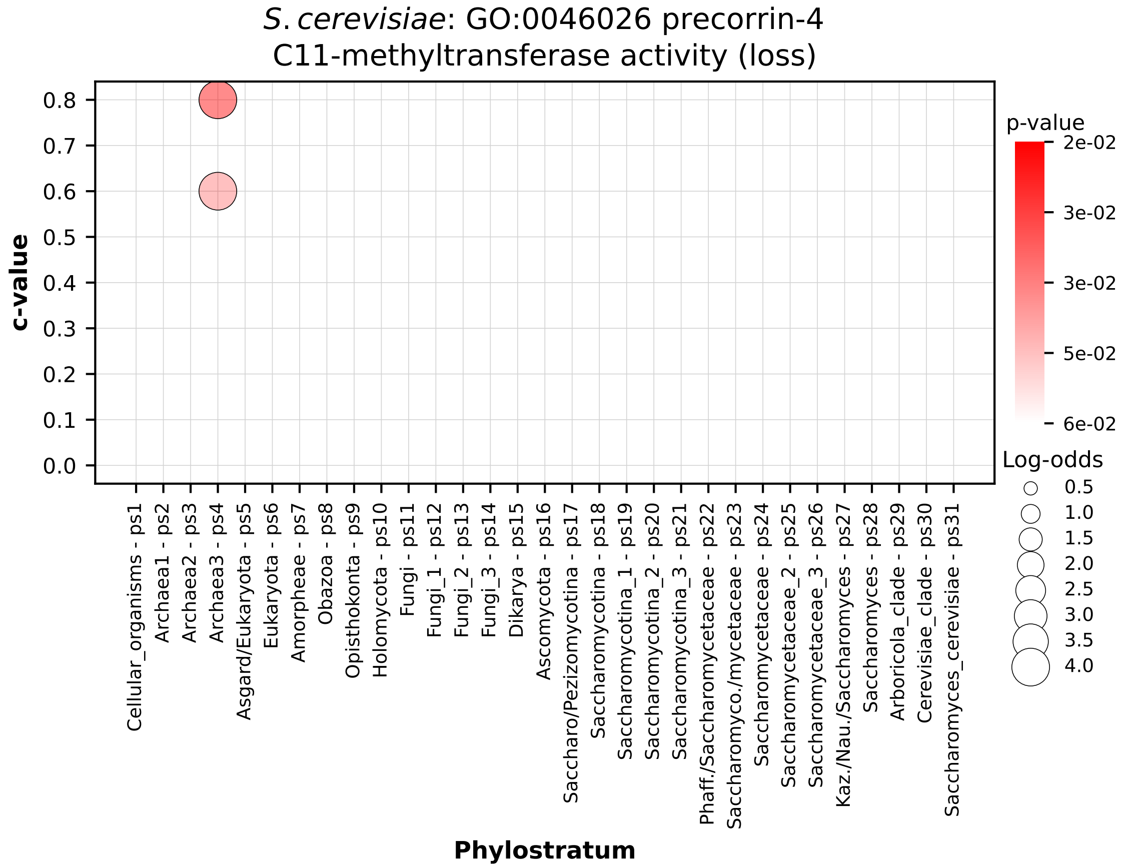

S. cerevisiae: GO:0046026 precorrin-4
C11-methyltransferase activity (loss)
c-value
0.0
0.1
0.2
0.3
0.4
0.5
0.6
0.7
0.8
p-value
2e-02
3e-02
3e-02
5e-02
6e-02
Log-odds
0.5
1.0
1.5
2.0
2.5
3.0
3.5
4.0
Cellular_organisms - ps1
Archaea1 - ps2
Archaea2 - ps3
Archaea3 - ps4
Asgard/Eukaryota - ps5
Eukaryota - ps6
Amorpheae - ps7
Obazoa - ps8
Opisthokonta - ps9
Holomycota - ps10
Fungi - ps11
Fungi_1 - ps12
Fungi_2 - ps13
Fungi_3 - ps14
Dikarya - ps15
Ascomycota - ps16
Saccharo/Pezizomycotina - ps17
Saccharomycotina - ps18
Saccharomycotina_1 - ps19
Saccharomycotina_2 - ps20
Saccharomycotina_3 - ps21
Phaff./Saccharomycetaceae - ps22
Saccharomyco./mycetaceae - ps23
Saccharomycetaceae - ps24
Saccharomycetaceae_2 - ps25
Saccharomycetaceae_3 - ps26
Kaz./Nau./Saccharomyces - ps27
Saccharomyces - ps28
Arboricola_clade - ps29
Cerevisiae_clade - ps30
Saccharomyces_cerevisiae - ps31
Phylostratum

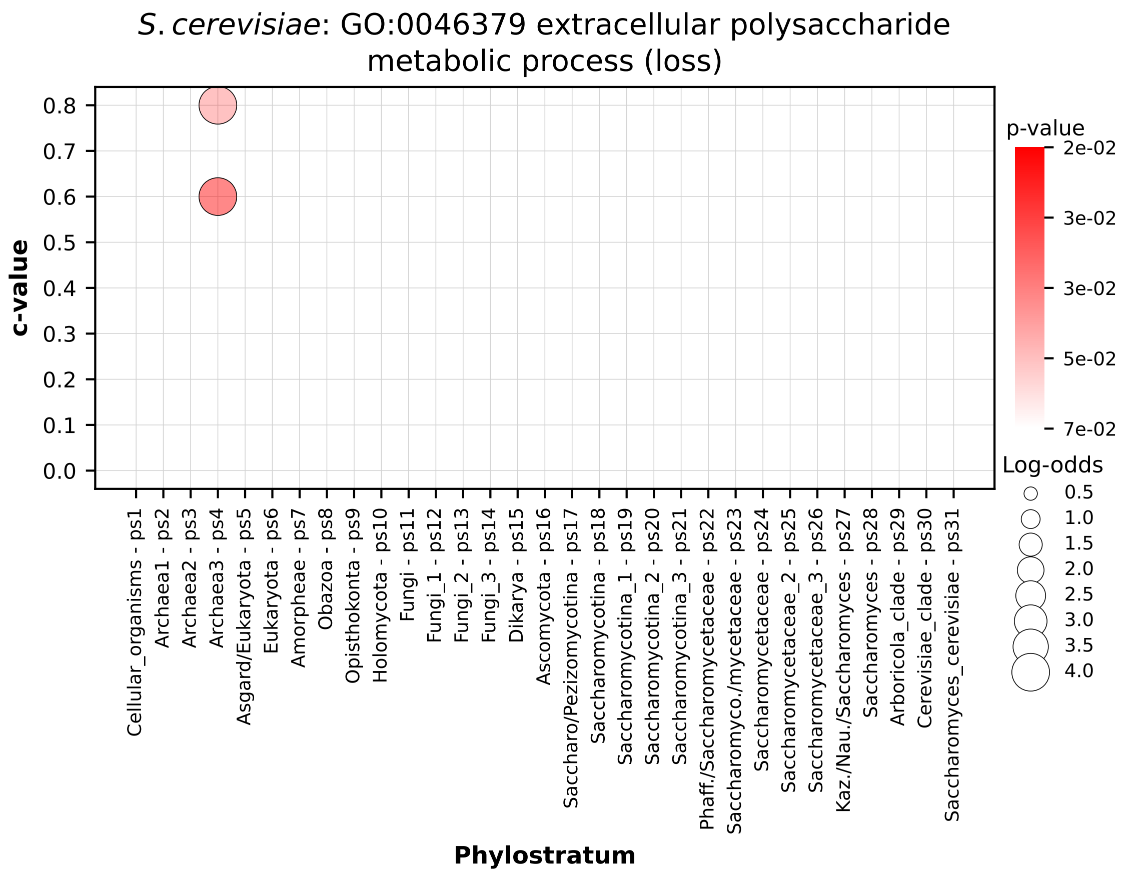

S. cerevisiae: GO:0046379 extracellular polysaccharide metabolic process (loss)
c-value
0.0
0.1
0.2
0.3
0.4
0.5
0.6
0.7
0.8
p-value
2e-02
3e-02
3e-02
5e-02
7e-02
Log-odds
0.5
1.0
1.5
2.0
2.5
3.0
3.5
4.0
Cellular_organisms - ps1
Archaea1 - ps2
Archaea2 - ps3
Archaea3 - ps4
Asgard/Eukaryota - ps5
Eukaryota - ps6
Amorpheae - ps7
Obazoa - ps8
Opisthokonta - ps9
Holomycota - ps10
Fungi - ps11
Fungi_1 - ps12
Fungi_2 - ps13
Fungi_3 - ps14
Dikarya - ps15
Ascomycota - ps16
Saccharo/Pezizomycotina - ps17
Saccharomycotina - ps18
Saccharomycotina_1 - ps19
Saccharomycotina_2 - ps20
Saccharomycotina_3 - ps21
Phaff./Saccharomycetaceae - ps22
Saccharomyco./mycetaceae - ps23
Saccharomycetaceae - ps24
Saccharomycetaceae_2 - ps25
Saccharomycetaceae_3 - ps26
Kaz./Nau./Saccharomyces - ps27
Saccharomyces - ps28
Arboricola_clade - ps29
Cerevisiae_clade - ps30
Saccharomyces_cerevisiae - ps31
Phylostratum

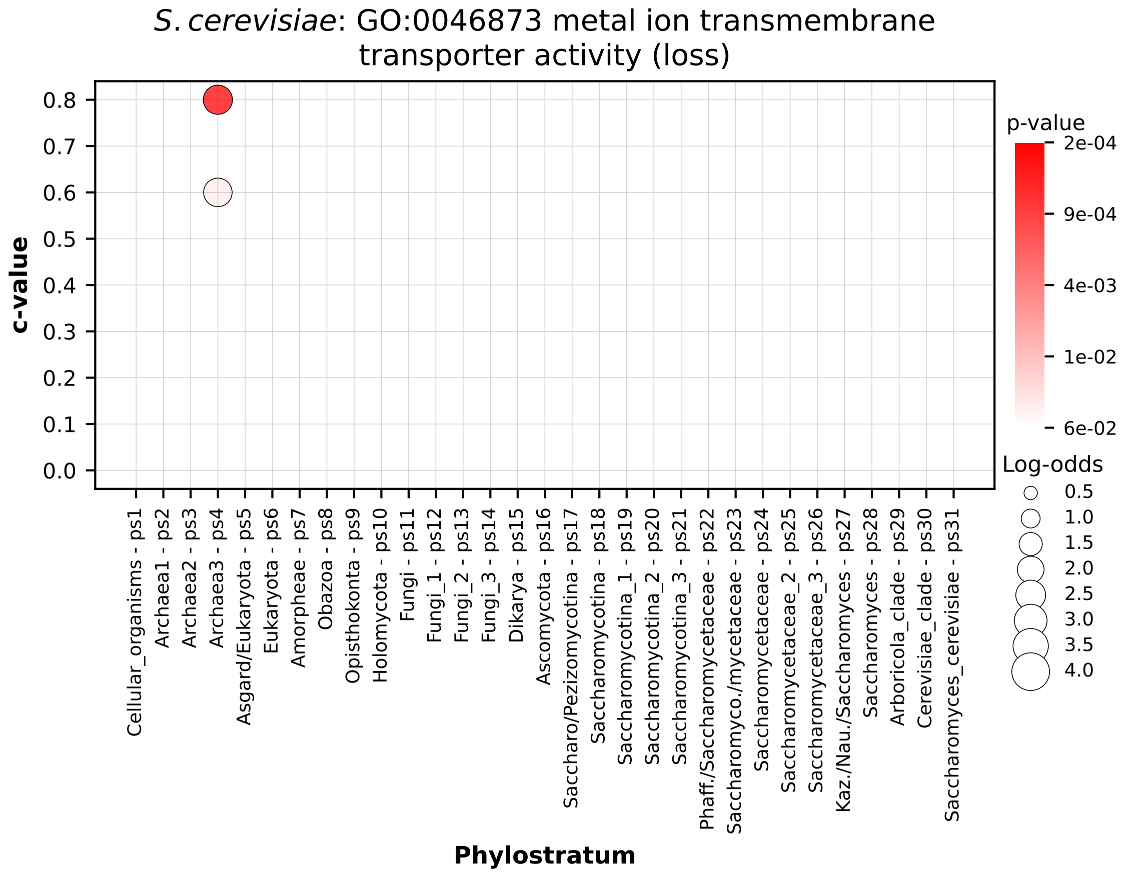

S. cerevisiae: GO:0046873 metal ion transmembrane transporter activity (loss)
c-value
0.8
0.7
0.6
0.5
0.4
0.3
0.2
0.1
0.0
p-value
2e-04
9e-04
4e-03
1e-02
6e-02
Log-odds
0.5
1.0
1.5
2.0
2.5
3.0
3.5
4.0
Cellular_organisms - ps1
Archaea1 - ps2
Archaea2 - ps3
Archaea3 - ps4
Asgard/Eukaryota - ps5
Eukaryota - ps6
Amorpheae - ps7
Obazoa - ps8
Opisthokonta - ps9
Holomycota - ps10
Fungi - ps11
Fungi_1 - ps12
Fungi_2 - ps13
Fungi_3 - ps14
Dikarya - ps15
Ascomycota - ps16
Saccharo/Pezizomycotina - ps17
Saccharomycotina - ps18
Saccharomycotina_1 - ps19
Saccharomycotina_2 - ps20
Saccharomycotina_3 - ps21
Phaff./Saccharomycetaceae - ps22
Saccharomyco./mycetaceae - ps23
Saccharomycetaceae - ps24
Saccharomycetaceae_2 - ps25
Saccharomycetaceae_3 - ps26
Kaz./Nau./Saccharomyces - ps27
Saccharomyces - ps28
Arboricola_clade - ps29
Cerevisiae_clade - ps30
Saccharomyces_cerevisiae - ps31
Phylostratum

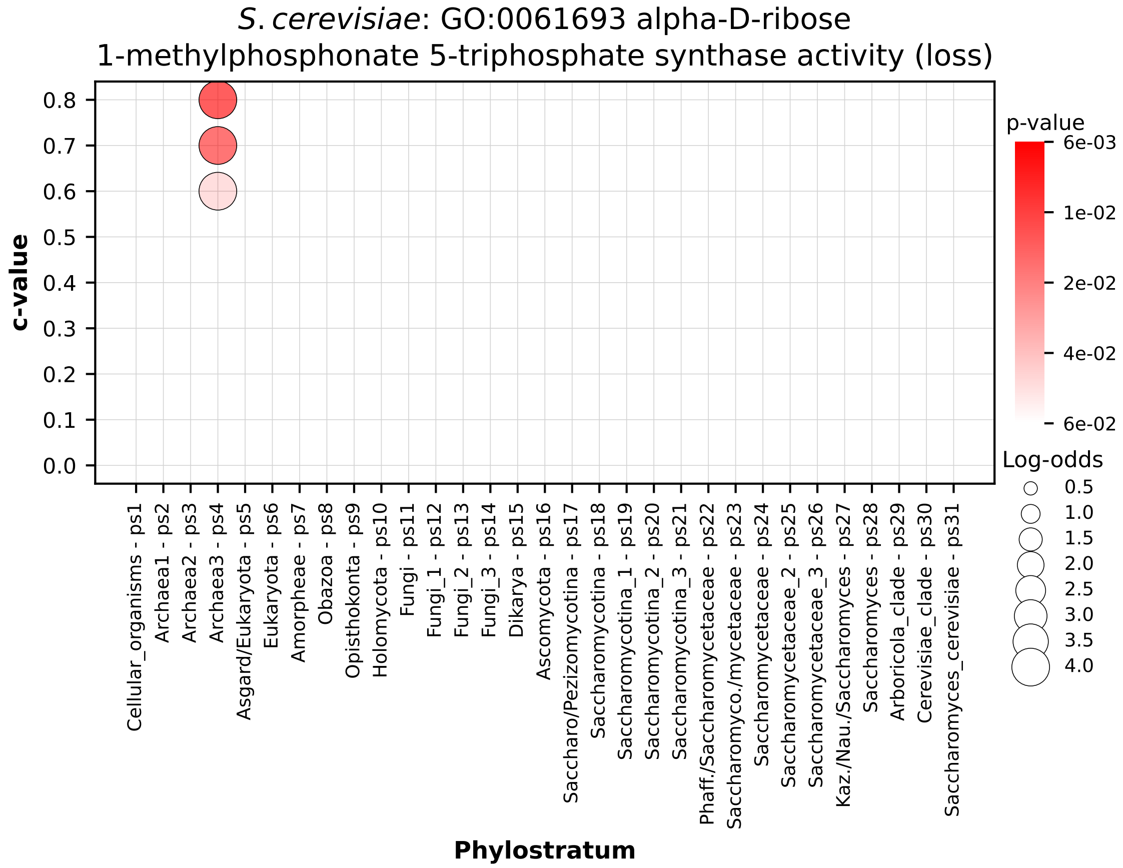
S. cerevisiae: GO:0061693 alpha-D-ribose
1-methylphosphonate 5-triphosphate synthase activity (loss)
c-value
0.8
0.7
0.6
0.5
0.4
0.3
0.2
0.1
0.0
p-value
6e-03
1e-02
2e-02
4e-02
6e-02
Log-odds
0.5
1.0
1.5
2.0
2.5
3.0
3.5
4.0
Cellular_organisms - ps1
Archaea1 - ps2
Archaea2 - ps3
Archaea3 - ps4
Asgard/Eukaryota - ps5
Eukaryota - ps6
Amorpheae - ps7
Obazoa - ps8
Opisthokonta - ps9
Holomycota - ps10
Fungi - ps11
Fungi_1 - ps12
Fungi_2 - ps13
Fungi_3 - ps14
Dikarya - ps15
Ascomycota - ps16
Saccharo/Pezizomycotina - ps17
Saccharomycotina - ps18
Saccharomycotina_1 - ps19
Saccharomycotina_2 - ps20
Saccharomycotina_3 - ps21
Phaff./Saccharomycetaceae - ps22
Saccharomyco./mycetaceae - ps23
Saccharomycetaceae - ps24
Saccharomycetaceae_2 - ps25
Saccharomycetaceae_3 - ps26
Kaz./Nau./Saccharomyces - ps27
Saccharomyces - ps28
Arboricola_clade - ps29
Cerevisiae_clade - ps30
Saccharomyces_cerevisiae - ps31
Phylostratum

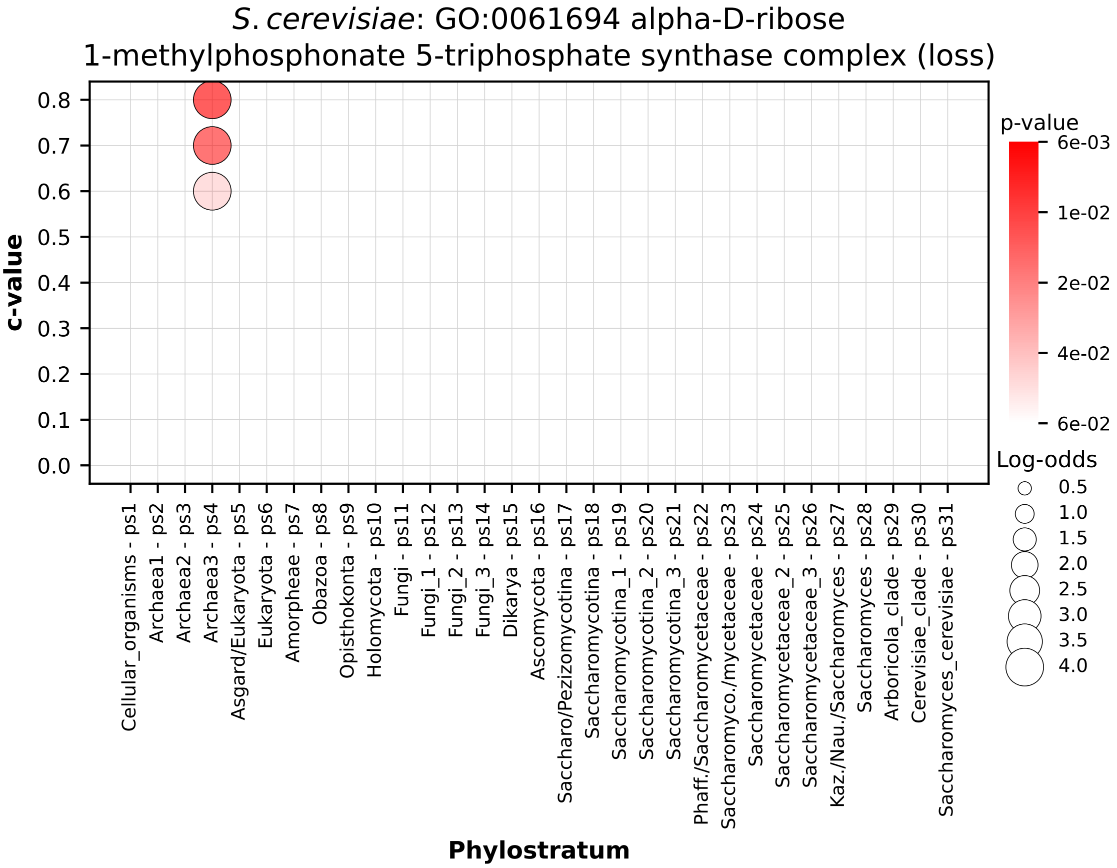

S. cerevisiae: GO:0061694 alpha-D-ribose
1-methylphosphonate 5-triphosphate synthase complex (loss)
c-value
0.0
0.1
0.2
0.3
0.4
0.5
0.6
0.7
0.8
Phylostratum
Cellular_organisms - ps1
Archaea1 - ps2
Archaea2 - ps3
Archaea3 - ps4
Asgard/Eukaryota - ps5
Eukaryota - ps6
Amorpheae - ps7
Obazoa - ps8
Opisthokonta - ps9
Holomycota - ps10
Fungi - ps11
Fungi_1 - ps12
Fungi_2 - ps13
Fungi_3 - ps14
Dikarya - ps15
Ascomycota - ps16
Saccharo/Pezizomycotina - ps17
Saccharomycotina - ps18
Saccharomycotina_1 - ps19
Saccharomycotina_2 - ps20
Saccharomycotina_3 - ps21
Phaff./Saccharomycetaceae - ps22
Saccharomyco./mycetaceae - ps23
Saccharomycetaceae - ps24
Saccharomycetaceae_2 - ps25
Saccharomycetaceae_3 - ps26
Kaz./Nau./Saccharomyces - ps27
Saccharomyces - ps28
Arboricola_clade - ps29
Cerevisiae_clade - ps30
Saccharomyces_cerevisiae - ps31
p-value
6e-03
1e-02
2e-02
4e-02
6e-02
Log-odds
0.5
1.0
1.5
2.0
2.5
3.0
3.5
4.0

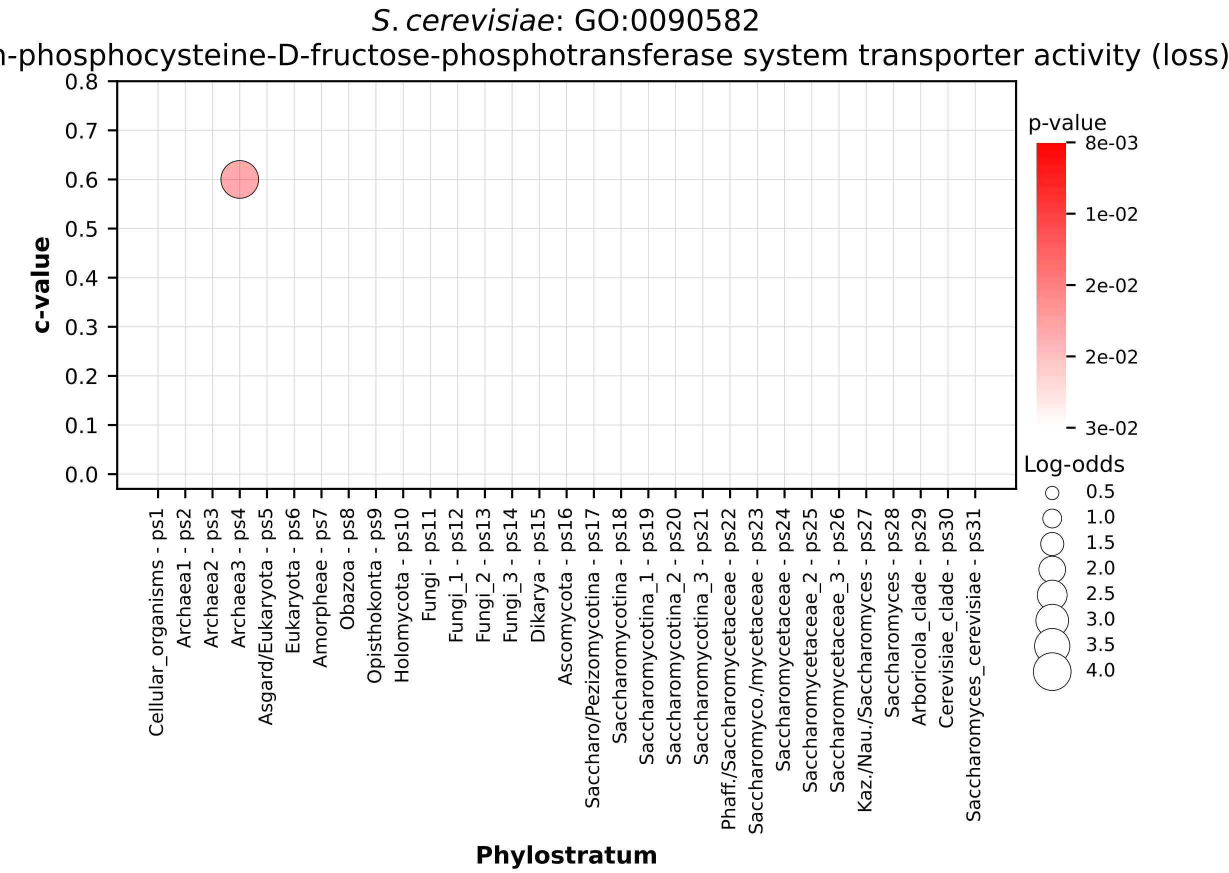

S. cerevisiae: GO:0090582
n-phosphocysteine-D-fructose-phosphotransferase system transporter activity (loss)
c-value
0.8
0.7
0.6
0.5
0.4
0.3
0.2
0.1
0.0
p-value
8e-03
1e-02
2e-02
2e-02
3e-02
Log-odds
0.5
1.0
1.5
2.0
2.5
3.0
3.5
4.0
Cellular_organisms - ps1
Archaea1 - ps2
Archaea2 - ps3
Archaea3 - ps4
Asgard/Eukaryota - ps5
Eukaryota - ps6
Amorpheae - ps7
Obazoa - ps8
Opisthokonta - ps9
Holomycota - ps10
Fungi - ps11
Fungi_1 - ps12
Fungi_2 - ps13
Fungi_3 - ps14
Dikarya - ps15
Ascomycota - ps16
Saccharo/Pezizomycotina - ps17
Saccharomycotina - ps18
Saccharomycotina_1 - ps19
Saccharomycotina_2 - ps20
Saccharomycotina_3 - ps21
Phaff./Saccharomycetaceae - ps22
Saccharomyco./mycetaceae - ps23
Saccharomycetaceae - ps24
Saccharomycetaceae_2 - ps25
Saccharomycetaceae_3 - ps26
Kaz./Nau./Saccharomyces - ps27
Saccharomyces - ps28
Arboricola_clade - ps29
Cerevisiae_clade - ps30
Saccharomyces_cerevisiae - ps31
Phylostratum

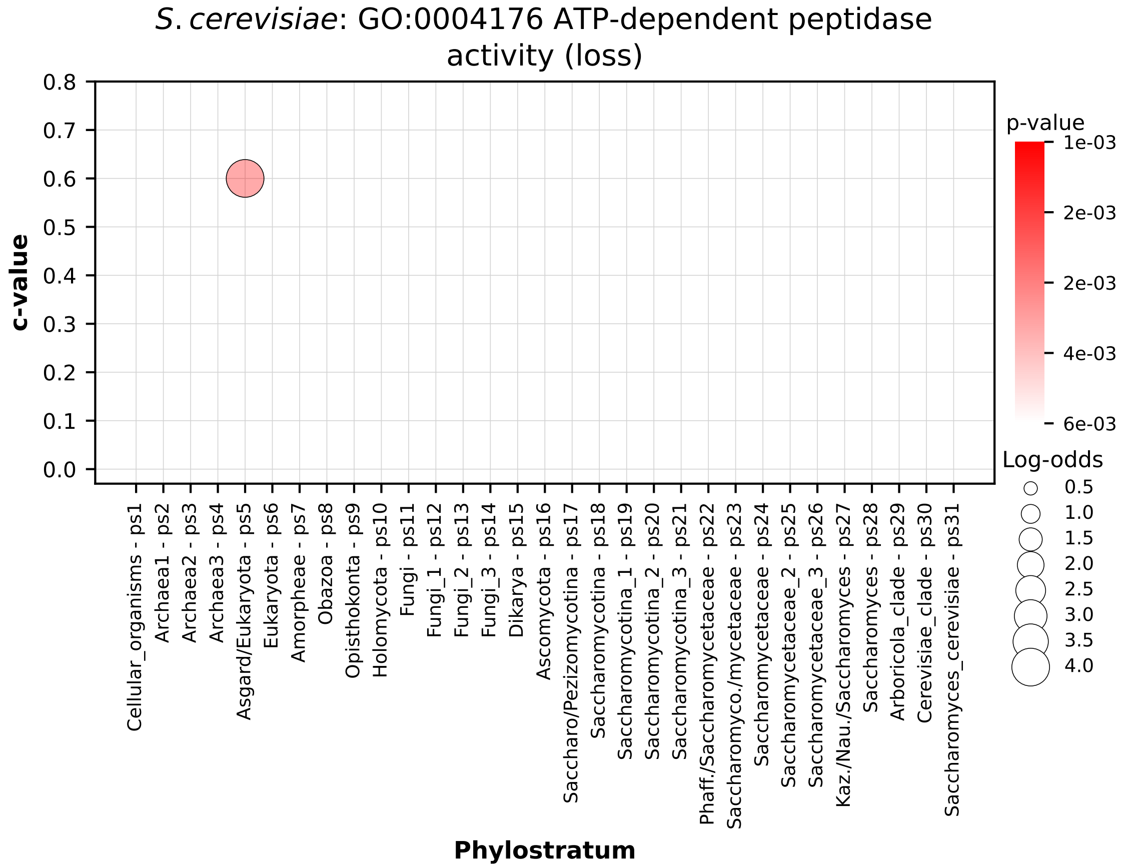

S. cerevisiae: GO:0004176 ATP-dependent peptidase activity (loss)
c-value
0.8
0.7
0.6
0.5
0.4
0.3
0.2
0.1
0.0
p-value
1e-03
2e-03
2e-03
4e-03
6e-03
Log-odds
0.5
1.0
1.5
2.0
2.5
3.0
3.5
4.0
Cellular_organisms - ps1
Archaea1 - ps2
Archaea2 - ps3
Archaea3 - ps4
Asgard/Eukaryota - ps5
Eukaryota - ps6
Amorpheae - ps7
Obazoa - ps8
Opisthokonta - ps9
Holomycota - ps10
Fungi - ps11
Fungi_1 - ps12
Fungi_2 - ps13
Fungi_3 - ps14
Dikarya - ps15
Ascomycota - ps16
Saccharo/Pezizomycotina - ps17
Saccharomycotina - ps18
Saccharomycotina_1 - ps19
Saccharomycotina_2 - ps20
Saccharomycotina_3 - ps21
Phaff./Saccharomycetaceae - ps22
Saccharomyco./mycetaceae - ps23
Saccharomycetaceae - ps24
Saccharomycetaceae_2 - ps25
Saccharomycetaceae_3 - ps26
Kaz./Nau./Saccharomyces - ps27
Saccharomyces - ps28
Arboricola_clade - ps29
Cerevisiae_clade - ps30
Saccharomyces_cerevisiae - ps31
Phylostratum

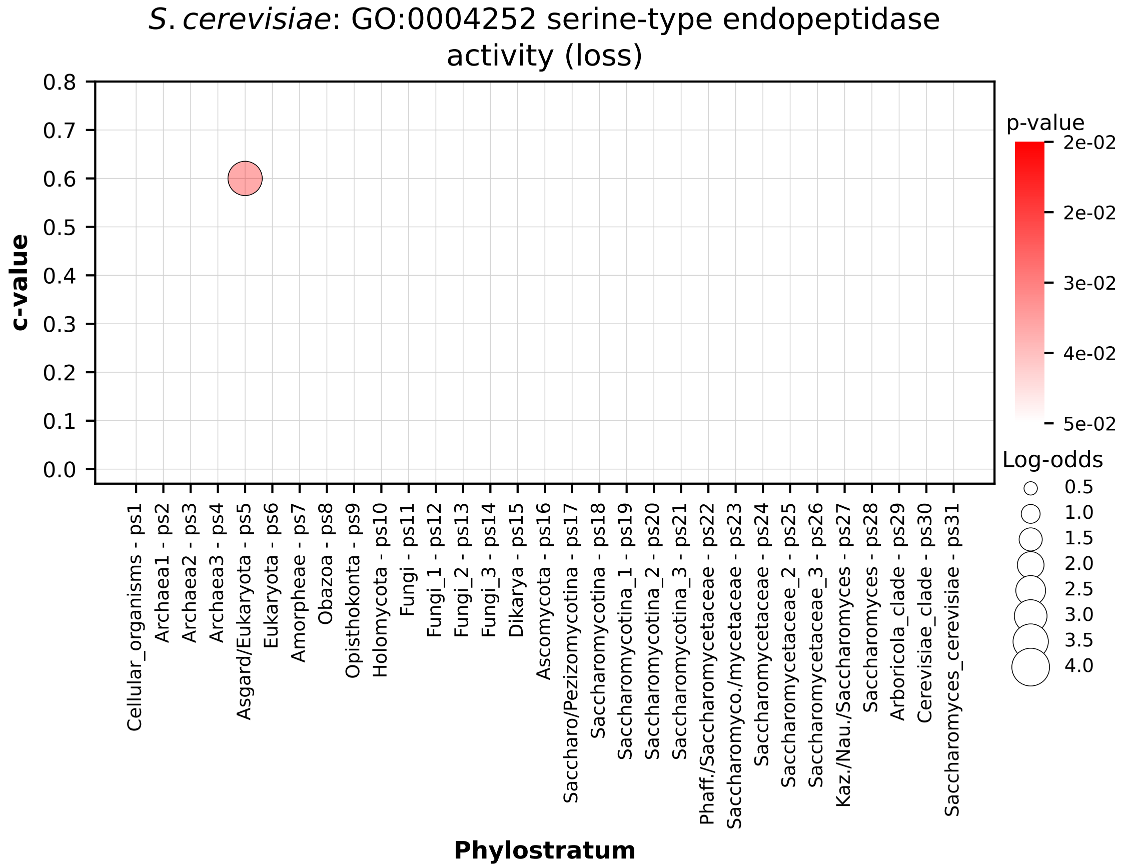

S. cerevisiae: GO:0004252 serine-type endopeptidase activity (loss)
c-value
0.8
0.7
0.6
0.5
0.4
0.3
0.2
0.1
0.0
Phylostratum
Cellular_organisms - ps1
Archaea1 - ps2
Archaea2 - ps3
Archaea3 - ps4
Asgard/Eukaryota - ps5
Eukaryota - ps6
Amorpheae - ps7
Obazoa - ps8
Opisthokonta - ps9
Holomycota - ps10
Fungi - ps11
Fungi_1 - ps12
Fungi_2 - ps13
Fungi_3 - ps14
Dikarya - ps15
Ascomycota - ps16
Saccharo/Pezizomycotina - ps17
Saccharomycotina - ps18
Saccharomycotina_1 - ps19
Saccharomycotina_2 - ps20
Saccharomycotina_3 - ps21
Phaff./Saccharomycetaceae - ps22
Saccharomyco./mycetaceae - ps23
Saccharomycetaceae - ps24
Saccharomycetaceae_2 - ps25
Saccharomycetaceae_3 - ps26
Kaz./Nau./Saccharomyces - ps27
Saccharomyces - ps28
Arboricola_clade - ps29
Cerevisiae_clade - ps30
Saccharomyces_cerevisiae - ps31
p-value
2e-02
2e-02
3e-02
4e-02
5e-02
Log-odds
0.5
1.0
1.5
2.0
2.5
3.0
3.5
4.0

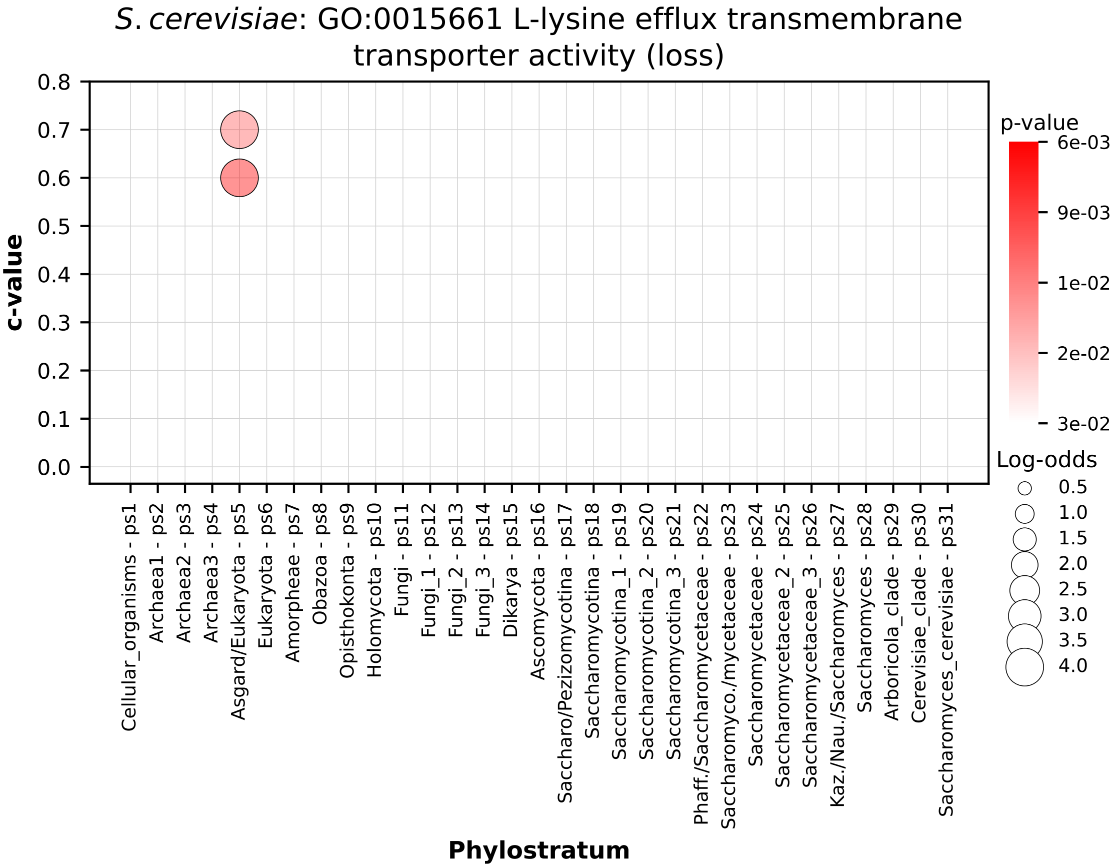

S. cerevisiae: GO:0015661 L-lysine efflux transmembrane transporter activity (loss)
c-value
0.8
0.7
0.6
0.5
0.4
0.3
0.2
0.1
0.0
p-value
6e-03
9e-03
1e-02
2e-02
3e-02
Log-odds
0.5
1.0
1.5
2.0
2.5
3.0
3.5
4.0
Cellular_organisms - ps1
Archaea1 - ps2
Archaea2 - ps3
Archaea3 - ps4
Asgard/Eukaryota - ps5
Eukaryota - ps6
Amorpheae - ps7
Obazoa - ps8
Opisthokonta - ps9
Holomycota - ps10
Fungi - ps11
Fungi_1 - ps12
Fungi_2 - ps13
Fungi_3 - ps14
Dikarya - ps15
Ascomycota - ps16
Saccharo/Pezizomycotina - ps17
Saccharomycotina - ps18
Saccharomycotina_1 - ps19
Saccharomycotina_2 - ps20
Saccharomycotina_3 - ps21
Phaff./Saccharomycetaceae - ps22
Saccharomyco./mycetaceae - ps23
Saccharomycetaceae - ps24
Saccharomycetaceae_2 - ps25
Saccharomycetaceae_3 - ps26
Kaz./Nau./Saccharomyces - ps27
Saccharomyces - ps28
Arboricola_clade - ps29
Cerevisiae_clade - ps30
Saccharomyces_cerevisiae - ps31
Phylostratum

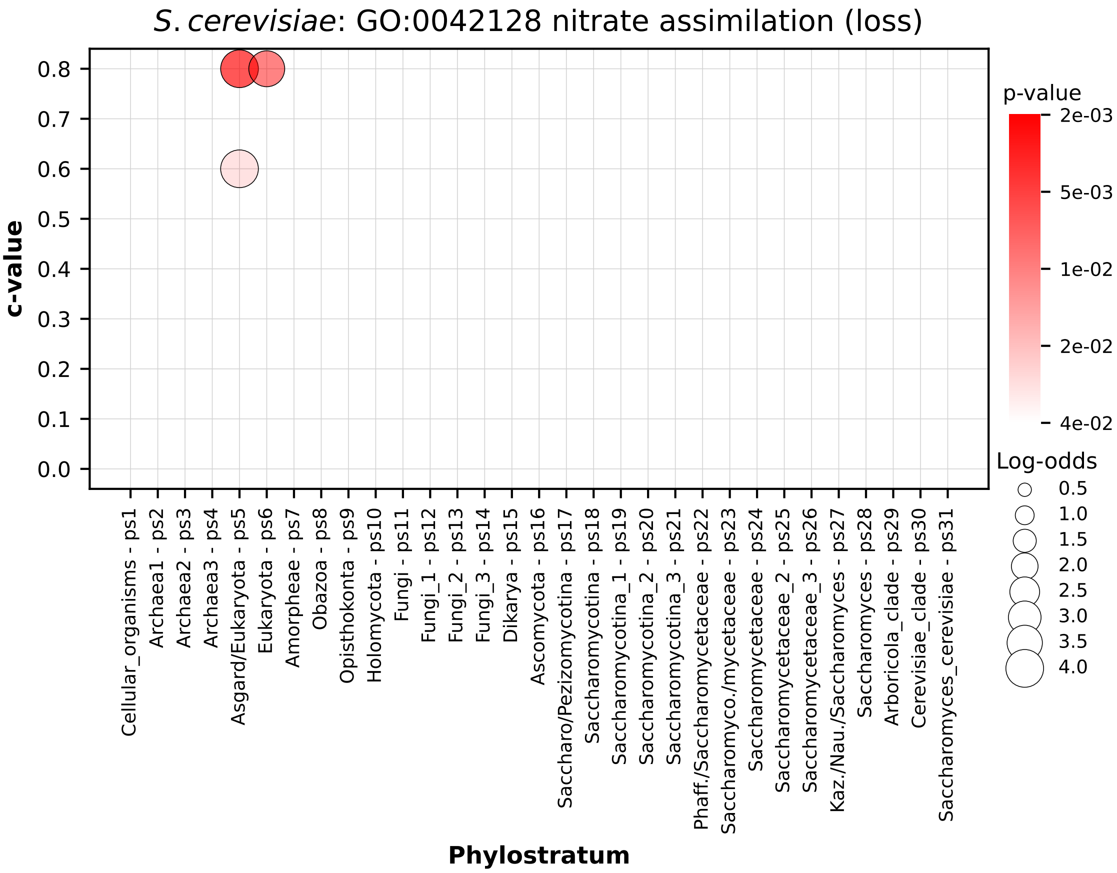

S. cerevisiae: GO:0042128 nitrate assimilation (loss)
c-value
0.8
0.7
0.6
0.5
0.4
0.3
0.2
0.1
0.0
p-value
2e-03
5e-03
1e-02
2e-02
4e-02
Log-odds
0.5
1.0
1.5
2.0
2.5
3.0
3.5
4.0
Cellular_organisms - ps1
Archaea1 - ps2
Archaea2 - ps3
Archaea3 - ps4
Asgard/Eukaryota - ps5
Eukaryota - ps6
Amorpheae - ps7
Obazoa - ps8
Opisthokonta - ps9
Holomycota - ps10
Fungi - ps11
Fungi_1 - ps12
Fungi_2 - ps13
Fungi_3 - ps14
Dikarya - ps15
Ascomycota - ps16
Saccharo/Pezizomycotina - ps17
Saccharomycotina - ps18
Saccharomycotina_1 - ps19
Saccharomycotina_2 - ps20
Saccharomycotina_3 - ps21
Phaff./Saccharomycetaceae - ps22
Saccharomyco./mycetaceae - ps23
Saccharomycetaceae - ps24
Saccharomycetaceae_2 - ps25
Saccharomycetaceae_3 - ps26
Kaz./Nau./Saccharomyces - ps27
Saccharomyces - ps28
Arboricola_clade - ps29
Cerevisiae_clade - ps30
Saccharomyces_cerevisiae - ps31
Phylostratum

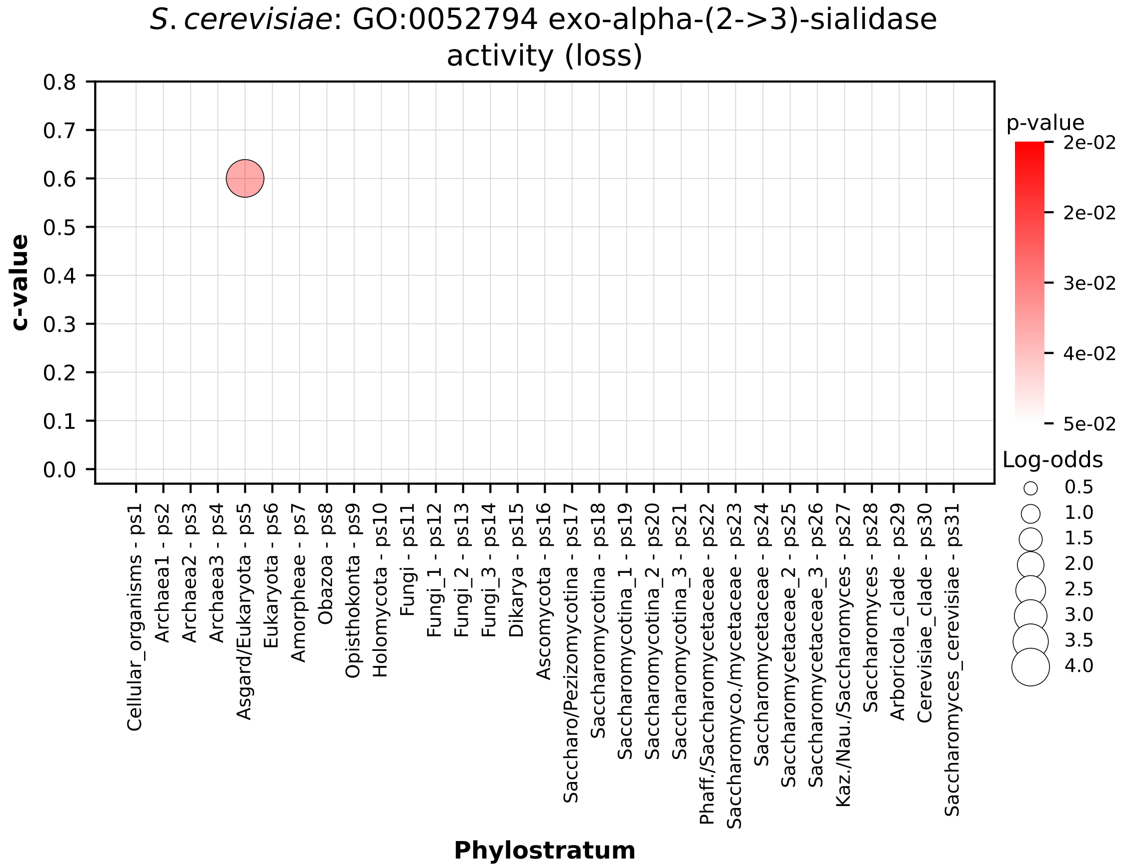

S. cerevisiae: GO:0052794 exo-alpha-(2->3)-sialidase activity (loss)
c-value
0.8
0.7
0.6
0.5
0.4
0.3
0.2
0.1
0.0
p-value
2e-02
2e-02
3e-02
4e-02
5e-02
Log-odds
0.5
1.0
1.5
2.0
2.5
3.0
3.5
4.0
Cellular_organisms - ps1
Archaea1 - ps2
Archaea2 - ps3
Archaea3 - ps4
Asgard/Eukaryota - ps5
Eukaryota - ps6
Amorpheae - ps7
Obazoa - ps8
Opisthokonta - ps9
Holomycota - ps10
Fungi - ps11
Fungi_1 - ps12
Fungi_2 - ps13
Fungi_3 - ps14
Dikarya - ps15
Ascomycota - ps16
Saccharo/Pezizomycotina - ps17
Saccharomycotina - ps18
Saccharomycotina_1 - ps19
Saccharomycotina_2 - ps20
Saccharomycotina_3 - ps21
Phaff./Saccharomycetaceae - ps22
Saccharomyco./mycetaceae - ps23
Saccharomycetaceae - ps24
Saccharomycetaceae_2 - ps25
Saccharomycetaceae_3 - ps26
Kaz./Nau./Saccharomyces - ps27
Saccharomyces - ps28
Arboricola_clade - ps29
Cerevisiae_clade - ps30
Saccharomyces_cerevisiae - ps31
Phylostratum

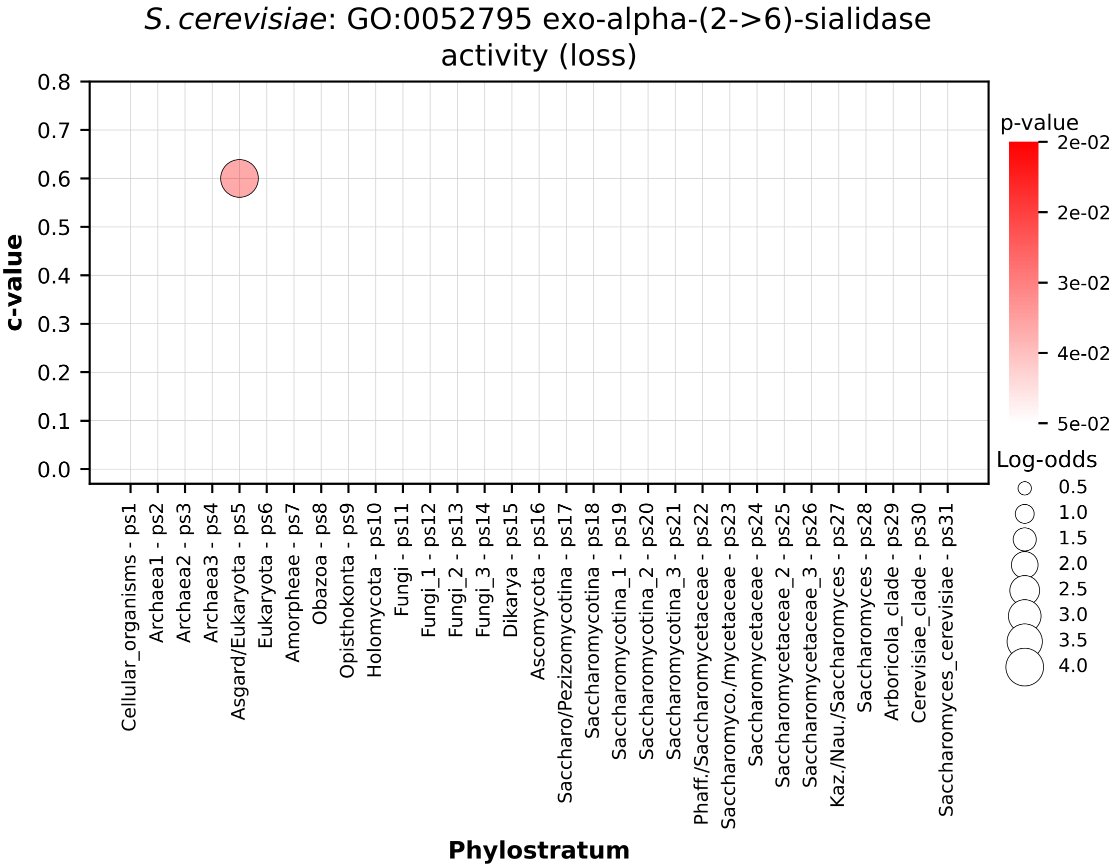

S. cerevisiae: GO:0052795 exo-alpha-(2->6)-sialidase activity (loss)
c-value
0.8
0.7
0.6
0.5
0.4
0.3
0.2
0.1
0.0
Phylostratum
Cellular_organisms - ps1
Archaea1 - ps2
Archaea2 - ps3
Archaea3 - ps4
Asgard/Eukaryota - ps5
Eukaryota - ps6
Amorpheae - ps7
Obazoa - ps8
Opisthokonta - ps9
Holomycota - ps10
Fungi - ps11
Fungi_1 - ps12
Fungi_2 - ps13
Fungi_3 - ps14
Dikarya - ps15
Ascomycota - ps16
Saccharo/Pezizomycotina - ps17
Saccharomycotina - ps18
Saccharomycotina_1 - ps19
Saccharomycotina_2 - ps20
Saccharomycotina_3 - ps21
Phaff./Saccharomycetaceae - ps22
Saccharomyco./mycetaceae - ps23
Saccharomycetaceae - ps24
Saccharomycetaceae_2 - ps25
Saccharomycetaceae_3 - ps26
Kaz./Nau./Saccharomyces - ps27
Saccharomyces - ps28
Arboricola_clade - ps29
Cerevisiae_clade - ps30
Saccharomyces_cerevisiae - ps31
p-value
2e-02
2e-02
3e-02
4e-02
5e-02
Log-odds
0.5
1.0
1.5
2.0
2.5
3.0
3.5
4.0

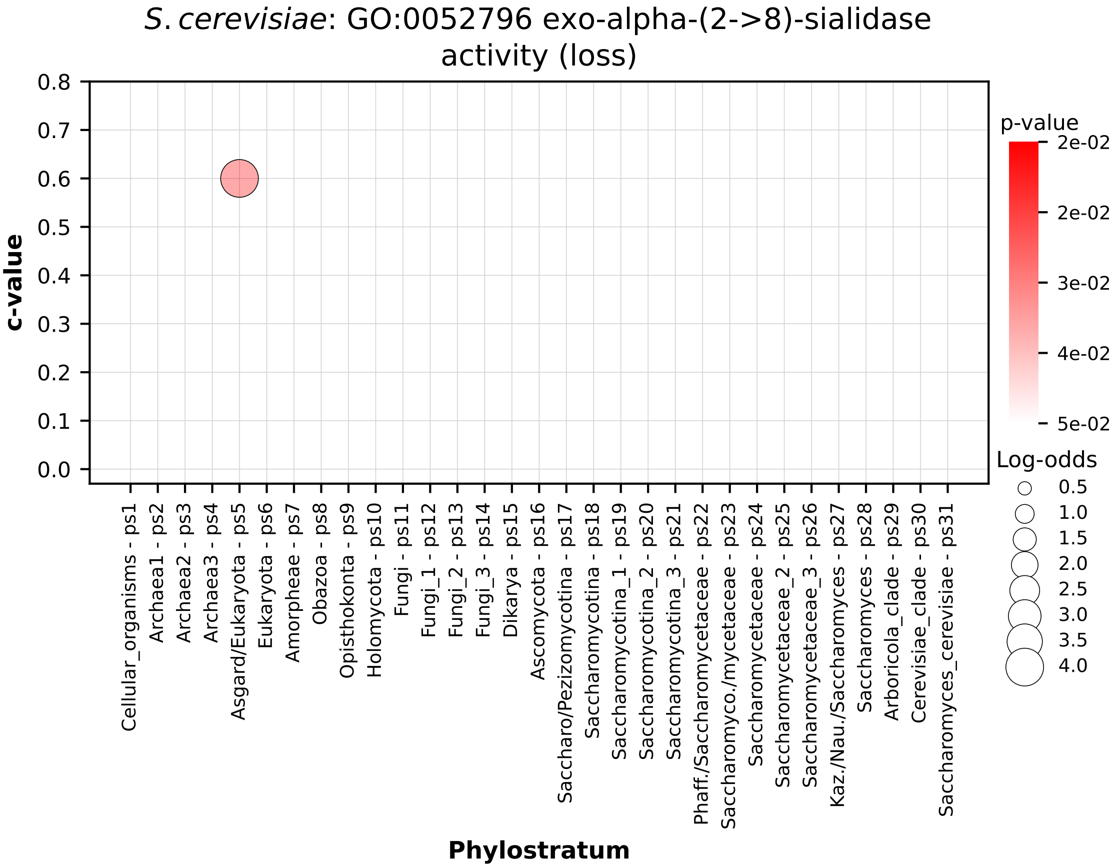

S. cerevisiae: GO:0052796 exo-alpha-(2->8)-sialidase activity (loss)
c-value
0.8
0.7
0.6
0.5
0.4
0.3
0.2
0.1
0.0
p-value
2e-02
2e-02
3e-02
4e-02
5e-02
Log-odds
0.5
1.0
1.5
2.0
2.5
3.0
3.5
4.0
Cellular_organisms - ps1
Archaea1 - ps2
Archaea2 - ps3
Archaea3 - ps4
Asgard/Eukaryota - ps5
Eukaryota - ps6
Amorpheae - ps7
Obazoa - ps8
Opisthokonta - ps9
Holomycota - ps10
Fungi - ps11
Fungi_1 - ps12
Fungi_2 - ps13
Fungi_3 - ps14
Dikarya - ps15
Ascomycota - ps16
Saccharo/Pezizomycotina - ps17
Saccharomycotina - ps18
Saccharomycotina_1 - ps19
Saccharomycotina_2 - ps20
Saccharomycotina_3 - ps21
Phaff./Saccharomycetaceae - ps22
Saccharomyco./mycetaceae - ps23
Saccharomycetaceae - ps24
Saccharomycetaceae_2 - ps25
Saccharomycetaceae_3 - ps26
Kaz./Nau./Saccharomyces - ps27
Saccharomyces - ps28
Arboricola_clade - ps29
Cerevisiae_clade - ps30
Saccharomyces_cerevisiae - ps31
Phylostratum

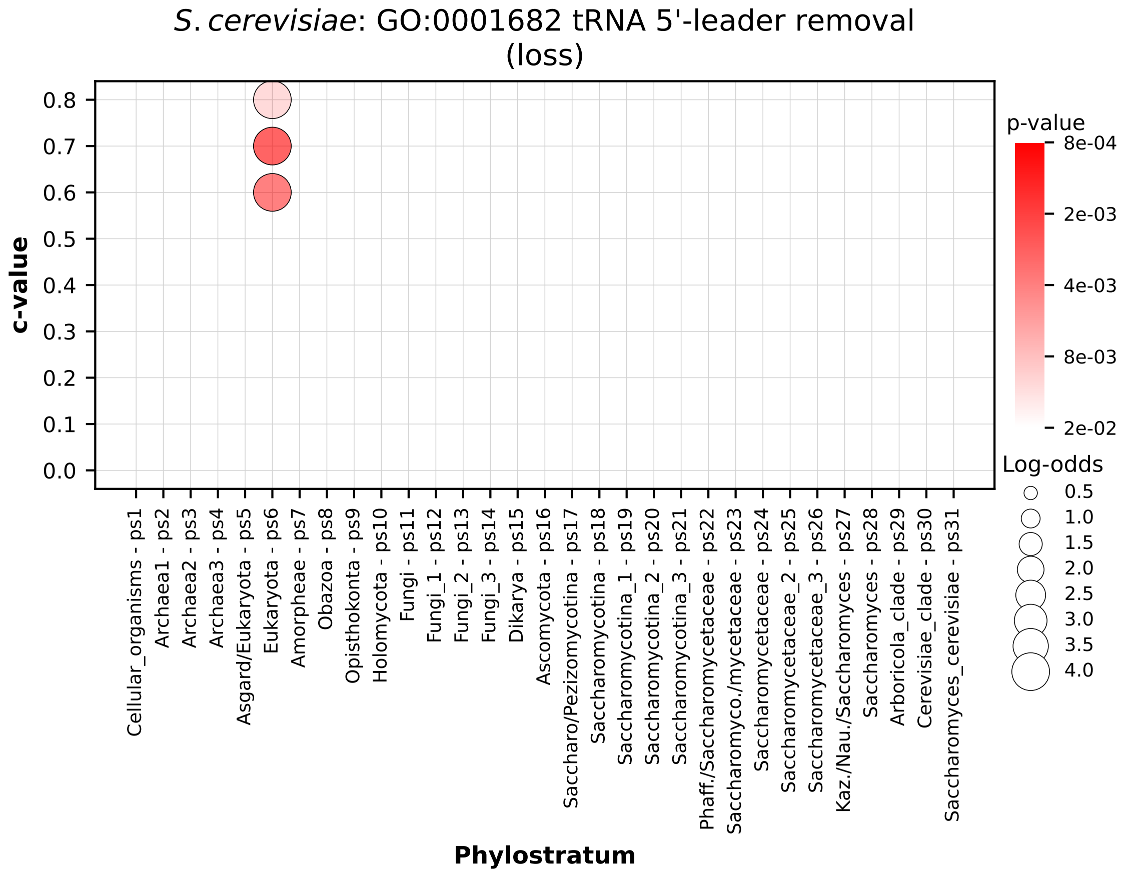
S. cerevisiae: GO:0001682 tRNA 5'-leader removal
(loss)
c-value
0.0
0.1
0.2
0.3
0.4
0.5
0.6
0.7
0.8
p-value
8e-04
2e-03
4e-03
8e-03
2e-02
Log-odds
0.5
1.0
1.5
2.0
2.5
3.0
3.5
4.0
Cellular_organisms - ps1
Archaea1 - ps2
Archaea2 - ps3
Archaea3 - ps4
Asgard/Eukaryota - ps5
Eukaryota - ps6
Amorpheae - ps7
Obazoa - ps8
Opisthokonta - ps9
Holomycota - ps10
Fungi - ps11
Fungi_1 - ps12
Fungi_2 - ps13
Fungi_3 - ps14
Dikarya - ps15
Ascomycota - ps16
Saccharo/Pezizomycotina - ps17
Saccharomycotina - ps18
Saccharomycotina_1 - ps19
Saccharomycotina_2 - ps20
Saccharomycotina_3 - ps21
Phaff./Saccharomycetaceae - ps22
Saccharomyco./mycetaceae - ps23
Saccharomycetaceae - ps24
Saccharomycetaceae_2 - ps25
Saccharomycetaceae_3 - ps26
Kaz./Nau./Saccharomyces - ps27
Saccharomyces - ps28
Arboricola_clade - ps29
Cerevisiae_clade - ps30
Saccharomyces_cerevisiae - ps31
Phylostratum

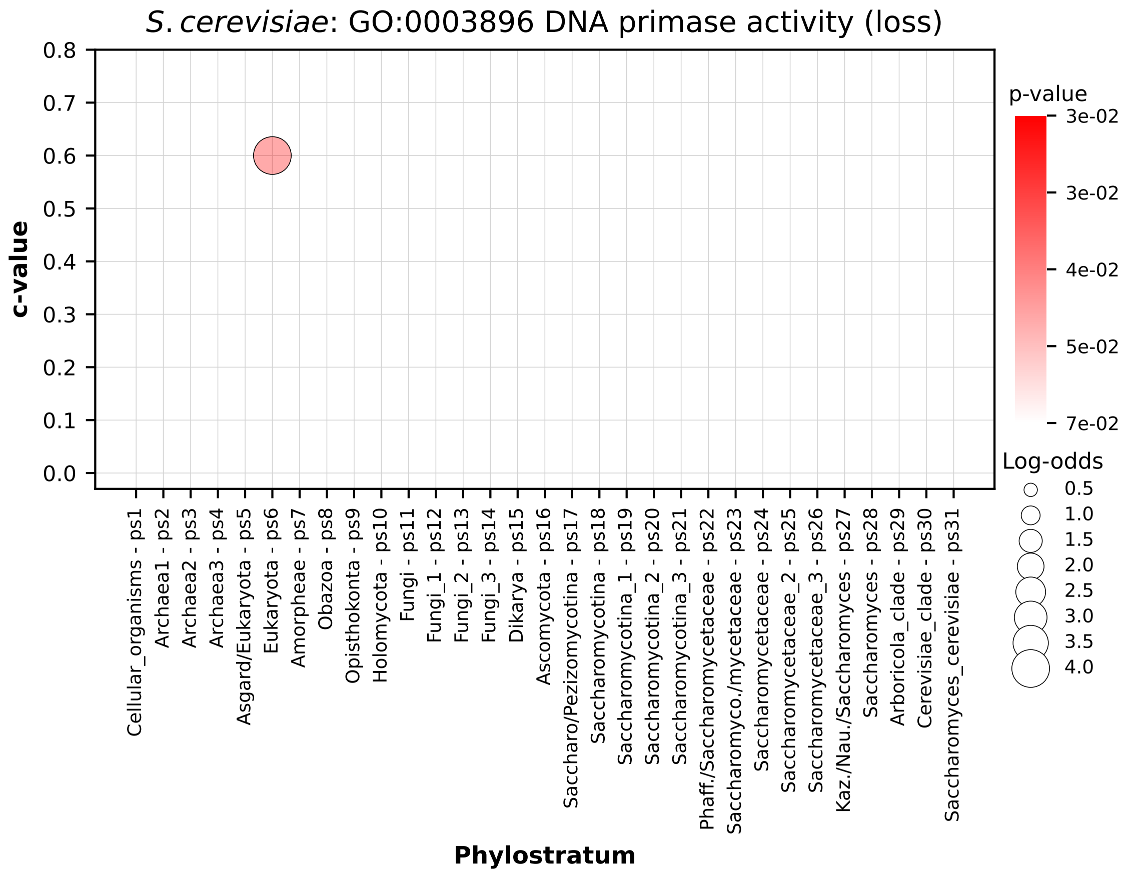
S. cerevisiae: GO:0003896 DNA primase activity (loss)
c-value
0.8
0.7
0.6
0.5
0.4
0.3
0.2
0.1
0.0
Phylostratum
Cellular_organisms - ps1
Archaea1 - ps2
Archaea2 - ps3
Archaea3 - ps4
Asgard/Eukaryota - ps5
Eukaryota - ps6
Amorpheae - ps7
Obazoa - ps8
Opisthokonta - ps9
Holomycota - ps10
Fungi - ps11
Fungi_1 - ps12
Fungi_2 - ps13
Fungi_3 - ps14
Dikarya - ps15
Ascomycota - ps16
Saccharo/Pezizomycotina - ps17
Saccharomycotina - ps18
Saccharomycotina_1 - ps19
Saccharomycotina_2 - ps20
Saccharomycotina_3 - ps21
Phaff./Saccharomycetaceae - ps22
Saccharomyco./mycetaceae - ps23
Saccharomycetaceae - ps24
Saccharomycetaceae_2 - ps25
Saccharomycetaceae_3 - ps26
Kaz./Nau./Saccharomyces - ps27
Saccharomyces - ps28
Arboricola_clade - ps29
Cerevisiae_clade - ps30
Saccharomyces_cerevisiae - ps31
p-value
3e-02
3e-02
4e-02
5e-02
7e-02
Log-odds
0.5
1.0
1.5
2.0
2.5
3.0
3.5
4.0

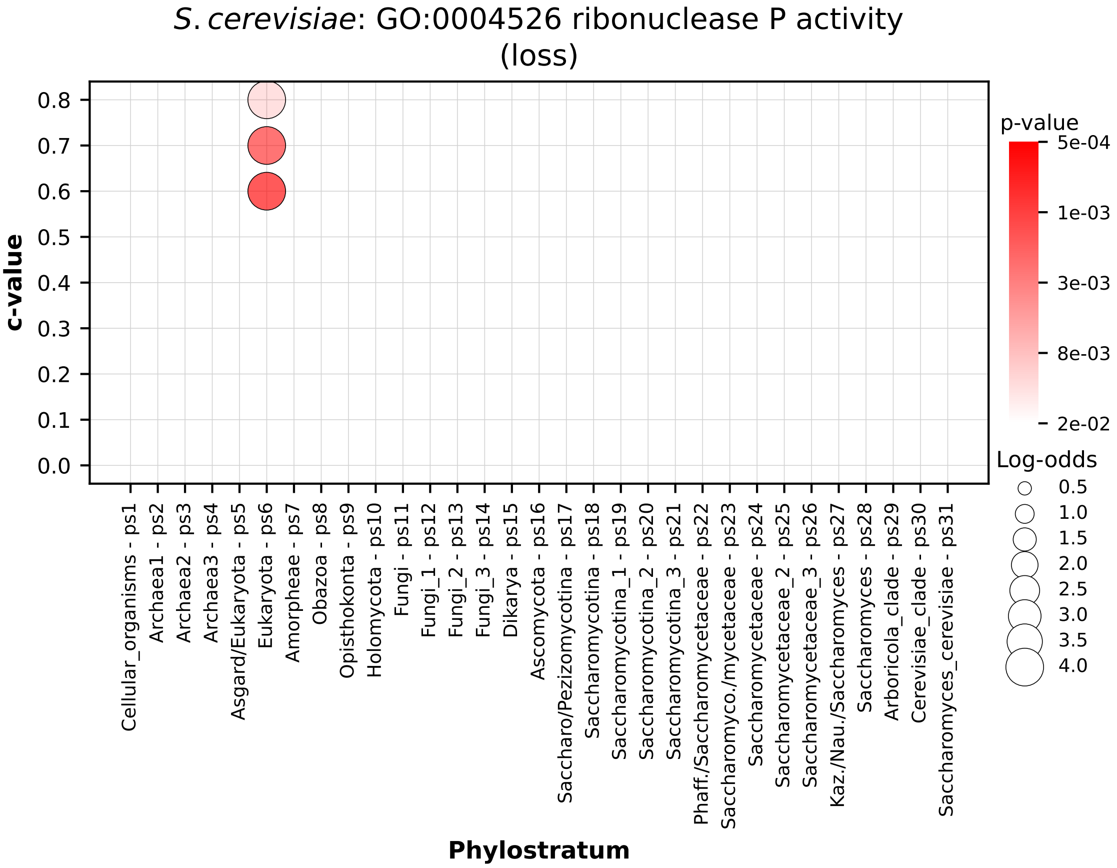

S. cerevisiae: GO:0004526 ribonuclease P activity
(loss)
c-value
0.0
0.1
0.2
0.3
0.4
0.5
0.6
0.7
0.8
p-value
5e-04
1e-03
3e-03
8e-03
2e-02
Log-odds
0.5
1.0
1.5
2.0
2.5
3.0
3.5
4.0
Cellular_organisms - ps1
Archaea1 - ps2
Archaea2 - ps3
Archaea3 - ps4
Asgard/Eukaryota - ps5
Eukaryota - ps6
Amorpheae - ps7
Obazoa - ps8
Opisthokonta - ps9
Holomycota - ps10
Fungi - ps11
Fungi_1 - ps12
Fungi_2 - ps13
Fungi_3 - ps14
Dikarya - ps15
Ascomycota - ps16
Saccharo/Pezizomycotina - ps17
Saccharomycotina - ps18
Saccharomycotina_1 - ps19
Saccharomycotina_2 - ps20
Saccharomycotina_3 - ps21
Phaff./Saccharomycetaceae - ps22
Saccharomyco./mycetaceae - ps23
Saccharomycetaceae - ps24
Saccharomycetaceae_2 - ps25
Saccharomycetaceae_3 - ps26
Kaz./Nau./Saccharomyces - ps27
Saccharomyces - ps28
Arboricola_clade - ps29
Cerevisiae_clade - ps30
Saccharomyces_cerevisiae - ps31
Phylostratum

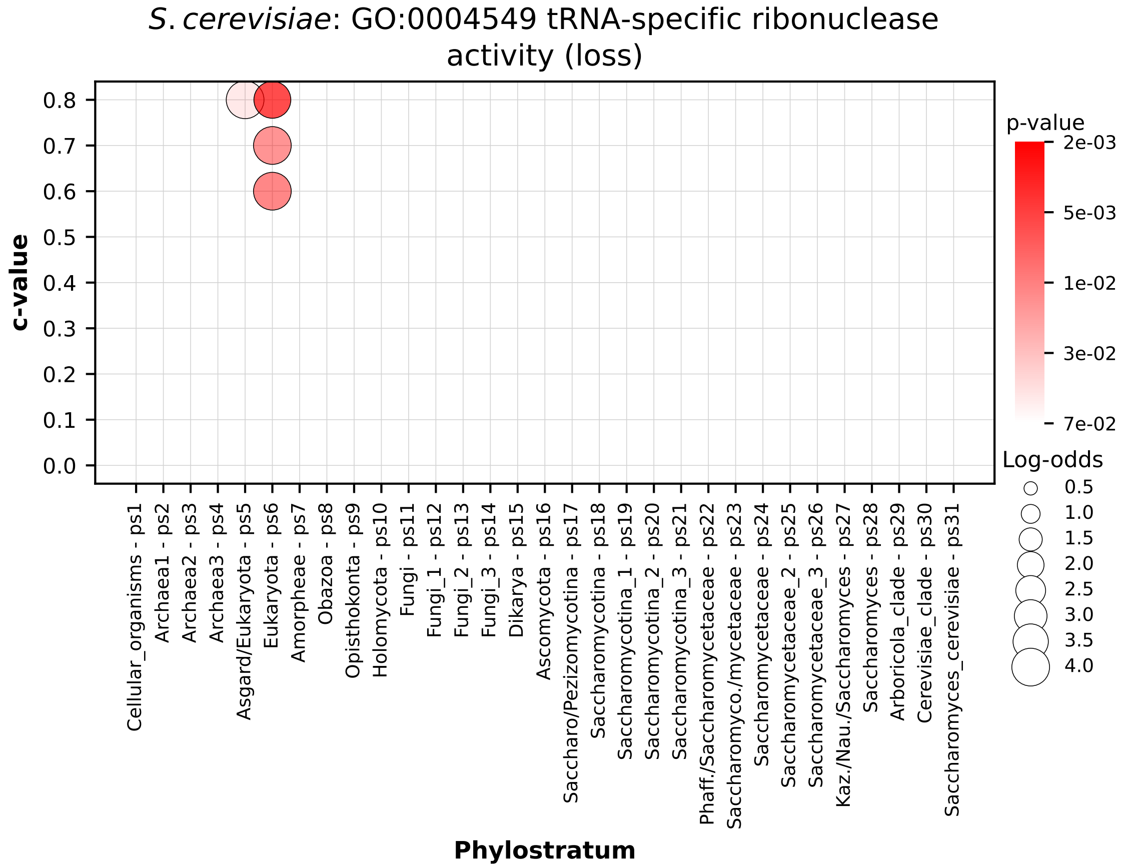

S. cerevisiae: GO:0004549 tRNA-specific ribonuclease activity (loss)
c-value
0.8
0.7
0.6
0.5
0.4
0.3
0.2
0.1
0.0
Phylostratum
Cellular_organisms - ps1
Archaea1 - ps2
Archaea2 - ps3
Archaea3 - ps4
Asgard/Eukaryota - ps5
Eukaryota - ps6
Amorpheae - ps7
Obazoa - ps8
Opisthokonta - ps9
Holomycota - ps10
Fungi - ps11
Fungi_1 - ps12
Fungi_2 - ps13
Fungi_3 - ps14
Dikarya - ps15
Ascomycota - ps16
Saccharo/Pezizomycotina - ps17
Saccharomycotina - ps18
Saccharomycotina_1 - ps19
Saccharomycotina_2 - ps20
Saccharomycotina_3 - ps21
Phaff./Saccharomycetaceae - ps22
Saccharomyco./mycetaceae - ps23
Saccharomycetaceae - ps24
Saccharomycetaceae_2 - ps25
Saccharomycetaceae_3 - ps26
Kaz./Nau./Saccharomyces - ps27
Saccharomyces - ps28
Arboricola_clade - ps29
Cerevisiae_clade - ps30
Saccharomyces_cerevisiae - ps31
p-value
2e-03
5e-03
1e-02
3e-02
7e-02
Log-odds
0.5
1.0
1.5
2.0
2.5
3.0
3.5
4.0

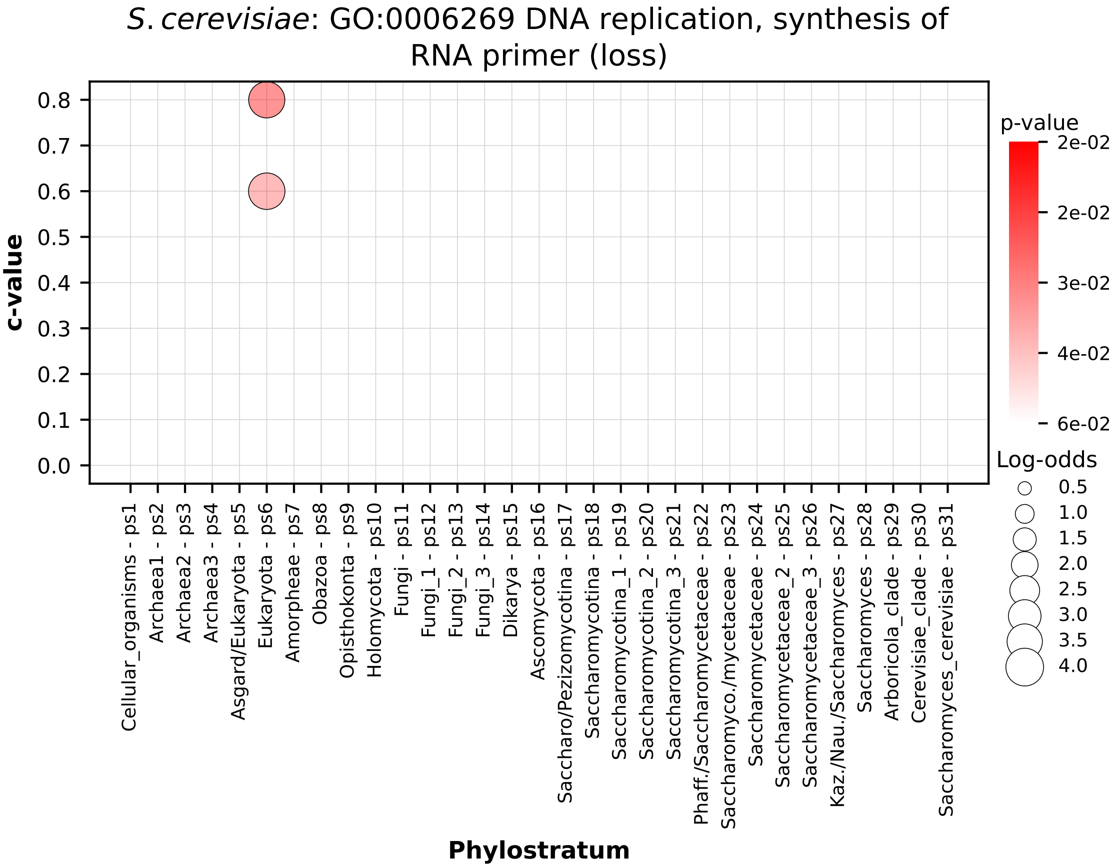

S. cerevisiae: GO:0006269 DNA replication, synthesis of RNA primer (loss)
c-value
0.0
0.1
0.2
0.3
0.4
0.5
0.6
0.7
0.8
Phylostratum
Cellular_organisms - ps1
Archaea1 - ps2
Archaea2 - ps3
Archaea3 - ps4
Asgard/Eukaryota - ps5
Eukaryota - ps6
Amorpheae - ps7
Obazoa - ps8
Opisthokonta - ps9
Holomycota - ps10
Fungi - ps11
Fungi_1 - ps12
Fungi_2 - ps13
Fungi_3 - ps14
Dikarya - ps15
Ascomycota - ps16
Saccharo/Pezizomycotina - ps17
Saccharomycotina - ps18
Saccharomycotina_1 - ps19
Saccharomycotina_2 - ps20
Saccharomycotina_3 - ps21
Phaff./Saccharomycetaceae - ps22
Saccharomyco./mycetaceae - ps23
Saccharomycetaceae - ps24
Saccharomycetaceae_2 - ps25
Saccharomycetaceae_3 - ps26
Kaz./Nau./Saccharomyces - ps27
Saccharomyces - ps28
Arboricola_clade - ps29
Cerevisiae_clade - ps30
Saccharomyces_cerevisiae - ps31
p-value
2e-02
2e-02
3e-02
4e-02
6e-02
Log-odds
0.5
1.0
1.5
2.0
2.5
3.0
3.5
4.0

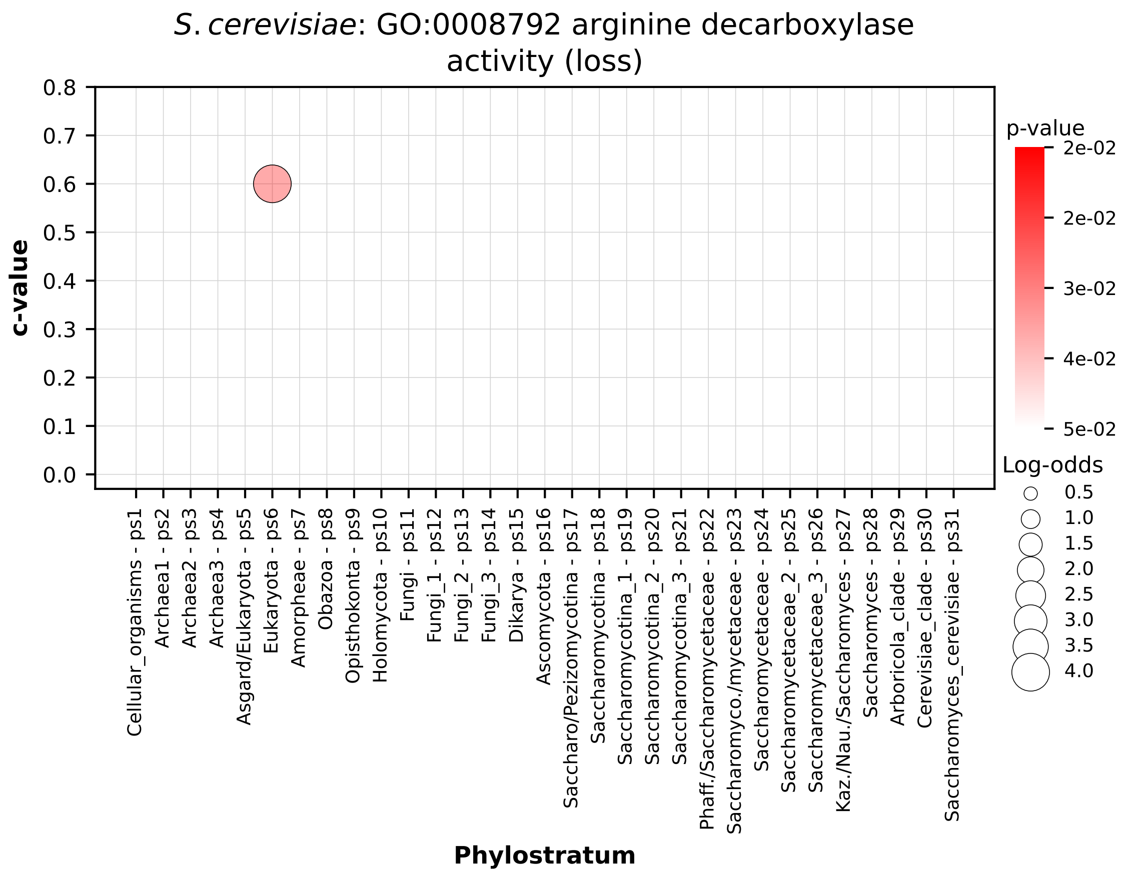

S. cerevisiae: GO:0008792 arginine decarboxylase activity (loss)
c-value
0.8
0.7
0.6
0.5
0.4
0.3
0.2
0.1
0.0
p-value
2e-02
2e-02
3e-02
4e-02
5e-02
Log-odds
0.5
1.0
1.5
2.0
2.5
3.0
3.5
4.0
Cellular_organisms - ps1
Archaea1 - ps2
Archaea2 - ps3
Archaea3 - ps4
Asgard/Eukaryota - ps5
Eukaryota - ps6
Amorpheae - ps7
Obazoa - ps8
Opisthokonta - ps9
Holomycota - ps10
Fungi - ps11
Fungi_1 - ps12
Fungi_2 - ps13
Fungi_3 - ps14
Dikarya - ps15
Ascomycota - ps16
Saccharo/Pezizomycotina - ps17
Saccharomycotina - ps18
Saccharomycotina_1 - ps19
Saccharomycotina_2 - ps20
Saccharomycotina_3 - ps21
Phaff./Saccharomycetaceae - ps22
Saccharomyco./mycetaceae - ps23
Saccharomycetaceae - ps24
Saccharomycetaceae_2 - ps25
Saccharomycetaceae_3 - ps26
Kaz./Nau./Saccharomyces - ps27
Saccharomyces - ps28
Arboricola_clade - ps29
Cerevisiae_clade - ps30
Saccharomyces_cerevisiae - ps31
Phylostratum

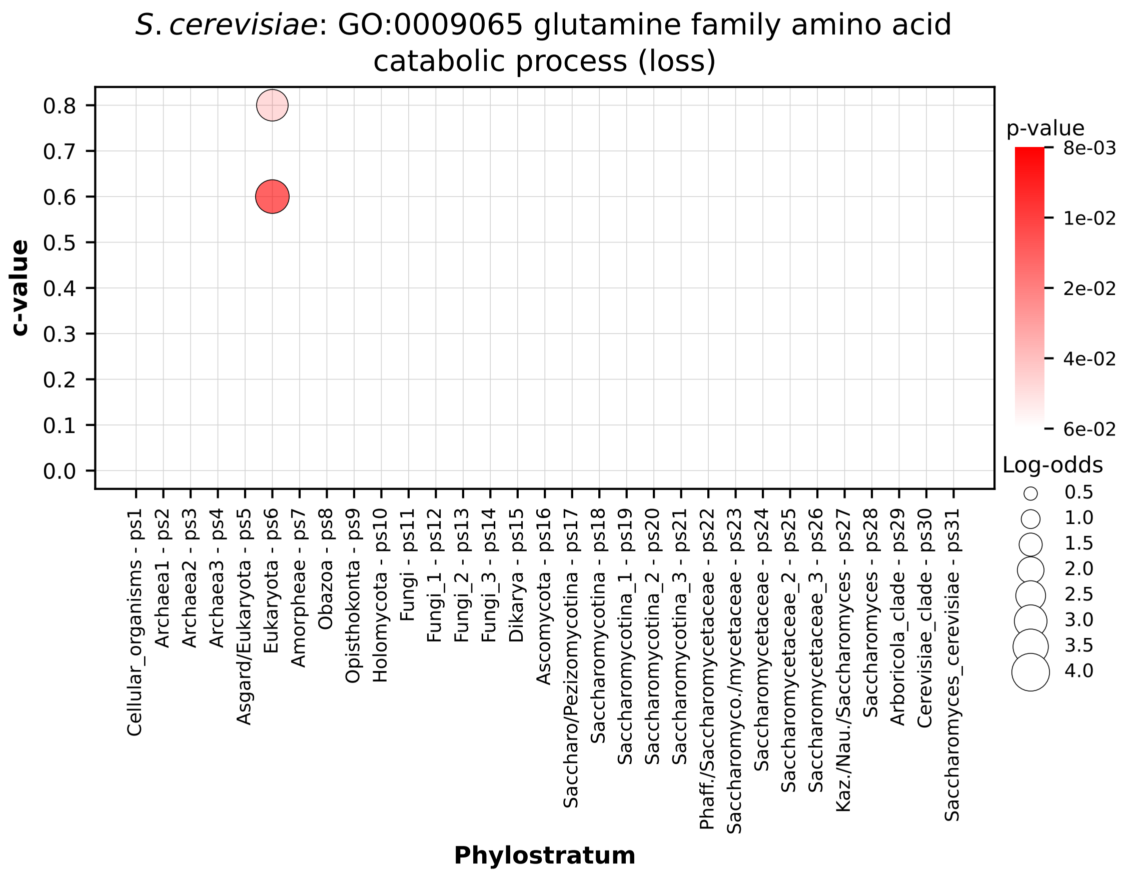

S. cerevisiae: GO:0009065 glutamine family amino acid catabolic process (loss)
c-value
0.0
0.1
0.2
0.3
0.4
0.5
0.6
0.7
0.8
p-value
8e-03
1e-02
2e-02
4e-02
6e-02
Log-odds
0.5
1.0
1.5
2.0
2.5
3.0
3.5
4.0
Cellular_organisms - ps1
Archaea1 - ps2
Archaea2 - ps3
Archaea3 - ps4
Asgard/Eukaryota - ps5
Eukaryota - ps6
Amorpheae - ps7
Obazoa - ps8
Opisthokonta - ps9
Holomycota - ps10
Fungi - ps11
Fungi_1 - ps12
Fungi_2 - ps13
Fungi_3 - ps14
Dikarya - ps15
Ascomycota - ps16
Saccharo/Pezizomycotina - ps17
Saccharomycotina - ps18
Saccharomycotina_1 - ps19
Saccharomycotina_2 - ps20
Saccharomycotina_3 - ps21
Phaff./Saccharomycetaceae - ps22
Saccharomyco./mycetaceae - ps23
Saccharomycetaceae - ps24
Saccharomycetaceae_2 - ps25
Saccharomycetaceae_3 - ps26
Kaz./Nau./Saccharomyces - ps27
Saccharomyces - ps28
Arboricola_clade - ps29
Cerevisiae_clade - ps30
Saccharomyces_cerevisiae - ps31
Phylostratum

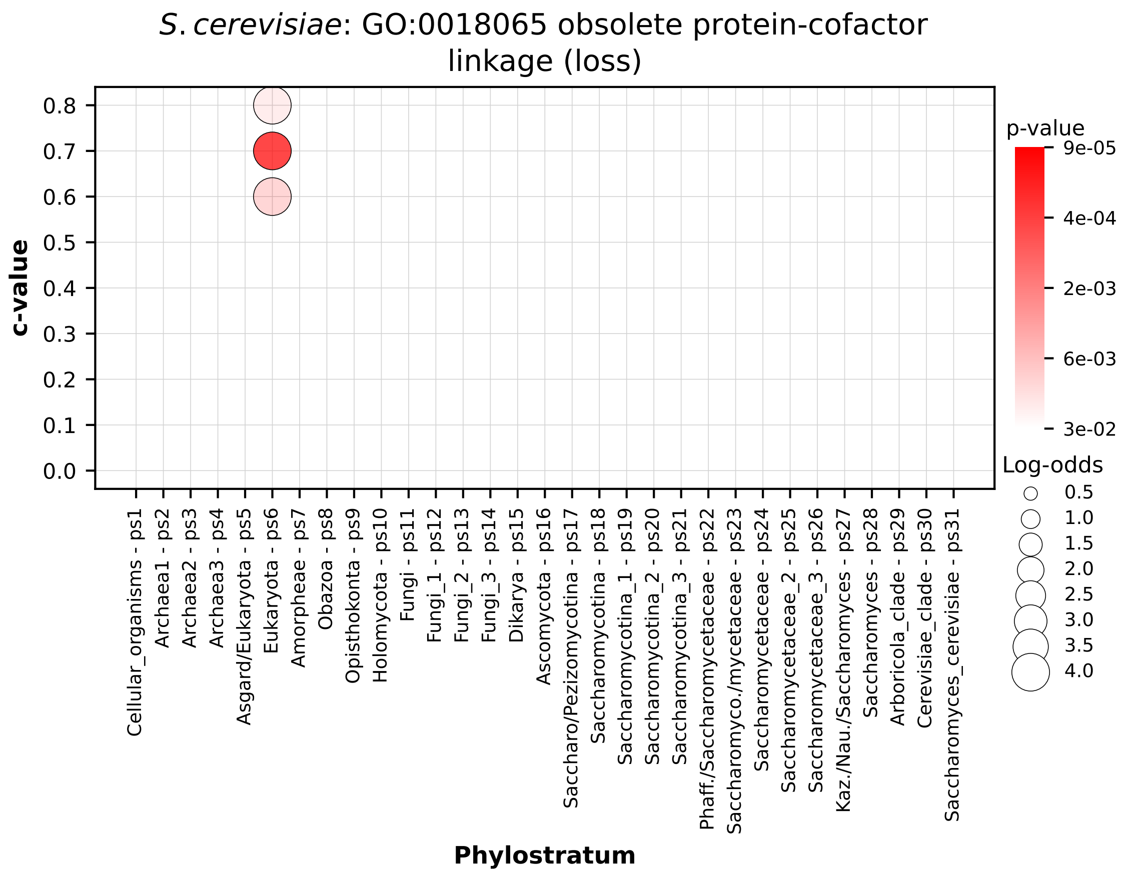

S. cerevisiae: GO:0018065 obsolete protein-cofactor linkage (loss)
c-value
0.0
0.1
0.2
0.3
0.4
0.5
0.6
0.7
0.8
Phylostratum
Cellular_organisms - ps1
Archaea1 - ps2
Archaea2 - ps3
Archaea3 - ps4
Asgard/Eukaryota - ps5
Eukaryota - ps6
Amorpheae - ps7
Obazoa - ps8
Opisthokonta - ps9
Holomycota - ps10
Fungi - ps11
Fungi_1 - ps12
Fungi_2 - ps13
Fungi_3 - ps14
Dikarya - ps15
Ascomycota - ps16
Saccharo/Pezizomycotina - ps17
Saccharomycotina - ps18
Saccharomycotina_1 - ps19
Saccharomycotina_2 - ps20
Saccharomycotina_3 - ps21
Phaff./Saccharomycetaceae - ps22
Saccharomyco./mycetaceae - ps23
Saccharomycetaceae - ps24
Saccharomycetaceae_2 - ps25
Saccharomycetaceae_3 - ps26
Kaz./Nau./Saccharomyces - ps27
Saccharomyces - ps28
Arboricola_clade - ps29
Cerevisiae_clade - ps30
Saccharomyces_cerevisiae - ps31
p-value
9e-05
4e-04
2e-03
6e-03
3e-02
Log-odds
0.5
1.0
1.5
2.0
2.5
3.0
3.5
4.0

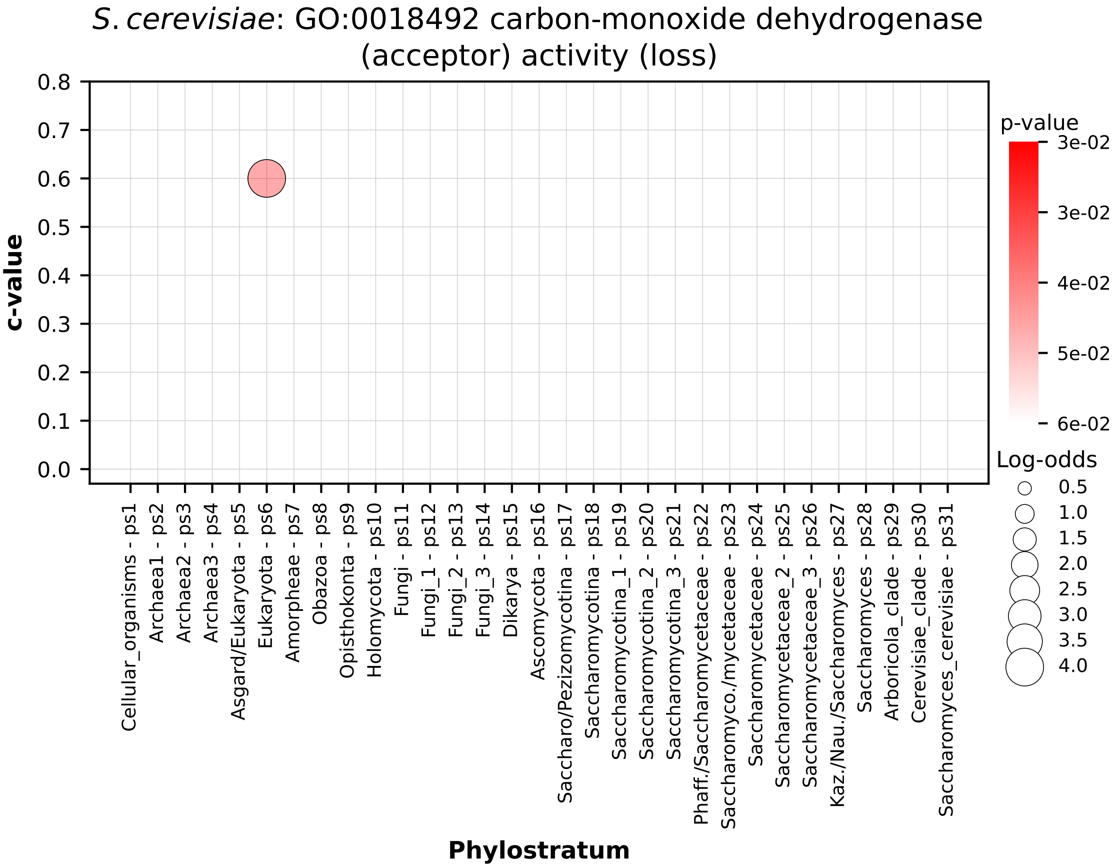

S. cerevisiae: GO:0018492 carbon-monoxide dehydrogenase (acceptor) activity (loss)
c-value
0.8
0.7
0.6
0.5
0.4
0.3
0.2
0.1
0.0
p-value
3e-02
3e-02
4e-02
5e-02
6e-02
Log-odds
0.5
1.0
1.5
2.0
2.5
3.0
3.5
4.0
Cellular_organisms - ps1
Archaea1 - ps2
Archaea2 - ps3
Archaea3 - ps4
Asgard/Eukaryota - ps5
Eukaryota - ps6
Amorpheae - ps7
Obazoa - ps8
Opisthokonta - ps9
Holomycota - ps10
Fungi - ps11
Fungi_1 - ps12
Fungi_2 - ps13
Fungi_3 - ps14
Dikarya - ps15
Ascomycota - ps16
Saccharo/Pezizomycotina - ps17
Saccharomycotina - ps18
Saccharomycotina_1 - ps19
Saccharomycotina_2 - ps20
Saccharomycotina_3 - ps21
Phaff./Saccharomycetaceae - ps22
Saccharomyco./mycetaceae - ps23
Saccharomycetaceae - ps24
Saccharomycetaceae_2 - ps25
Saccharomycetaceae_3 - ps26
Kaz./Nau./Saccharomyces - ps27
Saccharomyces - ps28
Arboricola_clade - ps29
Cerevisiae_clade - ps30
Saccharomyces_cerevisiae - ps31
Phylostratum

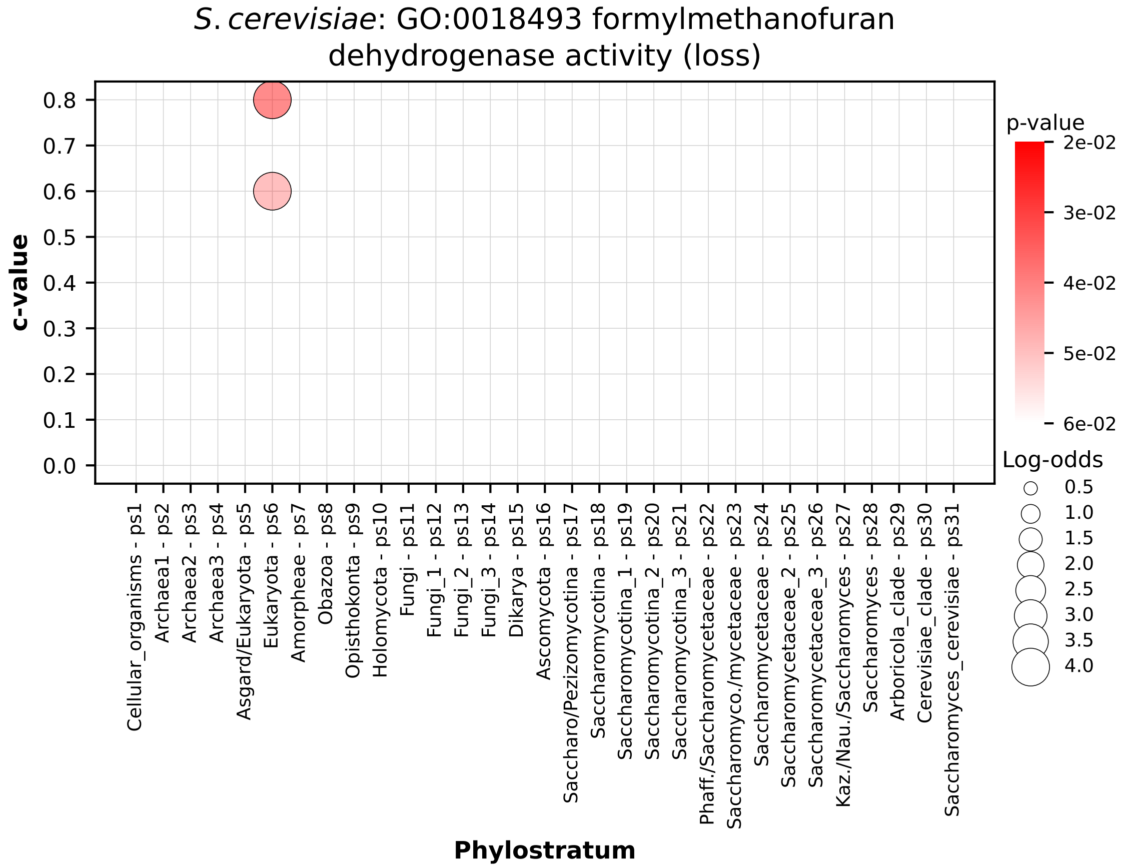
S. cerevisiae: GO:0018493 formylmethanofuran dehydrogenase activity (loss)
c-value
0.0
0.1
0.2
0.3
0.4
0.5
0.6
0.7
0.8
p-value
2e-02
3e-02
4e-02
5e-02
6e-02
Log-odds
0.5
1.0
1.5
2.0
2.5
3.0
3.5
4.0
Cellular_organisms - ps1
Archaea1 - ps2
Archaea2 - ps3
Archaea3 - ps4
Asgard/Eukaryota - ps5
Eukaryota - ps6
Amorpheae - ps7
Obazoa - ps8
Opisthokonta - ps9
Holomycota - ps10
Fungi - ps11
Fungi_1 - ps12
Fungi_2 - ps13
Fungi_3 - ps14
Dikarya - ps15
Ascomycota - ps16
Saccharo/Pezizomycotina - ps17
Saccharomycotina - ps18
Saccharomycotina_1 - ps19
Saccharomycotina_2 - ps20
Saccharomycotina_3 - ps21
Phaff./Saccharomycetaceae - ps22
Saccharomyco./mycetaceae - ps23
Saccharomycetaceae - ps24
Saccharomycetaceae_2 - ps25
Saccharomycetaceae_3 - ps26
Kaz./Nau./Saccharomyces - ps27
Saccharomyces - ps28
Arboricola_clade - ps29
Cerevisiae_clade - ps30
Saccharomyces_cerevisiae - ps31
Phylostratum

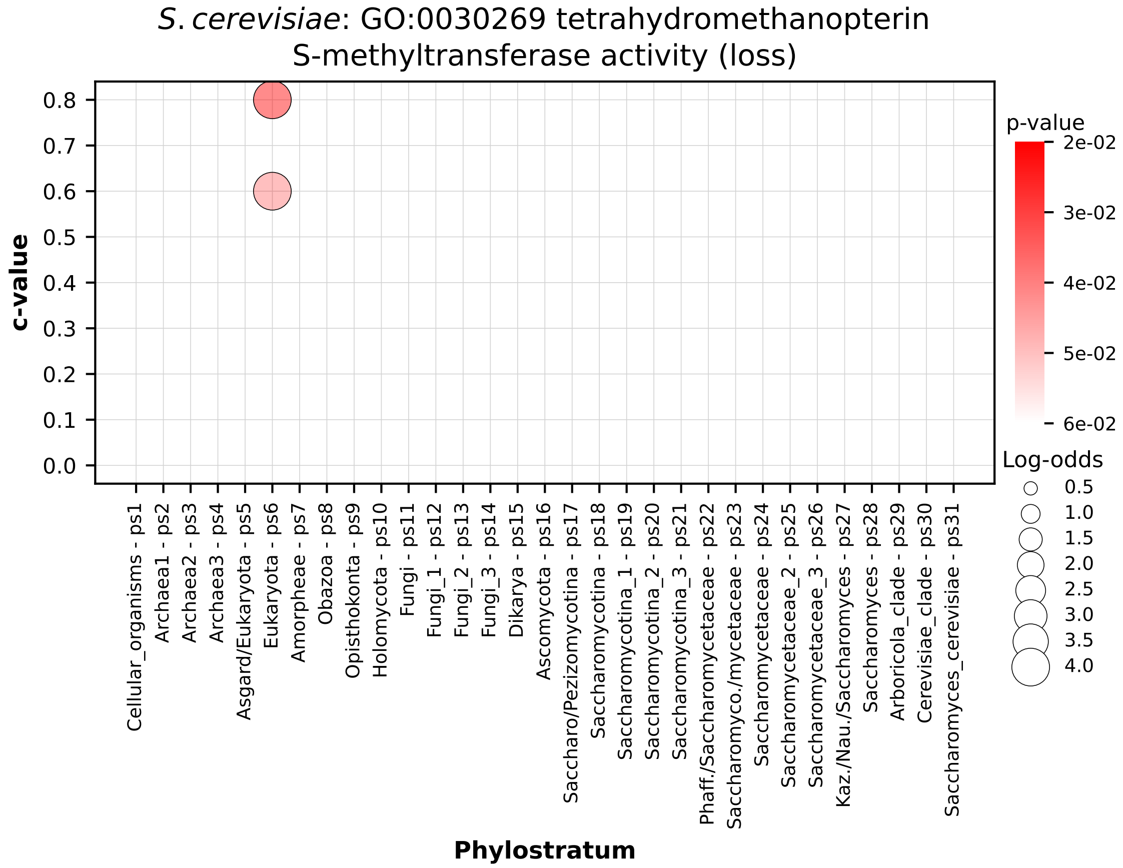
S. cerevisiae: GO:0030269 tetrahydromethanopterin S-methyltransferase activity (loss)
c-value
0.8
0.7
0.6
0.5
0.4
0.3
0.2
0.1
0.0
p-value
2e-02
3e-02
4e-02
5e-02
6e-02
Log-odds
0.5
1.0
1.5
2.0
2.5
3.0
3.5
4.0
Cellular_organisms - ps1
Archaea1 - ps2
Archaea2 - ps3
Archaea3 - ps4
Asgard/Eukaryota - ps5
Eukaryota - ps6
Amorpheae - ps7
Obazoa - ps8
Opisthokonta - ps9
Holomycota - ps10
Fungi - ps11
Fungi_1 - ps12
Fungi_2 - ps13
Fungi_3 - ps14
Dikarya - ps15
Ascomycota - ps16
Saccharo/Pezizomycotina - ps17
Saccharomycotina - ps18
Saccharomycotina_1 - ps19
Saccharomycotina_2 - ps20
Saccharomycotina_3 - ps21
Phaff./Saccharomycetaceae - ps22
Saccharomyco./mycetaceae - ps23
Saccharomycetaceae - ps24
Saccharomycetaceae_2 - ps25
Saccharomycetaceae_3 - ps26
Kaz./Nau./Saccharomyces - ps27
Saccharomyces - ps28
Arboricola_clade - ps29
Cerevisiae_clade - ps30
Saccharomyces_cerevisiae - ps31
Phylostratum

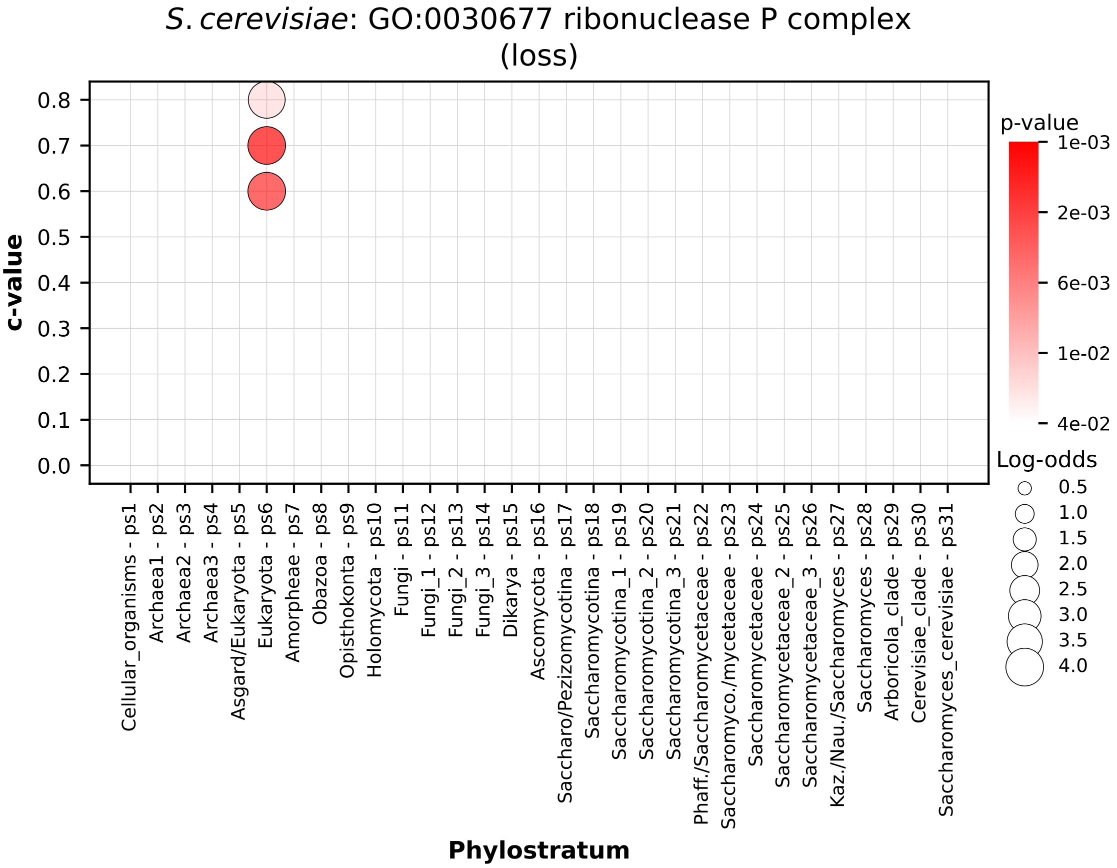

S. cerevisiae: GO:0030677 ribonuclease P complex
(loss)
c-value
0.8
0.7
0.6
0.5
0.4
0.3
0.2
0.1
0.0
p-value
1e-03
2e-03
6e-03
1e-02
4e-02
Log-odds
0.5
1.0
1.5
2.0
2.5
3.0
3.5
4.0
Cellular_organisms - ps1
Archaea1 - ps2
Archaea2 - ps3
Archaea3 - ps4
Asgard/Eukaryota - ps5
Eukaryota - ps6
Amorpheae - ps7
Obazoa - ps8
Opisthokonta - ps9
Holomycota - ps10
Fungi - ps11
Fungi_1 - ps12
Fungi_2 - ps13
Fungi_3 - ps14
Dikarya - ps15
Ascomycota - ps16
Saccharo/Pezizomycotina - ps17
Saccharomycotina - ps18
Saccharomycotina_1 - ps19
Saccharomycotina_2 - ps20
Saccharomycotina_3 - ps21
Phaff./Saccharomycetaceae - ps22
Saccharomyco./mycetaceae - ps23
Saccharomycetaceae - ps24
Saccharomycetaceae_2 - ps25
Saccharomycetaceae_3 - ps26
Kaz./Nau./Saccharomyces - ps27
Saccharomyces - ps28
Arboricola_clade - ps29
Cerevisiae_clade - ps30
Saccharomyces_cerevisiae - ps31
Phylostratum

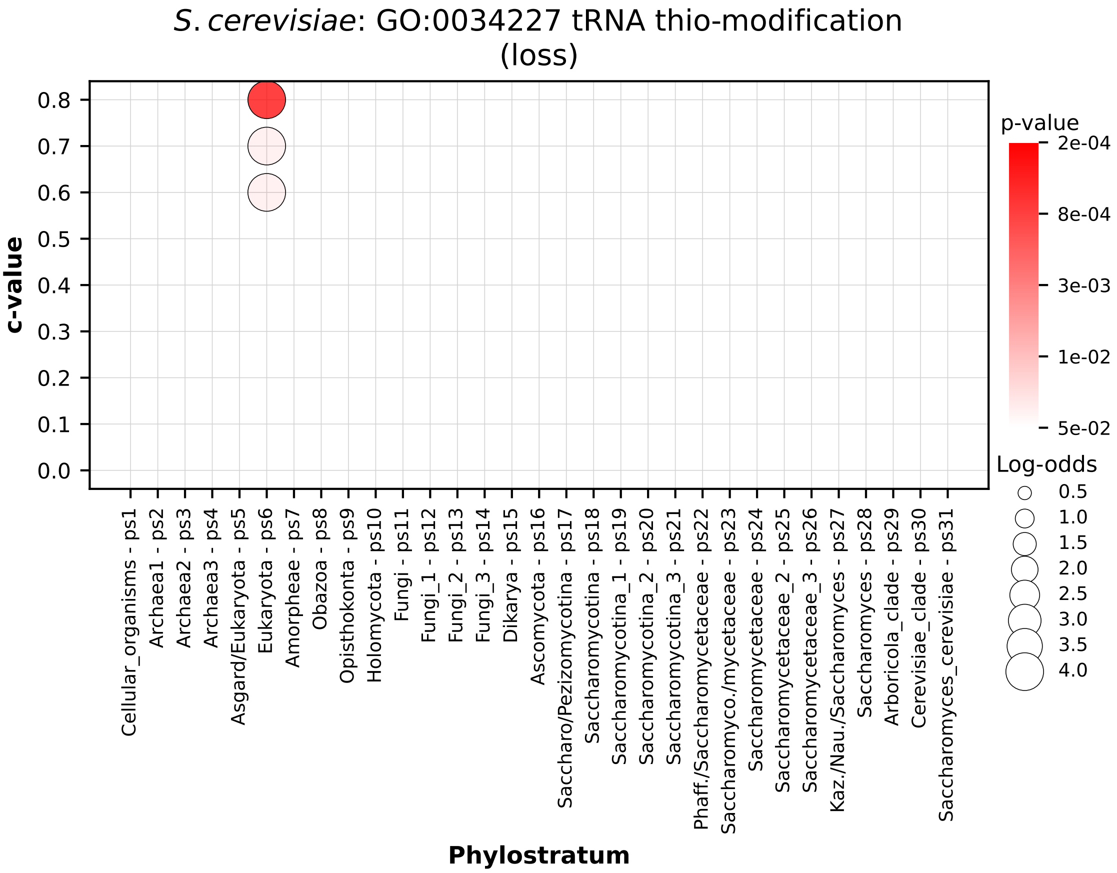
S. cerevisiae: GO:0034227 tRNA thio-modification (loss)
c-value
0.8
0.7
0.6
0.5
0.4
0.3
0.2
0.1
0.0
p-value
2e-04
8e-04
3e-03
1e-02
5e-02
Log-odds
0.5
1.0
1.5
2.0
2.5
3.0
3.5
4.0
Cellular_organisms - ps1
Archaea1 - ps2
Archaea2 - ps3
Archaea3 - ps4
Asgard/Eukaryota - ps5
Eukaryota - ps6
Amorpheae - ps7
Obazoa - ps8
Opisthokonta - ps9
Holomycota - ps10
Fungi - ps11
Fungi_1 - ps12
Fungi_2 - ps13
Fungi_3 - ps14
Dikarya - ps15
Ascomycota - ps16
Saccharo/Pezizomycotina - ps17
Saccharomycotina - ps18
Saccharomycotina_1 - ps19
Saccharomycotina_2 - ps20
Saccharomycotina_3 - ps21
Phaff./Saccharomycetaceae - ps22
Saccharomyco./mycetaceae - ps23
Saccharomycetaceae - ps24
Saccharomycetaceae_2 - ps25
Saccharomycetaceae_3 - ps26
Kaz./Nau./Saccharomyces - ps27
Saccharomyces - ps28
Arboricola_clade - ps29
Cerevisiae_clade - ps30
Saccharomyces_cerevisiae - ps31
Phylostratum

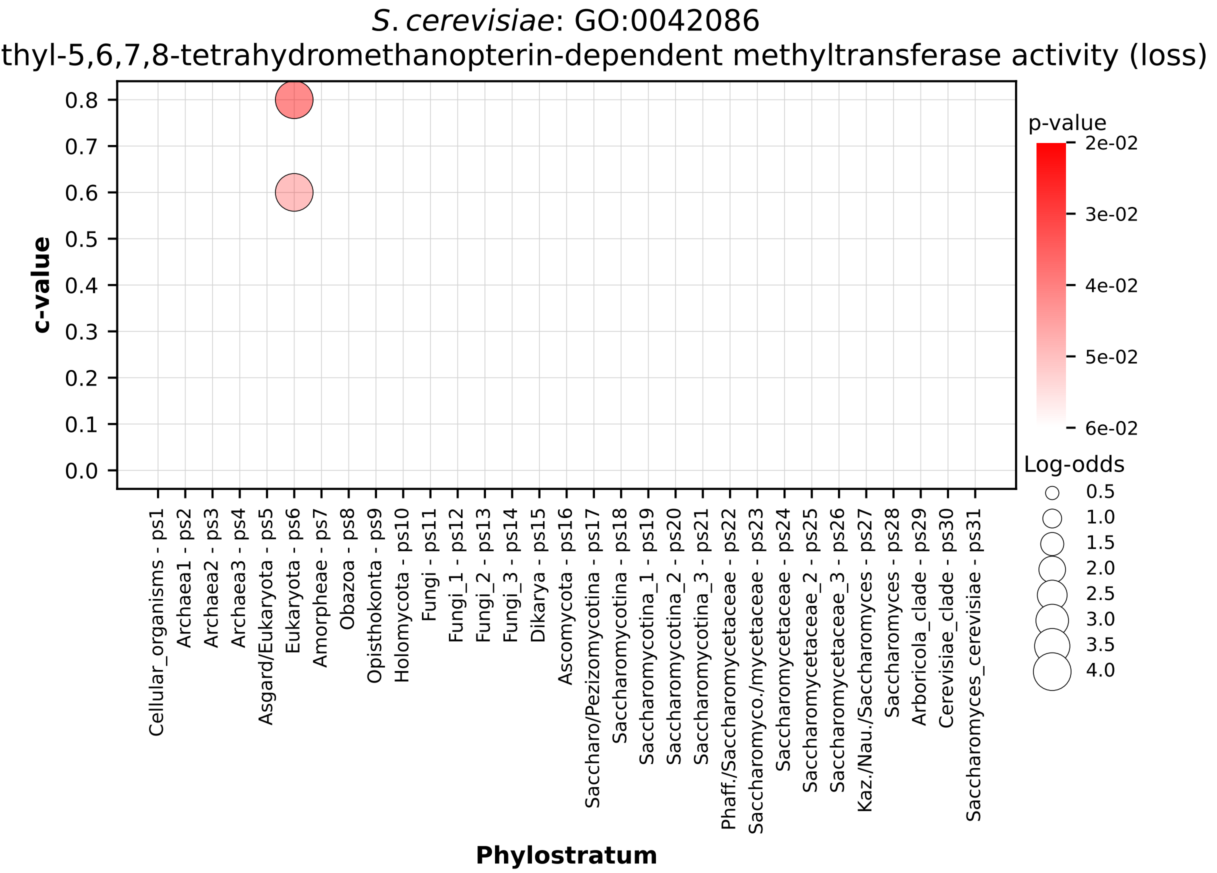

S. cerevisiae: GO:0042086
thyl-5,6,7,8-tetrahydromethanopterin-dependent methyltransferase activity (loss)
c-value
0.8
0.7
0.6
0.5
0.4
0.3
0.2
0.1
0.0
p-value
2e-02
3e-02
4e-02
5e-02
6e-02
Log-odds
0.5
1.0
1.5
2.0
2.5
3.0
3.5
4.0
Cellular_organisms - ps1
Archaea1 - ps2
Archaea2 - ps3
Archaea3 - ps4
Asgard/Eukaryota - ps5
Eukaryota - ps6
Amorpheae - ps7
Obazoa - ps8
Opisthokonta - ps9
Holomycota - ps10
Fungi - ps11
Fungi_1 - ps12
Fungi_2 - ps13
Fungi_3 - ps14
Dikarya - ps15
Ascomycota - ps16
Saccharo/Pezizomycotina - ps17
Saccharomycotina - ps18
Saccharomycotina_1 - ps19
Saccharomycotina_2 - ps20
Saccharomycotina_3 - ps21
Phaff./Saccharomycetaceae - ps22
Saccharomyco./mycetaceae - ps23
Saccharomycetaceae - ps24
Saccharomycetaceae_2 - ps25
Saccharomycetaceae_3 - ps26
Kaz./Nau./Saccharomyces - ps27
Saccharomyces - ps28
Arboricola_clade - ps29
Cerevisiae_clade - ps30
Saccharomyces_cerevisiae - ps31
Phylostratum

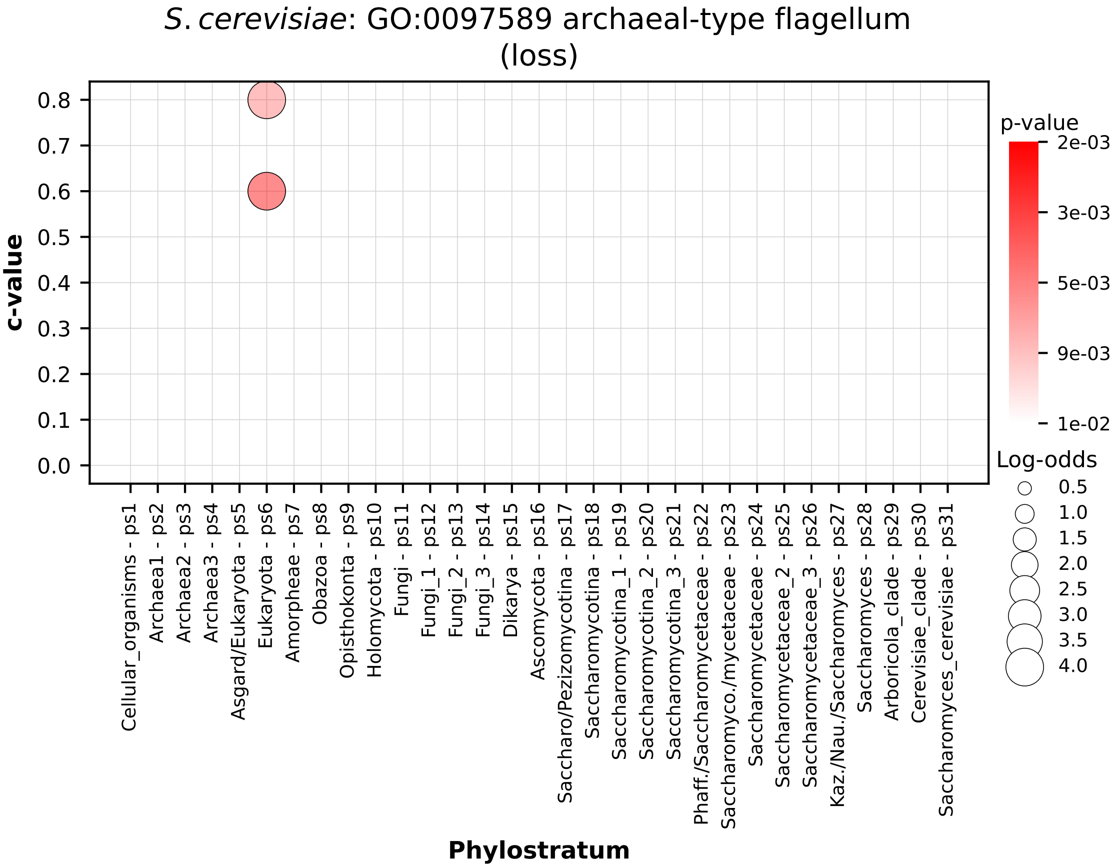
S. cerevisiae: GO:0097589 archaeal-type flagellum
(loss)
c-value
0.8
0.7
0.6
0.5
0.4
0.3
0.2
0.1
0.0
p-value
2e-03
3e-03
5e-03
9e-03
1e-02
Log-odds
0.5
1.0
1.5
2.0
2.5
3.0
3.5
4.0
Cellular_organisms - ps1
Archaea1 - ps2
Archaea2 - ps3
Archaea3 - ps4
Asgard/Eukaryota - ps5
Eukaryota - ps6
Amorpheae - ps7
Obazoa - ps8
Opisthokonta - ps9
Holomycota - ps10
Fungi - ps11
Fungi_1 - ps12
Fungi_2 - ps13
Fungi_3 - ps14
Dikarya - ps15
Ascomycota - ps16
Saccharo/Pezizomycotina - ps17
Saccharomycotina - ps18
Saccharomycotina_1 - ps19
Saccharomycotina_2 - ps20
Saccharomycotina_3 - ps21
Phaff./Saccharomycetaceae - ps22
Saccharomyco./mycetaceae - ps23
Saccharomycetaceae - ps24
Saccharomycetaceae_2 - ps25
Saccharomycetaceae_3 - ps26
Kaz./Nau./Saccharomyces - ps27
Saccharomyces - ps28
Arboricola_clade - ps29
Cerevisiae_clade - ps30
Saccharomyces_cerevisiae - ps31
Phylostratum

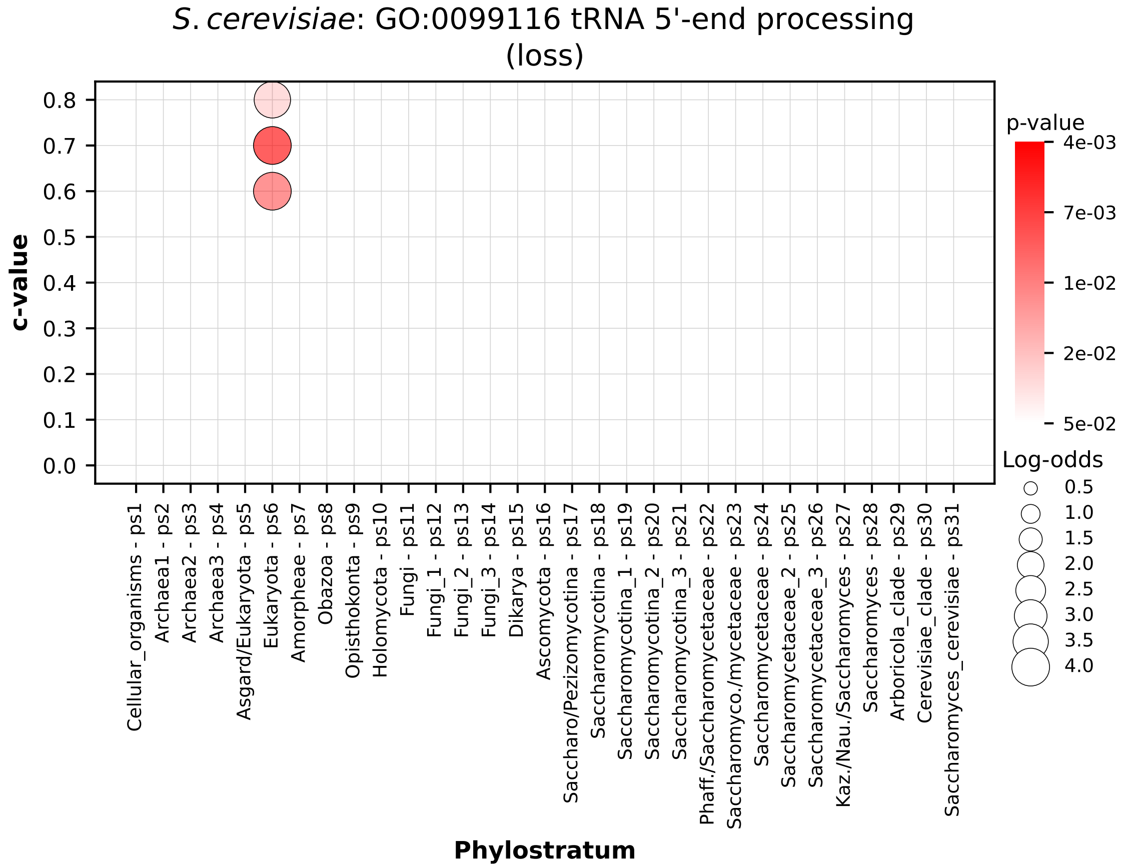

S. cerevisiae: GO:0099116 tRNA 5'-end processing
(loss)
c-value
0.8
0.7
0.6
0.5
0.4
0.3
0.2
0.1
0.0
p-value
4e-03
7e-03
1e-02
2e-02
5e-02
Log-odds
0.5
1.0
1.5
2.0
2.5
3.0
3.5
4.0
Cellular_organisms - ps1
Archaea1 - ps2
Archaea2 - ps3
Archaea3 - ps4
Asgard/Eukaryota - ps5
Eukaryota - ps6
Amorpheae - ps7
Obazoa - ps8
Opisthokonta - ps9
Holomycota - ps10
Fungi - ps11
Fungi_1 - ps12
Fungi_2 - ps13
Fungi_3 - ps14
Dikarya - ps15
Ascomycota - ps16
Saccharo/Pezizomycotina - ps17
Saccharomycotina - ps18
Saccharomycotina_1 - ps19
Saccharomycotina_2 - ps20
Saccharomycotina_3 - ps21
Phaff./Saccharomycetaceae - ps22
Saccharomyco./mycetaceae - ps23
Saccharomycetaceae - ps24
Saccharomycetaceae_2 - ps25
Saccharomycetaceae_3 - ps26
Kaz./Nau./Saccharomyces - ps27
Saccharomyces - ps28
Arboricola_clade - ps29
Cerevisiae_clade - ps30
Saccharomyces_cerevisiae - ps31
Phylostratum

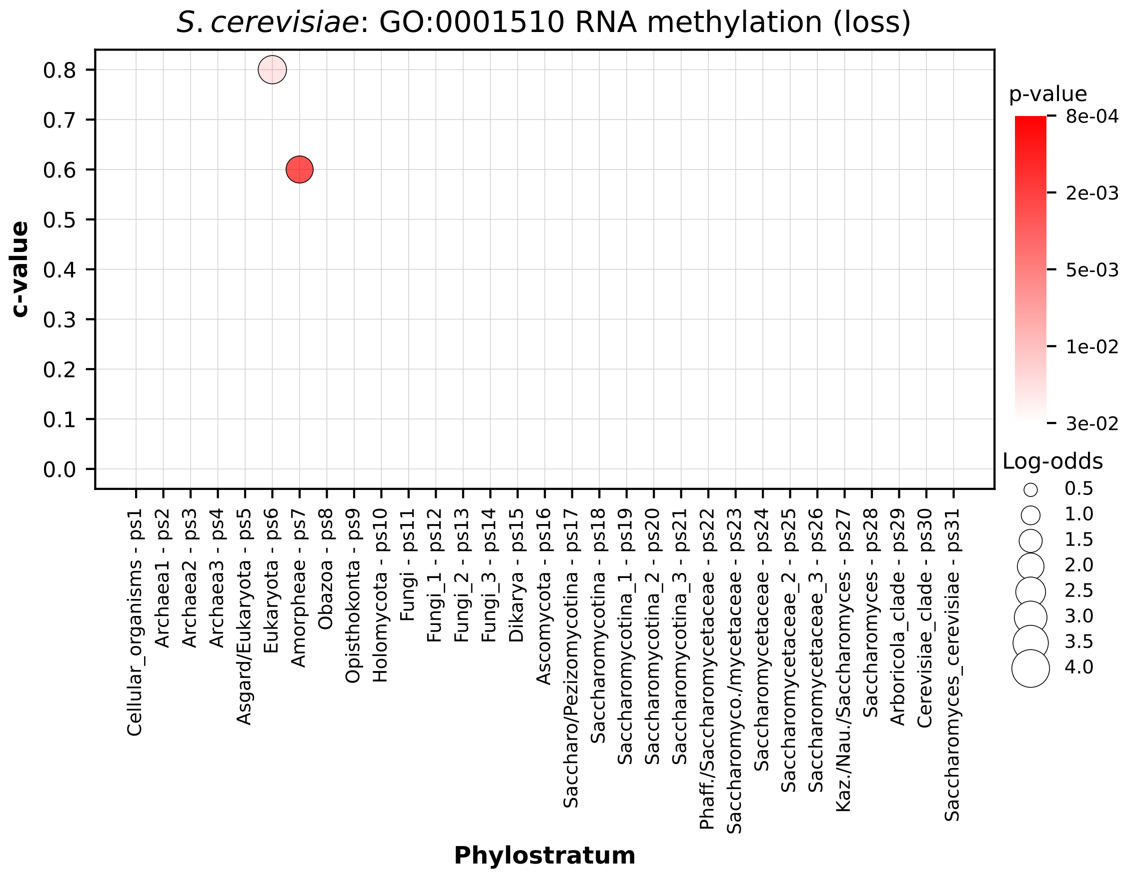

S. cerevisiae: GO:0001510 RNA methylation (loss)
c-value
0.0
0.1
0.2
0.3
0.4
0.5
0.6
0.7
0.8
Phylostratum
Cellular_organisms - ps1
Archaea1 - ps2
Archaea2 - ps3
Archaea3 - ps4
Asgard/Eukaryota - ps5
Eukaryota - ps6
Amorpheae - ps7
Obazoa - ps8
Opisthokonta - ps9
Holomycota - ps10
Fungi - ps11
Fungi_1 - ps12
Fungi_2 - ps13
Fungi_3 - ps14
Dikarya - ps15
Ascomycota - ps16
Saccharo/Pezizomycotina - ps17
Saccharomycotina - ps18
Saccharomycotina_1 - ps19
Saccharomycotina_2 - ps20
Saccharomycotina_3 - ps21
Phaff./Saccharomycetaceae - ps22
Saccharomyco./mycetaceae - ps23
Saccharomycetaceae - ps24
Saccharomycetaceae_2 - ps25
Saccharomycetaceae_3 - ps26
Kaz./Nau./Saccharomyces - ps27
Saccharomyces - ps28
Arboricola_clade - ps29
Cerevisiae_clade - ps30
Saccharomyces_cerevisiae - ps31
p-value
8e-04
2e-03
5e-03
1e-02
3e-02
Log-odds
0.5
1.0
1.5
2.0
2.5
3.0
3.5
4.0

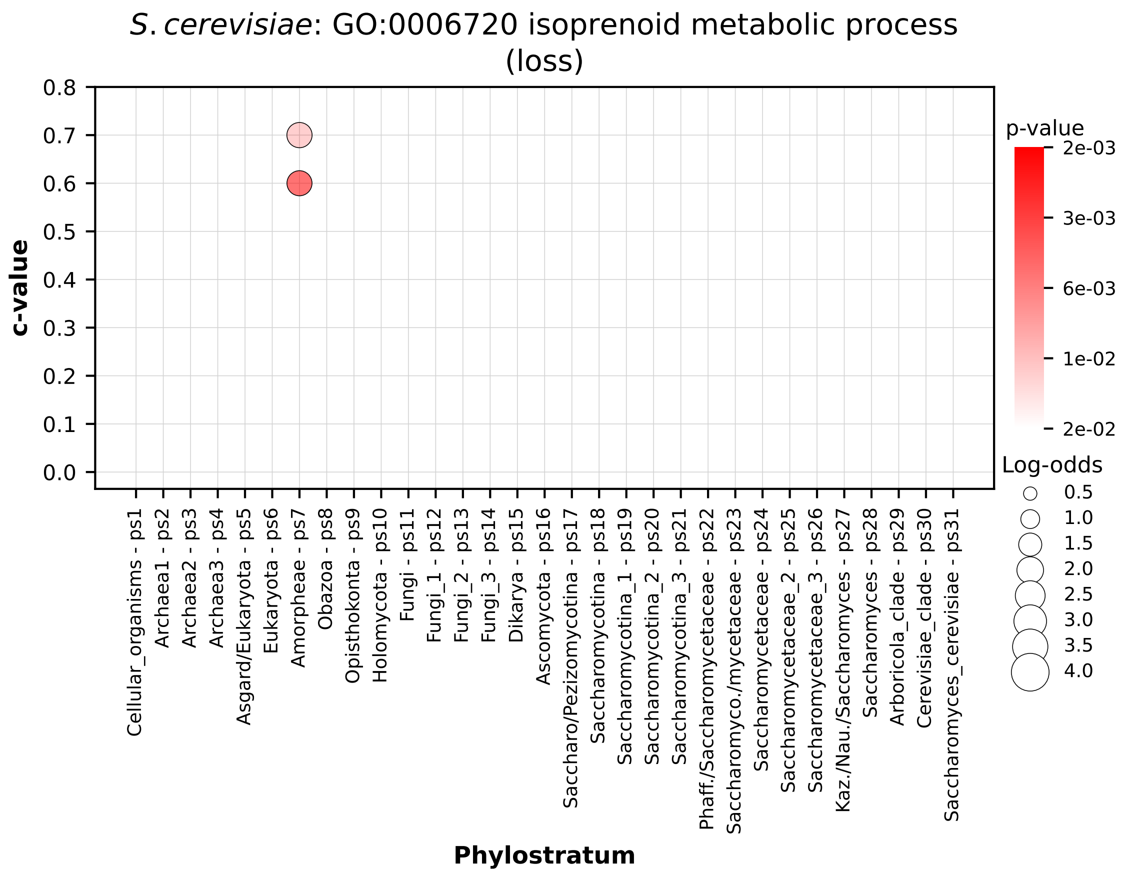

S. cerevisiae: GO:0006720 isoprenoid metabolic process (loss)
c-value
0.8
0.7
0.6
0.5
0.4
0.3
0.2
0.1
0.0
p-value
2e-03
3e-03
6e-03
1e-02
2e-02
Log-odds
0.5
1.0
1.5
2.0
2.5
3.0
3.5
4.0
Cellular_organisms - ps1
Archaea1 - ps2
Archaea2 - ps3
Archaea3 - ps4
Asgard/Eukaryota - ps5
Eukaryota - ps6
Amorpheae - ps7
Obazoa - ps8
Opisthokonta - ps9
Holomycota - ps10
Fungi - ps11
Fungi_1 - ps12
Fungi_2 - ps13
Fungi_3 - ps14
Dikarya - ps15
Ascomycota - ps16
Saccharo/Pezizomycotina - ps17
Saccharomycotina - ps18
Saccharomycotina_1 - ps19
Saccharomycotina_2 - ps20
Saccharomycotina_3 - ps21
Phaff./Saccharomycetaceae - ps22
Saccharomyco./mycetaceae - ps23
Saccharomycetaceae - ps24
Saccharomycetaceae_2 - ps25
Saccharomycetaceae_3 - ps26
Kaz./Nau./Saccharomyces - ps27
Saccharomyces - ps28
Arboricola_clade - ps29
Cerevisiae_clade - ps30
Saccharomyces_cerevisiae - ps31
Phylostratum

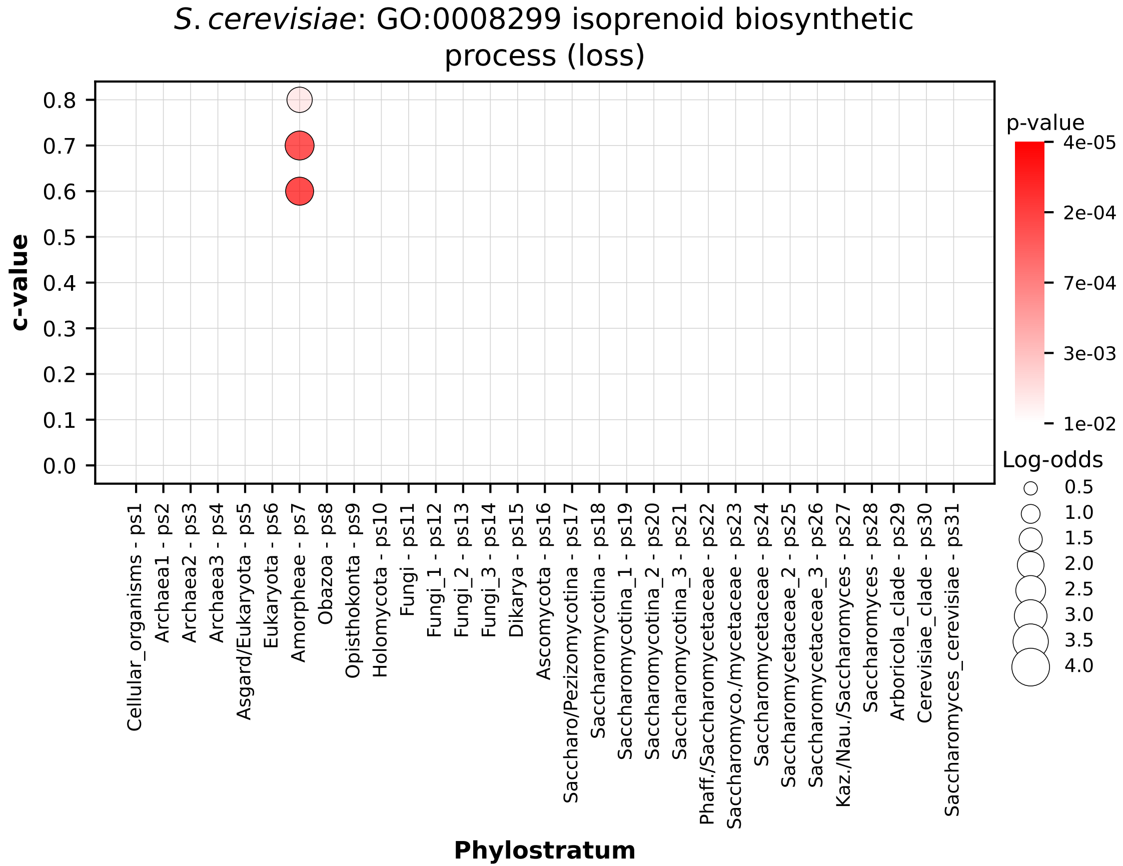

S. cerevisiae: GO:0008299 isoprenoid biosynthetic process (loss)
c-value
0.8
0.7
0.6
0.5
0.4
0.3
0.2
0.1
0.0
p-value
4e-05
2e-04
7e-04
3e-03
1e-02
Log-odds
0.5
1.0
1.5
2.0
2.5
3.0
3.5
4.0
Cellular_organisms - ps1
Archaea1 - ps2
Archaea2 - ps3
Archaea3 - ps4
Asgard/Eukaryota - ps5
Eukaryota - ps6
Amorpheae - ps7
Obazoa - ps8
Opisthokonta - ps9
Holomycota - ps10
Fungi - ps11
Fungi_1 - ps12
Fungi_2 - ps13
Fungi_3 - ps14
Dikarya - ps15
Ascomycota - ps16
Saccharo/Pezizomycotina - ps17
Saccharomycotina - ps18
Saccharomycotina_1 - ps19
Saccharomycotina_2 - ps20
Saccharomycotina_3 - ps21
Phaff./Saccharomycetaceae - ps22
Saccharomyco./mycetaceae - ps23
Saccharomycetaceae - ps24
Saccharomycetaceae_2 - ps25
Saccharomycetaceae_3 - ps26
Kaz./Nau./Saccharomyces - ps27
Saccharomyces - ps28
Arboricola_clade - ps29
Cerevisiae_clade - ps30
Saccharomyces_cerevisiae - ps31
Phylostratum

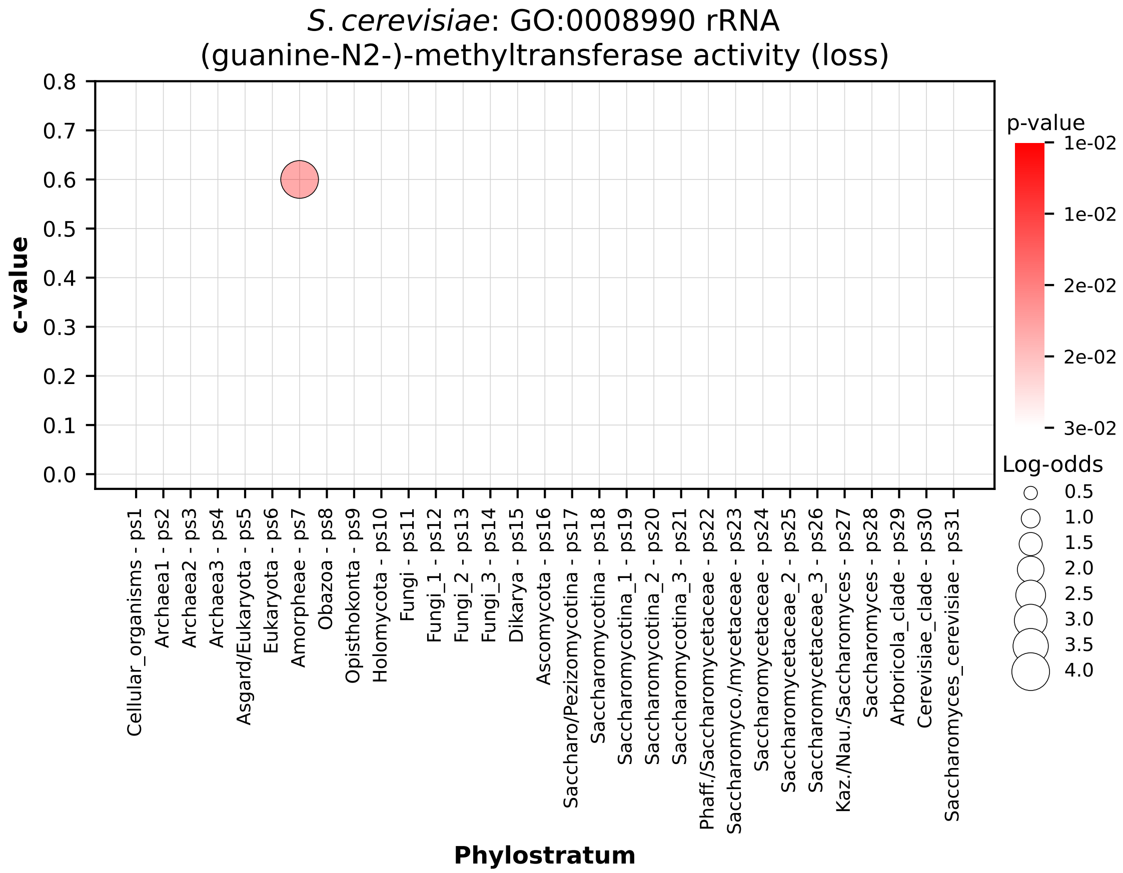

S. cerevisiae: GO:0008990 rRNA (guanine-N2-)-methyltransferase activity (loss)
c-value
0.0
0.1
0.2
0.3
0.4
0.5
0.6
0.7
0.8
p-value
1e-02
1e-02
2e-02
2e-02
3e-02
Log-odds
0.5
1.0
1.5
2.0
2.5
3.0
3.5
4.0
Cellular_organisms - ps1
Archaea1 - ps2
Archaea2 - ps3
Archaea3 - ps4
Asgard/Eukaryota - ps5
Eukaryota - ps6
Amorpheae - ps7
Obazoa - ps8
Opisthokonta - ps9
Holomycota - ps10
Fungi - ps11
Fungi_1 - ps12
Fungi_2 - ps13
Fungi_3 - ps14
Dikarya - ps15
Ascomycota - ps16
Saccharo/Pezizomycotina - ps17
Saccharomycotina - ps18
Saccharomycotina_1 - ps19
Saccharomycotina_2 - ps20
Saccharomycotina_3 - ps21
Phaff./Saccharomycetaceae - ps22
Saccharomyco./mycetaceae - ps23
Saccharomycetaceae - ps24
Saccharomycetaceae_2 - ps25
Saccharomycetaceae_3 - ps26
Kaz./Nau./Saccharomyces - ps27
Saccharomyces - ps28
Arboricola_clade - ps29
Cerevisiae_clade - ps30
Saccharomyces_cerevisiae - ps31
Phylostratum

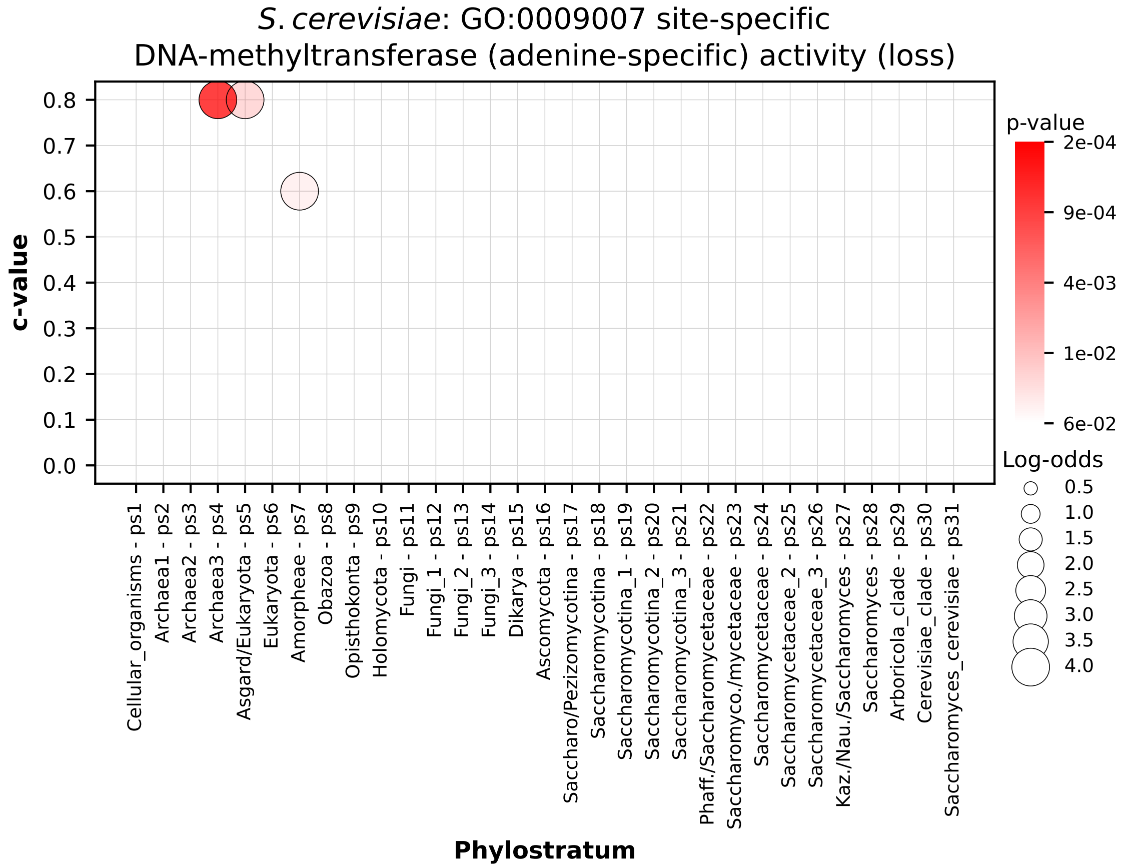
S. cerevisiae: GO:0009007 site-specific
DNA-methyltransferase (adenine-specific) activity (loss)
c-value
0.8
0.7
0.6
0.5
0.4
0.3
0.2
0.1
0.0
p-value
2e-04
9e-04
4e-03
1e-02
6e-02
Log-odds
0.5
1.0
1.5
2.0
2.5
3.0
3.5
4.0
Cellular_organisms - ps1
Archaea1 - ps2
Archaea2 - ps3
Archaea3 - ps4
Asgard/Eukaryota - ps5
Eukaryota - ps6
Amorpheae - ps7
Obazoa - ps8
Opisthokonta - ps9
Holomycota - ps10
Fungi - ps11
Fungi_1 - ps12
Fungi_2 - ps13
Fungi_3 - ps14
Dikarya - ps15
Ascomycota - ps16
Saccharo/Pezizomycotina - ps17
Saccharomycotina - ps18
Saccharomycotina_1 - ps19
Saccharomycotina_2 - ps20
Saccharomycotina_3 - ps21
Phaff./Saccharomycetaceae - ps22
Saccharomyco./mycetaceae - ps23
Saccharomycetaceae - ps24
Saccharomycetaceae_2 - ps25
Saccharomycetaceae_3 - ps26
Kaz./Nau./Saccharomyces - ps27
Saccharomyces - ps28
Arboricola_clade - ps29
Cerevisiae_clade - ps30
Saccharomyces_cerevisiae - ps31
Phylostratum

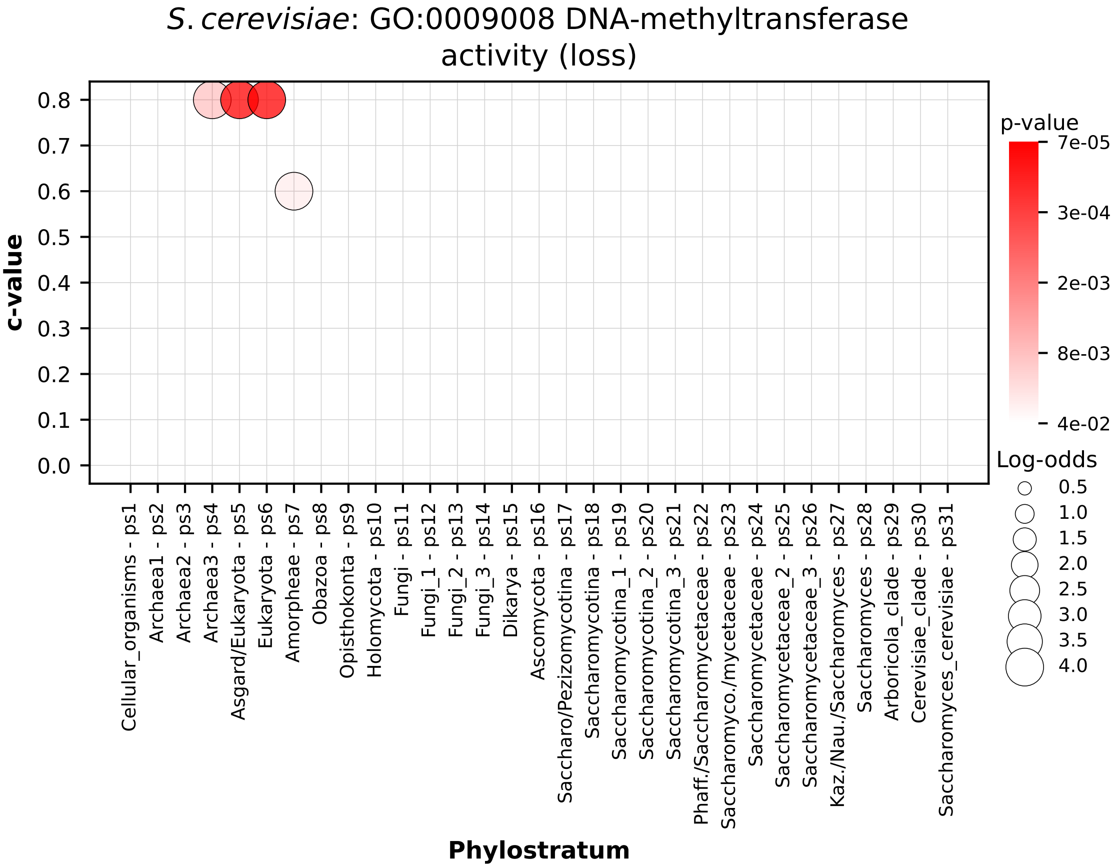

$S. cerevisiae$: GO:0009008 DNA-methyltransferase activity (loss)
c-value
0.0
0.1
0.2
0.3
0.4
0.5
0.6
0.7
0.8
Phylostratum
Cellular_organisms - ps1
Archaea1 - ps2
Archaea2 - ps3
Archaea3 - ps4
Asgard/Eukaryota - ps5
Eukaryota - ps6
Amorpheae - ps7
Obazoa - ps8
Opisthokonta - ps9
Holomycota - ps10
Fungi - ps11
Fungi_1 - ps12
Fungi_2 - ps13
Fungi_3 - ps14
Dikarya - ps15
Ascomycota - ps16
Saccharo/Pezizomycotina - ps17
Saccharomycotina - ps18
Saccharomycotina_1 - ps19
Saccharomycotina_2 - ps20
Saccharomycotina_3 - ps21
Phaff./Saccharomycetaceae - ps22
Saccharomyco./mycetaceae - ps23
Saccharomycetaceae - ps24
Saccharomycetaceae_2 - ps25
Saccharomycetaceae_3 - ps26
Kaz./Nau./Saccharomyces - ps27
Saccharomyces - ps28
Arboricola_clade - ps29
Cerevisiae_clade - ps30
Saccharomyces_cerevisiae - ps31
p-value
7e-05
3e-04
2e-03
8e-03
4e-02
Log-odds
0.5
1.0
1.5
2.0
2.5
3.0
3.5
4.0

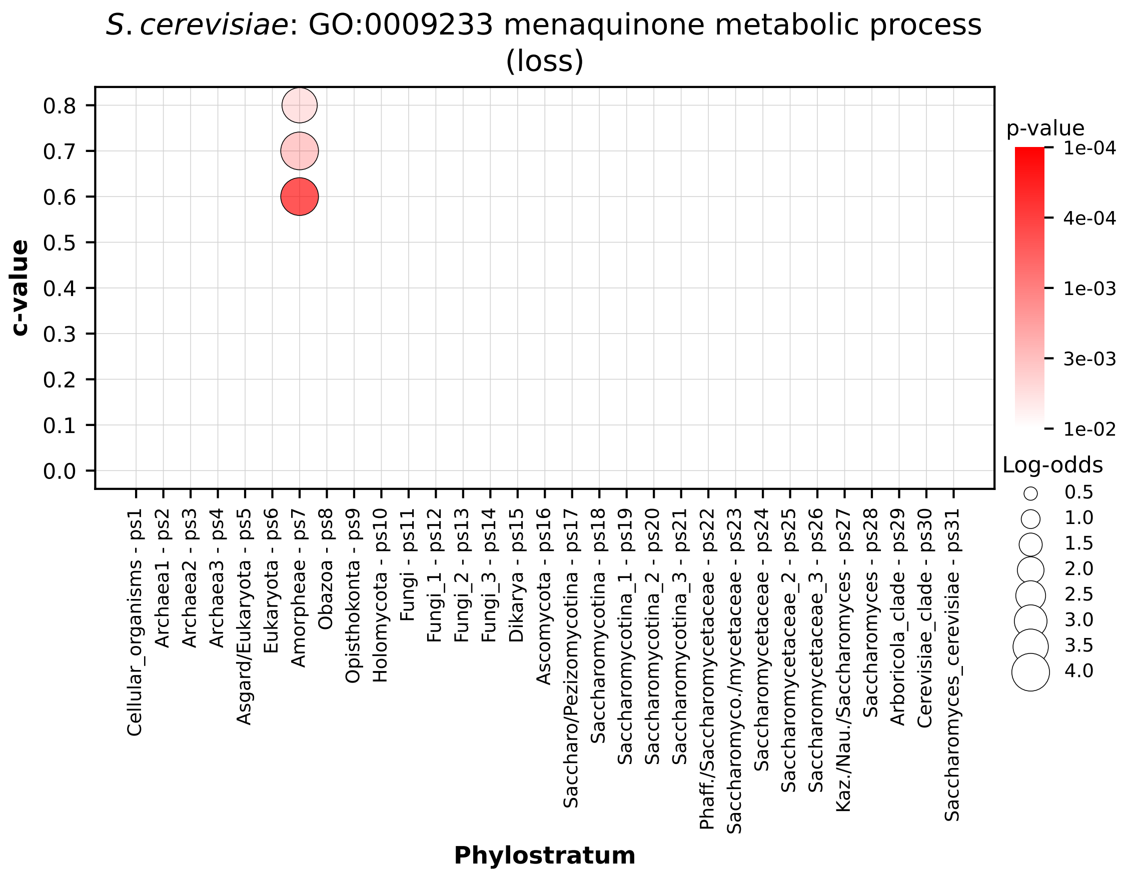

S. cerevisiae: GO:0009233 menaquinone metabolic process (loss)
c-value
0.8
0.7
0.6
0.5
0.4
0.3
0.2
0.1
0.0
p-value
1e-04
4e-04
1e-03
3e-03
1e-02
Log-odds
0.5
1.0
1.5
2.0
2.5
3.0
3.5
4.0
Cellular_organisms - ps1
Archaea1 - ps2
Archaea2 - ps3
Archaea3 - ps4
Asgard/Eukaryota - ps5
Eukaryota - ps6
Amorpheae - ps7
Obazoa - ps8
Opisthokonta - ps9
Holomycota - ps10
Fungi - ps11
Fungi_1 - ps12
Fungi_2 - ps13
Fungi_3 - ps14
Dikarya - ps15
Ascomycota - ps16
Saccharo/Pezizomycotina - ps17
Saccharomycotina - ps18
Saccharomycotina_1 - ps19
Saccharomycotina_2 - ps20
Saccharomycotina_3 - ps21
Phaff./Saccharomycetaceae - ps22
Saccharomyco./mycetaceae - ps23
Saccharomycetaceae - ps24
Saccharomycetaceae_2 - ps25
Saccharomycetaceae_3 - ps26
Kaz./Nau./Saccharomyces - ps27
Saccharomyces - ps28
Arboricola_clade - ps29
Cerevisiae_clade - ps30
Saccharomyces_cerevisiae - ps31
Phylostratum

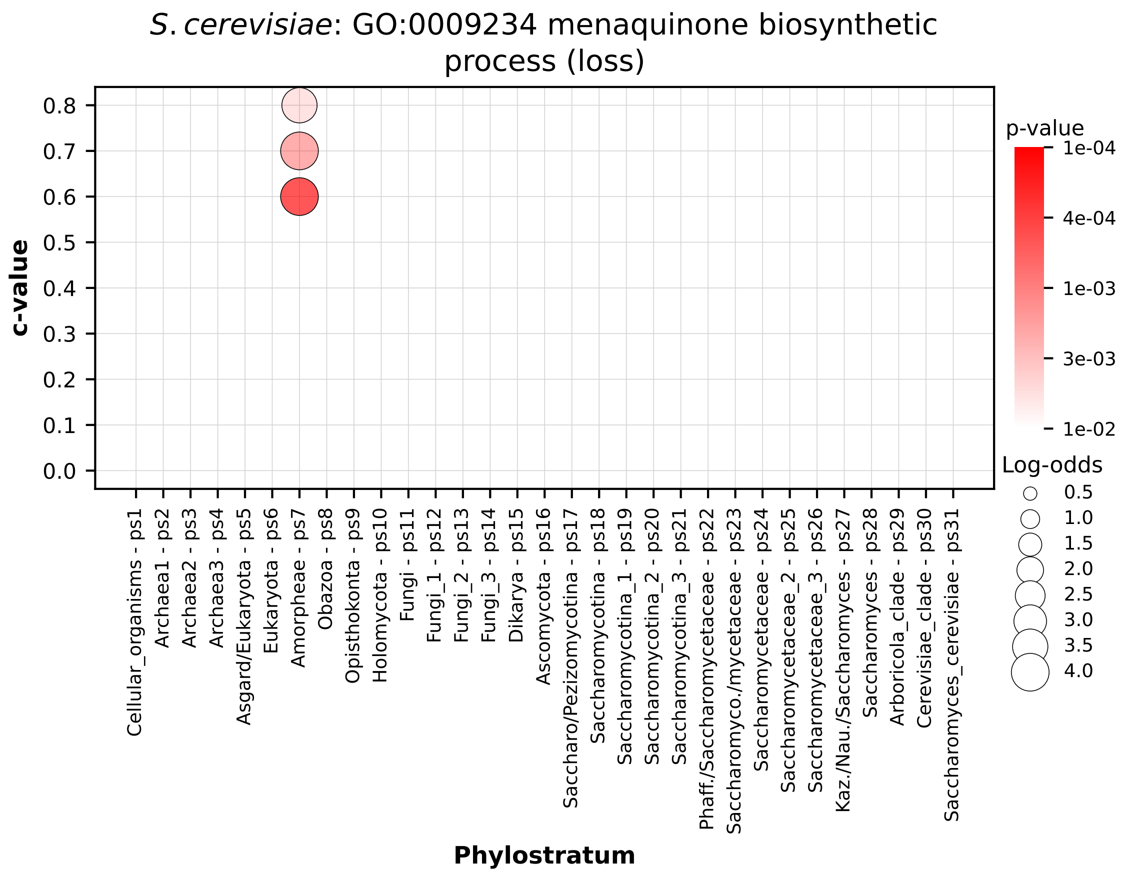

S. cerevisiae: GO:0009234 menaquinone biosynthetic process (loss)
c-value
0.0
0.1
0.2
0.3
0.4
0.5
0.6
0.7
0.8
p-value
1e-04
4e-04
1e-03
3e-03
1e-02
Log-odds
0.5
1.0
1.5
2.0
2.5
3.0
3.5
4.0
Cellular_organisms - ps1
Archaea1 - ps2
Archaea2 - ps3
Archaea3 - ps4
Asgard/Eukaryota - ps5
Eukaryota - ps6
Amorpheae - ps7
Obazoa - ps8
Opisthokonta - ps9
Holomycota - ps10
Fungi - ps11
Fungi_1 - ps12
Fungi_2 - ps13
Fungi_3 - ps14
Dikarya - ps15
Ascomycota - ps16
Saccharo/Pezizomycotina - ps17
Saccharomycotina - ps18
Saccharomycotina_1 - ps19
Saccharomycotina_2 - ps20
Saccharomycotina_3 - ps21
Phaff./Saccharomycetaceae - ps22
Saccharomyco./mycetaceae - ps23
Saccharomycetaceae - ps24
Saccharomycetaceae_2 - ps25
Saccharomycetaceae_3 - ps26
Kaz./Nau./Saccharomyces - ps27
Saccharomyces - ps28
Arboricola_clade - ps29
Cerevisiae_clade - ps30
Saccharomyces_cerevisiae - ps31
Phylostratum

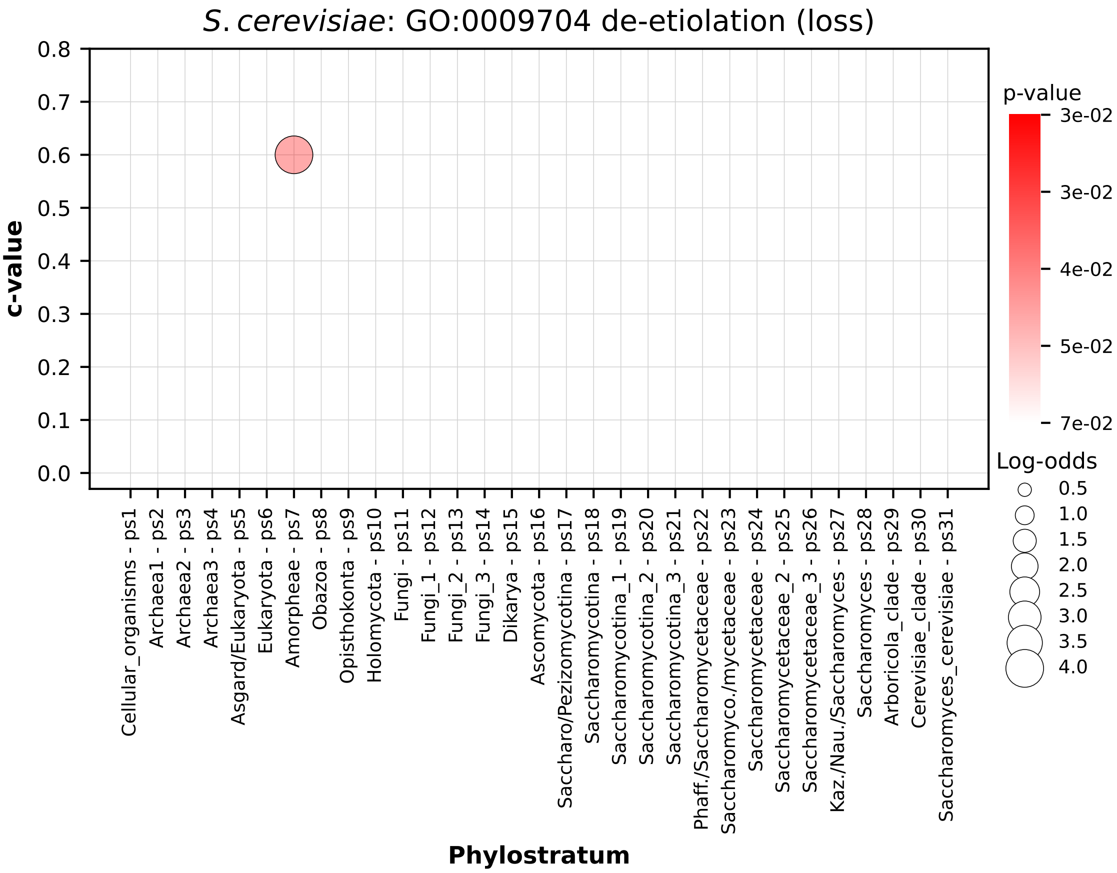

S. cerevisiae: GO:0009704 de-etiolation (loss)
c-value
0.8
0.7
0.6
0.5
0.4
0.3
0.2
0.1
0.0
p-value
3e-02
3e-02
4e-02
5e-02
7e-02
Log-odds
0.5
1.0
1.5
2.0
2.5
3.0
3.5
4.0
Cellular_organisms - ps1
Archaea1 - ps2
Archaea2 - ps3
Archaea3 - ps4
Asgard/Eukaryota - ps5
Eukaryota - ps6
Amorpheae - ps7
Obazoa - ps8
Opisthokonta - ps9
Holomycota - ps10
Fungi - ps11
Fungi_1 - ps12
Fungi_2 - ps13
Fungi_3 - ps14
Dikarya - ps15
Ascomycota - ps16
Saccharo/Pezizomycotina - ps17
Saccharomycotina - ps18
Saccharomycotina_1 - ps19
Saccharomycotina_2 - ps20
Saccharomycotina_3 - ps21
Phaff./Saccharomycetaceae - ps22
Saccharomyco./mycetaceae - ps23
Saccharomycetaceae - ps24
Saccharomycetaceae_2 - ps25
Saccharomycetaceae_3 - ps26
Kaz./Nau./Saccharomyces - ps27
Saccharomyces - ps28
Arboricola_clade - ps29
Cerevisiae_clade - ps30
Saccharomyces_cerevisiae - ps31
Phylostratum

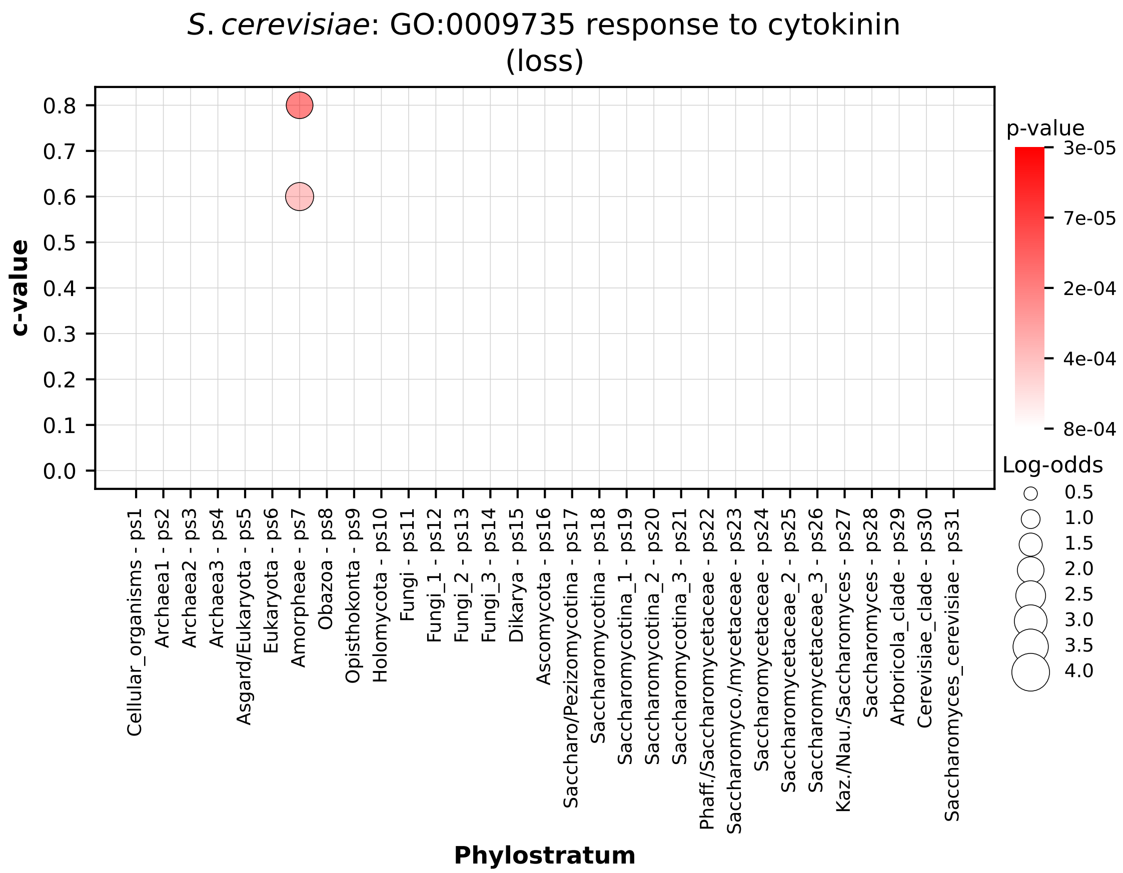

$S. cerevisiae$: GO:0009735 response to cytokinin
(loss)
c-value
0.0
0.1
0.2
0.3
0.4
0.5
0.6
0.7
0.8
Phylostratum
Cellular_organisms - ps1
Archaea1 - ps2
Archaea2 - ps3
Archaea3 - ps4
Asgard/Eukaryota - ps5
Eukaryota - ps6
Amorpheae - ps7
Obazoa - ps8
Opisthokonta - ps9
Holomycota - ps10
Fungi - ps11
Fungi_1 - ps12
Fungi_2 - ps13
Fungi_3 - ps14
Dikarya - ps15
Ascomycota - ps16
Saccharo/Pezizomycotina - ps17
Saccharomycotina - ps18
Saccharomycotina_1 - ps19
Saccharomycotina_2 - ps20
Saccharomycotina_3 - ps21
Phaff./Saccharomycetaceae - ps22
Saccharomyco./mycetaceae - ps23
Saccharomycetaceae - ps24
Saccharomycetaceae_2 - ps25
Saccharomycetaceae_3 - ps26
Kaz./Nau./Saccharomyces - ps27
Saccharomyces - ps28
Arboricola_clade - ps29
Cerevisiae_clade - ps30
Saccharomyces_cerevisiae - ps31
p-value
3e-05
7e-05
2e-04
4e-04
8e-04
Log-odds
0.5
1.0
1.5
2.0
2.5
3.0
3.5
4.0

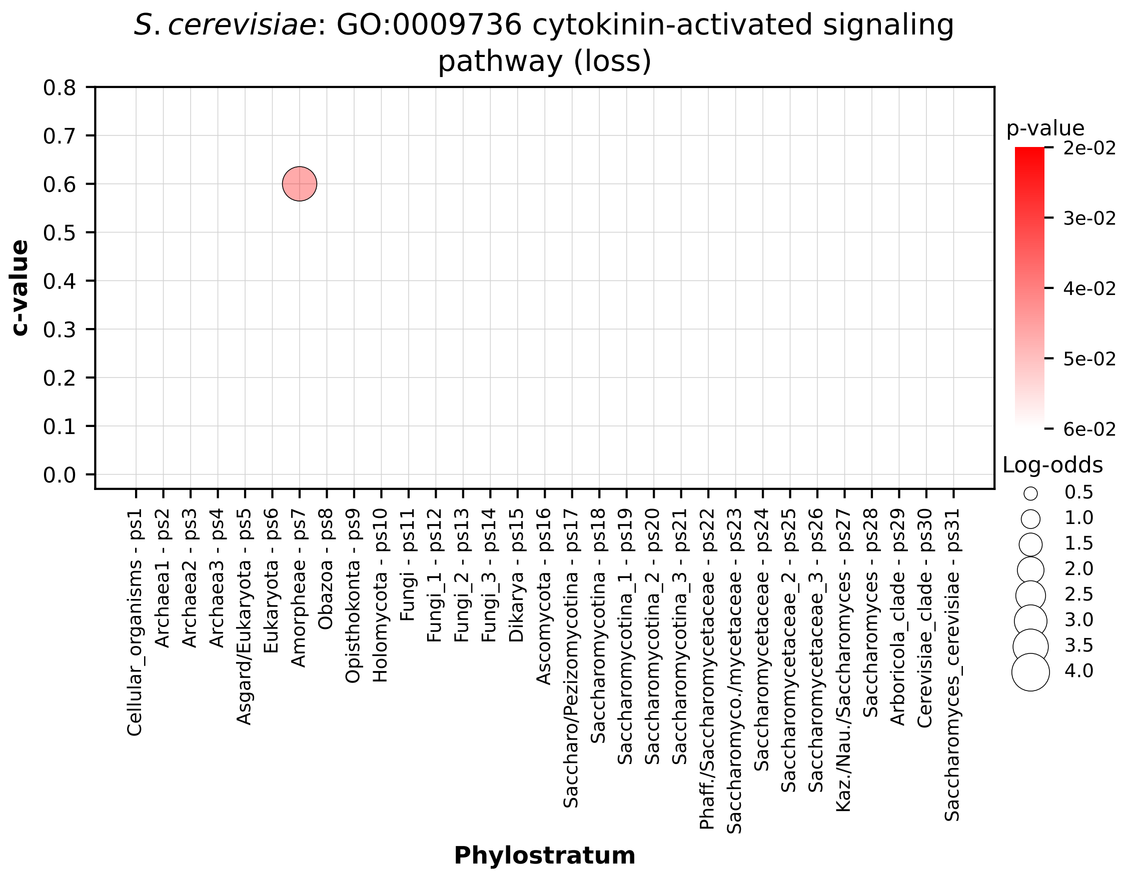
S. cerevisiae: GO:0009736 cytokinin-activated signaling pathway (loss)
c-value
0.8
0.7
0.6
0.5
0.4
0.3
0.2
0.1
0.0
p-value
2e-02
3e-02
4e-02
5e-02
6e-02
Log-odds
0.5
1.0
1.5
2.0
2.5
3.0
3.5
4.0
Cellular_organisms - ps1
Archaea1 - ps2
Archaea2 - ps3
Archaea3 - ps4
Asgard/Eukaryota - ps5
Eukaryota - ps6
Amorpheae - ps7
Obazoa - ps8
Opisthokonta - ps9
Holomycota - ps10
Fungi - ps11
Fungi_1 - ps12
Fungi_2 - ps13
Fungi_3 - ps14
Dikarya - ps15
Ascomycota - ps16
Saccharo/Pezizomycotina - ps17
Saccharomycotina - ps18
Saccharomycotina_1 - ps19
Saccharomycotina_2 - ps20
Saccharomycotina_3 - ps21
Phaff./Saccharomycetaceae - ps22
Saccharomyco./mycetaceae - ps23
Saccharomycetaceae - ps24
Saccharomycetaceae_2 - ps25
Saccharomycetaceae_3 - ps26
Kaz./Nau./Saccharomyces - ps27
Saccharomyces - ps28
Arboricola_clade - ps29
Cerevisiae_clade - ps30
Saccharomyces_cerevisiae - ps31
Phylostratum

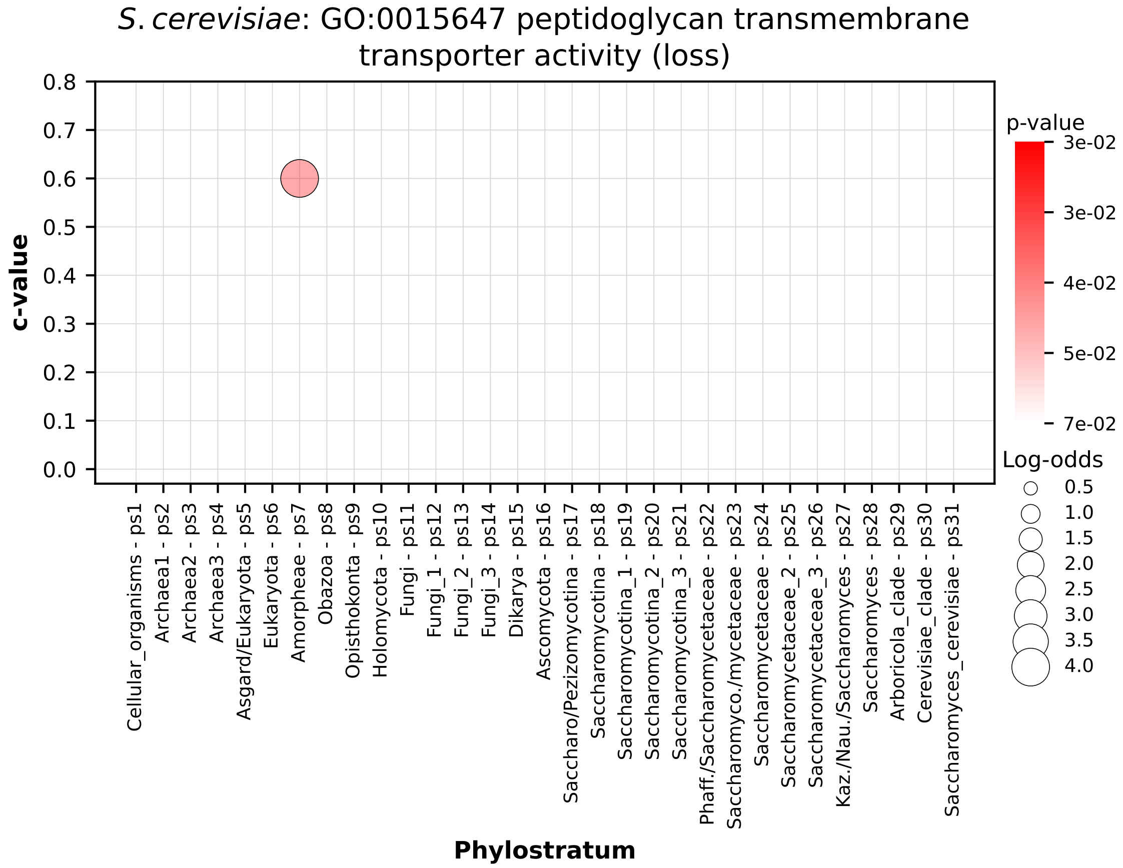

S. cerevisiae: GO:0015647 peptidoglycan transmembrane transporter activity (loss)
c-value
0.8
0.7
0.6
0.5
0.4
0.3
0.2
0.1
0.0
p-value
3e-02
3e-02
4e-02
5e-02
7e-02
Log-odds
0.5
1.0
1.5
2.0
2.5
3.0
3.5
4.0
Cellular_organisms - ps1
Archaea1 - ps2
Archaea2 - ps3
Archaea3 - ps4
Asgard/Eukaryota - ps5
Eukaryota - ps6
Amorpheae - ps7
Obazoa - ps8
Opisthokonta - ps9
Holomycota - ps10
Fungi - ps11
Fungi_1 - ps12
Fungi_2 - ps13
Fungi_3 - ps14
Dikarya - ps15
Ascomycota - ps16
Saccharo/Pezizomycotina - ps17
Saccharomycotina - ps18
Saccharomycotina_1 - ps19
Saccharomycotina_2 - ps20
Saccharomycotina_3 - ps21
Phaff./Saccharomycetaceae - ps22
Saccharomyco./mycetaceae - ps23
Saccharomycetaceae - ps24
Saccharomycetaceae_2 - ps25
Saccharomycetaceae_3 - ps26
Kaz./Nau./Saccharomyces - ps27
Saccharomyces - ps28
Arboricola_clade - ps29
Cerevisiae_clade - ps30
Saccharomyces_cerevisiae - ps31
Phylostratum

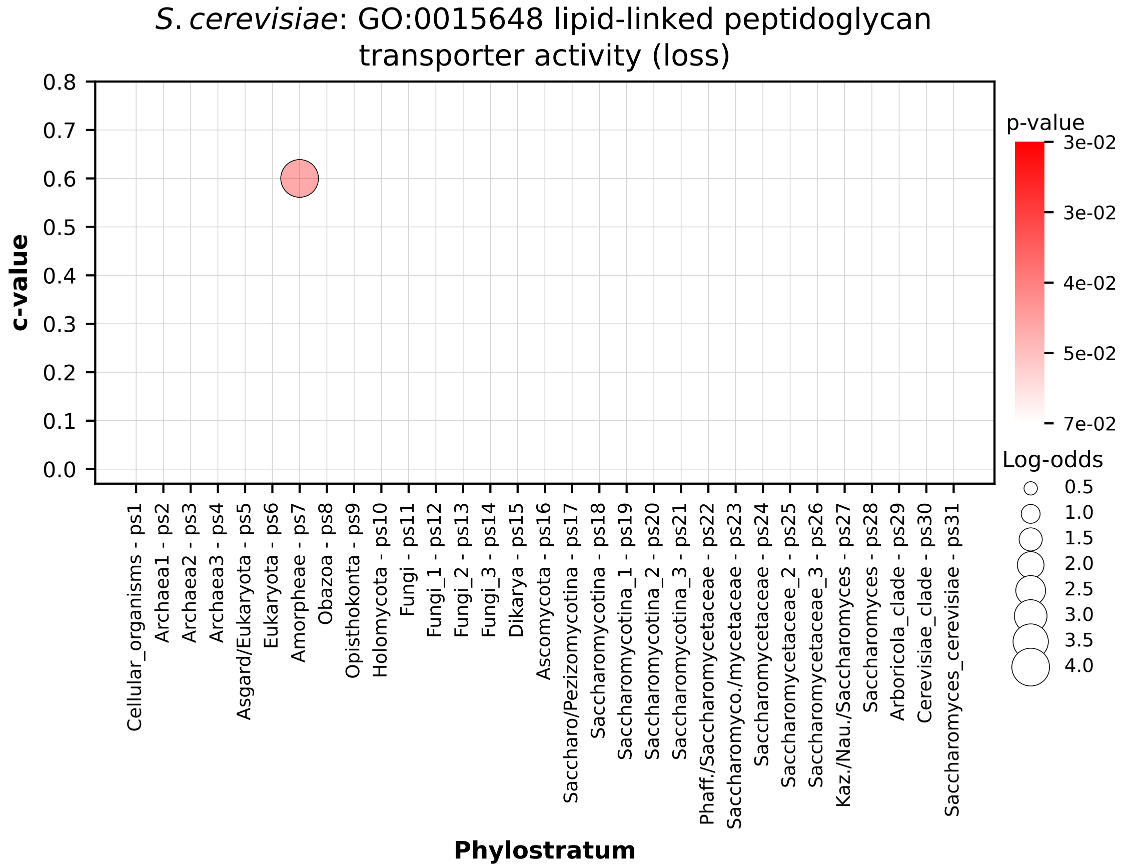

S. cerevisiae: GO:0015648 lipid-linked peptidoglycan transporter activity (loss)
c-value
0.8
0.7
0.6
0.5
0.4
0.3
0.2
0.1
0.0
p-value
3e-02
3e-02
4e-02
5e-02
7e-02
Log-odds
0.5
1.0
1.5
2.0
2.5
3.0
3.5
4.0
Cellular_organisms - ps1
Archaea1 - ps2
Archaea2 - ps3
Archaea3 - ps4
Asgard/Eukaryota - ps5
Eukaryota - ps6
Amorpheae - ps7
Obazoa - ps8
Opisthokonta - ps9
Holomycota - ps10
Fungi - ps11
Fungi_1 - ps12
Fungi_2 - ps13
Fungi_3 - ps14
Dikarya - ps15
Ascomycota - ps16
Saccharo/Pezizomycotina - ps17
Saccharomycotina - ps18
Saccharomycotina_1 - ps19
Saccharomycotina_2 - ps20
Saccharomycotina_3 - ps21
Phaff./Saccharomycetaceae - ps22
Saccharomyco./mycetaceae - ps23
Saccharomycetaceae - ps24
Saccharomycetaceae_2 - ps25
Saccharomycetaceae_3 - ps26
Kaz./Nau./Saccharomyces - ps27
Saccharomyces - ps28
Arboricola_clade - ps29
Cerevisiae_clade - ps30
Saccharomyces_cerevisiae - ps31
Phylostratum

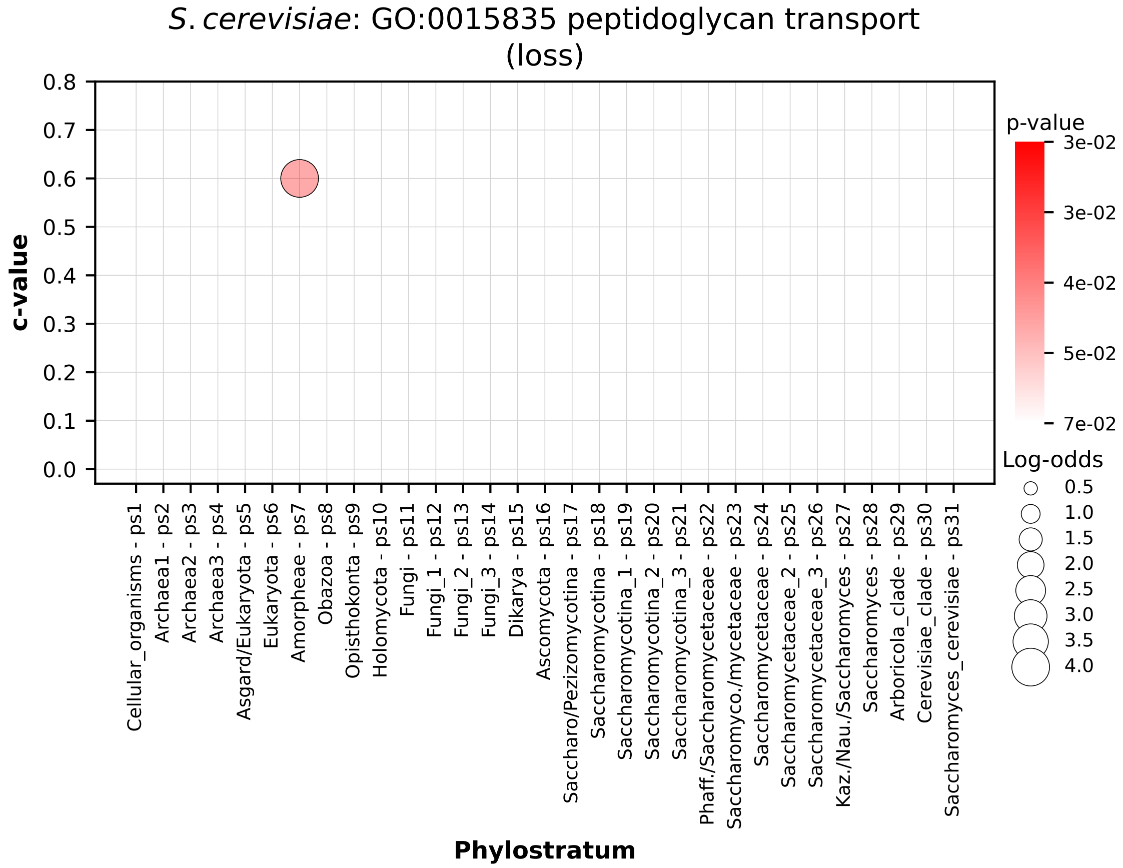

S. cerevisiae: GO:0015835 peptidoglycan transport (loss)
c-value
0.0
0.1
0.2
0.3
0.4
0.5
0.6
0.7
0.8
Phylostratum
Cellular_organisms - ps1
Archaea1 - ps2
Archaea2 - ps3
Archaea3 - ps4
Asgard/Eukaryota - ps5
Eukaryota - ps6
Amorpheae - ps7
Obazoa - ps8
Opisthokonta - ps9
Holomycota - ps10
Fungi - ps11
Fungi_1 - ps12
Fungi_2 - ps13
Fungi_3 - ps14
Dikarya - ps15
Ascomycota - ps16
Saccharo/Pezizomycotina - ps17
Saccharomycotina - ps18
Saccharomycotina_1 - ps19
Saccharomycotina_2 - ps20
Saccharomycotina_3 - ps21
Phaff./Saccharomycetaceae - ps22
Saccharomyco./mycetaceae - ps23
Saccharomycetaceae - ps24
Saccharomycetaceae_2 - ps25
Saccharomycetaceae_3 - ps26
Kaz./Nau./Saccharomyces - ps27
Saccharomyces - ps28
Arboricola_clade - ps29
Cerevisiae_clade - ps30
Saccharomyces_cerevisiae - ps31
p-value
3e-02
3e-02
4e-02
5e-02
7e-02
Log-odds
0.5
1.0
1.5
2.0
2.5
3.0
3.5
4.0

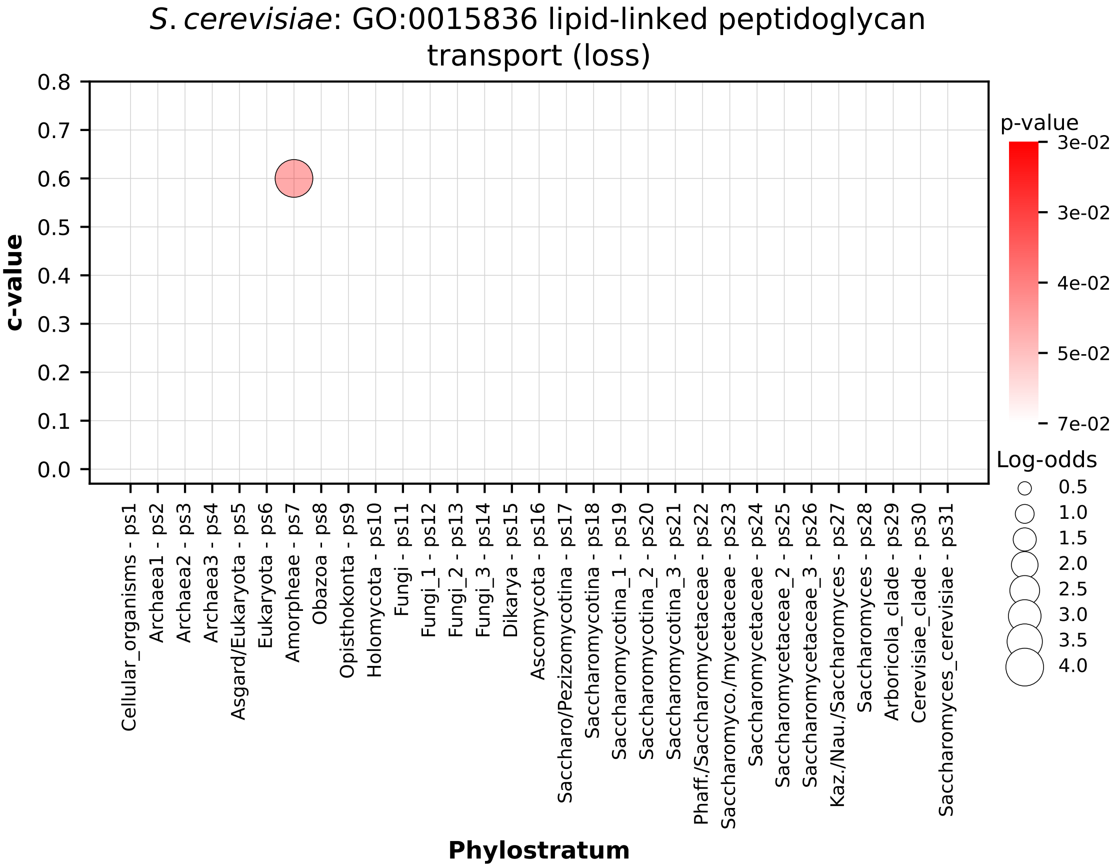

S. cerevisiae: GO:0015836 lipid-linked peptidoglycan transport (loss)
c-value
0.8
0.7
0.6
0.5
0.4
0.3
0.2
0.1
0.0
Phylostratum
Cellular_organisms - ps1
Archaea1 - ps2
Archaea2 - ps3
Archaea3 - ps4
Asgard/Eukaryota - ps5
Eukaryota - ps6
Amorpheae - ps7
Obazoa - ps8
Opisthokonta - ps9
Holomycota - ps10
Fungi - ps11
Fungi_1 - ps12
Fungi_2 - ps13
Fungi_3 - ps14
Dikarya - ps15
Ascomycota - ps16
Saccharo/Pezizomycotina - ps17
Saccharomycotina - ps18
Saccharomycotina_1 - ps19
Saccharomycotina_2 - ps20
Saccharomycotina_3 - ps21
Phaff./Saccharomycetaceae - ps22
Saccharomyco./mycetaceae - ps23
Saccharomycetaceae - ps24
Saccharomycetaceae_2 - ps25
Saccharomycetaceae_3 - ps26
Kaz./Nau./Saccharomyces - ps27
Saccharomyces - ps28
Arboricola_clade - ps29
Cerevisiae_clade - ps30
Saccharomyces_cerevisiae - ps31
p-value
3e-02
3e-02
4e-02
5e-02
7e-02
Log-odds
0.5
1.0
1.5
2.0
2.5
3.0
3.5
4.0

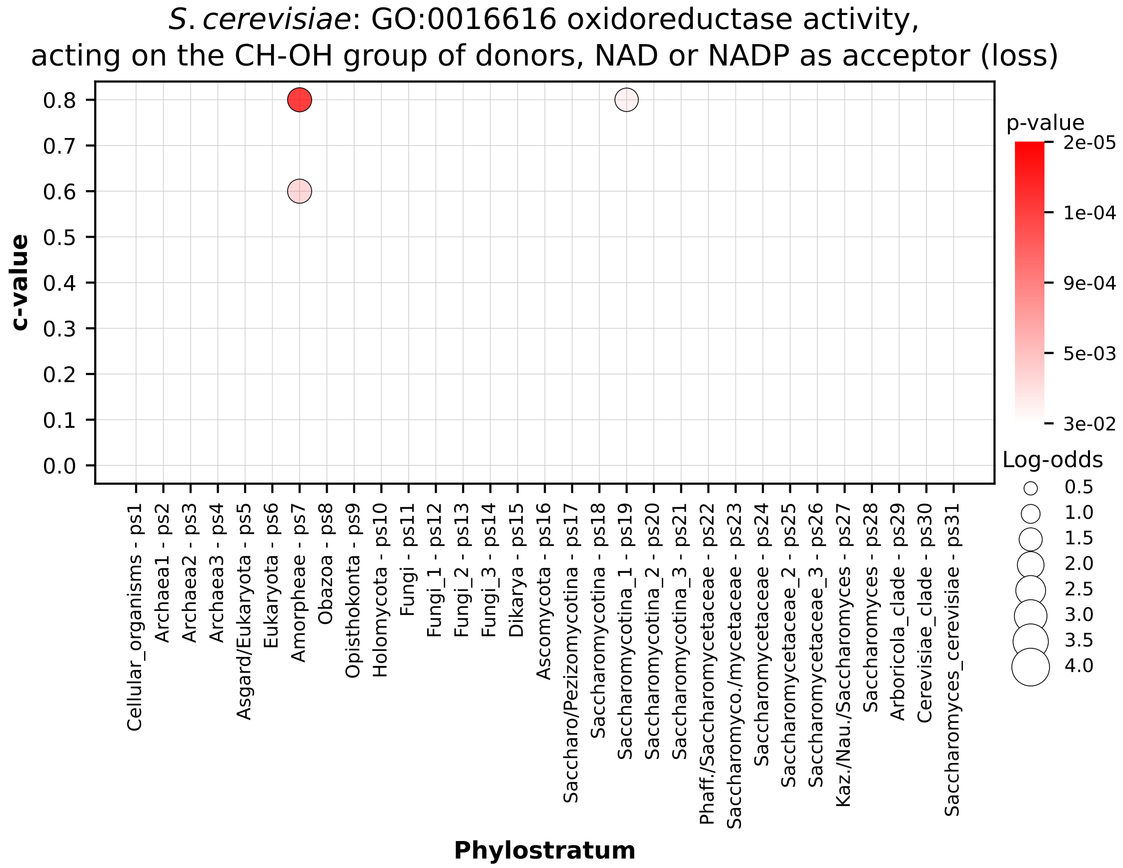
S. cerevisiae: GO:0016616 oxidoreductase activity,
acting on the CH-OH group of donors, NAD or NADP as acceptor (loss)
c-value
0.8
0.7
0.6
0.5
0.4
0.3
0.2
0.1
0.0
Phylostratum
Cellular_organisms - ps1
Archaea1 - ps2
Archaea2 - ps3
Archaea3 - ps4
Asgard/Eukaryota - ps5
Eukaryota - ps6
Amorpheae - ps7
Obazoa - ps8
Opisthokonta - ps9
Holomycota - ps10
Fungi - ps11
Fungi_1 - ps12
Fungi_2 - ps13
Fungi_3 - ps14
Dikarya - ps15
Ascomycota - ps16
Saccharo/Pezizomycotina - ps17
Saccharomycotina - ps18
Saccharomycotina_1 - ps19
Saccharomycotina_2 - ps20
Saccharomycotina_3 - ps21
Phaff./Saccharomycetaceae - ps22
Saccharomyco./mycetaceae - ps23
Saccharomycetaceae - ps24
Saccharomycetaceae_2 - ps25
Saccharomycetaceae_3 - ps26
Kaz./Nau./Saccharomyces - ps27
Saccharomyces - ps28
Arboricola_clade - ps29
Cerevisiae_clade - ps30
Saccharomyces_cerevisiae - ps31
p-value
2e-05
1e-04
9e-04
5e-03
3e-02
Log-odds
0.5
1.0
1.5
2.0
2.5
3.0
3.5
4.0

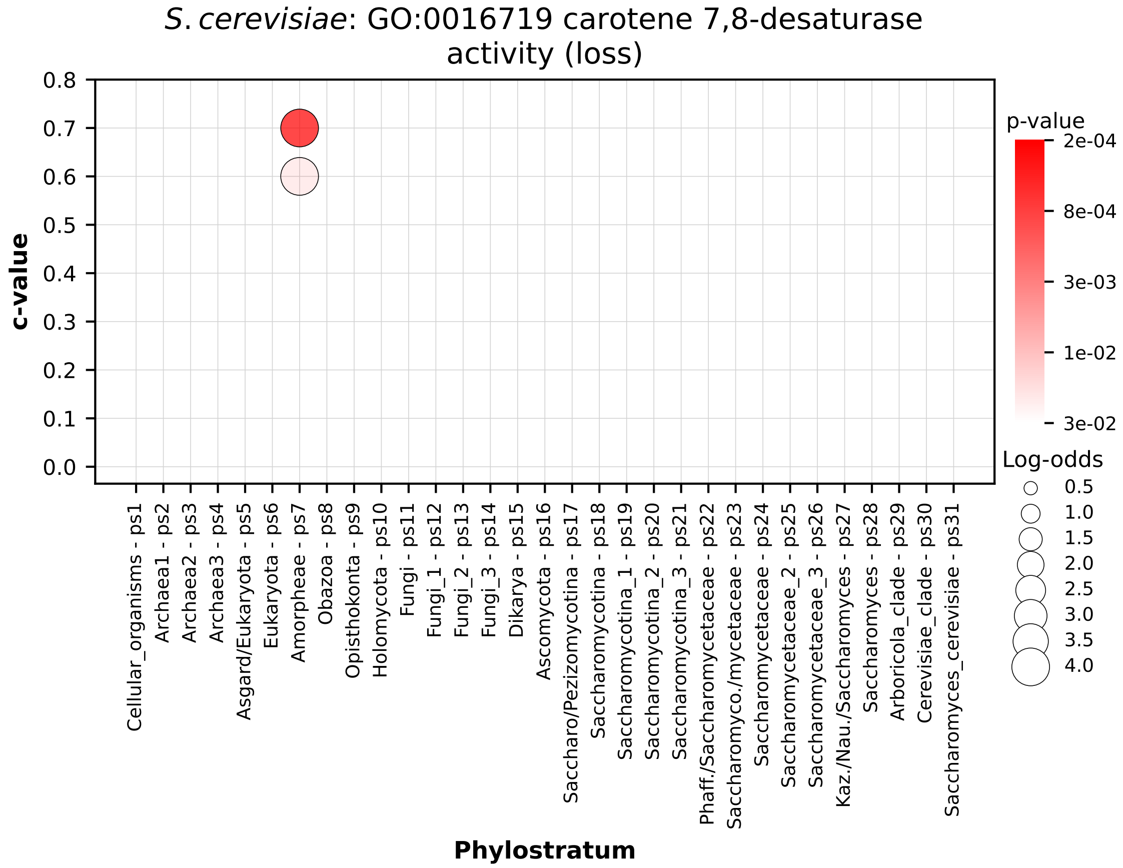
S. cerevisiae: GO:0016719 carotene 7,8-desaturase activity (loss)
c-value
0.8
0.7
0.6
0.5
0.4
0.3
0.2
0.1
0.0
p-value
2e-04
8e-04
3e-03
1e-02
3e-02
Log-odds
0.5
1.0
1.5
2.0
2.5
3.0
3.5
4.0
Cellular_organisms - ps1
Archaea1 - ps2
Archaea2 - ps3
Archaea3 - ps4
Asgard/Eukaryota - ps5
Eukaryota - ps6
Amorpheae - ps7
Obazoa - ps8
Opisthokonta - ps9
Holomycota - ps10
Fungi - ps11
Fungi_1 - ps12
Fungi_2 - ps13
Fungi_3 - ps14
Dikarya - ps15
Ascomycota - ps16
Saccharo/Pezizomycotina - ps17
Saccharomycotina - ps18
Saccharomycotina_1 - ps19
Saccharomycotina_2 - ps20
Saccharomycotina_3 - ps21
Phaff./Saccharomycetaceae - ps22
Saccharomyco./mycetaceae - ps23
Saccharomycetaceae - ps24
Saccharomycetaceae_2 - ps25
Saccharomycetaceae_3 - ps26
Kaz./Nau./Saccharomyces - ps27
Saccharomyces - ps28
Arboricola_clade - ps29
Cerevisiae_clade - ps30
Saccharomyces_cerevisiae - ps31
Phylostratum

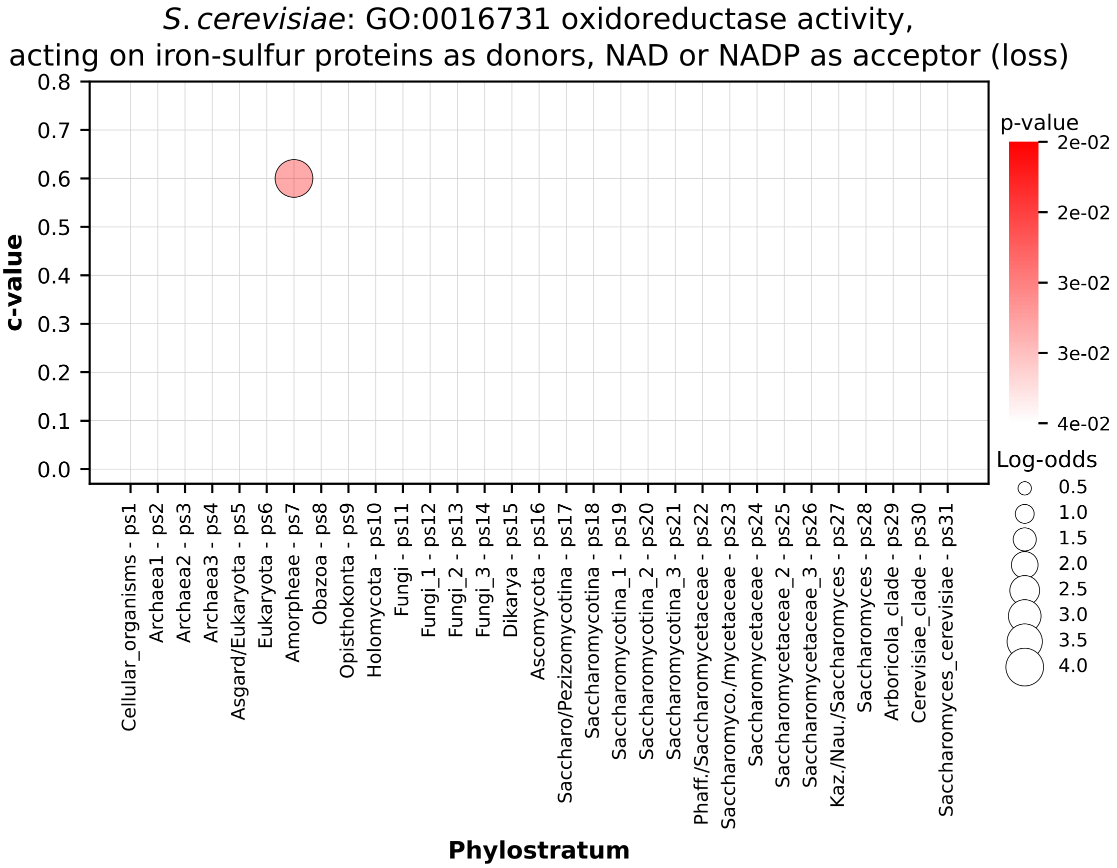

S. cerevisiae: GO:0016731 oxidoreductase activity,
acting on iron-sulfur proteins as donors, NAD or NADP as acceptor (loss)
c-value
0.8
0.7
0.6
0.5
0.4
0.3
0.2
0.1
0.0
p-value
2e-02
2e-02
3e-02
3e-02
4e-02
Log-odds
0.5
1.0
1.5
2.0
2.5
3.0
3.5
4.0
Cellular_organisms - ps1
Archaea1 - ps2
Archaea2 - ps3
Archaea3 - ps4
Asgard/Eukaryota - ps5
Eukaryota - ps6
Amorpheae - ps7
Obazoa - ps8
Opisthokonta - ps9
Holomycota - ps10
Fungi - ps11
Fungi_1 - ps12
Fungi_2 - ps13
Fungi_3 - ps14
Dikarya - ps15
Ascomycota - ps16
Saccharo/Pezizomycotina - ps17
Saccharomycotina - ps18
Saccharomycotina_1 - ps19
Saccharomycotina_2 - ps20
Saccharomycotina_3 - ps21
Phaff./Saccharomycetaceae - ps22
Saccharomyco./mycetaceae - ps23
Saccharomycetaceae - ps24
Saccharomycetaceae_2 - ps25
Saccharomycetaceae_3 - ps26
Kaz./Nau./Saccharomyces - ps27
Saccharomyces - ps28
Arboricola_clade - ps29
Cerevisiae_clade - ps30
Saccharomyces_cerevisiae - ps31
Phylostratum

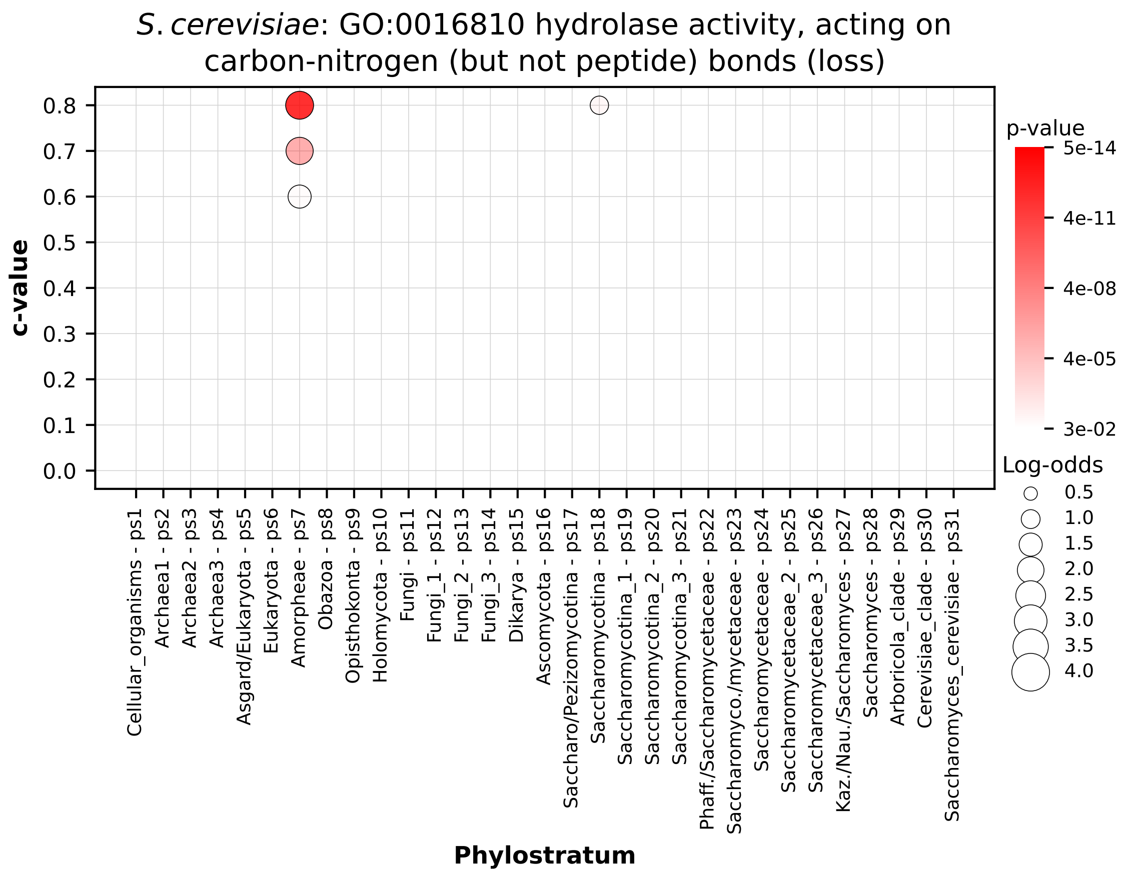

S. cerevisiae: GO:0016810 hydrolase activity, acting on carbon-nitrogen (but not peptide) bonds (loss)
c-value
0.8
0.7
0.6
0.5
0.4
0.3
0.2
0.1
0.0
Phylostratum
Cellular_organisms - ps1
Archaea1 - ps2
Archaea2 - ps3
Archaea3 - ps4
Asgard/Eukaryota - ps5
Eukaryota - ps6
Amorpheae - ps7
Obazoa - ps8
Opisthokonta - ps9
Holomycota - ps10
Fungi - ps11
Fungi_1 - ps12
Fungi_2 - ps13
Fungi_3 - ps14
Dikarya - ps15
Ascomycota - ps16
Saccharo/Pezizomycotina - ps17
Saccharomycotina - ps18
Saccharomycotina_1 - ps19
Saccharomycotina_2 - ps20
Saccharomycotina_3 - ps21
Phaff./Saccharomycetaceae - ps22
Saccharomyco./mycetaceae - ps23
Saccharomycetaceae - ps24
Saccharomycetaceae_2 - ps25
Saccharomycetaceae_3 - ps26
Kaz./Nau./Saccharomyces - ps27
Saccharomyces - ps28
Arboricola_clade - ps29
Cerevisiae_clade - ps30
Saccharomyces_cerevisiae - ps31
p-value
5e-14
4e-11
4e-08
4e-05
3e-02
Log-odds
0.5
1.0
1.5
2.0
2.5
3.0
3.5
4.0

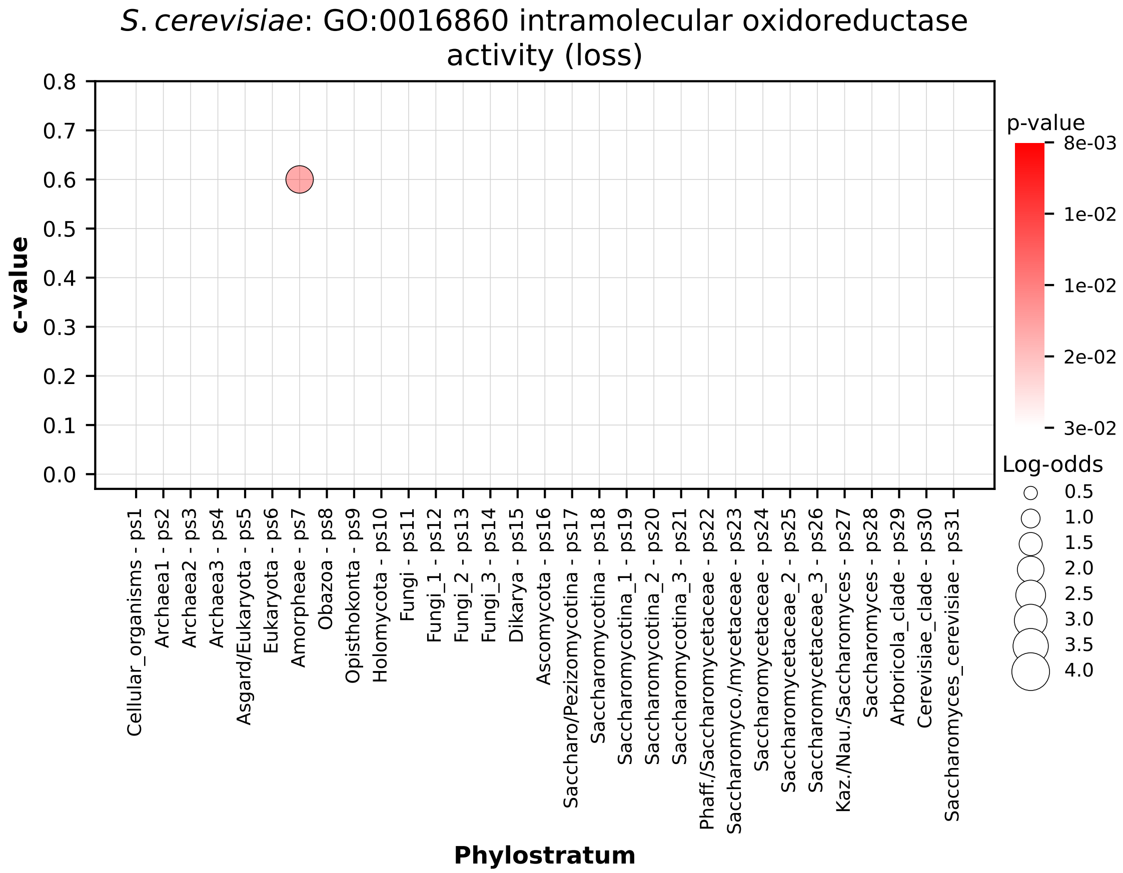

S. cerevisiae: GO:0016860 intramolecular oxidoreductase activity (loss)
c-value
0.8
0.7
0.6
0.5
0.4
0.3
0.2
0.1
0.0
p-value
8e-03
1e-02
1e-02
2e-02
3e-02
Log-odds
0.5
1.0
1.5
2.0
2.5
3.0
3.5
4.0
Cellular_organisms - ps1
Archaea1 - ps2
Archaea2 - ps3
Archaea3 - ps4
Asgard/Eukaryota - ps5
Eukaryota - ps6
Amorpheae - ps7
Obazoa - ps8
Opisthokonta - ps9
Holomycota - ps10
Fungi - ps11
Fungi_1 - ps12
Fungi_2 - ps13
Fungi_3 - ps14
Dikarya - ps15
Ascomycota - ps16
Saccharo/Pezizomycotina - ps17
Saccharomycotina - ps18
Saccharomycotina_1 - ps19
Saccharomycotina_2 - ps20
Saccharomycotina_3 - ps21
Phaff./Saccharomycetaceae - ps22
Saccharomyco./mycetaceae - ps23
Saccharomycetaceae - ps24
Saccharomycetaceae_2 - ps25
Saccharomycetaceae_3 - ps26
Kaz./Nau./Saccharomyces - ps27
Saccharomyces - ps28
Arboricola_clade - ps29
Cerevisiae_clade - ps30
Saccharomyces_cerevisiae - ps31
Phylostratum

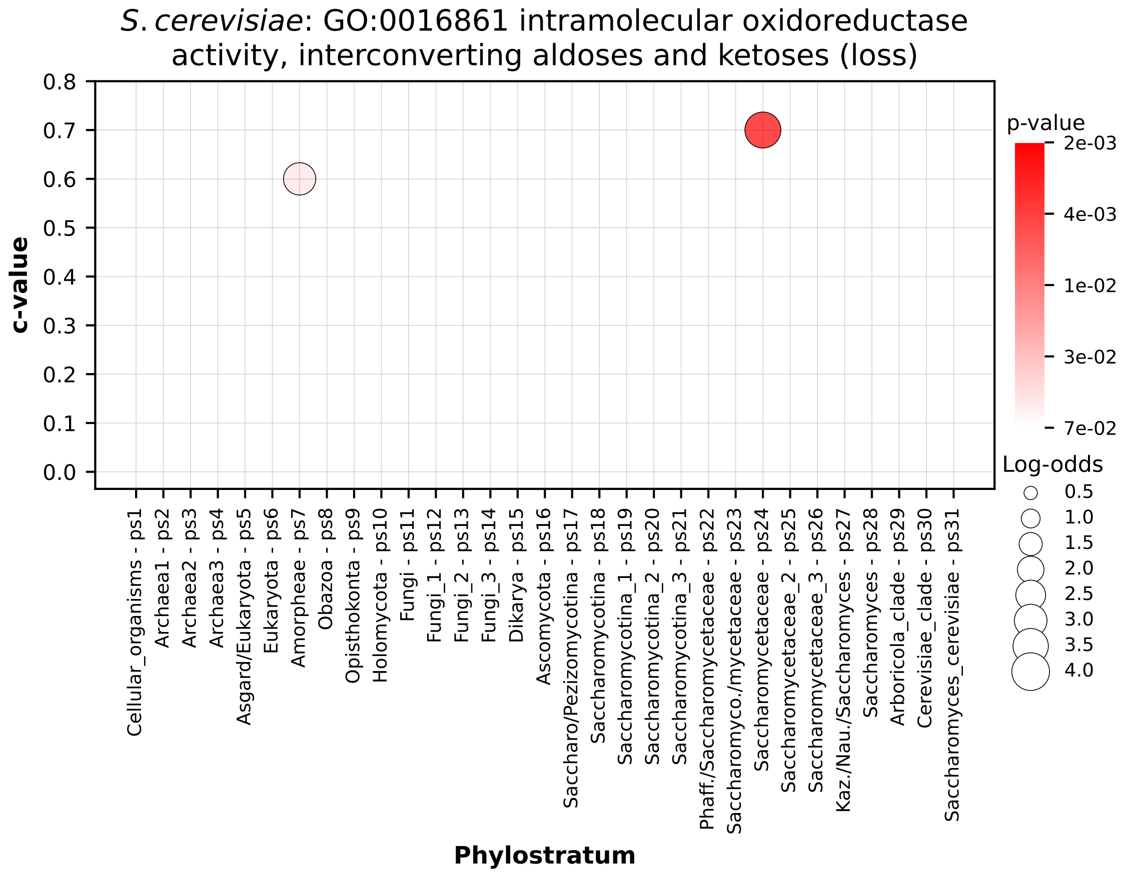

S. cerevisiae: GO:0016861 intramolecular oxidoreductase activity, interconverting aldoses and ketoses (loss)
c-value
0.8
0.7
0.6
0.5
0.4
0.3
0.2
0.1
0.0
p-value
2e-03
4e-03
1e-02
3e-02
7e-02
Log-odds
0.5
1.0
1.5
2.0
2.5
3.0
3.5
4.0
Cellular_organisms - ps1
Archaea1 - ps2
Archaea2 - ps3
Archaea3 - ps4
Asgard/Eukaryota - ps5
Eukaryota - ps6
Amorpheae - ps7
Obazoa - ps8
Opisthokonta - ps9
Holomycota - ps10
Fungi - ps11
Fungi_1 - ps12
Fungi_2 - ps13
Fungi_3 - ps14
Dikarya - ps15
Ascomycota - ps16
Saccharo/Pezizomycotina - ps17
Saccharomycotina - ps18
Saccharomycotina_1 - ps19
Saccharomycotina_2 - ps20
Saccharomycotina_3 - ps21
Phaff./Saccharomycetaceae - ps22
Saccharomyco./mycetaceae - ps23
Saccharomycetaceae - ps24
Saccharomycetaceae_2 - ps25
Saccharomycetaceae_3 - ps26
Kaz./Nau./Saccharomyces - ps27
Saccharomyces - ps28
Arboricola_clade - ps29
Cerevisiae_clade - ps30
Saccharomyces_cerevisiae - ps31
Phylostratum

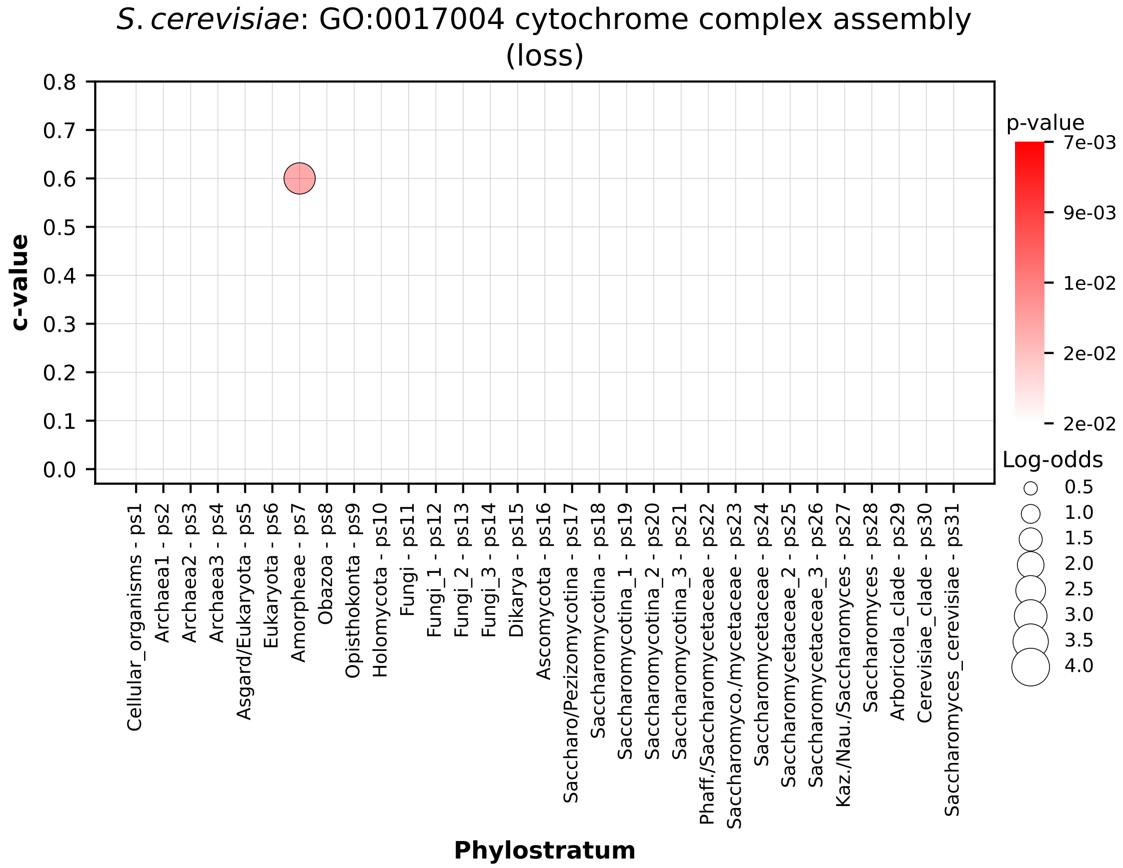
S. cerevisiae: GO:0017004 cytochrome complex assembly
(loss)
c-value
0.8
0.7
0.6
0.5
0.4
0.3
0.2
0.1
0.0
p-value
7e-03
9e-03
1e-02
2e-02
2e-02
Log-odds
0.5
1.0
1.5
2.0
2.5
3.0
3.5
4.0
Cellular_organisms - ps1
Archaea1 - ps2
Archaea2 - ps3
Archaea3 - ps4
Asgard/Eukaryota - ps5
Eukaryota - ps6
Amorpheae - ps7
Obazoa - ps8
Opisthokonta - ps9
Holomycota - ps10
Fungi - ps11
Fungi_1 - ps12
Fungi_2 - ps13
Fungi_3 - ps14
Dikarya - ps15
Ascomycota - ps16
Saccharo/Pezizomycotina - ps17
Saccharomycotina - ps18
Saccharomycotina_1 - ps19
Saccharomycotina_2 - ps20
Saccharomycotina_3 - ps21
Phaff./Saccharomycetaceae - ps22
Saccharomyco./mycetaceae - ps23
Saccharomycetaceae - ps24
Saccharomycetaceae_2 - ps25
Saccharomycetaceae_3 - ps26
Kaz./Nau./Saccharomyces - ps27
Saccharomyces - ps28
Arboricola_clade - ps29
Cerevisiae_clade - ps30
Saccharomyces_cerevisiae - ps31
Phylostratum

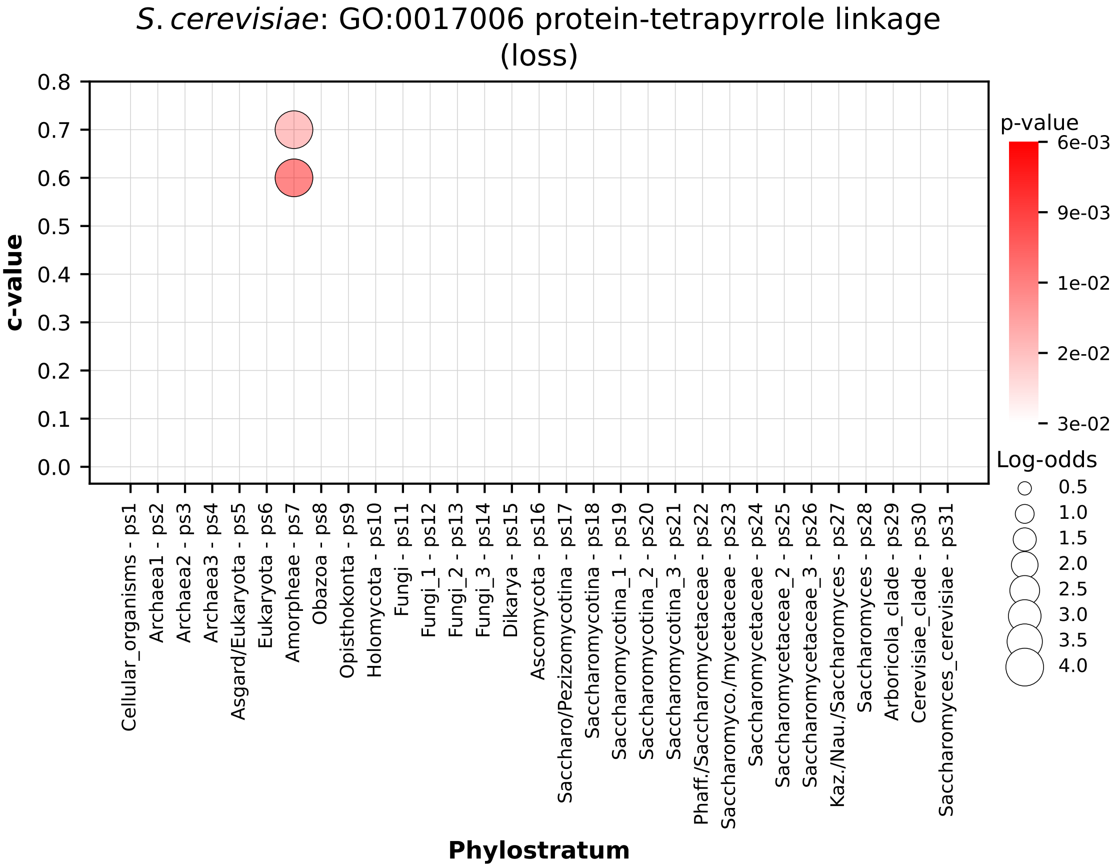
S. cerevisiae: GO:0017006 protein-tetrapyrrole linkage (loss)
c-value
0.8
0.7
0.6
0.5
0.4
0.3
0.2
0.1
0.0
p-value
6e-03
9e-03
1e-02
2e-02
3e-02
Log-odds
0.5
1.0
1.5
2.0
2.5
3.0
3.5
4.0
Cellular_organisms - ps1
Archaea1 - ps2
Archaea2 - ps3
Archaea3 - ps4
Asgard/Eukaryota - ps5
Eukaryota - ps6
Amorpheae - ps7
Obazoa - ps8
Opisthokonta - ps9
Holomycota - ps10
Fungi - ps11
Fungi_1 - ps12
Fungi_2 - ps13
Fungi_3 - ps14
Dikarya - ps15
Ascomycota - ps16
Saccharo/Pezizomycotina - ps17
Saccharomycotina - ps18
Saccharomycotina_1 - ps19
Saccharomycotina_2 - ps20
Saccharomycotina_3 - ps21
Phaff./Saccharomycetaceae - ps22
Saccharomyco./mycetaceae - ps23
Saccharomycetaceae - ps24
Saccharomycetaceae_2 - ps25
Saccharomycetaceae_3 - ps26
Kaz./Nau./Saccharomyces - ps27
Saccharomyces - ps28
Arboricola_clade - ps29
Cerevisiae_clade - ps30
Saccharomyces_cerevisiae - ps31
Phylostratum

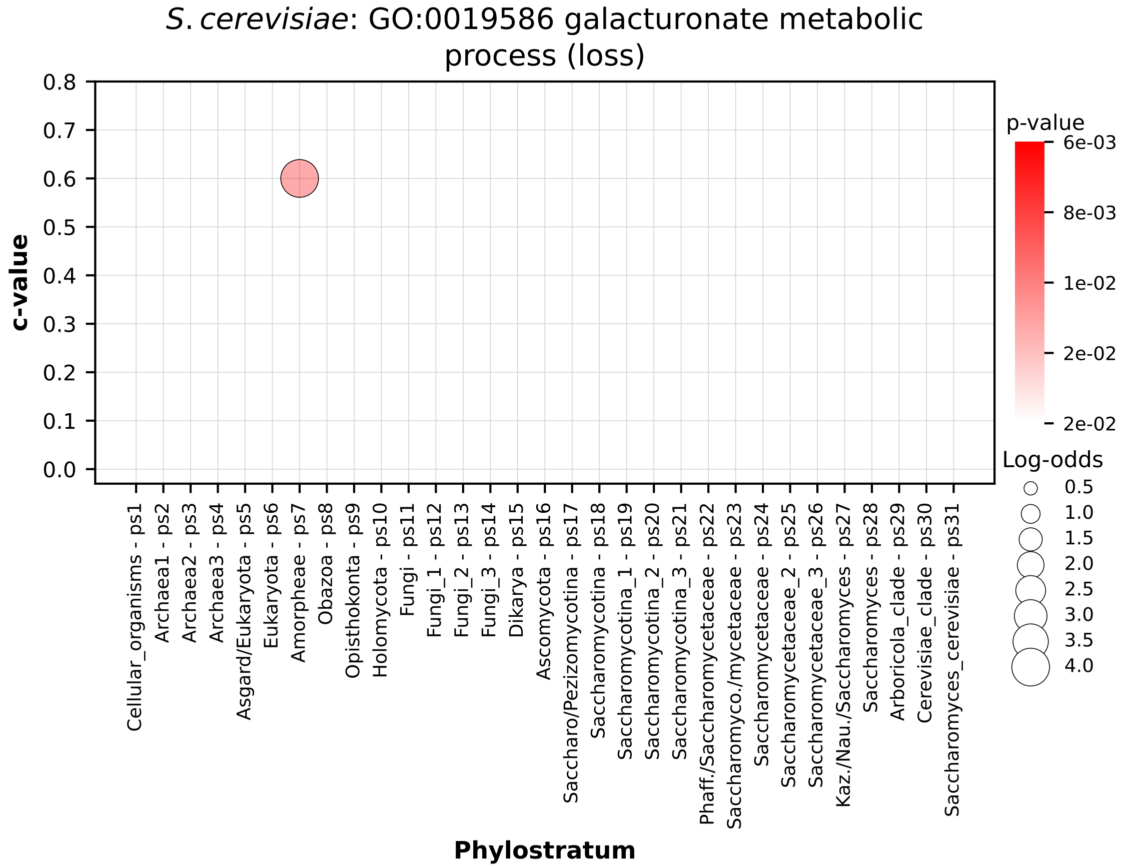

S. cerevisiae: GO:0019586 galacturonate metabolic process (loss)
c-value
0.8
0.7
0.6
0.5
0.4
0.3
0.2
0.1
0.0
p-value
6e-03
8e-03
1e-02
2e-02
2e-02
Log-odds
0.5
1.0
1.5
2.0
2.5
3.0
3.5
4.0
Cellular_organisms - ps1
Archaea1 - ps2
Archaea2 - ps3
Archaea3 - ps4
Asgard/Eukaryota - ps5
Eukaryota - ps6
Amorpheae - ps7
Obazoa - ps8
Opisthokonta - ps9
Holomycota - ps10
Fungi - ps11
Fungi_1 - ps12
Fungi_2 - ps13
Fungi_3 - ps14
Dikarya - ps15
Ascomycota - ps16
Saccharo/Pezizomycotina - ps17
Saccharomycotina - ps18
Saccharomycotina_1 - ps19
Saccharomycotina_2 - ps20
Saccharomycotina_3 - ps21
Phaff./Saccharomycetaceae - ps22
Saccharomyco./mycetaceae - ps23
Saccharomycetaceae - ps24
Saccharomycetaceae_2 - ps25
Saccharomycetaceae_3 - ps26
Kaz./Nau./Saccharomyces - ps27
Saccharomyces - ps28
Arboricola_clade - ps29
Cerevisiae_clade - ps30
Saccharomyces_cerevisiae - ps31
Phylostratum

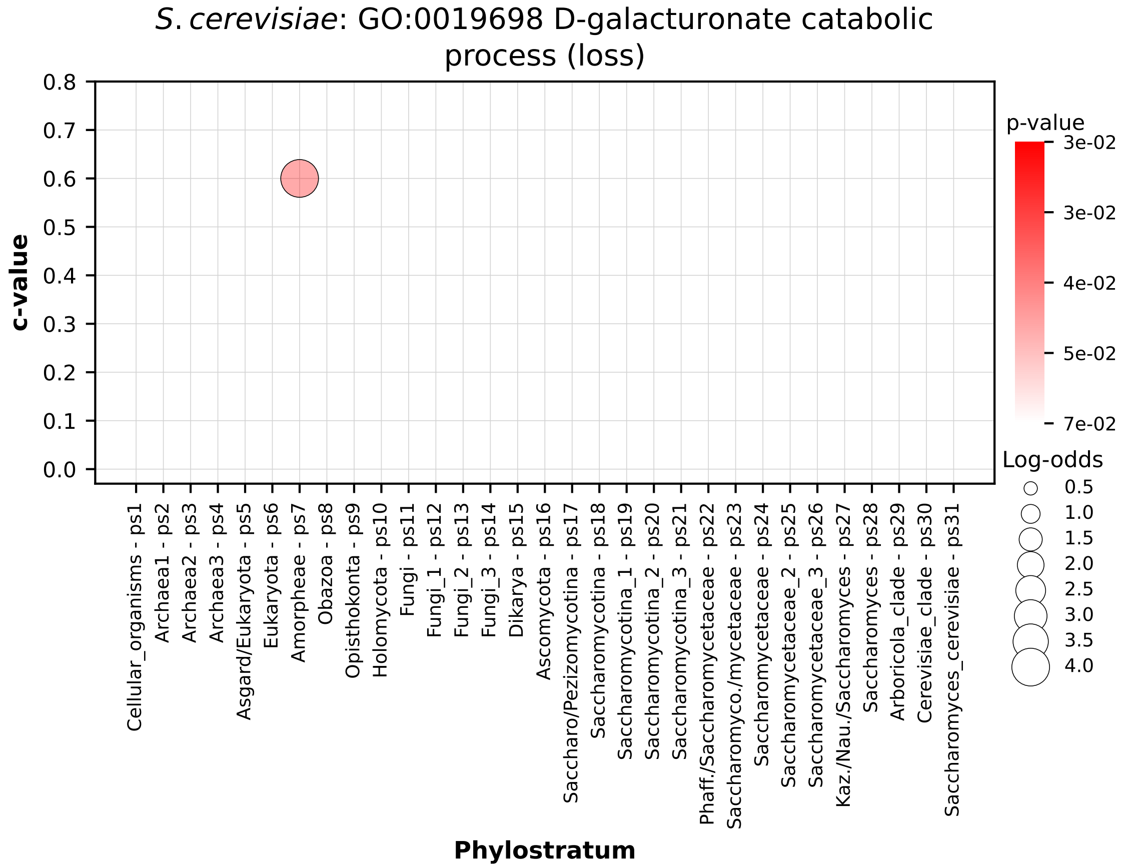

S. cerevisiae: GO:0019698 D-galacturonate catabolic process (loss)
c-value
0.8
0.7
0.6
0.5
0.4
0.3
0.2
0.1
0.0
p-value
3e-02
3e-02
4e-02
5e-02
7e-02
Log-odds
0.5
1.0
1.5
2.0
2.5
3.0
3.5
4.0
Cellular_organisms - ps1
Archaea1 - ps2
Archaea2 - ps3
Archaea3 - ps4
Asgard/Eukaryota - ps5
Eukaryota - ps6
Amorpheae - ps7
Obazoa - ps8
Opisthokonta - ps9
Holomycota - ps10
Fungi - ps11
Fungi_1 - ps12
Fungi_2 - ps13
Fungi_3 - ps14
Dikarya - ps15
Ascomycota - ps16
Saccharo/Pezizomycotina - ps17
Saccharomycotina - ps18
Saccharomycotina_1 - ps19
Saccharomycotina_2 - ps20
Saccharomycotina_3 - ps21
Phaff./Saccharomycetaceae - ps22
Saccharomyco./mycetaceae - ps23
Saccharomycetaceae - ps24
Saccharomycetaceae_2 - ps25
Saccharomycetaceae_3 - ps26
Kaz./Nau./Saccharomyces - ps27
Saccharomyces - ps28
Arboricola_clade - ps29
Cerevisiae_clade - ps30
Saccharomyces_cerevisiae - ps31
Phylostratum

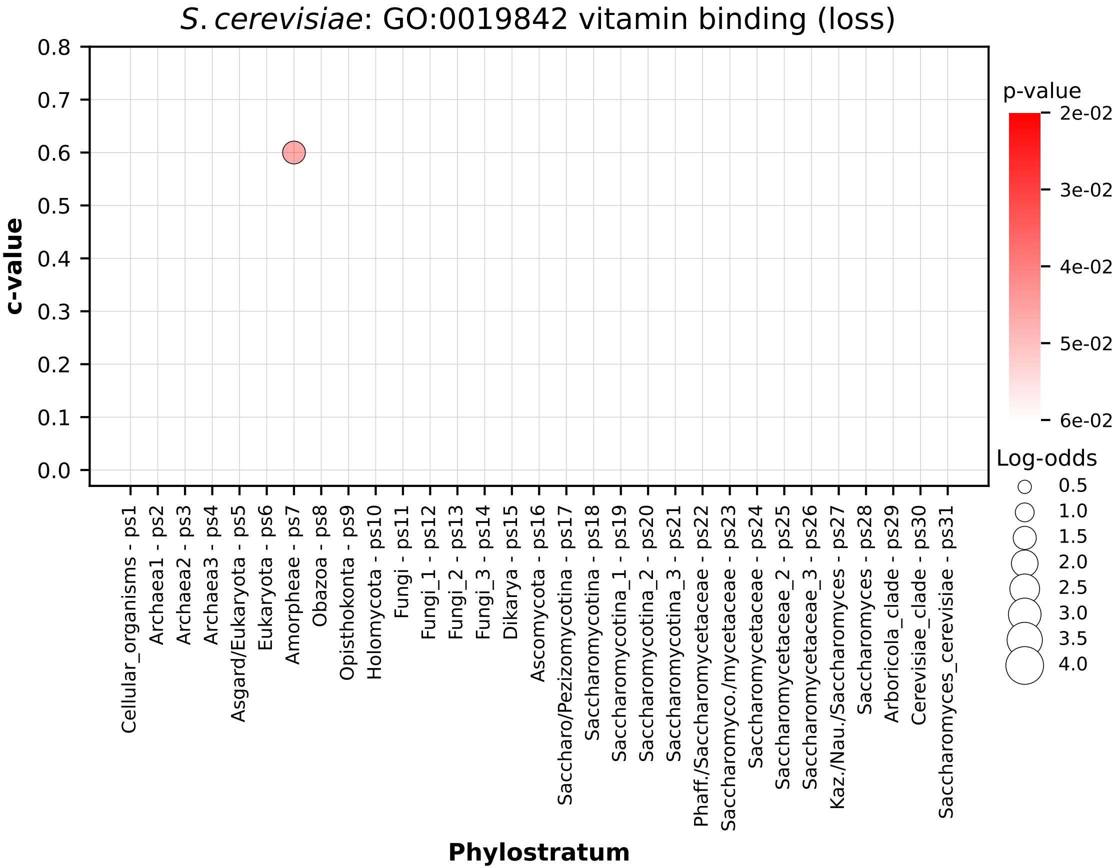
S. cerevisiae: GO:0019842 vitamin binding (loss)
c-value
0.8
0.7
0.6
0.5
0.4
0.3
0.2
0.1
0.0
Phylostratum
Cellular_organisms - ps1
Archaea1 - ps2
Archaea2 - ps3
Archaea3 - ps4
Asgard/Eukaryota - ps5
Eukaryota - ps6
Amorpheae - ps7
Obazoa - ps8
Opisthokonta - ps9
Holomycota - ps10
Fungi - ps11
Fungi_1 - ps12
Fungi_2 - ps13
Fungi_3 - ps14
Dikarya - ps15
Ascomycota - ps16
Saccharo/Pezizomycotina - ps17
Saccharomycotina - ps18
Saccharomycotina_1 - ps19
Saccharomycotina_2 - ps20
Saccharomycotina_3 - ps21
Phaff./Saccharomycetaceae - ps22
Saccharomyco./mycetaceae - ps23
Saccharomycetaceae - ps24
Saccharomycetaceae_2 - ps25
Saccharomycetaceae_3 - ps26
Kaz./Nau./Saccharomyces - ps27
Saccharomyces - ps28
Arboricola_clade - ps29
Cerevisiae_clade - ps30
Saccharomyces_cerevisiae - ps31
p-value
2e-02
3e-02
4e-02
5e-02
6e-02
Log-odds
0.5
1.0
1.5
2.0
2.5
3.0
3.5
4.0

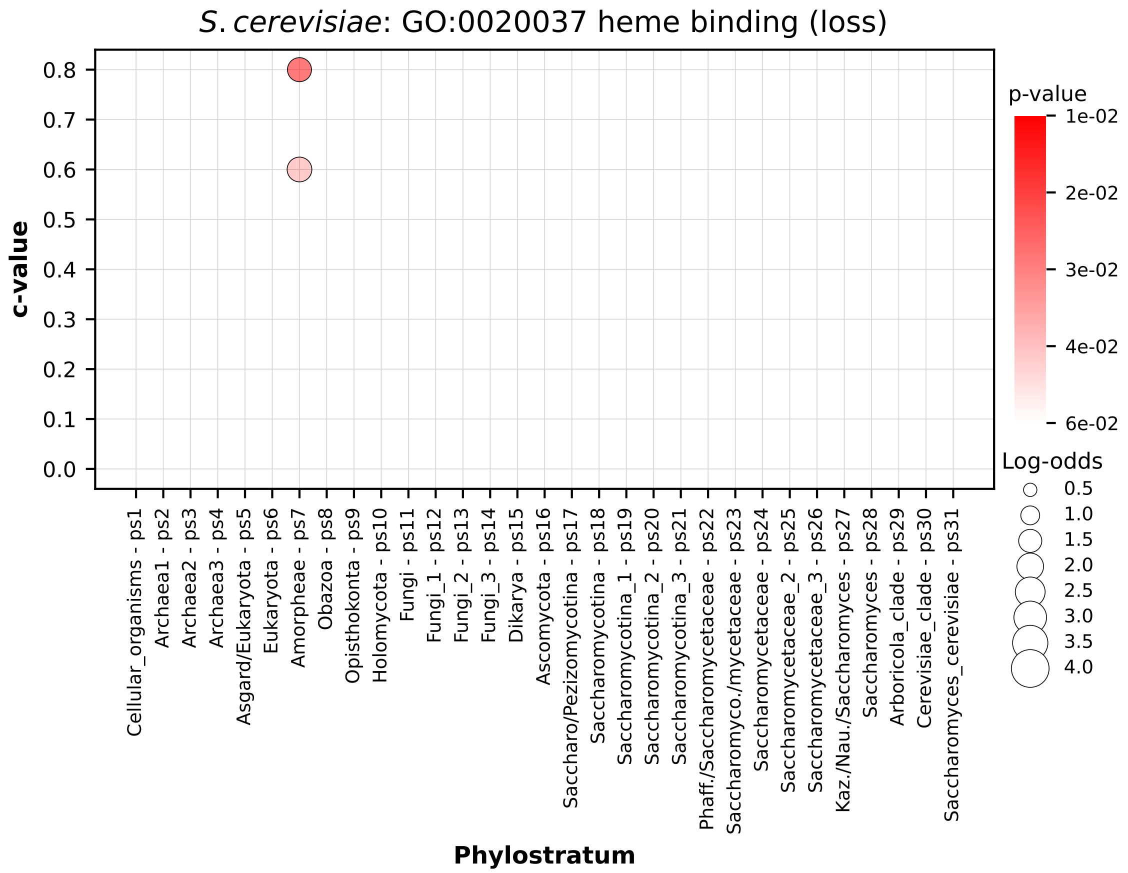

S. cerevisiae: GO:0020037 heme binding (loss)
c-value
0.0
0.1
0.2
0.3
0.4
0.5
0.6
0.7
0.8
p-value
1e-02
2e-02
3e-02
4e-02
6e-02
Log-odds
0.5
1.0
1.5
2.0
2.5
3.0
3.5
4.0
Cellular_organisms - ps1
Archaea1 - ps2
Archaea2 - ps3
Archaea3 - ps4
Asgard/Eukaryota - ps5
Eukaryota - ps6
Amorpheae - ps7
Obazoa - ps8
Opisthokonta - ps9
Holomycota - ps10
Fungi - ps11
Fungi_1 - ps12
Fungi_2 - ps13
Fungi_3 - ps14
Dikarya - ps15
Ascomycota - ps16
Saccharo/Pezizomycotina - ps17
Saccharomycotina - ps18
Saccharomycotina_1 - ps19
Saccharomycotina_2 - ps20
Saccharomycotina_3 - ps21
Phaff./Saccharomycetaceae - ps22
Saccharomyco./mycetaceae - ps23
Saccharomycetaceae - ps24
Saccharomycetaceae_2 - ps25
Saccharomycetaceae_3 - ps26
Kaz./Nau./Saccharomyces - ps27
Saccharomyces - ps28
Arboricola_clade - ps29
Cerevisiae_clade - ps30
Saccharomyces_cerevisiae - ps31
Phylostratum

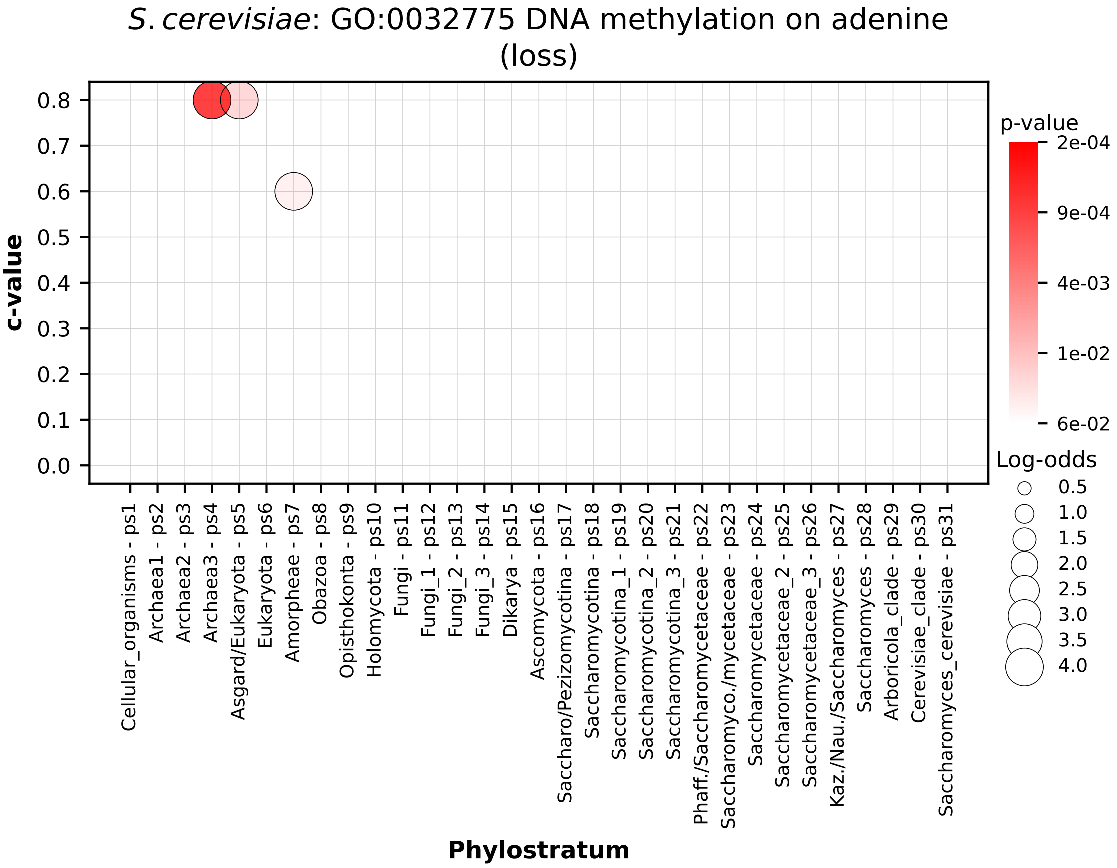
S. cerevisiae: GO:0032775 DNA methylation on adenine
(loss)
c-value
0.8
0.7
0.6
0.5
0.4
0.3
0.2
0.1
0.0
p-value
2e-04
9e-04
4e-03
1e-02
6e-02
Log-odds
0.5
1.0
1.5
2.0
2.5
3.0
3.5
4.0
Cellular_organisms - ps1
Archaea1 - ps2
Archaea2 - ps3
Archaea3 - ps4
Asgard/Eukaryota - ps5
Eukaryota - ps6
Amorpheae - ps7
Obazoa - ps8
Opisthokonta - ps9
Holomycota - ps10
Fungi - ps11
Fungi_1 - ps12
Fungi_2 - ps13
Fungi_3 - ps14
Dikarya - ps15
Ascomycota - ps16
Saccharo/Pezizomycotina - ps17
Saccharomycotina - ps18
Saccharomycotina_1 - ps19
Saccharomycotina_2 - ps20
Saccharomycotina_3 - ps21
Phaff./Saccharomycetaceae - ps22
Saccharomyco./mycetaceae - ps23
Saccharomycetaceae - ps24
Saccharomycetaceae_2 - ps25
Saccharomycetaceae_3 - ps26
Kaz./Nau./Saccharomyces - ps27
Saccharomyces - ps28
Arboricola_clade - ps29
Cerevisiae_clade - ps30
Saccharomyces_cerevisiae - ps31
Phylostratum

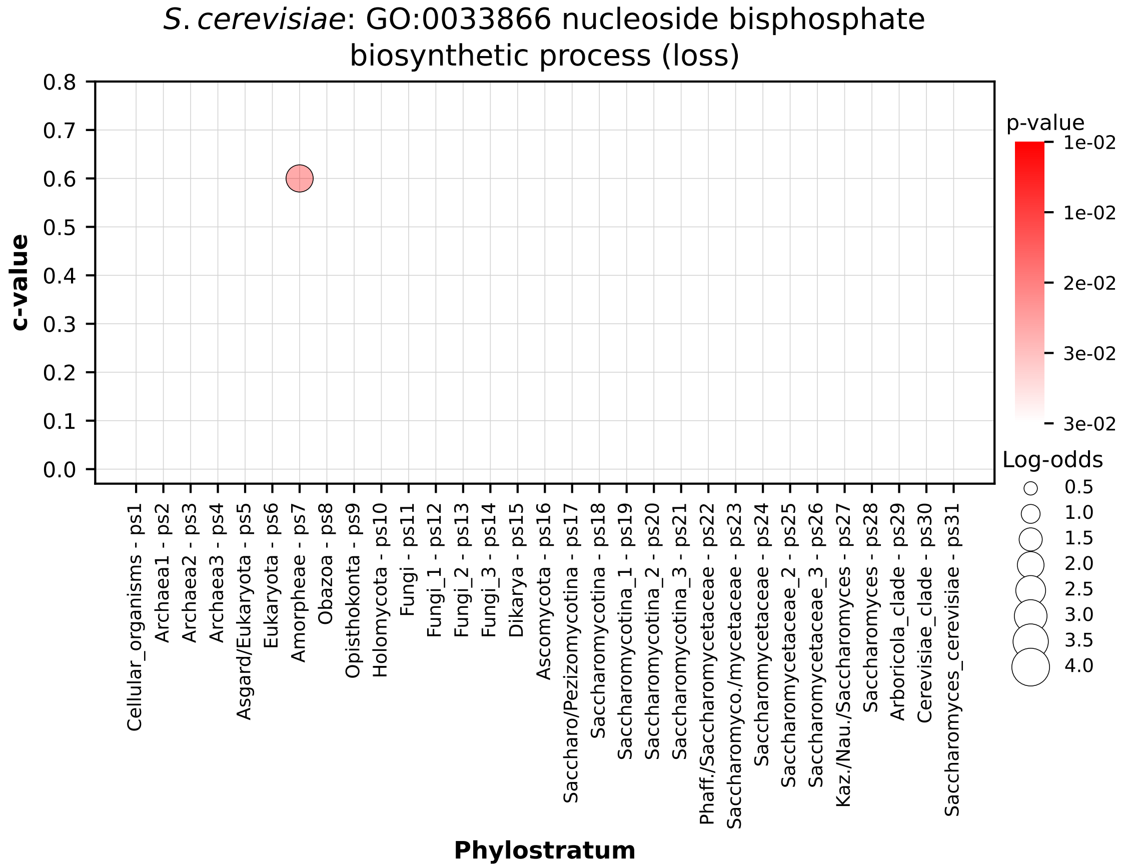
S. cerevisiae: GO:0033866 nucleoside bisphosphate biosynthetic process (loss)
c-value
p-value
1e-02
1e-02
2e-02
3e-02
3e-02
Log-odds
0.5
1.0
1.5
2.0
2.5
3.0
3.5
4.0
Cellular_organisms - ps1
Archaea1 - ps2
Archaea2 - ps3
Archaea3 - ps4
Asgard/Eukaryota - ps5
Eukaryota - ps6
Amorpheae - ps7
Obazoa - ps8
Opisthokonta - ps9
Holomycota - ps10
Fungi - ps11
Fungi_1 - ps12
Fungi_2 - ps13
Fungi_3 - ps14
Dikarya - ps15
Ascomycota - ps16
Saccharo/Pezizomycotina - ps17
Saccharomycotina - ps18
Saccharomycotina_1 - ps19
Saccharomycotina_2 - ps20
Saccharomycotina_3 - ps21
Phaff./Saccharomycetaceae - ps22
Saccharomyco./mycetaceae - ps23
Saccharomycetaceae - ps24
Saccharomycetaceae_2 - ps25
Saccharomycetaceae_3 - ps26
Kaz./Nau./Saccharomyces - ps27
Saccharomyces - ps28
Arboricola_clade - ps29
Cerevisiae_clade - ps30
Saccharomyces_cerevisiae - ps31
Phylostratum

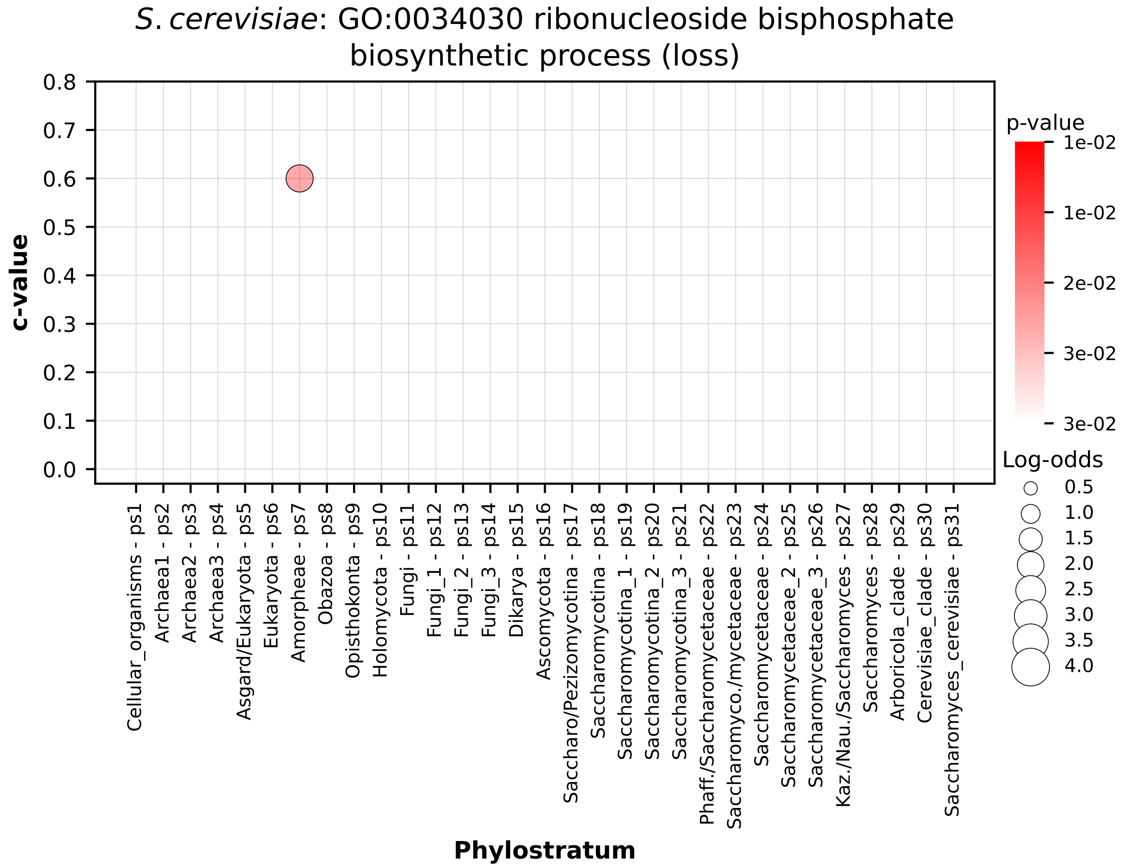

S. cerevisiae: GO:0034030 ribonucleoside bisphosphate biosynthetic process (loss)
c-value
0.8
0.7
0.6
0.5
0.4
0.3
0.2
0.1
0.0
p-value
1e-02
1e-02
2e-02
3e-02
3e-02
Log-odds
0.5
1.0
1.5
2.0
2.5
3.0
3.5
4.0
Cellular_organisms - ps1
Archaea1 - ps2
Archaea2 - ps3
Archaea3 - ps4
Asgard/Eukaryota - ps5
Eukaryota - ps6
Amorpheae - ps7
Obazoa - ps8
Opisthokonta - ps9
Holomycota - ps10
Fungi - ps11
Fungi_1 - ps12
Fungi_2 - ps13
Fungi_3 - ps14
Dikarya - ps15
Ascomycota - ps16
Saccharo/Pezizomycotina - ps17
Saccharomycotina - ps18
Saccharomycotina_1 - ps19
Saccharomycotina_2 - ps20
Saccharomycotina_3 - ps21
Phaff./Saccharomycetaceae - ps22
Saccharomyco./mycetaceae - ps23
Saccharomycetaceae - ps24
Saccharomycetaceae_2 - ps25
Saccharomycetaceae_3 - ps26
Kaz./Nau./Saccharomyces - ps27
Saccharomyces - ps28
Arboricola_clade - ps29
Cerevisiae_clade - ps30
Saccharomyces_cerevisiae - ps31
Phylostratum

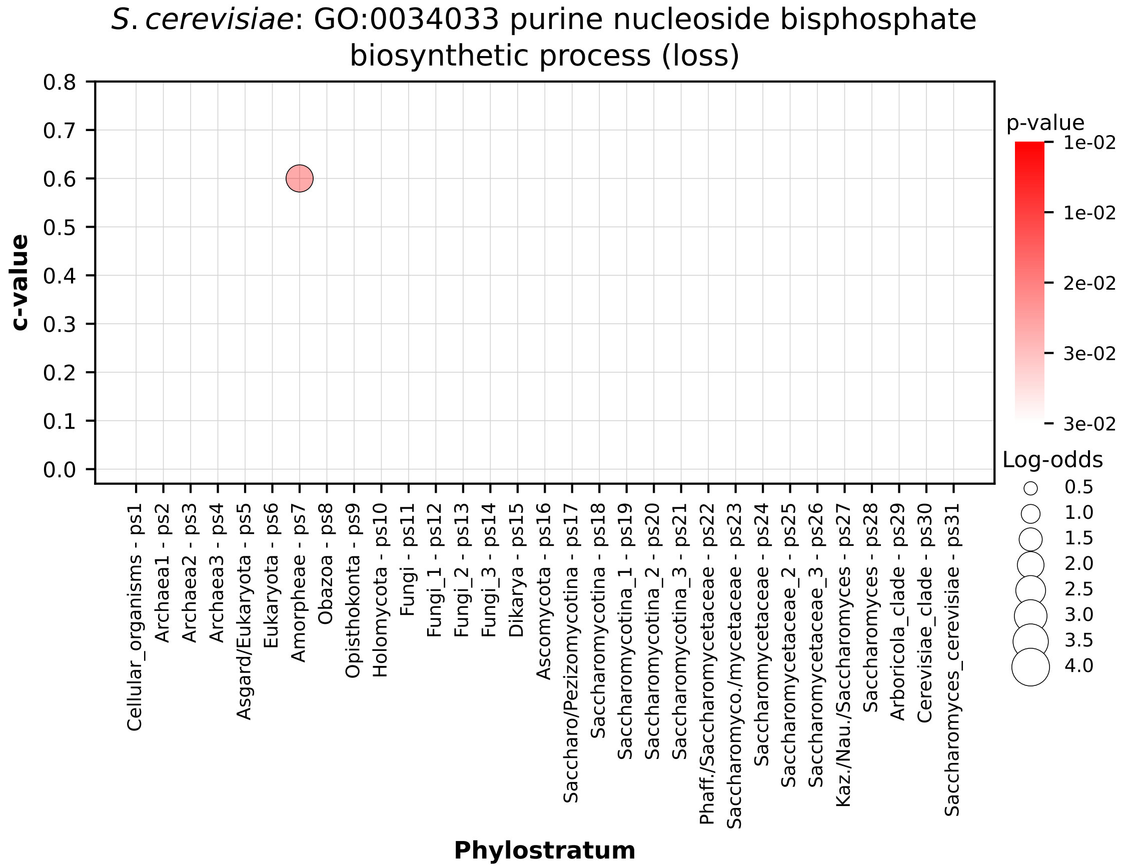

S. cerevisiae: GO:0034033 purine nucleoside bisphosphate biosynthetic process (loss)
c-value
0.8
0.7
0.6
0.5
0.4
0.3
0.2
0.1
0.0
p-value
1e-02
1e-02
2e-02
3e-02
3e-02
Log-odds
0.5
1.0
1.5
2.0
2.5
3.0
3.5
4.0
Cellular_organisms - ps1
Archaea1 - ps2
Archaea2 - ps3
Archaea3 - ps4
Asgard/Eukaryota - ps5
Eukaryota - ps6
Amorpheae - ps7
Obazoa - ps8
Opisthokonta - ps9
Holomycota - ps10
Fungi - ps11
Fungi_1 - ps12
Fungi_2 - ps13
Fungi_3 - ps14
Dikarya - ps15
Ascomycota - ps16
Saccharo/Pezizomycotina - ps17
Saccharomycotina - ps18
Saccharomycotina_1 - ps19
Saccharomycotina_2 - ps20
Saccharomycotina_3 - ps21
Phaff./Saccharomycetaceae - ps22
Saccharomyco./mycetaceae - ps23
Saccharomycetaceae - ps24
Saccharomycetaceae_2 - ps25
Saccharomycetaceae_3 - ps26
Kaz./Nau./Saccharomyces - ps27
Saccharomyces - ps28
Arboricola_clade - ps29
Cerevisiae_clade - ps30
Saccharomyces_cerevisiae - ps31
Phylostratum

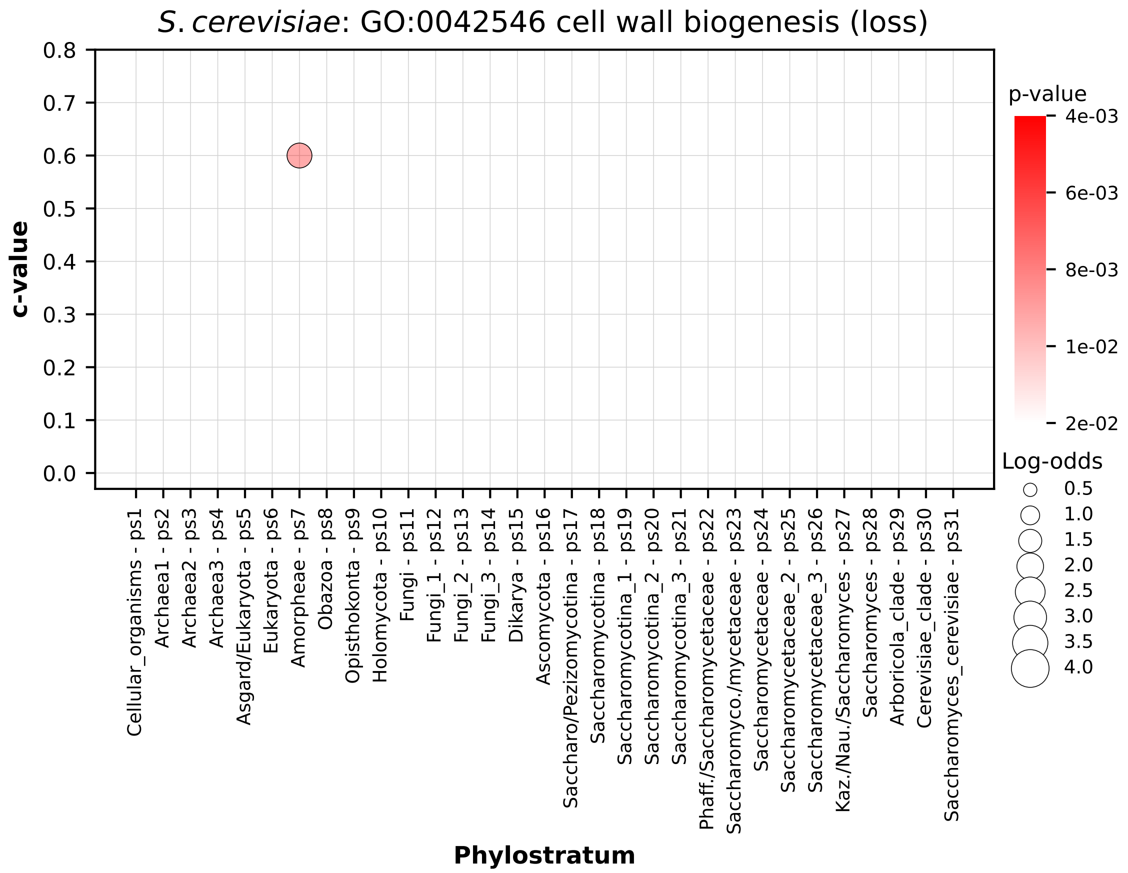
S. cerevisiae: GO:0042546 cell wall biogenesis (loss)
c-value
0.8
0.7
0.6
0.5
0.4
0.3
0.2
0.1
0.0
p-value
4e-03
6e-03
8e-03
1e-02
2e-02
Log-odds
0.5
1.0
1.5
2.0
2.5
3.0
3.5
4.0
Cellular_organisms - ps1
Archaea1 - ps2
Archaea2 - ps3
Archaea3 - ps4
Asgard/Eukaryota - ps5
Eukaryota - ps6
Amorpheae - ps7
Obazoa - ps8
Opisthokonta - ps9
Holomycota - ps10
Fungi - ps11
Fungi_1 - ps12
Fungi_2 - ps13
Fungi_3 - ps14
Dikarya - ps15
Ascomycota - ps16
Saccharo/Pezizomycotina - ps17
Saccharomycotina - ps18
Saccharomycotina_1 - ps19
Saccharomycotina_2 - ps20
Saccharomycotina_3 - ps21
Phaff./Saccharomycetaceae - ps22
Saccharomyco./mycetaceae - ps23
Saccharomycetaceae - ps24
Saccharomycetaceae_2 - ps25
Saccharomycetaceae_3 - ps26
Kaz./Nau./Saccharomyces - ps27
Saccharomyces - ps28
Arboricola_clade - ps29
Cerevisiae_clade - ps30
Saccharomyces_cerevisiae - ps31
Phylostratum

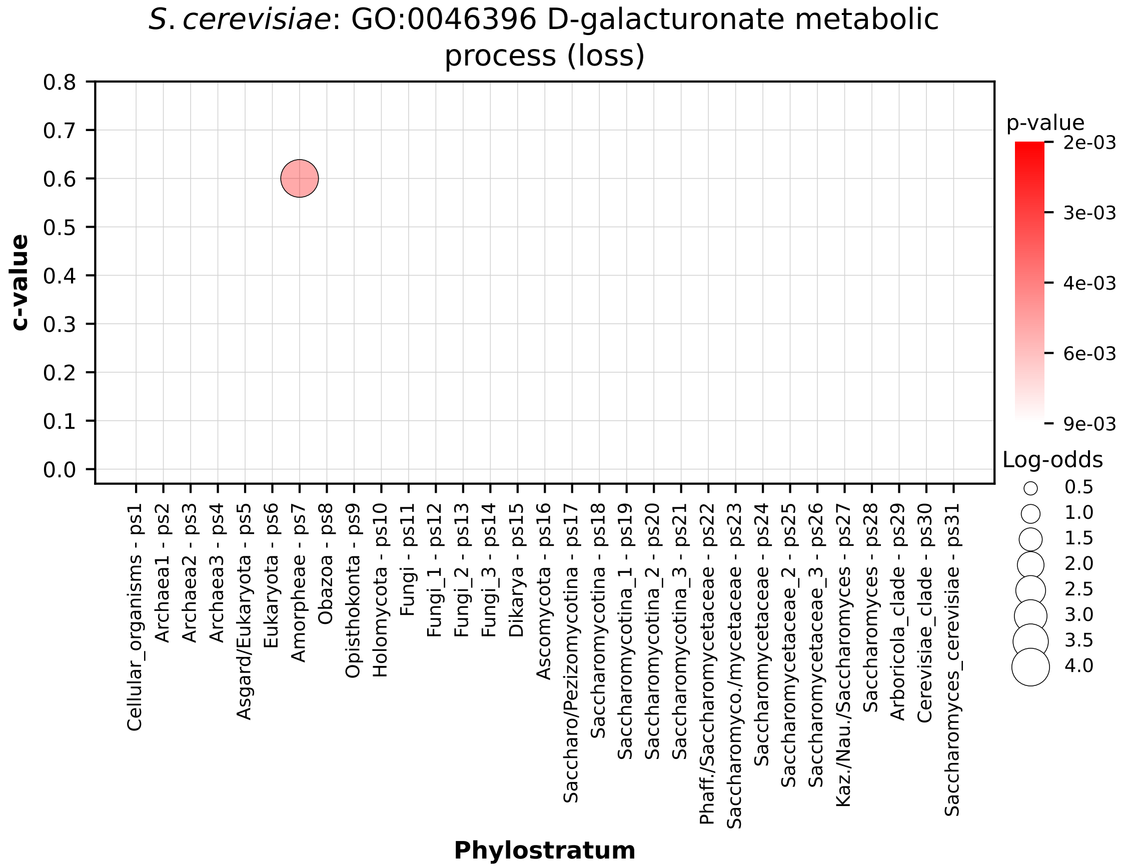

S. cerevisiae: GO:0046396 D-galacturonate metabolic process (loss)
c-value
0.8
0.7
0.6
0.5
0.4
0.3
0.2
0.1
0.0
Phylostratum
Cellular_organisms - ps1
Archaea1 - ps2
Archaea2 - ps3
Archaea3 - ps4
Asgard/Eukaryota - ps5
Eukaryota - ps6
Amorpheae - ps7
Obazoa - ps8
Opisthokonta - ps9
Holomycota - ps10
Fungi - ps11
Fungi_1 - ps12
Fungi_2 - ps13
Fungi_3 - ps14
Dikarya - ps15
Ascomycota - ps16
Saccharo/Pezizomycotina - ps17
Saccharomycotina - ps18
Saccharomycotina_1 - ps19
Saccharomycotina_2 - ps20
Saccharomycotina_3 - ps21
Phaff./Saccharomycetaceae - ps22
Saccharomyco./mycetaceae - ps23
Saccharomycetaceae - ps24
Saccharomycetaceae_2 - ps25
Saccharomycetaceae_3 - ps26
Kaz./Nau./Saccharomyces - ps27
Saccharomyces - ps28
Arboricola_clade - ps29
Cerevisiae_clade - ps30
Saccharomyces_cerevisiae - ps31
p-value
2e-03
3e-03
4e-03
6e-03
9e-03
Log-odds
0.5
1.0
1.5
2.0
2.5
3.0
3.5
4.0

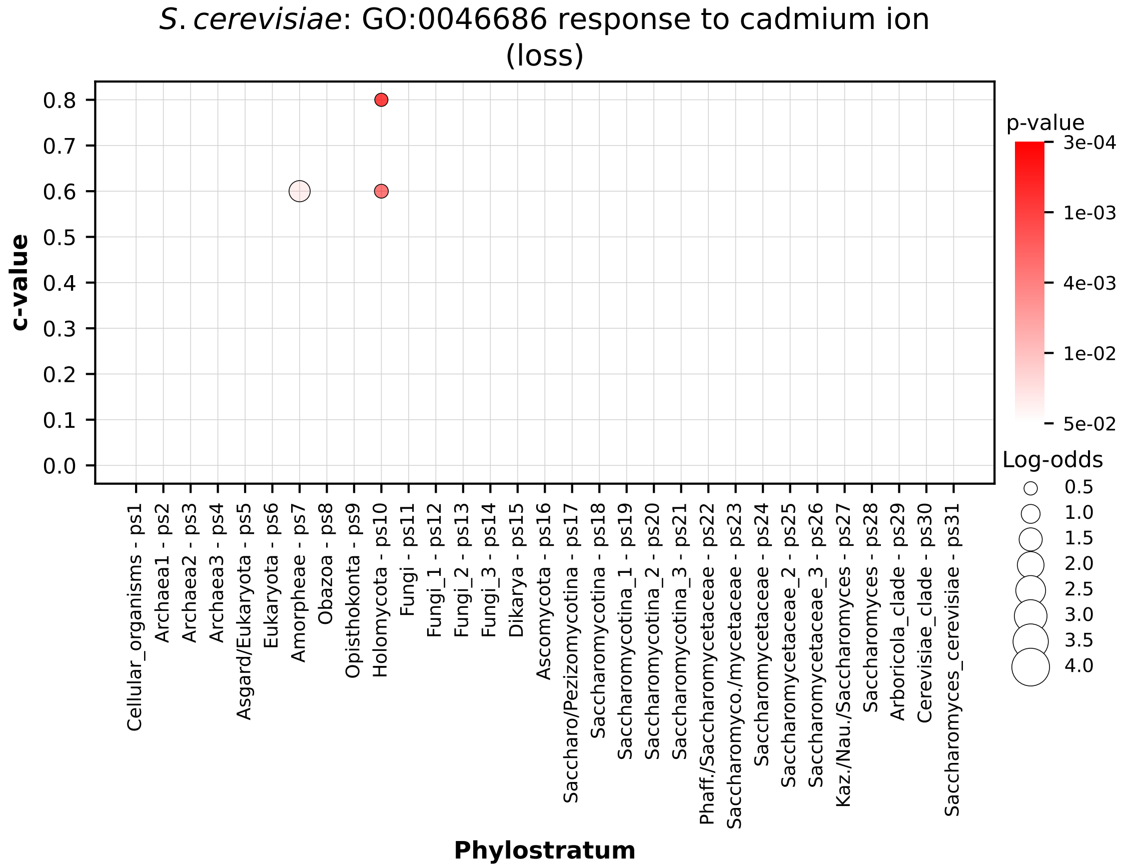

S. cerevisiae: GO:0046686 response to cadmium ion
(loss)
c-value
0.0
0.1
0.2
0.3
0.4
0.5
0.6
0.7
0.8
p-value
3e-04
1e-03
4e-03
1e-02
5e-02
Log-odds
0.5
1.0
1.5
2.0
2.5
3.0
3.5
4.0
Cellular_organisms - ps1
Archaea1 - ps2
Archaea2 - ps3
Archaea3 - ps4
Asgard/Eukaryota - ps5
Eukaryota - ps6
Amorpheae - ps7
Obazoa - ps8
Opisthokonta - ps9
Holomycota - ps10
Fungi - ps11
Fungi_1 - ps12
Fungi_2 - ps13
Fungi_3 - ps14
Dikarya - ps15
Ascomycota - ps16
Saccharo/Pezizomycotina - ps17
Saccharomycotina - ps18
Saccharomycotina_1 - ps19
Saccharomycotina_2 - ps20
Saccharomycotina_3 - ps21
Phaff./Saccharomycetaceae - ps22
Saccharomyco./mycetaceae - ps23
Saccharomycetaceae - ps24
Saccharomycetaceae_2 - ps25
Saccharomycetaceae_3 - ps26
Kaz./Nau./Saccharomyces - ps27
Saccharomyces - ps28
Arboricola_clade - ps29
Cerevisiae_clade - ps30
Saccharomyces_cerevisiae - ps31
Phylostratum

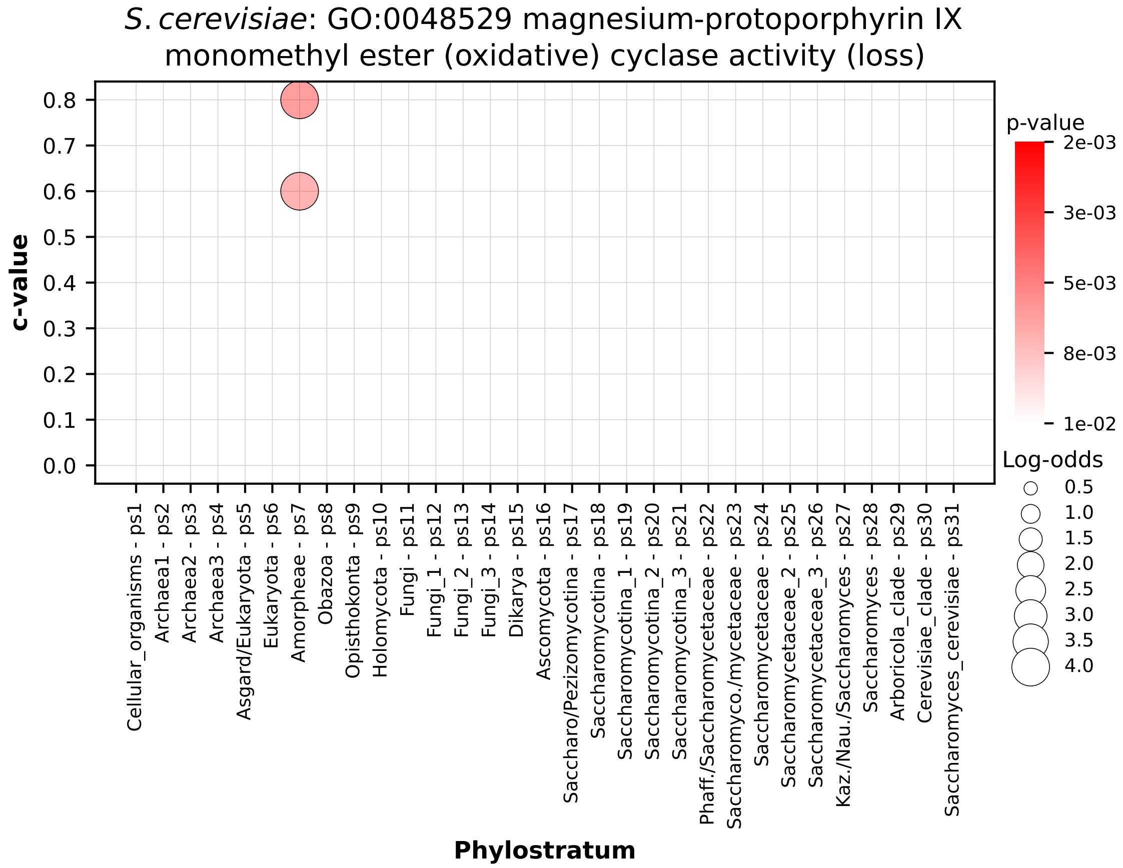

S. cerevisiae: GO:0048529 magnesium-protoporphyrin IX monomethyl ester (oxidative) cyclase activity (loss)
c-value
0.0
0.1
0.2
0.3
0.4
0.5
0.6
0.7
0.8
p-value
2e-03
3e-03
5e-03
8e-03
1e-02
Log-odds
0.5
1.0
1.5
2.0
2.5
3.0
3.5
4.0
Cellular_organisms - ps1
Archaea1 - ps2
Archaea2 - ps3
Archaea3 - ps4
Asgard/Eukaryota - ps5
Eukaryota - ps6
Amorpheae - ps7
Obazoa - ps8
Opisthokonta - ps9
Holomycota - ps10
Fungi - ps11
Fungi_1 - ps12
Fungi_2 - ps13
Fungi_3 - ps14
Dikarya - ps15
Ascomycota - ps16
Saccharo/Pezizomycotina - ps17
Saccharomycotina - ps18
Saccharomycotina_1 - ps19
Saccharomycotina_2 - ps20
Saccharomycotina_3 - ps21
Phaff./Saccharomycetaceae - ps22
Saccharomyco./mycetaceae - ps23
Saccharomycetaceae - ps24
Saccharomycetaceae_2 - ps25
Saccharomycetaceae_3 - ps26
Kaz./Nau./Saccharomyces - ps27
Saccharomyces - ps28
Arboricola_clade - ps29
Cerevisiae_clade - ps30
Saccharomyces_cerevisiae - ps31
Phylostratum

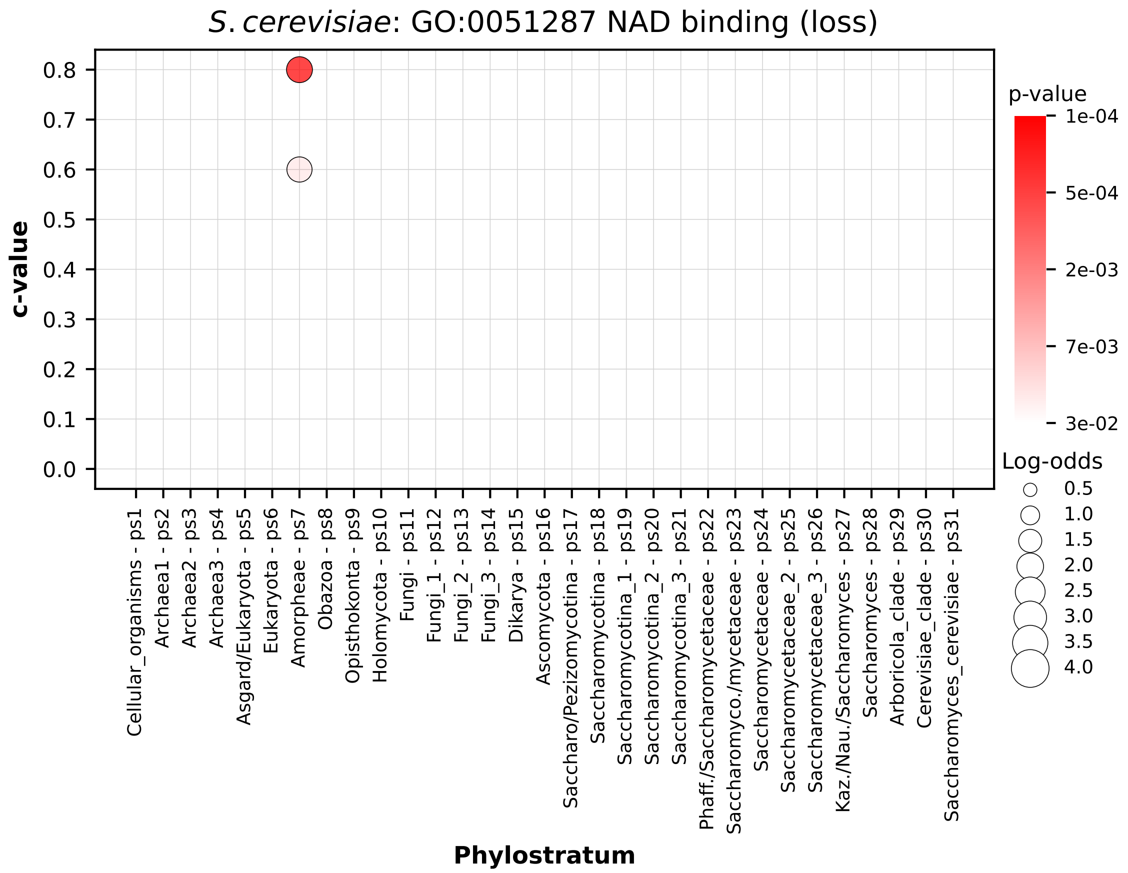

S. cerevisiae: GO:0051287 NAD binding (loss)
c-value
0.8
0.7
0.6
0.5
0.4
0.3
0.2
0.1
0.0
p-value
1e-04
5e-04
2e-03
7e-03
3e-02
Log-odds
0.5
1.0
1.5
2.0
2.5
3.0
3.5
4.0
Cellular_organisms - ps1
Archaea1 - ps2
Archaea2 - ps3
Archaea3 - ps4
Asgard/Eukaryota - ps5
Eukaryota - ps6
Amorpheae - ps7
Obazoa - ps8
Opisthokonta - ps9
Holomycota - ps10
Fungi - ps11
Fungi_1 - ps12
Fungi_2 - ps13
Fungi_3 - ps14
Dikarya - ps15
Ascomycota - ps16
Saccharo/Pezizomycotina - ps17
Saccharomycotina - ps18
Saccharomycotina_1 - ps19
Saccharomycotina_2 - ps20
Saccharomycotina_3 - ps21
Phaff./Saccharomycetaceae - ps22
Saccharomyco./mycetaceae - ps23
Saccharomycetaceae - ps24
Saccharomycetaceae_2 - ps25
Saccharomycetaceae_3 - ps26
Kaz./Nau./Saccharomyces - ps27
Saccharomyces - ps28
Arboricola_clade - ps29
Cerevisiae_clade - ps30
Saccharomyces_cerevisiae - ps31
Phylostratum

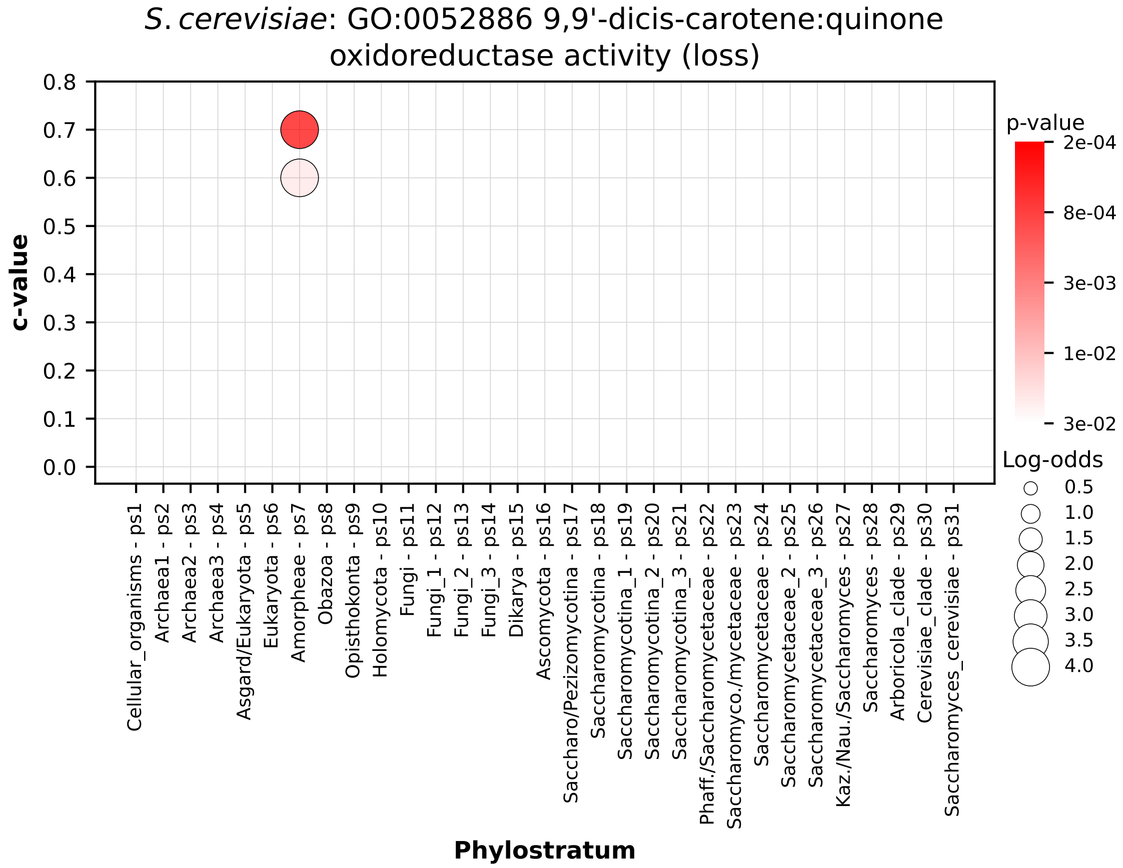

S. cerevisiae: GO:0052886 9,9'-dicis-carotene:quinone oxidoreductase activity (loss)
c-value
0.8
0.7
0.6
0.5
0.4
0.3
0.2
0.1
0.0
p-value
2e-04
8e-04
3e-03
1e-02
3e-02
Log-odds
0.5
1.0
1.5
2.0
2.5
3.0
3.5
4.0
Cellular_organisms - ps1
Archaea1 - ps2
Archaea2 - ps3
Archaea3 - ps4
Asgard/Eukaryota - ps5
Eukaryota - ps6
Amorpheae - ps7
Obazoa - ps8
Opisthokonta - ps9
Holomycota - ps10
Fungi - ps11
Fungi_1 - ps12
Fungi_2 - ps13
Fungi_3 - ps14
Dikarya - ps15
Ascomycota - ps16
Saccharo/Pezizomycotina - ps17
Saccharomycotina - ps18
Saccharomycotina_1 - ps19
Saccharomycotina_2 - ps20
Saccharomycotina_3 - ps21
Phaff./Saccharomycetaceae - ps22
Saccharomyco./mycetaceae - ps23
Saccharomycetaceae - ps24
Saccharomycetaceae_2 - ps25
Saccharomycetaceae_3 - ps26
Kaz./Nau./Saccharomyces - ps27
Saccharomyces - ps28
Arboricola_clade - ps29
Cerevisiae_clade - ps30
Saccharomyces_cerevisiae - ps31
Phylostratum

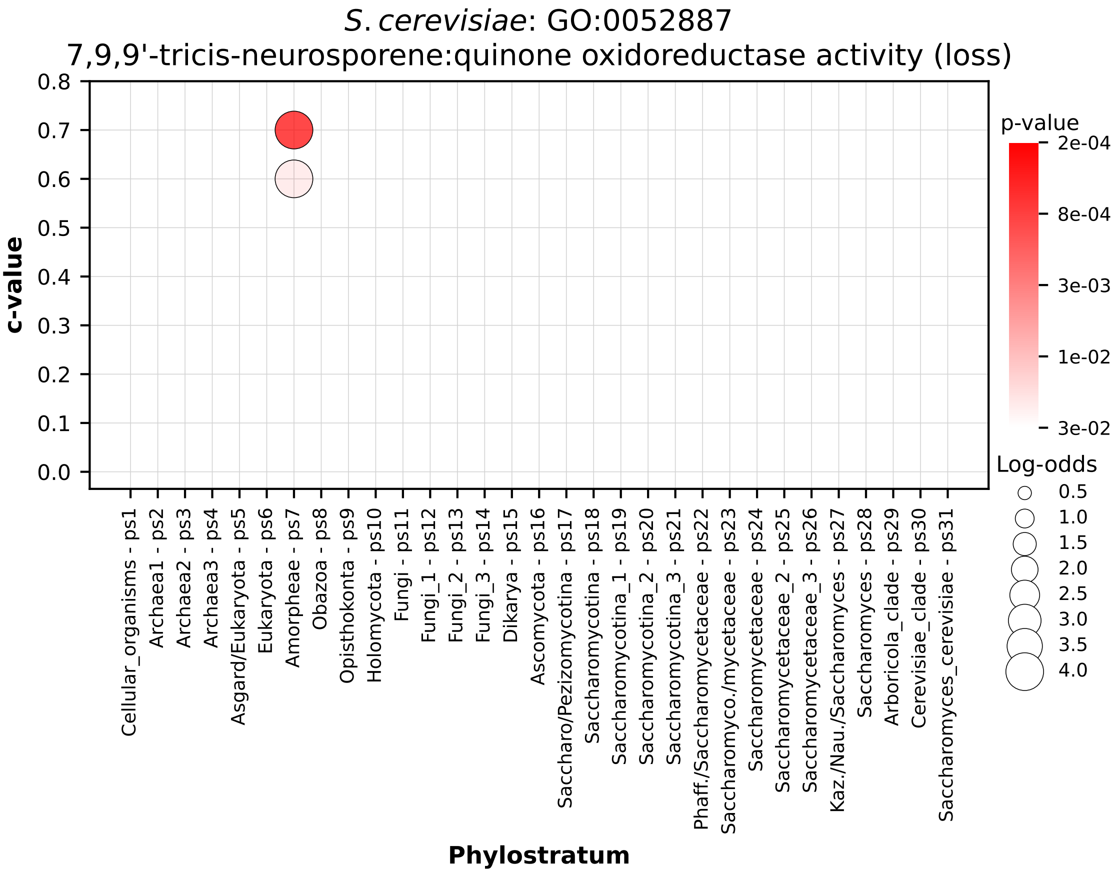

S. cerevisiae: GO:0052887
7,9,9'-tricis-neurosporene:quinone oxidoreductase activity (loss)
c-value
0.8
0.7
0.6
0.5
0.4
0.3
0.2
0.1
0.0
p-value
2e-04
8e-04
3e-03
1e-02
3e-02
Log-odds
0.5
1.0
1.5
2.0
2.5
3.0
3.5
4.0
Cellular_organisms - ps1
Archaea1 - ps2
Archaea2 - ps3
Archaea3 - ps4
Asgard/Eukaryota - ps5
Eukaryota - ps6
Amorpheae - ps7
Obazoa - ps8
Opisthokonta - ps9
Holomycota - ps10
Fungi - ps11
Fungi_1 - ps12
Fungi_2 - ps13
Fungi_3 - ps14
Dikarya - ps15
Ascomycota - ps16
Saccharo/Pezizomycotina - ps17
Saccharomycotina - ps18
Saccharomycotina_1 - ps19
Saccharomycotina_2 - ps20
Saccharomycotina_3 - ps21
Phaff./Saccharomycetaceae - ps22
Saccharomyco./mycetaceae - ps23
Saccharomycetaceae - ps24
Saccharomycetaceae_2 - ps25
Saccharomycetaceae_3 - ps26
Kaz./Nau./Saccharomyces - ps27
Saccharomyces - ps28
Arboricola_clade - ps29
Cerevisiae_clade - ps30
Saccharomyces_cerevisiae - ps31
Phylostratum

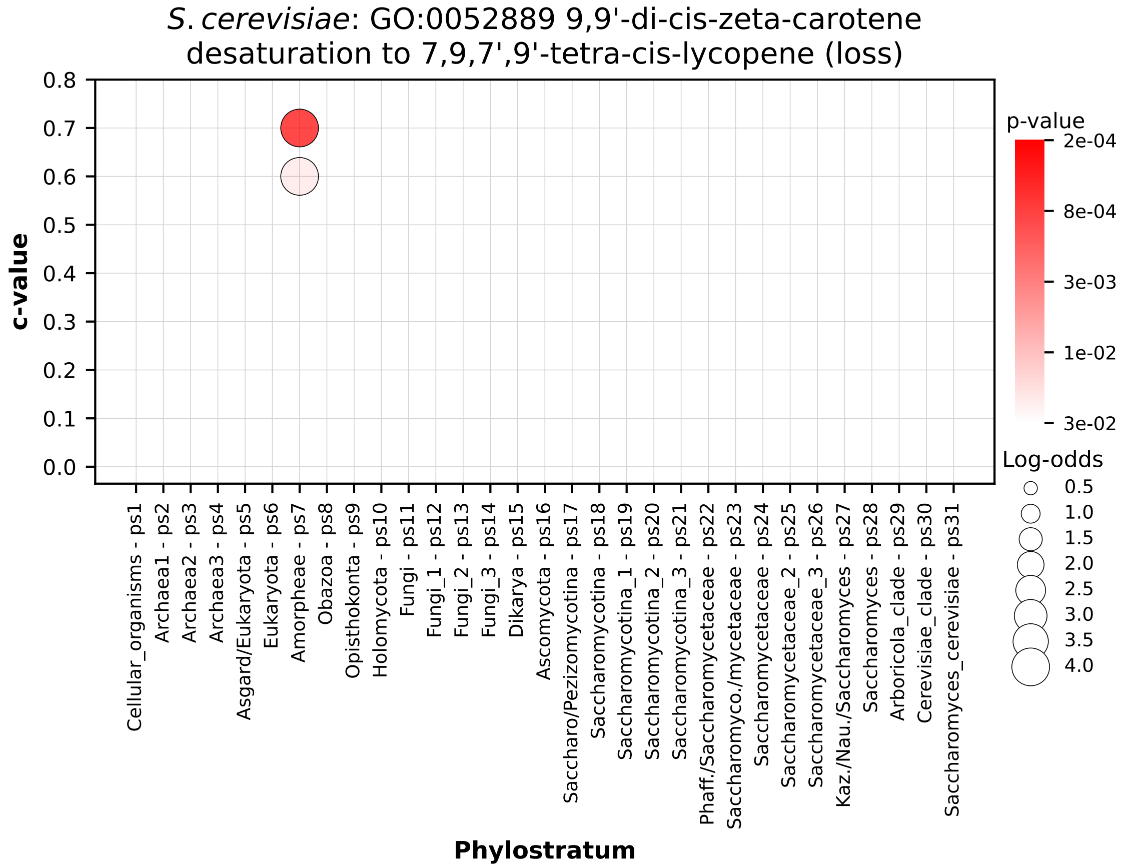

S. cerevisiae: GO:0052889 9,9'-di-cis-zeta-carotene desaturation to 7,9,7',9'-tetra-cis-lycopene (loss)
c-value
0.8
0.7
0.6
0.5
0.4
0.3
0.2
0.1
0.0
p-value
2e-04
8e-04
3e-03
1e-02
3e-02
Log-odds
0.5
1.0
1.5
2.0
2.5
3.0
3.5
4.0
Cellular_organisms - ps1
Archaea1 - ps2
Archaea2 - ps3
Archaea3 - ps4
Asgard/Eukaryota - ps5
Eukaryota - ps6
Amorpheae - ps7
Obazoa - ps8
Opisthokonta - ps9
Holomycota - ps10
Fungi - ps11
Fungi_1 - ps12
Fungi_2 - ps13
Fungi_3 - ps14
Dikarya - ps15
Ascomycota - ps16
Saccharo/Pezizomycotina - ps17
Saccharomycotina - ps18
Saccharomycotina_1 - ps19
Saccharomycotina_2 - ps20
Saccharomycotina_3 - ps21
Phaff./Saccharomycetaceae - ps22
Saccharomyco./mycetaceae - ps23
Saccharomycetaceae - ps24
Saccharomycetaceae_2 - ps25
Saccharomycetaceae_3 - ps26
Kaz./Nau./Saccharomyces - ps27
Saccharomyces - ps28
Arboricola_clade - ps29
Cerevisiae_clade - ps30
Saccharomyces_cerevisiae - ps31
Phylostratum

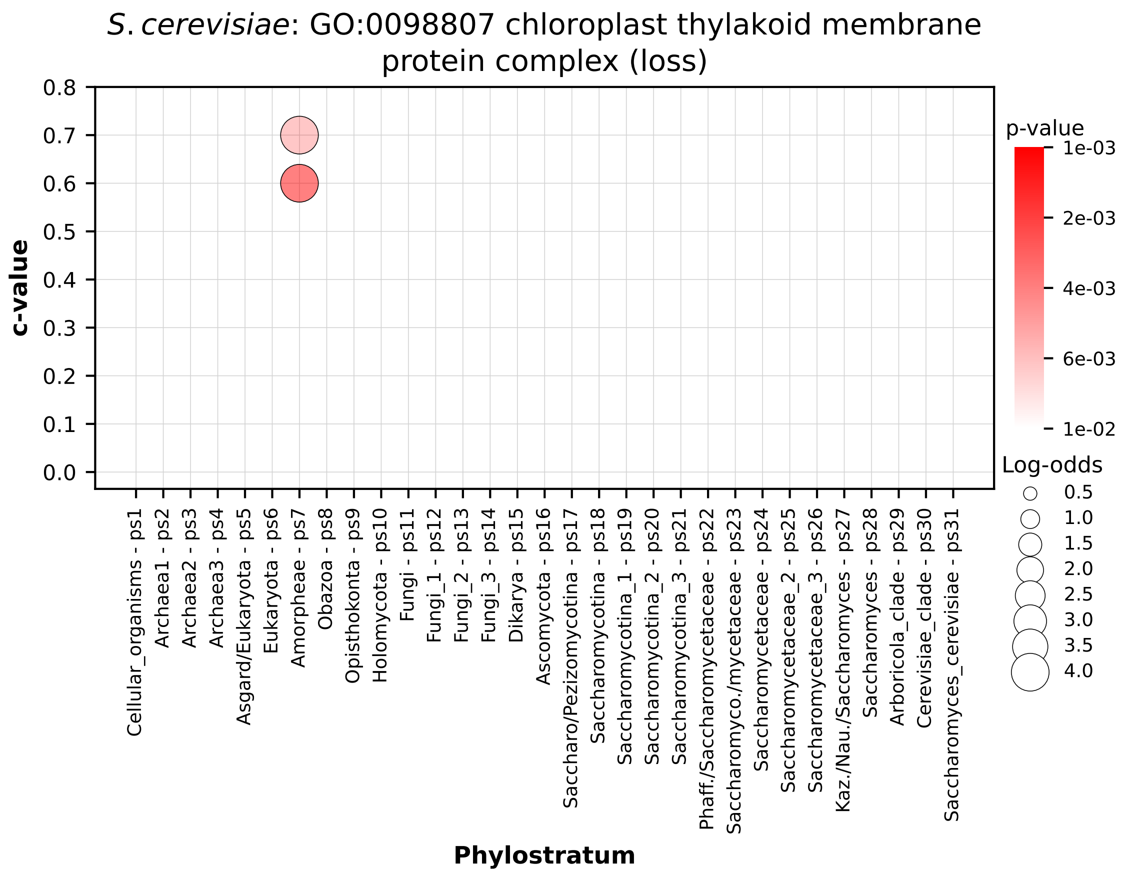

S. cerevisiae: GO:0098807 chloroplast thylakoid membrane protein complex (loss)
c-value
0.8
0.7
0.6
0.5
0.4
0.3
0.2
0.1
0.0
p-value
1e-03
2e-03
4e-03
6e-03
1e-02
Log-odds
0.5
1.0
1.5
2.0
2.5
3.0
3.5
4.0
Cellular_organisms - ps1
Archaea1 - ps2
Archaea2 - ps3
Archaea3 - ps4
Asgard/Eukaryota - ps5
Eukaryota - ps6
Amorpheae - ps7
Obazoa - ps8
Opisthokonta - ps9
Holomycota - ps10
Fungi - ps11
Fungi_1 - ps12
Fungi_2 - ps13
Fungi_3 - ps14
Dikarya - ps15
Ascomycota - ps16
Saccharo/Pezizomycotina - ps17
Saccharomycotina - ps18
Saccharomycotina_1 - ps19
Saccharomycotina_2 - ps20
Saccharomycotina_3 - ps21
Phaff./Saccharomycetaceae - ps22
Saccharomyco./mycetaceae - ps23
Saccharomycetaceae - ps24
Saccharomycetaceae_2 - ps25
Saccharomycetaceae_3 - ps26
Kaz./Nau./Saccharomyces - ps27
Saccharomyces - ps28
Arboricola_clade - ps29
Cerevisiae_clade - ps30
Saccharomyces_cerevisiae - ps31
Phylostratum

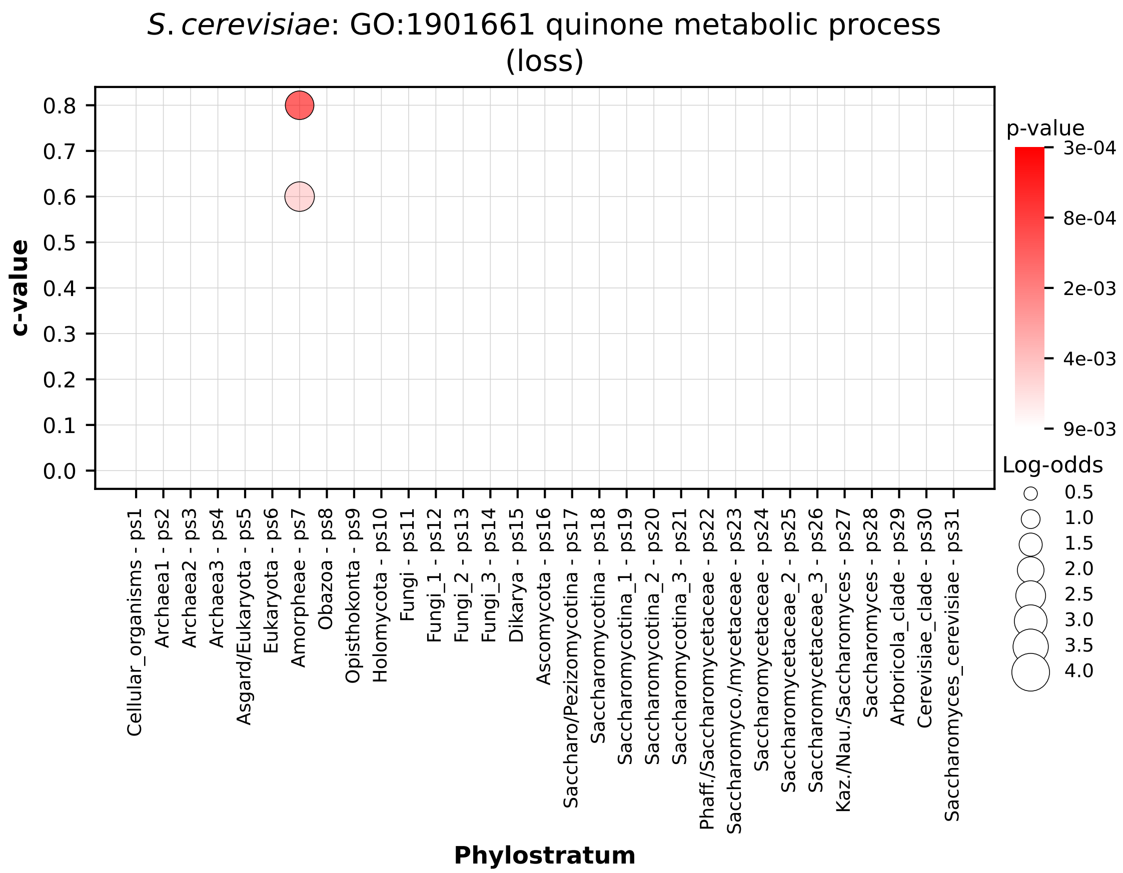

S. cerevisiae: GO:1901661 quinone metabolic process
(loss)
c-value
0.0
0.1
0.2
0.3
0.4
0.5
0.6
0.7
0.8
p-value
3e-04
8e-04
2e-03
4e-03
9e-03
Log-odds
0.5
1.0
1.5
2.0
2.5
3.0
3.5
4.0
Cellular_organisms - ps1
Archaea1 - ps2
Archaea2 - ps3
Archaea3 - ps4
Asgard/Eukaryota - ps5
Eukaryota - ps6
Amorpheae - ps7
Obazoa - ps8
Opisthokonta - ps9
Holomycota - ps10
Fungi - ps11
Fungi_1 - ps12
Fungi_2 - ps13
Fungi_3 - ps14
Dikarya - ps15
Ascomycota - ps16
Saccharo/Pezizomycotina - ps17
Saccharomycotina - ps18
Saccharomycotina_1 - ps19
Saccharomycotina_2 - ps20
Saccharomycotina_3 - ps21
Phaff./Saccharomycetaceae - ps22
Saccharomyco./mycetaceae - ps23
Saccharomycetaceae - ps24
Saccharomycetaceae_2 - ps25
Saccharomycetaceae_3 - ps26
Kaz./Nau./Saccharomyces - ps27
Saccharomyces - ps28
Arboricola_clade - ps29
Cerevisiae_clade - ps30
Saccharomyces_cerevisiae - ps31
Phylostratum

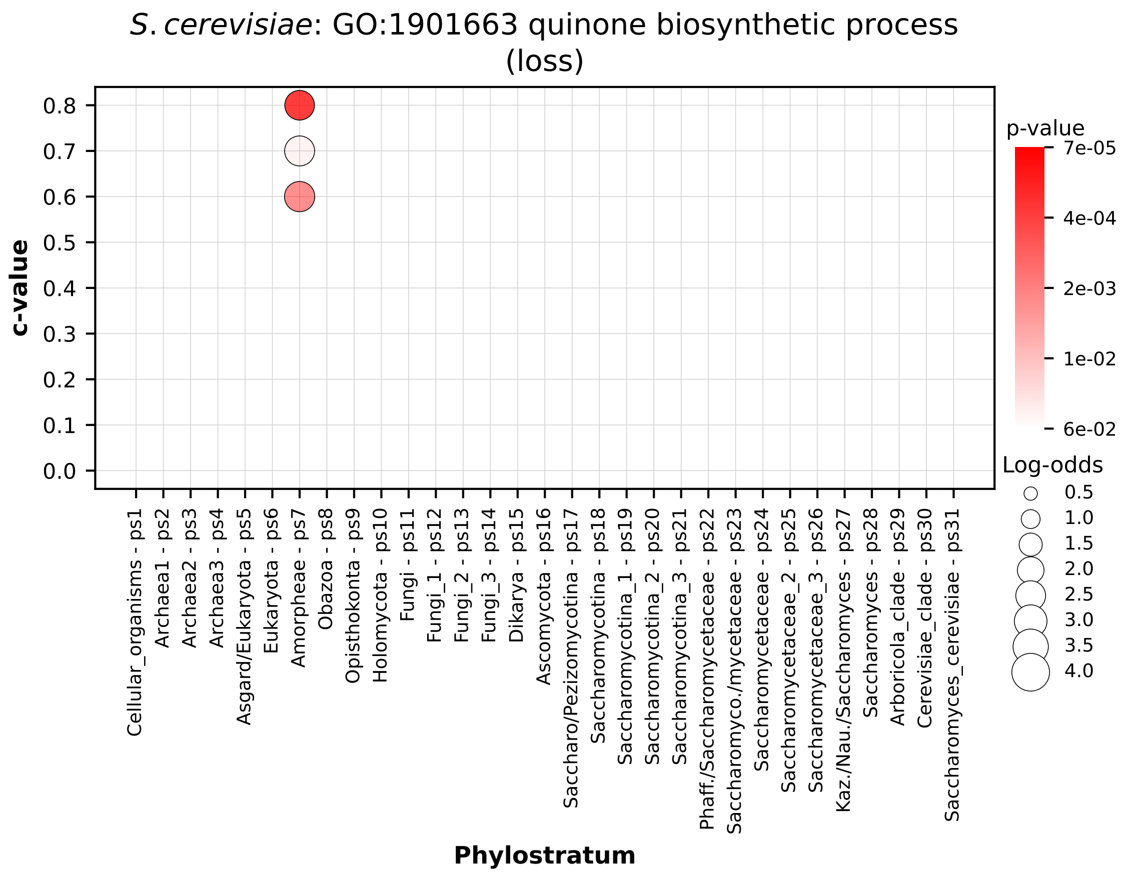

S. cerevisiae: GO:1901663 quinone biosynthetic process
(loss)
c-value
0.8
0.7
0.6
0.5
0.4
0.3
0.2
0.1
0.0
p-value
7e-05
4e-04
2e-03
1e-02
6e-02
Log-odds
0.5
1.0
1.5
2.0
2.5
3.0
3.5
4.0
Cellular_organisms - ps1
Archaea1 - ps2
Archaea2 - ps3
Archaea3 - ps4
Asgard/Eukaryota - ps5
Eukaryota - ps6
Amorpheae - ps7
Obazoa - ps8
Opisthokonta - ps9
Holomycota - ps10
Fungi - ps11
Fungi_1 - ps12
Fungi_2 - ps13
Fungi_3 - ps14
Dikarya - ps15
Ascomycota - ps16
Saccharo/Pezizomycotina - ps17
Saccharomycotina - ps18
Saccharomycotina_1 - ps19
Saccharomycotina_2 - ps20
Saccharomycotina_3 - ps21
Phaff./Saccharomycetaceae - ps22
Saccharomyco./mycetaceae - ps23
Saccharomycetaceae - ps24
Saccharomycetaceae_2 - ps25
Saccharomycetaceae_3 - ps26
Kaz./Nau./Saccharomyces - ps27
Saccharomyces - ps28
Arboricola_clade - ps29
Cerevisiae_clade - ps30
Saccharomyces_cerevisiae - ps31
Phylostratum

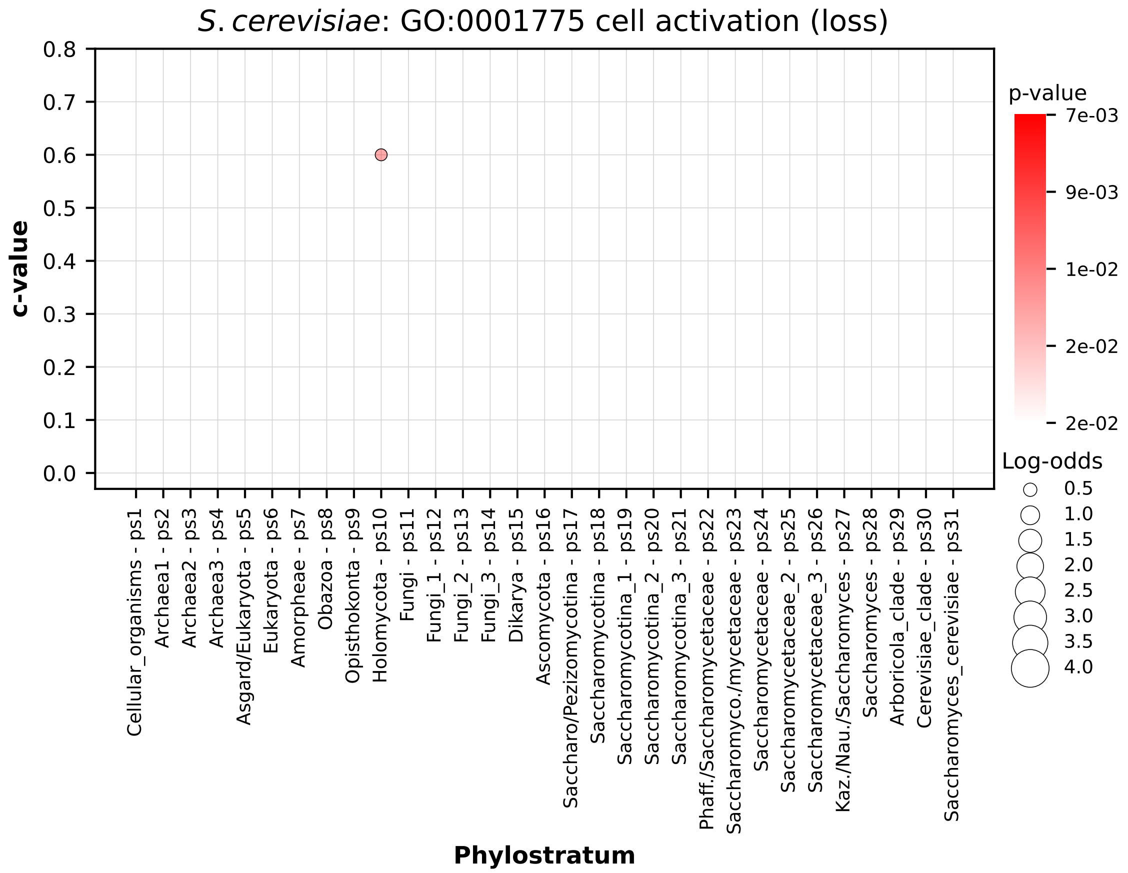

S. cerevisiae: GO:0001775 cell activation (loss)
c-value
0.8
0.7
0.6
0.5
0.4
0.3
0.2
0.1
0.0
Phylostratum
Cellular_organisms - ps1
Archaea1 - ps2
Archaea2 - ps3
Archaea3 - ps4
Asgard/Eukaryota - ps5
Eukaryota - ps6
Amorpheae - ps7
Obazoa - ps8
Opisthokonta - ps9
Holomycota - ps10
Fungi - ps11
Fungi_1 - ps12
Fungi_2 - ps13
Fungi_3 - ps14
Dikarya - ps15
Ascomycota - ps16
Saccharo/Pezizomycotina - ps17
Saccharomycotina - ps18
Saccharomycotina_1 - ps19
Saccharomycotina_2 - ps20
Saccharomycotina_3 - ps21
Phaff./Saccharomycetaceae - ps22
Saccharomyco./mycetaceae - ps23
Saccharomycetaceae - ps24
Saccharomycetaceae_2 - ps25
Saccharomycetaceae_3 - ps26
Kaz./Nau./Saccharomyces - ps27
Saccharomyces - ps28
Arboricola_clade - ps29
Cerevisiae_clade - ps30
Saccharomyces_cerevisiae - ps31
p-value
7e-03
9e-03
1e-02
2e-02
2e-02
Log-odds
0.5
1.0
1.5
2.0
2.5
3.0
3.5
4.0

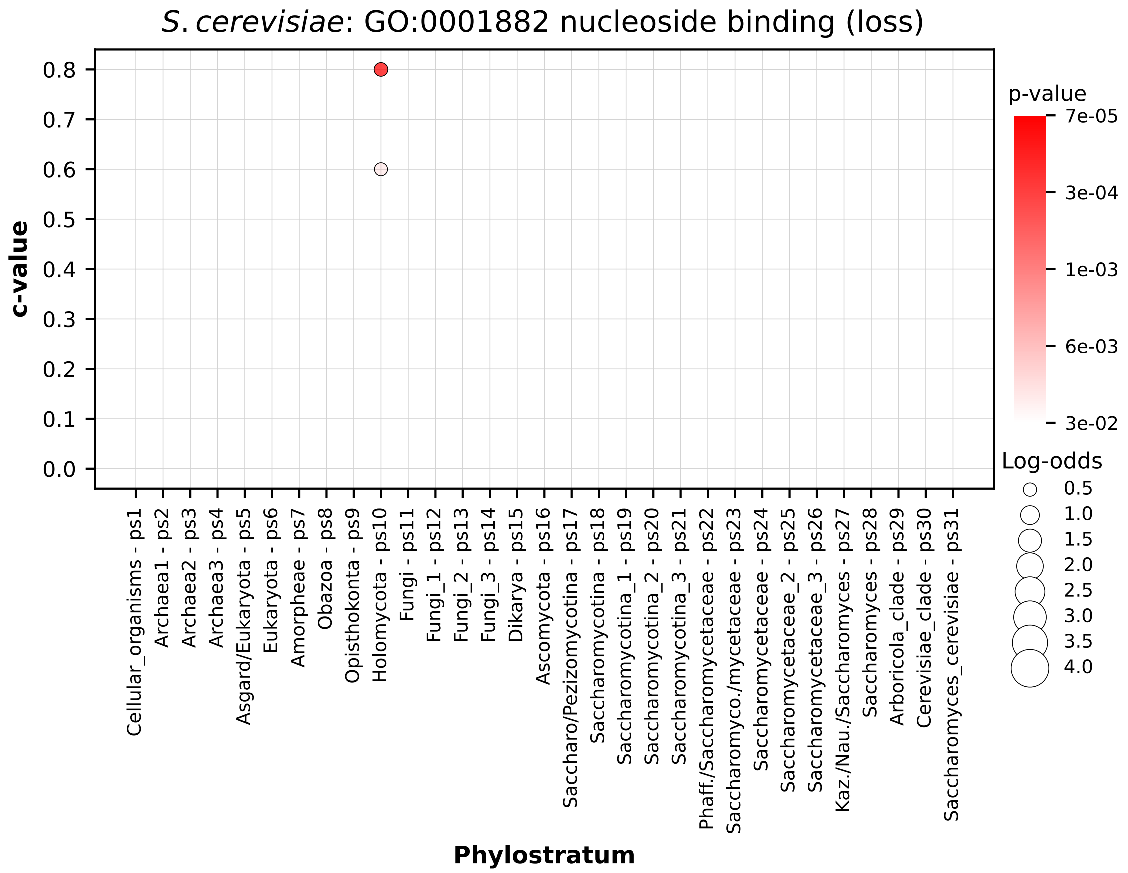

S. cerevisiae: GO:0001882 nucleoside binding (loss)
c-value
0.8
0.7
0.6
0.5
0.4
0.3
0.2
0.1
0.0
p-value
7e-05
3e-04
1e-03
6e-03
3e-02
Log-odds
0.5
1.0
1.5
2.0
2.5
3.0
3.5
4.0
Cellular_organisms - ps1
Archaea1 - ps2
Archaea2 - ps3
Archaea3 - ps4
Asgard/Eukaryota - ps5
Eukaryota - ps6
Amorpheae - ps7
Obazoa - ps8
Opisthokonta - ps9
Holomycota - ps10
Fungi - ps11
Fungi_1 - ps12
Fungi_2 - ps13
Fungi_3 - ps14
Dikarya - ps15
Ascomycota - ps16
Saccharo/Pezizomycotina - ps17
Saccharomycotina - ps18
Saccharomycotina_1 - ps19
Saccharomycotina_2 - ps20
Saccharomycotina_3 - ps21
Phaff./Saccharomycetaceae - ps22
Saccharomyco./mycetaceae - ps23
Saccharomycetaceae - ps24
Saccharomycetaceae_2 - ps25
Saccharomycetaceae_3 - ps26
Kaz./Nau./Saccharomyces - ps27
Saccharomyces - ps28
Arboricola_clade - ps29
Cerevisiae_clade - ps30
Saccharomyces_cerevisiae - ps31
Phylostratum

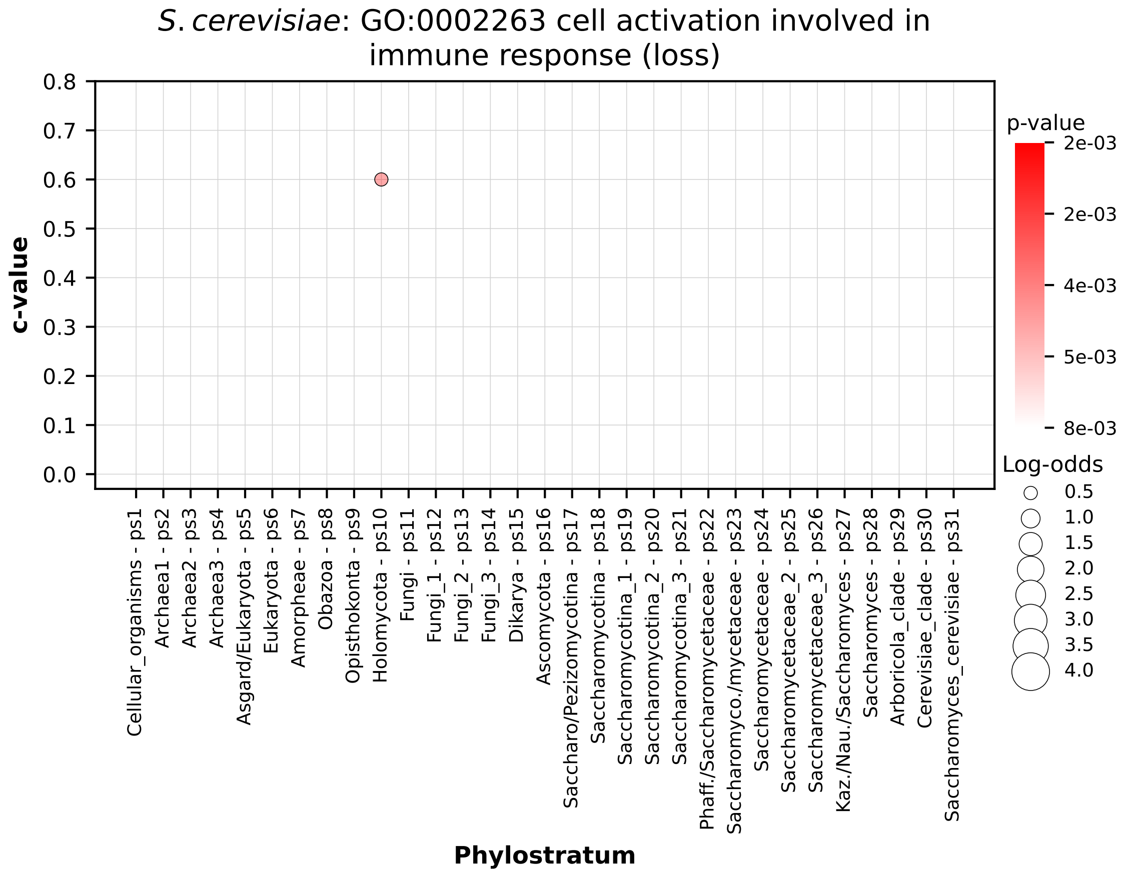

S. cerevisiae: GO:0002263 cell activation involved in immune response (loss)
c-value
0.8
0.7
0.6
0.5
0.4
0.3
0.2
0.1
0.0
Phylostratum
Cellular_organisms - ps1
Archaea1 - ps2
Archaea2 - ps3
Archaea3 - ps4
Asgard/Eukaryota - ps5
Eukaryota - ps6
Amorpheae - ps7
Obazoa - ps8
Opisthokonta - ps9
Holomycota - ps10
Fungi - ps11
Fungi_1 - ps12
Fungi_2 - ps13
Fungi_3 - ps14
Dikarya - ps15
Ascomycota - ps16
Saccharo/Pezizomycotina - ps17
Saccharomycotina - ps18
Saccharomycotina_1 - ps19
Saccharomycotina_2 - ps20
Saccharomycotina_3 - ps21
Phaff./Saccharomycetaceae - ps22
Saccharomyco./mycetaceae - ps23
Saccharomycetaceae - ps24
Saccharomycetaceae_2 - ps25
Saccharomycetaceae_3 - ps26
Kaz./Nau./Saccharomyces - ps27
Saccharomyces - ps28
Arboricola_clade - ps29
Cerevisiae_clade - ps30
Saccharomyces_cerevisiae - ps31
p-value
2e-03
2e-03
4e-03
5e-03
8e-03
Log-odds
0.5
1.0
1.5
2.0
2.5
3.0
3.5
4.0

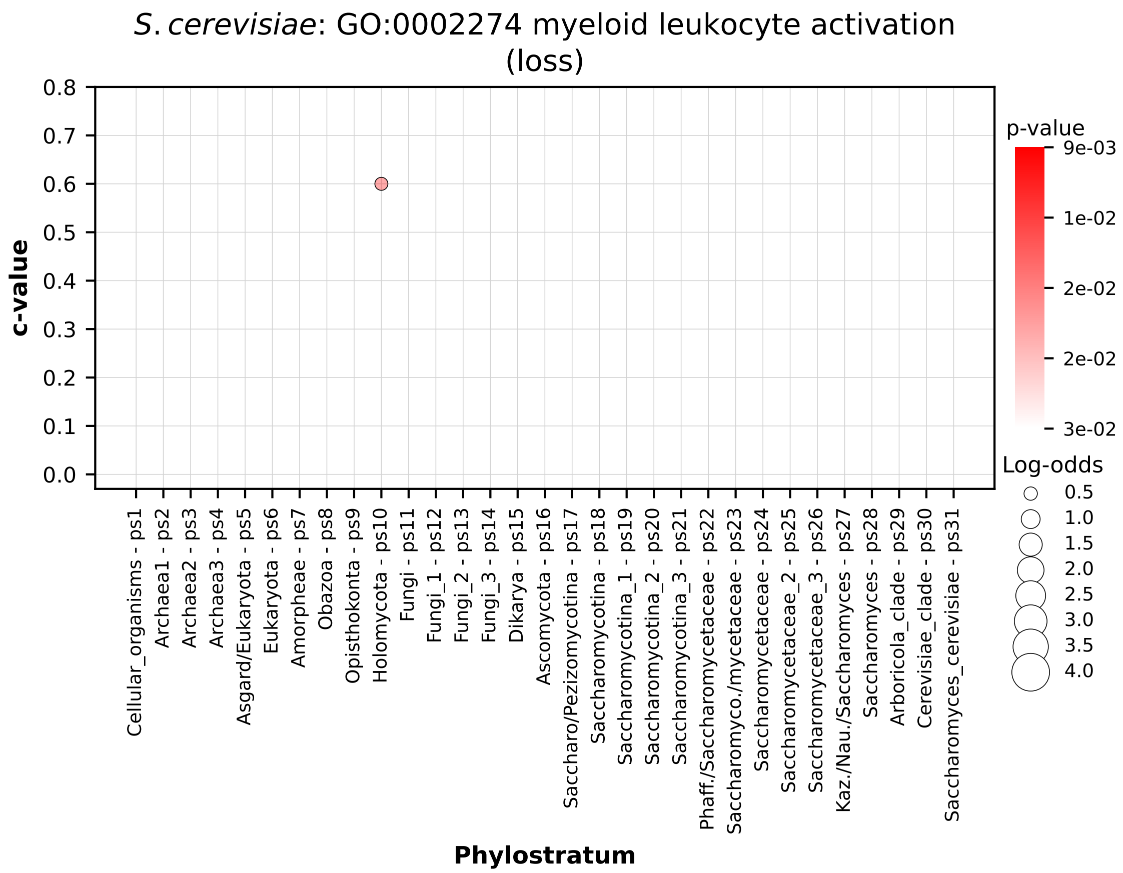
S. cerevisiae: GO:0002274 myeloid leukocyte activation (loss)
c-value
Phylostratum
p-value
9e-03
1e-02
2e-02
2e-02
3e-02
Log-odds
0.5
1.0
1.5
2.0
2.5
3.0
3.5
4.0
0.0
0.1
0.2
0.3
0.4
0.5
0.6
0.7
0.8
Cellular_organisms - ps1
Archaea1 - ps2
Archaea2 - ps3
Archaea3 - ps4
Asgard/Eukaryota - ps5
Eukaryota - ps6
Amorpheae - ps7
Obazoa - ps8
Opisthokonta - ps9
Holomycota - ps10
Fungi - ps11
Fungi_1 - ps12
Fungi_2 - ps13
Fungi_3 - ps14
Dikarya - ps15
Ascomycota - ps16
Saccharo/Pezizomycotina - ps17
Saccharomycotina - ps18
Saccharomycotina_1 - ps19
Saccharomycotina_2 - ps20
Saccharomycotina_3 - ps21
Phaff./Saccharomycetaceae - ps22
Saccharomyco./mycetaceae - ps23
Saccharomycetaceae - ps24
Saccharomycetaceae_2 - ps25
Saccharomycetaceae_3 - ps26
Kaz./Nau./Saccharomyces - ps27
Saccharomyces - ps28
Arboricola_clade - ps29
Cerevisiae_clade - ps30
Saccharomyces_cerevisiae - ps31

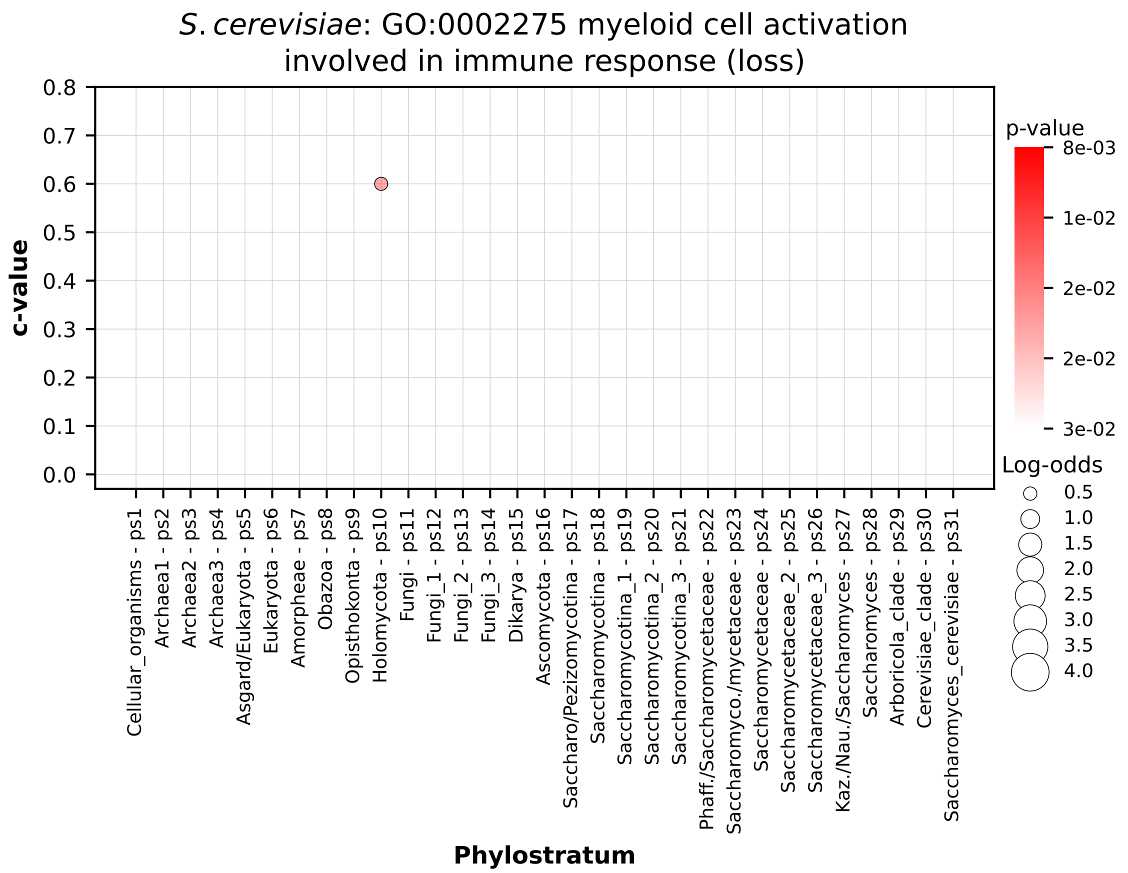
S. cerevisiae: GO:0002275 myeloid cell activation involved in immune response (loss)
c-value
0.8
0.7
0.6
0.5
0.4
0.3
0.2
0.1
0.0
p-value
8e-03
1e-02
2e-02
2e-02
3e-02
Log-odds
0.5
1.0
1.5
2.0
2.5
3.0
3.5
4.0
Cellular_organisms - ps1
Archaea1 - ps2
Archaea2 - ps3
Archaea3 - ps4
Asgard/Eukaryota - ps5
Eukaryota - ps6
Amorpheae - ps7
Obazoa - ps8
Opisthokonta - ps9
Holomycota - ps10
Fungi - ps11
Fungi_1 - ps12
Fungi_2 - ps13
Fungi_3 - ps14
Dikarya - ps15
Ascomycota - ps16
Saccharo/Pezizomycotina - ps17
Saccharomycotina - ps18
Saccharomycotina_1 - ps19
Saccharomycotina_2 - ps20
Saccharomycotina_3 - ps21
Phaff./Saccharomycetaceae - ps22
Saccharomyco./mycetaceae - ps23
Saccharomycetaceae - ps24
Saccharomycetaceae_2 - ps25
Saccharomycetaceae_3 - ps26
Kaz./Nau./Saccharomyces - ps27
Saccharomyces - ps28
Arboricola_clade - ps29
Cerevisiae_clade - ps30
Saccharomyces_cerevisiae - ps31
Phylostratum

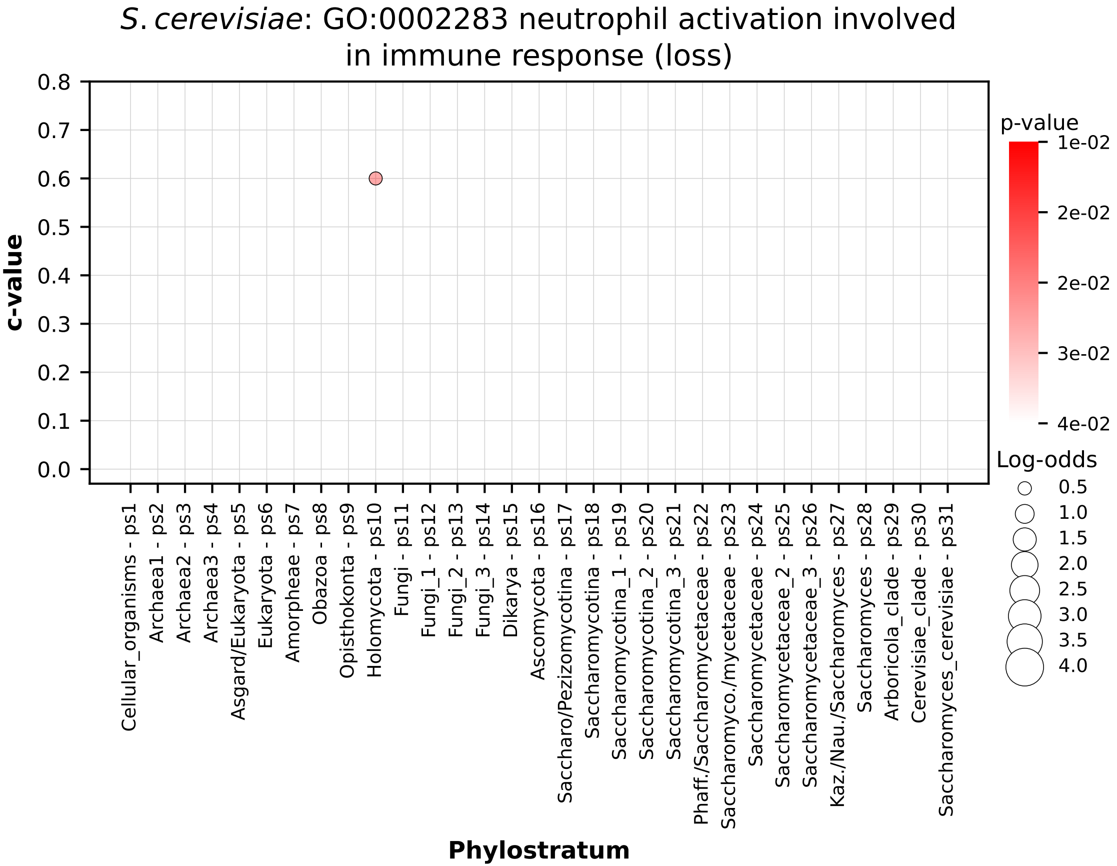

S. cerevisiae: GO:0002283 neutrophil activation involved in immune response (loss)
c-value
0.0
0.1
0.2
0.3
0.4
0.5
0.6
0.7
0.8
p-value
1e-02
2e-02
2e-02
3e-02
4e-02
Log-odds
0.5
1.0
1.5
2.0
2.5
3.0
3.5
4.0
Cellular_organisms - ps1
Archaea1 - ps2
Archaea2 - ps3
Archaea3 - ps4
Asgard/Eukaryota - ps5
Eukaryota - ps6
Amorpheae - ps7
Obazoa - ps8
Opisthokonta - ps9
Holomycota - ps10
Fungi - ps11
Fungi_1 - ps12
Fungi_2 - ps13
Fungi_3 - ps14
Dikarya - ps15
Ascomycota - ps16
Saccharo/Pezizomycotina - ps17
Saccharomycotina - ps18
Saccharomycotina_1 - ps19
Saccharomycotina_2 - ps20
Saccharomycotina_3 - ps21
Phaff./Saccharomycetaceae - ps22
Saccharomyco./mycetaceae - ps23
Saccharomycetaceae - ps24
Saccharomycetaceae_2 - ps25
Saccharomycetaceae_3 - ps26
Kaz./Nau./Saccharomyces - ps27
Saccharomyces - ps28
Arboricola_clade - ps29
Cerevisiae_clade - ps30
Saccharomyces_cerevisiae - ps31
Phylostratum

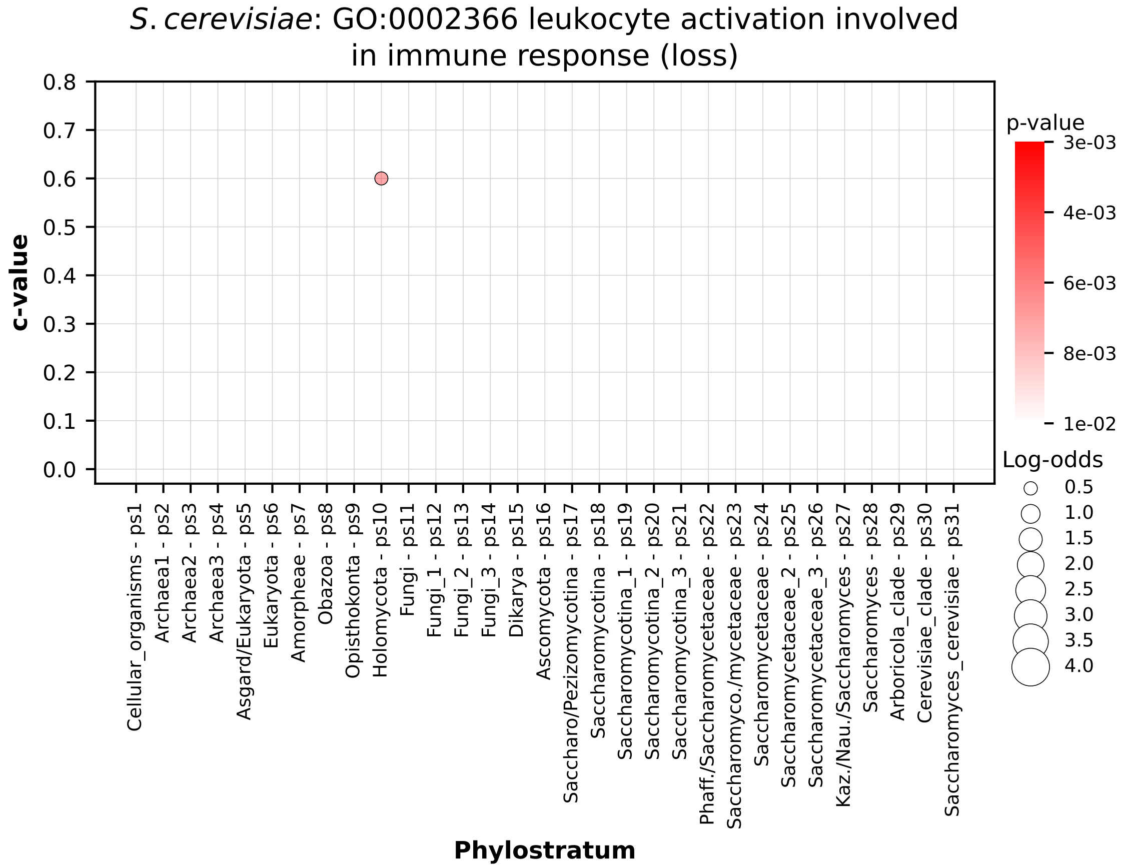

S. cerevisiae: GO:0002366 leukocyte activation involved in immune response (loss)
c-value
0.0
0.1
0.2
0.3
0.4
0.5
0.6
0.7
0.8
Phylostratum
Cellular_organisms - ps1
Archaea1 - ps2
Archaea2 - ps3
Archaea3 - ps4
Asgard/Eukaryota - ps5
Eukaryota - ps6
Amorpheae - ps7
Obazoa - ps8
Opisthokonta - ps9
Holomycota - ps10
Fungi - ps11
Fungi_1 - ps12
Fungi_2 - ps13
Fungi_3 - ps14
Dikarya - ps15
Ascomycota - ps16
Saccharo/Pezizomycotina - ps17
Saccharomycotina - ps18
Saccharomycotina_1 - ps19
Saccharomycotina_2 - ps20
Saccharomycotina_3 - ps21
Phaff./Saccharomycetaceae - ps22
Saccharomyco./mycetaceae - ps23
Saccharomycetaceae - ps24
Saccharomycetaceae_2 - ps25
Saccharomycetaceae_3 - ps26
Kaz./Nau./Saccharomyces - ps27
Saccharomyces - ps28
Arboricola_clade - ps29
Cerevisiae_clade - ps30
Saccharomyces_cerevisiae - ps31
p-value
3e-03
4e-03
6e-03
8e-03
1e-02
Log-odds
0.5
1.0
1.5
2.0
2.5
3.0
3.5
4.0

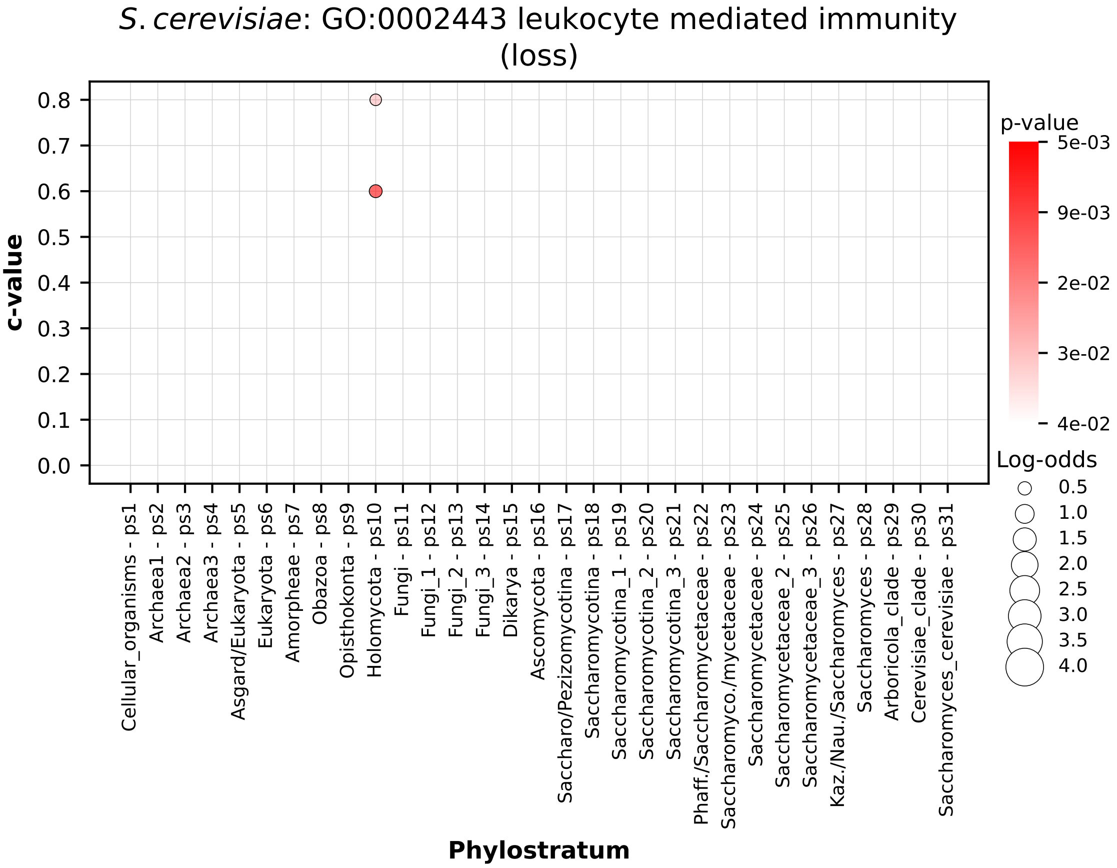

S. cerevisiae: GO:0002443 leukocyte mediated immunity (loss)
c-value
0.0
0.1
0.2
0.3
0.4
0.5
0.6
0.7
0.8
p-value
5e-03
9e-03
2e-02
3e-02
4e-02
Log-odds
0.5
1.0
1.5
2.0
2.5
3.0
3.5
4.0
Cellular_organisms - ps1
Archaea1 - ps2
Archaea2 - ps3
Archaea3 - ps4
Asgard/Eukaryota - ps5
Eukaryota - ps6
Amorpheae - ps7
Obazoa - ps8
Opisthokonta - ps9
Holomycota - ps10
Fungi - ps11
Fungi_1 - ps12
Fungi_2 - ps13
Fungi_3 - ps14
Dikarya - ps15
Ascomycota - ps16
Saccharo/Pezizomycotina - ps17
Saccharomycotina - ps18
Saccharomycotina_1 - ps19
Saccharomycotina_2 - ps20
Saccharomycotina_3 - ps21
Phaff./Saccharomycetaceae - ps22
Saccharomyco./mycetaceae - ps23
Saccharomycetaceae - ps24
Saccharomycetaceae_2 - ps25
Saccharomycetaceae_3 - ps26
Kaz./Nau./Saccharomyces - ps27
Saccharomyces - ps28
Arboricola_clade - ps29
Cerevisiae_clade - ps30
Saccharomyces_cerevisiae - ps31
Phylostratum

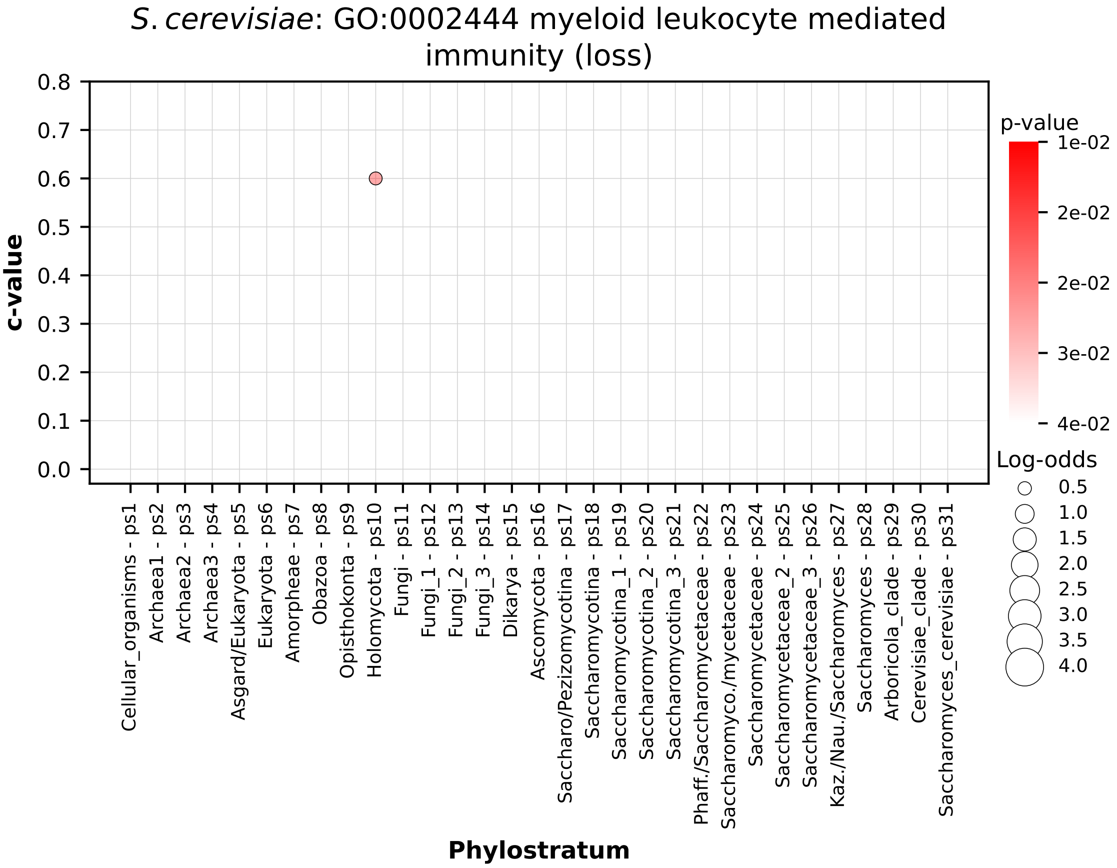
S. cerevisiae: GO:0002444 myeloid leukocyte mediated immunity (loss)
c-value
0.8
0.7
0.6
0.5
0.4
0.3
0.2
0.1
0.0
p-value
1e-02
2e-02
2e-02
3e-02
4e-02
Log-odds
0.5
1.0
1.5
2.0
2.5
3.0
3.5
4.0
Cellular_organisms - ps1
Archaea1 - ps2
Archaea2 - ps3
Archaea3 - ps4
Asgard/Eukaryota - ps5
Eukaryota - ps6
Amorpheae - ps7
Obazoa - ps8
Opisthokonta - ps9
Holomycota - ps10
Fungi - ps11
Fungi_1 - ps12
Fungi_2 - ps13
Fungi_3 - ps14
Dikarya - ps15
Ascomycota - ps16
Saccharo/Pezizomycotina - ps17
Saccharomycotina - ps18
Saccharomycotina_1 - ps19
Saccharomycotina_2 - ps20
Saccharomycotina_3 - ps21
Phaff./Saccharomycetaceae - ps22
Saccharomyco./mycetaceae - ps23
Saccharomycetaceae - ps24
Saccharomycetaceae_2 - ps25
Saccharomycetaceae_3 - ps26
Kaz./Nau./Saccharomyces - ps27
Saccharomyces - ps28
Arboricola_clade - ps29
Cerevisiae_clade - ps30
Saccharomyces_cerevisiae - ps31
Phylostratum

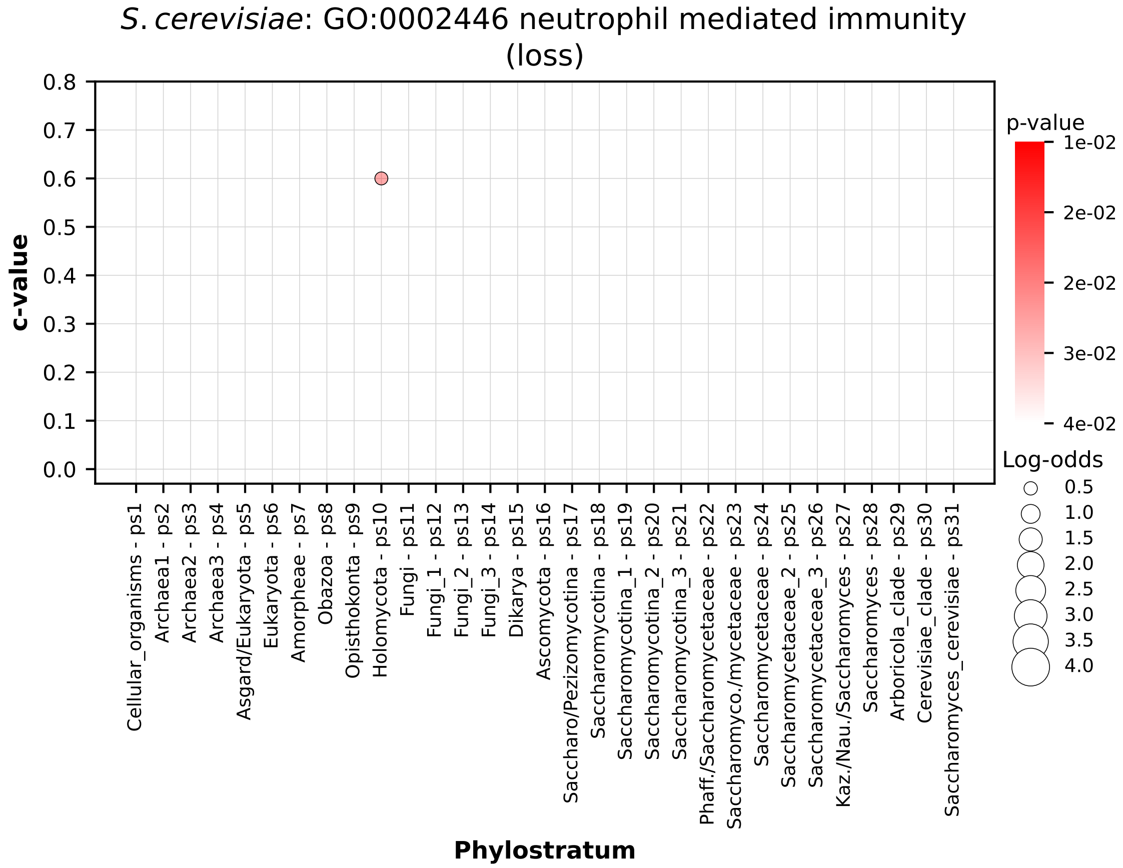
S. cerevisiae: GO:0002446 neutrophil mediated immunity
(loss)
c-value
0.0
0.1
0.2
0.3
0.4
0.5
0.6
0.7
0.8
Phylostratum
Cellular_organisms - ps1
Archaea1 - ps2
Archaea2 - ps3
Archaea3 - ps4
Asgard/Eukaryota - ps5
Eukaryota - ps6
Amorpheae - ps7
Obazoa - ps8
Opisthokonta - ps9
Holomycota - ps10
Fungi - ps11
Fungi_1 - ps12
Fungi_2 - ps13
Fungi_3 - ps14
Dikarya - ps15
Ascomycota - ps16
Saccharo/Pezizomycotina - ps17
Saccharomycotina - ps18
Saccharomycotina_1 - ps19
Saccharomycotina_2 - ps20
Saccharomycotina_3 - ps21
Phaff./Saccharomycetaceae - ps22
Saccharomyco./mycetaceae - ps23
Saccharomycetaceae - ps24
Saccharomycetaceae_2 - ps25
Saccharomycetaceae_3 - ps26
Kaz./Nau./Saccharomyces - ps27
Saccharomyces - ps28
Arboricola_clade - ps29
Cerevisiae_clade - ps30
Saccharomyces_cerevisiae - ps31
p-value
1e-02
2e-02
2e-02
3e-02
4e-02
Log-odds
0.5
1.0
1.5
2.0
2.5
3.0
3.5
4.0

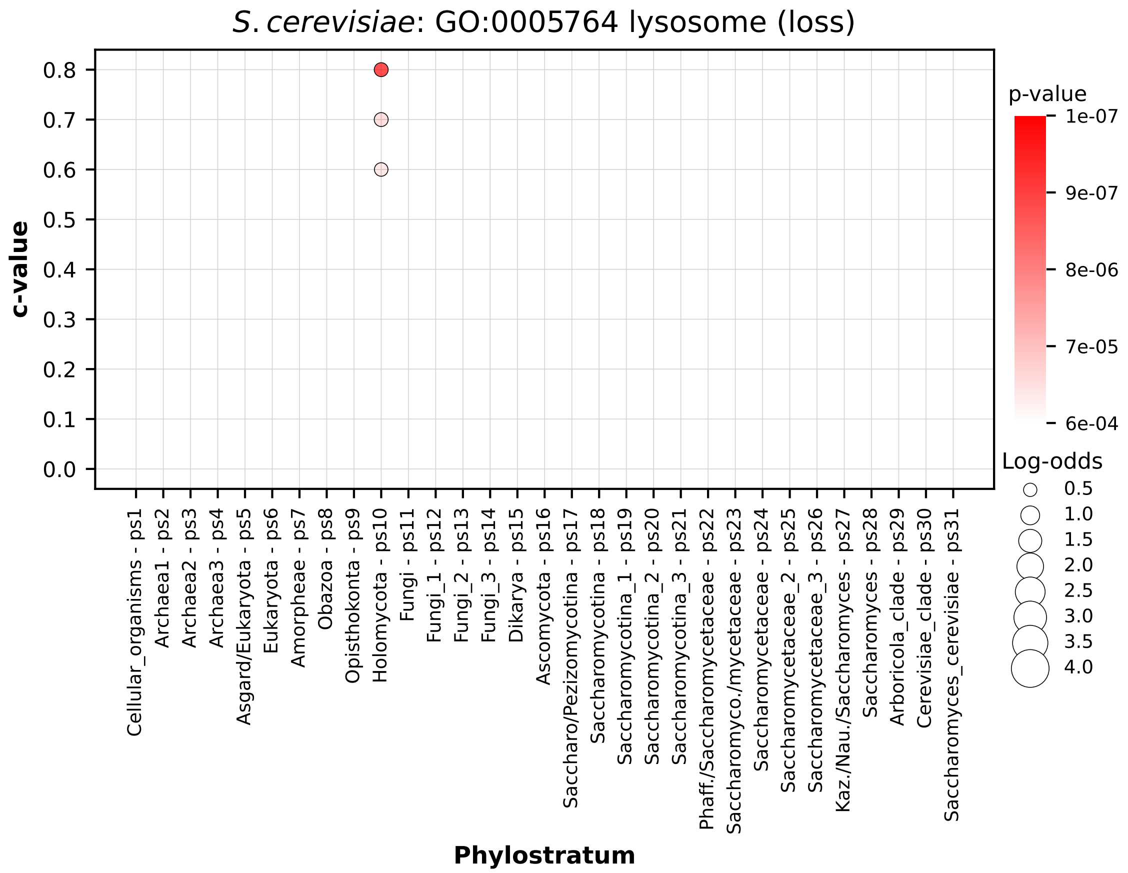

S. cerevisiae: GO:0005764 lysosome (loss)
c-value
0.0
0.1
0.2
0.3
0.4
0.5
0.6
0.7
0.8
Phylostratum
Cellular_organisms - ps1
Archaea1 - ps2
Archaea2 - ps3
Archaea3 - ps4
Asgard/Eukaryota - ps5
Eukaryota - ps6
Amorpheae - ps7
Obazoa - ps8
Opisthokonta - ps9
Holomycota - ps10
Fungi - ps11
Fungi_1 - ps12
Fungi_2 - ps13
Fungi_3 - ps14
Dikarya - ps15
Ascomycota - ps16
Saccharo/Pezizomycotina - ps17
Saccharomycotina - ps18
Saccharomycotina_1 - ps19
Saccharomycotina_2 - ps20
Saccharomycotina_3 - ps21
Phaff./Saccharomycetaceae - ps22
Saccharomyco./mycetaceae - ps23
Saccharomycetaceae - ps24
Saccharomycetaceae_2 - ps25
Saccharomycetaceae_3 - ps26
Kaz./Nau./Saccharomyces - ps27
Saccharomyces - ps28
Arboricola_clade - ps29
Cerevisiae_clade - ps30
Saccharomyces_cerevisiae - ps31
p-value
1e-07
9e-07
8e-06
7e-05
6e-04
Log-odds
0.5
1.0
1.5
2.0
2.5
3.0
3.5
4.0

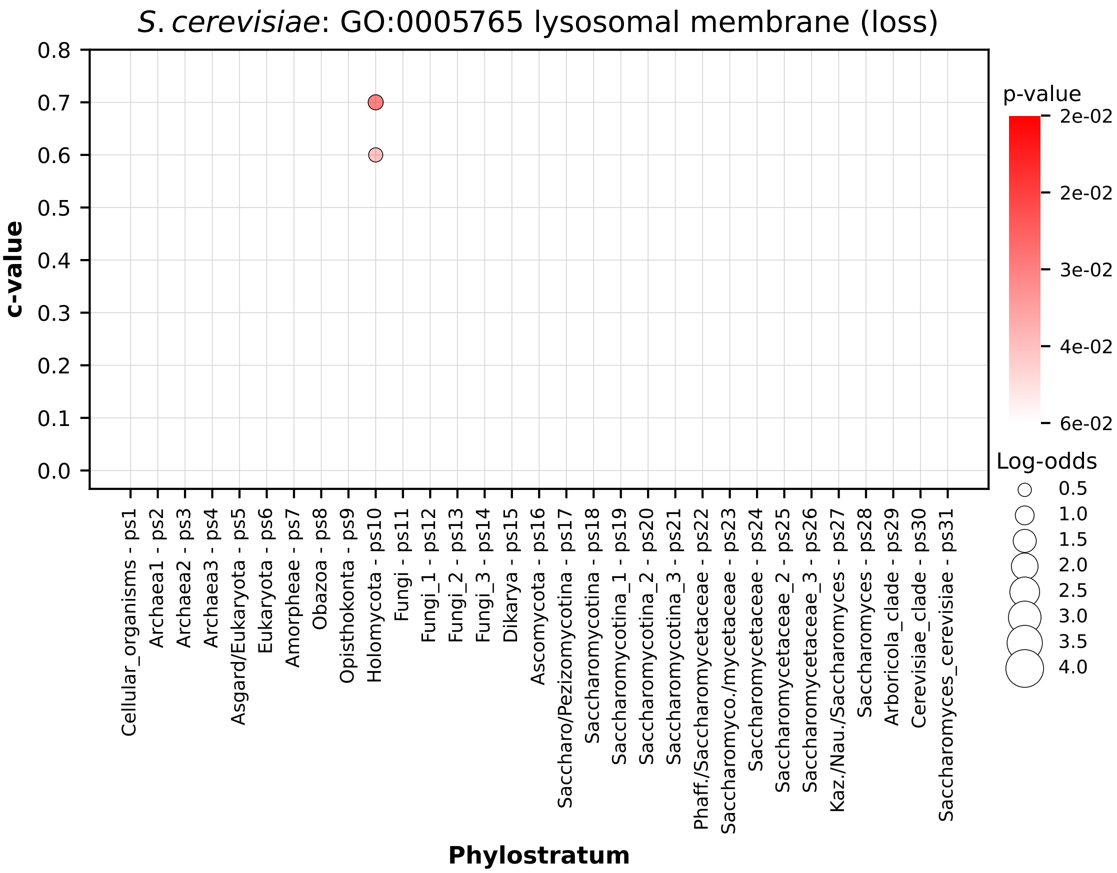

S. cerevisiae: GO:0005765 lysosomal membrane (loss)
c-value
0.8
0.7
0.6
0.5
0.4
0.3
0.2
0.1
0.0
p-value
2e-02
2e-02
3e-02
4e-02
6e-02
Log-odds
0.5
1.0
1.5
2.0
2.5
3.0
3.5
4.0
Cellular_organisms - ps1
Archaea1 - ps2
Archaea2 - ps3
Archaea3 - ps4
Asgard/Eukaryota - ps5
Eukaryota - ps6
Amorpheae - ps7
Obazoa - ps8
Opisthokonta - ps9
Holomycota - ps10
Fungi - ps11
Fungi_1 - ps12
Fungi_2 - ps13
Fungi_3 - ps14
Dikarya - ps15
Ascomycota - ps16
Saccharo/Pezizomycotina - ps17
Saccharomycotina - ps18
Saccharomycotina_1 - ps19
Saccharomycotina_2 - ps20
Saccharomycotina_3 - ps21
Phaff./Saccharomycetaceae - ps22
Saccharomyco./mycetaceae - ps23
Saccharomycetaceae - ps24
Saccharomycetaceae_2 - ps25
Saccharomycetaceae_3 - ps26
Kaz./Nau./Saccharomyces - ps27
Saccharomyces - ps28
Arboricola_clade - ps29
Cerevisiae_clade - ps30
Saccharomyces_cerevisiae - ps31
Phylostratum

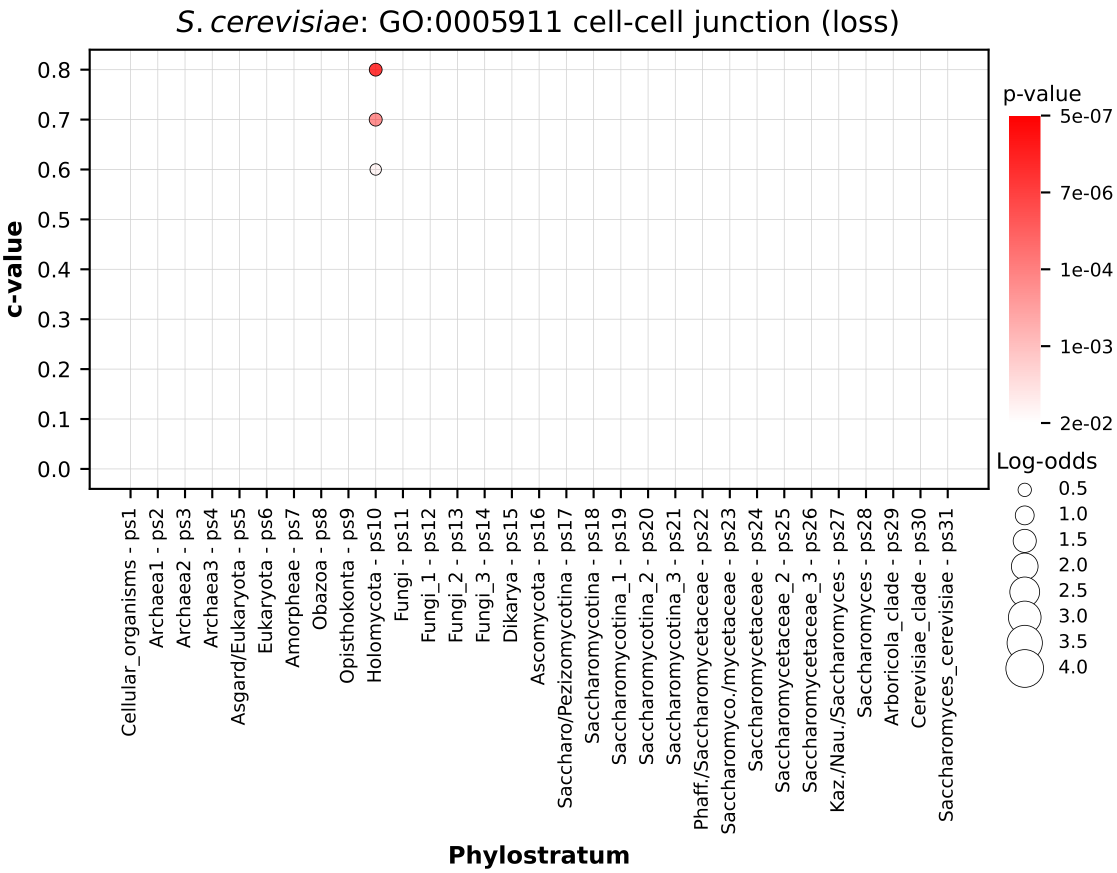

$S. cerevisiae$: GO:0005911 cell-cell junction (loss)
c-value
0.0
0.1
0.2
0.3
0.4
0.5
0.6
0.7
0.8
p-value
5e-07
7e-06
1e-04
1e-03
2e-02
Log-odds
0.5
1.0
1.5
2.0
2.5
3.0
3.5
4.0
Cellular_organisms - ps1
Archaea1 - ps2
Archaea2 - ps3
Archaea3 - ps4
Asgard/Eukaryota - ps5
Eukaryota - ps6
Amorpheae - ps7
Obazoa - ps8
Opisthokonta - ps9
Holomycota - ps10
Fungi - ps11
Fungi_1 - ps12
Fungi_2 - ps13
Fungi_3 - ps14
Dikarya - ps15
Ascomycota - ps16
Saccharo/Pezizomycotina - ps17
Saccharomycotina - ps18
Saccharomycotina_1 - ps19
Saccharomycotina_2 - ps20
Saccharomycotina_3 - ps21
Phaff./Saccharomycetaceae - ps22
Saccharomyco./mycetaceae - ps23
Saccharomycetaceae - ps24
Saccharomycetaceae_2 - ps25
Saccharomycetaceae_3 - ps26
Kaz./Nau./Saccharomyces - ps27
Saccharomyces - ps28
Arboricola_clade - ps29
Cerevisiae_clade - ps30
Saccharomyces_cerevisiae - ps31
Phylostratum

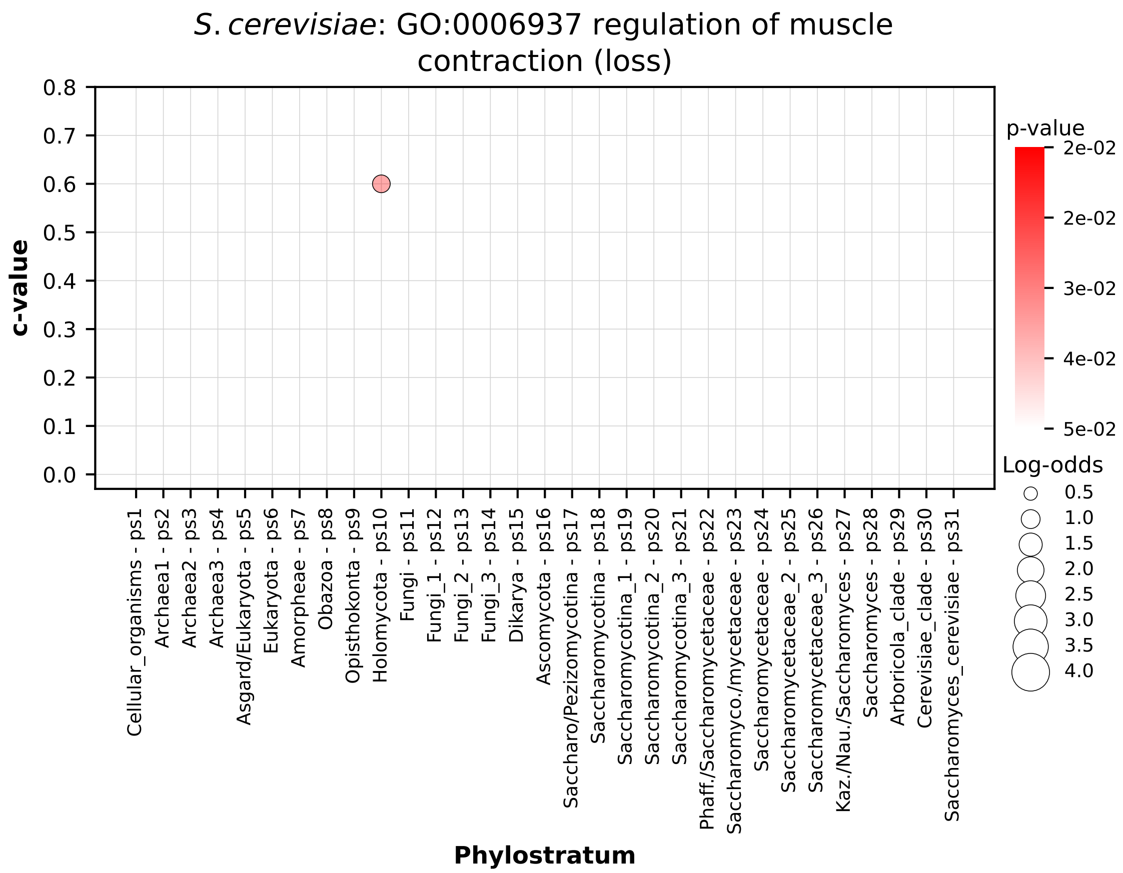
S. cerevisiae: GO:0006937 regulation of muscle contraction (loss)
c-value
0.8
0.7
0.6
0.5
0.4
0.3
0.2
0.1
0.0
Phylostratum
Cellular_organisms - ps1
Archaea1 - ps2
Archaea2 - ps3
Archaea3 - ps4
Asgard/Eukaryota - ps5
Eukaryota - ps6
Amorpheae - ps7
Obazoa - ps8
Opisthokonta - ps9
Holomycota - ps10
Fungi - ps11
Fungi_1 - ps12
Fungi_2 - ps13
Fungi_3 - ps14
Dikarya - ps15
Ascomycota - ps16
Saccharo/Pezizomycotina - ps17
Saccharomycotina - ps18
Saccharomycotina_1 - ps19
Saccharomycotina_2 - ps20
Saccharomycotina_3 - ps21
Phaff./Saccharomycetaceae - ps22
Saccharomyco./mycetaceae - ps23
Saccharomycetaceae - ps24
Saccharomycetaceae_2 - ps25
Saccharomycetaceae_3 - ps26
Kaz./Nau./Saccharomyces - ps27
Saccharomyces - ps28
Arboricola_clade - ps29
Cerevisiae_clade - ps30
Saccharomyces_cerevisiae - ps31
p-value
2e-02
2e-02
3e-02
4e-02
5e-02
Log-odds
0.5
1.0
1.5
2.0
2.5
3.0
3.5
4.0

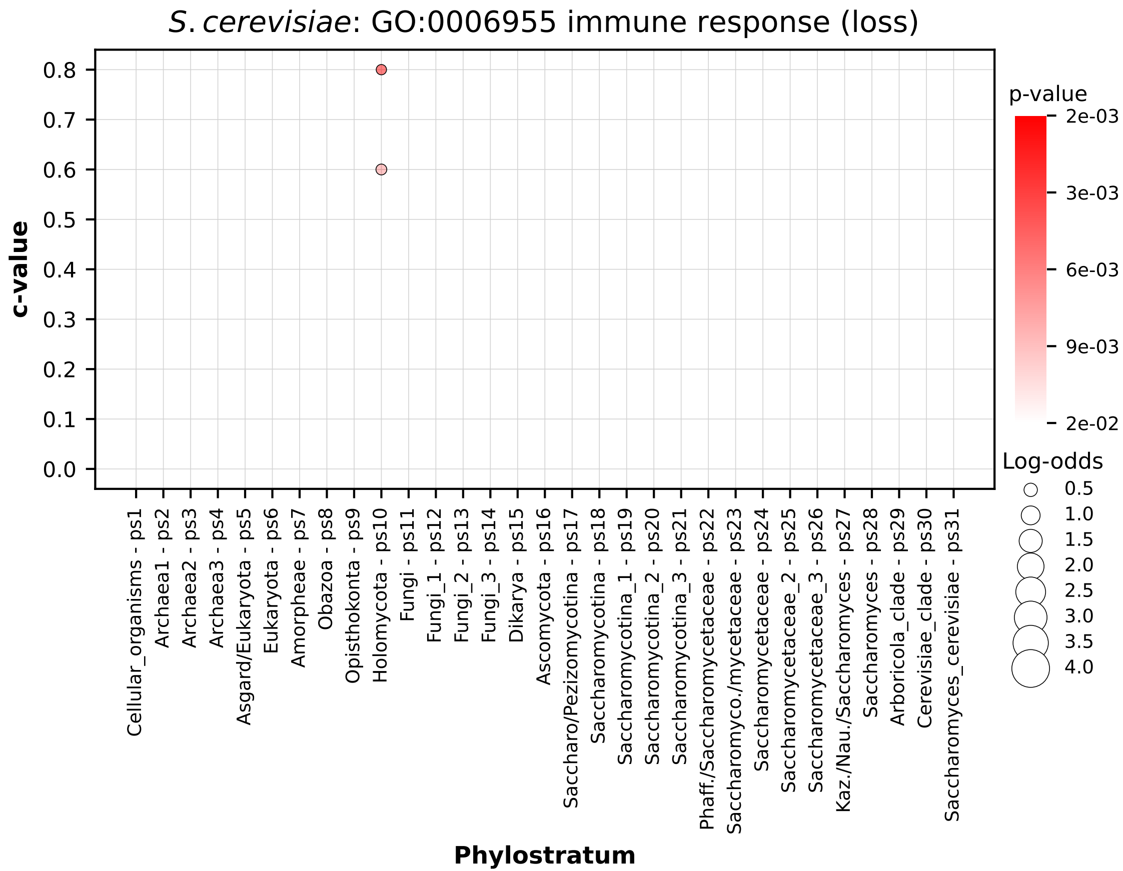

S. cerevisiae: GO:0006955 immune response (loss)
c-value
0.0
0.1
0.2
0.3
0.4
0.5
0.6
0.7
0.8
p-value
2e-03
3e-03
6e-03
9e-03
2e-02
Log-odds
0.5
1.0
1.5
2.0
2.5
3.0
3.5
4.0
Cellular_organisms - ps1
Archaea1 - ps2
Archaea2 - ps3
Archaea3 - ps4
Asgard/Eukaryota - ps5
Eukaryota - ps6
Amorpheae - ps7
Obazoa - ps8
Opisthokonta - ps9
Holomycota - ps10
Fungi - ps11
Fungi_1 - ps12
Fungi_2 - ps13
Fungi_3 - ps14
Dikarya - ps15
Ascomycota - ps16
Saccharo/Pezizomycotina - ps17
Saccharomycotina - ps18
Saccharomycotina_1 - ps19
Saccharomycotina_2 - ps20
Saccharomycotina_3 - ps21
Phaff./Saccharomycetaceae - ps22
Saccharomyco./mycetaceae - ps23
Saccharomycetaceae - ps24
Saccharomycetaceae_2 - ps25
Saccharomycetaceae_3 - ps26
Kaz./Nau./Saccharomyces - ps27
Saccharomyces - ps28
Arboricola_clade - ps29
Cerevisiae_clade - ps30
Saccharomyces_cerevisiae - ps31
Phylostratum

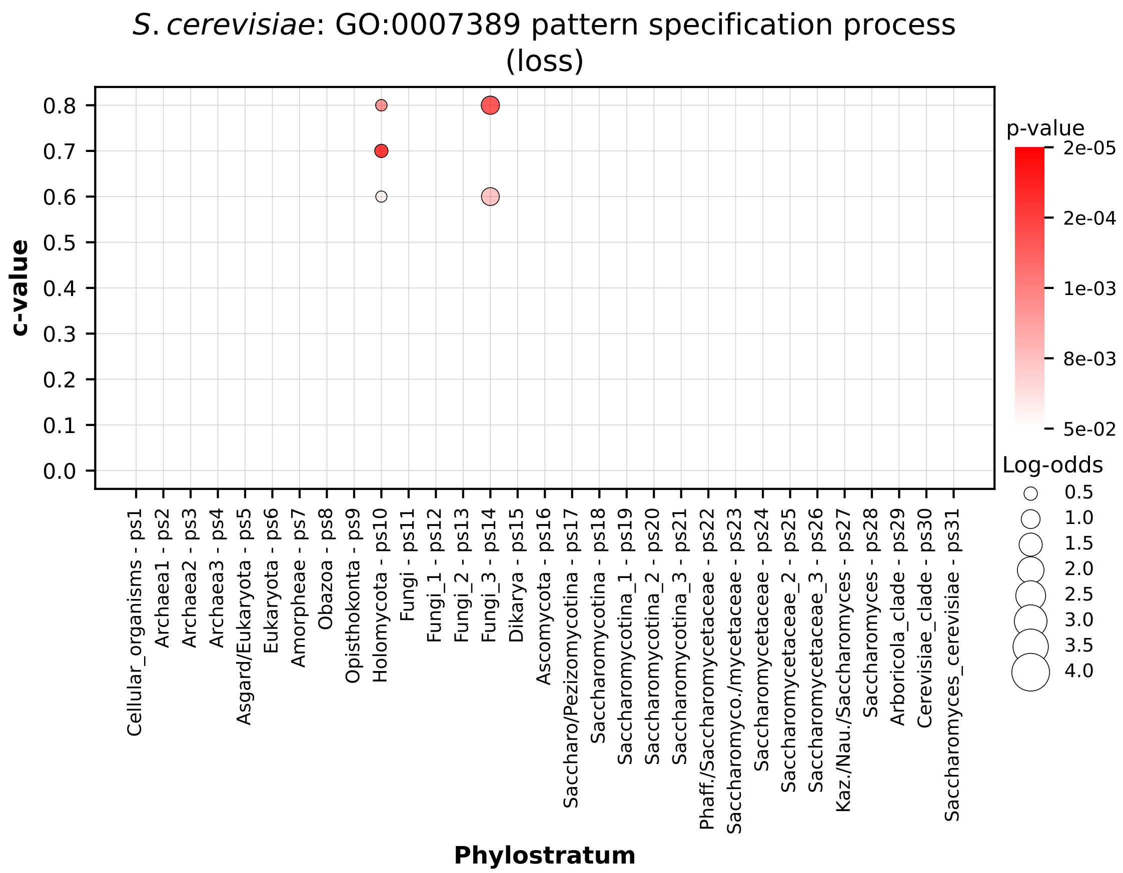

S. cerevisiae: GO:0007389 pattern specification process
(loss)
c-value
0.0
0.1
0.2
0.3
0.4
0.5
0.6
0.7
0.8
Phylostratum
Cellular_organisms - ps1
Archaea1 - ps2
Archaea2 - ps3
Archaea3 - ps4
Asgard/Eukaryota - ps5
Eukaryota - ps6
Amorpheae - ps7
Obazoa - ps8
Opisthokonta - ps9
Holomycota - ps10
Fungi - ps11
Fungi_1 - ps12
Fungi_2 - ps13
Fungi_3 - ps14
Dikarya - ps15
Ascomycota - ps16
Saccharo/Pezizomycotina - ps17
Saccharomycotina - ps18
Saccharomycotina_1 - ps19
Saccharomycotina_2 - ps20
Saccharomycotina_3 - ps21
Phaff./Saccharomycetaceae - ps22
Saccharomyco./mycetaceae - ps23
Saccharomycetaceae - ps24
Saccharomycetaceae_2 - ps25
Saccharomycetaceae_3 - ps26
Kaz./Nau./Saccharomyces - ps27
Saccharomyces - ps28
Arboricola_clade - ps29
Cerevisiae_clade - ps30
Saccharomyces_cerevisiae - ps31
p-value
2e-05
2e-04
1e-03
8e-03
5e-02
Log-odds
0.5
1.0
1.5
2.0
2.5
3.0
3.5
4.0

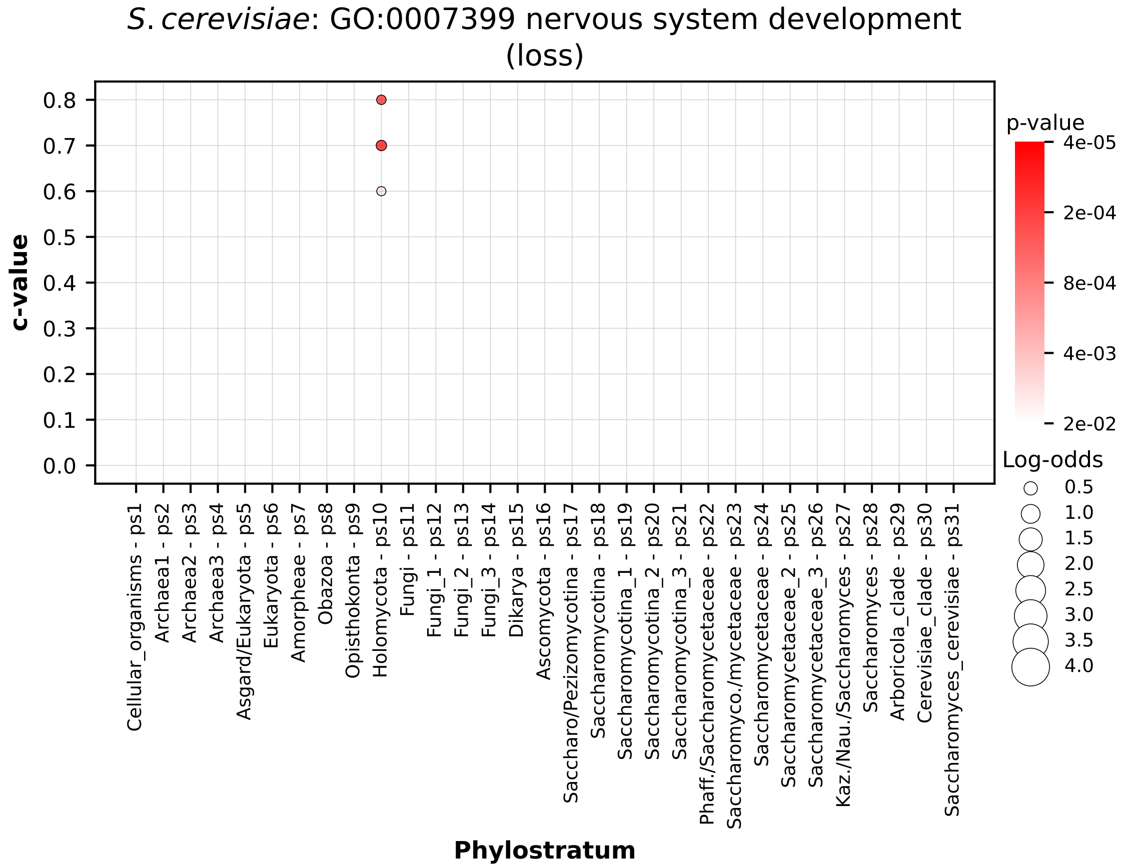
S. cerevisiae: GO:0007399 nervous system development (loss)
c-value
0.0
0.1
0.2
0.3
0.4
0.5
0.6
0.7
0.8
Phylostratum
Cellular_organisms - ps1
Archaea1 - ps2
Archaea2 - ps3
Archaea3 - ps4
Asgard/Eukaryota - ps5
Eukaryota - ps6
Amorpheae - ps7
Obazoa - ps8
Opisthokonta - ps9
Holomycota - ps10
Fungi - ps11
Fungi_1 - ps12
Fungi_2 - ps13
Fungi_3 - ps14
Dikarya - ps15
Ascomycota - ps16
Saccharo/Pezizomycotina - ps17
Saccharomycotina - ps18
Saccharomycotina_1 - ps19
Saccharomycotina_2 - ps20
Saccharomycotina_3 - ps21
Phaff./Saccharomycetaceae - ps22
Saccharomyco./mycetaceae - ps23
Saccharomycetaceae - ps24
Saccharomycetaceae_2 - ps25
Saccharomycetaceae_3 - ps26
Kaz./Nau./Saccharomyces - ps27
Saccharomyces - ps28
Arboricola_clade - ps29
Cerevisiae_clade - ps30
Saccharomyces_cerevisiae - ps31
p-value
4e-05
2e-04
8e-04
4e-03
2e-02
Log-odds
0.5
1.0
1.5
2.0
2.5
3.0
3.5
4.0

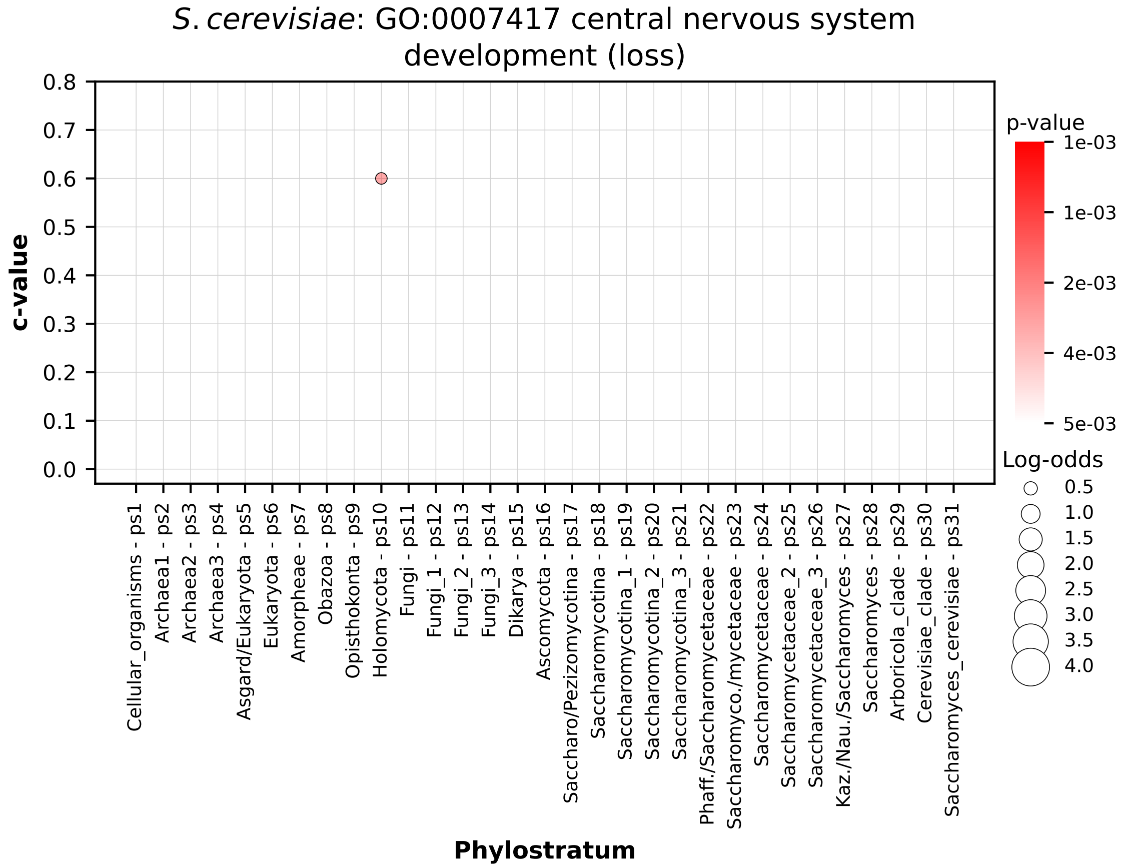

S. cerevisiae: GO:0007417 central nervous system development (loss)
c-value
0.8
0.7
0.6
0.5
0.4
0.3
0.2
0.1
0.0
p-value
1e-03
1e-03
2e-03
4e-03
5e-03
Log-odds
0.5
1.0
1.5
2.0
2.5
3.0
3.5
4.0
Cellular_organisms - ps1
Archaea1 - ps2
Archaea2 - ps3
Archaea3 - ps4
Asgard/Eukaryota - ps5
Eukaryota - ps6
Amorpheae - ps7
Obazoa - ps8
Opisthokonta - ps9
Holomycota - ps10
Fungi - ps11
Fungi_1 - ps12
Fungi_2 - ps13
Fungi_3 - ps14
Dikarya - ps15
Ascomycota - ps16
Saccharo/Pezizomycotina - ps17
Saccharomycotina - ps18
Saccharomycotina_1 - ps19
Saccharomycotina_2 - ps20
Saccharomycotina_3 - ps21
Phaff./Saccharomycetaceae - ps22
Saccharomyco./mycetaceae - ps23
Saccharomycetaceae - ps24
Saccharomycetaceae_2 - ps25
Saccharomycetaceae_3 - ps26
Kaz./Nau./Saccharomyces - ps27
Saccharomyces - ps28
Arboricola_clade - ps29
Cerevisiae_clade - ps30
Saccharomyces_cerevisiae - ps31
Phylostratum

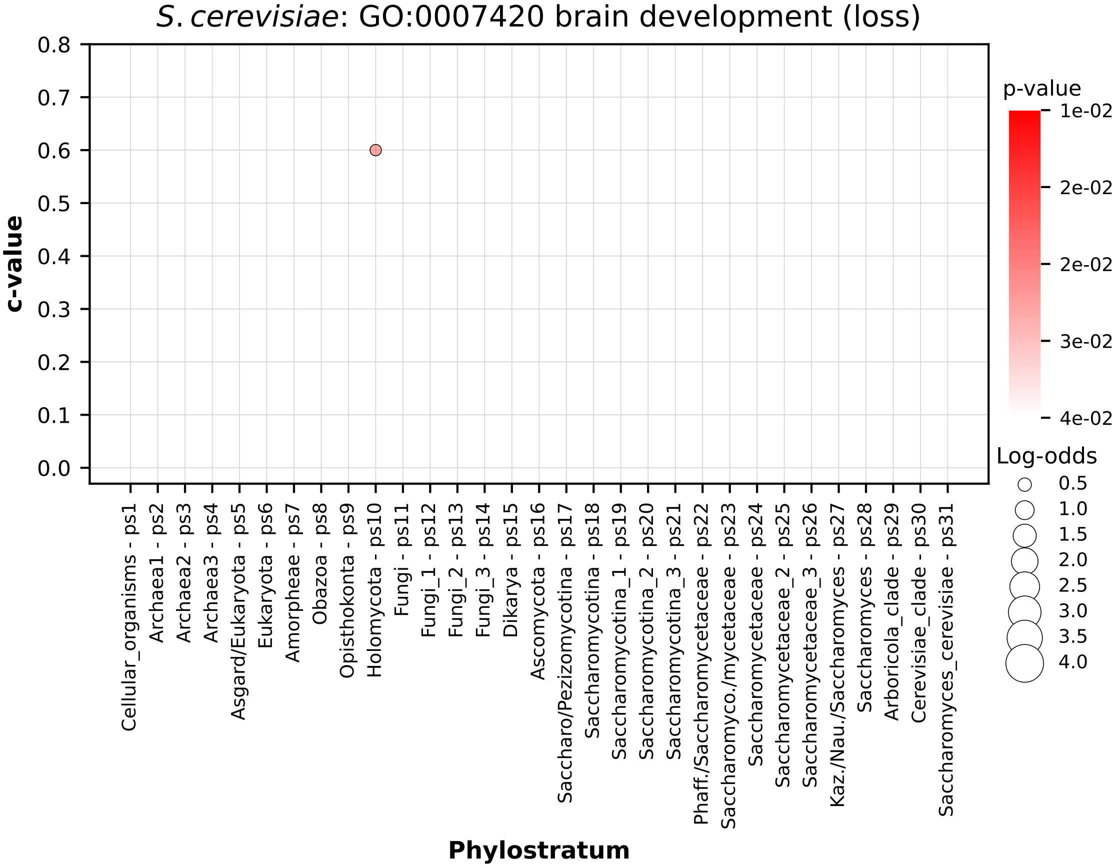
S. cerevisiae: GO:0007420 brain development (loss)
c-value
0.8
0.7
0.6
0.5
0.4
0.3
0.2
0.1
0.0
p-value
1e-02
2e-02
2e-02
3e-02
4e-02
Log-odds
0.5
1.0
1.5
2.0
2.5
3.0
3.5
4.0
Cellular_organisms - ps1
Archaea1 - ps2
Archaea2 - ps3
Archaea3 - ps4
Asgard/Eukaryota - ps5
Eukaryota - ps6
Amorpheae - ps7
Obazoa - ps8
Opisthokonta - ps9
Holomycota - ps10
Fungi - ps11
Fungi_1 - ps12
Fungi_2 - ps13
Fungi_3 - ps14
Dikarya - ps15
Ascomycota - ps16
Saccharo/Pezizomycotina - ps17
Saccharomycotina - ps18
Saccharomycotina_1 - ps19
Saccharomycotina_2 - ps20
Saccharomycotina_3 - ps21
Phaff./Saccharomycetaceae - ps22
Saccharomyco./mycetaceae - ps23
Saccharomycetaceae - ps24
Saccharomycetaceae_2 - ps25
Saccharomycetaceae_3 - ps26
Kaz./Nau./Saccharomyces - ps27
Saccharomyces - ps28
Arboricola_clade - ps29
Cerevisiae_clade - ps30
Saccharomyces_cerevisiae - ps31
Phylostratum

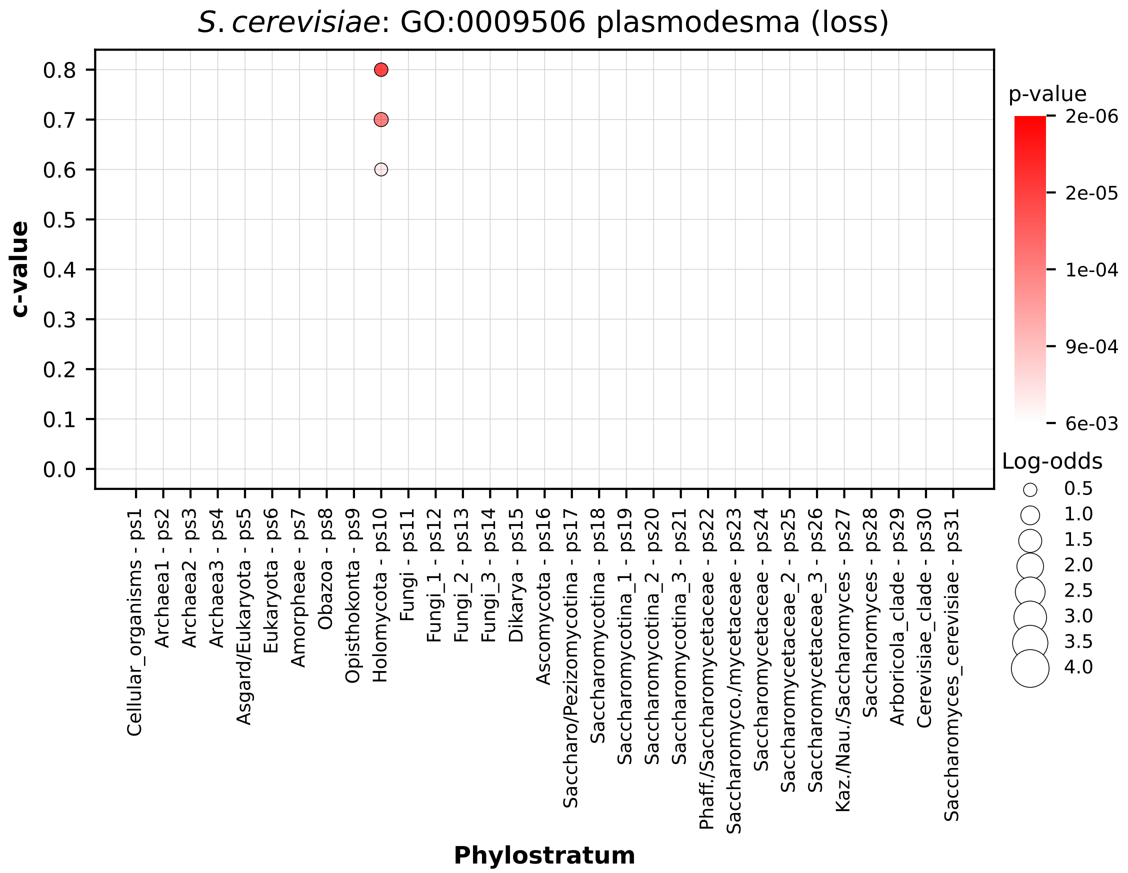

S. cerevisiae: GO:0009506 plasmodesma (loss)
c-value
0.8
0.7
0.6
0.5
0.4
0.3
0.2
0.1
0.0
Phylostratum
Cellular_organisms - ps1
Archaea1 - ps2
Archaea2 - ps3
Archaea3 - ps4
Asgard/Eukaryota - ps5
Eukaryota - ps6
Amorpheae - ps7
Obazoa - ps8
Opisthokonta - ps9
Holomycota - ps10
Fungi - ps11
Fungi_1 - ps12
Fungi_2 - ps13
Fungi_3 - ps14
Dikarya - ps15
Ascomycota - ps16
Saccharo/Pezizomycotina - ps17
Saccharomycotina - ps18
Saccharomycotina_1 - ps19
Saccharomycotina_2 - ps20
Saccharomycotina_3 - ps21
Phaff./Saccharomycetaceae - ps22
Saccharomyco./mycetaceae - ps23
Saccharomycetaceae - ps24
Saccharomycetaceae_2 - ps25
Saccharomycetaceae_3 - ps26
Kaz./Nau./Saccharomyces - ps27
Saccharomyces - ps28
Arboricola_clade - ps29
Cerevisiae_clade - ps30
Saccharomyces_cerevisiae - ps31
p-value
2e-06
2e-05
1e-04
9e-04
6e-03
Log-odds
0.5
1.0
1.5
2.0
2.5
3.0
3.5
4.0

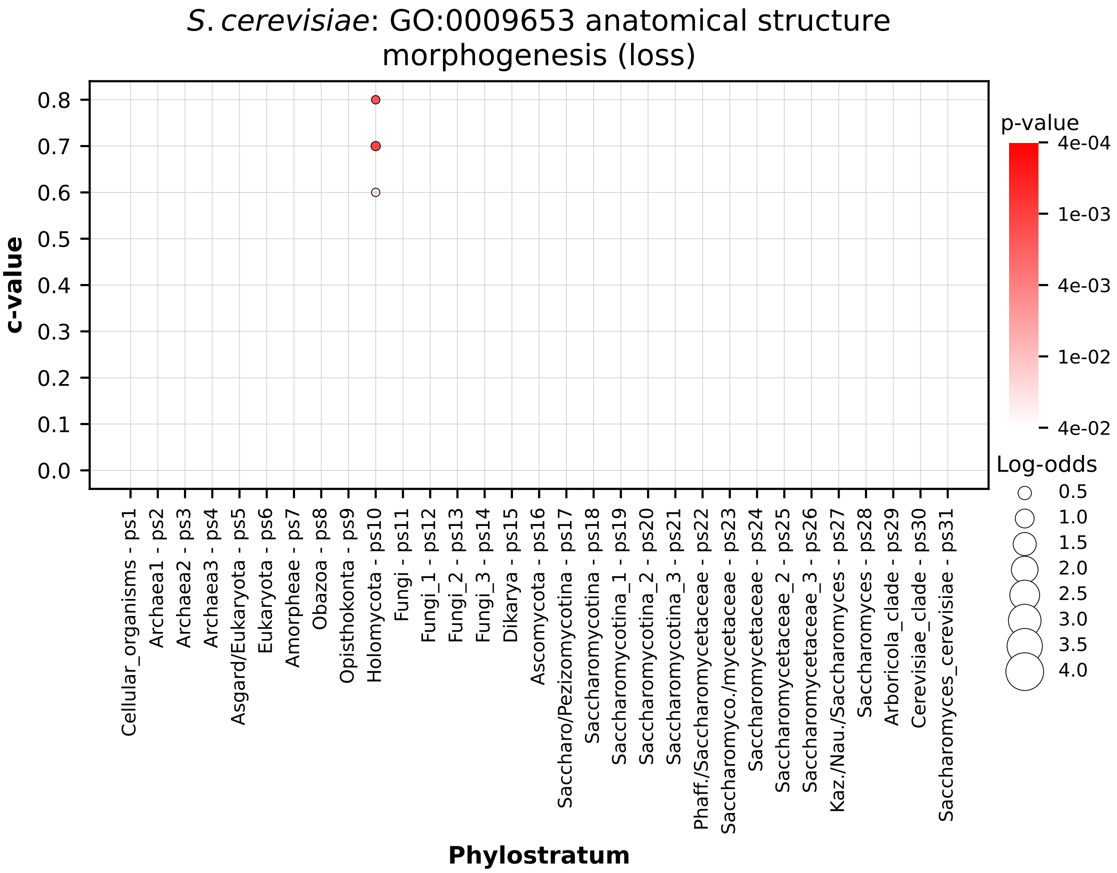
S. cerevisiae: GO:0009653 anatomical structure morphogenesis (loss)
c-value
0.0
0.1
0.2
0.3
0.4
0.5
0.6
0.7
0.8
Phylostratum
Cellular_organisms - ps1
Archaea1 - ps2
Archaea2 - ps3
Archaea3 - ps4
Asgard/Eukaryota - ps5
Eukaryota - ps6
Amorpheae - ps7
Obazoa - ps8
Opisthokonta - ps9
Holomycota - ps10
Fungi - ps11
Fungi_1 - ps12
Fungi_2 - ps13
Fungi_3 - ps14
Dikarya - ps15
Ascomycota - ps16
Saccharo/Pezizomycotina - ps17
Saccharomycotina - ps18
Saccharomycotina_1 - ps19
Saccharomycotina_2 - ps20
Saccharomycotina_3 - ps21
Phaff./Saccharomycetaceae - ps22
Saccharomyco./mycetaceae - ps23
Saccharomycetaceae - ps24
Saccharomycetaceae_2 - ps25
Saccharomycetaceae_3 - ps26
Kaz./Nau./Saccharomyces - ps27
Saccharomyces - ps28
Arboricola_clade - ps29
Cerevisiae_clade - ps30
Saccharomyces_cerevisiae - ps31
p-value
4e-04
1e-03
4e-03
1e-02
4e-02
Log-odds
0.5
1.0
1.5
2.0
2.5
3.0
3.5
4.0

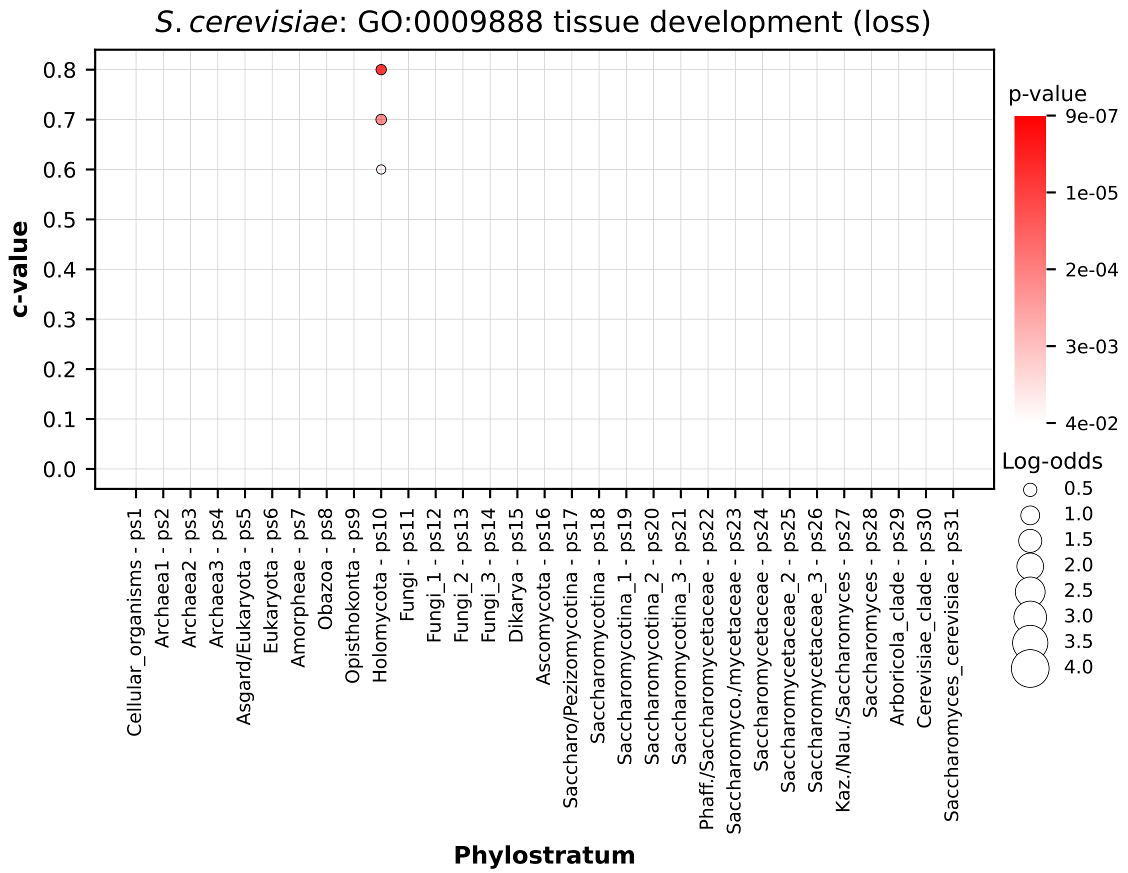
S. cerevisiae: GO:0009888 tissue development (loss)
c-value
0.8
0.7
0.6
0.5
0.4
0.3
0.2
0.1
0.0
p-value
9e-07
1e-05
2e-04
3e-03
4e-02
Log-odds
0.5
1.0
1.5
2.0
2.5
3.0
3.5
4.0
Cellular_organisms - ps1
Archaea1 - ps2
Archaea2 - ps3
Archaea3 - ps4
Asgard/Eukaryota - ps5
Eukaryota - ps6
Amorpheae - ps7
Obazoa - ps8
Opisthokonta - ps9
Holomycota - ps10
Fungi - ps11
Fungi_1 - ps12
Fungi_2 - ps13
Fungi_3 - ps14
Dikarya - ps15
Ascomycota - ps16
Saccharo/Pezizomycotina - ps17
Saccharomycotina - ps18
Saccharomycotina_1 - ps19
Saccharomycotina_2 - ps20
Saccharomycotina_3 - ps21
Phaff./Saccharomycetaceae - ps22
Saccharomyco./mycetaceae - ps23
Saccharomycetaceae - ps24
Saccharomycetaceae_2 - ps25
Saccharomycetaceae_3 - ps26
Kaz./Nau./Saccharomyces - ps27
Saccharomyces - ps28
Arboricola_clade - ps29
Cerevisiae_clade - ps30
Saccharomyces_cerevisiae - ps31
Phylostratum

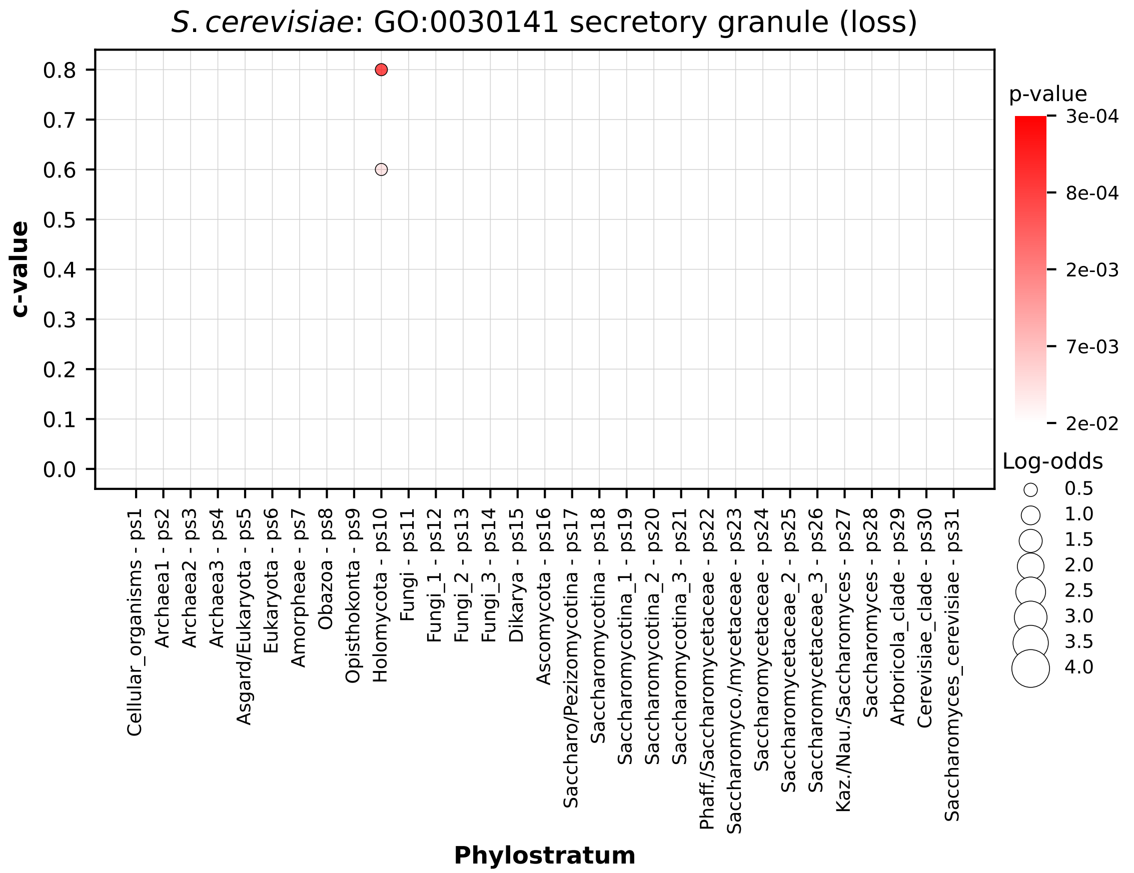

S. cerevisiae: GO:0030141 secretory granule (loss)
c-value
0.0
0.1
0.2
0.3
0.4
0.5
0.6
0.7
0.8
Phylostratum
Cellular_organisms - ps1
Archaea1 - ps2
Archaea2 - ps3
Archaea3 - ps4
Asgard/Eukaryota - ps5
Eukaryota - ps6
Amorpheae - ps7
Obazoa - ps8
Opisthokonta - ps9
Holomycota - ps10
Fungi - ps11
Fungi_1 - ps12
Fungi_2 - ps13
Fungi_3 - ps14
Dikarya - ps15
Ascomycota - ps16
Saccharo/Pezizomycotina - ps17
Saccharomycotina - ps18
Saccharomycotina_1 - ps19
Saccharomycotina_2 - ps20
Saccharomycotina_3 - ps21
Phaff./Saccharomycetaceae - ps22
Saccharomyco./mycetaceae - ps23
Saccharomycetaceae - ps24
Saccharomycetaceae_2 - ps25
Saccharomycetaceae_3 - ps26
Kaz./Nau./Saccharomyces - ps27
Saccharomyces - ps28
Arboricola_clade - ps29
Cerevisiae_clade - ps30
Saccharomyces_cerevisiae - ps31
p-value
3e-04
8e-04
2e-03
7e-03
2e-02
Log-odds
0.5
1.0
1.5
2.0
2.5
3.0
3.5
4.0

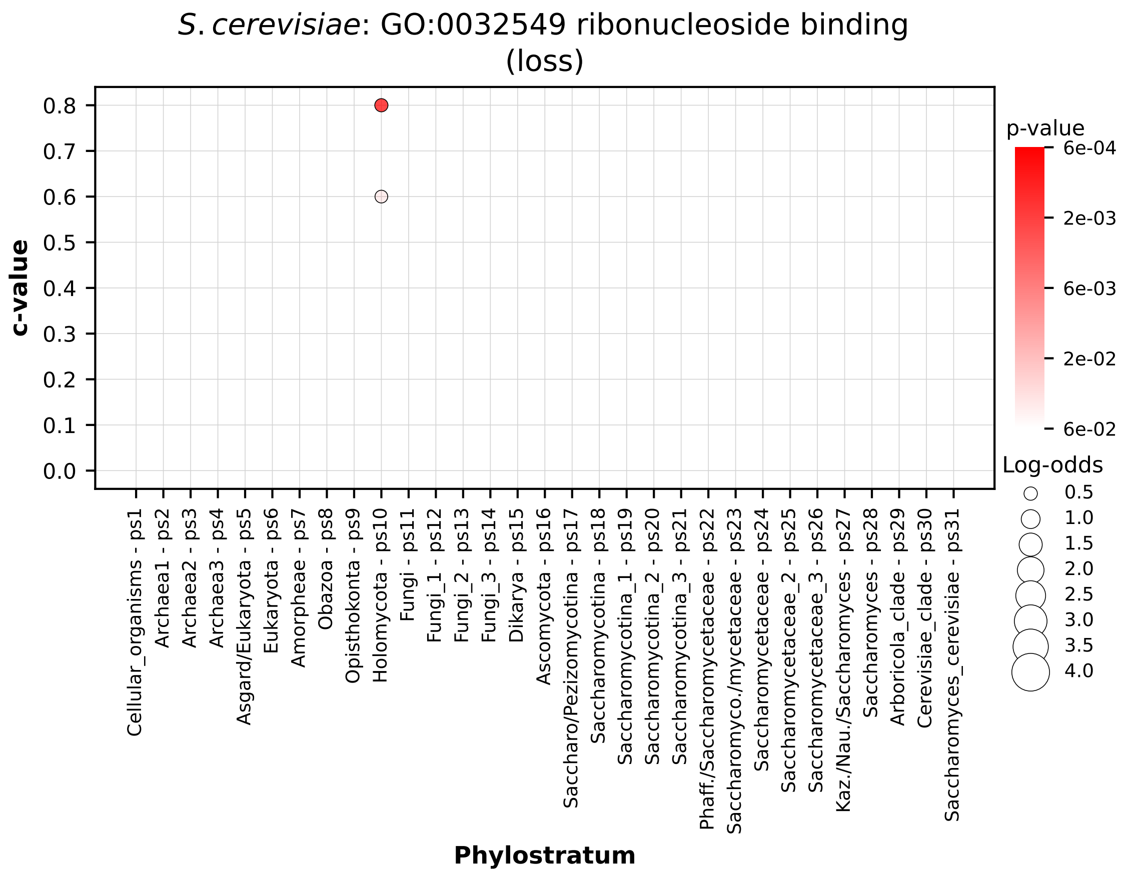

S. cerevisiae: GO:0032549 ribonucleoside binding
(loss)
c-value
0.0
0.1
0.2
0.3
0.4
0.5
0.6
0.7
0.8
p-value
6e-04
2e-03
6e-03
2e-02
6e-02
Log-odds
0.5
1.0
1.5
2.0
2.5
3.0
3.5
4.0
Cellular_organisms - ps1
Archaea1 - ps2
Archaea2 - ps3
Archaea3 - ps4
Asgard/Eukaryota - ps5
Eukaryota - ps6
Amorpheae - ps7
Obazoa - ps8
Opisthokonta - ps9
Holomycota - ps10
Fungi - ps11
Fungi_1 - ps12
Fungi_2 - ps13
Fungi_3 - ps14
Dikarya - ps15
Ascomycota - ps16
Saccharo/Pezizomycotina - ps17
Saccharomycotina - ps18
Saccharomycotina_1 - ps19
Saccharomycotina_2 - ps20
Saccharomycotina_3 - ps21
Phaff./Saccharomycetaceae - ps22
Saccharomyco./mycetaceae - ps23
Saccharomycetaceae - ps24
Saccharomycetaceae_2 - ps25
Saccharomycetaceae_3 - ps26
Kaz./Nau./Saccharomyces - ps27
Saccharomyces - ps28
Arboricola_clade - ps29
Cerevisiae_clade - ps30
Saccharomyces_cerevisiae - ps31
Phylostratum

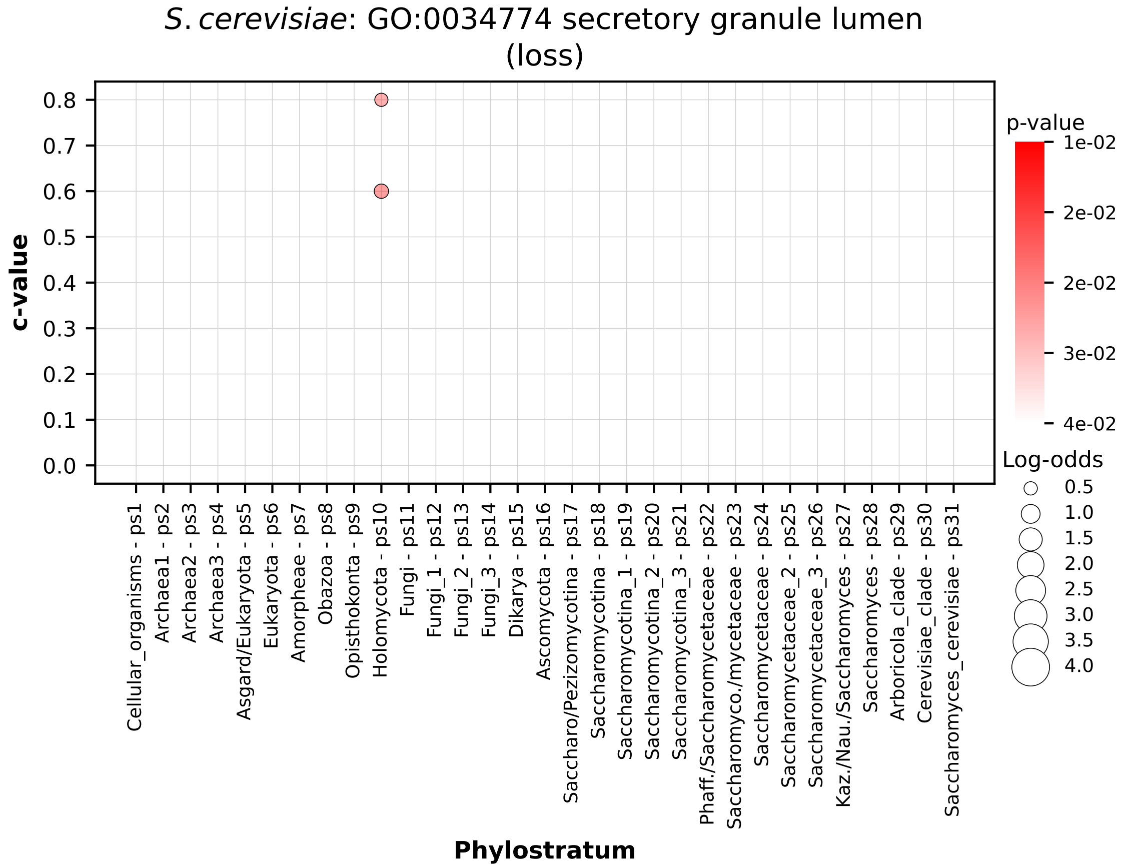

S. cerevisiae: GO:0034774 secretory granule lumen (loss)
c-value
0.0
0.1
0.2
0.3
0.4
0.5
0.6
0.7
0.8
Phylostratum
Cellular_organisms - ps1
Archaea1 - ps2
Archaea2 - ps3
Archaea3 - ps4
Asgard/Eukaryota - ps5
Eukaryota - ps6
Amorpheae - ps7
Obazoa - ps8
Opisthokonta - ps9
Holomycota - ps10
Fungi - ps11
Fungi_1 - ps12
Fungi_2 - ps13
Fungi_3 - ps14
Dikarya - ps15
Ascomycota - ps16
Saccharo/Pezizomycotina - ps17
Saccharomycotina - ps18
Saccharomycotina_1 - ps19
Saccharomycotina_2 - ps20
Saccharomycotina_3 - ps21
Phaff./Saccharomycetaceae - ps22
Saccharomyco./mycetaceae - ps23
Saccharomycetaceae - ps24
Saccharomycetaceae_2 - ps25
Saccharomycetaceae_3 - ps26
Kaz./Nau./Saccharomyces - ps27
Saccharomyces - ps28
Arboricola_clade - ps29
Cerevisiae_clade - ps30
Saccharomyces_cerevisiae - ps31
p-value
1e-02
2e-02
2e-02
3e-02
4e-02
Log-odds
0.5
1.0
1.5
2.0
2.5
3.0
3.5
4.0

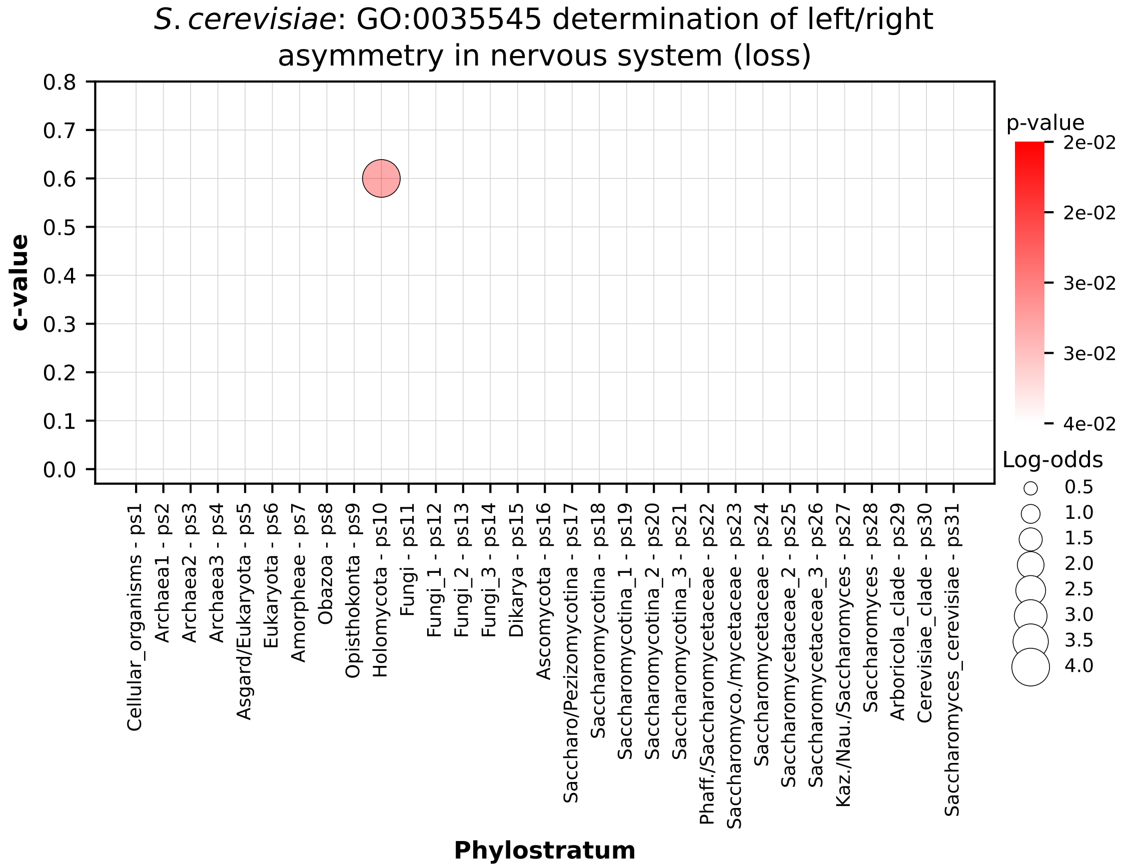
S. cerevisiae: GO:0035545 determination of left/right asymmetry in nervous system (loss)
c-value
0.8
0.7
0.6
0.5
0.4
0.3
0.2
0.1
0.0
p-value
2e-02
2e-02
3e-02
3e-02
4e-02
Log-odds
0.5
1.0
1.5
2.0
2.5
3.0
3.5
4.0
Cellular_organisms - ps1
Archaea1 - ps2
Archaea2 - ps3
Archaea3 - ps4
Asgard/Eukaryota - ps5
Eukaryota - ps6
Amorpheae - ps7
Obazoa - ps8
Opisthokonta - ps9
Holomycota - ps10
Fungi - ps11
Fungi_1 - ps12
Fungi_2 - ps13
Fungi_3 - ps14
Dikarya - ps15
Ascomycota - ps16
Saccharo/Pezizomycotina - ps17
Saccharomycotina - ps18
Saccharomycotina_1 - ps19
Saccharomycotina_2 - ps20
Saccharomycotina_3 - ps21
Phaff./Saccharomycetaceae - ps22
Saccharomyco./mycetaceae - ps23
Saccharomycetaceae - ps24
Saccharomycetaceae_2 - ps25
Saccharomycetaceae_3 - ps26
Kaz./Nau./Saccharomyces - ps27
Saccharomyces - ps28
Arboricola_clade - ps29
Cerevisiae_clade - ps30
Saccharomyces_cerevisiae - ps31
Phylostratum

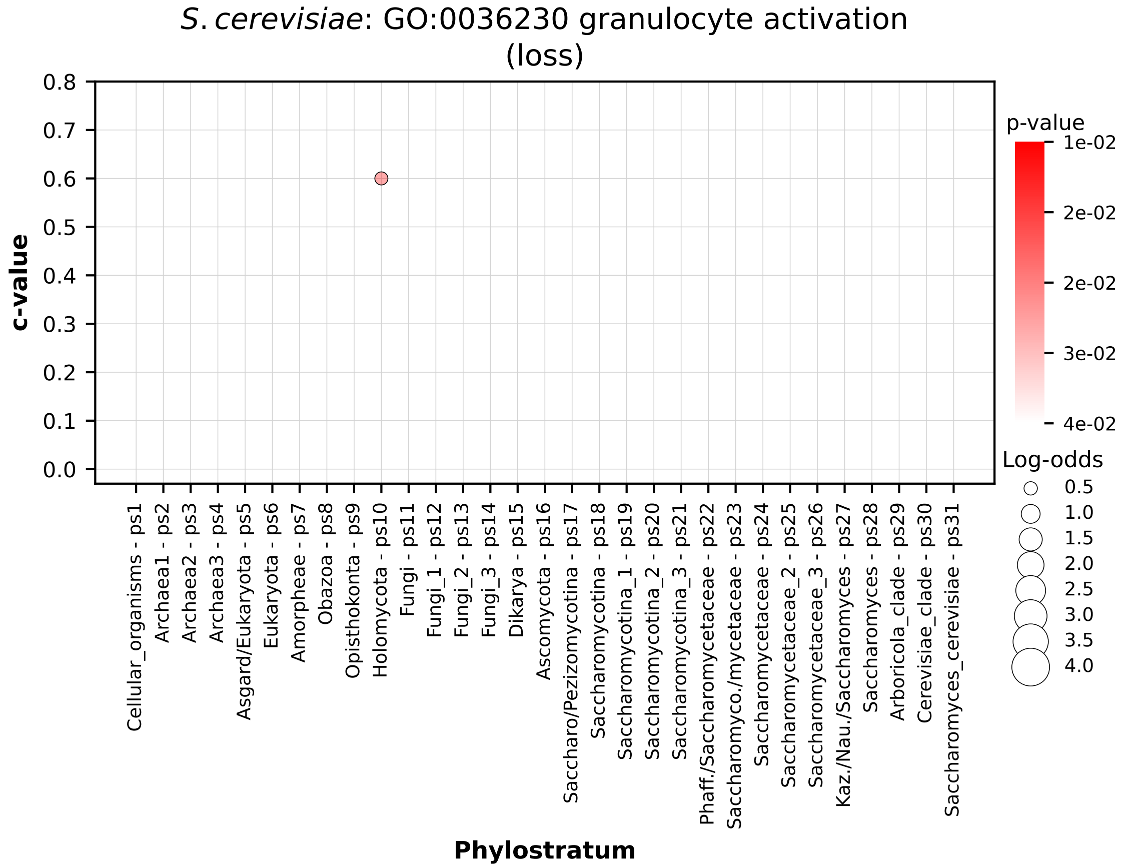

S. cerevisiae: GO:0036230 granulocyte activation
(loss)
c-value
0.8
0.7
0.6
0.5
0.4
0.3
0.2
0.1
0.0
p-value
1e-02
2e-02
2e-02
3e-02
4e-02
Log-odds
0.5
1.0
1.5
2.0
2.5
3.0
3.5
4.0
Cellular_organisms - ps1
Archaea1 - ps2
Archaea2 - ps3
Archaea3 - ps4
Asgard/Eukaryota - ps5
Eukaryota - ps6
Amorpheae - ps7
Obazoa - ps8
Opisthokonta - ps9
Holomycota - ps10
Fungi - ps11
Fungi_1 - ps12
Fungi_2 - ps13
Fungi_3 - ps14
Dikarya - ps15
Ascomycota - ps16
Saccharo/Pezizomycotina - ps17
Saccharomycotina - ps18
Saccharomycotina_1 - ps19
Saccharomycotina_2 - ps20
Saccharomycotina_3 - ps21
Phaff./Saccharomycetaceae - ps22
Saccharomyco./mycetaceae - ps23
Saccharomycetaceae - ps24
Saccharomycetaceae_2 - ps25
Saccharomycetaceae_3 - ps26
Kaz./Nau./Saccharomyces - ps27
Saccharomyces - ps28
Arboricola_clade - ps29
Cerevisiae_clade - ps30
Saccharomyces_cerevisiae - ps31
Phylostratum

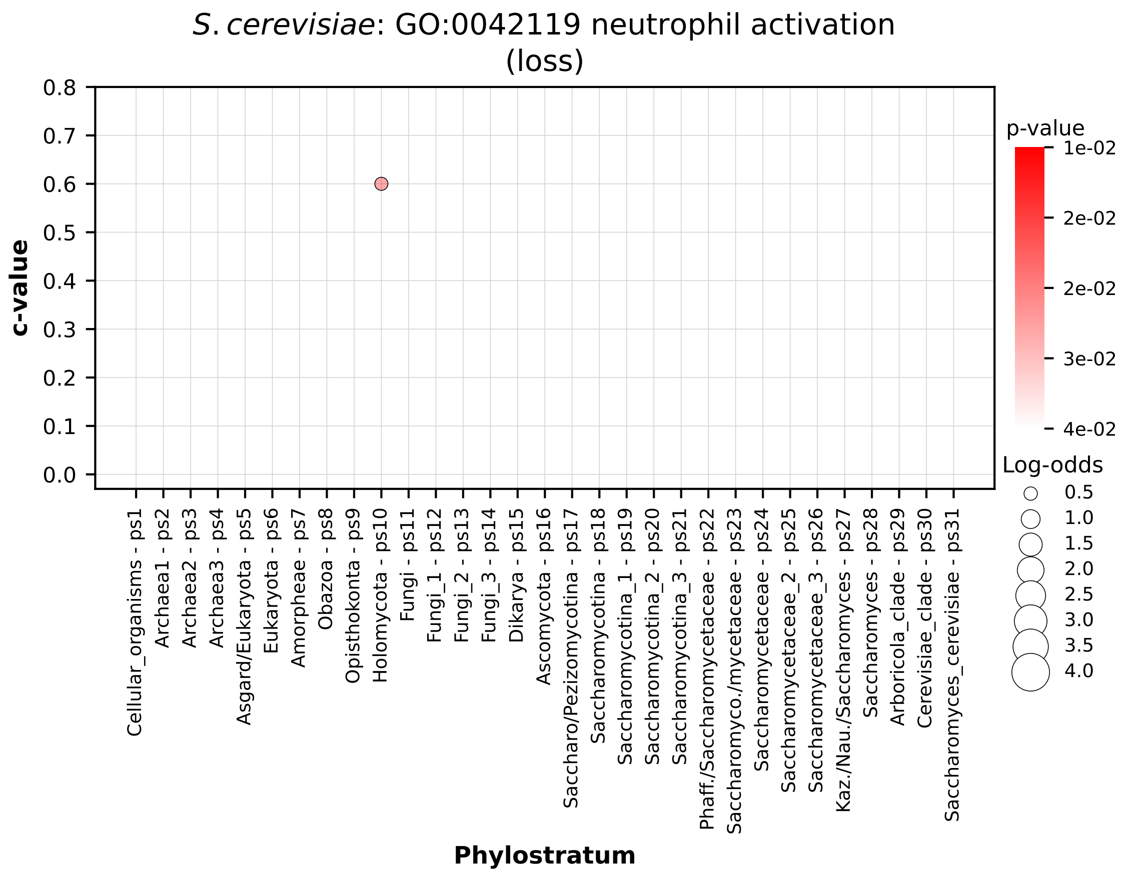
S. cerevisiae: GO:0042119 neutrophil activation
(loss)
c-value
0.0
0.1
0.2
0.3
0.4
0.5
0.6
0.7
0.8
Phylostratum
Cellular_organisms - ps1
Archaea1 - ps2
Archaea2 - ps3
Archaea3 - ps4
Asgard/Eukaryota - ps5
Eukaryota - ps6
Amorpheae - ps7
Obazoa - ps8
Opisthokonta - ps9
Holomycota - ps10
Fungi - ps11
Fungi_1 - ps12
Fungi_2 - ps13
Fungi_3 - ps14
Dikarya - ps15
Ascomycota - ps16
Saccharo/Pezizomycotina - ps17
Saccharomycotina - ps18
Saccharomycotina_1 - ps19
Saccharomycotina_2 - ps20
Saccharomycotina_3 - ps21
Phaff./Saccharomycetaceae - ps22
Saccharomyco./mycetaceae - ps23
Saccharomycetaceae - ps24
Saccharomycetaceae_2 - ps25
Saccharomycetaceae_3 - ps26
Kaz./Nau./Saccharomyces - ps27
Saccharomyces - ps28
Arboricola_clade - ps29
Cerevisiae_clade - ps30
Saccharomyces_cerevisiae - ps31
p-value
1e-02
2e-02
2e-02
3e-02
4e-02
Log-odds
0.5
1.0
1.5
2.0
2.5
3.0
3.5
4.0

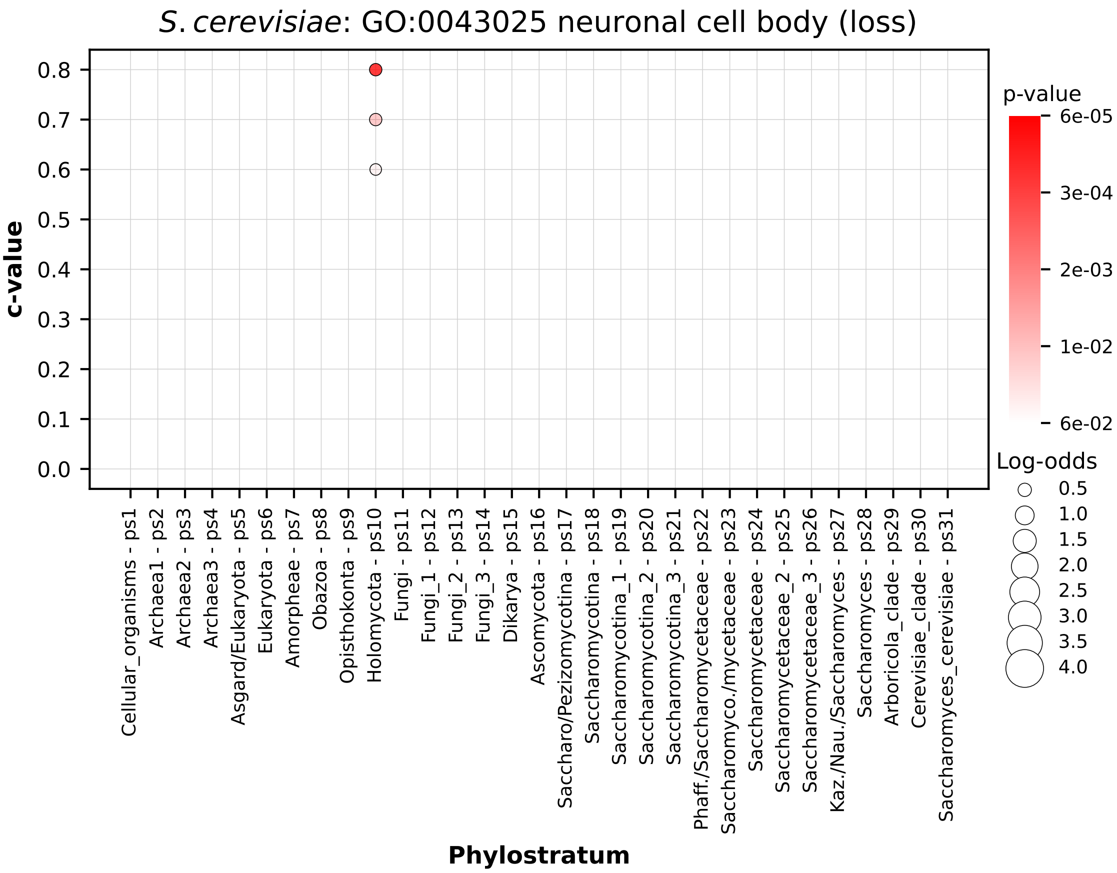

S. cerevisiae: GO:0043025 neuronal cell body (loss)
c-value
0.0
0.1
0.2
0.3
0.4
0.5
0.6
0.7
0.8
p-value
6e-05
3e-04
2e-03
1e-02
6e-02
Log-odds
0.5
1.0
1.5
2.0
2.5
3.0
3.5
4.0
Cellular_organisms - ps1
Archaea1 - ps2
Archaea2 - ps3
Archaea3 - ps4
Asgard/Eukaryota - ps5
Eukaryota - ps6
Amorpheae - ps7
Obazoa - ps8
Opisthokonta - ps9
Holomycota - ps10
Fungi - ps11
Fungi_1 - ps12
Fungi_2 - ps13
Fungi_3 - ps14
Dikarya - ps15
Ascomycota - ps16
Saccharo/Pezizomycotina - ps17
Saccharomycotina - ps18
Saccharomycotina_1 - ps19
Saccharomycotina_2 - ps20
Saccharomycotina_3 - ps21
Phaff./Saccharomycetaceae - ps22
Saccharomyco./mycetaceae - ps23
Saccharomycetaceae - ps24
Saccharomycetaceae_2 - ps25
Saccharomycetaceae_3 - ps26
Kaz./Nau./Saccharomyces - ps27
Saccharomyces - ps28
Arboricola_clade - ps29
Cerevisiae_clade - ps30
Saccharomyces_cerevisiae - ps31
Phylostratum

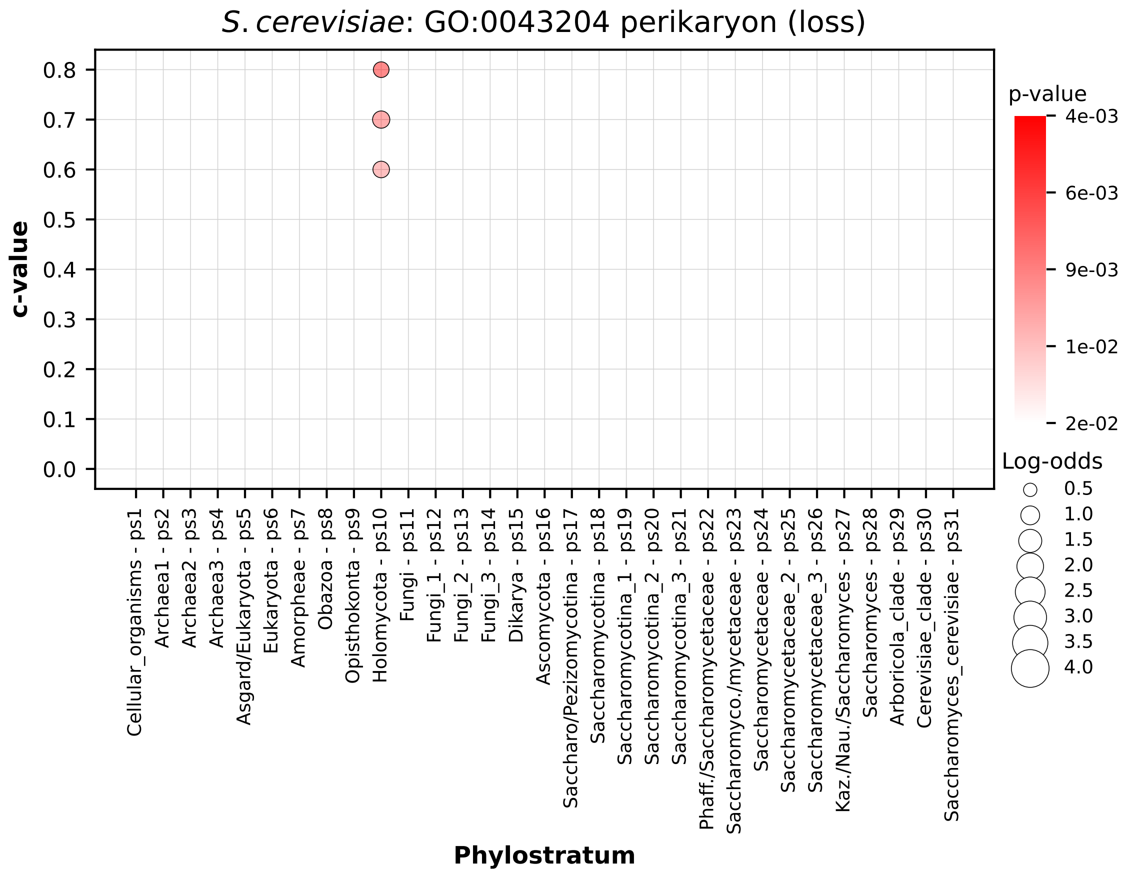
S. cerevisiae: GO:0043204 perikaryon (loss)
c-value
0.0
0.1
0.2
0.3
0.4
0.5
0.6
0.7
0.8
p-value
4e-03
6e-03
9e-03
1e-02
2e-02
Log-odds
0.5
1.0
1.5
2.0
2.5
3.0
3.5
4.0
Cellular_organisms - ps1
Archaea1 - ps2
Archaea2 - ps3
Archaea3 - ps4
Asgard/Eukaryota - ps5
Eukaryota - ps6
Amorpheae - ps7
Obazoa - ps8
Opisthokonta - ps9
Holomycota - ps10
Fungi - ps11
Fungi_1 - ps12
Fungi_2 - ps13
Fungi_3 - ps14
Dikarya - ps15
Ascomycota - ps16
Saccharo/Pezizomycotina - ps17
Saccharomycotina - ps18
Saccharomycotina_1 - ps19
Saccharomycotina_2 - ps20
Saccharomycotina_3 - ps21
Phaff./Saccharomycetaceae - ps22
Saccharomyco./mycetaceae - ps23
Saccharomycetaceae - ps24
Saccharomycetaceae_2 - ps25
Saccharomycetaceae_3 - ps26
Kaz./Nau./Saccharomyces - ps27
Saccharomyces - ps28
Arboricola_clade - ps29
Cerevisiae_clade - ps30
Saccharomyces_cerevisiae - ps31
Phylostratum

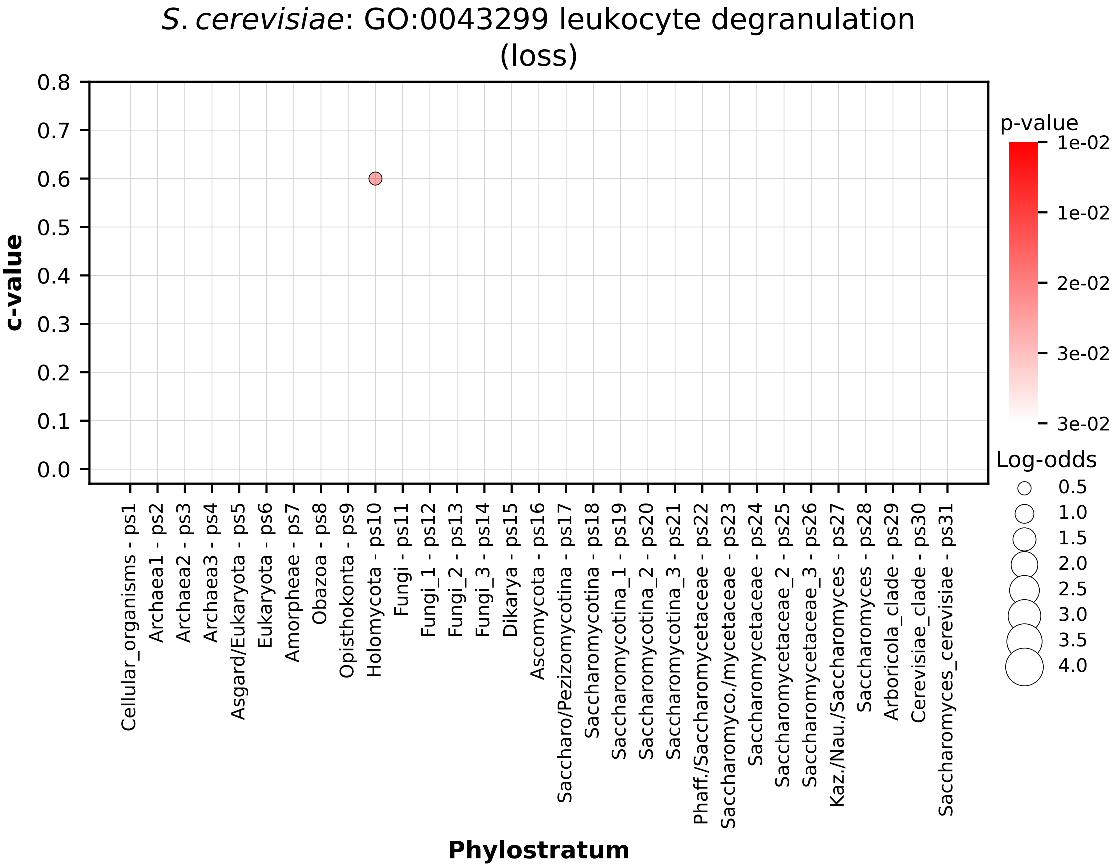

S. cerevisiae: GO:0043299 leukocyte degranulation
(loss)
c-value
0.8
0.7
0.6
0.5
0.4
0.3
0.2
0.1
0.0
p-value
1e-02
1e-02
2e-02
3e-02
3e-02
Log-odds
0.5
1.0
1.5
2.0
2.5
3.0
3.5
4.0
Cellular_organisms - ps1
Archaea1 - ps2
Archaea2 - ps3
Archaea3 - ps4
Asgard/Eukaryota - ps5
Eukaryota - ps6
Amorpheae - ps7
Obazoa - ps8
Opisthokonta - ps9
Holomycota - ps10
Fungi - ps11
Fungi_1 - ps12
Fungi_2 - ps13
Fungi_3 - ps14
Dikarya - ps15
Ascomycota - ps16
Saccharo/Pezizomycotina - ps17
Saccharomycotina - ps18
Saccharomycotina_1 - ps19
Saccharomycotina_2 - ps20
Saccharomycotina_3 - ps21
Phaff./Saccharomycetaceae - ps22
Saccharomyco./mycetaceae - ps23
Saccharomycetaceae - ps24
Saccharomycetaceae_2 - ps25
Saccharomycetaceae_3 - ps26
Kaz./Nau./Saccharomyces - ps27
Saccharomyces - ps28
Arboricola_clade - ps29
Cerevisiae_clade - ps30
Saccharomyces_cerevisiae - ps31
Phylostratum

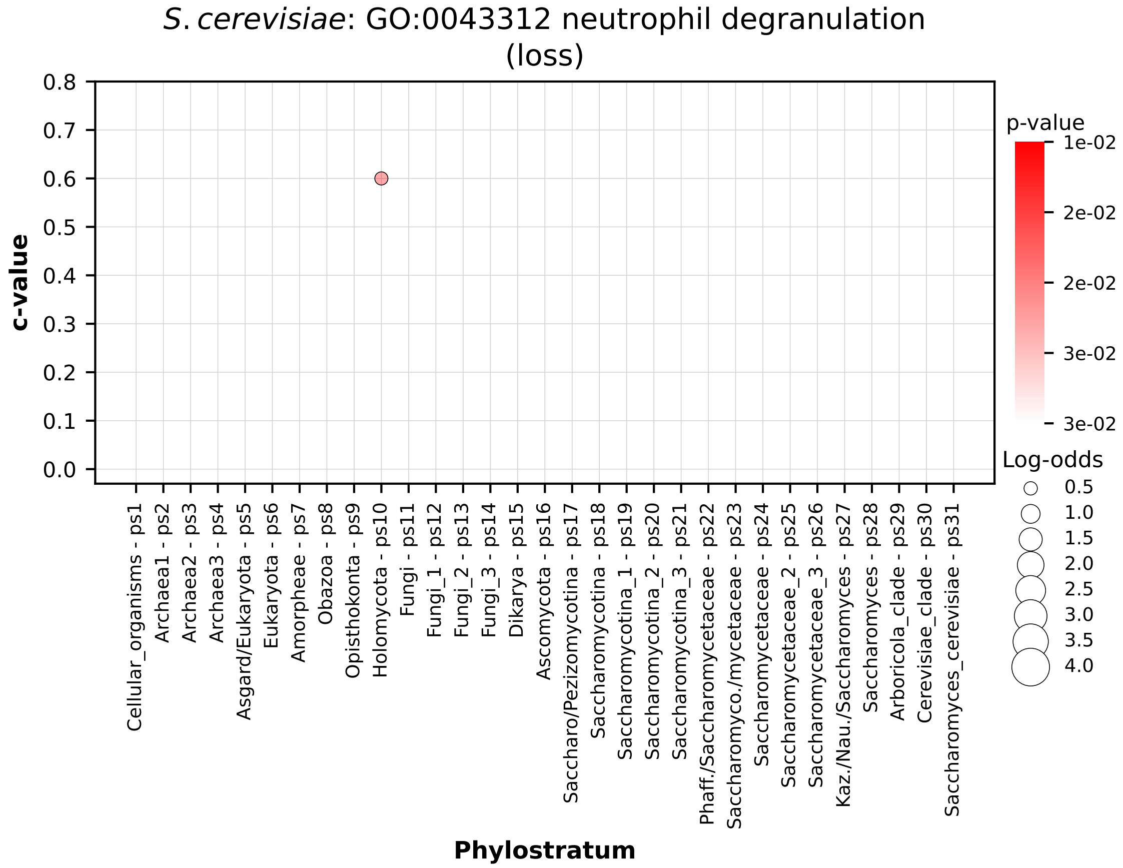
S. cerevisiae: GO:0043312 neutrophil degranulation
(loss)
c-value
0.8
0.7
0.6
0.5
0.4
0.3
0.2
0.1
0.0
p-value
1e-02
2e-02
2e-02
3e-02
3e-02
Log-odds
0.5
1.0
1.5
2.0
2.5
3.0
3.5
4.0
Cellular_organisms - ps1
Archaea1 - ps2
Archaea2 - ps3
Archaea3 - ps4
Asgard/Eukaryota - ps5
Eukaryota - ps6
Amorpheae - ps7
Obazoa - ps8
Opisthokonta - ps9
Holomycota - ps10
Fungi - ps11
Fungi_1 - ps12
Fungi_2 - ps13
Fungi_3 - ps14
Dikarya - ps15
Ascomycota - ps16
Saccharo/Pezizomycotina - ps17
Saccharomycotina - ps18
Saccharomycotina_1 - ps19
Saccharomycotina_2 - ps20
Saccharomycotina_3 - ps21
Phaff./Saccharomycetaceae - ps22
Saccharomyco./mycetaceae - ps23
Saccharomycetaceae - ps24
Saccharomycetaceae_2 - ps25
Saccharomycetaceae_3 - ps26
Kaz./Nau./Saccharomyces - ps27
Saccharomyces - ps28
Arboricola_clade - ps29
Cerevisiae_clade - ps30
Saccharomyces_cerevisiae - ps31
Phylostratum

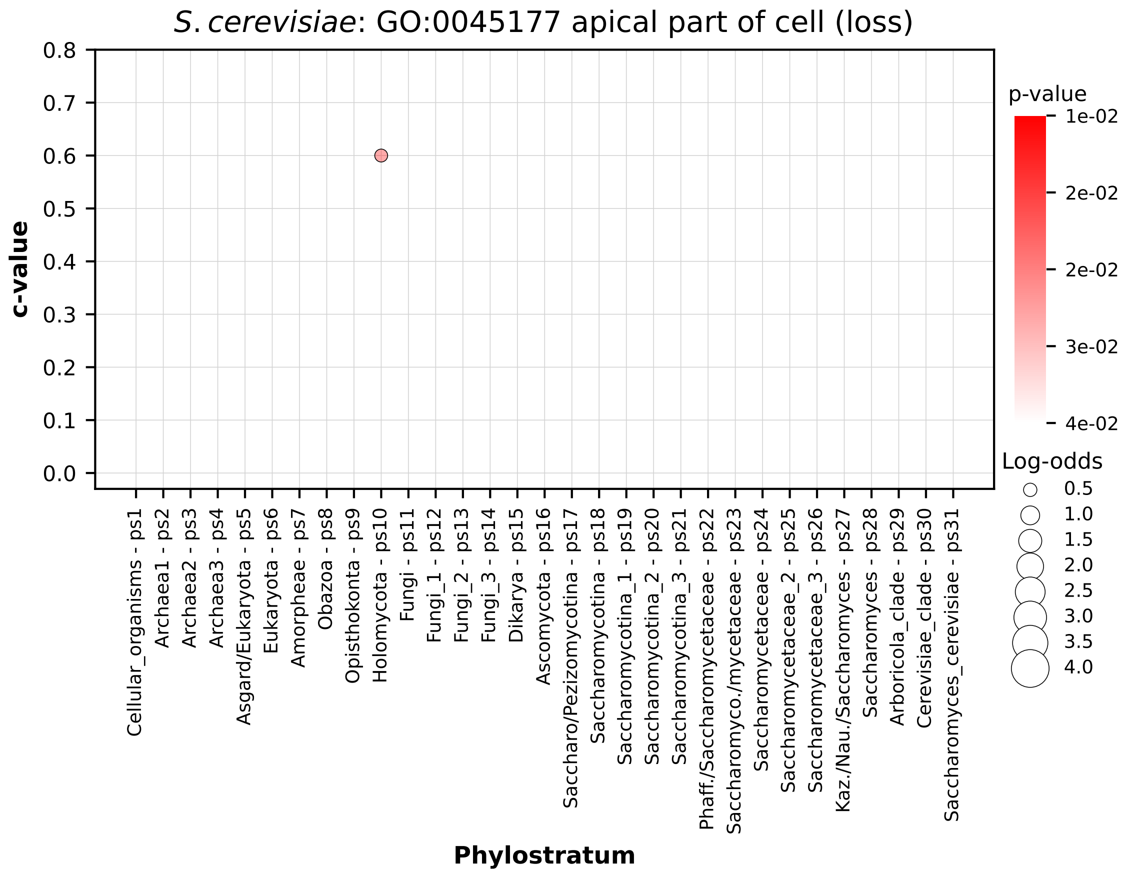

S. cerevisiae: GO:0045177 apical part of cell (loss)
c-value
0.8
0.7
0.6
0.5
0.4
0.3
0.2
0.1
0.0
Phylostratum
Cellular_organisms - ps1
Archaea1 - ps2
Archaea2 - ps3
Archaea3 - ps4
Asgard/Eukaryota - ps5
Eukaryota - ps6
Amorpheae - ps7
Obazoa - ps8
Opisthokonta - ps9
Holomycota - ps10
Fungi - ps11
Fungi_1 - ps12
Fungi_2 - ps13
Fungi_3 - ps14
Dikarya - ps15
Ascomycota - ps16
Saccharo/Pezizomycotina - ps17
Saccharomycotina - ps18
Saccharomycotina_1 - ps19
Saccharomycotina_2 - ps20
Saccharomycotina_3 - ps21
Phaff./Saccharomycetaceae - ps22
Saccharomyco./mycetaceae - ps23
Saccharomycetaceae - ps24
Saccharomycetaceae_2 - ps25
Saccharomycetaceae_3 - ps26
Kaz./Nau./Saccharomyces - ps27
Saccharomyces - ps28
Arboricola_clade - ps29
Cerevisiae_clade - ps30
Saccharomyces_cerevisiae - ps31
p-value
1e-02
2e-02
2e-02
3e-02
4e-02
Log-odds
0.5
1.0
1.5
2.0
2.5
3.0
3.5
4.0

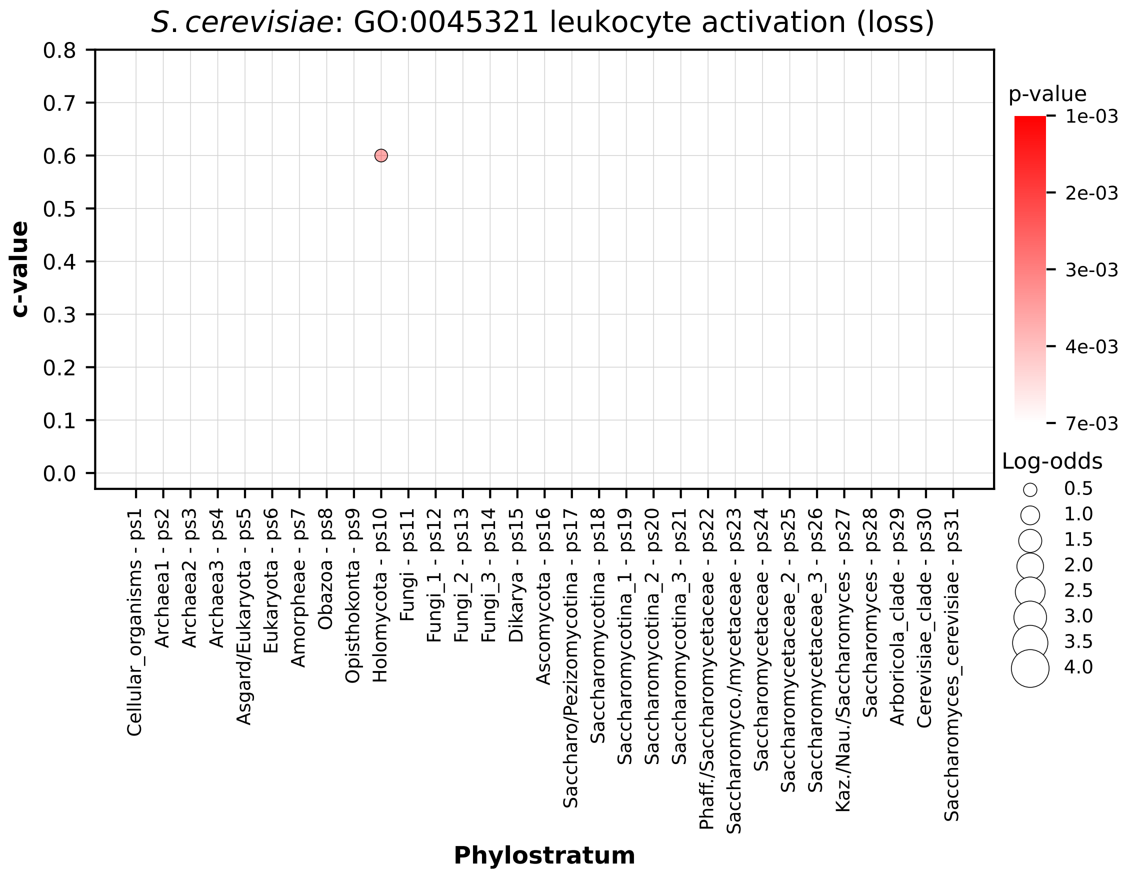
S. cerevisiae: GO:0045321 leukocyte activation (loss)
c-value
0.8
0.7
0.6
0.5
0.4
0.3
0.2
0.1
0.0
p-value
1e-03
2e-03
3e-03
4e-03
7e-03
Log-odds
0.5
1.0
1.5
2.0
2.5
3.0
3.5
4.0
Cellular_organisms - ps1
Archaea1 - ps2
Archaea2 - ps3
Archaea3 - ps4
Asgard/Eukaryota - ps5
Eukaryota - ps6
Amorpheae - ps7
Obazoa - ps8
Opisthokonta - ps9
Holomycota - ps10
Fungi - ps11
Fungi_1 - ps12
Fungi_2 - ps13
Fungi_3 - ps14
Dikarya - ps15
Ascomycota - ps16
Saccharo/Pezizomycotina - ps17
Saccharomycotina - ps18
Saccharomycotina_1 - ps19
Saccharomycotina_2 - ps20
Saccharomycotina_3 - ps21
Phaff./Saccharomycetaceae - ps22
Saccharomyco./mycetaceae - ps23
Saccharomycetaceae - ps24
Saccharomycetaceae_2 - ps25
Saccharomycetaceae_3 - ps26
Kaz./Nau./Saccharomyces - ps27
Saccharomyces - ps28
Arboricola_clade - ps29
Cerevisiae_clade - ps30
Saccharomyces_cerevisiae - ps31
Phylostratum

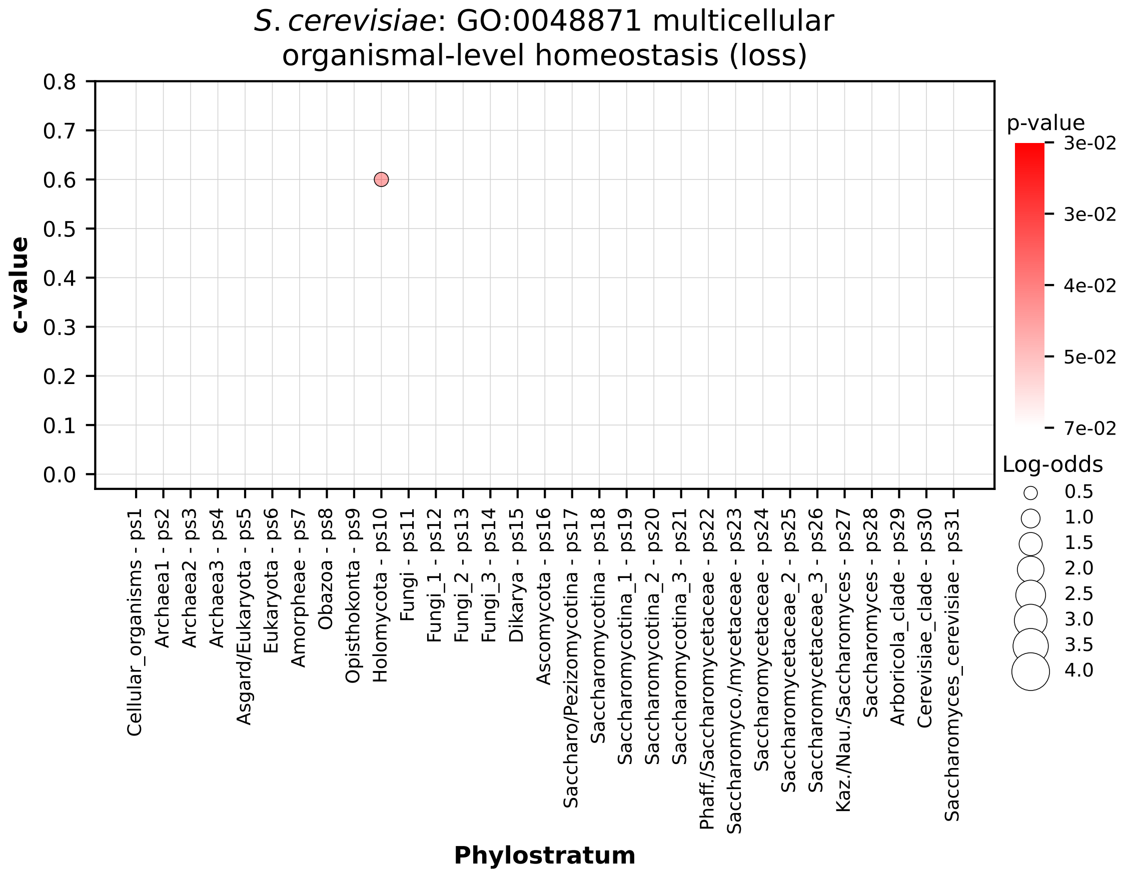
S. cerevisiae: GO:0048871 multicellular organismal-level homeostasis (loss)
c-value
0.8
0.7
0.6
0.5
0.4
0.3
0.2
0.1
0.0
Phylostratum
Cellular_organisms - ps1
Archaea1 - ps2
Archaea2 - ps3
Archaea3 - ps4
Asgard/Eukaryota - ps5
Eukaryota - ps6
Amorpheae - ps7
Obazoa - ps8
Opisthokonta - ps9
Holomycota - ps10
Fungi - ps11
Fungi_1 - ps12
Fungi_2 - ps13
Fungi_3 - ps14
Dikarya - ps15
Ascomycota - ps16
Saccharo/Pezizomycotina - ps17
Saccharomycotina - ps18
Saccharomycotina_1 - ps19
Saccharomycotina_2 - ps20
Saccharomycotina_3 - ps21
Phaff./Saccharomycetaceae - ps22
Saccharomyco./mycetaceae - ps23
Saccharomycetaceae - ps24
Saccharomycetaceae_2 - ps25
Saccharomycetaceae_3 - ps26
Kaz./Nau./Saccharomyces - ps27
Saccharomyces - ps28
Arboricola_clade - ps29
Cerevisiae_clade - ps30
Saccharomyces_cerevisiae - ps31
p-value
3e-02
3e-02
4e-02
5e-02
7e-02
Log-odds
0.5
1.0
1.5
2.0
2.5
3.0
3.5
4.0

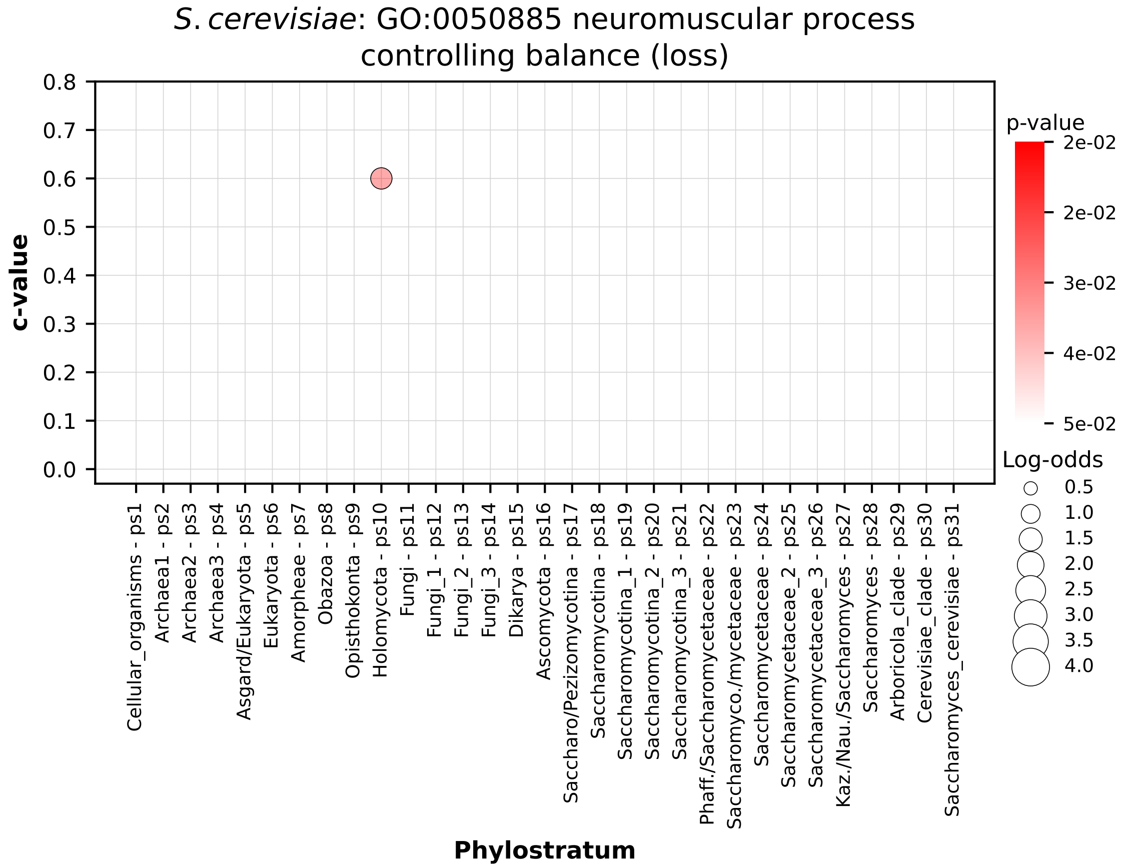

S. cerevisiae: GO:0050885 neuromuscular process controlling balance (loss)
c-value
0.8
0.7
0.6
0.5
0.4
0.3
0.2
0.1
0.0
p-value
2e-02
2e-02
3e-02
4e-02
5e-02
Log-odds
0.5
1.0
1.5
2.0
2.5
3.0
3.5
4.0
Cellular_organisms - ps1
Archaea1 - ps2
Archaea2 - ps3
Archaea3 - ps4
Asgard/Eukaryota - ps5
Eukaryota - ps6
Amorpheae - ps7
Obazoa - ps8
Opisthokonta - ps9
Holomycota - ps10
Fungi - ps11
Fungi_1 - ps12
Fungi_2 - ps13
Fungi_3 - ps14
Dikarya - ps15
Ascomycota - ps16
Saccharo/Pezizomycotina - ps17
Saccharomycotina - ps18
Saccharomycotina_1 - ps19
Saccharomycotina_2 - ps20
Saccharomycotina_3 - ps21
Phaff./Saccharomycetaceae - ps22
Saccharomyco./mycetaceae - ps23
Saccharomycetaceae - ps24
Saccharomycetaceae_2 - ps25
Saccharomycetaceae_3 - ps26
Kaz./Nau./Saccharomyces - ps27
Saccharomyces - ps28
Arboricola_clade - ps29
Cerevisiae_clade - ps30
Saccharomyces_cerevisiae - ps31
Phylostratum

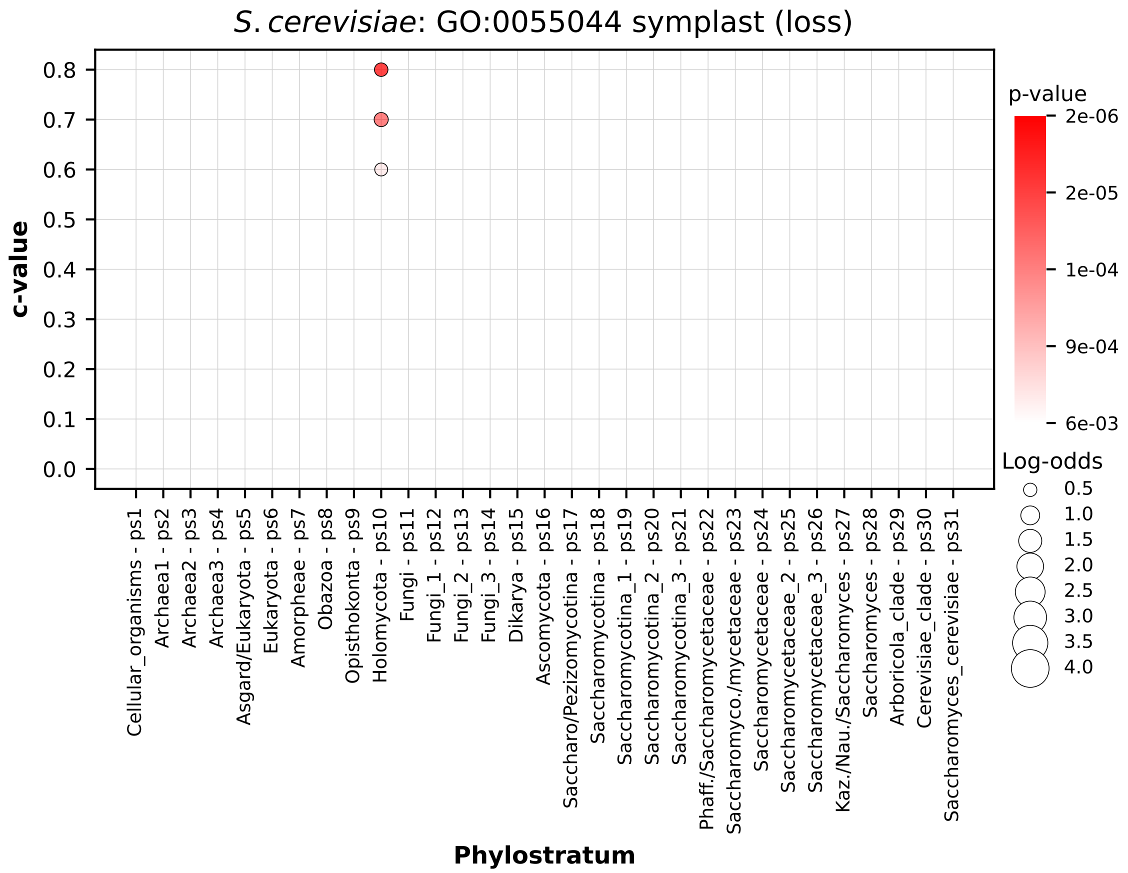

S. cerevisiae: GO:0055044 symplast (loss)
c-value
0.8
0.7
0.6
0.5
0.4
0.3
0.2
0.1
0.0
p-value
2e-06
2e-05
1e-04
9e-04
6e-03
Log-odds
0.5
1.0
1.5
2.0
2.5
3.0
3.5
4.0
Cellular_organisms - ps1
Archaea1 - ps2
Archaea2 - ps3
Archaea3 - ps4
Asgard/Eukaryota - ps5
Eukaryota - ps6
Amorpheae - ps7
Obazoa - ps8
Opisthokonta - ps9
Holomycota - ps10
Fungi - ps11
Fungi_1 - ps12
Fungi_2 - ps13
Fungi_3 - ps14
Dikarya - ps15
Ascomycota - ps16
Saccharo/Pezizomycotina - ps17
Saccharomycotina - ps18
Saccharomycotina_1 - ps19
Saccharomycotina_2 - ps20
Saccharomycotina_3 - ps21
Phaff./Saccharomycetaceae - ps22
Saccharomyco./mycetaceae - ps23
Saccharomycetaceae - ps24
Saccharomycetaceae_2 - ps25
Saccharomycetaceae_3 - ps26
Kaz./Nau./Saccharomyces - ps27
Saccharomyces - ps28
Arboricola_clade - ps29
Cerevisiae_clade - ps30
Saccharomyces_cerevisiae - ps31
Phylostratum

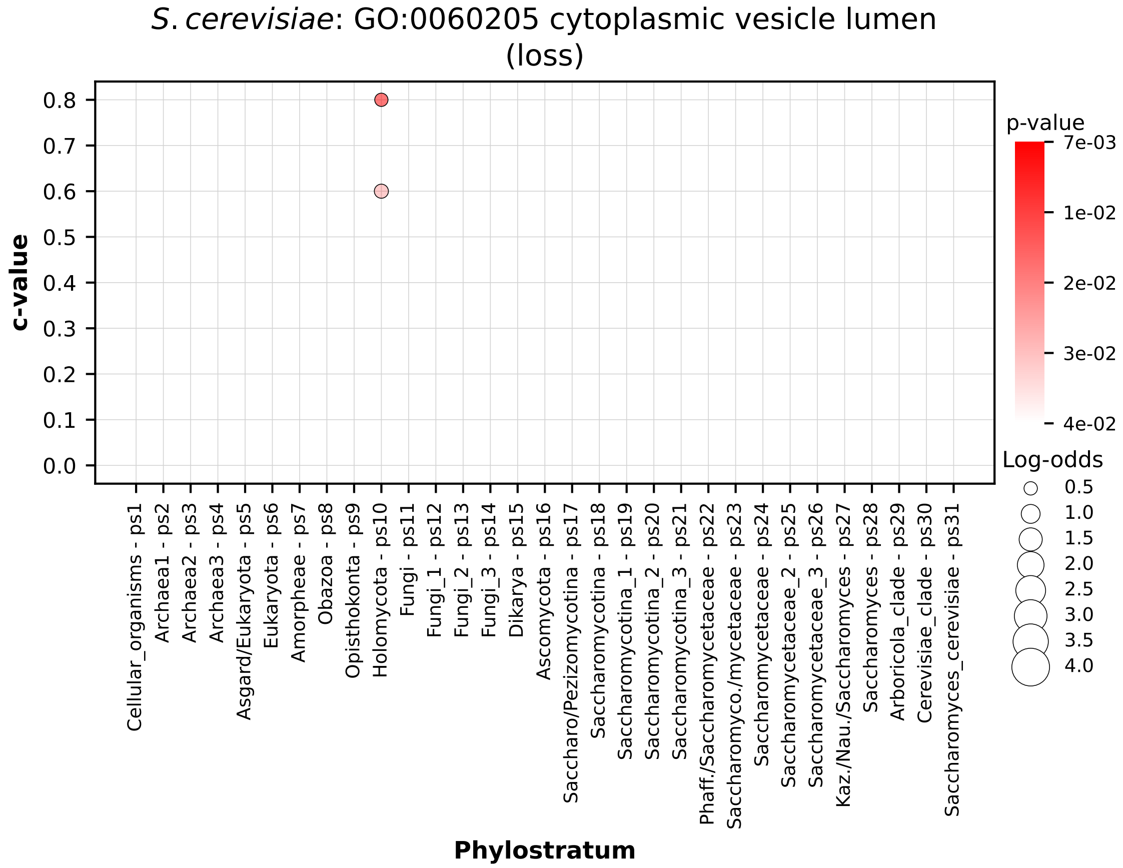
S. cerevisiae: GO:0060205 cytoplasmic vesicle lumen
(loss)
c-value
0.0
0.1
0.2
0.3
0.4
0.5
0.6
0.7
0.8
Phylostratum
Cellular_organisms - ps1
Archaea1 - ps2
Archaea2 - ps3
Archaea3 - ps4
Asgard/Eukaryota - ps5
Eukaryota - ps6
Amorpheae - ps7
Obazoa - ps8
Opisthokonta - ps9
Holomycota - ps10
Fungi - ps11
Fungi_1 - ps12
Fungi_2 - ps13
Fungi_3 - ps14
Dikarya - ps15
Ascomycota - ps16
Saccharo/Pezizomycotina - ps17
Saccharomycotina - ps18
Saccharomycotina_1 - ps19
Saccharomycotina_2 - ps20
Saccharomycotina_3 - ps21
Phaff./Saccharomycetaceae - ps22
Saccharomyco./mycetaceae - ps23
Saccharomycetaceae - ps24
Saccharomycetaceae_2 - ps25
Saccharomycetaceae_3 - ps26
Kaz./Nau./Saccharomyces - ps27
Saccharomyces - ps28
Arboricola_clade - ps29
Cerevisiae_clade - ps30
Saccharomyces_cerevisiae - ps31
p-value
7e-03
1e-02
2e-02
3e-02
4e-02
Log-odds
0.5
1.0
1.5
2.0
2.5
3.0
3.5
4.0

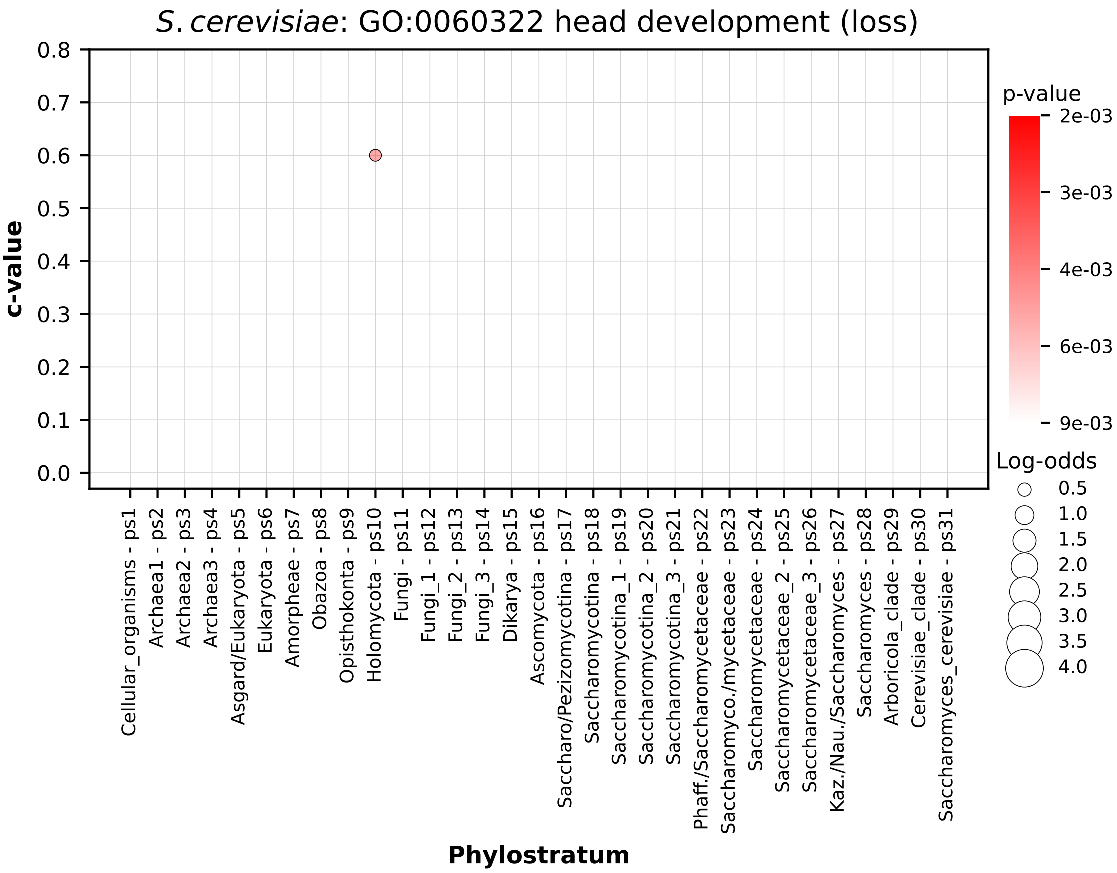

S. cerevisiae: GO:0060322 head development (loss)
c-value
0.8
0.7
0.6
0.5
0.4
0.3
0.2
0.1
0.0
p-value
2e-03
3e-03
4e-03
6e-03
9e-03
Log-odds
0.5
1.0
1.5
2.0
2.5
3.0
3.5
4.0
Cellular_organisms - ps1
Archaea1 - ps2
Archaea2 - ps3
Archaea3 - ps4
Asgard/Eukaryota - ps5
Eukaryota - ps6
Amorpheae - ps7
Obazoa - ps8
Opisthokonta - ps9
Holomycota - ps10
Fungi - ps11
Fungi_1 - ps12
Fungi_2 - ps13
Fungi_3 - ps14
Dikarya - ps15
Ascomycota - ps16
Saccharo/Pezizomycotina - ps17
Saccharomycotina - ps18
Saccharomycotina_1 - ps19
Saccharomycotina_2 - ps20
Saccharomycotina_3 - ps21
Phaff./Saccharomycetaceae - ps22
Saccharomyco./mycetaceae - ps23
Saccharomycetaceae - ps24
Saccharomycetaceae_2 - ps25
Saccharomycetaceae_3 - ps26
Kaz./Nau./Saccharomyces - ps27
Saccharomyces - ps28
Arboricola_clade - ps29
Cerevisiae_clade - ps30
Saccharomyces_cerevisiae - ps31
Phylostratum

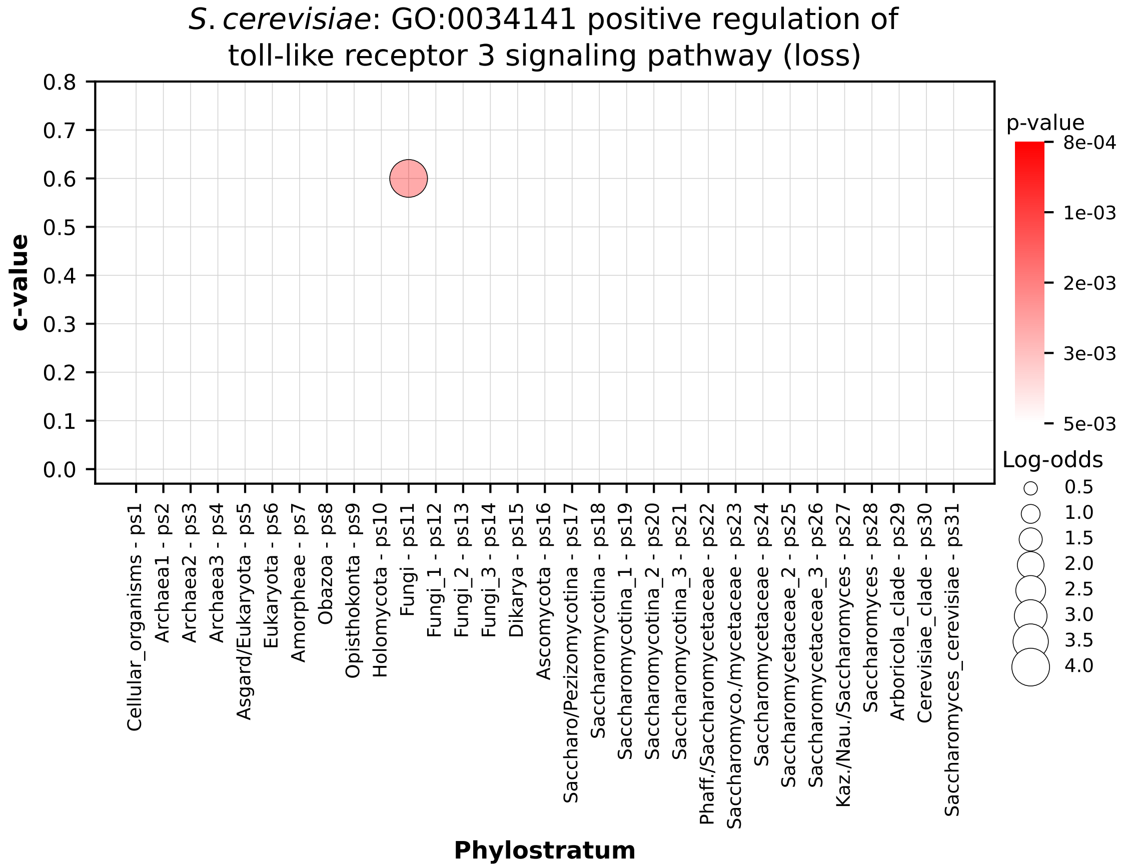
S. cerevisiae: GO:0034141 positive regulation of toll-like receptor 3 signaling pathway (loss)
c-value
0.0
0.1
0.2
0.3
0.4
0.5
0.6
0.7
0.8
Phylostratum
Cellular_organisms - ps1
Archaea1 - ps2
Archaea2 - ps3
Archaea3 - ps4
Asgard/Eukaryota - ps5
Eukaryota - ps6
Amorpheae - ps7
Obazoa - ps8
Opisthokonta - ps9
Holomycota - ps10
Fungi - ps11
Fungi_1 - ps12
Fungi_2 - ps13
Fungi_3 - ps14
Dikarya - ps15
Ascomycota - ps16
Saccharo/Pezizomycotina - ps17
Saccharomycotina - ps18
Saccharomycotina_1 - ps19
Saccharomycotina_2 - ps20
Saccharomycotina_3 - ps21
Phaff./Saccharomycetaceae - ps22
Saccharomyco./mycetaceae - ps23
Saccharomycetaceae - ps24
Saccharomycetaceae_2 - ps25
Saccharomycetaceae_3 - ps26
Kaz./Nau./Saccharomyces - ps27
Saccharomyces - ps28
Arboricola_clade - ps29
Cerevisiae_clade - ps30
Saccharomyces_cerevisiae - ps31
p-value
8e-04
1e-03
2e-03
3e-03
5e-03
Log-odds
0.5
1.0
1.5
2.0
2.5
3.0
3.5
4.0

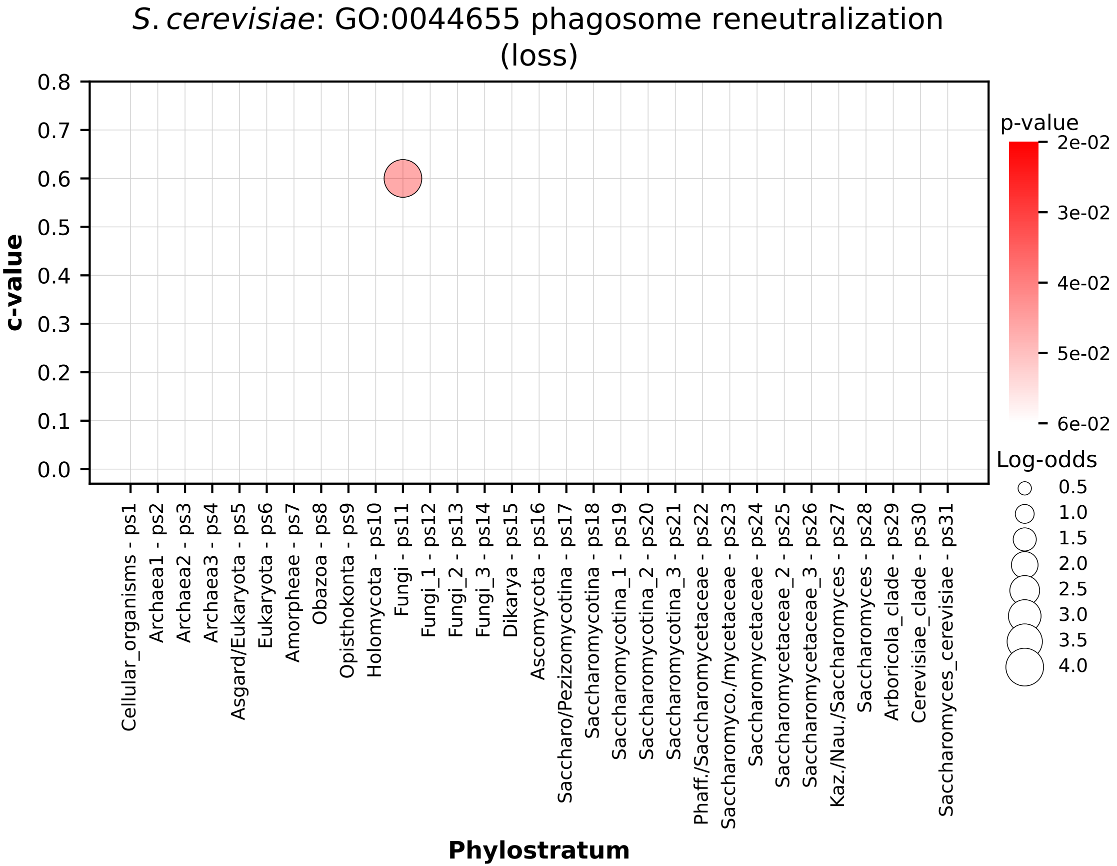
S. cerevisiae: GO:0044655 phagosome reneutralization (loss)
c-value
0.8
0.7
0.6
0.5
0.4
0.3
0.2
0.1
0.0
p-value
2e-02
3e-02
4e-02
5e-02
6e-02
Log-odds
0.5
1.0
1.5
2.0
2.5
3.0
3.5
4.0
Cellular_organisms - ps1
Archaea1 - ps2
Archaea2 - ps3
Archaea3 - ps4
Asgard/Eukaryota - ps5
Eukaryota - ps6
Amorpheae - ps7
Obazoa - ps8
Opisthokonta - ps9
Holomycota - ps10
Fungi - ps11
Fungi_1 - ps12
Fungi_2 - ps13
Fungi_3 - ps14
Dikarya - ps15
Ascomycota - ps16
Saccharo/Pezizomycotina - ps17
Saccharomycotina - ps18
Saccharomycotina_1 - ps19
Saccharomycotina_2 - ps20
Saccharomycotina_3 - ps21
Phaff./Saccharomycetaceae - ps22
Saccharomyco./mycetaceae - ps23
Saccharomycetaceae - ps24
Saccharomycetaceae_2 - ps25
Saccharomycetaceae_3 - ps26
Kaz./Nau./Saccharomyces - ps27
Saccharomyces - ps28
Arboricola_clade - ps29
Cerevisiae_clade - ps30
Saccharomyces_cerevisiae - ps31
Phylostratum

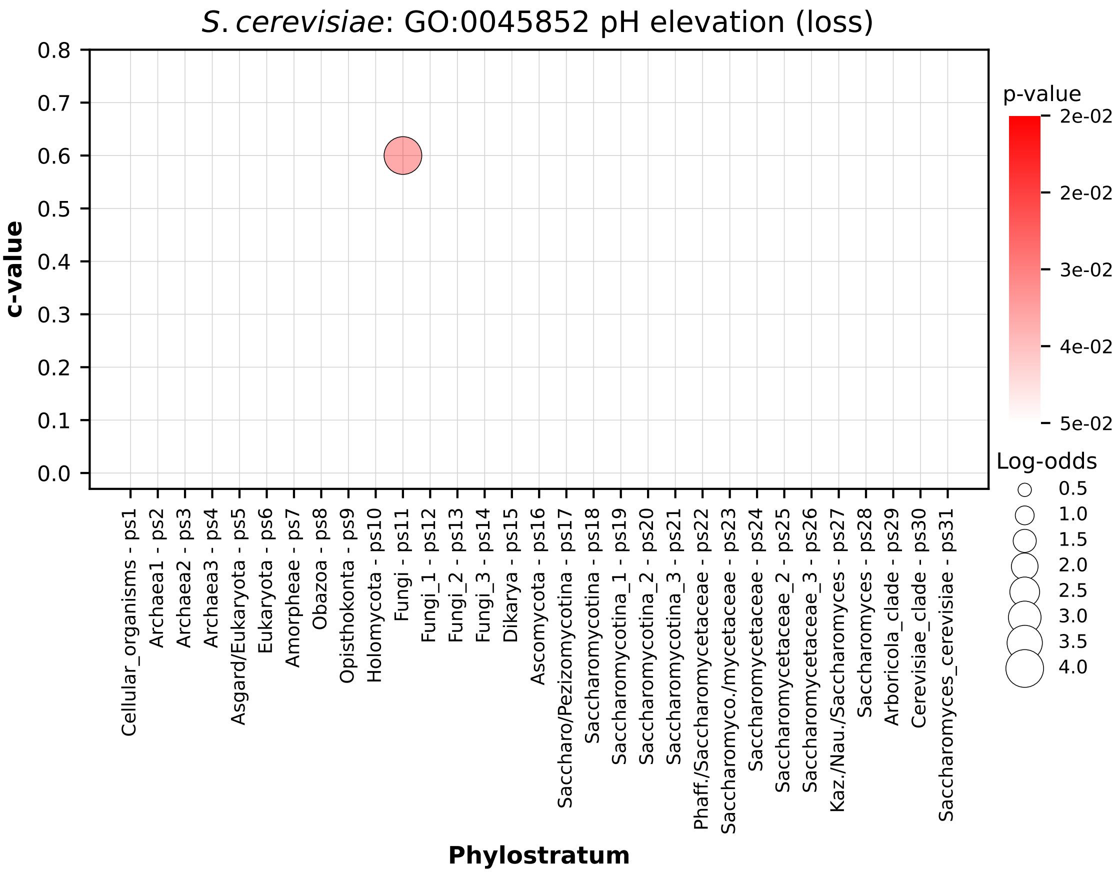

S. cerevisiae: GO:0045852 pH elevation (loss)
c-value
0.8
0.7
0.6
0.5
0.4
0.3
0.2
0.1
0.0
Phylostratum
Cellular_organisms - ps1
Archaea1 - ps2
Archaea2 - ps3
Archaea3 - ps4
Asgard/Eukaryota - ps5
Eukaryota - ps6
Amorpheae - ps7
Obazoa - ps8
Opisthokonta - ps9
Holomycota - ps10
Fungi - ps11
Fungi_1 - ps12
Fungi_2 - ps13
Fungi_3 - ps14
Dikarya - ps15
Ascomycota - ps16
Saccharo/Pezizomycotina - ps17
Saccharomycotina - ps18
Saccharomycotina_1 - ps19
Saccharomycotina_2 - ps20
Saccharomycotina_3 - ps21
Phaff./Saccharomycetaceae - ps22
Saccharomyco./mycetaceae - ps23
Saccharomycetaceae - ps24
Saccharomycetaceae_2 - ps25
Saccharomycetaceae_3 - ps26
Kaz./Nau./Saccharomyces - ps27
Saccharomyces - ps28
Arboricola_clade - ps29
Cerevisiae_clade - ps30
Saccharomyces_cerevisiae - ps31
p-value
2e-02
2e-02
3e-02
4e-02
5e-02
Log-odds
0.5
1.0
1.5
2.0
2.5
3.0
3.5
4.0

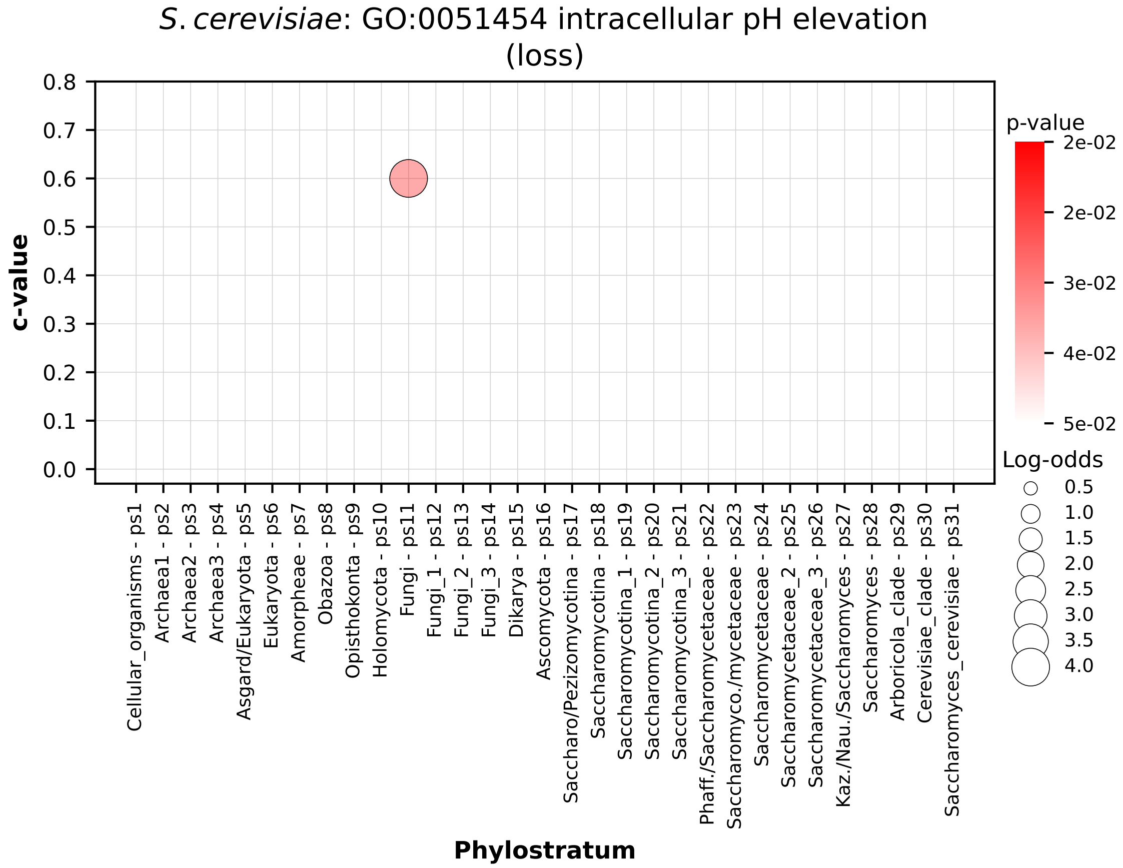

S. cerevisiae: GO:0051454 intracellular pH elevation (loss)
c-value
0.8
0.7
0.6
0.5
0.4
0.3
0.2
0.1
0.0
p-value
2e-02
2e-02
3e-02
4e-02
5e-02
Log-odds
0.5
1.0
1.5
2.0
2.5
3.0
3.5
4.0
Cellular_organisms - ps1
Archaea1 - ps2
Archaea2 - ps3
Archaea3 - ps4
Asgard/Eukaryota - ps5
Eukaryota - ps6
Amorpheae - ps7
Obazoa - ps8
Opisthokonta - ps9
Holomycota - ps10
Fungi - ps11
Fungi_1 - ps12
Fungi_2 - ps13
Fungi_3 - ps14
Dikarya - ps15
Ascomycota - ps16
Saccharo/Pezizomycotina - ps17
Saccharomycotina - ps18
Saccharomycotina_1 - ps19
Saccharomycotina_2 - ps20
Saccharomycotina_3 - ps21
Phaff./Saccharomycetaceae - ps22
Saccharomyco./mycetaceae - ps23
Saccharomycetaceae - ps24
Saccharomycetaceae_2 - ps25
Saccharomycetaceae_3 - ps26
Kaz./Nau./Saccharomyces - ps27
Saccharomyces - ps28
Arboricola_clade - ps29
Cerevisiae_clade - ps30
Saccharomyces_cerevisiae - ps31
Phylostratum

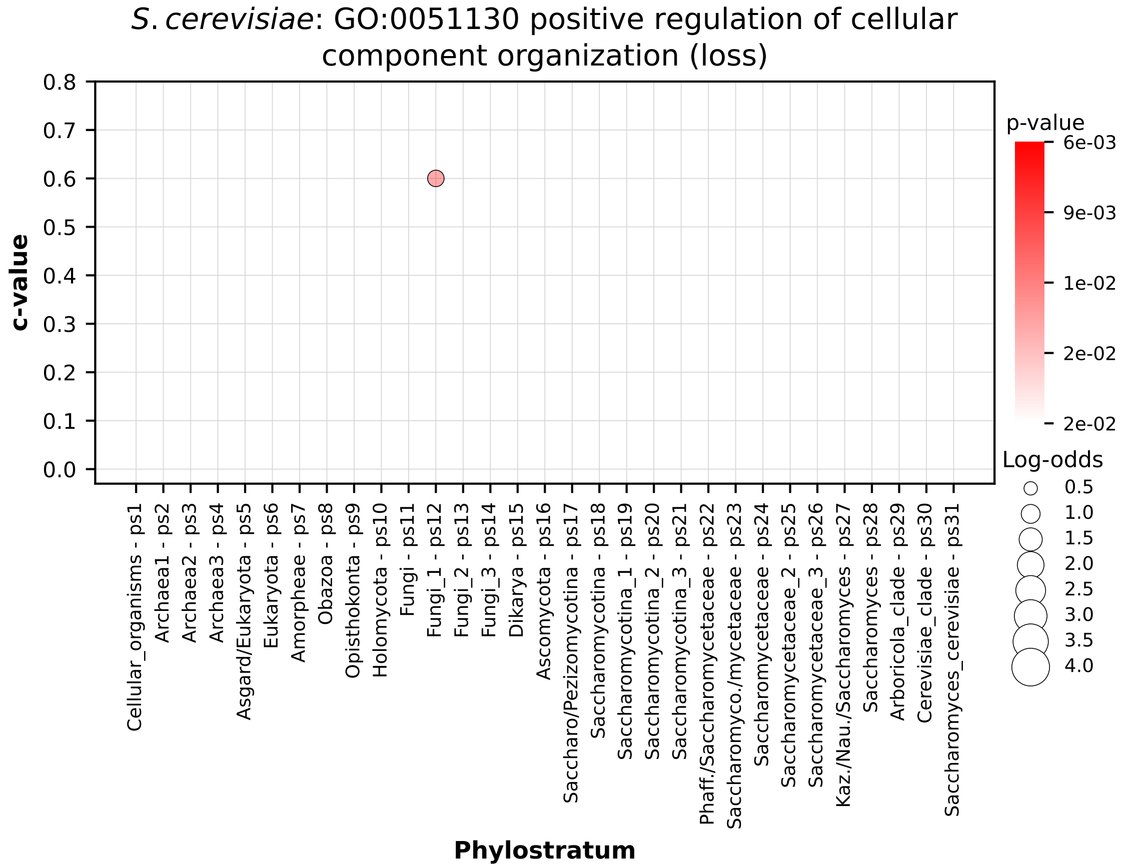

S. cerevisiae: GO:0051130 positive regulation of cellular component organization (loss)
c-value
0.8
0.7
0.6
0.5
0.4
0.3
0.2
0.1
0.0
p-value
6e-03
9e-03
1e-02
2e-02
2e-02
Log-odds
0.5
1.0
1.5
2.0
2.5
3.0
3.5
4.0
Cellular_organisms - ps1
Archaea1 - ps2
Archaea2 - ps3
Archaea3 - ps4
Asgard/Eukaryota - ps5
Eukaryota - ps6
Amorpheae - ps7
Obazoa - ps8
Opisthokonta - ps9
Holomycota - ps10
Fungi - ps11
Fungi_1 - ps12
Fungi_2 - ps13
Fungi_3 - ps14
Dikarya - ps15
Ascomycota - ps16
Saccharo/Pezizomycotina - ps17
Saccharomycotina - ps18
Saccharomycotina_1 - ps19
Saccharomycotina_2 - ps20
Saccharomycotina_3 - ps21
Phaff./Saccharomycetaceae - ps22
Saccharomyco./mycetaceae - ps23
Saccharomycetaceae - ps24
Saccharomycetaceae_2 - ps25
Saccharomycetaceae_3 - ps26
Kaz./Nau./Saccharomyces - ps27
Saccharomyces - ps28
Arboricola_clade - ps29
Cerevisiae_clade - ps30
Saccharomyces_cerevisiae - ps31
Phylostratum

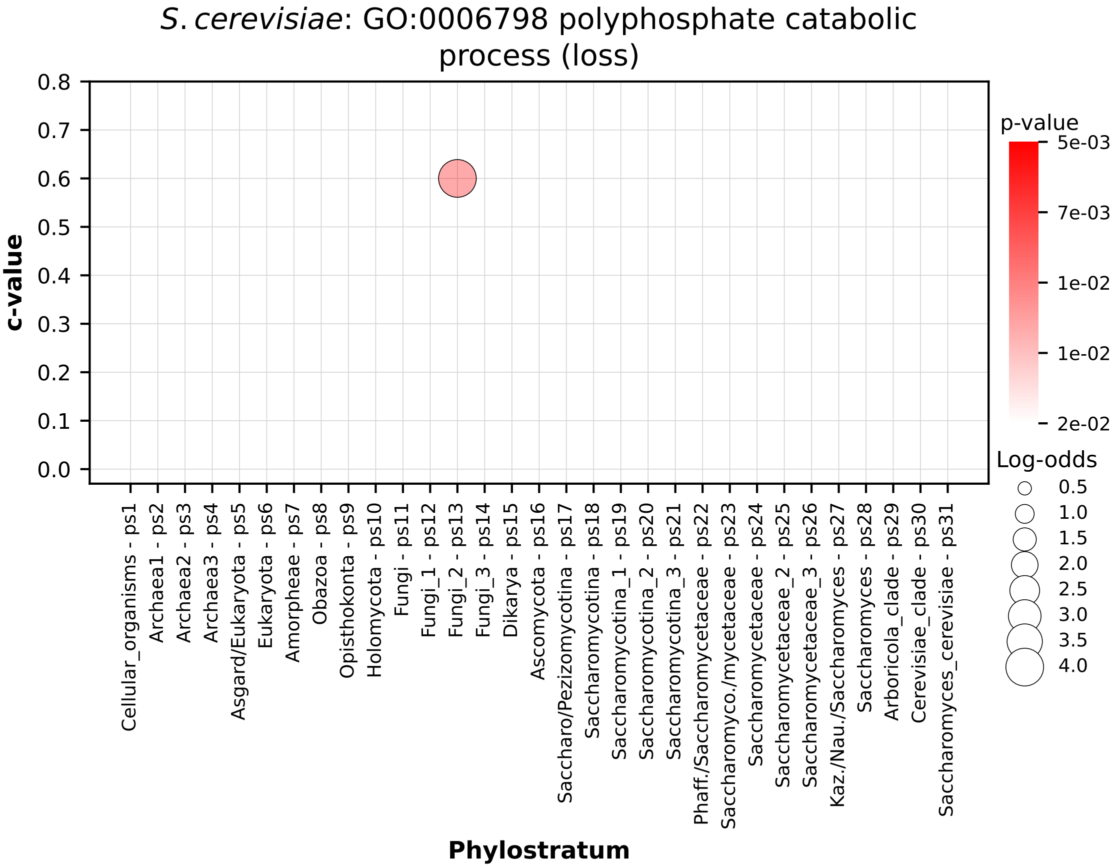

S. cerevisiae: GO:0006798 polyphosphate catabolic process (loss)
c-value
0.8
0.7
0.6
0.5
0.4
0.3
0.2
0.1
0.0
Cellular_organisms - ps1
Archaea1 - ps2
Archaea2 - ps3
Archaea3 - ps4
Asgard/Eukaryota - ps5
Eukaryota - ps6
Amorpheae - ps7
Obazoa - ps8
Opisthokonta - ps9
Holomycota - ps10
Fungi - ps11
Fungi_1 - ps12
Fungi_2 - ps13
Fungi_3 - ps14
Dikarya - ps15
Ascomycota - ps16
Saccharo/Pezizomycotina - ps17
Saccharomycotina - ps18
Saccharomycotina_1 - ps19
Saccharomycotina_2 - ps20
Saccharomycotina_3 - ps21
Phaff./Saccharomycetaceae - ps22
Saccharomyco./mycetaceae - ps23
Saccharomycetaceae - ps24
Saccharomycetaceae_2 - ps25
Saccharomycetaceae_3 - ps26
Kaz./Nau./Saccharomyces - ps27
Saccharomyces - ps28
Arboricola_clade - ps29
Cerevisiae_clade - ps30
Saccharomyces_cerevisiae - ps31
Phylostratum
p-value
5e-03
7e-03
1e-02
1e-02
2e-02
Log-odds
0.5
1.0
1.5
2.0
2.5
3.0
3.5
4.0

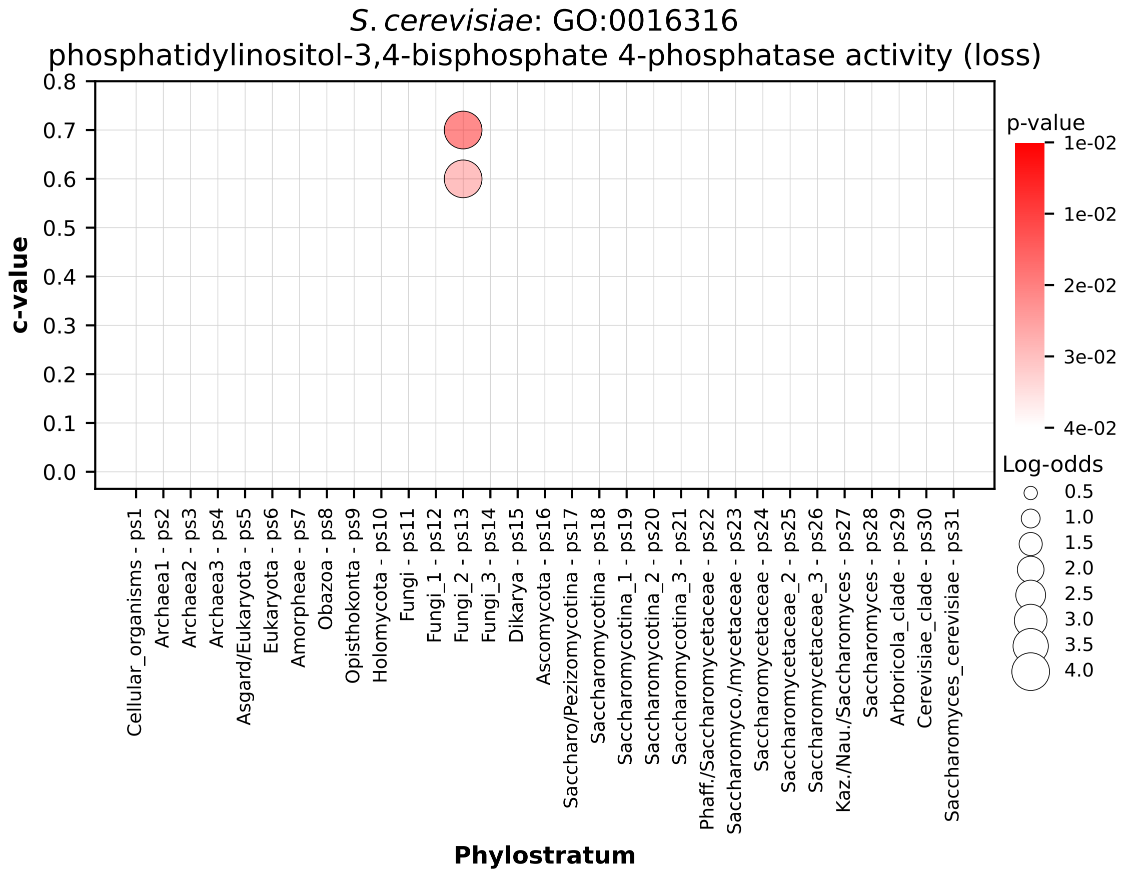

S. cerevisiae: GO:0016316
phosphatidylinositol-3,4-bisphosphate 4-phosphatase activity (loss)
c-value
0.8
0.7
0.6
0.5
0.4
0.3
0.2
0.1
0.0
p-value
1e-02
1e-02
2e-02
3e-02
4e-02
Log-odds
0.5
1.0
1.5
2.0
2.5
3.0
3.5
4.0
Cellular_organisms - ps1
Archaea1 - ps2
Archaea2 - ps3
Archaea3 - ps4
Asgard/Eukaryota - ps5
Eukaryota - ps6
Amorpheae - ps7
Obazoa - ps8
Opisthokonta - ps9
Holomycota - ps10
Fungi - ps11
Fungi_1 - ps12
Fungi_2 - ps13
Fungi_3 - ps14
Dikarya - ps15
Ascomycota - ps16
Saccharo/Pezizomycotina - ps17
Saccharomycotina - ps18
Saccharomycotina_1 - ps19
Saccharomycotina_2 - ps20
Saccharomycotina_3 - ps21
Phaff./Saccharomycetaceae - ps22
Saccharomyco./mycetaceae - ps23
Saccharomycetaceae - ps24
Saccharomycetaceae_2 - ps25
Saccharomycetaceae_3 - ps26
Kaz./Nau./Saccharomyces - ps27
Saccharomyces - ps28
Arboricola_clade - ps29
Cerevisiae_clade - ps30
Saccharomyces_cerevisiae - ps31
Phylostratum

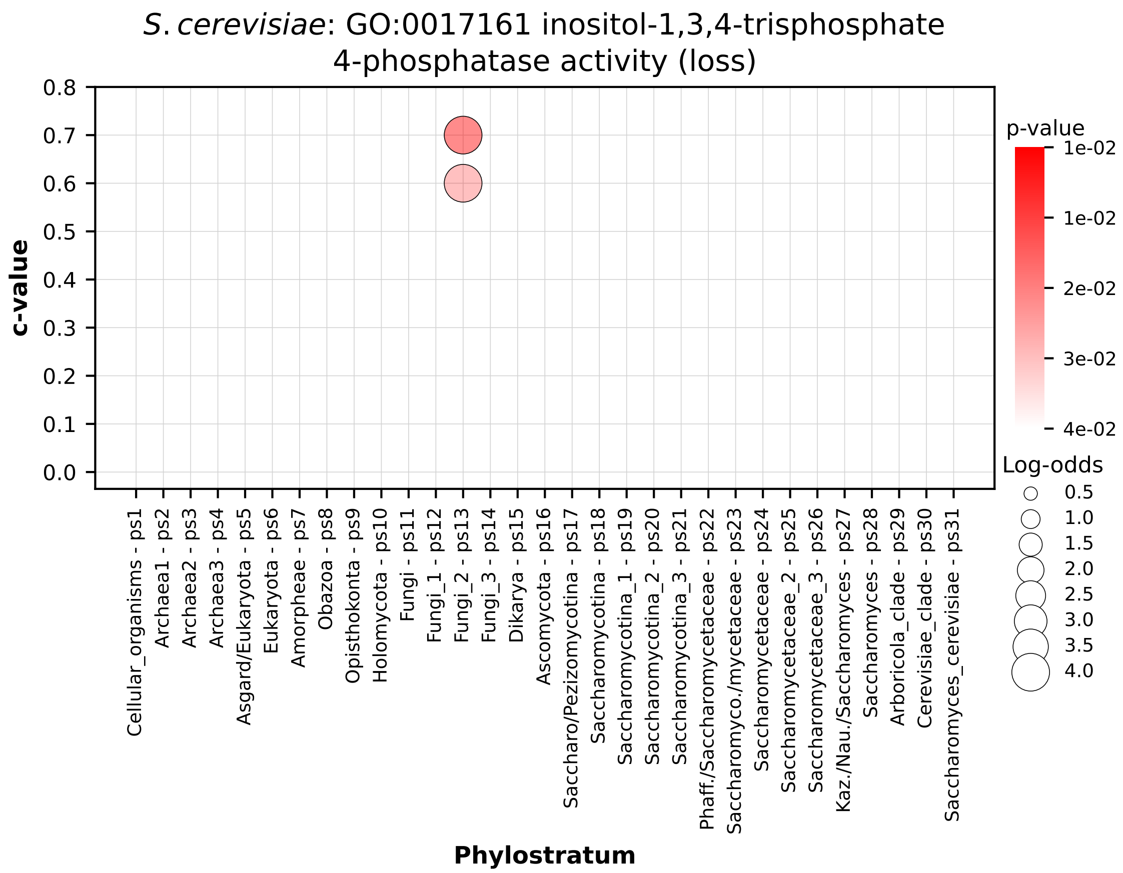
S. cerevisiae: GO:0017161 inositol-1,3,4-trisphosphate 4-phosphatase activity (loss)
c-value
0.8
0.7
0.6
0.5
0.4
0.3
0.2
0.1
0.0
p-value
1e-02
1e-02
2e-02
3e-02
4e-02
Log-odds
0.5
1.0
1.5
2.0
2.5
3.0
3.5
4.0
Cellular_organisms - ps1
Archaea1 - ps2
Archaea2 - ps3
Archaea3 - ps4
Asgard/Eukaryota - ps5
Eukaryota - ps6
Amorpheae - ps7
Obazoa - ps8
Opisthokonta - ps9
Holomycota - ps10
Fungi - ps11
Fungi_1 - ps12
Fungi_2 - ps13
Fungi_3 - ps14
Dikarya - ps15
Ascomycota - ps16
Saccharo/Pezizomycotina - ps17
Saccharomycotina - ps18
Saccharomycotina_1 - ps19
Saccharomycotina_2 - ps20
Saccharomycotina_3 - ps21
Phaff./Saccharomycetaceae - ps22
Saccharomyco./mycetaceae - ps23
Saccharomycetaceae - ps24
Saccharomycetaceae_2 - ps25
Saccharomycetaceae_3 - ps26
Kaz./Nau./Saccharomyces - ps27
Saccharomyces - ps28
Arboricola_clade - ps29
Cerevisiae_clade - ps30
Saccharomyces_cerevisiae - ps31
Phylostratum

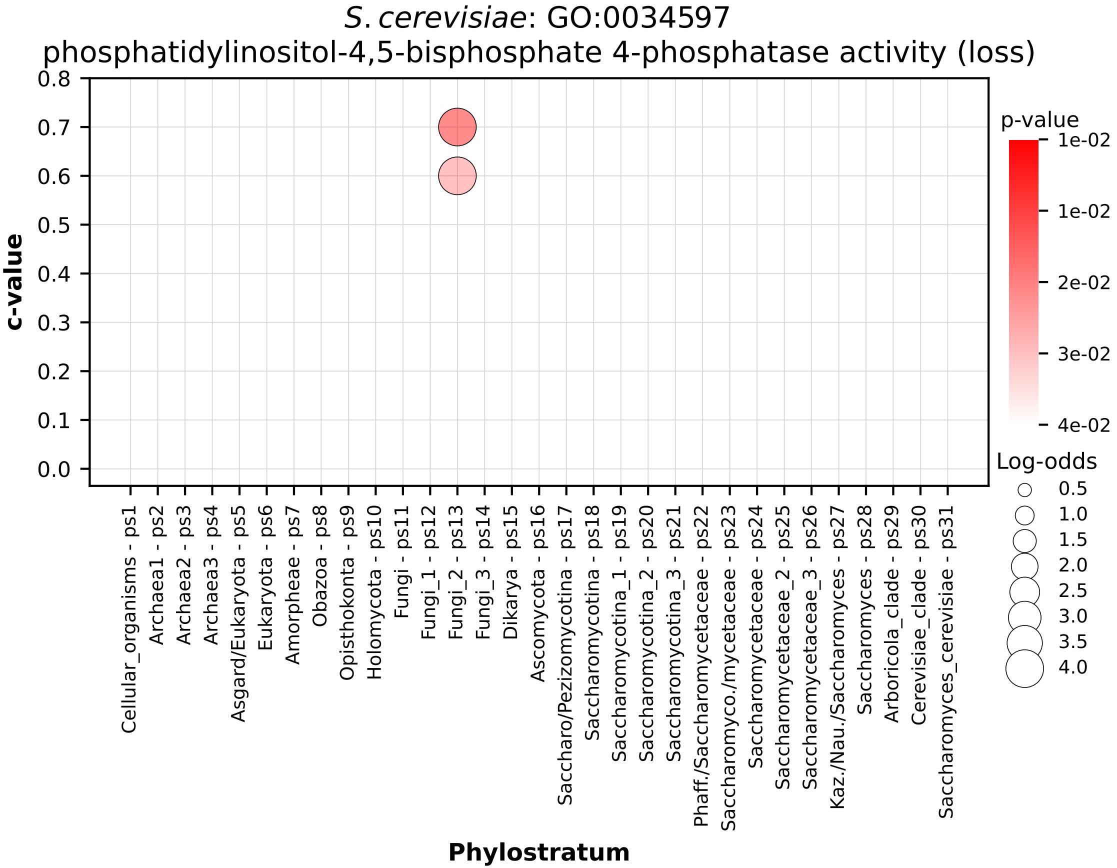

S. cerevisiae: GO:0034597
phosphatidylinositol-4,5-bisphosphate 4-phosphatase activity (loss)
c-value
0.8
0.7
0.6
0.5
0.4
0.3
0.2
0.1
0.0
p-value
1e-02
1e-02
2e-02
3e-02
4e-02
Log-odds
0.5
1.0
1.5
2.0
2.5
3.0
3.5
4.0
Cellular_organisms - ps1
Archaea1 - ps2
Archaea2 - ps3
Archaea3 - ps4
Asgard/Eukaryota - ps5
Eukaryota - ps6
Amorpheae - ps7
Obazoa - ps8
Opisthokonta - ps9
Holomycota - ps10
Fungi - ps11
Fungi_1 - ps12
Fungi_2 - ps13
Fungi_3 - ps14
Dikarya - ps15
Ascomycota - ps16
Saccharo/Pezizomycotina - ps17
Saccharomycotina - ps18
Saccharomycotina_1 - ps19
Saccharomycotina_2 - ps20
Saccharomycotina_3 - ps21
Phaff./Saccharomycetaceae - ps22
Saccharomyco./mycetaceae - ps23
Saccharomycetaceae - ps24
Saccharomycetaceae_2 - ps25
Saccharomycetaceae_3 - ps26
Kaz./Nau./Saccharomyces - ps27
Saccharomyces - ps28
Arboricola_clade - ps29
Cerevisiae_clade - ps30
Saccharomyces_cerevisiae - ps31
Phylostratum

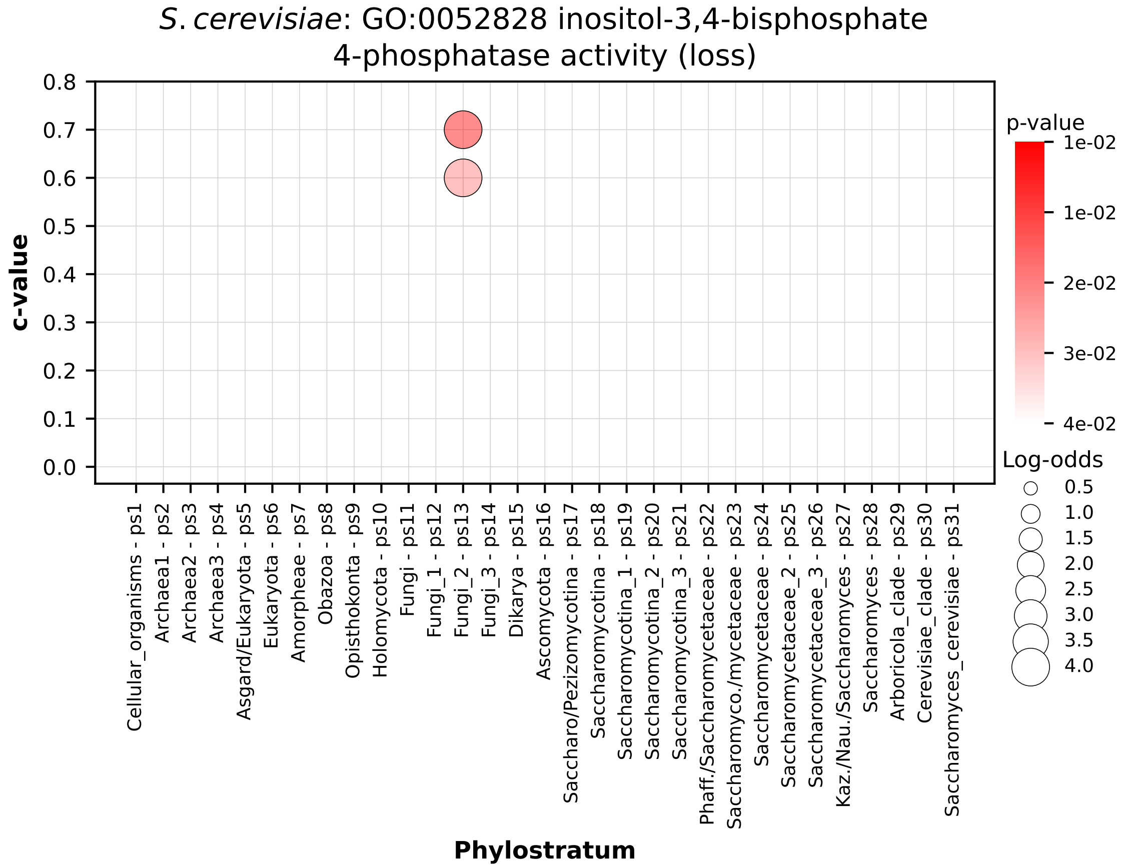
S. cerevisiae: GO:0052828 inositol-3,4-bisphosphate 4-phosphatase activity (loss)
c-value
0.8
0.7
0.6
0.5
0.4
0.3
0.2
0.1
0.0
p-value
1e-02
1e-02
2e-02
3e-02
4e-02
Log-odds
0.5
1.0
1.5
2.0
2.5
3.0
3.5
4.0
Cellular_organisms - ps1
Archaea1 - ps2
Archaea2 - ps3
Archaea3 - ps4
Asgard/Eukaryota - ps5
Eukaryota - ps6
Amorpheae - ps7
Obazoa - ps8
Opisthokonta - ps9
Holomycota - ps10
Fungi - ps11
Fungi_1 - ps12
Fungi_2 - ps13
Fungi_3 - ps14
Dikarya - ps15
Ascomycota - ps16
Saccharo/Pezizomycotina - ps17
Saccharomycotina - ps18
Saccharomycotina_1 - ps19
Saccharomycotina_2 - ps20
Saccharomycotina_3 - ps21
Phaff./Saccharomycetaceae - ps22
Saccharomyco./mycetaceae - ps23
Saccharomycetaceae - ps24
Saccharomycetaceae_2 - ps25
Saccharomycetaceae_3 - ps26
Kaz./Nau./Saccharomyces - ps27
Saccharomyces - ps28
Arboricola_clade - ps29
Cerevisiae_clade - ps30
Saccharomyces_cerevisiae - ps31
Phylostratum

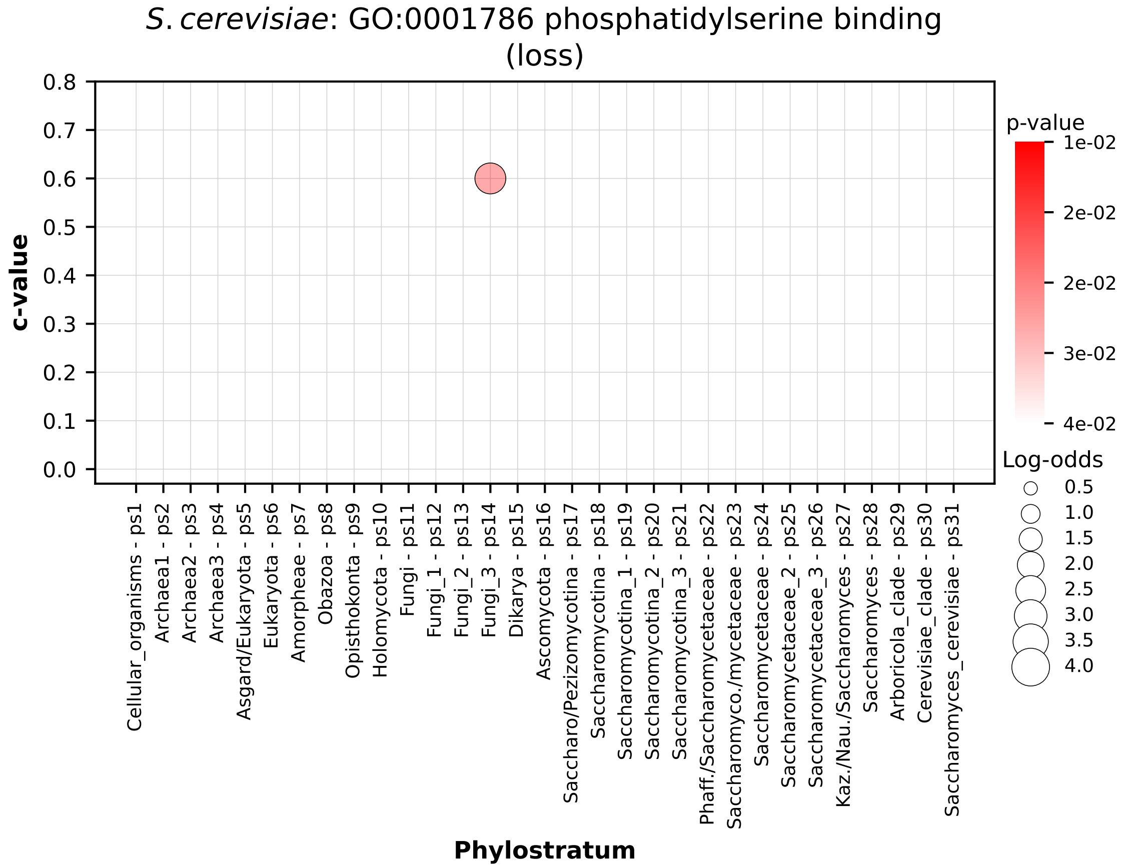
S. cerevisiae: GO:0001786 phosphatidylserine binding
(loss)
c-value
0.8
0.7
0.6
0.5
0.4
0.3
0.2
0.1
0.0
p-value
1e-02
2e-02
2e-02
3e-02
4e-02
Log-odds
0.5
1.0
1.5
2.0
2.5
3.0
3.5
4.0
Cellular_organisms - ps1
Archaea1 - ps2
Archaea2 - ps3
Archaea3 - ps4
Asgard/Eukaryota - ps5
Eukaryota - ps6
Amorpheae - ps7
Obazoa - ps8
Opisthokonta - ps9
Holomycota - ps10
Fungi - ps11
Fungi_1 - ps12
Fungi_2 - ps13
Fungi_3 - ps14
Dikarya - ps15
Ascomycota - ps16
Saccharo/Pezizomycotina - ps17
Saccharomycotina - ps18
Saccharomycotina_1 - ps19
Saccharomycotina_2 - ps20
Saccharomycotina_3 - ps21
Phaff./Saccharomycetaceae - ps22
Saccharomyco./mycetaceae - ps23
Saccharomycetaceae - ps24
Saccharomycetaceae_2 - ps25
Saccharomycetaceae_3 - ps26
Kaz./Nau./Saccharomyces - ps27
Saccharomyces - ps28
Arboricola_clade - ps29
Cerevisiae_clade - ps30
Saccharomyces_cerevisiae - ps31
Phylostratum

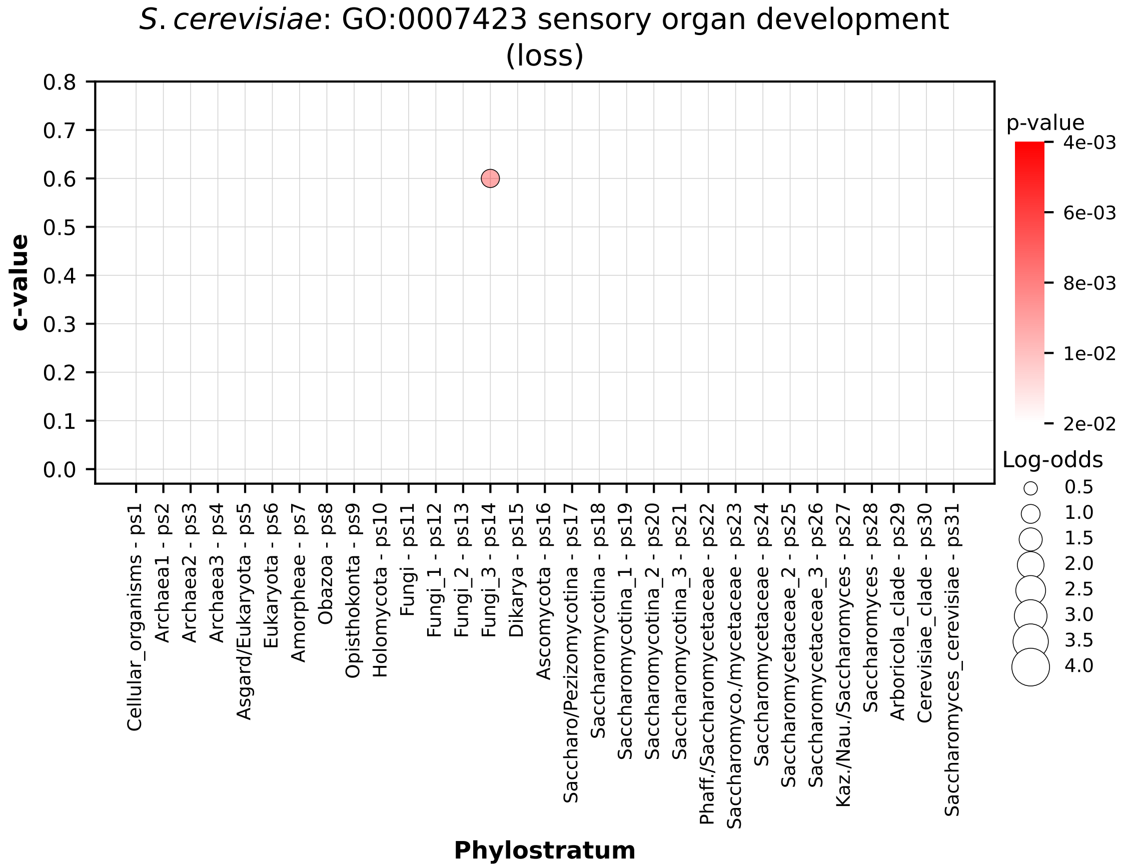

S. cerevisiae: GO:0007423 sensory organ development (loss)
c-value
0.0
0.1
0.2
0.3
0.4
0.5
0.6
0.7
0.8
Phylostratum
Cellular_organisms - ps1
Archaea1 - ps2
Archaea2 - ps3
Archaea3 - ps4
Asgard/Eukaryota - ps5
Eukaryota - ps6
Amorpheae - ps7
Obazoa - ps8
Opisthokonta - ps9
Holomycota - ps10
Fungi - ps11
Fungi_1 - ps12
Fungi_2 - ps13
Fungi_3 - ps14
Dikarya - ps15
Ascomycota - ps16
Saccharo/Pezizomycotina - ps17
Saccharomycotina - ps18
Saccharomycotina_1 - ps19
Saccharomycotina_2 - ps20
Saccharomycotina_3 - ps21
Phaff./Saccharomycetaceae - ps22
Saccharomyco./mycetaceae - ps23
Saccharomycetaceae - ps24
Saccharomycetaceae_2 - ps25
Saccharomycetaceae_3 - ps26
Kaz./Nau./Saccharomyces - ps27
Saccharomyces - ps28
Arboricola_clade - ps29
Cerevisiae_clade - ps30
Saccharomyces_cerevisiae - ps31
p-value
4e-03
6e-03
8e-03
1e-02
2e-02
Log-odds
0.5
1.0
1.5
2.0
2.5
3.0
3.5
4.0

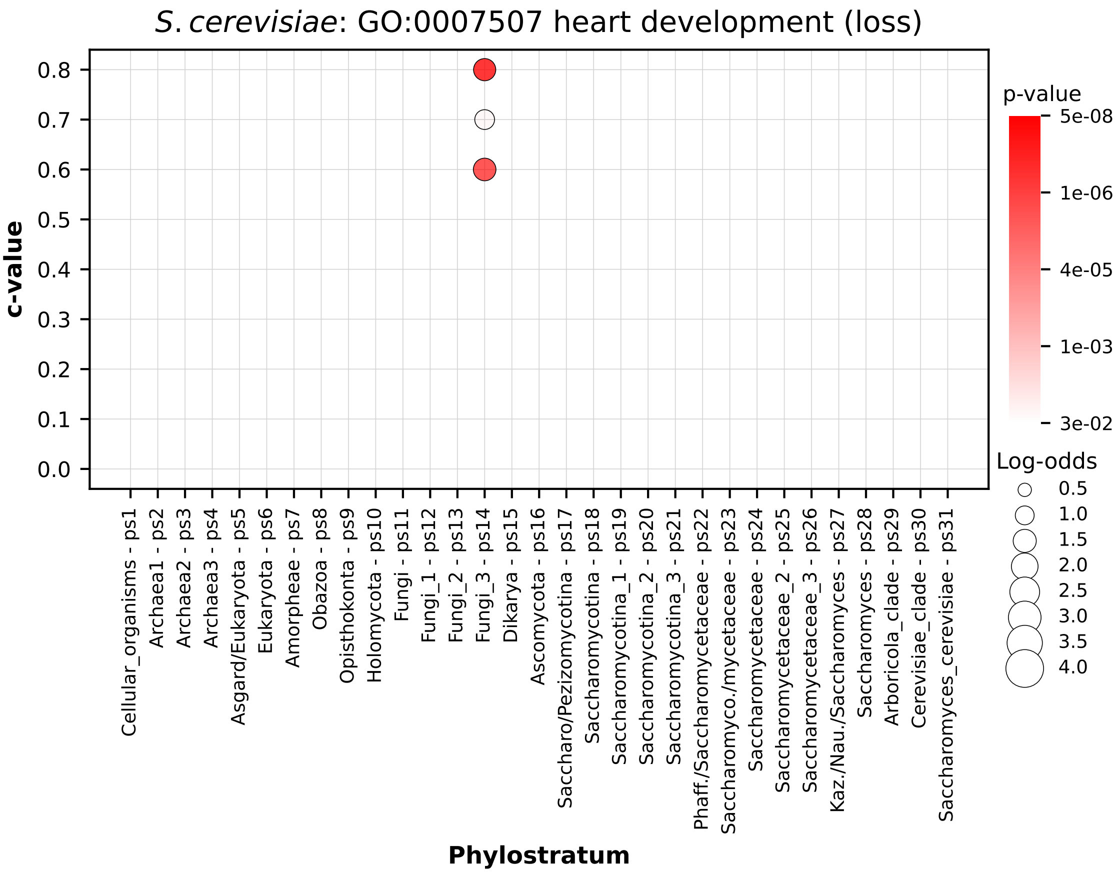

S. cerevisiae: GO:0007507 heart development (loss)
c-value
0.0
0.1
0.2
0.3
0.4
0.5
0.6
0.7
0.8
p-value
5e-08
1e-06
4e-05
1e-03
3e-02
Log-odds
0.5
1.0
1.5
2.0
2.5
3.0
3.5
4.0
Cellular_organisms - ps1
Archaea1 - ps2
Archaea2 - ps3
Archaea3 - ps4
Asgard/Eukaryota - ps5
Eukaryota - ps6
Amorpheae - ps7
Obazoa - ps8
Opisthokonta - ps9
Holomycota - ps10
Fungi - ps11
Fungi_1 - ps12
Fungi_2 - ps13
Fungi_3 - ps14
Dikarya - ps15
Ascomycota - ps16
Saccharo/Pezizomycotina - ps17
Saccharomycotina - ps18
Saccharomycotina_1 - ps19
Saccharomycotina_2 - ps20
Saccharomycotina_3 - ps21
Phaff./Saccharomycetaceae - ps22
Saccharomyco./mycetaceae - ps23
Saccharomycetaceae - ps24
Saccharomycetaceae_2 - ps25
Saccharomycetaceae_3 - ps26
Kaz./Nau./Saccharomyces - ps27
Saccharomyces - ps28
Arboricola_clade - ps29
Cerevisiae_clade - ps30
Saccharomyces_cerevisiae - ps31
Phylostratum

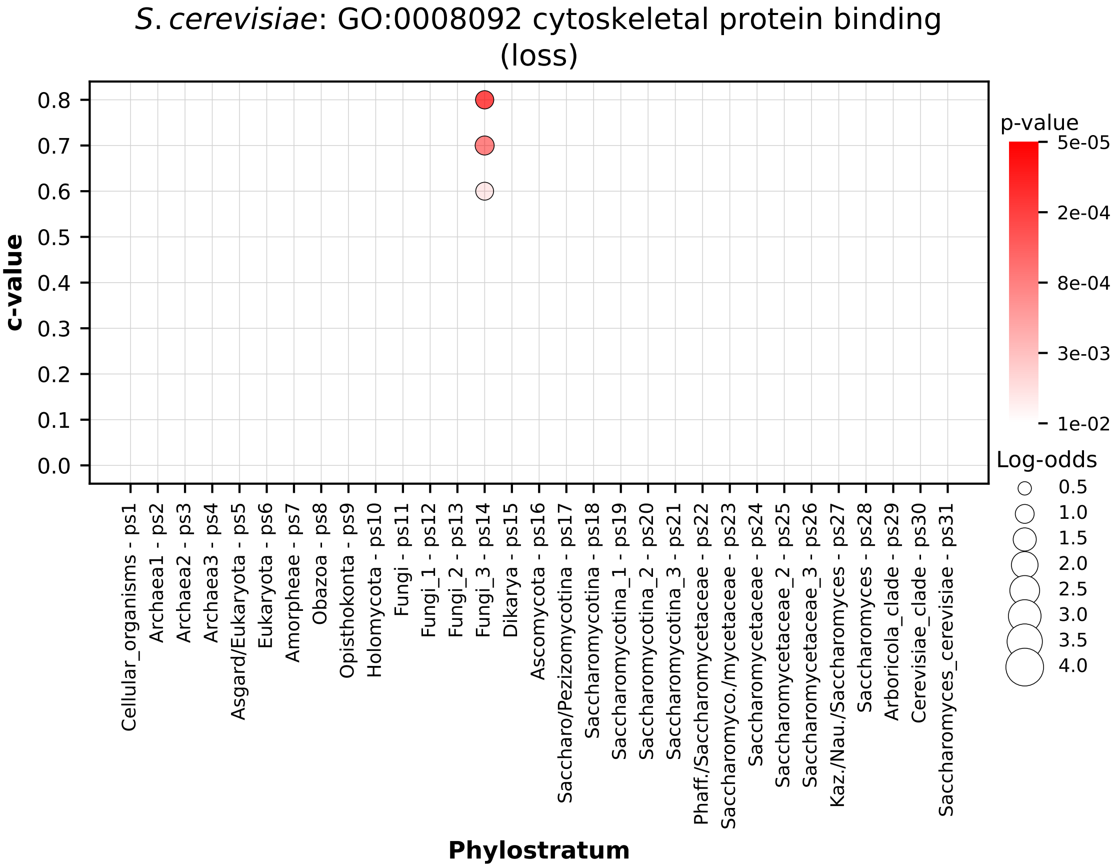

S. cerevisiae: GO:0008092 cytoskeletal protein binding
(loss)
c-value
0.8
0.7
0.6
0.5
0.4
0.3
0.2
0.1
0.0
p-value
5e-05
2e-04
8e-04
3e-03
1e-02
Log-odds
0.5
1.0
1.5
2.0
2.5
3.0
3.5
4.0
Cellular_organisms - ps1
Archaea1 - ps2
Archaea2 - ps3
Archaea3 - ps4
Asgard/Eukaryota - ps5
Eukaryota - ps6
Amorpheae - ps7
Obazoa - ps8
Opisthokonta - ps9
Holomycota - ps10
Fungi - ps11
Fungi_1 - ps12
Fungi_2 - ps13
Fungi_3 - ps14
Dikarya - ps15
Ascomycota - ps16
Saccharo/Pezizomycotina - ps17
Saccharomycotina - ps18
Saccharomycotina_1 - ps19
Saccharomycotina_2 - ps20
Saccharomycotina_3 - ps21
Phaff./Saccharomycetaceae - ps22
Saccharomyco./mycetaceae - ps23
Saccharomycetaceae - ps24
Saccharomycetaceae_2 - ps25
Saccharomycetaceae_3 - ps26
Kaz./Nau./Saccharomyces - ps27
Saccharomyces - ps28
Arboricola_clade - ps29
Cerevisiae_clade - ps30
Saccharomyces_cerevisiae - ps31
Phylostratum

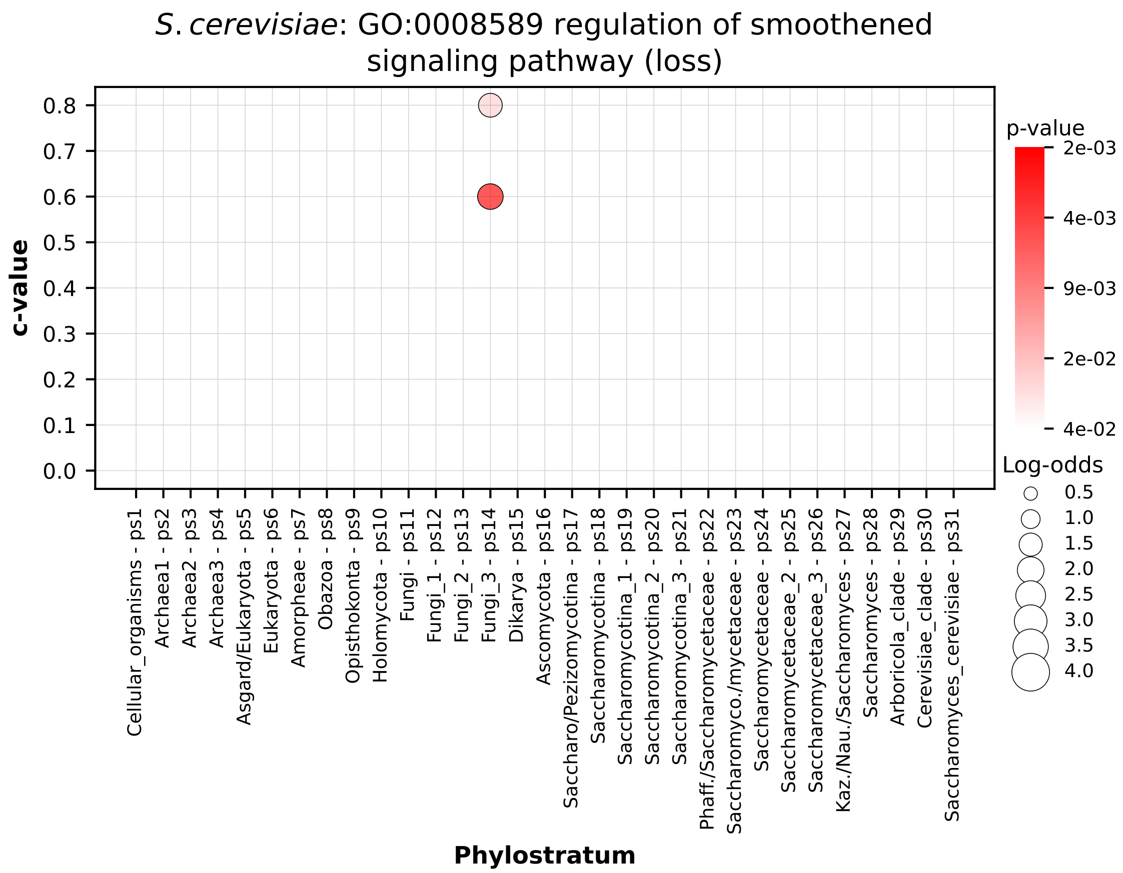
S. cerevisiae: GO:0008589 regulation of smoothened signaling pathway (loss)
c-value
0.0
0.1
0.2
0.3
0.4
0.5
0.6
0.7
0.8
p-value
2e-03
4e-03
9e-03
2e-02
4e-02
Log-odds
0.5
1.0
1.5
2.0
2.5
3.0
3.5
4.0
Cellular_organisms - ps1
Archaea1 - ps2
Archaea2 - ps3
Archaea3 - ps4
Asgard/Eukaryota - ps5
Eukaryota - ps6
Amorpheae - ps7
Obazoa - ps8
Opisthokonta - ps9
Holomycota - ps10
Fungi - ps11
Fungi_1 - ps12
Fungi_2 - ps13
Fungi_3 - ps14
Dikarya - ps15
Ascomycota - ps16
Saccharo/Pezizomycotina - ps17
Saccharomycotina - ps18
Saccharomycotina_1 - ps19
Saccharomycotina_2 - ps20
Saccharomycotina_3 - ps21
Phaff./Saccharomycetaceae - ps22
Saccharomyco./mycetaceae - ps23
Saccharomycetaceae - ps24
Saccharomycetaceae_2 - ps25
Saccharomycetaceae_3 - ps26
Kaz./Nau./Saccharomyces - ps27
Saccharomyces - ps28
Arboricola_clade - ps29
Cerevisiae_clade - ps30
Saccharomyces_cerevisiae - ps31
Phylostratum

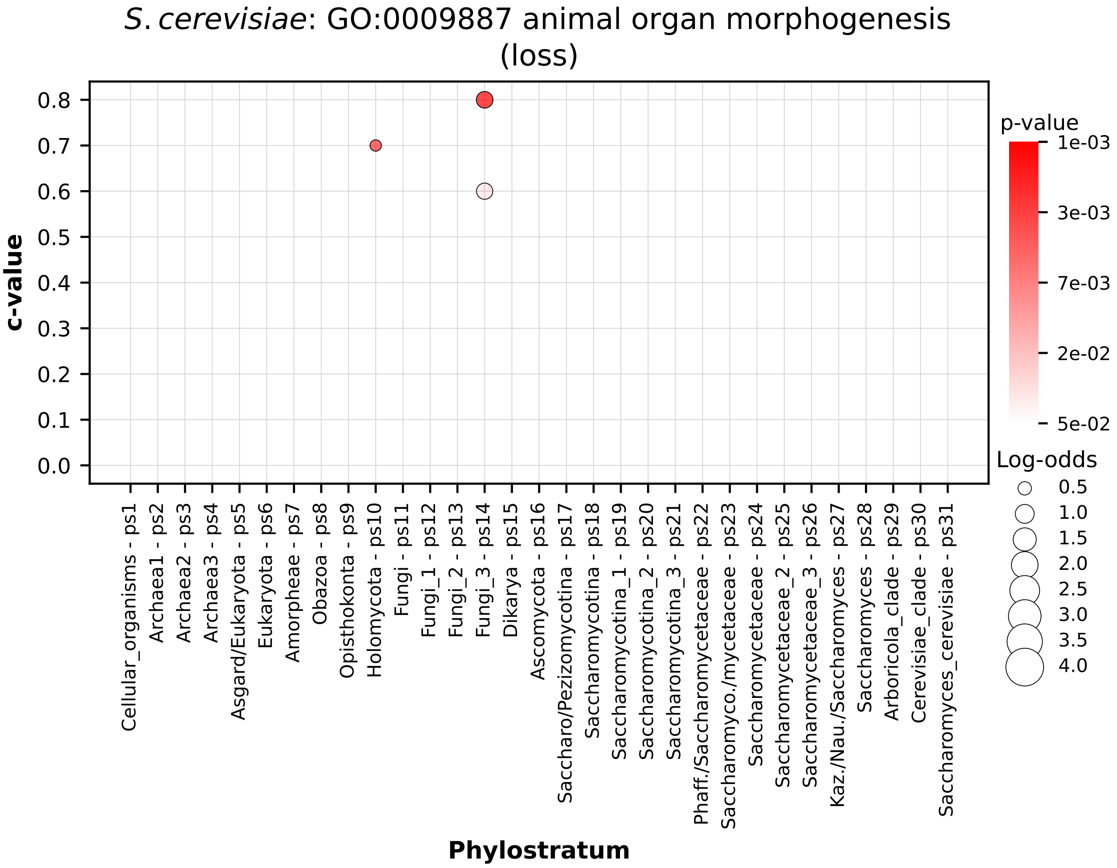
S. cerevisiae: GO:0009887 animal organ morphogenesis
(loss)
c-value
0.0
0.1
0.2
0.3
0.4
0.5
0.6
0.7
0.8
p-value
1e-03
3e-03
7e-03
2e-02
5e-02
Log-odds
0.5
1.0
1.5
2.0
2.5
3.0
3.5
4.0
Cellular_organisms - ps1
Archaea1 - ps2
Archaea2 - ps3
Archaea3 - ps4
Asgard/Eukaryota - ps5
Eukaryota - ps6
Amorpheae - ps7
Obazoa - ps8
Opisthokonta - ps9
Holomycota - ps10
Fungi - ps11
Fungi_1 - ps12
Fungi_2 - ps13
Fungi_3 - ps14
Dikarya - ps15
Ascomycota - ps16
Saccharo/Pezizomycotina - ps17
Saccharomycotina - ps18
Saccharomycotina_1 - ps19
Saccharomycotina_2 - ps20
Saccharomycotina_3 - ps21
Phaff./Saccharomycetaceae - ps22
Saccharomyco./mycetaceae - ps23
Saccharomycetaceae - ps24
Saccharomycetaceae_2 - ps25
Saccharomycetaceae_3 - ps26
Kaz./Nau./Saccharomyces - ps27
Saccharomyces - ps28
Arboricola_clade - ps29
Cerevisiae_clade - ps30
Saccharomyces_cerevisiae - ps31
Phylostratum

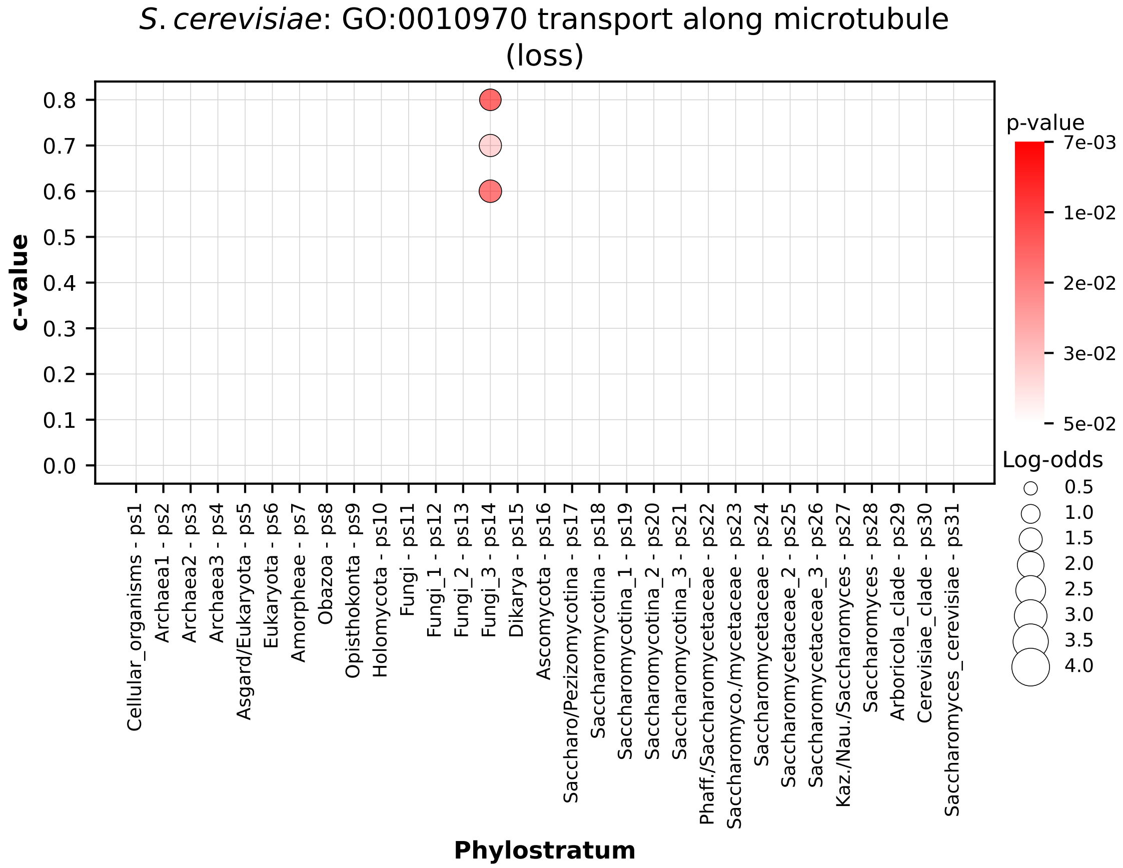

S. cerevisiae: GO:0010970 transport along microtubule (loss)
c-value
0.0
0.1
0.2
0.3
0.4
0.5
0.6
0.7
0.8
p-value
7e-03
1e-02
2e-02
3e-02
5e-02
Log-odds
0.5
1.0
1.5
2.0
2.5
3.0
3.5
4.0
Cellular_organisms - ps1
Archaea1 - ps2
Archaea2 - ps3
Archaea3 - ps4
Asgard/Eukaryota - ps5
Eukaryota - ps6
Amorpheae - ps7
Obazoa - ps8
Opisthokonta - ps9
Holomycota - ps10
Fungi - ps11
Fungi_1 - ps12
Fungi_2 - ps13
Fungi_3 - ps14
Dikarya - ps15
Ascomycota - ps16
Saccharo/Pezizomycotina - ps17
Saccharomycotina - ps18
Saccharomycotina_1 - ps19
Saccharomycotina_2 - ps20
Saccharomycotina_3 - ps21
Phaff./Saccharomycetaceae - ps22
Saccharomyco./mycetaceae - ps23
Saccharomycetaceae - ps24
Saccharomycetaceae_2 - ps25
Saccharomycetaceae_3 - ps26
Kaz./Nau./Saccharomyces - ps27
Saccharomyces - ps28
Arboricola_clade - ps29
Cerevisiae_clade - ps30
Saccharomyces_cerevisiae - ps31
Phylostratum

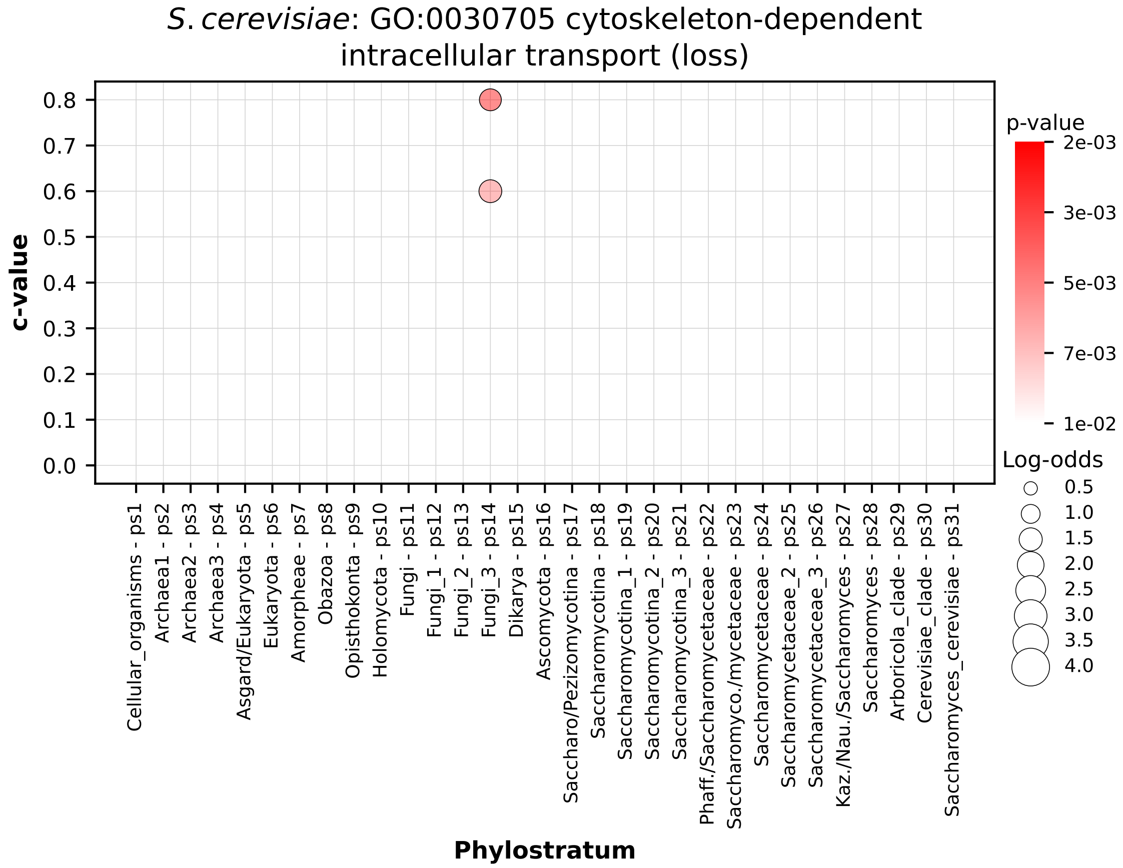

S. cerevisiae: GO:0030705 cytoskeleton-dependent intracellular transport (loss)
c-value
0.0
0.1
0.2
0.3
0.4
0.5
0.6
0.7
0.8
Phylostratum
Cellular_organisms - ps1
Archaea1 - ps2
Archaea2 - ps3
Archaea3 - ps4
Asgard/Eukaryota - ps5
Eukaryota - ps6
Amorpheae - ps7
Obazoa - ps8
Opisthokonta - ps9
Holomycota - ps10
Fungi - ps11
Fungi_1 - ps12
Fungi_2 - ps13
Fungi_3 - ps14
Dikarya - ps15
Ascomycota - ps16
Saccharo/Pezizomycotina - ps17
Saccharomycotina - ps18
Saccharomycotina_1 - ps19
Saccharomycotina_2 - ps20
Saccharomycotina_3 - ps21
Phaff./Saccharomycetaceae - ps22
Saccharomyco./mycetaceae - ps23
Saccharomycetaceae - ps24
Saccharomycetaceae_2 - ps25
Saccharomycetaceae_3 - ps26
Kaz./Nau./Saccharomyces - ps27
Saccharomyces - ps28
Arboricola_clade - ps29
Cerevisiae_clade - ps30
Saccharomyces_cerevisiae - ps31
p-value
2e-03
3e-03
5e-03
7e-03
1e-02
Log-odds
0.5
1.0
1.5
2.0
2.5
3.0
3.5
4.0

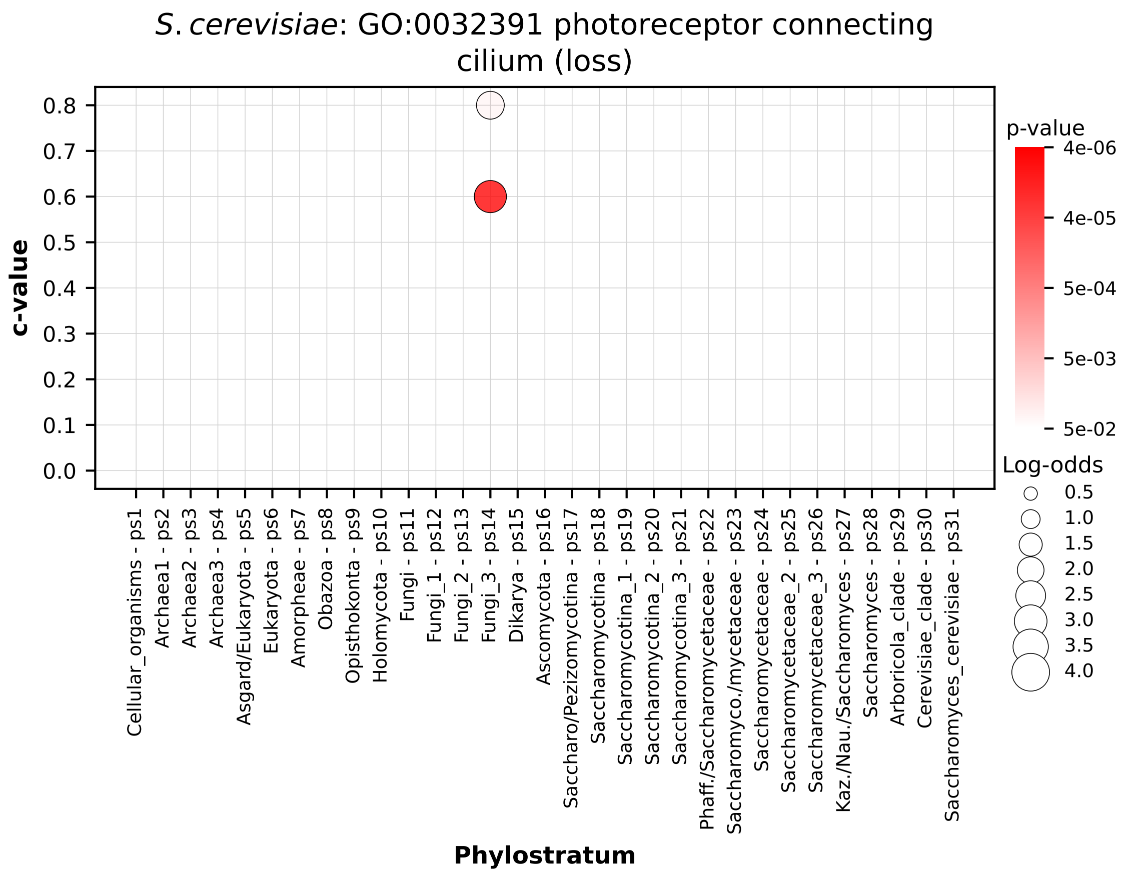

S. cerevisiae: GO:0032391 photoreceptor connecting cilium (loss)
c-value
0.0
0.1
0.2
0.3
0.4
0.5
0.6
0.7
0.8
Phylostratum
Cellular_organisms - ps1
Archaea1 - ps2
Archaea2 - ps3
Archaea3 - ps4
Asgard/Eukaryota - ps5
Eukaryota - ps6
Amorpheae - ps7
Obazoa - ps8
Opisthokonta - ps9
Holomycota - ps10
Fungi - ps11
Fungi_1 - ps12
Fungi_2 - ps13
Fungi_3 - ps14
Dikarya - ps15
Ascomycota - ps16
Saccharo/Pezizomycotina - ps17
Saccharomycotina - ps18
Saccharomycotina_1 - ps19
Saccharomycotina_2 - ps20
Saccharomycotina_3 - ps21
Phaff./Saccharomycetaceae - ps22
Saccharomyco./mycetaceae - ps23
Saccharomycetaceae - ps24
Saccharomycetaceae_2 - ps25
Saccharomycetaceae_3 - ps26
Kaz./Nau./Saccharomyces - ps27
Saccharomyces - ps28
Arboricola_clade - ps29
Cerevisiae_clade - ps30
Saccharomyces_cerevisiae - ps31
p-value
4e-06
4e-05
5e-04
5e-03
5e-02
Log-odds
0.5
1.0
1.5
2.0
2.5
3.0
3.5
4.0

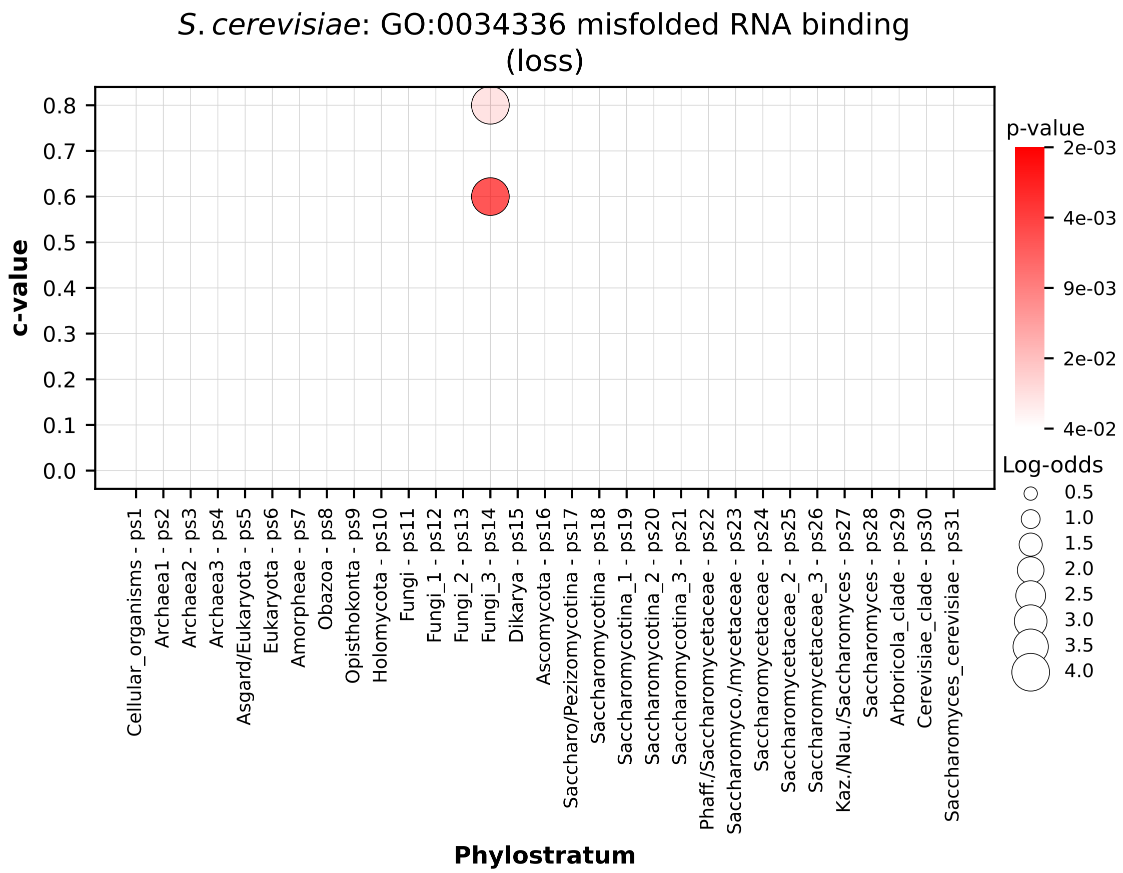

S. cerevisiae: GO:0034336 misfolded RNA binding (loss)
c-value
0.0
0.1
0.2
0.3
0.4
0.5
0.6
0.7
0.8
Phylostratum
Cellular_organisms - ps1
Archaea1 - ps2
Archaea2 - ps3
Archaea3 - ps4
Asgard/Eukaryota - ps5
Eukaryota - ps6
Amorpheae - ps7
Obazoa - ps8
Opisthokonta - ps9
Holomycota - ps10
Fungi - ps11
Fungi_1 - ps12
Fungi_2 - ps13
Fungi_3 - ps14
Dikarya - ps15
Ascomycota - ps16
Saccharo/Pezizomycotina - ps17
Saccharomycotina - ps18
Saccharomycotina_1 - ps19
Saccharomycotina_2 - ps20
Saccharomycotina_3 - ps21
Phaff./Saccharomycetaceae - ps22
Saccharomyco./mycetaceae - ps23
Saccharomycetaceae - ps24
Saccharomycetaceae_2 - ps25
Saccharomycetaceae_3 - ps26
Kaz./Nau./Saccharomyces - ps27
Saccharomyces - ps28
Arboricola_clade - ps29
Cerevisiae_clade - ps30
Saccharomyces_cerevisiae - ps31
p-value
2e-03
4e-03
9e-03
2e-02
4e-02
Log-odds
0.5
1.0
1.5
2.0
2.5
3.0
3.5
4.0

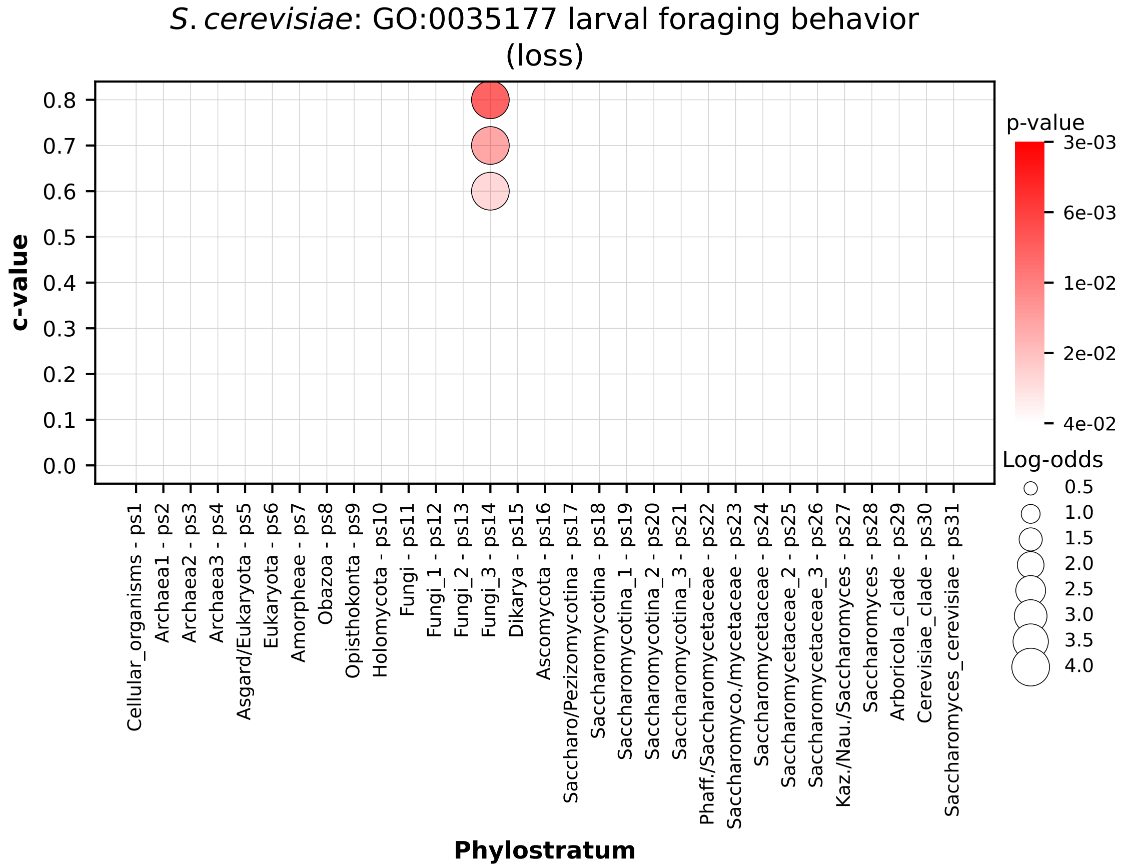

$S. cerevisiae$: GO:0035177 larval foraging behavior
(loss)
c-value
0.0
0.1
0.2
0.3
0.4
0.5
0.6
0.7
0.8
Phylostratum
Cellular_organisms - ps1
Archaea1 - ps2
Archaea2 - ps3
Archaea3 - ps4
Asgard/Eukaryota - ps5
Eukaryota - ps6
Amorpheae - ps7
Obazoa - ps8
Opisthokonta - ps9
Holomycota - ps10
Fungi - ps11
Fungi_1 - ps12
Fungi_2 - ps13
Fungi_3 - ps14
Dikarya - ps15
Ascomycota - ps16
Saccharo/Pezizomycotina - ps17
Saccharomycotina - ps18
Saccharomycotina_1 - ps19
Saccharomycotina_2 - ps20
Saccharomycotina_3 - ps21
Phaff./Saccharomycetaceae - ps22
Saccharomyco./mycetaceae - ps23
Saccharomycetaceae - ps24
Saccharomycetaceae_2 - ps25
Saccharomycetaceae_3 - ps26
Kaz./Nau./Saccharomyces - ps27
Saccharomyces - ps28
Arboricola_clade - ps29
Cerevisiae_clade - ps30
Saccharomyces_cerevisiae - ps31
p-value
3e-03
6e-03
1e-02
2e-02
4e-02
Log-odds
0.5
1.0
1.5
2.0
2.5
3.0
3.5
4.0

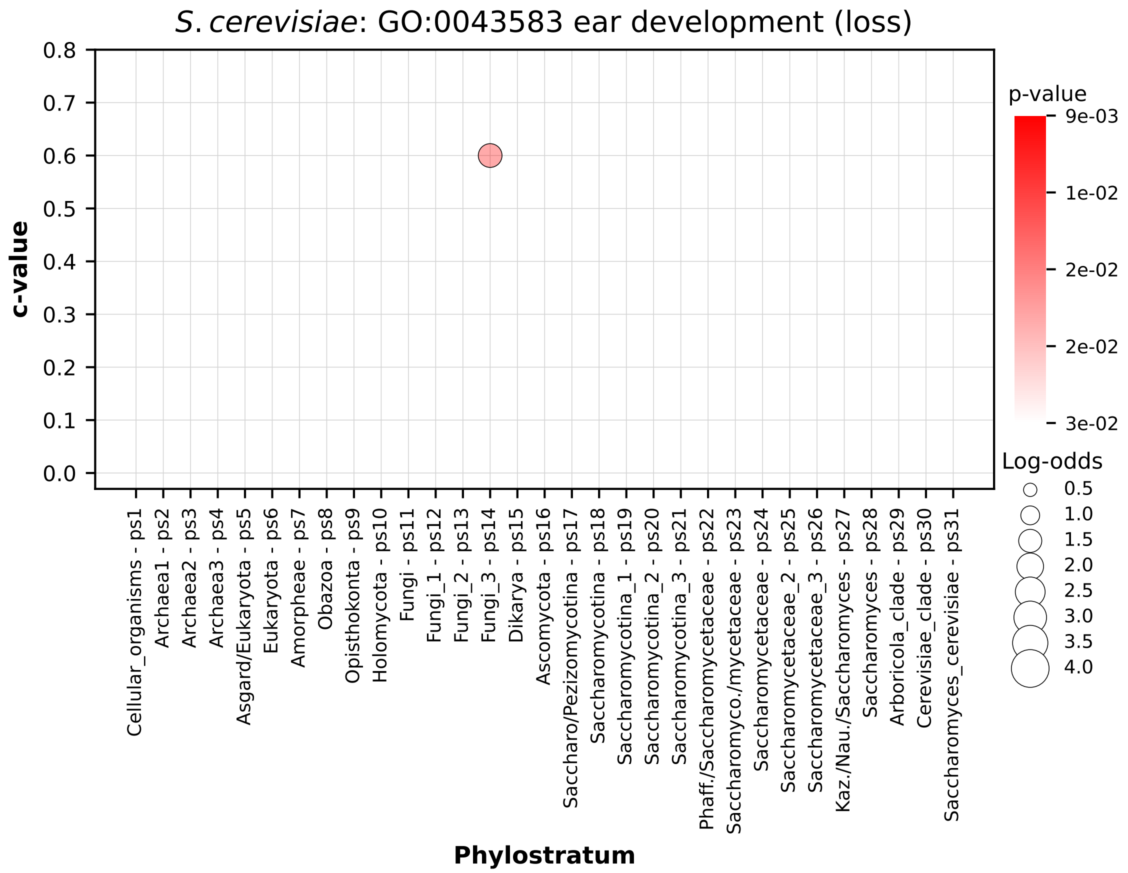
S. cerevisiae: GO:0043583 ear development (loss)
c-value
0.8
0.7
0.6
0.5
0.4
0.3
0.2
0.1
0.0
p-value
9e-03
1e-02
2e-02
2e-02
3e-02
Log-odds
0.5
1.0
1.5
2.0
2.5
3.0
3.5
4.0
Cellular_organisms - ps1
Archaea1 - ps2
Archaea2 - ps3
Archaea3 - ps4
Asgard/Eukaryota - ps5
Eukaryota - ps6
Amorpheae - ps7
Obazoa - ps8
Opisthokonta - ps9
Holomycota - ps10
Fungi - ps11
Fungi_1 - ps12
Fungi_2 - ps13
Fungi_3 - ps14
Dikarya - ps15
Ascomycota - ps16
Saccharo/Pezizomycotina - ps17
Saccharomycotina - ps18
Saccharomycotina_1 - ps19
Saccharomycotina_2 - ps20
Saccharomycotina_3 - ps21
Phaff./Saccharomycetaceae - ps22
Saccharomyco./mycetaceae - ps23
Saccharomycetaceae - ps24
Saccharomycetaceae_2 - ps25
Saccharomycetaceae_3 - ps26
Kaz./Nau./Saccharomyces - ps27
Saccharomyces - ps28
Arboricola_clade - ps29
Cerevisiae_clade - ps30
Saccharomyces_cerevisiae - ps31
Phylostratum

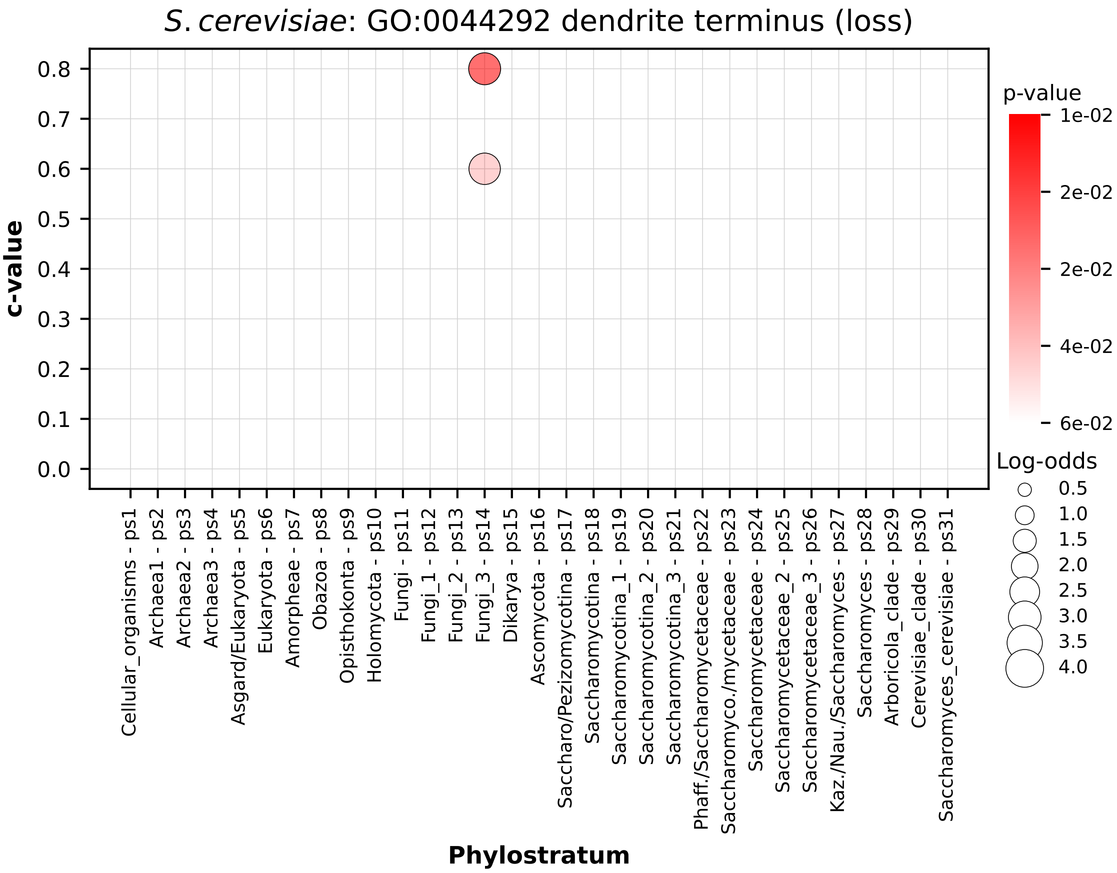

S. cerevisiae: GO:0044292 dendrite terminus (loss)
c-value
0.0
0.1
0.2
0.3
0.4
0.5
0.6
0.7
0.8
p-value
1e-02
2e-02
2e-02
4e-02
6e-02
Log-odds
0.5
1.0
1.5
2.0
2.5
3.0
3.5
4.0
Cellular_organisms - ps1
Archaea1 - ps2
Archaea2 - ps3
Archaea3 - ps4
Asgard/Eukaryota - ps5
Eukaryota - ps6
Amorpheae - ps7
Obazoa - ps8
Opisthokonta - ps9
Holomycota - ps10
Fungi - ps11
Fungi_1 - ps12
Fungi_2 - ps13
Fungi_3 - ps14
Dikarya - ps15
Ascomycota - ps16
Saccharo/Pezizomycotina - ps17
Saccharomycotina - ps18
Saccharomycotina_1 - ps19
Saccharomycotina_2 - ps20
Saccharomycotina_3 - ps21
Phaff./Saccharomycetaceae - ps22
Saccharomyco./mycetaceae - ps23
Saccharomycetaceae - ps24
Saccharomycetaceae_2 - ps25
Saccharomycetaceae_3 - ps26
Kaz./Nau./Saccharomyces - ps27
Saccharomyces - ps28
Arboricola_clade - ps29
Cerevisiae_clade - ps30
Saccharomyces_cerevisiae - ps31
Phylostratum

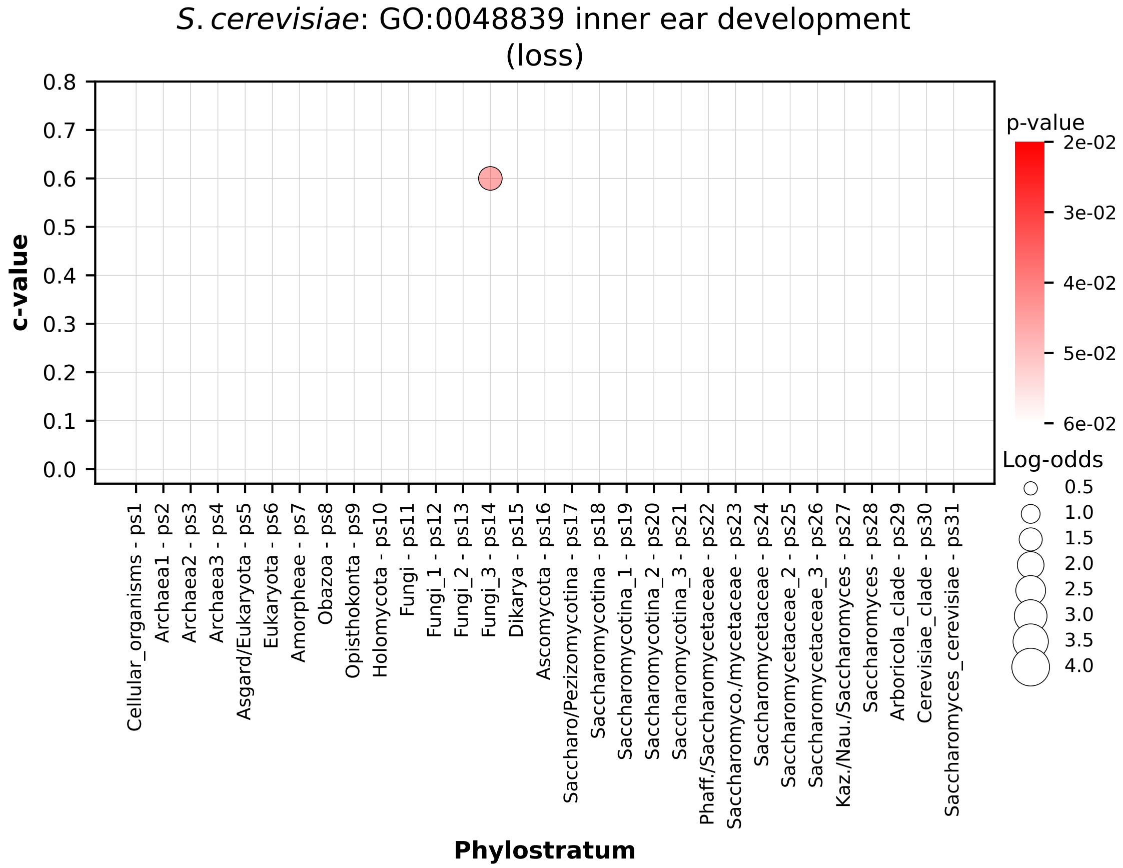

S. cerevisiae: GO:0048839 inner ear development
(loss)
c-value
0.0
0.1
0.2
0.3
0.4
0.5
0.6
0.7
0.8
Phylostratum
Cellular_organisms - ps1
Archaea1 - ps2
Archaea2 - ps3
Archaea3 - ps4
Asgard/Eukaryota - ps5
Eukaryota - ps6
Amorpheae - ps7
Obazoa - ps8
Opisthokonta - ps9
Holomycota - ps10
Fungi - ps11
Fungi_1 - ps12
Fungi_2 - ps13
Fungi_3 - ps14
Dikarya - ps15
Ascomycota - ps16
Saccharo/Pezizomycotina - ps17
Saccharomycotina - ps18
Saccharomycotina_1 - ps19
Saccharomycotina_2 - ps20
Saccharomycotina_3 - ps21
Phaff./Saccharomycetaceae - ps22
Saccharomyco./mycetaceae - ps23
Saccharomycetaceae - ps24
Saccharomycetaceae_2 - ps25
Saccharomycetaceae_3 - ps26
Kaz./Nau./Saccharomyces - ps27
Saccharomyces - ps28
Arboricola_clade - ps29
Cerevisiae_clade - ps30
Saccharomyces_cerevisiae - ps31
p-value
2e-02
3e-02
4e-02
5e-02
6e-02
Log-odds
0.5
1.0
1.5
2.0
2.5
3.0
3.5
4.0

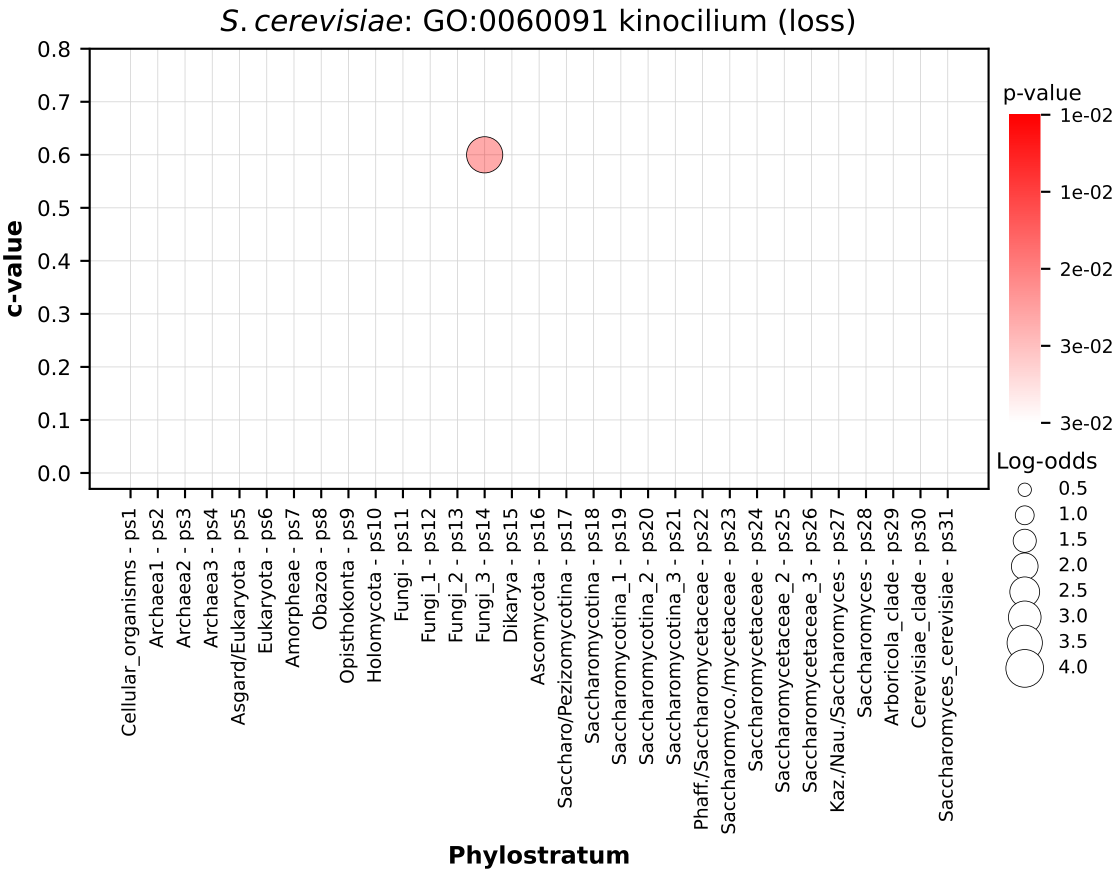
S. cerevisiae: GO:0060091 kinocilium (loss)
c-value
0.8
0.7
0.6
0.5
0.4
0.3
0.2
0.1
0.0
p-value
1e-02
1e-02
2e-02
3e-02
3e-02
Log-odds
0.5
1.0
1.5
2.0
2.5
3.0
3.5
4.0
Cellular_organisms - ps1
Archaea1 - ps2
Archaea2 - ps3
Archaea3 - ps4
Asgard/Eukaryota - ps5
Eukaryota - ps6
Amorpheae - ps7
Obazoa - ps8
Opisthokonta - ps9
Holomycota - ps10
Fungi - ps11
Fungi_1 - ps12
Fungi_2 - ps13
Fungi_3 - ps14
Dikarya - ps15
Ascomycota - ps16
Saccharo/Pezizomycotina - ps17
Saccharomycotina - ps18
Saccharomycotina_1 - ps19
Saccharomycotina_2 - ps20
Saccharomycotina_3 - ps21
Phaff./Saccharomycetaceae - ps22
Saccharomyco./mycetaceae - ps23
Saccharomycetaceae - ps24
Saccharomycetaceae_2 - ps25
Saccharomycetaceae_3 - ps26
Kaz./Nau./Saccharomyces - ps27
Saccharomyces - ps28
Arboricola_clade - ps29
Cerevisiae_clade - ps30
Saccharomyces_cerevisiae - ps31
Phylostratum

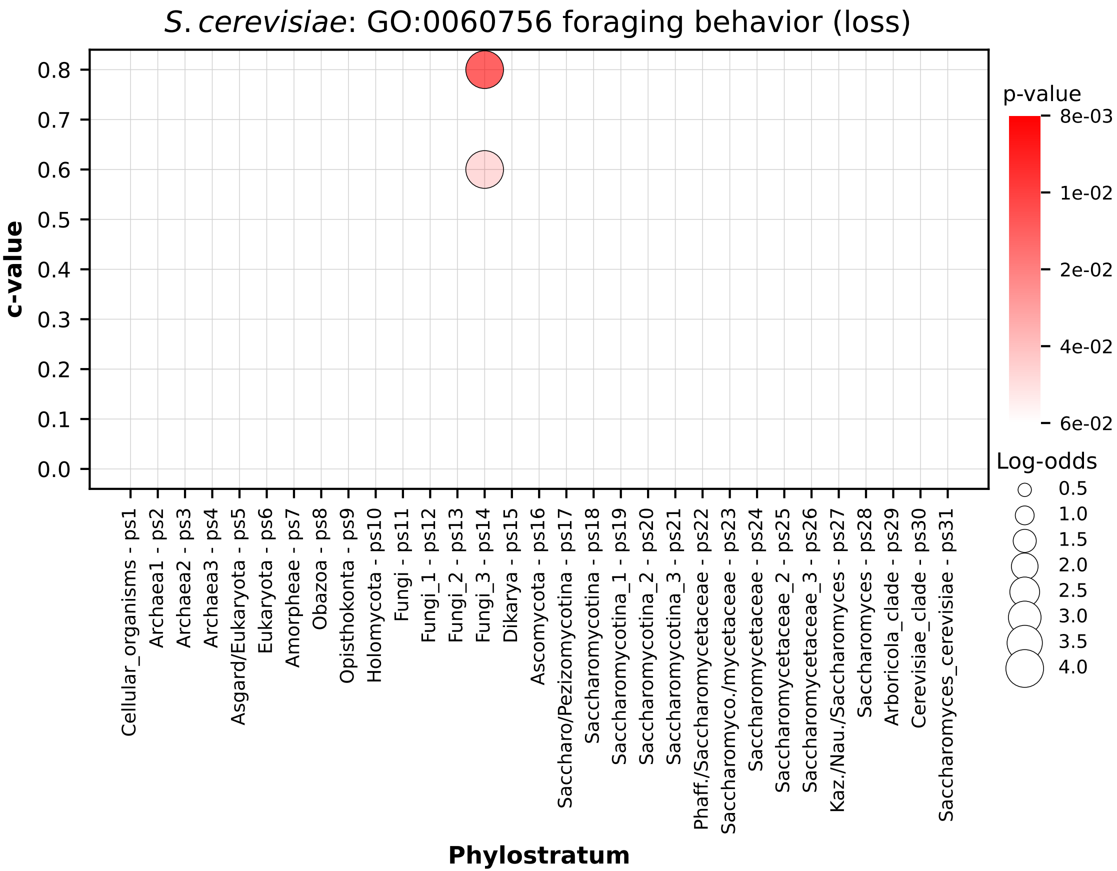

S. cerevisiae: GO:0060756 foraging behavior (loss)
c-value
0.8
0.7
0.6
0.5
0.4
0.3
0.2
0.1
0.0
p-value
8e-03
1e-02
2e-02
4e-02
6e-02
Log-odds
0.5
1.0
1.5
2.0
2.5
3.0
3.5
4.0
Cellular_organisms - ps1
Archaea1 - ps2
Archaea2 - ps3
Archaea3 - ps4
Asgard/Eukaryota - ps5
Eukaryota - ps6
Amorpheae - ps7
Obazoa - ps8
Opisthokonta - ps9
Holomycota - ps10
Fungi - ps11
Fungi_1 - ps12
Fungi_2 - ps13
Fungi_3 - ps14
Dikarya - ps15
Ascomycota - ps16
Saccharo/Pezizomycotina - ps17
Saccharomycotina - ps18
Saccharomycotina_1 - ps19
Saccharomycotina_2 - ps20
Saccharomycotina_3 - ps21
Phaff./Saccharomycetaceae - ps22
Saccharomyco./mycetaceae - ps23
Saccharomycetaceae - ps24
Saccharomycetaceae_2 - ps25
Saccharomycetaceae_3 - ps26
Kaz./Nau./Saccharomyces - ps27
Saccharomyces - ps28
Arboricola_clade - ps29
Cerevisiae_clade - ps30
Saccharomyces_cerevisiae - ps31
Phylostratum

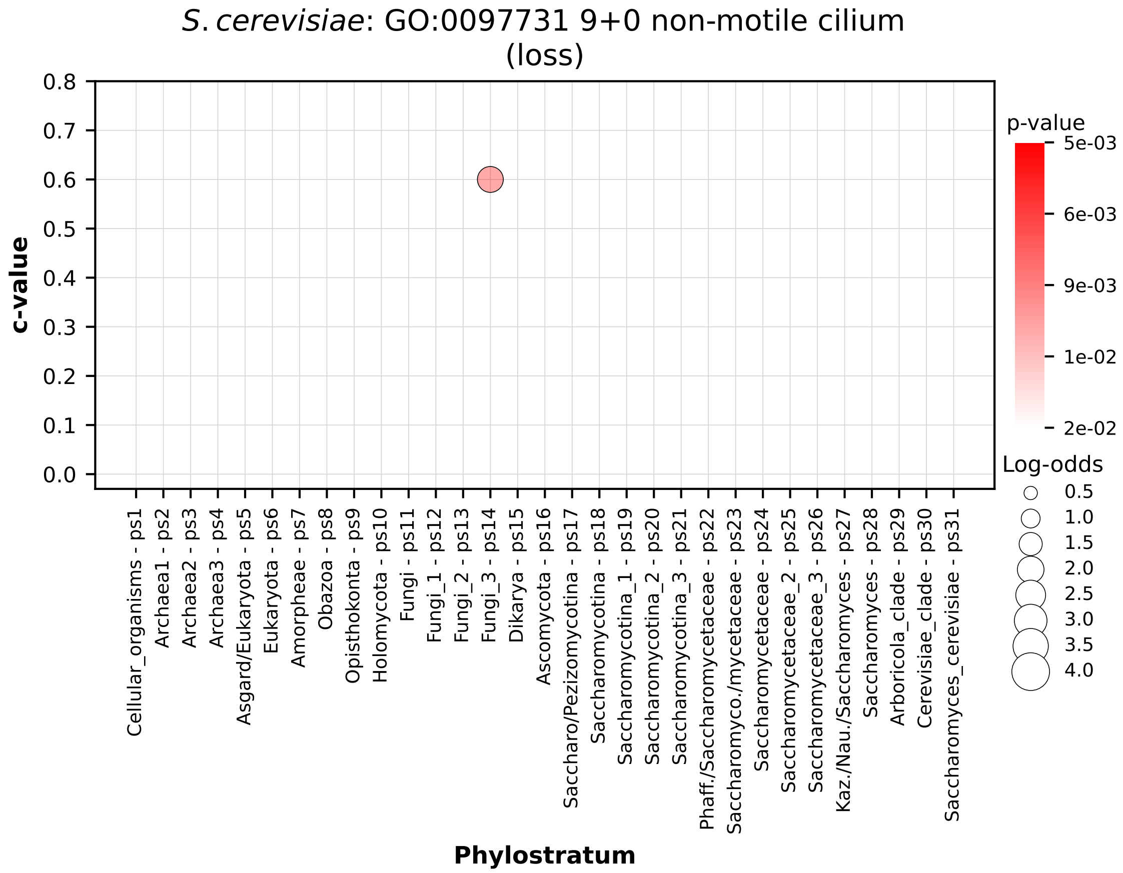
S. cerevisiae: GO:0097731 9+0 non-motile cilium
(loss)
c-value
0.8
0.7
0.6
0.5
0.4
0.3
0.2
0.1
0.0
p-value
5e-03
6e-03
9e-03
1e-02
2e-02
Log-odds
0.5
1.0
1.5
2.0
2.5
3.0
3.5
4.0
Cellular_organisms - ps1
Archaea1 - ps2
Archaea2 - ps3
Archaea3 - ps4
Asgard/Eukaryota - ps5
Eukaryota - ps6
Amorpheae - ps7
Obazoa - ps8
Opisthokonta - ps9
Holomycota - ps10
Fungi - ps11
Fungi_1 - ps12
Fungi_2 - ps13
Fungi_3 - ps14
Dikarya - ps15
Ascomycota - ps16
Saccharo/Pezizomycotina - ps17
Saccharomycotina - ps18
Saccharomycotina_1 - ps19
Saccharomycotina_2 - ps20
Saccharomycotina_3 - ps21
Phaff./Saccharomycetaceae - ps22
Saccharomyco./mycetaceae - ps23
Saccharomycetaceae - ps24
Saccharomycetaceae_2 - ps25
Saccharomycetaceae_3 - ps26
Kaz./Nau./Saccharomyces - ps27
Saccharomyces - ps28
Arboricola_clade - ps29
Cerevisiae_clade - ps30
Saccharomyces_cerevisiae - ps31
Phylostratum

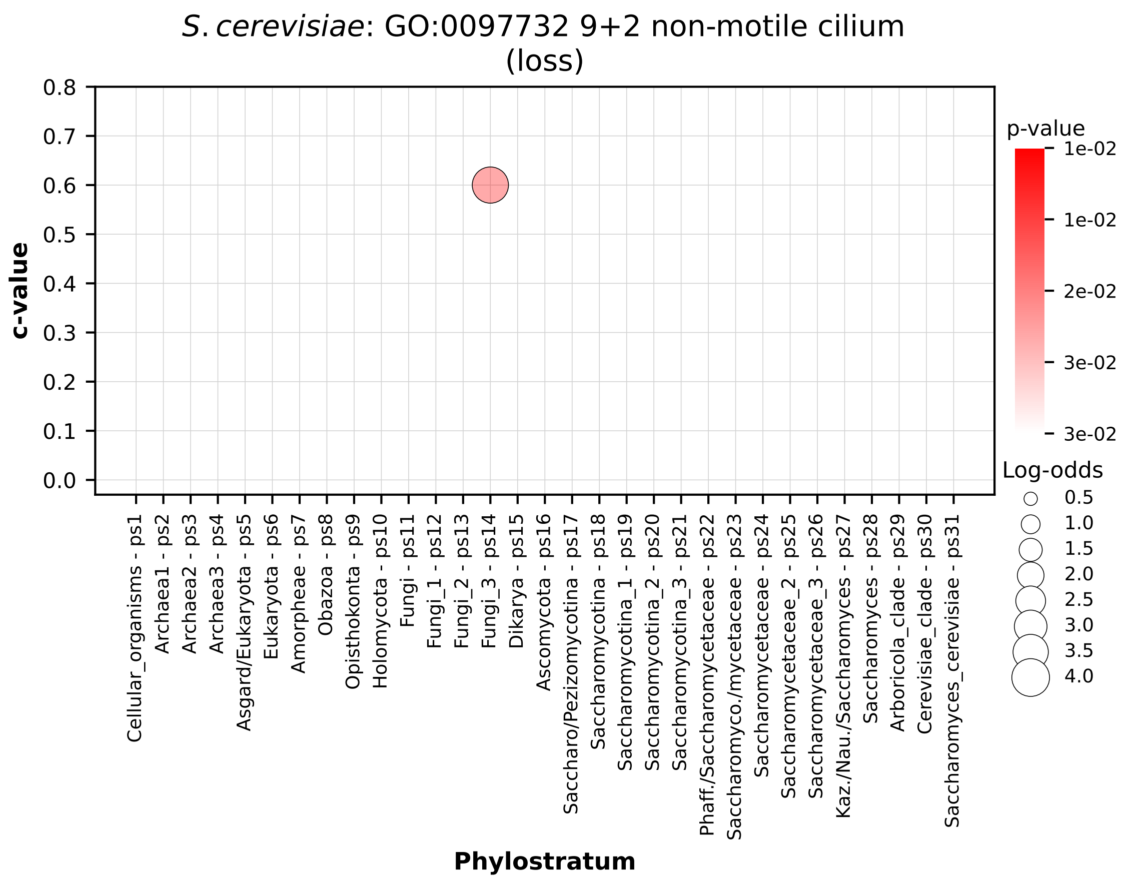

S. cerevisiae: GO:0097732 9+2 non-motile cilium
(loss)
c-value
0.8
0.7
0.6
0.5
0.4
0.3
0.2
0.1
0.0
p-value
1e-02
1e-02
2e-02
3e-02
3e-02
Log-odds
0.5
1.0
1.5
2.0
2.5
3.0
3.5
4.0
Cellular_organisms - ps1
Archaea1 - ps2
Archaea2 - ps3
Archaea3 - ps4
Asgard/Eukaryota - ps5
Eukaryota - ps6
Amorpheae - ps7
Obazoa - ps8
Opisthokonta - ps9
Holomycota - ps10
Fungi - ps11
Fungi_1 - ps12
Fungi_2 - ps13
Fungi_3 - ps14
Dikarya - ps15
Ascomycota - ps16
Saccharo/Pezizomycotina - ps17
Saccharomycotina - ps18
Saccharomycotina_1 - ps19
Saccharomycotina_2 - ps20
Saccharomycotina_3 - ps21
Phaff./Saccharomycetaceae - ps22
Saccharomyco./mycetaceae - ps23
Saccharomycetaceae - ps24
Saccharomycetaceae_2 - ps25
Saccharomycetaceae_3 - ps26
Kaz./Nau./Saccharomyces - ps27
Saccharomyces - ps28
Arboricola_clade - ps29
Cerevisiae_clade - ps30
Saccharomyces_cerevisiae - ps31
Phylostratum

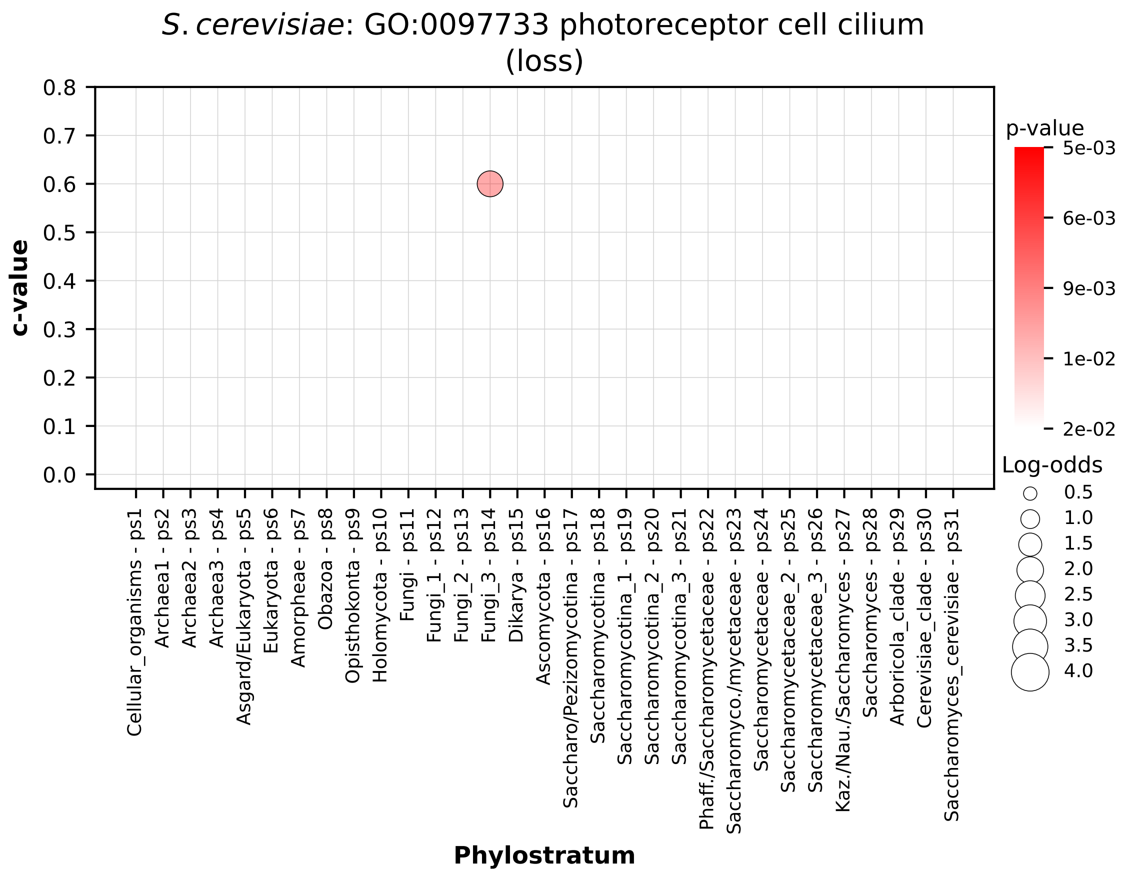

S. cerevisiae: GO:0097733 photoreceptor cell cilium
(loss)
c-value
0.8
0.7
0.6
0.5
0.4
0.3
0.2
0.1
0.0
p-value
5e-03
6e-03
9e-03
1e-02
2e-02
Log-odds
0.5
1.0
1.5
2.0
2.5
3.0
3.5
4.0
Cellular_organisms - ps1
Archaea1 - ps2
Archaea2 - ps3
Archaea3 - ps4
Asgard/Eukaryota - ps5
Eukaryota - ps6
Amorpheae - ps7
Obazoa - ps8
Opisthokonta - ps9
Holomycota - ps10
Fungi - ps11
Fungi_1 - ps12
Fungi_2 - ps13
Fungi_3 - ps14
Dikarya - ps15
Ascomycota - ps16
Saccharo/Pezizomycotina - ps17
Saccharomycotina - ps18
Saccharomycotina_1 - ps19
Saccharomycotina_2 - ps20
Saccharomycotina_3 - ps21
Phaff./Saccharomycetaceae - ps22
Saccharomyco./mycetaceae - ps23
Saccharomycetaceae - ps24
Saccharomycetaceae_2 - ps25
Saccharomycetaceae_3 - ps26
Kaz./Nau./Saccharomyces - ps27
Saccharomyces - ps28
Arboricola_clade - ps29
Cerevisiae_clade - ps30
Saccharomyces_cerevisiae - ps31
Phylostratum

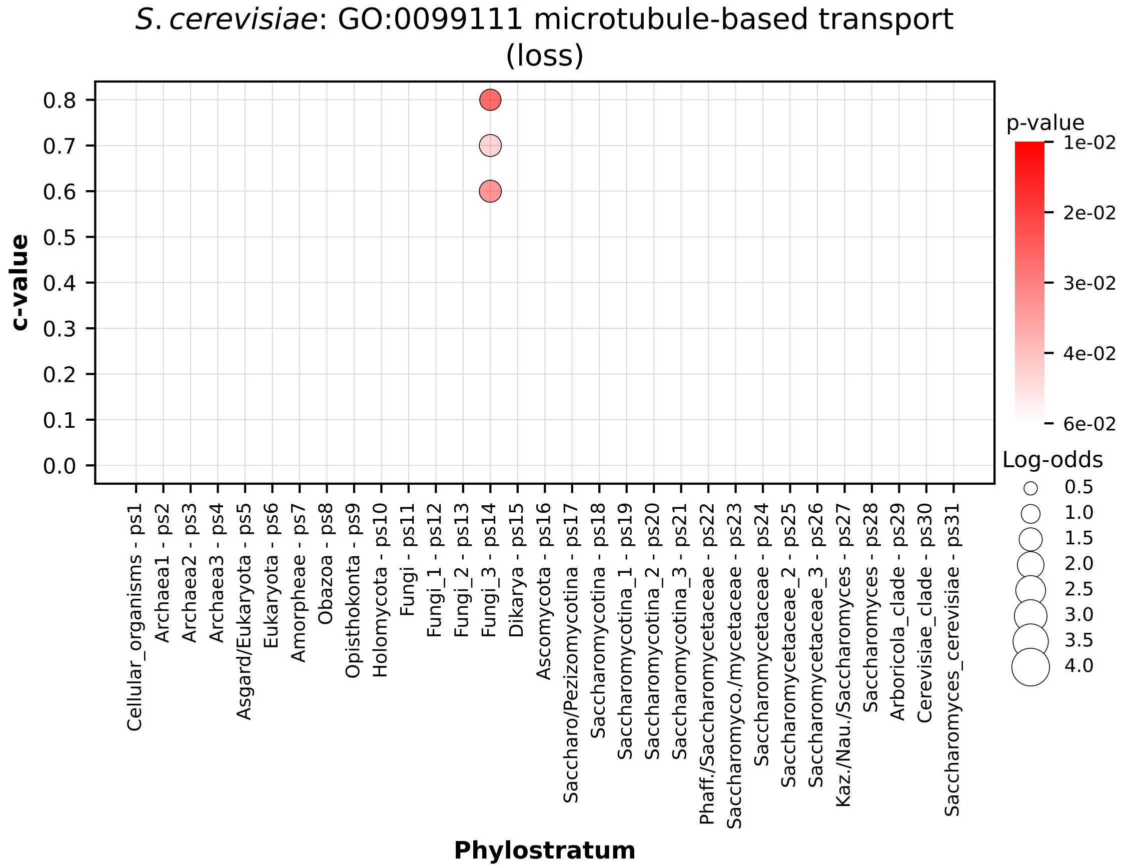

S. cerevisiae: GO:0099111 microtubule-based transport
(loss)
c-value
0.0
0.1
0.2
0.3
0.4
0.5
0.6
0.7
0.8
p-value
1e-02
2e-02
3e-02
4e-02
6e-02
Log-odds
0.5
1.0
1.5
2.0
2.5
3.0
3.5
4.0
Cellular_organisms - ps1
Archaea1 - ps2
Archaea2 - ps3
Archaea3 - ps4
Asgard/Eukaryota - ps5
Eukaryota - ps6
Amorpheae - ps7
Obazoa - ps8
Opisthokonta - ps9
Holomycota - ps10
Fungi - ps11
Fungi_1 - ps12
Fungi_2 - ps13
Fungi_3 - ps14
Dikarya - ps15
Ascomycota - ps16
Saccharo/Pezizomycotina - ps17
Saccharomycotina - ps18
Saccharomycotina_1 - ps19
Saccharomycotina_2 - ps20
Saccharomycotina_3 - ps21
Phaff./Saccharomycetaceae - ps22
Saccharomyco./mycetaceae - ps23
Saccharomycetaceae - ps24
Saccharomycetaceae_2 - ps25
Saccharomycetaceae_3 - ps26
Kaz./Nau./Saccharomyces - ps27
Saccharomyces - ps28
Arboricola_clade - ps29
Cerevisiae_clade - ps30
Saccharomyces_cerevisiae - ps31
Phylostratum

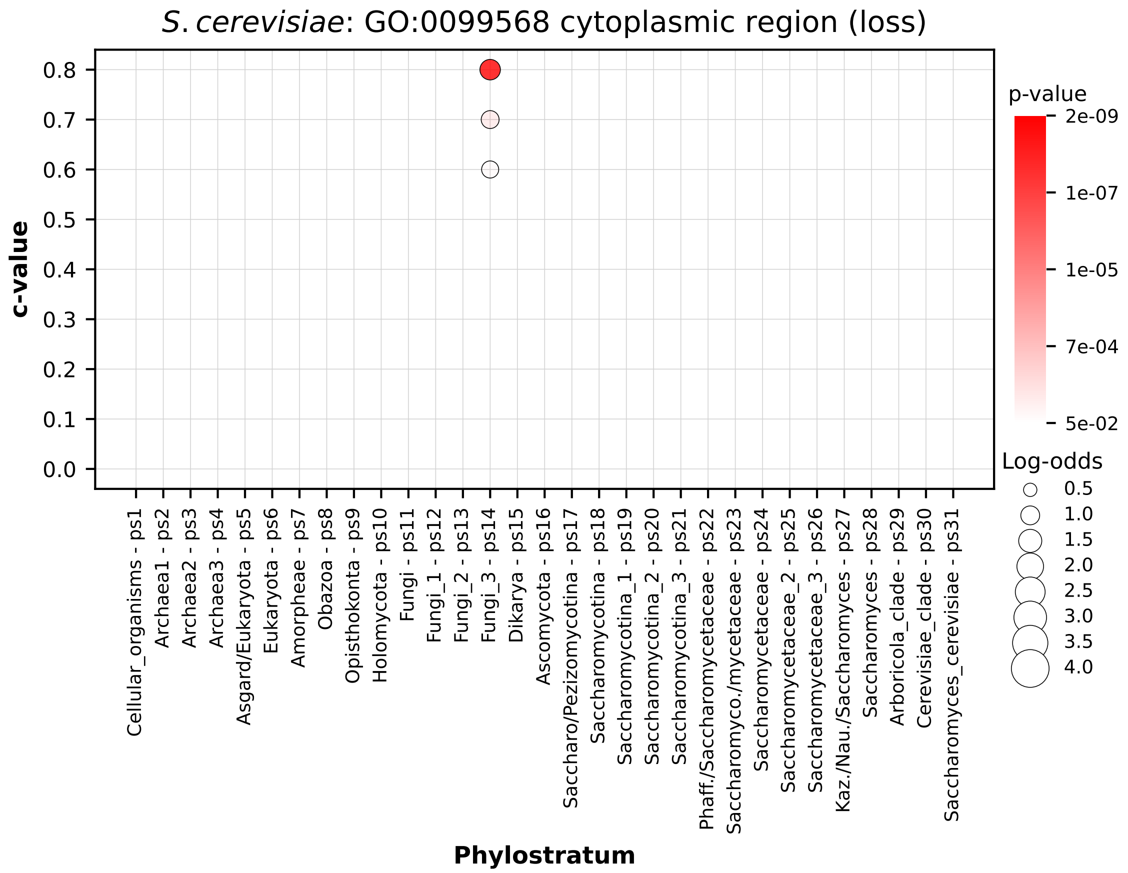

S. cerevisiae: GO:0099568 cytoplasmic region (loss)
c-value
0.0
0.1
0.2
0.3
0.4
0.5
0.6
0.7
0.8
p-value
2e-09
1e-07
1e-05
7e-04
5e-02
Log-odds
0.5
1.0
1.5
2.0
2.5
3.0
3.5
4.0
Cellular_organisms - ps1
Archaea1 - ps2
Archaea2 - ps3
Archaea3 - ps4
Asgard/Eukaryota - ps5
Eukaryota - ps6
Amorpheae - ps7
Obazoa - ps8
Opisthokonta - ps9
Holomycota - ps10
Fungi - ps11
Fungi_1 - ps12
Fungi_2 - ps13
Fungi_3 - ps14
Dikarya - ps15
Ascomycota - ps16
Saccharo/Pezizomycotina - ps17
Saccharomycotina - ps18
Saccharomycotina_1 - ps19
Saccharomycotina_2 - ps20
Saccharomycotina_3 - ps21
Phaff./Saccharomycetaceae - ps22
Saccharomyco./mycetaceae - ps23
Saccharomycetaceae - ps24
Saccharomycetaceae_2 - ps25
Saccharomycetaceae_3 - ps26
Kaz./Nau./Saccharomyces - ps27
Saccharomyces - ps28
Arboricola_clade - ps29
Cerevisiae_clade - ps30
Saccharomyces_cerevisiae - ps31
Phylostratum

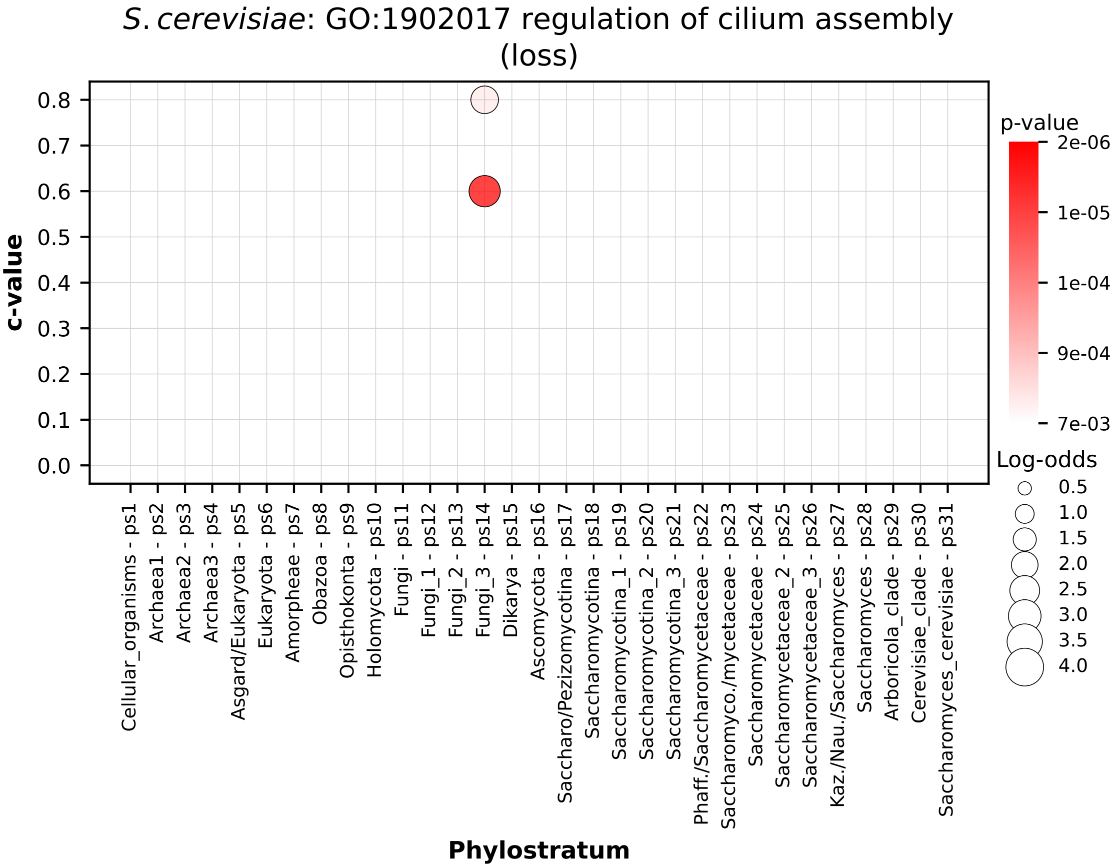
S. cerevisiae: GO:1902017 regulation of cilium assembly
(loss)
c-value
0.0
0.1
0.2
0.3
0.4
0.5
0.6
0.7
0.8
Phylostratum
Cellular_organisms - ps1
Archaea1 - ps2
Archaea2 - ps3
Archaea3 - ps4
Asgard/Eukaryota - ps5
Eukaryota - ps6
Amorpheae - ps7
Obazoa - ps8
Opisthokonta - ps9
Holomycota - ps10
Fungi - ps11
Fungi_1 - ps12
Fungi_2 - ps13
Fungi_3 - ps14
Dikarya - ps15
Ascomycota - ps16
Saccharo/Pezizomycotina - ps17
Saccharomycotina - ps18
Saccharomycotina_1 - ps19
Saccharomycotina_2 - ps20
Saccharomycotina_3 - ps21
Phaff./Saccharomycetaceae - ps22
Saccharomyco./mycetaceae - ps23
Saccharomycetaceae - ps24
Saccharomycetaceae_2 - ps25
Saccharomycetaceae_3 - ps26
Kaz./Nau./Saccharomyces - ps27
Saccharomyces - ps28
Arboricola_clade - ps29
Cerevisiae_clade - ps30
Saccharomyces_cerevisiae - ps31
p-value
2e-06
1e-05
1e-04
9e-04
7e-03
Log-odds
0.5
1.0
1.5
2.0
2.5
3.0
3.5
4.0

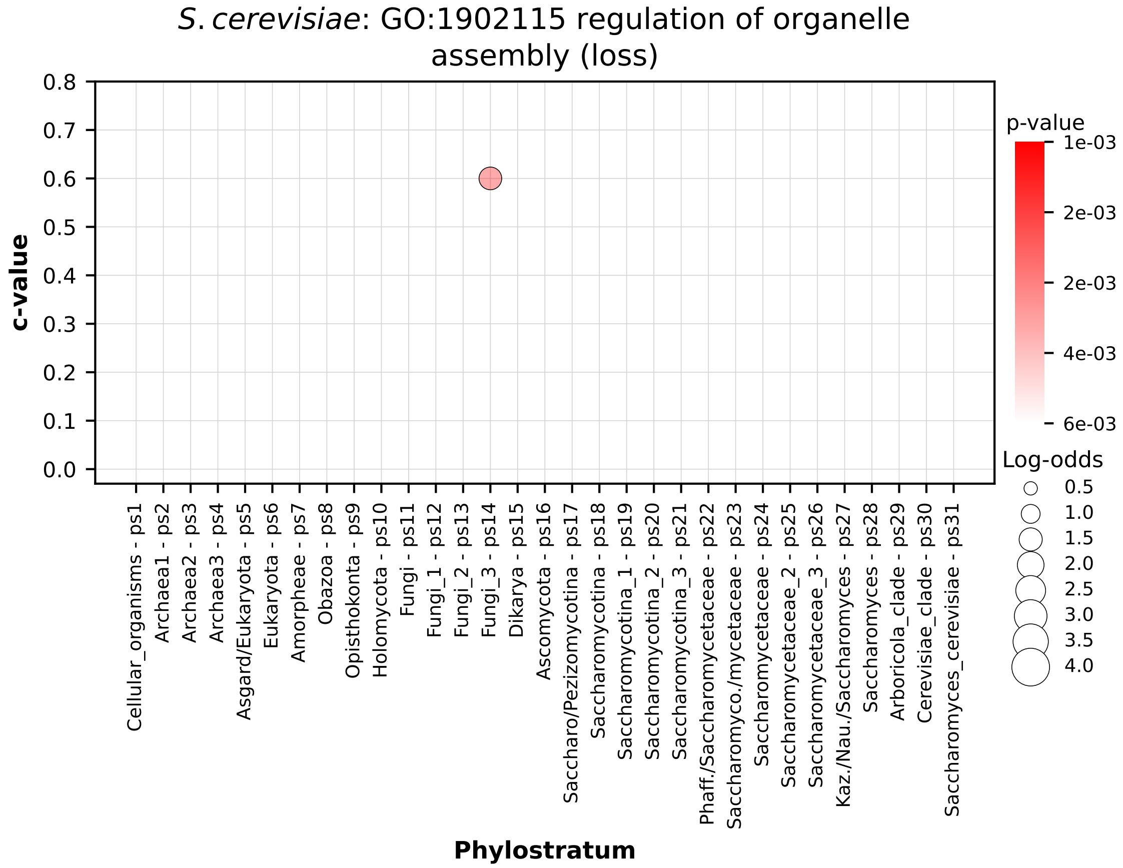

S. cerevisiae: GO:1902115 regulation of organelle assembly (loss)
c-value
0.8
0.7
0.6
0.5
0.4
0.3
0.2
0.1
0.0
p-value
1e-03
2e-03
2e-03
4e-03
6e-03
Log-odds
0.5
1.0
1.5
2.0
2.5
3.0
3.5
4.0
Cellular_organisms - ps1
Archaea1 - ps2
Archaea2 - ps3
Archaea3 - ps4
Asgard/Eukaryota - ps5
Eukaryota - ps6
Amorpheae - ps7
Obazoa - ps8
Opisthokonta - ps9
Holomycota - ps10
Fungi - ps11
Fungi_1 - ps12
Fungi_2 - ps13
Fungi_3 - ps14
Dikarya - ps15
Ascomycota - ps16
Saccharo/Pezizomycotina - ps17
Saccharomycotina - ps18
Saccharomycotina_1 - ps19
Saccharomycotina_2 - ps20
Saccharomycotina_3 - ps21
Phaff./Saccharomycetaceae - ps22
Saccharomyco./mycetaceae - ps23
Saccharomycetaceae - ps24
Saccharomycetaceae_2 - ps25
Saccharomycetaceae_3 - ps26
Kaz./Nau./Saccharomyces - ps27
Saccharomyces - ps28
Arboricola_clade - ps29
Cerevisiae_clade - ps30
Saccharomyces_cerevisiae - ps31
Phylostratum

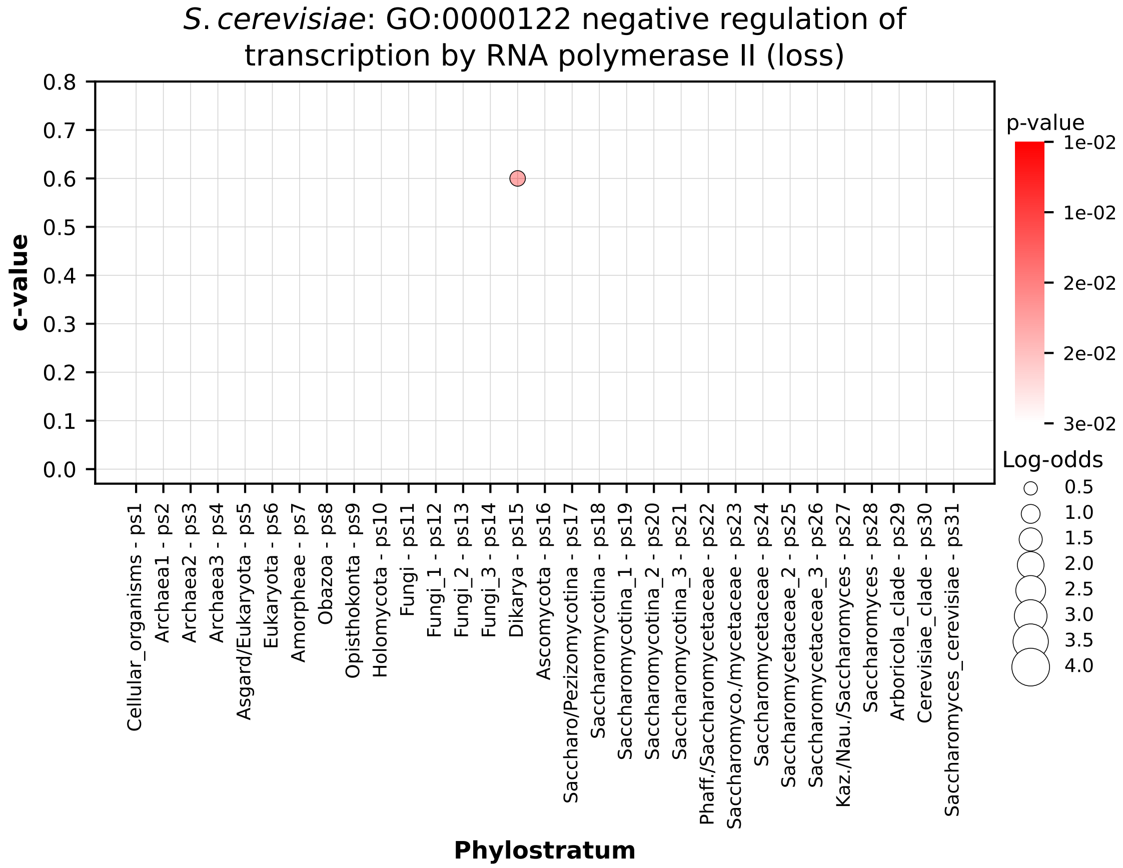

S. cerevisiae: GO:0000122 negative regulation of transcription by RNA polymerase II (loss)
c-value
0.8
0.7
0.6
0.5
0.4
0.3
0.2
0.1
0.0
p-value
1e-02
1e-02
2e-02
2e-02
3e-02
Log-odds
0.5
1.0
1.5
2.0
2.5
3.0
3.5
4.0
Cellular_organisms - ps1
Archaea1 - ps2
Archaea2 - ps3
Archaea3 - ps4
Asgard/Eukaryota - ps5
Eukaryota - ps6
Amorpheae - ps7
Obazoa - ps8
Opisthokonta - ps9
Holomycota - ps10
Fungi - ps11
Fungi_1 - ps12
Fungi_2 - ps13
Fungi_3 - ps14
Dikarya - ps15
Ascomycota - ps16
Saccharo/Pezizomycotina - ps17
Saccharomycotina - ps18
Saccharomycotina_1 - ps19
Saccharomycotina_2 - ps20
Saccharomycotina_3 - ps21
Phaff./Saccharomycetaceae - ps22
Saccharomyco./mycetaceae - ps23
Saccharomycetaceae - ps24
Saccharomycetaceae_2 - ps25
Saccharomycetaceae_3 - ps26
Kaz./Nau./Saccharomyces - ps27
Saccharomyces - ps28
Arboricola_clade - ps29
Cerevisiae_clade - ps30
Saccharomyces_cerevisiae - ps31
Phylostratum

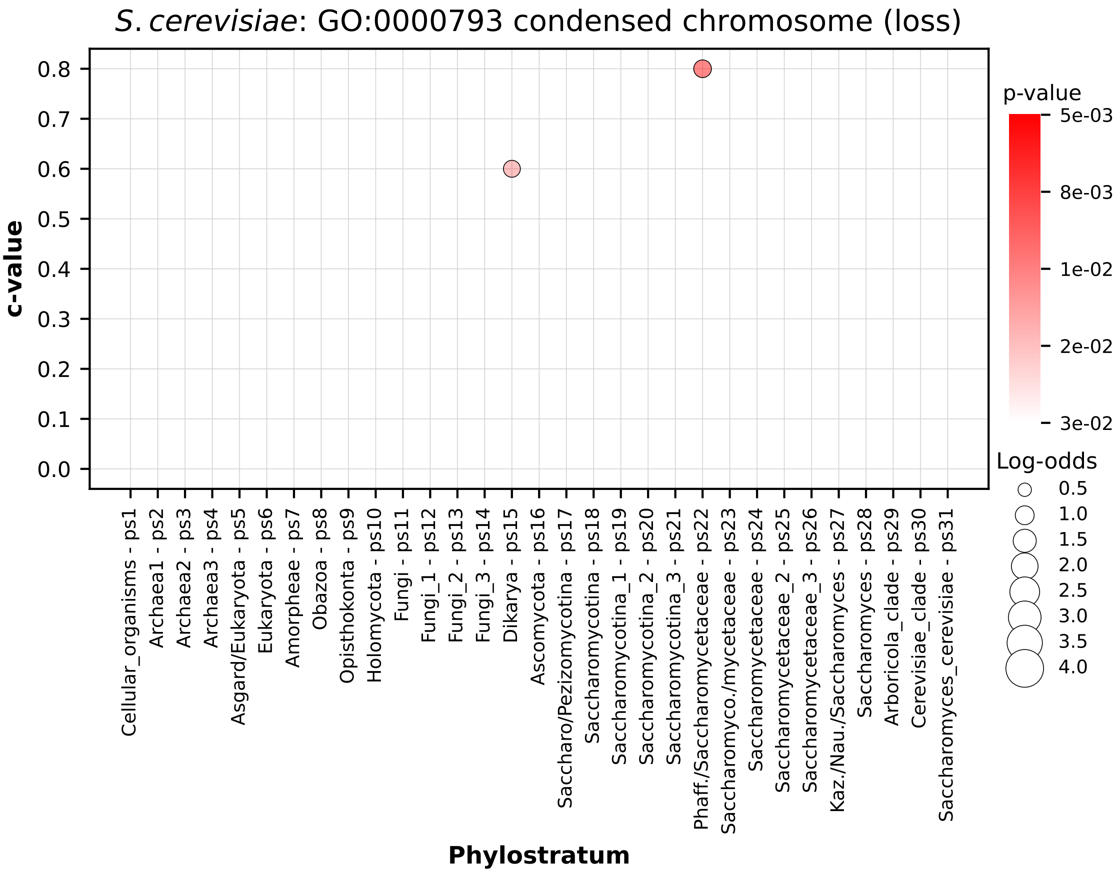

S. cerevisiae: GO:0000793 condensed chromosome (loss)
c-value
0.0
0.1
0.2
0.3
0.4
0.5
0.6
0.7
0.8
p-value
5e-03
8e-03
1e-02
2e-02
3e-02
Log-odds
0.5
1.0
1.5
2.0
2.5
3.0
3.5
4.0
Cellular_organisms - ps1
Archaea1 - ps2
Archaea2 - ps3
Archaea3 - ps4
Asgard/Eukaryota - ps5
Eukaryota - ps6
Amorpheae - ps7
Obazoa - ps8
Opisthokonta - ps9
Holomycota - ps10
Fungi - ps11
Fungi_1 - ps12
Fungi_2 - ps13
Fungi_3 - ps14
Dikarya - ps15
Ascomycota - ps16
Saccharo/Pezizomycotina - ps17
Saccharomycotina - ps18
Saccharomycotina_1 - ps19
Saccharomycotina_2 - ps20
Saccharomycotina_3 - ps21
Phaff./Saccharomycetaceae - ps22
Saccharomyco./mycetaceae - ps23
Saccharomycetaceae - ps24
Saccharomycetaceae_2 - ps25
Saccharomycetaceae_3 - ps26
Kaz./Nau./Saccharomyces - ps27
Saccharomyces - ps28
Arboricola_clade - ps29
Cerevisiae_clade - ps30
Saccharomyces_cerevisiae - ps31
Phylostratum

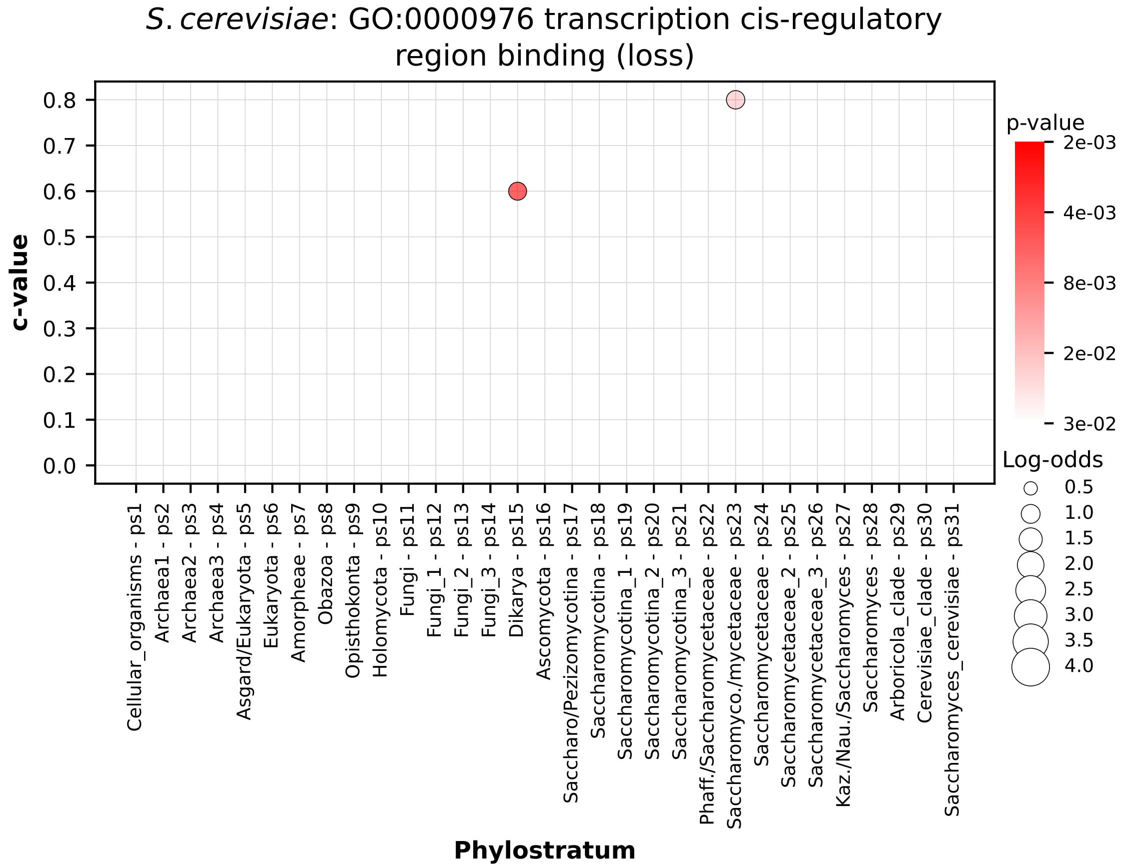

S. cerevisiae: GO:0000976 transcription cis-regulatory region binding (loss)
c-value
0.0
0.1
0.2
0.3
0.4
0.5
0.6
0.7
0.8
Phylostratum
Cellular_organisms - ps1
Archaea1 - ps2
Archaea2 - ps3
Archaea3 - ps4
Asgard/Eukaryota - ps5
Eukaryota - ps6
Amorpheae - ps7
Obazoa - ps8
Opisthokonta - ps9
Holomycota - ps10
Fungi - ps11
Fungi_1 - ps12
Fungi_2 - ps13
Fungi_3 - ps14
Dikarya - ps15
Ascomycota - ps16
Saccharo/Pezizomycotina - ps17
Saccharomycotina - ps18
Saccharomycotina_1 - ps19
Saccharomycotina_2 - ps20
Saccharomycotina_3 - ps21
Phaff./Saccharomycetaceae - ps22
Saccharomyco./mycetaceae - ps23
Saccharomycetaceae - ps24
Saccharomycetaceae_2 - ps25
Saccharomycetaceae_3 - ps26
Kaz./Nau./Saccharomyces - ps27
Saccharomyces - ps28
Arboricola_clade - ps29
Cerevisiae_clade - ps30
Saccharomyces_cerevisiae - ps31
p-value
2e-03
4e-03
8e-03
2e-02
3e-02
Log-odds
0.5
1.0
1.5
2.0
2.5
3.0
3.5
4.0

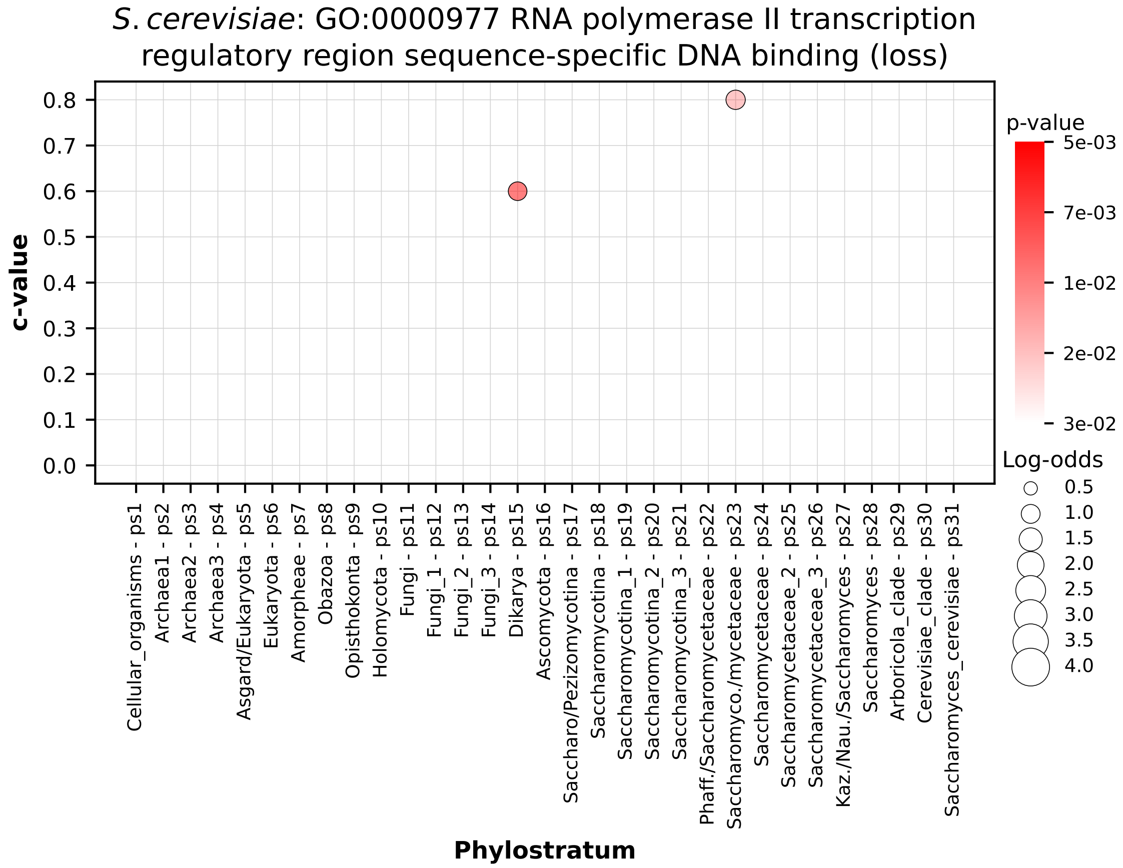

S. cerevisiae: GO:0000977 RNA polymerase II transcription regulatory region sequence-specific DNA binding (loss)
c-value
0.0
0.1
0.2
0.3
0.4
0.5
0.6
0.7
0.8
Phylostratum
Cellular_organisms - ps1
Archaea1 - ps2
Archaea2 - ps3
Archaea3 - ps4
Asgard/Eukaryota - ps5
Eukaryota - ps6
Amorpheae - ps7
Obazoa - ps8
Opisthokonta - ps9
Holomycota - ps10
Fungi - ps11
Fungi_1 - ps12
Fungi_2 - ps13
Fungi_3 - ps14
Dikarya - ps15
Ascomycota - ps16
Saccharo/Pezizomycotina - ps17
Saccharomycotina - ps18
Saccharomycotina_1 - ps19
Saccharomycotina_2 - ps20
Saccharomycotina_3 - ps21
Phaff./Saccharomycetaceae - ps22
Saccharomyco./mycetaceae - ps23
Saccharomycetaceae - ps24
Saccharomycetaceae_2 - ps25
Saccharomycetaceae_3 - ps26
Kaz./Nau./Saccharomyces - ps27
Saccharomyces - ps28
Arboricola_clade - ps29
Cerevisiae_clade - ps30
Saccharomyces_cerevisiae - ps31
p-value
5e-03
7e-03
1e-02
2e-02
3e-02
Log-odds
0.5
1.0
1.5
2.0
2.5
3.0
3.5
4.0

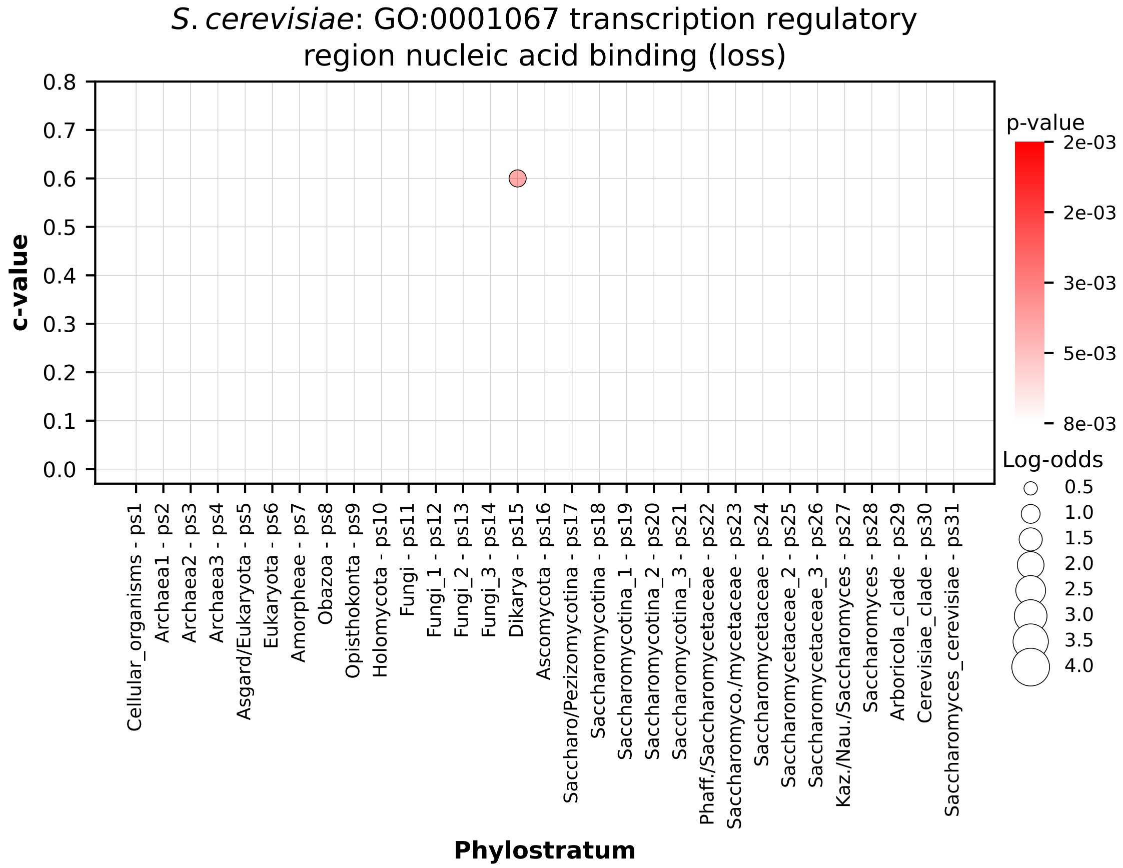
S. cerevisiae: GO:0001067 transcription regulatory region nucleic acid binding (loss)
c-value
0.8
0.7
0.6
0.5
0.4
0.3
0.2
0.1
0.0
p-value
2e-03
2e-03
3e-03
5e-03
8e-03
Log-odds
0.5
1.0
1.5
2.0
2.5
3.0
3.5
4.0
Cellular_organisms - ps1
Archaea1 - ps2
Archaea2 - ps3
Archaea3 - ps4
Asgard/Eukaryota - ps5
Eukaryota - ps6
Amorpheae - ps7
Obazoa - ps8
Opisthokonta - ps9
Holomycota - ps10
Fungi - ps11
Fungi_1 - ps12
Fungi_2 - ps13
Fungi_3 - ps14
Dikarya - ps15
Ascomycota - ps16
Saccharo/Pezizomycotina - ps17
Saccharomycotina - ps18
Saccharomycotina_1 - ps19
Saccharomycotina_2 - ps20
Saccharomycotina_3 - ps21
Phaff./Saccharomycetaceae - ps22
Saccharomyco./mycetaceae - ps23
Saccharomycetaceae - ps24
Saccharomycetaceae_2 - ps25
Saccharomycetaceae_3 - ps26
Kaz./Nau./Saccharomyces - ps27
Saccharomyces - ps28
Arboricola_clade - ps29
Cerevisiae_clade - ps30
Saccharomyces_cerevisiae - ps31
Phylostratum

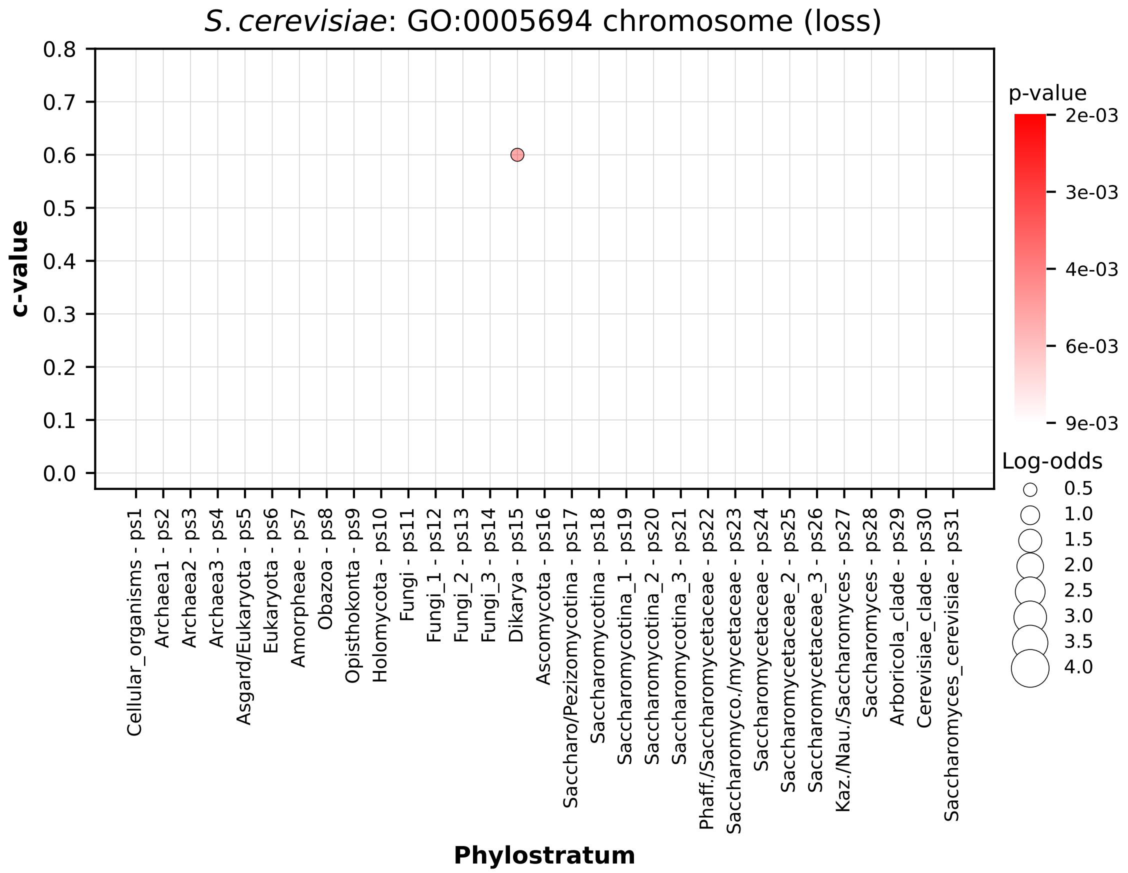

S. cerevisiae: GO:0005694 chromosome (loss)
c-value
0.8
0.7
0.6
0.5
0.4
0.3
0.2
0.1
0.0
p-value
2e-03
3e-03
4e-03
6e-03
9e-03
Log-odds
0.5
1.0
1.5
2.0
2.5
3.0
3.5
4.0
Cellular_organisms - ps1
Archaea1 - ps2
Archaea2 - ps3
Archaea3 - ps4
Asgard/Eukaryota - ps5
Eukaryota - ps6
Amorpheae - ps7
Obazoa - ps8
Opisthokonta - ps9
Holomycota - ps10
Fungi - ps11
Fungi_1 - ps12
Fungi_2 - ps13
Fungi_3 - ps14
Dikarya - ps15
Ascomycota - ps16
Saccharo/Pezizomycotina - ps17
Saccharomycotina - ps18
Saccharomycotina_1 - ps19
Saccharomycotina_2 - ps20
Saccharomycotina_3 - ps21
Phaff./Saccharomycetaceae - ps22
Saccharomyco./mycetaceae - ps23
Saccharomycetaceae - ps24
Saccharomycetaceae_2 - ps25
Saccharomycetaceae_3 - ps26
Kaz./Nau./Saccharomyces - ps27
Saccharomyces - ps28
Arboricola_clade - ps29
Cerevisiae_clade - ps30
Saccharomyces_cerevisiae - ps31
Phylostratum

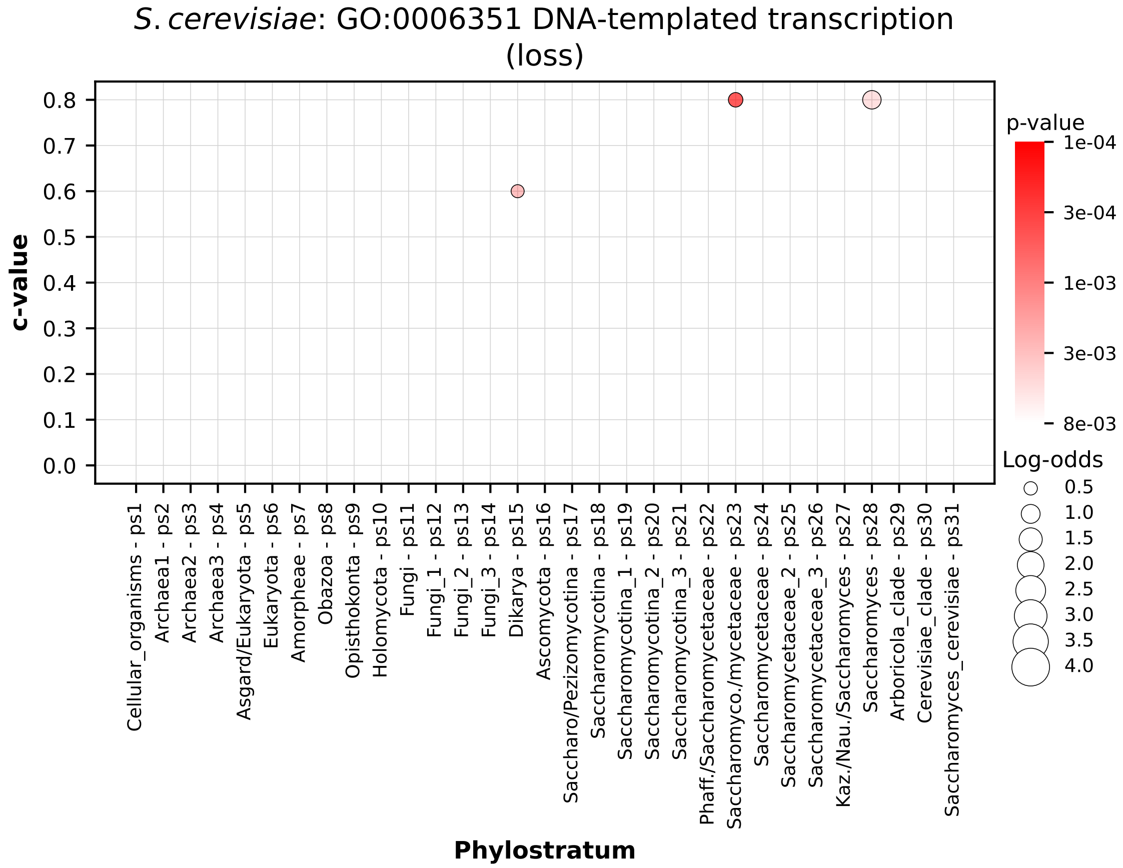
S. cerevisiae: GO:0006351 DNA-templated transcription
(loss)
c-value
0.0
0.1
0.2
0.3
0.4
0.5
0.6
0.7
0.8
Phylostratum
Cellular_organisms - ps1
Archaea1 - ps2
Archaea2 - ps3
Archaea3 - ps4
Asgard/Eukaryota - ps5
Eukaryota - ps6
Amorpheae - ps7
Obazoa - ps8
Opisthokonta - ps9
Holomycota - ps10
Fungi - ps11
Fungi_1 - ps12
Fungi_2 - ps13
Fungi_3 - ps14
Dikarya - ps15
Ascomycota - ps16
Saccharo/Pezizomycotina - ps17
Saccharomycotina - ps18
Saccharomycotina_1 - ps19
Saccharomycotina_2 - ps20
Saccharomycotina_3 - ps21
Phaff./Saccharomycetaceae - ps22
Saccharomyco./mycetaceae - ps23
Saccharomycetaceae - ps24
Saccharomycetaceae_2 - ps25
Saccharomycetaceae_3 - ps26
Kaz./Nau./Saccharomyces - ps27
Saccharomyces - ps28
Arboricola_clade - ps29
Cerevisiae_clade - ps30
Saccharomyces_cerevisiae - ps31
p-value
1e-04
3e-04
1e-03
3e-03
8e-03
Log-odds
0.5
1.0
1.5
2.0
2.5
3.0
3.5
4.0

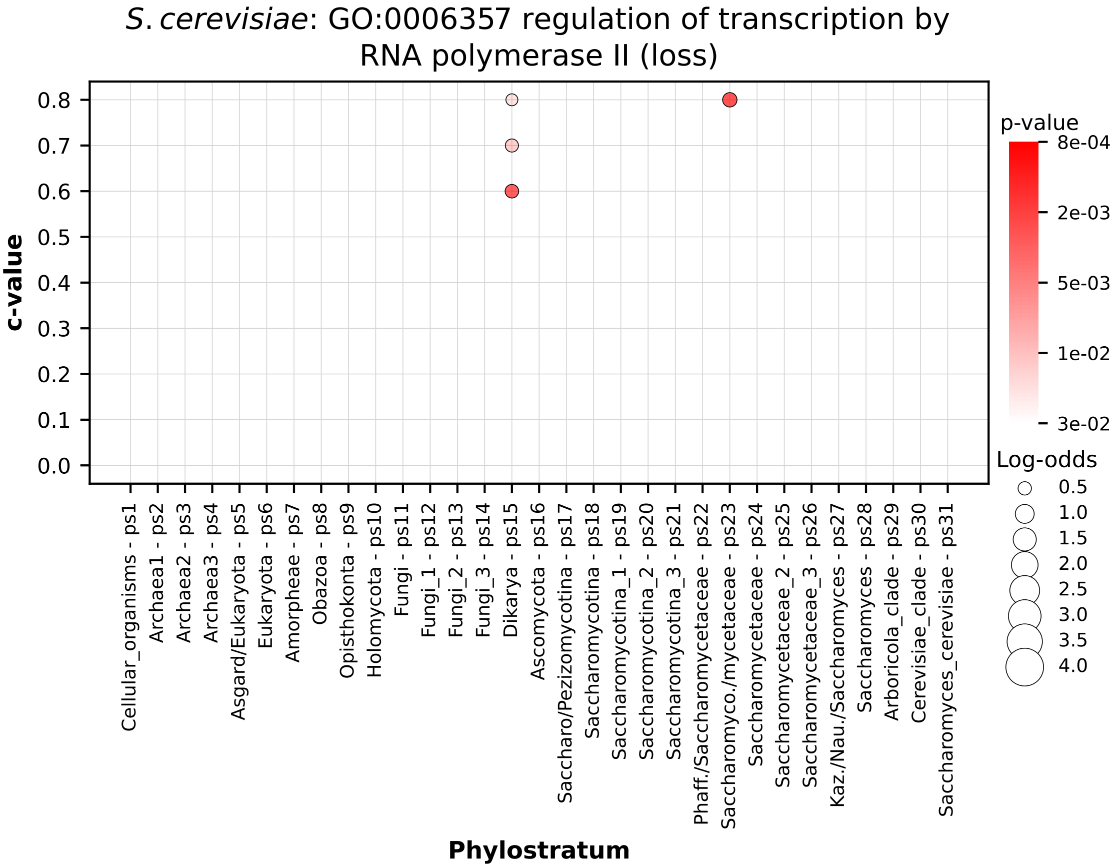

S. cerevisiae: GO:0006357 regulation of transcription by RNA polymerase II (loss)
c-value
0.8
0.7
0.6
0.5
0.4
0.3
0.2
0.1
0.0
p-value
8e-04
2e-03
5e-03
1e-02
3e-02
Log-odds
0.5
1.0
1.5
2.0
2.5
3.0
3.5
4.0
Cellular_organisms - ps1
Archaea1 - ps2
Archaea2 - ps3
Archaea3 - ps4
Asgard/Eukaryota - ps5
Eukaryota - ps6
Amorpheae - ps7
Obazoa - ps8
Opisthokonta - ps9
Holomycota - ps10
Fungi - ps11
Fungi_1 - ps12
Fungi_2 - ps13
Fungi_3 - ps14
Dikarya - ps15
Ascomycota - ps16
Saccharo/Pezizomycotina - ps17
Saccharomycotina - ps18
Saccharomycotina_1 - ps19
Saccharomycotina_2 - ps20
Saccharomycotina_3 - ps21
Phaff./Saccharomycetaceae - ps22
Saccharomyco./mycetaceae - ps23
Saccharomycetaceae - ps24
Saccharomycetaceae_2 - ps25
Saccharomycetaceae_3 - ps26
Kaz./Nau./Saccharomyces - ps27
Saccharomyces - ps28
Arboricola_clade - ps29
Cerevisiae_clade - ps30
Saccharomyces_cerevisiae - ps31
Phylostratum

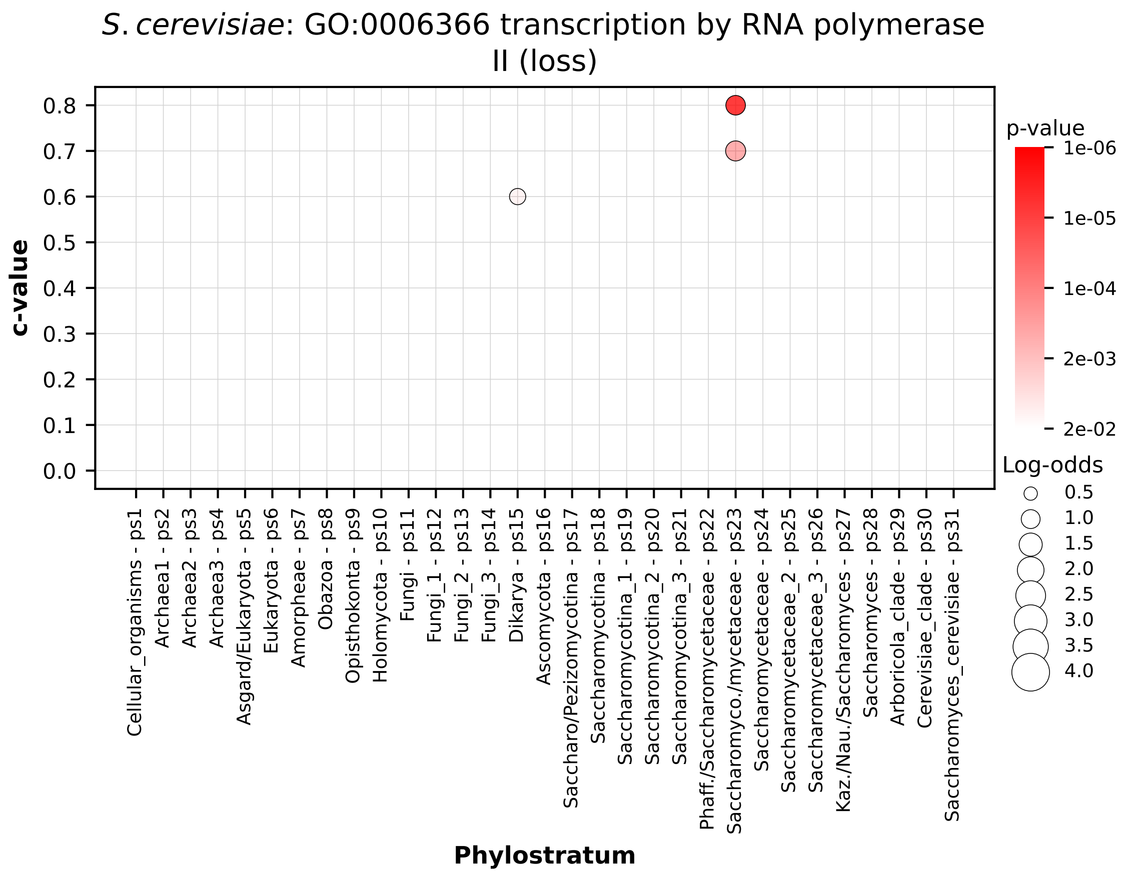

S. cerevisiae: GO:0006366 transcription by RNA polymerase II (loss)
c-value
0.8
0.7
0.6
0.5
0.4
0.3
0.2
0.1
0.0
Phylostratum
Cellular_organisms - ps1
Archaea1 - ps2
Archaea2 - ps3
Archaea3 - ps4
Asgard/Eukaryota - ps5
Eukaryota - ps6
Amorpheae - ps7
Obazoa - ps8
Opisthokonta - ps9
Holomycota - ps10
Fungi - ps11
Fungi_1 - ps12
Fungi_2 - ps13
Fungi_3 - ps14
Dikarya - ps15
Ascomycota - ps16
Saccharo/Pezizomycotina - ps17
Saccharomycotina - ps18
Saccharomycotina_1 - ps19
Saccharomycotina_2 - ps20
Saccharomycotina_3 - ps21
Phaff./Saccharomycetaceae - ps22
Saccharomyco./mycetaceae - ps23
Saccharomycetaceae - ps24
Saccharomycetaceae_2 - ps25
Saccharomycetaceae_3 - ps26
Kaz./Nau./Saccharomyces - ps27
Saccharomyces - ps28
Arboricola_clade - ps29
Cerevisiae_clade - ps30
Saccharomyces_cerevisiae - ps31
p-value
1e-06
1e-05
1e-04
2e-03
2e-02
Log-odds
0.5
1.0
1.5
2.0
2.5
3.0
3.5
4.0

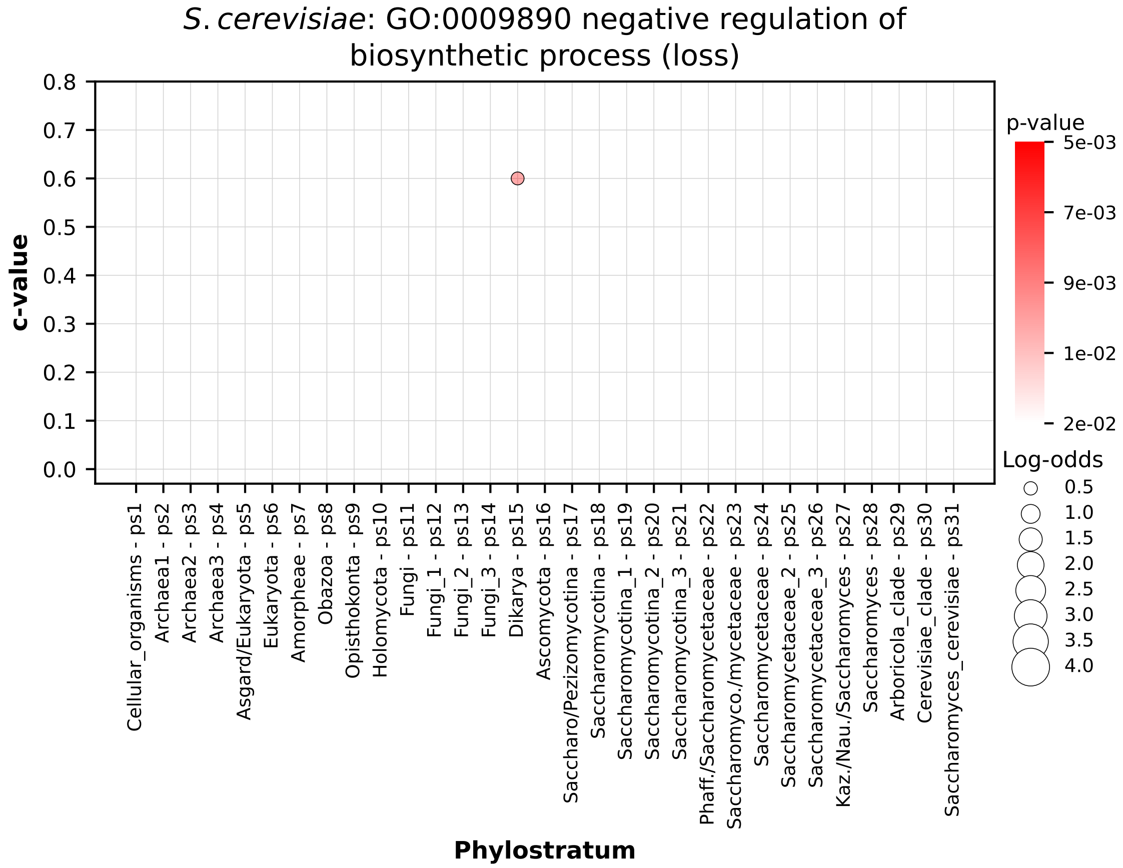

S. cerevisiae: GO:0009890 negative regulation of biosynthetic process (loss)
c-value
0.8
0.7
0.6
0.5
0.4
0.3
0.2
0.1
0.0
p-value
5e-03
7e-03
9e-03
1e-02
2e-02
Log-odds
0.5
1.0
1.5
2.0
2.5
3.0
3.5
4.0
Cellular_organisms - ps1
Archaea1 - ps2
Archaea2 - ps3
Archaea3 - ps4
Asgard/Eukaryota - ps5
Eukaryota - ps6
Amorpheae - ps7
Obazoa - ps8
Opisthokonta - ps9
Holomycota - ps10
Fungi - ps11
Fungi_1 - ps12
Fungi_2 - ps13
Fungi_3 - ps14
Dikarya - ps15
Ascomycota - ps16
Saccharo/Pezizomycotina - ps17
Saccharomycotina - ps18
Saccharomycotina_1 - ps19
Saccharomycotina_2 - ps20
Saccharomycotina_3 - ps21
Phaff./Saccharomycetaceae - ps22
Saccharomyco./mycetaceae - ps23
Saccharomycetaceae - ps24
Saccharomycetaceae_2 - ps25
Saccharomycetaceae_3 - ps26
Kaz./Nau./Saccharomyces - ps27
Saccharomyces - ps28
Arboricola_clade - ps29
Cerevisiae_clade - ps30
Saccharomyces_cerevisiae - ps31
Phylostratum

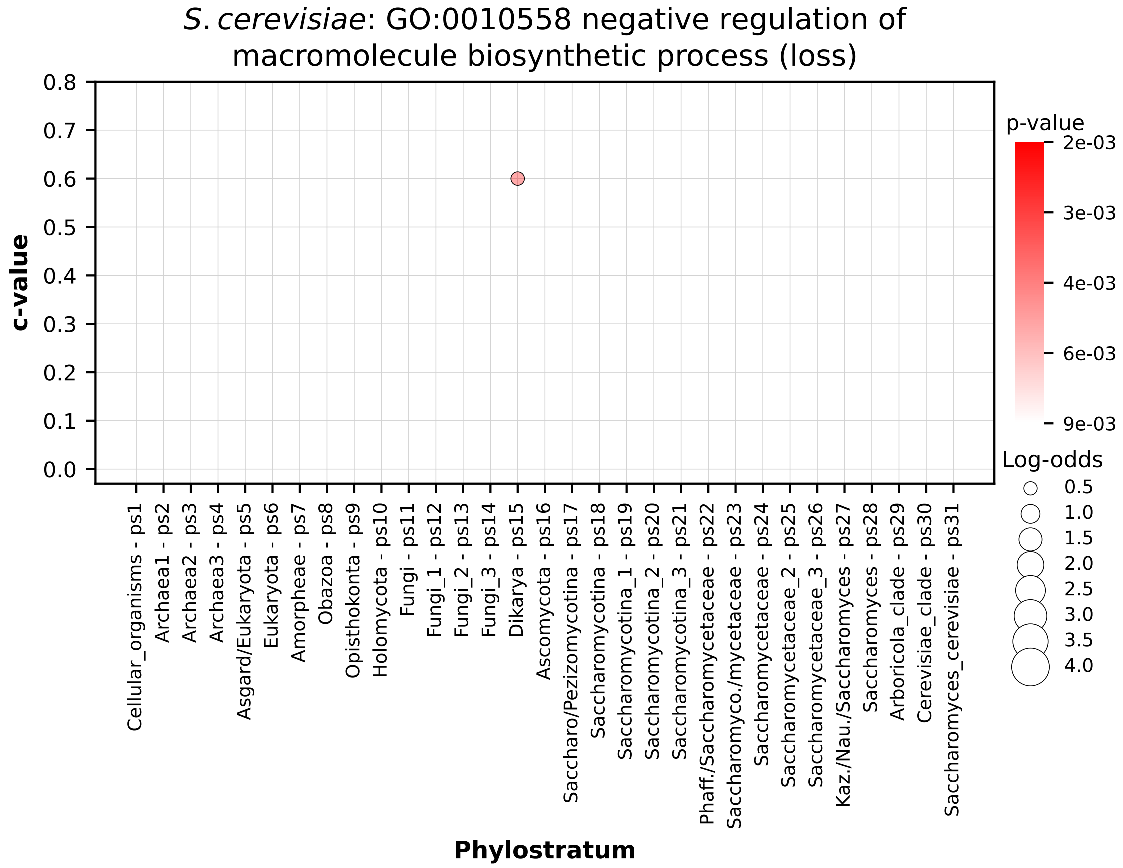

S. cerevisiae: GO:0010558 negative regulation of macromolecule biosynthetic process (loss)
c-value
0.8
0.7
0.6
0.5
0.4
0.3
0.2
0.1
0.0
Phylostratum
Cellular_organisms - ps1
Archaea1 - ps2
Archaea2 - ps3
Archaea3 - ps4
Asgard/Eukaryota - ps5
Eukaryota - ps6
Amorpheae - ps7
Obazoa - ps8
Opisthokonta - ps9
Holomycota - ps10
Fungi - ps11
Fungi_1 - ps12
Fungi_2 - ps13
Fungi_3 - ps14
Dikarya - ps15
Ascomycota - ps16
Saccharo/Pezizomycotina - ps17
Saccharomycotina - ps18
Saccharomycotina_1 - ps19
Saccharomycotina_2 - ps20
Saccharomycotina_3 - ps21
Phaff./Saccharomycetaceae - ps22
Saccharomyco./mycetaceae - ps23
Saccharomycetaceae - ps24
Saccharomycetaceae_2 - ps25
Saccharomycetaceae_3 - ps26
Kaz./Nau./Saccharomyces - ps27
Saccharomyces - ps28
Arboricola_clade - ps29
Cerevisiae_clade - ps30
Saccharomyces_cerevisiae - ps31
p-value
2e-03
3e-03
4e-03
6e-03
9e-03
Log-odds
0.5
1.0
1.5
2.0
2.5
3.0
3.5
4.0

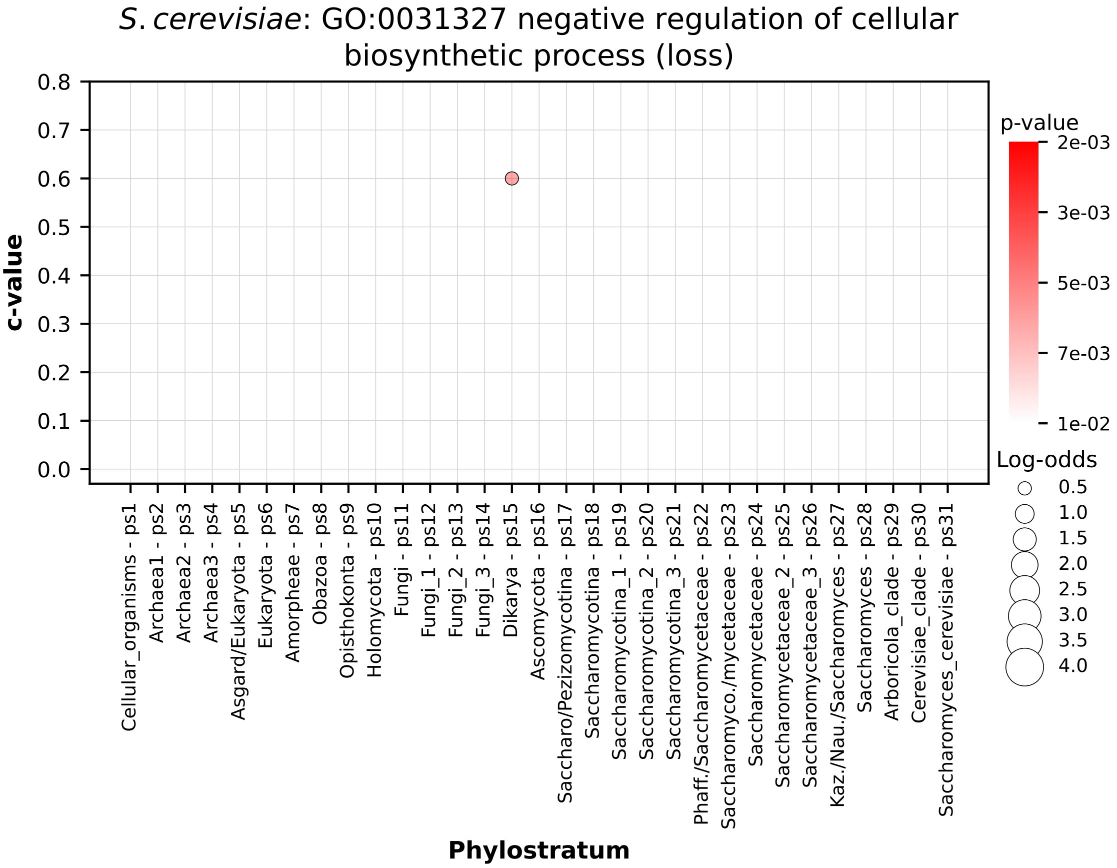

S. cerevisiae: GO:0031327 negative regulation of cellular biosynthetic process (loss)
c-value
0.8
0.7
0.6
0.5
0.4
0.3
0.2
0.1
0.0
p-value
2e-03
3e-03
5e-03
7e-03
1e-02
Log-odds
0.5
1.0
1.5
2.0
2.5
3.0
3.5
4.0
Cellular_organisms - ps1
Archaea1 - ps2
Archaea2 - ps3
Archaea3 - ps4
Asgard/Eukaryota - ps5
Eukaryota - ps6
Amorpheae - ps7
Obazoa - ps8
Opisthokonta - ps9
Holomycota - ps10
Fungi - ps11
Fungi_1 - ps12
Fungi_2 - ps13
Fungi_3 - ps14
Dikarya - ps15
Ascomycota - ps16
Saccharo/Pezizomycotina - ps17
Saccharomycotina - ps18
Saccharomycotina_1 - ps19
Saccharomycotina_2 - ps20
Saccharomycotina_3 - ps21
Phaff./Saccharomycetaceae - ps22
Saccharomyco./mycetaceae - ps23
Saccharomycetaceae - ps24
Saccharomycetaceae_2 - ps25
Saccharomycetaceae_3 - ps26
Kaz./Nau./Saccharomyces - ps27
Saccharomyces - ps28
Arboricola_clade - ps29
Cerevisiae_clade - ps30
Saccharomyces_cerevisiae - ps31
Phylostratum

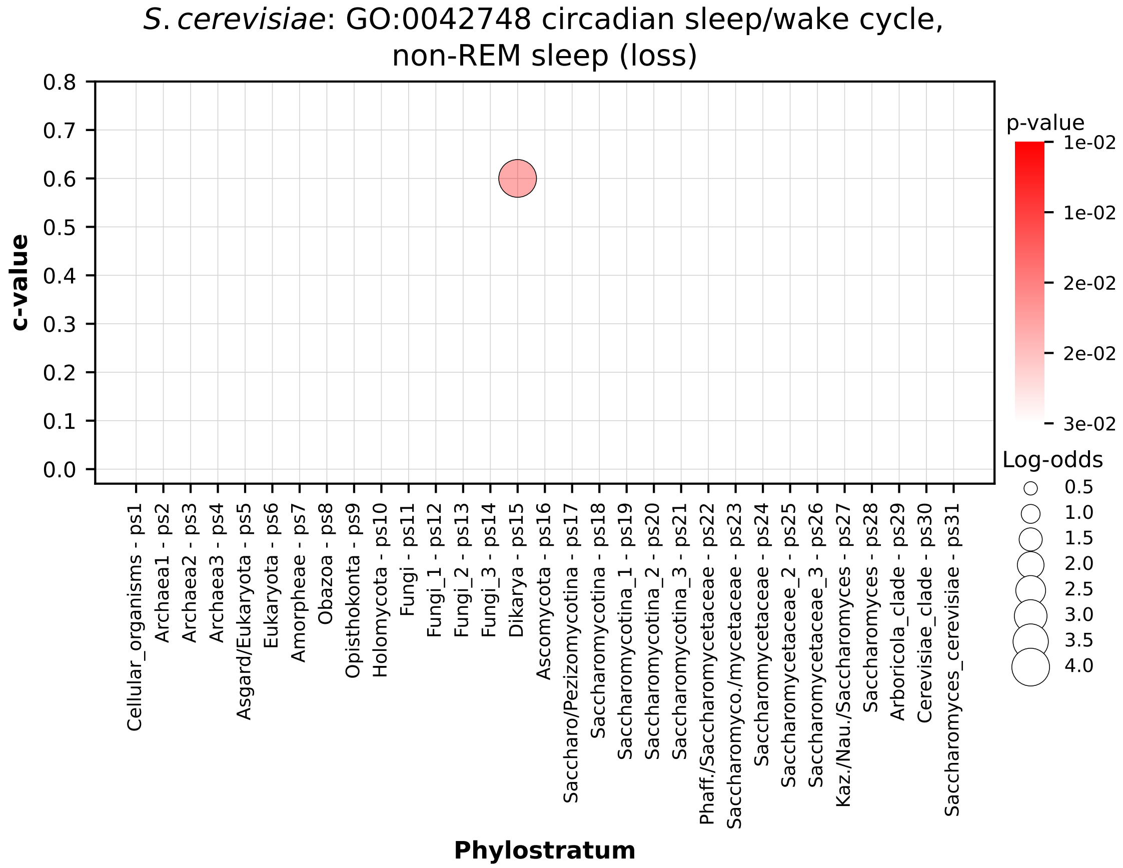
S. cerevisiae: GO:0042748 circadian sleep/wake cycle, non-REM sleep (loss)
c-value
0.0
0.1
0.2
0.3
0.4
0.5
0.6
0.7
0.8
Phylostratum
Cellular_organisms - ps1
Archaea1 - ps2
Archaea2 - ps3
Archaea3 - ps4
Asgard/Eukaryota - ps5
Eukaryota - ps6
Amorpheae - ps7
Obazoa - ps8
Opisthokonta - ps9
Holomycota - ps10
Fungi - ps11
Fungi_1 - ps12
Fungi_2 - ps13
Fungi_3 - ps14
Dikarya - ps15
Ascomycota - ps16
Saccharo/Pezizomycotina - ps17
Saccharomycotina - ps18
Saccharomycotina_1 - ps19
Saccharomycotina_2 - ps20
Saccharomycotina_3 - ps21
Phaff./Saccharomycetaceae - ps22
Saccharomyco./mycetaceae - ps23
Saccharomycetaceae - ps24
Saccharomycetaceae_2 - ps25
Saccharomycetaceae_3 - ps26
Kaz./Nau./Saccharomyces - ps27
Saccharomyces - ps28
Arboricola_clade - ps29
Cerevisiae_clade - ps30
Saccharomyces_cerevisiae - ps31
p-value
1e-02
1e-02
2e-02
2e-02
3e-02
Log-odds
0.5
1.0
1.5
2.0
2.5
3.0
3.5
4.0

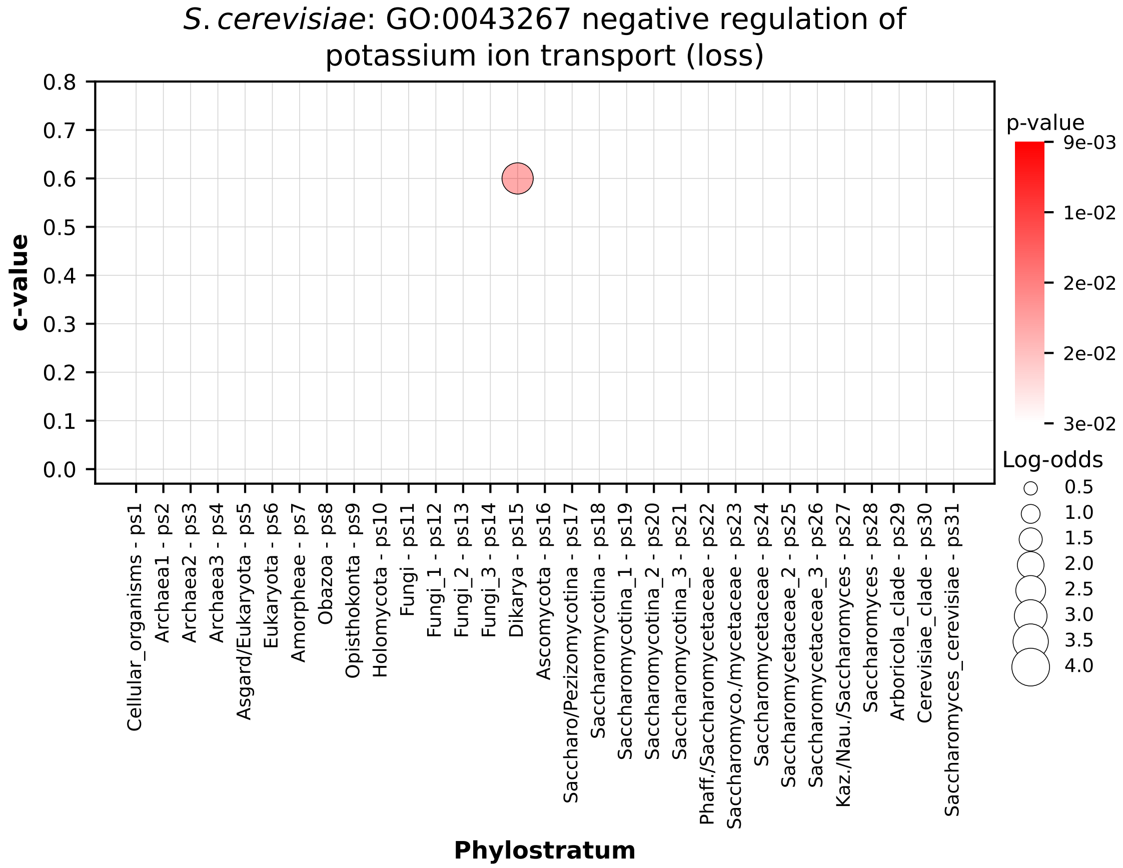

S. cerevisiae: GO:0043267 negative regulation of potassium ion transport (loss)
c-value
0.8
0.7
0.6
0.5
0.4
0.3
0.2
0.1
0.0
p-value
9e-03
1e-02
2e-02
2e-02
3e-02
Log-odds
0.5
1.0
1.5
2.0
2.5
3.0
3.5
4.0
Cellular_organisms - ps1
Archaea1 - ps2
Archaea2 - ps3
Archaea3 - ps4
Asgard/Eukaryota - ps5
Eukaryota - ps6
Amorpheae - ps7
Obazoa - ps8
Opisthokonta - ps9
Holomycota - ps10
Fungi - ps11
Fungi_1 - ps12
Fungi_2 - ps13
Fungi_3 - ps14
Dikarya - ps15
Ascomycota - ps16
Saccharo/Pezizomycotina - ps17
Saccharomycotina - ps18
Saccharomycotina_1 - ps19
Saccharomycotina_2 - ps20
Saccharomycotina_3 - ps21
Phaff./Saccharomycetaceae - ps22
Saccharomyco./mycetaceae - ps23
Saccharomycetaceae - ps24
Saccharomycetaceae_2 - ps25
Saccharomycetaceae_3 - ps26
Kaz./Nau./Saccharomyces - ps27
Saccharomyces - ps28
Arboricola_clade - ps29
Cerevisiae_clade - ps30
Saccharomyces_cerevisiae - ps31
Phylostratum

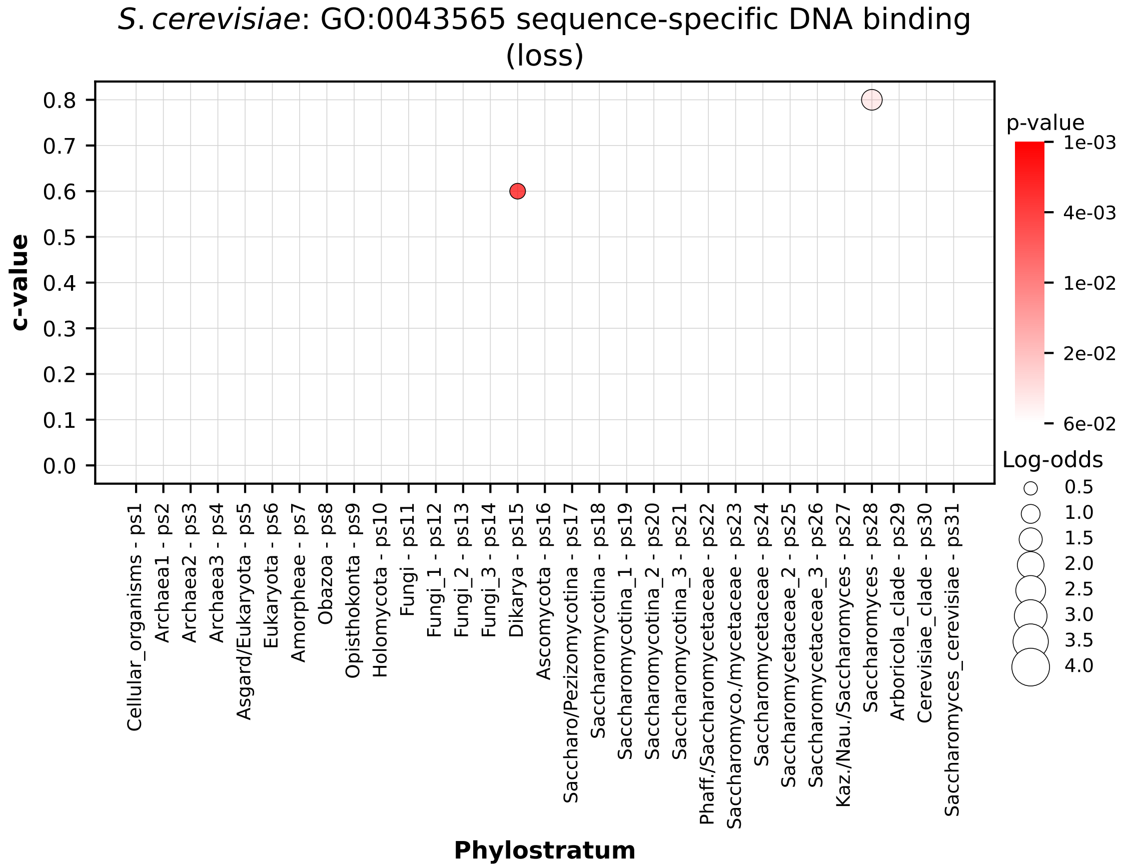

S. cerevisiae: GO:0043565 sequence-specific DNA binding
(loss)
c-value
0.0
0.1
0.2
0.3
0.4
0.5
0.6
0.7
0.8
p-value
1e-03
4e-03
1e-02
2e-02
6e-02
Log-odds
0.5
1.0
1.5
2.0
2.5
3.0
3.5
4.0
Cellular_organisms - ps1
Archaea1 - ps2
Archaea2 - ps3
Archaea3 - ps4
Asgard/Eukaryota - ps5
Eukaryota - ps6
Amorpheae - ps7
Obazoa - ps8
Opisthokonta - ps9
Holomycota - ps10
Fungi - ps11
Fungi_1 - ps12
Fungi_2 - ps13
Fungi_3 - ps14
Dikarya - ps15
Ascomycota - ps16
Saccharo/Pezizomycotina - ps17
Saccharomycotina - ps18
Saccharomycotina_1 - ps19
Saccharomycotina_2 - ps20
Saccharomycotina_3 - ps21
Phaff./Saccharomycetaceae - ps22
Saccharomyco./mycetaceae - ps23
Saccharomycetaceae - ps24
Saccharomycetaceae_2 - ps25
Saccharomycetaceae_3 - ps26
Kaz./Nau./Saccharomyces - ps27
Saccharomyces - ps28
Arboricola_clade - ps29
Cerevisiae_clade - ps30
Saccharomyces_cerevisiae - ps31
Phylostratum

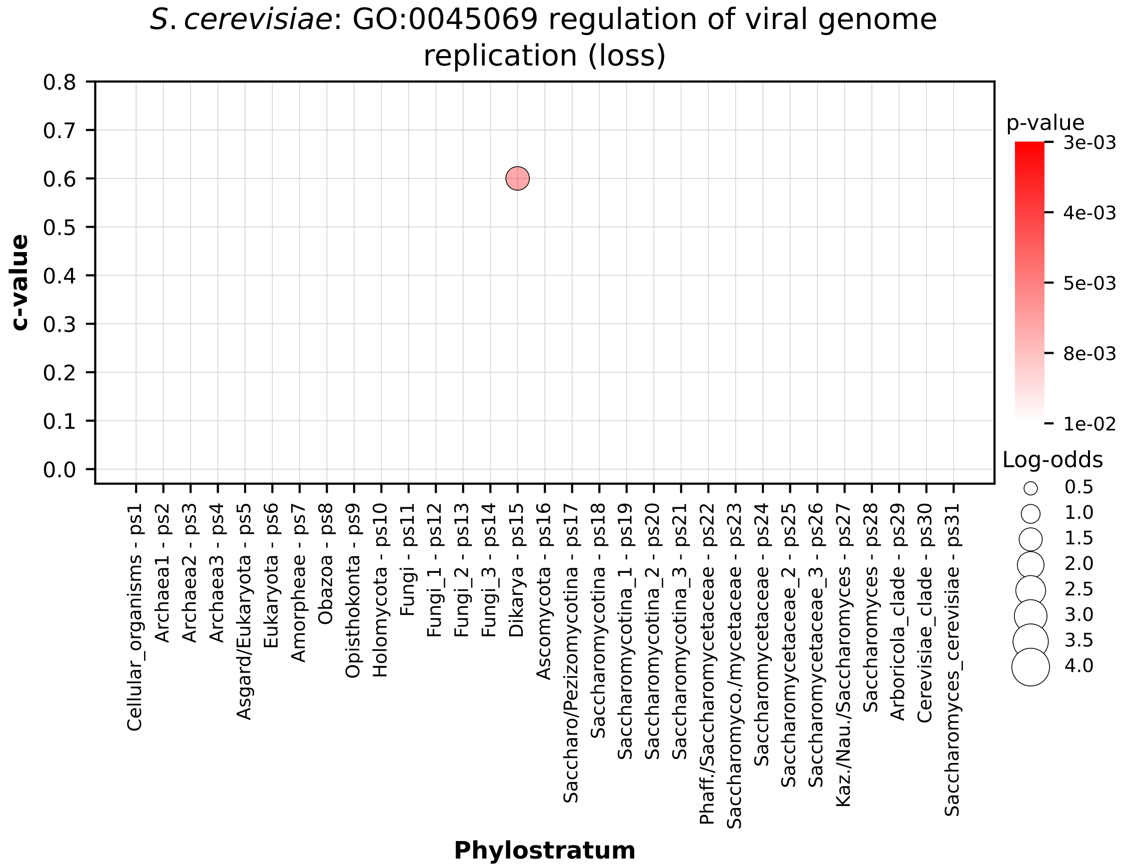

S. cerevisiae: GO:0045069 regulation of viral genome replication (loss)
c-value
0.8
0.7
0.6
0.5
0.4
0.3
0.2
0.1
0.0
p-value
3e-03
4e-03
5e-03
8e-03
1e-02
Log-odds
0.5
1.0
1.5
2.0
2.5
3.0
3.5
4.0
Cellular_organisms - ps1
Archaea1 - ps2
Archaea2 - ps3
Archaea3 - ps4
Asgard/Eukaryota - ps5
Eukaryota - ps6
Amorpheae - ps7
Obazoa - ps8
Opisthokonta - ps9
Holomycota - ps10
Fungi - ps11
Fungi_1 - ps12
Fungi_2 - ps13
Fungi_3 - ps14
Dikarya - ps15
Ascomycota - ps16
Saccharo/Pezizomycotina - ps17
Saccharomycotina - ps18
Saccharomycotina_1 - ps19
Saccharomycotina_2 - ps20
Saccharomycotina_3 - ps21
Phaff./Saccharomycetaceae - ps22
Saccharomyco./mycetaceae - ps23
Saccharomycetaceae - ps24
Saccharomycetaceae_2 - ps25
Saccharomycetaceae_3 - ps26
Kaz./Nau./Saccharomyces - ps27
Saccharomyces - ps28
Arboricola_clade - ps29
Cerevisiae_clade - ps30
Saccharomyces_cerevisiae - ps31
Phylostratum

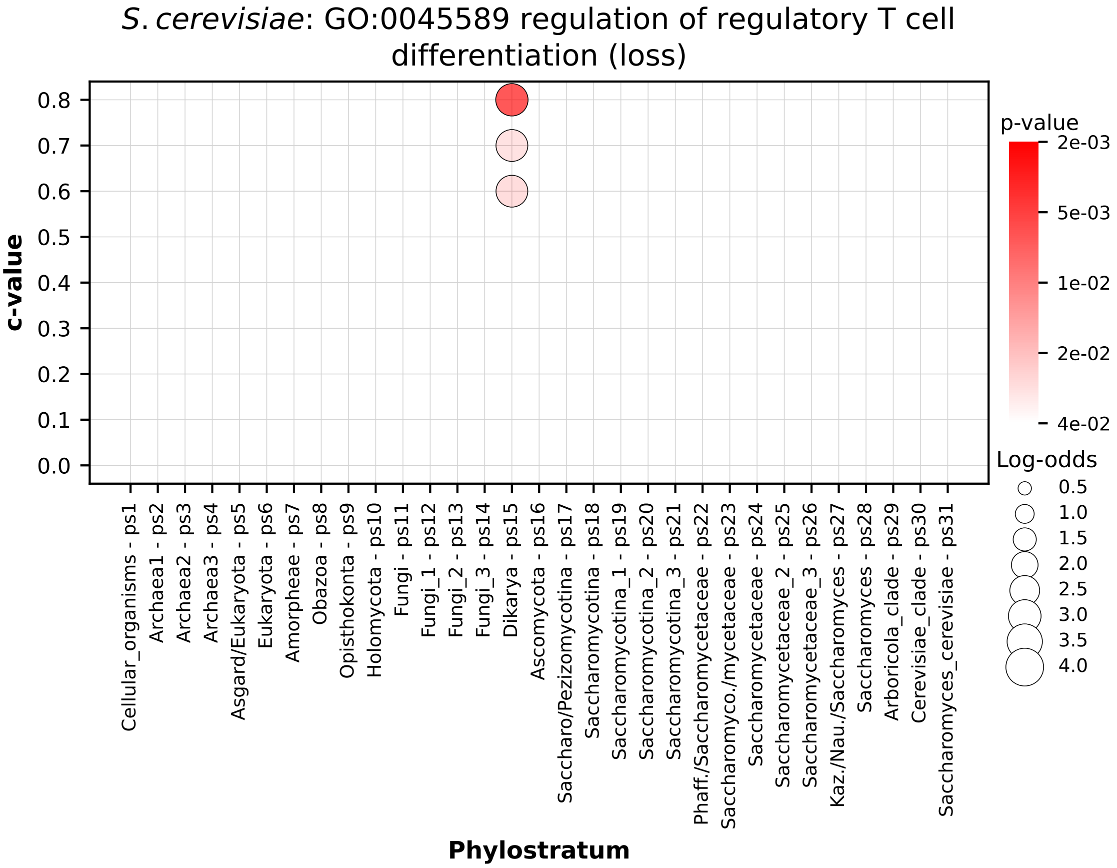

S. cerevisiae: GO:0045589 regulation of regulatory T cell differentiation (loss)
c-value
0.0
0.1
0.2
0.3
0.4
0.5
0.6
0.7
0.8
p-value
2e-03
5e-03
1e-02
2e-02
4e-02
Log-odds
0.5
1.0
1.5
2.0
2.5
3.0
3.5
4.0
Cellular_organisms - ps1
Archaea1 - ps2
Archaea2 - ps3
Archaea3 - ps4
Asgard/Eukaryota - ps5
Eukaryota - ps6
Amorpheae - ps7
Obazoa - ps8
Opisthokonta - ps9
Holomycota - ps10
Fungi - ps11
Fungi_1 - ps12
Fungi_2 - ps13
Fungi_3 - ps14
Dikarya - ps15
Ascomycota - ps16
Saccharo/Pezizomycotina - ps17
Saccharomycotina - ps18
Saccharomycotina_1 - ps19
Saccharomycotina_2 - ps20
Saccharomycotina_3 - ps21
Phaff./Saccharomycetaceae - ps22
Saccharomyco./mycetaceae - ps23
Saccharomycetaceae - ps24
Saccharomycetaceae_2 - ps25
Saccharomycetaceae_3 - ps26
Kaz./Nau./Saccharomyces - ps27
Saccharomyces - ps28
Arboricola_clade - ps29
Cerevisiae_clade - ps30
Saccharomyces_cerevisiae - ps31
Phylostratum

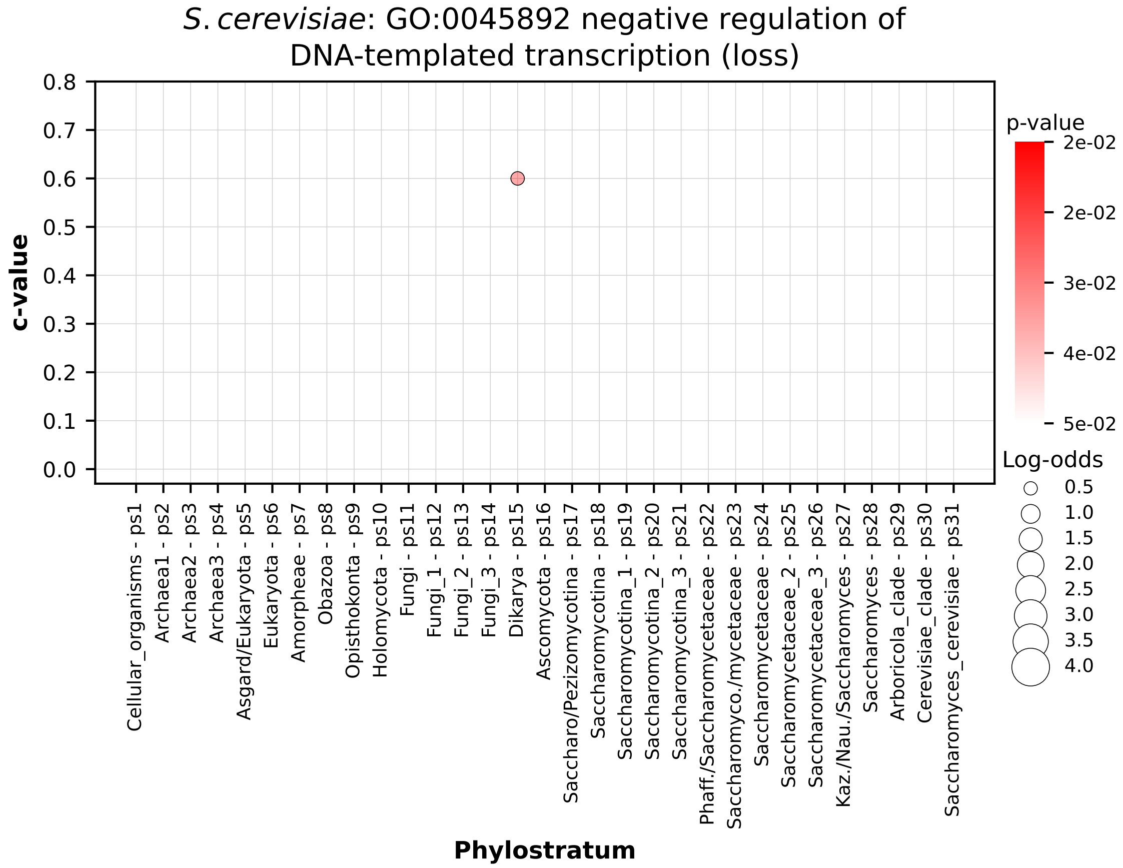
S. cerevisiae: GO:0045892 negative regulation of DNA-templated transcription (loss)
c-value
0.8
0.7
0.6
0.5
0.4
0.3
0.2
0.1
0.0
p-value
2e-02
2e-02
3e-02
4e-02
5e-02
Log-odds
0.5
1.0
1.5
2.0
2.5
3.0
3.5
4.0
Cellular_organisms - ps1
Archaea1 - ps2
Archaea2 - ps3
Archaea3 - ps4
Asgard/Eukaryota - ps5
Eukaryota - ps6
Amorpheae - ps7
Obazoa - ps8
Opisthokonta - ps9
Holomycota - ps10
Fungi - ps11
Fungi_1 - ps12
Fungi_2 - ps13
Fungi_3 - ps14
Dikarya - ps15
Ascomycota - ps16
Saccharo/Pezizomycotina - ps17
Saccharomycotina - ps18
Saccharomycotina_1 - ps19
Saccharomycotina_2 - ps20
Saccharomycotina_3 - ps21
Phaff./Saccharomycetaceae - ps22
Saccharomyco./mycetaceae - ps23
Saccharomycetaceae - ps24
Saccharomycetaceae_2 - ps25
Saccharomycetaceae_3 - ps26
Kaz./Nau./Saccharomyces - ps27
Saccharomyces - ps28
Arboricola_clade - ps29
Cerevisiae_clade - ps30
Saccharomyces_cerevisiae - ps31
Phylostratum

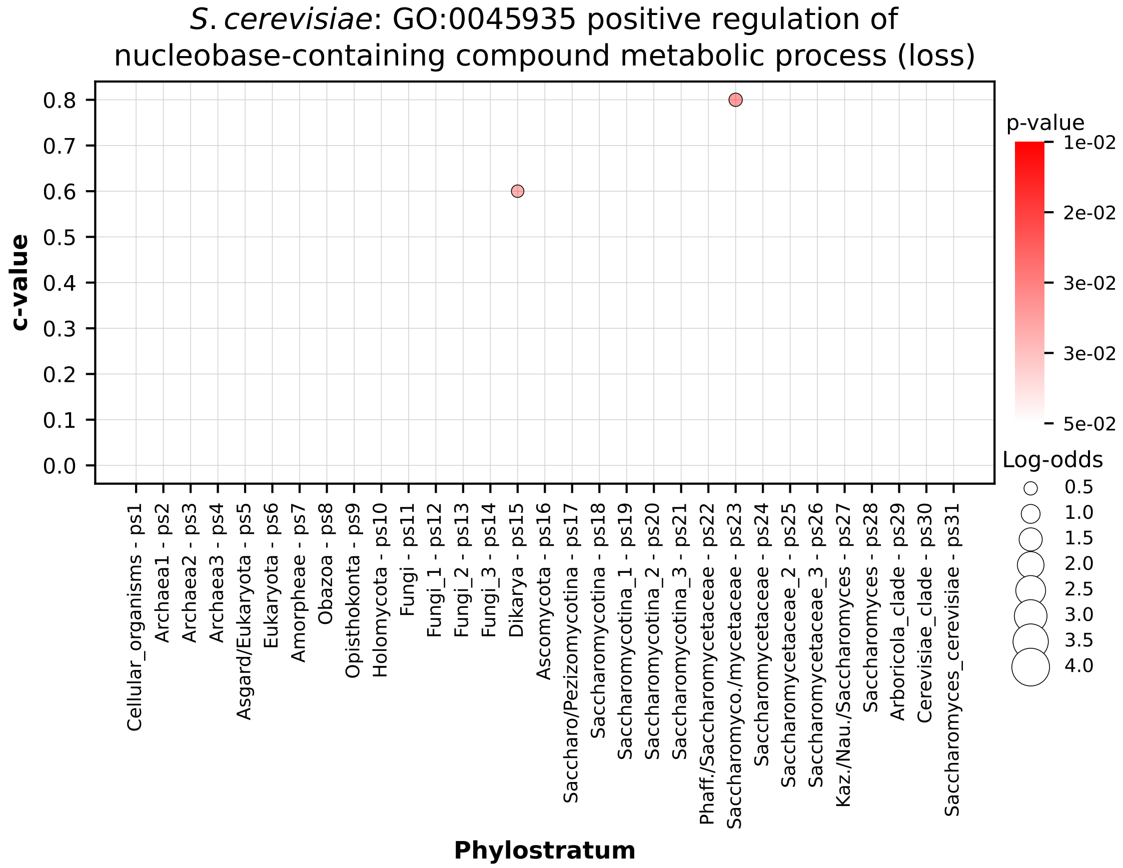

S. cerevisiae: GO:0045935 positive regulation of nucleobase-containing compound metabolic process (loss)
c-value
0.0
0.1
0.2
0.3
0.4
0.5
0.6
0.7
0.8
Phylostratum
Cellular_organisms - ps1
Archaea1 - ps2
Archaea2 - ps3
Archaea3 - ps4
Asgard/Eukaryota - ps5
Eukaryota - ps6
Amorpheae - ps7
Obazoa - ps8
Opisthokonta - ps9
Holomycota - ps10
Fungi - ps11
Fungi_1 - ps12
Fungi_2 - ps13
Fungi_3 - ps14
Dikarya - ps15
Ascomycota - ps16
Saccharo/Pezizomycotina - ps17
Saccharomycotina - ps18
Saccharomycotina_1 - ps19
Saccharomycotina_2 - ps20
Saccharomycotina_3 - ps21
Phaff./Saccharomycetaceae - ps22
Saccharomyco./mycetaceae - ps23
Saccharomycetaceae - ps24
Saccharomycetaceae_2 - ps25
Saccharomycetaceae_3 - ps26
Kaz./Nau./Saccharomyces - ps27
Saccharomyces - ps28
Arboricola_clade - ps29
Cerevisiae_clade - ps30
Saccharomyces_cerevisiae - ps31
p-value
1e-02
2e-02
3e-02
3e-02
5e-02
Log-odds
0.5
1.0
1.5
2.0
2.5
3.0
3.5
4.0

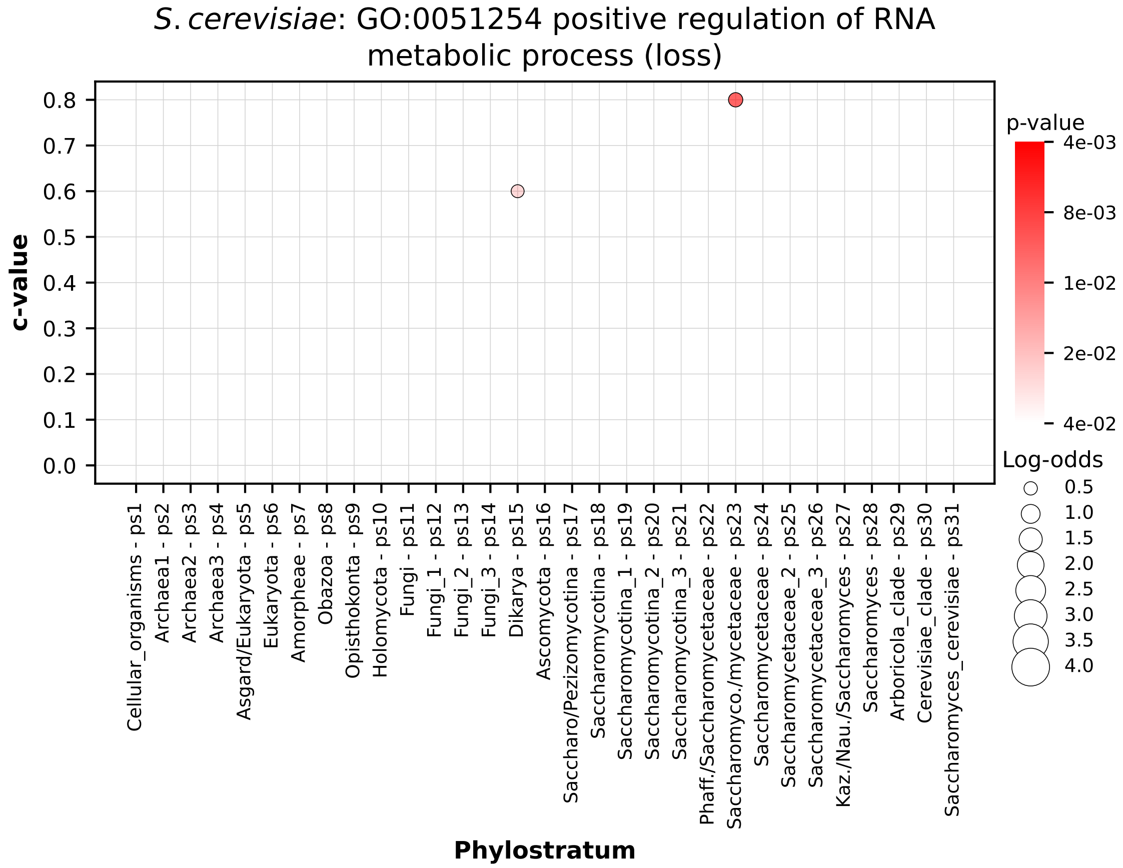
S. cerevisiae: GO:0051254 positive regulation of RNA metabolic process (loss)
c-value
0.0
0.1
0.2
0.3
0.4
0.5
0.6
0.7
0.8
p-value
4e-03
8e-03
1e-02
2e-02
4e-02
Log-odds
0.5
1.0
1.5
2.0
2.5
3.0
3.5
4.0
Cellular_organisms - ps1
Archaea1 - ps2
Archaea2 - ps3
Archaea3 - ps4
Asgard/Eukaryota - ps5
Eukaryota - ps6
Amorpheae - ps7
Obazoa - ps8
Opisthokonta - ps9
Holomycota - ps10
Fungi - ps11
Fungi_1 - ps12
Fungi_2 - ps13
Fungi_3 - ps14
Dikarya - ps15
Ascomycota - ps16
Saccharo/Pezizomycotina - ps17
Saccharomycotina - ps18
Saccharomycotina_1 - ps19
Saccharomycotina_2 - ps20
Saccharomycotina_3 - ps21
Phaff./Saccharomycetaceae - ps22
Saccharomyco./mycetaceae - ps23
Saccharomycetaceae - ps24
Saccharomycetaceae_2 - ps25
Saccharomycetaceae_3 - ps26
Kaz./Nau./Saccharomyces - ps27
Saccharomyces - ps28
Arboricola_clade - ps29
Cerevisiae_clade - ps30
Saccharomyces_cerevisiae - ps31
Phylostratum

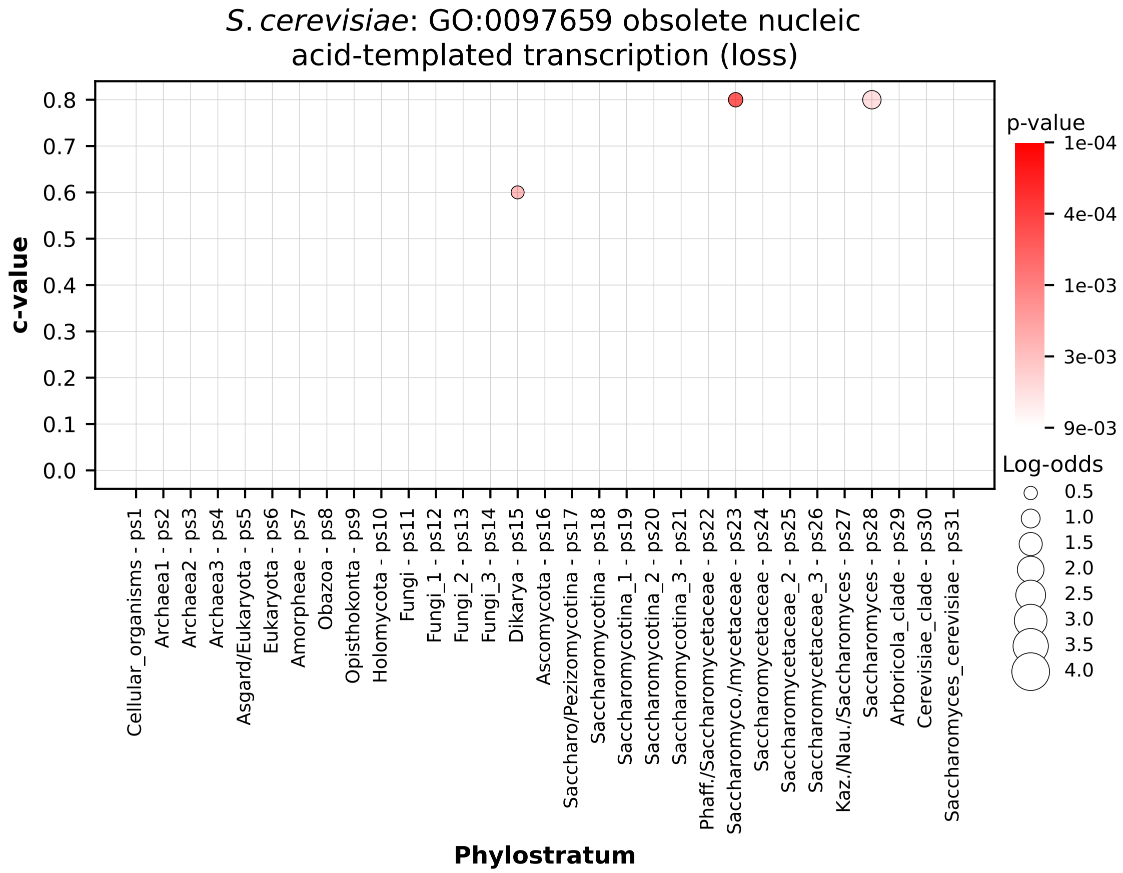
S. cerevisiae: GO:0097659 obsolete nucleic acid-templated transcription (loss)
c-value
0.0
0.1
0.2
0.3
0.4
0.5
0.6
0.7
0.8
p-value
1e-04
4e-04
1e-03
3e-03
9e-03
Log-odds
0.5
1.0
1.5
2.0
2.5
3.0
3.5
4.0
Cellular_organisms - ps1
Archaea1 - ps2
Archaea2 - ps3
Archaea3 - ps4
Asgard/Eukaryota - ps5
Eukaryota - ps6
Amorpheae - ps7
Obazoa - ps8
Opisthokonta - ps9
Holomycota - ps10
Fungi - ps11
Fungi_1 - ps12
Fungi_2 - ps13
Fungi_3 - ps14
Dikarya - ps15
Ascomycota - ps16
Saccharo/Pezizomycotina - ps17
Saccharomycotina - ps18
Saccharomycotina_1 - ps19
Saccharomycotina_2 - ps20
Saccharomycotina_3 - ps21
Phaff./Saccharomycetaceae - ps22
Saccharomyco./mycetaceae - ps23
Saccharomycetaceae - ps24
Saccharomycetaceae_2 - ps25
Saccharomycetaceae_3 - ps26
Kaz./Nau./Saccharomyces - ps27
Saccharomyces - ps28
Arboricola_clade - ps29
Cerevisiae_clade - ps30
Saccharomyces_cerevisiae - ps31
Phylostratum

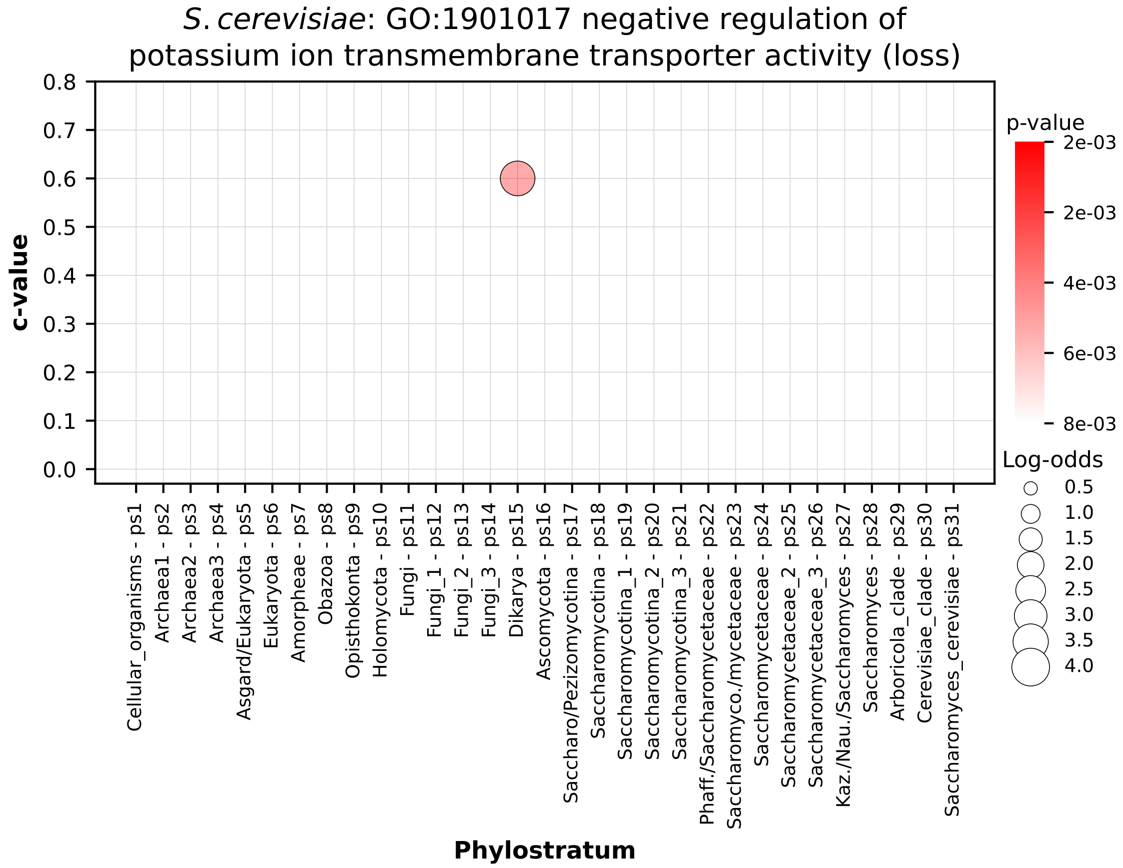
S. cerevisiae: GO:1901017 negative regulation of potassium ion transmembrane transporter activity (loss)
c-value
0.8
0.7
0.6
0.5
0.4
0.3
0.2
0.1
0.0
p-value
2e-03
2e-03
4e-03
6e-03
8e-03
Log-odds
0.5
1.0
1.5
2.0
2.5
3.0
3.5
4.0
Cellular_organisms - ps1
Archaea1 - ps2
Archaea2 - ps3
Archaea3 - ps4
Asgard/Eukaryota - ps5
Eukaryota - ps6
Amorpheae - ps7
Obazoa - ps8
Opisthokonta - ps9
Holomycota - ps10
Fungi - ps11
Fungi_1 - ps12
Fungi_2 - ps13
Fungi_3 - ps14
Dikarya - ps15
Ascomycota - ps16
Saccharo/Pezizomycotina - ps17
Saccharomycotina - ps18
Saccharomycotina_1 - ps19
Saccharomycotina_2 - ps20
Saccharomycotina_3 - ps21
Phaff./Saccharomycetaceae - ps22
Saccharomyco./mycetaceae - ps23
Saccharomycetaceae - ps24
Saccharomycetaceae_2 - ps25
Saccharomycetaceae_3 - ps26
Kaz./Nau./Saccharomyces - ps27
Saccharomyces - ps28
Arboricola_clade - ps29
Cerevisiae_clade - ps30
Saccharomyces_cerevisiae - ps31
Phylostratum

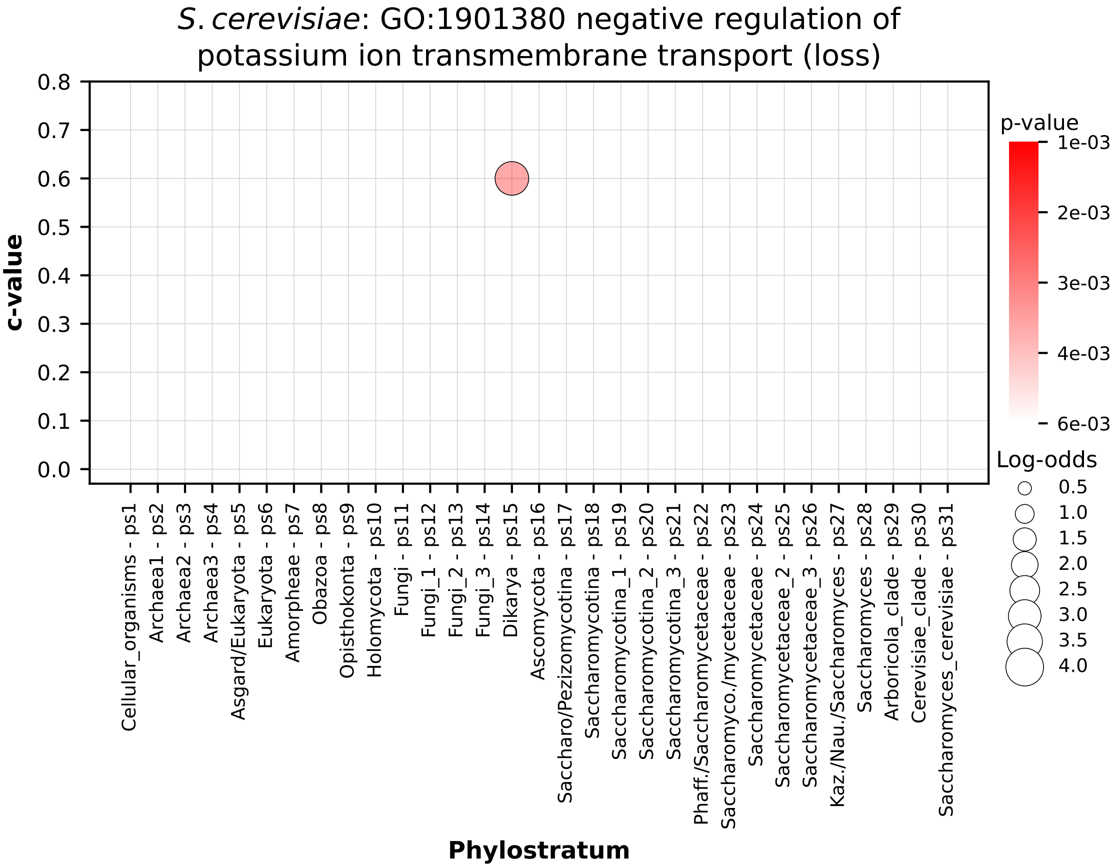
S. cerevisiae: GO:1901380 negative regulation of potassium ion transmembrane transport (loss)
c-value
0.8
0.7
0.6
0.5
0.4
0.3
0.2
0.1
0.0
p-value
1e-03
2e-03
3e-03
4e-03
6e-03
Log-odds
0.5
1.0
1.5
2.0
2.5
3.0
3.5
4.0
Cellular_organisms - ps1
Archaea1 - ps2
Archaea2 - ps3
Archaea3 - ps4
Asgard/Eukaryota - ps5
Eukaryota - ps6
Amorpheae - ps7
Obazoa - ps8
Opisthokonta - ps9
Holomycota - ps10
Fungi - ps11
Fungi_1 - ps12
Fungi_2 - ps13
Fungi_3 - ps14
Dikarya - ps15
Ascomycota - ps16
Saccharo/Pezizomycotina - ps17
Saccharomycotina - ps18
Saccharomycotina_1 - ps19
Saccharomycotina_2 - ps20
Saccharomycotina_3 - ps21
Phaff./Saccharomycetaceae - ps22
Saccharomyco./mycetaceae - ps23
Saccharomycetaceae - ps24
Saccharomycetaceae_2 - ps25
Saccharomycetaceae_3 - ps26
Kaz./Nau./Saccharomyces - ps27
Saccharomyces - ps28
Arboricola_clade - ps29
Cerevisiae_clade - ps30
Saccharomyces_cerevisiae - ps31
Phylostratum

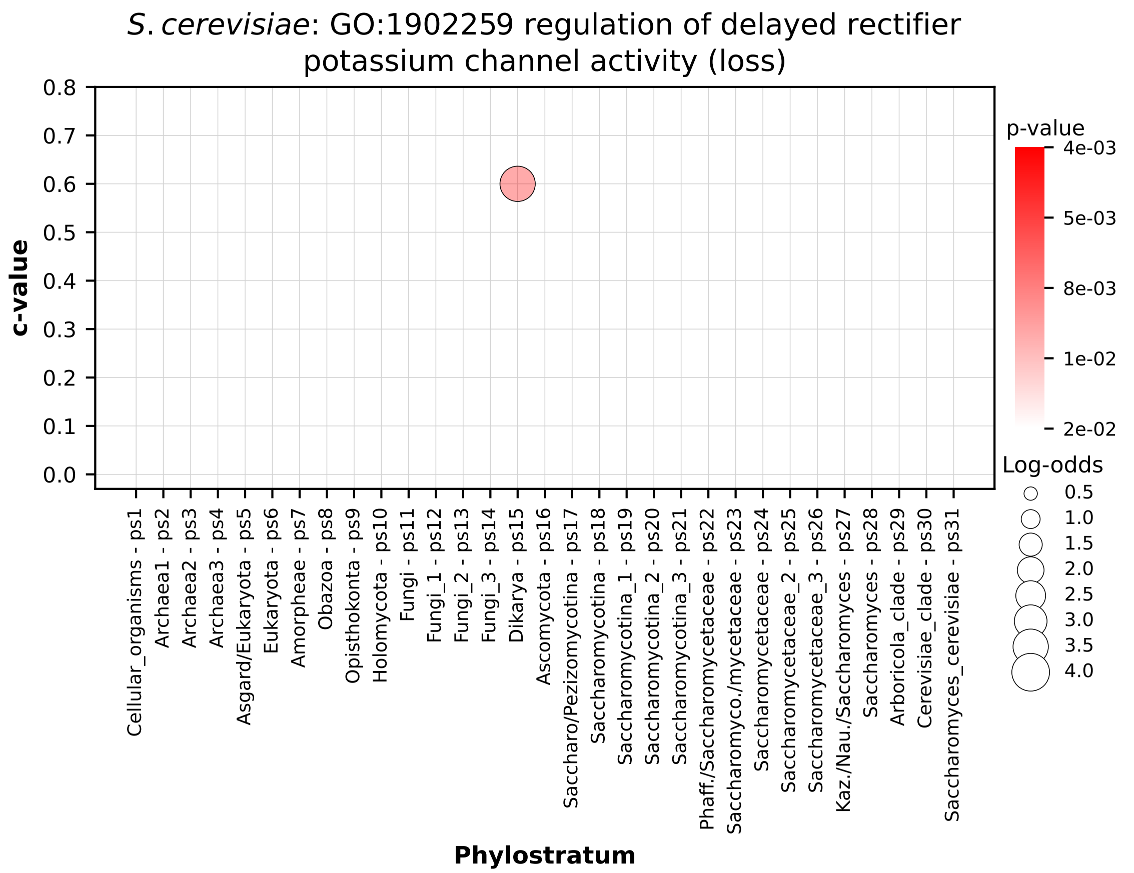

S. cerevisiae: GO:1902259 regulation of delayed rectifier potassium channel activity (loss)
c-value
0.8
0.7
0.6
0.5
0.4
0.3
0.2
0.1
0.0
p-value
4e-03
5e-03
8e-03
1e-02
2e-02
Log-odds
0.5
1.0
1.5
2.0
2.5
3.0
3.5
4.0
Cellular_organisms - ps1
Archaea1 - ps2
Archaea2 - ps3
Archaea3 - ps4
Asgard/Eukaryota - ps5
Eukaryota - ps6
Amorpheae - ps7
Obazoa - ps8
Opisthokonta - ps9
Holomycota - ps10
Fungi - ps11
Fungi_1 - ps12
Fungi_2 - ps13
Fungi_3 - ps14
Dikarya - ps15
Ascomycota - ps16
Saccharo/Pezizomycotina - ps17
Saccharomycotina - ps18
Saccharomycotina_1 - ps19
Saccharomycotina_2 - ps20
Saccharomycotina_3 - ps21
Phaff./Saccharomycetaceae - ps22
Saccharomyco./mycetaceae - ps23
Saccharomycetaceae - ps24
Saccharomycetaceae_2 - ps25
Saccharomycetaceae_3 - ps26
Kaz./Nau./Saccharomyces - ps27
Saccharomyces - ps28
Arboricola_clade - ps29
Cerevisiae_clade - ps30
Saccharomyces_cerevisiae - ps31
Phylostratum

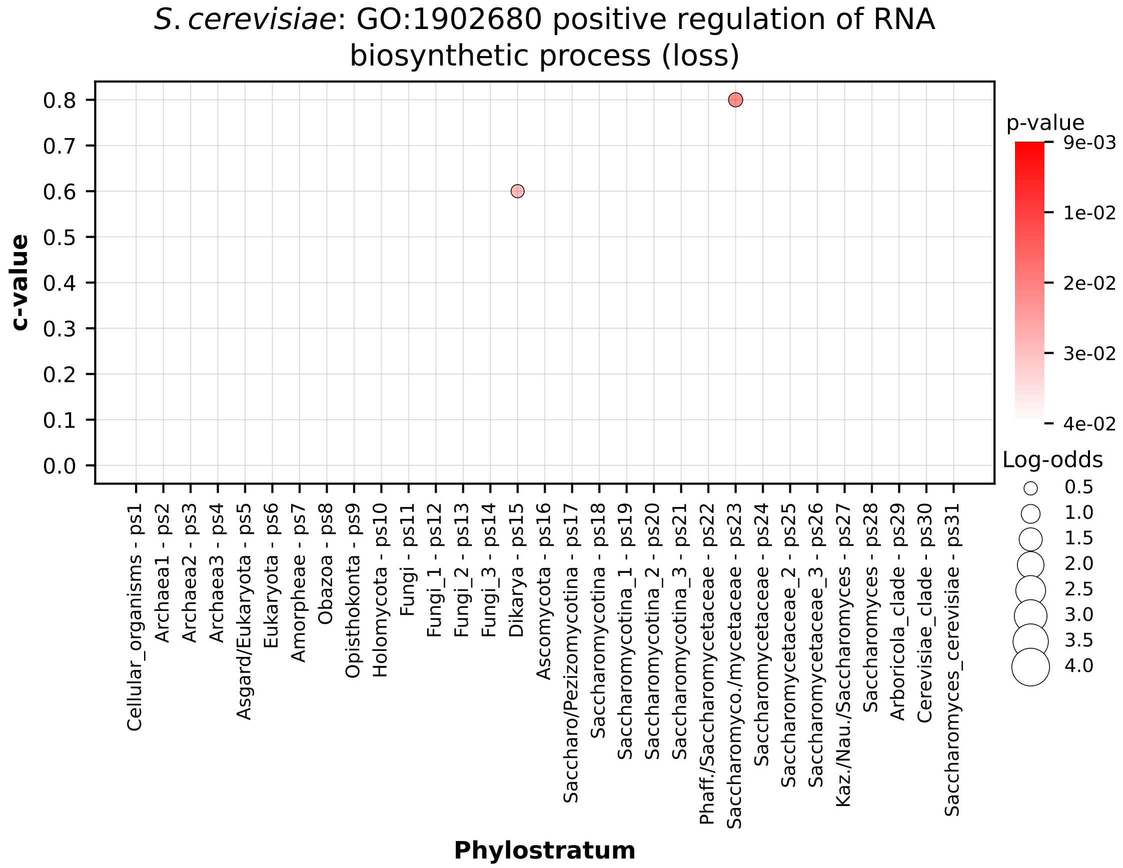

S. cerevisiae: GO:1902680 positive regulation of RNA biosynthetic process (loss)
c-value
0.8
0.7
0.6
0.5
0.4
0.3
0.2
0.1
0.0
p-value
9e-03
1e-02
2e-02
3e-02
4e-02
Log-odds
0.5
1.0
1.5
2.0
2.5
3.0
3.5
4.0
Cellular_organisms - ps1
Archaea1 - ps2
Archaea2 - ps3
Archaea3 - ps4
Asgard/Eukaryota - ps5
Eukaryota - ps6
Amorpheae - ps7
Obazoa - ps8
Opisthokonta - ps9
Holomycota - ps10
Fungi - ps11
Fungi_1 - ps12
Fungi_2 - ps13
Fungi_3 - ps14
Dikarya - ps15
Ascomycota - ps16
Saccharo/Pezizomycotina - ps17
Saccharomycotina - ps18
Saccharomycotina_1 - ps19
Saccharomycotina_2 - ps20
Saccharomycotina_3 - ps21
Phaff./Saccharomycetaceae - ps22
Saccharomyco./mycetaceae - ps23
Saccharomycetaceae - ps24
Saccharomycetaceae_2 - ps25
Saccharomycetaceae_3 - ps26
Kaz./Nau./Saccharomyces - ps27
Saccharomyces - ps28
Arboricola_clade - ps29
Cerevisiae_clade - ps30
Saccharomyces_cerevisiae - ps31
Phylostratum

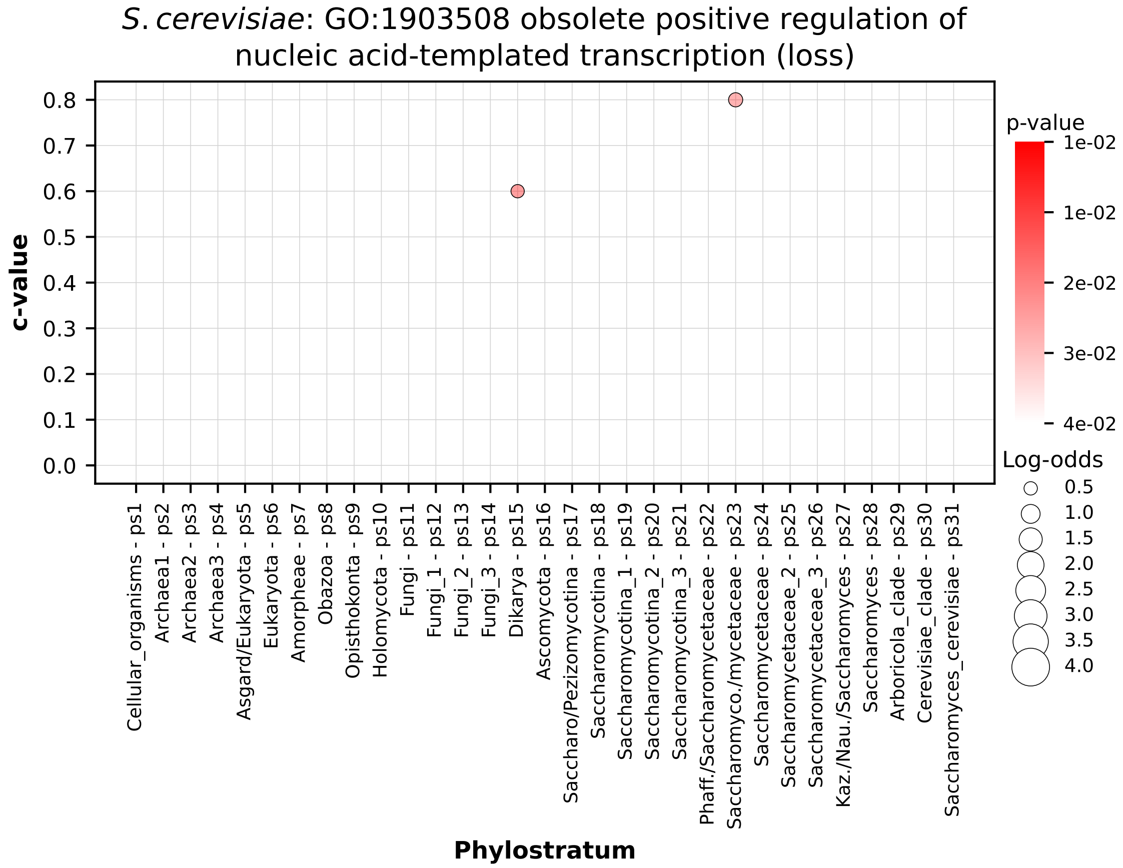
S. cerevisiae: GO:1903508 obsolete positive regulation of nucleic acid-templated transcription (loss)
c-value
0.8
0.7
0.6
0.5
0.4
0.3
0.2
0.1
0.0
Phylostratum
Cellular_organisms - ps1
Archaea1 - ps2
Archaea2 - ps3
Archaea3 - ps4
Asgard/Eukaryota - ps5
Eukaryota - ps6
Amorpheae - ps7
Obazoa - ps8
Opisthokonta - ps9
Holomycota - ps10
Fungi - ps11
Fungi_1 - ps12
Fungi_2 - ps13
Fungi_3 - ps14
Dikarya - ps15
Ascomycota - ps16
Saccharo/Pezizomycotina - ps17
Saccharomycotina - ps18
Saccharomycotina_1 - ps19
Saccharomycotina_2 - ps20
Saccharomycotina_3 - ps21
Phaff./Saccharomycetaceae - ps22
Saccharomyco./mycetaceae - ps23
Saccharomycetaceae - ps24
Saccharomycetaceae_2 - ps25
Saccharomycetaceae_3 - ps26
Kaz./Nau./Saccharomyces - ps27
Saccharomyces - ps28
Arboricola_clade - ps29
Cerevisiae_clade - ps30
Saccharomyces_cerevisiae - ps31
p-value
1e-02
1e-02
2e-02
3e-02
4e-02
Log-odds
0.5
1.0
1.5
2.0
2.5
3.0
3.5
4.0

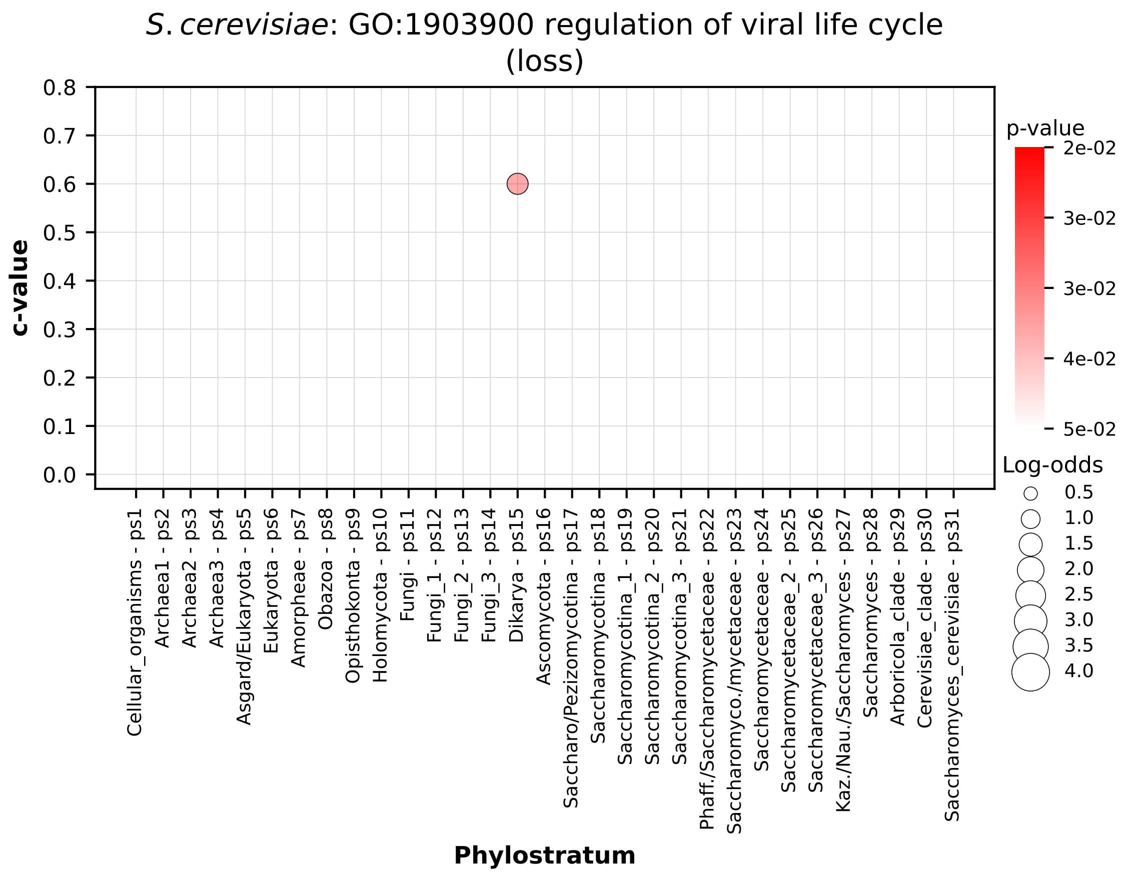

S. cerevisiae: GO:1903900 regulation of viral life cycle (loss)
c-value
0.8
0.7
0.6
0.5
0.4
0.3
0.2
0.1
0.0
Phylostratum
Cellular_organisms - ps1
Archaea1 - ps2
Archaea2 - ps3
Archaea3 - ps4
Asgard/Eukaryota - ps5
Eukaryota - ps6
Amorpheae - ps7
Obazoa - ps8
Opisthokonta - ps9
Holomycota - ps10
Fungi - ps11
Fungi_1 - ps12
Fungi_2 - ps13
Fungi_3 - ps14
Dikarya - ps15
Ascomycota - ps16
Saccharo/Pezizomycotina - ps17
Saccharomycotina - ps18
Saccharomycotina_1 - ps19
Saccharomycotina_2 - ps20
Saccharomycotina_3 - ps21
Phaff./Saccharomycetaceae - ps22
Saccharomyco./mycetaceae - ps23
Saccharomycetaceae - ps24
Saccharomycetaceae_2 - ps25
Saccharomycetaceae_3 - ps26
Kaz./Nau./Saccharomyces - ps27
Saccharomyces - ps28
Arboricola_clade - ps29
Cerevisiae_clade - ps30
Saccharomyces_cerevisiae - ps31
p-value
2e-02
3e-02
3e-02
4e-02
5e-02
Log-odds
0.5
1.0
1.5
2.0
2.5
3.0
3.5
4.0

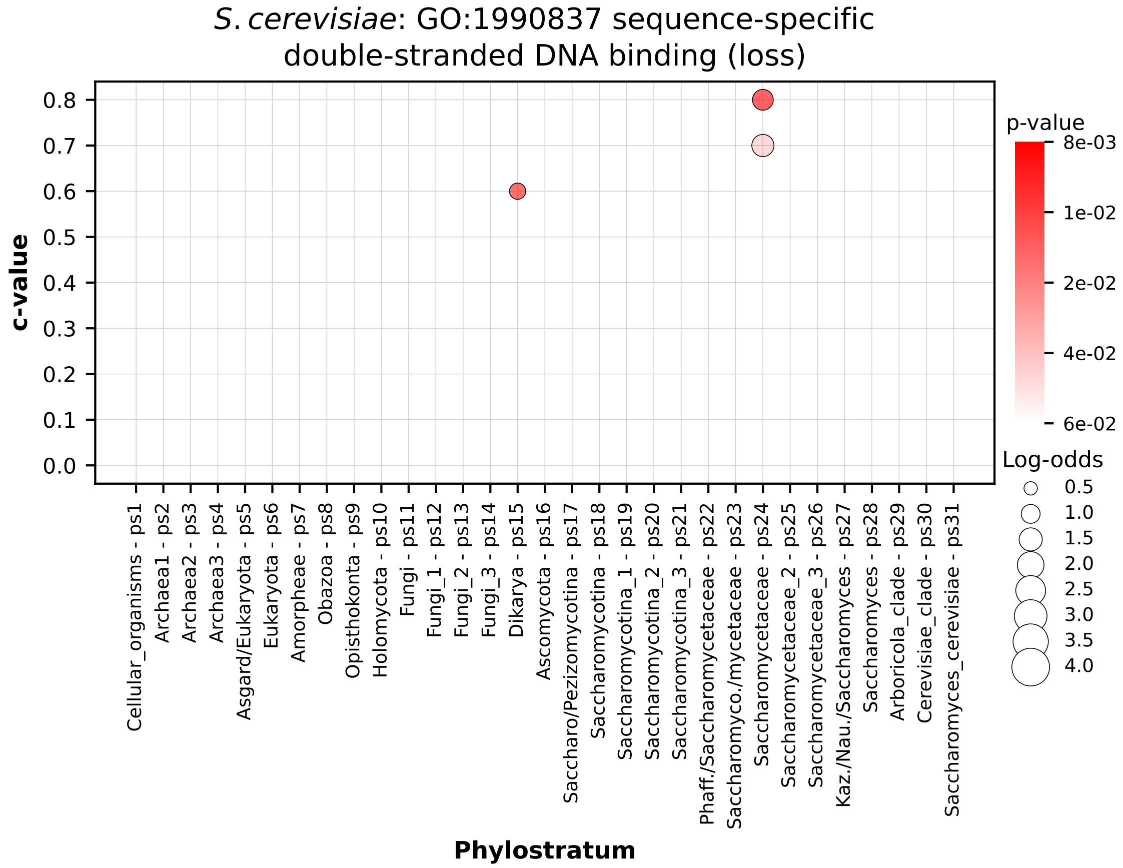

S. cerevisiae: GO:1990837 sequence-specific double-stranded DNA binding (loss)
c-value
0.0
0.1
0.2
0.3
0.4
0.5
0.6
0.7
0.8
p-value
8e-03
1e-02
2e-02
4e-02
6e-02
Log-odds
0.5
1.0
1.5
2.0
2.5
3.0
3.5
4.0
Cellular_organisms - ps1
Archaea1 - ps2
Archaea2 - ps3
Archaea3 - ps4
Asgard/Eukaryota - ps5
Eukaryota - ps6
Amorpheae - ps7
Obazoa - ps8
Opisthokonta - ps9
Holomycota - ps10
Fungi - ps11
Fungi_1 - ps12
Fungi_2 - ps13
Fungi_3 - ps14
Dikarya - ps15
Ascomycota - ps16
Saccharo/Pezizomycotina - ps17
Saccharomycotina - ps18
Saccharomycotina_1 - ps19
Saccharomycotina_2 - ps20
Saccharomycotina_3 - ps21
Phaff./Saccharomycetaceae - ps22
Saccharomyco./mycetaceae - ps23
Saccharomycetaceae - ps24
Saccharomycetaceae_2 - ps25
Saccharomycetaceae_3 - ps26
Kaz./Nau./Saccharomyces - ps27
Saccharomyces - ps28
Arboricola_clade - ps29
Cerevisiae_clade - ps30
Saccharomyces_cerevisiae - ps31
Phylostratum

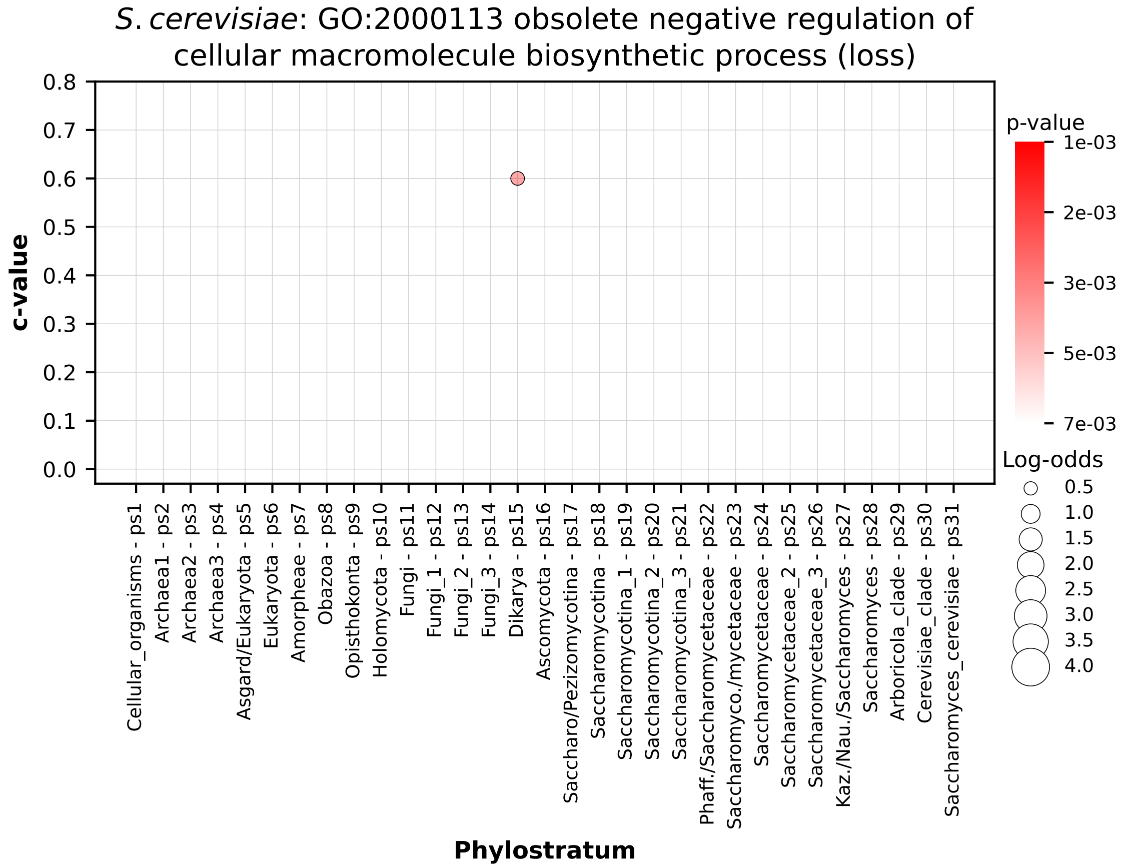

S. cerevisiae: GO:2000113 obsolete negative regulation of cellular macromolecule biosynthetic process (loss)
c-value
0.8
0.7
0.6
0.5
0.4
0.3
0.2
0.1
0.0
Phylostratum
Cellular_organisms - ps1
Archaea1 - ps2
Archaea2 - ps3
Archaea3 - ps4
Asgard/Eukaryota - ps5
Eukaryota - ps6
Amorpheae - ps7
Obazoa - ps8
Opisthokonta - ps9
Holomycota - ps10
Fungi - ps11
Fungi_1 - ps12
Fungi_2 - ps13
Fungi_3 - ps14
Dikarya - ps15
Ascomycota - ps16
Saccharo/Pezizomycotina - ps17
Saccharomycotina - ps18
Saccharomycotina_1 - ps19
Saccharomycotina_2 - ps20
Saccharomycotina_3 - ps21
Phaff./Saccharomycetaceae - ps22
Saccharomyco./mycetaceae - ps23
Saccharomycetaceae - ps24
Saccharomycetaceae_2 - ps25
Saccharomycetaceae_3 - ps26
Kaz./Nau./Saccharomyces - ps27
Saccharomyces - ps28
Arboricola_clade - ps29
Cerevisiae_clade - ps30
Saccharomyces_cerevisiae - ps31
p-value
1e-03
2e-03
3e-03
5e-03
7e-03
Log-odds
0.5
1.0
1.5
2.0
2.5
3.0
3.5
4.0

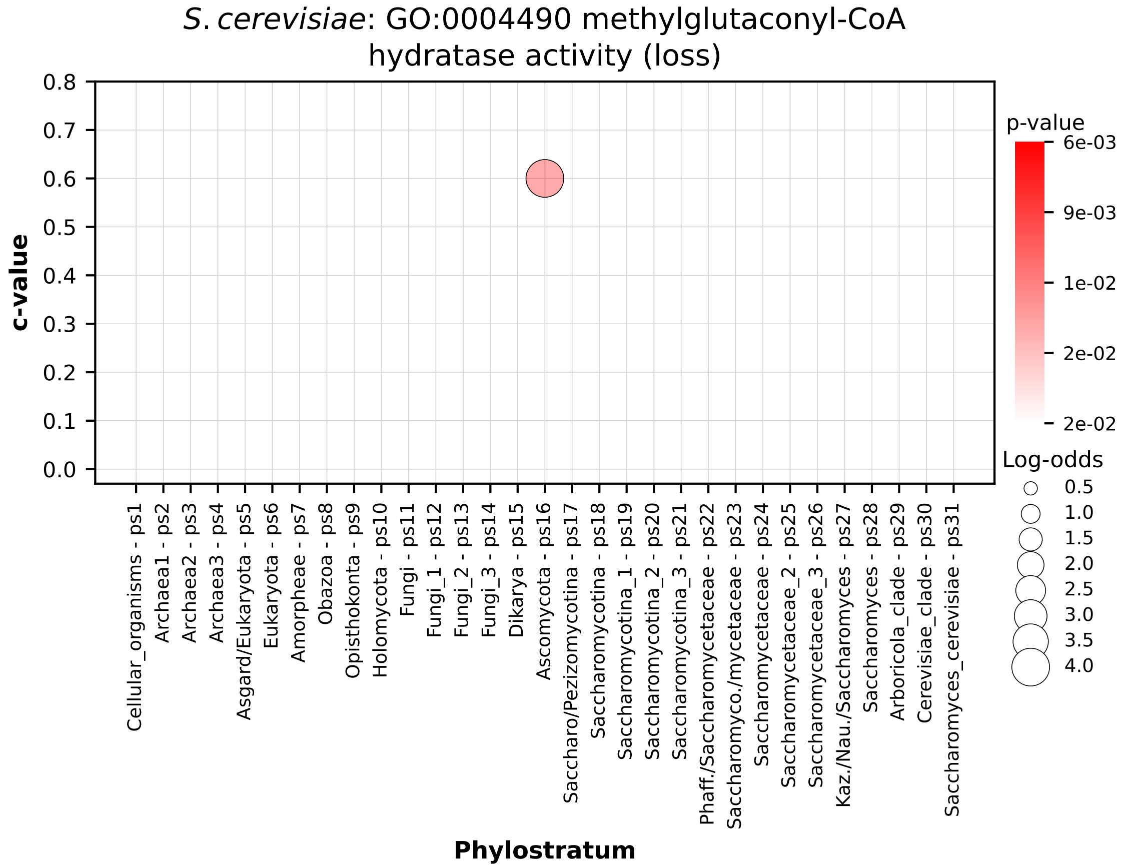

S. cerevisiae: GO:0004490 methylglutaconyl-CoA hydratase activity (loss)
c-value
0.8
0.7
0.6
0.5
0.4
0.3
0.2
0.1
0.0
p-value
6e-03
9e-03
1e-02
2e-02
2e-02
Log-odds
0.5
1.0
1.5
2.0
2.5
3.0
3.5
4.0
Cellular_organisms - ps1
Archaea1 - ps2
Archaea2 - ps3
Archaea3 - ps4
Asgard/Eukaryota - ps5
Eukaryota - ps6
Amorpheae - ps7
Obazoa - ps8
Opisthokonta - ps9
Holomycota - ps10
Fungi - ps11
Fungi_1 - ps12
Fungi_2 - ps13
Fungi_3 - ps14
Dikarya - ps15
Ascomycota - ps16
Saccharo/Pezizomycotina - ps17
Saccharomycotina - ps18
Saccharomycotina_1 - ps19
Saccharomycotina_2 - ps20
Saccharomycotina_3 - ps21
Phaff./Saccharomycetaceae - ps22
Saccharomyco./mycetaceae - ps23
Saccharomycetaceae - ps24
Saccharomycetaceae_2 - ps25
Saccharomycetaceae_3 - ps26
Kaz./Nau./Saccharomyces - ps27
Saccharomyces - ps28
Arboricola_clade - ps29
Cerevisiae_clade - ps30
Saccharomyces_cerevisiae - ps31
Phylostratum

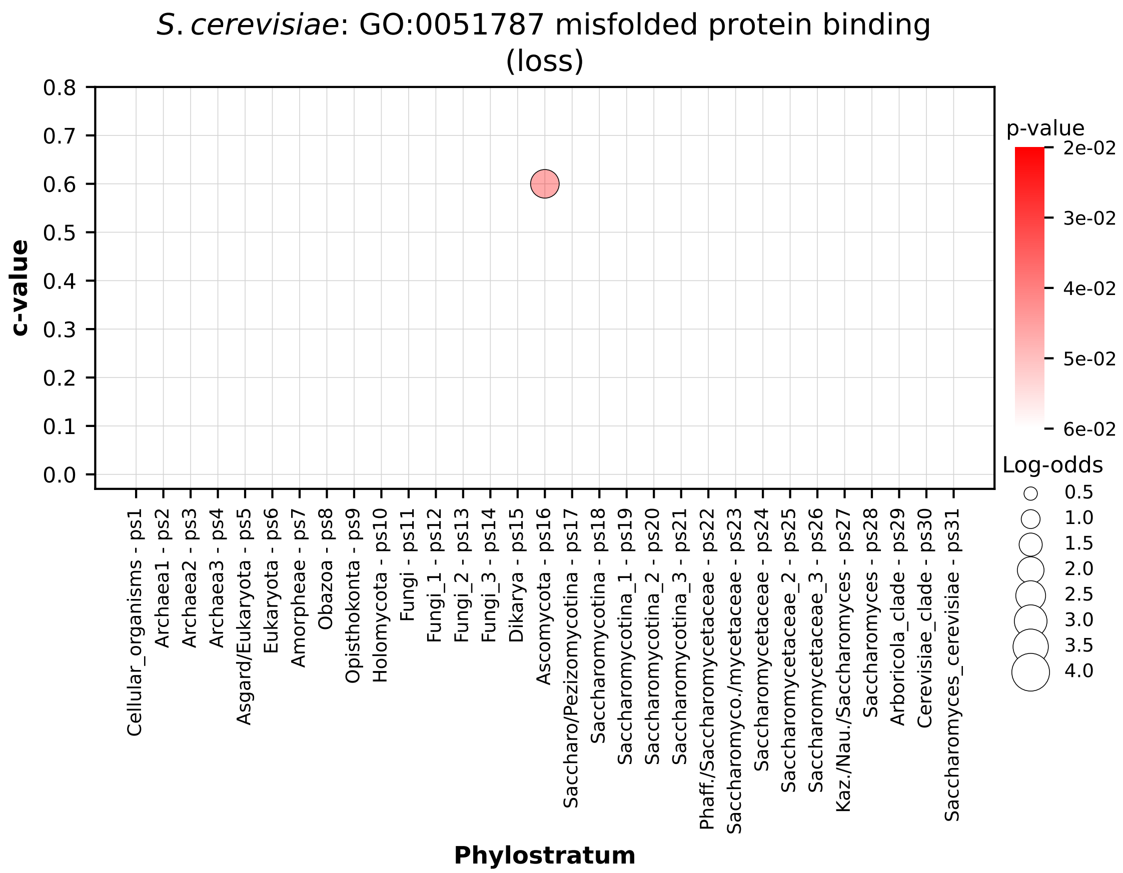
S. cerevisiae: GO:0051787 misfolded protein binding (loss)
c-value
0.8
0.7
0.6
0.5
0.4
0.3
0.2
0.1
0.0
p-value
2e-02
3e-02
4e-02
5e-02
6e-02
Log-odds
0.5
1.0
1.5
2.0
2.5
3.0
3.5
4.0
Cellular_organisms - ps1
Archaea1 - ps2
Archaea2 - ps3
Archaea3 - ps4
Asgard/Eukaryota - ps5
Eukaryota - ps6
Amorpheae - ps7
Obazoa - ps8
Opisthokonta - ps9
Holomycota - ps10
Fungi - ps11
Fungi_1 - ps12
Fungi_2 - ps13
Fungi_3 - ps14
Dikarya - ps15
Ascomycota - ps16
Saccharo/Pezizomycotina - ps17
Saccharomycotina - ps18
Saccharomycotina_1 - ps19
Saccharomycotina_2 - ps20
Saccharomycotina_3 - ps21
Phaff./Saccharomycetaceae - ps22
Saccharomyco./mycetaceae - ps23
Saccharomycetaceae - ps24
Saccharomycetaceae_2 - ps25
Saccharomycetaceae_3 - ps26
Kaz./Nau./Saccharomyces - ps27
Saccharomyces - ps28
Arboricola_clade - ps29
Cerevisiae_clade - ps30
Saccharomyces_cerevisiae - ps31
Phylostratum

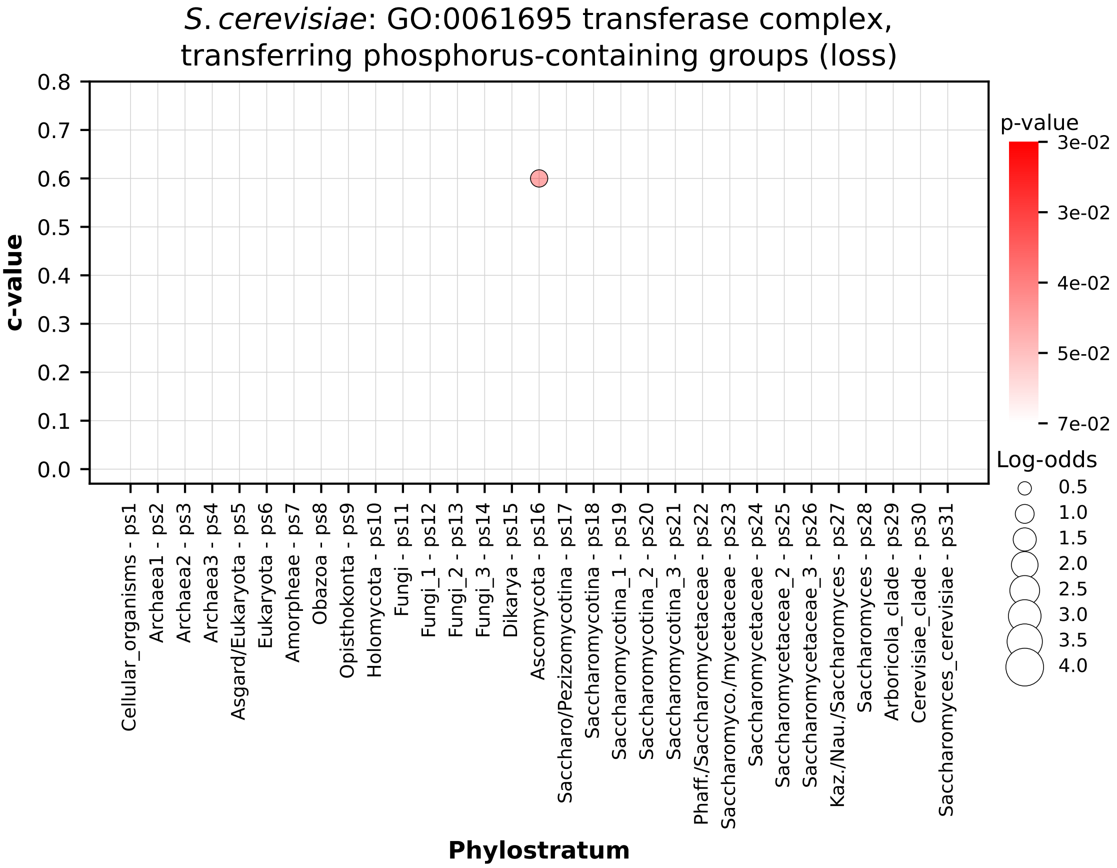

S. cerevisiae: GO:0061695 transferase complex, transferring phosphorus-containing groups (loss)
c-value
0.8
0.7
0.6
0.5
0.4
0.3
0.2
0.1
0.0
p-value
3e-02
3e-02
4e-02
5e-02
7e-02
Log-odds
0.5
1.0
1.5
2.0
2.5
3.0
3.5
4.0
Cellular_organisms - ps1
Archaea1 - ps2
Archaea2 - ps3
Archaea3 - ps4
Asgard/Eukaryota - ps5
Eukaryota - ps6
Amorpheae - ps7
Obazoa - ps8
Opisthokonta - ps9
Holomycota - ps10
Fungi - ps11
Fungi_1 - ps12
Fungi_2 - ps13
Fungi_3 - ps14
Dikarya - ps15
Ascomycota - ps16
Saccharo/Pezizomycotina - ps17
Saccharomycotina - ps18
Saccharomycotina_1 - ps19
Saccharomycotina_2 - ps20
Saccharomycotina_3 - ps21
Phaff./Saccharomycetaceae - ps22
Saccharomyco./mycetaceae - ps23
Saccharomycetaceae - ps24
Saccharomycetaceae_2 - ps25
Saccharomycetaceae_3 - ps26
Kaz./Nau./Saccharomyces - ps27
Saccharomyces - ps28
Arboricola_clade - ps29
Cerevisiae_clade - ps30
Saccharomyces_cerevisiae - ps31
Phylostratum

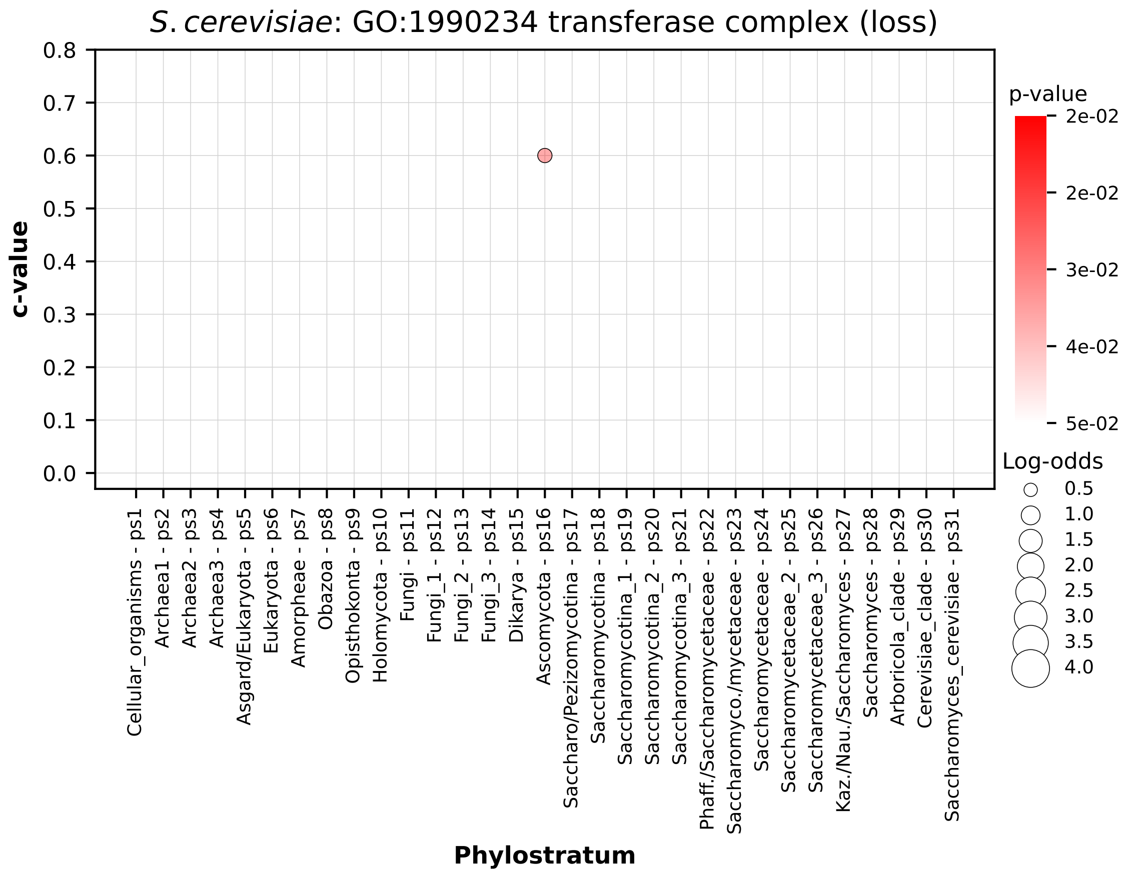
S. cerevisiae: GO:1990234 transferase complex (loss)
c-value
0.8
0.7
0.6
0.5
0.4
0.3
0.2
0.1
0.0
p-value
2e-02
2e-02
3e-02
4e-02
5e-02
Log-odds
0.5
1.0
1.5
2.0
2.5
3.0
3.5
4.0
Cellular_organisms - ps1
Archaea1 - ps2
Archaea2 - ps3
Archaea3 - ps4
Asgard/Eukaryota - ps5
Eukaryota - ps6
Amorpheae - ps7
Obazoa - ps8
Opisthokonta - ps9
Holomycota - ps10
Fungi - ps11
Fungi_1 - ps12
Fungi_2 - ps13
Fungi_3 - ps14
Dikarya - ps15
Ascomycota - ps16
Saccharo/Pezizomycotina - ps17
Saccharomycotina - ps18
Saccharomycotina_1 - ps19
Saccharomycotina_2 - ps20
Saccharomycotina_3 - ps21
Phaff./Saccharomycetaceae - ps22
Saccharomyco./mycetaceae - ps23
Saccharomycetaceae - ps24
Saccharomycetaceae_2 - ps25
Saccharomycetaceae_3 - ps26
Kaz./Nau./Saccharomyces - ps27
Saccharomyces - ps28
Arboricola_clade - ps29
Cerevisiae_clade - ps30
Saccharomyces_cerevisiae - ps31
Phylostratum

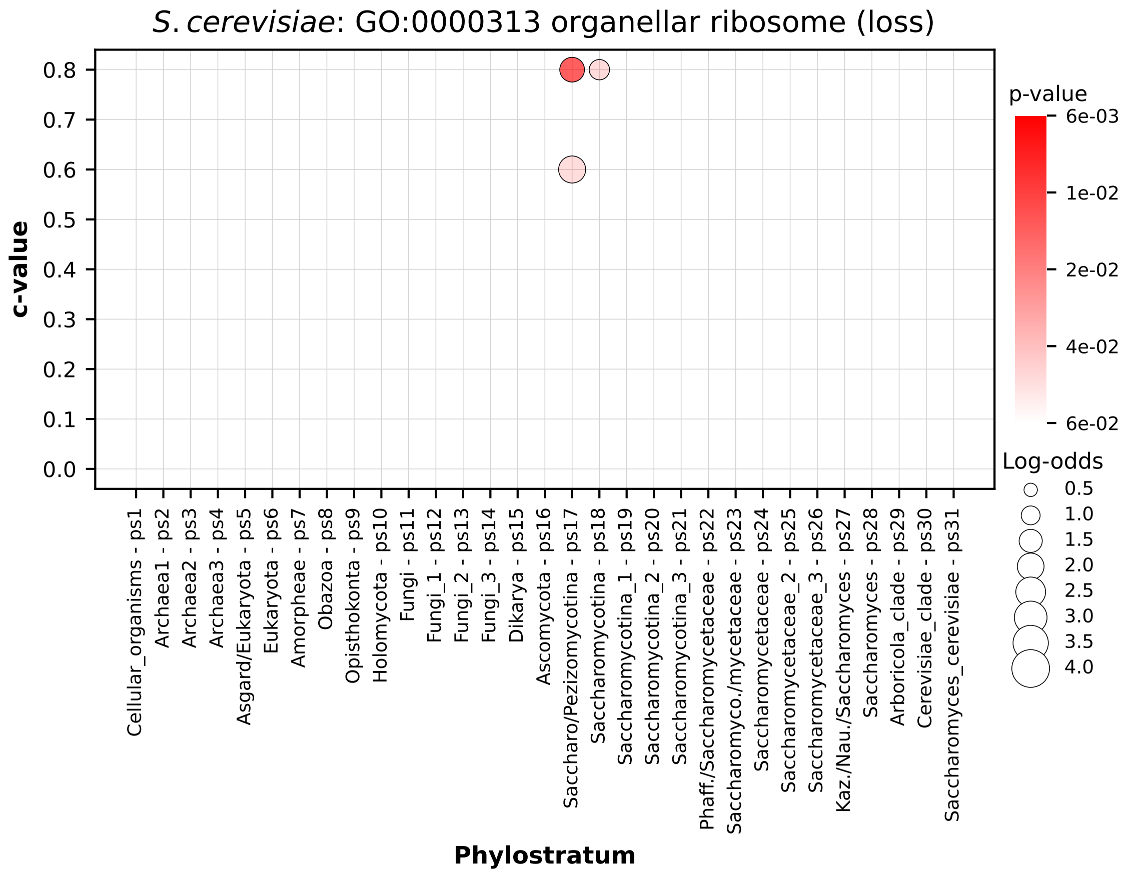

S. cerevisiae: GO:0000313 organellar ribosome (loss)
c-value
0.0
0.1
0.2
0.3
0.4
0.5
0.6
0.7
0.8
p-value
6e-03
1e-02
2e-02
4e-02
6e-02
Log-odds
0.5
1.0
1.5
2.0
2.5
3.0
3.5
4.0
Cellular_organisms - ps1
Archaea1 - ps2
Archaea2 - ps3
Archaea3 - ps4
Asgard/Eukaryota - ps5
Eukaryota - ps6
Amorpheae - ps7
Obazoa - ps8
Opisthokonta - ps9
Holomycota - ps10
Fungi - ps11
Fungi_1 - ps12
Fungi_2 - ps13
Fungi_3 - ps14
Dikarya - ps15
Ascomycota - ps16
Saccharo/Pezizomycotina - ps17
Saccharomycotina - ps18
Saccharomycotina_1 - ps19
Saccharomycotina_2 - ps20
Saccharomycotina_3 - ps21
Phaff./Saccharomycetaceae - ps22
Saccharomyco./mycetaceae - ps23
Saccharomycetaceae - ps24
Saccharomycetaceae_2 - ps25
Saccharomycetaceae_3 - ps26
Kaz./Nau./Saccharomyces - ps27
Saccharomyces - ps28
Arboricola_clade - ps29
Cerevisiae_clade - ps30
Saccharomyces_cerevisiae - ps31
Phylostratum

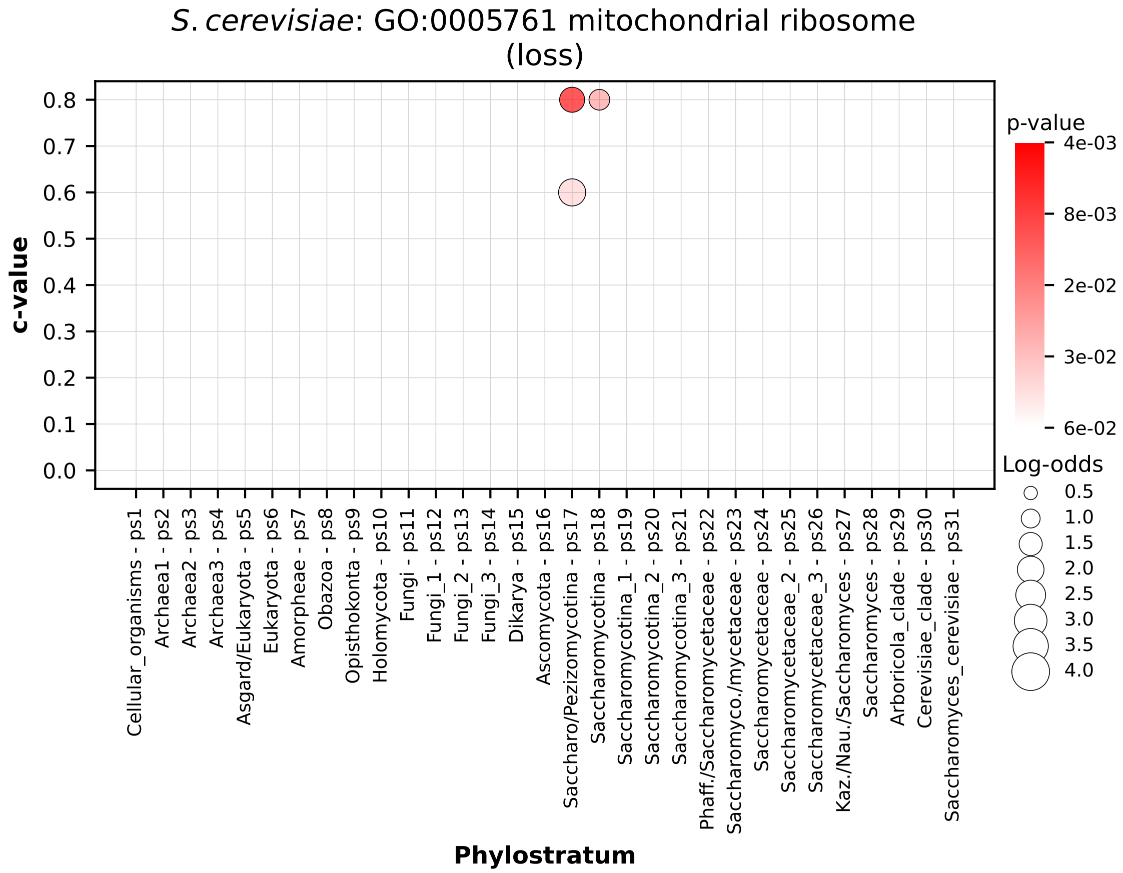
S. cerevisiae: GO:0005761 mitochondrial ribosome
(loss)
c-value
0.8
0.7
0.6
0.5
0.4
0.3
0.2
0.1
0.0
p-value
4e-03
8e-03
2e-02
3e-02
6e-02
Log-odds
0.5
1.0
1.5
2.0
2.5
3.0
3.5
4.0
Cellular_organisms - ps1
Archaea1 - ps2
Archaea2 - ps3
Archaea3 - ps4
Asgard/Eukaryota - ps5
Eukaryota - ps6
Amorpheae - ps7
Obazoa - ps8
Opisthokonta - ps9
Holomycota - ps10
Fungi - ps11
Fungi_1 - ps12
Fungi_2 - ps13
Fungi_3 - ps14
Dikarya - ps15
Ascomycota - ps16
Saccharo/Pezizomycotina - ps17
Saccharomycotina - ps18
Saccharomycotina_1 - ps19
Saccharomycotina_2 - ps20
Saccharomycotina_3 - ps21
Phaff./Saccharomycetaceae - ps22
Saccharomyco./mycetaceae - ps23
Saccharomycetaceae - ps24
Saccharomycetaceae_2 - ps25
Saccharomycetaceae_3 - ps26
Kaz./Nau./Saccharomyces - ps27
Saccharomyces - ps28
Arboricola_clade - ps29
Cerevisiae_clade - ps30
Saccharomyces_cerevisiae - ps31
Phylostratum

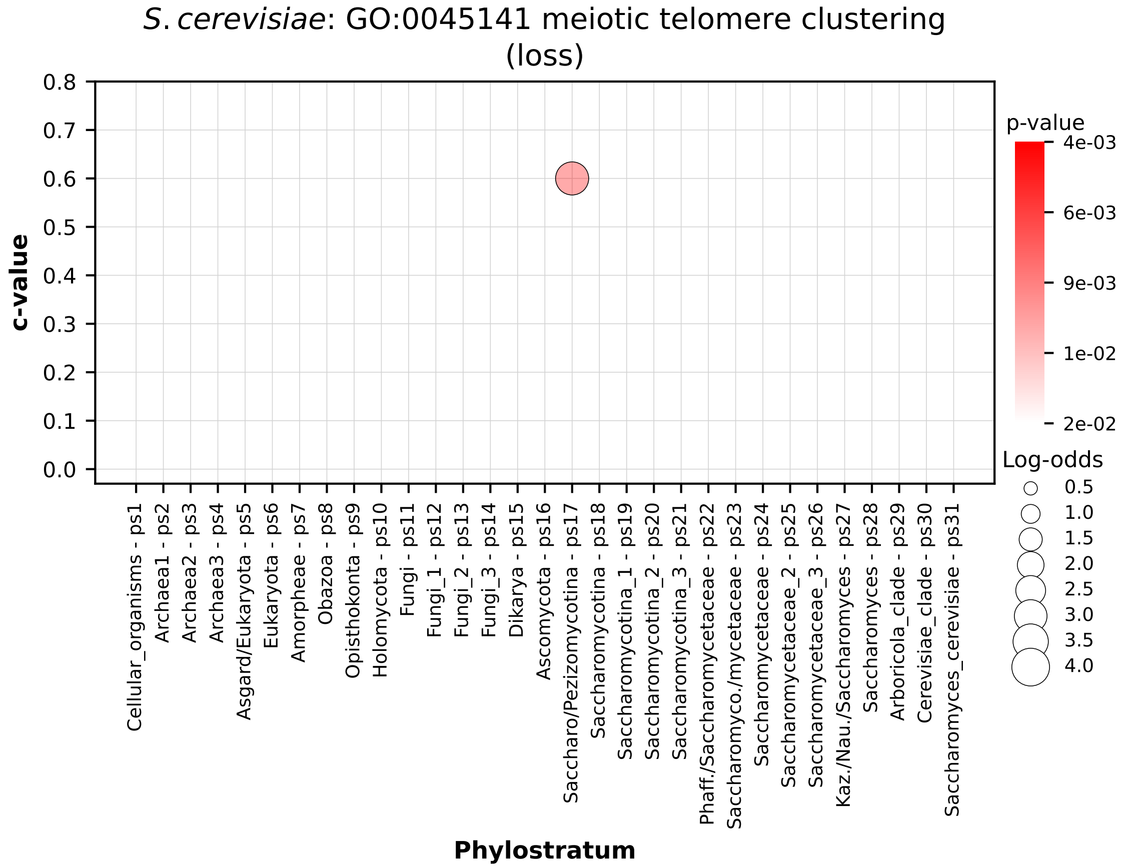
S. cerevisiae: GO:0045141 meiotic telomere clustering
(loss)
c-value
0.0
0.1
0.2
0.3
0.4
0.5
0.6
0.7
0.8
Phylostratum
Cellular_organisms - ps1
Archaea1 - ps2
Archaea2 - ps3
Archaea3 - ps4
Asgard/Eukaryota - ps5
Eukaryota - ps6
Amorpheae - ps7
Obazoa - ps8
Opisthokonta - ps9
Holomycota - ps10
Fungi - ps11
Fungi_1 - ps12
Fungi_2 - ps13
Fungi_3 - ps14
Dikarya - ps15
Ascomycota - ps16
Saccharo/Pezizomycotina - ps17
Saccharomycotina - ps18
Saccharomycotina_1 - ps19
Saccharomycotina_2 - ps20
Saccharomycotina_3 - ps21
Phaff./Saccharomycetaceae - ps22
Saccharomyco./mycetaceae - ps23
Saccharomycetaceae - ps24
Saccharomycetaceae_2 - ps25
Saccharomycetaceae_3 - ps26
Kaz./Nau./Saccharomyces - ps27
Saccharomyces - ps28
Arboricola_clade - ps29
Cerevisiae_clade - ps30
Saccharomyces_cerevisiae - ps31
p-value
4e-03
6e-03
9e-03
1e-02
2e-02
Log-odds
0.5
1.0
1.5
2.0
2.5
3.0
3.5
4.0

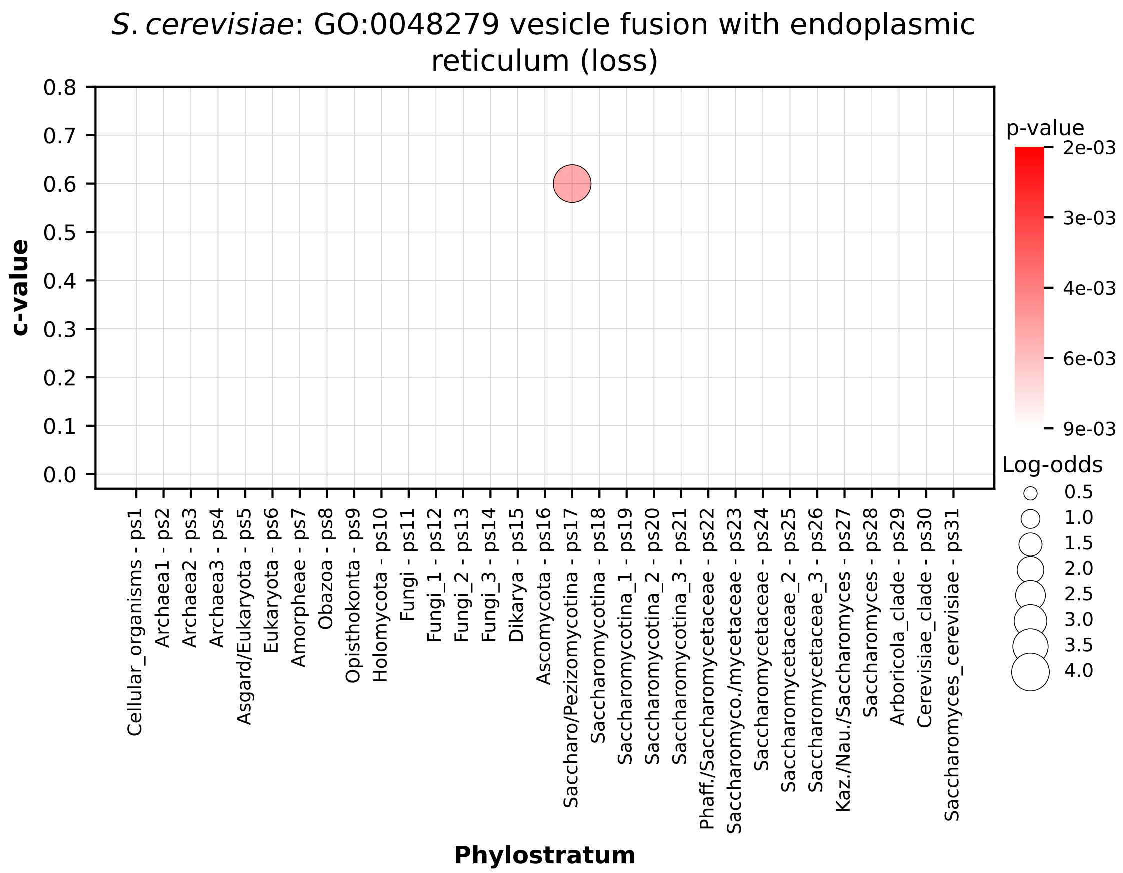

S. cerevisiae: GO:0048279 vesicle fusion with endoplasmic reticulum (loss)
c-value
0.8
0.7
0.6
0.5
0.4
0.3
0.2
0.1
0.0
p-value
2e-03
3e-03
4e-03
6e-03
9e-03
Log-odds
0.5
1.0
1.5
2.0
2.5
3.0
3.5
4.0
Cellular_organisms - ps1
Archaea1 - ps2
Archaea2 - ps3
Archaea3 - ps4
Asgard/Eukaryota - ps5
Eukaryota - ps6
Amorpheae - ps7
Obazoa - ps8
Opisthokonta - ps9
Holomycota - ps10
Fungi - ps11
Fungi_1 - ps12
Fungi_2 - ps13
Fungi_3 - ps14
Dikarya - ps15
Ascomycota - ps16
Saccharo/Pezizomycotina - ps17
Saccharomycotina - ps18
Saccharomycotina_1 - ps19
Saccharomycotina_2 - ps20
Saccharomycotina_3 - ps21
Phaff./Saccharomycetaceae - ps22
Saccharomyco./mycetaceae - ps23
Saccharomycetaceae - ps24
Saccharomycetaceae_2 - ps25
Saccharomycetaceae_3 - ps26
Kaz./Nau./Saccharomyces - ps27
Saccharomyces - ps28
Arboricola_clade - ps29
Cerevisiae_clade - ps30
Saccharomyces_cerevisiae - ps31
Phylostratum

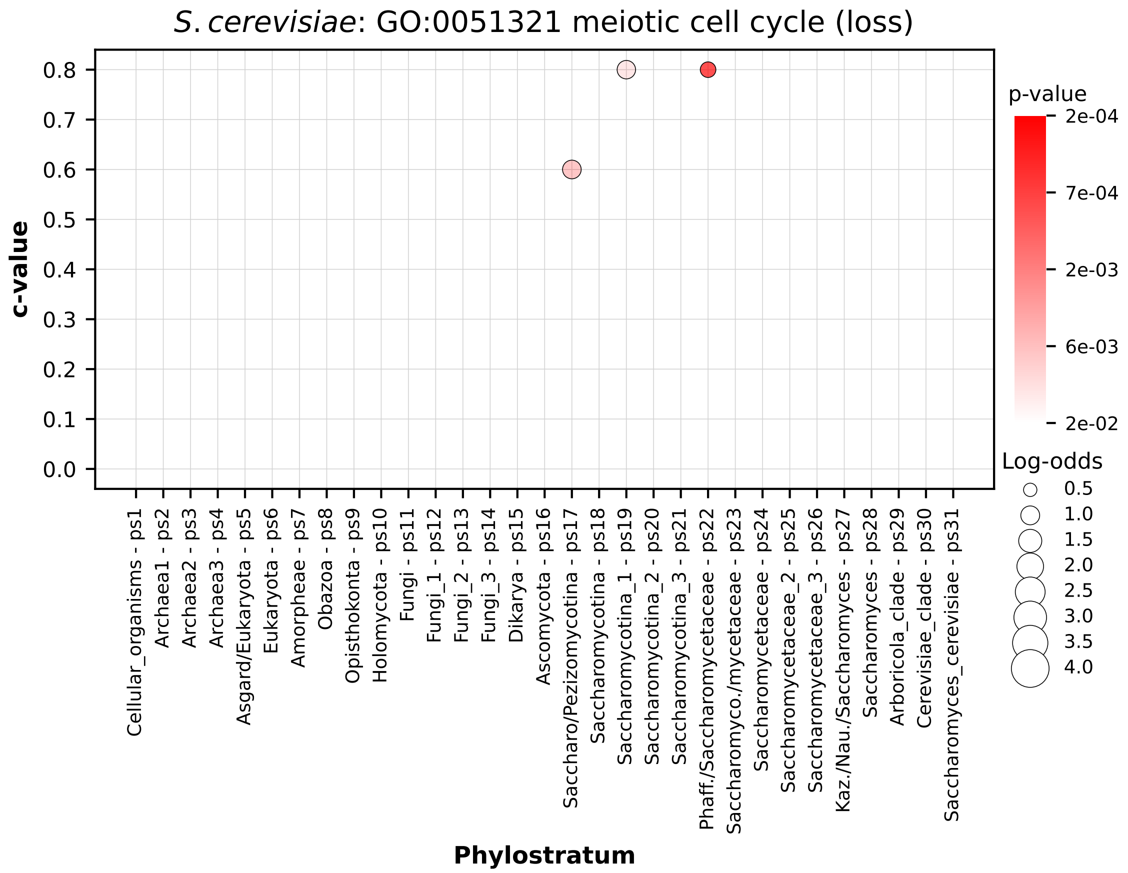
S. cerevisiae: GO:0051321 meiotic cell cycle (loss)
c-value
0.8
0.7
0.6
0.5
0.4
0.3
0.2
0.1
0.0
p-value
2e-04
7e-04
2e-03
6e-03
2e-02
Log-odds
0.5
1.0
1.5
2.0
2.5
3.0
3.5
4.0
Cellular_organisms - ps1
Archaea1 - ps2
Archaea2 - ps3
Archaea3 - ps4
Asgard/Eukaryota - ps5
Eukaryota - ps6
Amorpheae - ps7
Obazoa - ps8
Opisthokonta - ps9
Holomycota - ps10
Fungi - ps11
Fungi_1 - ps12
Fungi_2 - ps13
Fungi_3 - ps14
Dikarya - ps15
Ascomycota - ps16
Saccharo/Pezizomycotina - ps17
Saccharomycotina - ps18
Saccharomycotina_1 - ps19
Saccharomycotina_2 - ps20
Saccharomycotina_3 - ps21
Phaff./Saccharomycetaceae - ps22
Saccharomyco./mycetaceae - ps23
Saccharomycetaceae - ps24
Saccharomycetaceae_2 - ps25
Saccharomycetaceae_3 - ps26
Kaz./Nau./Saccharomyces - ps27
Saccharomyces - ps28
Arboricola_clade - ps29
Cerevisiae_clade - ps30
Saccharomyces_cerevisiae - ps31
Phylostratum

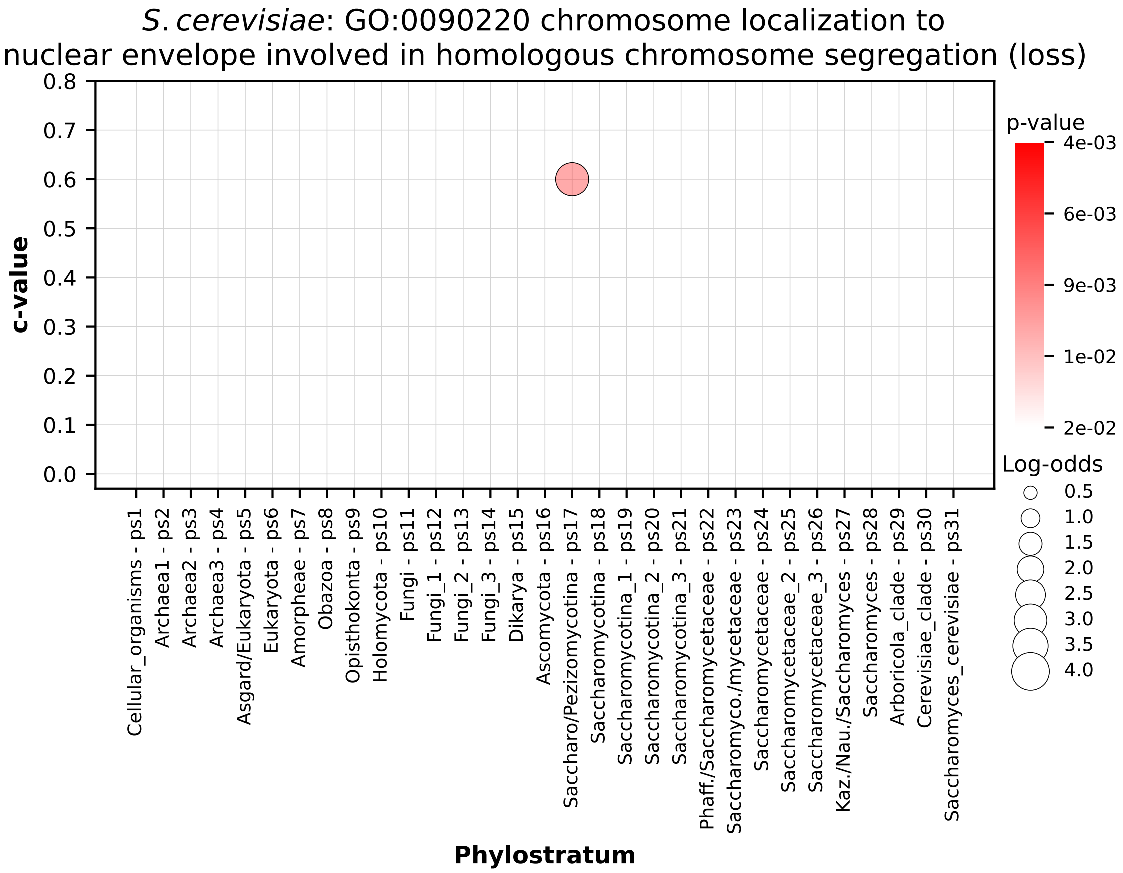
S. cerevisiae: GO:0090220 chromosome localization to nuclear envelope involved in homologous chromosome segregation (loss)
c-value
0.8
0.7
0.6
0.5
0.4
0.3
0.2
0.1
0.0
Phylostratum
Cellular_organisms - ps1
Archaea1 - ps2
Archaea2 - ps3
Archaea3 - ps4
Asgard/Eukaryota - ps5
Eukaryota - ps6
Amorpheae - ps7
Obazoa - ps8
Opisthokonta - ps9
Holomycota - ps10
Fungi - ps11
Fungi_1 - ps12
Fungi_2 - ps13
Fungi_3 - ps14
Dikarya - ps15
Ascomycota - ps16
Saccharo/Pezizomycotina - ps17
Saccharomycotina - ps18
Saccharomycotina_1 - ps19
Saccharomycotina_2 - ps20
Saccharomycotina_3 - ps21
Phaff./Saccharomycetaceae - ps22
Saccharomyco./mycetaceae - ps23
Saccharomycetaceae - ps24
Saccharomycetaceae_2 - ps25
Saccharomycetaceae_3 - ps26
Kaz./Nau./Saccharomyces - ps27
Saccharomyces - ps28
Arboricola_clade - ps29
Cerevisiae_clade - ps30
Saccharomyces_cerevisiae - ps31
p-value
4e-03
6e-03
9e-03
1e-02
2e-02
Log-odds
0.5
1.0
1.5
2.0
2.5
3.0
3.5
4.0

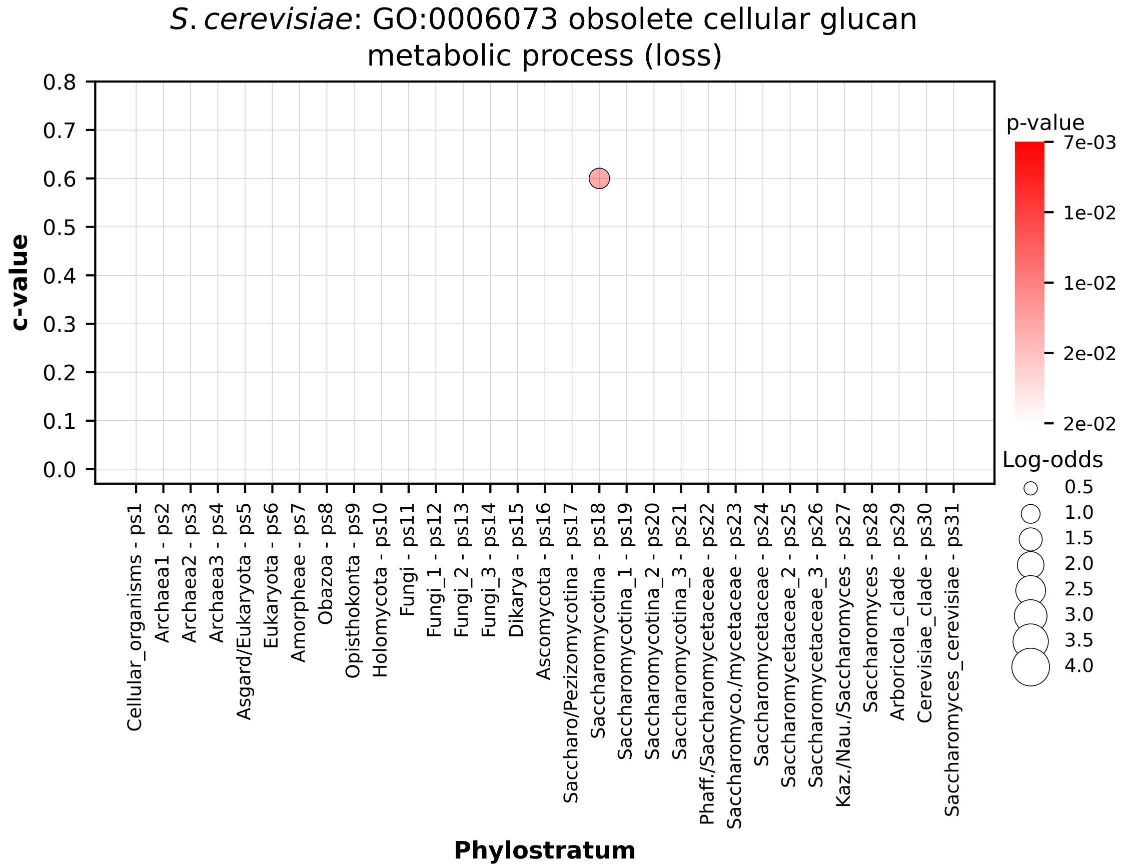
S. cerevisiae: GO:0006073 obsolete cellular glucan metabolic process (loss)
c-value
0.8
0.7
0.6
0.5
0.4
0.3
0.2
0.1
0.0
p-value
7e-03
1e-02
1e-02
2e-02
2e-02
Log-odds
0.5
1.0
1.5
2.0
2.5
3.0
3.5
4.0
Cellular_organisms - ps1
Archaea1 - ps2
Archaea2 - ps3
Archaea3 - ps4
Asgard/Eukaryota - ps5
Eukaryota - ps6
Amorpheae - ps7
Obazoa - ps8
Opisthokonta - ps9
Holomycota - ps10
Fungi - ps11
Fungi_1 - ps12
Fungi_2 - ps13
Fungi_3 - ps14
Dikarya - ps15
Ascomycota - ps16
Saccharo/Pezizomycotina - ps17
Saccharomycotina - ps18
Saccharomycotina_1 - ps19
Saccharomycotina_2 - ps20
Saccharomycotina_3 - ps21
Phaff./Saccharomycetaceae - ps22
Saccharomyco./mycetaceae - ps23
Saccharomycetaceae - ps24
Saccharomycetaceae_2 - ps25
Saccharomycetaceae_3 - ps26
Kaz./Nau./Saccharomyces - ps27
Saccharomyces - ps28
Arboricola_clade - ps29
Cerevisiae_clade - ps30
Saccharomyces_cerevisiae - ps31
Phylostratum

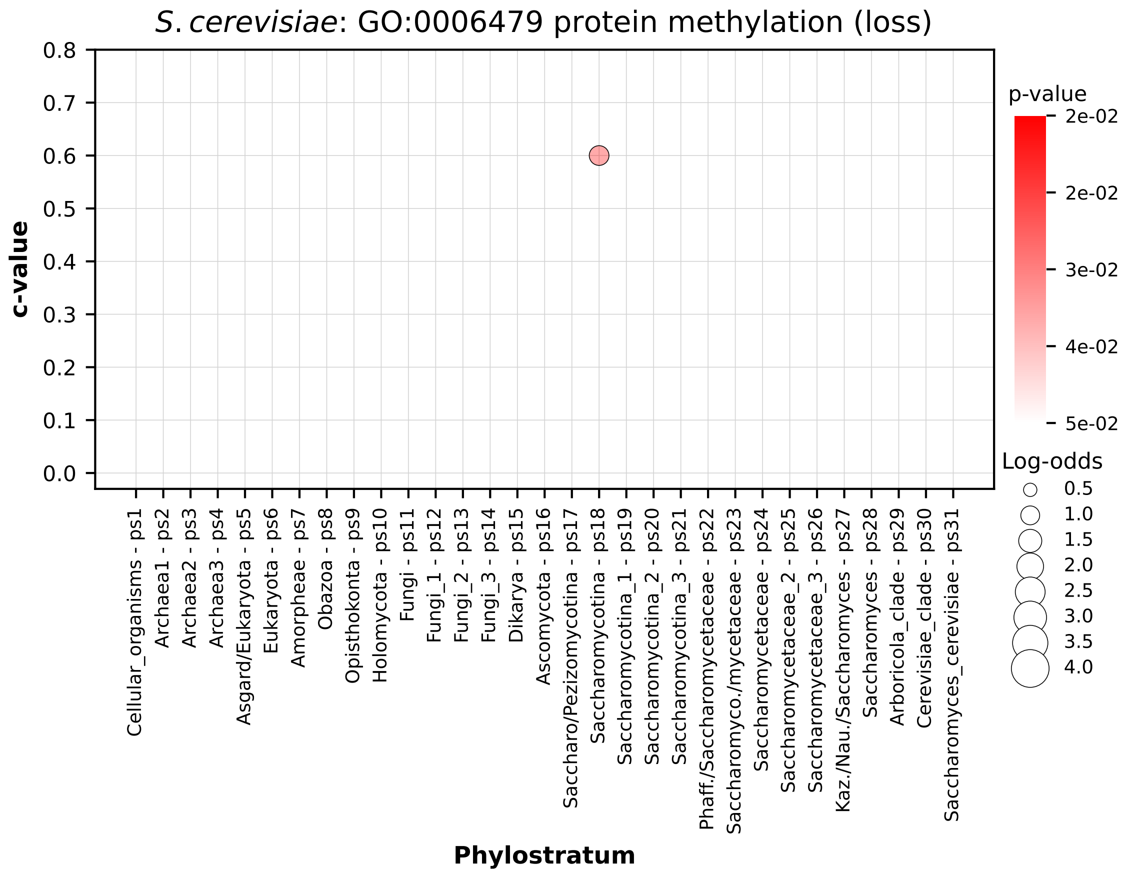
S. cerevisiae: GO:0006479 protein methylation (loss)
c-value
0.8
0.7
0.6
0.5
0.4
0.3
0.2
0.1
0.0
Phylostratum
Cellular_organisms - ps1
Archaea1 - ps2
Archaea2 - ps3
Archaea3 - ps4
Asgard/Eukaryota - ps5
Eukaryota - ps6
Amorpheae - ps7
Obazoa - ps8
Opisthokonta - ps9
Holomycota - ps10
Fungi - ps11
Fungi_1 - ps12
Fungi_2 - ps13
Fungi_3 - ps14
Dikarya - ps15
Ascomycota - ps16
Saccharo/Pezizomycotina - ps17
Saccharomycotina - ps18
Saccharomycotina_1 - ps19
Saccharomycotina_2 - ps20
Saccharomycotina_3 - ps21
Phaff./Saccharomycetaceae - ps22
Saccharomyco./mycetaceae - ps23
Saccharomycetaceae - ps24
Saccharomycetaceae_2 - ps25
Saccharomycetaceae_3 - ps26
Kaz./Nau./Saccharomyces - ps27
Saccharomyces - ps28
Arboricola_clade - ps29
Cerevisiae_clade - ps30
Saccharomyces_cerevisiae - ps31
p-value
2e-02
2e-02
3e-02
4e-02
5e-02
Log-odds
0.5
1.0
1.5
2.0
2.5
3.0
3.5
4.0

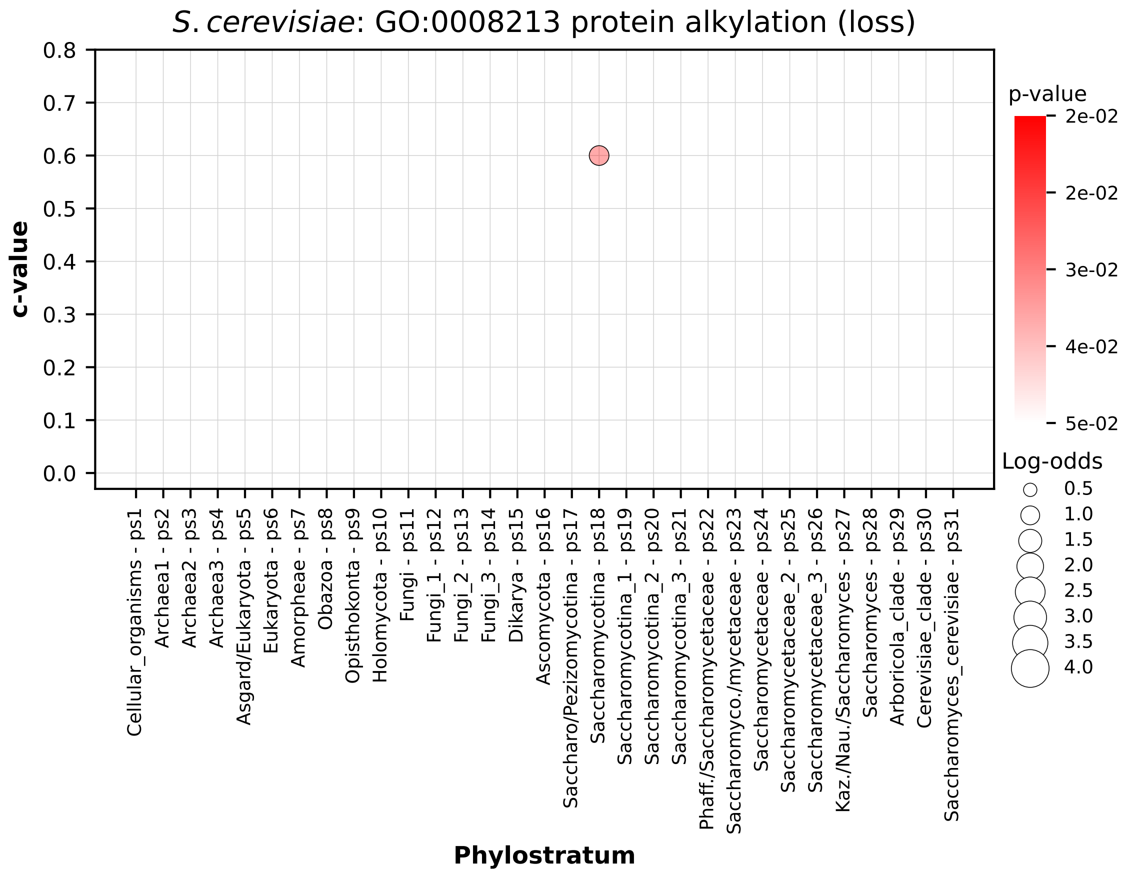

S. cerevisiae: GO:0008213 protein alkylation (loss)
c-value
0.8
0.7
0.6
0.5
0.4
0.3
0.2
0.1
0.0
p-value
2e-02
2e-02
3e-02
4e-02
5e-02
Log-odds
0.5
1.0
1.5
2.0
2.5
3.0
3.5
4.0
Cellular_organisms - ps1
Archaea1 - ps2
Archaea2 - ps3
Archaea3 - ps4
Asgard/Eukaryota - ps5
Eukaryota - ps6
Amorpheae - ps7
Obazoa - ps8
Opisthokonta - ps9
Holomycota - ps10
Fungi - ps11
Fungi_1 - ps12
Fungi_2 - ps13
Fungi_3 - ps14
Dikarya - ps15
Ascomycota - ps16
Saccharo/Pezizomycotina - ps17
Saccharomycotina - ps18
Saccharomycotina_1 - ps19
Saccharomycotina_2 - ps20
Saccharomycotina_3 - ps21
Phaff./Saccharomycetaceae - ps22
Saccharomyco./mycetaceae - ps23
Saccharomycetaceae - ps24
Saccharomycetaceae_2 - ps25
Saccharomycetaceae_3 - ps26
Kaz./Nau./Saccharomyces - ps27
Saccharomyces - ps28
Arboricola_clade - ps29
Cerevisiae_clade - ps30
Saccharomyces_cerevisiae - ps31
Phylostratum

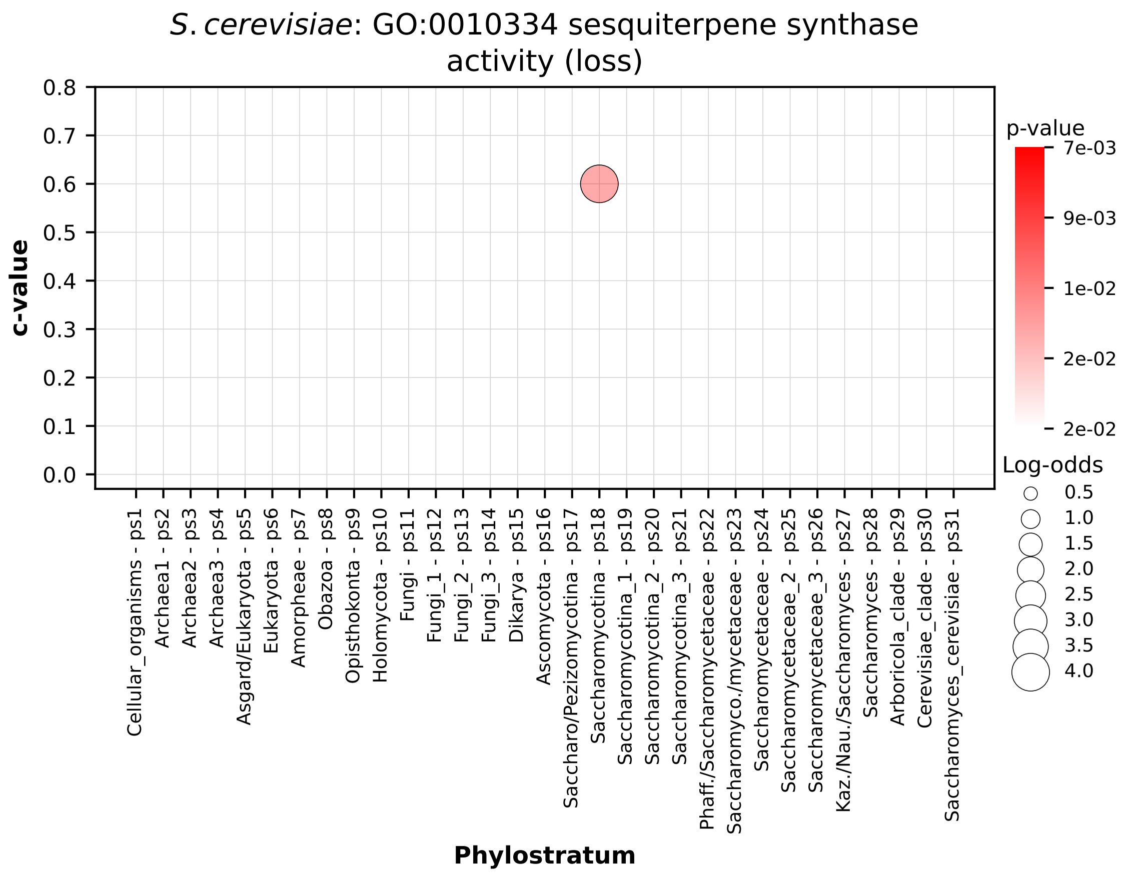

S. cerevisiae: GO:0010334 sesquiterpene synthase activity (loss)
c-value
0.8
0.7
0.6
0.5
0.4
0.3
0.2
0.1
0.0
Phylostratum
Cellular_organisms - ps1
Archaea1 - ps2
Archaea2 - ps3
Archaea3 - ps4
Asgard/Eukaryota - ps5
Eukaryota - ps6
Amorpheae - ps7
Obazoa - ps8
Opisthokonta - ps9
Holomycota - ps10
Fungi - ps11
Fungi_1 - ps12
Fungi_2 - ps13
Fungi_3 - ps14
Dikarya - ps15
Ascomycota - ps16
Saccharo/Pezizomycotina - ps17
Saccharomycotina - ps18
Saccharomycotina_1 - ps19
Saccharomycotina_2 - ps20
Saccharomycotina_3 - ps21
Phaff./Saccharomycetaceae - ps22
Saccharomyco./mycetaceae - ps23
Saccharomycetaceae - ps24
Saccharomycetaceae_2 - ps25
Saccharomycetaceae_3 - ps26
Kaz./Nau./Saccharomyces - ps27
Saccharomyces - ps28
Arboricola_clade - ps29
Cerevisiae_clade - ps30
Saccharomyces_cerevisiae - ps31
p-value
7e-03
9e-03
1e-02
2e-02
2e-02
Log-odds
0.5
1.0
1.5
2.0
2.5
3.0
3.5
4.0

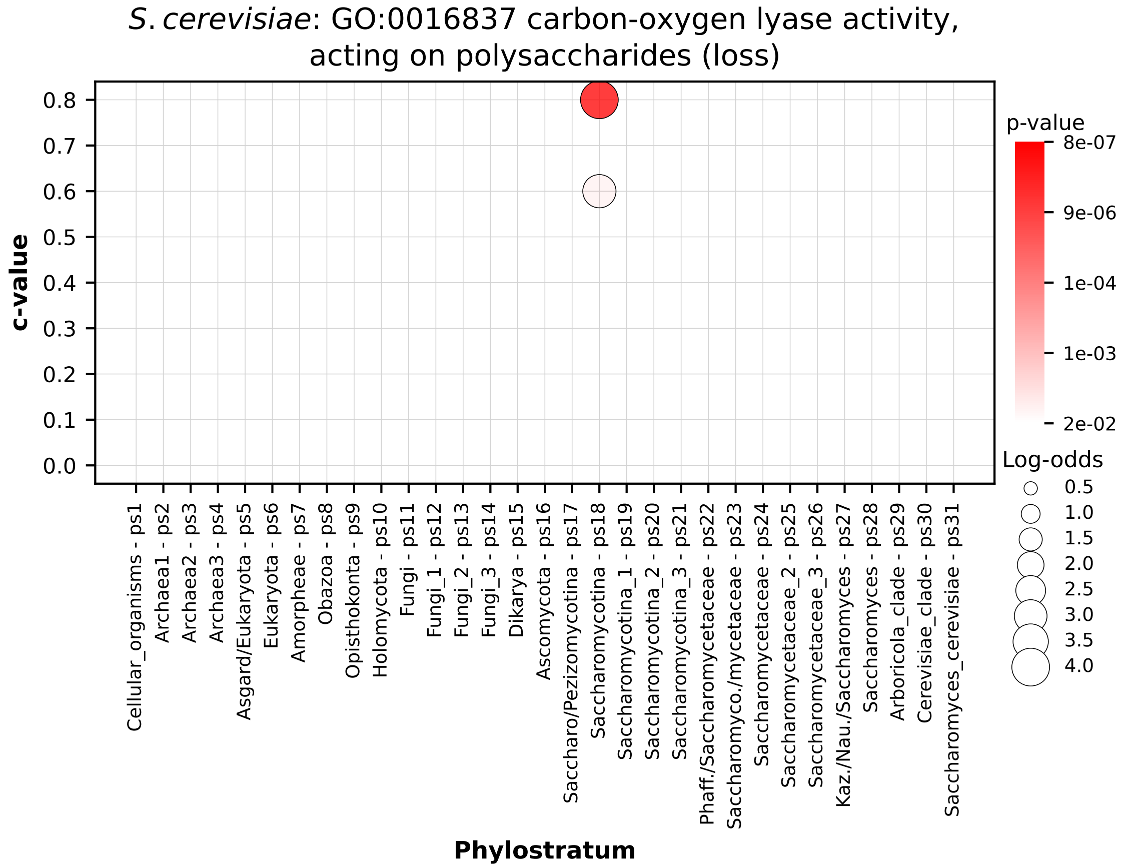
S. cerevisiae: GO:0016837 carbon-oxygen lyase activity, acting on polysaccharides (loss)
c-value
0.8
0.7
0.6
0.5
0.4
0.3
0.2
0.1
0.0
Phylostratum
Cellular_organisms - ps1
Archaea1 - ps2
Archaea2 - ps3
Archaea3 - ps4
Asgard/Eukaryota - ps5
Eukaryota - ps6
Amorpheae - ps7
Obazoa - ps8
Opisthokonta - ps9
Holomycota - ps10
Fungi - ps11
Fungi_1 - ps12
Fungi_2 - ps13
Fungi_3 - ps14
Dikarya - ps15
Ascomycota - ps16
Saccharo/Pezizomycotina - ps17
Saccharomycotina - ps18
Saccharomycotina_1 - ps19
Saccharomycotina_2 - ps20
Saccharomycotina_3 - ps21
Phaff./Saccharomycetaceae - ps22
Saccharomyco./mycetaceae - ps23
Saccharomycetaceae - ps24
Saccharomycetaceae_2 - ps25
Saccharomycetaceae_3 - ps26
Kaz./Nau./Saccharomyces - ps27
Saccharomyces - ps28
Arboricola_clade - ps29
Cerevisiae_clade - ps30
Saccharomyces_cerevisiae - ps31
p-value
8e-07
9e-06
1e-04
1e-03
2e-02
Log-odds
0.5
1.0
1.5
2.0
2.5
3.0
3.5
4.0

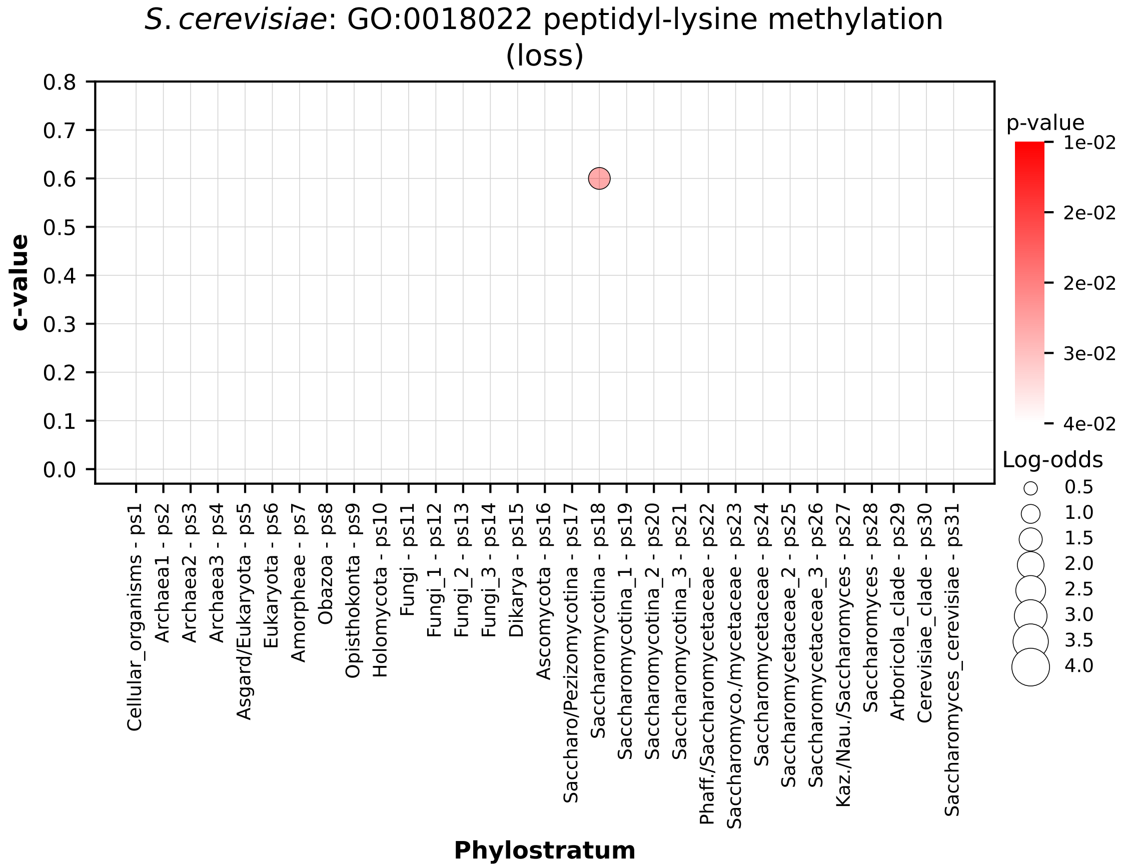
S. cerevisiae: GO:0018022 peptidyl-lysine methylation
(loss)
c-value
0.0
0.1
0.2
0.3
0.4
0.5
0.6
0.7
0.8
Phylostratum
Cellular_organisms - ps1
Archaea1 - ps2
Archaea2 - ps3
Archaea3 - ps4
Asgard/Eukaryota - ps5
Eukaryota - ps6
Amorpheae - ps7
Obazoa - ps8
Opisthokonta - ps9
Holomycota - ps10
Fungi - ps11
Fungi_1 - ps12
Fungi_2 - ps13
Fungi_3 - ps14
Dikarya - ps15
Ascomycota - ps16
Saccharo/Pezizomycotina - ps17
Saccharomycotina - ps18
Saccharomycotina_1 - ps19
Saccharomycotina_2 - ps20
Saccharomycotina_3 - ps21
Phaff./Saccharomycetaceae - ps22
Saccharomyco./mycetaceae - ps23
Saccharomycetaceae - ps24
Saccharomycetaceae_2 - ps25
Saccharomycetaceae_3 - ps26
Kaz./Nau./Saccharomyces - ps27
Saccharomyces - ps28
Arboricola_clade - ps29
Cerevisiae_clade - ps30
Saccharomyces_cerevisiae - ps31
p-value
1e-02
2e-02
2e-02
3e-02
4e-02
Log-odds
0.5
1.0
1.5
2.0
2.5
3.0
3.5
4.0

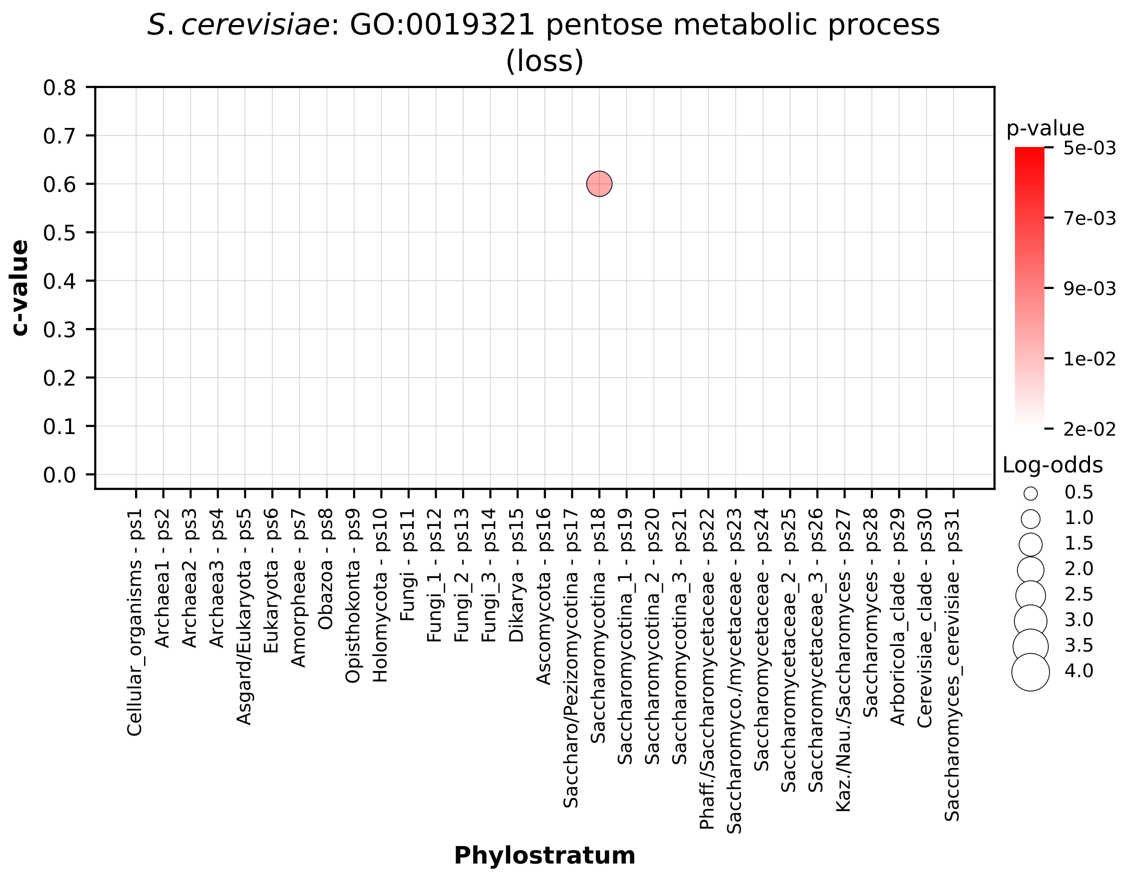

S. cerevisiae: GO:0019321 pentose metabolic process
(loss)
c-value
0.0
0.1
0.2
0.3
0.4
0.5
0.6
0.7
0.8
p-value
5e-03
7e-03
9e-03
1e-02
2e-02
Log-odds
0.5
1.0
1.5
2.0
2.5
3.0
3.5
4.0
Cellular_organisms - ps1
Archaea1 - ps2
Archaea2 - ps3
Archaea3 - ps4
Asgard/Eukaryota - ps5
Eukaryota - ps6
Amorpheae - ps7
Obazoa - ps8
Opisthokonta - ps9
Holomycota - ps10
Fungi - ps11
Fungi_1 - ps12
Fungi_2 - ps13
Fungi_3 - ps14
Dikarya - ps15
Ascomycota - ps16
Saccharo/Pezizomycotina - ps17
Saccharomycotina - ps18
Saccharomycotina_1 - ps19
Saccharomycotina_2 - ps20
Saccharomycotina_3 - ps21
Phaff./Saccharomycetaceae - ps22
Saccharomyco./mycetaceae - ps23
Saccharomycetaceae - ps24
Saccharomycetaceae_2 - ps25
Saccharomycetaceae_3 - ps26
Kaz./Nau./Saccharomyces - ps27
Saccharomyces - ps28
Arboricola_clade - ps29
Cerevisiae_clade - ps30
Saccharomyces_cerevisiae - ps31
Phylostratum

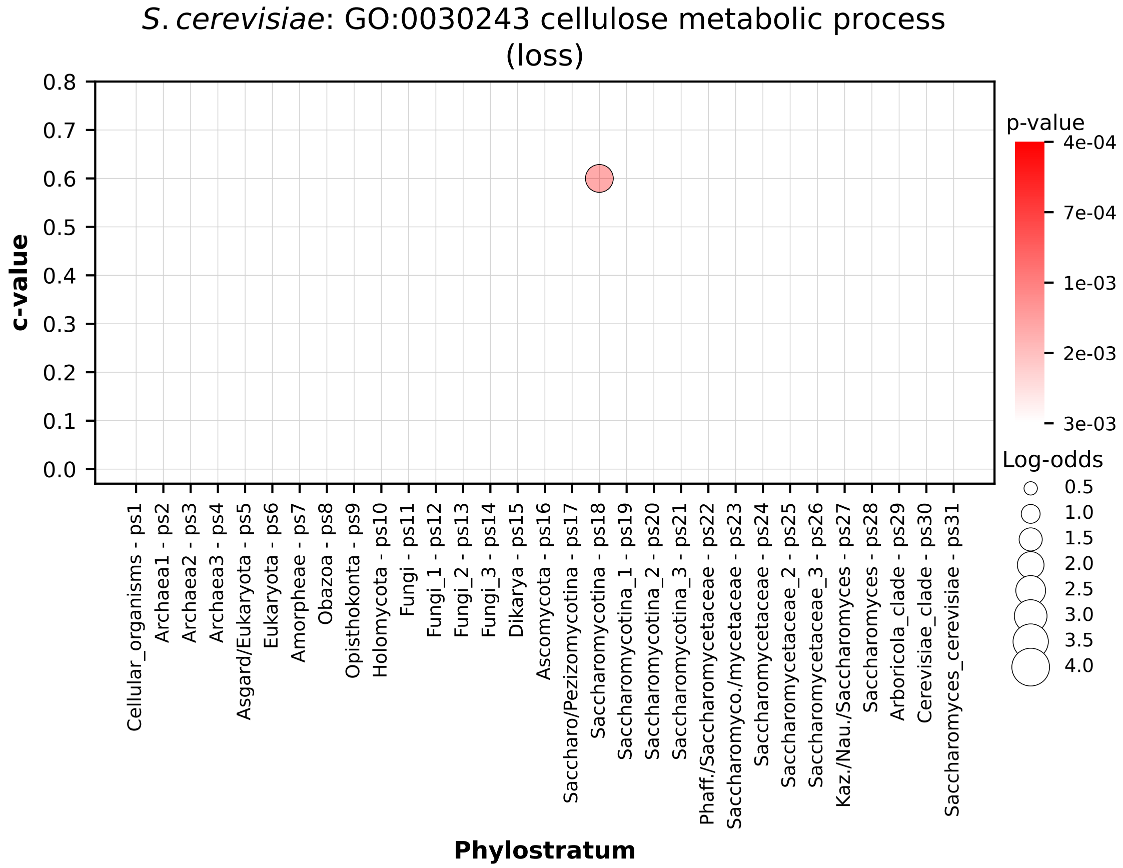

S. cerevisiae: GO:0030243 cellulose metabolic process
(loss)
c-value
0.8
0.7
0.6
0.5
0.4
0.3
0.2
0.1
0.0
p-value
4e-04
7e-04
1e-03
2e-03
3e-03
Log-odds
0.5
1.0
1.5
2.0
2.5
3.0
3.5
4.0
Cellular_organisms - ps1
Archaea1 - ps2
Archaea2 - ps3
Archaea3 - ps4
Asgard/Eukaryota - ps5
Eukaryota - ps6
Amorpheae - ps7
Obazoa - ps8
Opisthokonta - ps9
Holomycota - ps10
Fungi - ps11
Fungi_1 - ps12
Fungi_2 - ps13
Fungi_3 - ps14
Dikarya - ps15
Ascomycota - ps16
Saccharo/Pezizomycotina - ps17
Saccharomycotina - ps18
Saccharomycotina_1 - ps19
Saccharomycotina_2 - ps20
Saccharomycotina_3 - ps21
Phaff./Saccharomycetaceae - ps22
Saccharomyco./mycetaceae - ps23
Saccharomycetaceae - ps24
Saccharomycetaceae_2 - ps25
Saccharomycetaceae_3 - ps26
Kaz./Nau./Saccharomyces - ps27
Saccharomyces - ps28
Arboricola_clade - ps29
Cerevisiae_clade - ps30
Saccharomyces_cerevisiae - ps31
Phylostratum

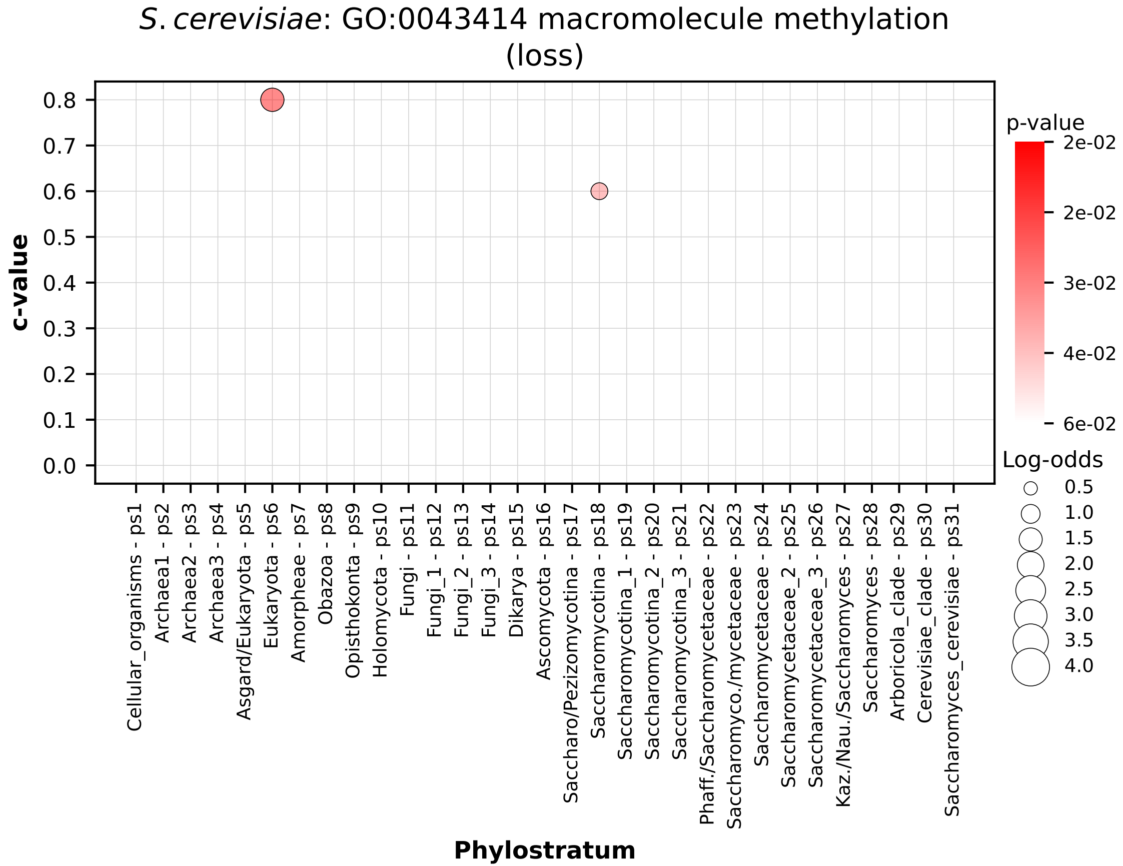

S. cerevisiae: GO:0043414 macromolecule methylation (loss)
c-value
0.0
0.1
0.2
0.3
0.4
0.5
0.6
0.7
0.8
Phylostratum
Cellular_organisms - ps1
Archaea1 - ps2
Archaea2 - ps3
Archaea3 - ps4
Asgard/Eukaryota - ps5
Eukaryota - ps6
Amorpheae - ps7
Obazoa - ps8
Opisthokonta - ps9
Holomycota - ps10
Fungi - ps11
Fungi_1 - ps12
Fungi_2 - ps13
Fungi_3 - ps14
Dikarya - ps15
Ascomycota - ps16
Saccharo/Pezizomycotina - ps17
Saccharomycotina - ps18
Saccharomycotina_1 - ps19
Saccharomycotina_2 - ps20
Saccharomycotina_3 - ps21
Phaff./Saccharomycetaceae - ps22
Saccharomyco./mycetaceae - ps23
Saccharomycetaceae - ps24
Saccharomycetaceae_2 - ps25
Saccharomycetaceae_3 - ps26
Kaz./Nau./Saccharomyces - ps27
Saccharomyces - ps28
Arboricola_clade - ps29
Cerevisiae_clade - ps30
Saccharomyces_cerevisiae - ps31
p-value
2e-02
2e-02
3e-02
4e-02
6e-02
Log-odds
0.5
1.0
1.5
2.0
2.5
3.0
3.5
4.0

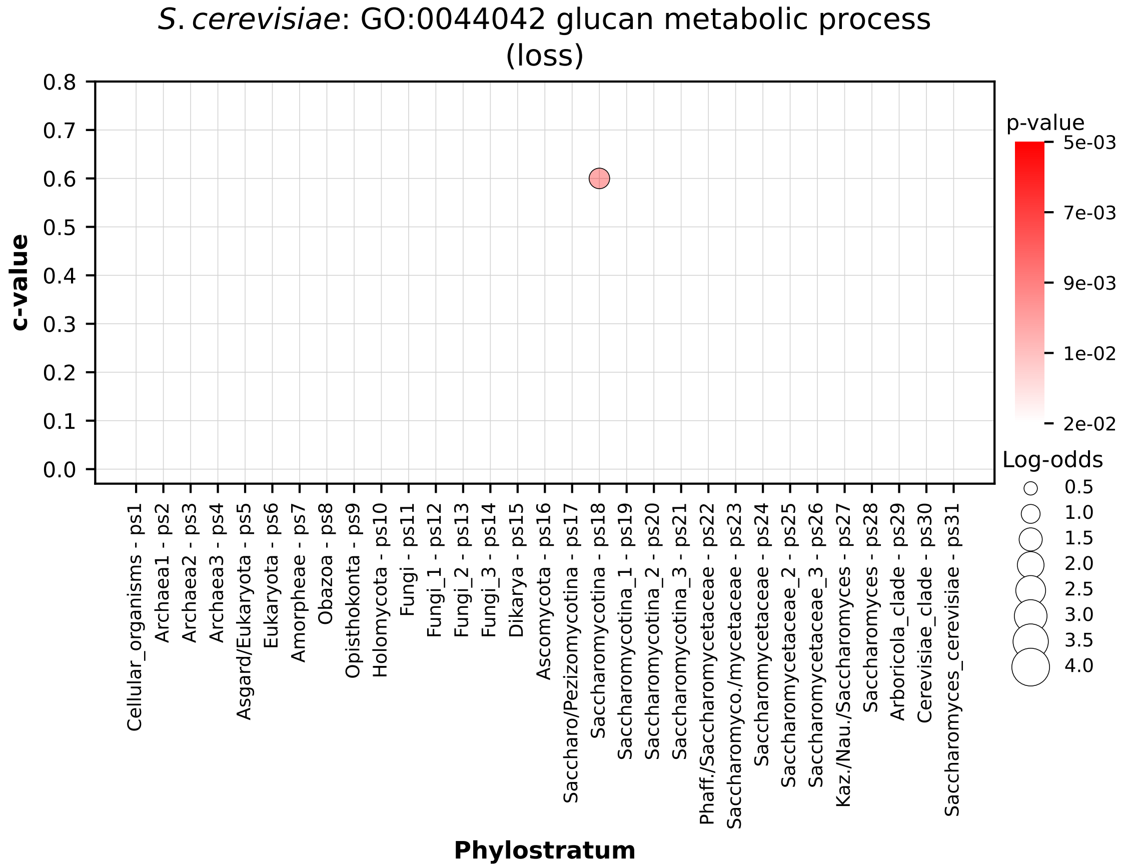
S. cerevisiae: GO:0044042 glucan metabolic process
(loss)
c-value
0.8
0.7
0.6
0.5
0.4
0.3
0.2
0.1
0.0
p-value
5e-03
7e-03
9e-03
1e-02
2e-02
Log-odds
0.5
1.0
1.5
2.0
2.5
3.0
3.5
4.0
Cellular_organisms - ps1
Archaea1 - ps2
Archaea2 - ps3
Archaea3 - ps4
Asgard/Eukaryota - ps5
Eukaryota - ps6
Amorpheae - ps7
Obazoa - ps8
Opisthokonta - ps9
Holomycota - ps10
Fungi - ps11
Fungi_1 - ps12
Fungi_2 - ps13
Fungi_3 - ps14
Dikarya - ps15
Ascomycota - ps16
Saccharo/Pezizomycotina - ps17
Saccharomycotina - ps18
Saccharomycotina_1 - ps19
Saccharomycotina_2 - ps20
Saccharomycotina_3 - ps21
Phaff./Saccharomycetaceae - ps22
Saccharomyco./mycetaceae - ps23
Saccharomycetaceae - ps24
Saccharomycetaceae_2 - ps25
Saccharomycetaceae_3 - ps26
Kaz./Nau./Saccharomyces - ps27
Saccharomyces - ps28
Arboricola_clade - ps29
Cerevisiae_clade - ps30
Saccharomyces_cerevisiae - ps31
Phylostratum

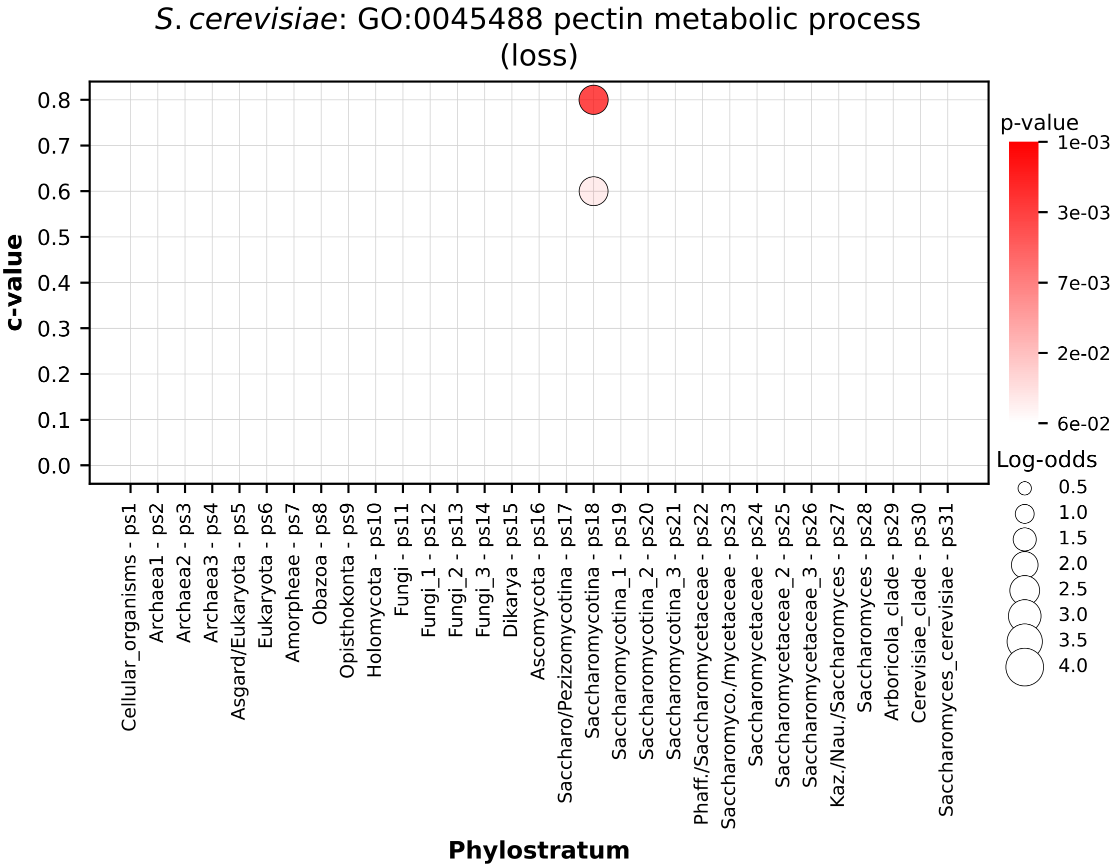

S. cerevisiae: GO:0045488 pectin metabolic process
(loss)
c-value
0.0
0.1
0.2
0.3
0.4
0.5
0.6
0.7
0.8
p-value
1e-03
3e-03
7e-03
2e-02
6e-02
Log-odds
0.5
1.0
1.5
2.0
2.5
3.0
3.5
4.0
Cellular_organisms - ps1
Archaea1 - ps2
Archaea2 - ps3
Archaea3 - ps4
Asgard/Eukaryota - ps5
Eukaryota - ps6
Amorpheae - ps7
Obazoa - ps8
Opisthokonta - ps9
Holomycota - ps10
Fungi - ps11
Fungi_1 - ps12
Fungi_2 - ps13
Fungi_3 - ps14
Dikarya - ps15
Ascomycota - ps16
Saccharo/Pezizomycotina - ps17
Saccharomycotina - ps18
Saccharomycotina_1 - ps19
Saccharomycotina_2 - ps20
Saccharomycotina_3 - ps21
Phaff./Saccharomycetaceae - ps22
Saccharomyco./mycetaceae - ps23
Saccharomycetaceae - ps24
Saccharomycetaceae_2 - ps25
Saccharomycetaceae_3 - ps26
Kaz./Nau./Saccharomyces - ps27
Saccharomyces - ps28
Arboricola_clade - ps29
Cerevisiae_clade - ps30
Saccharomyces_cerevisiae - ps31
Phylostratum

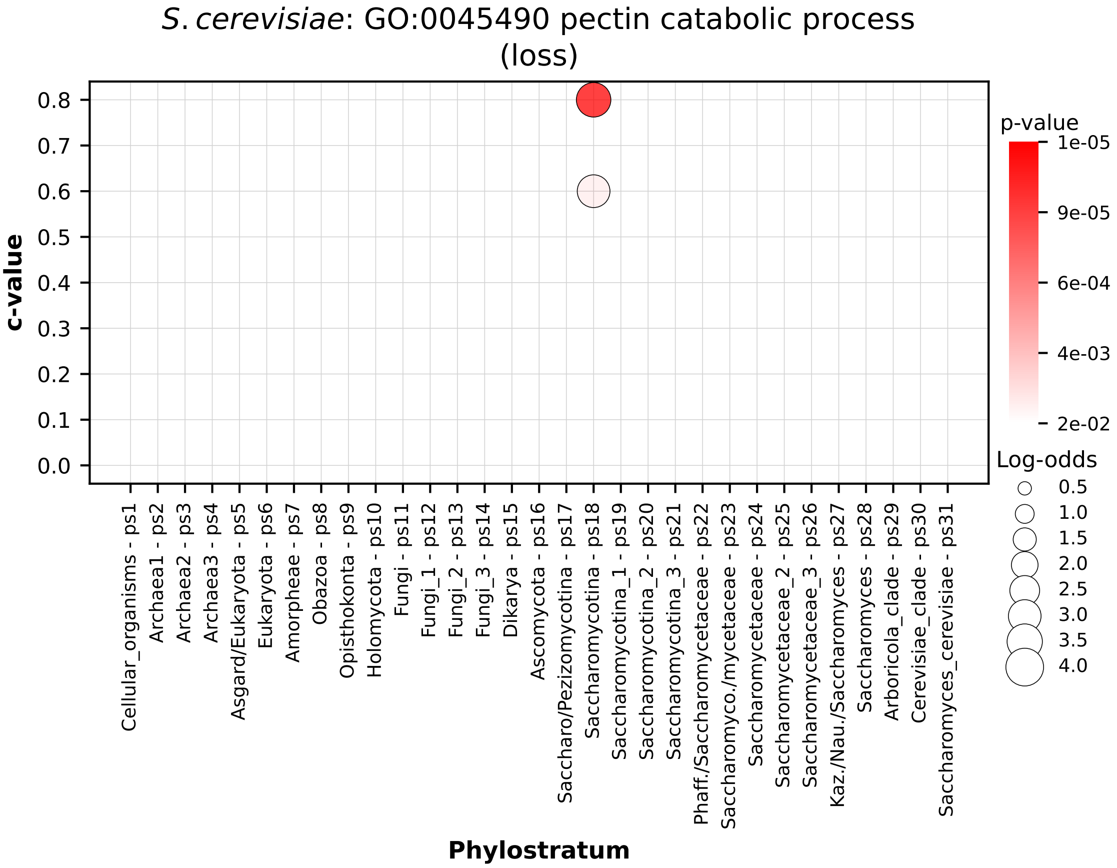
S. cerevisiae: GO:0045490 pectin catabolic process
(loss)
c-value
0.8
0.7
0.6
0.5
0.4
0.3
0.2
0.1
0.0
p-value
1e-05
9e-05
6e-04
4e-03
2e-02
Log-odds
0.5
1.0
1.5
2.0
2.5
3.0
3.5
4.0
Cellular_organisms - ps1
Archaea1 - ps2
Archaea2 - ps3
Archaea3 - ps4
Asgard/Eukaryota - ps5
Eukaryota - ps6
Amorpheae - ps7
Obazoa - ps8
Opisthokonta - ps9
Holomycota - ps10
Fungi - ps11
Fungi_1 - ps12
Fungi_2 - ps13
Fungi_3 - ps14
Dikarya - ps15
Ascomycota - ps16
Saccharo/Pezizomycotina - ps17
Saccharomycotina - ps18
Saccharomycotina_1 - ps19
Saccharomycotina_2 - ps20
Saccharomycotina_3 - ps21
Phaff./Saccharomycetaceae - ps22
Saccharomyco./mycetaceae - ps23
Saccharomycetaceae - ps24
Saccharomycetaceae_2 - ps25
Saccharomycetaceae_3 - ps26
Kaz./Nau./Saccharomyces - ps27
Saccharomyces - ps28
Arboricola_clade - ps29
Cerevisiae_clade - ps30
Saccharomyces_cerevisiae - ps31
Phylostratum

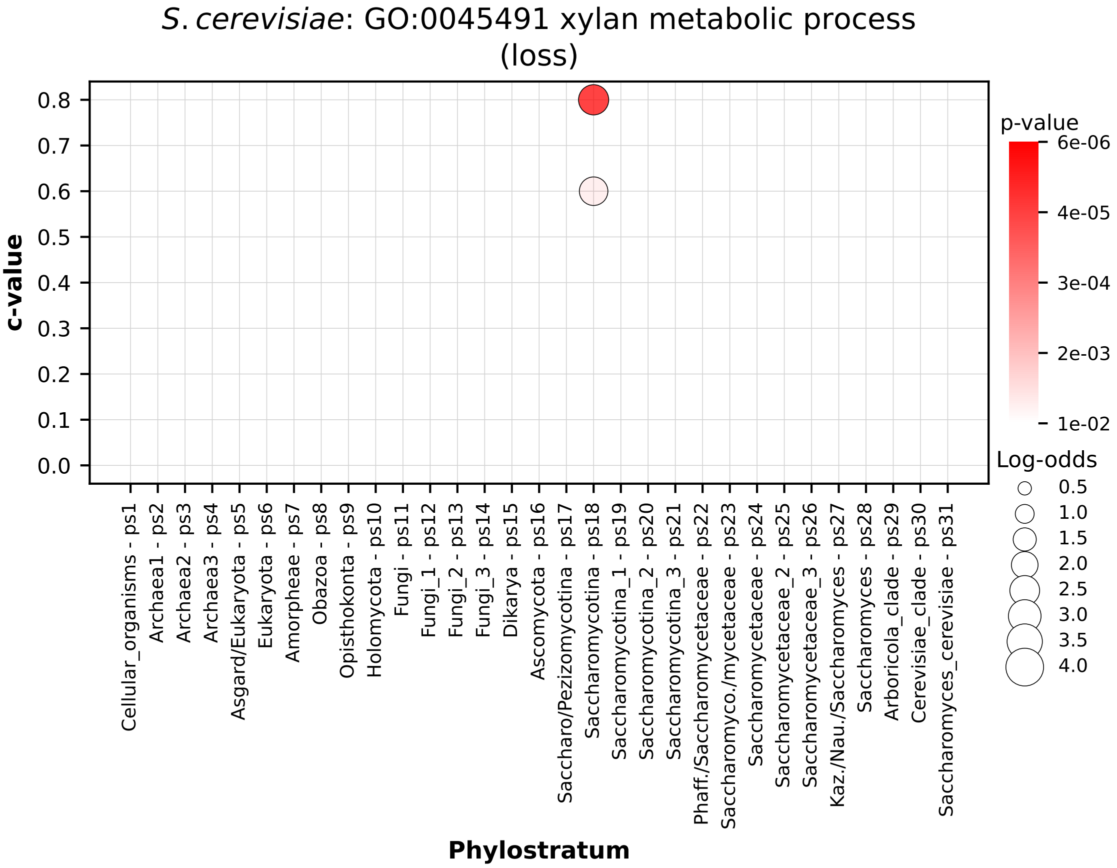
S. cerevisiae: GO:0045491 xylan metabolic process
(loss)
c-value
0.0
0.1
0.2
0.3
0.4
0.5
0.6
0.7
0.8
p-value
6e-06
4e-05
3e-04
2e-03
1e-02
Log-odds
0.5
1.0
1.5
2.0
2.5
3.0
3.5
4.0
Cellular_organisms - ps1
Archaea1 - ps2
Archaea2 - ps3
Archaea3 - ps4
Asgard/Eukaryota - ps5
Eukaryota - ps6
Amorpheae - ps7
Obazoa - ps8
Opisthokonta - ps9
Holomycota - ps10
Fungi - ps11
Fungi_1 - ps12
Fungi_2 - ps13
Fungi_3 - ps14
Dikarya - ps15
Ascomycota - ps16
Saccharo/Pezizomycotina - ps17
Saccharomycotina - ps18
Saccharomycotina_1 - ps19
Saccharomycotina_2 - ps20
Saccharomycotina_3 - ps21
Phaff./Saccharomycetaceae - ps22
Saccharomyco./mycetaceae - ps23
Saccharomycetaceae - ps24
Saccharomycetaceae_2 - ps25
Saccharomycetaceae_3 - ps26
Kaz./Nau./Saccharomyces - ps27
Saccharomyces - ps28
Arboricola_clade - ps29
Cerevisiae_clade - ps30
Saccharomyces_cerevisiae - ps31
Phylostratum

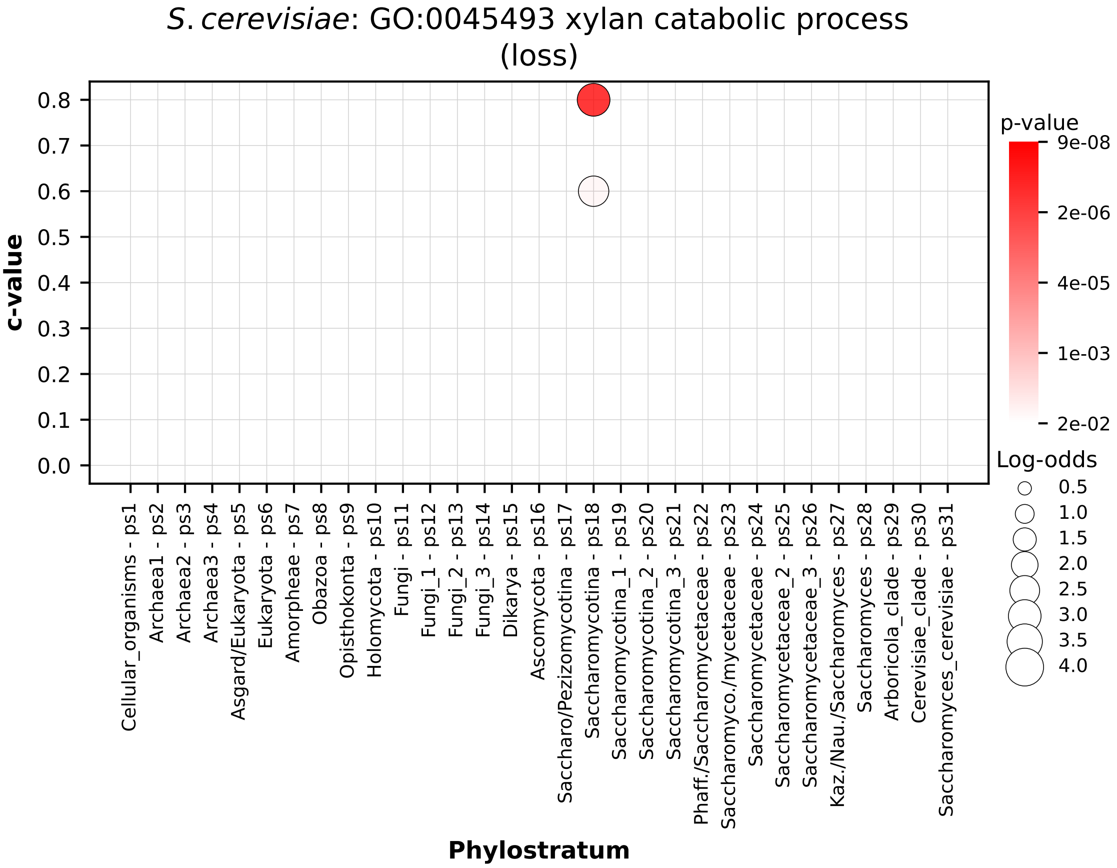

S. cerevisiae: GO:0045493 xylan catabolic process
(loss)
c-value
0.8
0.7
0.6
0.5
0.4
0.3
0.2
0.1
0.0
p-value
9e-08
2e-06
4e-05
1e-03
2e-02
Log-odds
0.5
1.0
1.5
2.0
2.5
3.0
3.5
4.0
Cellular_organisms - ps1
Archaea1 - ps2
Archaea2 - ps3
Archaea3 - ps4
Asgard/Eukaryota - ps5
Eukaryota - ps6
Amorpheae - ps7
Obazoa - ps8
Opisthokonta - ps9
Holomycota - ps10
Fungi - ps11
Fungi_1 - ps12
Fungi_2 - ps13
Fungi_3 - ps14
Dikarya - ps15
Ascomycota - ps16
Saccharo/Pezizomycotina - ps17
Saccharomycotina - ps18
Saccharomycotina_1 - ps19
Saccharomycotina_2 - ps20
Saccharomycotina_3 - ps21
Phaff./Saccharomycetaceae - ps22
Saccharomyco./mycetaceae - ps23
Saccharomycetaceae - ps24
Saccharomycetaceae_2 - ps25
Saccharomycetaceae_3 - ps26
Kaz./Nau./Saccharomyces - ps27
Saccharomyces - ps28
Arboricola_clade - ps29
Cerevisiae_clade - ps30
Saccharomyces_cerevisiae - ps31
Phylostratum

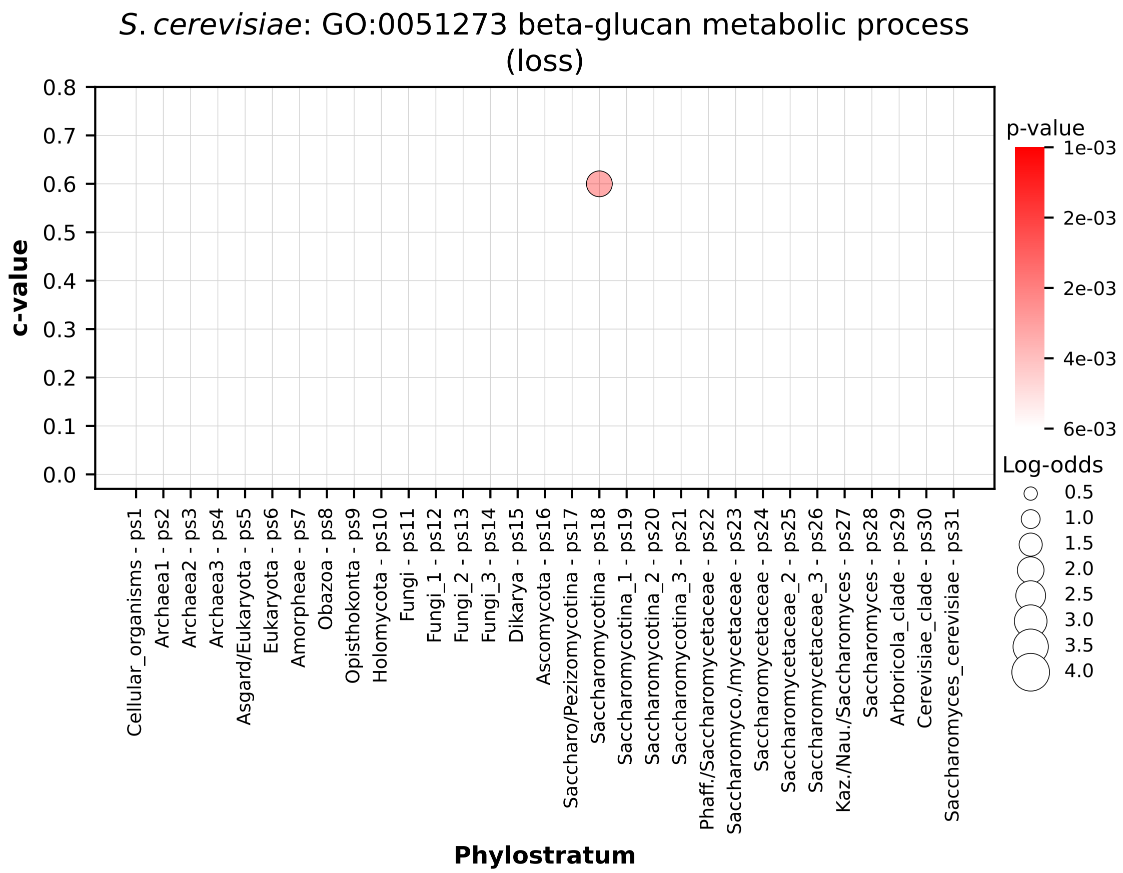

S. cerevisiae: GO:0051273 beta-glucan metabolic process
(loss)
c-value
0.8
0.7
0.6
0.5
0.4
0.3
0.2
0.1
0.0
Phylostratum
Cellular_organisms - ps1
Archaea1 - ps2
Archaea2 - ps3
Archaea3 - ps4
Asgard/Eukaryota - ps5
Eukaryota - ps6
Amorpheae - ps7
Obazoa - ps8
Opisthokonta - ps9
Holomycota - ps10
Fungi - ps11
Fungi_1 - ps12
Fungi_2 - ps13
Fungi_3 - ps14
Dikarya - ps15
Ascomycota - ps16
Saccharo/Pezizomycotina - ps17
Saccharomycotina - ps18
Saccharomycotina_1 - ps19
Saccharomycotina_2 - ps20
Saccharomycotina_3 - ps21
Phaff./Saccharomycetaceae - ps22
Saccharomyco./mycetaceae - ps23
Saccharomycetaceae - ps24
Saccharomycetaceae_2 - ps25
Saccharomycetaceae_3 - ps26
Kaz./Nau./Saccharomyces - ps27
Saccharomyces - ps28
Arboricola_clade - ps29
Cerevisiae_clade - ps30
Saccharomyces_cerevisiae - ps31
p-value
1e-03
2e-03
2e-03
4e-03
6e-03
Log-odds
0.5
1.0
1.5
2.0
2.5
3.0
3.5
4.0

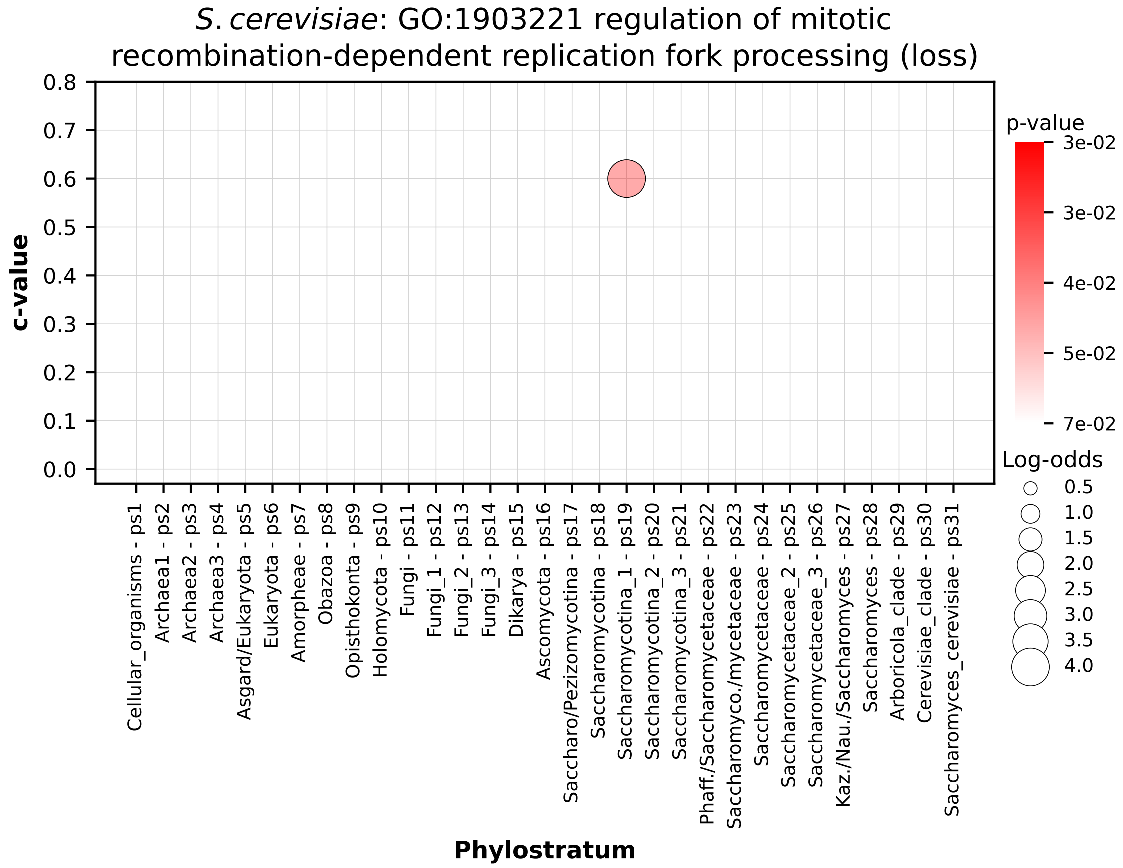

S. cerevisiae: GO:1903221 regulation of mitotic recombination-dependent replication fork processing (loss)
c-value
0.8
0.7
0.6
0.5
0.4
0.3
0.2
0.1
0.0
Phylostratum
Cellular_organisms - ps1
Archaea1 - ps2
Archaea2 - ps3
Archaea3 - ps4
Asgard/Eukaryota - ps5
Eukaryota - ps6
Amorpheae - ps7
Obazoa - ps8
Opisthokonta - ps9
Holomycota - ps10
Fungi - ps11
Fungi_1 - ps12
Fungi_2 - ps13
Fungi_3 - ps14
Dikarya - ps15
Ascomycota - ps16
Saccharo/Pezizomycotina - ps17
Saccharomycotina - ps18
Saccharomycotina_1 - ps19
Saccharomycotina_2 - ps20
Saccharomycotina_3 - ps21
Phaff./Saccharomycetaceae - ps22
Saccharomyco./mycetaceae - ps23
Saccharomycetaceae - ps24
Saccharomycetaceae_2 - ps25
Saccharomycetaceae_3 - ps26
Kaz./Nau./Saccharomyces - ps27
Saccharomyces - ps28
Arboricola_clade - ps29
Cerevisiae_clade - ps30
Saccharomyces_cerevisiae - ps31
p-value
3e-02
3e-02
4e-02
5e-02
7e-02
Log-odds
0.5
1.0
1.5
2.0
2.5
3.0
3.5
4.0

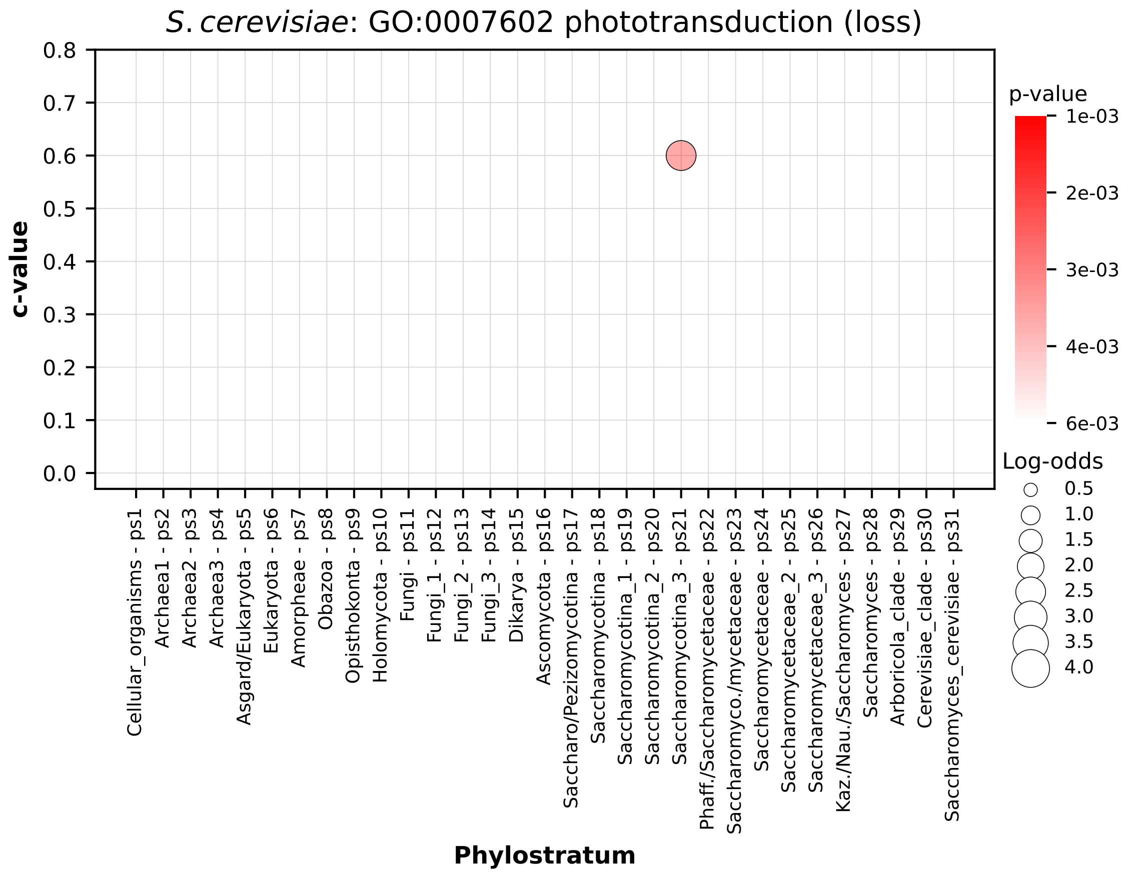
S. cerevisiae: GO:0007602 phototransduction (loss)
c-value
0.8
0.7
0.6
0.5
0.4
0.3
0.2
0.1
0.0
p-value
1e-03
2e-03
3e-03
4e-03
6e-03
Log-odds
0.5
1.0
1.5
2.0
2.5
3.0
3.5
4.0
Cellular_organisms - ps1
Archaea1 - ps2
Archaea2 - ps3
Archaea3 - ps4
Asgard/Eukaryota - ps5
Eukaryota - ps6
Amorpheae - ps7
Obazoa - ps8
Opisthokonta - ps9
Holomycota - ps10
Fungi - ps11
Fungi_1 - ps12
Fungi_2 - ps13
Fungi_3 - ps14
Dikarya - ps15
Ascomycota - ps16
Saccharo/Pezizomycotina - ps17
Saccharomycotina - ps18
Saccharomycotina_1 - ps19
Saccharomycotina_2 - ps20
Saccharomycotina_3 - ps21
Phaff./Saccharomycetaceae - ps22
Saccharomyco./mycetaceae - ps23
Saccharomycetaceae - ps24
Saccharomycetaceae_2 - ps25
Saccharomycetaceae_3 - ps26
Kaz./Nau./Saccharomyces - ps27
Saccharomyces - ps28
Arboricola_clade - ps29
Cerevisiae_clade - ps30
Saccharomyces_cerevisiae - ps31
Phylostratum

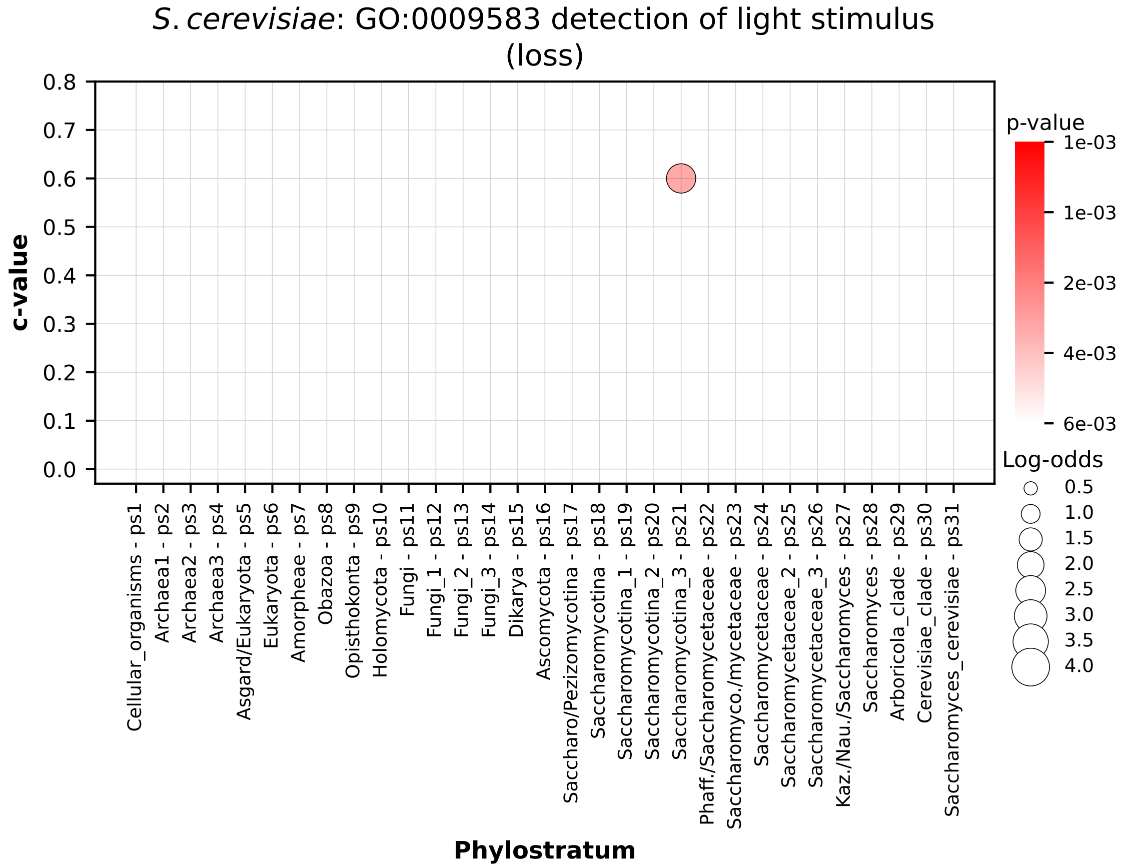

S. cerevisiae: GO:0009583 detection of light stimulus (loss)
c-value
0.8
0.7
0.6
0.5
0.4
0.3
0.2
0.1
0.0
p-value
1e-03
1e-03
2e-03
4e-03
6e-03
Log-odds
0.5
1.0
1.5
2.0
2.5
3.0
3.5
4.0
Cellular_organisms - ps1
Archaea1 - ps2
Archaea2 - ps3
Archaea3 - ps4
Asgard/Eukaryota - ps5
Eukaryota - ps6
Amorpheae - ps7
Obazoa - ps8
Opisthokonta - ps9
Holomycota - ps10
Fungi - ps11
Fungi_1 - ps12
Fungi_2 - ps13
Fungi_3 - ps14
Dikarya - ps15
Ascomycota - ps16
Saccharo/Pezizomycotina - ps17
Saccharomycotina - ps18
Saccharomycotina_1 - ps19
Saccharomycotina_2 - ps20
Saccharomycotina_3 - ps21
Phaff./Saccharomycetaceae - ps22
Saccharomyco./mycetaceae - ps23
Saccharomycetaceae - ps24
Saccharomycetaceae_2 - ps25
Saccharomycetaceae_3 - ps26
Kaz./Nau./Saccharomyces - ps27
Saccharomyces - ps28
Arboricola_clade - ps29
Cerevisiae_clade - ps30
Saccharomyces_cerevisiae - ps31
Phylostratum

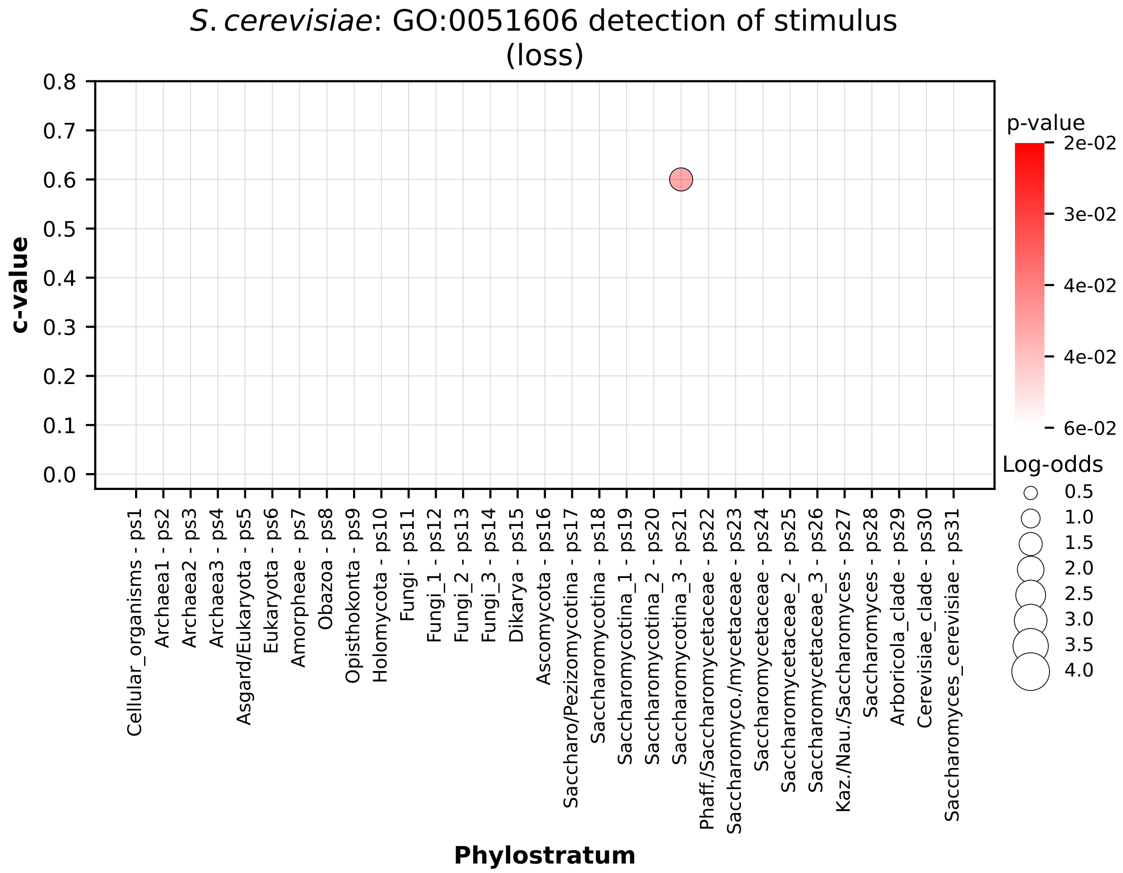

S. cerevisiae: GO:0051606 detection of stimulus
(loss)
c-value
0.8
0.7
0.6
0.5
0.4
0.3
0.2
0.1
0.0
p-value
2e-02
3e-02
4e-02
4e-02
6e-02
Log-odds
0.5
1.0
1.5
2.0
2.5
3.0
3.5
4.0
Cellular_organisms - ps1
Archaea1 - ps2
Archaea2 - ps3
Archaea3 - ps4
Asgard/Eukaryota - ps5
Eukaryota - ps6
Amorpheae - ps7
Obazoa - ps8
Opisthokonta - ps9
Holomycota - ps10
Fungi - ps11
Fungi_1 - ps12
Fungi_2 - ps13
Fungi_3 - ps14
Dikarya - ps15
Ascomycota - ps16
Saccharo/Pezizomycotina - ps17
Saccharomycotina - ps18
Saccharomycotina_1 - ps19
Saccharomycotina_2 - ps20
Saccharomycotina_3 - ps21
Phaff./Saccharomycetaceae - ps22
Saccharomyco./mycetaceae - ps23
Saccharomycetaceae - ps24
Saccharomycetaceae_2 - ps25
Saccharomycetaceae_3 - ps26
Kaz./Nau./Saccharomyces - ps27
Saccharomyces - ps28
Arboricola_clade - ps29
Cerevisiae_clade - ps30
Saccharomyces_cerevisiae - ps31
Phylostratum

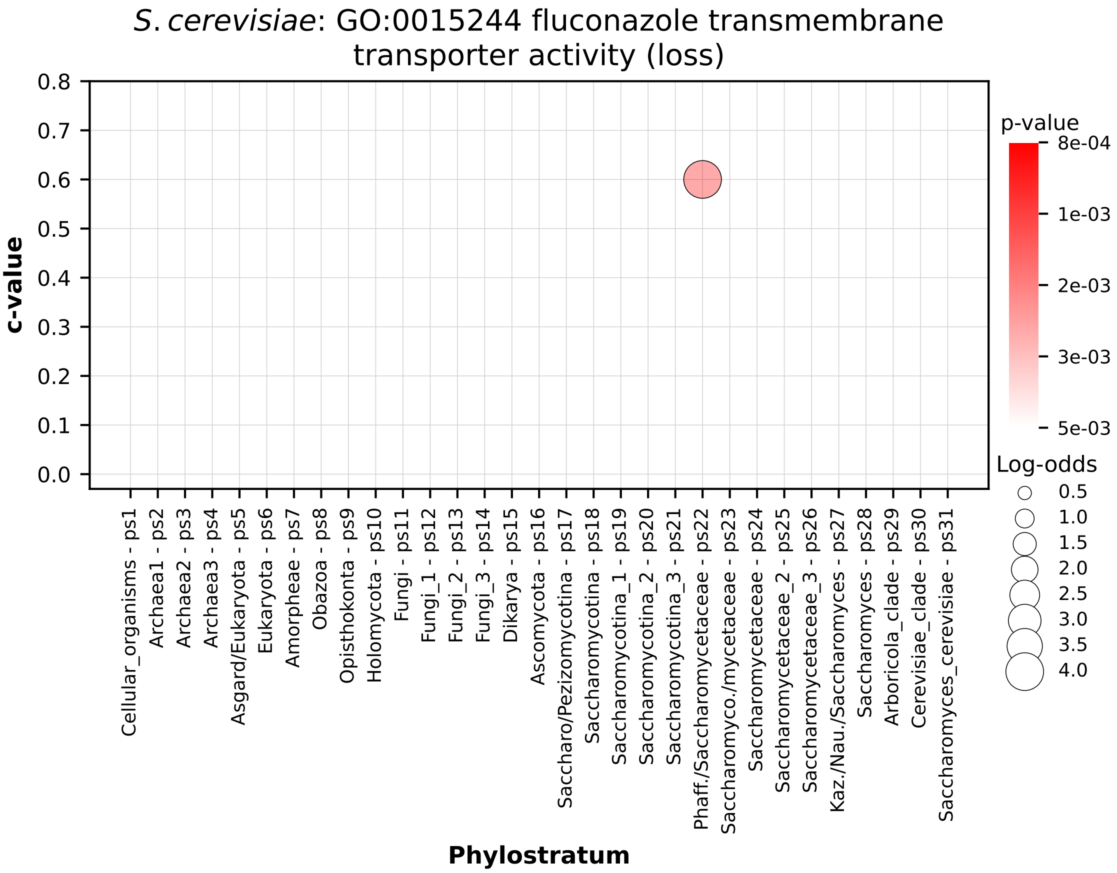

S. cerevisiae: GO:0015244 fluconazole transmembrane transporter activity (loss)
c-value
0.8
0.7
0.6
0.5
0.4
0.3
0.2
0.1
0.0
p-value
8e-04
1e-03
2e-03
3e-03
5e-03
Log-odds
0.5
1.0
1.5
2.0
2.5
3.0
3.5
4.0
Cellular_organisms - ps1
Archaea1 - ps2
Archaea2 - ps3
Archaea3 - ps4
Asgard/Eukaryota - ps5
Eukaryota - ps6
Amorpheae - ps7
Obazoa - ps8
Opisthokonta - ps9
Holomycota - ps10
Fungi - ps11
Fungi_1 - ps12
Fungi_2 - ps13
Fungi_3 - ps14
Dikarya - ps15
Ascomycota - ps16
Saccharo/Pezizomycotina - ps17
Saccharomycotina - ps18
Saccharomycotina_1 - ps19
Saccharomycotina_2 - ps20
Saccharomycotina_3 - ps21
Phaff./Saccharomycetaceae - ps22
Saccharomyco./mycetaceae - ps23
Saccharomycetaceae - ps24
Saccharomycetaceae_2 - ps25
Saccharomycetaceae_3 - ps26
Kaz./Nau./Saccharomyces - ps27
Saccharomyces - ps28
Arboricola_clade - ps29
Cerevisiae_clade - ps30
Saccharomyces_cerevisiae - ps31
Phylostratum

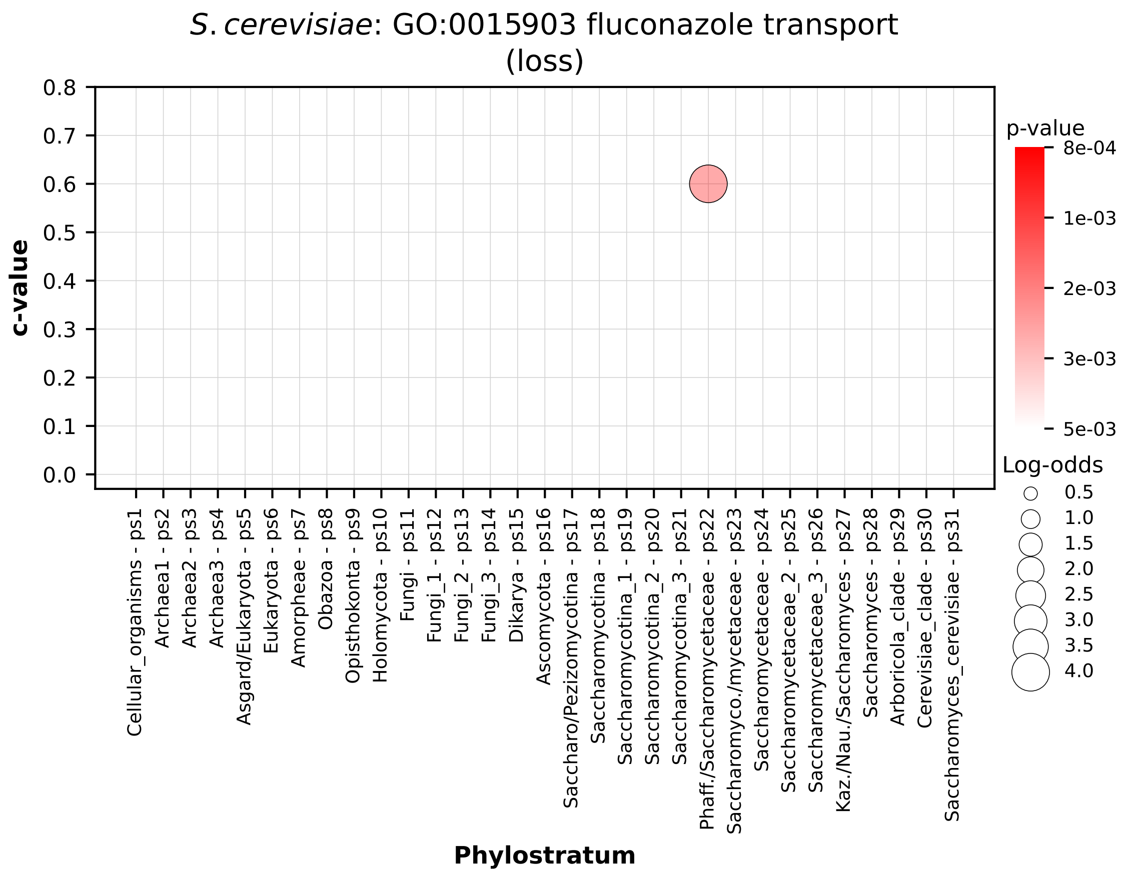
S. cerevisiae: GO:0015903 fluconazole transport
(loss)
c-value
0.8
0.7
0.6
0.5
0.4
0.3
0.2
0.1
0.0
p-value
8e-04
1e-03
2e-03
3e-03
5e-03
Log-odds
0.5
1.0
1.5
2.0
2.5
3.0
3.5
4.0
Cellular_organisms - ps1
Archaea1 - ps2
Archaea2 - ps3
Archaea3 - ps4
Asgard/Eukaryota - ps5
Eukaryota - ps6
Amorpheae - ps7
Obazoa - ps8
Opisthokonta - ps9
Holomycota - ps10
Fungi - ps11
Fungi_1 - ps12
Fungi_2 - ps13
Fungi_3 - ps14
Dikarya - ps15
Ascomycota - ps16
Saccharo/Pezizomycotina - ps17
Saccharomycotina - ps18
Saccharomycotina_1 - ps19
Saccharomycotina_2 - ps20
Saccharomycotina_3 - ps21
Phaff./Saccharomycetaceae - ps22
Saccharomyco./mycetaceae - ps23
Saccharomycetaceae - ps24
Saccharomycetaceae_2 - ps25
Saccharomycetaceae_3 - ps26
Kaz./Nau./Saccharomyces - ps27
Saccharomyces - ps28
Arboricola_clade - ps29
Cerevisiae_clade - ps30
Saccharomyces_cerevisiae - ps31
Phylostratum

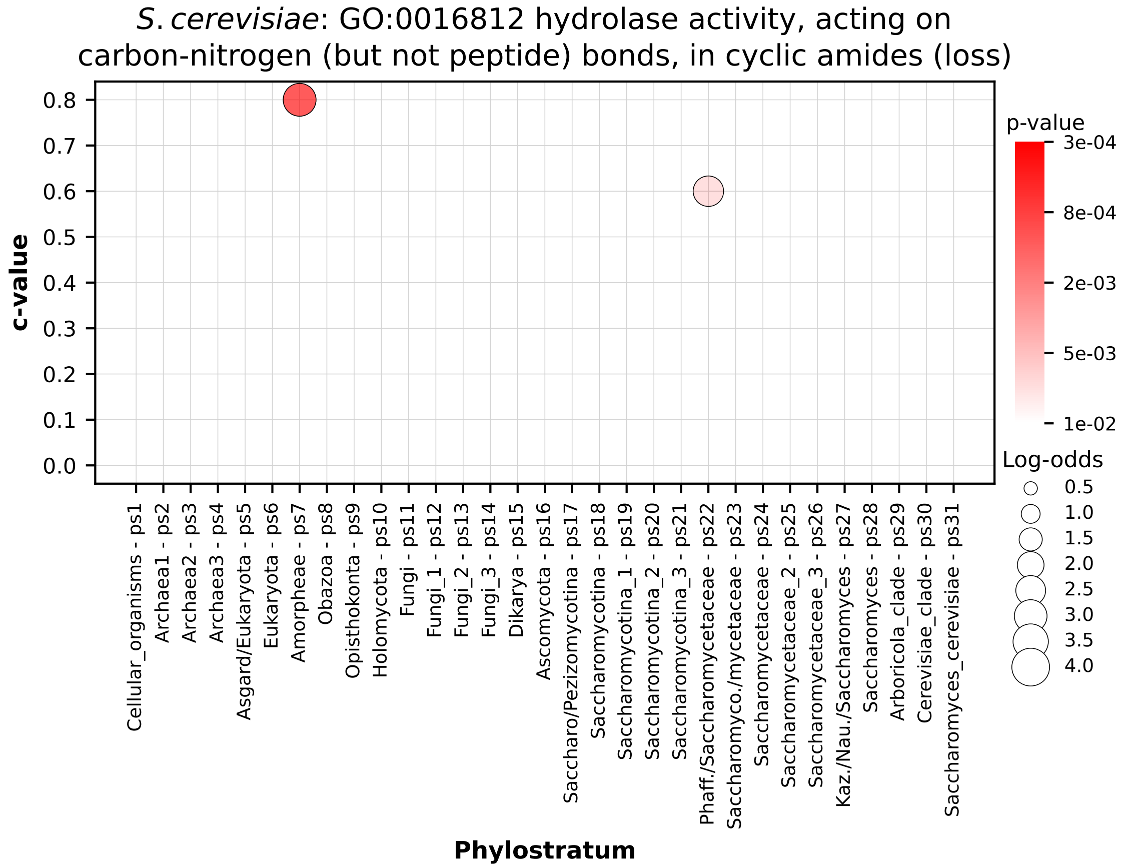
S. cerevisiae: GO:0016812 hydrolase activity, acting on carbon-nitrogen (but not peptide) bonds, in cyclic amides (loss)
c-value
0.0
0.1
0.2
0.3
0.4
0.5
0.6
0.7
0.8
p-value
3e-04
8e-04
2e-03
5e-03
1e-02
Log-odds
0.5
1.0
1.5
2.0
2.5
3.0
3.5
4.0
Cellular_organisms - ps1
Archaea1 - ps2
Archaea2 - ps3
Archaea3 - ps4
Asgard/Eukaryota - ps5
Eukaryota - ps6
Amorpheae - ps7
Obazoa - ps8
Opisthokonta - ps9
Holomycota - ps10
Fungi - ps11
Fungi_1 - ps12
Fungi_2 - ps13
Fungi_3 - ps14
Dikarya - ps15
Ascomycota - ps16
Saccharo/Pezizomycotina - ps17
Saccharomycotina - ps18
Saccharomycotina_1 - ps19
Saccharomycotina_2 - ps20
Saccharomycotina_3 - ps21
Phaff./Saccharomycetaceae - ps22
Saccharomyco./mycetaceae - ps23
Saccharomycetaceae - ps24
Saccharomycetaceae_2 - ps25
Saccharomycetaceae_3 - ps26
Kaz./Nau./Saccharomyces - ps27
Saccharomyces - ps28
Arboricola_clade - ps29
Cerevisiae_clade - ps30
Saccharomyces_cerevisiae - ps31
Phylostratum

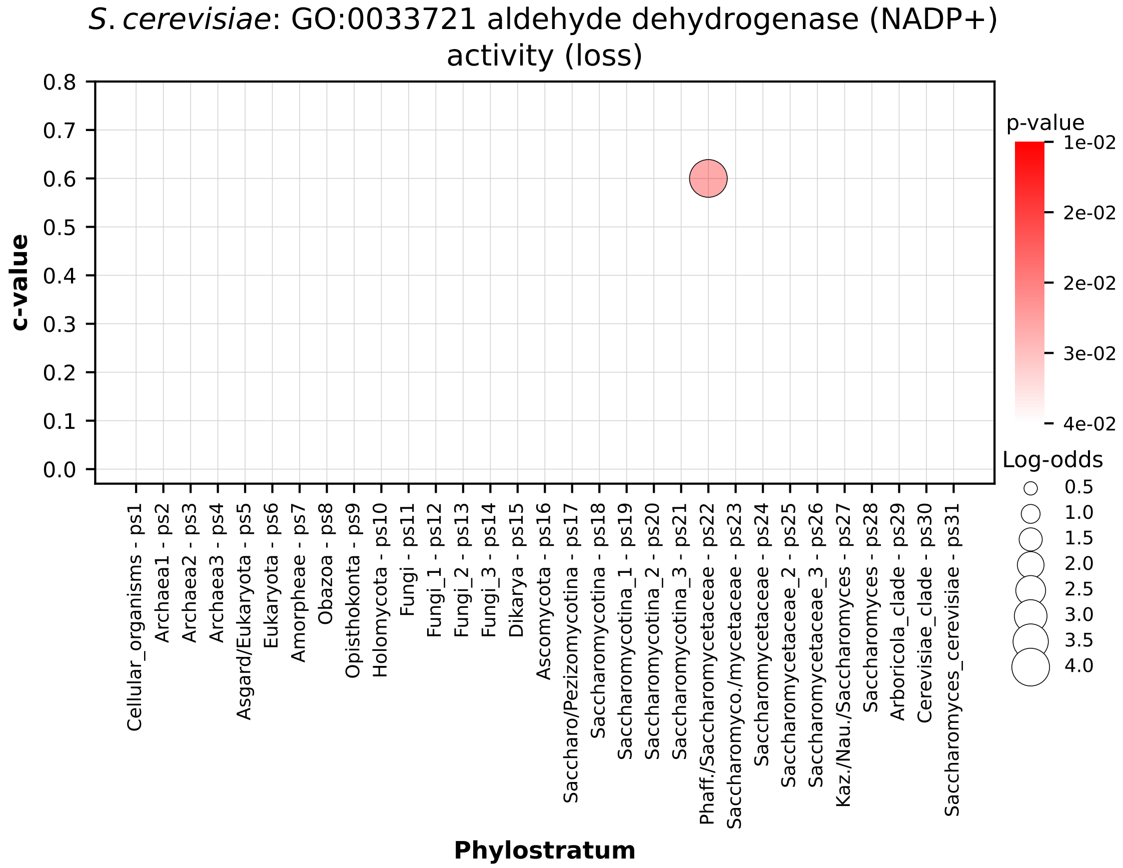
S. cerevisiae: GO:0033721 aldehyde dehydrogenase (NADP+) activity (loss)
c-value
0.8
0.7
0.6
0.5
0.4
0.3
0.2
0.1
0.0
p-value
1e-02
2e-02
2e-02
3e-02
4e-02
Log-odds
0.5
1.0
1.5
2.0
2.5
3.0
3.5
4.0
Cellular_organisms - ps1
Archaea1 - ps2
Archaea2 - ps3
Archaea3 - ps4
Asgard/Eukaryota - ps5
Eukaryota - ps6
Amorpheae - ps7
Obazoa - ps8
Opisthokonta - ps9
Holomycota - ps10
Fungi - ps11
Fungi_1 - ps12
Fungi_2 - ps13
Fungi_3 - ps14
Dikarya - ps15
Ascomycota - ps16
Saccharo/Pezizomycotina - ps17
Saccharomycotina - ps18
Saccharomycotina_1 - ps19
Saccharomycotina_2 - ps20
Saccharomycotina_3 - ps21
Phaff./Saccharomycetaceae - ps22
Saccharomyco./mycetaceae - ps23
Saccharomycetaceae - ps24
Saccharomycetaceae_2 - ps25
Saccharomycetaceae_3 - ps26
Kaz./Nau./Saccharomyces - ps27
Saccharomyces - ps28
Arboricola_clade - ps29
Cerevisiae_clade - ps30
Saccharomyces_cerevisiae - ps31
Phylostratum

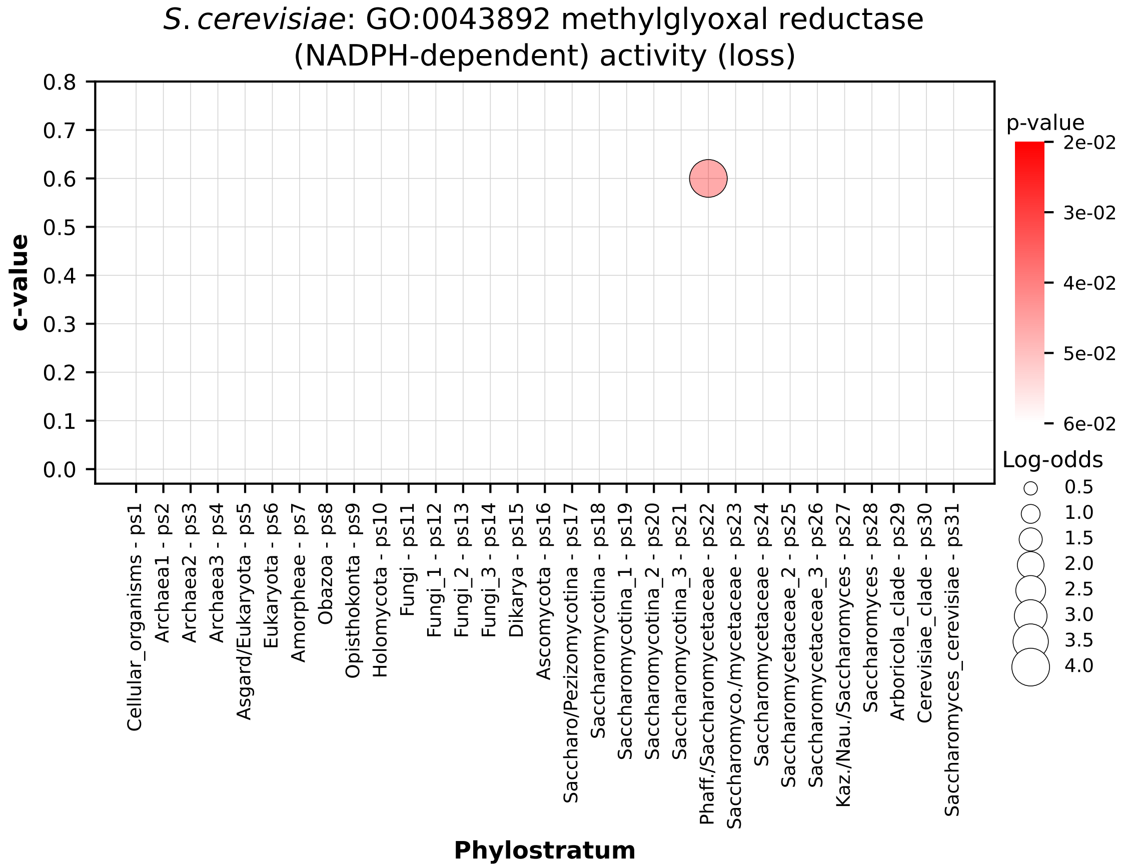

S. cerevisiae: GO:0043892 methylglyoxal reductase (NADPH-dependent) activity (loss)
c-value
0.8
0.7
0.6
0.5
0.4
0.3
0.2
0.1
0.0
p-value
2e-02
3e-02
4e-02
5e-02
6e-02
Log-odds
0.5
1.0
1.5
2.0
2.5
3.0
3.5
4.0
Cellular_organisms - ps1
Archaea1 - ps2
Archaea2 - ps3
Archaea3 - ps4
Asgard/Eukaryota - ps5
Eukaryota - ps6
Amorpheae - ps7
Obazoa - ps8
Opisthokonta - ps9
Holomycota - ps10
Fungi - ps11
Fungi_1 - ps12
Fungi_2 - ps13
Fungi_3 - ps14
Dikarya - ps15
Ascomycota - ps16
Saccharo/Pezizomycotina - ps17
Saccharomycotina - ps18
Saccharomycotina_1 - ps19
Saccharomycotina_2 - ps20
Saccharomycotina_3 - ps21
Phaff./Saccharomycetaceae - ps22
Saccharomyco./mycetaceae - ps23
Saccharomycetaceae - ps24
Saccharomycetaceae_2 - ps25
Saccharomycetaceae_3 - ps26
Kaz./Nau./Saccharomyces - ps27
Saccharomyces - ps28
Arboricola_clade - ps29
Cerevisiae_clade - ps30
Saccharomyces_cerevisiae - ps31
Phylostratum

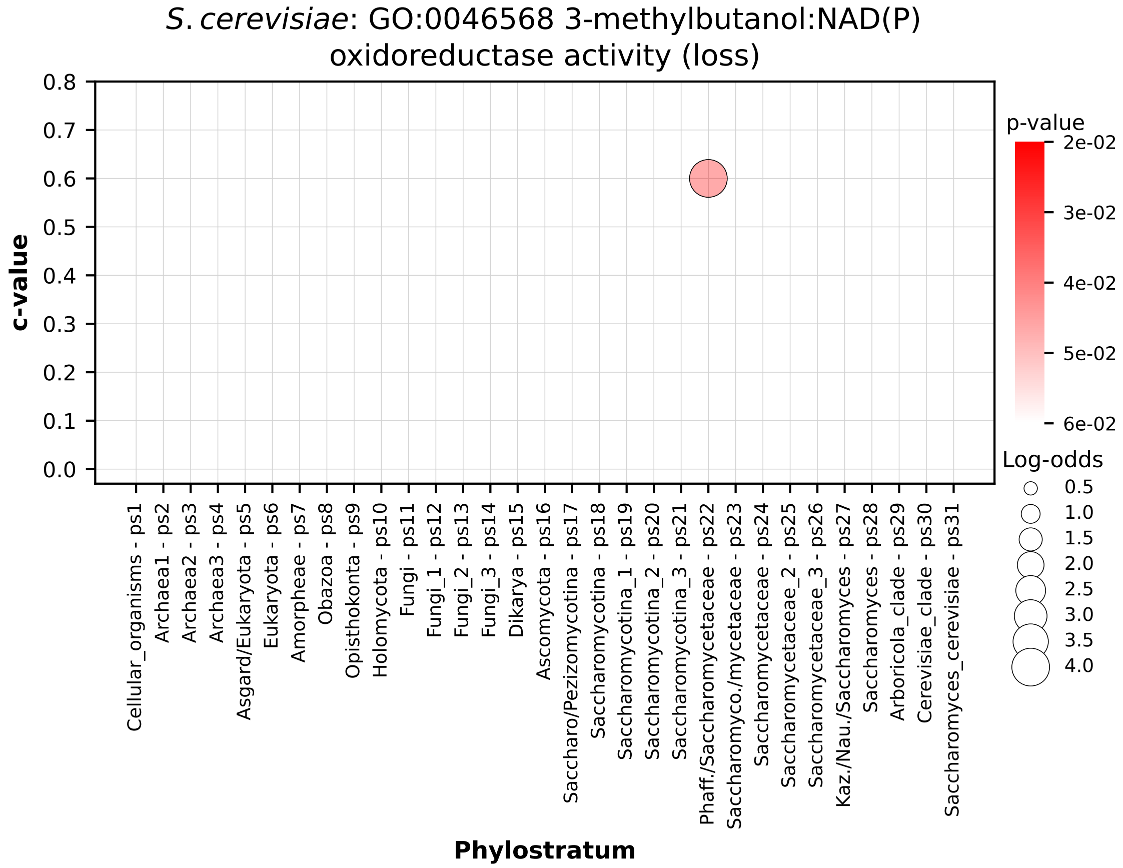
S. cerevisiae: GO:0046568 3-methylbutanol:NAD(P) oxidoreductase activity (loss)
c-value
0.8
0.7
0.6
0.5
0.4
0.3
0.2
0.1
0.0
p-value
2e-02
3e-02
4e-02
5e-02
6e-02
Log-odds
0.5
1.0
1.5
2.0
2.5
3.0
3.5
4.0
Cellular_organisms - ps1
Archaea1 - ps2
Archaea2 - ps3
Archaea3 - ps4
Asgard/Eukaryota - ps5
Eukaryota - ps6
Amorpheae - ps7
Obazoa - ps8
Opisthokonta - ps9
Holomycota - ps10
Fungi - ps11
Fungi_1 - ps12
Fungi_2 - ps13
Fungi_3 - ps14
Dikarya - ps15
Ascomycota - ps16
Saccharo/Pezizomycotina - ps17
Saccharomycotina - ps18
Saccharomycotina_1 - ps19
Saccharomycotina_2 - ps20
Saccharomycotina_3 - ps21
Phaff./Saccharomycetaceae - ps22
Saccharomyco./mycetaceae - ps23
Saccharomycetaceae - ps24
Saccharomycetaceae_2 - ps25
Saccharomycetaceae_3 - ps26
Kaz./Nau./Saccharomyces - ps27
Saccharomyces - ps28
Arboricola_clade - ps29
Cerevisiae_clade - ps30
Saccharomyces_cerevisiae - ps31
Phylostratum

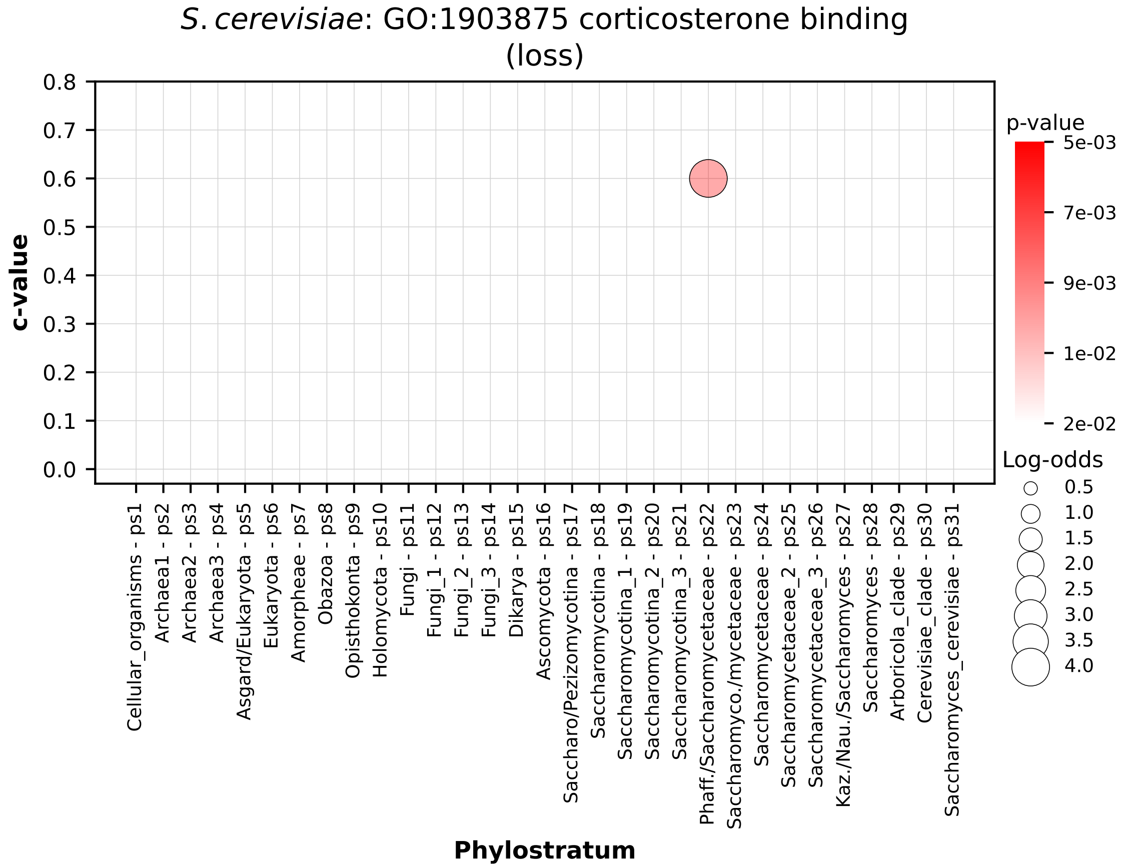
S. cerevisiae: GO:1903875 corticosterone binding
(loss)
c-value
0.8
0.7
0.6
0.5
0.4
0.3
0.2
0.1
0.0
p-value
5e-03
7e-03
9e-03
1e-02
2e-02
Log-odds
0.5
1.0
1.5
2.0
2.5
3.0
3.5
4.0
Cellular_organisms - ps1
Archaea1 - ps2
Archaea2 - ps3
Archaea3 - ps4
Asgard/Eukaryota - ps5
Eukaryota - ps6
Amorpheae - ps7
Obazoa - ps8
Opisthokonta - ps9
Holomycota - ps10
Fungi - ps11
Fungi_1 - ps12
Fungi_2 - ps13
Fungi_3 - ps14
Dikarya - ps15
Ascomycota - ps16
Saccharo/Pezizomycotina - ps17
Saccharomycotina - ps18
Saccharomycotina_1 - ps19
Saccharomycotina_2 - ps20
Saccharomycotina_3 - ps21
Phaff./Saccharomycetaceae - ps22
Saccharomyco./mycetaceae - ps23
Saccharomycetaceae - ps24
Saccharomycetaceae_2 - ps25
Saccharomycetaceae_3 - ps26
Kaz./Nau./Saccharomyces - ps27
Saccharomyces - ps28
Arboricola_clade - ps29
Cerevisiae_clade - ps30
Saccharomyces_cerevisiae - ps31
Phylostratum

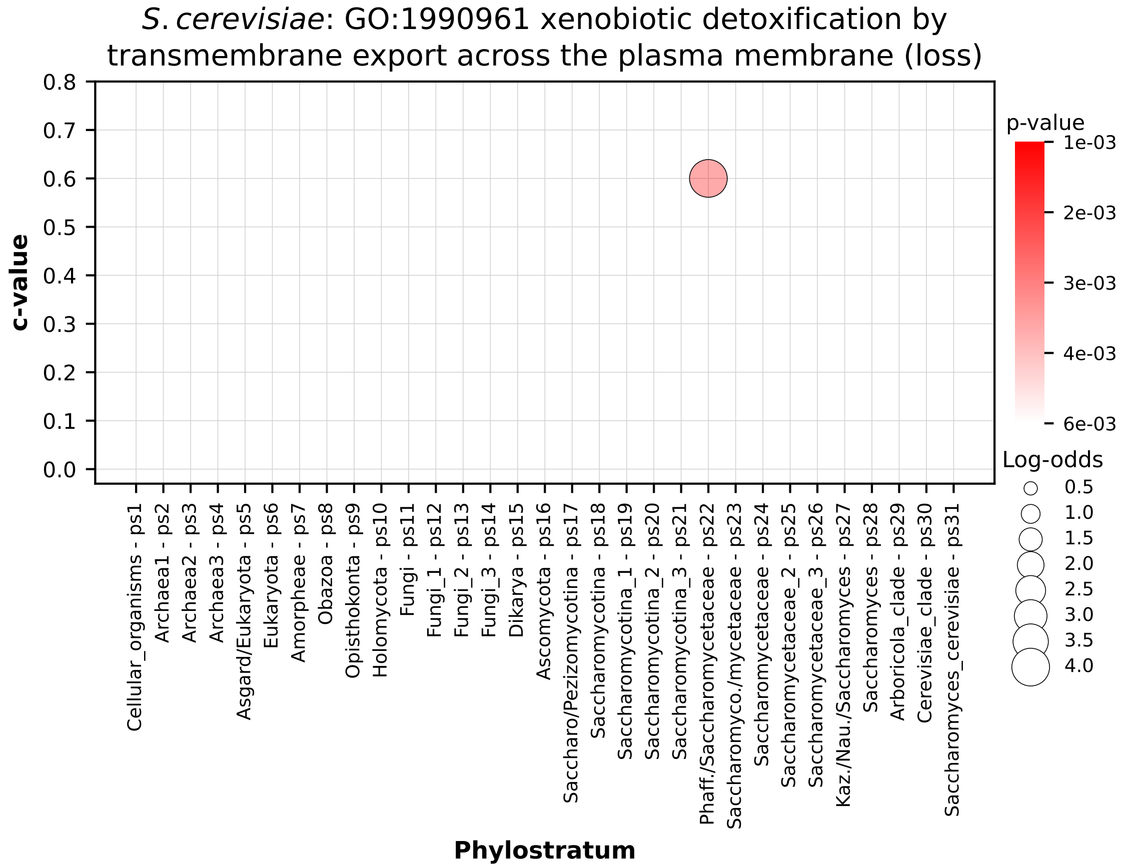

S. cerevisiae: GO:1990961 xenobiotic detoxification by transmembrane export across the plasma membrane (loss)
c-value
0.8
0.7
0.6
0.5
0.4
0.3
0.2
0.1
0.0
p-value
1e-03
2e-03
3e-03
4e-03
6e-03
Log-odds
0.5
1.0
1.5
2.0
2.5
3.0
3.5
4.0
Cellular_organisms - ps1
Archaea1 - ps2
Archaea2 - ps3
Archaea3 - ps4
Asgard/Eukaryota - ps5
Eukaryota - ps6
Amorpheae - ps7
Obazoa - ps8
Opisthokonta - ps9
Holomycota - ps10
Fungi - ps11
Fungi_1 - ps12
Fungi_2 - ps13
Fungi_3 - ps14
Dikarya - ps15
Ascomycota - ps16
Saccharo/Pezizomycotina - ps17
Saccharomycotina - ps18
Saccharomycotina_1 - ps19
Saccharomycotina_2 - ps20
Saccharomycotina_3 - ps21
Phaff./Saccharomycetaceae - ps22
Saccharomyco./mycetaceae - ps23
Saccharomycetaceae - ps24
Saccharomycetaceae_2 - ps25
Saccharomycetaceae_3 - ps26
Kaz./Nau./Saccharomyces - ps27
Saccharomyces - ps28
Arboricola_clade - ps29
Cerevisiae_clade - ps30
Saccharomyces_cerevisiae - ps31
Phylostratum

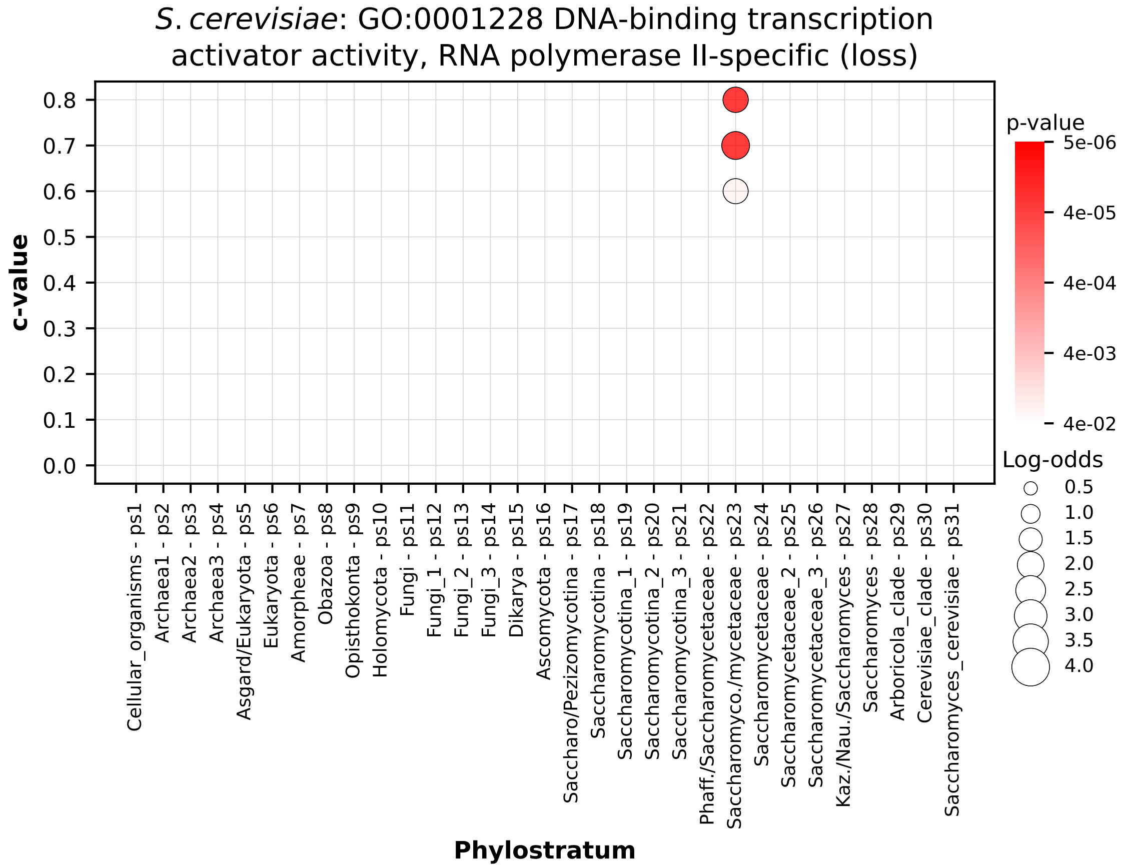

S. cerevisiae: GO:0001228 DNA-binding transcription activator activity, RNA polymerase II-specific (loss)
c-value
0.0
0.1
0.2
0.3
0.4
0.5
0.6
0.7
0.8
Phylostratum
Cellular_organisms - ps1
Archaea1 - ps2
Archaea2 - ps3
Archaea3 - ps4
Asgard/Eukaryota - ps5
Eukaryota - ps6
Amorpheae - ps7
Obazoa - ps8
Opisthokonta - ps9
Holomycota - ps10
Fungi - ps11
Fungi_1 - ps12
Fungi_2 - ps13
Fungi_3 - ps14
Dikarya - ps15
Ascomycota - ps16
Saccharo/Pezizomycotina - ps17
Saccharomycotina - ps18
Saccharomycotina_1 - ps19
Saccharomycotina_2 - ps20
Saccharomycotina_3 - ps21
Phaff./Saccharomycetaceae - ps22
Saccharomyco./mycetaceae - ps23
Saccharomycetaceae - ps24
Saccharomycetaceae_2 - ps25
Saccharomycetaceae_3 - ps26
Kaz./Nau./Saccharomyces - ps27
Saccharomyces - ps28
Arboricola_clade - ps29
Cerevisiae_clade - ps30
Saccharomyces_cerevisiae - ps31
p-value
5e-06
4e-05
4e-04
4e-03
4e-02
Log-odds
0.5
1.0
1.5
2.0
2.5
3.0
3.5
4.0

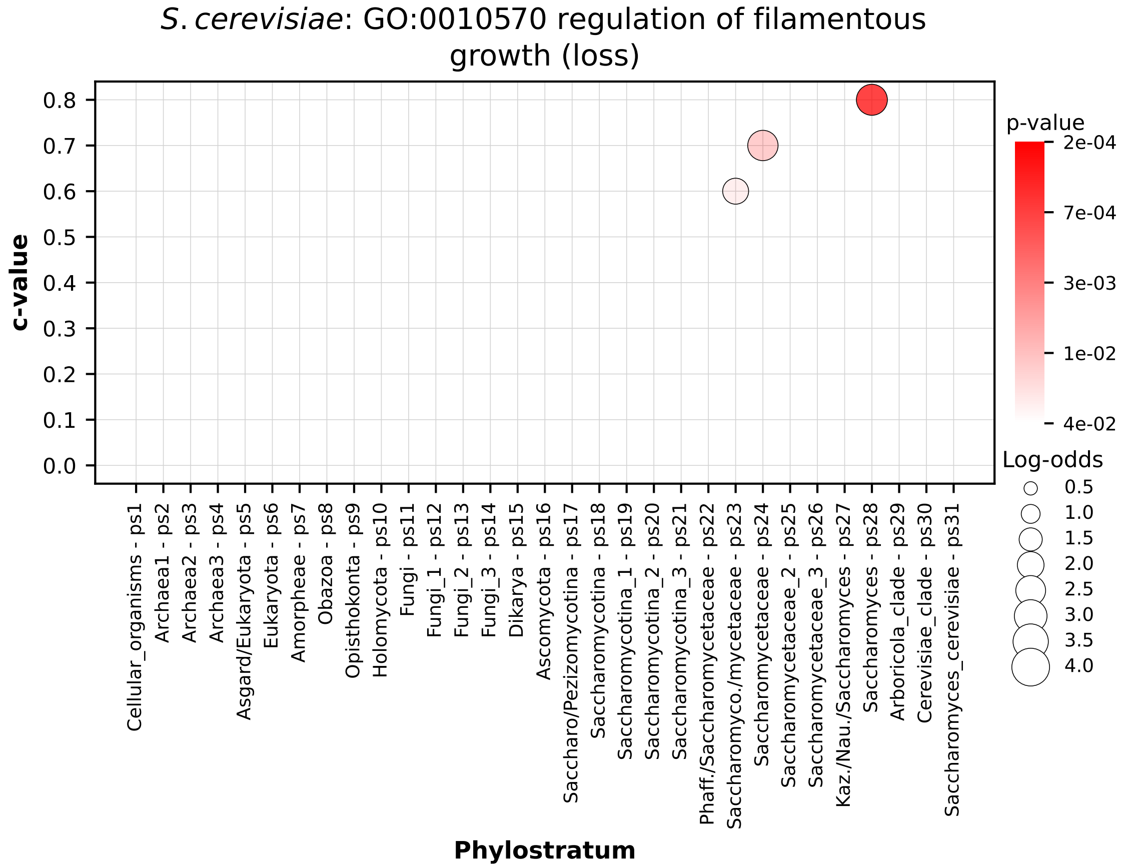

S. cerevisiae: GO:0010570 regulation of filamentous growth (loss)
c-value
0.8
0.7
0.6
0.5
0.4
0.3
0.2
0.1
0.0
p-value
2e-04
7e-04
3e-03
1e-02
4e-02
Log-odds
0.5
1.0
1.5
2.0
2.5
3.0
3.5
4.0
Cellular_organisms - ps1
Archaea1 - ps2
Archaea2 - ps3
Archaea3 - ps4
Asgard/Eukaryota - ps5
Eukaryota - ps6
Amorpheae - ps7
Obazoa - ps8
Opisthokonta - ps9
Holomycota - ps10
Fungi - ps11
Fungi_1 - ps12
Fungi_2 - ps13
Fungi_3 - ps14
Dikarya - ps15
Ascomycota - ps16
Saccharo/Pezizomycotina - ps17
Saccharomycotina - ps18
Saccharomycotina_1 - ps19
Saccharomycotina_2 - ps20
Saccharomycotina_3 - ps21
Phaff./Saccharomycetaceae - ps22
Saccharomyco./mycetaceae - ps23
Saccharomycetaceae - ps24
Saccharomycetaceae_2 - ps25
Saccharomycetaceae_3 - ps26
Kaz./Nau./Saccharomyces - ps27
Saccharomyces - ps28
Arboricola_clade - ps29
Cerevisiae_clade - ps30
Saccharomyces_cerevisiae - ps31
Phylostratum

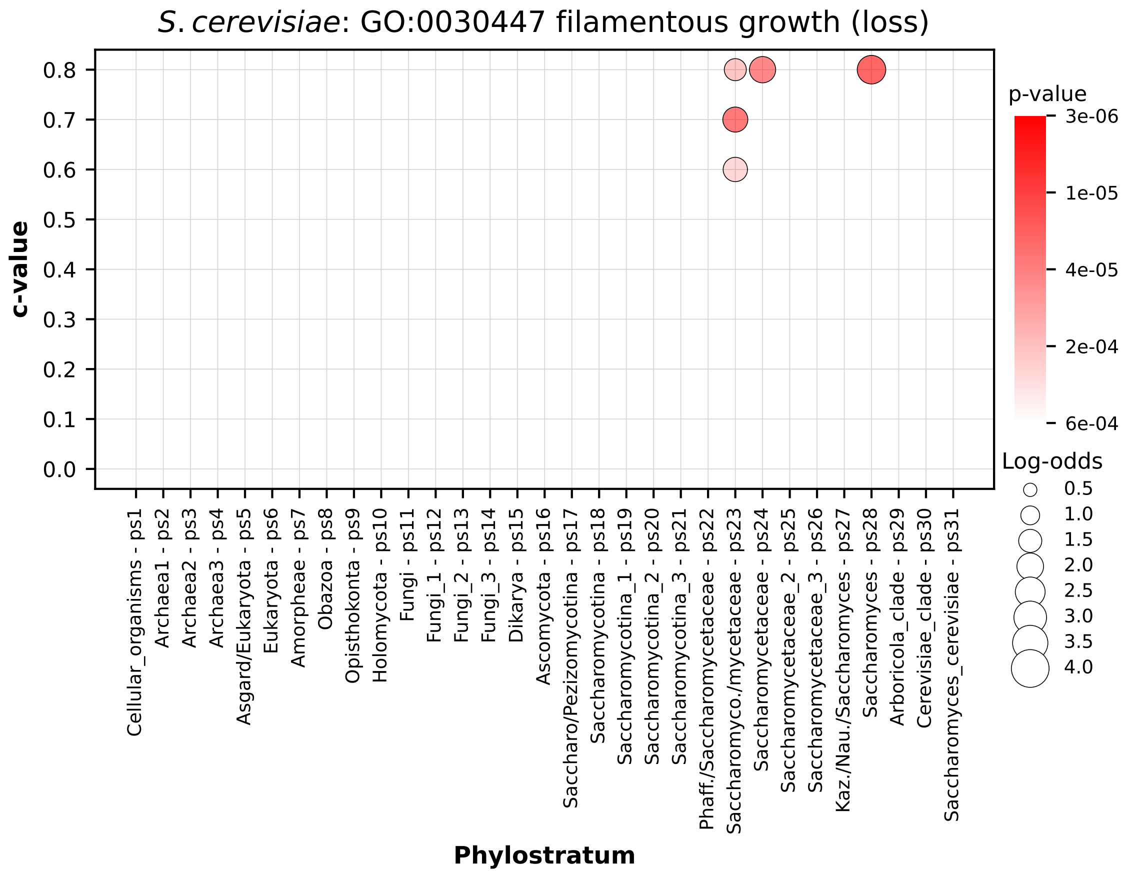
S. cerevisiae: GO:0030447 filamentous growth (loss)
c-value
0.8
0.7
0.6
0.5
0.4
0.3
0.2
0.1
0.0
p-value
3e-06
1e-05
4e-05
2e-04
6e-04
Log-odds
0.5
1.0
1.5
2.0
2.5
3.0
3.5
4.0
Cellular_organisms - ps1
Archaea1 - ps2
Archaea2 - ps3
Archaea3 - ps4
Asgard/Eukaryota - ps5
Eukaryota - ps6
Amorpheae - ps7
Obazoa - ps8
Opisthokonta - ps9
Holomycota - ps10
Fungi - ps11
Fungi_1 - ps12
Fungi_2 - ps13
Fungi_3 - ps14
Dikarya - ps15
Ascomycota - ps16
Saccharo/Pezizomycotina - ps17
Saccharomycotina - ps18
Saccharomycotina_1 - ps19
Saccharomycotina_2 - ps20
Saccharomycotina_3 - ps21
Phaff./Saccharomycetaceae - ps22
Saccharomyco./mycetaceae - ps23
Saccharomycetaceae - ps24
Saccharomycetaceae_2 - ps25
Saccharomycetaceae_3 - ps26
Kaz./Nau./Saccharomyces - ps27
Saccharomyces - ps28
Arboricola_clade - ps29
Cerevisiae_clade - ps30
Saccharomyces_cerevisiae - ps31
Phylostratum

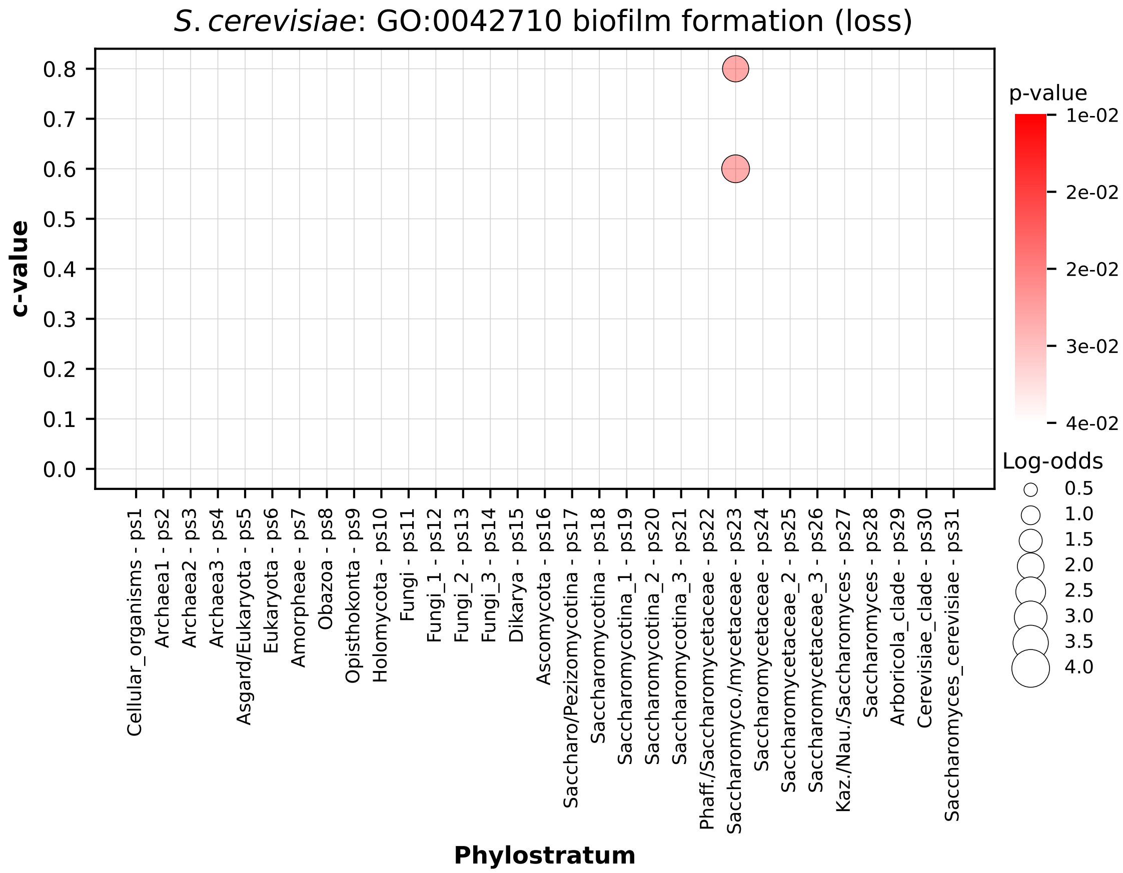
S. cerevisiae: GO:0042710 biofilm formation (loss)
c-value
0.8
0.7
0.6
0.5
0.4
0.3
0.2
0.1
0.0
p-value
1e-02
2e-02
2e-02
3e-02
4e-02
Log-odds
0.5
1.0
1.5
2.0
2.5
3.0
3.5
4.0
Cellular_organisms - ps1
Archaea1 - ps2
Archaea2 - ps3
Archaea3 - ps4
Asgard/Eukaryota - ps5
Eukaryota - ps6
Amorpheae - ps7
Obazoa - ps8
Opisthokonta - ps9
Holomycota - ps10
Fungi - ps11
Fungi_1 - ps12
Fungi_2 - ps13
Fungi_3 - ps14
Dikarya - ps15
Ascomycota - ps16
Saccharo/Pezizomycotina - ps17
Saccharomycotina - ps18
Saccharomycotina_1 - ps19
Saccharomycotina_2 - ps20
Saccharomycotina_3 - ps21
Phaff./Saccharomycetaceae - ps22
Saccharomyco./mycetaceae - ps23
Saccharomycetaceae - ps24
Saccharomycetaceae_2 - ps25
Saccharomycetaceae_3 - ps26
Kaz./Nau./Saccharomyces - ps27
Saccharomyces - ps28
Arboricola_clade - ps29
Cerevisiae_clade - ps30
Saccharomyces_cerevisiae - ps31
Phylostratum

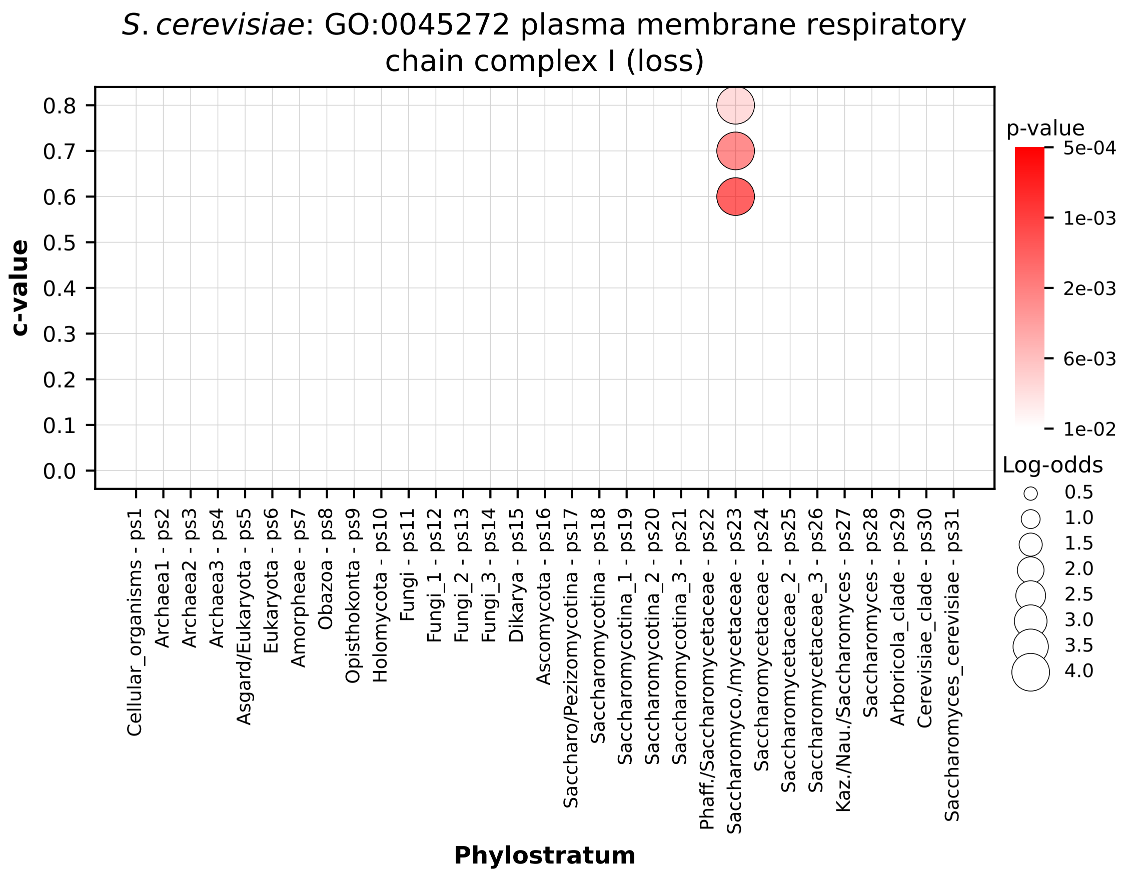

S. cerevisiae: GO:0045272 plasma membrane respiratory chain complex I (loss)
c-value
0.0
0.1
0.2
0.3
0.4
0.5
0.6
0.7
0.8
p-value
5e-04
1e-03
2e-03
6e-03
1e-02
Log-odds
0.5
1.0
1.5
2.0
2.5
3.0
3.5
4.0
Cellular_organisms - ps1
Archaea1 - ps2
Archaea2 - ps3
Archaea3 - ps4
Asgard/Eukaryota - ps5
Eukaryota - ps6
Amorpheae - ps7
Obazoa - ps8
Opisthokonta - ps9
Holomycota - ps10
Fungi - ps11
Fungi_1 - ps12
Fungi_2 - ps13
Fungi_3 - ps14
Dikarya - ps15
Ascomycota - ps16
Saccharo/Pezizomycotina - ps17
Saccharomycotina - ps18
Saccharomycotina_1 - ps19
Saccharomycotina_2 - ps20
Saccharomycotina_3 - ps21
Phaff./Saccharomycetaceae - ps22
Saccharomyco./mycetaceae - ps23
Saccharomycetaceae - ps24
Saccharomycetaceae_2 - ps25
Saccharomycetaceae_3 - ps26
Kaz./Nau./Saccharomyces - ps27
Saccharomyces - ps28
Arboricola_clade - ps29
Cerevisiae_clade - ps30
Saccharomyces_cerevisiae - ps31
Phylostratum

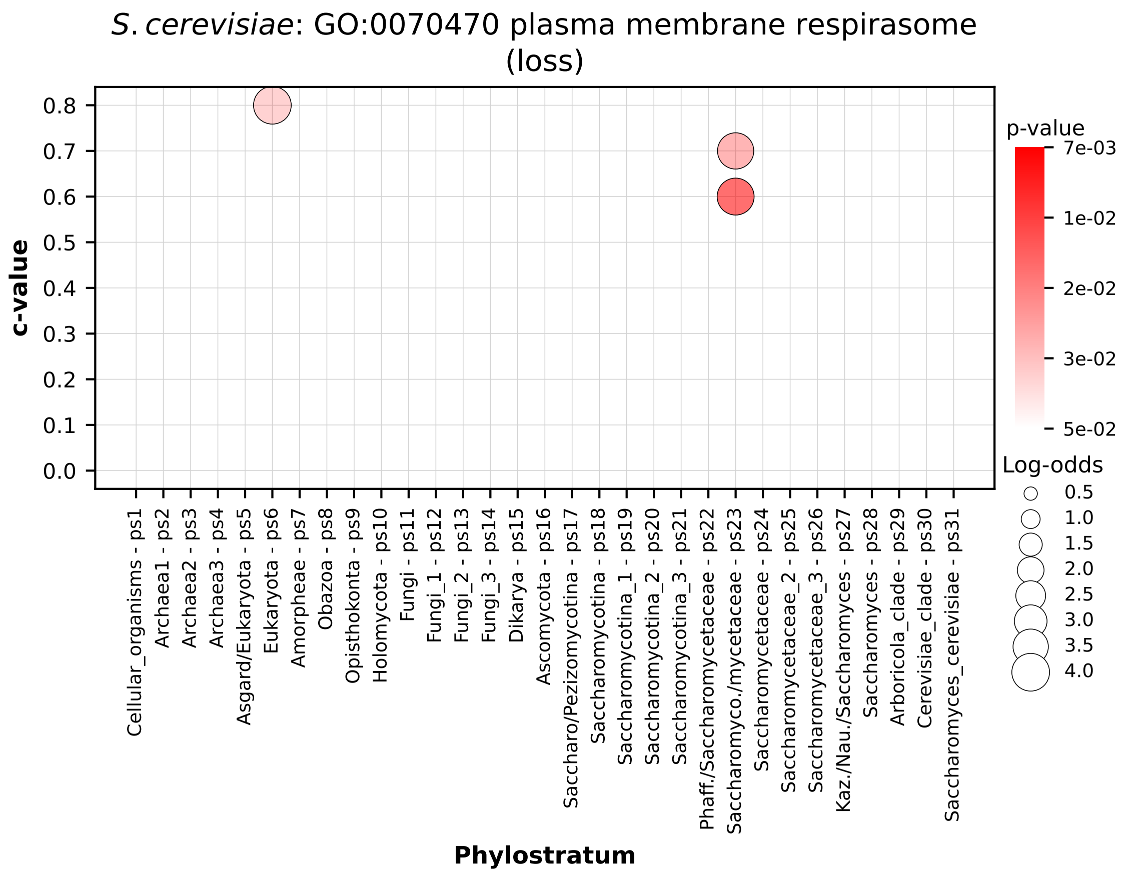

S. cerevisiae: GO:0070470 plasma membrane respirasome
(loss)
c-value
0.8
0.7
0.6
0.5
0.4
0.3
0.2
0.1
0.0
p-value
7e-03
1e-02
2e-02
3e-02
5e-02
Log-odds
0.5
1.0
1.5
2.0
2.5
3.0
3.5
4.0
Cellular_organisms - ps1
Archaea1 - ps2
Archaea2 - ps3
Archaea3 - ps4
Asgard/Eukaryota - ps5
Eukaryota - ps6
Amorpheae - ps7
Obazoa - ps8
Opisthokonta - ps9
Holomycota - ps10
Fungi - ps11
Fungi_1 - ps12
Fungi_2 - ps13
Fungi_3 - ps14
Dikarya - ps15
Ascomycota - ps16
Saccharo/Pezizomycotina - ps17
Saccharomycotina - ps18
Saccharomycotina_1 - ps19
Saccharomycotina_2 - ps20
Saccharomycotina_3 - ps21
Phaff./Saccharomycetaceae - ps22
Saccharomyco./mycetaceae - ps23
Saccharomycetaceae - ps24
Saccharomycetaceae_2 - ps25
Saccharomycetaceae_3 - ps26
Kaz./Nau./Saccharomyces - ps27
Saccharomyces - ps28
Arboricola_clade - ps29
Cerevisiae_clade - ps30
Saccharomyces_cerevisiae - ps31
Phylostratum

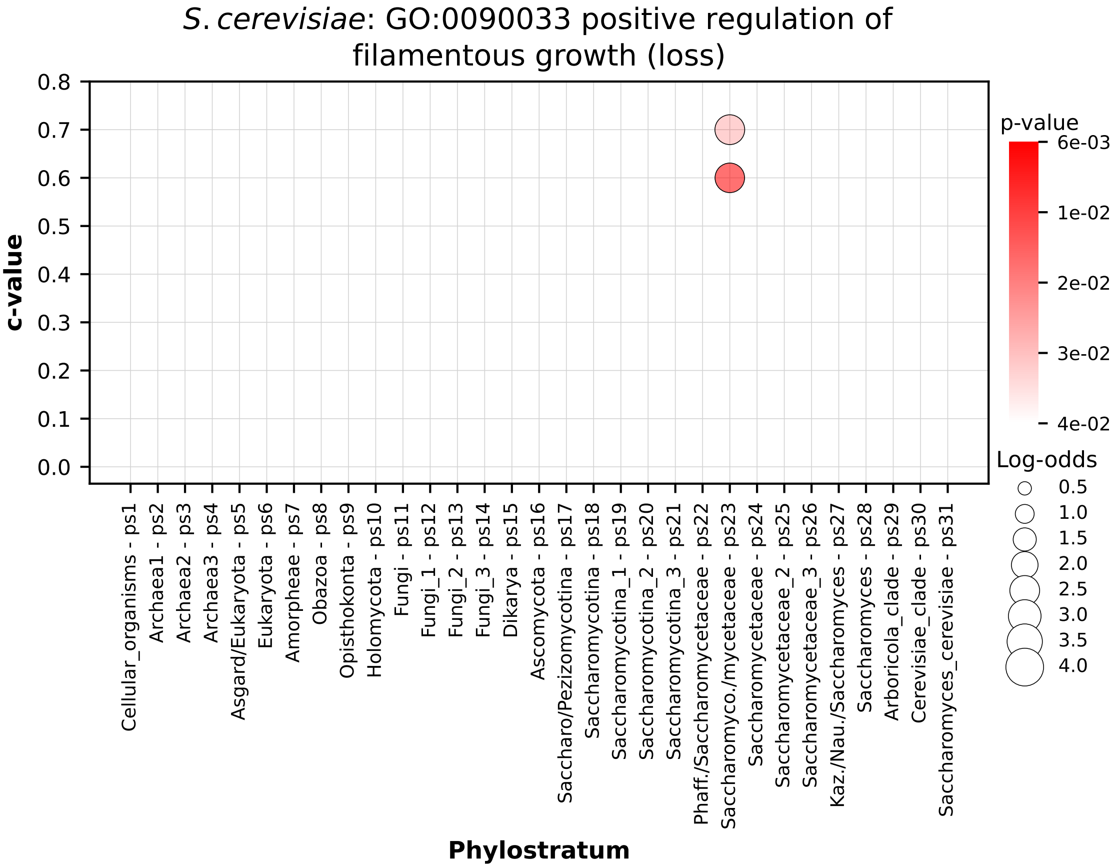

S. cerevisiae: GO:0090033 positive regulation of filamentous growth (loss)
c-value
0.8
0.7
0.6
0.5
0.4
0.3
0.2
0.1
0.0
p-value
6e-03
1e-02
2e-02
3e-02
4e-02
Log-odds
0.5
1.0
1.5
2.0
2.5
3.0
3.5
4.0
Cellular_organisms - ps1
Archaea1 - ps2
Archaea2 - ps3
Archaea3 - ps4
Asgard/Eukaryota - ps5
Eukaryota - ps6
Amorpheae - ps7
Obazoa - ps8
Opisthokonta - ps9
Holomycota - ps10
Fungi - ps11
Fungi_1 - ps12
Fungi_2 - ps13
Fungi_3 - ps14
Dikarya - ps15
Ascomycota - ps16
Saccharo/Pezizomycotina - ps17
Saccharomycotina - ps18
Saccharomycotina_1 - ps19
Saccharomycotina_2 - ps20
Saccharomycotina_3 - ps21
Phaff./Saccharomycetaceae - ps22
Saccharomyco./mycetaceae - ps23
Saccharomycetaceae - ps24
Saccharomycetaceae_2 - ps25
Saccharomycetaceae_3 - ps26
Kaz./Nau./Saccharomyces - ps27
Saccharomyces - ps28
Arboricola_clade - ps29
Cerevisiae_clade - ps30
Saccharomyces_cerevisiae - ps31
Phylostratum

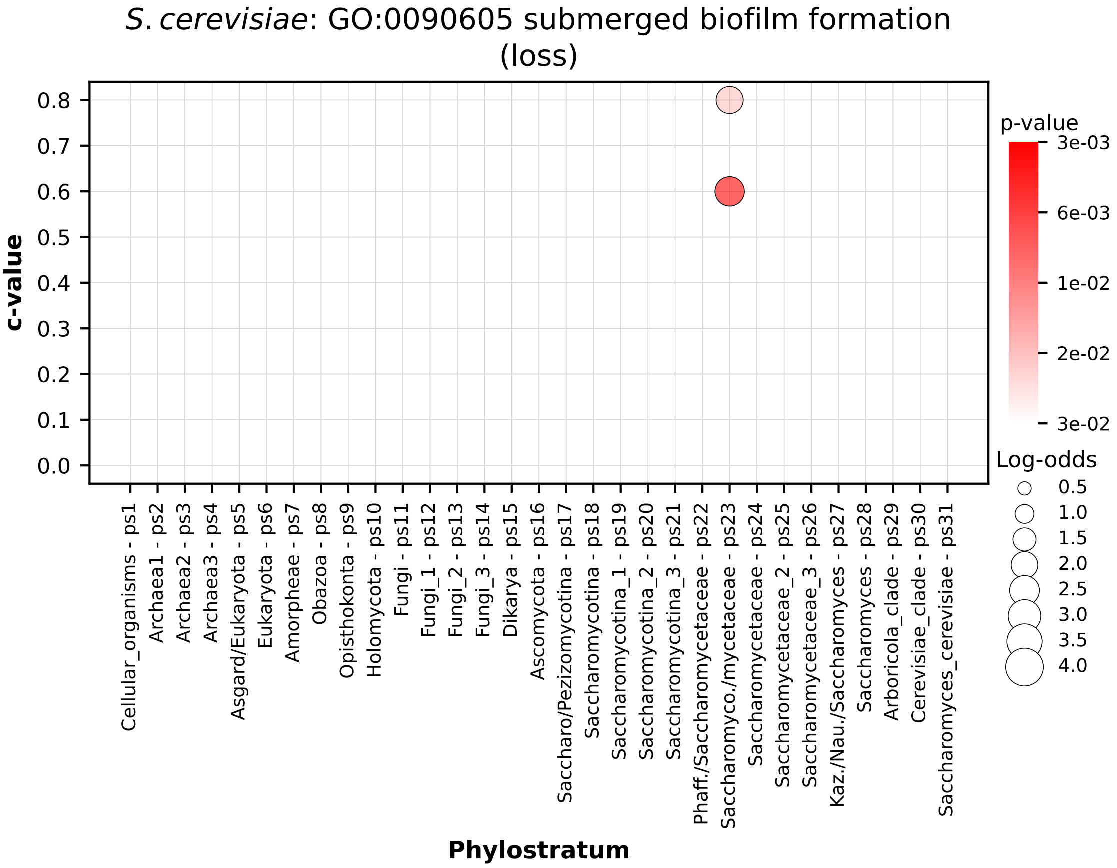

S. cerevisiae: GO:0090605 submerged biofilm formation
(loss)
c-value
0.0
0.1
0.2
0.3
0.4
0.5
0.6
0.7
0.8
p-value
3e-03
6e-03
1e-02
2e-02
3e-02
Log-odds
0.5
1.0
1.5
2.0
2.5
3.0
3.5
4.0
Cellular_organisms - ps1
Archaea1 - ps2
Archaea2 - ps3
Archaea3 - ps4
Asgard/Eukaryota - ps5
Eukaryota - ps6
Amorpheae - ps7
Obazoa - ps8
Opisthokonta - ps9
Holomycota - ps10
Fungi - ps11
Fungi_1 - ps12
Fungi_2 - ps13
Fungi_3 - ps14
Dikarya - ps15
Ascomycota - ps16
Saccharo/Pezizomycotina - ps17
Saccharomycotina - ps18
Saccharomycotina_1 - ps19
Saccharomycotina_2 - ps20
Saccharomycotina_3 - ps21
Phaff./Saccharomycetaceae - ps22
Saccharomyco./mycetaceae - ps23
Saccharomycetaceae - ps24
Saccharomycetaceae_2 - ps25
Saccharomycetaceae_3 - ps26
Kaz./Nau./Saccharomyces - ps27
Saccharomyces - ps28
Arboricola_clade - ps29
Cerevisiae_clade - ps30
Saccharomyces_cerevisiae - ps31
Phylostratum

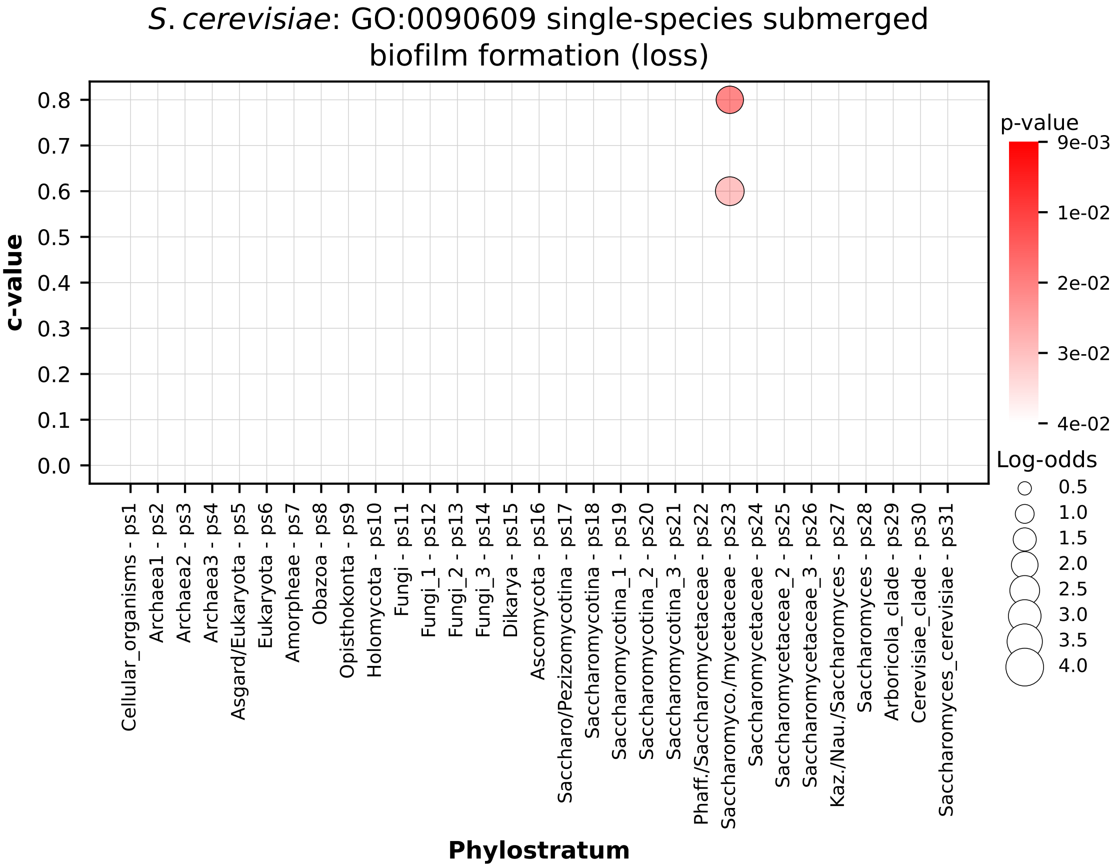

S. cerevisiae: GO:0090609 single-species submerged biofilm formation (loss)
c-value
0.0
0.1
0.2
0.3
0.4
0.5
0.6
0.7
0.8
p-value
9e-03
1e-02
2e-02
3e-02
4e-02
Log-odds
0.5
1.0
1.5
2.0
2.5
3.0
3.5
4.0
Cellular_organisms - ps1
Archaea1 - ps2
Archaea2 - ps3
Archaea3 - ps4
Asgard/Eukaryota - ps5
Eukaryota - ps6
Amorpheae - ps7
Obazoa - ps8
Opisthokonta - ps9
Holomycota - ps10
Fungi - ps11
Fungi_1 - ps12
Fungi_2 - ps13
Fungi_3 - ps14
Dikarya - ps15
Ascomycota - ps16
Saccharo/Pezizomycotina - ps17
Saccharomycotina - ps18
Saccharomycotina_1 - ps19
Saccharomycotina_2 - ps20
Saccharomycotina_3 - ps21
Phaff./Saccharomycetaceae - ps22
Saccharomyco./mycetaceae - ps23
Saccharomycetaceae - ps24
Saccharomycetaceae_2 - ps25
Saccharomycetaceae_3 - ps26
Kaz./Nau./Saccharomyces - ps27
Saccharomyces - ps28
Arboricola_clade - ps29
Cerevisiae_clade - ps30
Saccharomyces_cerevisiae - ps31
Phylostratum

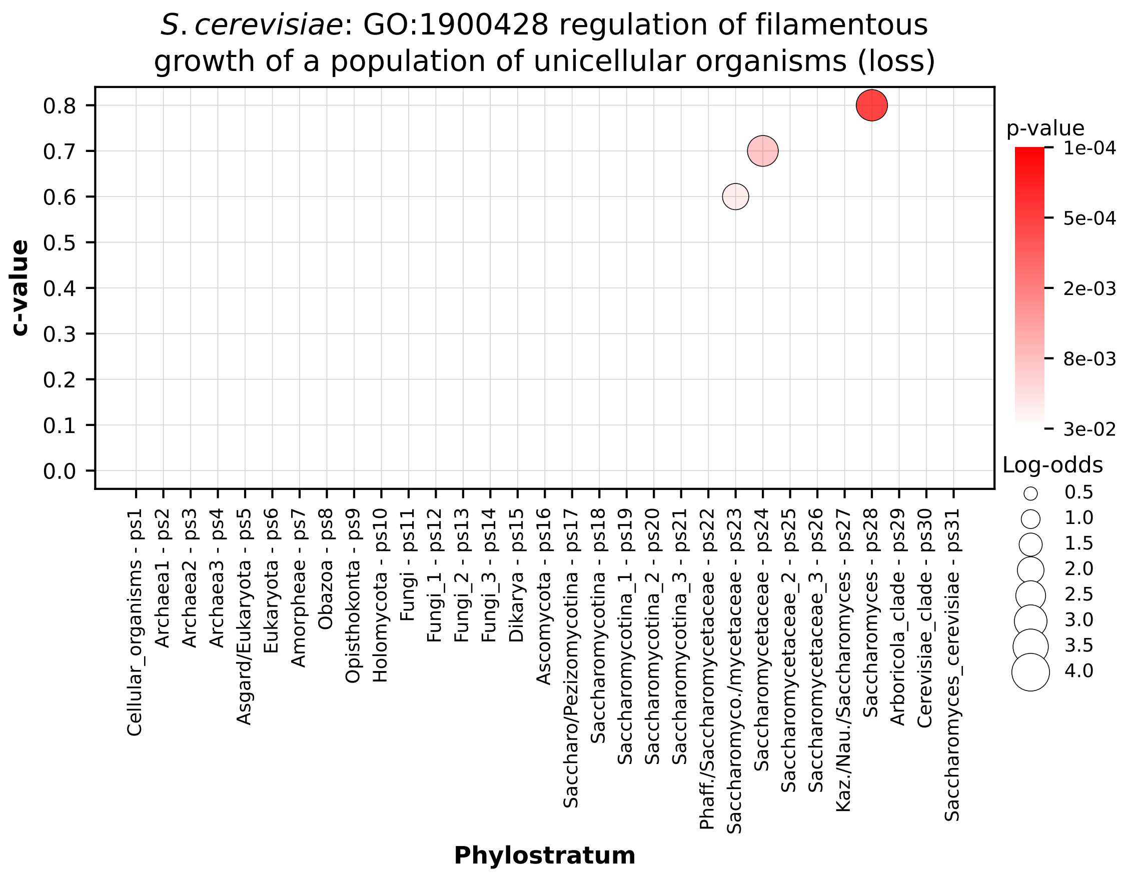
S. cerevisiae: GO:1900428 regulation of filamentous growth of a population of unicellular organisms (loss)
c-value
0.0
0.1
0.2
0.3
0.4
0.5
0.6
0.7
0.8
Phylostratum
Cellular_organisms - ps1
Archaea1 - ps2
Archaea2 - ps3
Archaea3 - ps4
Asgard/Eukaryota - ps5
Eukaryota - ps6
Amorpheae - ps7
Obazoa - ps8
Opisthokonta - ps9
Holomycota - ps10
Fungi - ps11
Fungi_1 - ps12
Fungi_2 - ps13
Fungi_3 - ps14
Dikarya - ps15
Ascomycota - ps16
Saccharo/Pezizomycotina - ps17
Saccharomycotina - ps18
Saccharomycotina_1 - ps19
Saccharomycotina_2 - ps20
Saccharomycotina_3 - ps21
Phaff./Saccharomycetaceae - ps22
Saccharomyco./mycetaceae - ps23
Saccharomycetaceae - ps24
Saccharomycetaceae_2 - ps25
Saccharomycetaceae_3 - ps26
Kaz./Nau./Saccharomyces - ps27
Saccharomyces - ps28
Arboricola_clade - ps29
Cerevisiae_clade - ps30
Saccharomyces_cerevisiae - ps31
p-value
1e-04
5e-04
2e-03
8e-03
3e-02
Log-odds
0.5
1.0
1.5
2.0
2.5
3.0
3.5
4.0

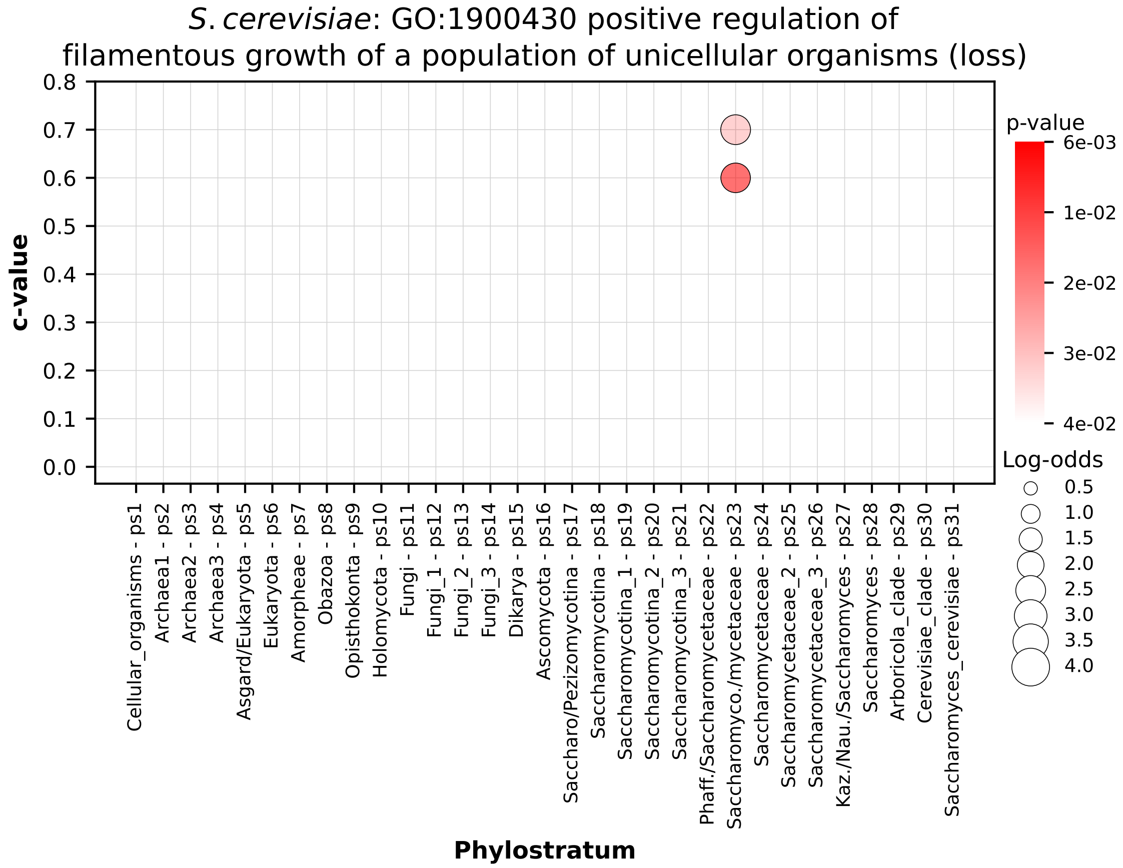
S. cerevisiae: GO:1900430 positive regulation of filamentous growth of a population of unicellular organisms (loss)
c-value
Phylostratum
p-value
6e-03
1e-02
2e-02
3e-02
4e-02
Log-odds
0.5
1.0
1.5
2.0
2.5
3.0
3.5
4.0
Cellular_organisms - ps1
Archaea1 - ps2
Archaea2 - ps3
Archaea3 - ps4
Asgard/Eukaryota - ps5
Eukaryota - ps6
Amorpheae - ps7
Obazoa - ps8
Opisthokonta - ps9
Holomycota - ps10
Fungi - ps11
Fungi_1 - ps12
Fungi_2 - ps13
Fungi_3 - ps14
Dikarya - ps15
Ascomycota - ps16
Saccharo/Pezizomycotina - ps17
Saccharomycotina - ps18
Saccharomycotina_1 - ps19
Saccharomycotina_2 - ps20
Saccharomycotina_3 - ps21
Phaff./Saccharomycetaceae - ps22
Saccharomyco./mycetaceae - ps23
Saccharomycetaceae - ps24
Saccharomycetaceae_2 - ps25
Saccharomycetaceae_3 - ps26
Kaz./Nau./Saccharomyces - ps27
Saccharomyces - ps28
Arboricola_clade - ps29
Cerevisiae_clade - ps30
Saccharomyces_cerevisiae - ps31

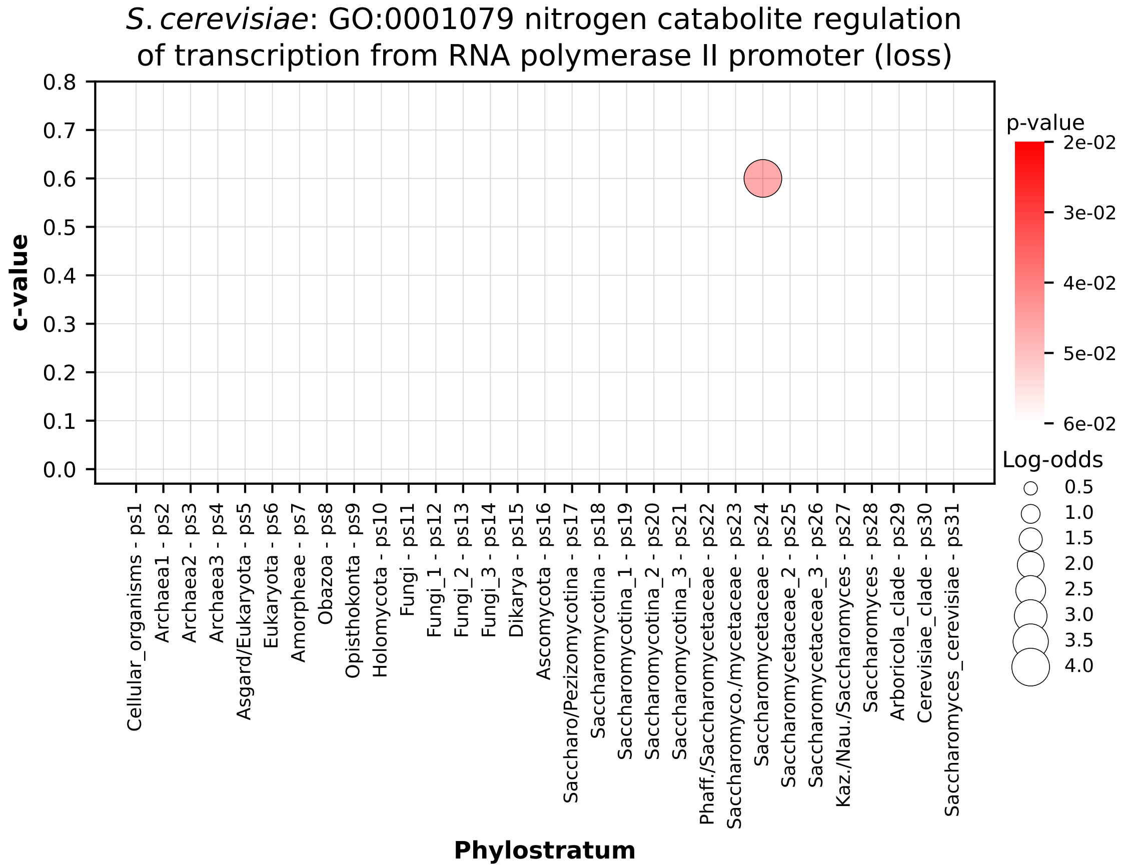

S. cerevisiae: GO:0001079 nitrogen catabolite regulation of transcription from RNA polymerase II promoter (loss)
c-value
0.0
0.1
0.2
0.3
0.4
0.5
0.6
0.7
0.8
Phylostratum
Cellular_organisms - ps1
Archaea1 - ps2
Archaea2 - ps3
Archaea3 - ps4
Asgard/Eukaryota - ps5
Eukaryota - ps6
Amorpheae - ps7
Obazoa - ps8
Opisthokonta - ps9
Holomycota - ps10
Fungi - ps11
Fungi_1 - ps12
Fungi_2 - ps13
Fungi_3 - ps14
Dikarya - ps15
Ascomycota - ps16
Saccharo/Pezizomycotina - ps17
Saccharomycotina - ps18
Saccharomycotina_1 - ps19
Saccharomycotina_2 - ps20
Saccharomycotina_3 - ps21
Phaff./Saccharomycetaceae - ps22
Saccharomyco./mycetaceae - ps23
Saccharomycetaceae - ps24
Saccharomycetaceae_2 - ps25
Saccharomycetaceae_3 - ps26
Kaz./Nau./Saccharomyces - ps27
Saccharomyces - ps28
Arboricola_clade - ps29
Cerevisiae_clade - ps30
Saccharomyces_cerevisiae - ps31
p-value
2e-02
3e-02
4e-02
5e-02
6e-02
Log-odds
0.5
1.0
1.5
2.0
2.5
3.0
3.5
4.0

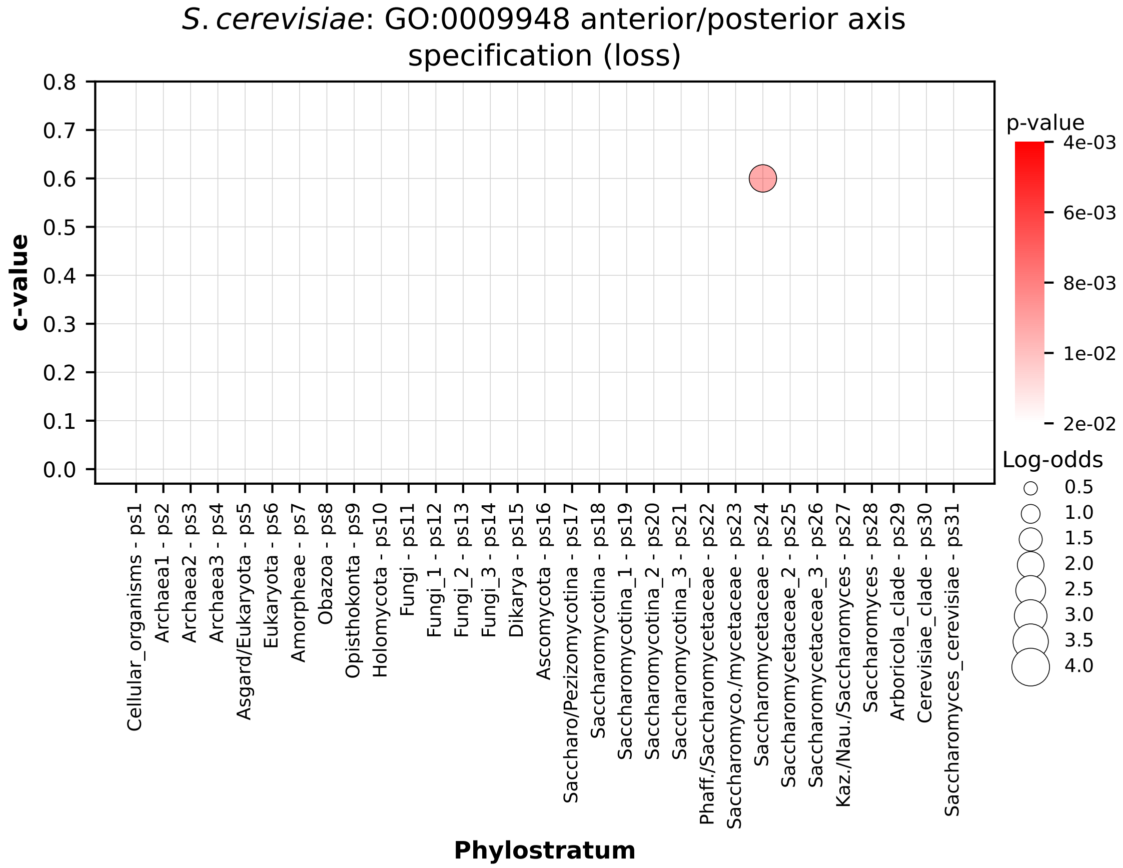

S. cerevisiae: GO:0009948 anterior/posterior axis specification (loss)
c-value
0.8
0.7
0.6
0.5
0.4
0.3
0.2
0.1
0.0
Phylostratum
Cellular_organisms - ps1
Archaea1 - ps2
Archaea2 - ps3
Archaea3 - ps4
Asgard/Eukaryota - ps5
Eukaryota - ps6
Amorpheae - ps7
Obazoa - ps8
Opisthokonta - ps9
Holomycota - ps10
Fungi - ps11
Fungi_1 - ps12
Fungi_2 - ps13
Fungi_3 - ps14
Dikarya - ps15
Ascomycota - ps16
Saccharo/Pezizomycotina - ps17
Saccharomycotina - ps18
Saccharomycotina_1 - ps19
Saccharomycotina_2 - ps20
Saccharomycotina_3 - ps21
Phaff./Saccharomycetaceae - ps22
Saccharomyco./mycetaceae - ps23
Saccharomycetaceae - ps24
Saccharomycetaceae_2 - ps25
Saccharomycetaceae_3 - ps26
Kaz./Nau./Saccharomyces - ps27
Saccharomyces - ps28
Arboricola_clade - ps29
Cerevisiae_clade - ps30
Saccharomyces_cerevisiae - ps31
p-value
4e-03
6e-03
8e-03
1e-02
2e-02
Log-odds
0.5
1.0
1.5
2.0
2.5
3.0
3.5
4.0

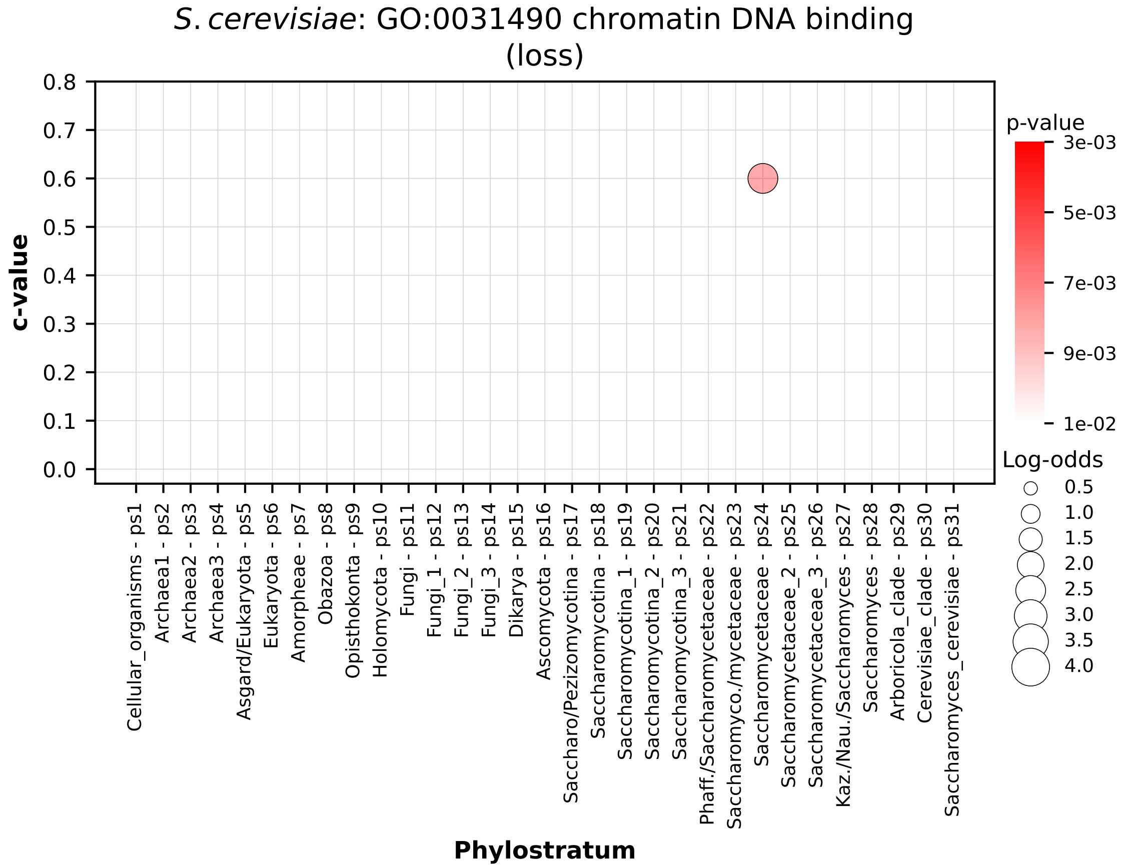

S. cerevisiae: GO:0031490 chromatin DNA binding
(loss)
c-value
0.8
0.7
0.6
0.5
0.4
0.3
0.2
0.1
0.0
p-value
3e-03
5e-03
7e-03
9e-03
1e-02
Log-odds
0.5
1.0
1.5
2.0
2.5
3.0
3.5
4.0
Cellular_organisms - ps1
Archaea1 - ps2
Archaea2 - ps3
Archaea3 - ps4
Asgard/Eukaryota - ps5
Eukaryota - ps6
Amorpheae - ps7
Obazoa - ps8
Opisthokonta - ps9
Holomycota - ps10
Fungi - ps11
Fungi_1 - ps12
Fungi_2 - ps13
Fungi_3 - ps14
Dikarya - ps15
Ascomycota - ps16
Saccharo/Pezizomycotina - ps17
Saccharomycotina - ps18
Saccharomycotina_1 - ps19
Saccharomycotina_2 - ps20
Saccharomycotina_3 - ps21
Phaff./Saccharomycetaceae - ps22
Saccharomyco./mycetaceae - ps23
Saccharomycetaceae - ps24
Saccharomycetaceae_2 - ps25
Saccharomycetaceae_3 - ps26
Kaz./Nau./Saccharomyces - ps27
Saccharomyces - ps28
Arboricola_clade - ps29
Cerevisiae_clade - ps30
Saccharomyces_cerevisiae - ps31
Phylostratum

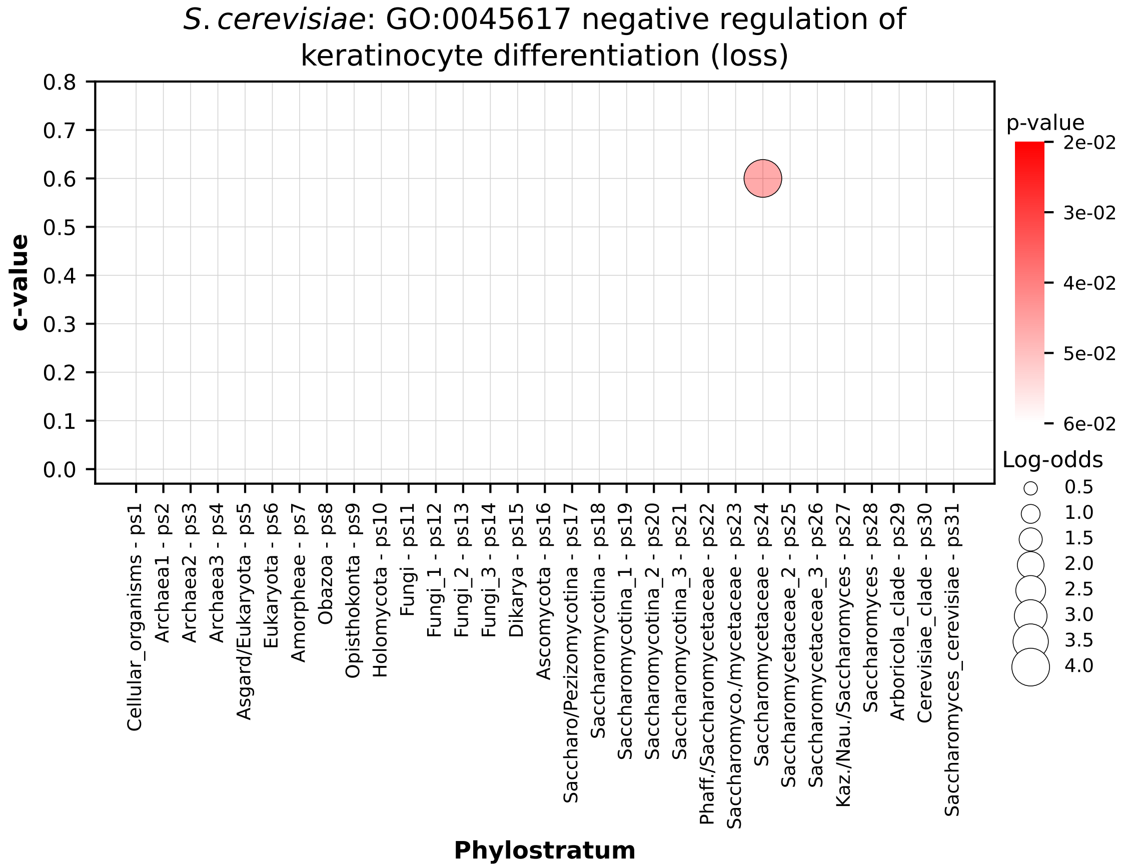
S. cerevisiae: GO:0045617 negative regulation of keratinocyte differentiation (loss)
c-value
0.8
0.7
0.6
0.5
0.4
0.3
0.2
0.1
0.0
p-value
2e-02
3e-02
4e-02
5e-02
6e-02
Log-odds
0.5
1.0
1.5
2.0
2.5
3.0
3.5
4.0
Cellular_organisms - ps1
Archaea1 - ps2
Archaea2 - ps3
Archaea3 - ps4
Asgard/Eukaryota - ps5
Eukaryota - ps6
Amorpheae - ps7
Obazoa - ps8
Opisthokonta - ps9
Holomycota - ps10
Fungi - ps11
Fungi_1 - ps12
Fungi_2 - ps13
Fungi_3 - ps14
Dikarya - ps15
Ascomycota - ps16
Saccharo/Pezizomycotina - ps17
Saccharomycotina - ps18
Saccharomycotina_1 - ps19
Saccharomycotina_2 - ps20
Saccharomycotina_3 - ps21
Phaff./Saccharomycetaceae - ps22
Saccharomyco./mycetaceae - ps23
Saccharomycetaceae - ps24
Saccharomycetaceae_2 - ps25
Saccharomycetaceae_3 - ps26
Kaz./Nau./Saccharomyces - ps27
Saccharomyces - ps28
Arboricola_clade - ps29
Cerevisiae_clade - ps30
Saccharomyces_cerevisiae - ps31
Phylostratum

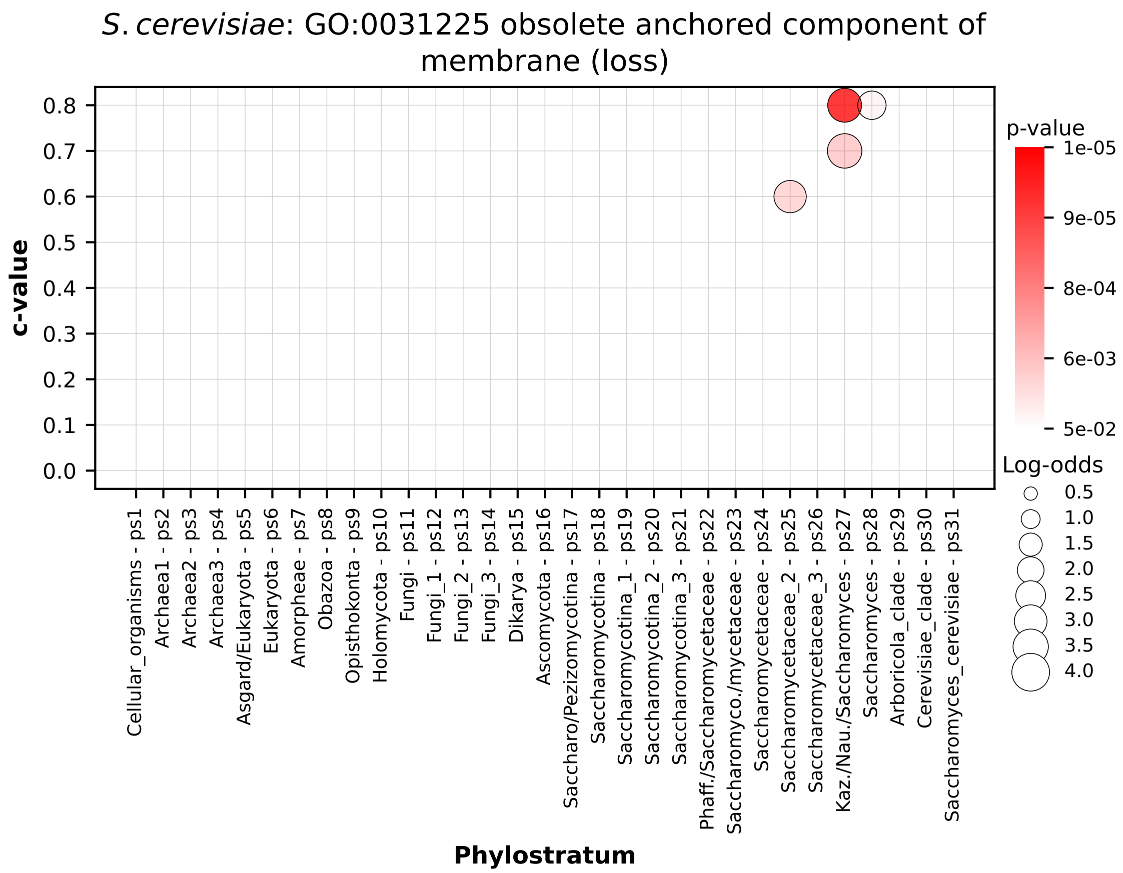

S. cerevisiae: GO:0031225 obsolete anchored component of membrane (loss)
c-value
0.8
0.7
0.6
0.5
0.4
0.3
0.2
0.1
0.0
p-value
1e-05
9e-05
8e-04
6e-03
5e-02
Log-odds
0.5
1.0
1.5
2.0
2.5
3.0
3.5
4.0
Cellular_organisms - ps1
Archaea1 - ps2
Archaea2 - ps3
Archaea3 - ps4
Asgard/Eukaryota - ps5
Eukaryota - ps6
Amorpheae - ps7
Obazoa - ps8
Opisthokonta - ps9
Holomycota - ps10
Fungi - ps11
Fungi_1 - ps12
Fungi_2 - ps13
Fungi_3 - ps14
Dikarya - ps15
Ascomycota - ps16
Saccharo/Pezizomycotina - ps17
Saccharomycotina - ps18
Saccharomycotina_1 - ps19
Saccharomycotina_2 - ps20
Saccharomycotina_3 - ps21
Phaff./Saccharomycetaceae - ps22
Saccharomyco./mycetaceae - ps23
Saccharomycetaceae - ps24
Saccharomycetaceae_2 - ps25
Saccharomycetaceae_3 - ps26
Kaz./Nau./Saccharomyces - ps27
Saccharomyces - ps28
Arboricola_clade - ps29
Cerevisiae_clade - ps30
Saccharomyces_cerevisiae - ps31
Phylostratum

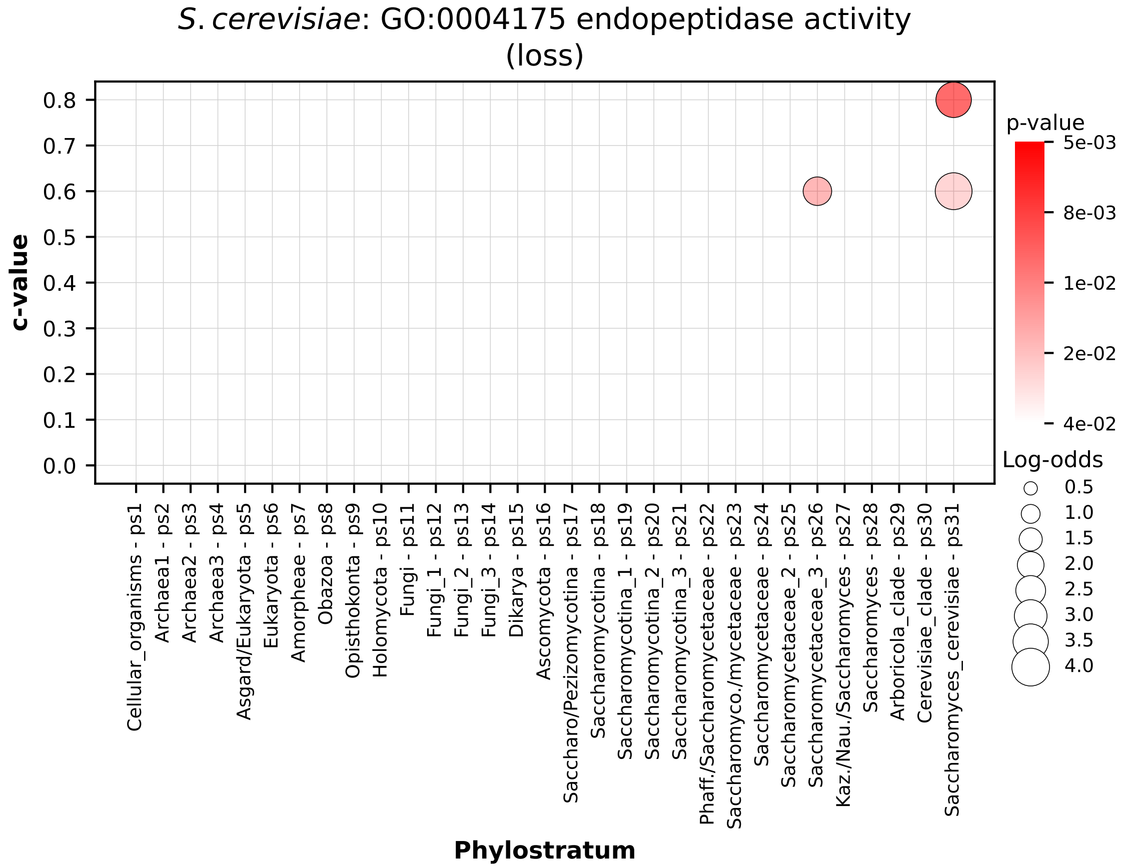
S. cerevisiae: GO:0004175 endopeptidase activity
(loss)
c-value
0.0
0.1
0.2
0.3
0.4
0.5
0.6
0.7
0.8
Phylostratum
Cellular_organisms - ps1
Archaea1 - ps2
Archaea2 - ps3
Archaea3 - ps4
Asgard/Eukaryota - ps5
Eukaryota - ps6
Amorpheae - ps7
Obazoa - ps8
Opisthokonta - ps9
Holomycota - ps10
Fungi - ps11
Fungi_1 - ps12
Fungi_2 - ps13
Fungi_3 - ps14
Dikarya - ps15
Ascomycota - ps16
Saccharo/Pezizomycotina - ps17
Saccharomycotina - ps18
Saccharomycotina_1 - ps19
Saccharomycotina_2 - ps20
Saccharomycotina_3 - ps21
Phaff./Saccharomycetaceae - ps22
Saccharomyco./mycetaceae - ps23
Saccharomycetaceae - ps24
Saccharomycetaceae_2 - ps25
Saccharomycetaceae_3 - ps26
Kaz./Nau./Saccharomyces - ps27
Saccharomyces - ps28
Arboricola_clade - ps29
Cerevisiae_clade - ps30
Saccharomyces_cerevisiae - ps31
p-value
5e-03
8e-03
1e-02
2e-02
4e-02
Log-odds
0.5
1.0
1.5
2.0
2.5
3.0
3.5
4.0

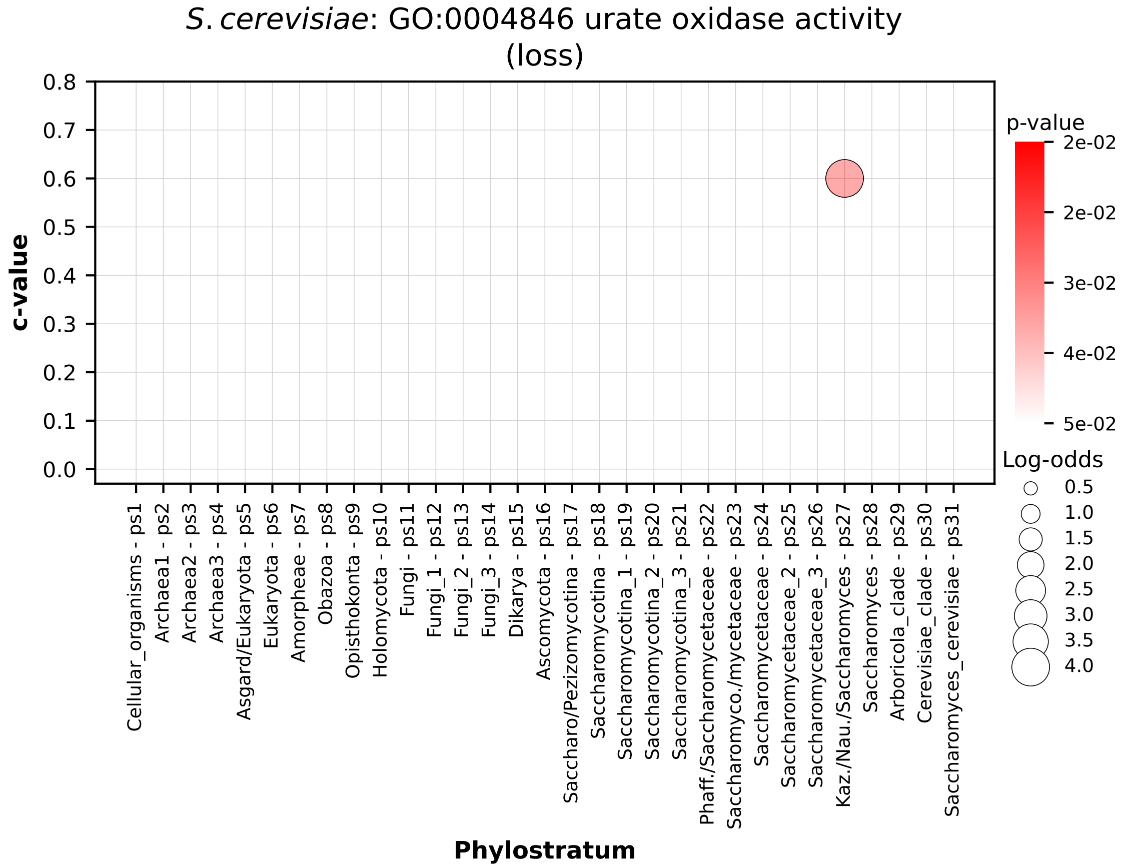
S. cerevisiae: GO:0004846 urate oxidase activity
(loss)
c-value
0.8
0.7
0.6
0.5
0.4
0.3
0.2
0.1
0.0
p-value
2e-02
2e-02
3e-02
4e-02
5e-02
Log-odds
0.5
1.0
1.5
2.0
2.5
3.0
3.5
4.0
Cellular_organisms - ps1
Archaea1 - ps2
Archaea2 - ps3
Archaea3 - ps4
Asgard/Eukaryota - ps5
Eukaryota - ps6
Amorpheae - ps7
Obazoa - ps8
Opisthokonta - ps9
Holomycota - ps10
Fungi - ps11
Fungi_1 - ps12
Fungi_2 - ps13
Fungi_3 - ps14
Dikarya - ps15
Ascomycota - ps16
Saccharo/Pezizomycotina - ps17
Saccharomycotina - ps18
Saccharomycotina_1 - ps19
Saccharomycotina_2 - ps20
Saccharomycotina_3 - ps21
Phaff./Saccharomycetaceae - ps22
Saccharomyco./mycetaceae - ps23
Saccharomycetaceae - ps24
Saccharomycetaceae_2 - ps25
Saccharomycetaceae_3 - ps26
Kaz./Nau./Saccharomyces - ps27
Saccharomyces - ps28
Arboricola_clade - ps29
Cerevisiae_clade - ps30
Saccharomyces_cerevisiae - ps31
Phylostratum

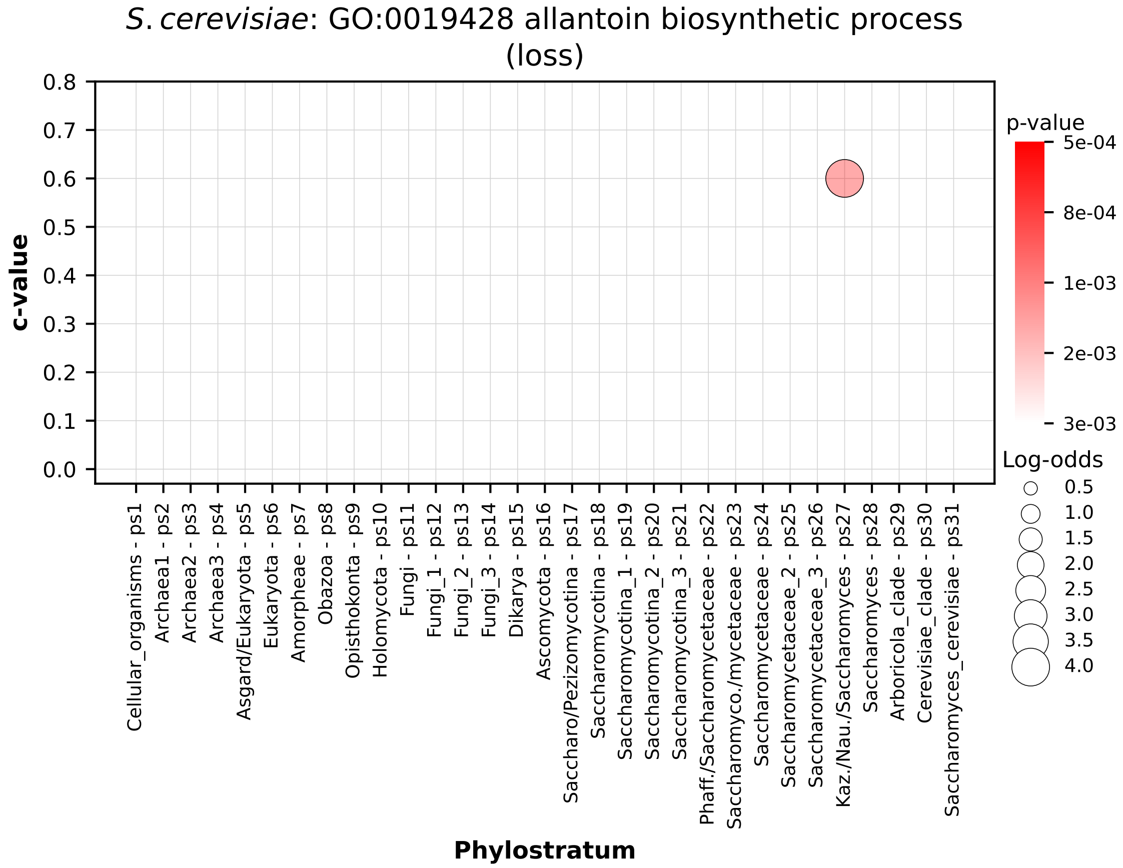

S. cerevisiae: GO:0019428 allantoin biosynthetic process (loss)
c-value
0.8
0.7
0.6
0.5
0.4
0.3
0.2
0.1
0.0
Phylostratum
Cellular_organisms - ps1
Archaea1 - ps2
Archaea2 - ps3
Archaea3 - ps4
Asgard/Eukaryota - ps5
Eukaryota - ps6
Amorpheae - ps7
Obazoa - ps8
Opisthokonta - ps9
Holomycota - ps10
Fungi - ps11
Fungi_1 - ps12
Fungi_2 - ps13
Fungi_3 - ps14
Dikarya - ps15
Ascomycota - ps16
Saccharo/Pezizomycotina - ps17
Saccharomycotina - ps18
Saccharomycotina_1 - ps19
Saccharomycotina_2 - ps20
Saccharomycotina_3 - ps21
Phaff./Saccharomycetaceae - ps22
Saccharomyco./mycetaceae - ps23
Saccharomycetaceae - ps24
Saccharomycetaceae_2 - ps25
Saccharomycetaceae_3 - ps26
Kaz./Nau./Saccharomyces - ps27
Saccharomyces - ps28
Arboricola_clade - ps29
Cerevisiae_clade - ps30
Saccharomyces_cerevisiae - ps31
p-value
5e-04
8e-04
1e-03
2e-03
3e-03
Log-odds
0.5
1.0
1.5
2.0
2.5
3.0
3.5
4.0

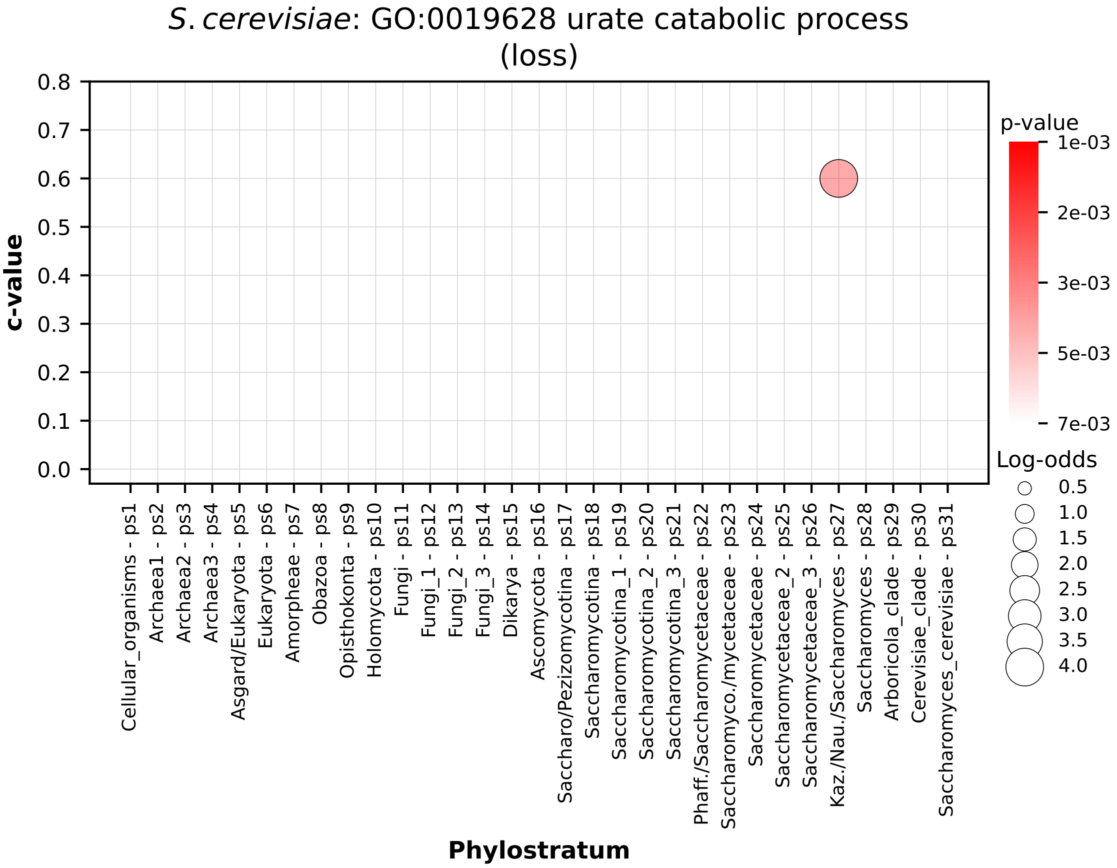

S. cerevisiae: GO:0019628 urate catabolic process
(loss)
c-value
0.8
0.7
0.6
0.5
0.4
0.3
0.2
0.1
0.0
Phylostratum
Cellular_organisms - ps1
Archaea1 - ps2
Archaea2 - ps3
Archaea3 - ps4
Asgard/Eukaryota - ps5
Eukaryota - ps6
Amorpheae - ps7
Obazoa - ps8
Opisthokonta - ps9
Holomycota - ps10
Fungi - ps11
Fungi_1 - ps12
Fungi_2 - ps13
Fungi_3 - ps14
Dikarya - ps15
Ascomycota - ps16
Saccharo/Pezizomycotina - ps17
Saccharomycotina - ps18
Saccharomycotina_1 - ps19
Saccharomycotina_2 - ps20
Saccharomycotina_3 - ps21
Phaff./Saccharomycetaceae - ps22
Saccharomyco./mycetaceae - ps23
Saccharomycetaceae - ps24
Saccharomycetaceae_2 - ps25
Saccharomycetaceae_3 - ps26
Kaz./Nau./Saccharomyces - ps27
Saccharomyces - ps28
Arboricola_clade - ps29
Cerevisiae_clade - ps30
Saccharomyces_cerevisiae - ps31
p-value
1e-03
2e-03
3e-03
5e-03
7e-03
Log-odds
0.5
1.0
1.5
2.0
2.5
3.0
3.5
4.0

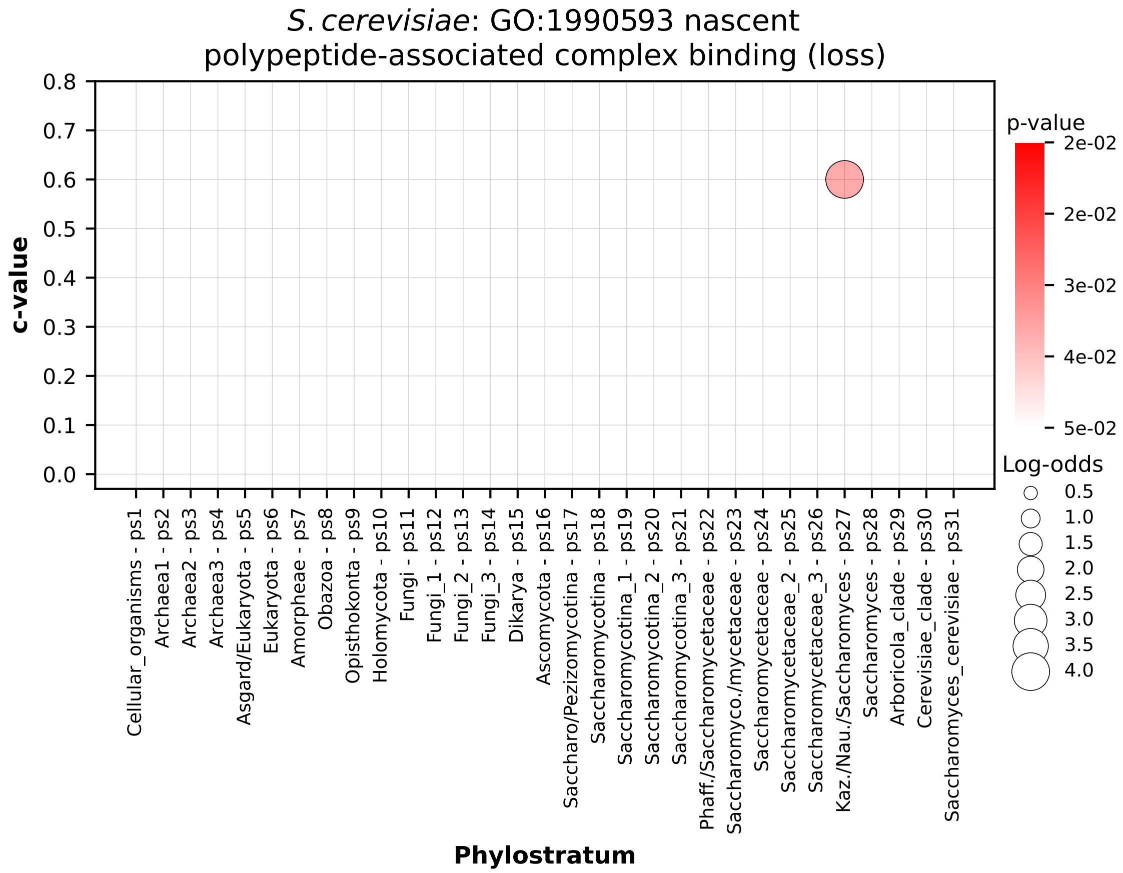

S. cerevisiae: GO:1990593 nascent polypeptide-associated complex binding (loss)
c-value
0.0
0.1
0.2
0.3
0.4
0.5
0.6
0.7
0.8
p-value
2e-02
2e-02
3e-02
4e-02
5e-02
Log-odds
0.5
1.0
1.5
2.0
2.5
3.0
3.5
4.0
Cellular_organisms - ps1
Archaea1 - ps2
Archaea2 - ps3
Archaea3 - ps4
Asgard/Eukaryota - ps5
Eukaryota - ps6
Amorpheae - ps7
Obazoa - ps8
Opisthokonta - ps9
Holomycota - ps10
Fungi - ps11
Fungi_1 - ps12
Fungi_2 - ps13
Fungi_3 - ps14
Dikarya - ps15
Ascomycota - ps16
Saccharo/Pezizomycotina - ps17
Saccharomycotina - ps18
Saccharomycotina_1 - ps19
Saccharomycotina_2 - ps20
Saccharomycotina_3 - ps21
Phaff./Saccharomycetaceae - ps22
Saccharomyco./mycetaceae - ps23
Saccharomycetaceae - ps24
Saccharomycetaceae_2 - ps25
Saccharomycetaceae_3 - ps26
Kaz./Nau./Saccharomyces - ps27
Saccharomyces - ps28
Arboricola_clade - ps29
Cerevisiae_clade - ps30
Saccharomyces_cerevisiae - ps31
Phylostratum

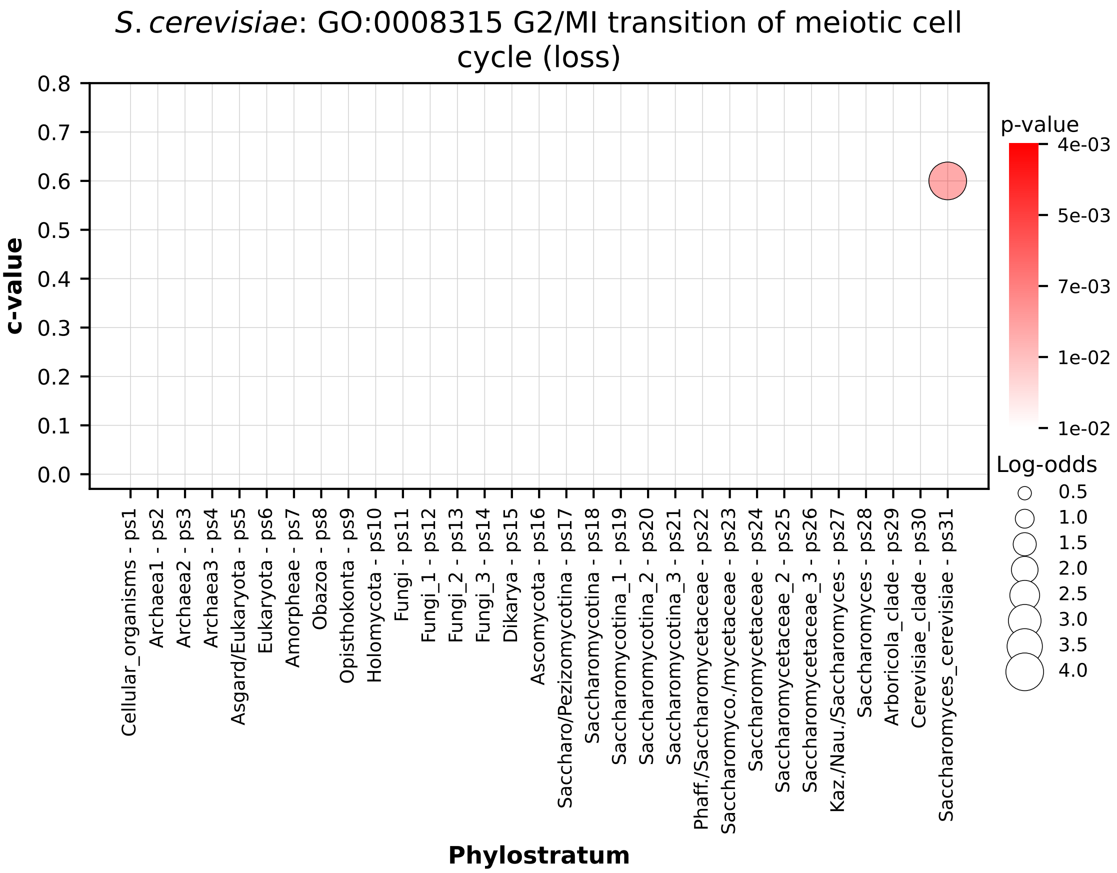

S. cerevisiae: GO:0008315 G2/MI transition of meiotic cell cycle (loss)
c-value
0.0
0.1
0.2
0.3
0.4
0.5
0.6
0.7
0.8
Phylostratum
Cellular_organisms - ps1
Archaea1 - ps2
Archaea2 - ps3
Archaea3 - ps4
Asgard/Eukaryota - ps5
Eukaryota - ps6
Amorpheae - ps7
Obazoa - ps8
Opisthokonta - ps9
Holomycota - ps10
Fungi - ps11
Fungi_1 - ps12
Fungi_2 - ps13
Fungi_3 - ps14
Dikarya - ps15
Ascomycota - ps16
Saccharo/Pezizomycotina - ps17
Saccharomycotina - ps18
Saccharomycotina_1 - ps19
Saccharomycotina_2 - ps20
Saccharomycotina_3 - ps21
Phaff./Saccharomycetaceae - ps22
Saccharomyco./mycetaceae - ps23
Saccharomycetaceae - ps24
Saccharomycetaceae_2 - ps25
Saccharomycetaceae_3 - ps26
Kaz./Nau./Saccharomyces - ps27
Saccharomyces - ps28
Arboricola_clade - ps29
Cerevisiae_clade - ps30
Saccharomyces_cerevisiae - ps31
p-value
4e-03
5e-03
7e-03
1e-02
1e-02
Log-odds
0.5
1.0
1.5
2.0
2.5
3.0
3.5
4.0

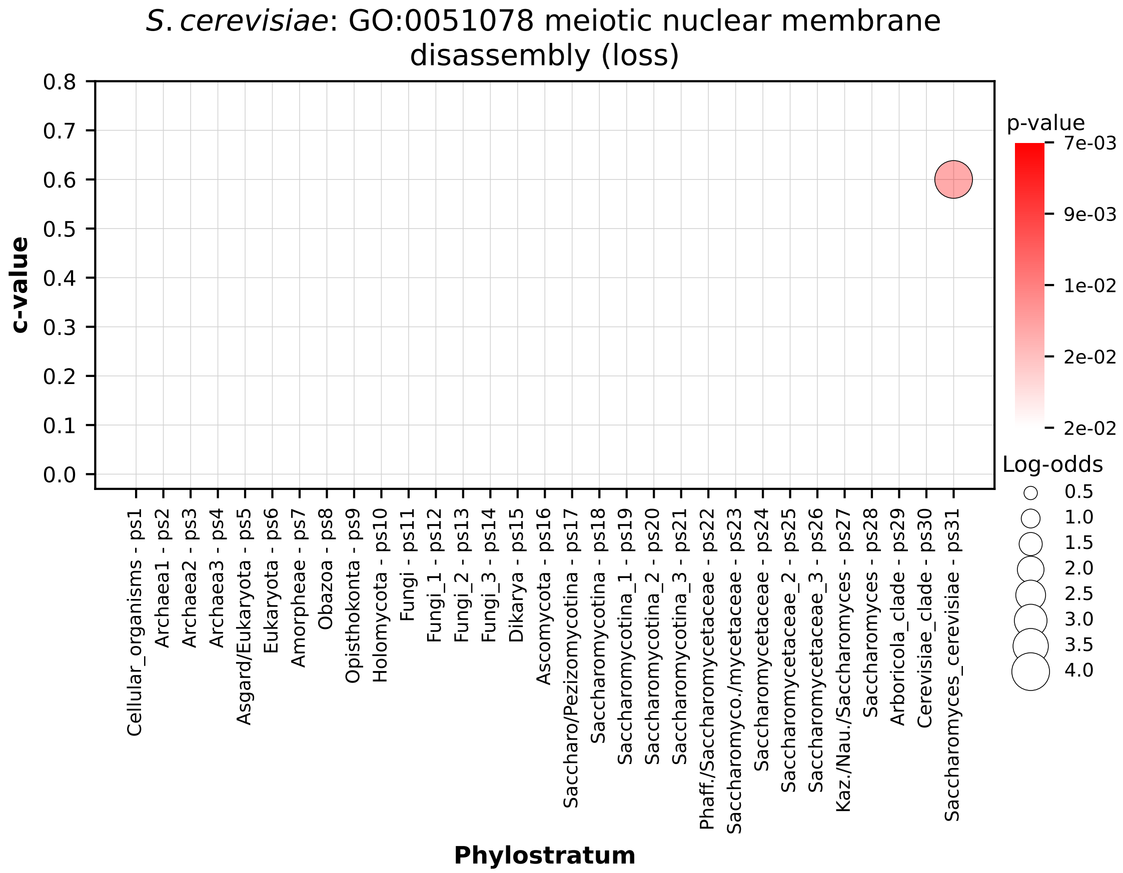

S. cerevisiae: GO:0051078 meiotic nuclear membrane disassembly (loss)
c-value
0.8
0.7
0.6
0.5
0.4
0.3
0.2
0.1
0.0
Phylostratum
Cellular_organisms - ps1
Archaea1 - ps2
Archaea2 - ps3
Archaea3 - ps4
Asgard/Eukaryota - ps5
Eukaryota - ps6
Amorpheae - ps7
Obazoa - ps8
Opisthokonta - ps9
Holomycota - ps10
Fungi - ps11
Fungi_1 - ps12
Fungi_2 - ps13
Fungi_3 - ps14
Dikarya - ps15
Ascomycota - ps16
Saccharo/Pezizomycotina - ps17
Saccharomycotina - ps18
Saccharomycotina_1 - ps19
Saccharomycotina_2 - ps20
Saccharomycotina_3 - ps21
Phaff./Saccharomycetaceae - ps22
Saccharomyco./mycetaceae - ps23
Saccharomycetaceae - ps24
Saccharomycetaceae_2 - ps25
Saccharomycetaceae_3 - ps26
Kaz./Nau./Saccharomyces - ps27
Saccharomyces - ps28
Arboricola_clade - ps29
Cerevisiae_clade - ps30
Saccharomyces_cerevisiae - ps31
p-value
7e-03
9e-03
1e-02
2e-02
2e-02
Log-odds
0.5
1.0
1.5
2.0
2.5
3.0
3.5
4.0

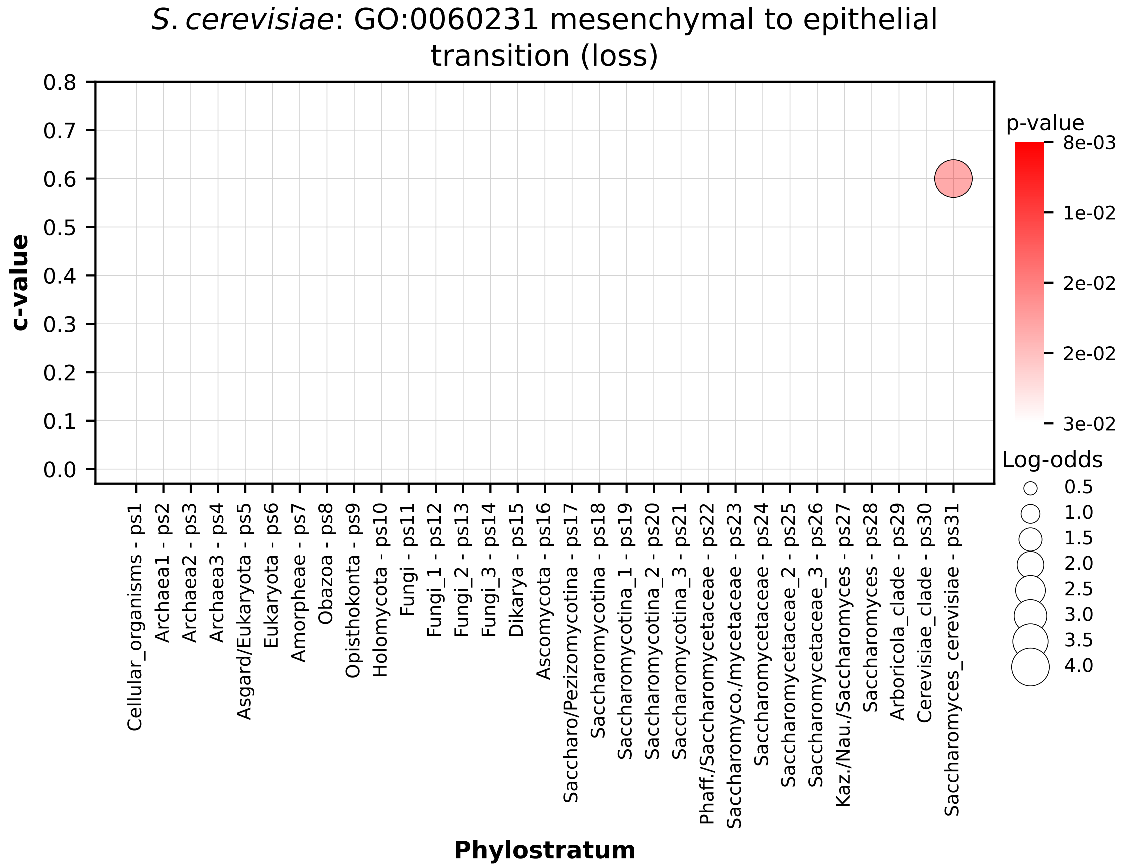

S. cerevisiae: GO:0060231 mesenchymal to epithelial transition (loss)
c-value
0.8
0.7
0.6
0.5
0.4
0.3
0.2
0.1
0.0
p-value
8e-03
1e-02
2e-02
2e-02
3e-02
Log-odds
0.5
1.0
1.5
2.0
2.5
3.0
3.5
4.0
Cellular_organisms - ps1
Archaea1 - ps2
Archaea2 - ps3
Archaea3 - ps4
Asgard/Eukaryota - ps5
Eukaryota - ps6
Amorpheae - ps7
Obazoa - ps8
Opisthokonta - ps9
Holomycota - ps10
Fungi - ps11
Fungi_1 - ps12
Fungi_2 - ps13
Fungi_3 - ps14
Dikarya - ps15
Ascomycota - ps16
Saccharo/Pezizomycotina - ps17
Saccharomycotina - ps18
Saccharomycotina_1 - ps19
Saccharomycotina_2 - ps20
Saccharomycotina_3 - ps21
Phaff./Saccharomycetaceae - ps22
Saccharomyco./mycetaceae - ps23
Saccharomycetaceae - ps24
Saccharomycetaceae_2 - ps25
Saccharomycetaceae_3 - ps26
Kaz./Nau./Saccharomyces - ps27
Saccharomyces - ps28
Arboricola_clade - ps29
Cerevisiae_clade - ps30
Saccharomyces_cerevisiae - ps31
Phylostratum

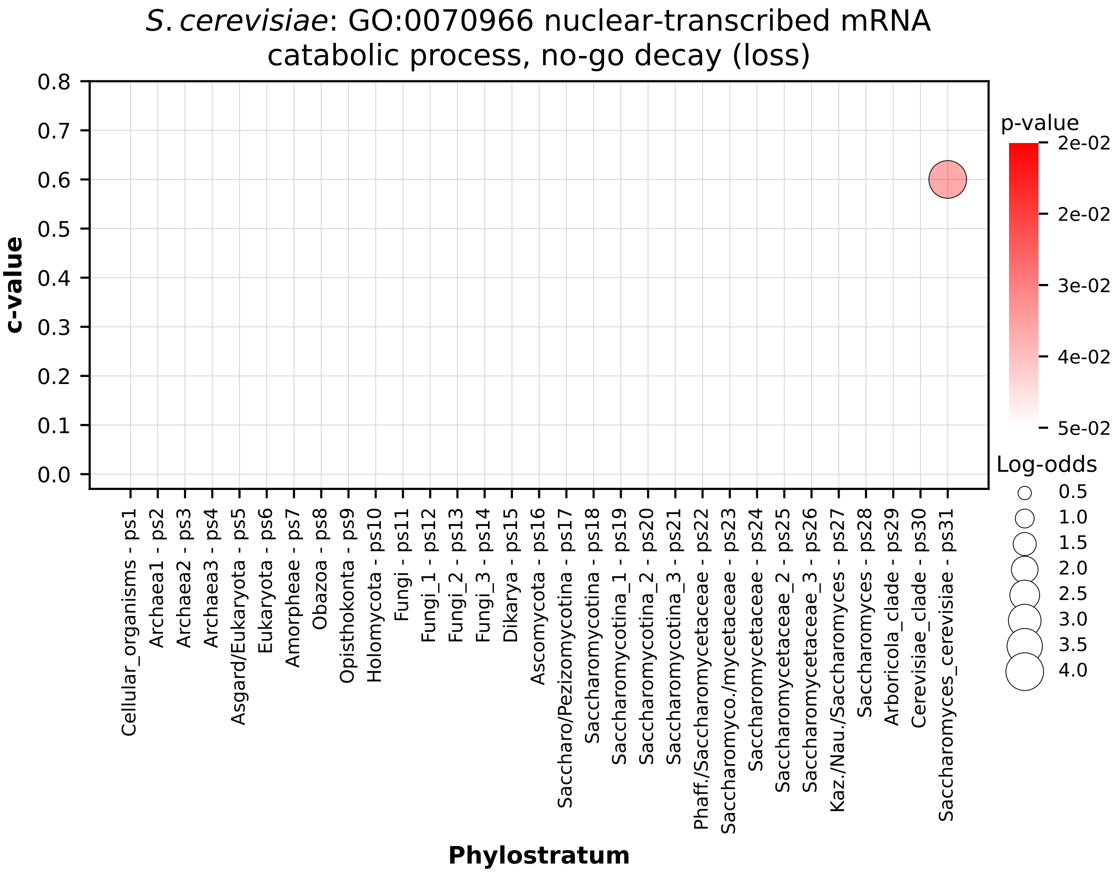

S. cerevisiae: GO:0070966 nuclear-transcribed mRNA catabolic process, no-go decay (loss)
c-value
0.0
0.1
0.2
0.3
0.4
0.5
0.6
0.7
0.8
p-value
2e-02
2e-02
3e-02
4e-02
5e-02
Log-odds
0.5
1.0
1.5
2.0
2.5
3.0
3.5
4.0
Cellular_organisms - ps1
Archaea1 - ps2
Archaea2 - ps3
Archaea3 - ps4
Asgard/Eukaryota - ps5
Eukaryota - ps6
Amorpheae - ps7
Obazoa - ps8
Opisthokonta - ps9
Holomycota - ps10
Fungi - ps11
Fungi_1 - ps12
Fungi_2 - ps13
Fungi_3 - ps14
Dikarya - ps15
Ascomycota - ps16
Saccharo/Pezizomycotina - ps17
Saccharomycotina - ps18
Saccharomycotina_1 - ps19
Saccharomycotina_2 - ps20
Saccharomycotina_3 - ps21
Phaff./Saccharomycetaceae - ps22
Saccharomyco./mycetaceae - ps23
Saccharomycetaceae - ps24
Saccharomycetaceae_2 - ps25
Saccharomycetaceae_3 - ps26
Kaz./Nau./Saccharomyces - ps27
Saccharomyces - ps28
Arboricola_clade - ps29
Cerevisiae_clade - ps30
Saccharomyces_cerevisiae - ps31
Phylostratum

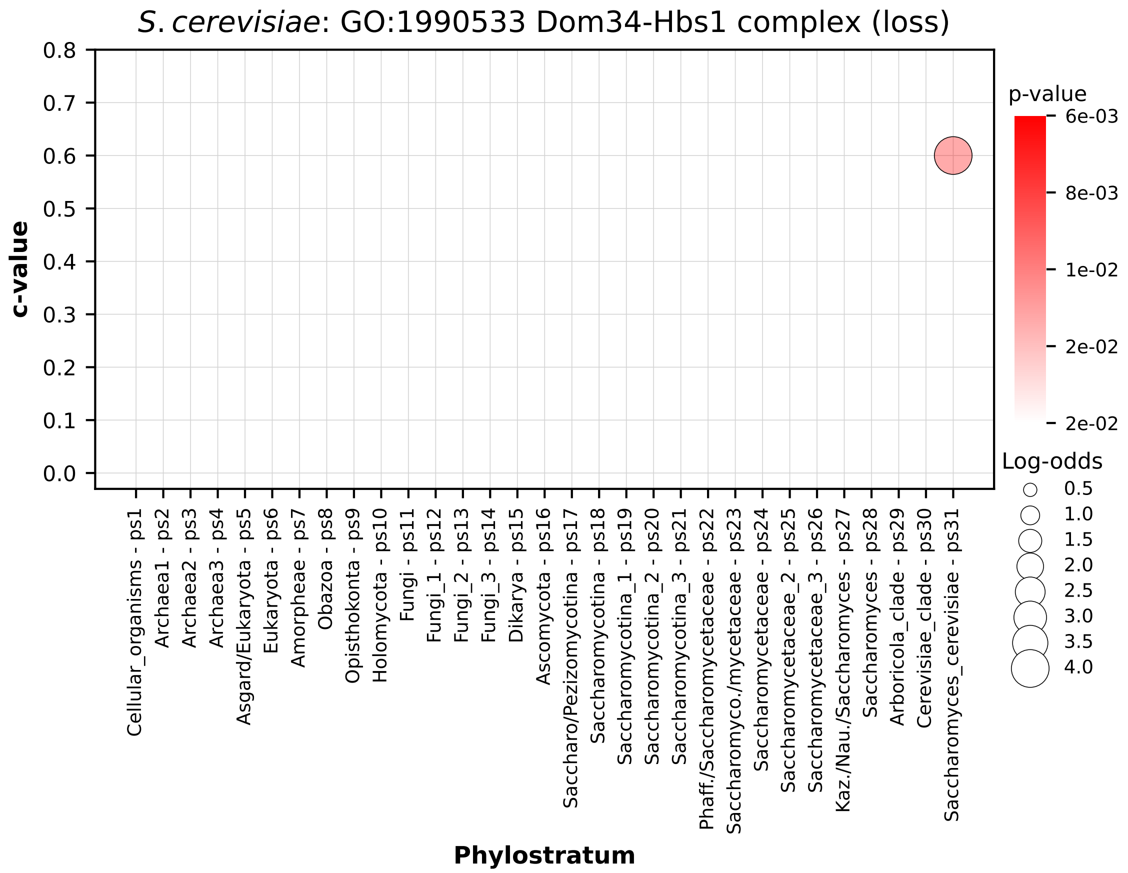

S. cerevisiae: GO:1990533 Dom34-Hbs1 complex (loss)
c-value
0.8
0.7
0.6
0.5
0.4
0.3
0.2
0.1
0.0
p-value
6e-03
8e-03
1e-02
2e-02
2e-02
Log-odds
0.5
1.0
1.5
2.0
2.5
3.0
3.5
4.0
Cellular_organisms - ps1
Archaea1 - ps2
Archaea2 - ps3
Archaea3 - ps4
Asgard/Eukaryota - ps5
Eukaryota - ps6
Amorpheae - ps7
Obazoa - ps8
Opisthokonta - ps9
Holomycota - ps10
Fungi - ps11
Fungi_1 - ps12
Fungi_2 - ps13
Fungi_3 - ps14
Dikarya - ps15
Ascomycota - ps16
Saccharo/Pezizomycotina - ps17
Saccharomycotina - ps18
Saccharomycotina_1 - ps19
Saccharomycotina_2 - ps20
Saccharomycotina_3 - ps21
Phaff./Saccharomycetaceae - ps22
Saccharomyco./mycetaceae - ps23
Saccharomycetaceae - ps24
Saccharomycetaceae_2 - ps25
Saccharomycetaceae_3 - ps26
Kaz./Nau./Saccharomyces - ps27
Saccharomyces - ps28
Arboricola_clade - ps29
Cerevisiae_clade - ps30
Saccharomyces_cerevisiae - ps31
Phylostratum

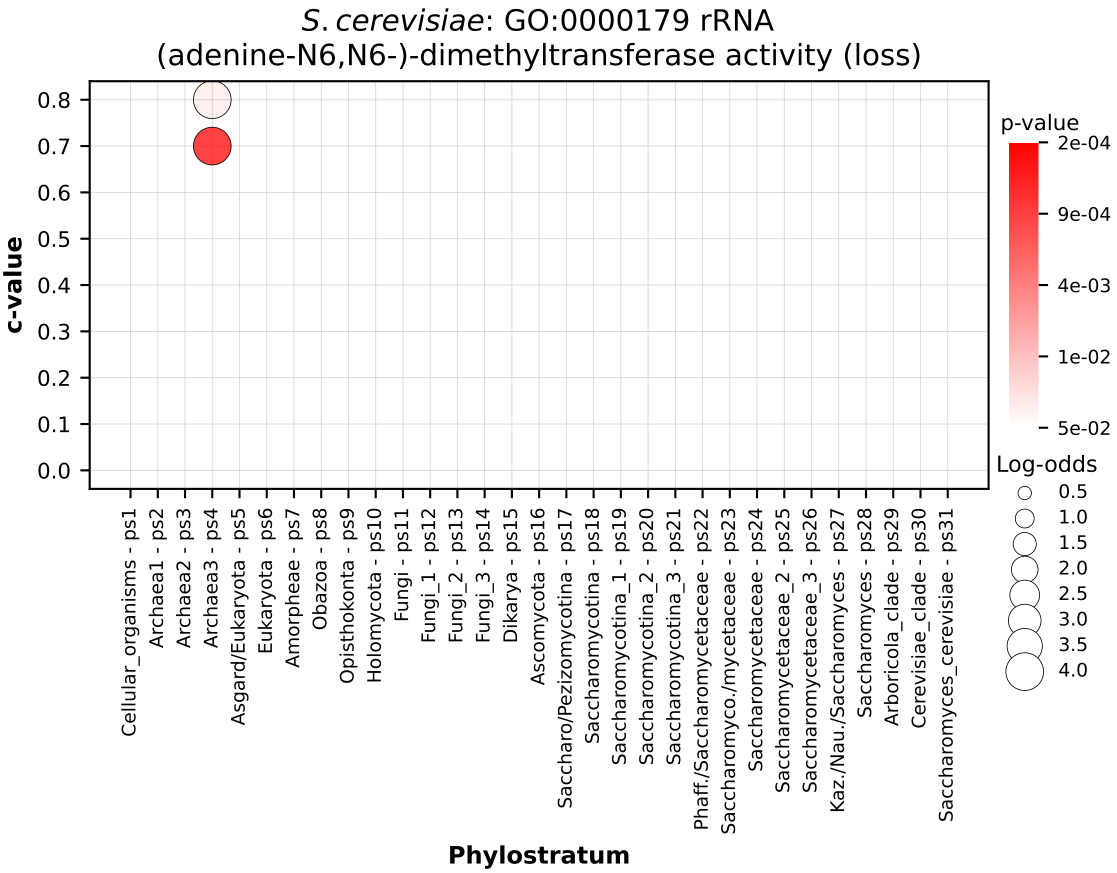
S. cerevisiae: GO:0000179 rRNA
(adenine-N6,N6-)-dimethyltransferase activity (loss)
c-value
0.8
0.7
0.6
0.5
0.4
0.3
0.2
0.1
0.0
p-value
2e-04
9e-04
4e-03
1e-02
5e-02
Log-odds
0.5
1.0
1.5
2.0
2.5
3.0
3.5
4.0
Cellular_organisms - ps1
Archaea1 - ps2
Archaea2 - ps3
Archaea3 - ps4
Asgard/Eukaryota - ps5
Eukaryota - ps6
Amorpheae - ps7
Obazoa - ps8
Opisthokonta - ps9
Holomycota - ps10
Fungi - ps11
Fungi_1 - ps12
Fungi_2 - ps13
Fungi_3 - ps14
Dikarya - ps15
Ascomycota - ps16
Saccharo/Pezizomycotina - ps17
Saccharomycotina - ps18
Saccharomycotina_1 - ps19
Saccharomycotina_2 - ps20
Saccharomycotina_3 - ps21
Phaff./Saccharomycetaceae - ps22
Saccharomyco./mycetaceae - ps23
Saccharomycetaceae - ps24
Saccharomycetaceae_2 - ps25
Saccharomycetaceae_3 - ps26
Kaz./Nau./Saccharomyces - ps27
Saccharomyces - ps28
Arboricola_clade - ps29
Cerevisiae_clade - ps30
Saccharomyces_cerevisiae - ps31
Phylostratum

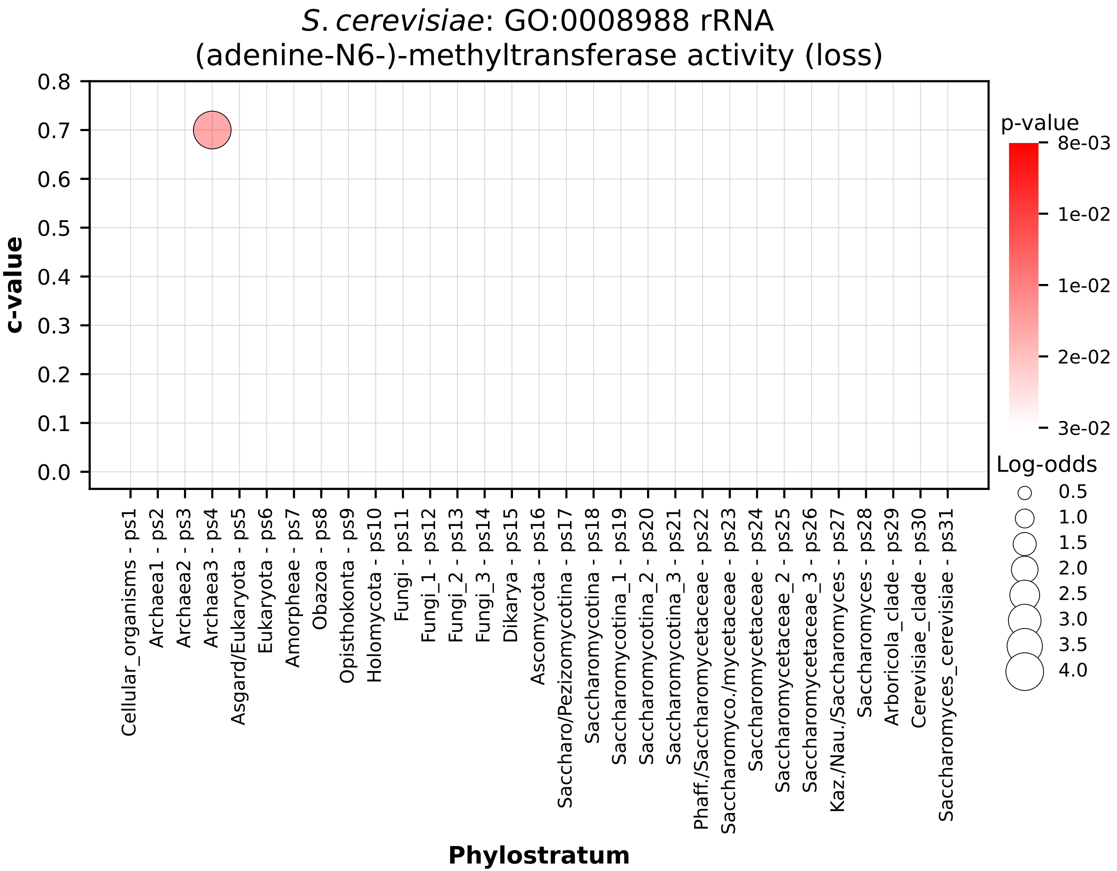

S. cerevisiae: GO:0008988 rRNA
(adenine-N6-)-methyltransferase activity (loss)
c-value
0.8
0.7
0.6
0.5
0.4
0.3
0.2
0.1
0.0
p-value
8e-03
1e-02
1e-02
2e-02
3e-02
Log-odds
0.5
1.0
1.5
2.0
2.5
3.0
3.5
4.0
Cellular_organisms - ps1
Archaea1 - ps2
Archaea2 - ps3
Archaea3 - ps4
Asgard/Eukaryota - ps5
Eukaryota - ps6
Amorpheae - ps7
Obazoa - ps8
Opisthokonta - ps9
Holomycota - ps10
Fungi - ps11
Fungi_1 - ps12
Fungi_2 - ps13
Fungi_3 - ps14
Dikarya - ps15
Ascomycota - ps16
Saccharo/Pezizomycotina - ps17
Saccharomycotina - ps18
Saccharomycotina_1 - ps19
Saccharomycotina_2 - ps20
Saccharomycotina_3 - ps21
Phaff./Saccharomycetaceae - ps22
Saccharomyco./mycetaceae - ps23
Saccharomycetaceae - ps24
Saccharomycetaceae_2 - ps25
Saccharomycetaceae_3 - ps26
Kaz./Nau./Saccharomyces - ps27
Saccharomyces - ps28
Arboricola_clade - ps29
Cerevisiae_clade - ps30
Saccharomyces_cerevisiae - ps31
Phylostratum

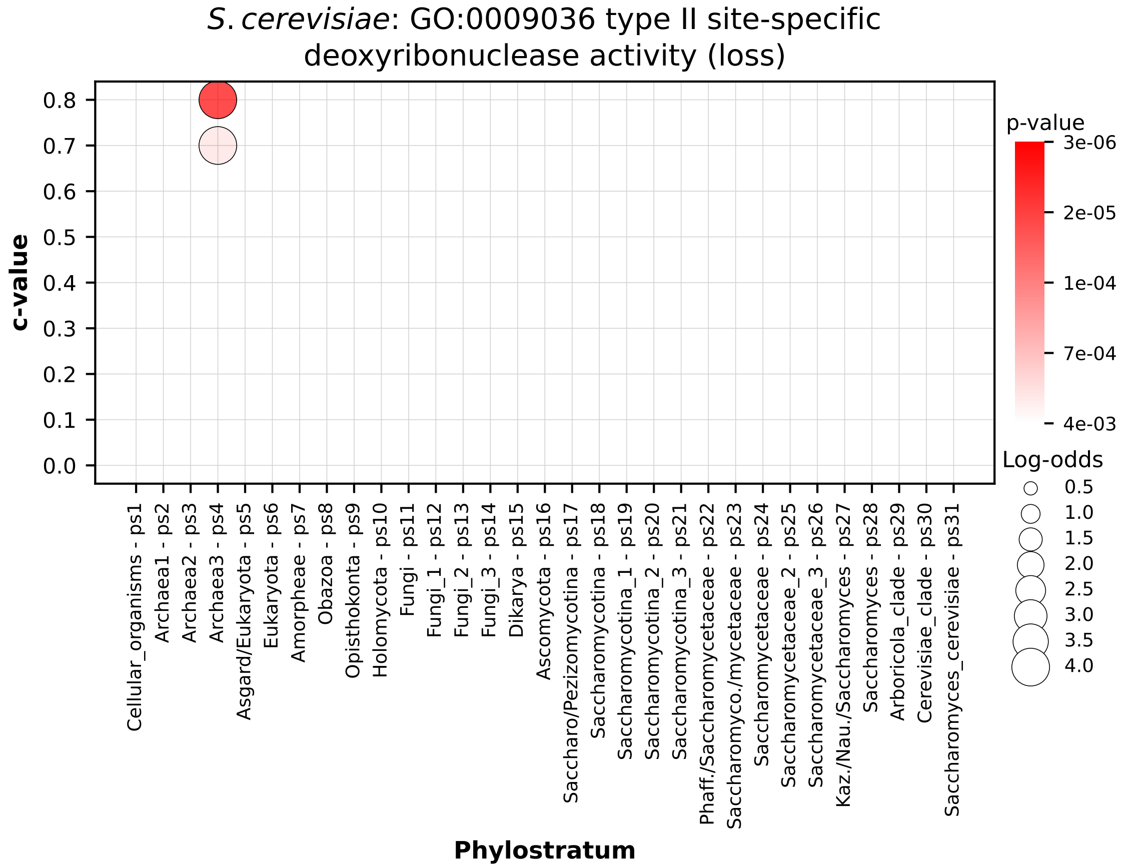
S. cerevisiae: GO:0009036 type II site-specific deoxyribonuclease activity (loss)
c-value
0.8
0.7
0.6
0.5
0.4
0.3
0.2
0.1
0.0
p-value
3e-06
2e-05
1e-04
7e-04
4e-03
Log-odds
0.5
1.0
1.5
2.0
2.5
3.0
3.5
4.0
Cellular_organisms - ps1
Archaea1 - ps2
Archaea2 - ps3
Archaea3 - ps4
Asgard/Eukaryota - ps5
Eukaryota - ps6
Amorpheae - ps7
Obazoa - ps8
Opisthokonta - ps9
Holomycota - ps10
Fungi - ps11
Fungi_1 - ps12
Fungi_2 - ps13
Fungi_3 - ps14
Dikarya - ps15
Ascomycota - ps16
Saccharo/Pezizomycotina - ps17
Saccharomycotina - ps18
Saccharomycotina_1 - ps19
Saccharomycotina_2 - ps20
Saccharomycotina_3 - ps21
Phaff./Saccharomycetaceae - ps22
Saccharomyco./mycetaceae - ps23
Saccharomycetaceae - ps24
Saccharomycetaceae_2 - ps25
Saccharomycetaceae_3 - ps26
Kaz./Nau./Saccharomyces - ps27
Saccharomyces - ps28
Arboricola_clade - ps29
Cerevisiae_clade - ps30
Saccharomyces_cerevisiae - ps31
Phylostratum

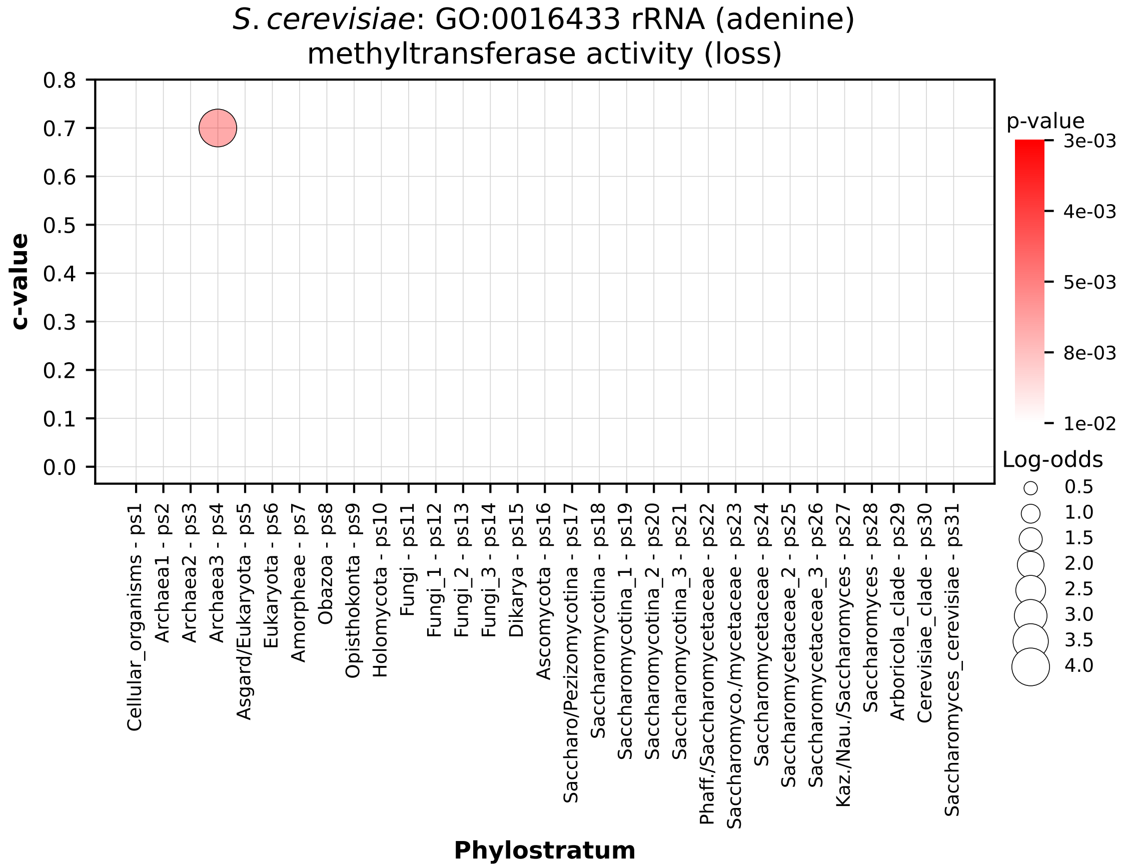

S. cerevisiae: GO:0016433 rRNA (adenine) methyltransferase activity (loss)
c-value
0.8
0.7
0.6
0.5
0.4
0.3
0.2
0.1
0.0
p-value
3e-03
4e-03
5e-03
8e-03
1e-02
Log-odds
0.5
1.0
1.5
2.0
2.5
3.0
3.5
4.0
Cellular_organisms - ps1
Archaea1 - ps2
Archaea2 - ps3
Archaea3 - ps4
Asgard/Eukaryota - ps5
Eukaryota - ps6
Amorpheae - ps7
Obazoa - ps8
Opisthokonta - ps9
Holomycota - ps10
Fungi - ps11
Fungi_1 - ps12
Fungi_2 - ps13
Fungi_3 - ps14
Dikarya - ps15
Ascomycota - ps16
Saccharo/Pezizomycotina - ps17
Saccharomycotina - ps18
Saccharomycotina_1 - ps19
Saccharomycotina_2 - ps20
Saccharomycotina_3 - ps21
Phaff./Saccharomycetaceae - ps22
Saccharomyco./mycetaceae - ps23
Saccharomycetaceae - ps24
Saccharomycetaceae_2 - ps25
Saccharomycetaceae_3 - ps26
Kaz./Nau./Saccharomyces - ps27
Saccharomyces - ps28
Arboricola_clade - ps29
Cerevisiae_clade - ps30
Saccharomyces_cerevisiae - ps31
Phylostratum

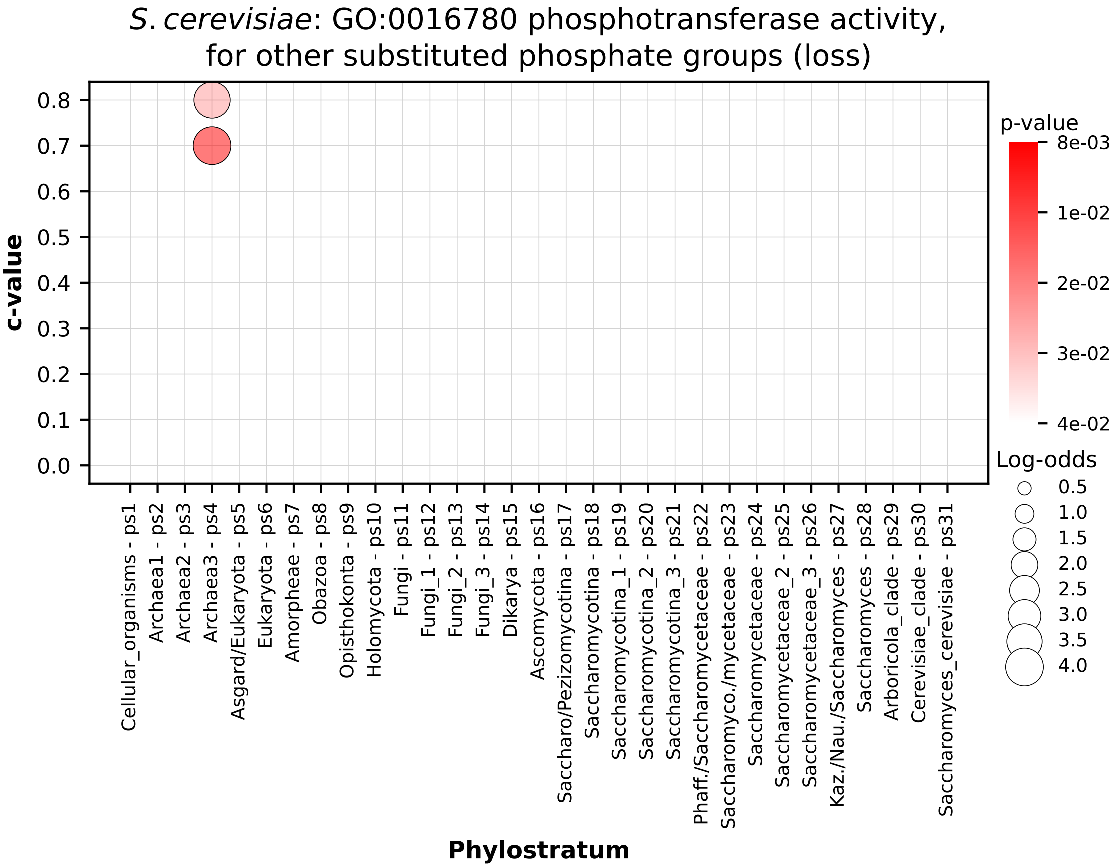

S. cerevisiae: GO:0016780 phosphotransferase activity, for other substituted phosphate groups (loss)
c-value
0.0
0.1
0.2
0.3
0.4
0.5
0.6
0.7
0.8
Phylostratum
Cellular_organisms - ps1
Archaea1 - ps2
Archaea2 - ps3
Archaea3 - ps4
Asgard/Eukaryota - ps5
Eukaryota - ps6
Amorpheae - ps7
Obazoa - ps8
Opisthokonta - ps9
Holomycota - ps10
Fungi - ps11
Fungi_1 - ps12
Fungi_2 - ps13
Fungi_3 - ps14
Dikarya - ps15
Ascomycota - ps16
Saccharo/Pezizomycotina - ps17
Saccharomycotina - ps18
Saccharomycotina_1 - ps19
Saccharomycotina_2 - ps20
Saccharomycotina_3 - ps21
Phaff./Saccharomycetaceae - ps22
Saccharomyco./mycetaceae - ps23
Saccharomycetaceae - ps24
Saccharomycetaceae_2 - ps25
Saccharomycetaceae_3 - ps26
Kaz./Nau./Saccharomyces - ps27
Saccharomyces - ps28
Arboricola_clade - ps29
Cerevisiae_clade - ps30
Saccharomyces_cerevisiae - ps31
p-value
8e-03
1e-02
2e-02
3e-02
4e-02
Log-odds
0.5
1.0
1.5
2.0
2.5
3.0
3.5
4.0

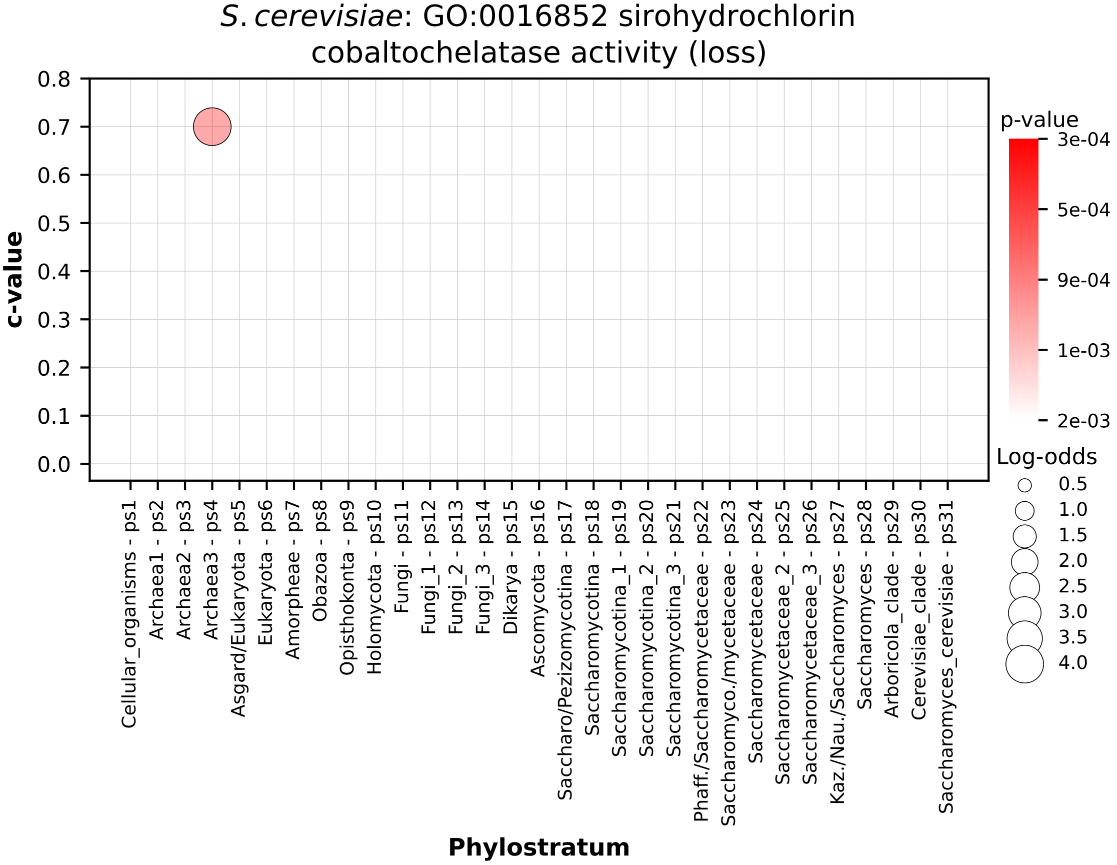
S. cerevisiae: GO:0016852 sirohydrochlorin cobaltochelatase activity (loss)
c-value
0.8
0.7
0.6
0.5
0.4
0.3
0.2
0.1
0.0
p-value
3e-04
5e-04
9e-04
1e-03
2e-03
Log-odds
0.5
1.0
1.5
2.0
2.5
3.0
3.5
4.0
Cellular_organisms - ps1
Archaea1 - ps2
Archaea2 - ps3
Archaea3 - ps4
Asgard/Eukaryota - ps5
Eukaryota - ps6
Amorpheae - ps7
Obazoa - ps8
Opisthokonta - ps9
Holomycota - ps10
Fungi - ps11
Fungi_1 - ps12
Fungi_2 - ps13
Fungi_3 - ps14
Dikarya - ps15
Ascomycota - ps16
Saccharo/Pezizomycotina - ps17
Saccharomycotina - ps18
Saccharomycotina_1 - ps19
Saccharomycotina_2 - ps20
Saccharomycotina_3 - ps21
Phaff./Saccharomycetaceae - ps22
Saccharomyco./mycetaceae - ps23
Saccharomycetaceae - ps24
Saccharomycetaceae_2 - ps25
Saccharomycetaceae_3 - ps26
Kaz./Nau./Saccharomyces - ps27
Saccharomyces - ps28
Arboricola_clade - ps29
Cerevisiae_clade - ps30
Saccharomyces_cerevisiae - ps31
Phylostratum

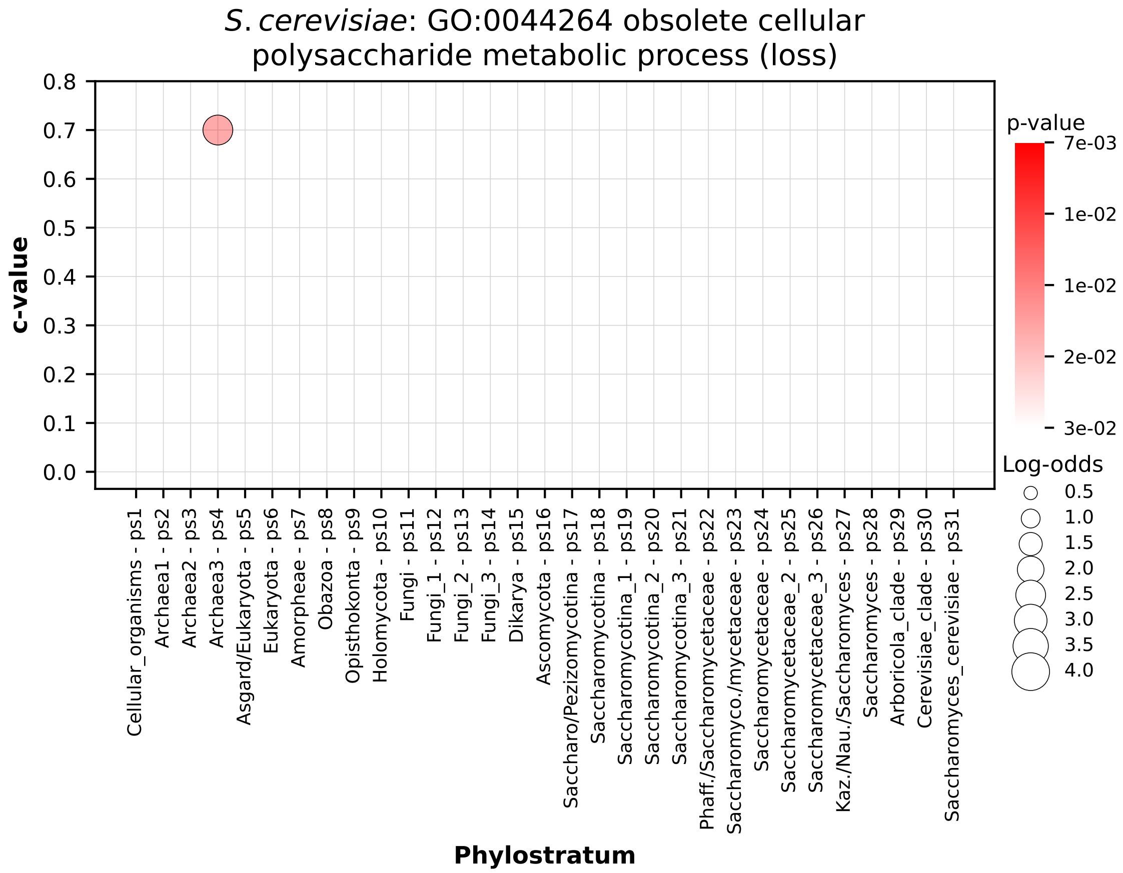

S. cerevisiae: GO:0044264 obsolete cellular polysaccharide metabolic process (loss)
c-value
0.0
0.1
0.2
0.3
0.4
0.5
0.6
0.7
0.8
p-value
7e-03
1e-02
1e-02
2e-02
3e-02
Log-odds
0.5
1.0
1.5
2.0
2.5
3.0
3.5
4.0
Cellular_organisms - ps1
Archaea1 - ps2
Archaea2 - ps3
Archaea3 - ps4
Asgard/Eukaryota - ps5
Eukaryota - ps6
Amorpheae - ps7
Obazoa - ps8
Opisthokonta - ps9
Holomycota - ps10
Fungi - ps11
Fungi_1 - ps12
Fungi_2 - ps13
Fungi_3 - ps14
Dikarya - ps15
Ascomycota - ps16
Saccharo/Pezizomycotina - ps17
Saccharomycotina - ps18
Saccharomycotina_1 - ps19
Saccharomycotina_2 - ps20
Saccharomycotina_3 - ps21
Phaff./Saccharomycetaceae - ps22
Saccharomyco./mycetaceae - ps23
Saccharomycetaceae - ps24
Saccharomycetaceae_2 - ps25
Saccharomycetaceae_3 - ps26
Kaz./Nau./Saccharomyces - ps27
Saccharomyces - ps28
Arboricola_clade - ps29
Cerevisiae_clade - ps30
Saccharomyces_cerevisiae - ps31
Phylostratum

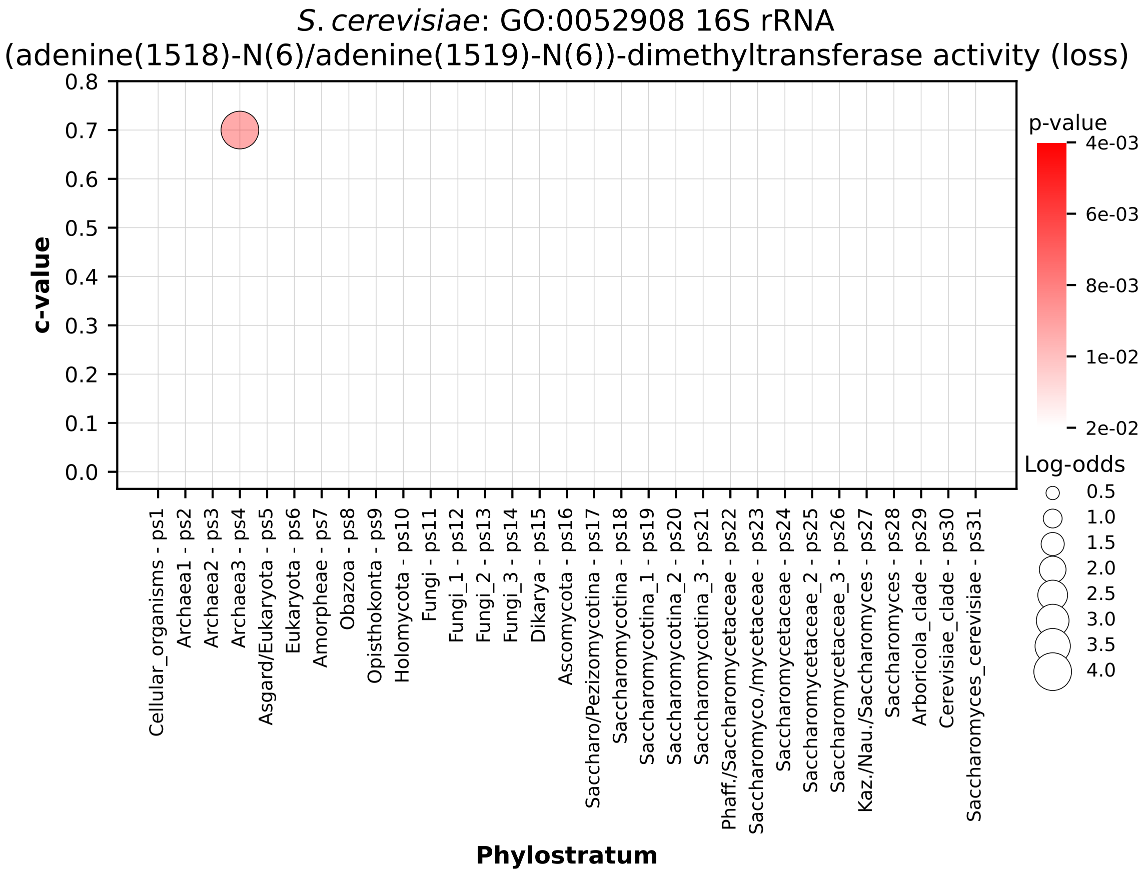

S. cerevisiae: GO:0052908 16S rRNA (adenine(1518)-N(6)/adenine(1519)-N(6))-dimethyltransferase activity (loss)
c-value
0.8
0.7
0.6
0.5
0.4
0.3
0.2
0.1
0.0
Phylostratum
Cellular_organisms - ps1
Archaea1 - ps2
Archaea2 - ps3
Archaea3 - ps4
Asgard/Eukaryota - ps5
Eukaryota - ps6
Amorpheae - ps7
Obazoa - ps8
Opisthokonta - ps9
Holomycota - ps10
Fungi - ps11
Fungi_1 - ps12
Fungi_2 - ps13
Fungi_3 - ps14
Dikarya - ps15
Ascomycota - ps16
Saccharo/Pezizomycotina - ps17
Saccharomycotina - ps18
Saccharomycotina_1 - ps19
Saccharomycotina_2 - ps20
Saccharomycotina_3 - ps21
Phaff./Saccharomycetaceae - ps22
Saccharomyco./mycetaceae - ps23
Saccharomycetaceae - ps24
Saccharomycetaceae_2 - ps25
Saccharomycetaceae_3 - ps26
Kaz./Nau./Saccharomyces - ps27
Saccharomyces - ps28
Arboricola_clade - ps29
Cerevisiae_clade - ps30
Saccharomyces_cerevisiae - ps31
p-value
4e-03
6e-03
8e-03
1e-02
2e-02
Log-odds
0.5
1.0
1.5
2.0
2.5
3.0
3.5
4.0

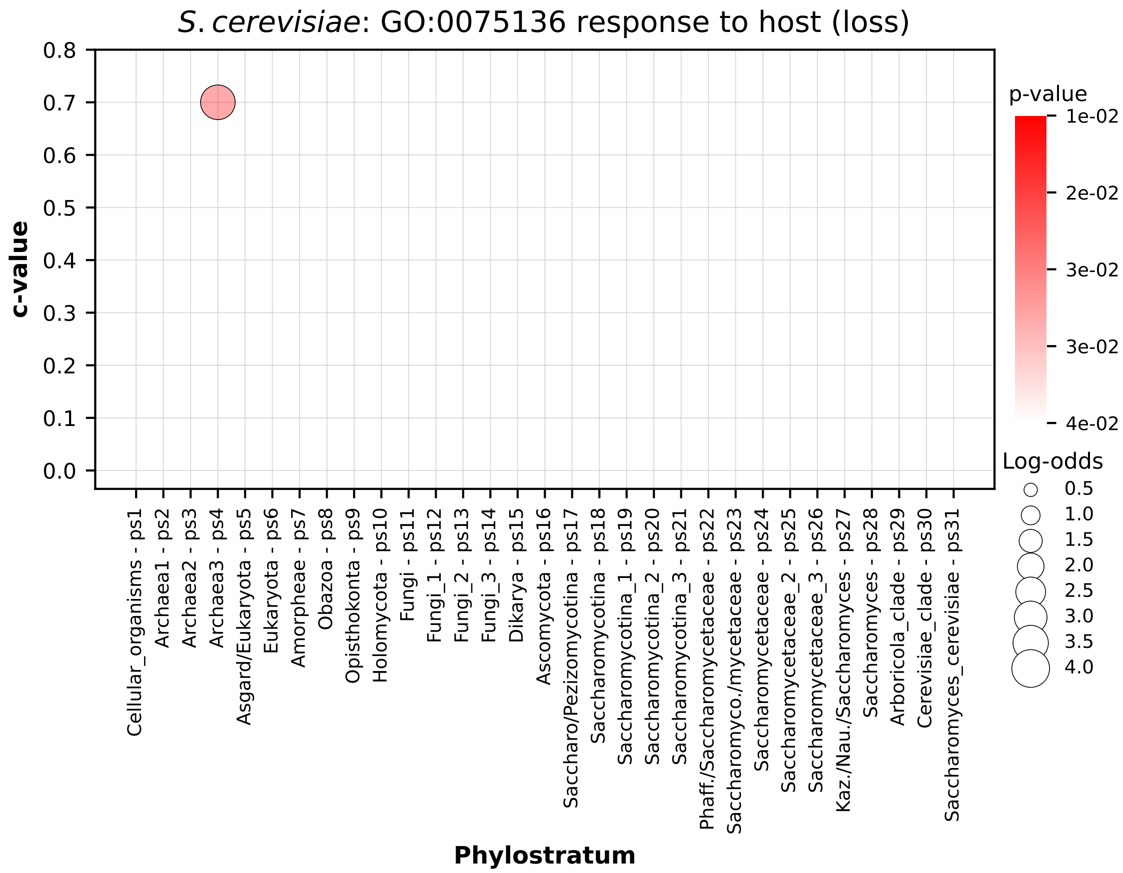

S. cerevisiae: GO:0075136 response to host (loss)
c-value
0.8
0.7
0.6
0.5
0.4
0.3
0.2
0.1
0.0
p-value
1e-02
2e-02
3e-02
3e-02
4e-02
Log-odds
0.5
1.0
1.5
2.0
2.5
3.0
3.5
4.0
Cellular_organisms - ps1
Archaea1 - ps2
Archaea2 - ps3
Archaea3 - ps4
Asgard/Eukaryota - ps5
Eukaryota - ps6
Amorpheae - ps7
Obazoa - ps8
Opisthokonta - ps9
Holomycota - ps10
Fungi - ps11
Fungi_1 - ps12
Fungi_2 - ps13
Fungi_3 - ps14
Dikarya - ps15
Ascomycota - ps16
Saccharo/Pezizomycotina - ps17
Saccharomycotina - ps18
Saccharomycotina_1 - ps19
Saccharomycotina_2 - ps20
Saccharomycotina_3 - ps21
Phaff./Saccharomycetaceae - ps22
Saccharomyco./mycetaceae - ps23
Saccharomycetaceae - ps24
Saccharomycetaceae_2 - ps25
Saccharomycetaceae_3 - ps26
Kaz./Nau./Saccharomyces - ps27
Saccharomyces - ps28
Arboricola_clade - ps29
Cerevisiae_clade - ps30
Saccharomyces_cerevisiae - ps31
Phylostratum

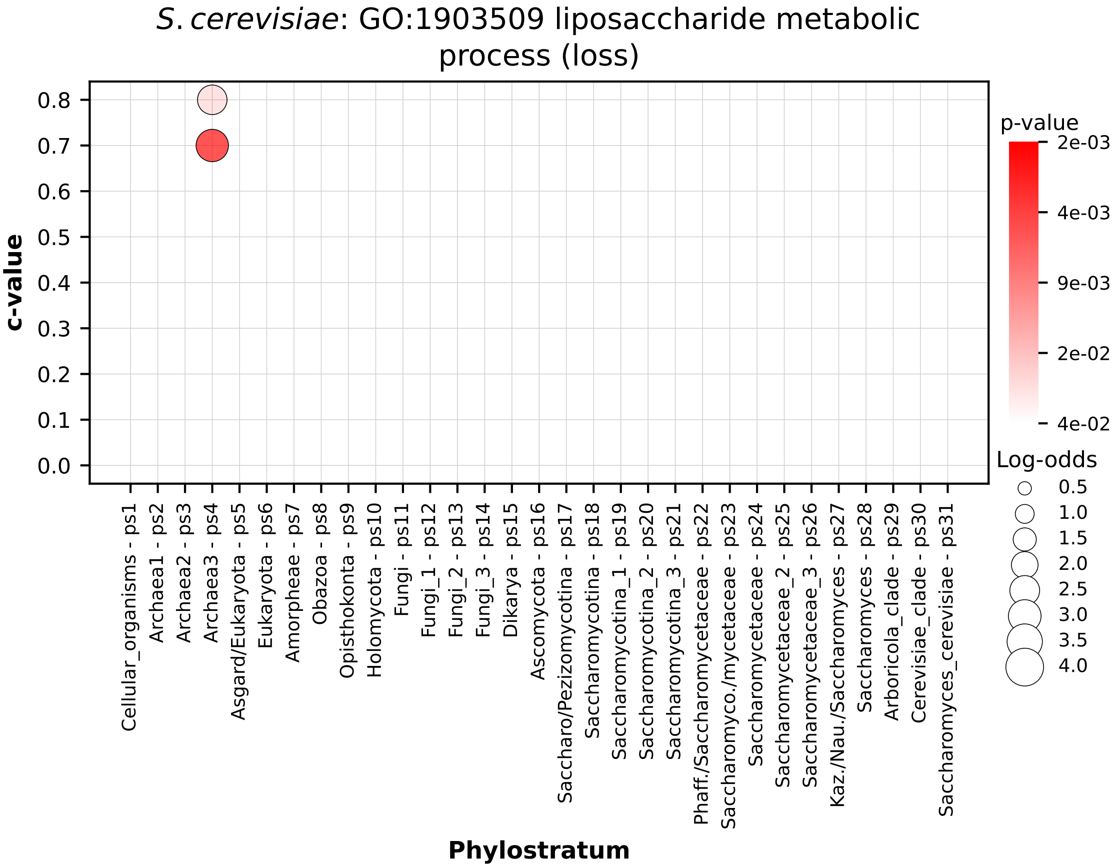

S. cerevisiae: GO:1903509 liposaccharide metabolic process (loss)
c-value
0.8
0.7
0.6
0.5
0.4
0.3
0.2
0.1
0.0
p-value
2e-03
4e-03
9e-03
2e-02
4e-02
Log-odds
0.5
1.0
1.5
2.0
2.5
3.0
3.5
4.0
Cellular_organisms - ps1
Archaea1 - ps2
Archaea2 - ps3
Archaea3 - ps4
Asgard/Eukaryota - ps5
Eukaryota - ps6
Amorpheae - ps7
Obazoa - ps8
Opisthokonta - ps9
Holomycota - ps10
Fungi - ps11
Fungi_1 - ps12
Fungi_2 - ps13
Fungi_3 - ps14
Dikarya - ps15
Ascomycota - ps16
Saccharo/Pezizomycotina - ps17
Saccharomycotina - ps18
Saccharomycotina_1 - ps19
Saccharomycotina_2 - ps20
Saccharomycotina_3 - ps21
Phaff./Saccharomycetaceae - ps22
Saccharomyco./mycetaceae - ps23
Saccharomycetaceae - ps24
Saccharomycetaceae_2 - ps25
Saccharomycetaceae_3 - ps26
Kaz./Nau./Saccharomyces - ps27
Saccharomyces - ps28
Arboricola_clade - ps29
Cerevisiae_clade - ps30
Saccharomyces_cerevisiae - ps31
Phylostratum

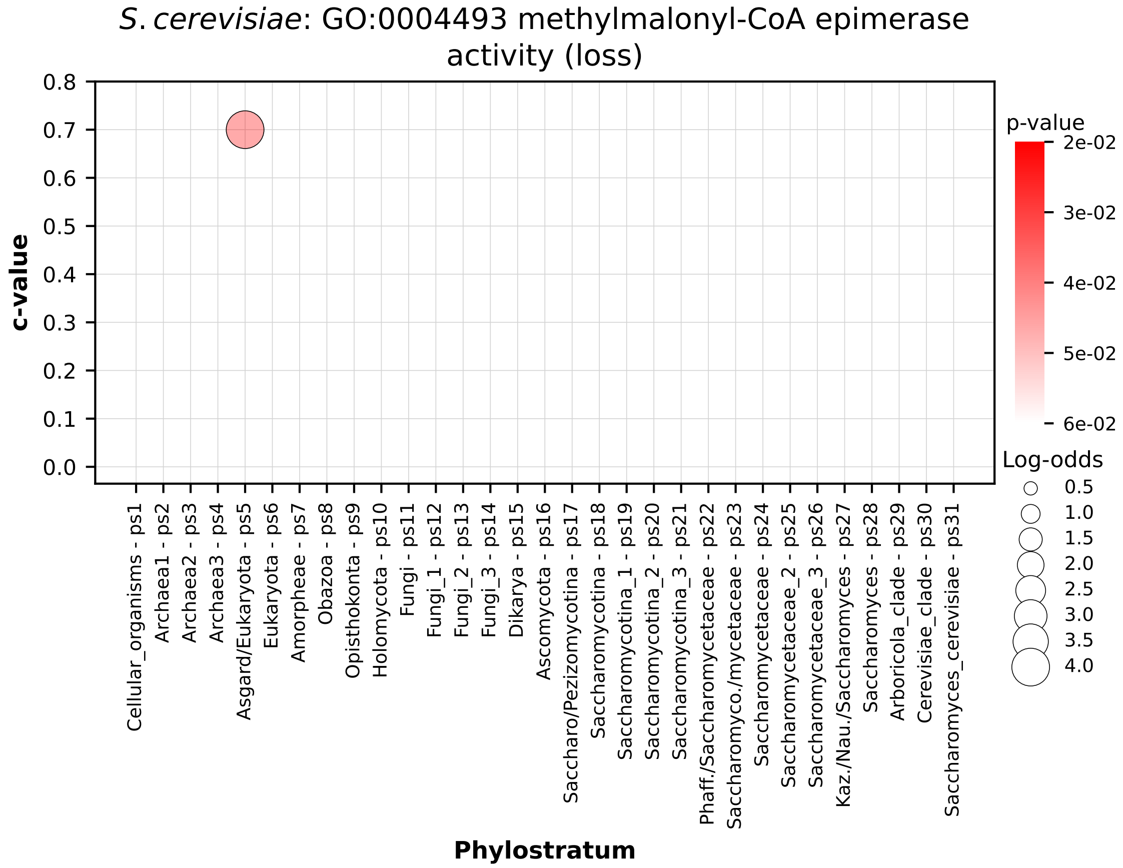

S. cerevisiae: GO:0004493 methylmalonyl-CoA epimerase activity (loss)
c-value
0.8
0.7
0.6
0.5
0.4
0.3
0.2
0.1
0.0
p-value
2e-02
3e-02
4e-02
5e-02
6e-02
Log-odds
0.5
1.0
1.5
2.0
2.5
3.0
3.5
4.0
Cellular_organisms - ps1
Archaea1 - ps2
Archaea2 - ps3
Archaea3 - ps4
Asgard/Eukaryota - ps5
Eukaryota - ps6
Amorpheae - ps7
Obazoa - ps8
Opisthokonta - ps9
Holomycota - ps10
Fungi - ps11
Fungi_1 - ps12
Fungi_2 - ps13
Fungi_3 - ps14
Dikarya - ps15
Ascomycota - ps16
Saccharo/Pezizomycotina - ps17
Saccharomycotina - ps18
Saccharomycotina_1 - ps19
Saccharomycotina_2 - ps20
Saccharomycotina_3 - ps21
Phaff./Saccharomycetaceae - ps22
Saccharomyco./mycetaceae - ps23
Saccharomycetaceae - ps24
Saccharomycetaceae_2 - ps25
Saccharomycetaceae_3 - ps26
Kaz./Nau./Saccharomyces - ps27
Saccharomyces - ps28
Arboricola_clade - ps29
Cerevisiae_clade - ps30
Saccharomyces_cerevisiae - ps31
Phylostratum

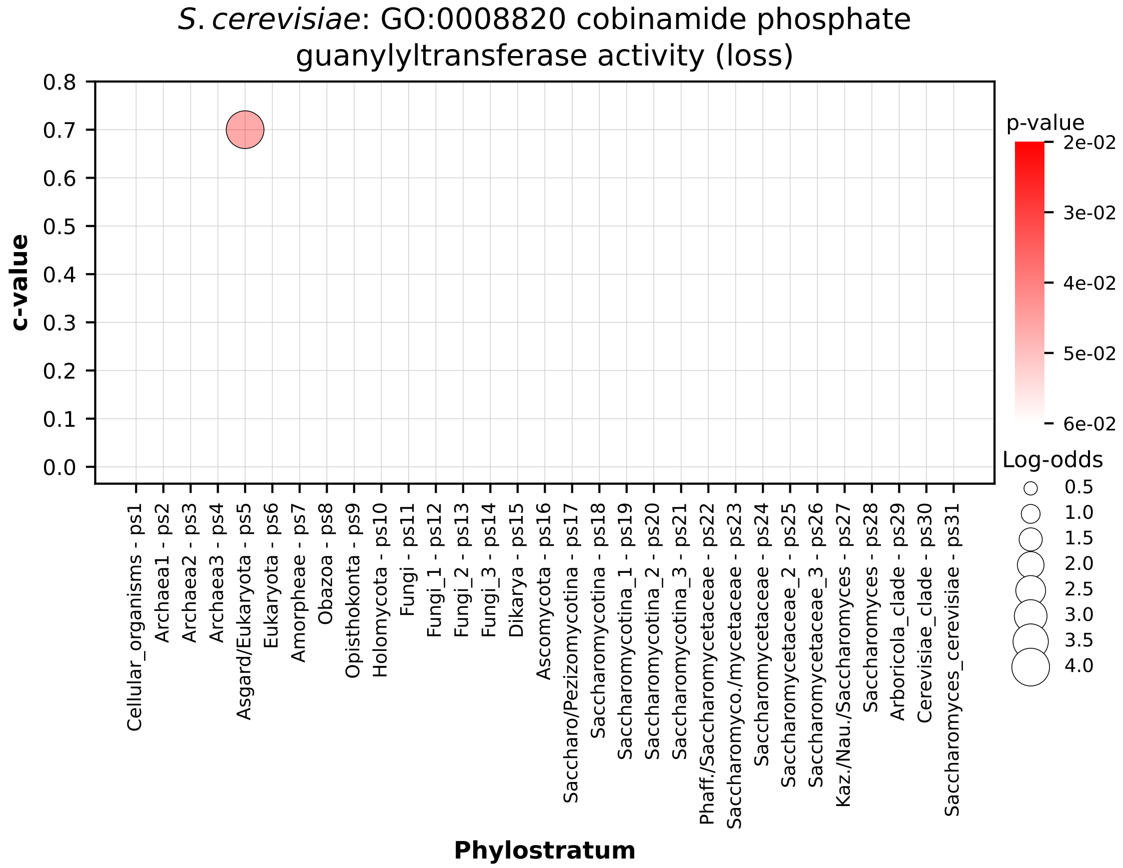

S. cerevisiae: GO:0008820 cobinamide phosphate guanylyltransferase activity (loss)
c-value
0.0
0.1
0.2
0.3
0.4
0.5
0.6
0.7
0.8
p-value
2e-02
3e-02
4e-02
5e-02
6e-02
Log-odds
0.5
1.0
1.5
2.0
2.5
3.0
3.5
4.0
Cellular_organisms - ps1
Archaea1 - ps2
Archaea2 - ps3
Archaea3 - ps4
Asgard/Eukaryota - ps5
Eukaryota - ps6
Amorpheae - ps7
Obazoa - ps8
Opisthokonta - ps9
Holomycota - ps10
Fungi - ps11
Fungi_1 - ps12
Fungi_2 - ps13
Fungi_3 - ps14
Dikarya - ps15
Ascomycota - ps16
Saccharo/Pezizomycotina - ps17
Saccharomycotina - ps18
Saccharomycotina_1 - ps19
Saccharomycotina_2 - ps20
Saccharomycotina_3 - ps21
Phaff./Saccharomycetaceae - ps22
Saccharomyco./mycetaceae - ps23
Saccharomycetaceae - ps24
Saccharomycetaceae_2 - ps25
Saccharomycetaceae_3 - ps26
Kaz./Nau./Saccharomyces - ps27
Saccharomyces - ps28
Arboricola_clade - ps29
Cerevisiae_clade - ps30
Saccharomyces_cerevisiae - ps31
Phylostratum

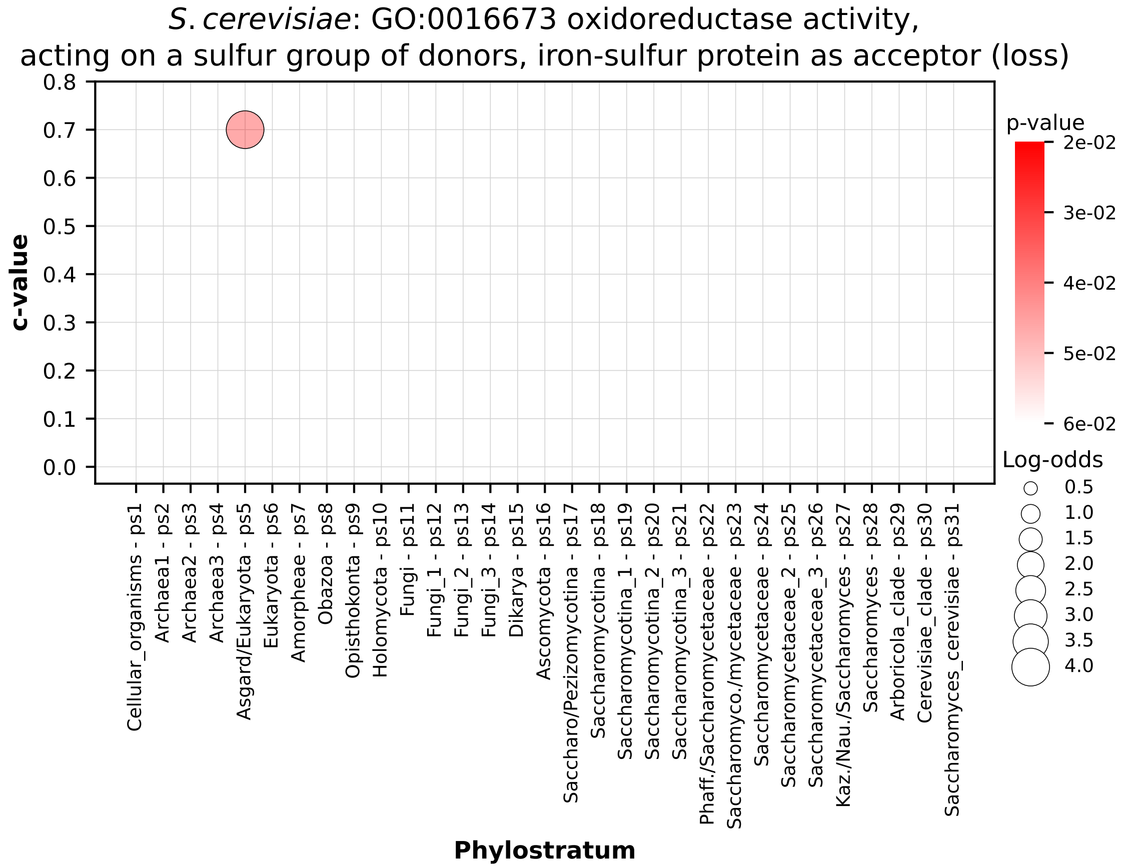
S. cerevisiae: GO:0016673 oxidoreductase activity,
acting on a sulfur group of donors, iron-sulfur protein as acceptor (loss)
c-value
0.8
0.7
0.6
0.5
0.4
0.3
0.2
0.1
0.0
p-value
2e-02
3e-02
4e-02
5e-02
6e-02
Log-odds
0.5
1.0
1.5
2.0
2.5
3.0
3.5
4.0
Cellular_organisms - ps1
Archaea1 - ps2
Archaea2 - ps3
Archaea3 - ps4
Asgard/Eukaryota - ps5
Eukaryota - ps6
Amorpheae - ps7
Obazoa - ps8
Opisthokonta - ps9
Holomycota - ps10
Fungi - ps11
Fungi_1 - ps12
Fungi_2 - ps13
Fungi_3 - ps14
Dikarya - ps15
Ascomycota - ps16
Saccharo/Pezizomycotina - ps17
Saccharomycotina - ps18
Saccharomycotina_1 - ps19
Saccharomycotina_2 - ps20
Saccharomycotina_3 - ps21
Phaff./Saccharomycetaceae - ps22
Saccharomyco./mycetaceae - ps23
Saccharomycetaceae - ps24
Saccharomycetaceae_2 - ps25
Saccharomycetaceae_3 - ps26
Kaz./Nau./Saccharomyces - ps27
Saccharomyces - ps28
Arboricola_clade - ps29
Cerevisiae_clade - ps30
Saccharomyces_cerevisiae - ps31
Phylostratum

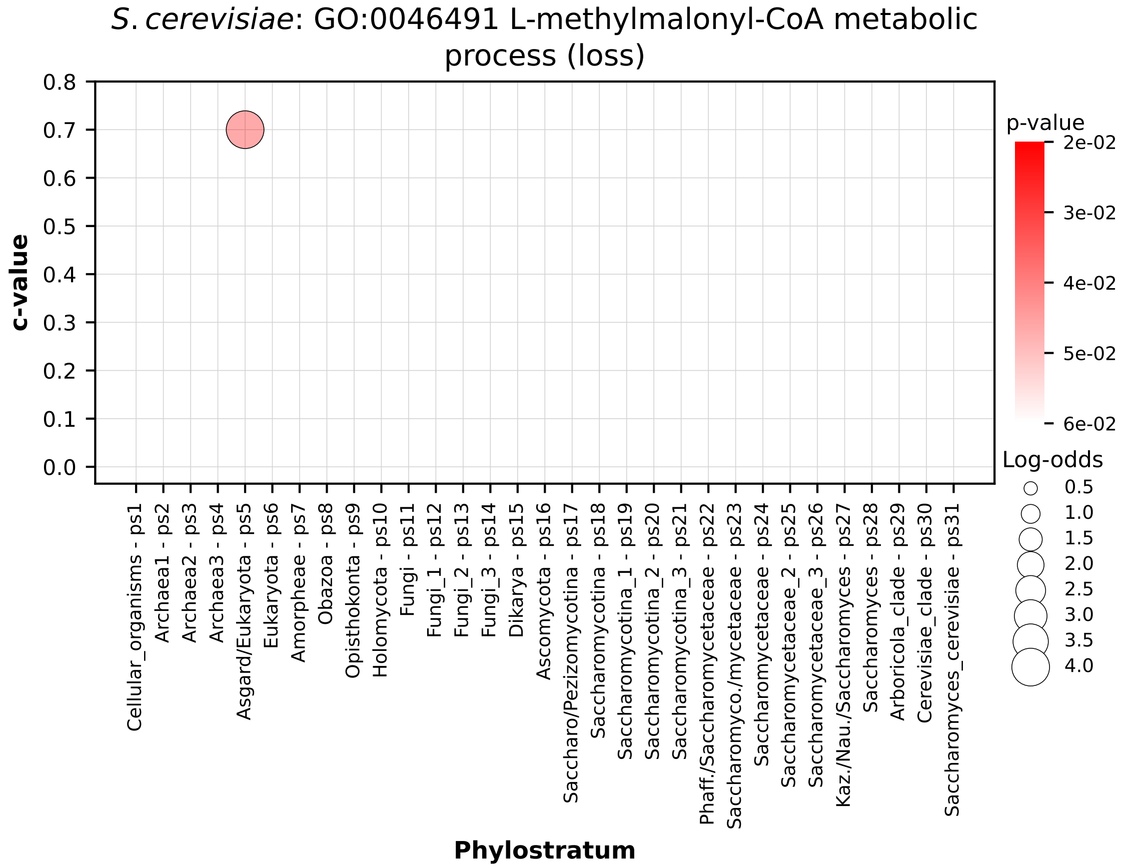

S. cerevisiae: GO:0046491 L-methylmalonyl-CoA metabolic process (loss)
c-value
0.8
0.7
0.6
0.5
0.4
0.3
0.2
0.1
0.0
Phylostratum
Cellular_organisms - ps1
Archaea1 - ps2
Archaea2 - ps3
Archaea3 - ps4
Asgard/Eukaryota - ps5
Eukaryota - ps6
Amorpheae - ps7
Obazoa - ps8
Opisthokonta - ps9
Holomycota - ps10
Fungi - ps11
Fungi_1 - ps12
Fungi_2 - ps13
Fungi_3 - ps14
Dikarya - ps15
Ascomycota - ps16
Saccharo/Pezizomycotina - ps17
Saccharomycotina - ps18
Saccharomycotina_1 - ps19
Saccharomycotina_2 - ps20
Saccharomycotina_3 - ps21
Phaff./Saccharomycetaceae - ps22
Saccharomyco./mycetaceae - ps23
Saccharomycetaceae - ps24
Saccharomycetaceae_2 - ps25
Saccharomycetaceae_3 - ps26
Kaz./Nau./Saccharomyces - ps27
Saccharomyces - ps28
Arboricola_clade - ps29
Cerevisiae_clade - ps30
Saccharomyces_cerevisiae - ps31
p-value
2e-02
3e-02
4e-02
5e-02
6e-02
Log-odds
0.5
1.0
1.5
2.0
2.5
3.0
3.5
4.0

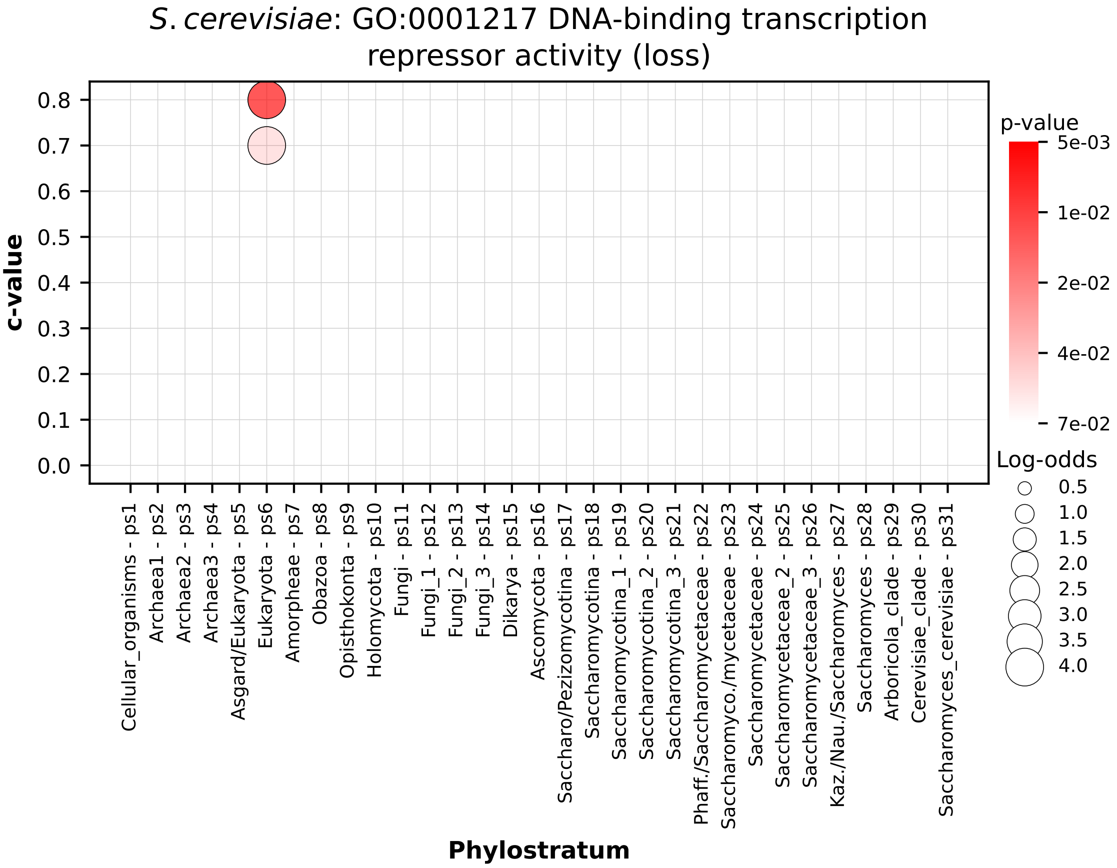

S. cerevisiae: GO:0001217 DNA-binding transcription repressor activity (loss)
c-value
0.8
0.7
0.6
0.5
0.4
0.3
0.2
0.1
0.0
p-value
5e-03
1e-02
2e-02
4e-02
7e-02
Log-odds
0.5
1.0
1.5
2.0
2.5
3.0
3.5
4.0
Cellular_organisms - ps1
Archaea1 - ps2
Archaea2 - ps3
Archaea3 - ps4
Asgard/Eukaryota - ps5
Eukaryota - ps6
Amorpheae - ps7
Obazoa - ps8
Opisthokonta - ps9
Holomycota - ps10
Fungi - ps11
Fungi_1 - ps12
Fungi_2 - ps13
Fungi_3 - ps14
Dikarya - ps15
Ascomycota - ps16
Saccharo/Pezizomycotina - ps17
Saccharomycotina - ps18
Saccharomycotina_1 - ps19
Saccharomycotina_2 - ps20
Saccharomycotina_3 - ps21
Phaff./Saccharomycetaceae - ps22
Saccharomyco./mycetaceae - ps23
Saccharomycetaceae - ps24
Saccharomycetaceae_2 - ps25
Saccharomycetaceae_3 - ps26
Kaz./Nau./Saccharomyces - ps27
Saccharomyces - ps28
Arboricola_clade - ps29
Cerevisiae_clade - ps30
Saccharomyces_cerevisiae - ps31
Phylostratum

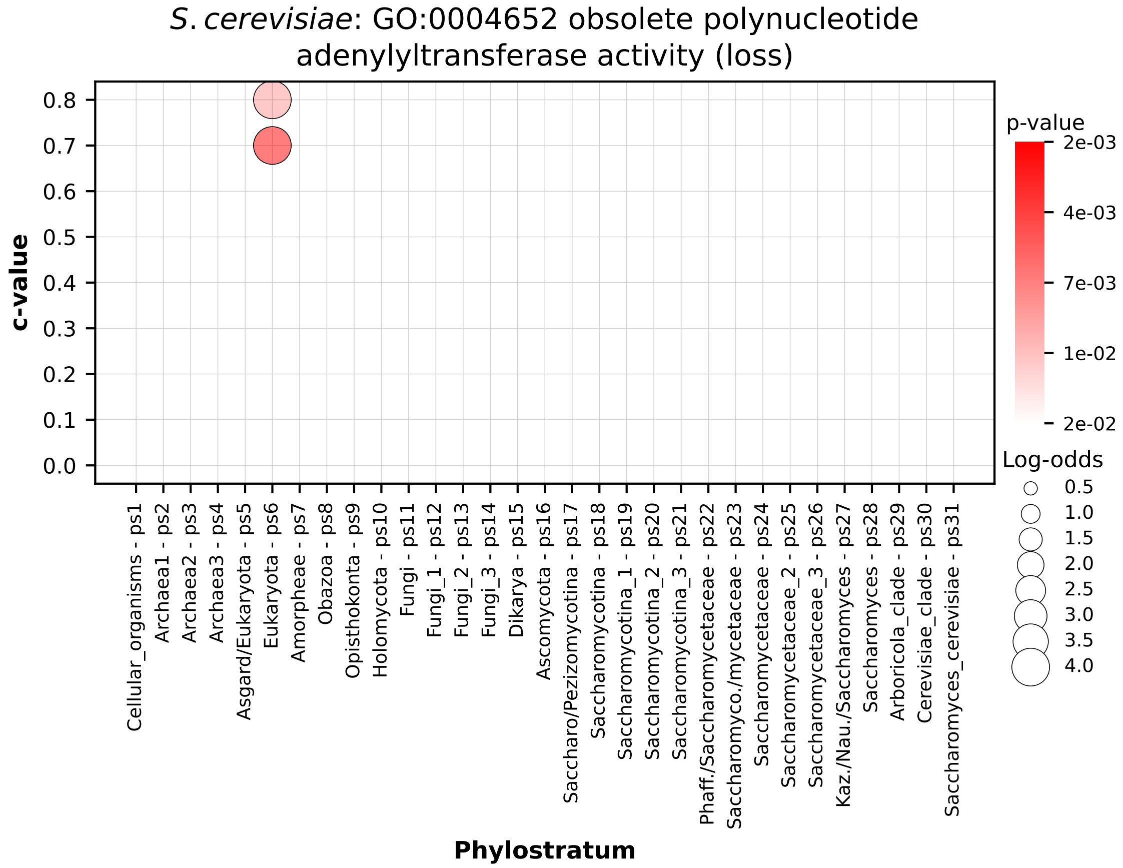

S. cerevisiae: GO:0004652 obsolete polynucleotide adenylyltransferase activity (loss)
c-value
0.0
0.1
0.2
0.3
0.4
0.5
0.6
0.7
0.8
Phylostratum
Cellular_organisms - ps1
Archaea1 - ps2
Archaea2 - ps3
Archaea3 - ps4
Asgard/Eukaryota - ps5
Eukaryota - ps6
Amorpheae - ps7
Obazoa - ps8
Opisthokonta - ps9
Holomycota - ps10
Fungi - ps11
Fungi_1 - ps12
Fungi_2 - ps13
Fungi_3 - ps14
Dikarya - ps15
Ascomycota - ps16
Saccharo/Pezizomycotina - ps17
Saccharomycotina - ps18
Saccharomycotina_1 - ps19
Saccharomycotina_2 - ps20
Saccharomycotina_3 - ps21
Phaff./Saccharomycetaceae - ps22
Saccharomyco./mycetaceae - ps23
Saccharomycetaceae - ps24
Saccharomycetaceae_2 - ps25
Saccharomycetaceae_3 - ps26
Kaz./Nau./Saccharomyces - ps27
Saccharomyces - ps28
Arboricola_clade - ps29
Cerevisiae_clade - ps30
Saccharomyces_cerevisiae - ps31
p-value
2e-03
4e-03
7e-03
1e-02
2e-02
Log-odds
0.5
1.0
1.5
2.0
2.5
3.0
3.5
4.0

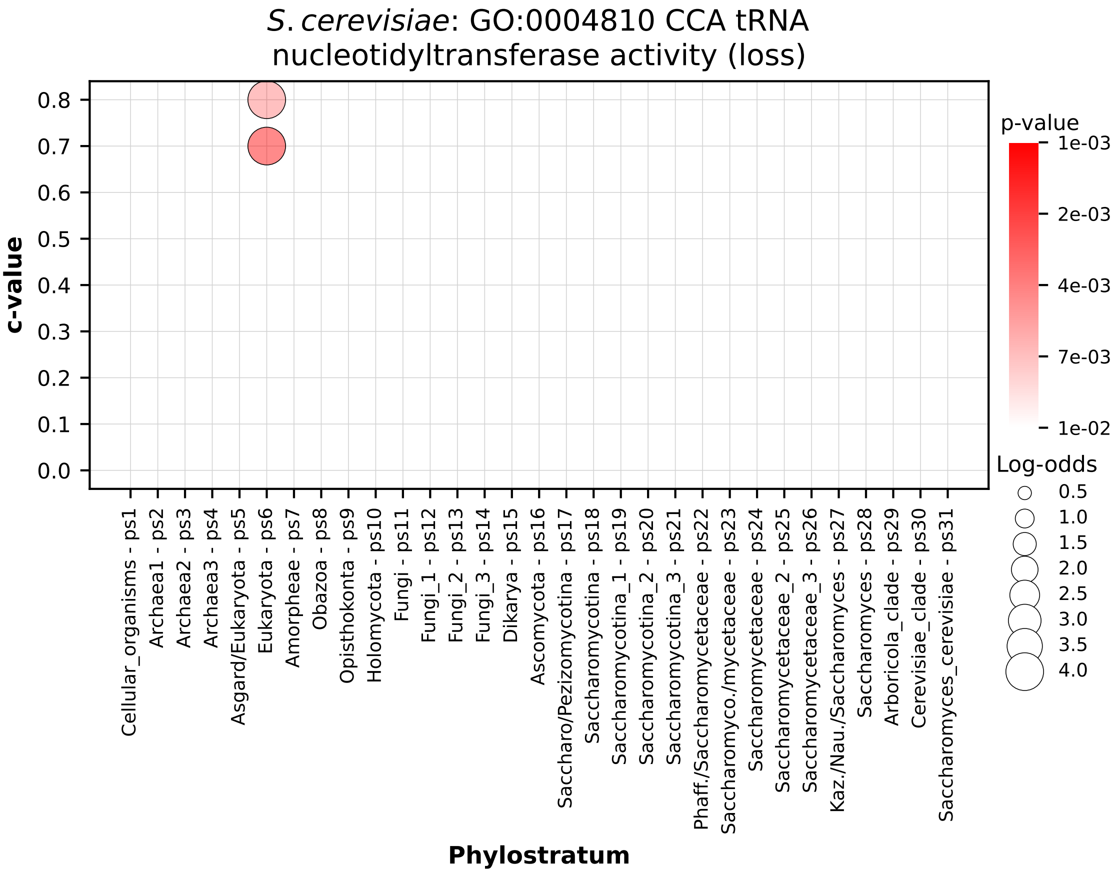

S. cerevisiae: GO:0004810 CCA tRNA nucleotidyltransferase activity (loss)
c-value
0.8
0.7
0.6
0.5
0.4
0.3
0.2
0.1
0.0
Phylostratum
Cellular_organisms - ps1
Archaea1 - ps2
Archaea2 - ps3
Archaea3 - ps4
Asgard/Eukaryota - ps5
Eukaryota - ps6
Amorpheae - ps7
Obazoa - ps8
Opisthokonta - ps9
Holomycota - ps10
Fungi - ps11
Fungi_1 - ps12
Fungi_2 - ps13
Fungi_3 - ps14
Dikarya - ps15
Ascomycota - ps16
Saccharo/Pezizomycotina - ps17
Saccharomycotina - ps18
Saccharomycotina_1 - ps19
Saccharomycotina_2 - ps20
Saccharomycotina_3 - ps21
Phaff./Saccharomycetaceae - ps22
Saccharomyco./mycetaceae - ps23
Saccharomycetaceae - ps24
Saccharomycetaceae_2 - ps25
Saccharomycetaceae_3 - ps26
Kaz./Nau./Saccharomyces - ps27
Saccharomyces - ps28
Arboricola_clade - ps29
Cerevisiae_clade - ps30
Saccharomyces_cerevisiae - ps31
p-value
1e-03
2e-03
4e-03
7e-03
1e-02
Log-odds
0.5
1.0
1.5
2.0
2.5
3.0
3.5
4.0

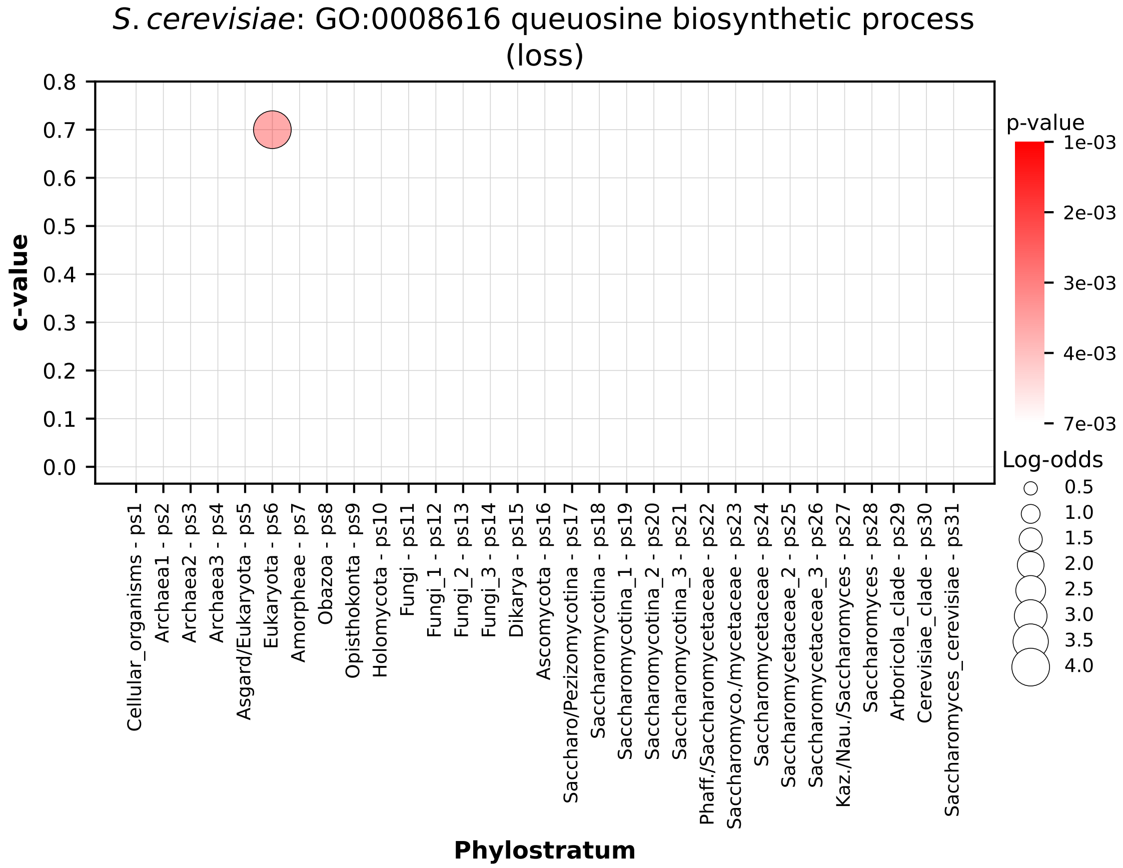

S. cerevisiae: GO:0008616 queuosine biosynthetic process (loss)
c-value
0.8
0.7
0.6
0.5
0.4
0.3
0.2
0.1
0.0
Phylostratum
Cellular_organisms - ps1
Archaea1 - ps2
Archaea2 - ps3
Archaea3 - ps4
Asgard/Eukaryota - ps5
Eukaryota - ps6
Amorpheae - ps7
Obazoa - ps8
Opisthokonta - ps9
Holomycota - ps10
Fungi - ps11
Fungi_1 - ps12
Fungi_2 - ps13
Fungi_3 - ps14
Dikarya - ps15
Ascomycota - ps16
Saccharo/Pezizomycotina - ps17
Saccharomycotina - ps18
Saccharomycotina_1 - ps19
Saccharomycotina_2 - ps20
Saccharomycotina_3 - ps21
Phaff./Saccharomycetaceae - ps22
Saccharomyco./mycetaceae - ps23
Saccharomycetaceae - ps24
Saccharomycetaceae_2 - ps25
Saccharomycetaceae_3 - ps26
Kaz./Nau./Saccharomyces - ps27
Saccharomyces - ps28
Arboricola_clade - ps29
Cerevisiae_clade - ps30
Saccharomyces_cerevisiae - ps31
p-value
1e-03
2e-03
3e-03
4e-03
7e-03
Log-odds
0.5
1.0
1.5
2.0
2.5
3.0
3.5
4.0

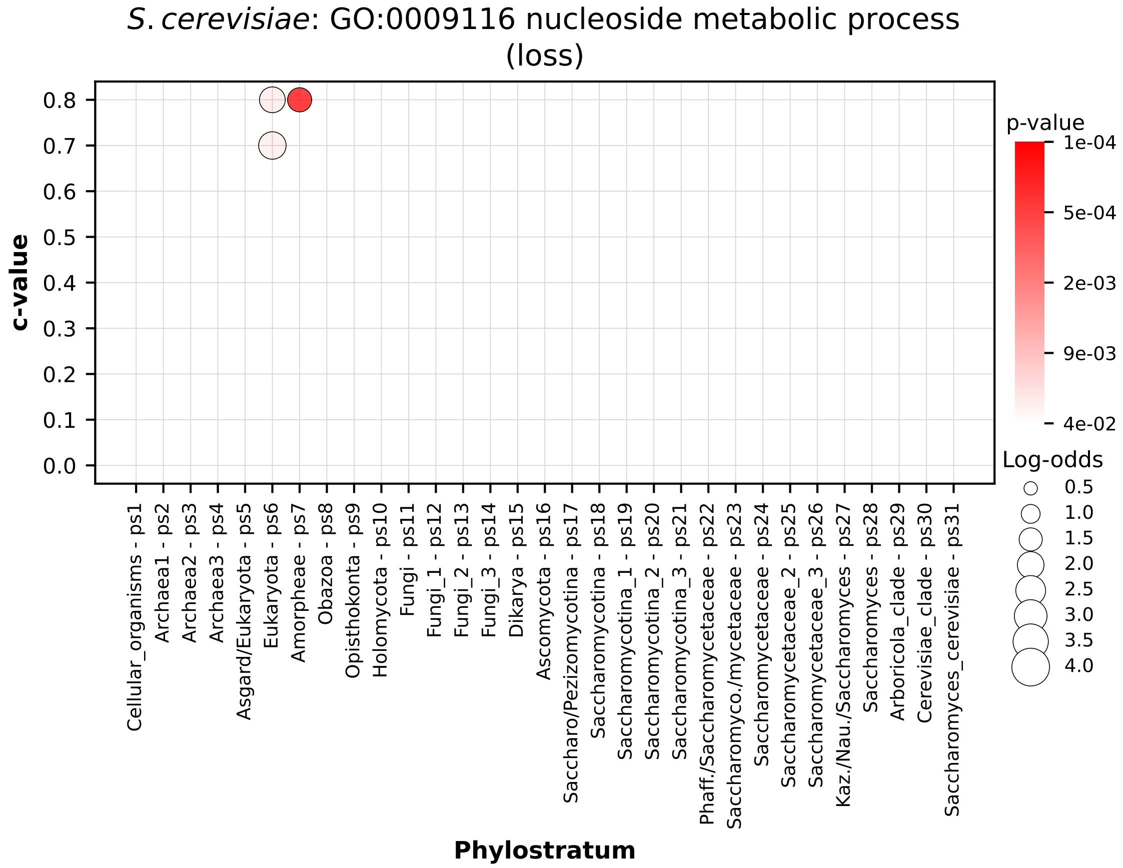

S. cerevisiae: GO:0009116 nucleoside metabolic process
(loss)
c-value
0.8
0.7
0.6
0.5
0.4
0.3
0.2
0.1
0.0
p-value
1e-04
5e-04
2e-03
9e-03
4e-02
Log-odds
0.5
1.0
1.5
2.0
2.5
3.0
3.5
4.0
Cellular_organisms - ps1
Archaea1 - ps2
Archaea2 - ps3
Archaea3 - ps4
Asgard/Eukaryota - ps5
Eukaryota - ps6
Amorpheae - ps7
Obazoa - ps8
Opisthokonta - ps9
Holomycota - ps10
Fungi - ps11
Fungi_1 - ps12
Fungi_2 - ps13
Fungi_3 - ps14
Dikarya - ps15
Ascomycota - ps16
Saccharo/Pezizomycotina - ps17
Saccharomycotina - ps18
Saccharomycotina_1 - ps19
Saccharomycotina_2 - ps20
Saccharomycotina_3 - ps21
Phaff./Saccharomycetaceae - ps22
Saccharomyco./mycetaceae - ps23
Saccharomycetaceae - ps24
Saccharomycetaceae_2 - ps25
Saccharomycetaceae_3 - ps26
Kaz./Nau./Saccharomyces - ps27
Saccharomyces - ps28
Arboricola_clade - ps29
Cerevisiae_clade - ps30
Saccharomyces_cerevisiae - ps31
Phylostratum

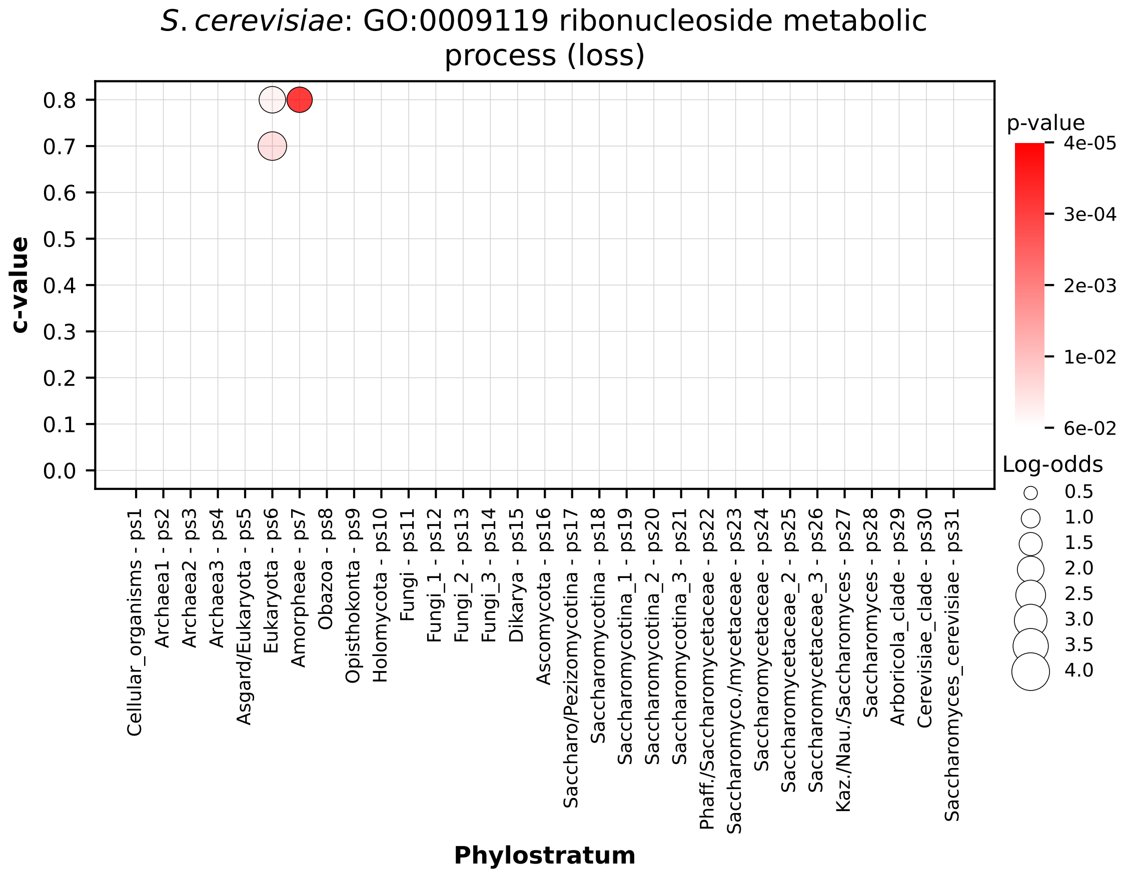

S. cerevisiae: GO:0009119 ribonucleoside metabolic process (loss)
c-value
0.8
0.7
0.6
0.5
0.4
0.3
0.2
0.1
0.0
p-value
4e-05
3e-04
2e-03
1e-02
6e-02
Log-odds
0.5
1.0
1.5
2.0
2.5
3.0
3.5
4.0
Cellular_organisms - ps1
Archaea1 - ps2
Archaea2 - ps3
Archaea3 - ps4
Asgard/Eukaryota - ps5
Eukaryota - ps6
Amorpheae - ps7
Obazoa - ps8
Opisthokonta - ps9
Holomycota - ps10
Fungi - ps11
Fungi_1 - ps12
Fungi_2 - ps13
Fungi_3 - ps14
Dikarya - ps15
Ascomycota - ps16
Saccharo/Pezizomycotina - ps17
Saccharomycotina - ps18
Saccharomycotina_1 - ps19
Saccharomycotina_2 - ps20
Saccharomycotina_3 - ps21
Phaff./Saccharomycetaceae - ps22
Saccharomyco./mycetaceae - ps23
Saccharomycetaceae - ps24
Saccharomycetaceae_2 - ps25
Saccharomycetaceae_3 - ps26
Kaz./Nau./Saccharomyces - ps27
Saccharomyces - ps28
Arboricola_clade - ps29
Cerevisiae_clade - ps30
Saccharomyces_cerevisiae - ps31
Phylostratum

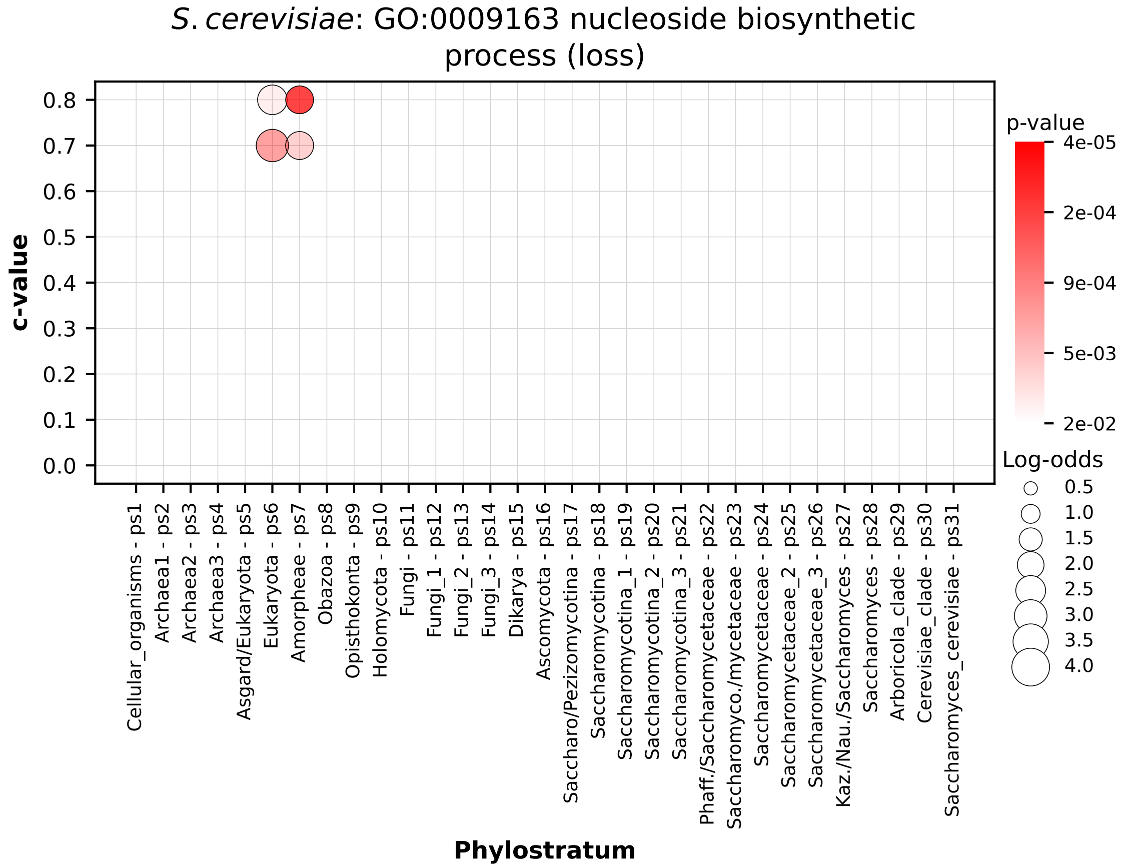
S. cerevisiae: GO:0009163 nucleoside biosynthetic process (loss)
c-value
Phylostratum
p-value
4e-05
2e-04
9e-04
5e-03
2e-02
Log-odds
0.5
1.0
1.5
2.0
2.5
3.0
3.5
4.0
Cellular_organisms - ps1
Archaea1 - ps2
Archaea2 - ps3
Archaea3 - ps4
Asgard/Eukaryota - ps5
Eukaryota - ps6
Amorpheae - ps7
Obazoa - ps8
Opisthokonta - ps9
Holomycota - ps10
Fungi - ps11
Fungi_1 - ps12
Fungi_2 - ps13
Fungi_3 - ps14
Dikarya - ps15
Ascomycota - ps16
Saccharo/Pezizomycotina - ps17
Saccharomycotina - ps18
Saccharomycotina_1 - ps19
Saccharomycotina_2 - ps20
Saccharomycotina_3 - ps21
Phaff./Saccharomycetaceae - ps22
Saccharomyco./mycetaceae - ps23
Saccharomycetaceae - ps24
Saccharomycetaceae_2 - ps25
Saccharomycetaceae_3 - ps26
Kaz./Nau./Saccharomyces - ps27
Saccharomyces - ps28
Arboricola_clade - ps29
Cerevisiae_clade - ps30
Saccharomyces_cerevisiae - ps31

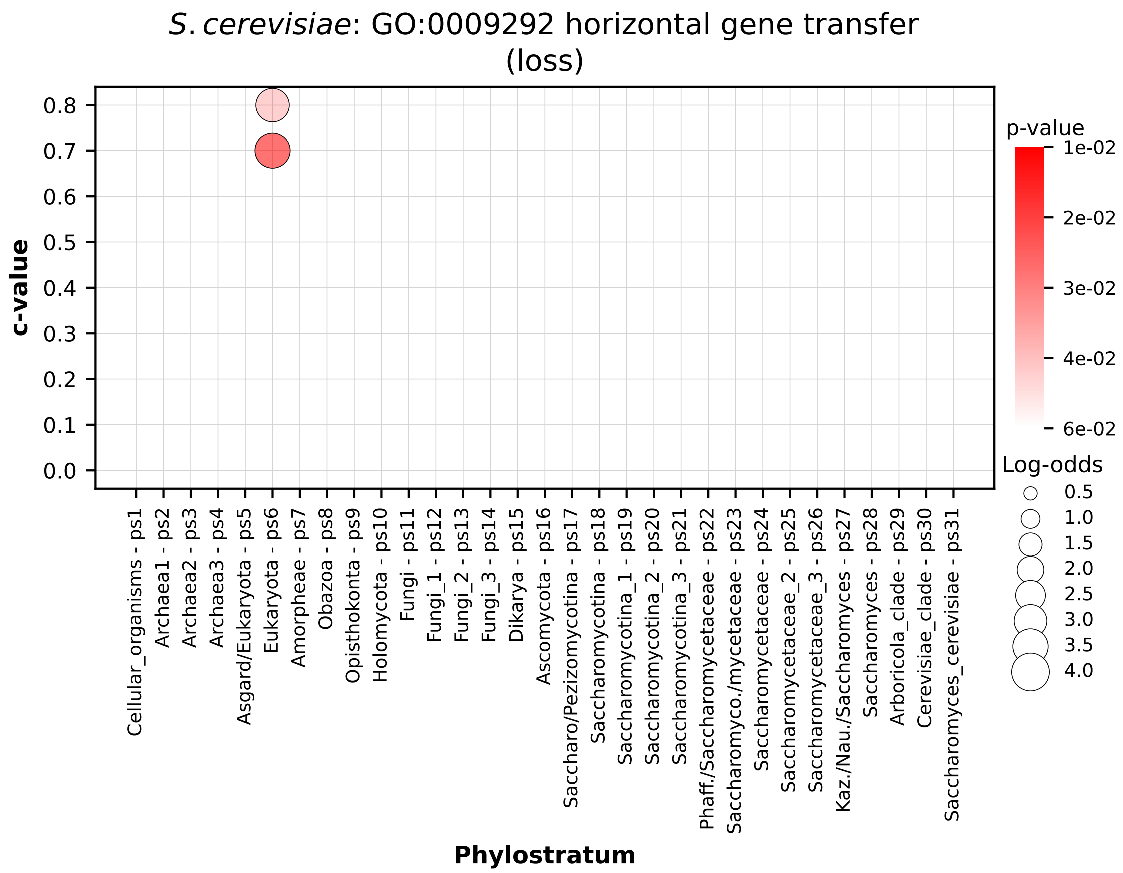
S. cerevisiae: GO:0009292 horizontal gene transfer
(loss)
c-value
0.0
0.1
0.2
0.3
0.4
0.5
0.6
0.7
0.8
p-value
1e-02
2e-02
3e-02
4e-02
6e-02
Log-odds
0.5
1.0
1.5
2.0
2.5
3.0
3.5
4.0
Cellular_organisms - ps1
Archaea1 - ps2
Archaea2 - ps3
Archaea3 - ps4
Asgard/Eukaryota - ps5
Eukaryota - ps6
Amorpheae - ps7
Obazoa - ps8
Opisthokonta - ps9
Holomycota - ps10
Fungi - ps11
Fungi_1 - ps12
Fungi_2 - ps13
Fungi_3 - ps14
Dikarya - ps15
Ascomycota - ps16
Saccharo/Pezizomycotina - ps17
Saccharomycotina - ps18
Saccharomycotina_1 - ps19
Saccharomycotina_2 - ps20
Saccharomycotina_3 - ps21
Phaff./Saccharomycetaceae - ps22
Saccharomyco./mycetaceae - ps23
Saccharomycetaceae - ps24
Saccharomycetaceae_2 - ps25
Saccharomycetaceae_3 - ps26
Kaz./Nau./Saccharomyces - ps27
Saccharomyces - ps28
Arboricola_clade - ps29
Cerevisiae_clade - ps30
Saccharomyces_cerevisiae - ps31
Phylostratum

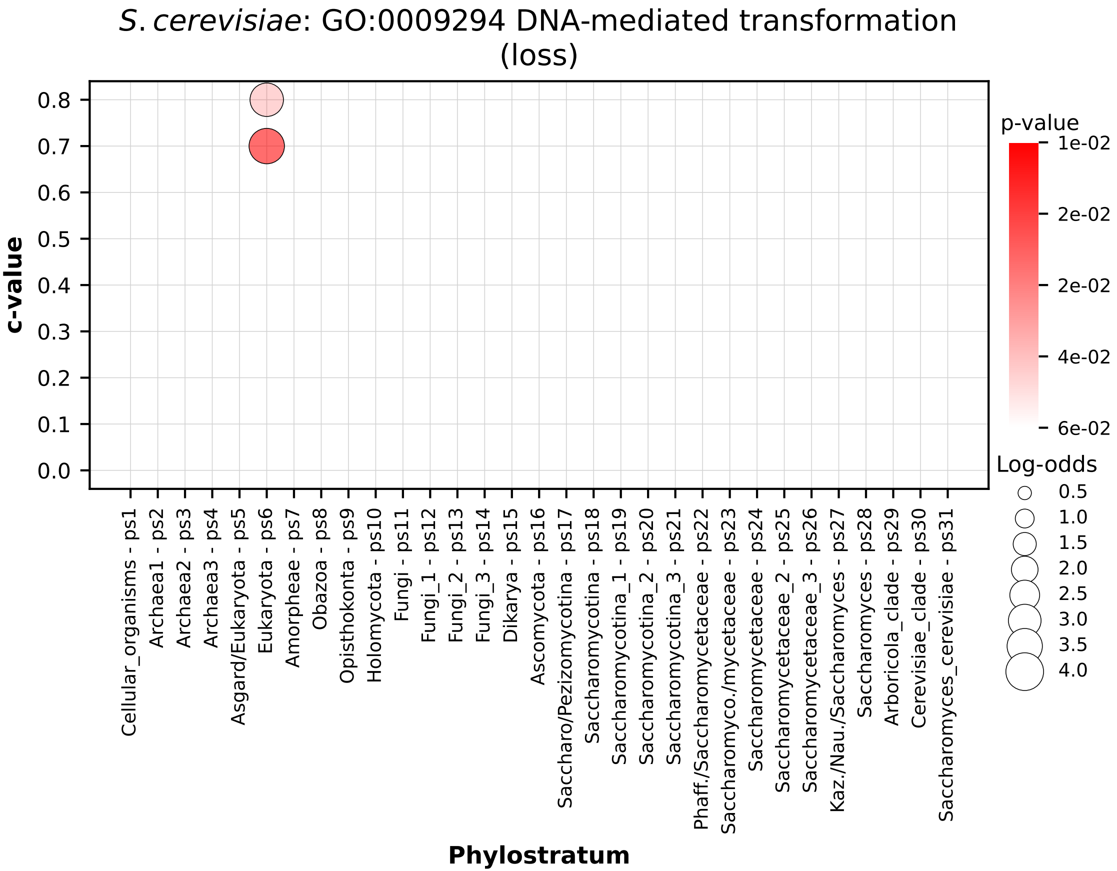

S. cerevisiae: GO:0009294 DNA-mediated transformation (loss)
c-value
0.8
0.7
0.6
0.5
0.4
0.3
0.2
0.1
0.0
p-value
1e-02
2e-02
2e-02
4e-02
6e-02
Log-odds
0.5
1.0
1.5
2.0
2.5
3.0
3.5
4.0
Cellular_organisms - ps1
Archaea1 - ps2
Archaea2 - ps3
Archaea3 - ps4
Asgard/Eukaryota - ps5
Eukaryota - ps6
Amorpheae - ps7
Obazoa - ps8
Opisthokonta - ps9
Holomycota - ps10
Fungi - ps11
Fungi_1 - ps12
Fungi_2 - ps13
Fungi_3 - ps14
Dikarya - ps15
Ascomycota - ps16
Saccharo/Pezizomycotina - ps17
Saccharomycotina - ps18
Saccharomycotina_1 - ps19
Saccharomycotina_2 - ps20
Saccharomycotina_3 - ps21
Phaff./Saccharomycetaceae - ps22
Saccharomyco./mycetaceae - ps23
Saccharomycetaceae - ps24
Saccharomycetaceae_2 - ps25
Saccharomycetaceae_3 - ps26
Kaz./Nau./Saccharomyces - ps27
Saccharomyces - ps28
Arboricola_clade - ps29
Cerevisiae_clade - ps30
Saccharomyces_cerevisiae - ps31
Phylostratum

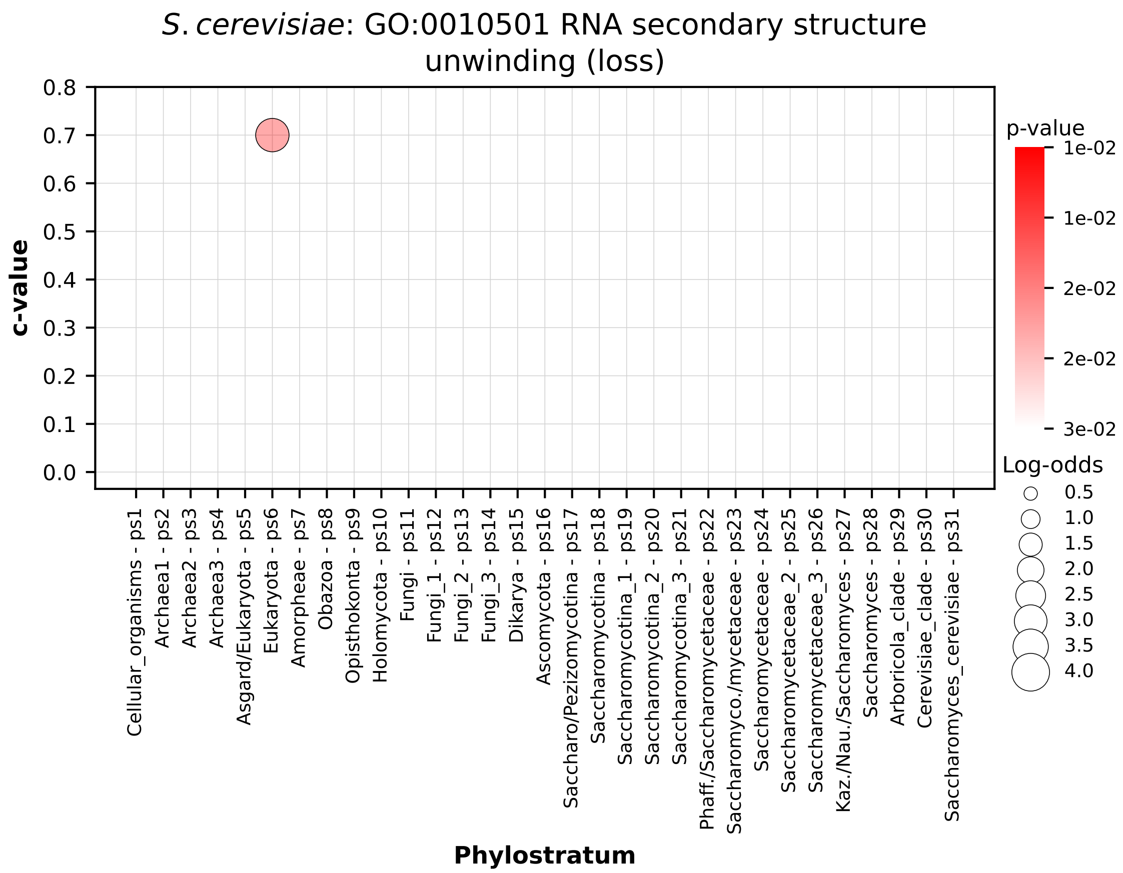

S. cerevisiae: GO:0010501 RNA secondary structure unwinding (loss)
c-value
0.8
0.7
0.6
0.5
0.4
0.3
0.2
0.1
0.0
p-value
1e-02
1e-02
2e-02
2e-02
3e-02
Log-odds
0.5
1.0
1.5
2.0
2.5
3.0
3.5
4.0
Cellular_organisms - ps1
Archaea1 - ps2
Archaea2 - ps3
Archaea3 - ps4
Asgard/Eukaryota - ps5
Eukaryota - ps6
Amorpheae - ps7
Obazoa - ps8
Opisthokonta - ps9
Holomycota - ps10
Fungi - ps11
Fungi_1 - ps12
Fungi_2 - ps13
Fungi_3 - ps14
Dikarya - ps15
Ascomycota - ps16
Saccharo/Pezizomycotina - ps17
Saccharomycotina - ps18
Saccharomycotina_1 - ps19
Saccharomycotina_2 - ps20
Saccharomycotina_3 - ps21
Phaff./Saccharomycetaceae - ps22
Saccharomyco./mycetaceae - ps23
Saccharomycetaceae - ps24
Saccharomycetaceae_2 - ps25
Saccharomycetaceae_3 - ps26
Kaz./Nau./Saccharomyces - ps27
Saccharomyces - ps28
Arboricola_clade - ps29
Cerevisiae_clade - ps30
Saccharomyces_cerevisiae - ps31
Phylostratum

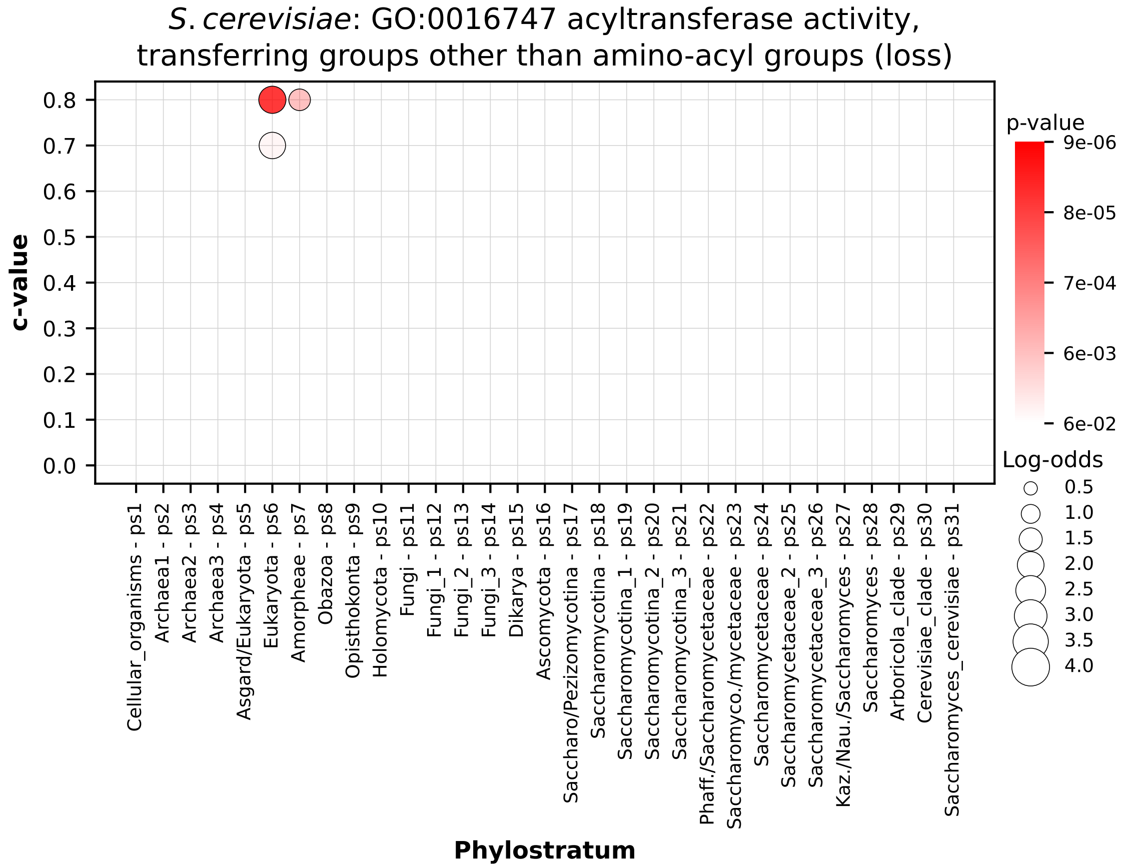

S. cerevisiae: GO:0016747 acyltransferase activity, transferring groups other than amino-acyl groups (loss)
c-value
0.0
0.1
0.2
0.3
0.4
0.5
0.6
0.7
0.8
Phylostratum
Cellular_organisms - ps1
Archaea1 - ps2
Archaea2 - ps3
Archaea3 - ps4
Asgard/Eukaryota - ps5
Eukaryota - ps6
Amorpheae - ps7
Obazoa - ps8
Opisthokonta - ps9
Holomycota - ps10
Fungi - ps11
Fungi_1 - ps12
Fungi_2 - ps13
Fungi_3 - ps14
Dikarya - ps15
Ascomycota - ps16
Saccharo/Pezizomycotina - ps17
Saccharomycotina - ps18
Saccharomycotina_1 - ps19
Saccharomycotina_2 - ps20
Saccharomycotina_3 - ps21
Phaff./Saccharomycetaceae - ps22
Saccharomyco./mycetaceae - ps23
Saccharomycetaceae - ps24
Saccharomycetaceae_2 - ps25
Saccharomycetaceae_3 - ps26
Kaz./Nau./Saccharomyces - ps27
Saccharomyces - ps28
Arboricola_clade - ps29
Cerevisiae_clade - ps30
Saccharomyces_cerevisiae - ps31
p-value
9e-06
8e-05
7e-04
6e-03
6e-02
Log-odds
0.5
1.0
1.5
2.0
2.5
3.0
3.5
4.0

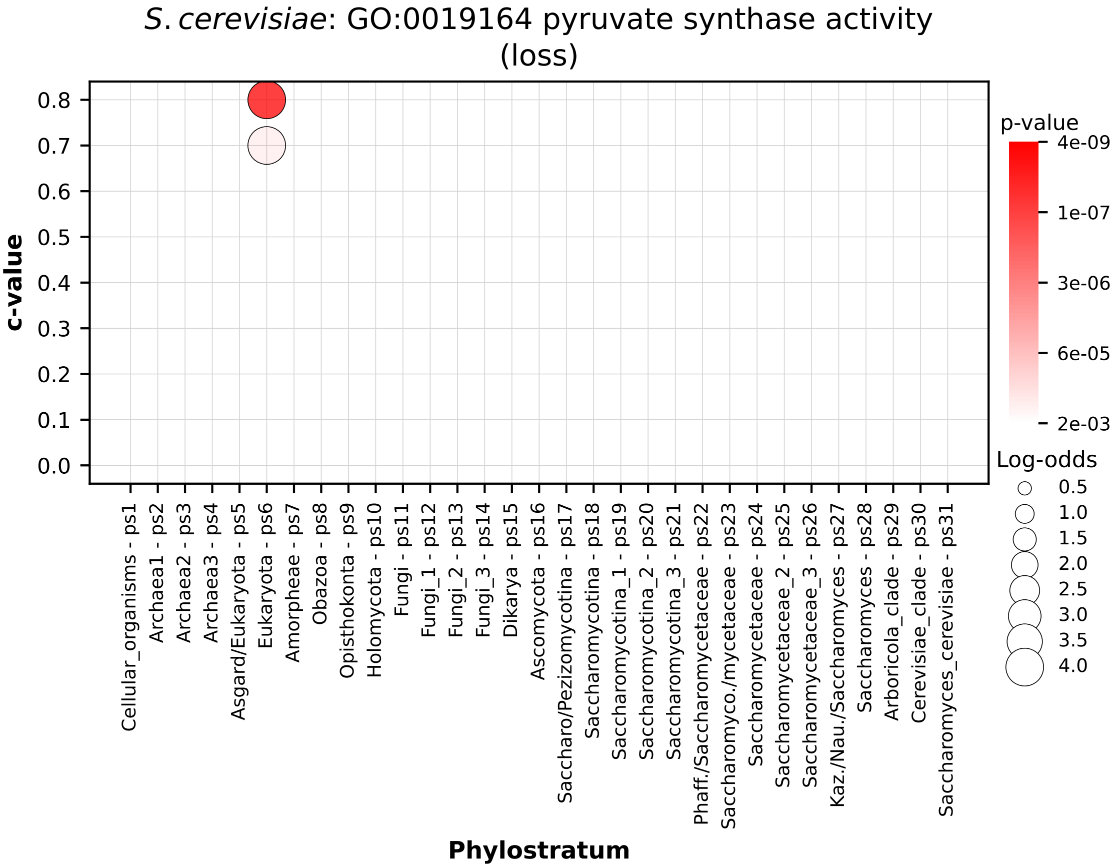

S. cerevisiae: GO:0019164 pyruvate synthase activity
(loss)
c-value
0.0
0.1
0.2
0.3
0.4
0.5
0.6
0.7
0.8
p-value
4e-09
1e-07
3e-06
6e-05
2e-03
Log-odds
0.5
1.0
1.5
2.0
2.5
3.0
3.5
4.0
Cellular_organisms - ps1
Archaea1 - ps2
Archaea2 - ps3
Archaea3 - ps4
Asgard/Eukaryota - ps5
Eukaryota - ps6
Amorpheae - ps7
Obazoa - ps8
Opisthokonta - ps9
Holomycota - ps10
Fungi - ps11
Fungi_1 - ps12
Fungi_2 - ps13
Fungi_3 - ps14
Dikarya - ps15
Ascomycota - ps16
Saccharo/Pezizomycotina - ps17
Saccharomycotina - ps18
Saccharomycotina_1 - ps19
Saccharomycotina_2 - ps20
Saccharomycotina_3 - ps21
Phaff./Saccharomycetaceae - ps22
Saccharomyco./mycetaceae - ps23
Saccharomycetaceae - ps24
Saccharomycetaceae_2 - ps25
Saccharomycetaceae_3 - ps26
Kaz./Nau./Saccharomyces - ps27
Saccharomyces - ps28
Arboricola_clade - ps29
Cerevisiae_clade - ps30
Saccharomyces_cerevisiae - ps31
Phylostratum

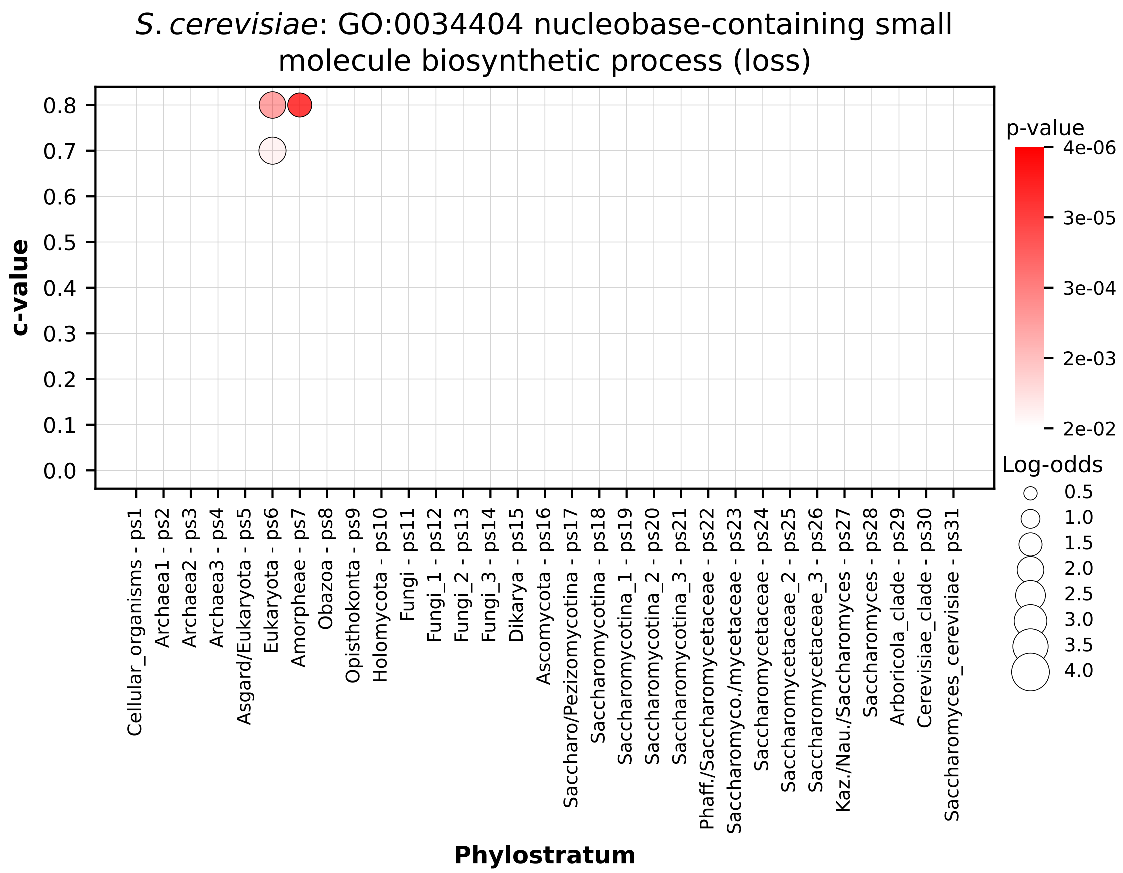

S. cerevisiae: GO:0034404 nucleobase-containing small molecule biosynthetic process (loss)
c-value
0.0
0.1
0.2
0.3
0.4
0.5
0.6
0.7
0.8
Phylostratum
Cellular_organisms - ps1
Archaea1 - ps2
Archaea2 - ps3
Archaea3 - ps4
Asgard/Eukaryota - ps5
Eukaryota - ps6
Amorpheae - ps7
Obazoa - ps8
Opisthokonta - ps9
Holomycota - ps10
Fungi - ps11
Fungi_1 - ps12
Fungi_2 - ps13
Fungi_3 - ps14
Dikarya - ps15
Ascomycota - ps16
Saccharo/Pezizomycotina - ps17
Saccharomycotina - ps18
Saccharomycotina_1 - ps19
Saccharomycotina_2 - ps20
Saccharomycotina_3 - ps21
Phaff./Saccharomycetaceae - ps22
Saccharomyco./mycetaceae - ps23
Saccharomycetaceae - ps24
Saccharomycetaceae_2 - ps25
Saccharomycetaceae_3 - ps26
Kaz./Nau./Saccharomyces - ps27
Saccharomyces - ps28
Arboricola_clade - ps29
Cerevisiae_clade - ps30
Saccharomyces_cerevisiae - ps31
p-value
4e-06
3e-05
3e-04
2e-03
2e-02
Log-odds
0.5
1.0
1.5
2.0
2.5
3.0
3.5
4.0

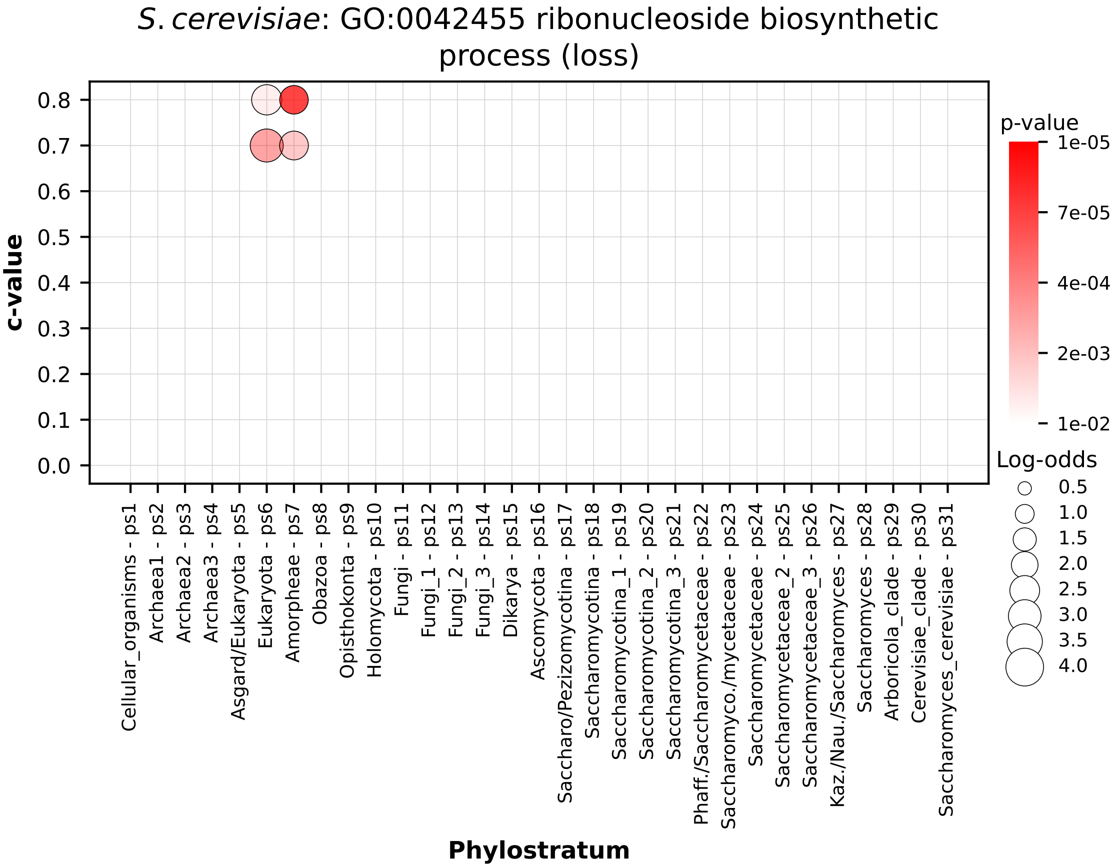

S. cerevisiae: GO:0042455 ribonucleoside biosynthetic process (loss)
c-value
0.0
0.1
0.2
0.3
0.4
0.5
0.6
0.7
0.8
Phylostratum
Cellular_organisms - ps1
Archaea1 - ps2
Archaea2 - ps3
Archaea3 - ps4
Asgard/Eukaryota - ps5
Eukaryota - ps6
Amorpheae - ps7
Obazoa - ps8
Opisthokonta - ps9
Holomycota - ps10
Fungi - ps11
Fungi_1 - ps12
Fungi_2 - ps13
Fungi_3 - ps14
Dikarya - ps15
Ascomycota - ps16
Saccharo/Pezizomycotina - ps17
Saccharomycotina - ps18
Saccharomycotina_1 - ps19
Saccharomycotina_2 - ps20
Saccharomycotina_3 - ps21
Phaff./Saccharomycetaceae - ps22
Saccharomyco./mycetaceae - ps23
Saccharomycetaceae - ps24
Saccharomycetaceae_2 - ps25
Saccharomycetaceae_3 - ps26
Kaz./Nau./Saccharomyces - ps27
Saccharomyces - ps28
Arboricola_clade - ps29
Cerevisiae_clade - ps30
Saccharomyces_cerevisiae - ps31
p-value
1e-05
7e-05
4e-04
2e-03
1e-02
Log-odds
0.5
1.0
1.5
2.0
2.5
3.0
3.5
4.0

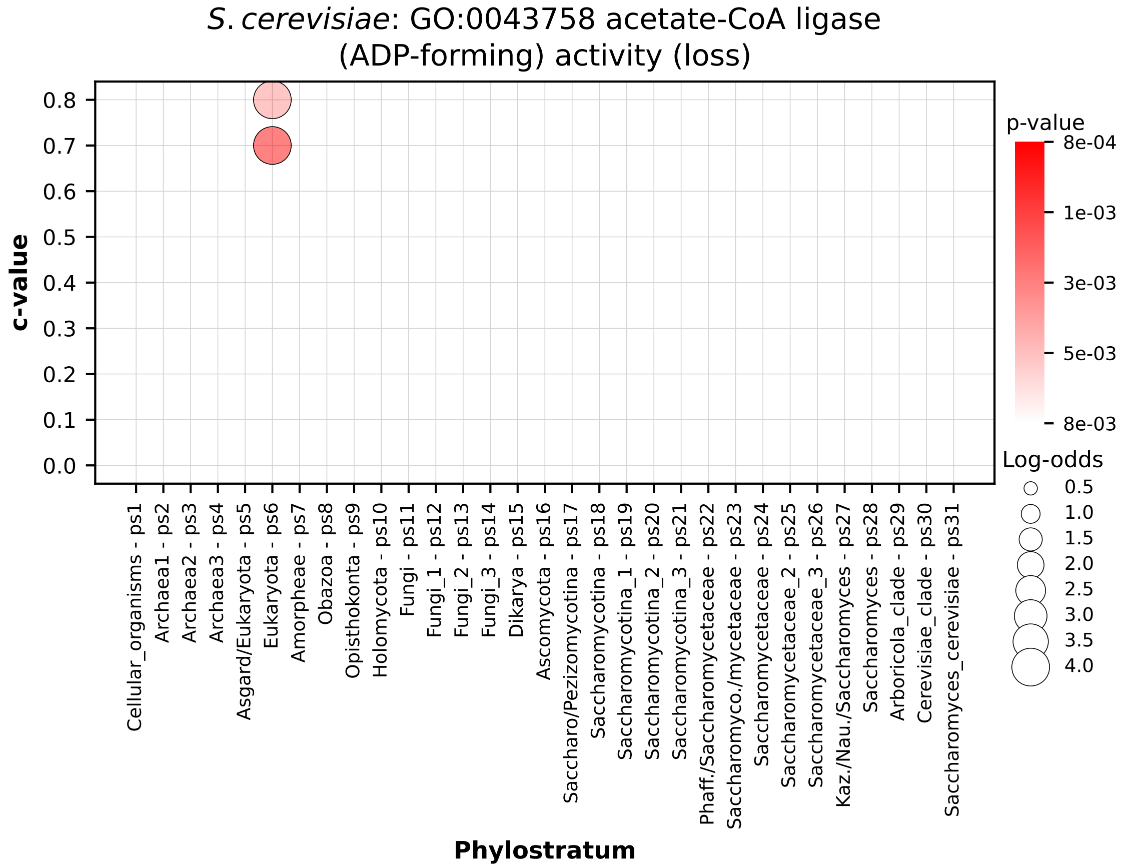
S. cerevisiae: GO:0043758 acetate-CoA ligase (ADP-forming) activity (loss)
c-value
0.8
0.7
0.6
0.5
0.4
0.3
0.2
0.1
0.0
p-value
8e-04
1e-03
3e-03
5e-03
8e-03
Log-odds
0.5
1.0
1.5
2.0
2.5
3.0
3.5
4.0
Cellular_organisms - ps1
Archaea1 - ps2
Archaea2 - ps3
Archaea3 - ps4
Asgard/Eukaryota - ps5
Eukaryota - ps6
Amorpheae - ps7
Obazoa - ps8
Opisthokonta - ps9
Holomycota - ps10
Fungi - ps11
Fungi_1 - ps12
Fungi_2 - ps13
Fungi_3 - ps14
Dikarya - ps15
Ascomycota - ps16
Saccharo/Pezizomycotina - ps17
Saccharomycotina - ps18
Saccharomycotina_1 - ps19
Saccharomycotina_2 - ps20
Saccharomycotina_3 - ps21
Phaff./Saccharomycetaceae - ps22
Saccharomyco./mycetaceae - ps23
Saccharomycetaceae - ps24
Saccharomycetaceae_2 - ps25
Saccharomycetaceae_3 - ps26
Kaz./Nau./Saccharomyces - ps27
Saccharomyces - ps28
Arboricola_clade - ps29
Cerevisiae_clade - ps30
Saccharomyces_cerevisiae - ps31
Phylostratum

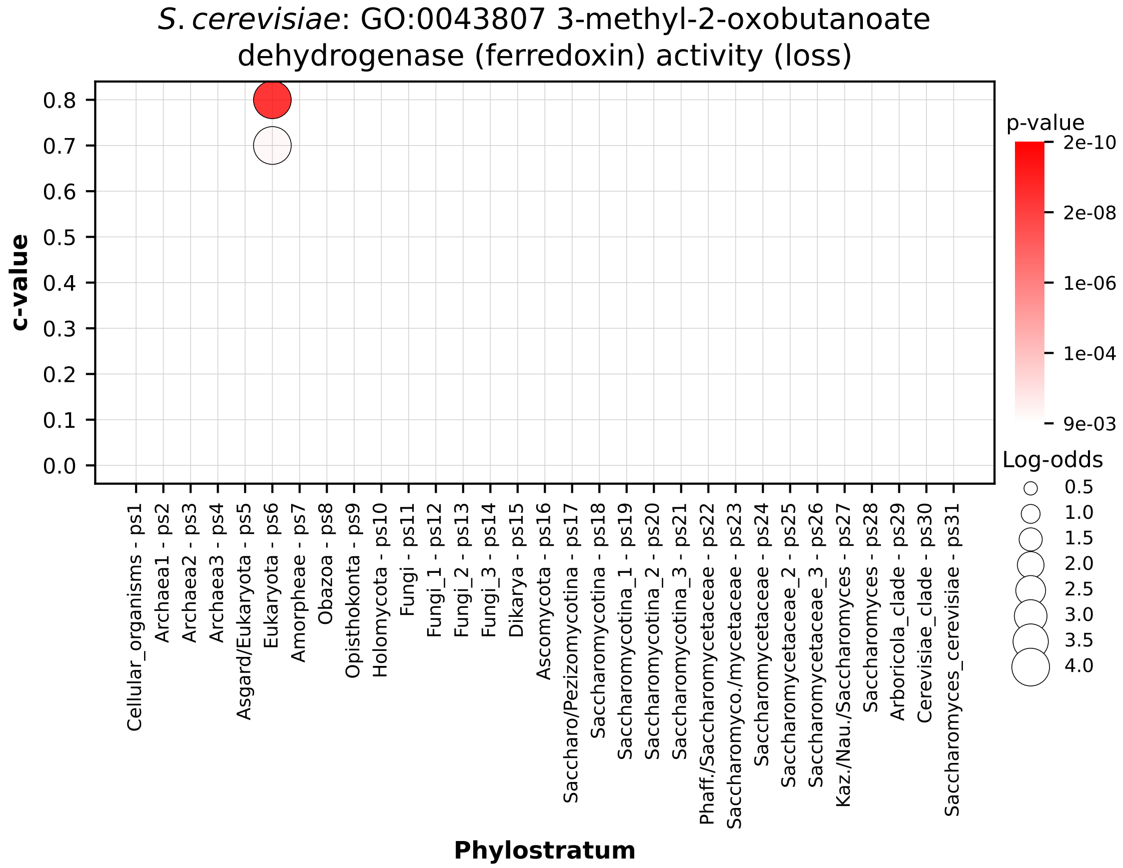
S. cerevisiae: GO:0043807 3-methyl-2-oxobutanoate dehydrogenase (ferredoxin) activity (loss)
c-value
0.8
0.7
0.6
0.5
0.4
0.3
0.2
0.1
0.0
p-value
2e-10
2e-08
1e-06
1e-04
9e-03
Log-odds
0.5
1.0
1.5
2.0
2.5
3.0
3.5
4.0
Cellular_organisms - ps1
Archaea1 - ps2
Archaea2 - ps3
Archaea3 - ps4
Asgard/Eukaryota - ps5
Eukaryota - ps6
Amorpheae - ps7
Obazoa - ps8
Opisthokonta - ps9
Holomycota - ps10
Fungi - ps11
Fungi_1 - ps12
Fungi_2 - ps13
Fungi_3 - ps14
Dikarya - ps15
Ascomycota - ps16
Saccharo/Pezizomycotina - ps17
Saccharomycotina - ps18
Saccharomycotina_1 - ps19
Saccharomycotina_2 - ps20
Saccharomycotina_3 - ps21
Phaff./Saccharomycetaceae - ps22
Saccharomyco./mycetaceae - ps23
Saccharomycetaceae - ps24
Saccharomycetaceae_2 - ps25
Saccharomycetaceae_3 - ps26
Kaz./Nau./Saccharomyces - ps27
Saccharomyces - ps28
Arboricola_clade - ps29
Cerevisiae_clade - ps30
Saccharomyces_cerevisiae - ps31
Phylostratum

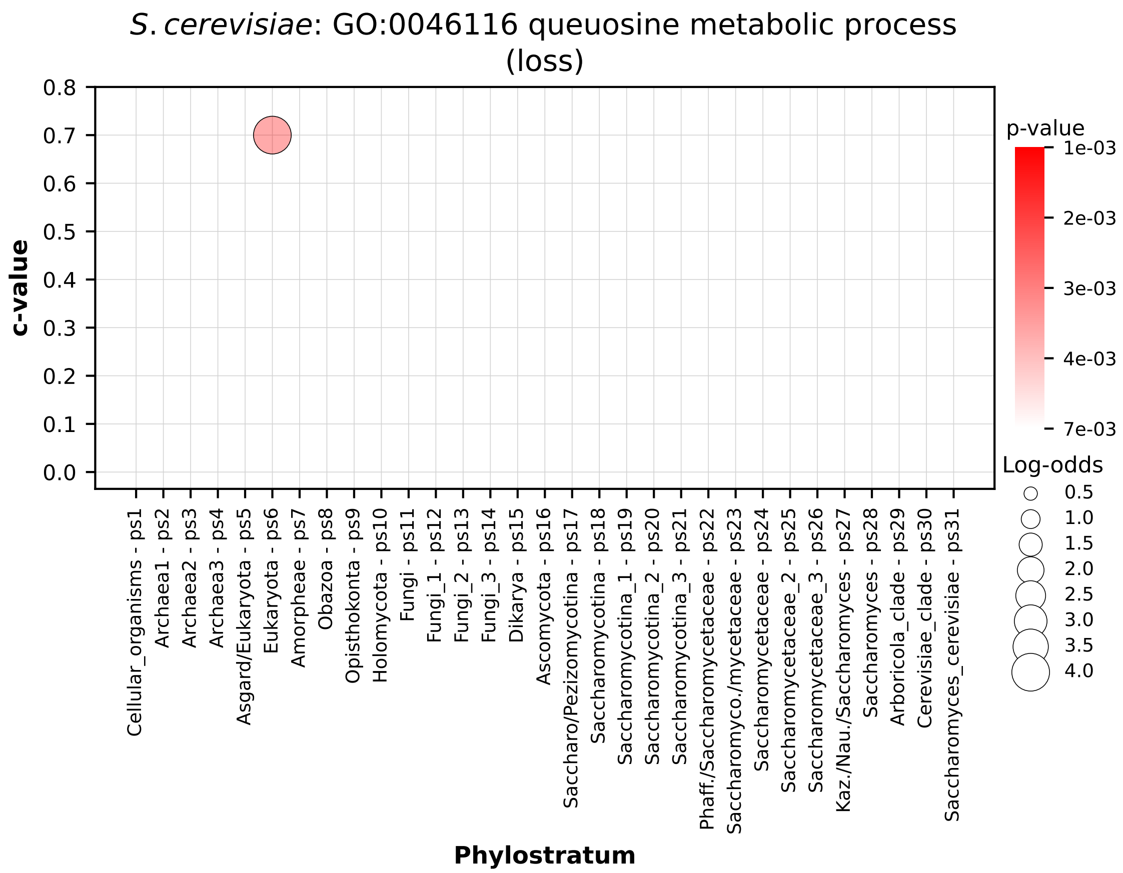

S. cerevisiae: GO:0046116 queuosine metabolic process
(loss)
c-value
0.8
0.7
0.6
0.5
0.4
0.3
0.2
0.1
0.0
p-value
1e-03
2e-03
3e-03
4e-03
7e-03
Log-odds
0.5
1.0
1.5
2.0
2.5
3.0
3.5
4.0
Cellular_organisms - ps1
Archaea1 - ps2
Archaea2 - ps3
Archaea3 - ps4
Asgard/Eukaryota - ps5
Eukaryota - ps6
Amorpheae - ps7
Obazoa - ps8
Opisthokonta - ps9
Holomycota - ps10
Fungi - ps11
Fungi_1 - ps12
Fungi_2 - ps13
Fungi_3 - ps14
Dikarya - ps15
Ascomycota - ps16
Saccharo/Pezizomycotina - ps17
Saccharomycotina - ps18
Saccharomycotina_1 - ps19
Saccharomycotina_2 - ps20
Saccharomycotina_3 - ps21
Phaff./Saccharomycetaceae - ps22
Saccharomyco./mycetaceae - ps23
Saccharomycetaceae - ps24
Saccharomycetaceae_2 - ps25
Saccharomycetaceae_3 - ps26
Kaz./Nau./Saccharomyces - ps27
Saccharomyces - ps28
Arboricola_clade - ps29
Cerevisiae_clade - ps30
Saccharomyces_cerevisiae - ps31
Phylostratum

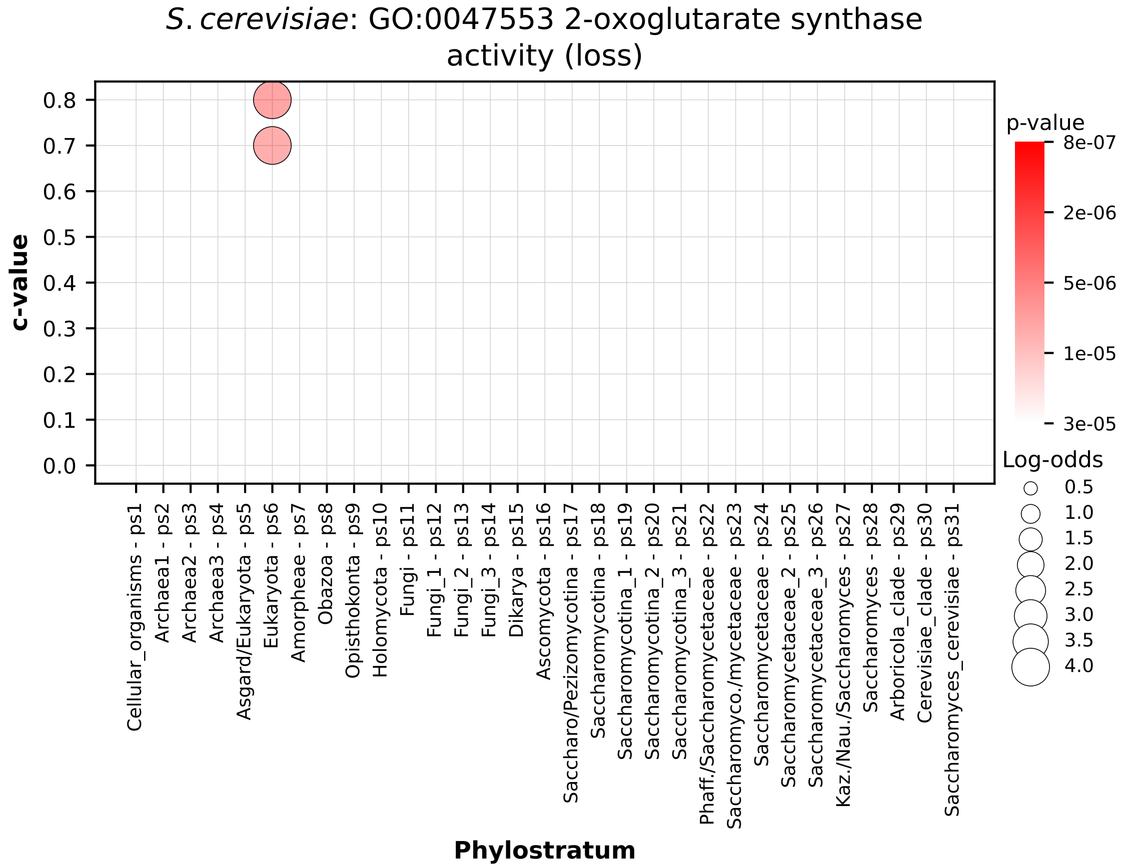
S. cerevisiae: GO:0047553 2-oxoglutarate synthase activity (loss)
c-value
0.0
0.1
0.2
0.3
0.4
0.5
0.6
0.7
0.8
p-value
8e-07
2e-06
5e-06
1e-05
3e-05
Log-odds
0.5
1.0
1.5
2.0
2.5
3.0
3.5
4.0
Cellular_organisms - ps1
Archaea1 - ps2
Archaea2 - ps3
Archaea3 - ps4
Asgard/Eukaryota - ps5
Eukaryota - ps6
Amorpheae - ps7
Obazoa - ps8
Opisthokonta - ps9
Holomycota - ps10
Fungi - ps11
Fungi_1 - ps12
Fungi_2 - ps13
Fungi_3 - ps14
Dikarya - ps15
Ascomycota - ps16
Saccharo/Pezizomycotina - ps17
Saccharomycotina - ps18
Saccharomycotina_1 - ps19
Saccharomycotina_2 - ps20
Saccharomycotina_3 - ps21
Phaff./Saccharomycetaceae - ps22
Saccharomyco./mycetaceae - ps23
Saccharomycetaceae - ps24
Saccharomycetaceae_2 - ps25
Saccharomycetaceae_3 - ps26
Kaz./Nau./Saccharomyces - ps27
Saccharomyces - ps28
Arboricola_clade - ps29
Cerevisiae_clade - ps30
Saccharomyces_cerevisiae - ps31
Phylostratum

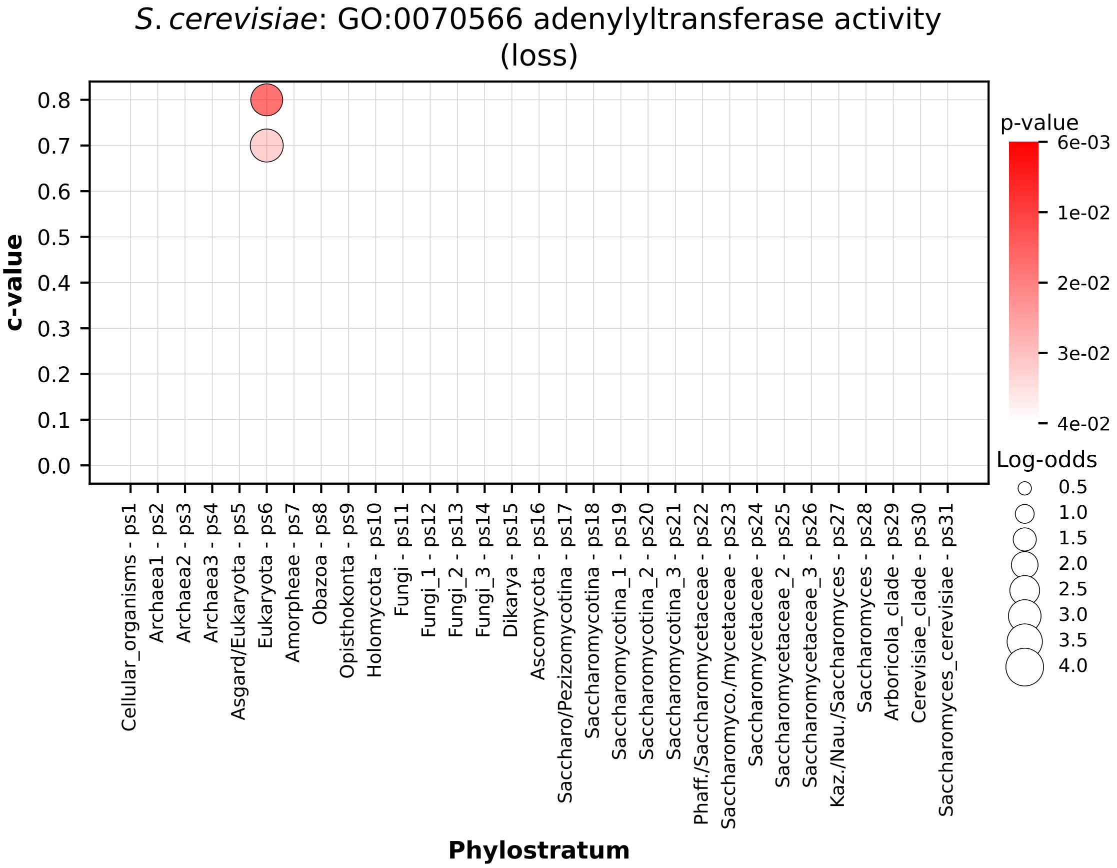
S. cerevisiae: GO:0070566 adenylyltransferase activity
(loss)
c-value
0.8
0.7
0.6
0.5
0.4
0.3
0.2
0.1
0.0
p-value
6e-03
1e-02
2e-02
3e-02
4e-02
Log-odds
0.5
1.0
1.5
2.0
2.5
3.0
3.5
4.0
Cellular_organisms - ps1
Archaea1 - ps2
Archaea2 - ps3
Archaea3 - ps4
Asgard/Eukaryota - ps5
Eukaryota - ps6
Amorpheae - ps7
Obazoa - ps8
Opisthokonta - ps9
Holomycota - ps10
Fungi - ps11
Fungi_1 - ps12
Fungi_2 - ps13
Fungi_3 - ps14
Dikarya - ps15
Ascomycota - ps16
Saccharo/Pezizomycotina - ps17
Saccharomycotina - ps18
Saccharomycotina_1 - ps19
Saccharomycotina_2 - ps20
Saccharomycotina_3 - ps21
Phaff./Saccharomycetaceae - ps22
Saccharomyco./mycetaceae - ps23
Saccharomycetaceae - ps24
Saccharomycetaceae_2 - ps25
Saccharomycetaceae_3 - ps26
Kaz./Nau./Saccharomyces - ps27
Saccharomyces - ps28
Arboricola_clade - ps29
Cerevisiae_clade - ps30
Saccharomyces_cerevisiae - ps31
Phylostratum

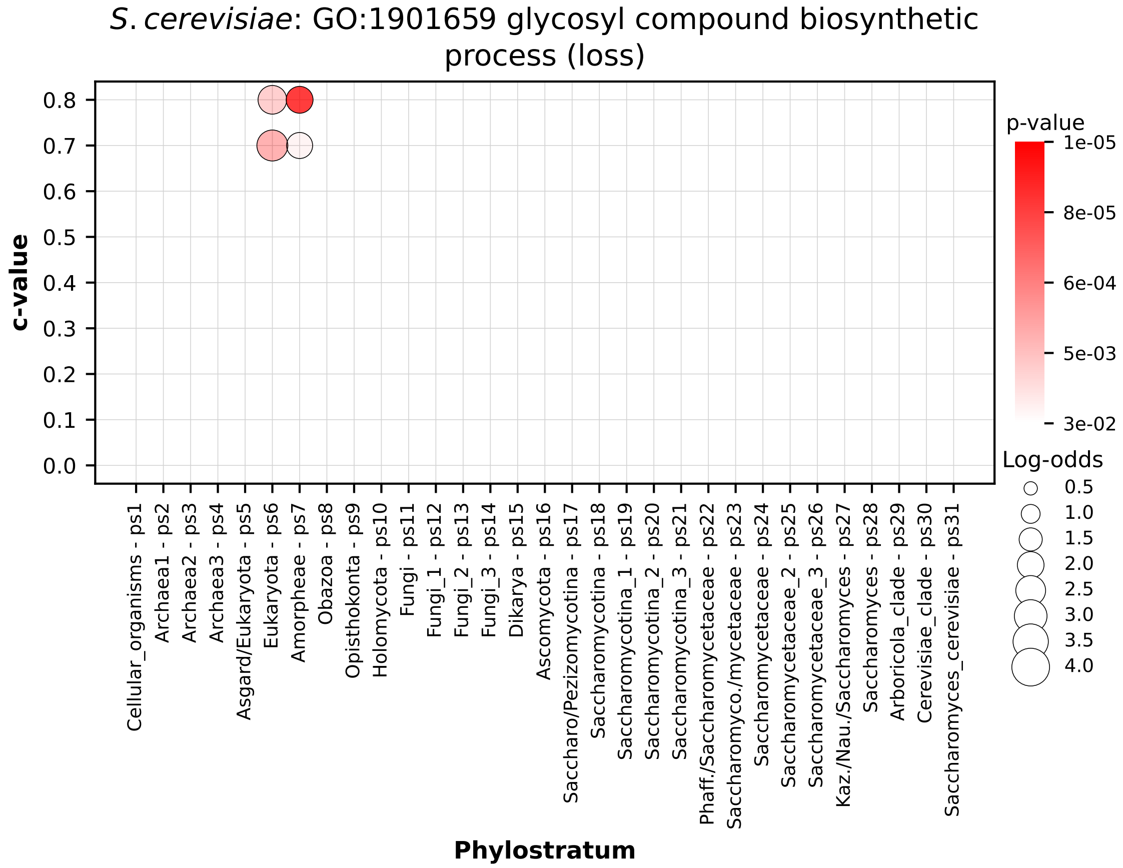

S. cerevisiae: GO:1901659 glycosyl compound biosynthetic process (loss)
c-value
0.8
0.7
0.6
0.5
0.4
0.3
0.2
0.1
0.0
Phylostratum
Cellular_organisms - ps1
Archaea1 - ps2
Archaea2 - ps3
Archaea3 - ps4
Asgard/Eukaryota - ps5
Eukaryota - ps6
Amorpheae - ps7
Obazoa - ps8
Opisthokonta - ps9
Holomycota - ps10
Fungi - ps11
Fungi_1 - ps12
Fungi_2 - ps13
Fungi_3 - ps14
Dikarya - ps15
Ascomycota - ps16
Saccharo/Pezizomycotina - ps17
Saccharomycotina - ps18
Saccharomycotina_1 - ps19
Saccharomycotina_2 - ps20
Saccharomycotina_3 - ps21
Phaff./Saccharomycetaceae - ps22
Saccharomyco./mycetaceae - ps23
Saccharomycetaceae - ps24
Saccharomycetaceae_2 - ps25
Saccharomycetaceae_3 - ps26
Kaz./Nau./Saccharomyces - ps27
Saccharomyces - ps28
Arboricola_clade - ps29
Cerevisiae_clade - ps30
Saccharomyces_cerevisiae - ps31
p-value
1e-05
8e-05
6e-04
5e-03
3e-02
Log-odds
0.5
1.0
1.5
2.0
2.5
3.0
3.5
4.0

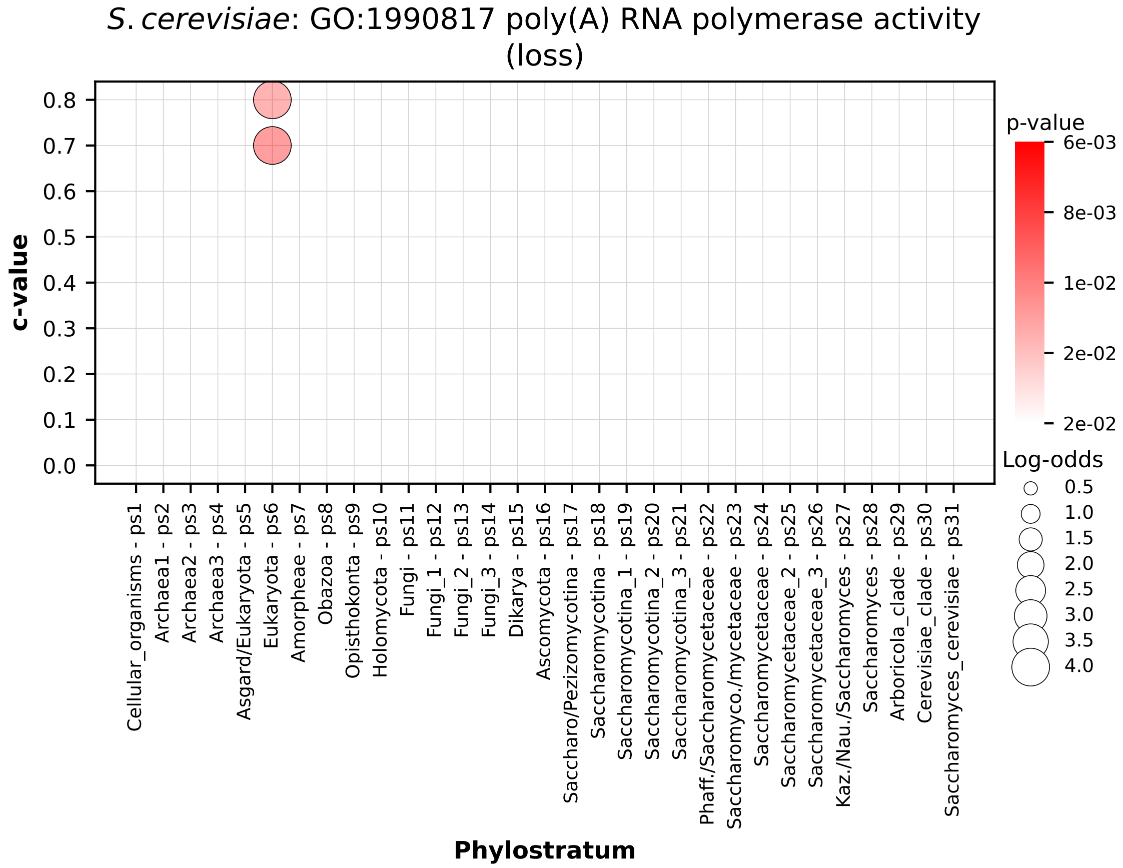
S. cerevisiae: GO:1990817 poly(A) RNA polymerase activity (loss)
c-value
0.0
0.1
0.2
0.3
0.4
0.5
0.6
0.7
0.8
Phylostratum
Cellular_organisms - ps1
Archaea1 - ps2
Archaea2 - ps3
Archaea3 - ps4
Asgard/Eukaryota - ps5
Eukaryota - ps6
Amorpheae - ps7
Obazoa - ps8
Opisthokonta - ps9
Holomycota - ps10
Fungi - ps11
Fungi_1 - ps12
Fungi_2 - ps13
Fungi_3 - ps14
Dikarya - ps15
Ascomycota - ps16
Saccharo/Pezizomycotina - ps17
Saccharomycotina - ps18
Saccharomycotina_1 - ps19
Saccharomycotina_2 - ps20
Saccharomycotina_3 - ps21
Phaff./Saccharomycetaceae - ps22
Saccharomyco./mycetaceae - ps23
Saccharomycetaceae - ps24
Saccharomycetaceae_2 - ps25
Saccharomycetaceae_3 - ps26
Kaz./Nau./Saccharomyces - ps27
Saccharomyces - ps28
Arboricola_clade - ps29
Cerevisiae_clade - ps30
Saccharomyces_cerevisiae - ps31
p-value
6e-03
8e-03
1e-02
2e-02
2e-02
Log-odds
0.5
1.0
1.5
2.0
2.5
3.0
3.5
4.0

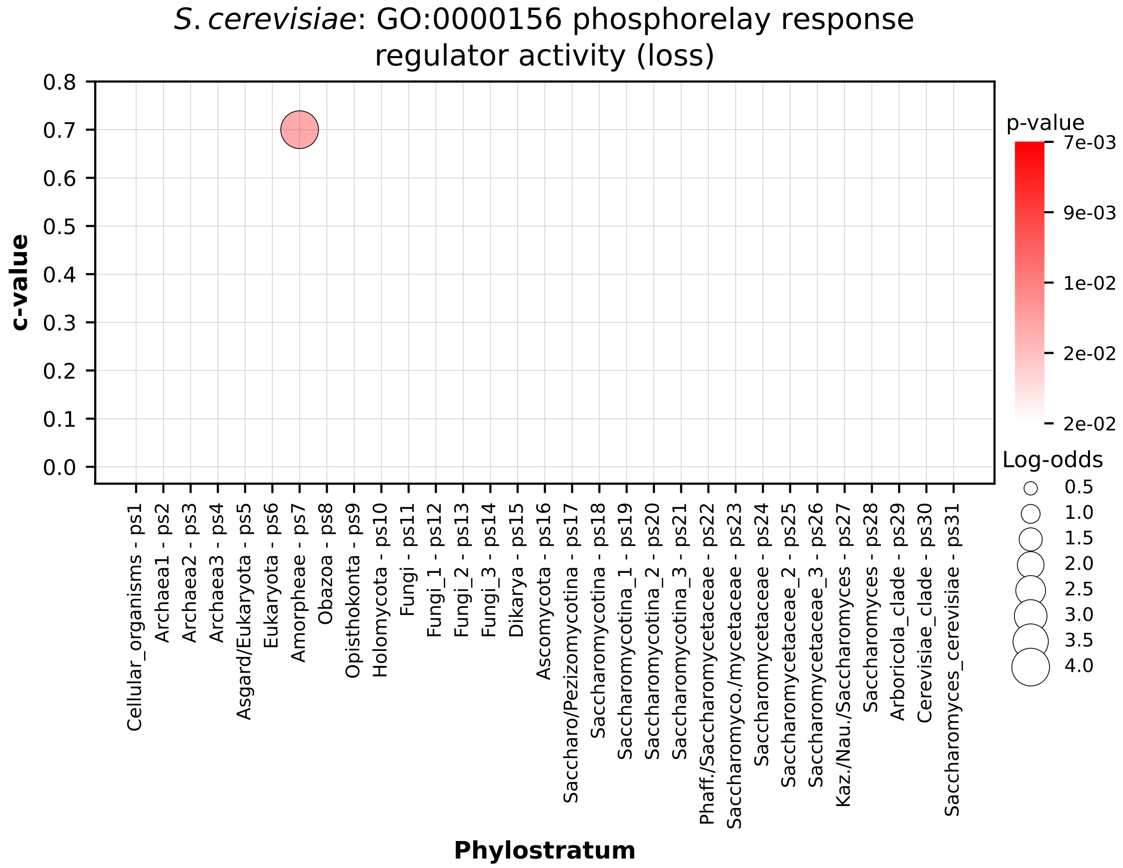

S. cerevisiae: GO:0000156 phosphorelay response regulator activity (loss)
c-value
0.8
0.7
0.6
0.5
0.4
0.3
0.2
0.1
0.0
p-value
7e-03
9e-03
1e-02
2e-02
2e-02
Log-odds
0.5
1.0
1.5
2.0
2.5
3.0
3.5
4.0
Cellular_organisms - ps1
Archaea1 - ps2
Archaea2 - ps3
Archaea3 - ps4
Asgard/Eukaryota - ps5
Eukaryota - ps6
Amorpheae - ps7
Obazoa - ps8
Opisthokonta - ps9
Holomycota - ps10
Fungi - ps11
Fungi_1 - ps12
Fungi_2 - ps13
Fungi_3 - ps14
Dikarya - ps15
Ascomycota - ps16
Saccharo/Pezizomycotina - ps17
Saccharomycotina - ps18
Saccharomycotina_1 - ps19
Saccharomycotina_2 - ps20
Saccharomycotina_3 - ps21
Phaff./Saccharomycetaceae - ps22
Saccharomyco./mycetaceae - ps23
Saccharomycetaceae - ps24
Saccharomycetaceae_2 - ps25
Saccharomycetaceae_3 - ps26
Kaz./Nau./Saccharomyces - ps27
Saccharomyces - ps28
Arboricola_clade - ps29
Cerevisiae_clade - ps30
Saccharomyces_cerevisiae - ps31
Phylostratum

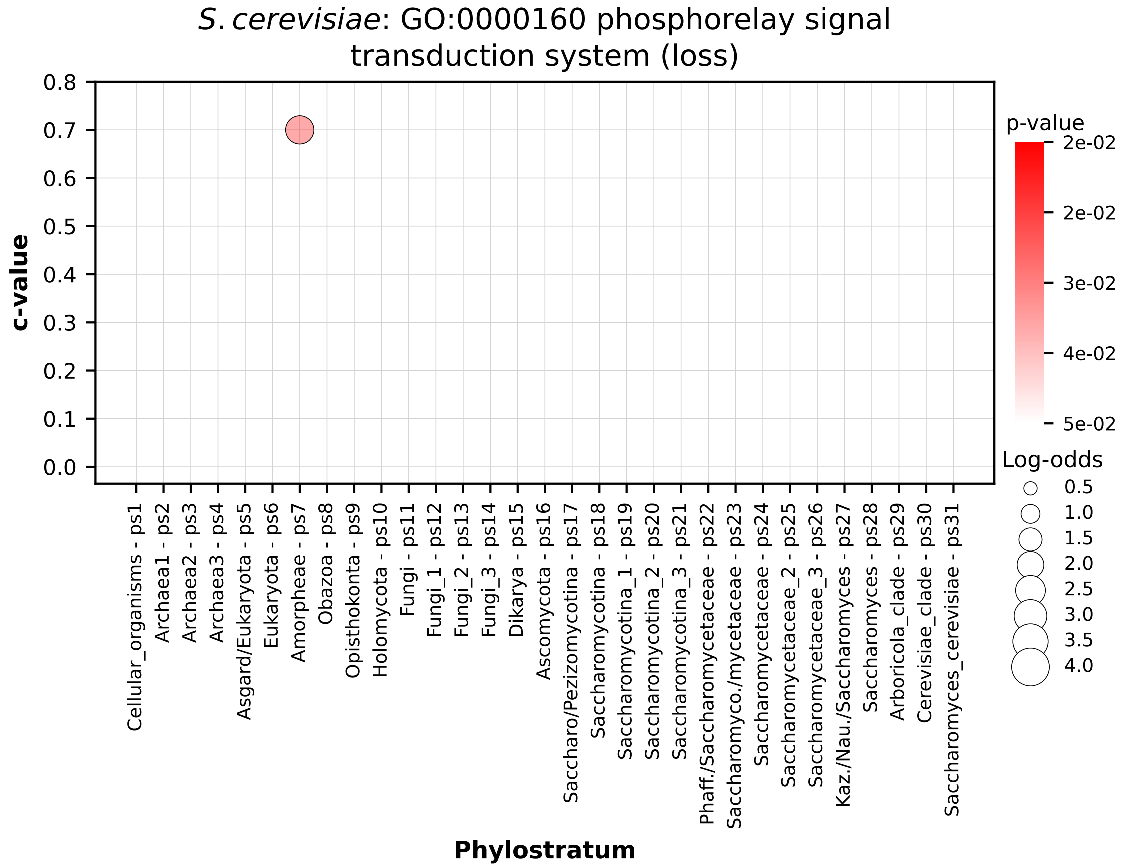
S. cerevisiae: GO:0000160 phosphorelay signal transduction system (loss)
c-value
0.8
0.7
0.6
0.5
0.4
0.3
0.2
0.1
0.0
p-value
2e-02
2e-02
3e-02
4e-02
5e-02
Log-odds
0.5
1.0
1.5
2.0
2.5
3.0
3.5
4.0
Cellular_organisms - ps1
Archaea1 - ps2
Archaea2 - ps3
Archaea3 - ps4
Asgard/Eukaryota - ps5
Eukaryota - ps6
Amorpheae - ps7
Obazoa - ps8
Opisthokonta - ps9
Holomycota - ps10
Fungi - ps11
Fungi_1 - ps12
Fungi_2 - ps13
Fungi_3 - ps14
Dikarya - ps15
Ascomycota - ps16
Saccharo/Pezizomycotina - ps17
Saccharomycotina - ps18
Saccharomycotina_1 - ps19
Saccharomycotina_2 - ps20
Saccharomycotina_3 - ps21
Phaff./Saccharomycetaceae - ps22
Saccharomyco./mycetaceae - ps23
Saccharomycetaceae - ps24
Saccharomycetaceae_2 - ps25
Saccharomycetaceae_3 - ps26
Kaz./Nau./Saccharomyces - ps27
Saccharomyces - ps28
Arboricola_clade - ps29
Cerevisiae_clade - ps30
Saccharomyces_cerevisiae - ps31
Phylostratum

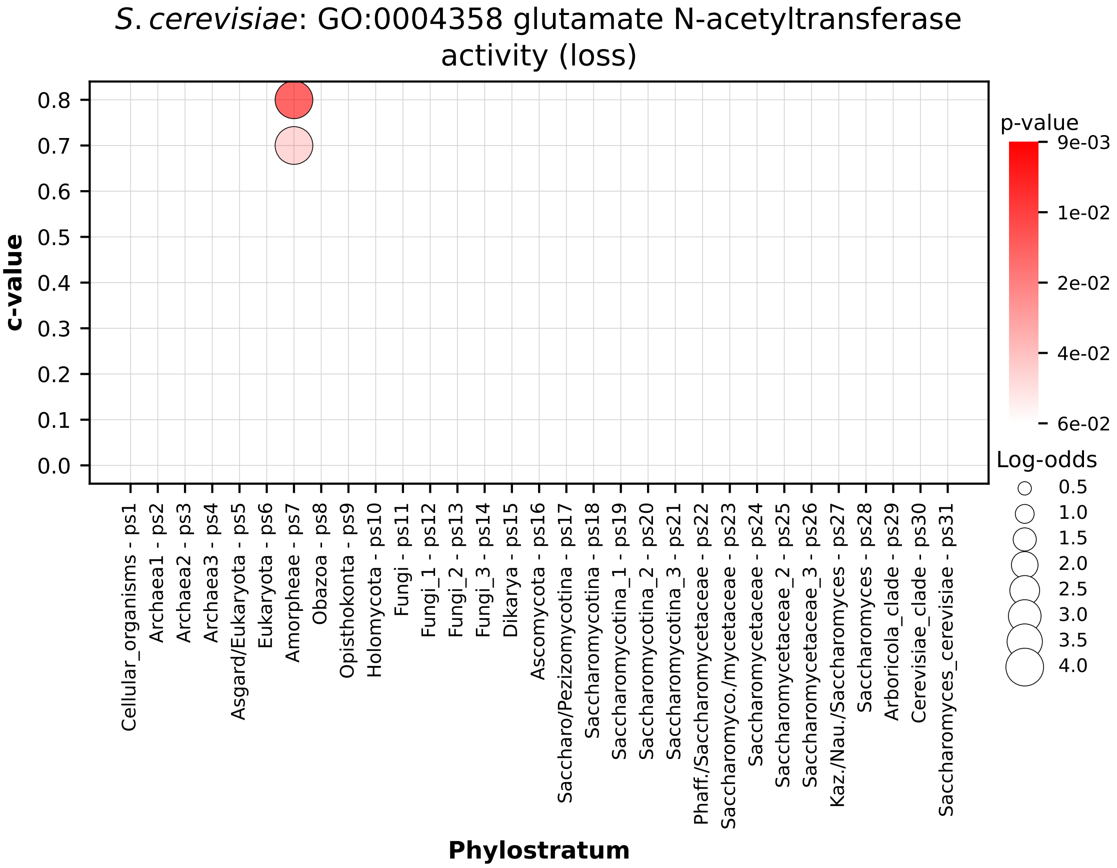

S. cerevisiae: GO:0004358 glutamate N-acetyltransferase activity (loss)
c-value
0.8
0.7
0.6
0.5
0.4
0.3
0.2
0.1
0.0
Phylostratum
Cellular_organisms - ps1
Archaea1 - ps2
Archaea2 - ps3
Archaea3 - ps4
Asgard/Eukaryota - ps5
Eukaryota - ps6
Amorpheae - ps7
Obazoa - ps8
Opisthokonta - ps9
Holomycota - ps10
Fungi - ps11
Fungi_1 - ps12
Fungi_2 - ps13
Fungi_3 - ps14
Dikarya - ps15
Ascomycota - ps16
Saccharo/Pezizomycotina - ps17
Saccharomycotina - ps18
Saccharomycotina_1 - ps19
Saccharomycotina_2 - ps20
Saccharomycotina_3 - ps21
Phaff./Saccharomycetaceae - ps22
Saccharomyco./mycetaceae - ps23
Saccharomycetaceae - ps24
Saccharomycetaceae_2 - ps25
Saccharomycetaceae_3 - ps26
Kaz./Nau./Saccharomyces - ps27
Saccharomyces - ps28
Arboricola_clade - ps29
Cerevisiae_clade - ps30
Saccharomyces_cerevisiae - ps31
p-value
9e-03
1e-02
2e-02
4e-02
6e-02
Log-odds
0.5
1.0
1.5
2.0
2.5
3.0
3.5
4.0

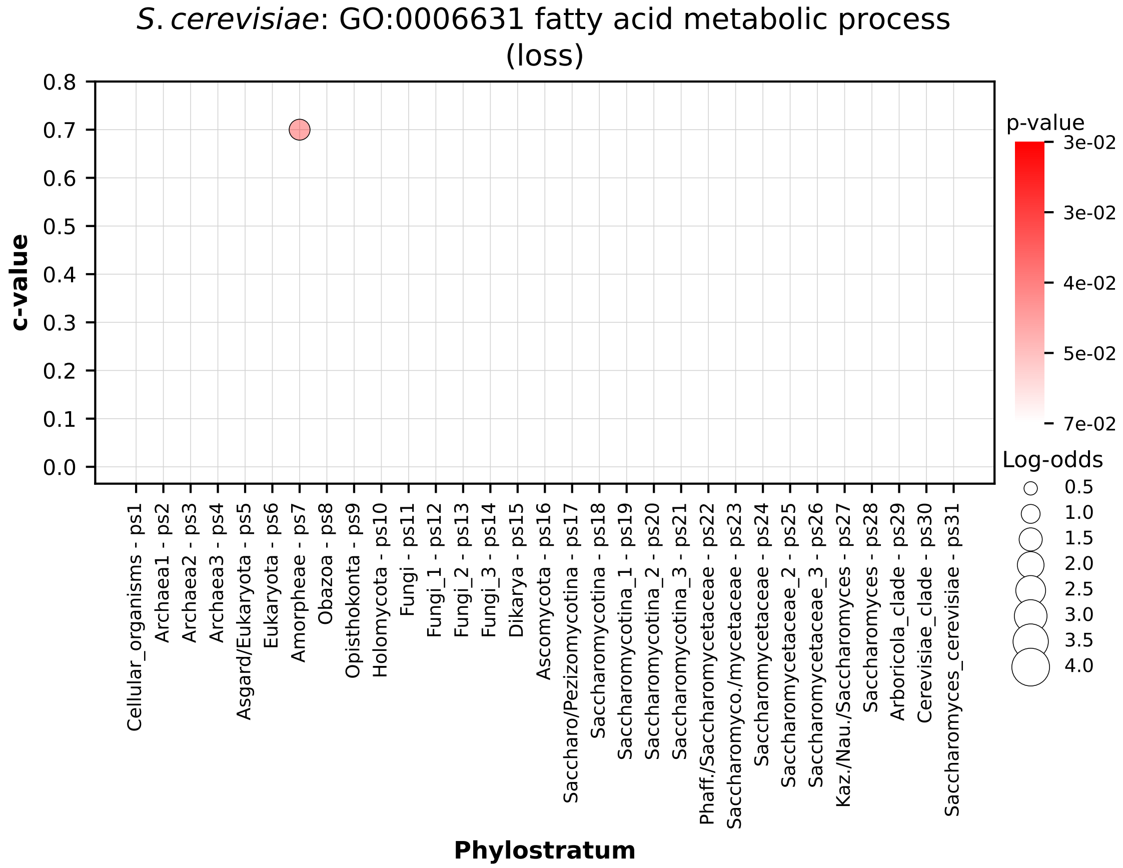
S. cerevisiae: GO:0006631 fatty acid metabolic process
(loss)
c-value
0.8
0.7
0.6
0.5
0.4
0.3
0.2
0.1
0.0
Phylostratum
Cellular_organisms - ps1
Archaea1 - ps2
Archaea2 - ps3
Archaea3 - ps4
Asgard/Eukaryota - ps5
Eukaryota - ps6
Amorpheae - ps7
Obazoa - ps8
Opisthokonta - ps9
Holomycota - ps10
Fungi - ps11
Fungi_1 - ps12
Fungi_2 - ps13
Fungi_3 - ps14
Dikarya - ps15
Ascomycota - ps16
Saccharo/Pezizomycotina - ps17
Saccharomycotina - ps18
Saccharomycotina_1 - ps19
Saccharomycotina_2 - ps20
Saccharomycotina_3 - ps21
Phaff./Saccharomycetaceae - ps22
Saccharomyco./mycetaceae - ps23
Saccharomycetaceae - ps24
Saccharomycetaceae_2 - ps25
Saccharomycetaceae_3 - ps26
Kaz./Nau./Saccharomyces - ps27
Saccharomyces - ps28
Arboricola_clade - ps29
Cerevisiae_clade - ps30
Saccharomyces_cerevisiae - ps31
p-value
3e-02
3e-02
4e-02
5e-02
7e-02
Log-odds
0.5
1.0
1.5
2.0
2.5
3.0
3.5
4.0

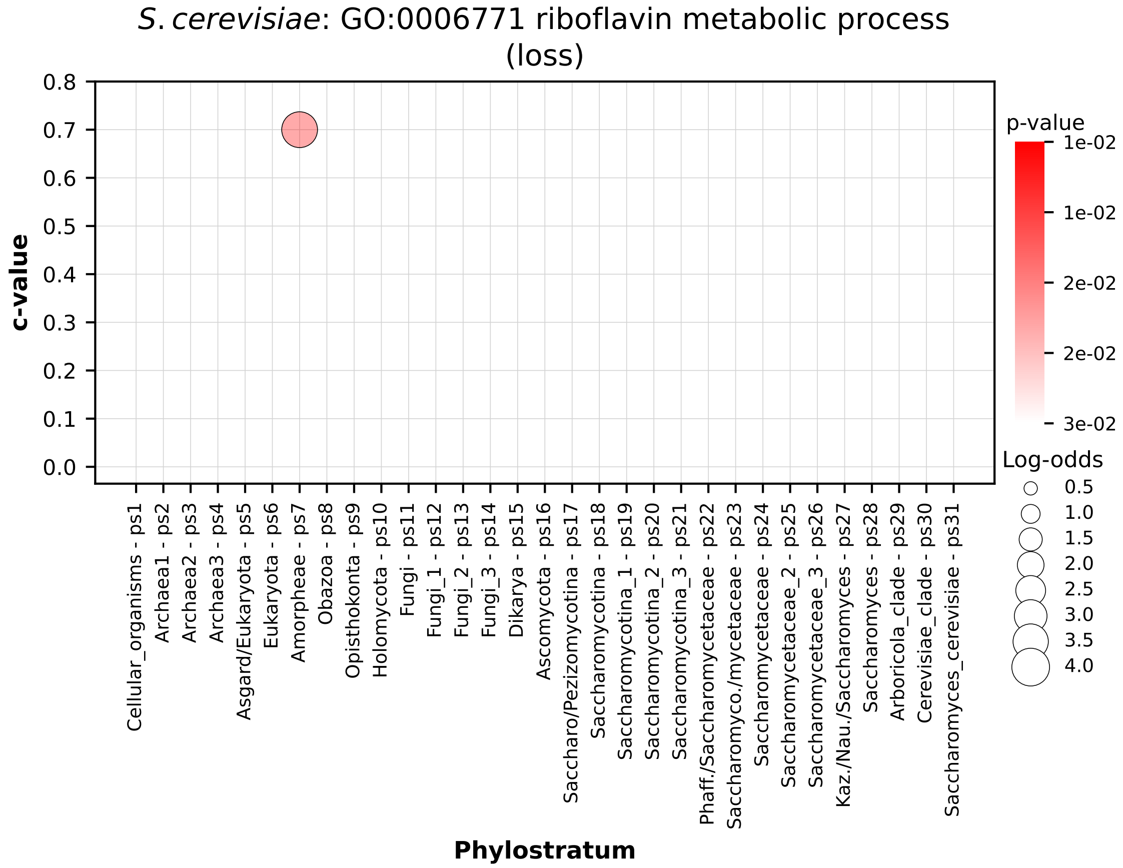
S. cerevisiae: GO:0006771 riboflavin metabolic process
(loss)
c-value
0.8
0.7
0.6
0.5
0.4
0.3
0.2
0.1
0.0
p-value
1e-02
1e-02
2e-02
2e-02
3e-02
Log-odds
0.5
1.0
1.5
2.0
2.5
3.0
3.5
4.0
Cellular_organisms - ps1
Archaea1 - ps2
Archaea2 - ps3
Archaea3 - ps4
Asgard/Eukaryota - ps5
Eukaryota - ps6
Amorpheae - ps7
Obazoa - ps8
Opisthokonta - ps9
Holomycota - ps10
Fungi - ps11
Fungi_1 - ps12
Fungi_2 - ps13
Fungi_3 - ps14
Dikarya - ps15
Ascomycota - ps16
Saccharo/Pezizomycotina - ps17
Saccharomycotina - ps18
Saccharomycotina_1 - ps19
Saccharomycotina_2 - ps20
Saccharomycotina_3 - ps21
Phaff./Saccharomycetaceae - ps22
Saccharomyco./mycetaceae - ps23
Saccharomycetaceae - ps24
Saccharomycetaceae_2 - ps25
Saccharomycetaceae_3 - ps26
Kaz./Nau./Saccharomyces - ps27
Saccharomyces - ps28
Arboricola_clade - ps29
Cerevisiae_clade - ps30
Saccharomyces_cerevisiae - ps31
Phylostratum

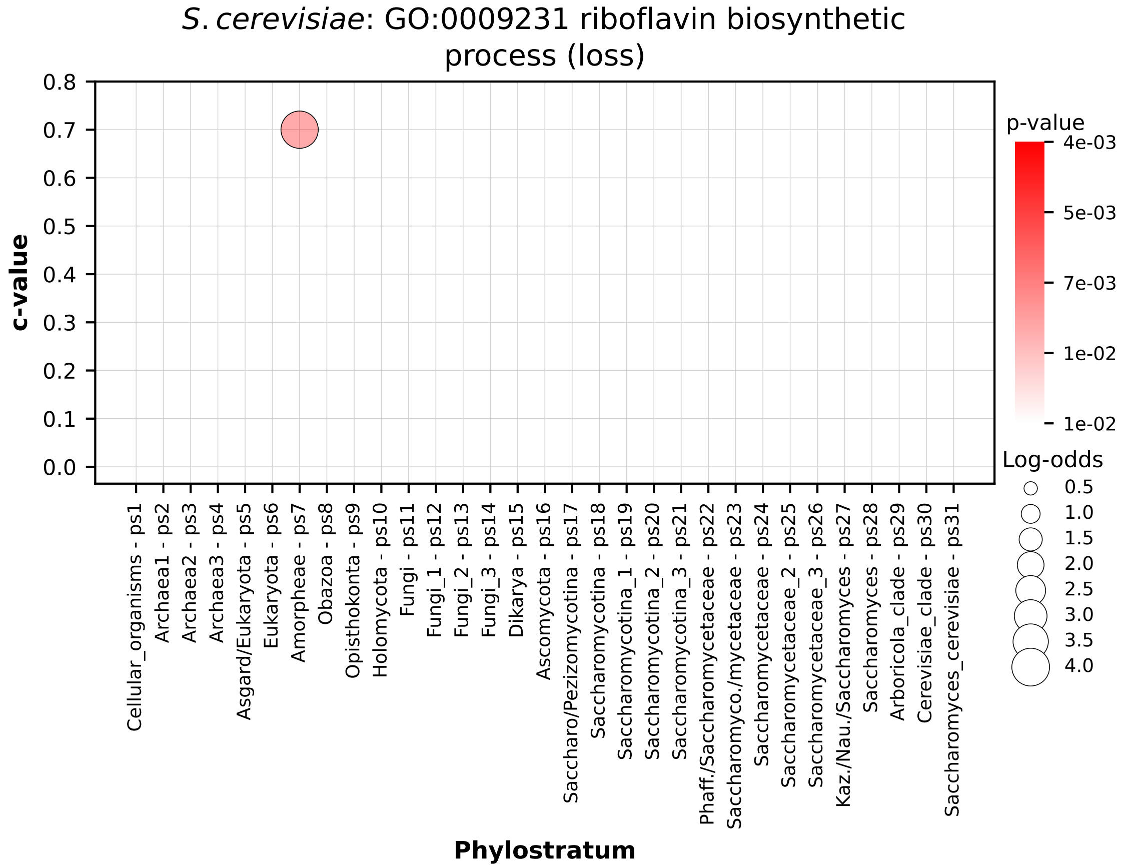
S. cerevisiae: GO:0009231 riboflavin biosynthetic process (loss)
c-value
0.8
0.7
0.6
0.5
0.4
0.3
0.2
0.1
0.0
p-value
4e-03
5e-03
7e-03
1e-02
1e-02
Log-odds
0.5
1.0
1.5
2.0
2.5
3.0
3.5
4.0
Cellular_organisms - ps1
Archaea1 - ps2
Archaea2 - ps3
Archaea3 - ps4
Asgard/Eukaryota - ps5
Eukaryota - ps6
Amorpheae - ps7
Obazoa - ps8
Opisthokonta - ps9
Holomycota - ps10
Fungi - ps11
Fungi_1 - ps12
Fungi_2 - ps13
Fungi_3 - ps14
Dikarya - ps15
Ascomycota - ps16
Saccharo/Pezizomycotina - ps17
Saccharomycotina - ps18
Saccharomycotina_1 - ps19
Saccharomycotina_2 - ps20
Saccharomycotina_3 - ps21
Phaff./Saccharomycetaceae - ps22
Saccharomyco./mycetaceae - ps23
Saccharomycetaceae - ps24
Saccharomycetaceae_2 - ps25
Saccharomycetaceae_3 - ps26
Kaz./Nau./Saccharomyces - ps27
Saccharomyces - ps28
Arboricola_clade - ps29
Cerevisiae_clade - ps30
Saccharomyces_cerevisiae - ps31
Phylostratum

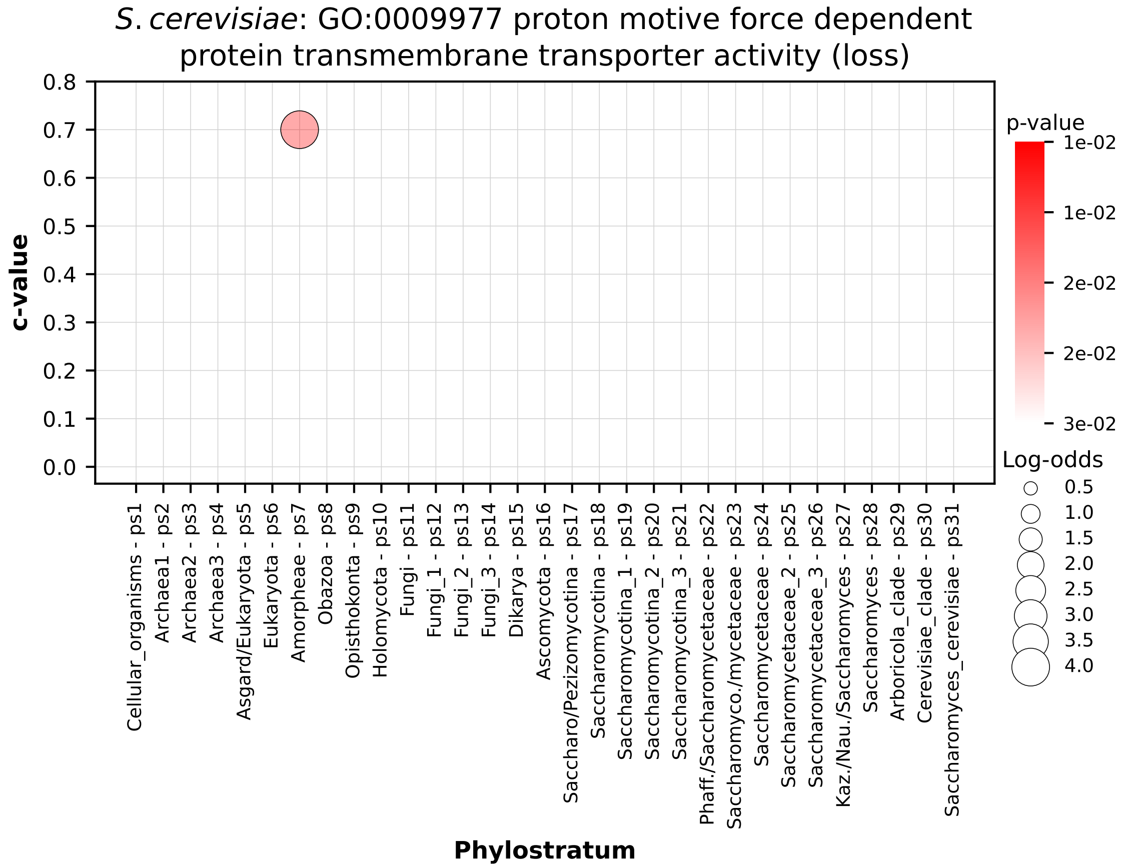
S. cerevisiae: GO:0009977 proton motive force dependent protein transmembrane transporter activity (loss)
c-value
0.8
0.7
0.6
0.5
0.4
0.3
0.2
0.1
0.0
p-value
1e-02
1e-02
2e-02
2e-02
3e-02
Log-odds
0.5
1.0
1.5
2.0
2.5
3.0
3.5
4.0
Cellular_organisms - ps1
Archaea1 - ps2
Archaea2 - ps3
Archaea3 - ps4
Asgard/Eukaryota - ps5
Eukaryota - ps6
Amorpheae - ps7
Obazoa - ps8
Opisthokonta - ps9
Holomycota - ps10
Fungi - ps11
Fungi_1 - ps12
Fungi_2 - ps13
Fungi_3 - ps14
Dikarya - ps15
Ascomycota - ps16
Saccharo/Pezizomycotina - ps17
Saccharomycotina - ps18
Saccharomycotina_1 - ps19
Saccharomycotina_2 - ps20
Saccharomycotina_3 - ps21
Phaff./Saccharomycetaceae - ps22
Saccharomyco./mycetaceae - ps23
Saccharomycetaceae - ps24
Saccharomycetaceae_2 - ps25
Saccharomycetaceae_3 - ps26
Kaz./Nau./Saccharomyces - ps27
Saccharomyces - ps28
Arboricola_clade - ps29
Cerevisiae_clade - ps30
Saccharomyces_cerevisiae - ps31
Phylostratum

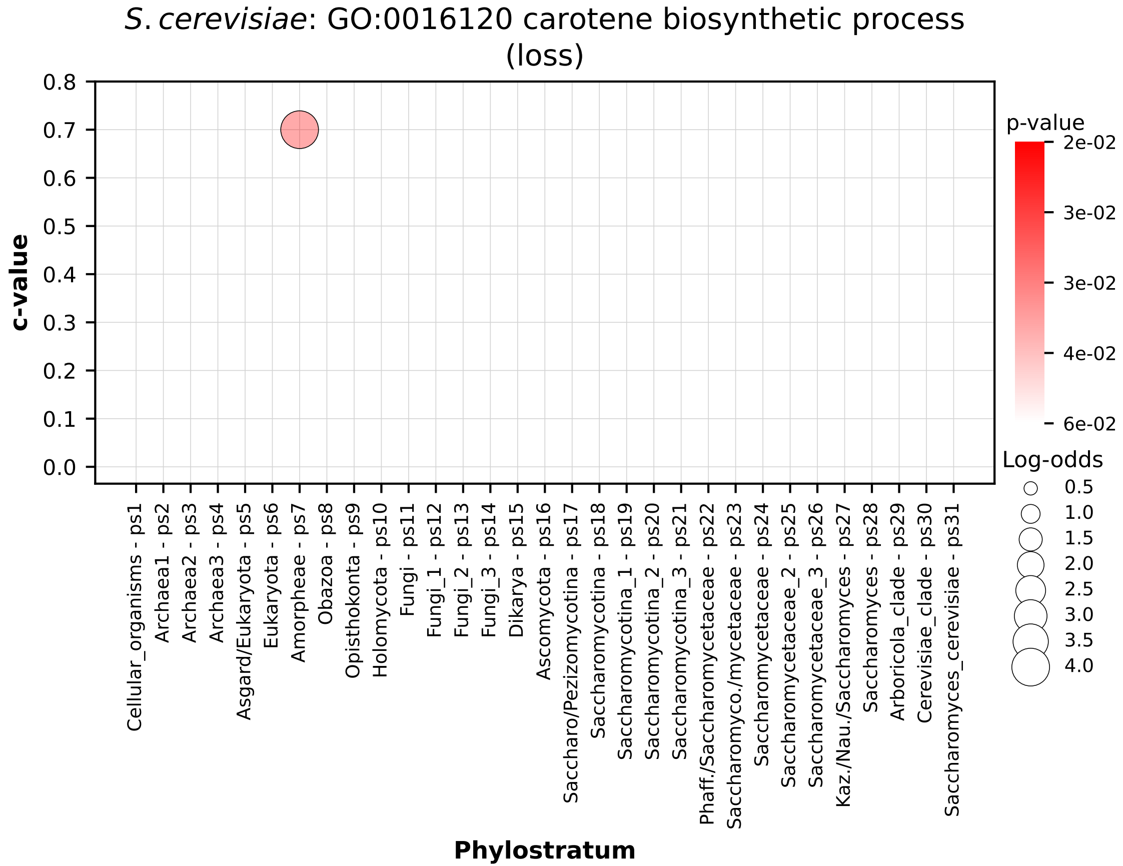

S. cerevisiae: GO:0016120 carotene biosynthetic process
(loss)
c-value
0.8
0.7
0.6
0.5
0.4
0.3
0.2
0.1
0.0
p-value
2e-02
3e-02
3e-02
4e-02
6e-02
Log-odds
0.5
1.0
1.5
2.0
2.5
3.0
3.5
4.0
Cellular_organisms - ps1
Archaea1 - ps2
Archaea2 - ps3
Archaea3 - ps4
Asgard/Eukaryota - ps5
Eukaryota - ps6
Amorpheae - ps7
Obazoa - ps8
Opisthokonta - ps9
Holomycota - ps10
Fungi - ps11
Fungi_1 - ps12
Fungi_2 - ps13
Fungi_3 - ps14
Dikarya - ps15
Ascomycota - ps16
Saccharo/Pezizomycotina - ps17
Saccharomycotina - ps18
Saccharomycotina_1 - ps19
Saccharomycotina_2 - ps20
Saccharomycotina_3 - ps21
Phaff./Saccharomycetaceae - ps22
Saccharomyco./mycetaceae - ps23
Saccharomycetaceae - ps24
Saccharomycetaceae_2 - ps25
Saccharomycetaceae_3 - ps26
Kaz./Nau./Saccharomyces - ps27
Saccharomyces - ps28
Arboricola_clade - ps29
Cerevisiae_clade - ps30
Saccharomyces_cerevisiae - ps31
Phylostratum

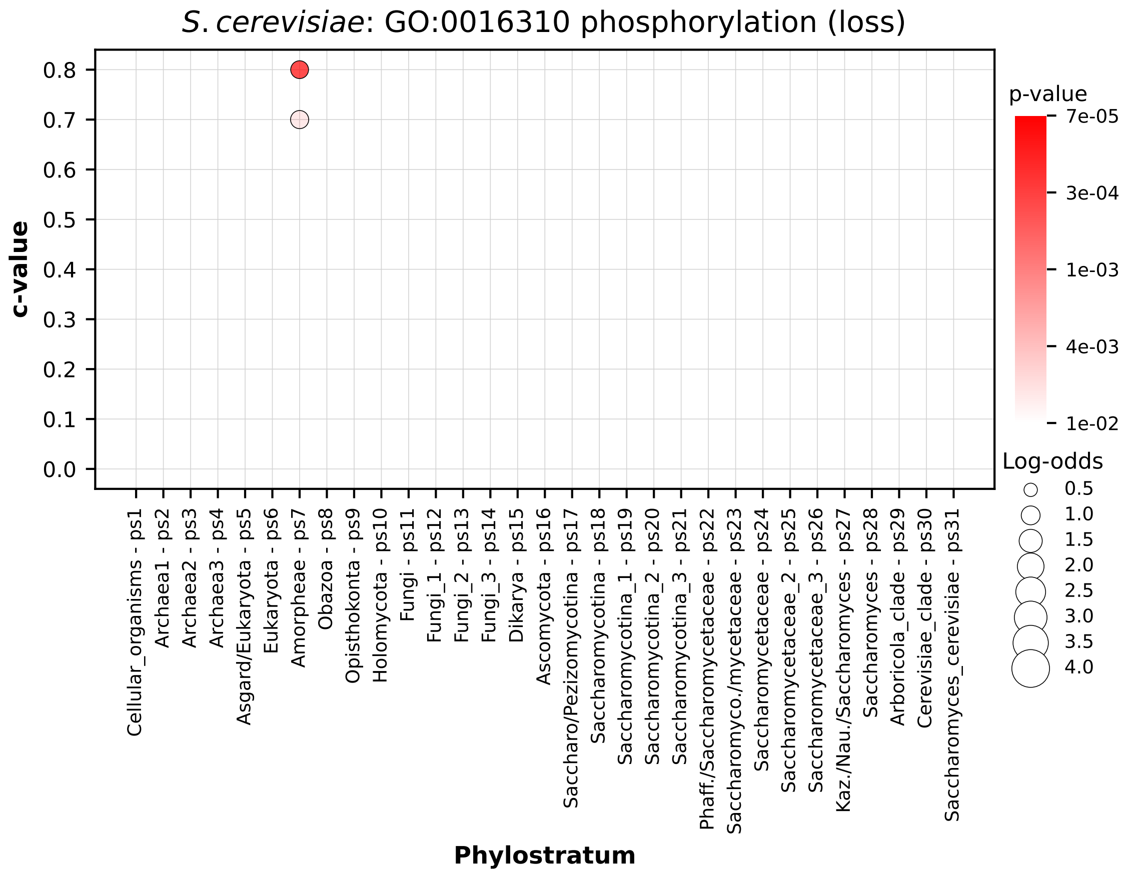

S. cerevisiae: GO:0016310 phosphorylation (loss)
c-value
0.8
0.7
0.6
0.5
0.4
0.3
0.2
0.1
0.0
Phylostratum
Cellular_organisms - ps1
Archaea1 - ps2
Archaea2 - ps3
Archaea3 - ps4
Asgard/Eukaryota - ps5
Eukaryota - ps6
Amorpheae - ps7
Obazoa - ps8
Opisthokonta - ps9
Holomycota - ps10
Fungi - ps11
Fungi_1 - ps12
Fungi_2 - ps13
Fungi_3 - ps14
Dikarya - ps15
Ascomycota - ps16
Saccharo/Pezizomycotina - ps17
Saccharomycotina - ps18
Saccharomycotina_1 - ps19
Saccharomycotina_2 - ps20
Saccharomycotina_3 - ps21
Phaff./Saccharomycetaceae - ps22
Saccharomyco./mycetaceae - ps23
Saccharomycetaceae - ps24
Saccharomycetaceae_2 - ps25
Saccharomycetaceae_3 - ps26
Kaz./Nau./Saccharomyces - ps27
Saccharomyces - ps28
Arboricola_clade - ps29
Cerevisiae_clade - ps30
Saccharomyces_cerevisiae - ps31
p-value
7e-05
3e-04
1e-03
4e-03
1e-02
Log-odds
0.5
1.0
1.5
2.0
2.5
3.0
3.5
4.0

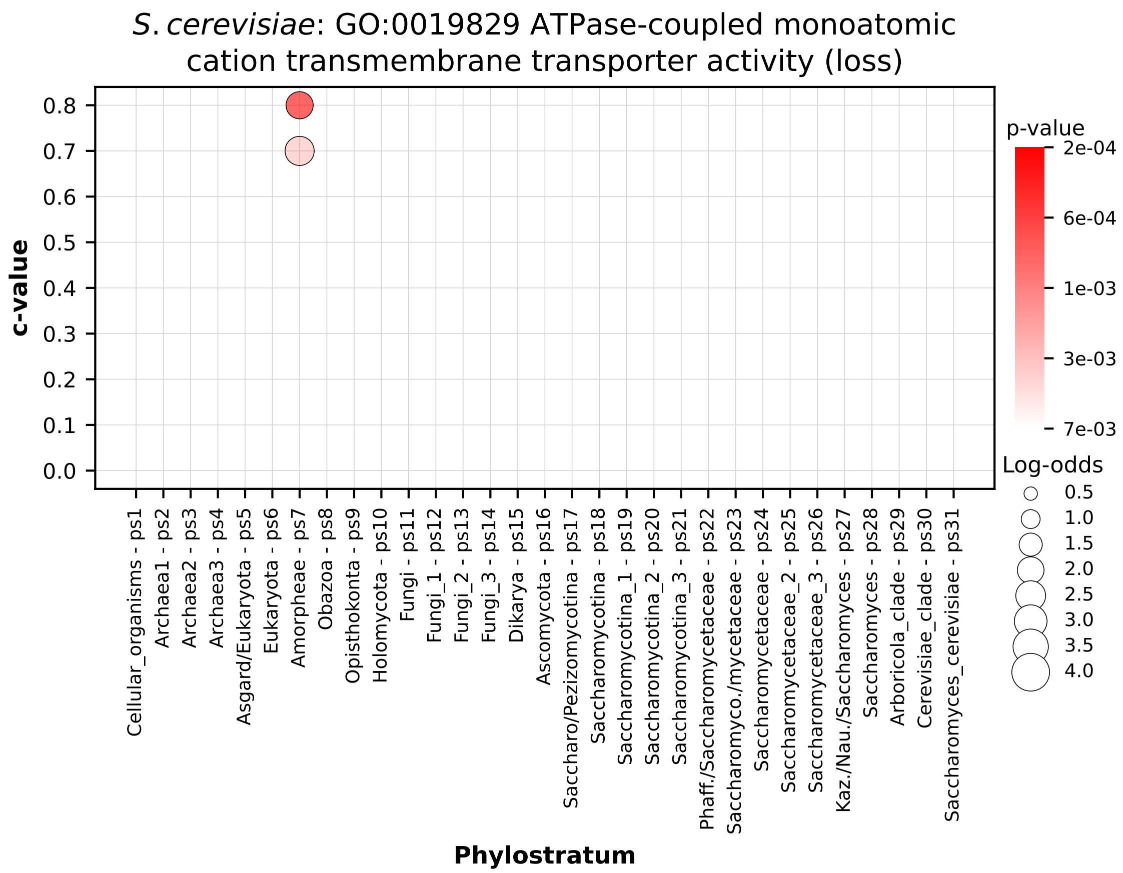
S. cerevisiae: GO:0019829 ATPase-coupled monoatomic cation transmembrane transporter activity (loss)
c-value
0.0
0.1
0.2
0.3
0.4
0.5
0.6
0.7
0.8
p-value
2e-04
6e-04
1e-03
3e-03
7e-03
Log-odds
0.5
1.0
1.5
2.0
2.5
3.0
3.5
4.0
Cellular_organisms - ps1
Archaea1 - ps2
Archaea2 - ps3
Archaea3 - ps4
Asgard/Eukaryota - ps5
Eukaryota - ps6
Amorpheae - ps7
Obazoa - ps8
Opisthokonta - ps9
Holomycota - ps10
Fungi - ps11
Fungi_1 - ps12
Fungi_2 - ps13
Fungi_3 - ps14
Dikarya - ps15
Ascomycota - ps16
Saccharo/Pezizomycotina - ps17
Saccharomycotina - ps18
Saccharomycotina_1 - ps19
Saccharomycotina_2 - ps20
Saccharomycotina_3 - ps21
Phaff./Saccharomycetaceae - ps22
Saccharomyco./mycetaceae - ps23
Saccharomycetaceae - ps24
Saccharomycetaceae_2 - ps25
Saccharomycetaceae_3 - ps26
Kaz./Nau./Saccharomyces - ps27
Saccharomyces - ps28
Arboricola_clade - ps29
Cerevisiae_clade - ps30
Saccharomyces_cerevisiae - ps31
Phylostratum

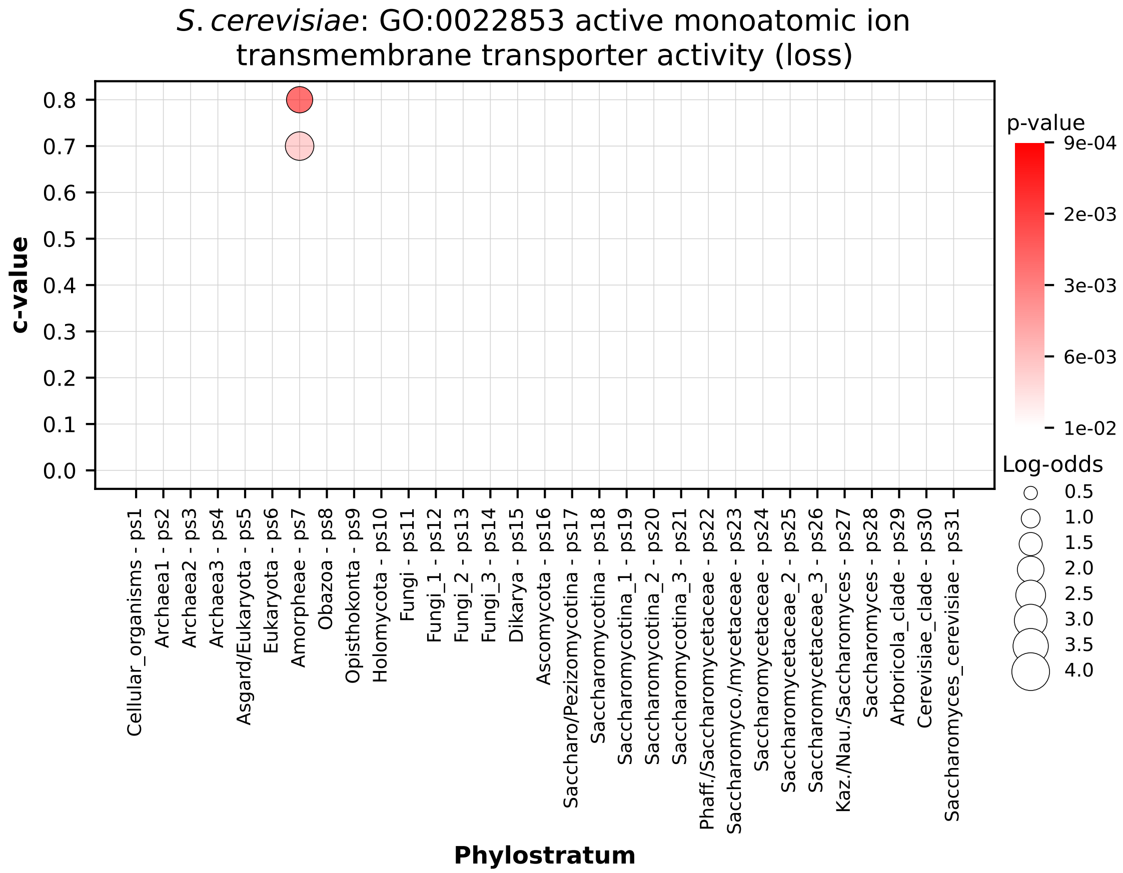

S. cerevisiae: GO:0022853 active monoatomic ion transmembrane transporter activity (loss)
c-value
0.8
0.7
0.6
0.5
0.4
0.3
0.2
0.1
0.0
p-value
9e-04
2e-03
3e-03
6e-03
1e-02
Log-odds
0.5
1.0
1.5
2.0
2.5
3.0
3.5
4.0
Cellular_organisms - ps1
Archaea1 - ps2
Archaea2 - ps3
Archaea3 - ps4
Asgard/Eukaryota - ps5
Eukaryota - ps6
Amorpheae - ps7
Obazoa - ps8
Opisthokonta - ps9
Holomycota - ps10
Fungi - ps11
Fungi_1 - ps12
Fungi_2 - ps13
Fungi_3 - ps14
Dikarya - ps15
Ascomycota - ps16
Saccharo/Pezizomycotina - ps17
Saccharomycotina - ps18
Saccharomycotina_1 - ps19
Saccharomycotina_2 - ps20
Saccharomycotina_3 - ps21
Phaff./Saccharomycetaceae - ps22
Saccharomyco./mycetaceae - ps23
Saccharomycetaceae - ps24
Saccharomycetaceae_2 - ps25
Saccharomycetaceae_3 - ps26
Kaz./Nau./Saccharomyces - ps27
Saccharomyces - ps28
Arboricola_clade - ps29
Cerevisiae_clade - ps30
Saccharomyces_cerevisiae - ps31
Phylostratum

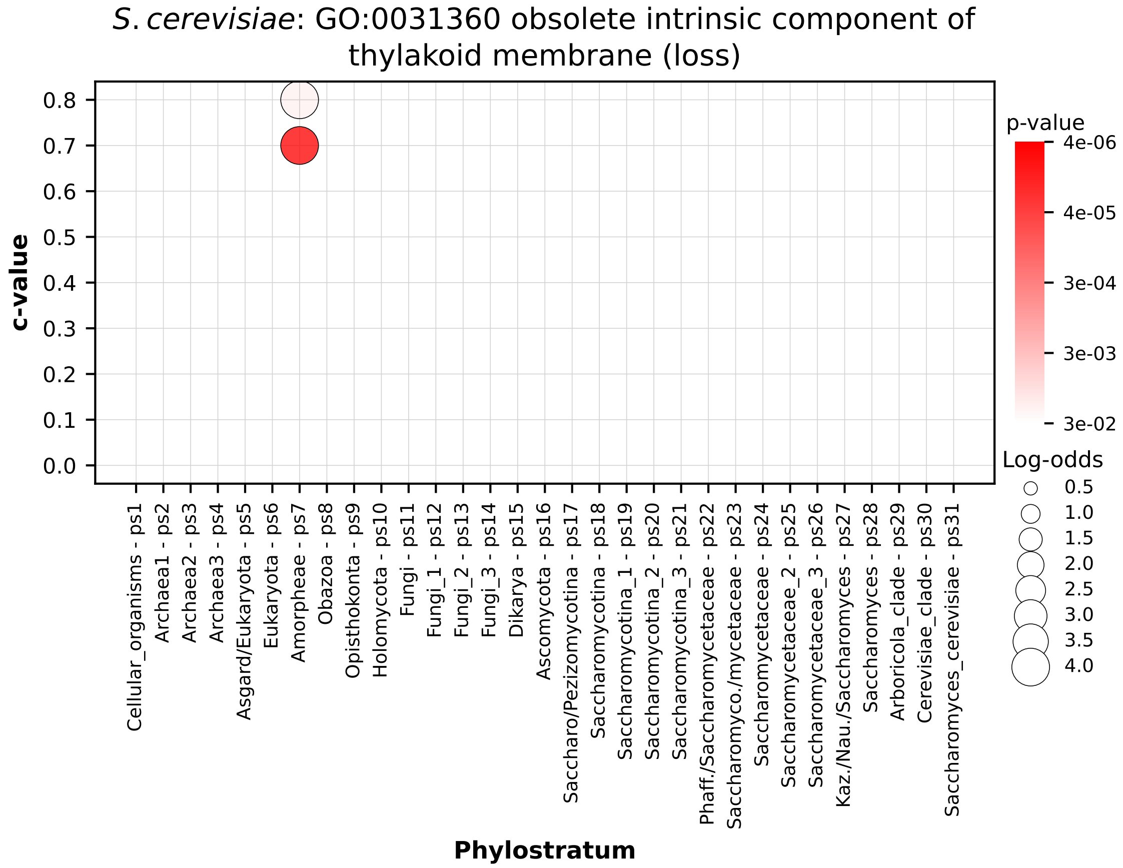
S. cerevisiae: GO:0031360 obsolete intrinsic component of thylakoid membrane (loss)
c-value
0.0
0.1
0.2
0.3
0.4
0.5
0.6
0.7
0.8
p-value
4e-06
4e-05
3e-04
3e-03
3e-02
Log-odds
0.5
1.0
1.5
2.0
2.5
3.0
3.5
4.0
Cellular_organisms - ps1
Archaea1 - ps2
Archaea2 - ps3
Archaea3 - ps4
Asgard/Eukaryota - ps5
Eukaryota - ps6
Amorpheae - ps7
Obazoa - ps8
Opisthokonta - ps9
Holomycota - ps10
Fungi - ps11
Fungi_1 - ps12
Fungi_2 - ps13
Fungi_3 - ps14
Dikarya - ps15
Ascomycota - ps16
Saccharo/Pezizomycotina - ps17
Saccharomycotina - ps18
Saccharomycotina_1 - ps19
Saccharomycotina_2 - ps20
Saccharomycotina_3 - ps21
Phaff./Saccharomycetaceae - ps22
Saccharomyco./mycetaceae - ps23
Saccharomycetaceae - ps24
Saccharomycetaceae_2 - ps25
Saccharomycetaceae_3 - ps26
Kaz./Nau./Saccharomyces - ps27
Saccharomyces - ps28
Arboricola_clade - ps29
Cerevisiae_clade - ps30
Saccharomyces_cerevisiae - ps31
Phylostratum

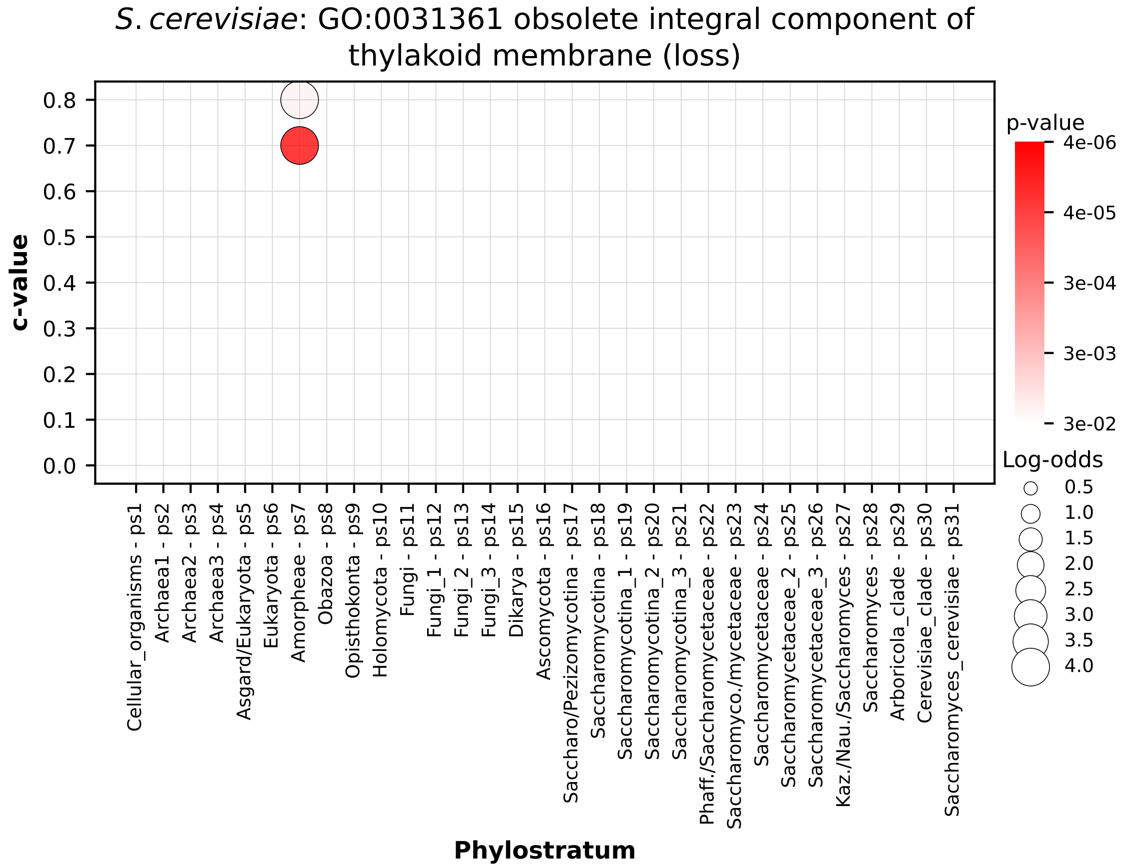
S. cerevisiae: GO:0031361 obsolete integral component of thylakoid membrane (loss)
c-value
0.0
0.1
0.2
0.3
0.4
0.5
0.6
0.7
0.8
p-value
4e-06
4e-05
3e-04
3e-03
3e-02
Log-odds
0.5
1.0
1.5
2.0
2.5
3.0
3.5
4.0
Cellular_organisms - ps1
Archaea1 - ps2
Archaea2 - ps3
Archaea3 - ps4
Asgard/Eukaryota - ps5
Eukaryota - ps6
Amorpheae - ps7
Obazoa - ps8
Opisthokonta - ps9
Holomycota - ps10
Fungi - ps11
Fungi_1 - ps12
Fungi_2 - ps13
Fungi_3 - ps14
Dikarya - ps15
Ascomycota - ps16
Saccharo/Pezizomycotina - ps17
Saccharomycotina - ps18
Saccharomycotina_1 - ps19
Saccharomycotina_2 - ps20
Saccharomycotina_3 - ps21
Phaff./Saccharomycetaceae - ps22
Saccharomyco./mycetaceae - ps23
Saccharomycetaceae - ps24
Saccharomycetaceae_2 - ps25
Saccharomycetaceae_3 - ps26
Kaz./Nau./Saccharomyces - ps27
Saccharomyces - ps28
Arboricola_clade - ps29
Cerevisiae_clade - ps30
Saccharomyces_cerevisiae - ps31
Phylostratum

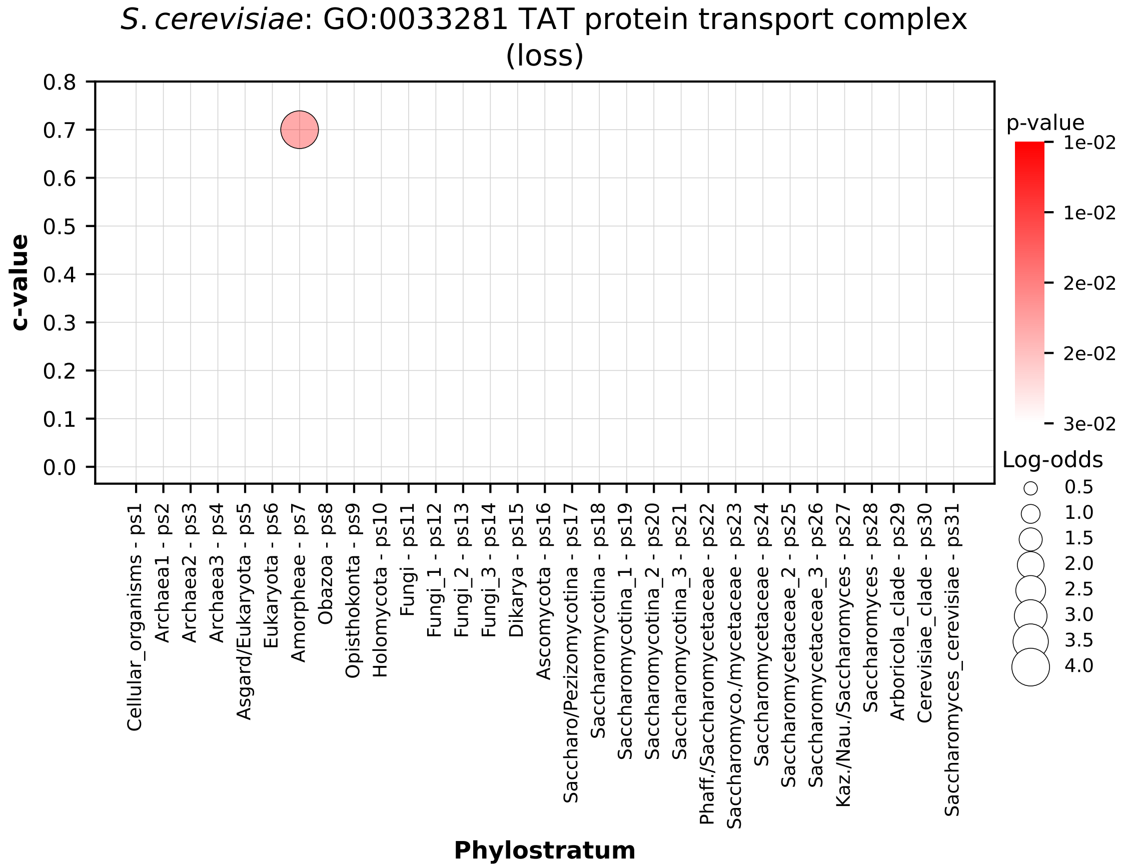

S. cerevisiae: GO:0033281 TAT protein transport complex (loss)
c-value
0.8
0.7
0.6
0.5
0.4
0.3
0.2
0.1
0.0
p-value
1e-02
1e-02
2e-02
2e-02
3e-02
Log-odds
0.5
1.0
1.5
2.0
2.5
3.0
3.5
4.0
Cellular_organisms - ps1
Archaea1 - ps2
Archaea2 - ps3
Archaea3 - ps4
Asgard/Eukaryota - ps5
Eukaryota - ps6
Amorpheae - ps7
Obazoa - ps8
Opisthokonta - ps9
Holomycota - ps10
Fungi - ps11
Fungi_1 - ps12
Fungi_2 - ps13
Fungi_3 - ps14
Dikarya - ps15
Ascomycota - ps16
Saccharo/Pezizomycotina - ps17
Saccharomycotina - ps18
Saccharomycotina_1 - ps19
Saccharomycotina_2 - ps20
Saccharomycotina_3 - ps21
Phaff./Saccharomycetaceae - ps22
Saccharomyco./mycetaceae - ps23
Saccharomycetaceae - ps24
Saccharomycetaceae_2 - ps25
Saccharomycetaceae_3 - ps26
Kaz./Nau./Saccharomyces - ps27
Saccharomyces - ps28
Arboricola_clade - ps29
Cerevisiae_clade - ps30
Saccharomyces_cerevisiae - ps31
Phylostratum

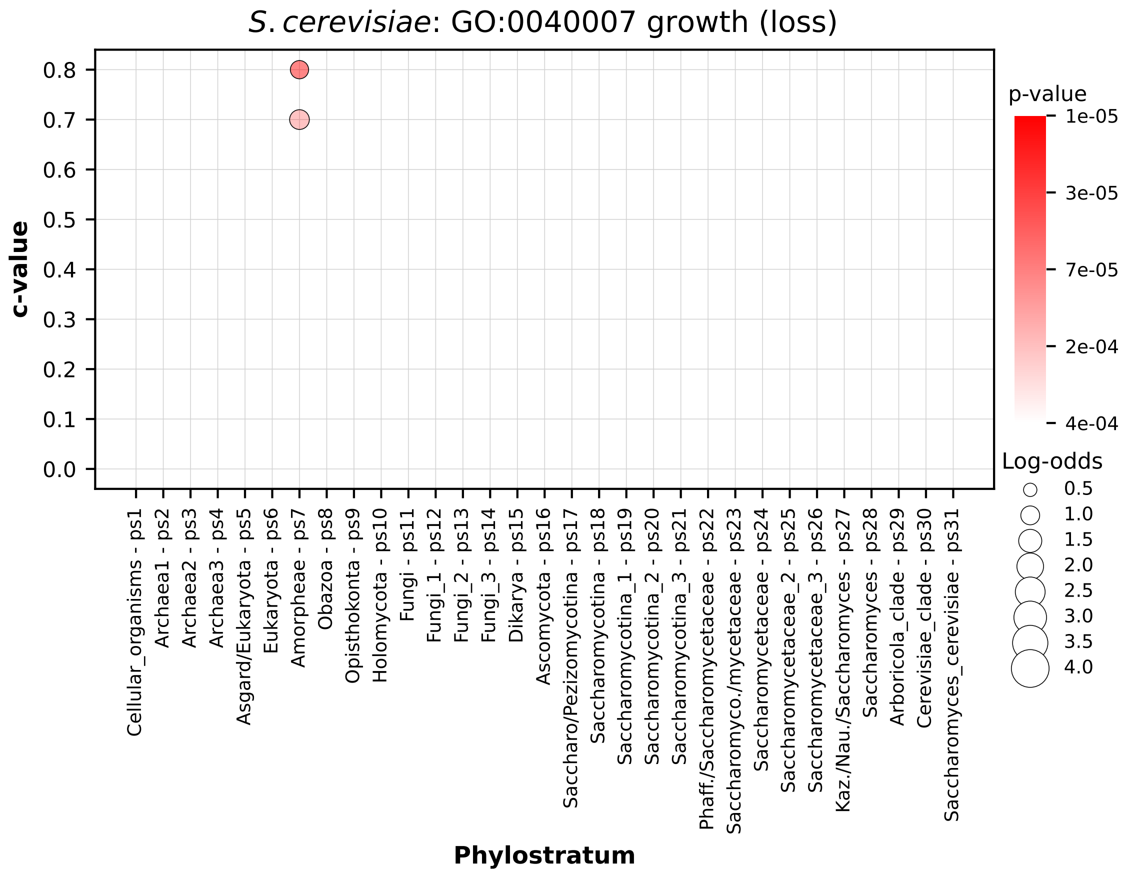

S. cerevisiae: GO:0040007 growth (loss)
c-value
0.0
0.1
0.2
0.3
0.4
0.5
0.6
0.7
0.8
Phylostratum
Cellular_organisms - ps1
Archaea1 - ps2
Archaea2 - ps3
Archaea3 - ps4
Asgard/Eukaryota - ps5
Eukaryota - ps6
Amorpheae - ps7
Obazoa - ps8
Opisthokonta - ps9
Holomycota - ps10
Fungi - ps11
Fungi_1 - ps12
Fungi_2 - ps13
Fungi_3 - ps14
Dikarya - ps15
Ascomycota - ps16
Saccharo/Pezizomycotina - ps17
Saccharomycotina - ps18
Saccharomycotina_1 - ps19
Saccharomycotina_2 - ps20
Saccharomycotina_3 - ps21
Phaff./Saccharomycetaceae - ps22
Saccharomyco./mycetaceae - ps23
Saccharomycetaceae - ps24
Saccharomycetaceae_2 - ps25
Saccharomycetaceae_3 - ps26
Kaz./Nau./Saccharomyces - ps27
Saccharomyces - ps28
Arboricola_clade - ps29
Cerevisiae_clade - ps30
Saccharomyces_cerevisiae - ps31
p-value
1e-05
3e-05
7e-05
2e-04
4e-04
Log-odds
0.5
1.0
1.5
2.0
2.5
3.0
3.5
4.0

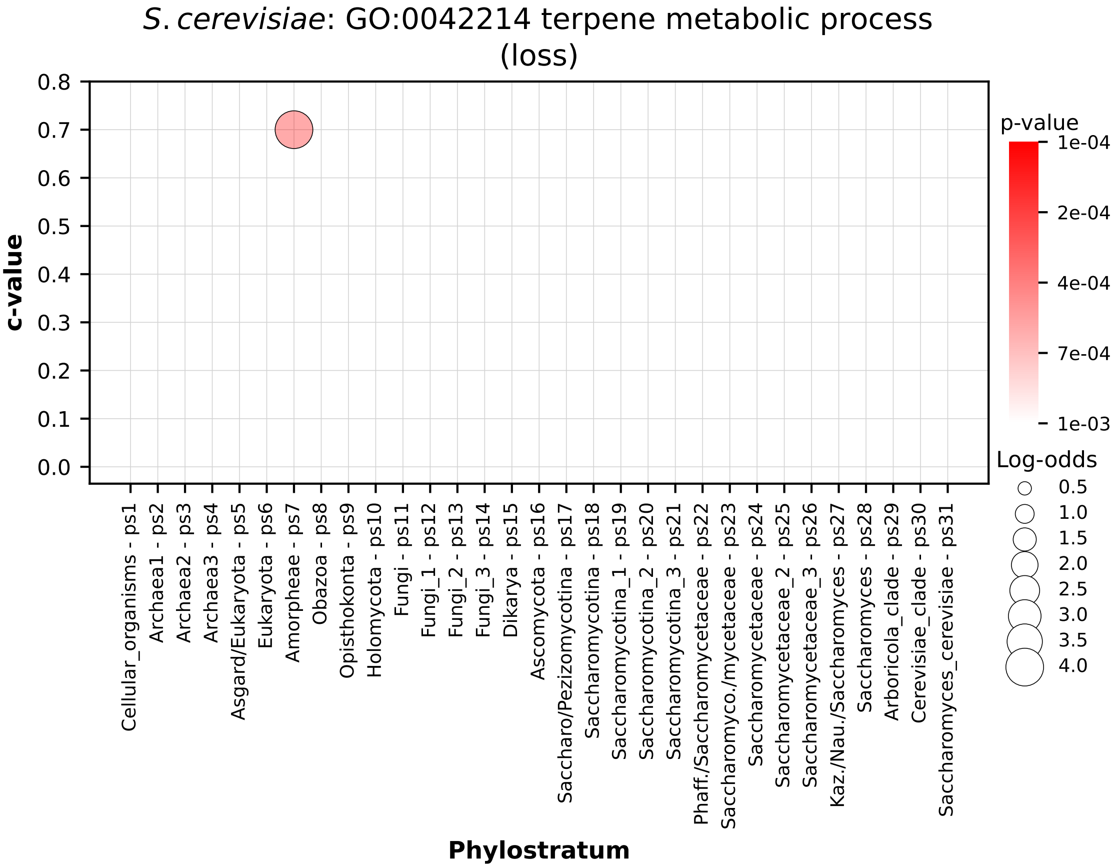

S. cerevisiae: GO:0042214 terpene metabolic process
(loss)
c-value
0.8
0.7
0.6
0.5
0.4
0.3
0.2
0.1
0.0
p-value
1e-04
2e-04
4e-04
7e-04
1e-03
Log-odds
0.5
1.0
1.5
2.0
2.5
3.0
3.5
4.0
Cellular_organisms - ps1
Archaea1 - ps2
Archaea2 - ps3
Archaea3 - ps4
Asgard/Eukaryota - ps5
Eukaryota - ps6
Amorpheae - ps7
Obazoa - ps8
Opisthokonta - ps9
Holomycota - ps10
Fungi - ps11
Fungi_1 - ps12
Fungi_2 - ps13
Fungi_3 - ps14
Dikarya - ps15
Ascomycota - ps16
Saccharo/Pezizomycotina - ps17
Saccharomycotina - ps18
Saccharomycotina_1 - ps19
Saccharomycotina_2 - ps20
Saccharomycotina_3 - ps21
Phaff./Saccharomycetaceae - ps22
Saccharomyco./mycetaceae - ps23
Saccharomycetaceae - ps24
Saccharomycetaceae_2 - ps25
Saccharomycetaceae_3 - ps26
Kaz./Nau./Saccharomyces - ps27
Saccharomyces - ps28
Arboricola_clade - ps29
Cerevisiae_clade - ps30
Saccharomyces_cerevisiae - ps31
Phylostratum

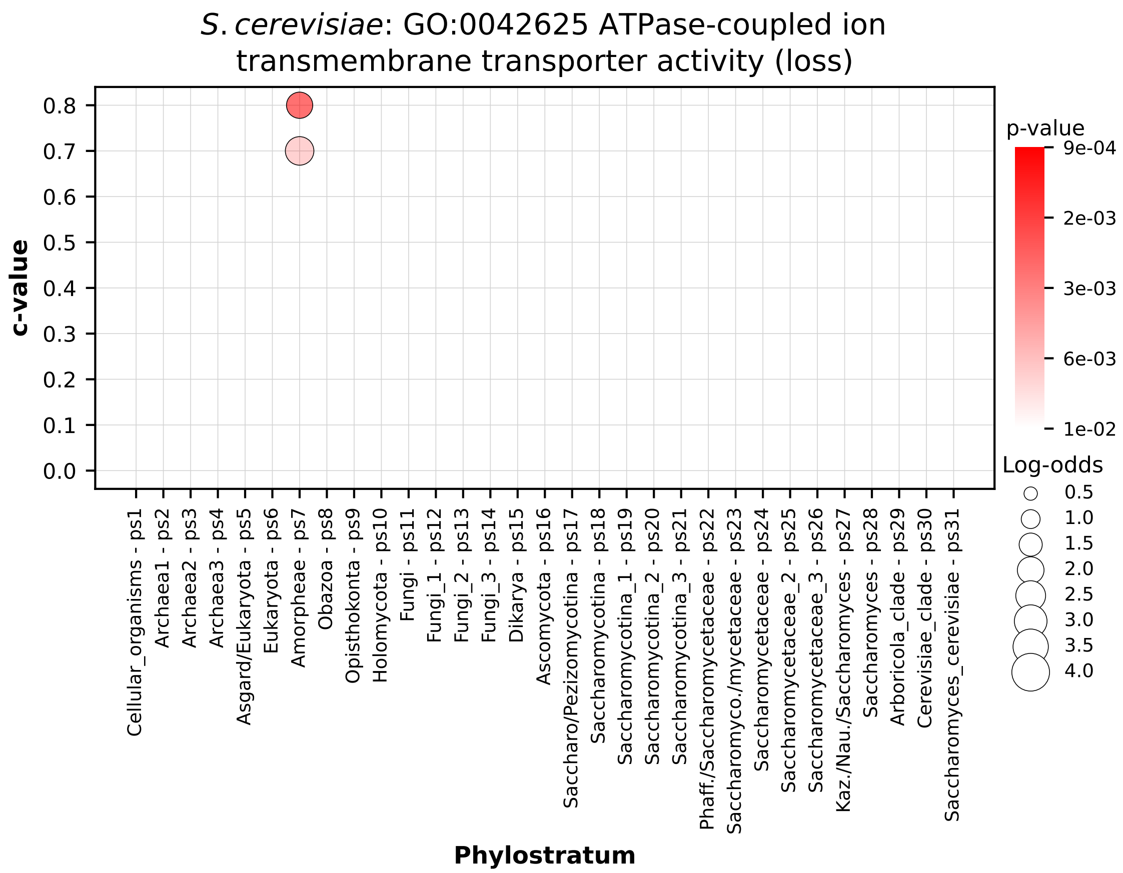

S. cerevisiae: GO:0042625 ATPase-coupled ion transmembrane transporter activity (loss)
c-value
0.0
0.1
0.2
0.3
0.4
0.5
0.6
0.7
0.8
p-value
9e-04
2e-03
3e-03
6e-03
1e-02
Log-odds
0.5
1.0
1.5
2.0
2.5
3.0
3.5
4.0
Cellular_organisms - ps1
Archaea1 - ps2
Archaea2 - ps3
Archaea3 - ps4
Asgard/Eukaryota - ps5
Eukaryota - ps6
Amorpheae - ps7
Obazoa - ps8
Opisthokonta - ps9
Holomycota - ps10
Fungi - ps11
Fungi_1 - ps12
Fungi_2 - ps13
Fungi_3 - ps14
Dikarya - ps15
Ascomycota - ps16
Saccharo/Pezizomycotina - ps17
Saccharomycotina - ps18
Saccharomycotina_1 - ps19
Saccharomycotina_2 - ps20
Saccharomycotina_3 - ps21
Phaff./Saccharomycetaceae - ps22
Saccharomyco./mycetaceae - ps23
Saccharomycetaceae - ps24
Saccharomycetaceae_2 - ps25
Saccharomycetaceae_3 - ps26
Kaz./Nau./Saccharomyces - ps27
Saccharomyces - ps28
Arboricola_clade - ps29
Cerevisiae_clade - ps30
Saccharomyces_cerevisiae - ps31
Phylostratum

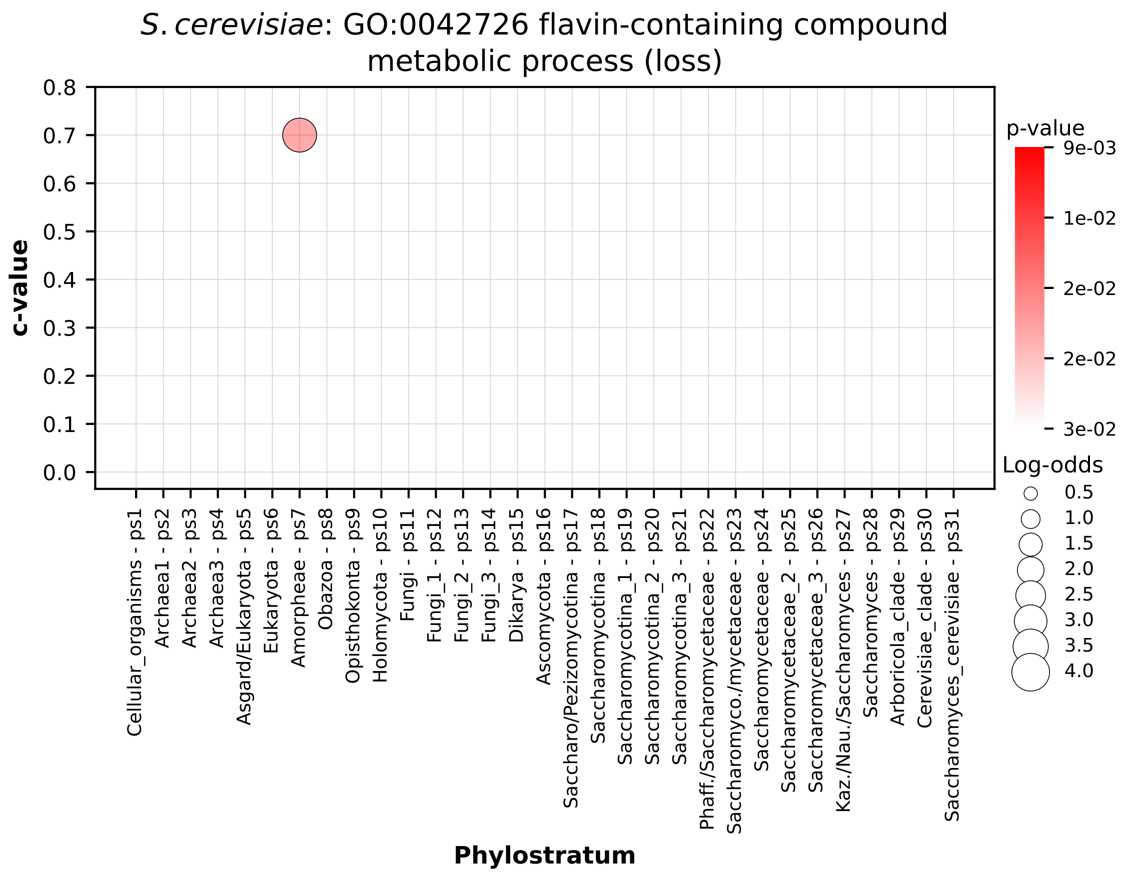

S. cerevisiae: GO:0042726 flavin-containing compound metabolic process (loss)
c-value
0.8
0.7
0.6
0.5
0.4
0.3
0.2
0.1
0.0
p-value
9e-03
1e-02
2e-02
2e-02
3e-02
Log-odds
0.5
1.0
1.5
2.0
2.5
3.0
3.5
4.0
Cellular_organisms - ps1
Archaea1 - ps2
Archaea2 - ps3
Archaea3 - ps4
Asgard/Eukaryota - ps5
Eukaryota - ps6
Amorpheae - ps7
Obazoa - ps8
Opisthokonta - ps9
Holomycota - ps10
Fungi - ps11
Fungi_1 - ps12
Fungi_2 - ps13
Fungi_3 - ps14
Dikarya - ps15
Ascomycota - ps16
Saccharo/Pezizomycotina - ps17
Saccharomycotina - ps18
Saccharomycotina_1 - ps19
Saccharomycotina_2 - ps20
Saccharomycotina_3 - ps21
Phaff./Saccharomycetaceae - ps22
Saccharomyco./mycetaceae - ps23
Saccharomycetaceae - ps24
Saccharomycetaceae_2 - ps25
Saccharomycetaceae_3 - ps26
Kaz./Nau./Saccharomyces - ps27
Saccharomyces - ps28
Arboricola_clade - ps29
Cerevisiae_clade - ps30
Saccharomyces_cerevisiae - ps31
Phylostratum

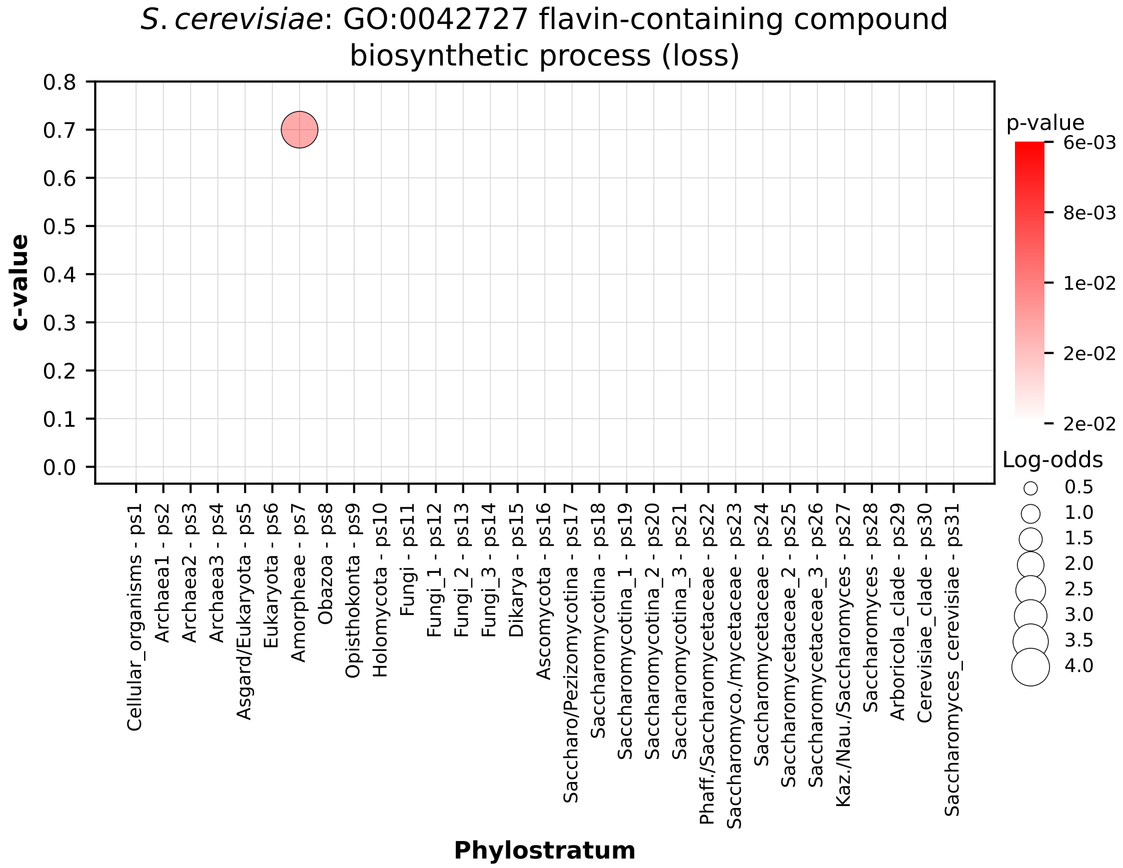
S. cerevisiae: GO:0042727 flavin-containing compound biosynthetic process (loss)
c-value
0.8
0.7
0.6
0.5
0.4
0.3
0.2
0.1
0.0
Phylostratum
Cellular_organisms - ps1
Archaea1 - ps2
Archaea2 - ps3
Archaea3 - ps4
Asgard/Eukaryota - ps5
Eukaryota - ps6
Amorpheae - ps7
Obazoa - ps8
Opisthokonta - ps9
Holomycota - ps10
Fungi - ps11
Fungi_1 - ps12
Fungi_2 - ps13
Fungi_3 - ps14
Dikarya - ps15
Ascomycota - ps16
Saccharo/Pezizomycotina - ps17
Saccharomycotina - ps18
Saccharomycotina_1 - ps19
Saccharomycotina_2 - ps20
Saccharomycotina_3 - ps21
Phaff./Saccharomycetaceae - ps22
Saccharomyco./mycetaceae - ps23
Saccharomycetaceae - ps24
Saccharomycetaceae_2 - ps25
Saccharomycetaceae_3 - ps26
Kaz./Nau./Saccharomyces - ps27
Saccharomyces - ps28
Arboricola_clade - ps29
Cerevisiae_clade - ps30
Saccharomyces_cerevisiae - ps31
p-value
6e-03
8e-03
1e-02
2e-02
2e-02
Log-odds
0.5
1.0
1.5
2.0
2.5
3.0
3.5
4.0

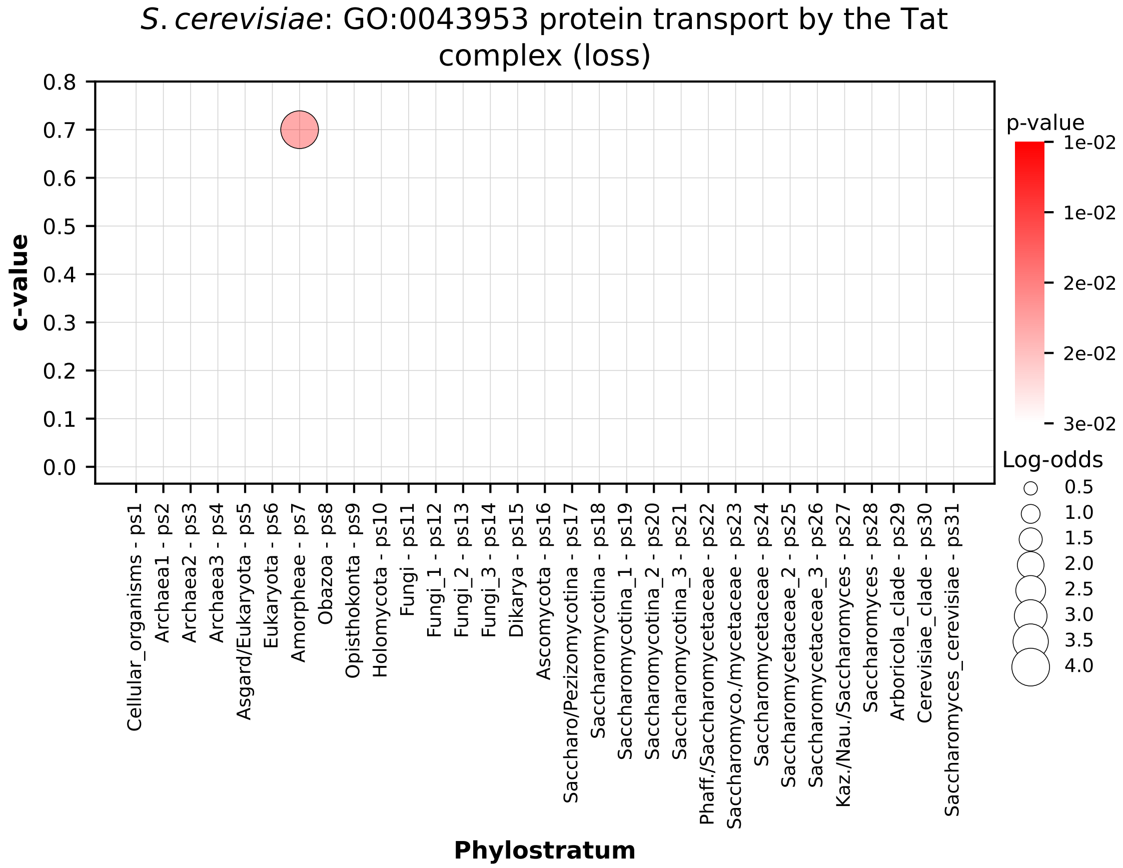

S. cerevisiae: GO:0043953 protein transport by the Tat complex (loss)
c-value
0.8
0.7
0.6
0.5
0.4
0.3
0.2
0.1
0.0
Phylostratum
Cellular_organisms - ps1
Archaea1 - ps2
Archaea2 - ps3
Archaea3 - ps4
Asgard/Eukaryota - ps5
Eukaryota - ps6
Amorpheae - ps7
Obazoa - ps8
Opisthokonta - ps9
Holomycota - ps10
Fungi - ps11
Fungi_1 - ps12
Fungi_2 - ps13
Fungi_3 - ps14
Dikarya - ps15
Ascomycota - ps16
Saccharo/Pezizomycotina - ps17
Saccharomycotina - ps18
Saccharomycotina_1 - ps19
Saccharomycotina_2 - ps20
Saccharomycotina_3 - ps21
Phaff./Saccharomycetaceae - ps22
Saccharomyco./mycetaceae - ps23
Saccharomycetaceae - ps24
Saccharomycetaceae_2 - ps25
Saccharomycetaceae_3 - ps26
Kaz./Nau./Saccharomyces - ps27
Saccharomyces - ps28
Arboricola_clade - ps29
Cerevisiae_clade - ps30
Saccharomyces_cerevisiae - ps31
p-value
1e-02
1e-02
2e-02
2e-02
3e-02
Log-odds
0.5
1.0
1.5
2.0
2.5
3.0
3.5
4.0

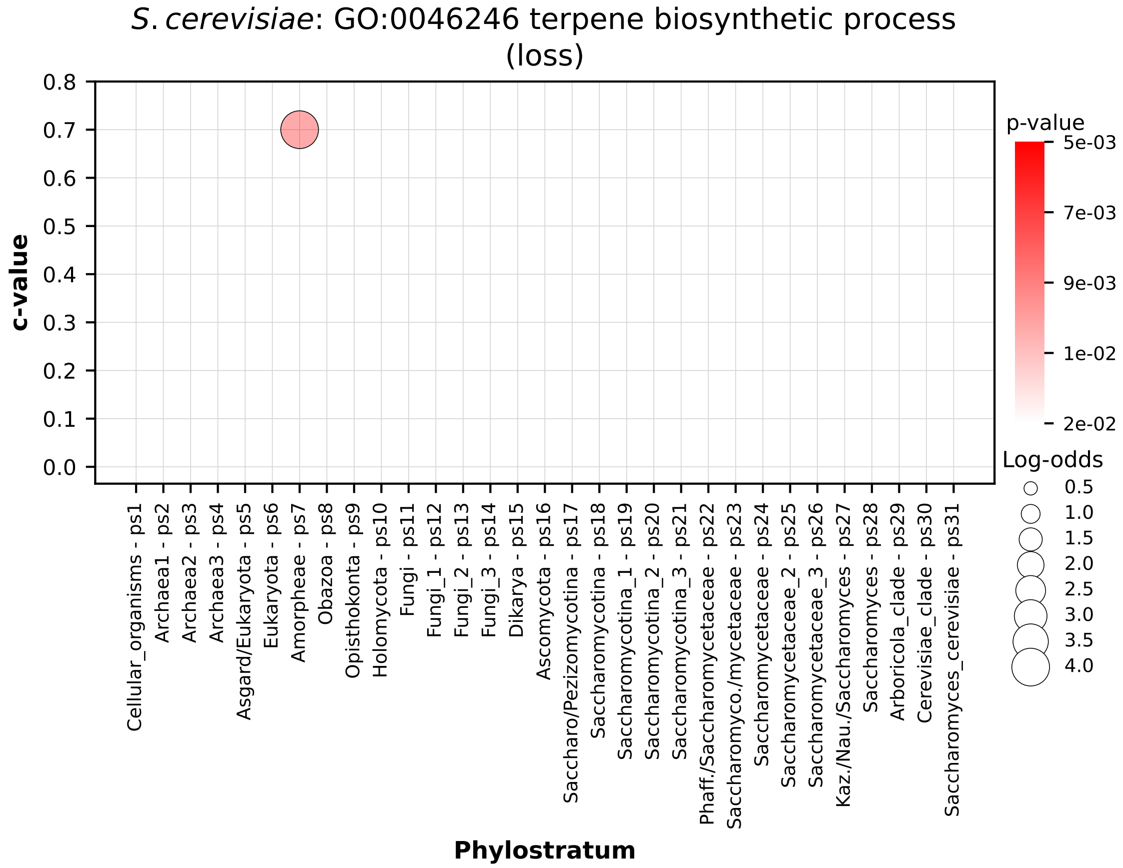
S. cerevisiae: GO:0046246 terpene biosynthetic process
(loss)
c-value
0.8
0.7
0.6
0.5
0.4
0.3
0.2
0.1
0.0
p-value
5e-03
7e-03
9e-03
1e-02
2e-02
Log-odds
0.5
1.0
1.5
2.0
2.5
3.0
3.5
4.0
Cellular_organisms - ps1
Archaea1 - ps2
Archaea2 - ps3
Archaea3 - ps4
Asgard/Eukaryota - ps5
Eukaryota - ps6
Amorpheae - ps7
Obazoa - ps8
Opisthokonta - ps9
Holomycota - ps10
Fungi - ps11
Fungi_1 - ps12
Fungi_2 - ps13
Fungi_3 - ps14
Dikarya - ps15
Ascomycota - ps16
Saccharo/Pezizomycotina - ps17
Saccharomycotina - ps18
Saccharomycotina_1 - ps19
Saccharomycotina_2 - ps20
Saccharomycotina_3 - ps21
Phaff./Saccharomycetaceae - ps22
Saccharomyco./mycetaceae - ps23
Saccharomycetaceae - ps24
Saccharomycetaceae_2 - ps25
Saccharomycetaceae_3 - ps26
Kaz./Nau./Saccharomyces - ps27
Saccharomyces - ps28
Arboricola_clade - ps29
Cerevisiae_clade - ps30
Saccharomyces_cerevisiae - ps31
Phylostratum

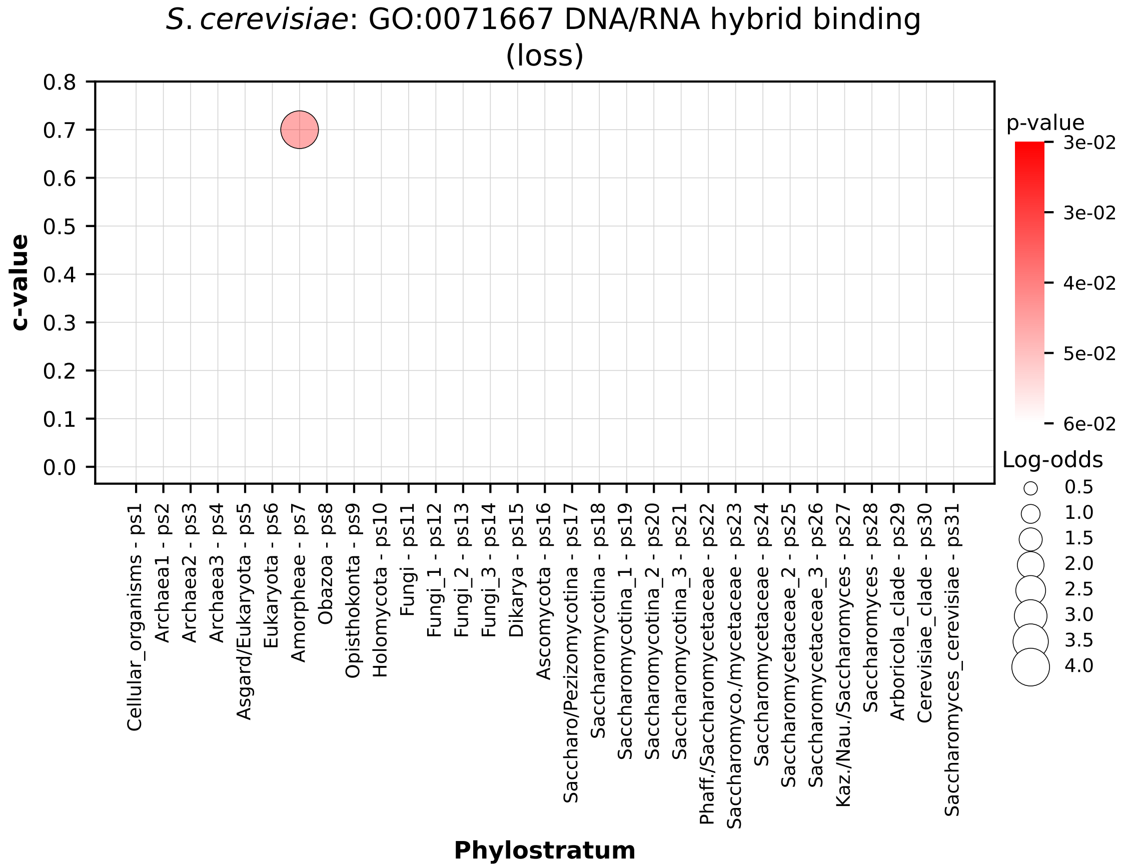
S. cerevisiae: GO:0071667 DNA/RNA hybrid binding (loss)
c-value
0.8
0.7
0.6
0.5
0.4
0.3
0.2
0.1
0.0
Phylostratum
Cellular_organisms - ps1
Archaea1 - ps2
Archaea2 - ps3
Archaea3 - ps4
Asgard/Eukaryota - ps5
Eukaryota - ps6
Amorpheae - ps7
Obazoa - ps8
Opisthokonta - ps9
Holomycota - ps10
Fungi - ps11
Fungi_1 - ps12
Fungi_2 - ps13
Fungi_3 - ps14
Dikarya - ps15
Ascomycota - ps16
Saccharo/Pezizomycotina - ps17
Saccharomycotina - ps18
Saccharomycotina_1 - ps19
Saccharomycotina_2 - ps20
Saccharomycotina_3 - ps21
Phaff./Saccharomycetaceae - ps22
Saccharomyco./mycetaceae - ps23
Saccharomycetaceae - ps24
Saccharomycetaceae_2 - ps25
Saccharomycetaceae_3 - ps26
Kaz./Nau./Saccharomyces - ps27
Saccharomyces - ps28
Arboricola_clade - ps29
Cerevisiae_clade - ps30
Saccharomyces_cerevisiae - ps31
p-value
3e-02
3e-02
4e-02
5e-02
6e-02
Log-odds
0.5
1.0
1.5
2.0
2.5
3.0
3.5
4.0

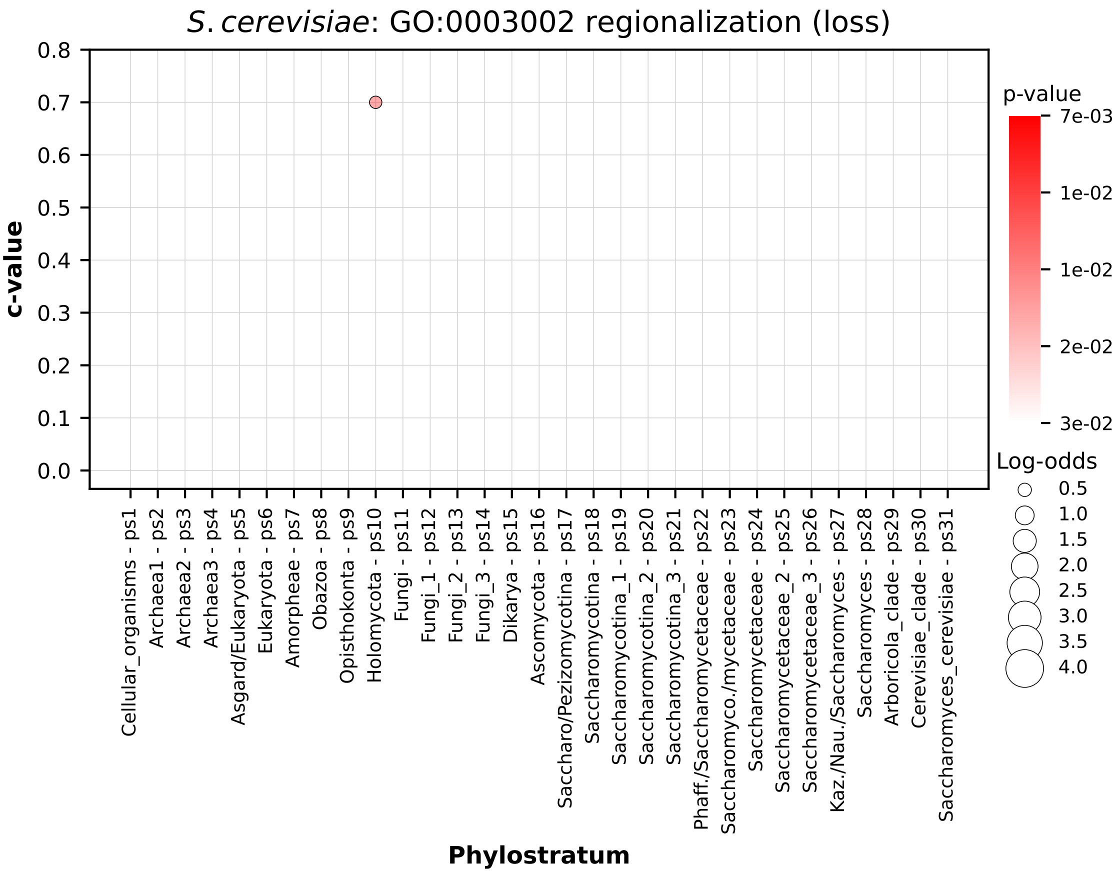
S. cerevisiae: GO:0003002 regionalization (loss)
c-value
0.0
0.1
0.2
0.3
0.4
0.5
0.6
0.7
0.8
Phylostratum
Cellular_organisms - ps1
Archaea1 - ps2
Archaea2 - ps3
Archaea3 - ps4
Asgard/Eukaryota - ps5
Eukaryota - ps6
Amorpheae - ps7
Obazoa - ps8
Opisthokonta - ps9
Holomycota - ps10
Fungi - ps11
Fungi_1 - ps12
Fungi_2 - ps13
Fungi_3 - ps14
Dikarya - ps15
Ascomycota - ps16
Saccharo/Pezizomycotina - ps17
Saccharomycotina - ps18
Saccharomycotina_1 - ps19
Saccharomycotina_2 - ps20
Saccharomycotina_3 - ps21
Phaff./Saccharomycetaceae - ps22
Saccharomyco./mycetaceae - ps23
Saccharomycetaceae - ps24
Saccharomycetaceae_2 - ps25
Saccharomycetaceae_3 - ps26
Kaz./Nau./Saccharomyces - ps27
Saccharomyces - ps28
Arboricola_clade - ps29
Cerevisiae_clade - ps30
Saccharomyces_cerevisiae - ps31
p-value
7e-03
1e-02
1e-02
2e-02
3e-02
Log-odds
0.5
1.0
1.5
2.0
2.5
3.0
3.5
4.0

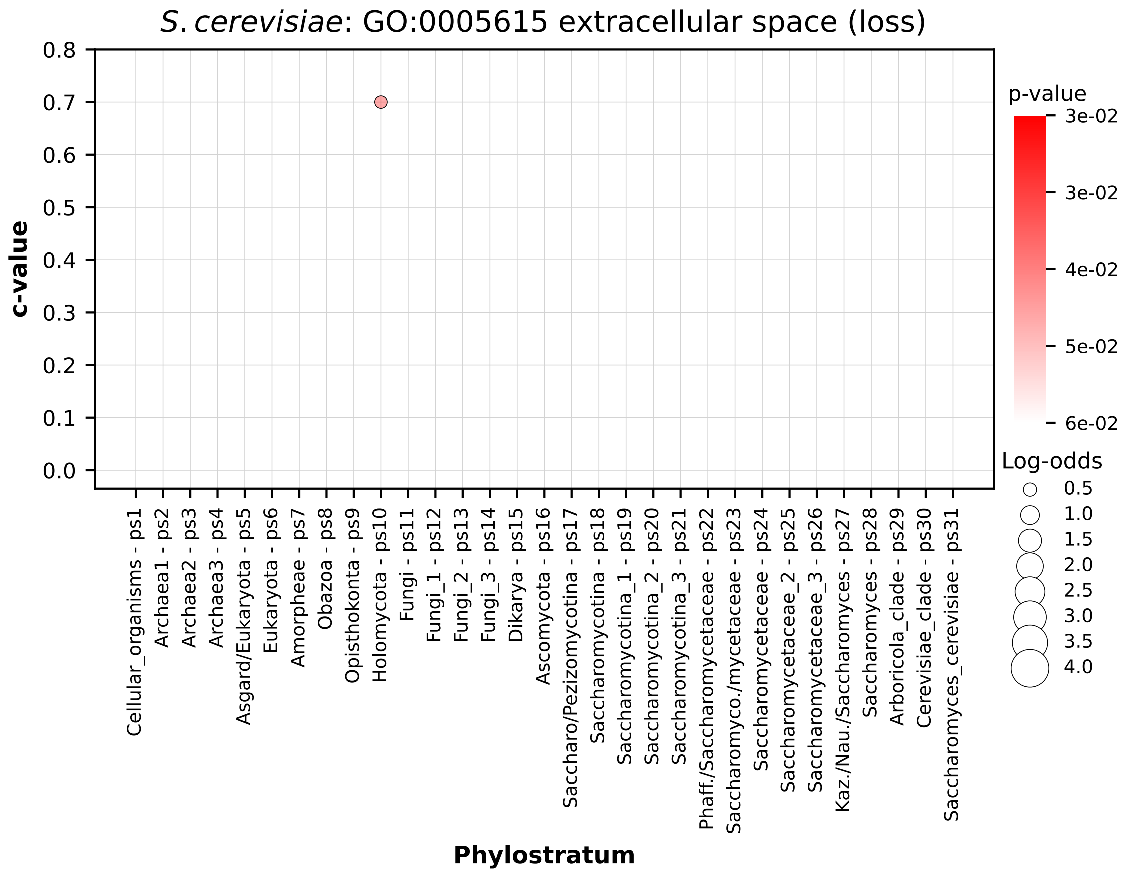
S. cerevisiae: GO:0005615 extracellular space (loss)
c-value
0.8
0.7
0.6
0.5
0.4
0.3
0.2
0.1
0.0
p-value
3e-02
3e-02
4e-02
5e-02
6e-02
Log-odds
0.5
1.0
1.5
2.0
2.5
3.0
3.5
4.0
Cellular_organisms - ps1
Archaea1 - ps2
Archaea2 - ps3
Archaea3 - ps4
Asgard/Eukaryota - ps5
Eukaryota - ps6
Amorpheae - ps7
Obazoa - ps8
Opisthokonta - ps9
Holomycota - ps10
Fungi - ps11
Fungi_1 - ps12
Fungi_2 - ps13
Fungi_3 - ps14
Dikarya - ps15
Ascomycota - ps16
Saccharo/Pezizomycotina - ps17
Saccharomycotina - ps18
Saccharomycotina_1 - ps19
Saccharomycotina_2 - ps20
Saccharomycotina_3 - ps21
Phaff./Saccharomycetaceae - ps22
Saccharomyco./mycetaceae - ps23
Saccharomycetaceae - ps24
Saccharomycetaceae_2 - ps25
Saccharomycetaceae_3 - ps26
Kaz./Nau./Saccharomyces - ps27
Saccharomyces - ps28
Arboricola_clade - ps29
Cerevisiae_clade - ps30
Saccharomyces_cerevisiae - ps31
Phylostratum

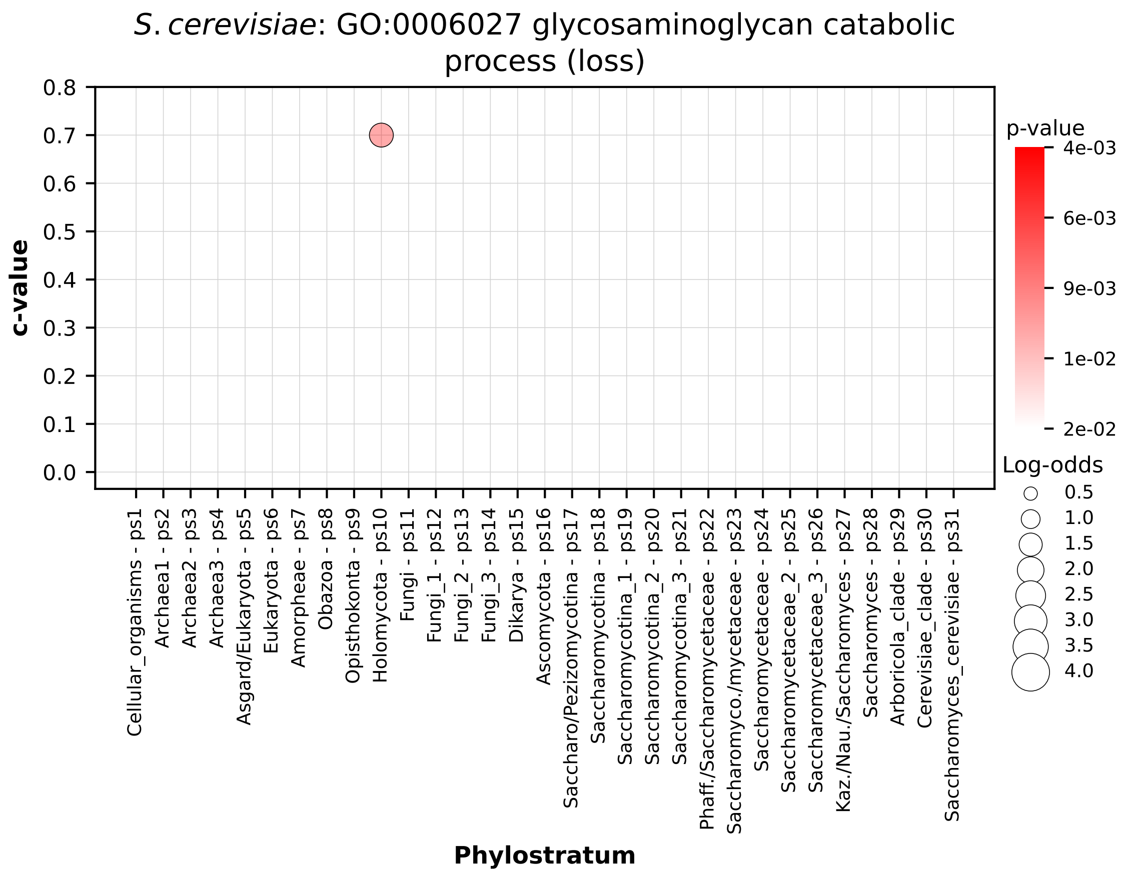
S. cerevisiae: GO:0006027 glycosaminoglycan catabolic process (loss)
c-value
0.8
0.7
0.6
0.5
0.4
0.3
0.2
0.1
0.0
p-value
4e-03
6e-03
9e-03
1e-02
2e-02
Log-odds
0.5
1.0
1.5
2.0
2.5
3.0
3.5
4.0
Cellular_organisms - ps1
Archaea1 - ps2
Archaea2 - ps3
Archaea3 - ps4
Asgard/Eukaryota - ps5
Eukaryota - ps6
Amorpheae - ps7
Obazoa - ps8
Opisthokonta - ps9
Holomycota - ps10
Fungi - ps11
Fungi_1 - ps12
Fungi_2 - ps13
Fungi_3 - ps14
Dikarya - ps15
Ascomycota - ps16
Saccharo/Pezizomycotina - ps17
Saccharomycotina - ps18
Saccharomycotina_1 - ps19
Saccharomycotina_2 - ps20
Saccharomycotina_3 - ps21
Phaff./Saccharomycetaceae - ps22
Saccharomyco./mycetaceae - ps23
Saccharomycetaceae - ps24
Saccharomycetaceae_2 - ps25
Saccharomycetaceae_3 - ps26
Kaz./Nau./Saccharomyces - ps27
Saccharomyces - ps28
Arboricola_clade - ps29
Cerevisiae_clade - ps30
Saccharomyces_cerevisiae - ps31
Phylostratum

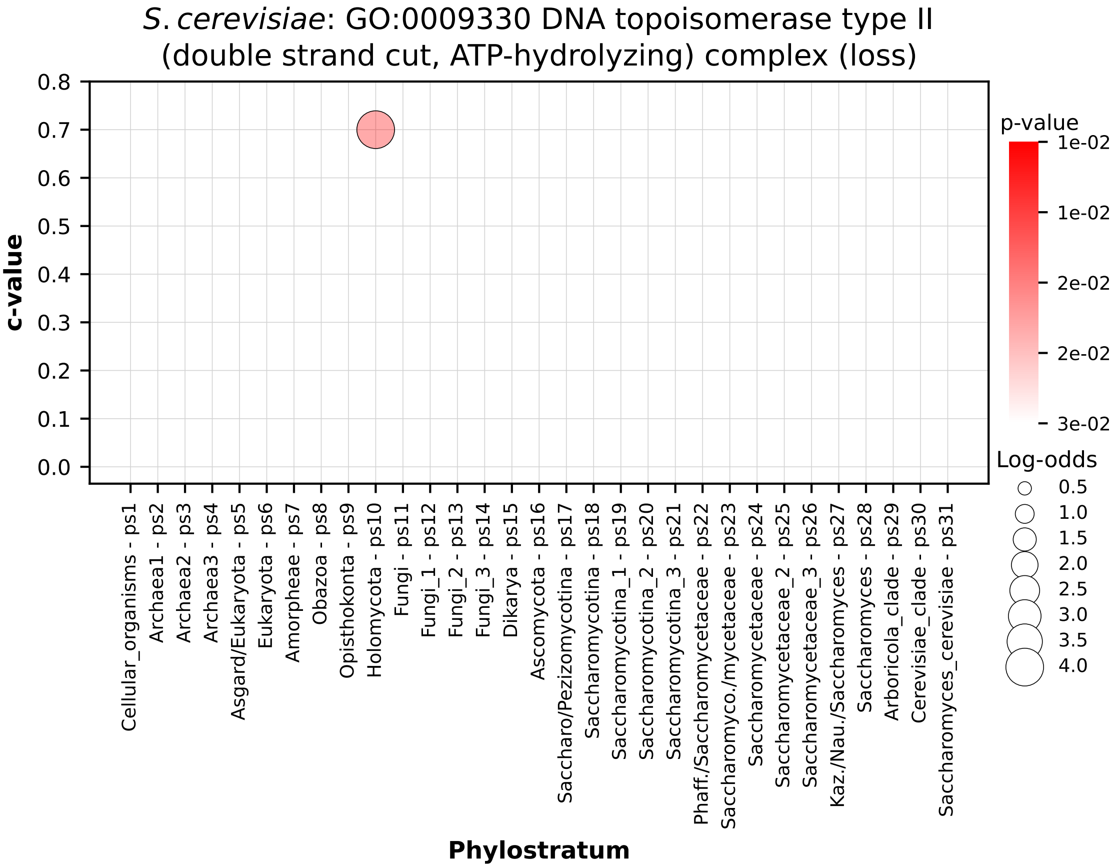
S. cerevisiae: GO:0009330 DNA topoisomerase type II (double strand cut, ATP-hydrolyzing) complex (loss)
c-value
0.8
0.7
0.6
0.5
0.4
0.3
0.2
0.1
0.0
Phylostratum
Cellular_organisms - ps1
Archaea1 - ps2
Archaea2 - ps3
Archaea3 - ps4
Asgard/Eukaryota - ps5
Eukaryota - ps6
Amorpheae - ps7
Obazoa - ps8
Opisthokonta - ps9
Holomycota - ps10
Fungi - ps11
Fungi_1 - ps12
Fungi_2 - ps13
Fungi_3 - ps14
Dikarya - ps15
Ascomycota - ps16
Saccharo/Pezizomycotina - ps17
Saccharomycotina - ps18
Saccharomycotina_1 - ps19
Saccharomycotina_2 - ps20
Saccharomycotina_3 - ps21
Phaff./Saccharomycetaceae - ps22
Saccharomyco./mycetaceae - ps23
Saccharomycetaceae - ps24
Saccharomycetaceae_2 - ps25
Saccharomycetaceae_3 - ps26
Kaz./Nau./Saccharomyces - ps27
Saccharomyces - ps28
Arboricola_clade - ps29
Cerevisiae_clade - ps30
Saccharomyces_cerevisiae - ps31
p-value
1e-02
1e-02
2e-02
2e-02
3e-02
Log-odds
0.5
1.0
1.5
2.0
2.5
3.0
3.5
4.0

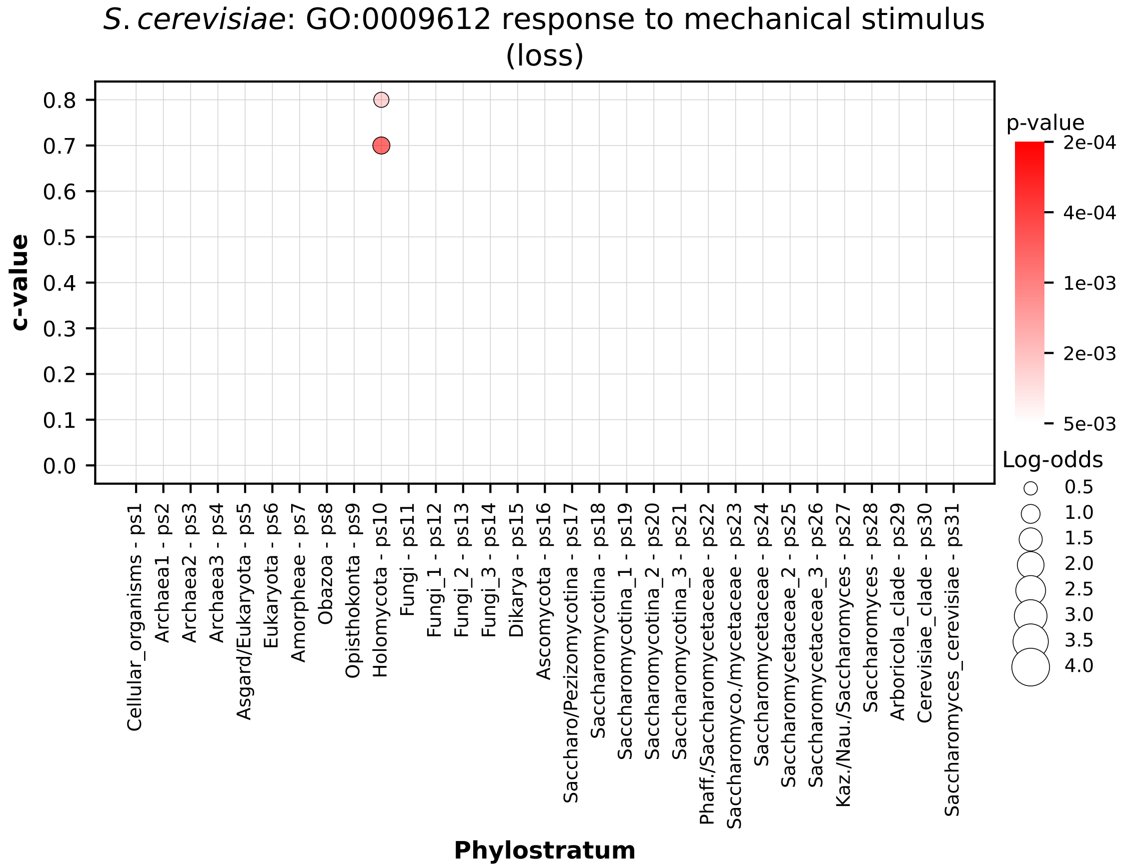
S. cerevisiae: GO:0009612 response to mechanical stimulus (loss)
c-value
0.0
0.1
0.2
0.3
0.4
0.5
0.6
0.7
0.8
Phylostratum
Cellular_organisms - ps1
Archaea1 - ps2
Archaea2 - ps3
Archaea3 - ps4
Asgard/Eukaryota - ps5
Eukaryota - ps6
Amorpheae - ps7
Obazoa - ps8
Opisthokonta - ps9
Holomycota - ps10
Fungi - ps11
Fungi_1 - ps12
Fungi_2 - ps13
Fungi_3 - ps14
Dikarya - ps15
Ascomycota - ps16
Saccharo/Pezizomycotina - ps17
Saccharomycotina - ps18
Saccharomycotina_1 - ps19
Saccharomycotina_2 - ps20
Saccharomycotina_3 - ps21
Phaff./Saccharomycetaceae - ps22
Saccharomyco./mycetaceae - ps23
Saccharomycetaceae - ps24
Saccharomycetaceae_2 - ps25
Saccharomycetaceae_3 - ps26
Kaz./Nau./Saccharomyces - ps27
Saccharomyces - ps28
Arboricola_clade - ps29
Cerevisiae_clade - ps30
Saccharomyces_cerevisiae - ps31
p-value
2e-04
4e-04
1e-03
2e-03
5e-03
Log-odds
0.5
1.0
1.5
2.0
2.5
3.0
3.5
4.0

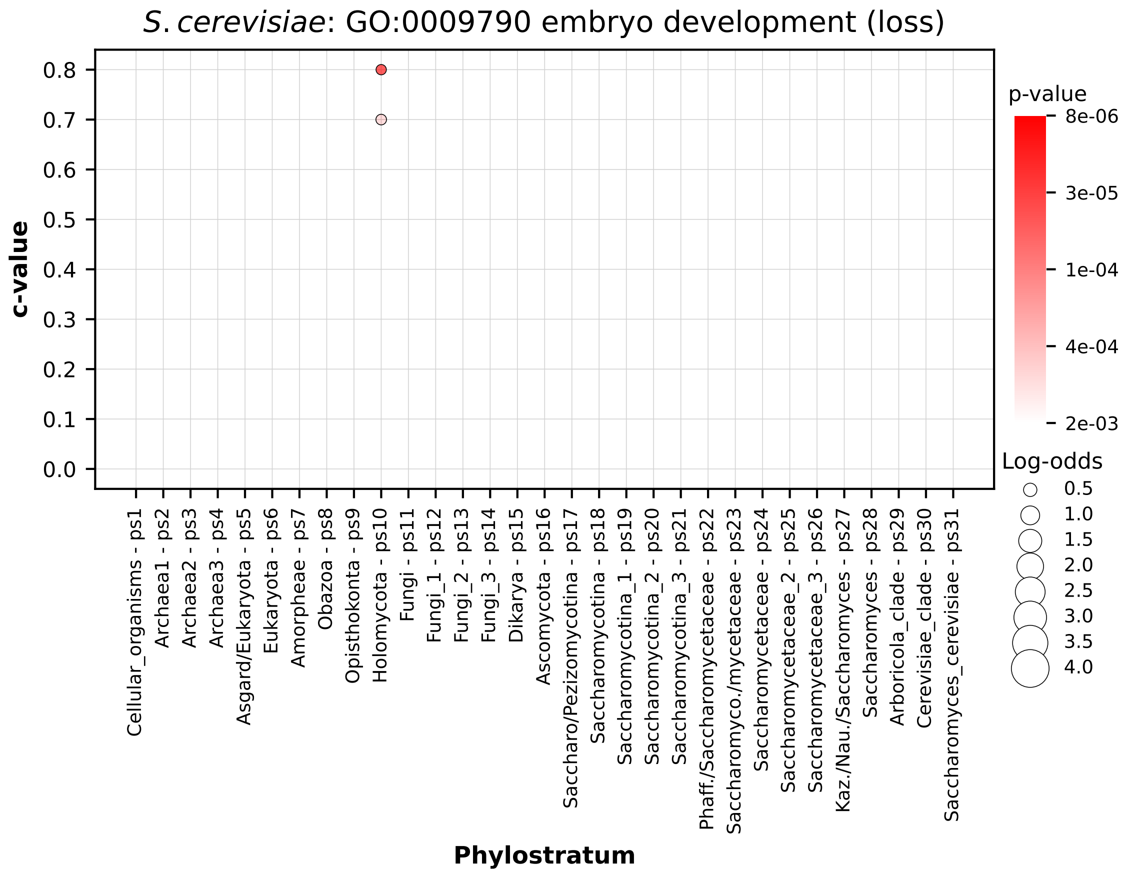
S. cerevisiae: GO:0009790 embryo development (loss)
c-value
0.0
0.1
0.2
0.3
0.4
0.5
0.6
0.7
0.8
p-value
8e-06
3e-05
1e-04
4e-04
2e-03
Log-odds
0.5
1.0
1.5
2.0
2.5
3.0
3.5
4.0
Cellular_organisms - ps1
Archaea1 - ps2
Archaea2 - ps3
Archaea3 - ps4
Asgard/Eukaryota - ps5
Eukaryota - ps6
Amorpheae - ps7
Obazoa - ps8
Opisthokonta - ps9
Holomycota - ps10
Fungi - ps11
Fungi_1 - ps12
Fungi_2 - ps13
Fungi_3 - ps14
Dikarya - ps15
Ascomycota - ps16
Saccharo/Pezizomycotina - ps17
Saccharomycotina - ps18
Saccharomycotina_1 - ps19
Saccharomycotina_2 - ps20
Saccharomycotina_3 - ps21
Phaff./Saccharomycetaceae - ps22
Saccharomyco./mycetaceae - ps23
Saccharomycetaceae - ps24
Saccharomycetaceae_2 - ps25
Saccharomycetaceae_3 - ps26
Kaz./Nau./Saccharomyces - ps27
Saccharomyces - ps28
Arboricola_clade - ps29
Cerevisiae_clade - ps30
Saccharomyces_cerevisiae - ps31
Phylostratum

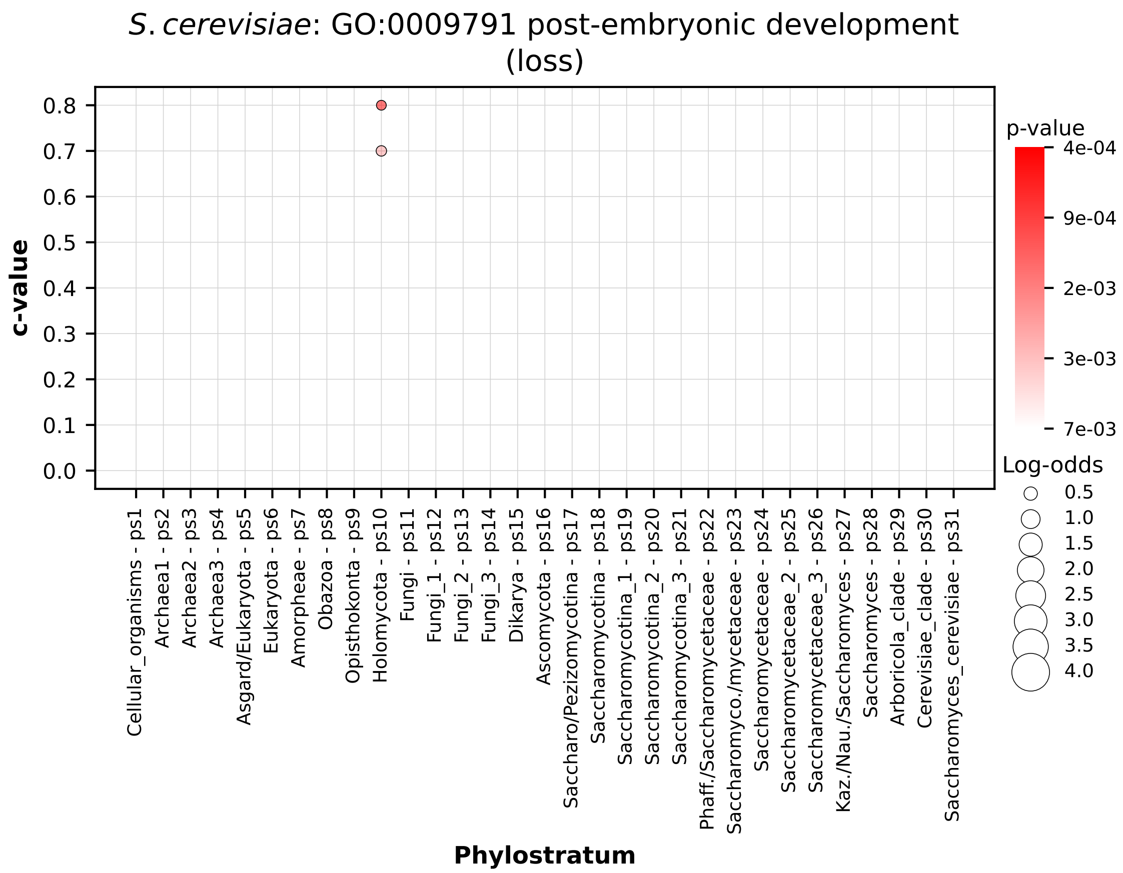

S. cerevisiae: GO:0009791 post-embryonic development (loss)
c-value
0.8
0.7
0.6
0.5
0.4
0.3
0.2
0.1
0.0
Phylostratum
Cellular_organisms - ps1
Archaea1 - ps2
Archaea2 - ps3
Archaea3 - ps4
Asgard/Eukaryota - ps5
Eukaryota - ps6
Amorpheae - ps7
Obazoa - ps8
Opisthokonta - ps9
Holomycota - ps10
Fungi - ps11
Fungi_1 - ps12
Fungi_2 - ps13
Fungi_3 - ps14
Dikarya - ps15
Ascomycota - ps16
Saccharo/Pezizomycotina - ps17
Saccharomycotina - ps18
Saccharomycotina_1 - ps19
Saccharomycotina_2 - ps20
Saccharomycotina_3 - ps21
Phaff./Saccharomycetaceae - ps22
Saccharomyco./mycetaceae - ps23
Saccharomycetaceae - ps24
Saccharomycetaceae_2 - ps25
Saccharomycetaceae_3 - ps26
Kaz./Nau./Saccharomyces - ps27
Saccharomyces - ps28
Arboricola_clade - ps29
Cerevisiae_clade - ps30
Saccharomyces_cerevisiae - ps31
p-value
4e-04
9e-04
2e-03
3e-03
7e-03
Log-odds
0.5
1.0
1.5
2.0
2.5
3.0
3.5
4.0

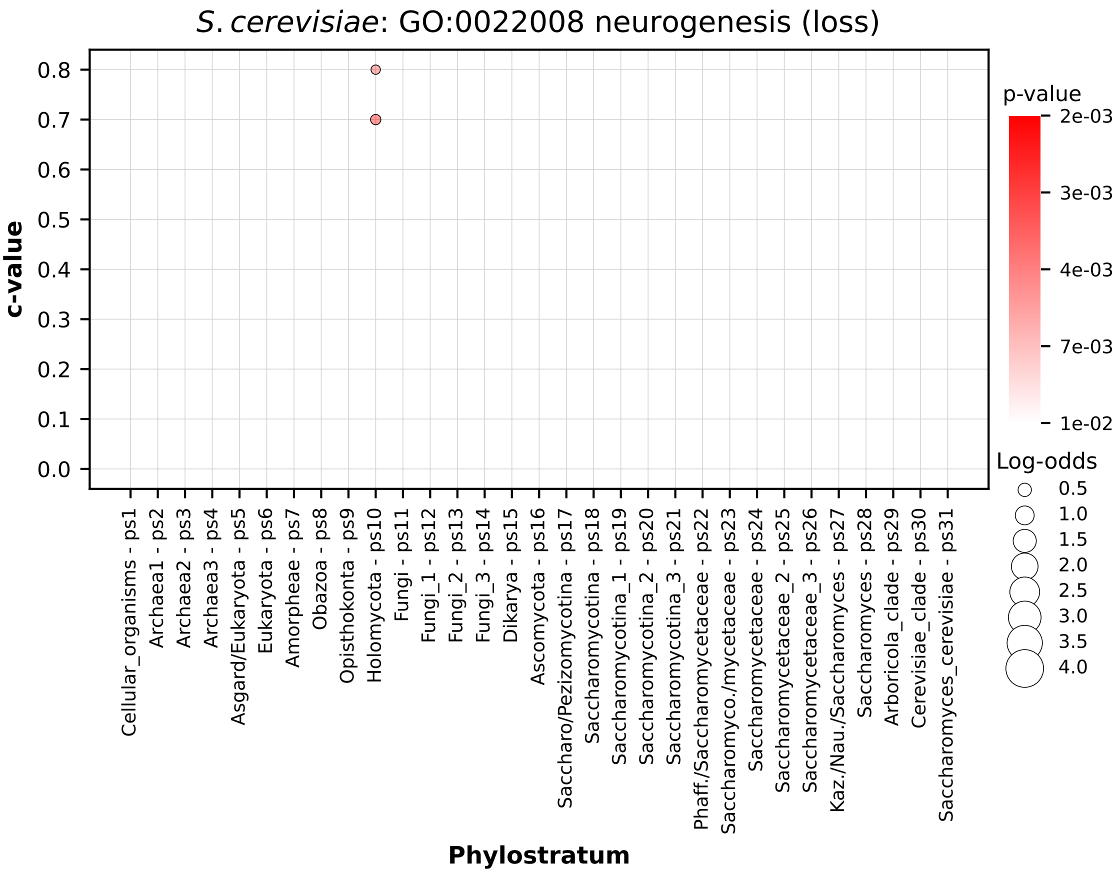

S. cerevisiae: GO:0022008 neurogenesis (loss)
c-value
0.0
0.1
0.2
0.3
0.4
0.5
0.6
0.7
0.8
p-value
2e-03
3e-03
4e-03
7e-03
1e-02
Log-odds
0.5
1.0
1.5
2.0
2.5
3.0
3.5
4.0
Cellular_organisms - ps1
Archaea1 - ps2
Archaea2 - ps3
Archaea3 - ps4
Asgard/Eukaryota - ps5
Eukaryota - ps6
Amorpheae - ps7
Obazoa - ps8
Opisthokonta - ps9
Holomycota - ps10
Fungi - ps11
Fungi_1 - ps12
Fungi_2 - ps13
Fungi_3 - ps14
Dikarya - ps15
Ascomycota - ps16
Saccharo/Pezizomycotina - ps17
Saccharomycotina - ps18
Saccharomycotina_1 - ps19
Saccharomycotina_2 - ps20
Saccharomycotina_3 - ps21
Phaff./Saccharomycetaceae - ps22
Saccharomyco./mycetaceae - ps23
Saccharomycetaceae - ps24
Saccharomycetaceae_2 - ps25
Saccharomycetaceae_3 - ps26
Kaz./Nau./Saccharomyces - ps27
Saccharomyces - ps28
Arboricola_clade - ps29
Cerevisiae_clade - ps30
Saccharomyces_cerevisiae - ps31
Phylostratum

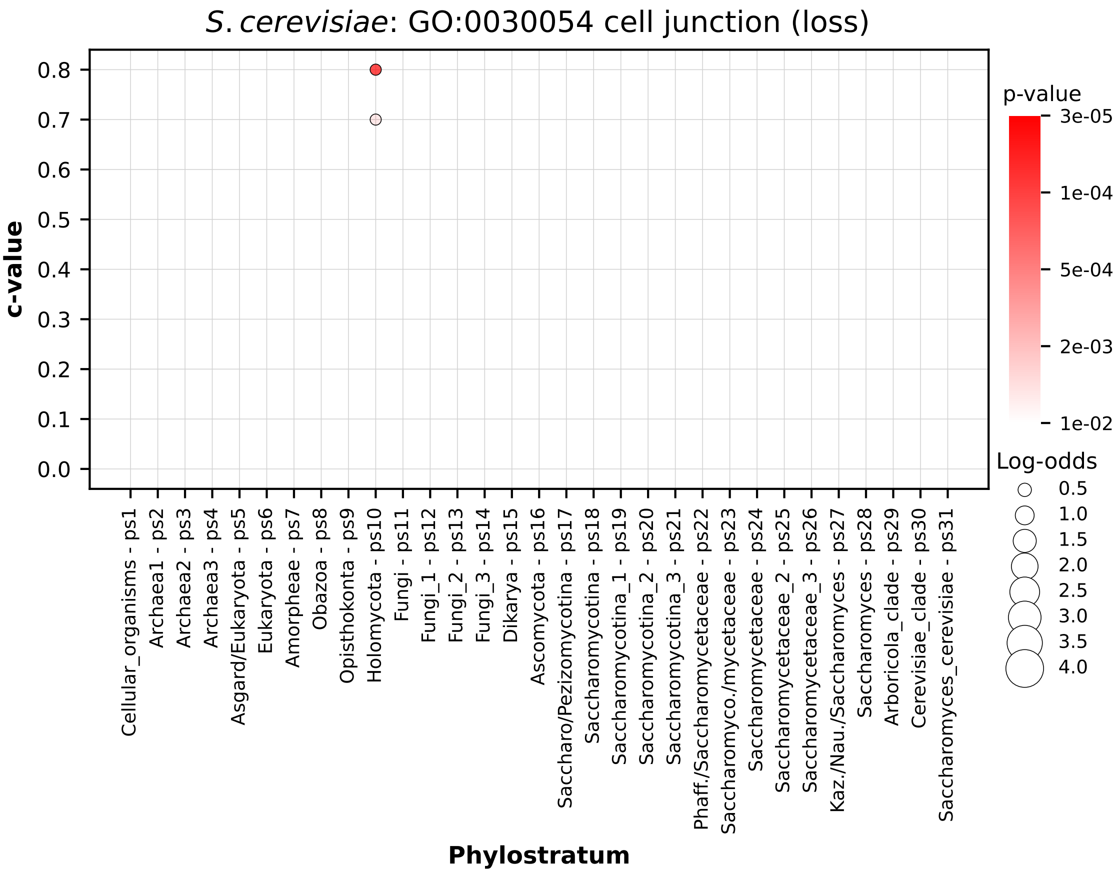

S. cerevisiae: GO:0030054 cell junction (loss)
c-value
0.8
0.7
0.6
0.5
0.4
0.3
0.2
0.1
0.0
p-value
3e-05
1e-04
5e-04
2e-03
1e-02
Log-odds
0.5
1.0
1.5
2.0
2.5
3.0
3.5
4.0
Cellular_organisms - ps1
Archaea1 - ps2
Archaea2 - ps3
Archaea3 - ps4
Asgard/Eukaryota - ps5
Eukaryota - ps6
Amorpheae - ps7
Obazoa - ps8
Opisthokonta - ps9
Holomycota - ps10
Fungi - ps11
Fungi_1 - ps12
Fungi_2 - ps13
Fungi_3 - ps14
Dikarya - ps15
Ascomycota - ps16
Saccharo/Pezizomycotina - ps17
Saccharomycotina - ps18
Saccharomycotina_1 - ps19
Saccharomycotina_2 - ps20
Saccharomycotina_3 - ps21
Phaff./Saccharomycetaceae - ps22
Saccharomyco./mycetaceae - ps23
Saccharomycetaceae - ps24
Saccharomycetaceae_2 - ps25
Saccharomycetaceae_3 - ps26
Kaz./Nau./Saccharomyces - ps27
Saccharomyces - ps28
Arboricola_clade - ps29
Cerevisiae_clade - ps30
Saccharomyces_cerevisiae - ps31
Phylostratum

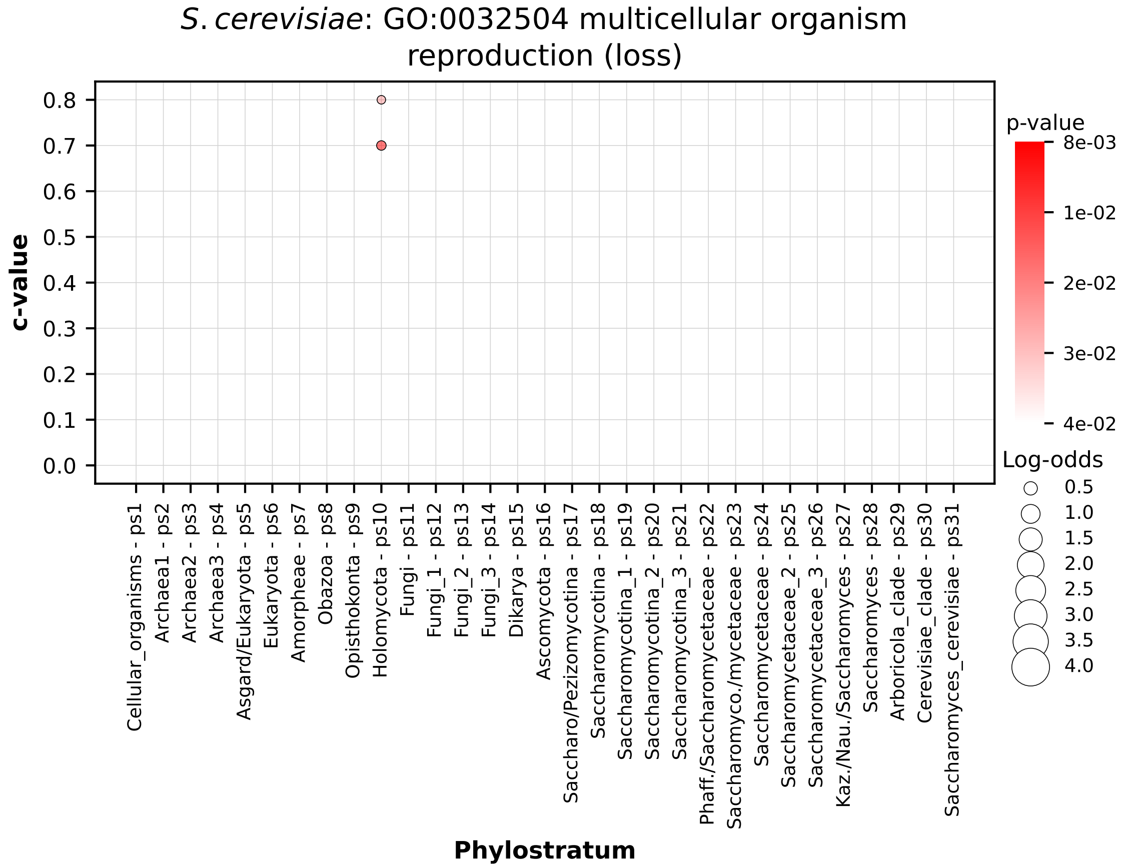

S. cerevisiae: GO:0032504 multicellular organism reproduction (loss)
c-value
0.0
0.1
0.2
0.3
0.4
0.5
0.6
0.7
0.8
p-value
8e-03
1e-02
2e-02
3e-02
4e-02
Log-odds
0.5
1.0
1.5
2.0
2.5
3.0
3.5
4.0
Cellular_organisms - ps1
Archaea1 - ps2
Archaea2 - ps3
Archaea3 - ps4
Asgard/Eukaryota - ps5
Eukaryota - ps6
Amorpheae - ps7
Obazoa - ps8
Opisthokonta - ps9
Holomycota - ps10
Fungi - ps11
Fungi_1 - ps12
Fungi_2 - ps13
Fungi_3 - ps14
Dikarya - ps15
Ascomycota - ps16
Saccharo/Pezizomycotina - ps17
Saccharomycotina - ps18
Saccharomycotina_1 - ps19
Saccharomycotina_2 - ps20
Saccharomycotina_3 - ps21
Phaff./Saccharomycetaceae - ps22
Saccharomyco./mycetaceae - ps23
Saccharomycetaceae - ps24
Saccharomycetaceae_2 - ps25
Saccharomycetaceae_3 - ps26
Kaz./Nau./Saccharomyces - ps27
Saccharomyces - ps28
Arboricola_clade - ps29
Cerevisiae_clade - ps30
Saccharomyces_cerevisiae - ps31
Phylostratum

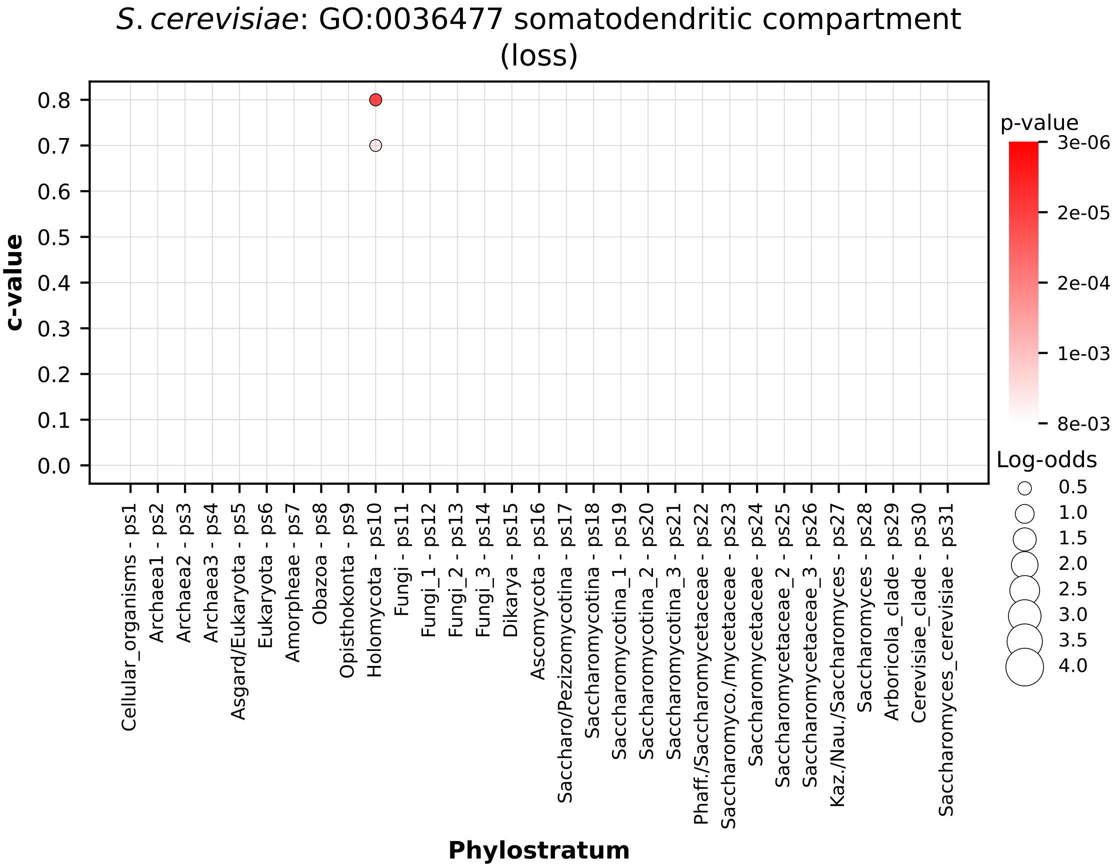

S. cerevisiae: GO:0036477 somatodendritic compartment (loss)
c-value
0.8
0.7
0.6
0.5
0.4
0.3
0.2
0.1
0.0
p-value
3e-06
2e-05
2e-04
1e-03
8e-03
Log-odds
0.5
1.0
1.5
2.0
2.5
3.0
3.5
4.0
Cellular_organisms - ps1
Archaea1 - ps2
Archaea2 - ps3
Archaea3 - ps4
Asgard/Eukaryota - ps5
Eukaryota - ps6
Amorpheae - ps7
Obazoa - ps8
Opisthokonta - ps9
Holomycota - ps10
Fungi - ps11
Fungi_1 - ps12
Fungi_2 - ps13
Fungi_3 - ps14
Dikarya - ps15
Ascomycota - ps16
Saccharo/Pezizomycotina - ps17
Saccharomycotina - ps18
Saccharomycotina_1 - ps19
Saccharomycotina_2 - ps20
Saccharomycotina_3 - ps21
Phaff./Saccharomycetaceae - ps22
Saccharomyco./mycetaceae - ps23
Saccharomycetaceae - ps24
Saccharomycetaceae_2 - ps25
Saccharomycetaceae_3 - ps26
Kaz./Nau./Saccharomyces - ps27
Saccharomyces - ps28
Arboricola_clade - ps29
Cerevisiae_clade - ps30
Saccharomyces_cerevisiae - ps31
Phylostratum

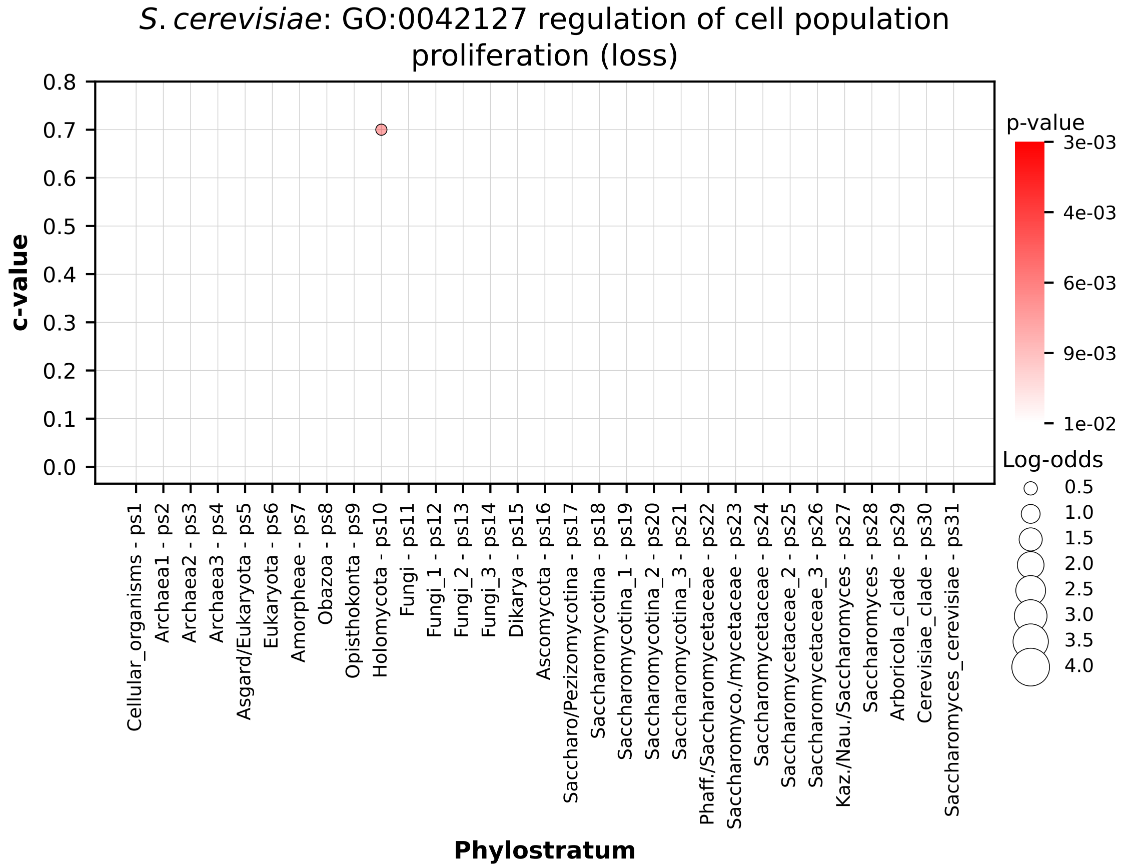

S. cerevisiae: GO:0042127 regulation of cell population proliferation (loss)
c-value
0.0
0.1
0.2
0.3
0.4
0.5
0.6
0.7
0.8
Phylostratum
Cellular_organisms - ps1
Archaea1 - ps2
Archaea2 - ps3
Archaea3 - ps4
Asgard/Eukaryota - ps5
Eukaryota - ps6
Amorpheae - ps7
Obazoa - ps8
Opisthokonta - ps9
Holomycota - ps10
Fungi - ps11
Fungi_1 - ps12
Fungi_2 - ps13
Fungi_3 - ps14
Dikarya - ps15
Ascomycota - ps16
Saccharo/Pezizomycotina - ps17
Saccharomycotina - ps18
Saccharomycotina_1 - ps19
Saccharomycotina_2 - ps20
Saccharomycotina_3 - ps21
Phaff./Saccharomycetaceae - ps22
Saccharomyco./mycetaceae - ps23
Saccharomycetaceae - ps24
Saccharomycetaceae_2 - ps25
Saccharomycetaceae_3 - ps26
Kaz./Nau./Saccharomyces - ps27
Saccharomyces - ps28
Arboricola_clade - ps29
Cerevisiae_clade - ps30
Saccharomyces_cerevisiae - ps31
p-value
3e-03
4e-03
6e-03
9e-03
1e-02
Log-odds
0.5
1.0
1.5
2.0
2.5
3.0
3.5
4.0

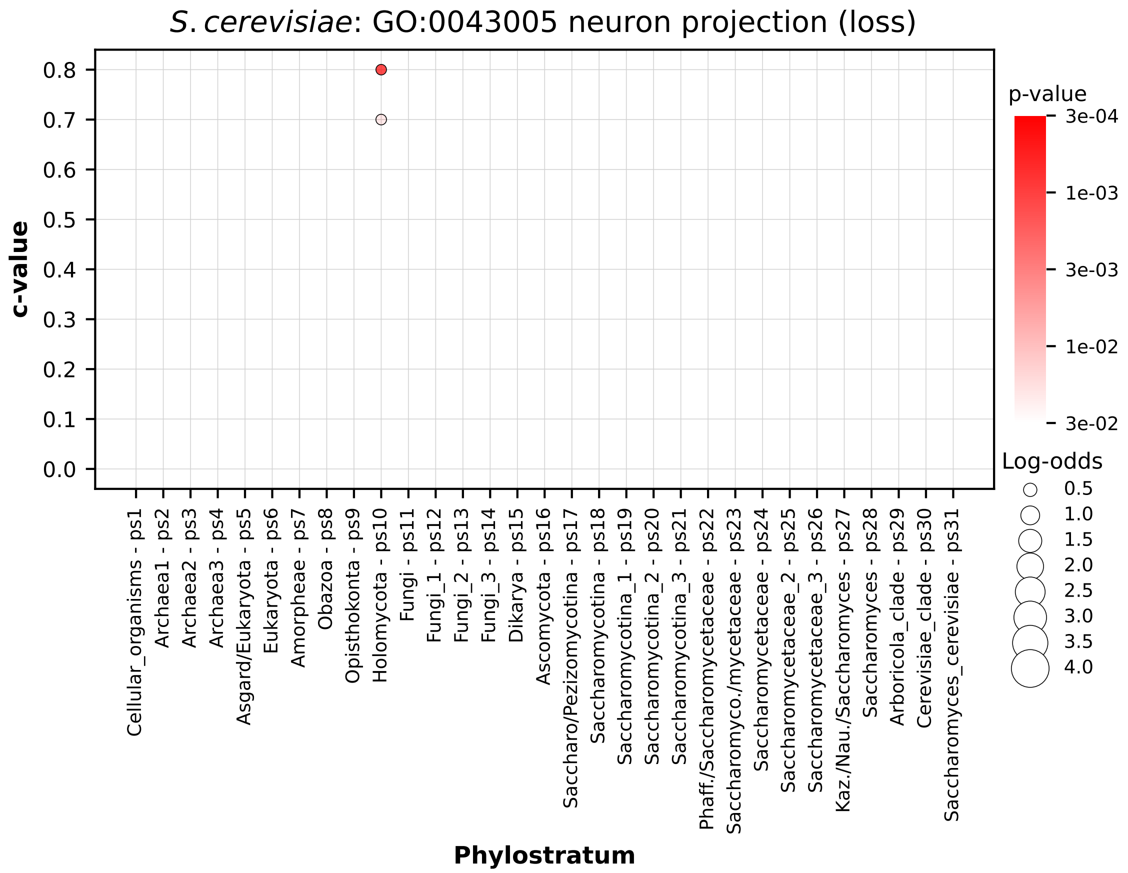

S. cerevisiae: GO:0043005 neuron projection (loss)
c-value
0.0
0.1
0.2
0.3
0.4
0.5
0.6
0.7
0.8
p-value
3e-04
1e-03
3e-03
1e-02
3e-02
Log-odds
0.5
1.0
1.5
2.0
2.5
3.0
3.5
4.0
Cellular_organisms - ps1
Archaea1 - ps2
Archaea2 - ps3
Archaea3 - ps4
Asgard/Eukaryota - ps5
Eukaryota - ps6
Amorpheae - ps7
Obazoa - ps8
Opisthokonta - ps9
Holomycota - ps10
Fungi - ps11
Fungi_1 - ps12
Fungi_2 - ps13
Fungi_3 - ps14
Dikarya - ps15
Ascomycota - ps16
Saccharo/Pezizomycotina - ps17
Saccharomycotina - ps18
Saccharomycotina_1 - ps19
Saccharomycotina_2 - ps20
Saccharomycotina_3 - ps21
Phaff./Saccharomycetaceae - ps22
Saccharomyco./mycetaceae - ps23
Saccharomycetaceae - ps24
Saccharomycetaceae_2 - ps25
Saccharomycetaceae_3 - ps26
Kaz./Nau./Saccharomyces - ps27
Saccharomyces - ps28
Arboricola_clade - ps29
Cerevisiae_clade - ps30
Saccharomyces_cerevisiae - ps31
Phylostratum

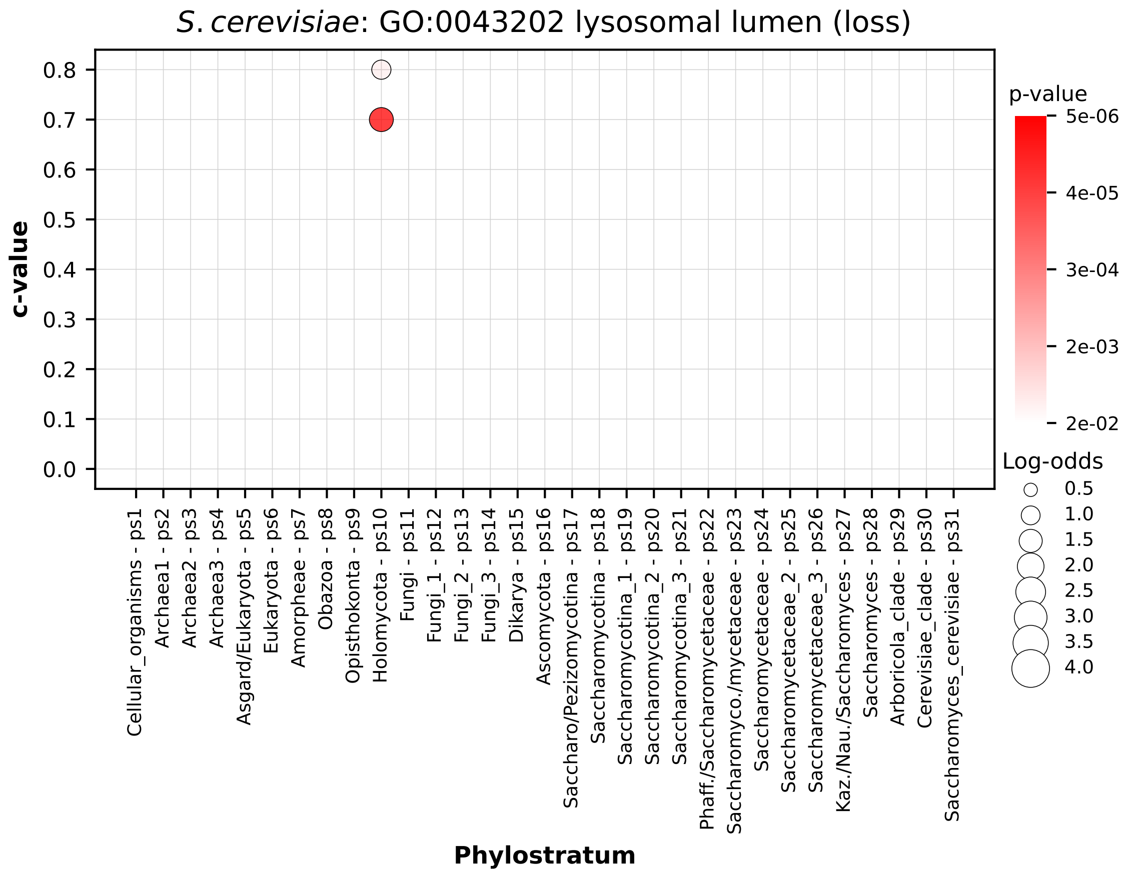

S. cerevisiae: GO:0043202 lysosomal lumen (loss)
c-value
0.0
0.1
0.2
0.3
0.4
0.5
0.6
0.7
0.8
p-value
5e-06
4e-05
3e-04
2e-03
2e-02
Log-odds
0.5
1.0
1.5
2.0
2.5
3.0
3.5
4.0
Cellular_organisms - ps1
Archaea1 - ps2
Archaea2 - ps3
Archaea3 - ps4
Asgard/Eukaryota - ps5
Eukaryota - ps6
Amorpheae - ps7
Obazoa - ps8
Opisthokonta - ps9
Holomycota - ps10
Fungi - ps11
Fungi_1 - ps12
Fungi_2 - ps13
Fungi_3 - ps14
Dikarya - ps15
Ascomycota - ps16
Saccharo/Pezizomycotina - ps17
Saccharomycotina - ps18
Saccharomycotina_1 - ps19
Saccharomycotina_2 - ps20
Saccharomycotina_3 - ps21
Phaff./Saccharomycetaceae - ps22
Saccharomyco./mycetaceae - ps23
Saccharomycetaceae - ps24
Saccharomycetaceae_2 - ps25
Saccharomycetaceae_3 - ps26
Kaz./Nau./Saccharomyces - ps27
Saccharomyces - ps28
Arboricola_clade - ps29
Cerevisiae_clade - ps30
Saccharomyces_cerevisiae - ps31
Phylostratum

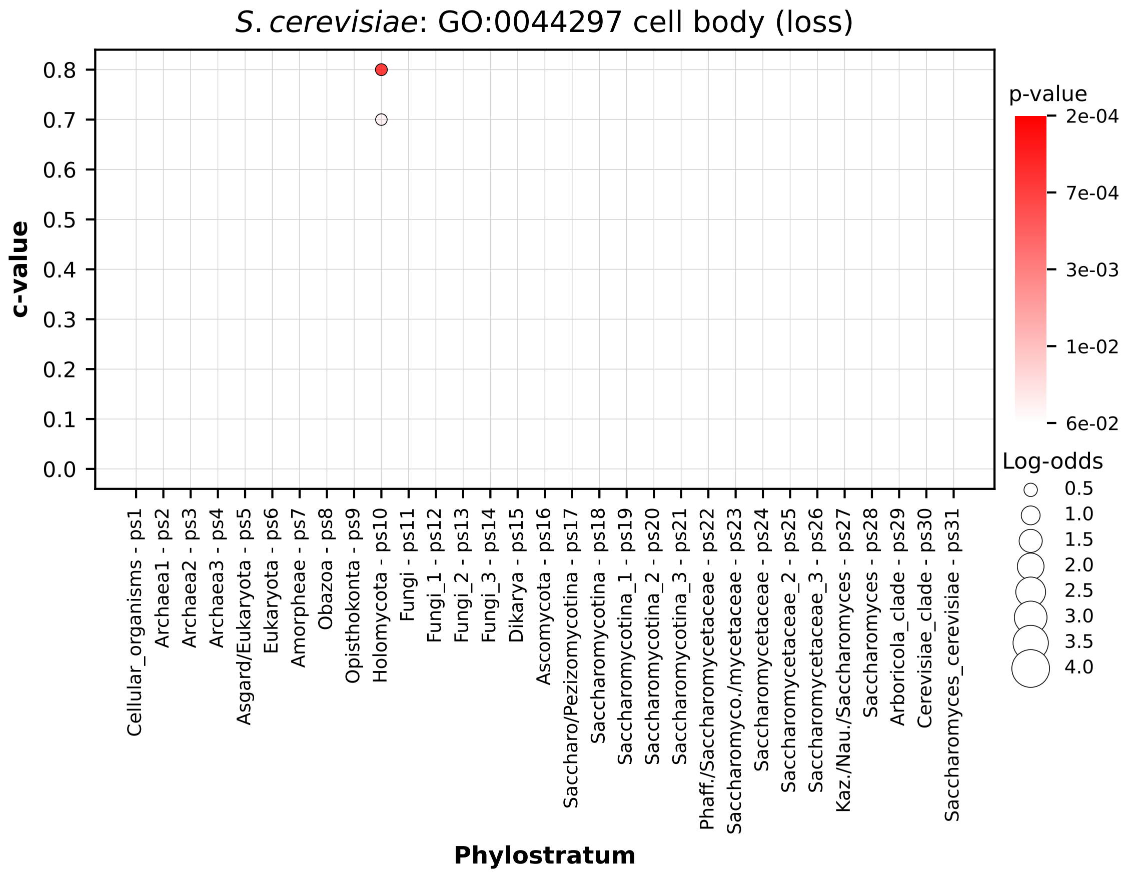
S. cerevisiae: GO:0044297 cell body (loss)
c-value
0.8
0.7
0.6
0.5
0.4
0.3
0.2
0.1
0.0
p-value
2e-04
7e-04
3e-03
1e-02
6e-02
Log-odds
0.5
1.0
1.5
2.0
2.5
3.0
3.5
4.0
Cellular_organisms - ps1
Archaea1 - ps2
Archaea2 - ps3
Archaea3 - ps4
Asgard/Eukaryota - ps5
Eukaryota - ps6
Amorpheae - ps7
Obazoa - ps8
Opisthokonta - ps9
Holomycota - ps10
Fungi - ps11
Fungi_1 - ps12
Fungi_2 - ps13
Fungi_3 - ps14
Dikarya - ps15
Ascomycota - ps16
Saccharo/Pezizomycotina - ps17
Saccharomycotina - ps18
Saccharomycotina_1 - ps19
Saccharomycotina_2 - ps20
Saccharomycotina_3 - ps21
Phaff./Saccharomycetaceae - ps22
Saccharomyco./mycetaceae - ps23
Saccharomycetaceae - ps24
Saccharomycetaceae_2 - ps25
Saccharomycetaceae_3 - ps26
Kaz./Nau./Saccharomyces - ps27
Saccharomyces - ps28
Arboricola_clade - ps29
Cerevisiae_clade - ps30
Saccharomyces_cerevisiae - ps31
Phylostratum

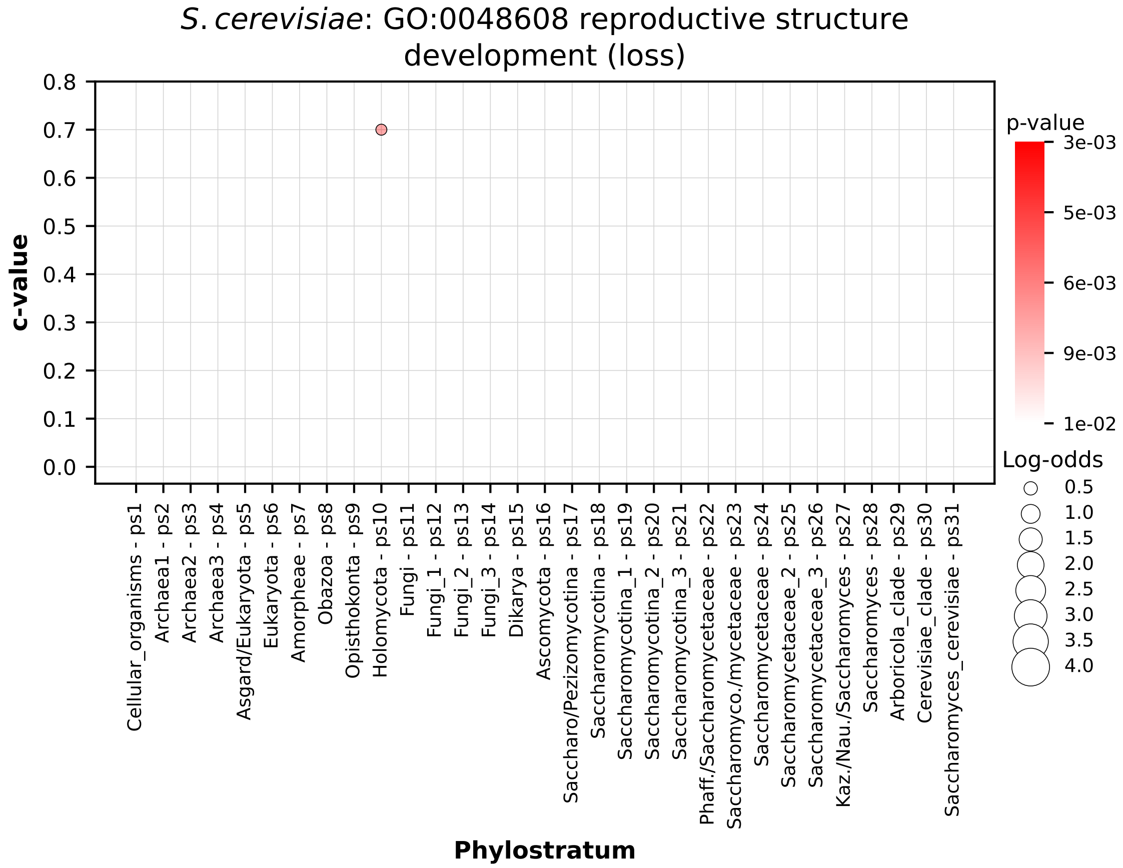

S. cerevisiae: GO:0048608 reproductive structure development (loss)
c-value
0.8
0.7
0.6
0.5
0.4
0.3
0.2
0.1
0.0
p-value
3e-03
5e-03
6e-03
9e-03
1e-02
Log-odds
0.5
1.0
1.5
2.0
2.5
3.0
3.5
4.0
Cellular_organisms - ps1
Archaea1 - ps2
Archaea2 - ps3
Archaea3 - ps4
Asgard/Eukaryota - ps5
Eukaryota - ps6
Amorpheae - ps7
Obazoa - ps8
Opisthokonta - ps9
Holomycota - ps10
Fungi - ps11
Fungi_1 - ps12
Fungi_2 - ps13
Fungi_3 - ps14
Dikarya - ps15
Ascomycota - ps16
Saccharo/Pezizomycotina - ps17
Saccharomycotina - ps18
Saccharomycotina_1 - ps19
Saccharomycotina_2 - ps20
Saccharomycotina_3 - ps21
Phaff./Saccharomycetaceae - ps22
Saccharomyco./mycetaceae - ps23
Saccharomycetaceae - ps24
Saccharomycetaceae_2 - ps25
Saccharomycetaceae_3 - ps26
Kaz./Nau./Saccharomyces - ps27
Saccharomyces - ps28
Arboricola_clade - ps29
Cerevisiae_clade - ps30
Saccharomyces_cerevisiae - ps31
Phylostratum

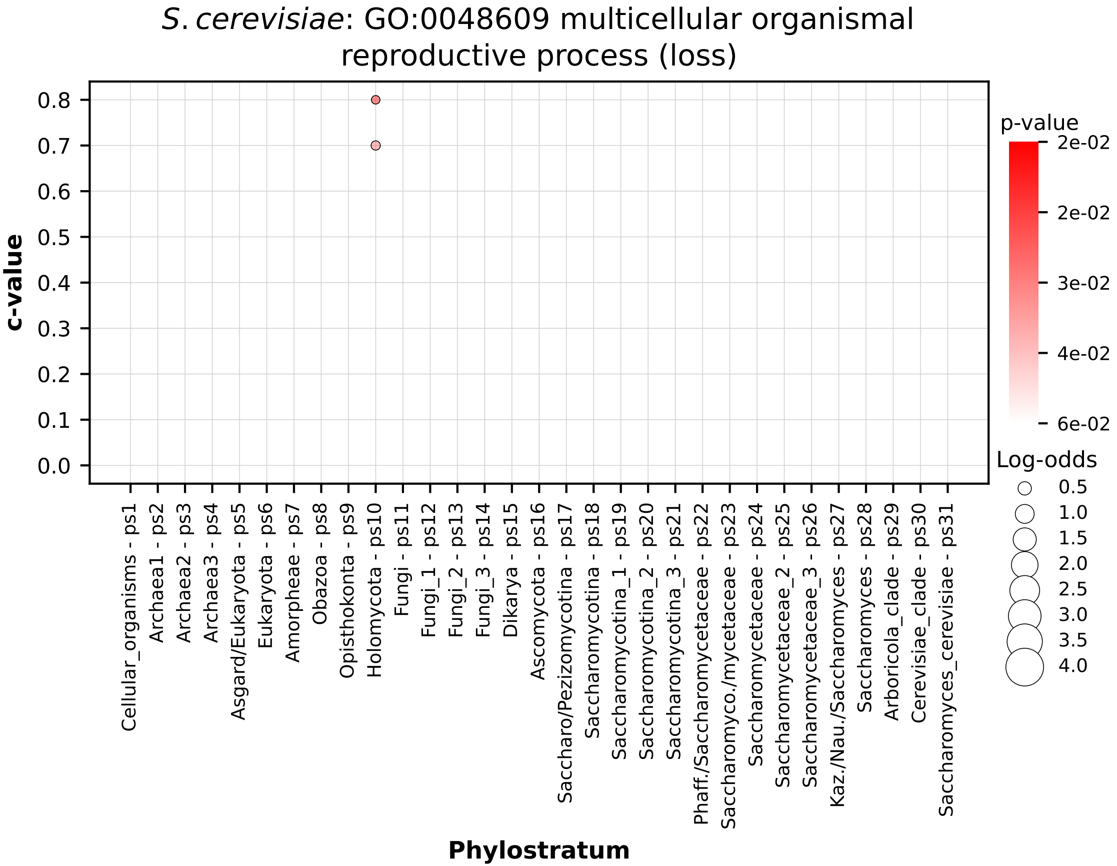

S. cerevisiae: GO:0048609 multicellular organismal reproductive process (loss)
c-value
0.0
0.1
0.2
0.3
0.4
0.5
0.6
0.7
0.8
Phylostratum
Cellular_organisms - ps1
Archaea1 - ps2
Archaea2 - ps3
Archaea3 - ps4
Asgard/Eukaryota - ps5
Eukaryota - ps6
Amorpheae - ps7
Obazoa - ps8
Opisthokonta - ps9
Holomycota - ps10
Fungi - ps11
Fungi_1 - ps12
Fungi_2 - ps13
Fungi_3 - ps14
Dikarya - ps15
Ascomycota - ps16
Saccharo/Pezizomycotina - ps17
Saccharomycotina - ps18
Saccharomycotina_1 - ps19
Saccharomycotina_2 - ps20
Saccharomycotina_3 - ps21
Phaff./Saccharomycetaceae - ps22
Saccharomyco./mycetaceae - ps23
Saccharomycetaceae - ps24
Saccharomycetaceae_2 - ps25
Saccharomycetaceae_3 - ps26
Kaz./Nau./Saccharomyces - ps27
Saccharomyces - ps28
Arboricola_clade - ps29
Cerevisiae_clade - ps30
Saccharomyces_cerevisiae - ps31
p-value
2e-02
2e-02
3e-02
4e-02
6e-02
Log-odds
0.5
1.0
1.5
2.0
2.5
3.0
3.5
4.0

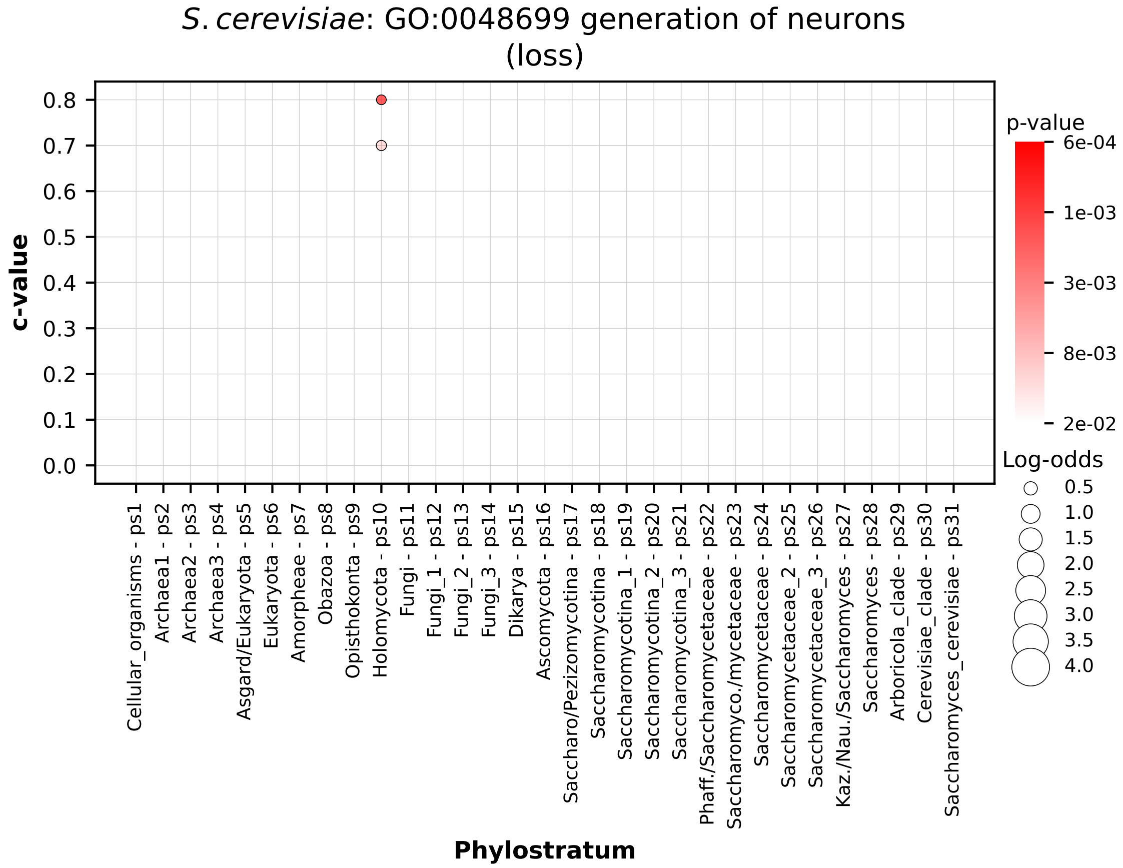

S. cerevisiae: GO:0048699 generation of neurons (loss)
c-value
0.0
0.1
0.2
0.3
0.4
0.5
0.6
0.7
0.8
Phylostratum
Cellular_organisms - ps1
Archaea1 - ps2
Archaea2 - ps3
Archaea3 - ps4
Asgard/Eukaryota - ps5
Eukaryota - ps6
Amorpheae - ps7
Obazoa - ps8
Opisthokonta - ps9
Holomycota - ps10
Fungi - ps11
Fungi_1 - ps12
Fungi_2 - ps13
Fungi_3 - ps14
Dikarya - ps15
Ascomycota - ps16
Saccharo/Pezizomycotina - ps17
Saccharomycotina - ps18
Saccharomycotina_1 - ps19
Saccharomycotina_2 - ps20
Saccharomycotina_3 - ps21
Phaff./Saccharomycetaceae - ps22
Saccharomyco./mycetaceae - ps23
Saccharomycetaceae - ps24
Saccharomycetaceae_2 - ps25
Saccharomycetaceae_3 - ps26
Kaz./Nau./Saccharomyces - ps27
Saccharomyces - ps28
Arboricola_clade - ps29
Cerevisiae_clade - ps30
Saccharomyces_cerevisiae - ps31
p-value
6e-04
1e-03
3e-03
8e-03
2e-02
Log-odds
0.5
1.0
1.5
2.0
2.5
3.0
3.5
4.0

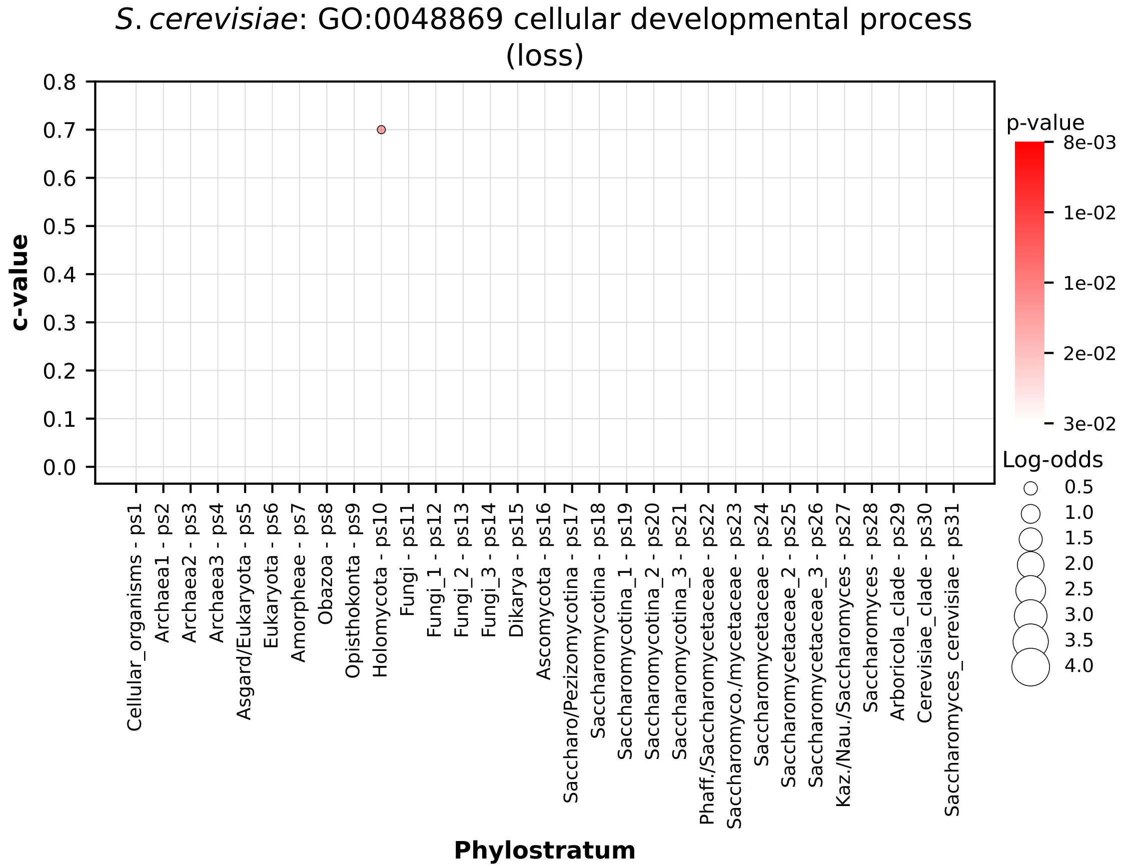
S. cerevisiae: GO:0048869 cellular developmental process
(loss)
c-value
0.8
0.7
0.6
0.5
0.4
0.3
0.2
0.1
0.0
p-value
8e-03
1e-02
1e-02
2e-02
3e-02
Log-odds
0.5
1.0
1.5
2.0
2.5
3.0
3.5
4.0
Cellular_organisms - ps1
Archaea1 - ps2
Archaea2 - ps3
Archaea3 - ps4
Asgard/Eukaryota - ps5
Eukaryota - ps6
Amorpheae - ps7
Obazoa - ps8
Opisthokonta - ps9
Holomycota - ps10
Fungi - ps11
Fungi_1 - ps12
Fungi_2 - ps13
Fungi_3 - ps14
Dikarya - ps15
Ascomycota - ps16
Saccharo/Pezizomycotina - ps17
Saccharomycotina - ps18
Saccharomycotina_1 - ps19
Saccharomycotina_2 - ps20
Saccharomycotina_3 - ps21
Phaff./Saccharomycetaceae - ps22
Saccharomyco./mycetaceae - ps23
Saccharomycetaceae - ps24
Saccharomycetaceae_2 - ps25
Saccharomycetaceae_3 - ps26
Kaz./Nau./Saccharomyces - ps27
Saccharomyces - ps28
Arboricola_clade - ps29
Cerevisiae_clade - ps30
Saccharomyces_cerevisiae - ps31
Phylostratum

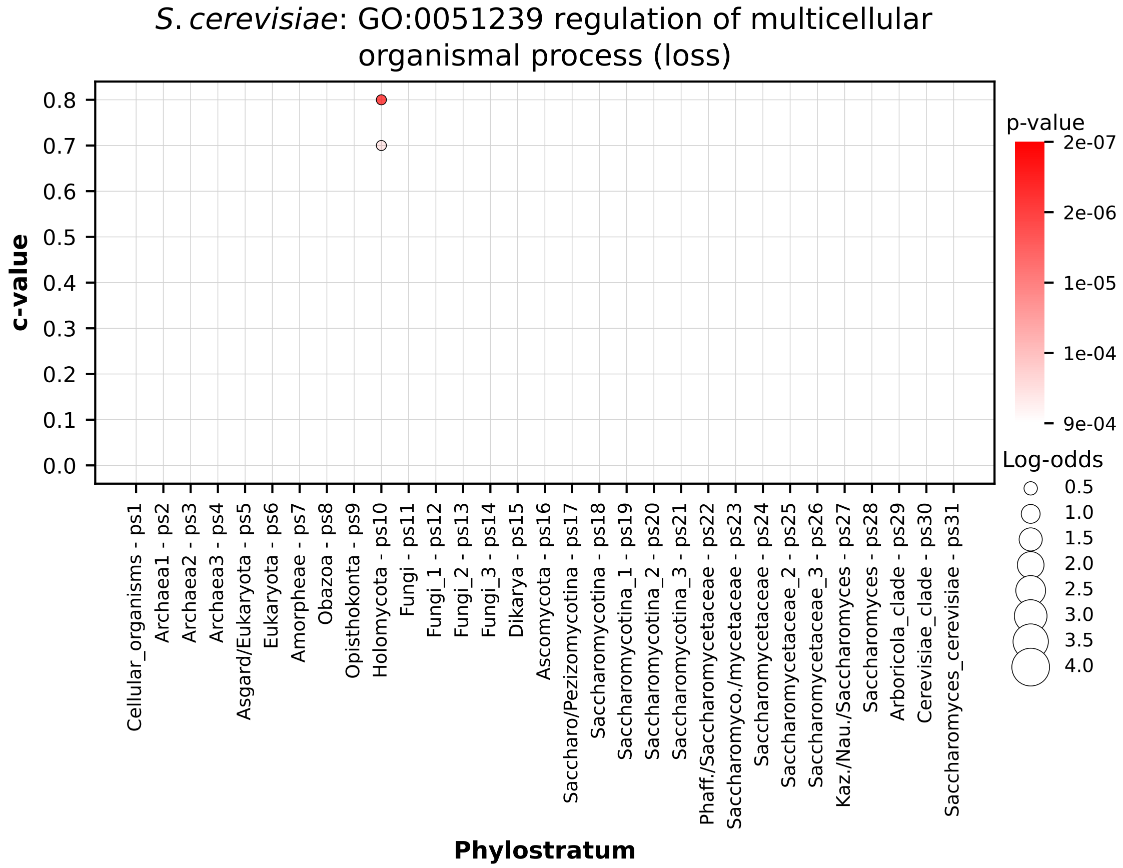
S. cerevisiae: GO:0051239 regulation of multicellular organismal process (loss)
c-value
0.0
0.1
0.2
0.3
0.4
0.5
0.6
0.7
0.8
Phylostratum
Cellular_organisms - ps1
Archaea1 - ps2
Archaea2 - ps3
Archaea3 - ps4
Asgard/Eukaryota - ps5
Eukaryota - ps6
Amorpheae - ps7
Obazoa - ps8
Opisthokonta - ps9
Holomycota - ps10
Fungi - ps11
Fungi_1 - ps12
Fungi_2 - ps13
Fungi_3 - ps14
Dikarya - ps15
Ascomycota - ps16
Saccharo/Pezizomycotina - ps17
Saccharomycotina - ps18
Saccharomycotina_1 - ps19
Saccharomycotina_2 - ps20
Saccharomycotina_3 - ps21
Phaff./Saccharomycetaceae - ps22
Saccharomyco./mycetaceae - ps23
Saccharomycetaceae - ps24
Saccharomycetaceae_2 - ps25
Saccharomycetaceae_3 - ps26
Kaz./Nau./Saccharomyces - ps27
Saccharomyces - ps28
Arboricola_clade - ps29
Cerevisiae_clade - ps30
Saccharomyces_cerevisiae - ps31
p-value
2e-07
2e-06
1e-05
1e-04
9e-04
Log-odds
0.5
1.0
1.5
2.0
2.5
3.0
3.5
4.0

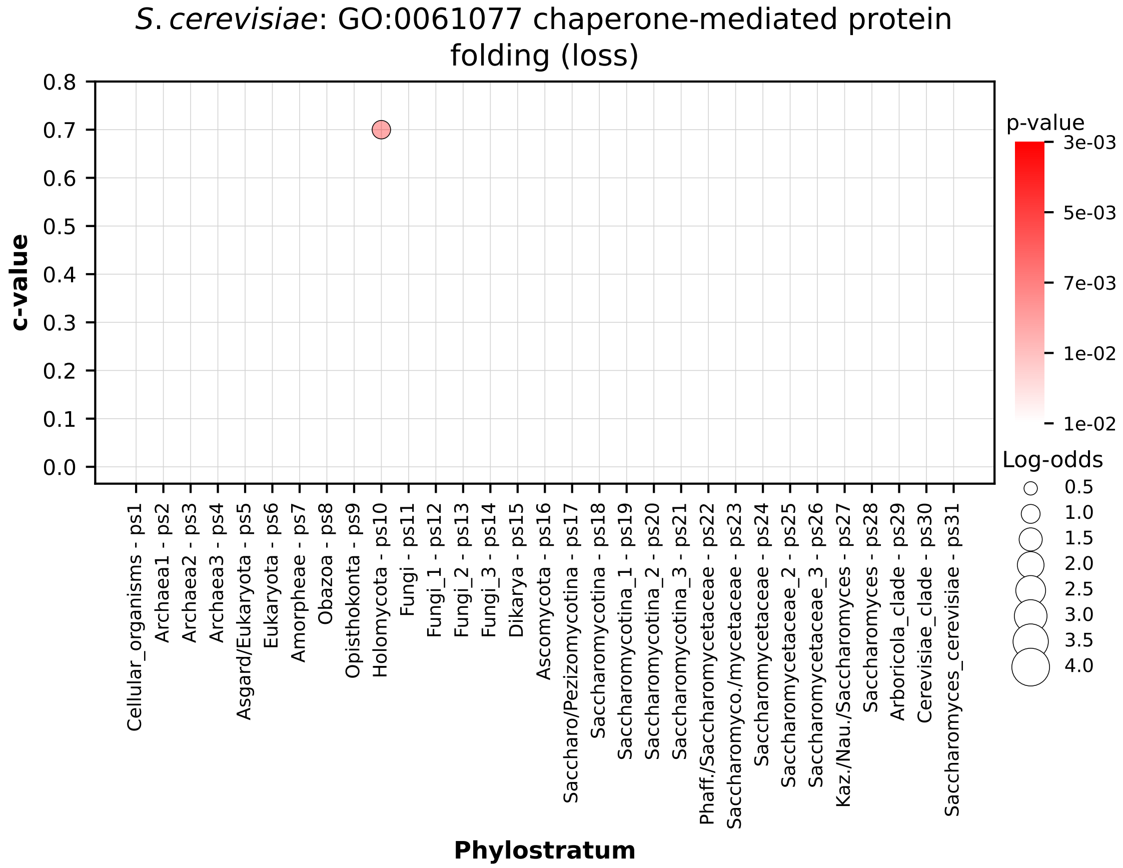
S. cerevisiae: GO:0061077 chaperone-mediated protein folding (loss)
c-value
0.8
0.7
0.6
0.5
0.4
0.3
0.2
0.1
0.0
p-value
3e-03
5e-03
7e-03
1e-02
1e-02
Log-odds
0.5
1.0
1.5
2.0
2.5
3.0
3.5
4.0
Cellular_organisms - ps1
Archaea1 - ps2
Archaea2 - ps3
Archaea3 - ps4
Asgard/Eukaryota - ps5
Eukaryota - ps6
Amorpheae - ps7
Obazoa - ps8
Opisthokonta - ps9
Holomycota - ps10
Fungi - ps11
Fungi_1 - ps12
Fungi_2 - ps13
Fungi_3 - ps14
Dikarya - ps15
Ascomycota - ps16
Saccharo/Pezizomycotina - ps17
Saccharomycotina - ps18
Saccharomycotina_1 - ps19
Saccharomycotina_2 - ps20
Saccharomycotina_3 - ps21
Phaff./Saccharomycetaceae - ps22
Saccharomyco./mycetaceae - ps23
Saccharomycetaceae - ps24
Saccharomycetaceae_2 - ps25
Saccharomycetaceae_3 - ps26
Kaz./Nau./Saccharomyces - ps27
Saccharomyces - ps28
Arboricola_clade - ps29
Cerevisiae_clade - ps30
Saccharomyces_cerevisiae - ps31
Phylostratum

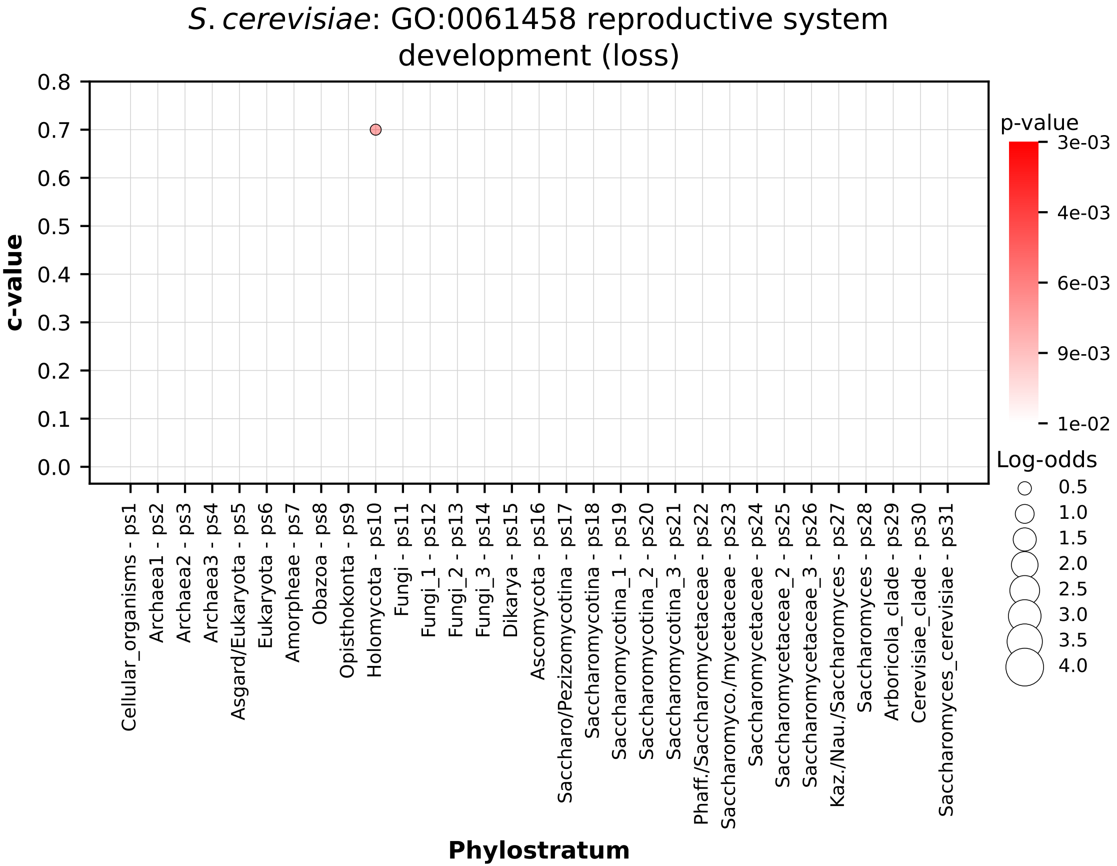

S. cerevisiae: GO:0061458 reproductive system development (loss)
c-value
0.0
0.1
0.2
0.3
0.4
0.5
0.6
0.7
0.8
p-value
3e-03
4e-03
6e-03
9e-03
1e-02
Log-odds
0.5
1.0
1.5
2.0
2.5
3.0
3.5
4.0
Cellular_organisms - ps1
Archaea1 - ps2
Archaea2 - ps3
Archaea3 - ps4
Asgard/Eukaryota - ps5
Eukaryota - ps6
Amorpheae - ps7
Obazoa - ps8
Opisthokonta - ps9
Holomycota - ps10
Fungi - ps11
Fungi_1 - ps12
Fungi_2 - ps13
Fungi_3 - ps14
Dikarya - ps15
Ascomycota - ps16
Saccharo/Pezizomycotina - ps17
Saccharomycotina - ps18
Saccharomycotina_1 - ps19
Saccharomycotina_2 - ps20
Saccharomycotina_3 - ps21
Phaff./Saccharomycetaceae - ps22
Saccharomyco./mycetaceae - ps23
Saccharomycetaceae - ps24
Saccharomycetaceae_2 - ps25
Saccharomycetaceae_3 - ps26
Kaz./Nau./Saccharomyces - ps27
Saccharomyces - ps28
Arboricola_clade - ps29
Cerevisiae_clade - ps30
Saccharomyces_cerevisiae - ps31
Phylostratum

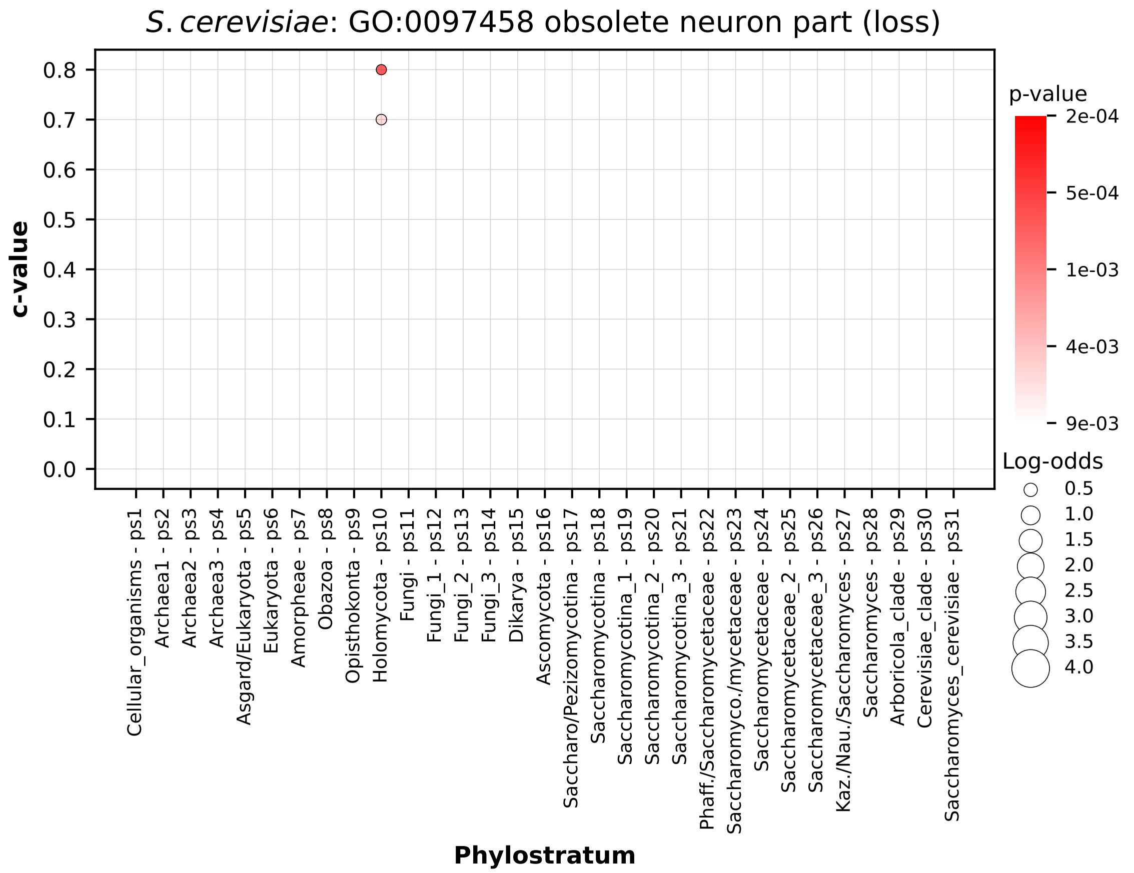

S. cerevisiae: GO:0097458 obsolete neuron part (loss)
c-value
0.8
0.7
0.6
0.5
0.4
0.3
0.2
0.1
0.0
p-value
2e-04
5e-04
1e-03
4e-03
9e-03
Log-odds
0.5
1.0
1.5
2.0
2.5
3.0
3.5
4.0
Cellular_organisms - ps1
Archaea1 - ps2
Archaea2 - ps3
Archaea3 - ps4
Asgard/Eukaryota - ps5
Eukaryota - ps6
Amorpheae - ps7
Obazoa - ps8
Opisthokonta - ps9
Holomycota - ps10
Fungi - ps11
Fungi_1 - ps12
Fungi_2 - ps13
Fungi_3 - ps14
Dikarya - ps15
Ascomycota - ps16
Saccharo/Pezizomycotina - ps17
Saccharomycotina - ps18
Saccharomycotina_1 - ps19
Saccharomycotina_2 - ps20
Saccharomycotina_3 - ps21
Phaff./Saccharomycetaceae - ps22
Saccharomyco./mycetaceae - ps23
Saccharomycetaceae - ps24
Saccharomycetaceae_2 - ps25
Saccharomycetaceae_3 - ps26
Kaz./Nau./Saccharomyces - ps27
Saccharomyces - ps28
Arboricola_clade - ps29
Cerevisiae_clade - ps30
Saccharomyces_cerevisiae - ps31
Phylostratum

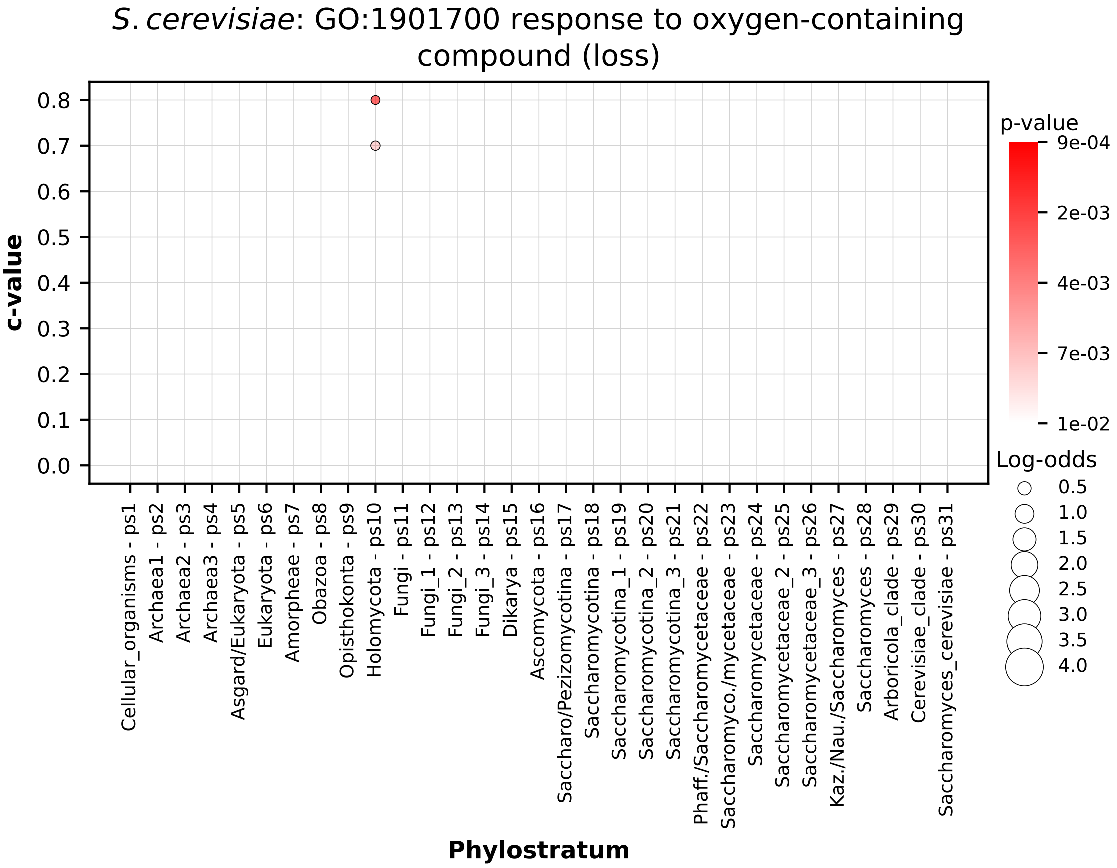

S. cerevisiae: GO:1901700 response to oxygen-containing compound (loss)
c-value
0.0
0.1
0.2
0.3
0.4
0.5
0.6
0.7
0.8
Phylostratum
Cellular_organisms - ps1
Archaea1 - ps2
Archaea2 - ps3
Archaea3 - ps4
Asgard/Eukaryota - ps5
Eukaryota - ps6
Amorpheae - ps7
Obazoa - ps8
Opisthokonta - ps9
Holomycota - ps10
Fungi - ps11
Fungi_1 - ps12
Fungi_2 - ps13
Fungi_3 - ps14
Dikarya - ps15
Ascomycota - ps16
Saccharo/Pezizomycotina - ps17
Saccharomycotina - ps18
Saccharomycotina_1 - ps19
Saccharomycotina_2 - ps20
Saccharomycotina_3 - ps21
Phaff./Saccharomycetaceae - ps22
Saccharomyco./mycetaceae - ps23
Saccharomycetaceae - ps24
Saccharomycetaceae_2 - ps25
Saccharomycetaceae_3 - ps26
Kaz./Nau./Saccharomyces - ps27
Saccharomyces - ps28
Arboricola_clade - ps29
Cerevisiae_clade - ps30
Saccharomyces_cerevisiae - ps31
p-value
9e-04
2e-03
4e-03
7e-03
1e-02
Log-odds
0.5
1.0
1.5
2.0
2.5
3.0
3.5
4.0

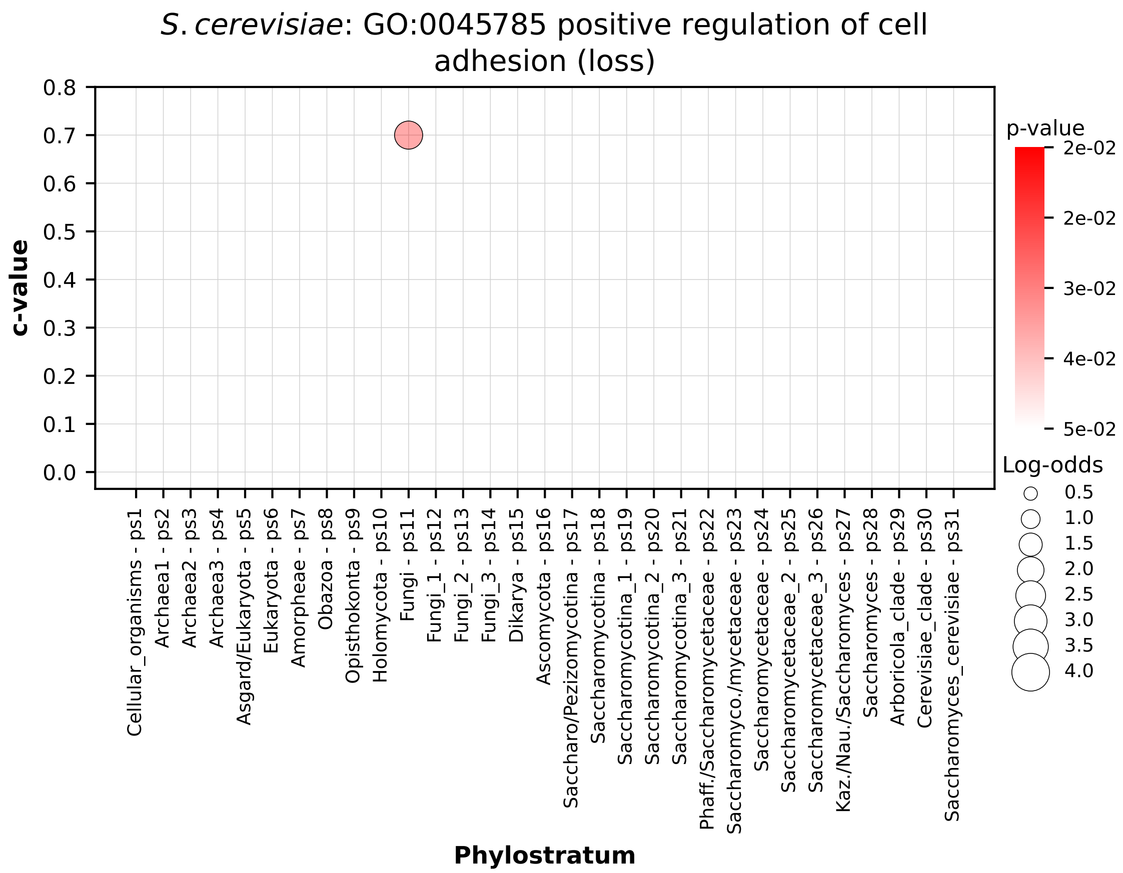

S. cerevisiae: GO:0045785 positive regulation of cell adhesion (loss)
c-value
0.8
0.7
0.6
0.5
0.4
0.3
0.2
0.1
0.0
p-value
2e-02
2e-02
3e-02
4e-02
5e-02
Log-odds
0.5
1.0
1.5
2.0
2.5
3.0
3.5
4.0
Cellular_organisms - ps1
Archaea1 - ps2
Archaea2 - ps3
Archaea3 - ps4
Asgard/Eukaryota - ps5
Eukaryota - ps6
Amorpheae - ps7
Obazoa - ps8
Opisthokonta - ps9
Holomycota - ps10
Fungi - ps11
Fungi_1 - ps12
Fungi_2 - ps13
Fungi_3 - ps14
Dikarya - ps15
Ascomycota - ps16
Saccharo/Pezizomycotina - ps17
Saccharomycotina - ps18
Saccharomycotina_1 - ps19
Saccharomycotina_2 - ps20
Saccharomycotina_3 - ps21
Phaff./Saccharomycetaceae - ps22
Saccharomyco./mycetaceae - ps23
Saccharomycetaceae - ps24
Saccharomycetaceae_2 - ps25
Saccharomycetaceae_3 - ps26
Kaz./Nau./Saccharomyces - ps27
Saccharomyces - ps28
Arboricola_clade - ps29
Cerevisiae_clade - ps30
Saccharomyces_cerevisiae - ps31
Phylostratum

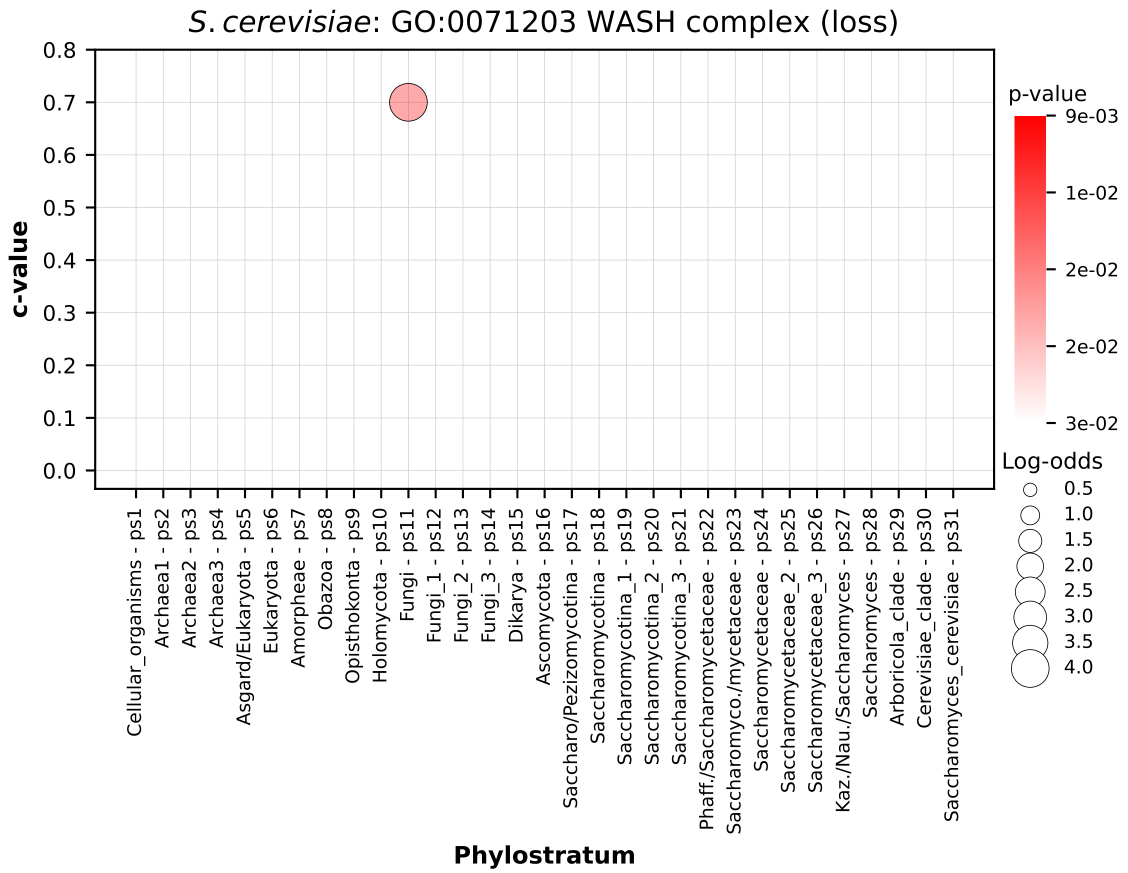

S. cerevisiae: GO:0071203 WASH complex (loss)
c-value
0.8
0.7
0.6
0.5
0.4
0.3
0.2
0.1
0.0
Phylostratum
Cellular_organisms - ps1
Archaea1 - ps2
Archaea2 - ps3
Archaea3 - ps4
Asgard/Eukaryota - ps5
Eukaryota - ps6
Amorpheae - ps7
Obazoa - ps8
Opisthokonta - ps9
Holomycota - ps10
Fungi - ps11
Fungi_1 - ps12
Fungi_2 - ps13
Fungi_3 - ps14
Dikarya - ps15
Ascomycota - ps16
Saccharo/Pezizomycotina - ps17
Saccharomycotina - ps18
Saccharomycotina_1 - ps19
Saccharomycotina_2 - ps20
Saccharomycotina_3 - ps21
Phaff./Saccharomycetaceae - ps22
Saccharomyco./mycetaceae - ps23
Saccharomycetaceae - ps24
Saccharomycetaceae_2 - ps25
Saccharomycetaceae_3 - ps26
Kaz./Nau./Saccharomyces - ps27
Saccharomyces - ps28
Arboricola_clade - ps29
Cerevisiae_clade - ps30
Saccharomyces_cerevisiae - ps31
p-value
9e-03
1e-02
2e-02
2e-02
3e-02
Log-odds
0.5
1.0
1.5
2.0
2.5
3.0
3.5
4.0

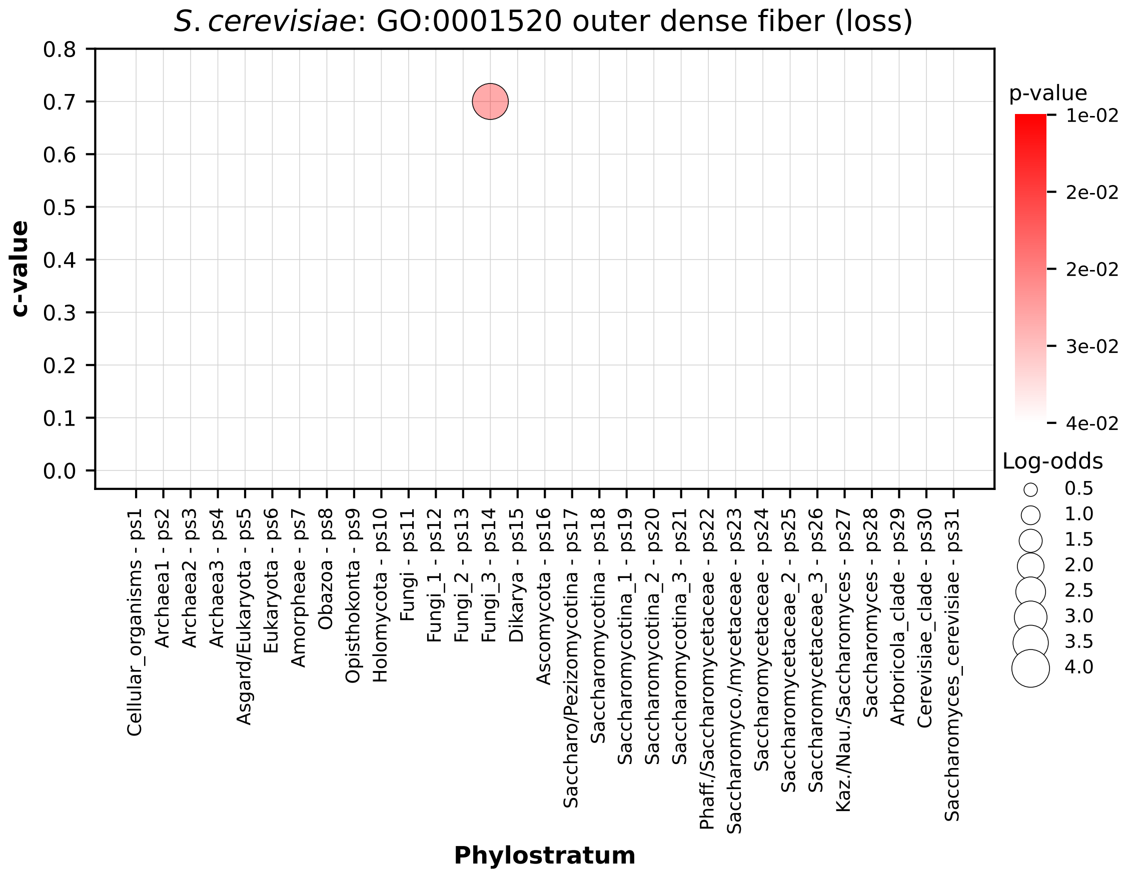

S. cerevisiae: GO:0001520 outer dense fiber (loss)
c-value
0.0
0.1
0.2
0.3
0.4
0.5
0.6
0.7
0.8
Phylostratum
Cellular_organisms - ps1
Archaea1 - ps2
Archaea2 - ps3
Archaea3 - ps4
Asgard/Eukaryota - ps5
Eukaryota - ps6
Amorpheae - ps7
Obazoa - ps8
Opisthokonta - ps9
Holomycota - ps10
Fungi - ps11
Fungi_1 - ps12
Fungi_2 - ps13
Fungi_3 - ps14
Dikarya - ps15
Ascomycota - ps16
Saccharo/Pezizomycotina - ps17
Saccharomycotina - ps18
Saccharomycotina_1 - ps19
Saccharomycotina_2 - ps20
Saccharomycotina_3 - ps21
Phaff./Saccharomycetaceae - ps22
Saccharomyco./mycetaceae - ps23
Saccharomycetaceae - ps24
Saccharomycetaceae_2 - ps25
Saccharomycetaceae_3 - ps26
Kaz./Nau./Saccharomyces - ps27
Saccharomyces - ps28
Arboricola_clade - ps29
Cerevisiae_clade - ps30
Saccharomyces_cerevisiae - ps31
p-value
1e-02
2e-02
2e-02
3e-02
4e-02
Log-odds
0.5
1.0
1.5
2.0
2.5
3.0
3.5
4.0

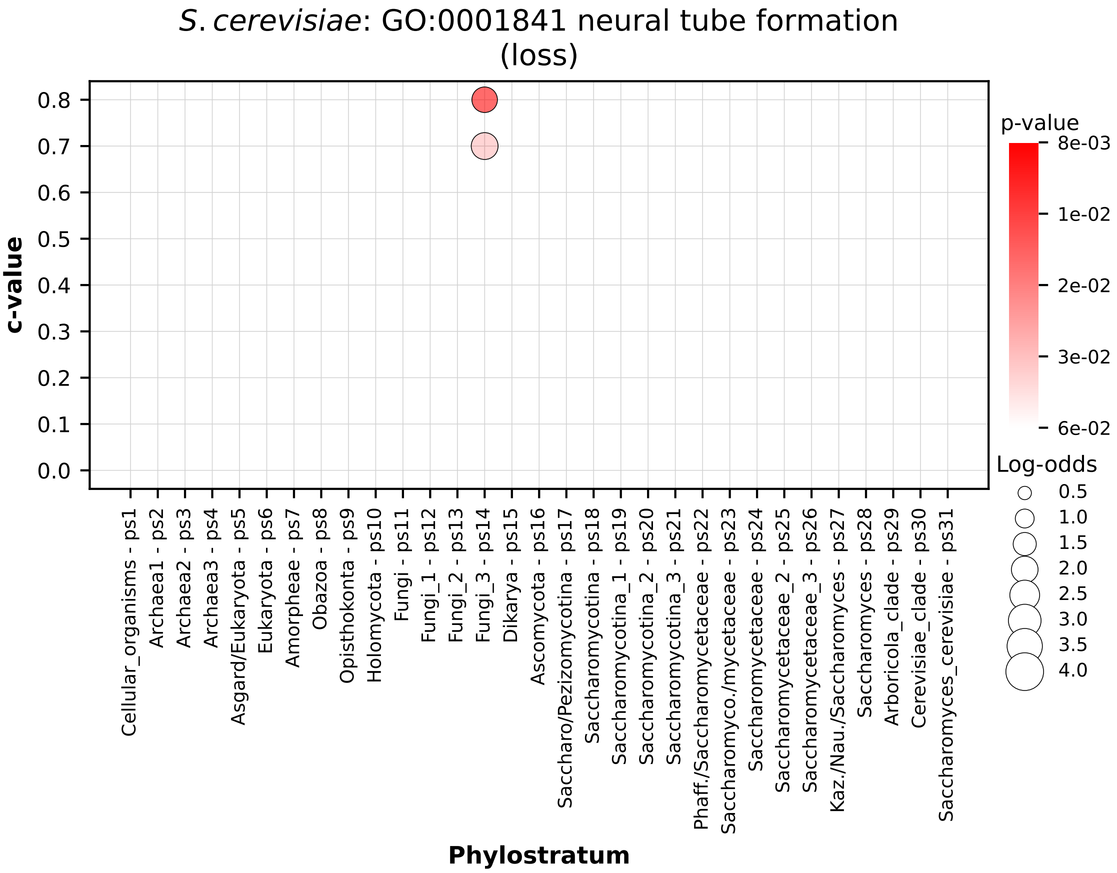
S. cerevisiae: GO:0001841 neural tube formation
(loss)
c-value
0.8
0.7
0.6
0.5
0.4
0.3
0.2
0.1
0.0
p-value
8e-03
1e-02
2e-02
3e-02
6e-02
Log-odds
0.5
1.0
1.5
2.0
2.5
3.0
3.5
4.0
Cellular_organisms - ps1
Archaea1 - ps2
Archaea2 - ps3
Archaea3 - ps4
Asgard/Eukaryota - ps5
Eukaryota - ps6
Amorpheae - ps7
Obazoa - ps8
Opisthokonta - ps9
Holomycota - ps10
Fungi - ps11
Fungi_1 - ps12
Fungi_2 - ps13
Fungi_3 - ps14
Dikarya - ps15
Ascomycota - ps16
Saccharo/Pezizomycotina - ps17
Saccharomycotina - ps18
Saccharomycotina_1 - ps19
Saccharomycotina_2 - ps20
Saccharomycotina_3 - ps21
Phaff./Saccharomycetaceae - ps22
Saccharomyco./mycetaceae - ps23
Saccharomycetaceae - ps24
Saccharomycetaceae_2 - ps25
Saccharomycetaceae_3 - ps26
Kaz./Nau./Saccharomyces - ps27
Saccharomyces - ps28
Arboricola_clade - ps29
Cerevisiae_clade - ps30
Saccharomyces_cerevisiae - ps31
Phylostratum

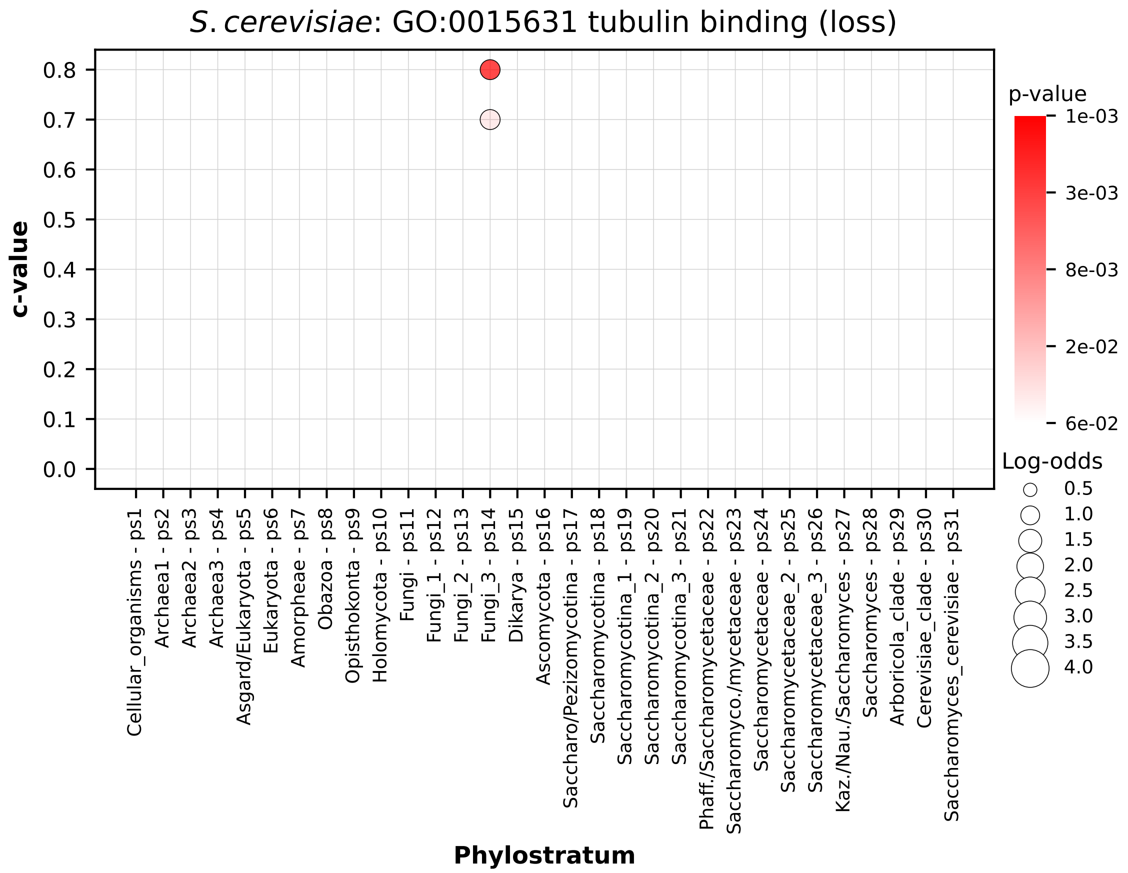
S. cerevisiae: GO:0015631 tubulin binding (loss)
c-value
0.8
0.7
0.6
0.5
0.4
0.3
0.2
0.1
0.0
Phylostratum
Cellular_organisms - ps1
Archaea1 - ps2
Archaea2 - ps3
Archaea3 - ps4
Asgard/Eukaryota - ps5
Eukaryota - ps6
Amorpheae - ps7
Obazoa - ps8
Opisthokonta - ps9
Holomycota - ps10
Fungi - ps11
Fungi_1 - ps12
Fungi_2 - ps13
Fungi_3 - ps14
Dikarya - ps15
Ascomycota - ps16
Saccharo/Pezizomycotina - ps17
Saccharomycotina - ps18
Saccharomycotina_1 - ps19
Saccharomycotina_2 - ps20
Saccharomycotina_3 - ps21
Phaff./Saccharomycetaceae - ps22
Saccharomyco./mycetaceae - ps23
Saccharomycetaceae - ps24
Saccharomycetaceae_2 - ps25
Saccharomycetaceae_3 - ps26
Kaz./Nau./Saccharomyces - ps27
Saccharomyces - ps28
Arboricola_clade - ps29
Cerevisiae_clade - ps30
Saccharomyces_cerevisiae - ps31
p-value
1e-03
3e-03
8e-03
2e-02
6e-02
Log-odds
0.5
1.0
1.5
2.0
2.5
3.0
3.5
4.0

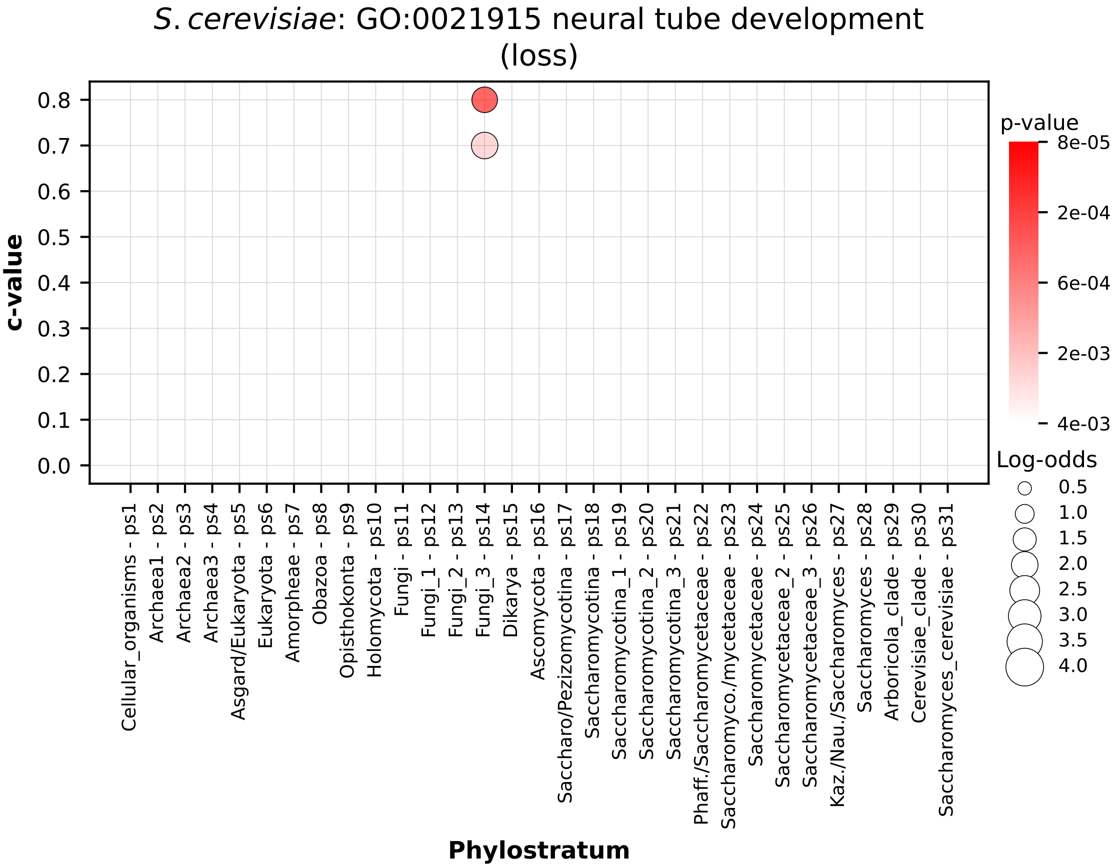

S. cerevisiae: GO:0021915 neural tube development
(loss)
c-value
0.8
0.7
0.6
0.5
0.4
0.3
0.2
0.1
0.0
p-value
8e-05
2e-04
6e-04
2e-03
4e-03
Log-odds
0.5
1.0
1.5
2.0
2.5
3.0
3.5
4.0
Cellular_organisms - ps1
Archaea1 - ps2
Archaea2 - ps3
Archaea3 - ps4
Asgard/Eukaryota - ps5
Eukaryota - ps6
Amorpheae - ps7
Obazoa - ps8
Opisthokonta - ps9
Holomycota - ps10
Fungi - ps11
Fungi_1 - ps12
Fungi_2 - ps13
Fungi_3 - ps14
Dikarya - ps15
Ascomycota - ps16
Saccharo/Pezizomycotina - ps17
Saccharomycotina - ps18
Saccharomycotina_1 - ps19
Saccharomycotina_2 - ps20
Saccharomycotina_3 - ps21
Phaff./Saccharomycetaceae - ps22
Saccharomyco./mycetaceae - ps23
Saccharomycetaceae - ps24
Saccharomycetaceae_2 - ps25
Saccharomycetaceae_3 - ps26
Kaz./Nau./Saccharomyces - ps27
Saccharomyces - ps28
Arboricola_clade - ps29
Cerevisiae_clade - ps30
Saccharomyces_cerevisiae - ps31
Phylostratum

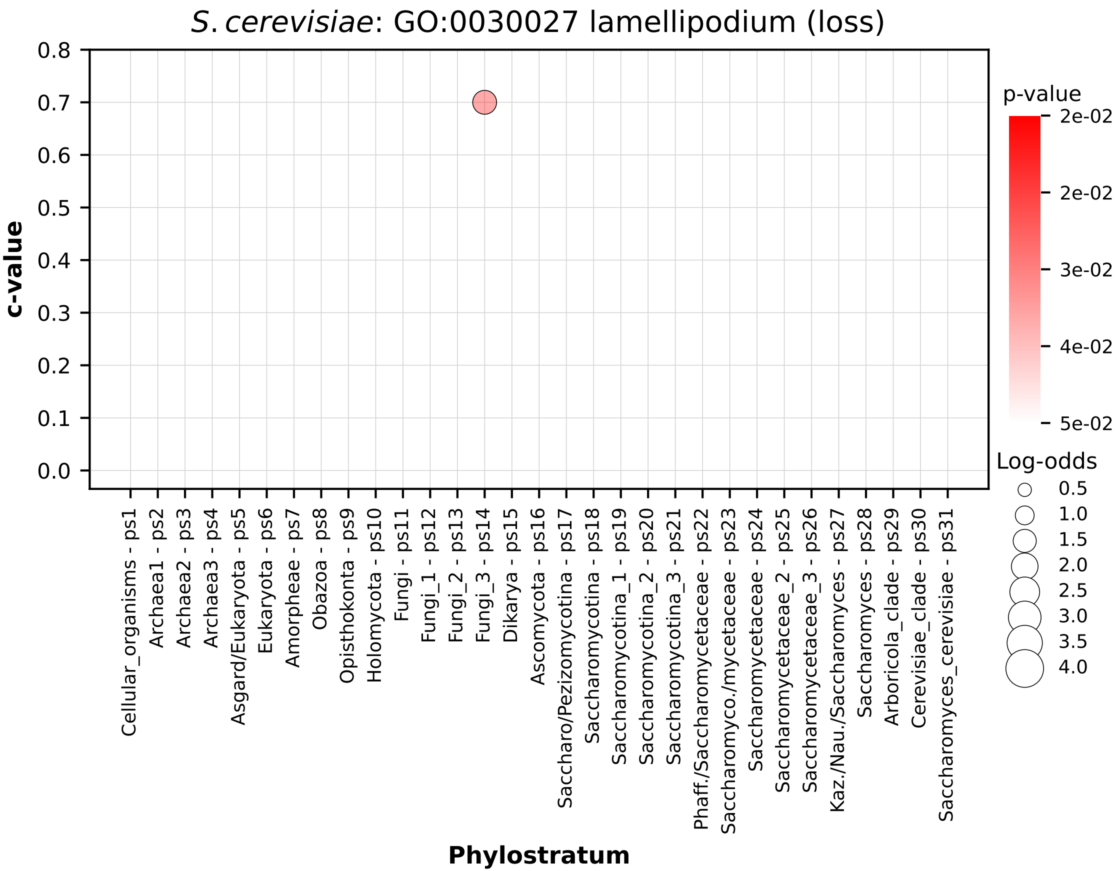

S. cerevisiae: GO:0030027 lamellipodium (loss)
c-value
0.8
0.7
0.6
0.5
0.4
0.3
0.2
0.1
0.0
Phylostratum
Cellular_organisms - ps1
Archaea1 - ps2
Archaea2 - ps3
Archaea3 - ps4
Asgard/Eukaryota - ps5
Eukaryota - ps6
Amorpheae - ps7
Obazoa - ps8
Opisthokonta - ps9
Holomycota - ps10
Fungi - ps11
Fungi_1 - ps12
Fungi_2 - ps13
Fungi_3 - ps14
Dikarya - ps15
Ascomycota - ps16
Saccharo/Pezizomycotina - ps17
Saccharomycotina - ps18
Saccharomycotina_1 - ps19
Saccharomycotina_2 - ps20
Saccharomycotina_3 - ps21
Phaff./Saccharomycetaceae - ps22
Saccharomyco./mycetaceae - ps23
Saccharomycetaceae - ps24
Saccharomycetaceae_2 - ps25
Saccharomycetaceae_3 - ps26
Kaz./Nau./Saccharomyces - ps27
Saccharomyces - ps28
Arboricola_clade - ps29
Cerevisiae_clade - ps30
Saccharomyces_cerevisiae - ps31
p-value
2e-02
2e-02
3e-02
4e-02
5e-02
Log-odds
0.5
1.0
1.5
2.0
2.5
3.0
3.5
4.0

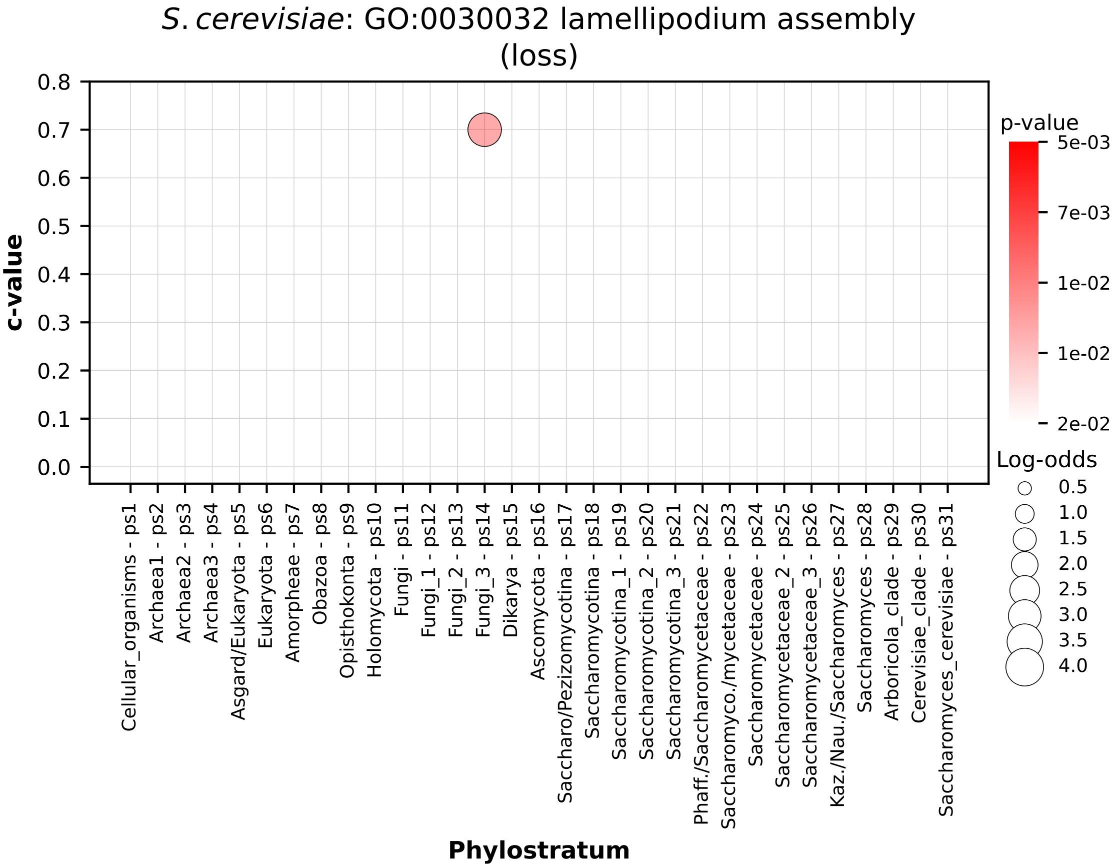

S. cerevisiae: GO:0030032 lamellipodium assembly
(loss)
c-value
0.8
0.7
0.6
0.5
0.4
0.3
0.2
0.1
0.0
p-value
5e-03
7e-03
1e-02
1e-02
2e-02
Log-odds
0.5
1.0
1.5
2.0
2.5
3.0
3.5
4.0
Cellular_organisms - ps1
Archaea1 - ps2
Archaea2 - ps3
Archaea3 - ps4
Asgard/Eukaryota - ps5
Eukaryota - ps6
Amorpheae - ps7
Obazoa - ps8
Opisthokonta - ps9
Holomycota - ps10
Fungi - ps11
Fungi_1 - ps12
Fungi_2 - ps13
Fungi_3 - ps14
Dikarya - ps15
Ascomycota - ps16
Saccharo/Pezizomycotina - ps17
Saccharomycotina - ps18
Saccharomycotina_1 - ps19
Saccharomycotina_2 - ps20
Saccharomycotina_3 - ps21
Phaff./Saccharomycetaceae - ps22
Saccharomyco./mycetaceae - ps23
Saccharomycetaceae - ps24
Saccharomycetaceae_2 - ps25
Saccharomycetaceae_3 - ps26
Kaz./Nau./Saccharomyces - ps27
Saccharomyces - ps28
Arboricola_clade - ps29
Cerevisiae_clade - ps30
Saccharomyces_cerevisiae - ps31
Phylostratum

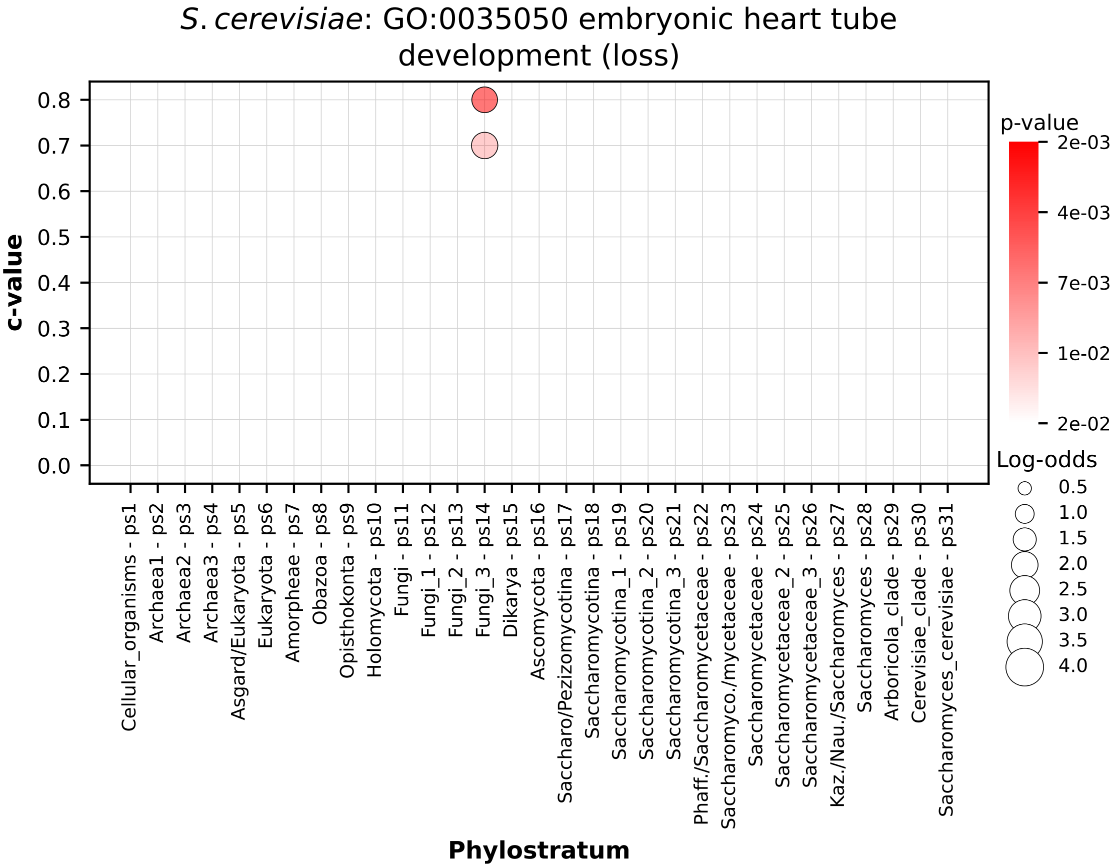
S. cerevisiae: GO:0035050 embryonic heart tube development (loss)
c-value
0.8
0.7
0.6
0.5
0.4
0.3
0.2
0.1
0.0
Phylostratum
Cellular_organisms - ps1
Archaea1 - ps2
Archaea2 - ps3
Archaea3 - ps4
Asgard/Eukaryota - ps5
Eukaryota - ps6
Amorpheae - ps7
Obazoa - ps8
Opisthokonta - ps9
Holomycota - ps10
Fungi - ps11
Fungi_1 - ps12
Fungi_2 - ps13
Fungi_3 - ps14
Dikarya - ps15
Ascomycota - ps16
Saccharo/Pezizomycotina - ps17
Saccharomycotina - ps18
Saccharomycotina_1 - ps19
Saccharomycotina_2 - ps20
Saccharomycotina_3 - ps21
Phaff./Saccharomycetaceae - ps22
Saccharomyco./mycetaceae - ps23
Saccharomycetaceae - ps24
Saccharomycetaceae_2 - ps25
Saccharomycetaceae_3 - ps26
Kaz./Nau./Saccharomyces - ps27
Saccharomyces - ps28
Arboricola_clade - ps29
Cerevisiae_clade - ps30
Saccharomyces_cerevisiae - ps31
p-value
2e-03
4e-03
7e-03
1e-02
2e-02
Log-odds
0.5
1.0
1.5
2.0
2.5
3.0
3.5
4.0

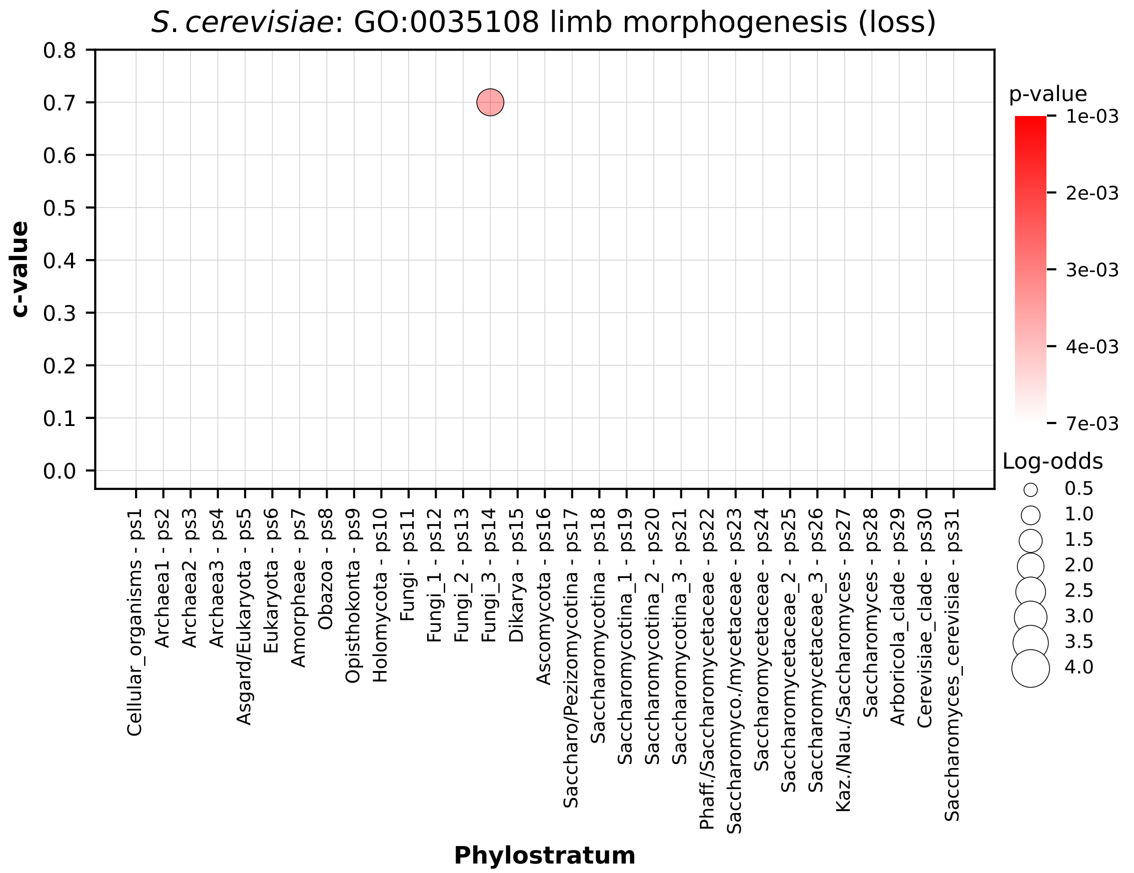

S. cerevisiae: GO:0035108 limb morphogenesis (loss)
c-value
0.8
0.7
0.6
0.5
0.4
0.3
0.2
0.1
0.0
p-value
1e-03
2e-03
3e-03
4e-03
7e-03
Log-odds
0.5
1.0
1.5
2.0
2.5
3.0
3.5
4.0
Cellular_organisms - ps1
Archaea1 - ps2
Archaea2 - ps3
Archaea3 - ps4
Asgard/Eukaryota - ps5
Eukaryota - ps6
Amorpheae - ps7
Obazoa - ps8
Opisthokonta - ps9
Holomycota - ps10
Fungi - ps11
Fungi_1 - ps12
Fungi_2 - ps13
Fungi_3 - ps14
Dikarya - ps15
Ascomycota - ps16
Saccharo/Pezizomycotina - ps17
Saccharomycotina - ps18
Saccharomycotina_1 - ps19
Saccharomycotina_2 - ps20
Saccharomycotina_3 - ps21
Phaff./Saccharomycetaceae - ps22
Saccharomyco./mycetaceae - ps23
Saccharomycetaceae - ps24
Saccharomycetaceae_2 - ps25
Saccharomycetaceae_3 - ps26
Kaz./Nau./Saccharomyces - ps27
Saccharomyces - ps28
Arboricola_clade - ps29
Cerevisiae_clade - ps30
Saccharomyces_cerevisiae - ps31
Phylostratum

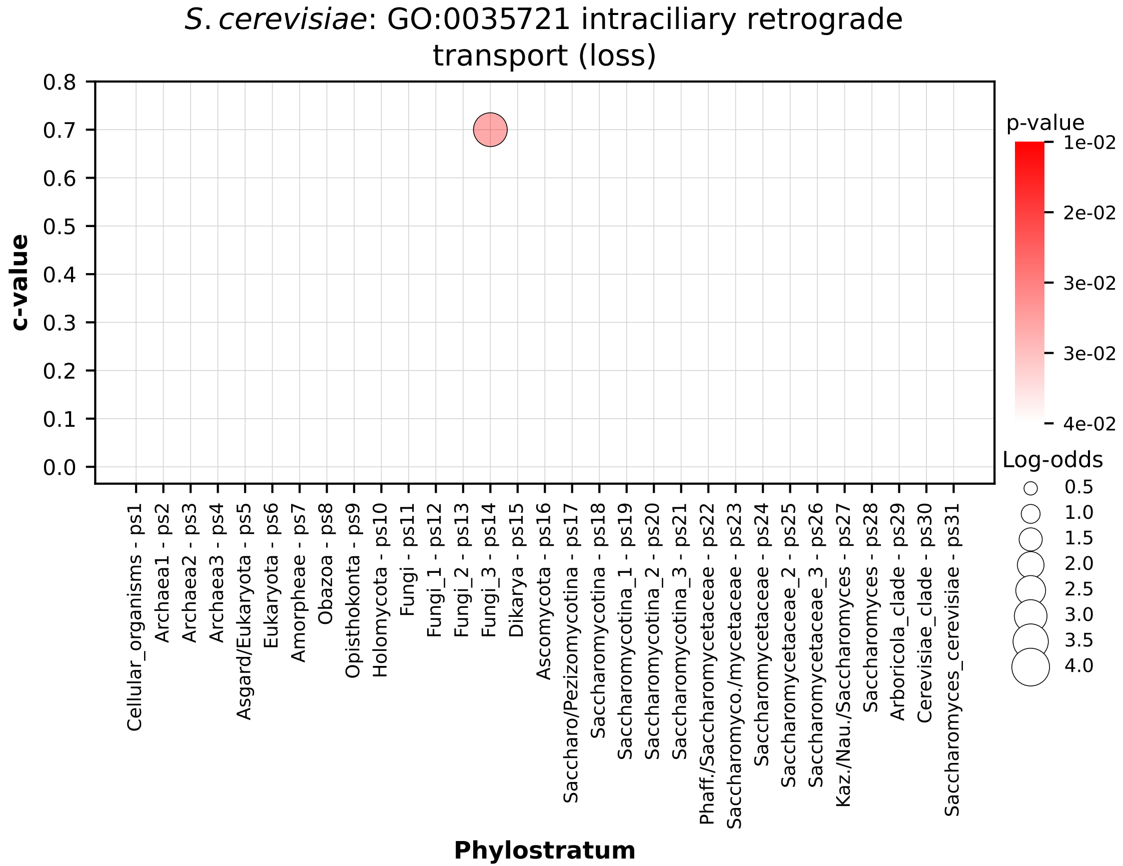

S. cerevisiae: GO:0035721 intraciliary retrograde transport (loss)
c-value
0.8
0.7
0.6
0.5
0.4
0.3
0.2
0.1
0.0
Phylostratum
Cellular_organisms - ps1
Archaea1 - ps2
Archaea2 - ps3
Archaea3 - ps4
Asgard/Eukaryota - ps5
Eukaryota - ps6
Amorpheae - ps7
Obazoa - ps8
Opisthokonta - ps9
Holomycota - ps10
Fungi - ps11
Fungi_1 - ps12
Fungi_2 - ps13
Fungi_3 - ps14
Dikarya - ps15
Ascomycota - ps16
Saccharo/Pezizomycotina - ps17
Saccharomycotina - ps18
Saccharomycotina_1 - ps19
Saccharomycotina_2 - ps20
Saccharomycotina_3 - ps21
Phaff./Saccharomycetaceae - ps22
Saccharomyco./mycetaceae - ps23
Saccharomycetaceae - ps24
Saccharomycetaceae_2 - ps25
Saccharomycetaceae_3 - ps26
Kaz./Nau./Saccharomyces - ps27
Saccharomyces - ps28
Arboricola_clade - ps29
Cerevisiae_clade - ps30
Saccharomyces_cerevisiae - ps31
p-value
1e-02
2e-02
3e-02
3e-02
4e-02
Log-odds
0.5
1.0
1.5
2.0
2.5
3.0
3.5
4.0

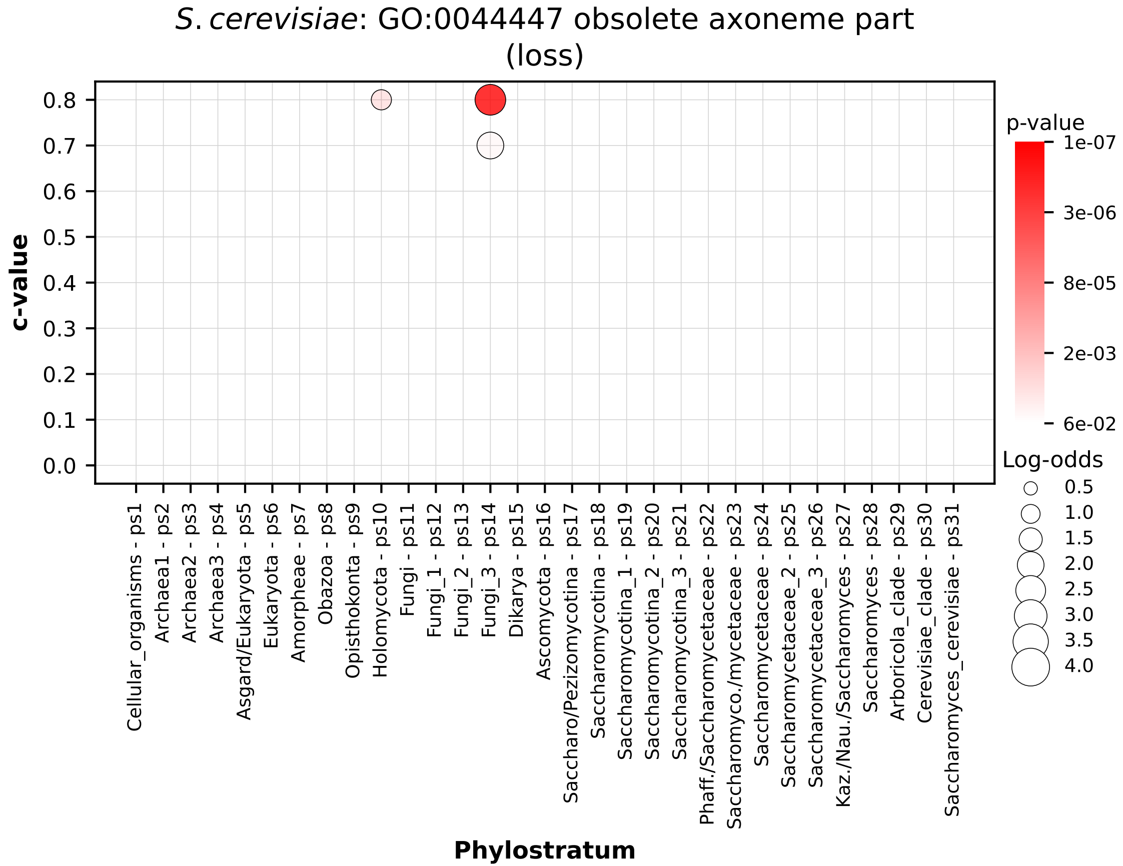

S. cerevisiae: GO:0044447 obsolete axoneme part
(loss)
c-value
0.0
0.1
0.2
0.3
0.4
0.5
0.6
0.7
0.8
Phylostratum
Cellular_organisms - ps1
Archaea1 - ps2
Archaea2 - ps3
Archaea3 - ps4
Asgard/Eukaryota - ps5
Eukaryota - ps6
Amorpheae - ps7
Obazoa - ps8
Opisthokonta - ps9
Holomycota - ps10
Fungi - ps11
Fungi_1 - ps12
Fungi_2 - ps13
Fungi_3 - ps14
Dikarya - ps15
Ascomycota - ps16
Saccharo/Pezizomycotina - ps17
Saccharomycotina - ps18
Saccharomycotina_1 - ps19
Saccharomycotina_2 - ps20
Saccharomycotina_3 - ps21
Phaff./Saccharomycetaceae - ps22
Saccharomyco./mycetaceae - ps23
Saccharomycetaceae - ps24
Saccharomycetaceae_2 - ps25
Saccharomycetaceae_3 - ps26
Kaz./Nau./Saccharomyces - ps27
Saccharomyces - ps28
Arboricola_clade - ps29
Cerevisiae_clade - ps30
Saccharomyces_cerevisiae - ps31
p-value
1e-07
3e-06
8e-05
2e-03
6e-02
Log-odds
0.5
1.0
1.5
2.0
2.5
3.0
3.5
4.0

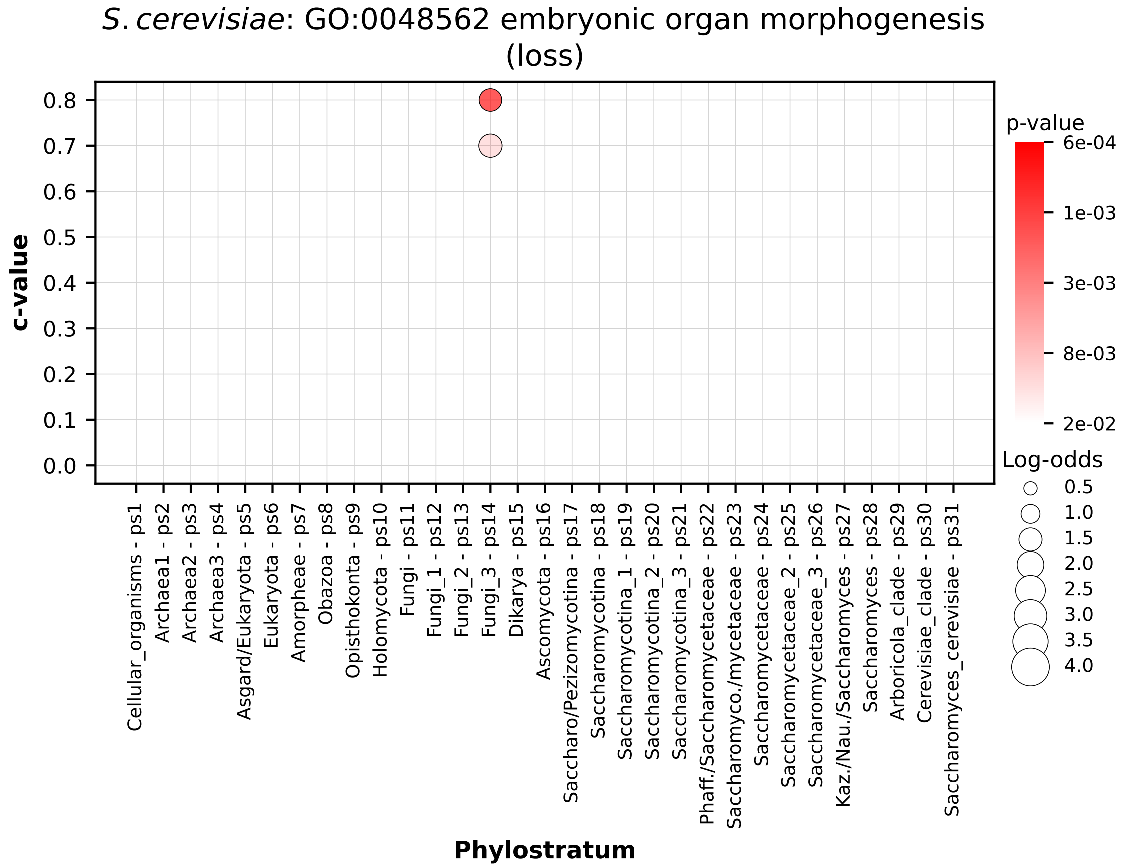

S. cerevisiae: GO:0048562 embryonic organ morphogenesis (loss)
c-value
0.8
0.7
0.6
0.5
0.4
0.3
0.2
0.1
0.0
p-value
6e-04
1e-03
3e-03
8e-03
2e-02
Log-odds
0.5
1.0
1.5
2.0
2.5
3.0
3.5
4.0
Cellular_organisms - ps1
Archaea1 - ps2
Archaea2 - ps3
Archaea3 - ps4
Asgard/Eukaryota - ps5
Eukaryota - ps6
Amorpheae - ps7
Obazoa - ps8
Opisthokonta - ps9
Holomycota - ps10
Fungi - ps11
Fungi_1 - ps12
Fungi_2 - ps13
Fungi_3 - ps14
Dikarya - ps15
Ascomycota - ps16
Saccharo/Pezizomycotina - ps17
Saccharomycotina - ps18
Saccharomycotina_1 - ps19
Saccharomycotina_2 - ps20
Saccharomycotina_3 - ps21
Phaff./Saccharomycetaceae - ps22
Saccharomyco./mycetaceae - ps23
Saccharomycetaceae - ps24
Saccharomycetaceae_2 - ps25
Saccharomycetaceae_3 - ps26
Kaz./Nau./Saccharomyces - ps27
Saccharomyces - ps28
Arboricola_clade - ps29
Cerevisiae_clade - ps30
Saccharomyces_cerevisiae - ps31
Phylostratum

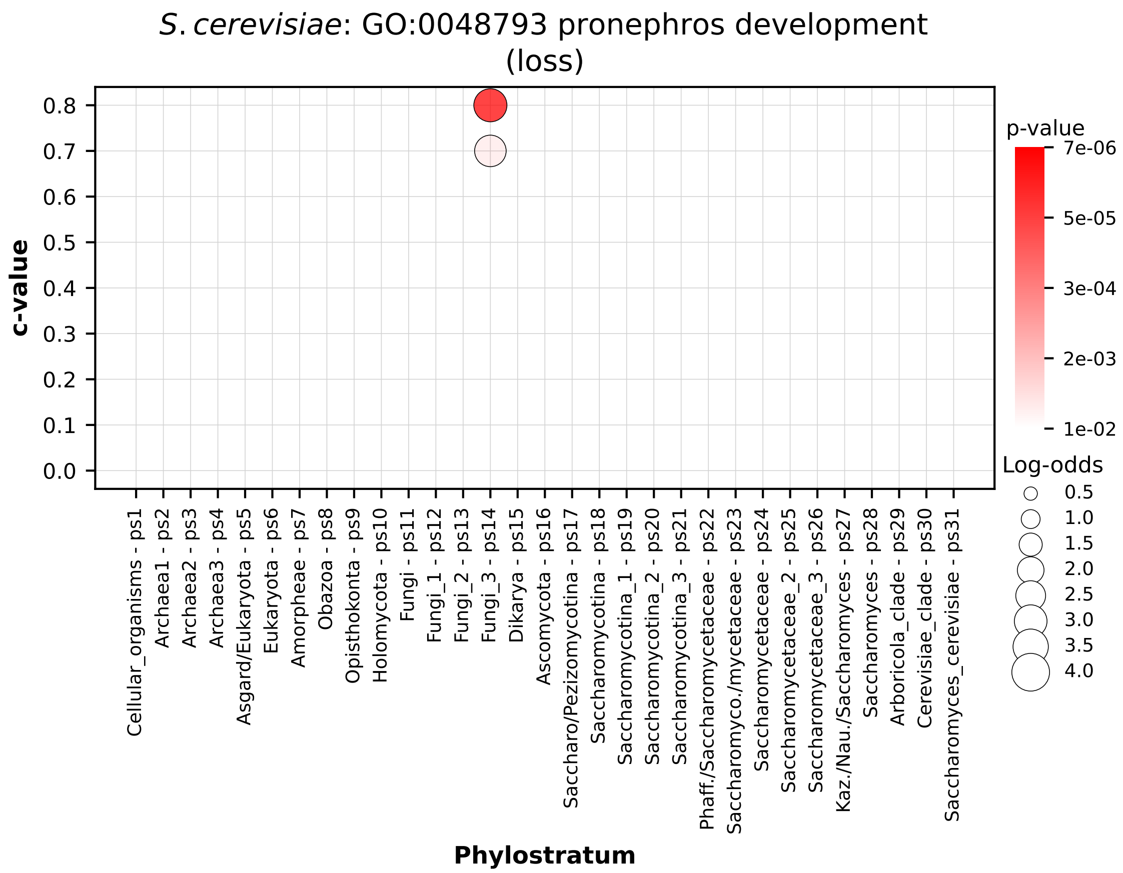
S. cerevisiae: GO:0048793 pronephros development
(loss)
c-value
0.8
0.7
0.6
0.5
0.4
0.3
0.2
0.1
0.0
p-value
7e-06
5e-05
3e-04
2e-03
1e-02
Log-odds
0.5
1.0
1.5
2.0
2.5
3.0
3.5
4.0
Cellular_organisms - ps1
Archaea1 - ps2
Archaea2 - ps3
Archaea3 - ps4
Asgard/Eukaryota - ps5
Eukaryota - ps6
Amorpheae - ps7
Obazoa - ps8
Opisthokonta - ps9
Holomycota - ps10
Fungi - ps11
Fungi_1 - ps12
Fungi_2 - ps13
Fungi_3 - ps14
Dikarya - ps15
Ascomycota - ps16
Saccharo/Pezizomycotina - ps17
Saccharomycotina - ps18
Saccharomycotina_1 - ps19
Saccharomycotina_2 - ps20
Saccharomycotina_3 - ps21
Phaff./Saccharomycetaceae - ps22
Saccharomyco./mycetaceae - ps23
Saccharomycetaceae - ps24
Saccharomycetaceae_2 - ps25
Saccharomycetaceae_3 - ps26
Kaz./Nau./Saccharomyces - ps27
Saccharomyces - ps28
Arboricola_clade - ps29
Cerevisiae_clade - ps30
Saccharomyces_cerevisiae - ps31
Phylostratum

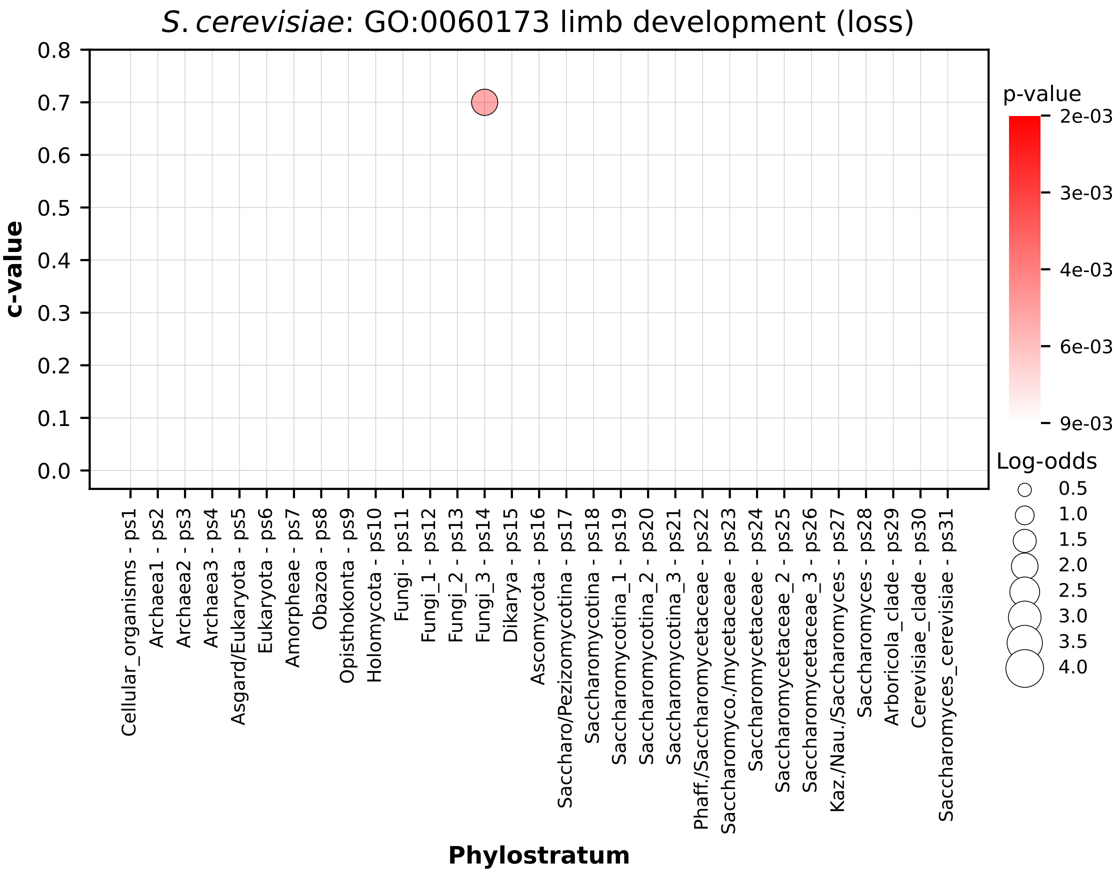

S. cerevisiae: GO:0060173 limb development (loss)
c-value
0.8
0.7
0.6
0.5
0.4
0.3
0.2
0.1
0.0
p-value
2e-03
3e-03
4e-03
6e-03
9e-03
Log-odds
0.5
1.0
1.5
2.0
2.5
3.0
3.5
4.0
Cellular_organisms - ps1
Archaea1 - ps2
Archaea2 - ps3
Archaea3 - ps4
Asgard/Eukaryota - ps5
Eukaryota - ps6
Amorpheae - ps7
Obazoa - ps8
Opisthokonta - ps9
Holomycota - ps10
Fungi - ps11
Fungi_1 - ps12
Fungi_2 - ps13
Fungi_3 - ps14
Dikarya - ps15
Ascomycota - ps16
Saccharo/Pezizomycotina - ps17
Saccharomycotina - ps18
Saccharomycotina_1 - ps19
Saccharomycotina_2 - ps20
Saccharomycotina_3 - ps21
Phaff./Saccharomycetaceae - ps22
Saccharomyco./mycetaceae - ps23
Saccharomycetaceae - ps24
Saccharomycetaceae_2 - ps25
Saccharomycetaceae_3 - ps26
Kaz./Nau./Saccharomyces - ps27
Saccharomyces - ps28
Arboricola_clade - ps29
Cerevisiae_clade - ps30
Saccharomyces_cerevisiae - ps31
Phylostratum

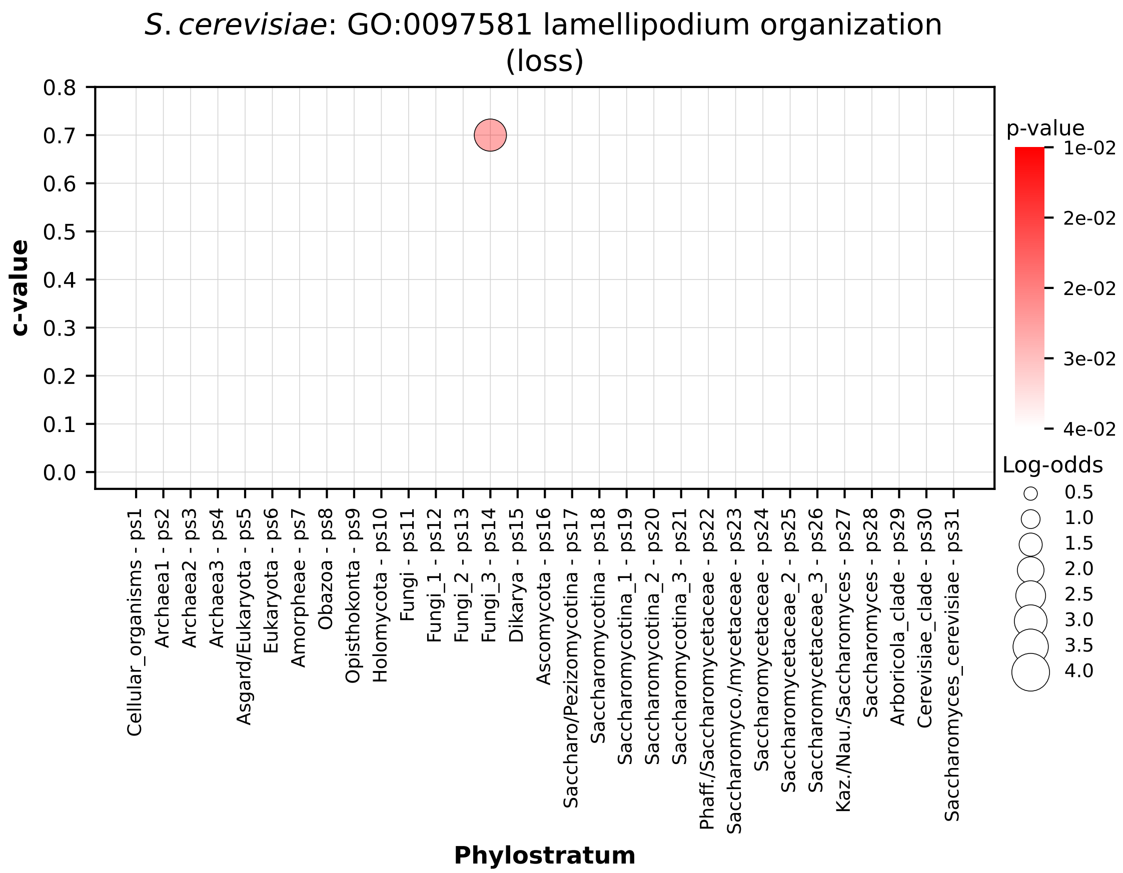
S. cerevisiae: GO:0097581 lamellipodium organization (loss)
c-value
0.8
0.7
0.6
0.5
0.4
0.3
0.2
0.1
0.0
Phylostratum
Cellular_organisms - ps1
Archaea1 - ps2
Archaea2 - ps3
Archaea3 - ps4
Asgard/Eukaryota - ps5
Eukaryota - ps6
Amorpheae - ps7
Obazoa - ps8
Opisthokonta - ps9
Holomycota - ps10
Fungi - ps11
Fungi_1 - ps12
Fungi_2 - ps13
Fungi_3 - ps14
Dikarya - ps15
Ascomycota - ps16
Saccharo/Pezizomycotina - ps17
Saccharomycotina - ps18
Saccharomycotina_1 - ps19
Saccharomycotina_2 - ps20
Saccharomycotina_3 - ps21
Phaff./Saccharomycetaceae - ps22
Saccharomyco./mycetaceae - ps23
Saccharomycetaceae - ps24
Saccharomycetaceae_2 - ps25
Saccharomycetaceae_3 - ps26
Kaz./Nau./Saccharomyces - ps27
Saccharomyces - ps28
Arboricola_clade - ps29
Cerevisiae_clade - ps30
Saccharomyces_cerevisiae - ps31
p-value
1e-02
2e-02
2e-02
3e-02
4e-02
Log-odds
0.5
1.0
1.5
2.0
2.5
3.0
3.5
4.0

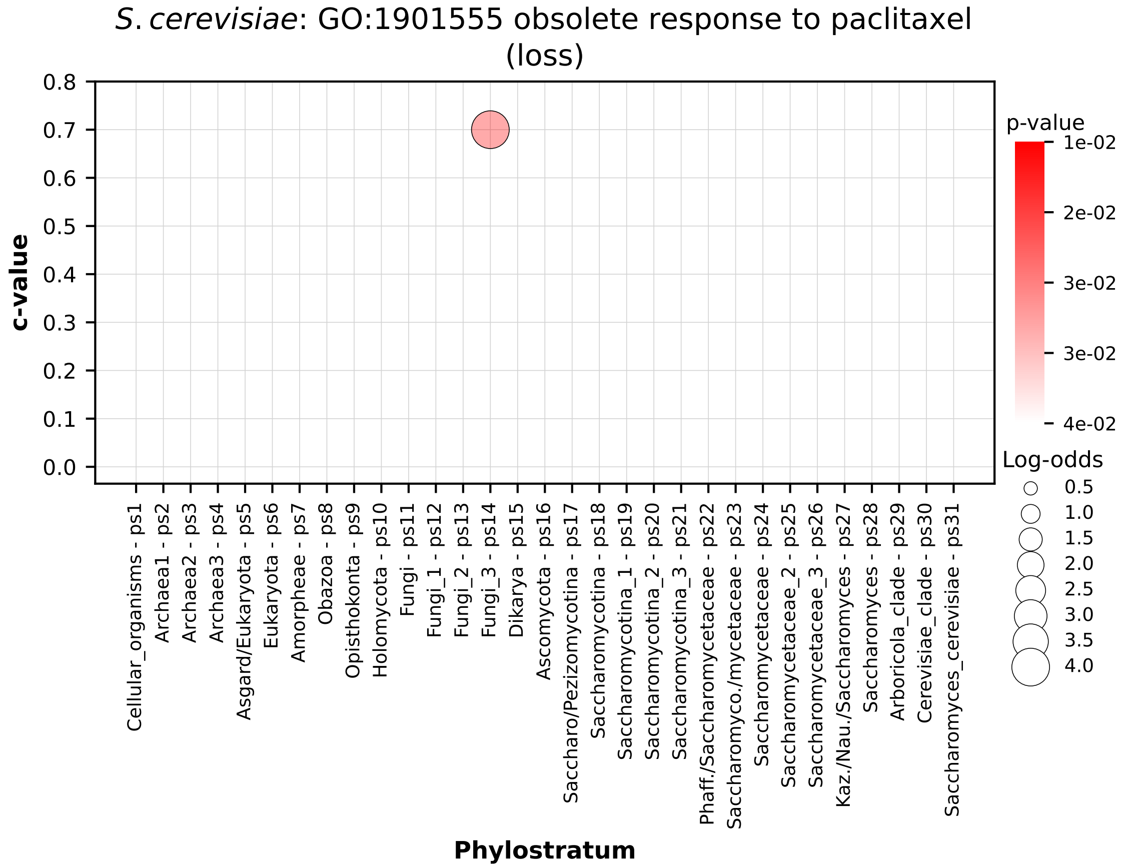

S. cerevisiae: GO:1901555 obsolete response to paclitaxel (loss)
c-value
0.8
0.7
0.6
0.5
0.4
0.3
0.2
0.1
0.0
p-value
1e-02
2e-02
3e-02
3e-02
4e-02
Log-odds
0.5
1.0
1.5
2.0
2.5
3.0
3.5
4.0
Cellular_organisms - ps1
Archaea1 - ps2
Archaea2 - ps3
Archaea3 - ps4
Asgard/Eukaryota - ps5
Eukaryota - ps6
Amorpheae - ps7
Obazoa - ps8
Opisthokonta - ps9
Holomycota - ps10
Fungi - ps11
Fungi_1 - ps12
Fungi_2 - ps13
Fungi_3 - ps14
Dikarya - ps15
Ascomycota - ps16
Saccharo/Pezizomycotina - ps17
Saccharomycotina - ps18
Saccharomycotina_1 - ps19
Saccharomycotina_2 - ps20
Saccharomycotina_3 - ps21
Phaff./Saccharomycetaceae - ps22
Saccharomyco./mycetaceae - ps23
Saccharomycetaceae - ps24
Saccharomycetaceae_2 - ps25
Saccharomycetaceae_3 - ps26
Kaz./Nau./Saccharomyces - ps27
Saccharomyces - ps28
Arboricola_clade - ps29
Cerevisiae_clade - ps30
Saccharomyces_cerevisiae - ps31
Phylostratum

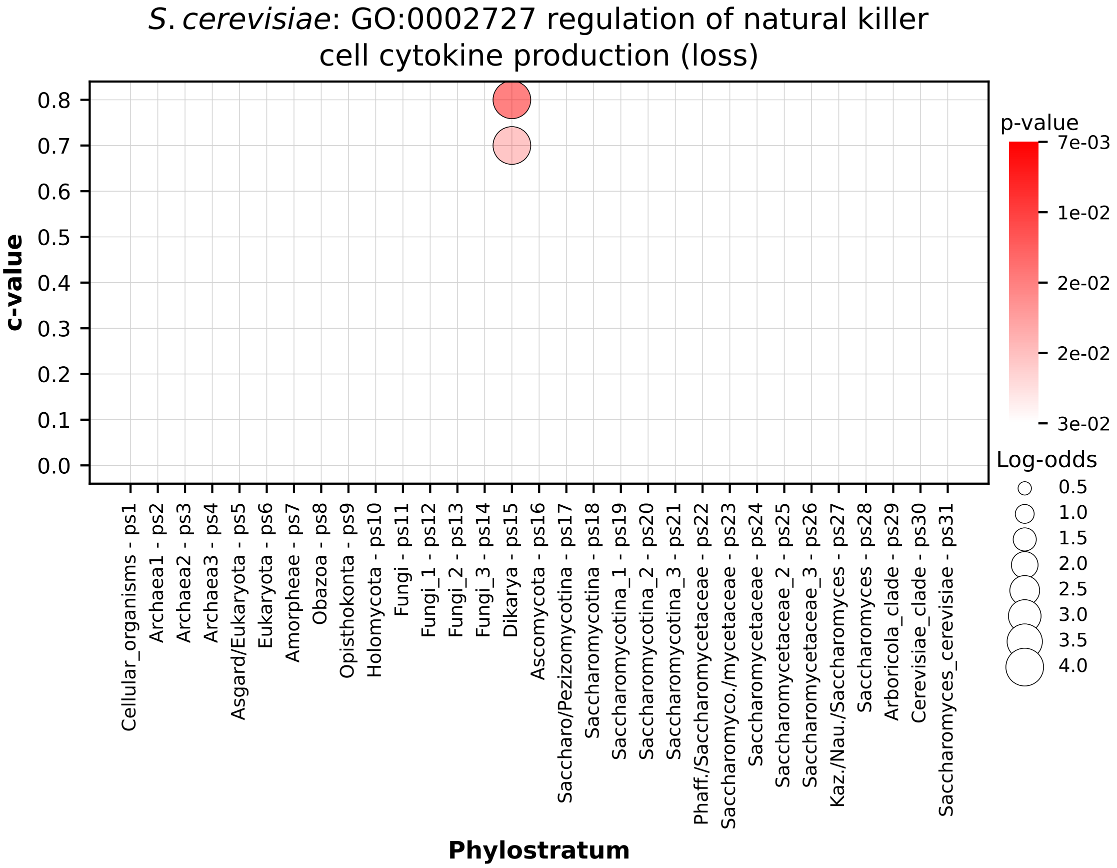

S. cerevisiae: GO:0002727 regulation of natural killer cell cytokine production (loss)
c-value
0.0
0.1
0.2
0.3
0.4
0.5
0.6
0.7
0.8
Phylostratum
Cellular_organisms - ps1
Archaea1 - ps2
Archaea2 - ps3
Archaea3 - ps4
Asgard/Eukaryota - ps5
Eukaryota - ps6
Amorpheae - ps7
Obazoa - ps8
Opisthokonta - ps9
Holomycota - ps10
Fungi - ps11
Fungi_1 - ps12
Fungi_2 - ps13
Fungi_3 - ps14
Dikarya - ps15
Ascomycota - ps16
Saccharo/Pezizomycotina - ps17
Saccharomycotina - ps18
Saccharomycotina_1 - ps19
Saccharomycotina_2 - ps20
Saccharomycotina_3 - ps21
Phaff./Saccharomycetaceae - ps22
Saccharomyco./mycetaceae - ps23
Saccharomycetaceae - ps24
Saccharomycetaceae_2 - ps25
Saccharomycetaceae_3 - ps26
Kaz./Nau./Saccharomyces - ps27
Saccharomyces - ps28
Arboricola_clade - ps29
Cerevisiae_clade - ps30
Saccharomyces_cerevisiae - ps31
p-value
7e-03
1e-02
2e-02
2e-02
3e-02
Log-odds
0.5
1.0
1.5
2.0
2.5
3.0
3.5
4.0

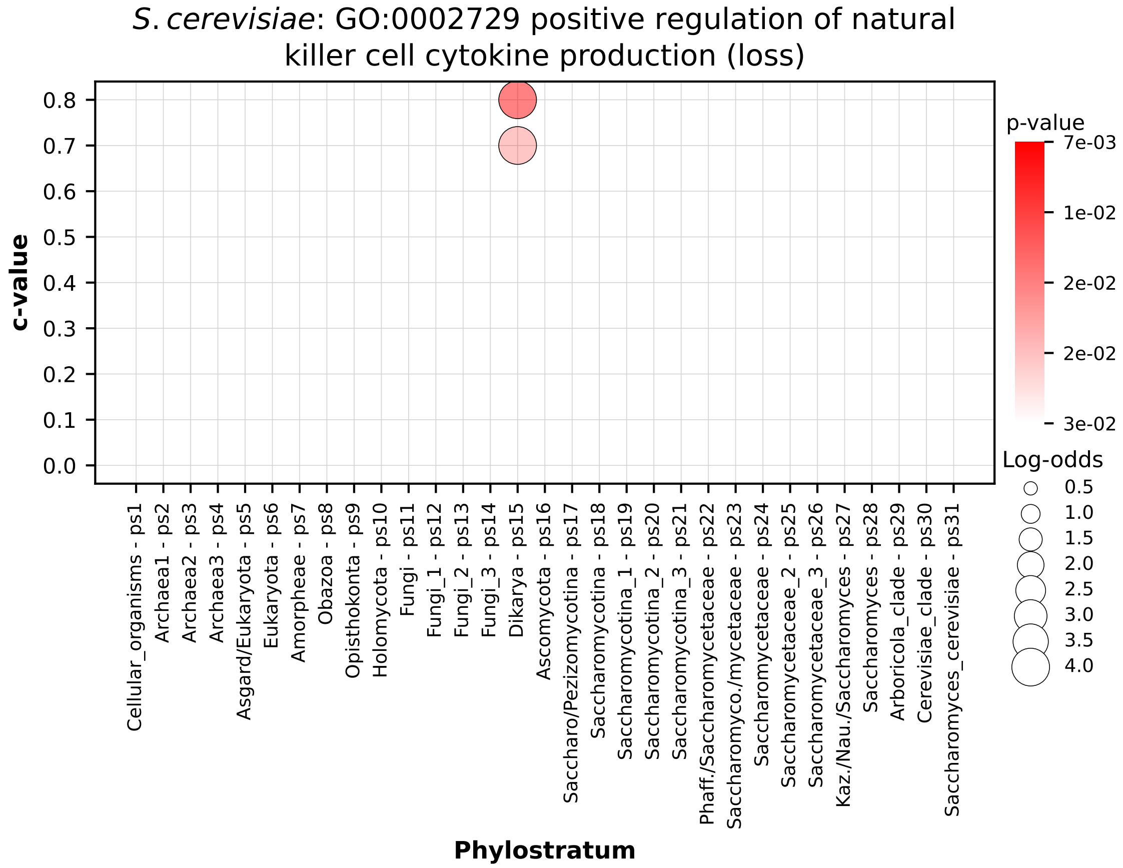

S. cerevisiae: GO:0002729 positive regulation of natural killer cell cytokine production (loss)
c-value
0.8
0.7
0.6
0.5
0.4
0.3
0.2
0.1
0.0
p-value
7e-03
1e-02
2e-02
2e-02
3e-02
Log-odds
0.5
1.0
1.5
2.0
2.5
3.0
3.5
4.0
Cellular_organisms - ps1
Archaea1 - ps2
Archaea2 - ps3
Archaea3 - ps4
Asgard/Eukaryota - ps5
Eukaryota - ps6
Amorpheae - ps7
Obazoa - ps8
Opisthokonta - ps9
Holomycota - ps10
Fungi - ps11
Fungi_1 - ps12
Fungi_2 - ps13
Fungi_3 - ps14
Dikarya - ps15
Ascomycota - ps16
Saccharo/Pezizomycotina - ps17
Saccharomycotina - ps18
Saccharomycotina_1 - ps19
Saccharomycotina_2 - ps20
Saccharomycotina_3 - ps21
Phaff./Saccharomycetaceae - ps22
Saccharomyco./mycetaceae - ps23
Saccharomycetaceae - ps24
Saccharomycetaceae_2 - ps25
Saccharomycetaceae_3 - ps26
Kaz./Nau./Saccharomyces - ps27
Saccharomyces - ps28
Arboricola_clade - ps29
Cerevisiae_clade - ps30
Saccharomyces_cerevisiae - ps31
Phylostratum

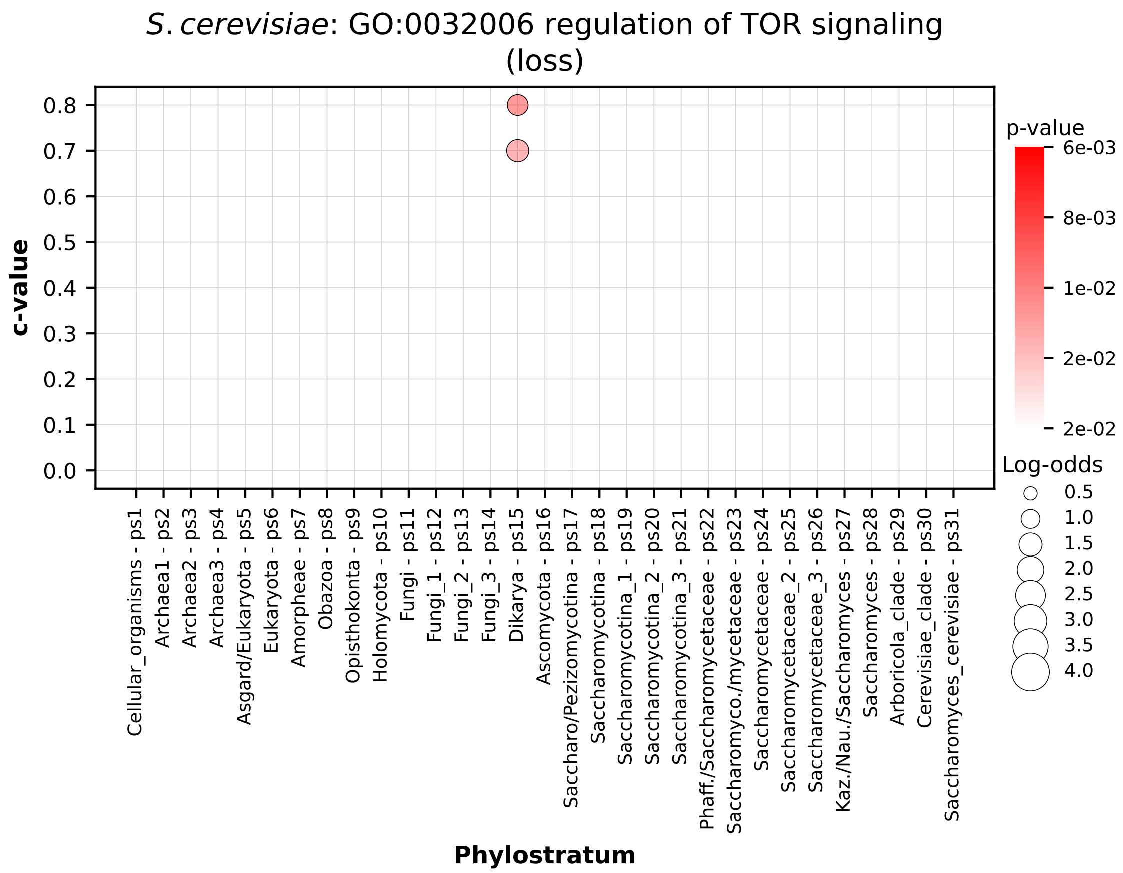
S. cerevisiae: GO:0032006 regulation of TOR signaling
(loss)
c-value
0.8
0.7
0.6
0.5
0.4
0.3
0.2
0.1
0.0
Phylostratum
Cellular_organisms - ps1
Archaea1 - ps2
Archaea2 - ps3
Archaea3 - ps4
Asgard/Eukaryota - ps5
Eukaryota - ps6
Amorpheae - ps7
Obazoa - ps8
Opisthokonta - ps9
Holomycota - ps10
Fungi - ps11
Fungi_1 - ps12
Fungi_2 - ps13
Fungi_3 - ps14
Dikarya - ps15
Ascomycota - ps16
Saccharo/Pezizomycotina - ps17
Saccharomycotina - ps18
Saccharomycotina_1 - ps19
Saccharomycotina_2 - ps20
Saccharomycotina_3 - ps21
Phaff./Saccharomycetaceae - ps22
Saccharomyco./mycetaceae - ps23
Saccharomycetaceae - ps24
Saccharomycetaceae_2 - ps25
Saccharomycetaceae_3 - ps26
Kaz./Nau./Saccharomyces - ps27
Saccharomyces - ps28
Arboricola_clade - ps29
Cerevisiae_clade - ps30
Saccharomyces_cerevisiae - ps31
p-value
6e-03
8e-03
1e-02
2e-02
2e-02
Log-odds
0.5
1.0
1.5
2.0
2.5
3.0
3.5
4.0

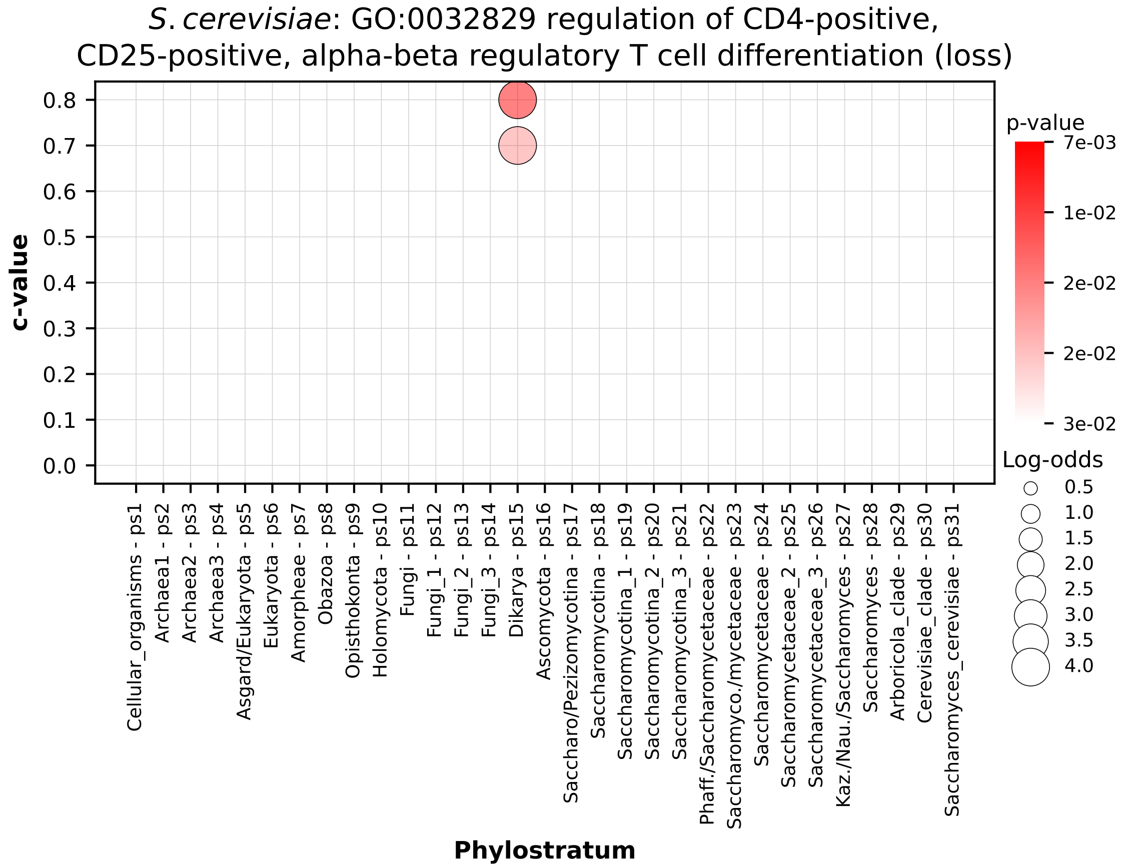

S. cerevisiae: GO:0032829 regulation of CD4-positive, CD25-positive, alpha-beta regulatory T cell differentiation (loss)
c-value
0.8
0.7
0.6
0.5
0.4
0.3
0.2
0.1
0.0
p-value
7e-03
1e-02
2e-02
2e-02
3e-02
Log-odds
0.5
1.0
1.5
2.0
2.5
3.0
3.5
4.0
Cellular_organisms - ps1
Archaea1 - ps2
Archaea2 - ps3
Archaea3 - ps4
Asgard/Eukaryota - ps5
Eukaryota - ps6
Amorpheae - ps7
Obazoa - ps8
Opisthokonta - ps9
Holomycota - ps10
Fungi - ps11
Fungi_1 - ps12
Fungi_2 - ps13
Fungi_3 - ps14
Dikarya - ps15
Ascomycota - ps16
Saccharo/Pezizomycotina - ps17
Saccharomycotina - ps18
Saccharomycotina_1 - ps19
Saccharomycotina_2 - ps20
Saccharomycotina_3 - ps21
Phaff./Saccharomycetaceae - ps22
Saccharomyco./mycetaceae - ps23
Saccharomycetaceae - ps24
Saccharomycetaceae_2 - ps25
Saccharomycetaceae_3 - ps26
Kaz./Nau./Saccharomyces - ps27
Saccharomyces - ps28
Arboricola_clade - ps29
Cerevisiae_clade - ps30
Saccharomyces_cerevisiae - ps31
Phylostratum

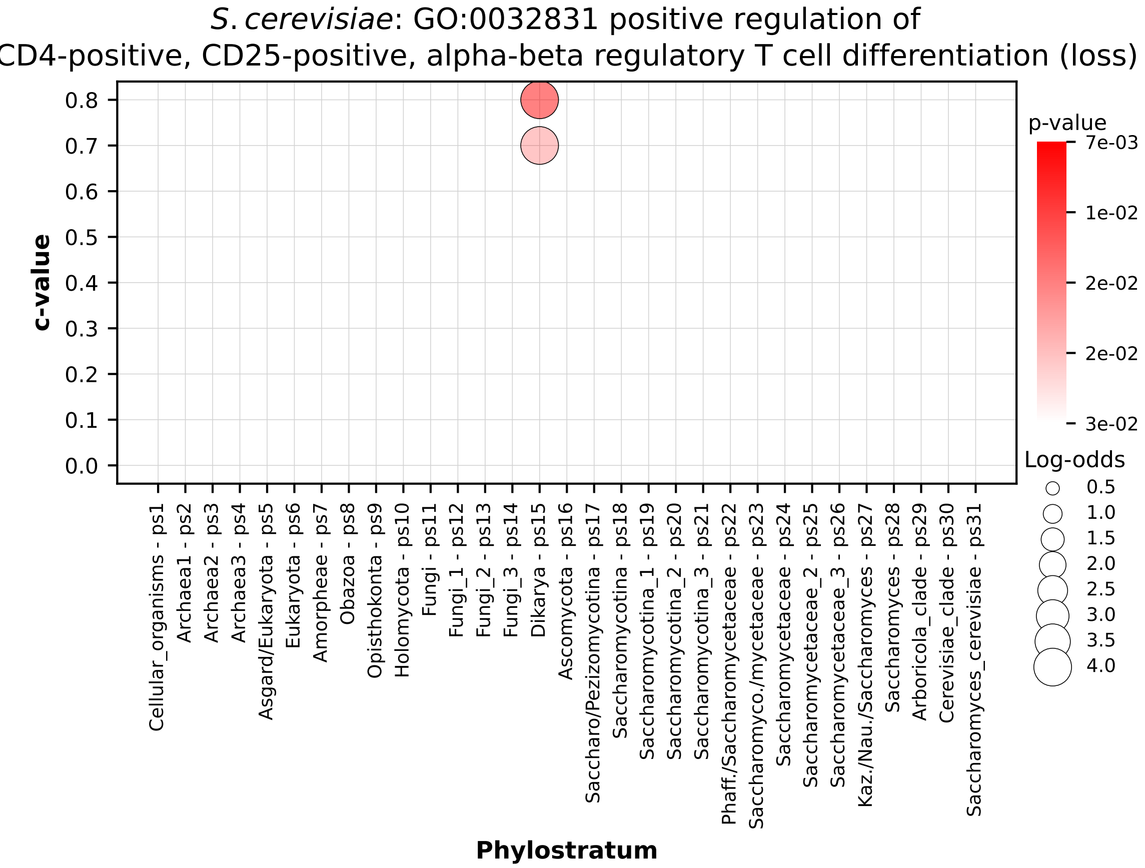

S. cerevisiae: GO:0032831 positive regulation of
CD4-positive, CD25-positive, alpha-beta regulatory T cell differentiation (loss)
c-value
0.8
0.7
0.6
0.5
0.4
0.3
0.2
0.1
0.0
p-value
7e-03
1e-02
2e-02
2e-02
3e-02
Log-odds
0.5
1.0
1.5
2.0
2.5
3.0
3.5
4.0
Cellular_organisms - ps1
Archaea1 - ps2
Archaea2 - ps3
Archaea3 - ps4
Asgard/Eukaryota - ps5
Eukaryota - ps6
Amorpheae - ps7
Obazoa - ps8
Opisthokonta - ps9
Holomycota - ps10
Fungi - ps11
Fungi_1 - ps12
Fungi_2 - ps13
Fungi_3 - ps14
Dikarya - ps15
Ascomycota - ps16
Saccharo/Pezizomycotina - ps17
Saccharomycotina - ps18
Saccharomycotina_1 - ps19
Saccharomycotina_2 - ps20
Saccharomycotina_3 - ps21
Phaff./Saccharomycetaceae - ps22
Saccharomyco./mycetaceae - ps23
Saccharomycetaceae - ps24
Saccharomycetaceae_2 - ps25
Saccharomycetaceae_3 - ps26
Kaz./Nau./Saccharomyces - ps27
Saccharomyces - ps28
Arboricola_clade - ps29
Cerevisiae_clade - ps30
Saccharomyces_cerevisiae - ps31
Phylostratum

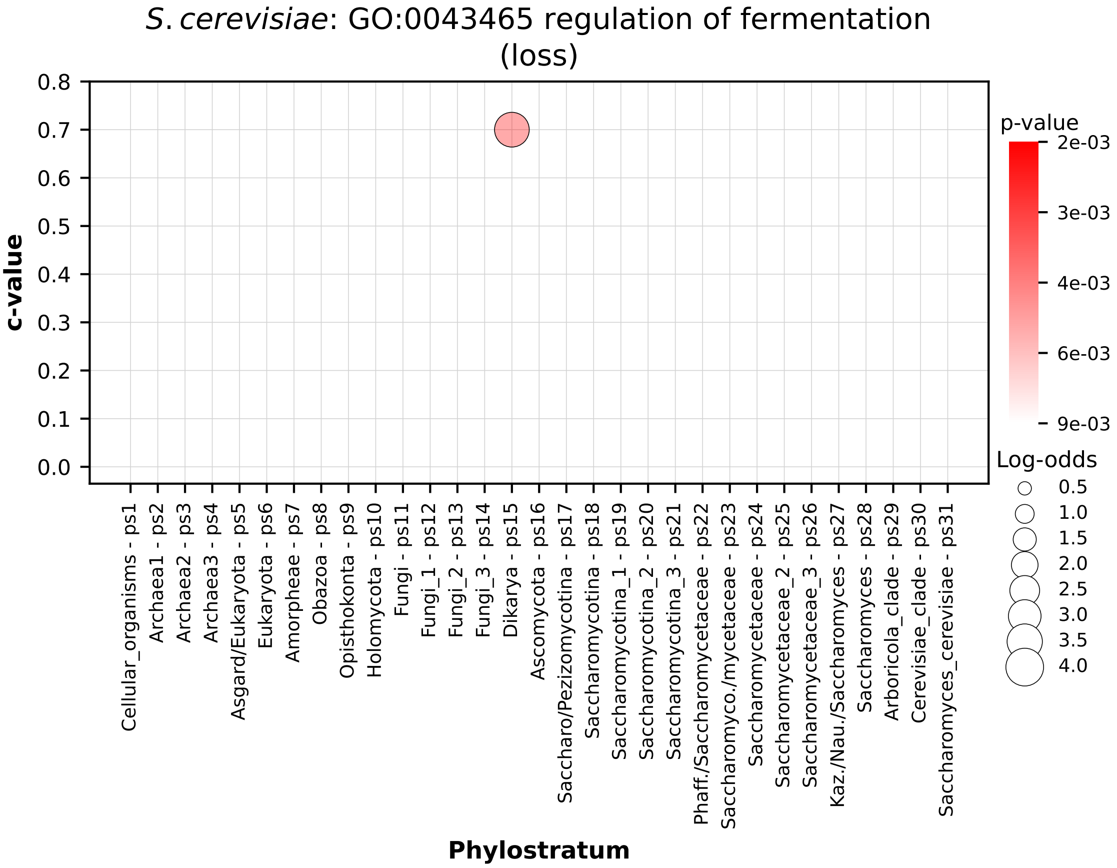

S. cerevisiae: GO:0043465 regulation of fermentation (loss)
c-value
0.8
0.7
0.6
0.5
0.4
0.3
0.2
0.1
0.0
p-value
2e-03
3e-03
4e-03
6e-03
9e-03
Log-odds
0.5
1.0
1.5
2.0
2.5
3.0
3.5
4.0
Cellular_organisms - ps1
Archaea1 - ps2
Archaea2 - ps3
Archaea3 - ps4
Asgard/Eukaryota - ps5
Eukaryota - ps6
Amorpheae - ps7
Obazoa - ps8
Opisthokonta - ps9
Holomycota - ps10
Fungi - ps11
Fungi_1 - ps12
Fungi_2 - ps13
Fungi_3 - ps14
Dikarya - ps15
Ascomycota - ps16
Saccharo/Pezizomycotina - ps17
Saccharomycotina - ps18
Saccharomycotina_1 - ps19
Saccharomycotina_2 - ps20
Saccharomycotina_3 - ps21
Phaff./Saccharomycetaceae - ps22
Saccharomyco./mycetaceae - ps23
Saccharomycetaceae - ps24
Saccharomycetaceae_2 - ps25
Saccharomycetaceae_3 - ps26
Kaz./Nau./Saccharomyces - ps27
Saccharomyces - ps28
Arboricola_clade - ps29
Cerevisiae_clade - ps30
Saccharomyces_cerevisiae - ps31
Phylostratum

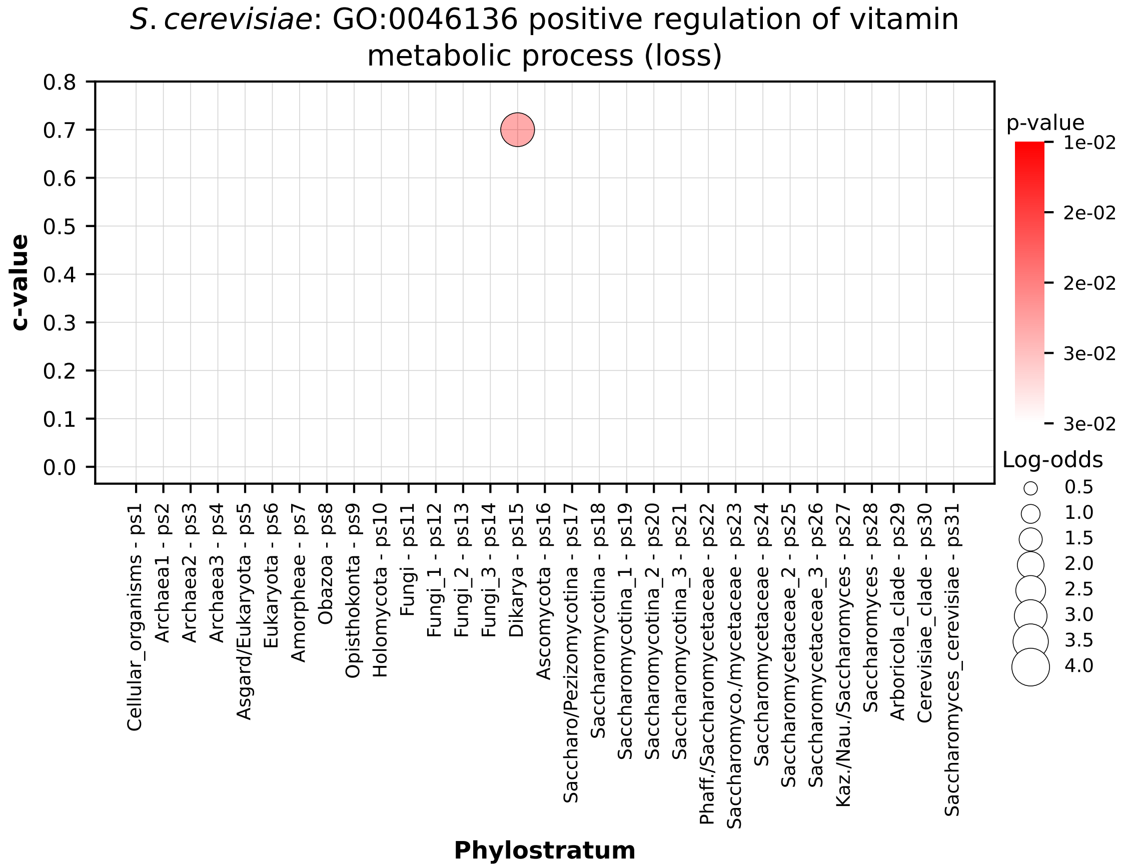

S. cerevisiae: GO:0046136 positive regulation of vitamin metabolic process (loss)
c-value
0.0
0.1
0.2
0.3
0.4
0.5
0.6
0.7
0.8
p-value
1e-02
2e-02
2e-02
3e-02
3e-02
Log-odds
0.5
1.0
1.5
2.0
2.5
3.0
3.5
4.0
Cellular_organisms - ps1
Archaea1 - ps2
Archaea2 - ps3
Archaea3 - ps4
Asgard/Eukaryota - ps5
Eukaryota - ps6
Amorpheae - ps7
Obazoa - ps8
Opisthokonta - ps9
Holomycota - ps10
Fungi - ps11
Fungi_1 - ps12
Fungi_2 - ps13
Fungi_3 - ps14
Dikarya - ps15
Ascomycota - ps16
Saccharo/Pezizomycotina - ps17
Saccharomycotina - ps18
Saccharomycotina_1 - ps19
Saccharomycotina_2 - ps20
Saccharomycotina_3 - ps21
Phaff./Saccharomycetaceae - ps22
Saccharomyco./mycetaceae - ps23
Saccharomycetaceae - ps24
Saccharomycetaceae_2 - ps25
Saccharomycetaceae_3 - ps26
Kaz./Nau./Saccharomyces - ps27
Saccharomyces - ps28
Arboricola_clade - ps29
Cerevisiae_clade - ps30
Saccharomyces_cerevisiae - ps31
Phylostratum

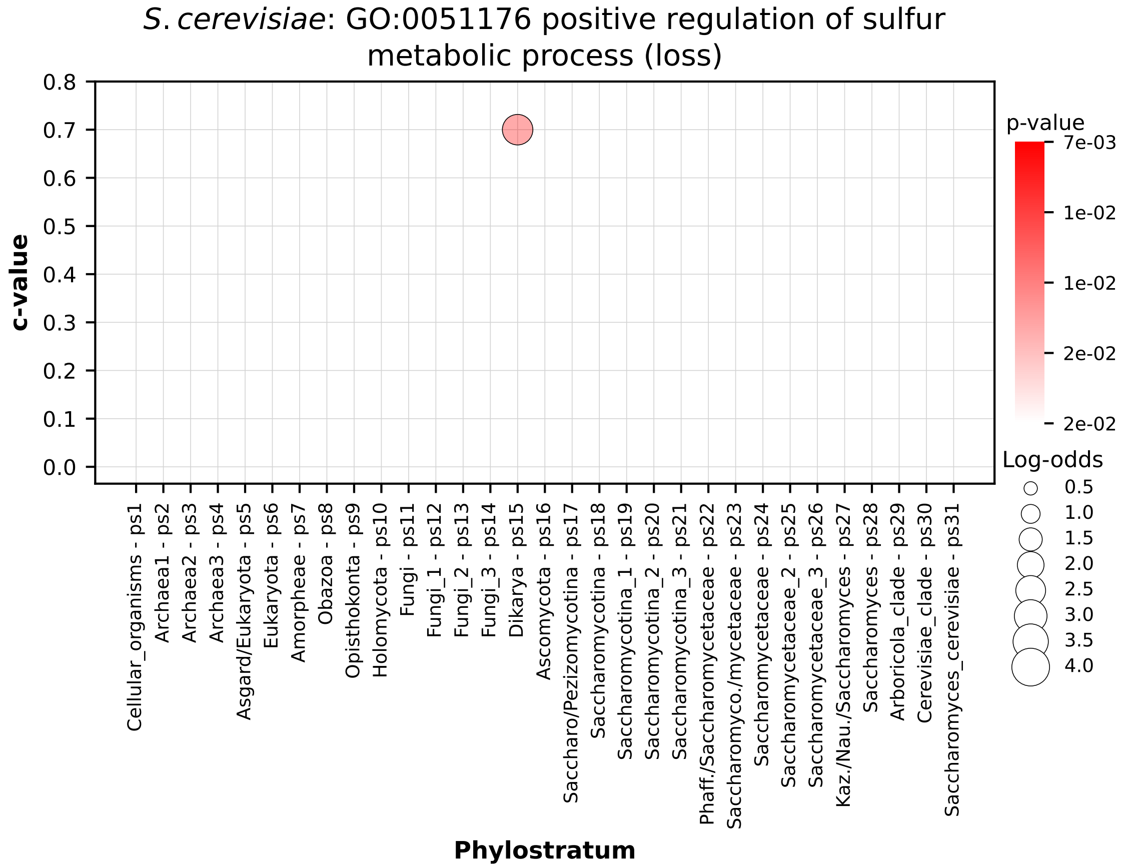
S. cerevisiae: GO:0051176 positive regulation of sulfur metabolic process (loss)
c-value
0.8
0.7
0.6
0.5
0.4
0.3
0.2
0.1
0.0
p-value
7e-03
1e-02
1e-02
2e-02
2e-02
Log-odds
0.5
1.0
1.5
2.0
2.5
3.0
3.5
4.0
Cellular_organisms - ps1
Archaea1 - ps2
Archaea2 - ps3
Archaea3 - ps4
Asgard/Eukaryota - ps5
Eukaryota - ps6
Amorpheae - ps7
Obazoa - ps8
Opisthokonta - ps9
Holomycota - ps10
Fungi - ps11
Fungi_1 - ps12
Fungi_2 - ps13
Fungi_3 - ps14
Dikarya - ps15
Ascomycota - ps16
Saccharo/Pezizomycotina - ps17
Saccharomycotina - ps18
Saccharomycotina_1 - ps19
Saccharomycotina_2 - ps20
Saccharomycotina_3 - ps21
Phaff./Saccharomycetaceae - ps22
Saccharomyco./mycetaceae - ps23
Saccharomycetaceae - ps24
Saccharomycetaceae_2 - ps25
Saccharomycetaceae_3 - ps26
Kaz./Nau./Saccharomyces - ps27
Saccharomyces - ps28
Arboricola_clade - ps29
Cerevisiae_clade - ps30
Saccharomyces_cerevisiae - ps31
Phylostratum

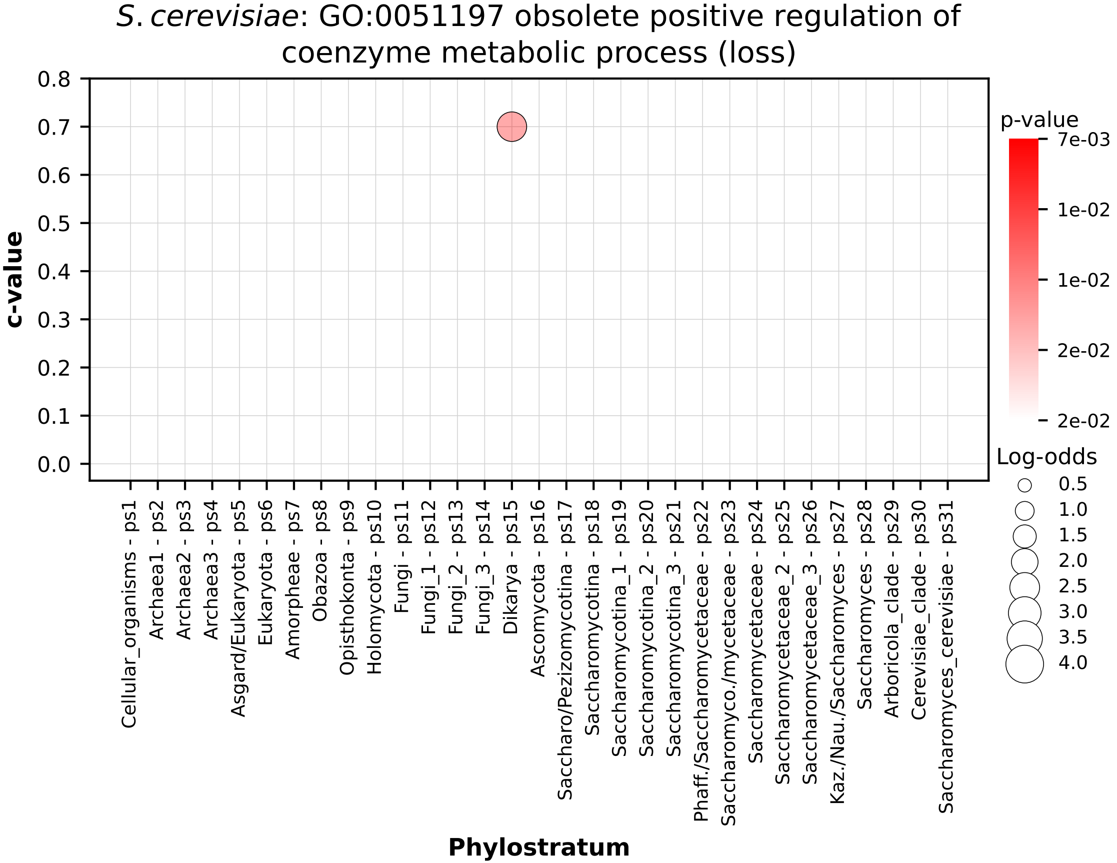

S. cerevisiae: GO:0051197 obsolete positive regulation of coenzyme metabolic process (loss)
c-value
0.8
0.7
0.6
0.5
0.4
0.3
0.2
0.1
0.0
p-value
7e-03
1e-02
1e-02
2e-02
2e-02
Log-odds
0.5
1.0
1.5
2.0
2.5
3.0
3.5
4.0
Cellular_organisms - ps1
Archaea1 - ps2
Archaea2 - ps3
Archaea3 - ps4
Asgard/Eukaryota - ps5
Eukaryota - ps6
Amorpheae - ps7
Obazoa - ps8
Opisthokonta - ps9
Holomycota - ps10
Fungi - ps11
Fungi_1 - ps12
Fungi_2 - ps13
Fungi_3 - ps14
Dikarya - ps15
Ascomycota - ps16
Saccharo/Pezizomycotina - ps17
Saccharomycotina - ps18
Saccharomycotina_1 - ps19
Saccharomycotina_2 - ps20
Saccharomycotina_3 - ps21
Phaff./Saccharomycetaceae - ps22
Saccharomyco./mycetaceae - ps23
Saccharomycetaceae - ps24
Saccharomycetaceae_2 - ps25
Saccharomycetaceae_3 - ps26
Kaz./Nau./Saccharomyces - ps27
Saccharomyces - ps28
Arboricola_clade - ps29
Cerevisiae_clade - ps30
Saccharomyces_cerevisiae - ps31
Phylostratum

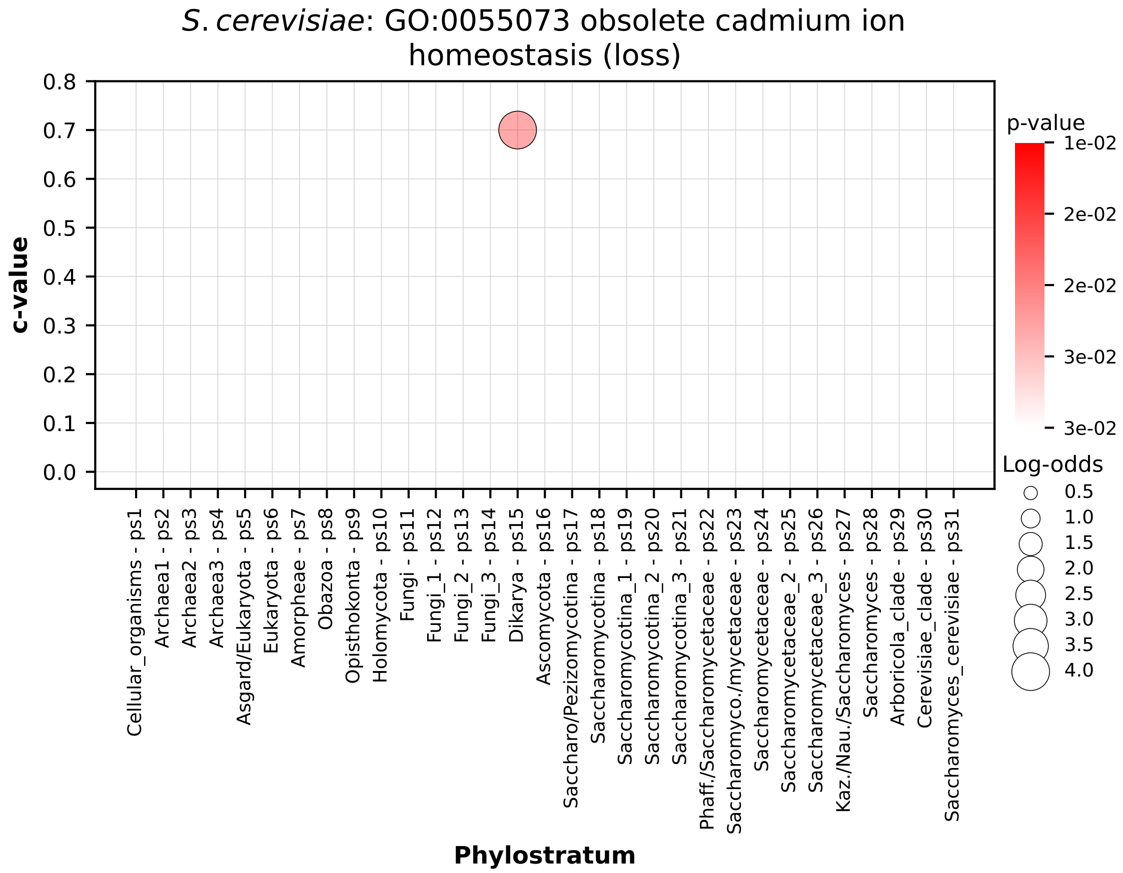

S. cerevisiae: GO:0055073 obsolete cadmium ion homeostasis (loss)
c-value
0.0
0.1
0.2
0.3
0.4
0.5
0.6
0.7
0.8
p-value
1e-02
2e-02
2e-02
3e-02
3e-02
Log-odds
0.5
1.0
1.5
2.0
2.5
3.0
3.5
4.0
Cellular_organisms - ps1
Archaea1 - ps2
Archaea2 - ps3
Archaea3 - ps4
Asgard/Eukaryota - ps5
Eukaryota - ps6
Amorpheae - ps7
Obazoa - ps8
Opisthokonta - ps9
Holomycota - ps10
Fungi - ps11
Fungi_1 - ps12
Fungi_2 - ps13
Fungi_3 - ps14
Dikarya - ps15
Ascomycota - ps16
Saccharo/Pezizomycotina - ps17
Saccharomycotina - ps18
Saccharomycotina_1 - ps19
Saccharomycotina_2 - ps20
Saccharomycotina_3 - ps21
Phaff./Saccharomycetaceae - ps22
Saccharomyco./mycetaceae - ps23
Saccharomycetaceae - ps24
Saccharomycetaceae_2 - ps25
Saccharomycetaceae_3 - ps26
Kaz./Nau./Saccharomyces - ps27
Saccharomyces - ps28
Arboricola_clade - ps29
Cerevisiae_clade - ps30
Saccharomyces_cerevisiae - ps31
Phylostratum

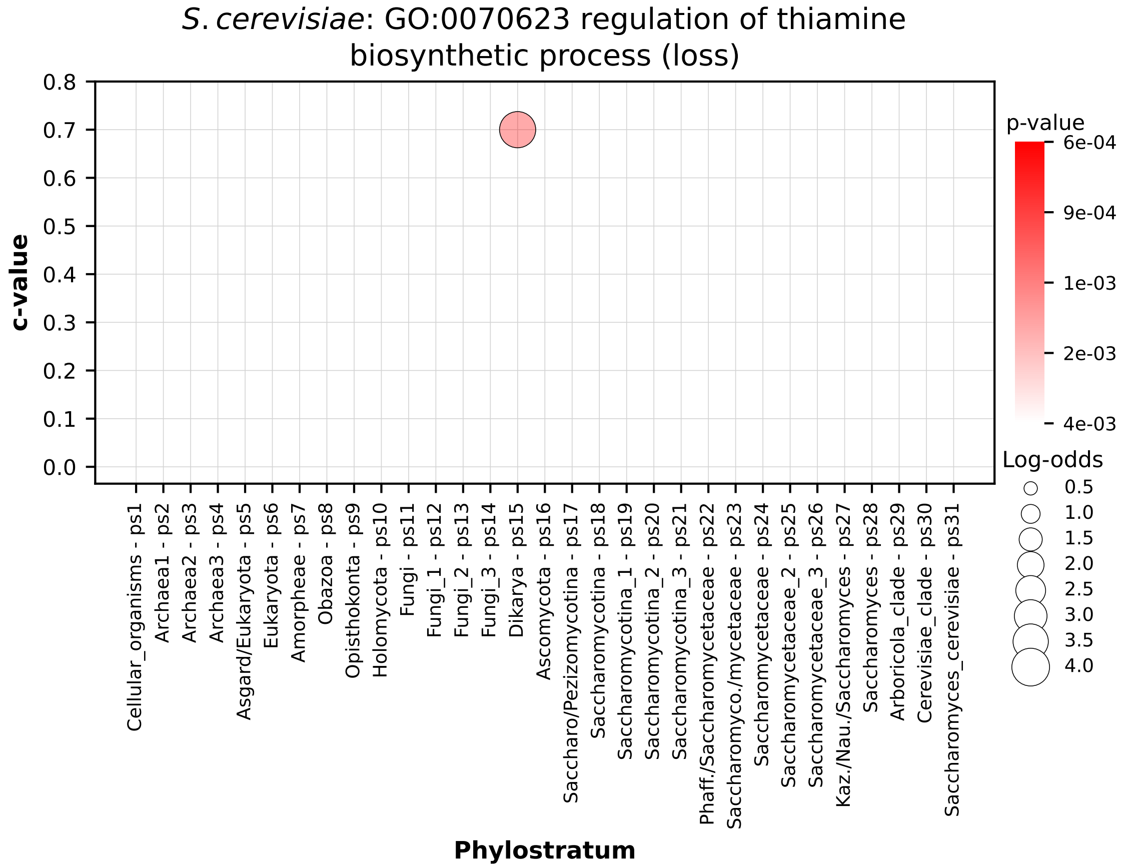

S. cerevisiae: GO:0070623 regulation of thiamine biosynthetic process (loss)
c-value
0.8
0.7
0.6
0.5
0.4
0.3
0.2
0.1
0.0
p-value
6e-04
9e-04
1e-03
2e-03
4e-03
Log-odds
0.5
1.0
1.5
2.0
2.5
3.0
3.5
4.0
Cellular_organisms - ps1
Archaea1 - ps2
Archaea2 - ps3
Archaea3 - ps4
Asgard/Eukaryota - ps5
Eukaryota - ps6
Amorpheae - ps7
Obazoa - ps8
Opisthokonta - ps9
Holomycota - ps10
Fungi - ps11
Fungi_1 - ps12
Fungi_2 - ps13
Fungi_3 - ps14
Dikarya - ps15
Ascomycota - ps16
Saccharo/Pezizomycotina - ps17
Saccharomycotina - ps18
Saccharomycotina_1 - ps19
Saccharomycotina_2 - ps20
Saccharomycotina_3 - ps21
Phaff./Saccharomycetaceae - ps22
Saccharomyco./mycetaceae - ps23
Saccharomycetaceae - ps24
Saccharomycetaceae_2 - ps25
Saccharomycetaceae_3 - ps26
Kaz./Nau./Saccharomyces - ps27
Saccharomyces - ps28
Arboricola_clade - ps29
Cerevisiae_clade - ps30
Saccharomyces_cerevisiae - ps31
Phylostratum

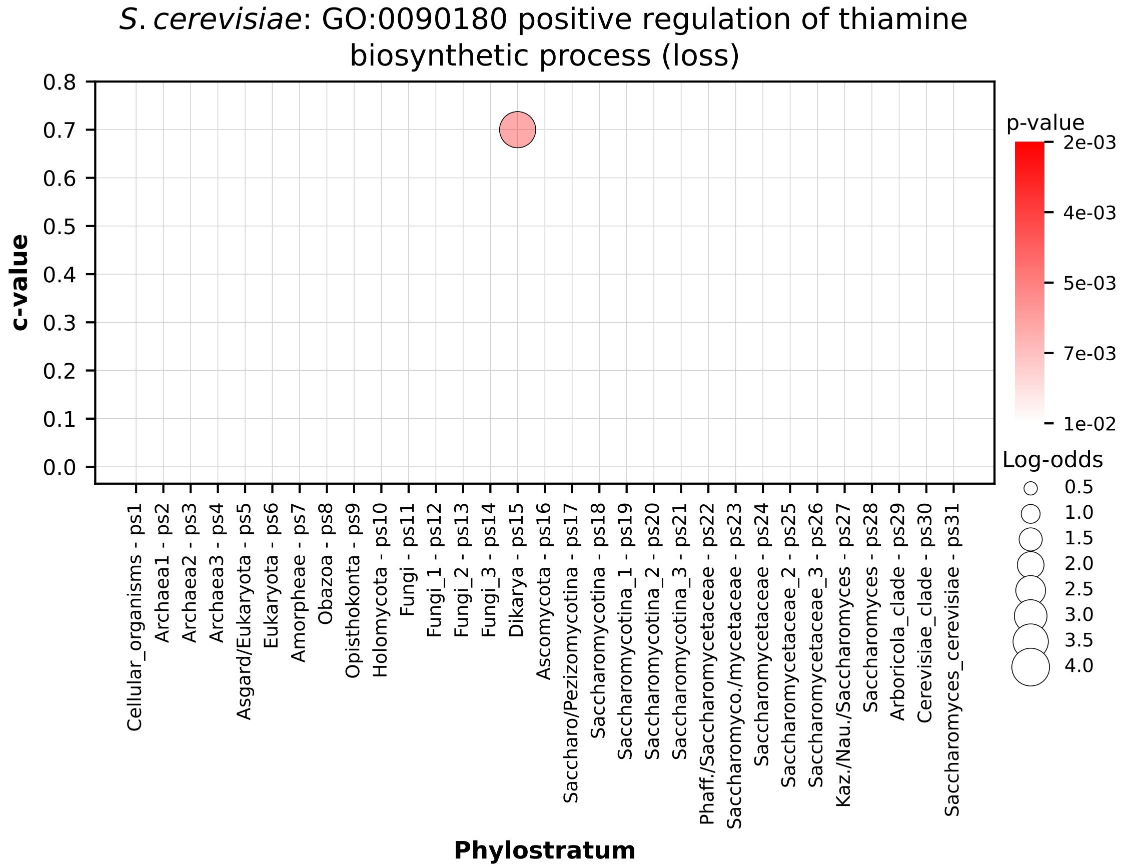

S. cerevisiae: GO:0090180 positive regulation of thiamine biosynthetic process (loss)
c-value
0.8
0.7
0.6
0.5
0.4
0.3
0.2
0.1
0.0
p-value
2e-03
4e-03
5e-03
7e-03
1e-02
Log-odds
0.5
1.0
1.5
2.0
2.5
3.0
3.5
4.0
Cellular_organisms - ps1
Archaea1 - ps2
Archaea2 - ps3
Archaea3 - ps4
Asgard/Eukaryota - ps5
Eukaryota - ps6
Amorpheae - ps7
Obazoa - ps8
Opisthokonta - ps9
Holomycota - ps10
Fungi - ps11
Fungi_1 - ps12
Fungi_2 - ps13
Fungi_3 - ps14
Dikarya - ps15
Ascomycota - ps16
Saccharo/Pezizomycotina - ps17
Saccharomycotina - ps18
Saccharomycotina_1 - ps19
Saccharomycotina_2 - ps20
Saccharomycotina_3 - ps21
Phaff./Saccharomycetaceae - ps22
Saccharomyco./mycetaceae - ps23
Saccharomycetaceae - ps24
Saccharomycetaceae_2 - ps25
Saccharomycetaceae_3 - ps26
Kaz./Nau./Saccharomyces - ps27
Saccharomyces - ps28
Arboricola_clade - ps29
Cerevisiae_clade - ps30
Saccharomyces_cerevisiae - ps31
Phylostratum

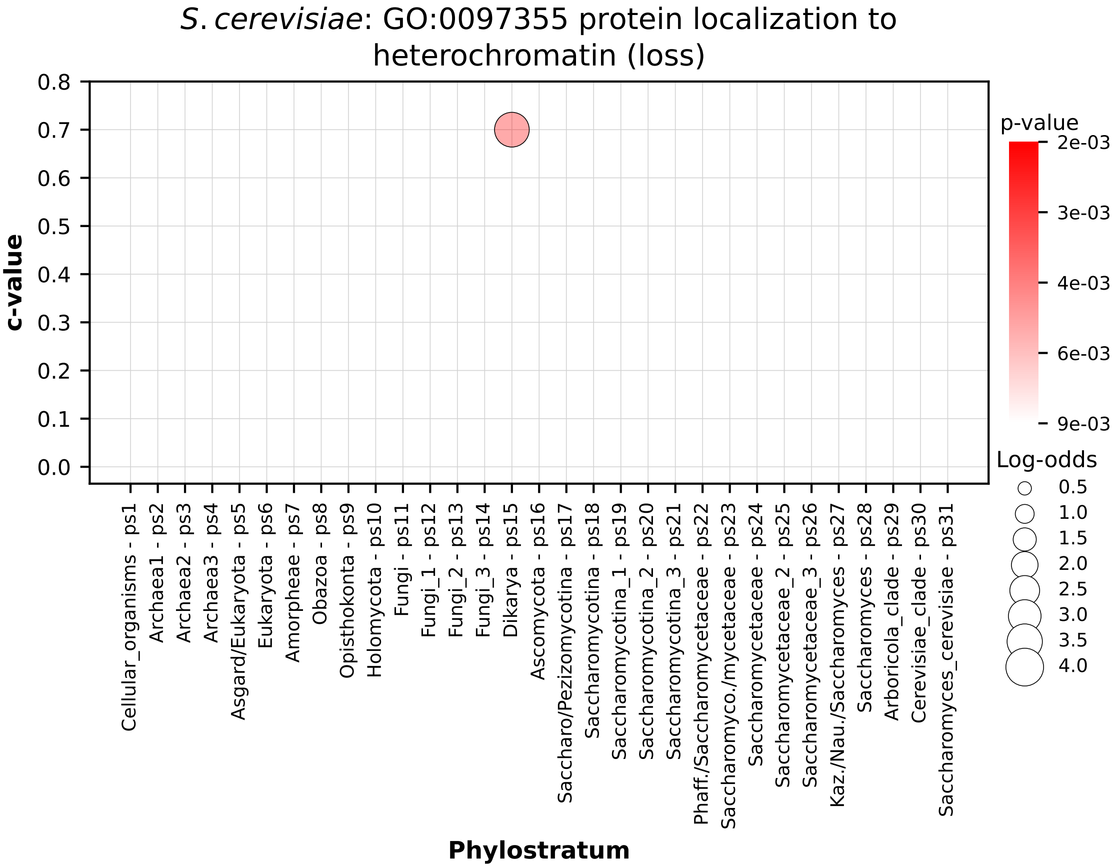

S. cerevisiae: GO:0097355 protein localization to heterochromatin (loss)
c-value
0.8
0.7
0.6
0.5
0.4
0.3
0.2
0.1
0.0
p-value
2e-03
3e-03
4e-03
6e-03
9e-03
Log-odds
0.5
1.0
1.5
2.0
2.5
3.0
3.5
4.0
Cellular_organisms - ps1
Archaea1 - ps2
Archaea2 - ps3
Archaea3 - ps4
Asgard/Eukaryota - ps5
Eukaryota - ps6
Amorpheae - ps7
Obazoa - ps8
Opisthokonta - ps9
Holomycota - ps10
Fungi - ps11
Fungi_1 - ps12
Fungi_2 - ps13
Fungi_3 - ps14
Dikarya - ps15
Ascomycota - ps16
Saccharo/Pezizomycotina - ps17
Saccharomycotina - ps18
Saccharomycotina_1 - ps19
Saccharomycotina_2 - ps20
Saccharomycotina_3 - ps21
Phaff./Saccharomycetaceae - ps22
Saccharomyco./mycetaceae - ps23
Saccharomycetaceae - ps24
Saccharomycetaceae_2 - ps25
Saccharomycetaceae_3 - ps26
Kaz./Nau./Saccharomyces - ps27
Saccharomyces - ps28
Arboricola_clade - ps29
Cerevisiae_clade - ps30
Saccharomyces_cerevisiae - ps31
Phylostratum

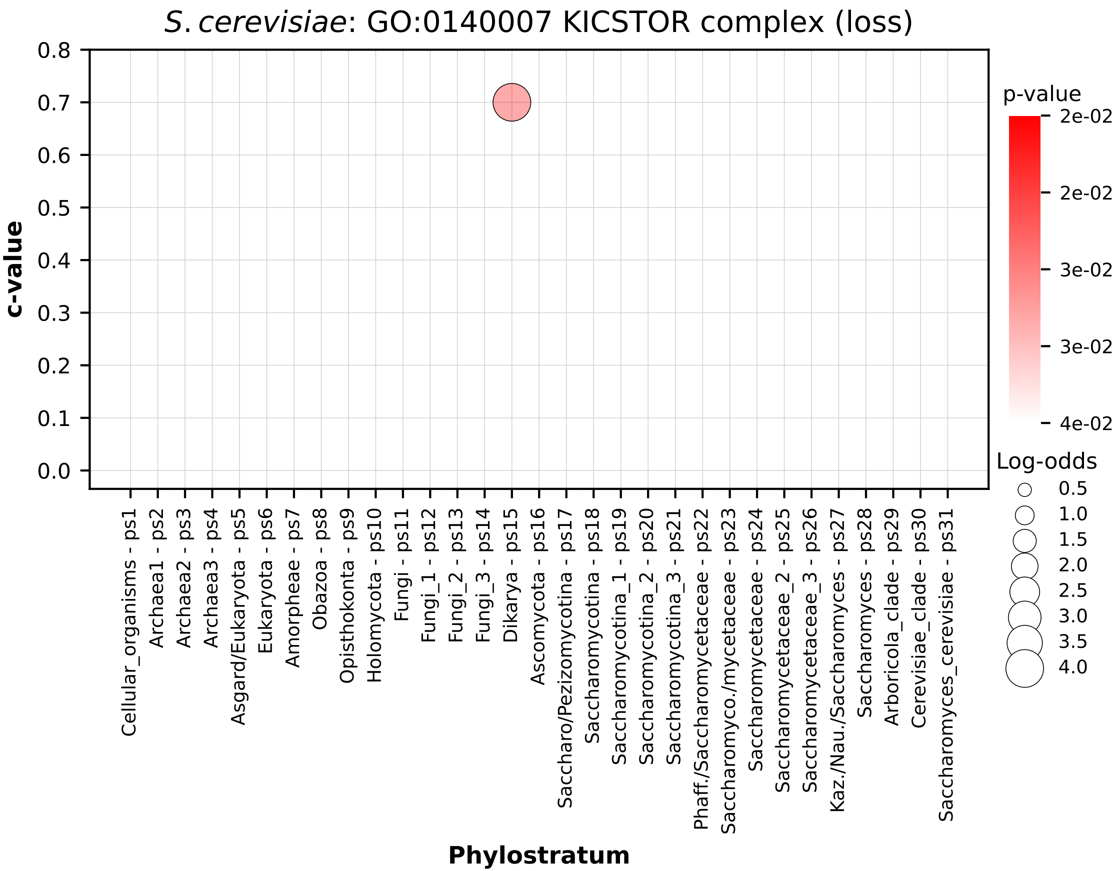
S. cerevisiae: GO:0140007 KICSTOR complex (loss)
c-value
0.8
0.7
0.6
0.5
0.4
0.3
0.2
0.1
0.0
p-value
2e-02
2e-02
3e-02
3e-02
4e-02
Log-odds
0.5
1.0
1.5
2.0
2.5
3.0
3.5
4.0
Cellular_organisms - ps1
Archaea1 - ps2
Archaea2 - ps3
Archaea3 - ps4
Asgard/Eukaryota - ps5
Eukaryota - ps6
Amorpheae - ps7
Obazoa - ps8
Opisthokonta - ps9
Holomycota - ps10
Fungi - ps11
Fungi_1 - ps12
Fungi_2 - ps13
Fungi_3 - ps14
Dikarya - ps15
Ascomycota - ps16
Saccharo/Pezizomycotina - ps17
Saccharomycotina - ps18
Saccharomycotina_1 - ps19
Saccharomycotina_2 - ps20
Saccharomycotina_3 - ps21
Phaff./Saccharomycetaceae - ps22
Saccharomyco./mycetaceae - ps23
Saccharomycetaceae - ps24
Saccharomycetaceae_2 - ps25
Saccharomycetaceae_3 - ps26
Kaz./Nau./Saccharomyces - ps27
Saccharomyces - ps28
Arboricola_clade - ps29
Cerevisiae_clade - ps30
Saccharomyces_cerevisiae - ps31
Phylostratum

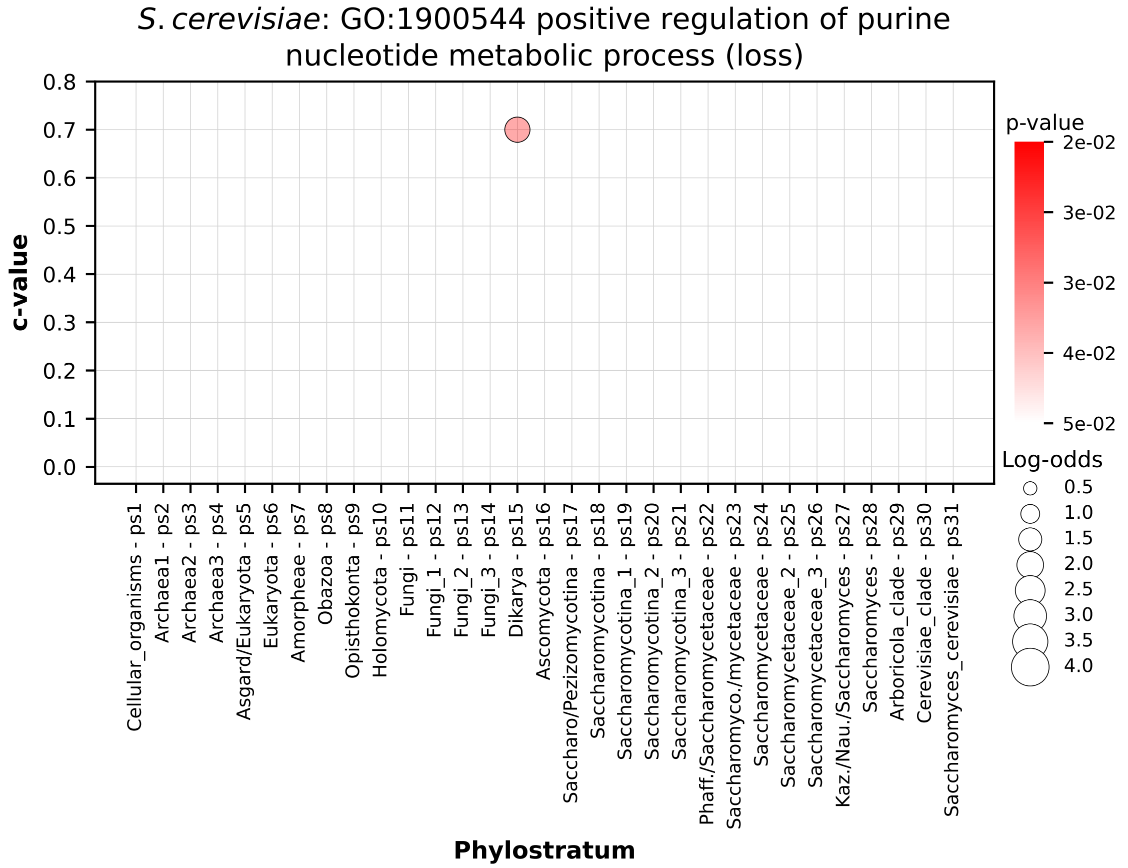

S. cerevisiae: GO:1900544 positive regulation of purine nucleotide metabolic process (loss)
c-value
0.8
0.7
0.6
0.5
0.4
0.3
0.2
0.1
0.0
Phylostratum
Cellular_organisms - ps1
Archaea1 - ps2
Archaea2 - ps3
Archaea3 - ps4
Asgard/Eukaryota - ps5
Eukaryota - ps6
Amorpheae - ps7
Obazoa - ps8
Opisthokonta - ps9
Holomycota - ps10
Fungi - ps11
Fungi_1 - ps12
Fungi_2 - ps13
Fungi_3 - ps14
Dikarya - ps15
Ascomycota - ps16
Saccharo/Pezizomycotina - ps17
Saccharomycotina - ps18
Saccharomycotina_1 - ps19
Saccharomycotina_2 - ps20
Saccharomycotina_3 - ps21
Phaff./Saccharomycetaceae - ps22
Saccharomyco./mycetaceae - ps23
Saccharomycetaceae - ps24
Saccharomycetaceae_2 - ps25
Saccharomycetaceae_3 - ps26
Kaz./Nau./Saccharomyces - ps27
Saccharomyces - ps28
Arboricola_clade - ps29
Cerevisiae_clade - ps30
Saccharomyces_cerevisiae - ps31
p-value
2e-02
3e-02
3e-02
4e-02
5e-02
Log-odds
0.5
1.0
1.5
2.0
2.5
3.0
3.5
4.0

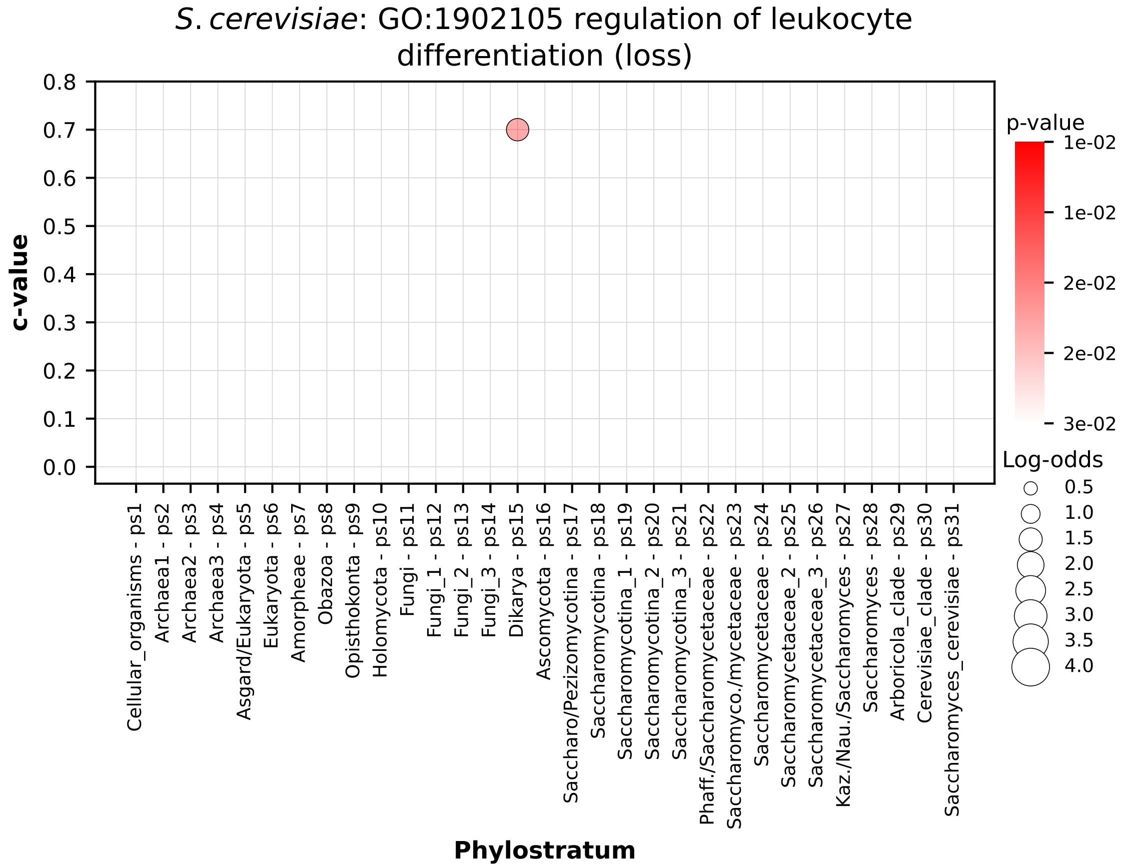

S. cerevisiae: GO:1902105 regulation of leukocyte differentiation (loss)
c-value
0.8
0.7
0.6
0.5
0.4
0.3
0.2
0.1
0.0
p-value
1e-02
1e-02
2e-02
2e-02
3e-02
Log-odds
0.5
1.0
1.5
2.0
2.5
3.0
3.5
4.0
Cellular_organisms - ps1
Archaea1 - ps2
Archaea2 - ps3
Archaea3 - ps4
Asgard/Eukaryota - ps5
Eukaryota - ps6
Amorpheae - ps7
Obazoa - ps8
Opisthokonta - ps9
Holomycota - ps10
Fungi - ps11
Fungi_1 - ps12
Fungi_2 - ps13
Fungi_3 - ps14
Dikarya - ps15
Ascomycota - ps16
Saccharo/Pezizomycotina - ps17
Saccharomycotina - ps18
Saccharomycotina_1 - ps19
Saccharomycotina_2 - ps20
Saccharomycotina_3 - ps21
Phaff./Saccharomycetaceae - ps22
Saccharomyco./mycetaceae - ps23
Saccharomycetaceae - ps24
Saccharomycetaceae_2 - ps25
Saccharomycetaceae_3 - ps26
Kaz./Nau./Saccharomyces - ps27
Saccharomyces - ps28
Arboricola_clade - ps29
Cerevisiae_clade - ps30
Saccharomyces_cerevisiae - ps31
Phylostratum

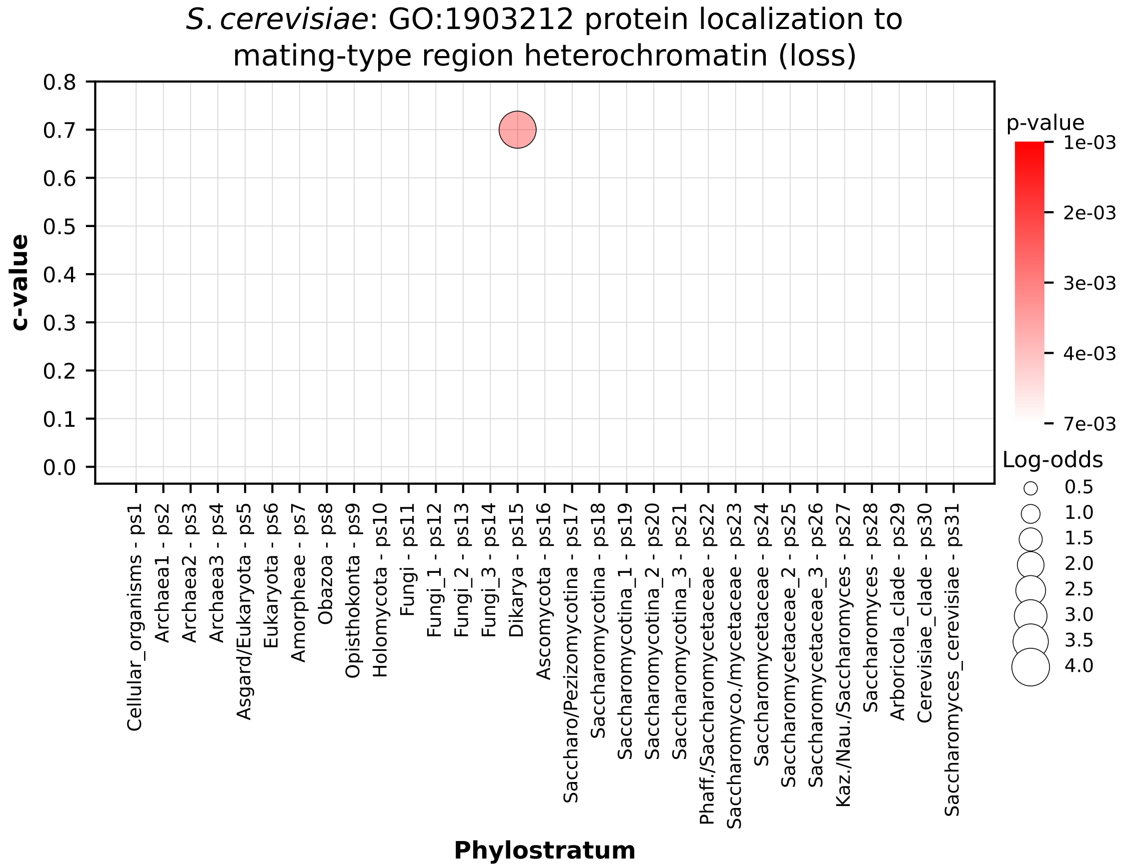

S. cerevisiae: GO:1903212 protein localization to mating-type region heterochromatin (loss)
c-value
0.8
0.7
0.6
0.5
0.4
0.3
0.2
0.1
0.0
p-value
1e-03
2e-03
3e-03
4e-03
7e-03
Log-odds
0.5
1.0
1.5
2.0
2.5
3.0
3.5
4.0
Cellular_organisms - ps1
Archaea1 - ps2
Archaea2 - ps3
Archaea3 - ps4
Asgard/Eukaryota - ps5
Eukaryota - ps6
Amorpheae - ps7
Obazoa - ps8
Opisthokonta - ps9
Holomycota - ps10
Fungi - ps11
Fungi_1 - ps12
Fungi_2 - ps13
Fungi_3 - ps14
Dikarya - ps15
Ascomycota - ps16
Saccharo/Pezizomycotina - ps17
Saccharomycotina - ps18
Saccharomycotina_1 - ps19
Saccharomycotina_2 - ps20
Saccharomycotina_3 - ps21
Phaff./Saccharomycetaceae - ps22
Saccharomyco./mycetaceae - ps23
Saccharomycetaceae - ps24
Saccharomycetaceae_2 - ps25
Saccharomycetaceae_3 - ps26
Kaz./Nau./Saccharomyces - ps27
Saccharomyces - ps28
Arboricola_clade - ps29
Cerevisiae_clade - ps30
Saccharomyces_cerevisiae - ps31
Phylostratum

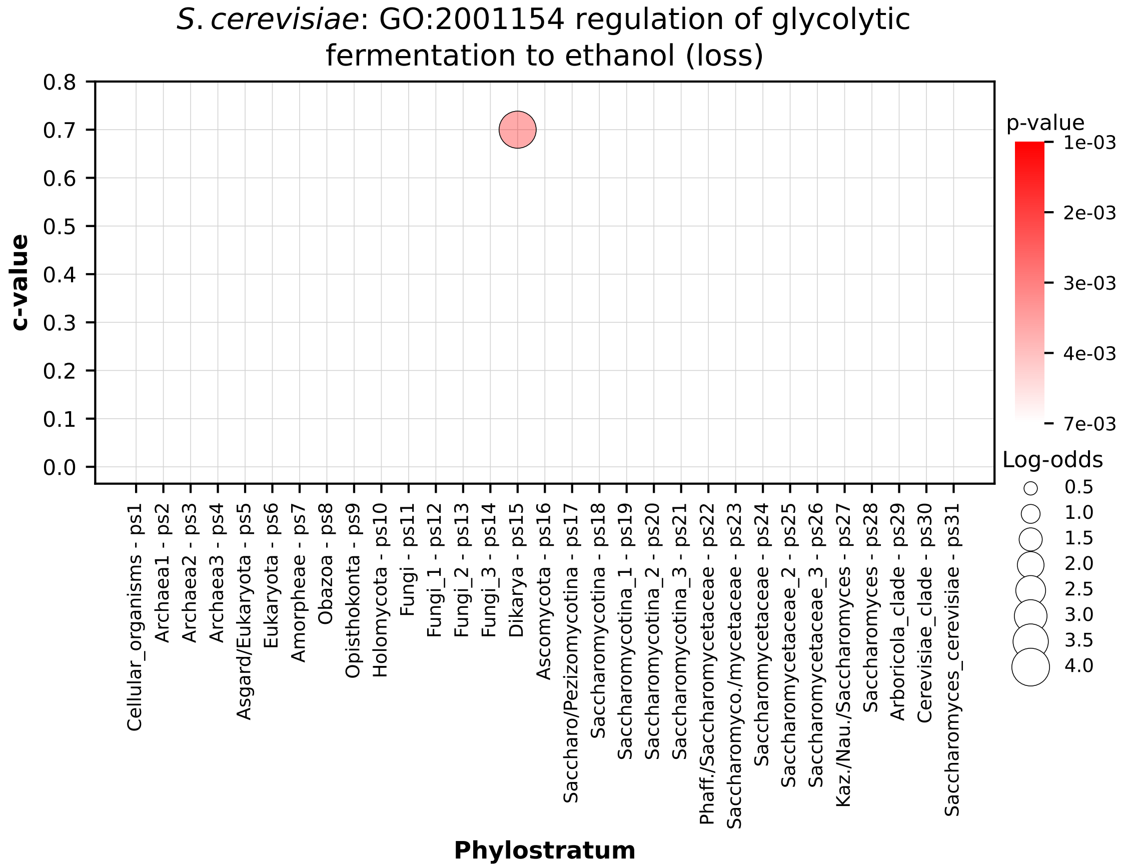

S. cerevisiae: GO:2001154 regulation of glycolytic fermentation to ethanol (loss)
c-value
0.8
0.7
0.6
0.5
0.4
0.3
0.2
0.1
0.0
p-value
1e-03
2e-03
3e-03
4e-03
7e-03
Log-odds
0.5
1.0
1.5
2.0
2.5
3.0
3.5
4.0
Cellular_organisms - ps1
Archaea1 - ps2
Archaea2 - ps3
Archaea3 - ps4
Asgard/Eukaryota - ps5
Eukaryota - ps6
Amorpheae - ps7
Obazoa - ps8
Opisthokonta - ps9
Holomycota - ps10
Fungi - ps11
Fungi_1 - ps12
Fungi_2 - ps13
Fungi_3 - ps14
Dikarya - ps15
Ascomycota - ps16
Saccharo/Pezizomycotina - ps17
Saccharomycotina - ps18
Saccharomycotina_1 - ps19
Saccharomycotina_2 - ps20
Saccharomycotina_3 - ps21
Phaff./Saccharomycetaceae - ps22
Saccharomyco./mycetaceae - ps23
Saccharomycetaceae - ps24
Saccharomycetaceae_2 - ps25
Saccharomycetaceae_3 - ps26
Kaz./Nau./Saccharomyces - ps27
Saccharomyces - ps28
Arboricola_clade - ps29
Cerevisiae_clade - ps30
Saccharomyces_cerevisiae - ps31
Phylostratum

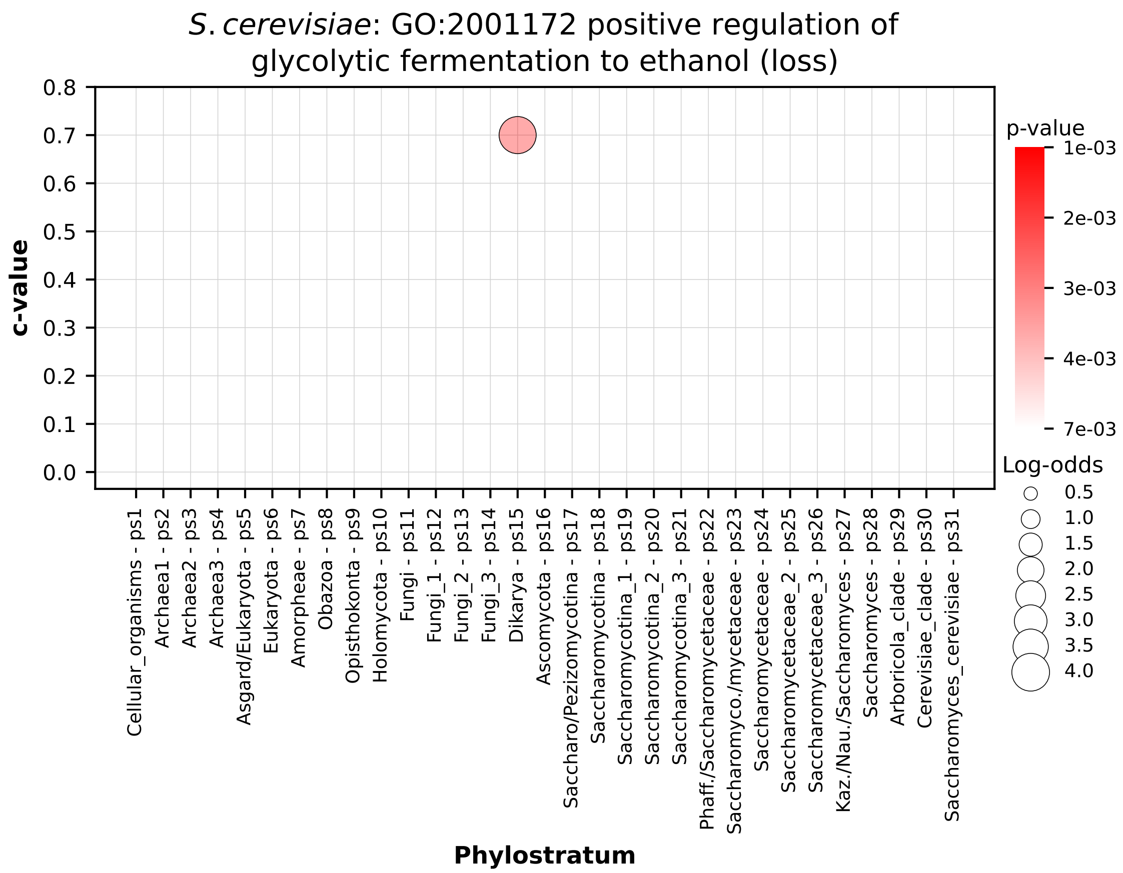

S. cerevisiae: GO:2001172 positive regulation of glycolytic fermentation to ethanol (loss)
c-value
0.8
0.7
0.6
0.5
0.4
0.3
0.2
0.1
0.0
Phylostratum
Cellular_organisms - ps1
Archaea1 - ps2
Archaea2 - ps3
Archaea3 - ps4
Asgard/Eukaryota - ps5
Eukaryota - ps6
Amorpheae - ps7
Obazoa - ps8
Opisthokonta - ps9
Holomycota - ps10
Fungi - ps11
Fungi_1 - ps12
Fungi_2 - ps13
Fungi_3 - ps14
Dikarya - ps15
Ascomycota - ps16
Saccharo/Pezizomycotina - ps17
Saccharomycotina - ps18
Saccharomycotina_1 - ps19
Saccharomycotina_2 - ps20
Saccharomycotina_3 - ps21
Phaff./Saccharomycetaceae - ps22
Saccharomyco./mycetaceae - ps23
Saccharomycetaceae - ps24
Saccharomycetaceae_2 - ps25
Saccharomycetaceae_3 - ps26
Kaz./Nau./Saccharomyces - ps27
Saccharomyces - ps28
Arboricola_clade - ps29
Cerevisiae_clade - ps30
Saccharomyces_cerevisiae - ps31
p-value
1e-03
2e-03
3e-03
4e-03
7e-03
Log-odds
0.5
1.0
1.5
2.0
2.5
3.0
3.5
4.0

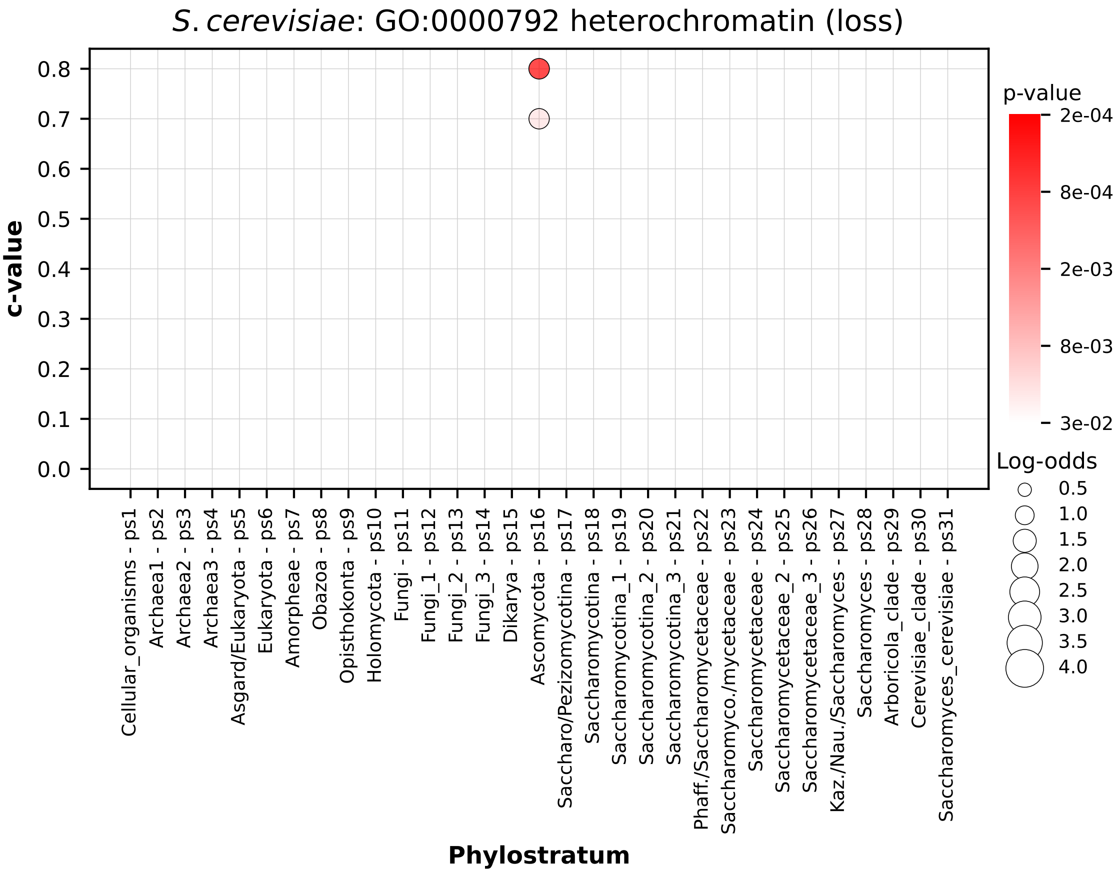

S. cerevisiae: GO:0000792 heterochromatin (loss)
c-value
0.0
0.1
0.2
0.3
0.4
0.5
0.6
0.7
0.8
p-value
2e-04
8e-04
2e-03
8e-03
3e-02
Log-odds
0.5
1.0
1.5
2.0
2.5
3.0
3.5
4.0
Cellular_organisms - ps1
Archaea1 - ps2
Archaea2 - ps3
Archaea3 - ps4
Asgard/Eukaryota - ps5
Eukaryota - ps6
Amorpheae - ps7
Obazoa - ps8
Opisthokonta - ps9
Holomycota - ps10
Fungi - ps11
Fungi_1 - ps12
Fungi_2 - ps13
Fungi_3 - ps14
Dikarya - ps15
Ascomycota - ps16
Saccharo/Pezizomycotina - ps17
Saccharomycotina - ps18
Saccharomycotina_1 - ps19
Saccharomycotina_2 - ps20
Saccharomycotina_3 - ps21
Phaff./Saccharomycetaceae - ps22
Saccharomyco./mycetaceae - ps23
Saccharomycetaceae - ps24
Saccharomycetaceae_2 - ps25
Saccharomycetaceae_3 - ps26
Kaz./Nau./Saccharomyces - ps27
Saccharomyces - ps28
Arboricola_clade - ps29
Cerevisiae_clade - ps30
Saccharomyces_cerevisiae - ps31
Phylostratum

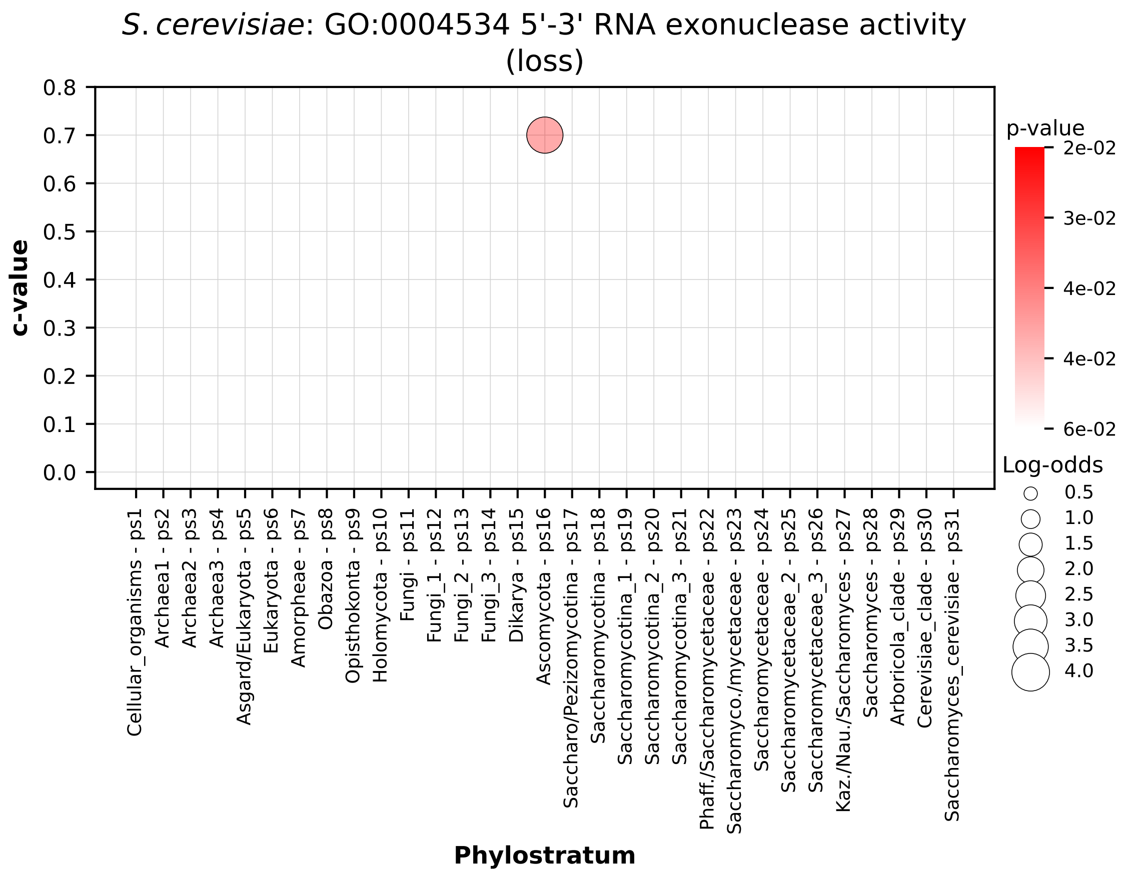
S. cerevisiae: GO:0004534 5'-3' RNA exonuclease activity (loss)
c-value
0.8
0.7
0.6
0.5
0.4
0.3
0.2
0.1
0.0
Phylostratum
Cellular_organisms - ps1
Archaea1 - ps2
Archaea2 - ps3
Archaea3 - ps4
Asgard/Eukaryota - ps5
Eukaryota - ps6
Amorpheae - ps7
Obazoa - ps8
Opisthokonta - ps9
Holomycota - ps10
Fungi - ps11
Fungi_1 - ps12
Fungi_2 - ps13
Fungi_3 - ps14
Dikarya - ps15
Ascomycota - ps16
Saccharo/Pezizomycotina - ps17
Saccharomycotina - ps18
Saccharomycotina_1 - ps19
Saccharomycotina_2 - ps20
Saccharomycotina_3 - ps21
Phaff./Saccharomycetaceae - ps22
Saccharomyco./mycetaceae - ps23
Saccharomycetaceae - ps24
Saccharomycetaceae_2 - ps25
Saccharomycetaceae_3 - ps26
Kaz./Nau./Saccharomyces - ps27
Saccharomyces - ps28
Arboricola_clade - ps29
Cerevisiae_clade - ps30
Saccharomyces_cerevisiae - ps31
p-value
2e-02
3e-02
4e-02
4e-02
6e-02
Log-odds
0.5
1.0
1.5
2.0
2.5
3.0
3.5
4.0

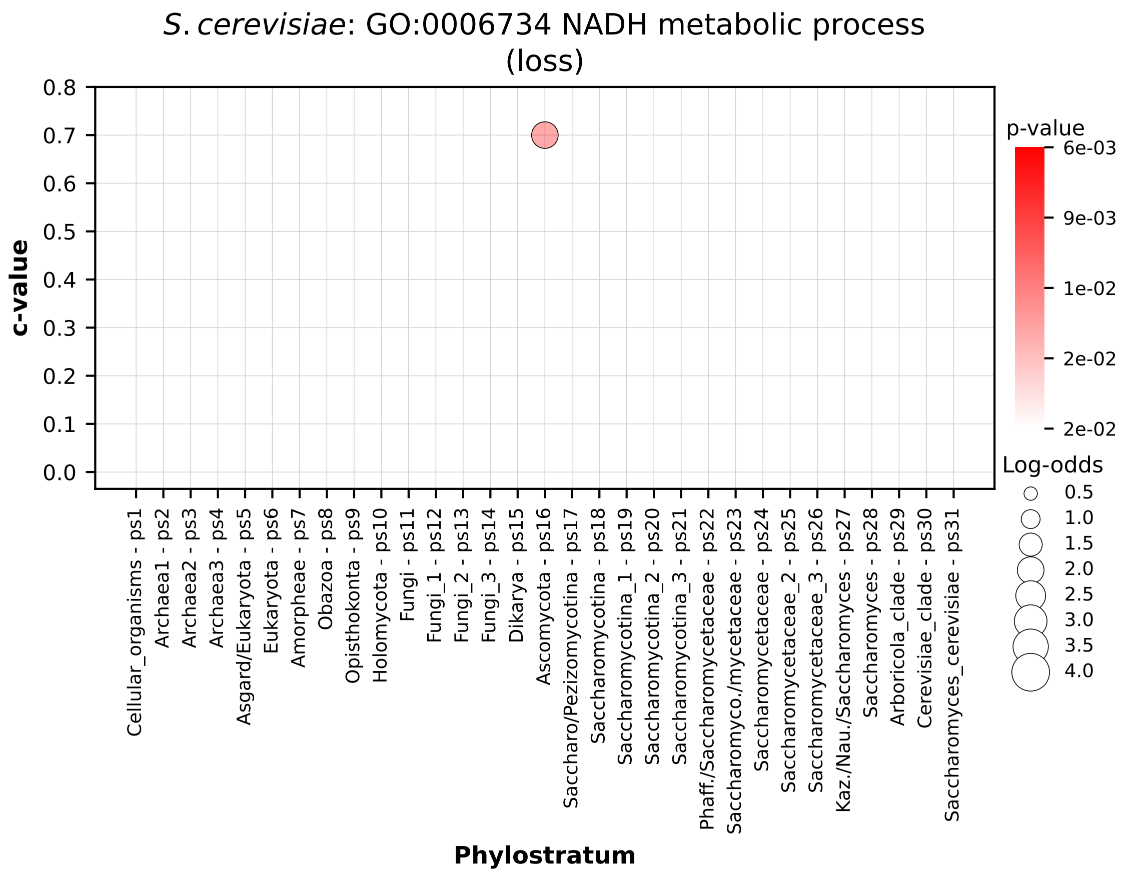
S. cerevisiae: GO:0006734 NADH metabolic process
(loss)
c-value
0.8
0.7
0.6
0.5
0.4
0.3
0.2
0.1
0.0
p-value
6e-03
9e-03
1e-02
2e-02
2e-02
Log-odds
0.5
1.0
1.5
2.0
2.5
3.0
3.5
4.0
Cellular_organisms - ps1
Archaea1 - ps2
Archaea2 - ps3
Archaea3 - ps4
Asgard/Eukaryota - ps5
Eukaryota - ps6
Amorpheae - ps7
Obazoa - ps8
Opisthokonta - ps9
Holomycota - ps10
Fungi - ps11
Fungi_1 - ps12
Fungi_2 - ps13
Fungi_3 - ps14
Dikarya - ps15
Ascomycota - ps16
Saccharo/Pezizomycotina - ps17
Saccharomycotina - ps18
Saccharomycotina_1 - ps19
Saccharomycotina_2 - ps20
Saccharomycotina_3 - ps21
Phaff./Saccharomycetaceae - ps22
Saccharomyco./mycetaceae - ps23
Saccharomycetaceae - ps24
Saccharomycetaceae_2 - ps25
Saccharomycetaceae_3 - ps26
Kaz./Nau./Saccharomyces - ps27
Saccharomyces - ps28
Arboricola_clade - ps29
Cerevisiae_clade - ps30
Saccharomyces_cerevisiae - ps31
Phylostratum

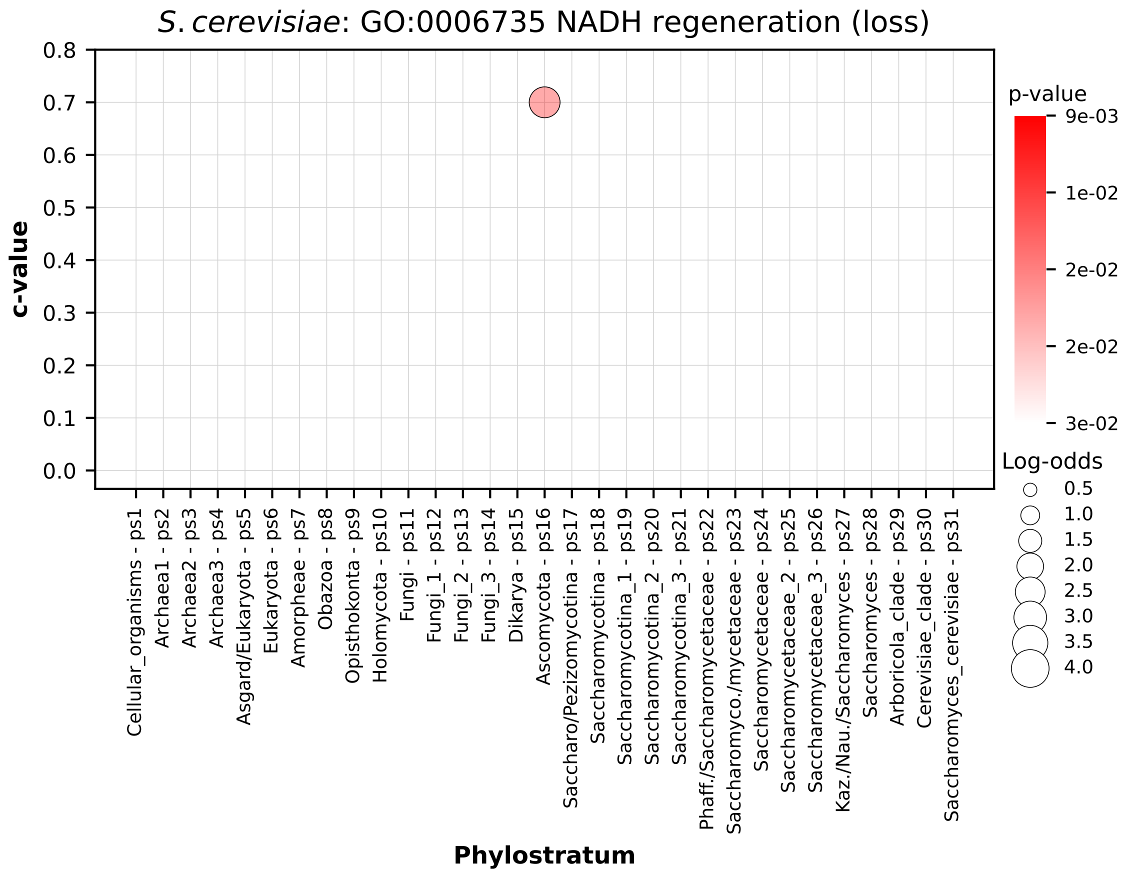

S. cerevisiae: GO:0006735 NADH regeneration (loss)
c-value
0.8
0.7
0.6
0.5
0.4
0.3
0.2
0.1
0.0
p-value
9e-03
1e-02
2e-02
2e-02
3e-02
Log-odds
0.5
1.0
1.5
2.0
2.5
3.0
3.5
4.0
Cellular_organisms - ps1
Archaea1 - ps2
Archaea2 - ps3
Archaea3 - ps4
Asgard/Eukaryota - ps5
Eukaryota - ps6
Amorpheae - ps7
Obazoa - ps8
Opisthokonta - ps9
Holomycota - ps10
Fungi - ps11
Fungi_1 - ps12
Fungi_2 - ps13
Fungi_3 - ps14
Dikarya - ps15
Ascomycota - ps16
Saccharo/Pezizomycotina - ps17
Saccharomycotina - ps18
Saccharomycotina_1 - ps19
Saccharomycotina_2 - ps20
Saccharomycotina_3 - ps21
Phaff./Saccharomycetaceae - ps22
Saccharomyco./mycetaceae - ps23
Saccharomycetaceae - ps24
Saccharomycetaceae_2 - ps25
Saccharomycetaceae_3 - ps26
Kaz./Nau./Saccharomyces - ps27
Saccharomyces - ps28
Arboricola_clade - ps29
Cerevisiae_clade - ps30
Saccharomyces_cerevisiae - ps31
Phylostratum

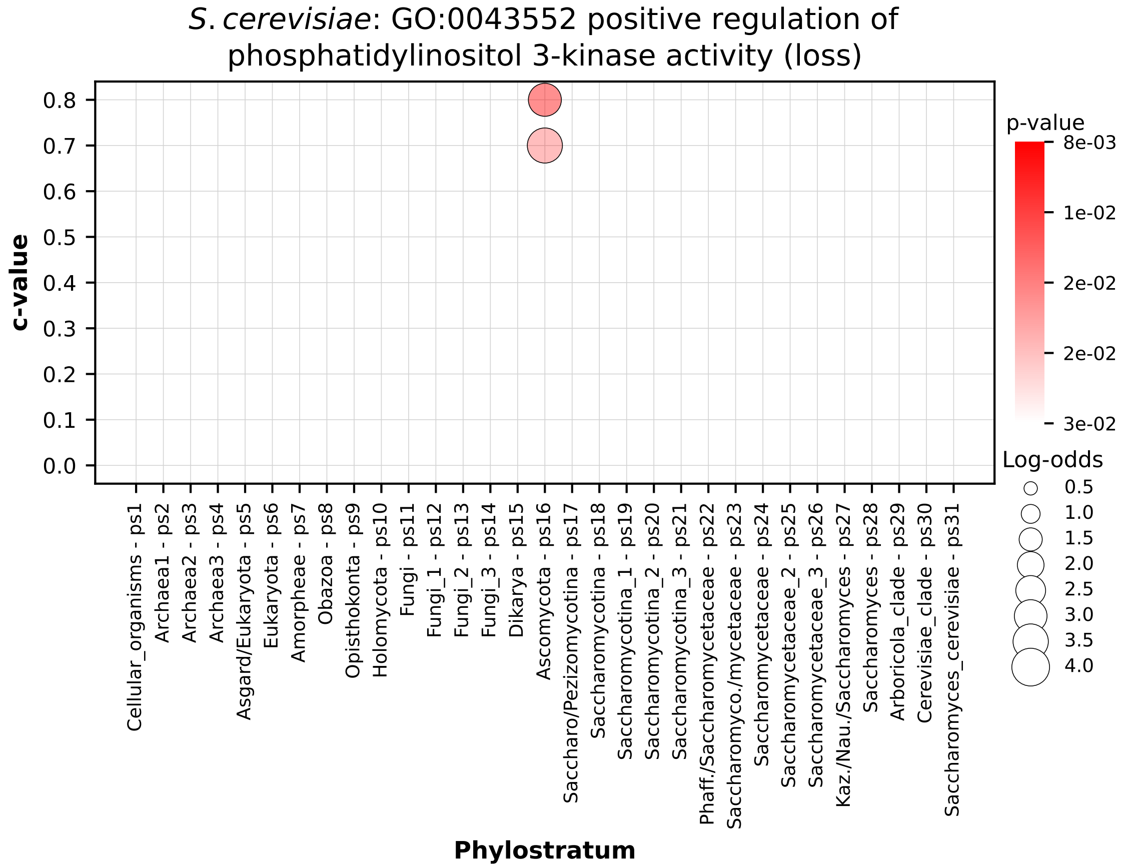

S. cerevisiae: GO:0043552 positive regulation of phosphatidylinositol 3-kinase activity (loss)
c-value
0.8
0.7
0.6
0.5
0.4
0.3
0.2
0.1
0.0
p-value
8e-03
1e-02
2e-02
2e-02
3e-02
Log-odds
0.5
1.0
1.5
2.0
2.5
3.0
3.5
4.0
Cellular_organisms - ps1
Archaea1 - ps2
Archaea2 - ps3
Archaea3 - ps4
Asgard/Eukaryota - ps5
Eukaryota - ps6
Amorpheae - ps7
Obazoa - ps8
Opisthokonta - ps9
Holomycota - ps10
Fungi - ps11
Fungi_1 - ps12
Fungi_2 - ps13
Fungi_3 - ps14
Dikarya - ps15
Ascomycota - ps16
Saccharo/Pezizomycotina - ps17
Saccharomycotina - ps18
Saccharomycotina_1 - ps19
Saccharomycotina_2 - ps20
Saccharomycotina_3 - ps21
Phaff./Saccharomycetaceae - ps22
Saccharomyco./mycetaceae - ps23
Saccharomycetaceae - ps24
Saccharomycetaceae_2 - ps25
Saccharomycetaceae_3 - ps26
Kaz./Nau./Saccharomyces - ps27
Saccharomyces - ps28
Arboricola_clade - ps29
Cerevisiae_clade - ps30
Saccharomyces_cerevisiae - ps31
Phylostratum

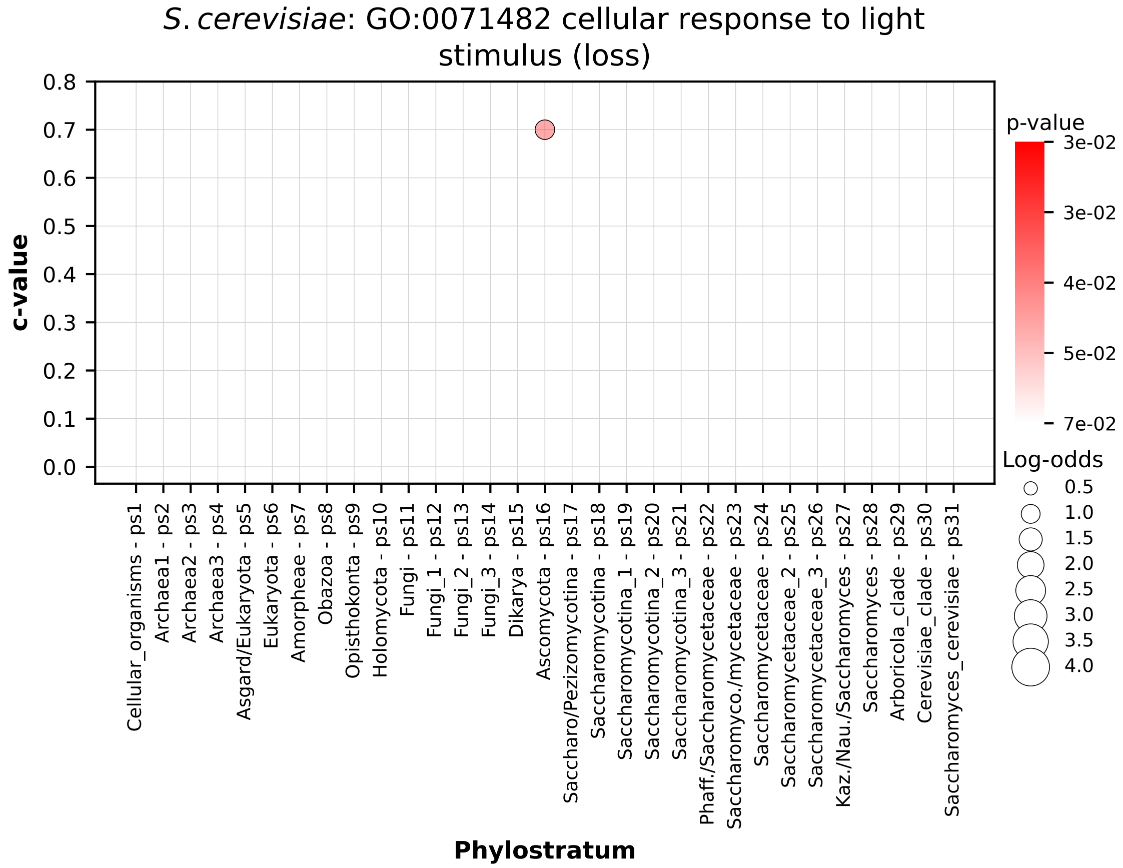

S. cerevisiae: GO:0071482 cellular response to light stimulus (loss)
c-value
0.8
0.7
0.6
0.5
0.4
0.3
0.2
0.1
0.0
p-value
3e-02
3e-02
4e-02
5e-02
7e-02
Log-odds
0.5
1.0
1.5
2.0
2.5
3.0
3.5
4.0
Cellular_organisms - ps1
Archaea1 - ps2
Archaea2 - ps3
Archaea3 - ps4
Asgard/Eukaryota - ps5
Eukaryota - ps6
Amorpheae - ps7
Obazoa - ps8
Opisthokonta - ps9
Holomycota - ps10
Fungi - ps11
Fungi_1 - ps12
Fungi_2 - ps13
Fungi_3 - ps14
Dikarya - ps15
Ascomycota - ps16
Saccharo/Pezizomycotina - ps17
Saccharomycotina - ps18
Saccharomycotina_1 - ps19
Saccharomycotina_2 - ps20
Saccharomycotina_3 - ps21
Phaff./Saccharomycetaceae - ps22
Saccharomyco./mycetaceae - ps23
Saccharomycetaceae - ps24
Saccharomycetaceae_2 - ps25
Saccharomycetaceae_3 - ps26
Kaz./Nau./Saccharomyces - ps27
Saccharomyces - ps28
Arboricola_clade - ps29
Cerevisiae_clade - ps30
Saccharomyces_cerevisiae - ps31
Phylostratum

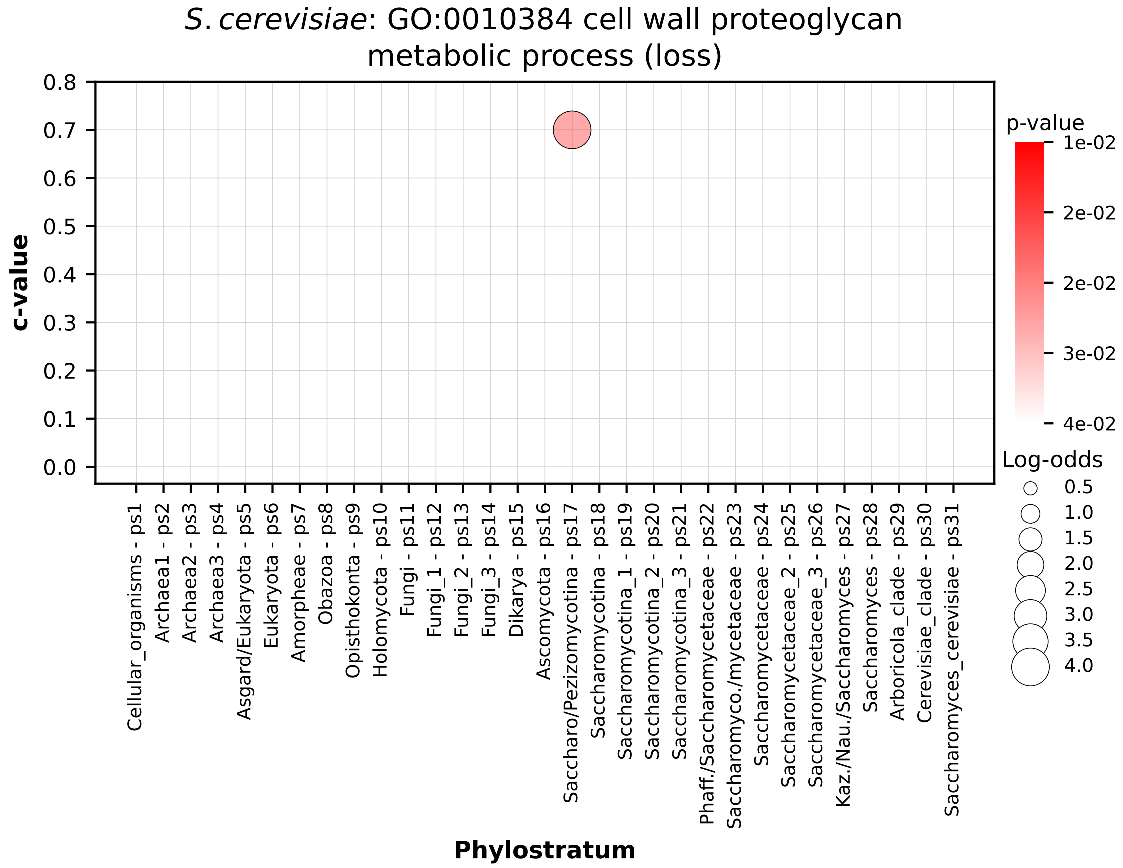

S. cerevisiae: GO:0010384 cell wall proteoglycan metabolic process (loss)
c-value
0.8
0.7
0.6
0.5
0.4
0.3
0.2
0.1
0.0
Phylostratum
Cellular_organisms - ps1
Archaea1 - ps2
Archaea2 - ps3
Archaea3 - ps4
Asgard/Eukaryota - ps5
Eukaryota - ps6
Amorpheae - ps7
Obazoa - ps8
Opisthokonta - ps9
Holomycota - ps10
Fungi - ps11
Fungi_1 - ps12
Fungi_2 - ps13
Fungi_3 - ps14
Dikarya - ps15
Ascomycota - ps16
Saccharo/Pezizomycotina - ps17
Saccharomycotina - ps18
Saccharomycotina_1 - ps19
Saccharomycotina_2 - ps20
Saccharomycotina_3 - ps21
Phaff./Saccharomycetaceae - ps22
Saccharomyco./mycetaceae - ps23
Saccharomycetaceae - ps24
Saccharomycetaceae_2 - ps25
Saccharomycetaceae_3 - ps26
Kaz./Nau./Saccharomyces - ps27
Saccharomyces - ps28
Arboricola_clade - ps29
Cerevisiae_clade - ps30
Saccharomyces_cerevisiae - ps31
p-value
1e-02
2e-02
2e-02
3e-02
4e-02
Log-odds
0.5
1.0
1.5
2.0
2.5
3.0
3.5
4.0

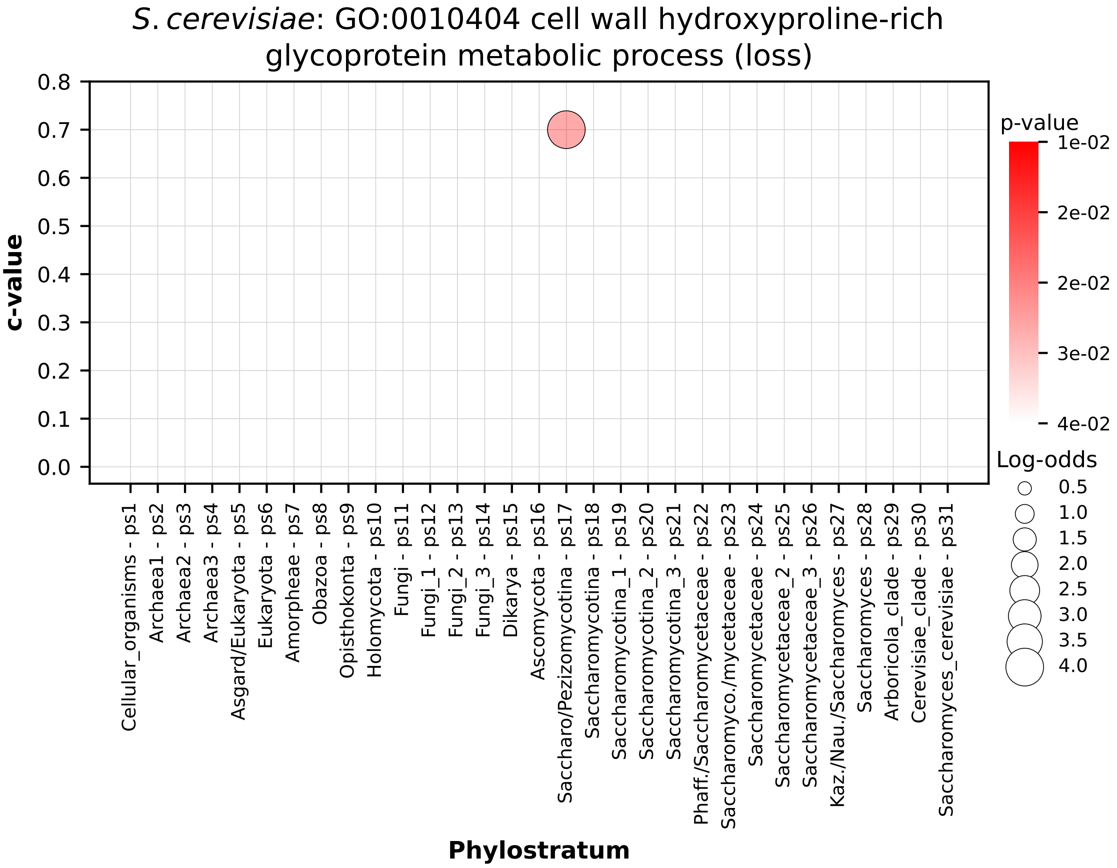
S. cerevisiae: GO:0010404 cell wall hydroxyproline-rich glycoprotein metabolic process (loss)
c-value
0.8
0.7
0.6
0.5
0.4
0.3
0.2
0.1
0.0
p-value
1e-02
2e-02
2e-02
3e-02
4e-02
Log-odds
0.5
1.0
1.5
2.0
2.5
3.0
3.5
4.0
Cellular_organisms - ps1
Archaea1 - ps2
Archaea2 - ps3
Archaea3 - ps4
Asgard/Eukaryota - ps5
Eukaryota - ps6
Amorpheae - ps7
Obazoa - ps8
Opisthokonta - ps9
Holomycota - ps10
Fungi - ps11
Fungi_1 - ps12
Fungi_2 - ps13
Fungi_3 - ps14
Dikarya - ps15
Ascomycota - ps16
Saccharo/Pezizomycotina - ps17
Saccharomycotina - ps18
Saccharomycotina_1 - ps19
Saccharomycotina_2 - ps20
Saccharomycotina_3 - ps21
Phaff./Saccharomycetaceae - ps22
Saccharomyco./mycetaceae - ps23
Saccharomycetaceae - ps24
Saccharomycetaceae_2 - ps25
Saccharomycetaceae_3 - ps26
Kaz./Nau./Saccharomyces - ps27
Saccharomyces - ps28
Arboricola_clade - ps29
Cerevisiae_clade - ps30
Saccharomyces_cerevisiae - ps31
Phylostratum

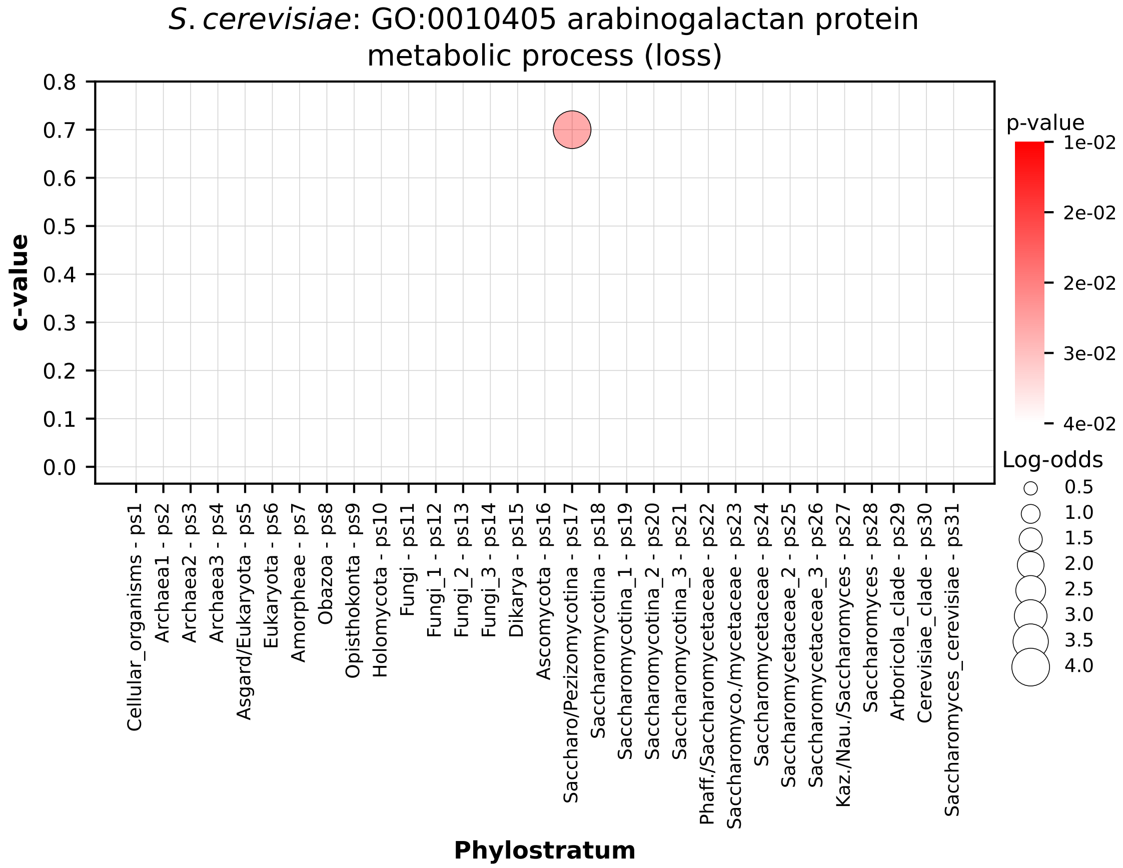

S. cerevisiae: GO:0010405 arabinogalactan protein metabolic process (loss)
c-value
0.8
0.7
0.6
0.5
0.4
0.3
0.2
0.1
0.0
p-value
1e-02
2e-02
2e-02
3e-02
4e-02
Log-odds
0.5
1.0
1.5
2.0
2.5
3.0
3.5
4.0
Cellular_organisms - ps1
Archaea1 - ps2
Archaea2 - ps3
Archaea3 - ps4
Asgard/Eukaryota - ps5
Eukaryota - ps6
Amorpheae - ps7
Obazoa - ps8
Opisthokonta - ps9
Holomycota - ps10
Fungi - ps11
Fungi_1 - ps12
Fungi_2 - ps13
Fungi_3 - ps14
Dikarya - ps15
Ascomycota - ps16
Saccharo/Pezizomycotina - ps17
Saccharomycotina - ps18
Saccharomycotina_1 - ps19
Saccharomycotina_2 - ps20
Saccharomycotina_3 - ps21
Phaff./Saccharomycetaceae - ps22
Saccharomyco./mycetaceae - ps23
Saccharomycetaceae - ps24
Saccharomycetaceae_2 - ps25
Saccharomycetaceae_3 - ps26
Kaz./Nau./Saccharomyces - ps27
Saccharomyces - ps28
Arboricola_clade - ps29
Cerevisiae_clade - ps30
Saccharomyces_cerevisiae - ps31
Phylostratum

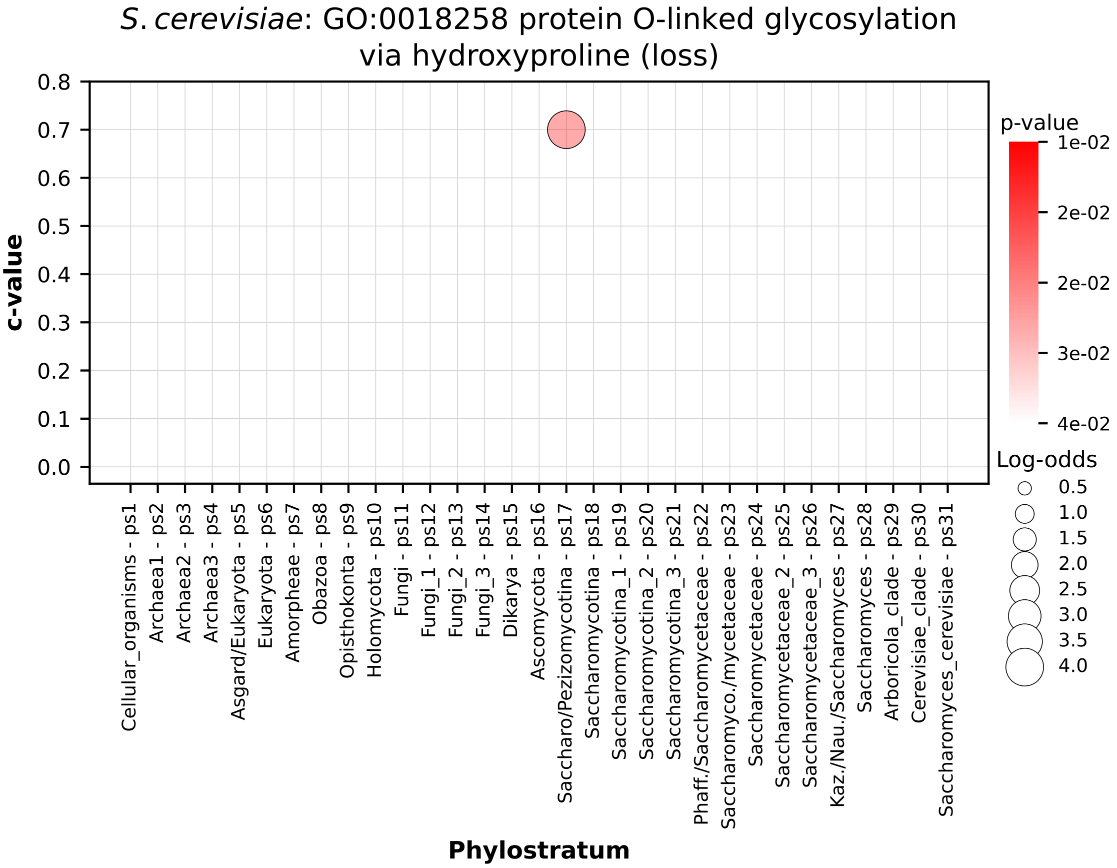
S. cerevisiae: GO:0018258 protein O-linked glycosylation via hydroxyproline (loss)
c-value
0.8
0.7
0.6
0.5
0.4
0.3
0.2
0.1
0.0
p-value
1e-02
2e-02
2e-02
3e-02
4e-02
Log-odds
0.5
1.0
1.5
2.0
2.5
3.0
3.5
4.0
Cellular_organisms - ps1
Archaea1 - ps2
Archaea2 - ps3
Archaea3 - ps4
Asgard/Eukaryota - ps5
Eukaryota - ps6
Amorpheae - ps7
Obazoa - ps8
Opisthokonta - ps9
Holomycota - ps10
Fungi - ps11
Fungi_1 - ps12
Fungi_2 - ps13
Fungi_3 - ps14
Dikarya - ps15
Ascomycota - ps16
Saccharo/Pezizomycotina - ps17
Saccharomycotina - ps18
Saccharomycotina_1 - ps19
Saccharomycotina_2 - ps20
Saccharomycotina_3 - ps21
Phaff./Saccharomycetaceae - ps22
Saccharomyco./mycetaceae - ps23
Saccharomycetaceae - ps24
Saccharomycetaceae_2 - ps25
Saccharomycetaceae_3 - ps26
Kaz./Nau./Saccharomyces - ps27
Saccharomyces - ps28
Arboricola_clade - ps29
Cerevisiae_clade - ps30
Saccharomyces_cerevisiae - ps31
Phylostratum

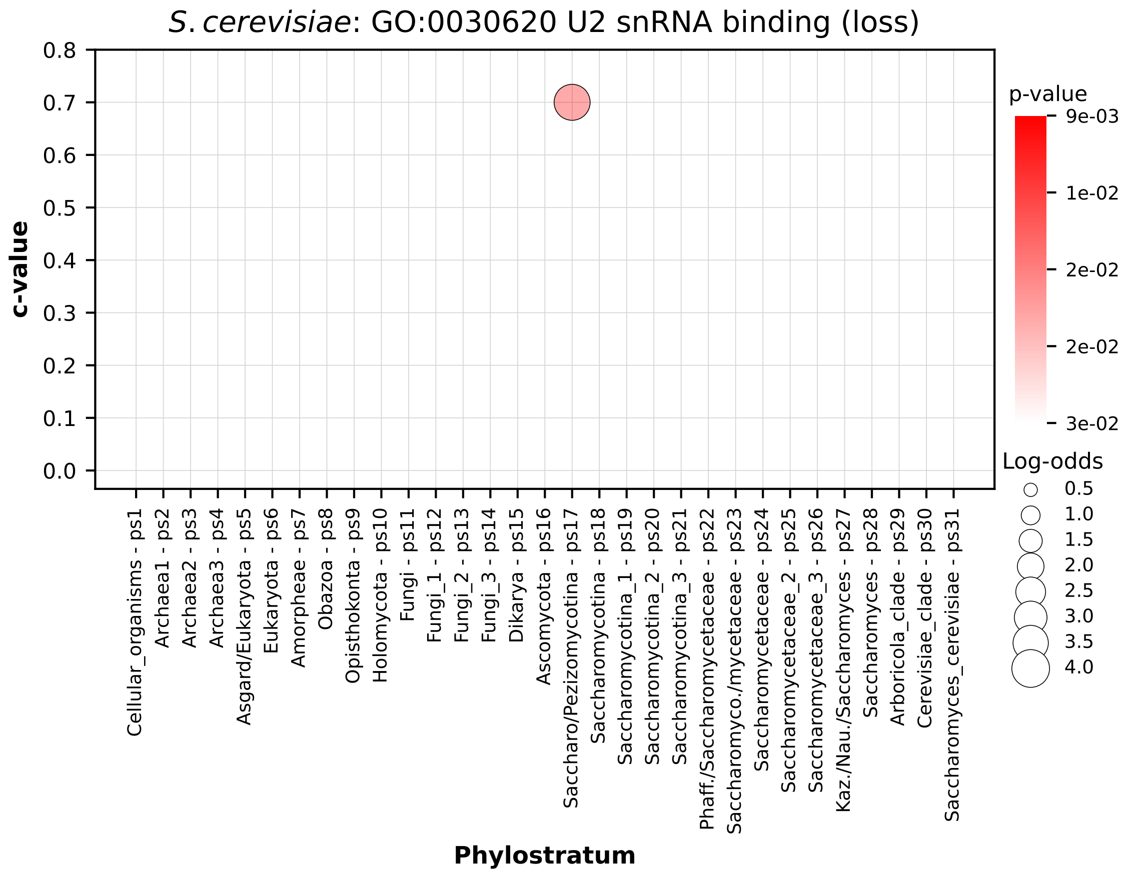

S. cerevisiae: GO:0030620 U2 snRNA binding (loss)
c-value
0.8
0.7
0.6
0.5
0.4
0.3
0.2
0.1
0.0
p-value
9e-03
1e-02
2e-02
2e-02
3e-02
Log-odds
0.5
1.0
1.5
2.0
2.5
3.0
3.5
4.0
Cellular_organisms - ps1
Archaea1 - ps2
Archaea2 - ps3
Archaea3 - ps4
Asgard/Eukaryota - ps5
Eukaryota - ps6
Amorpheae - ps7
Obazoa - ps8
Opisthokonta - ps9
Holomycota - ps10
Fungi - ps11
Fungi_1 - ps12
Fungi_2 - ps13
Fungi_3 - ps14
Dikarya - ps15
Ascomycota - ps16
Saccharo/Pezizomycotina - ps17
Saccharomycotina - ps18
Saccharomycotina_1 - ps19
Saccharomycotina_2 - ps20
Saccharomycotina_3 - ps21
Phaff./Saccharomycetaceae - ps22
Saccharomyco./mycetaceae - ps23
Saccharomycetaceae - ps24
Saccharomycetaceae_2 - ps25
Saccharomycetaceae_3 - ps26
Kaz./Nau./Saccharomyces - ps27
Saccharomyces - ps28
Arboricola_clade - ps29
Cerevisiae_clade - ps30
Saccharomyces_cerevisiae - ps31
Phylostratum

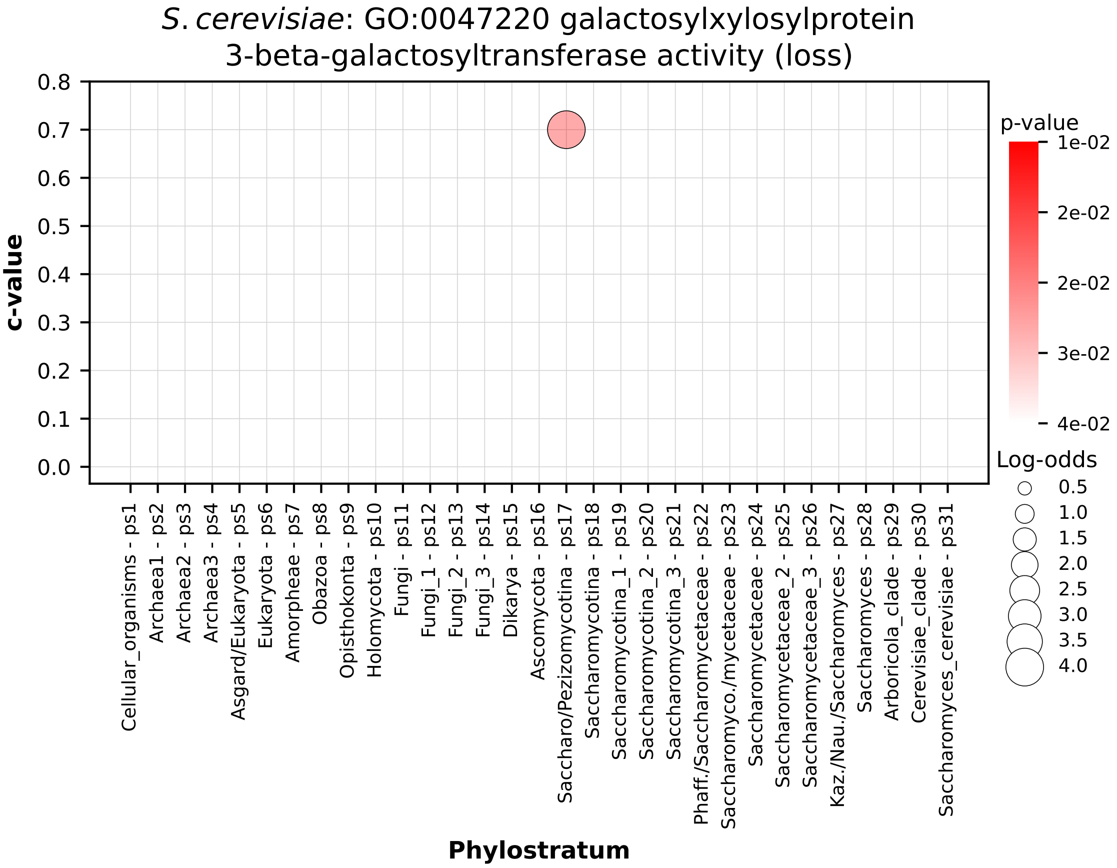
S. cerevisiae: GO:0047220 galactosylxylosylprotein 3-beta-galactosyltransferase activity (loss)
c-value
0.8
0.7
0.6
0.5
0.4
0.3
0.2
0.1
0.0
p-value
1e-02
2e-02
2e-02
3e-02
4e-02
Log-odds
0.5
1.0
1.5
2.0
2.5
3.0
3.5
4.0
Cellular_organisms - ps1
Archaea1 - ps2
Archaea2 - ps3
Archaea3 - ps4
Asgard/Eukaryota - ps5
Eukaryota - ps6
Amorpheae - ps7
Obazoa - ps8
Opisthokonta - ps9
Holomycota - ps10
Fungi - ps11
Fungi_1 - ps12
Fungi_2 - ps13
Fungi_3 - ps14
Dikarya - ps15
Ascomycota - ps16
Saccharo/Pezizomycotina - ps17
Saccharomycotina - ps18
Saccharomycotina_1 - ps19
Saccharomycotina_2 - ps20
Saccharomycotina_3 - ps21
Phaff./Saccharomycetaceae - ps22
Saccharomyco./mycetaceae - ps23
Saccharomycetaceae - ps24
Saccharomycetaceae_2 - ps25
Saccharomycetaceae_3 - ps26
Kaz./Nau./Saccharomyces - ps27
Saccharomyces - ps28
Arboricola_clade - ps29
Cerevisiae_clade - ps30
Saccharomyces_cerevisiae - ps31
Phylostratum

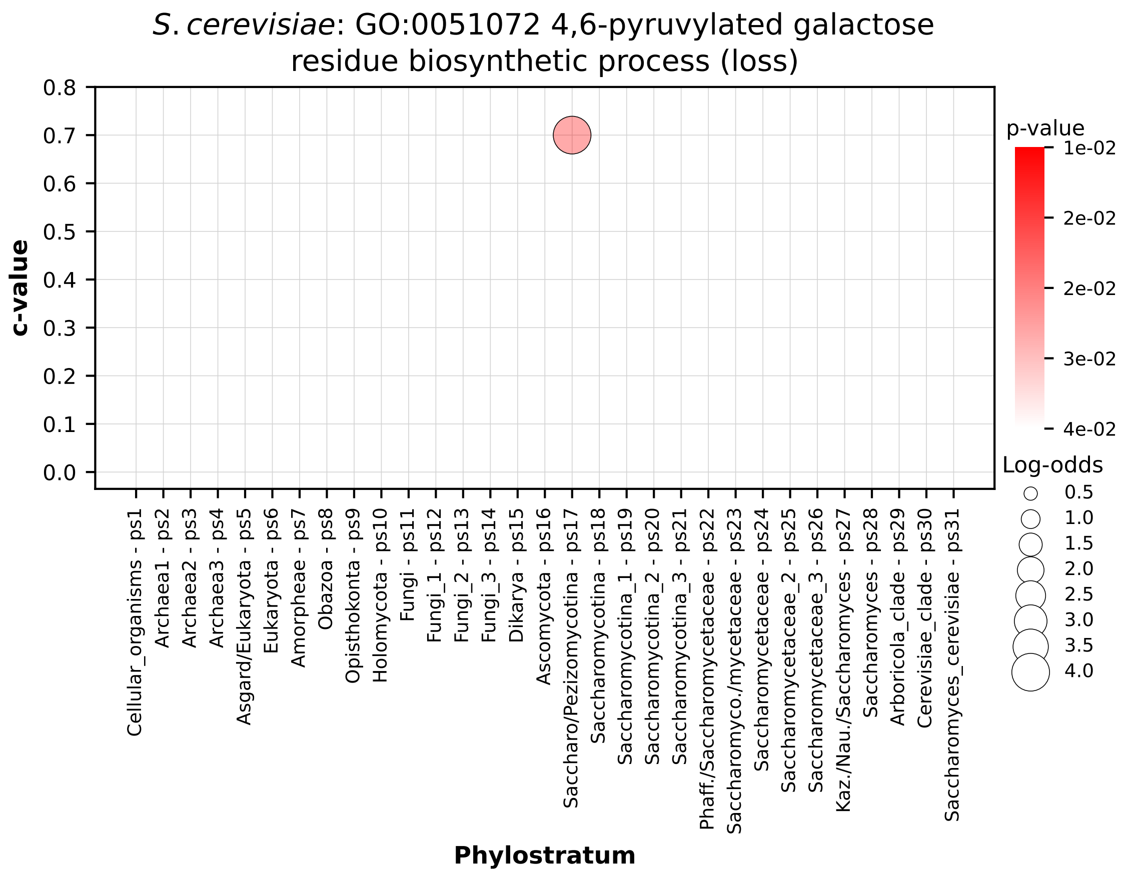
S. cerevisiae: GO:0051072 4,6-pyruvylated galactose residue biosynthetic process (loss)
c-value
0.8
0.7
0.6
0.5
0.4
0.3
0.2
0.1
0.0
Phylostratum
Cellular_organisms - ps1
Archaea1 - ps2
Archaea2 - ps3
Archaea3 - ps4
Asgard/Eukaryota - ps5
Eukaryota - ps6
Amorpheae - ps7
Obazoa - ps8
Opisthokonta - ps9
Holomycota - ps10
Fungi - ps11
Fungi_1 - ps12
Fungi_2 - ps13
Fungi_3 - ps14
Dikarya - ps15
Ascomycota - ps16
Saccharo/Pezizomycotina - ps17
Saccharomycotina - ps18
Saccharomycotina_1 - ps19
Saccharomycotina_2 - ps20
Saccharomycotina_3 - ps21
Phaff./Saccharomycetaceae - ps22
Saccharomyco./mycetaceae - ps23
Saccharomycetaceae - ps24
Saccharomycetaceae_2 - ps25
Saccharomycetaceae_3 - ps26
Kaz./Nau./Saccharomyces - ps27
Saccharomyces - ps28
Arboricola_clade - ps29
Cerevisiae_clade - ps30
Saccharomyces_cerevisiae - ps31
p-value
1e-02
2e-02
2e-02
3e-02
4e-02
Log-odds
0.5
1.0
1.5
2.0
2.5
3.0
3.5
4.0

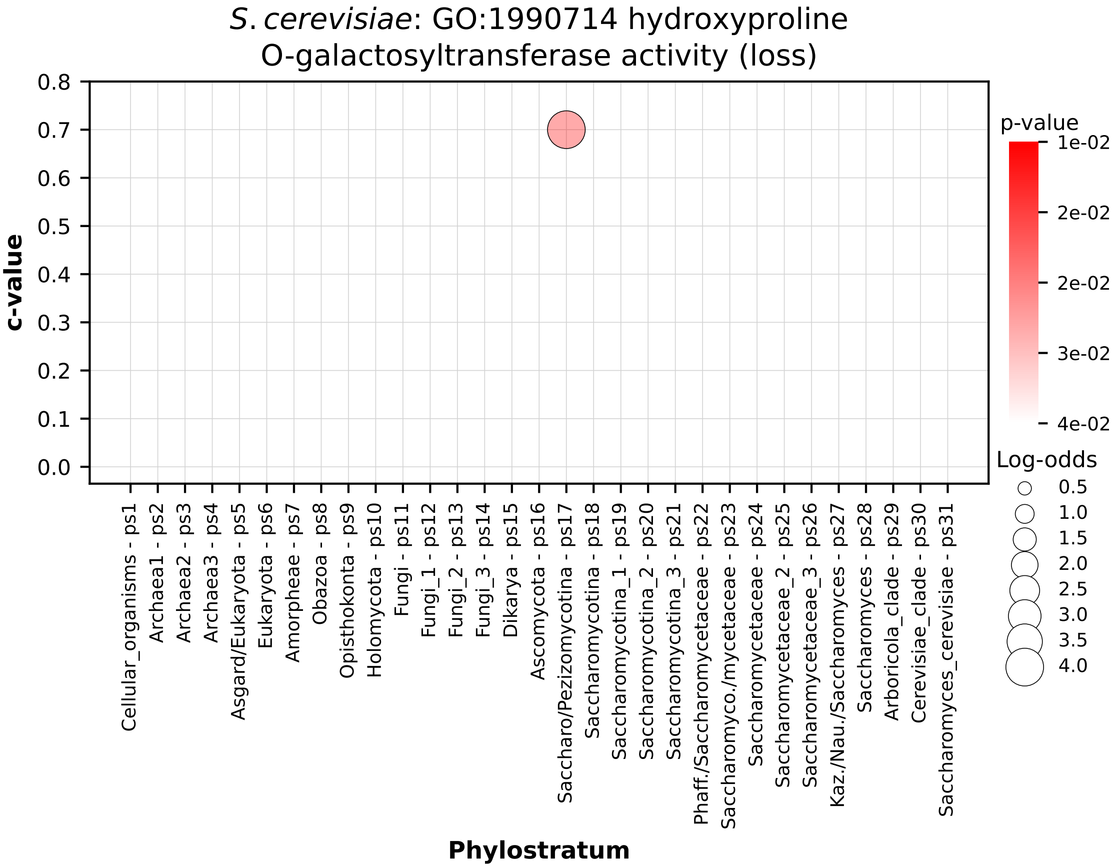

S. cerevisiae: GO:1990714 hydroxyproline O-galactosyltransferase activity (loss)
c-value
0.0
0.1
0.2
0.3
0.4
0.5
0.6
0.7
0.8
p-value
1e-02
2e-02
2e-02
3e-02
4e-02
Log-odds
0.5
1.0
1.5
2.0
2.5
3.0
3.5
4.0
Cellular_organisms - ps1
Archaea1 - ps2
Archaea2 - ps3
Archaea3 - ps4
Asgard/Eukaryota - ps5
Eukaryota - ps6
Amorpheae - ps7
Obazoa - ps8
Opisthokonta - ps9
Holomycota - ps10
Fungi - ps11
Fungi_1 - ps12
Fungi_2 - ps13
Fungi_3 - ps14
Dikarya - ps15
Ascomycota - ps16
Saccharo/Pezizomycotina - ps17
Saccharomycotina - ps18
Saccharomycotina_1 - ps19
Saccharomycotina_2 - ps20
Saccharomycotina_3 - ps21
Phaff./Saccharomycetaceae - ps22
Saccharomyco./mycetaceae - ps23
Saccharomycetaceae - ps24
Saccharomycetaceae_2 - ps25
Saccharomycetaceae_3 - ps26
Kaz./Nau./Saccharomyces - ps27
Saccharomyces - ps28
Arboricola_clade - ps29
Cerevisiae_clade - ps30
Saccharomyces_cerevisiae - ps31
Phylostratum

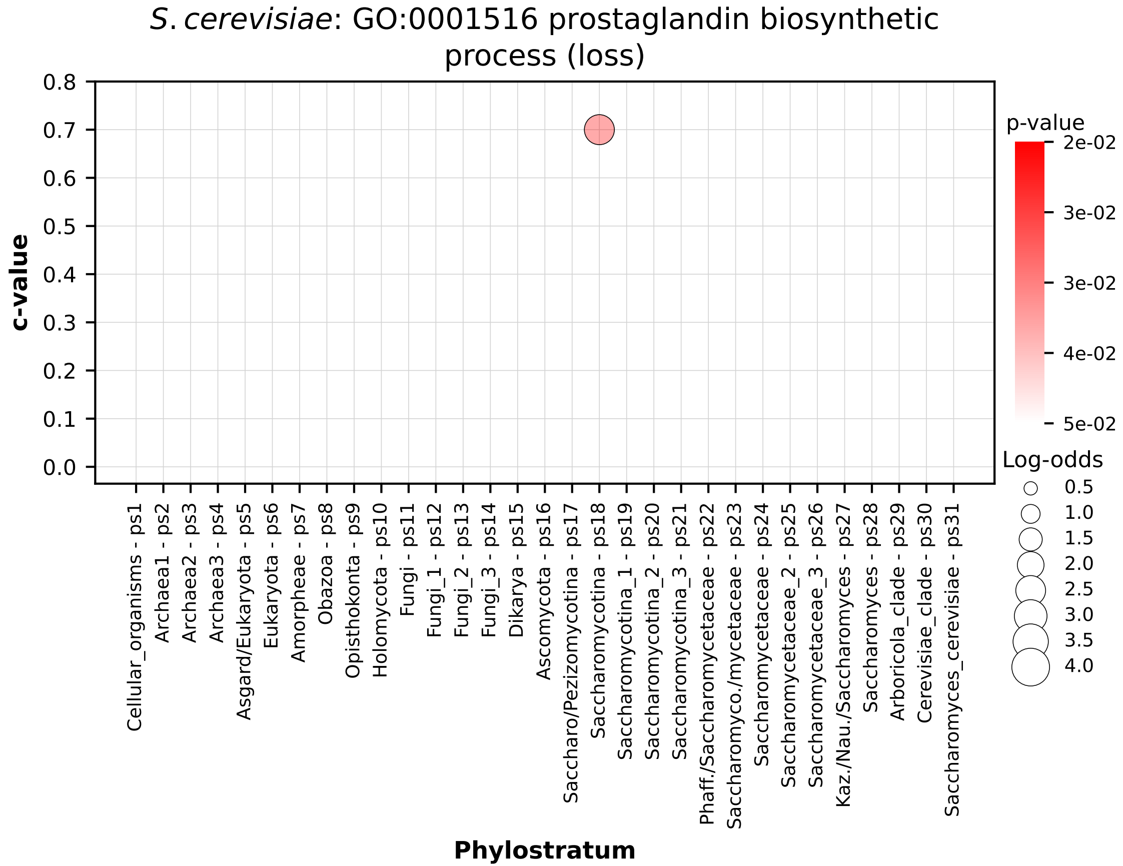
S. cerevisiae: GO:0001516 prostaglandin biosynthetic process (loss)
c-value
0.8
0.7
0.6
0.5
0.4
0.3
0.2
0.1
0.0
Phylostratum
Cellular_organisms - ps1
Archaea1 - ps2
Archaea2 - ps3
Archaea3 - ps4
Asgard/Eukaryota - ps5
Eukaryota - ps6
Amorpheae - ps7
Obazoa - ps8
Opisthokonta - ps9
Holomycota - ps10
Fungi - ps11
Fungi_1 - ps12
Fungi_2 - ps13
Fungi_3 - ps14
Dikarya - ps15
Ascomycota - ps16
Saccharo/Pezizomycotina - ps17
Saccharomycotina - ps18
Saccharomycotina_1 - ps19
Saccharomycotina_2 - ps20
Saccharomycotina_3 - ps21
Phaff./Saccharomycetaceae - ps22
Saccharomyco./mycetaceae - ps23
Saccharomycetaceae - ps24
Saccharomycetaceae_2 - ps25
Saccharomycetaceae_3 - ps26
Kaz./Nau./Saccharomyces - ps27
Saccharomyces - ps28
Arboricola_clade - ps29
Cerevisiae_clade - ps30
Saccharomyces_cerevisiae - ps31
p-value
2e-02
3e-02
3e-02
4e-02
5e-02
Log-odds
0.5
1.0
1.5
2.0
2.5
3.0
3.5
4.0

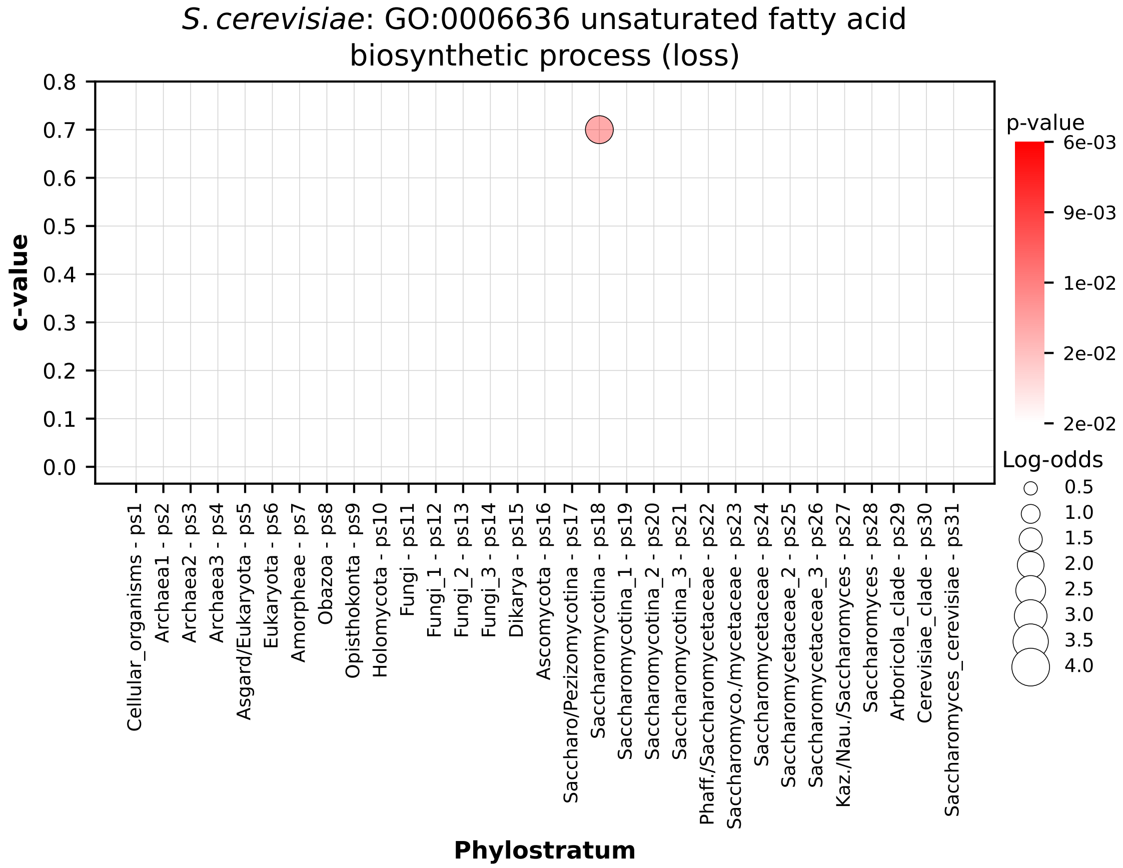
S. cerevisiae: GO:0006636 unsaturated fatty acid biosynthetic process (loss)
c-value
0.8
0.7
0.6
0.5
0.4
0.3
0.2
0.1
0.0
p-value
6e-03
9e-03
1e-02
2e-02
2e-02
Log-odds
0.5
1.0
1.5
2.0
2.5
3.0
3.5
4.0
Cellular_organisms - ps1
Archaea1 - ps2
Archaea2 - ps3
Archaea3 - ps4
Asgard/Eukaryota - ps5
Eukaryota - ps6
Amorpheae - ps7
Obazoa - ps8
Opisthokonta - ps9
Holomycota - ps10
Fungi - ps11
Fungi_1 - ps12
Fungi_2 - ps13
Fungi_3 - ps14
Dikarya - ps15
Ascomycota - ps16
Saccharo/Pezizomycotina - ps17
Saccharomycotina - ps18
Saccharomycotina_1 - ps19
Saccharomycotina_2 - ps20
Saccharomycotina_3 - ps21
Phaff./Saccharomycetaceae - ps22
Saccharomyco./mycetaceae - ps23
Saccharomycetaceae - ps24
Saccharomycetaceae_2 - ps25
Saccharomycetaceae_3 - ps26
Kaz./Nau./Saccharomyces - ps27
Saccharomyces - ps28
Arboricola_clade - ps29
Cerevisiae_clade - ps30
Saccharomyces_cerevisiae - ps31
Phylostratum

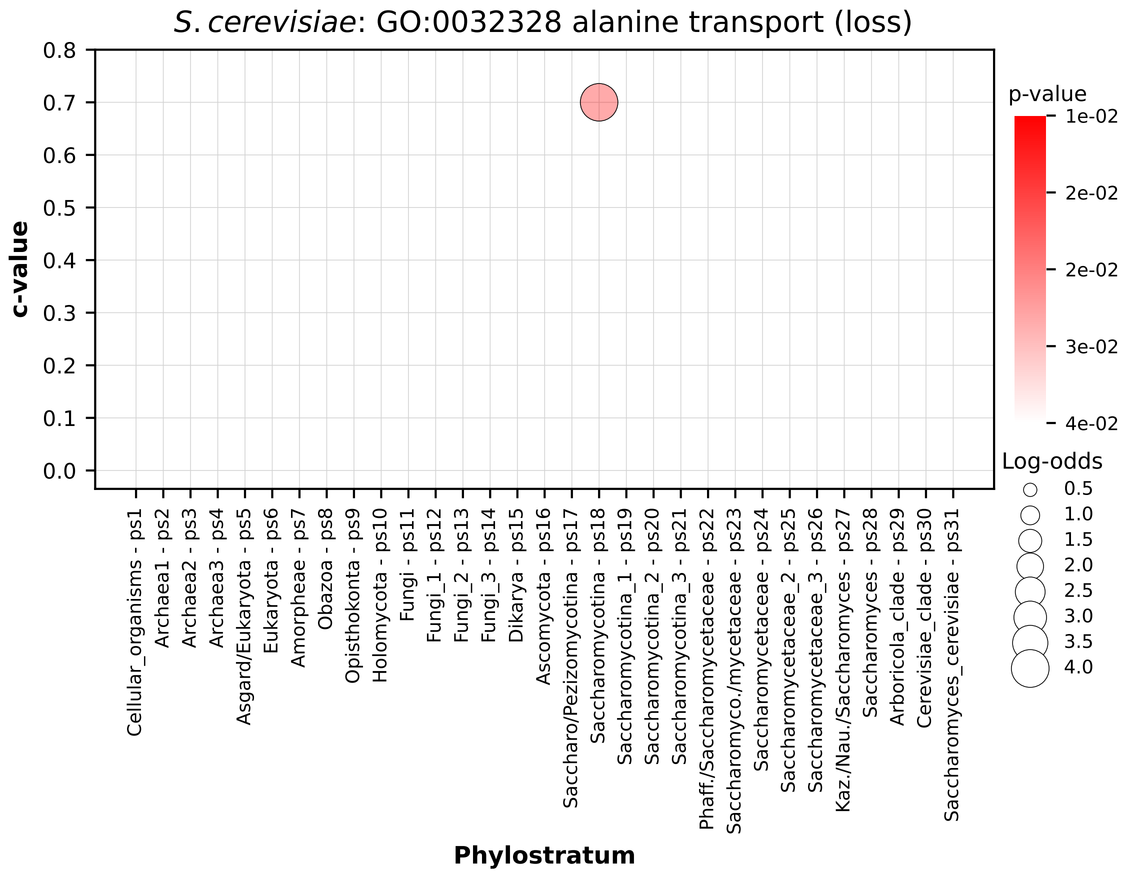
S. cerevisiae: GO:0032328 alanine transport (loss)
c-value
0.8
0.7
0.6
0.5
0.4
0.3
0.2
0.1
0.0
p-value
1e-02
2e-02
2e-02
3e-02
4e-02
Log-odds
0.5
1.0
1.5
2.0
2.5
3.0
3.5
4.0
Cellular_organisms - ps1
Archaea1 - ps2
Archaea2 - ps3
Archaea3 - ps4
Asgard/Eukaryota - ps5
Eukaryota - ps6
Amorpheae - ps7
Obazoa - ps8
Opisthokonta - ps9
Holomycota - ps10
Fungi - ps11
Fungi_1 - ps12
Fungi_2 - ps13
Fungi_3 - ps14
Dikarya - ps15
Ascomycota - ps16
Saccharo/Pezizomycotina - ps17
Saccharomycotina - ps18
Saccharomycotina_1 - ps19
Saccharomycotina_2 - ps20
Saccharomycotina_3 - ps21
Phaff./Saccharomycetaceae - ps22
Saccharomyco./mycetaceae - ps23
Saccharomycetaceae - ps24
Saccharomycetaceae_2 - ps25
Saccharomycetaceae_3 - ps26
Kaz./Nau./Saccharomyces - ps27
Saccharomyces - ps28
Arboricola_clade - ps29
Cerevisiae_clade - ps30
Saccharomyces_cerevisiae - ps31
Phylostratum

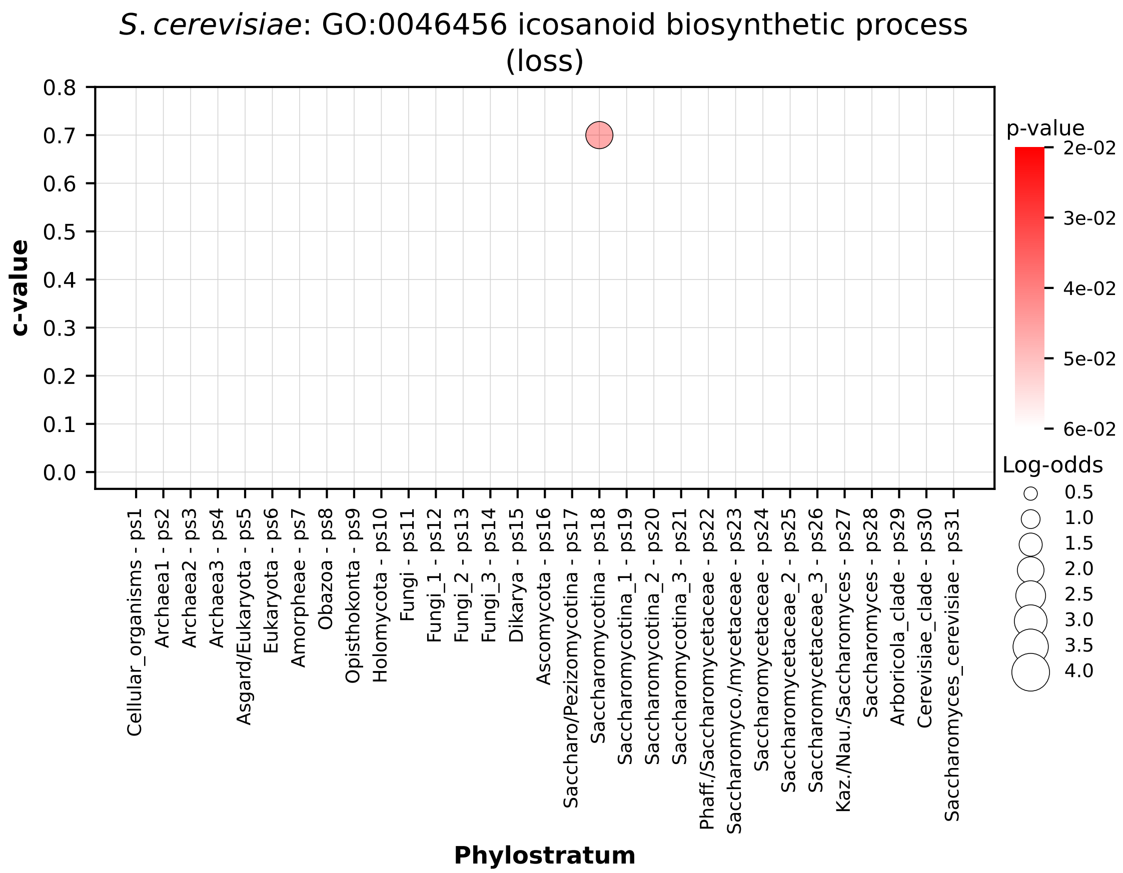

S. cerevisiae: GO:0046456 icosanoid biosynthetic process
(loss)
c-value
0.8
0.7
0.6
0.5
0.4
0.3
0.2
0.1
0.0
p-value
2e-02
3e-02
4e-02
5e-02
6e-02
Log-odds
0.5
1.0
1.5
2.0
2.5
3.0
3.5
4.0
Cellular_organisms - ps1
Archaea1 - ps2
Archaea2 - ps3
Archaea3 - ps4
Asgard/Eukaryota - ps5
Eukaryota - ps6
Amorpheae - ps7
Obazoa - ps8
Opisthokonta - ps9
Holomycota - ps10
Fungi - ps11
Fungi_1 - ps12
Fungi_2 - ps13
Fungi_3 - ps14
Dikarya - ps15
Ascomycota - ps16
Saccharo/Pezizomycotina - ps17
Saccharomycotina - ps18
Saccharomycotina_1 - ps19
Saccharomycotina_2 - ps20
Saccharomycotina_3 - ps21
Phaff./Saccharomycetaceae - ps22
Saccharomyco./mycetaceae - ps23
Saccharomycetaceae - ps24
Saccharomycetaceae_2 - ps25
Saccharomycetaceae_3 - ps26
Kaz./Nau./Saccharomyces - ps27
Saccharomyces - ps28
Arboricola_clade - ps29
Cerevisiae_clade - ps30
Saccharomyces_cerevisiae - ps31
Phylostratum

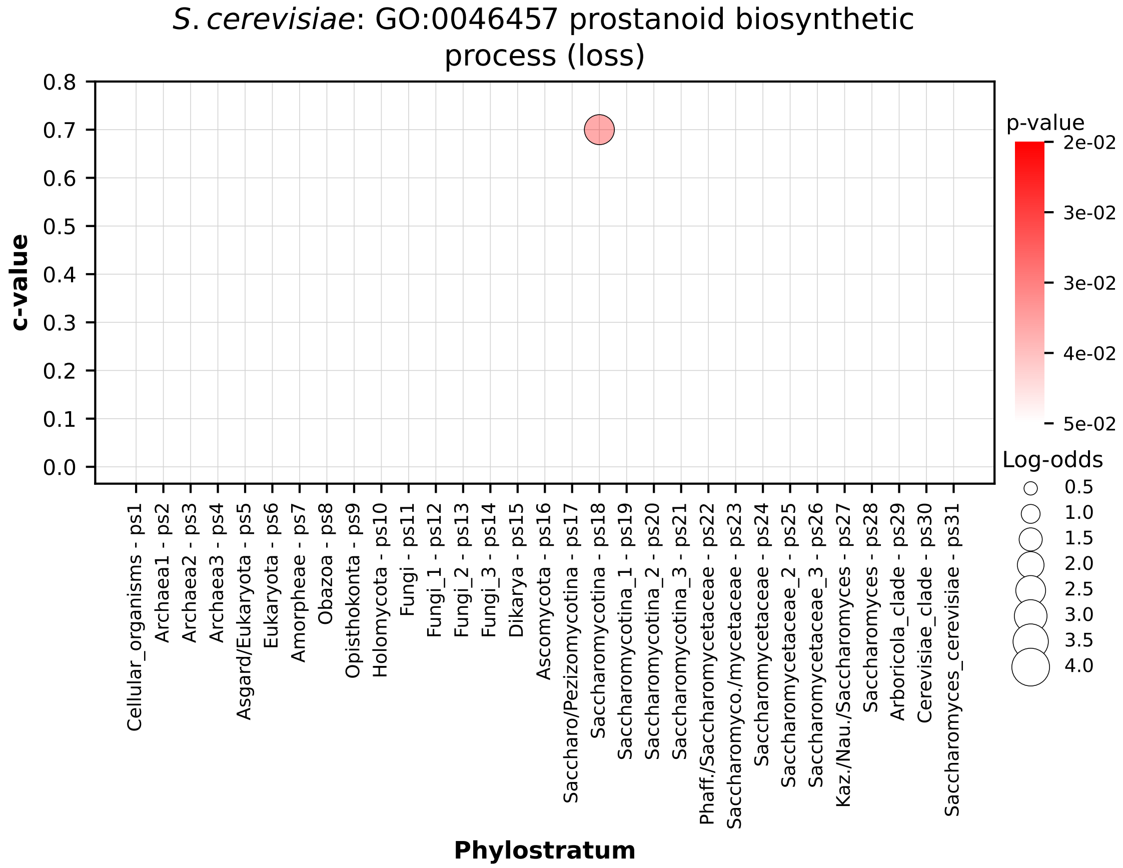
S. cerevisiae: GO:0046457 prostanoid biosynthetic process (loss)
c-value
0.8
0.7
0.6
0.5
0.4
0.3
0.2
0.1
0.0
Phylostratum
Cellular_organisms - ps1
Archaea1 - ps2
Archaea2 - ps3
Archaea3 - ps4
Asgard/Eukaryota - ps5
Eukaryota - ps6
Amorpheae - ps7
Obazoa - ps8
Opisthokonta - ps9
Holomycota - ps10
Fungi - ps11
Fungi_1 - ps12
Fungi_2 - ps13
Fungi_3 - ps14
Dikarya - ps15
Ascomycota - ps16
Saccharo/Pezizomycotina - ps17
Saccharomycotina - ps18
Saccharomycotina_1 - ps19
Saccharomycotina_2 - ps20
Saccharomycotina_3 - ps21
Phaff./Saccharomycetaceae - ps22
Saccharomyco./mycetaceae - ps23
Saccharomycetaceae - ps24
Saccharomycetaceae_2 - ps25
Saccharomycetaceae_3 - ps26
Kaz./Nau./Saccharomyces - ps27
Saccharomyces - ps28
Arboricola_clade - ps29
Cerevisiae_clade - ps30
Saccharomyces_cerevisiae - ps31
p-value
2e-02
3e-02
3e-02
4e-02
5e-02
Log-odds
0.5
1.0
1.5
2.0
2.5
3.0
3.5
4.0

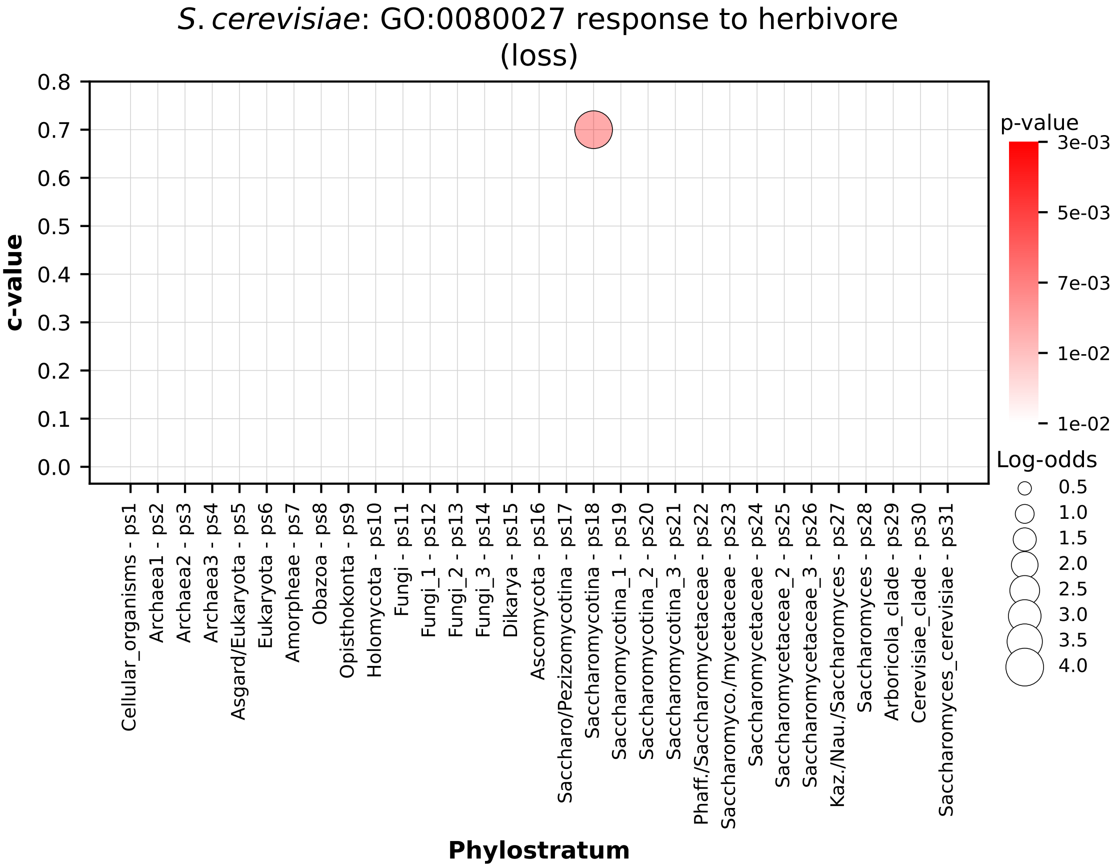

S. cerevisiae: GO:0080027 response to herbivore
(loss)
c-value
0.8
0.7
0.6
0.5
0.4
0.3
0.2
0.1
0.0
p-value
3e-03
5e-03
7e-03
1e-02
1e-02
Log-odds
0.5
1.0
1.5
2.0
2.5
3.0
3.5
4.0
Cellular_organisms - ps1
Archaea1 - ps2
Archaea2 - ps3
Archaea3 - ps4
Asgard/Eukaryota - ps5
Eukaryota - ps6
Amorpheae - ps7
Obazoa - ps8
Opisthokonta - ps9
Holomycota - ps10
Fungi - ps11
Fungi_1 - ps12
Fungi_2 - ps13
Fungi_3 - ps14
Dikarya - ps15
Ascomycota - ps16
Saccharo/Pezizomycotina - ps17
Saccharomycotina - ps18
Saccharomycotina_1 - ps19
Saccharomycotina_2 - ps20
Saccharomycotina_3 - ps21
Phaff./Saccharomycetaceae - ps22
Saccharomyco./mycetaceae - ps23
Saccharomycetaceae - ps24
Saccharomycetaceae_2 - ps25
Saccharomycetaceae_3 - ps26
Kaz./Nau./Saccharomyces - ps27
Saccharomyces - ps28
Arboricola_clade - ps29
Cerevisiae_clade - ps30
Saccharomyces_cerevisiae - ps31
Phylostratum

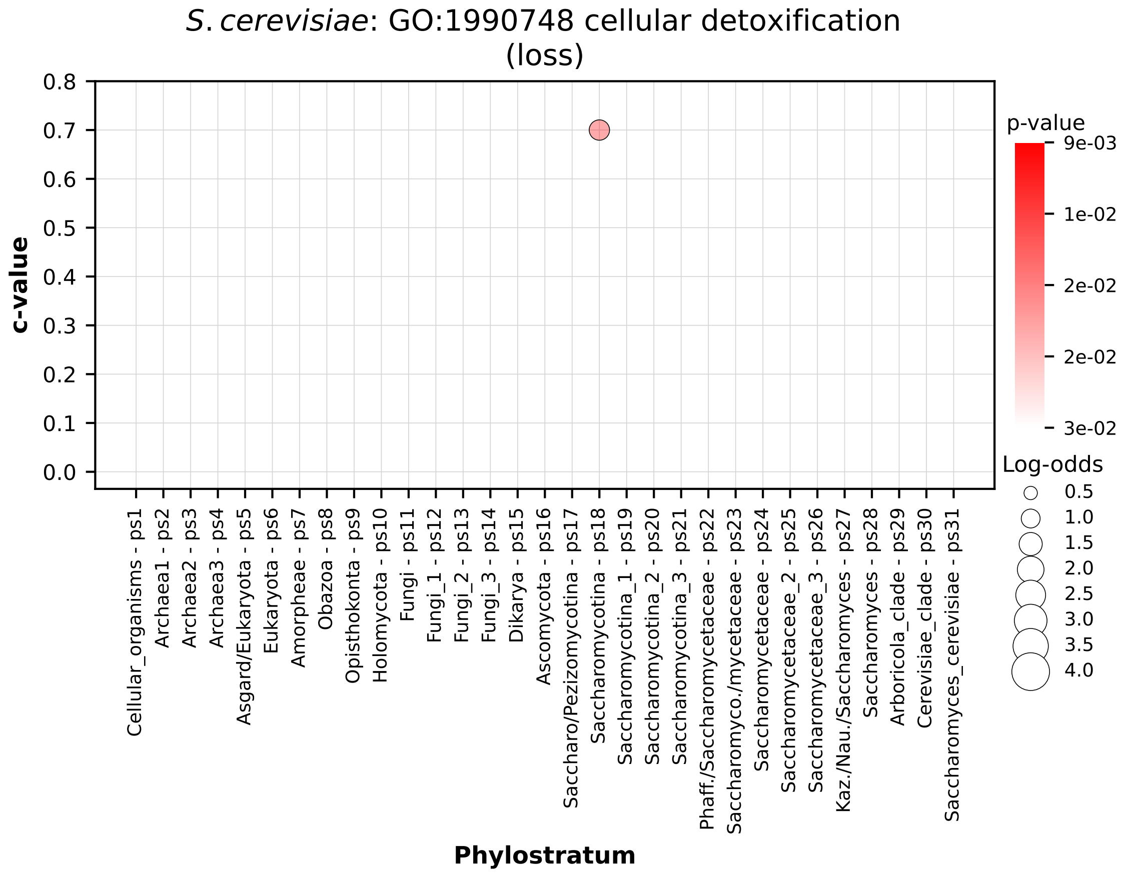
S. cerevisiae: GO:1990748 cellular detoxification
(loss)
c-value
0.8
0.7
0.6
0.5
0.4
0.3
0.2
0.1
0.0
p-value
9e-03
1e-02
2e-02
2e-02
3e-02
Log-odds
0.5
1.0
1.5
2.0
2.5
3.0
3.5
4.0
Cellular_organisms - ps1
Archaea1 - ps2
Archaea2 - ps3
Archaea3 - ps4
Asgard/Eukaryota - ps5
Eukaryota - ps6
Amorpheae - ps7
Obazoa - ps8
Opisthokonta - ps9
Holomycota - ps10
Fungi - ps11
Fungi_1 - ps12
Fungi_2 - ps13
Fungi_3 - ps14
Dikarya - ps15
Ascomycota - ps16
Saccharo/Pezizomycotina - ps17
Saccharomycotina - ps18
Saccharomycotina_1 - ps19
Saccharomycotina_2 - ps20
Saccharomycotina_3 - ps21
Phaff./Saccharomycetaceae - ps22
Saccharomyco./mycetaceae - ps23
Saccharomycetaceae - ps24
Saccharomycetaceae_2 - ps25
Saccharomycetaceae_3 - ps26
Kaz./Nau./Saccharomyces - ps27
Saccharomyces - ps28
Arboricola_clade - ps29
Cerevisiae_clade - ps30
Saccharomyces_cerevisiae - ps31
Phylostratum

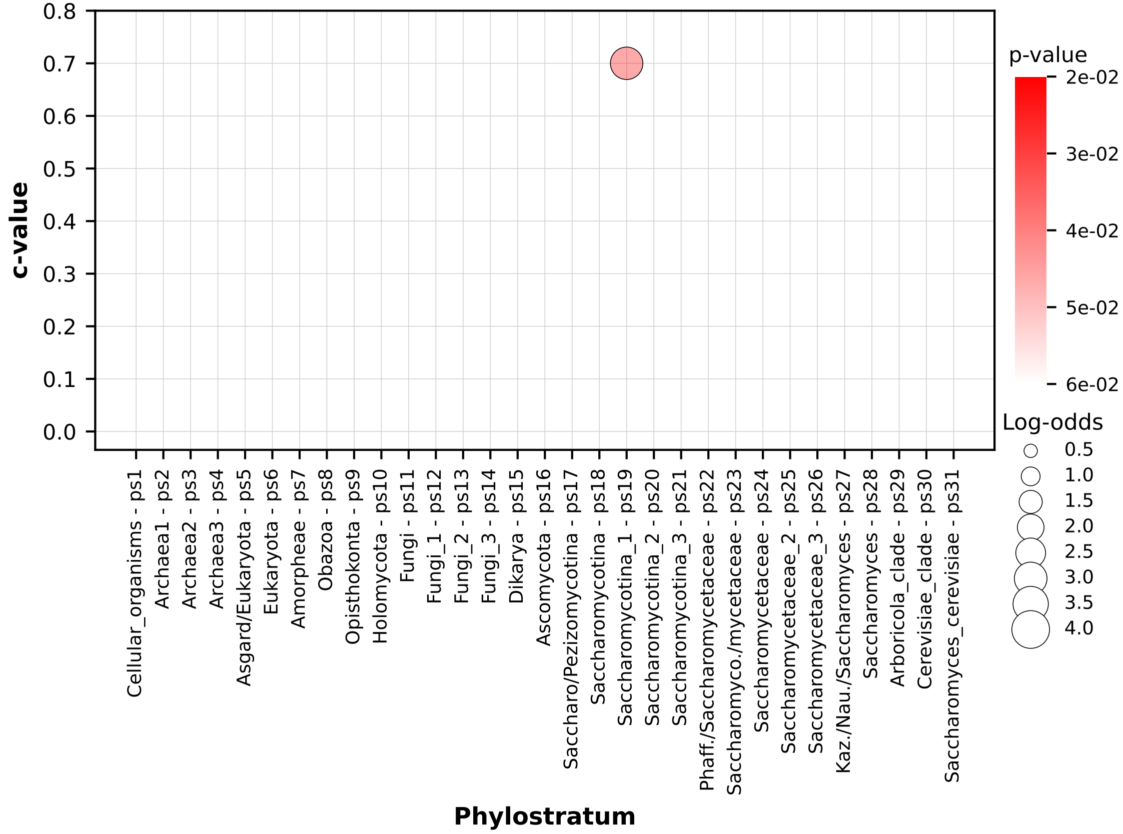
S. cerevisiae: GO:0016925 protein sumoylation (loss)
c-value
0.8
0.7
0.6
0.5
0.4
0.3
0.2
0.1
0.0
p-value
2e-02
3e-02
4e-02
5e-02
6e-02
Log-odds
0.5
1.0
1.5
2.0
2.5
3.0
3.5
4.0
Cellular_organisms - ps1
Archaea1 - ps2
Archaea2 - ps3
Archaea3 - ps4
Asgard/Eukaryota - ps5
Eukaryota - ps6
Amorpheae - ps7
Obazoa - ps8
Opisthokonta - ps9
Holomycota - ps10
Fungi - ps11
Fungi_1 - ps12
Fungi_2 - ps13
Fungi_3 - ps14
Dikarya - ps15
Ascomycota - ps16
Saccharo/Pezizomycotina - ps17
Saccharomycotina - ps18
Saccharomycotina_1 - ps19
Saccharomycotina_2 - ps20
Saccharomycotina_3 - ps21
Phaff./Saccharomycetaceae - ps22
Saccharomyco./mycetaceae - ps23
Saccharomycetaceae - ps24
Saccharomycetaceae_2 - ps25
Saccharomycetaceae_3 - ps26
Kaz./Nau./Saccharomyces - ps27
Saccharomyces - ps28
Arboricola_clade - ps29
Cerevisiae_clade - ps30
Saccharomyces_cerevisiae - ps31
Phylostratum

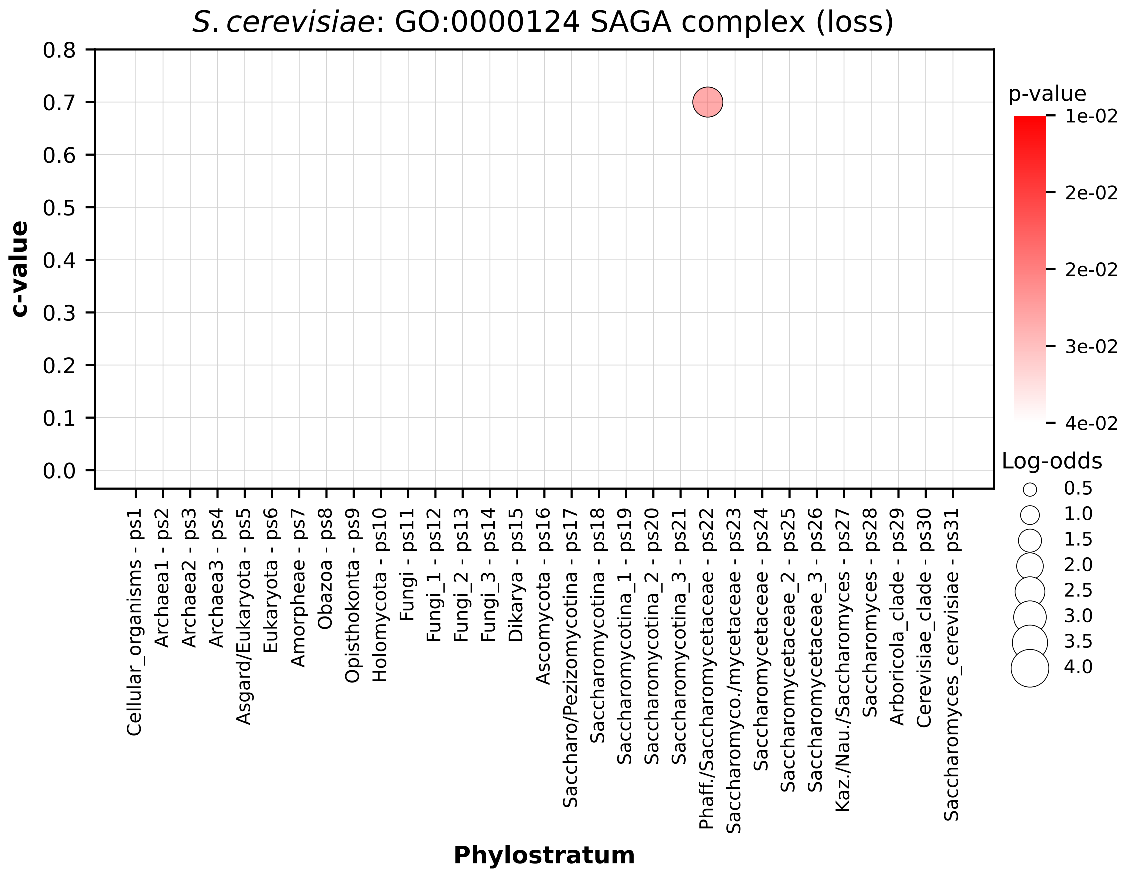
S. cerevisiae: GO:0000124 SAGA complex (loss)
c-value
0.8
0.7
0.6
0.5
0.4
0.3
0.2
0.1
0.0
Phylostratum
Cellular_organisms - ps1
Archaea1 - ps2
Archaea2 - ps3
Archaea3 - ps4
Asgard/Eukaryota - ps5
Eukaryota - ps6
Amorpheae - ps7
Obazoa - ps8
Opisthokonta - ps9
Holomycota - ps10
Fungi - ps11
Fungi_1 - ps12
Fungi_2 - ps13
Fungi_3 - ps14
Dikarya - ps15
Ascomycota - ps16
Saccharo/Pezizomycotina - ps17
Saccharomycotina - ps18
Saccharomycotina_1 - ps19
Saccharomycotina_2 - ps20
Saccharomycotina_3 - ps21
Phaff./Saccharomycetaceae - ps22
Saccharomyco./mycetaceae - ps23
Saccharomycetaceae - ps24
Saccharomycetaceae_2 - ps25
Saccharomycetaceae_3 - ps26
Kaz./Nau./Saccharomyces - ps27
Saccharomyces - ps28
Arboricola_clade - ps29
Cerevisiae_clade - ps30
Saccharomyces_cerevisiae - ps31
p-value
1e-02
2e-02
2e-02
3e-02
4e-02
Log-odds
0.5
1.0
1.5
2.0
2.5
3.0
3.5
4.0

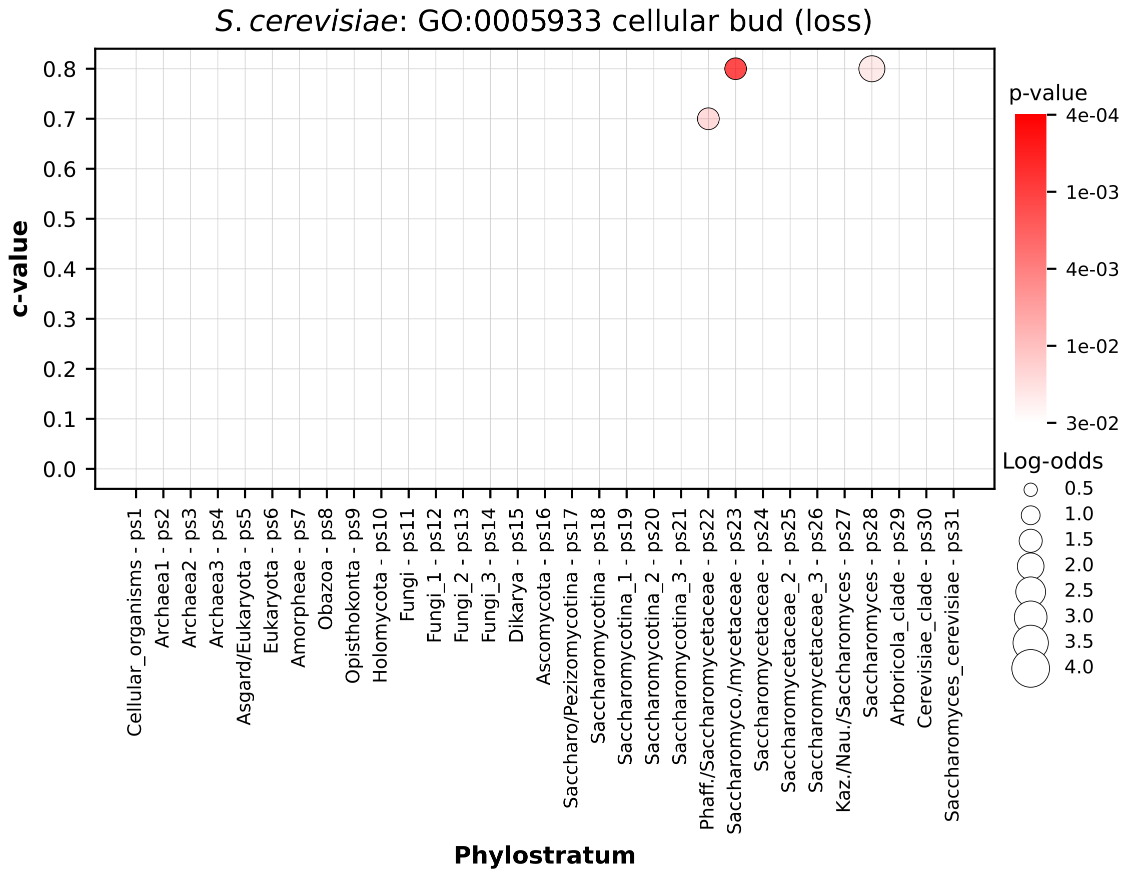
S. cerevisiae: GO:0005933 cellular bud (loss)
c-value
0.8
0.7
0.6
0.5
0.4
0.3
0.2
0.1
0.0
p-value
4e-04
1e-03
4e-03
1e-02
3e-02
Log-odds
0.5
1.0
1.5
2.0
2.5
3.0
3.5
4.0
Cellular_organisms - ps1
Archaea1 - ps2
Archaea2 - ps3
Archaea3 - ps4
Asgard/Eukaryota - ps5
Eukaryota - ps6
Amorpheae - ps7
Obazoa - ps8
Opisthokonta - ps9
Holomycota - ps10
Fungi - ps11
Fungi_1 - ps12
Fungi_2 - ps13
Fungi_3 - ps14
Dikarya - ps15
Ascomycota - ps16
Saccharo/Pezizomycotina - ps17
Saccharomycotina - ps18
Saccharomycotina_1 - ps19
Saccharomycotina_2 - ps20
Saccharomycotina_3 - ps21
Phaff./Saccharomycetaceae - ps22
Saccharomyco./mycetaceae - ps23
Saccharomycetaceae - ps24
Saccharomycetaceae_2 - ps25
Saccharomycetaceae_3 - ps26
Kaz./Nau./Saccharomyces - ps27
Saccharomyces - ps28
Arboricola_clade - ps29
Cerevisiae_clade - ps30
Saccharomyces_cerevisiae - ps31
Phylostratum

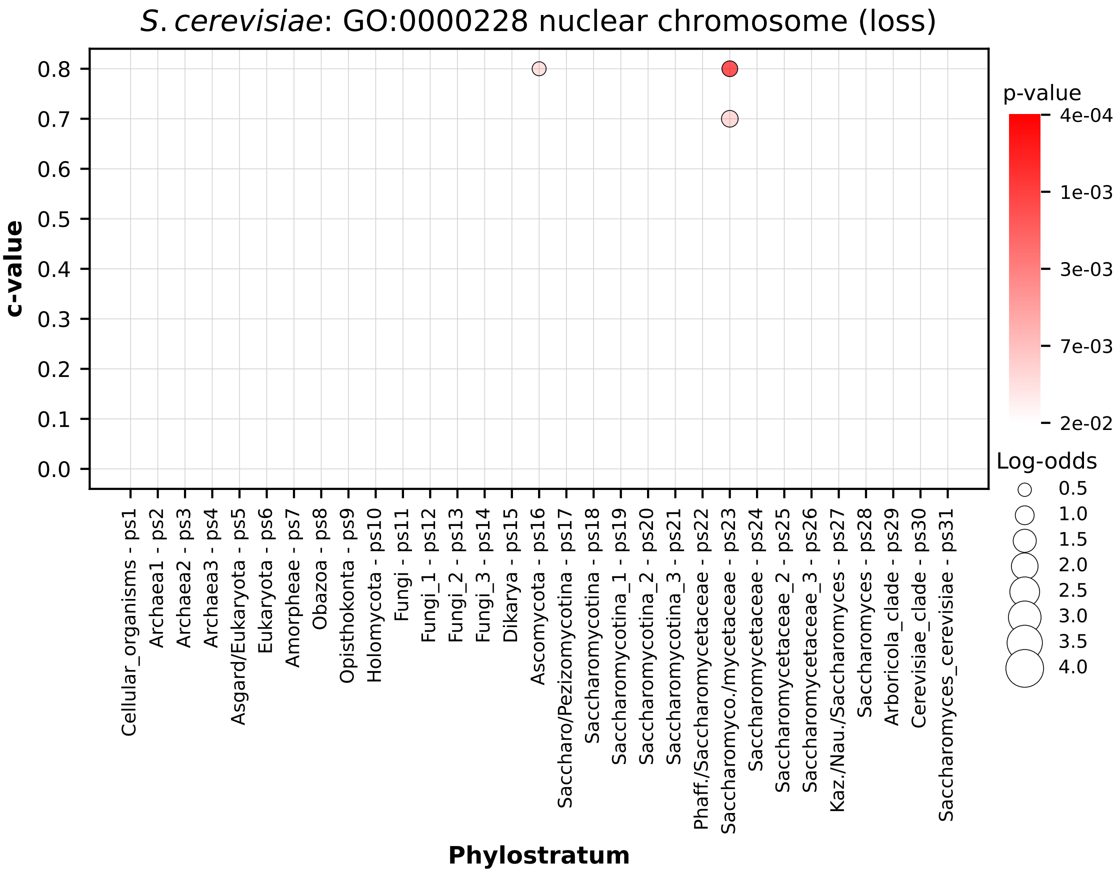

S. cerevisiae: GO:0000228 nuclear chromosome (loss)
c-value
0.0
0.1
0.2
0.3
0.4
0.5
0.6
0.7
0.8
p-value
4e-04
1e-03
3e-03
7e-03
2e-02
Log-odds
0.5
1.0
1.5
2.0
2.5
3.0
3.5
4.0
Cellular_organisms - ps1
Archaea1 - ps2
Archaea2 - ps3
Archaea3 - ps4
Asgard/Eukaryota - ps5
Eukaryota - ps6
Amorpheae - ps7
Obazoa - ps8
Opisthokonta - ps9
Holomycota - ps10
Fungi - ps11
Fungi_1 - ps12
Fungi_2 - ps13
Fungi_3 - ps14
Dikarya - ps15
Ascomycota - ps16
Saccharo/Pezizomycotina - ps17
Saccharomycotina - ps18
Saccharomycotina_1 - ps19
Saccharomycotina_2 - ps20
Saccharomycotina_3 - ps21
Phaff./Saccharomycetaceae - ps22
Saccharomyco./mycetaceae - ps23
Saccharomycetaceae - ps24
Saccharomycetaceae_2 - ps25
Saccharomycetaceae_3 - ps26
Kaz./Nau./Saccharomyces - ps27
Saccharomyces - ps28
Arboricola_clade - ps29
Cerevisiae_clade - ps30
Saccharomyces_cerevisiae - ps31
Phylostratum

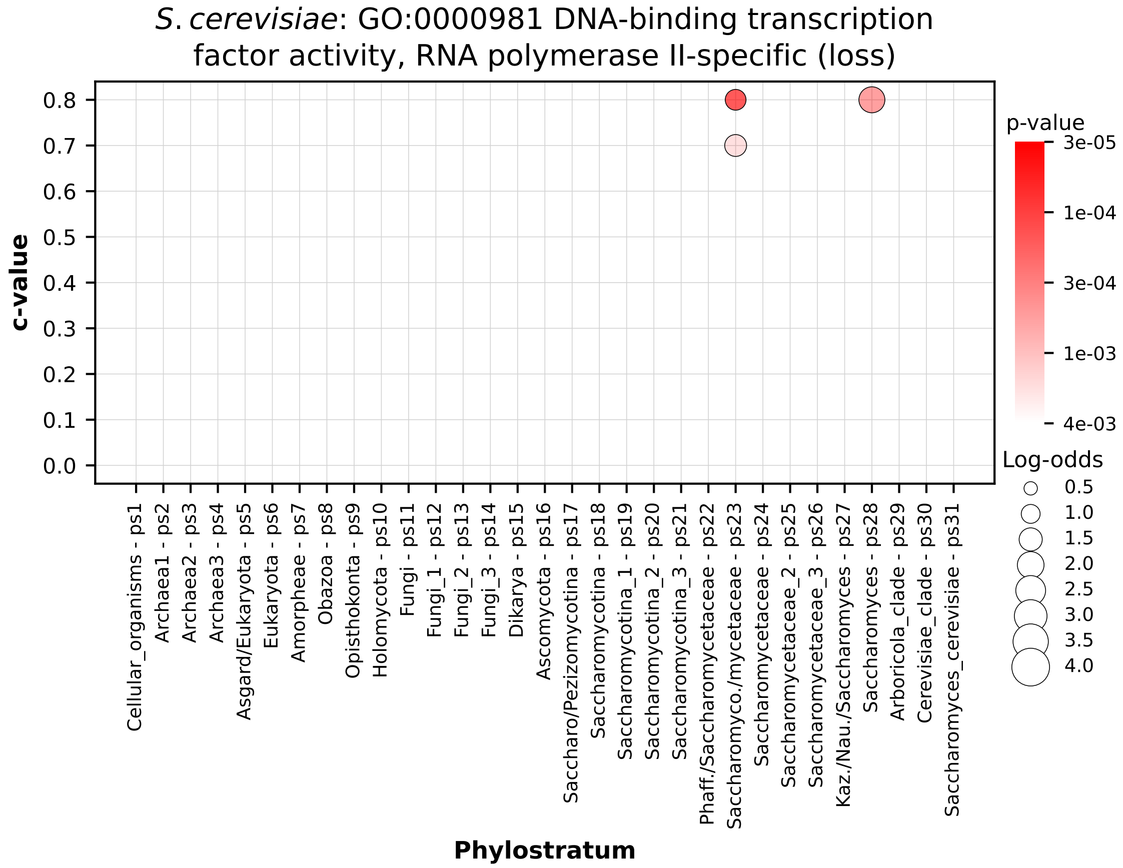

S. cerevisiae: GO:0000981 DNA-binding transcription factor activity, RNA polymerase II-specific (loss)
c-value
0.0
0.1
0.2
0.3
0.4
0.5
0.6
0.7
0.8
Phylostratum
Cellular_organisms - ps1
Archaea1 - ps2
Archaea2 - ps3
Archaea3 - ps4
Asgard/Eukaryota - ps5
Eukaryota - ps6
Amorpheae - ps7
Obazoa - ps8
Opisthokonta - ps9
Holomycota - ps10
Fungi - ps11
Fungi_1 - ps12
Fungi_2 - ps13
Fungi_3 - ps14
Dikarya - ps15
Ascomycota - ps16
Saccharo/Pezizomycotina - ps17
Saccharomycotina - ps18
Saccharomycotina_1 - ps19
Saccharomycotina_2 - ps20
Saccharomycotina_3 - ps21
Phaff./Saccharomycetaceae - ps22
Saccharomyco./mycetaceae - ps23
Saccharomycetaceae - ps24
Saccharomycetaceae_2 - ps25
Saccharomycetaceae_3 - ps26
Kaz./Nau./Saccharomyces - ps27
Saccharomyces - ps28
Arboricola_clade - ps29
Cerevisiae_clade - ps30
Saccharomyces_cerevisiae - ps31
p-value
3e-05
1e-04
3e-04
1e-03
4e-03
Log-odds
0.5
1.0
1.5
2.0
2.5
3.0
3.5
4.0

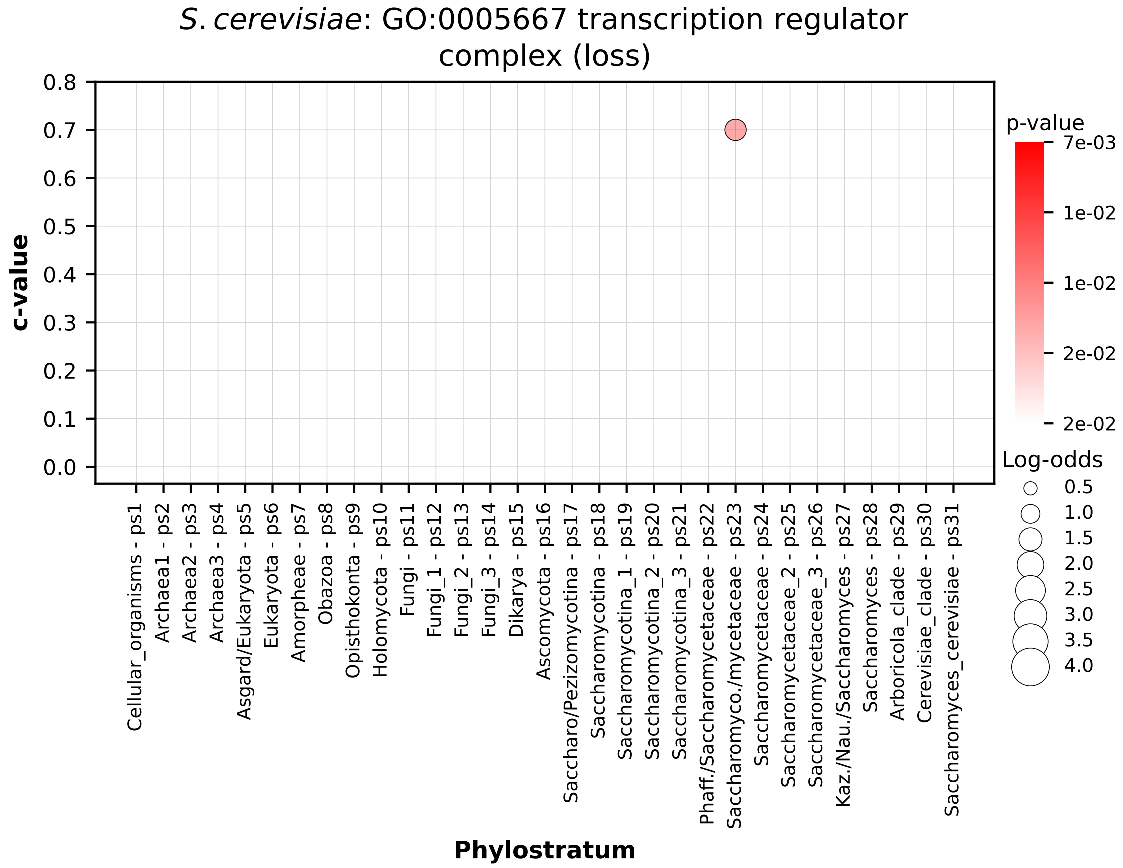
S. cerevisiae: GO:0005667 transcription regulator complex (loss)
c-value
0.8
0.7
0.6
0.5
0.4
0.3
0.2
0.1
0.0
p-value
7e-03
1e-02
1e-02
2e-02
2e-02
Log-odds
0.5
1.0
1.5
2.0
2.5
3.0
3.5
4.0
Cellular_organisms - ps1
Archaea1 - ps2
Archaea2 - ps3
Archaea3 - ps4
Asgard/Eukaryota - ps5
Eukaryota - ps6
Amorpheae - ps7
Obazoa - ps8
Opisthokonta - ps9
Holomycota - ps10
Fungi - ps11
Fungi_1 - ps12
Fungi_2 - ps13
Fungi_3 - ps14
Dikarya - ps15
Ascomycota - ps16
Saccharo/Pezizomycotina - ps17
Saccharomycotina - ps18
Saccharomycotina_1 - ps19
Saccharomycotina_2 - ps20
Saccharomycotina_3 - ps21
Phaff./Saccharomycetaceae - ps22
Saccharomyco./mycetaceae - ps23
Saccharomycetaceae - ps24
Saccharomycetaceae_2 - ps25
Saccharomycetaceae_3 - ps26
Kaz./Nau./Saccharomyces - ps27
Saccharomyces - ps28
Arboricola_clade - ps29
Cerevisiae_clade - ps30
Saccharomyces_cerevisiae - ps31
Phylostratum

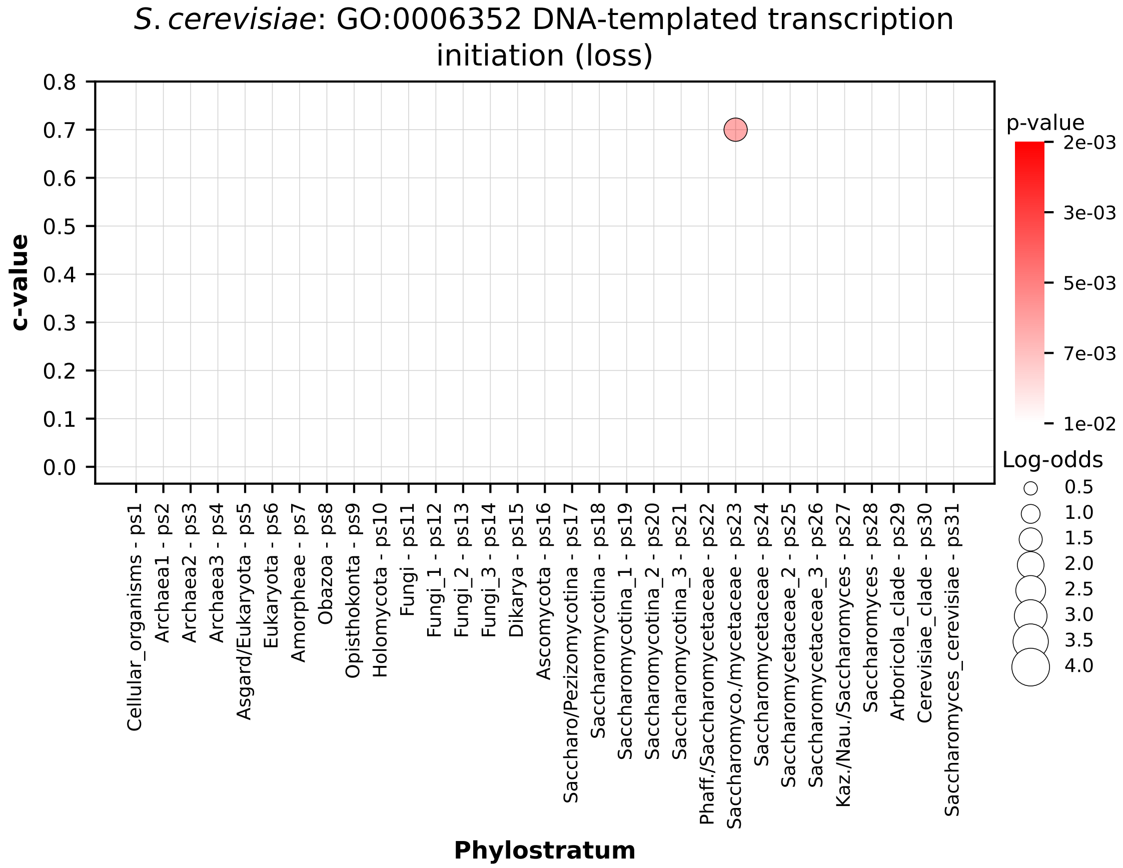

S. cerevisiae: GO:0006352 DNA-templated transcription initiation (loss)
c-value
0.8
0.7
0.6
0.5
0.4
0.3
0.2
0.1
0.0
p-value
2e-03
3e-03
5e-03
7e-03
1e-02
Log-odds
0.5
1.0
1.5
2.0
2.5
3.0
3.5
4.0
Cellular_organisms - ps1
Archaea1 - ps2
Archaea2 - ps3
Archaea3 - ps4
Asgard/Eukaryota - ps5
Eukaryota - ps6
Amorpheae - ps7
Obazoa - ps8
Opisthokonta - ps9
Holomycota - ps10
Fungi - ps11
Fungi_1 - ps12
Fungi_2 - ps13
Fungi_3 - ps14
Dikarya - ps15
Ascomycota - ps16
Saccharo/Pezizomycotina - ps17
Saccharomycotina - ps18
Saccharomycotina_1 - ps19
Saccharomycotina_2 - ps20
Saccharomycotina_3 - ps21
Phaff./Saccharomycetaceae - ps22
Saccharomyco./mycetaceae - ps23
Saccharomycetaceae - ps24
Saccharomycetaceae_2 - ps25
Saccharomycetaceae_3 - ps26
Kaz./Nau./Saccharomyces - ps27
Saccharomyces - ps28
Arboricola_clade - ps29
Cerevisiae_clade - ps30
Saccharomyces_cerevisiae - ps31
Phylostratum

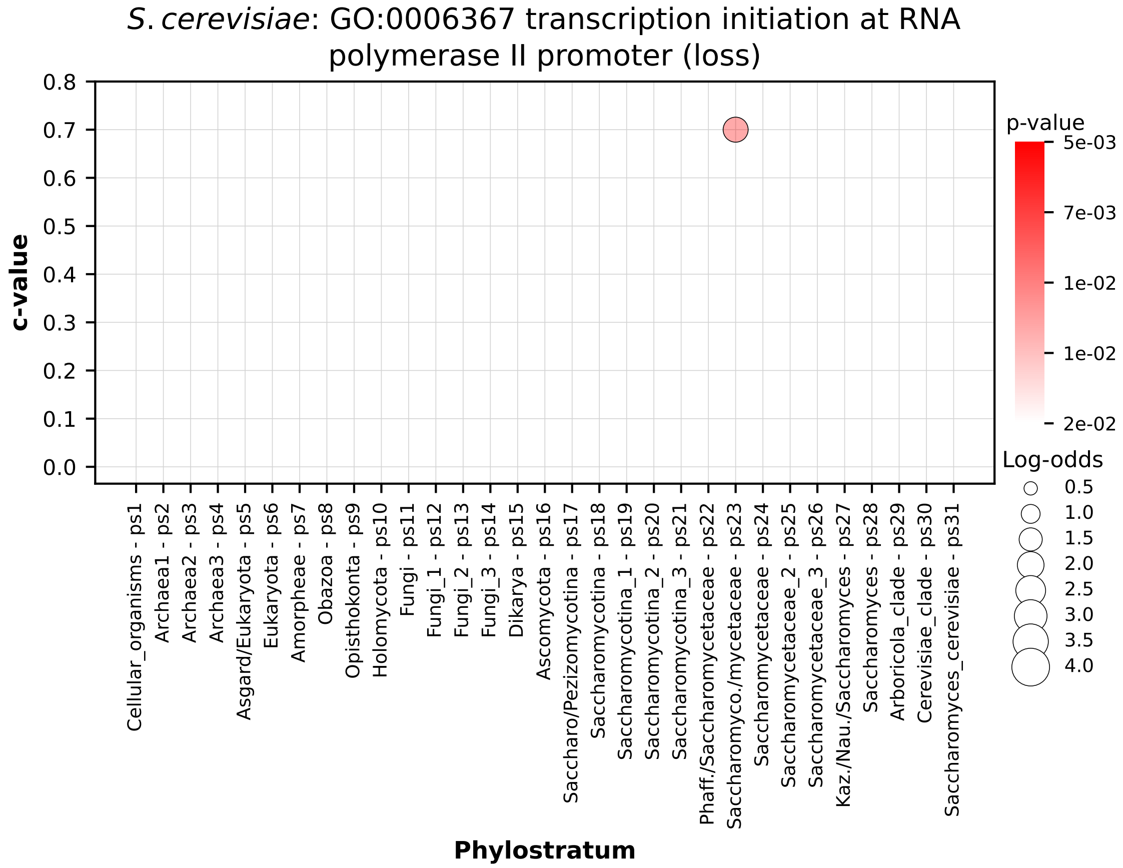

S. cerevisiae: GO:0006367 transcription initiation at RNA polymerase II promoter (loss)
c-value
0.8
0.7
0.6
0.5
0.4
0.3
0.2
0.1
0.0
p-value
5e-03
7e-03
1e-02
1e-02
2e-02
Log-odds
0.5
1.0
1.5
2.0
2.5
3.0
3.5
4.0
Cellular_organisms - ps1
Archaea1 - ps2
Archaea2 - ps3
Archaea3 - ps4
Asgard/Eukaryota - ps5
Eukaryota - ps6
Amorpheae - ps7
Obazoa - ps8
Opisthokonta - ps9
Holomycota - ps10
Fungi - ps11
Fungi_1 - ps12
Fungi_2 - ps13
Fungi_3 - ps14
Dikarya - ps15
Ascomycota - ps16
Saccharo/Pezizomycotina - ps17
Saccharomycotina - ps18
Saccharomycotina_1 - ps19
Saccharomycotina_2 - ps20
Saccharomycotina_3 - ps21
Phaff./Saccharomycetaceae - ps22
Saccharomyco./mycetaceae - ps23
Saccharomycetaceae - ps24
Saccharomycetaceae_2 - ps25
Saccharomycetaceae_3 - ps26
Kaz./Nau./Saccharomyces - ps27
Saccharomyces - ps28
Arboricola_clade - ps29
Cerevisiae_clade - ps30
Saccharomyces_cerevisiae - ps31
Phylostratum

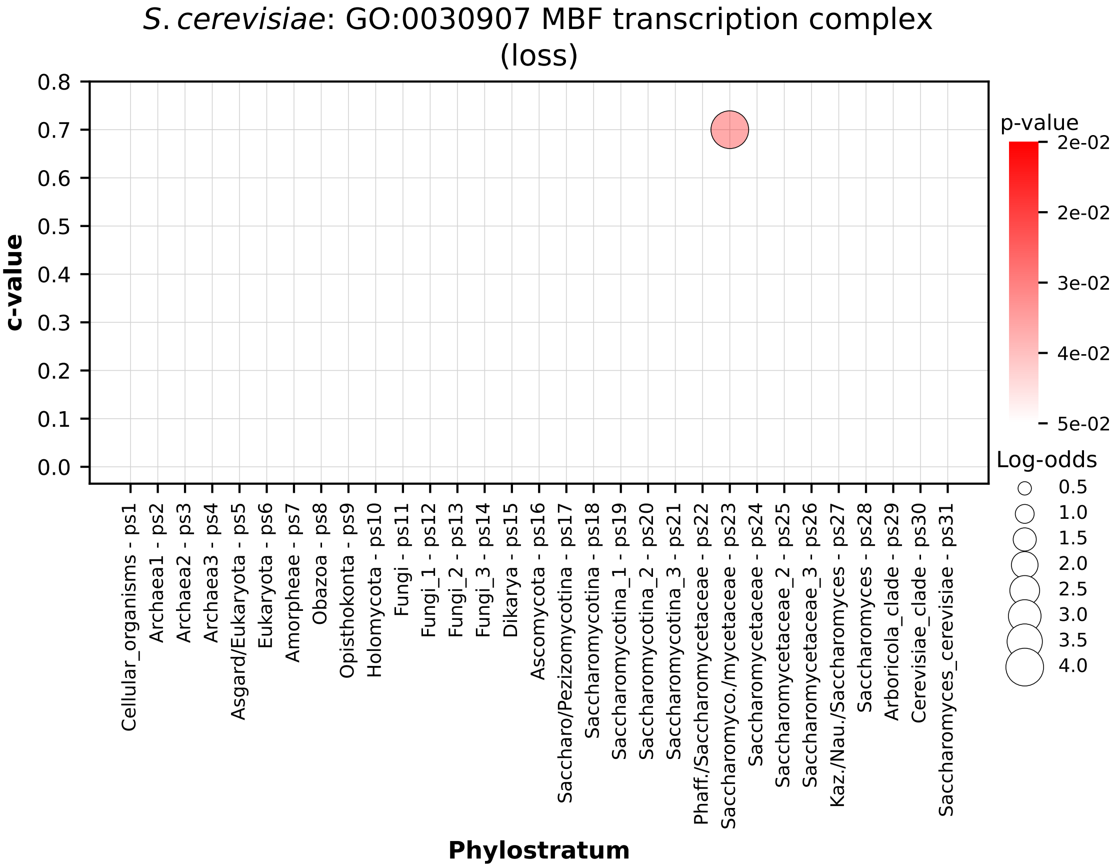
S. cerevisiae: GO:0030907 MBF transcription complex
(loss)
c-value
0.8
0.7
0.6
0.5
0.4
0.3
0.2
0.1
0.0
p-value
2e-02
2e-02
3e-02
4e-02
5e-02
Log-odds
0.5
1.0
1.5
2.0
2.5
3.0
3.5
4.0
Cellular_organisms - ps1
Archaea1 - ps2
Archaea2 - ps3
Archaea3 - ps4
Asgard/Eukaryota - ps5
Eukaryota - ps6
Amorpheae - ps7
Obazoa - ps8
Opisthokonta - ps9
Holomycota - ps10
Fungi - ps11
Fungi_1 - ps12
Fungi_2 - ps13
Fungi_3 - ps14
Dikarya - ps15
Ascomycota - ps16
Saccharo/Pezizomycotina - ps17
Saccharomycotina - ps18
Saccharomycotina_1 - ps19
Saccharomycotina_2 - ps20
Saccharomycotina_3 - ps21
Phaff./Saccharomycetaceae - ps22
Saccharomyco./mycetaceae - ps23
Saccharomycetaceae - ps24
Saccharomycetaceae_2 - ps25
Saccharomycetaceae_3 - ps26
Kaz./Nau./Saccharomyces - ps27
Saccharomyces - ps28
Arboricola_clade - ps29
Cerevisiae_clade - ps30
Saccharomyces_cerevisiae - ps31
Phylostratum

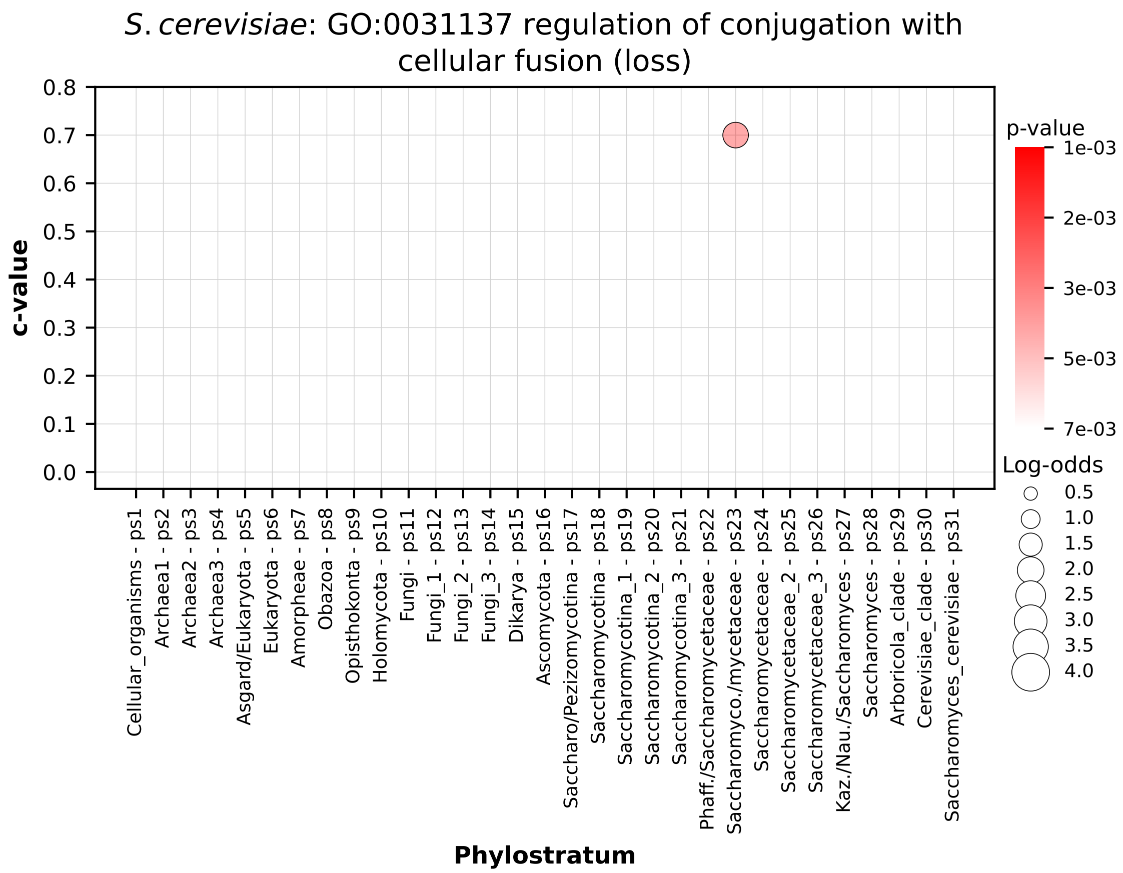
S. cerevisiae: GO:0031137 regulation of conjugation with cellular fusion (loss)
c-value
0.0
0.1
0.2
0.3
0.4
0.5
0.6
0.7
0.8
Phylostratum
Cellular_organisms - ps1
Archaea1 - ps2
Archaea2 - ps3
Archaea3 - ps4
Asgard/Eukaryota - ps5
Eukaryota - ps6
Amorpheae - ps7
Obazoa - ps8
Opisthokonta - ps9
Holomycota - ps10
Fungi - ps11
Fungi_1 - ps12
Fungi_2 - ps13
Fungi_3 - ps14
Dikarya - ps15
Ascomycota - ps16
Saccharo/Pezizomycotina - ps17
Saccharomycotina - ps18
Saccharomycotina_1 - ps19
Saccharomycotina_2 - ps20
Saccharomycotina_3 - ps21
Phaff./Saccharomycetaceae - ps22
Saccharomyco./mycetaceae - ps23
Saccharomycetaceae - ps24
Saccharomycetaceae_2 - ps25
Saccharomycetaceae_3 - ps26
Kaz./Nau./Saccharomyces - ps27
Saccharomyces - ps28
Arboricola_clade - ps29
Cerevisiae_clade - ps30
Saccharomyces_cerevisiae - ps31
p-value
1e-03
2e-03
3e-03
5e-03
7e-03
Log-odds
0.5
1.0
1.5
2.0
2.5
3.0
3.5
4.0

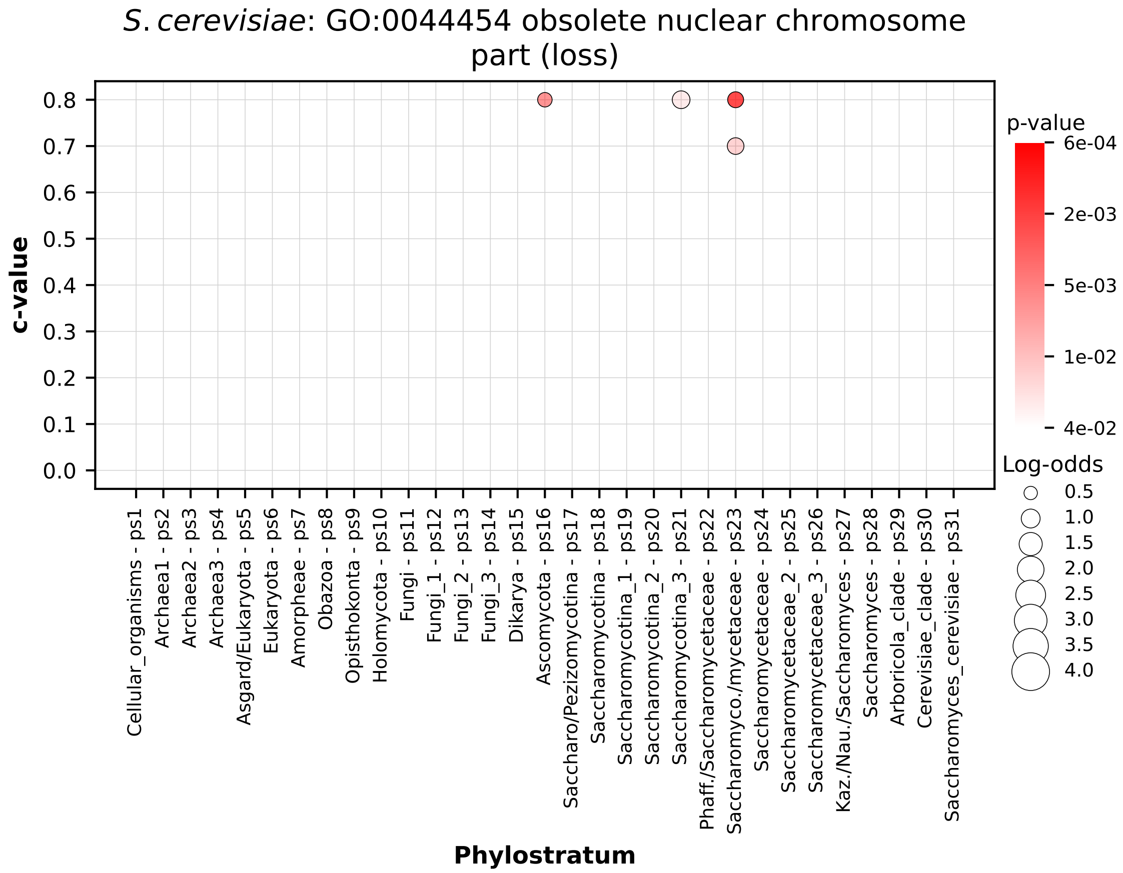

S. cerevisiae: GO:0044454 obsolete nuclear chromosome part (loss)
c-value
0.8
0.7
0.6
0.5
0.4
0.3
0.2
0.1
0.0
p-value
6e-04
2e-03
5e-03
1e-02
4e-02
Log-odds
0.5
1.0
1.5
2.0
2.5
3.0
3.5
4.0
Cellular_organisms - ps1
Archaea1 - ps2
Archaea2 - ps3
Archaea3 - ps4
Asgard/Eukaryota - ps5
Eukaryota - ps6
Amorpheae - ps7
Obazoa - ps8
Opisthokonta - ps9
Holomycota - ps10
Fungi - ps11
Fungi_1 - ps12
Fungi_2 - ps13
Fungi_3 - ps14
Dikarya - ps15
Ascomycota - ps16
Saccharo/Pezizomycotina - ps17
Saccharomycotina - ps18
Saccharomycotina_1 - ps19
Saccharomycotina_2 - ps20
Saccharomycotina_3 - ps21
Phaff./Saccharomycetaceae - ps22
Saccharomyco./mycetaceae - ps23
Saccharomycetaceae - ps24
Saccharomycetaceae_2 - ps25
Saccharomycetaceae_3 - ps26
Kaz./Nau./Saccharomyces - ps27
Saccharomyces - ps28
Arboricola_clade - ps29
Cerevisiae_clade - ps30
Saccharomyces_cerevisiae - ps31
Phylostratum

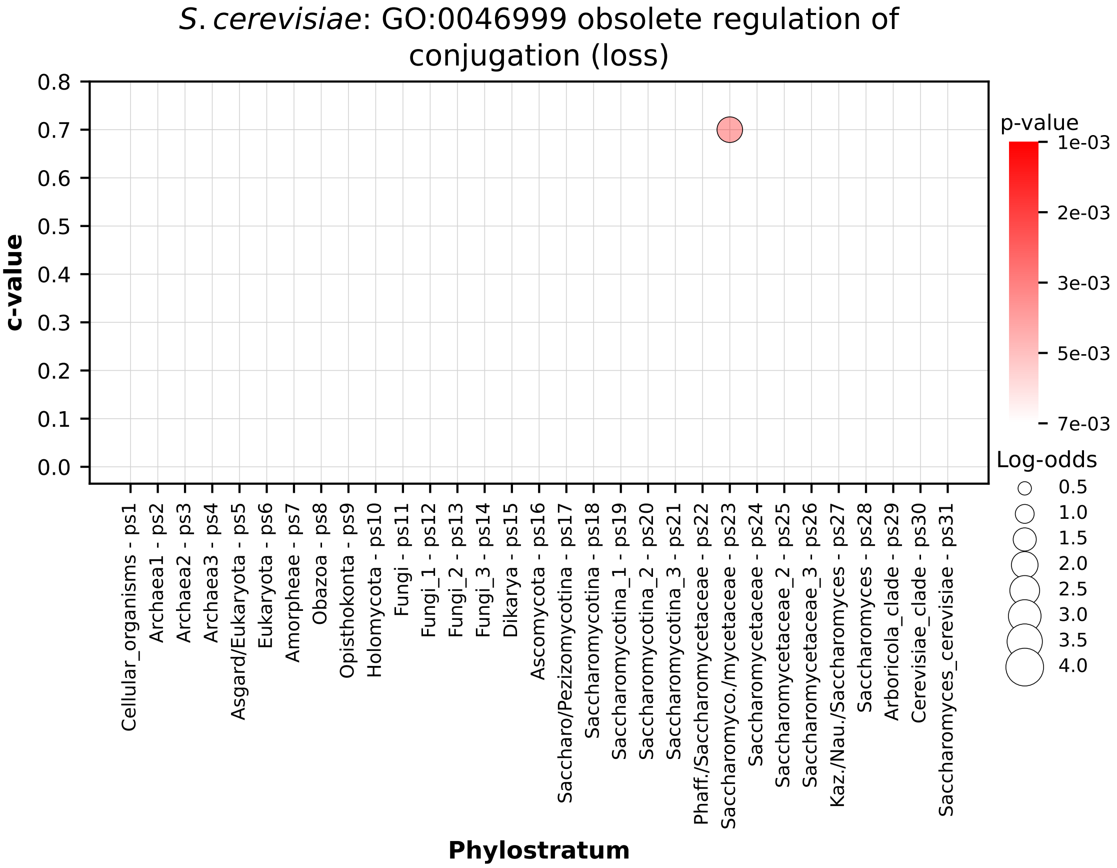

S. cerevisiae: GO:0046999 obsolete regulation of conjugation (loss)
c-value
0.8
0.7
0.6
0.5
0.4
0.3
0.2
0.1
0.0
p-value
1e-03
2e-03
3e-03
5e-03
7e-03
Log-odds
0.5
1.0
1.5
2.0
2.5
3.0
3.5
4.0
Cellular_organisms - ps1
Archaea1 - ps2
Archaea2 - ps3
Archaea3 - ps4
Asgard/Eukaryota - ps5
Eukaryota - ps6
Amorpheae - ps7
Obazoa - ps8
Opisthokonta - ps9
Holomycota - ps10
Fungi - ps11
Fungi_1 - ps12
Fungi_2 - ps13
Fungi_3 - ps14
Dikarya - ps15
Ascomycota - ps16
Saccharo/Pezizomycotina - ps17
Saccharomycotina - ps18
Saccharomycotina_1 - ps19
Saccharomycotina_2 - ps20
Saccharomycotina_3 - ps21
Phaff./Saccharomycetaceae - ps22
Saccharomyco./mycetaceae - ps23
Saccharomycetaceae - ps24
Saccharomycetaceae_2 - ps25
Saccharomycetaceae_3 - ps26
Kaz./Nau./Saccharomyces - ps27
Saccharomyces - ps28
Arboricola_clade - ps29
Cerevisiae_clade - ps30
Saccharomyces_cerevisiae - ps31
Phylostratum

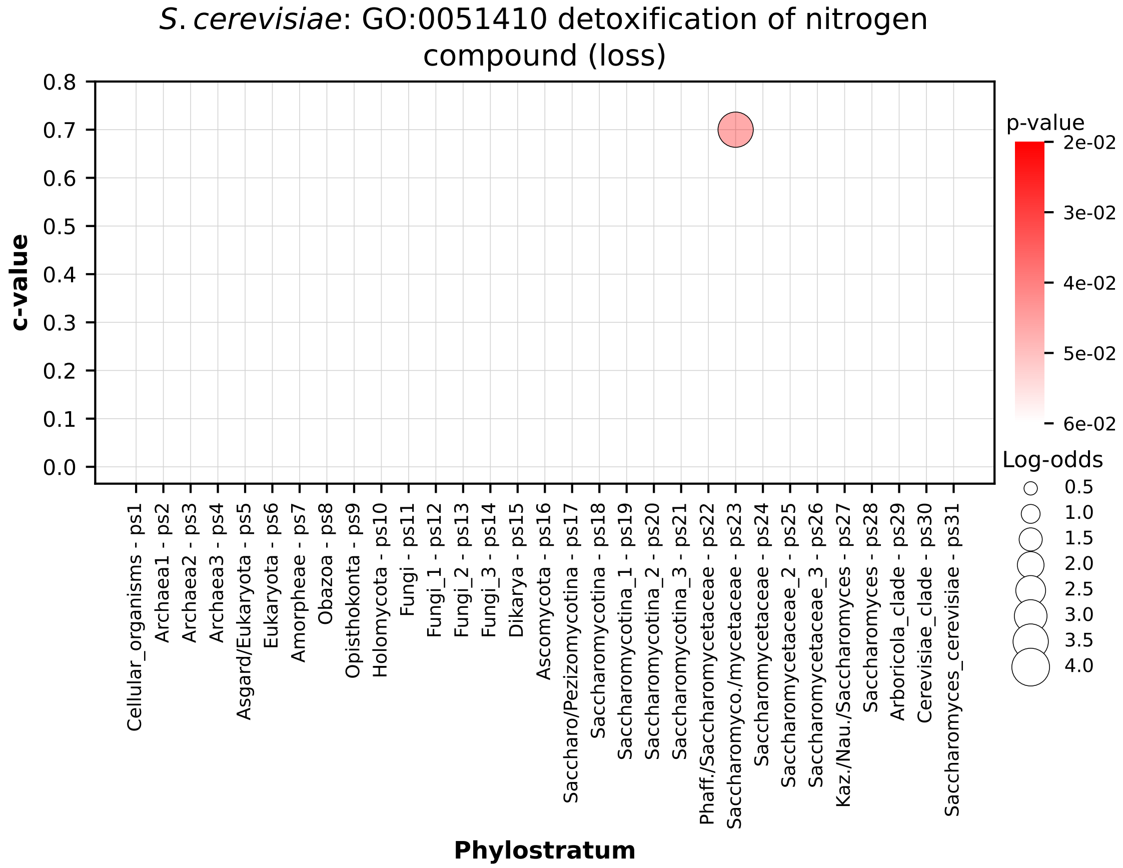

S. cerevisiae: GO:0051410 detoxification of nitrogen compound (loss)
c-value
0.8
0.7
0.6
0.5
0.4
0.3
0.2
0.1
0.0
p-value
2e-02
3e-02
4e-02
5e-02
6e-02
Log-odds
0.5
1.0
1.5
2.0
2.5
3.0
3.5
4.0
Cellular_organisms - ps1
Archaea1 - ps2
Archaea2 - ps3
Archaea3 - ps4
Asgard/Eukaryota - ps5
Eukaryota - ps6
Amorpheae - ps7
Obazoa - ps8
Opisthokonta - ps9
Holomycota - ps10
Fungi - ps11
Fungi_1 - ps12
Fungi_2 - ps13
Fungi_3 - ps14
Dikarya - ps15
Ascomycota - ps16
Saccharo/Pezizomycotina - ps17
Saccharomycotina - ps18
Saccharomycotina_1 - ps19
Saccharomycotina_2 - ps20
Saccharomycotina_3 - ps21
Phaff./Saccharomycetaceae - ps22
Saccharomyco./mycetaceae - ps23
Saccharomycetaceae - ps24
Saccharomycetaceae_2 - ps25
Saccharomycetaceae_3 - ps26
Kaz./Nau./Saccharomyces - ps27
Saccharomyces - ps28
Arboricola_clade - ps29
Cerevisiae_clade - ps30
Saccharomyces_cerevisiae - ps31
Phylostratum

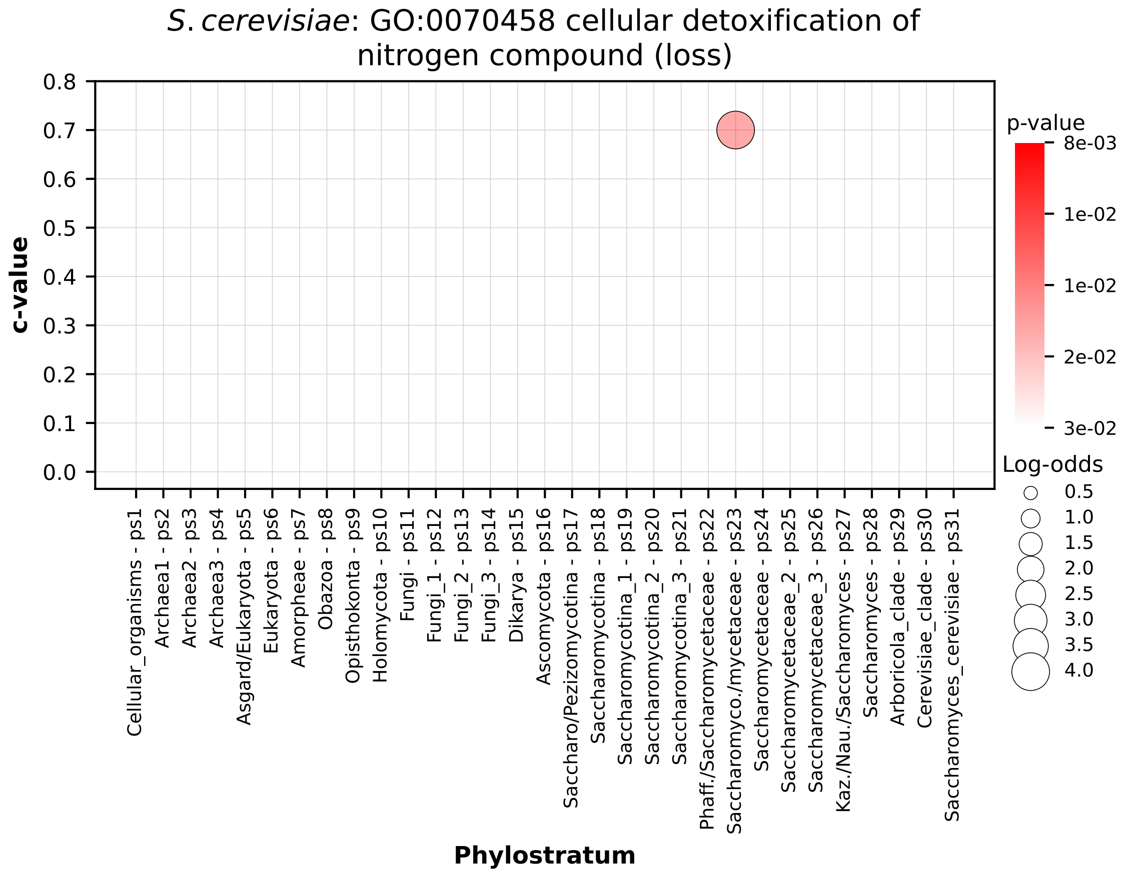
S. cerevisiae: GO:0070458 cellular detoxification of nitrogen compound (loss)
c-value
0.0
0.1
0.2
0.3
0.4
0.5
0.6
0.7
0.8
Phylostratum
Cellular_organisms - ps1
Archaea1 - ps2
Archaea2 - ps3
Archaea3 - ps4
Asgard/Eukaryota - ps5
Eukaryota - ps6
Amorpheae - ps7
Obazoa - ps8
Opisthokonta - ps9
Holomycota - ps10
Fungi - ps11
Fungi_1 - ps12
Fungi_2 - ps13
Fungi_3 - ps14
Dikarya - ps15
Ascomycota - ps16
Saccharo/Pezizomycotina - ps17
Saccharomycotina - ps18
Saccharomycotina_1 - ps19
Saccharomycotina_2 - ps20
Saccharomycotina_3 - ps21
Phaff./Saccharomycetaceae - ps22
Saccharomyco./mycetaceae - ps23
Saccharomycetaceae - ps24
Saccharomycetaceae_2 - ps25
Saccharomycetaceae_3 - ps26
Kaz./Nau./Saccharomyces - ps27
Saccharomyces - ps28
Arboricola_clade - ps29
Cerevisiae_clade - ps30
Saccharomyces_cerevisiae - ps31
p-value
8e-03
1e-02
1e-02
2e-02
3e-02
Log-odds
0.5
1.0
1.5
2.0
2.5
3.0
3.5
4.0

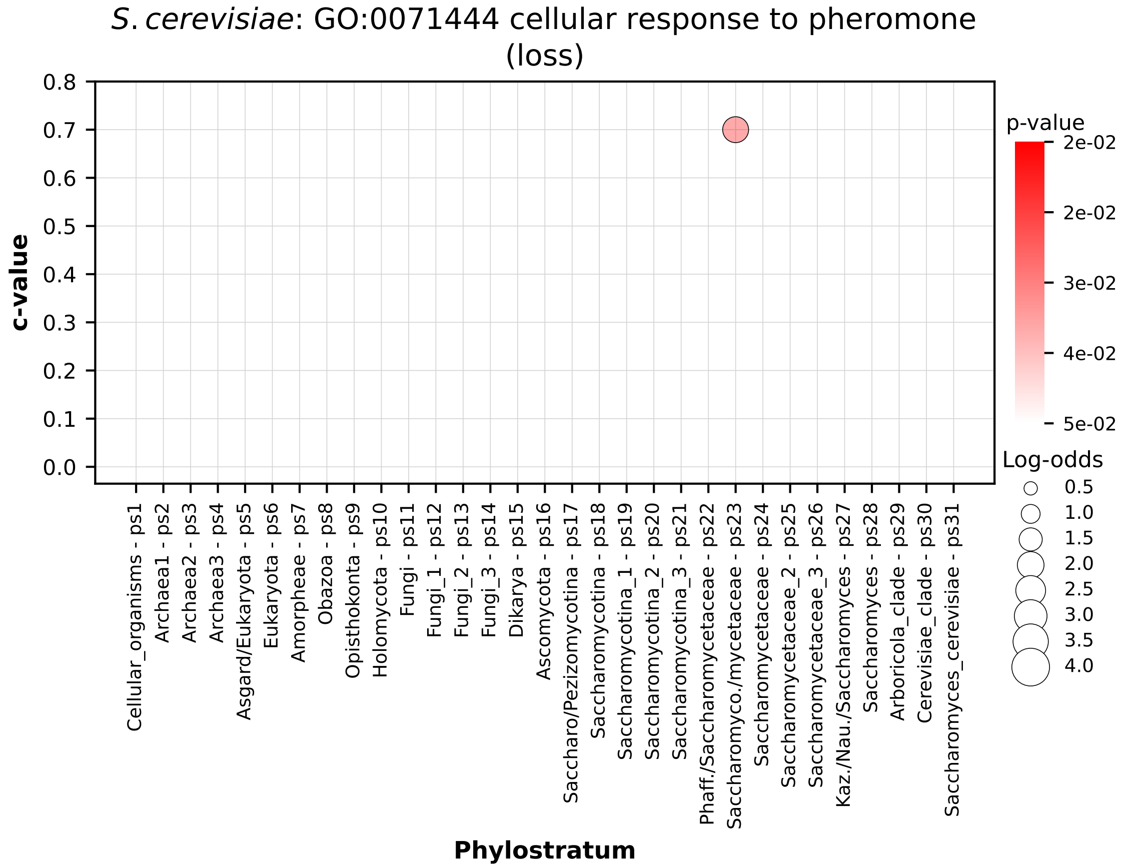
S. cerevisiae: GO:0071444 cellular response to pheromone
(loss)
c-value
0.8
0.7
0.6
0.5
0.4
0.3
0.2
0.1
0.0
p-value
2e-02
2e-02
3e-02
4e-02
5e-02
Log-odds
0.5
1.0
1.5
2.0
2.5
3.0
3.5
4.0
Cellular_organisms - ps1
Archaea1 - ps2
Archaea2 - ps3
Archaea3 - ps4
Asgard/Eukaryota - ps5
Eukaryota - ps6
Amorpheae - ps7
Obazoa - ps8
Opisthokonta - ps9
Holomycota - ps10
Fungi - ps11
Fungi_1 - ps12
Fungi_2 - ps13
Fungi_3 - ps14
Dikarya - ps15
Ascomycota - ps16
Saccharo/Pezizomycotina - ps17
Saccharomycotina - ps18
Saccharomycotina_1 - ps19
Saccharomycotina_2 - ps20
Saccharomycotina_3 - ps21
Phaff./Saccharomycetaceae - ps22
Saccharomyco./mycetaceae - ps23
Saccharomycetaceae - ps24
Saccharomycetaceae_2 - ps25
Saccharomycetaceae_3 - ps26
Kaz./Nau./Saccharomyces - ps27
Saccharomyces - ps28
Arboricola_clade - ps29
Cerevisiae_clade - ps30
Saccharomyces_cerevisiae - ps31
Phylostratum

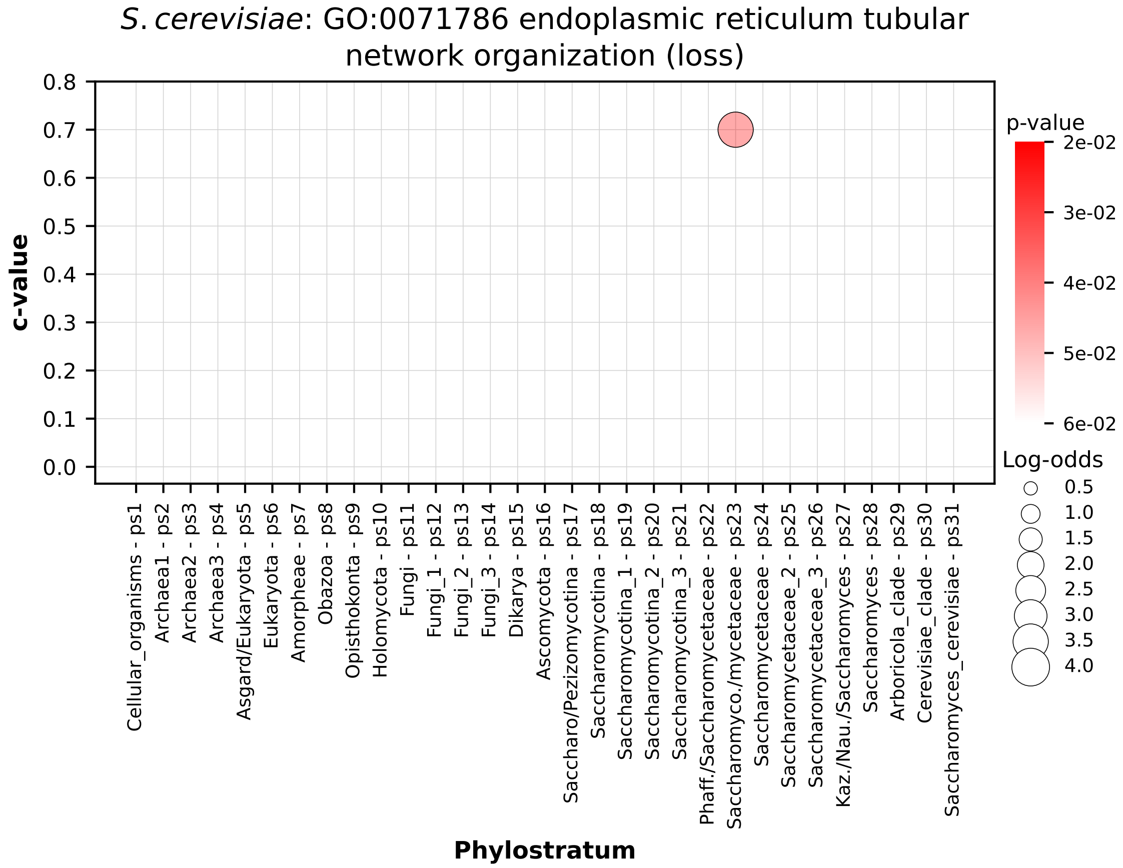

S. cerevisiae: GO:0071786 endoplasmic reticulum tubular network organization (loss)
c-value
0.8
0.7
0.6
0.5
0.4
0.3
0.2
0.1
0.0
p-value
2e-02
3e-02
4e-02
5e-02
6e-02
Log-odds
0.5
1.0
1.5
2.0
2.5
3.0
3.5
4.0
Cellular_organisms - ps1
Archaea1 - ps2
Archaea2 - ps3
Archaea3 - ps4
Asgard/Eukaryota - ps5
Eukaryota - ps6
Amorpheae - ps7
Obazoa - ps8
Opisthokonta - ps9
Holomycota - ps10
Fungi - ps11
Fungi_1 - ps12
Fungi_2 - ps13
Fungi_3 - ps14
Dikarya - ps15
Ascomycota - ps16
Saccharo/Pezizomycotina - ps17
Saccharomycotina - ps18
Saccharomycotina_1 - ps19
Saccharomycotina_2 - ps20
Saccharomycotina_3 - ps21
Phaff./Saccharomycetaceae - ps22
Saccharomyco./mycetaceae - ps23
Saccharomycetaceae - ps24
Saccharomycetaceae_2 - ps25
Saccharomycetaceae_3 - ps26
Kaz./Nau./Saccharomyces - ps27
Saccharomyces - ps28
Arboricola_clade - ps29
Cerevisiae_clade - ps30
Saccharomyces_cerevisiae - ps31
Phylostratum

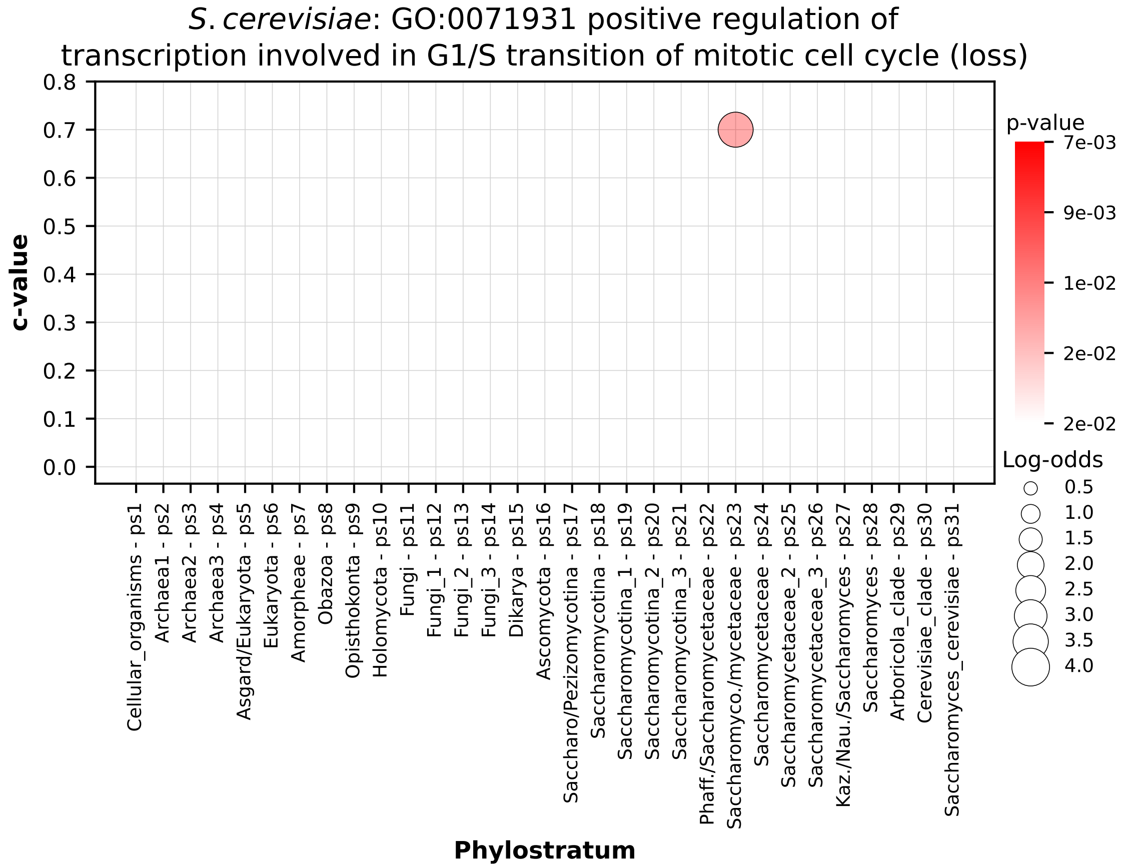
S. cerevisiae: GO:0071931 positive regulation of transcription involved in G1/S transition of mitotic cell cycle (loss)
c-value
0.8
0.7
0.6
0.5
0.4
0.3
0.2
0.1
0.0
p-value
7e-03
9e-03
1e-02
2e-02
2e-02
Log-odds
0.5
1.0
1.5
2.0
2.5
3.0
3.5
4.0
Cellular_organisms - ps1
Archaea1 - ps2
Archaea2 - ps3
Archaea3 - ps4
Asgard/Eukaryota - ps5
Eukaryota - ps6
Amorpheae - ps7
Obazoa - ps8
Opisthokonta - ps9
Holomycota - ps10
Fungi - ps11
Fungi_1 - ps12
Fungi_2 - ps13
Fungi_3 - ps14
Dikarya - ps15
Ascomycota - ps16
Saccharo/Pezizomycotina - ps17
Saccharomycotina - ps18
Saccharomycotina_1 - ps19
Saccharomycotina_2 - ps20
Saccharomycotina_3 - ps21
Phaff./Saccharomycetaceae - ps22
Saccharomyco./mycetaceae - ps23
Saccharomycetaceae - ps24
Saccharomycetaceae_2 - ps25
Saccharomycetaceae_3 - ps26
Kaz./Nau./Saccharomyces - ps27
Saccharomyces - ps28
Arboricola_clade - ps29
Cerevisiae_clade - ps30
Saccharomyces_cerevisiae - ps31
Phylostratum

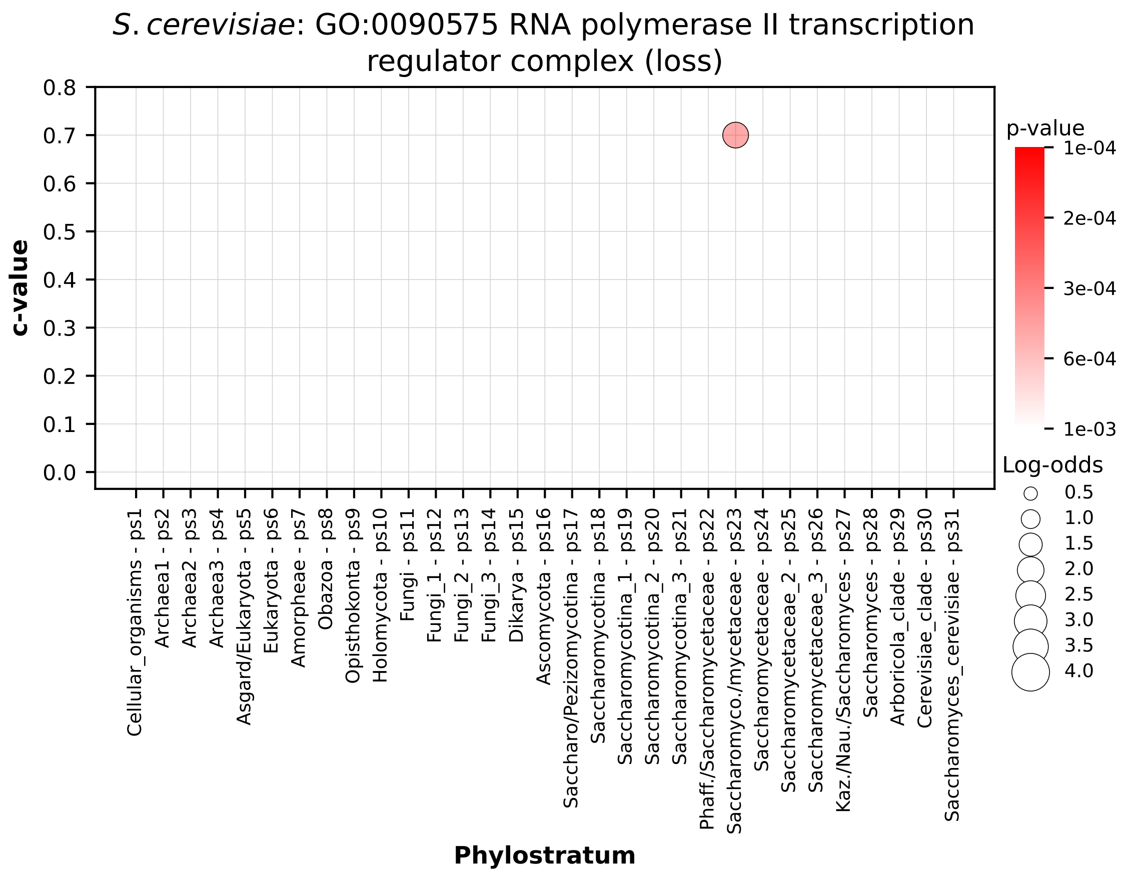

S. cerevisiae: GO:0090575 RNA polymerase II transcription regulator complex (loss)
c-value
0.8
0.7
0.6
0.5
0.4
0.3
0.2
0.1
0.0
Phylostratum
Cellular_organisms - ps1
Archaea1 - ps2
Archaea2 - ps3
Archaea3 - ps4
Asgard/Eukaryota - ps5
Eukaryota - ps6
Amorpheae - ps7
Obazoa - ps8
Opisthokonta - ps9
Holomycota - ps10
Fungi - ps11
Fungi_1 - ps12
Fungi_2 - ps13
Fungi_3 - ps14
Dikarya - ps15
Ascomycota - ps16
Saccharo/Pezizomycotina - ps17
Saccharomycotina - ps18
Saccharomycotina_1 - ps19
Saccharomycotina_2 - ps20
Saccharomycotina_3 - ps21
Phaff./Saccharomycetaceae - ps22
Saccharomyco./mycetaceae - ps23
Saccharomycetaceae - ps24
Saccharomycetaceae_2 - ps25
Saccharomycetaceae_3 - ps26
Kaz./Nau./Saccharomyces - ps27
Saccharomyces - ps28
Arboricola_clade - ps29
Cerevisiae_clade - ps30
Saccharomyces_cerevisiae - ps31
p-value
1e-04
2e-04
3e-04
6e-04
1e-03
Log-odds
0.5
1.0
1.5
2.0
2.5
3.0
3.5
4.0

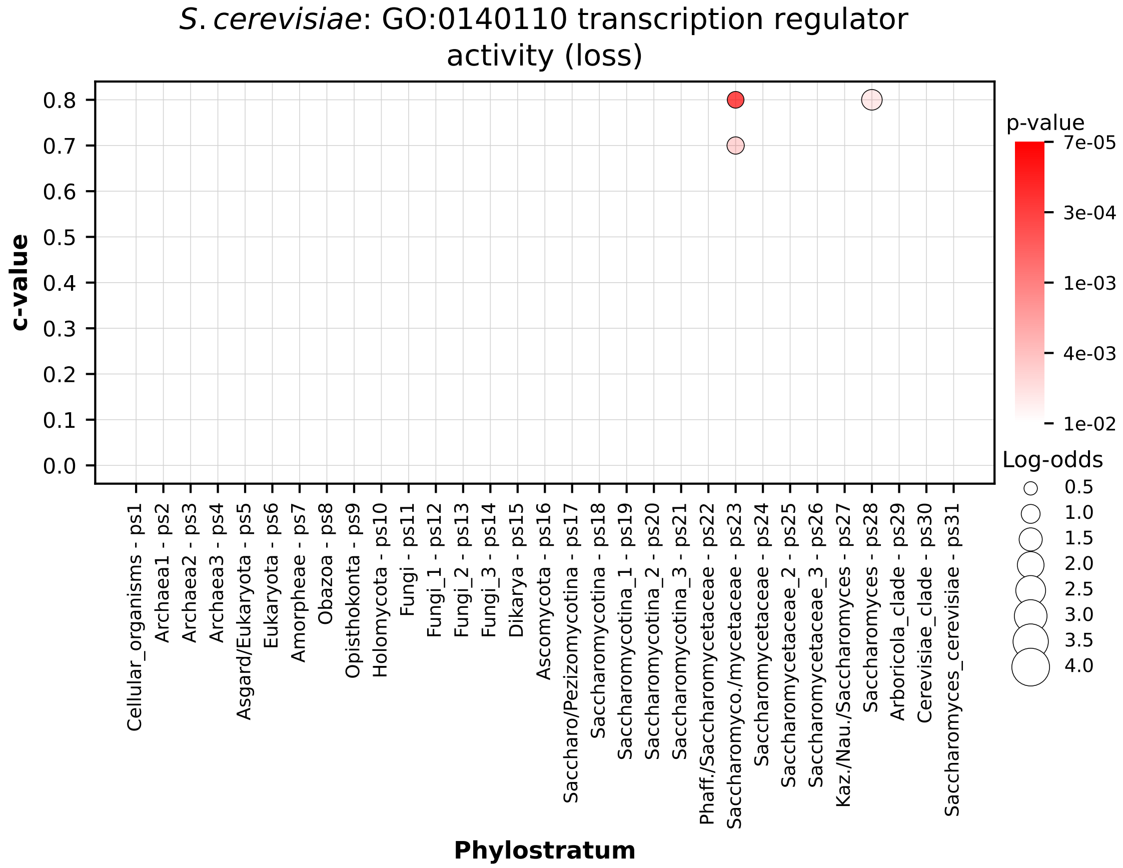

S. cerevisiae: GO:0140110 transcription regulator activity (loss)
c-value
0.0
0.1
0.2
0.3
0.4
0.5
0.6
0.7
0.8
p-value
7e-05
3e-04
1e-03
4e-03
1e-02
Log-odds
0.5
1.0
1.5
2.0
2.5
3.0
3.5
4.0
Cellular_organisms - ps1
Archaea1 - ps2
Archaea2 - ps3
Archaea3 - ps4
Asgard/Eukaryota - ps5
Eukaryota - ps6
Amorpheae - ps7
Obazoa - ps8
Opisthokonta - ps9
Holomycota - ps10
Fungi - ps11
Fungi_1 - ps12
Fungi_2 - ps13
Fungi_3 - ps14
Dikarya - ps15
Ascomycota - ps16
Saccharo/Pezizomycotina - ps17
Saccharomycotina - ps18
Saccharomycotina_1 - ps19
Saccharomycotina_2 - ps20
Saccharomycotina_3 - ps21
Phaff./Saccharomycetaceae - ps22
Saccharomyco./mycetaceae - ps23
Saccharomycetaceae - ps24
Saccharomycetaceae_2 - ps25
Saccharomycetaceae_3 - ps26
Kaz./Nau./Saccharomyces - ps27
Saccharomyces - ps28
Arboricola_clade - ps29
Cerevisiae_clade - ps30
Saccharomyces_cerevisiae - ps31
Phylostratum

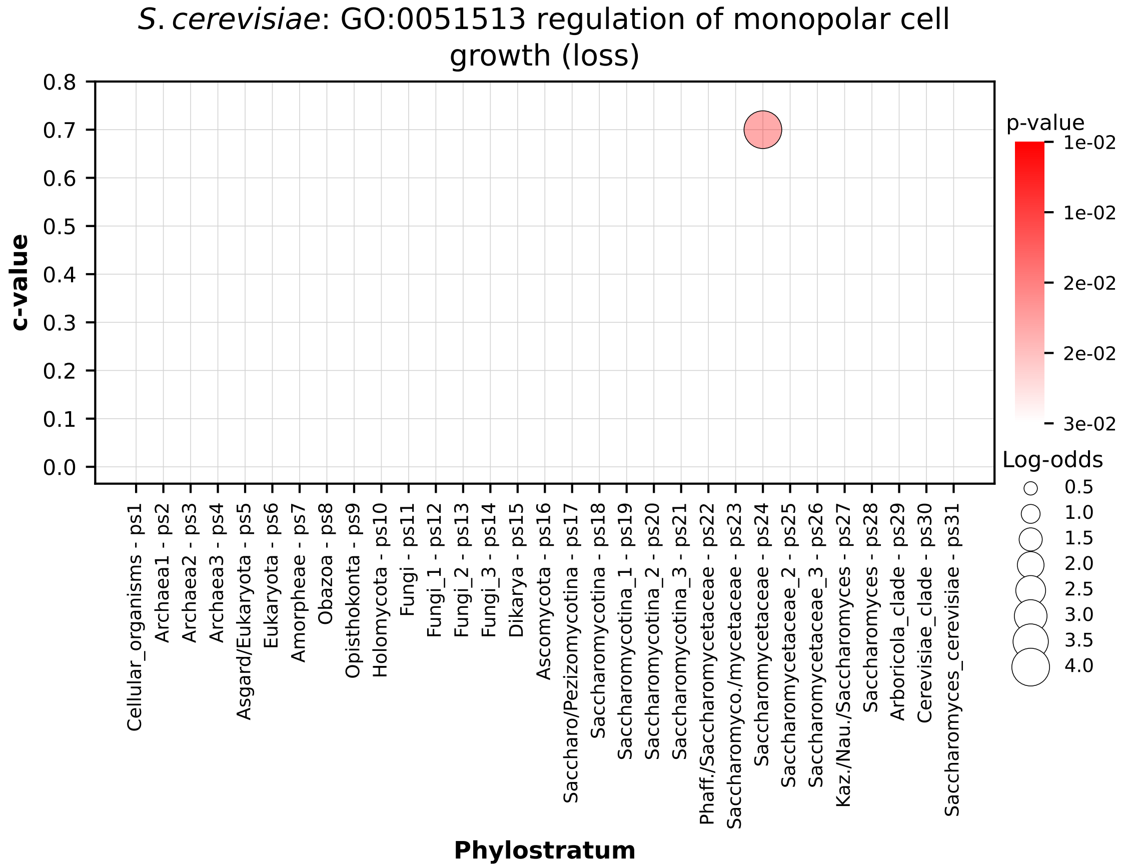

S. cerevisiae: GO:0051513 regulation of monopolar cell growth (loss)
c-value
0.8
0.7
0.6
0.5
0.4
0.3
0.2
0.1
0.0
Phylostratum
Cellular_organisms - ps1
Archaea1 - ps2
Archaea2 - ps3
Archaea3 - ps4
Asgard/Eukaryota - ps5
Eukaryota - ps6
Amorpheae - ps7
Obazoa - ps8
Opisthokonta - ps9
Holomycota - ps10
Fungi - ps11
Fungi_1 - ps12
Fungi_2 - ps13
Fungi_3 - ps14
Dikarya - ps15
Ascomycota - ps16
Saccharo/Pezizomycotina - ps17
Saccharomycotina - ps18
Saccharomycotina_1 - ps19
Saccharomycotina_2 - ps20
Saccharomycotina_3 - ps21
Phaff./Saccharomycetaceae - ps22
Saccharomyco./mycetaceae - ps23
Saccharomycetaceae - ps24
Saccharomycetaceae_2 - ps25
Saccharomycetaceae_3 - ps26
Kaz./Nau./Saccharomyces - ps27
Saccharomyces - ps28
Arboricola_clade - ps29
Cerevisiae_clade - ps30
Saccharomyces_cerevisiae - ps31
p-value
1e-02
1e-02
2e-02
2e-02
3e-02
Log-odds
0.5
1.0
1.5
2.0
2.5
3.0
3.5
4.0

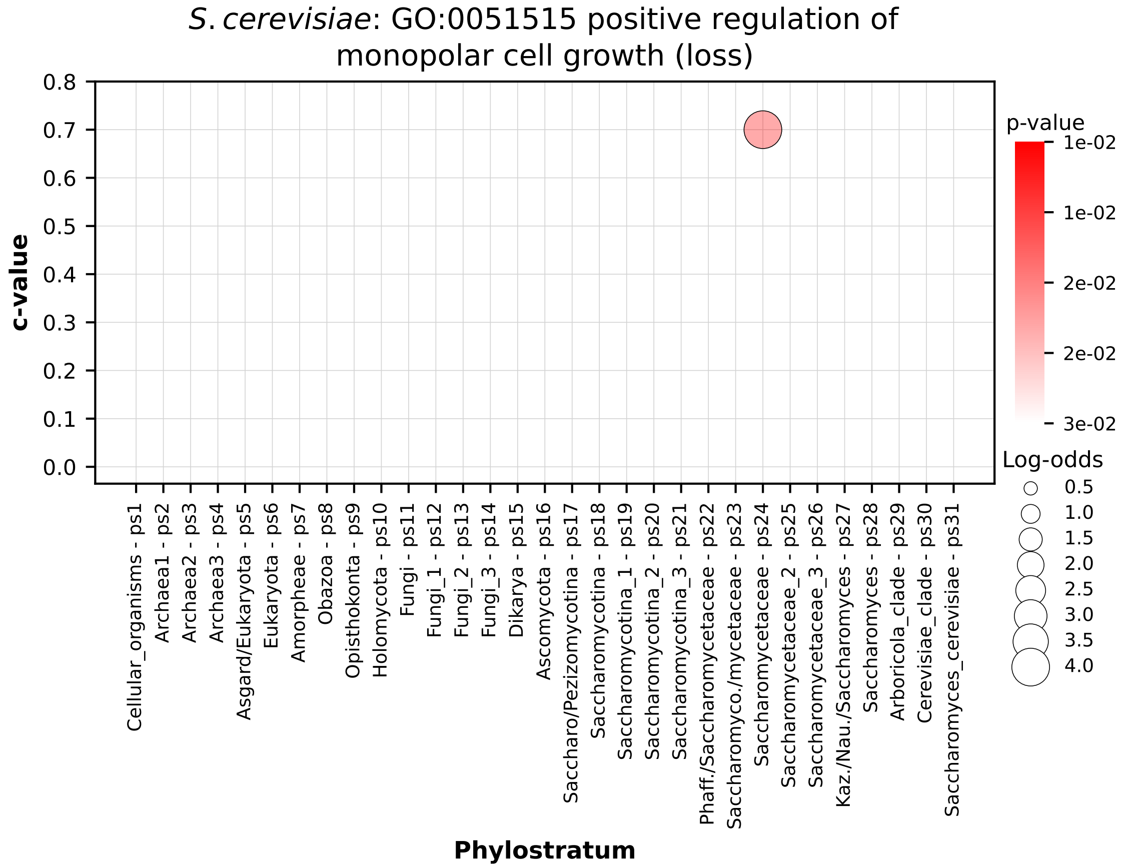

S. cerevisiae: GO:0051515 positive regulation of monopolar cell growth (loss)
c-value
0.8
0.7
0.6
0.5
0.4
0.3
0.2
0.1
0.0
p-value
1e-02
1e-02
2e-02
2e-02
3e-02
Log-odds
0.5
1.0
1.5
2.0
2.5
3.0
3.5
4.0
Cellular_organisms - ps1
Archaea1 - ps2
Archaea2 - ps3
Archaea3 - ps4
Asgard/Eukaryota - ps5
Eukaryota - ps6
Amorpheae - ps7
Obazoa - ps8
Opisthokonta - ps9
Holomycota - ps10
Fungi - ps11
Fungi_1 - ps12
Fungi_2 - ps13
Fungi_3 - ps14
Dikarya - ps15
Ascomycota - ps16
Saccharo/Pezizomycotina - ps17
Saccharomycotina - ps18
Saccharomycotina_1 - ps19
Saccharomycotina_2 - ps20
Saccharomycotina_3 - ps21
Phaff./Saccharomycetaceae - ps22
Saccharomyco./mycetaceae - ps23
Saccharomycetaceae - ps24
Saccharomycetaceae_2 - ps25
Saccharomycetaceae_3 - ps26
Kaz./Nau./Saccharomyces - ps27
Saccharomyces - ps28
Arboricola_clade - ps29
Cerevisiae_clade - ps30
Saccharomyces_cerevisiae - ps31
Phylostratum

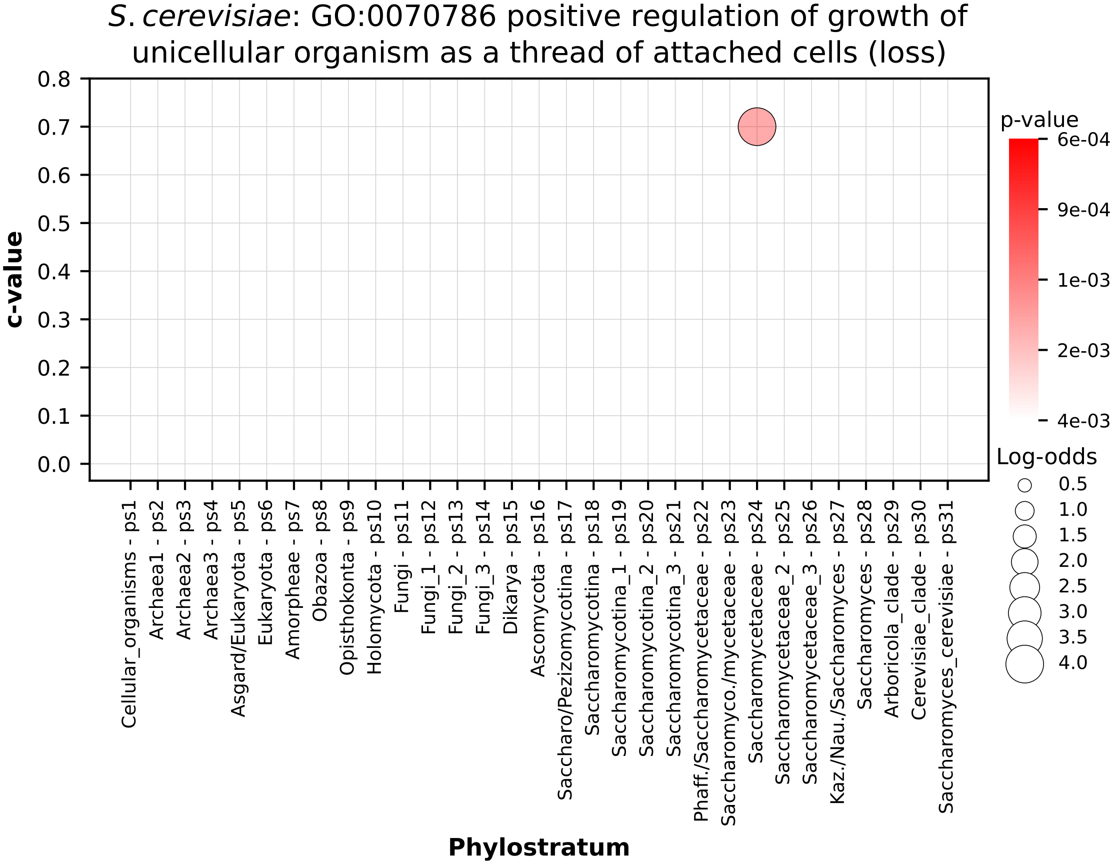

S. cerevisiae: GO:0070786 positive regulation of growth of unicellular organism as a thread of attached cells (loss)
c-value
0.8
0.7
0.6
0.5
0.4
0.3
0.2
0.1
0.0
p-value
6e-04
9e-04
1e-03
2e-03
4e-03
Log-odds
0.5
1.0
1.5
2.0
2.5
3.0
3.5
4.0
Cellular_organisms - ps1
Archaea1 - ps2
Archaea2 - ps3
Archaea3 - ps4
Asgard/Eukaryota - ps5
Eukaryota - ps6
Amorpheae - ps7
Obazoa - ps8
Opisthokonta - ps9
Holomycota - ps10
Fungi - ps11
Fungi_1 - ps12
Fungi_2 - ps13
Fungi_3 - ps14
Dikarya - ps15
Ascomycota - ps16
Saccharo/Pezizomycotina - ps17
Saccharomycotina - ps18
Saccharomycotina_1 - ps19
Saccharomycotina_2 - ps20
Saccharomycotina_3 - ps21
Phaff./Saccharomycetaceae - ps22
Saccharomyco./mycetaceae - ps23
Saccharomycetaceae - ps24
Saccharomycetaceae_2 - ps25
Saccharomycetaceae_3 - ps26
Kaz./Nau./Saccharomyces - ps27
Saccharomyces - ps28
Arboricola_clade - ps29
Cerevisiae_clade - ps30
Saccharomyces_cerevisiae - ps31
Phylostratum

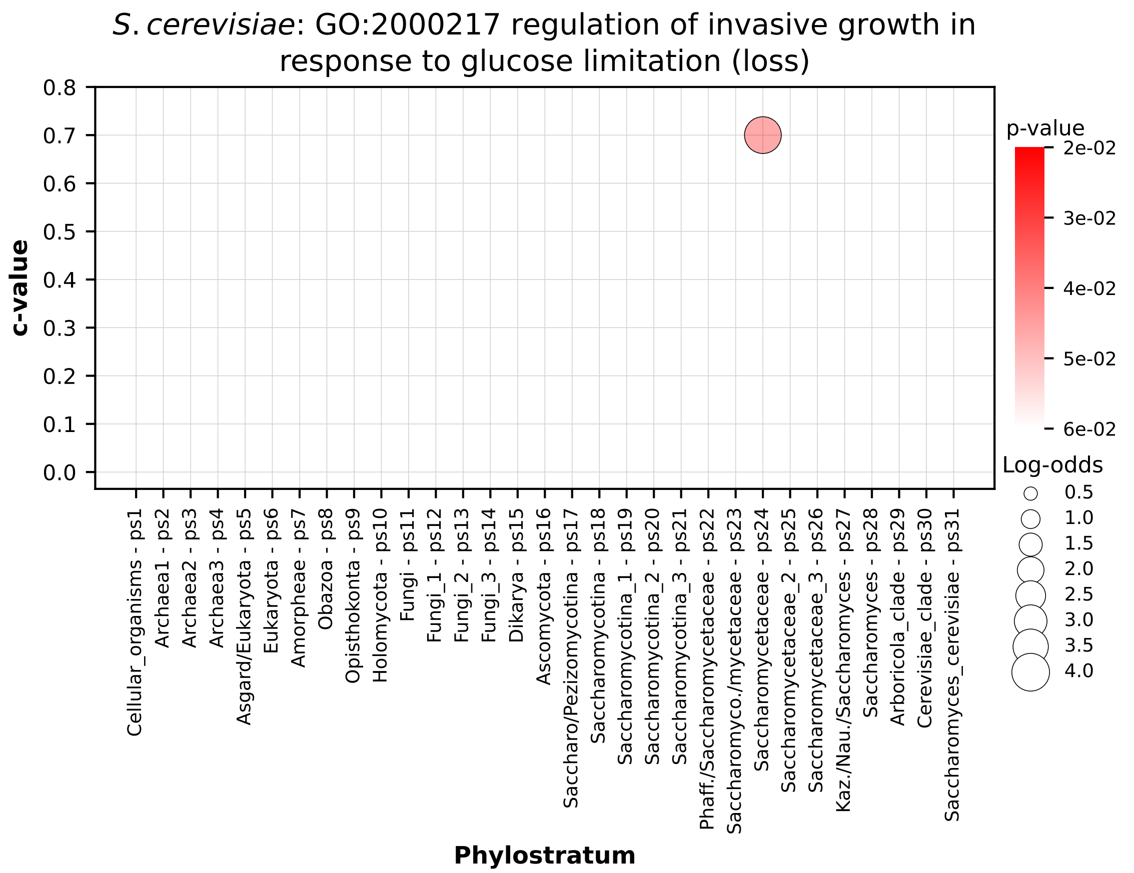
S. cerevisiae: GO:2000217 regulation of invasive growth in response to glucose limitation (loss)
c-value
0.8
0.7
0.6
0.5
0.4
0.3
0.2
0.1
0.0
p-value
2e-02
3e-02
4e-02
5e-02
6e-02
Log-odds
0.5
1.0
1.5
2.0
2.5
3.0
3.5
4.0
Cellular_organisms - ps1
Archaea1 - ps2
Archaea2 - ps3
Archaea3 - ps4
Asgard/Eukaryota - ps5
Eukaryota - ps6
Amorpheae - ps7
Obazoa - ps8
Opisthokonta - ps9
Holomycota - ps10
Fungi - ps11
Fungi_1 - ps12
Fungi_2 - ps13
Fungi_3 - ps14
Dikarya - ps15
Ascomycota - ps16
Saccharo/Pezizomycotina - ps17
Saccharomycotina - ps18
Saccharomycotina_1 - ps19
Saccharomycotina_2 - ps20
Saccharomycotina_3 - ps21
Phaff./Saccharomycetaceae - ps22
Saccharomyco./mycetaceae - ps23
Saccharomycetaceae - ps24
Saccharomycetaceae_2 - ps25
Saccharomycetaceae_3 - ps26
Kaz./Nau./Saccharomyces - ps27
Saccharomyces - ps28
Arboricola_clade - ps29
Cerevisiae_clade - ps30
Saccharomyces_cerevisiae - ps31
Phylostratum

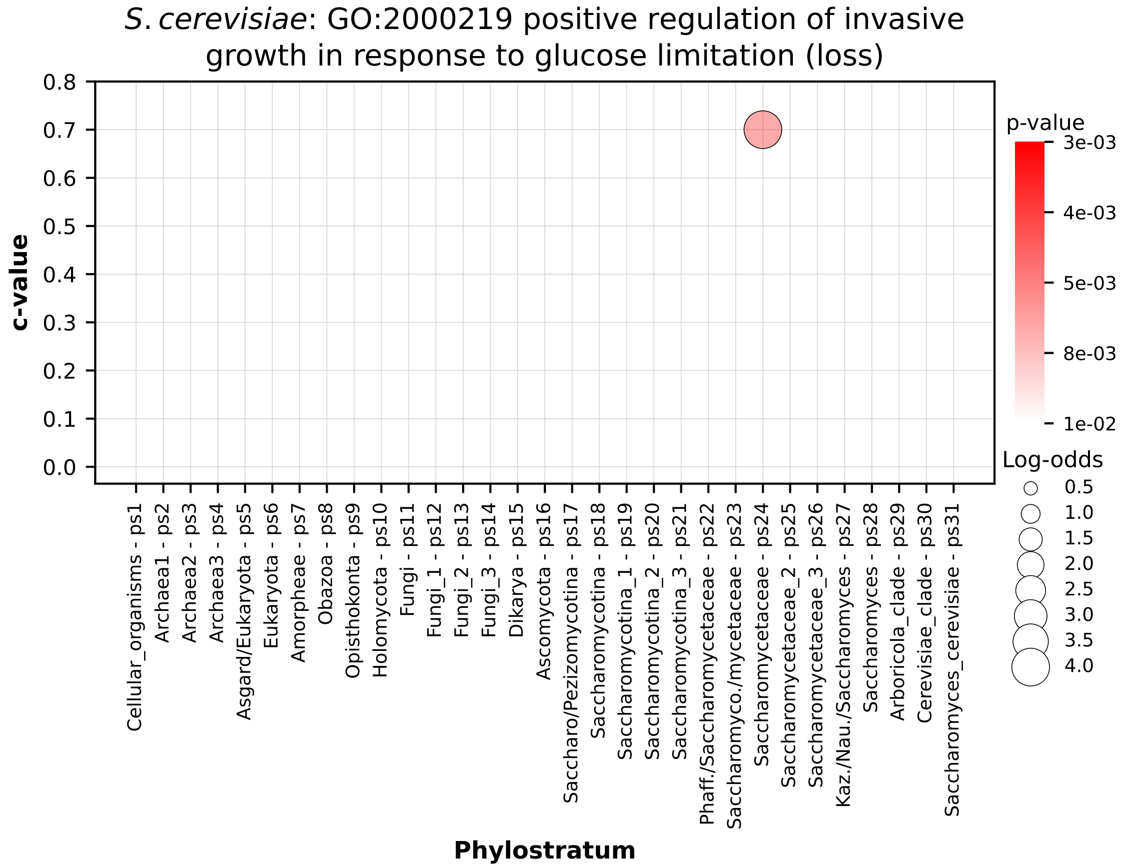
S. cerevisiae: GO:2000219 positive regulation of invasive growth in response to glucose limitation (loss)
c-value
Phylostratum
p-value
3e-03
4e-03
5e-03
8e-03
1e-02
Log-odds
0.5
1.0
1.5
2.0
2.5
3.0
3.5
4.0
Cellular_organisms - ps1
Archaea1 - ps2
Archaea2 - ps3
Archaea3 - ps4
Asgard/Eukaryota - ps5
Eukaryota - ps6
Amorpheae - ps7
Obazoa - ps8
Opisthokonta - ps9
Holomycota - ps10
Fungi - ps11
Fungi_1 - ps12
Fungi_2 - ps13
Fungi_3 - ps14
Dikarya - ps15
Ascomycota - ps16
Saccharo/Pezizomycotina - ps17
Saccharomycotina - ps18
Saccharomycotina_1 - ps19
Saccharomycotina_2 - ps20
Saccharomycotina_3 - ps21
Phaff./Saccharomycetaceae - ps22
Saccharomyco./mycetaceae - ps23
Saccharomycetaceae - ps24
Saccharomycetaceae_2 - ps25
Saccharomycetaceae_3 - ps26
Kaz./Nau./Saccharomyces - ps27
Saccharomyces - ps28
Arboricola_clade - ps29
Cerevisiae_clade - ps30
Saccharomyces_cerevisiae - ps31

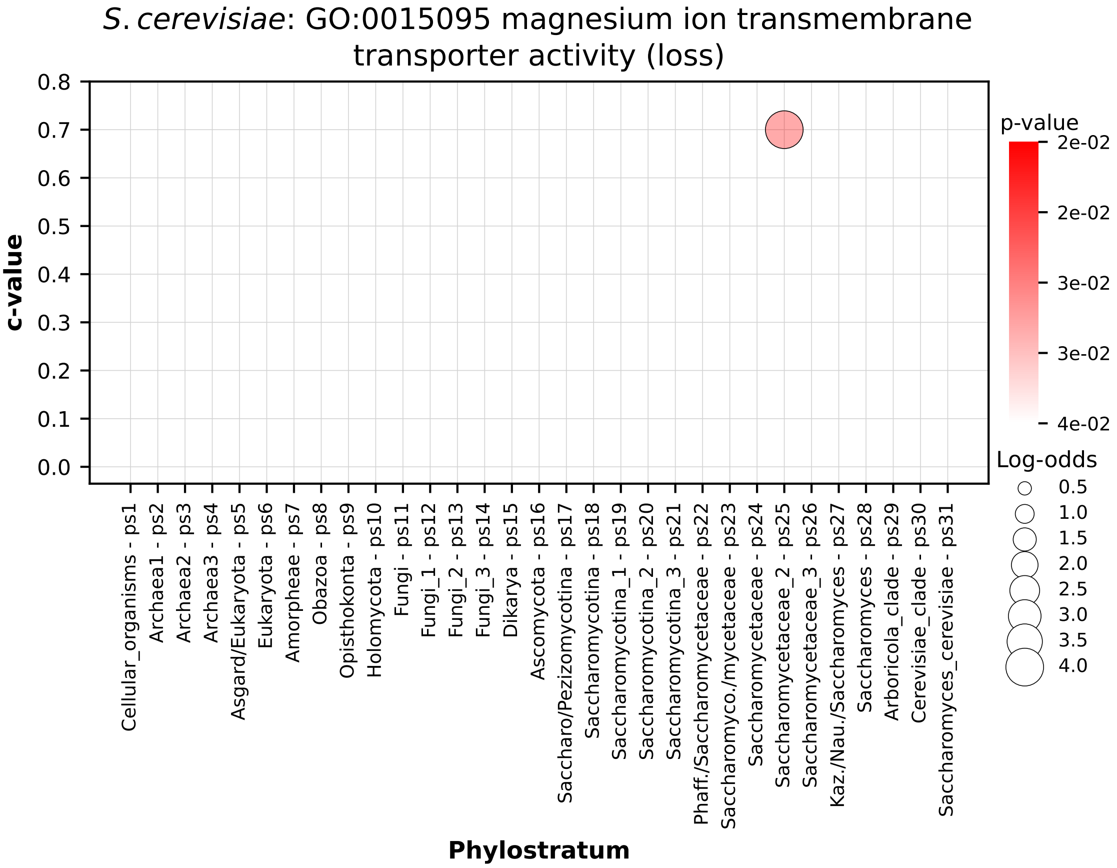
S. cerevisiae: GO:0015095 magnesium ion transmembrane transporter activity (loss)
c-value
0.8
0.7
0.6
0.5
0.4
0.3
0.2
0.1
0.0
p-value
2e-02
2e-02
3e-02
3e-02
4e-02
Log-odds
0.5
1.0
1.5
2.0
2.5
3.0
3.5
4.0
Cellular_organisms - ps1
Archaea1 - ps2
Archaea2 - ps3
Archaea3 - ps4
Asgard/Eukaryota - ps5
Eukaryota - ps6
Amorpheae - ps7
Obazoa - ps8
Opisthokonta - ps9
Holomycota - ps10
Fungi - ps11
Fungi_1 - ps12
Fungi_2 - ps13
Fungi_3 - ps14
Dikarya - ps15
Ascomycota - ps16
Saccharo/Pezizomycotina - ps17
Saccharomycotina - ps18
Saccharomycotina_1 - ps19
Saccharomycotina_2 - ps20
Saccharomycotina_3 - ps21
Phaff./Saccharomycetaceae - ps22
Saccharomyco./mycetaceae - ps23
Saccharomycetaceae - ps24
Saccharomycetaceae_2 - ps25
Saccharomycetaceae_3 - ps26
Kaz./Nau./Saccharomyces - ps27
Saccharomyces - ps28
Arboricola_clade - ps29
Cerevisiae_clade - ps30
Saccharomyces_cerevisiae - ps31
Phylostratum

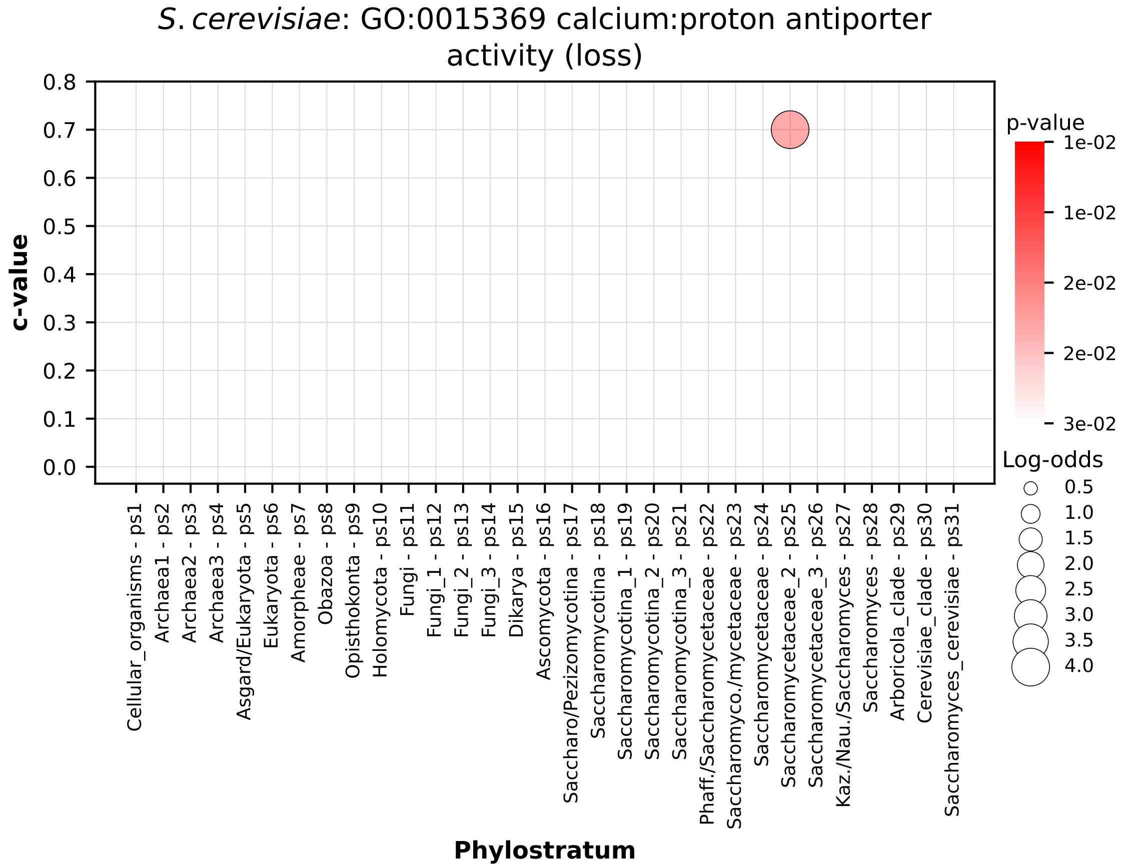

S. cerevisiae: GO:0015369 calcium:proton antiporter activity (loss)
c-value
0.8
0.7
0.6
0.5
0.4
0.3
0.2
0.1
0.0
p-value
1e-02
1e-02
2e-02
2e-02
3e-02
Log-odds
0.5
1.0
1.5
2.0
2.5
3.0
3.5
4.0
Cellular_organisms - ps1
Archaea1 - ps2
Archaea2 - ps3
Archaea3 - ps4
Asgard/Eukaryota - ps5
Eukaryota - ps6
Amorpheae - ps7
Obazoa - ps8
Opisthokonta - ps9
Holomycota - ps10
Fungi - ps11
Fungi_1 - ps12
Fungi_2 - ps13
Fungi_3 - ps14
Dikarya - ps15
Ascomycota - ps16
Saccharo/Pezizomycotina - ps17
Saccharomycotina - ps18
Saccharomycotina_1 - ps19
Saccharomycotina_2 - ps20
Saccharomycotina_3 - ps21
Phaff./Saccharomycetaceae - ps22
Saccharomyco./mycetaceae - ps23
Saccharomycetaceae - ps24
Saccharomycetaceae_2 - ps25
Saccharomycetaceae_3 - ps26
Kaz./Nau./Saccharomyces - ps27
Saccharomyces - ps28
Arboricola_clade - ps29
Cerevisiae_clade - ps30
Saccharomyces_cerevisiae - ps31
Phylostratum

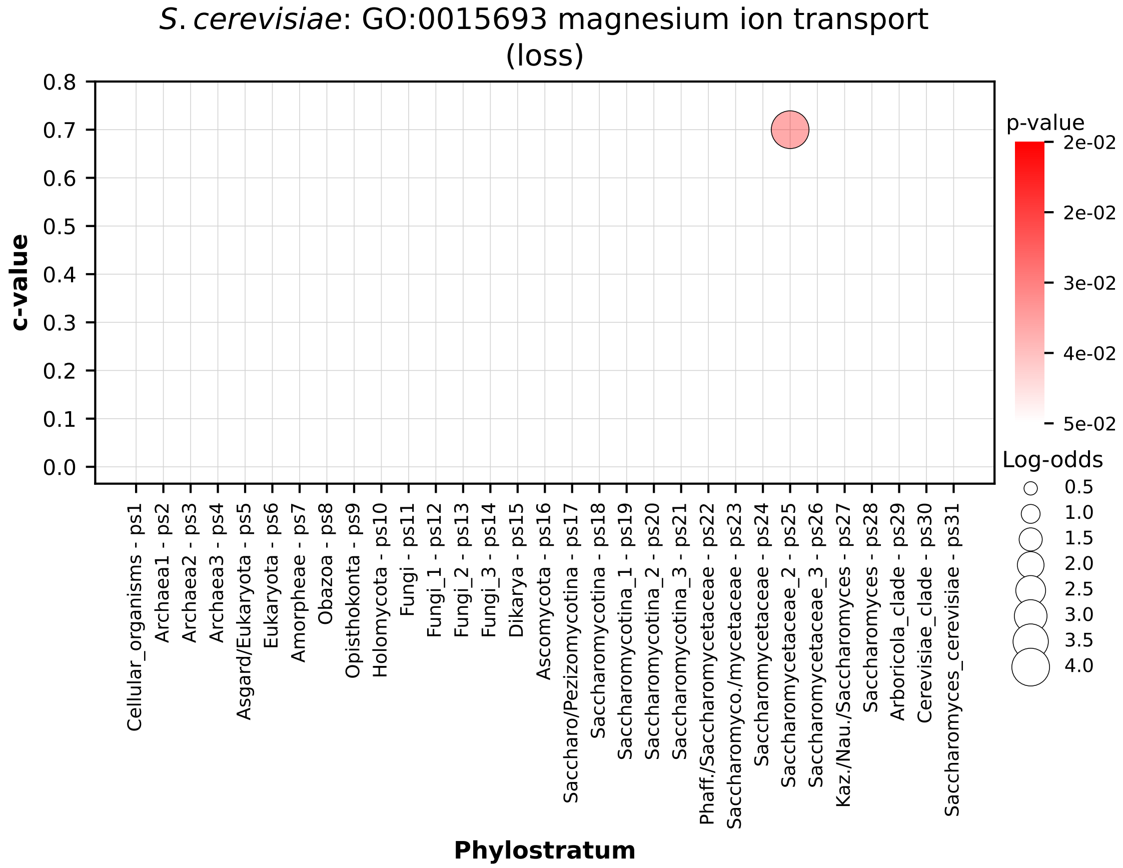
S. cerevisiae: GO:0015693 magnesium ion transport
(loss)
c-value
0.0
0.1
0.2
0.3
0.4
0.5
0.6
0.7
0.8
p-value
2e-02
2e-02
3e-02
4e-02
5e-02
Log-odds
0.5
1.0
1.5
2.0
2.5
3.0
3.5
4.0
Cellular_organisms - ps1
Archaea1 - ps2
Archaea2 - ps3
Archaea3 - ps4
Asgard/Eukaryota - ps5
Eukaryota - ps6
Amorpheae - ps7
Obazoa - ps8
Opisthokonta - ps9
Holomycota - ps10
Fungi - ps11
Fungi_1 - ps12
Fungi_2 - ps13
Fungi_3 - ps14
Dikarya - ps15
Ascomycota - ps16
Saccharo/Pezizomycotina - ps17
Saccharomycotina - ps18
Saccharomycotina_1 - ps19
Saccharomycotina_2 - ps20
Saccharomycotina_3 - ps21
Phaff./Saccharomycetaceae - ps22
Saccharomyco./mycetaceae - ps23
Saccharomycetaceae - ps24
Saccharomycetaceae_2 - ps25
Saccharomycetaceae_3 - ps26
Kaz./Nau./Saccharomyces - ps27
Saccharomyces - ps28
Arboricola_clade - ps29
Cerevisiae_clade - ps30
Saccharomyces_cerevisiae - ps31
Phylostratum

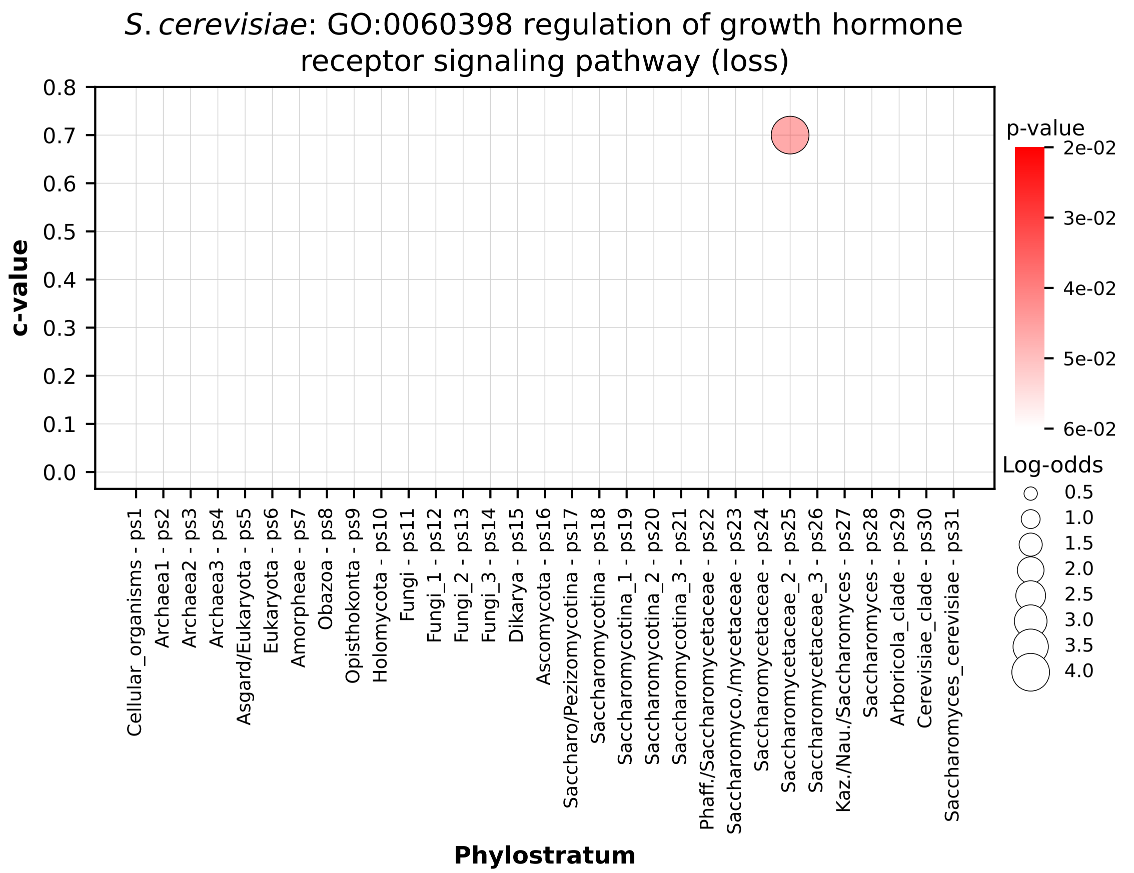

S. cerevisiae: GO:0060398 regulation of growth hormone receptor signaling pathway (loss)
c-value
0.8
0.7
0.6
0.5
0.4
0.3
0.2
0.1
0.0
p-value
2e-02
3e-02
4e-02
5e-02
6e-02
Log-odds
0.5
1.0
1.5
2.0
2.5
3.0
3.5
4.0
Cellular_organisms - ps1
Archaea1 - ps2
Archaea2 - ps3
Archaea3 - ps4
Asgard/Eukaryota - ps5
Eukaryota - ps6
Amorpheae - ps7
Obazoa - ps8
Opisthokonta - ps9
Holomycota - ps10
Fungi - ps11
Fungi_1 - ps12
Fungi_2 - ps13
Fungi_3 - ps14
Dikarya - ps15
Ascomycota - ps16
Saccharo/Pezizomycotina - ps17
Saccharomycotina - ps18
Saccharomycotina_1 - ps19
Saccharomycotina_2 - ps20
Saccharomycotina_3 - ps21
Phaff./Saccharomycetaceae - ps22
Saccharomyco./mycetaceae - ps23
Saccharomycetaceae - ps24
Saccharomycetaceae_2 - ps25
Saccharomycetaceae_3 - ps26
Kaz./Nau./Saccharomyces - ps27
Saccharomyces - ps28
Arboricola_clade - ps29
Cerevisiae_clade - ps30
Saccharomyces_cerevisiae - ps31
Phylostratum

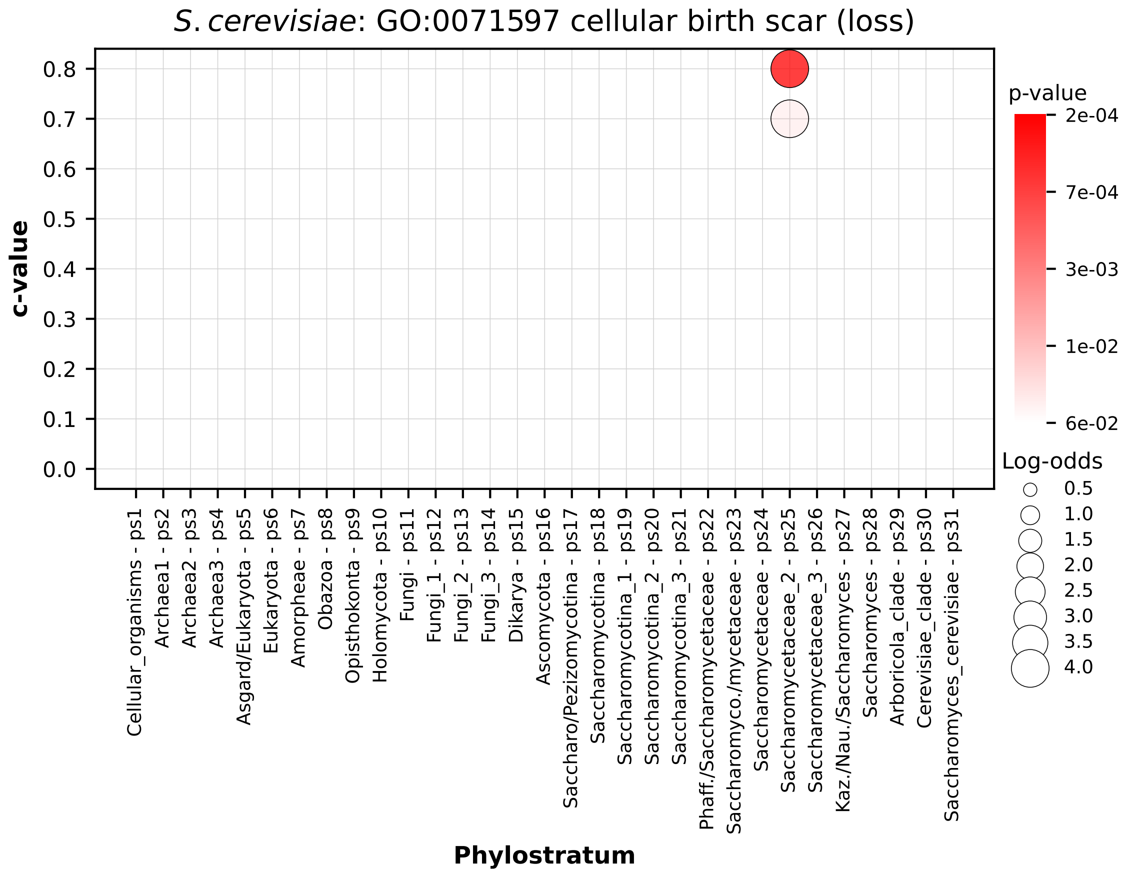

S. cerevisiae: GO:0071597 cellular birth scar (loss)
c-value
0.8
0.7
0.6
0.5
0.4
0.3
0.2
0.1
0.0
Phylostratum
Cellular_organisms - ps1
Archaea1 - ps2
Archaea2 - ps3
Archaea3 - ps4
Asgard/Eukaryota - ps5
Eukaryota - ps6
Amorpheae - ps7
Obazoa - ps8
Opisthokonta - ps9
Holomycota - ps10
Fungi - ps11
Fungi_1 - ps12
Fungi_2 - ps13
Fungi_3 - ps14
Dikarya - ps15
Ascomycota - ps16
Saccharo/Pezizomycotina - ps17
Saccharomycotina - ps18
Saccharomycotina_1 - ps19
Saccharomycotina_2 - ps20
Saccharomycotina_3 - ps21
Phaff./Saccharomycetaceae - ps22
Saccharomyco./mycetaceae - ps23
Saccharomycetaceae - ps24
Saccharomycetaceae_2 - ps25
Saccharomycetaceae_3 - ps26
Kaz./Nau./Saccharomyces - ps27
Saccharomyces - ps28
Arboricola_clade - ps29
Cerevisiae_clade - ps30
Saccharomyces_cerevisiae - ps31
p-value
2e-04
7e-04
3e-03
1e-02
6e-02
Log-odds
0.5
1.0
1.5
2.0
2.5
3.0
3.5
4.0

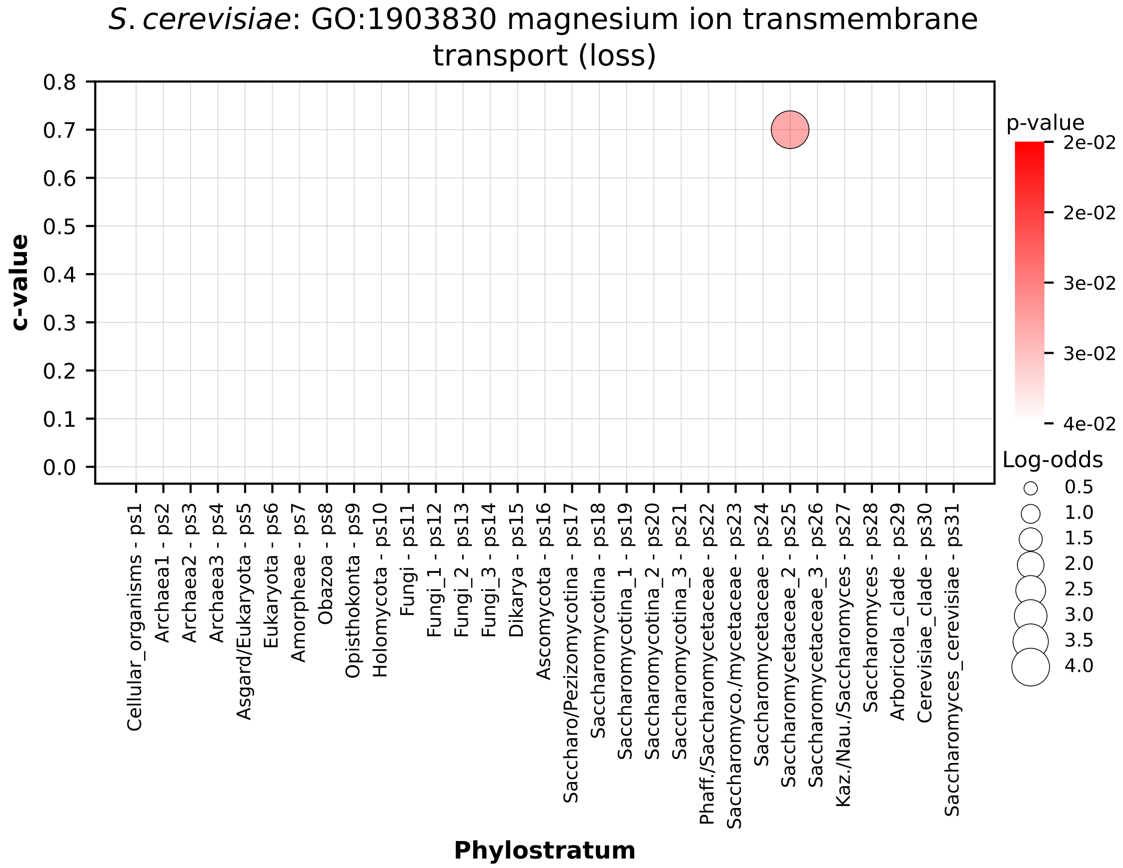
S. cerevisiae: GO:1903830 magnesium ion transmembrane transport (loss)
c-value
0.8
0.7
0.6
0.5
0.4
0.3
0.2
0.1
0.0
p-value
2e-02
2e-02
3e-02
3e-02
4e-02
Log-odds
0.5
1.0
1.5
2.0
2.5
3.0
3.5
4.0
Cellular_organisms - ps1
Archaea1 - ps2
Archaea2 - ps3
Archaea3 - ps4
Asgard/Eukaryota - ps5
Eukaryota - ps6
Amorpheae - ps7
Obazoa - ps8
Opisthokonta - ps9
Holomycota - ps10
Fungi - ps11
Fungi_1 - ps12
Fungi_2 - ps13
Fungi_3 - ps14
Dikarya - ps15
Ascomycota - ps16
Saccharo/Pezizomycotina - ps17
Saccharomycotina - ps18
Saccharomycotina_1 - ps19
Saccharomycotina_2 - ps20
Saccharomycotina_3 - ps21
Phaff./Saccharomycetaceae - ps22
Saccharomyco./mycetaceae - ps23
Saccharomycetaceae - ps24
Saccharomycetaceae_2 - ps25
Saccharomycetaceae_3 - ps26
Kaz./Nau./Saccharomyces - ps27
Saccharomyces - ps28
Arboricola_clade - ps29
Cerevisiae_clade - ps30
Saccharomyces_cerevisiae - ps31
Phylostratum

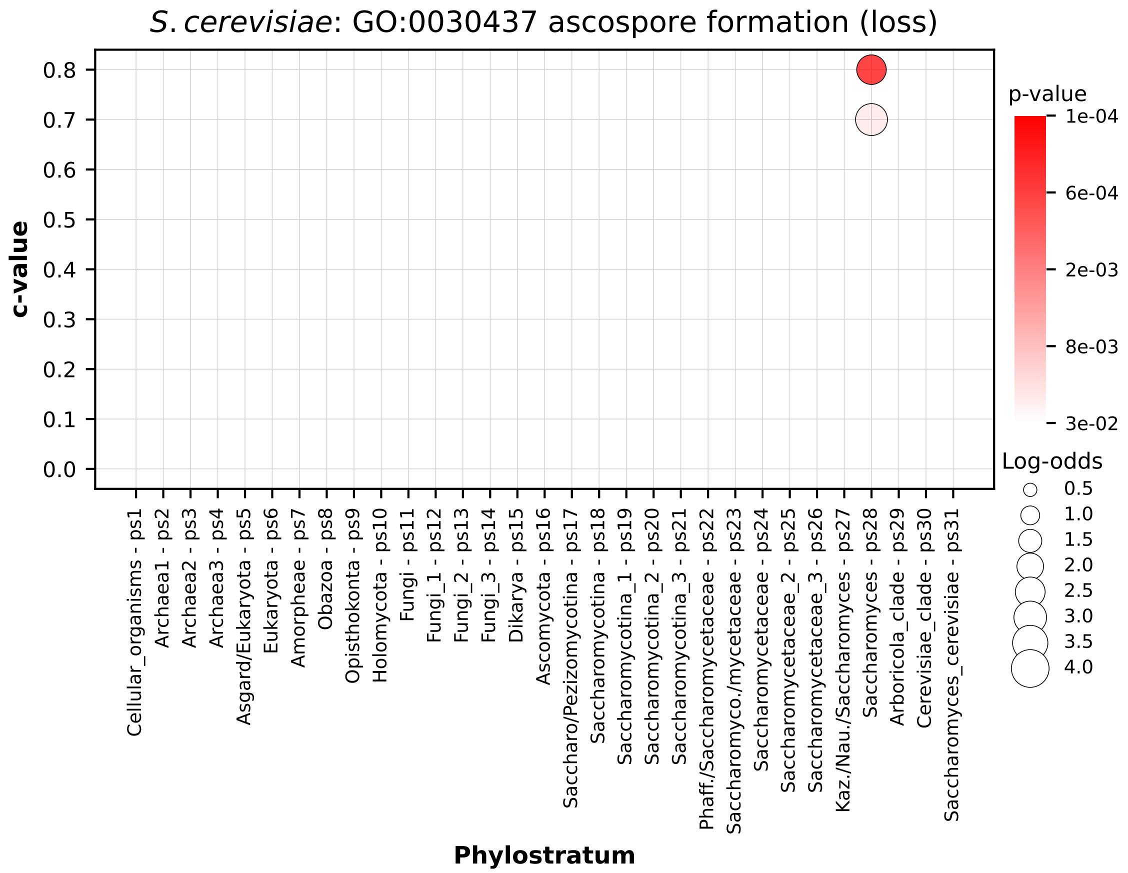

S. cerevisiae: GO:0030437 ascospore formation (loss)
c-value
0.8
0.7
0.6
0.5
0.4
0.3
0.2
0.1
0.0
p-value
1e-04
6e-04
2e-03
8e-03
3e-02
Log-odds
0.5
1.0
1.5
2.0
2.5
3.0
3.5
4.0
Cellular_organisms - ps1
Archaea1 - ps2
Archaea2 - ps3
Archaea3 - ps4
Asgard/Eukaryota - ps5
Eukaryota - ps6
Amorpheae - ps7
Obazoa - ps8
Opisthokonta - ps9
Holomycota - ps10
Fungi - ps11
Fungi_1 - ps12
Fungi_2 - ps13
Fungi_3 - ps14
Dikarya - ps15
Ascomycota - ps16
Saccharo/Pezizomycotina - ps17
Saccharomycotina - ps18
Saccharomycotina_1 - ps19
Saccharomycotina_2 - ps20
Saccharomycotina_3 - ps21
Phaff./Saccharomycetaceae - ps22
Saccharomyco./mycetaceae - ps23
Saccharomycetaceae - ps24
Saccharomycetaceae_2 - ps25
Saccharomycetaceae_3 - ps26
Kaz./Nau./Saccharomyces - ps27
Saccharomyces - ps28
Arboricola_clade - ps29
Cerevisiae_clade - ps30
Saccharomyces_cerevisiae - ps31
Phylostratum

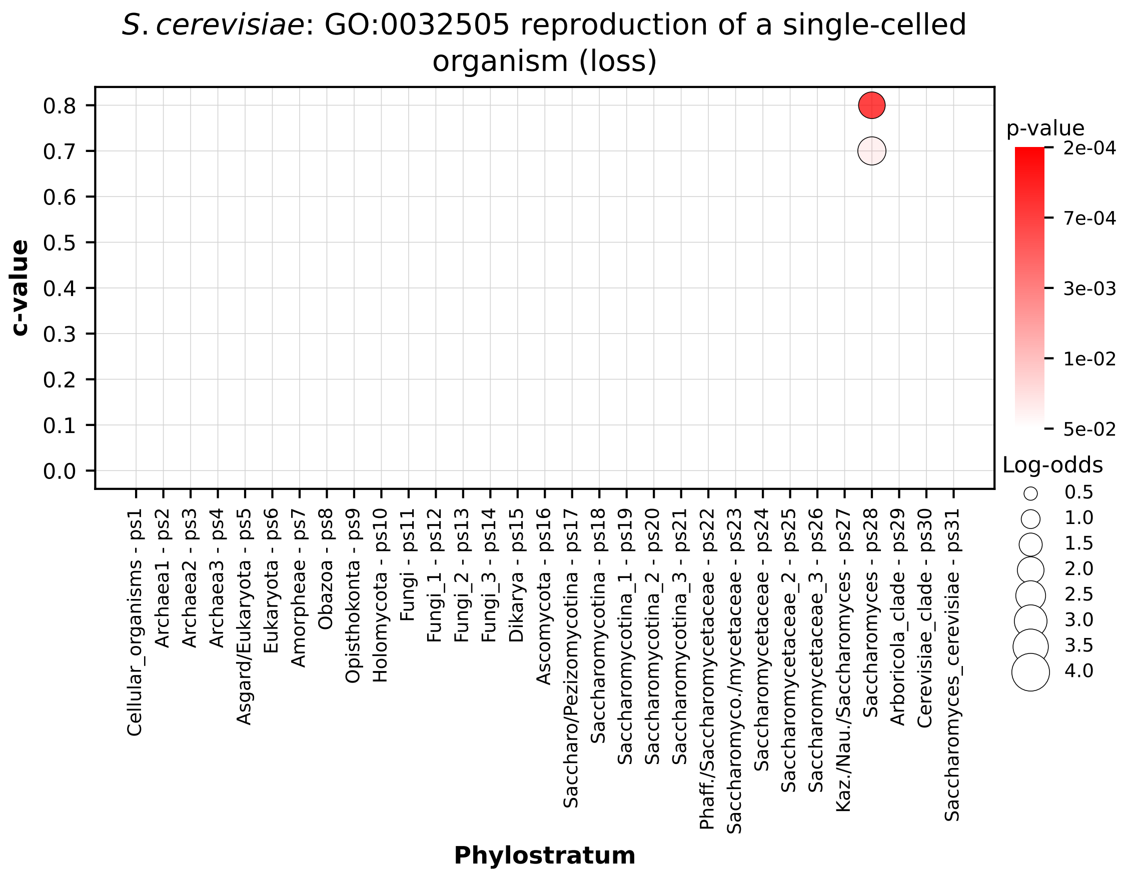

S. cerevisiae: GO:0032505 reproduction of a single-celled organism (loss)
c-value
0.0
0.1
0.2
0.3
0.4
0.5
0.6
0.7
0.8
Phylostratum
Cellular_organisms - ps1
Archaea1 - ps2
Archaea2 - ps3
Archaea3 - ps4
Asgard/Eukaryota - ps5
Eukaryota - ps6
Amorpheae - ps7
Obazoa - ps8
Opisthokonta - ps9
Holomycota - ps10
Fungi - ps11
Fungi_1 - ps12
Fungi_2 - ps13
Fungi_3 - ps14
Dikarya - ps15
Ascomycota - ps16
Saccharo/Pezizomycotina - ps17
Saccharomycotina - ps18
Saccharomycotina_1 - ps19
Saccharomycotina_2 - ps20
Saccharomycotina_3 - ps21
Phaff./Saccharomycetaceae - ps22
Saccharomyco./mycetaceae - ps23
Saccharomycetaceae - ps24
Saccharomycetaceae_2 - ps25
Saccharomycetaceae_3 - ps26
Kaz./Nau./Saccharomyces - ps27
Saccharomyces - ps28
Arboricola_clade - ps29
Cerevisiae_clade - ps30
Saccharomyces_cerevisiae - ps31
p-value
2e-04
7e-04
3e-03
1e-02
5e-02
Log-odds
0.5
1.0
1.5
2.0
2.5
3.0
3.5
4.0

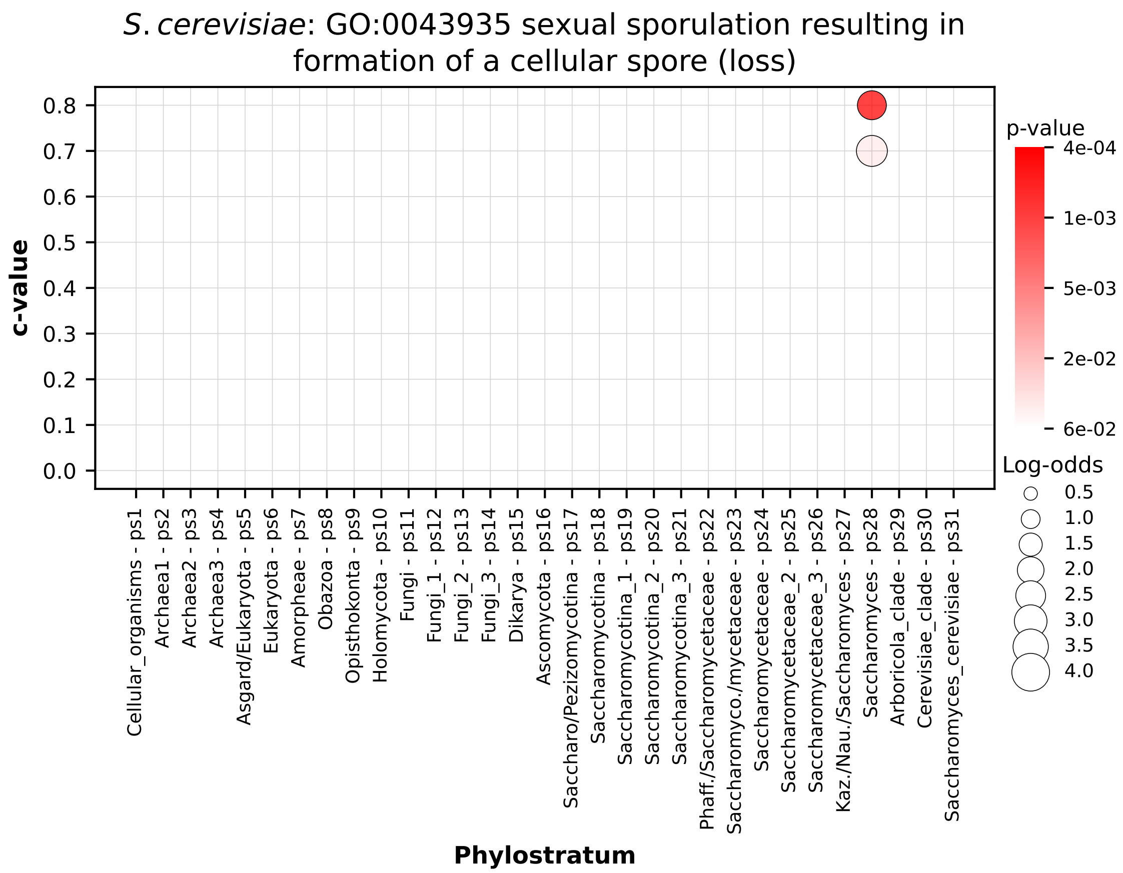
S. cerevisiae: GO:0043935 sexual sporulation resulting in formation of a cellular spore (loss)
c-value
0.8
0.7
0.6
0.5
0.4
0.3
0.2
0.1
0.0
p-value
4e-04
1e-03
5e-03
2e-02
6e-02
Log-odds
0.5
1.0
1.5
2.0
2.5
3.0
3.5
4.0
Cellular_organisms - ps1
Archaea1 - ps2
Archaea2 - ps3
Archaea3 - ps4
Asgard/Eukaryota - ps5
Eukaryota - ps6
Amorpheae - ps7
Obazoa - ps8
Opisthokonta - ps9
Holomycota - ps10
Fungi - ps11
Fungi_1 - ps12
Fungi_2 - ps13
Fungi_3 - ps14
Dikarya - ps15
Ascomycota - ps16
Saccharo/Pezizomycotina - ps17
Saccharomycotina - ps18
Saccharomycotina_1 - ps19
Saccharomycotina_2 - ps20
Saccharomycotina_3 - ps21
Phaff./Saccharomycetaceae - ps22
Saccharomyco./mycetaceae - ps23
Saccharomycetaceae - ps24
Saccharomycetaceae_2 - ps25
Saccharomycetaceae_3 - ps26
Kaz./Nau./Saccharomyces - ps27
Saccharomyces - ps28
Arboricola_clade - ps29
Cerevisiae_clade - ps30
Saccharomyces_cerevisiae - ps31
Phylostratum

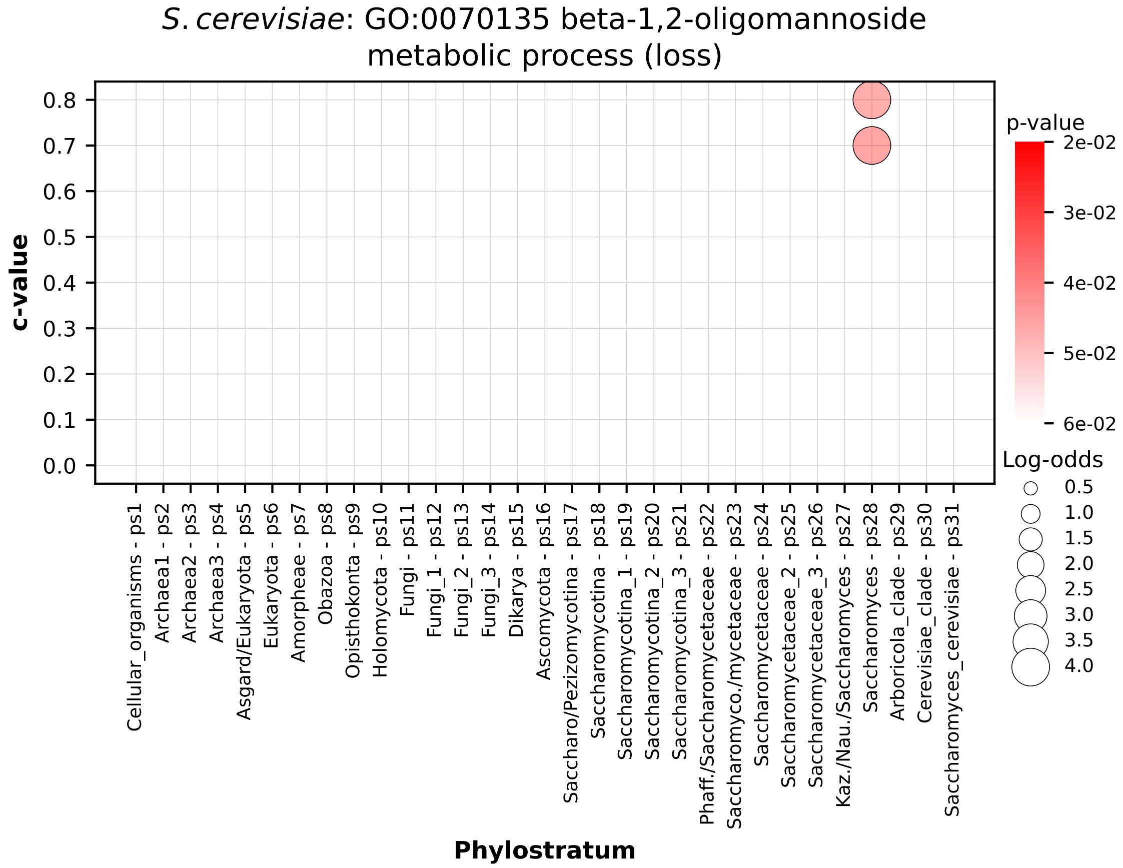
S. cerevisiae: GO:0070135 beta-1,2-oligomannoside metabolic process (loss)
c-value
0.0
0.1
0.2
0.3
0.4
0.5
0.6
0.7
0.8
p-value
2e-02
3e-02
4e-02
5e-02
6e-02
Log-odds
0.5
1.0
1.5
2.0
2.5
3.0
3.5
4.0
Cellular_organisms - ps1
Archaea1 - ps2
Archaea2 - ps3
Archaea3 - ps4
Asgard/Eukaryota - ps5
Eukaryota - ps6
Amorpheae - ps7
Obazoa - ps8
Opisthokonta - ps9
Holomycota - ps10
Fungi - ps11
Fungi_1 - ps12
Fungi_2 - ps13
Fungi_3 - ps14
Dikarya - ps15
Ascomycota - ps16
Saccharo/Pezizomycotina - ps17
Saccharomycotina - ps18
Saccharomycotina_1 - ps19
Saccharomycotina_2 - ps20
Saccharomycotina_3 - ps21
Phaff./Saccharomycetaceae - ps22
Saccharomyco./mycetaceae - ps23
Saccharomycetaceae - ps24
Saccharomycetaceae_2 - ps25
Saccharomycetaceae_3 - ps26
Kaz./Nau./Saccharomyces - ps27
Saccharomyces - ps28
Arboricola_clade - ps29
Cerevisiae_clade - ps30
Saccharomyces_cerevisiae - ps31
Phylostratum

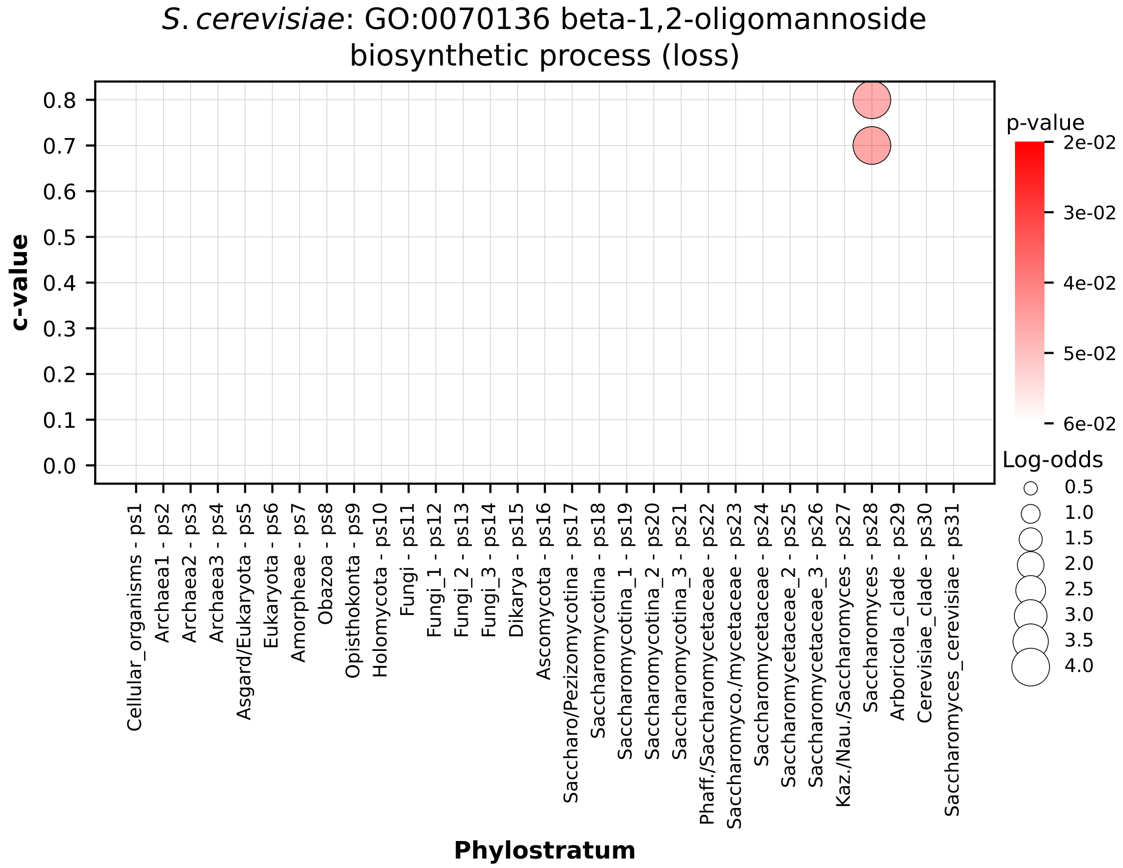

S. cerevisiae: GO:0070136 beta-1,2-oligomannoside biosynthetic process (loss)
c-value
0.8
0.7
0.6
0.5
0.4
0.3
0.2
0.1
0.0
p-value
2e-02
3e-02
4e-02
5e-02
6e-02
Log-odds
0.5
1.0
1.5
2.0
2.5
3.0
3.5
4.0
Cellular_organisms - ps1
Archaea1 - ps2
Archaea2 - ps3
Archaea3 - ps4
Asgard/Eukaryota - ps5
Eukaryota - ps6
Amorpheae - ps7
Obazoa - ps8
Opisthokonta - ps9
Holomycota - ps10
Fungi - ps11
Fungi_1 - ps12
Fungi_2 - ps13
Fungi_3 - ps14
Dikarya - ps15
Ascomycota - ps16
Saccharo/Pezizomycotina - ps17
Saccharomycotina - ps18
Saccharomycotina_1 - ps19
Saccharomycotina_2 - ps20
Saccharomycotina_3 - ps21
Phaff./Saccharomycetaceae - ps22
Saccharomyco./mycetaceae - ps23
Saccharomycetaceae - ps24
Saccharomycetaceae_2 - ps25
Saccharomycetaceae_3 - ps26
Kaz./Nau./Saccharomyces - ps27
Saccharomyces - ps28
Arboricola_clade - ps29
Cerevisiae_clade - ps30
Saccharomyces_cerevisiae - ps31
Phylostratum

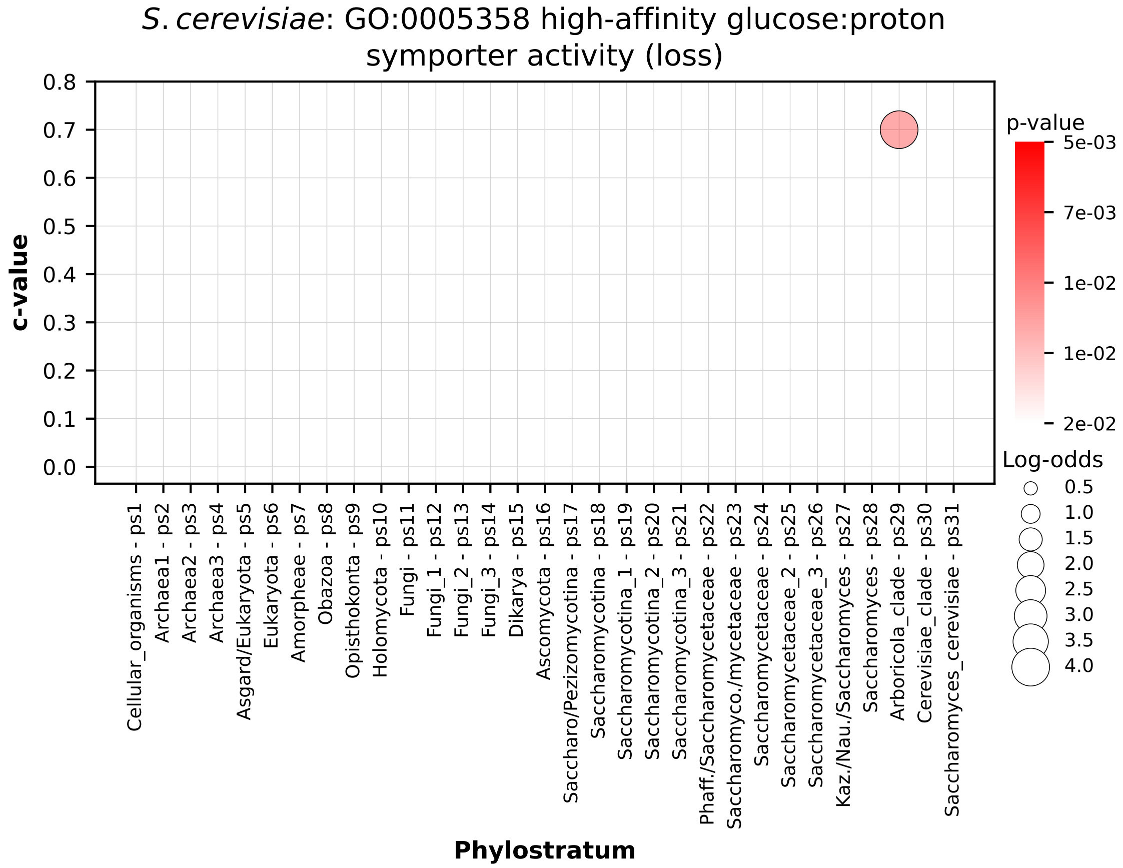
S. cerevisiae: GO:0005358 high-affinity glucose:proton symporter activity (loss)
c-value
0.8
0.7
0.6
0.5
0.4
0.3
0.2
0.1
0.0
p-value
5e-03
7e-03
1e-02
1e-02
2e-02
Log-odds
0.5
1.0
1.5
2.0
2.5
3.0
3.5
4.0
Cellular_organisms - ps1
Archaea1 - ps2
Archaea2 - ps3
Archaea3 - ps4
Asgard/Eukaryota - ps5
Eukaryota - ps6
Amorpheae - ps7
Obazoa - ps8
Opisthokonta - ps9
Holomycota - ps10
Fungi - ps11
Fungi_1 - ps12
Fungi_2 - ps13
Fungi_3 - ps14
Dikarya - ps15
Ascomycota - ps16
Saccharo/Pezizomycotina - ps17
Saccharomycotina - ps18
Saccharomycotina_1 - ps19
Saccharomycotina_2 - ps20
Saccharomycotina_3 - ps21
Phaff./Saccharomycetaceae - ps22
Saccharomyco./mycetaceae - ps23
Saccharomycetaceae - ps24
Saccharomycetaceae_2 - ps25
Saccharomycetaceae_3 - ps26
Kaz./Nau./Saccharomyces - ps27
Saccharomyces - ps28
Arboricola_clade - ps29
Cerevisiae_clade - ps30
Saccharomyces_cerevisiae - ps31
Phylostratum

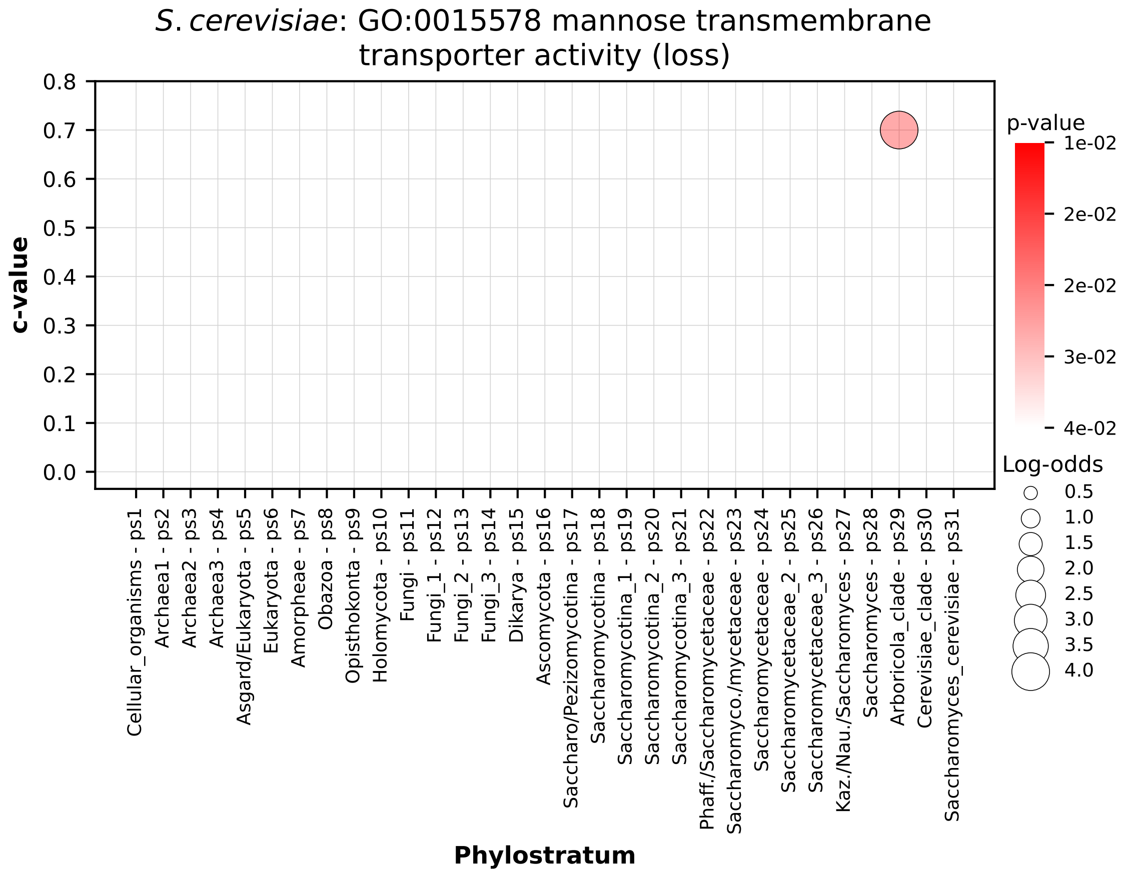

S. cerevisiae: GO:0015578 mannose transmembrane transporter activity (loss)
c-value
0.8
0.7
0.6
0.5
0.4
0.3
0.2
0.1
0.0
Phylostratum
Cellular_organisms - ps1
Archaea1 - ps2
Archaea2 - ps3
Archaea3 - ps4
Asgard/Eukaryota - ps5
Eukaryota - ps6
Amorpheae - ps7
Obazoa - ps8
Opisthokonta - ps9
Holomycota - ps10
Fungi - ps11
Fungi_1 - ps12
Fungi_2 - ps13
Fungi_3 - ps14
Dikarya - ps15
Ascomycota - ps16
Saccharo/Pezizomycotina - ps17
Saccharomycotina - ps18
Saccharomycotina_1 - ps19
Saccharomycotina_2 - ps20
Saccharomycotina_3 - ps21
Phaff./Saccharomycetaceae - ps22
Saccharomyco./mycetaceae - ps23
Saccharomycetaceae - ps24
Saccharomycetaceae_2 - ps25
Saccharomycetaceae_3 - ps26
Kaz./Nau./Saccharomyces - ps27
Saccharomyces - ps28
Arboricola_clade - ps29
Cerevisiae_clade - ps30
Saccharomyces_cerevisiae - ps31
p-value
1e-02
2e-02
2e-02
3e-02
4e-02
Log-odds
0.5
1.0
1.5
2.0
2.5
3.0
3.5
4.0

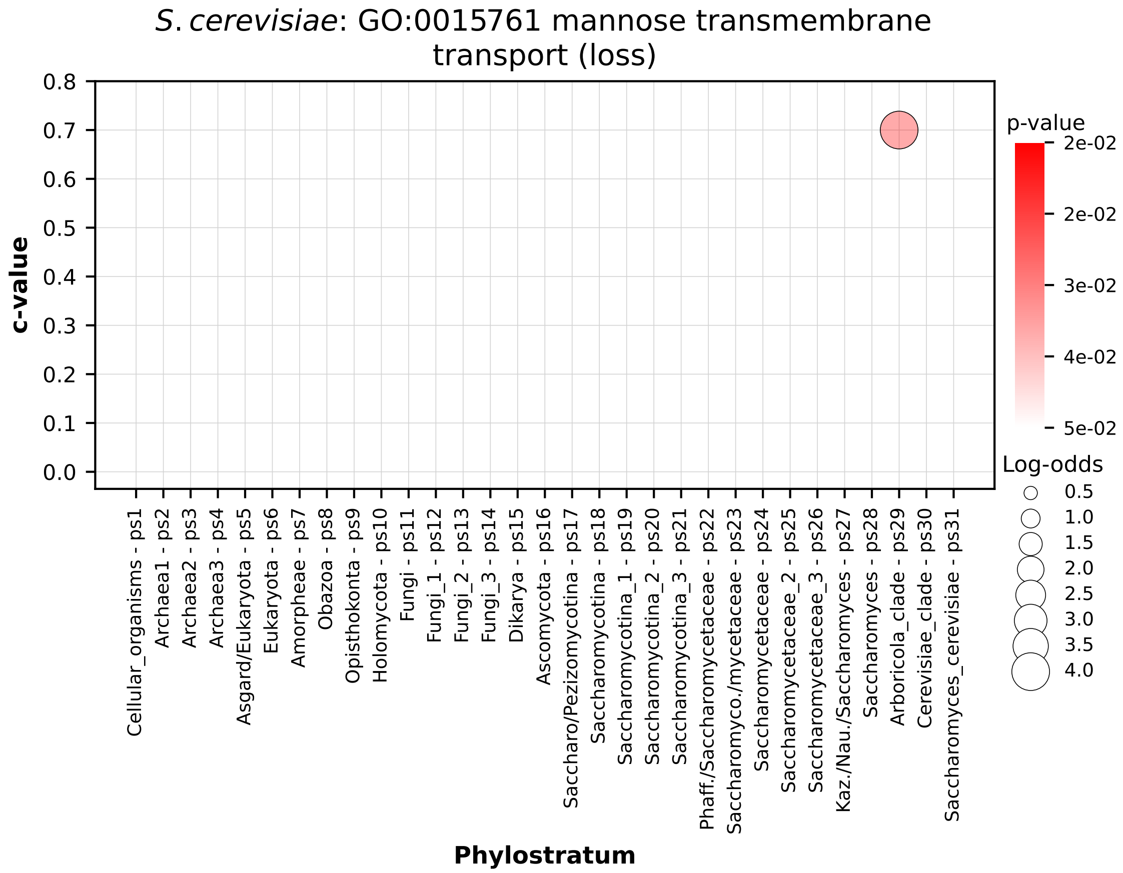

S. cerevisiae: GO:0015761 mannose transmembrane transport (loss)
c-value
0.8
0.7
0.6
0.5
0.4
0.3
0.2
0.1
0.0
p-value
2e-02
2e-02
3e-02
4e-02
5e-02
Log-odds
0.5
1.0
1.5
2.0
2.5
3.0
3.5
4.0
Cellular_organisms - ps1
Archaea1 - ps2
Archaea2 - ps3
Archaea3 - ps4
Asgard/Eukaryota - ps5
Eukaryota - ps6
Amorpheae - ps7
Obazoa - ps8
Opisthokonta - ps9
Holomycota - ps10
Fungi - ps11
Fungi_1 - ps12
Fungi_2 - ps13
Fungi_3 - ps14
Dikarya - ps15
Ascomycota - ps16
Saccharo/Pezizomycotina - ps17
Saccharomycotina - ps18
Saccharomycotina_1 - ps19
Saccharomycotina_2 - ps20
Saccharomycotina_3 - ps21
Phaff./Saccharomycetaceae - ps22
Saccharomyco./mycetaceae - ps23
Saccharomycetaceae - ps24
Saccharomycetaceae_2 - ps25
Saccharomycetaceae_3 - ps26
Kaz./Nau./Saccharomyces - ps27
Saccharomyces - ps28
Arboricola_clade - ps29
Cerevisiae_clade - ps30
Saccharomyces_cerevisiae - ps31
Phylostratum

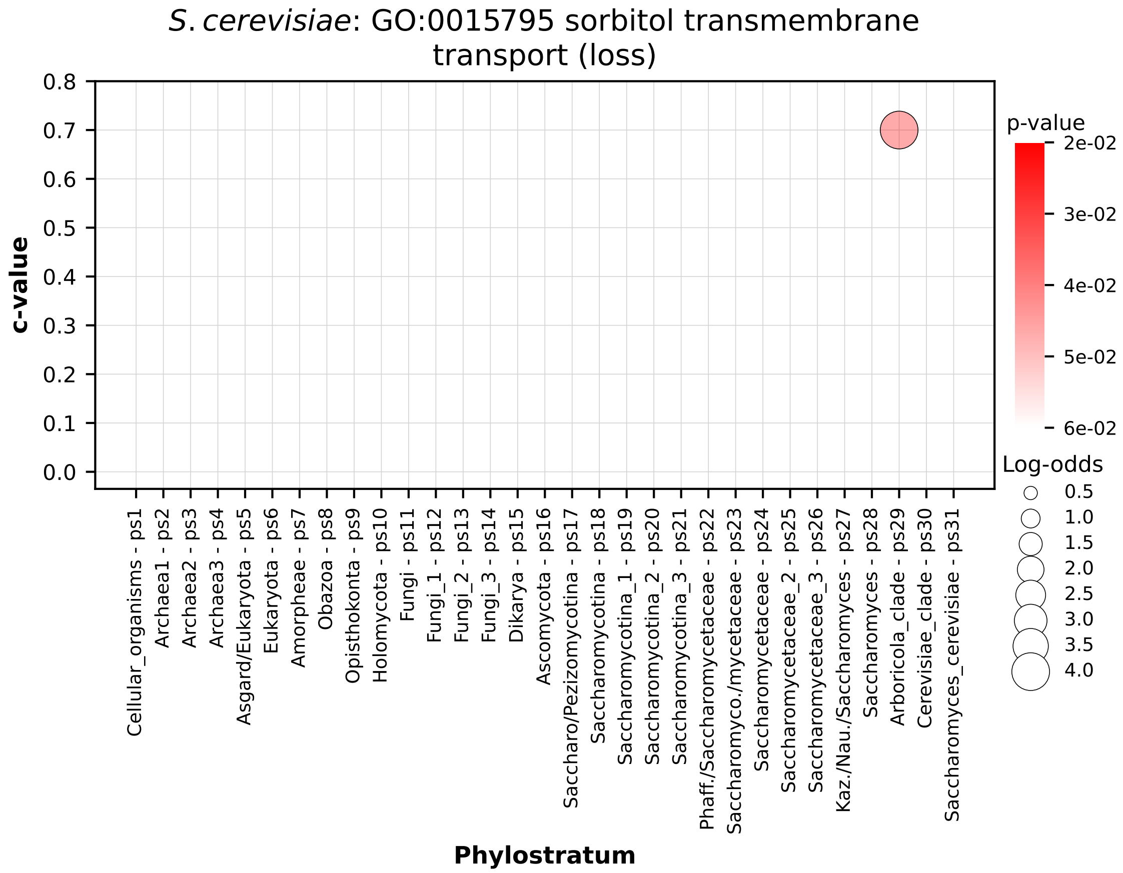

S. cerevisiae: GO:0015795 sorbitol transmembrane transport (loss)
c-value
0.0
0.1
0.2
0.3
0.4
0.5
0.6
0.7
0.8
Phylostratum
Cellular_organisms - ps1
Archaea1 - ps2
Archaea2 - ps3
Archaea3 - ps4
Asgard/Eukaryota - ps5
Eukaryota - ps6
Amorpheae - ps7
Obazoa - ps8
Opisthokonta - ps9
Holomycota - ps10
Fungi - ps11
Fungi_1 - ps12
Fungi_2 - ps13
Fungi_3 - ps14
Dikarya - ps15
Ascomycota - ps16
Saccharo/Pezizomycotina - ps17
Saccharomycotina - ps18
Saccharomycotina_1 - ps19
Saccharomycotina_2 - ps20
Saccharomycotina_3 - ps21
Phaff./Saccharomycetaceae - ps22
Saccharomyco./mycetaceae - ps23
Saccharomycetaceae - ps24
Saccharomycetaceae_2 - ps25
Saccharomycetaceae_3 - ps26
Kaz./Nau./Saccharomyces - ps27
Saccharomyces - ps28
Arboricola_clade - ps29
Cerevisiae_clade - ps30
Saccharomyces_cerevisiae - ps31
p-value
2e-02
3e-02
4e-02
5e-02
6e-02
Log-odds
0.5
1.0
1.5
2.0
2.5
3.0
3.5
4.0

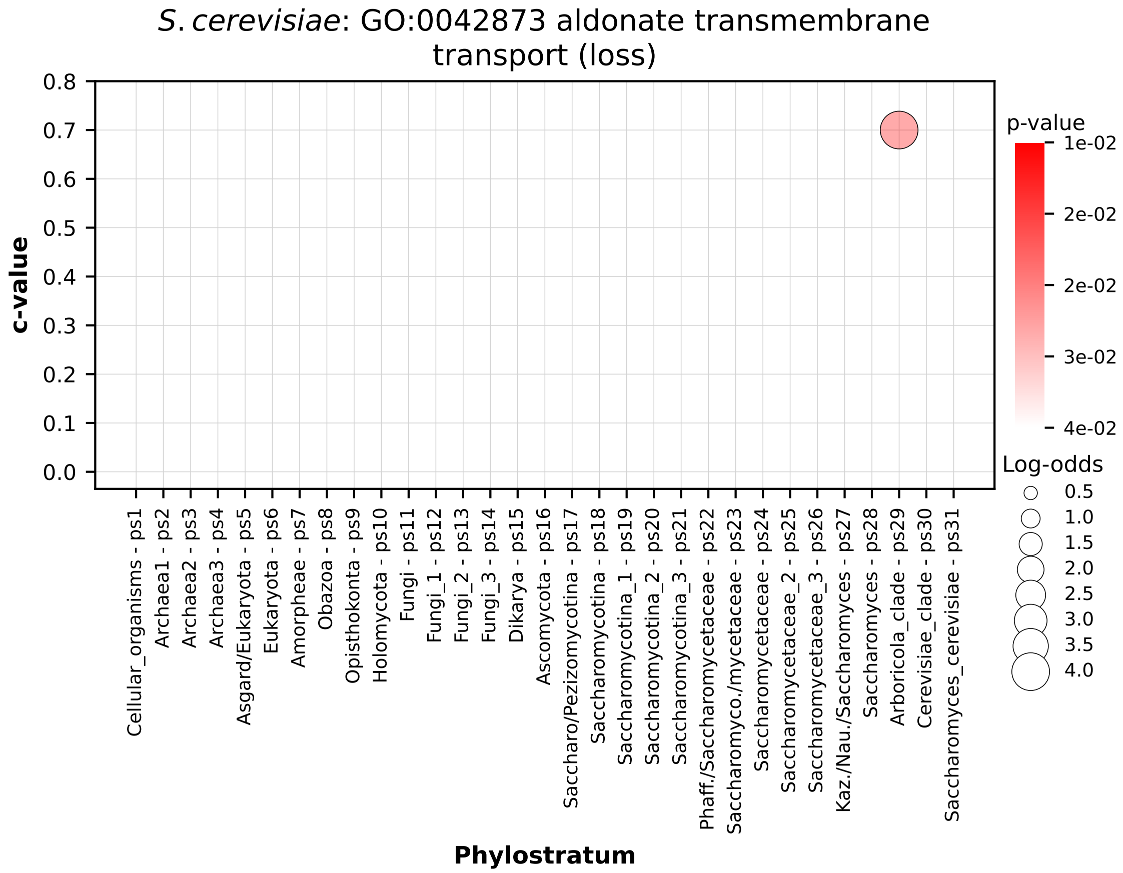

S. cerevisiae: GO:0042873 aldonate transmembrane transport (loss)
c-value
0.8
0.7
0.6
0.5
0.4
0.3
0.2
0.1
0.0
p-value
1e-02
2e-02
2e-02
3e-02
4e-02
Log-odds
0.5
1.0
1.5
2.0
2.5
3.0
3.5
4.0
Cellular_organisms - ps1
Archaea1 - ps2
Archaea2 - ps3
Archaea3 - ps4
Asgard/Eukaryota - ps5
Eukaryota - ps6
Amorpheae - ps7
Obazoa - ps8
Opisthokonta - ps9
Holomycota - ps10
Fungi - ps11
Fungi_1 - ps12
Fungi_2 - ps13
Fungi_3 - ps14
Dikarya - ps15
Ascomycota - ps16
Saccharo/Pezizomycotina - ps17
Saccharomycotina - ps18
Saccharomycotina_1 - ps19
Saccharomycotina_2 - ps20
Saccharomycotina_3 - ps21
Phaff./Saccharomycetaceae - ps22
Saccharomyco./mycetaceae - ps23
Saccharomycetaceae - ps24
Saccharomycetaceae_2 - ps25
Saccharomycetaceae_3 - ps26
Kaz./Nau./Saccharomyces - ps27
Saccharomyces - ps28
Arboricola_clade - ps29
Cerevisiae_clade - ps30
Saccharomyces_cerevisiae - ps31
Phylostratum

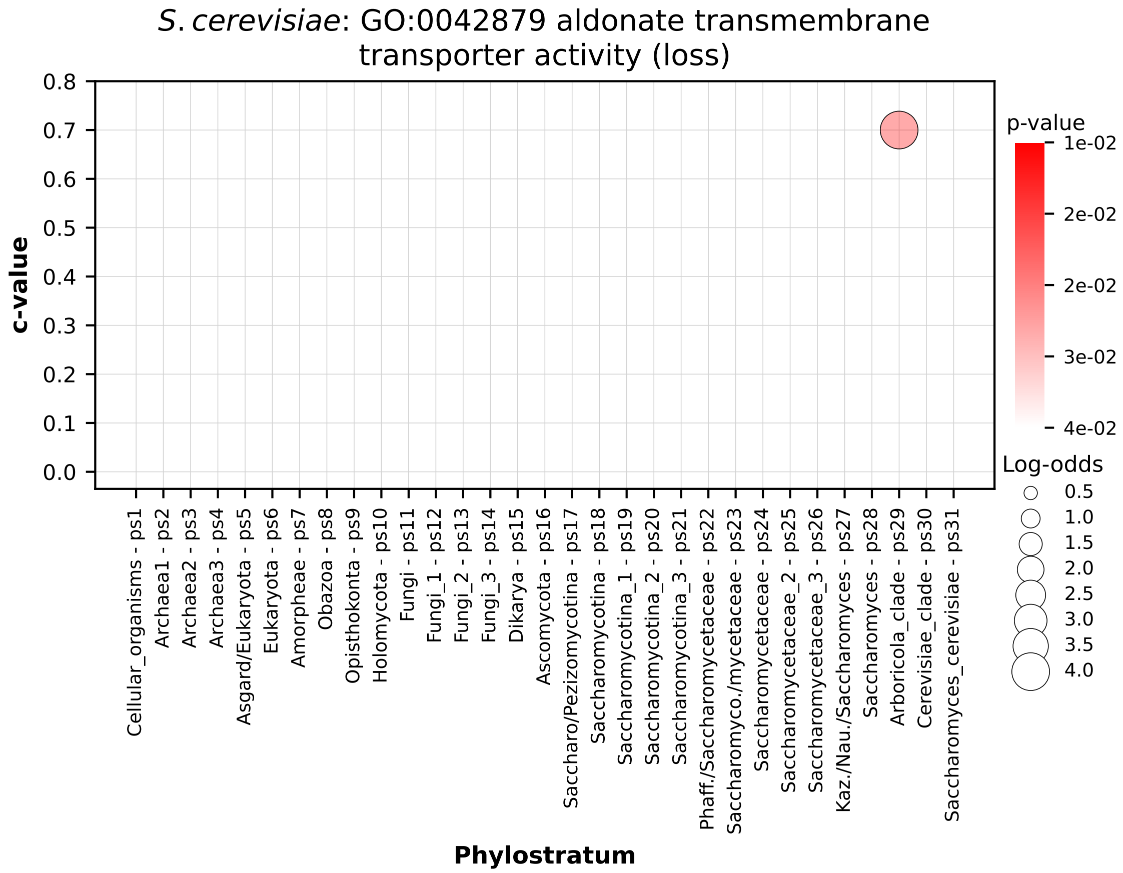
S. cerevisiae: GO:0042879 aldonate transmembrane transporter activity (loss)
c-value
0.0
0.1
0.2
0.3
0.4
0.5
0.6
0.7
0.8
p-value
1e-02
2e-02
2e-02
3e-02
4e-02
Log-odds
0.5
1.0
1.5
2.0
2.5
3.0
3.5
4.0
Cellular_organisms - ps1
Archaea1 - ps2
Archaea2 - ps3
Archaea3 - ps4
Asgard/Eukaryota - ps5
Eukaryota - ps6
Amorpheae - ps7
Obazoa - ps8
Opisthokonta - ps9
Holomycota - ps10
Fungi - ps11
Fungi_1 - ps12
Fungi_2 - ps13
Fungi_3 - ps14
Dikarya - ps15
Ascomycota - ps16
Saccharo/Pezizomycotina - ps17
Saccharomycotina - ps18
Saccharomycotina_1 - ps19
Saccharomycotina_2 - ps20
Saccharomycotina_3 - ps21
Phaff./Saccharomycetaceae - ps22
Saccharomyco./mycetaceae - ps23
Saccharomycetaceae - ps24
Saccharomycetaceae_2 - ps25
Saccharomycetaceae_3 - ps26
Kaz./Nau./Saccharomyces - ps27
Saccharomyces - ps28
Arboricola_clade - ps29
Cerevisiae_clade - ps30
Saccharomyces_cerevisiae - ps31
Phylostratum

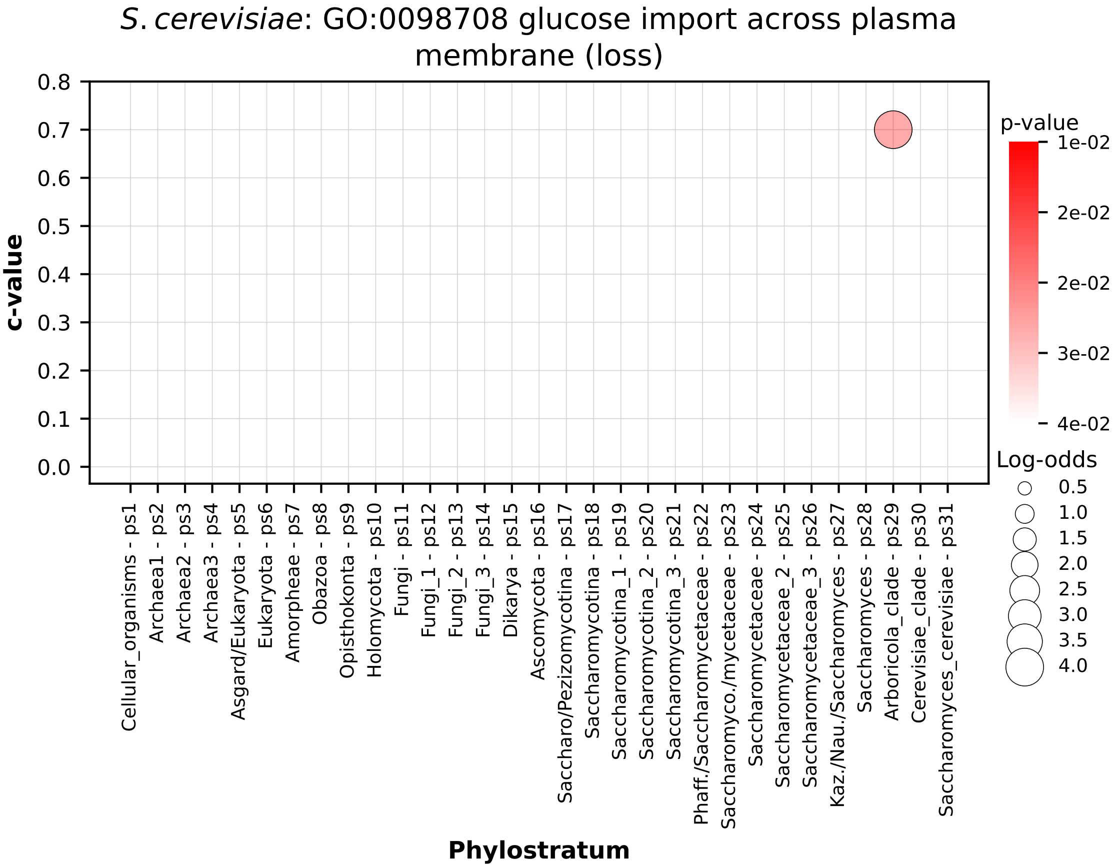

S. cerevisiae: GO:0098708 glucose import across plasma membrane (loss)
c-value
0.8
0.7
0.6
0.5
0.4
0.3
0.2
0.1
0.0
p-value
1e-02
2e-02
2e-02
3e-02
4e-02
Log-odds
0.5
1.0
1.5
2.0
2.5
3.0
3.5
4.0
Cellular_organisms - ps1
Archaea1 - ps2
Archaea2 - ps3
Archaea3 - ps4
Asgard/Eukaryota - ps5
Eukaryota - ps6
Amorpheae - ps7
Obazoa - ps8
Opisthokonta - ps9
Holomycota - ps10
Fungi - ps11
Fungi_1 - ps12
Fungi_2 - ps13
Fungi_3 - ps14
Dikarya - ps15
Ascomycota - ps16
Saccharo/Pezizomycotina - ps17
Saccharomycotina - ps18
Saccharomycotina_1 - ps19
Saccharomycotina_2 - ps20
Saccharomycotina_3 - ps21
Phaff./Saccharomycetaceae - ps22
Saccharomyco./mycetaceae - ps23
Saccharomycetaceae - ps24
Saccharomycetaceae_2 - ps25
Saccharomycetaceae_3 - ps26
Kaz./Nau./Saccharomyces - ps27
Saccharomyces - ps28
Arboricola_clade - ps29
Cerevisiae_clade - ps30
Saccharomyces_cerevisiae - ps31
Phylostratum

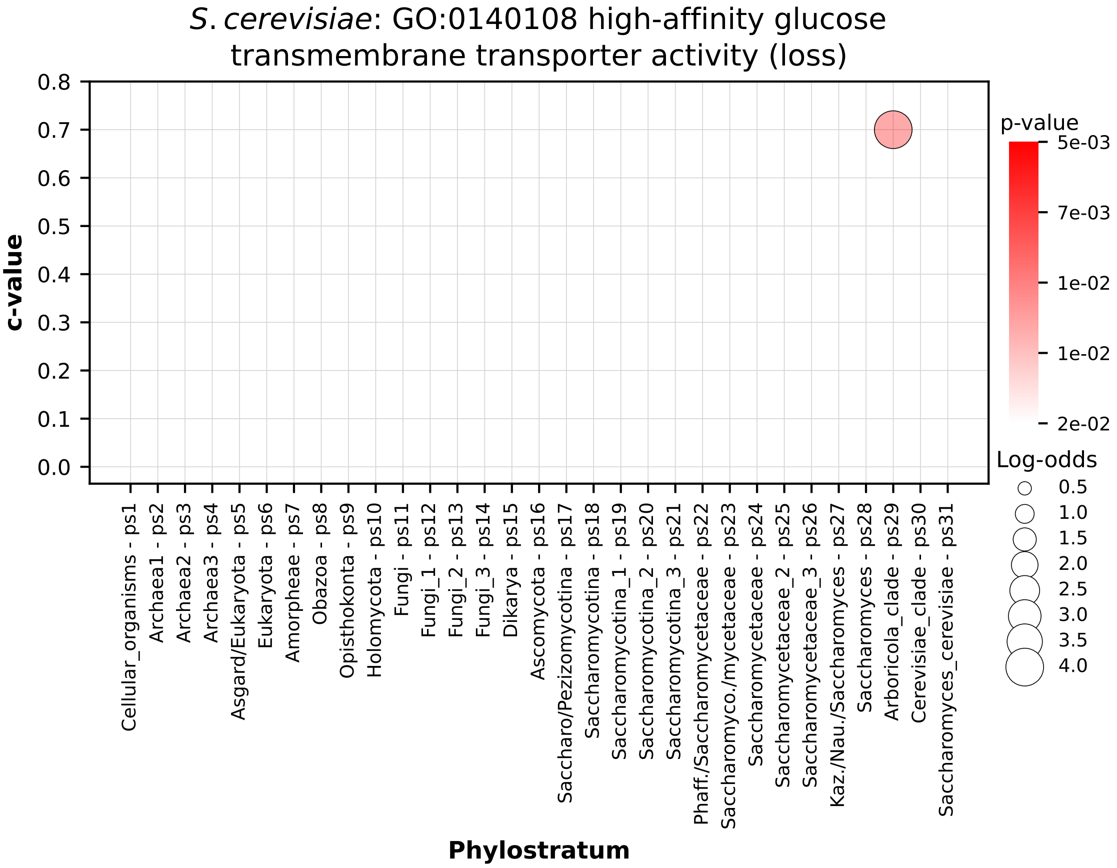
S. cerevisiae: GO:0140108 high-affinity glucose transmembrane transporter activity (loss)
c-value
0.8
0.7
0.6
0.5
0.4
0.3
0.2
0.1
0.0
p-value
5e-03
7e-03
1e-02
1e-02
2e-02
Log-odds
0.5
1.0
1.5
2.0
2.5
3.0
3.5
4.0
Cellular_organisms - ps1
Archaea1 - ps2
Archaea2 - ps3
Archaea3 - ps4
Asgard/Eukaryota - ps5
Eukaryota - ps6
Amorpheae - ps7
Obazoa - ps8
Opisthokonta - ps9
Holomycota - ps10
Fungi - ps11
Fungi_1 - ps12
Fungi_2 - ps13
Fungi_3 - ps14
Dikarya - ps15
Ascomycota - ps16
Saccharo/Pezizomycotina - ps17
Saccharomycotina - ps18
Saccharomycotina_1 - ps19
Saccharomycotina_2 - ps20
Saccharomycotina_3 - ps21
Phaff./Saccharomycetaceae - ps22
Saccharomyco./mycetaceae - ps23
Saccharomycetaceae - ps24
Saccharomycetaceae_2 - ps25
Saccharomycetaceae_3 - ps26
Kaz./Nau./Saccharomyces - ps27
Saccharomyces - ps28
Arboricola_clade - ps29
Cerevisiae_clade - ps30
Saccharomyces_cerevisiae - ps31
Phylostratum

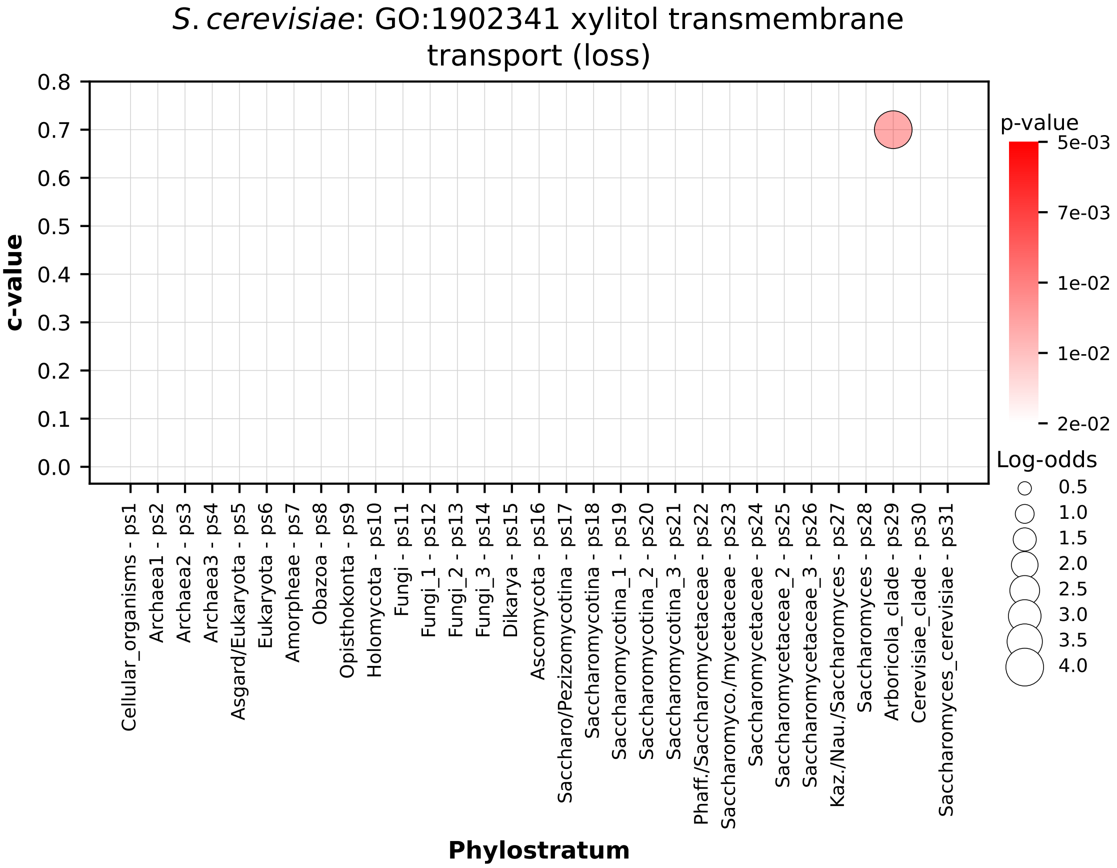
S. cerevisiae: GO:1902341 xylitol transmembrane transport (loss)
c-value
0.8
0.7
0.6
0.5
0.4
0.3
0.2
0.1
0.0
Phylostratum
Cellular_organisms - ps1
Archaea1 - ps2
Archaea2 - ps3
Archaea3 - ps4
Asgard/Eukaryota - ps5
Eukaryota - ps6
Amorpheae - ps7
Obazoa - ps8
Opisthokonta - ps9
Holomycota - ps10
Fungi - ps11
Fungi_1 - ps12
Fungi_2 - ps13
Fungi_3 - ps14
Dikarya - ps15
Ascomycota - ps16
Saccharo/Pezizomycotina - ps17
Saccharomycotina - ps18
Saccharomycotina_1 - ps19
Saccharomycotina_2 - ps20
Saccharomycotina_3 - ps21
Phaff./Saccharomycetaceae - ps22
Saccharomyco./mycetaceae - ps23
Saccharomycetaceae - ps24
Saccharomycetaceae_2 - ps25
Saccharomycetaceae_3 - ps26
Kaz./Nau./Saccharomyces - ps27
Saccharomyces - ps28
Arboricola_clade - ps29
Cerevisiae_clade - ps30
Saccharomyces_cerevisiae - ps31
p-value
5e-03
7e-03
1e-02
1e-02
2e-02
Log-odds
0.5
1.0
1.5
2.0
2.5
3.0
3.5
4.0

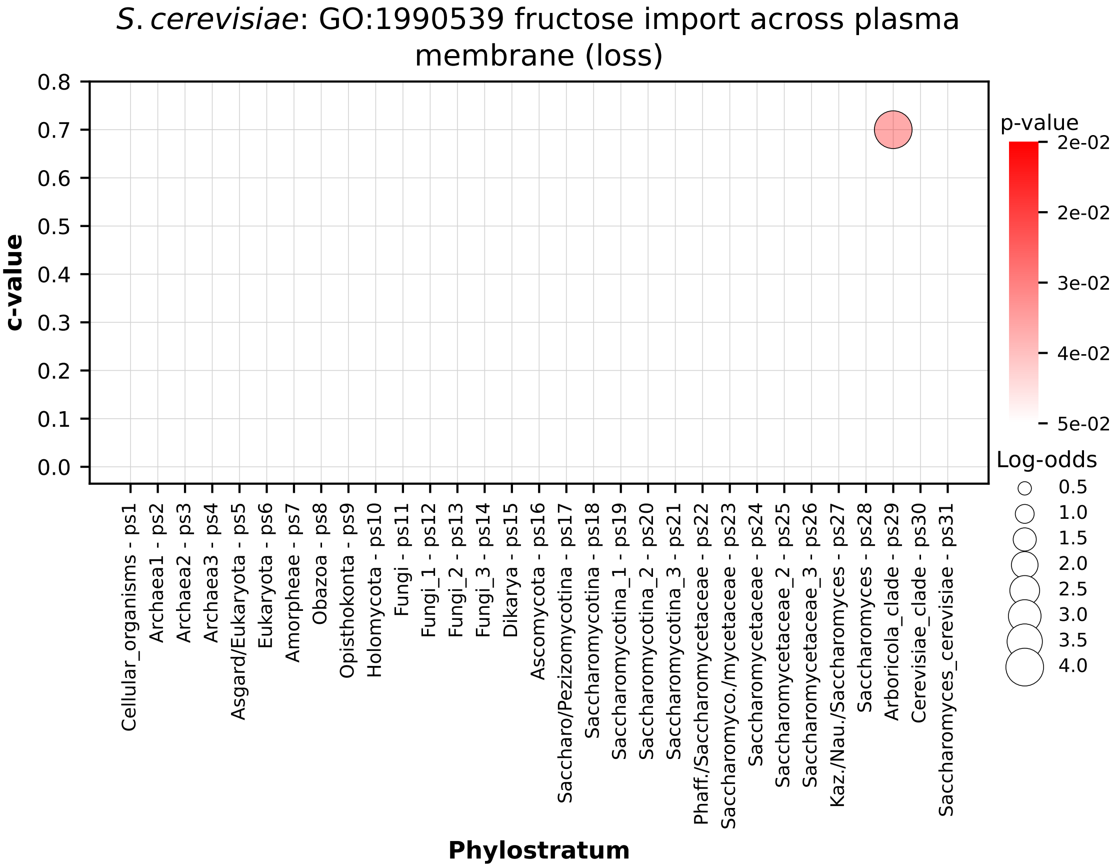

S. cerevisiae: GO:1990539 fructose import across plasma membrane (loss)
c-value
0.8
0.7
0.6
0.5
0.4
0.3
0.2
0.1
0.0
p-value
2e-02
2e-02
3e-02
4e-02
5e-02
Log-odds
0.5
1.0
1.5
2.0
2.5
3.0
3.5
4.0
Cellular_organisms - ps1
Archaea1 - ps2
Archaea2 - ps3
Archaea3 - ps4
Asgard/Eukaryota - ps5
Eukaryota - ps6
Amorpheae - ps7
Obazoa - ps8
Opisthokonta - ps9
Holomycota - ps10
Fungi - ps11
Fungi_1 - ps12
Fungi_2 - ps13
Fungi_3 - ps14
Dikarya - ps15
Ascomycota - ps16
Saccharo/Pezizomycotina - ps17
Saccharomycotina - ps18
Saccharomycotina_1 - ps19
Saccharomycotina_2 - ps20
Saccharomycotina_3 - ps21
Phaff./Saccharomycetaceae - ps22
Saccharomyco./mycetaceae - ps23
Saccharomycetaceae - ps24
Saccharomycetaceae_2 - ps25
Saccharomycetaceae_3 - ps26
Kaz./Nau./Saccharomyces - ps27
Saccharomyces - ps28
Arboricola_clade - ps29
Cerevisiae_clade - ps30
Saccharomyces_cerevisiae - ps31
Phylostratum

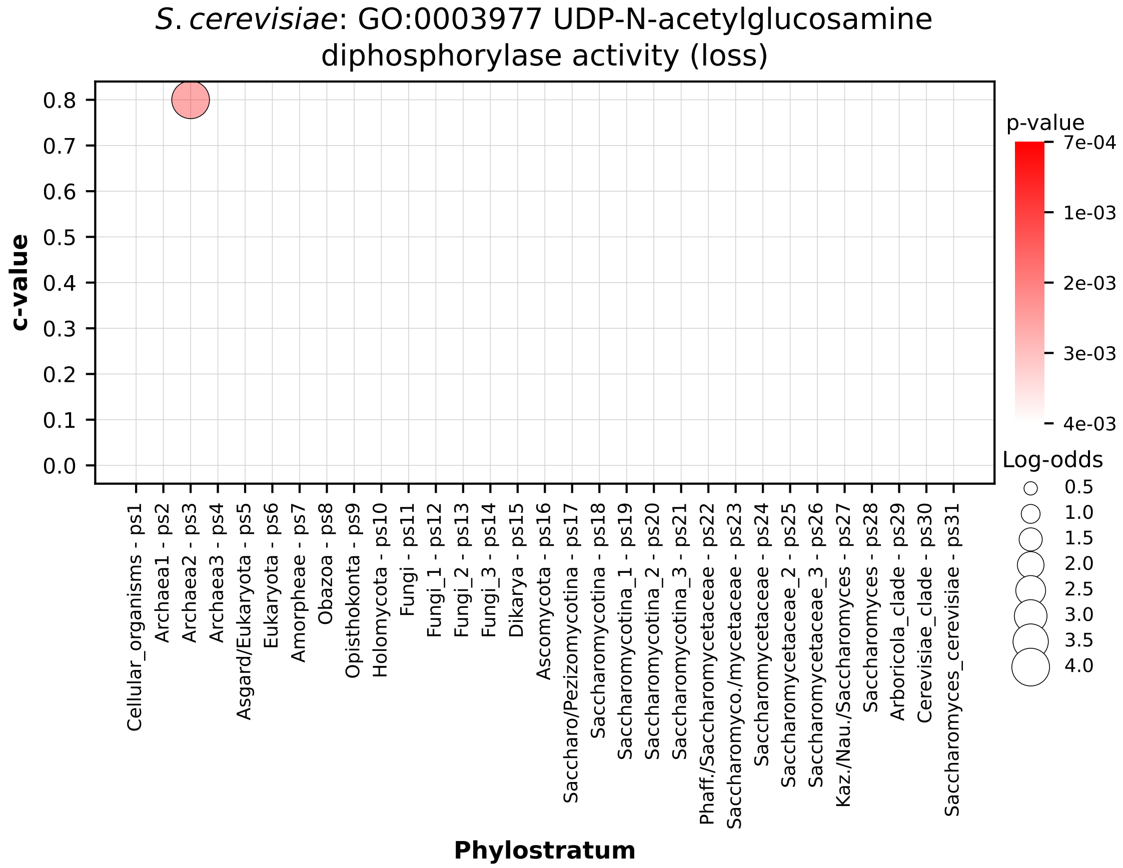

S. cerevisiae: GO:0003977 UDP-N-acetylglucosamine diphosphorylase activity (loss)
c-value
0.8
0.7
0.6
0.5
0.4
0.3
0.2
0.1
0.0
p-value
7e-04
1e-03
2e-03
3e-03
4e-03
Log-odds
0.5
1.0
1.5
2.0
2.5
3.0
3.5
4.0
Cellular_organisms - ps1
Archaea1 - ps2
Archaea2 - ps3
Archaea3 - ps4
Asgard/Eukaryota - ps5
Eukaryota - ps6
Amorpheae - ps7
Obazoa - ps8
Opisthokonta - ps9
Holomycota - ps10
Fungi - ps11
Fungi_1 - ps12
Fungi_2 - ps13
Fungi_3 - ps14
Dikarya - ps15
Ascomycota - ps16
Saccharo/Pezizomycotina - ps17
Saccharomycotina - ps18
Saccharomycotina_1 - ps19
Saccharomycotina_2 - ps20
Saccharomycotina_3 - ps21
Phaff./Saccharomycetaceae - ps22
Saccharomyco./mycetaceae - ps23
Saccharomycetaceae - ps24
Saccharomycetaceae_2 - ps25
Saccharomycetaceae_3 - ps26
Kaz./Nau./Saccharomyces - ps27
Saccharomyces - ps28
Arboricola_clade - ps29
Cerevisiae_clade - ps30
Saccharomyces_cerevisiae - ps31
Phylostratum

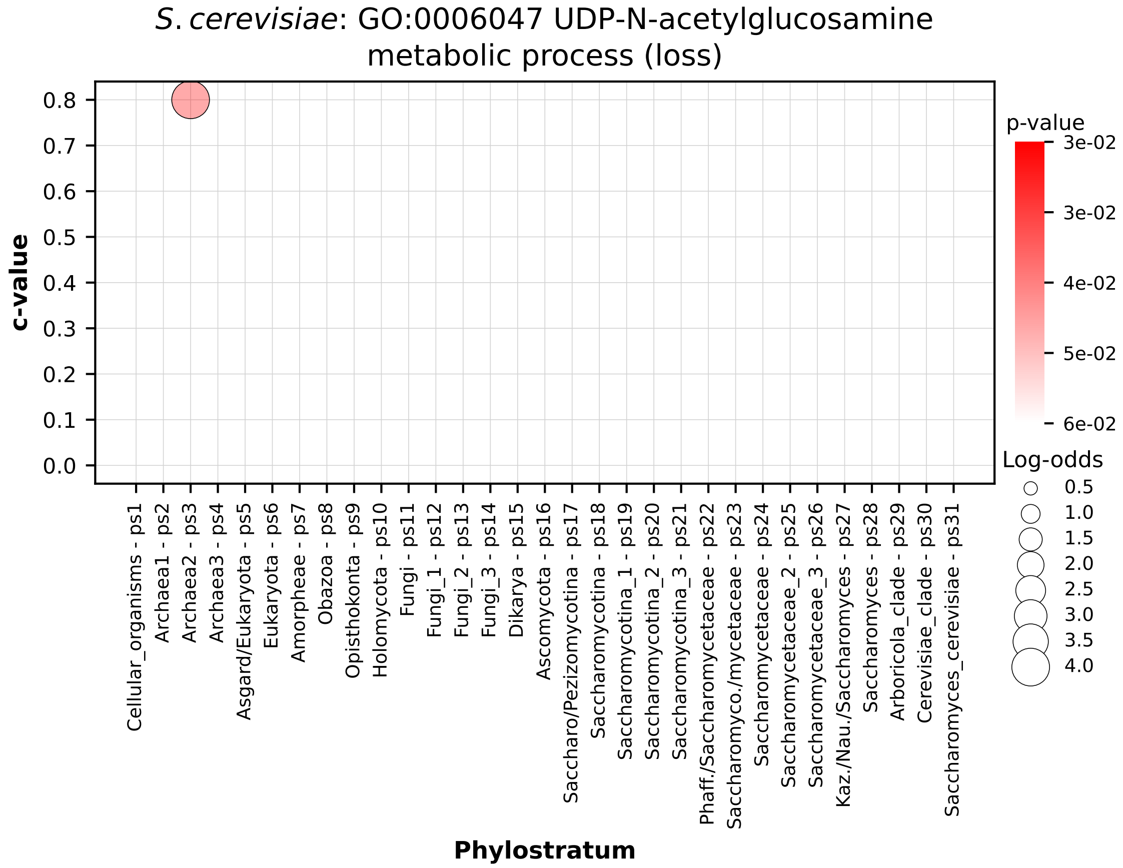

S. cerevisiae: GO:0006047 UDP-N-acetylglucosamine metabolic process (loss)
c-value
0.0
0.1
0.2
0.3
0.4
0.5
0.6
0.7
0.8
Phylostratum
Cellular_organisms - ps1
Archaea1 - ps2
Archaea2 - ps3
Archaea3 - ps4
Asgard/Eukaryota - ps5
Eukaryota - ps6
Amorpheae - ps7
Obazoa - ps8
Opisthokonta - ps9
Holomycota - ps10
Fungi - ps11
Fungi_1 - ps12
Fungi_2 - ps13
Fungi_3 - ps14
Dikarya - ps15
Ascomycota - ps16
Saccharo/Pezizomycotina - ps17
Saccharomycotina - ps18
Saccharomycotina_1 - ps19
Saccharomycotina_2 - ps20
Saccharomycotina_3 - ps21
Phaff./Saccharomycetaceae - ps22
Saccharomyco./mycetaceae - ps23
Saccharomycetaceae - ps24
Saccharomycetaceae_2 - ps25
Saccharomycetaceae_3 - ps26
Kaz./Nau./Saccharomyces - ps27
Saccharomyces - ps28
Arboricola_clade - ps29
Cerevisiae_clade - ps30
Saccharomyces_cerevisiae - ps31
p-value
3e-02
3e-02
4e-02
5e-02
6e-02
Log-odds
0.5
1.0
1.5
2.0
2.5
3.0
3.5
4.0

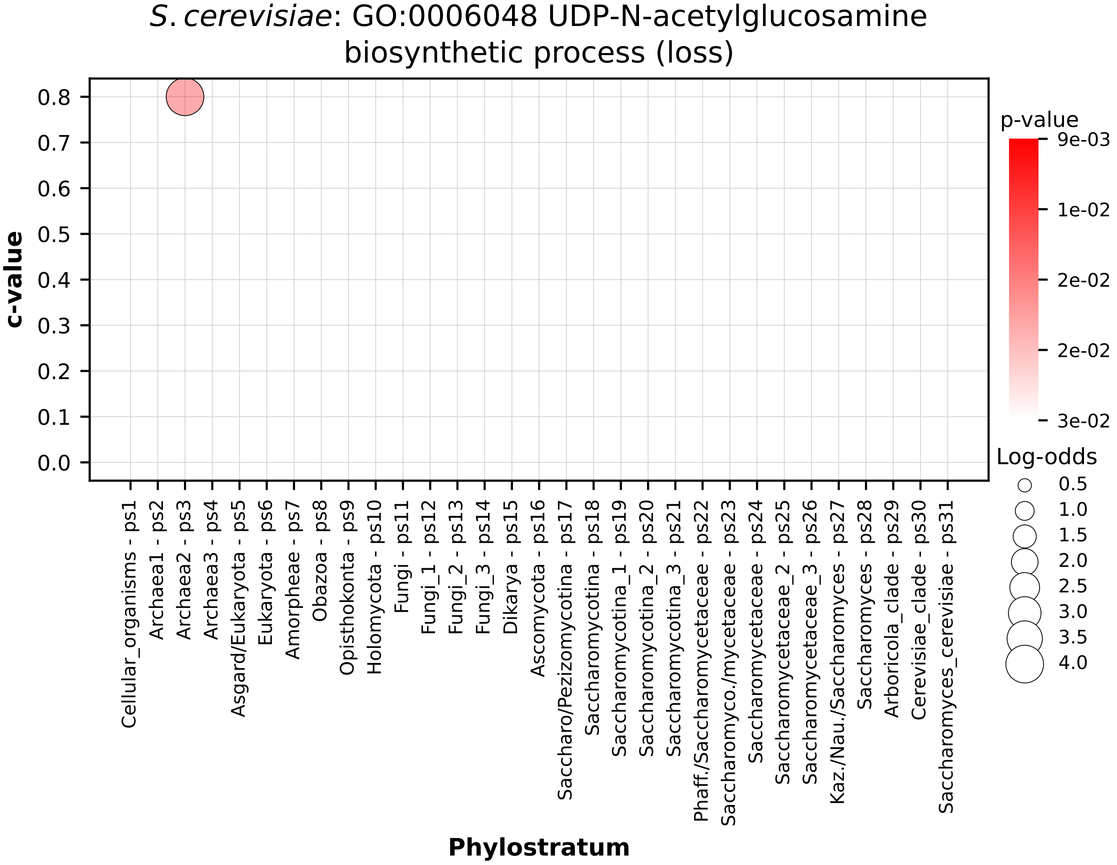
S. cerevisiae: GO:0006048 UDP-N-acetylglucosamine biosynthetic process (loss)
c-value
Phylostratum
p-value
9e-03
1e-02
2e-02
2e-02
3e-02
Log-odds
0.5
1.0
1.5
2.0
2.5
3.0
3.5
4.0
Cellular_organisms - ps1
Archaea1 - ps2
Archaea2 - ps3
Archaea3 - ps4
Asgard/Eukaryota - ps5
Eukaryota - ps6
Amorpheae - ps7
Obazoa - ps8
Opisthokonta - ps9
Holomycota - ps10
Fungi - ps11
Fungi_1 - ps12
Fungi_2 - ps13
Fungi_3 - ps14
Dikarya - ps15
Ascomycota - ps16
Saccharo/Pezizomycotina - ps17
Saccharomycotina - ps18
Saccharomycotina_1 - ps19
Saccharomycotina_2 - ps20
Saccharomycotina_3 - ps21
Phaff./Saccharomycetaceae - ps22
Saccharomyco./mycetaceae - ps23
Saccharomycetaceae - ps24
Saccharomycetaceae_2 - ps25
Saccharomycetaceae_3 - ps26
Kaz./Nau./Saccharomyces - ps27
Saccharomyces - ps28
Arboricola_clade - ps29
Cerevisiae_clade - ps30
Saccharomyces_cerevisiae - ps31

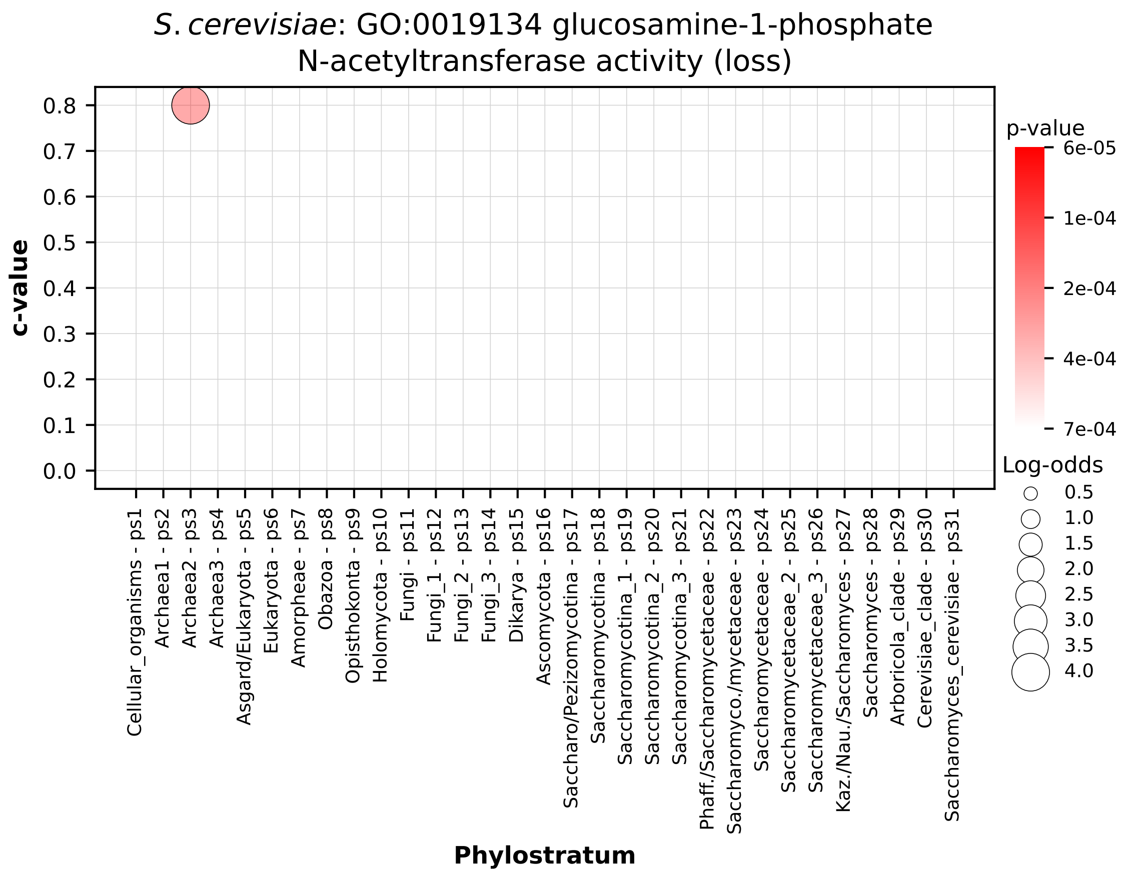

S. cerevisiae: GO:0019134 glucosamine-1-phosphate N-acetyltransferase activity (loss)
c-value
0.0
0.1
0.2
0.3
0.4
0.5
0.6
0.7
0.8
p-value
6e-05
1e-04
2e-04
4e-04
7e-04
Log-odds
0.5
1.0
1.5
2.0
2.5
3.0
3.5
4.0
Cellular_organisms - ps1
Archaea1 - ps2
Archaea2 - ps3
Archaea3 - ps4
Asgard/Eukaryota - ps5
Eukaryota - ps6
Amorpheae - ps7
Obazoa - ps8
Opisthokonta - ps9
Holomycota - ps10
Fungi - ps11
Fungi_1 - ps12
Fungi_2 - ps13
Fungi_3 - ps14
Dikarya - ps15
Ascomycota - ps16
Saccharo/Pezizomycotina - ps17
Saccharomycotina - ps18
Saccharomycotina_1 - ps19
Saccharomycotina_2 - ps20
Saccharomycotina_3 - ps21
Phaff./Saccharomycetaceae - ps22
Saccharomyco./mycetaceae - ps23
Saccharomycetaceae - ps24
Saccharomycetaceae_2 - ps25
Saccharomycetaceae_3 - ps26
Kaz./Nau./Saccharomyces - ps27
Saccharomyces - ps28
Arboricola_clade - ps29
Cerevisiae_clade - ps30
Saccharomyces_cerevisiae - ps31
Phylostratum

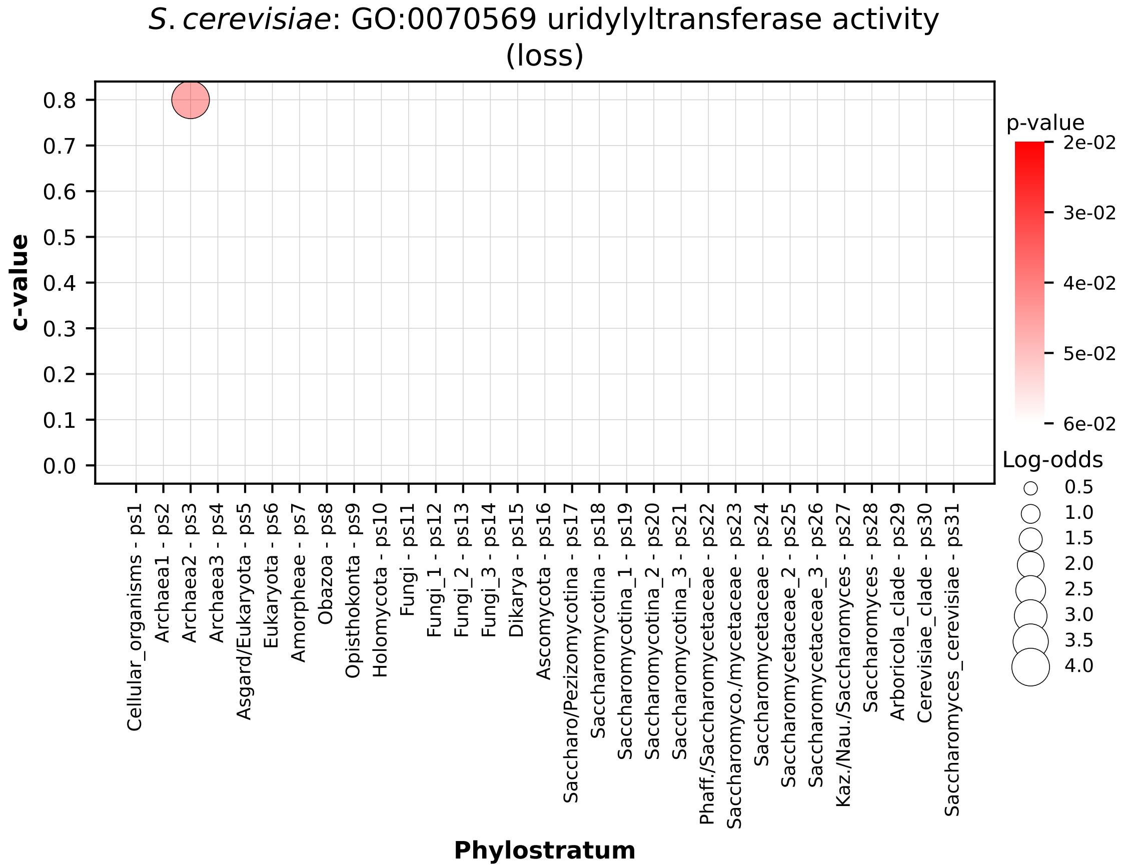

S. cerevisiae: GO:0070569 uridylyltransferase activity (loss)
c-value
0.8
0.7
0.6
0.5
0.4
0.3
0.2
0.1
0.0
p-value
2e-02
3e-02
4e-02
5e-02
6e-02
Log-odds
0.5
1.0
1.5
2.0
2.5
3.0
3.5
4.0
Cellular_organisms - ps1
Archaea1 - ps2
Archaea2 - ps3
Archaea3 - ps4
Asgard/Eukaryota - ps5
Eukaryota - ps6
Amorpheae - ps7
Obazoa - ps8
Opisthokonta - ps9
Holomycota - ps10
Fungi - ps11
Fungi_1 - ps12
Fungi_2 - ps13
Fungi_3 - ps14
Dikarya - ps15
Ascomycota - ps16
Saccharo/Pezizomycotina - ps17
Saccharomycotina - ps18
Saccharomycotina_1 - ps19
Saccharomycotina_2 - ps20
Saccharomycotina_3 - ps21
Phaff./Saccharomycetaceae - ps22
Saccharomyco./mycetaceae - ps23
Saccharomycetaceae - ps24
Saccharomycetaceae_2 - ps25
Saccharomycetaceae_3 - ps26
Kaz./Nau./Saccharomyces - ps27
Saccharomyces - ps28
Arboricola_clade - ps29
Cerevisiae_clade - ps30
Saccharomyces_cerevisiae - ps31
Phylostratum

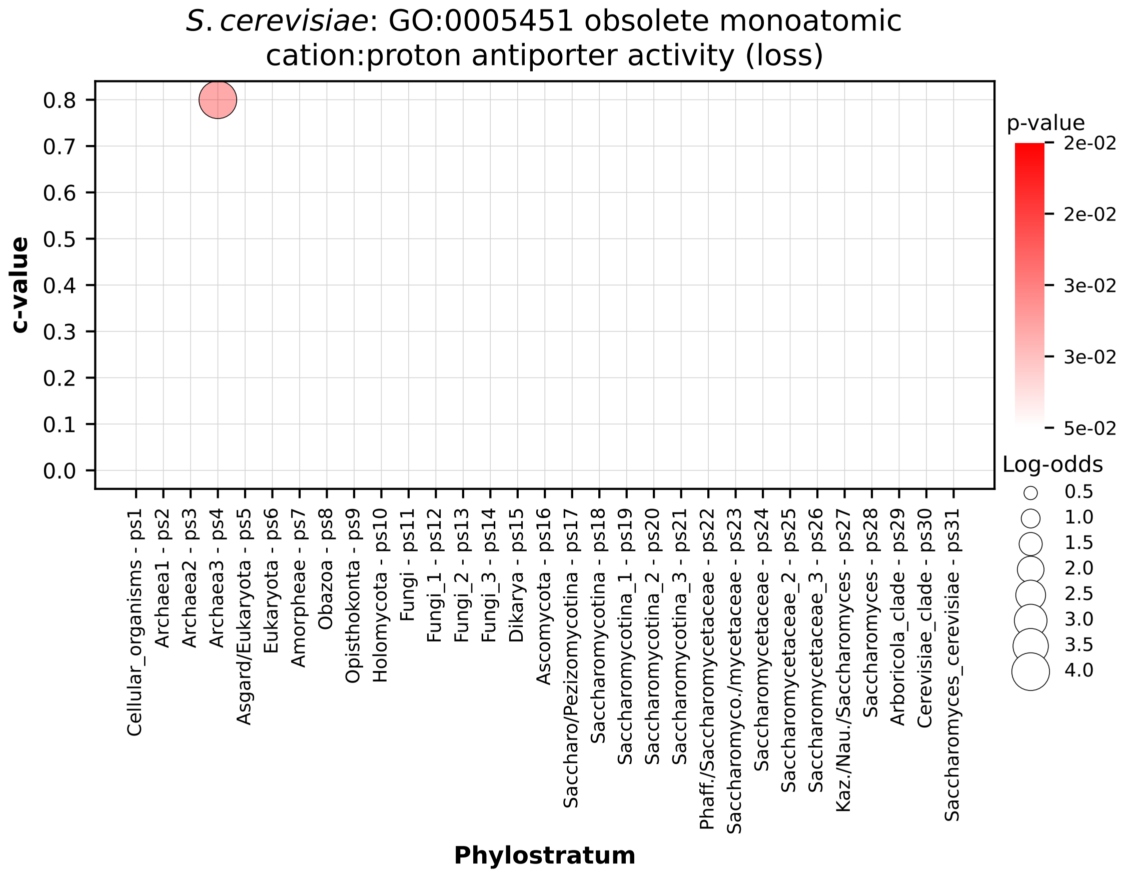

S. cerevisiae: GO:0005451 obsolete monoatomic cation:proton antiporter activity (loss)
c-value
0.8
0.7
0.6
0.5
0.4
0.3
0.2
0.1
0.0
Phylostratum
Cellular_organisms - ps1
Archaea1 - ps2
Archaea2 - ps3
Archaea3 - ps4
Asgard/Eukaryota - ps5
Eukaryota - ps6
Amorpheae - ps7
Obazoa - ps8
Opisthokonta - ps9
Holomycota - ps10
Fungi - ps11
Fungi_1 - ps12
Fungi_2 - ps13
Fungi_3 - ps14
Dikarya - ps15
Ascomycota - ps16
Saccharo/Pezizomycotina - ps17
Saccharomycotina - ps18
Saccharomycotina_1 - ps19
Saccharomycotina_2 - ps20
Saccharomycotina_3 - ps21
Phaff./Saccharomycetaceae - ps22
Saccharomyco./mycetaceae - ps23
Saccharomycetaceae - ps24
Saccharomycetaceae_2 - ps25
Saccharomycetaceae_3 - ps26
Kaz./Nau./Saccharomyces - ps27
Saccharomyces - ps28
Arboricola_clade - ps29
Cerevisiae_clade - ps30
Saccharomyces_cerevisiae - ps31
p-value
2e-02
2e-02
3e-02
3e-02
5e-02
Log-odds
0.5
1.0
1.5
2.0
2.5
3.0
3.5
4.0

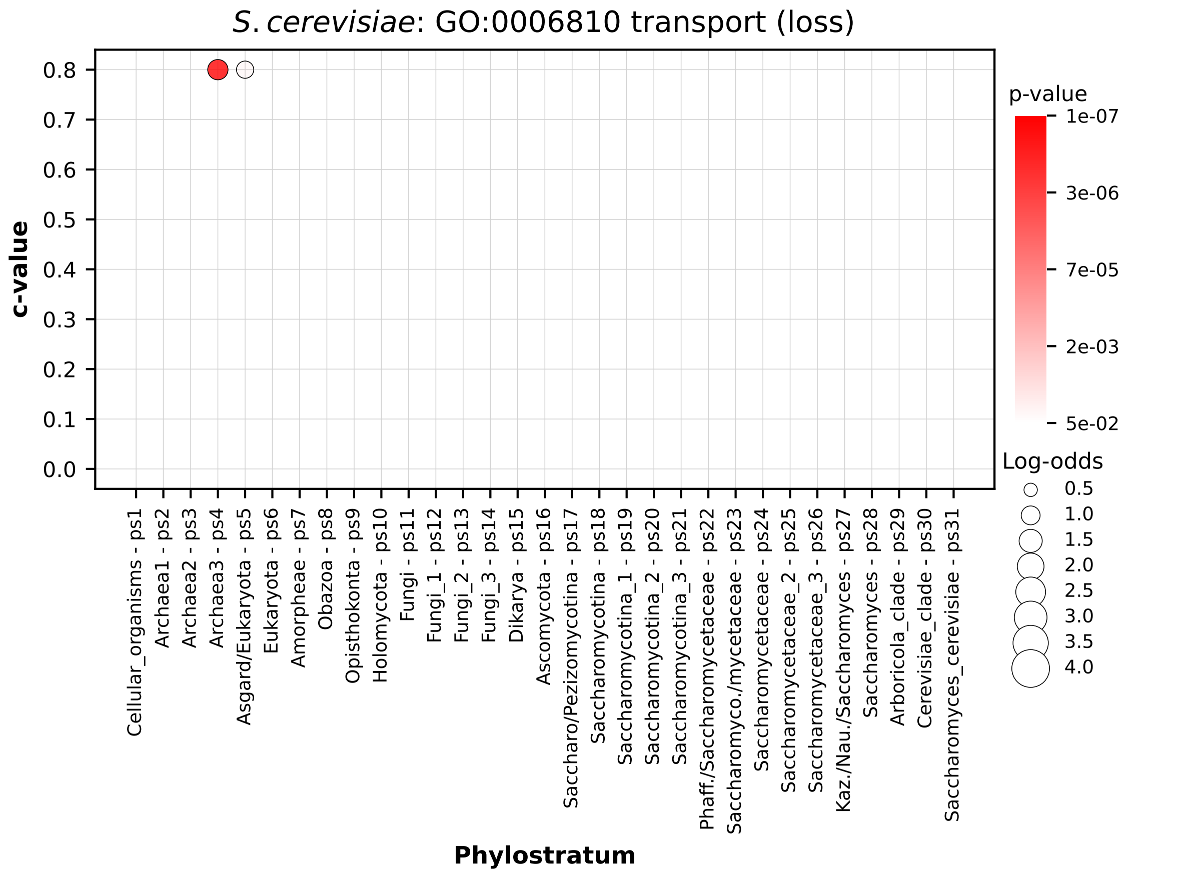

S. cerevisiae: GO:0006810 transport (loss)
c-value
0.8
0.7
0.6
0.5
0.4
0.3
0.2
0.1
0.0
p-value
1e-07
3e-06
7e-05
2e-03
5e-02
Log-odds
0.5
1.0
1.5
2.0
2.5
3.0
3.5
4.0
Cellular_organisms - ps1
Archaea1 - ps2
Archaea2 - ps3
Archaea3 - ps4
Asgard/Eukaryota - ps5
Eukaryota - ps6
Amorpheae - ps7
Obazoa - ps8
Opisthokonta - ps9
Holomycota - ps10
Fungi - ps11
Fungi_1 - ps12
Fungi_2 - ps13
Fungi_3 - ps14
Dikarya - ps15
Ascomycota - ps16
Saccharo/Pezizomycotina - ps17
Saccharomycotina - ps18
Saccharomycotina_1 - ps19
Saccharomycotina_2 - ps20
Saccharomycotina_3 - ps21
Phaff./Saccharomycetaceae - ps22
Saccharomyco./mycetaceae - ps23
Saccharomycetaceae - ps24
Saccharomycetaceae_2 - ps25
Saccharomycetaceae_3 - ps26
Kaz./Nau./Saccharomyces - ps27
Saccharomyces - ps28
Arboricola_clade - ps29
Cerevisiae_clade - ps30
Saccharomyces_cerevisiae - ps31
Phylostratum

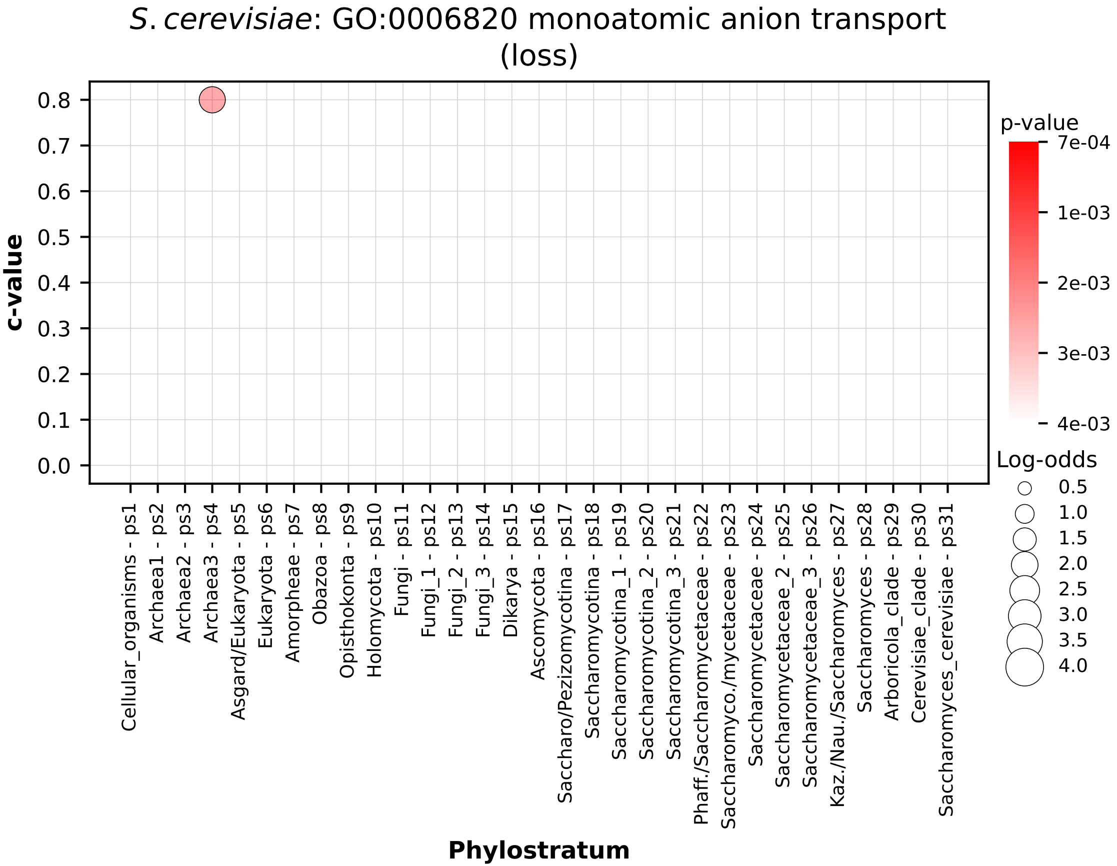
S. cerevisiae: GO:0006820 monoatomic anion transport
(loss)
c-value
0.8
0.7
0.6
0.5
0.4
0.3
0.2
0.1
0.0
Phylostratum
Cellular_organisms - ps1
Archaea1 - ps2
Archaea2 - ps3
Archaea3 - ps4
Asgard/Eukaryota - ps5
Eukaryota - ps6
Amorpheae - ps7
Obazoa - ps8
Opisthokonta - ps9
Holomycota - ps10
Fungi - ps11
Fungi_1 - ps12
Fungi_2 - ps13
Fungi_3 - ps14
Dikarya - ps15
Ascomycota - ps16
Saccharo/Pezizomycotina - ps17
Saccharomycotina - ps18
Saccharomycotina_1 - ps19
Saccharomycotina_2 - ps20
Saccharomycotina_3 - ps21
Phaff./Saccharomycetaceae - ps22
Saccharomyco./mycetaceae - ps23
Saccharomycetaceae - ps24
Saccharomycetaceae_2 - ps25
Saccharomycetaceae_3 - ps26
Kaz./Nau./Saccharomyces - ps27
Saccharomyces - ps28
Arboricola_clade - ps29
Cerevisiae_clade - ps30
Saccharomyces_cerevisiae - ps31
p-value
7e-04
1e-03
2e-03
3e-03
4e-03
Log-odds
0.5
1.0
1.5
2.0
2.5
3.0
3.5
4.0

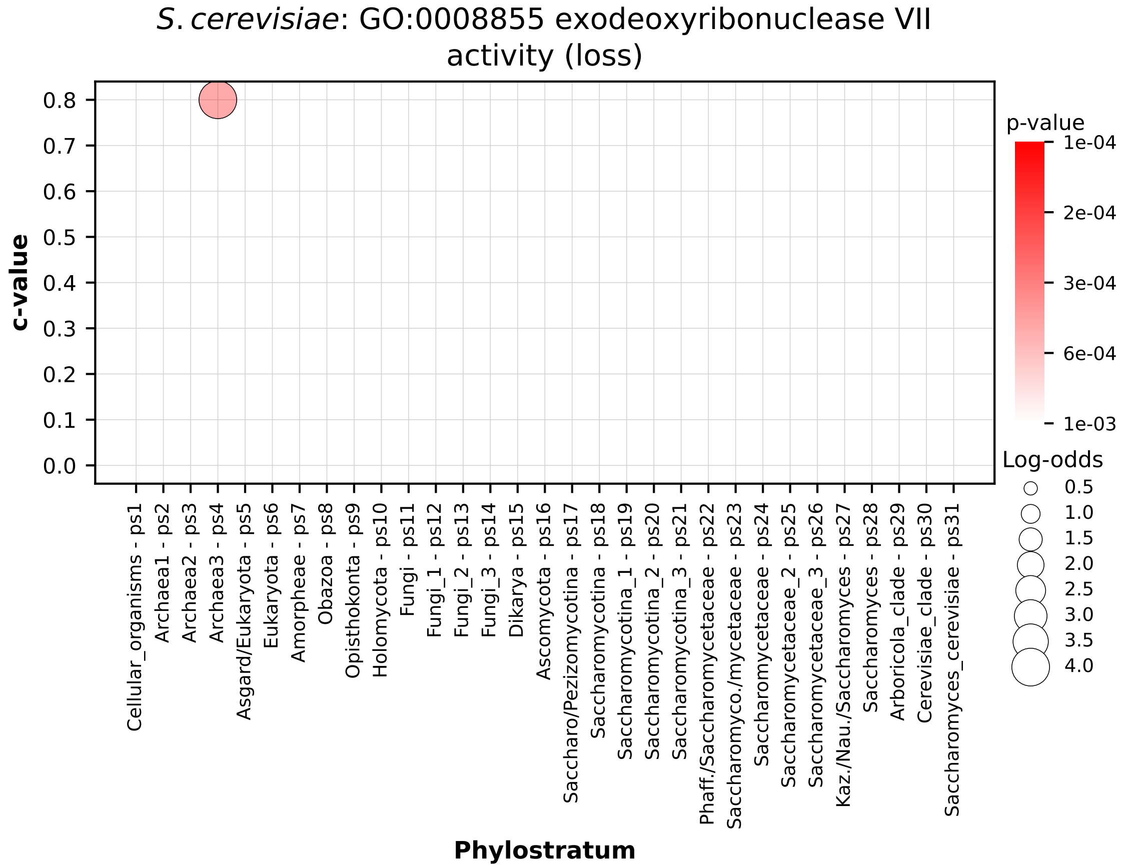
S. cerevisiae: GO:0008855 exodeoxyribonuclease VII activity (loss)
c-value
0.0
0.1
0.2
0.3
0.4
0.5
0.6
0.7
0.8
p-value
1e-04
2e-04
3e-04
6e-04
1e-03
Log-odds
0.5
1.0
1.5
2.0
2.5
3.0
3.5
4.0
Cellular_organisms - ps1
Archaea1 - ps2
Archaea2 - ps3
Archaea3 - ps4
Asgard/Eukaryota - ps5
Eukaryota - ps6
Amorpheae - ps7
Obazoa - ps8
Opisthokonta - ps9
Holomycota - ps10
Fungi - ps11
Fungi_1 - ps12
Fungi_2 - ps13
Fungi_3 - ps14
Dikarya - ps15
Ascomycota - ps16
Saccharo/Pezizomycotina - ps17
Saccharomycotina - ps18
Saccharomycotina_1 - ps19
Saccharomycotina_2 - ps20
Saccharomycotina_3 - ps21
Phaff./Saccharomycetaceae - ps22
Saccharomyco./mycetaceae - ps23
Saccharomycetaceae - ps24
Saccharomycetaceae_2 - ps25
Saccharomycetaceae_3 - ps26
Kaz./Nau./Saccharomyces - ps27
Saccharomyces - ps28
Arboricola_clade - ps29
Cerevisiae_clade - ps30
Saccharomyces_cerevisiae - ps31
Phylostratum

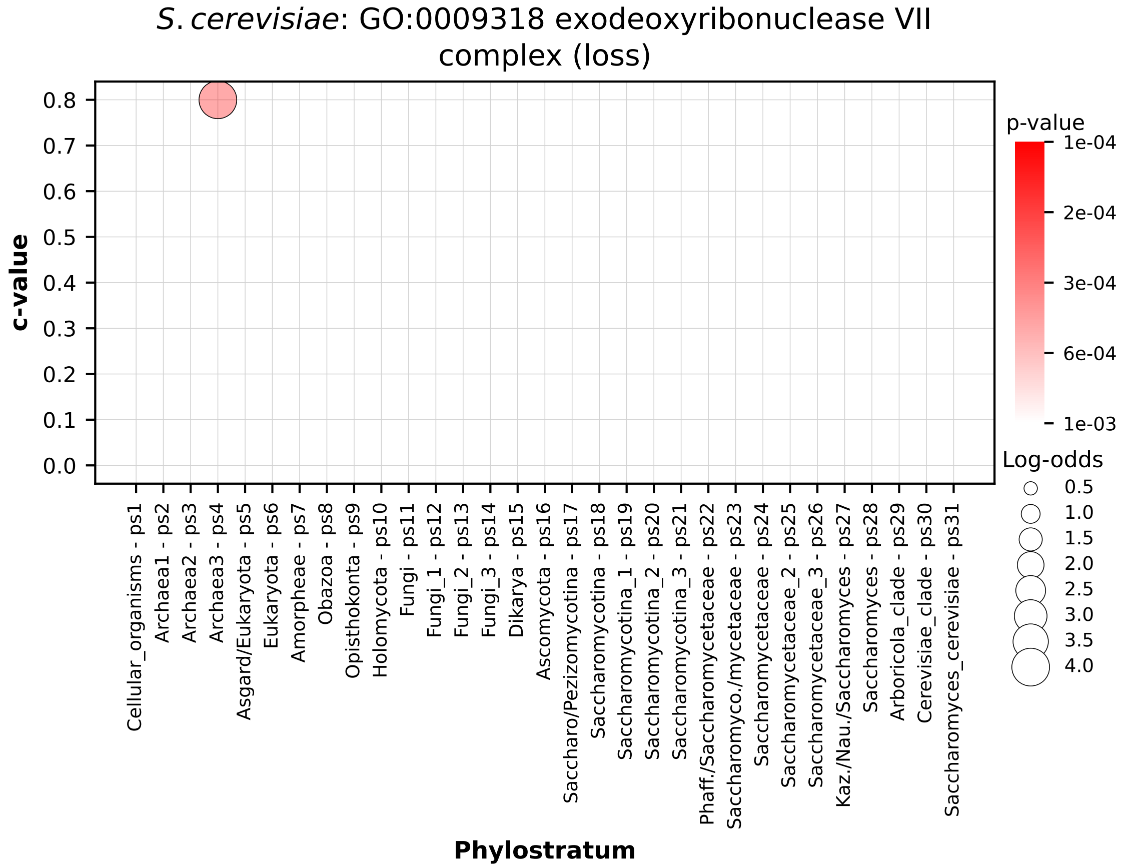

S. cerevisiae: GO:0009318 exodeoxyribonuclease VII complex (loss)
c-value
0.8
0.7
0.6
0.5
0.4
0.3
0.2
0.1
0.0
p-value
1e-04
2e-04
3e-04
6e-04
1e-03
Log-odds
0.5
1.0
1.5
2.0
2.5
3.0
3.5
4.0
Cellular_organisms - ps1
Archaea1 - ps2
Archaea2 - ps3
Archaea3 - ps4
Asgard/Eukaryota - ps5
Eukaryota - ps6
Amorpheae - ps7
Obazoa - ps8
Opisthokonta - ps9
Holomycota - ps10
Fungi - ps11
Fungi_1 - ps12
Fungi_2 - ps13
Fungi_3 - ps14
Dikarya - ps15
Ascomycota - ps16
Saccharo/Pezizomycotina - ps17
Saccharomycotina - ps18
Saccharomycotina_1 - ps19
Saccharomycotina_2 - ps20
Saccharomycotina_3 - ps21
Phaff./Saccharomycetaceae - ps22
Saccharomyco./mycetaceae - ps23
Saccharomycetaceae - ps24
Saccharomycetaceae_2 - ps25
Saccharomycetaceae_3 - ps26
Kaz./Nau./Saccharomyces - ps27
Saccharomyces - ps28
Arboricola_clade - ps29
Cerevisiae_clade - ps30
Saccharomyces_cerevisiae - ps31
Phylostratum

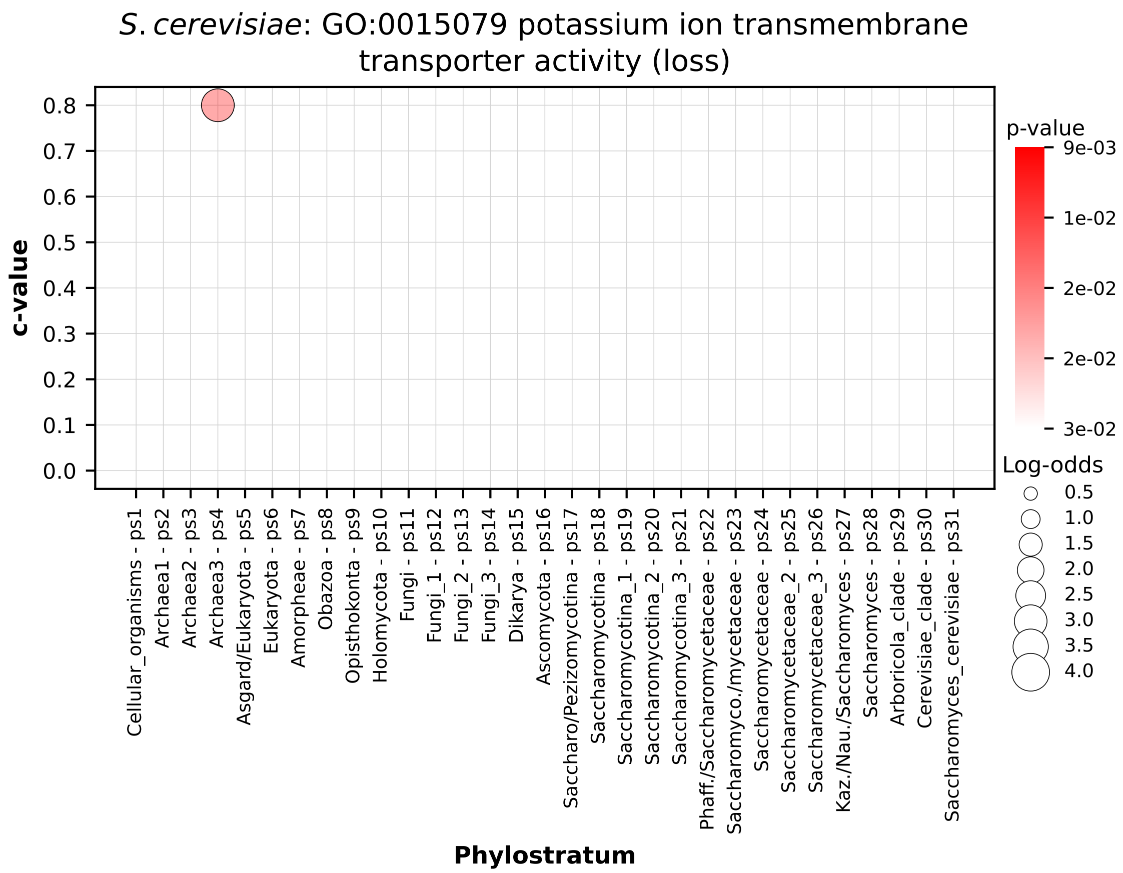

S. cerevisiae: GO:0015079 potassium ion transmembrane transporter activity (loss)
c-value
0.0
0.1
0.2
0.3
0.4
0.5
0.6
0.7
0.8
p-value
9e-03
1e-02
2e-02
2e-02
3e-02
Log-odds
0.5
1.0
1.5
2.0
2.5
3.0
3.5
4.0
Cellular_organisms - ps1
Archaea1 - ps2
Archaea2 - ps3
Archaea3 - ps4
Asgard/Eukaryota - ps5
Eukaryota - ps6
Amorpheae - ps7
Obazoa - ps8
Opisthokonta - ps9
Holomycota - ps10
Fungi - ps11
Fungi_1 - ps12
Fungi_2 - ps13
Fungi_3 - ps14
Dikarya - ps15
Ascomycota - ps16
Saccharo/Pezizomycotina - ps17
Saccharomycotina - ps18
Saccharomycotina_1 - ps19
Saccharomycotina_2 - ps20
Saccharomycotina_3 - ps21
Phaff./Saccharomycetaceae - ps22
Saccharomyco./mycetaceae - ps23
Saccharomycetaceae - ps24
Saccharomycetaceae_2 - ps25
Saccharomycetaceae_3 - ps26
Kaz./Nau./Saccharomyces - ps27
Saccharomyces - ps28
Arboricola_clade - ps29
Cerevisiae_clade - ps30
Saccharomyces_cerevisiae - ps31
Phylostratum

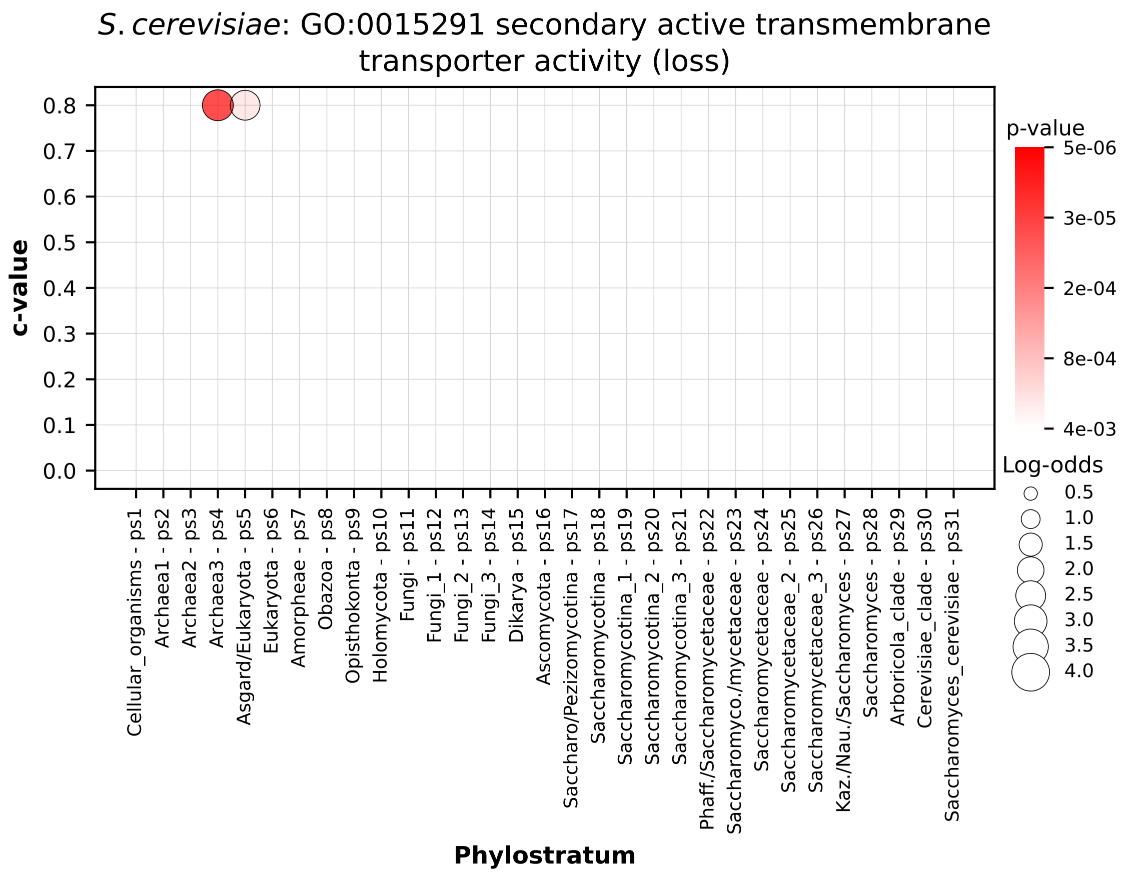

S. cerevisiae: GO:0015291 secondary active transmembrane transporter activity (loss)
c-value
0.0
0.1
0.2
0.3
0.4
0.5
0.6
0.7
0.8
Phylostratum
Cellular_organisms - ps1
Archaea1 - ps2
Archaea2 - ps3
Archaea3 - ps4
Asgard/Eukaryota - ps5
Eukaryota - ps6
Amorpheae - ps7
Obazoa - ps8
Opisthokonta - ps9
Holomycota - ps10
Fungi - ps11
Fungi_1 - ps12
Fungi_2 - ps13
Fungi_3 - ps14
Dikarya - ps15
Ascomycota - ps16
Saccharo/Pezizomycotina - ps17
Saccharomycotina - ps18
Saccharomycotina_1 - ps19
Saccharomycotina_2 - ps20
Saccharomycotina_3 - ps21
Phaff./Saccharomycetaceae - ps22
Saccharomyco./mycetaceae - ps23
Saccharomycetaceae - ps24
Saccharomycetaceae_2 - ps25
Saccharomycetaceae_3 - ps26
Kaz./Nau./Saccharomyces - ps27
Saccharomyces - ps28
Arboricola_clade - ps29
Cerevisiae_clade - ps30
Saccharomyces_cerevisiae - ps31
p-value
5e-06
3e-05
2e-04
8e-04
4e-03
Log-odds
0.5
1.0
1.5
2.0
2.5
3.0
3.5
4.0

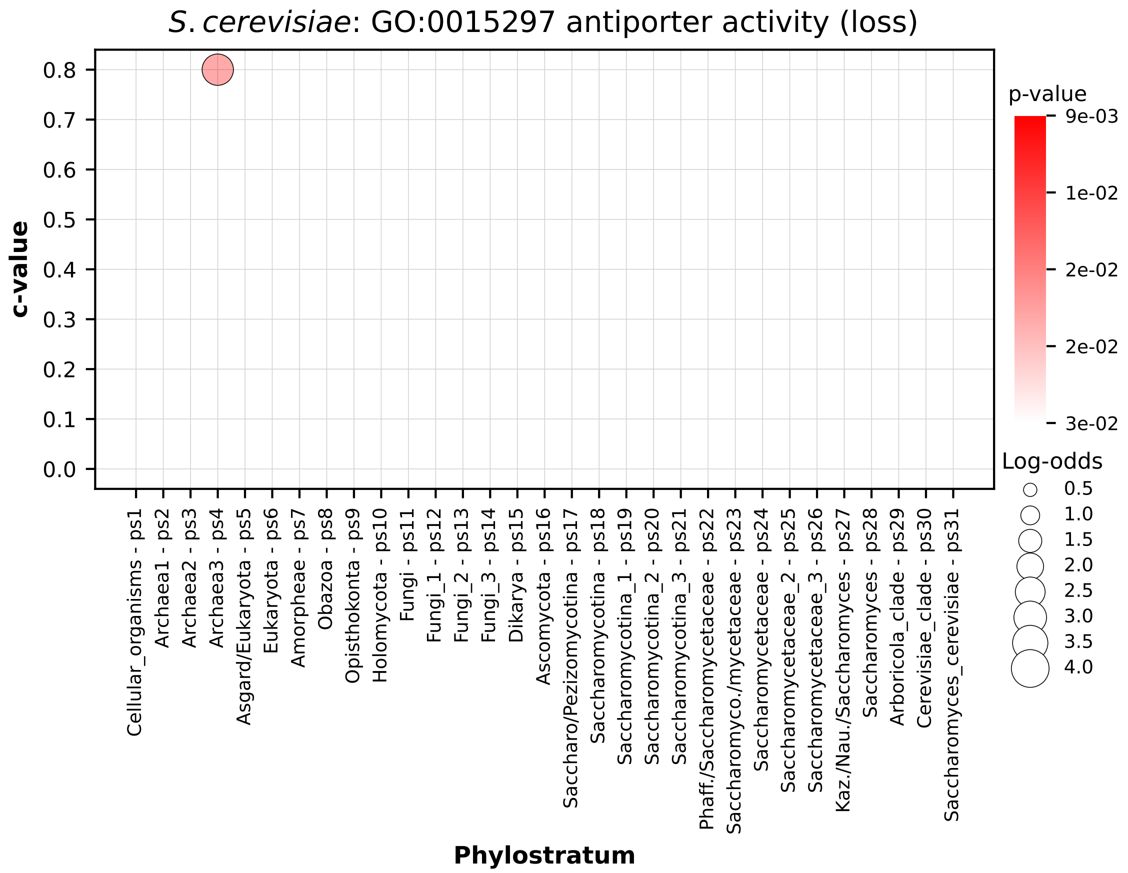

S. cerevisiae: GO:0015297 antiporter activity (loss)
c-value
0.0
0.1
0.2
0.3
0.4
0.5
0.6
0.7
0.8
Phylostratum
Cellular_organisms - ps1
Archaea1 - ps2
Archaea2 - ps3
Archaea3 - ps4
Asgard/Eukaryota - ps5
Eukaryota - ps6
Amorpheae - ps7
Obazoa - ps8
Opisthokonta - ps9
Holomycota - ps10
Fungi - ps11
Fungi_1 - ps12
Fungi_2 - ps13
Fungi_3 - ps14
Dikarya - ps15
Ascomycota - ps16
Saccharo/Pezizomycotina - ps17
Saccharomycotina - ps18
Saccharomycotina_1 - ps19
Saccharomycotina_2 - ps20
Saccharomycotina_3 - ps21
Phaff./Saccharomycetaceae - ps22
Saccharomyco./mycetaceae - ps23
Saccharomycetaceae - ps24
Saccharomycetaceae_2 - ps25
Saccharomycetaceae_3 - ps26
Kaz./Nau./Saccharomyces - ps27
Saccharomyces - ps28
Arboricola_clade - ps29
Cerevisiae_clade - ps30
Saccharomyces_cerevisiae - ps31
p-value
9e-03
1e-02
2e-02
2e-02
3e-02
Log-odds
0.5
1.0
1.5
2.0
2.5
3.0
3.5
4.0

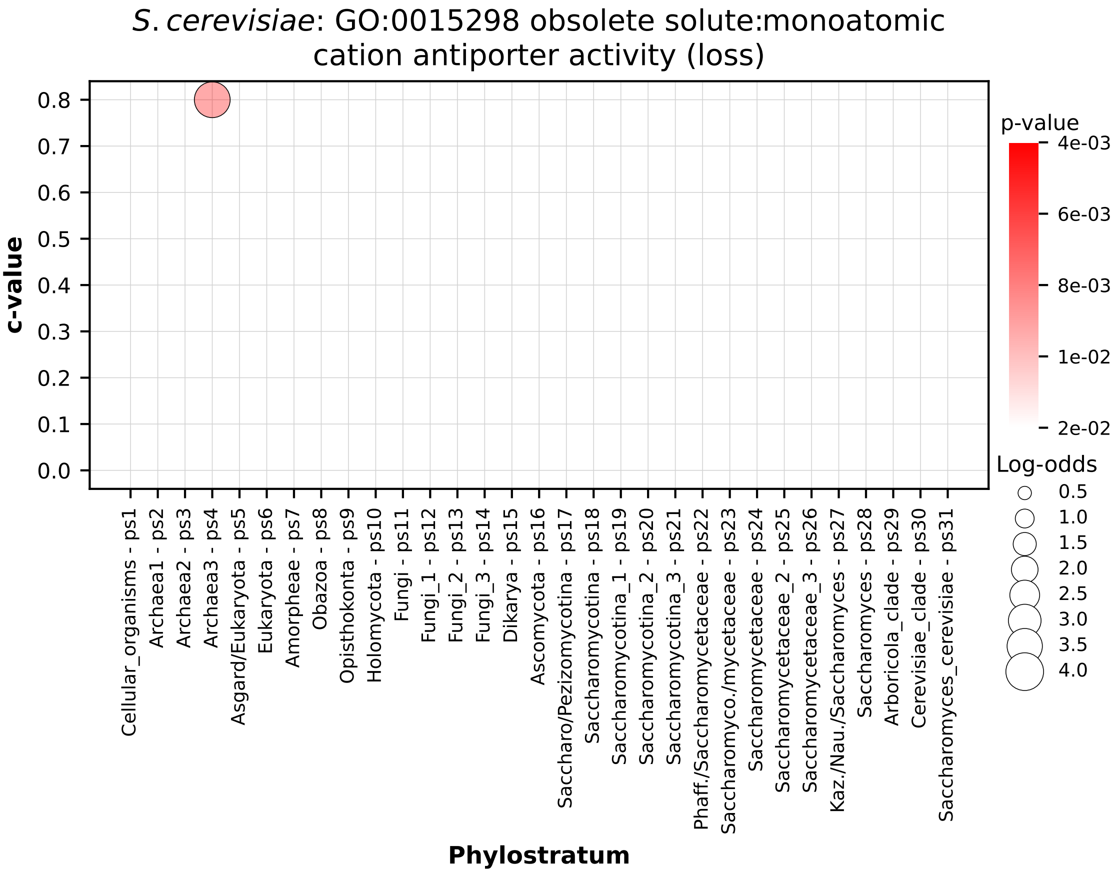
S. cerevisiae: GO:0015298 obsolete solute:monoatomic cation antiporter activity (loss)
c-value
0.0
0.1
0.2
0.3
0.4
0.5
0.6
0.7
0.8
Phylostratum
Cellular_organisms - ps1
Archaea1 - ps2
Archaea2 - ps3
Archaea3 - ps4
Asgard/Eukaryota - ps5
Eukaryota - ps6
Amorpheae - ps7
Obazoa - ps8
Opisthokonta - ps9
Holomycota - ps10
Fungi - ps11
Fungi_1 - ps12
Fungi_2 - ps13
Fungi_3 - ps14
Dikarya - ps15
Ascomycota - ps16
Saccharo/Pezizomycotina - ps17
Saccharomycotina - ps18
Saccharomycotina_1 - ps19
Saccharomycotina_2 - ps20
Saccharomycotina_3 - ps21
Phaff./Saccharomycetaceae - ps22
Saccharomyco./mycetaceae - ps23
Saccharomycetaceae - ps24
Saccharomycetaceae_2 - ps25
Saccharomycetaceae_3 - ps26
Kaz./Nau./Saccharomyces - ps27
Saccharomyces - ps28
Arboricola_clade - ps29
Cerevisiae_clade - ps30
Saccharomyces_cerevisiae - ps31
p-value
4e-03
6e-03
8e-03
1e-02
2e-02
Log-odds
0.5
1.0
1.5
2.0
2.5
3.0
3.5
4.0

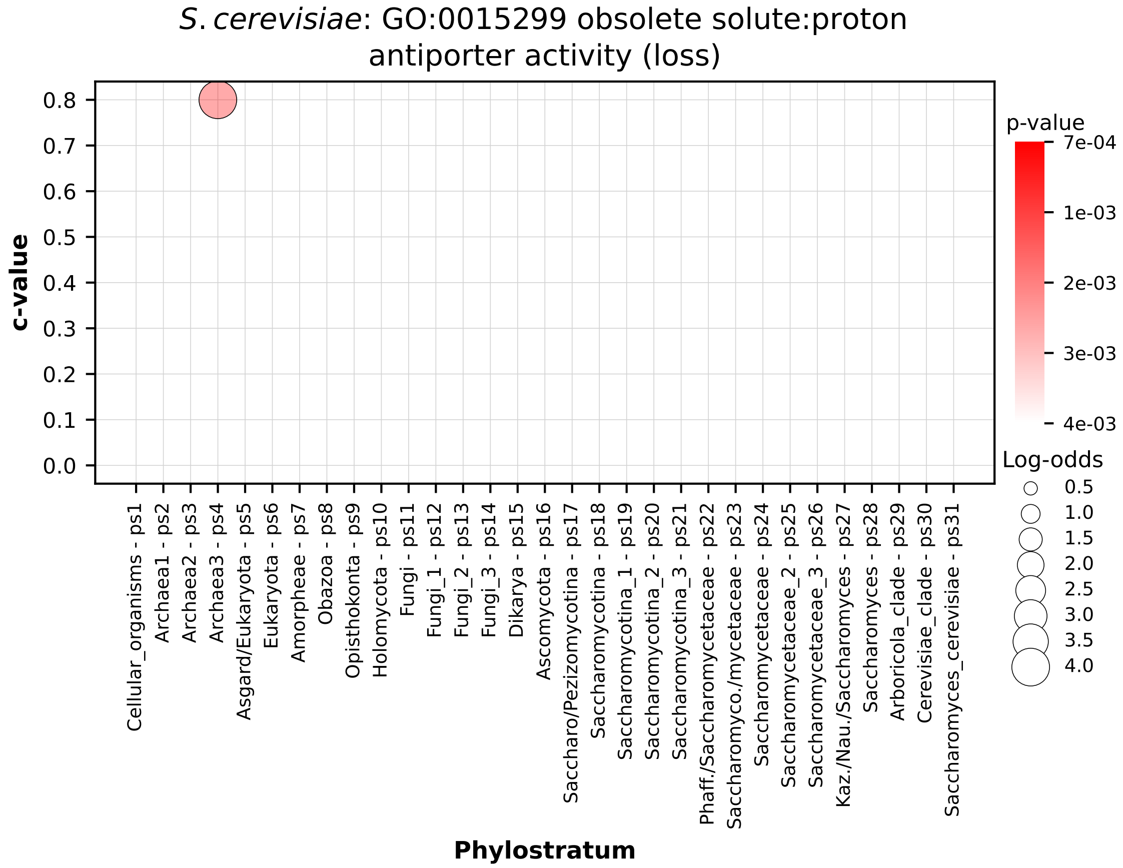

S. cerevisiae: GO:0015299 obsolete solute:proton antiporter activity (loss)
c-value
0.0
0.1
0.2
0.3
0.4
0.5
0.6
0.7
0.8
p-value
7e-04
1e-03
2e-03
3e-03
4e-03
Log-odds
0.5
1.0
1.5
2.0
2.5
3.0
3.5
4.0
Cellular_organisms - ps1
Archaea1 - ps2
Archaea2 - ps3
Archaea3 - ps4
Asgard/Eukaryota - ps5
Eukaryota - ps6
Amorpheae - ps7
Obazoa - ps8
Opisthokonta - ps9
Holomycota - ps10
Fungi - ps11
Fungi_1 - ps12
Fungi_2 - ps13
Fungi_3 - ps14
Dikarya - ps15
Ascomycota - ps16
Saccharo/Pezizomycotina - ps17
Saccharomycotina - ps18
Saccharomycotina_1 - ps19
Saccharomycotina_2 - ps20
Saccharomycotina_3 - ps21
Phaff./Saccharomycetaceae - ps22
Saccharomyco./mycetaceae - ps23
Saccharomycetaceae - ps24
Saccharomycetaceae_2 - ps25
Saccharomycetaceae_3 - ps26
Kaz./Nau./Saccharomyces - ps27
Saccharomyces - ps28
Arboricola_clade - ps29
Cerevisiae_clade - ps30
Saccharomyces_cerevisiae - ps31
Phylostratum

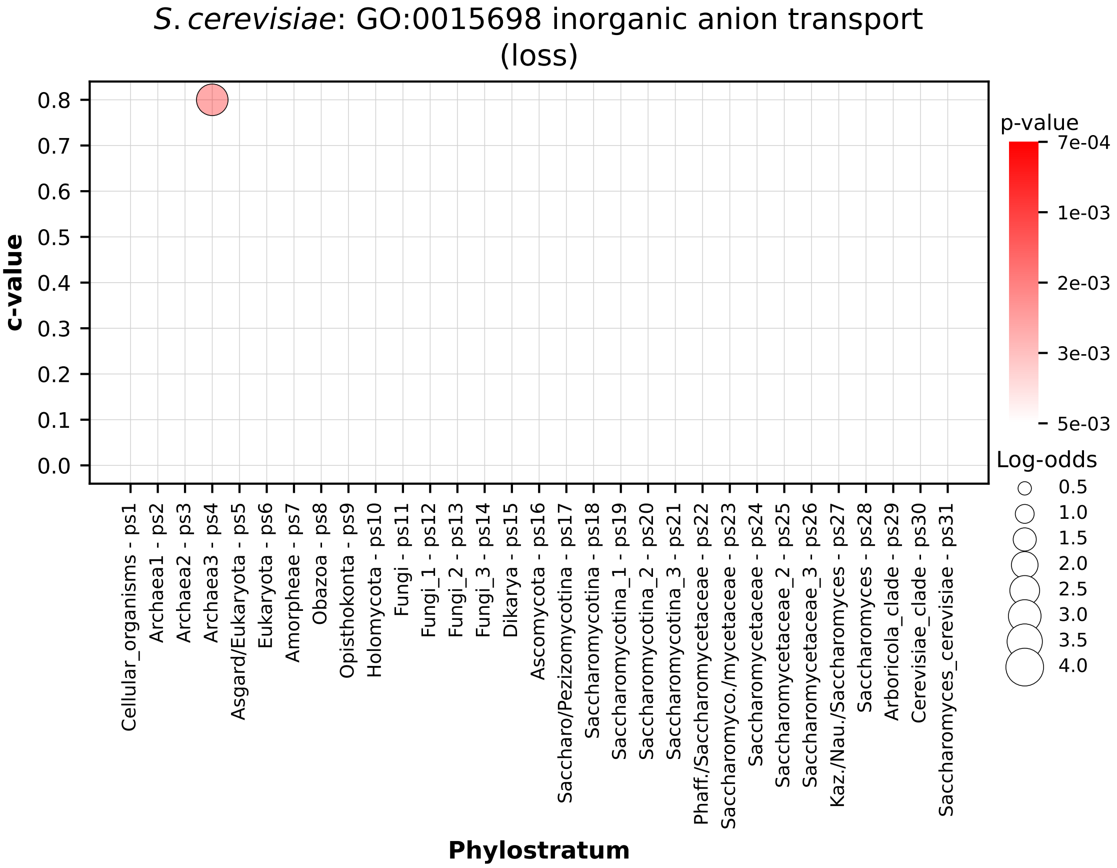

S. cerevisiae: GO:0015698 inorganic anion transport
(loss)
c-value
0.0
0.1
0.2
0.3
0.4
0.5
0.6
0.7
0.8
p-value
7e-04
1e-03
2e-03
3e-03
5e-03
Log-odds
0.5
1.0
1.5
2.0
2.5
3.0
3.5
4.0
Cellular_organisms - ps1
Archaea1 - ps2
Archaea2 - ps3
Archaea3 - ps4
Asgard/Eukaryota - ps5
Eukaryota - ps6
Amorpheae - ps7
Obazoa - ps8
Opisthokonta - ps9
Holomycota - ps10
Fungi - ps11
Fungi_1 - ps12
Fungi_2 - ps13
Fungi_3 - ps14
Dikarya - ps15
Ascomycota - ps16
Saccharo/Pezizomycotina - ps17
Saccharomycotina - ps18
Saccharomycotina_1 - ps19
Saccharomycotina_2 - ps20
Saccharomycotina_3 - ps21
Phaff./Saccharomycetaceae - ps22
Saccharomyco./mycetaceae - ps23
Saccharomycetaceae - ps24
Saccharomycetaceae_2 - ps25
Saccharomycetaceae_3 - ps26
Kaz./Nau./Saccharomyces - ps27
Saccharomyces - ps28
Arboricola_clade - ps29
Cerevisiae_clade - ps30
Saccharomyces_cerevisiae - ps31
Phylostratum

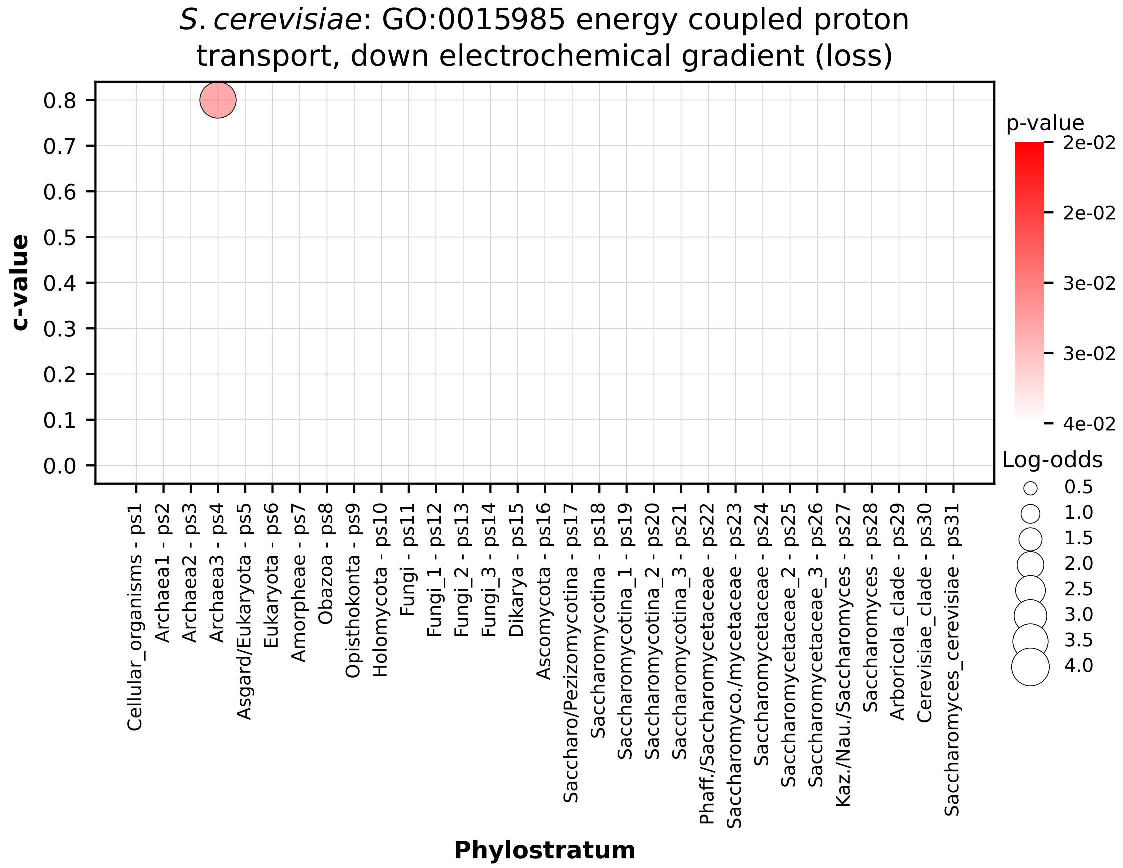

S. cerevisiae: GO:0015985 energy coupled proton transport, down electrochemical gradient (loss)
c-value
0.0
0.1
0.2
0.3
0.4
0.5
0.6
0.7
0.8
p-value
2e-02
2e-02
3e-02
3e-02
4e-02
Log-odds
0.5
1.0
1.5
2.0
2.5
3.0
3.5
4.0
Cellular_organisms - ps1
Archaea1 - ps2
Archaea2 - ps3
Archaea3 - ps4
Asgard/Eukaryota - ps5
Eukaryota - ps6
Amorpheae - ps7
Obazoa - ps8
Opisthokonta - ps9
Holomycota - ps10
Fungi - ps11
Fungi_1 - ps12
Fungi_2 - ps13
Fungi_3 - ps14
Dikarya - ps15
Ascomycota - ps16
Saccharo/Pezizomycotina - ps17
Saccharomycotina - ps18
Saccharomycotina_1 - ps19
Saccharomycotina_2 - ps20
Saccharomycotina_3 - ps21
Phaff./Saccharomycetaceae - ps22
Saccharomyco./mycetaceae - ps23
Saccharomycetaceae - ps24
Saccharomycetaceae_2 - ps25
Saccharomycetaceae_3 - ps26
Kaz./Nau./Saccharomyces - ps27
Saccharomyces - ps28
Arboricola_clade - ps29
Cerevisiae_clade - ps30
Saccharomyces_cerevisiae - ps31
Phylostratum

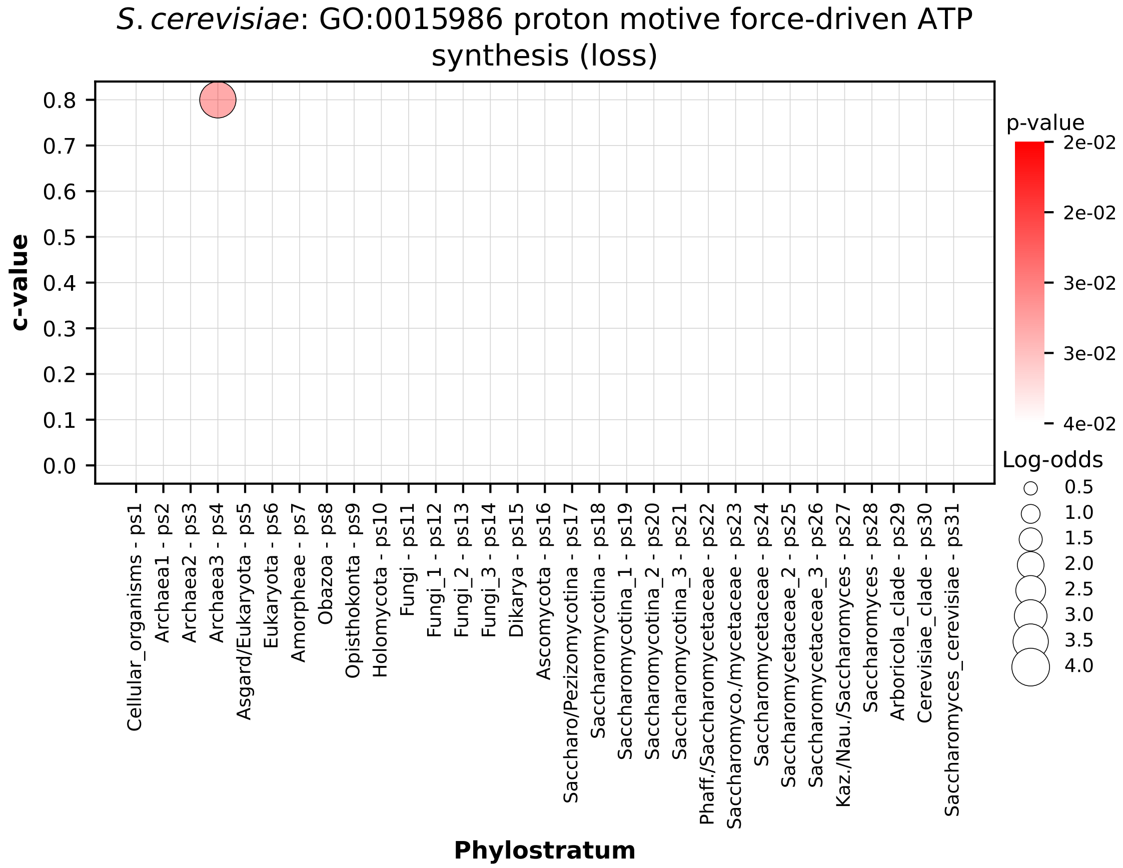

S. cerevisiae: GO:0015986 proton motive force-driven ATP synthesis (loss)
c-value
0.0
0.1
0.2
0.3
0.4
0.5
0.6
0.7
0.8
p-value
2e-02
2e-02
3e-02
3e-02
4e-02
Log-odds
0.5
1.0
1.5
2.0
2.5
3.0
3.5
4.0
Cellular_organisms - ps1
Archaea1 - ps2
Archaea2 - ps3
Archaea3 - ps4
Asgard/Eukaryota - ps5
Eukaryota - ps6
Amorpheae - ps7
Obazoa - ps8
Opisthokonta - ps9
Holomycota - ps10
Fungi - ps11
Fungi_1 - ps12
Fungi_2 - ps13
Fungi_3 - ps14
Dikarya - ps15
Ascomycota - ps16
Saccharo/Pezizomycotina - ps17
Saccharomycotina - ps18
Saccharomycotina_1 - ps19
Saccharomycotina_2 - ps20
Saccharomycotina_3 - ps21
Phaff./Saccharomycetaceae - ps22
Saccharomyco./mycetaceae - ps23
Saccharomycetaceae - ps24
Saccharomycetaceae_2 - ps25
Saccharomycetaceae_3 - ps26
Kaz./Nau./Saccharomyces - ps27
Saccharomyces - ps28
Arboricola_clade - ps29
Cerevisiae_clade - ps30
Saccharomyces_cerevisiae - ps31
Phylostratum

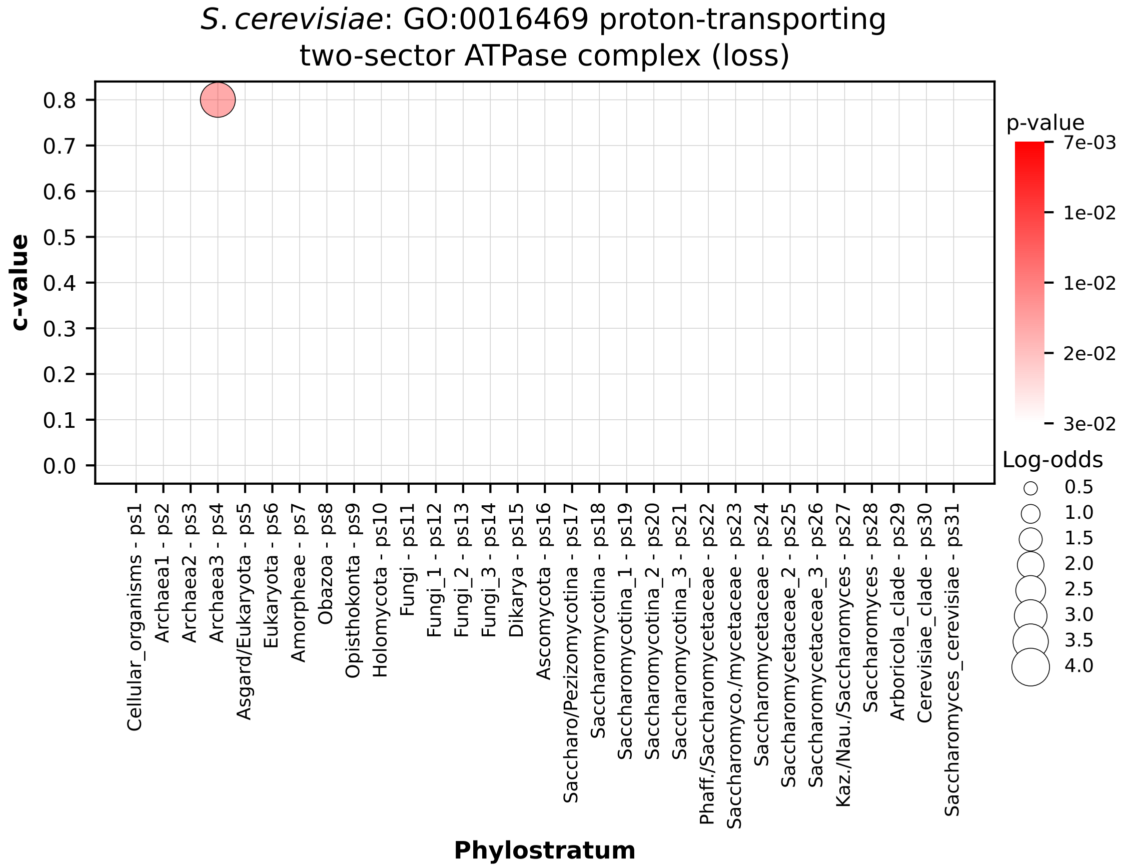

S. cerevisiae: GO:0016469 proton-transporting two-sector ATPase complex (loss)
c-value
0.0
0.1
0.2
0.3
0.4
0.5
0.6
0.7
0.8
p-value
7e-03
1e-02
1e-02
2e-02
3e-02
Log-odds
0.5
1.0
1.5
2.0
2.5
3.0
3.5
4.0
Cellular_organisms - ps1
Archaea1 - ps2
Archaea2 - ps3
Archaea3 - ps4
Asgard/Eukaryota - ps5
Eukaryota - ps6
Amorpheae - ps7
Obazoa - ps8
Opisthokonta - ps9
Holomycota - ps10
Fungi - ps11
Fungi_1 - ps12
Fungi_2 - ps13
Fungi_3 - ps14
Dikarya - ps15
Ascomycota - ps16
Saccharo/Pezizomycotina - ps17
Saccharomycotina - ps18
Saccharomycotina_1 - ps19
Saccharomycotina_2 - ps20
Saccharomycotina_3 - ps21
Phaff./Saccharomycetaceae - ps22
Saccharomyco./mycetaceae - ps23
Saccharomycetaceae - ps24
Saccharomycetaceae_2 - ps25
Saccharomycetaceae_3 - ps26
Kaz./Nau./Saccharomyces - ps27
Saccharomyces - ps28
Arboricola_clade - ps29
Cerevisiae_clade - ps30
Saccharomyces_cerevisiae - ps31
Phylostratum

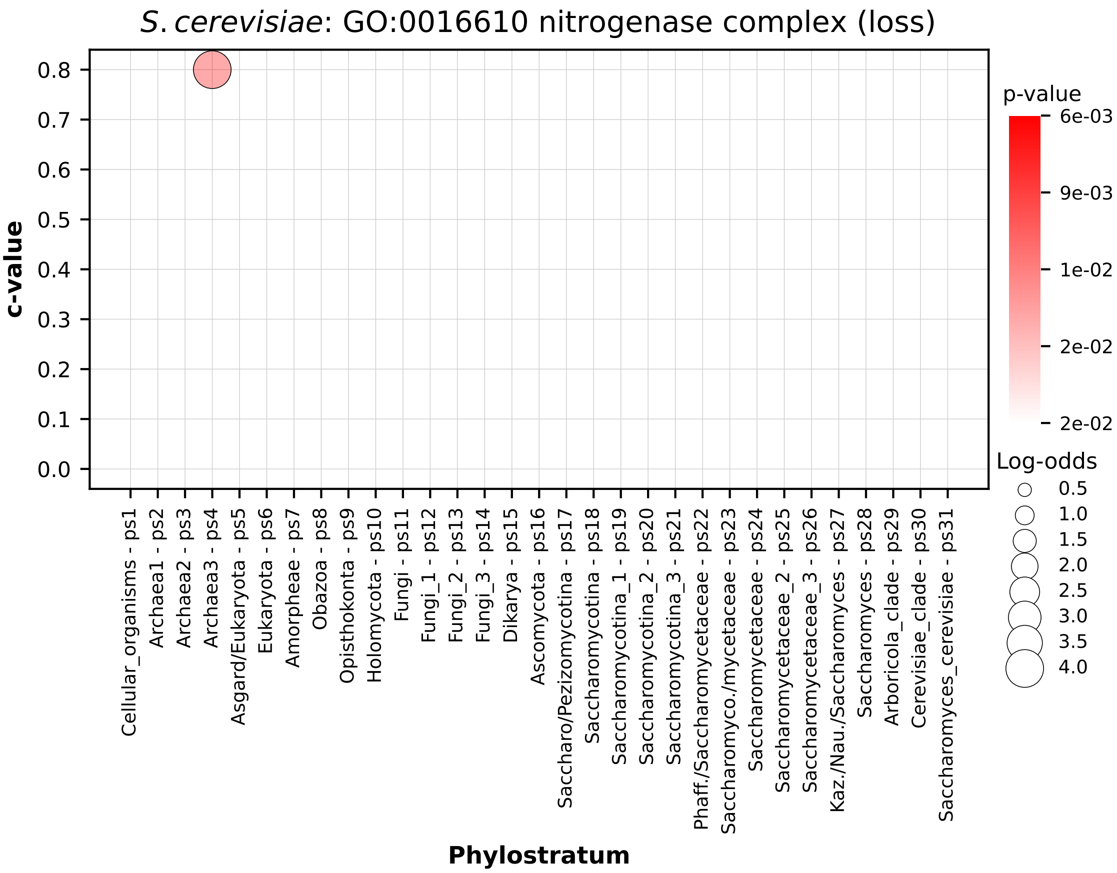
S. cerevisiae: GO:0016610 nitrogenase complex (loss)
c-value
0.0
0.1
0.2
0.3
0.4
0.5
0.6
0.7
0.8
Phylostratum
Cellular_organisms - ps1
Archaea1 - ps2
Archaea2 - ps3
Archaea3 - ps4
Asgard/Eukaryota - ps5
Eukaryota - ps6
Amorpheae - ps7
Obazoa - ps8
Opisthokonta - ps9
Holomycota - ps10
Fungi - ps11
Fungi_1 - ps12
Fungi_2 - ps13
Fungi_3 - ps14
Dikarya - ps15
Ascomycota - ps16
Saccharo/Pezizomycotina - ps17
Saccharomycotina - ps18
Saccharomycotina_1 - ps19
Saccharomycotina_2 - ps20
Saccharomycotina_3 - ps21
Phaff./Saccharomycetaceae - ps22
Saccharomyco./mycetaceae - ps23
Saccharomycetaceae - ps24
Saccharomycetaceae_2 - ps25
Saccharomycetaceae_3 - ps26
Kaz./Nau./Saccharomyces - ps27
Saccharomyces - ps28
Arboricola_clade - ps29
Cerevisiae_clade - ps30
Saccharomyces_cerevisiae - ps31
p-value
6e-03
9e-03
1e-02
2e-02
2e-02
Log-odds
0.5
1.0
1.5
2.0
2.5
3.0
3.5
4.0

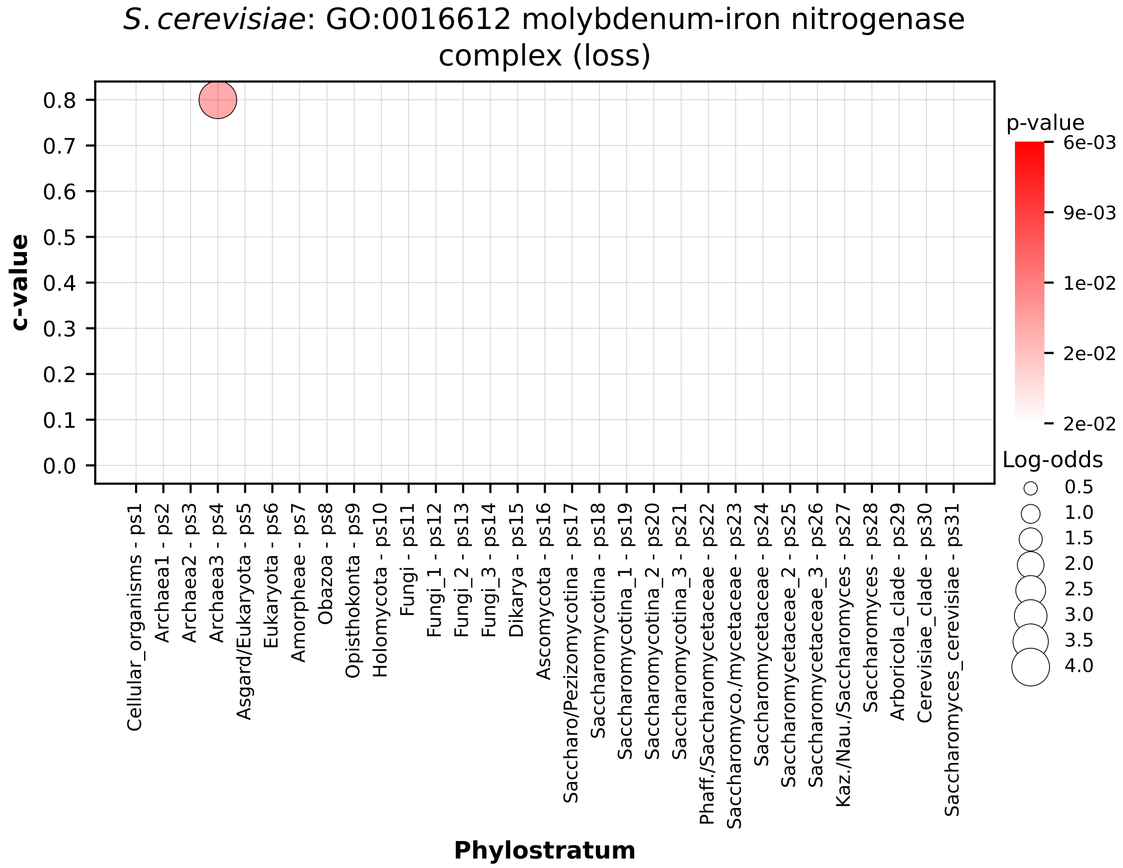

S. cerevisiae: GO:0016612 molybdenum-iron nitrogenase complex (loss)
c-value
0.0
0.1
0.2
0.3
0.4
0.5
0.6
0.7
0.8
Phylostratum
Cellular_organisms - ps1
Archaea1 - ps2
Archaea2 - ps3
Archaea3 - ps4
Asgard/Eukaryota - ps5
Eukaryota - ps6
Amorpheae - ps7
Obazoa - ps8
Opisthokonta - ps9
Holomycota - ps10
Fungi - ps11
Fungi_1 - ps12
Fungi_2 - ps13
Fungi_3 - ps14
Dikarya - ps15
Ascomycota - ps16
Saccharo/Pezizomycotina - ps17
Saccharomycotina - ps18
Saccharomycotina_1 - ps19
Saccharomycotina_2 - ps20
Saccharomycotina_3 - ps21
Phaff./Saccharomycetaceae - ps22
Saccharomyco./mycetaceae - ps23
Saccharomycetaceae - ps24
Saccharomycetaceae_2 - ps25
Saccharomycetaceae_3 - ps26
Kaz./Nau./Saccharomyces - ps27
Saccharomyces - ps28
Arboricola_clade - ps29
Cerevisiae_clade - ps30
Saccharomyces_cerevisiae - ps31
p-value
6e-03
9e-03
1e-02
2e-02
2e-02
Log-odds
0.5
1.0
1.5
2.0
2.5
3.0
3.5
4.0

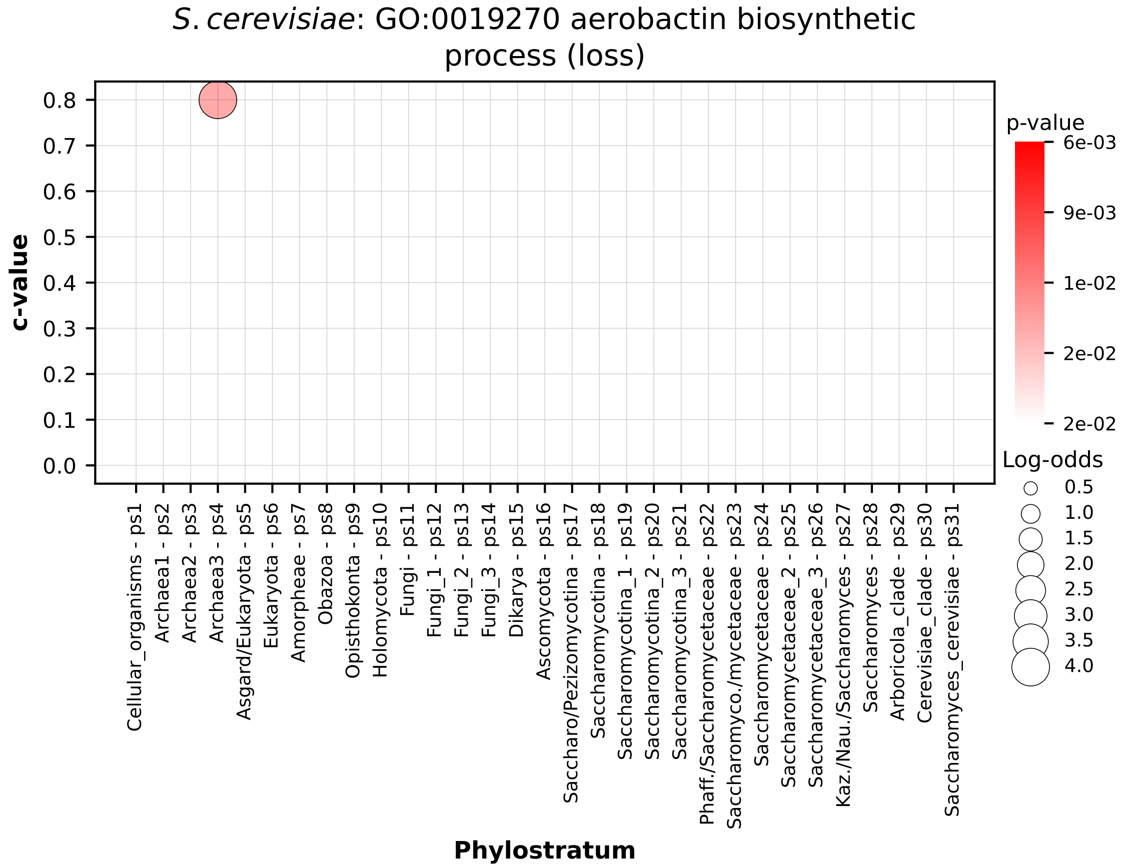

S. cerevisiae: GO:0019270 aerobactin biosynthetic process (loss)
c-value
0.0
0.1
0.2
0.3
0.4
0.5
0.6
0.7
0.8
Phylostratum
Cellular_organisms - ps1
Archaea1 - ps2
Archaea2 - ps3
Archaea3 - ps4
Asgard/Eukaryota - ps5
Eukaryota - ps6
Amorpheae - ps7
Obazoa - ps8
Opisthokonta - ps9
Holomycota - ps10
Fungi - ps11
Fungi_1 - ps12
Fungi_2 - ps13
Fungi_3 - ps14
Dikarya - ps15
Ascomycota - ps16
Saccharo/Pezizomycotina - ps17
Saccharomycotina - ps18
Saccharomycotina_1 - ps19
Saccharomycotina_2 - ps20
Saccharomycotina_3 - ps21
Phaff./Saccharomycetaceae - ps22
Saccharomyco./mycetaceae - ps23
Saccharomycetaceae - ps24
Saccharomycetaceae_2 - ps25
Saccharomycetaceae_3 - ps26
Kaz./Nau./Saccharomyces - ps27
Saccharomyces - ps28
Arboricola_clade - ps29
Cerevisiae_clade - ps30
Saccharomyces_cerevisiae - ps31
p-value
6e-03
9e-03
1e-02
2e-02
2e-02
Log-odds
0.5
1.0
1.5
2.0
2.5
3.0
3.5
4.0

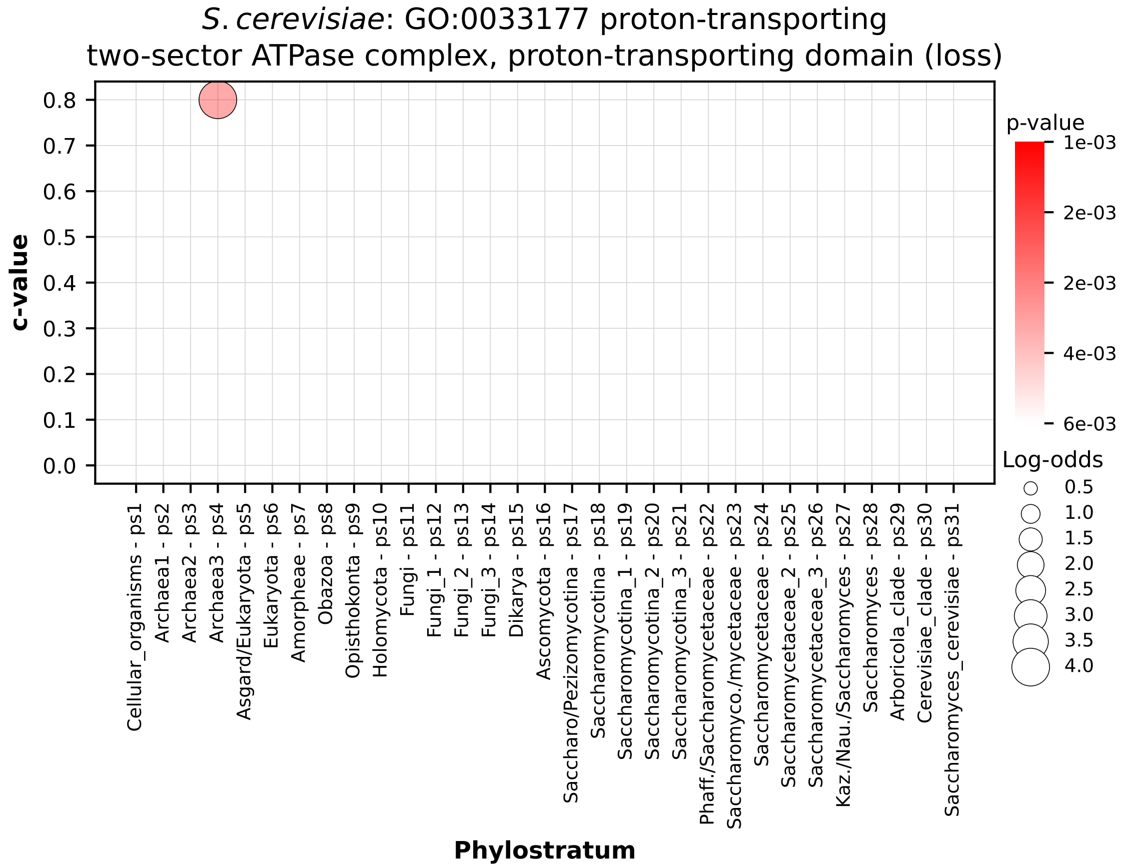

S. cerevisiae: GO:0033177 proton-transporting two-sector ATPase complex, proton-transporting domain (loss)
c-value
0.0
0.1
0.2
0.3
0.4
0.5
0.6
0.7
0.8
p-value
1e-03
2e-03
2e-03
4e-03
6e-03
Log-odds
0.5
1.0
1.5
2.0
2.5
3.0
3.5
4.0
Cellular_organisms - ps1
Archaea1 - ps2
Archaea2 - ps3
Archaea3 - ps4
Asgard/Eukaryota - ps5
Eukaryota - ps6
Amorpheae - ps7
Obazoa - ps8
Opisthokonta - ps9
Holomycota - ps10
Fungi - ps11
Fungi_1 - ps12
Fungi_2 - ps13
Fungi_3 - ps14
Dikarya - ps15
Ascomycota - ps16
Saccharo/Pezizomycotina - ps17
Saccharomycotina - ps18
Saccharomycotina_1 - ps19
Saccharomycotina_2 - ps20
Saccharomycotina_3 - ps21
Phaff./Saccharomycetaceae - ps22
Saccharomyco./mycetaceae - ps23
Saccharomycetaceae - ps24
Saccharomycetaceae_2 - ps25
Saccharomycetaceae_3 - ps26
Kaz./Nau./Saccharomyces - ps27
Saccharomyces - ps28
Arboricola_clade - ps29
Cerevisiae_clade - ps30
Saccharomyces_cerevisiae - ps31
Phylostratum

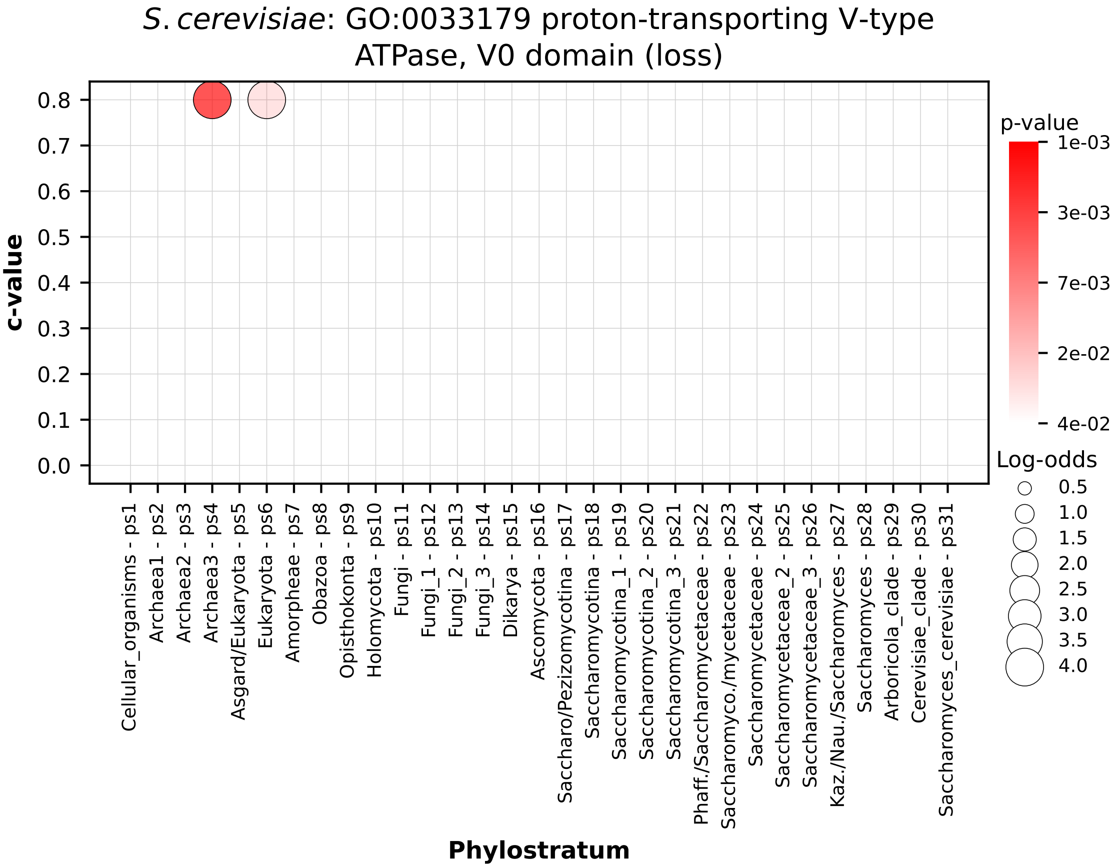

S. cerevisiae: GO:0033179 proton-transporting V-type ATPase, V0 domain (loss)
c-value
0.8
0.7
0.6
0.5
0.4
0.3
0.2
0.1
0.0
p-value
1e-03
3e-03
7e-03
2e-02
4e-02
Log-odds
0.5
1.0
1.5
2.0
2.5
3.0
3.5
4.0
Cellular_organisms - ps1
Archaea1 - ps2
Archaea2 - ps3
Archaea3 - ps4
Asgard/Eukaryota - ps5
Eukaryota - ps6
Amorpheae - ps7
Obazoa - ps8
Opisthokonta - ps9
Holomycota - ps10
Fungi - ps11
Fungi_1 - ps12
Fungi_2 - ps13
Fungi_3 - ps14
Dikarya - ps15
Ascomycota - ps16
Saccharo/Pezizomycotina - ps17
Saccharomycotina - ps18
Saccharomycotina_1 - ps19
Saccharomycotina_2 - ps20
Saccharomycotina_3 - ps21
Phaff./Saccharomycetaceae - ps22
Saccharomyco./mycetaceae - ps23
Saccharomycetaceae - ps24
Saccharomycetaceae_2 - ps25
Saccharomycetaceae_3 - ps26
Kaz./Nau./Saccharomyces - ps27
Saccharomyces - ps28
Arboricola_clade - ps29
Cerevisiae_clade - ps30
Saccharomyces_cerevisiae - ps31
Phylostratum

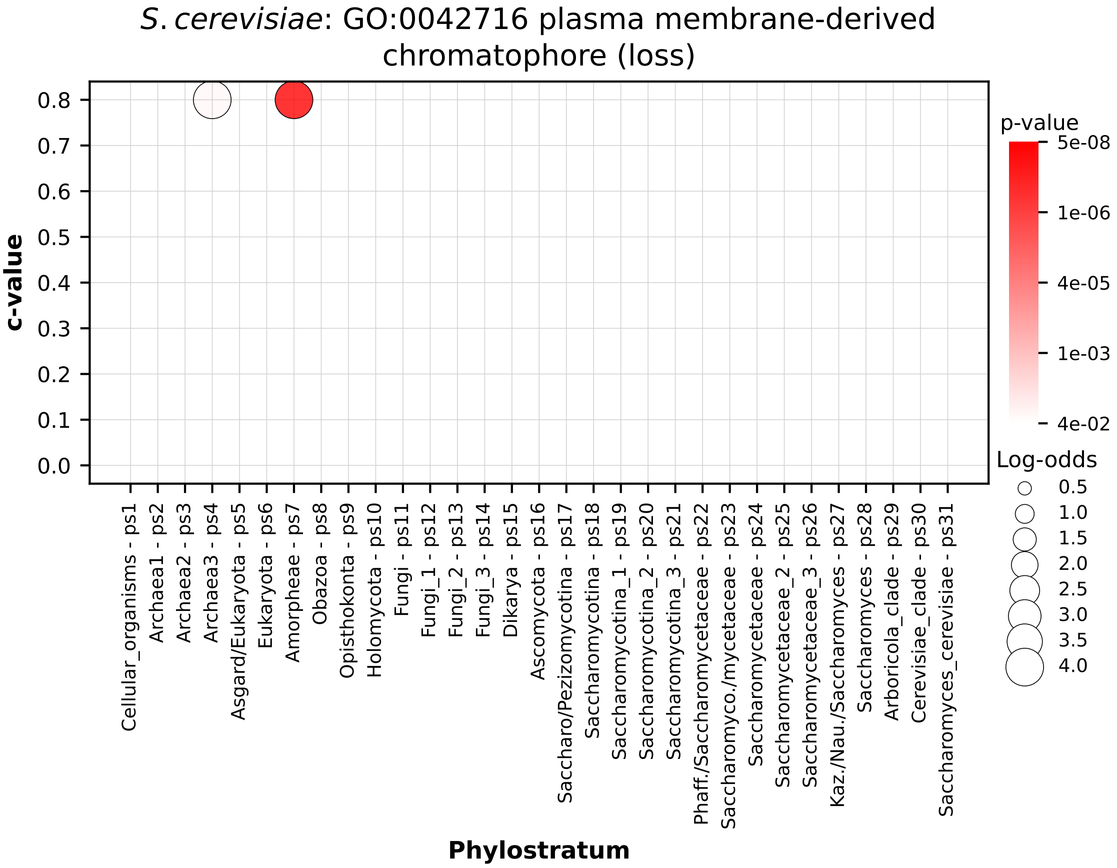

S. cerevisiae: GO:0042716 plasma membrane-derived chromatophore (loss)
c-value
0.0
0.1
0.2
0.3
0.4
0.5
0.6
0.7
0.8
Phylostratum
Cellular_organisms - ps1
Archaea1 - ps2
Archaea2 - ps3
Archaea3 - ps4
Asgard/Eukaryota - ps5
Eukaryota - ps6
Amorpheae - ps7
Obazoa - ps8
Opisthokonta - ps9
Holomycota - ps10
Fungi - ps11
Fungi_1 - ps12
Fungi_2 - ps13
Fungi_3 - ps14
Dikarya - ps15
Ascomycota - ps16
Saccharo/Pezizomycotina - ps17
Saccharomycotina - ps18
Saccharomycotina_1 - ps19
Saccharomycotina_2 - ps20
Saccharomycotina_3 - ps21
Phaff./Saccharomycetaceae - ps22
Saccharomyco./mycetaceae - ps23
Saccharomycetaceae - ps24
Saccharomycetaceae_2 - ps25
Saccharomycetaceae_3 - ps26
Kaz./Nau./Saccharomyces - ps27
Saccharomyces - ps28
Arboricola_clade - ps29
Cerevisiae_clade - ps30
Saccharomyces_cerevisiae - ps31
p-value
5e-08
1e-06
4e-05
1e-03
4e-02
Log-odds
0.5
1.0
1.5
2.0
2.5
3.0
3.5
4.0

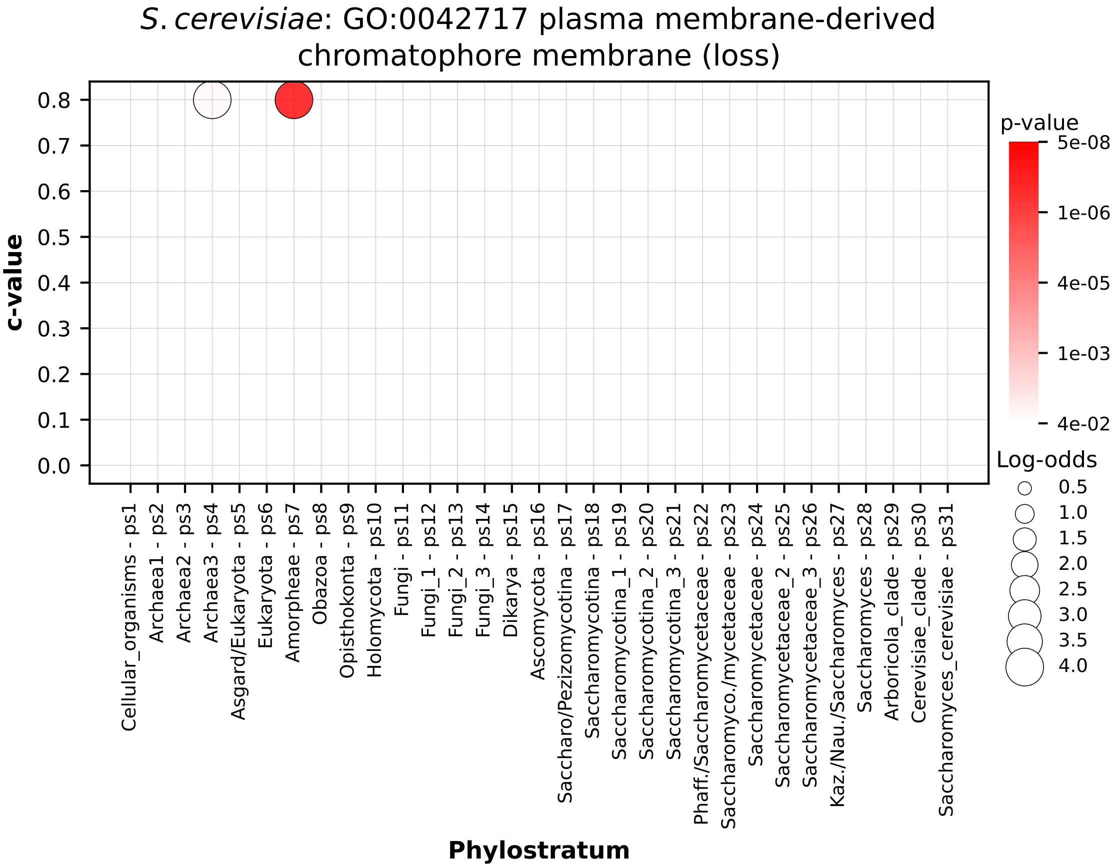
S. cerevisiae: GO:0042717 plasma membrane-derived chromatophore membrane (loss)
c-value
0.0
0.1
0.2
0.3
0.4
0.5
0.6
0.7
0.8
Phylostratum
Cellular_organisms - ps1
Archaea1 - ps2
Archaea2 - ps3
Archaea3 - ps4
Asgard/Eukaryota - ps5
Eukaryota - ps6
Amorpheae - ps7
Obazoa - ps8
Opisthokonta - ps9
Holomycota - ps10
Fungi - ps11
Fungi_1 - ps12
Fungi_2 - ps13
Fungi_3 - ps14
Dikarya - ps15
Ascomycota - ps16
Saccharo/Pezizomycotina - ps17
Saccharomycotina - ps18
Saccharomycotina_1 - ps19
Saccharomycotina_2 - ps20
Saccharomycotina_3 - ps21
Phaff./Saccharomycetaceae - ps22
Saccharomyco./mycetaceae - ps23
Saccharomycetaceae - ps24
Saccharomycetaceae_2 - ps25
Saccharomycetaceae_3 - ps26
Kaz./Nau./Saccharomyces - ps27
Saccharomyces - ps28
Arboricola_clade - ps29
Cerevisiae_clade - ps30
Saccharomyces_cerevisiae - ps31
p-value
5e-08
1e-06
4e-05
1e-03
4e-02
Log-odds
0.5
1.0
1.5
2.0
2.5
3.0
3.5
4.0

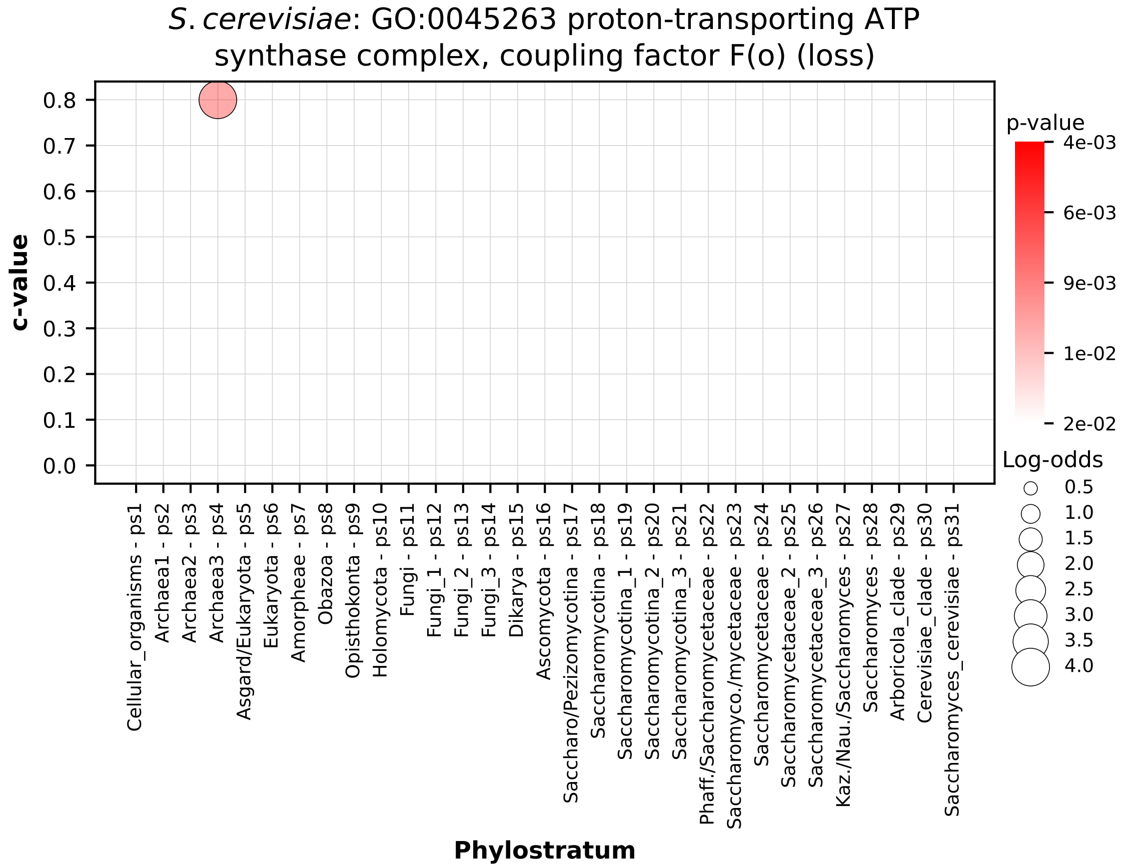

S. cerevisiae: GO:0045263 proton-transporting ATP synthase complex, coupling factor F(o) (loss)
c-value
0.0
0.1
0.2
0.3
0.4
0.5
0.6
0.7
0.8
Phylostratum
Cellular_organisms - ps1
Archaea1 - ps2
Archaea2 - ps3
Archaea3 - ps4
Asgard/Eukaryota - ps5
Eukaryota - ps6
Amorpheae - ps7
Obazoa - ps8
Opisthokonta - ps9
Holomycota - ps10
Fungi - ps11
Fungi_1 - ps12
Fungi_2 - ps13
Fungi_3 - ps14
Dikarya - ps15
Ascomycota - ps16
Saccharo/Pezizomycotina - ps17
Saccharomycotina - ps18
Saccharomycotina_1 - ps19
Saccharomycotina_2 - ps20
Saccharomycotina_3 - ps21
Phaff./Saccharomycetaceae - ps22
Saccharomyco./mycetaceae - ps23
Saccharomycetaceae - ps24
Saccharomycetaceae_2 - ps25
Saccharomycetaceae_3 - ps26
Kaz./Nau./Saccharomyces - ps27
Saccharomyces - ps28
Arboricola_clade - ps29
Cerevisiae_clade - ps30
Saccharomyces_cerevisiae - ps31
p-value
4e-03
6e-03
9e-03
1e-02
2e-02
Log-odds
0.5
1.0
1.5
2.0
2.5
3.0
3.5
4.0

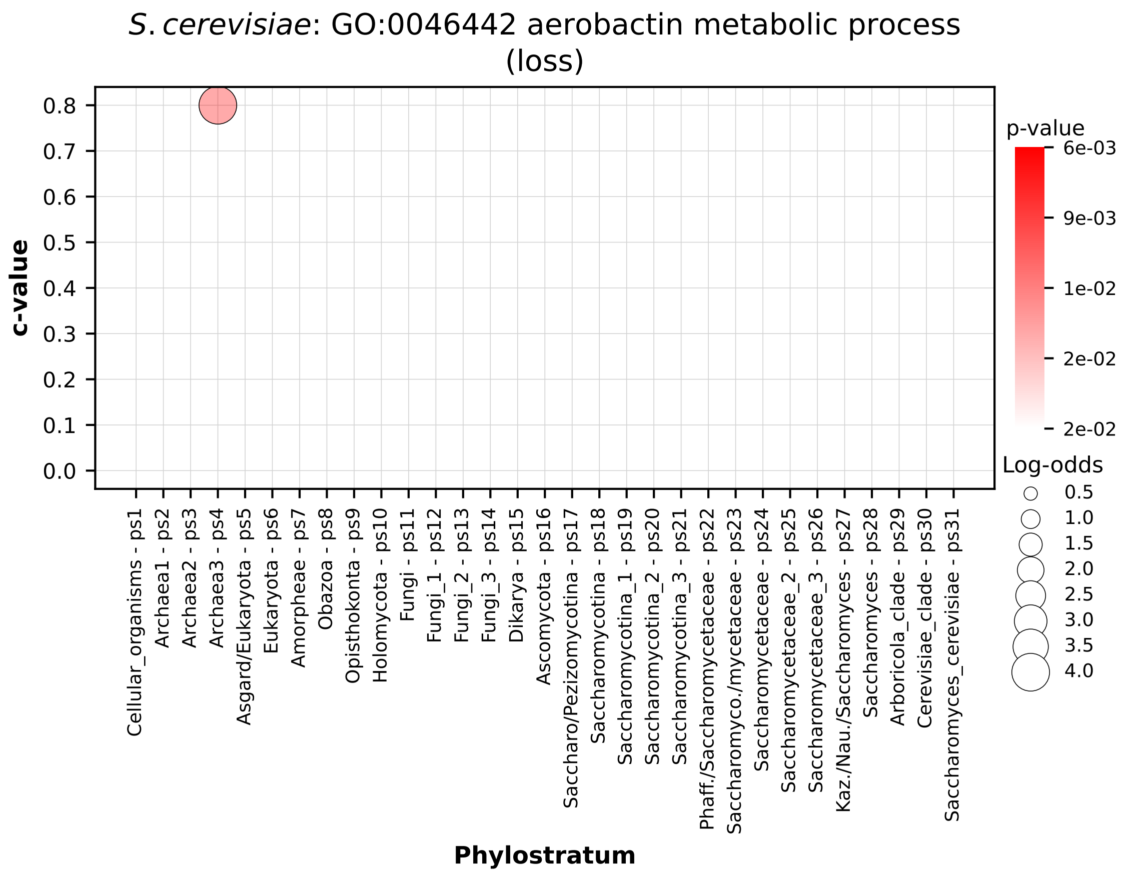
S. cerevisiae: GO:0046442 aerobactin metabolic process
(loss)
c-value
0.0
0.1
0.2
0.3
0.4
0.5
0.6
0.7
0.8
p-value
6e-03
9e-03
1e-02
2e-02
2e-02
Log-odds
0.5
1.0
1.5
2.0
2.5
3.0
3.5
4.0
Cellular_organisms - ps1
Archaea1 - ps2
Archaea2 - ps3
Archaea3 - ps4
Asgard/Eukaryota - ps5
Eukaryota - ps6
Amorpheae - ps7
Obazoa - ps8
Opisthokonta - ps9
Holomycota - ps10
Fungi - ps11
Fungi_1 - ps12
Fungi_2 - ps13
Fungi_3 - ps14
Dikarya - ps15
Ascomycota - ps16
Saccharo/Pezizomycotina - ps17
Saccharomycotina - ps18
Saccharomycotina_1 - ps19
Saccharomycotina_2 - ps20
Saccharomycotina_3 - ps21
Phaff./Saccharomycetaceae - ps22
Saccharomyco./mycetaceae - ps23
Saccharomycetaceae - ps24
Saccharomycetaceae_2 - ps25
Saccharomycetaceae_3 - ps26
Kaz./Nau./Saccharomyces - ps27
Saccharomyces - ps28
Arboricola_clade - ps29
Cerevisiae_clade - ps30
Saccharomyces_cerevisiae - ps31
Phylostratum

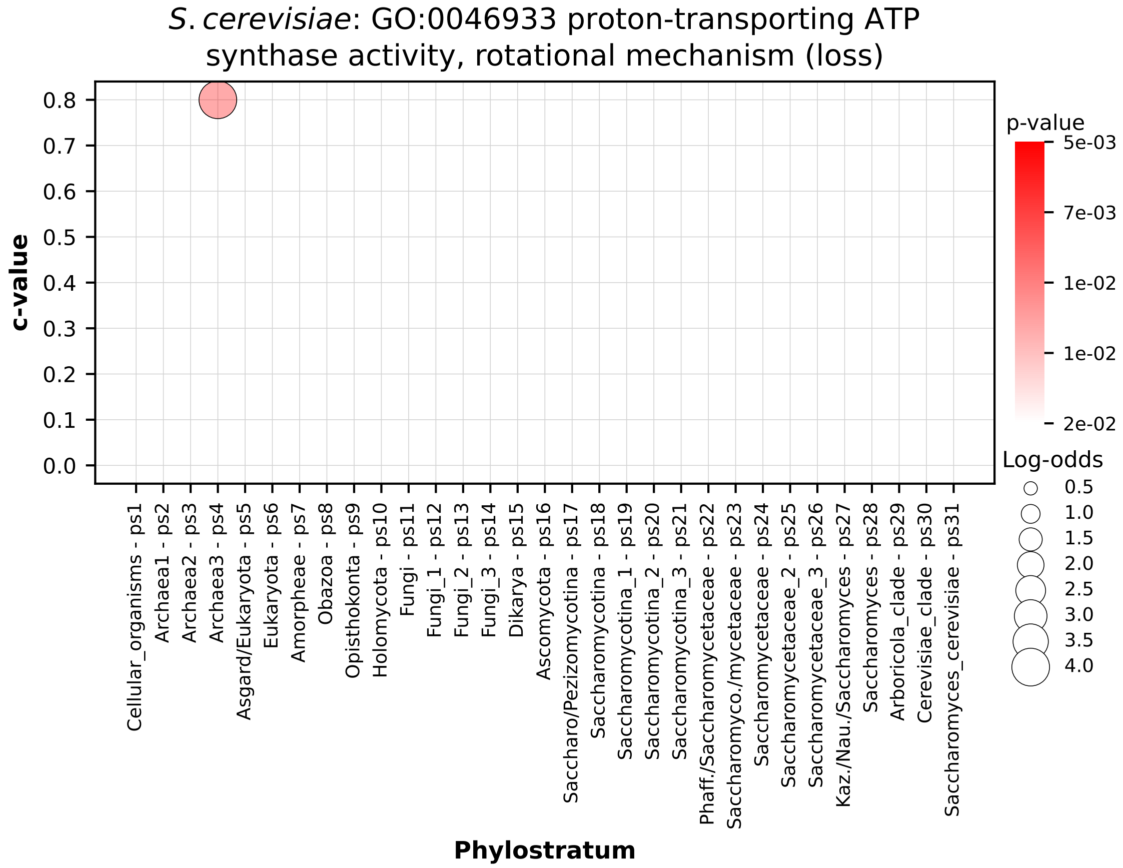
S. cerevisiae: GO:0046933 proton-transporting ATP synthase activity, rotational mechanism (loss)
c-value
0.0
0.1
0.2
0.3
0.4
0.5
0.6
0.7
0.8
Phylostratum
Cellular_organisms - ps1
Archaea1 - ps2
Archaea2 - ps3
Archaea3 - ps4
Asgard/Eukaryota - ps5
Eukaryota - ps6
Amorpheae - ps7
Obazoa - ps8
Opisthokonta - ps9
Holomycota - ps10
Fungi - ps11
Fungi_1 - ps12
Fungi_2 - ps13
Fungi_3 - ps14
Dikarya - ps15
Ascomycota - ps16
Saccharo/Pezizomycotina - ps17
Saccharomycotina - ps18
Saccharomycotina_1 - ps19
Saccharomycotina_2 - ps20
Saccharomycotina_3 - ps21
Phaff./Saccharomycetaceae - ps22
Saccharomyco./mycetaceae - ps23
Saccharomycetaceae - ps24
Saccharomycetaceae_2 - ps25
Saccharomycetaceae_3 - ps26
Kaz./Nau./Saccharomyces - ps27
Saccharomyces - ps28
Arboricola_clade - ps29
Cerevisiae_clade - ps30
Saccharomyces_cerevisiae - ps31
p-value
5e-03
7e-03
1e-02
1e-02
2e-02
Log-odds
0.5
1.0
1.5
2.0
2.5
3.0
3.5
4.0

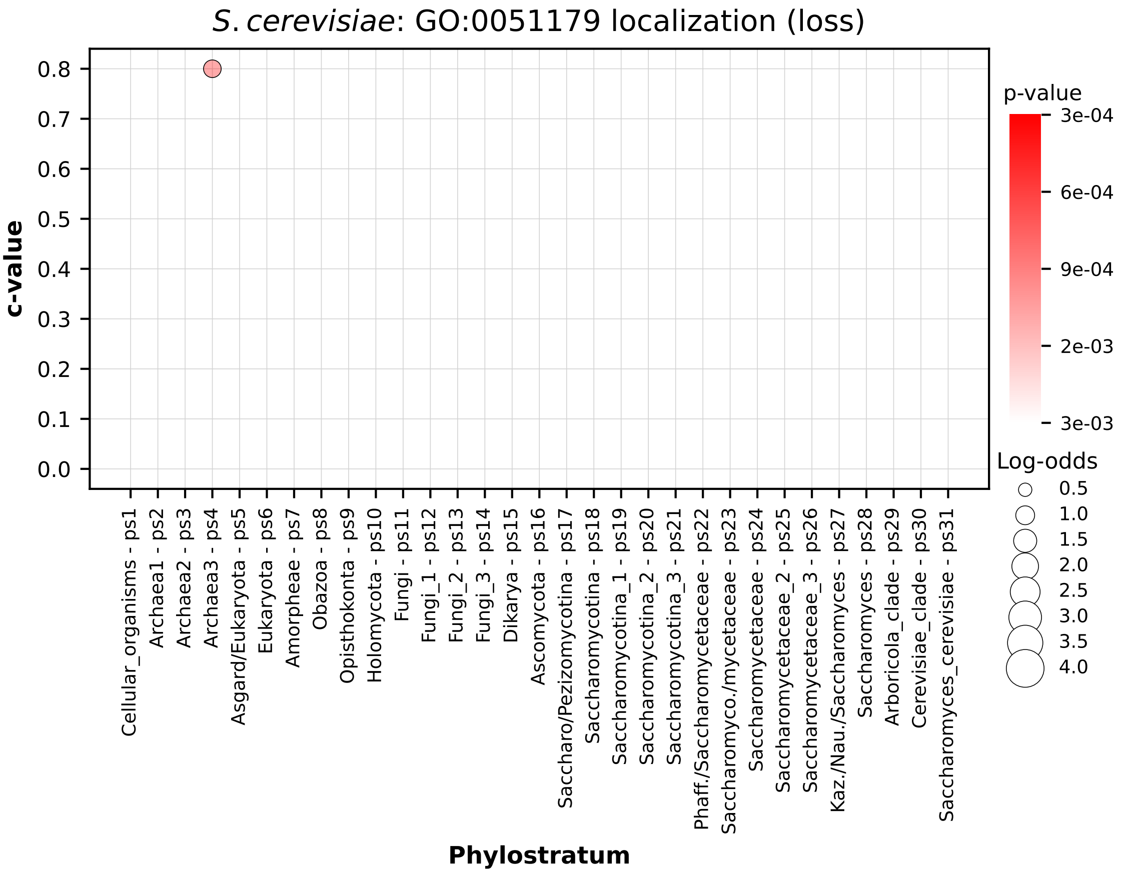

S. cerevisiae: GO:0051179 localization (loss)
c-value
0.8
0.7
0.6
0.5
0.4
0.3
0.2
0.1
0.0
p-value
3e-04
6e-04
9e-04
2e-03
3e-03
Log-odds
0.5
1.0
1.5
2.0
2.5
3.0
3.5
4.0
Cellular_organisms - ps1
Archaea1 - ps2
Archaea2 - ps3
Archaea3 - ps4
Asgard/Eukaryota - ps5
Eukaryota - ps6
Amorpheae - ps7
Obazoa - ps8
Opisthokonta - ps9
Holomycota - ps10
Fungi - ps11
Fungi_1 - ps12
Fungi_2 - ps13
Fungi_3 - ps14
Dikarya - ps15
Ascomycota - ps16
Saccharo/Pezizomycotina - ps17
Saccharomycotina - ps18
Saccharomycotina_1 - ps19
Saccharomycotina_2 - ps20
Saccharomycotina_3 - ps21
Phaff./Saccharomycetaceae - ps22
Saccharomyco./mycetaceae - ps23
Saccharomycetaceae - ps24
Saccharomycetaceae_2 - ps25
Saccharomycetaceae_3 - ps26
Kaz./Nau./Saccharomyces - ps27
Saccharomyces - ps28
Arboricola_clade - ps29
Cerevisiae_clade - ps30
Saccharomyces_cerevisiae - ps31
Phylostratum

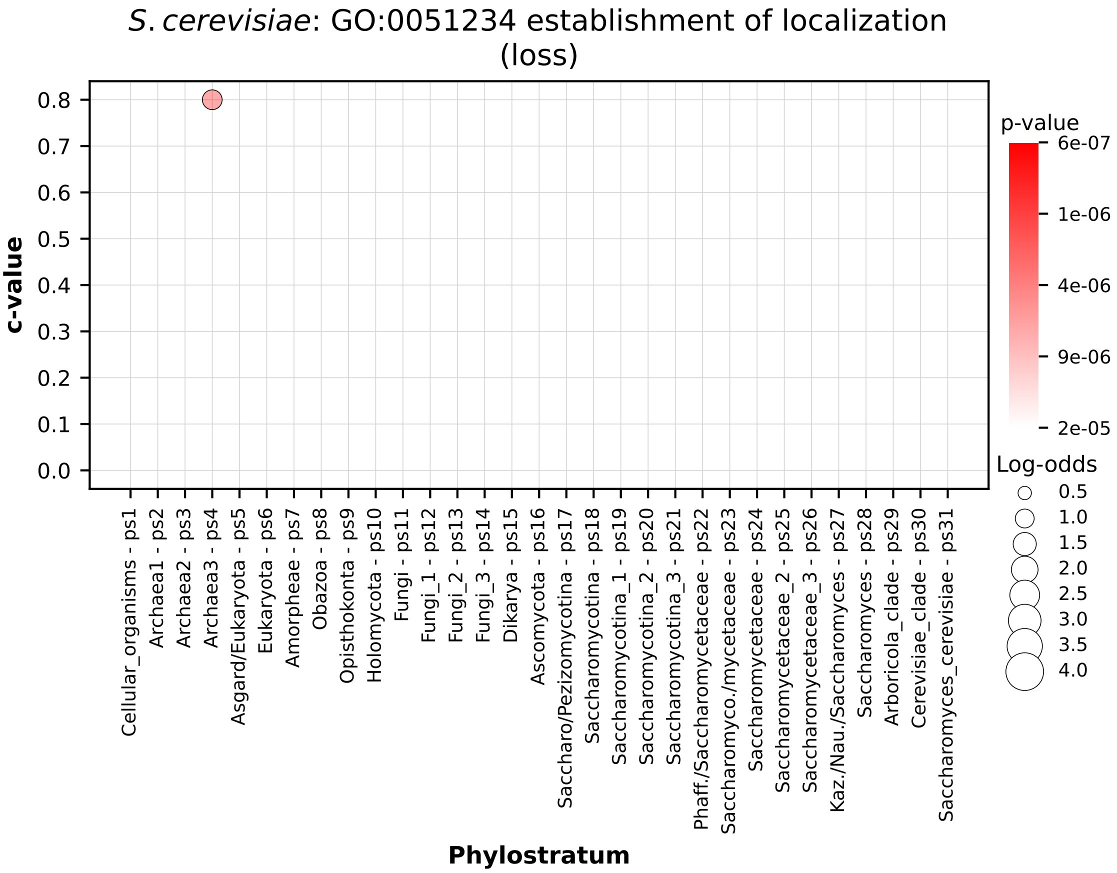

S. cerevisiae: GO:0051234 establishment of localization (loss)
c-value
0.0
0.1
0.2
0.3
0.4
0.5
0.6
0.7
0.8
p-value
6e-07
1e-06
4e-06
9e-06
2e-05
Log-odds
0.5
1.0
1.5
2.0
2.5
3.0
3.5
4.0
Cellular_organisms - ps1
Archaea1 - ps2
Archaea2 - ps3
Archaea3 - ps4
Asgard/Eukaryota - ps5
Eukaryota - ps6
Amorpheae - ps7
Obazoa - ps8
Opisthokonta - ps9
Holomycota - ps10
Fungi - ps11
Fungi_1 - ps12
Fungi_2 - ps13
Fungi_3 - ps14
Dikarya - ps15
Ascomycota - ps16
Saccharo/Pezizomycotina - ps17
Saccharomycotina - ps18
Saccharomycotina_1 - ps19
Saccharomycotina_2 - ps20
Saccharomycotina_3 - ps21
Phaff./Saccharomycetaceae - ps22
Saccharomyco./mycetaceae - ps23
Saccharomycetaceae - ps24
Saccharomycetaceae_2 - ps25
Saccharomycetaceae_3 - ps26
Kaz./Nau./Saccharomyces - ps27
Saccharomyces - ps28
Arboricola_clade - ps29
Cerevisiae_clade - ps30
Saccharomyces_cerevisiae - ps31
Phylostratum

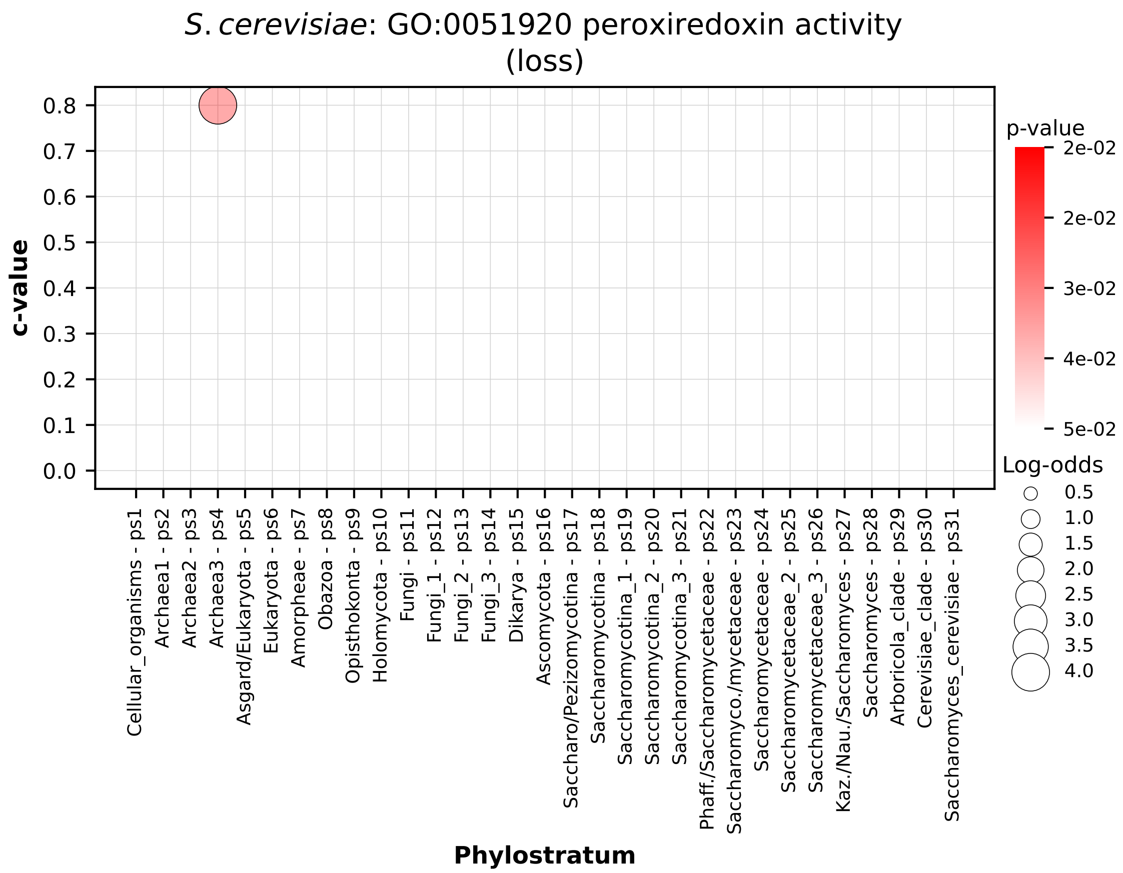
S. cerevisiae: GO:0051920 peroxiredoxin activity
(loss)
c-value
0.8
0.7
0.6
0.5
0.4
0.3
0.2
0.1
0.0
p-value
2e-02
2e-02
3e-02
4e-02
5e-02
Log-odds
0.5
1.0
1.5
2.0
2.5
3.0
3.5
4.0
Cellular_organisms - ps1
Archaea1 - ps2
Archaea2 - ps3
Archaea3 - ps4
Asgard/Eukaryota - ps5
Eukaryota - ps6
Amorpheae - ps7
Obazoa - ps8
Opisthokonta - ps9
Holomycota - ps10
Fungi - ps11
Fungi_1 - ps12
Fungi_2 - ps13
Fungi_3 - ps14
Dikarya - ps15
Ascomycota - ps16
Saccharo/Pezizomycotina - ps17
Saccharomycotina - ps18
Saccharomycotina_1 - ps19
Saccharomycotina_2 - ps20
Saccharomycotina_3 - ps21
Phaff./Saccharomycetaceae - ps22
Saccharomyco./mycetaceae - ps23
Saccharomycetaceae - ps24
Saccharomycetaceae_2 - ps25
Saccharomycetaceae_3 - ps26
Kaz./Nau./Saccharomyces - ps27
Saccharomyces - ps28
Arboricola_clade - ps29
Cerevisiae_clade - ps30
Saccharomyces_cerevisiae - ps31
Phylostratum

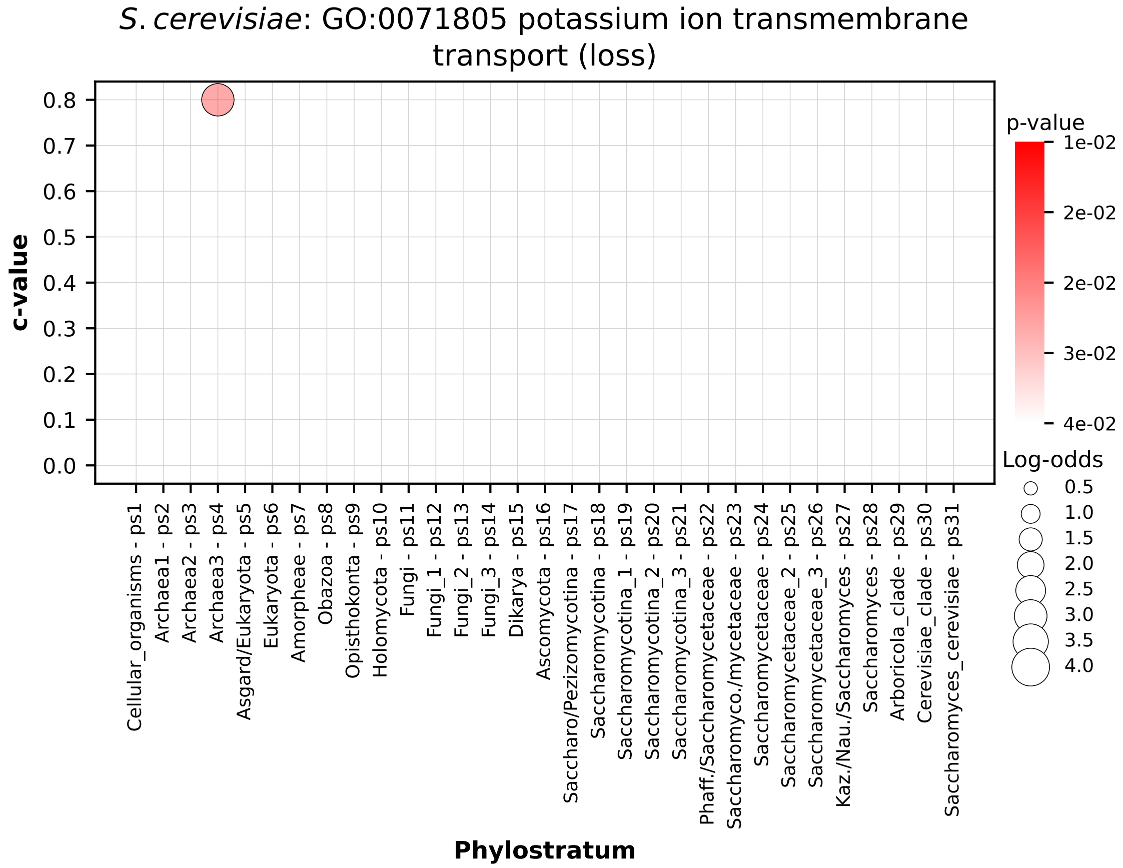
S. cerevisiae: GO:0071805 potassium ion transmembrane transport (loss)
c-value
0.8
0.7
0.6
0.5
0.4
0.3
0.2
0.1
0.0
p-value
1e-02
2e-02
2e-02
3e-02
4e-02
Log-odds
0.5
1.0
1.5
2.0
2.5
3.0
3.5
4.0
Cellular_organisms - ps1
Archaea1 - ps2
Archaea2 - ps3
Archaea3 - ps4
Asgard/Eukaryota - ps5
Eukaryota - ps6
Amorpheae - ps7
Obazoa - ps8
Opisthokonta - ps9
Holomycota - ps10
Fungi - ps11
Fungi_1 - ps12
Fungi_2 - ps13
Fungi_3 - ps14
Dikarya - ps15
Ascomycota - ps16
Saccharo/Pezizomycotina - ps17
Saccharomycotina - ps18
Saccharomycotina_1 - ps19
Saccharomycotina_2 - ps20
Saccharomycotina_3 - ps21
Phaff./Saccharomycetaceae - ps22
Saccharomyco./mycetaceae - ps23
Saccharomycetaceae - ps24
Saccharomycetaceae_2 - ps25
Saccharomycetaceae_3 - ps26
Kaz./Nau./Saccharomyces - ps27
Saccharomyces - ps28
Arboricola_clade - ps29
Cerevisiae_clade - ps30
Saccharomyces_cerevisiae - ps31
Phylostratum

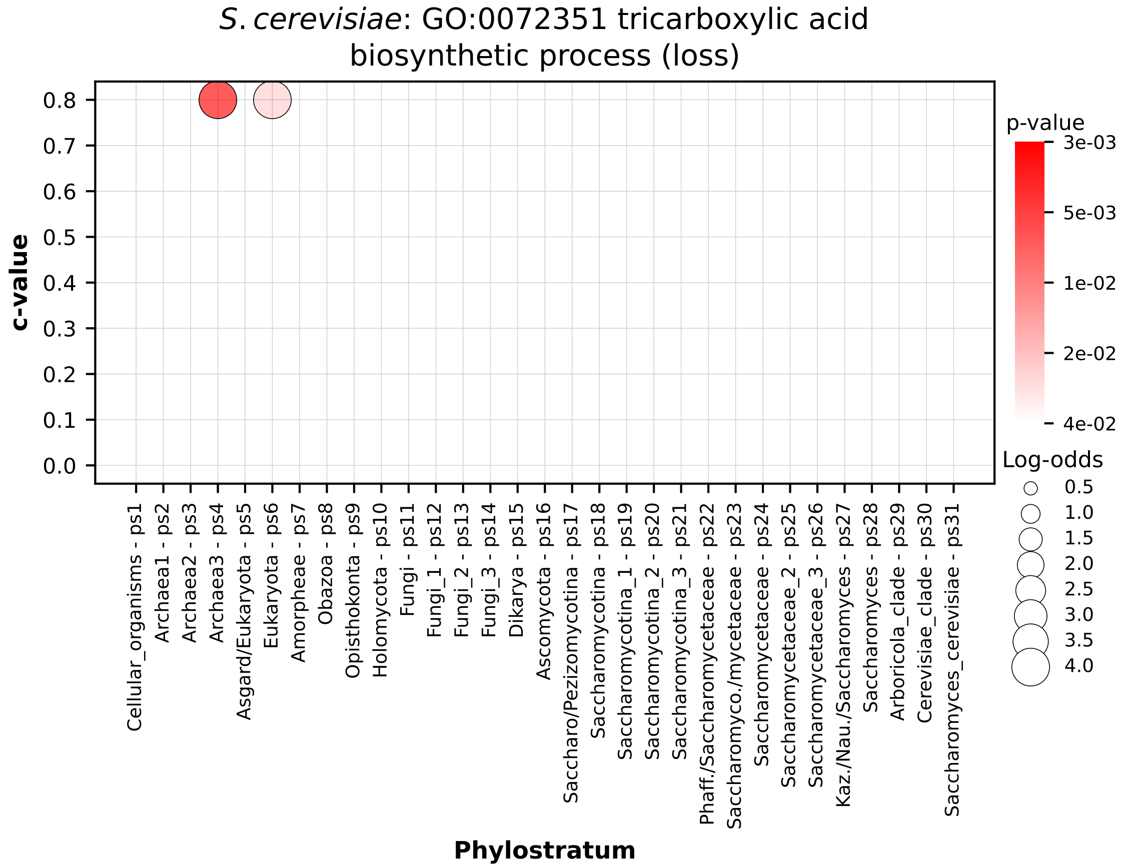

S. cerevisiae: GO:0072351 tricarboxylic acid biosynthetic process (loss)
c-value
0.0
0.1
0.2
0.3
0.4
0.5
0.6
0.7
0.8
p-value
3e-03
5e-03
1e-02
2e-02
4e-02
Log-odds
0.5
1.0
1.5
2.0
2.5
3.0
3.5
4.0
Cellular_organisms - ps1
Archaea1 - ps2
Archaea2 - ps3
Archaea3 - ps4
Asgard/Eukaryota - ps5
Eukaryota - ps6
Amorpheae - ps7
Obazoa - ps8
Opisthokonta - ps9
Holomycota - ps10
Fungi - ps11
Fungi_1 - ps12
Fungi_2 - ps13
Fungi_3 - ps14
Dikarya - ps15
Ascomycota - ps16
Saccharo/Pezizomycotina - ps17
Saccharomycotina - ps18
Saccharomycotina_1 - ps19
Saccharomycotina_2 - ps20
Saccharomycotina_3 - ps21
Phaff./Saccharomycetaceae - ps22
Saccharomyco./mycetaceae - ps23
Saccharomycetaceae - ps24
Saccharomycetaceae_2 - ps25
Saccharomycetaceae_3 - ps26
Kaz./Nau./Saccharomyces - ps27
Saccharomyces - ps28
Arboricola_clade - ps29
Cerevisiae_clade - ps30
Saccharomyces_cerevisiae - ps31
Phylostratum

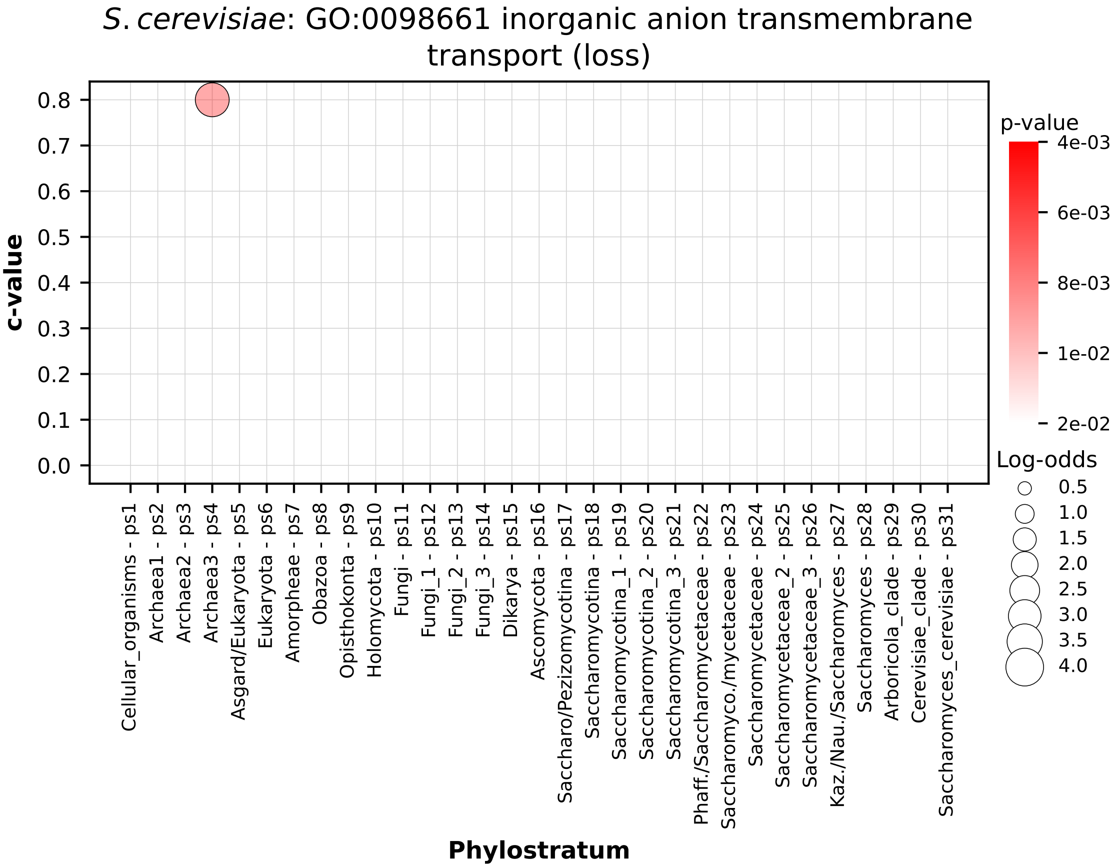
S. cerevisiae: GO:0098661 inorganic anion transmembrane transport (loss)
c-value
0.0
0.1
0.2
0.3
0.4
0.5
0.6
0.7
0.8
p-value
4e-03
6e-03
8e-03
1e-02
2e-02
Log-odds
0.5
1.0
1.5
2.0
2.5
3.0
3.5
4.0
Cellular_organisms - ps1
Archaea1 - ps2
Archaea2 - ps3
Archaea3 - ps4
Asgard/Eukaryota - ps5
Eukaryota - ps6
Amorpheae - ps7
Obazoa - ps8
Opisthokonta - ps9
Holomycota - ps10
Fungi - ps11
Fungi_1 - ps12
Fungi_2 - ps13
Fungi_3 - ps14
Dikarya - ps15
Ascomycota - ps16
Saccharo/Pezizomycotina - ps17
Saccharomycotina - ps18
Saccharomycotina_1 - ps19
Saccharomycotina_2 - ps20
Saccharomycotina_3 - ps21
Phaff./Saccharomycetaceae - ps22
Saccharomyco./mycetaceae - ps23
Saccharomycetaceae - ps24
Saccharomycetaceae_2 - ps25
Saccharomycetaceae_3 - ps26
Kaz./Nau./Saccharomyces - ps27
Saccharomyces - ps28
Arboricola_clade - ps29
Cerevisiae_clade - ps30
Saccharomyces_cerevisiae - ps31
Phylostratum

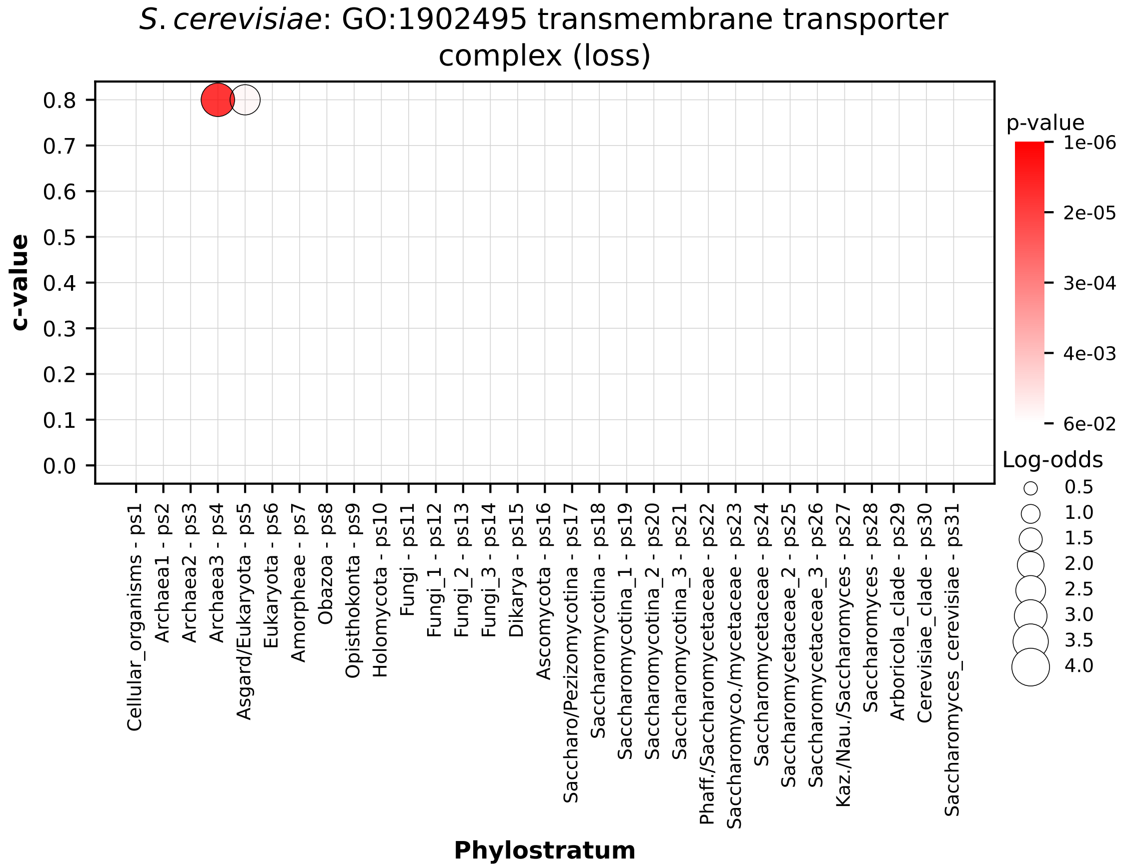
S. cerevisiae: GO:1902495 transmembrane transporter complex (loss)
c-value
0.0
0.1
0.2
0.3
0.4
0.5
0.6
0.7
0.8
Phylostratum
Cellular_organisms - ps1
Archaea1 - ps2
Archaea2 - ps3
Archaea3 - ps4
Asgard/Eukaryota - ps5
Eukaryota - ps6
Amorpheae - ps7
Obazoa - ps8
Opisthokonta - ps9
Holomycota - ps10
Fungi - ps11
Fungi_1 - ps12
Fungi_2 - ps13
Fungi_3 - ps14
Dikarya - ps15
Ascomycota - ps16
Saccharo/Pezizomycotina - ps17
Saccharomycotina - ps18
Saccharomycotina_1 - ps19
Saccharomycotina_2 - ps20
Saccharomycotina_3 - ps21
Phaff./Saccharomycetaceae - ps22
Saccharomyco./mycetaceae - ps23
Saccharomycetaceae - ps24
Saccharomycetaceae_2 - ps25
Saccharomycetaceae_3 - ps26
Kaz./Nau./Saccharomyces - ps27
Saccharomyces - ps28
Arboricola_clade - ps29
Cerevisiae_clade - ps30
Saccharomyces_cerevisiae - ps31
p-value
1e-06
2e-05
3e-04
4e-03
6e-02
Log-odds
0.5
1.0
1.5
2.0
2.5
3.0
3.5
4.0

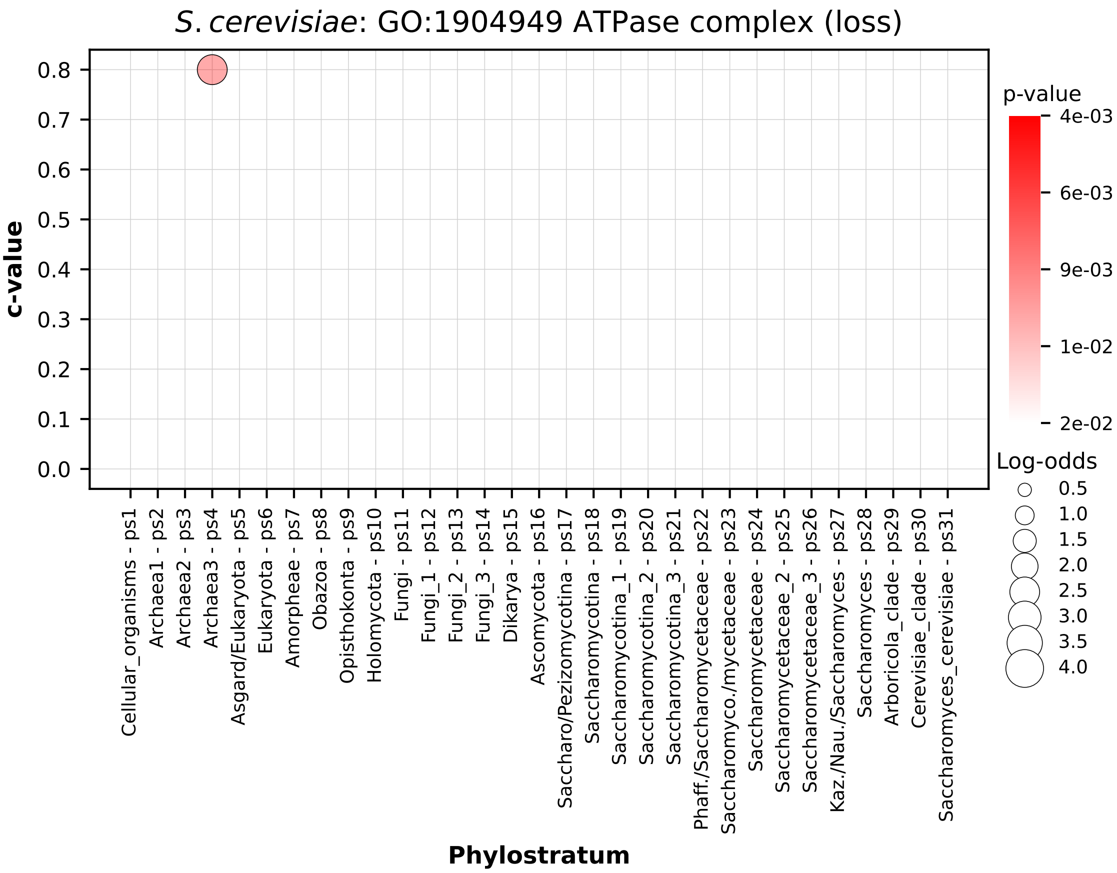

S. cerevisiae: GO:1904949 ATPase complex (loss)
c-value
0.8
0.7
0.6
0.5
0.4
0.3
0.2
0.1
0.0
p-value
4e-03
6e-03
9e-03
1e-02
2e-02
Log-odds
0.5
1.0
1.5
2.0
2.5
3.0
3.5
4.0
Cellular_organisms - ps1
Archaea1 - ps2
Archaea2 - ps3
Archaea3 - ps4
Asgard/Eukaryota - ps5
Eukaryota - ps6
Amorpheae - ps7
Obazoa - ps8
Opisthokonta - ps9
Holomycota - ps10
Fungi - ps11
Fungi_1 - ps12
Fungi_2 - ps13
Fungi_3 - ps14
Dikarya - ps15
Ascomycota - ps16
Saccharo/Pezizomycotina - ps17
Saccharomycotina - ps18
Saccharomycotina_1 - ps19
Saccharomycotina_2 - ps20
Saccharomycotina_3 - ps21
Phaff./Saccharomycetaceae - ps22
Saccharomyco./mycetaceae - ps23
Saccharomycetaceae - ps24
Saccharomycetaceae_2 - ps25
Saccharomycetaceae_3 - ps26
Kaz./Nau./Saccharomyces - ps27
Saccharomyces - ps28
Arboricola_clade - ps29
Cerevisiae_clade - ps30
Saccharomyces_cerevisiae - ps31
Phylostratum

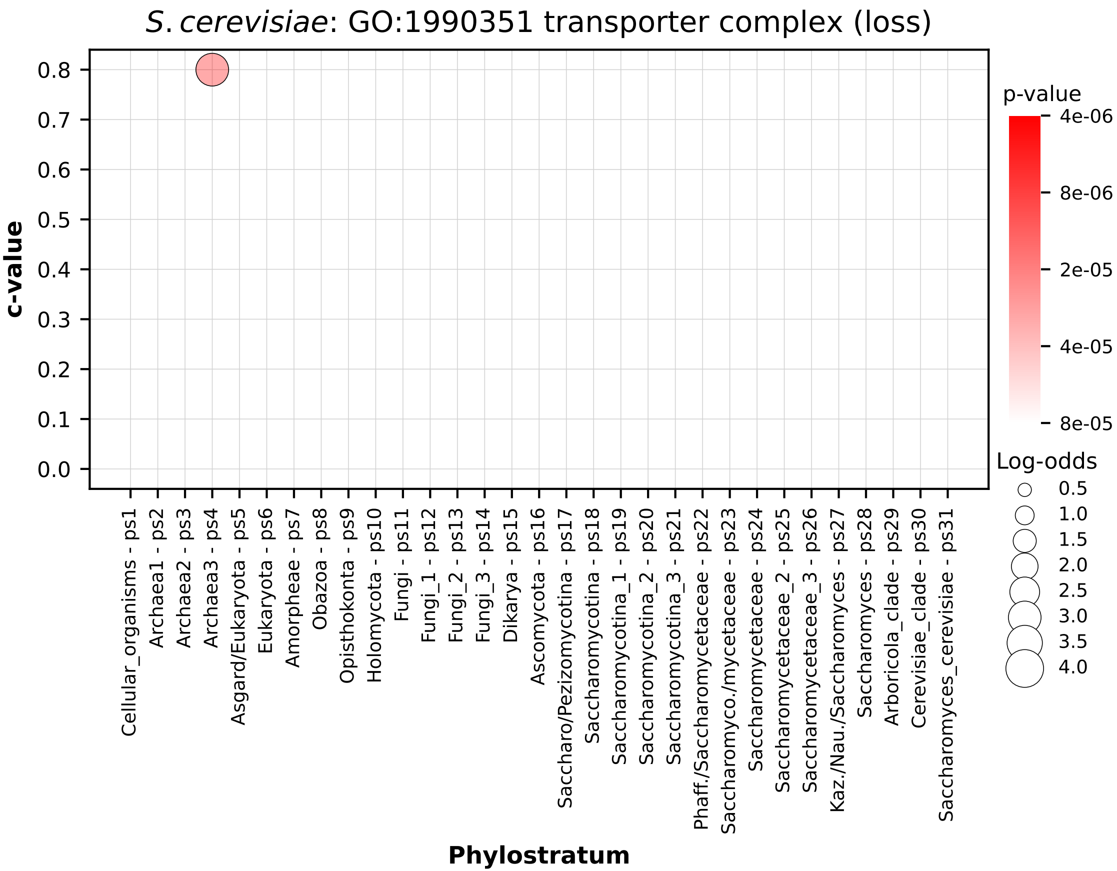

S. cerevisiae: GO:1990351 transporter complex (loss)
c-value
0.0
0.1
0.2
0.3
0.4
0.5
0.6
0.7
0.8
Phylostratum
Cellular_organisms - ps1
Archaea1 - ps2
Archaea2 - ps3
Archaea3 - ps4
Asgard/Eukaryota - ps5
Eukaryota - ps6
Amorpheae - ps7
Obazoa - ps8
Opisthokonta - ps9
Holomycota - ps10
Fungi - ps11
Fungi_1 - ps12
Fungi_2 - ps13
Fungi_3 - ps14
Dikarya - ps15
Ascomycota - ps16
Saccharo/Pezizomycotina - ps17
Saccharomycotina - ps18
Saccharomycotina_1 - ps19
Saccharomycotina_2 - ps20
Saccharomycotina_3 - ps21
Phaff./Saccharomycetaceae - ps22
Saccharomyco./mycetaceae - ps23
Saccharomycetaceae - ps24
Saccharomycetaceae_2 - ps25
Saccharomycetaceae_3 - ps26
Kaz./Nau./Saccharomyces - ps27
Saccharomyces - ps28
Arboricola_clade - ps29
Cerevisiae_clade - ps30
Saccharomyces_cerevisiae - ps31
p-value
4e-06
8e-06
2e-05
4e-05
8e-05
Log-odds
0.5
1.0
1.5
2.0
2.5
3.0
3.5
4.0

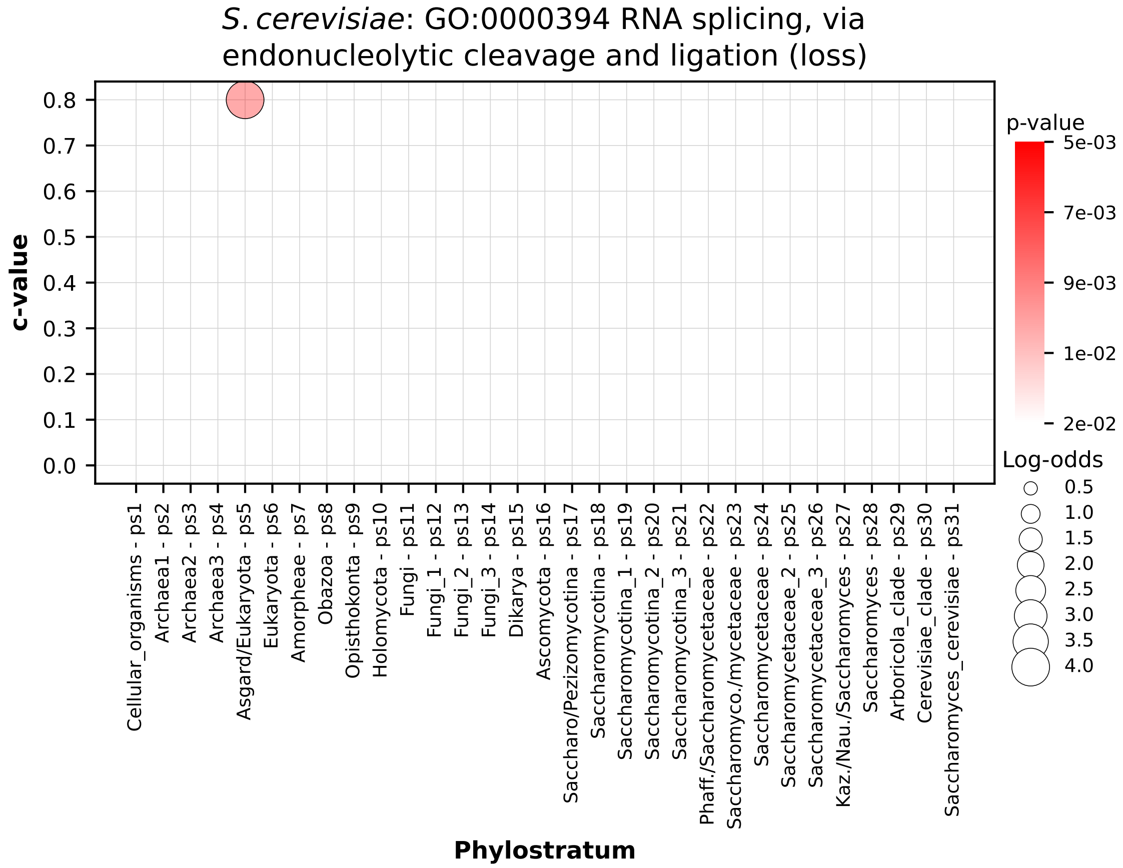

S. cerevisiae: GO:0000394 RNA splicing, via endonucleolytic cleavage and ligation (loss)
c-value
0.0
0.1
0.2
0.3
0.4
0.5
0.6
0.7
0.8
Phylostratum
Cellular_organisms - ps1
Archaea1 - ps2
Archaea2 - ps3
Archaea3 - ps4
Asgard/Eukaryota - ps5
Eukaryota - ps6
Amorpheae - ps7
Obazoa - ps8
Opisthokonta - ps9
Holomycota - ps10
Fungi - ps11
Fungi_1 - ps12
Fungi_2 - ps13
Fungi_3 - ps14
Dikarya - ps15
Ascomycota - ps16
Saccharo/Pezizomycotina - ps17
Saccharomycotina - ps18
Saccharomycotina_1 - ps19
Saccharomycotina_2 - ps20
Saccharomycotina_3 - ps21
Phaff./Saccharomycetaceae - ps22
Saccharomyco./mycetaceae - ps23
Saccharomycetaceae - ps24
Saccharomycetaceae_2 - ps25
Saccharomycetaceae_3 - ps26
Kaz./Nau./Saccharomyces - ps27
Saccharomyces - ps28
Arboricola_clade - ps29
Cerevisiae_clade - ps30
Saccharomyces_cerevisiae - ps31
p-value
5e-03
7e-03
9e-03
1e-02
2e-02
Log-odds
0.5
1.0
1.5
2.0
2.5
3.0
3.5
4.0

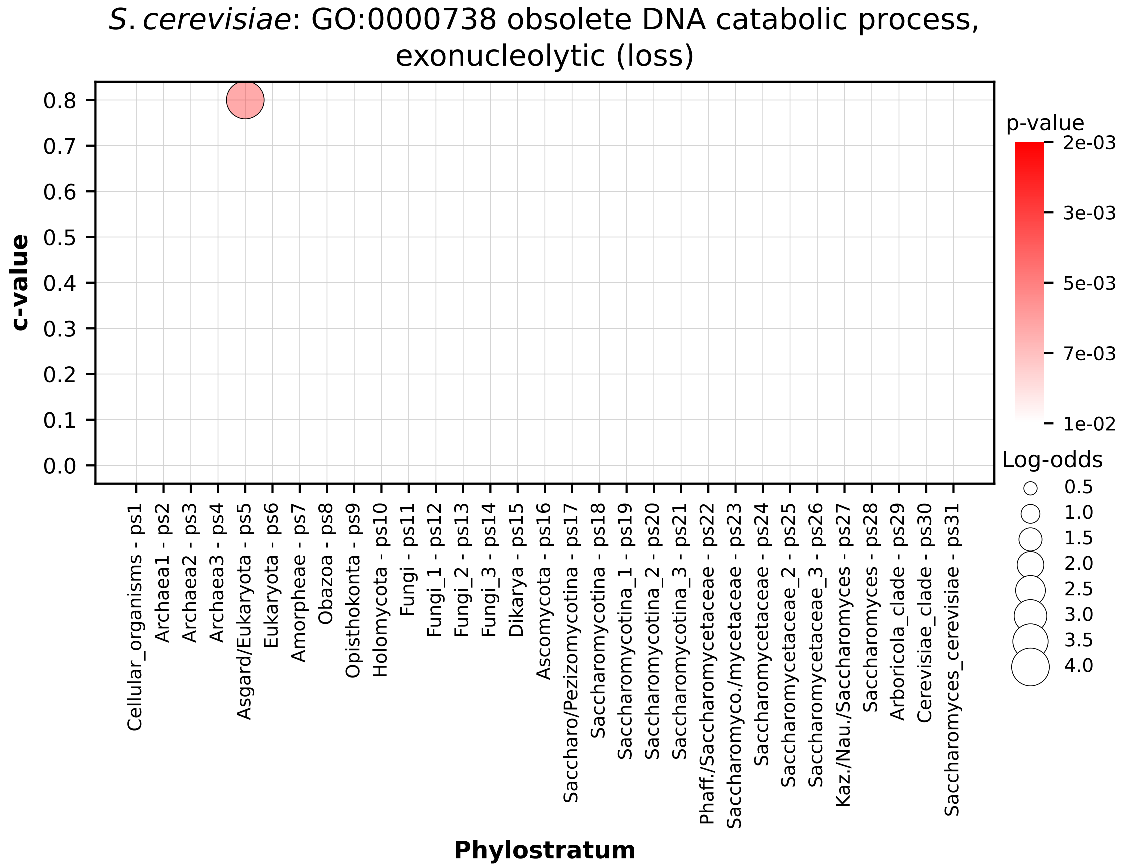
S. cerevisiae: GO:0000738 obsolete DNA catabolic process, exonucleolytic (loss)
c-value
0.8
0.7
0.6
0.5
0.4
0.3
0.2
0.1
0.0
p-value
2e-03
3e-03
5e-03
7e-03
1e-02
Log-odds
0.5
1.0
1.5
2.0
2.5
3.0
3.5
4.0
Cellular_organisms - ps1
Archaea1 - ps2
Archaea2 - ps3
Archaea3 - ps4
Asgard/Eukaryota - ps5
Eukaryota - ps6
Amorpheae - ps7
Obazoa - ps8
Opisthokonta - ps9
Holomycota - ps10
Fungi - ps11
Fungi_1 - ps12
Fungi_2 - ps13
Fungi_3 - ps14
Dikarya - ps15
Ascomycota - ps16
Saccharo/Pezizomycotina - ps17
Saccharomycotina - ps18
Saccharomycotina_1 - ps19
Saccharomycotina_2 - ps20
Saccharomycotina_3 - ps21
Phaff./Saccharomycetaceae - ps22
Saccharomyco./mycetaceae - ps23
Saccharomycetaceae - ps24
Saccharomycetaceae_2 - ps25
Saccharomycetaceae_3 - ps26
Kaz./Nau./Saccharomyces - ps27
Saccharomyces - ps28
Arboricola_clade - ps29
Cerevisiae_clade - ps30
Saccharomyces_cerevisiae - ps31
Phylostratum

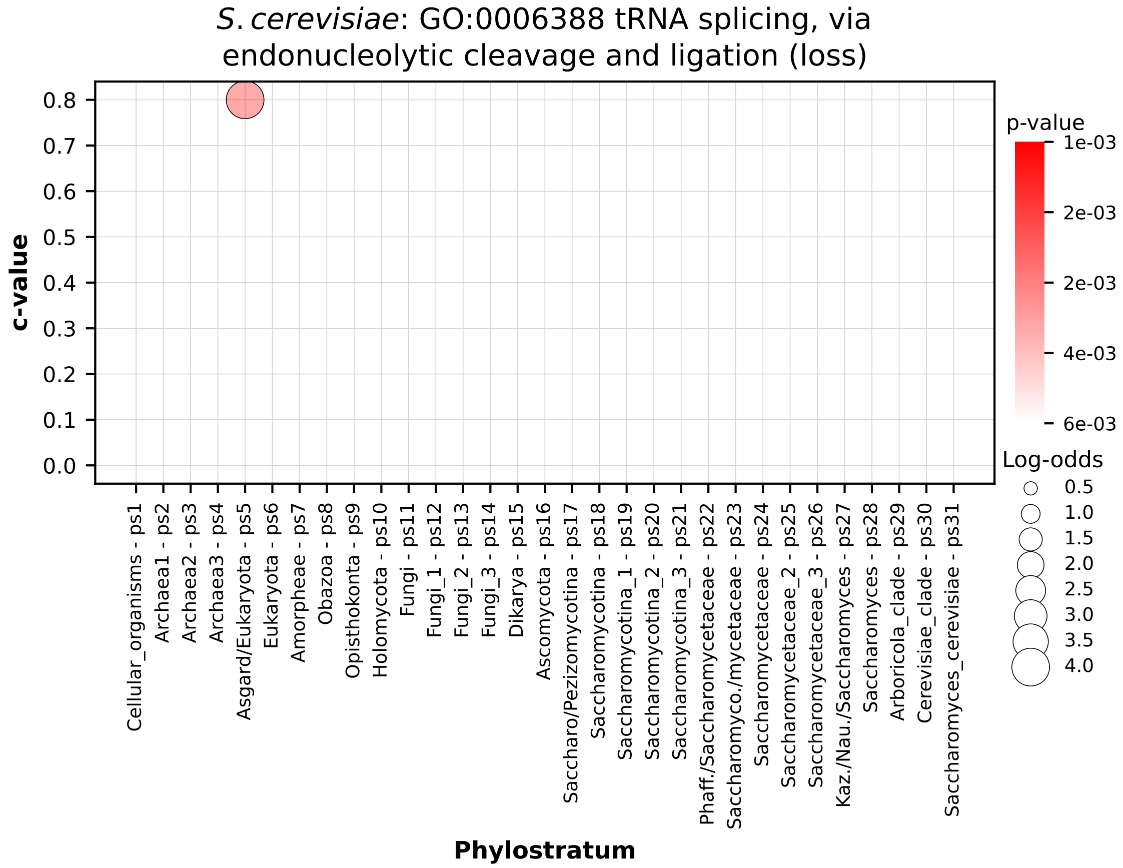
S. cerevisiae: GO:0006388 tRNA splicing, via endonucleolytic cleavage and ligation (loss)
c-value
0.0
0.1
0.2
0.3
0.4
0.5
0.6
0.7
0.8
p-value
1e-03
2e-03
2e-03
4e-03
6e-03
Log-odds
0.5
1.0
1.5
2.0
2.5
3.0
3.5
4.0
Cellular_organisms - ps1
Archaea1 - ps2
Archaea2 - ps3
Archaea3 - ps4
Asgard/Eukaryota - ps5
Eukaryota - ps6
Amorpheae - ps7
Obazoa - ps8
Opisthokonta - ps9
Holomycota - ps10
Fungi - ps11
Fungi_1 - ps12
Fungi_2 - ps13
Fungi_3 - ps14
Dikarya - ps15
Ascomycota - ps16
Saccharo/Pezizomycotina - ps17
Saccharomycotina - ps18
Saccharomycotina_1 - ps19
Saccharomycotina_2 - ps20
Saccharomycotina_3 - ps21
Phaff./Saccharomycetaceae - ps22
Saccharomyco./mycetaceae - ps23
Saccharomycetaceae - ps24
Saccharomycetaceae_2 - ps25
Saccharomycetaceae_3 - ps26
Kaz./Nau./Saccharomyces - ps27
Saccharomyces - ps28
Arboricola_clade - ps29
Cerevisiae_clade - ps30
Saccharomyces_cerevisiae - ps31
Phylostratum

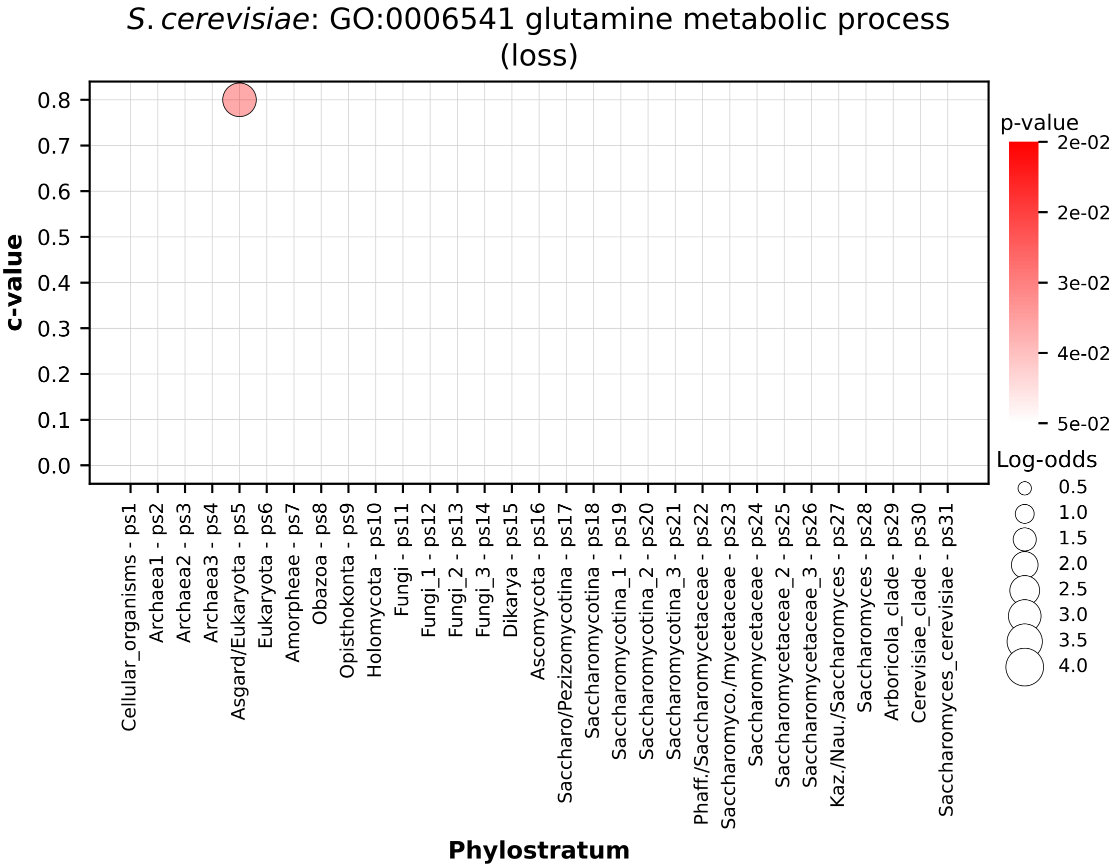
S. cerevisiae: GO:0006541 glutamine metabolic process
(loss)
c-value
0.0
0.1
0.2
0.3
0.4
0.5
0.6
0.7
0.8
p-value
2e-02
2e-02
3e-02
4e-02
5e-02
Log-odds
0.5
1.0
1.5
2.0
2.5
3.0
3.5
4.0
Cellular_organisms - ps1
Archaea1 - ps2
Archaea2 - ps3
Archaea3 - ps4
Asgard/Eukaryota - ps5
Eukaryota - ps6
Amorpheae - ps7
Obazoa - ps8
Opisthokonta - ps9
Holomycota - ps10
Fungi - ps11
Fungi_1 - ps12
Fungi_2 - ps13
Fungi_3 - ps14
Dikarya - ps15
Ascomycota - ps16
Saccharo/Pezizomycotina - ps17
Saccharomycotina - ps18
Saccharomycotina_1 - ps19
Saccharomycotina_2 - ps20
Saccharomycotina_3 - ps21
Phaff./Saccharomycetaceae - ps22
Saccharomyco./mycetaceae - ps23
Saccharomycetaceae - ps24
Saccharomycetaceae_2 - ps25
Saccharomycetaceae_3 - ps26
Kaz./Nau./Saccharomyces - ps27
Saccharomyces - ps28
Arboricola_clade - ps29
Cerevisiae_clade - ps30
Saccharomyces_cerevisiae - ps31
Phylostratum

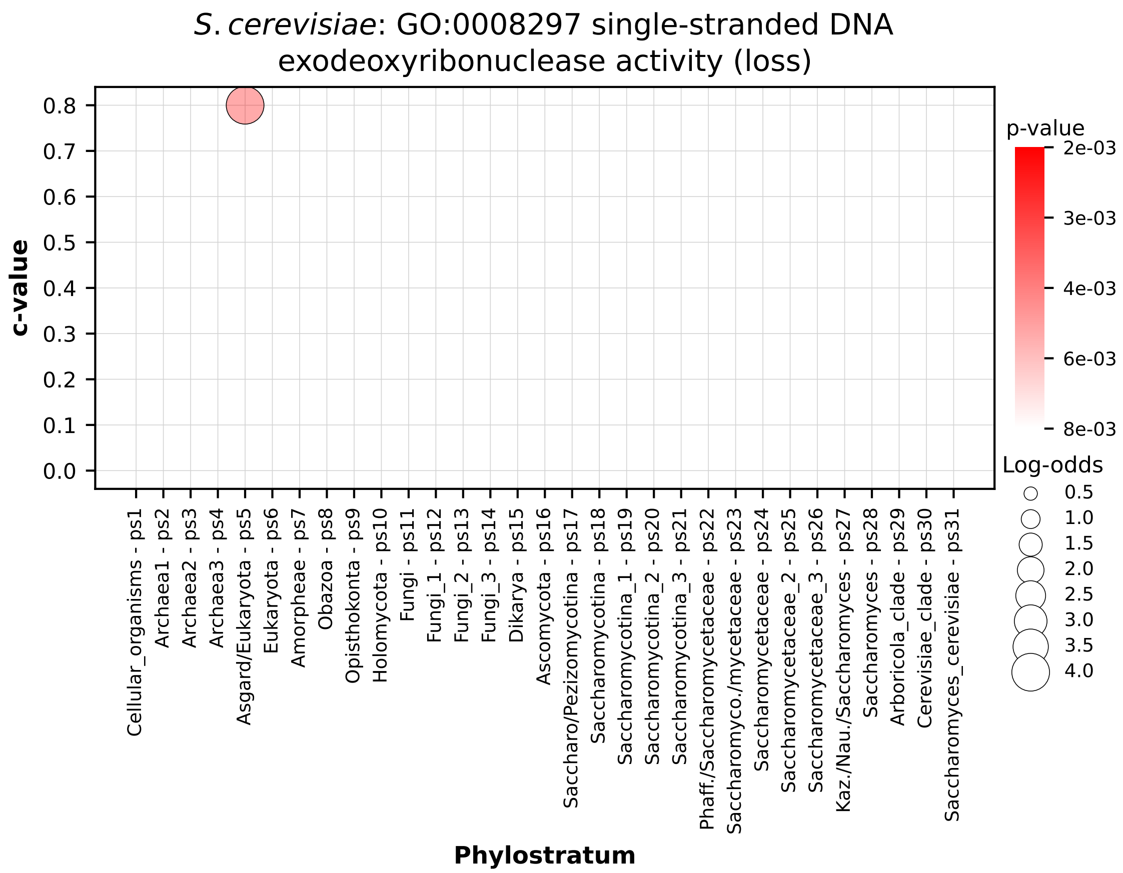

S. cerevisiae: GO:0008297 single-stranded DNA exodeoxyribonuclease activity (loss)
c-value
0.0
0.1
0.2
0.3
0.4
0.5
0.6
0.7
0.8
Phylostratum
Cellular_organisms - ps1
Archaea1 - ps2
Archaea2 - ps3
Archaea3 - ps4
Asgard/Eukaryota - ps5
Eukaryota - ps6
Amorpheae - ps7
Obazoa - ps8
Opisthokonta - ps9
Holomycota - ps10
Fungi - ps11
Fungi_1 - ps12
Fungi_2 - ps13
Fungi_3 - ps14
Dikarya - ps15
Ascomycota - ps16
Saccharo/Pezizomycotina - ps17
Saccharomycotina - ps18
Saccharomycotina_1 - ps19
Saccharomycotina_2 - ps20
Saccharomycotina_3 - ps21
Phaff./Saccharomycetaceae - ps22
Saccharomyco./mycetaceae - ps23
Saccharomycetaceae - ps24
Saccharomycetaceae_2 - ps25
Saccharomycetaceae_3 - ps26
Kaz./Nau./Saccharomyces - ps27
Saccharomyces - ps28
Arboricola_clade - ps29
Cerevisiae_clade - ps30
Saccharomyces_cerevisiae - ps31
p-value
2e-03
3e-03
4e-03
6e-03
8e-03
Log-odds
0.5
1.0
1.5
2.0
2.5
3.0
3.5
4.0

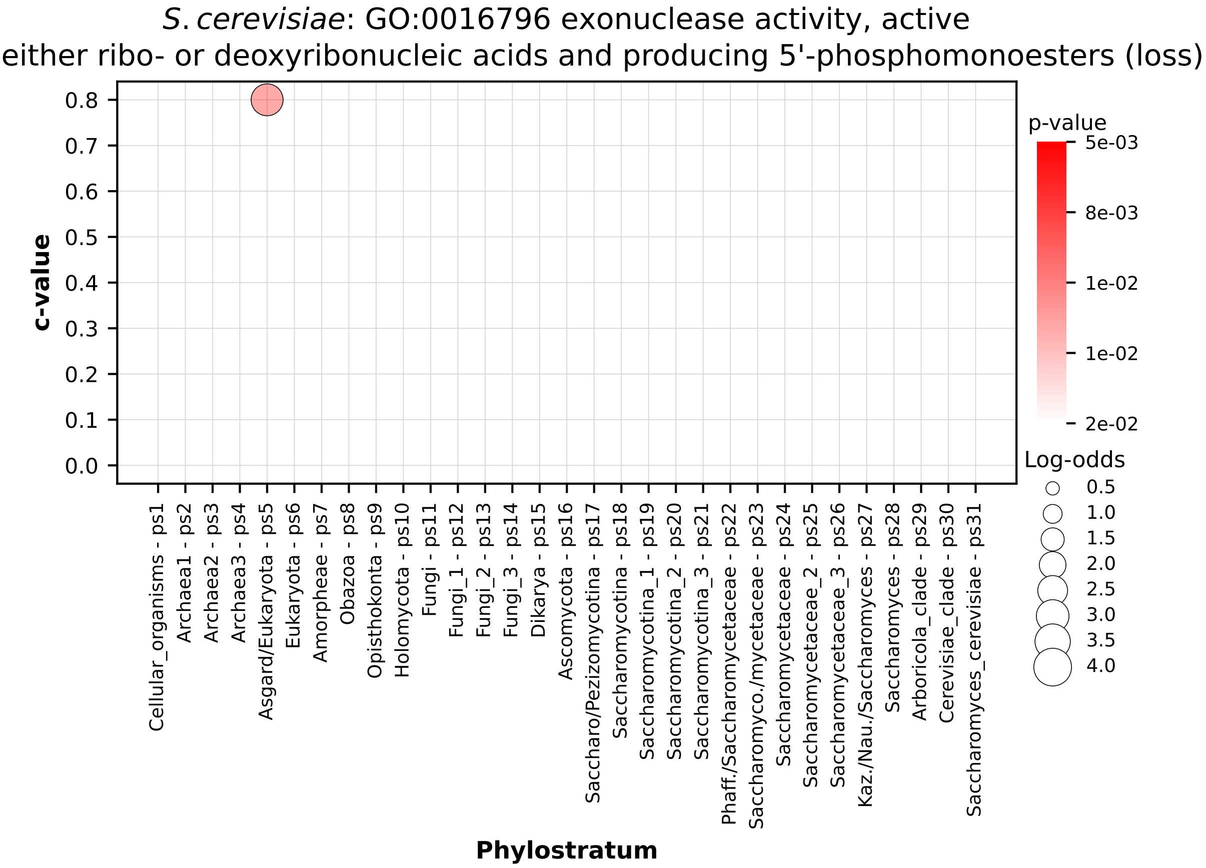

$S. cerevisiae$: GO:0016796 exonuclease activity, active
either ribo- or deoxyribonucleic acids and producing 5'-phosphomonoesters (loss)
c-value
0.0
0.1
0.2
0.3
0.4
0.5
0.6
0.7
0.8
p-value
5e-03
8e-03
1e-02
1e-02
2e-02
Log-odds
0.5
1.0
1.5
2.0
2.5
3.0
3.5
4.0
Cellular_organisms - ps1
Archaea1 - ps2
Archaea2 - ps3
Archaea3 - ps4
Asgard/Eukaryota - ps5
Eukaryota - ps6
Amorpheae - ps7
Obazoa - ps8
Opisthokonta - ps9
Holomycota - ps10
Fungi - ps11
Fungi_1 - ps12
Fungi_2 - ps13
Fungi_3 - ps14
Dikarya - ps15
Ascomycota - ps16
Saccharo/Pezizomycotina - ps17
Saccharomycotina - ps18
Saccharomycotina_1 - ps19
Saccharomycotina_2 - ps20
Saccharomycotina_3 - ps21
Phaff./Saccharomycetaceae - ps22
Saccharomyco./mycetaceae - ps23
Saccharomycetaceae - ps24
Saccharomycetaceae_2 - ps25
Saccharomycetaceae_3 - ps26
Kaz./Nau./Saccharomyces - ps27
Saccharomyces - ps28
Arboricola_clade - ps29
Cerevisiae_clade - ps30
Saccharomyces_cerevisiae - ps31
Phylostratum

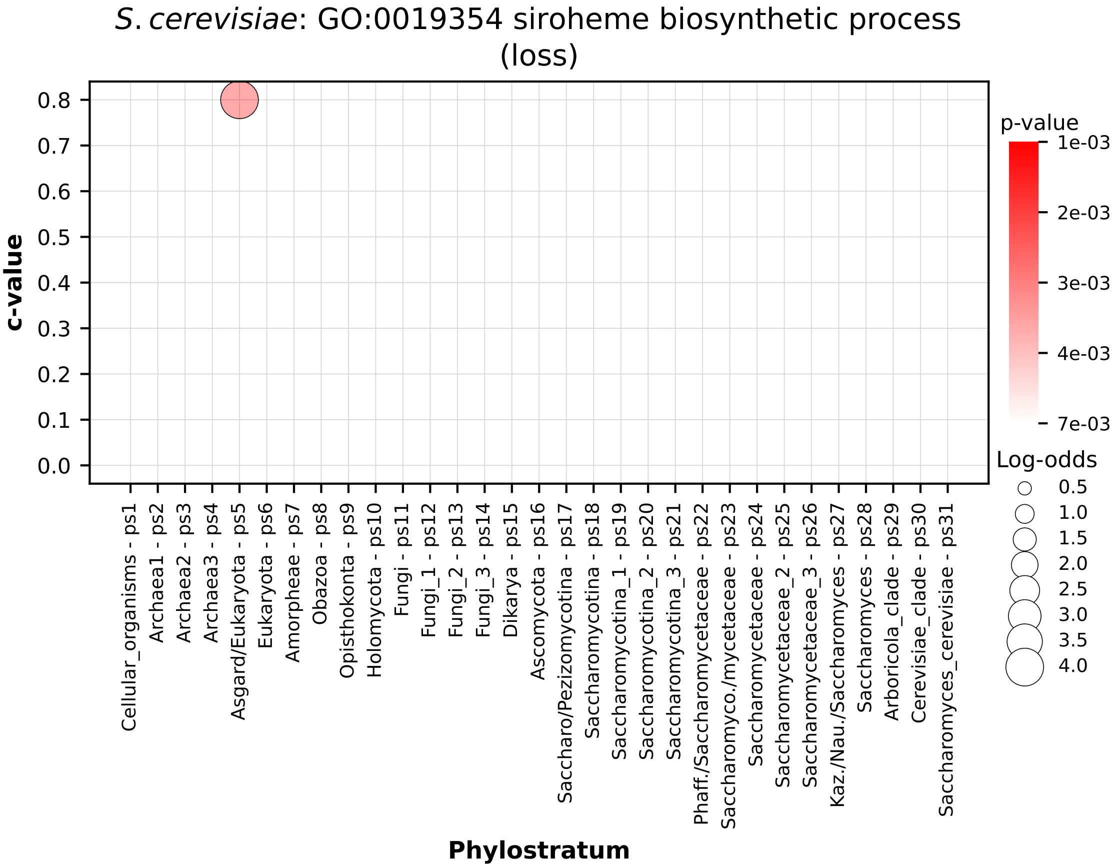

S. cerevisiae: GO:0019354 siroheme biosynthetic process (loss)
c-value
0.8
0.7
0.6
0.5
0.4
0.3
0.2
0.1
0.0
p-value
1e-03
2e-03
3e-03
4e-03
7e-03
Log-odds
0.5
1.0
1.5
2.0
2.5
3.0
3.5
4.0
Cellular_organisms - ps1
Archaea1 - ps2
Archaea2 - ps3
Archaea3 - ps4
Asgard/Eukaryota - ps5
Eukaryota - ps6
Amorpheae - ps7
Obazoa - ps8
Opisthokonta - ps9
Holomycota - ps10
Fungi - ps11
Fungi_1 - ps12
Fungi_2 - ps13
Fungi_3 - ps14
Dikarya - ps15
Ascomycota - ps16
Saccharo/Pezizomycotina - ps17
Saccharomycotina - ps18
Saccharomycotina_1 - ps19
Saccharomycotina_2 - ps20
Saccharomycotina_3 - ps21
Phaff./Saccharomycetaceae - ps22
Saccharomyco./mycetaceae - ps23
Saccharomycetaceae - ps24
Saccharomycetaceae_2 - ps25
Saccharomycetaceae_3 - ps26
Kaz./Nau./Saccharomyces - ps27
Saccharomyces - ps28
Arboricola_clade - ps29
Cerevisiae_clade - ps30
Saccharomyces_cerevisiae - ps31
Phylostratum

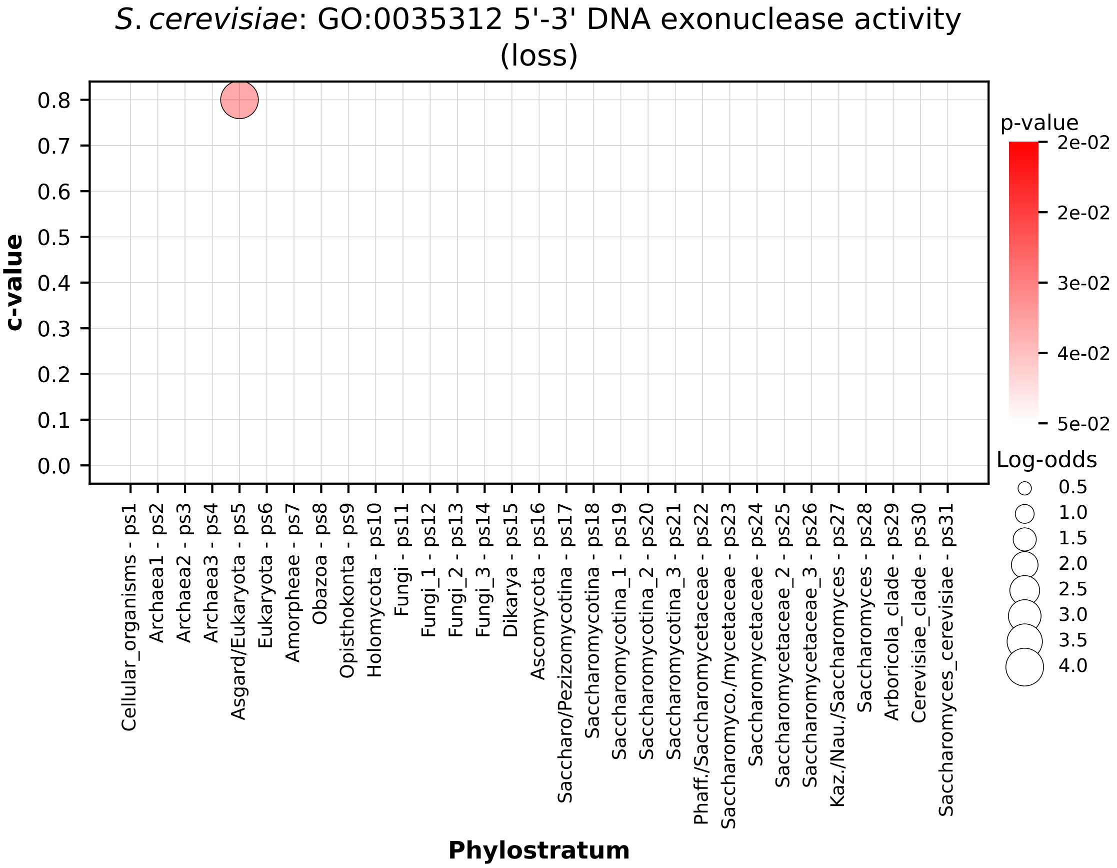

S. cerevisiae: GO:0035312 5'-3' DNA exonuclease activity
(loss)
c-value
0.8
0.7
0.6
0.5
0.4
0.3
0.2
0.1
0.0
p-value
2e-02
2e-02
3e-02
4e-02
5e-02
Log-odds
0.5
1.0
1.5
2.0
2.5
3.0
3.5
4.0
Cellular_organisms - ps1
Archaea1 - ps2
Archaea2 - ps3
Archaea3 - ps4
Asgard/Eukaryota - ps5
Eukaryota - ps6
Amorpheae - ps7
Obazoa - ps8
Opisthokonta - ps9
Holomycota - ps10
Fungi - ps11
Fungi_1 - ps12
Fungi_2 - ps13
Fungi_3 - ps14
Dikarya - ps15
Ascomycota - ps16
Saccharo/Pezizomycotina - ps17
Saccharomycotina - ps18
Saccharomycotina_1 - ps19
Saccharomycotina_2 - ps20
Saccharomycotina_3 - ps21
Phaff./Saccharomycetaceae - ps22
Saccharomyco./mycetaceae - ps23
Saccharomycetaceae - ps24
Saccharomycetaceae_2 - ps25
Saccharomycetaceae_3 - ps26
Kaz./Nau./Saccharomyces - ps27
Saccharomyces - ps28
Arboricola_clade - ps29
Cerevisiae_clade - ps30
Saccharomyces_cerevisiae - ps31
Phylostratum

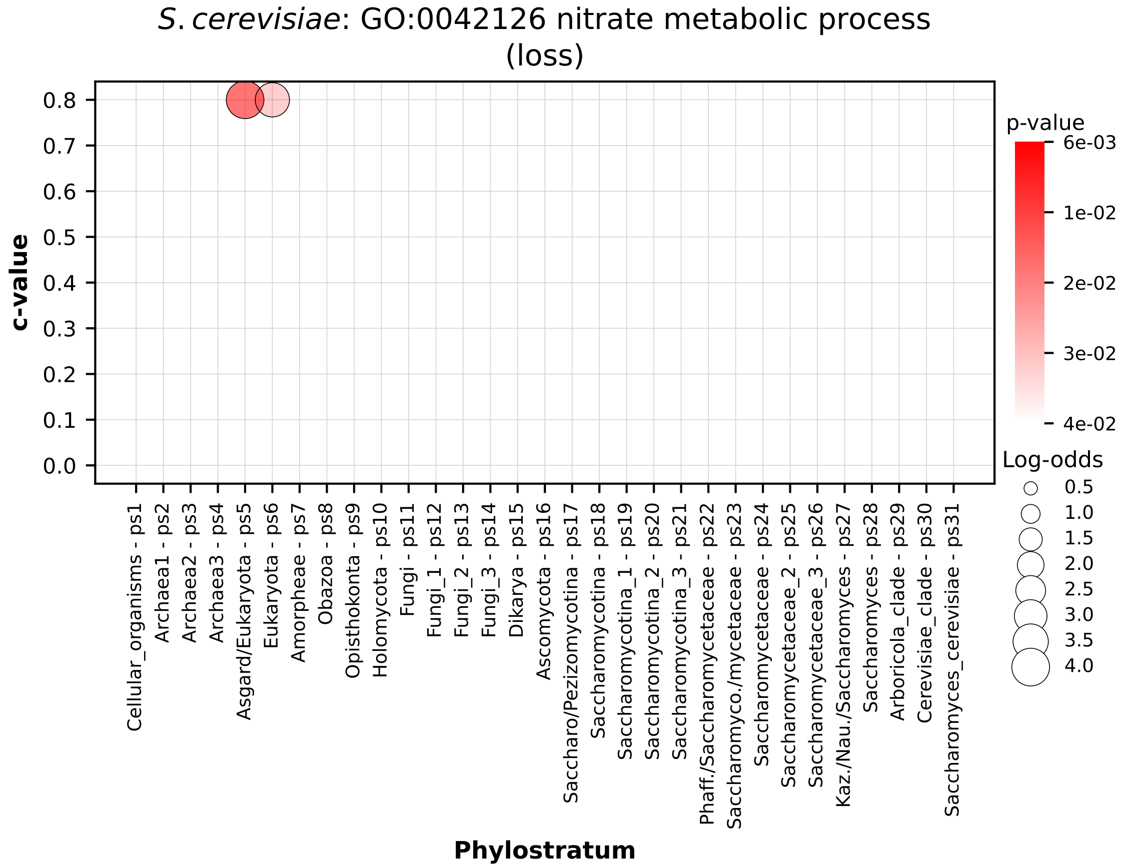

S. cerevisiae: GO:0042126 nitrate metabolic process
(loss)
c-value
0.0
0.1
0.2
0.3
0.4
0.5
0.6
0.7
0.8
p-value
6e-03
1e-02
2e-02
3e-02
4e-02
Log-odds
0.5
1.0
1.5
2.0
2.5
3.0
3.5
4.0
Cellular_organisms - ps1
Archaea1 - ps2
Archaea2 - ps3
Archaea3 - ps4
Asgard/Eukaryota - ps5
Eukaryota - ps6
Amorpheae - ps7
Obazoa - ps8
Opisthokonta - ps9
Holomycota - ps10
Fungi - ps11
Fungi_1 - ps12
Fungi_2 - ps13
Fungi_3 - ps14
Dikarya - ps15
Ascomycota - ps16
Saccharo/Pezizomycotina - ps17
Saccharomycotina - ps18
Saccharomycotina_1 - ps19
Saccharomycotina_2 - ps20
Saccharomycotina_3 - ps21
Phaff./Saccharomycetaceae - ps22
Saccharomyco./mycetaceae - ps23
Saccharomycetaceae - ps24
Saccharomycetaceae_2 - ps25
Saccharomycetaceae_3 - ps26
Kaz./Nau./Saccharomyces - ps27
Saccharomyces - ps28
Arboricola_clade - ps29
Cerevisiae_clade - ps30
Saccharomyces_cerevisiae - ps31
Phylostratum

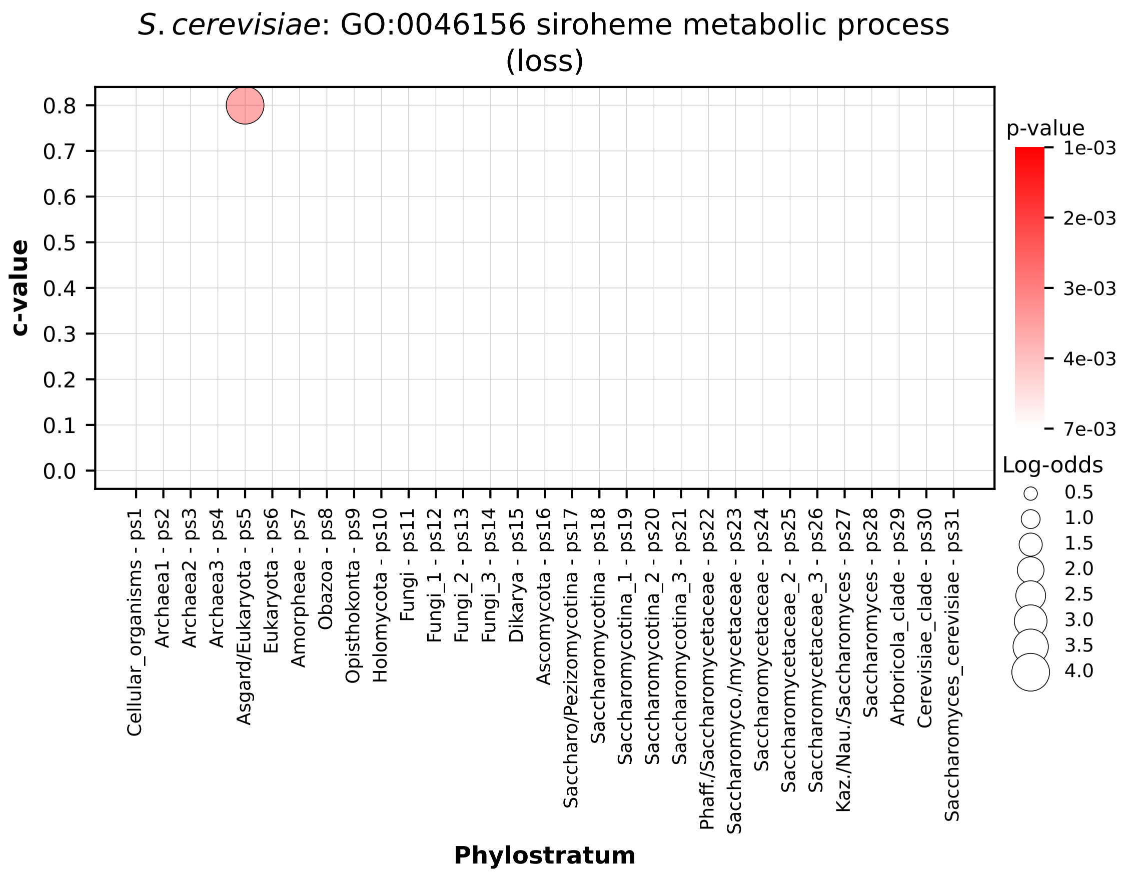
S. cerevisiae: GO:0046156 siroheme metabolic process
(loss)
c-value
0.0
0.1
0.2
0.3
0.4
0.5
0.6
0.7
0.8
p-value
1e-03
2e-03
3e-03
4e-03
7e-03
Log-odds
0.5
1.0
1.5
2.0
2.5
3.0
3.5
4.0
Cellular_organisms - ps1
Archaea1 - ps2
Archaea2 - ps3
Archaea3 - ps4
Asgard/Eukaryota - ps5
Eukaryota - ps6
Amorpheae - ps7
Obazoa - ps8
Opisthokonta - ps9
Holomycota - ps10
Fungi - ps11
Fungi_1 - ps12
Fungi_2 - ps13
Fungi_3 - ps14
Dikarya - ps15
Ascomycota - ps16
Saccharo/Pezizomycotina - ps17
Saccharomycotina - ps18
Saccharomycotina_1 - ps19
Saccharomycotina_2 - ps20
Saccharomycotina_3 - ps21
Phaff./Saccharomycetaceae - ps22
Saccharomyco./mycetaceae - ps23
Saccharomycetaceae - ps24
Saccharomycetaceae_2 - ps25
Saccharomycetaceae_3 - ps26
Kaz./Nau./Saccharomyces - ps27
Saccharomyces - ps28
Arboricola_clade - ps29
Cerevisiae_clade - ps30
Saccharomyces_cerevisiae - ps31
Phylostratum

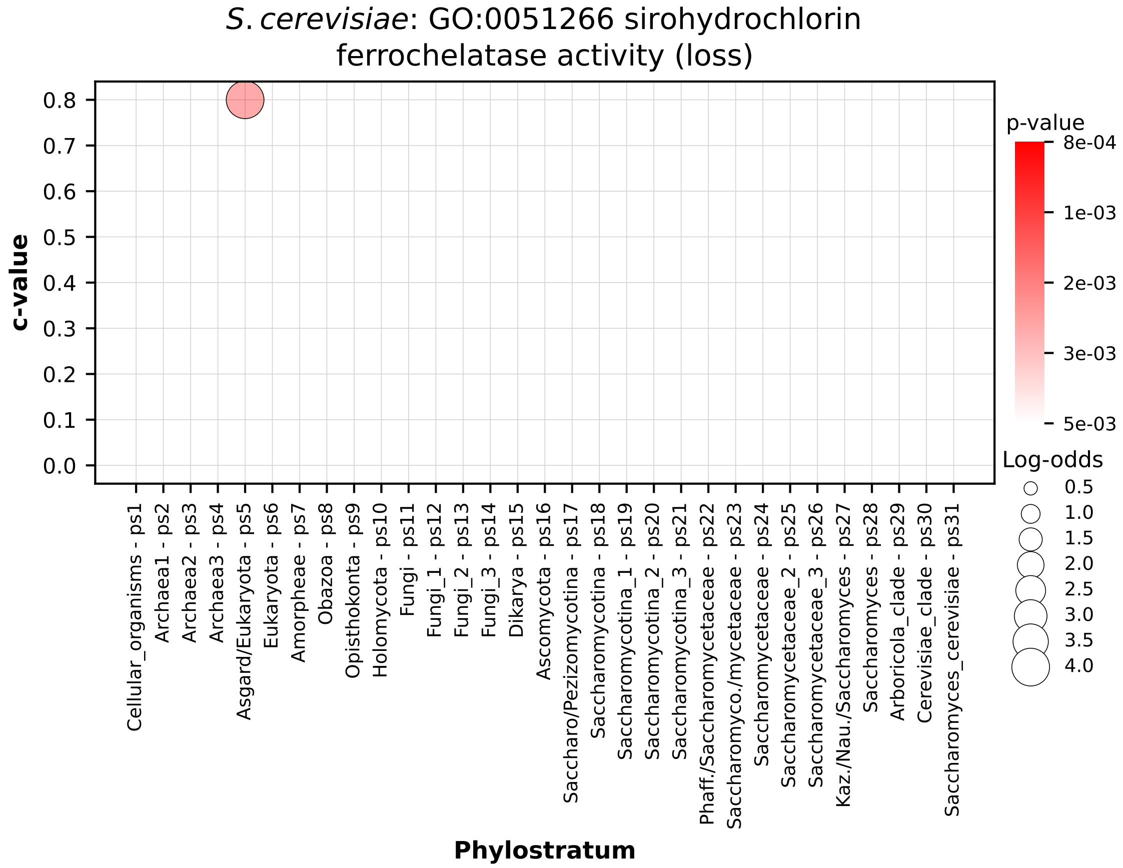

S. cerevisiae: GO:0051266 sirohydrochlorin ferrochelatase activity (loss)
c-value
0.8
0.7
0.6
0.5
0.4
0.3
0.2
0.1
0.0
p-value
8e-04
1e-03
2e-03
3e-03
5e-03
Log-odds
0.5
1.0
1.5
2.0
2.5
3.0
3.5
4.0
Cellular_organisms - ps1
Archaea1 - ps2
Archaea2 - ps3
Archaea3 - ps4
Asgard/Eukaryota - ps5
Eukaryota - ps6
Amorpheae - ps7
Obazoa - ps8
Opisthokonta - ps9
Holomycota - ps10
Fungi - ps11
Fungi_1 - ps12
Fungi_2 - ps13
Fungi_3 - ps14
Dikarya - ps15
Ascomycota - ps16
Saccharo/Pezizomycotina - ps17
Saccharomycotina - ps18
Saccharomycotina_1 - ps19
Saccharomycotina_2 - ps20
Saccharomycotina_3 - ps21
Phaff./Saccharomycetaceae - ps22
Saccharomyco./mycetaceae - ps23
Saccharomycetaceae - ps24
Saccharomycetaceae_2 - ps25
Saccharomycetaceae_3 - ps26
Kaz./Nau./Saccharomyces - ps27
Saccharomyces - ps28
Arboricola_clade - ps29
Cerevisiae_clade - ps30
Saccharomyces_cerevisiae - ps31
Phylostratum

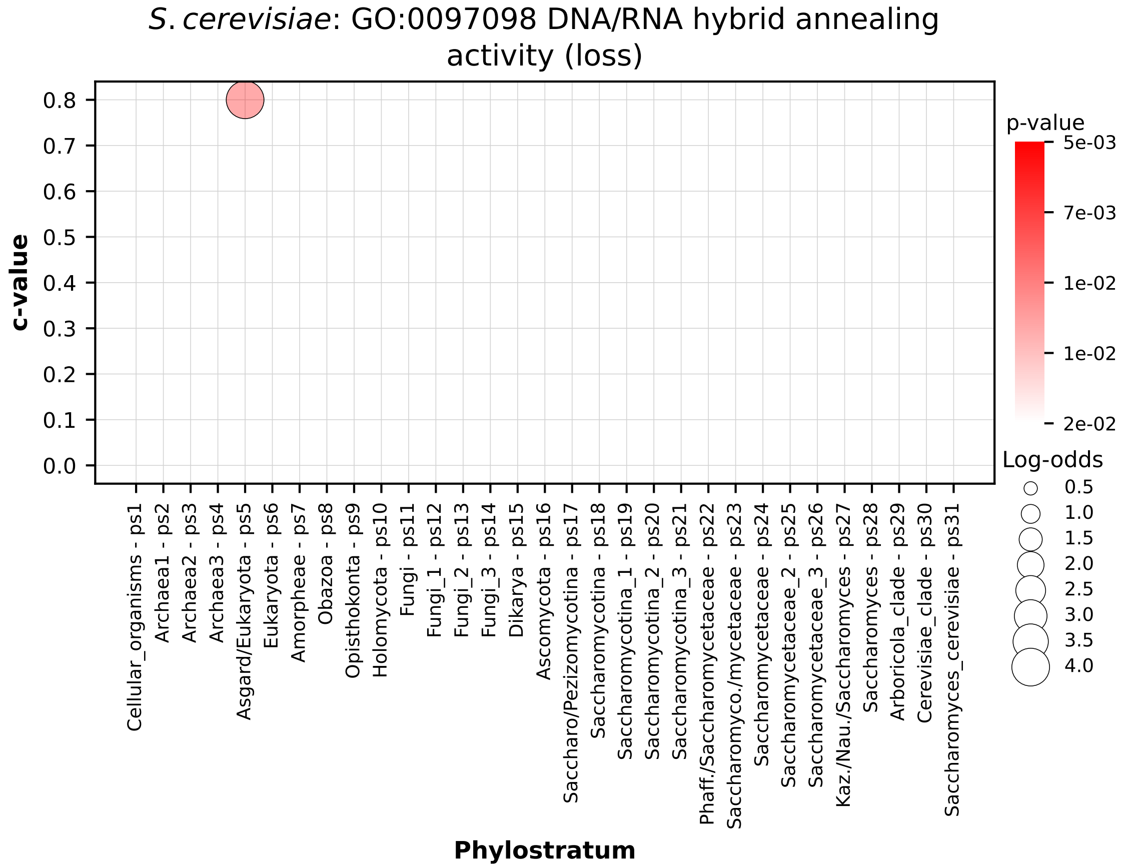

S. cerevisiae: GO:0097098 DNA/RNA hybrid annealing activity (loss)
c-value
0.0
0.1
0.2
0.3
0.4
0.5
0.6
0.7
0.8
Phylostratum
Cellular_organisms - ps1
Archaea1 - ps2
Archaea2 - ps3
Archaea3 - ps4
Asgard/Eukaryota - ps5
Eukaryota - ps6
Amorpheae - ps7
Obazoa - ps8
Opisthokonta - ps9
Holomycota - ps10
Fungi - ps11
Fungi_1 - ps12
Fungi_2 - ps13
Fungi_3 - ps14
Dikarya - ps15
Ascomycota - ps16
Saccharo/Pezizomycotina - ps17
Saccharomycotina - ps18
Saccharomycotina_1 - ps19
Saccharomycotina_2 - ps20
Saccharomycotina_3 - ps21
Phaff./Saccharomycetaceae - ps22
Saccharomyco./mycetaceae - ps23
Saccharomycetaceae - ps24
Saccharomycetaceae_2 - ps25
Saccharomycetaceae_3 - ps26
Kaz./Nau./Saccharomyces - ps27
Saccharomyces - ps28
Arboricola_clade - ps29
Cerevisiae_clade - ps30
Saccharomyces_cerevisiae - ps31
p-value
5e-03
7e-03
1e-02
1e-02
2e-02
Log-odds
0.5
1.0
1.5
2.0
2.5
3.0
3.5
4.0

S. cerevisiae: GO:0002143 tRNA wobble position uridine thiolation (loss)

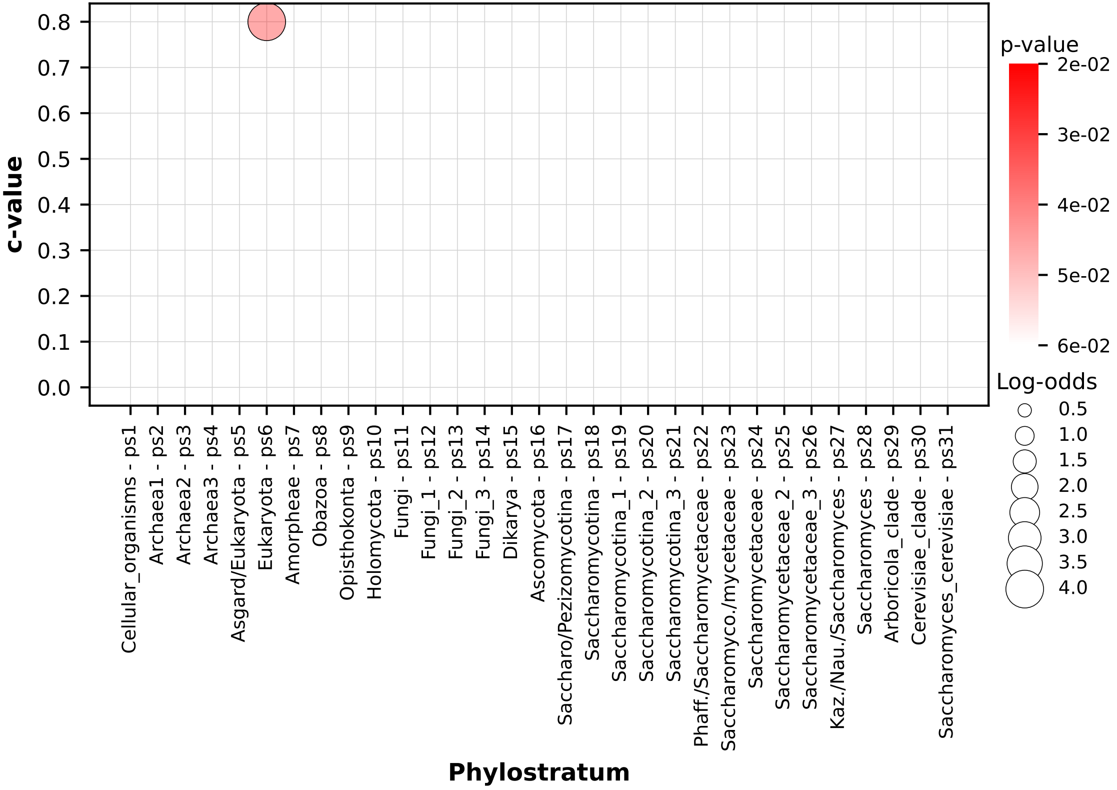

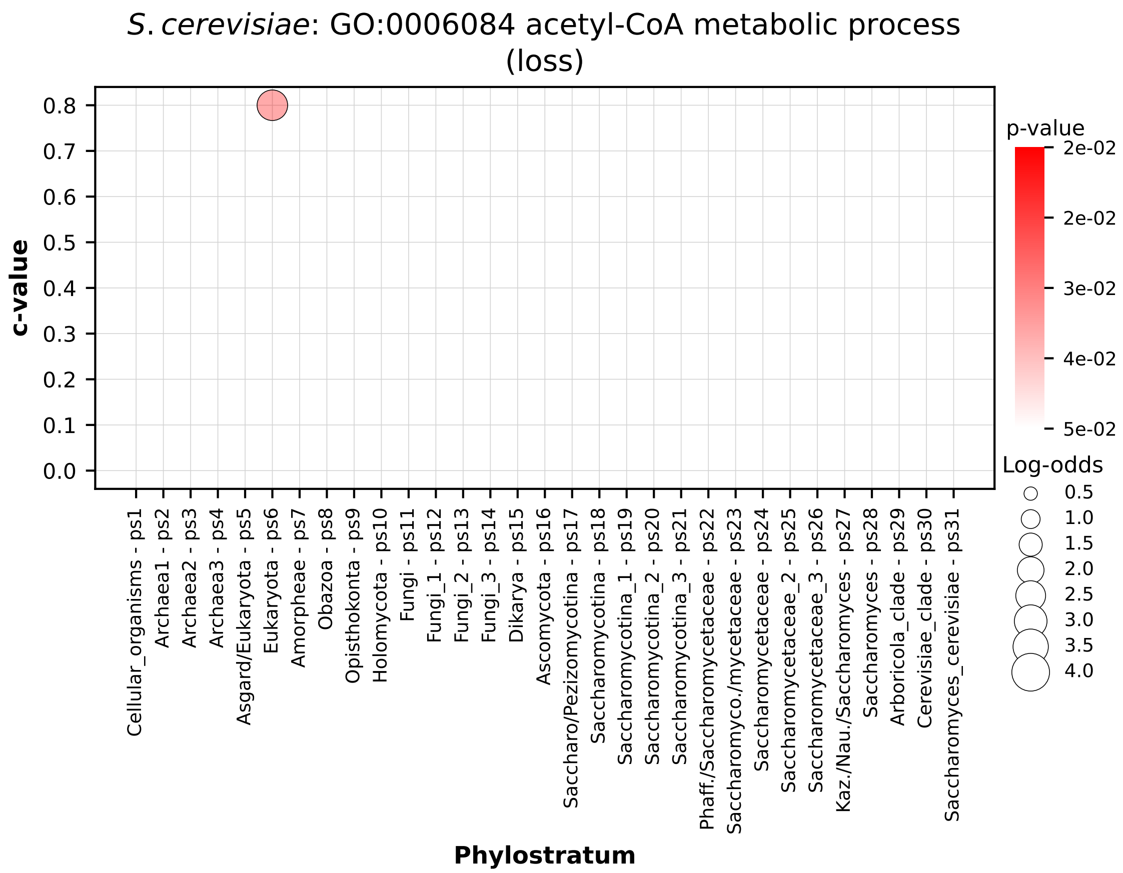
S. cerevisiae: GO:0006084 acetyl-CoA metabolic process
(loss)
c-value
0.0
0.1
0.2
0.3
0.4
0.5
0.6
0.7
0.8
p-value
2e-02
2e-02
3e-02
4e-02
5e-02
Log-odds
0.5
1.0
1.5
2.0
2.5
3.0
3.5
4.0
Cellular_organisms - ps1
Archaea1 - ps2
Archaea2 - ps3
Archaea3 - ps4
Asgard/Eukaryota - ps5
Eukaryota - ps6
Amorpheae - ps7
Obazoa - ps8
Opisthokonta - ps9
Holomycota - ps10
Fungi - ps11
Fungi_1 - ps12
Fungi_2 - ps13
Fungi_3 - ps14
Dikarya - ps15
Ascomycota - ps16
Saccharo/Pezizomycotina - ps17
Saccharomycotina - ps18
Saccharomycotina_1 - ps19
Saccharomycotina_2 - ps20
Saccharomycotina_3 - ps21
Phaff./Saccharomycetaceae - ps22
Saccharomyco./mycetaceae - ps23
Saccharomycetaceae - ps24
Saccharomycetaceae_2 - ps25
Saccharomycetaceae_3 - ps26
Kaz./Nau./Saccharomyces - ps27
Saccharomyces - ps28
Arboricola_clade - ps29
Cerevisiae_clade - ps30
Saccharomyces_cerevisiae - ps31
Phylostratum

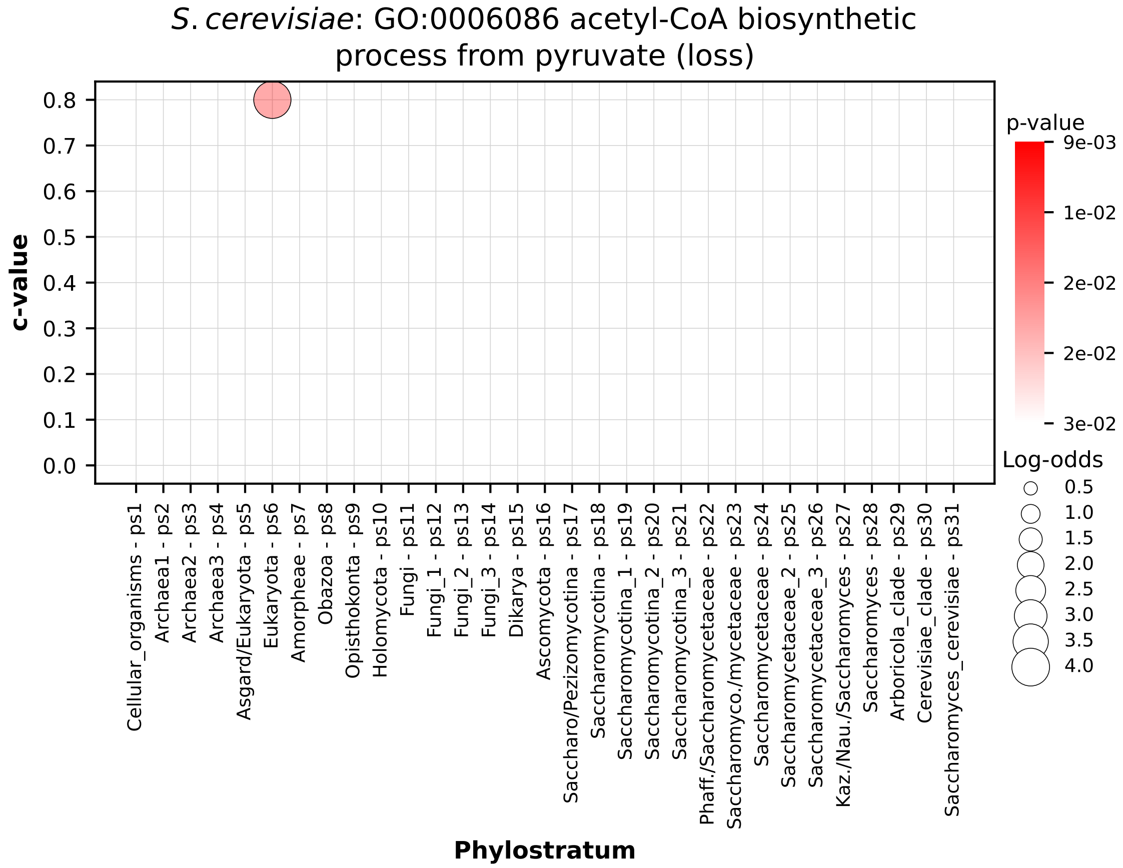

S. cerevisiae: GO:0006086 acetyl-CoA biosynthetic process from pyruvate (loss)
c-value
0.0
0.1
0.2
0.3
0.4
0.5
0.6
0.7
0.8
Phylostratum
Cellular_organisms - ps1
Archaea1 - ps2
Archaea2 - ps3
Archaea3 - ps4
Asgard/Eukaryota - ps5
Eukaryota - ps6
Amorpheae - ps7
Obazoa - ps8
Opisthokonta - ps9
Holomycota - ps10
Fungi - ps11
Fungi_1 - ps12
Fungi_2 - ps13
Fungi_3 - ps14
Dikarya - ps15
Ascomycota - ps16
Saccharo/Pezizomycotina - ps17
Saccharomycotina - ps18
Saccharomycotina_1 - ps19
Saccharomycotina_2 - ps20
Saccharomycotina_3 - ps21
Phaff./Saccharomycetaceae - ps22
Saccharomyco./mycetaceae - ps23
Saccharomycetaceae - ps24
Saccharomycetaceae_2 - ps25
Saccharomycetaceae_3 - ps26
Kaz./Nau./Saccharomyces - ps27
Saccharomyces - ps28
Arboricola_clade - ps29
Cerevisiae_clade - ps30
Saccharomyces_cerevisiae - ps31
p-value
9e-03
1e-02
2e-02
2e-02
3e-02
Log-odds
0.5
1.0
1.5
2.0
2.5
3.0
3.5
4.0

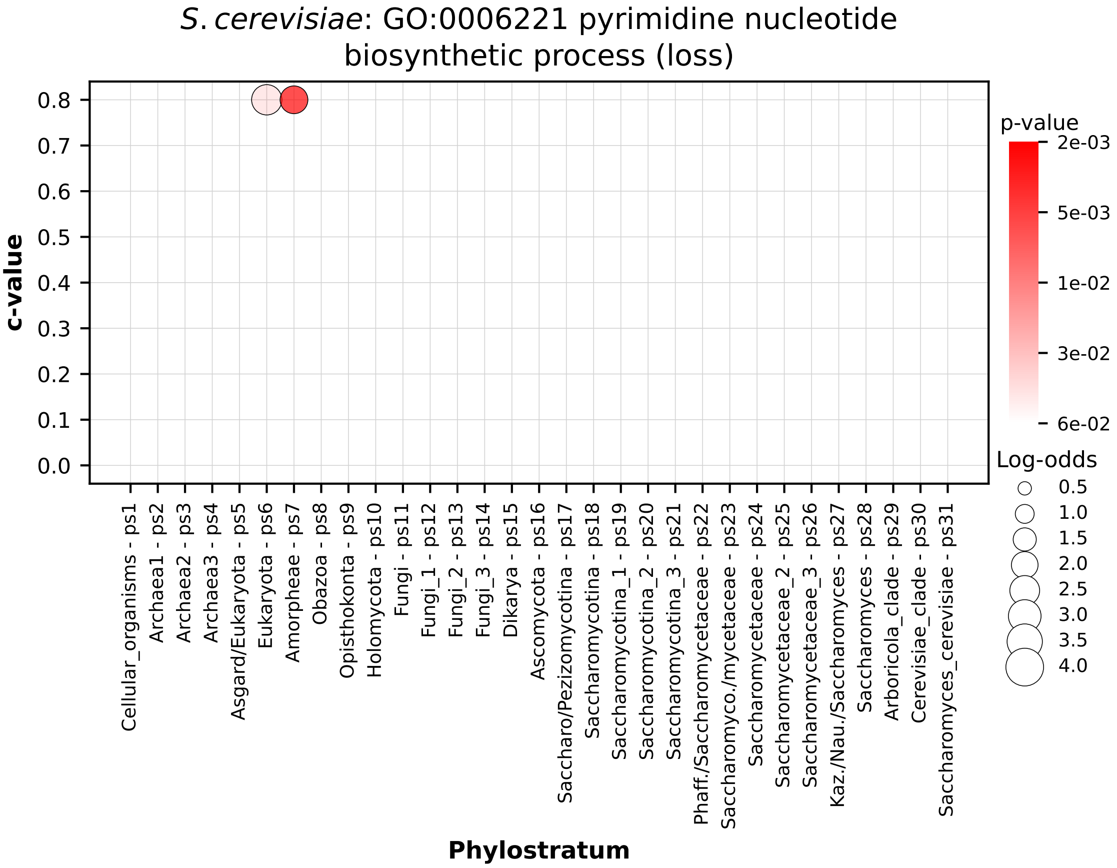
S. cerevisiae: GO:0006221 pyrimidine nucleotide biosynthetic process (loss)
c-value
0.0
0.1
0.2
0.3
0.4
0.5
0.6
0.7
0.8
Phylostratum
Cellular_organisms - ps1
Archaea1 - ps2
Archaea2 - ps3
Archaea3 - ps4
Asgard/Eukaryota - ps5
Eukaryota - ps6
Amorpheae - ps7
Obazoa - ps8
Opisthokonta - ps9
Holomycota - ps10
Fungi - ps11
Fungi_1 - ps12
Fungi_2 - ps13
Fungi_3 - ps14
Dikarya - ps15
Ascomycota - ps16
Saccharo/Pezizomycotina - ps17
Saccharomycotina - ps18
Saccharomycotina_1 - ps19
Saccharomycotina_2 - ps20
Saccharomycotina_3 - ps21
Phaff./Saccharomycetaceae - ps22
Saccharomyco./mycetaceae - ps23
Saccharomycetaceae - ps24
Saccharomycetaceae_2 - ps25
Saccharomycetaceae_3 - ps26
Kaz./Nau./Saccharomyces - ps27
Saccharomyces - ps28
Arboricola_clade - ps29
Cerevisiae_clade - ps30
Saccharomyces_cerevisiae - ps31
p-value
2e-03
5e-03
1e-02
3e-02
6e-02
Log-odds
0.5
1.0
1.5
2.0
2.5
3.0
3.5
4.0

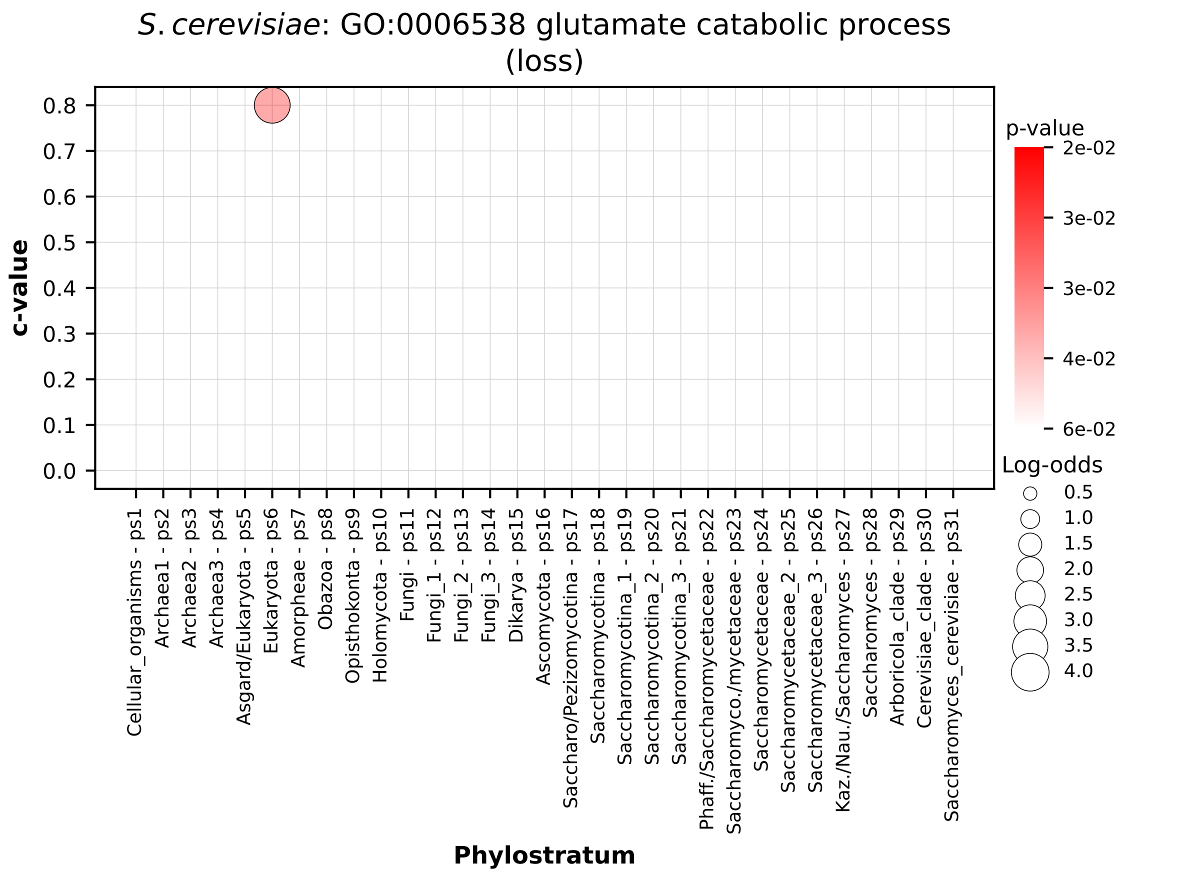
S. cerevisiae: GO:0006538 glutamate catabolic process
(loss)
c-value
0.0
0.1
0.2
0.3
0.4
0.5
0.6
0.7
0.8
p-value
2e-02
3e-02
3e-02
4e-02
6e-02
Log-odds
0.5
1.0
1.5
2.0
2.5
3.0
3.5
4.0
Cellular_organisms - ps1
Archaea1 - ps2
Archaea2 - ps3
Archaea3 - ps4
Asgard/Eukaryota - ps5
Eukaryota - ps6
Amorpheae - ps7
Obazoa - ps8
Opisthokonta - ps9
Holomycota - ps10
Fungi - ps11
Fungi_1 - ps12
Fungi_2 - ps13
Fungi_3 - ps14
Dikarya - ps15
Ascomycota - ps16
Saccharo/Pezizomycotina - ps17
Saccharomycotina - ps18
Saccharomycotina_1 - ps19
Saccharomycotina_2 - ps20
Saccharomycotina_3 - ps21
Phaff./Saccharomycetaceae - ps22
Saccharomyco./mycetaceae - ps23
Saccharomycetaceae - ps24
Saccharomycetaceae_2 - ps25
Saccharomycetaceae_3 - ps26
Kaz./Nau./Saccharomyces - ps27
Saccharomyces - ps28
Arboricola_clade - ps29
Cerevisiae_clade - ps30
Saccharomyces_cerevisiae - ps31
Phylostratum

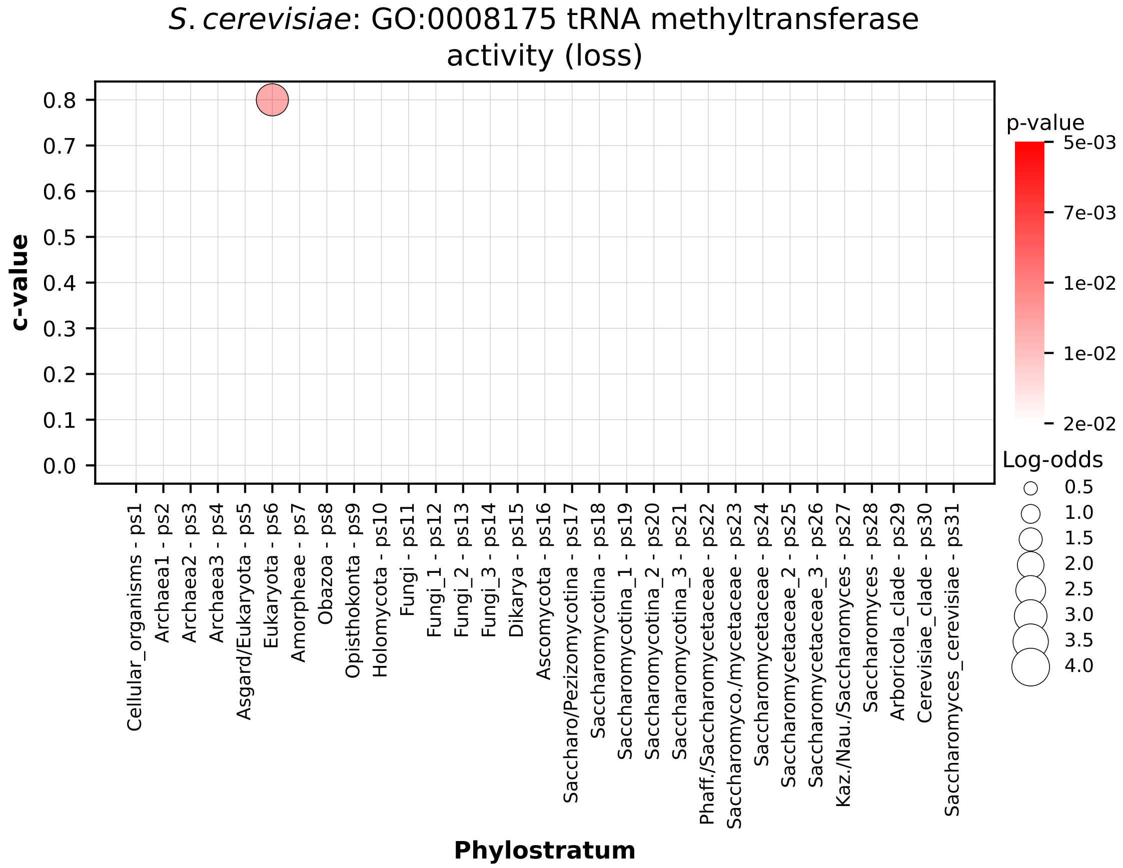

S. cerevisiae: GO:0008175 tRNA methyltransferase activity (loss)
c-value
0.8
0.7
0.6
0.5
0.4
0.3
0.2
0.1
0.0
p-value
5e-03
7e-03
1e-02
1e-02
2e-02
Log-odds
0.5
1.0
1.5
2.0
2.5
3.0
3.5
4.0
Cellular_organisms - ps1
Archaea1 - ps2
Archaea2 - ps3
Archaea3 - ps4
Asgard/Eukaryota - ps5
Eukaryota - ps6
Amorpheae - ps7
Obazoa - ps8
Opisthokonta - ps9
Holomycota - ps10
Fungi - ps11
Fungi_1 - ps12
Fungi_2 - ps13
Fungi_3 - ps14
Dikarya - ps15
Ascomycota - ps16
Saccharo/Pezizomycotina - ps17
Saccharomycotina - ps18
Saccharomycotina_1 - ps19
Saccharomycotina_2 - ps20
Saccharomycotina_3 - ps21
Phaff./Saccharomycetaceae - ps22
Saccharomyco./mycetaceae - ps23
Saccharomycetaceae - ps24
Saccharomycetaceae_2 - ps25
Saccharomycetaceae_3 - ps26
Kaz./Nau./Saccharomyces - ps27
Saccharomyces - ps28
Arboricola_clade - ps29
Cerevisiae_clade - ps30
Saccharomyces_cerevisiae - ps31
Phylostratum

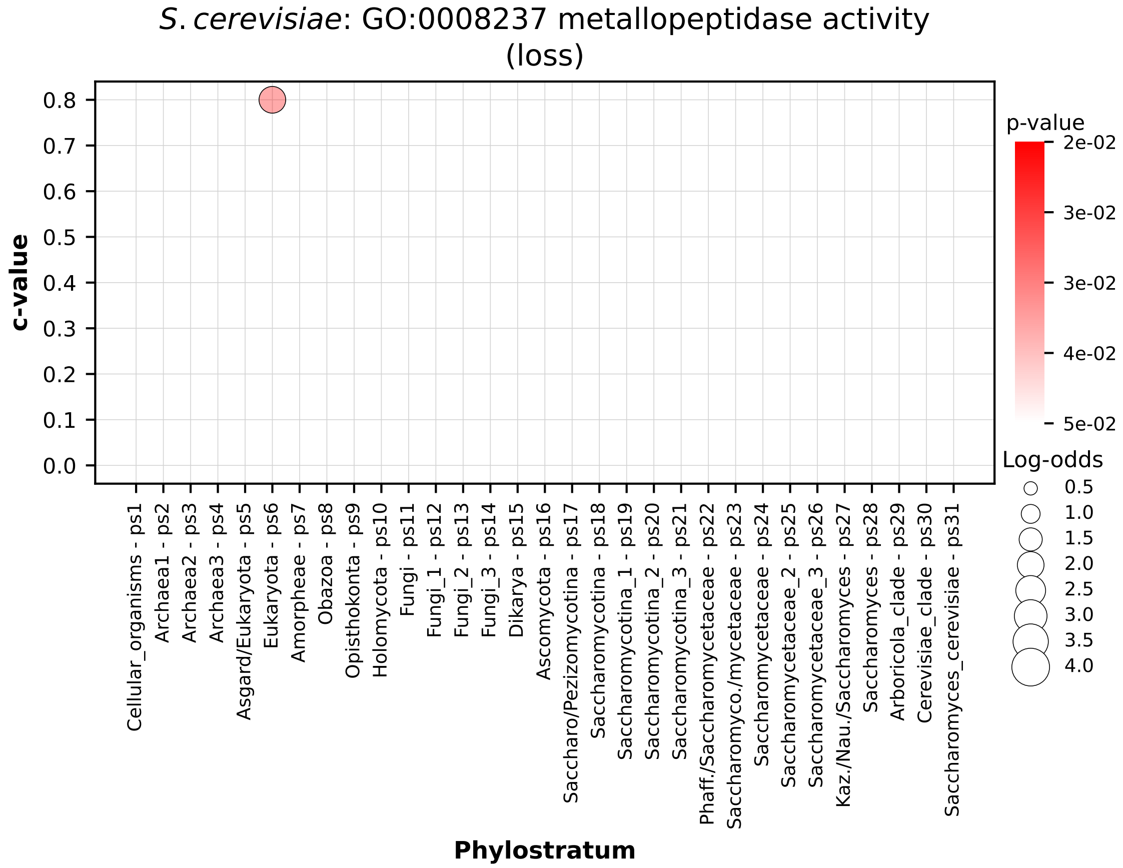

S. cerevisiae: GO:0008237 metallopeptidase activity (loss)
c-value
0.0
0.1
0.2
0.3
0.4
0.5
0.6
0.7
0.8
Phylostratum
Cellular_organisms - ps1
Archaea1 - ps2
Archaea2 - ps3
Archaea3 - ps4
Asgard/Eukaryota - ps5
Eukaryota - ps6
Amorpheae - ps7
Obazoa - ps8
Opisthokonta - ps9
Holomycota - ps10
Fungi - ps11
Fungi_1 - ps12
Fungi_2 - ps13
Fungi_3 - ps14
Dikarya - ps15
Ascomycota - ps16
Saccharo/Pezizomycotina - ps17
Saccharomycotina - ps18
Saccharomycotina_1 - ps19
Saccharomycotina_2 - ps20
Saccharomycotina_3 - ps21
Phaff./Saccharomycetaceae - ps22
Saccharomyco./mycetaceae - ps23
Saccharomycetaceae - ps24
Saccharomycetaceae_2 - ps25
Saccharomycetaceae_3 - ps26
Kaz./Nau./Saccharomyces - ps27
Saccharomyces - ps28
Arboricola_clade - ps29
Cerevisiae_clade - ps30
Saccharomyces_cerevisiae - ps31
p-value
2e-02
3e-02
3e-02
4e-02
5e-02
Log-odds
0.5
1.0
1.5
2.0
2.5
3.0
3.5
4.0

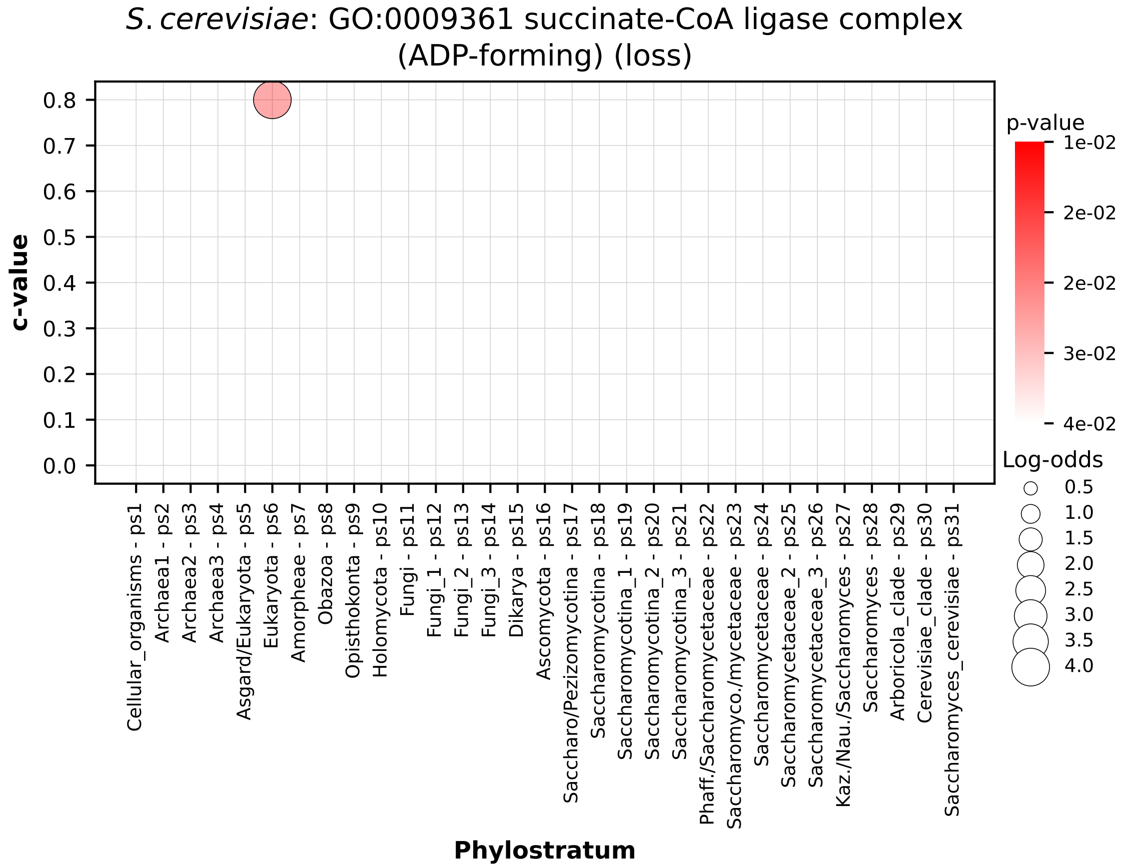
S. cerevisiae: GO:0009361 succinate-CoA ligase complex (ADP-forming) (loss)
c-value
0.0
0.1
0.2
0.3
0.4
0.5
0.6
0.7
0.8
p-value
1e-02
2e-02
2e-02
3e-02
4e-02
Log-odds
0.5
1.0
1.5
2.0
2.5
3.0
3.5
4.0
Cellular_organisms - ps1
Archaea1 - ps2
Archaea2 - ps3
Archaea3 - ps4
Asgard/Eukaryota - ps5
Eukaryota - ps6
Amorpheae - ps7
Obazoa - ps8
Opisthokonta - ps9
Holomycota - ps10
Fungi - ps11
Fungi_1 - ps12
Fungi_2 - ps13
Fungi_3 - ps14
Dikarya - ps15
Ascomycota - ps16
Saccharo/Pezizomycotina - ps17
Saccharomycotina - ps18
Saccharomycotina_1 - ps19
Saccharomycotina_2 - ps20
Saccharomycotina_3 - ps21
Phaff./Saccharomycetaceae - ps22
Saccharomyco./mycetaceae - ps23
Saccharomycetaceae - ps24
Saccharomycetaceae_2 - ps25
Saccharomycetaceae_3 - ps26
Kaz./Nau./Saccharomyces - ps27
Saccharomyces - ps28
Arboricola_clade - ps29
Cerevisiae_clade - ps30
Saccharomyces_cerevisiae - ps31
Phylostratum

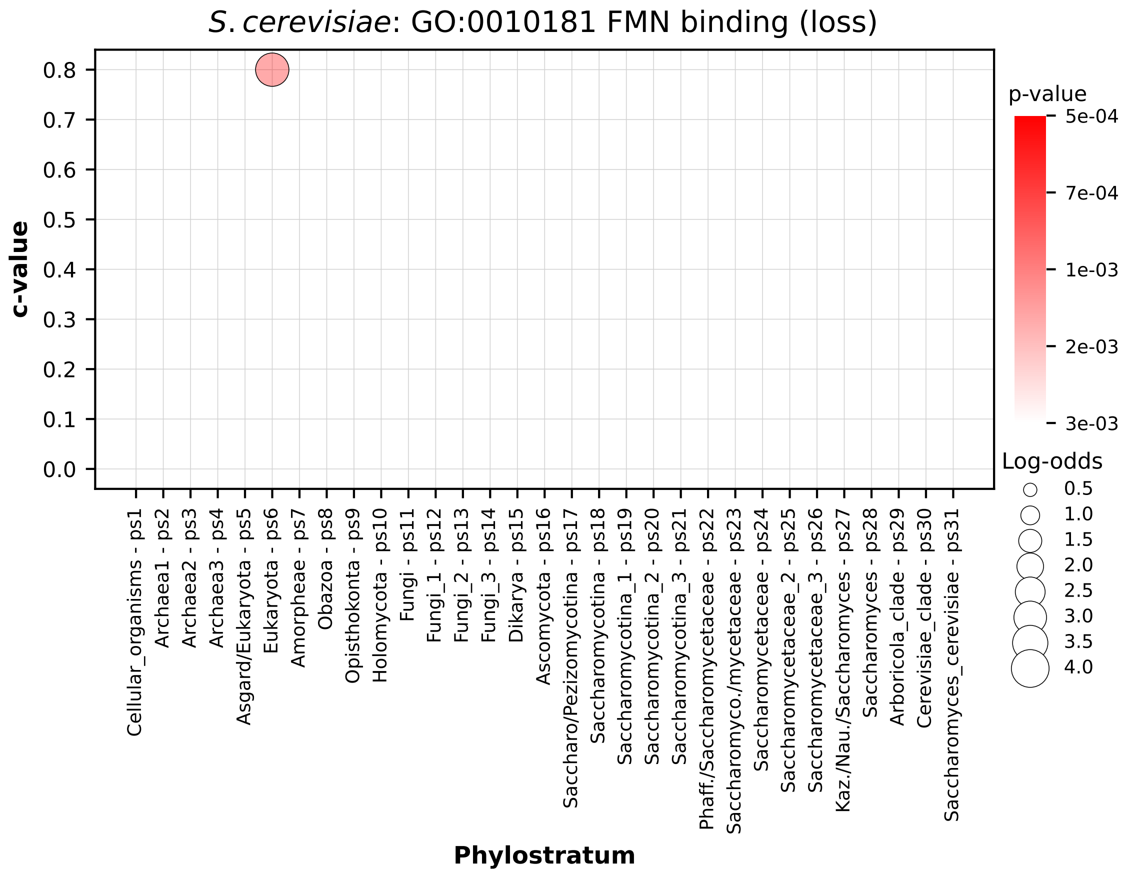
S. cerevisiae: GO:0010181 FMN binding (loss)
c-value
0.0
0.1
0.2
0.3
0.4
0.5
0.6
0.7
0.8
p-value
5e-04
7e-04
1e-03
2e-03
3e-03
Log-odds
0.5
1.0
1.5
2.0
2.5
3.0
3.5
4.0
Cellular_organisms - ps1
Archaea1 - ps2
Archaea2 - ps3
Archaea3 - ps4
Asgard/Eukaryota - ps5
Eukaryota - ps6
Amorpheae - ps7
Obazoa - ps8
Opisthokonta - ps9
Holomycota - ps10
Fungi - ps11
Fungi_1 - ps12
Fungi_2 - ps13
Fungi_3 - ps14
Dikarya - ps15
Ascomycota - ps16
Saccharo/Pezizomycotina - ps17
Saccharomycotina - ps18
Saccharomycotina_1 - ps19
Saccharomycotina_2 - ps20
Saccharomycotina_3 - ps21
Phaff./Saccharomycetaceae - ps22
Saccharomyco./mycetaceae - ps23
Saccharomycetaceae - ps24
Saccharomycetaceae_2 - ps25
Saccharomycetaceae_3 - ps26
Kaz./Nau./Saccharomyces - ps27
Saccharomyces - ps28
Arboricola_clade - ps29
Cerevisiae_clade - ps30
Saccharomyces_cerevisiae - ps31
Phylostratum

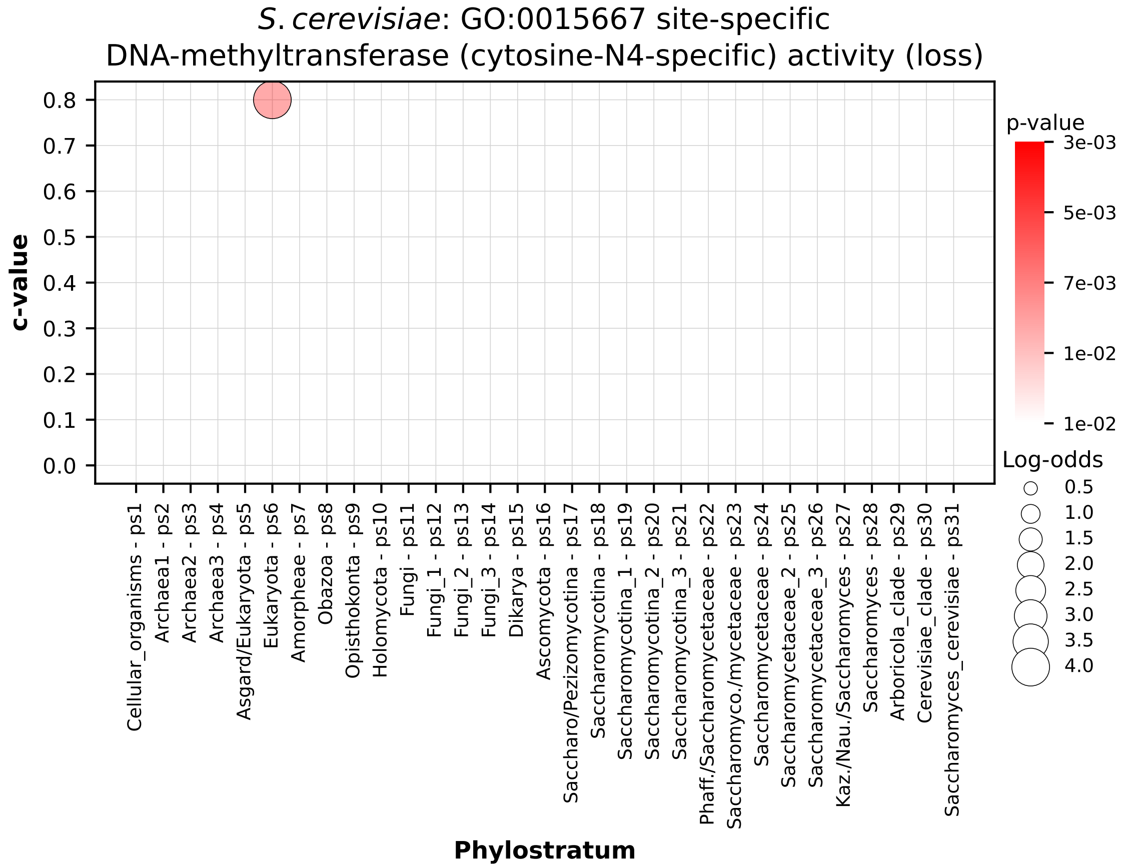

S. cerevisiae: GO:0015667 site-specific
DNA-methyltransferase (cytosine-N4-specific) activity (loss)
c-value
0.8
0.7
0.6
0.5
0.4
0.3
0.2
0.1
0.0
p-value
3e-03
5e-03
7e-03
1e-02
1e-02
Log-odds
0.5
1.0
1.5
2.0
2.5
3.0
3.5
4.0
Cellular_organisms - ps1
Archaea1 - ps2
Archaea2 - ps3
Archaea3 - ps4
Asgard/Eukaryota - ps5
Eukaryota - ps6
Amorpheae - ps7
Obazoa - ps8
Opisthokonta - ps9
Holomycota - ps10
Fungi - ps11
Fungi_1 - ps12
Fungi_2 - ps13
Fungi_3 - ps14
Dikarya - ps15
Ascomycota - ps16
Saccharo/Pezizomycotina - ps17
Saccharomycotina - ps18
Saccharomycotina_1 - ps19
Saccharomycotina_2 - ps20
Saccharomycotina_3 - ps21
Phaff./Saccharomycetaceae - ps22
Saccharomyco./mycetaceae - ps23
Saccharomycetaceae - ps24
Saccharomycetaceae_2 - ps25
Saccharomycetaceae_3 - ps26
Kaz./Nau./Saccharomyces - ps27
Saccharomyces - ps28
Arboricola_clade - ps29
Cerevisiae_clade - ps30
Saccharomyces_cerevisiae - ps31
Phylostratum

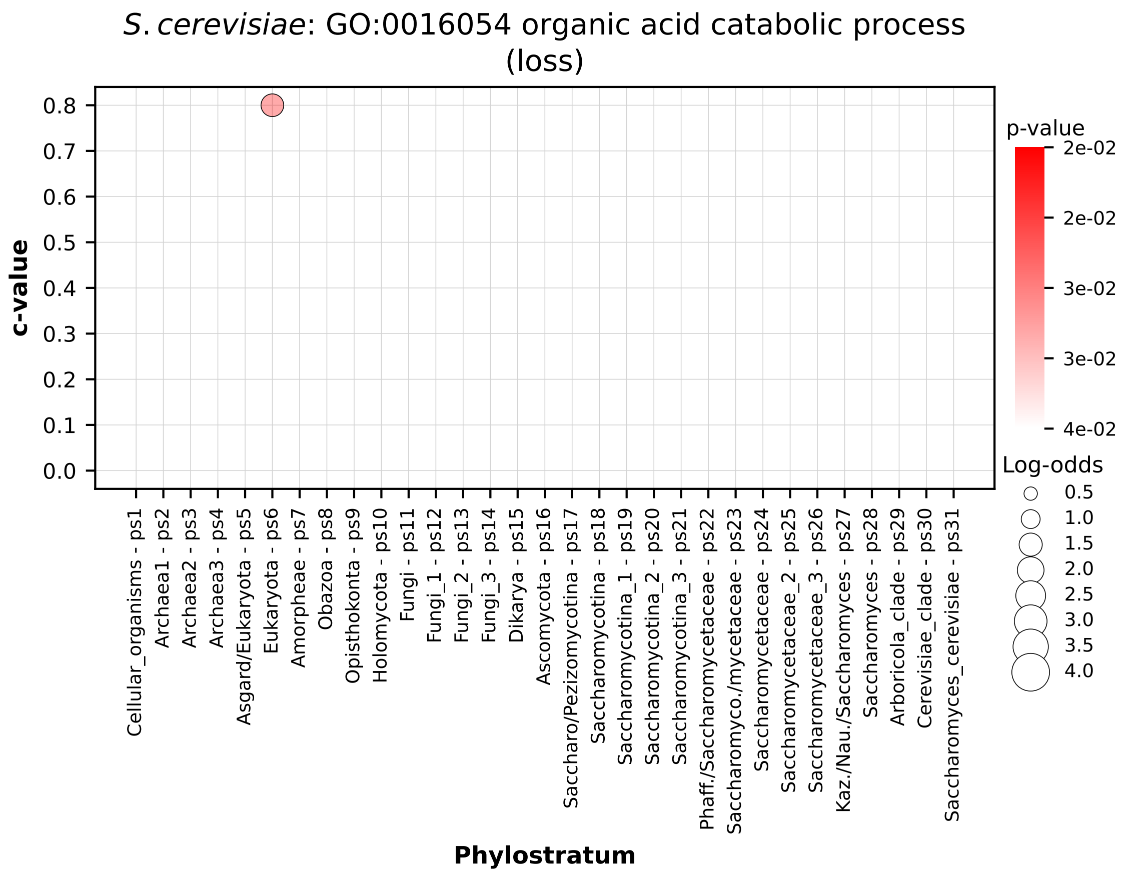
S. cerevisiae: GO:0016054 organic acid catabolic process (loss)
c-value
0.0
0.1
0.2
0.3
0.4
0.5
0.6
0.7
0.8
p-value
2e-02
2e-02
3e-02
3e-02
4e-02
Log-odds
0.5
1.0
1.5
2.0
2.5
3.0
3.5
4.0
Cellular_organisms - ps1
Archaea1 - ps2
Archaea2 - ps3
Archaea3 - ps4
Asgard/Eukaryota - ps5
Eukaryota - ps6
Amorpheae - ps7
Obazoa - ps8
Opisthokonta - ps9
Holomycota - ps10
Fungi - ps11
Fungi_1 - ps12
Fungi_2 - ps13
Fungi_3 - ps14
Dikarya - ps15
Ascomycota - ps16
Saccharo/Pezizomycotina - ps17
Saccharomycotina - ps18
Saccharomycotina_1 - ps19
Saccharomycotina_2 - ps20
Saccharomycotina_3 - ps21
Phaff./Saccharomycetaceae - ps22
Saccharomyco./mycetaceae - ps23
Saccharomycetaceae - ps24
Saccharomycetaceae_2 - ps25
Saccharomycetaceae_3 - ps26
Kaz./Nau./Saccharomyces - ps27
Saccharomyces - ps28
Arboricola_clade - ps29
Cerevisiae_clade - ps30
Saccharomyces_cerevisiae - ps31
Phylostratum

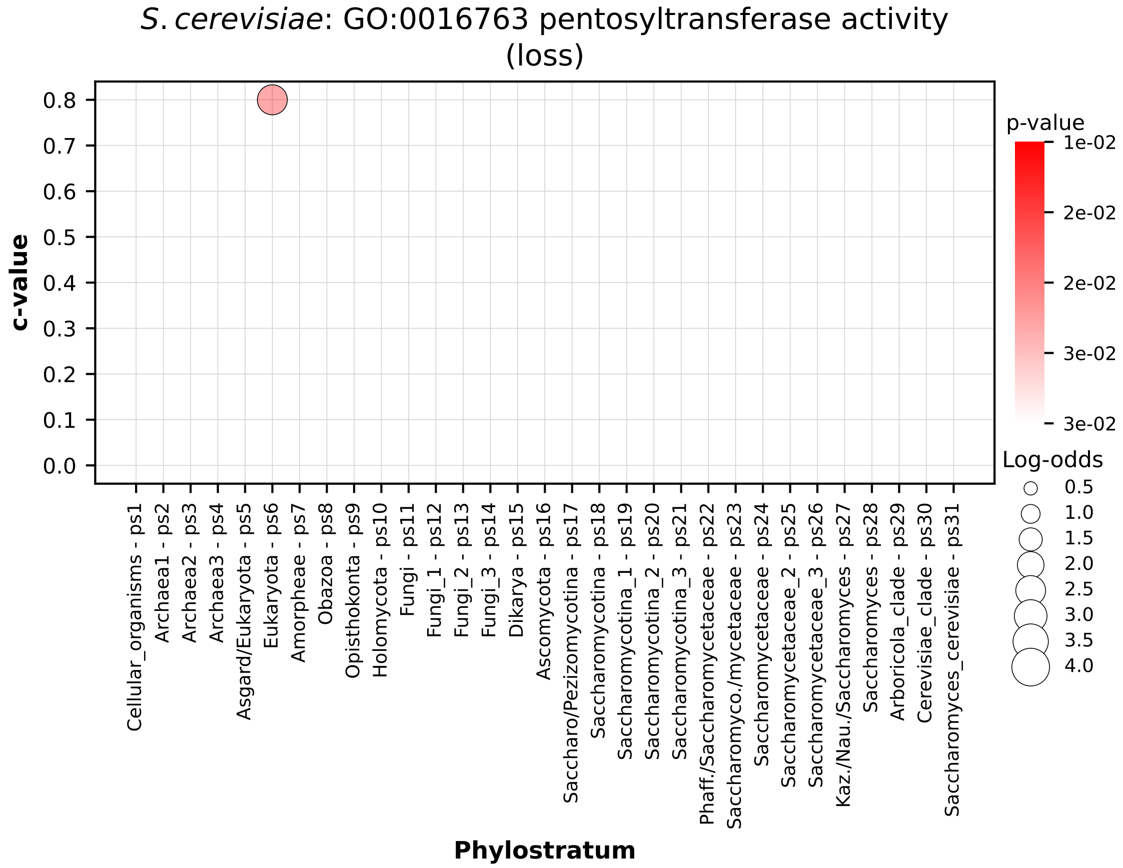

S. cerevisiae: GO:0016763 pentosyltransferase activity
(loss)
c-value
0.8
0.7
0.6
0.5
0.4
0.3
0.2
0.1
0.0
p-value
1e-02
2e-02
2e-02
3e-02
3e-02
Log-odds
0.5
1.0
1.5
2.0
2.5
3.0
3.5
4.0
Cellular_organisms - ps1
Archaea1 - ps2
Archaea2 - ps3
Archaea3 - ps4
Asgard/Eukaryota - ps5
Eukaryota - ps6
Amorpheae - ps7
Obazoa - ps8
Opisthokonta - ps9
Holomycota - ps10
Fungi - ps11
Fungi_1 - ps12
Fungi_2 - ps13
Fungi_3 - ps14
Dikarya - ps15
Ascomycota - ps16
Saccharo/Pezizomycotina - ps17
Saccharomycotina - ps18
Saccharomycotina_1 - ps19
Saccharomycotina_2 - ps20
Saccharomycotina_3 - ps21
Phaff./Saccharomycetaceae - ps22
Saccharomyco./mycetaceae - ps23
Saccharomycetaceae - ps24
Saccharomycetaceae_2 - ps25
Saccharomycetaceae_3 - ps26
Kaz./Nau./Saccharomyces - ps27
Saccharomyces - ps28
Arboricola_clade - ps29
Cerevisiae_clade - ps30
Saccharomyces_cerevisiae - ps31
Phylostratum

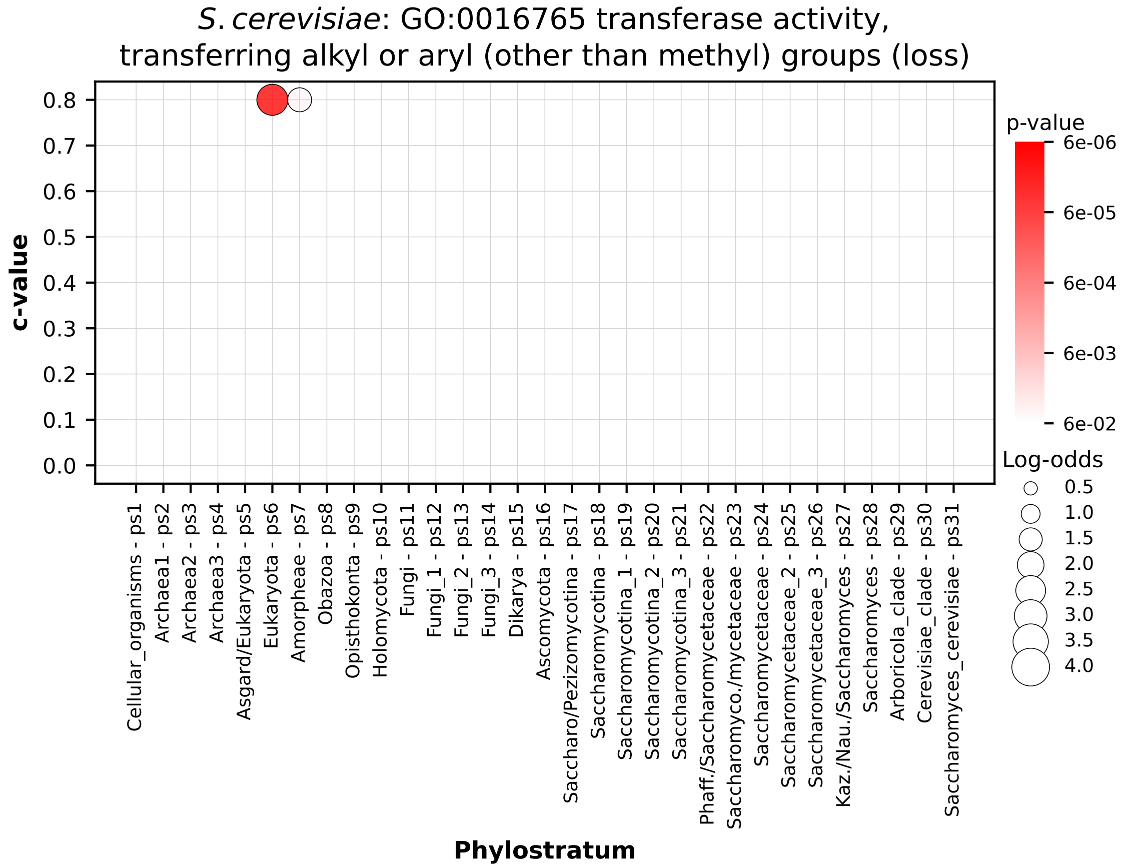

S. cerevisiae: GO:0016765 transferase activity, transferring alkyl or aryl (other than methyl) groups (loss)
c-value
0.0
0.1
0.2
0.3
0.4
0.5
0.6
0.7
0.8
p-value
6e-06
6e-05
6e-04
6e-03
6e-02
Log-odds
0.5
1.0
1.5
2.0
2.5
3.0
3.5
4.0
Cellular_organisms - ps1
Archaea1 - ps2
Archaea2 - ps3
Archaea3 - ps4
Asgard/Eukaryota - ps5
Eukaryota - ps6
Amorpheae - ps7
Obazoa - ps8
Opisthokonta - ps9
Holomycota - ps10
Fungi - ps11
Fungi_1 - ps12
Fungi_2 - ps13
Fungi_3 - ps14
Dikarya - ps15
Ascomycota - ps16
Saccharo/Pezizomycotina - ps17
Saccharomycotina - ps18
Saccharomycotina_1 - ps19
Saccharomycotina_2 - ps20
Saccharomycotina_3 - ps21
Phaff./Saccharomycetaceae - ps22
Saccharomyco./mycetaceae - ps23
Saccharomycetaceae - ps24
Saccharomycetaceae_2 - ps25
Saccharomycetaceae_3 - ps26
Kaz./Nau./Saccharomyces - ps27
Saccharomyces - ps28
Arboricola_clade - ps29
Cerevisiae_clade - ps30
Saccharomyces_cerevisiae - ps31
Phylostratum

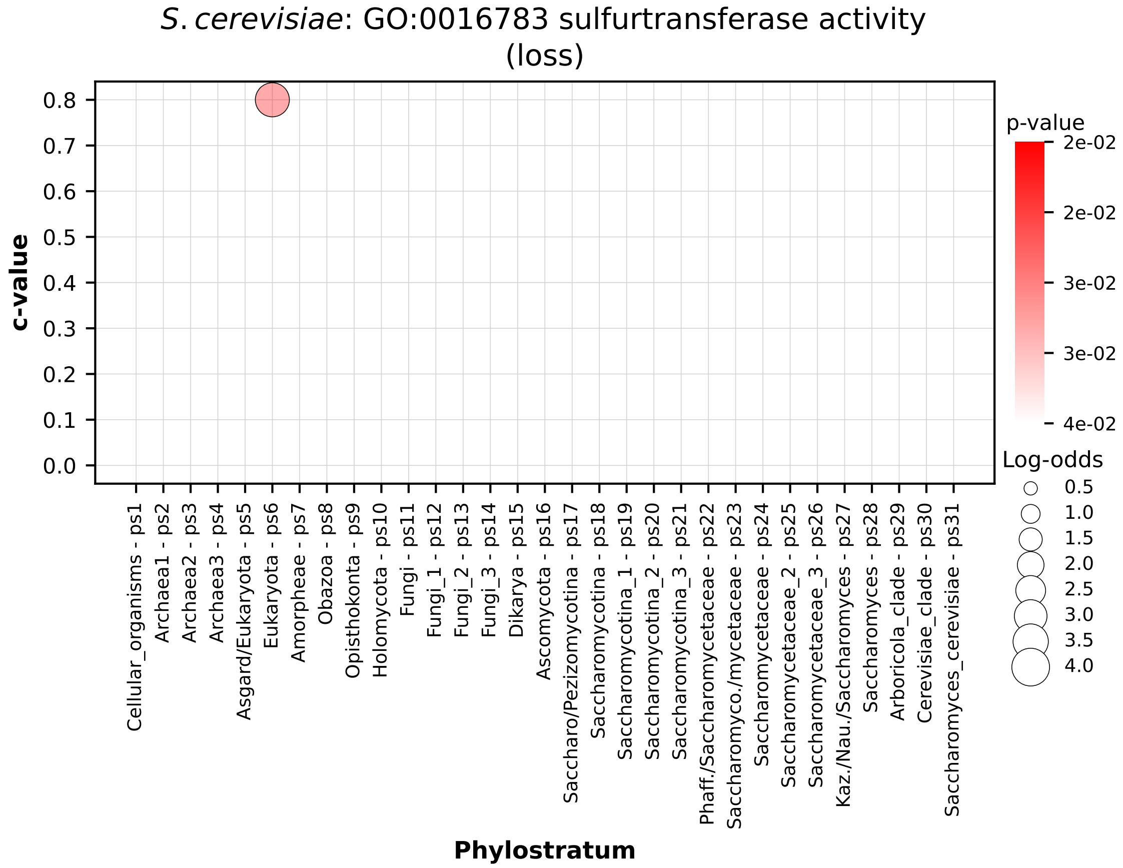

S. cerevisiae: GO:0016783 sulfurtransferase activity
(loss)
c-value
0.8
0.7
0.6
0.5
0.4
0.3
0.2
0.1
0.0
p-value
2e-02
2e-02
3e-02
3e-02
4e-02
Log-odds
0.5
1.0
1.5
2.0
2.5
3.0
3.5
4.0
Cellular_organisms - ps1
Archaea1 - ps2
Archaea2 - ps3
Archaea3 - ps4
Asgard/Eukaryota - ps5
Eukaryota - ps6
Amorpheae - ps7
Obazoa - ps8
Opisthokonta - ps9
Holomycota - ps10
Fungi - ps11
Fungi_1 - ps12
Fungi_2 - ps13
Fungi_3 - ps14
Dikarya - ps15
Ascomycota - ps16
Saccharo/Pezizomycotina - ps17
Saccharomycotina - ps18
Saccharomycotina_1 - ps19
Saccharomycotina_2 - ps20
Saccharomycotina_3 - ps21
Phaff./Saccharomycetaceae - ps22
Saccharomyco./mycetaceae - ps23
Saccharomycetaceae - ps24
Saccharomycetaceae_2 - ps25
Saccharomycetaceae_3 - ps26
Kaz./Nau./Saccharomyces - ps27
Saccharomyces - ps28
Arboricola_clade - ps29
Cerevisiae_clade - ps30
Saccharomyces_cerevisiae - ps31
Phylostratum

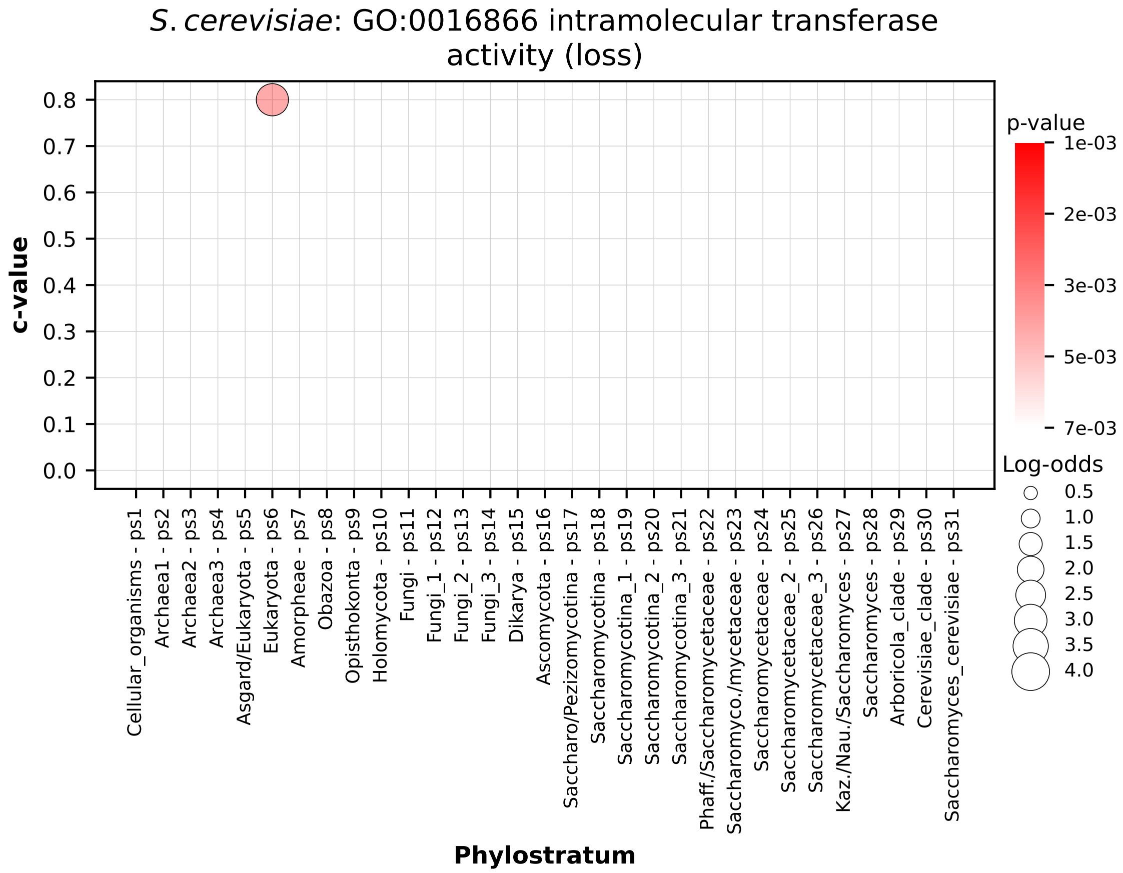

S. cerevisiae: GO:0016866 intramolecular transferase activity (loss)
c-value
0.0
0.1
0.2
0.3
0.4
0.5
0.6
0.7
0.8
p-value
1e-03
2e-03
3e-03
5e-03
7e-03
Log-odds
0.5
1.0
1.5
2.0
2.5
3.0
3.5
4.0
Cellular_organisms - ps1
Archaea1 - ps2
Archaea2 - ps3
Archaea3 - ps4
Asgard/Eukaryota - ps5
Eukaryota - ps6
Amorpheae - ps7
Obazoa - ps8
Opisthokonta - ps9
Holomycota - ps10
Fungi - ps11
Fungi_1 - ps12
Fungi_2 - ps13
Fungi_3 - ps14
Dikarya - ps15
Ascomycota - ps16
Saccharo/Pezizomycotina - ps17
Saccharomycotina - ps18
Saccharomycotina_1 - ps19
Saccharomycotina_2 - ps20
Saccharomycotina_3 - ps21
Phaff./Saccharomycetaceae - ps22
Saccharomyco./mycetaceae - ps23
Saccharomycetaceae - ps24
Saccharomycetaceae_2 - ps25
Saccharomycetaceae_3 - ps26
Kaz./Nau./Saccharomyces - ps27
Saccharomyces - ps28
Arboricola_clade - ps29
Cerevisiae_clade - ps30
Saccharomyces_cerevisiae - ps31
Phylostratum

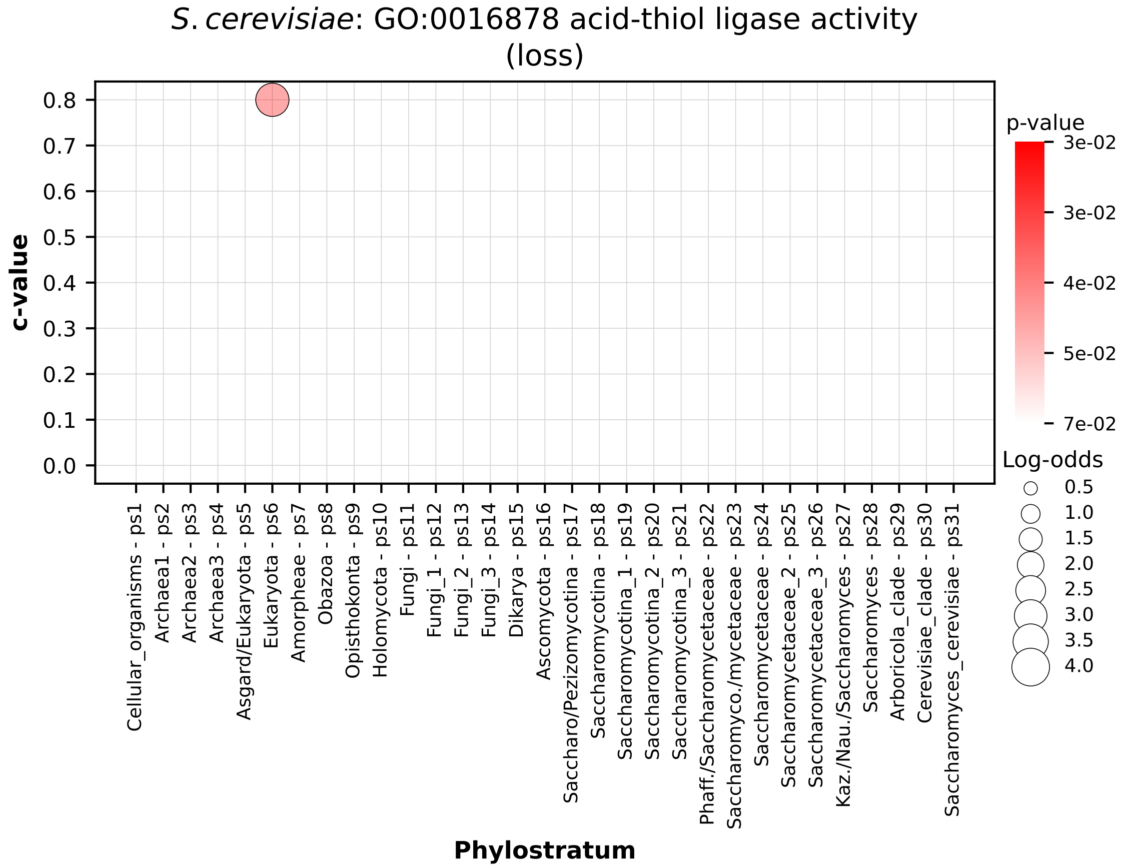

S. cerevisiae: GO:0016878 acid-thiol ligase activity
(loss)
c-value
0.0
0.1
0.2
0.3
0.4
0.5
0.6
0.7
0.8
p-value
3e-02
3e-02
4e-02
5e-02
7e-02
Log-odds
0.5
1.0
1.5
2.0
2.5
3.0
3.5
4.0
Cellular_organisms - ps1
Archaea1 - ps2
Archaea2 - ps3
Archaea3 - ps4
Asgard/Eukaryota - ps5
Eukaryota - ps6
Amorpheae - ps7
Obazoa - ps8
Opisthokonta - ps9
Holomycota - ps10
Fungi - ps11
Fungi_1 - ps12
Fungi_2 - ps13
Fungi_3 - ps14
Dikarya - ps15
Ascomycota - ps16
Saccharo/Pezizomycotina - ps17
Saccharomycotina - ps18
Saccharomycotina_1 - ps19
Saccharomycotina_2 - ps20
Saccharomycotina_3 - ps21
Phaff./Saccharomycetaceae - ps22
Saccharomyco./mycetaceae - ps23
Saccharomycetaceae - ps24
Saccharomycetaceae_2 - ps25
Saccharomycetaceae_3 - ps26
Kaz./Nau./Saccharomyces - ps27
Saccharomyces - ps28
Arboricola_clade - ps29
Cerevisiae_clade - ps30
Saccharomyces_cerevisiae - ps31
Phylostratum

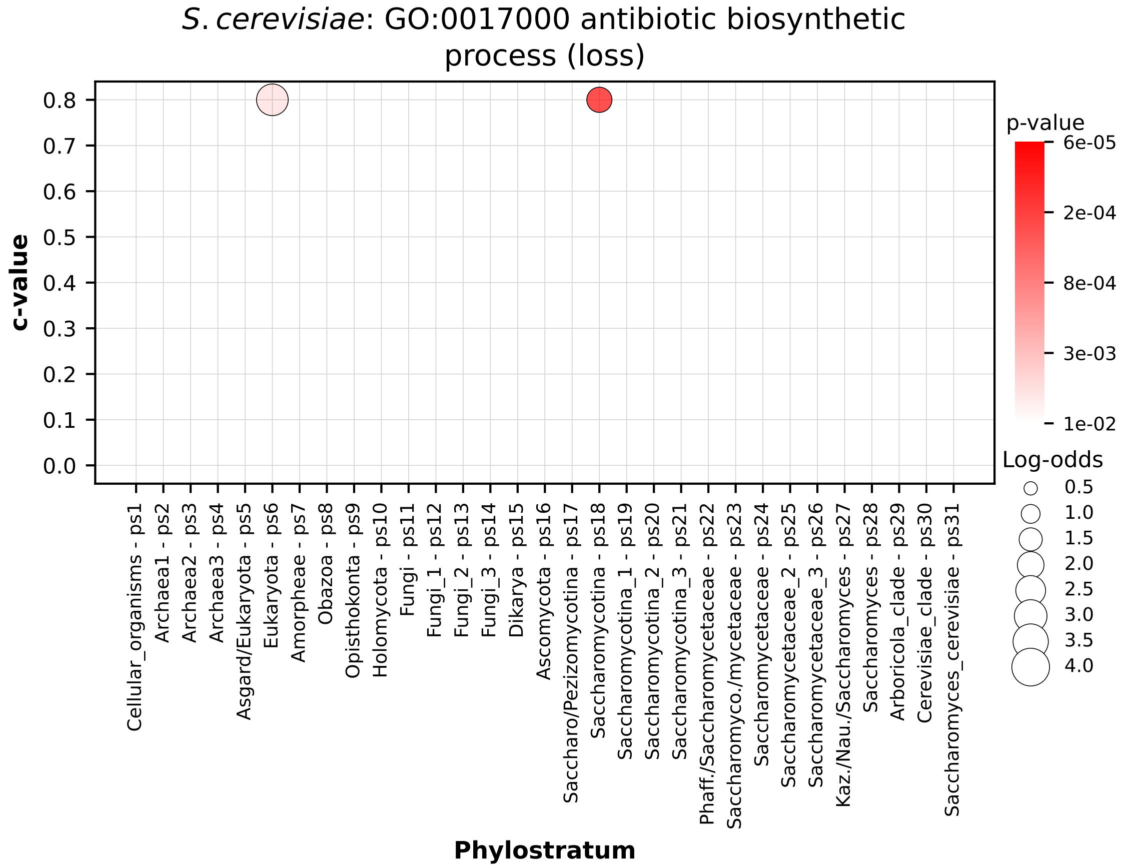

S. cerevisiae: GO:0017000 antibiotic biosynthetic process (loss)
c-value
0.8
0.7
0.6
0.5
0.4
0.3
0.2
0.1
0.0
p-value
6e-05
2e-04
8e-04
3e-03
1e-02
Log-odds
0.5
1.0
1.5
2.0
2.5
3.0
3.5
4.0
Cellular_organisms - ps1
Archaea1 - ps2
Archaea2 - ps3
Archaea3 - ps4
Asgard/Eukaryota - ps5
Eukaryota - ps6
Amorpheae - ps7
Obazoa - ps8
Opisthokonta - ps9
Holomycota - ps10
Fungi - ps11
Fungi_1 - ps12
Fungi_2 - ps13
Fungi_3 - ps14
Dikarya - ps15
Ascomycota - ps16
Saccharo/Pezizomycotina - ps17
Saccharomycotina - ps18
Saccharomycotina_1 - ps19
Saccharomycotina_2 - ps20
Saccharomycotina_3 - ps21
Phaff./Saccharomycetaceae - ps22
Saccharomyco./mycetaceae - ps23
Saccharomycetaceae - ps24
Saccharomycetaceae_2 - ps25
Saccharomycetaceae_3 - ps26
Kaz./Nau./Saccharomyces - ps27
Saccharomyces - ps28
Arboricola_clade - ps29
Cerevisiae_clade - ps30
Saccharomyces_cerevisiae - ps31
Phylostratum

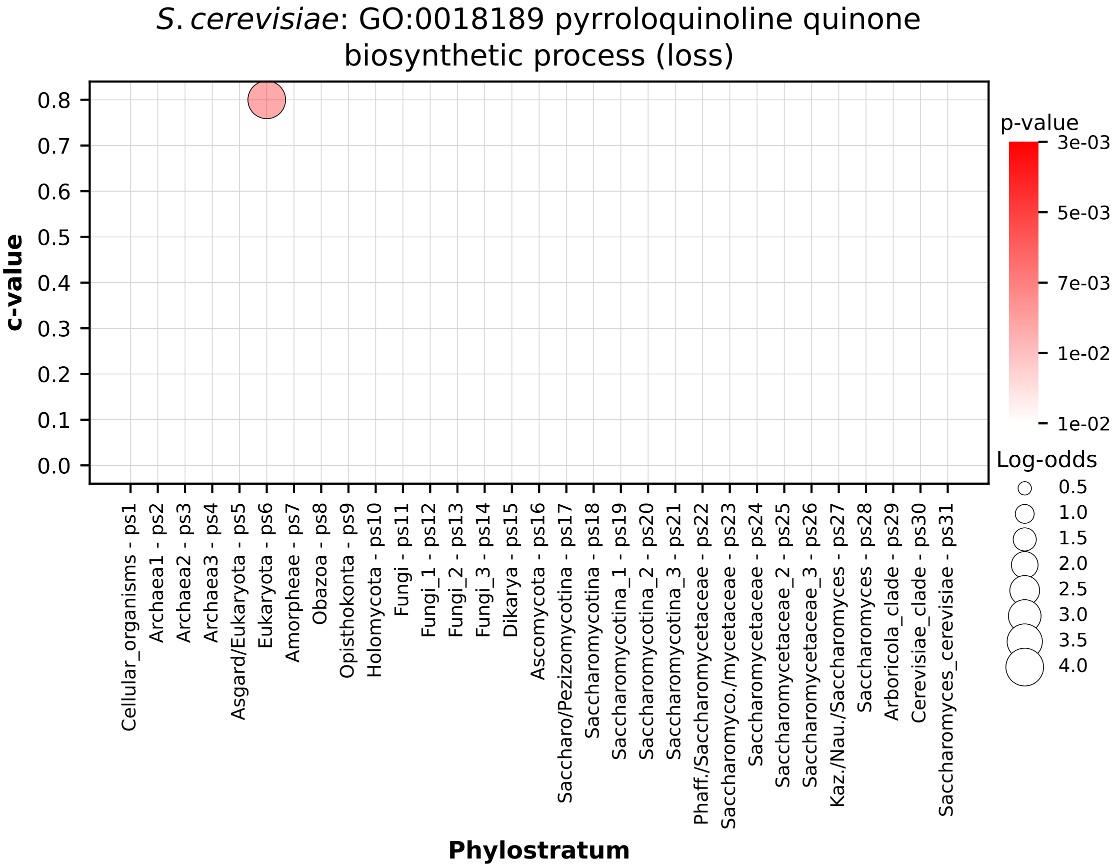

S. cerevisiae: GO:0018189 pyrroloquinoline quinone biosynthetic process (loss)
c-value
0.0
0.1
0.2
0.3
0.4
0.5
0.6
0.7
0.8
p-value
3e-03
5e-03
7e-03
1e-02
1e-02
Log-odds
0.5
1.0
1.5
2.0
2.5
3.0
3.5
4.0
Cellular_organisms - ps1
Archaea1 - ps2
Archaea2 - ps3
Archaea3 - ps4
Asgard/Eukaryota - ps5
Eukaryota - ps6
Amorpheae - ps7
Obazoa - ps8
Opisthokonta - ps9
Holomycota - ps10
Fungi - ps11
Fungi_1 - ps12
Fungi_2 - ps13
Fungi_3 - ps14
Dikarya - ps15
Ascomycota - ps16
Saccharo/Pezizomycotina - ps17
Saccharomycotina - ps18
Saccharomycotina_1 - ps19
Saccharomycotina_2 - ps20
Saccharomycotina_3 - ps21
Phaff./Saccharomycetaceae - ps22
Saccharomyco./mycetaceae - ps23
Saccharomycetaceae - ps24
Saccharomycetaceae_2 - ps25
Saccharomycetaceae_3 - ps26
Kaz./Nau./Saccharomyces - ps27
Saccharomyces - ps28
Arboricola_clade - ps29
Cerevisiae_clade - ps30
Saccharomyces_cerevisiae - ps31
Phylostratum

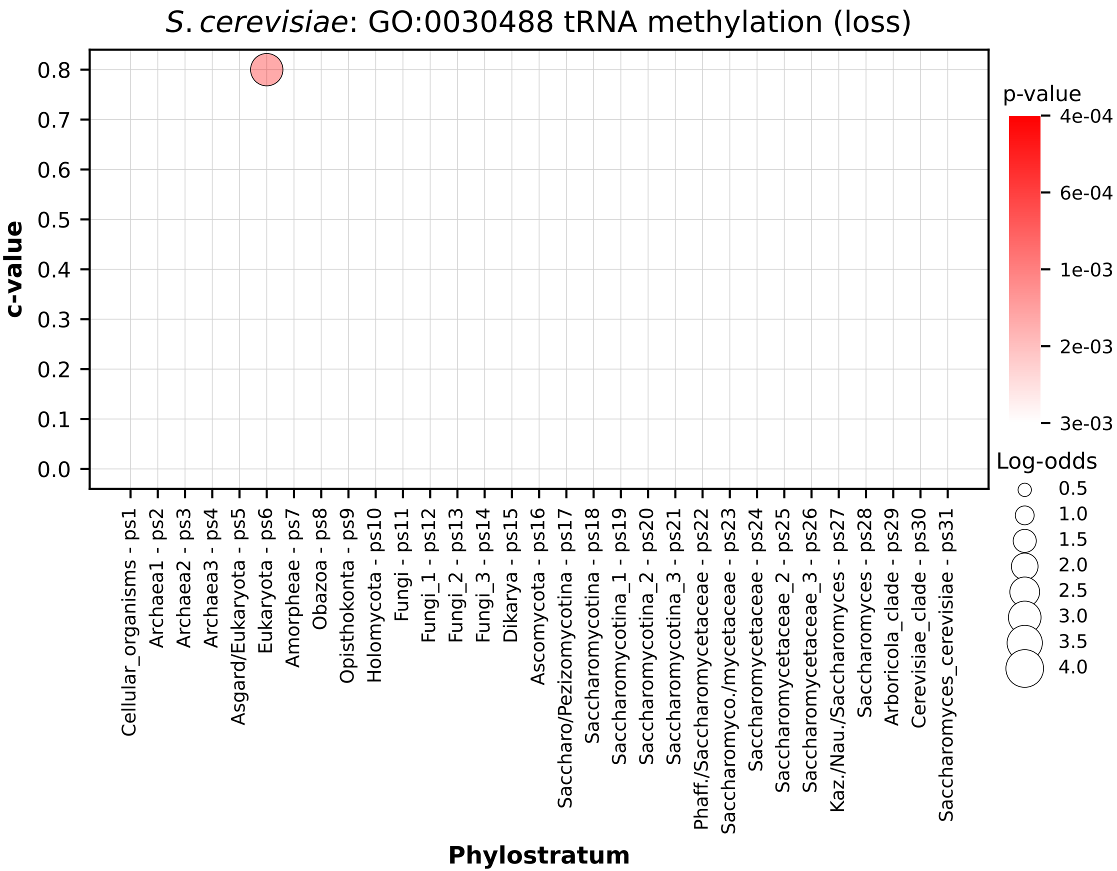

S. cerevisiae: GO:0030488 tRNA methylation (loss)
c-value
0.8
0.7
0.6
0.5
0.4
0.3
0.2
0.1
0.0
p-value
4e-04
6e-04
1e-03
2e-03
3e-03
Log-odds
0.5
1.0
1.5
2.0
2.5
3.0
3.5
4.0
Cellular_organisms - ps1
Archaea1 - ps2
Archaea2 - ps3
Archaea3 - ps4
Asgard/Eukaryota - ps5
Eukaryota - ps6
Amorpheae - ps7
Obazoa - ps8
Opisthokonta - ps9
Holomycota - ps10
Fungi - ps11
Fungi_1 - ps12
Fungi_2 - ps13
Fungi_3 - ps14
Dikarya - ps15
Ascomycota - ps16
Saccharo/Pezizomycotina - ps17
Saccharomycotina - ps18
Saccharomycotina_1 - ps19
Saccharomycotina_2 - ps20
Saccharomycotina_3 - ps21
Phaff./Saccharomycetaceae - ps22
Saccharomyco./mycetaceae - ps23
Saccharomycetaceae - ps24
Saccharomycetaceae_2 - ps25
Saccharomycetaceae_3 - ps26
Kaz./Nau./Saccharomyces - ps27
Saccharomyces - ps28
Arboricola_clade - ps29
Cerevisiae_clade - ps30
Saccharomyces_cerevisiae - ps31
Phylostratum

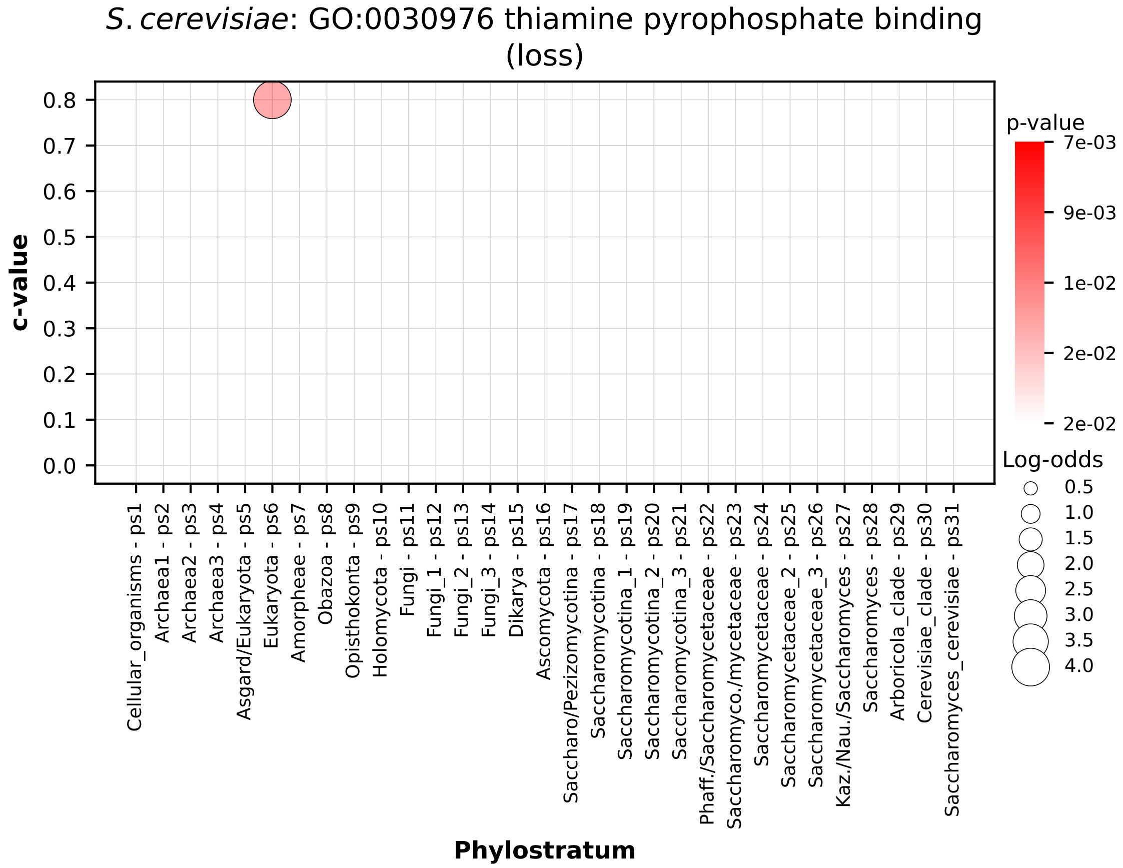

S. cerevisiae: GO:0030976 thiamine pyrophosphate binding (loss)
c-value
0.8
0.7
0.6
0.5
0.4
0.3
0.2
0.1
0.0
p-value
7e-03
9e-03
1e-02
2e-02
2e-02
Log-odds
0.5
1.0
1.5
2.0
2.5
3.0
3.5
4.0
Cellular_organisms - ps1
Archaea1 - ps2
Archaea2 - ps3
Archaea3 - ps4
Asgard/Eukaryota - ps5
Eukaryota - ps6
Amorpheae - ps7
Obazoa - ps8
Opisthokonta - ps9
Holomycota - ps10
Fungi - ps11
Fungi_1 - ps12
Fungi_2 - ps13
Fungi_3 - ps14
Dikarya - ps15
Ascomycota - ps16
Saccharo/Pezizomycotina - ps17
Saccharomycotina - ps18
Saccharomycotina_1 - ps19
Saccharomycotina_2 - ps20
Saccharomycotina_3 - ps21
Phaff./Saccharomycetaceae - ps22
Saccharomyco./mycetaceae - ps23
Saccharomycetaceae - ps24
Saccharomycetaceae_2 - ps25
Saccharomycetaceae_3 - ps26
Kaz./Nau./Saccharomyces - ps27
Saccharomyces - ps28
Arboricola_clade - ps29
Cerevisiae_clade - ps30
Saccharomyces_cerevisiae - ps31
Phylostratum

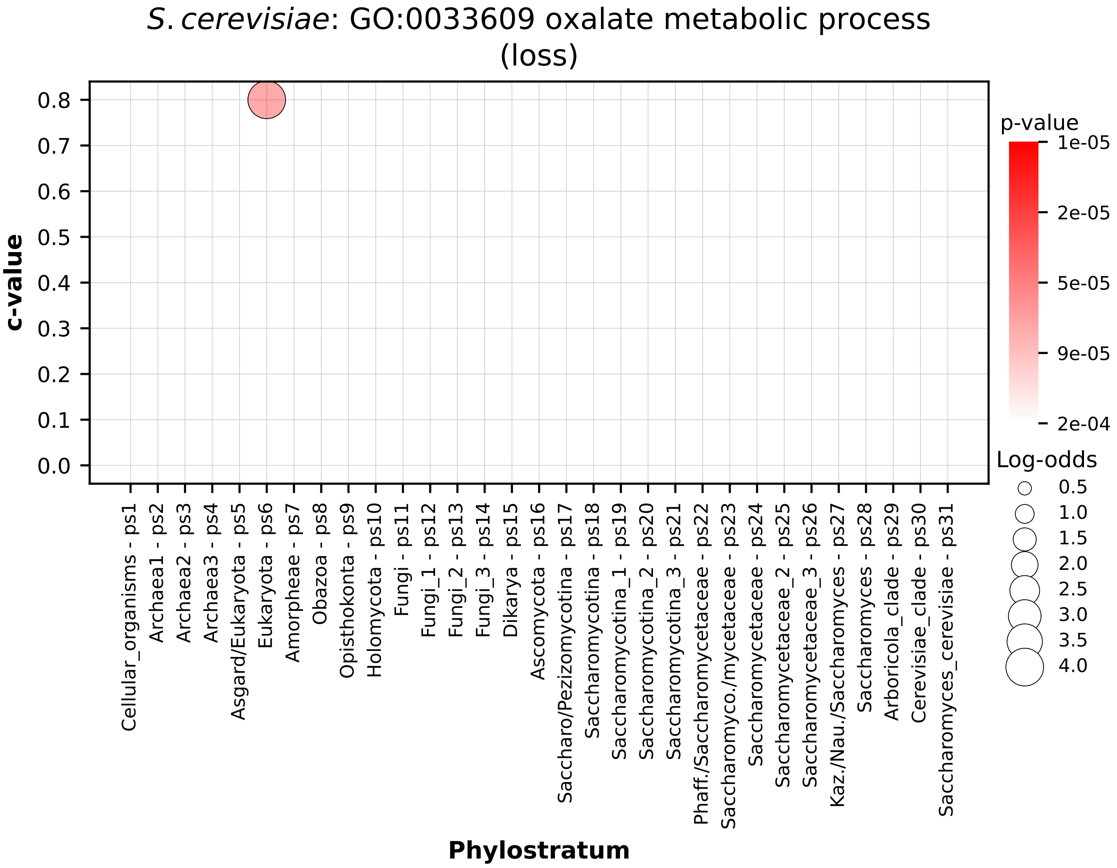

S. cerevisiae: GO:0033609 oxalate metabolic process
(loss)
c-value
0.0
0.1
0.2
0.3
0.4
0.5
0.6
0.7
0.8
p-value
1e-05
2e-05
5e-05
9e-05
2e-04
Log-odds
0.5
1.0
1.5
2.0
2.5
3.0
3.5
4.0
Cellular_organisms - ps1
Archaea1 - ps2
Archaea2 - ps3
Archaea3 - ps4
Asgard/Eukaryota - ps5
Eukaryota - ps6
Amorpheae - ps7
Obazoa - ps8
Opisthokonta - ps9
Holomycota - ps10
Fungi - ps11
Fungi_1 - ps12
Fungi_2 - ps13
Fungi_3 - ps14
Dikarya - ps15
Ascomycota - ps16
Saccharo/Pezizomycotina - ps17
Saccharomycotina - ps18
Saccharomycotina_1 - ps19
Saccharomycotina_2 - ps20
Saccharomycotina_3 - ps21
Phaff./Saccharomycetaceae - ps22
Saccharomyco./mycetaceae - ps23
Saccharomycetaceae - ps24
Saccharomycetaceae_2 - ps25
Saccharomycetaceae_3 - ps26
Kaz./Nau./Saccharomyces - ps27
Saccharomyces - ps28
Arboricola_clade - ps29
Cerevisiae_clade - ps30
Saccharomyces_cerevisiae - ps31
Phylostratum

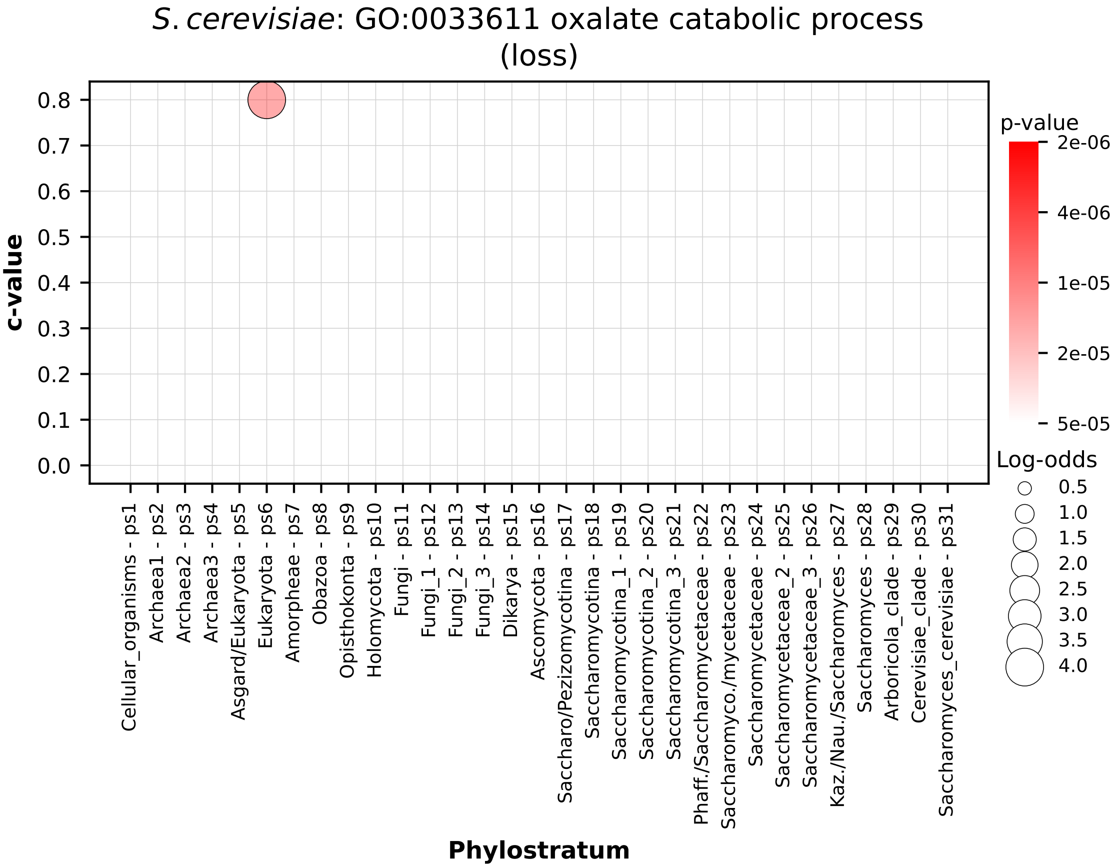

S. cerevisiae: GO:0033611 oxalate catabolic process
(loss)
c-value
0.0
0.1
0.2
0.3
0.4
0.5
0.6
0.7
0.8
p-value
2e-06
4e-06
1e-05
2e-05
5e-05
Log-odds
0.5
1.0
1.5
2.0
2.5
3.0
3.5
4.0
Cellular_organisms - ps1
Archaea1 - ps2
Archaea2 - ps3
Archaea3 - ps4
Asgard/Eukaryota - ps5
Eukaryota - ps6
Amorpheae - ps7
Obazoa - ps8
Opisthokonta - ps9
Holomycota - ps10
Fungi - ps11
Fungi_1 - ps12
Fungi_2 - ps13
Fungi_3 - ps14
Dikarya - ps15
Ascomycota - ps16
Saccharo/Pezizomycotina - ps17
Saccharomycotina - ps18
Saccharomycotina_1 - ps19
Saccharomycotina_2 - ps20
Saccharomycotina_3 - ps21
Phaff./Saccharomycetaceae - ps22
Saccharomyco./mycetaceae - ps23
Saccharomycetaceae - ps24
Saccharomycetaceae_2 - ps25
Saccharomycetaceae_3 - ps26
Kaz./Nau./Saccharomyces - ps27
Saccharomyces - ps28
Arboricola_clade - ps29
Cerevisiae_clade - ps30
Saccharomyces_cerevisiae - ps31
Phylostratum

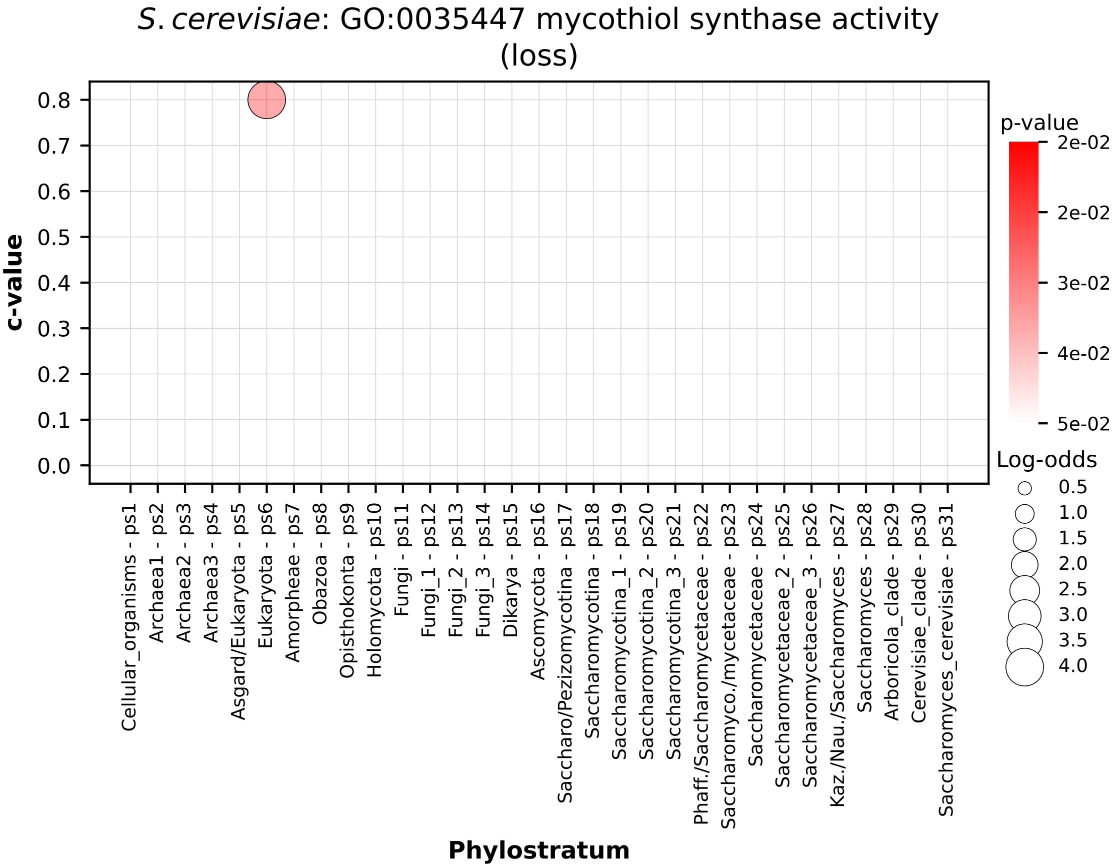

S. cerevisiae: GO:0035447 mycothiol synthase activity
(loss)
c-value
0.0
0.1
0.2
0.3
0.4
0.5
0.6
0.7
0.8
p-value
2e-02
2e-02
3e-02
4e-02
5e-02
Log-odds
0.5
1.0
1.5
2.0
2.5
3.0
3.5
4.0
Cellular_organisms - ps1
Archaea1 - ps2
Archaea2 - ps3
Archaea3 - ps4
Asgard/Eukaryota - ps5
Eukaryota - ps6
Amorpheae - ps7
Obazoa - ps8
Opisthokonta - ps9
Holomycota - ps10
Fungi - ps11
Fungi_1 - ps12
Fungi_2 - ps13
Fungi_3 - ps14
Dikarya - ps15
Ascomycota - ps16
Saccharo/Pezizomycotina - ps17
Saccharomycotina - ps18
Saccharomycotina_1 - ps19
Saccharomycotina_2 - ps20
Saccharomycotina_3 - ps21
Phaff./Saccharomycetaceae - ps22
Saccharomyco./mycetaceae - ps23
Saccharomycetaceae - ps24
Saccharomycetaceae_2 - ps25
Saccharomycetaceae_3 - ps26
Kaz./Nau./Saccharomyces - ps27
Saccharomyces - ps28
Arboricola_clade - ps29
Cerevisiae_clade - ps30
Saccharomyces_cerevisiae - ps31
Phylostratum

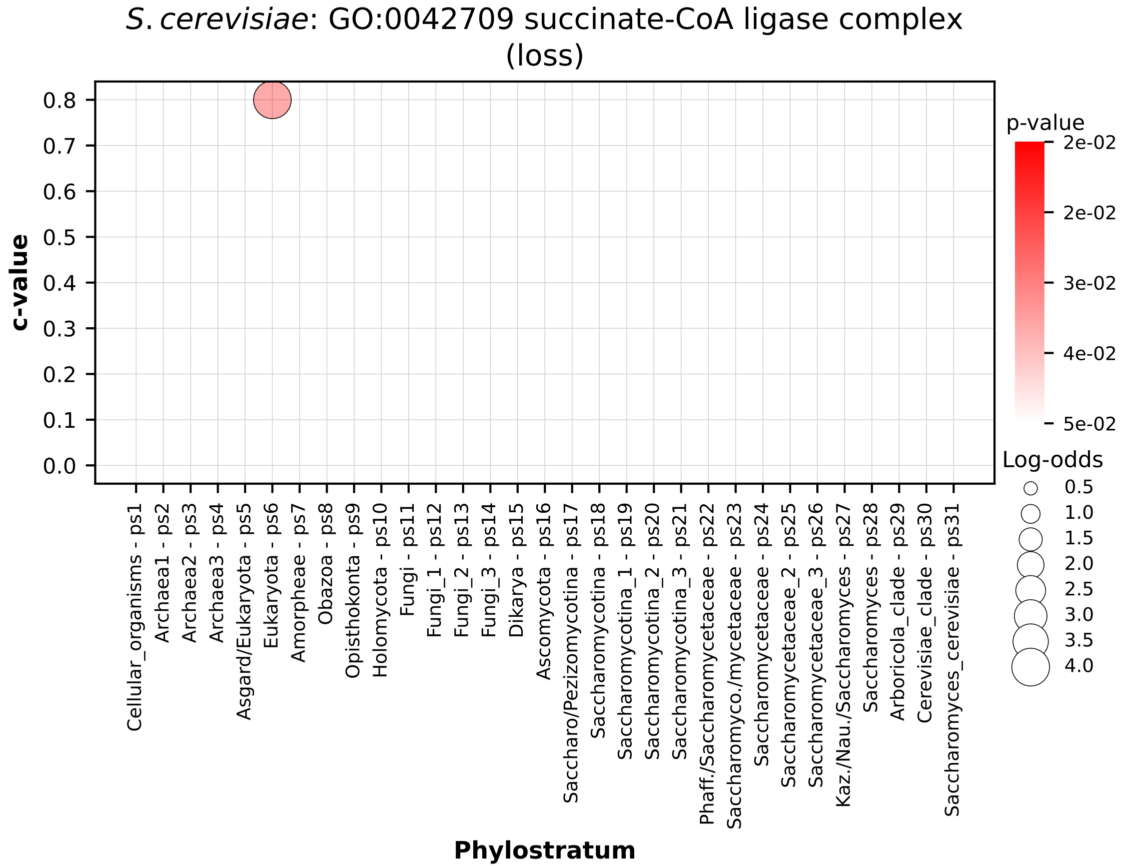

S. cerevisiae: GO:0042709 succinate-CoA ligase complex (loss)
c-value
0.8
0.7
0.6
0.5
0.4
0.3
0.2
0.1
0.0
p-value
2e-02
2e-02
3e-02
4e-02
5e-02
Log-odds
0.5
1.0
1.5
2.0
2.5
3.0
3.5
4.0
Cellular_organisms - ps1
Archaea1 - ps2
Archaea2 - ps3
Archaea3 - ps4
Asgard/Eukaryota - ps5
Eukaryota - ps6
Amorpheae - ps7
Obazoa - ps8
Opisthokonta - ps9
Holomycota - ps10
Fungi - ps11
Fungi_1 - ps12
Fungi_2 - ps13
Fungi_3 - ps14
Dikarya - ps15
Ascomycota - ps16
Saccharo/Pezizomycotina - ps17
Saccharomycotina - ps18
Saccharomycotina_1 - ps19
Saccharomycotina_2 - ps20
Saccharomycotina_3 - ps21
Phaff./Saccharomycetaceae - ps22
Saccharomyco./mycetaceae - ps23
Saccharomycetaceae - ps24
Saccharomycetaceae_2 - ps25
Saccharomycetaceae_3 - ps26
Kaz./Nau./Saccharomyces - ps27
Saccharomyces - ps28
Arboricola_clade - ps29
Cerevisiae_clade - ps30
Saccharomyces_cerevisiae - ps31
Phylostratum

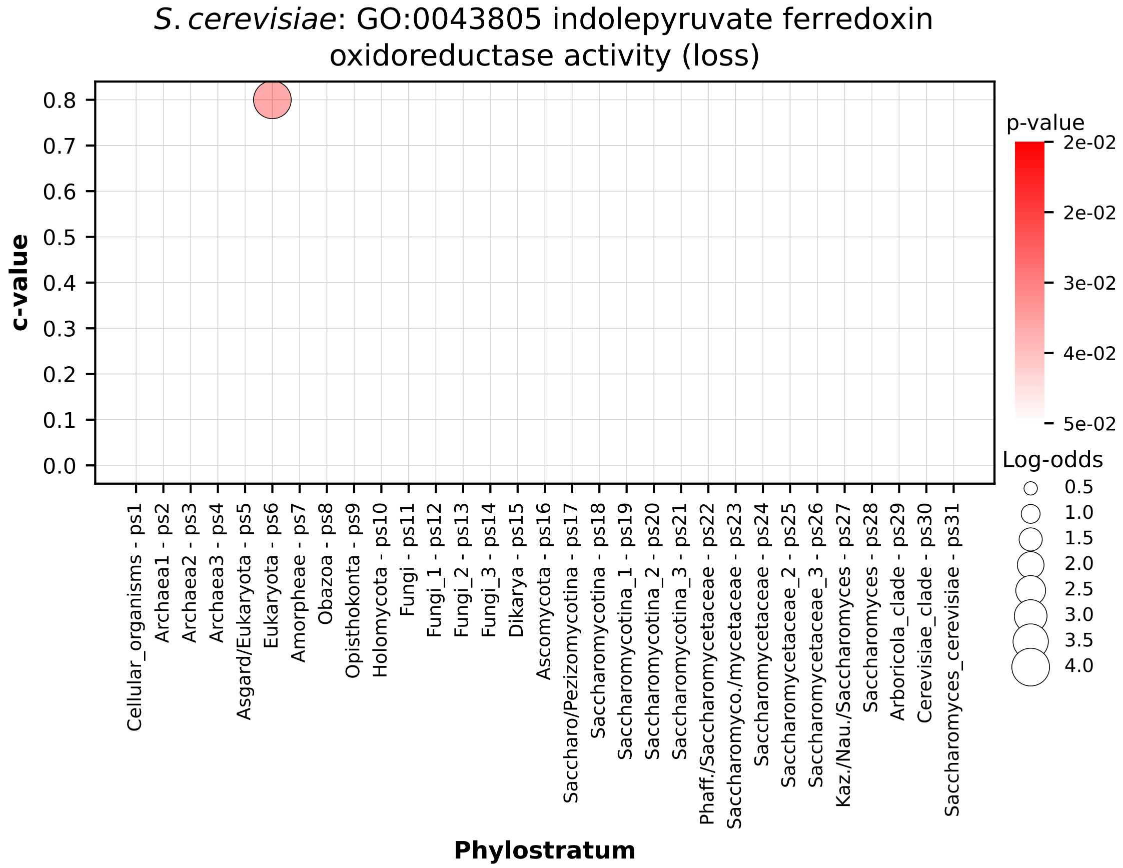

S. cerevisiae: GO:0043805 indolepyruvate ferredoxin oxidoreductase activity (loss)
c-value
0.8
0.7
0.6
0.5
0.4
0.3
0.2
0.1
0.0
p-value
2e-02
2e-02
3e-02
4e-02
5e-02
Log-odds
0.5
1.0
1.5
2.0
2.5
3.0
3.5
4.0
Cellular_organisms - ps1
Archaea1 - ps2
Archaea2 - ps3
Archaea3 - ps4
Asgard/Eukaryota - ps5
Eukaryota - ps6
Amorpheae - ps7
Obazoa - ps8
Opisthokonta - ps9
Holomycota - ps10
Fungi - ps11
Fungi_1 - ps12
Fungi_2 - ps13
Fungi_3 - ps14
Dikarya - ps15
Ascomycota - ps16
Saccharo/Pezizomycotina - ps17
Saccharomycotina - ps18
Saccharomycotina_1 - ps19
Saccharomycotina_2 - ps20
Saccharomycotina_3 - ps21
Phaff./Saccharomycetaceae - ps22
Saccharomyco./mycetaceae - ps23
Saccharomycetaceae - ps24
Saccharomycetaceae_2 - ps25
Saccharomycetaceae_3 - ps26
Kaz./Nau./Saccharomyces - ps27
Saccharomyces - ps28
Arboricola_clade - ps29
Cerevisiae_clade - ps30
Saccharomyces_cerevisiae - ps31
Phylostratum

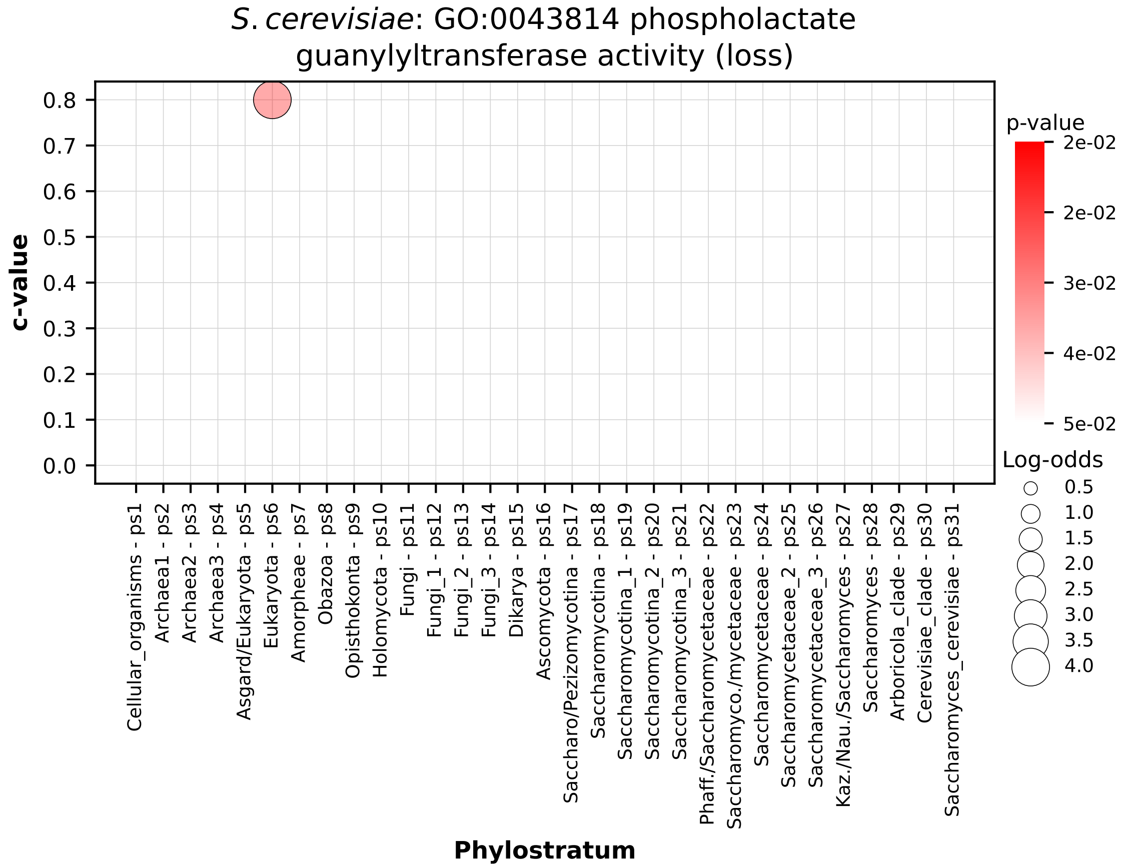
S. cerevisiae: GO:0043814 phospholactate guanylyltransferase activity (loss)
c-value
0.8
0.7
0.6
0.5
0.4
0.3
0.2
0.1
0.0
Phylostratum
Cellular_organisms - ps1
Archaea1 - ps2
Archaea2 - ps3
Archaea3 - ps4
Asgard/Eukaryota - ps5
Eukaryota - ps6
Amorpheae - ps7
Obazoa - ps8
Opisthokonta - ps9
Holomycota - ps10
Fungi - ps11
Fungi_1 - ps12
Fungi_2 - ps13
Fungi_3 - ps14
Dikarya - ps15
Ascomycota - ps16
Saccharo/Pezizomycotina - ps17
Saccharomycotina - ps18
Saccharomycotina_1 - ps19
Saccharomycotina_2 - ps20
Saccharomycotina_3 - ps21
Phaff./Saccharomycetaceae - ps22
Saccharomyco./mycetaceae - ps23
Saccharomycetaceae - ps24
Saccharomycetaceae_2 - ps25
Saccharomycetaceae_3 - ps26
Kaz./Nau./Saccharomyces - ps27
Saccharomyces - ps28
Arboricola_clade - ps29
Cerevisiae_clade - ps30
Saccharomyces_cerevisiae - ps31
p-value
2e-02
2e-02
3e-02
4e-02
5e-02
Log-odds
0.5
1.0
1.5
2.0
2.5
3.0
3.5
4.0

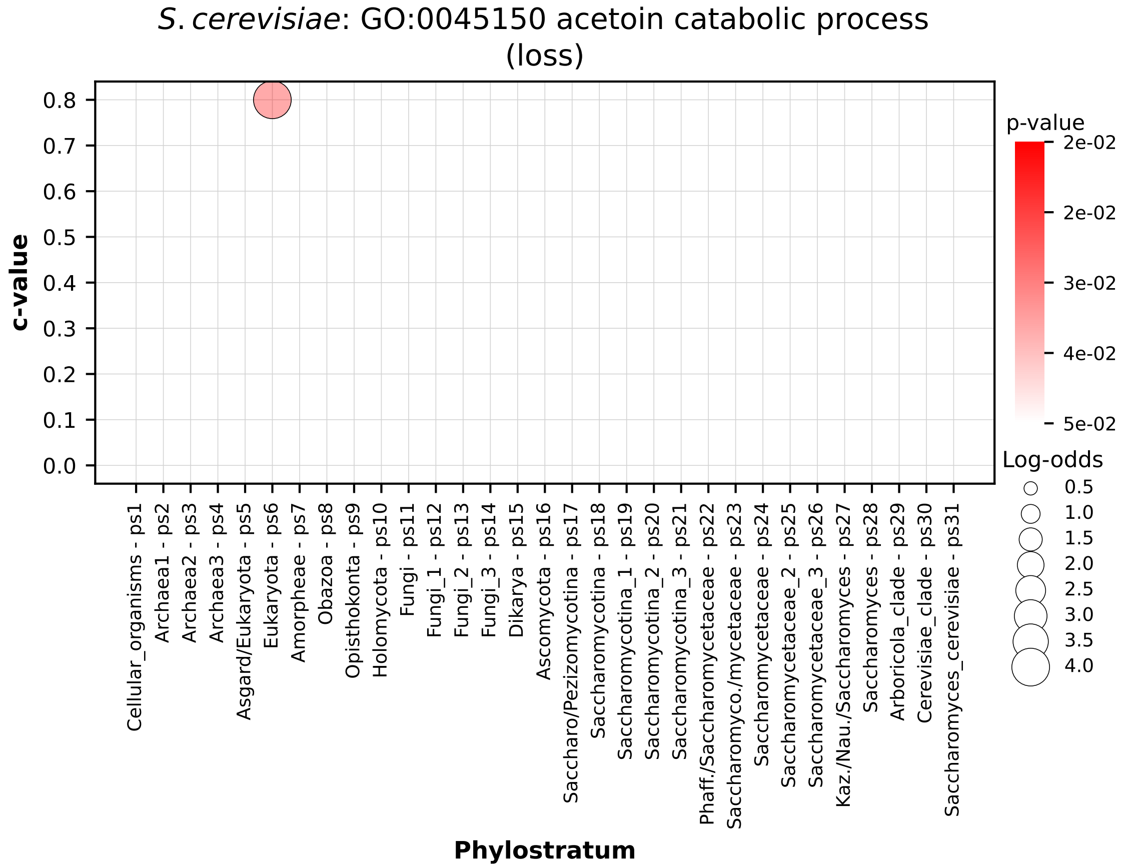

S. cerevisiae: GO:0045150 acetoin catabolic process
(loss)
c-value
0.8
0.7
0.6
0.5
0.4
0.3
0.2
0.1
0.0
p-value
2e-02
2e-02
3e-02
4e-02
5e-02
Log-odds
0.5
1.0
1.5
2.0
2.5
3.0
3.5
4.0
Cellular_organisms - ps1
Archaea1 - ps2
Archaea2 - ps3
Archaea3 - ps4
Asgard/Eukaryota - ps5
Eukaryota - ps6
Amorpheae - ps7
Obazoa - ps8
Opisthokonta - ps9
Holomycota - ps10
Fungi - ps11
Fungi_1 - ps12
Fungi_2 - ps13
Fungi_3 - ps14
Dikarya - ps15
Ascomycota - ps16
Saccharo/Pezizomycotina - ps17
Saccharomycotina - ps18
Saccharomycotina_1 - ps19
Saccharomycotina_2 - ps20
Saccharomycotina_3 - ps21
Phaff./Saccharomycetaceae - ps22
Saccharomyco./mycetaceae - ps23
Saccharomycetaceae - ps24
Saccharomycetaceae_2 - ps25
Saccharomycetaceae_3 - ps26
Kaz./Nau./Saccharomyces - ps27
Saccharomyces - ps28
Arboricola_clade - ps29
Cerevisiae_clade - ps30
Saccharomyces_cerevisiae - ps31
Phylostratum

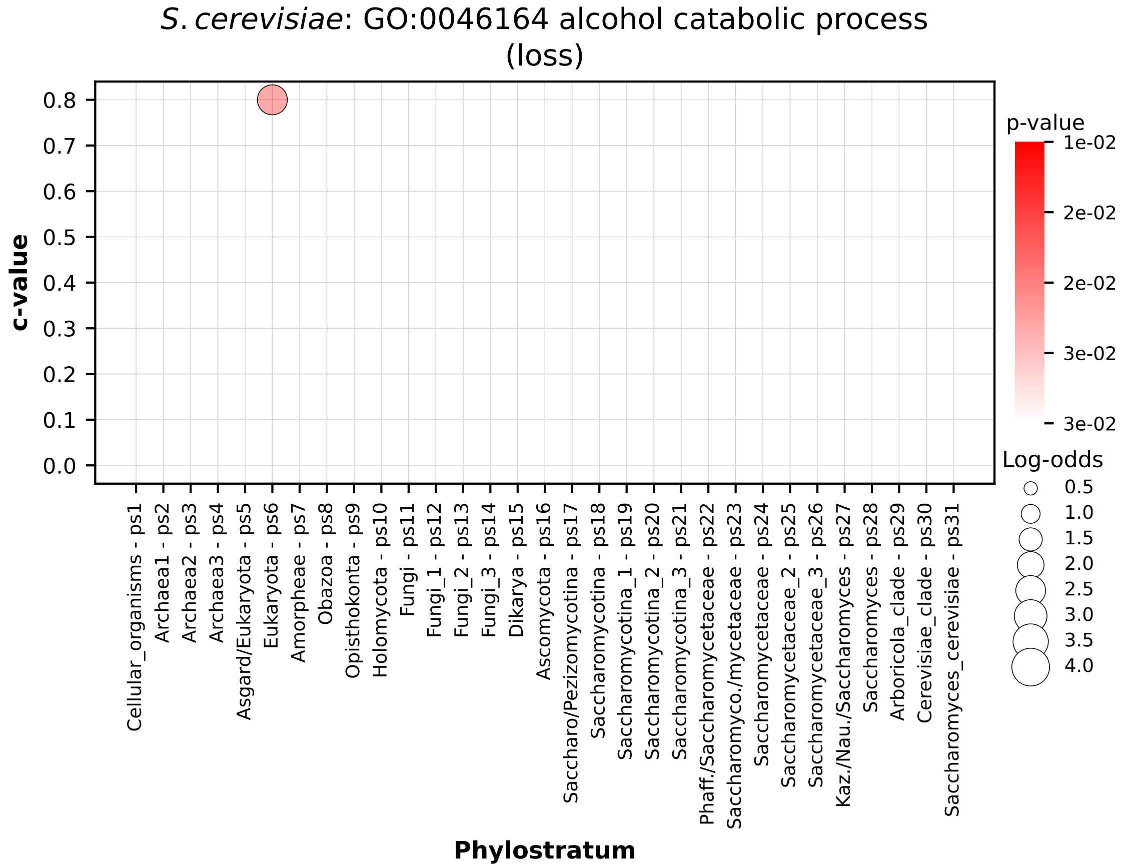

S. cerevisiae: GO:0046164 alcohol catabolic process
(loss)
c-value
0.8
0.7
0.6
0.5
0.4
0.3
0.2
0.1
0.0
p-value
1e-02
2e-02
2e-02
3e-02
3e-02
Log-odds
0.5
1.0
1.5
2.0
2.5
3.0
3.5
4.0
Cellular_organisms - ps1
Archaea1 - ps2
Archaea2 - ps3
Archaea3 - ps4
Asgard/Eukaryota - ps5
Eukaryota - ps6
Amorpheae - ps7
Obazoa - ps8
Opisthokonta - ps9
Holomycota - ps10
Fungi - ps11
Fungi_1 - ps12
Fungi_2 - ps13
Fungi_3 - ps14
Dikarya - ps15
Ascomycota - ps16
Saccharo/Pezizomycotina - ps17
Saccharomycotina - ps18
Saccharomycotina_1 - ps19
Saccharomycotina_2 - ps20
Saccharomycotina_3 - ps21
Phaff./Saccharomycetaceae - ps22
Saccharomyco./mycetaceae - ps23
Saccharomycetaceae - ps24
Saccharomycetaceae_2 - ps25
Saccharomycetaceae_3 - ps26
Kaz./Nau./Saccharomyces - ps27
Saccharomyces - ps28
Arboricola_clade - ps29
Cerevisiae_clade - ps30
Saccharomyces_cerevisiae - ps31
Phylostratum

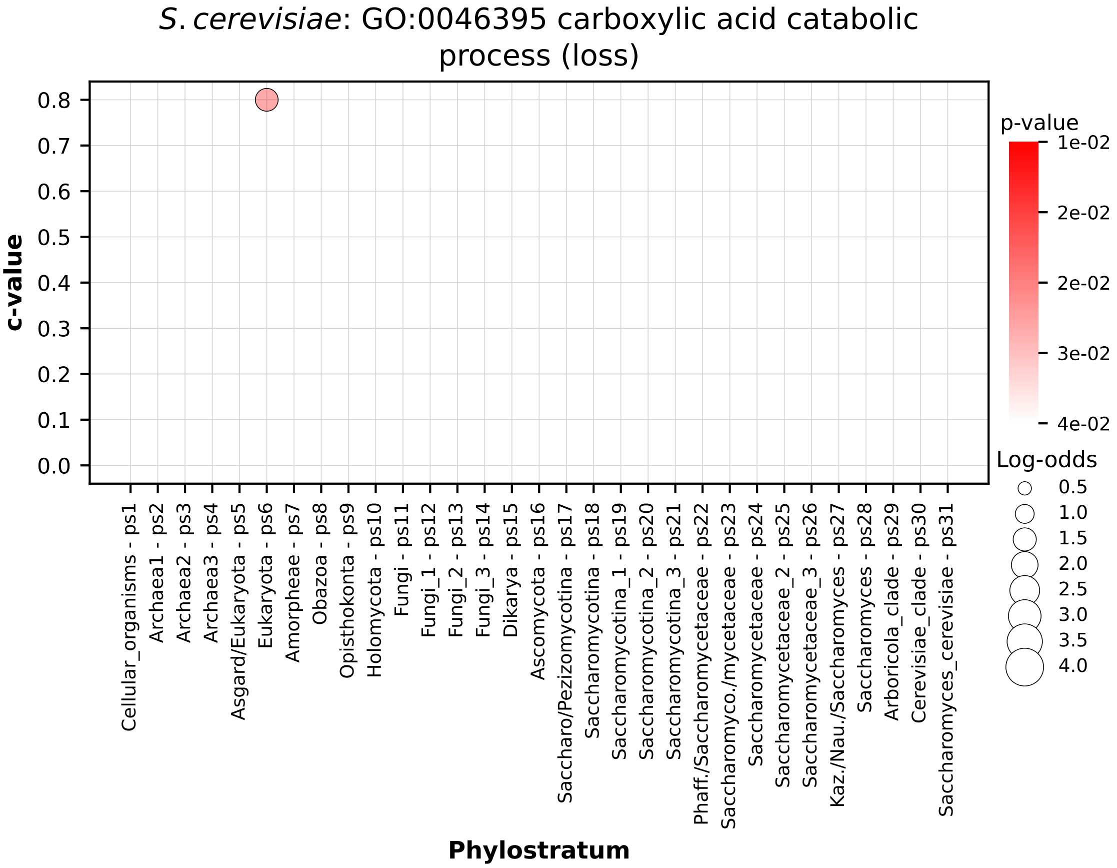

S. cerevisiae: GO:0046395 carboxylic acid catabolic process (loss)
c-value
0.8
0.7
0.6
0.5
0.4
0.3
0.2
0.1
0.0
Phylostratum
Cellular_organisms - ps1
Archaea1 - ps2
Archaea2 - ps3
Archaea3 - ps4
Asgard/Eukaryota - ps5
Eukaryota - ps6
Amorpheae - ps7
Obazoa - ps8
Opisthokonta - ps9
Holomycota - ps10
Fungi - ps11
Fungi_1 - ps12
Fungi_2 - ps13
Fungi_3 - ps14
Dikarya - ps15
Ascomycota - ps16
Saccharo/Pezizomycotina - ps17
Saccharomycotina - ps18
Saccharomycotina_1 - ps19
Saccharomycotina_2 - ps20
Saccharomycotina_3 - ps21
Phaff./Saccharomycetaceae - ps22
Saccharomyco./mycetaceae - ps23
Saccharomycetaceae - ps24
Saccharomycetaceae_2 - ps25
Saccharomycetaceae_3 - ps26
Kaz./Nau./Saccharomyces - ps27
Saccharomyces - ps28
Arboricola_clade - ps29
Cerevisiae_clade - ps30
Saccharomyces_cerevisiae - ps31
p-value
1e-02
2e-02
2e-02
3e-02
4e-02
Log-odds
0.5
1.0
1.5
2.0
2.5
3.0
3.5
4.0

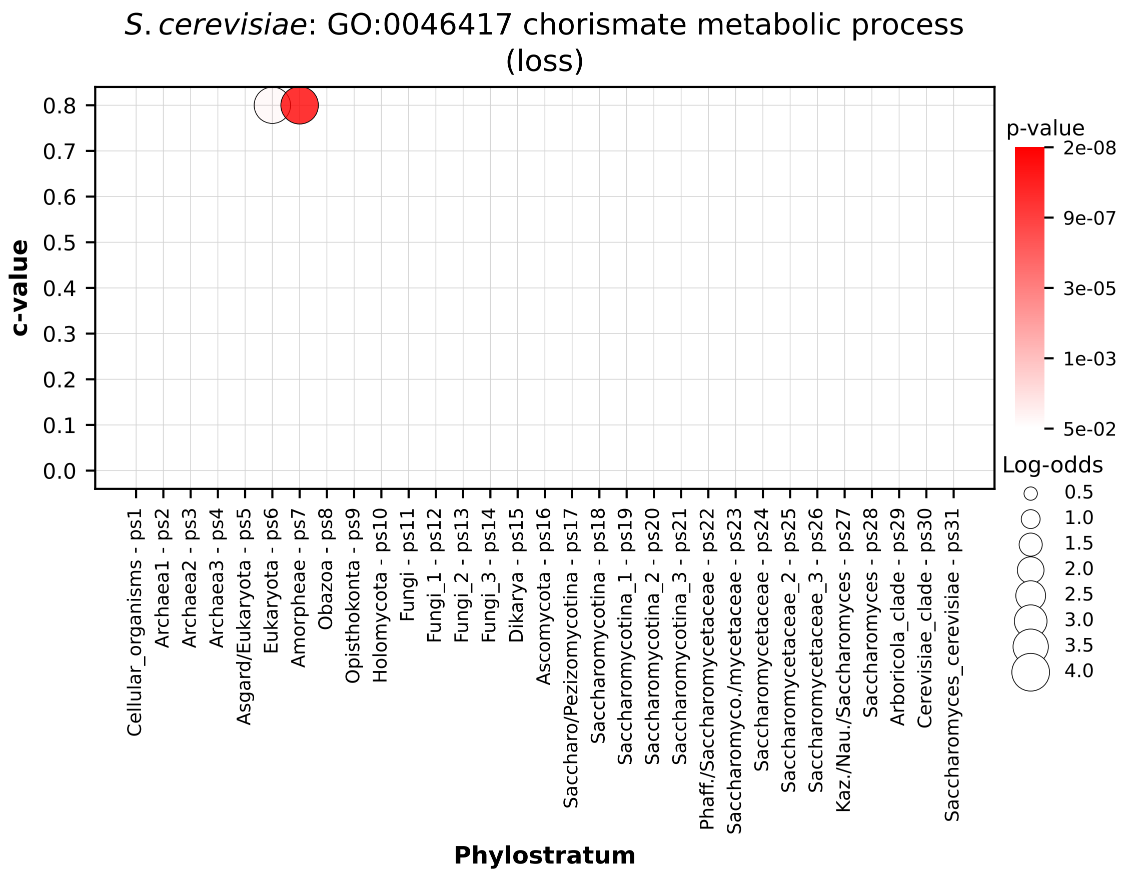
S. cerevisiae: GO:0046417 chorismate metabolic process
(loss)
c-value
0.8
0.7
0.6
0.5
0.4
0.3
0.2
0.1
0.0
p-value
2e-08
9e-07
3e-05
1e-03
5e-02
Log-odds
0.5
1.0
1.5
2.0
2.5
3.0
3.5
4.0
Cellular_organisms - ps1
Archaea1 - ps2
Archaea2 - ps3
Archaea3 - ps4
Asgard/Eukaryota - ps5
Eukaryota - ps6
Amorpheae - ps7
Obazoa - ps8
Opisthokonta - ps9
Holomycota - ps10
Fungi - ps11
Fungi_1 - ps12
Fungi_2 - ps13
Fungi_3 - ps14
Dikarya - ps15
Ascomycota - ps16
Saccharo/Pezizomycotina - ps17
Saccharomycotina - ps18
Saccharomycotina_1 - ps19
Saccharomycotina_2 - ps20
Saccharomycotina_3 - ps21
Phaff./Saccharomycetaceae - ps22
Saccharomyco./mycetaceae - ps23
Saccharomycetaceae - ps24
Saccharomycetaceae_2 - ps25
Saccharomycetaceae_3 - ps26
Kaz./Nau./Saccharomyces - ps27
Saccharomyces - ps28
Arboricola_clade - ps29
Cerevisiae_clade - ps30
Saccharomyces_cerevisiae - ps31
Phylostratum

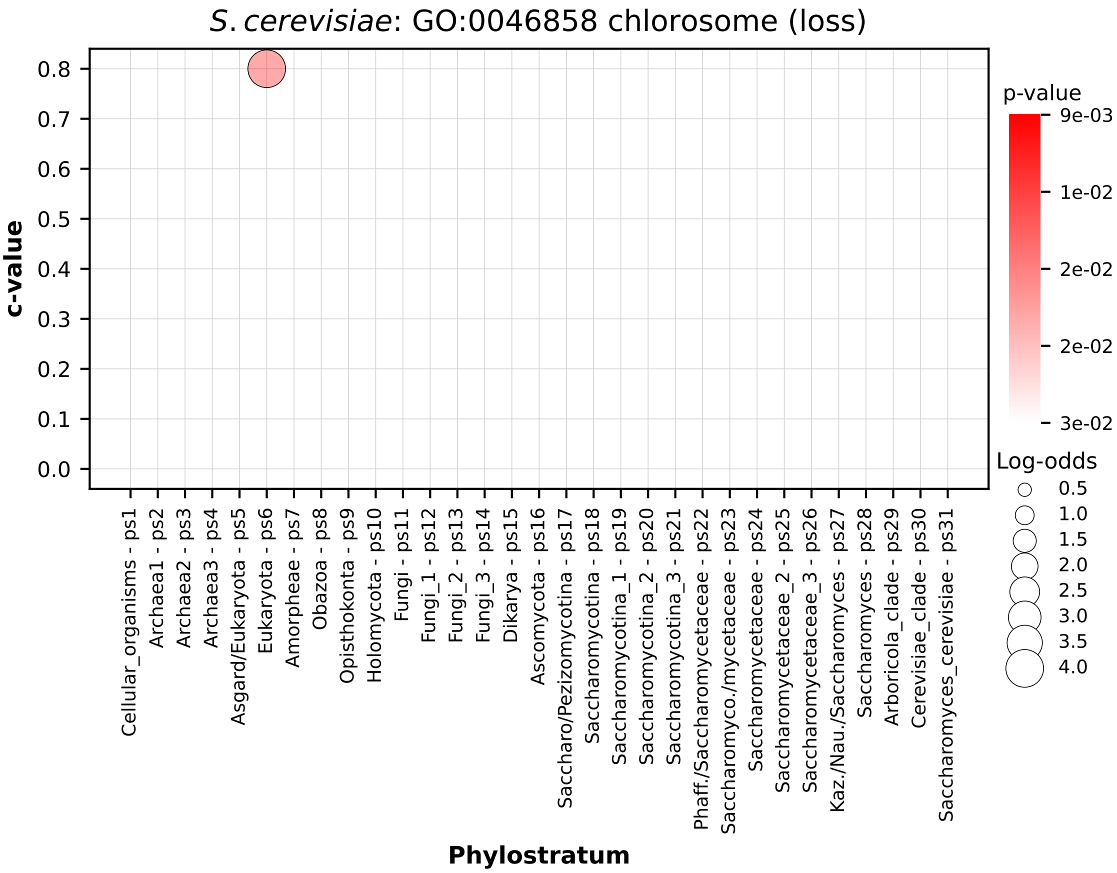

S. cerevisiae: GO:0046858 chlorosome (loss)
c-value
0.0
0.1
0.2
0.3
0.4
0.5
0.6
0.7
0.8
p-value
9e-03
1e-02
2e-02
2e-02
3e-02
Log-odds
0.5
1.0
1.5
2.0
2.5
3.0
3.5
4.0
Cellular_organisms - ps1
Archaea1 - ps2
Archaea2 - ps3
Archaea3 - ps4
Asgard/Eukaryota - ps5
Eukaryota - ps6
Amorpheae - ps7
Obazoa - ps8
Opisthokonta - ps9
Holomycota - ps10
Fungi - ps11
Fungi_1 - ps12
Fungi_2 - ps13
Fungi_3 - ps14
Dikarya - ps15
Ascomycota - ps16
Saccharo/Pezizomycotina - ps17
Saccharomycotina - ps18
Saccharomycotina_1 - ps19
Saccharomycotina_2 - ps20
Saccharomycotina_3 - ps21
Phaff./Saccharomycetaceae - ps22
Saccharomyco./mycetaceae - ps23
Saccharomycetaceae - ps24
Saccharomycetaceae_2 - ps25
Saccharomycetaceae_3 - ps26
Kaz./Nau./Saccharomyces - ps27
Saccharomyces - ps28
Arboricola_clade - ps29
Cerevisiae_clade - ps30
Saccharomyces_cerevisiae - ps31
Phylostratum

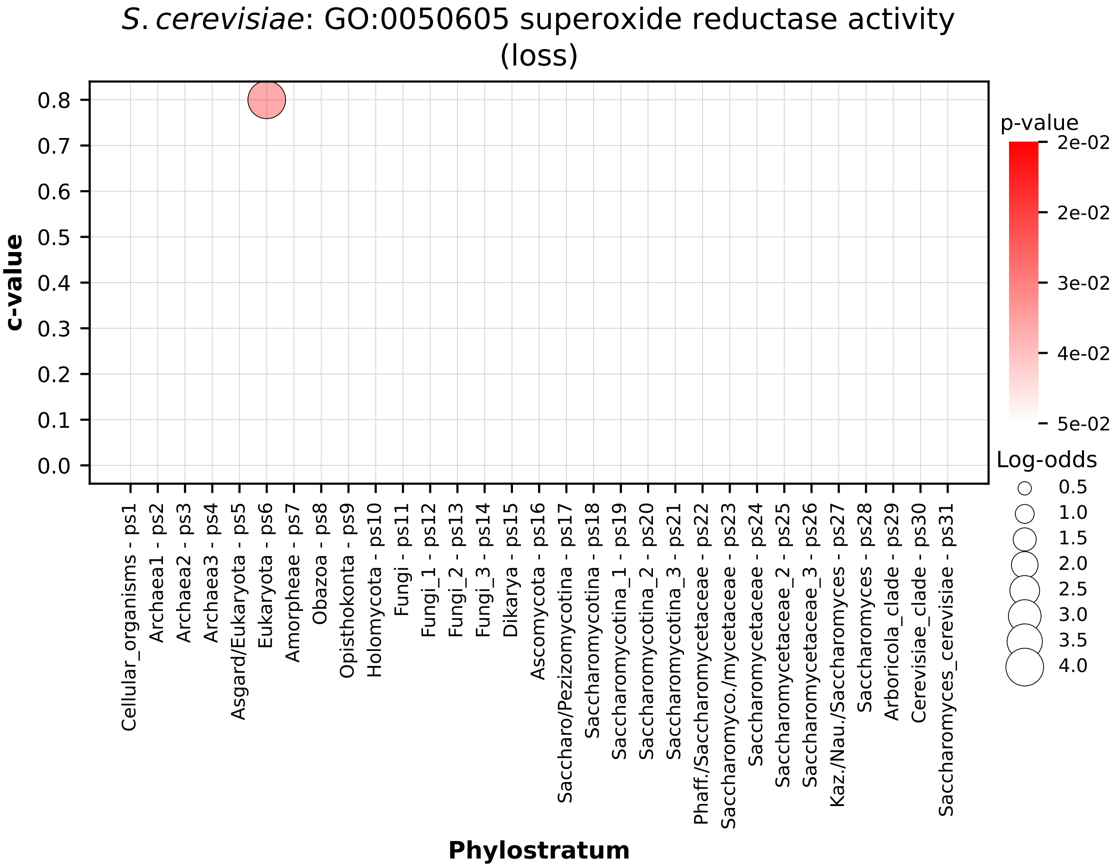

S. cerevisiae: GO:0050605 superoxide reductase activity (loss)
c-value
0.8
0.7
0.6
0.5
0.4
0.3
0.2
0.1
0.0
p-value
2e-02
2e-02
3e-02
4e-02
5e-02
Log-odds
0.5
1.0
1.5
2.0
2.5
3.0
3.5
4.0
Cellular_organisms - ps1
Archaea1 - ps2
Archaea2 - ps3
Archaea3 - ps4
Asgard/Eukaryota - ps5
Eukaryota - ps6
Amorpheae - ps7
Obazoa - ps8
Opisthokonta - ps9
Holomycota - ps10
Fungi - ps11
Fungi_1 - ps12
Fungi_2 - ps13
Fungi_3 - ps14
Dikarya - ps15
Ascomycota - ps16
Saccharo/Pezizomycotina - ps17
Saccharomycotina - ps18
Saccharomycotina_1 - ps19
Saccharomycotina_2 - ps20
Saccharomycotina_3 - ps21
Phaff./Saccharomycetaceae - ps22
Saccharomyco./mycetaceae - ps23
Saccharomycetaceae - ps24
Saccharomycetaceae_2 - ps25
Saccharomycetaceae_3 - ps26
Kaz./Nau./Saccharomyces - ps27
Saccharomyces - ps28
Arboricola_clade - ps29
Cerevisiae_clade - ps30
Saccharomyces_cerevisiae - ps31
Phylostratum

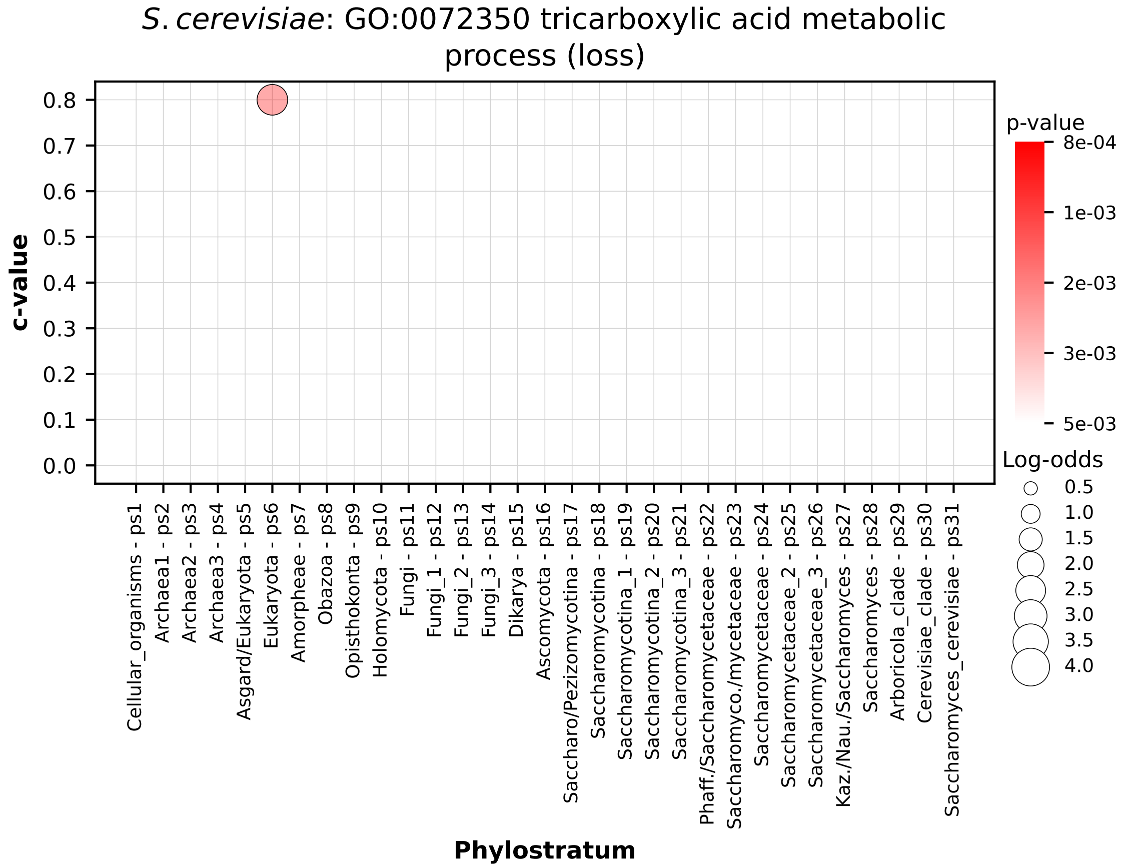

S. cerevisiae: GO:0072350 tricarboxylic acid metabolic process (loss)
c-value
0.8
0.7
0.6
0.5
0.4
0.3
0.2
0.1
0.0
p-value
8e-04
1e-03
2e-03
3e-03
5e-03
Log-odds
0.5
1.0
1.5
2.0
2.5
3.0
3.5
4.0
Cellular_organisms - ps1
Archaea1 - ps2
Archaea2 - ps3
Archaea3 - ps4
Asgard/Eukaryota - ps5
Eukaryota - ps6
Amorpheae - ps7
Obazoa - ps8
Opisthokonta - ps9
Holomycota - ps10
Fungi - ps11
Fungi_1 - ps12
Fungi_2 - ps13
Fungi_3 - ps14
Dikarya - ps15
Ascomycota - ps16
Saccharo/Pezizomycotina - ps17
Saccharomycotina - ps18
Saccharomycotina_1 - ps19
Saccharomycotina_2 - ps20
Saccharomycotina_3 - ps21
Phaff./Saccharomycetaceae - ps22
Saccharomyco./mycetaceae - ps23
Saccharomycetaceae - ps24
Saccharomycetaceae_2 - ps25
Saccharomycetaceae_3 - ps26
Kaz./Nau./Saccharomyces - ps27
Saccharomyces - ps28
Arboricola_clade - ps29
Cerevisiae_clade - ps30
Saccharomyces_cerevisiae - ps31
Phylostratum

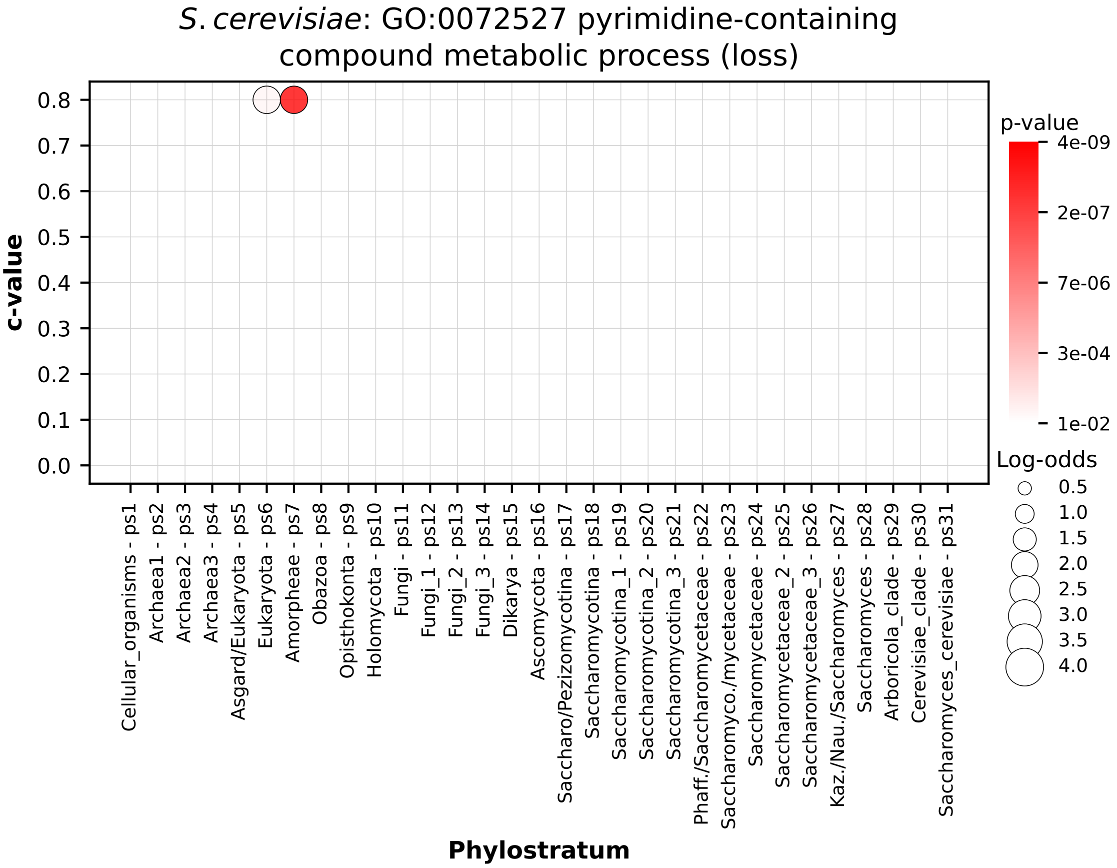
S. cerevisiae: GO:0072527 pyrimidine-containing compound metabolic process (loss)
c-value
0.8
0.7
0.6
0.5
0.4
0.3
0.2
0.1
0.0
p-value
4e-09
2e-07
7e-06
3e-04
1e-02
Log-odds
0.5
1.0
1.5
2.0
2.5
3.0
3.5
4.0
Cellular_organisms - ps1
Archaea1 - ps2
Archaea2 - ps3
Archaea3 - ps4
Asgard/Eukaryota - ps5
Eukaryota - ps6
Amorpheae - ps7
Obazoa - ps8
Opisthokonta - ps9
Holomycota - ps10
Fungi - ps11
Fungi_1 - ps12
Fungi_2 - ps13
Fungi_3 - ps14
Dikarya - ps15
Ascomycota - ps16
Saccharo/Pezizomycotina - ps17
Saccharomycotina - ps18
Saccharomycotina_1 - ps19
Saccharomycotina_2 - ps20
Saccharomycotina_3 - ps21
Phaff./Saccharomycetaceae - ps22
Saccharomyco./mycetaceae - ps23
Saccharomycetaceae - ps24
Saccharomycetaceae_2 - ps25
Saccharomycetaceae_3 - ps26
Kaz./Nau./Saccharomyces - ps27
Saccharomyces - ps28
Arboricola_clade - ps29
Cerevisiae_clade - ps30
Saccharomyces_cerevisiae - ps31
Phylostratum

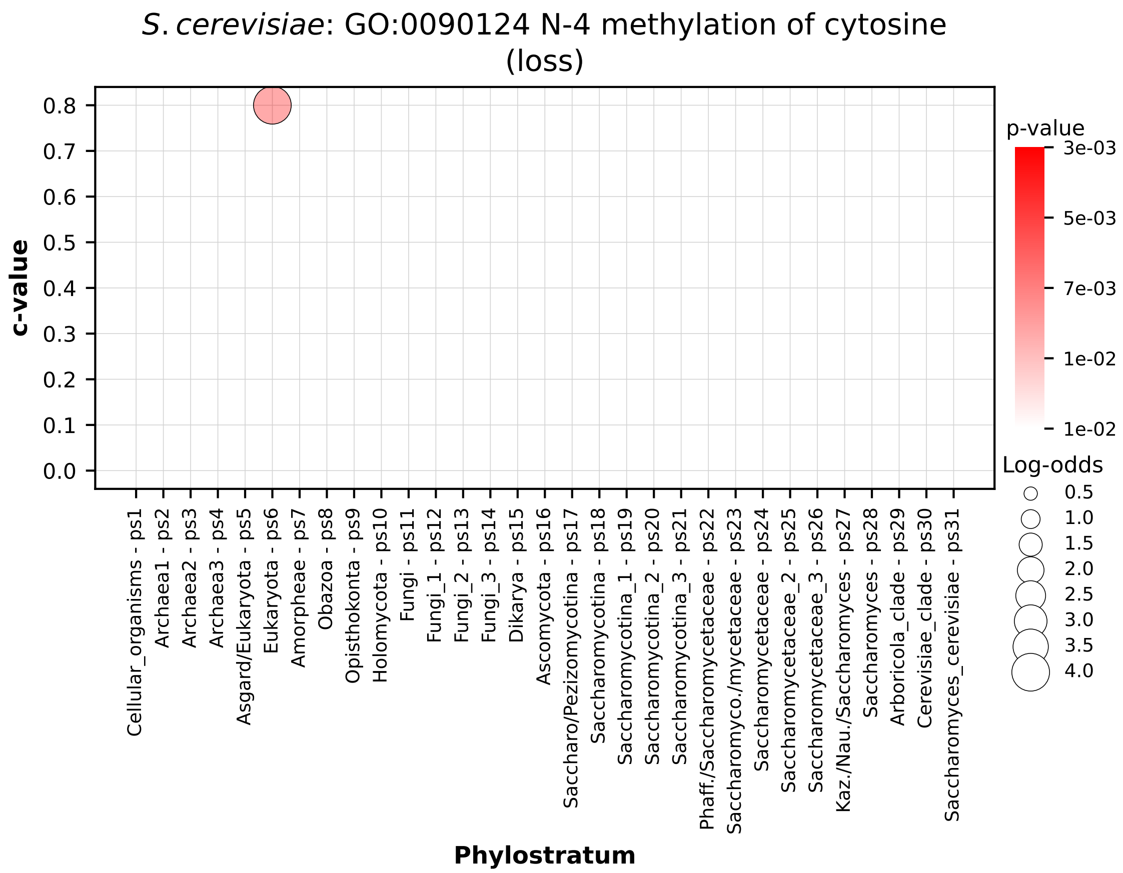

S. cerevisiae: GO:0090124 N-4 methylation of cytosine
(loss)
c-value
0.8
0.7
0.6
0.5
0.4
0.3
0.2
0.1
0.0
p-value
3e-03
5e-03
7e-03
1e-02
1e-02
Log-odds
0.5
1.0
1.5
2.0
2.5
3.0
3.5
4.0
Cellular_organisms - ps1
Archaea1 - ps2
Archaea2 - ps3
Archaea3 - ps4
Asgard/Eukaryota - ps5
Eukaryota - ps6
Amorpheae - ps7
Obazoa - ps8
Opisthokonta - ps9
Holomycota - ps10
Fungi - ps11
Fungi_1 - ps12
Fungi_2 - ps13
Fungi_3 - ps14
Dikarya - ps15
Ascomycota - ps16
Saccharo/Pezizomycotina - ps17
Saccharomycotina - ps18
Saccharomycotina_1 - ps19
Saccharomycotina_2 - ps20
Saccharomycotina_3 - ps21
Phaff./Saccharomycetaceae - ps22
Saccharomyco./mycetaceae - ps23
Saccharomycetaceae - ps24
Saccharomycetaceae_2 - ps25
Saccharomycetaceae_3 - ps26
Kaz./Nau./Saccharomyces - ps27
Saccharomyces - ps28
Arboricola_clade - ps29
Cerevisiae_clade - ps30
Saccharomyces_cerevisiae - ps31
Phylostratum

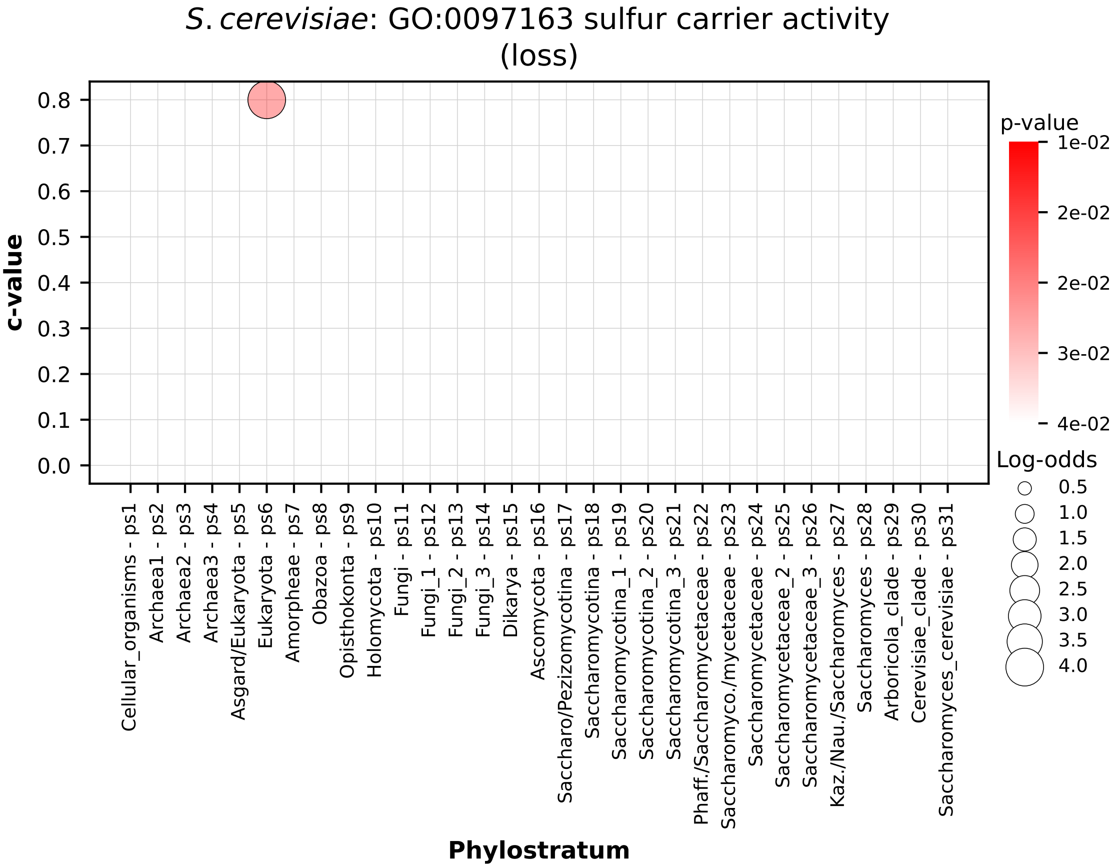

S. cerevisiae: GO:0097163 sulfur carrier activity
(loss)
c-value
0.8
0.7
0.6
0.5
0.4
0.3
0.2
0.1
0.0
p-value
1e-02
2e-02
2e-02
3e-02
4e-02
Log-odds
0.5
1.0
1.5
2.0
2.5
3.0
3.5
4.0
Cellular_organisms - ps1
Archaea1 - ps2
Archaea2 - ps3
Archaea3 - ps4
Asgard/Eukaryota - ps5
Eukaryota - ps6
Amorpheae - ps7
Obazoa - ps8
Opisthokonta - ps9
Holomycota - ps10
Fungi - ps11
Fungi_1 - ps12
Fungi_2 - ps13
Fungi_3 - ps14
Dikarya - ps15
Ascomycota - ps16
Saccharo/Pezizomycotina - ps17
Saccharomycotina - ps18
Saccharomycotina_1 - ps19
Saccharomycotina_2 - ps20
Saccharomycotina_3 - ps21
Phaff./Saccharomycetaceae - ps22
Saccharomyco./mycetaceae - ps23
Saccharomycetaceae - ps24
Saccharomycetaceae_2 - ps25
Saccharomycetaceae_3 - ps26
Kaz./Nau./Saccharomyces - ps27
Saccharomyces - ps28
Arboricola_clade - ps29
Cerevisiae_clade - ps30
Saccharomyces_cerevisiae - ps31
Phylostratum

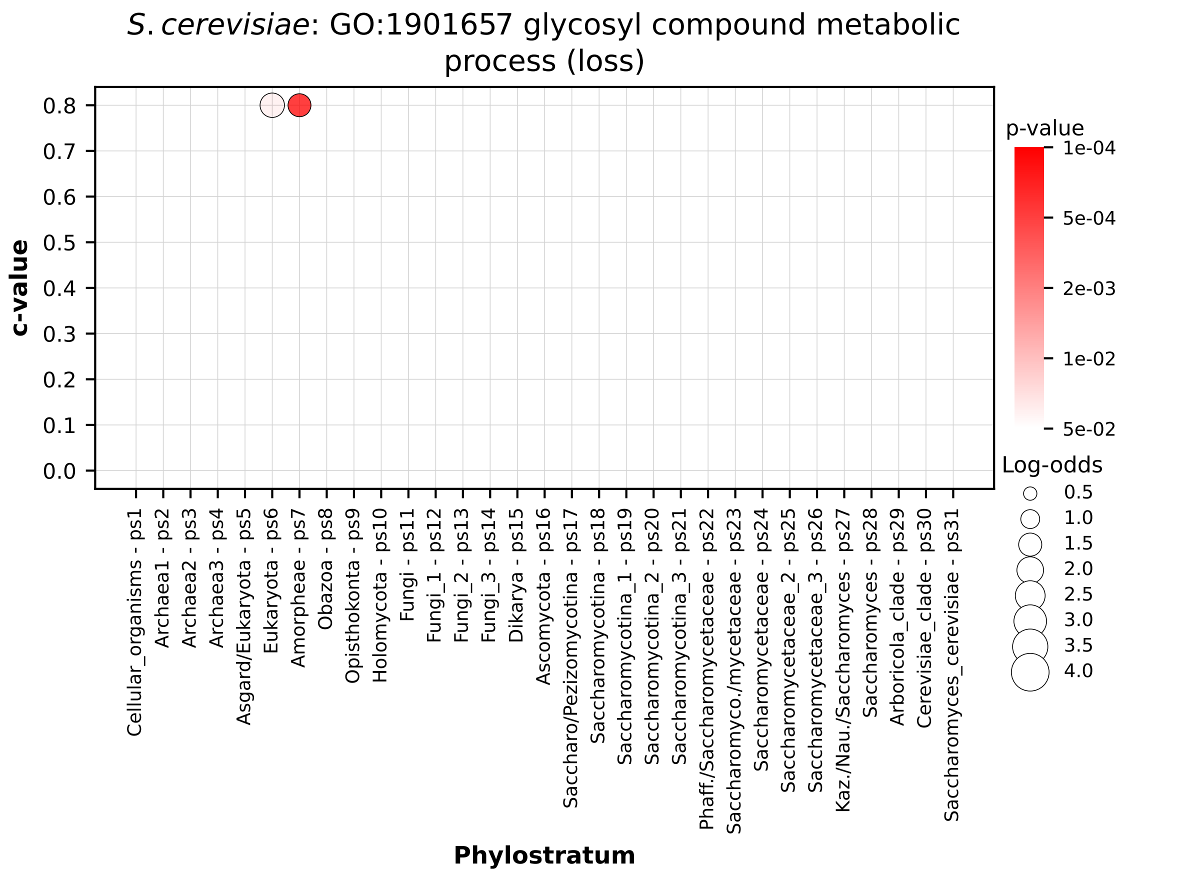

S. cerevisiae: GO:1901657 glycosyl compound metabolic process (loss)
c-value
0.0
0.1
0.2
0.3
0.4
0.5
0.6
0.7
0.8
Phylostratum
Cellular_organisms - ps1
Archaea1 - ps2
Archaea2 - ps3
Archaea3 - ps4
Asgard/Eukaryota - ps5
Eukaryota - ps6
Amorpheae - ps7
Obazoa - ps8
Opisthokonta - ps9
Holomycota - ps10
Fungi - ps11
Fungi_1 - ps12
Fungi_2 - ps13
Fungi_3 - ps14
Dikarya - ps15
Ascomycota - ps16
Saccharo/Pezizomycotina - ps17
Saccharomycotina - ps18
Saccharomycotina_1 - ps19
Saccharomycotina_2 - ps20
Saccharomycotina_3 - ps21
Phaff./Saccharomycetaceae - ps22
Saccharomyco./mycetaceae - ps23
Saccharomycetaceae - ps24
Saccharomycetaceae_2 - ps25
Saccharomycetaceae_3 - ps26
Kaz./Nau./Saccharomyces - ps27
Saccharomyces - ps28
Arboricola_clade - ps29
Cerevisiae_clade - ps30
Saccharomyces_cerevisiae - ps31
p-value
1e-04
5e-04
2e-03
1e-02
5e-02
Log-odds
0.5
1.0
1.5
2.0
2.5
3.0
3.5
4.0

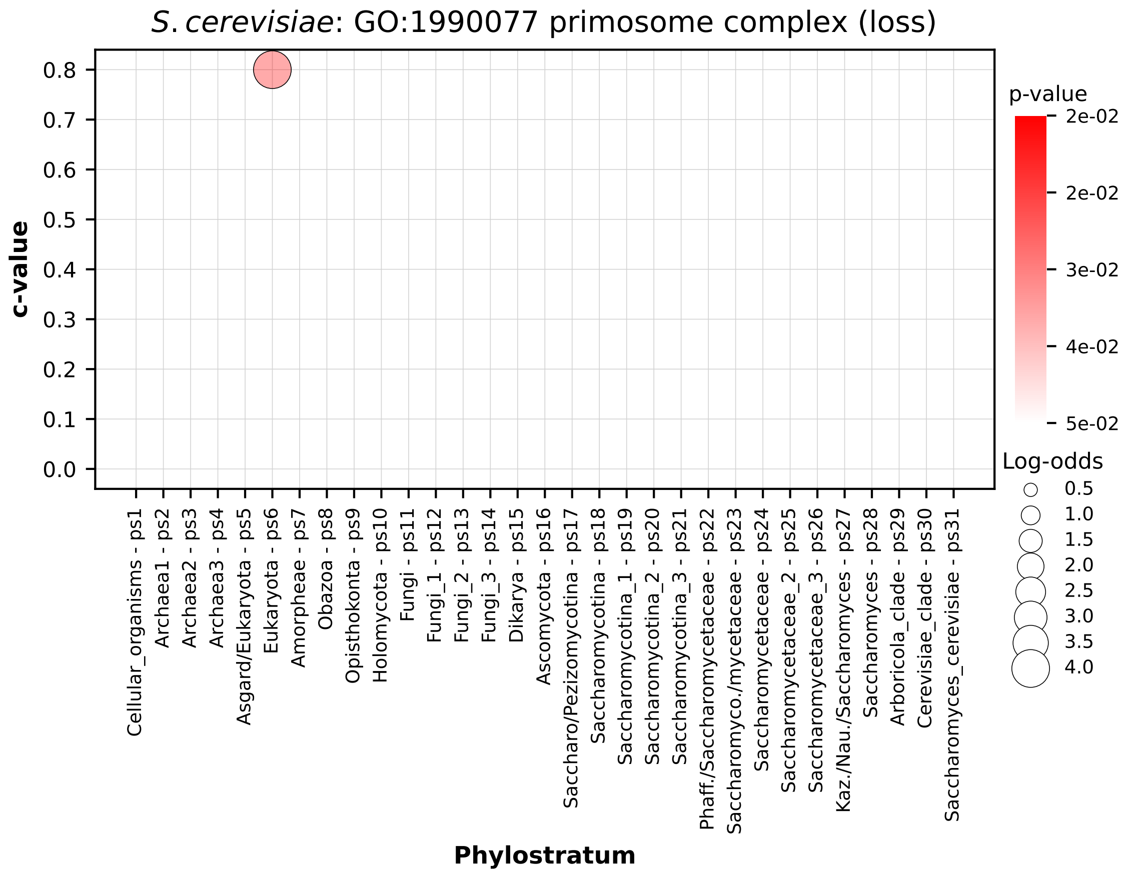
S. cerevisiae: GO:1990077 primosome complex (loss)
c-value
0.0
0.1
0.2
0.3
0.4
0.5
0.6
0.7
0.8
Phylostratum
Cellular_organisms - ps1
Archaea1 - ps2
Archaea2 - ps3
Archaea3 - ps4
Asgard/Eukaryota - ps5
Eukaryota - ps6
Amorpheae - ps7
Obazoa - ps8
Opisthokonta - ps9
Holomycota - ps10
Fungi - ps11
Fungi_1 - ps12
Fungi_2 - ps13
Fungi_3 - ps14
Dikarya - ps15
Ascomycota - ps16
Saccharo/Pezizomycotina - ps17
Saccharomycotina - ps18
Saccharomycotina_1 - ps19
Saccharomycotina_2 - ps20
Saccharomycotina_3 - ps21
Phaff./Saccharomycetaceae - ps22
Saccharomyco./mycetaceae - ps23
Saccharomycetaceae - ps24
Saccharomycetaceae_2 - ps25
Saccharomycetaceae_3 - ps26
Kaz./Nau./Saccharomyces - ps27
Saccharomyces - ps28
Arboricola_clade - ps29
Cerevisiae_clade - ps30
Saccharomyces_cerevisiae - ps31
p-value
2e-02
2e-02
3e-02
4e-02
5e-02
Log-odds
0.5
1.0
1.5
2.0
2.5
3.0
3.5
4.0

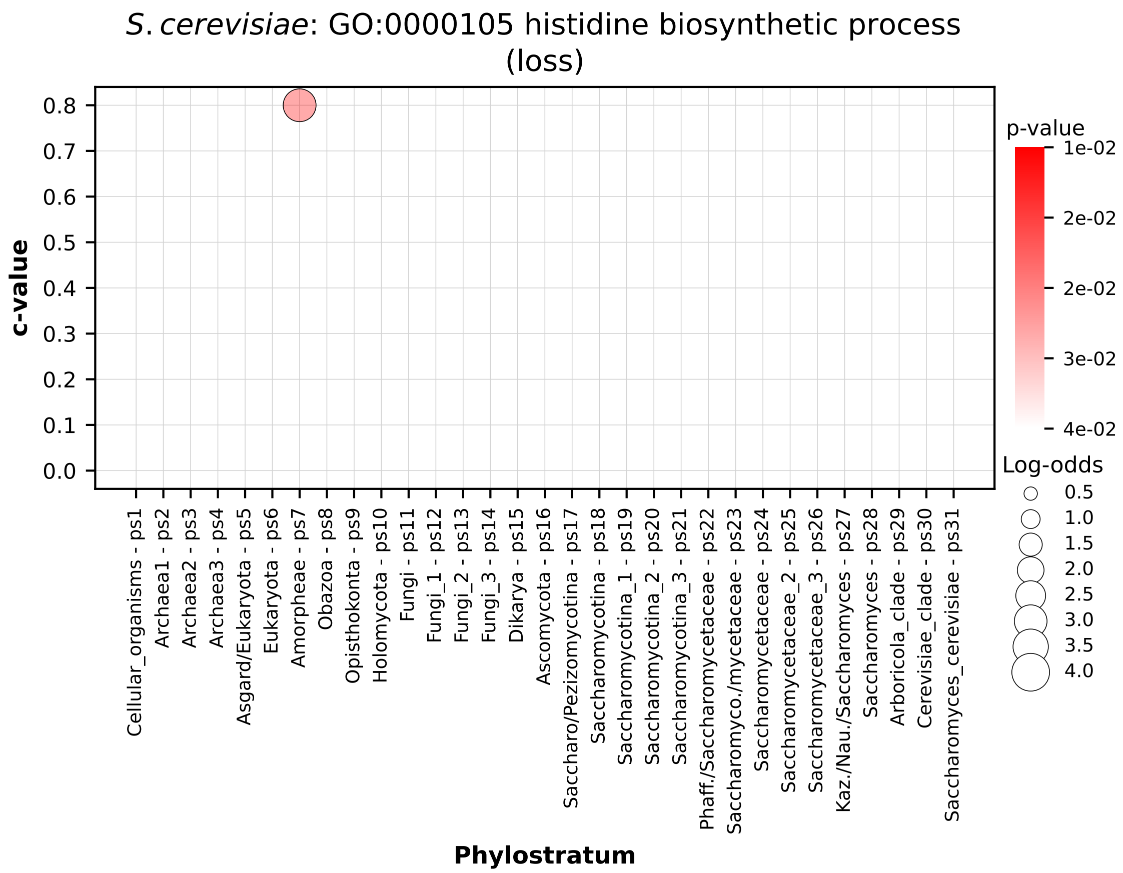
S. cerevisiae: GO:0000105 histidine biosynthetic process
(loss)
c-value
0.0
0.1
0.2
0.3
0.4
0.5
0.6
0.7
0.8
p-value
1e-02
2e-02
2e-02
3e-02
4e-02
Log-odds
0.5
1.0
1.5
2.0
2.5
3.0
3.5
4.0
Cellular_organisms - ps1
Archaea1 - ps2
Archaea2 - ps3
Archaea3 - ps4
Asgard/Eukaryota - ps5
Eukaryota - ps6
Amorpheae - ps7
Obazoa - ps8
Opisthokonta - ps9
Holomycota - ps10
Fungi - ps11
Fungi_1 - ps12
Fungi_2 - ps13
Fungi_3 - ps14
Dikarya - ps15
Ascomycota - ps16
Saccharo/Pezizomycotina - ps17
Saccharomycotina - ps18
Saccharomycotina_1 - ps19
Saccharomycotina_2 - ps20
Saccharomycotina_3 - ps21
Phaff./Saccharomycetaceae - ps22
Saccharomyco./mycetaceae - ps23
Saccharomycetaceae - ps24
Saccharomycetaceae_2 - ps25
Saccharomycetaceae_3 - ps26
Kaz./Nau./Saccharomyces - ps27
Saccharomyces - ps28
Arboricola_clade - ps29
Cerevisiae_clade - ps30
Saccharomyces_cerevisiae - ps31
Phylostratum

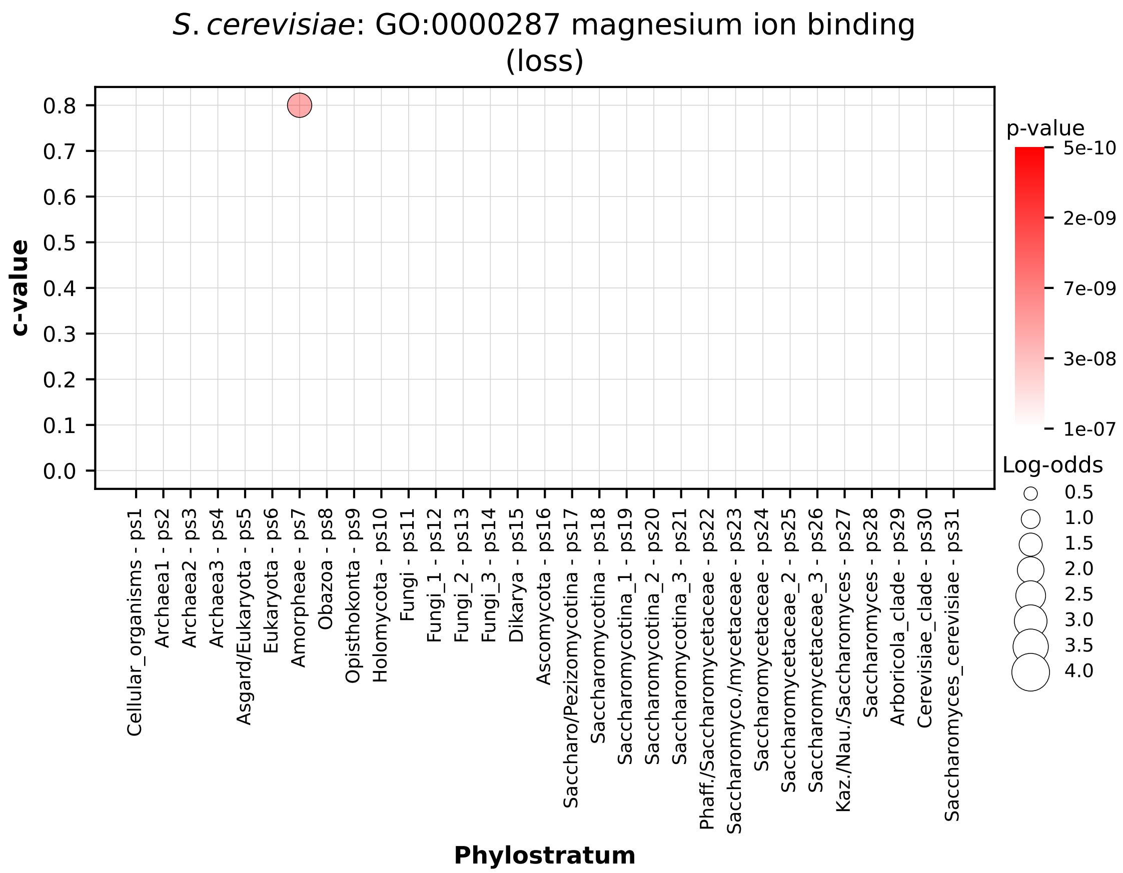
S. cerevisiae: GO:0000287 magnesium ion binding
(loss)
c-value
0.8
0.7
0.6
0.5
0.4
0.3
0.2
0.1
0.0
p-value
5e-10
2e-09
7e-09
3e-08
1e-07
Log-odds
0.5
1.0
1.5
2.0
2.5
3.0
3.5
4.0
Cellular_organisms - ps1
Archaea1 - ps2
Archaea2 - ps3
Archaea3 - ps4
Asgard/Eukaryota - ps5
Eukaryota - ps6
Amorpheae - ps7
Obazoa - ps8
Opisthokonta - ps9
Holomycota - ps10
Fungi - ps11
Fungi_1 - ps12
Fungi_2 - ps13
Fungi_3 - ps14
Dikarya - ps15
Ascomycota - ps16
Saccharo/Pezizomycotina - ps17
Saccharomycotina - ps18
Saccharomycotina_1 - ps19
Saccharomycotina_2 - ps20
Saccharomycotina_3 - ps21
Phaff./Saccharomycetaceae - ps22
Saccharomyco./mycetaceae - ps23
Saccharomycetaceae - ps24
Saccharomycetaceae_2 - ps25
Saccharomycetaceae_3 - ps26
Kaz./Nau./Saccharomyces - ps27
Saccharomyces - ps28
Arboricola_clade - ps29
Cerevisiae_clade - ps30
Saccharomyces_cerevisiae - ps31
Phylostratum

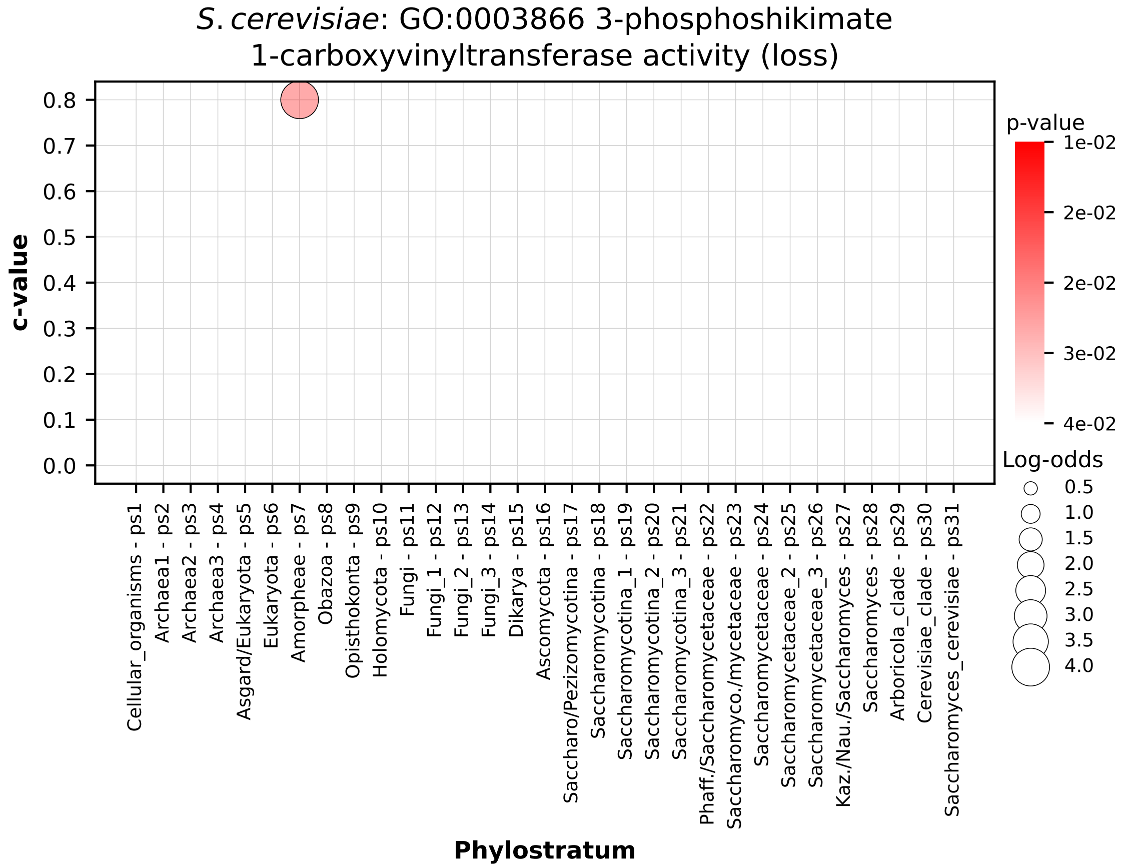

S. cerevisiae: GO:0003866 3-phosphoshikimate 1-carboxyvinyltransferase activity (loss)
c-value
0.0
0.1
0.2
0.3
0.4
0.5
0.6
0.7
0.8
Phylostratum
Cellular_organisms - ps1
Archaea1 - ps2
Archaea2 - ps3
Archaea3 - ps4
Asgard/Eukaryota - ps5
Eukaryota - ps6
Amorpheae - ps7
Obazoa - ps8
Opisthokonta - ps9
Holomycota - ps10
Fungi - ps11
Fungi_1 - ps12
Fungi_2 - ps13
Fungi_3 - ps14
Dikarya - ps15
Ascomycota - ps16
Saccharo/Pezizomycotina - ps17
Saccharomycotina - ps18
Saccharomycotina_1 - ps19
Saccharomycotina_2 - ps20
Saccharomycotina_3 - ps21
Phaff./Saccharomycetaceae - ps22
Saccharomyco./mycetaceae - ps23
Saccharomycetaceae - ps24
Saccharomycetaceae_2 - ps25
Saccharomycetaceae_3 - ps26
Kaz./Nau./Saccharomyces - ps27
Saccharomyces - ps28
Arboricola_clade - ps29
Cerevisiae_clade - ps30
Saccharomyces_cerevisiae - ps31
p-value
1e-02
2e-02
2e-02
3e-02
4e-02
Log-odds
0.5
1.0
1.5
2.0
2.5
3.0
3.5
4.0

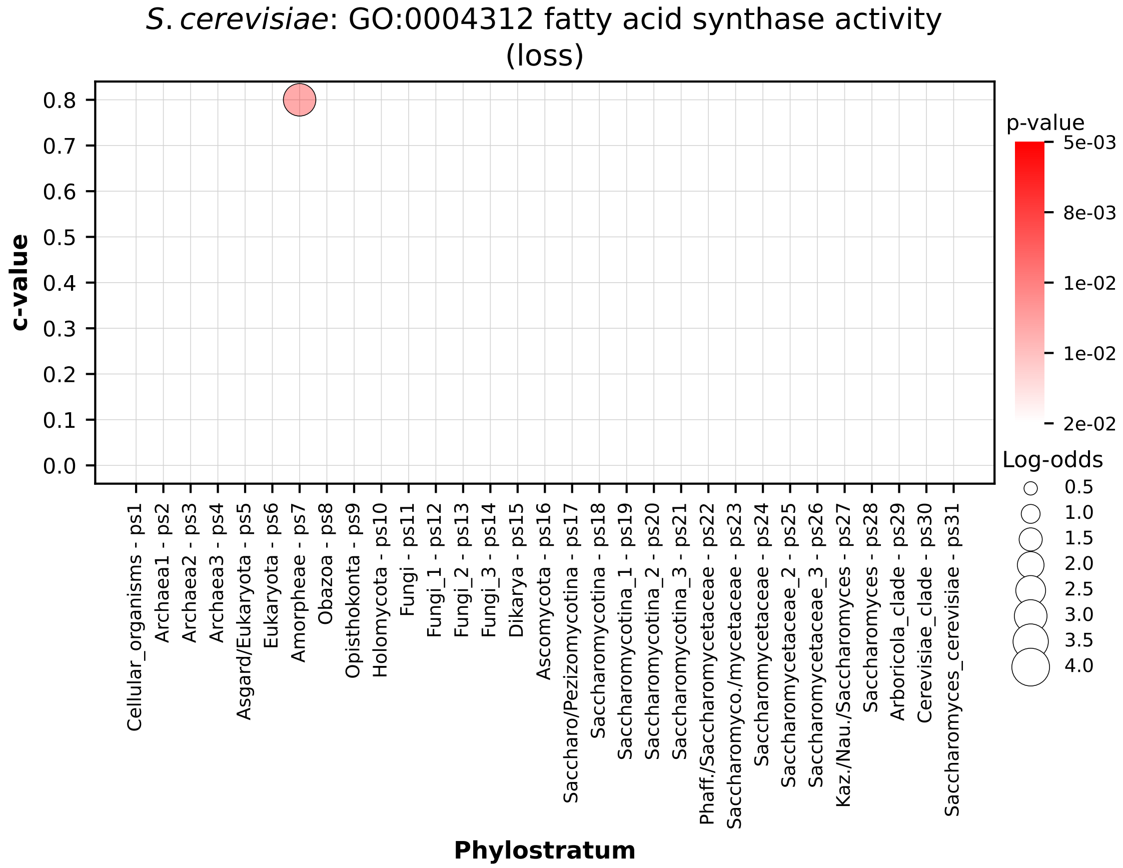
S. cerevisiae: GO:0004312 fatty acid synthase activity
(loss)
c-value
0.0
0.1
0.2
0.3
0.4
0.5
0.6
0.7
0.8
Phylostratum
Cellular_organisms - ps1
Archaea1 - ps2
Archaea2 - ps3
Archaea3 - ps4
Asgard/Eukaryota - ps5
Eukaryota - ps6
Amorpheae - ps7
Obazoa - ps8
Opisthokonta - ps9
Holomycota - ps10
Fungi - ps11
Fungi_1 - ps12
Fungi_2 - ps13
Fungi_3 - ps14
Dikarya - ps15
Ascomycota - ps16
Saccharo/Pezizomycotina - ps17
Saccharomycotina - ps18
Saccharomycotina_1 - ps19
Saccharomycotina_2 - ps20
Saccharomycotina_3 - ps21
Phaff./Saccharomycetaceae - ps22
Saccharomyco./mycetaceae - ps23
Saccharomycetaceae - ps24
Saccharomycetaceae_2 - ps25
Saccharomycetaceae_3 - ps26
Kaz./Nau./Saccharomyces - ps27
Saccharomyces - ps28
Arboricola_clade - ps29
Cerevisiae_clade - ps30
Saccharomyces_cerevisiae - ps31
p-value
5e-03
8e-03
1e-02
1e-02
2e-02
Log-odds
0.5
1.0
1.5
2.0
2.5
3.0
3.5
4.0

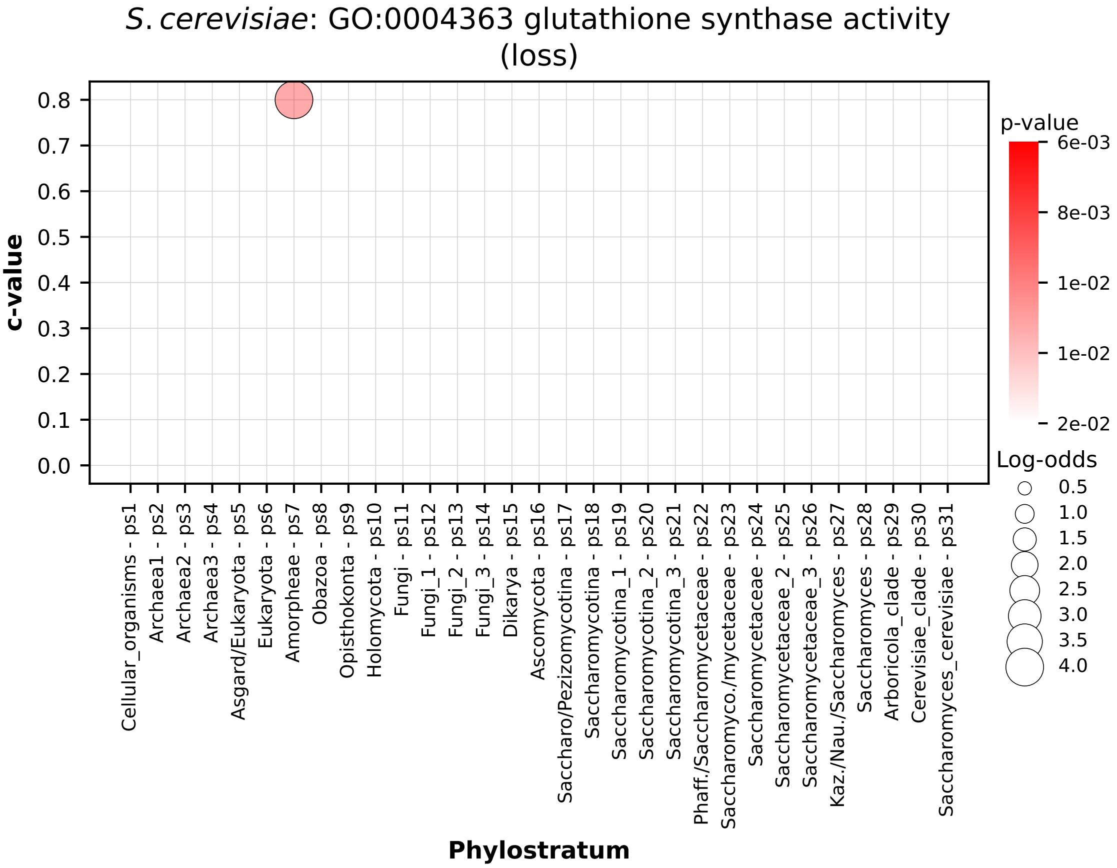

S. cerevisiae: GO:0004363 glutathione synthase activity (loss)
c-value
0.0
0.1
0.2
0.3
0.4
0.5
0.6
0.7
0.8
p-value
6e-03
8e-03
1e-02
1e-02
2e-02
Log-odds
0.5
1.0
1.5
2.0
2.5
3.0
3.5
4.0
Cellular_organisms - ps1
Archaea1 - ps2
Archaea2 - ps3
Archaea3 - ps4
Asgard/Eukaryota - ps5
Eukaryota - ps6
Amorpheae - ps7
Obazoa - ps8
Opisthokonta - ps9
Holomycota - ps10
Fungi - ps11
Fungi_1 - ps12
Fungi_2 - ps13
Fungi_3 - ps14
Dikarya - ps15
Ascomycota - ps16
Saccharo/Pezizomycotina - ps17
Saccharomycotina - ps18
Saccharomycotina_1 - ps19
Saccharomycotina_2 - ps20
Saccharomycotina_3 - ps21
Phaff./Saccharomycetaceae - ps22
Saccharomyco./mycetaceae - ps23
Saccharomycetaceae - ps24
Saccharomycetaceae_2 - ps25
Saccharomycetaceae_3 - ps26
Kaz./Nau./Saccharomyces - ps27
Saccharomyces - ps28
Arboricola_clade - ps29
Cerevisiae_clade - ps30
Saccharomyces_cerevisiae - ps31
Phylostratum

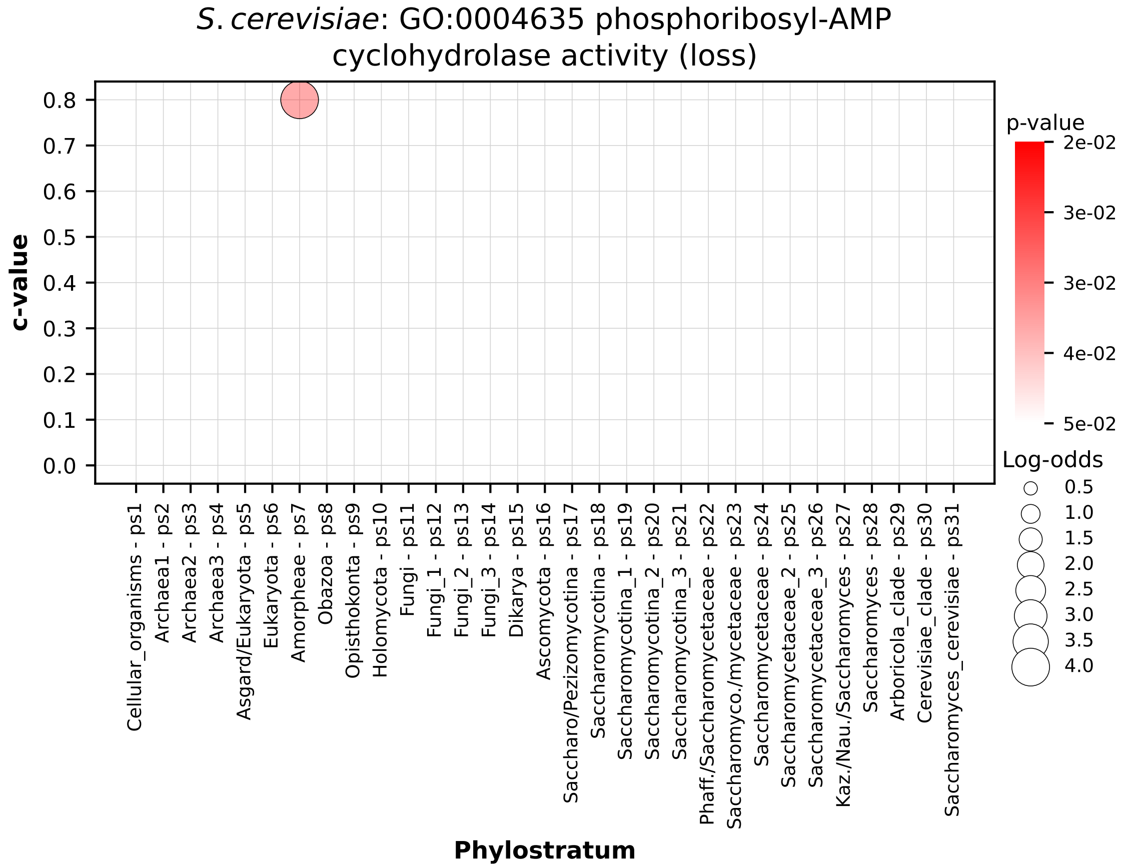

S. cerevisiae: GO:0004635 phosphoribosyl-AMP cyclohydrolase activity (loss)
c-value
0.0
0.1
0.2
0.3
0.4
0.5
0.6
0.7
0.8
Phylostratum
Cellular_organisms - ps1
Archaea1 - ps2
Archaea2 - ps3
Archaea3 - ps4
Asgard/Eukaryota - ps5
Eukaryota - ps6
Amorpheae - ps7
Obazoa - ps8
Opisthokonta - ps9
Holomycota - ps10
Fungi - ps11
Fungi_1 - ps12
Fungi_2 - ps13
Fungi_3 - ps14
Dikarya - ps15
Ascomycota - ps16
Saccharo/Pezizomycotina - ps17
Saccharomycotina - ps18
Saccharomycotina_1 - ps19
Saccharomycotina_2 - ps20
Saccharomycotina_3 - ps21
Phaff./Saccharomycetaceae - ps22
Saccharomyco./mycetaceae - ps23
Saccharomycetaceae - ps24
Saccharomycetaceae_2 - ps25
Saccharomycetaceae_3 - ps26
Kaz./Nau./Saccharomyces - ps27
Saccharomyces - ps28
Arboricola_clade - ps29
Cerevisiae_clade - ps30
Saccharomyces_cerevisiae - ps31
p-value
2e-02
3e-02
3e-02
4e-02
5e-02
Log-odds
0.5
1.0
1.5
2.0
2.5
3.0
3.5
4.0

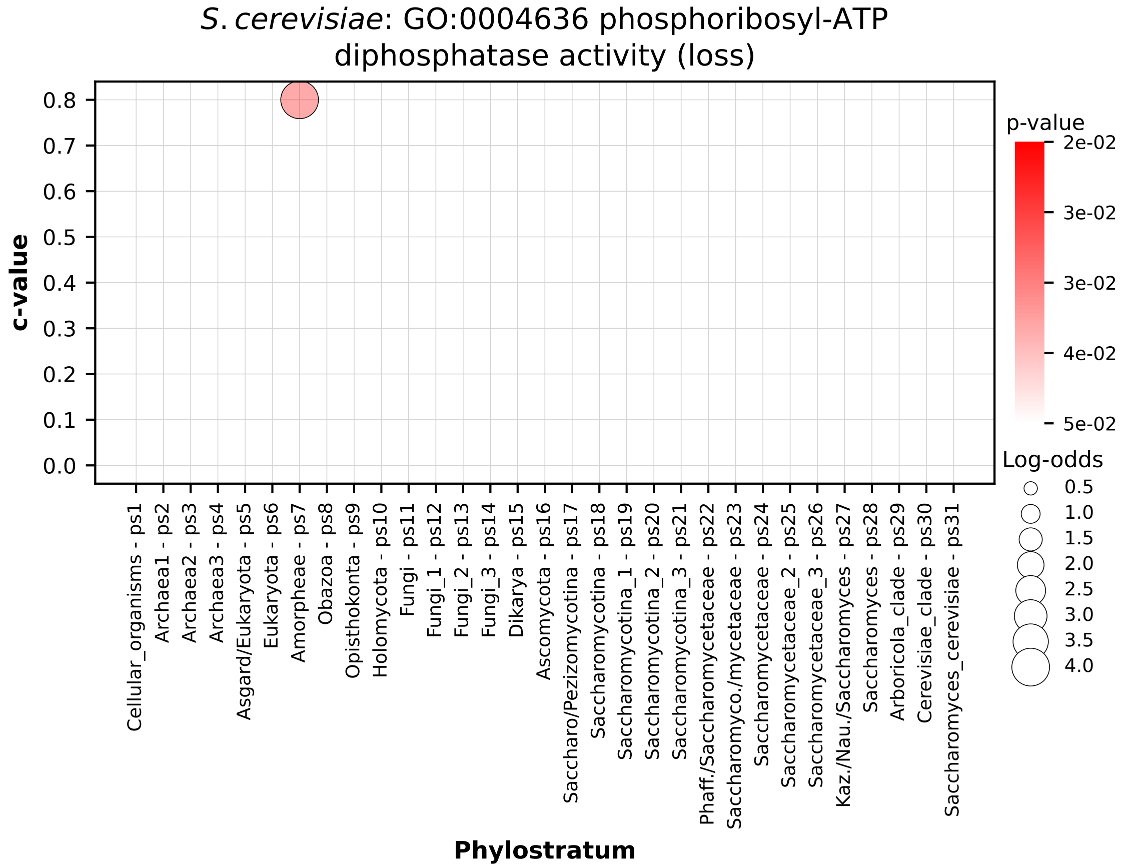
S. cerevisiae: GO:0004636 phosphoribosyl-ATP diphosphatase activity (loss)
c-value
0.0
0.1
0.2
0.3
0.4
0.5
0.6
0.7
0.8
Phylostratum
Cellular_organisms - ps1
Archaea1 - ps2
Archaea2 - ps3
Archaea3 - ps4
Asgard/Eukaryota - ps5
Eukaryota - ps6
Amorpheae - ps7
Obazoa - ps8
Opisthokonta - ps9
Holomycota - ps10
Fungi - ps11
Fungi_1 - ps12
Fungi_2 - ps13
Fungi_3 - ps14
Dikarya - ps15
Ascomycota - ps16
Saccharo/Pezizomycotina - ps17
Saccharomycotina - ps18
Saccharomycotina_1 - ps19
Saccharomycotina_2 - ps20
Saccharomycotina_3 - ps21
Phaff./Saccharomycetaceae - ps22
Saccharomyco./mycetaceae - ps23
Saccharomycetaceae - ps24
Saccharomycetaceae_2 - ps25
Saccharomycetaceae_3 - ps26
Kaz./Nau./Saccharomyces - ps27
Saccharomyces - ps28
Arboricola_clade - ps29
Cerevisiae_clade - ps30
Saccharomyces_cerevisiae - ps31
p-value
2e-02
3e-02
3e-02
4e-02
5e-02
Log-odds
0.5
1.0
1.5
2.0
2.5
3.0
3.5
4.0

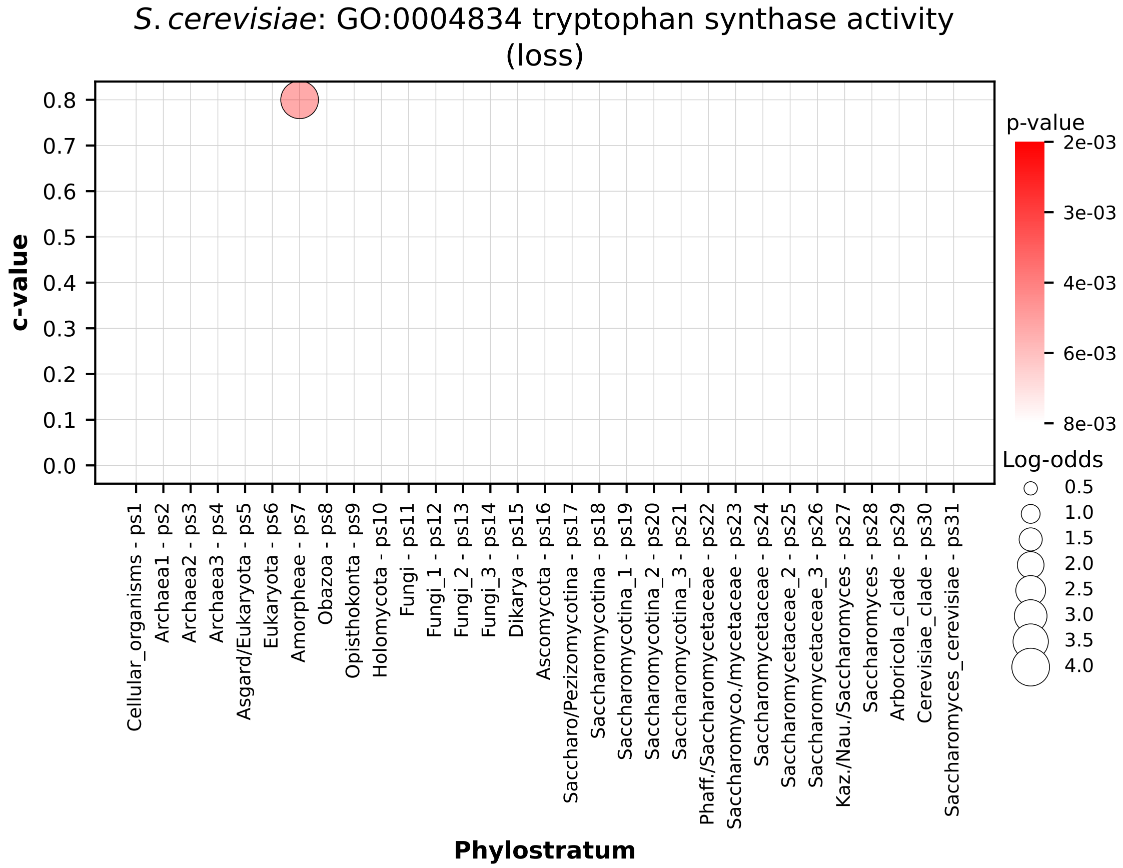
S. cerevisiae: GO:0004834 tryptophan synthase activity (loss)
c-value
0.0
0.1
0.2
0.3
0.4
0.5
0.6
0.7
0.8
p-value
2e-03
3e-03
4e-03
6e-03
8e-03
Log-odds
0.5
1.0
1.5
2.0
2.5
3.0
3.5
4.0
Cellular_organisms - ps1
Archaea1 - ps2
Archaea2 - ps3
Archaea3 - ps4
Asgard/Eukaryota - ps5
Eukaryota - ps6
Amorpheae - ps7
Obazoa - ps8
Opisthokonta - ps9
Holomycota - ps10
Fungi - ps11
Fungi_1 - ps12
Fungi_2 - ps13
Fungi_3 - ps14
Dikarya - ps15
Ascomycota - ps16
Saccharo/Pezizomycotina - ps17
Saccharomycotina - ps18
Saccharomycotina_1 - ps19
Saccharomycotina_2 - ps20
Saccharomycotina_3 - ps21
Phaff./Saccharomycetaceae - ps22
Saccharomyco./mycetaceae - ps23
Saccharomycetaceae - ps24
Saccharomycetaceae_2 - ps25
Saccharomycetaceae_3 - ps26
Kaz./Nau./Saccharomyces - ps27
Saccharomyces - ps28
Arboricola_clade - ps29
Cerevisiae_clade - ps30
Saccharomyces_cerevisiae - ps31
Phylostratum

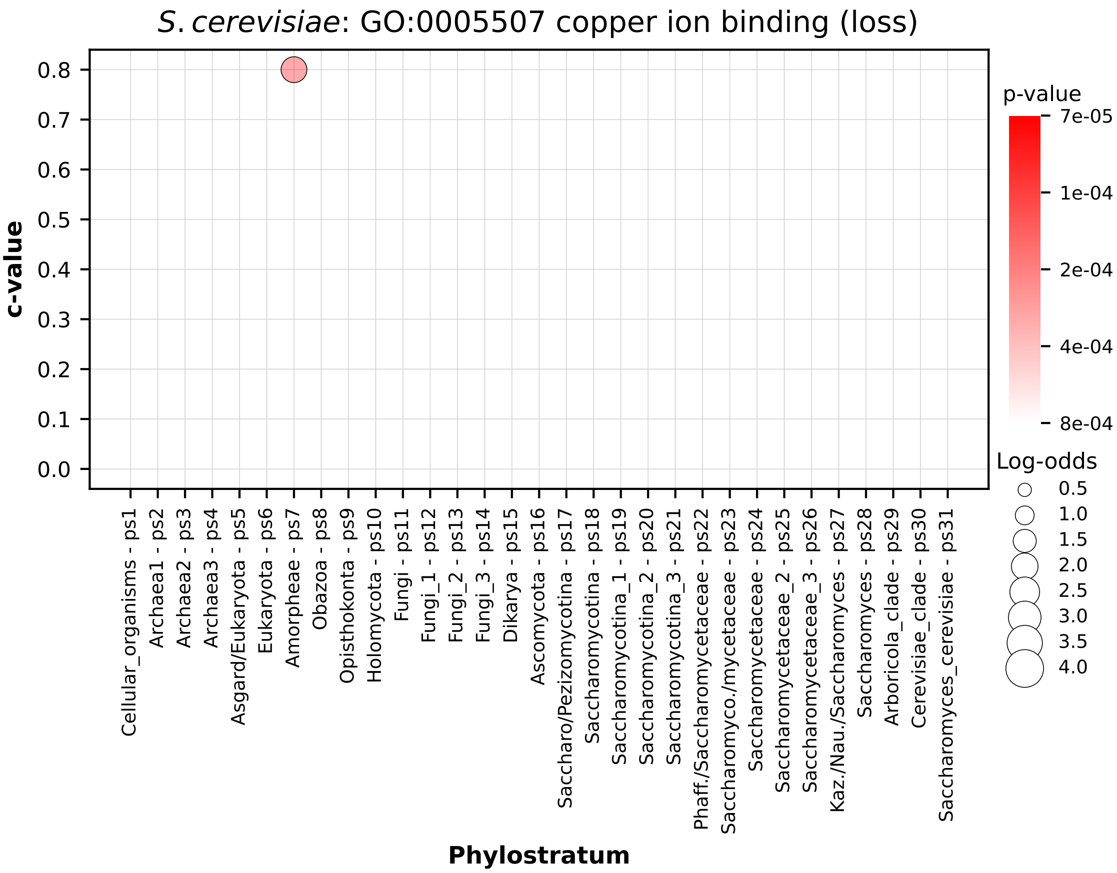
S. cerevisiae: GO:0005507 copper ion binding (loss)
c-value
0.8
0.7
0.6
0.5
0.4
0.3
0.2
0.1
0.0
Phylostratum
Cellular_organisms - ps1
Archaea1 - ps2
Archaea2 - ps3
Archaea3 - ps4
Asgard/Eukaryota - ps5
Eukaryota - ps6
Amorpheae - ps7
Obazoa - ps8
Opisthokonta - ps9
Holomycota - ps10
Fungi - ps11
Fungi_1 - ps12
Fungi_2 - ps13
Fungi_3 - ps14
Dikarya - ps15
Ascomycota - ps16
Saccharo/Pezizomycotina - ps17
Saccharomycotina - ps18
Saccharomycotina_1 - ps19
Saccharomycotina_2 - ps20
Saccharomycotina_3 - ps21
Phaff./Saccharomycetaceae - ps22
Saccharomyco./mycetaceae - ps23
Saccharomycetaceae - ps24
Saccharomycetaceae_2 - ps25
Saccharomycetaceae_3 - ps26
Kaz./Nau./Saccharomyces - ps27
Saccharomyces - ps28
Arboricola_clade - ps29
Cerevisiae_clade - ps30
Saccharomyces_cerevisiae - ps31
p-value
7e-05
1e-04
2e-04
4e-04
8e-04
Log-odds
0.5
1.0
1.5
2.0
2.5
3.0
3.5
4.0

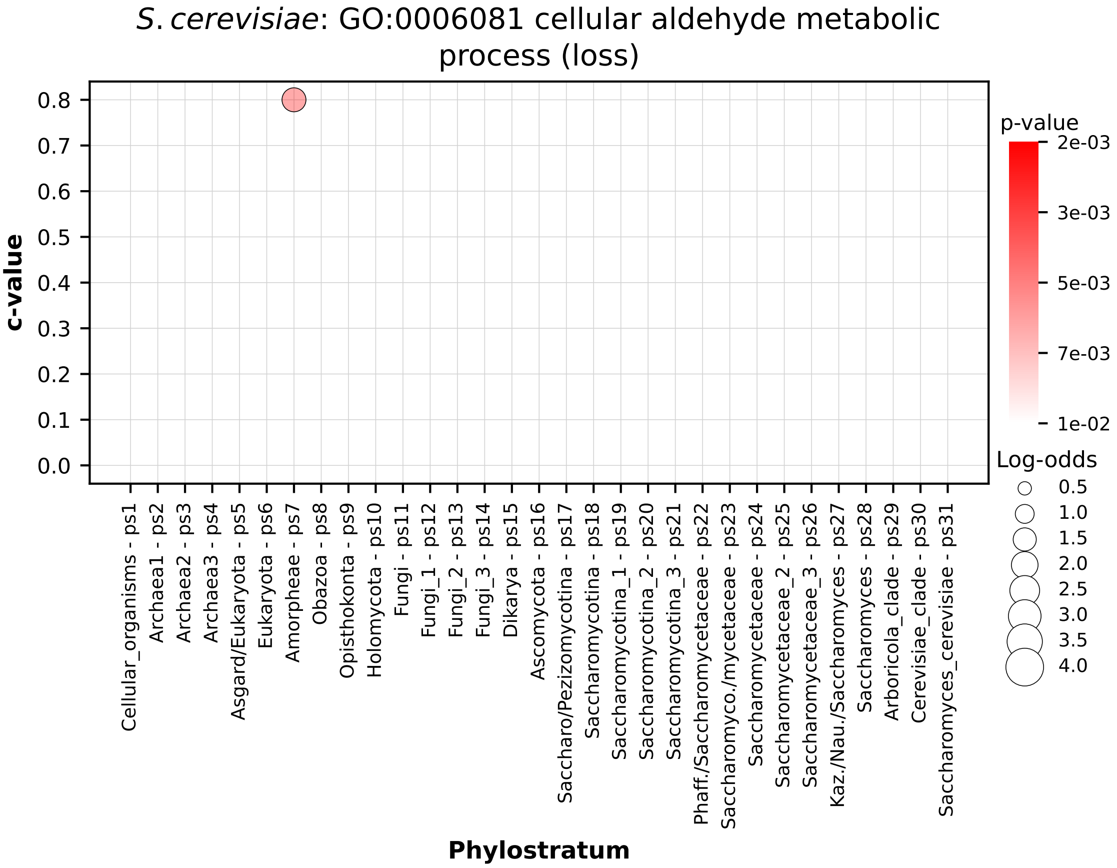

S. cerevisiae: GO:0006081 cellular aldehyde metabolic process (loss)
c-value
0.8
0.7
0.6
0.5
0.4
0.3
0.2
0.1
0.0
p-value
2e-03
3e-03
5e-03
7e-03
1e-02
Log-odds
0.5
1.0
1.5
2.0
2.5
3.0
3.5
4.0
Cellular_organisms - ps1
Archaea1 - ps2
Archaea2 - ps3
Archaea3 - ps4
Asgard/Eukaryota - ps5
Eukaryota - ps6
Amorpheae - ps7
Obazoa - ps8
Opisthokonta - ps9
Holomycota - ps10
Fungi - ps11
Fungi_1 - ps12
Fungi_2 - ps13
Fungi_3 - ps14
Dikarya - ps15
Ascomycota - ps16
Saccharo/Pezizomycotina - ps17
Saccharomycotina - ps18
Saccharomycotina_1 - ps19
Saccharomycotina_2 - ps20
Saccharomycotina_3 - ps21
Phaff./Saccharomycetaceae - ps22
Saccharomyco./mycetaceae - ps23
Saccharomycetaceae - ps24
Saccharomycetaceae_2 - ps25
Saccharomycetaceae_3 - ps26
Kaz./Nau./Saccharomyces - ps27
Saccharomyces - ps28
Arboricola_clade - ps29
Cerevisiae_clade - ps30
Saccharomyces_cerevisiae - ps31
Phylostratum

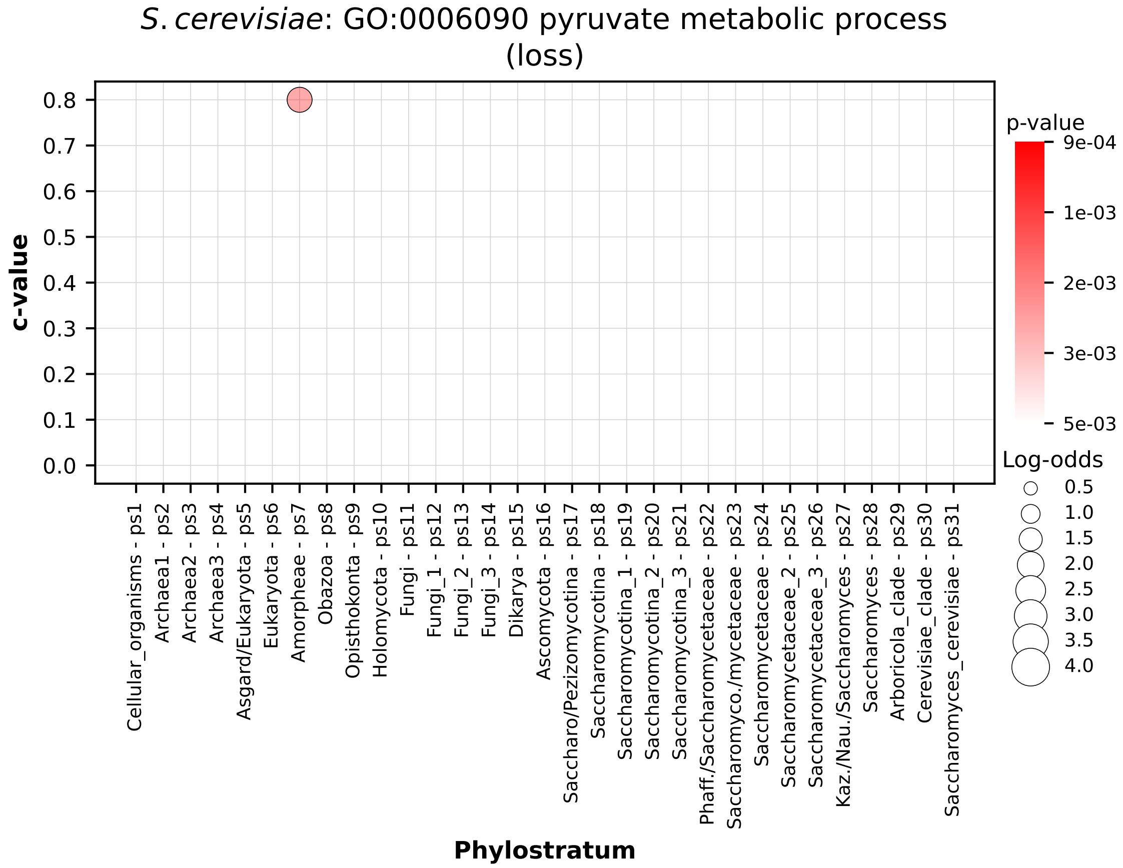

S. cerevisiae: GO:0006090 pyruvate metabolic process
(loss)
c-value
0.0
0.1
0.2
0.3
0.4
0.5
0.6
0.7
0.8
p-value
9e-04
1e-03
2e-03
3e-03
5e-03
Log-odds
0.5
1.0
1.5
2.0
2.5
3.0
3.5
4.0
Cellular_organisms - ps1
Archaea1 - ps2
Archaea2 - ps3
Archaea3 - ps4
Asgard/Eukaryota - ps5
Eukaryota - ps6
Amorpheae - ps7
Obazoa - ps8
Opisthokonta - ps9
Holomycota - ps10
Fungi - ps11
Fungi_1 - ps12
Fungi_2 - ps13
Fungi_3 - ps14
Dikarya - ps15
Ascomycota - ps16
Saccharo/Pezizomycotina - ps17
Saccharomycotina - ps18
Saccharomycotina_1 - ps19
Saccharomycotina_2 - ps20
Saccharomycotina_3 - ps21
Phaff./Saccharomycetaceae - ps22
Saccharomyco./mycetaceae - ps23
Saccharomycetaceae - ps24
Saccharomycetaceae_2 - ps25
Saccharomycetaceae_3 - ps26
Kaz./Nau./Saccharomyces - ps27
Saccharomyces - ps28
Arboricola_clade - ps29
Cerevisiae_clade - ps30
Saccharomyces_cerevisiae - ps31
Phylostratum

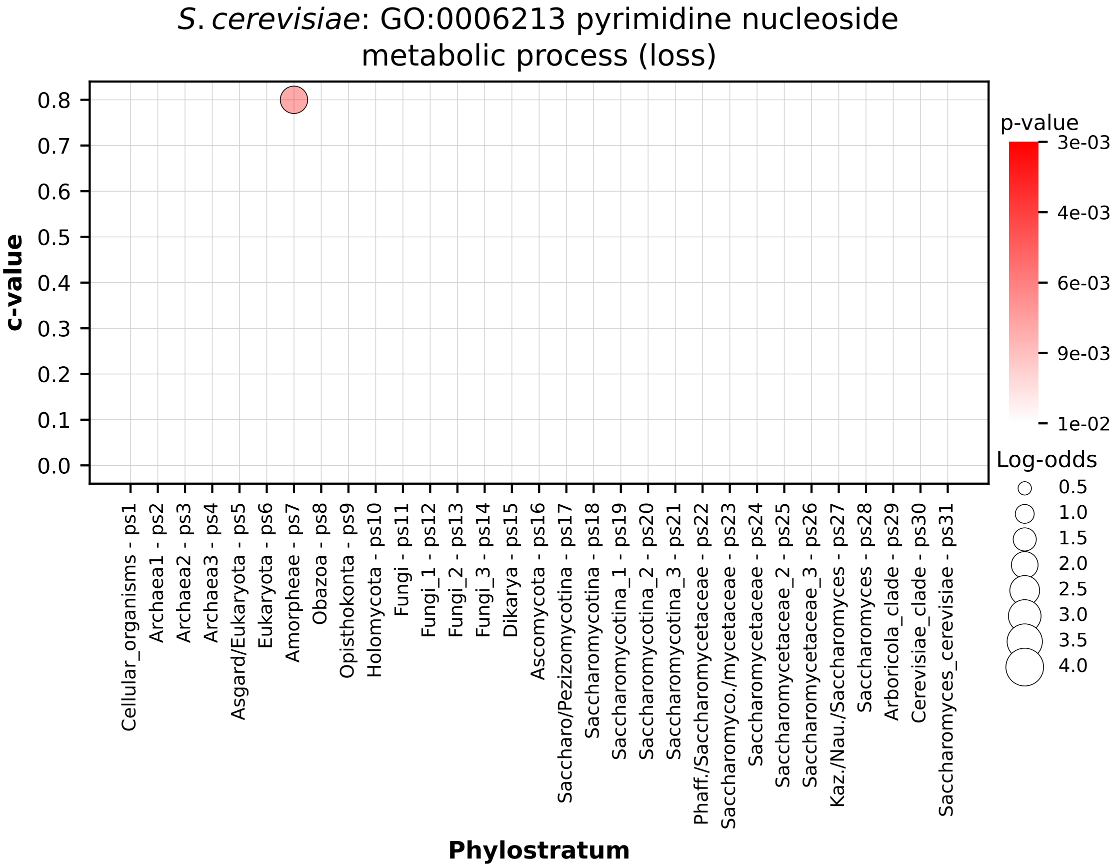

S. cerevisiae: GO:0006213 pyrimidine nucleoside metabolic process (loss)
c-value
0.0
0.1
0.2
0.3
0.4
0.5
0.6
0.7
0.8
p-value
3e-03
4e-03
6e-03
9e-03
1e-02
Log-odds
0.5
1.0
1.5
2.0
2.5
3.0
3.5
4.0
Cellular_organisms - ps1
Archaea1 - ps2
Archaea2 - ps3
Archaea3 - ps4
Asgard/Eukaryota - ps5
Eukaryota - ps6
Amorpheae - ps7
Obazoa - ps8
Opisthokonta - ps9
Holomycota - ps10
Fungi - ps11
Fungi_1 - ps12
Fungi_2 - ps13
Fungi_3 - ps14
Dikarya - ps15
Ascomycota - ps16
Saccharo/Pezizomycotina - ps17
Saccharomycotina - ps18
Saccharomycotina_1 - ps19
Saccharomycotina_2 - ps20
Saccharomycotina_3 - ps21
Phaff./Saccharomycetaceae - ps22
Saccharomyco./mycetaceae - ps23
Saccharomycetaceae - ps24
Saccharomycetaceae_2 - ps25
Saccharomycetaceae_3 - ps26
Kaz./Nau./Saccharomyces - ps27
Saccharomyces - ps28
Arboricola_clade - ps29
Cerevisiae_clade - ps30
Saccharomyces_cerevisiae - ps31
Phylostratum

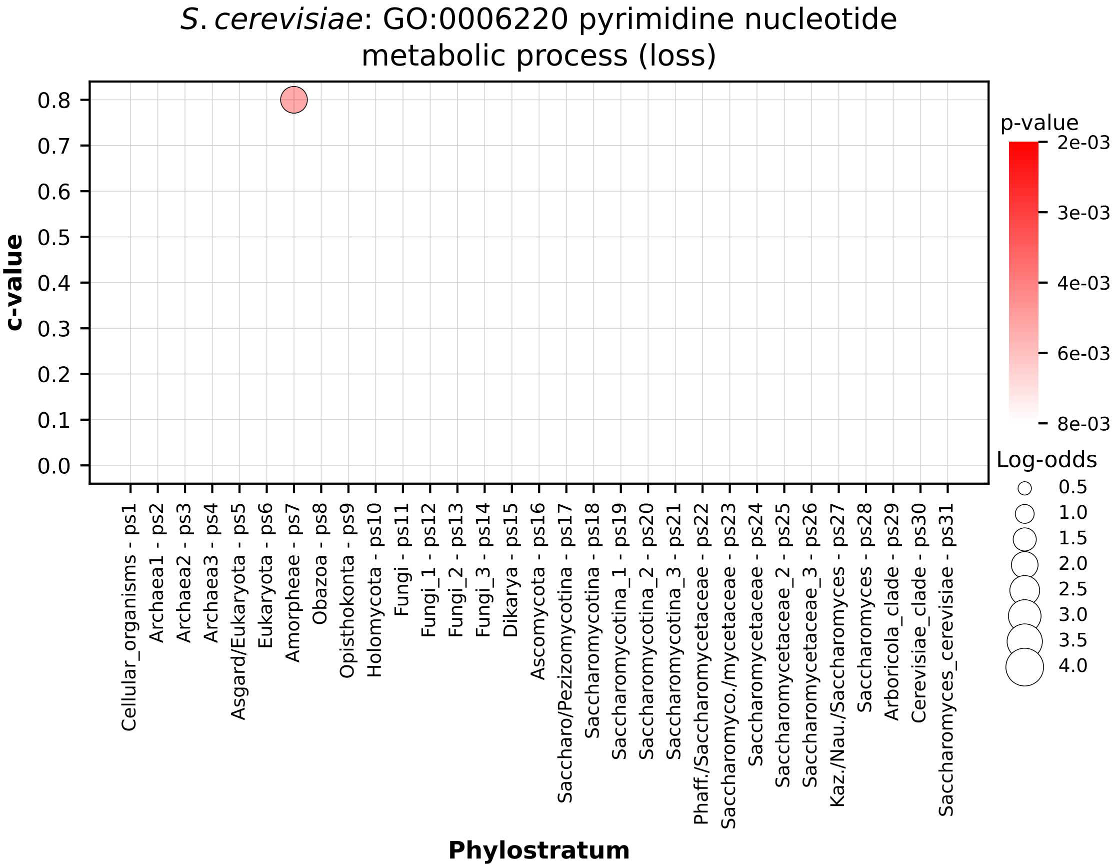
S. cerevisiae: GO:0006220 pyrimidine nucleotide metabolic process (loss)
c-value
0.0
0.1
0.2
0.3
0.4
0.5
0.6
0.7
0.8
Phylostratum
Cellular_organisms - ps1
Archaea1 - ps2
Archaea2 - ps3
Archaea3 - ps4
Asgard/Eukaryota - ps5
Eukaryota - ps6
Amorpheae - ps7
Obazoa - ps8
Opisthokonta - ps9
Holomycota - ps10
Fungi - ps11
Fungi_1 - ps12
Fungi_2 - ps13
Fungi_3 - ps14
Dikarya - ps15
Ascomycota - ps16
Saccharo/Pezizomycotina - ps17
Saccharomycotina - ps18
Saccharomycotina_1 - ps19
Saccharomycotina_2 - ps20
Saccharomycotina_3 - ps21
Phaff./Saccharomycetaceae - ps22
Saccharomyco./mycetaceae - ps23
Saccharomycetaceae - ps24
Saccharomycetaceae_2 - ps25
Saccharomycetaceae_3 - ps26
Kaz./Nau./Saccharomyces - ps27
Saccharomyces - ps28
Arboricola_clade - ps29
Cerevisiae_clade - ps30
Saccharomyces_cerevisiae - ps31
p-value
2e-03
3e-03
4e-03
6e-03
8e-03
Log-odds
0.5
1.0
1.5
2.0
2.5
3.0
3.5
4.0

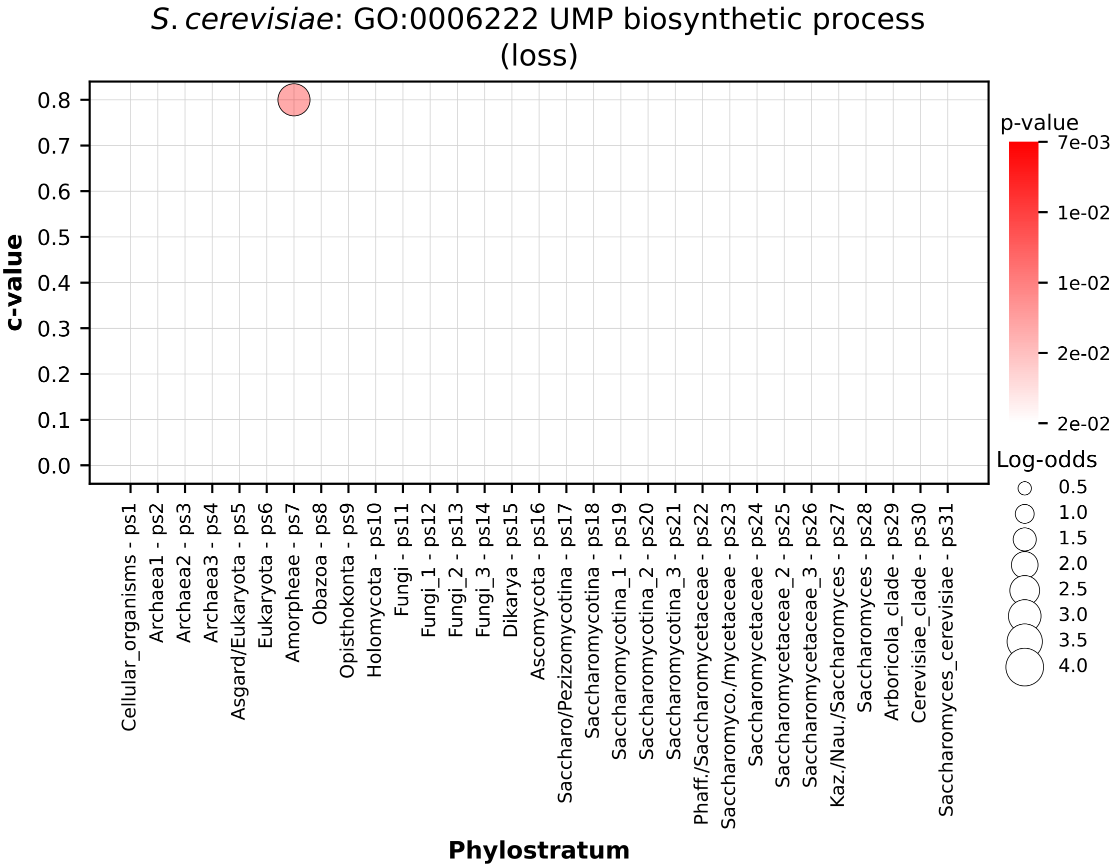
S. cerevisiae: GO:0006222 UMP biosynthetic process
(loss)
c-value
0.0
0.1
0.2
0.3
0.4
0.5
0.6
0.7
0.8
p-value
7e-03
1e-02
1e-02
2e-02
2e-02
Log-odds
0.5
1.0
1.5
2.0
2.5
3.0
3.5
4.0
Cellular_organisms - ps1
Archaea1 - ps2
Archaea2 - ps3
Archaea3 - ps4
Asgard/Eukaryota - ps5
Eukaryota - ps6
Amorpheae - ps7
Obazoa - ps8
Opisthokonta - ps9
Holomycota - ps10
Fungi - ps11
Fungi_1 - ps12
Fungi_2 - ps13
Fungi_3 - ps14
Dikarya - ps15
Ascomycota - ps16
Saccharo/Pezizomycotina - ps17
Saccharomycotina - ps18
Saccharomycotina_1 - ps19
Saccharomycotina_2 - ps20
Saccharomycotina_3 - ps21
Phaff./Saccharomycetaceae - ps22
Saccharomyco./mycetaceae - ps23
Saccharomycetaceae - ps24
Saccharomycetaceae_2 - ps25
Saccharomycetaceae_3 - ps26
Kaz./Nau./Saccharomyces - ps27
Saccharomyces - ps28
Arboricola_clade - ps29
Cerevisiae_clade - ps30
Saccharomyces_cerevisiae - ps31
Phylostratum

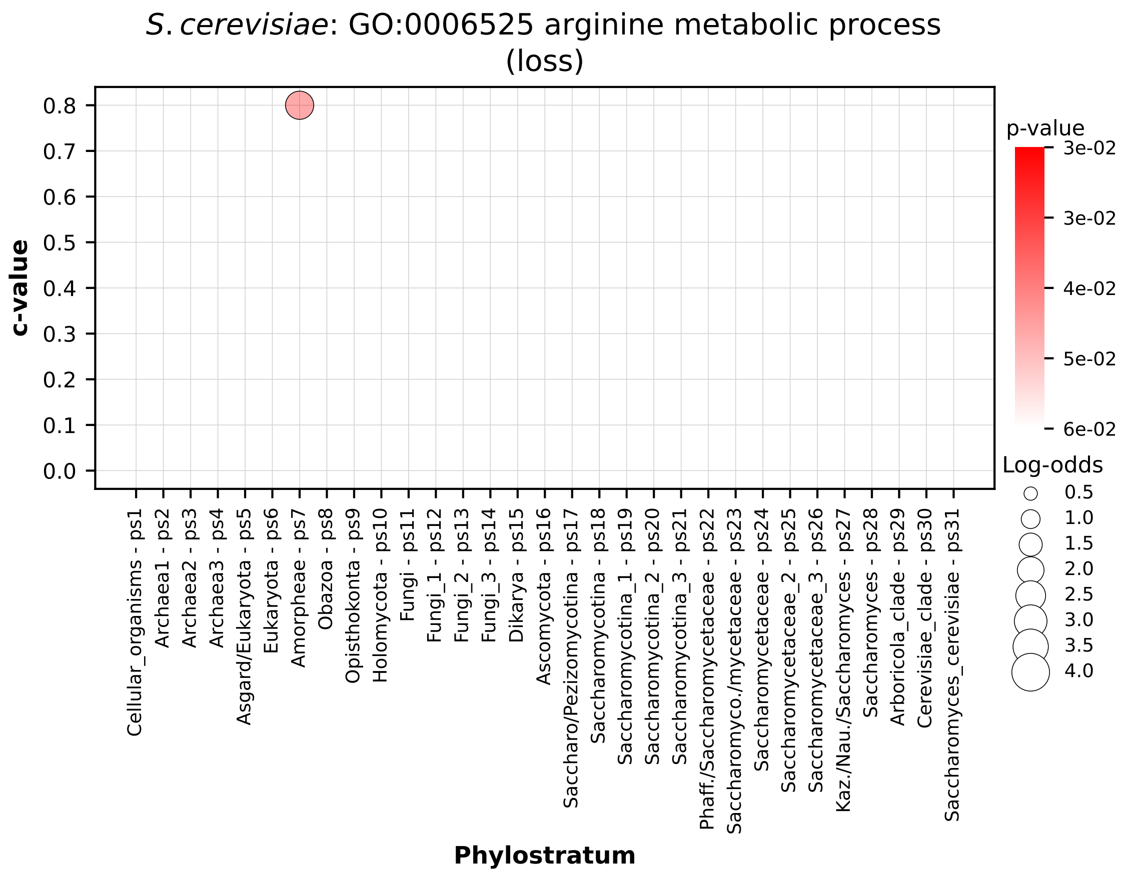
S. cerevisiae: GO:0006525 arginine metabolic process
(loss)
c-value
0.0
0.1
0.2
0.3
0.4
0.5
0.6
0.7
0.8
Phylostratum
Cellular_organisms - ps1
Archaea1 - ps2
Archaea2 - ps3
Archaea3 - ps4
Asgard/Eukaryota - ps5
Eukaryota - ps6
Amorpheae - ps7
Obazoa - ps8
Opisthokonta - ps9
Holomycota - ps10
Fungi - ps11
Fungi_1 - ps12
Fungi_2 - ps13
Fungi_3 - ps14
Dikarya - ps15
Ascomycota - ps16
Saccharo/Pezizomycotina - ps17
Saccharomycotina - ps18
Saccharomycotina_1 - ps19
Saccharomycotina_2 - ps20
Saccharomycotina_3 - ps21
Phaff./Saccharomycetaceae - ps22
Saccharomyco./mycetaceae - ps23
Saccharomycetaceae - ps24
Saccharomycetaceae_2 - ps25
Saccharomycetaceae_3 - ps26
Kaz./Nau./Saccharomyces - ps27
Saccharomyces - ps28
Arboricola_clade - ps29
Cerevisiae_clade - ps30
Saccharomyces_cerevisiae - ps31
p-value
3e-02
3e-02
4e-02
5e-02
6e-02
Log-odds
0.5
1.0
1.5
2.0
2.5
3.0
3.5
4.0

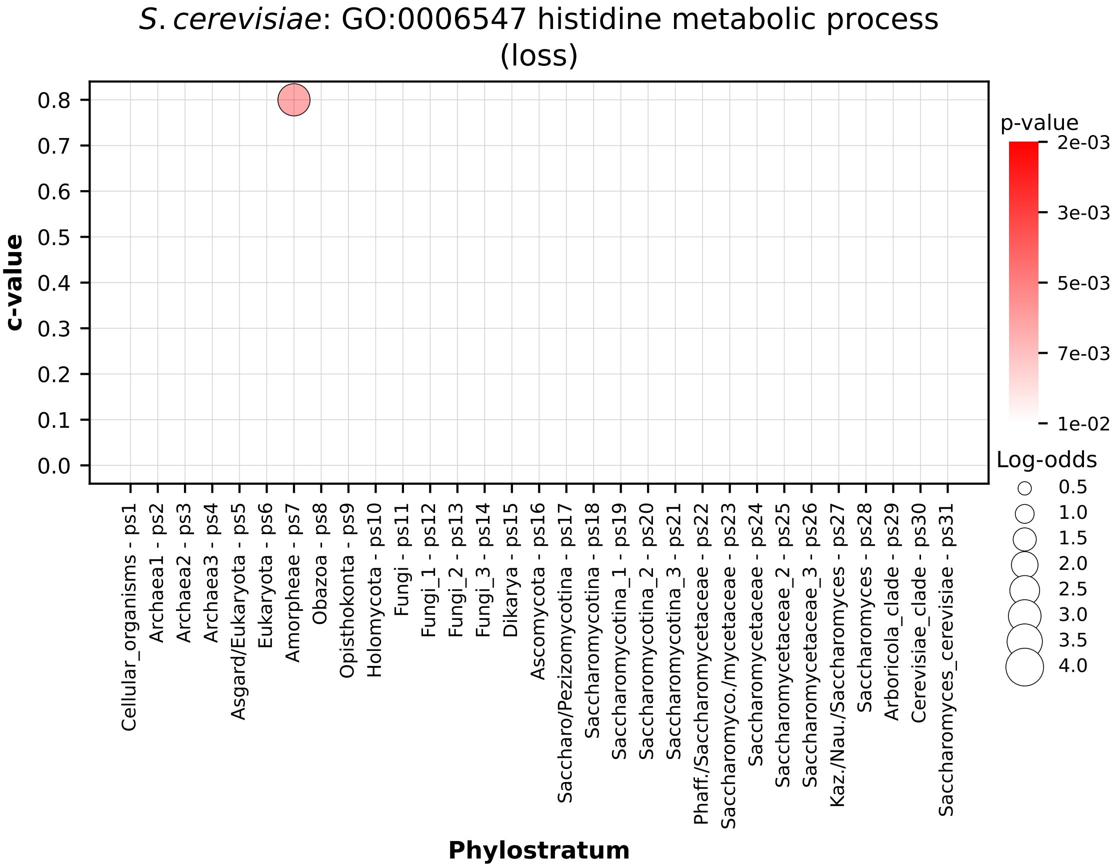
S. cerevisiae: GO:0006547 histidine metabolic process
(loss)
c-value
0.0
0.1
0.2
0.3
0.4
0.5
0.6
0.7
0.8
Phylostratum
Cellular_organisms - ps1
Archaea1 - ps2
Archaea2 - ps3
Archaea3 - ps4
Asgard/Eukaryota - ps5
Eukaryota - ps6
Amorpheae - ps7
Obazoa - ps8
Opisthokonta - ps9
Holomycota - ps10
Fungi - ps11
Fungi_1 - ps12
Fungi_2 - ps13
Fungi_3 - ps14
Dikarya - ps15
Ascomycota - ps16
Saccharo/Pezizomycotina - ps17
Saccharomycotina - ps18
Saccharomycotina_1 - ps19
Saccharomycotina_2 - ps20
Saccharomycotina_3 - ps21
Phaff./Saccharomycetaceae - ps22
Saccharomyco./mycetaceae - ps23
Saccharomycetaceae - ps24
Saccharomycetaceae_2 - ps25
Saccharomycetaceae_3 - ps26
Kaz./Nau./Saccharomyces - ps27
Saccharomyces - ps28
Arboricola_clade - ps29
Cerevisiae_clade - ps30
Saccharomyces_cerevisiae - ps31
p-value
2e-03
3e-03
5e-03
7e-03
1e-02
Log-odds
0.5
1.0
1.5
2.0
2.5
3.0
3.5
4.0

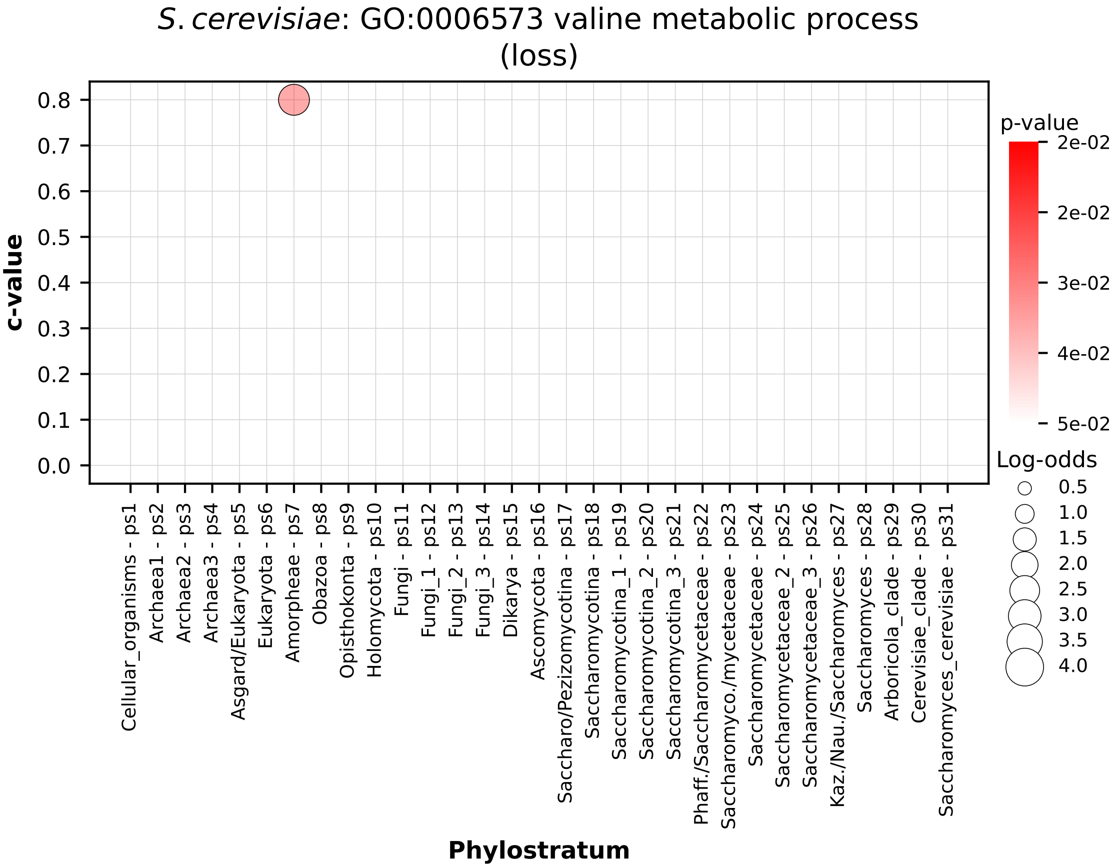

S. cerevisiae: GO:0006573 valine metabolic process
(loss)
c-value
0.0
0.1
0.2
0.3
0.4
0.5
0.6
0.7
0.8
p-value
2e-02
2e-02
3e-02
4e-02
5e-02
Log-odds
0.5
1.0
1.5
2.0
2.5
3.0
3.5
4.0
Cellular_organisms - ps1
Archaea1 - ps2
Archaea2 - ps3
Archaea3 - ps4
Asgard/Eukaryota - ps5
Eukaryota - ps6
Amorpheae - ps7
Obazoa - ps8
Opisthokonta - ps9
Holomycota - ps10
Fungi - ps11
Fungi_1 - ps12
Fungi_2 - ps13
Fungi_3 - ps14
Dikarya - ps15
Ascomycota - ps16
Saccharo/Pezizomycotina - ps17
Saccharomycotina - ps18
Saccharomycotina_1 - ps19
Saccharomycotina_2 - ps20
Saccharomycotina_3 - ps21
Phaff./Saccharomycetaceae - ps22
Saccharomyco./mycetaceae - ps23
Saccharomycetaceae - ps24
Saccharomycetaceae_2 - ps25
Saccharomycetaceae_3 - ps26
Kaz./Nau./Saccharomyces - ps27
Saccharomyces - ps28
Arboricola_clade - ps29
Cerevisiae_clade - ps30
Saccharomyces_cerevisiae - ps31
Phylostratum

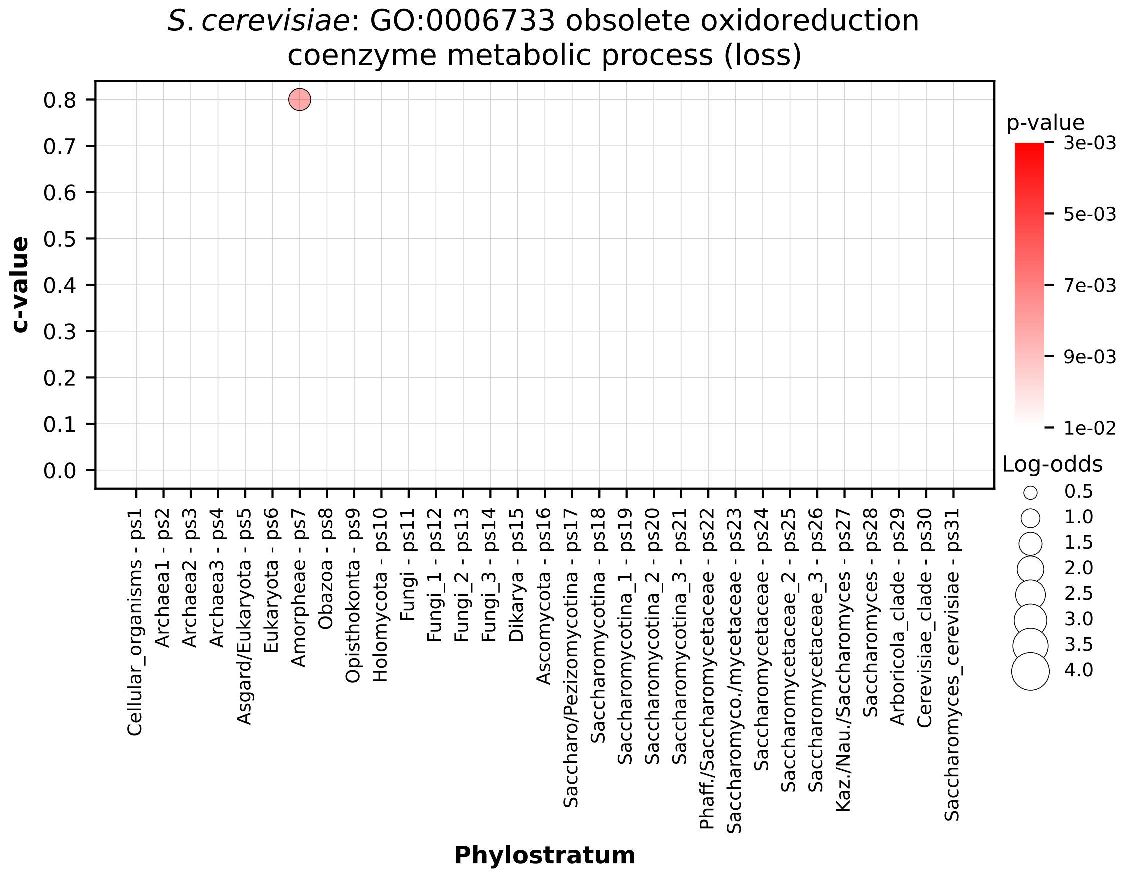
S. cerevisiae: GO:0006733 obsolete oxidoreduction coenzyme metabolic process (loss)
c-value
0.8
0.7
0.6
0.5
0.4
0.3
0.2
0.1
0.0
p-value
3e-03
5e-03
7e-03
9e-03
1e-02
Log-odds
0.5
1.0
1.5
2.0
2.5
3.0
3.5
4.0
Cellular_organisms - ps1
Archaea1 - ps2
Archaea2 - ps3
Archaea3 - ps4
Asgard/Eukaryota - ps5
Eukaryota - ps6
Amorpheae - ps7
Obazoa - ps8
Opisthokonta - ps9
Holomycota - ps10
Fungi - ps11
Fungi_1 - ps12
Fungi_2 - ps13
Fungi_3 - ps14
Dikarya - ps15
Ascomycota - ps16
Saccharo/Pezizomycotina - ps17
Saccharomycotina - ps18
Saccharomycotina_1 - ps19
Saccharomycotina_2 - ps20
Saccharomycotina_3 - ps21
Phaff./Saccharomycetaceae - ps22
Saccharomyco./mycetaceae - ps23
Saccharomycetaceae - ps24
Saccharomycetaceae_2 - ps25
Saccharomycetaceae_3 - ps26
Kaz./Nau./Saccharomyces - ps27
Saccharomyces - ps28
Arboricola_clade - ps29
Cerevisiae_clade - ps30
Saccharomyces_cerevisiae - ps31
Phylostratum

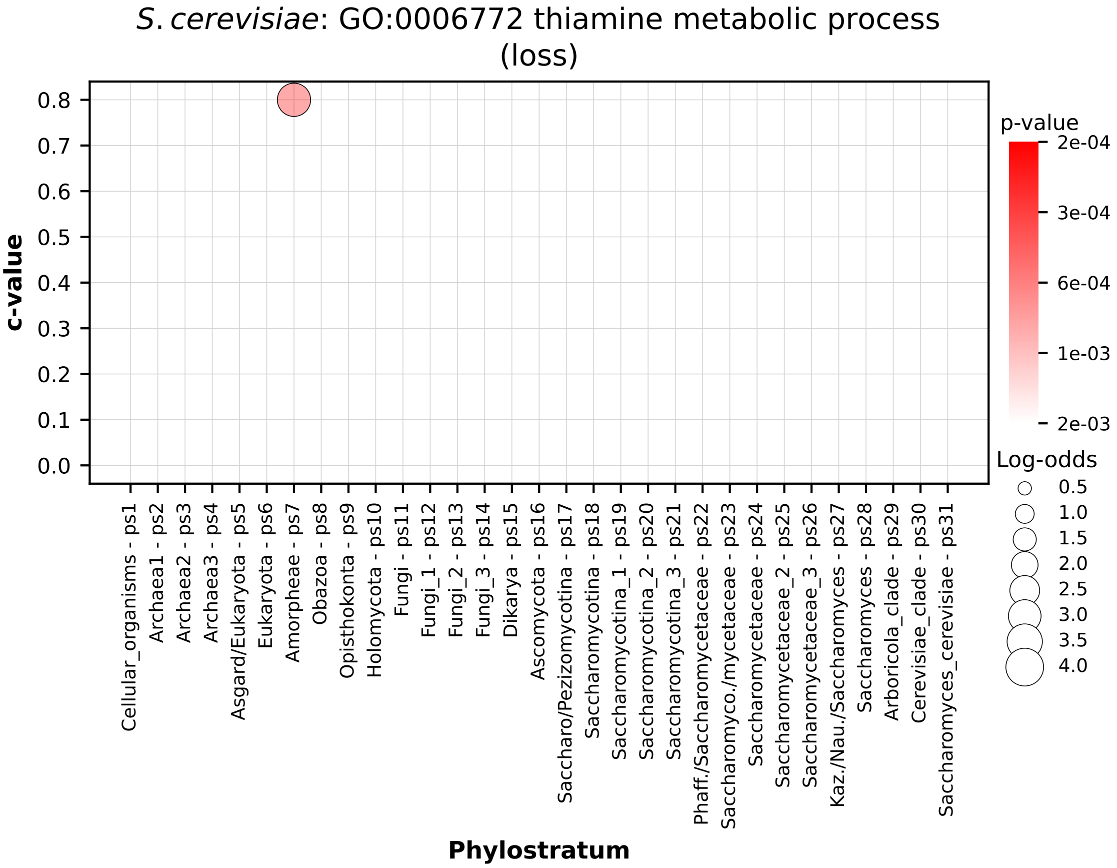

S. cerevisiae: GO:0006772 thiamine metabolic process
(loss)
c-value
0.8
0.7
0.6
0.5
0.4
0.3
0.2
0.1
0.0
p-value
2e-04
3e-04
6e-04
1e-03
2e-03
Log-odds
0.5
1.0
1.5
2.0
2.5
3.0
3.5
4.0
Cellular_organisms - ps1
Archaea1 - ps2
Archaea2 - ps3
Archaea3 - ps4
Asgard/Eukaryota - ps5
Eukaryota - ps6
Amorpheae - ps7
Obazoa - ps8
Opisthokonta - ps9
Holomycota - ps10
Fungi - ps11
Fungi_1 - ps12
Fungi_2 - ps13
Fungi_3 - ps14
Dikarya - ps15
Ascomycota - ps16
Saccharo/Pezizomycotina - ps17
Saccharomycotina - ps18
Saccharomycotina_1 - ps19
Saccharomycotina_2 - ps20
Saccharomycotina_3 - ps21
Phaff./Saccharomycetaceae - ps22
Saccharomyco./mycetaceae - ps23
Saccharomycetaceae - ps24
Saccharomycetaceae_2 - ps25
Saccharomycetaceae_3 - ps26
Kaz./Nau./Saccharomyces - ps27
Saccharomyces - ps28
Arboricola_clade - ps29
Cerevisiae_clade - ps30
Saccharomyces_cerevisiae - ps31
Phylostratum

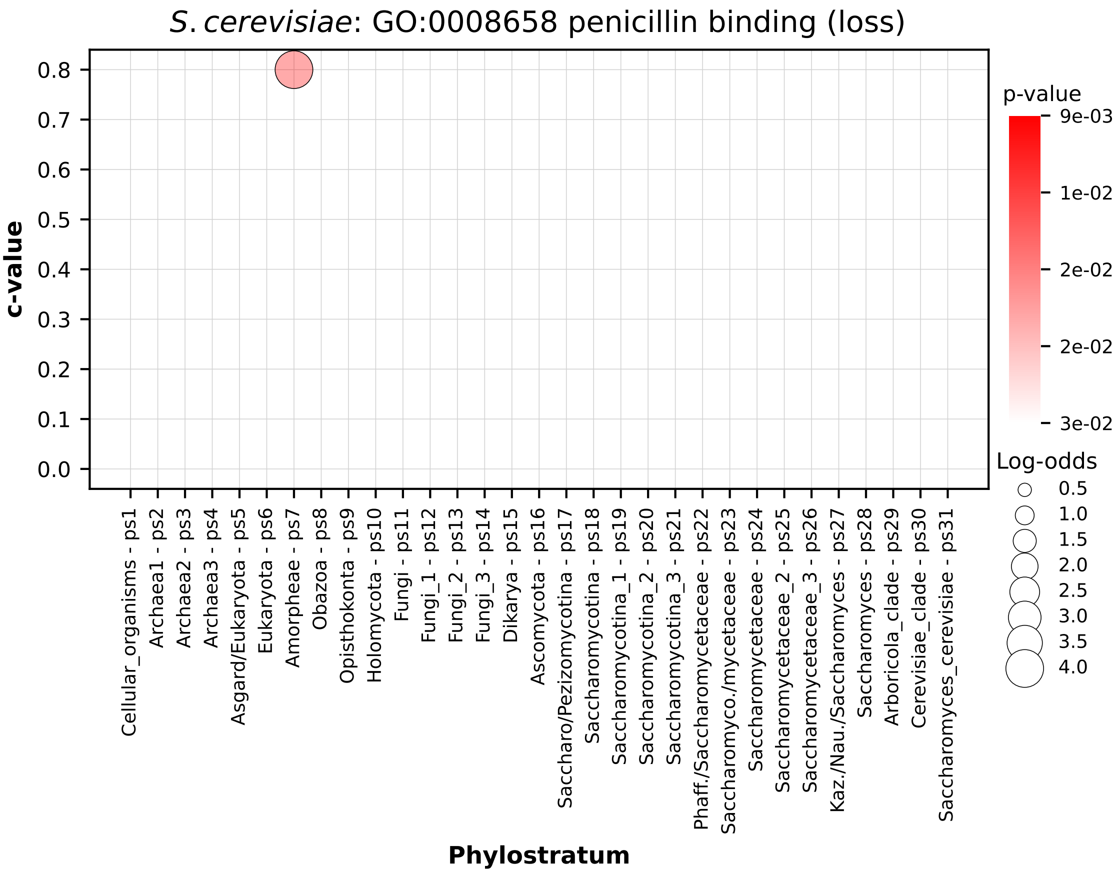
S. cerevisiae: GO:0008658 penicillin binding (loss)
c-value
0.0
0.1
0.2
0.3
0.4
0.5
0.6
0.7
0.8
p-value
9e-03
1e-02
2e-02
2e-02
3e-02
Log-odds
0.5
1.0
1.5
2.0
2.5
3.0
3.5
4.0
Cellular_organisms - ps1
Archaea1 - ps2
Archaea2 - ps3
Archaea3 - ps4
Asgard/Eukaryota - ps5
Eukaryota - ps6
Amorpheae - ps7
Obazoa - ps8
Opisthokonta - ps9
Holomycota - ps10
Fungi - ps11
Fungi_1 - ps12
Fungi_2 - ps13
Fungi_3 - ps14
Dikarya - ps15
Ascomycota - ps16
Saccharo/Pezizomycotina - ps17
Saccharomycotina - ps18
Saccharomycotina_1 - ps19
Saccharomycotina_2 - ps20
Saccharomycotina_3 - ps21
Phaff./Saccharomycetaceae - ps22
Saccharomyco./mycetaceae - ps23
Saccharomycetaceae - ps24
Saccharomycetaceae_2 - ps25
Saccharomycetaceae_3 - ps26
Kaz./Nau./Saccharomyces - ps27
Saccharomyces - ps28
Arboricola_clade - ps29
Cerevisiae_clade - ps30
Saccharomyces_cerevisiae - ps31
Phylostratum

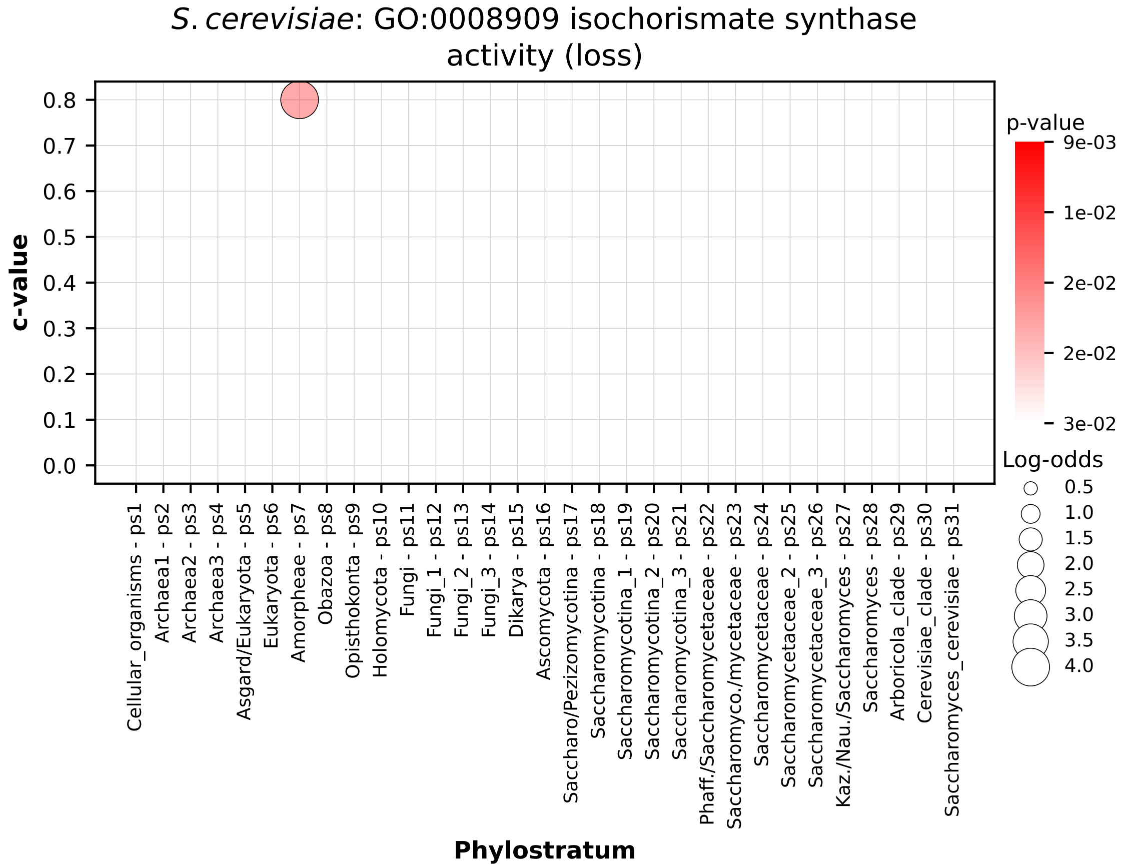

S. cerevisiae: GO:0008909 isochorismate synthase activity (loss)
c-value
0.0
0.1
0.2
0.3
0.4
0.5
0.6
0.7
0.8
p-value
9e-03
1e-02
2e-02
2e-02
3e-02
Log-odds
0.5
1.0
1.5
2.0
2.5
3.0
3.5
4.0
Cellular_organisms - ps1
Archaea1 - ps2
Archaea2 - ps3
Archaea3 - ps4
Asgard/Eukaryota - ps5
Eukaryota - ps6
Amorpheae - ps7
Obazoa - ps8
Opisthokonta - ps9
Holomycota - ps10
Fungi - ps11
Fungi_1 - ps12
Fungi_2 - ps13
Fungi_3 - ps14
Dikarya - ps15
Ascomycota - ps16
Saccharo/Pezizomycotina - ps17
Saccharomycotina - ps18
Saccharomycotina_1 - ps19
Saccharomycotina_2 - ps20
Saccharomycotina_3 - ps21
Phaff./Saccharomycetaceae - ps22
Saccharomyco./mycetaceae - ps23
Saccharomycetaceae - ps24
Saccharomycetaceae_2 - ps25
Saccharomycetaceae_3 - ps26
Kaz./Nau./Saccharomyces - ps27
Saccharomyces - ps28
Arboricola_clade - ps29
Cerevisiae_clade - ps30
Saccharomyces_cerevisiae - ps31
Phylostratum

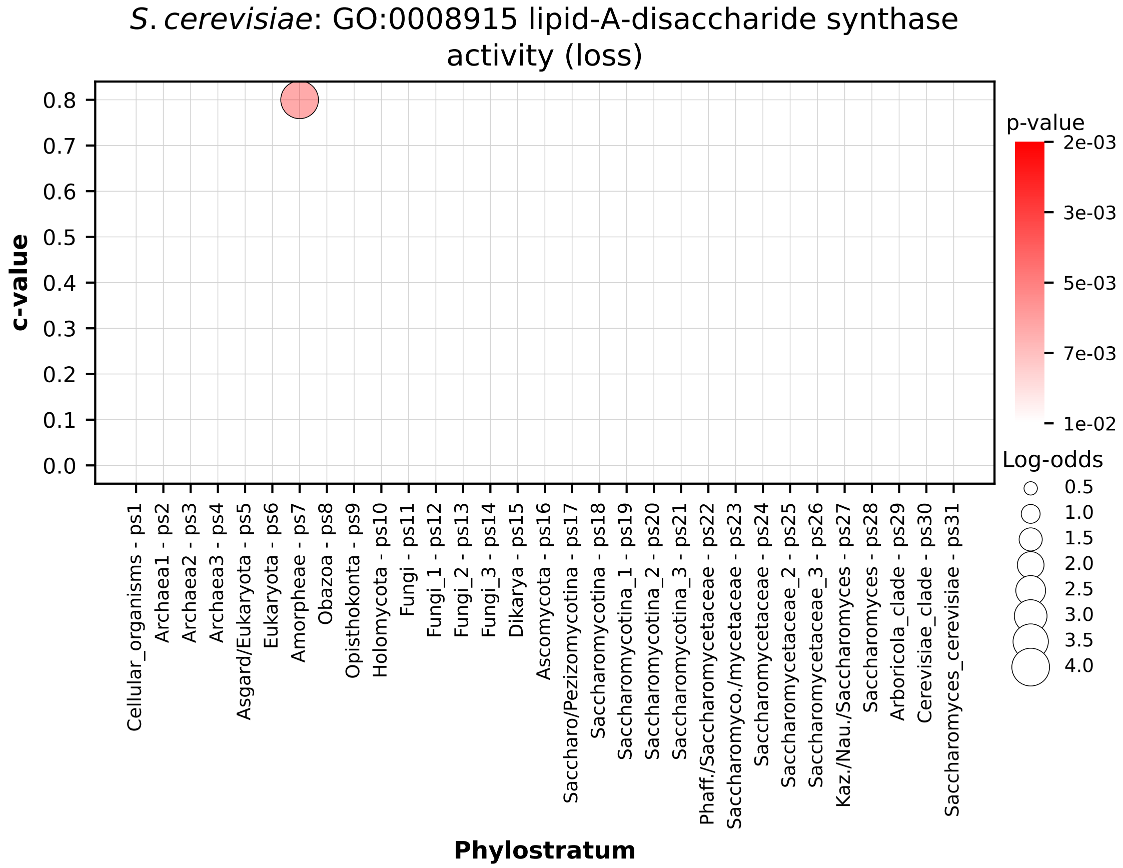

S. cerevisiae: GO:0008915 lipid-A-disaccharide synthase activity (loss)
c-value
0.8
0.7
0.6
0.5
0.4
0.3
0.2
0.1
0.0
p-value
2e-03
3e-03
5e-03
7e-03
1e-02
Log-odds
0.5
1.0
1.5
2.0
2.5
3.0
3.5
4.0
Cellular_organisms - ps1
Archaea1 - ps2
Archaea2 - ps3
Archaea3 - ps4
Asgard/Eukaryota - ps5
Eukaryota - ps6
Amorpheae - ps7
Obazoa - ps8
Opisthokonta - ps9
Holomycota - ps10
Fungi - ps11
Fungi_1 - ps12
Fungi_2 - ps13
Fungi_3 - ps14
Dikarya - ps15
Ascomycota - ps16
Saccharo/Pezizomycotina - ps17
Saccharomycotina - ps18
Saccharomycotina_1 - ps19
Saccharomycotina_2 - ps20
Saccharomycotina_3 - ps21
Phaff./Saccharomycetaceae - ps22
Saccharomyco./mycetaceae - ps23
Saccharomycetaceae - ps24
Saccharomycetaceae_2 - ps25
Saccharomycetaceae_3 - ps26
Kaz./Nau./Saccharomyces - ps27
Saccharomyces - ps28
Arboricola_clade - ps29
Cerevisiae_clade - ps30
Saccharomyces_cerevisiae - ps31
Phylostratum

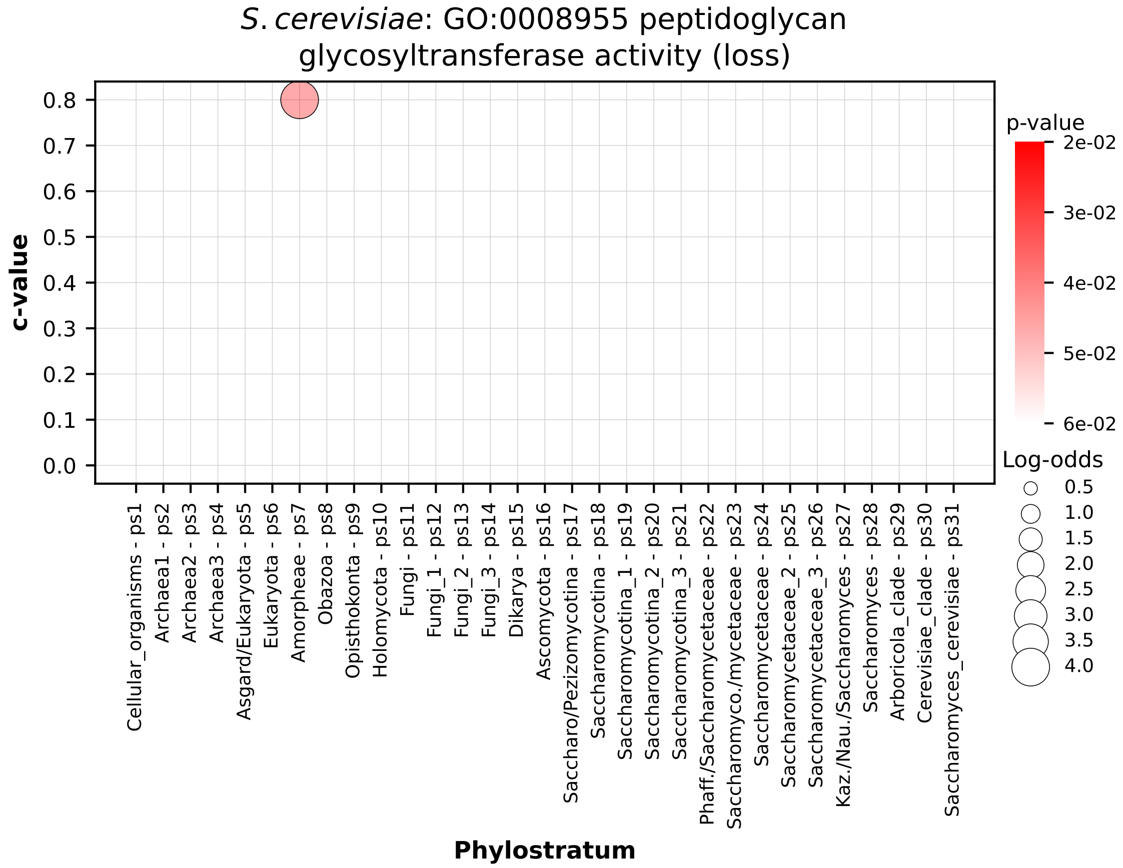
S. cerevisiae: GO:0008955 peptidoglycan glycosyltransferase activity (loss)
c-value
0.8
0.7
0.6
0.5
0.4
0.3
0.2
0.1
0.0
Phylostratum
Cellular_organisms - ps1
Archaea1 - ps2
Archaea2 - ps3
Archaea3 - ps4
Asgard/Eukaryota - ps5
Eukaryota - ps6
Amorpheae - ps7
Obazoa - ps8
Opisthokonta - ps9
Holomycota - ps10
Fungi - ps11
Fungi_1 - ps12
Fungi_2 - ps13
Fungi_3 - ps14
Dikarya - ps15
Ascomycota - ps16
Saccharo/Pezizomycotina - ps17
Saccharomycotina - ps18
Saccharomycotina_1 - ps19
Saccharomycotina_2 - ps20
Saccharomycotina_3 - ps21
Phaff./Saccharomycetaceae - ps22
Saccharomyco./mycetaceae - ps23
Saccharomycetaceae - ps24
Saccharomycetaceae_2 - ps25
Saccharomycetaceae_3 - ps26
Kaz./Nau./Saccharomyces - ps27
Saccharomyces - ps28
Arboricola_clade - ps29
Cerevisiae_clade - ps30
Saccharomyces_cerevisiae - ps31
p-value
2e-02
3e-02
4e-02
5e-02
6e-02
Log-odds
0.5
1.0
1.5
2.0
2.5
3.0
3.5
4.0

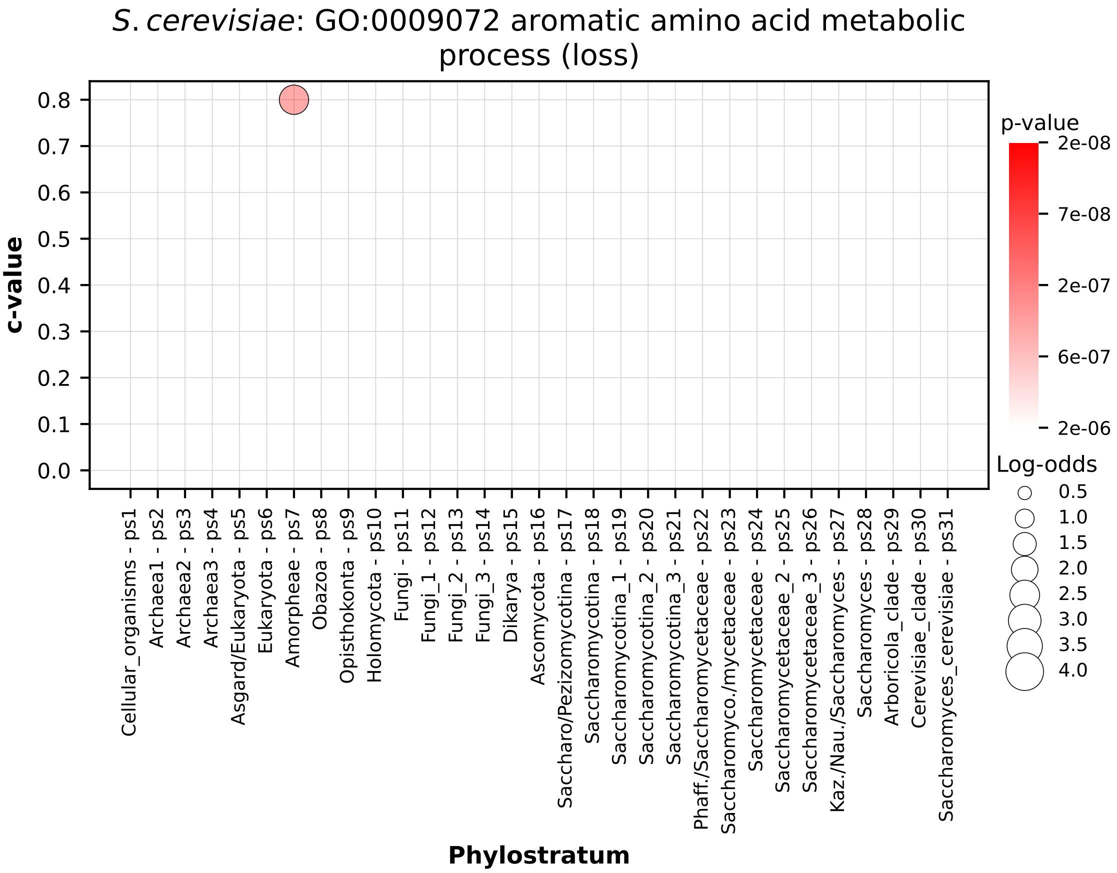

S. cerevisiae: GO:0009072 aromatic amino acid metabolic process (loss)
c-value
0.0
0.1
0.2
0.3
0.4
0.5
0.6
0.7
0.8
p-value
2e-08
7e-08
2e-07
6e-07
2e-06
Log-odds
0.5
1.0
1.5
2.0
2.5
3.0
3.5
4.0
Cellular_organisms - ps1
Archaea1 - ps2
Archaea2 - ps3
Archaea3 - ps4
Asgard/Eukaryota - ps5
Eukaryota - ps6
Amorpheae - ps7
Obazoa - ps8
Opisthokonta - ps9
Holomycota - ps10
Fungi - ps11
Fungi_1 - ps12
Fungi_2 - ps13
Fungi_3 - ps14
Dikarya - ps15
Ascomycota - ps16
Saccharo/Pezizomycotina - ps17
Saccharomycotina - ps18
Saccharomycotina_1 - ps19
Saccharomycotina_2 - ps20
Saccharomycotina_3 - ps21
Phaff./Saccharomycetaceae - ps22
Saccharomyco./mycetaceae - ps23
Saccharomycetaceae - ps24
Saccharomycetaceae_2 - ps25
Saccharomycetaceae_3 - ps26
Kaz./Nau./Saccharomyces - ps27
Saccharomyces - ps28
Arboricola_clade - ps29
Cerevisiae_clade - ps30
Saccharomyces_cerevisiae - ps31
Phylostratum

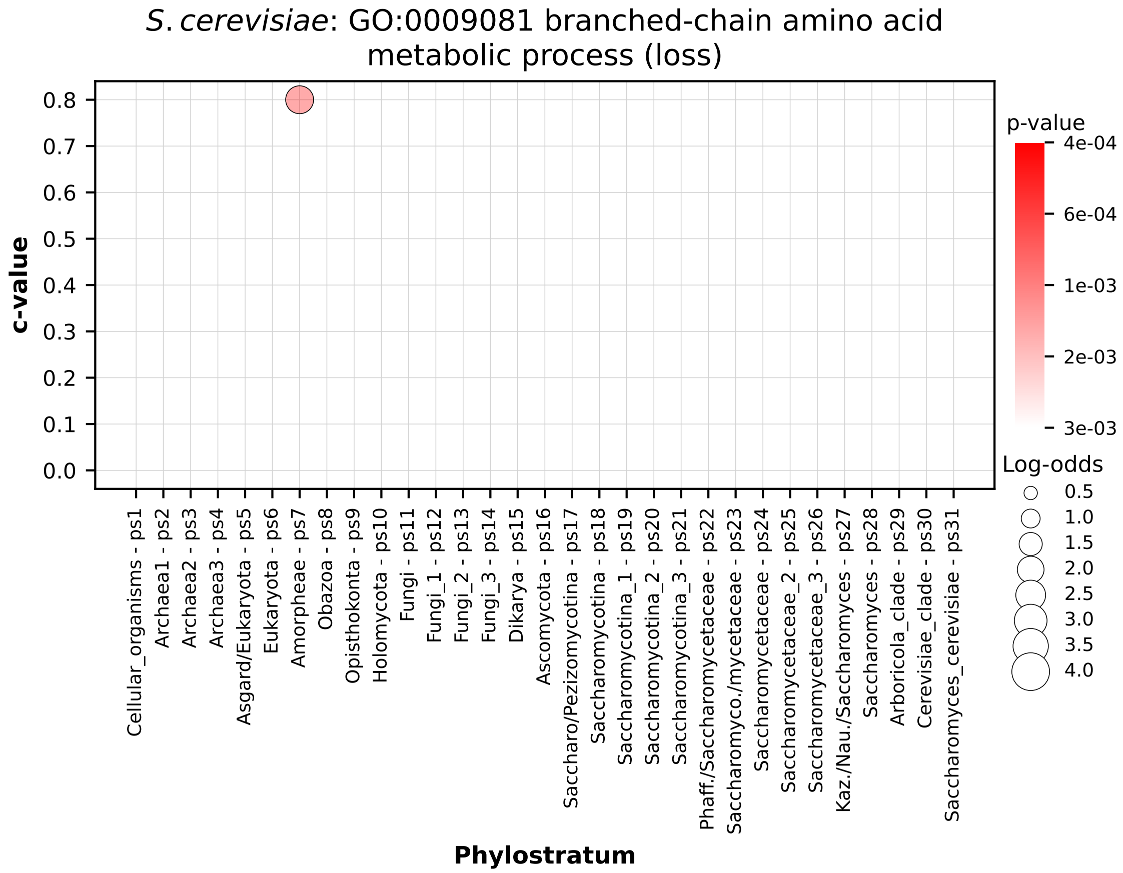

S. cerevisiae: GO:0009081 branched-chain amino acid metabolic process (loss)
c-value
0.0
0.1
0.2
0.3
0.4
0.5
0.6
0.7
0.8
Phylostratum
Cellular_organisms - ps1
Archaea1 - ps2
Archaea2 - ps3
Archaea3 - ps4
Asgard/Eukaryota - ps5
Eukaryota - ps6
Amorpheae - ps7
Obazoa - ps8
Opisthokonta - ps9
Holomycota - ps10
Fungi - ps11
Fungi_1 - ps12
Fungi_2 - ps13
Fungi_3 - ps14
Dikarya - ps15
Ascomycota - ps16
Saccharo/Pezizomycotina - ps17
Saccharomycotina - ps18
Saccharomycotina_1 - ps19
Saccharomycotina_2 - ps20
Saccharomycotina_3 - ps21
Phaff./Saccharomycetaceae - ps22
Saccharomyco./mycetaceae - ps23
Saccharomycetaceae - ps24
Saccharomycetaceae_2 - ps25
Saccharomycetaceae_3 - ps26
Kaz./Nau./Saccharomyces - ps27
Saccharomyces - ps28
Arboricola_clade - ps29
Cerevisiae_clade - ps30
Saccharomyces_cerevisiae - ps31
p-value
4e-04
6e-04
1e-03
2e-03
3e-03
Log-odds
0.5
1.0
1.5
2.0
2.5
3.0
3.5
4.0

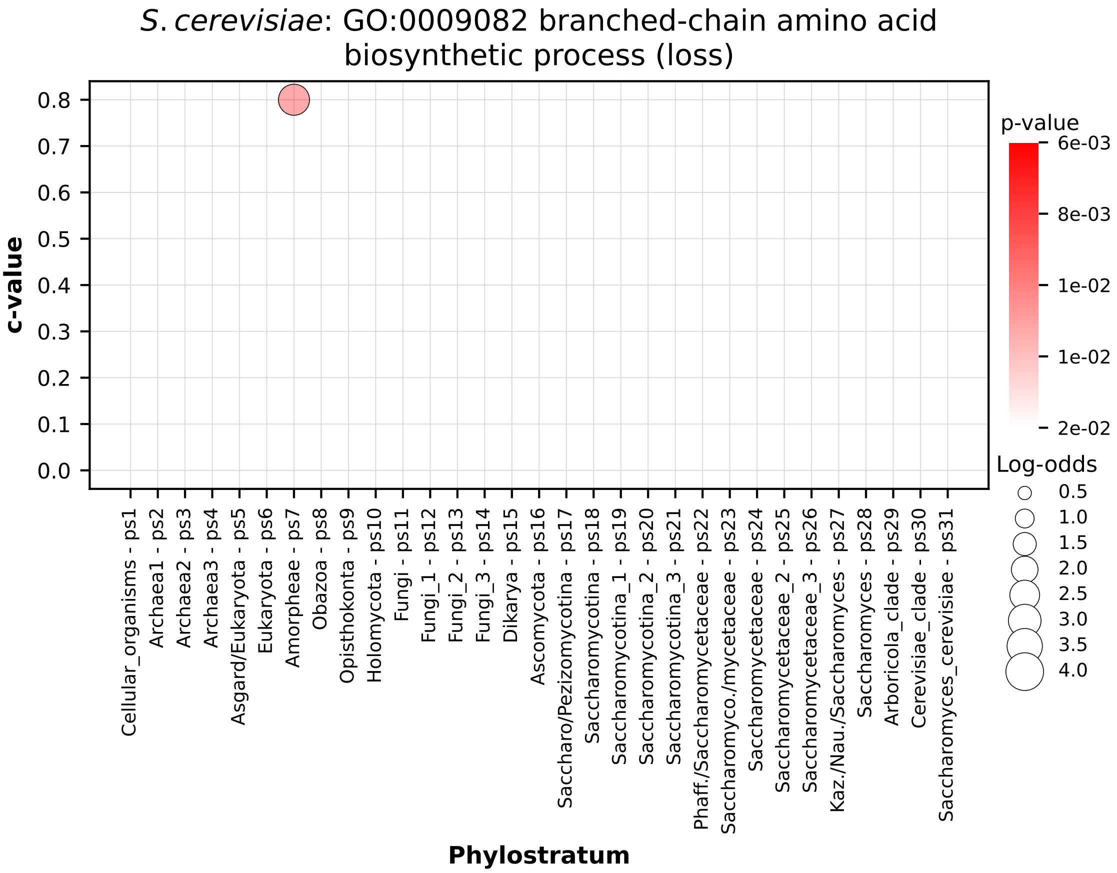

S. cerevisiae: GO:0009082 branched-chain amino acid biosynthetic process (loss)
c-value
0.8
0.7
0.6
0.5
0.4
0.3
0.2
0.1
0.0
p-value
6e-03
8e-03
1e-02
1e-02
2e-02
Log-odds
0.5
1.0
1.5
2.0
2.5
3.0
3.5
4.0
Cellular_organisms - ps1
Archaea1 - ps2
Archaea2 - ps3
Archaea3 - ps4
Asgard/Eukaryota - ps5
Eukaryota - ps6
Amorpheae - ps7
Obazoa - ps8
Opisthokonta - ps9
Holomycota - ps10
Fungi - ps11
Fungi_1 - ps12
Fungi_2 - ps13
Fungi_3 - ps14
Dikarya - ps15
Ascomycota - ps16
Saccharo/Pezizomycotina - ps17
Saccharomycotina - ps18
Saccharomycotina_1 - ps19
Saccharomycotina_2 - ps20
Saccharomycotina_3 - ps21
Phaff./Saccharomycetaceae - ps22
Saccharomyco./mycetaceae - ps23
Saccharomycetaceae - ps24
Saccharomycetaceae_2 - ps25
Saccharomycetaceae_3 - ps26
Kaz./Nau./Saccharomyces - ps27
Saccharomyces - ps28
Arboricola_clade - ps29
Cerevisiae_clade - ps30
Saccharomyces_cerevisiae - ps31
Phylostratum

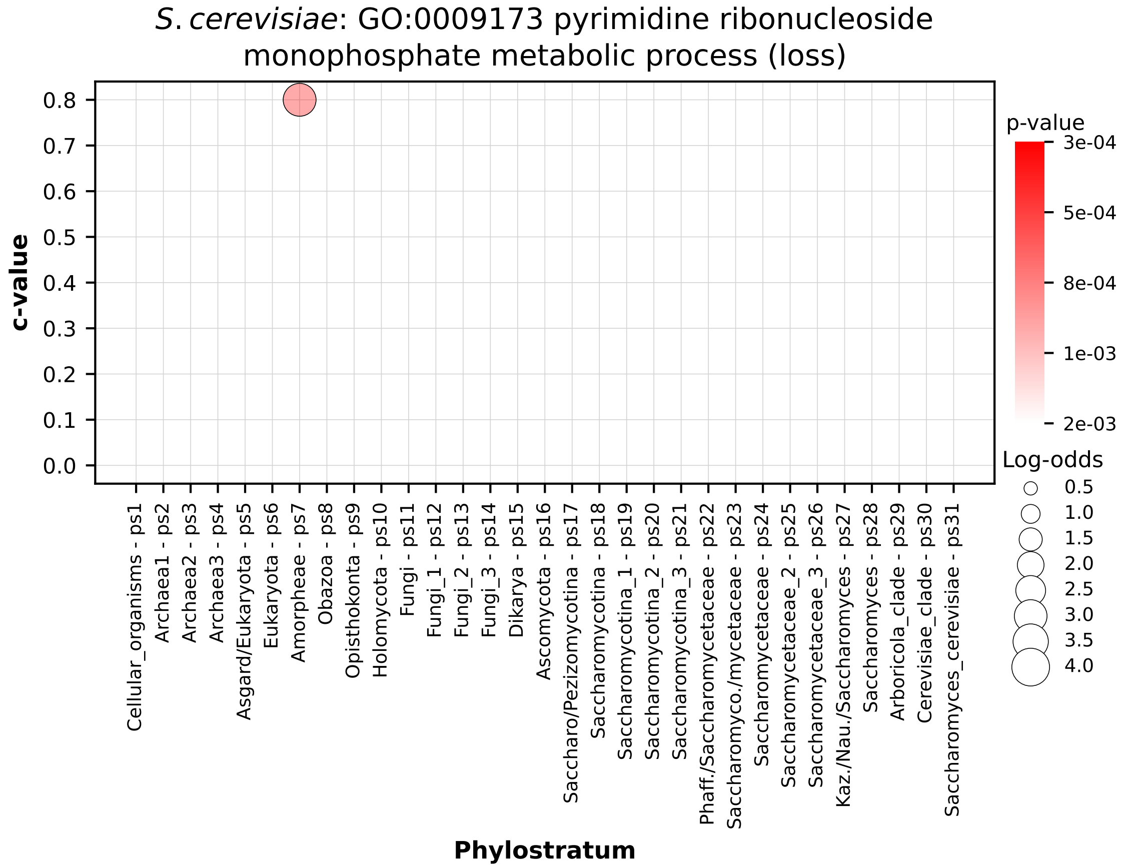

S. cerevisiae: GO:0009173 pyrimidine ribonucleoside monophosphate metabolic process (loss)
c-value
0.0
0.1
0.2
0.3
0.4
0.5
0.6
0.7
0.8
p-value
3e-04
5e-04
8e-04
1e-03
2e-03
Log-odds
0.5
1.0
1.5
2.0
2.5
3.0
3.5
4.0
Cellular_organisms - ps1
Archaea1 - ps2
Archaea2 - ps3
Archaea3 - ps4
Asgard/Eukaryota - ps5
Eukaryota - ps6
Amorpheae - ps7
Obazoa - ps8
Opisthokonta - ps9
Holomycota - ps10
Fungi - ps11
Fungi_1 - ps12
Fungi_2 - ps13
Fungi_3 - ps14
Dikarya - ps15
Ascomycota - ps16
Saccharo/Pezizomycotina - ps17
Saccharomycotina - ps18
Saccharomycotina_1 - ps19
Saccharomycotina_2 - ps20
Saccharomycotina_3 - ps21
Phaff./Saccharomycetaceae - ps22
Saccharomyco./mycetaceae - ps23
Saccharomycetaceae - ps24
Saccharomycetaceae_2 - ps25
Saccharomycetaceae_3 - ps26
Kaz./Nau./Saccharomyces - ps27
Saccharomyces - ps28
Arboricola_clade - ps29
Cerevisiae_clade - ps30
Saccharomyces_cerevisiae - ps31
Phylostratum

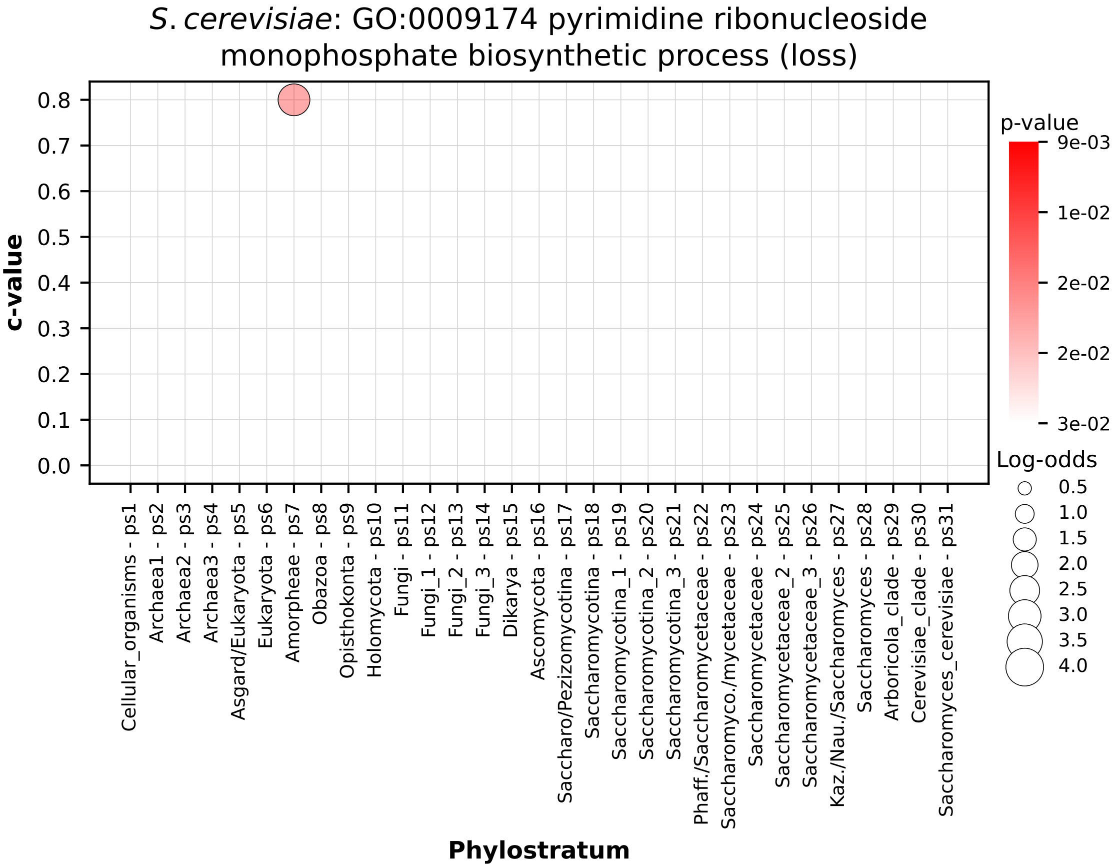

S. cerevisiae: GO:0009174 pyrimidine ribonucleoside monophosphate biosynthetic process (loss)
c-value
0.8
0.7
0.6
0.5
0.4
0.3
0.2
0.1
0.0
p-value
9e-03
1e-02
2e-02
2e-02
3e-02
Log-odds
0.5
1.0
1.5
2.0
2.5
3.0
3.5
4.0
Cellular_organisms - ps1
Archaea1 - ps2
Archaea2 - ps3
Archaea3 - ps4
Asgard/Eukaryota - ps5
Eukaryota - ps6
Amorpheae - ps7
Obazoa - ps8
Opisthokonta - ps9
Holomycota - ps10
Fungi - ps11
Fungi_1 - ps12
Fungi_2 - ps13
Fungi_3 - ps14
Dikarya - ps15
Ascomycota - ps16
Saccharo/Pezizomycotina - ps17
Saccharomycotina - ps18
Saccharomycotina_1 - ps19
Saccharomycotina_2 - ps20
Saccharomycotina_3 - ps21
Phaff./Saccharomycetaceae - ps22
Saccharomyco./mycetaceae - ps23
Saccharomycetaceae - ps24
Saccharomycetaceae_2 - ps25
Saccharomycetaceae_3 - ps26
Kaz./Nau./Saccharomyces - ps27
Saccharomyces - ps28
Arboricola_clade - ps29
Cerevisiae_clade - ps30
Saccharomyces_cerevisiae - ps31
Phylostratum

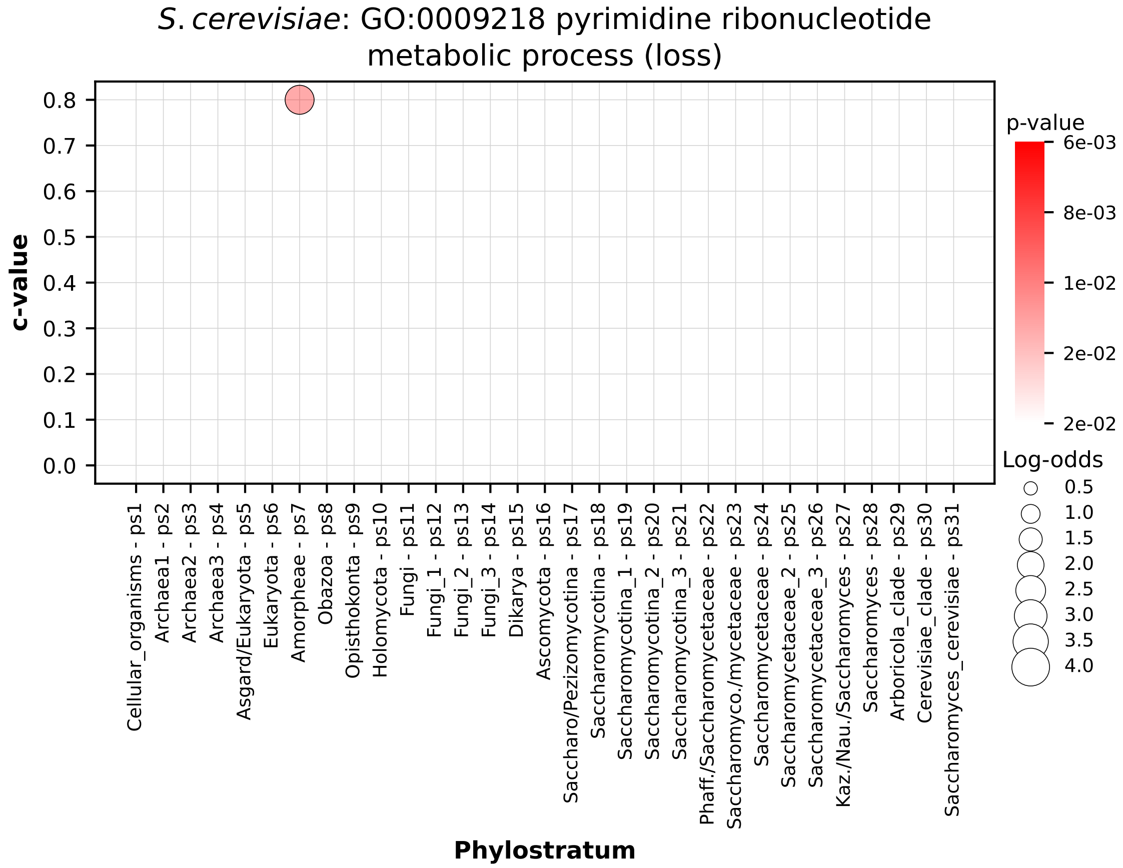

S. cerevisiae: GO:0009218 pyrimidine ribonucleotide metabolic process (loss)
c-value
0.0
0.1
0.2
0.3
0.4
0.5
0.6
0.7
0.8
Phylostratum
Cellular_organisms - ps1
Archaea1 - ps2
Archaea2 - ps3
Archaea3 - ps4
Asgard/Eukaryota - ps5
Eukaryota - ps6
Amorpheae - ps7
Obazoa - ps8
Opisthokonta - ps9
Holomycota - ps10
Fungi - ps11
Fungi_1 - ps12
Fungi_2 - ps13
Fungi_3 - ps14
Dikarya - ps15
Ascomycota - ps16
Saccharo/Pezizomycotina - ps17
Saccharomycotina - ps18
Saccharomycotina_1 - ps19
Saccharomycotina_2 - ps20
Saccharomycotina_3 - ps21
Phaff./Saccharomycetaceae - ps22
Saccharomyco./mycetaceae - ps23
Saccharomycetaceae - ps24
Saccharomycetaceae_2 - ps25
Saccharomycetaceae_3 - ps26
Kaz./Nau./Saccharomyces - ps27
Saccharomyces - ps28
Arboricola_clade - ps29
Cerevisiae_clade - ps30
Saccharomyces_cerevisiae - ps31
p-value
6e-03
8e-03
1e-02
2e-02
2e-02
Log-odds
0.5
1.0
1.5
2.0
2.5
3.0
3.5
4.0

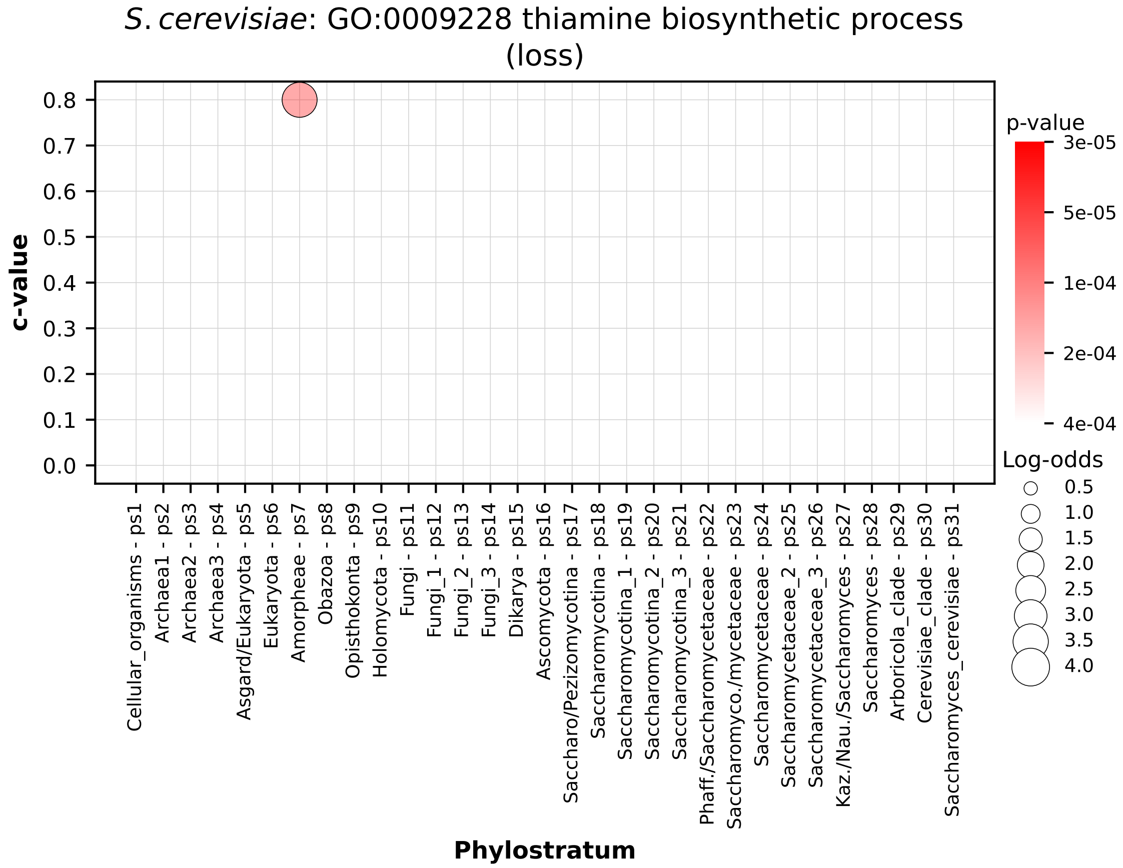
S. cerevisiae: GO:0009228 thiamine biosynthetic process
(loss)
c-value
0.0
0.1
0.2
0.3
0.4
0.5
0.6
0.7
0.8
Phylostratum
Cellular_organisms - ps1
Archaea1 - ps2
Archaea2 - ps3
Archaea3 - ps4
Asgard/Eukaryota - ps5
Eukaryota - ps6
Amorpheae - ps7
Obazoa - ps8
Opisthokonta - ps9
Holomycota - ps10
Fungi - ps11
Fungi_1 - ps12
Fungi_2 - ps13
Fungi_3 - ps14
Dikarya - ps15
Ascomycota - ps16
Saccharo/Pezizomycotina - ps17
Saccharomycotina - ps18
Saccharomycotina_1 - ps19
Saccharomycotina_2 - ps20
Saccharomycotina_3 - ps21
Phaff./Saccharomycetaceae - ps22
Saccharomyco./mycetaceae - ps23
Saccharomycetaceae - ps24
Saccharomycetaceae_2 - ps25
Saccharomycetaceae_3 - ps26
Kaz./Nau./Saccharomyces - ps27
Saccharomyces - ps28
Arboricola_clade - ps29
Cerevisiae_clade - ps30
Saccharomyces_cerevisiae - ps31
p-value
3e-05
5e-05
1e-04
2e-04
4e-04
Log-odds
0.5
1.0
1.5
2.0
2.5
3.0
3.5
4.0

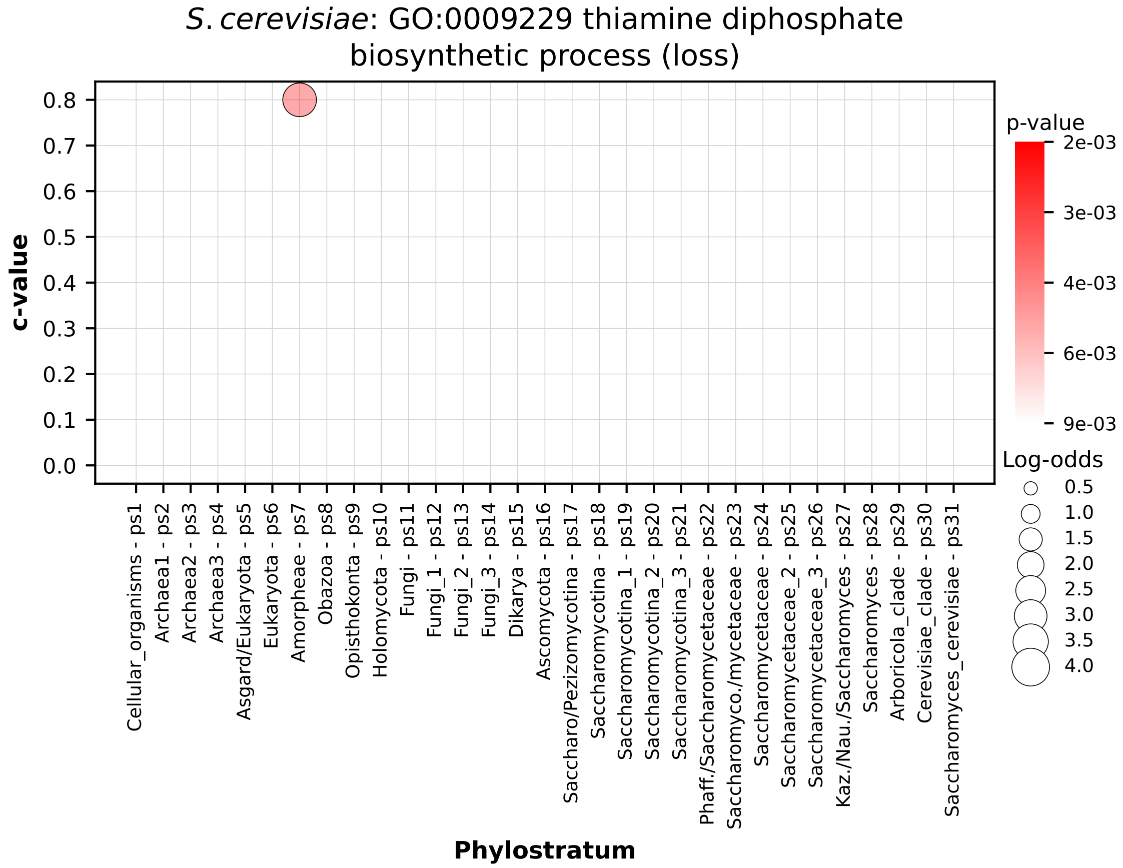

S. cerevisiae: GO:0009229 thiamine diphosphate biosynthetic process (loss)
c-value
0.8
0.7
0.6
0.5
0.4
0.3
0.2
0.1
0.0
p-value
2e-03
3e-03
4e-03
6e-03
9e-03
Log-odds
0.5
1.0
1.5
2.0
2.5
3.0
3.5
4.0
Cellular_organisms - ps1
Archaea1 - ps2
Archaea2 - ps3
Archaea3 - ps4
Asgard/Eukaryota - ps5
Eukaryota - ps6
Amorpheae - ps7
Obazoa - ps8
Opisthokonta - ps9
Holomycota - ps10
Fungi - ps11
Fungi_1 - ps12
Fungi_2 - ps13
Fungi_3 - ps14
Dikarya - ps15
Ascomycota - ps16
Saccharo/Pezizomycotina - ps17
Saccharomycotina - ps18
Saccharomycotina_1 - ps19
Saccharomycotina_2 - ps20
Saccharomycotina_3 - ps21
Phaff./Saccharomycetaceae - ps22
Saccharomyco./mycetaceae - ps23
Saccharomycetaceae - ps24
Saccharomycetaceae_2 - ps25
Saccharomycetaceae_3 - ps26
Kaz./Nau./Saccharomyces - ps27
Saccharomyces - ps28
Arboricola_clade - ps29
Cerevisiae_clade - ps30
Saccharomyces_cerevisiae - ps31
Phylostratum

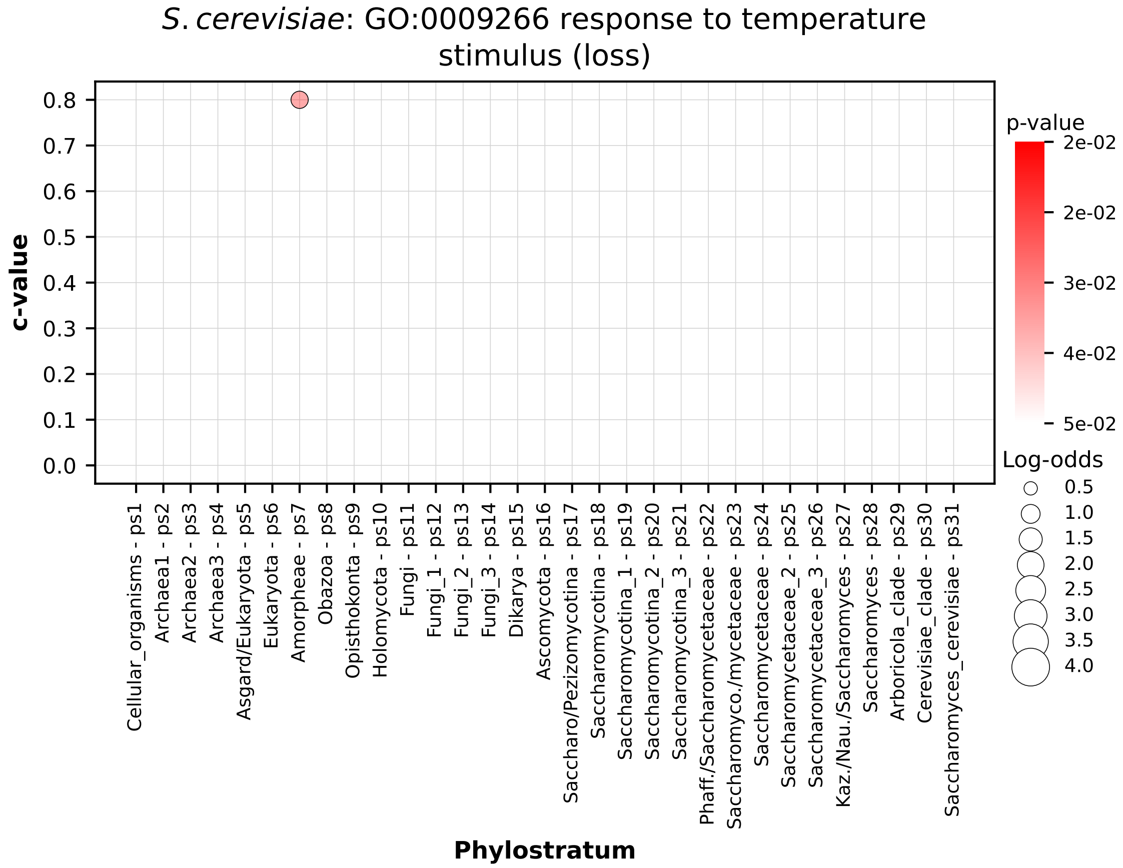

S. cerevisiae: GO:0009266 response to temperature stimulus (loss)
c-value
0.0
0.1
0.2
0.3
0.4
0.5
0.6
0.7
0.8
p-value
2e-02
2e-02
3e-02
4e-02
5e-02
Log-odds
0.5
1.0
1.5
2.0
2.5
3.0
3.5
4.0
Cellular_organisms - ps1
Archaea1 - ps2
Archaea2 - ps3
Archaea3 - ps4
Asgard/Eukaryota - ps5
Eukaryota - ps6
Amorpheae - ps7
Obazoa - ps8
Opisthokonta - ps9
Holomycota - ps10
Fungi - ps11
Fungi_1 - ps12
Fungi_2 - ps13
Fungi_3 - ps14
Dikarya - ps15
Ascomycota - ps16
Saccharo/Pezizomycotina - ps17
Saccharomycotina - ps18
Saccharomycotina_1 - ps19
Saccharomycotina_2 - ps20
Saccharomycotina_3 - ps21
Phaff./Saccharomycetaceae - ps22
Saccharomyco./mycetaceae - ps23
Saccharomycetaceae - ps24
Saccharomycetaceae_2 - ps25
Saccharomycetaceae_3 - ps26
Kaz./Nau./Saccharomyces - ps27
Saccharomyces - ps28
Arboricola_clade - ps29
Cerevisiae_clade - ps30
Saccharomyces_cerevisiae - ps31
Phylostratum

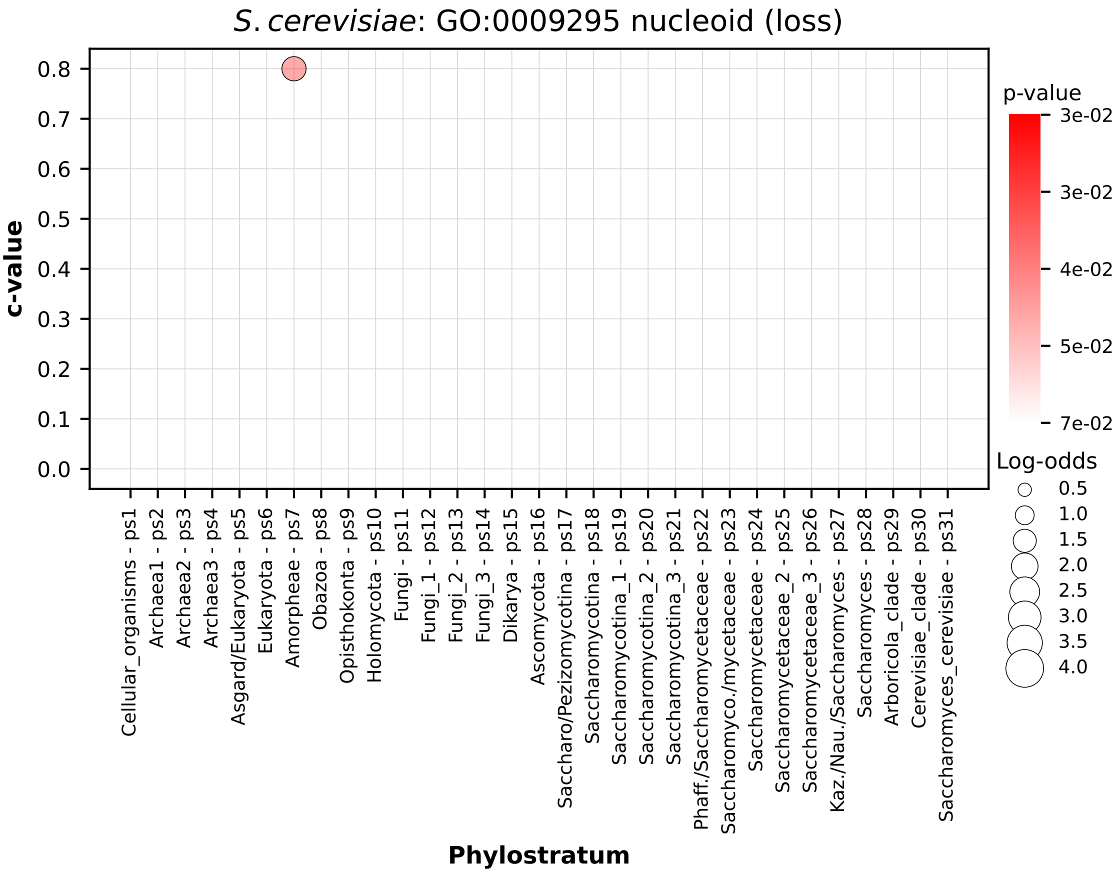
S. cerevisiae: GO:0009295 nucleoid (loss)
c-value
0.0
0.1
0.2
0.3
0.4
0.5
0.6
0.7
0.8
Phylostratum
Cellular_organisms - ps1
Archaea1 - ps2
Archaea2 - ps3
Archaea3 - ps4
Asgard/Eukaryota - ps5
Eukaryota - ps6
Amorpheae - ps7
Obazoa - ps8
Opisthokonta - ps9
Holomycota - ps10
Fungi - ps11
Fungi_1 - ps12
Fungi_2 - ps13
Fungi_3 - ps14
Dikarya - ps15
Ascomycota - ps16
Saccharo/Pezizomycotina - ps17
Saccharomycotina - ps18
Saccharomycotina_1 - ps19
Saccharomycotina_2 - ps20
Saccharomycotina_3 - ps21
Phaff./Saccharomycetaceae - ps22
Saccharomyco./mycetaceae - ps23
Saccharomycetaceae - ps24
Saccharomycetaceae_2 - ps25
Saccharomycetaceae_3 - ps26
Kaz./Nau./Saccharomyces - ps27
Saccharomyces - ps28
Arboricola_clade - ps29
Cerevisiae_clade - ps30
Saccharomyces_cerevisiae - ps31
p-value
3e-02
3e-02
4e-02
5e-02
7e-02
Log-odds
0.5
1.0
1.5
2.0
2.5
3.0
3.5
4.0

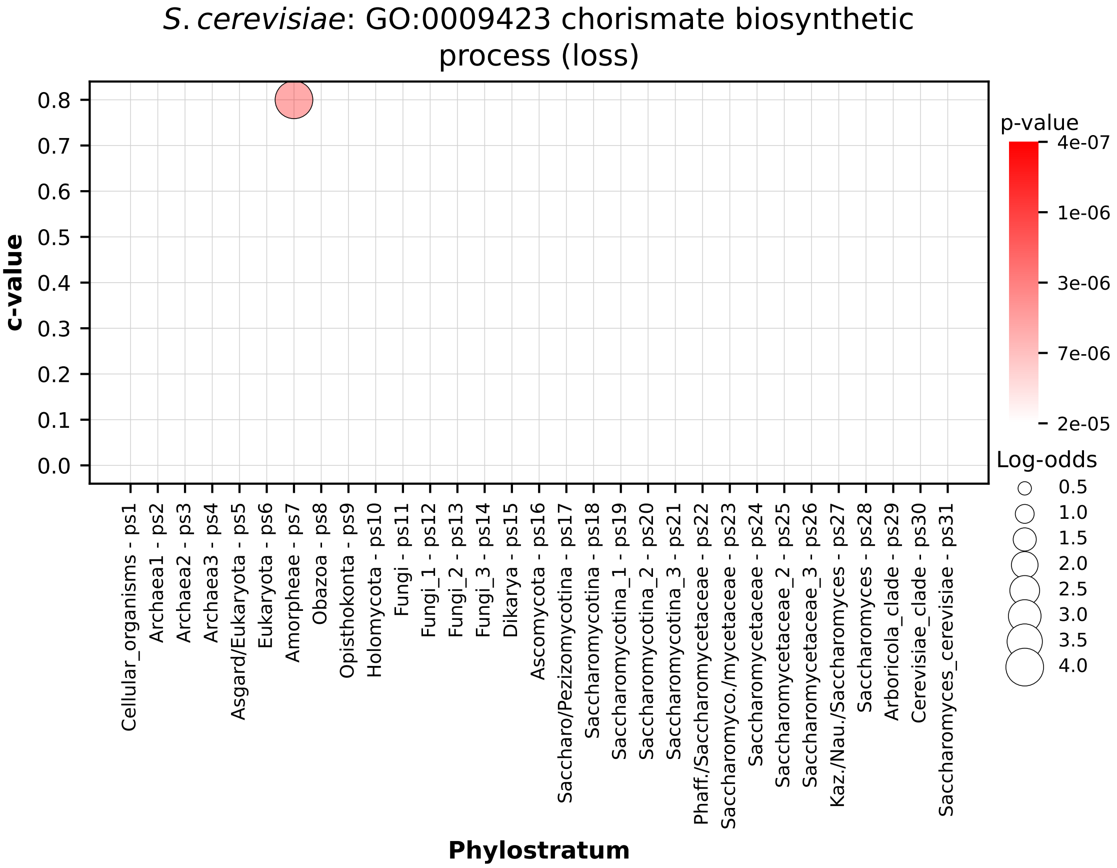

S. cerevisiae: GO:0009423 chorismate biosynthetic process (loss)
c-value
0.0
0.1
0.2
0.3
0.4
0.5
0.6
0.7
0.8
p-value
4e-07
1e-06
3e-06
7e-06
2e-05
Log-odds
0.5
1.0
1.5
2.0
2.5
3.0
3.5
4.0
Cellular_organisms - ps1
Archaea1 - ps2
Archaea2 - ps3
Archaea3 - ps4
Asgard/Eukaryota - ps5
Eukaryota - ps6
Amorpheae - ps7
Obazoa - ps8
Opisthokonta - ps9
Holomycota - ps10
Fungi - ps11
Fungi_1 - ps12
Fungi_2 - ps13
Fungi_3 - ps14
Dikarya - ps15
Ascomycota - ps16
Saccharo/Pezizomycotina - ps17
Saccharomycotina - ps18
Saccharomycotina_1 - ps19
Saccharomycotina_2 - ps20
Saccharomycotina_3 - ps21
Phaff./Saccharomycetaceae - ps22
Saccharomyco./mycetaceae - ps23
Saccharomycetaceae - ps24
Saccharomycetaceae_2 - ps25
Saccharomycetaceae_3 - ps26
Kaz./Nau./Saccharomyces - ps27
Saccharomyces - ps28
Arboricola_clade - ps29
Cerevisiae_clade - ps30
Saccharomyces_cerevisiae - ps31
Phylostratum

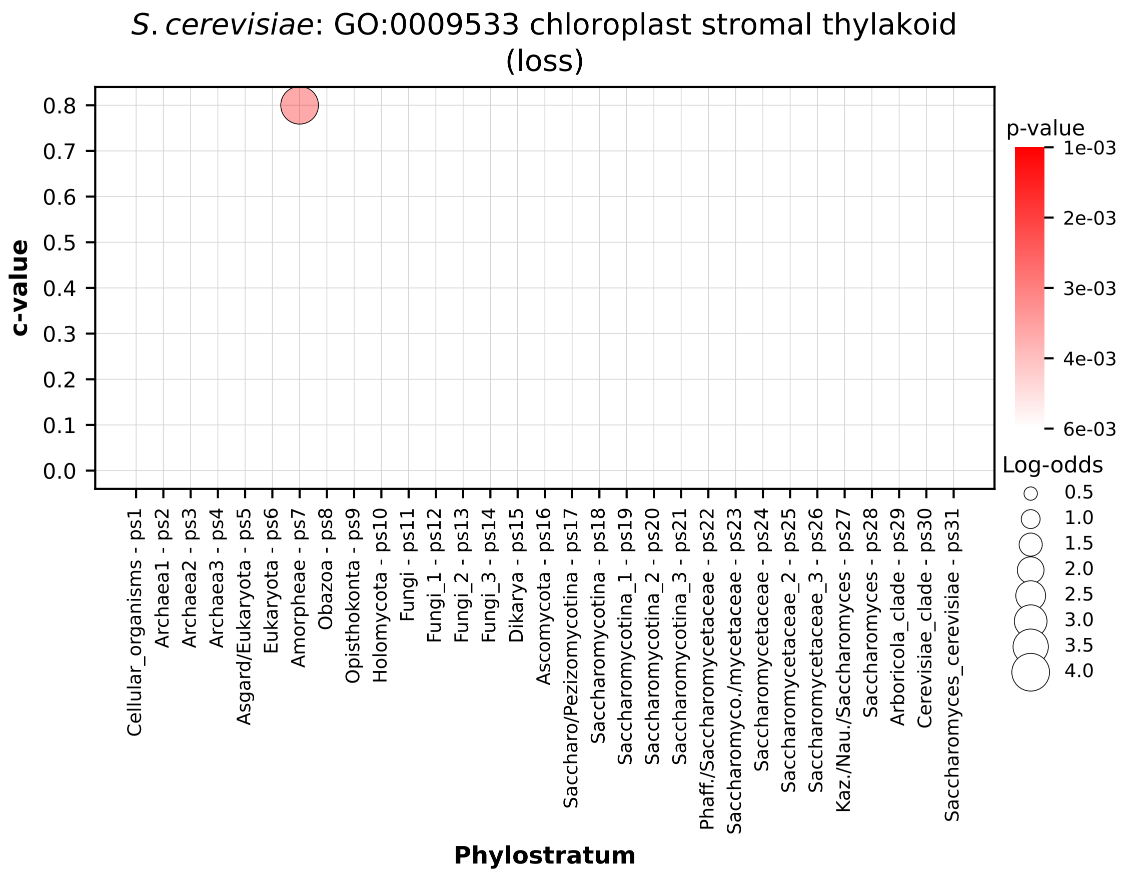

S. cerevisiae: GO:0009533 chloroplast stromal thylakoid (loss)
c-value
0.0
0.1
0.2
0.3
0.4
0.5
0.6
0.7
0.8
p-value
1e-03
2e-03
3e-03
4e-03
6e-03
Log-odds
0.5
1.0
1.5
2.0
2.5
3.0
3.5
4.0
Cellular_organisms - ps1
Archaea1 - ps2
Archaea2 - ps3
Archaea3 - ps4
Asgard/Eukaryota - ps5
Eukaryota - ps6
Amorpheae - ps7
Obazoa - ps8
Opisthokonta - ps9
Holomycota - ps10
Fungi - ps11
Fungi_1 - ps12
Fungi_2 - ps13
Fungi_3 - ps14
Dikarya - ps15
Ascomycota - ps16
Saccharo/Pezizomycotina - ps17
Saccharomycotina - ps18
Saccharomycotina_1 - ps19
Saccharomycotina_2 - ps20
Saccharomycotina_3 - ps21
Phaff./Saccharomycetaceae - ps22
Saccharomyco./mycetaceae - ps23
Saccharomycetaceae - ps24
Saccharomycetaceae_2 - ps25
Saccharomycetaceae_3 - ps26
Kaz./Nau./Saccharomyces - ps27
Saccharomyces - ps28
Arboricola_clade - ps29
Cerevisiae_clade - ps30
Saccharomyces_cerevisiae - ps31
Phylostratum

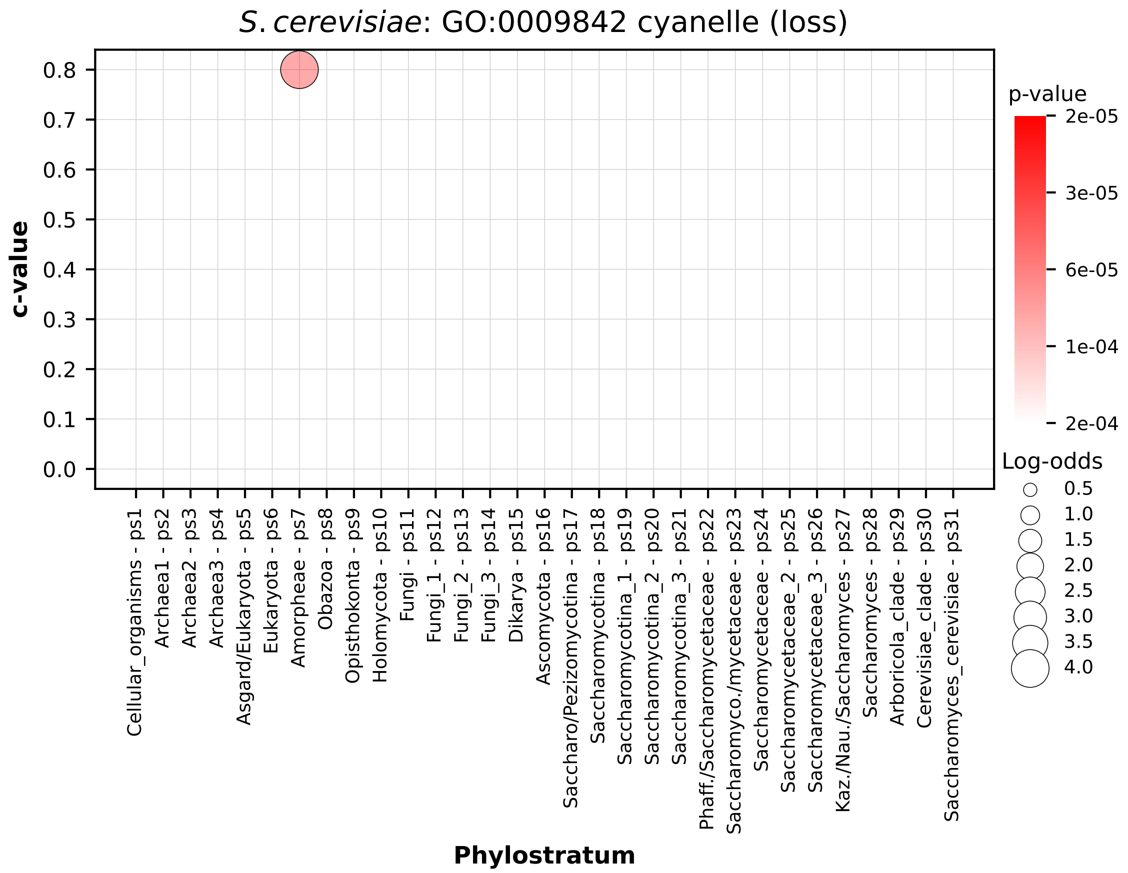
S. cerevisiae: GO:0009842 cyanelle (loss)
c-value
0.0
0.1
0.2
0.3
0.4
0.5
0.6
0.7
0.8
p-value
2e-05
3e-05
6e-05
1e-04
2e-04
Log-odds
0.5
1.0
1.5
2.0
2.5
3.0
3.5
4.0
Cellular_organisms - ps1
Archaea1 - ps2
Archaea2 - ps3
Archaea3 - ps4
Asgard/Eukaryota - ps5
Eukaryota - ps6
Amorpheae - ps7
Obazoa - ps8
Opisthokonta - ps9
Holomycota - ps10
Fungi - ps11
Fungi_1 - ps12
Fungi_2 - ps13
Fungi_3 - ps14
Dikarya - ps15
Ascomycota - ps16
Saccharo/Pezizomycotina - ps17
Saccharomycotina - ps18
Saccharomycotina_1 - ps19
Saccharomycotina_2 - ps20
Saccharomycotina_3 - ps21
Phaff./Saccharomycetaceae - ps22
Saccharomyco./mycetaceae - ps23
Saccharomycetaceae - ps24
Saccharomycetaceae_2 - ps25
Saccharomycetaceae_3 - ps26
Kaz./Nau./Saccharomyces - ps27
Saccharomyces - ps28
Arboricola_clade - ps29
Cerevisiae_clade - ps30
Saccharomyces_cerevisiae - ps31
Phylostratum

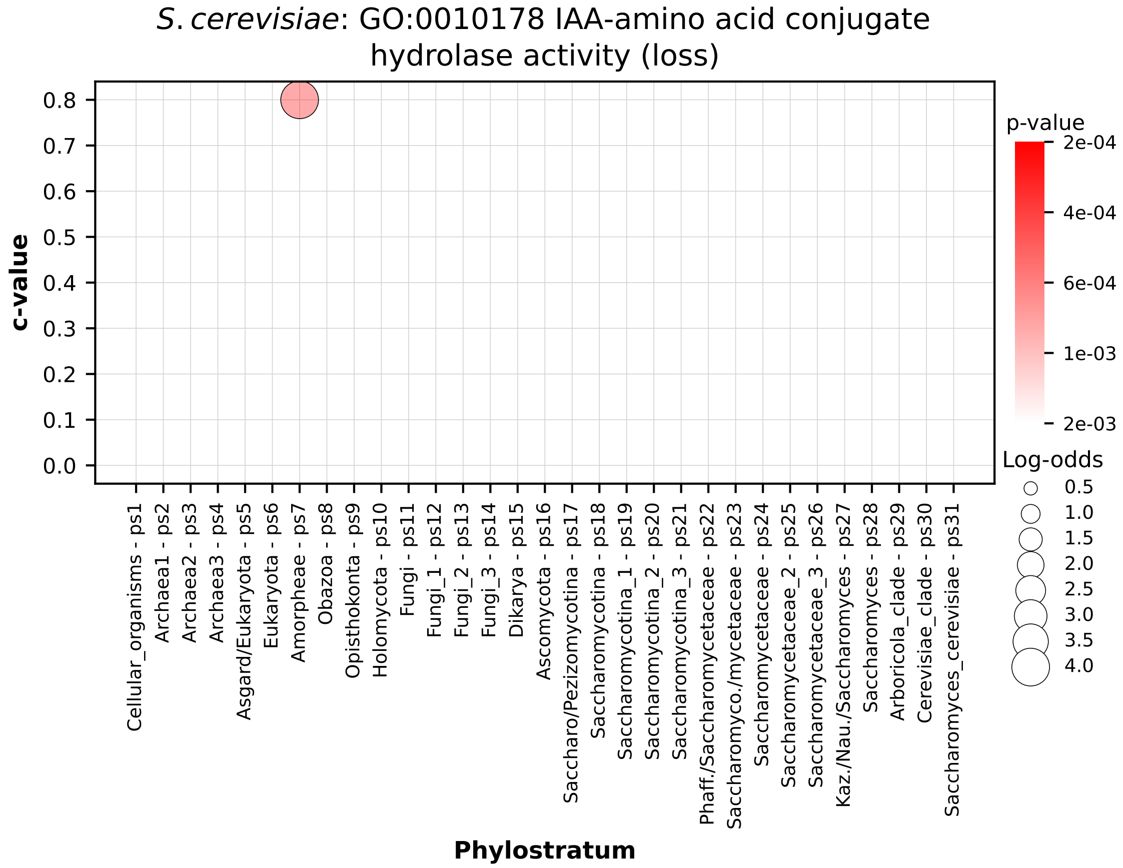
S. cerevisiae: GO:0010178 IAA-amino acid conjugate hydrolase activity (loss)
c-value
0.0
0.1
0.2
0.3
0.4
0.5
0.6
0.7
0.8
p-value
2e-04
4e-04
6e-04
1e-03
2e-03
Log-odds
0.5
1.0
1.5
2.0
2.5
3.0
3.5
4.0
Cellular_organisms - ps1
Archaea1 - ps2
Archaea2 - ps3
Archaea3 - ps4
Asgard/Eukaryota - ps5
Eukaryota - ps6
Amorpheae - ps7
Obazoa - ps8
Opisthokonta - ps9
Holomycota - ps10
Fungi - ps11
Fungi_1 - ps12
Fungi_2 - ps13
Fungi_3 - ps14
Dikarya - ps15
Ascomycota - ps16
Saccharo/Pezizomycotina - ps17
Saccharomycotina - ps18
Saccharomycotina_1 - ps19
Saccharomycotina_2 - ps20
Saccharomycotina_3 - ps21
Phaff./Saccharomycetaceae - ps22
Saccharomyco./mycetaceae - ps23
Saccharomycetaceae - ps24
Saccharomycetaceae_2 - ps25
Saccharomycetaceae_3 - ps26
Kaz./Nau./Saccharomyces - ps27
Saccharomyces - ps28
Arboricola_clade - ps29
Cerevisiae_clade - ps30
Saccharomyces_cerevisiae - ps31
Phylostratum

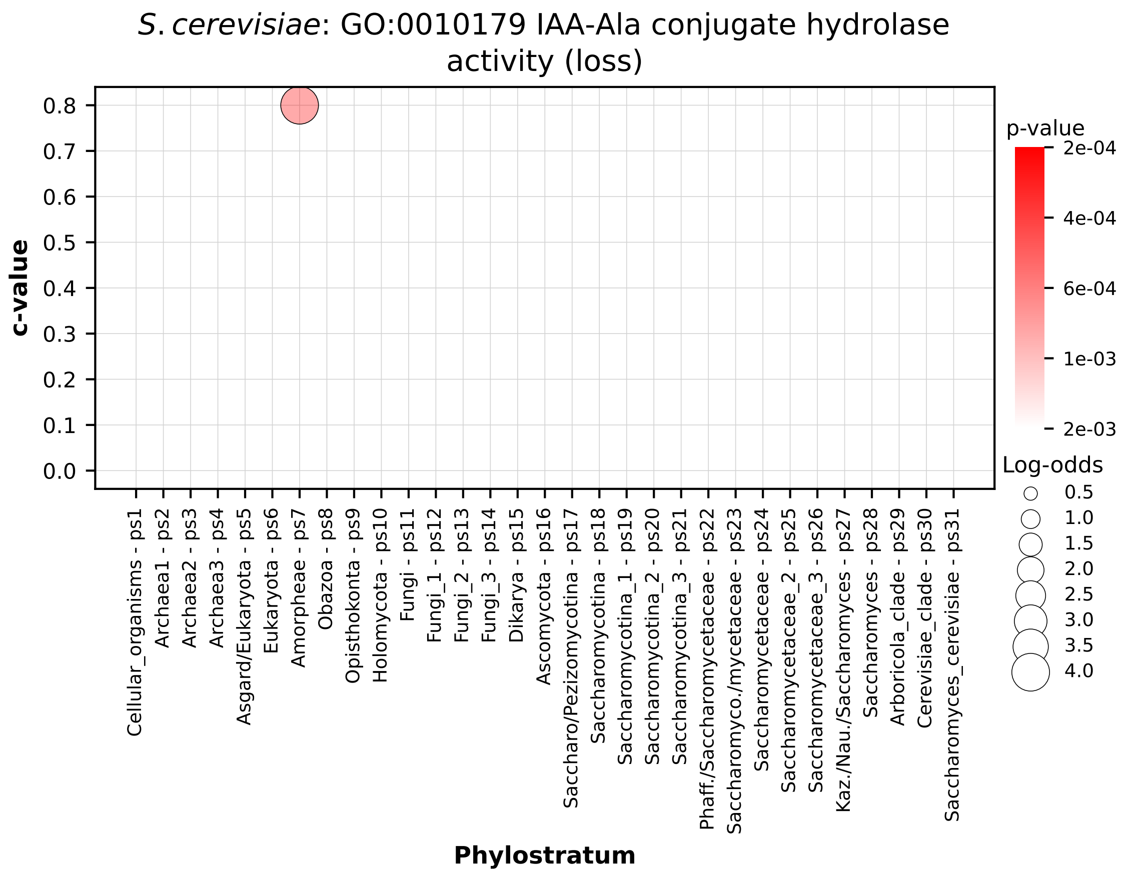

S. cerevisiae: GO:0010179 IAA-Ala conjugate hydrolase activity (loss)
c-value
0.0
0.1
0.2
0.3
0.4
0.5
0.6
0.7
0.8
p-value
2e-04
4e-04
6e-04
1e-03
2e-03
Log-odds
0.5
1.0
1.5
2.0
2.5
3.0
3.5
4.0
Cellular_organisms - ps1
Archaea1 - ps2
Archaea2 - ps3
Archaea3 - ps4
Asgard/Eukaryota - ps5
Eukaryota - ps6
Amorpheae - ps7
Obazoa - ps8
Opisthokonta - ps9
Holomycota - ps10
Fungi - ps11
Fungi_1 - ps12
Fungi_2 - ps13
Fungi_3 - ps14
Dikarya - ps15
Ascomycota - ps16
Saccharo/Pezizomycotina - ps17
Saccharomycotina - ps18
Saccharomycotina_1 - ps19
Saccharomycotina_2 - ps20
Saccharomycotina_3 - ps21
Phaff./Saccharomycetaceae - ps22
Saccharomyco./mycetaceae - ps23
Saccharomycetaceae - ps24
Saccharomycetaceae_2 - ps25
Saccharomycetaceae_3 - ps26
Kaz./Nau./Saccharomyces - ps27
Saccharomyces - ps28
Arboricola_clade - ps29
Cerevisiae_clade - ps30
Saccharomyces_cerevisiae - ps31
Phylostratum

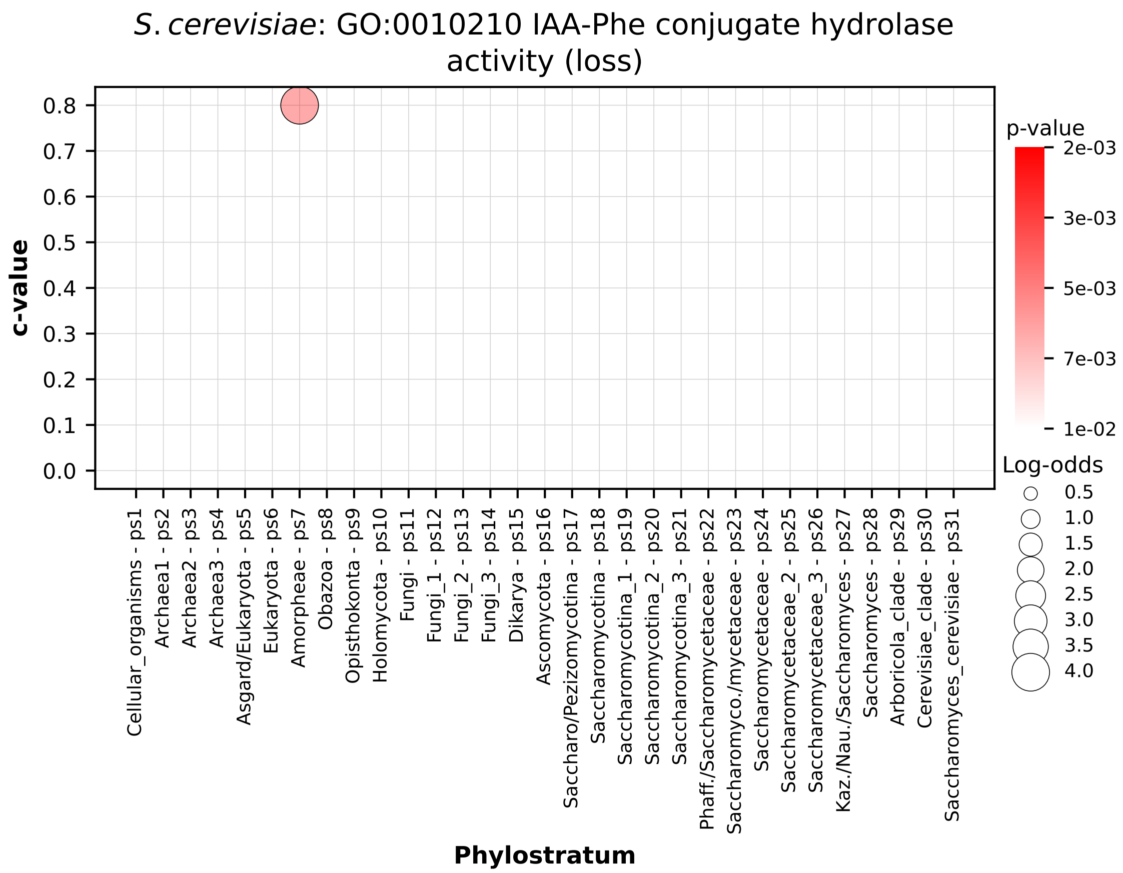
S. cerevisiae: GO:0010210 IAA-Phe conjugate hydrolase activity (loss)
c-value
0.0
0.1
0.2
0.3
0.4
0.5
0.6
0.7
0.8
Phylostratum
Cellular_organisms - ps1
Archaea1 - ps2
Archaea2 - ps3
Archaea3 - ps4
Asgard/Eukaryota - ps5
Eukaryota - ps6
Amorpheae - ps7
Obazoa - ps8
Opisthokonta - ps9
Holomycota - ps10
Fungi - ps11
Fungi_1 - ps12
Fungi_2 - ps13
Fungi_3 - ps14
Dikarya - ps15
Ascomycota - ps16
Saccharo/Pezizomycotina - ps17
Saccharomycotina - ps18
Saccharomycotina_1 - ps19
Saccharomycotina_2 - ps20
Saccharomycotina_3 - ps21
Phaff./Saccharomycetaceae - ps22
Saccharomyco./mycetaceae - ps23
Saccharomycetaceae - ps24
Saccharomycetaceae_2 - ps25
Saccharomycetaceae_3 - ps26
Kaz./Nau./Saccharomyces - ps27
Saccharomyces - ps28
Arboricola_clade - ps29
Cerevisiae_clade - ps30
Saccharomyces_cerevisiae - ps31
p-value
2e-03
3e-03
5e-03
7e-03
1e-02
Log-odds
0.5
1.0
1.5
2.0
2.5
3.0
3.5
4.0

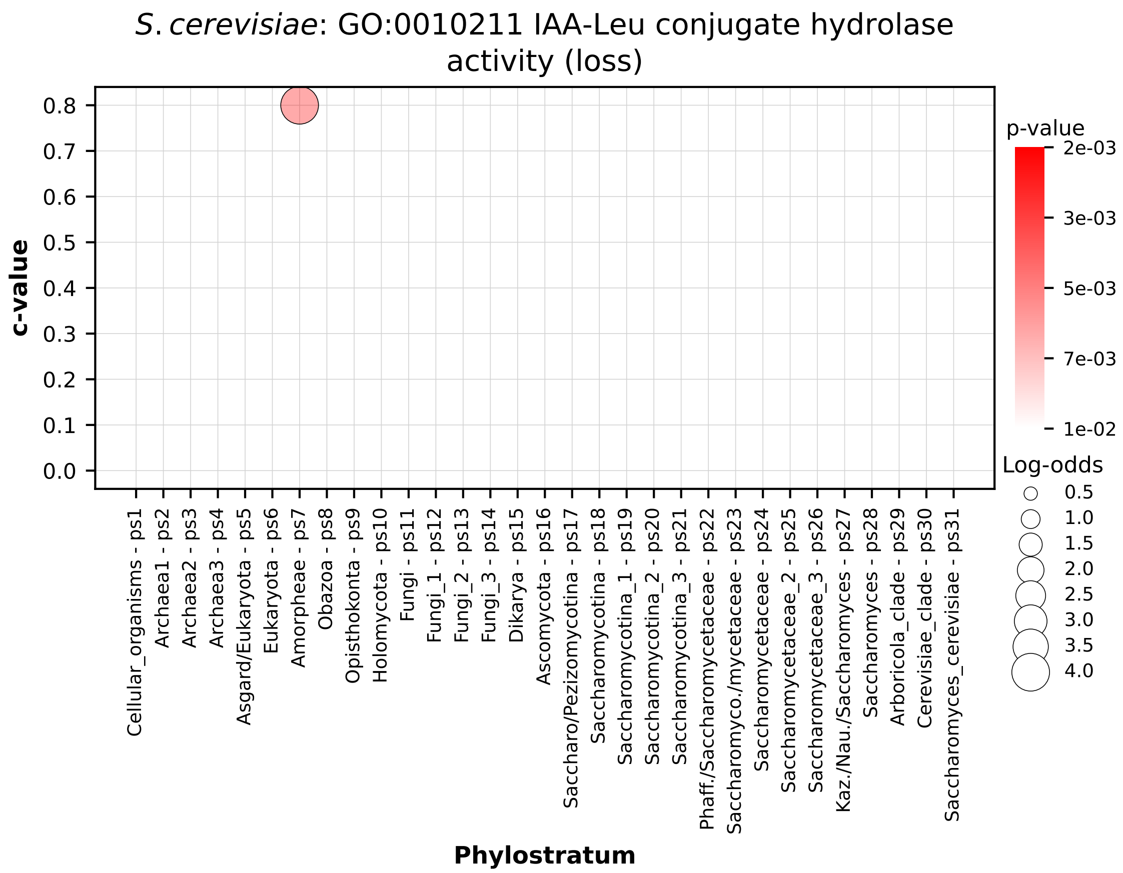
S. cerevisiae: GO:0010211 IAA-Leu conjugate hydrolase activity (loss)
c-value
0.0
0.1
0.2
0.3
0.4
0.5
0.6
0.7
0.8
p-value
2e-03
3e-03
5e-03
7e-03
1e-02
Log-odds
0.5
1.0
1.5
2.0
2.5
3.0
3.5
4.0
Cellular_organisms - ps1
Archaea1 - ps2
Archaea2 - ps3
Archaea3 - ps4
Asgard/Eukaryota - ps5
Eukaryota - ps6
Amorpheae - ps7
Obazoa - ps8
Opisthokonta - ps9
Holomycota - ps10
Fungi - ps11
Fungi_1 - ps12
Fungi_2 - ps13
Fungi_3 - ps14
Dikarya - ps15
Ascomycota - ps16
Saccharo/Pezizomycotina - ps17
Saccharomycotina - ps18
Saccharomycotina_1 - ps19
Saccharomycotina_2 - ps20
Saccharomycotina_3 - ps21
Phaff./Saccharomycetaceae - ps22
Saccharomyco./mycetaceae - ps23
Saccharomycetaceae - ps24
Saccharomycetaceae_2 - ps25
Saccharomycetaceae_3 - ps26
Kaz./Nau./Saccharomyces - ps27
Saccharomyces - ps28
Arboricola_clade - ps29
Cerevisiae_clade - ps30
Saccharomyces_cerevisiae - ps31
Phylostratum

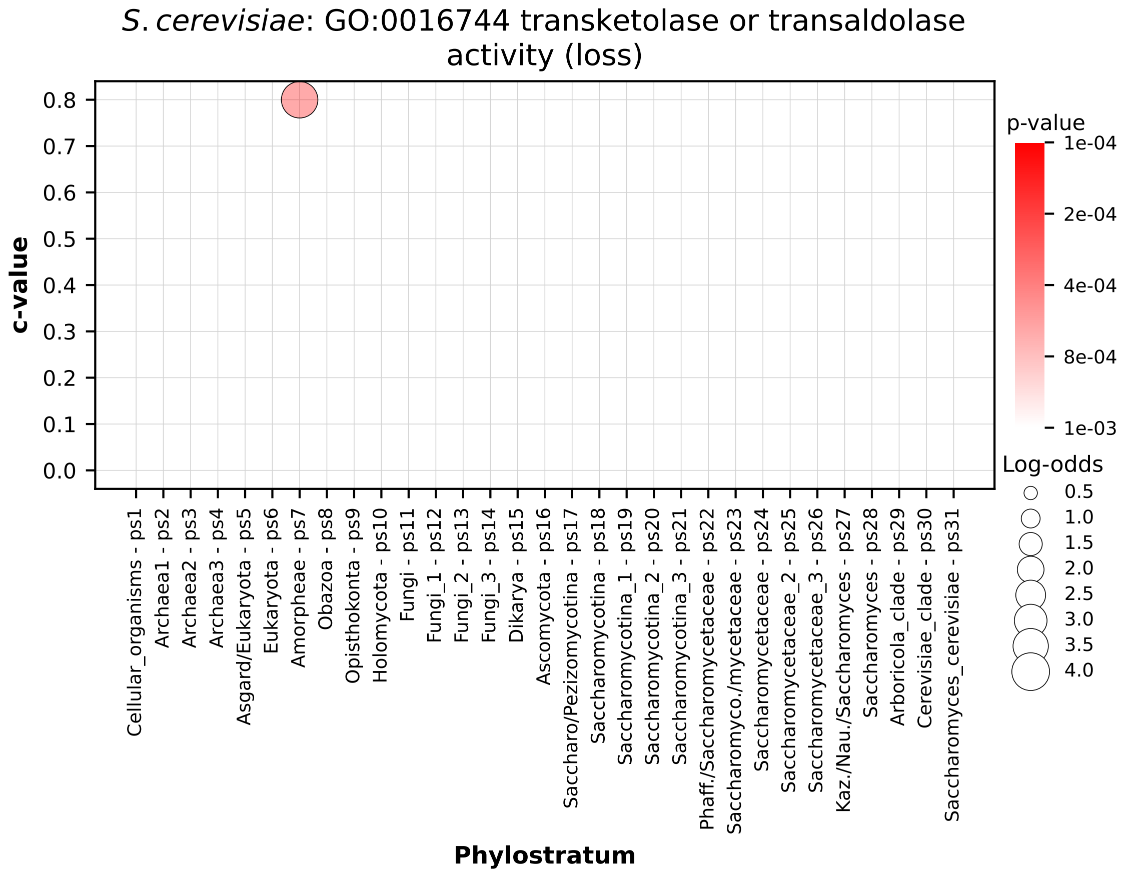
S. cerevisiae: GO:0016744 transketolase or transaldolase activity (loss)
c-value
0.0
0.1
0.2
0.3
0.4
0.5
0.6
0.7
0.8
Phylostratum
Cellular_organisms - ps1
Archaea1 - ps2
Archaea2 - ps3
Archaea3 - ps4
Asgard/Eukaryota - ps5
Eukaryota - ps6
Amorpheae - ps7
Obazoa - ps8
Opisthokonta - ps9
Holomycota - ps10
Fungi - ps11
Fungi_1 - ps12
Fungi_2 - ps13
Fungi_3 - ps14
Dikarya - ps15
Ascomycota - ps16
Saccharo/Pezizomycotina - ps17
Saccharomycotina - ps18
Saccharomycotina_1 - ps19
Saccharomycotina_2 - ps20
Saccharomycotina_3 - ps21
Phaff./Saccharomycetaceae - ps22
Saccharomyco./mycetaceae - ps23
Saccharomycetaceae - ps24
Saccharomycetaceae_2 - ps25
Saccharomycetaceae_3 - ps26
Kaz./Nau./Saccharomyces - ps27
Saccharomyces - ps28
Arboricola_clade - ps29
Cerevisiae_clade - ps30
Saccharomyces_cerevisiae - ps31
p-value
1e-04
2e-04
4e-04
8e-04
1e-03
Log-odds
0.5
1.0
1.5
2.0
2.5
3.0
3.5
4.0

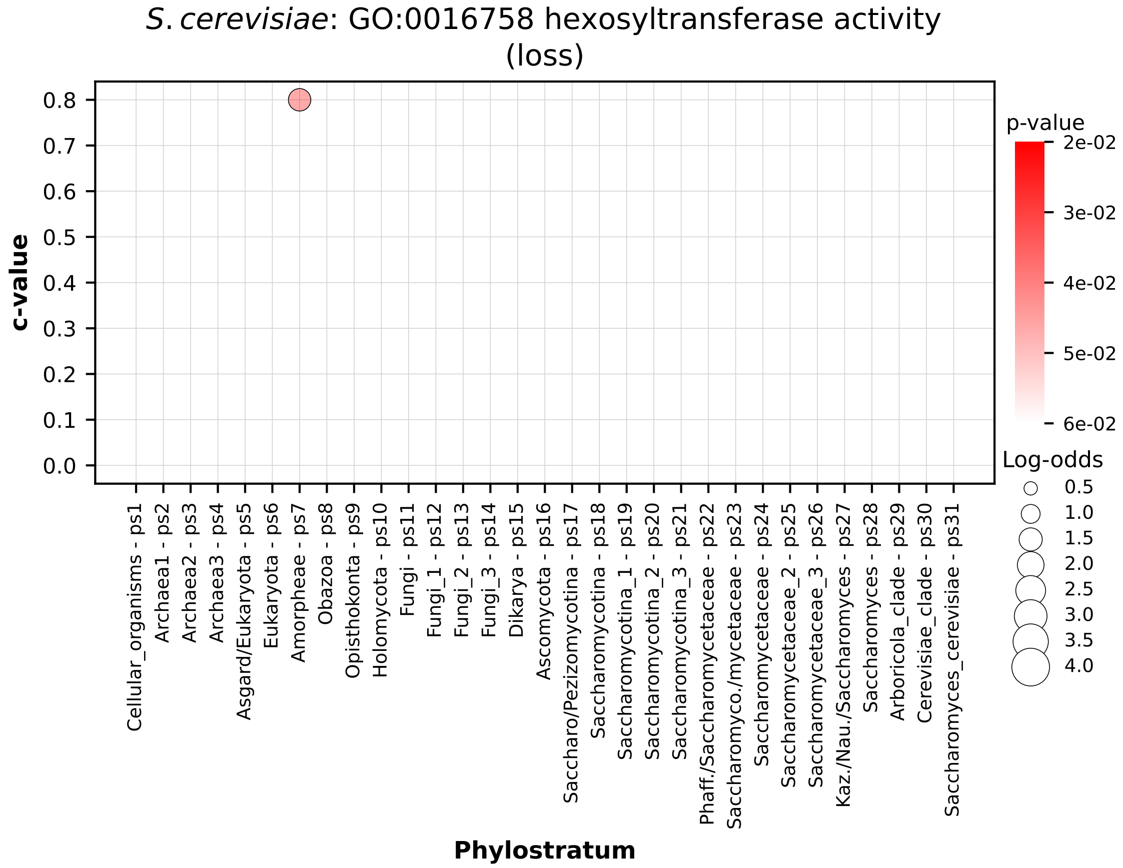

S. cerevisiae: GO:0016758 hexosyltransferase activity
(loss)
c-value
0.8
0.7
0.6
0.5
0.4
0.3
0.2
0.1
0.0
p-value
2e-02
3e-02
4e-02
5e-02
6e-02
Log-odds
0.5
1.0
1.5
2.0
2.5
3.0
3.5
4.0
Cellular_organisms - ps1
Archaea1 - ps2
Archaea2 - ps3
Archaea3 - ps4
Asgard/Eukaryota - ps5
Eukaryota - ps6
Amorpheae - ps7
Obazoa - ps8
Opisthokonta - ps9
Holomycota - ps10
Fungi - ps11
Fungi_1 - ps12
Fungi_2 - ps13
Fungi_3 - ps14
Dikarya - ps15
Ascomycota - ps16
Saccharo/Pezizomycotina - ps17
Saccharomycotina - ps18
Saccharomycotina_1 - ps19
Saccharomycotina_2 - ps20
Saccharomycotina_3 - ps21
Phaff./Saccharomycetaceae - ps22
Saccharomyco./mycetaceae - ps23
Saccharomycetaceae - ps24
Saccharomycetaceae_2 - ps25
Saccharomycetaceae_3 - ps26
Kaz./Nau./Saccharomyces - ps27
Saccharomyces - ps28
Arboricola_clade - ps29
Cerevisiae_clade - ps30
Saccharomyces_cerevisiae - ps31
Phylostratum

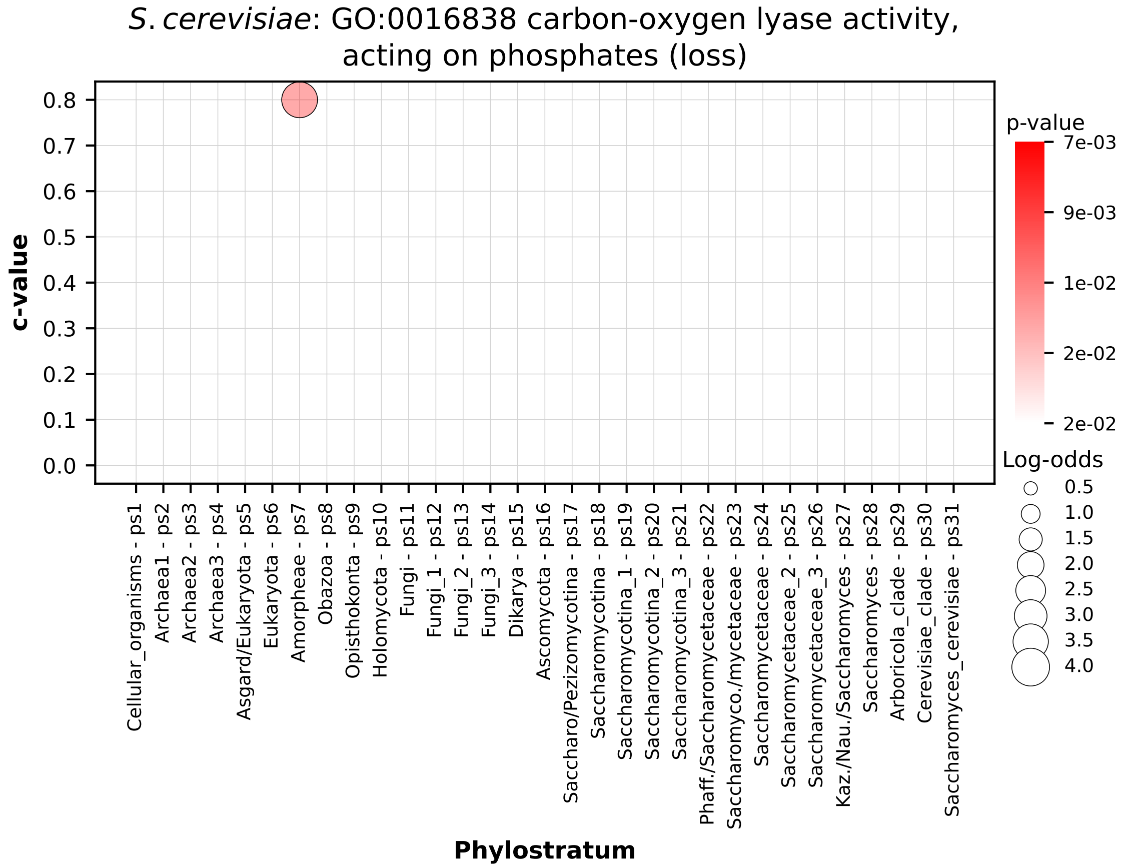

S. cerevisiae: GO:0016838 carbon-oxygen lyase activity, acting on phosphates (loss)
c-value
0.0
0.1
0.2
0.3
0.4
0.5
0.6
0.7
0.8
Phylostratum
Cellular_organisms - ps1
Archaea1 - ps2
Archaea2 - ps3
Archaea3 - ps4
Asgard/Eukaryota - ps5
Eukaryota - ps6
Amorpheae - ps7
Obazoa - ps8
Opisthokonta - ps9
Holomycota - ps10
Fungi - ps11
Fungi_1 - ps12
Fungi_2 - ps13
Fungi_3 - ps14
Dikarya - ps15
Ascomycota - ps16
Saccharo/Pezizomycotina - ps17
Saccharomycotina - ps18
Saccharomycotina_1 - ps19
Saccharomycotina_2 - ps20
Saccharomycotina_3 - ps21
Phaff./Saccharomycetaceae - ps22
Saccharomyco./mycetaceae - ps23
Saccharomycetaceae - ps24
Saccharomycetaceae_2 - ps25
Saccharomycetaceae_3 - ps26
Kaz./Nau./Saccharomyces - ps27
Saccharomyces - ps28
Arboricola_clade - ps29
Cerevisiae_clade - ps30
Saccharomyces_cerevisiae - ps31
p-value
7e-03
9e-03
1e-02
2e-02
2e-02
Log-odds
0.5
1.0
1.5
2.0
2.5
3.0
3.5
4.0

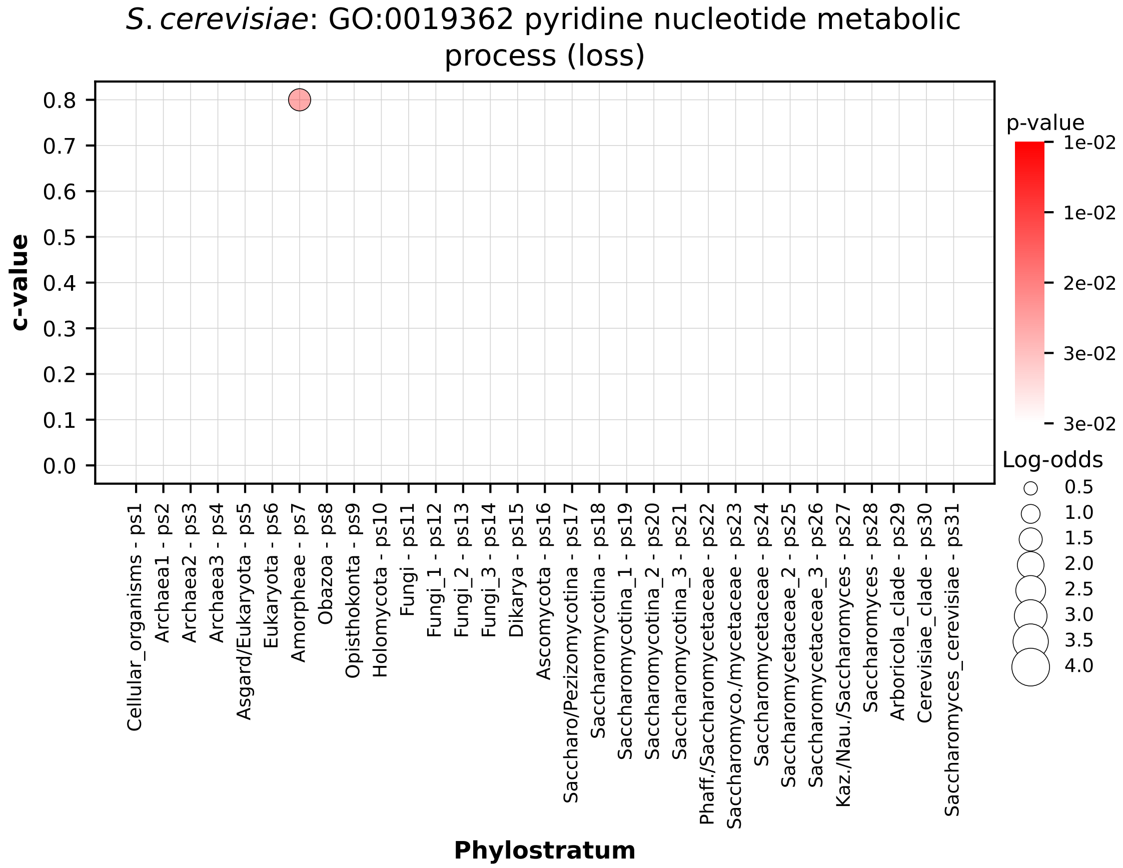
S. cerevisiae: GO:0019362 pyridine nucleotide metabolic process (loss)
c-value
0.0
0.1
0.2
0.3
0.4
0.5
0.6
0.7
0.8
p-value
1e-02
1e-02
2e-02
3e-02
3e-02
Log-odds
0.5
1.0
1.5
2.0
2.5
3.0
3.5
4.0
Cellular_organisms - ps1
Archaea1 - ps2
Archaea2 - ps3
Archaea3 - ps4
Asgard/Eukaryota - ps5
Eukaryota - ps6
Amorpheae - ps7
Obazoa - ps8
Opisthokonta - ps9
Holomycota - ps10
Fungi - ps11
Fungi_1 - ps12
Fungi_2 - ps13
Fungi_3 - ps14
Dikarya - ps15
Ascomycota - ps16
Saccharo/Pezizomycotina - ps17
Saccharomycotina - ps18
Saccharomycotina_1 - ps19
Saccharomycotina_2 - ps20
Saccharomycotina_3 - ps21
Phaff./Saccharomycetaceae - ps22
Saccharomyco./mycetaceae - ps23
Saccharomycetaceae - ps24
Saccharomycetaceae_2 - ps25
Saccharomycetaceae_3 - ps26
Kaz./Nau./Saccharomyces - ps27
Saccharomyces - ps28
Arboricola_clade - ps29
Cerevisiae_clade - ps30
Saccharomyces_cerevisiae - ps31
Phylostratum

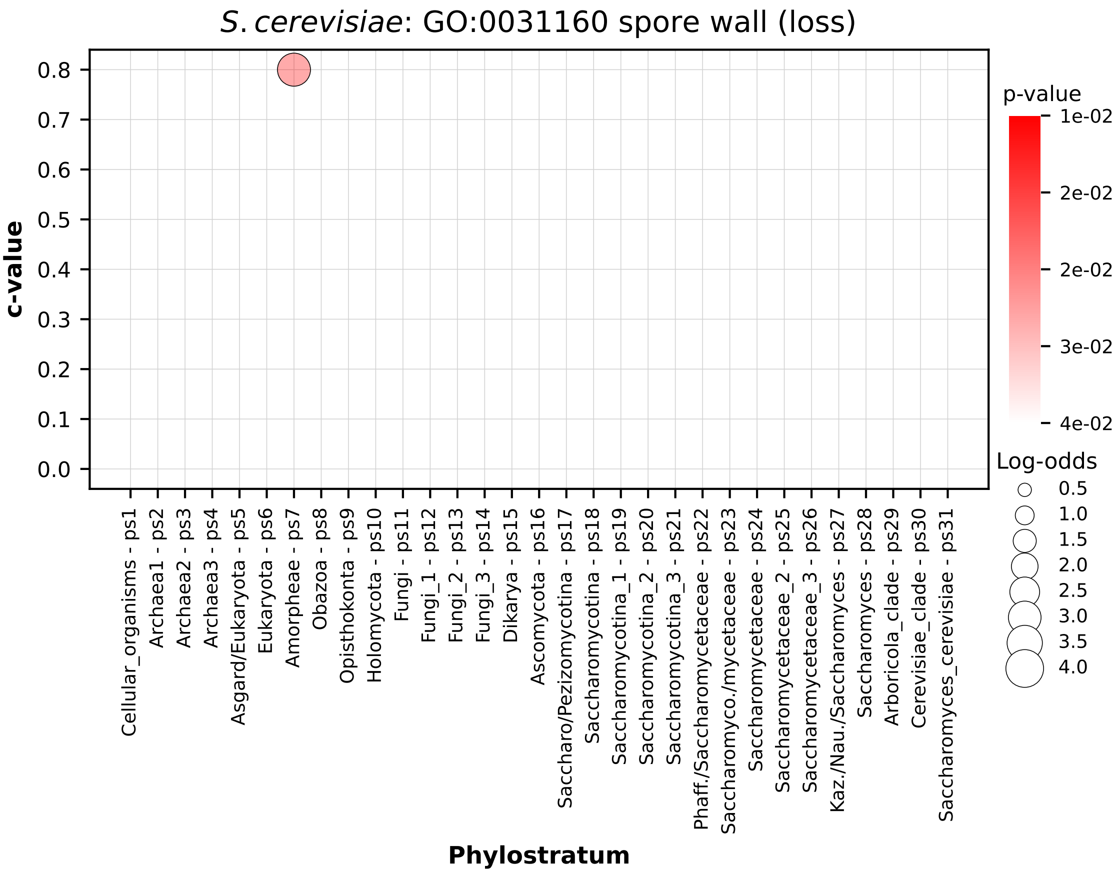
S. cerevisiae: GO:0031160 spore wall (loss)
c-value
0.0
0.1
0.2
0.3
0.4
0.5
0.6
0.7
0.8
p-value
1e-02
2e-02
2e-02
3e-02
4e-02
Log-odds
0.5
1.0
1.5
2.0
2.5
3.0
3.5
4.0
Cellular_organisms - ps1
Archaea1 - ps2
Archaea2 - ps3
Archaea3 - ps4
Asgard/Eukaryota - ps5
Eukaryota - ps6
Amorpheae - ps7
Obazoa - ps8
Opisthokonta - ps9
Holomycota - ps10
Fungi - ps11
Fungi_1 - ps12
Fungi_2 - ps13
Fungi_3 - ps14
Dikarya - ps15
Ascomycota - ps16
Saccharo/Pezizomycotina - ps17
Saccharomycotina - ps18
Saccharomycotina_1 - ps19
Saccharomycotina_2 - ps20
Saccharomycotina_3 - ps21
Phaff./Saccharomycetaceae - ps22
Saccharomyco./mycetaceae - ps23
Saccharomycetaceae - ps24
Saccharomycetaceae_2 - ps25
Saccharomycetaceae_3 - ps26
Kaz./Nau./Saccharomyces - ps27
Saccharomyces - ps28
Arboricola_clade - ps29
Cerevisiae_clade - ps30
Saccharomyces_cerevisiae - ps31
Phylostratum

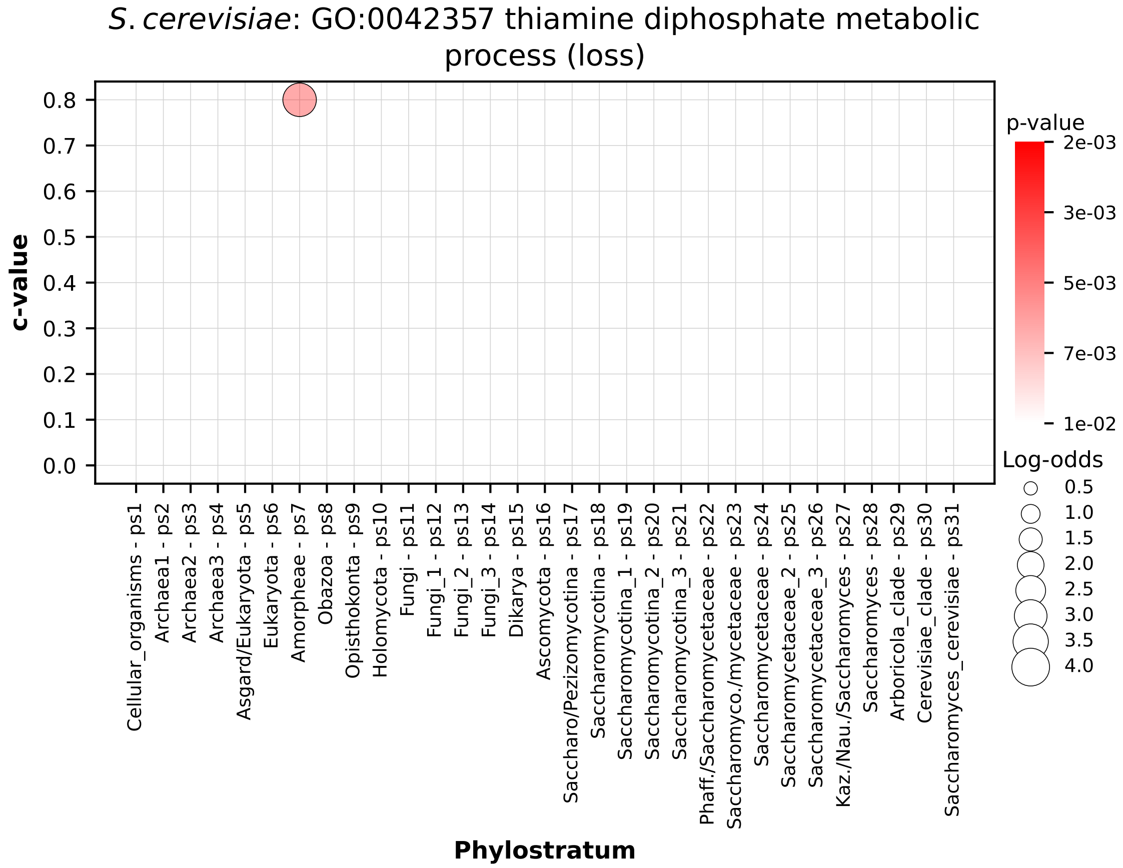

S. cerevisiae: GO:0042357 thiamine diphosphate metabolic process (loss)
c-value
0.0
0.1
0.2
0.3
0.4
0.5
0.6
0.7
0.8
Phylostratum
Cellular_organisms - ps1
Archaea1 - ps2
Archaea2 - ps3
Archaea3 - ps4
Asgard/Eukaryota - ps5
Eukaryota - ps6
Amorpheae - ps7
Obazoa - ps8
Opisthokonta - ps9
Holomycota - ps10
Fungi - ps11
Fungi_1 - ps12
Fungi_2 - ps13
Fungi_3 - ps14
Dikarya - ps15
Ascomycota - ps16
Saccharo/Pezizomycotina - ps17
Saccharomycotina - ps18
Saccharomycotina_1 - ps19
Saccharomycotina_2 - ps20
Saccharomycotina_3 - ps21
Phaff./Saccharomycetaceae - ps22
Saccharomyco./mycetaceae - ps23
Saccharomycetaceae - ps24
Saccharomycetaceae_2 - ps25
Saccharomycetaceae_3 - ps26
Kaz./Nau./Saccharomyces - ps27
Saccharomyces - ps28
Arboricola_clade - ps29
Cerevisiae_clade - ps30
Saccharomyces_cerevisiae - ps31
p-value
2e-03
3e-03
5e-03
7e-03
1e-02
Log-odds
0.5
1.0
1.5
2.0
2.5
3.0
3.5
4.0

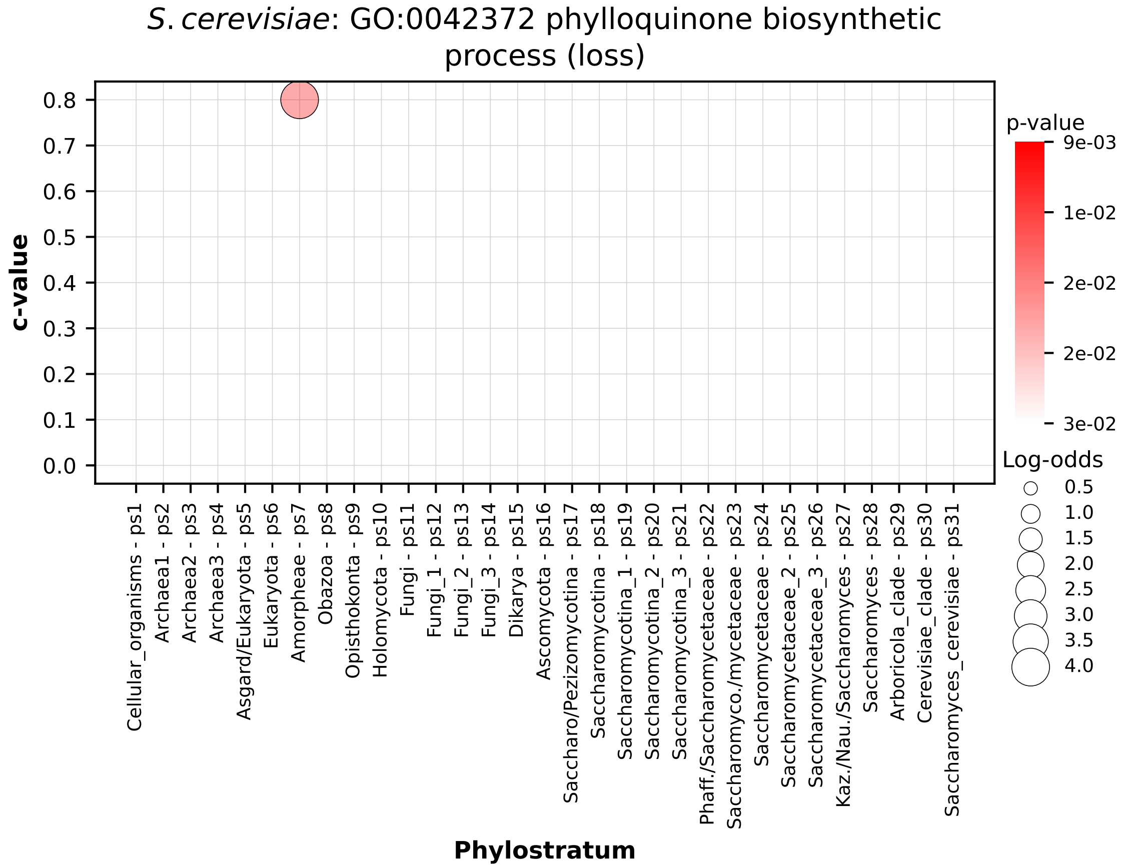

S. cerevisiae: GO:0042372 phylloquinone biosynthetic process (loss)
c-value
0.0
0.1
0.2
0.3
0.4
0.5
0.6
0.7
0.8
Phylostratum
Cellular_organisms - ps1
Archaea1 - ps2
Archaea2 - ps3
Archaea3 - ps4
Asgard/Eukaryota - ps5
Eukaryota - ps6
Amorpheae - ps7
Obazoa - ps8
Opisthokonta - ps9
Holomycota - ps10
Fungi - ps11
Fungi_1 - ps12
Fungi_2 - ps13
Fungi_3 - ps14
Dikarya - ps15
Ascomycota - ps16
Saccharo/Pezizomycotina - ps17
Saccharomycotina - ps18
Saccharomycotina_1 - ps19
Saccharomycotina_2 - ps20
Saccharomycotina_3 - ps21
Phaff./Saccharomycetaceae - ps22
Saccharomyco./mycetaceae - ps23
Saccharomycetaceae - ps24
Saccharomycetaceae_2 - ps25
Saccharomycetaceae_3 - ps26
Kaz./Nau./Saccharomyces - ps27
Saccharomyces - ps28
Arboricola_clade - ps29
Cerevisiae_clade - ps30
Saccharomyces_cerevisiae - ps31
p-value
9e-03
1e-02
2e-02
2e-02
3e-02
Log-odds
0.5
1.0
1.5
2.0
2.5
3.0
3.5
4.0

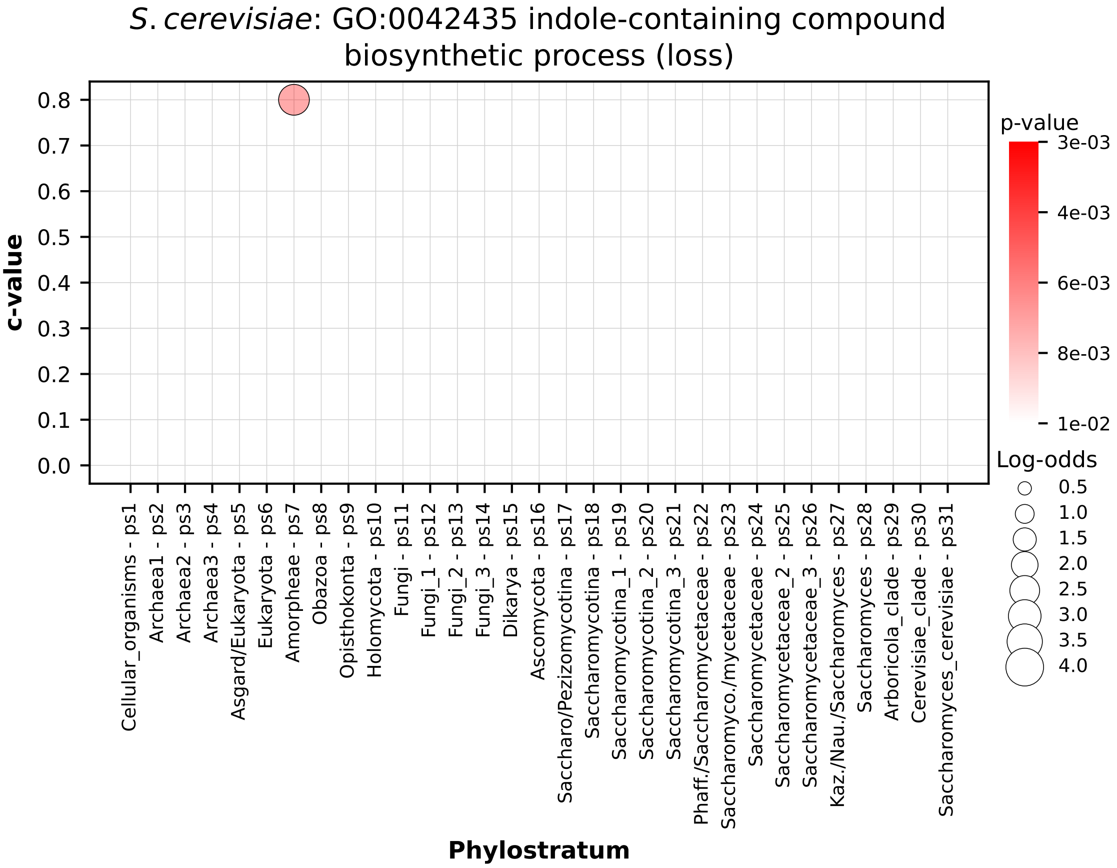

S. cerevisiae: GO:0042435 indole-containing compound biosynthetic process (loss)
c-value
0.8
0.7
0.6
0.5
0.4
0.3
0.2
0.1
0.0
p-value
3e-03
4e-03
6e-03
8e-03
1e-02
Log-odds
0.5
1.0
1.5
2.0
2.5
3.0
3.5
4.0
Cellular_organisms - ps1
Archaea1 - ps2
Archaea2 - ps3
Archaea3 - ps4
Asgard/Eukaryota - ps5
Eukaryota - ps6
Amorpheae - ps7
Obazoa - ps8
Opisthokonta - ps9
Holomycota - ps10
Fungi - ps11
Fungi_1 - ps12
Fungi_2 - ps13
Fungi_3 - ps14
Dikarya - ps15
Ascomycota - ps16
Saccharo/Pezizomycotina - ps17
Saccharomycotina - ps18
Saccharomycotina_1 - ps19
Saccharomycotina_2 - ps20
Saccharomycotina_3 - ps21
Phaff./Saccharomycetaceae - ps22
Saccharomyco./mycetaceae - ps23
Saccharomycetaceae - ps24
Saccharomycetaceae_2 - ps25
Saccharomycetaceae_3 - ps26
Kaz./Nau./Saccharomyces - ps27
Saccharomyces - ps28
Arboricola_clade - ps29
Cerevisiae_clade - ps30
Saccharomyces_cerevisiae - ps31
Phylostratum

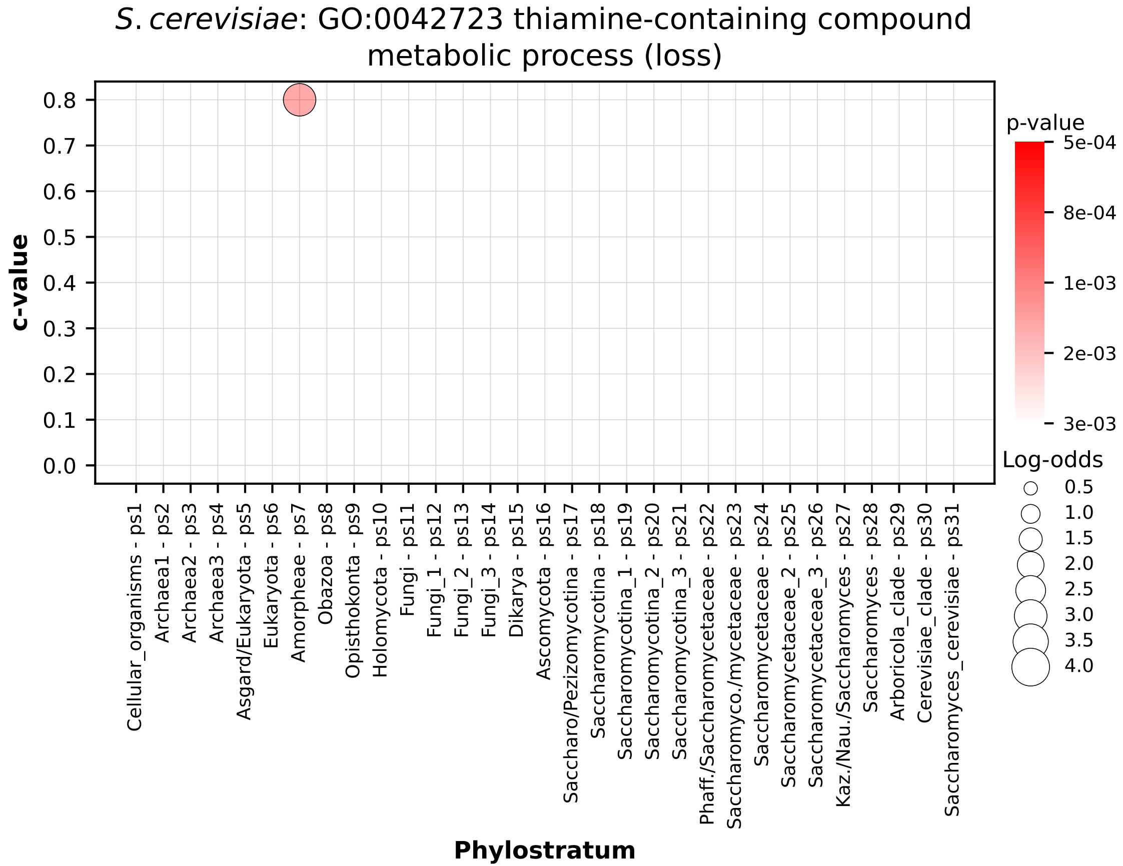
S. cerevisiae: GO:0042723 thiamine-containing compound metabolic process (loss)
c-value
0.0
0.1
0.2
0.3
0.4
0.5
0.6
0.7
0.8
p-value
5e-04
8e-04
1e-03
2e-03
3e-03
Log-odds
0.5
1.0
1.5
2.0
2.5
3.0
3.5
4.0
Cellular_organisms - ps1
Archaea1 - ps2
Archaea2 - ps3
Archaea3 - ps4
Asgard/Eukaryota - ps5
Eukaryota - ps6
Amorpheae - ps7
Obazoa - ps8
Opisthokonta - ps9
Holomycota - ps10
Fungi - ps11
Fungi_1 - ps12
Fungi_2 - ps13
Fungi_3 - ps14
Dikarya - ps15
Ascomycota - ps16
Saccharo/Pezizomycotina - ps17
Saccharomycotina - ps18
Saccharomycotina_1 - ps19
Saccharomycotina_2 - ps20
Saccharomycotina_3 - ps21
Phaff./Saccharomycetaceae - ps22
Saccharomyco./mycetaceae - ps23
Saccharomycetaceae - ps24
Saccharomycetaceae_2 - ps25
Saccharomycetaceae_3 - ps26
Kaz./Nau./Saccharomyces - ps27
Saccharomyces - ps28
Arboricola_clade - ps29
Cerevisiae_clade - ps30
Saccharomyces_cerevisiae - ps31
Phylostratum

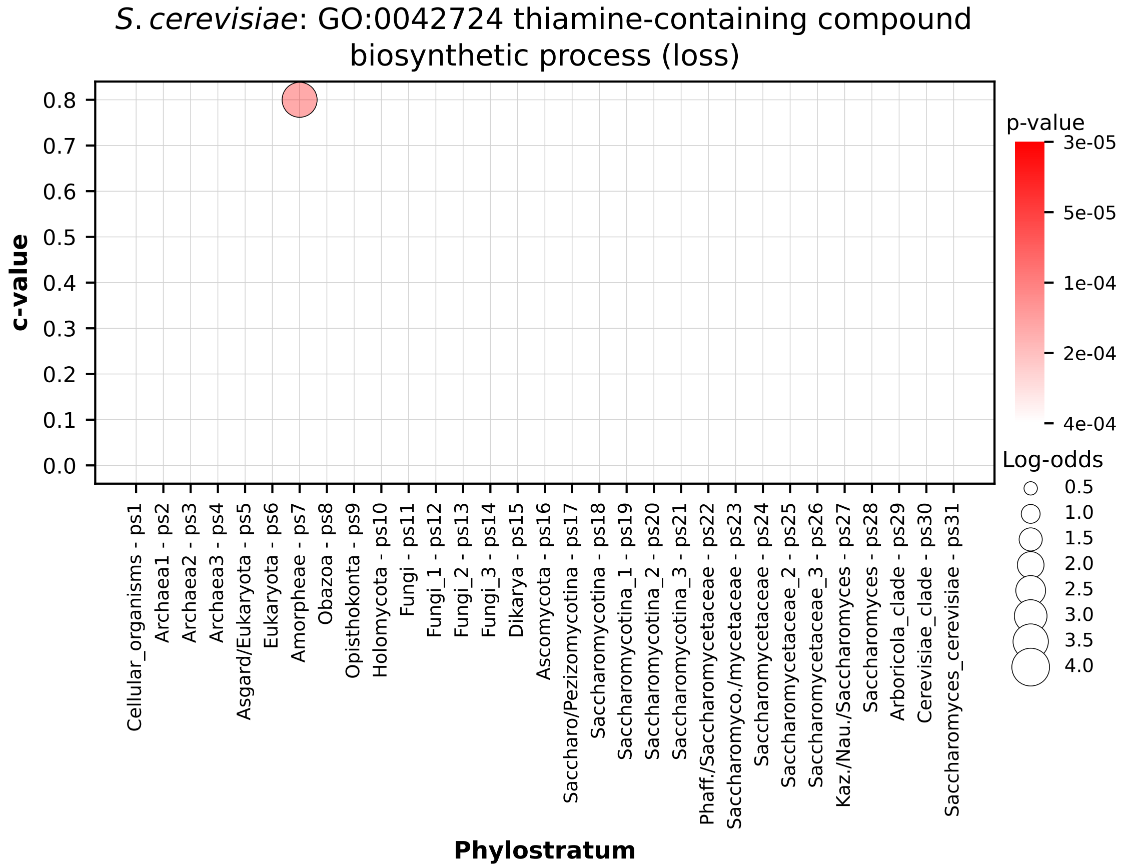

S. cerevisiae: GO:0042724 thiamine-containing compound biosynthetic process (loss)
c-value
0.0
0.1
0.2
0.3
0.4
0.5
0.6
0.7
0.8
Phylostratum
Cellular_organisms - ps1
Archaea1 - ps2
Archaea2 - ps3
Archaea3 - ps4
Asgard/Eukaryota - ps5
Eukaryota - ps6
Amorpheae - ps7
Obazoa - ps8
Opisthokonta - ps9
Holomycota - ps10
Fungi - ps11
Fungi_1 - ps12
Fungi_2 - ps13
Fungi_3 - ps14
Dikarya - ps15
Ascomycota - ps16
Saccharo/Pezizomycotina - ps17
Saccharomycotina - ps18
Saccharomycotina_1 - ps19
Saccharomycotina_2 - ps20
Saccharomycotina_3 - ps21
Phaff./Saccharomycetaceae - ps22
Saccharomyco./mycetaceae - ps23
Saccharomycetaceae - ps24
Saccharomycetaceae_2 - ps25
Saccharomycetaceae_3 - ps26
Kaz./Nau./Saccharomyces - ps27
Saccharomyces - ps28
Arboricola_clade - ps29
Cerevisiae_clade - ps30
Saccharomyces_cerevisiae - ps31
p-value
3e-05
5e-05
1e-04
2e-04
4e-04
Log-odds
0.5
1.0
1.5
2.0
2.5
3.0
3.5
4.0

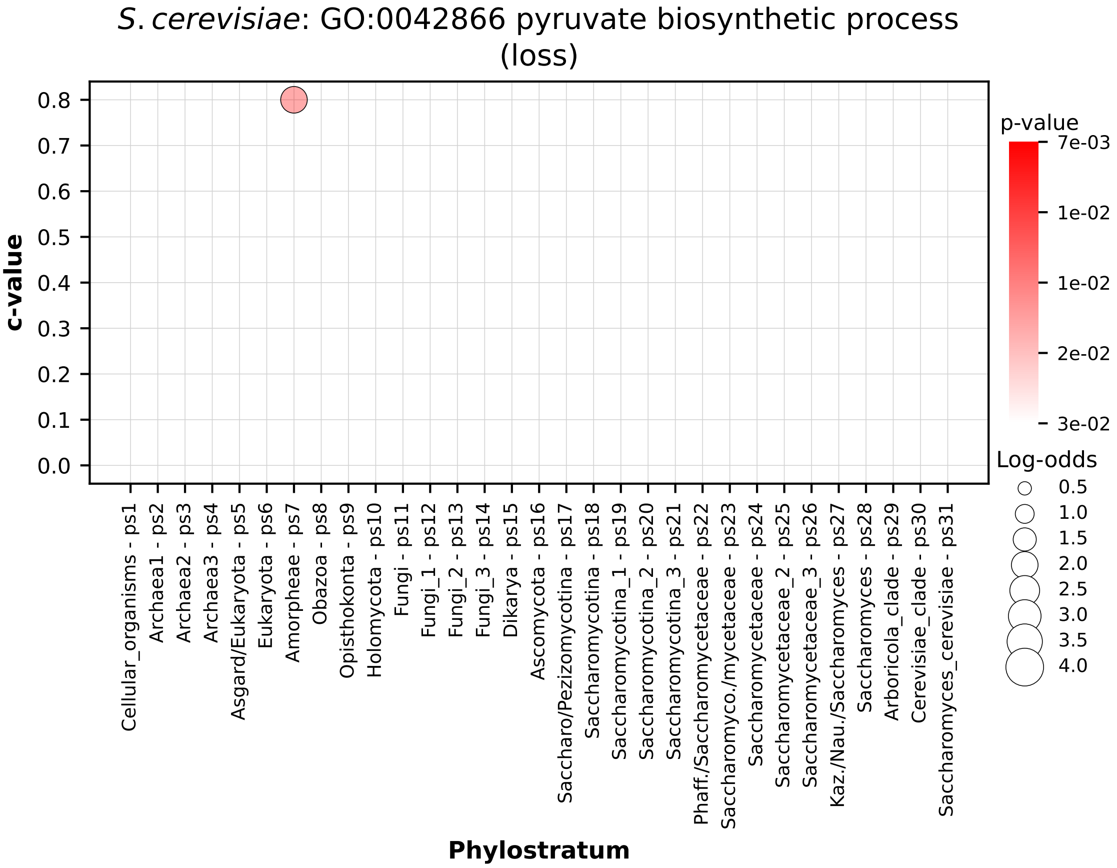

S. cerevisiae: GO:0042866 pyruvate biosynthetic process
(loss)
c-value
0.0
0.1
0.2
0.3
0.4
0.5
0.6
0.7
0.8
Phylostratum
Cellular_organisms - ps1
Archaea1 - ps2
Archaea2 - ps3
Archaea3 - ps4
Asgard/Eukaryota - ps5
Eukaryota - ps6
Amorpheae - ps7
Obazoa - ps8
Opisthokonta - ps9
Holomycota - ps10
Fungi - ps11
Fungi_1 - ps12
Fungi_2 - ps13
Fungi_3 - ps14
Dikarya - ps15
Ascomycota - ps16
Saccharo/Pezizomycotina - ps17
Saccharomycotina - ps18
Saccharomycotina_1 - ps19
Saccharomycotina_2 - ps20
Saccharomycotina_3 - ps21
Phaff./Saccharomycetaceae - ps22
Saccharomyco./mycetaceae - ps23
Saccharomycetaceae - ps24
Saccharomycetaceae_2 - ps25
Saccharomycetaceae_3 - ps26
Kaz./Nau./Saccharomyces - ps27
Saccharomyces - ps28
Arboricola_clade - ps29
Cerevisiae_clade - ps30
Saccharomyces_cerevisiae - ps31
p-value
7e-03
1e-02
1e-02
2e-02
3e-02
Log-odds
0.5
1.0
1.5
2.0
2.5
3.0
3.5
4.0

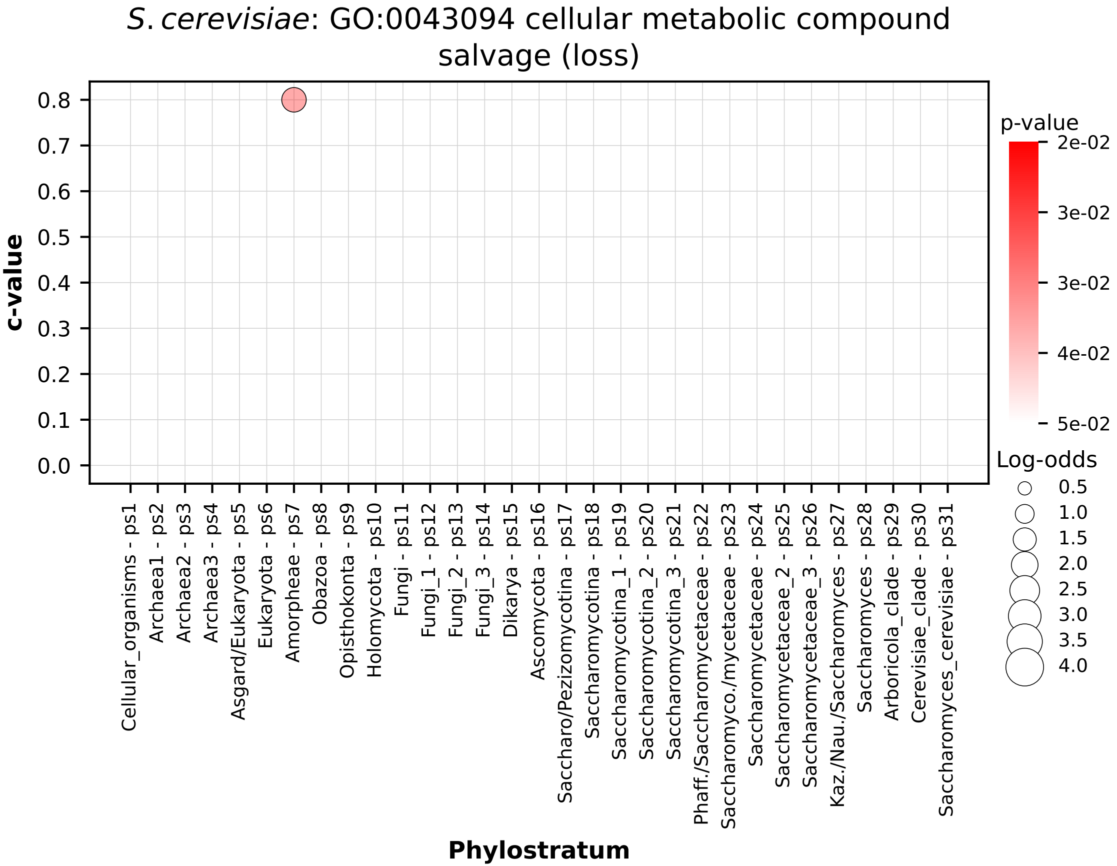

S. cerevisiae: GO:0043094 cellular metabolic compound salvage (loss)
c-value
0.0
0.1
0.2
0.3
0.4
0.5
0.6
0.7
0.8
Phylostratum
Cellular_organisms - ps1
Archaea1 - ps2
Archaea2 - ps3
Archaea3 - ps4
Asgard/Eukaryota - ps5
Eukaryota - ps6
Amorpheae - ps7
Obazoa - ps8
Opisthokonta - ps9
Holomycota - ps10
Fungi - ps11
Fungi_1 - ps12
Fungi_2 - ps13
Fungi_3 - ps14
Dikarya - ps15
Ascomycota - ps16
Saccharo/Pezizomycotina - ps17
Saccharomycotina - ps18
Saccharomycotina_1 - ps19
Saccharomycotina_2 - ps20
Saccharomycotina_3 - ps21
Phaff./Saccharomycetaceae - ps22
Saccharomyco./mycetaceae - ps23
Saccharomycetaceae - ps24
Saccharomycetaceae_2 - ps25
Saccharomycetaceae_3 - ps26
Kaz./Nau./Saccharomyces - ps27
Saccharomyces - ps28
Arboricola_clade - ps29
Cerevisiae_clade - ps30
Saccharomyces_cerevisiae - ps31
p-value
2e-02
3e-02
3e-02
4e-02
5e-02
Log-odds
0.5
1.0
1.5
2.0
2.5
3.0
3.5
4.0

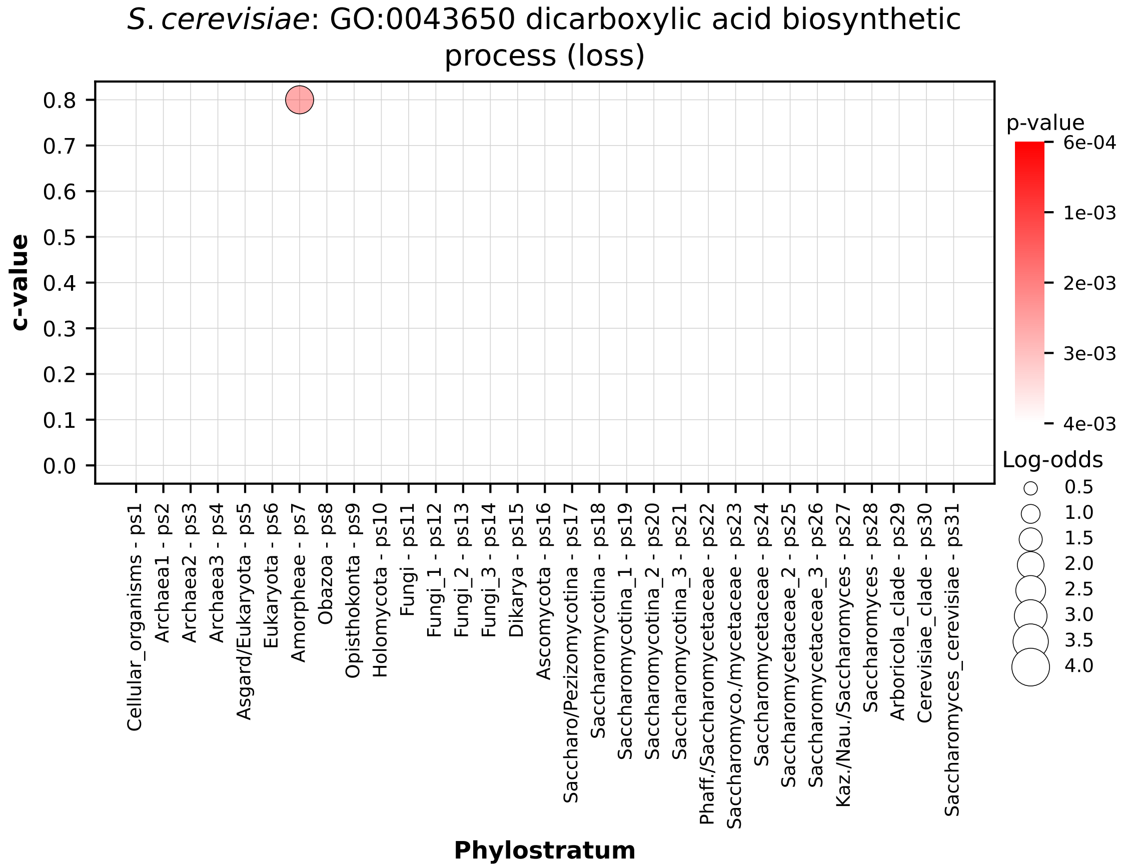
S. cerevisiae: GO:0043650 dicarboxylic acid biosynthetic process (loss)
c-value
0.0
0.1
0.2
0.3
0.4
0.5
0.6
0.7
0.8
p-value
6e-04
1e-03
2e-03
3e-03
4e-03
Log-odds
0.5
1.0
1.5
2.0
2.5
3.0
3.5
4.0
Cellular_organisms - ps1
Archaea1 - ps2
Archaea2 - ps3
Archaea3 - ps4
Asgard/Eukaryota - ps5
Eukaryota - ps6
Amorpheae - ps7
Obazoa - ps8
Opisthokonta - ps9
Holomycota - ps10
Fungi - ps11
Fungi_1 - ps12
Fungi_2 - ps13
Fungi_3 - ps14
Dikarya - ps15
Ascomycota - ps16
Saccharo/Pezizomycotina - ps17
Saccharomycotina - ps18
Saccharomycotina_1 - ps19
Saccharomycotina_2 - ps20
Saccharomycotina_3 - ps21
Phaff./Saccharomycetaceae - ps22
Saccharomyco./mycetaceae - ps23
Saccharomycetaceae - ps24
Saccharomycetaceae_2 - ps25
Saccharomycetaceae_3 - ps26
Kaz./Nau./Saccharomyces - ps27
Saccharomyces - ps28
Arboricola_clade - ps29
Cerevisiae_clade - ps30
Saccharomyces_cerevisiae - ps31
Phylostratum

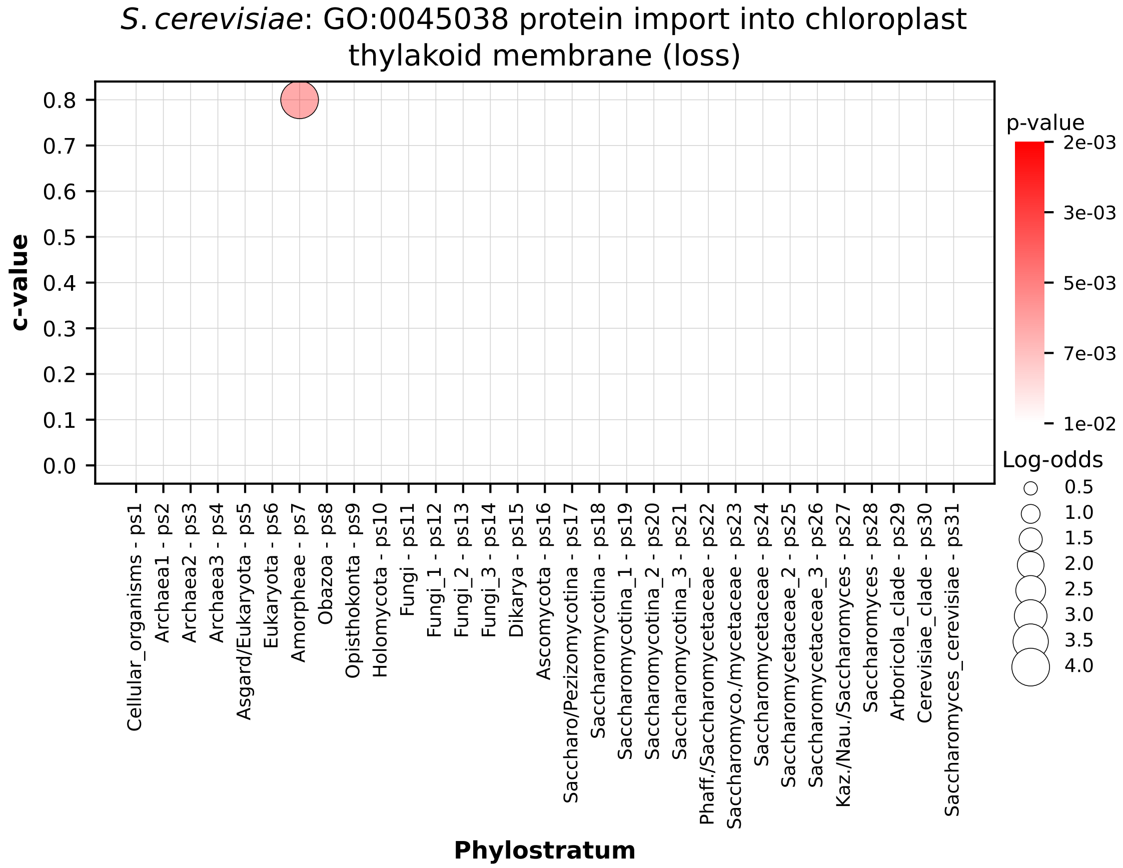

S. cerevisiae: GO:0045038 protein import into chloroplast thylakoid membrane (loss)
c-value
0.0
0.1
0.2
0.3
0.4
0.5
0.6
0.7
0.8
Phylostratum
Cellular_organisms - ps1
Archaea1 - ps2
Archaea2 - ps3
Archaea3 - ps4
Asgard/Eukaryota - ps5
Eukaryota - ps6
Amorpheae - ps7
Obazoa - ps8
Opisthokonta - ps9
Holomycota - ps10
Fungi - ps11
Fungi_1 - ps12
Fungi_2 - ps13
Fungi_3 - ps14
Dikarya - ps15
Ascomycota - ps16
Saccharo/Pezizomycotina - ps17
Saccharomycotina - ps18
Saccharomycotina_1 - ps19
Saccharomycotina_2 - ps20
Saccharomycotina_3 - ps21
Phaff./Saccharomycetaceae - ps22
Saccharomyco./mycetaceae - ps23
Saccharomycetaceae - ps24
Saccharomycetaceae_2 - ps25
Saccharomycetaceae_3 - ps26
Kaz./Nau./Saccharomyces - ps27
Saccharomyces - ps28
Arboricola_clade - ps29
Cerevisiae_clade - ps30
Saccharomyces_cerevisiae - ps31
p-value
2e-03
3e-03
5e-03
7e-03
1e-02
Log-odds
0.5
1.0
1.5
2.0
2.5
3.0
3.5
4.0

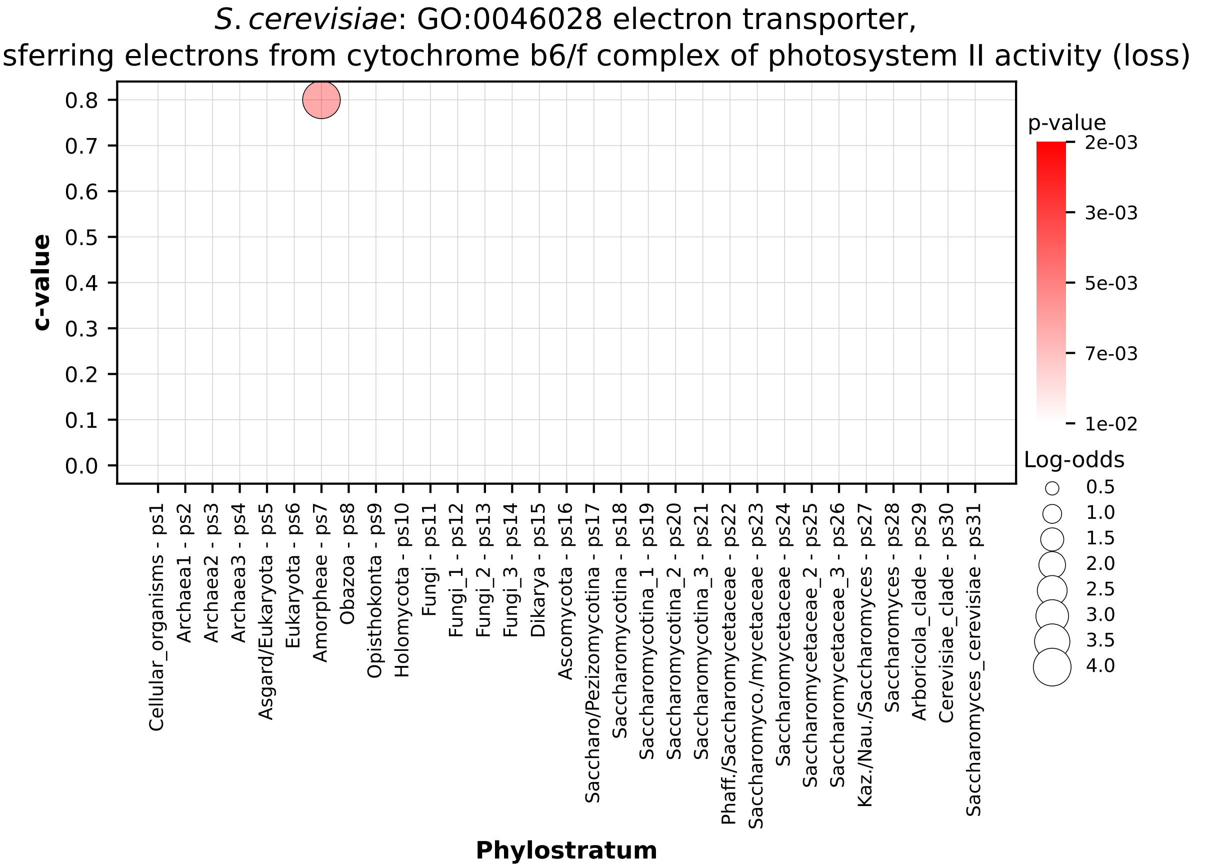

S. cerevisiae: GO:0046028 electron transporter,
sferring electrons from cytochrome b6/f complex of photosystem II activity (loss)
c-value
0.8
0.7
0.6
0.5
0.4
0.3
0.2
0.1
0.0
p-value
2e-03
3e-03
5e-03
7e-03
1e-02
Log-odds
0.5
1.0
1.5
2.0
2.5
3.0
3.5
4.0
Cellular_organisms - ps1
Archaea1 - ps2
Archaea2 - ps3
Archaea3 - ps4
Asgard/Eukaryota - ps5
Eukaryota - ps6
Amorpheae - ps7
Obazoa - ps8
Opisthokonta - ps9
Holomycota - ps10
Fungi - ps11
Fungi_1 - ps12
Fungi_2 - ps13
Fungi_3 - ps14
Dikarya - ps15
Ascomycota - ps16
Saccharo/Pezizomycotina - ps17
Saccharomycotina - ps18
Saccharomycotina_1 - ps19
Saccharomycotina_2 - ps20
Saccharomycotina_3 - ps21
Phaff./Saccharomycetaceae - ps22
Saccharomyco./mycetaceae - ps23
Saccharomycetaceae - ps24
Saccharomycetaceae_2 - ps25
Saccharomycetaceae_3 - ps26
Kaz./Nau./Saccharomyces - ps27
Saccharomyces - ps28
Arboricola_clade - ps29
Cerevisiae_clade - ps30
Saccharomyces_cerevisiae - ps31
Phylostratum

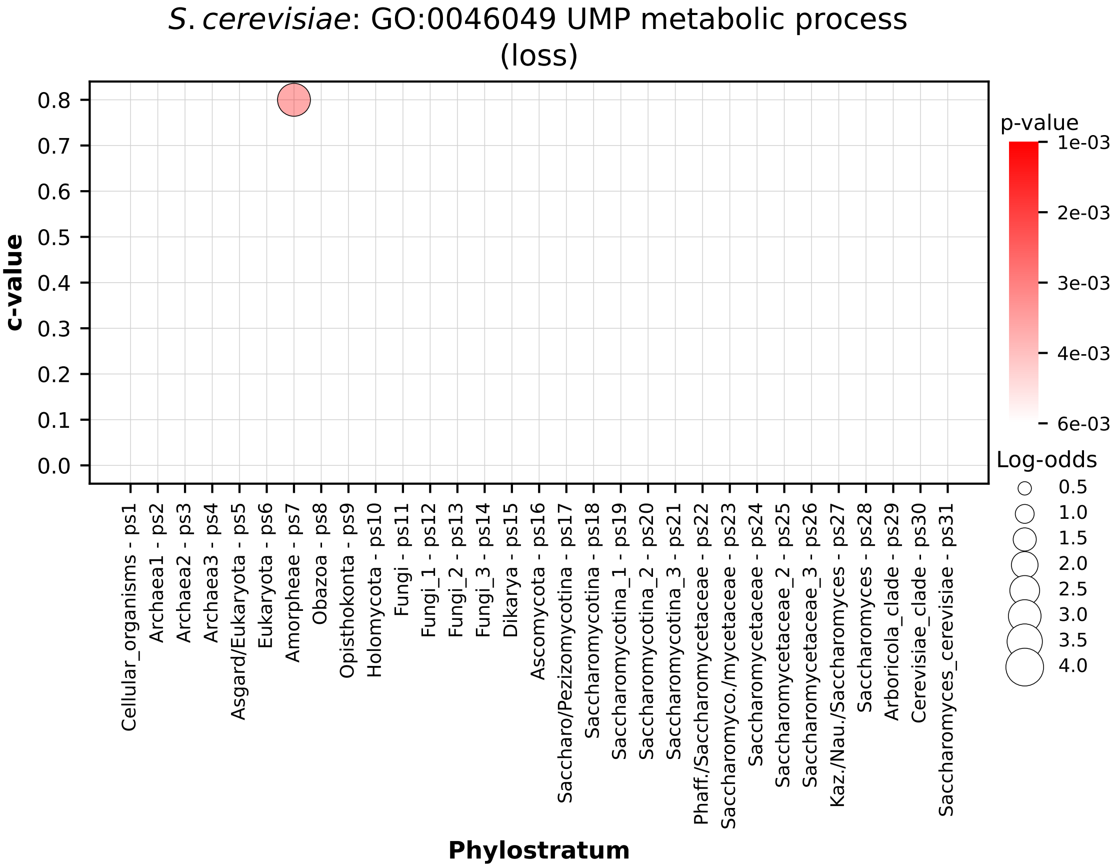

S. cerevisiae: GO:0046049 UMP metabolic process (loss)
c-value
0.8
0.7
0.6
0.5
0.4
0.3
0.2
0.1
0.0
p-value
1e-03
2e-03
3e-03
4e-03
6e-03
Log-odds
0.5
1.0
1.5
2.0
2.5
3.0
3.5
4.0
Cellular_organisms - ps1
Archaea1 - ps2
Archaea2 - ps3
Archaea3 - ps4
Asgard/Eukaryota - ps5
Eukaryota - ps6
Amorpheae - ps7
Obazoa - ps8
Opisthokonta - ps9
Holomycota - ps10
Fungi - ps11
Fungi_1 - ps12
Fungi_2 - ps13
Fungi_3 - ps14
Dikarya - ps15
Ascomycota - ps16
Saccharo/Pezizomycotina - ps17
Saccharomycotina - ps18
Saccharomycotina_1 - ps19
Saccharomycotina_2 - ps20
Saccharomycotina_3 - ps21
Phaff./Saccharomycetaceae - ps22
Saccharomyco./mycetaceae - ps23
Saccharomycetaceae - ps24
Saccharomycetaceae_2 - ps25
Saccharomycetaceae_3 - ps26
Kaz./Nau./Saccharomyces - ps27
Saccharomyces - ps28
Arboricola_clade - ps29
Cerevisiae_clade - ps30
Saccharomyces_cerevisiae - ps31
Phylostratum

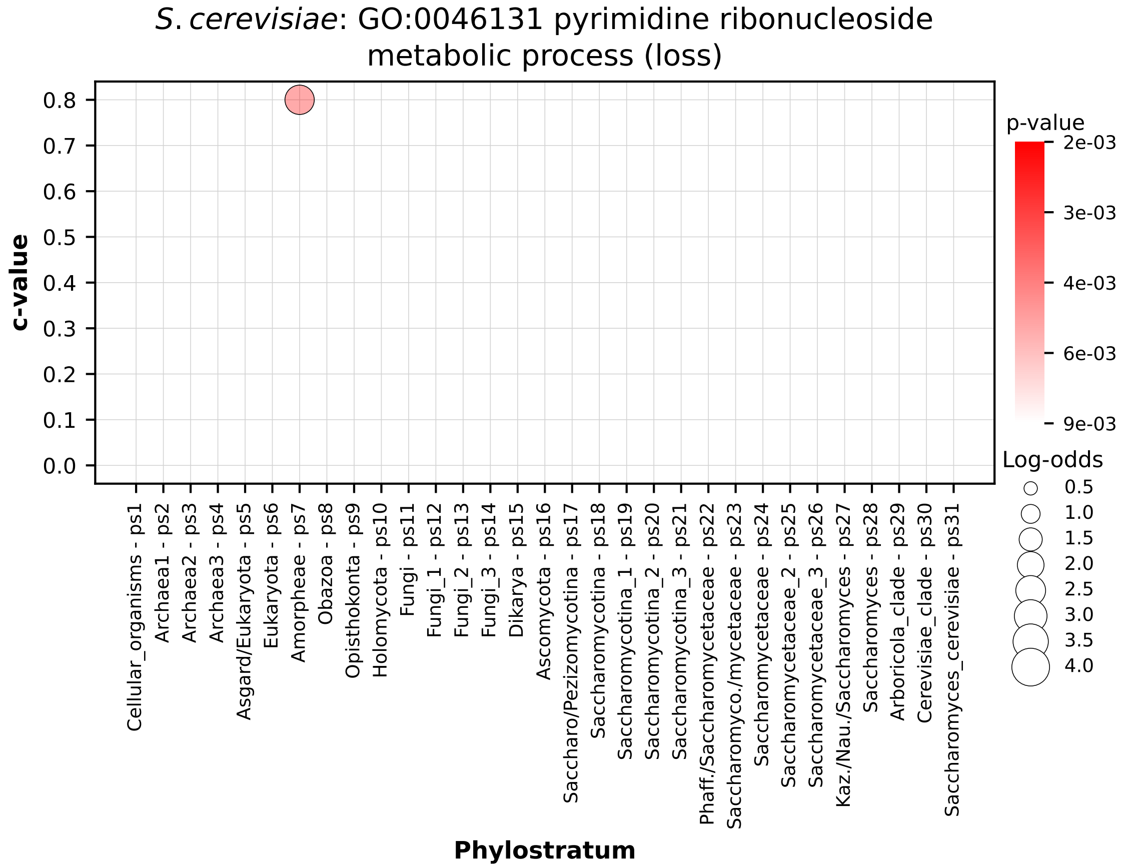

$S. cerevisiae$: GO:0046131 pyrimidine ribonucleoside metabolic process (loss)
c-value
0.0
0.1
0.2
0.3
0.4
0.5
0.6
0.7
0.8
p-value
2e-03
3e-03
4e-03
6e-03
9e-03
Log-odds
0.5
1.0
1.5
2.0
2.5
3.0
3.5
4.0
Cellular_organisms - ps1
Archaea1 - ps2
Archaea2 - ps3
Archaea3 - ps4
Asgard/Eukaryota - ps5
Eukaryota - ps6
Amorpheae - ps7
Obazoa - ps8
Opisthokonta - ps9
Holomycota - ps10
Fungi - ps11
Fungi_1 - ps12
Fungi_2 - ps13
Fungi_3 - ps14
Dikarya - ps15
Ascomycota - ps16
Saccharo/Pezizomycotina - ps17
Saccharomycotina - ps18
Saccharomycotina_1 - ps19
Saccharomycotina_2 - ps20
Saccharomycotina_3 - ps21
Phaff./Saccharomycetaceae - ps22
Saccharomyco./mycetaceae - ps23
Saccharomycetaceae - ps24
Saccharomycetaceae_2 - ps25
Saccharomycetaceae_3 - ps26
Kaz./Nau./Saccharomyces - ps27
Saccharomyces - ps28
Arboricola_clade - ps29
Cerevisiae_clade - ps30
Saccharomyces_cerevisiae - ps31
Phylostratum

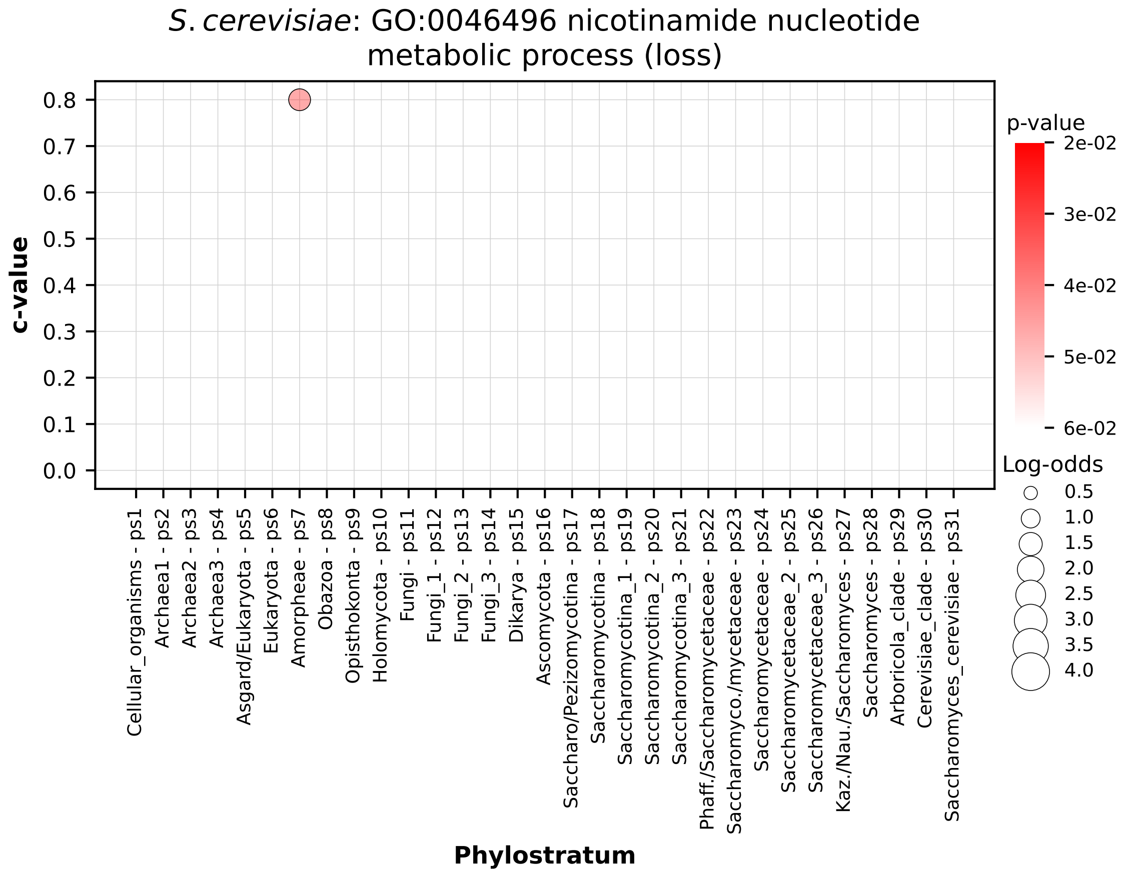

S. cerevisiae: GO:0046496 nicotinamide nucleotide metabolic process (loss)
c-value
0.0
0.1
0.2
0.3
0.4
0.5
0.6
0.7
0.8
p-value
2e-02
3e-02
4e-02
5e-02
6e-02
Log-odds
0.5
1.0
1.5
2.0
2.5
3.0
3.5
4.0
Cellular_organisms - ps1
Archaea1 - ps2
Archaea2 - ps3
Archaea3 - ps4
Asgard/Eukaryota - ps5
Eukaryota - ps6
Amorpheae - ps7
Obazoa - ps8
Opisthokonta - ps9
Holomycota - ps10
Fungi - ps11
Fungi_1 - ps12
Fungi_2 - ps13
Fungi_3 - ps14
Dikarya - ps15
Ascomycota - ps16
Saccharo/Pezizomycotina - ps17
Saccharomycotina - ps18
Saccharomycotina_1 - ps19
Saccharomycotina_2 - ps20
Saccharomycotina_3 - ps21
Phaff./Saccharomycetaceae - ps22
Saccharomyco./mycetaceae - ps23
Saccharomycetaceae - ps24
Saccharomycetaceae_2 - ps25
Saccharomycetaceae_3 - ps26
Kaz./Nau./Saccharomyces - ps27
Saccharomyces - ps28
Arboricola_clade - ps29
Cerevisiae_clade - ps30
Saccharomyces_cerevisiae - ps31
Phylostratum

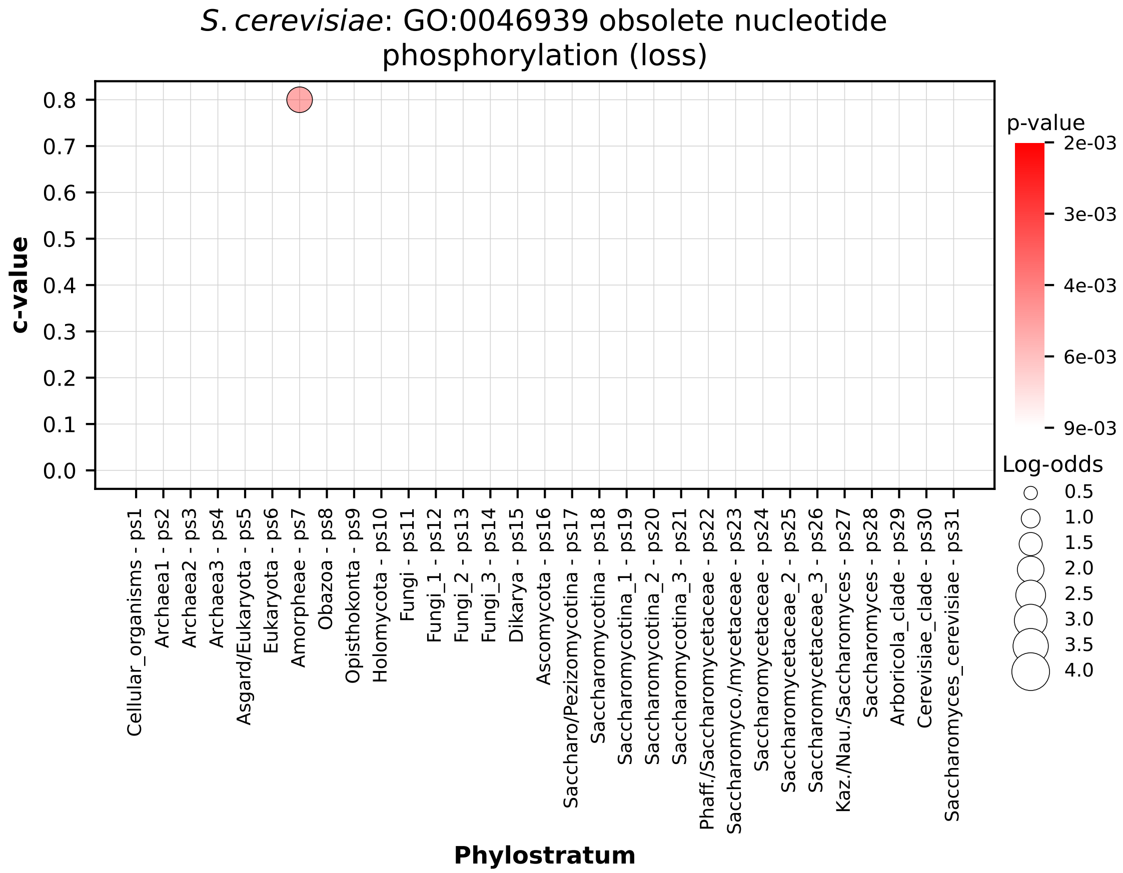

S. cerevisiae: GO:0046939 obsolete nucleotide phosphorylation (loss)
c-value
0.0
0.1
0.2
0.3
0.4
0.5
0.6
0.7
0.8
Phylostratum
Cellular_organisms - ps1
Archaea1 - ps2
Archaea2 - ps3
Archaea3 - ps4
Asgard/Eukaryota - ps5
Eukaryota - ps6
Amorpheae - ps7
Obazoa - ps8
Opisthokonta - ps9
Holomycota - ps10
Fungi - ps11
Fungi_1 - ps12
Fungi_2 - ps13
Fungi_3 - ps14
Dikarya - ps15
Ascomycota - ps16
Saccharo/Pezizomycotina - ps17
Saccharomycotina - ps18
Saccharomycotina_1 - ps19
Saccharomycotina_2 - ps20
Saccharomycotina_3 - ps21
Phaff./Saccharomycetaceae - ps22
Saccharomyco./mycetaceae - ps23
Saccharomycetaceae - ps24
Saccharomycetaceae_2 - ps25
Saccharomycetaceae_3 - ps26
Kaz./Nau./Saccharomyces - ps27
Saccharomyces - ps28
Arboricola_clade - ps29
Cerevisiae_clade - ps30
Saccharomyces_cerevisiae - ps31
p-value
2e-03
3e-03
4e-03
6e-03
9e-03
Log-odds
0.5
1.0
1.5
2.0
2.5
3.0
3.5
4.0

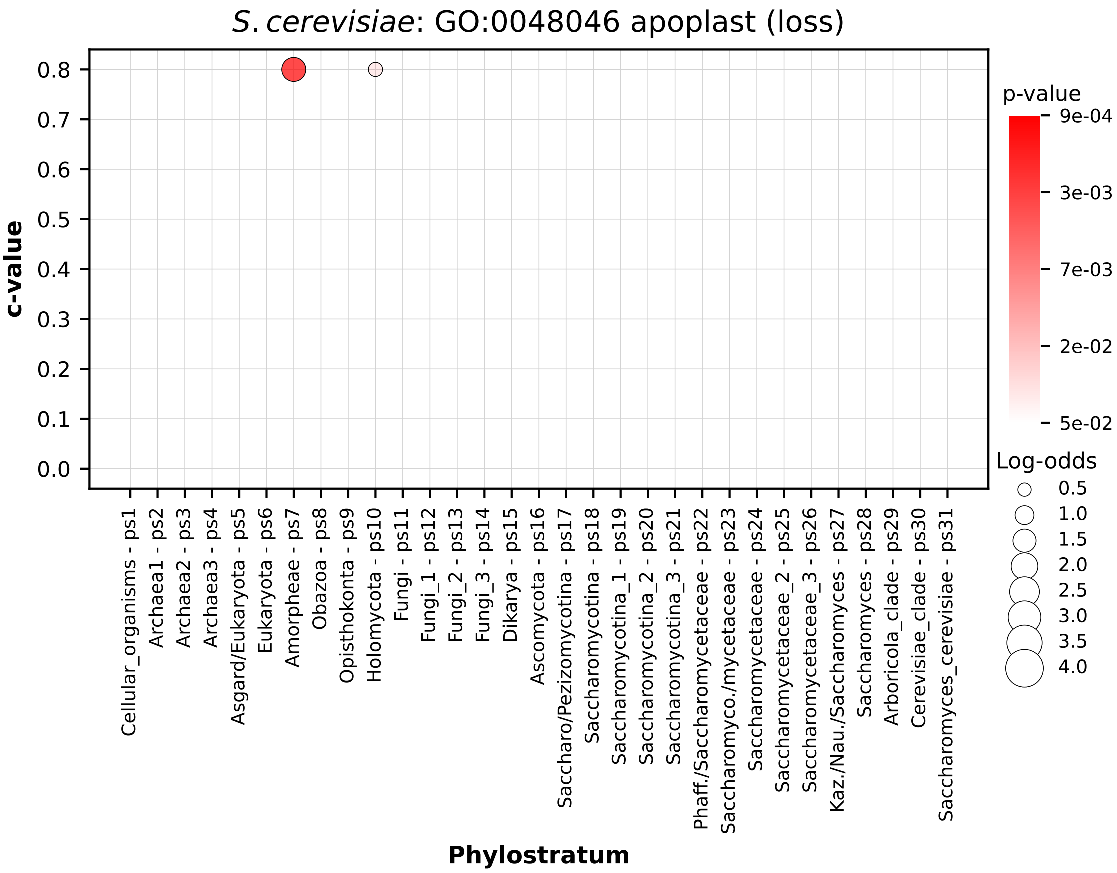

S. cerevisiae: GO:0048046 apoplast (loss)
c-value
0.0
0.1
0.2
0.3
0.4
0.5
0.6
0.7
0.8
Phylostratum
Cellular_organisms - ps1
Archaea1 - ps2
Archaea2 - ps3
Archaea3 - ps4
Asgard/Eukaryota - ps5
Eukaryota - ps6
Amorpheae - ps7
Obazoa - ps8
Opisthokonta - ps9
Holomycota - ps10
Fungi - ps11
Fungi_1 - ps12
Fungi_2 - ps13
Fungi_3 - ps14
Dikarya - ps15
Ascomycota - ps16
Saccharo/Pezizomycotina - ps17
Saccharomycotina - ps18
Saccharomycotina_1 - ps19
Saccharomycotina_2 - ps20
Saccharomycotina_3 - ps21
Phaff./Saccharomycetaceae - ps22
Saccharomyco./mycetaceae - ps23
Saccharomycetaceae - ps24
Saccharomycetaceae_2 - ps25
Saccharomycetaceae_3 - ps26
Kaz./Nau./Saccharomyces - ps27
Saccharomyces - ps28
Arboricola_clade - ps29
Cerevisiae_clade - ps30
Saccharomyces_cerevisiae - ps31
p-value
9e-04
3e-03
7e-03
2e-02
5e-02
Log-odds
0.5
1.0
1.5
2.0
2.5
3.0
3.5
4.0

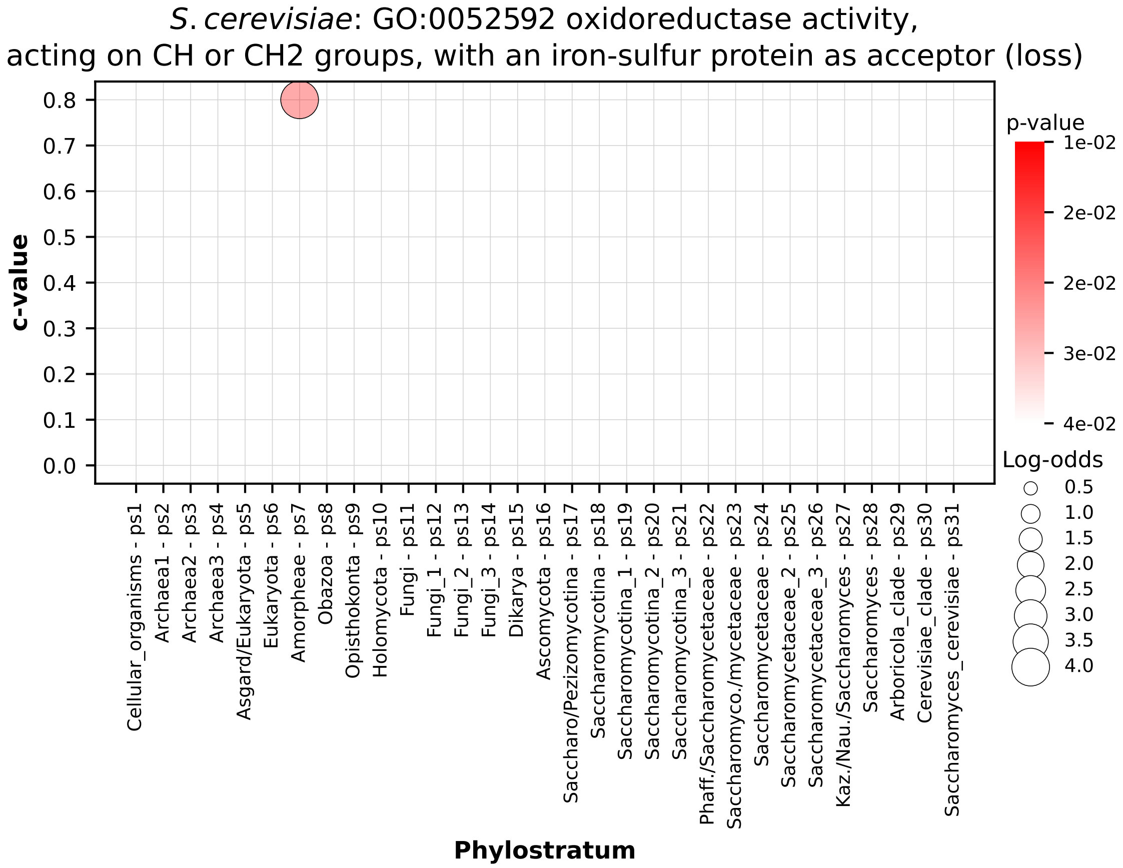

S. cerevisiae: GO:0052592 oxidoreductase activity,
acting on CH or CH2 groups, with an iron-sulfur protein as acceptor (loss)
c-value
0.8
0.7
0.6
0.5
0.4
0.3
0.2
0.1
0.0
p-value
1e-02
2e-02
2e-02
3e-02
4e-02
Log-odds
0.5
1.0
1.5
2.0
2.5
3.0
3.5
4.0
Cellular_organisms - ps1
Archaea1 - ps2
Archaea2 - ps3
Archaea3 - ps4
Asgard/Eukaryota - ps5
Eukaryota - ps6
Amorpheae - ps7
Obazoa - ps8
Opisthokonta - ps9
Holomycota - ps10
Fungi - ps11
Fungi_1 - ps12
Fungi_2 - ps13
Fungi_3 - ps14
Dikarya - ps15
Ascomycota - ps16
Saccharo/Pezizomycotina - ps17
Saccharomycotina - ps18
Saccharomycotina_1 - ps19
Saccharomycotina_2 - ps20
Saccharomycotina_3 - ps21
Phaff./Saccharomycetaceae - ps22
Saccharomyco./mycetaceae - ps23
Saccharomycetaceae - ps24
Saccharomycetaceae_2 - ps25
Saccharomycetaceae_3 - ps26
Kaz./Nau./Saccharomyces - ps27
Saccharomyces - ps28
Arboricola_clade - ps29
Cerevisiae_clade - ps30
Saccharomyces_cerevisiae - ps31
Phylostratum

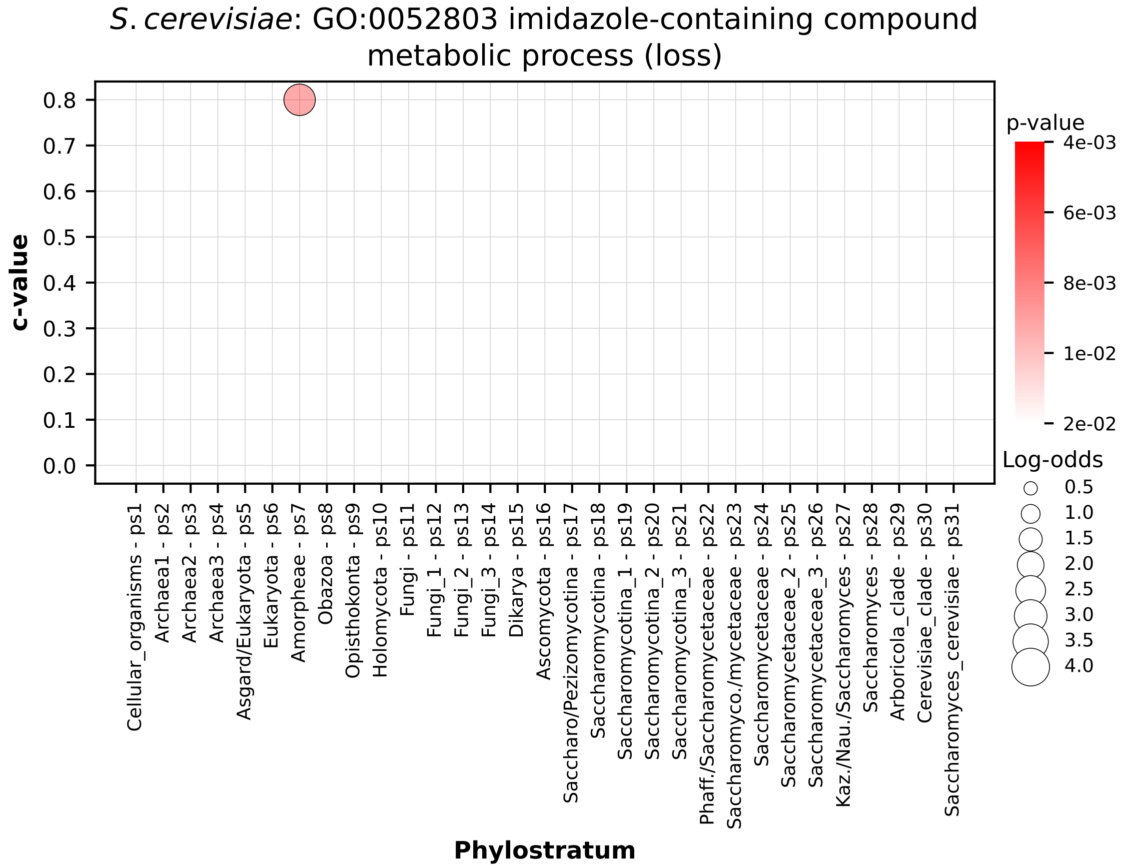
S. cerevisiae: GO:0052803 imidazole-containing compound metabolic process (loss)
c-value
Phylostratum
p-value
4e-03
6e-03
8e-03
1e-02
2e-02
Log-odds
0.5
1.0
1.5
2.0
2.5
3.0
3.5
4.0
0.0
0.1
0.2
0.3
0.4
0.5
0.6
0.7
0.8
Cellular_organisms - ps1
Archaea1 - ps2
Archaea2 - ps3
Archaea3 - ps4
Asgard/Eukaryota - ps5
Eukaryota - ps6
Amorpheae - ps7
Obazoa - ps8
Opisthokonta - ps9
Holomycota - ps10
Fungi - ps11
Fungi_1 - ps12
Fungi_2 - ps13
Fungi_3 - ps14
Dikarya - ps15
Ascomycota - ps16
Saccharo/Pezizomycotina - ps17
Saccharomycotina - ps18
Saccharomycotina_1 - ps19
Saccharomycotina_2 - ps20
Saccharomycotina_3 - ps21
Phaff./Saccharomycetaceae - ps22
Saccharomyco./mycetaceae - ps23
Saccharomycetaceae - ps24
Saccharomycetaceae_2 - ps25
Saccharomycetaceae_3 - ps26
Kaz./Nau./Saccharomyces - ps27
Saccharomyces - ps28
Arboricola_clade - ps29
Cerevisiae_clade - ps30
Saccharomyces_cerevisiae - ps31

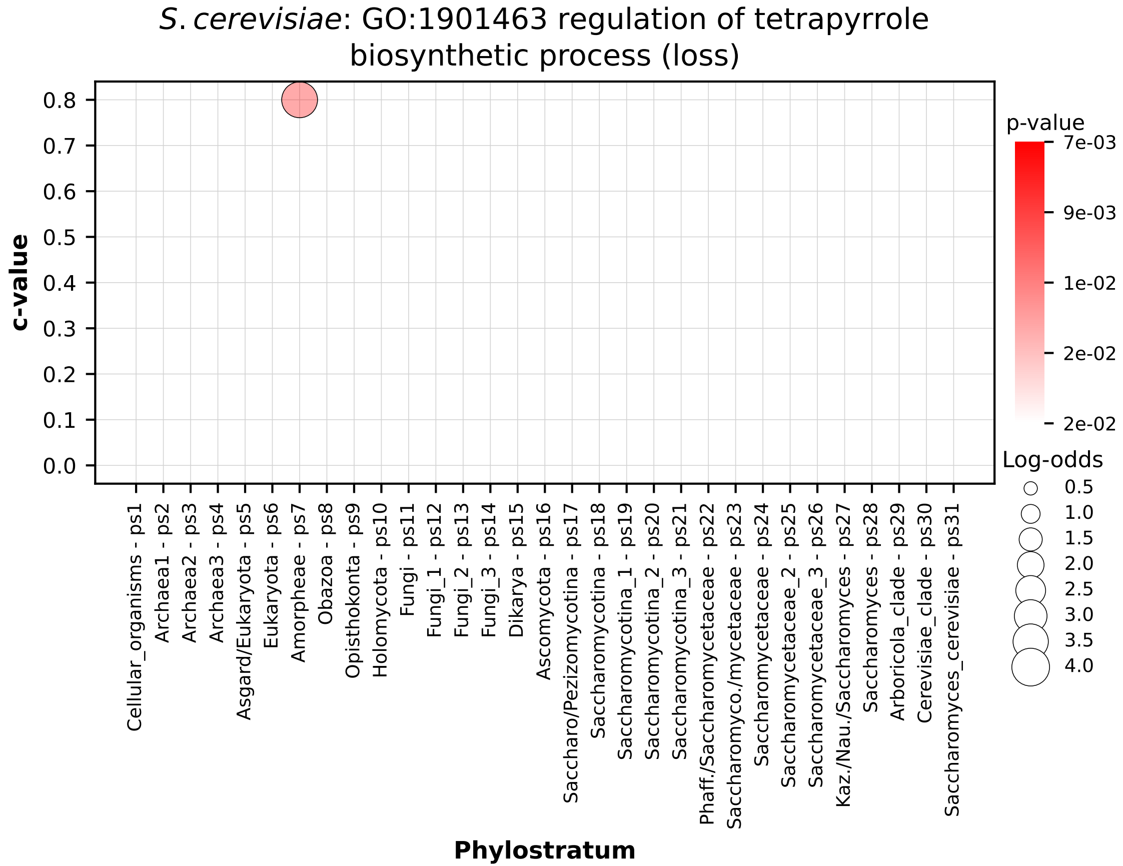

S. cerevisiae: GO:1901463 regulation of tetrapyrrole biosynthetic process (loss)
c-value
0.0
0.1
0.2
0.3
0.4
0.5
0.6
0.7
0.8
p-value
7e-03
9e-03
1e-02
2e-02
2e-02
Log-odds
0.5
1.0
1.5
2.0
2.5
3.0
3.5
4.0
Cellular_organisms - ps1
Archaea1 - ps2
Archaea2 - ps3
Archaea3 - ps4
Asgard/Eukaryota - ps5
Eukaryota - ps6
Amorpheae - ps7
Obazoa - ps8
Opisthokonta - ps9
Holomycota - ps10
Fungi - ps11
Fungi_1 - ps12
Fungi_2 - ps13
Fungi_3 - ps14
Dikarya - ps15
Ascomycota - ps16
Saccharo/Pezizomycotina - ps17
Saccharomycotina - ps18
Saccharomycotina_1 - ps19
Saccharomycotina_2 - ps20
Saccharomycotina_3 - ps21
Phaff./Saccharomycetaceae - ps22
Saccharomyco./mycetaceae - ps23
Saccharomycetaceae - ps24
Saccharomycetaceae_2 - ps25
Saccharomycetaceae_3 - ps26
Kaz./Nau./Saccharomyces - ps27
Saccharomyces - ps28
Arboricola_clade - ps29
Cerevisiae_clade - ps30
Saccharomyces_cerevisiae - ps31
Phylostratum

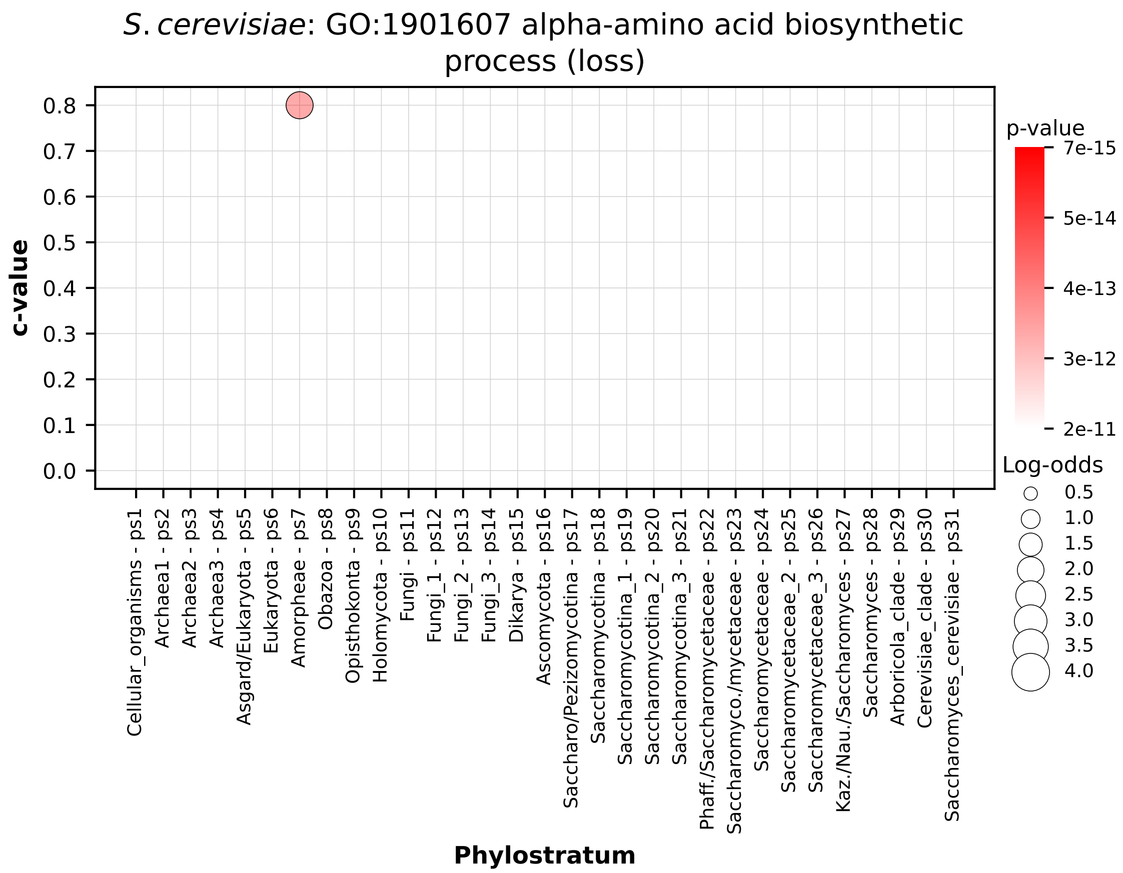

S. cerevisiae: GO:1901607 alpha-amino acid biosynthetic process (loss)
c-value
0.0
0.1
0.2
0.3
0.4
0.5
0.6
0.7
0.8
Phylostratum
Cellular_organisms - ps1
Archaea1 - ps2
Archaea2 - ps3
Archaea3 - ps4
Asgard/Eukaryota - ps5
Eukaryota - ps6
Amorpheae - ps7
Obazoa - ps8
Opisthokonta - ps9
Holomycota - ps10
Fungi - ps11
Fungi_1 - ps12
Fungi_2 - ps13
Fungi_3 - ps14
Dikarya - ps15
Ascomycota - ps16
Saccharo/Pezizomycotina - ps17
Saccharomycotina - ps18
Saccharomycotina_1 - ps19
Saccharomycotina_2 - ps20
Saccharomycotina_3 - ps21
Phaff./Saccharomycetaceae - ps22
Saccharomyco./mycetaceae - ps23
Saccharomycetaceae - ps24
Saccharomycetaceae_2 - ps25
Saccharomycetaceae_3 - ps26
Kaz./Nau./Saccharomyces - ps27
Saccharomyces - ps28
Arboricola_clade - ps29
Cerevisiae_clade - ps30
Saccharomyces_cerevisiae - ps31
p-value
7e-15
5e-14
4e-13
3e-12
2e-11
Log-odds
0.5
1.0
1.5
2.0
2.5
3.0
3.5
4.0

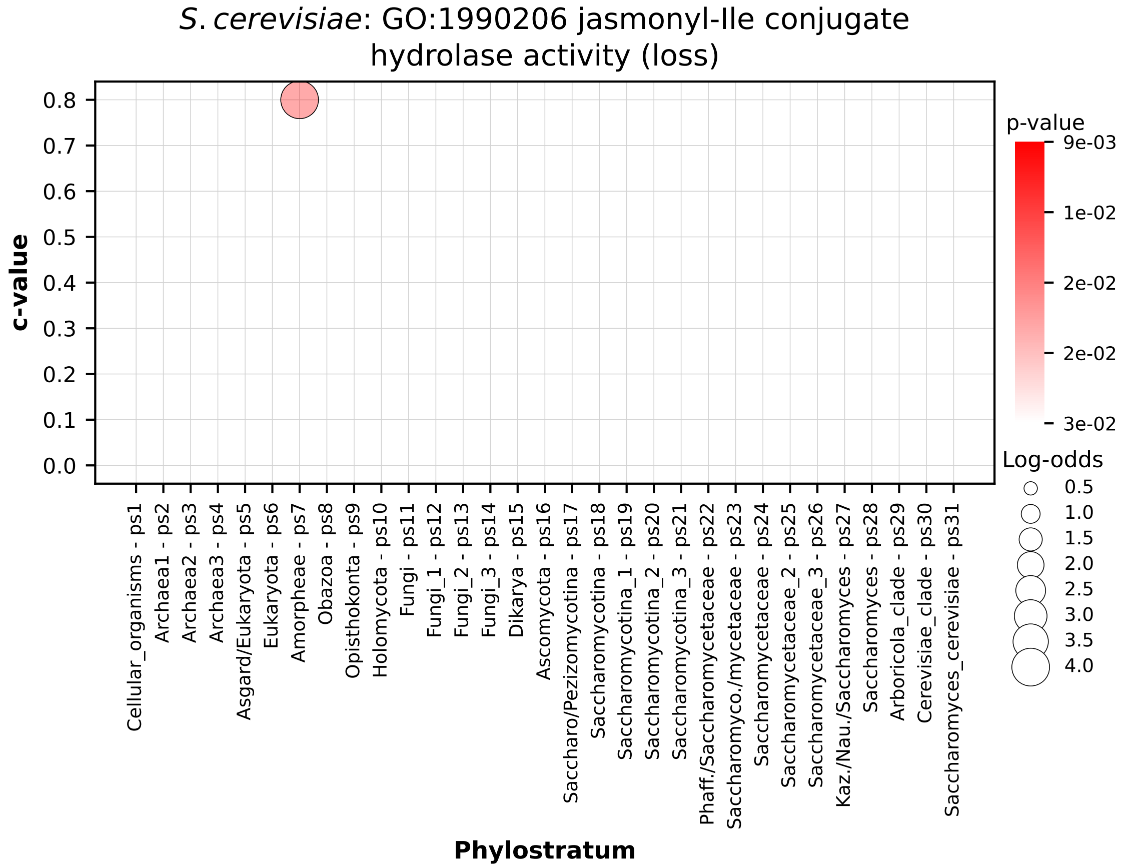

S. cerevisiae: GO:1990206 jasmonyl-Ile conjugate hydrolase activity (loss)
c-value
0.8
0.7
0.6
0.5
0.4
0.3
0.2
0.1
0.0
p-value
9e-03
1e-02
2e-02
2e-02
3e-02
Log-odds
0.5
1.0
1.5
2.0
2.5
3.0
3.5
4.0
Cellular_organisms - ps1
Archaea1 - ps2
Archaea2 - ps3
Archaea3 - ps4
Asgard/Eukaryota - ps5
Eukaryota - ps6
Amorpheae - ps7
Obazoa - ps8
Opisthokonta - ps9
Holomycota - ps10
Fungi - ps11
Fungi_1 - ps12
Fungi_2 - ps13
Fungi_3 - ps14
Dikarya - ps15
Ascomycota - ps16
Saccharo/Pezizomycotina - ps17
Saccharomycotina - ps18
Saccharomycotina_1 - ps19
Saccharomycotina_2 - ps20
Saccharomycotina_3 - ps21
Phaff./Saccharomycetaceae - ps22
Saccharomyco./mycetaceae - ps23
Saccharomycetaceae - ps24
Saccharomycetaceae_2 - ps25
Saccharomycetaceae_3 - ps26
Kaz./Nau./Saccharomyces - ps27
Saccharomyces - ps28
Arboricola_clade - ps29
Cerevisiae_clade - ps30
Saccharomyces_cerevisiae - ps31
Phylostratum

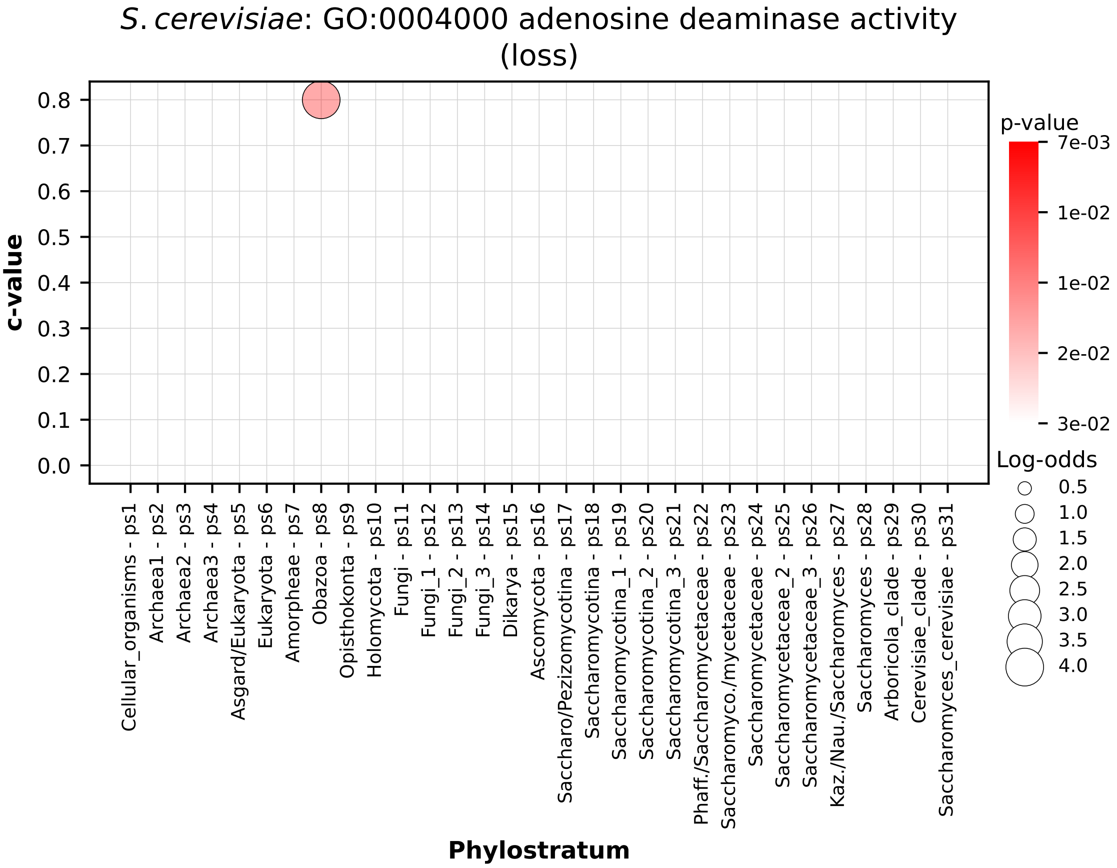

S. cerevisiae: GO:0004000 adenosine deaminase activity (loss)
c-value
0.0
0.1
0.2
0.3
0.4
0.5
0.6
0.7
0.8
Phylostratum
Cellular_organisms - ps1
Archaea1 - ps2
Archaea2 - ps3
Archaea3 - ps4
Asgard/Eukaryota - ps5
Eukaryota - ps6
Amorpheae - ps7
Obazoa - ps8
Opisthokonta - ps9
Holomycota - ps10
Fungi - ps11
Fungi_1 - ps12
Fungi_2 - ps13
Fungi_3 - ps14
Dikarya - ps15
Ascomycota - ps16
Saccharo/Pezizomycotina - ps17
Saccharomycotina - ps18
Saccharomycotina_1 - ps19
Saccharomycotina_2 - ps20
Saccharomycotina_3 - ps21
Phaff./Saccharomycetaceae - ps22
Saccharomyco./mycetaceae - ps23
Saccharomycetaceae - ps24
Saccharomycetaceae_2 - ps25
Saccharomycetaceae_3 - ps26
Kaz./Nau./Saccharomyces - ps27
Saccharomyces - ps28
Arboricola_clade - ps29
Cerevisiae_clade - ps30
Saccharomyces_cerevisiae - ps31
p-value
7e-03
1e-02
1e-02
2e-02
3e-02
Log-odds
0.5
1.0
1.5
2.0
2.5
3.0
3.5
4.0

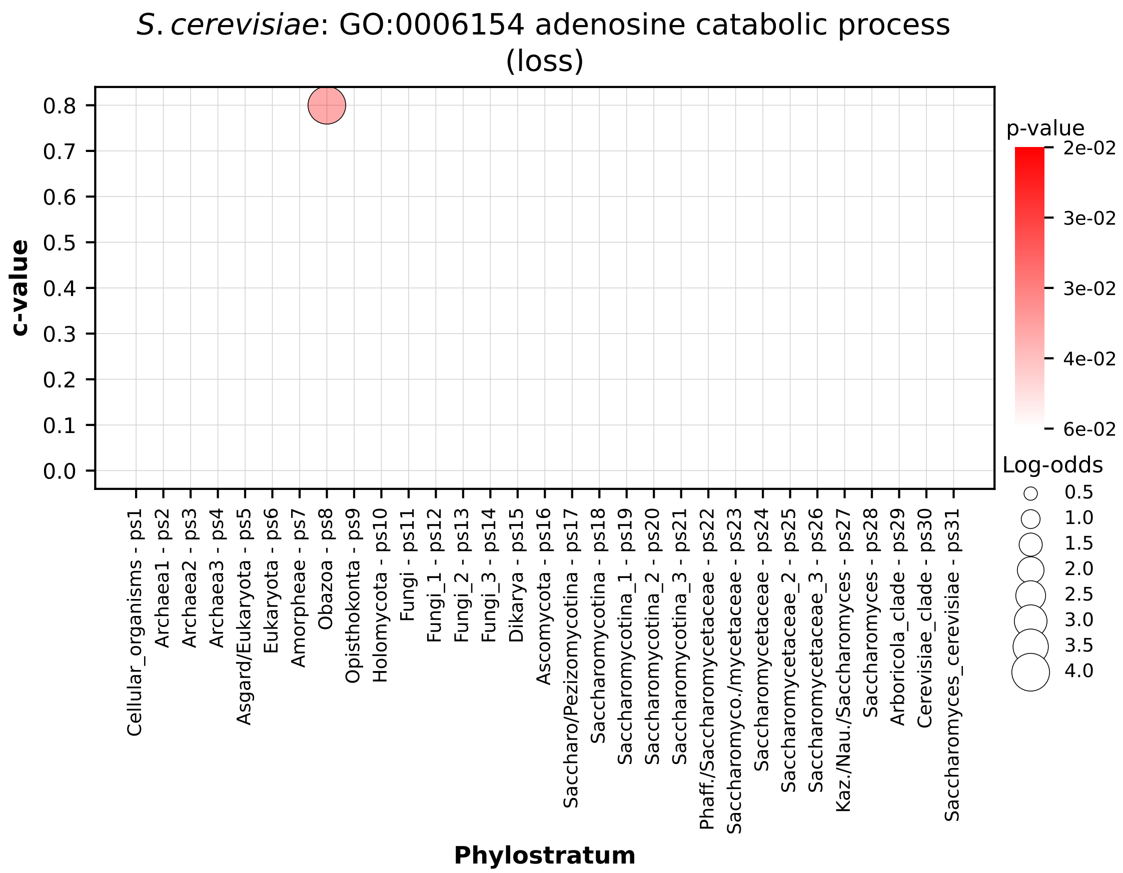
S. cerevisiae: GO:0006154 adenosine catabolic process
(loss)
c-value
0.8
0.7
0.6
0.5
0.4
0.3
0.2
0.1
0.0
p-value
2e-02
3e-02
3e-02
4e-02
6e-02
Log-odds
0.5
1.0
1.5
2.0
2.5
3.0
3.5
4.0
Cellular_organisms - ps1
Archaea1 - ps2
Archaea2 - ps3
Archaea3 - ps4
Asgard/Eukaryota - ps5
Eukaryota - ps6
Amorpheae - ps7
Obazoa - ps8
Opisthokonta - ps9
Holomycota - ps10
Fungi - ps11
Fungi_1 - ps12
Fungi_2 - ps13
Fungi_3 - ps14
Dikarya - ps15
Ascomycota - ps16
Saccharo/Pezizomycotina - ps17
Saccharomycotina - ps18
Saccharomycotina_1 - ps19
Saccharomycotina_2 - ps20
Saccharomycotina_3 - ps21
Phaff./Saccharomycetaceae - ps22
Saccharomyco./mycetaceae - ps23
Saccharomycetaceae - ps24
Saccharomycetaceae_2 - ps25
Saccharomycetaceae_3 - ps26
Kaz./Nau./Saccharomyces - ps27
Saccharomyces - ps28
Arboricola_clade - ps29
Cerevisiae_clade - ps30
Saccharomyces_cerevisiae - ps31
Phylostratum

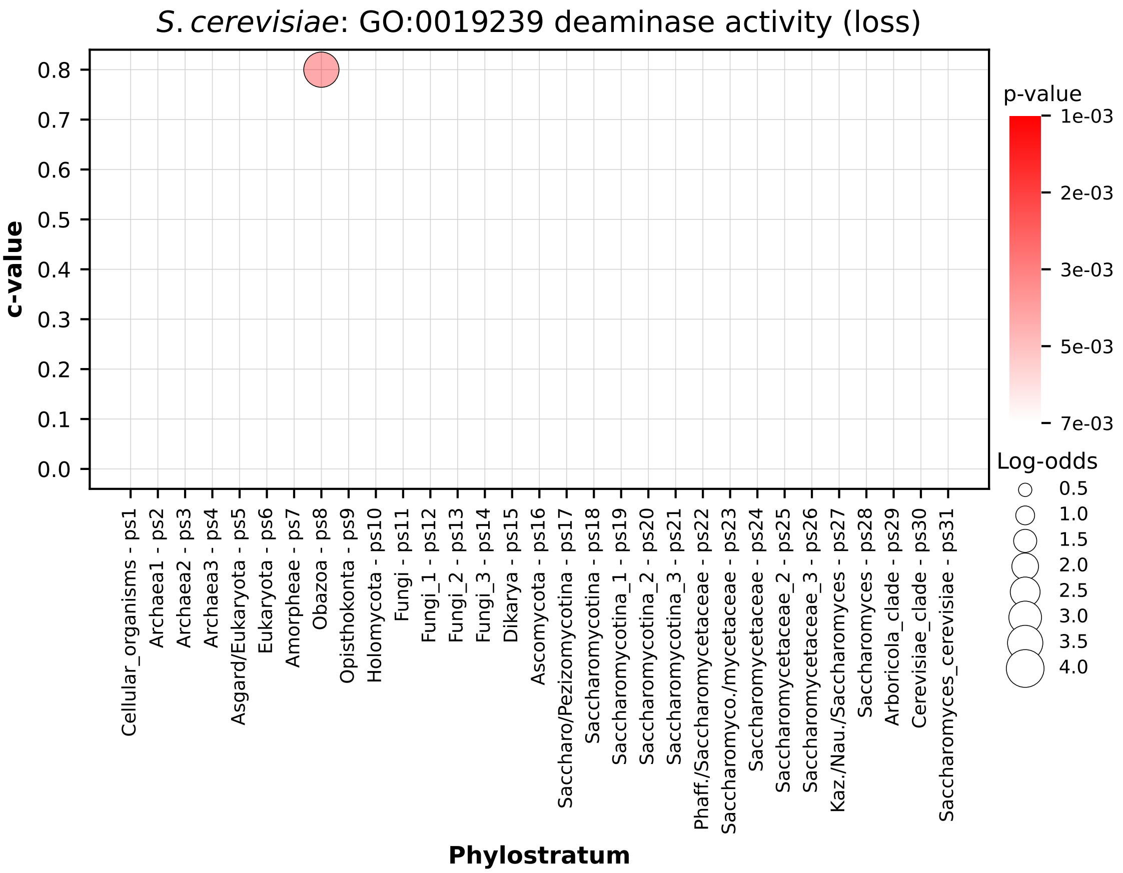

S. cerevisiae: GO:0019239 deaminase activity (loss)
c-value
0.8
0.7
0.6
0.5
0.4
0.3
0.2
0.1
0.0
p-value
1e-03
2e-03
3e-03
5e-03
7e-03
Log-odds
0.5
1.0
1.5
2.0
2.5
3.0
3.5
4.0
Cellular_organisms - ps1
Archaea1 - ps2
Archaea2 - ps3
Archaea3 - ps4
Asgard/Eukaryota - ps5
Eukaryota - ps6
Amorpheae - ps7
Obazoa - ps8
Opisthokonta - ps9
Holomycota - ps10
Fungi - ps11
Fungi_1 - ps12
Fungi_2 - ps13
Fungi_3 - ps14
Dikarya - ps15
Ascomycota - ps16
Saccharo/Pezizomycotina - ps17
Saccharomycotina - ps18
Saccharomycotina_1 - ps19
Saccharomycotina_2 - ps20
Saccharomycotina_3 - ps21
Phaff./Saccharomycetaceae - ps22
Saccharomyco./mycetaceae - ps23
Saccharomycetaceae - ps24
Saccharomycetaceae_2 - ps25
Saccharomycetaceae_3 - ps26
Kaz./Nau./Saccharomyces - ps27
Saccharomyces - ps28
Arboricola_clade - ps29
Cerevisiae_clade - ps30
Saccharomyces_cerevisiae - ps31
Phylostratum

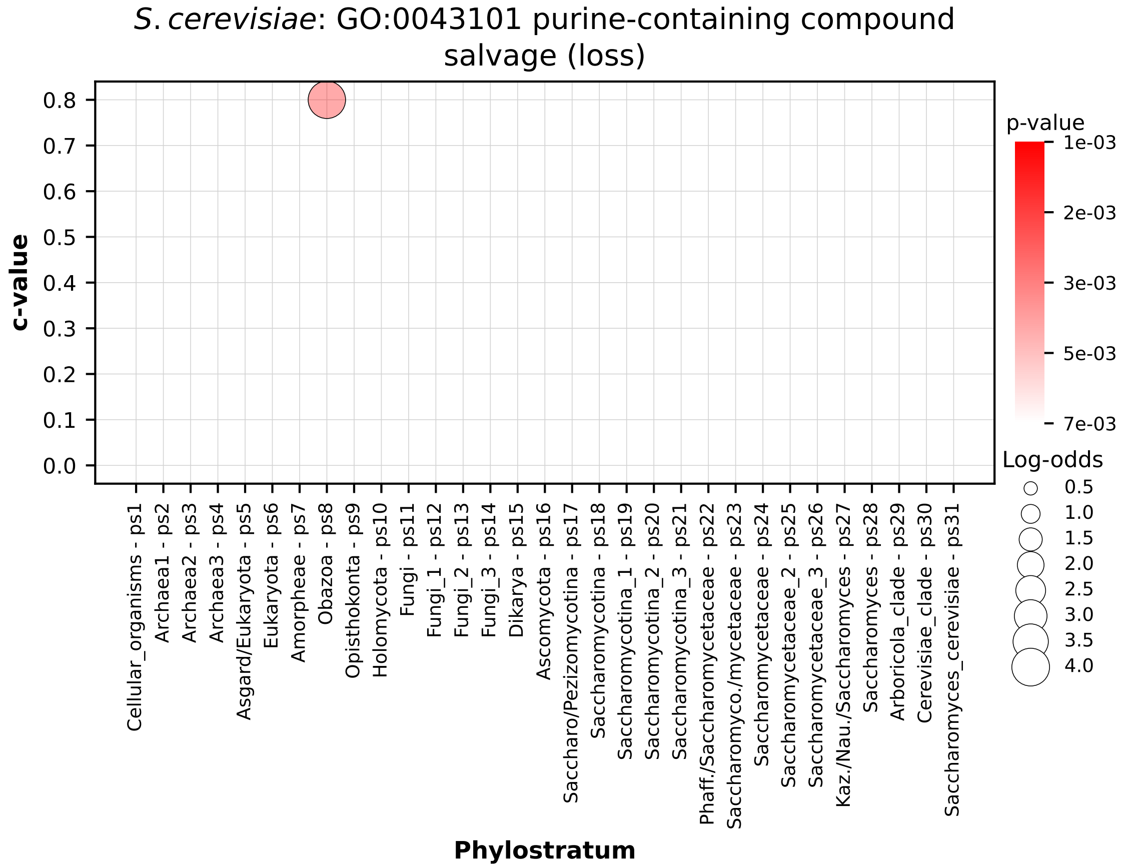

S. cerevisiae: GO:0043101 purine-containing compound salvage (loss)
c-value
0.8
0.7
0.6
0.5
0.4
0.3
0.2
0.1
0.0
p-value
1e-03
2e-03
3e-03
5e-03
7e-03
Log-odds
0.5
1.0
1.5
2.0
2.5
3.0
3.5
4.0
Cellular_organisms - ps1
Archaea1 - ps2
Archaea2 - ps3
Archaea3 - ps4
Asgard/Eukaryota - ps5
Eukaryota - ps6
Amorpheae - ps7
Obazoa - ps8
Opisthokonta - ps9
Holomycota - ps10
Fungi - ps11
Fungi_1 - ps12
Fungi_2 - ps13
Fungi_3 - ps14
Dikarya - ps15
Ascomycota - ps16
Saccharo/Pezizomycotina - ps17
Saccharomycotina - ps18
Saccharomycotina_1 - ps19
Saccharomycotina_2 - ps20
Saccharomycotina_3 - ps21
Phaff./Saccharomycetaceae - ps22
Saccharomyco./mycetaceae - ps23
Saccharomycetaceae - ps24
Saccharomycetaceae_2 - ps25
Saccharomycetaceae_3 - ps26
Kaz./Nau./Saccharomyces - ps27
Saccharomyces - ps28
Arboricola_clade - ps29
Cerevisiae_clade - ps30
Saccharomyces_cerevisiae - ps31
Phylostratum

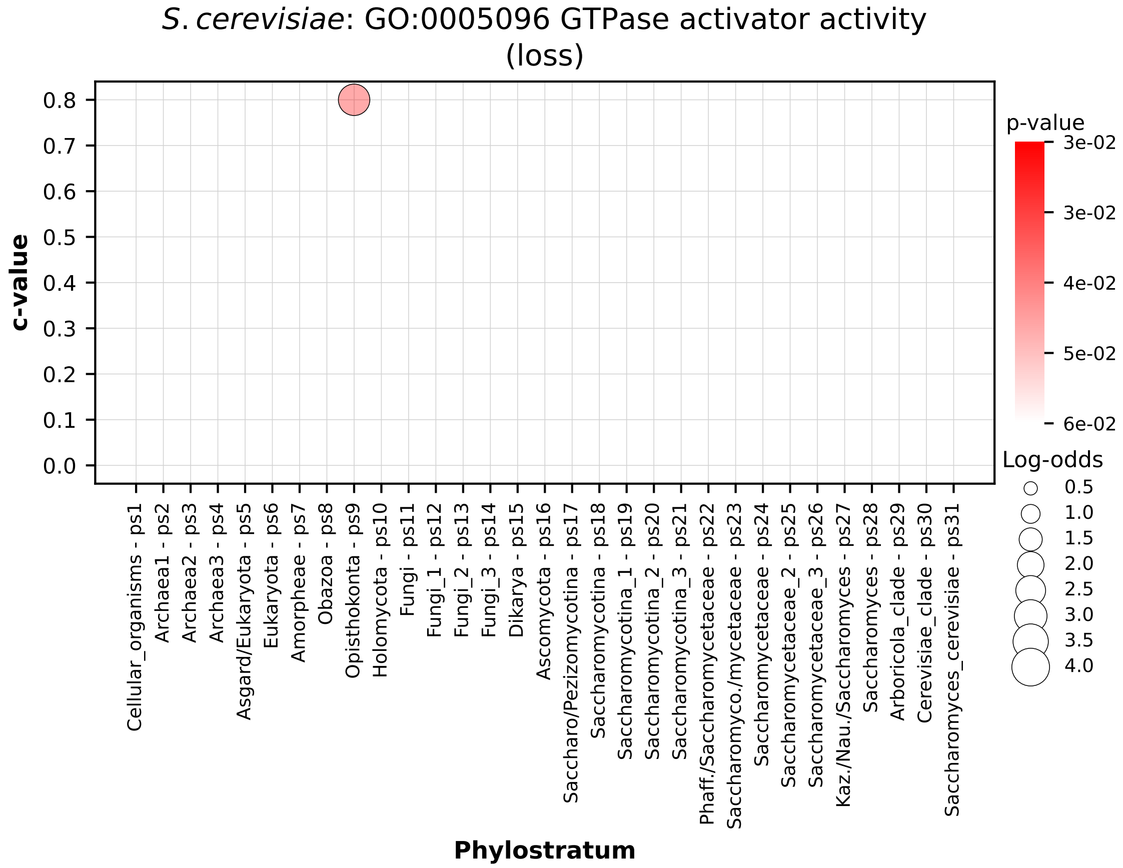

S. cerevisiae: GO:0005096 GTPase activator activity
(loss)
c-value
0.0
0.1
0.2
0.3
0.4
0.5
0.6
0.7
0.8
p-value
3e-02
3e-02
4e-02
5e-02
6e-02
Log-odds
0.5
1.0
1.5
2.0
2.5
3.0
3.5
4.0
Cellular_organisms - ps1
Archaea1 - ps2
Archaea2 - ps3
Archaea3 - ps4
Asgard/Eukaryota - ps5
Eukaryota - ps6
Amorpheae - ps7
Obazoa - ps8
Opisthokonta - ps9
Holomycota - ps10
Fungi - ps11
Fungi_1 - ps12
Fungi_2 - ps13
Fungi_3 - ps14
Dikarya - ps15
Ascomycota - ps16
Saccharo/Pezizomycotina - ps17
Saccharomycotina - ps18
Saccharomycotina_1 - ps19
Saccharomycotina_2 - ps20
Saccharomycotina_3 - ps21
Phaff./Saccharomycetaceae - ps22
Saccharomyco./mycetaceae - ps23
Saccharomycetaceae - ps24
Saccharomycetaceae_2 - ps25
Saccharomycetaceae_3 - ps26
Kaz./Nau./Saccharomyces - ps27
Saccharomyces - ps28
Arboricola_clade - ps29
Cerevisiae_clade - ps30
Saccharomyces_cerevisiae - ps31
Phylostratum

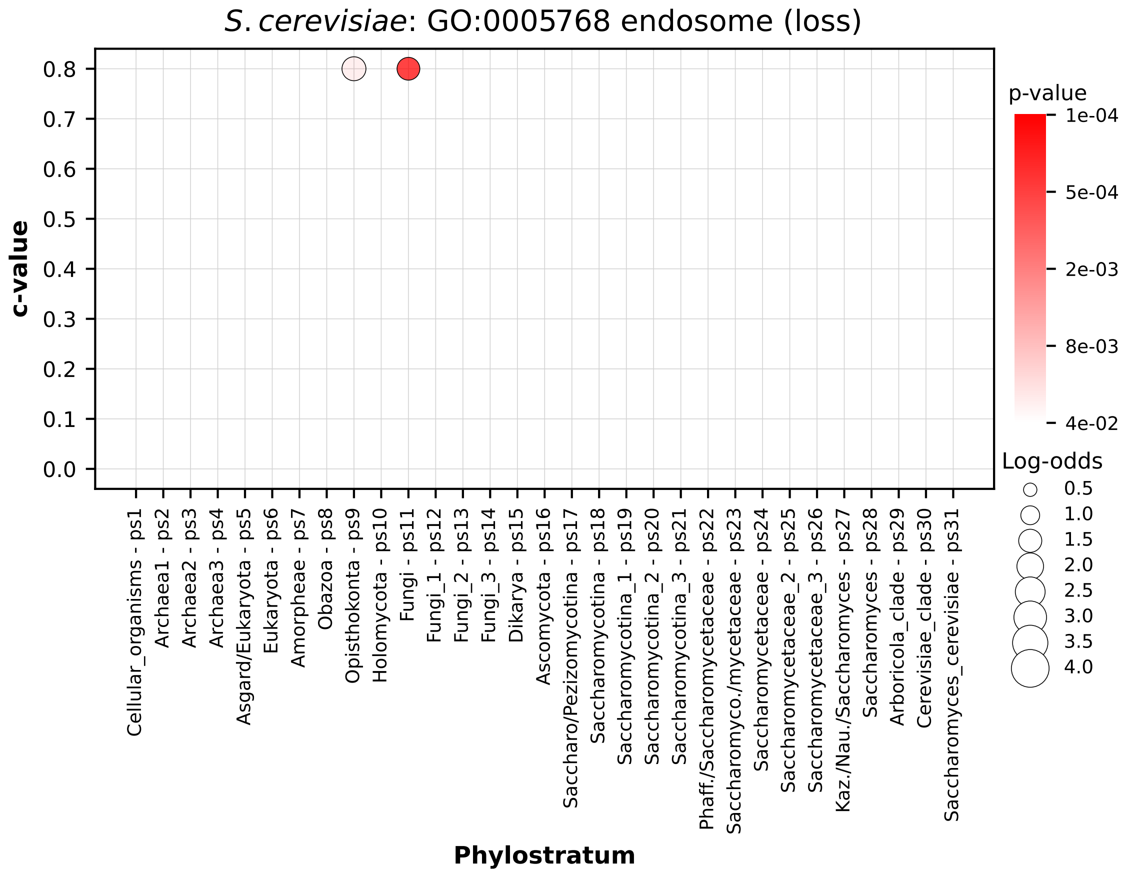

S. cerevisiae: GO:0005768 endosome (loss)
c-value
0.0
0.1
0.2
0.3
0.4
0.5
0.6
0.7
0.8
p-value
1e-04
5e-04
2e-03
8e-03
4e-02
Log-odds
0.5
1.0
1.5
2.0
2.5
3.0
3.5
4.0
Cellular_organisms - ps1
Archaea1 - ps2
Archaea2 - ps3
Archaea3 - ps4
Asgard/Eukaryota - ps5
Eukaryota - ps6
Amorpheae - ps7
Obazoa - ps8
Opisthokonta - ps9
Holomycota - ps10
Fungi - ps11
Fungi_1 - ps12
Fungi_2 - ps13
Fungi_3 - ps14
Dikarya - ps15
Ascomycota - ps16
Saccharo/Pezizomycotina - ps17
Saccharomycotina - ps18
Saccharomycotina_1 - ps19
Saccharomycotina_2 - ps20
Saccharomycotina_3 - ps21
Phaff./Saccharomycetaceae - ps22
Saccharomyco./mycetaceae - ps23
Saccharomycetaceae - ps24
Saccharomycetaceae_2 - ps25
Saccharomycetaceae_3 - ps26
Kaz./Nau./Saccharomyces - ps27
Saccharomyces - ps28
Arboricola_clade - ps29
Cerevisiae_clade - ps30
Saccharomyces_cerevisiae - ps31
Phylostratum

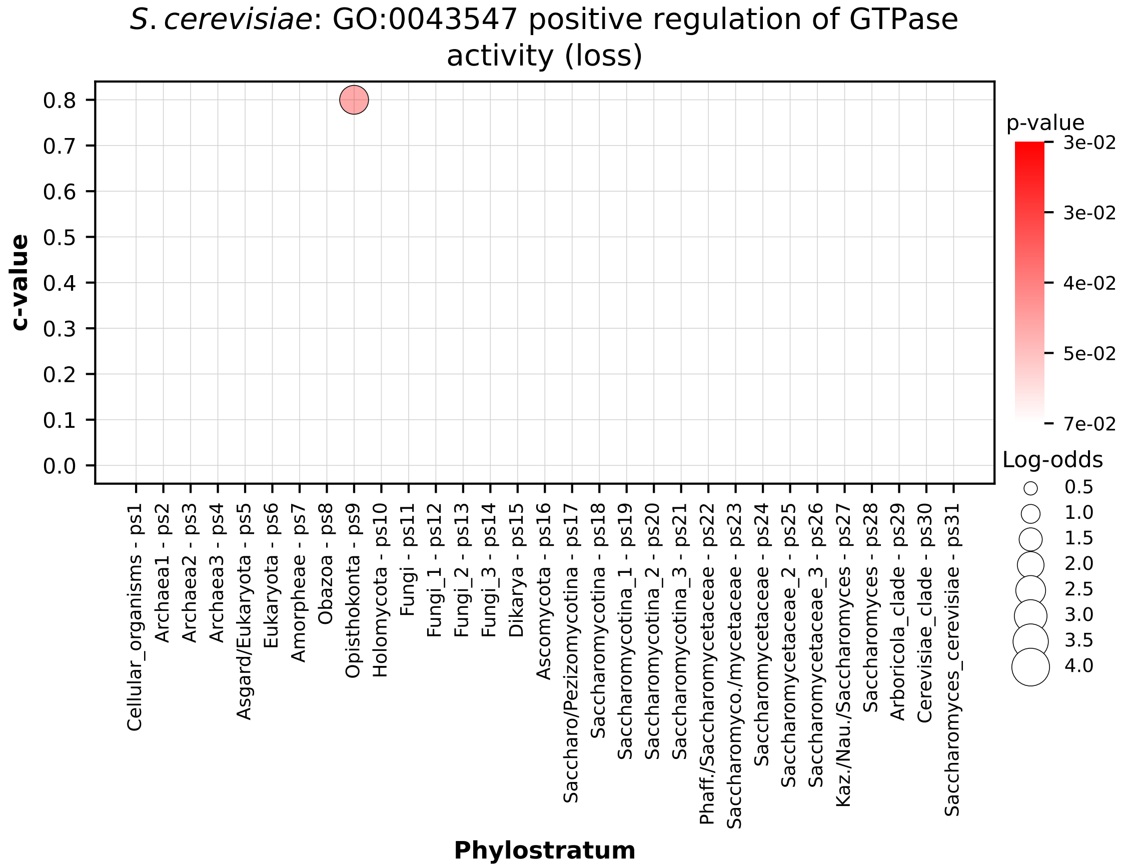

S. cerevisiae: GO:0043547 positive regulation of GTPase activity (loss)
c-value
0.0
0.1
0.2
0.3
0.4
0.5
0.6
0.7
0.8
p-value
3e-02
3e-02
4e-02
5e-02
7e-02
Log-odds
0.5
1.0
1.5
2.0
2.5
3.0
3.5
4.0
Cellular_organisms - ps1
Archaea1 - ps2
Archaea2 - ps3
Archaea3 - ps4
Asgard/Eukaryota - ps5
Eukaryota - ps6
Amorpheae - ps7
Obazoa - ps8
Opisthokonta - ps9
Holomycota - ps10
Fungi - ps11
Fungi_1 - ps12
Fungi_2 - ps13
Fungi_3 - ps14
Dikarya - ps15
Ascomycota - ps16
Saccharo/Pezizomycotina - ps17
Saccharomycotina - ps18
Saccharomycotina_1 - ps19
Saccharomycotina_2 - ps20
Saccharomycotina_3 - ps21
Phaff./Saccharomycetaceae - ps22
Saccharomyco./mycetaceae - ps23
Saccharomycetaceae - ps24
Saccharomycetaceae_2 - ps25
Saccharomycetaceae_3 - ps26
Kaz./Nau./Saccharomyces - ps27
Saccharomyces - ps28
Arboricola_clade - ps29
Cerevisiae_clade - ps30
Saccharomyces_cerevisiae - ps31
Phylostratum

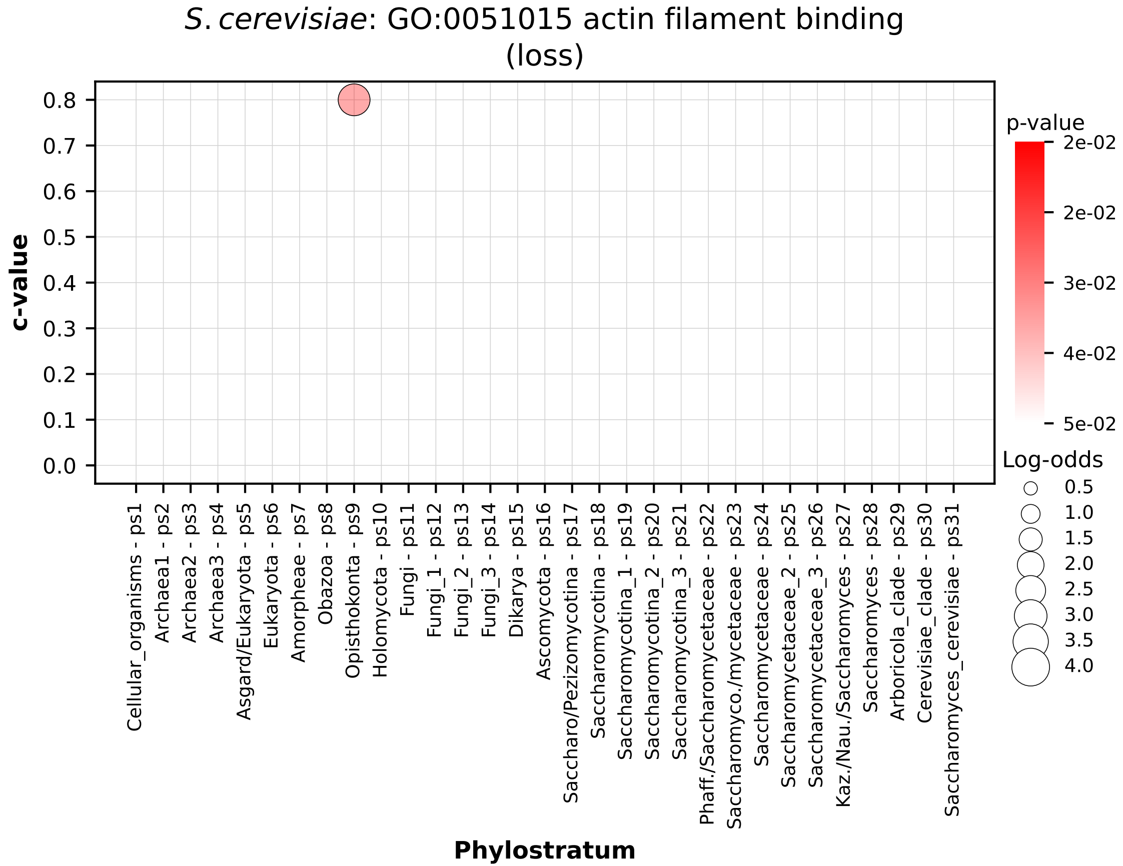
S. cerevisiae: GO:0051015 actin filament binding
(loss)
c-value
0.0
0.1
0.2
0.3
0.4
0.5
0.6
0.7
0.8
Phylostratum
Cellular_organisms - ps1
Archaea1 - ps2
Archaea2 - ps3
Archaea3 - ps4
Asgard/Eukaryota - ps5
Eukaryota - ps6
Amorpheae - ps7
Obazoa - ps8
Opisthokonta - ps9
Holomycota - ps10
Fungi - ps11
Fungi_1 - ps12
Fungi_2 - ps13
Fungi_3 - ps14
Dikarya - ps15
Ascomycota - ps16
Saccharo/Pezizomycotina - ps17
Saccharomycotina - ps18
Saccharomycotina_1 - ps19
Saccharomycotina_2 - ps20
Saccharomycotina_3 - ps21
Phaff./Saccharomycetaceae - ps22
Saccharomyco./mycetaceae - ps23
Saccharomycetaceae - ps24
Saccharomycetaceae_2 - ps25
Saccharomycetaceae_3 - ps26
Kaz./Nau./Saccharomyces - ps27
Saccharomyces - ps28
Arboricola_clade - ps29
Cerevisiae_clade - ps30
Saccharomyces_cerevisiae - ps31
p-value
2e-02
2e-02
3e-02
4e-02
5e-02
Log-odds
0.5
1.0
1.5
2.0
2.5
3.0
3.5
4.0

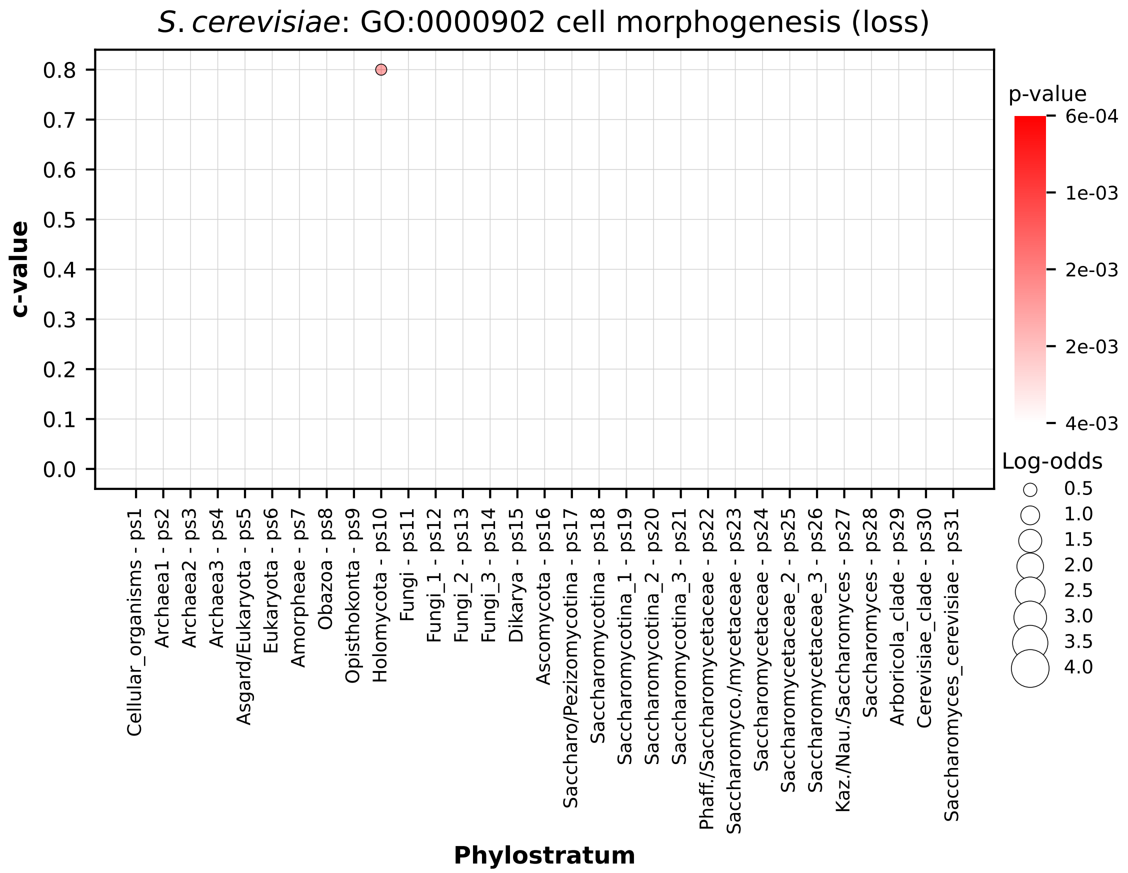
S. cerevisiae: GO:0000902 cell morphogenesis (loss)
c-value
0.0
0.1
0.2
0.3
0.4
0.5
0.6
0.7
0.8
p-value
6e-04
1e-03
2e-03
2e-03
4e-03
Log-odds
0.5
1.0
1.5
2.0
2.5
3.0
3.5
4.0
Cellular_organisms - ps1
Archaea1 - ps2
Archaea2 - ps3
Archaea3 - ps4
Asgard/Eukaryota - ps5
Eukaryota - ps6
Amorpheae - ps7
Obazoa - ps8
Opisthokonta - ps9
Holomycota - ps10
Fungi - ps11
Fungi_1 - ps12
Fungi_2 - ps13
Fungi_3 - ps14
Dikarya - ps15
Ascomycota - ps16
Saccharo/Pezizomycotina - ps17
Saccharomycotina - ps18
Saccharomycotina_1 - ps19
Saccharomycotina_2 - ps20
Saccharomycotina_3 - ps21
Phaff./Saccharomycetaceae - ps22
Saccharomyco./mycetaceae - ps23
Saccharomycetaceae - ps24
Saccharomycetaceae_2 - ps25
Saccharomycetaceae_3 - ps26
Kaz./Nau./Saccharomyces - ps27
Saccharomyces - ps28
Arboricola_clade - ps29
Cerevisiae_clade - ps30
Saccharomyces_cerevisiae - ps31
Phylostratum

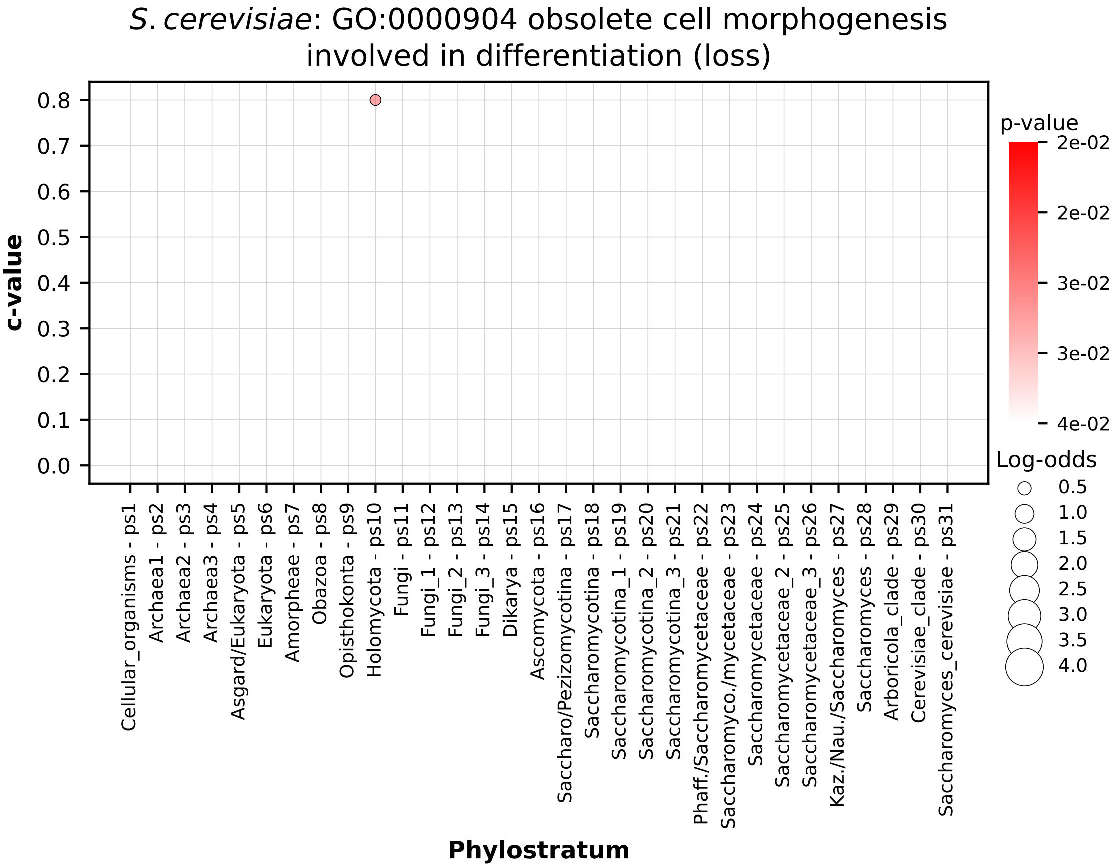
S. cerevisiae: GO:0000904 obsolete cell morphogenesis involved in differentiation (loss)
c-value
0.0
0.1
0.2
0.3
0.4
0.5
0.6
0.7
0.8
p-value
2e-02
2e-02
3e-02
3e-02
4e-02
Log-odds
0.5
1.0
1.5
2.0
2.5
3.0
3.5
4.0
Cellular_organisms - ps1
Archaea1 - ps2
Archaea2 - ps3
Archaea3 - ps4
Asgard/Eukaryota - ps5
Eukaryota - ps6
Amorpheae - ps7
Obazoa - ps8
Opisthokonta - ps9
Holomycota - ps10
Fungi - ps11
Fungi_1 - ps12
Fungi_2 - ps13
Fungi_3 - ps14
Dikarya - ps15
Ascomycota - ps16
Saccharo/Pezizomycotina - ps17
Saccharomycotina - ps18
Saccharomycotina_1 - ps19
Saccharomycotina_2 - ps20
Saccharomycotina_3 - ps21
Phaff./Saccharomycetaceae - ps22
Saccharomyco./mycetaceae - ps23
Saccharomycetaceae - ps24
Saccharomycetaceae_2 - ps25
Saccharomycetaceae_3 - ps26
Kaz./Nau./Saccharomyces - ps27
Saccharomyces - ps28
Arboricola_clade - ps29
Cerevisiae_clade - ps30
Saccharomyces_cerevisiae - ps31
Phylostratum

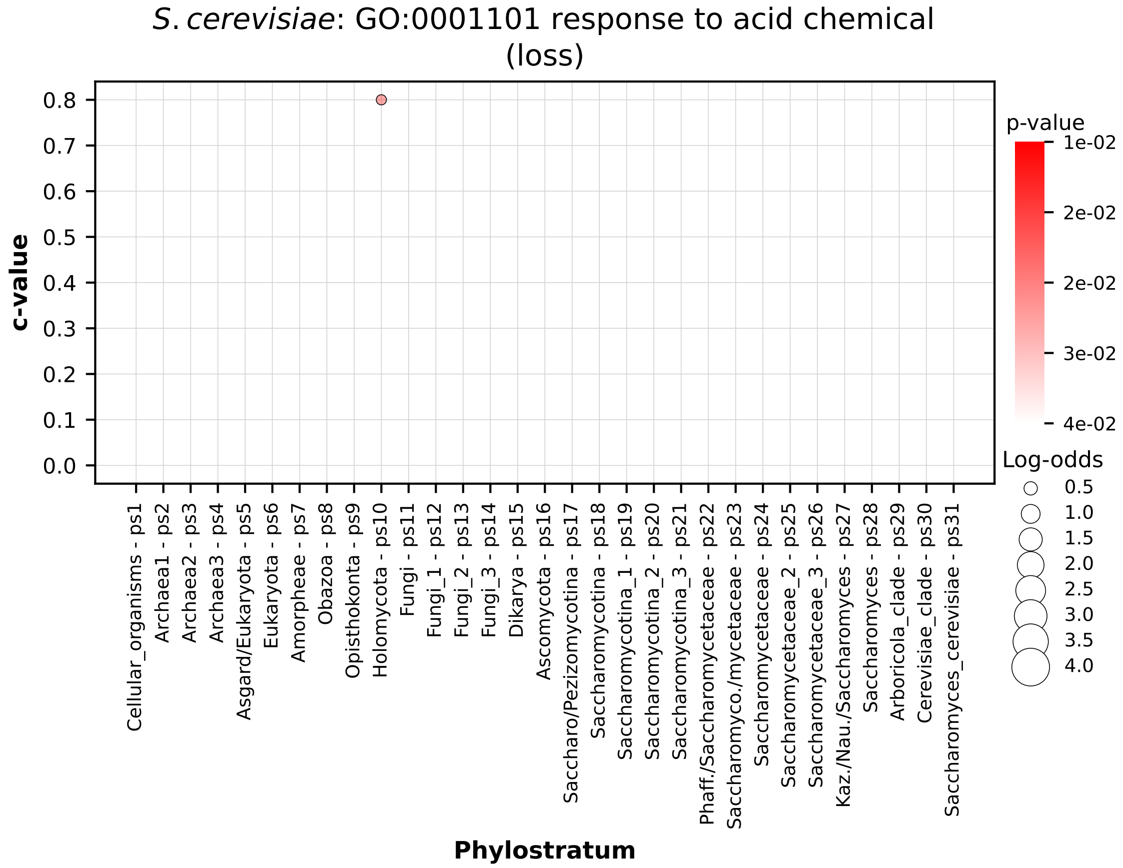

S. cerevisiae: GO:0001101 response to acid chemical
(loss)
c-value
0.8
0.7
0.6
0.5
0.4
0.3
0.2
0.1
0.0
p-value
1e-02
2e-02
2e-02
3e-02
4e-02
Log-odds
0.5
1.0
1.5
2.0
2.5
3.0
3.5
4.0
Cellular_organisms - ps1
Archaea1 - ps2
Archaea2 - ps3
Archaea3 - ps4
Asgard/Eukaryota - ps5
Eukaryota - ps6
Amorpheae - ps7
Obazoa - ps8
Opisthokonta - ps9
Holomycota - ps10
Fungi - ps11
Fungi_1 - ps12
Fungi_2 - ps13
Fungi_3 - ps14
Dikarya - ps15
Ascomycota - ps16
Saccharo/Pezizomycotina - ps17
Saccharomycotina - ps18
Saccharomycotina_1 - ps19
Saccharomycotina_2 - ps20
Saccharomycotina_3 - ps21
Phaff./Saccharomycetaceae - ps22
Saccharomyco./mycetaceae - ps23
Saccharomycetaceae - ps24
Saccharomycetaceae_2 - ps25
Saccharomycetaceae_3 - ps26
Kaz./Nau./Saccharomyces - ps27
Saccharomyces - ps28
Arboricola_clade - ps29
Cerevisiae_clade - ps30
Saccharomyces_cerevisiae - ps31
Phylostratum

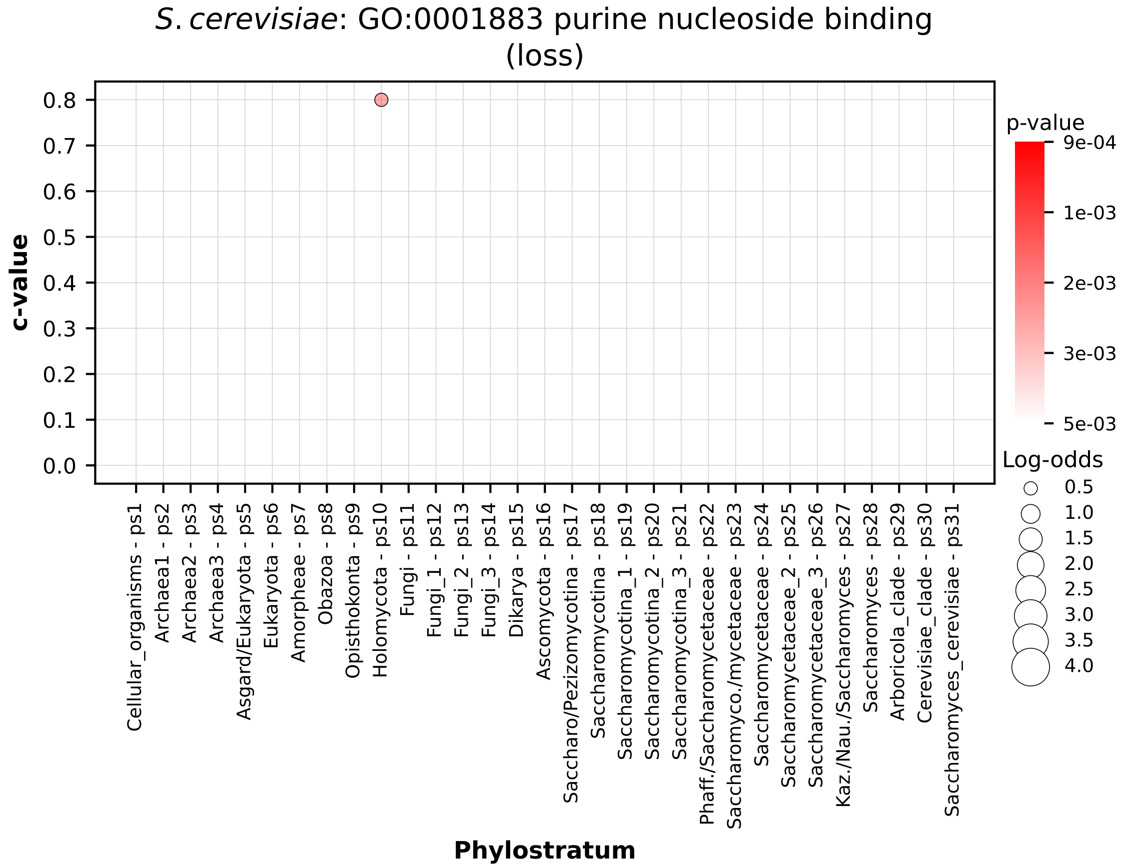

S. cerevisiae: GO:0001883 purine nucleoside binding
(loss)
c-value
0.0
0.1
0.2
0.3
0.4
0.5
0.6
0.7
0.8
Phylostratum
Cellular_organisms - ps1
Archaea1 - ps2
Archaea2 - ps3
Archaea3 - ps4
Asgard/Eukaryota - ps5
Eukaryota - ps6
Amorpheae - ps7
Obazoa - ps8
Opisthokonta - ps9
Holomycota - ps10
Fungi - ps11
Fungi_1 - ps12
Fungi_2 - ps13
Fungi_3 - ps14
Dikarya - ps15
Ascomycota - ps16
Saccharo/Pezizomycotina - ps17
Saccharomycotina - ps18
Saccharomycotina_1 - ps19
Saccharomycotina_2 - ps20
Saccharomycotina_3 - ps21
Phaff./Saccharomycetaceae - ps22
Saccharomyco./mycetaceae - ps23
Saccharomycetaceae - ps24
Saccharomycetaceae_2 - ps25
Saccharomycetaceae_3 - ps26
Kaz./Nau./Saccharomyces - ps27
Saccharomyces - ps28
Arboricola_clade - ps29
Cerevisiae_clade - ps30
Saccharomyces_cerevisiae - ps31
p-value
9e-04
1e-03
2e-03
3e-03
5e-03
Log-odds
0.5
1.0
1.5
2.0
2.5
3.0
3.5
4.0

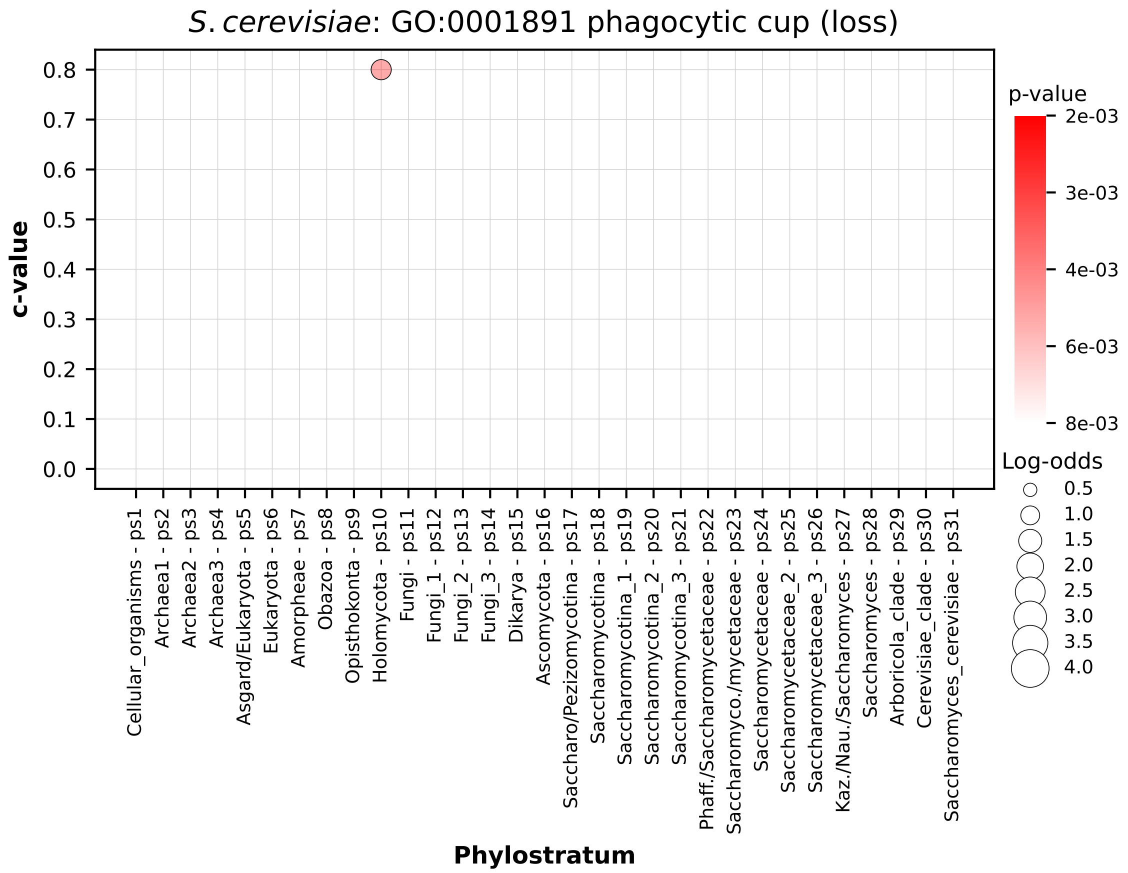

S. cerevisiae: GO:0001891 phagocytic cup (loss)
c-value
0.0
0.1
0.2
0.3
0.4
0.5
0.6
0.7
0.8
p-value
2e-03
3e-03
4e-03
6e-03
8e-03
Log-odds
0.5
1.0
1.5
2.0
2.5
3.0
3.5
4.0
Cellular_organisms - ps1
Archaea1 - ps2
Archaea2 - ps3
Archaea3 - ps4
Asgard/Eukaryota - ps5
Eukaryota - ps6
Amorpheae - ps7
Obazoa - ps8
Opisthokonta - ps9
Holomycota - ps10
Fungi - ps11
Fungi_1 - ps12
Fungi_2 - ps13
Fungi_3 - ps14
Dikarya - ps15
Ascomycota - ps16
Saccharo/Pezizomycotina - ps17
Saccharomycotina - ps18
Saccharomycotina_1 - ps19
Saccharomycotina_2 - ps20
Saccharomycotina_3 - ps21
Phaff./Saccharomycetaceae - ps22
Saccharomyco./mycetaceae - ps23
Saccharomycetaceae - ps24
Saccharomycetaceae_2 - ps25
Saccharomycetaceae_3 - ps26
Kaz./Nau./Saccharomyces - ps27
Saccharomyces - ps28
Arboricola_clade - ps29
Cerevisiae_clade - ps30
Saccharomyces_cerevisiae - ps31
Phylostratum

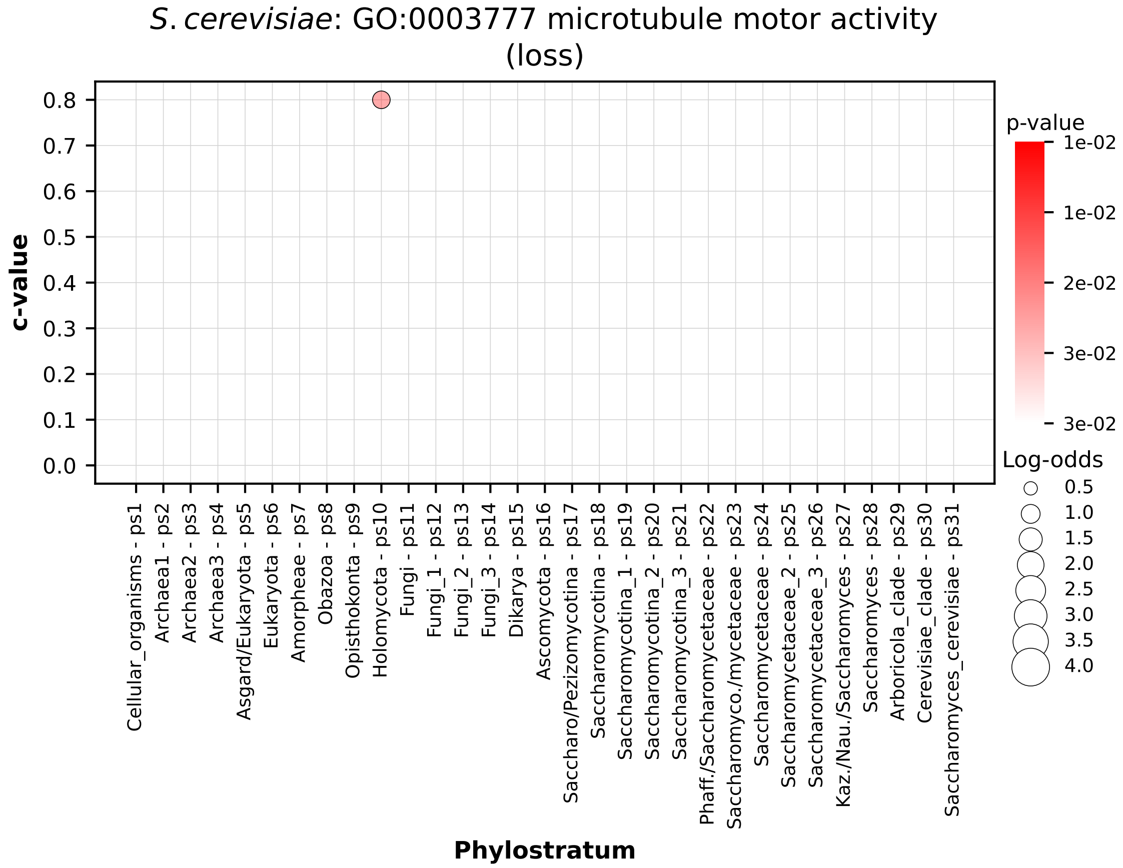

S. cerevisiae: GO:0003777 microtubule motor activity
(loss)
c-value
0.0
0.1
0.2
0.3
0.4
0.5
0.6
0.7
0.8
Phylostratum
Cellular_organisms - ps1
Archaea1 - ps2
Archaea2 - ps3
Archaea3 - ps4
Asgard/Eukaryota - ps5
Eukaryota - ps6
Amorpheae - ps7
Obazoa - ps8
Opisthokonta - ps9
Holomycota - ps10
Fungi - ps11
Fungi_1 - ps12
Fungi_2 - ps13
Fungi_3 - ps14
Dikarya - ps15
Ascomycota - ps16
Saccharo/Pezizomycotina - ps17
Saccharomycotina - ps18
Saccharomycotina_1 - ps19
Saccharomycotina_2 - ps20
Saccharomycotina_3 - ps21
Phaff./Saccharomycetaceae - ps22
Saccharomyco./mycetaceae - ps23
Saccharomycetaceae - ps24
Saccharomycetaceae_2 - ps25
Saccharomycetaceae_3 - ps26
Kaz./Nau./Saccharomyces - ps27
Saccharomyces - ps28
Arboricola_clade - ps29
Cerevisiae_clade - ps30
Saccharomyces_cerevisiae - ps31
p-value
1e-02
1e-02
2e-02
3e-02
3e-02
Log-odds
0.5
1.0
1.5
2.0
2.5
3.0
3.5
4.0

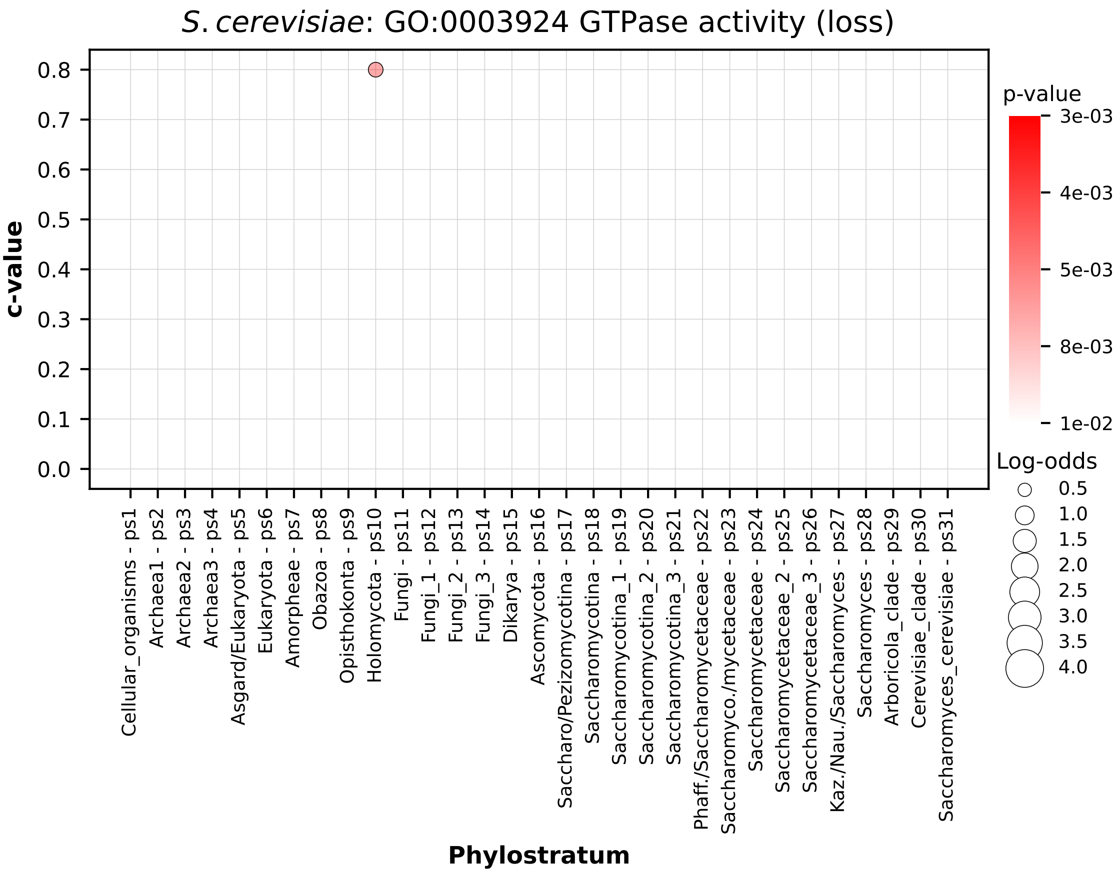

S. cerevisiae: GO:0003924 GTPase activity (loss)
c-value
0.0
0.1
0.2
0.3
0.4
0.5
0.6
0.7
0.8
Phylostratum
Cellular_organisms - ps1
Archaea1 - ps2
Archaea2 - ps3
Archaea3 - ps4
Asgard/Eukaryota - ps5
Eukaryota - ps6
Amorpheae - ps7
Obazoa - ps8
Opisthokonta - ps9
Holomycota - ps10
Fungi - ps11
Fungi_1 - ps12
Fungi_2 - ps13
Fungi_3 - ps14
Dikarya - ps15
Ascomycota - ps16
Saccharo/Pezizomycotina - ps17
Saccharomycotina - ps18
Saccharomycotina_1 - ps19
Saccharomycotina_2 - ps20
Saccharomycotina_3 - ps21
Phaff./Saccharomycetaceae - ps22
Saccharomyco./mycetaceae - ps23
Saccharomycetaceae - ps24
Saccharomycetaceae_2 - ps25
Saccharomycetaceae_3 - ps26
Kaz./Nau./Saccharomyces - ps27
Saccharomyces - ps28
Arboricola_clade - ps29
Cerevisiae_clade - ps30
Saccharomyces_cerevisiae - ps31
p-value
3e-03
4e-03
5e-03
8e-03
1e-02
Log-odds
0.5
1.0
1.5
2.0
2.5
3.0
3.5
4.0

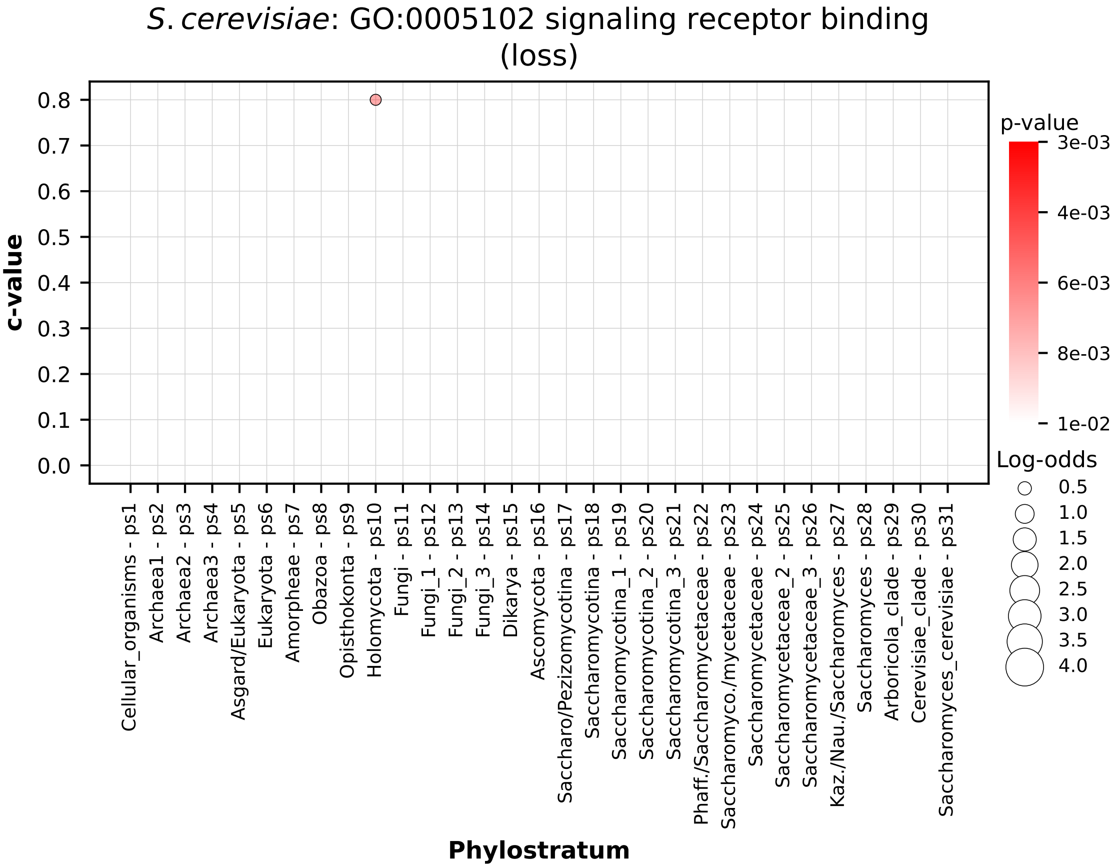

S. cerevisiae: GO:0005102 signaling receptor binding
(loss)
c-value
0.0
0.1
0.2
0.3
0.4
0.5
0.6
0.7
0.8
p-value
3e-03
4e-03
6e-03
8e-03
1e-02
Log-odds
0.5
1.0
1.5
2.0
2.5
3.0
3.5
4.0
Cellular_organisms - ps1
Archaea1 - ps2
Archaea2 - ps3
Archaea3 - ps4
Asgard/Eukaryota - ps5
Eukaryota - ps6
Amorpheae - ps7
Obazoa - ps8
Opisthokonta - ps9
Holomycota - ps10
Fungi - ps11
Fungi_1 - ps12
Fungi_2 - ps13
Fungi_3 - ps14
Dikarya - ps15
Ascomycota - ps16
Saccharo/Pezizomycotina - ps17
Saccharomycotina - ps18
Saccharomycotina_1 - ps19
Saccharomycotina_2 - ps20
Saccharomycotina_3 - ps21
Phaff./Saccharomycetaceae - ps22
Saccharomyco./mycetaceae - ps23
Saccharomycetaceae - ps24
Saccharomycetaceae_2 - ps25
Saccharomycetaceae_3 - ps26
Kaz./Nau./Saccharomyces - ps27
Saccharomyces - ps28
Arboricola_clade - ps29
Cerevisiae_clade - ps30
Saccharomyces_cerevisiae - ps31
Phylostratum

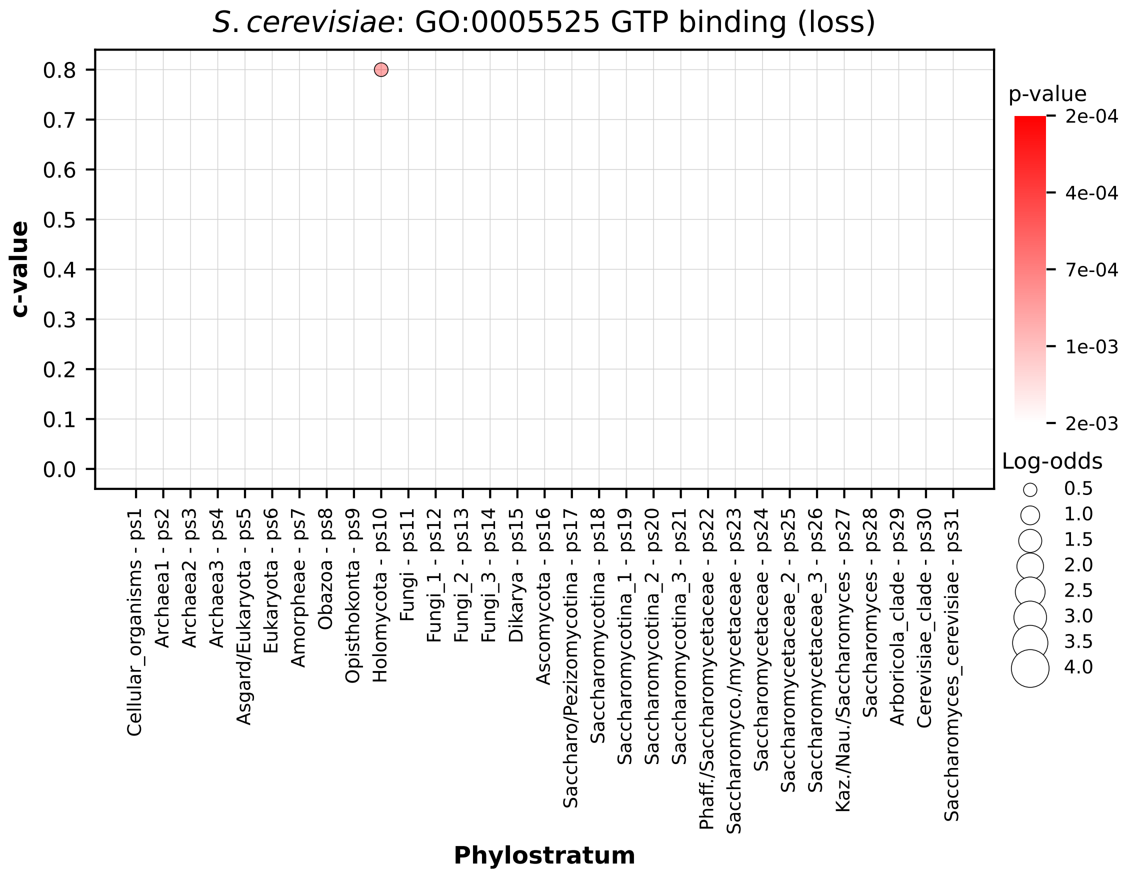
S. cerevisiae: GO:0005525 GTP binding (loss)
c-value
0.8
0.7
0.6
0.5
0.4
0.3
0.2
0.1
0.0
Phylostratum
Cellular_organisms - ps1
Archaea1 - ps2
Archaea2 - ps3
Archaea3 - ps4
Asgard/Eukaryota - ps5
Eukaryota - ps6
Amorpheae - ps7
Obazoa - ps8
Opisthokonta - ps9
Holomycota - ps10
Fungi - ps11
Fungi_1 - ps12
Fungi_2 - ps13
Fungi_3 - ps14
Dikarya - ps15
Ascomycota - ps16
Saccharo/Pezizomycotina - ps17
Saccharomycotina - ps18
Saccharomycotina_1 - ps19
Saccharomycotina_2 - ps20
Saccharomycotina_3 - ps21
Phaff./Saccharomycetaceae - ps22
Saccharomyco./mycetaceae - ps23
Saccharomycetaceae - ps24
Saccharomycetaceae_2 - ps25
Saccharomycetaceae_3 - ps26
Kaz./Nau./Saccharomyces - ps27
Saccharomyces - ps28
Arboricola_clade - ps29
Cerevisiae_clade - ps30
Saccharomyces_cerevisiae - ps31
p-value
2e-04
4e-04
7e-04
1e-03
2e-03
Log-odds
0.5
1.0
1.5
2.0
2.5
3.0
3.5
4.0

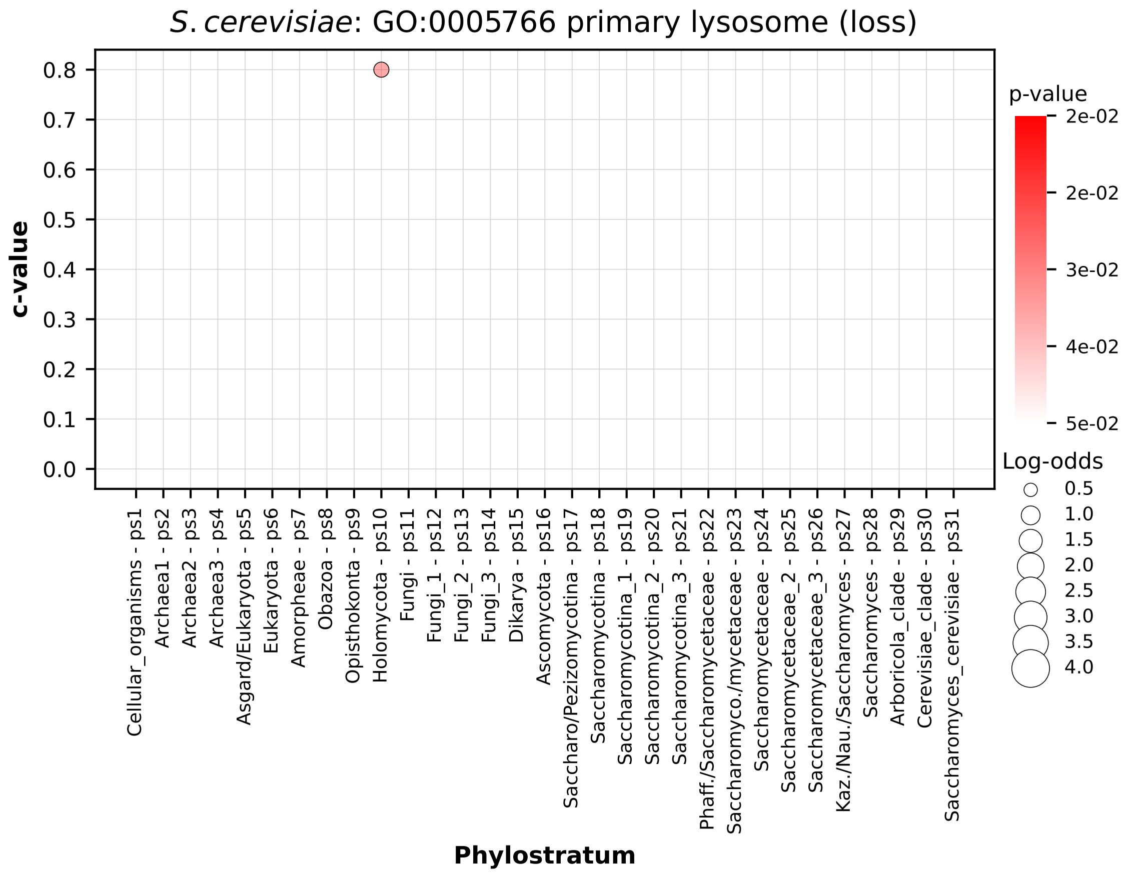

S. cerevisiae: GO:0005766 primary lysosome (loss)
c-value
0.0
0.1
0.2
0.3
0.4
0.5
0.6
0.7
0.8
Phylostratum
Cellular_organisms - ps1
Archaea1 - ps2
Archaea2 - ps3
Archaea3 - ps4
Asgard/Eukaryota - ps5
Eukaryota - ps6
Amorpheae - ps7
Obazoa - ps8
Opisthokonta - ps9
Holomycota - ps10
Fungi - ps11
Fungi_1 - ps12
Fungi_2 - ps13
Fungi_3 - ps14
Dikarya - ps15
Ascomycota - ps16
Saccharo/Pezizomycotina - ps17
Saccharomycotina - ps18
Saccharomycotina_1 - ps19
Saccharomycotina_2 - ps20
Saccharomycotina_3 - ps21
Phaff./Saccharomycetaceae - ps22
Saccharomyco./mycetaceae - ps23
Saccharomycetaceae - ps24
Saccharomycetaceae_2 - ps25
Saccharomycetaceae_3 - ps26
Kaz./Nau./Saccharomyces - ps27
Saccharomyces - ps28
Arboricola_clade - ps29
Cerevisiae_clade - ps30
Saccharomyces_cerevisiae - ps31
p-value
2e-02
2e-02
3e-02
4e-02
5e-02
Log-odds
0.5
1.0
1.5
2.0
2.5
3.0
3.5
4.0

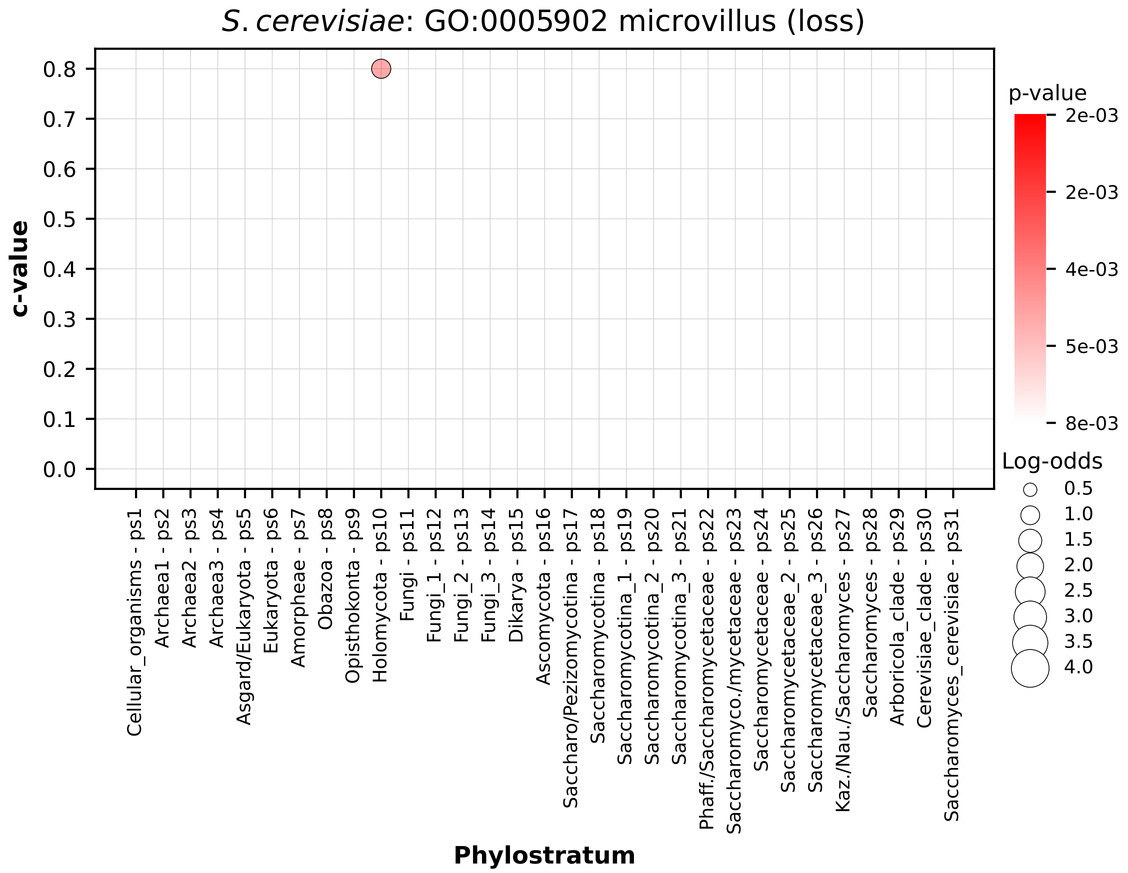
S. cerevisiae: GO:0005902 microvillus (loss)
c-value
0.0
0.1
0.2
0.3
0.4
0.5
0.6
0.7
0.8
p-value
2e-03
2e-03
4e-03
5e-03
8e-03
Log-odds
0.5
1.0
1.5
2.0
2.5
3.0
3.5
4.0
Cellular_organisms - ps1
Archaea1 - ps2
Archaea2 - ps3
Archaea3 - ps4
Asgard/Eukaryota - ps5
Eukaryota - ps6
Amorpheae - ps7
Obazoa - ps8
Opisthokonta - ps9
Holomycota - ps10
Fungi - ps11
Fungi_1 - ps12
Fungi_2 - ps13
Fungi_3 - ps14
Dikarya - ps15
Ascomycota - ps16
Saccharo/Pezizomycotina - ps17
Saccharomycotina - ps18
Saccharomycotina_1 - ps19
Saccharomycotina_2 - ps20
Saccharomycotina_3 - ps21
Phaff./Saccharomycetaceae - ps22
Saccharomyco./mycetaceae - ps23
Saccharomycetaceae - ps24
Saccharomycetaceae_2 - ps25
Saccharomycetaceae_3 - ps26
Kaz./Nau./Saccharomyces - ps27
Saccharomyces - ps28
Arboricola_clade - ps29
Cerevisiae_clade - ps30
Saccharomyces_cerevisiae - ps31
Phylostratum

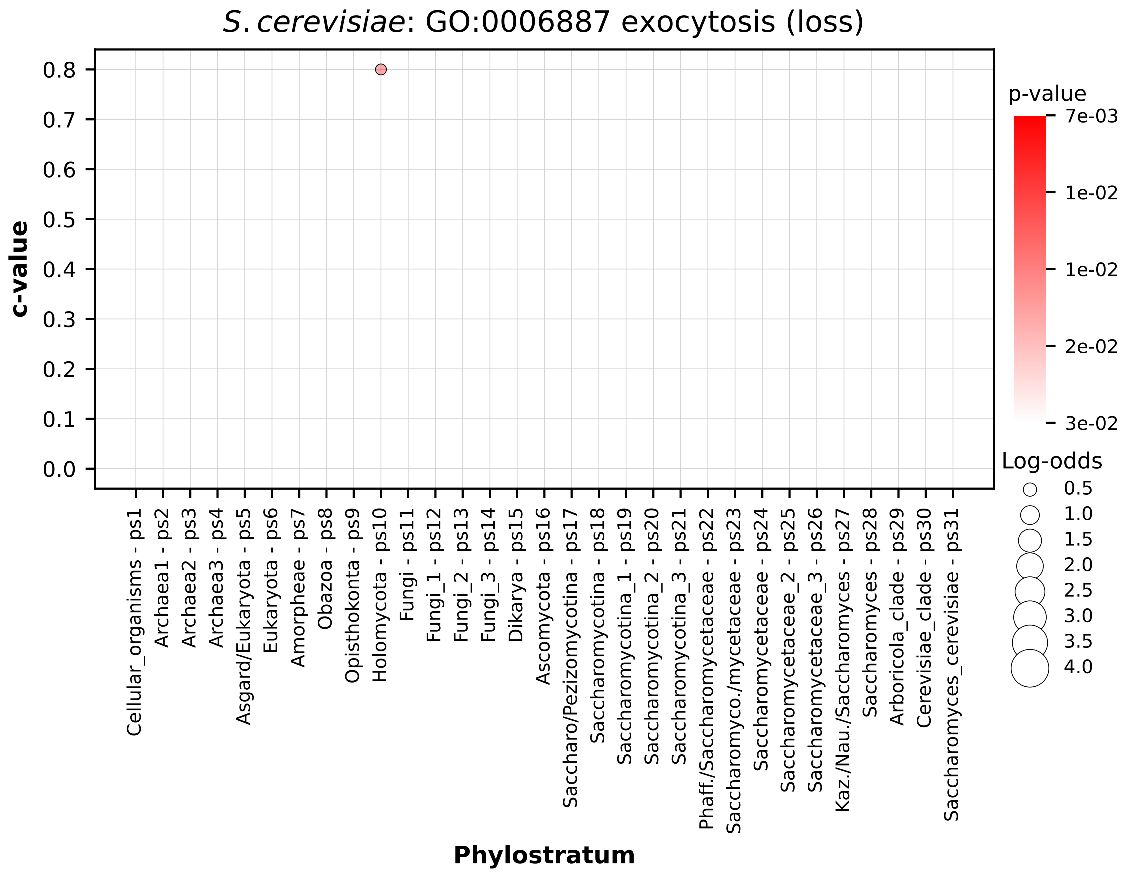
S. cerevisiae: GO:0006887 exocytosis (loss)
c-value
0.0
0.1
0.2
0.3
0.4
0.5
0.6
0.7
0.8
p-value
7e-03
1e-02
1e-02
2e-02
3e-02
Log-odds
0.5
1.0
1.5
2.0
2.5
3.0
3.5
4.0
Cellular_organisms - ps1
Archaea1 - ps2
Archaea2 - ps3
Archaea3 - ps4
Asgard/Eukaryota - ps5
Eukaryota - ps6
Amorpheae - ps7
Obazoa - ps8
Opisthokonta - ps9
Holomycota - ps10
Fungi - ps11
Fungi_1 - ps12
Fungi_2 - ps13
Fungi_3 - ps14
Dikarya - ps15
Ascomycota - ps16
Saccharo/Pezizomycotina - ps17
Saccharomycotina - ps18
Saccharomycotina_1 - ps19
Saccharomycotina_2 - ps20
Saccharomycotina_3 - ps21
Phaff./Saccharomycetaceae - ps22
Saccharomyco./mycetaceae - ps23
Saccharomycetaceae - ps24
Saccharomycetaceae_2 - ps25
Saccharomycetaceae_3 - ps26
Kaz./Nau./Saccharomyces - ps27
Saccharomyces - ps28
Arboricola_clade - ps29
Cerevisiae_clade - ps30
Saccharomyces_cerevisiae - ps31
Phylostratum

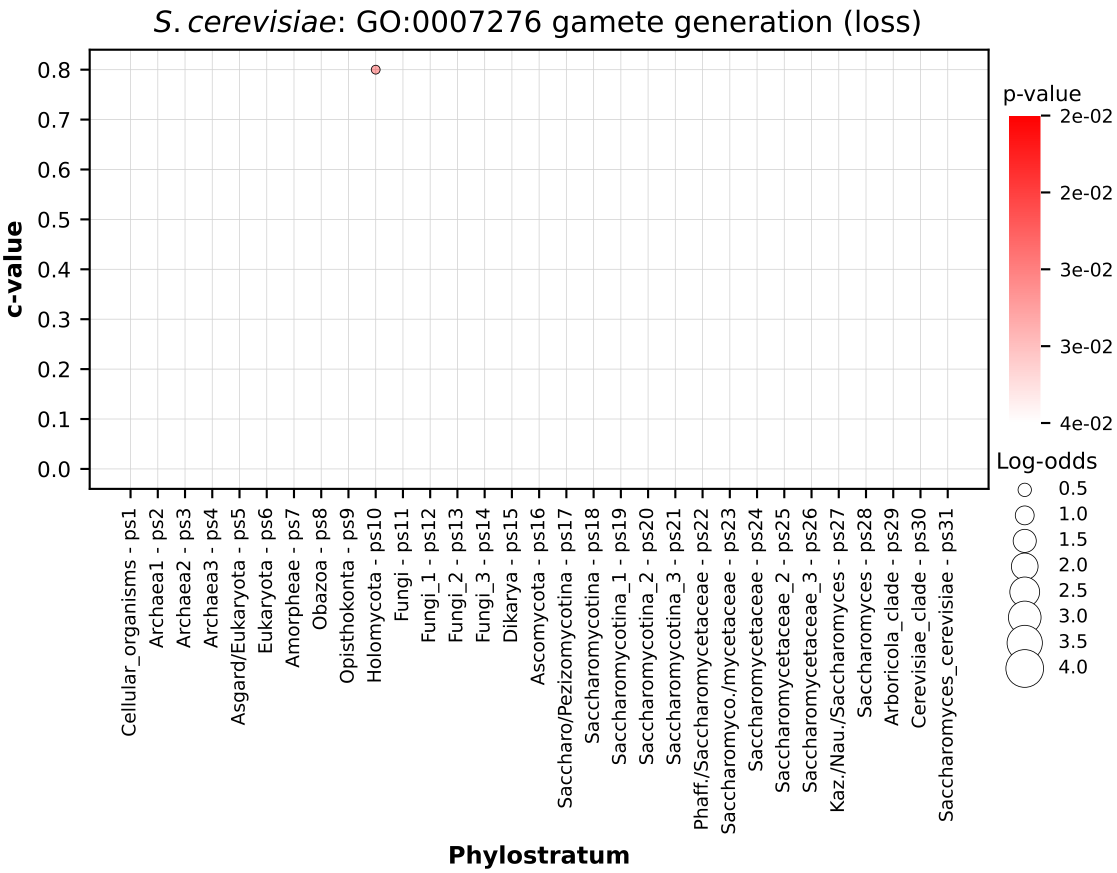
S. cerevisiae: GO:0007276 gamete generation (loss)
c-value
0.0
0.1
0.2
0.3
0.4
0.5
0.6
0.7
0.8
p-value
2e-02
2e-02
3e-02
3e-02
4e-02
Log-odds
0.5
1.0
1.5
2.0
2.5
3.0
3.5
4.0
Cellular_organisms - ps1
Archaea1 - ps2
Archaea2 - ps3
Archaea3 - ps4
Asgard/Eukaryota - ps5
Eukaryota - ps6
Amorpheae - ps7
Obazoa - ps8
Opisthokonta - ps9
Holomycota - ps10
Fungi - ps11
Fungi_1 - ps12
Fungi_2 - ps13
Fungi_3 - ps14
Dikarya - ps15
Ascomycota - ps16
Saccharo/Pezizomycotina - ps17
Saccharomycotina - ps18
Saccharomycotina_1 - ps19
Saccharomycotina_2 - ps20
Saccharomycotina_3 - ps21
Phaff./Saccharomycetaceae - ps22
Saccharomyco./mycetaceae - ps23
Saccharomycetaceae - ps24
Saccharomycetaceae_2 - ps25
Saccharomycetaceae_3 - ps26
Kaz./Nau./Saccharomyces - ps27
Saccharomyces - ps28
Arboricola_clade - ps29
Cerevisiae_clade - ps30
Saccharomyces_cerevisiae - ps31
Phylostratum

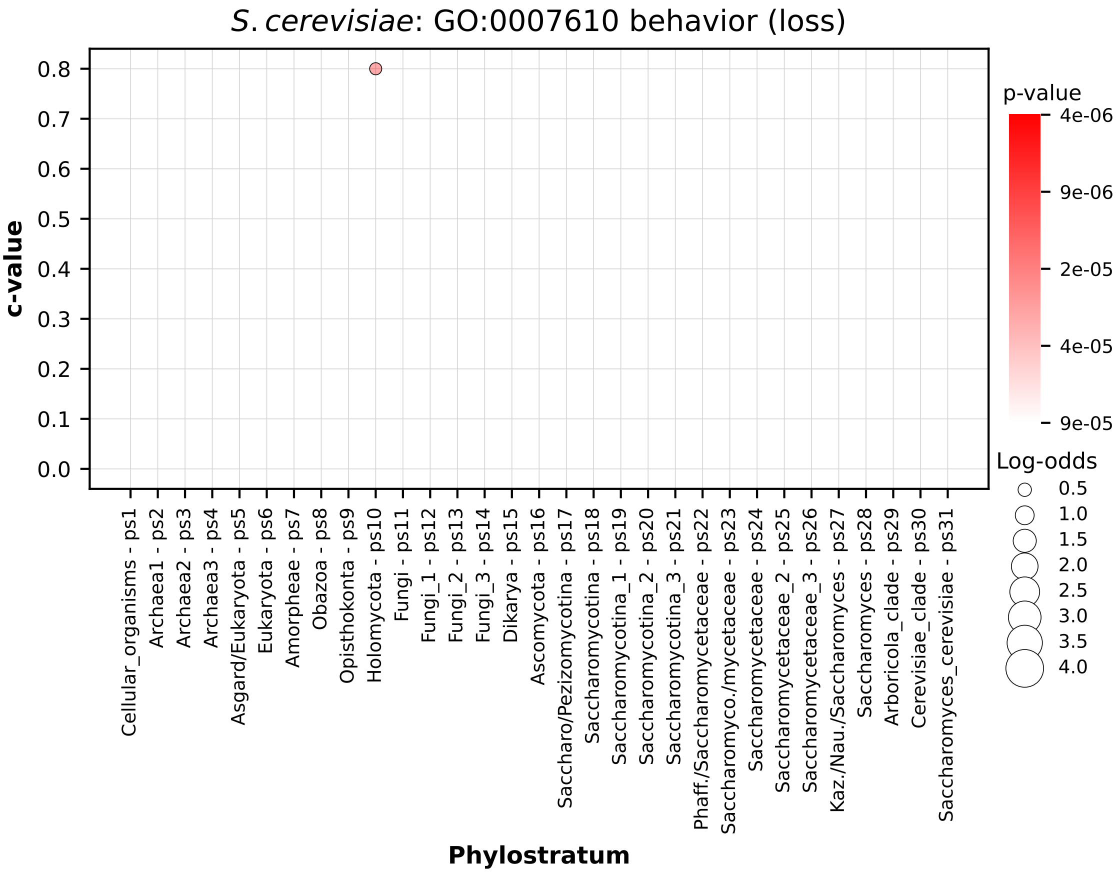
S. cerevisiae: GO:0007610 behavior (loss)
c-value
0.0
0.1
0.2
0.3
0.4
0.5
0.6
0.7
0.8
Phylostratum
Cellular_organisms - ps1
Archaea1 - ps2
Archaea2 - ps3
Archaea3 - ps4
Asgard/Eukaryota - ps5
Eukaryota - ps6
Amorpheae - ps7
Obazoa - ps8
Opisthokonta - ps9
Holomycota - ps10
Fungi - ps11
Fungi_1 - ps12
Fungi_2 - ps13
Fungi_3 - ps14
Dikarya - ps15
Ascomycota - ps16
Saccharo/Pezizomycotina - ps17
Saccharomycotina - ps18
Saccharomycotina_1 - ps19
Saccharomycotina_2 - ps20
Saccharomycotina_3 - ps21
Phaff./Saccharomycetaceae - ps22
Saccharomyco./mycetaceae - ps23
Saccharomycetaceae - ps24
Saccharomycetaceae_2 - ps25
Saccharomycetaceae_3 - ps26
Kaz./Nau./Saccharomyces - ps27
Saccharomyces - ps28
Arboricola_clade - ps29
Cerevisiae_clade - ps30
Saccharomyces_cerevisiae - ps31
p-value
4e-06
9e-06
2e-05
4e-05
9e-05
Log-odds
0.5
1.0
1.5
2.0
2.5
3.0
3.5
4.0

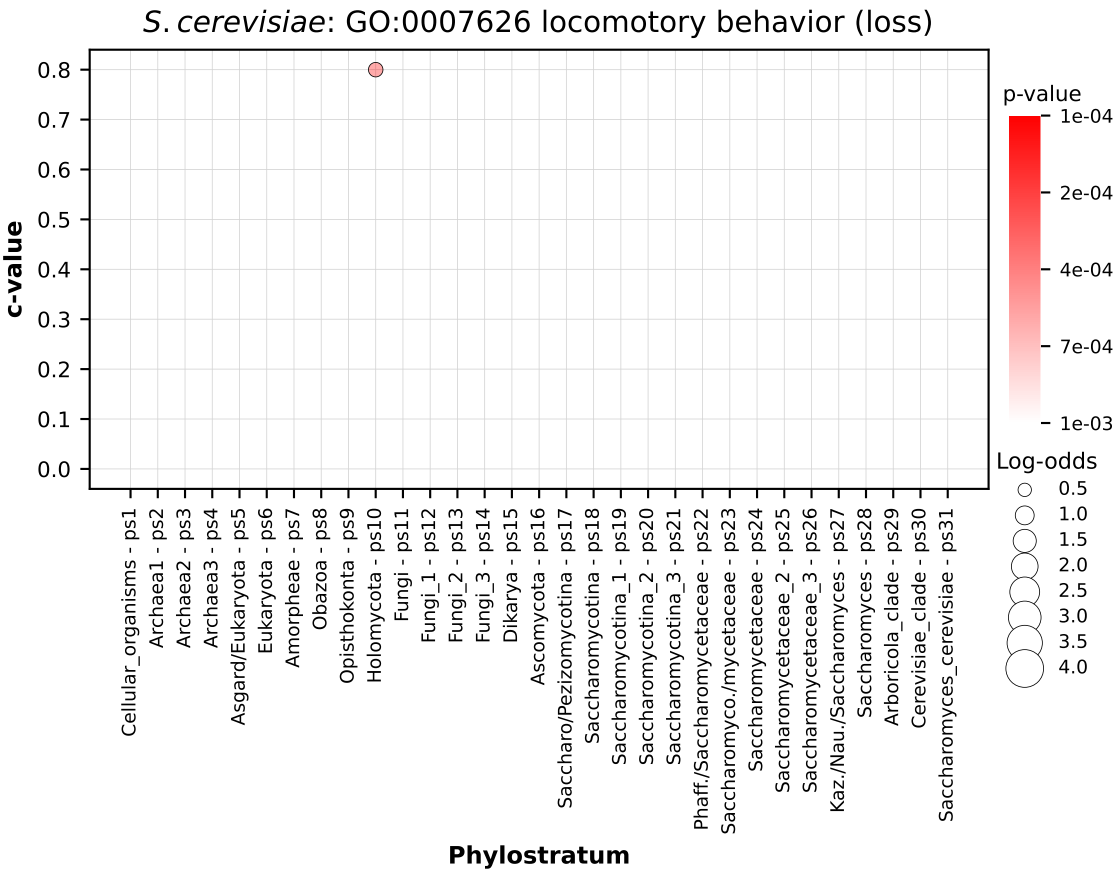
S. cerevisiae: GO:0007626 locomotory behavior (loss)
c-value
0.0
0.1
0.2
0.3
0.4
0.5
0.6
0.7
0.8
Phylostratum
Cellular_organisms - ps1
Archaea1 - ps2
Archaea2 - ps3
Archaea3 - ps4
Asgard/Eukaryota - ps5
Eukaryota - ps6
Amorpheae - ps7
Obazoa - ps8
Opisthokonta - ps9
Holomycota - ps10
Fungi - ps11
Fungi_1 - ps12
Fungi_2 - ps13
Fungi_3 - ps14
Dikarya - ps15
Ascomycota - ps16
Saccharo/Pezizomycotina - ps17
Saccharomycotina - ps18
Saccharomycotina_1 - ps19
Saccharomycotina_2 - ps20
Saccharomycotina_3 - ps21
Phaff./Saccharomycetaceae - ps22
Saccharomyco./mycetaceae - ps23
Saccharomycetaceae - ps24
Saccharomycetaceae_2 - ps25
Saccharomycetaceae_3 - ps26
Kaz./Nau./Saccharomyces - ps27
Saccharomyces - ps28
Arboricola_clade - ps29
Cerevisiae_clade - ps30
Saccharomyces_cerevisiae - ps31
p-value
1e-04
2e-04
4e-04
7e-04
1e-03
Log-odds
0.5
1.0
1.5
2.0
2.5
3.0
3.5
4.0

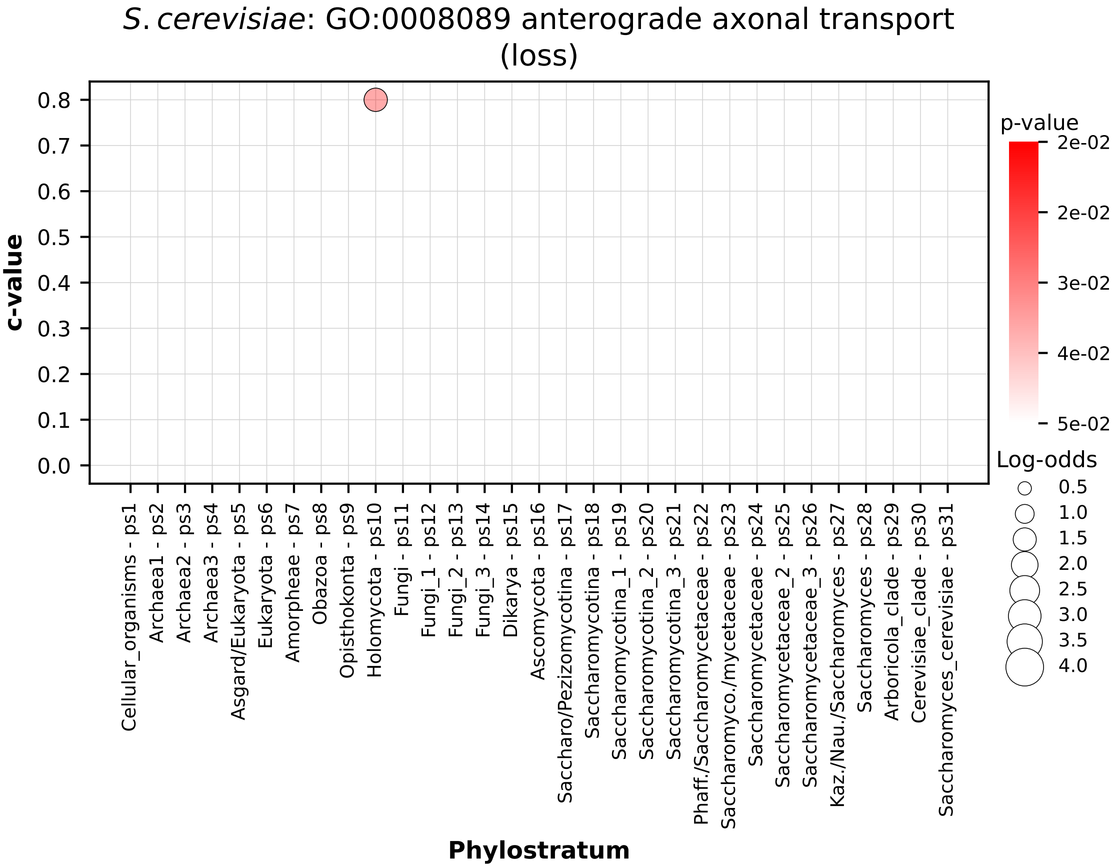

S. cerevisiae: GO:0008089 anterograde axonal transport
(loss)
c-value
0.0
0.1
0.2
0.3
0.4
0.5
0.6
0.7
0.8
p-value
2e-02
2e-02
3e-02
4e-02
5e-02
Log-odds
0.5
1.0
1.5
2.0
2.5
3.0
3.5
4.0
Cellular_organisms - ps1
Archaea1 - ps2
Archaea2 - ps3
Archaea3 - ps4
Asgard/Eukaryota - ps5
Eukaryota - ps6
Amorpheae - ps7
Obazoa - ps8
Opisthokonta - ps9
Holomycota - ps10
Fungi - ps11
Fungi_1 - ps12
Fungi_2 - ps13
Fungi_3 - ps14
Dikarya - ps15
Ascomycota - ps16
Saccharo/Pezizomycotina - ps17
Saccharomycotina - ps18
Saccharomycotina_1 - ps19
Saccharomycotina_2 - ps20
Saccharomycotina_3 - ps21
Phaff./Saccharomycetaceae - ps22
Saccharomyco./mycetaceae - ps23
Saccharomycetaceae - ps24
Saccharomycetaceae_2 - ps25
Saccharomycetaceae_3 - ps26
Kaz./Nau./Saccharomyces - ps27
Saccharomyces - ps28
Arboricola_clade - ps29
Cerevisiae_clade - ps30
Saccharomyces_cerevisiae - ps31
Phylostratum

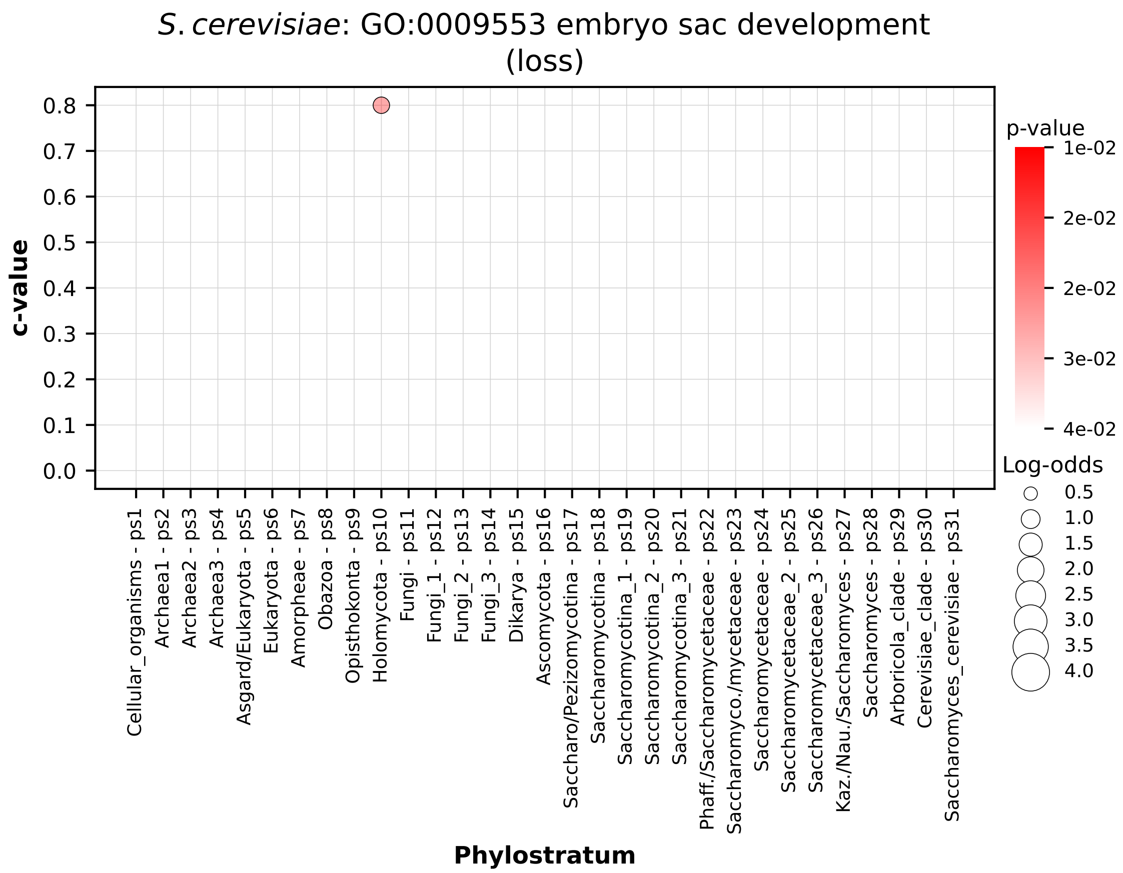

S. cerevisiae: GO:0009553 embryo sac development
(loss)
c-value
0.0
0.1
0.2
0.3
0.4
0.5
0.6
0.7
0.8
Phylostratum
Cellular_organisms - ps1
Archaea1 - ps2
Archaea2 - ps3
Archaea3 - ps4
Asgard/Eukaryota - ps5
Eukaryota - ps6
Amorpheae - ps7
Obazoa - ps8
Opisthokonta - ps9
Holomycota - ps10
Fungi - ps11
Fungi_1 - ps12
Fungi_2 - ps13
Fungi_3 - ps14
Dikarya - ps15
Ascomycota - ps16
Saccharo/Pezizomycotina - ps17
Saccharomycotina - ps18
Saccharomycotina_1 - ps19
Saccharomycotina_2 - ps20
Saccharomycotina_3 - ps21
Phaff./Saccharomycetaceae - ps22
Saccharomyco./mycetaceae - ps23
Saccharomycetaceae - ps24
Saccharomycetaceae_2 - ps25
Saccharomycetaceae_3 - ps26
Kaz./Nau./Saccharomyces - ps27
Saccharomyces - ps28
Arboricola_clade - ps29
Cerevisiae_clade - ps30
Saccharomyces_cerevisiae - ps31
p-value
1e-02
2e-02
2e-02
3e-02
4e-02
Log-odds
0.5
1.0
1.5
2.0
2.5
3.0
3.5
4.0

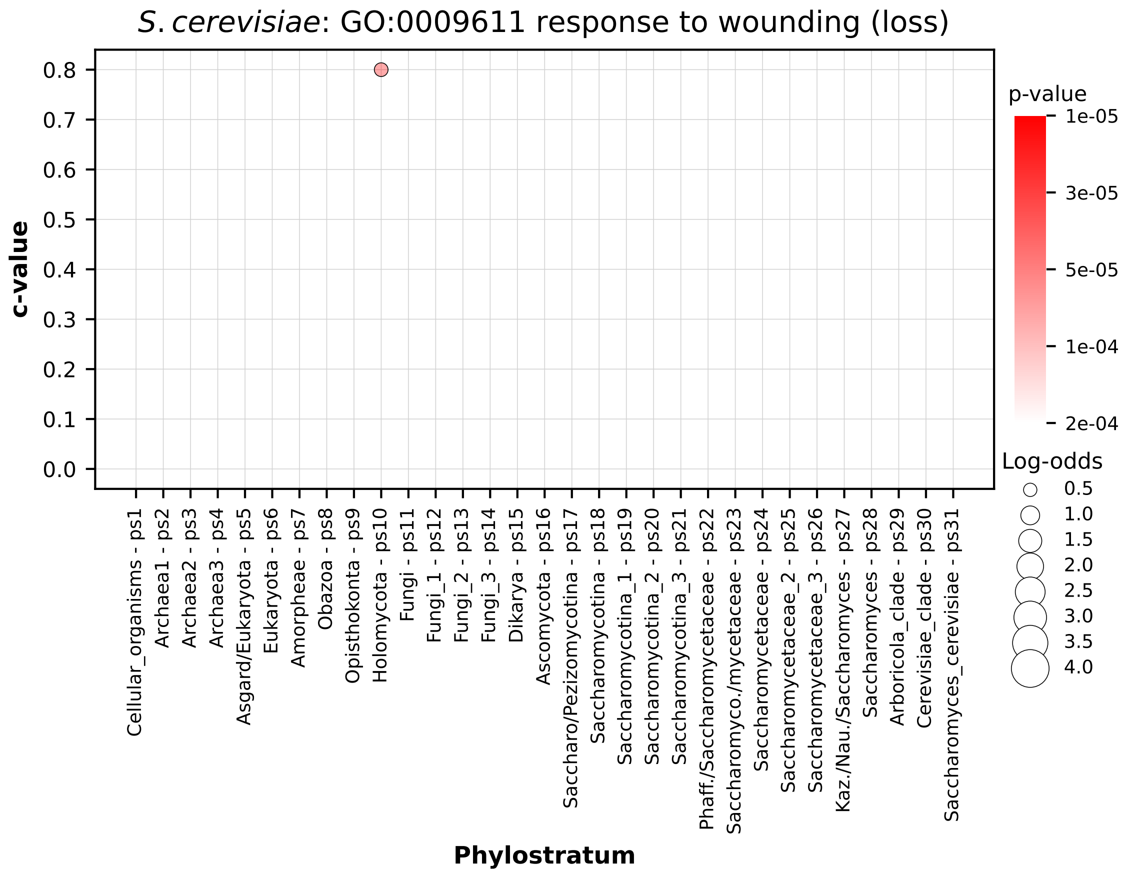

S. cerevisiae: GO:0009611 response to wounding (loss)
c-value
0.0
0.1
0.2
0.3
0.4
0.5
0.6
0.7
0.8
p-value
1e-05
3e-05
5e-05
1e-04
2e-04
Log-odds
0.5
1.0
1.5
2.0
2.5
3.0
3.5
4.0
Cellular_organisms - ps1
Archaea1 - ps2
Archaea2 - ps3
Archaea3 - ps4
Asgard/Eukaryota - ps5
Eukaryota - ps6
Amorpheae - ps7
Obazoa - ps8
Opisthokonta - ps9
Holomycota - ps10
Fungi - ps11
Fungi_1 - ps12
Fungi_2 - ps13
Fungi_3 - ps14
Dikarya - ps15
Ascomycota - ps16
Saccharo/Pezizomycotina - ps17
Saccharomycotina - ps18
Saccharomycotina_1 - ps19
Saccharomycotina_2 - ps20
Saccharomycotina_3 - ps21
Phaff./Saccharomycetaceae - ps22
Saccharomyco./mycetaceae - ps23
Saccharomycetaceae - ps24
Saccharomycetaceae_2 - ps25
Saccharomycetaceae_3 - ps26
Kaz./Nau./Saccharomyces - ps27
Saccharomyces - ps28
Arboricola_clade - ps29
Cerevisiae_clade - ps30
Saccharomyces_cerevisiae - ps31
Phylostratum

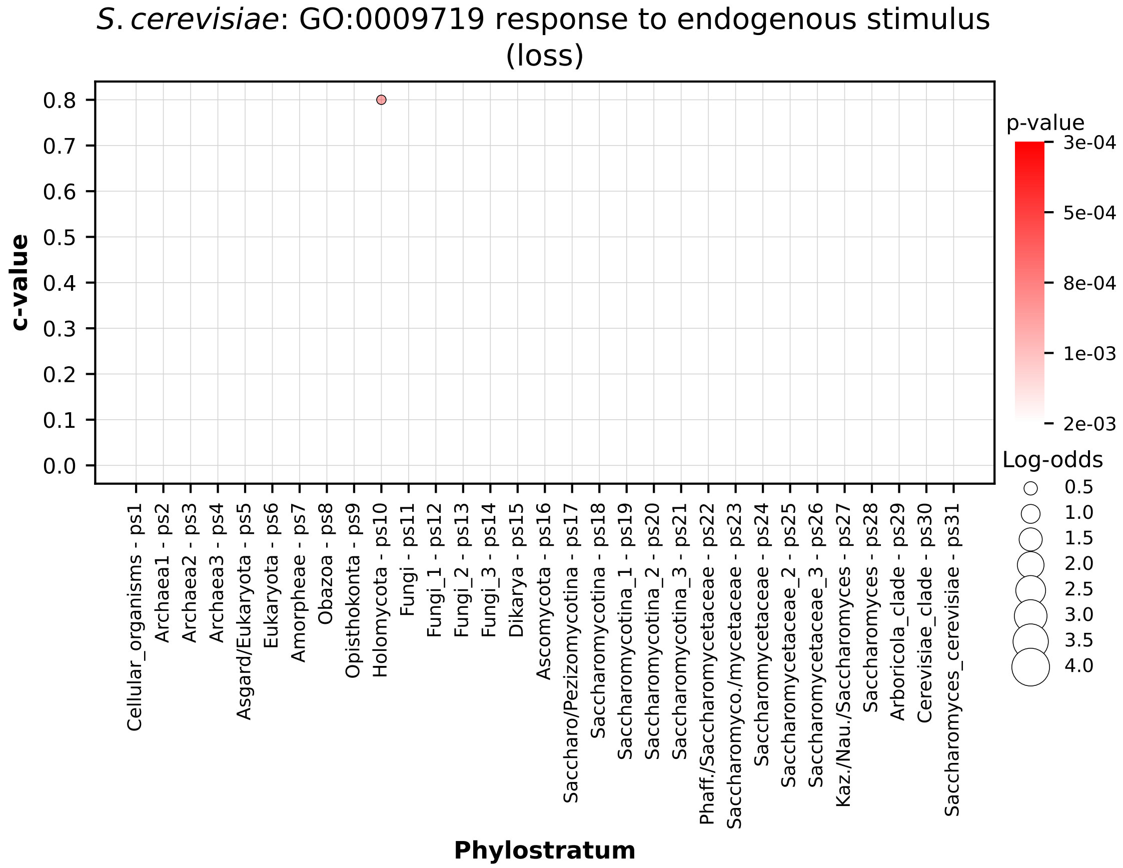
S. cerevisiae: GO:0009719 response to endogenous stimulus (loss)
c-value
0.8
0.7
0.6
0.5
0.4
0.3
0.2
0.1
0.0
p-value
3e-04
5e-04
8e-04
1e-03
2e-03
Log-odds
0.5
1.0
1.5
2.0
2.5
3.0
3.5
4.0
Cellular_organisms - ps1
Archaea1 - ps2
Archaea2 - ps3
Archaea3 - ps4
Asgard/Eukaryota - ps5
Eukaryota - ps6
Amorpheae - ps7
Obazoa - ps8
Opisthokonta - ps9
Holomycota - ps10
Fungi - ps11
Fungi_1 - ps12
Fungi_2 - ps13
Fungi_3 - ps14
Dikarya - ps15
Ascomycota - ps16
Saccharo/Pezizomycotina - ps17
Saccharomycotina - ps18
Saccharomycotina_1 - ps19
Saccharomycotina_2 - ps20
Saccharomycotina_3 - ps21
Phaff./Saccharomycetaceae - ps22
Saccharomyco./mycetaceae - ps23
Saccharomycetaceae - ps24
Saccharomycetaceae_2 - ps25
Saccharomycetaceae_3 - ps26
Kaz./Nau./Saccharomyces - ps27
Saccharomyces - ps28
Arboricola_clade - ps29
Cerevisiae_clade - ps30
Saccharomyces_cerevisiae - ps31
Phylostratum

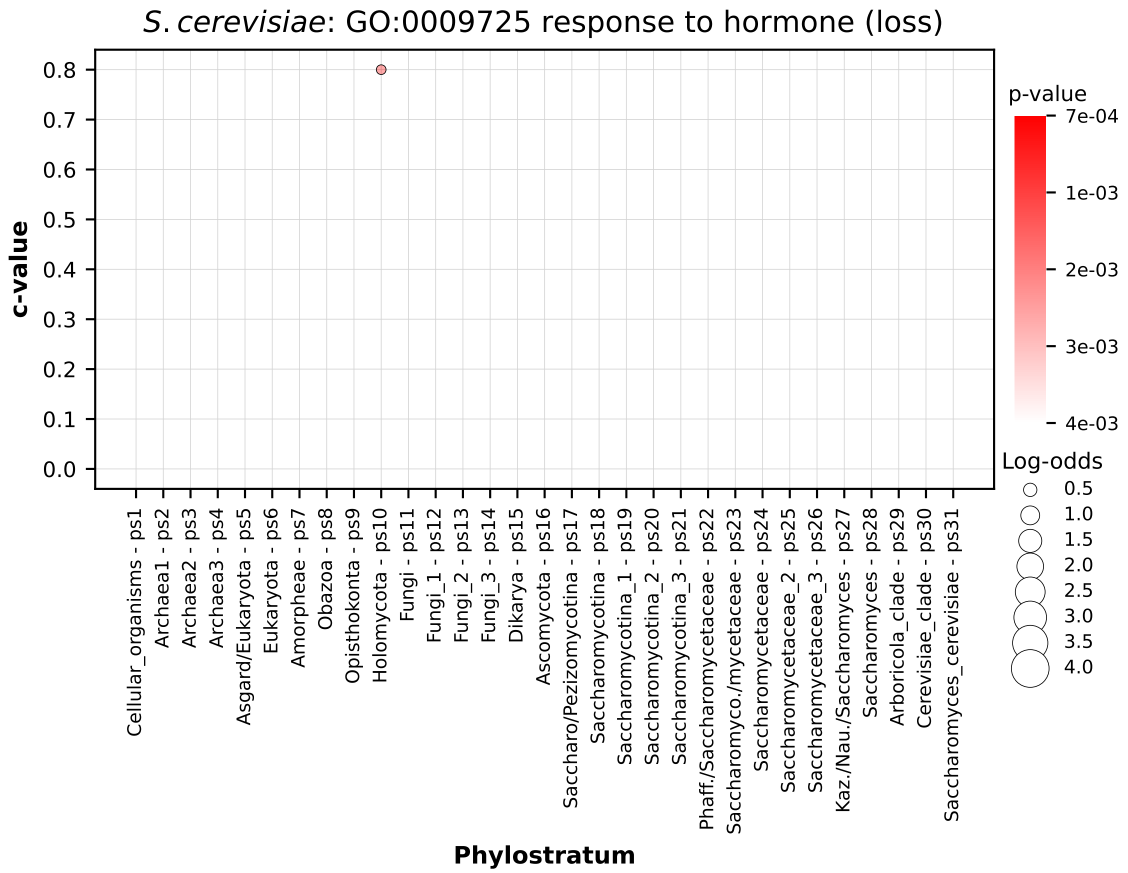

S. cerevisiae: GO:0009725 response to hormone (loss)
c-value
0.0
0.1
0.2
0.3
0.4
0.5
0.6
0.7
0.8
p-value
7e-04
1e-03
2e-03
3e-03
4e-03
Log-odds
0.5
1.0
1.5
2.0
2.5
3.0
3.5
4.0
Cellular_organisms - ps1
Archaea1 - ps2
Archaea2 - ps3
Archaea3 - ps4
Asgard/Eukaryota - ps5
Eukaryota - ps6
Amorpheae - ps7
Obazoa - ps8
Opisthokonta - ps9
Holomycota - ps10
Fungi - ps11
Fungi_1 - ps12
Fungi_2 - ps13
Fungi_3 - ps14
Dikarya - ps15
Ascomycota - ps16
Saccharo/Pezizomycotina - ps17
Saccharomycotina - ps18
Saccharomycotina_1 - ps19
Saccharomycotina_2 - ps20
Saccharomycotina_3 - ps21
Phaff./Saccharomycetaceae - ps22
Saccharomyco./mycetaceae - ps23
Saccharomycetaceae - ps24
Saccharomycetaceae_2 - ps25
Saccharomycetaceae_3 - ps26
Kaz./Nau./Saccharomyces - ps27
Saccharomyces - ps28
Arboricola_clade - ps29
Cerevisiae_clade - ps30
Saccharomyces_cerevisiae - ps31
Phylostratum

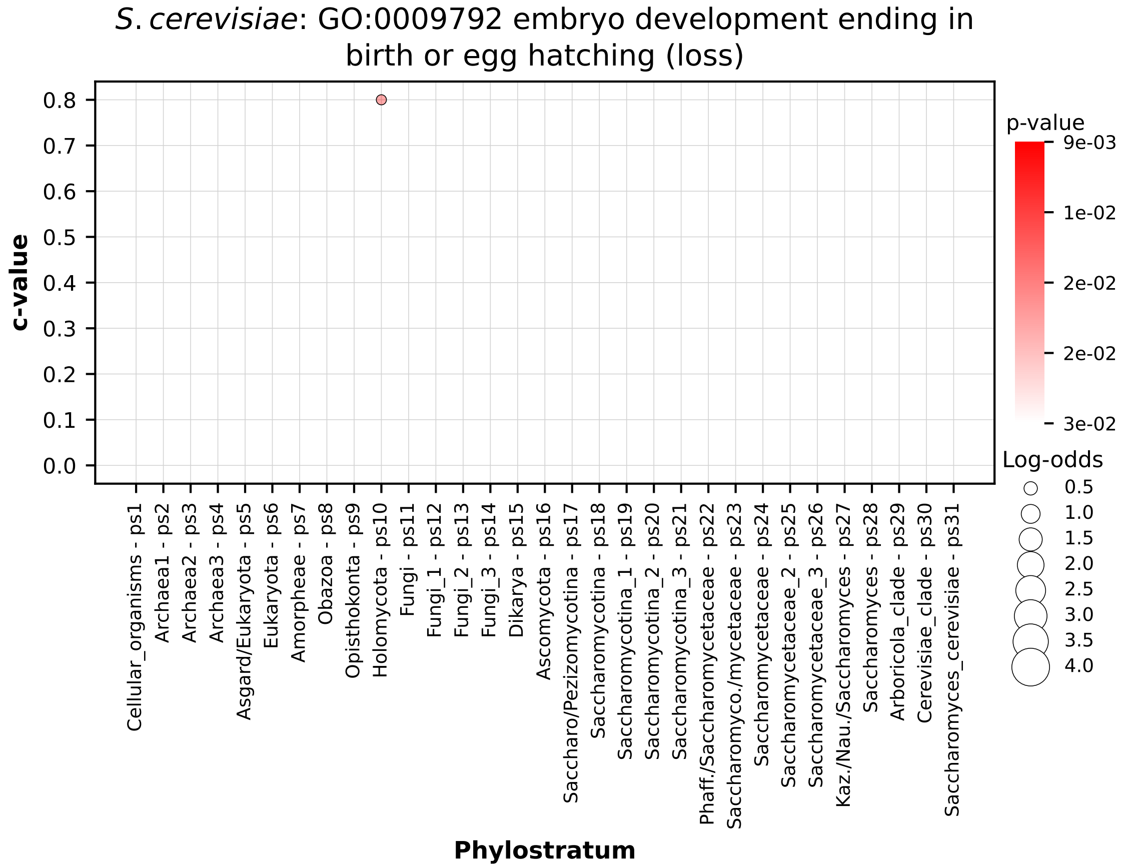

S. cerevisiae: GO:0009792 embryo development ending in birth or egg hatching (loss)
c-value
0.0
0.1
0.2
0.3
0.4
0.5
0.6
0.7
0.8
Phylostratum
Cellular_organisms - ps1
Archaea1 - ps2
Archaea2 - ps3
Archaea3 - ps4
Asgard/Eukaryota - ps5
Eukaryota - ps6
Amorpheae - ps7
Obazoa - ps8
Opisthokonta - ps9
Holomycota - ps10
Fungi - ps11
Fungi_1 - ps12
Fungi_2 - ps13
Fungi_3 - ps14
Dikarya - ps15
Ascomycota - ps16
Saccharo/Pezizomycotina - ps17
Saccharomycotina - ps18
Saccharomycotina_1 - ps19
Saccharomycotina_2 - ps20
Saccharomycotina_3 - ps21
Phaff./Saccharomycetaceae - ps22
Saccharomyco./mycetaceae - ps23
Saccharomycetaceae - ps24
Saccharomycetaceae_2 - ps25
Saccharomycetaceae_3 - ps26
Kaz./Nau./Saccharomyces - ps27
Saccharomyces - ps28
Arboricola_clade - ps29
Cerevisiae_clade - ps30
Saccharomyces_cerevisiae - ps31
p-value
9e-03
1e-02
2e-02
2e-02
3e-02
Log-odds
0.5
1.0
1.5
2.0
2.5
3.0
3.5
4.0

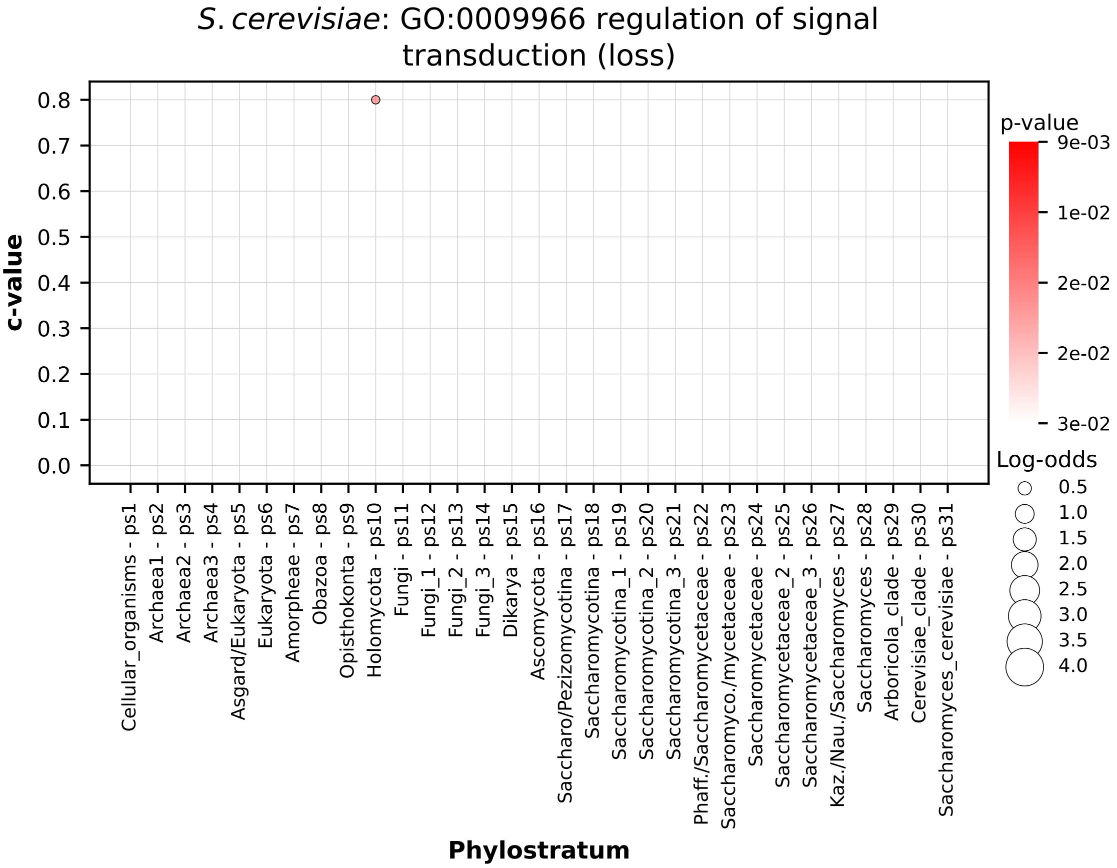

S. cerevisiae: GO:0009966 regulation of signal transduction (loss)
c-value
0.0
0.1
0.2
0.3
0.4
0.5
0.6
0.7
0.8
p-value
9e-03
1e-02
2e-02
2e-02
3e-02
Log-odds
0.5
1.0
1.5
2.0
2.5
3.0
3.5
4.0
Cellular_organisms - ps1
Archaea1 - ps2
Archaea2 - ps3
Archaea3 - ps4
Asgard/Eukaryota - ps5
Eukaryota - ps6
Amorpheae - ps7
Obazoa - ps8
Opisthokonta - ps9
Holomycota - ps10
Fungi - ps11
Fungi_1 - ps12
Fungi_2 - ps13
Fungi_3 - ps14
Dikarya - ps15
Ascomycota - ps16
Saccharo/Pezizomycotina - ps17
Saccharomycotina - ps18
Saccharomycotina_1 - ps19
Saccharomycotina_2 - ps20
Saccharomycotina_3 - ps21
Phaff./Saccharomycetaceae - ps22
Saccharomyco./mycetaceae - ps23
Saccharomycetaceae - ps24
Saccharomycetaceae_2 - ps25
Saccharomycetaceae_3 - ps26
Kaz./Nau./Saccharomyces - ps27
Saccharomyces - ps28
Arboricola_clade - ps29
Cerevisiae_clade - ps30
Saccharomyces_cerevisiae - ps31
Phylostratum

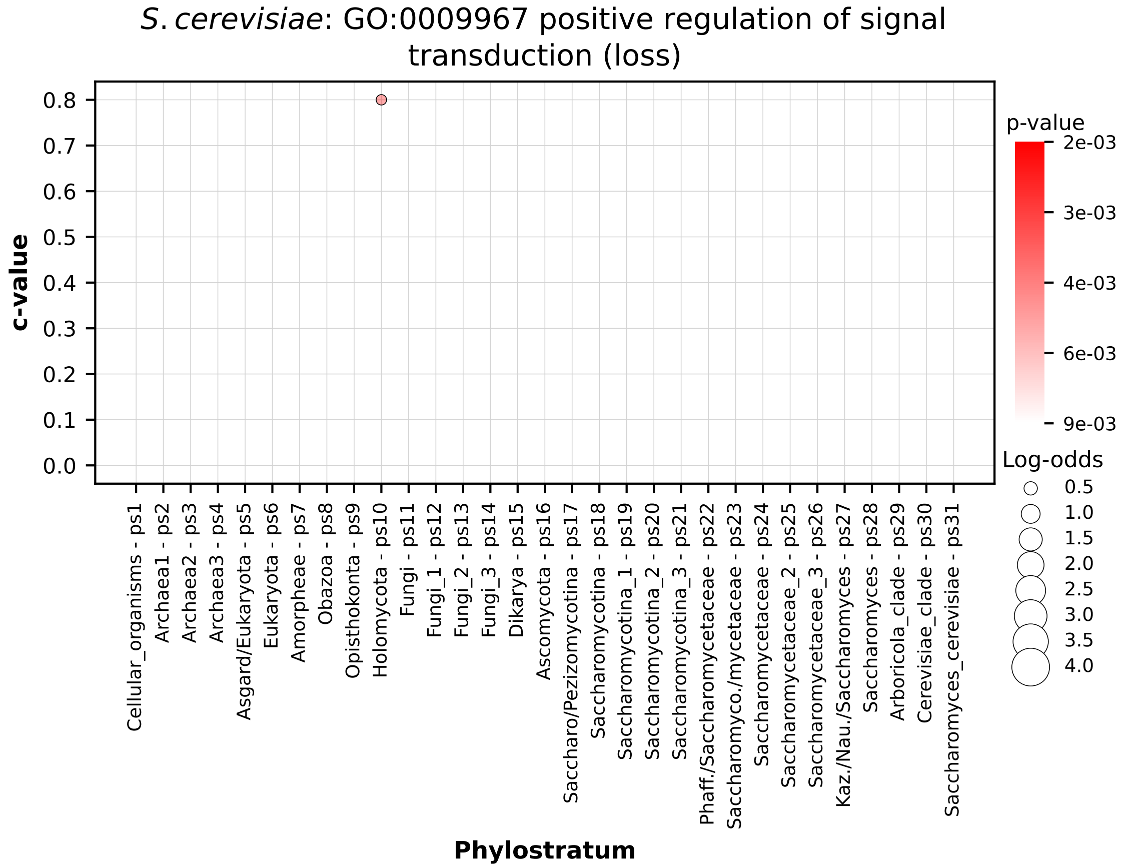
S. cerevisiae: GO:0009967 positive regulation of signal transduction (loss)
c-value
0.0
0.1
0.2
0.3
0.4
0.5
0.6
0.7
0.8
p-value
2e-03
3e-03
4e-03
6e-03
9e-03
Log-odds
0.5
1.0
1.5
2.0
2.5
3.0
3.5
4.0
Cellular_organisms - ps1
Archaea1 - ps2
Archaea2 - ps3
Archaea3 - ps4
Asgard/Eukaryota - ps5
Eukaryota - ps6
Amorpheae - ps7
Obazoa - ps8
Opisthokonta - ps9
Holomycota - ps10
Fungi - ps11
Fungi_1 - ps12
Fungi_2 - ps13
Fungi_3 - ps14
Dikarya - ps15
Ascomycota - ps16
Saccharo/Pezizomycotina - ps17
Saccharomycotina - ps18
Saccharomycotina_1 - ps19
Saccharomycotina_2 - ps20
Saccharomycotina_3 - ps21
Phaff./Saccharomycetaceae - ps22
Saccharomyco./mycetaceae - ps23
Saccharomycetaceae - ps24
Saccharomycetaceae_2 - ps25
Saccharomycetaceae_3 - ps26
Kaz./Nau./Saccharomyces - ps27
Saccharomyces - ps28
Arboricola_clade - ps29
Cerevisiae_clade - ps30
Saccharomyces_cerevisiae - ps31
Phylostratum

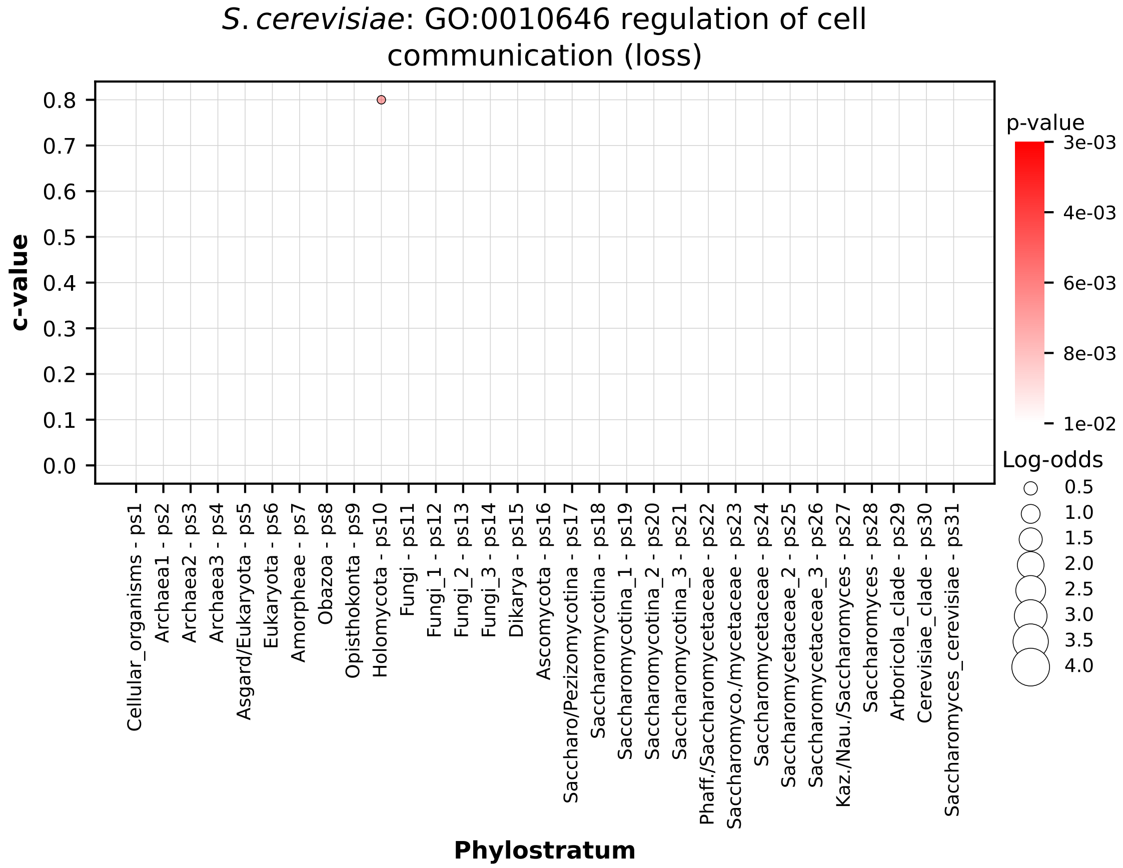
S. cerevisiae: GO:0010646 regulation of cell communication (loss)
c-value
0.0
0.1
0.2
0.3
0.4
0.5
0.6
0.7
0.8
Phylostratum
Cellular_organisms - ps1
Archaea1 - ps2
Archaea2 - ps3
Archaea3 - ps4
Asgard/Eukaryota - ps5
Eukaryota - ps6
Amorpheae - ps7
Obazoa - ps8
Opisthokonta - ps9
Holomycota - ps10
Fungi - ps11
Fungi_1 - ps12
Fungi_2 - ps13
Fungi_3 - ps14
Dikarya - ps15
Ascomycota - ps16
Saccharo/Pezizomycotina - ps17
Saccharomycotina - ps18
Saccharomycotina_1 - ps19
Saccharomycotina_2 - ps20
Saccharomycotina_3 - ps21
Phaff./Saccharomycetaceae - ps22
Saccharomyco./mycetaceae - ps23
Saccharomycetaceae - ps24
Saccharomycetaceae_2 - ps25
Saccharomycetaceae_3 - ps26
Kaz./Nau./Saccharomyces - ps27
Saccharomyces - ps28
Arboricola_clade - ps29
Cerevisiae_clade - ps30
Saccharomyces_cerevisiae - ps31
p-value
3e-03
4e-03
6e-03
8e-03
1e-02
Log-odds
0.5
1.0
1.5
2.0
2.5
3.0
3.5
4.0

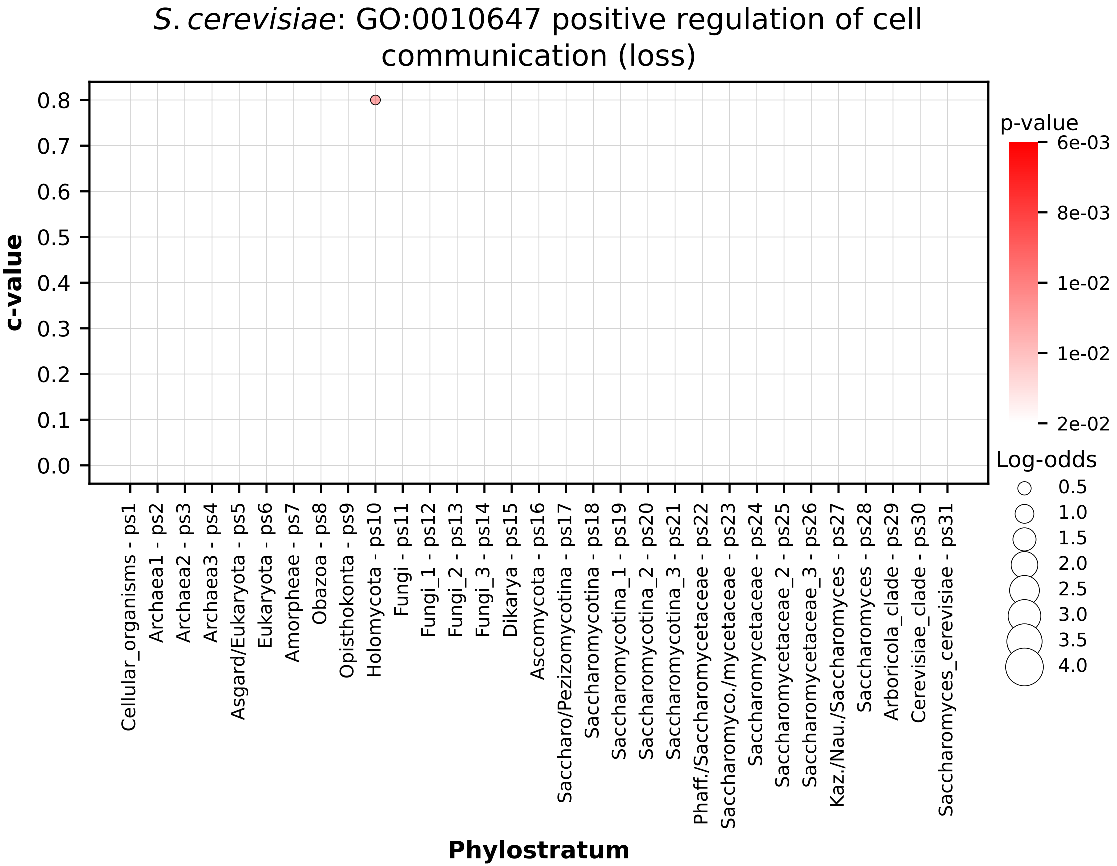

S. cerevisiae: GO:0010647 positive regulation of cell communication (loss)
c-value
0.0
0.1
0.2
0.3
0.4
0.5
0.6
0.7
0.8
p-value
6e-03
8e-03
1e-02
1e-02
2e-02
Log-odds
0.5
1.0
1.5
2.0
2.5
3.0
3.5
4.0
Cellular_organisms - ps1
Archaea1 - ps2
Archaea2 - ps3
Archaea3 - ps4
Asgard/Eukaryota - ps5
Eukaryota - ps6
Amorpheae - ps7
Obazoa - ps8
Opisthokonta - ps9
Holomycota - ps10
Fungi - ps11
Fungi_1 - ps12
Fungi_2 - ps13
Fungi_3 - ps14
Dikarya - ps15
Ascomycota - ps16
Saccharo/Pezizomycotina - ps17
Saccharomycotina - ps18
Saccharomycotina_1 - ps19
Saccharomycotina_2 - ps20
Saccharomycotina_3 - ps21
Phaff./Saccharomycetaceae - ps22
Saccharomyco./mycetaceae - ps23
Saccharomycetaceae - ps24
Saccharomycetaceae_2 - ps25
Saccharomycetaceae_3 - ps26
Kaz./Nau./Saccharomyces - ps27
Saccharomyces - ps28
Arboricola_clade - ps29
Cerevisiae_clade - ps30
Saccharomyces_cerevisiae - ps31
Phylostratum

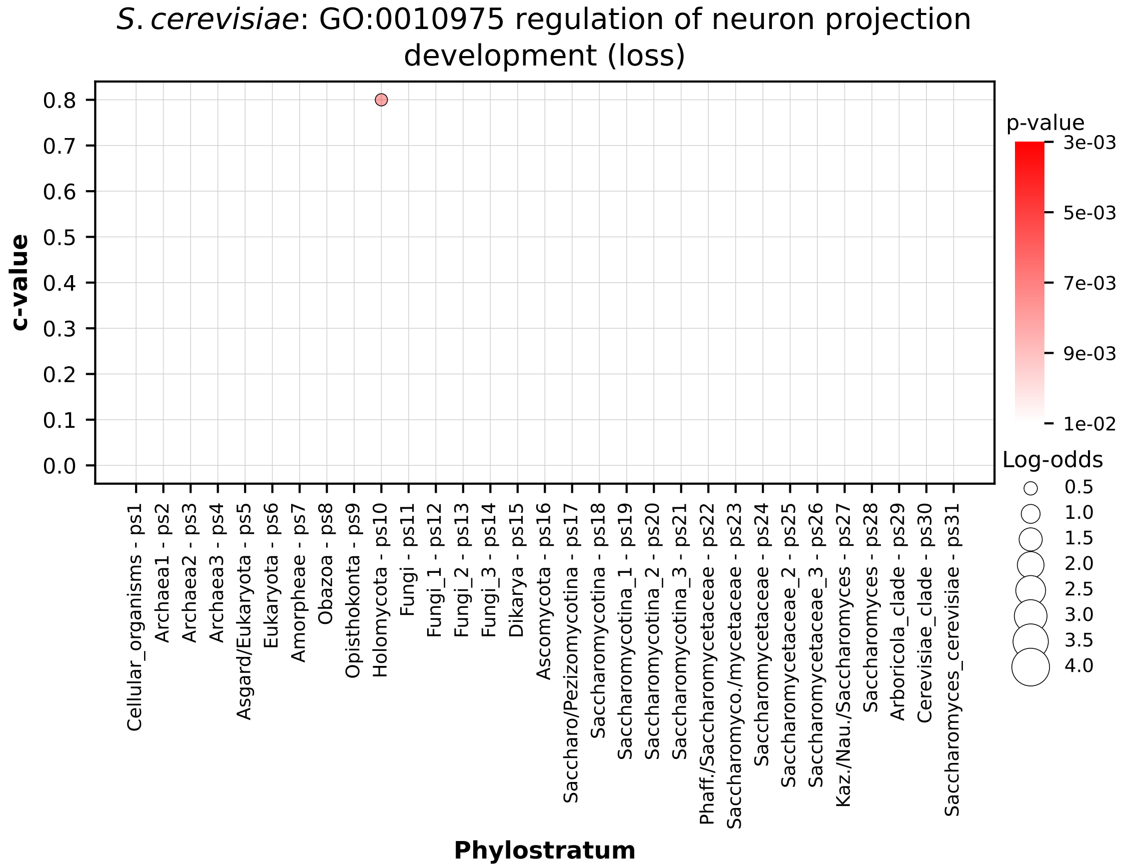

S. cerevisiae: GO:0010975 regulation of neuron projection development (loss)
c-value
0.0
0.1
0.2
0.3
0.4
0.5
0.6
0.7
0.8
p-value
3e-03
5e-03
7e-03
9e-03
1e-02
Log-odds
0.5
1.0
1.5
2.0
2.5
3.0
3.5
4.0
Cellular_organisms - ps1
Archaea1 - ps2
Archaea2 - ps3
Archaea3 - ps4
Asgard/Eukaryota - ps5
Eukaryota - ps6
Amorpheae - ps7
Obazoa - ps8
Opisthokonta - ps9
Holomycota - ps10
Fungi - ps11
Fungi_1 - ps12
Fungi_2 - ps13
Fungi_3 - ps14
Dikarya - ps15
Ascomycota - ps16
Saccharo/Pezizomycotina - ps17
Saccharomycotina - ps18
Saccharomycotina_1 - ps19
Saccharomycotina_2 - ps20
Saccharomycotina_3 - ps21
Phaff./Saccharomycetaceae - ps22
Saccharomyco./mycetaceae - ps23
Saccharomycetaceae - ps24
Saccharomycetaceae_2 - ps25
Saccharomycetaceae_3 - ps26
Kaz./Nau./Saccharomyces - ps27
Saccharomyces - ps28
Arboricola_clade - ps29
Cerevisiae_clade - ps30
Saccharomyces_cerevisiae - ps31
Phylostratum

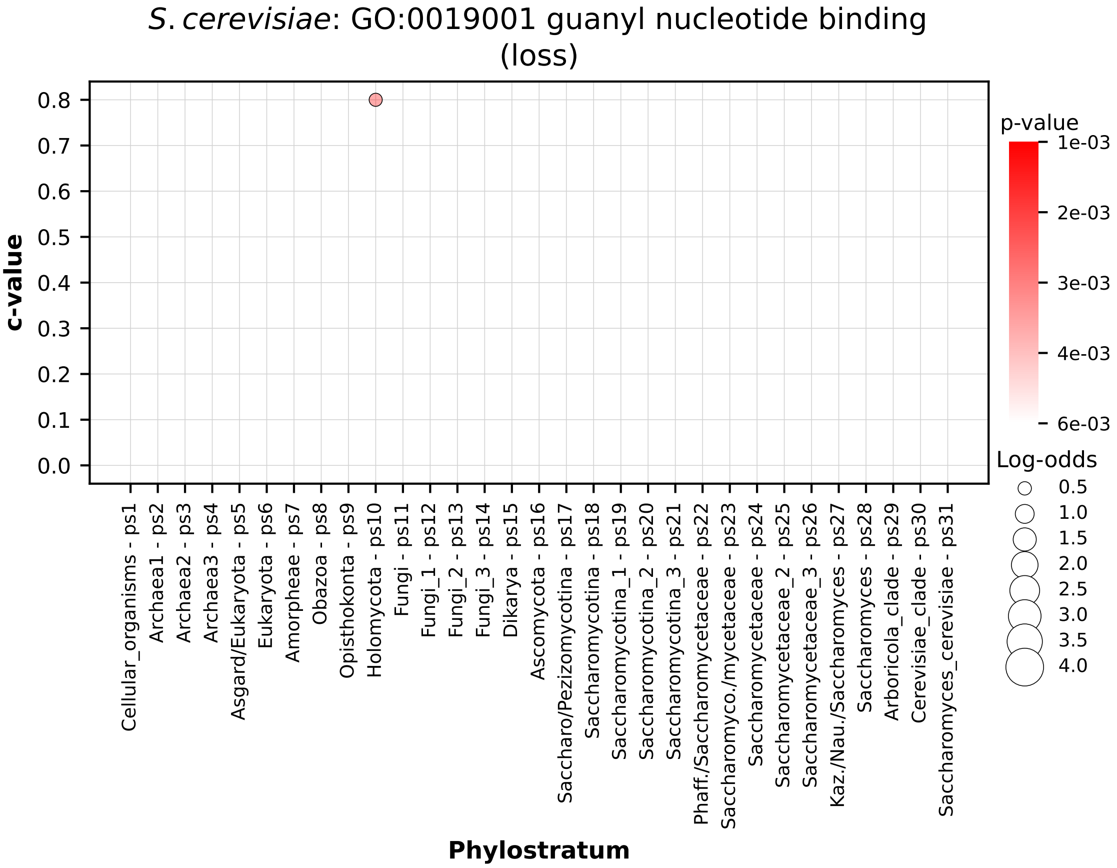
S. cerevisiae: GO:0019001 guanyl nucleotide binding
(loss)
c-value
0.0
0.1
0.2
0.3
0.4
0.5
0.6
0.7
0.8
Phylostratum
Cellular_organisms - ps1
Archaea1 - ps2
Archaea2 - ps3
Archaea3 - ps4
Asgard/Eukaryota - ps5
Eukaryota - ps6
Amorpheae - ps7
Obazoa - ps8
Opisthokonta - ps9
Holomycota - ps10
Fungi - ps11
Fungi_1 - ps12
Fungi_2 - ps13
Fungi_3 - ps14
Dikarya - ps15
Ascomycota - ps16
Saccharo/Pezizomycotina - ps17
Saccharomycotina - ps18
Saccharomycotina_1 - ps19
Saccharomycotina_2 - ps20
Saccharomycotina_3 - ps21
Phaff./Saccharomycetaceae - ps22
Saccharomyco./mycetaceae - ps23
Saccharomycetaceae - ps24
Saccharomycetaceae_2 - ps25
Saccharomycetaceae_3 - ps26
Kaz./Nau./Saccharomyces - ps27
Saccharomyces - ps28
Arboricola_clade - ps29
Cerevisiae_clade - ps30
Saccharomyces_cerevisiae - ps31
p-value
1e-03
2e-03
3e-03
4e-03
6e-03
Log-odds
0.5
1.0
1.5
2.0
2.5
3.0
3.5
4.0

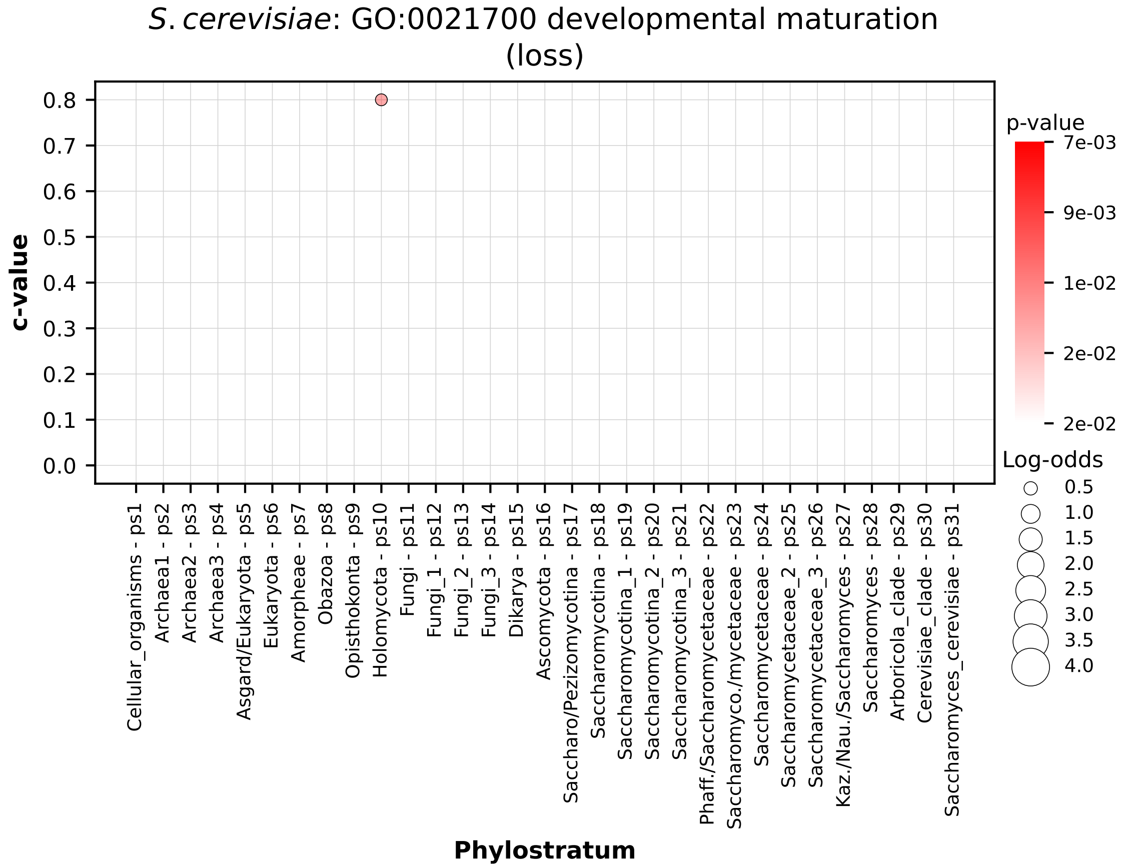
S. cerevisiae: GO:0021700 developmental maturation
(loss)
c-value
0.0
0.1
0.2
0.3
0.4
0.5
0.6
0.7
0.8
Phylostratum
Cellular_organisms - ps1
Archaea1 - ps2
Archaea2 - ps3
Archaea3 - ps4
Asgard/Eukaryota - ps5
Eukaryota - ps6
Amorpheae - ps7
Obazoa - ps8
Opisthokonta - ps9
Holomycota - ps10
Fungi - ps11
Fungi_1 - ps12
Fungi_2 - ps13
Fungi_3 - ps14
Dikarya - ps15
Ascomycota - ps16
Saccharo/Pezizomycotina - ps17
Saccharomycotina - ps18
Saccharomycotina_1 - ps19
Saccharomycotina_2 - ps20
Saccharomycotina_3 - ps21
Phaff./Saccharomycetaceae - ps22
Saccharomyco./mycetaceae - ps23
Saccharomycetaceae - ps24
Saccharomycetaceae_2 - ps25
Saccharomycetaceae_3 - ps26
Kaz./Nau./Saccharomyces - ps27
Saccharomyces - ps28
Arboricola_clade - ps29
Cerevisiae_clade - ps30
Saccharomyces_cerevisiae - ps31
p-value
7e-03
9e-03
1e-02
2e-02
2e-02
Log-odds
0.5
1.0
1.5
2.0
2.5
3.0
3.5
4.0

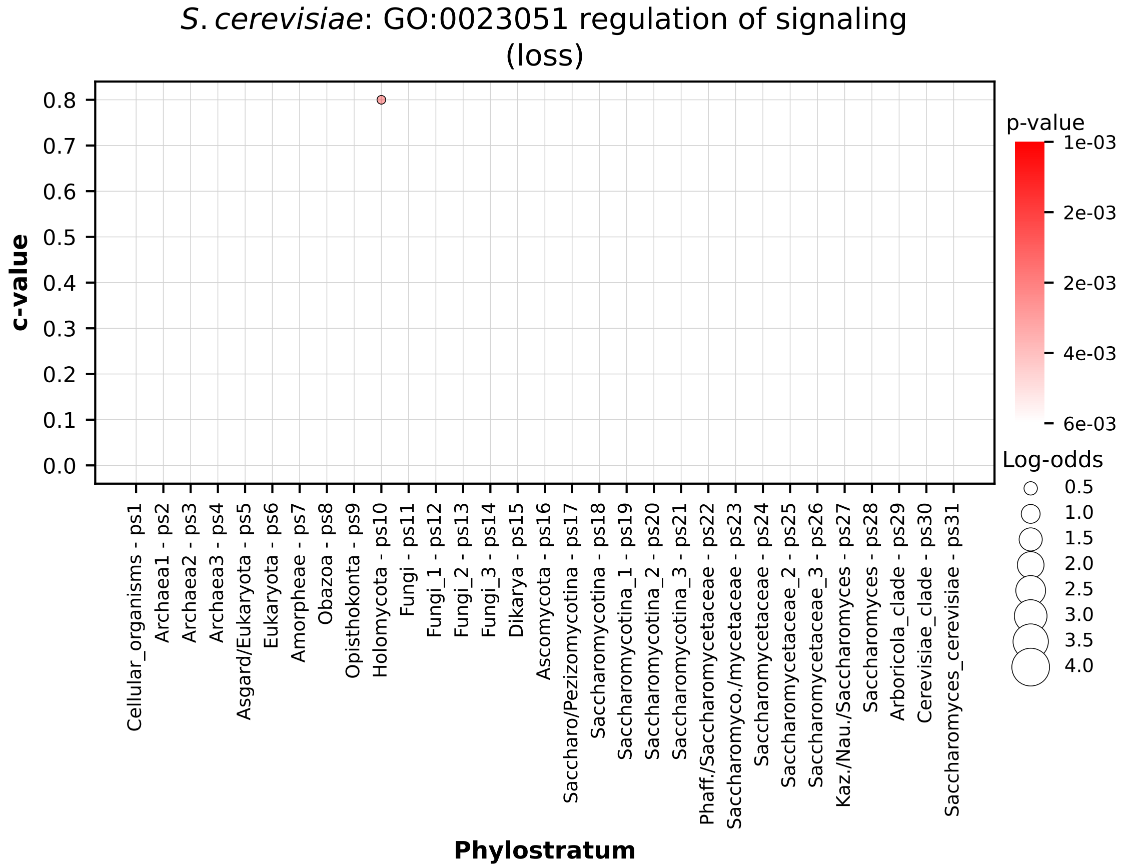

S. cerevisiae: GO:0023051 regulation of signaling
(loss)
c-value
0.0
0.1
0.2
0.3
0.4
0.5
0.6
0.7
0.8
p-value
1e-03
2e-03
2e-03
4e-03
6e-03
Log-odds
0.5
1.0
1.5
2.0
2.5
3.0
3.5
4.0
Cellular_organisms - ps1
Archaea1 - ps2
Archaea2 - ps3
Archaea3 - ps4
Asgard/Eukaryota - ps5
Eukaryota - ps6
Amorpheae - ps7
Obazoa - ps8
Opisthokonta - ps9
Holomycota - ps10
Fungi - ps11
Fungi_1 - ps12
Fungi_2 - ps13
Fungi_3 - ps14
Dikarya - ps15
Ascomycota - ps16
Saccharo/Pezizomycotina - ps17
Saccharomycotina - ps18
Saccharomycotina_1 - ps19
Saccharomycotina_2 - ps20
Saccharomycotina_3 - ps21
Phaff./Saccharomycetaceae - ps22
Saccharomyco./mycetaceae - ps23
Saccharomycetaceae - ps24
Saccharomycetaceae_2 - ps25
Saccharomycetaceae_3 - ps26
Kaz./Nau./Saccharomyces - ps27
Saccharomyces - ps28
Arboricola_clade - ps29
Cerevisiae_clade - ps30
Saccharomyces_cerevisiae - ps31
Phylostratum

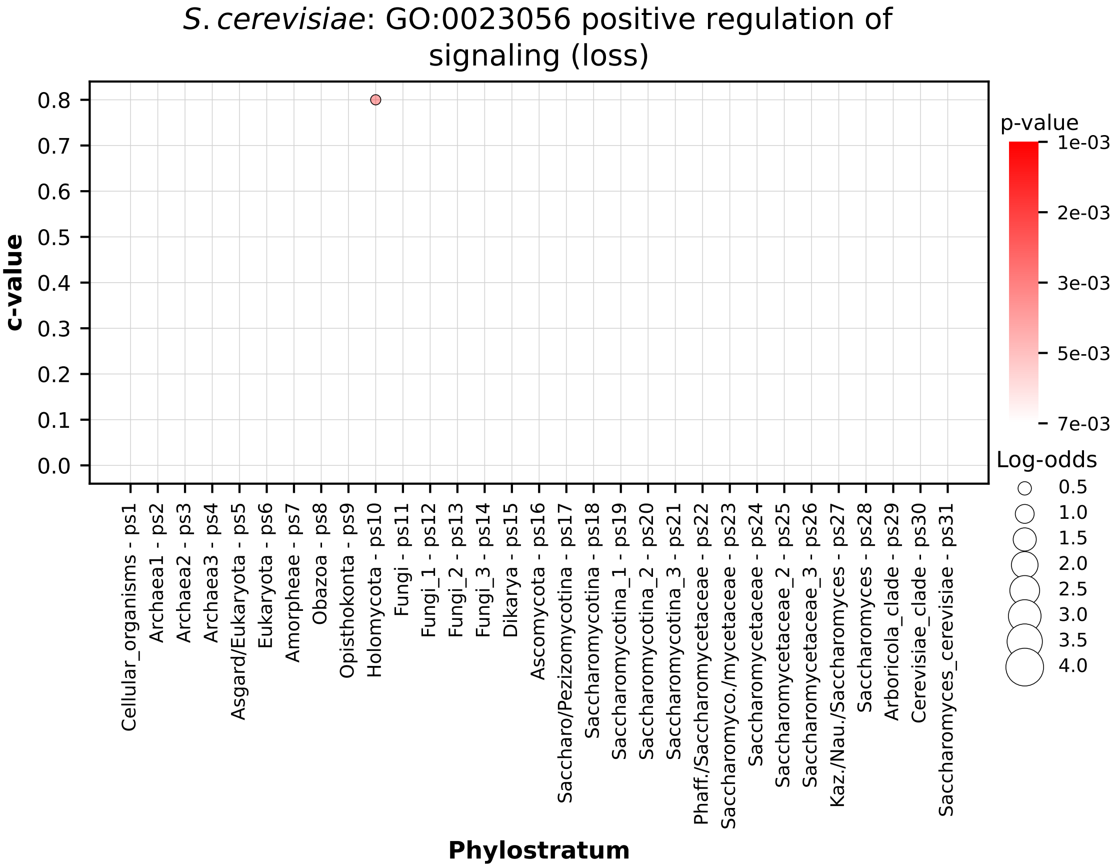
S. cerevisiae: GO:0023056 positive regulation of signaling (loss)
c-value
0.0
0.1
0.2
0.3
0.4
0.5
0.6
0.7
0.8
Phylostratum
Cellular_organisms - ps1
Archaea1 - ps2
Archaea2 - ps3
Archaea3 - ps4
Asgard/Eukaryota - ps5
Eukaryota - ps6
Amorpheae - ps7
Obazoa - ps8
Opisthokonta - ps9
Holomycota - ps10
Fungi - ps11
Fungi_1 - ps12
Fungi_2 - ps13
Fungi_3 - ps14
Dikarya - ps15
Ascomycota - ps16
Saccharo/Pezizomycotina - ps17
Saccharomycotina - ps18
Saccharomycotina_1 - ps19
Saccharomycotina_2 - ps20
Saccharomycotina_3 - ps21
Phaff./Saccharomycetaceae - ps22
Saccharomyco./mycetaceae - ps23
Saccharomycetaceae - ps24
Saccharomycetaceae_2 - ps25
Saccharomycetaceae_3 - ps26
Kaz./Nau./Saccharomyces - ps27
Saccharomyces - ps28
Arboricola_clade - ps29
Cerevisiae_clade - ps30
Saccharomyces_cerevisiae - ps31
p-value
1e-03
2e-03
3e-03
5e-03
7e-03
Log-odds
0.5
1.0
1.5
2.0
2.5
3.0
3.5
4.0

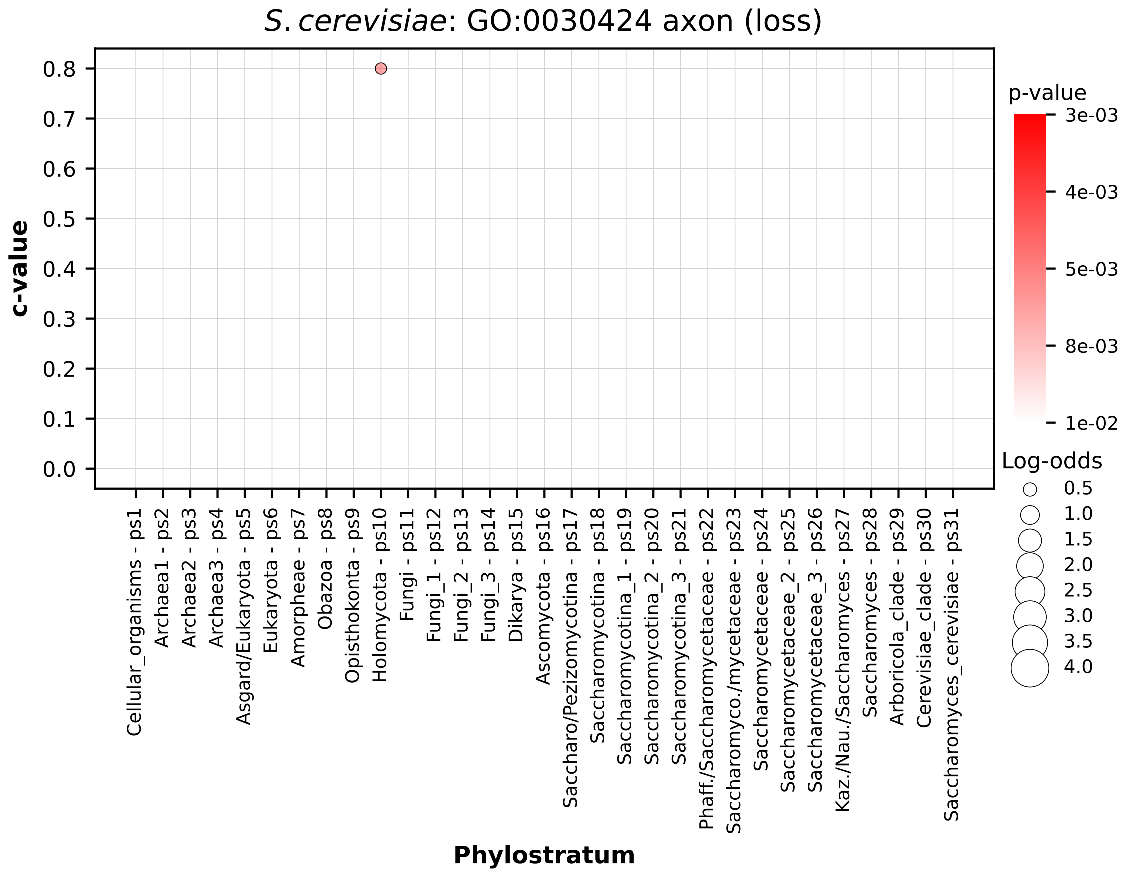

S. cerevisiae: GO:0030424 axon (loss)
c-value
0.8
0.7
0.6
0.5
0.4
0.3
0.2
0.1
0.0
p-value
3e-03
4e-03
5e-03
8e-03
1e-02
Log-odds
0.5
1.0
1.5
2.0
2.5
3.0
3.5
4.0
Cellular_organisms - ps1
Archaea1 - ps2
Archaea2 - ps3
Archaea3 - ps4
Asgard/Eukaryota - ps5
Eukaryota - ps6
Amorpheae - ps7
Obazoa - ps8
Opisthokonta - ps9
Holomycota - ps10
Fungi - ps11
Fungi_1 - ps12
Fungi_2 - ps13
Fungi_3 - ps14
Dikarya - ps15
Ascomycota - ps16
Saccharo/Pezizomycotina - ps17
Saccharomycotina - ps18
Saccharomycotina_1 - ps19
Saccharomycotina_2 - ps20
Saccharomycotina_3 - ps21
Phaff./Saccharomycetaceae - ps22
Saccharomyco./mycetaceae - ps23
Saccharomycetaceae - ps24
Saccharomycetaceae_2 - ps25
Saccharomycetaceae_3 - ps26
Kaz./Nau./Saccharomyces - ps27
Saccharomyces - ps28
Arboricola_clade - ps29
Cerevisiae_clade - ps30
Saccharomyces_cerevisiae - ps31
Phylostratum

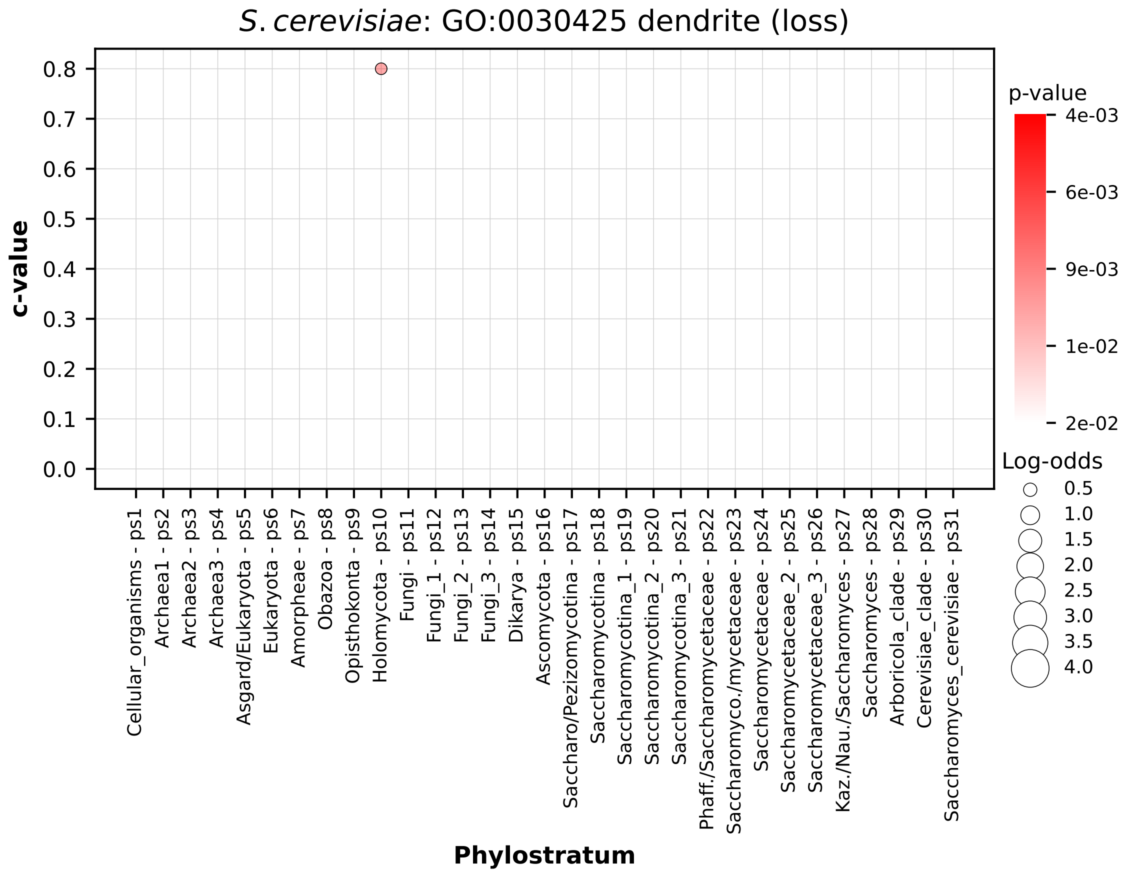
S. cerevisiae: GO:0030425 dendrite (loss)
c-value
0.0
0.1
0.2
0.3
0.4
0.5
0.6
0.7
0.8
Phylostratum
Cellular_organisms - ps1
Archaea1 - ps2
Archaea2 - ps3
Archaea3 - ps4
Asgard/Eukaryota - ps5
Eukaryota - ps6
Amorpheae - ps7
Obazoa - ps8
Opisthokonta - ps9
Holomycota - ps10
Fungi - ps11
Fungi_1 - ps12
Fungi_2 - ps13
Fungi_3 - ps14
Dikarya - ps15
Ascomycota - ps16
Saccharo/Pezizomycotina - ps17
Saccharomycotina - ps18
Saccharomycotina_1 - ps19
Saccharomycotina_2 - ps20
Saccharomycotina_3 - ps21
Phaff./Saccharomycetaceae - ps22
Saccharomyco./mycetaceae - ps23
Saccharomycetaceae - ps24
Saccharomycetaceae_2 - ps25
Saccharomycetaceae_3 - ps26
Kaz./Nau./Saccharomyces - ps27
Saccharomyces - ps28
Arboricola_clade - ps29
Cerevisiae_clade - ps30
Saccharomyces_cerevisiae - ps31
p-value
4e-03
6e-03
9e-03
1e-02
2e-02
Log-odds
0.5
1.0
1.5
2.0
2.5
3.0
3.5
4.0

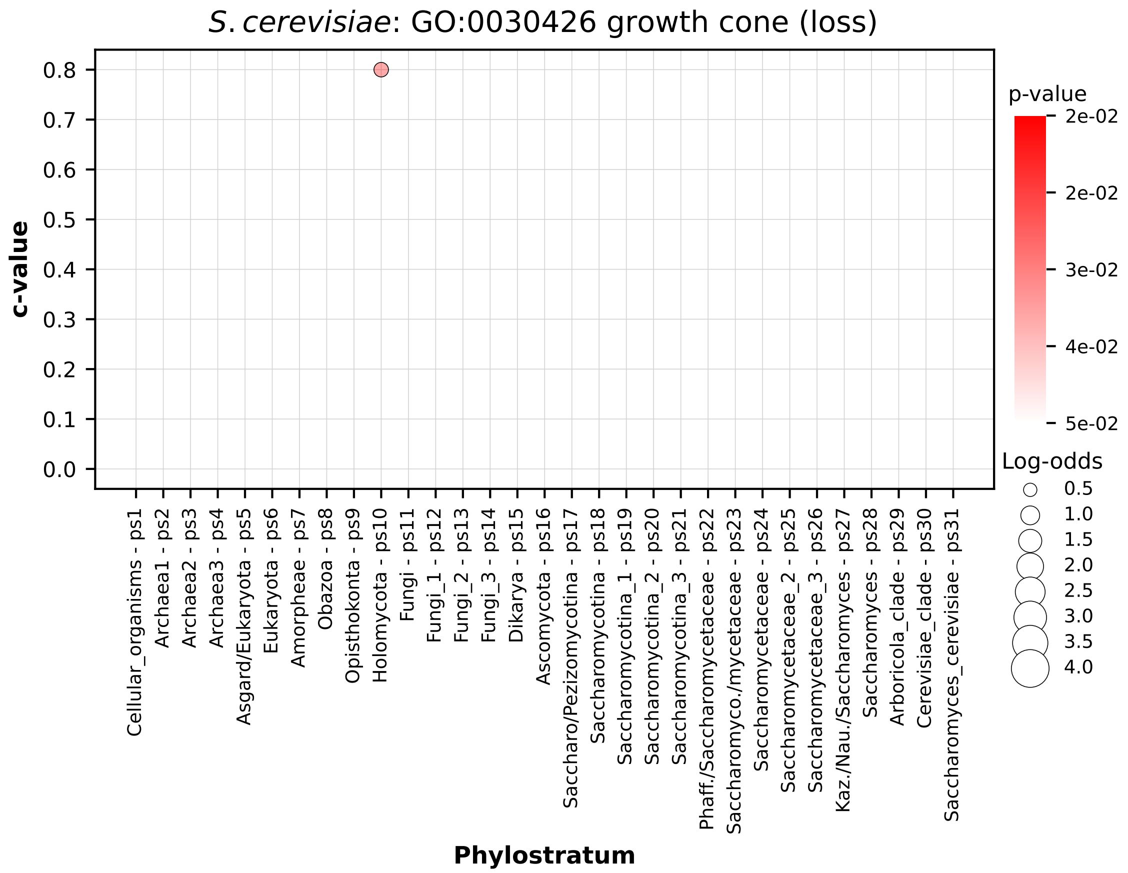

S. cerevisiae: GO:0030426 growth cone (loss)
c-value
0.8
0.7
0.6
0.5
0.4
0.3
0.2
0.1
0.0
p-value
2e-02
2e-02
3e-02
4e-02
5e-02
Log-odds
0.5
1.0
1.5
2.0
2.5
3.0
3.5
4.0
Cellular_organisms - ps1
Archaea1 - ps2
Archaea2 - ps3
Archaea3 - ps4
Asgard/Eukaryota - ps5
Eukaryota - ps6
Amorpheae - ps7
Obazoa - ps8
Opisthokonta - ps9
Holomycota - ps10
Fungi - ps11
Fungi_1 - ps12
Fungi_2 - ps13
Fungi_3 - ps14
Dikarya - ps15
Ascomycota - ps16
Saccharo/Pezizomycotina - ps17
Saccharomycotina - ps18
Saccharomycotina_1 - ps19
Saccharomycotina_2 - ps20
Saccharomycotina_3 - ps21
Phaff./Saccharomycetaceae - ps22
Saccharomyco./mycetaceae - ps23
Saccharomycetaceae - ps24
Saccharomycetaceae_2 - ps25
Saccharomycetaceae_3 - ps26
Kaz./Nau./Saccharomyces - ps27
Saccharomyces - ps28
Arboricola_clade - ps29
Cerevisiae_clade - ps30
Saccharomyces_cerevisiae - ps31
Phylostratum

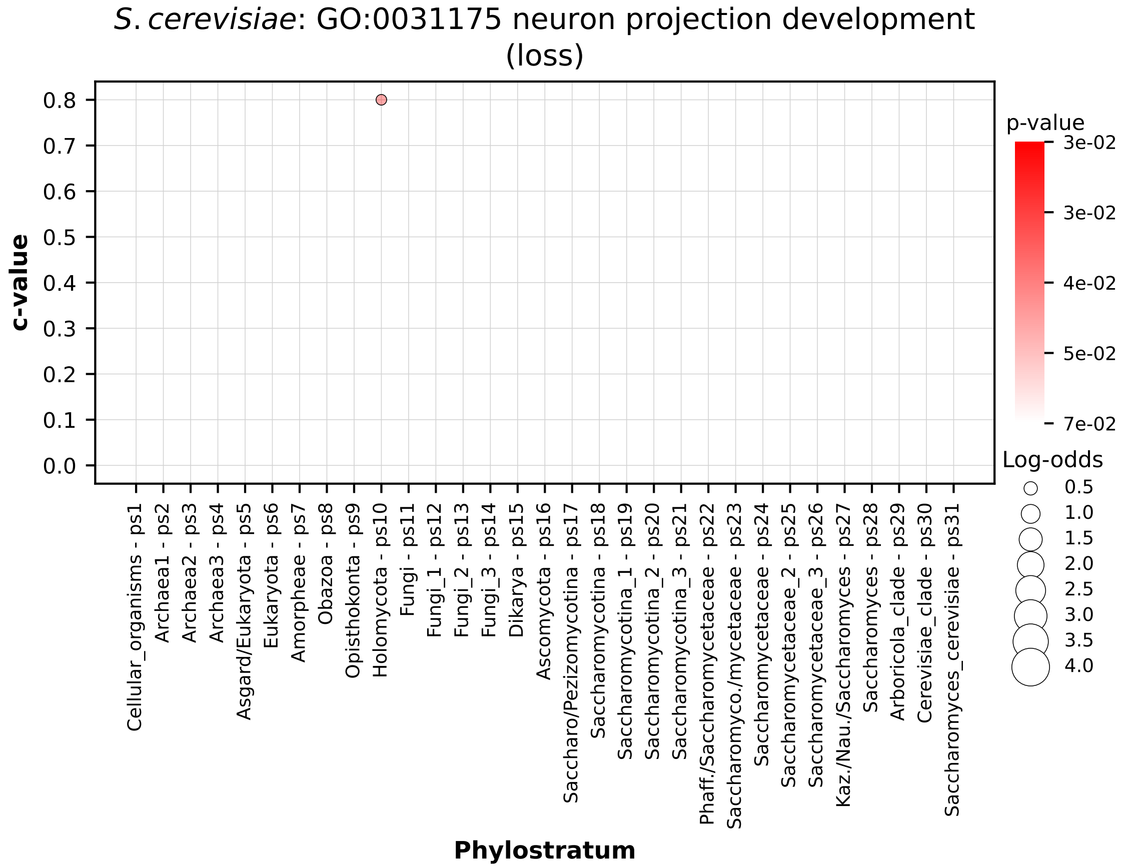

S. cerevisiae: GO:0031175 neuron projection development (loss)
c-value
0.8
0.7
0.6
0.5
0.4
0.3
0.2
0.1
0.0
p-value
3e-02
3e-02
4e-02
5e-02
7e-02
Log-odds
0.5
1.0
1.5
2.0
2.5
3.0
3.5
4.0
Cellular_organisms - ps1
Archaea1 - ps2
Archaea2 - ps3
Archaea3 - ps4
Asgard/Eukaryota - ps5
Eukaryota - ps6
Amorpheae - ps7
Obazoa - ps8
Opisthokonta - ps9
Holomycota - ps10
Fungi - ps11
Fungi_1 - ps12
Fungi_2 - ps13
Fungi_3 - ps14
Dikarya - ps15
Ascomycota - ps16
Saccharo/Pezizomycotina - ps17
Saccharomycotina - ps18
Saccharomycotina_1 - ps19
Saccharomycotina_2 - ps20
Saccharomycotina_3 - ps21
Phaff./Saccharomycetaceae - ps22
Saccharomyco./mycetaceae - ps23
Saccharomycetaceae - ps24
Saccharomycetaceae_2 - ps25
Saccharomycetaceae_3 - ps26
Kaz./Nau./Saccharomyces - ps27
Saccharomyces - ps28
Arboricola_clade - ps29
Cerevisiae_clade - ps30
Saccharomyces_cerevisiae - ps31
Phylostratum

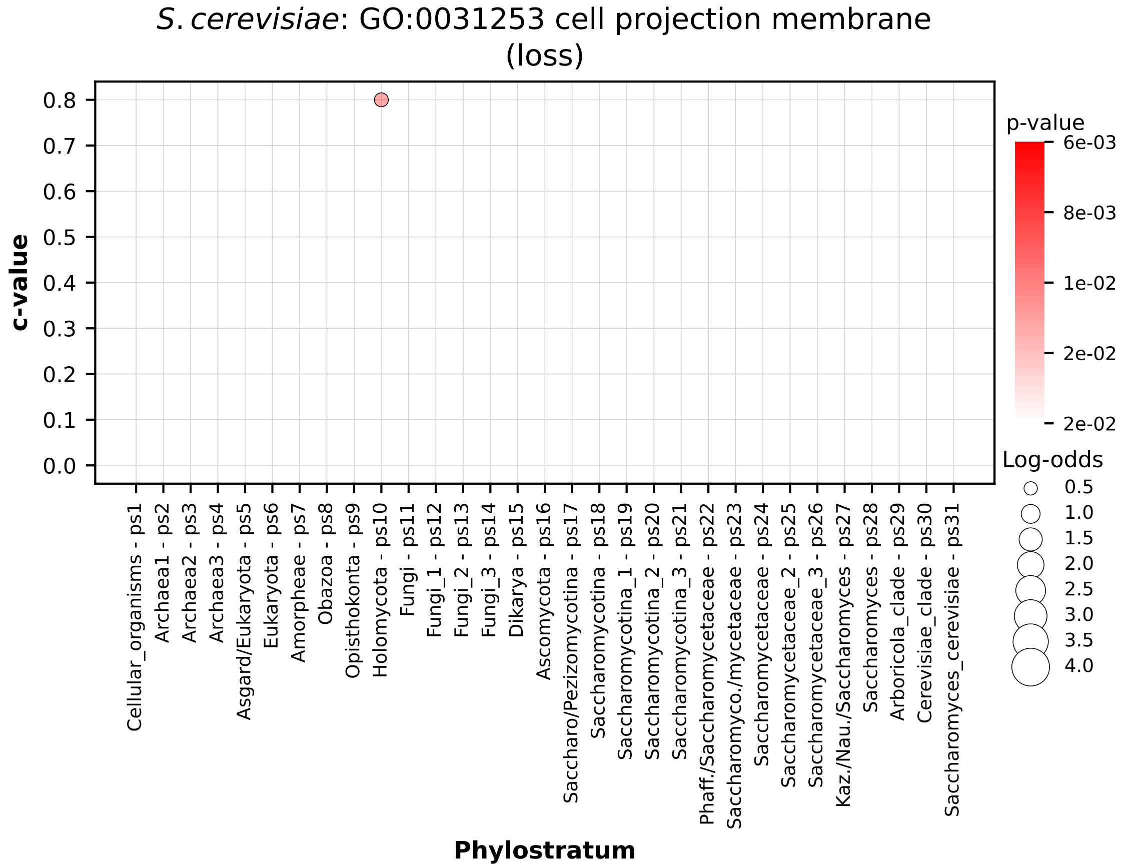

S. cerevisiae: GO:0031253 cell projection membrane
(loss)
c-value
0.0
0.1
0.2
0.3
0.4
0.5
0.6
0.7
0.8
p-value
6e-03
8e-03
1e-02
2e-02
2e-02
Log-odds
0.5
1.0
1.5
2.0
2.5
3.0
3.5
4.0
Cellular_organisms - ps1
Archaea1 - ps2
Archaea2 - ps3
Archaea3 - ps4
Asgard/Eukaryota - ps5
Eukaryota - ps6
Amorpheae - ps7
Obazoa - ps8
Opisthokonta - ps9
Holomycota - ps10
Fungi - ps11
Fungi_1 - ps12
Fungi_2 - ps13
Fungi_3 - ps14
Dikarya - ps15
Ascomycota - ps16
Saccharo/Pezizomycotina - ps17
Saccharomycotina - ps18
Saccharomycotina_1 - ps19
Saccharomycotina_2 - ps20
Saccharomycotina_3 - ps21
Phaff./Saccharomycetaceae - ps22
Saccharomyco./mycetaceae - ps23
Saccharomycetaceae - ps24
Saccharomycetaceae_2 - ps25
Saccharomycetaceae_3 - ps26
Kaz./Nau./Saccharomyces - ps27
Saccharomyces - ps28
Arboricola_clade - ps29
Cerevisiae_clade - ps30
Saccharomyces_cerevisiae - ps31
Phylostratum

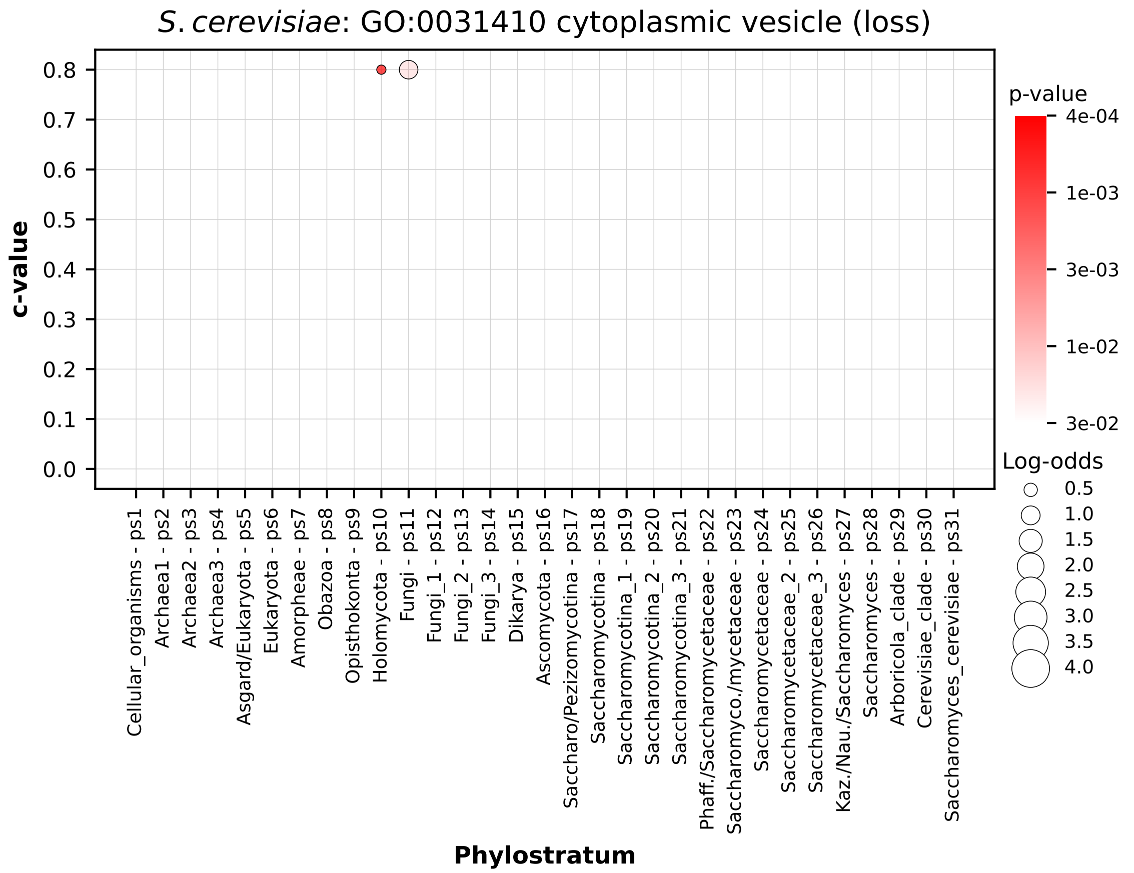

S. cerevisiae: GO:0031410 cytoplasmic vesicle (loss)
c-value
0.8
0.7
0.6
0.5
0.4
0.3
0.2
0.1
0.0
p-value
4e-04
1e-03
3e-03
1e-02
3e-02
Log-odds
0.5
1.0
1.5
2.0
2.5
3.0
3.5
4.0
Cellular_organisms - ps1
Archaea1 - ps2
Archaea2 - ps3
Archaea3 - ps4
Asgard/Eukaryota - ps5
Eukaryota - ps6
Amorpheae - ps7
Obazoa - ps8
Opisthokonta - ps9
Holomycota - ps10
Fungi - ps11
Fungi_1 - ps12
Fungi_2 - ps13
Fungi_3 - ps14
Dikarya - ps15
Ascomycota - ps16
Saccharo/Pezizomycotina - ps17
Saccharomycotina - ps18
Saccharomycotina_1 - ps19
Saccharomycotina_2 - ps20
Saccharomycotina_3 - ps21
Phaff./Saccharomycetaceae - ps22
Saccharomyco./mycetaceae - ps23
Saccharomycetaceae - ps24
Saccharomycetaceae_2 - ps25
Saccharomycetaceae_3 - ps26
Kaz./Nau./Saccharomyces - ps27
Saccharomyces - ps28
Arboricola_clade - ps29
Cerevisiae_clade - ps30
Saccharomyces_cerevisiae - ps31
Phylostratum

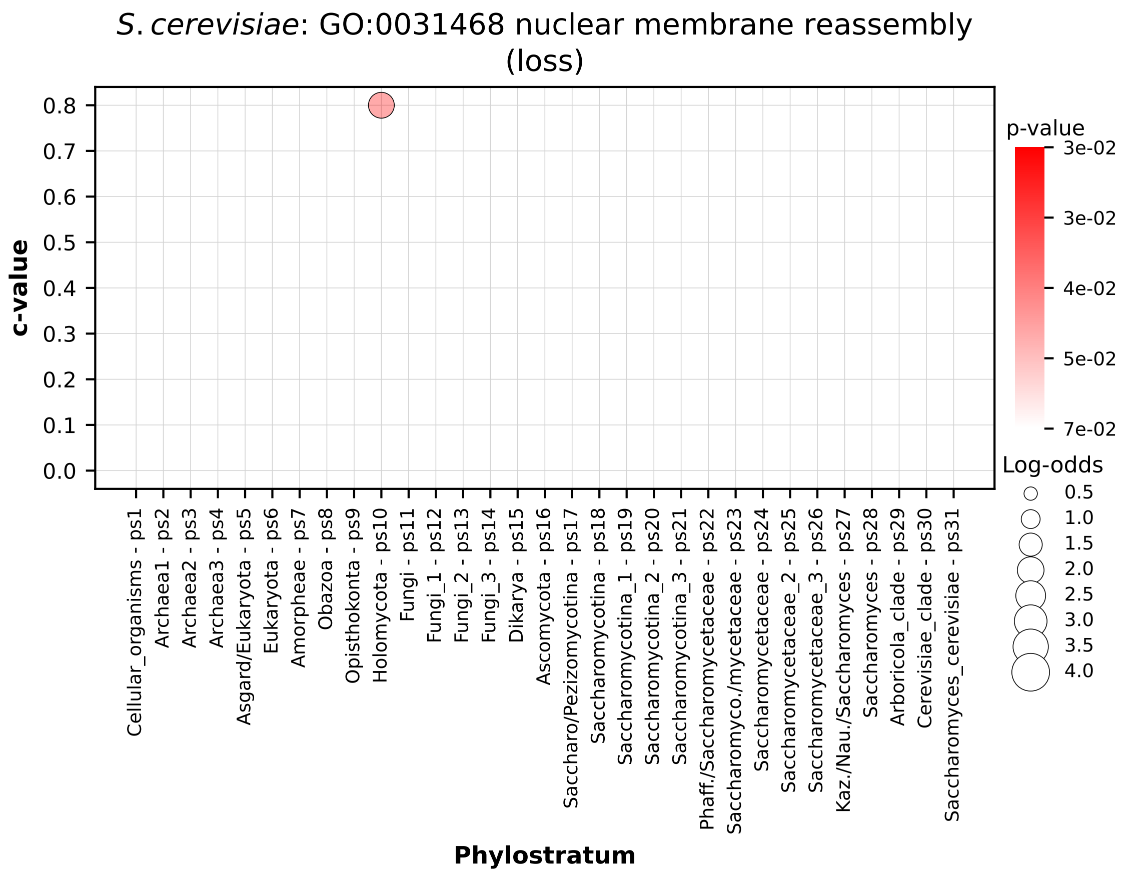

S. cerevisiae: GO:0031468 nuclear membrane reassembly
(loss)
c-value
0.8
0.7
0.6
0.5
0.4
0.3
0.2
0.1
0.0
p-value
3e-02
3e-02
4e-02
5e-02
7e-02
Log-odds
0.5
1.0
1.5
2.0
2.5
3.0
3.5
4.0
Cellular_organisms - ps1
Archaea1 - ps2
Archaea2 - ps3
Archaea3 - ps4
Asgard/Eukaryota - ps5
Eukaryota - ps6
Amorpheae - ps7
Obazoa - ps8
Opisthokonta - ps9
Holomycota - ps10
Fungi - ps11
Fungi_1 - ps12
Fungi_2 - ps13
Fungi_3 - ps14
Dikarya - ps15
Ascomycota - ps16
Saccharo/Pezizomycotina - ps17
Saccharomycotina - ps18
Saccharomycotina_1 - ps19
Saccharomycotina_2 - ps20
Saccharomycotina_3 - ps21
Phaff./Saccharomycetaceae - ps22
Saccharomyco./mycetaceae - ps23
Saccharomycetaceae - ps24
Saccharomycetaceae_2 - ps25
Saccharomycetaceae_3 - ps26
Kaz./Nau./Saccharomyces - ps27
Saccharomyces - ps28
Arboricola_clade - ps29
Cerevisiae_clade - ps30
Saccharomyces_cerevisiae - ps31
Phylostratum

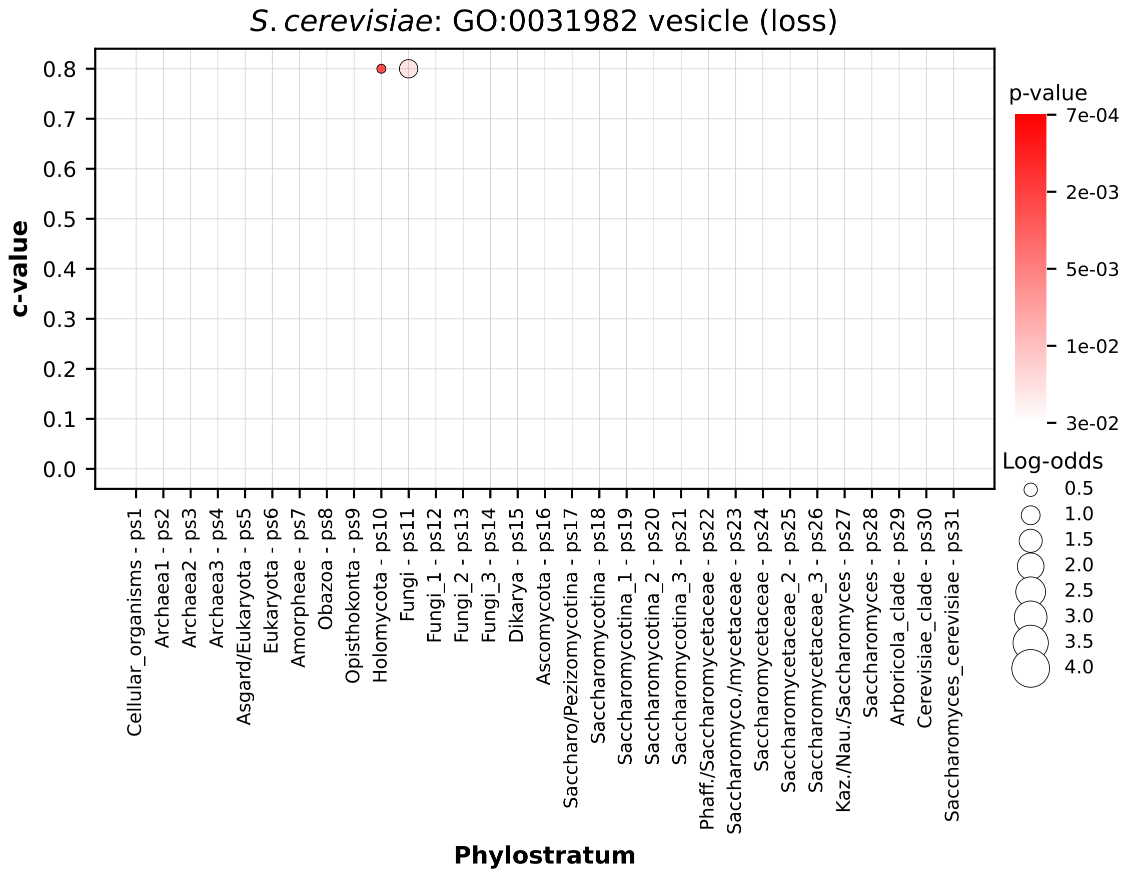

S. cerevisiae: GO:0031982 vesicle (loss)
c-value
0.0
0.1
0.2
0.3
0.4
0.5
0.6
0.7
0.8
p-value
7e-04
2e-03
5e-03
1e-02
3e-02
Log-odds
0.5
1.0
1.5
2.0
2.5
3.0
3.5
4.0
Cellular_organisms - ps1
Archaea1 - ps2
Archaea2 - ps3
Archaea3 - ps4
Asgard/Eukaryota - ps5
Eukaryota - ps6
Amorpheae - ps7
Obazoa - ps8
Opisthokonta - ps9
Holomycota - ps10
Fungi - ps11
Fungi_1 - ps12
Fungi_2 - ps13
Fungi_3 - ps14
Dikarya - ps15
Ascomycota - ps16
Saccharo/Pezizomycotina - ps17
Saccharomycotina - ps18
Saccharomycotina_1 - ps19
Saccharomycotina_2 - ps20
Saccharomycotina_3 - ps21
Phaff./Saccharomycetaceae - ps22
Saccharomyco./mycetaceae - ps23
Saccharomycetaceae - ps24
Saccharomycetaceae_2 - ps25
Saccharomycetaceae_3 - ps26
Kaz./Nau./Saccharomyces - ps27
Saccharomyces - ps28
Arboricola_clade - ps29
Cerevisiae_clade - ps30
Saccharomyces_cerevisiae - ps31
Phylostratum

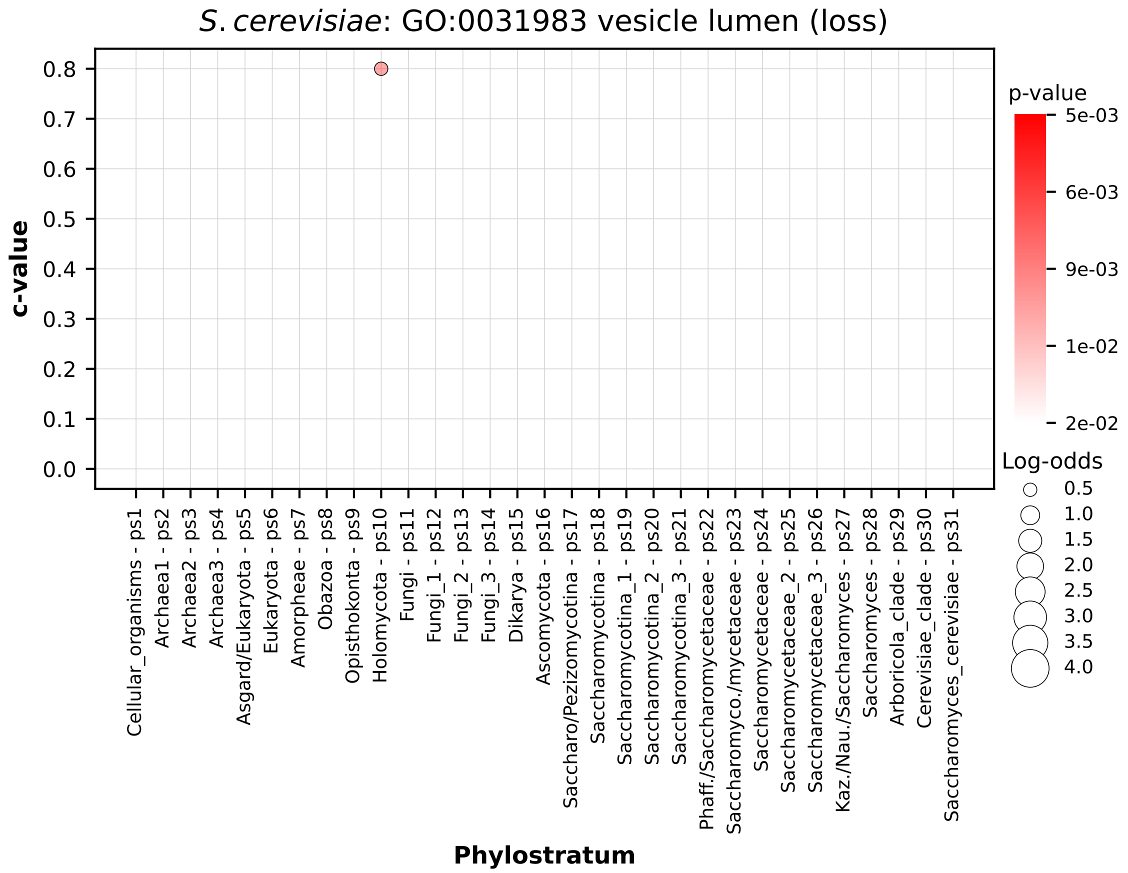
S. cerevisiae: GO:0031983 vesicle lumen (loss)
c-value
0.0
0.1
0.2
0.3
0.4
0.5
0.6
0.7
0.8
p-value
5e-03
6e-03
9e-03
1e-02
2e-02
Log-odds
0.5
1.0
1.5
2.0
2.5
3.0
3.5
4.0
Cellular_organisms - ps1
Archaea1 - ps2
Archaea2 - ps3
Archaea3 - ps4
Asgard/Eukaryota - ps5
Eukaryota - ps6
Amorpheae - ps7
Obazoa - ps8
Opisthokonta - ps9
Holomycota - ps10
Fungi - ps11
Fungi_1 - ps12
Fungi_2 - ps13
Fungi_3 - ps14
Dikarya - ps15
Ascomycota - ps16
Saccharo/Pezizomycotina - ps17
Saccharomycotina - ps18
Saccharomycotina_1 - ps19
Saccharomycotina_2 - ps20
Saccharomycotina_3 - ps21
Phaff./Saccharomycetaceae - ps22
Saccharomyco./mycetaceae - ps23
Saccharomycetaceae - ps24
Saccharomycetaceae_2 - ps25
Saccharomycetaceae_3 - ps26
Kaz./Nau./Saccharomyces - ps27
Saccharomyces - ps28
Arboricola_clade - ps29
Cerevisiae_clade - ps30
Saccharomyces_cerevisiae - ps31
Phylostratum

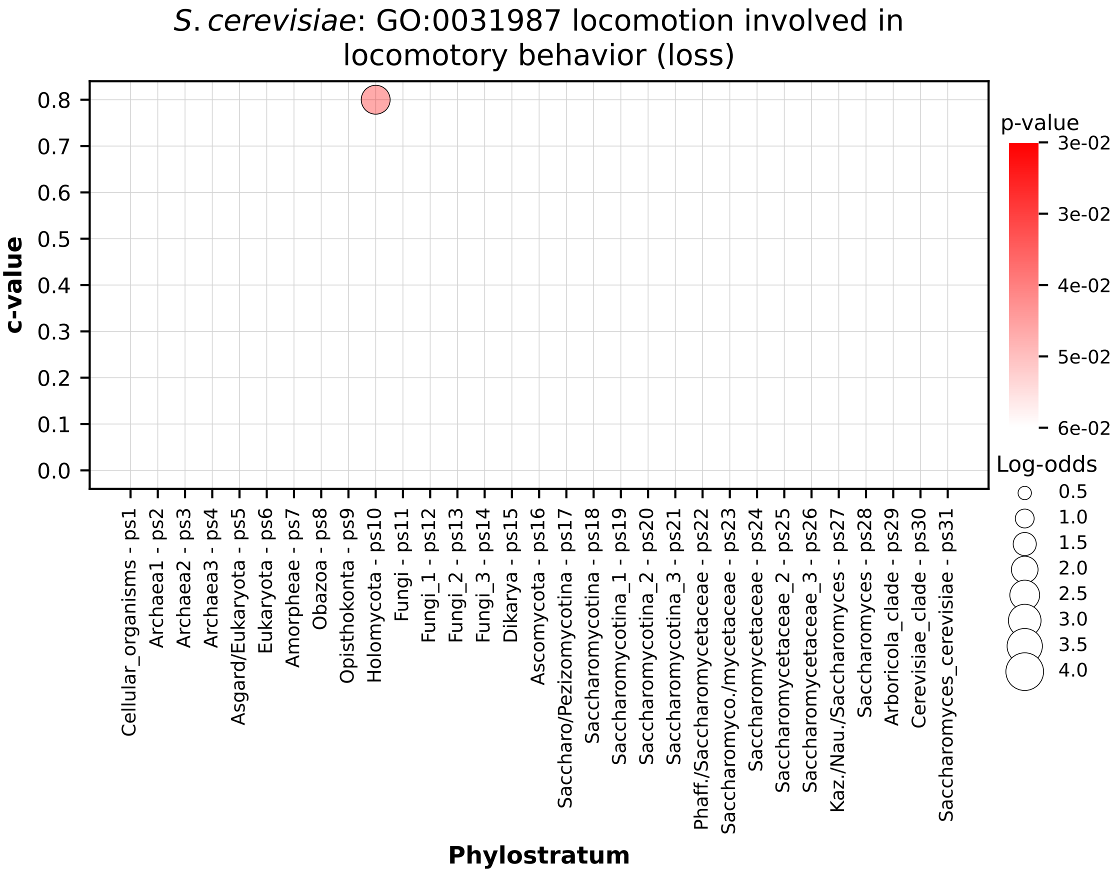
S. cerevisiae: GO:0031987 locomotion involved in locomotory behavior (loss)
c-value
0.0
0.1
0.2
0.3
0.4
0.5
0.6
0.7
0.8
p-value
3e-02
3e-02
4e-02
5e-02
6e-02
Log-odds
0.5
1.0
1.5
2.0
2.5
3.0
3.5
4.0
Cellular_organisms - ps1
Archaea1 - ps2
Archaea2 - ps3
Archaea3 - ps4
Asgard/Eukaryota - ps5
Eukaryota - ps6
Amorpheae - ps7
Obazoa - ps8
Opisthokonta - ps9
Holomycota - ps10
Fungi - ps11
Fungi_1 - ps12
Fungi_2 - ps13
Fungi_3 - ps14
Dikarya - ps15
Ascomycota - ps16
Saccharo/Pezizomycotina - ps17
Saccharomycotina - ps18
Saccharomycotina_1 - ps19
Saccharomycotina_2 - ps20
Saccharomycotina_3 - ps21
Phaff./Saccharomycetaceae - ps22
Saccharomyco./mycetaceae - ps23
Saccharomycetaceae - ps24
Saccharomycetaceae_2 - ps25
Saccharomycetaceae_3 - ps26
Kaz./Nau./Saccharomyces - ps27
Saccharomyces - ps28
Arboricola_clade - ps29
Cerevisiae_clade - ps30
Saccharomyces_cerevisiae - ps31
Phylostratum

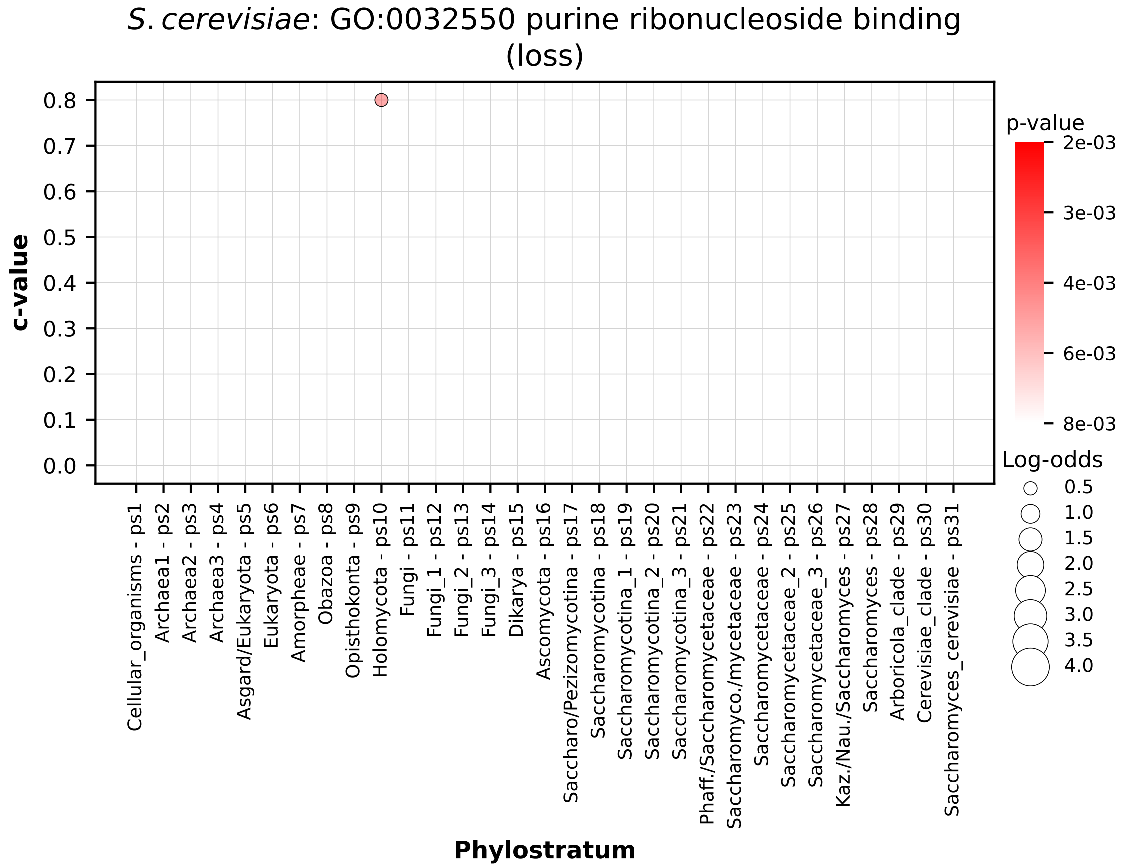

S. cerevisiae: GO:0032550 purine ribonucleoside binding
(loss)
c-value
0.0
0.1
0.2
0.3
0.4
0.5
0.6
0.7
0.8
Phylostratum
Cellular_organisms - ps1
Archaea1 - ps2
Archaea2 - ps3
Archaea3 - ps4
Asgard/Eukaryota - ps5
Eukaryota - ps6
Amorpheae - ps7
Obazoa - ps8
Opisthokonta - ps9
Holomycota - ps10
Fungi - ps11
Fungi_1 - ps12
Fungi_2 - ps13
Fungi_3 - ps14
Dikarya - ps15
Ascomycota - ps16
Saccharo/Pezizomycotina - ps17
Saccharomycotina - ps18
Saccharomycotina_1 - ps19
Saccharomycotina_2 - ps20
Saccharomycotina_3 - ps21
Phaff./Saccharomycetaceae - ps22
Saccharomyco./mycetaceae - ps23
Saccharomycetaceae - ps24
Saccharomycetaceae_2 - ps25
Saccharomycetaceae_3 - ps26
Kaz./Nau./Saccharomyces - ps27
Saccharomyces - ps28
Arboricola_clade - ps29
Cerevisiae_clade - ps30
Saccharomyces_cerevisiae - ps31
p-value
2e-03
3e-03
4e-03
6e-03
8e-03
Log-odds
0.5
1.0
1.5
2.0
2.5
3.0
3.5
4.0

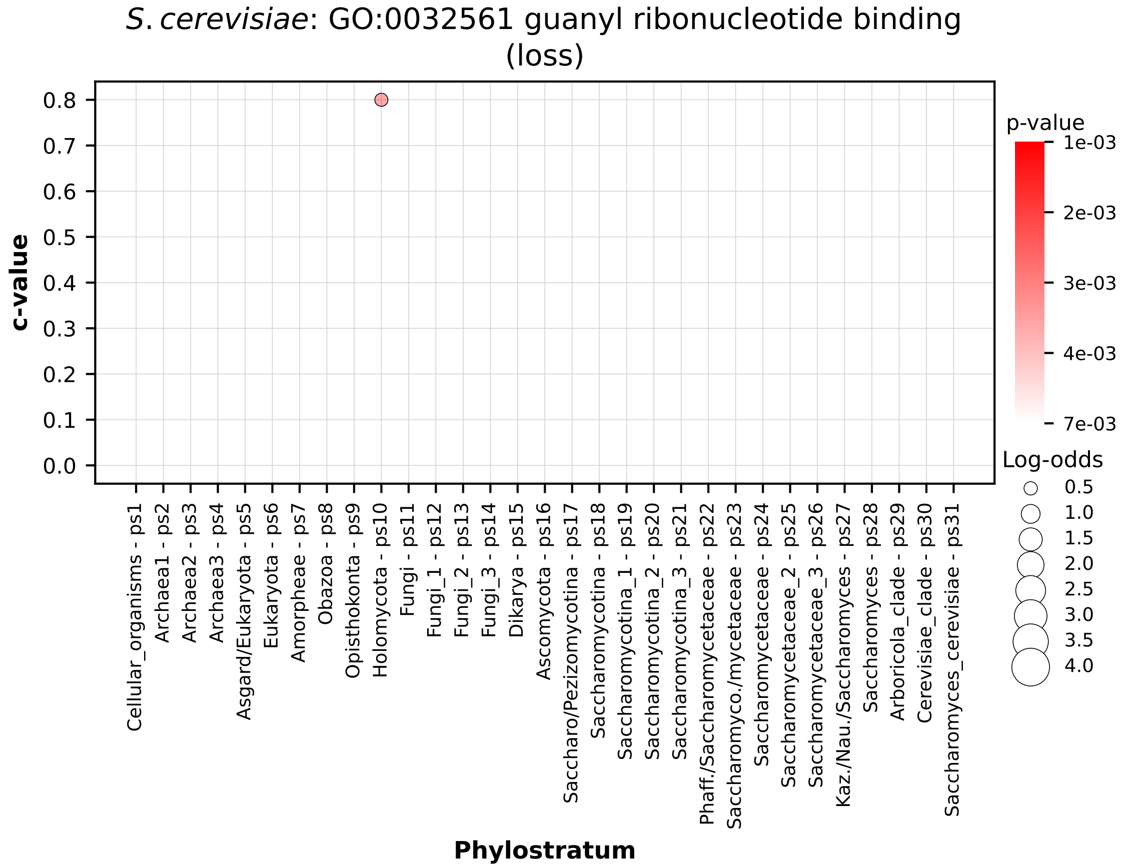
S. cerevisiae: GO:0032561 guanyl ribonucleotide binding
(loss)
c-value
0.0
0.1
0.2
0.3
0.4
0.5
0.6
0.7
0.8
p-value
1e-03
2e-03
3e-03
4e-03
7e-03
Log-odds
0.5
1.0
1.5
2.0
2.5
3.0
3.5
4.0
Cellular_organisms - ps1
Archaea1 - ps2
Archaea2 - ps3
Archaea3 - ps4
Asgard/Eukaryota - ps5
Eukaryota - ps6
Amorpheae - ps7
Obazoa - ps8
Opisthokonta - ps9
Holomycota - ps10
Fungi - ps11
Fungi_1 - ps12
Fungi_2 - ps13
Fungi_3 - ps14
Dikarya - ps15
Ascomycota - ps16
Saccharo/Pezizomycotina - ps17
Saccharomycotina - ps18
Saccharomycotina_1 - ps19
Saccharomycotina_2 - ps20
Saccharomycotina_3 - ps21
Phaff./Saccharomycetaceae - ps22
Saccharomyco./mycetaceae - ps23
Saccharomycetaceae - ps24
Saccharomycetaceae_2 - ps25
Saccharomycetaceae_3 - ps26
Kaz./Nau./Saccharomyces - ps27
Saccharomyces - ps28
Arboricola_clade - ps29
Cerevisiae_clade - ps30
Saccharomyces_cerevisiae - ps31
Phylostratum

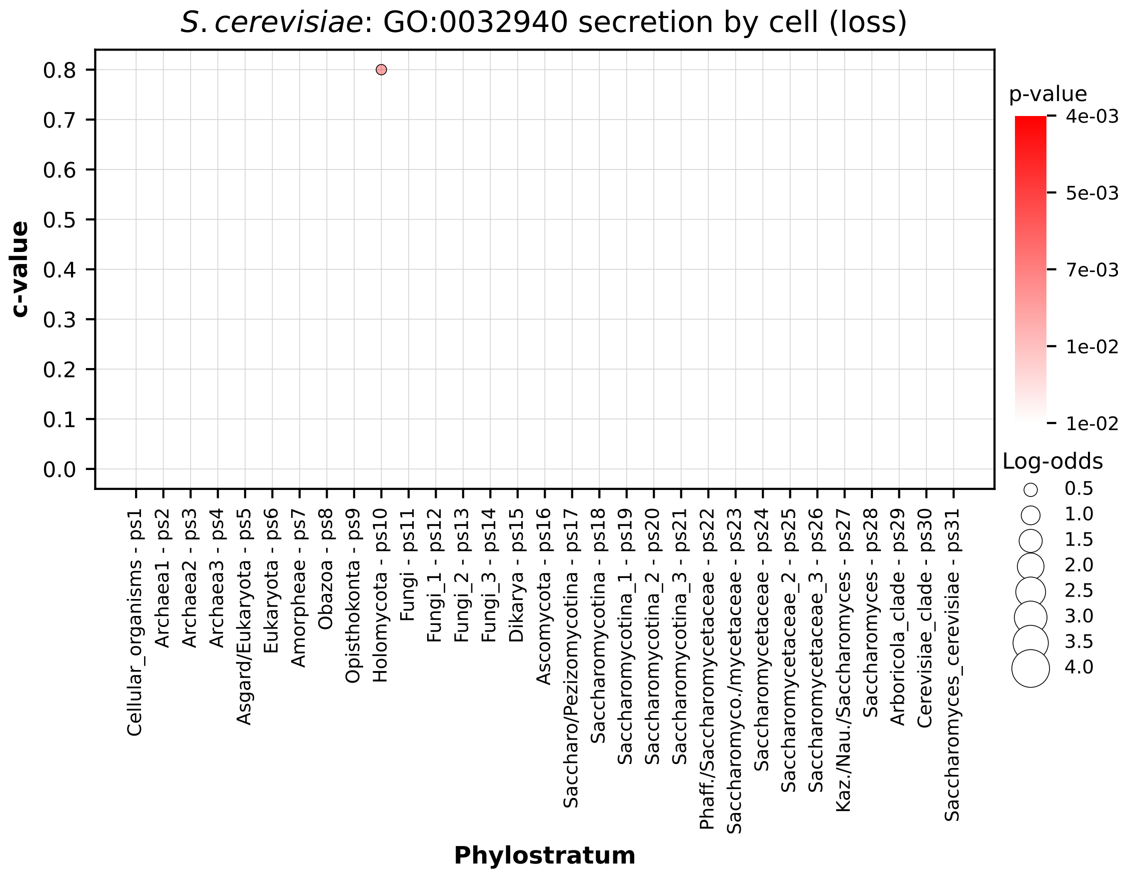

S. cerevisiae: GO:0032940 secretion by cell (loss)
c-value
0.0
0.1
0.2
0.3
0.4
0.5
0.6
0.7
0.8
p-value
4e-03
5e-03
7e-03
1e-02
1e-02
Log-odds
0.5
1.0
1.5
2.0
2.5
3.0
3.5
4.0
Cellular_organisms - ps1
Archaea1 - ps2
Archaea2 - ps3
Archaea3 - ps4
Asgard/Eukaryota - ps5
Eukaryota - ps6
Amorpheae - ps7
Obazoa - ps8
Opisthokonta - ps9
Holomycota - ps10
Fungi - ps11
Fungi_1 - ps12
Fungi_2 - ps13
Fungi_3 - ps14
Dikarya - ps15
Ascomycota - ps16
Saccharo/Pezizomycotina - ps17
Saccharomycotina - ps18
Saccharomycotina_1 - ps19
Saccharomycotina_2 - ps20
Saccharomycotina_3 - ps21
Phaff./Saccharomycetaceae - ps22
Saccharomyco./mycetaceae - ps23
Saccharomycetaceae - ps24
Saccharomycetaceae_2 - ps25
Saccharomycetaceae_3 - ps26
Kaz./Nau./Saccharomyces - ps27
Saccharomyces - ps28
Arboricola_clade - ps29
Cerevisiae_clade - ps30
Saccharomyces_cerevisiae - ps31
Phylostratum

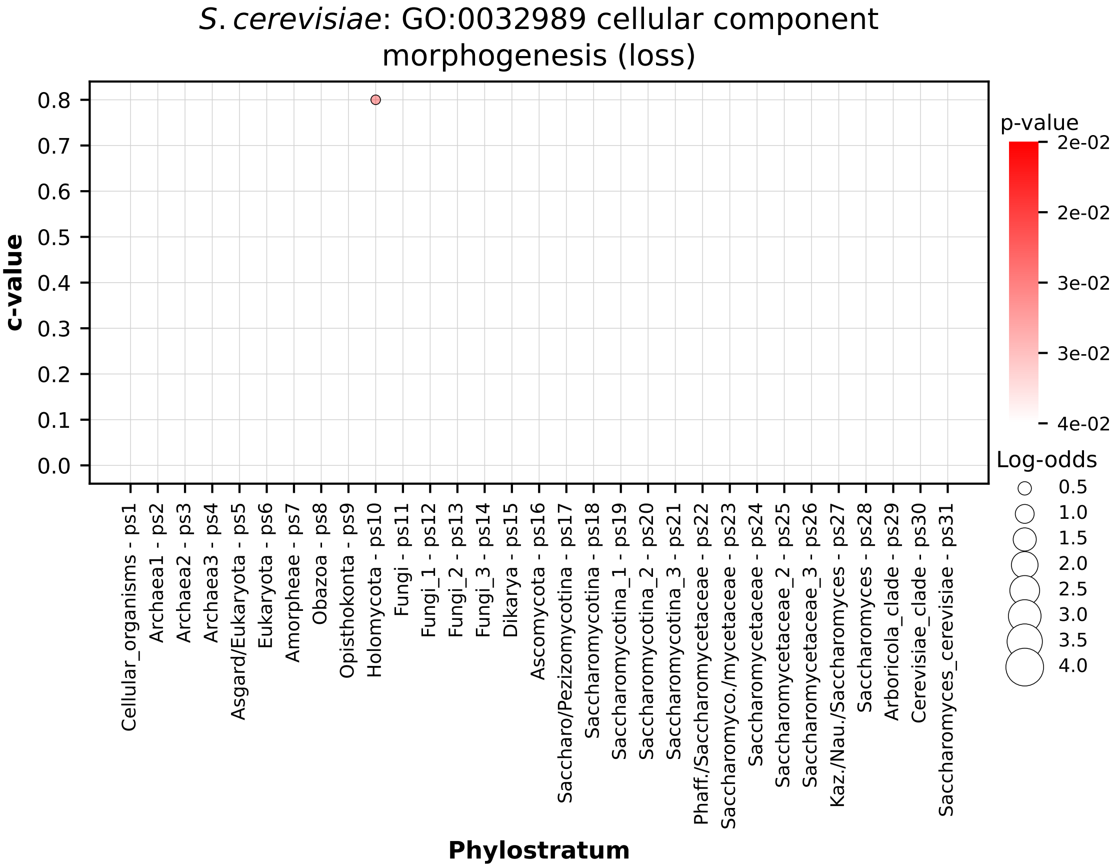

S. cerevisiae: GO:0032989 cellular component morphogenesis (loss)
c-value
0.0
0.1
0.2
0.3
0.4
0.5
0.6
0.7
0.8
p-value
2e-02
2e-02
3e-02
3e-02
4e-02
Log-odds
0.5
1.0
1.5
2.0
2.5
3.0
3.5
4.0
Cellular_organisms - ps1
Archaea1 - ps2
Archaea2 - ps3
Archaea3 - ps4
Asgard/Eukaryota - ps5
Eukaryota - ps6
Amorpheae - ps7
Obazoa - ps8
Opisthokonta - ps9
Holomycota - ps10
Fungi - ps11
Fungi_1 - ps12
Fungi_2 - ps13
Fungi_3 - ps14
Dikarya - ps15
Ascomycota - ps16
Saccharo/Pezizomycotina - ps17
Saccharomycotina - ps18
Saccharomycotina_1 - ps19
Saccharomycotina_2 - ps20
Saccharomycotina_3 - ps21
Phaff./Saccharomycetaceae - ps22
Saccharomyco./mycetaceae - ps23
Saccharomycetaceae - ps24
Saccharomycetaceae_2 - ps25
Saccharomycetaceae_3 - ps26
Kaz./Nau./Saccharomyces - ps27
Saccharomyces - ps28
Arboricola_clade - ps29
Cerevisiae_clade - ps30
Saccharomyces_cerevisiae - ps31
Phylostratum

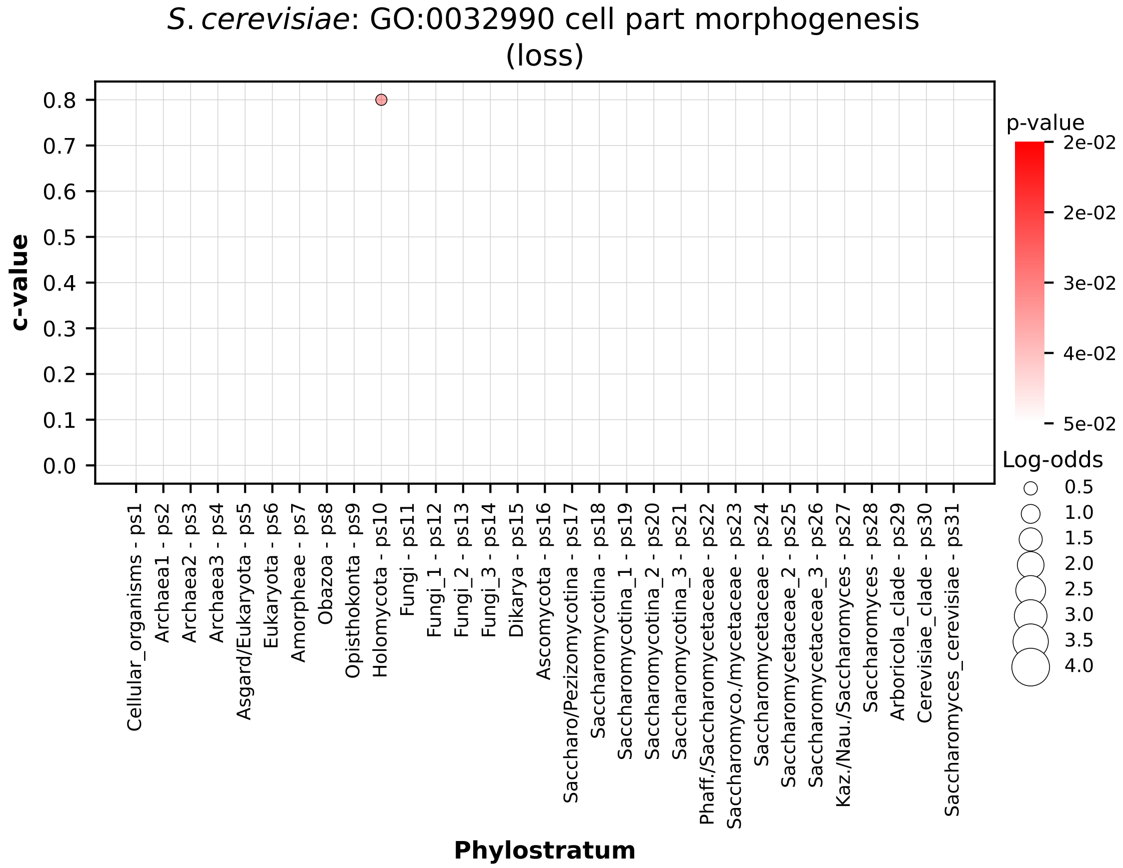

S. cerevisiae: GO:0032990 cell part morphogenesis
(loss)
c-value
0.8
0.7
0.6
0.5
0.4
0.3
0.2
0.1
0.0
Phylostratum
Cellular_organisms - ps1
Archaea1 - ps2
Archaea2 - ps3
Archaea3 - ps4
Asgard/Eukaryota - ps5
Eukaryota - ps6
Amorpheae - ps7
Obazoa - ps8
Opisthokonta - ps9
Holomycota - ps10
Fungi - ps11
Fungi_1 - ps12
Fungi_2 - ps13
Fungi_3 - ps14
Dikarya - ps15
Ascomycota - ps16
Saccharo/Pezizomycotina - ps17
Saccharomycotina - ps18
Saccharomycotina_1 - ps19
Saccharomycotina_2 - ps20
Saccharomycotina_3 - ps21
Phaff./Saccharomycetaceae - ps22
Saccharomyco./mycetaceae - ps23
Saccharomycetaceae - ps24
Saccharomycetaceae_2 - ps25
Saccharomycetaceae_3 - ps26
Kaz./Nau./Saccharomyces - ps27
Saccharomyces - ps28
Arboricola_clade - ps29
Cerevisiae_clade - ps30
Saccharomyces_cerevisiae - ps31
p-value
2e-02
2e-02
3e-02
4e-02
5e-02
Log-odds
0.5
1.0
1.5
2.0
2.5
3.0
3.5
4.0

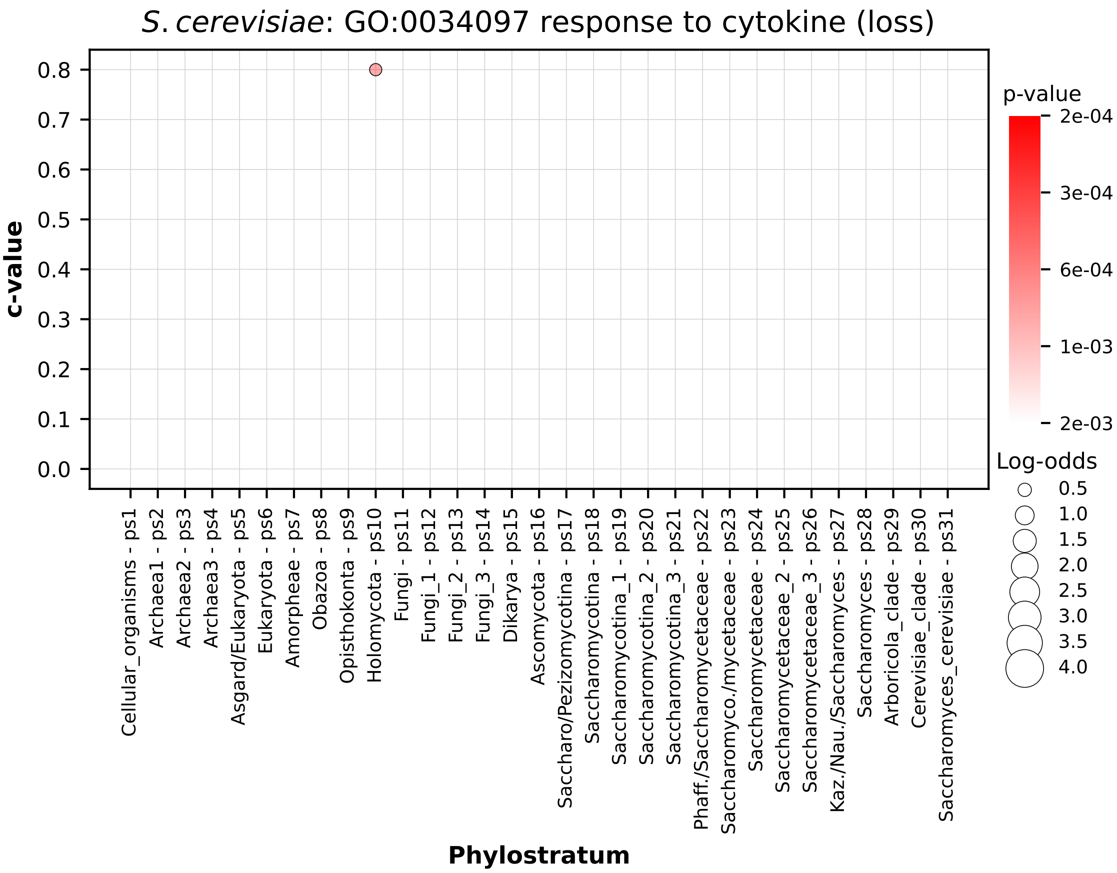

S. cerevisiae: GO:0034097 response to cytokine (loss)
c-value
0.8
0.7
0.6
0.5
0.4
0.3
0.2
0.1
0.0
p-value
2e-04
3e-04
6e-04
1e-03
2e-03
Log-odds
0.5
1.0
1.5
2.0
2.5
3.0
3.5
4.0
Cellular_organisms - ps1
Archaea1 - ps2
Archaea2 - ps3
Archaea3 - ps4
Asgard/Eukaryota - ps5
Eukaryota - ps6
Amorpheae - ps7
Obazoa - ps8
Opisthokonta - ps9
Holomycota - ps10
Fungi - ps11
Fungi_1 - ps12
Fungi_2 - ps13
Fungi_3 - ps14
Dikarya - ps15
Ascomycota - ps16
Saccharo/Pezizomycotina - ps17
Saccharomycotina - ps18
Saccharomycotina_1 - ps19
Saccharomycotina_2 - ps20
Saccharomycotina_3 - ps21
Phaff./Saccharomycetaceae - ps22
Saccharomyco./mycetaceae - ps23
Saccharomycetaceae - ps24
Saccharomycetaceae_2 - ps25
Saccharomycetaceae_3 - ps26
Kaz./Nau./Saccharomyces - ps27
Saccharomyces - ps28
Arboricola_clade - ps29
Cerevisiae_clade - ps30
Saccharomyces_cerevisiae - ps31
Phylostratum

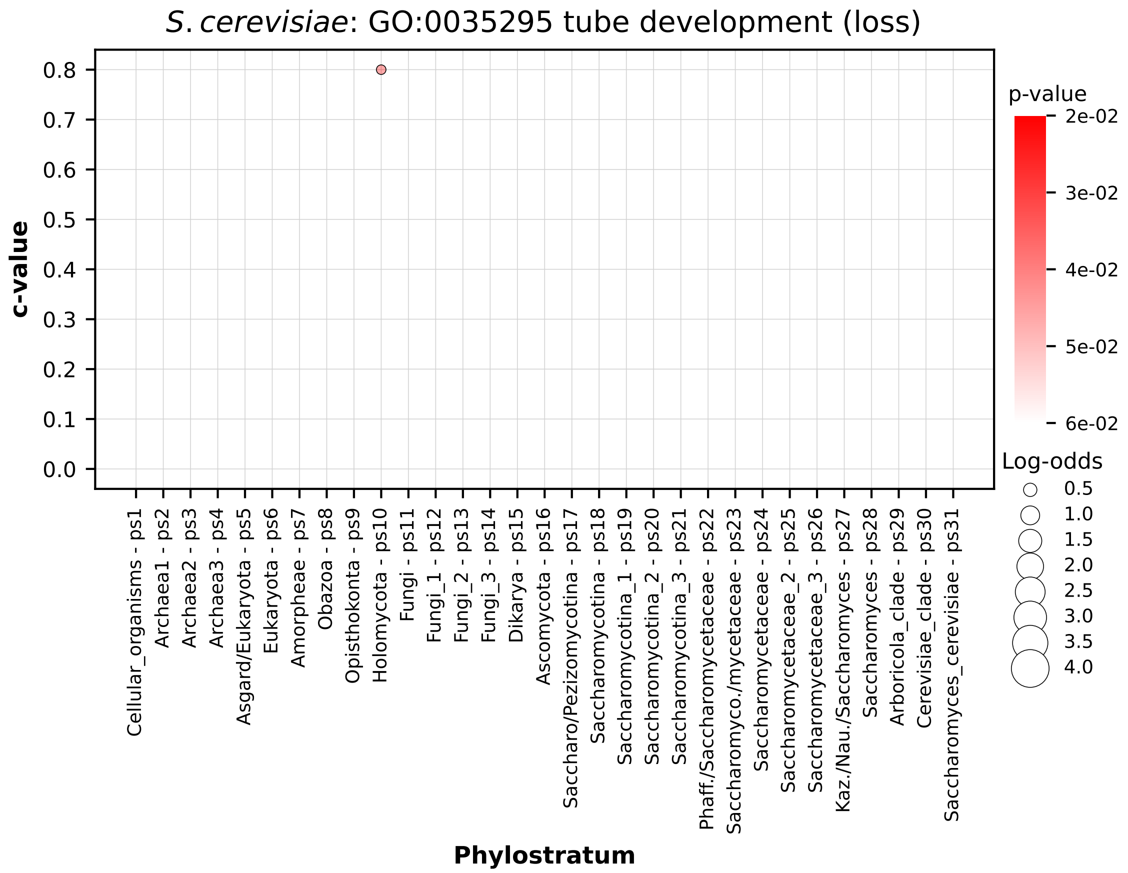
S. cerevisiae: GO:0035295 tube development (loss)
c-value
0.8
0.7
0.6
0.5
0.4
0.3
0.2
0.1
0.0
p-value
2e-02
3e-02
4e-02
5e-02
6e-02
Log-odds
0.5
1.0
1.5
2.0
2.5
3.0
3.5
4.0
Cellular_organisms - ps1
Archaea1 - ps2
Archaea2 - ps3
Archaea3 - ps4
Asgard/Eukaryota - ps5
Eukaryota - ps6
Amorpheae - ps7
Obazoa - ps8
Opisthokonta - ps9
Holomycota - ps10
Fungi - ps11
Fungi_1 - ps12
Fungi_2 - ps13
Fungi_3 - ps14
Dikarya - ps15
Ascomycota - ps16
Saccharo/Pezizomycotina - ps17
Saccharomycotina - ps18
Saccharomycotina_1 - ps19
Saccharomycotina_2 - ps20
Saccharomycotina_3 - ps21
Phaff./Saccharomycetaceae - ps22
Saccharomyco./mycetaceae - ps23
Saccharomycetaceae - ps24
Saccharomycetaceae_2 - ps25
Saccharomycetaceae_3 - ps26
Kaz./Nau./Saccharomyces - ps27
Saccharomyces - ps28
Arboricola_clade - ps29
Cerevisiae_clade - ps30
Saccharomyces_cerevisiae - ps31
Phylostratum

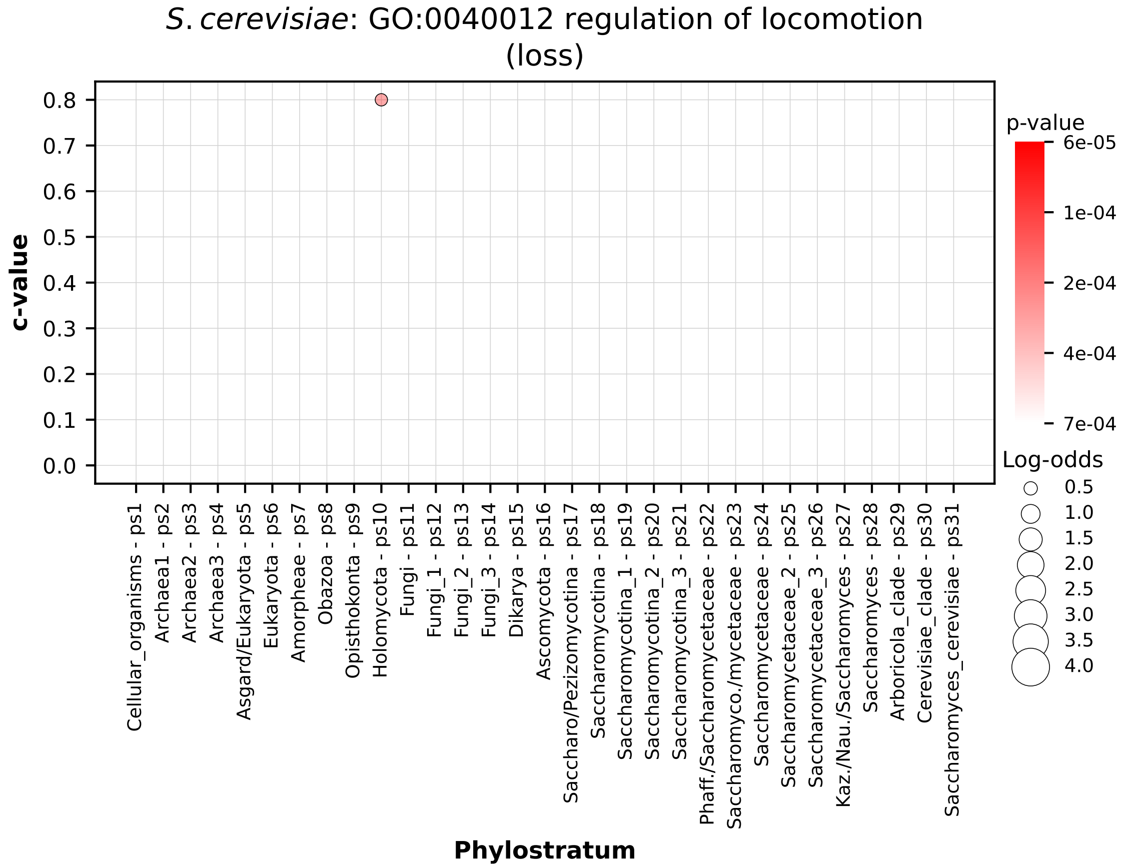
S. cerevisiae: GO:0040012 regulation of locomotion
(loss)
c-value
0.0
0.1
0.2
0.3
0.4
0.5
0.6
0.7
0.8
p-value
6e-05
1e-04
2e-04
4e-04
7e-04
Log-odds
0.5
1.0
1.5
2.0
2.5
3.0
3.5
4.0
Cellular_organisms - ps1
Archaea1 - ps2
Archaea2 - ps3
Archaea3 - ps4
Asgard/Eukaryota - ps5
Eukaryota - ps6
Amorpheae - ps7
Obazoa - ps8
Opisthokonta - ps9
Holomycota - ps10
Fungi - ps11
Fungi_1 - ps12
Fungi_2 - ps13
Fungi_3 - ps14
Dikarya - ps15
Ascomycota - ps16
Saccharo/Pezizomycotina - ps17
Saccharomycotina - ps18
Saccharomycotina_1 - ps19
Saccharomycotina_2 - ps20
Saccharomycotina_3 - ps21
Phaff./Saccharomycetaceae - ps22
Saccharomyco./mycetaceae - ps23
Saccharomycetaceae - ps24
Saccharomycetaceae_2 - ps25
Saccharomycetaceae_3 - ps26
Kaz./Nau./Saccharomyces - ps27
Saccharomyces - ps28
Arboricola_clade - ps29
Cerevisiae_clade - ps30
Saccharomyces_cerevisiae - ps31
Phylostratum

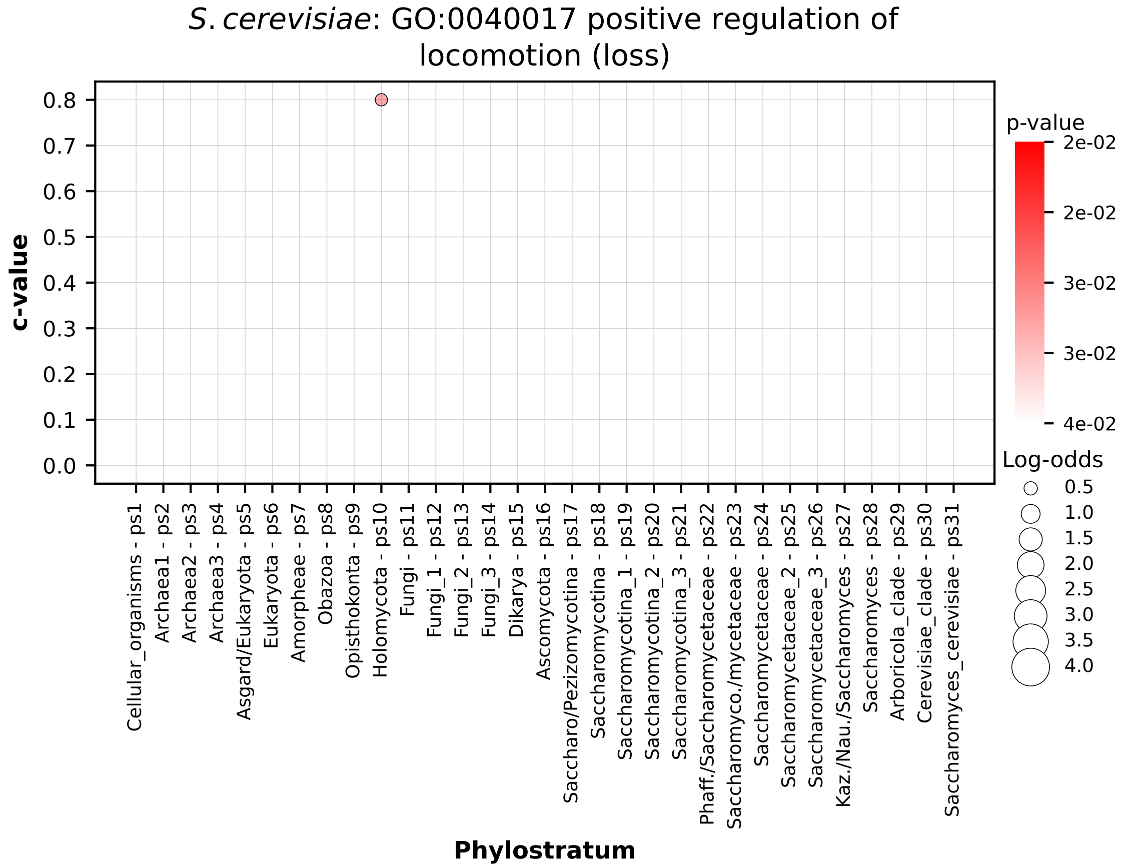
S. cerevisiae: GO:0040017 positive regulation of locomotion (loss)
c-value
0.8
0.7
0.6
0.5
0.4
0.3
0.2
0.1
0.0
p-value
2e-02
2e-02
3e-02
3e-02
4e-02
Log-odds
0.5
1.0
1.5
2.0
2.5
3.0
3.5
4.0
Cellular_organisms - ps1
Archaea1 - ps2
Archaea2 - ps3
Archaea3 - ps4
Asgard/Eukaryota - ps5
Eukaryota - ps6
Amorpheae - ps7
Obazoa - ps8
Opisthokonta - ps9
Holomycota - ps10
Fungi - ps11
Fungi_1 - ps12
Fungi_2 - ps13
Fungi_3 - ps14
Dikarya - ps15
Ascomycota - ps16
Saccharo/Pezizomycotina - ps17
Saccharomycotina - ps18
Saccharomycotina_1 - ps19
Saccharomycotina_2 - ps20
Saccharomycotina_3 - ps21
Phaff./Saccharomycetaceae - ps22
Saccharomyco./mycetaceae - ps23
Saccharomycetaceae - ps24
Saccharomycetaceae_2 - ps25
Saccharomycetaceae_3 - ps26
Kaz./Nau./Saccharomyces - ps27
Saccharomyces - ps28
Arboricola_clade - ps29
Cerevisiae_clade - ps30
Saccharomyces_cerevisiae - ps31
Phylostratum

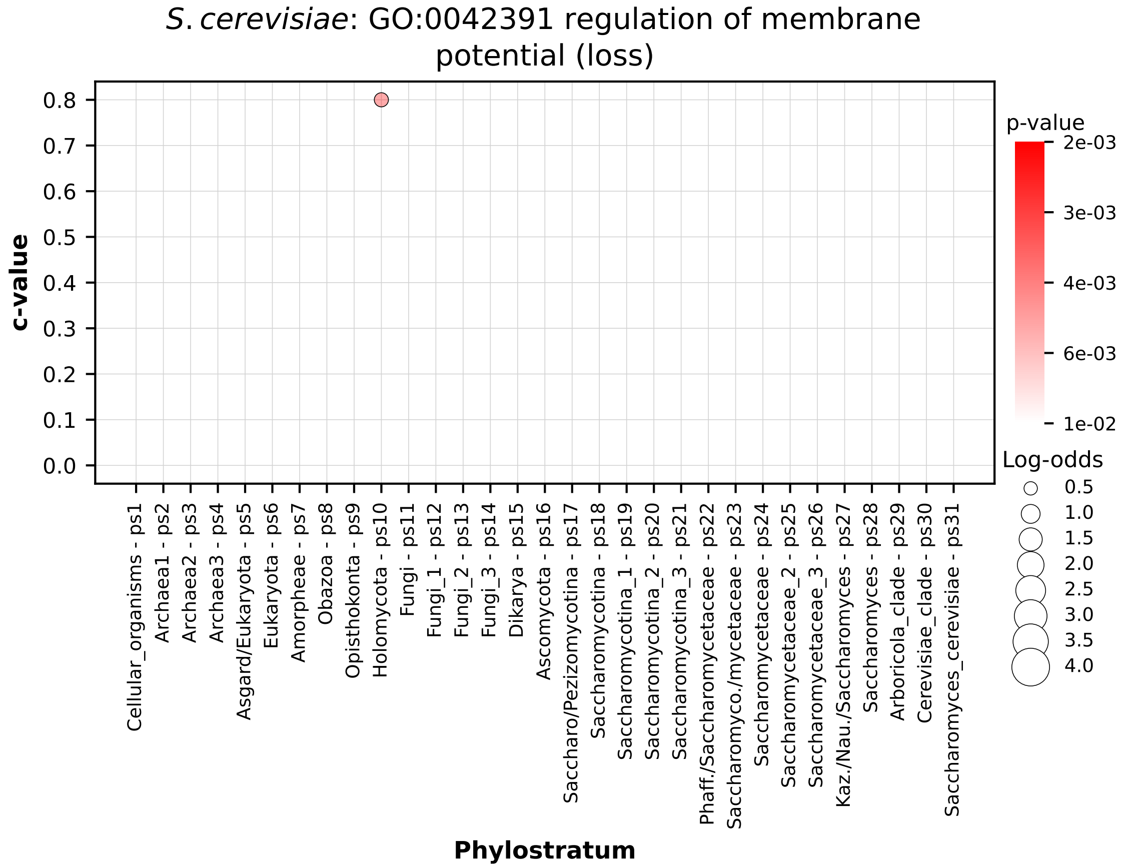
S. cerevisiae: GO:0042391 regulation of membrane potential (loss)
c-value
0.0
0.1
0.2
0.3
0.4
0.5
0.6
0.7
0.8
p-value
2e-03
3e-03
4e-03
6e-03
1e-02
Log-odds
0.5
1.0
1.5
2.0
2.5
3.0
3.5
4.0
Cellular_organisms - ps1
Archaea1 - ps2
Archaea2 - ps3
Archaea3 - ps4
Asgard/Eukaryota - ps5
Eukaryota - ps6
Amorpheae - ps7
Obazoa - ps8
Opisthokonta - ps9
Holomycota - ps10
Fungi - ps11
Fungi_1 - ps12
Fungi_2 - ps13
Fungi_3 - ps14
Dikarya - ps15
Ascomycota - ps16
Saccharo/Pezizomycotina - ps17
Saccharomycotina - ps18
Saccharomycotina_1 - ps19
Saccharomycotina_2 - ps20
Saccharomycotina_3 - ps21
Phaff./Saccharomycetaceae - ps22
Saccharomyco./mycetaceae - ps23
Saccharomycetaceae - ps24
Saccharomycetaceae_2 - ps25
Saccharomycetaceae_3 - ps26
Kaz./Nau./Saccharomyces - ps27
Saccharomyces - ps28
Arboricola_clade - ps29
Cerevisiae_clade - ps30
Saccharomyces_cerevisiae - ps31
Phylostratum

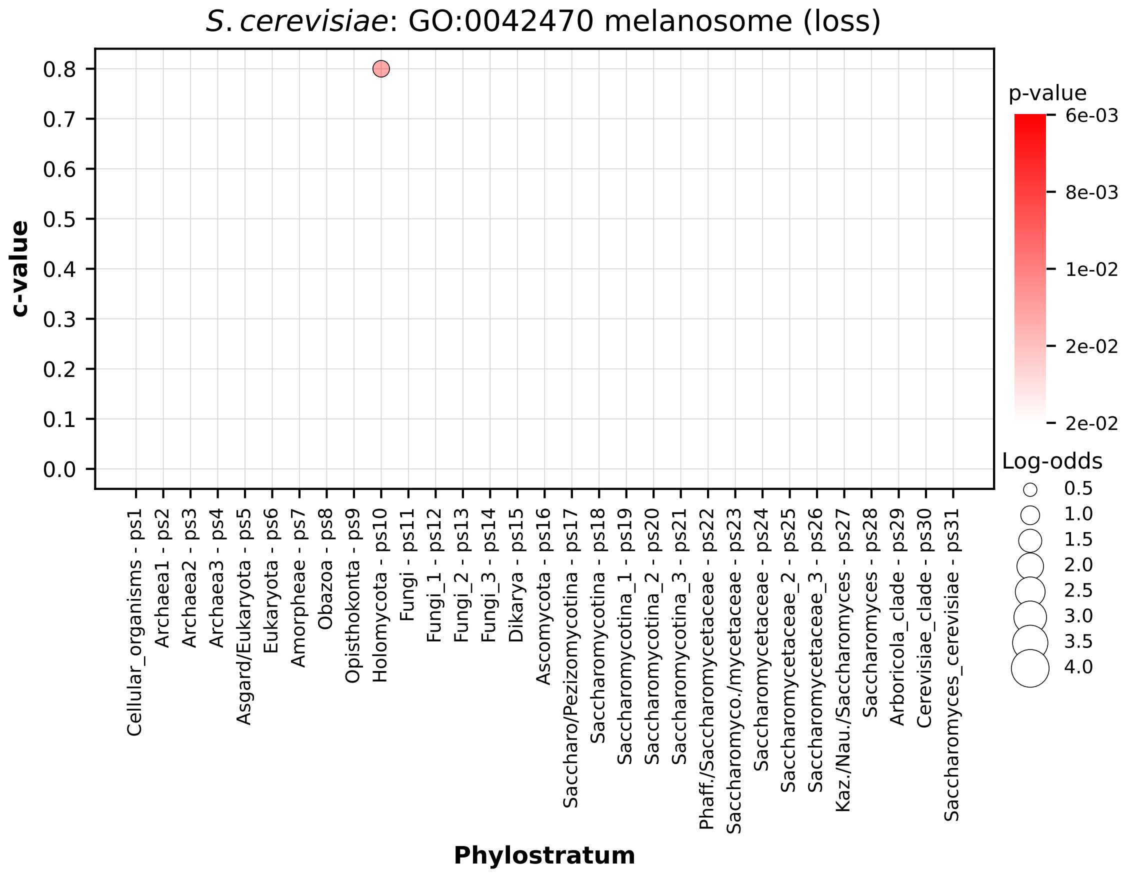
S. cerevisiae: GO:0042470 melanosome (loss)
c-value
0.0
0.1
0.2
0.3
0.4
0.5
0.6
0.7
0.8
p-value
6e-03
8e-03
1e-02
2e-02
2e-02
Log-odds
0.5
1.0
1.5
2.0
2.5
3.0
3.5
4.0
Cellular_organisms - ps1
Archaea1 - ps2
Archaea2 - ps3
Archaea3 - ps4
Asgard/Eukaryota - ps5
Eukaryota - ps6
Amorpheae - ps7
Obazoa - ps8
Opisthokonta - ps9
Holomycota - ps10
Fungi - ps11
Fungi_1 - ps12
Fungi_2 - ps13
Fungi_3 - ps14
Dikarya - ps15
Ascomycota - ps16
Saccharo/Pezizomycotina - ps17
Saccharomycotina - ps18
Saccharomycotina_1 - ps19
Saccharomycotina_2 - ps20
Saccharomycotina_3 - ps21
Phaff./Saccharomycetaceae - ps22
Saccharomyco./mycetaceae - ps23
Saccharomycetaceae - ps24
Saccharomycetaceae_2 - ps25
Saccharomycetaceae_3 - ps26
Kaz./Nau./Saccharomyces - ps27
Saccharomyces - ps28
Arboricola_clade - ps29
Cerevisiae_clade - ps30
Saccharomyces_cerevisiae - ps31
Phylostratum

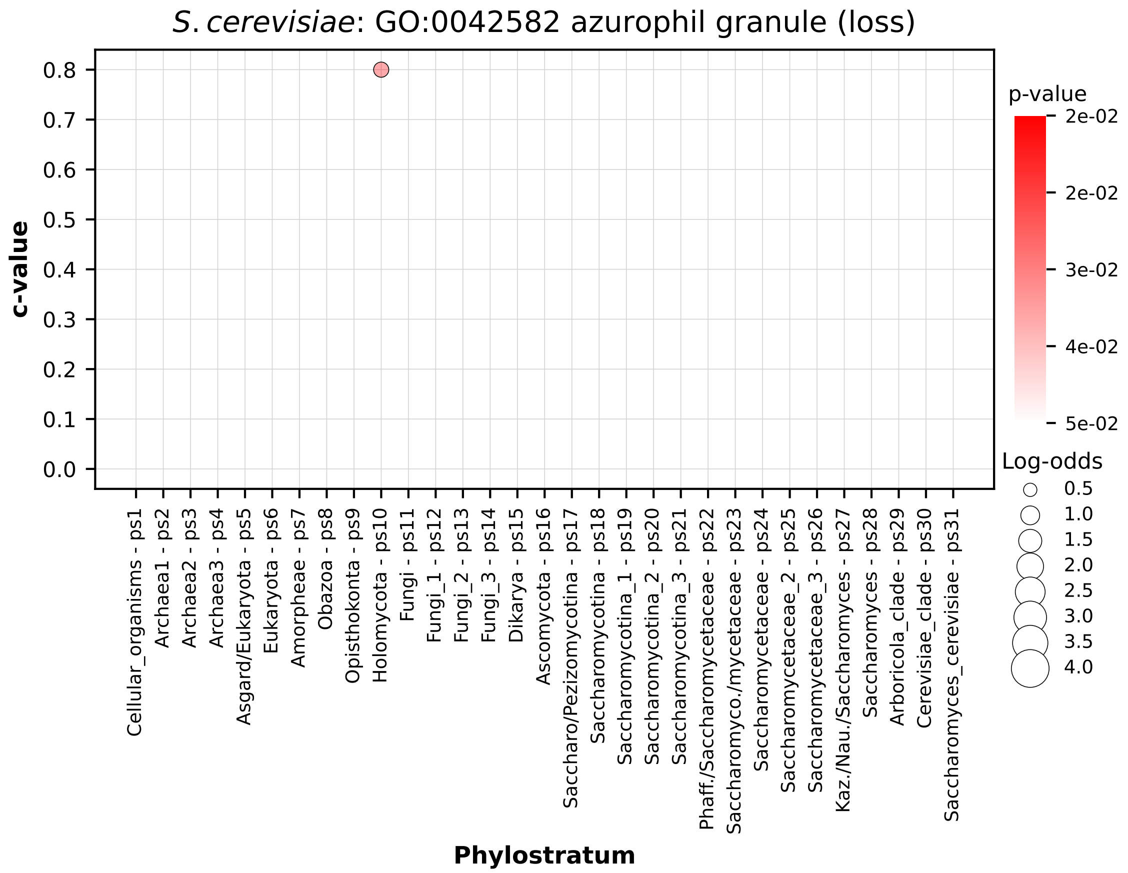

S. cerevisiae: GO:0042582 azurophil granule (loss)
c-value
0.0
0.1
0.2
0.3
0.4
0.5
0.6
0.7
0.8
p-value
2e-02
2e-02
3e-02
4e-02
5e-02
Log-odds
0.5
1.0
1.5
2.0
2.5
3.0
3.5
4.0
Cellular_organisms - ps1
Archaea1 - ps2
Archaea2 - ps3
Archaea3 - ps4
Asgard/Eukaryota - ps5
Eukaryota - ps6
Amorpheae - ps7
Obazoa - ps8
Opisthokonta - ps9
Holomycota - ps10
Fungi - ps11
Fungi_1 - ps12
Fungi_2 - ps13
Fungi_3 - ps14
Dikarya - ps15
Ascomycota - ps16
Saccharo/Pezizomycotina - ps17
Saccharomycotina - ps18
Saccharomycotina_1 - ps19
Saccharomycotina_2 - ps20
Saccharomycotina_3 - ps21
Phaff./Saccharomycetaceae - ps22
Saccharomyco./mycetaceae - ps23
Saccharomycetaceae - ps24
Saccharomycetaceae_2 - ps25
Saccharomycetaceae_3 - ps26
Kaz./Nau./Saccharomyces - ps27
Saccharomyces - ps28
Arboricola_clade - ps29
Cerevisiae_clade - ps30
Saccharomyces_cerevisiae - ps31
Phylostratum

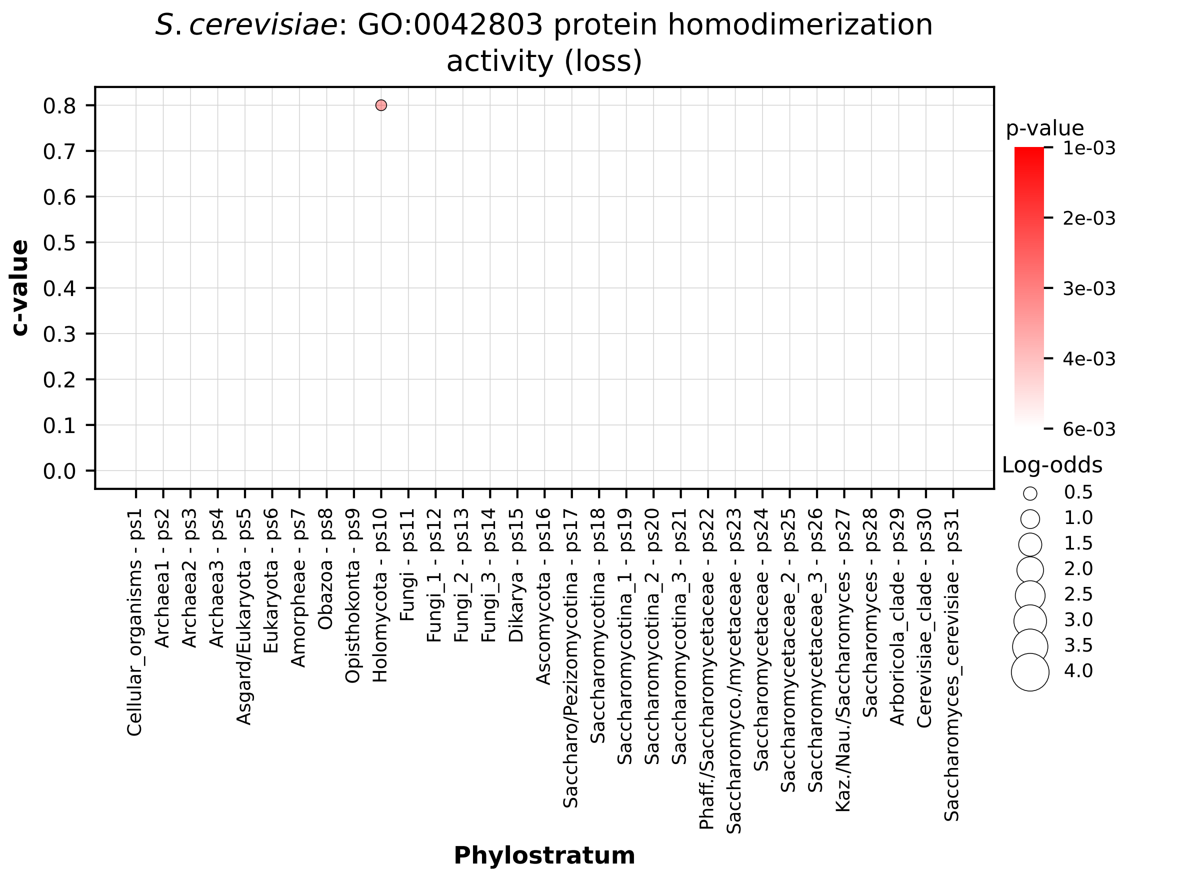

S. cerevisiae: GO:0042803 protein homodimerization activity (loss)
c-value
0.0
0.1
0.2
0.3
0.4
0.5
0.6
0.7
0.8
p-value
1e-03
2e-03
3e-03
4e-03
6e-03
Log-odds
0.5
1.0
1.5
2.0
2.5
3.0
3.5
4.0
Cellular_organisms - ps1
Archaea1 - ps2
Archaea2 - ps3
Archaea3 - ps4
Asgard/Eukaryota - ps5
Eukaryota - ps6
Amorpheae - ps7
Obazoa - ps8
Opisthokonta - ps9
Holomycota - ps10
Fungi - ps11
Fungi_1 - ps12
Fungi_2 - ps13
Fungi_3 - ps14
Dikarya - ps15
Ascomycota - ps16
Saccharo/Pezizomycotina - ps17
Saccharomycotina - ps18
Saccharomycotina_1 - ps19
Saccharomycotina_2 - ps20
Saccharomycotina_3 - ps21
Phaff./Saccharomycetaceae - ps22
Saccharomyco./mycetaceae - ps23
Saccharomycetaceae - ps24
Saccharomycetaceae_2 - ps25
Saccharomycetaceae_3 - ps26
Kaz./Nau./Saccharomyces - ps27
Saccharomyces - ps28
Arboricola_clade - ps29
Cerevisiae_clade - ps30
Saccharomyces_cerevisiae - ps31
Phylostratum

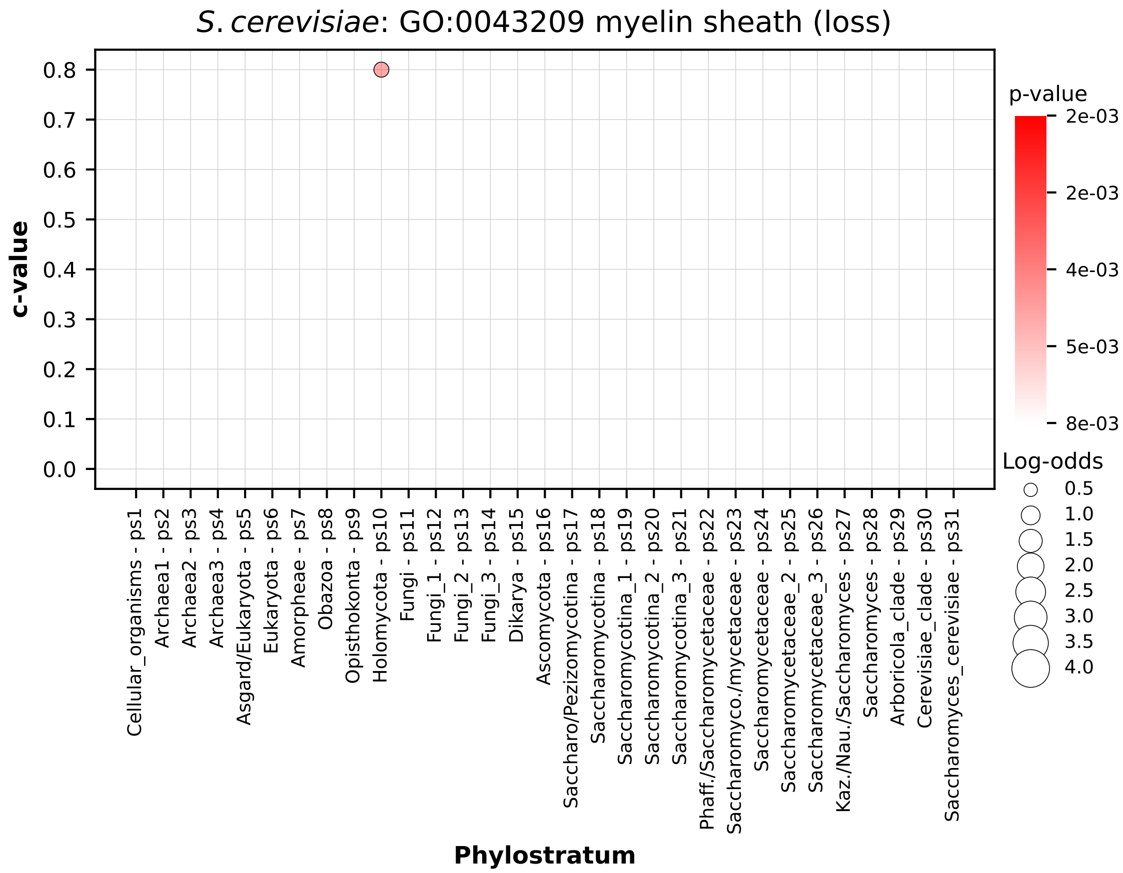
S. cerevisiae: GO:0043209 myelin sheath (loss)
c-value
0.0
0.1
0.2
0.3
0.4
0.5
0.6
0.7
0.8
Phylostratum
Cellular_organisms - ps1
Archaea1 - ps2
Archaea2 - ps3
Archaea3 - ps4
Asgard/Eukaryota - ps5
Eukaryota - ps6
Amorpheae - ps7
Obazoa - ps8
Opisthokonta - ps9
Holomycota - ps10
Fungi - ps11
Fungi_1 - ps12
Fungi_2 - ps13
Fungi_3 - ps14
Dikarya - ps15
Ascomycota - ps16
Saccharo/Pezizomycotina - ps17
Saccharomycotina - ps18
Saccharomycotina_1 - ps19
Saccharomycotina_2 - ps20
Saccharomycotina_3 - ps21
Phaff./Saccharomycetaceae - ps22
Saccharomyco./mycetaceae - ps23
Saccharomycetaceae - ps24
Saccharomycetaceae_2 - ps25
Saccharomycetaceae_3 - ps26
Kaz./Nau./Saccharomyces - ps27
Saccharomyces - ps28
Arboricola_clade - ps29
Cerevisiae_clade - ps30
Saccharomyces_cerevisiae - ps31
p-value
2e-03
2e-03
4e-03
5e-03
8e-03
Log-odds
0.5
1.0
1.5
2.0
2.5
3.0
3.5
4.0

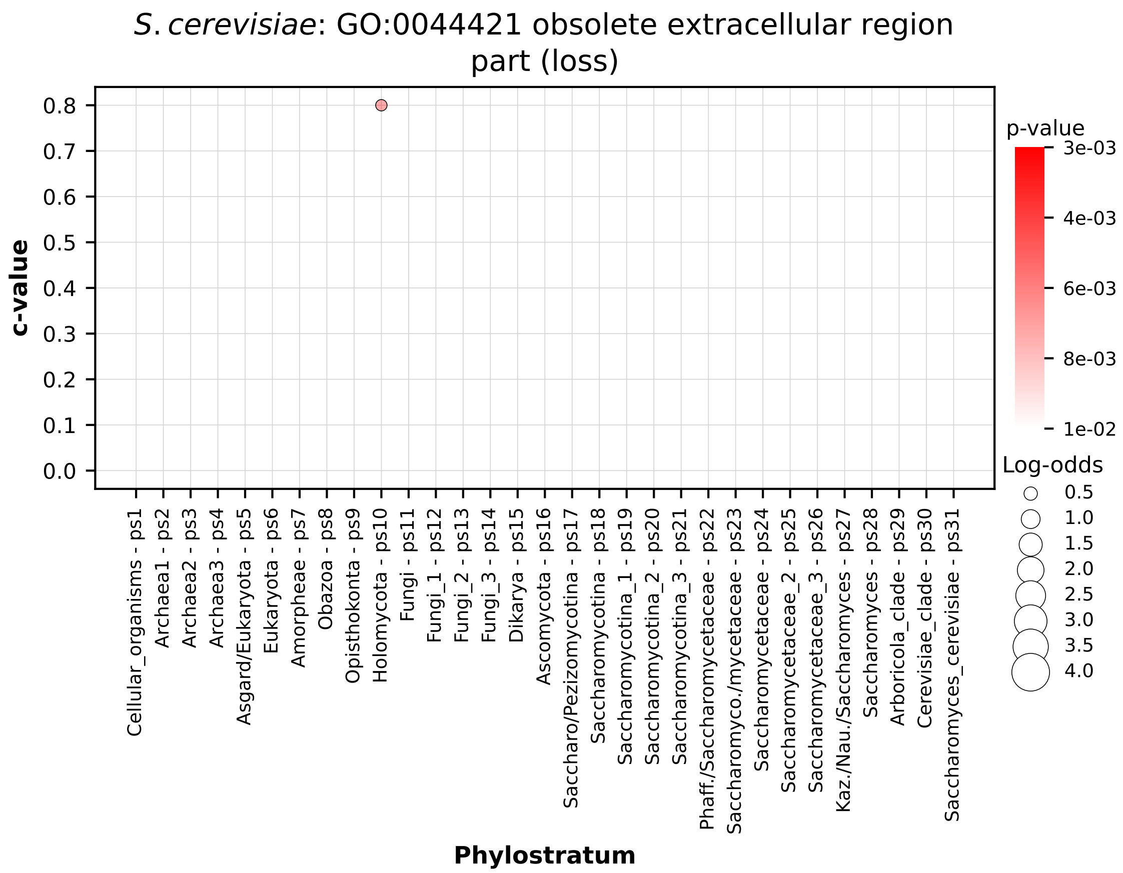

S. cerevisiae: GO:0044421 obsolete extracellular region part (loss)
c-value
0.0
0.1
0.2
0.3
0.4
0.5
0.6
0.7
0.8
p-value
3e-03
4e-03
6e-03
8e-03
1e-02
Log-odds
0.5
1.0
1.5
2.0
2.5
3.0
3.5
4.0
Cellular_organisms - ps1
Archaea1 - ps2
Archaea2 - ps3
Archaea3 - ps4
Asgard/Eukaryota - ps5
Eukaryota - ps6
Amorpheae - ps7
Obazoa - ps8
Opisthokonta - ps9
Holomycota - ps10
Fungi - ps11
Fungi_1 - ps12
Fungi_2 - ps13
Fungi_3 - ps14
Dikarya - ps15
Ascomycota - ps16
Saccharo/Pezizomycotina - ps17
Saccharomycotina - ps18
Saccharomycotina_1 - ps19
Saccharomycotina_2 - ps20
Saccharomycotina_3 - ps21
Phaff./Saccharomycetaceae - ps22
Saccharomyco./mycetaceae - ps23
Saccharomycetaceae - ps24
Saccharomycetaceae_2 - ps25
Saccharomycetaceae_3 - ps26
Kaz./Nau./Saccharomyces - ps27
Saccharomyces - ps28
Arboricola_clade - ps29
Cerevisiae_clade - ps30
Saccharomyces_cerevisiae - ps31
Phylostratum

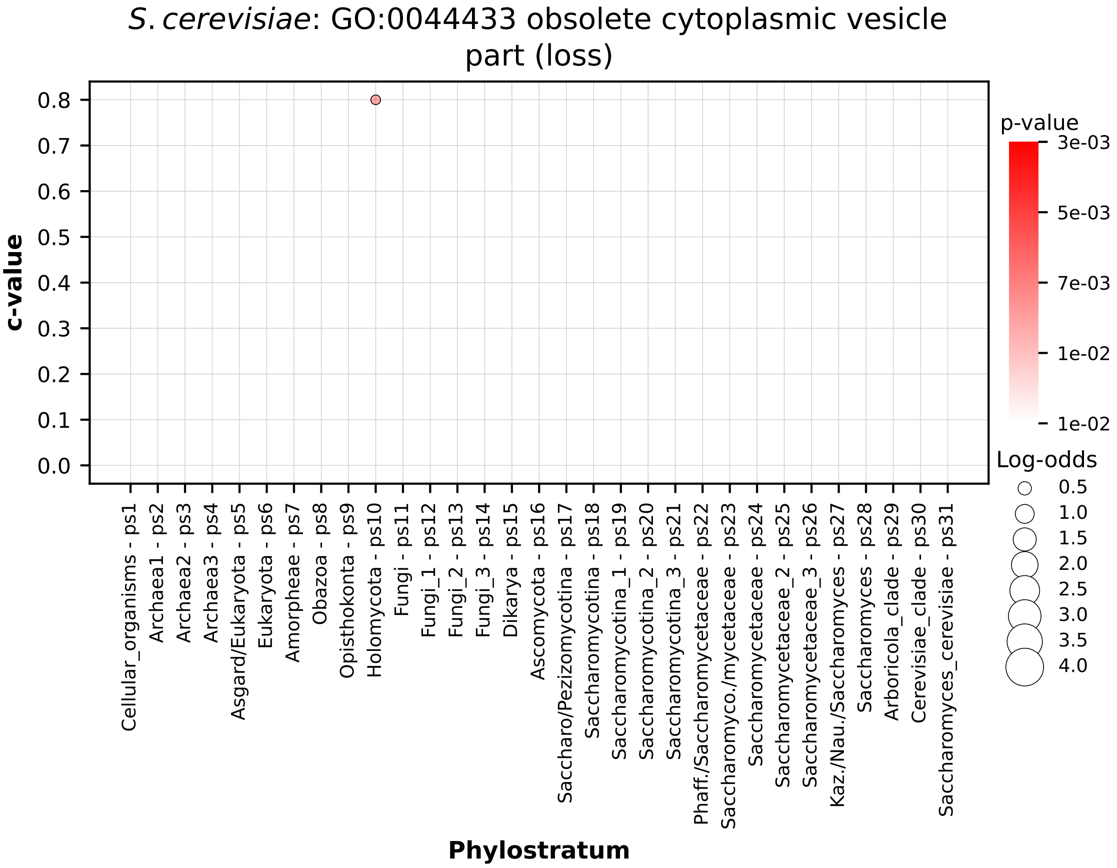

$S. cerevisiae$: GO:0044433 obsolete cytoplasmic vesicle part (loss)
c-value
0.0
0.1
0.2
0.3
0.4
0.5
0.6
0.7
0.8
p-value
3e-03
5e-03
7e-03
1e-02
1e-02
Log-odds
0.5
1.0
1.5
2.0
2.5
3.0
3.5
4.0
Cellular_organisms - ps1
Archaea1 - ps2
Archaea2 - ps3
Archaea3 - ps4
Asgard/Eukaryota - ps5
Eukaryota - ps6
Amorpheae - ps7
Obazoa - ps8
Opisthokonta - ps9
Holomycota - ps10
Fungi - ps11
Fungi_1 - ps12
Fungi_2 - ps13
Fungi_3 - ps14
Dikarya - ps15
Ascomycota - ps16
Saccharo/Pezizomycotina - ps17
Saccharomycotina - ps18
Saccharomycotina_1 - ps19
Saccharomycotina_2 - ps20
Saccharomycotina_3 - ps21
Phaff./Saccharomycetaceae - ps22
Saccharomyco./mycetaceae - ps23
Saccharomycetaceae - ps24
Saccharomycetaceae_2 - ps25
Saccharomycetaceae_3 - ps26
Kaz./Nau./Saccharomyces - ps27
Saccharomyces - ps28
Arboricola_clade - ps29
Cerevisiae_clade - ps30
Saccharomyces_cerevisiae - ps31
Phylostratum

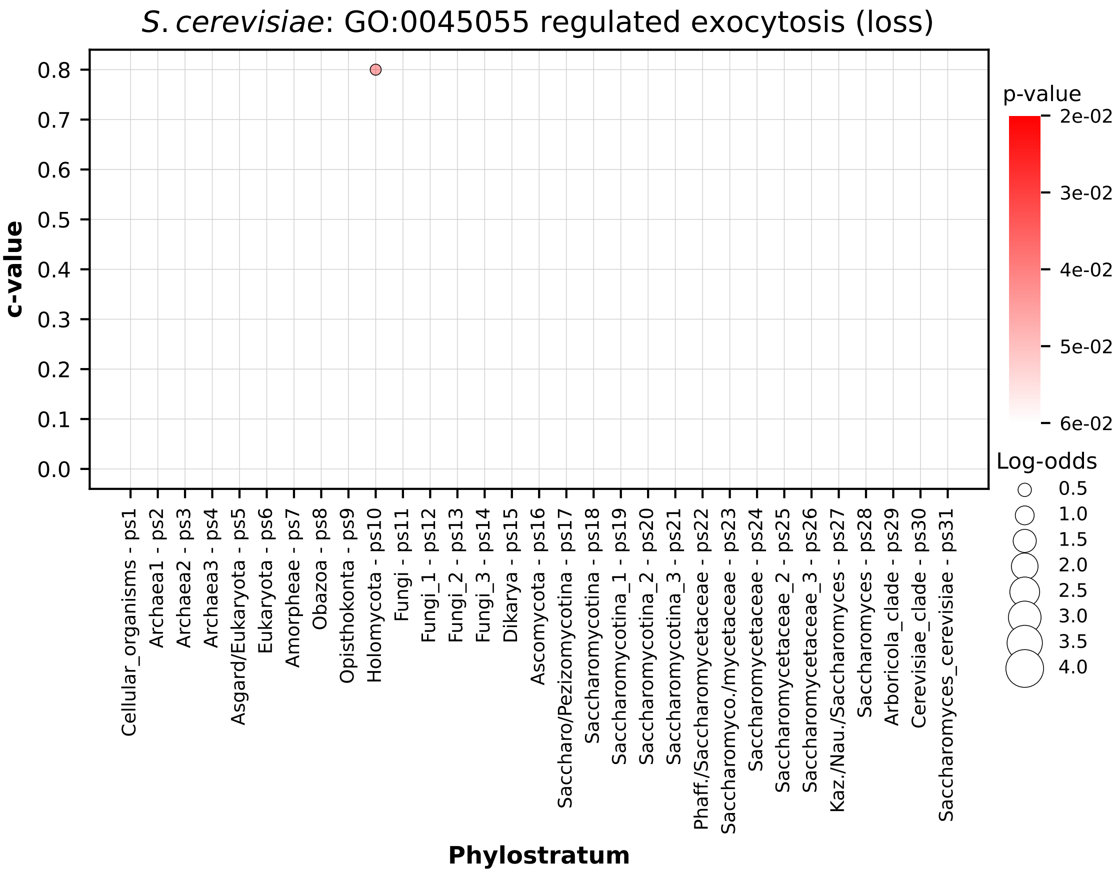
S. cerevisiae: GO:0045055 regulated exocytosis (loss)
c-value
0.8
0.7
0.6
0.5
0.4
0.3
0.2
0.1
0.0
p-value
2e-02
3e-02
4e-02
5e-02
6e-02
Log-odds
0.5
1.0
1.5
2.0
2.5
3.0
3.5
4.0
Cellular_organisms - ps1
Archaea1 - ps2
Archaea2 - ps3
Archaea3 - ps4
Asgard/Eukaryota - ps5
Eukaryota - ps6
Amorpheae - ps7
Obazoa - ps8
Opisthokonta - ps9
Holomycota - ps10
Fungi - ps11
Fungi_1 - ps12
Fungi_2 - ps13
Fungi_3 - ps14
Dikarya - ps15
Ascomycota - ps16
Saccharo/Pezizomycotina - ps17
Saccharomycotina - ps18
Saccharomycotina_1 - ps19
Saccharomycotina_2 - ps20
Saccharomycotina_3 - ps21
Phaff./Saccharomycetaceae - ps22
Saccharomyco./mycetaceae - ps23
Saccharomycetaceae - ps24
Saccharomycetaceae_2 - ps25
Saccharomycetaceae_3 - ps26
Kaz./Nau./Saccharomyces - ps27
Saccharomyces - ps28
Arboricola_clade - ps29
Cerevisiae_clade - ps30
Saccharomyces_cerevisiae - ps31
Phylostratum

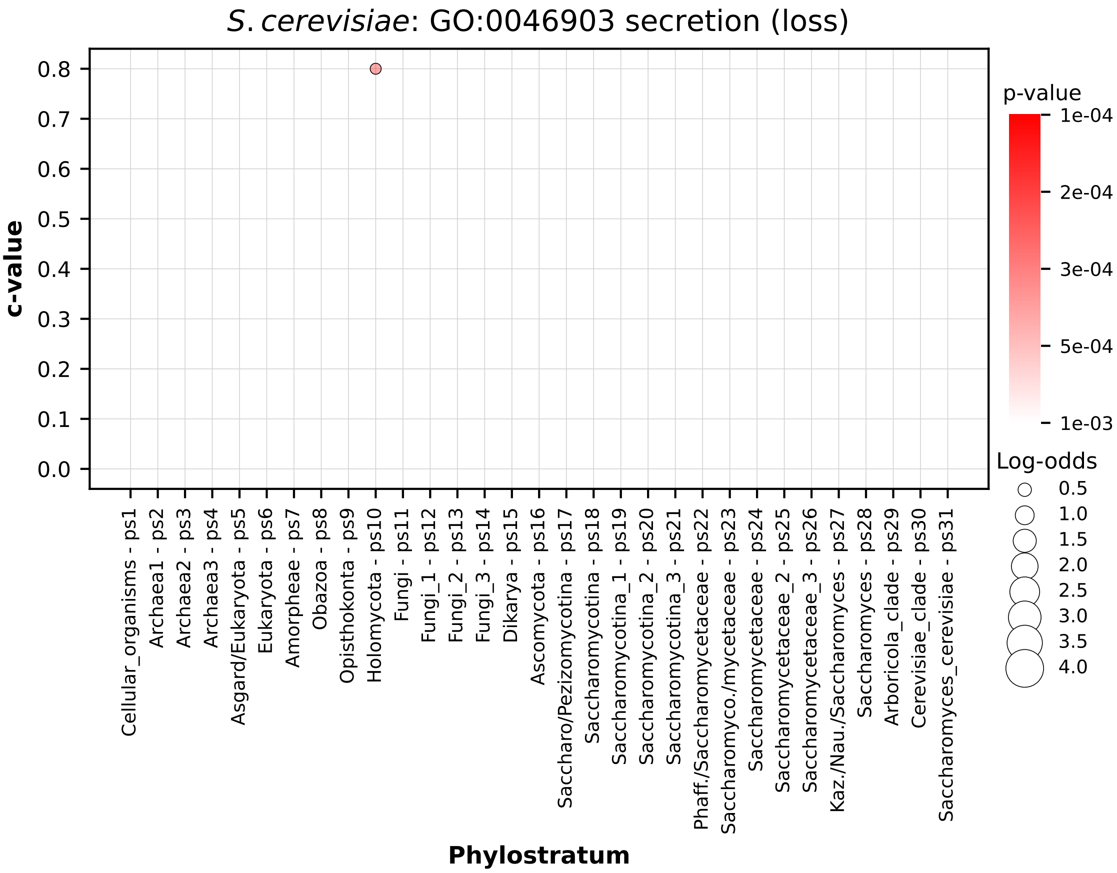

S. cerevisiae: GO:0046903 secretion (loss)
c-value
0.8
0.7
0.6
0.5
0.4
0.3
0.2
0.1
0.0
p-value
1e-04
2e-04
3e-04
5e-04
1e-03
Log-odds
0.5
1.0
1.5
2.0
2.5
3.0
3.5
4.0
Cellular_organisms - ps1
Archaea1 - ps2
Archaea2 - ps3
Archaea3 - ps4
Asgard/Eukaryota - ps5
Eukaryota - ps6
Amorpheae - ps7
Obazoa - ps8
Opisthokonta - ps9
Holomycota - ps10
Fungi - ps11
Fungi_1 - ps12
Fungi_2 - ps13
Fungi_3 - ps14
Dikarya - ps15
Ascomycota - ps16
Saccharo/Pezizomycotina - ps17
Saccharomycotina - ps18
Saccharomycotina_1 - ps19
Saccharomycotina_2 - ps20
Saccharomycotina_3 - ps21
Phaff./Saccharomycetaceae - ps22
Saccharomyco./mycetaceae - ps23
Saccharomycetaceae - ps24
Saccharomycetaceae_2 - ps25
Saccharomycetaceae_3 - ps26
Kaz./Nau./Saccharomyces - ps27
Saccharomyces - ps28
Arboricola_clade - ps29
Cerevisiae_clade - ps30
Saccharomyces_cerevisiae - ps31
Phylostratum

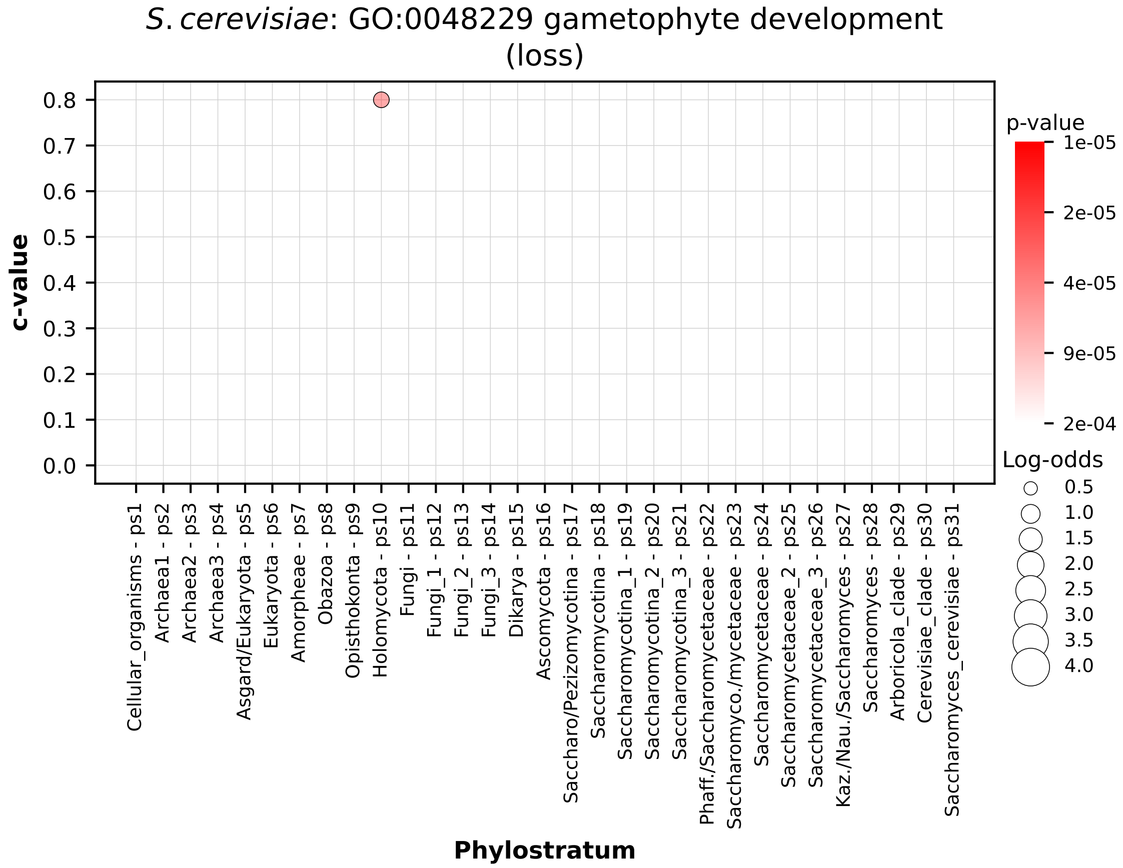

S. cerevisiae: GO:0048229 gametophyte development (loss)
c-value
0.0
0.1
0.2
0.3
0.4
0.5
0.6
0.7
0.8
Phylostratum
Cellular_organisms - ps1
Archaea1 - ps2
Archaea2 - ps3
Archaea3 - ps4
Asgard/Eukaryota - ps5
Eukaryota - ps6
Amorpheae - ps7
Obazoa - ps8
Opisthokonta - ps9
Holomycota - ps10
Fungi - ps11
Fungi_1 - ps12
Fungi_2 - ps13
Fungi_3 - ps14
Dikarya - ps15
Ascomycota - ps16
Saccharo/Pezizomycotina - ps17
Saccharomycotina - ps18
Saccharomycotina_1 - ps19
Saccharomycotina_2 - ps20
Saccharomycotina_3 - ps21
Phaff./Saccharomycetaceae - ps22
Saccharomyco./mycetaceae - ps23
Saccharomycetaceae - ps24
Saccharomycetaceae_2 - ps25
Saccharomycetaceae_3 - ps26
Kaz./Nau./Saccharomyces - ps27
Saccharomyces - ps28
Arboricola_clade - ps29
Cerevisiae_clade - ps30
Saccharomyces_cerevisiae - ps31
p-value
1e-05
2e-05
4e-05
9e-05
2e-04
Log-odds
0.5
1.0
1.5
2.0
2.5
3.0
3.5
4.0

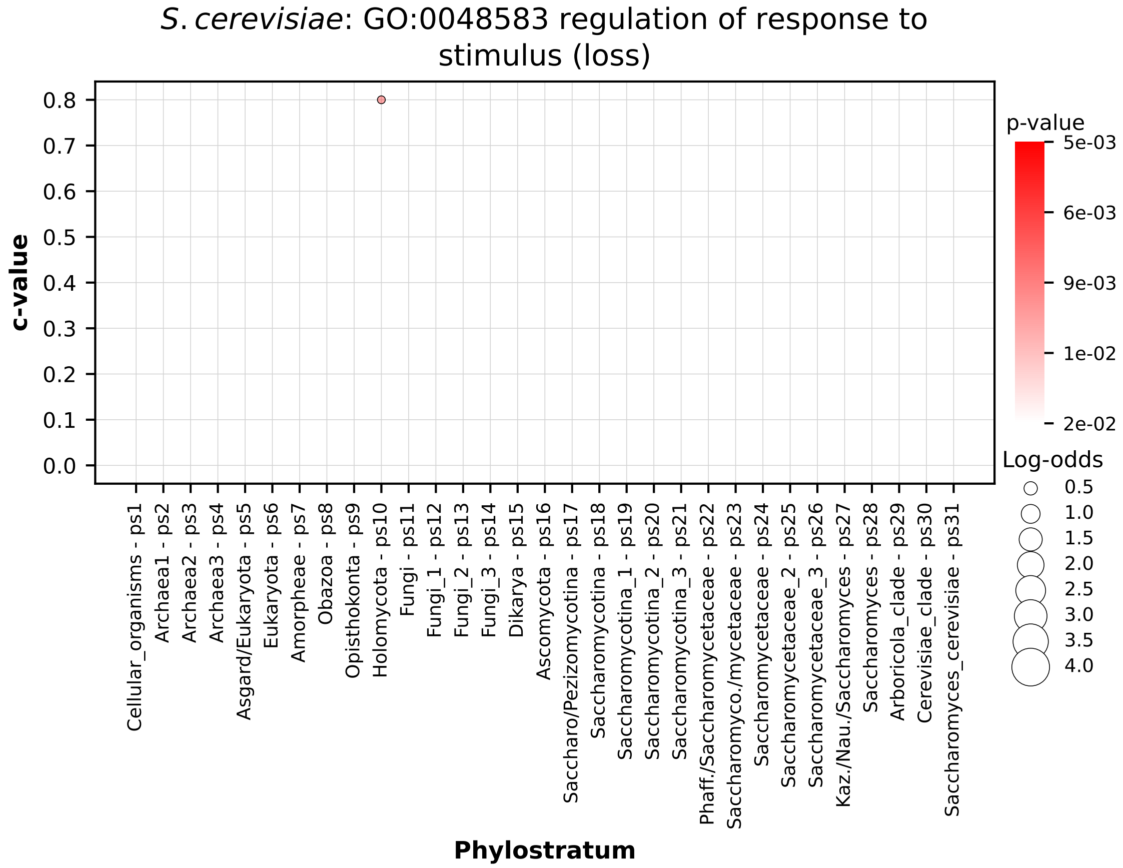
S. cerevisiae: GO:0048583 regulation of response to stimulus (loss)
c-value
0.8
0.7
0.6
0.5
0.4
0.3
0.2
0.1
0.0
p-value
5e-03
6e-03
9e-03
1e-02
2e-02
Log-odds
0.5
1.0
1.5
2.0
2.5
3.0
3.5
4.0
Cellular_organisms - ps1
Archaea1 - ps2
Archaea2 - ps3
Archaea3 - ps4
Asgard/Eukaryota - ps5
Eukaryota - ps6
Amorpheae - ps7
Obazoa - ps8
Opisthokonta - ps9
Holomycota - ps10
Fungi - ps11
Fungi_1 - ps12
Fungi_2 - ps13
Fungi_3 - ps14
Dikarya - ps15
Ascomycota - ps16
Saccharo/Pezizomycotina - ps17
Saccharomycotina - ps18
Saccharomycotina_1 - ps19
Saccharomycotina_2 - ps20
Saccharomycotina_3 - ps21
Phaff./Saccharomycetaceae - ps22
Saccharomyco./mycetaceae - ps23
Saccharomycetaceae - ps24
Saccharomycetaceae_2 - ps25
Saccharomycetaceae_3 - ps26
Kaz./Nau./Saccharomyces - ps27
Saccharomyces - ps28
Arboricola_clade - ps29
Cerevisiae_clade - ps30
Saccharomyces_cerevisiae - ps31
Phylostratum

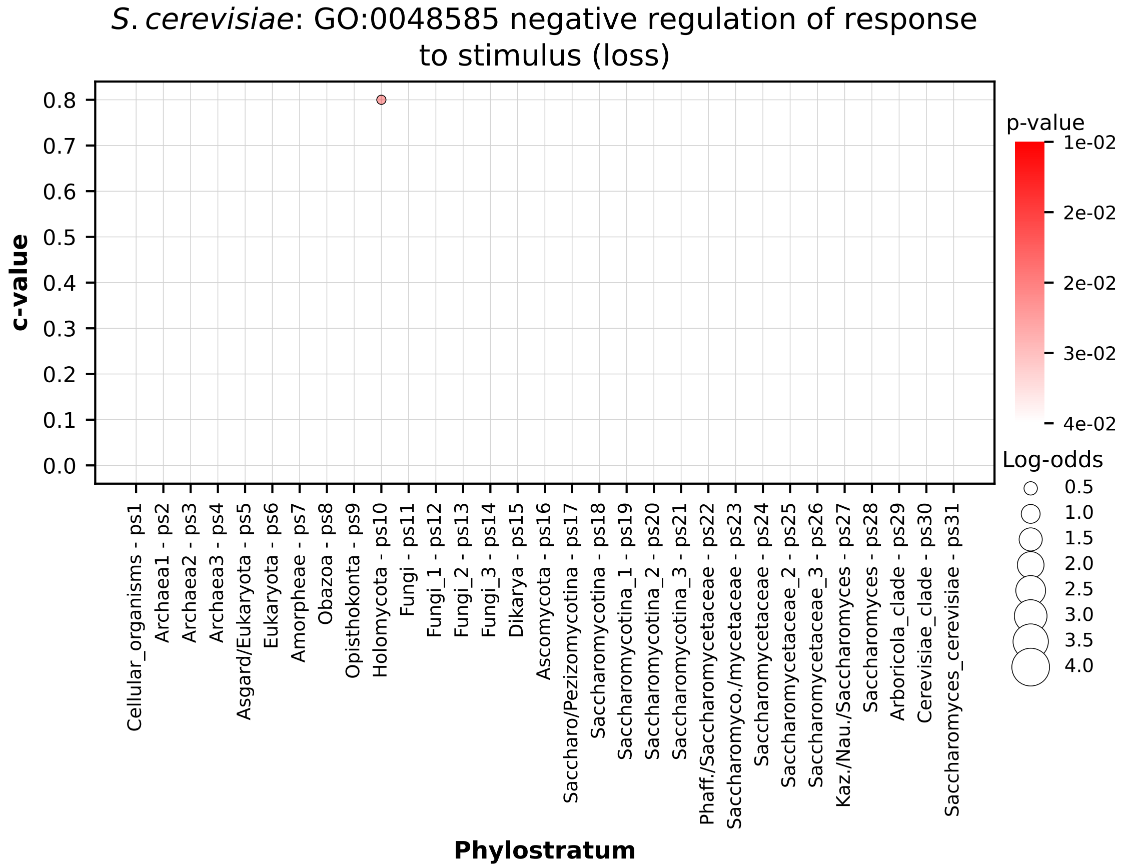
S. cerevisiae: GO:0048585 negative regulation of response to stimulus (loss)
c-value
0.0
0.1
0.2
0.3
0.4
0.5
0.6
0.7
0.8
p-value
1e-02
2e-02
2e-02
3e-02
4e-02
Log-odds
0.5
1.0
1.5
2.0
2.5
3.0
3.5
4.0
Cellular_organisms - ps1
Archaea1 - ps2
Archaea2 - ps3
Archaea3 - ps4
Asgard/Eukaryota - ps5
Eukaryota - ps6
Amorpheae - ps7
Obazoa - ps8
Opisthokonta - ps9
Holomycota - ps10
Fungi - ps11
Fungi_1 - ps12
Fungi_2 - ps13
Fungi_3 - ps14
Dikarya - ps15
Ascomycota - ps16
Saccharo/Pezizomycotina - ps17
Saccharomycotina - ps18
Saccharomycotina_1 - ps19
Saccharomycotina_2 - ps20
Saccharomycotina_3 - ps21
Phaff./Saccharomycetaceae - ps22
Saccharomyco./mycetaceae - ps23
Saccharomycetaceae - ps24
Saccharomycetaceae_2 - ps25
Saccharomycetaceae_3 - ps26
Kaz./Nau./Saccharomyces - ps27
Saccharomyces - ps28
Arboricola_clade - ps29
Cerevisiae_clade - ps30
Saccharomyces_cerevisiae - ps31
Phylostratum

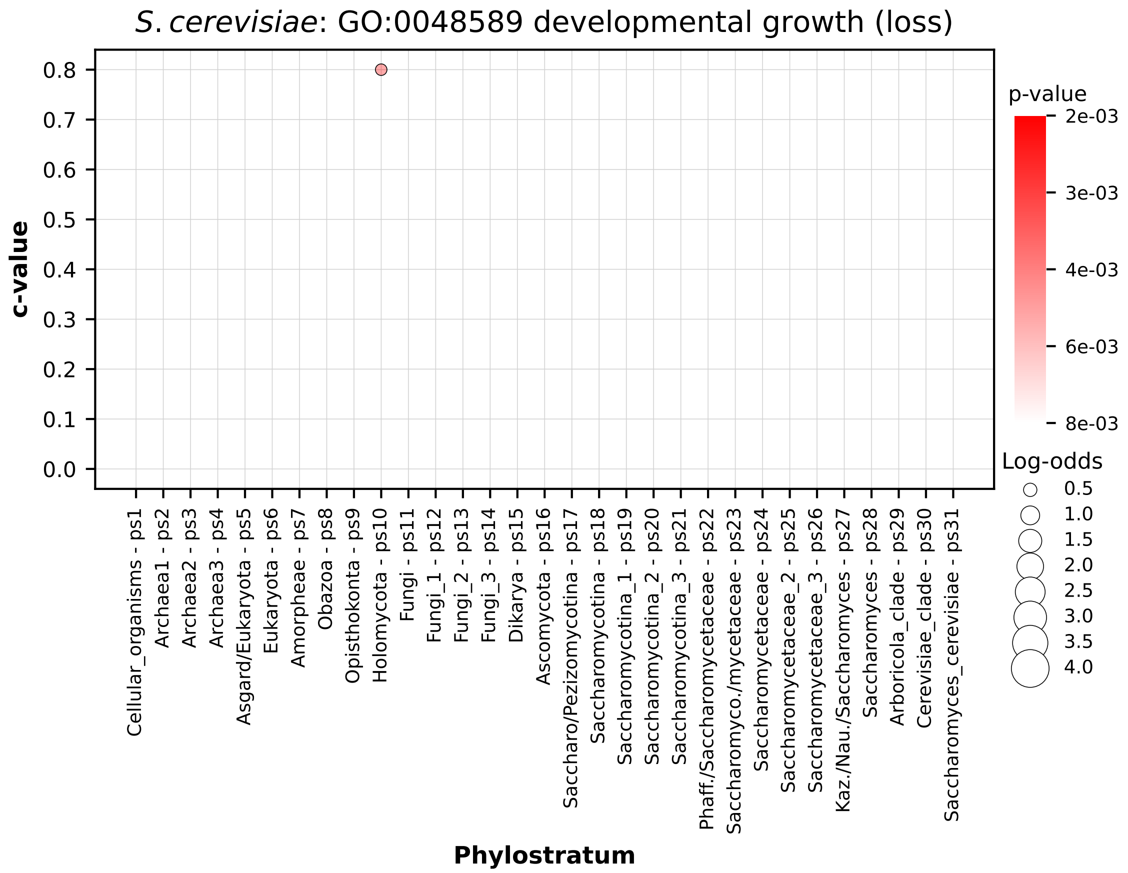

S. cerevisiae: GO:0048589 developmental growth (loss)
c-value
0.0
0.1
0.2
0.3
0.4
0.5
0.6
0.7
0.8
p-value
2e-03
3e-03
4e-03
6e-03
8e-03
Log-odds
0.5
1.0
1.5
2.0
2.5
3.0
3.5
4.0
Phylostratum
Cellular_organisms - ps1
Archaea1 - ps2
Archaea2 - ps3
Archaea3 - ps4
Asgard/Eukaryota - ps5
Eukaryota - ps6
Amorpheae - ps7
Obazoa - ps8
Opisthokonta - ps9
Holomycota - ps10
Fungi - ps11
Fungi_1 - ps12
Fungi_2 - ps13
Fungi_3 - ps14
Dikarya - ps15
Ascomycota - ps16
Saccharo/Pezizomycotina - ps17
Saccharomycotina - ps18
Saccharomycotina_1 - ps19
Saccharomycotina_2 - ps20
Saccharomycotina_3 - ps21
Phaff./Saccharomycetaceae - ps22
Saccharomyco./mycetaceae - ps23
Saccharomycetaceae - ps24
Saccharomycetaceae_2 - ps25
Saccharomycetaceae_3 - ps26
Kaz./Nau./Saccharomyces - ps27
Saccharomyces - ps28
Arboricola_clade - ps29
Cerevisiae_clade - ps30
Saccharomyces_cerevisiae - ps31

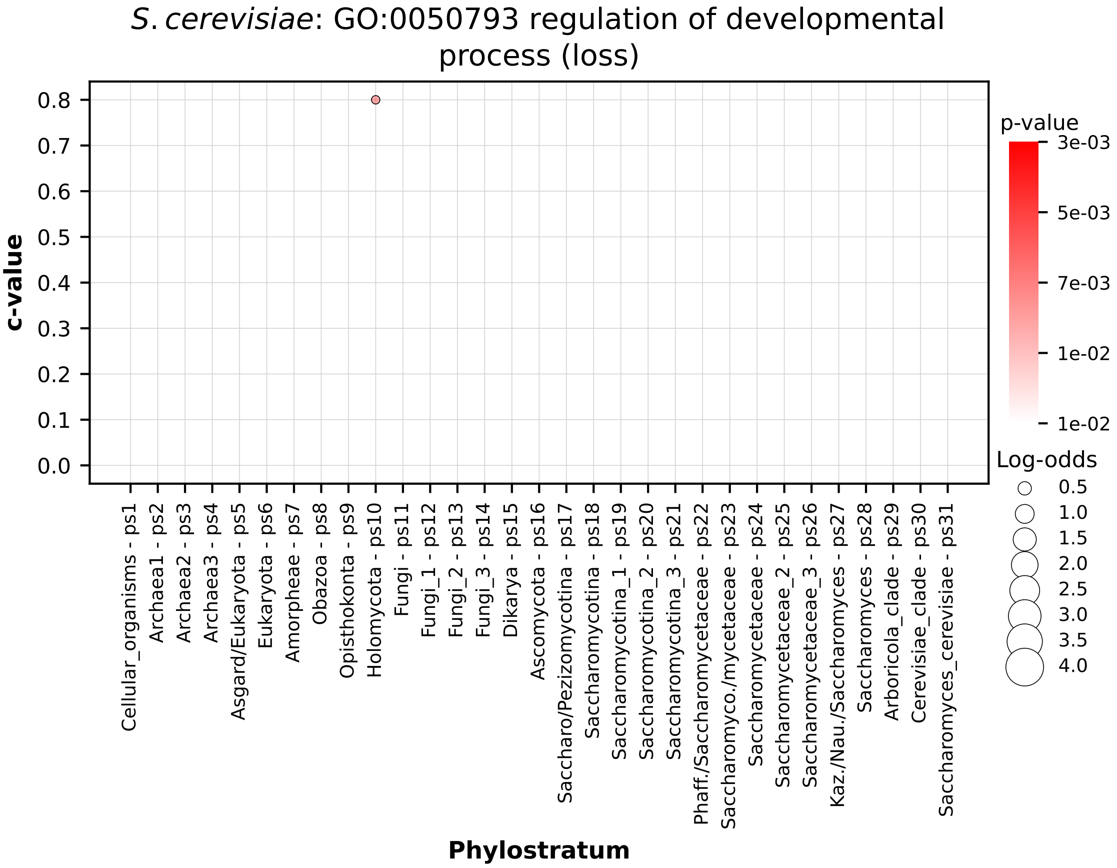
S. cerevisiae: GO:0050793 regulation of developmental process (loss)
c-value
0.0
0.1
0.2
0.3
0.4
0.5
0.6
0.7
0.8
Phylostratum
Cellular_organisms - ps1
Archaea1 - ps2
Archaea2 - ps3
Archaea3 - ps4
Asgard/Eukaryota - ps5
Eukaryota - ps6
Amorpheae - ps7
Obazoa - ps8
Opisthokonta - ps9
Holomycota - ps10
Fungi - ps11
Fungi_1 - ps12
Fungi_2 - ps13
Fungi_3 - ps14
Dikarya - ps15
Ascomycota - ps16
Saccharo/Pezizomycotina - ps17
Saccharomycotina - ps18
Saccharomycotina_1 - ps19
Saccharomycotina_2 - ps20
Saccharomycotina_3 - ps21
Phaff./Saccharomycetaceae - ps22
Saccharomyco./mycetaceae - ps23
Saccharomycetaceae - ps24
Saccharomycetaceae_2 - ps25
Saccharomycetaceae_3 - ps26
Kaz./Nau./Saccharomyces - ps27
Saccharomyces - ps28
Arboricola_clade - ps29
Cerevisiae_clade - ps30
Saccharomyces_cerevisiae - ps31
p-value
3e-03
5e-03
7e-03
1e-02
1e-02
Log-odds
0.5
1.0
1.5
2.0
2.5
3.0
3.5
4.0

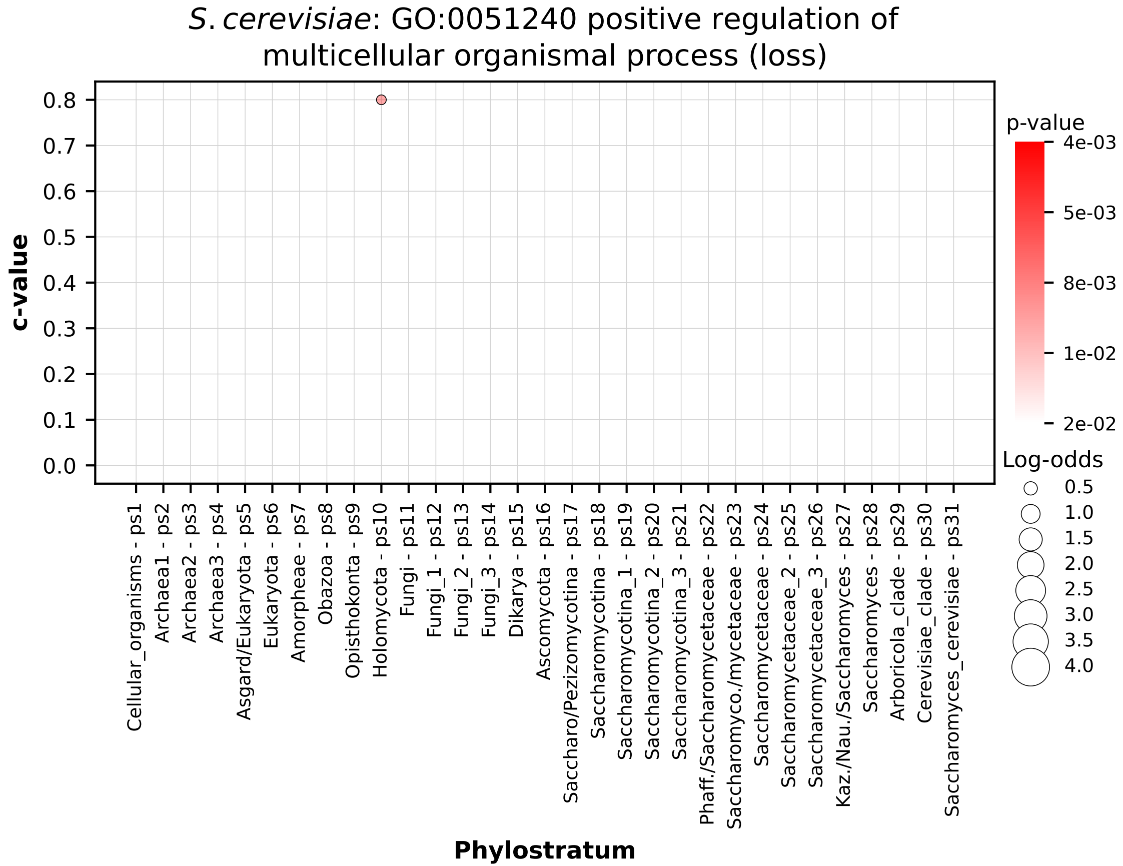
S. cerevisiae: GO:0051240 positive regulation of multicellular organismal process (loss)
c-value
0.0
0.1
0.2
0.3
0.4
0.5
0.6
0.7
0.8
Phylostratum
Cellular_organisms - ps1
Archaea1 - ps2
Archaea2 - ps3
Archaea3 - ps4
Asgard/Eukaryota - ps5
Eukaryota - ps6
Amorpheae - ps7
Obazoa - ps8
Opisthokonta - ps9
Holomycota - ps10
Fungi - ps11
Fungi_1 - ps12
Fungi_2 - ps13
Fungi_3 - ps14
Dikarya - ps15
Ascomycota - ps16
Saccharo/Pezizomycotina - ps17
Saccharomycotina - ps18
Saccharomycotina_1 - ps19
Saccharomycotina_2 - ps20
Saccharomycotina_3 - ps21
Phaff./Saccharomycetaceae - ps22
Saccharomyco./mycetaceae - ps23
Saccharomycetaceae - ps24
Saccharomycetaceae_2 - ps25
Saccharomycetaceae_3 - ps26
Kaz./Nau./Saccharomyces - ps27
Saccharomyces - ps28
Arboricola_clade - ps29
Cerevisiae_clade - ps30
Saccharomyces_cerevisiae - ps31
p-value
4e-03
5e-03
8e-03
1e-02
2e-02
Log-odds
0.5
1.0
1.5
2.0
2.5
3.0
3.5
4.0

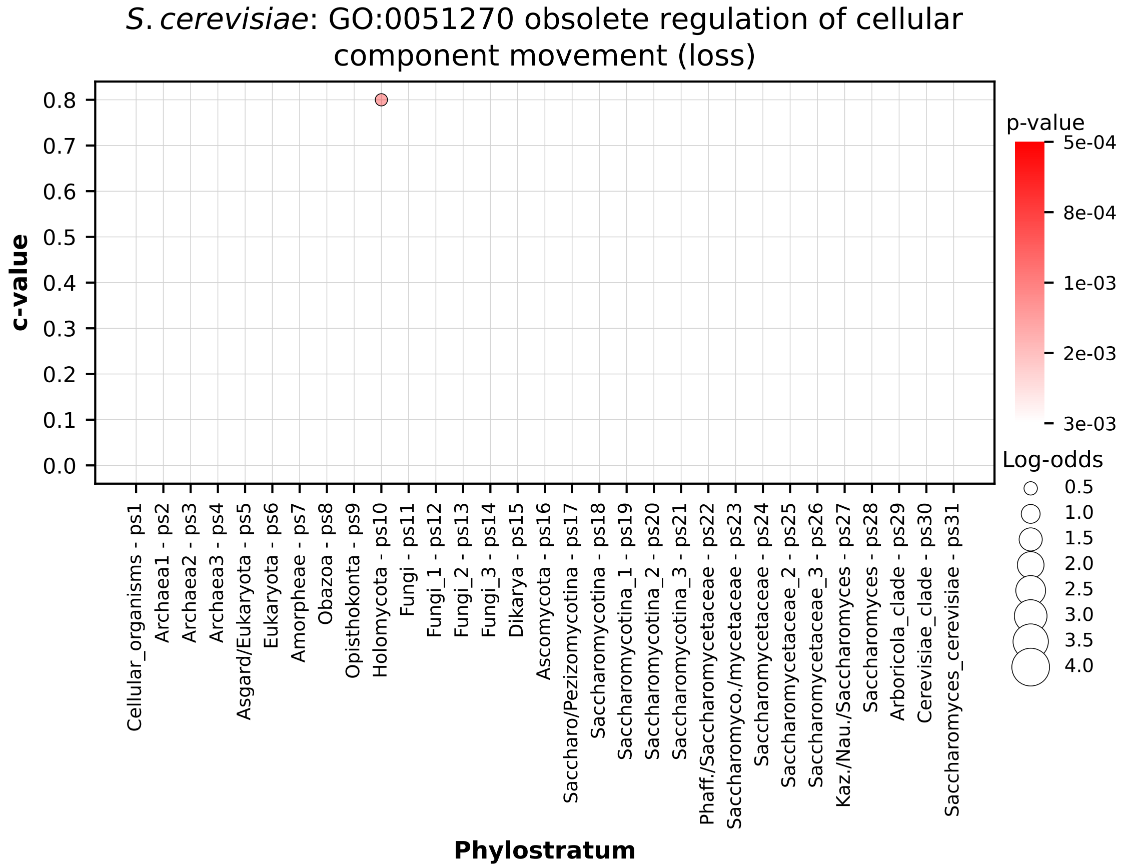

S. cerevisiae: GO:0051270 obsolete regulation of cellular component movement (loss)
c-value
0.8
0.7
0.6
0.5
0.4
0.3
0.2
0.1
0.0
p-value
5e-04
8e-04
1e-03
2e-03
3e-03
Log-odds
0.5
1.0
1.5
2.0
2.5
3.0
3.5
4.0
Cellular_organisms - ps1
Archaea1 - ps2
Archaea2 - ps3
Archaea3 - ps4
Asgard/Eukaryota - ps5
Eukaryota - ps6
Amorpheae - ps7
Obazoa - ps8
Opisthokonta - ps9
Holomycota - ps10
Fungi - ps11
Fungi_1 - ps12
Fungi_2 - ps13
Fungi_3 - ps14
Dikarya - ps15
Ascomycota - ps16
Saccharo/Pezizomycotina - ps17
Saccharomycotina - ps18
Saccharomycotina_1 - ps19
Saccharomycotina_2 - ps20
Saccharomycotina_3 - ps21
Phaff./Saccharomycetaceae - ps22
Saccharomyco./mycetaceae - ps23
Saccharomycetaceae - ps24
Saccharomycetaceae_2 - ps25
Saccharomycetaceae_3 - ps26
Kaz./Nau./Saccharomyces - ps27
Saccharomyces - ps28
Arboricola_clade - ps29
Cerevisiae_clade - ps30
Saccharomyces_cerevisiae - ps31
Phylostratum

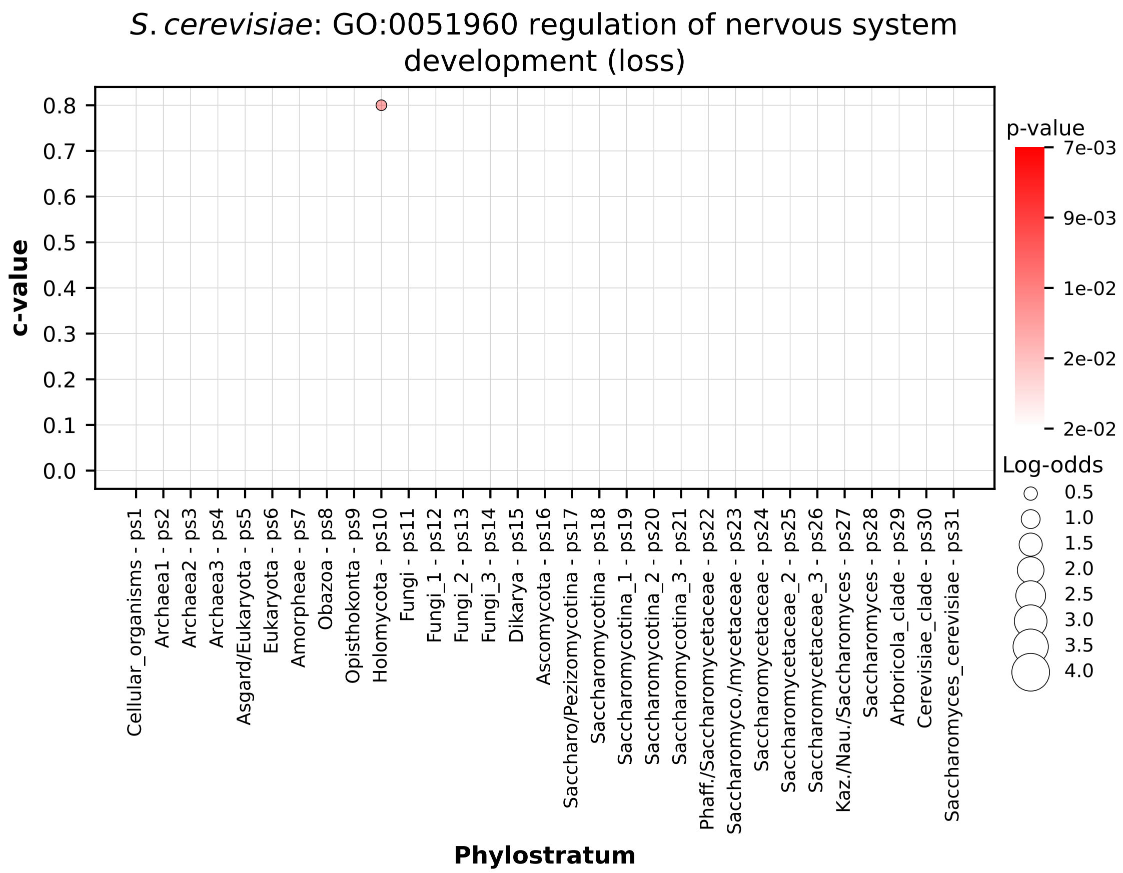

S. cerevisiae: GO:0051960 regulation of nervous system development (loss)
c-value
0.8
0.7
0.6
0.5
0.4
0.3
0.2
0.1
0.0
p-value
7e-03
9e-03
1e-02
2e-02
2e-02
Log-odds
0.5
1.0
1.5
2.0
2.5
3.0
3.5
4.0
Cellular_organisms - ps1
Archaea1 - ps2
Archaea2 - ps3
Archaea3 - ps4
Asgard/Eukaryota - ps5
Eukaryota - ps6
Amorpheae - ps7
Obazoa - ps8
Opisthokonta - ps9
Holomycota - ps10
Fungi - ps11
Fungi_1 - ps12
Fungi_2 - ps13
Fungi_3 - ps14
Dikarya - ps15
Ascomycota - ps16
Saccharo/Pezizomycotina - ps17
Saccharomycotina - ps18
Saccharomycotina_1 - ps19
Saccharomycotina_2 - ps20
Saccharomycotina_3 - ps21
Phaff./Saccharomycetaceae - ps22
Saccharomyco./mycetaceae - ps23
Saccharomycetaceae - ps24
Saccharomycetaceae_2 - ps25
Saccharomycetaceae_3 - ps26
Kaz./Nau./Saccharomyces - ps27
Saccharomyces - ps28
Arboricola_clade - ps29
Cerevisiae_clade - ps30
Saccharomyces_cerevisiae - ps31
Phylostratum

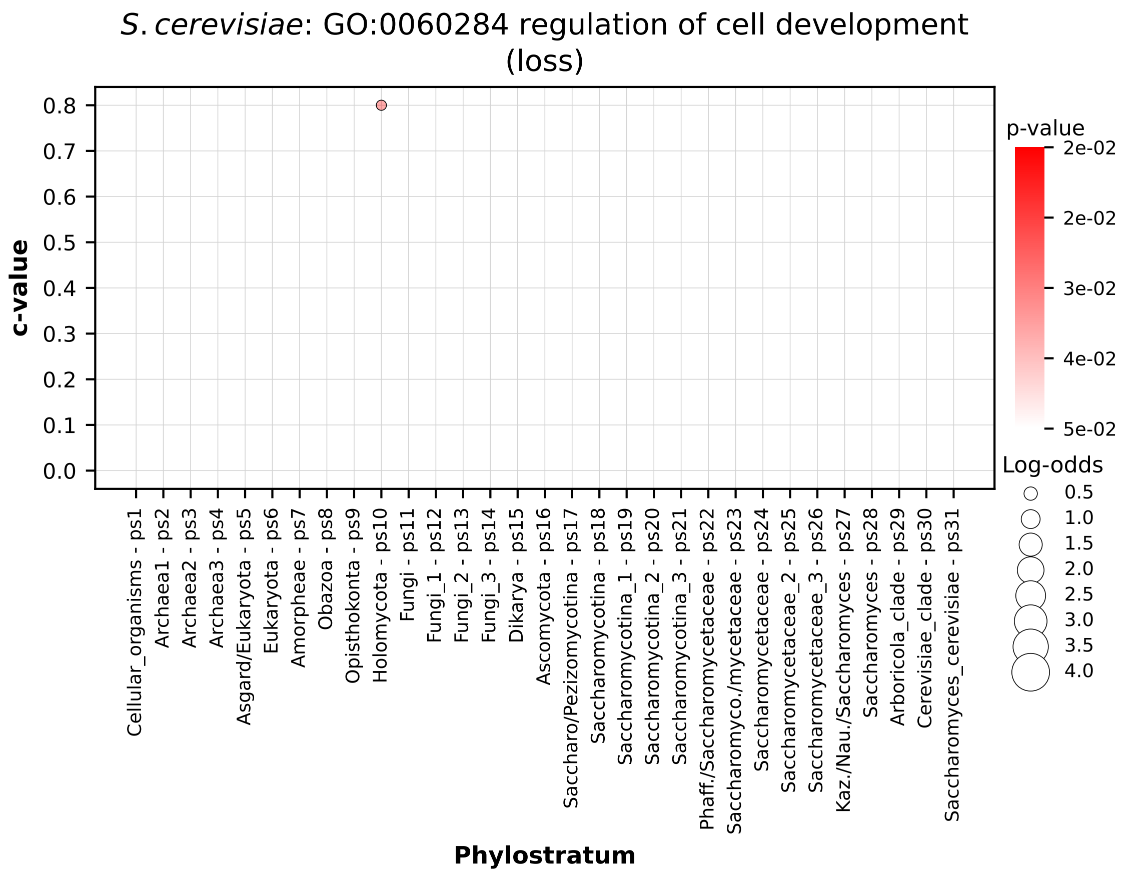
S. cerevisiae: GO:0060284 regulation of cell development
(loss)
c-value
0.8
0.7
0.6
0.5
0.4
0.3
0.2
0.1
0.0
p-value
2e-02
2e-02
3e-02
4e-02
5e-02
Log-odds
0.5
1.0
1.5
2.0
2.5
3.0
3.5
4.0
Cellular_organisms - ps1
Archaea1 - ps2
Archaea2 - ps3
Archaea3 - ps4
Asgard/Eukaryota - ps5
Eukaryota - ps6
Amorpheae - ps7
Obazoa - ps8
Opisthokonta - ps9
Holomycota - ps10
Fungi - ps11
Fungi_1 - ps12
Fungi_2 - ps13
Fungi_3 - ps14
Dikarya - ps15
Ascomycota - ps16
Saccharo/Pezizomycotina - ps17
Saccharomycotina - ps18
Saccharomycotina_1 - ps19
Saccharomycotina_2 - ps20
Saccharomycotina_3 - ps21
Phaff./Saccharomycetaceae - ps22
Saccharomyco./mycetaceae - ps23
Saccharomycetaceae - ps24
Saccharomycetaceae_2 - ps25
Saccharomycetaceae_3 - ps26
Kaz./Nau./Saccharomyces - ps27
Saccharomyces - ps28
Arboricola_clade - ps29
Cerevisiae_clade - ps30
Saccharomyces_cerevisiae - ps31
Phylostratum

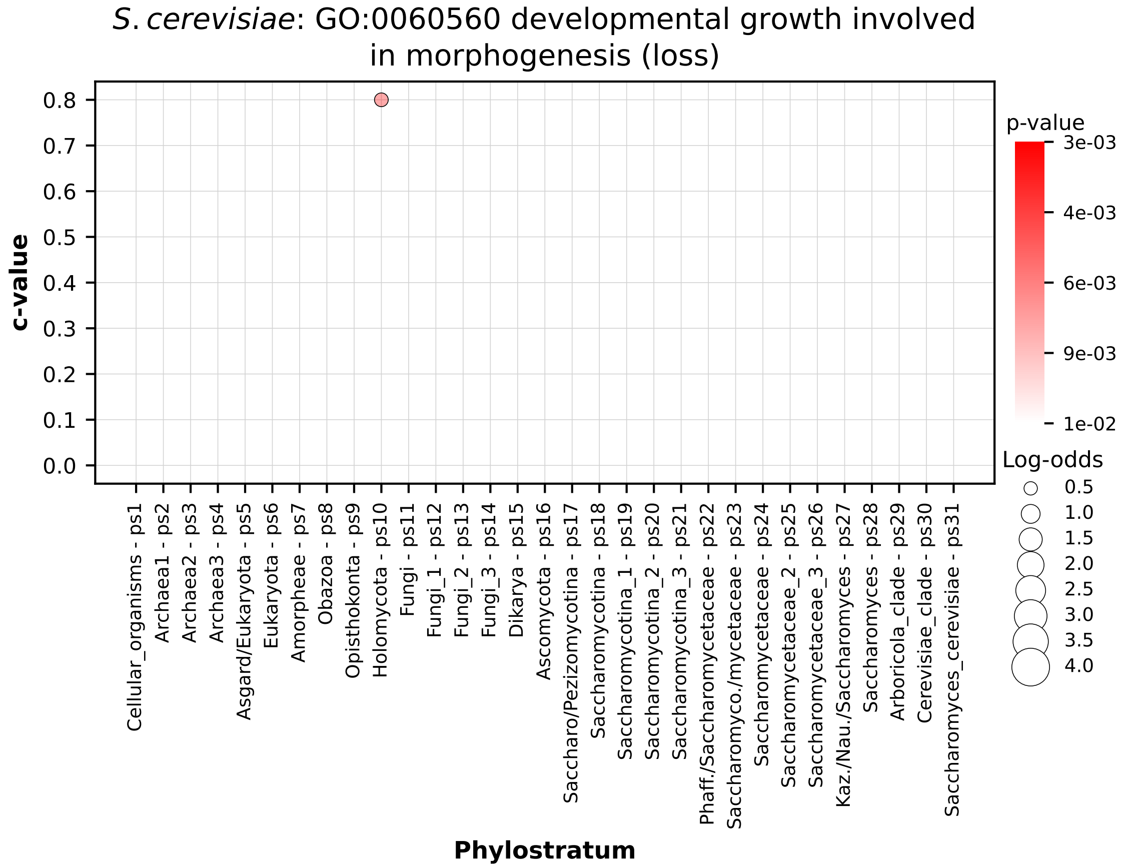
S. cerevisiae: GO:0060560 developmental growth involved in morphogenesis (loss)
c-value
0.8
0.7
0.6
0.5
0.4
0.3
0.2
0.1
0.0
p-value
3e-03
4e-03
6e-03
9e-03
1e-02
Log-odds
0.5
1.0
1.5
2.0
2.5
3.0
3.5
4.0
Cellular_organisms - ps1
Archaea1 - ps2
Archaea2 - ps3
Archaea3 - ps4
Asgard/Eukaryota - ps5
Eukaryota - ps6
Amorpheae - ps7
Obazoa - ps8
Opisthokonta - ps9
Holomycota - ps10
Fungi - ps11
Fungi_1 - ps12
Fungi_2 - ps13
Fungi_3 - ps14
Dikarya - ps15
Ascomycota - ps16
Saccharo/Pezizomycotina - ps17
Saccharomycotina - ps18
Saccharomycotina_1 - ps19
Saccharomycotina_2 - ps20
Saccharomycotina_3 - ps21
Phaff./Saccharomycetaceae - ps22
Saccharomyco./mycetaceae - ps23
Saccharomycetaceae - ps24
Saccharomycetaceae_2 - ps25
Saccharomycetaceae_3 - ps26
Kaz./Nau./Saccharomyces - ps27
Saccharomyces - ps28
Arboricola_clade - ps29
Cerevisiae_clade - ps30
Saccharomyces_cerevisiae - ps31
Phylostratum

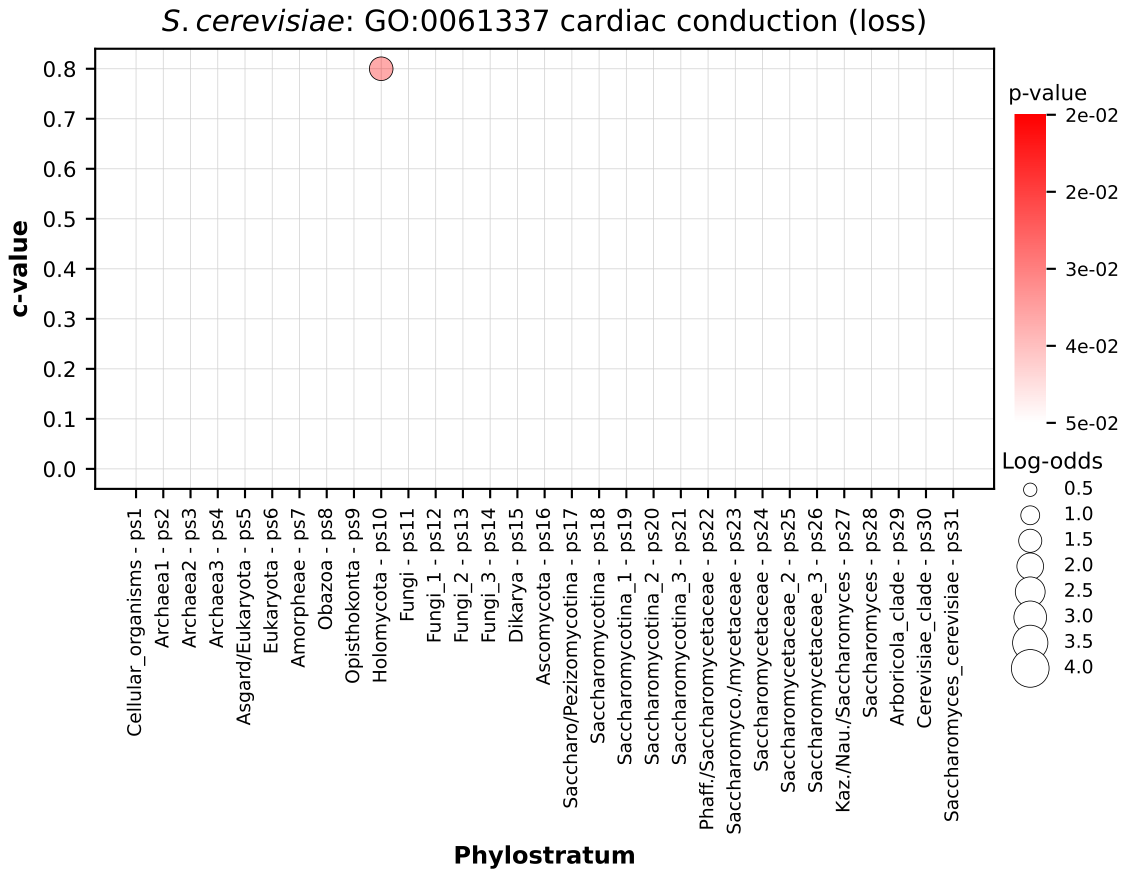

S. cerevisiae: GO:0061337 cardiac conduction (loss)
c-value
0.0
0.1
0.2
0.3
0.4
0.5
0.6
0.7
0.8
Phylostratum
Cellular_organisms - ps1
Archaea1 - ps2
Archaea2 - ps3
Archaea3 - ps4
Asgard/Eukaryota - ps5
Eukaryota - ps6
Amorpheae - ps7
Obazoa - ps8
Opisthokonta - ps9
Holomycota - ps10
Fungi - ps11
Fungi_1 - ps12
Fungi_2 - ps13
Fungi_3 - ps14
Dikarya - ps15
Ascomycota - ps16
Saccharo/Pezizomycotina - ps17
Saccharomycotina - ps18
Saccharomycotina_1 - ps19
Saccharomycotina_2 - ps20
Saccharomycotina_3 - ps21
Phaff./Saccharomycetaceae - ps22
Saccharomyco./mycetaceae - ps23
Saccharomycetaceae - ps24
Saccharomycetaceae_2 - ps25
Saccharomycetaceae_3 - ps26
Kaz./Nau./Saccharomyces - ps27
Saccharomyces - ps28
Arboricola_clade - ps29
Cerevisiae_clade - ps30
Saccharomyces_cerevisiae - ps31
p-value
2e-02
2e-02
3e-02
4e-02
5e-02
Log-odds
0.5
1.0
1.5
2.0
2.5
3.0
3.5
4.0

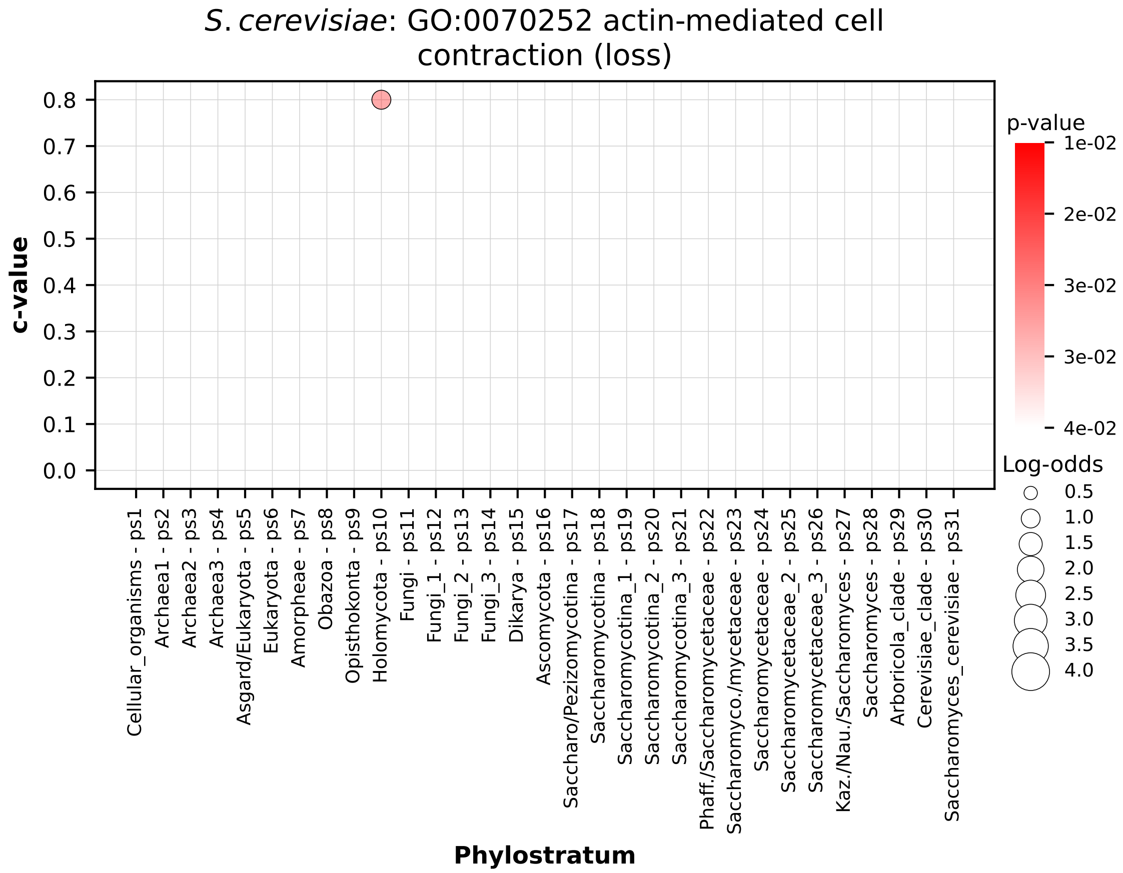
S. cerevisiae: GO:0070252 actin-mediated cell contraction (loss)
c-value
0.0
0.1
0.2
0.3
0.4
0.5
0.6
0.7
0.8
Phylostratum
Cellular_organisms - ps1
Archaea1 - ps2
Archaea2 - ps3
Archaea3 - ps4
Asgard/Eukaryota - ps5
Eukaryota - ps6
Amorpheae - ps7
Obazoa - ps8
Opisthokonta - ps9
Holomycota - ps10
Fungi - ps11
Fungi_1 - ps12
Fungi_2 - ps13
Fungi_3 - ps14
Dikarya - ps15
Ascomycota - ps16
Saccharo/Pezizomycotina - ps17
Saccharomycotina - ps18
Saccharomycotina_1 - ps19
Saccharomycotina_2 - ps20
Saccharomycotina_3 - ps21
Phaff./Saccharomycetaceae - ps22
Saccharomyco./mycetaceae - ps23
Saccharomycetaceae - ps24
Saccharomycetaceae_2 - ps25
Saccharomycetaceae_3 - ps26
Kaz./Nau./Saccharomyces - ps27
Saccharomyces - ps28
Arboricola_clade - ps29
Cerevisiae_clade - ps30
Saccharomyces_cerevisiae - ps31
p-value
1e-02
2e-02
3e-02
3e-02
4e-02
Log-odds
0.5
1.0
1.5
2.0
2.5
3.0
3.5
4.0

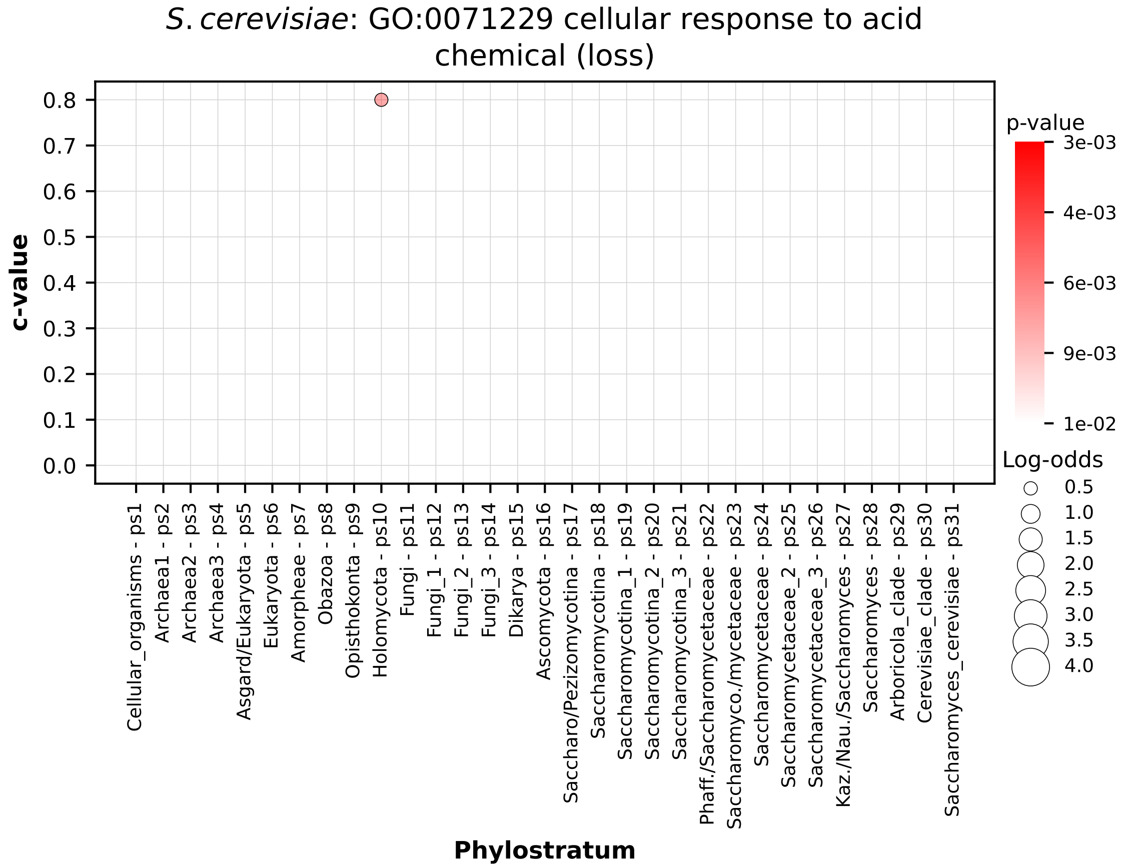
S. cerevisiae: GO:0071229 cellular response to acid chemical (loss)
c-value
0.8
0.7
0.6
0.5
0.4
0.3
0.2
0.1
0.0
p-value
3e-03
4e-03
6e-03
9e-03
1e-02
Log-odds
0.5
1.0
1.5
2.0
2.5
3.0
3.5
4.0
Cellular_organisms - ps1
Archaea1 - ps2
Archaea2 - ps3
Archaea3 - ps4
Asgard/Eukaryota - ps5
Eukaryota - ps6
Amorpheae - ps7
Obazoa - ps8
Opisthokonta - ps9
Holomycota - ps10
Fungi - ps11
Fungi_1 - ps12
Fungi_2 - ps13
Fungi_3 - ps14
Dikarya - ps15
Ascomycota - ps16
Saccharo/Pezizomycotina - ps17
Saccharomycotina - ps18
Saccharomycotina_1 - ps19
Saccharomycotina_2 - ps20
Saccharomycotina_3 - ps21
Phaff./Saccharomycetaceae - ps22
Saccharomyco./mycetaceae - ps23
Saccharomycetaceae - ps24
Saccharomycetaceae_2 - ps25
Saccharomycetaceae_3 - ps26
Kaz./Nau./Saccharomyces - ps27
Saccharomyces - ps28
Arboricola_clade - ps29
Cerevisiae_clade - ps30
Saccharomyces_cerevisiae - ps31
Phylostratum

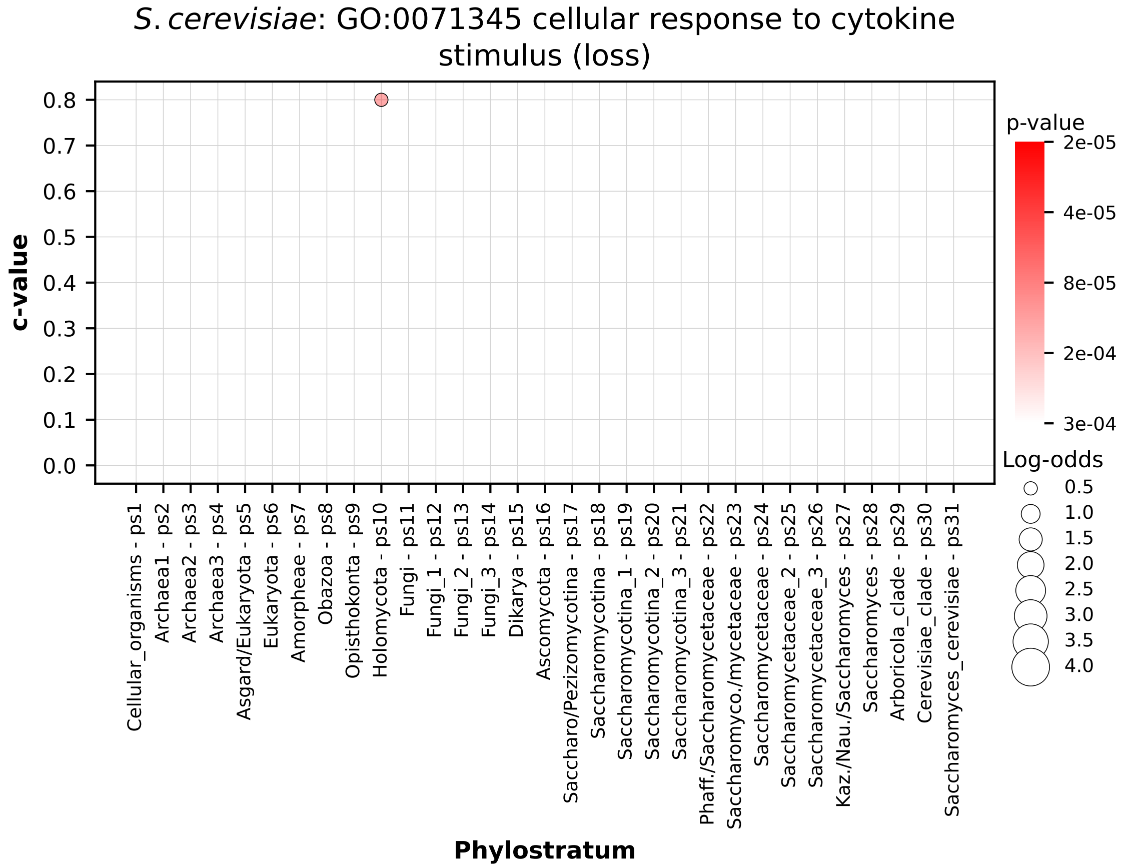
S. cerevisiae: GO:0071345 cellular response to cytokine stimulus (loss)
c-value
0.0
0.1
0.2
0.3
0.4
0.5
0.6
0.7
0.8
p-value
2e-05
4e-05
8e-05
2e-04
3e-04
Log-odds
0.5
1.0
1.5
2.0
2.5
3.0
3.5
4.0
Cellular_organisms - ps1
Archaea1 - ps2
Archaea2 - ps3
Archaea3 - ps4
Asgard/Eukaryota - ps5
Eukaryota - ps6
Amorpheae - ps7
Obazoa - ps8
Opisthokonta - ps9
Holomycota - ps10
Fungi - ps11
Fungi_1 - ps12
Fungi_2 - ps13
Fungi_3 - ps14
Dikarya - ps15
Ascomycota - ps16
Saccharo/Pezizomycotina - ps17
Saccharomycotina - ps18
Saccharomycotina_1 - ps19
Saccharomycotina_2 - ps20
Saccharomycotina_3 - ps21
Phaff./Saccharomycetaceae - ps22
Saccharomyco./mycetaceae - ps23
Saccharomycetaceae - ps24
Saccharomycetaceae_2 - ps25
Saccharomycetaceae_3 - ps26
Kaz./Nau./Saccharomyces - ps27
Saccharomyces - ps28
Arboricola_clade - ps29
Cerevisiae_clade - ps30
Saccharomyces_cerevisiae - ps31
Phylostratum

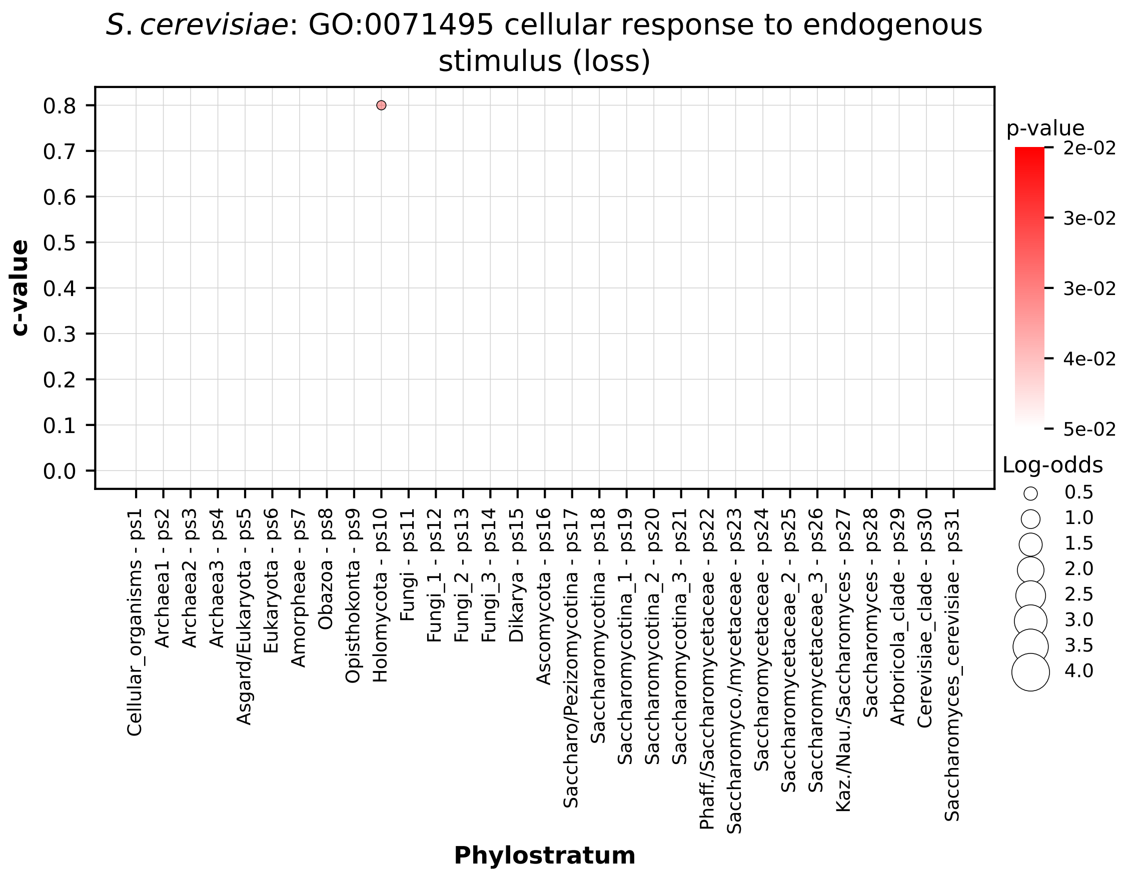

S. cerevisiae: GO:0071495 cellular response to endogenous stimulus (loss)
c-value
0.8
0.7
0.6
0.5
0.4
0.3
0.2
0.1
0.0
p-value
2e-02
3e-02
3e-02
4e-02
5e-02
Log-odds
0.5
1.0
1.5
2.0
2.5
3.0
3.5
4.0
Cellular_organisms - ps1
Archaea1 - ps2
Archaea2 - ps3
Archaea3 - ps4
Asgard/Eukaryota - ps5
Eukaryota - ps6
Amorpheae - ps7
Obazoa - ps8
Opisthokonta - ps9
Holomycota - ps10
Fungi - ps11
Fungi_1 - ps12
Fungi_2 - ps13
Fungi_3 - ps14
Dikarya - ps15
Ascomycota - ps16
Saccharo/Pezizomycotina - ps17
Saccharomycotina - ps18
Saccharomycotina_1 - ps19
Saccharomycotina_2 - ps20
Saccharomycotina_3 - ps21
Phaff./Saccharomycetaceae - ps22
Saccharomyco./mycetaceae - ps23
Saccharomycetaceae - ps24
Saccharomycetaceae_2 - ps25
Saccharomycetaceae_3 - ps26
Kaz./Nau./Saccharomyces - ps27
Saccharomyces - ps28
Arboricola_clade - ps29
Cerevisiae_clade - ps30
Saccharomyces_cerevisiae - ps31
Phylostratum

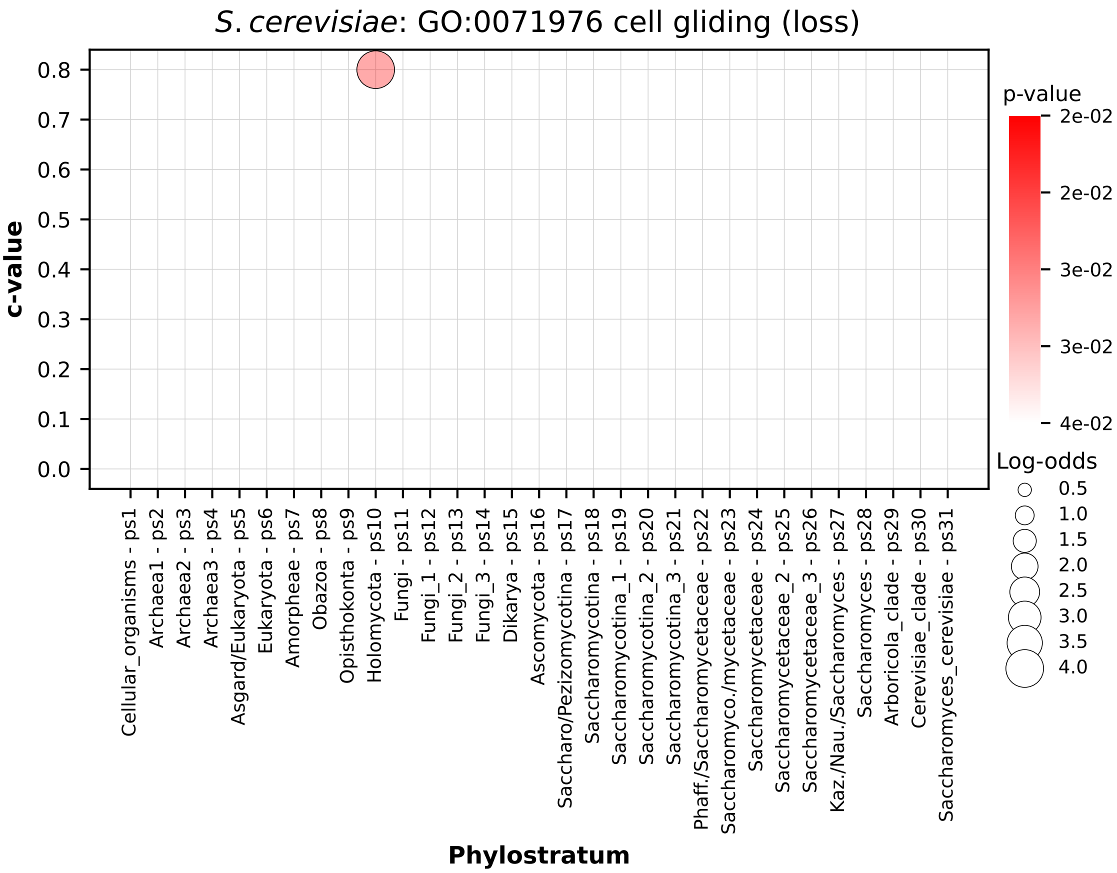

S. cerevisiae: GO:0071976 cell gliding (loss)
c-value
0.8
0.7
0.6
0.5
0.4
0.3
0.2
0.1
0.0
p-value
2e-02
2e-02
3e-02
3e-02
4e-02
Log-odds
0.5
1.0
1.5
2.0
2.5
3.0
3.5
4.0
Cellular_organisms - ps1
Archaea1 - ps2
Archaea2 - ps3
Archaea3 - ps4
Asgard/Eukaryota - ps5
Eukaryota - ps6
Amorpheae - ps7
Obazoa - ps8
Opisthokonta - ps9
Holomycota - ps10
Fungi - ps11
Fungi_1 - ps12
Fungi_2 - ps13
Fungi_3 - ps14
Dikarya - ps15
Ascomycota - ps16
Saccharo/Pezizomycotina - ps17
Saccharomycotina - ps18
Saccharomycotina_1 - ps19
Saccharomycotina_2 - ps20
Saccharomycotina_3 - ps21
Phaff./Saccharomycetaceae - ps22
Saccharomyco./mycetaceae - ps23
Saccharomycetaceae - ps24
Saccharomycetaceae_2 - ps25
Saccharomycetaceae_3 - ps26
Kaz./Nau./Saccharomyces - ps27
Saccharomyces - ps28
Arboricola_clade - ps29
Cerevisiae_clade - ps30
Saccharomyces_cerevisiae - ps31
Phylostratum

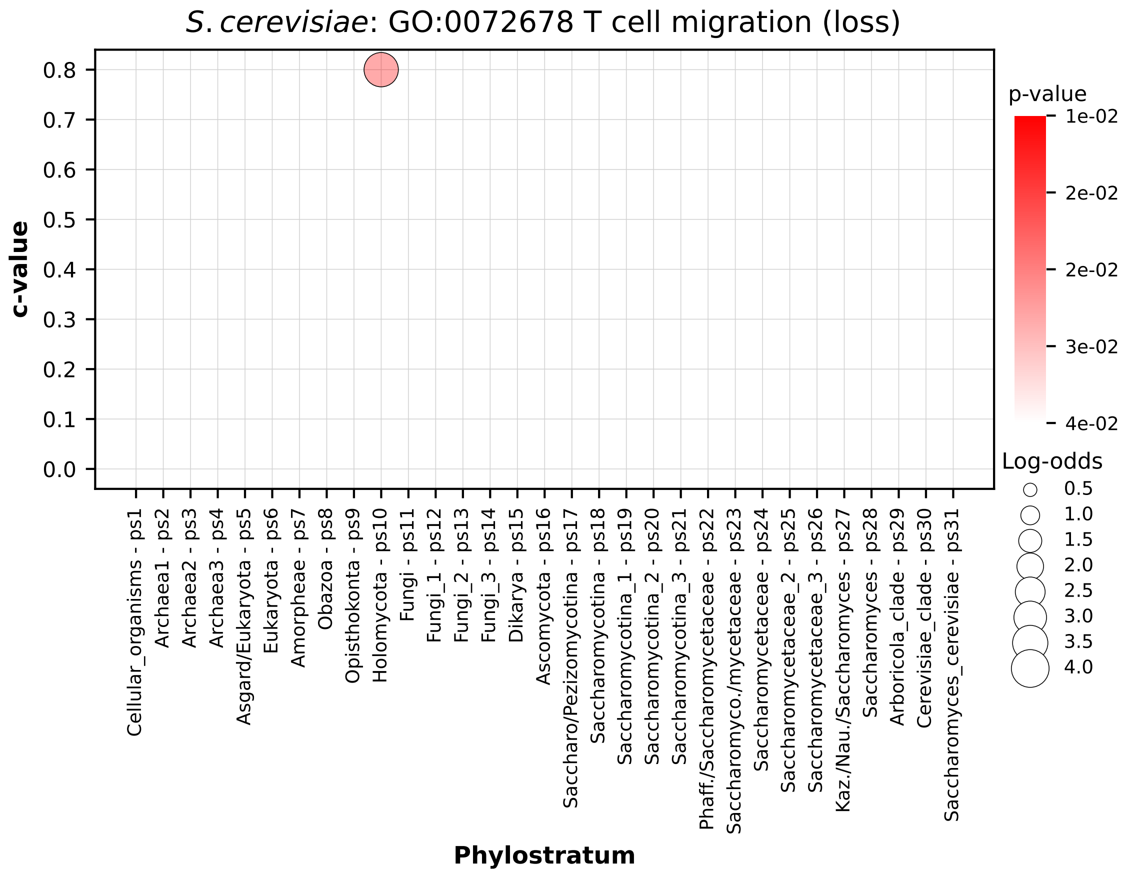

S. cerevisiae: GO:0072678 T cell migration (loss)
c-value
0.8
0.7
0.6
0.5
0.4
0.3
0.2
0.1
0.0
p-value
1e-02
2e-02
2e-02
3e-02
4e-02
Log-odds
0.5
1.0
1.5
2.0
2.5
3.0
3.5
4.0
Cellular_organisms - ps1
Archaea1 - ps2
Archaea2 - ps3
Archaea3 - ps4
Asgard/Eukaryota - ps5
Eukaryota - ps6
Amorpheae - ps7
Obazoa - ps8
Opisthokonta - ps9
Holomycota - ps10
Fungi - ps11
Fungi_1 - ps12
Fungi_2 - ps13
Fungi_3 - ps14
Dikarya - ps15
Ascomycota - ps16
Saccharo/Pezizomycotina - ps17
Saccharomycotina - ps18
Saccharomycotina_1 - ps19
Saccharomycotina_2 - ps20
Saccharomycotina_3 - ps21
Phaff./Saccharomycetaceae - ps22
Saccharomyco./mycetaceae - ps23
Saccharomycetaceae - ps24
Saccharomycetaceae_2 - ps25
Saccharomycetaceae_3 - ps26
Kaz./Nau./Saccharomyces - ps27
Saccharomyces - ps28
Arboricola_clade - ps29
Cerevisiae_clade - ps30
Saccharomyces_cerevisiae - ps31
Phylostratum

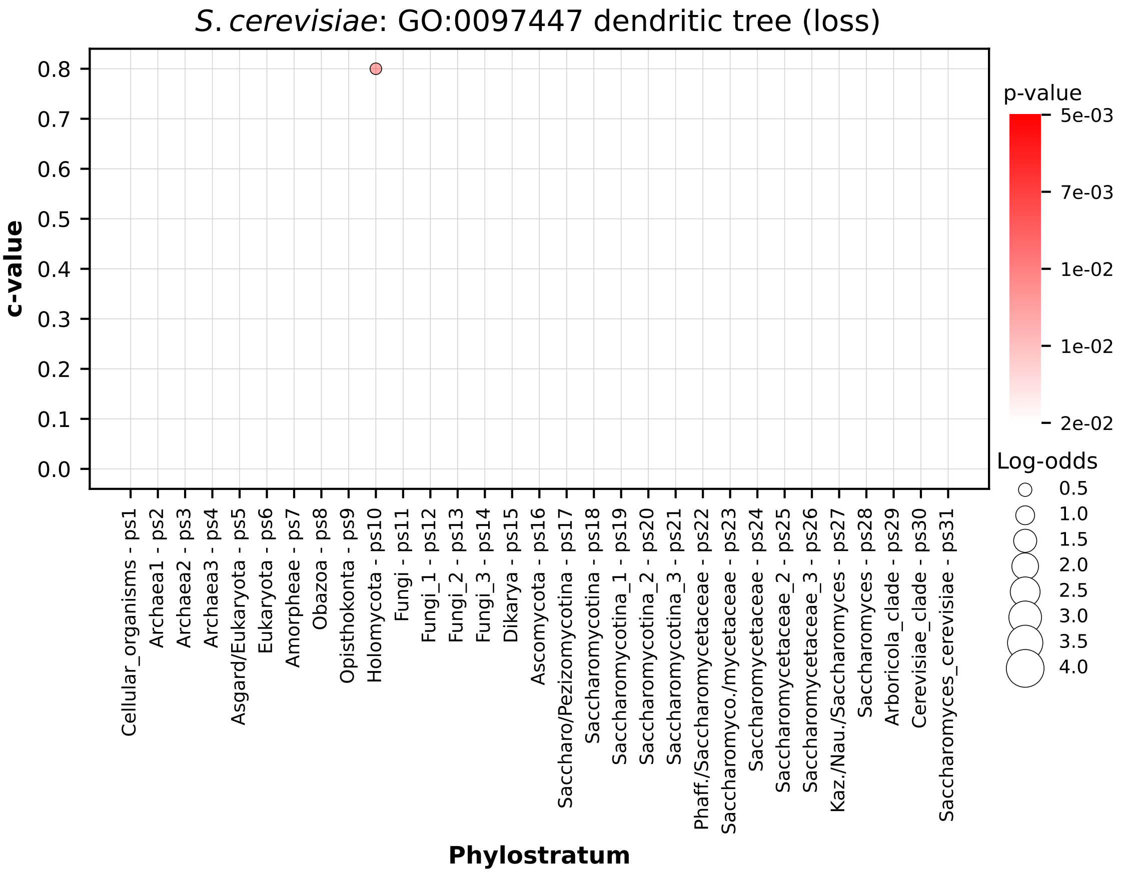
S. cerevisiae: GO:0097447 dendritic tree (loss)
c-value
0.8
0.7
0.6
0.5
0.4
0.3
0.2
0.1
0.0
p-value
5e-03
7e-03
1e-02
1e-02
2e-02
Log-odds
0.5
1.0
1.5
2.0
2.5
3.0
3.5
4.0
Cellular_organisms - ps1
Archaea1 - ps2
Archaea2 - ps3
Archaea3 - ps4
Asgard/Eukaryota - ps5
Eukaryota - ps6
Amorpheae - ps7
Obazoa - ps8
Opisthokonta - ps9
Holomycota - ps10
Fungi - ps11
Fungi_1 - ps12
Fungi_2 - ps13
Fungi_3 - ps14
Dikarya - ps15
Ascomycota - ps16
Saccharo/Pezizomycotina - ps17
Saccharomycotina - ps18
Saccharomycotina_1 - ps19
Saccharomycotina_2 - ps20
Saccharomycotina_3 - ps21
Phaff./Saccharomycetaceae - ps22
Saccharomyco./mycetaceae - ps23
Saccharomycetaceae - ps24
Saccharomycetaceae_2 - ps25
Saccharomycetaceae_3 - ps26
Kaz./Nau./Saccharomyces - ps27
Saccharomyces - ps28
Arboricola_clade - ps29
Cerevisiae_clade - ps30
Saccharomyces_cerevisiae - ps31
Phylostratum

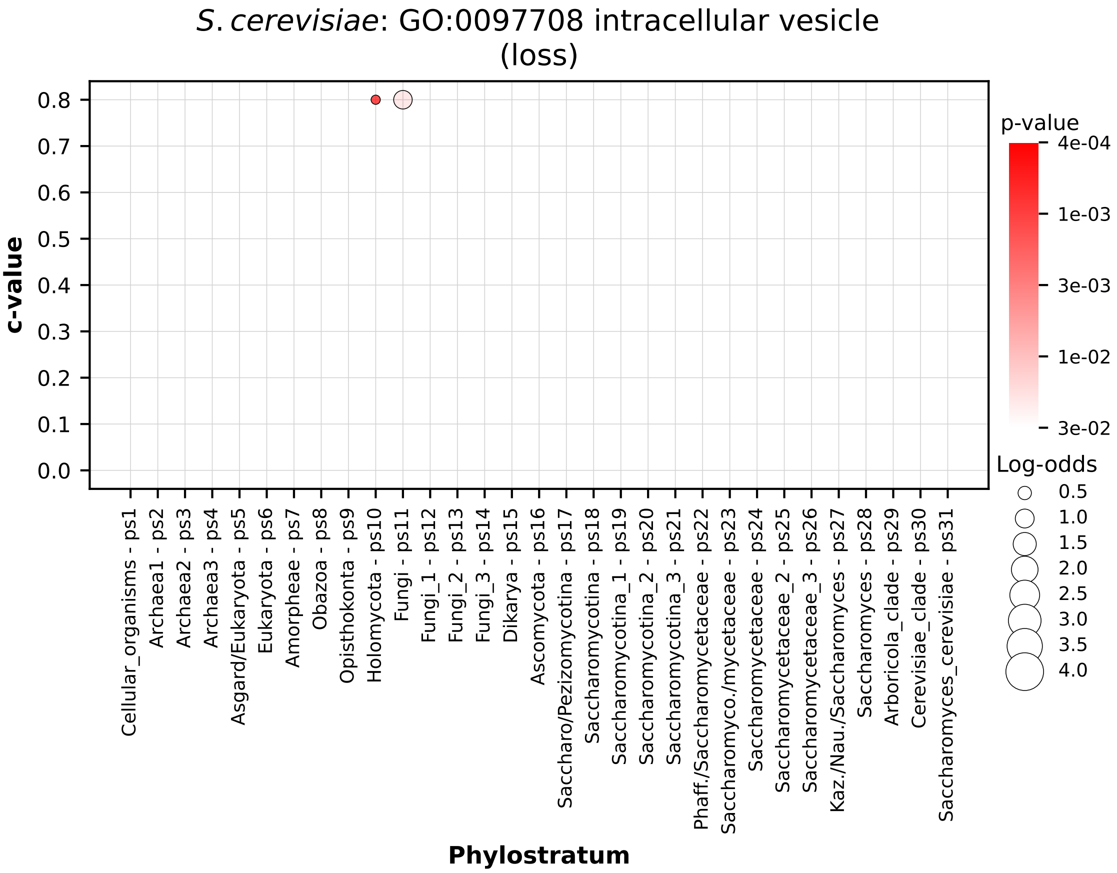
S. cerevisiae: GO:0097708 intracellular vesicle
(loss)
c-value
0.0
0.1
0.2
0.3
0.4
0.5
0.6
0.7
0.8
p-value
4e-04
1e-03
3e-03
1e-02
3e-02
Log-odds
0.5
1.0
1.5
2.0
2.5
3.0
3.5
4.0
Cellular_organisms - ps1
Archaea1 - ps2
Archaea2 - ps3
Archaea3 - ps4
Asgard/Eukaryota - ps5
Eukaryota - ps6
Amorpheae - ps7
Obazoa - ps8
Opisthokonta - ps9
Holomycota - ps10
Fungi - ps11
Fungi_1 - ps12
Fungi_2 - ps13
Fungi_3 - ps14
Dikarya - ps15
Ascomycota - ps16
Saccharo/Pezizomycotina - ps17
Saccharomycotina - ps18
Saccharomycotina_1 - ps19
Saccharomycotina_2 - ps20
Saccharomycotina_3 - ps21
Phaff./Saccharomycetaceae - ps22
Saccharomyco./mycetaceae - ps23
Saccharomycetaceae - ps24
Saccharomycetaceae_2 - ps25
Saccharomycetaceae_3 - ps26
Kaz./Nau./Saccharomyces - ps27
Saccharomyces - ps28
Arboricola_clade - ps29
Cerevisiae_clade - ps30
Saccharomyces_cerevisiae - ps31
Phylostratum

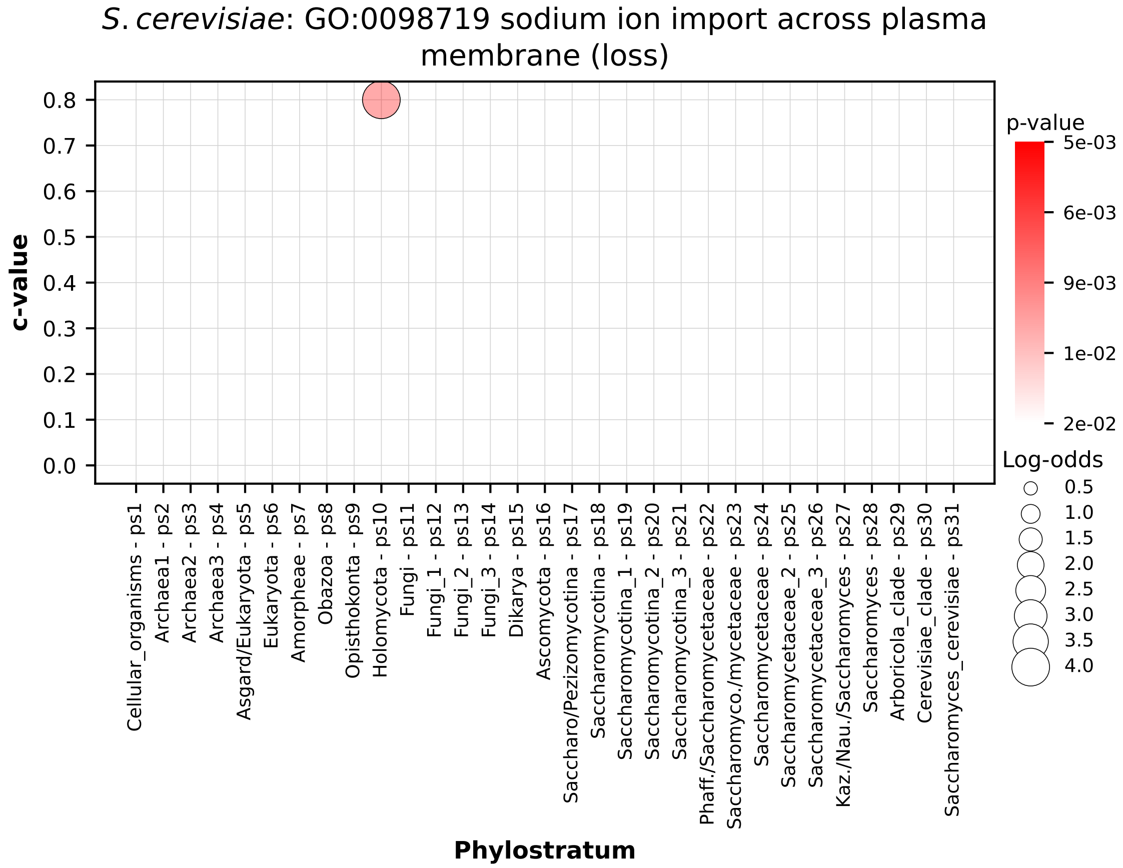

S. cerevisiae: GO:0098719 sodium ion import across plasma membrane (loss)
c-value
0.8
0.7
0.6
0.5
0.4
0.3
0.2
0.1
0.0
p-value
5e-03
6e-03
9e-03
1e-02
2e-02
Log-odds
0.5
1.0
1.5
2.0
2.5
3.0
3.5
4.0
Cellular_organisms - ps1
Archaea1 - ps2
Archaea2 - ps3
Archaea3 - ps4
Asgard/Eukaryota - ps5
Eukaryota - ps6
Amorpheae - ps7
Obazoa - ps8
Opisthokonta - ps9
Holomycota - ps10
Fungi - ps11
Fungi_1 - ps12
Fungi_2 - ps13
Fungi_3 - ps14
Dikarya - ps15
Ascomycota - ps16
Saccharo/Pezizomycotina - ps17
Saccharomycotina - ps18
Saccharomycotina_1 - ps19
Saccharomycotina_2 - ps20
Saccharomycotina_3 - ps21
Phaff./Saccharomycetaceae - ps22
Saccharomyco./mycetaceae - ps23
Saccharomycetaceae - ps24
Saccharomycetaceae_2 - ps25
Saccharomycetaceae_3 - ps26
Kaz./Nau./Saccharomyces - ps27
Saccharomyces - ps28
Arboricola_clade - ps29
Cerevisiae_clade - ps30
Saccharomyces_cerevisiae - ps31
Phylostratum

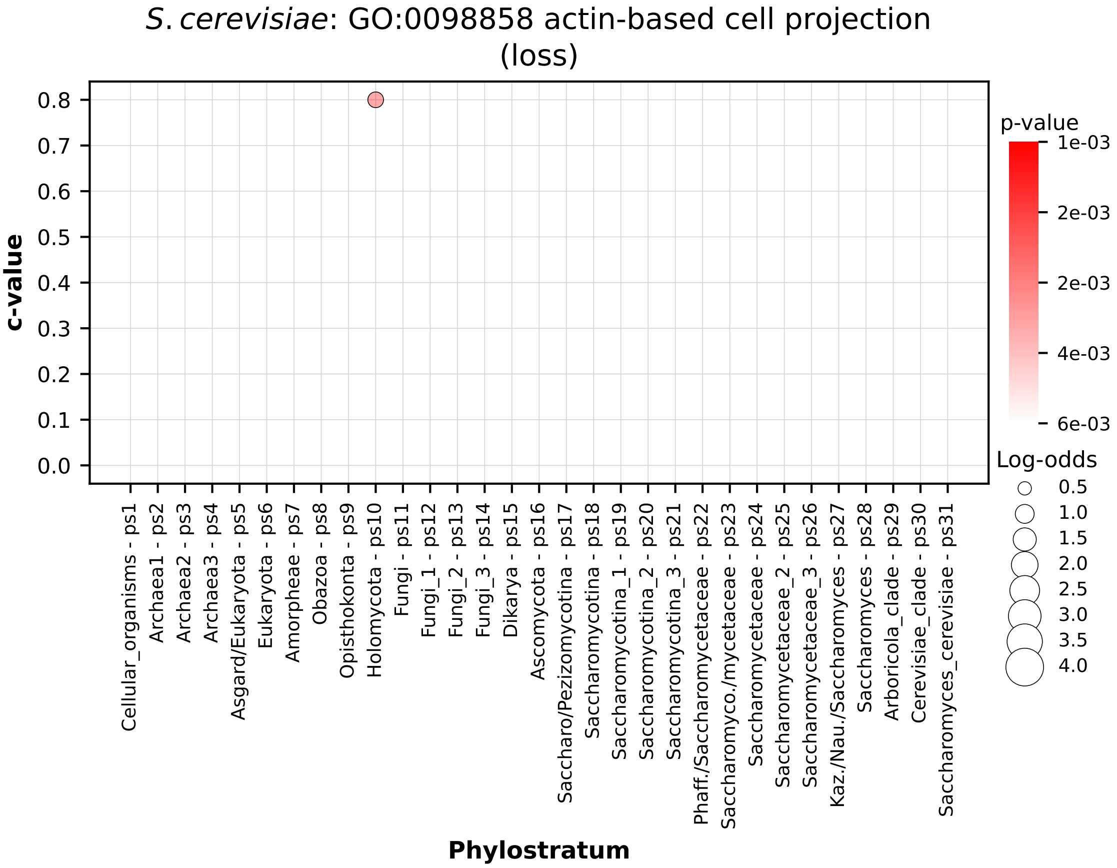

S. cerevisiae: GO:0098858 actin-based cell projection
(loss)
c-value
0.0
0.1
0.2
0.3
0.4
0.5
0.6
0.7
0.8
p-value
1e-03
2e-03
2e-03
4e-03
6e-03
Log-odds
0.5
1.0
1.5
2.0
2.5
3.0
3.5
4.0
Cellular_organisms - ps1
Archaea1 - ps2
Archaea2 - ps3
Archaea3 - ps4
Asgard/Eukaryota - ps5
Eukaryota - ps6
Amorpheae - ps7
Obazoa - ps8
Opisthokonta - ps9
Holomycota - ps10
Fungi - ps11
Fungi_1 - ps12
Fungi_2 - ps13
Fungi_3 - ps14
Dikarya - ps15
Ascomycota - ps16
Saccharo/Pezizomycotina - ps17
Saccharomycotina - ps18
Saccharomycotina_1 - ps19
Saccharomycotina_2 - ps20
Saccharomycotina_3 - ps21
Phaff./Saccharomycetaceae - ps22
Saccharomyco./mycetaceae - ps23
Saccharomycetaceae - ps24
Saccharomycetaceae_2 - ps25
Saccharomycetaceae_3 - ps26
Kaz./Nau./Saccharomyces - ps27
Saccharomyces - ps28
Arboricola_clade - ps29
Cerevisiae_clade - ps30
Saccharomyces_cerevisiae - ps31
Phylostratum

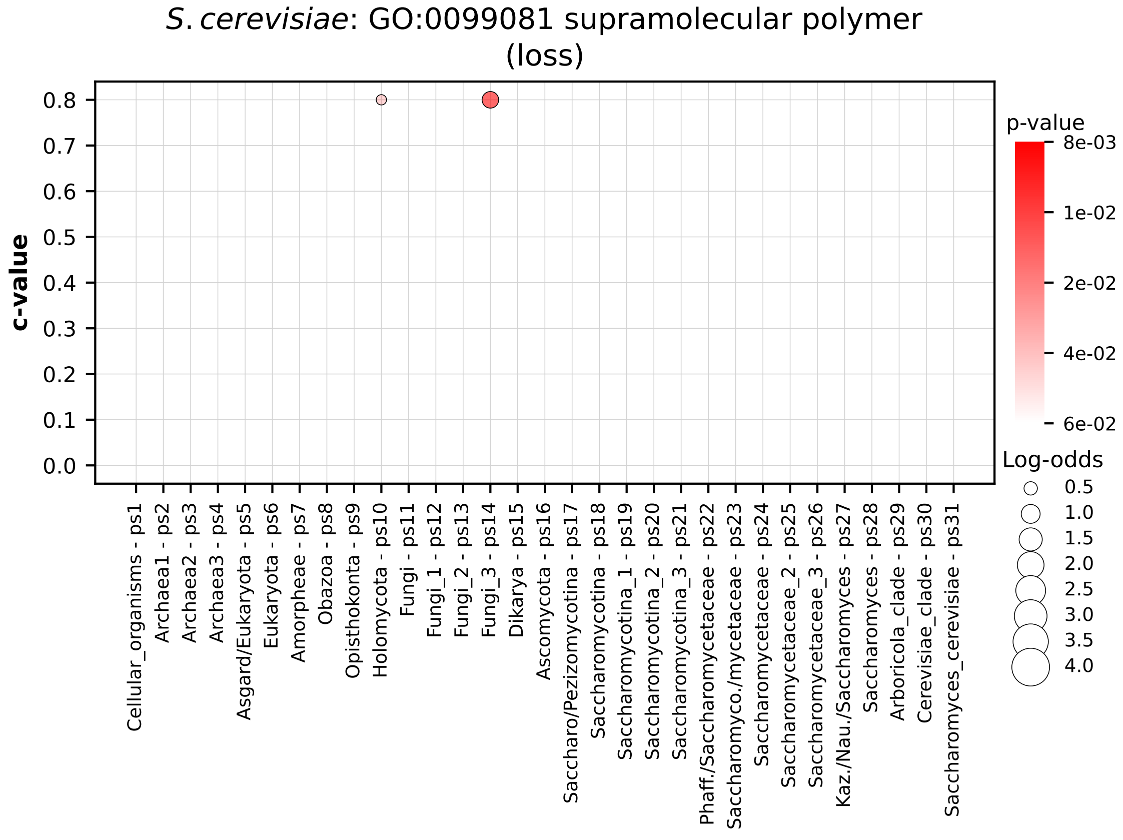

S. cerevisiae: GO:0099081 supramolecular polymer (loss)
c-value
0.0
0.1
0.2
0.3
0.4
0.5
0.6
0.7
0.8
Phylostratum
Cellular_organisms - ps1
Archaea1 - ps2
Archaea2 - ps3
Archaea3 - ps4
Asgard/Eukaryota - ps5
Eukaryota - ps6
Amorpheae - ps7
Obazoa - ps8
Opisthokonta - ps9
Holomycota - ps10
Fungi - ps11
Fungi_1 - ps12
Fungi_2 - ps13
Fungi_3 - ps14
Dikarya - ps15
Ascomycota - ps16
Saccharo/Pezizomycotina - ps17
Saccharomycotina - ps18
Saccharomycotina_1 - ps19
Saccharomycotina_2 - ps20
Saccharomycotina_3 - ps21
Phaff./Saccharomycetaceae - ps22
Saccharomyco./mycetaceae - ps23
Saccharomycetaceae - ps24
Saccharomycetaceae_2 - ps25
Saccharomycetaceae_3 - ps26
Kaz./Nau./Saccharomyces - ps27
Saccharomyces - ps28
Arboricola_clade - ps29
Cerevisiae_clade - ps30
Saccharomyces_cerevisiae - ps31
p-value
8e-03
1e-02
2e-02
4e-02
6e-02
Log-odds
0.5
1.0
1.5
2.0
2.5
3.0
3.5
4.0

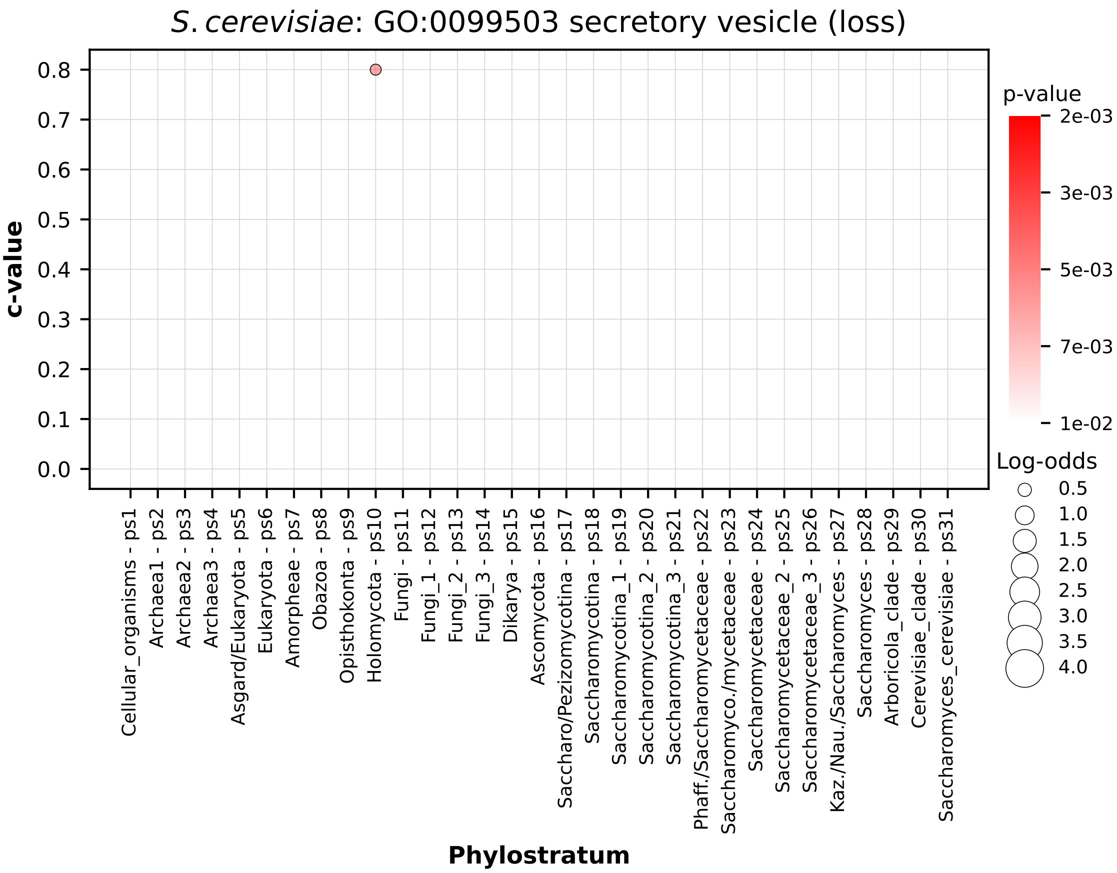
S. cerevisiae: GO:0099503 secretory vesicle (loss)
c-value
0.0
0.1
0.2
0.3
0.4
0.5
0.6
0.7
0.8
Phylostratum
Cellular_organisms - ps1
Archaea1 - ps2
Archaea2 - ps3
Archaea3 - ps4
Asgard/Eukaryota - ps5
Eukaryota - ps6
Amorpheae - ps7
Obazoa - ps8
Opisthokonta - ps9
Holomycota - ps10
Fungi - ps11
Fungi_1 - ps12
Fungi_2 - ps13
Fungi_3 - ps14
Dikarya - ps15
Ascomycota - ps16
Saccharo/Pezizomycotina - ps17
Saccharomycotina - ps18
Saccharomycotina_1 - ps19
Saccharomycotina_2 - ps20
Saccharomycotina_3 - ps21
Phaff./Saccharomycetaceae - ps22
Saccharomyco./mycetaceae - ps23
Saccharomycetaceae - ps24
Saccharomycetaceae_2 - ps25
Saccharomycetaceae_3 - ps26
Kaz./Nau./Saccharomyces - ps27
Saccharomyces - ps28
Arboricola_clade - ps29
Cerevisiae_clade - ps30
Saccharomyces_cerevisiae - ps31
p-value
2e-03
3e-03
5e-03
7e-03
1e-02
Log-odds
0.5
1.0
1.5
2.0
2.5
3.0
3.5
4.0

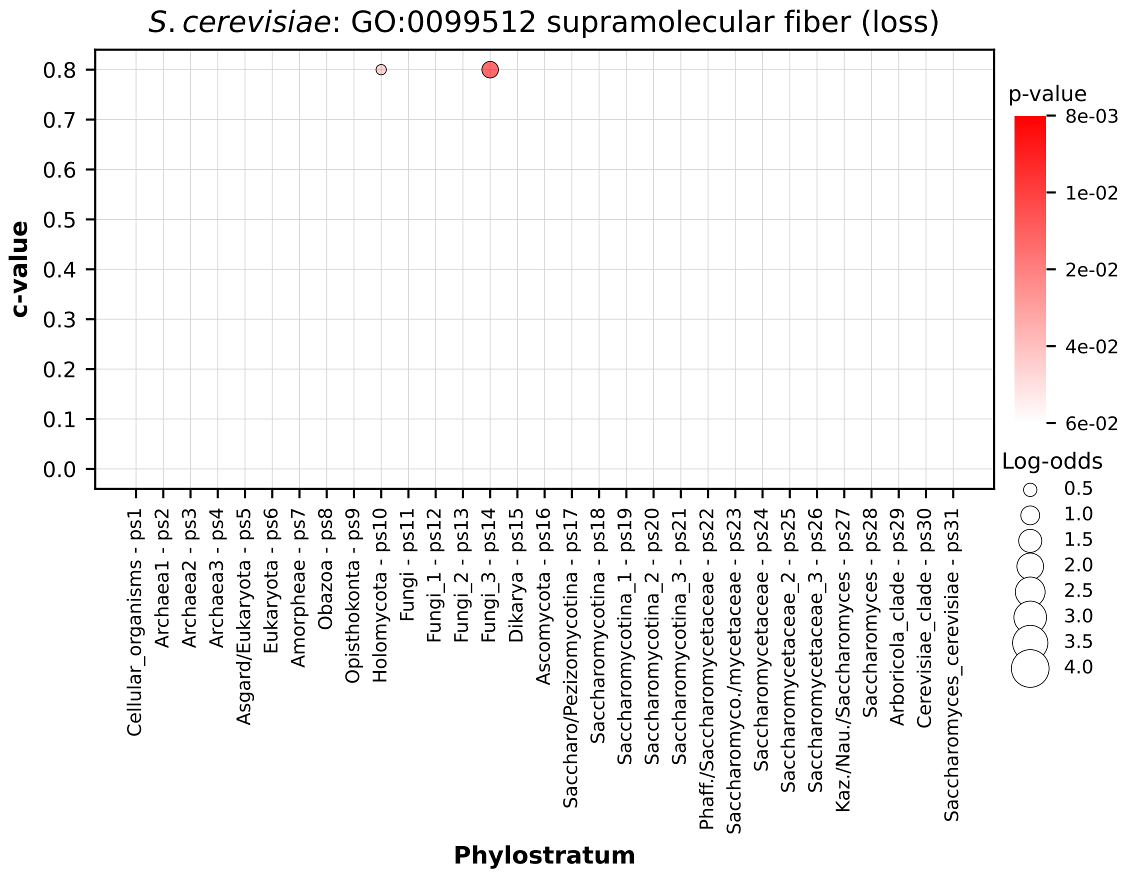

S. cerevisiae: GO:0099512 supramolecular fiber (loss)
c-value
0.0
0.1
0.2
0.3
0.4
0.5
0.6
0.7
0.8
p-value
8e-03
1e-02
2e-02
4e-02
6e-02
Log-odds
0.5
1.0
1.5
2.0
2.5
3.0
3.5
4.0
Cellular_organisms - ps1
Archaea1 - ps2
Archaea2 - ps3
Archaea3 - ps4
Asgard/Eukaryota - ps5
Eukaryota - ps6
Amorpheae - ps7
Obazoa - ps8
Opisthokonta - ps9
Holomycota - ps10
Fungi - ps11
Fungi_1 - ps12
Fungi_2 - ps13
Fungi_3 - ps14
Dikarya - ps15
Ascomycota - ps16
Saccharo/Pezizomycotina - ps17
Saccharomycotina - ps18
Saccharomycotina_1 - ps19
Saccharomycotina_2 - ps20
Saccharomycotina_3 - ps21
Phaff./Saccharomycetaceae - ps22
Saccharomyco./mycetaceae - ps23
Saccharomycetaceae - ps24
Saccharomycetaceae_2 - ps25
Saccharomycetaceae_3 - ps26
Kaz./Nau./Saccharomyces - ps27
Saccharomyces - ps28
Arboricola_clade - ps29
Cerevisiae_clade - ps30
Saccharomyces_cerevisiae - ps31
Phylostratum

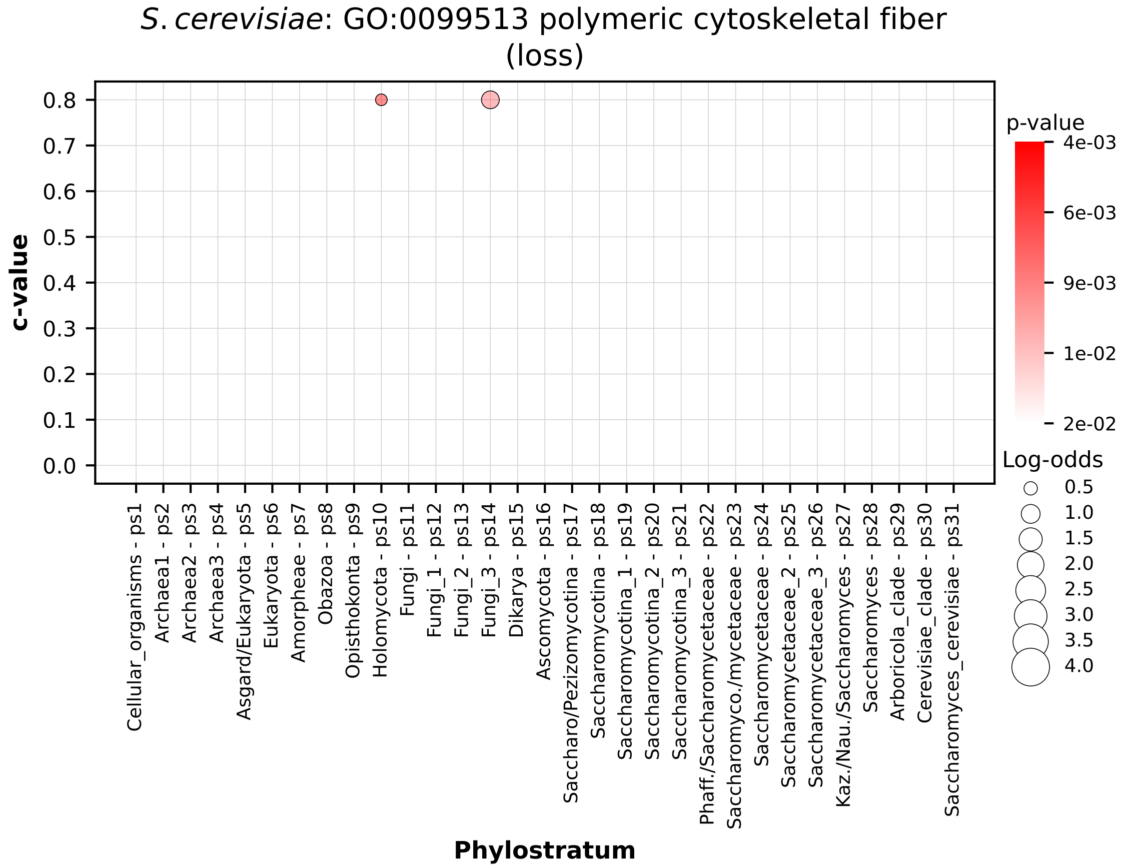

S. cerevisiae: GO:0099513 polymeric cytoskeletal fiber
(loss)
c-value
0.0
0.1
0.2
0.3
0.4
0.5
0.6
0.7
0.8
Phylostratum
Cellular_organisms - ps1
Archaea1 - ps2
Archaea2 - ps3
Archaea3 - ps4
Asgard/Eukaryota - ps5
Eukaryota - ps6
Amorpheae - ps7
Obazoa - ps8
Opisthokonta - ps9
Holomycota - ps10
Fungi - ps11
Fungi_1 - ps12
Fungi_2 - ps13
Fungi_3 - ps14
Dikarya - ps15
Ascomycota - ps16
Saccharo/Pezizomycotina - ps17
Saccharomycotina - ps18
Saccharomycotina_1 - ps19
Saccharomycotina_2 - ps20
Saccharomycotina_3 - ps21
Phaff./Saccharomycetaceae - ps22
Saccharomyco./mycetaceae - ps23
Saccharomycetaceae - ps24
Saccharomycetaceae_2 - ps25
Saccharomycetaceae_3 - ps26
Kaz./Nau./Saccharomyces - ps27
Saccharomyces - ps28
Arboricola_clade - ps29
Cerevisiae_clade - ps30
Saccharomyces_cerevisiae - ps31
p-value
4e-03
6e-03
9e-03
1e-02
2e-02
Log-odds
0.5
1.0
1.5
2.0
2.5
3.0
3.5
4.0

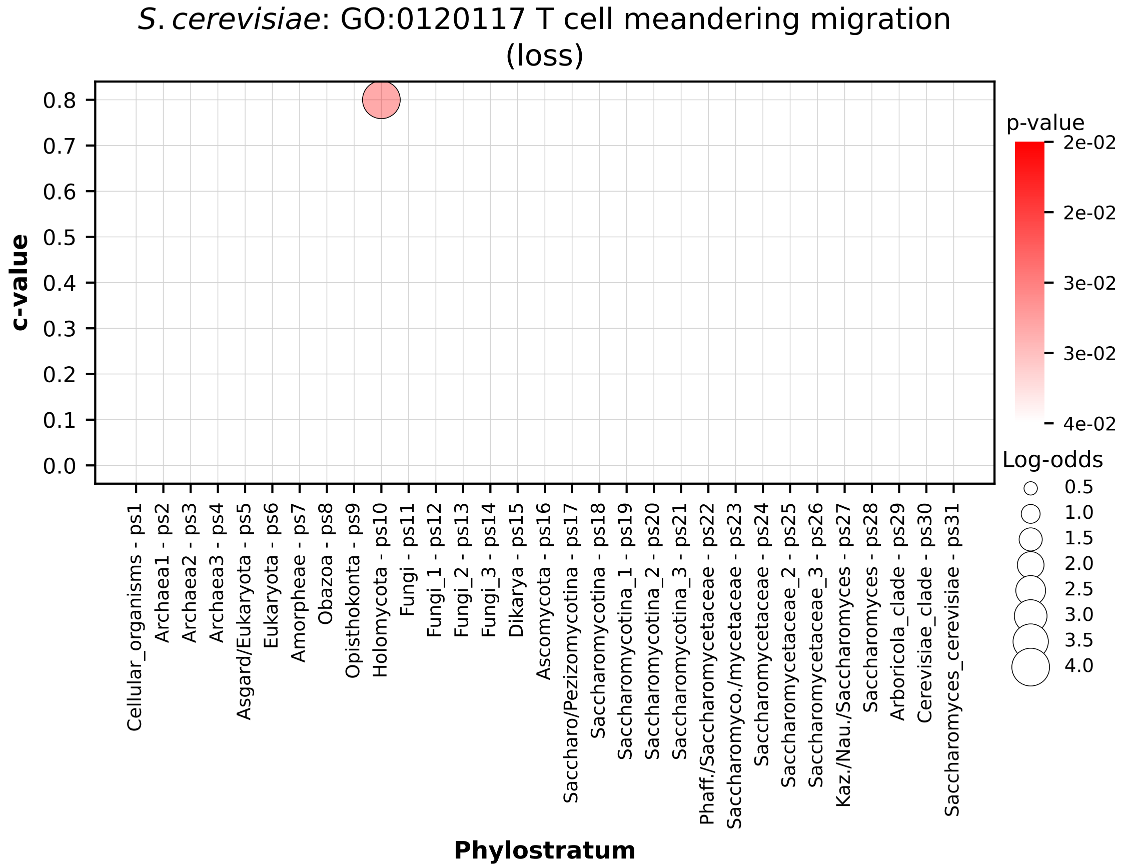

S. cerevisiae: GO:0120117 T cell meandering migration
(loss)
c-value
0.0
0.1
0.2
0.3
0.4
0.5
0.6
0.7
0.8
p-value
2e-02
2e-02
3e-02
3e-02
4e-02
Log-odds
0.5
1.0
1.5
2.0
2.5
3.0
3.5
4.0
Cellular_organisms - ps1
Archaea1 - ps2
Archaea2 - ps3
Archaea3 - ps4
Asgard/Eukaryota - ps5
Eukaryota - ps6
Amorpheae - ps7
Obazoa - ps8
Opisthokonta - ps9
Holomycota - ps10
Fungi - ps11
Fungi_1 - ps12
Fungi_2 - ps13
Fungi_3 - ps14
Dikarya - ps15
Ascomycota - ps16
Saccharo/Pezizomycotina - ps17
Saccharomycotina - ps18
Saccharomycotina_1 - ps19
Saccharomycotina_2 - ps20
Saccharomycotina_3 - ps21
Phaff./Saccharomycetaceae - ps22
Saccharomyco./mycetaceae - ps23
Saccharomycetaceae - ps24
Saccharomycetaceae_2 - ps25
Saccharomycetaceae_3 - ps26
Kaz./Nau./Saccharomyces - ps27
Saccharomyces - ps28
Arboricola_clade - ps29
Cerevisiae_clade - ps30
Saccharomyces_cerevisiae - ps31
Phylostratum

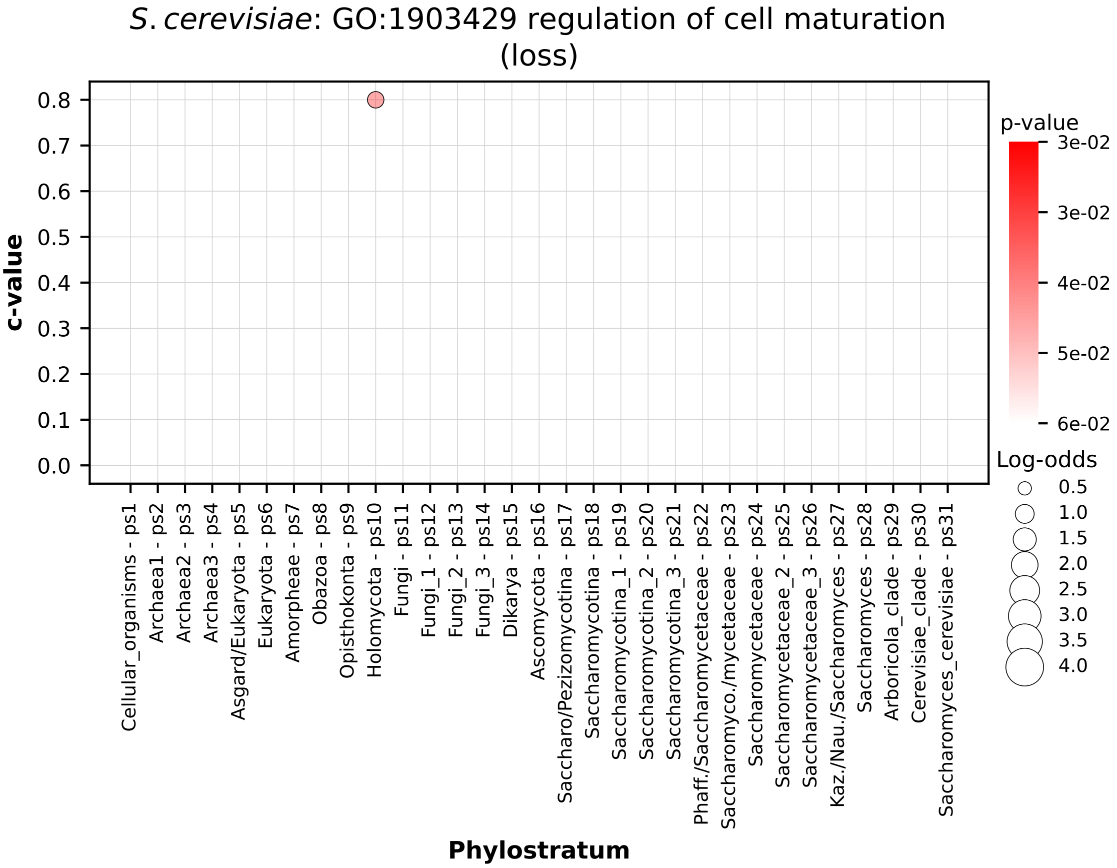

S. cerevisiae: GO:1903429 regulation of cell maturation
(loss)
c-value
0.0
0.1
0.2
0.3
0.4
0.5
0.6
0.7
0.8
Phylostratum
Cellular_organisms - ps1
Archaea1 - ps2
Archaea2 - ps3
Archaea3 - ps4
Asgard/Eukaryota - ps5
Eukaryota - ps6
Amorpheae - ps7
Obazoa - ps8
Opisthokonta - ps9
Holomycota - ps10
Fungi - ps11
Fungi_1 - ps12
Fungi_2 - ps13
Fungi_3 - ps14
Dikarya - ps15
Ascomycota - ps16
Saccharo/Pezizomycotina - ps17
Saccharomycotina - ps18
Saccharomycotina_1 - ps19
Saccharomycotina_2 - ps20
Saccharomycotina_3 - ps21
Phaff./Saccharomycetaceae - ps22
Saccharomyco./mycetaceae - ps23
Saccharomycetaceae - ps24
Saccharomycetaceae_2 - ps25
Saccharomycetaceae_3 - ps26
Kaz./Nau./Saccharomyces - ps27
Saccharomyces - ps28
Arboricola_clade - ps29
Cerevisiae_clade - ps30
Saccharomyces_cerevisiae - ps31
p-value
3e-02
3e-02
4e-02
5e-02
6e-02
Log-odds
0.5
1.0
1.5
2.0
2.5
3.0
3.5
4.0

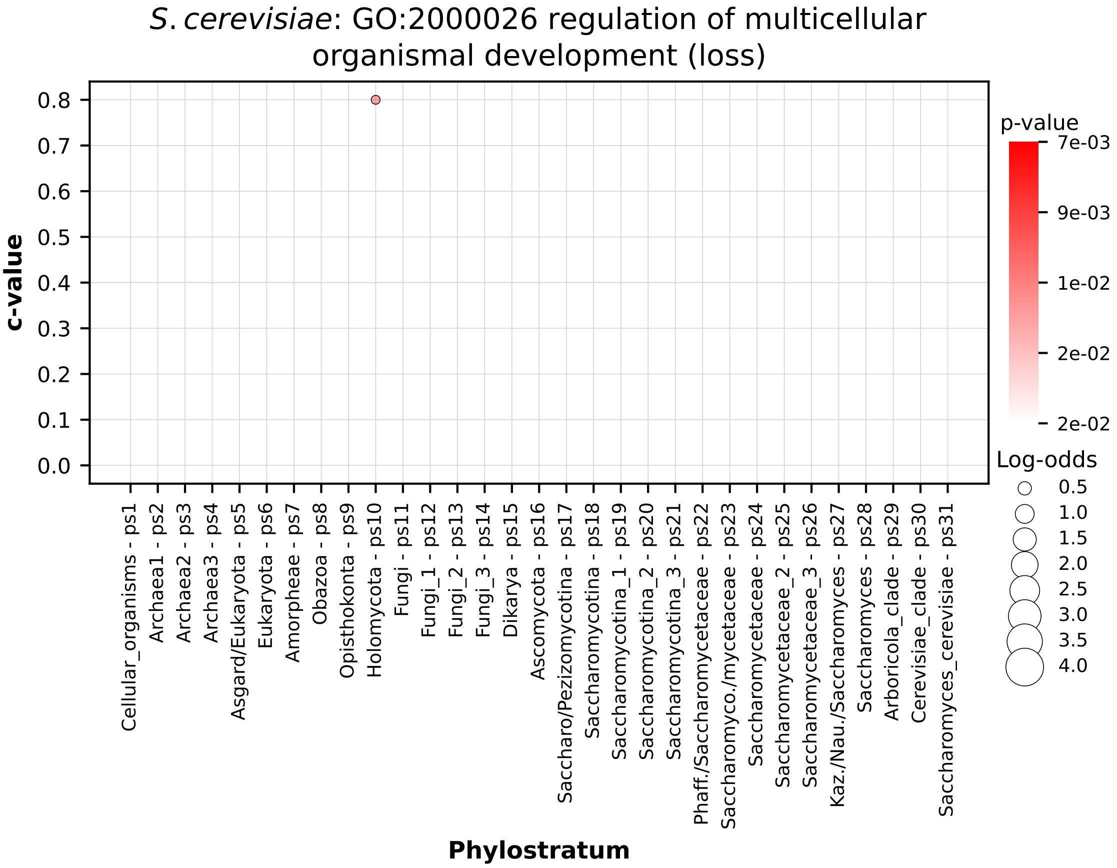

S. cerevisiae: GO:2000026 regulation of multicellular organismal development (loss)
c-value
0.0
0.1
0.2
0.3
0.4
0.5
0.6
0.7
0.8
p-value
7e-03
9e-03
1e-02
2e-02
2e-02
Log-odds
0.5
1.0
1.5
2.0
2.5
3.0
3.5
4.0
Cellular_organisms - ps1
Archaea1 - ps2
Archaea2 - ps3
Archaea3 - ps4
Asgard/Eukaryota - ps5
Eukaryota - ps6
Amorpheae - ps7
Obazoa - ps8
Opisthokonta - ps9
Holomycota - ps10
Fungi - ps11
Fungi_1 - ps12
Fungi_2 - ps13
Fungi_3 - ps14
Dikarya - ps15
Ascomycota - ps16
Saccharo/Pezizomycotina - ps17
Saccharomycotina - ps18
Saccharomycotina_1 - ps19
Saccharomycotina_2 - ps20
Saccharomycotina_3 - ps21
Phaff./Saccharomycetaceae - ps22
Saccharomyco./mycetaceae - ps23
Saccharomycetaceae - ps24
Saccharomycetaceae_2 - ps25
Saccharomycetaceae_3 - ps26
Kaz./Nau./Saccharomyces - ps27
Saccharomyces - ps28
Arboricola_clade - ps29
Cerevisiae_clade - ps30
Saccharomyces_cerevisiae - ps31
Phylostratum

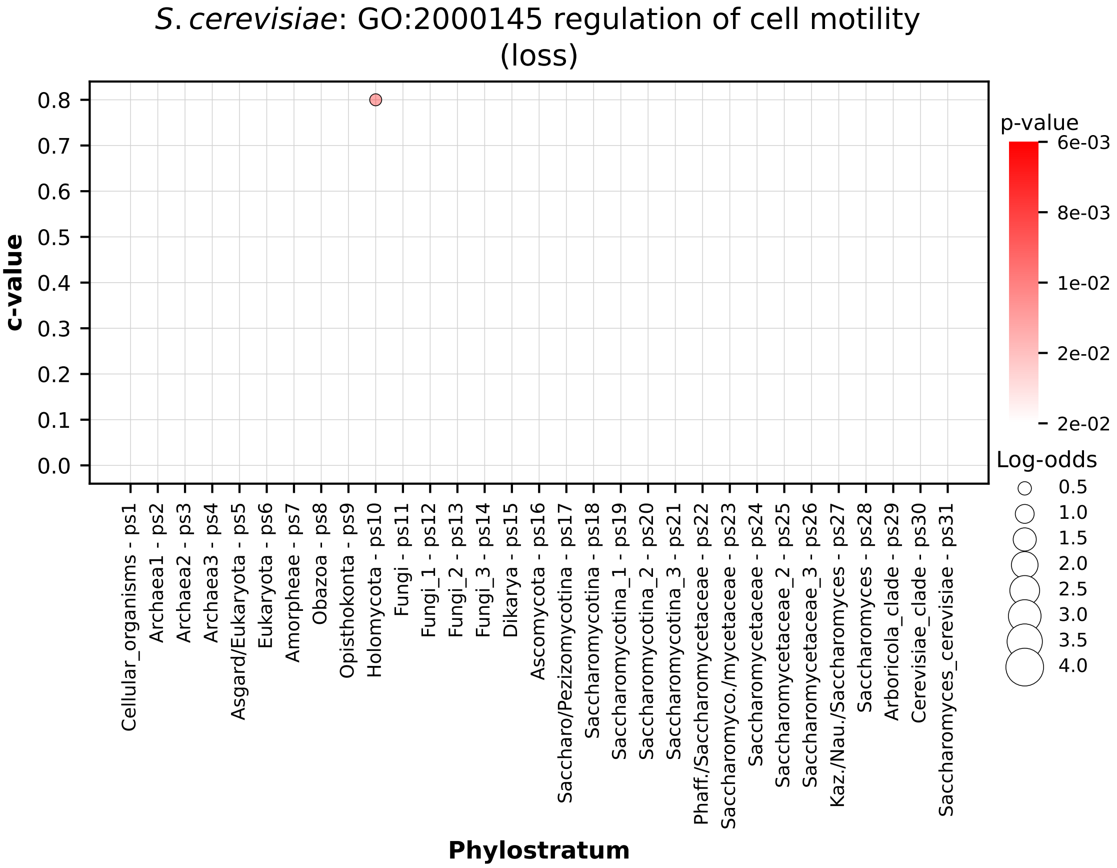

S. cerevisiae: GO:2000145 regulation of cell motility
(loss)
c-value
0.0
0.1
0.2
0.3
0.4
0.5
0.6
0.7
0.8
p-value
6e-03
8e-03
1e-02
2e-02
2e-02
Log-odds
0.5
1.0
1.5
2.0
2.5
3.0
3.5
4.0
Cellular_organisms - ps1
Archaea1 - ps2
Archaea2 - ps3
Archaea3 - ps4
Asgard/Eukaryota - ps5
Eukaryota - ps6
Amorpheae - ps7
Obazoa - ps8
Opisthokonta - ps9
Holomycota - ps10
Fungi - ps11
Fungi_1 - ps12
Fungi_2 - ps13
Fungi_3 - ps14
Dikarya - ps15
Ascomycota - ps16
Saccharo/Pezizomycotina - ps17
Saccharomycotina - ps18
Saccharomycotina_1 - ps19
Saccharomycotina_2 - ps20
Saccharomycotina_3 - ps21
Phaff./Saccharomycetaceae - ps22
Saccharomyco./mycetaceae - ps23
Saccharomycetaceae - ps24
Saccharomycetaceae_2 - ps25
Saccharomycetaceae_3 - ps26
Kaz./Nau./Saccharomyces - ps27
Saccharomyces - ps28
Arboricola_clade - ps29
Cerevisiae_clade - ps30
Saccharomyces_cerevisiae - ps31
Phylostratum

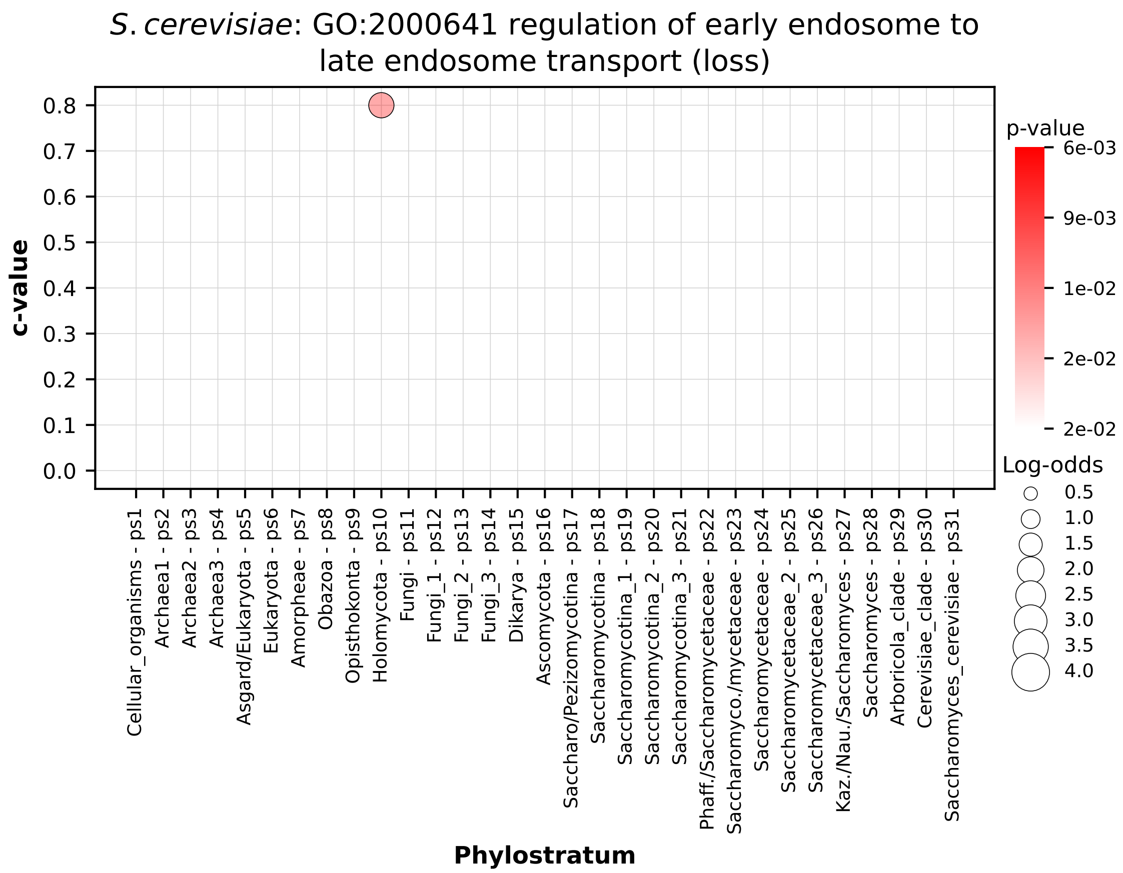

S. cerevisiae: GO:2000641 regulation of early endosome to late endosome transport (loss)
c-value
0.0
0.1
0.2
0.3
0.4
0.5
0.6
0.7
0.8
Phylostratum
Cellular_organisms - ps1
Archaea1 - ps2
Archaea2 - ps3
Archaea3 - ps4
Asgard/Eukaryota - ps5
Eukaryota - ps6
Amorpheae - ps7
Obazoa - ps8
Opisthokonta - ps9
Holomycota - ps10
Fungi - ps11
Fungi_1 - ps12
Fungi_2 - ps13
Fungi_3 - ps14
Dikarya - ps15
Ascomycota - ps16
Saccharo/Pezizomycotina - ps17
Saccharomycotina - ps18
Saccharomycotina_1 - ps19
Saccharomycotina_2 - ps20
Saccharomycotina_3 - ps21
Phaff./Saccharomycetaceae - ps22
Saccharomyco./mycetaceae - ps23
Saccharomycetaceae - ps24
Saccharomycetaceae_2 - ps25
Saccharomycetaceae_3 - ps26
Kaz./Nau./Saccharomyces - ps27
Saccharomyces - ps28
Arboricola_clade - ps29
Cerevisiae_clade - ps30
Saccharomyces_cerevisiae - ps31
p-value
6e-03
9e-03
1e-02
2e-02
2e-02
Log-odds
0.5
1.0
1.5
2.0
2.5
3.0
3.5
4.0

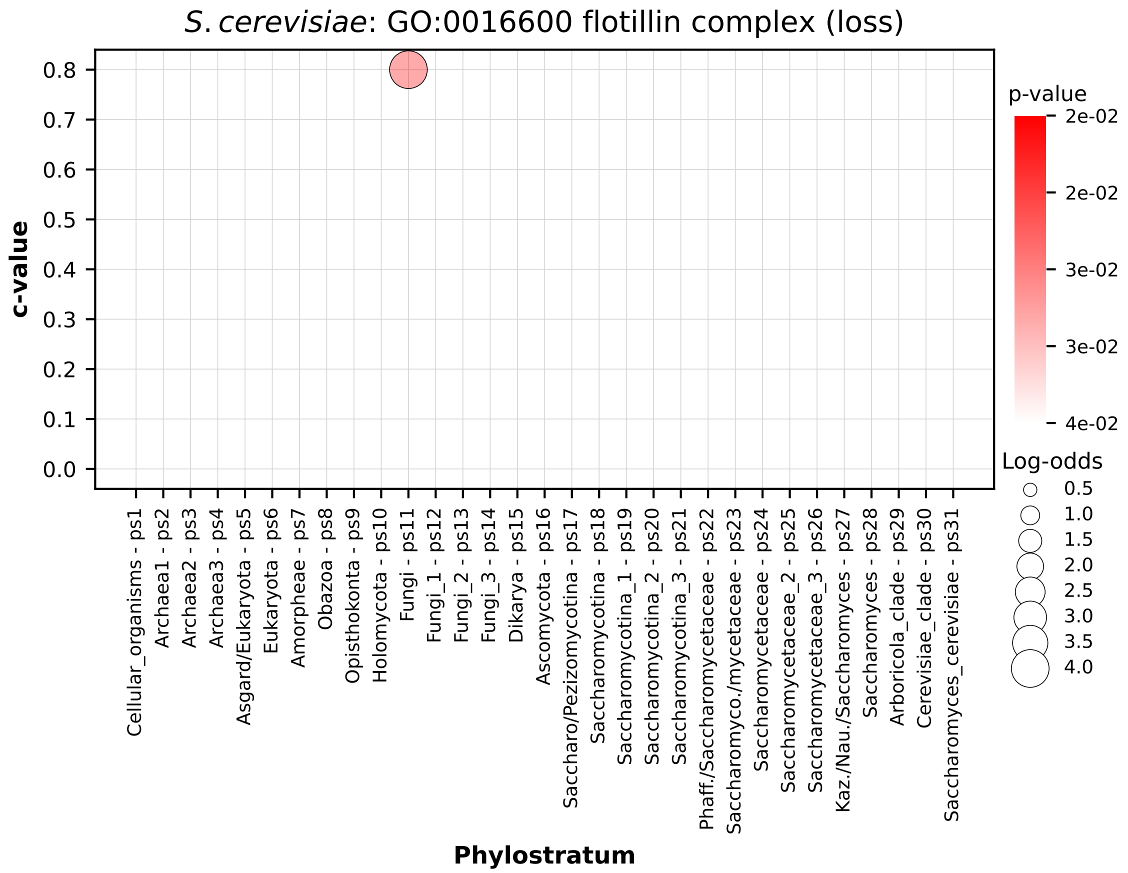

S. cerevisiae: GO:0016600 flotillin complex (loss)
c-value
0.8
0.7
0.6
0.5
0.4
0.3
0.2
0.1
0.0
p-value
2e-02
2e-02
3e-02
3e-02
4e-02
Log-odds
0.5
1.0
1.5
2.0
2.5
3.0
3.5
4.0
Cellular_organisms - ps1
Archaea1 - ps2
Archaea2 - ps3
Archaea3 - ps4
Asgard/Eukaryota - ps5
Eukaryota - ps6
Amorpheae - ps7
Obazoa - ps8
Opisthokonta - ps9
Holomycota - ps10
Fungi - ps11
Fungi_1 - ps12
Fungi_2 - ps13
Fungi_3 - ps14
Dikarya - ps15
Ascomycota - ps16
Saccharo/Pezizomycotina - ps17
Saccharomycotina - ps18
Saccharomycotina_1 - ps19
Saccharomycotina_2 - ps20
Saccharomycotina_3 - ps21
Phaff./Saccharomycetaceae - ps22
Saccharomyco./mycetaceae - ps23
Saccharomycetaceae - ps24
Saccharomycetaceae_2 - ps25
Saccharomycetaceae_3 - ps26
Kaz./Nau./Saccharomyces - ps27
Saccharomyces - ps28
Arboricola_clade - ps29
Cerevisiae_clade - ps30
Saccharomyces_cerevisiae - ps31
Phylostratum

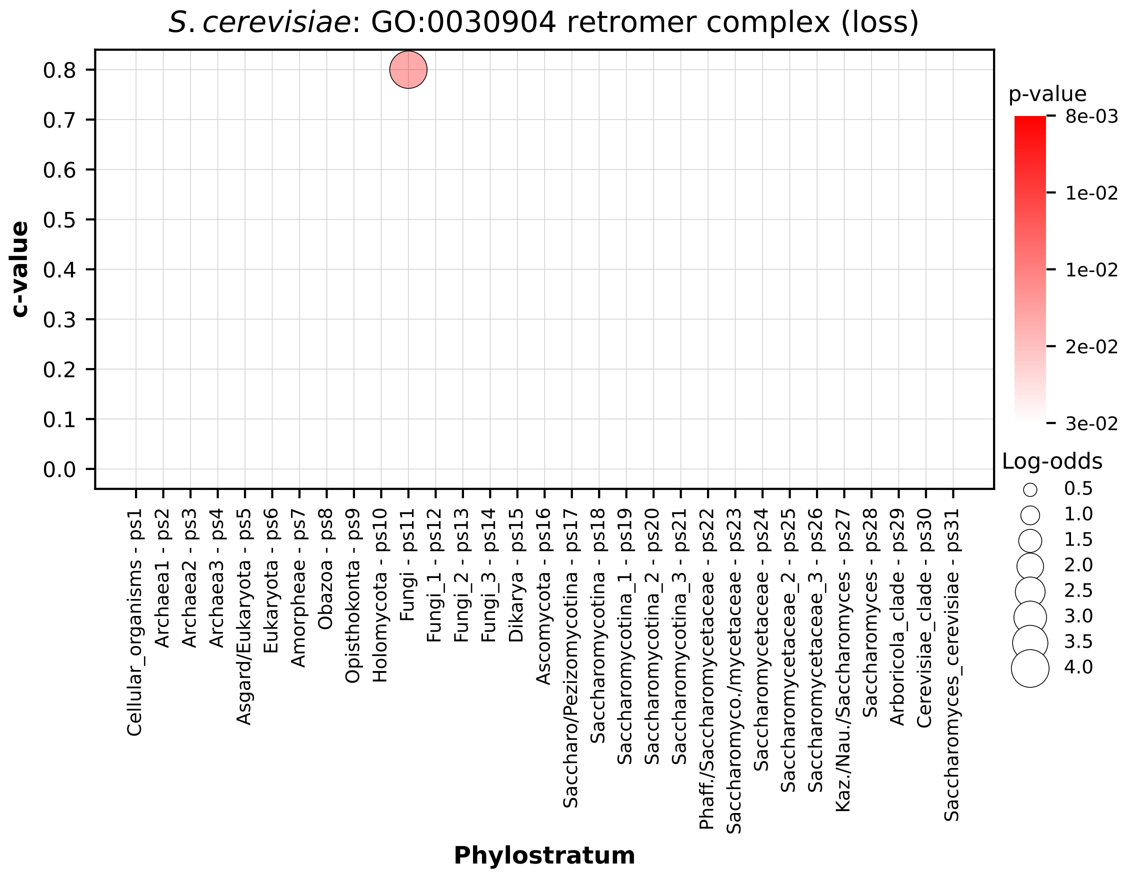

S. cerevisiae: GO:0030904 retromer complex (loss)
c-value
0.8
0.7
0.6
0.5
0.4
0.3
0.2
0.1
0.0
Phylostratum
Cellular_organisms - ps1
Archaea1 - ps2
Archaea2 - ps3
Archaea3 - ps4
Asgard/Eukaryota - ps5
Eukaryota - ps6
Amorpheae - ps7
Obazoa - ps8
Opisthokonta - ps9
Holomycota - ps10
Fungi - ps11
Fungi_1 - ps12
Fungi_2 - ps13
Fungi_3 - ps14
Dikarya - ps15
Ascomycota - ps16
Saccharo/Pezizomycotina - ps17
Saccharomycotina - ps18
Saccharomycotina_1 - ps19
Saccharomycotina_2 - ps20
Saccharomycotina_3 - ps21
Phaff./Saccharomycetaceae - ps22
Saccharomyco./mycetaceae - ps23
Saccharomycetaceae - ps24
Saccharomycetaceae_2 - ps25
Saccharomycetaceae_3 - ps26
Kaz./Nau./Saccharomyces - ps27
Saccharomyces - ps28
Arboricola_clade - ps29
Cerevisiae_clade - ps30
Saccharomyces_cerevisiae - ps31
p-value
8e-03
1e-02
1e-02
2e-02
3e-02
Log-odds
0.5
1.0
1.5
2.0
2.5
3.0
3.5
4.0

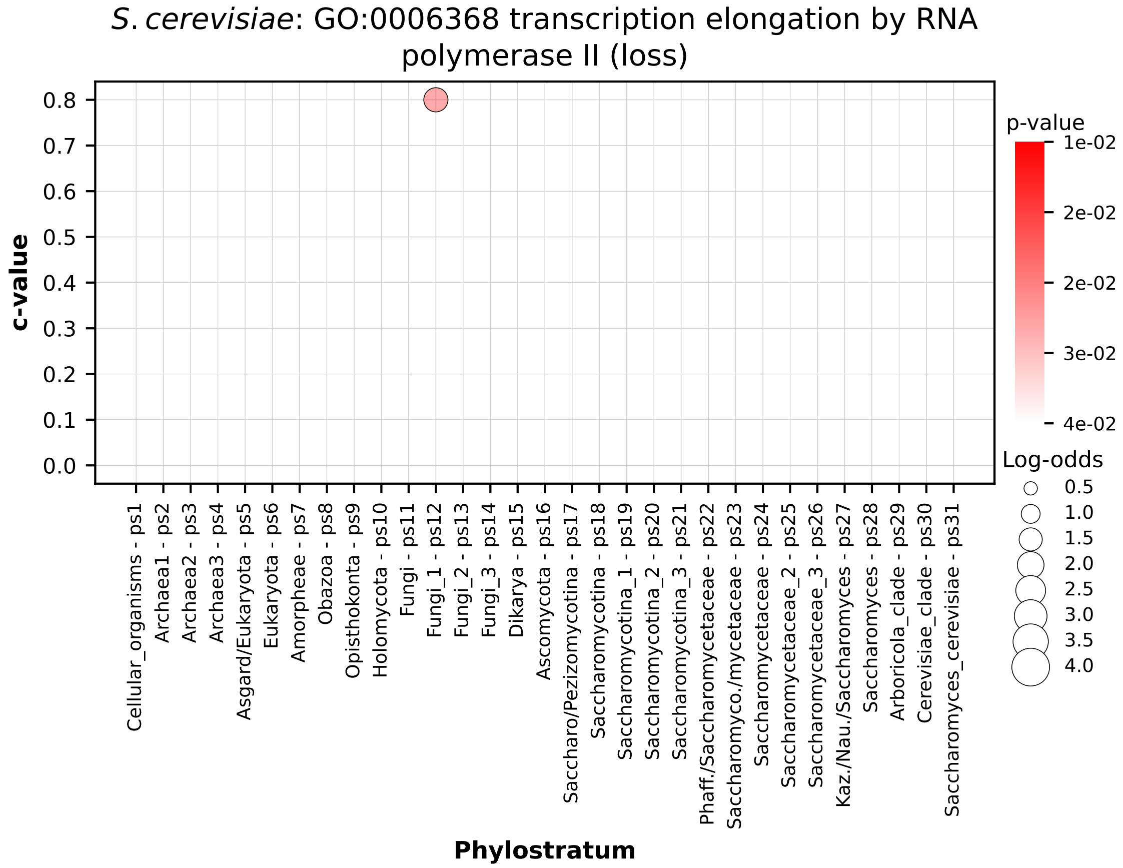
S. cerevisiae: GO:0006368 transcription elongation by RNA polymerase II (loss)
c-value
0.8
0.7
0.6
0.5
0.4
0.3
0.2
0.1
0.0
p-value
1e-02
2e-02
2e-02
3e-02
4e-02
Log-odds
0.5
1.0
1.5
2.0
2.5
3.0
3.5
4.0
Cellular_organisms - ps1
Archaea1 - ps2
Archaea2 - ps3
Archaea3 - ps4
Asgard/Eukaryota - ps5
Eukaryota - ps6
Amorpheae - ps7
Obazoa - ps8
Opisthokonta - ps9
Holomycota - ps10
Fungi - ps11
Fungi_1 - ps12
Fungi_2 - ps13
Fungi_3 - ps14
Dikarya - ps15
Ascomycota - ps16
Saccharo/Pezizomycotina - ps17
Saccharomycotina - ps18
Saccharomycotina_1 - ps19
Saccharomycotina_2 - ps20
Saccharomycotina_3 - ps21
Phaff./Saccharomycetaceae - ps22
Saccharomyco./mycetaceae - ps23
Saccharomycetaceae - ps24
Saccharomycetaceae_2 - ps25
Saccharomycetaceae_3 - ps26
Kaz./Nau./Saccharomyces - ps27
Saccharomyces - ps28
Arboricola_clade - ps29
Cerevisiae_clade - ps30
Saccharomyces_cerevisiae - ps31
Phylostratum

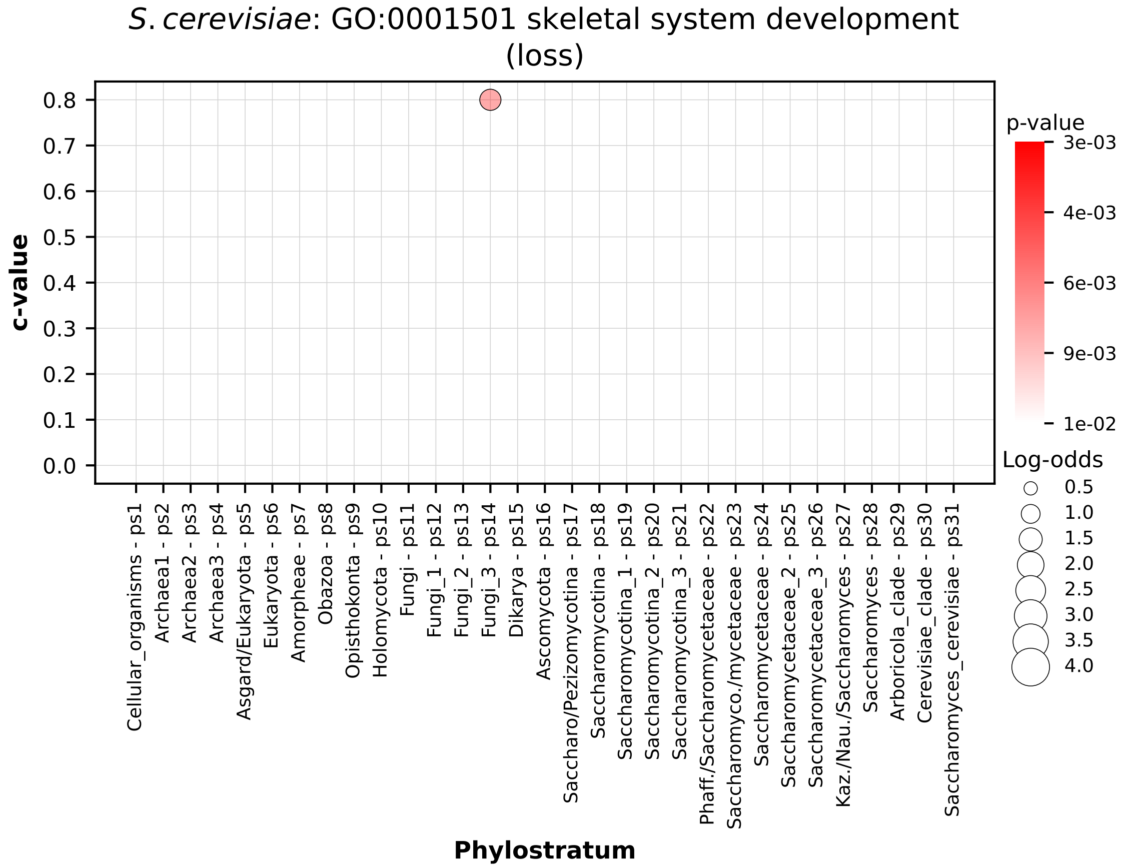
S. cerevisiae: GO:0001501 skeletal system development
(loss)
c-value
0.0
0.1
0.2
0.3
0.4
0.5
0.6
0.7
0.8
Phylostratum
Cellular_organisms - ps1
Archaea1 - ps2
Archaea2 - ps3
Archaea3 - ps4
Asgard/Eukaryota - ps5
Eukaryota - ps6
Amorpheae - ps7
Obazoa - ps8
Opisthokonta - ps9
Holomycota - ps10
Fungi - ps11
Fungi_1 - ps12
Fungi_2 - ps13
Fungi_3 - ps14
Dikarya - ps15
Ascomycota - ps16
Saccharo/Pezizomycotina - ps17
Saccharomycotina - ps18
Saccharomycotina_1 - ps19
Saccharomycotina_2 - ps20
Saccharomycotina_3 - ps21
Phaff./Saccharomycetaceae - ps22
Saccharomyco./mycetaceae - ps23
Saccharomycetaceae - ps24
Saccharomycetaceae_2 - ps25
Saccharomycetaceae_3 - ps26
Kaz./Nau./Saccharomyces - ps27
Saccharomyces - ps28
Arboricola_clade - ps29
Cerevisiae_clade - ps30
Saccharomyces_cerevisiae - ps31
p-value
3e-03
4e-03
6e-03
9e-03
1e-02
Log-odds
0.5
1.0
1.5
2.0
2.5
3.0
3.5
4.0

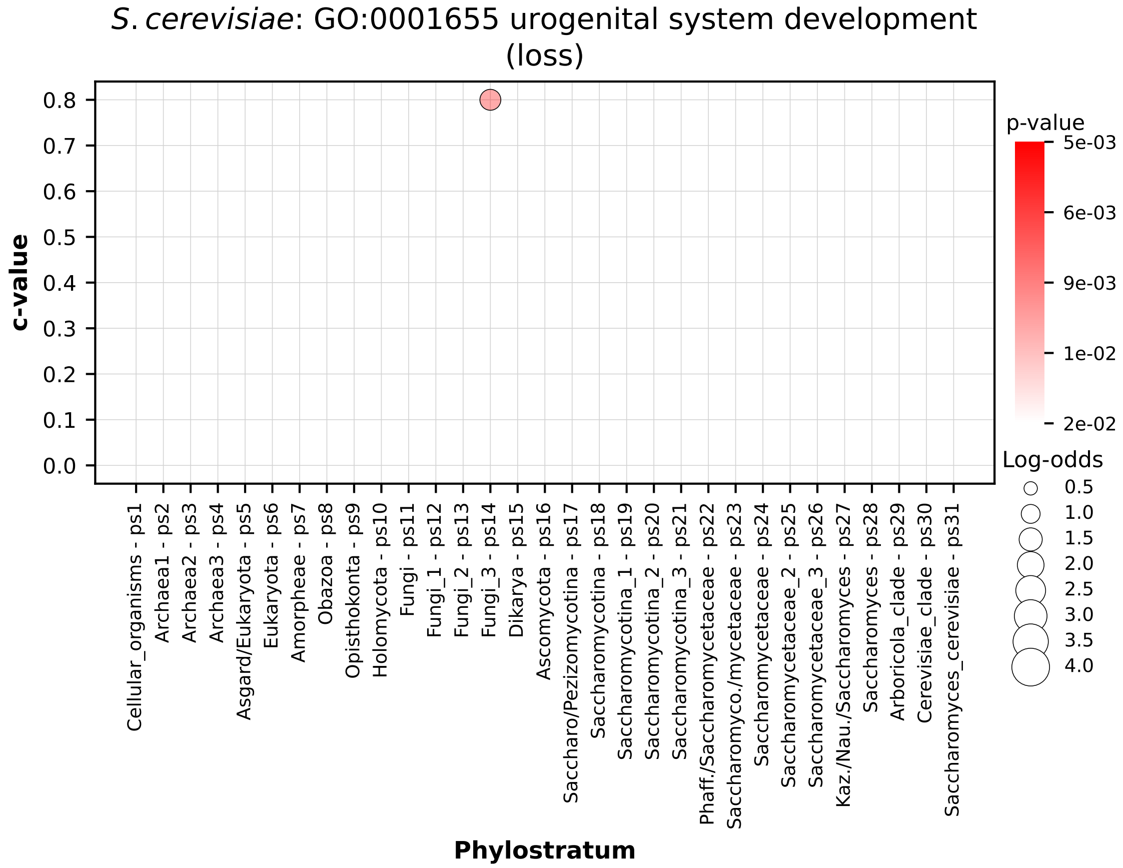

S. cerevisiae: GO:0001655 urogenital system development
(loss)
c-value
0.0
0.1
0.2
0.3
0.4
0.5
0.6
0.7
0.8
p-value
5e-03
6e-03
9e-03
1e-02
2e-02
Log-odds
0.5
1.0
1.5
2.0
2.5
3.0
3.5
4.0
Cellular_organisms - ps1
Archaea1 - ps2
Archaea2 - ps3
Archaea3 - ps4
Asgard/Eukaryota - ps5
Eukaryota - ps6
Amorpheae - ps7
Obazoa - ps8
Opisthokonta - ps9
Holomycota - ps10
Fungi - ps11
Fungi_1 - ps12
Fungi_2 - ps13
Fungi_3 - ps14
Dikarya - ps15
Ascomycota - ps16
Saccharo/Pezizomycotina - ps17
Saccharomycotina - ps18
Saccharomycotina_1 - ps19
Saccharomycotina_2 - ps20
Saccharomycotina_3 - ps21
Phaff./Saccharomycetaceae - ps22
Saccharomyco./mycetaceae - ps23
Saccharomycetaceae - ps24
Saccharomycetaceae_2 - ps25
Saccharomycetaceae_3 - ps26
Kaz./Nau./Saccharomyces - ps27
Saccharomyces - ps28
Arboricola_clade - ps29
Cerevisiae_clade - ps30
Saccharomyces_cerevisiae - ps31
Phylostratum

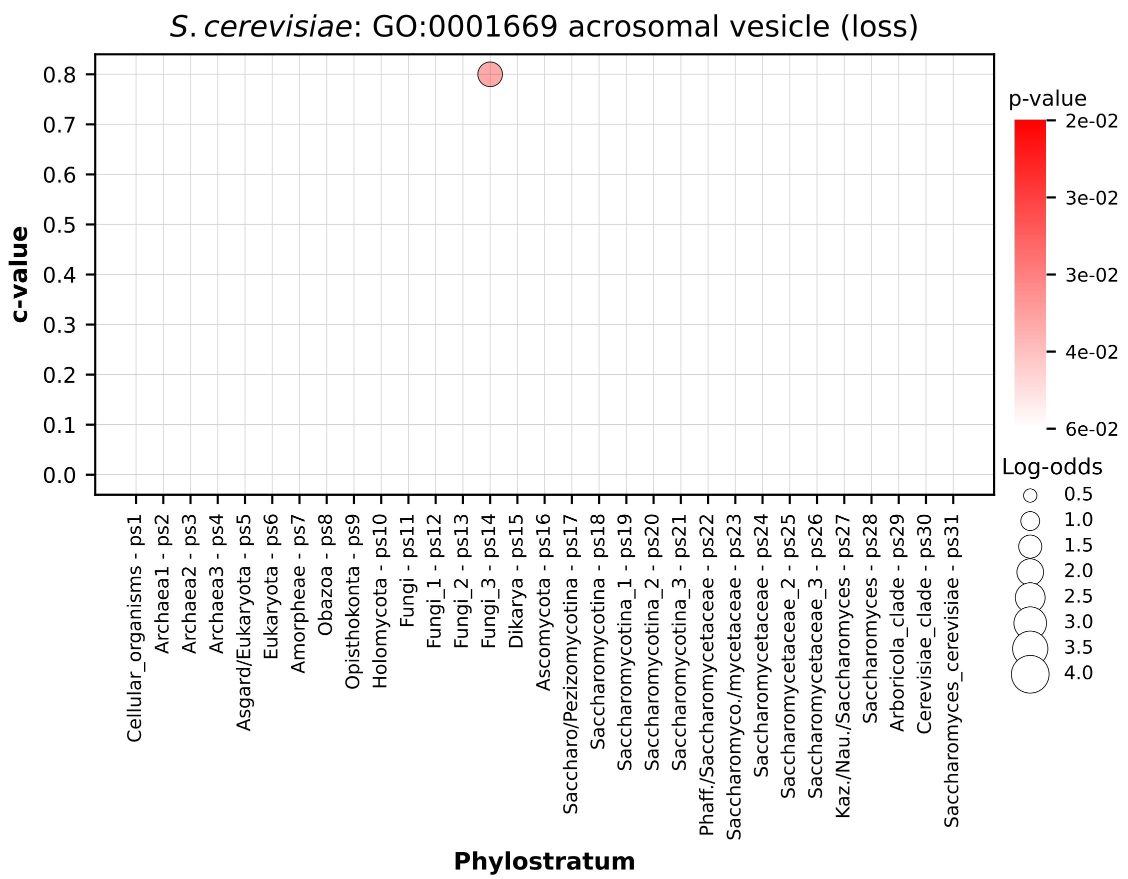
S. cerevisiae: GO:0001669 acrosomal vesicle (loss)
c-value
0.0
0.1
0.2
0.3
0.4
0.5
0.6
0.7
0.8
Phylostratum
Cellular_organisms - ps1
Archaea1 - ps2
Archaea2 - ps3
Archaea3 - ps4
Asgard/Eukaryota - ps5
Eukaryota - ps6
Amorpheae - ps7
Obazoa - ps8
Opisthokonta - ps9
Holomycota - ps10
Fungi - ps11
Fungi_1 - ps12
Fungi_2 - ps13
Fungi_3 - ps14
Dikarya - ps15
Ascomycota - ps16
Saccharo/Pezizomycotina - ps17
Saccharomycotina - ps18
Saccharomycotina_1 - ps19
Saccharomycotina_2 - ps20
Saccharomycotina_3 - ps21
Phaff./Saccharomycetaceae - ps22
Saccharomyco./mycetaceae - ps23
Saccharomycetaceae - ps24
Saccharomycetaceae_2 - ps25
Saccharomycetaceae_3 - ps26
Kaz./Nau./Saccharomyces - ps27
Saccharomyces - ps28
Arboricola_clade - ps29
Cerevisiae_clade - ps30
Saccharomyces_cerevisiae - ps31
p-value
2e-02
3e-02
3e-02
4e-02
6e-02
Log-odds
0.5
1.0
1.5
2.0
2.5
3.0
3.5
4.0

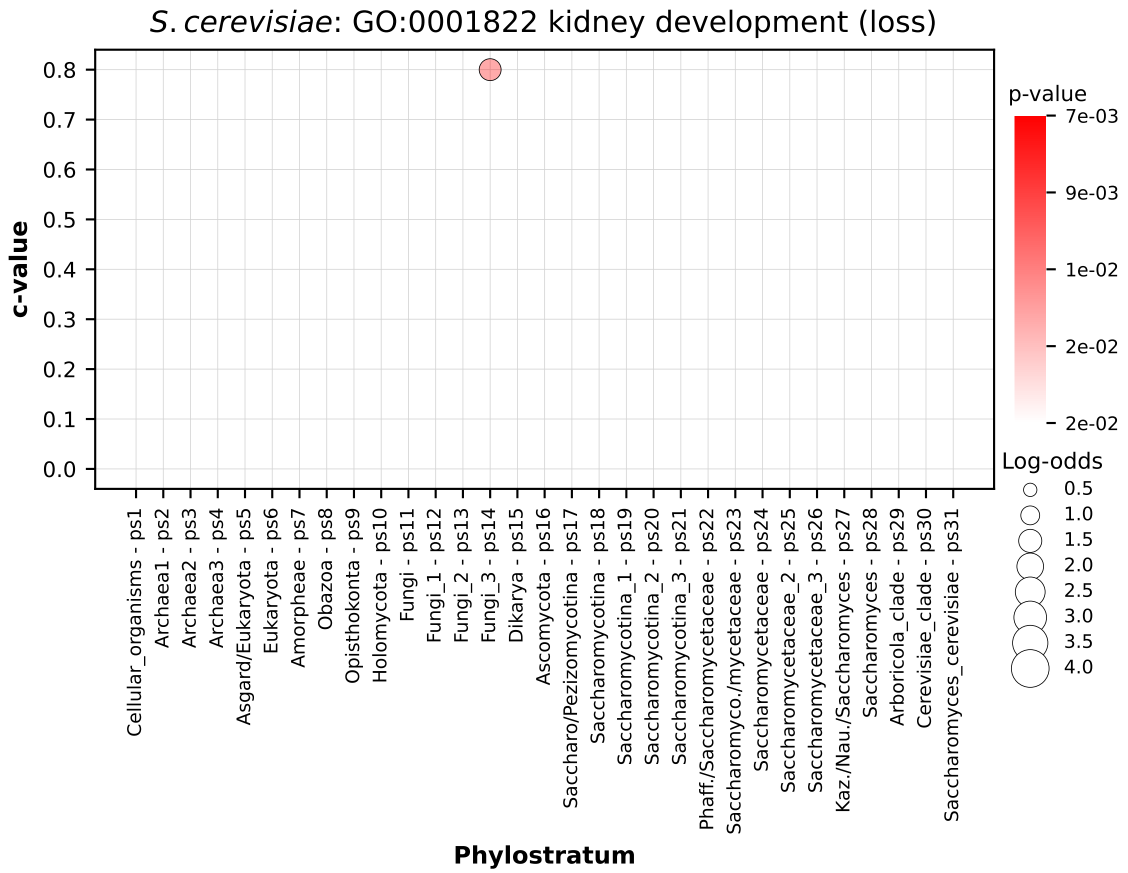

S. cerevisiae: GO:0001822 kidney development (loss)
c-value
0.8
0.7
0.6
0.5
0.4
0.3
0.2
0.1
0.0
p-value
7e-03
9e-03
1e-02
2e-02
2e-02
Log-odds
0.5
1.0
1.5
2.0
2.5
3.0
3.5
4.0
Cellular_organisms - ps1
Archaea1 - ps2
Archaea2 - ps3
Archaea3 - ps4
Asgard/Eukaryota - ps5
Eukaryota - ps6
Amorpheae - ps7
Obazoa - ps8
Opisthokonta - ps9
Holomycota - ps10
Fungi - ps11
Fungi_1 - ps12
Fungi_2 - ps13
Fungi_3 - ps14
Dikarya - ps15
Ascomycota - ps16
Saccharo/Pezizomycotina - ps17
Saccharomycotina - ps18
Saccharomycotina_1 - ps19
Saccharomycotina_2 - ps20
Saccharomycotina_3 - ps21
Phaff./Saccharomycetaceae - ps22
Saccharomyco./mycetaceae - ps23
Saccharomycetaceae - ps24
Saccharomycetaceae_2 - ps25
Saccharomycetaceae_3 - ps26
Kaz./Nau./Saccharomyces - ps27
Saccharomyces - ps28
Arboricola_clade - ps29
Cerevisiae_clade - ps30
Saccharomyces_cerevisiae - ps31
Phylostratum

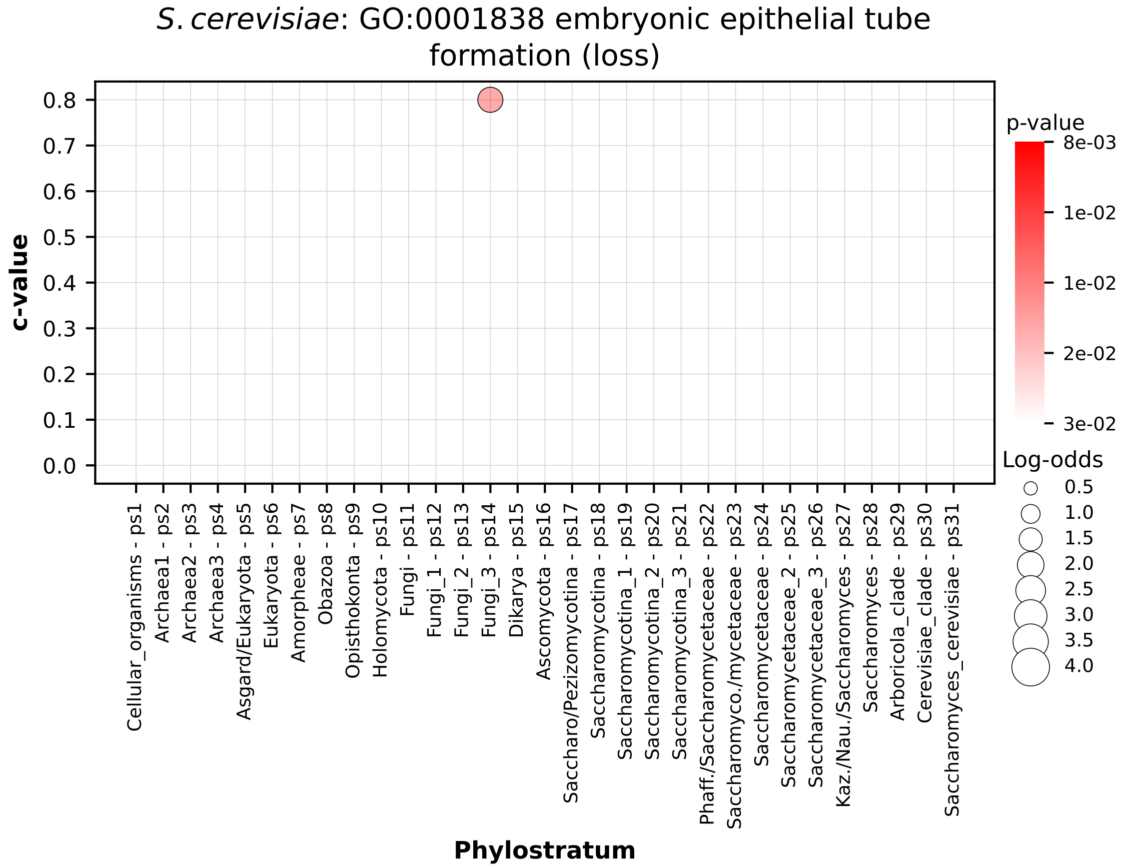

S. cerevisiae: GO:0001838 embryonic epithelial tube formation (loss)
c-value
0.0
0.1
0.2
0.3
0.4
0.5
0.6
0.7
0.8
Phylostratum
Cellular_organisms - ps1
Archaea1 - ps2
Archaea2 - ps3
Archaea3 - ps4
Asgard/Eukaryota - ps5
Eukaryota - ps6
Amorpheae - ps7
Obazoa - ps8
Opisthokonta - ps9
Holomycota - ps10
Fungi - ps11
Fungi_1 - ps12
Fungi_2 - ps13
Fungi_3 - ps14
Dikarya - ps15
Ascomycota - ps16
Saccharo/Pezizomycotina - ps17
Saccharomycotina - ps18
Saccharomycotina_1 - ps19
Saccharomycotina_2 - ps20
Saccharomycotina_3 - ps21
Phaff./Saccharomycetaceae - ps22
Saccharomyco./mycetaceae - ps23
Saccharomycetaceae - ps24
Saccharomycetaceae_2 - ps25
Saccharomycetaceae_3 - ps26
Kaz./Nau./Saccharomyces - ps27
Saccharomyces - ps28
Arboricola_clade - ps29
Cerevisiae_clade - ps30
Saccharomyces_cerevisiae - ps31
p-value
8e-03
1e-02
1e-02
2e-02
3e-02
Log-odds
0.5
1.0
1.5
2.0
2.5
3.0
3.5
4.0

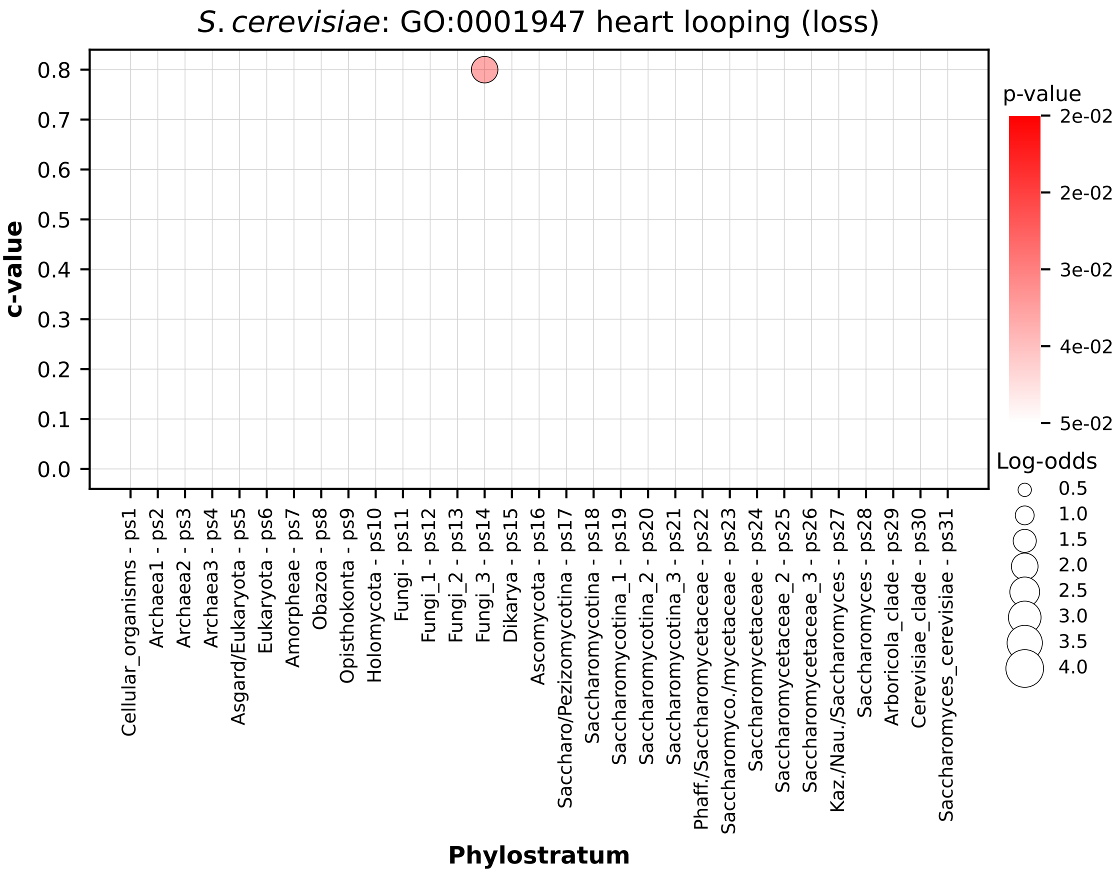

S. cerevisiae: GO:0001947 heart looping (loss)
c-value
0.0
0.1
0.2
0.3
0.4
0.5
0.6
0.7
0.8
p-value
2e-02
2e-02
3e-02
4e-02
5e-02
Log-odds
0.5
1.0
1.5
2.0
2.5
3.0
3.5
4.0
Cellular_organisms - ps1
Archaea1 - ps2
Archaea2 - ps3
Archaea3 - ps4
Asgard/Eukaryota - ps5
Eukaryota - ps6
Amorpheae - ps7
Obazoa - ps8
Opisthokonta - ps9
Holomycota - ps10
Fungi - ps11
Fungi_1 - ps12
Fungi_2 - ps13
Fungi_3 - ps14
Dikarya - ps15
Ascomycota - ps16
Saccharo/Pezizomycotina - ps17
Saccharomycotina - ps18
Saccharomycotina_1 - ps19
Saccharomycotina_2 - ps20
Saccharomycotina_3 - ps21
Phaff./Saccharomycetaceae - ps22
Saccharomyco./mycetaceae - ps23
Saccharomycetaceae - ps24
Saccharomycetaceae_2 - ps25
Saccharomycetaceae_3 - ps26
Kaz./Nau./Saccharomyces - ps27
Saccharomyces - ps28
Arboricola_clade - ps29
Cerevisiae_clade - ps30
Saccharomyces_cerevisiae - ps31
Phylostratum

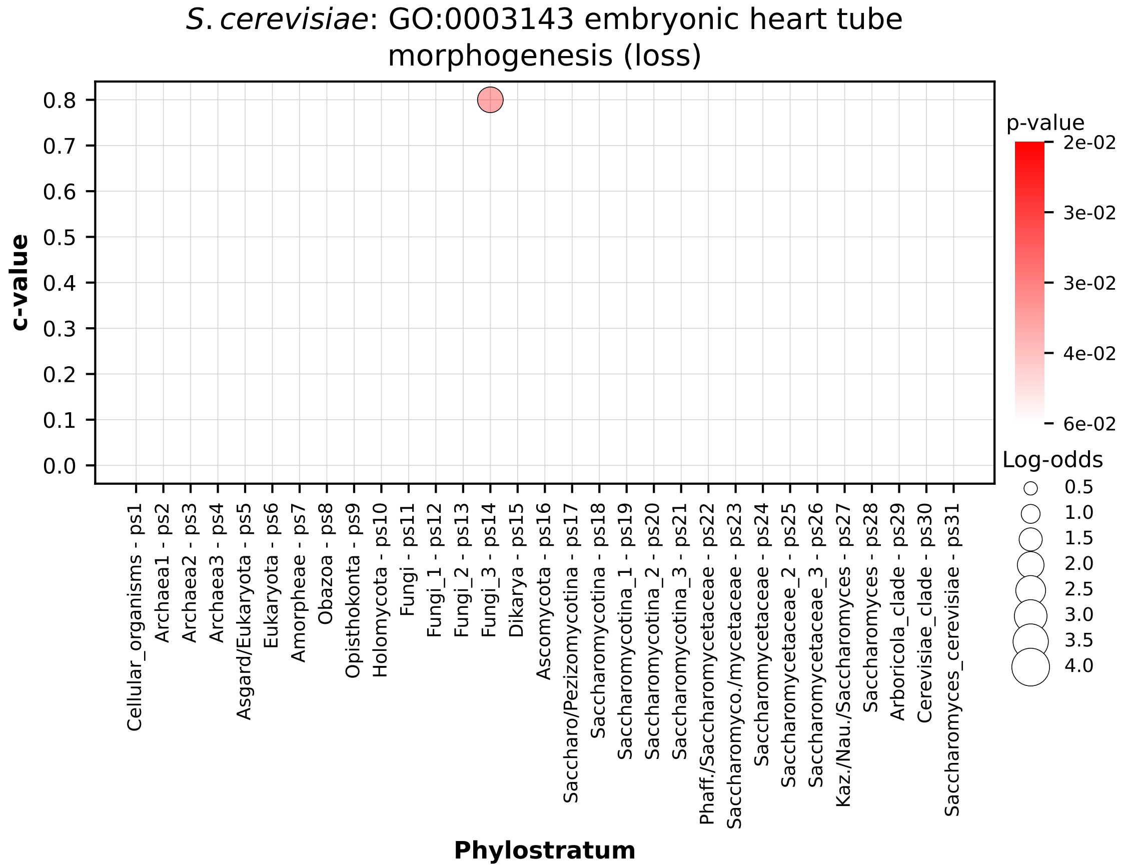

S. cerevisiae: GO:0003143 embryonic heart tube morphogenesis (loss)
c-value
0.0
0.1
0.2
0.3
0.4
0.5
0.6
0.7
0.8
Phylostratum
Cellular_organisms - ps1
Archaea1 - ps2
Archaea2 - ps3
Archaea3 - ps4
Asgard/Eukaryota - ps5
Eukaryota - ps6
Amorpheae - ps7
Obazoa - ps8
Opisthokonta - ps9
Holomycota - ps10
Fungi - ps11
Fungi_1 - ps12
Fungi_2 - ps13
Fungi_3 - ps14
Dikarya - ps15
Ascomycota - ps16
Saccharo/Pezizomycotina - ps17
Saccharomycotina - ps18
Saccharomycotina_1 - ps19
Saccharomycotina_2 - ps20
Saccharomycotina_3 - ps21
Phaff./Saccharomycetaceae - ps22
Saccharomyco./mycetaceae - ps23
Saccharomycetaceae - ps24
Saccharomycetaceae_2 - ps25
Saccharomycetaceae_3 - ps26
Kaz./Nau./Saccharomyces - ps27
Saccharomyces - ps28
Arboricola_clade - ps29
Cerevisiae_clade - ps30
Saccharomyces_cerevisiae - ps31
p-value
2e-02
3e-02
3e-02
4e-02
6e-02
Log-odds
0.5
1.0
1.5
2.0
2.5
3.0
3.5
4.0

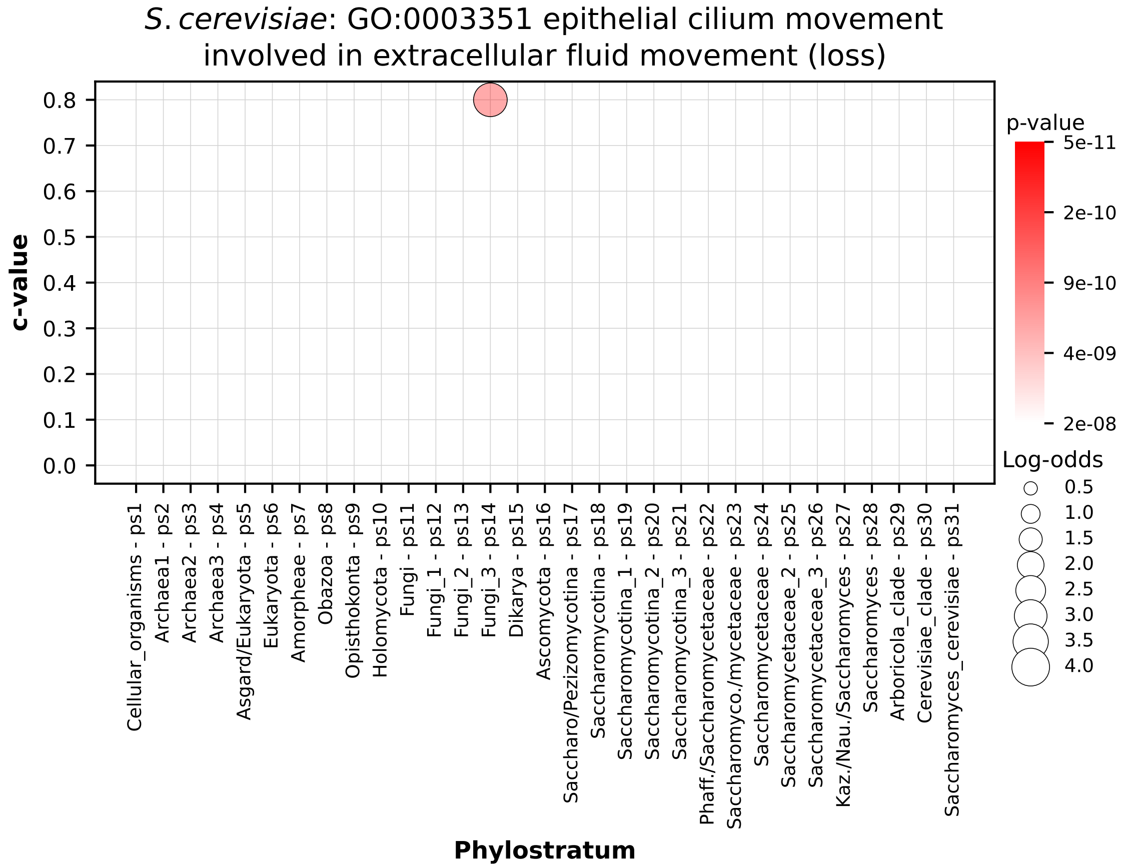
S. cerevisiae: GO:0003351 epithelial cilium movement involved in extracellular fluid movement (loss)
c-value
0.0
0.1
0.2
0.3
0.4
0.5
0.6
0.7
0.8
Phylostratum
Cellular_organisms - ps1
Archaea1 - ps2
Archaea2 - ps3
Archaea3 - ps4
Asgard/Eukaryota - ps5
Eukaryota - ps6
Amorpheae - ps7
Obazoa - ps8
Opisthokonta - ps9
Holomycota - ps10
Fungi - ps11
Fungi_1 - ps12
Fungi_2 - ps13
Fungi_3 - ps14
Dikarya - ps15
Ascomycota - ps16
Saccharo/Pezizomycotina - ps17
Saccharomycotina - ps18
Saccharomycotina_1 - ps19
Saccharomycotina_2 - ps20
Saccharomycotina_3 - ps21
Phaff./Saccharomycetaceae - ps22
Saccharomyco./mycetaceae - ps23
Saccharomycetaceae - ps24
Saccharomycetaceae_2 - ps25
Saccharomycetaceae_3 - ps26
Kaz./Nau./Saccharomyces - ps27
Saccharomyces - ps28
Arboricola_clade - ps29
Cerevisiae_clade - ps30
Saccharomyces_cerevisiae - ps31
p-value
5e-11
2e-10
9e-10
4e-09
2e-08
Log-odds
0.5
1.0
1.5
2.0
2.5
3.0
3.5
4.0

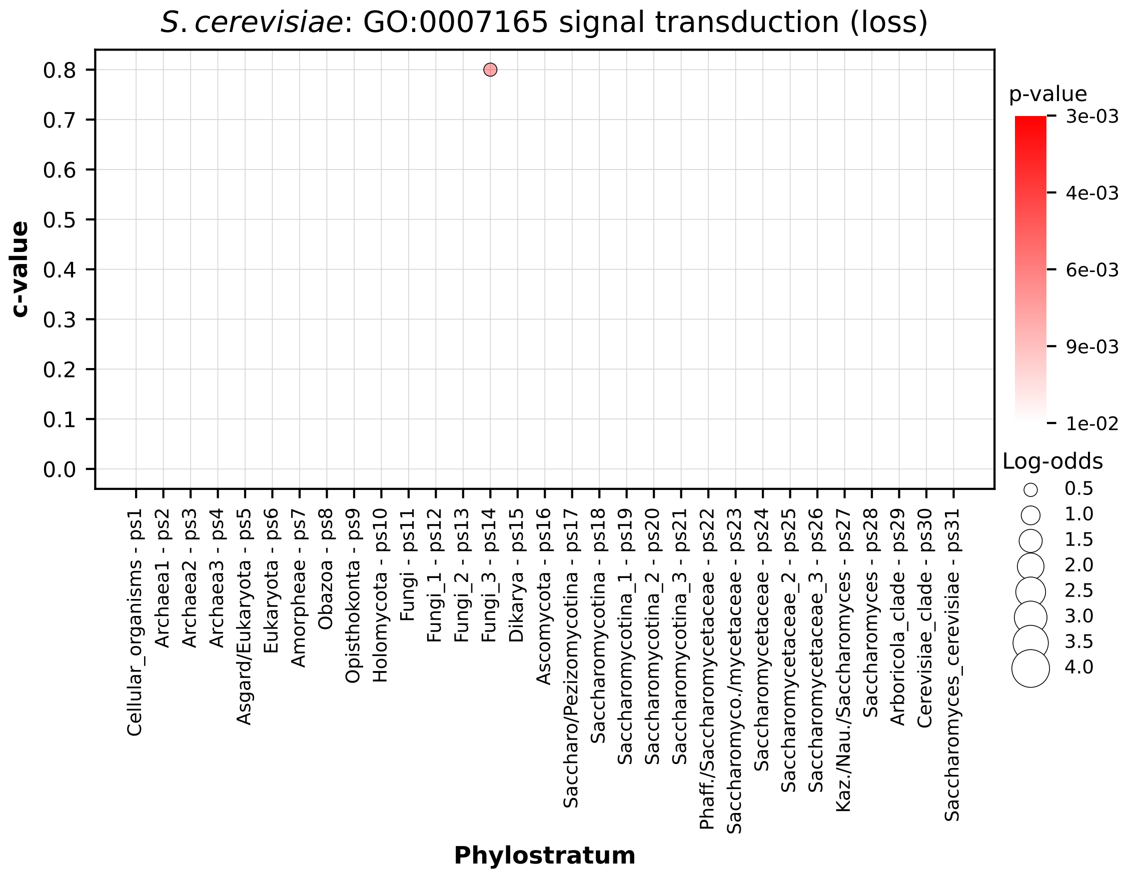
S. cerevisiae: GO:0007165 signal transduction (loss)
c-value
0.0
0.1
0.2
0.3
0.4
0.5
0.6
0.7
0.8
p-value
3e-03
4e-03
6e-03
9e-03
1e-02
Log-odds
0.5
1.0
1.5
2.0
2.5
3.0
3.5
4.0
Cellular_organisms - ps1
Archaea1 - ps2
Archaea2 - ps3
Archaea3 - ps4
Asgard/Eukaryota - ps5
Eukaryota - ps6
Amorpheae - ps7
Obazoa - ps8
Opisthokonta - ps9
Holomycota - ps10
Fungi - ps11
Fungi_1 - ps12
Fungi_2 - ps13
Fungi_3 - ps14
Dikarya - ps15
Ascomycota - ps16
Saccharo/Pezizomycotina - ps17
Saccharomycotina - ps18
Saccharomycotina_1 - ps19
Saccharomycotina_2 - ps20
Saccharomycotina_3 - ps21
Phaff./Saccharomycetaceae - ps22
Saccharomyco./mycetaceae - ps23
Saccharomycetaceae - ps24
Saccharomycetaceae_2 - ps25
Saccharomycetaceae_3 - ps26
Kaz./Nau./Saccharomyces - ps27
Saccharomyces - ps28
Arboricola_clade - ps29
Cerevisiae_clade - ps30
Saccharomyces_cerevisiae - ps31
Phylostratum

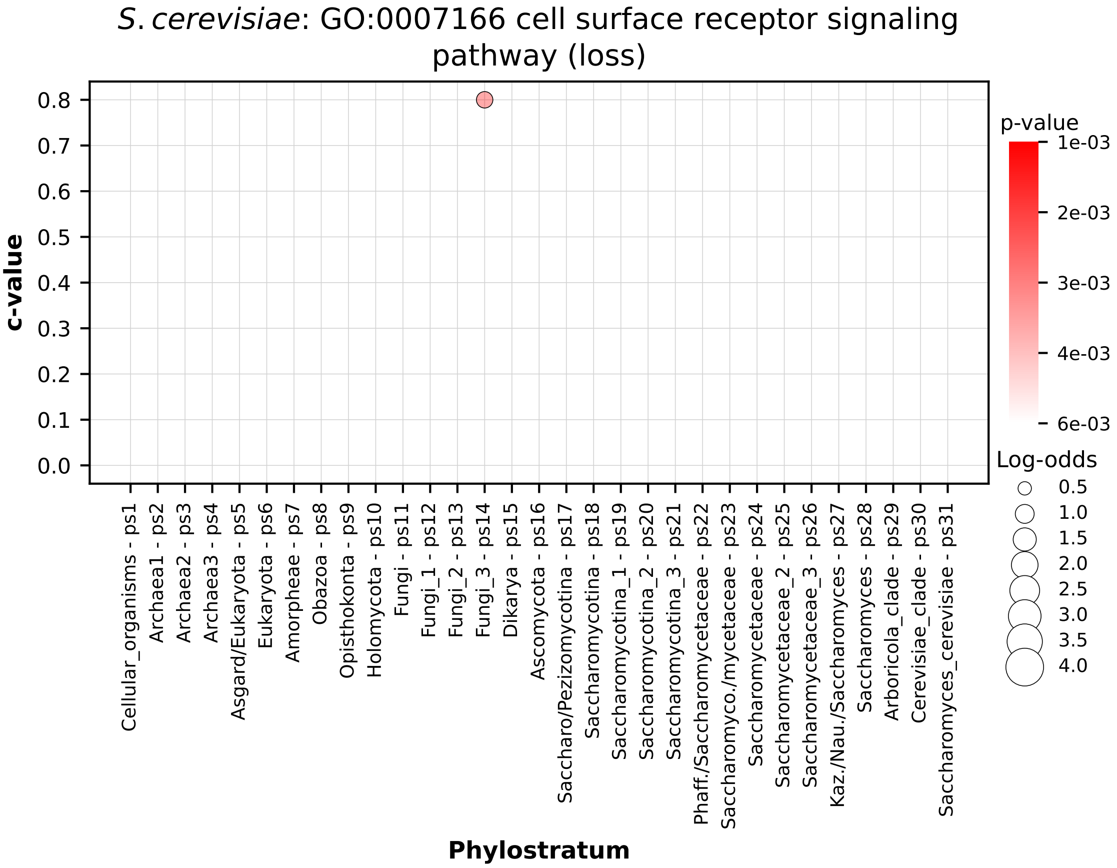

S. cerevisiae: GO:0007166 cell surface receptor signaling pathway (loss)
c-value
0.0
0.1
0.2
0.3
0.4
0.5
0.6
0.7
0.8
p-value
1e-03
2e-03
3e-03
4e-03
6e-03
Log-odds
0.5
1.0
1.5
2.0
2.5
3.0
3.5
4.0
Cellular_organisms - ps1
Archaea1 - ps2
Archaea2 - ps3
Archaea3 - ps4
Asgard/Eukaryota - ps5
Eukaryota - ps6
Amorpheae - ps7
Obazoa - ps8
Opisthokonta - ps9
Holomycota - ps10
Fungi - ps11
Fungi_1 - ps12
Fungi_2 - ps13
Fungi_3 - ps14
Dikarya - ps15
Ascomycota - ps16
Saccharo/Pezizomycotina - ps17
Saccharomycotina - ps18
Saccharomycotina_1 - ps19
Saccharomycotina_2 - ps20
Saccharomycotina_3 - ps21
Phaff./Saccharomycetaceae - ps22
Saccharomyco./mycetaceae - ps23
Saccharomycetaceae - ps24
Saccharomycetaceae_2 - ps25
Saccharomycetaceae_3 - ps26
Kaz./Nau./Saccharomyces - ps27
Saccharomyces - ps28
Arboricola_clade - ps29
Cerevisiae_clade - ps30
Saccharomyces_cerevisiae - ps31
Phylostratum

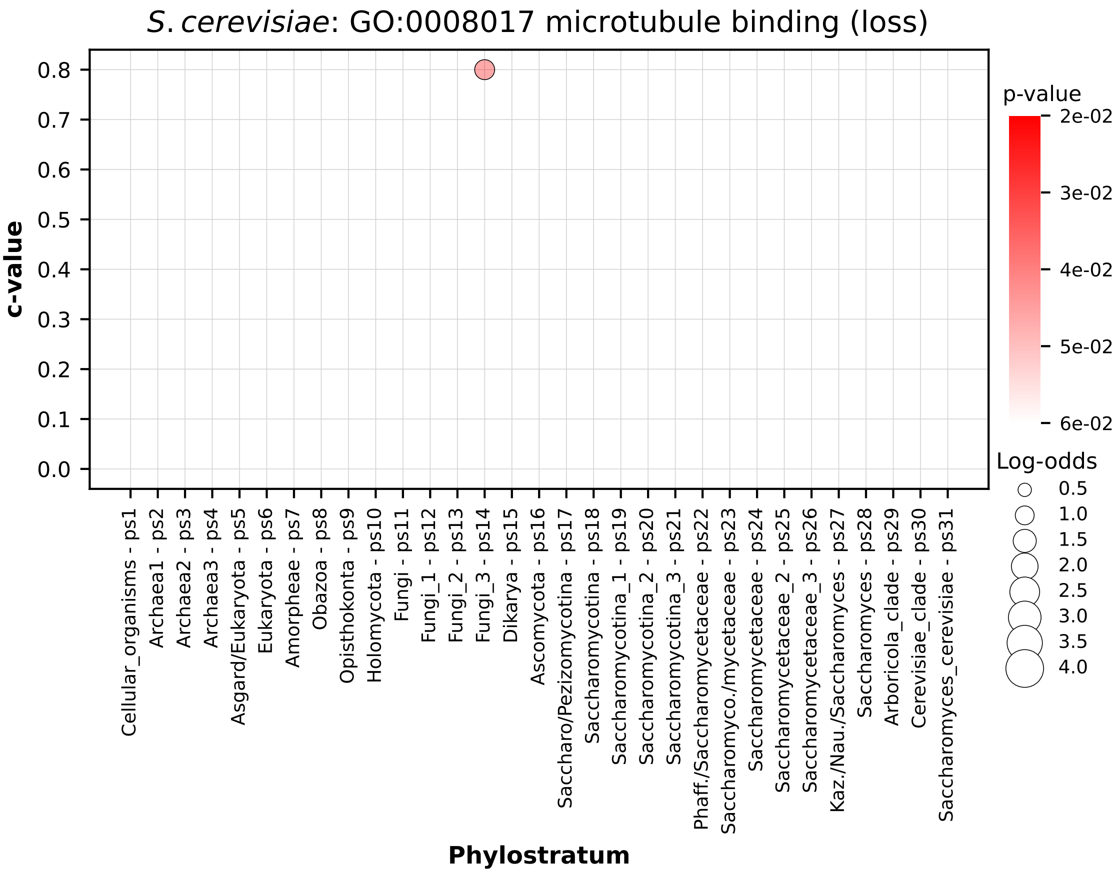
S. cerevisiae: GO:0008017 microtubule binding (loss)
c-value
0.0
0.1
0.2
0.3
0.4
0.5
0.6
0.7
0.8
p-value
2e-02
3e-02
4e-02
5e-02
6e-02
Log-odds
0.5
1.0
1.5
2.0
2.5
3.0
3.5
4.0
Cellular_organisms - ps1
Archaea1 - ps2
Archaea2 - ps3
Archaea3 - ps4
Asgard/Eukaryota - ps5
Eukaryota - ps6
Amorpheae - ps7
Obazoa - ps8
Opisthokonta - ps9
Holomycota - ps10
Fungi - ps11
Fungi_1 - ps12
Fungi_2 - ps13
Fungi_3 - ps14
Dikarya - ps15
Ascomycota - ps16
Saccharo/Pezizomycotina - ps17
Saccharomycotina - ps18
Saccharomycotina_1 - ps19
Saccharomycotina_2 - ps20
Saccharomycotina_3 - ps21
Phaff./Saccharomycetaceae - ps22
Saccharomyco./mycetaceae - ps23
Saccharomycetaceae - ps24
Saccharomycetaceae_2 - ps25
Saccharomycetaceae_3 - ps26
Kaz./Nau./Saccharomyces - ps27
Saccharomyces - ps28
Arboricola_clade - ps29
Cerevisiae_clade - ps30
Saccharomyces_cerevisiae - ps31
Phylostratum

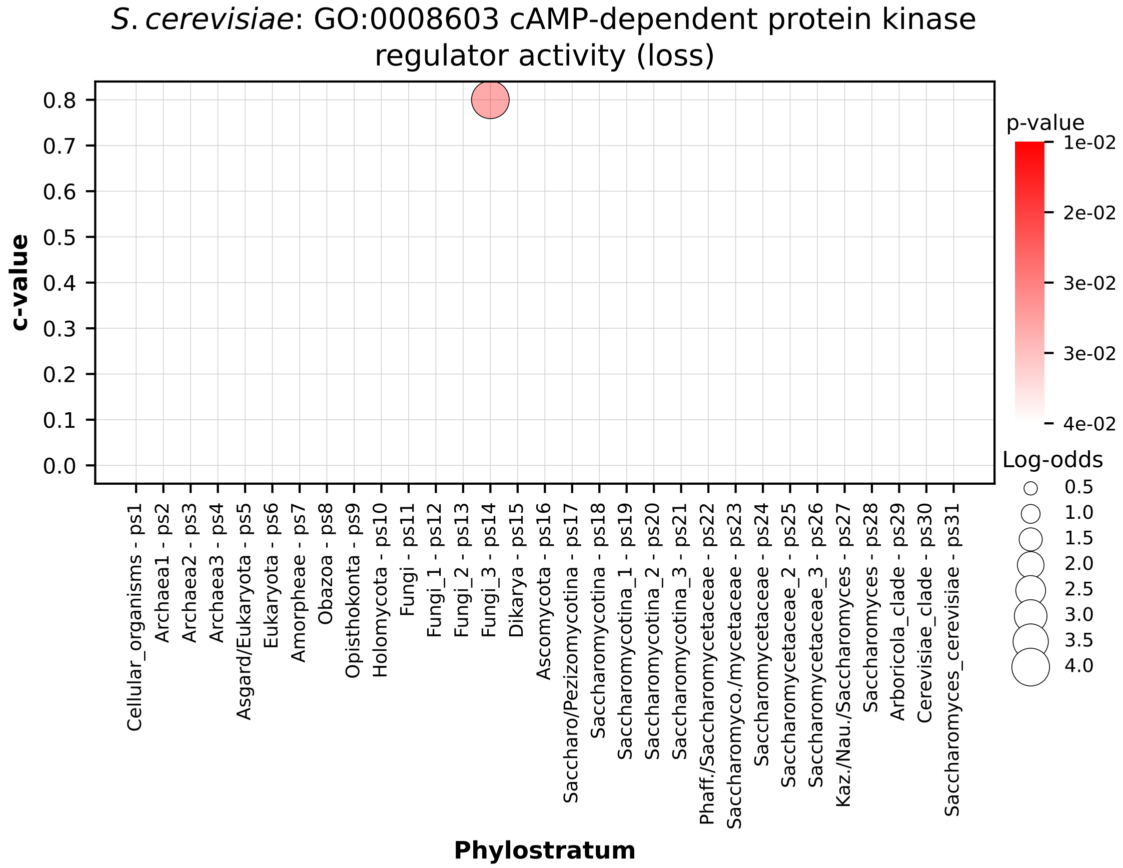
S. cerevisiae: GO:0008603 cAMP-dependent protein kinase regulator activity (loss)
c-value
0.8
0.7
0.6
0.5
0.4
0.3
0.2
0.1
0.0
p-value
1e-02
2e-02
3e-02
3e-02
4e-02
Log-odds
0.5
1.0
1.5
2.0
2.5
3.0
3.5
4.0
Cellular_organisms - ps1
Archaea1 - ps2
Archaea2 - ps3
Archaea3 - ps4
Asgard/Eukaryota - ps5
Eukaryota - ps6
Amorpheae - ps7
Obazoa - ps8
Opisthokonta - ps9
Holomycota - ps10
Fungi - ps11
Fungi_1 - ps12
Fungi_2 - ps13
Fungi_3 - ps14
Dikarya - ps15
Ascomycota - ps16
Saccharo/Pezizomycotina - ps17
Saccharomycotina - ps18
Saccharomycotina_1 - ps19
Saccharomycotina_2 - ps20
Saccharomycotina_3 - ps21
Phaff./Saccharomycetaceae - ps22
Saccharomyco./mycetaceae - ps23
Saccharomycetaceae - ps24
Saccharomycetaceae_2 - ps25
Saccharomycetaceae_3 - ps26
Kaz./Nau./Saccharomyces - ps27
Saccharomyces - ps28
Arboricola_clade - ps29
Cerevisiae_clade - ps30
Saccharomyces_cerevisiae - ps31
Phylostratum

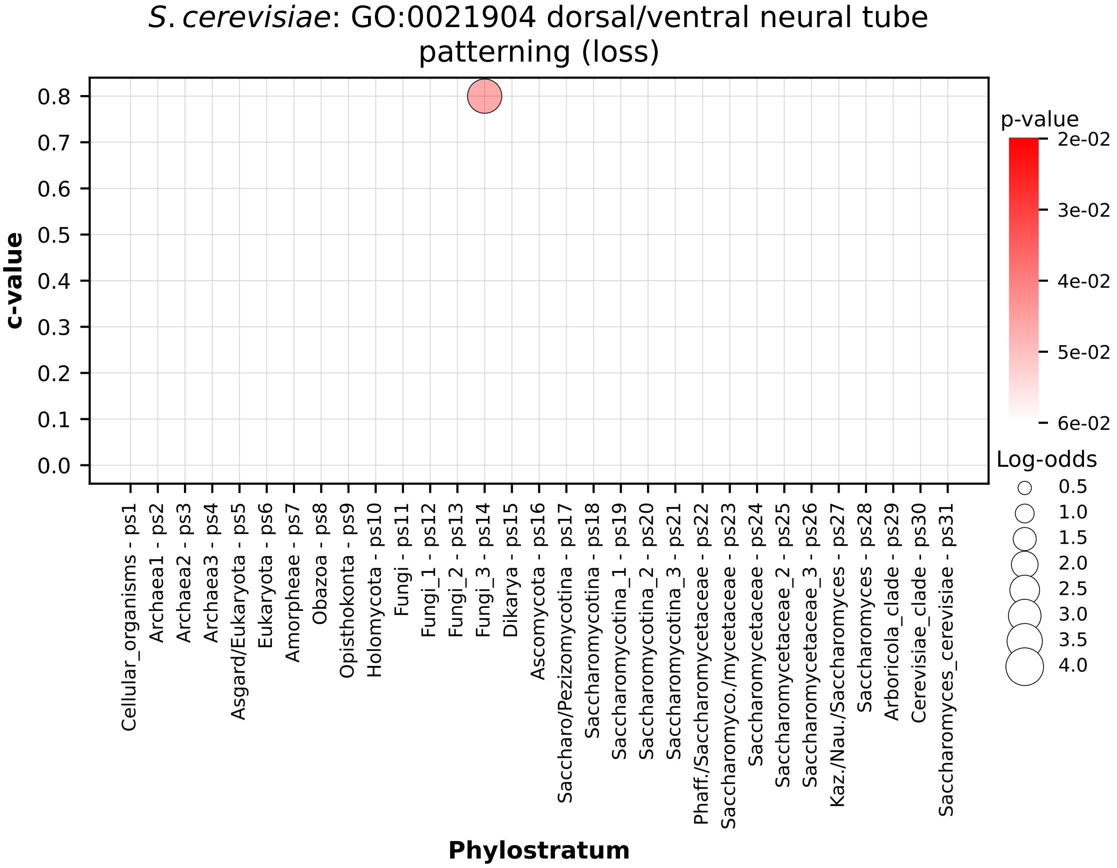
S. cerevisiae: GO:0021904 dorsal/ventral neural tube patterning (loss)
c-value
0.0
0.1
0.2
0.3
0.4
0.5
0.6
0.7
0.8
p-value
2e-02
3e-02
4e-02
5e-02
6e-02
Log-odds
0.5
1.0
1.5
2.0
2.5
3.0
3.5
4.0
Cellular_organisms - ps1
Archaea1 - ps2
Archaea2 - ps3
Archaea3 - ps4
Asgard/Eukaryota - ps5
Eukaryota - ps6
Amorpheae - ps7
Obazoa - ps8
Opisthokonta - ps9
Holomycota - ps10
Fungi - ps11
Fungi_1 - ps12
Fungi_2 - ps13
Fungi_3 - ps14
Dikarya - ps15
Ascomycota - ps16
Saccharo/Pezizomycotina - ps17
Saccharomycotina - ps18
Saccharomycotina_1 - ps19
Saccharomycotina_2 - ps20
Saccharomycotina_3 - ps21
Phaff./Saccharomycetaceae - ps22
Saccharomyco./mycetaceae - ps23
Saccharomycetaceae - ps24
Saccharomycetaceae_2 - ps25
Saccharomycetaceae_3 - ps26
Kaz./Nau./Saccharomyces - ps27
Saccharomyces - ps28
Arboricola_clade - ps29
Cerevisiae_clade - ps30
Saccharomyces_cerevisiae - ps31
Phylostratum

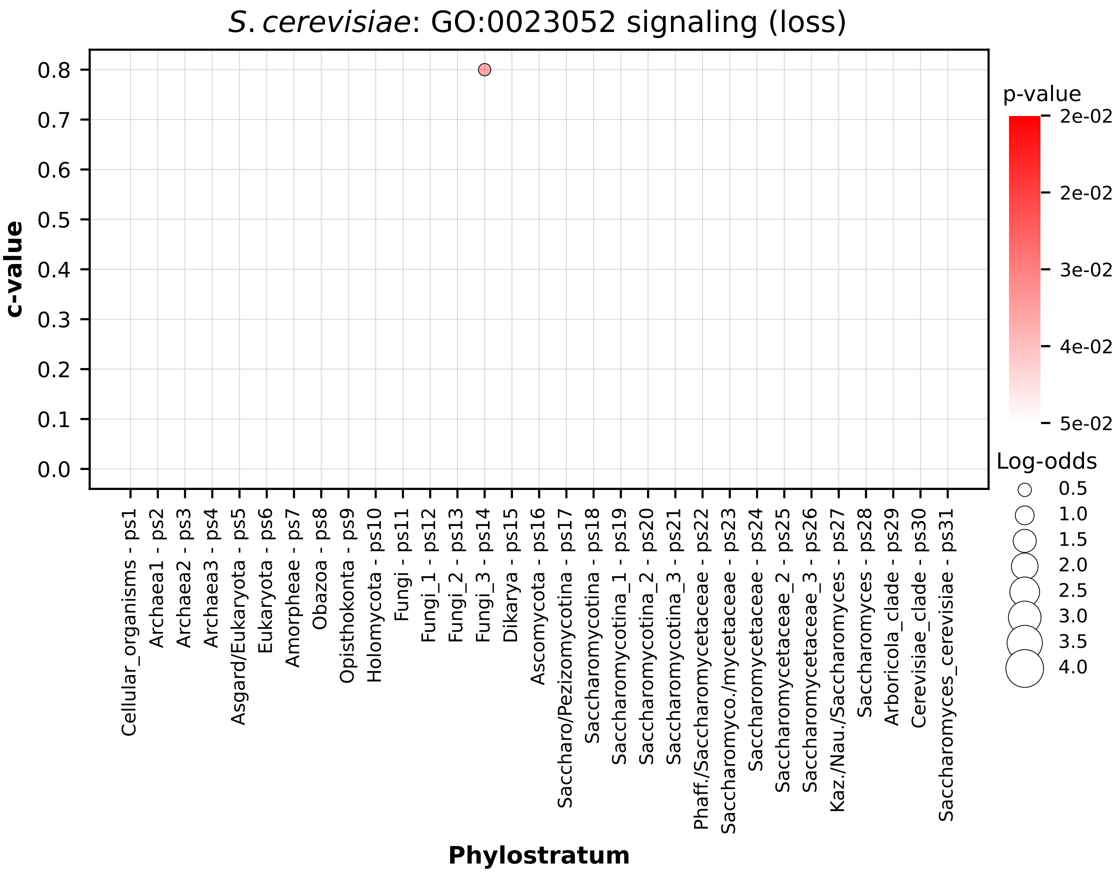
S. cerevisiae: GO:0023052 signaling (loss)
c-value
0.0
0.1
0.2
0.3
0.4
0.5
0.6
0.7
0.8
p-value
2e-02
2e-02
3e-02
4e-02
5e-02
Log-odds
0.5
1.0
1.5
2.0
2.5
3.0
3.5
4.0
Cellular_organisms - ps1
Archaea1 - ps2
Archaea2 - ps3
Archaea3 - ps4
Asgard/Eukaryota - ps5
Eukaryota - ps6
Amorpheae - ps7
Obazoa - ps8
Opisthokonta - ps9
Holomycota - ps10
Fungi - ps11
Fungi_1 - ps12
Fungi_2 - ps13
Fungi_3 - ps14
Dikarya - ps15
Ascomycota - ps16
Saccharo/Pezizomycotina - ps17
Saccharomycotina - ps18
Saccharomycotina_1 - ps19
Saccharomycotina_2 - ps20
Saccharomycotina_3 - ps21
Phaff./Saccharomycetaceae - ps22
Saccharomyco./mycetaceae - ps23
Saccharomycetaceae - ps24
Saccharomycetaceae_2 - ps25
Saccharomycetaceae_3 - ps26
Kaz./Nau./Saccharomyces - ps27
Saccharomyces - ps28
Arboricola_clade - ps29
Cerevisiae_clade - ps30
Saccharomyces_cerevisiae - ps31
Phylostratum

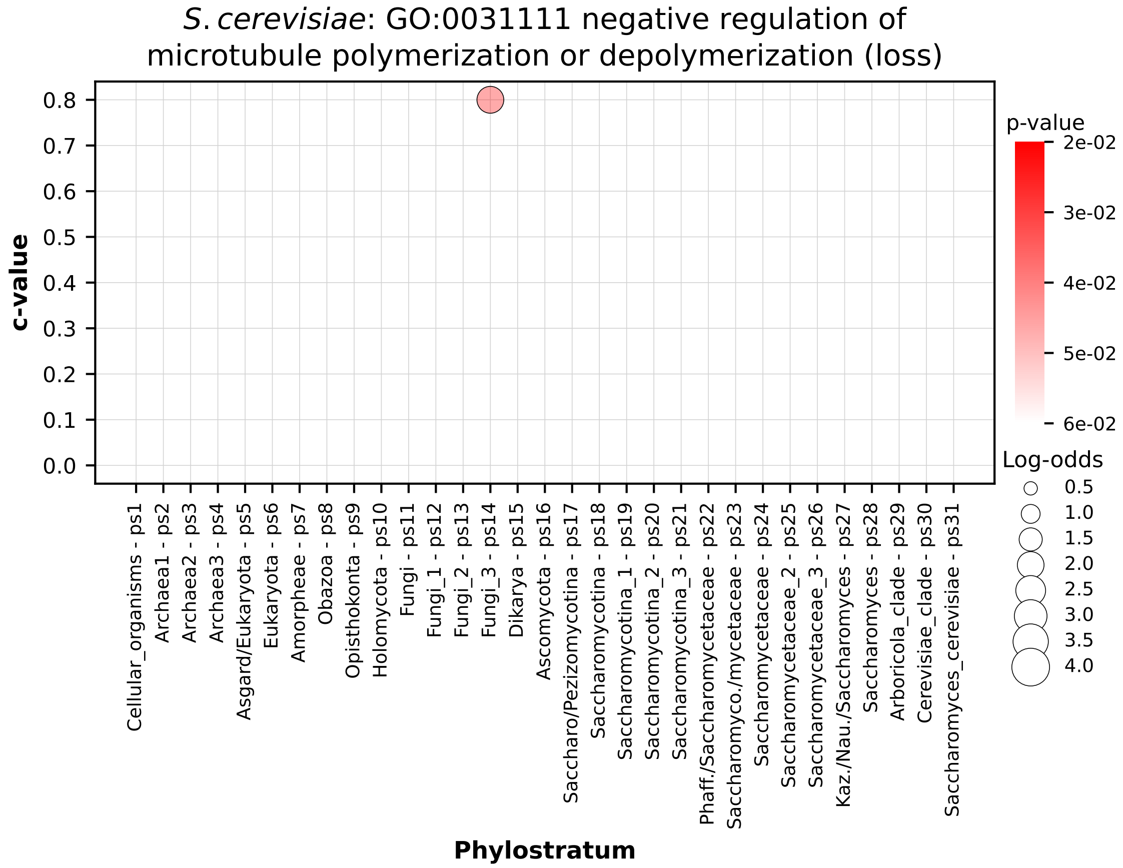
S. cerevisiae: GO:0031111 negative regulation of microtubule polymerization or depolymerization (loss)
c-value
0.0
0.1
0.2
0.3
0.4
0.5
0.6
0.7
0.8
p-value
2e-02
3e-02
4e-02
5e-02
6e-02
Log-odds
0.5
1.0
1.5
2.0
2.5
3.0
3.5
4.0
Cellular_organisms - ps1
Archaea1 - ps2
Archaea2 - ps3
Archaea3 - ps4
Asgard/Eukaryota - ps5
Eukaryota - ps6
Amorpheae - ps7
Obazoa - ps8
Opisthokonta - ps9
Holomycota - ps10
Fungi - ps11
Fungi_1 - ps12
Fungi_2 - ps13
Fungi_3 - ps14
Dikarya - ps15
Ascomycota - ps16
Saccharo/Pezizomycotina - ps17
Saccharomycotina - ps18
Saccharomycotina_1 - ps19
Saccharomycotina_2 - ps20
Saccharomycotina_3 - ps21
Phaff./Saccharomycetaceae - ps22
Saccharomyco./mycetaceae - ps23
Saccharomycetaceae - ps24
Saccharomycetaceae_2 - ps25
Saccharomycetaceae_3 - ps26
Kaz./Nau./Saccharomyces - ps27
Saccharomyces - ps28
Arboricola_clade - ps29
Cerevisiae_clade - ps30
Saccharomyces_cerevisiae - ps31
Phylostratum

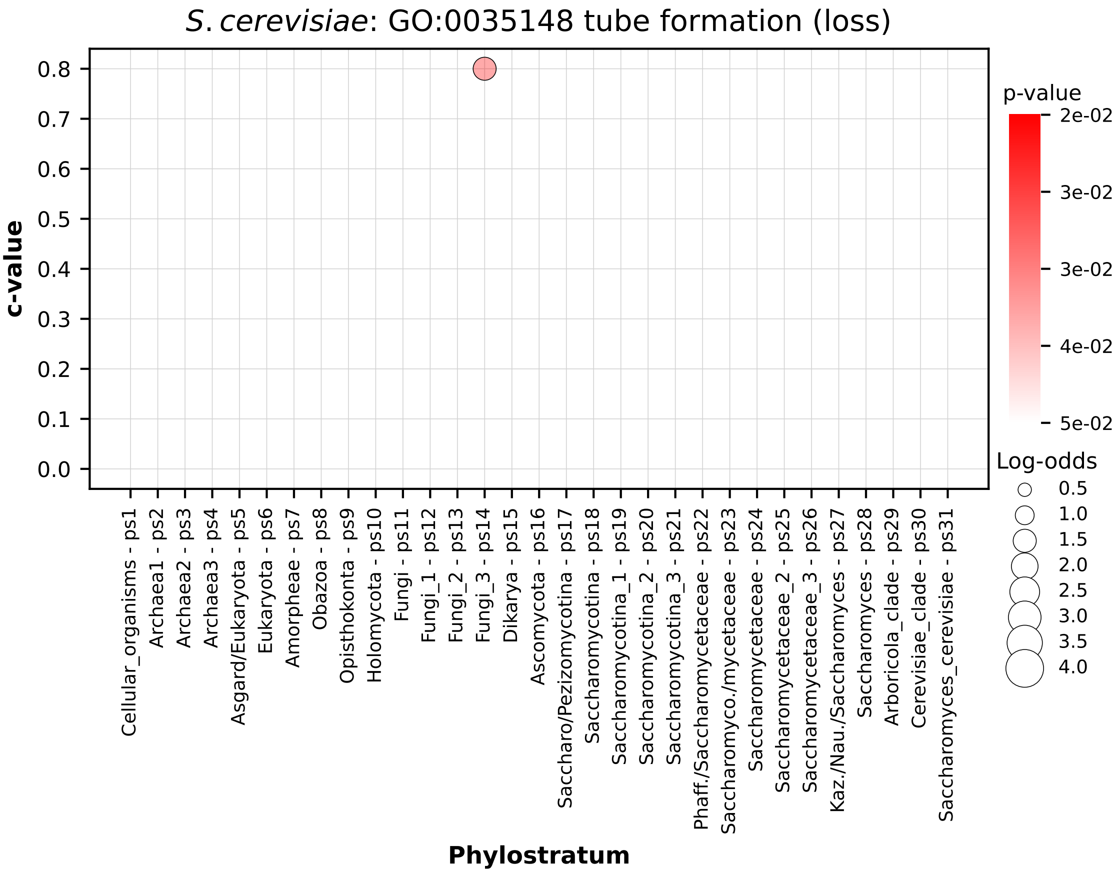
S. cerevisiae: GO:0035148 tube formation (loss)
c-value
0.8
0.7
0.6
0.5
0.4
0.3
0.2
0.1
0.0
p-value
2e-02
3e-02
3e-02
4e-02
5e-02
Log-odds
0.5
1.0
1.5
2.0
2.5
3.0
3.5
4.0
Cellular_organisms - ps1
Archaea1 - ps2
Archaea2 - ps3
Archaea3 - ps4
Asgard/Eukaryota - ps5
Eukaryota - ps6
Amorpheae - ps7
Obazoa - ps8
Opisthokonta - ps9
Holomycota - ps10
Fungi - ps11
Fungi_1 - ps12
Fungi_2 - ps13
Fungi_3 - ps14
Dikarya - ps15
Ascomycota - ps16
Saccharo/Pezizomycotina - ps17
Saccharomycotina - ps18
Saccharomycotina_1 - ps19
Saccharomycotina_2 - ps20
Saccharomycotina_3 - ps21
Phaff./Saccharomycetaceae - ps22
Saccharomyco./mycetaceae - ps23
Saccharomycetaceae - ps24
Saccharomycetaceae_2 - ps25
Saccharomycetaceae_3 - ps26
Kaz./Nau./Saccharomyces - ps27
Saccharomyces - ps28
Arboricola_clade - ps29
Cerevisiae_clade - ps30
Saccharomyces_cerevisiae - ps31
Phylostratum

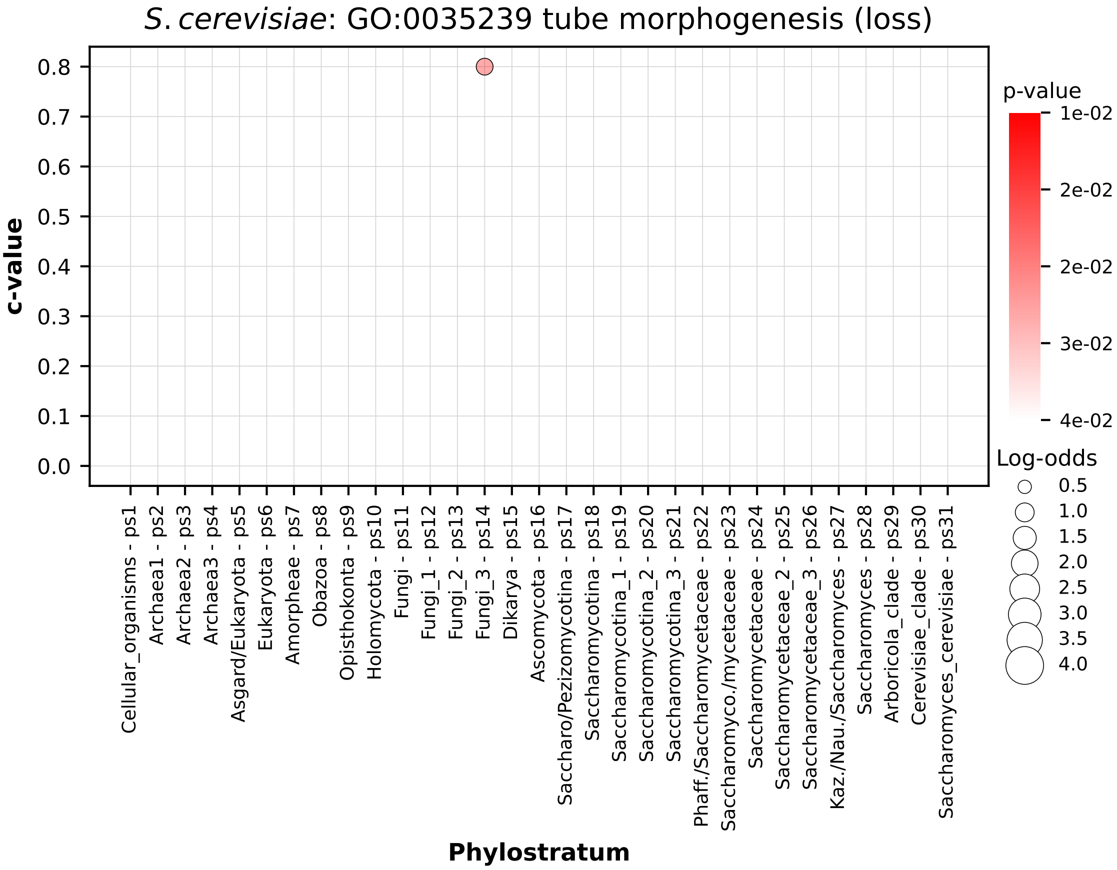
S. cerevisiae: GO:0035239 tube morphogenesis (loss)
c-value
0.0
0.1
0.2
0.3
0.4
0.5
0.6
0.7
0.8
Phylostratum
Cellular_organisms - ps1
Archaea1 - ps2
Archaea2 - ps3
Archaea3 - ps4
Asgard/Eukaryota - ps5
Eukaryota - ps6
Amorpheae - ps7
Obazoa - ps8
Opisthokonta - ps9
Holomycota - ps10
Fungi - ps11
Fungi_1 - ps12
Fungi_2 - ps13
Fungi_3 - ps14
Dikarya - ps15
Ascomycota - ps16
Saccharo/Pezizomycotina - ps17
Saccharomycotina - ps18
Saccharomycotina_1 - ps19
Saccharomycotina_2 - ps20
Saccharomycotina_3 - ps21
Phaff./Saccharomycetaceae - ps22
Saccharomyco./mycetaceae - ps23
Saccharomycetaceae - ps24
Saccharomycetaceae_2 - ps25
Saccharomycetaceae_3 - ps26
Kaz./Nau./Saccharomyces - ps27
Saccharomyces - ps28
Arboricola_clade - ps29
Cerevisiae_clade - ps30
Saccharomyces_cerevisiae - ps31
p-value
1e-02
2e-02
2e-02
3e-02
4e-02
Log-odds
0.5
1.0
1.5
2.0
2.5
3.0
3.5
4.0

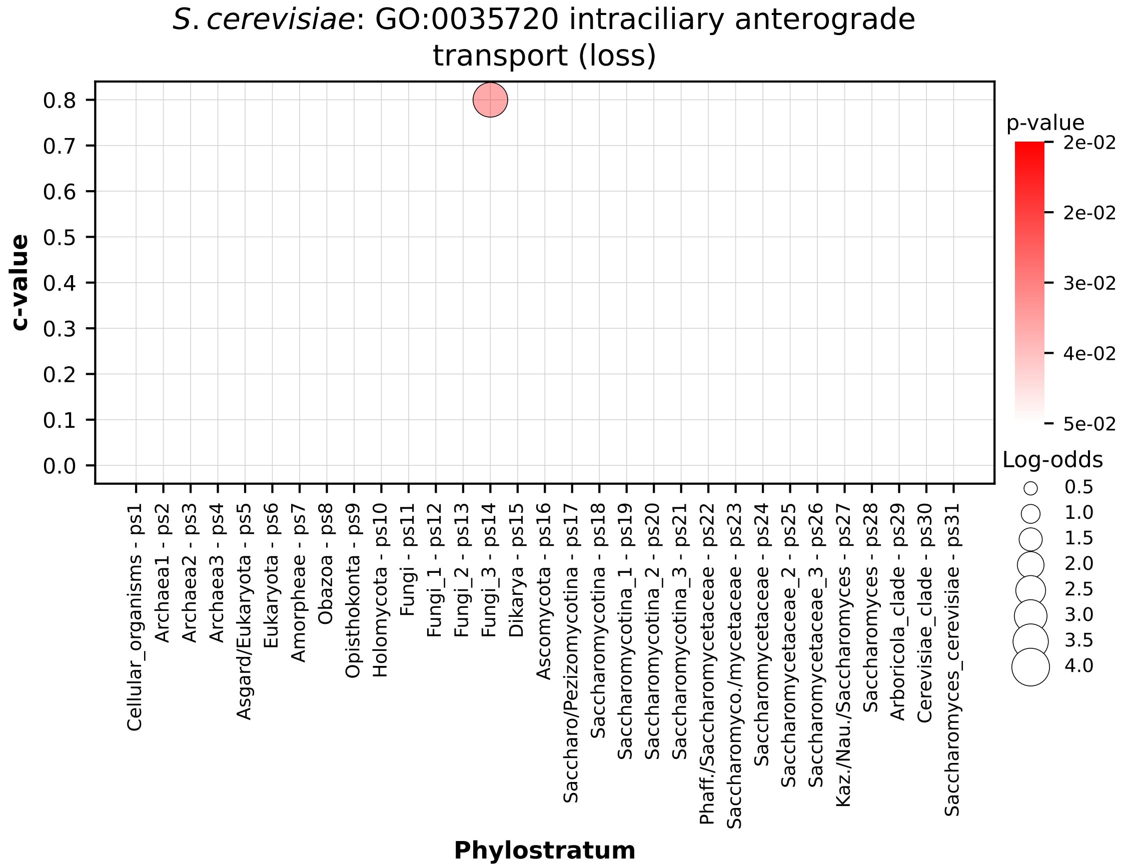
S. cerevisiae: GO:0035720 intraciliary anterograde transport (loss)
c-value
0.0
0.1
0.2
0.3
0.4
0.5
0.6
0.7
0.8
p-value
2e-02
2e-02
3e-02
4e-02
5e-02
Log-odds
0.5
1.0
1.5
2.0
2.5
3.0
3.5
4.0
Cellular_organisms - ps1
Archaea1 - ps2
Archaea2 - ps3
Archaea3 - ps4
Asgard/Eukaryota - ps5
Eukaryota - ps6
Amorpheae - ps7
Obazoa - ps8
Opisthokonta - ps9
Holomycota - ps10
Fungi - ps11
Fungi_1 - ps12
Fungi_2 - ps13
Fungi_3 - ps14
Dikarya - ps15
Ascomycota - ps16
Saccharo/Pezizomycotina - ps17
Saccharomycotina - ps18
Saccharomycotina_1 - ps19
Saccharomycotina_2 - ps20
Saccharomycotina_3 - ps21
Phaff./Saccharomycetaceae - ps22
Saccharomyco./mycetaceae - ps23
Saccharomycetaceae - ps24
Saccharomycetaceae_2 - ps25
Saccharomycetaceae_3 - ps26
Kaz./Nau./Saccharomyces - ps27
Saccharomyces - ps28
Arboricola_clade - ps29
Cerevisiae_clade - ps30
Saccharomyces_cerevisiae - ps31
Phylostratum

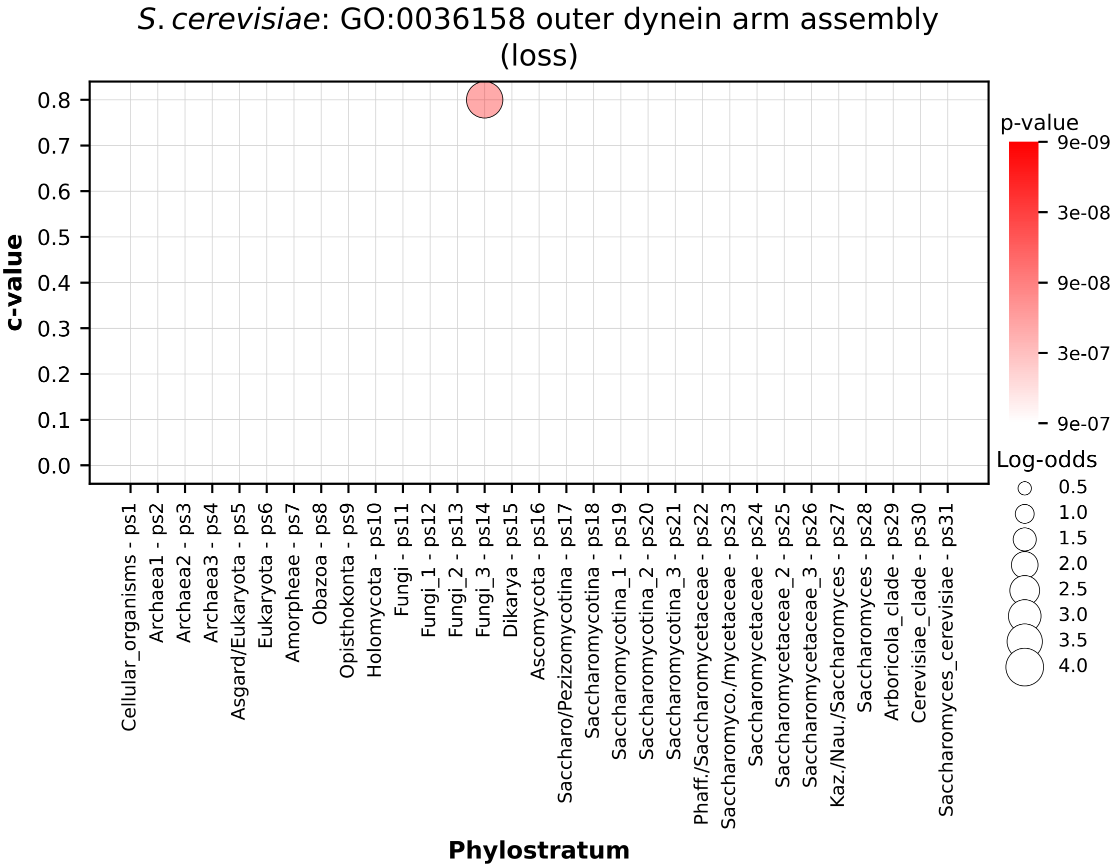

S. cerevisiae: GO:0036158 outer dynein arm assembly
(loss)
c-value
0.8
0.7
0.6
0.5
0.4
0.3
0.2
0.1
0.0
p-value
9e-09
3e-08
9e-08
3e-07
9e-07
Log-odds
0.5
1.0
1.5
2.0
2.5
3.0
3.5
4.0
Cellular_organisms - ps1
Archaea1 - ps2
Archaea2 - ps3
Archaea3 - ps4
Asgard/Eukaryota - ps5
Eukaryota - ps6
Amorpheae - ps7
Obazoa - ps8
Opisthokonta - ps9
Holomycota - ps10
Fungi - ps11
Fungi_1 - ps12
Fungi_2 - ps13
Fungi_3 - ps14
Dikarya - ps15
Ascomycota - ps16
Saccharo/Pezizomycotina - ps17
Saccharomycotina - ps18
Saccharomycotina_1 - ps19
Saccharomycotina_2 - ps20
Saccharomycotina_3 - ps21
Phaff./Saccharomycetaceae - ps22
Saccharomyco./mycetaceae - ps23
Saccharomycetaceae - ps24
Saccharomycetaceae_2 - ps25
Saccharomycetaceae_3 - ps26
Kaz./Nau./Saccharomyces - ps27
Saccharomyces - ps28
Arboricola_clade - ps29
Cerevisiae_clade - ps30
Saccharomyces_cerevisiae - ps31
Phylostratum

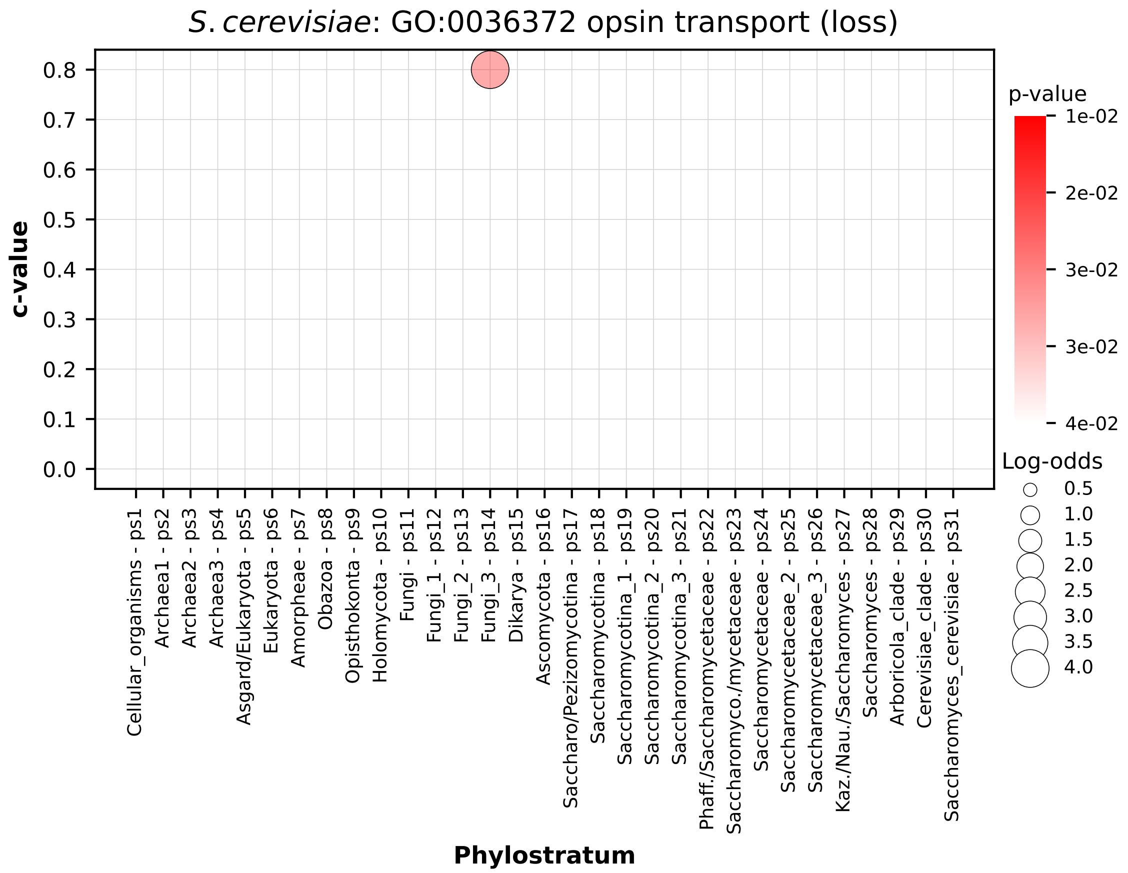
S. cerevisiae: GO:0036372 opsin transport (loss)
c-value
0.8
0.7
0.6
0.5
0.4
0.3
0.2
0.1
0.0
p-value
1e-02
2e-02
3e-02
3e-02
4e-02
Log-odds
0.5
1.0
1.5
2.0
2.5
3.0
3.5
4.0
Cellular_organisms - ps1
Archaea1 - ps2
Archaea2 - ps3
Archaea3 - ps4
Asgard/Eukaryota - ps5
Eukaryota - ps6
Amorpheae - ps7
Obazoa - ps8
Opisthokonta - ps9
Holomycota - ps10
Fungi - ps11
Fungi_1 - ps12
Fungi_2 - ps13
Fungi_3 - ps14
Dikarya - ps15
Ascomycota - ps16
Saccharo/Pezizomycotina - ps17
Saccharomycotina - ps18
Saccharomycotina_1 - ps19
Saccharomycotina_2 - ps20
Saccharomycotina_3 - ps21
Phaff./Saccharomycetaceae - ps22
Saccharomyco./mycetaceae - ps23
Saccharomycetaceae - ps24
Saccharomycetaceae_2 - ps25
Saccharomycetaceae_3 - ps26
Kaz./Nau./Saccharomyces - ps27
Saccharomyces - ps28
Arboricola_clade - ps29
Cerevisiae_clade - ps30
Saccharomyces_cerevisiae - ps31
Phylostratum

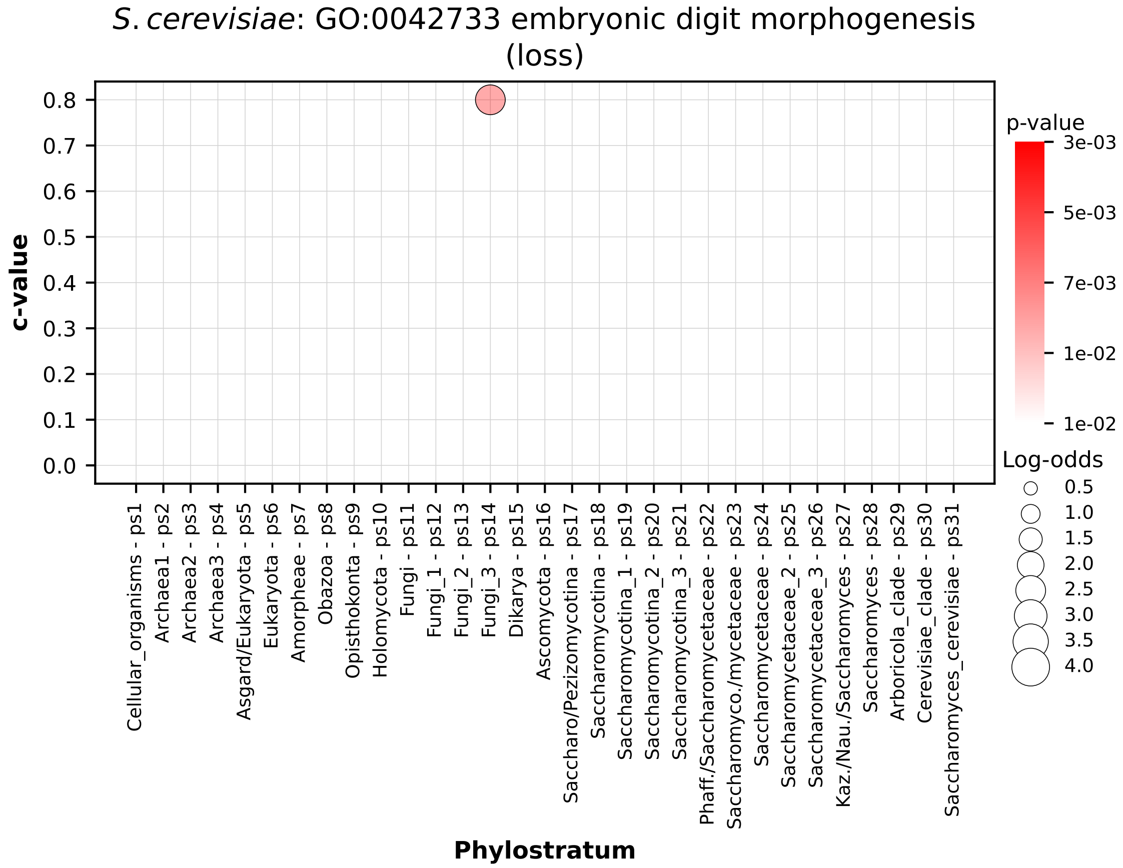
S. cerevisiae: GO:0042733 embryonic digit morphogenesis
(loss)
c-value
0.0
0.1
0.2
0.3
0.4
0.5
0.6
0.7
0.8
p-value
3e-03
5e-03
7e-03
1e-02
1e-02
Log-odds
0.5
1.0
1.5
2.0
2.5
3.0
3.5
4.0
Cellular_organisms - ps1
Archaea1 - ps2
Archaea2 - ps3
Archaea3 - ps4
Asgard/Eukaryota - ps5
Eukaryota - ps6
Amorpheae - ps7
Obazoa - ps8
Opisthokonta - ps9
Holomycota - ps10
Fungi - ps11
Fungi_1 - ps12
Fungi_2 - ps13
Fungi_3 - ps14
Dikarya - ps15
Ascomycota - ps16
Saccharo/Pezizomycotina - ps17
Saccharomycotina - ps18
Saccharomycotina_1 - ps19
Saccharomycotina_2 - ps20
Saccharomycotina_3 - ps21
Phaff./Saccharomycetaceae - ps22
Saccharomyco./mycetaceae - ps23
Saccharomycetaceae - ps24
Saccharomycetaceae_2 - ps25
Saccharomycetaceae_3 - ps26
Kaz./Nau./Saccharomyces - ps27
Saccharomyces - ps28
Arboricola_clade - ps29
Cerevisiae_clade - ps30
Saccharomyces_cerevisiae - ps31
Phylostratum

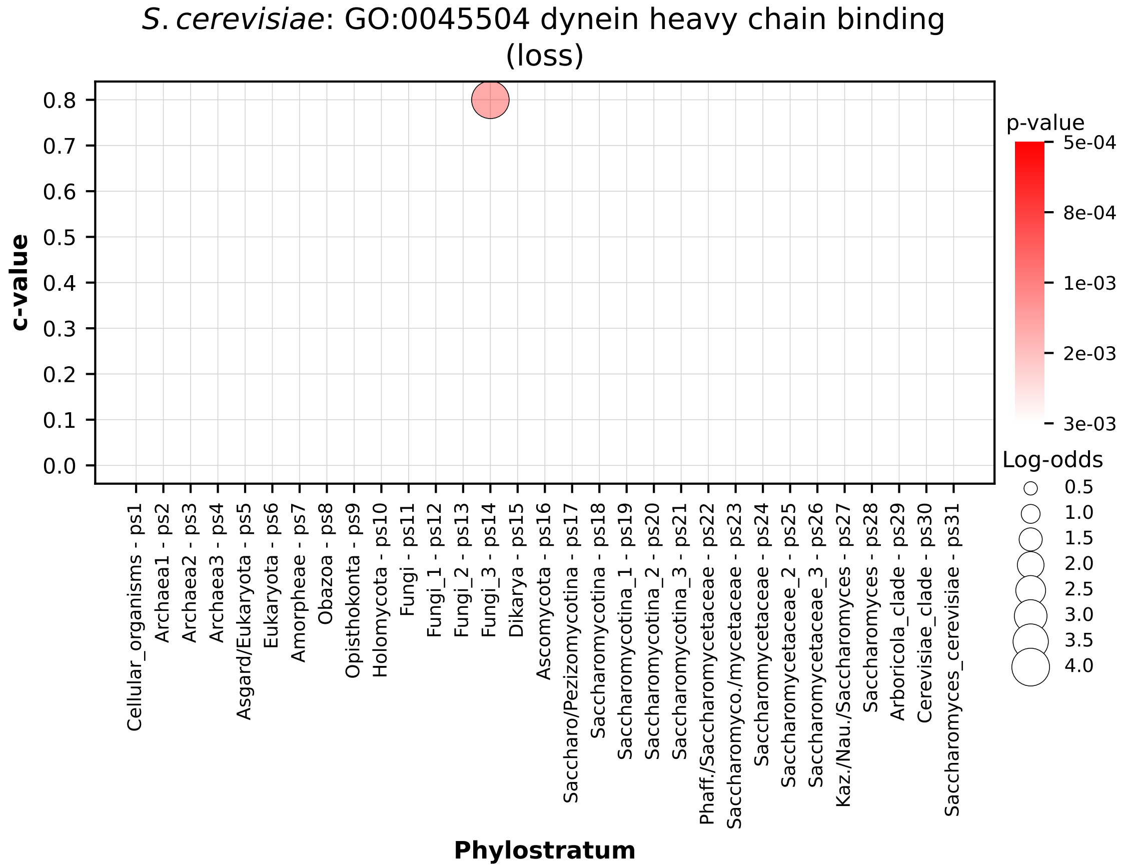

S. cerevisiae: GO:0045504 dynein heavy chain binding
(loss)
c-value
0.8
0.7
0.6
0.5
0.4
0.3
0.2
0.1
0.0
p-value
5e-04
8e-04
1e-03
2e-03
3e-03
Log-odds
0.5
1.0
1.5
2.0
2.5
3.0
3.5
4.0
Cellular_organisms - ps1
Archaea1 - ps2
Archaea2 - ps3
Archaea3 - ps4
Asgard/Eukaryota - ps5
Eukaryota - ps6
Amorpheae - ps7
Obazoa - ps8
Opisthokonta - ps9
Holomycota - ps10
Fungi - ps11
Fungi_1 - ps12
Fungi_2 - ps13
Fungi_3 - ps14
Dikarya - ps15
Ascomycota - ps16
Saccharo/Pezizomycotina - ps17
Saccharomycotina - ps18
Saccharomycotina_1 - ps19
Saccharomycotina_2 - ps20
Saccharomycotina_3 - ps21
Phaff./Saccharomycetaceae - ps22
Saccharomyco./mycetaceae - ps23
Saccharomycetaceae - ps24
Saccharomycetaceae_2 - ps25
Saccharomycetaceae_3 - ps26
Kaz./Nau./Saccharomyces - ps27
Saccharomyces - ps28
Arboricola_clade - ps29
Cerevisiae_clade - ps30
Saccharomyces_cerevisiae - ps31
Phylostratum

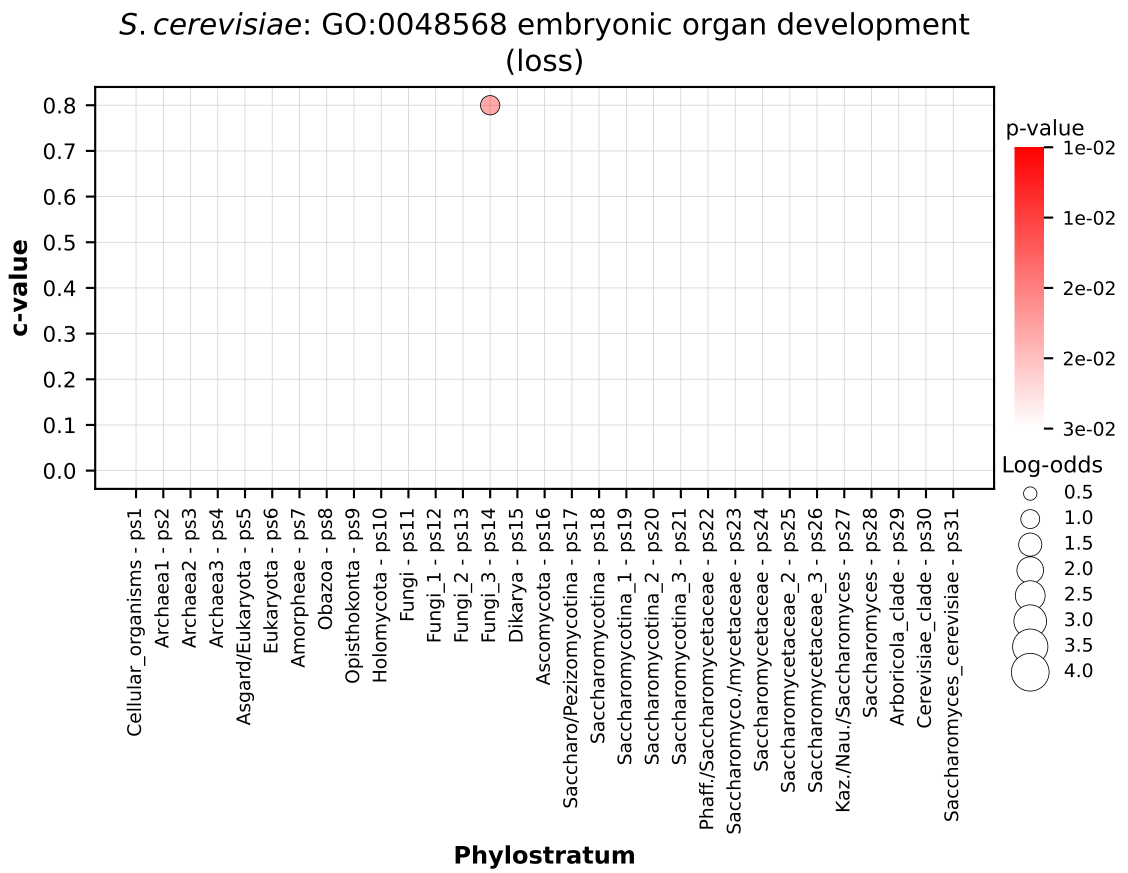
S. cerevisiae: GO:0048568 embryonic organ development
(loss)
c-value
0.0
0.1
0.2
0.3
0.4
0.5
0.6
0.7
0.8
Phylostratum
Cellular_organisms - ps1
Archaea1 - ps2
Archaea2 - ps3
Archaea3 - ps4
Asgard/Eukaryota - ps5
Eukaryota - ps6
Amorpheae - ps7
Obazoa - ps8
Opisthokonta - ps9
Holomycota - ps10
Fungi - ps11
Fungi_1 - ps12
Fungi_2 - ps13
Fungi_3 - ps14
Dikarya - ps15
Ascomycota - ps16
Saccharo/Pezizomycotina - ps17
Saccharomycotina - ps18
Saccharomycotina_1 - ps19
Saccharomycotina_2 - ps20
Saccharomycotina_3 - ps21
Phaff./Saccharomycetaceae - ps22
Saccharomyco./mycetaceae - ps23
Saccharomycetaceae - ps24
Saccharomycetaceae_2 - ps25
Saccharomycetaceae_3 - ps26
Kaz./Nau./Saccharomyces - ps27
Saccharomyces - ps28
Arboricola_clade - ps29
Cerevisiae_clade - ps30
Saccharomyces_cerevisiae - ps31
p-value
1e-02
1e-02
2e-02
2e-02
3e-02
Log-odds
0.5
1.0
1.5
2.0
2.5
3.0
3.5
4.0

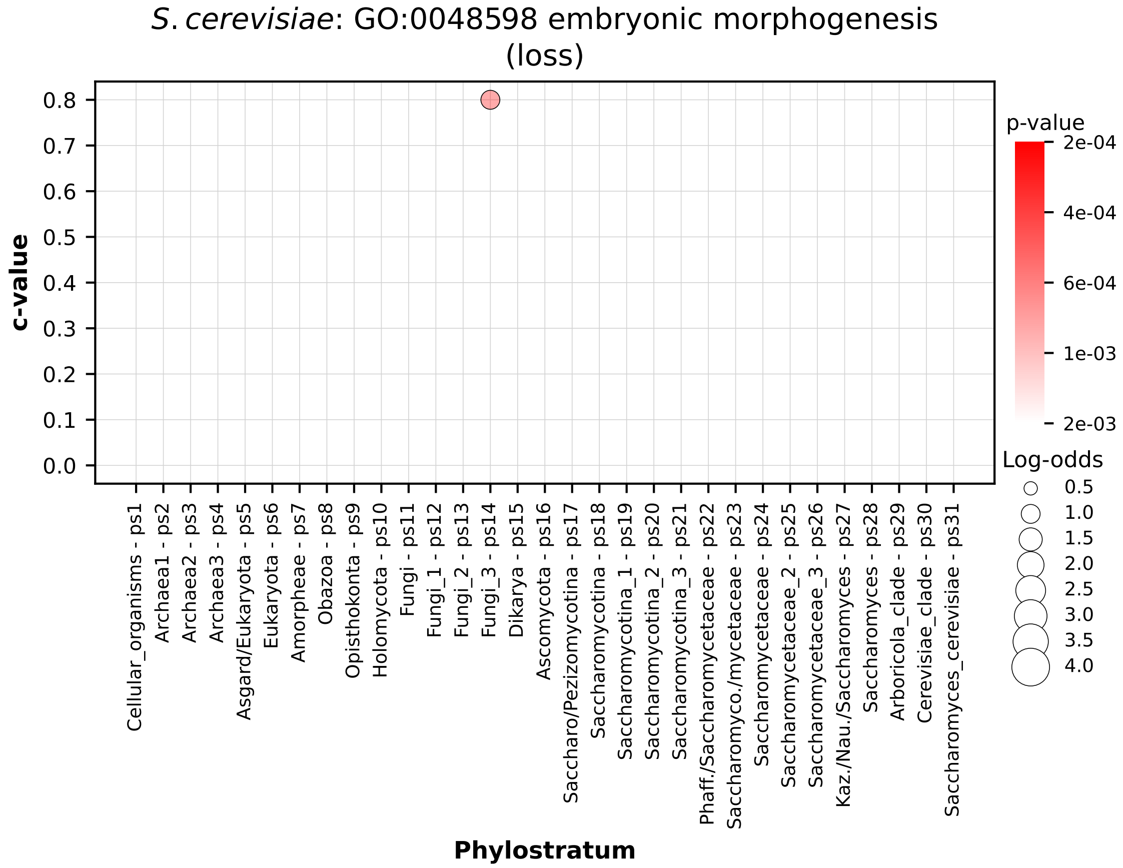

S. cerevisiae: GO:0048598 embryonic morphogenesis
(loss)
c-value
0.8
0.7
0.6
0.5
0.4
0.3
0.2
0.1
0.0
p-value
2e-04
4e-04
6e-04
1e-03
2e-03
Log-odds
0.5
1.0
1.5
2.0
2.5
3.0
3.5
4.0
Cellular_organisms - ps1
Archaea1 - ps2
Archaea2 - ps3
Archaea3 - ps4
Asgard/Eukaryota - ps5
Eukaryota - ps6
Amorpheae - ps7
Obazoa - ps8
Opisthokonta - ps9
Holomycota - ps10
Fungi - ps11
Fungi_1 - ps12
Fungi_2 - ps13
Fungi_3 - ps14
Dikarya - ps15
Ascomycota - ps16
Saccharo/Pezizomycotina - ps17
Saccharomycotina - ps18
Saccharomycotina_1 - ps19
Saccharomycotina_2 - ps20
Saccharomycotina_3 - ps21
Phaff./Saccharomycetaceae - ps22
Saccharomyco./mycetaceae - ps23
Saccharomycetaceae - ps24
Saccharomycetaceae_2 - ps25
Saccharomycetaceae_3 - ps26
Kaz./Nau./Saccharomyces - ps27
Saccharomyces - ps28
Arboricola_clade - ps29
Cerevisiae_clade - ps30
Saccharomyces_cerevisiae - ps31
Phylostratum

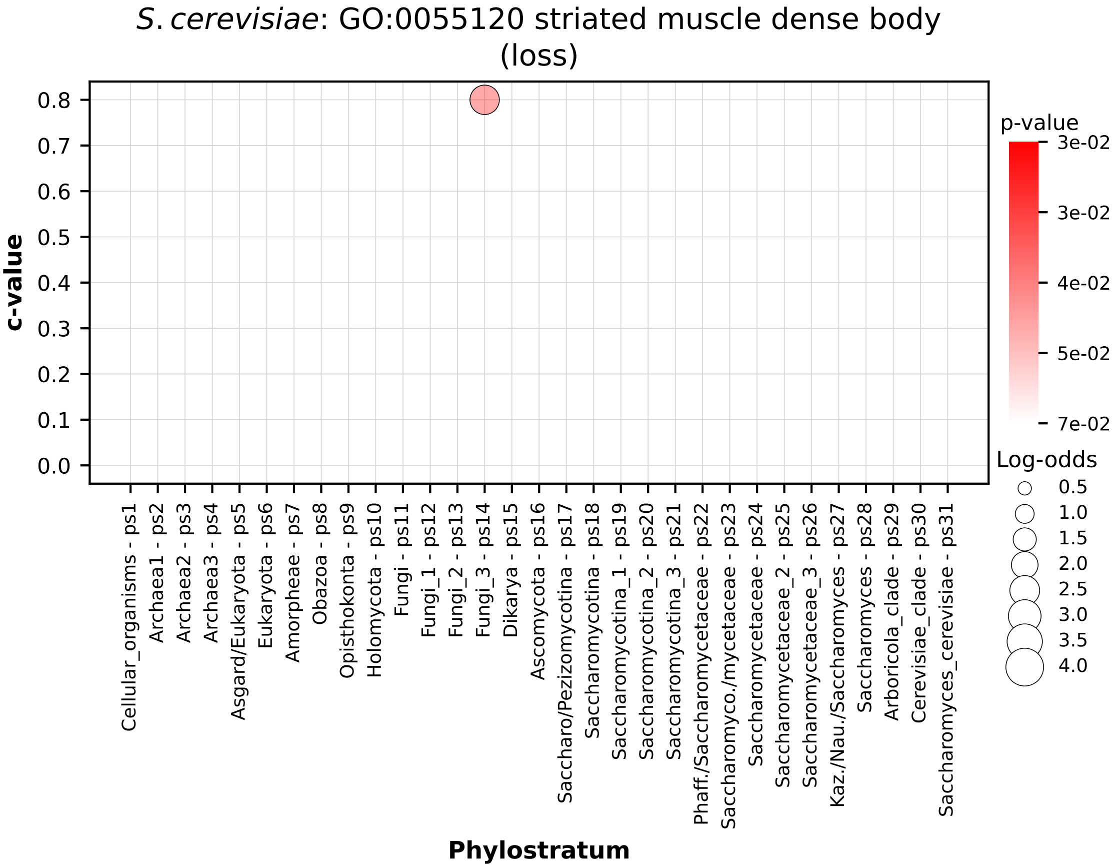

S. cerevisiae: GO:0055120 striated muscle dense body (loss)
c-value
0.0
0.1
0.2
0.3
0.4
0.5
0.6
0.7
0.8
p-value
3e-02
3e-02
4e-02
5e-02
7e-02
Log-odds
0.5
1.0
1.5
2.0
2.5
3.0
3.5
4.0
Cellular_organisms - ps1
Archaea1 - ps2
Archaea2 - ps3
Archaea3 - ps4
Asgard/Eukaryota - ps5
Eukaryota - ps6
Amorpheae - ps7
Obazoa - ps8
Opisthokonta - ps9
Holomycota - ps10
Fungi - ps11
Fungi_1 - ps12
Fungi_2 - ps13
Fungi_3 - ps14
Dikarya - ps15
Ascomycota - ps16
Saccharo/Pezizomycotina - ps17
Saccharomycotina - ps18
Saccharomycotina_1 - ps19
Saccharomycotina_2 - ps20
Saccharomycotina_3 - ps21
Phaff./Saccharomycetaceae - ps22
Saccharomyco./mycetaceae - ps23
Saccharomycetaceae - ps24
Saccharomycetaceae_2 - ps25
Saccharomycetaceae_3 - ps26
Kaz./Nau./Saccharomyces - ps27
Saccharomyces - ps28
Arboricola_clade - ps29
Cerevisiae_clade - ps30
Saccharomyces_cerevisiae - ps31
Phylostratum

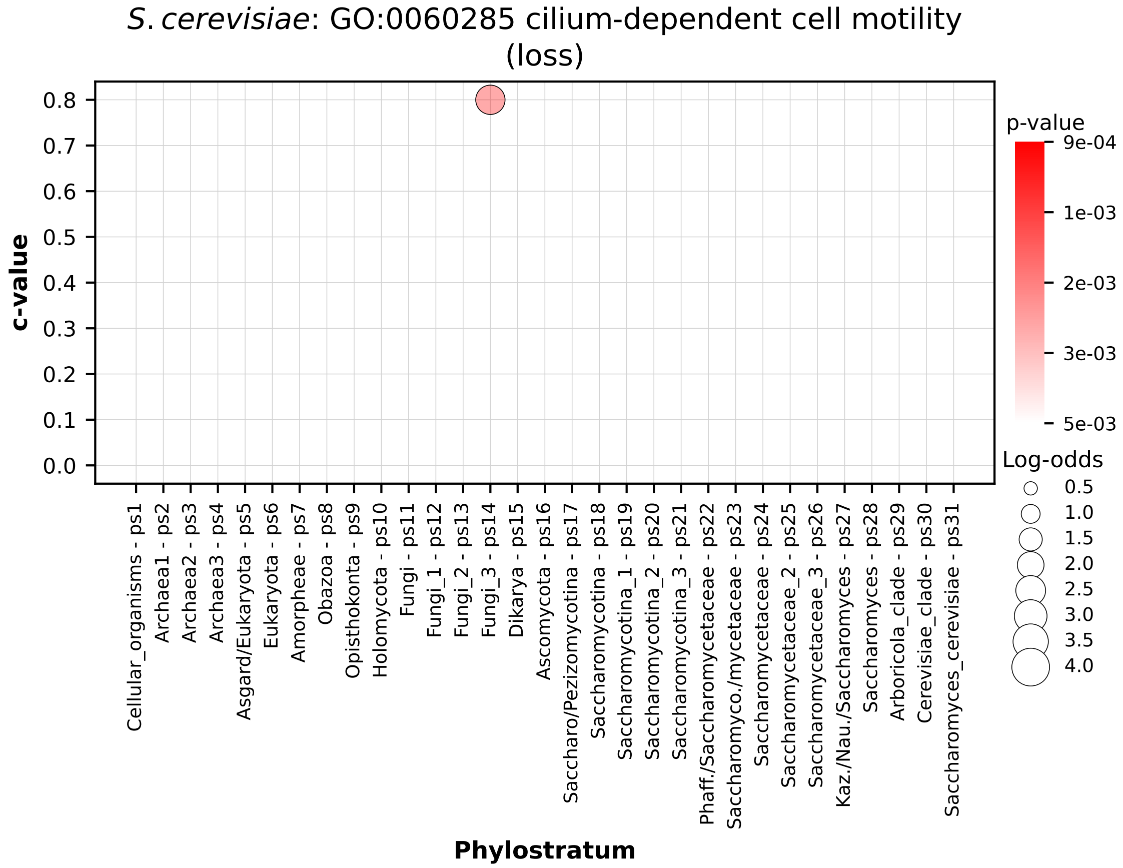
S. cerevisiae: GO:0060285 cilium-dependent cell motility
(loss)
c-value
0.0
0.1
0.2
0.3
0.4
0.5
0.6
0.7
0.8
Phylostratum
Cellular_organisms - ps1
Archaea1 - ps2
Archaea2 - ps3
Archaea3 - ps4
Asgard/Eukaryota - ps5
Eukaryota - ps6
Amorpheae - ps7
Obazoa - ps8
Opisthokonta - ps9
Holomycota - ps10
Fungi - ps11
Fungi_1 - ps12
Fungi_2 - ps13
Fungi_3 - ps14
Dikarya - ps15
Ascomycota - ps16
Saccharo/Pezizomycotina - ps17
Saccharomycotina - ps18
Saccharomycotina_1 - ps19
Saccharomycotina_2 - ps20
Saccharomycotina_3 - ps21
Phaff./Saccharomycetaceae - ps22
Saccharomyco./mycetaceae - ps23
Saccharomycetaceae - ps24
Saccharomycetaceae_2 - ps25
Saccharomycetaceae_3 - ps26
Kaz./Nau./Saccharomyces - ps27
Saccharomyces - ps28
Arboricola_clade - ps29
Cerevisiae_clade - ps30
Saccharomyces_cerevisiae - ps31
p-value
9e-04
1e-03
2e-03
3e-03
5e-03
Log-odds
0.5
1.0
1.5
2.0
2.5
3.0
3.5
4.0

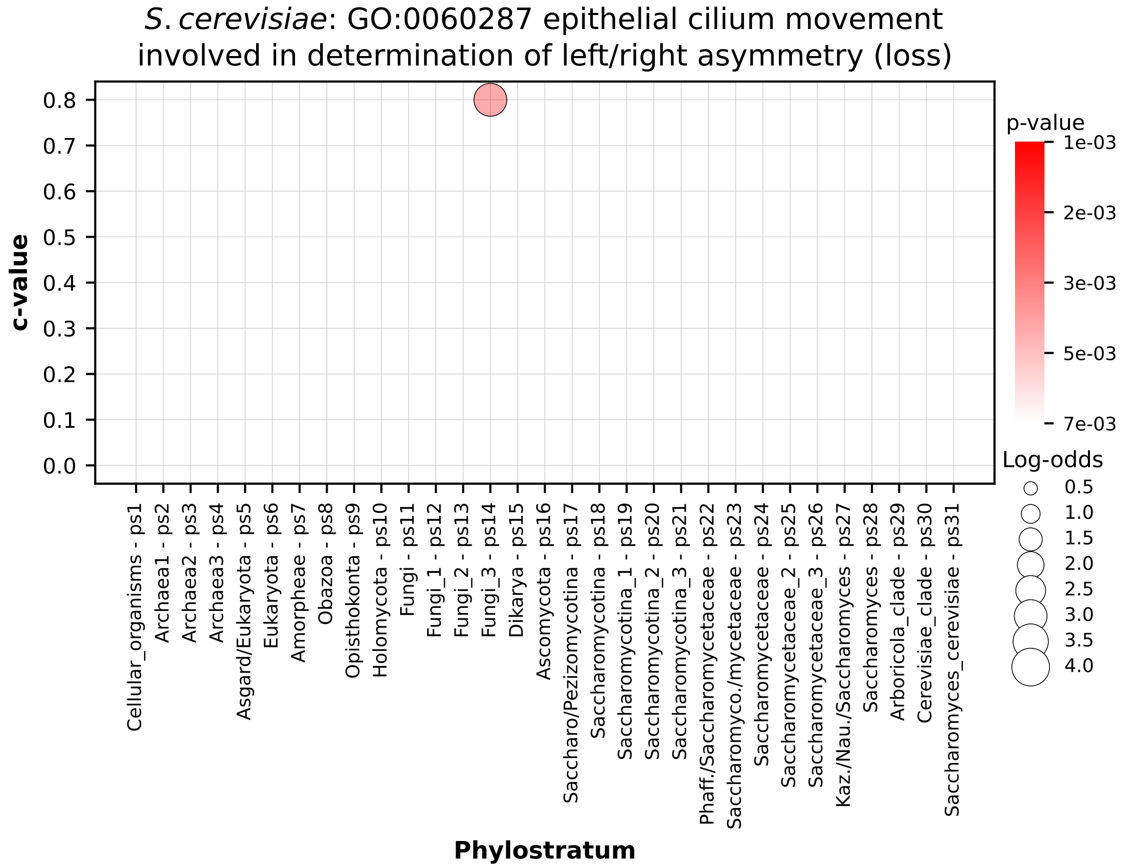
S. cerevisiae: GO:0060287 epithelial cilium movement involved in determination of left/right asymmetry (loss)
c-value
0.0
0.1
0.2
0.3
0.4
0.5
0.6
0.7
0.8
p-value
1e-03
2e-03
3e-03
5e-03
7e-03
Log-odds
0.5
1.0
1.5
2.0
2.5
3.0
3.5
4.0
Cellular_organisms - ps1
Archaea1 - ps2
Archaea2 - ps3
Archaea3 - ps4
Asgard/Eukaryota - ps5
Eukaryota - ps6
Amorpheae - ps7
Obazoa - ps8
Opisthokonta - ps9
Holomycota - ps10
Fungi - ps11
Fungi_1 - ps12
Fungi_2 - ps13
Fungi_3 - ps14
Dikarya - ps15
Ascomycota - ps16
Saccharo/Pezizomycotina - ps17
Saccharomycotina - ps18
Saccharomycotina_1 - ps19
Saccharomycotina_2 - ps20
Saccharomycotina_3 - ps21
Phaff./Saccharomycetaceae - ps22
Saccharomyco./mycetaceae - ps23
Saccharomycetaceae - ps24
Saccharomycetaceae_2 - ps25
Saccharomycetaceae_3 - ps26
Kaz./Nau./Saccharomyces - ps27
Saccharomyces - ps28
Arboricola_clade - ps29
Cerevisiae_clade - ps30
Saccharomyces_cerevisiae - ps31
Phylostratum

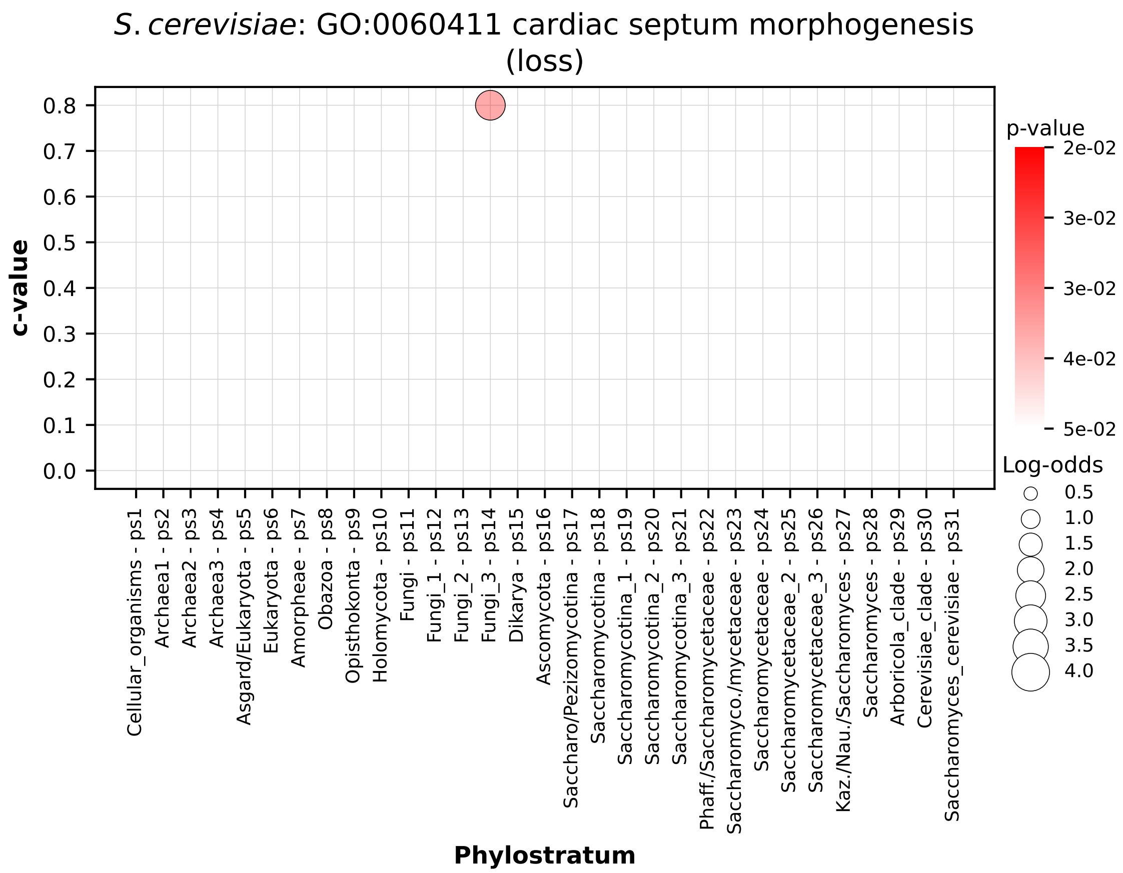

S. cerevisiae: GO:0060411 cardiac septum morphogenesis (loss)
c-value
0.0
0.1
0.2
0.3
0.4
0.5
0.6
0.7
0.8
Phylostratum
Cellular_organisms - ps1
Archaea1 - ps2
Archaea2 - ps3
Archaea3 - ps4
Asgard/Eukaryota - ps5
Eukaryota - ps6
Amorpheae - ps7
Obazoa - ps8
Opisthokonta - ps9
Holomycota - ps10
Fungi - ps11
Fungi_1 - ps12
Fungi_2 - ps13
Fungi_3 - ps14
Dikarya - ps15
Ascomycota - ps16
Saccharo/Pezizomycotina - ps17
Saccharomycotina - ps18
Saccharomycotina_1 - ps19
Saccharomycotina_2 - ps20
Saccharomycotina_3 - ps21
Phaff./Saccharomycetaceae - ps22
Saccharomyco./mycetaceae - ps23
Saccharomycetaceae - ps24
Saccharomycetaceae_2 - ps25
Saccharomycetaceae_3 - ps26
Kaz./Nau./Saccharomyces - ps27
Saccharomyces - ps28
Arboricola_clade - ps29
Cerevisiae_clade - ps30
Saccharomyces_cerevisiae - ps31
p-value
2e-02
3e-02
3e-02
4e-02
5e-02
Log-odds
0.5
1.0
1.5
2.0
2.5
3.0
3.5
4.0

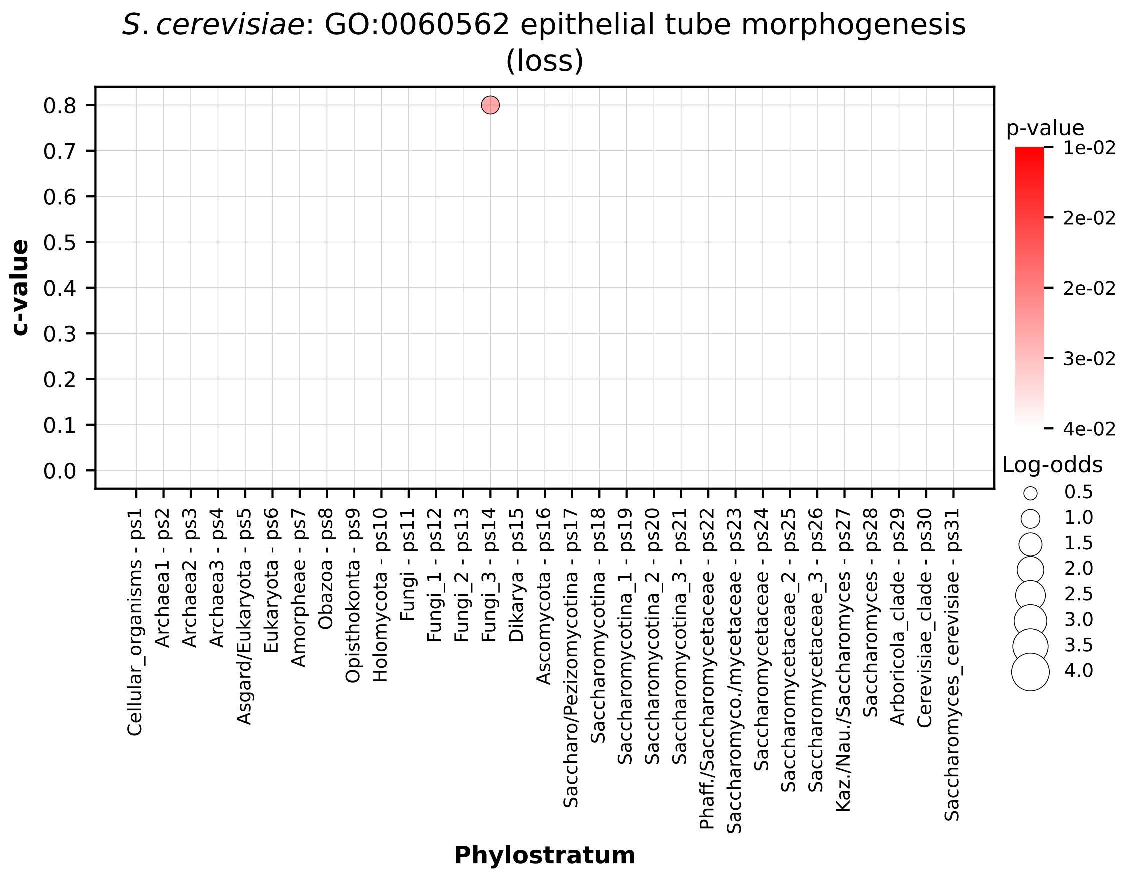
S. cerevisiae: GO:0060562 epithelial tube morphogenesis
(loss)
c-value
0.8
0.7
0.6
0.5
0.4
0.3
0.2
0.1
0.0
p-value
1e-02
2e-02
2e-02
3e-02
4e-02
Log-odds
0.5
1.0
1.5
2.0
2.5
3.0
3.5
4.0
Cellular_organisms - ps1
Archaea1 - ps2
Archaea2 - ps3
Archaea3 - ps4
Asgard/Eukaryota - ps5
Eukaryota - ps6
Amorpheae - ps7
Obazoa - ps8
Opisthokonta - ps9
Holomycota - ps10
Fungi - ps11
Fungi_1 - ps12
Fungi_2 - ps13
Fungi_3 - ps14
Dikarya - ps15
Ascomycota - ps16
Saccharo/Pezizomycotina - ps17
Saccharomycotina - ps18
Saccharomycotina_1 - ps19
Saccharomycotina_2 - ps20
Saccharomycotina_3 - ps21
Phaff./Saccharomycetaceae - ps22
Saccharomyco./mycetaceae - ps23
Saccharomycetaceae - ps24
Saccharomycetaceae_2 - ps25
Saccharomycetaceae_3 - ps26
Kaz./Nau./Saccharomyces - ps27
Saccharomyces - ps28
Arboricola_clade - ps29
Cerevisiae_clade - ps30
Saccharomyces_cerevisiae - ps31
Phylostratum

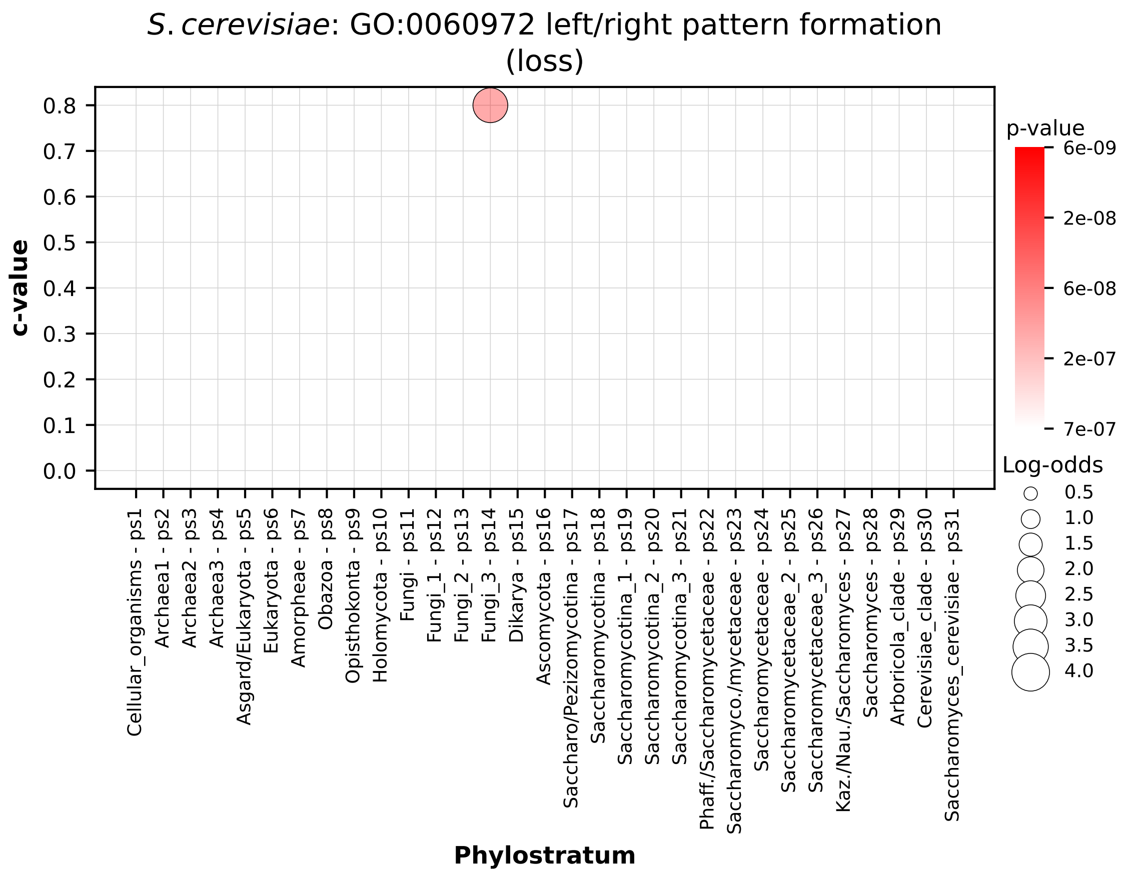

S. cerevisiae: GO:0060972 left/right pattern formation
(loss)
c-value
0.8
0.7
0.6
0.5
0.4
0.3
0.2
0.1
0.0
p-value
6e-09
2e-08
6e-08
2e-07
7e-07
Log-odds
0.5
1.0
1.5
2.0
2.5
3.0
3.5
4.0
Cellular_organisms - ps1
Archaea1 - ps2
Archaea2 - ps3
Archaea3 - ps4
Asgard/Eukaryota - ps5
Eukaryota - ps6
Amorpheae - ps7
Obazoa - ps8
Opisthokonta - ps9
Holomycota - ps10
Fungi - ps11
Fungi_1 - ps12
Fungi_2 - ps13
Fungi_3 - ps14
Dikarya - ps15
Ascomycota - ps16
Saccharo/Pezizomycotina - ps17
Saccharomycotina - ps18
Saccharomycotina_1 - ps19
Saccharomycotina_2 - ps20
Saccharomycotina_3 - ps21
Phaff./Saccharomycetaceae - ps22
Saccharomyco./mycetaceae - ps23
Saccharomycetaceae - ps24
Saccharomycetaceae_2 - ps25
Saccharomycetaceae_3 - ps26
Kaz./Nau./Saccharomyces - ps27
Saccharomyces - ps28
Arboricola_clade - ps29
Cerevisiae_clade - ps30
Saccharomyces_cerevisiae - ps31
Phylostratum

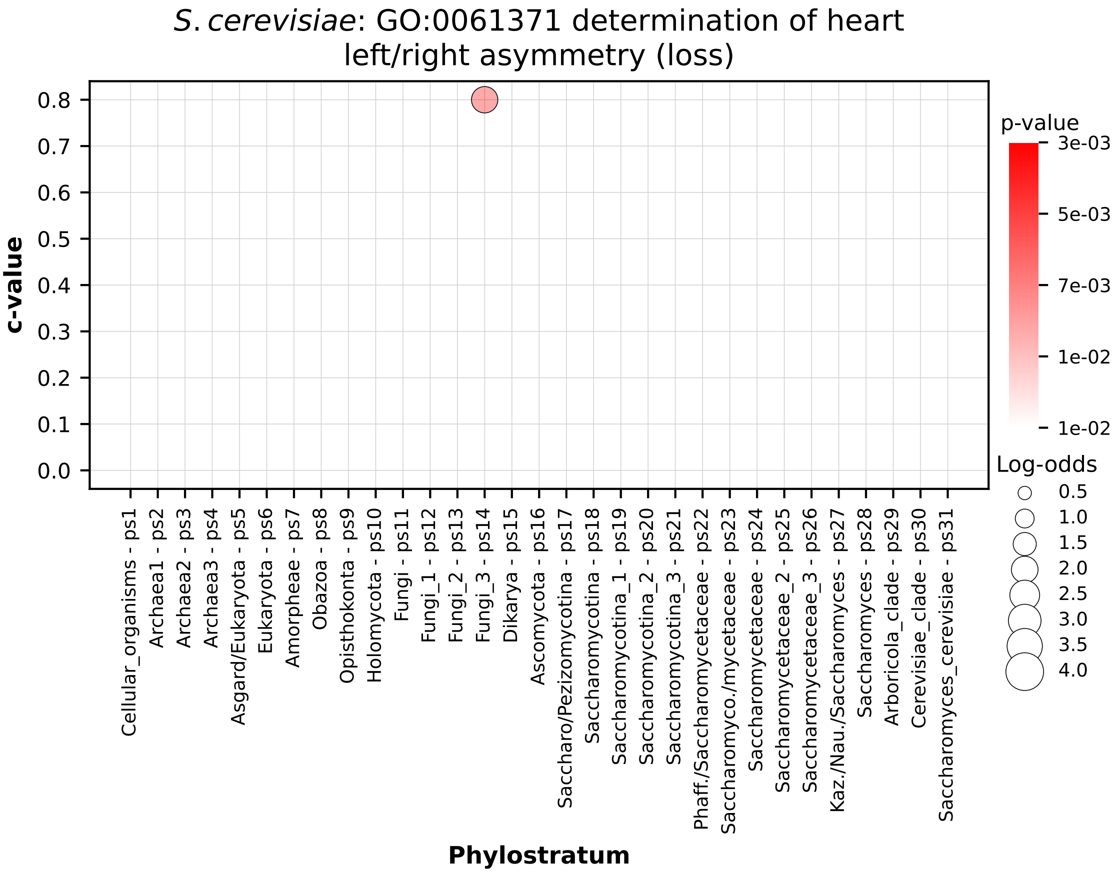

S. cerevisiae: GO:0061371 determination of heart left/right asymmetry (loss)
c-value
0.0
0.1
0.2
0.3
0.4
0.5
0.6
0.7
0.8
p-value
3e-03
5e-03
7e-03
1e-02
1e-02
Log-odds
0.5
1.0
1.5
2.0
2.5
3.0
3.5
4.0
Cellular_organisms - ps1
Archaea1 - ps2
Archaea2 - ps3
Archaea3 - ps4
Asgard/Eukaryota - ps5
Eukaryota - ps6
Amorpheae - ps7
Obazoa - ps8
Opisthokonta - ps9
Holomycota - ps10
Fungi - ps11
Fungi_1 - ps12
Fungi_2 - ps13
Fungi_3 - ps14
Dikarya - ps15
Ascomycota - ps16
Saccharo/Pezizomycotina - ps17
Saccharomycotina - ps18
Saccharomycotina_1 - ps19
Saccharomycotina_2 - ps20
Saccharomycotina_3 - ps21
Phaff./Saccharomycetaceae - ps22
Saccharomyco./mycetaceae - ps23
Saccharomycetaceae - ps24
Saccharomycetaceae_2 - ps25
Saccharomycetaceae_3 - ps26
Kaz./Nau./Saccharomyces - ps27
Saccharomyces - ps28
Arboricola_clade - ps29
Cerevisiae_clade - ps30
Saccharomyces_cerevisiae - ps31
Phylostratum

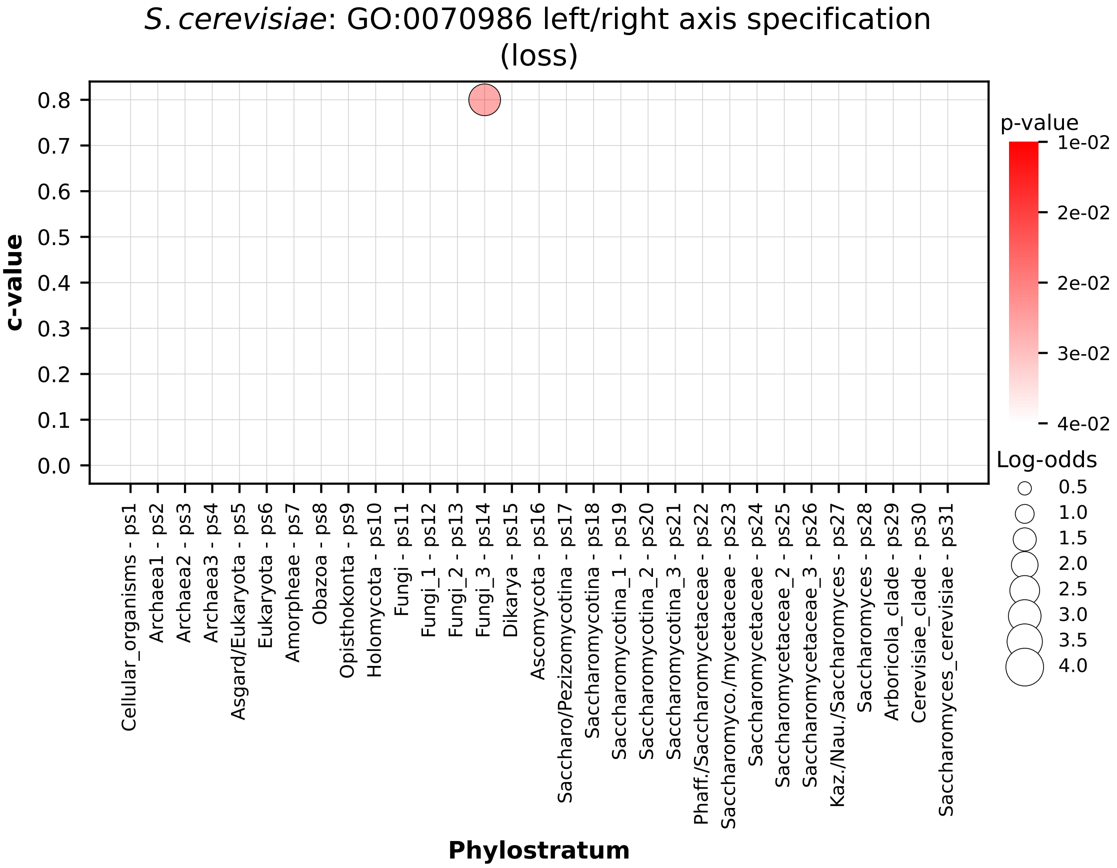

S. cerevisiae: GO:0070986 left/right axis specification
(loss)
c-value
0.8
0.7
0.6
0.5
0.4
0.3
0.2
0.1
0.0
p-value
1e-02
2e-02
2e-02
3e-02
4e-02
Log-odds
0.5
1.0
1.5
2.0
2.5
3.0
3.5
4.0
Cellular_organisms - ps1
Archaea1 - ps2
Archaea2 - ps3
Archaea3 - ps4
Asgard/Eukaryota - ps5
Eukaryota - ps6
Amorpheae - ps7
Obazoa - ps8
Opisthokonta - ps9
Holomycota - ps10
Fungi - ps11
Fungi_1 - ps12
Fungi_2 - ps13
Fungi_3 - ps14
Dikarya - ps15
Ascomycota - ps16
Saccharo/Pezizomycotina - ps17
Saccharomycotina - ps18
Saccharomycotina_1 - ps19
Saccharomycotina_2 - ps20
Saccharomycotina_3 - ps21
Phaff./Saccharomycetaceae - ps22
Saccharomyco./mycetaceae - ps23
Saccharomycetaceae - ps24
Saccharomycetaceae_2 - ps25
Saccharomycetaceae_3 - ps26
Kaz./Nau./Saccharomyces - ps27
Saccharomyces - ps28
Arboricola_clade - ps29
Cerevisiae_clade - ps30
Saccharomyces_cerevisiae - ps31
Phylostratum

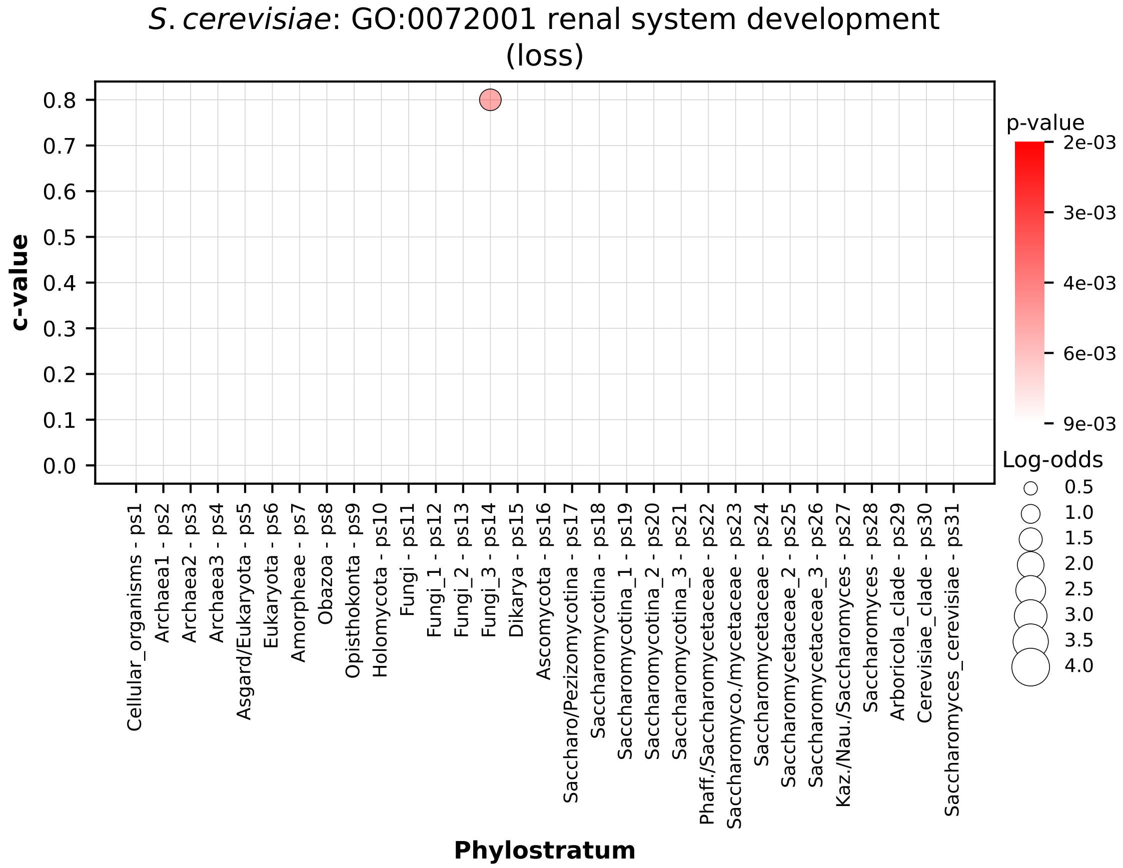

S. cerevisiae: GO:0072001 renal system development
(loss)
c-value
0.0
0.1
0.2
0.3
0.4
0.5
0.6
0.7
0.8
p-value
2e-03
3e-03
4e-03
6e-03
9e-03
Log-odds
0.5
1.0
1.5
2.0
2.5
3.0
3.5
4.0
Cellular_organisms - ps1
Archaea1 - ps2
Archaea2 - ps3
Archaea3 - ps4
Asgard/Eukaryota - ps5
Eukaryota - ps6
Amorpheae - ps7
Obazoa - ps8
Opisthokonta - ps9
Holomycota - ps10
Fungi - ps11
Fungi_1 - ps12
Fungi_2 - ps13
Fungi_3 - ps14
Dikarya - ps15
Ascomycota - ps16
Saccharo/Pezizomycotina - ps17
Saccharomycotina - ps18
Saccharomycotina_1 - ps19
Saccharomycotina_2 - ps20
Saccharomycotina_3 - ps21
Phaff./Saccharomycetaceae - ps22
Saccharomyco./mycetaceae - ps23
Saccharomycetaceae - ps24
Saccharomycetaceae_2 - ps25
Saccharomycetaceae_3 - ps26
Kaz./Nau./Saccharomyces - ps27
Saccharomyces - ps28
Arboricola_clade - ps29
Cerevisiae_clade - ps30
Saccharomyces_cerevisiae - ps31
Phylostratum

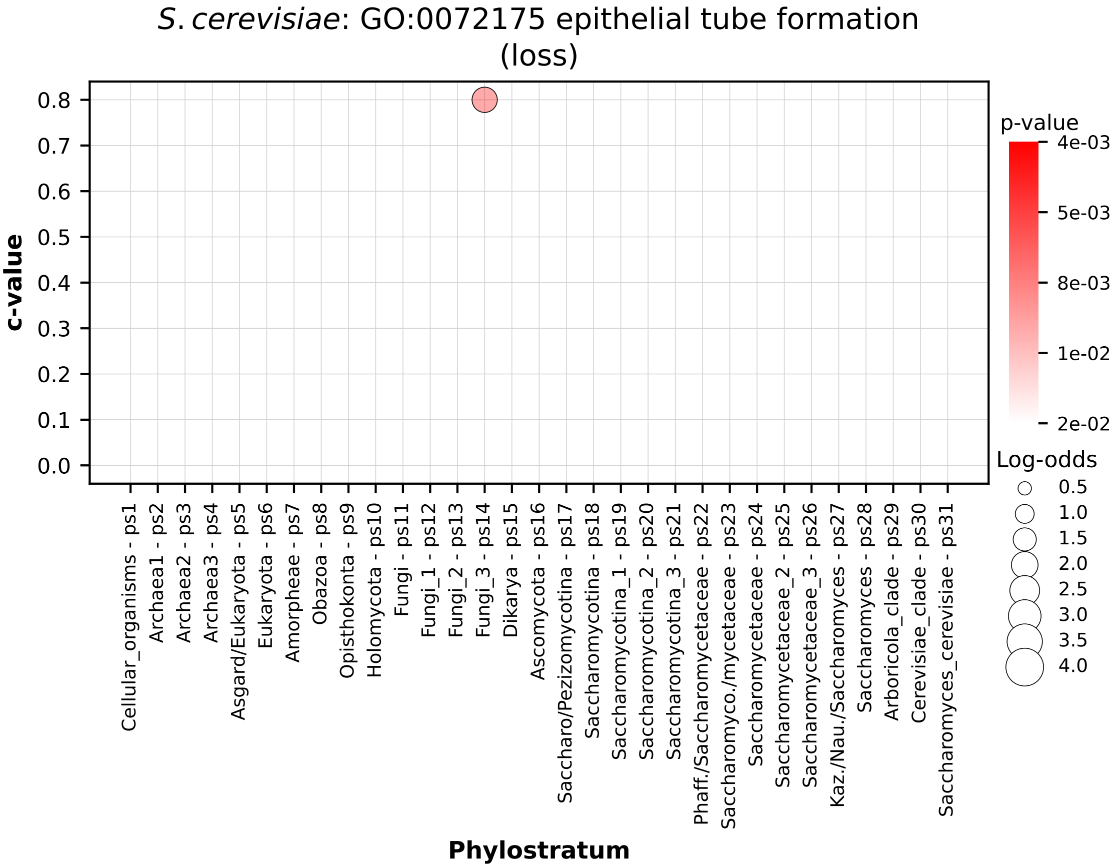

S. cerevisiae: GO:0072175 epithelial tube formation
(loss)
c-value
0.0
0.1
0.2
0.3
0.4
0.5
0.6
0.7
0.8
p-value
4e-03
5e-03
8e-03
1e-02
2e-02
Log-odds
0.5
1.0
1.5
2.0
2.5
3.0
3.5
4.0
Cellular_organisms - ps1
Archaea1 - ps2
Archaea2 - ps3
Archaea3 - ps4
Asgard/Eukaryota - ps5
Eukaryota - ps6
Amorpheae - ps7
Obazoa - ps8
Opisthokonta - ps9
Holomycota - ps10
Fungi - ps11
Fungi_1 - ps12
Fungi_2 - ps13
Fungi_3 - ps14
Dikarya - ps15
Ascomycota - ps16
Saccharo/Pezizomycotina - ps17
Saccharomycotina - ps18
Saccharomycotina_1 - ps19
Saccharomycotina_2 - ps20
Saccharomycotina_3 - ps21
Phaff./Saccharomycetaceae - ps22
Saccharomyco./mycetaceae - ps23
Saccharomycetaceae - ps24
Saccharomycetaceae_2 - ps25
Saccharomycetaceae_3 - ps26
Kaz./Nau./Saccharomyces - ps27
Saccharomyces - ps28
Arboricola_clade - ps29
Cerevisiae_clade - ps30
Saccharomyces_cerevisiae - ps31
Phylostratum

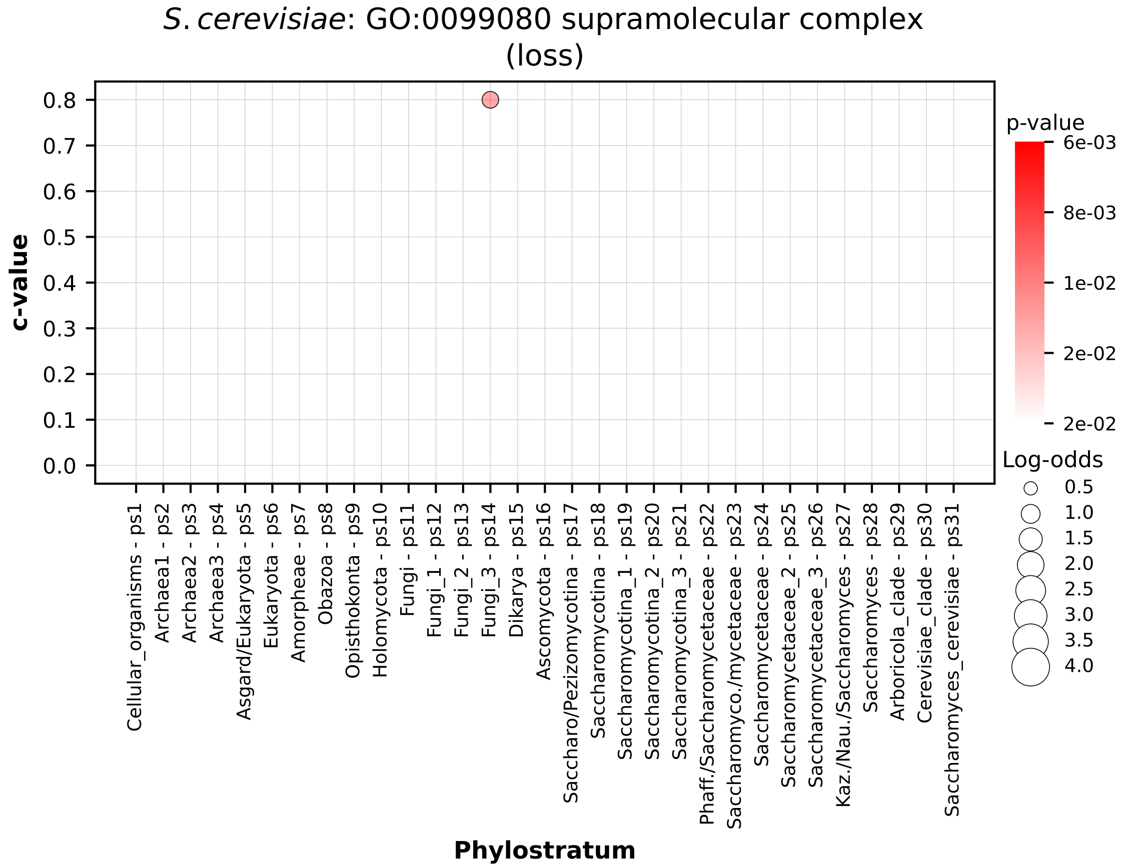
S. cerevisiae: GO:0099080 supramolecular complex
(loss)
c-value
0.0
0.1
0.2
0.3
0.4
0.5
0.6
0.7
0.8
p-value
6e-03
8e-03
1e-02
2e-02
2e-02
Log-odds
0.5
1.0
1.5
2.0
2.5
3.0
3.5
4.0
Cellular_organisms - ps1
Archaea1 - ps2
Archaea2 - ps3
Archaea3 - ps4
Asgard/Eukaryota - ps5
Eukaryota - ps6
Amorpheae - ps7
Obazoa - ps8
Opisthokonta - ps9
Holomycota - ps10
Fungi - ps11
Fungi_1 - ps12
Fungi_2 - ps13
Fungi_3 - ps14
Dikarya - ps15
Ascomycota - ps16
Saccharo/Pezizomycotina - ps17
Saccharomycotina - ps18
Saccharomycotina_1 - ps19
Saccharomycotina_2 - ps20
Saccharomycotina_3 - ps21
Phaff./Saccharomycetaceae - ps22
Saccharomyco./mycetaceae - ps23
Saccharomycetaceae - ps24
Saccharomycetaceae_2 - ps25
Saccharomycetaceae_3 - ps26
Kaz./Nau./Saccharomyces - ps27
Saccharomyces - ps28
Arboricola_clade - ps29
Cerevisiae_clade - ps30
Saccharomyces_cerevisiae - ps31
Phylostratum

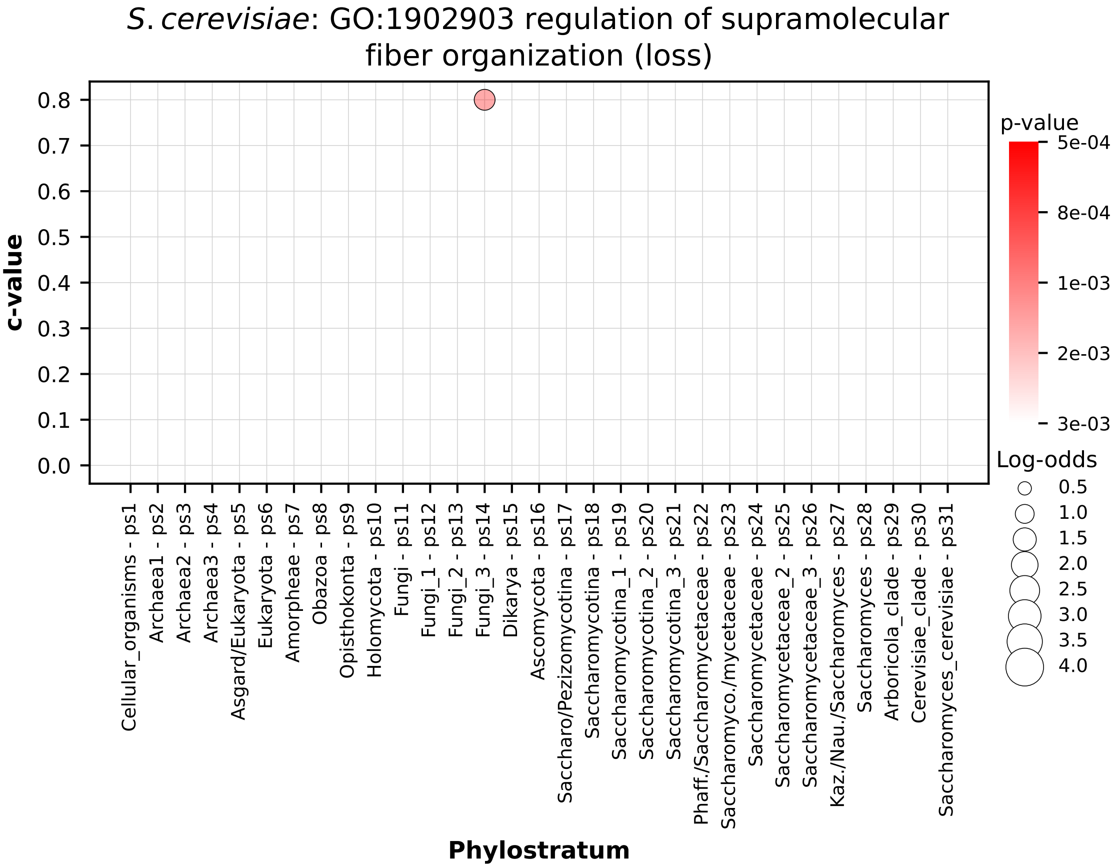

S. cerevisiae: GO:1902903 regulation of supramolecular fiber organization (loss)
c-value
0.8
0.7
0.6
0.5
0.4
0.3
0.2
0.1
0.0
Phylostratum
Cellular_organisms - ps1
Archaea1 - ps2
Archaea2 - ps3
Archaea3 - ps4
Asgard/Eukaryota - ps5
Eukaryota - ps6
Amorpheae - ps7
Obazoa - ps8
Opisthokonta - ps9
Holomycota - ps10
Fungi - ps11
Fungi_1 - ps12
Fungi_2 - ps13
Fungi_3 - ps14
Dikarya - ps15
Ascomycota - ps16
Saccharo/Pezizomycotina - ps17
Saccharomycotina - ps18
Saccharomycotina_1 - ps19
Saccharomycotina_2 - ps20
Saccharomycotina_3 - ps21
Phaff./Saccharomycetaceae - ps22
Saccharomyco./mycetaceae - ps23
Saccharomycetaceae - ps24
Saccharomycetaceae_2 - ps25
Saccharomycetaceae_3 - ps26
Kaz./Nau./Saccharomyces - ps27
Saccharomyces - ps28
Arboricola_clade - ps29
Cerevisiae_clade - ps30
Saccharomyces_cerevisiae - ps31
p-value
5e-04
8e-04
1e-03
2e-03
3e-03
Log-odds
0.5
1.0
1.5
2.0
2.5
3.0
3.5
4.0

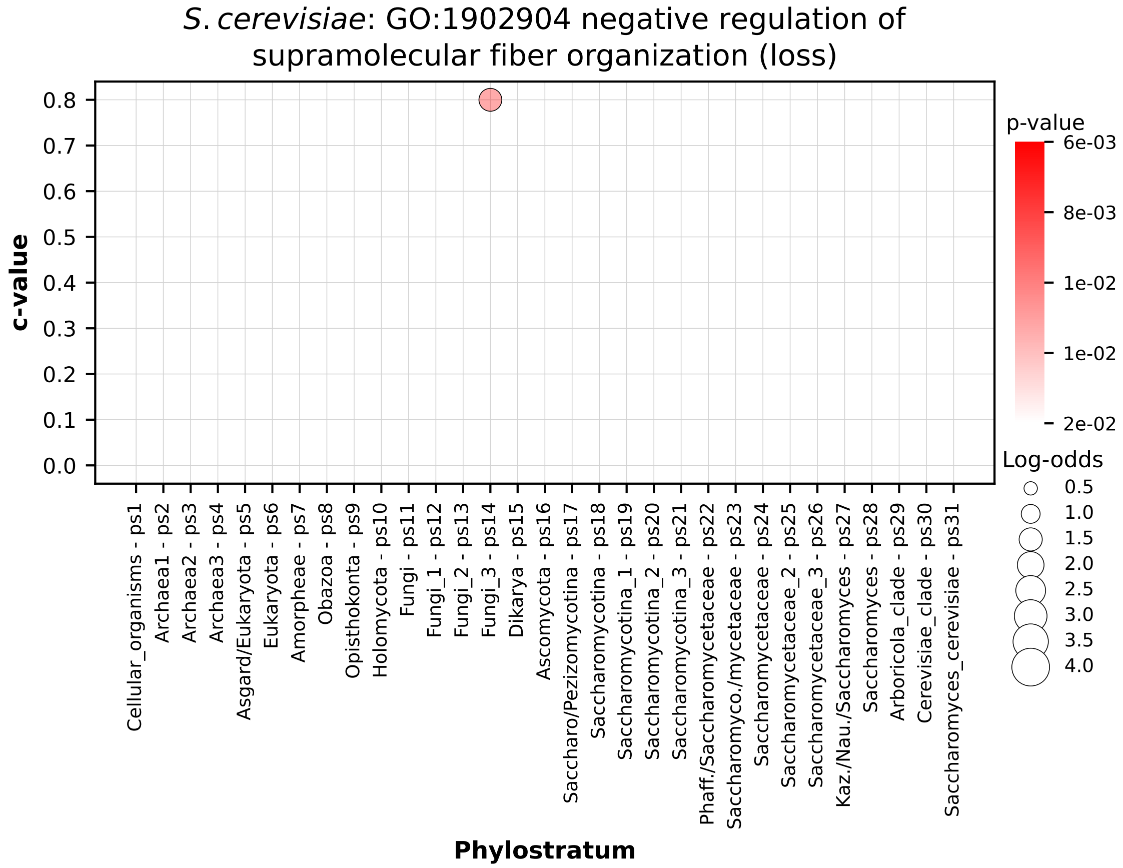

S. cerevisiae: GO:1902904 negative regulation of supramolecular fiber organization (loss)
c-value
0.0
0.1
0.2
0.3
0.4
0.5
0.6
0.7
0.8
Phylostratum
Cellular_organisms - ps1
Archaea1 - ps2
Archaea2 - ps3
Archaea3 - ps4
Asgard/Eukaryota - ps5
Eukaryota - ps6
Amorpheae - ps7
Obazoa - ps8
Opisthokonta - ps9
Holomycota - ps10
Fungi - ps11
Fungi_1 - ps12
Fungi_2 - ps13
Fungi_3 - ps14
Dikarya - ps15
Ascomycota - ps16
Saccharo/Pezizomycotina - ps17
Saccharomycotina - ps18
Saccharomycotina_1 - ps19
Saccharomycotina_2 - ps20
Saccharomycotina_3 - ps21
Phaff./Saccharomycetaceae - ps22
Saccharomyco./mycetaceae - ps23
Saccharomycetaceae - ps24
Saccharomycetaceae_2 - ps25
Saccharomycetaceae_3 - ps26
Kaz./Nau./Saccharomyces - ps27
Saccharomyces - ps28
Arboricola_clade - ps29
Cerevisiae_clade - ps30
Saccharomyces_cerevisiae - ps31
p-value
6e-03
8e-03
1e-02
1e-02
2e-02
Log-odds
0.5
1.0
1.5
2.0
2.5
3.0
3.5
4.0

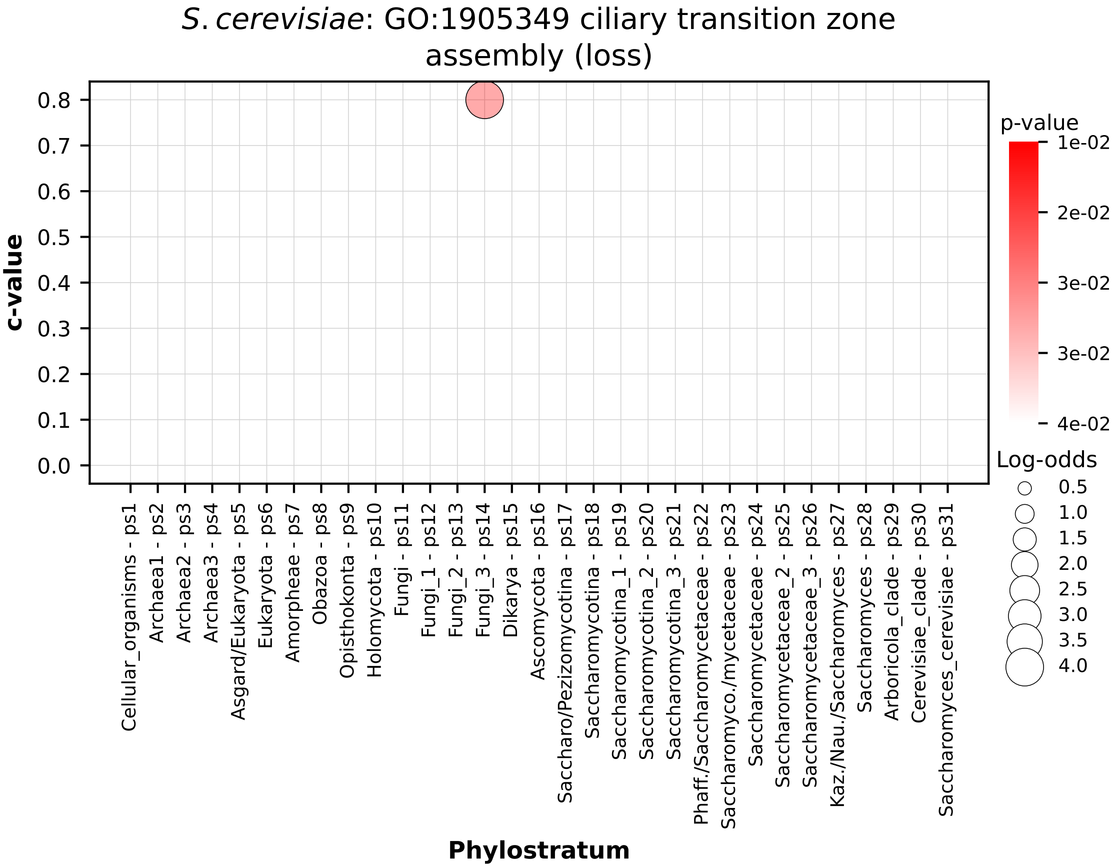

S. cerevisiae: GO:1905349 ciliary transition zone assembly (loss)
c-value
0.8
0.7
0.6
0.5
0.4
0.3
0.2
0.1
0.0
p-value
1e-02
2e-02
3e-02
3e-02
4e-02
Log-odds
0.5
1.0
1.5
2.0
2.5
3.0
3.5
4.0
Cellular_organisms - ps1
Archaea1 - ps2
Archaea2 - ps3
Archaea3 - ps4
Asgard/Eukaryota - ps5
Eukaryota - ps6
Amorpheae - ps7
Obazoa - ps8
Opisthokonta - ps9
Holomycota - ps10
Fungi - ps11
Fungi_1 - ps12
Fungi_2 - ps13
Fungi_3 - ps14
Dikarya - ps15
Ascomycota - ps16
Saccharo/Pezizomycotina - ps17
Saccharomycotina - ps18
Saccharomycotina_1 - ps19
Saccharomycotina_2 - ps20
Saccharomycotina_3 - ps21
Phaff./Saccharomycetaceae - ps22
Saccharomyco./mycetaceae - ps23
Saccharomycetaceae - ps24
Saccharomycetaceae_2 - ps25
Saccharomycetaceae_3 - ps26
Kaz./Nau./Saccharomyces - ps27
Saccharomyces - ps28
Arboricola_clade - ps29
Cerevisiae_clade - ps30
Saccharomyces_cerevisiae - ps31
Phylostratum

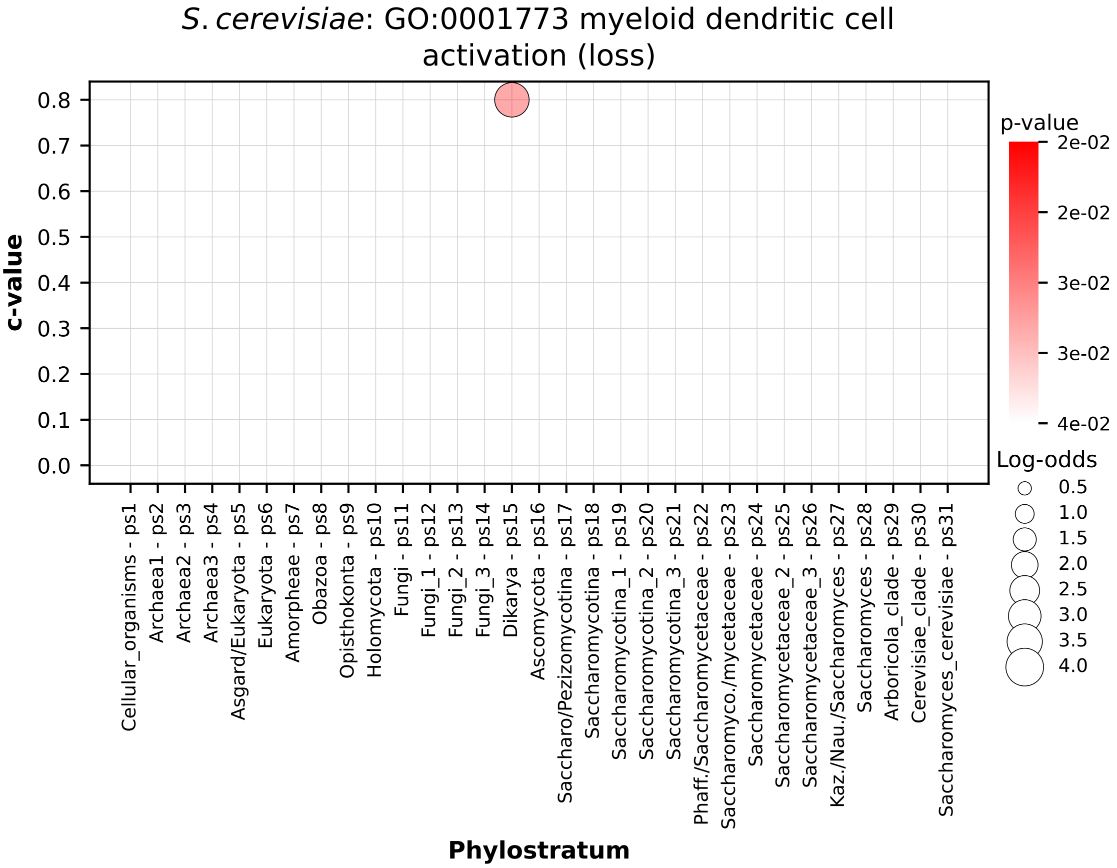
S. cerevisiae: GO:0001773 myeloid dendritic cell activation (loss)
c-value
0.0
0.1
0.2
0.3
0.4
0.5
0.6
0.7
0.8
p-value
2e-02
2e-02
3e-02
3e-02
4e-02
Log-odds
0.5
1.0
1.5
2.0
2.5
3.0
3.5
4.0
Cellular_organisms - ps1
Archaea1 - ps2
Archaea2 - ps3
Archaea3 - ps4
Asgard/Eukaryota - ps5
Eukaryota - ps6
Amorpheae - ps7
Obazoa - ps8
Opisthokonta - ps9
Holomycota - ps10
Fungi - ps11
Fungi_1 - ps12
Fungi_2 - ps13
Fungi_3 - ps14
Dikarya - ps15
Ascomycota - ps16
Saccharo/Pezizomycotina - ps17
Saccharomycotina - ps18
Saccharomycotina_1 - ps19
Saccharomycotina_2 - ps20
Saccharomycotina_3 - ps21
Phaff./Saccharomycetaceae - ps22
Saccharomyco./mycetaceae - ps23
Saccharomycetaceae - ps24
Saccharomycetaceae_2 - ps25
Saccharomycetaceae_3 - ps26
Kaz./Nau./Saccharomyces - ps27
Saccharomyces - ps28
Arboricola_clade - ps29
Cerevisiae_clade - ps30
Saccharomyces_cerevisiae - ps31
Phylostratum

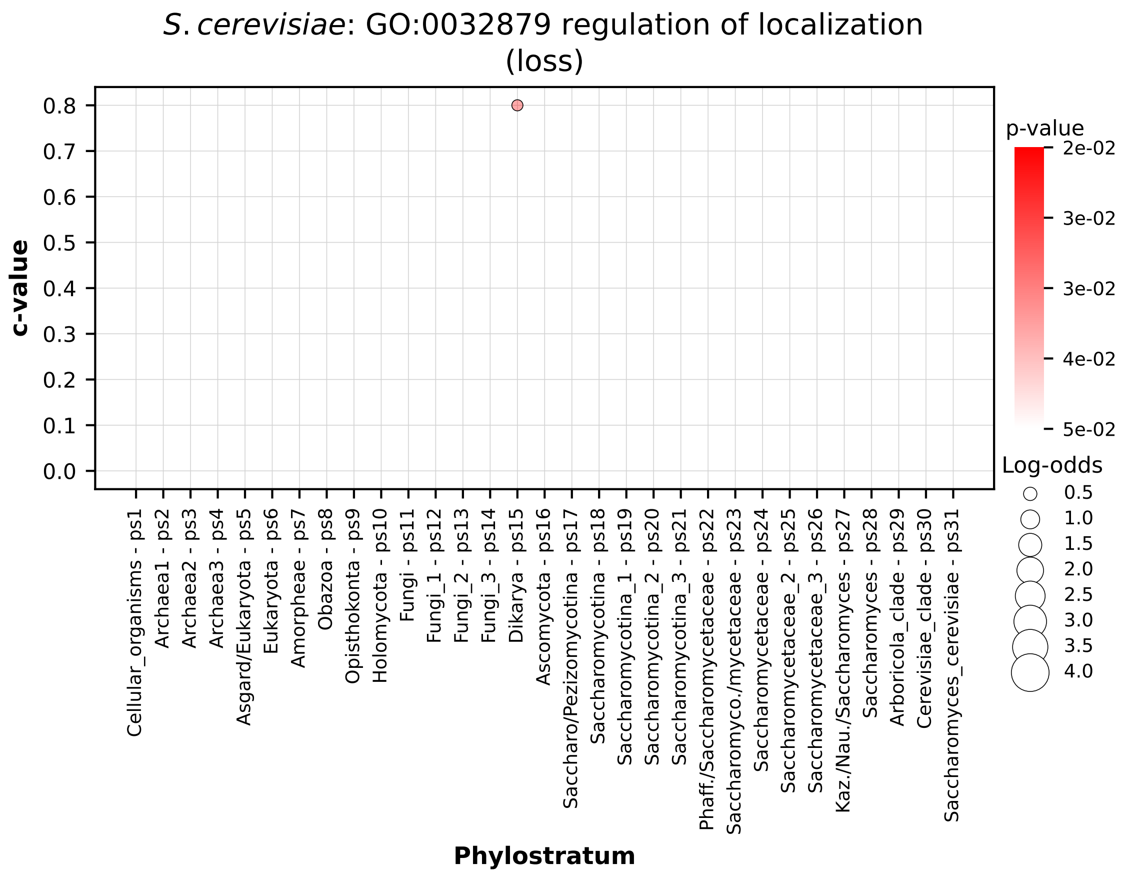

S. cerevisiae: GO:0032879 regulation of localization (loss)
c-value
0.8
0.7
0.6
0.5
0.4
0.3
0.2
0.1
0.0
p-value
2e-02
3e-02
3e-02
4e-02
5e-02
Log-odds
0.5
1.0
1.5
2.0
2.5
3.0
3.5
4.0
Cellular_organisms - ps1
Archaea1 - ps2
Archaea2 - ps3
Archaea3 - ps4
Asgard/Eukaryota - ps5
Eukaryota - ps6
Amorpheae - ps7
Obazoa - ps8
Opisthokonta - ps9
Holomycota - ps10
Fungi - ps11
Fungi_1 - ps12
Fungi_2 - ps13
Fungi_3 - ps14
Dikarya - ps15
Ascomycota - ps16
Saccharo/Pezizomycotina - ps17
Saccharomycotina - ps18
Saccharomycotina_1 - ps19
Saccharomycotina_2 - ps20
Saccharomycotina_3 - ps21
Phaff./Saccharomycetaceae - ps22
Saccharomyco./mycetaceae - ps23
Saccharomycetaceae - ps24
Saccharomycetaceae_2 - ps25
Saccharomycetaceae_3 - ps26
Kaz./Nau./Saccharomyces - ps27
Saccharomyces - ps28
Arboricola_clade - ps29
Cerevisiae_clade - ps30
Saccharomyces_cerevisiae - ps31
Phylostratum

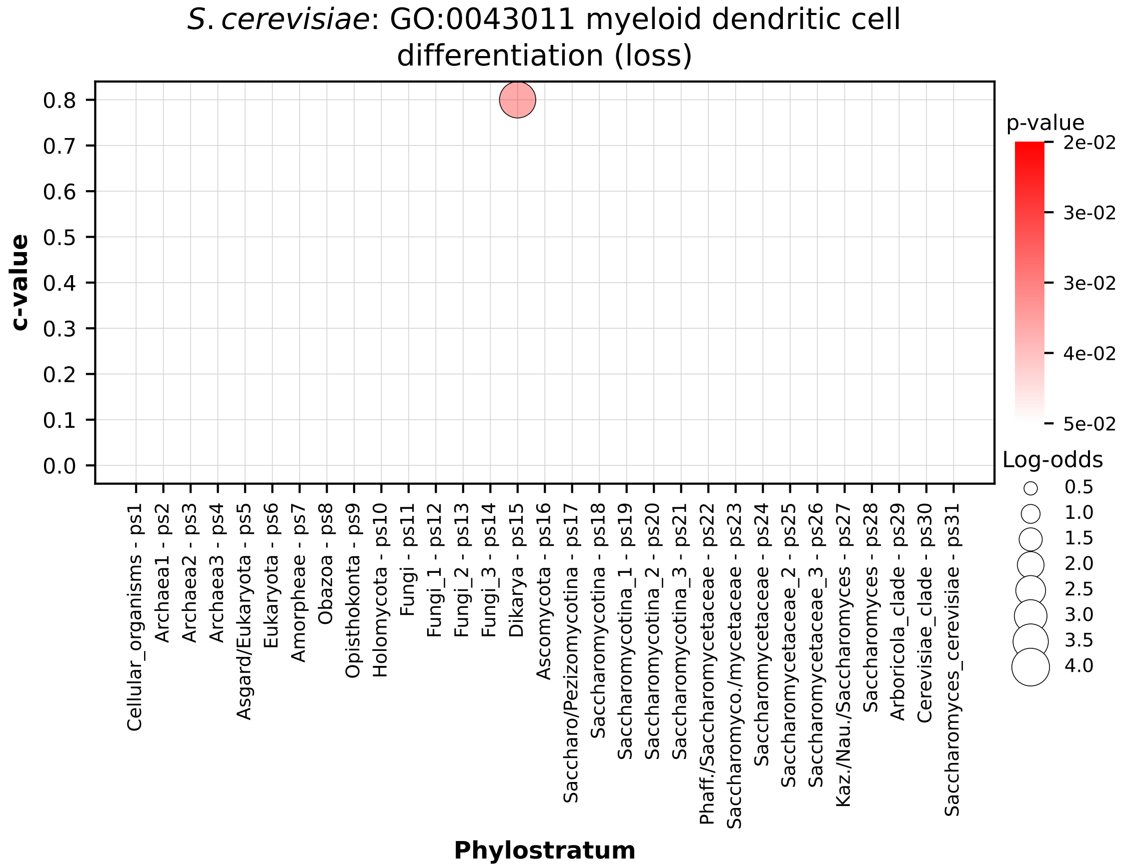
S. cerevisiae: GO:0043011 myeloid dendritic cell differentiation (loss)
c-value
0.8
0.7
0.6
0.5
0.4
0.3
0.2
0.1
0.0
p-value
2e-02
3e-02
3e-02
4e-02
5e-02
Log-odds
0.5
1.0
1.5
2.0
2.5
3.0
3.5
4.0
Cellular_organisms - ps1
Archaea1 - ps2
Archaea2 - ps3
Archaea3 - ps4
Asgard/Eukaryota - ps5
Eukaryota - ps6
Amorpheae - ps7
Obazoa - ps8
Opisthokonta - ps9
Holomycota - ps10
Fungi - ps11
Fungi_1 - ps12
Fungi_2 - ps13
Fungi_3 - ps14
Dikarya - ps15
Ascomycota - ps16
Saccharo/Pezizomycotina - ps17
Saccharomycotina - ps18
Saccharomycotina_1 - ps19
Saccharomycotina_2 - ps20
Saccharomycotina_3 - ps21
Phaff./Saccharomycetaceae - ps22
Saccharomyco./mycetaceae - ps23
Saccharomycetaceae - ps24
Saccharomycetaceae_2 - ps25
Saccharomycetaceae_3 - ps26
Kaz./Nau./Saccharomyces - ps27
Saccharomyces - ps28
Arboricola_clade - ps29
Cerevisiae_clade - ps30
Saccharomyces_cerevisiae - ps31
Phylostratum

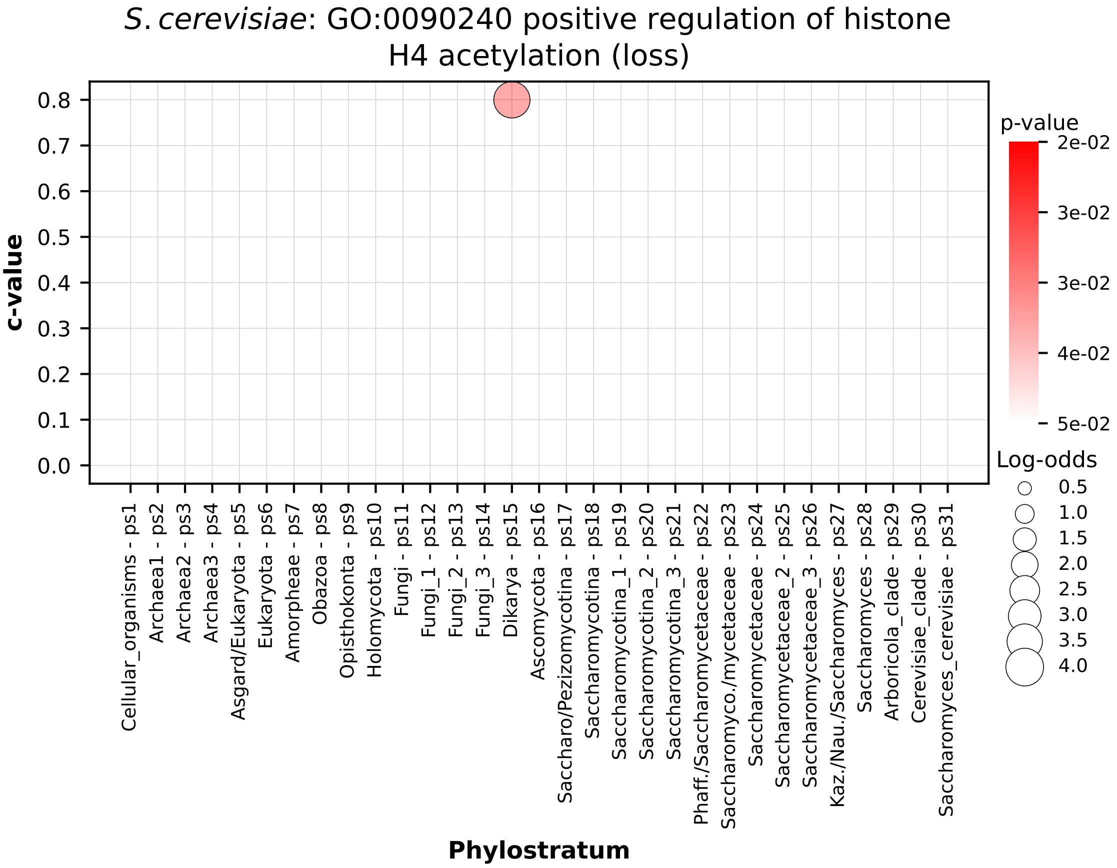

S. cerevisiae: GO:0090240 positive regulation of histone H4 acetylation (loss)
c-value
0.0
0.1
0.2
0.3
0.4
0.5
0.6
0.7
0.8
p-value
2e-02
3e-02
3e-02
4e-02
5e-02
Log-odds
0.5
1.0
1.5
2.0
2.5
3.0
3.5
4.0
Cellular_organisms - ps1
Archaea1 - ps2
Archaea2 - ps3
Archaea3 - ps4
Asgard/Eukaryota - ps5
Eukaryota - ps6
Amorpheae - ps7
Obazoa - ps8
Opisthokonta - ps9
Holomycota - ps10
Fungi - ps11
Fungi_1 - ps12
Fungi_2 - ps13
Fungi_3 - ps14
Dikarya - ps15
Ascomycota - ps16
Saccharo/Pezizomycotina - ps17
Saccharomycotina - ps18
Saccharomycotina_1 - ps19
Saccharomycotina_2 - ps20
Saccharomycotina_3 - ps21
Phaff./Saccharomycetaceae - ps22
Saccharomyco./mycetaceae - ps23
Saccharomycetaceae - ps24
Saccharomycetaceae_2 - ps25
Saccharomycetaceae_3 - ps26
Kaz./Nau./Saccharomyces - ps27
Saccharomyces - ps28
Arboricola_clade - ps29
Cerevisiae_clade - ps30
Saccharomyces_cerevisiae - ps31
Phylostratum

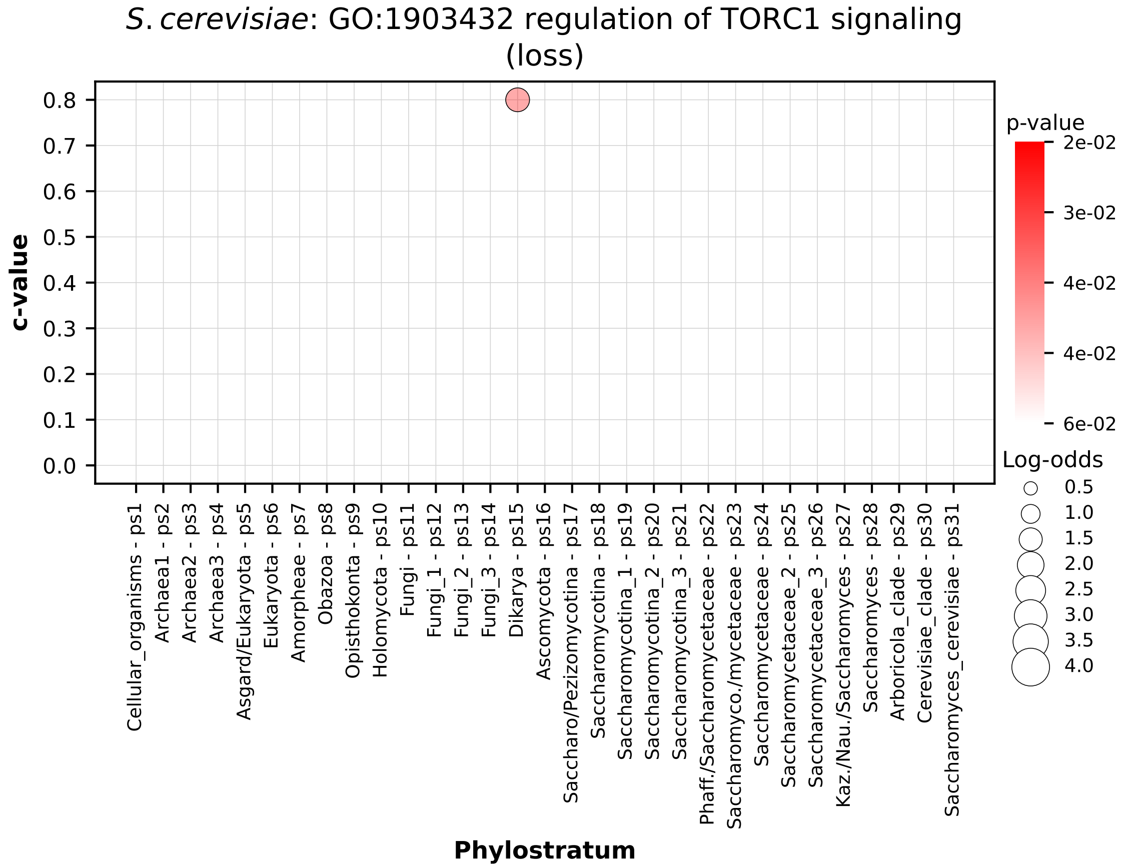

S. cerevisiae: GO:1903432 regulation of TORC1 signaling (loss)
c-value
0.8
0.7
0.6
0.5
0.4
0.3
0.2
0.1
0.0
p-value
2e-02
3e-02
4e-02
4e-02
6e-02
Log-odds
0.5
1.0
1.5
2.0
2.5
3.0
3.5
4.0
Cellular_organisms - ps1
Archaea1 - ps2
Archaea2 - ps3
Archaea3 - ps4
Asgard/Eukaryota - ps5
Eukaryota - ps6
Amorpheae - ps7
Obazoa - ps8
Opisthokonta - ps9
Holomycota - ps10
Fungi - ps11
Fungi_1 - ps12
Fungi_2 - ps13
Fungi_3 - ps14
Dikarya - ps15
Ascomycota - ps16
Saccharo/Pezizomycotina - ps17
Saccharomycotina - ps18
Saccharomycotina_1 - ps19
Saccharomycotina_2 - ps20
Saccharomycotina_3 - ps21
Phaff./Saccharomycetaceae - ps22
Saccharomyco./mycetaceae - ps23
Saccharomycetaceae - ps24
Saccharomycetaceae_2 - ps25
Saccharomycetaceae_3 - ps26
Kaz./Nau./Saccharomyces - ps27
Saccharomyces - ps28
Arboricola_clade - ps29
Cerevisiae_clade - ps30
Saccharomyces_cerevisiae - ps31
Phylostratum

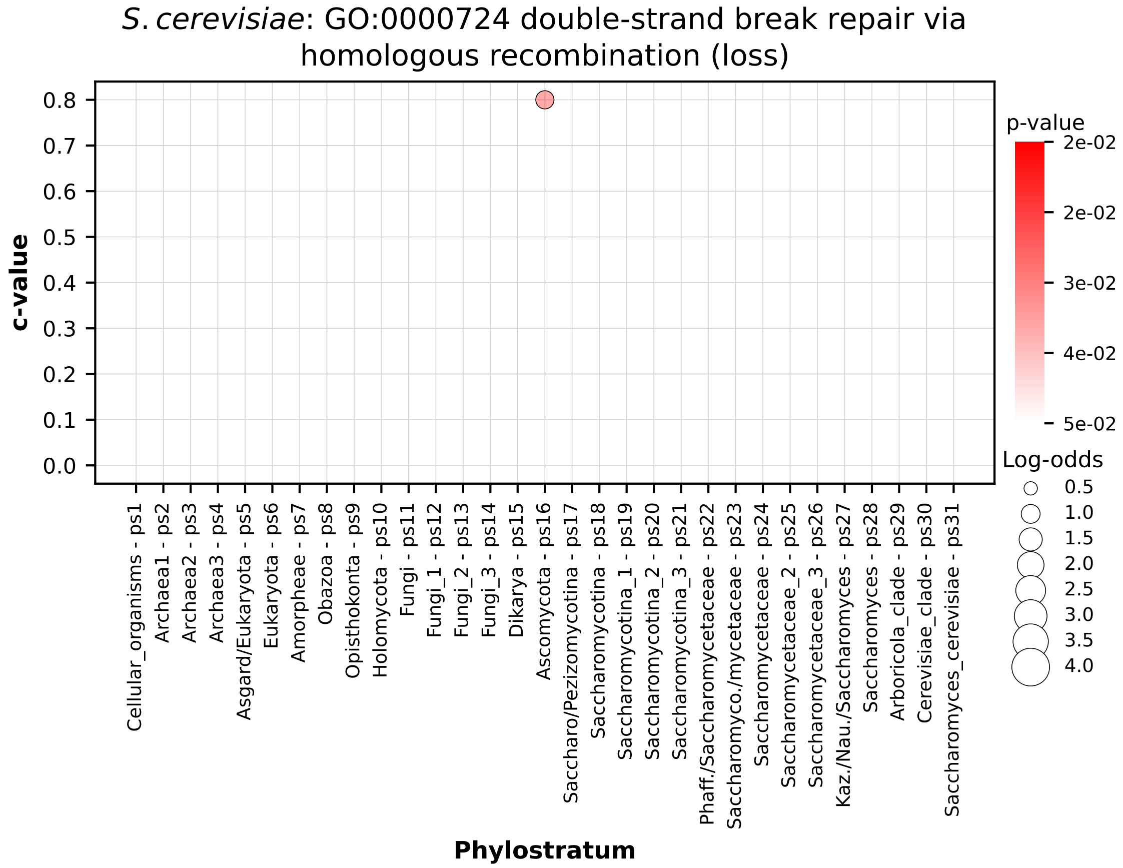
S. cerevisiae: GO:0000724 double-strand break repair via homologous recombination (loss)
c-value
0.0
0.1
0.2
0.3
0.4
0.5
0.6
0.7
0.8
Phylostratum
Cellular_organisms - ps1
Archaea1 - ps2
Archaea2 - ps3
Archaea3 - ps4
Asgard/Eukaryota - ps5
Eukaryota - ps6
Amorpheae - ps7
Obazoa - ps8
Opisthokonta - ps9
Holomycota - ps10
Fungi - ps11
Fungi_1 - ps12
Fungi_2 - ps13
Fungi_3 - ps14
Dikarya - ps15
Ascomycota - ps16
Saccharo/Pezizomycotina - ps17
Saccharomycotina - ps18
Saccharomycotina_1 - ps19
Saccharomycotina_2 - ps20
Saccharomycotina_3 - ps21
Phaff./Saccharomycetaceae - ps22
Saccharomyco./mycetaceae - ps23
Saccharomycetaceae - ps24
Saccharomycetaceae_2 - ps25
Saccharomycetaceae_3 - ps26
Kaz./Nau./Saccharomyces - ps27
Saccharomyces - ps28
Arboricola_clade - ps29
Cerevisiae_clade - ps30
Saccharomyces_cerevisiae - ps31
p-value
2e-02
2e-02
3e-02
4e-02
5e-02
Log-odds
0.5
1.0
1.5
2.0
2.5
3.0
3.5
4.0

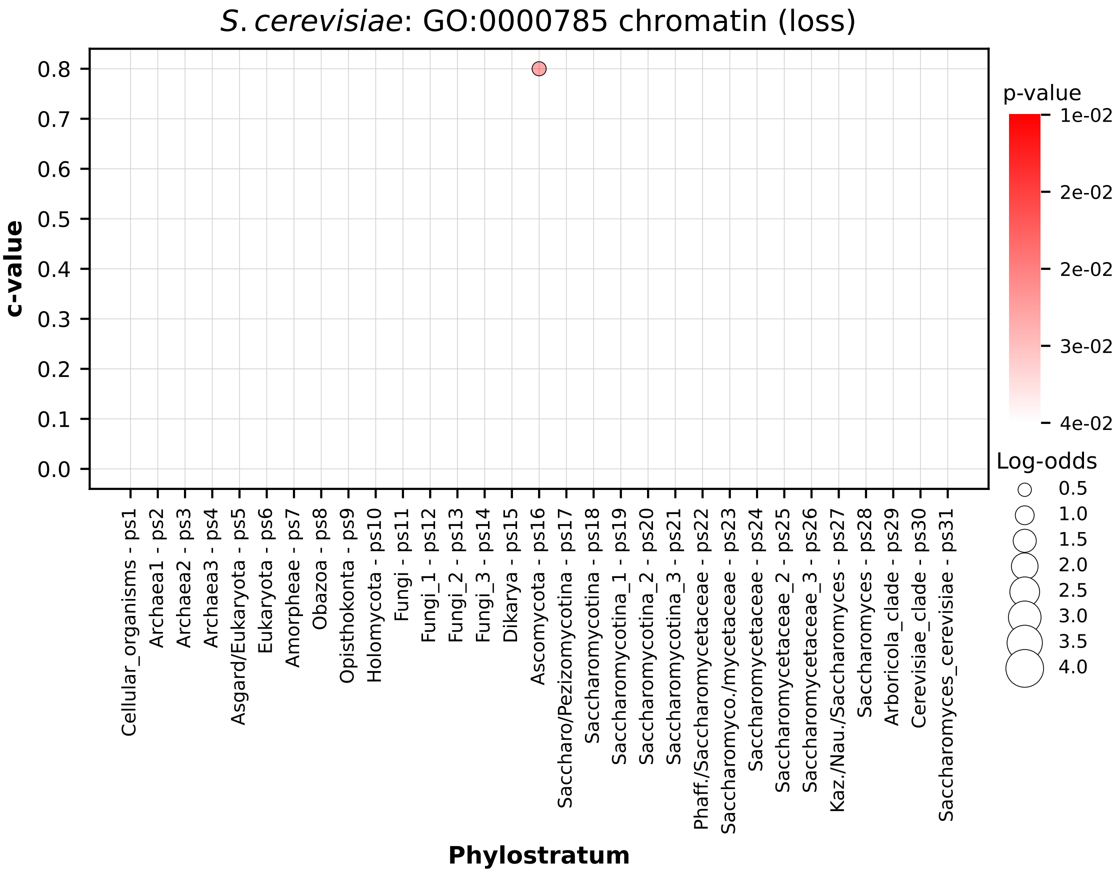

$S. cerevisiae$: GO:0000785 chromatin (loss)
c-value
0.0
0.1
0.2
0.3
0.4
0.5
0.6
0.7
0.8
Phylostratum
Cellular_organisms - ps1
Archaea1 - ps2
Archaea2 - ps3
Archaea3 - ps4
Asgard/Eukaryota - ps5
Eukaryota - ps6
Amorpheae - ps7
Obazoa - ps8
Opisthokonta - ps9
Holomycota - ps10
Fungi - ps11
Fungi_1 - ps12
Fungi_2 - ps13
Fungi_3 - ps14
Dikarya - ps15
Ascomycota - ps16
Saccharo/Pezizomycotina - ps17
Saccharomycotina - ps18
Saccharomycotina_1 - ps19
Saccharomycotina_2 - ps20
Saccharomycotina_3 - ps21
Phaff./Saccharomycetaceae - ps22
Saccharomyco./mycetaceae - ps23
Saccharomycetaceae - ps24
Saccharomycetaceae_2 - ps25
Saccharomycetaceae_3 - ps26
Kaz./Nau./Saccharomyces - ps27
Saccharomyces - ps28
Arboricola_clade - ps29
Cerevisiae_clade - ps30
Saccharomyces_cerevisiae - ps31
p-value
1e-02
2e-02
2e-02
3e-02
4e-02
Log-odds
0.5
1.0
1.5
2.0
2.5
3.0
3.5
4.0

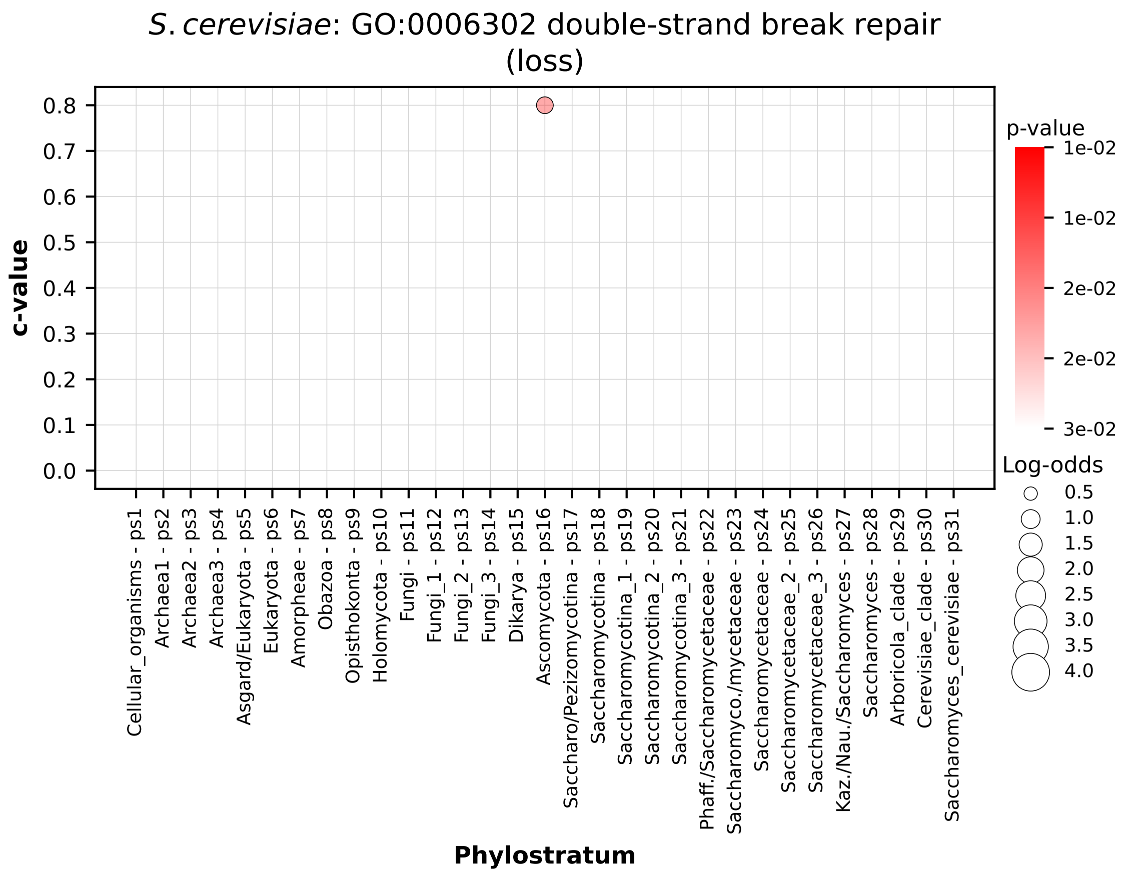

S. cerevisiae: GO:0006302 double-strand break repair
(loss)
c-value
0.8
0.7
0.6
0.5
0.4
0.3
0.2
0.1
0.0
p-value
1e-02
1e-02
2e-02
2e-02
3e-02
Log-odds
0.5
1.0
1.5
2.0
2.5
3.0
3.5
4.0
Cellular_organisms - ps1
Archaea1 - ps2
Archaea2 - ps3
Archaea3 - ps4
Asgard/Eukaryota - ps5
Eukaryota - ps6
Amorpheae - ps7
Obazoa - ps8
Opisthokonta - ps9
Holomycota - ps10
Fungi - ps11
Fungi_1 - ps12
Fungi_2 - ps13
Fungi_3 - ps14
Dikarya - ps15
Ascomycota - ps16
Saccharo/Pezizomycotina - ps17
Saccharomycotina - ps18
Saccharomycotina_1 - ps19
Saccharomycotina_2 - ps20
Saccharomycotina_3 - ps21
Phaff./Saccharomycetaceae - ps22
Saccharomyco./mycetaceae - ps23
Saccharomycetaceae - ps24
Saccharomycetaceae_2 - ps25
Saccharomycetaceae_3 - ps26
Kaz./Nau./Saccharomyces - ps27
Saccharomyces - ps28
Arboricola_clade - ps29
Cerevisiae_clade - ps30
Saccharomyces_cerevisiae - ps31
Phylostratum

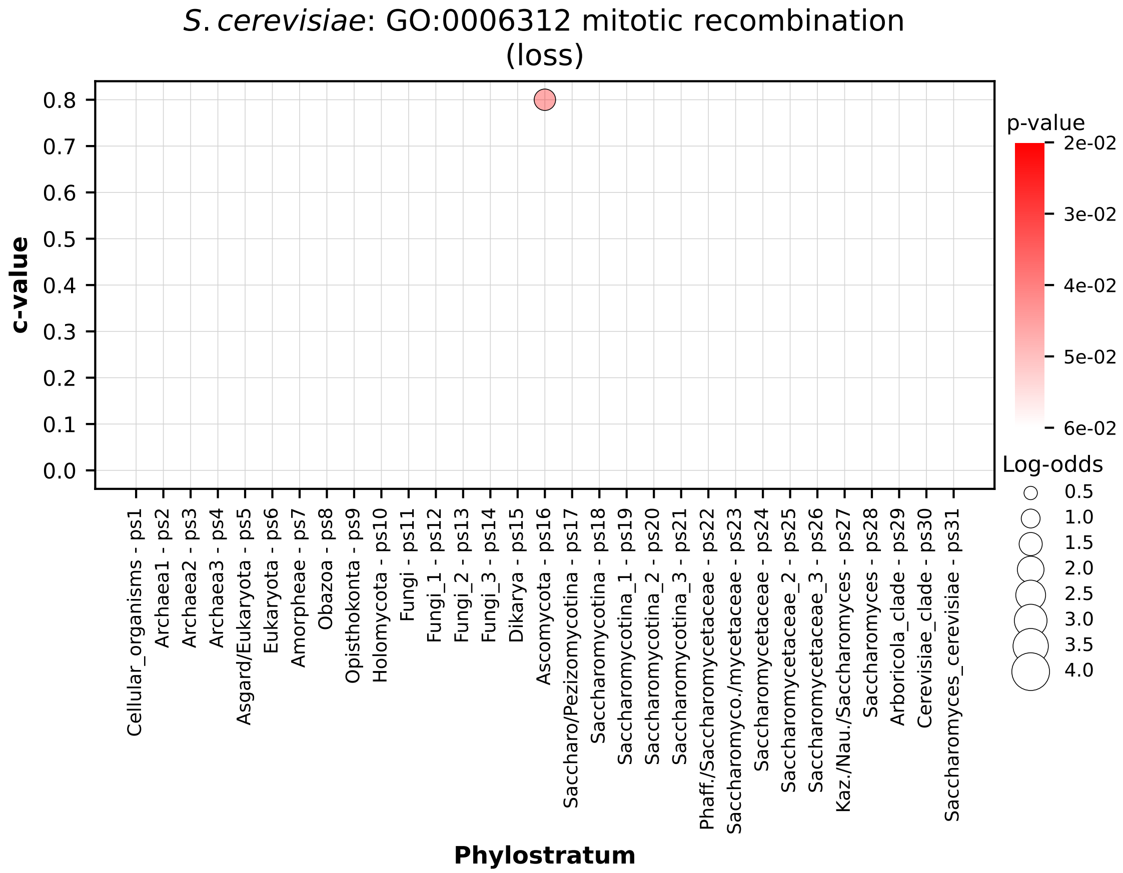

S. cerevisiae: GO:0006312 mitotic recombination (loss)
c-value
0.0
0.1
0.2
0.3
0.4
0.5
0.6
0.7
0.8
p-value
2e-02
3e-02
4e-02
5e-02
6e-02
Log-odds
0.5
1.0
1.5
2.0
2.5
3.0
3.5
4.0
Cellular_organisms - ps1
Archaea1 - ps2
Archaea2 - ps3
Archaea3 - ps4
Asgard/Eukaryota - ps5
Eukaryota - ps6
Amorpheae - ps7
Obazoa - ps8
Opisthokonta - ps9
Holomycota - ps10
Fungi - ps11
Fungi_1 - ps12
Fungi_2 - ps13
Fungi_3 - ps14
Dikarya - ps15
Ascomycota - ps16
Saccharo/Pezizomycotina - ps17
Saccharomycotina - ps18
Saccharomycotina_1 - ps19
Saccharomycotina_2 - ps20
Saccharomycotina_3 - ps21
Phaff./Saccharomycetaceae - ps22
Saccharomyco./mycetaceae - ps23
Saccharomycetaceae - ps24
Saccharomycetaceae_2 - ps25
Saccharomycetaceae_3 - ps26
Kaz./Nau./Saccharomyces - ps27
Saccharomyces - ps28
Arboricola_clade - ps29
Cerevisiae_clade - ps30
Saccharomyces_cerevisiae - ps31
Phylostratum

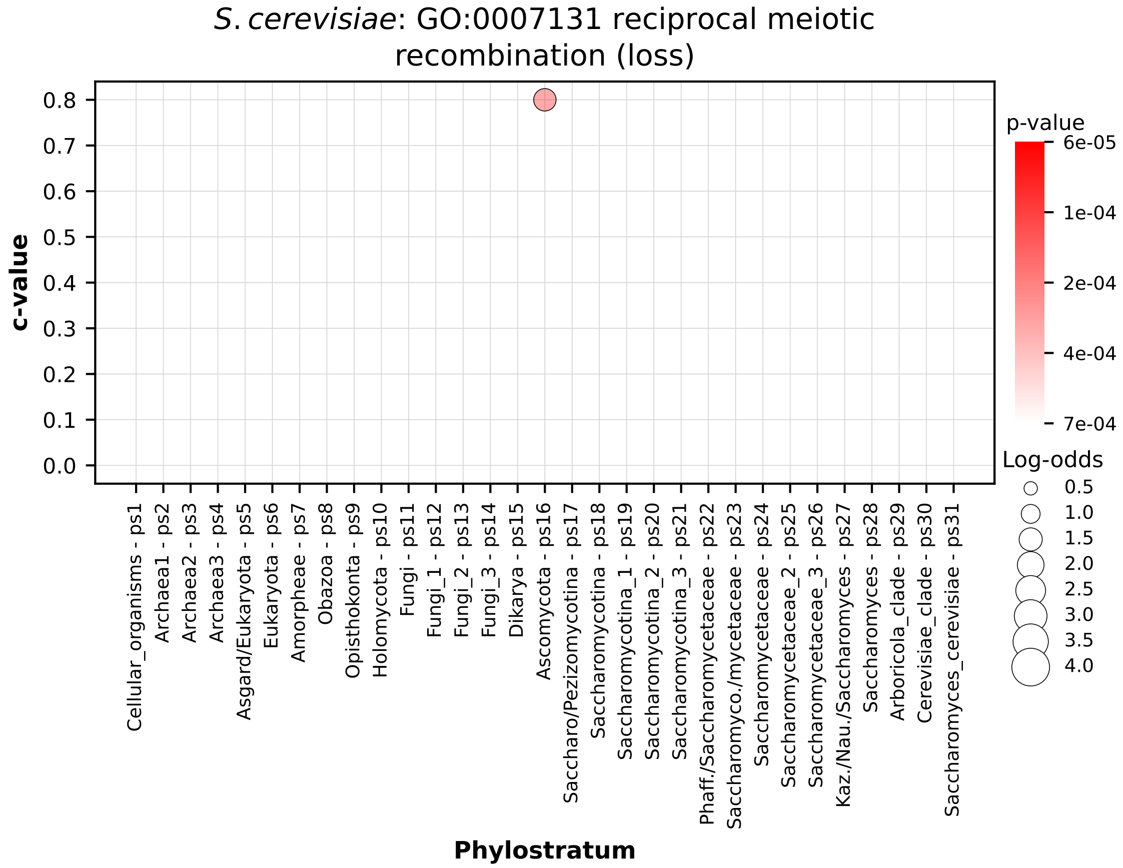
S. cerevisiae: GO:0007131 reciprocal meiotic recombination (loss)
c-value
0.0
0.1
0.2
0.3
0.4
0.5
0.6
0.7
0.8
Phylostratum
Cellular_organisms - ps1
Archaea1 - ps2
Archaea2 - ps3
Archaea3 - ps4
Asgard/Eukaryota - ps5
Eukaryota - ps6
Amorpheae - ps7
Obazoa - ps8
Opisthokonta - ps9
Holomycota - ps10
Fungi - ps11
Fungi_1 - ps12
Fungi_2 - ps13
Fungi_3 - ps14
Dikarya - ps15
Ascomycota - ps16
Saccharo/Pezizomycotina - ps17
Saccharomycotina - ps18
Saccharomycotina_1 - ps19
Saccharomycotina_2 - ps20
Saccharomycotina_3 - ps21
Phaff./Saccharomycetaceae - ps22
Saccharomyco./mycetaceae - ps23
Saccharomycetaceae - ps24
Saccharomycetaceae_2 - ps25
Saccharomycetaceae_3 - ps26
Kaz./Nau./Saccharomyces - ps27
Saccharomyces - ps28
Arboricola_clade - ps29
Cerevisiae_clade - ps30
Saccharomyces_cerevisiae - ps31
p-value
6e-05
1e-04
2e-04
4e-04
7e-04
Log-odds
0.5
1.0
1.5
2.0
2.5
3.0
3.5
4.0

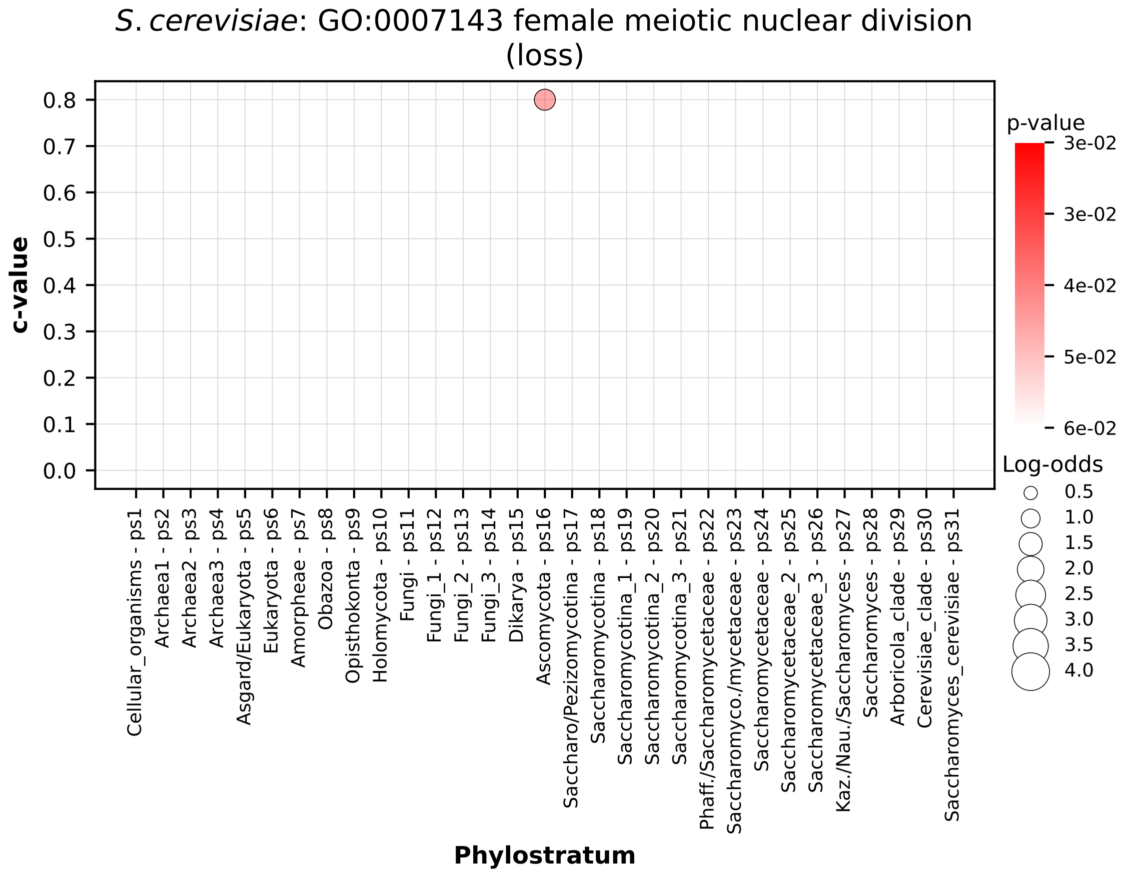
S. cerevisiae: GO:0007143 female meiotic nuclear division
(loss)
c-value
0.0
0.1
0.2
0.3
0.4
0.5
0.6
0.7
0.8
p-value
3e-02
3e-02
4e-02
5e-02
6e-02
Log-odds
0.5
1.0
1.5
2.0
2.5
3.0
3.5
4.0
Cellular_organisms - ps1
Archaea1 - ps2
Archaea2 - ps3
Archaea3 - ps4
Asgard/Eukaryota - ps5
Eukaryota - ps6
Amorpheae - ps7
Obazoa - ps8
Opisthokonta - ps9
Holomycota - ps10
Fungi - ps11
Fungi_1 - ps12
Fungi_2 - ps13
Fungi_3 - ps14
Dikarya - ps15
Ascomycota - ps16
Saccharo/Pezizomycotina - ps17
Saccharomycotina - ps18
Saccharomycotina_1 - ps19
Saccharomycotina_2 - ps20
Saccharomycotina_3 - ps21
Phaff./Saccharomycetaceae - ps22
Saccharomyco./mycetaceae - ps23
Saccharomycetaceae - ps24
Saccharomycetaceae_2 - ps25
Saccharomycetaceae_3 - ps26
Kaz./Nau./Saccharomyces - ps27
Saccharomyces - ps28
Arboricola_clade - ps29
Cerevisiae_clade - ps30
Saccharomyces_cerevisiae - ps31
Phylostratum

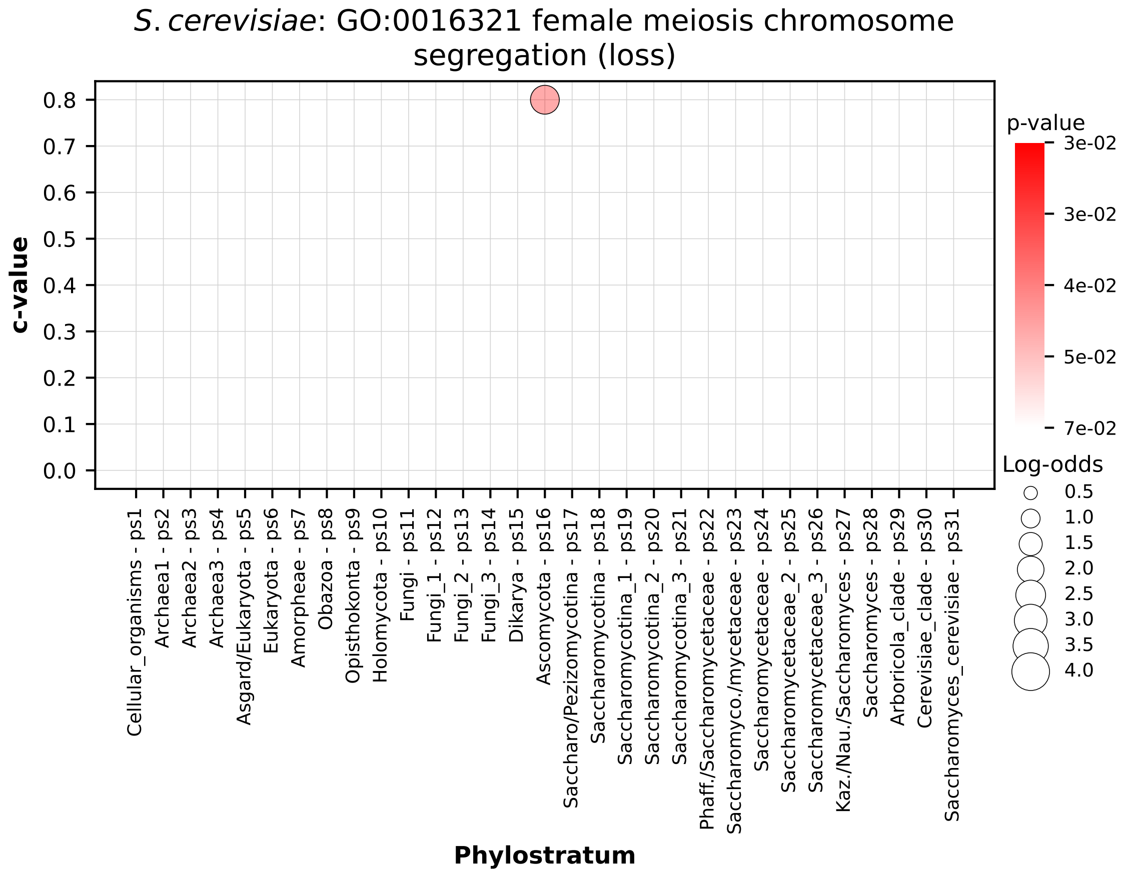
S. cerevisiae: GO:0016321 female meiosis chromosome segregation (loss)
c-value
0.0
0.1
0.2
0.3
0.4
0.5
0.6
0.7
0.8
p-value
3e-02
3e-02
4e-02
5e-02
7e-02
Log-odds
0.5
1.0
1.5
2.0
2.5
3.0
3.5
4.0
Cellular_organisms - ps1
Archaea1 - ps2
Archaea2 - ps3
Archaea3 - ps4
Asgard/Eukaryota - ps5
Eukaryota - ps6
Amorpheae - ps7
Obazoa - ps8
Opisthokonta - ps9
Holomycota - ps10
Fungi - ps11
Fungi_1 - ps12
Fungi_2 - ps13
Fungi_3 - ps14
Dikarya - ps15
Ascomycota - ps16
Saccharo/Pezizomycotina - ps17
Saccharomycotina - ps18
Saccharomycotina_1 - ps19
Saccharomycotina_2 - ps20
Saccharomycotina_3 - ps21
Phaff./Saccharomycetaceae - ps22
Saccharomyco./mycetaceae - ps23
Saccharomycetaceae - ps24
Saccharomycetaceae_2 - ps25
Saccharomycetaceae_3 - ps26
Kaz./Nau./Saccharomyces - ps27
Saccharomyces - ps28
Arboricola_clade - ps29
Cerevisiae_clade - ps30
Saccharomyces_cerevisiae - ps31
Phylostratum

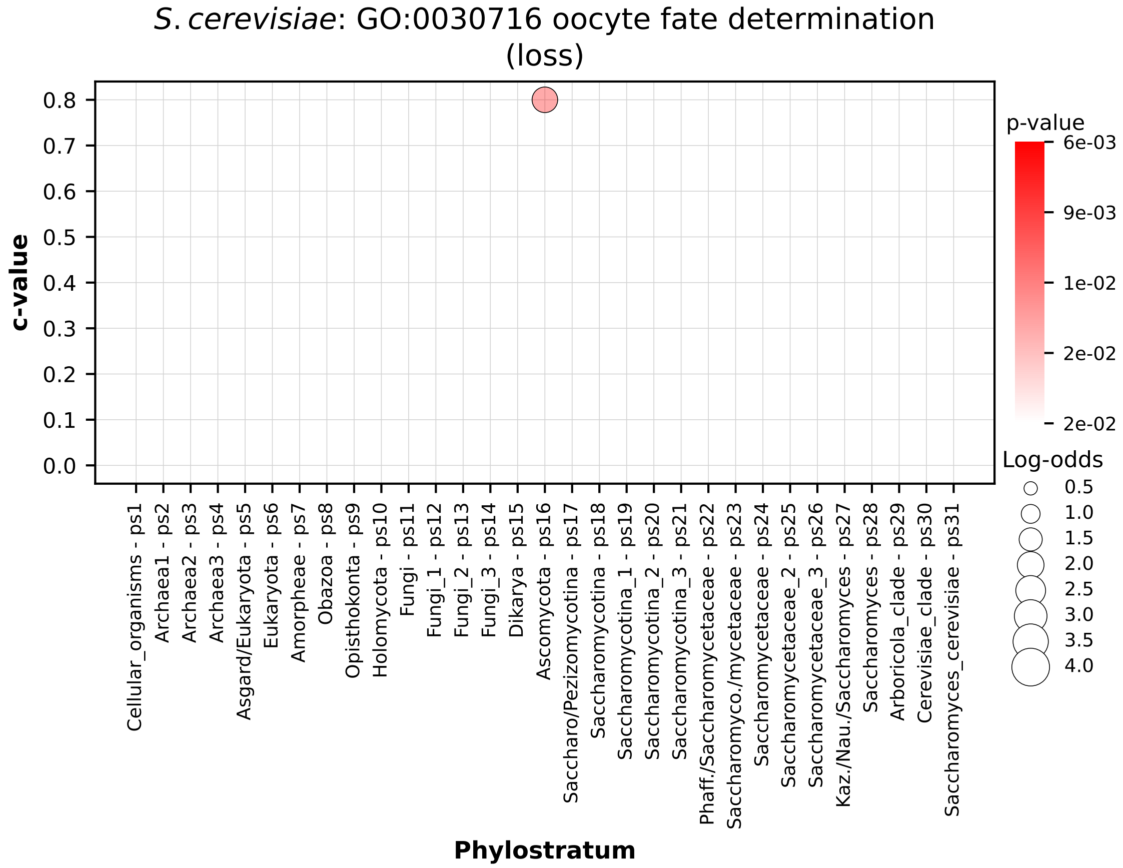

S. cerevisiae: GO:0030716 oocyte fate determination
(loss)
c-value
0.0
0.1
0.2
0.3
0.4
0.5
0.6
0.7
0.8
Phylostratum
Cellular_organisms - ps1
Archaea1 - ps2
Archaea2 - ps3
Archaea3 - ps4
Asgard/Eukaryota - ps5
Eukaryota - ps6
Amorpheae - ps7
Obazoa - ps8
Opisthokonta - ps9
Holomycota - ps10
Fungi - ps11
Fungi_1 - ps12
Fungi_2 - ps13
Fungi_3 - ps14
Dikarya - ps15
Ascomycota - ps16
Saccharo/Pezizomycotina - ps17
Saccharomycotina - ps18
Saccharomycotina_1 - ps19
Saccharomycotina_2 - ps20
Saccharomycotina_3 - ps21
Phaff./Saccharomycetaceae - ps22
Saccharomyco./mycetaceae - ps23
Saccharomycetaceae - ps24
Saccharomycetaceae_2 - ps25
Saccharomycetaceae_3 - ps26
Kaz./Nau./Saccharomyces - ps27
Saccharomyces - ps28
Arboricola_clade - ps29
Cerevisiae_clade - ps30
Saccharomyces_cerevisiae - ps31
p-value
6e-03
9e-03
1e-02
2e-02
2e-02
Log-odds
0.5
1.0
1.5
2.0
2.5
3.0
3.5
4.0

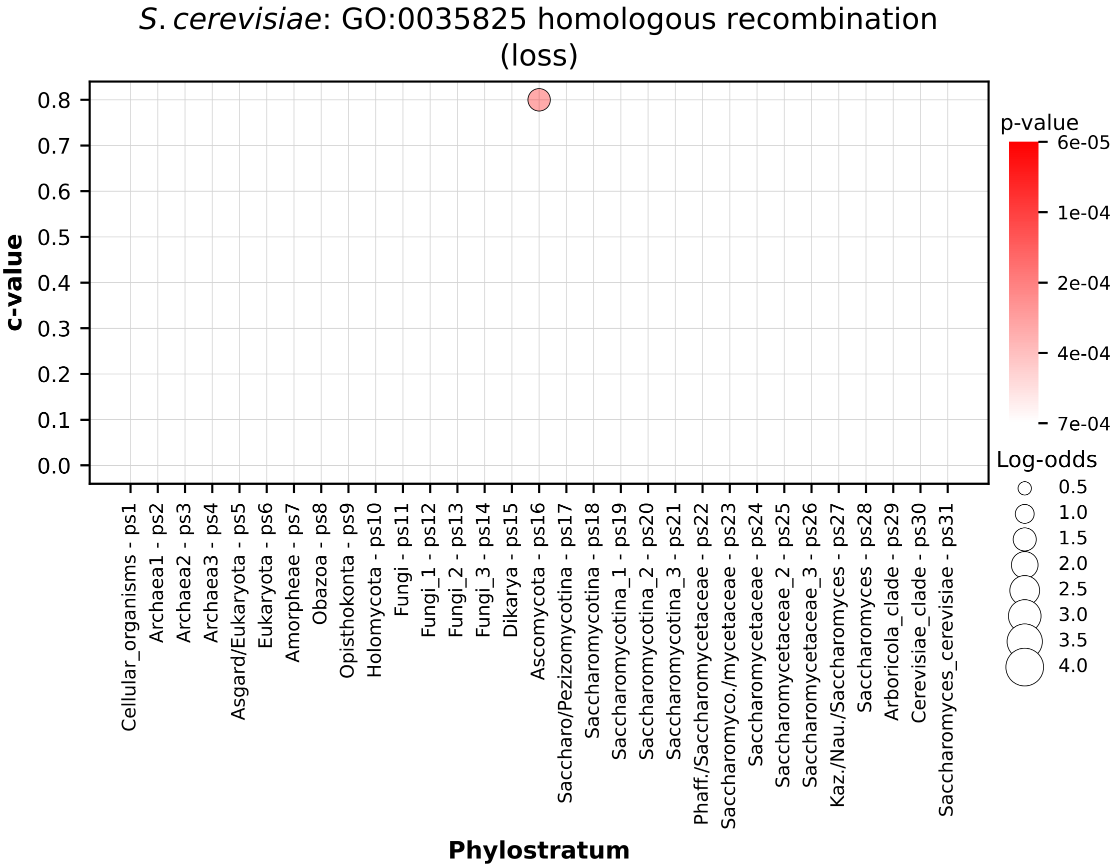

S. cerevisiae: GO:0035825 homologous recombination
(loss)
c-value
0.0
0.1
0.2
0.3
0.4
0.5
0.6
0.7
0.8
p-value
6e-05
1e-04
2e-04
4e-04
7e-04
Log-odds
0.5
1.0
1.5
2.0
2.5
3.0
3.5
4.0
Cellular_organisms - ps1
Archaea1 - ps2
Archaea2 - ps3
Archaea3 - ps4
Asgard/Eukaryota - ps5
Eukaryota - ps6
Amorpheae - ps7
Obazoa - ps8
Opisthokonta - ps9
Holomycota - ps10
Fungi - ps11
Fungi_1 - ps12
Fungi_2 - ps13
Fungi_3 - ps14
Dikarya - ps15
Ascomycota - ps16
Saccharo/Pezizomycotina - ps17
Saccharomycotina - ps18
Saccharomycotina_1 - ps19
Saccharomycotina_2 - ps20
Saccharomycotina_3 - ps21
Phaff./Saccharomycetaceae - ps22
Saccharomyco./mycetaceae - ps23
Saccharomycetaceae - ps24
Saccharomycetaceae_2 - ps25
Saccharomycetaceae_3 - ps26
Kaz./Nau./Saccharomyces - ps27
Saccharomyces - ps28
Arboricola_clade - ps29
Cerevisiae_clade - ps30
Saccharomyces_cerevisiae - ps31
Phylostratum

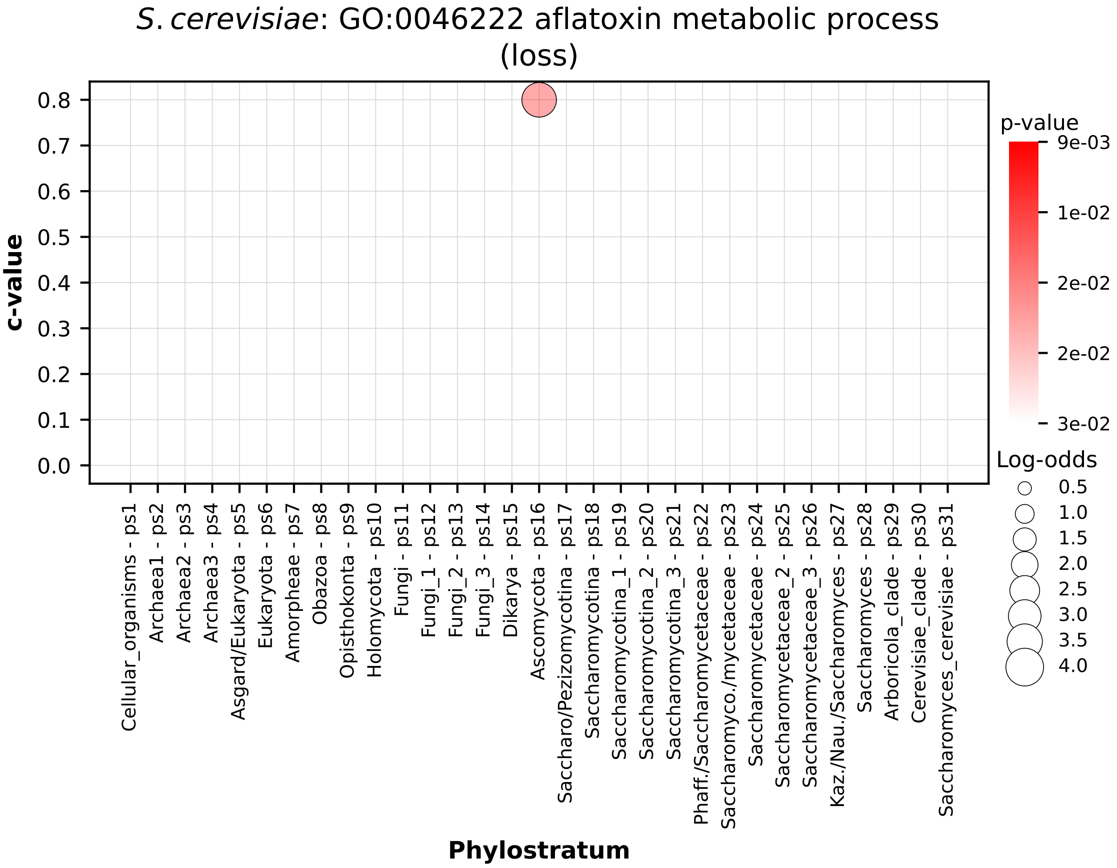

S. cerevisiae: GO:0046222 aflatoxin metabolic process
(loss)
c-value
0.0
0.1
0.2
0.3
0.4
0.5
0.6
0.7
0.8
p-value
9e-03
1e-02
2e-02
2e-02
3e-02
Log-odds
0.5
1.0
1.5
2.0
2.5
3.0
3.5
4.0
Cellular_organisms - ps1
Archaea1 - ps2
Archaea2 - ps3
Archaea3 - ps4
Asgard/Eukaryota - ps5
Eukaryota - ps6
Amorpheae - ps7
Obazoa - ps8
Opisthokonta - ps9
Holomycota - ps10
Fungi - ps11
Fungi_1 - ps12
Fungi_2 - ps13
Fungi_3 - ps14
Dikarya - ps15
Ascomycota - ps16
Saccharo/Pezizomycotina - ps17
Saccharomycotina - ps18
Saccharomycotina_1 - ps19
Saccharomycotina_2 - ps20
Saccharomycotina_3 - ps21
Phaff./Saccharomycetaceae - ps22
Saccharomyco./mycetaceae - ps23
Saccharomycetaceae - ps24
Saccharomycetaceae_2 - ps25
Saccharomycetaceae_3 - ps26
Kaz./Nau./Saccharomyces - ps27
Saccharomyces - ps28
Arboricola_clade - ps29
Cerevisiae_clade - ps30
Saccharomyces_cerevisiae - ps31
Phylostratum

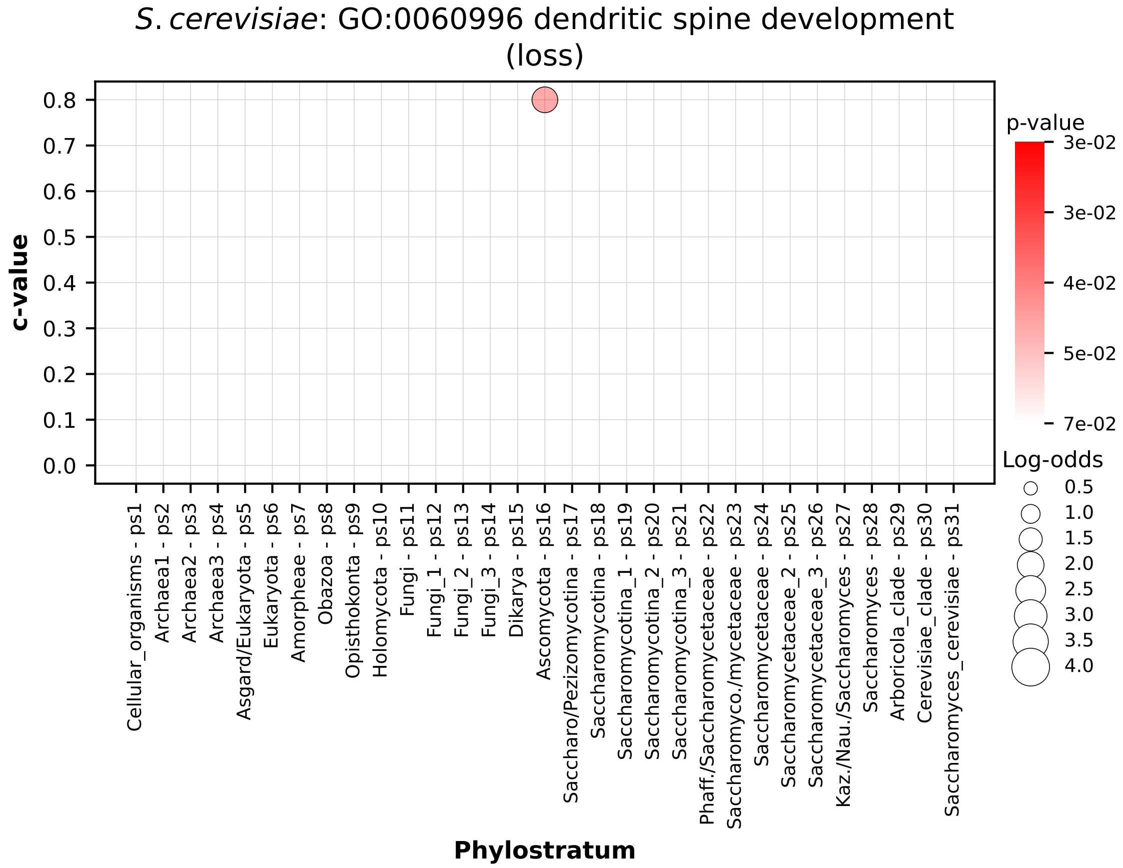

S. cerevisiae: GO:0060996 dendritic spine development (loss)
c-value
0.0
0.1
0.2
0.3
0.4
0.5
0.6
0.7
0.8
p-value
3e-02
3e-02
4e-02
5e-02
7e-02
Log-odds
0.5
1.0
1.5
2.0
2.5
3.0
3.5
4.0
Cellular_organisms - ps1
Archaea1 - ps2
Archaea2 - ps3
Archaea3 - ps4
Asgard/Eukaryota - ps5
Eukaryota - ps6
Amorpheae - ps7
Obazoa - ps8
Opisthokonta - ps9
Holomycota - ps10
Fungi - ps11
Fungi_1 - ps12
Fungi_2 - ps13
Fungi_3 - ps14
Dikarya - ps15
Ascomycota - ps16
Saccharo/Pezizomycotina - ps17
Saccharomycotina - ps18
Saccharomycotina_1 - ps19
Saccharomycotina_2 - ps20
Saccharomycotina_3 - ps21
Phaff./Saccharomycetaceae - ps22
Saccharomyco./mycetaceae - ps23
Saccharomycetaceae - ps24
Saccharomycetaceae_2 - ps25
Saccharomycetaceae_3 - ps26
Kaz./Nau./Saccharomyces - ps27
Saccharomyces - ps28
Arboricola_clade - ps29
Cerevisiae_clade - ps30
Saccharomyces_cerevisiae - ps31
Phylostratum

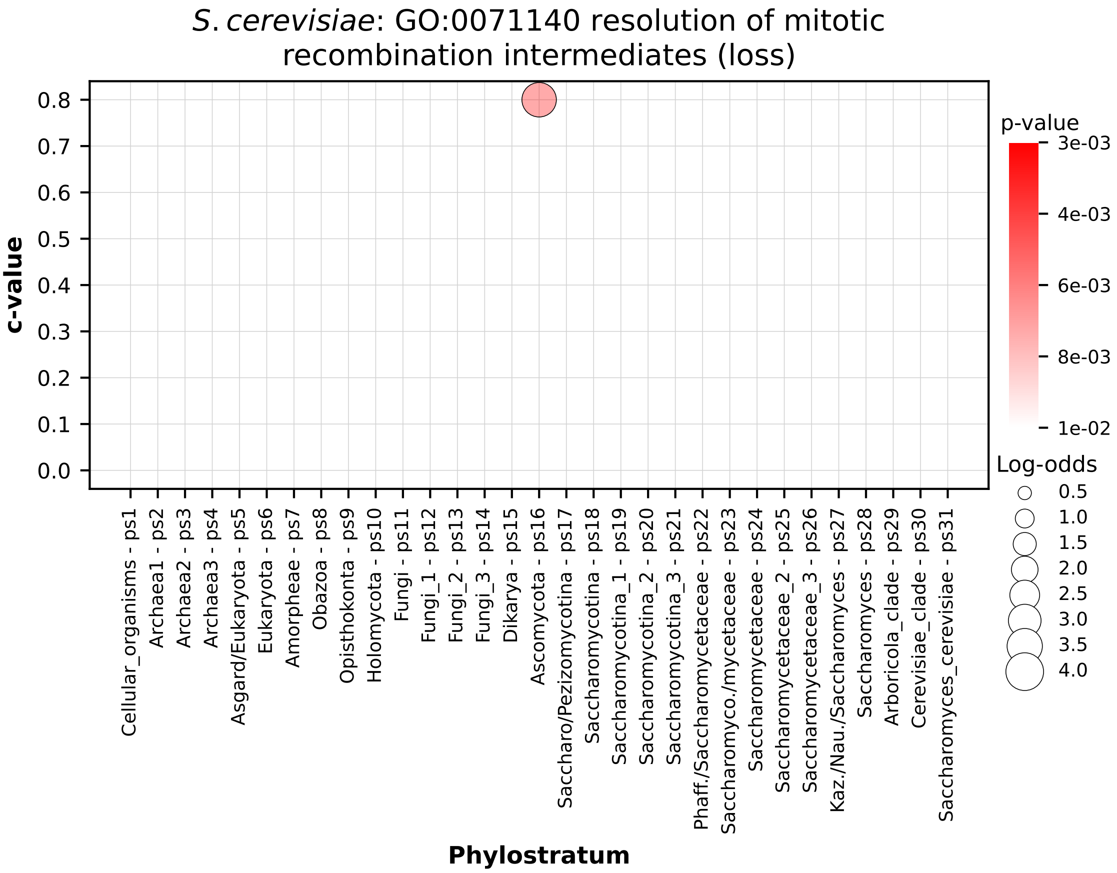
S. cerevisiae: GO:0071140 resolution of mitotic recombination intermediates (loss)
c-value
0.0
0.1
0.2
0.3
0.4
0.5
0.6
0.7
0.8
Phylostratum
Cellular_organisms - ps1
Archaea1 - ps2
Archaea2 - ps3
Archaea3 - ps4
Asgard/Eukaryota - ps5
Eukaryota - ps6
Amorpheae - ps7
Obazoa - ps8
Opisthokonta - ps9
Holomycota - ps10
Fungi - ps11
Fungi_1 - ps12
Fungi_2 - ps13
Fungi_3 - ps14
Dikarya - ps15
Ascomycota - ps16
Saccharo/Pezizomycotina - ps17
Saccharomycotina - ps18
Saccharomycotina_1 - ps19
Saccharomycotina_2 - ps20
Saccharomycotina_3 - ps21
Phaff./Saccharomycetaceae - ps22
Saccharomyco./mycetaceae - ps23
Saccharomycetaceae - ps24
Saccharomycetaceae_2 - ps25
Saccharomycetaceae_3 - ps26
Kaz./Nau./Saccharomyces - ps27
Saccharomyces - ps28
Arboricola_clade - ps29
Cerevisiae_clade - ps30
Saccharomyces_cerevisiae - ps31
p-value
3e-03
4e-03
6e-03
8e-03
1e-02
Log-odds
0.5
1.0
1.5
2.0
2.5
3.0
3.5
4.0

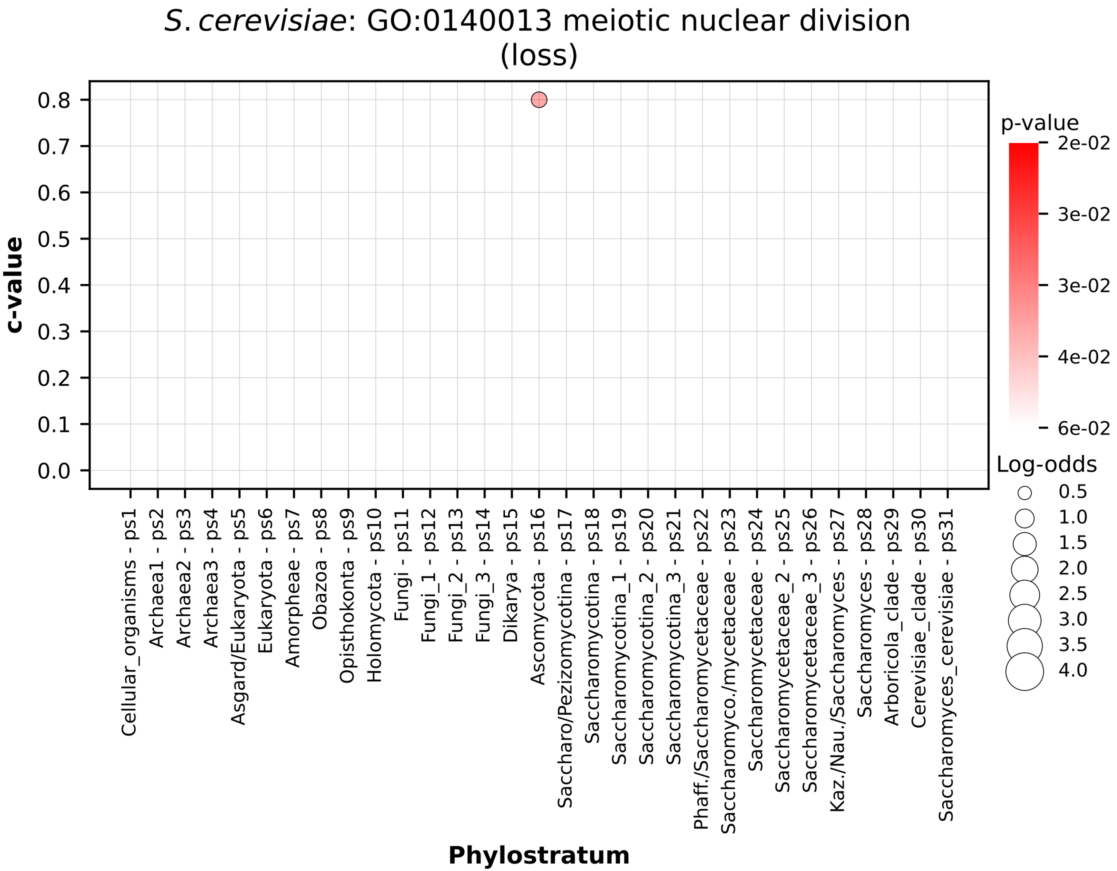

S. cerevisiae: GO:0140013 meiotic nuclear division
(loss)
c-value
0.0
0.1
0.2
0.3
0.4
0.5
0.6
0.7
0.8
Phylostratum
Cellular_organisms - ps1
Archaea1 - ps2
Archaea2 - ps3
Archaea3 - ps4
Asgard/Eukaryota - ps5
Eukaryota - ps6
Amorpheae - ps7
Obazoa - ps8
Opisthokonta - ps9
Holomycota - ps10
Fungi - ps11
Fungi_1 - ps12
Fungi_2 - ps13
Fungi_3 - ps14
Dikarya - ps15
Ascomycota - ps16
Saccharo/Pezizomycotina - ps17
Saccharomycotina - ps18
Saccharomycotina_1 - ps19
Saccharomycotina_2 - ps20
Saccharomycotina_3 - ps21
Phaff./Saccharomycetaceae - ps22
Saccharomyco./mycetaceae - ps23
Saccharomycetaceae - ps24
Saccharomycetaceae_2 - ps25
Saccharomycetaceae_3 - ps26
Kaz./Nau./Saccharomyces - ps27
Saccharomyces - ps28
Arboricola_clade - ps29
Cerevisiae_clade - ps30
Saccharomyces_cerevisiae - ps31
p-value
2e-02
3e-02
3e-02
4e-02
6e-02
Log-odds
0.5
1.0
1.5
2.0
2.5
3.0
3.5
4.0

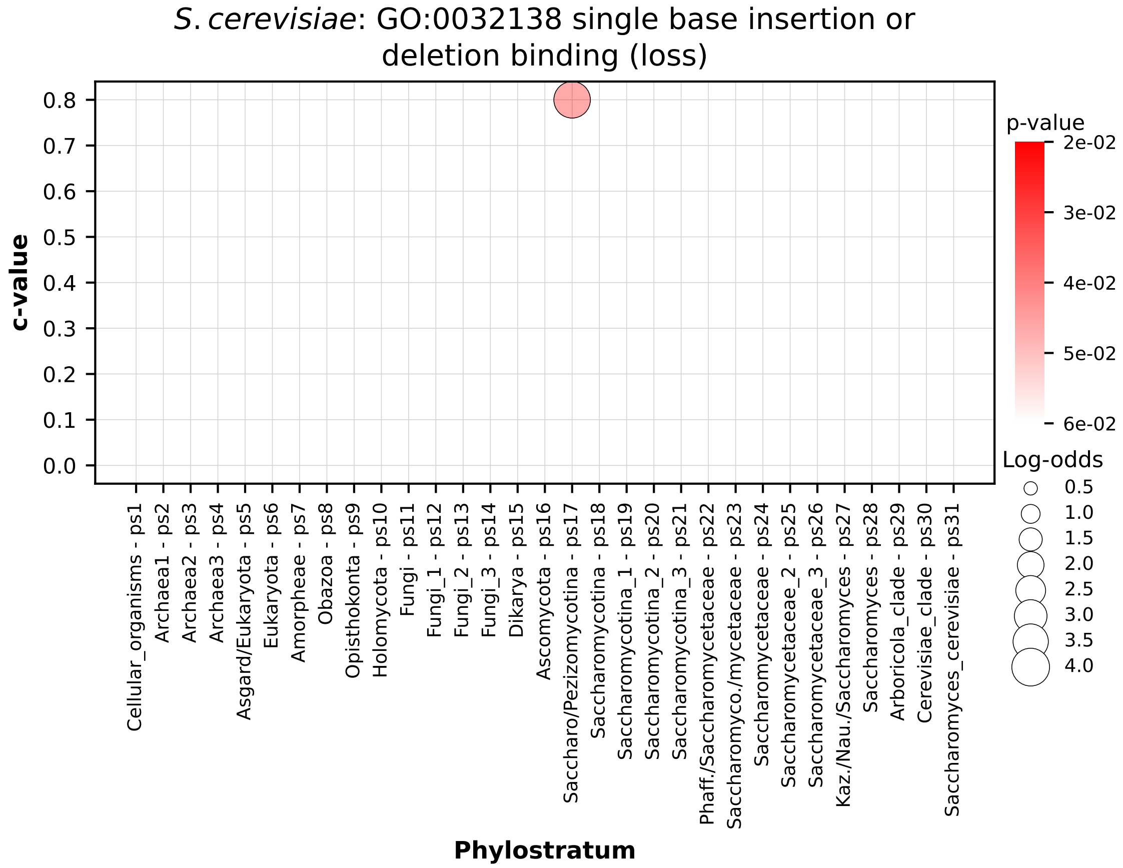

S. cerevisiae: GO:0032138 single base insertion or deletion binding (loss)
c-value
0.8
0.7
0.6
0.5
0.4
0.3
0.2
0.1
0.0
p-value
2e-02
3e-02
4e-02
5e-02
6e-02
Log-odds
0.5
1.0
1.5
2.0
2.5
3.0
3.5
4.0
Cellular_organisms - ps1
Archaea1 - ps2
Archaea2 - ps3
Archaea3 - ps4
Asgard/Eukaryota - ps5
Eukaryota - ps6
Amorpheae - ps7
Obazoa - ps8
Opisthokonta - ps9
Holomycota - ps10
Fungi - ps11
Fungi_1 - ps12
Fungi_2 - ps13
Fungi_3 - ps14
Dikarya - ps15
Ascomycota - ps16
Saccharo/Pezizomycotina - ps17
Saccharomycotina - ps18
Saccharomycotina_1 - ps19
Saccharomycotina_2 - ps20
Saccharomycotina_3 - ps21
Phaff./Saccharomycetaceae - ps22
Saccharomyco./mycetaceae - ps23
Saccharomycetaceae - ps24
Saccharomycetaceae_2 - ps25
Saccharomycetaceae_3 - ps26
Kaz./Nau./Saccharomyces - ps27
Saccharomyces - ps28
Arboricola_clade - ps29
Cerevisiae_clade - ps30
Saccharomyces_cerevisiae - ps31
Phylostratum

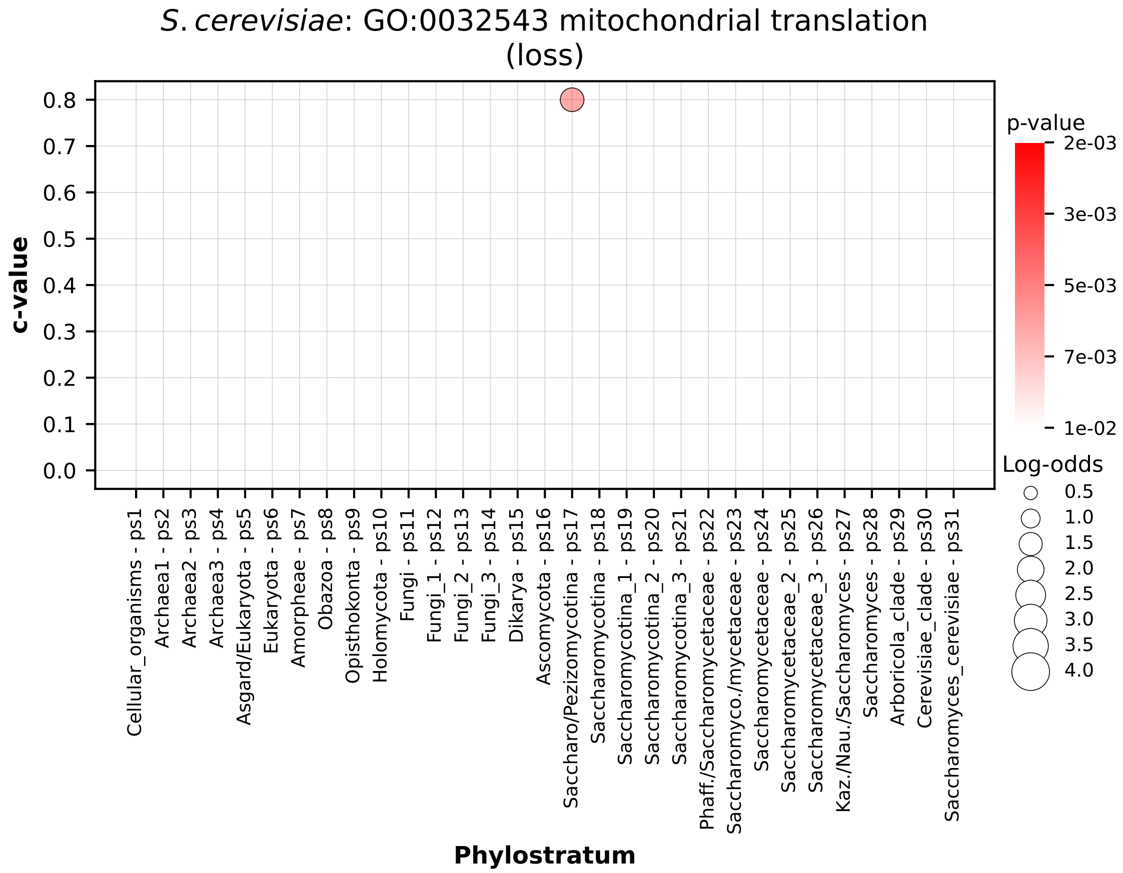
S. cerevisiae: GO:0032543 mitochondrial translation
(loss)
c-value
0.8
0.7
0.6
0.5
0.4
0.3
0.2
0.1
0.0
p-value
2e-03
3e-03
5e-03
7e-03
1e-02
Log-odds
0.5
1.0
1.5
2.0
2.5
3.0
3.5
4.0
Cellular_organisms - ps1
Archaea1 - ps2
Archaea2 - ps3
Archaea3 - ps4
Asgard/Eukaryota - ps5
Eukaryota - ps6
Amorpheae - ps7
Obazoa - ps8
Opisthokonta - ps9
Holomycota - ps10
Fungi - ps11
Fungi_1 - ps12
Fungi_2 - ps13
Fungi_3 - ps14
Dikarya - ps15
Ascomycota - ps16
Saccharo/Pezizomycotina - ps17
Saccharomycotina - ps18
Saccharomycotina_1 - ps19
Saccharomycotina_2 - ps20
Saccharomycotina_3 - ps21
Phaff./Saccharomycetaceae - ps22
Saccharomyco./mycetaceae - ps23
Saccharomycetaceae - ps24
Saccharomycetaceae_2 - ps25
Saccharomycetaceae_3 - ps26
Kaz./Nau./Saccharomyces - ps27
Saccharomyces - ps28
Arboricola_clade - ps29
Cerevisiae_clade - ps30
Saccharomyces_cerevisiae - ps31
Phylostratum

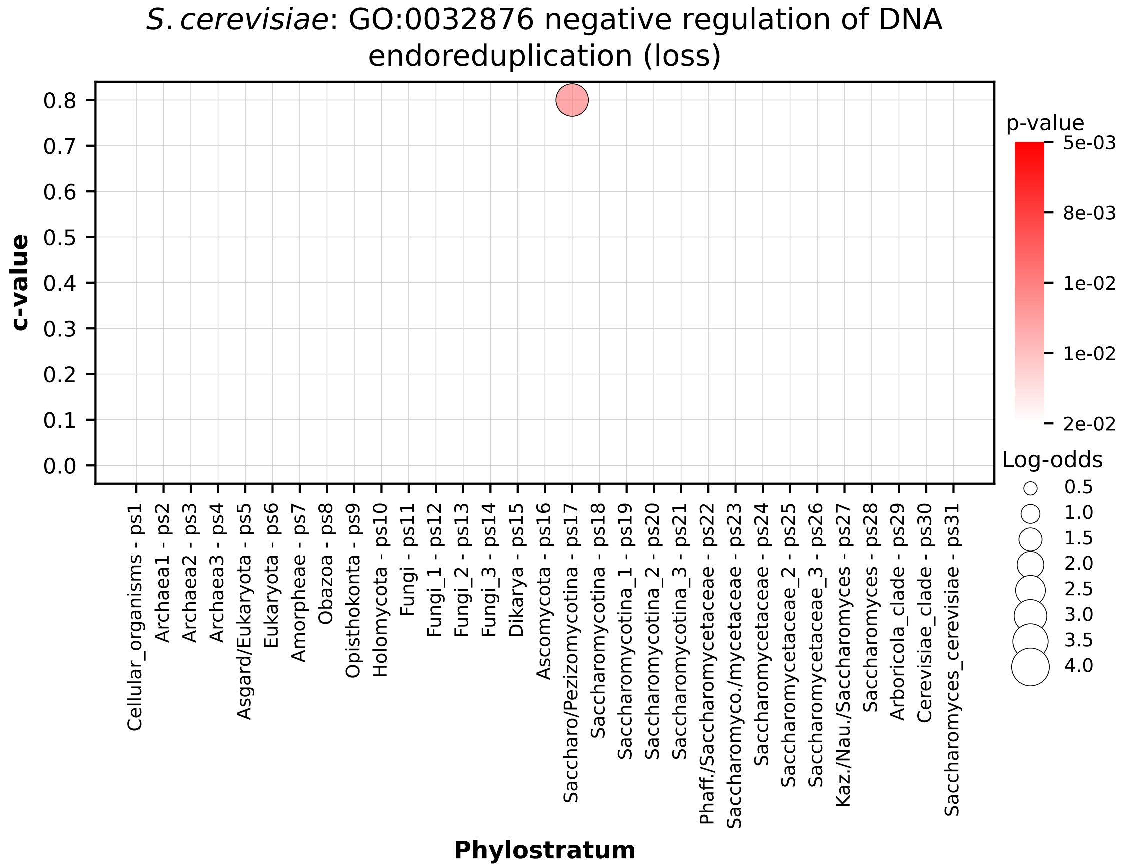

S. cerevisiae: GO:0032876 negative regulation of DNA endoreduplication (loss)
c-value
0.0
0.1
0.2
0.3
0.4
0.5
0.6
0.7
0.8
p-value
5e-03
8e-03
1e-02
1e-02
2e-02
Log-odds
0.5
1.0
1.5
2.0
2.5
3.0
3.5
4.0
Cellular_organisms - ps1
Archaea1 - ps2
Archaea2 - ps3
Archaea3 - ps4
Asgard/Eukaryota - ps5
Eukaryota - ps6
Amorpheae - ps7
Obazoa - ps8
Opisthokonta - ps9
Holomycota - ps10
Fungi - ps11
Fungi_1 - ps12
Fungi_2 - ps13
Fungi_3 - ps14
Dikarya - ps15
Ascomycota - ps16
Saccharo/Pezizomycotina - ps17
Saccharomycotina - ps18
Saccharomycotina_1 - ps19
Saccharomycotina_2 - ps20
Saccharomycotina_3 - ps21
Phaff./Saccharomycetaceae - ps22
Saccharomyco./mycetaceae - ps23
Saccharomycetaceae - ps24
Saccharomycetaceae_2 - ps25
Saccharomycetaceae_3 - ps26
Kaz./Nau./Saccharomyces - ps27
Saccharomyces - ps28
Arboricola_clade - ps29
Cerevisiae_clade - ps30
Saccharomyces_cerevisiae - ps31
Phylostratum

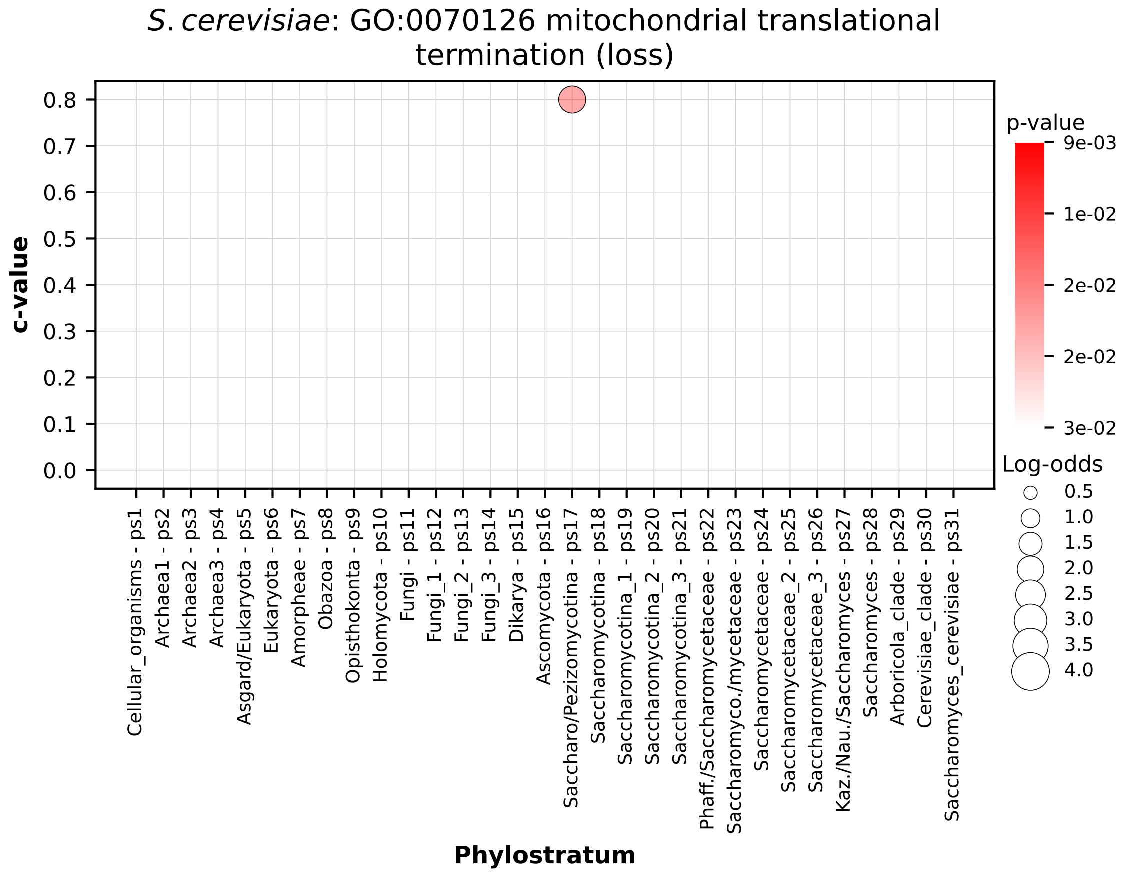
S. cerevisiae: GO:0070126 mitochondrial translational termination (loss)
c-value
0.8
0.7
0.6
0.5
0.4
0.3
0.2
0.1
0.0
Phylostratum
Cellular_organisms - ps1
Archaea1 - ps2
Archaea2 - ps3
Archaea3 - ps4
Asgard/Eukaryota - ps5
Eukaryota - ps6
Amorpheae - ps7
Obazoa - ps8
Opisthokonta - ps9
Holomycota - ps10
Fungi - ps11
Fungi_1 - ps12
Fungi_2 - ps13
Fungi_3 - ps14
Dikarya - ps15
Ascomycota - ps16
Saccharo/Pezizomycotina - ps17
Saccharomycotina - ps18
Saccharomycotina_1 - ps19
Saccharomycotina_2 - ps20
Saccharomycotina_3 - ps21
Phaff./Saccharomycetaceae - ps22
Saccharomyco./mycetaceae - ps23
Saccharomycetaceae - ps24
Saccharomycetaceae_2 - ps25
Saccharomycetaceae_3 - ps26
Kaz./Nau./Saccharomyces - ps27
Saccharomyces - ps28
Arboricola_clade - ps29
Cerevisiae_clade - ps30
Saccharomyces_cerevisiae - ps31
p-value
9e-03
1e-02
2e-02
2e-02
3e-02
Log-odds
0.5
1.0
1.5
2.0
2.5
3.0
3.5
4.0

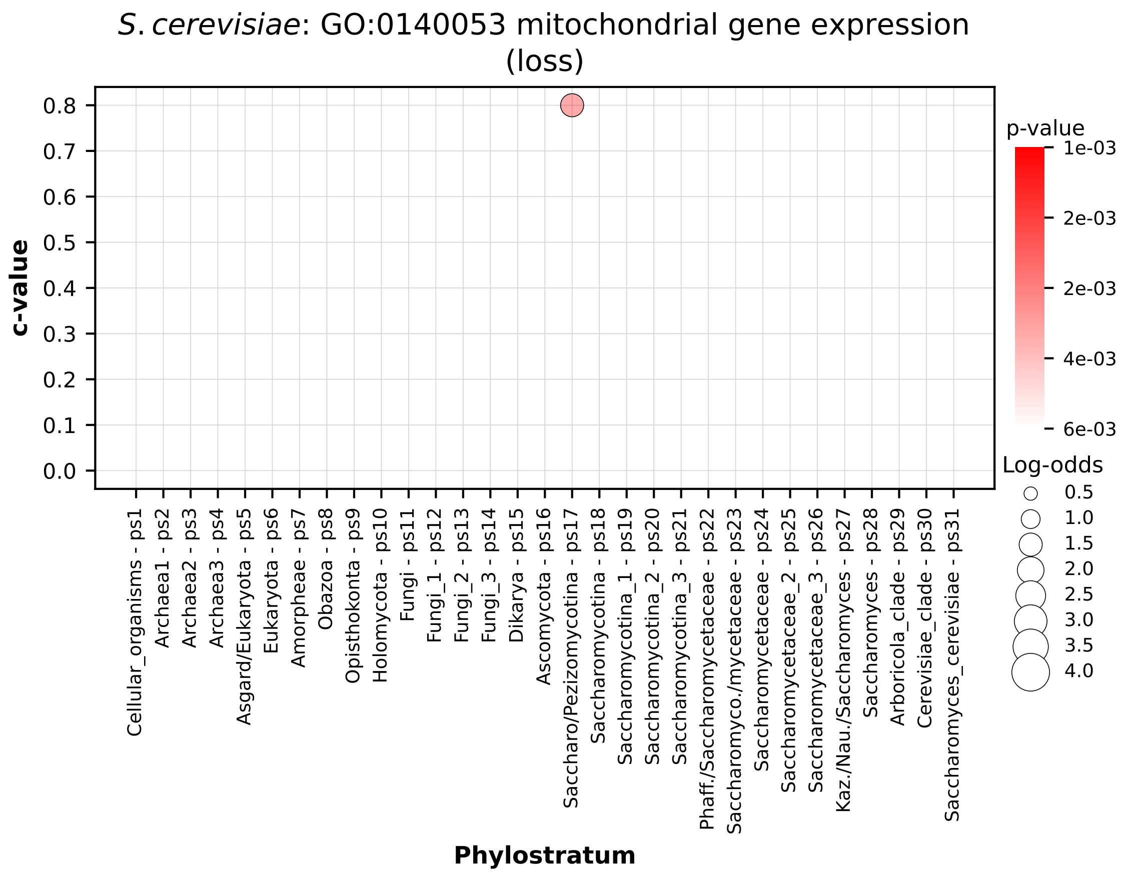

S. cerevisiae: GO:0140053 mitochondrial gene expression (loss)
c-value
0.8
0.7
0.6
0.5
0.4
0.3
0.2
0.1
0.0
p-value
1e-03
2e-03
2e-03
4e-03
6e-03
Log-odds
0.5
1.0
1.5
2.0
2.5
3.0
3.5
4.0
Cellular_organisms - ps1
Archaea1 - ps2
Archaea2 - ps3
Archaea3 - ps4
Asgard/Eukaryota - ps5
Eukaryota - ps6
Amorpheae - ps7
Obazoa - ps8
Opisthokonta - ps9
Holomycota - ps10
Fungi - ps11
Fungi_1 - ps12
Fungi_2 - ps13
Fungi_3 - ps14
Dikarya - ps15
Ascomycota - ps16
Saccharo/Pezizomycotina - ps17
Saccharomycotina - ps18
Saccharomycotina_1 - ps19
Saccharomycotina_2 - ps20
Saccharomycotina_3 - ps21
Phaff./Saccharomycetaceae - ps22
Saccharomyco./mycetaceae - ps23
Saccharomycetaceae - ps24
Saccharomycetaceae_2 - ps25
Saccharomycetaceae_3 - ps26
Kaz./Nau./Saccharomyces - ps27
Saccharomyces - ps28
Arboricola_clade - ps29
Cerevisiae_clade - ps30
Saccharomyces_cerevisiae - ps31
Phylostratum

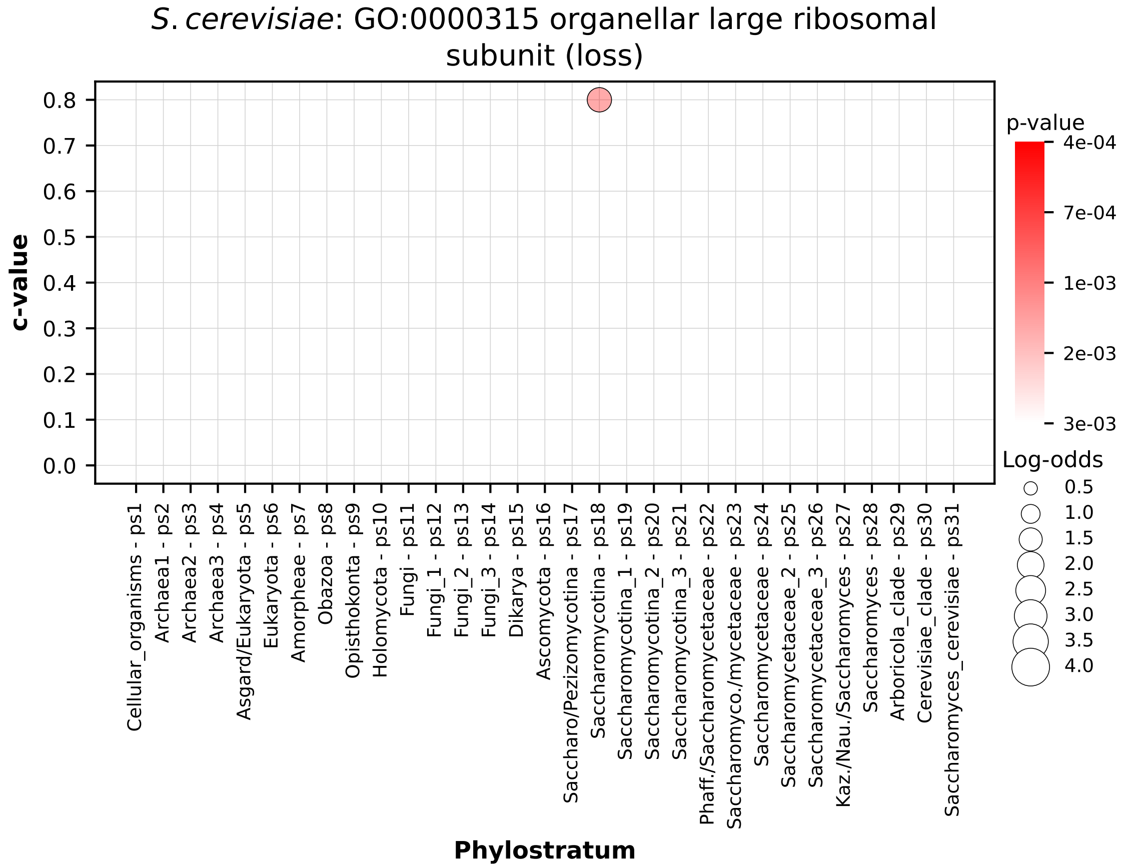
S. cerevisiae: GO:0000315 organellar large ribosomal subunit (loss)
c-value
0.0
0.1
0.2
0.3
0.4
0.5
0.6
0.7
0.8
p-value
4e-04
7e-04
1e-03
2e-03
3e-03
Log-odds
0.5
1.0
1.5
2.0
2.5
3.0
3.5
4.0
Cellular_organisms - ps1
Archaea1 - ps2
Archaea2 - ps3
Archaea3 - ps4
Asgard/Eukaryota - ps5
Eukaryota - ps6
Amorpheae - ps7
Obazoa - ps8
Opisthokonta - ps9
Holomycota - ps10
Fungi - ps11
Fungi_1 - ps12
Fungi_2 - ps13
Fungi_3 - ps14
Dikarya - ps15
Ascomycota - ps16
Saccharo/Pezizomycotina - ps17
Saccharomycotina - ps18
Saccharomycotina_1 - ps19
Saccharomycotina_2 - ps20
Saccharomycotina_3 - ps21
Phaff./Saccharomycetaceae - ps22
Saccharomyco./mycetaceae - ps23
Saccharomycetaceae - ps24
Saccharomycetaceae_2 - ps25
Saccharomycetaceae_3 - ps26
Kaz./Nau./Saccharomyces - ps27
Saccharomyces - ps28
Arboricola_clade - ps29
Cerevisiae_clade - ps30
Saccharomyces_cerevisiae - ps31
Phylostratum

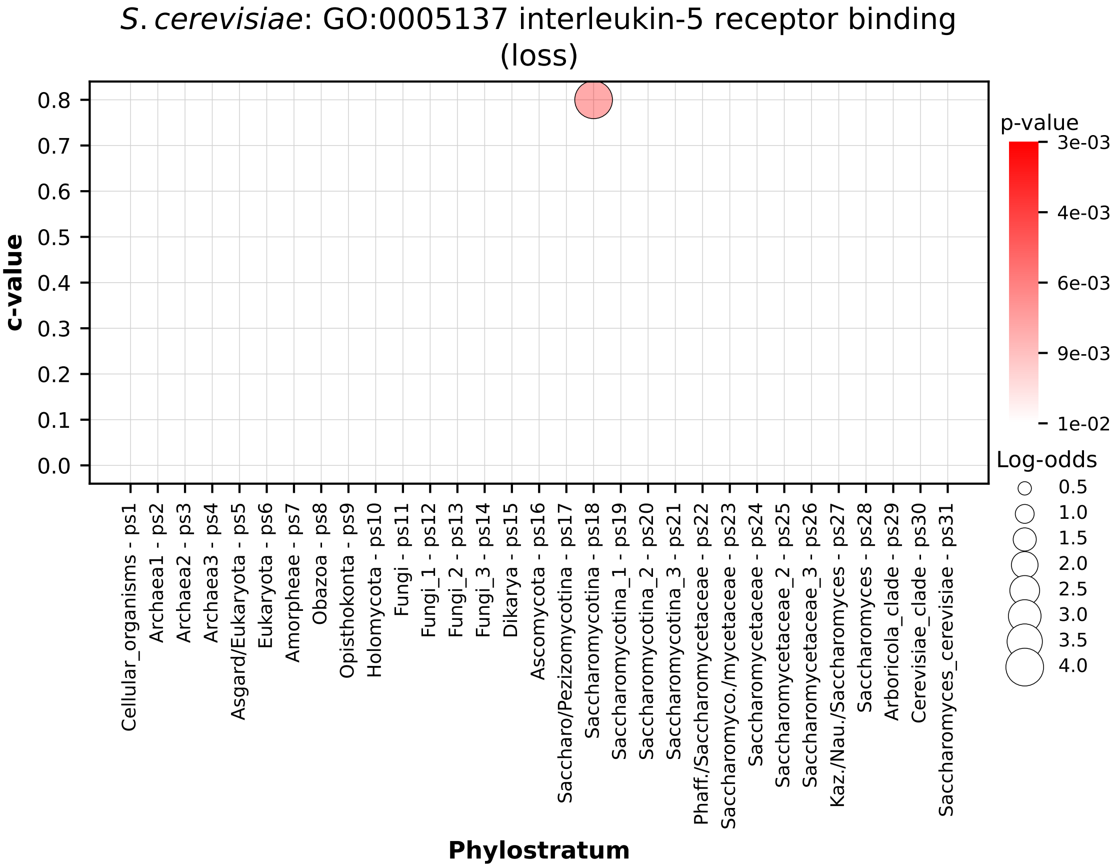

S. cerevisiae: GO:0005137 interleukin-5 receptor binding
(loss)
c-value
0.8
0.7
0.6
0.5
0.4
0.3
0.2
0.1
0.0
p-value
3e-03
4e-03
6e-03
9e-03
1e-02
Log-odds
0.5
1.0
1.5
2.0
2.5
3.0
3.5
4.0
Cellular_organisms - ps1
Archaea1 - ps2
Archaea2 - ps3
Archaea3 - ps4
Asgard/Eukaryota - ps5
Eukaryota - ps6
Amorpheae - ps7
Obazoa - ps8
Opisthokonta - ps9
Holomycota - ps10
Fungi - ps11
Fungi_1 - ps12
Fungi_2 - ps13
Fungi_3 - ps14
Dikarya - ps15
Ascomycota - ps16
Saccharo/Pezizomycotina - ps17
Saccharomycotina - ps18
Saccharomycotina_1 - ps19
Saccharomycotina_2 - ps20
Saccharomycotina_3 - ps21
Phaff./Saccharomycetaceae - ps22
Saccharomyco./mycetaceae - ps23
Saccharomycetaceae - ps24
Saccharomycetaceae_2 - ps25
Saccharomycetaceae_3 - ps26
Kaz./Nau./Saccharomyces - ps27
Saccharomyces - ps28
Arboricola_clade - ps29
Cerevisiae_clade - ps30
Saccharomyces_cerevisiae - ps31
Phylostratum

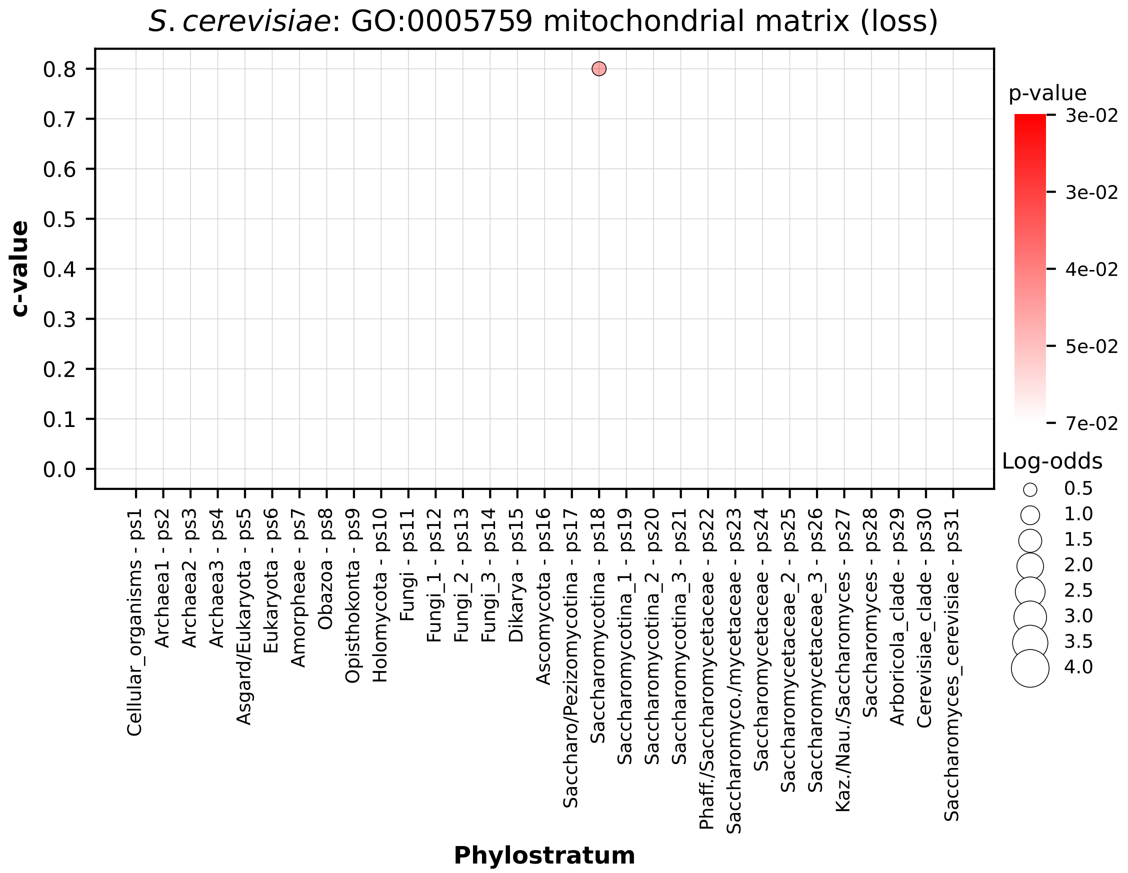

S. cerevisiae: GO:0005759 mitochondrial matrix (loss)
c-value
0.0
0.1
0.2
0.3
0.4
0.5
0.6
0.7
0.8
p-value
3e-02
3e-02
4e-02
5e-02
7e-02
Log-odds
0.5
1.0
1.5
2.0
2.5
3.0
3.5
4.0
Cellular_organisms - ps1
Archaea1 - ps2
Archaea2 - ps3
Archaea3 - ps4
Asgard/Eukaryota - ps5
Eukaryota - ps6
Amorpheae - ps7
Obazoa - ps8
Opisthokonta - ps9
Holomycota - ps10
Fungi - ps11
Fungi_1 - ps12
Fungi_2 - ps13
Fungi_3 - ps14
Dikarya - ps15
Ascomycota - ps16
Saccharo/Pezizomycotina - ps17
Saccharomycotina - ps18
Saccharomycotina_1 - ps19
Saccharomycotina_2 - ps20
Saccharomycotina_3 - ps21
Phaff./Saccharomycetaceae - ps22
Saccharomyco./mycetaceae - ps23
Saccharomycetaceae - ps24
Saccharomycetaceae_2 - ps25
Saccharomycetaceae_3 - ps26
Kaz./Nau./Saccharomyces - ps27
Saccharomyces - ps28
Arboricola_clade - ps29
Cerevisiae_clade - ps30
Saccharomyces_cerevisiae - ps31
Phylostratum

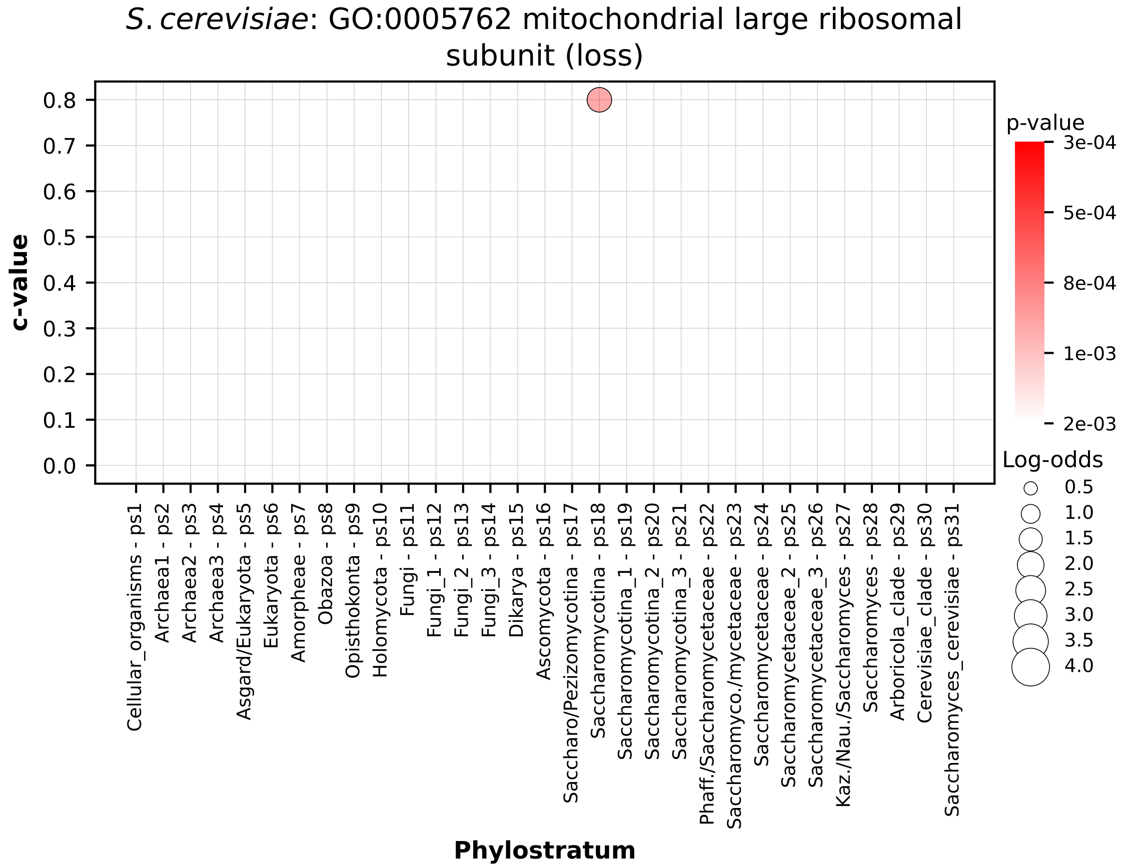
S. cerevisiae: GO:0005762 mitochondrial large ribosomal subunit (loss)
c-value
0.8
0.7
0.6
0.5
0.4
0.3
0.2
0.1
0.0
p-value
3e-04
5e-04
8e-04
1e-03
2e-03
Log-odds
0.5
1.0
1.5
2.0
2.5
3.0
3.5
4.0
Cellular_organisms - ps1
Archaea1 - ps2
Archaea2 - ps3
Archaea3 - ps4
Asgard/Eukaryota - ps5
Eukaryota - ps6
Amorpheae - ps7
Obazoa - ps8
Opisthokonta - ps9
Holomycota - ps10
Fungi - ps11
Fungi_1 - ps12
Fungi_2 - ps13
Fungi_3 - ps14
Dikarya - ps15
Ascomycota - ps16
Saccharo/Pezizomycotina - ps17
Saccharomycotina - ps18
Saccharomycotina_1 - ps19
Saccharomycotina_2 - ps20
Saccharomycotina_3 - ps21
Phaff./Saccharomycetaceae - ps22
Saccharomyco./mycetaceae - ps23
Saccharomycetaceae - ps24
Saccharomycetaceae_2 - ps25
Saccharomycetaceae_3 - ps26
Kaz./Nau./Saccharomyces - ps27
Saccharomyces - ps28
Arboricola_clade - ps29
Cerevisiae_clade - ps30
Saccharomyces_cerevisiae - ps31
Phylostratum

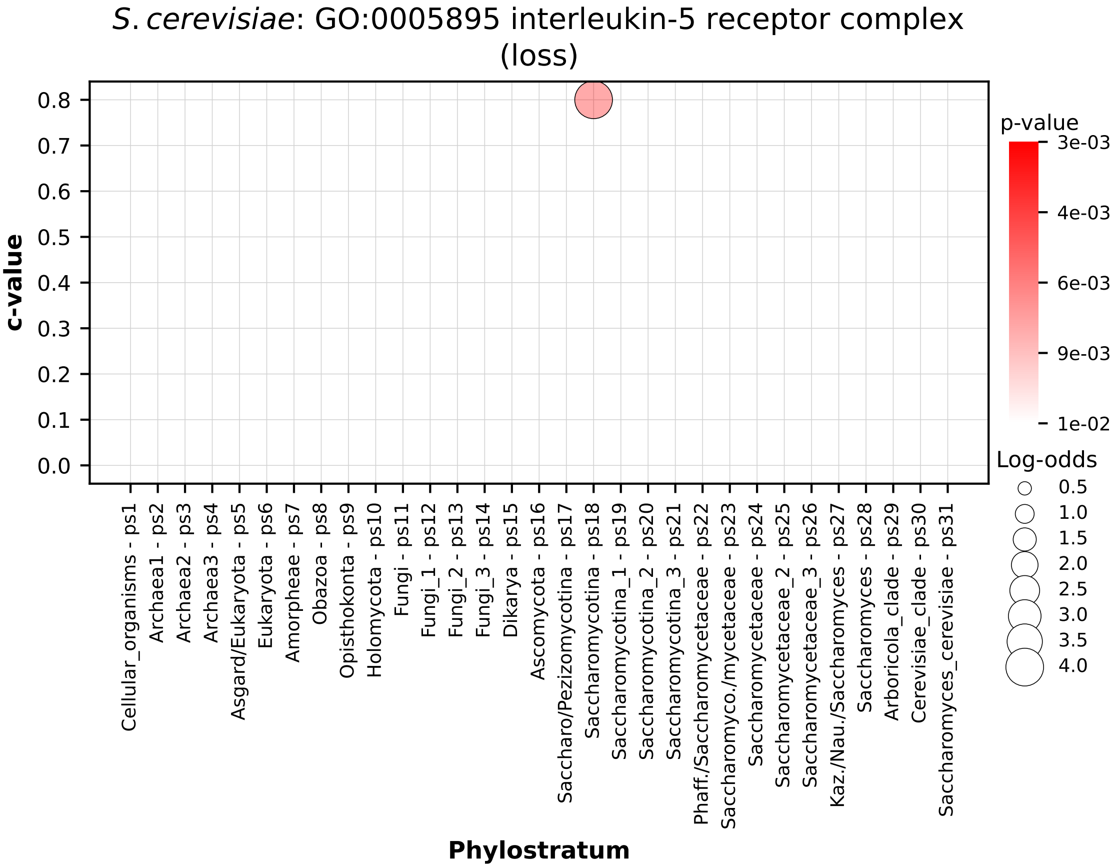

S. cerevisiae: GO:0005895 interleukin-5 receptor complex
(loss)
c-value
0.0
0.1
0.2
0.3
0.4
0.5
0.6
0.7
0.8
Phylostratum
Cellular_organisms - ps1
Archaea1 - ps2
Archaea2 - ps3
Archaea3 - ps4
Asgard/Eukaryota - ps5
Eukaryota - ps6
Amorpheae - ps7
Obazoa - ps8
Opisthokonta - ps9
Holomycota - ps10
Fungi - ps11
Fungi_1 - ps12
Fungi_2 - ps13
Fungi_3 - ps14
Dikarya - ps15
Ascomycota - ps16
Saccharo/Pezizomycotina - ps17
Saccharomycotina - ps18
Saccharomycotina_1 - ps19
Saccharomycotina_2 - ps20
Saccharomycotina_3 - ps21
Phaff./Saccharomycetaceae - ps22
Saccharomyco./mycetaceae - ps23
Saccharomycetaceae - ps24
Saccharomycetaceae_2 - ps25
Saccharomycetaceae_3 - ps26
Kaz./Nau./Saccharomyces - ps27
Saccharomyces - ps28
Arboricola_clade - ps29
Cerevisiae_clade - ps30
Saccharomyces_cerevisiae - ps31
p-value
3e-03
4e-03
6e-03
9e-03
1e-02
Log-odds
0.5
1.0
1.5
2.0
2.5
3.0
3.5
4.0

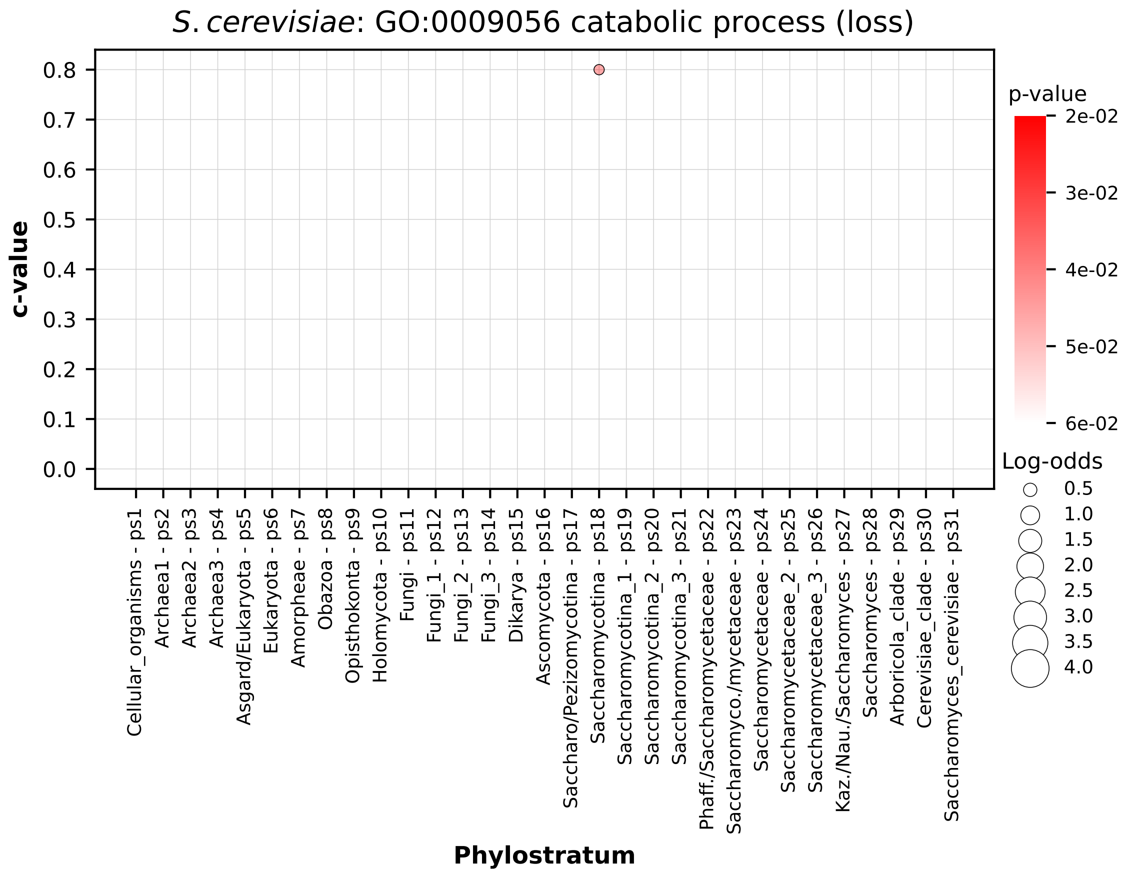
S. cerevisiae: GO:0009056 catabolic process (loss)
c-value
0.0
0.1
0.2
0.3
0.4
0.5
0.6
0.7
0.8
Phylostratum
Cellular_organisms - ps1
Archaea1 - ps2
Archaea2 - ps3
Archaea3 - ps4
Asgard/Eukaryota - ps5
Eukaryota - ps6
Amorpheae - ps7
Obazoa - ps8
Opisthokonta - ps9
Holomycota - ps10
Fungi - ps11
Fungi_1 - ps12
Fungi_2 - ps13
Fungi_3 - ps14
Dikarya - ps15
Ascomycota - ps16
Saccharo/Pezizomycotina - ps17
Saccharomycotina - ps18
Saccharomycotina_1 - ps19
Saccharomycotina_2 - ps20
Saccharomycotina_3 - ps21
Phaff./Saccharomycetaceae - ps22
Saccharomyco./mycetaceae - ps23
Saccharomycetaceae - ps24
Saccharomycetaceae_2 - ps25
Saccharomycetaceae_3 - ps26
Kaz./Nau./Saccharomyces - ps27
Saccharomyces - ps28
Arboricola_clade - ps29
Cerevisiae_clade - ps30
Saccharomyces_cerevisiae - ps31
p-value
2e-02
3e-02
4e-02
5e-02
6e-02
Log-odds
0.5
1.0
1.5
2.0
2.5
3.0
3.5
4.0

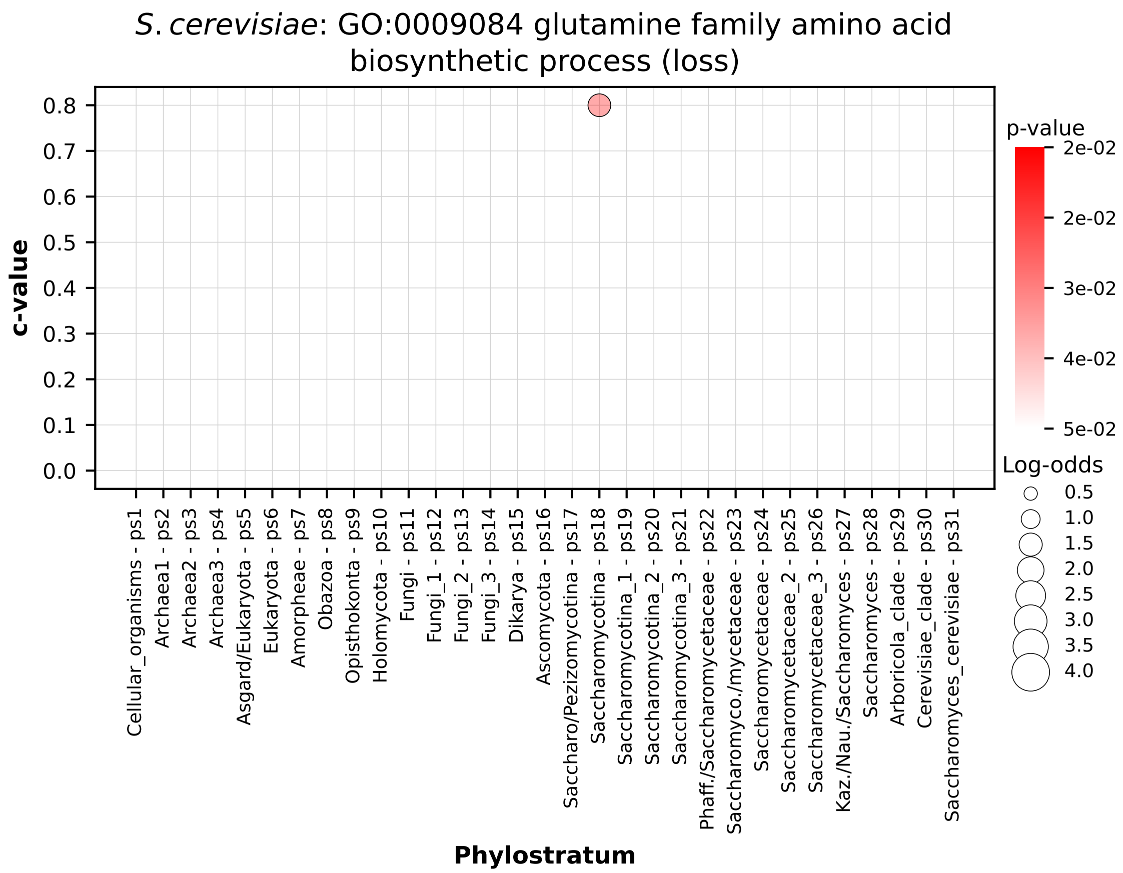

S. cerevisiae: GO:0009084 glutamine family amino acid biosynthetic process (loss)
c-value
0.8
0.7
0.6
0.5
0.4
0.3
0.2
0.1
0.0
p-value
2e-02
2e-02
3e-02
4e-02
5e-02
Log-odds
0.5
1.0
1.5
2.0
2.5
3.0
3.5
4.0
Cellular_organisms - ps1
Archaea1 - ps2
Archaea2 - ps3
Archaea3 - ps4
Asgard/Eukaryota - ps5
Eukaryota - ps6
Amorpheae - ps7
Obazoa - ps8
Opisthokonta - ps9
Holomycota - ps10
Fungi - ps11
Fungi_1 - ps12
Fungi_2 - ps13
Fungi_3 - ps14
Dikarya - ps15
Ascomycota - ps16
Saccharo/Pezizomycotina - ps17
Saccharomycotina - ps18
Saccharomycotina_1 - ps19
Saccharomycotina_2 - ps20
Saccharomycotina_3 - ps21
Phaff./Saccharomycetaceae - ps22
Saccharomyco./mycetaceae - ps23
Saccharomycetaceae - ps24
Saccharomycetaceae_2 - ps25
Saccharomycetaceae_3 - ps26
Kaz./Nau./Saccharomyces - ps27
Saccharomyces - ps28
Arboricola_clade - ps29
Cerevisiae_clade - ps30
Saccharomyces_cerevisiae - ps31
Phylostratum

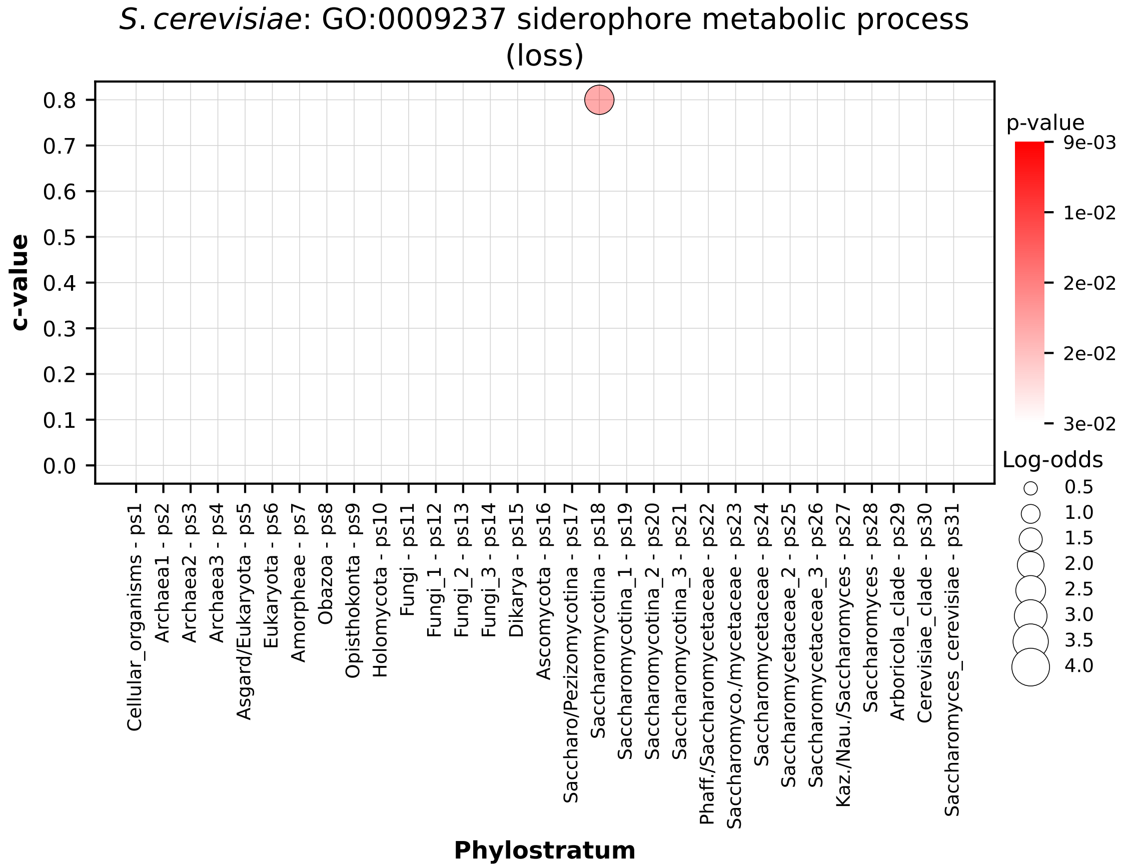
S. cerevisiae: GO:0009237 siderophore metabolic process
(loss)
c-value
0.0
0.1
0.2
0.3
0.4
0.5
0.6
0.7
0.8
p-value
9e-03
1e-02
2e-02
2e-02
3e-02
Log-odds
0.5
1.0
1.5
2.0
2.5
3.0
3.5
4.0
Cellular_organisms - ps1
Archaea1 - ps2
Archaea2 - ps3
Archaea3 - ps4
Asgard/Eukaryota - ps5
Eukaryota - ps6
Amorpheae - ps7
Obazoa - ps8
Opisthokonta - ps9
Holomycota - ps10
Fungi - ps11
Fungi_1 - ps12
Fungi_2 - ps13
Fungi_3 - ps14
Dikarya - ps15
Ascomycota - ps16
Saccharo/Pezizomycotina - ps17
Saccharomycotina - ps18
Saccharomycotina_1 - ps19
Saccharomycotina_2 - ps20
Saccharomycotina_3 - ps21
Phaff./Saccharomycetaceae - ps22
Saccharomyco./mycetaceae - ps23
Saccharomycetaceae - ps24
Saccharomycetaceae_2 - ps25
Saccharomycetaceae_3 - ps26
Kaz./Nau./Saccharomyces - ps27
Saccharomyces - ps28
Arboricola_clade - ps29
Cerevisiae_clade - ps30
Saccharomyces_cerevisiae - ps31
Phylostratum

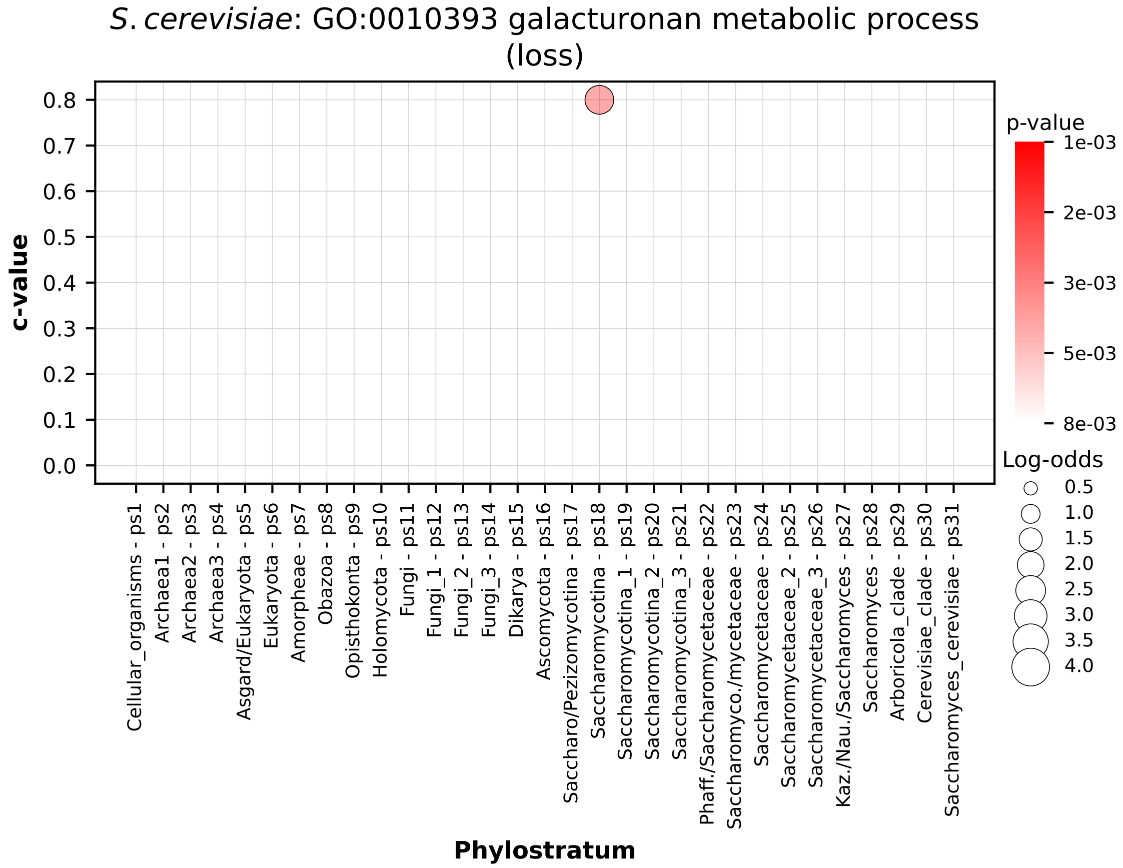

S. cerevisiae: GO:0010393 galacturonan metabolic process
(loss)
c-value
0.8
0.7
0.6
0.5
0.4
0.3
0.2
0.1
0.0
p-value
1e-03
2e-03
3e-03
5e-03
8e-03
Log-odds
0.5
1.0
1.5
2.0
2.5
3.0
3.5
4.0
Cellular_organisms - ps1
Archaea1 - ps2
Archaea2 - ps3
Archaea3 - ps4
Asgard/Eukaryota - ps5
Eukaryota - ps6
Amorpheae - ps7
Obazoa - ps8
Opisthokonta - ps9
Holomycota - ps10
Fungi - ps11
Fungi_1 - ps12
Fungi_2 - ps13
Fungi_3 - ps14
Dikarya - ps15
Ascomycota - ps16
Saccharo/Pezizomycotina - ps17
Saccharomycotina - ps18
Saccharomycotina_1 - ps19
Saccharomycotina_2 - ps20
Saccharomycotina_3 - ps21
Phaff./Saccharomycetaceae - ps22
Saccharomyco./mycetaceae - ps23
Saccharomycetaceae - ps24
Saccharomycetaceae_2 - ps25
Saccharomycetaceae_3 - ps26
Kaz./Nau./Saccharomyces - ps27
Saccharomyces - ps28
Arboricola_clade - ps29
Cerevisiae_clade - ps30
Saccharomyces_cerevisiae - ps31
Phylostratum

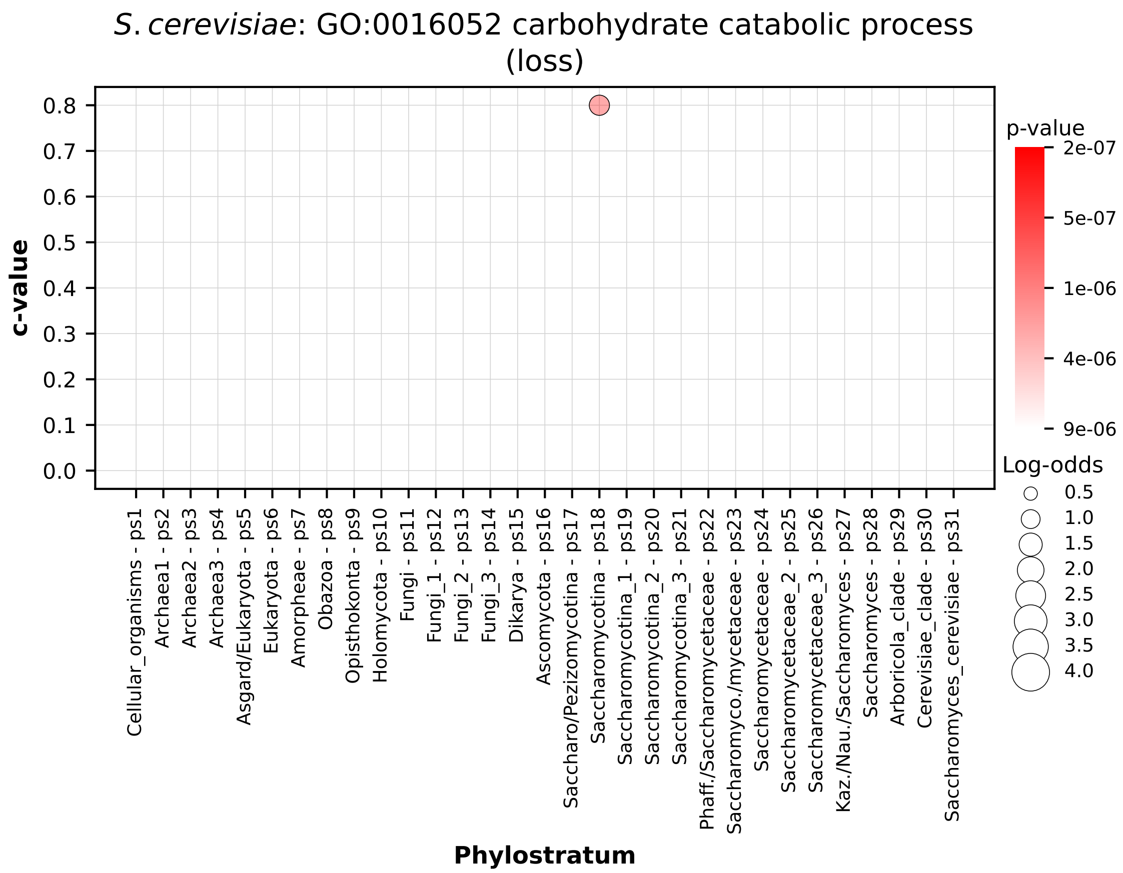

S. cerevisiae: GO:0016052 carbohydrate catabolic process (loss)
c-value
0.0
0.1
0.2
0.3
0.4
0.5
0.6
0.7
0.8
p-value
2e-07
5e-07
1e-06
4e-06
9e-06
Log-odds
0.5
1.0
1.5
2.0
2.5
3.0
3.5
4.0
Cellular_organisms - ps1
Archaea1 - ps2
Archaea2 - ps3
Archaea3 - ps4
Asgard/Eukaryota - ps5
Eukaryota - ps6
Amorpheae - ps7
Obazoa - ps8
Opisthokonta - ps9
Holomycota - ps10
Fungi - ps11
Fungi_1 - ps12
Fungi_2 - ps13
Fungi_3 - ps14
Dikarya - ps15
Ascomycota - ps16
Saccharo/Pezizomycotina - ps17
Saccharomycotina - ps18
Saccharomycotina_1 - ps19
Saccharomycotina_2 - ps20
Saccharomycotina_3 - ps21
Phaff./Saccharomycetaceae - ps22
Saccharomyco./mycetaceae - ps23
Saccharomycetaceae - ps24
Saccharomycetaceae_2 - ps25
Saccharomycetaceae_3 - ps26
Kaz./Nau./Saccharomyces - ps27
Saccharomyces - ps28
Arboricola_clade - ps29
Cerevisiae_clade - ps30
Saccharomyces_cerevisiae - ps31
Phylostratum

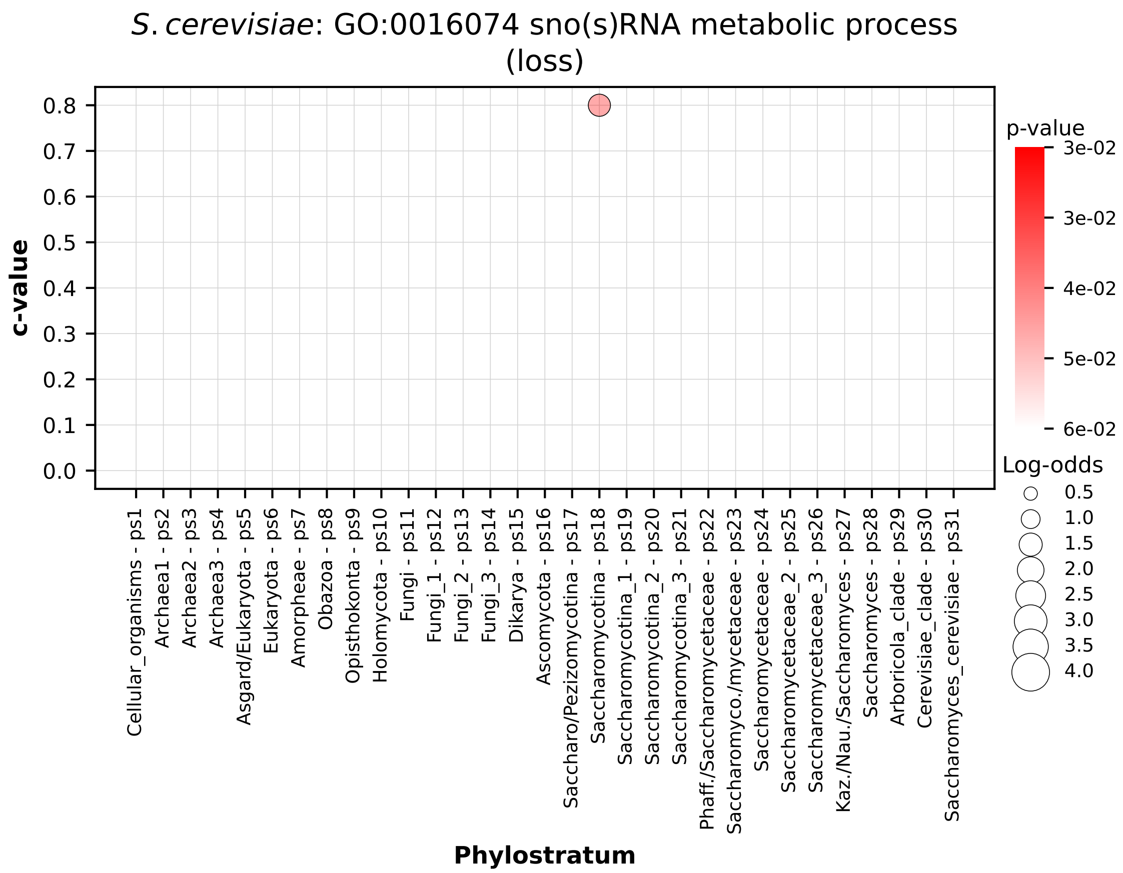
S. cerevisiae: GO:0016074 sno(s)RNA metabolic process
(loss)
c-value
0.8
0.7
0.6
0.5
0.4
0.3
0.2
0.1
0.0
p-value
3e-02
3e-02
4e-02
5e-02
6e-02
Log-odds
0.5
1.0
1.5
2.0
2.5
3.0
3.5
4.0
Cellular_organisms - ps1
Archaea1 - ps2
Archaea2 - ps3
Archaea3 - ps4
Asgard/Eukaryota - ps5
Eukaryota - ps6
Amorpheae - ps7
Obazoa - ps8
Opisthokonta - ps9
Holomycota - ps10
Fungi - ps11
Fungi_1 - ps12
Fungi_2 - ps13
Fungi_3 - ps14
Dikarya - ps15
Ascomycota - ps16
Saccharo/Pezizomycotina - ps17
Saccharomycotina - ps18
Saccharomycotina_1 - ps19
Saccharomycotina_2 - ps20
Saccharomycotina_3 - ps21
Phaff./Saccharomycetaceae - ps22
Saccharomyco./mycetaceae - ps23
Saccharomycetaceae - ps24
Saccharomycetaceae_2 - ps25
Saccharomycetaceae_3 - ps26
Kaz./Nau./Saccharomyces - ps27
Saccharomyces - ps28
Arboricola_clade - ps29
Cerevisiae_clade - ps30
Saccharomyces_cerevisiae - ps31
Phylostratum

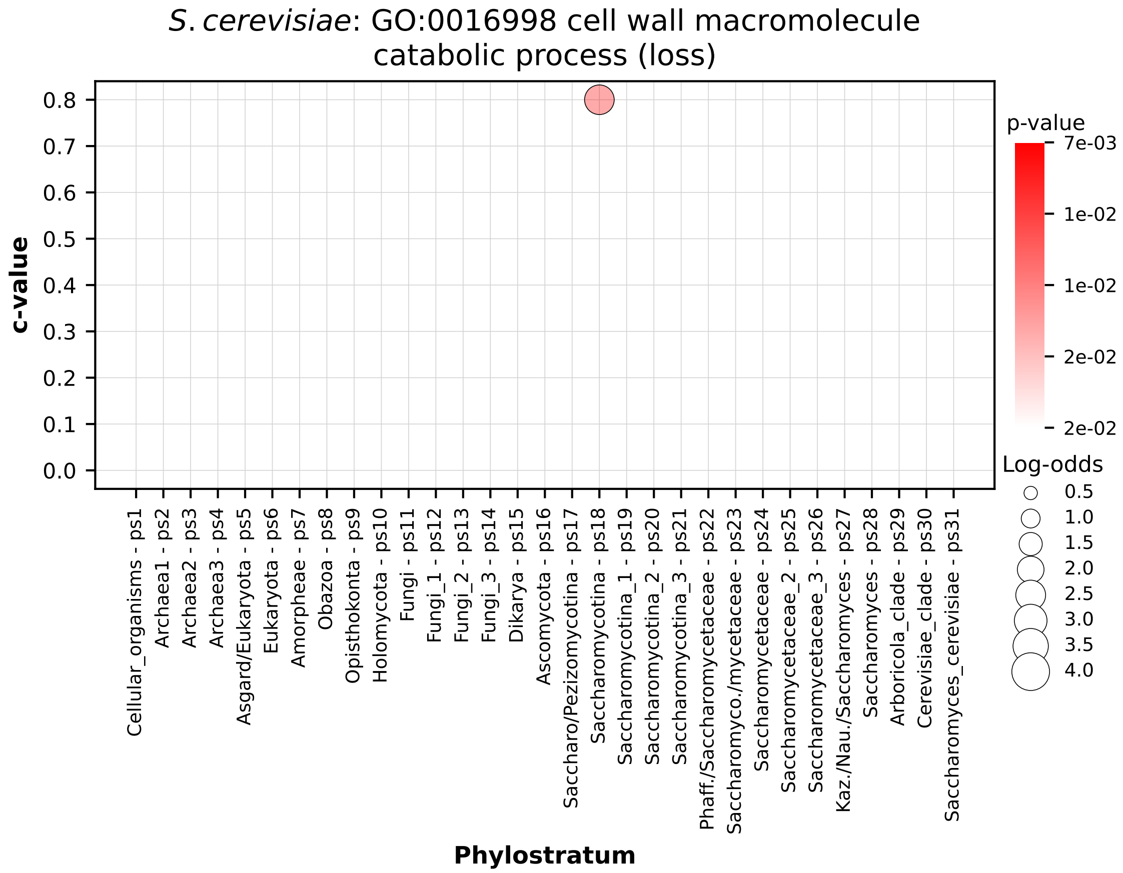
S. cerevisiae: GO:0016998 cell wall macromolecule catabolic process (loss)
c-value
0.0
0.1
0.2
0.3
0.4
0.5
0.6
0.7
0.8
Phylostratum
Cellular_organisms - ps1
Archaea1 - ps2
Archaea2 - ps3
Archaea3 - ps4
Asgard/Eukaryota - ps5
Eukaryota - ps6
Amorpheae - ps7
Obazoa - ps8
Opisthokonta - ps9
Holomycota - ps10
Fungi - ps11
Fungi_1 - ps12
Fungi_2 - ps13
Fungi_3 - ps14
Dikarya - ps15
Ascomycota - ps16
Saccharo/Pezizomycotina - ps17
Saccharomycotina - ps18
Saccharomycotina_1 - ps19
Saccharomycotina_2 - ps20
Saccharomycotina_3 - ps21
Phaff./Saccharomycetaceae - ps22
Saccharomyco./mycetaceae - ps23
Saccharomycetaceae - ps24
Saccharomycetaceae_2 - ps25
Saccharomycetaceae_3 - ps26
Kaz./Nau./Saccharomyces - ps27
Saccharomyces - ps28
Arboricola_clade - ps29
Cerevisiae_clade - ps30
Saccharomyces_cerevisiae - ps31
p-value
7e-03
1e-02
1e-02
2e-02
2e-02
Log-odds
0.5
1.0
1.5
2.0
2.5
3.0
3.5
4.0

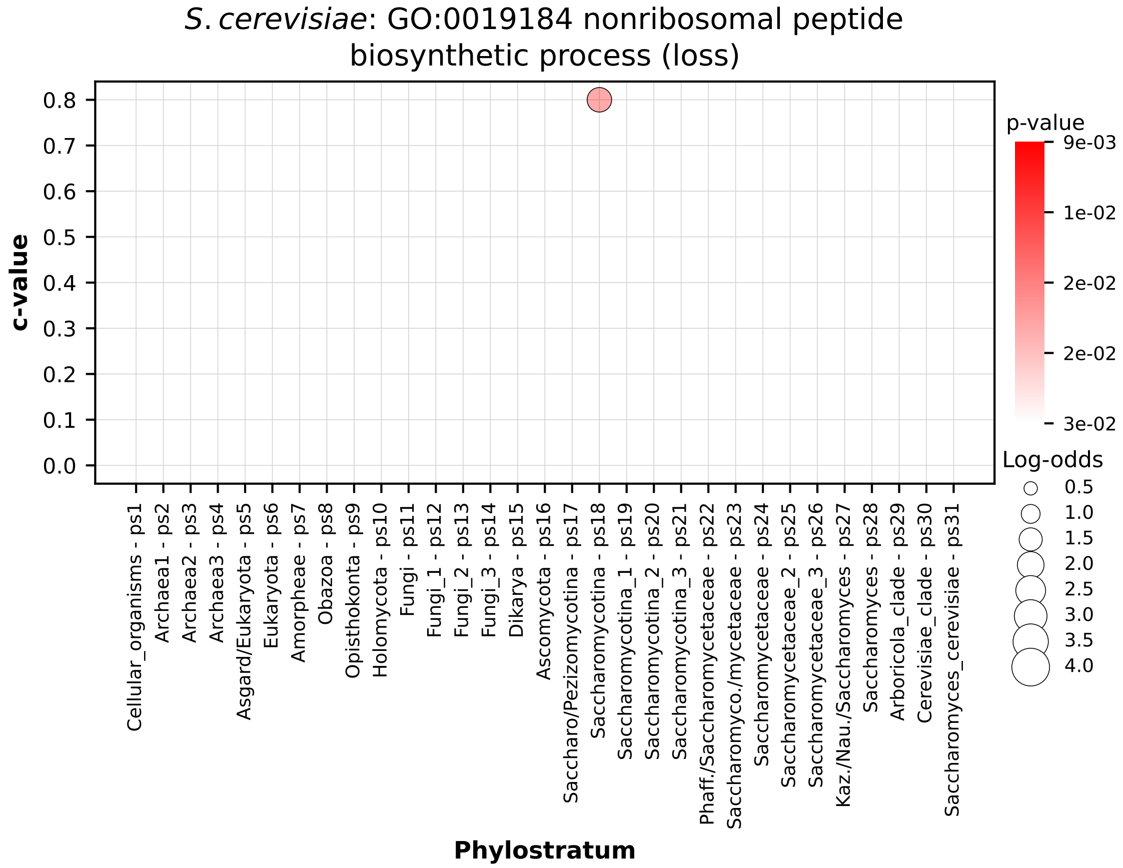
S. cerevisiae: GO:0019184 nonribosomal peptide biosynthetic process (loss)
c-value
0.0
0.1
0.2
0.3
0.4
0.5
0.6
0.7
0.8
p-value
9e-03
1e-02
2e-02
2e-02
3e-02
Log-odds
0.5
1.0
1.5
2.0
2.5
3.0
3.5
4.0
Cellular_organisms - ps1
Archaea1 - ps2
Archaea2 - ps3
Archaea3 - ps4
Asgard/Eukaryota - ps5
Eukaryota - ps6
Amorpheae - ps7
Obazoa - ps8
Opisthokonta - ps9
Holomycota - ps10
Fungi - ps11
Fungi_1 - ps12
Fungi_2 - ps13
Fungi_3 - ps14
Dikarya - ps15
Ascomycota - ps16
Saccharo/Pezizomycotina - ps17
Saccharomycotina - ps18
Saccharomycotina_1 - ps19
Saccharomycotina_2 - ps20
Saccharomycotina_3 - ps21
Phaff./Saccharomycetaceae - ps22
Saccharomyco./mycetaceae - ps23
Saccharomycetaceae - ps24
Saccharomycetaceae_2 - ps25
Saccharomycetaceae_3 - ps26
Kaz./Nau./Saccharomyces - ps27
Saccharomyces - ps28
Arboricola_clade - ps29
Cerevisiae_clade - ps30
Saccharomyces_cerevisiae - ps31
Phylostratum

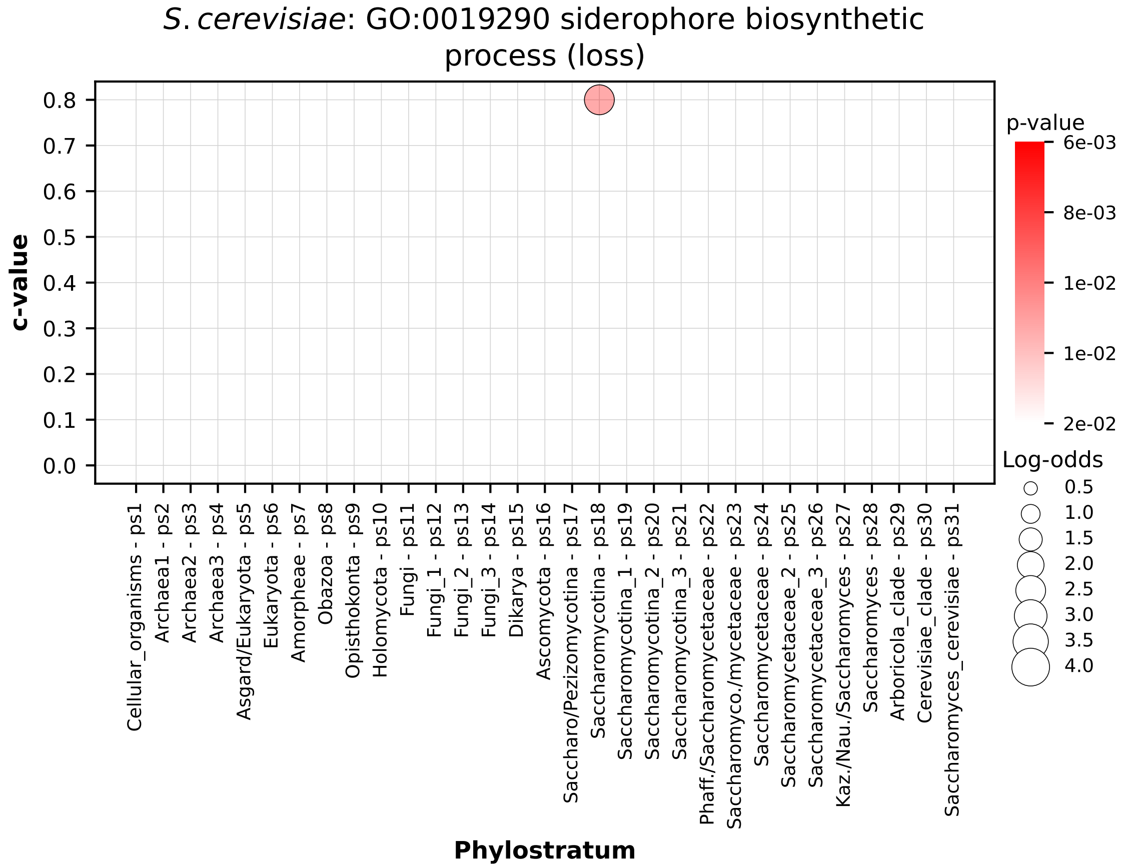

S. cerevisiae: GO:0019290 siderophore biosynthetic process (loss)
c-value
0.0
0.1
0.2
0.3
0.4
0.5
0.6
0.7
0.8
Phylostratum
Cellular_organisms - ps1
Archaea1 - ps2
Archaea2 - ps3
Archaea3 - ps4
Asgard/Eukaryota - ps5
Eukaryota - ps6
Amorpheae - ps7
Obazoa - ps8
Opisthokonta - ps9
Holomycota - ps10
Fungi - ps11
Fungi_1 - ps12
Fungi_2 - ps13
Fungi_3 - ps14
Dikarya - ps15
Ascomycota - ps16
Saccharo/Pezizomycotina - ps17
Saccharomycotina - ps18
Saccharomycotina_1 - ps19
Saccharomycotina_2 - ps20
Saccharomycotina_3 - ps21
Phaff./Saccharomycetaceae - ps22
Saccharomyco./mycetaceae - ps23
Saccharomycetaceae - ps24
Saccharomycetaceae_2 - ps25
Saccharomycetaceae_3 - ps26
Kaz./Nau./Saccharomyces - ps27
Saccharomyces - ps28
Arboricola_clade - ps29
Cerevisiae_clade - ps30
Saccharomyces_cerevisiae - ps31
p-value
6e-03
8e-03
1e-02
1e-02
2e-02
Log-odds
0.5
1.0
1.5
2.0
2.5
3.0
3.5
4.0

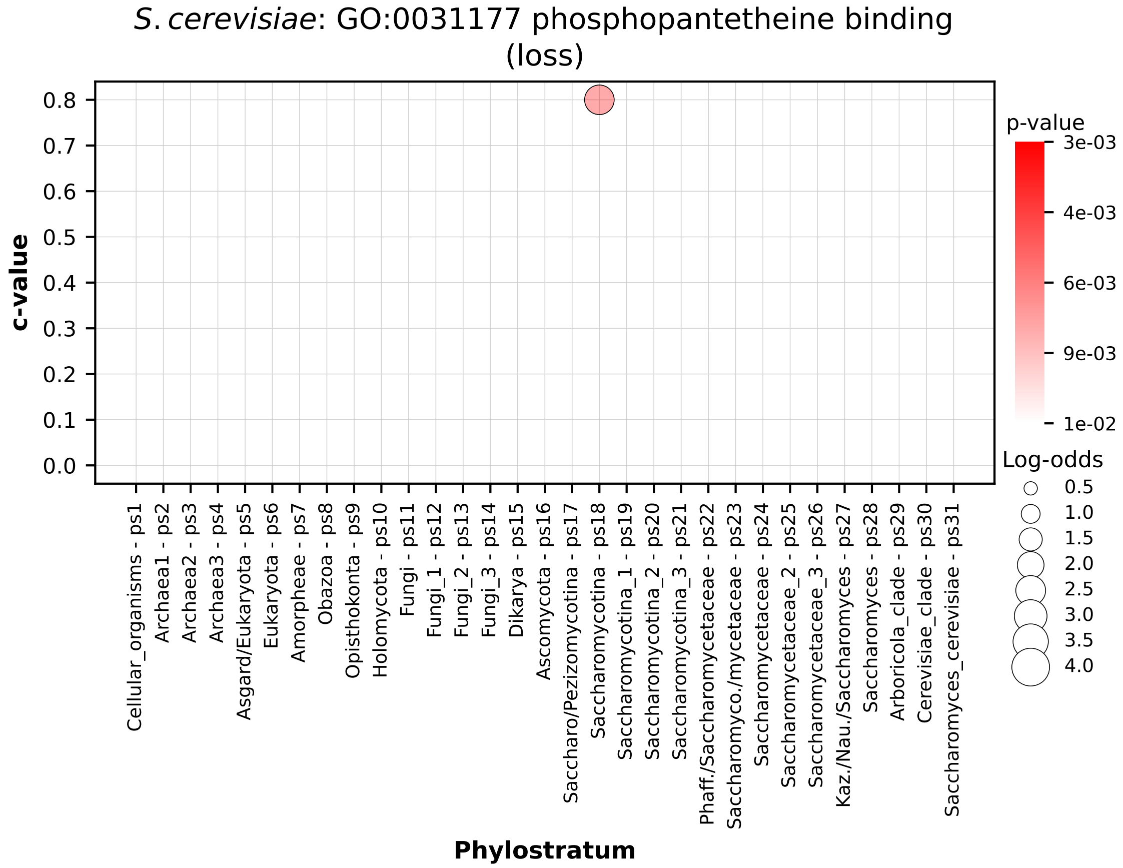
S. cerevisiae: GO:0031177 phosphopantetheine binding
(loss)
c-value
0.0
0.1
0.2
0.3
0.4
0.5
0.6
0.7
0.8
p-value
3e-03
4e-03
6e-03
9e-03
1e-02
Log-odds
0.5
1.0
1.5
2.0
2.5
3.0
3.5
4.0
Cellular_organisms - ps1
Archaea1 - ps2
Archaea2 - ps3
Archaea3 - ps4
Asgard/Eukaryota - ps5
Eukaryota - ps6
Amorpheae - ps7
Obazoa - ps8
Opisthokonta - ps9
Holomycota - ps10
Fungi - ps11
Fungi_1 - ps12
Fungi_2 - ps13
Fungi_3 - ps14
Dikarya - ps15
Ascomycota - ps16
Saccharo/Pezizomycotina - ps17
Saccharomycotina - ps18
Saccharomycotina_1 - ps19
Saccharomycotina_2 - ps20
Saccharomycotina_3 - ps21
Phaff./Saccharomycetaceae - ps22
Saccharomyco./mycetaceae - ps23
Saccharomycetaceae - ps24
Saccharomycetaceae_2 - ps25
Saccharomycetaceae_3 - ps26
Kaz./Nau./Saccharomyces - ps27
Saccharomyces - ps28
Arboricola_clade - ps29
Cerevisiae_clade - ps30
Saccharomyces_cerevisiae - ps31
Phylostratum

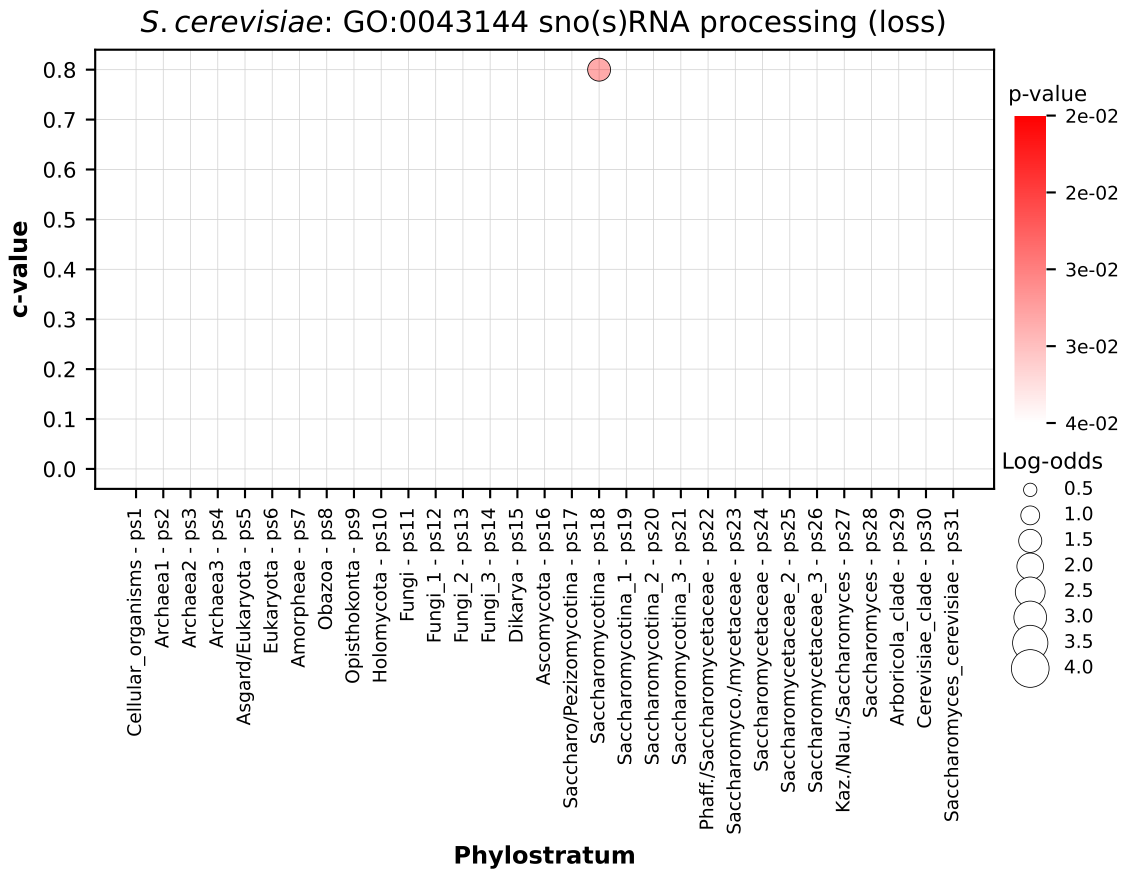

S. cerevisiae: GO:0043144 sno(s)RNA processing (loss)
c-value
0.8
0.7
0.6
0.5
0.4
0.3
0.2
0.1
0.0
Phylostratum
Cellular_organisms - ps1
Archaea1 - ps2
Archaea2 - ps3
Archaea3 - ps4
Asgard/Eukaryota - ps5
Eukaryota - ps6
Amorpheae - ps7
Obazoa - ps8
Opisthokonta - ps9
Holomycota - ps10
Fungi - ps11
Fungi_1 - ps12
Fungi_2 - ps13
Fungi_3 - ps14
Dikarya - ps15
Ascomycota - ps16
Saccharo/Pezizomycotina - ps17
Saccharomycotina - ps18
Saccharomycotina_1 - ps19
Saccharomycotina_2 - ps20
Saccharomycotina_3 - ps21
Phaff./Saccharomycetaceae - ps22
Saccharomyco./mycetaceae - ps23
Saccharomycetaceae - ps24
Saccharomycetaceae_2 - ps25
Saccharomycetaceae_3 - ps26
Kaz./Nau./Saccharomyces - ps27
Saccharomyces - ps28
Arboricola_clade - ps29
Cerevisiae_clade - ps30
Saccharomyces_cerevisiae - ps31
p-value
2e-02
2e-02
3e-02
3e-02
4e-02
Log-odds
0.5
1.0
1.5
2.0
2.5
3.0
3.5
4.0

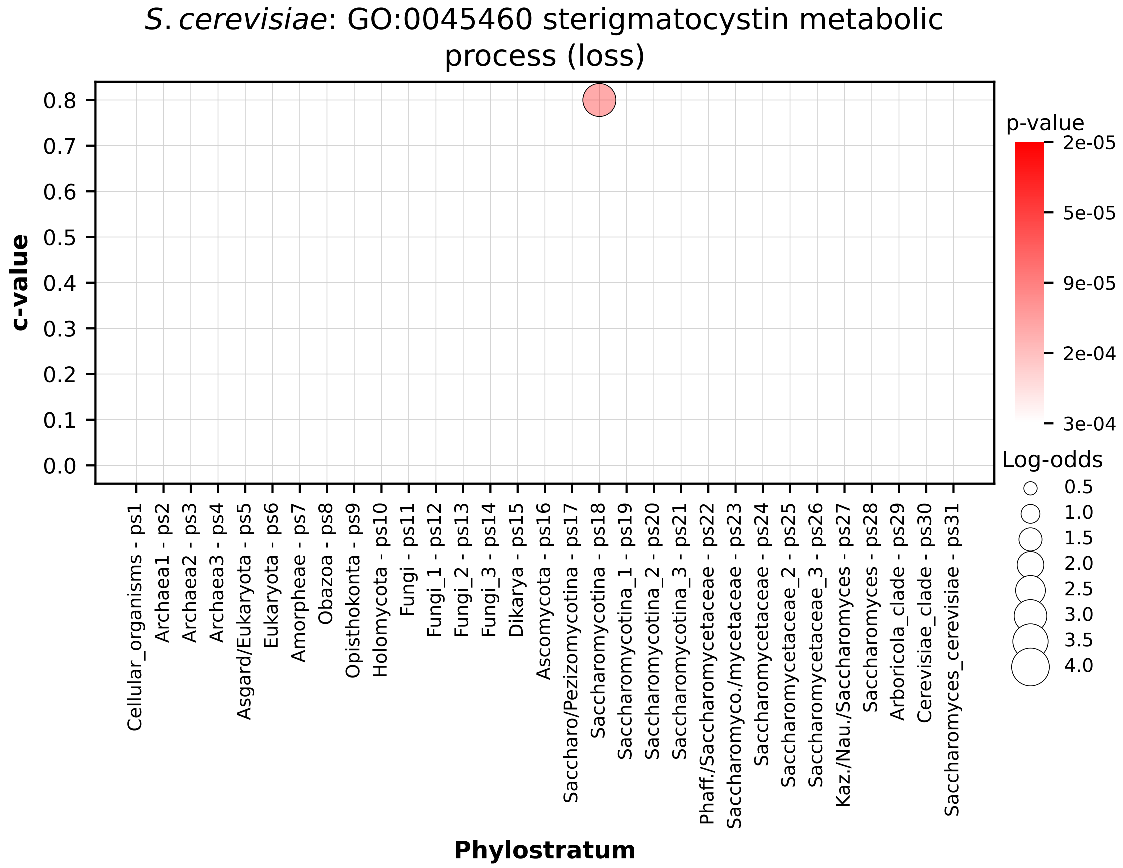

S. cerevisiae: GO:0045460 sterigmatocystin metabolic process (loss)
c-value
0.0
0.1
0.2
0.3
0.4
0.5
0.6
0.7
0.8
Phylostratum
Cellular_organisms - ps1
Archaea1 - ps2
Archaea2 - ps3
Archaea3 - ps4
Asgard/Eukaryota - ps5
Eukaryota - ps6
Amorpheae - ps7
Obazoa - ps8
Opisthokonta - ps9
Holomycota - ps10
Fungi - ps11
Fungi_1 - ps12
Fungi_2 - ps13
Fungi_3 - ps14
Dikarya - ps15
Ascomycota - ps16
Saccharo/Pezizomycotina - ps17
Saccharomycotina - ps18
Saccharomycotina_1 - ps19
Saccharomycotina_2 - ps20
Saccharomycotina_3 - ps21
Phaff./Saccharomycetaceae - ps22
Saccharomyco./mycetaceae - ps23
Saccharomycetaceae - ps24
Saccharomycetaceae_2 - ps25
Saccharomycetaceae_3 - ps26
Kaz./Nau./Saccharomyces - ps27
Saccharomyces - ps28
Arboricola_clade - ps29
Cerevisiae_clade - ps30
Saccharomyces_cerevisiae - ps31
p-value
2e-05
5e-05
9e-05
2e-04
3e-04
Log-odds
0.5
1.0
1.5
2.0
2.5
3.0
3.5
4.0

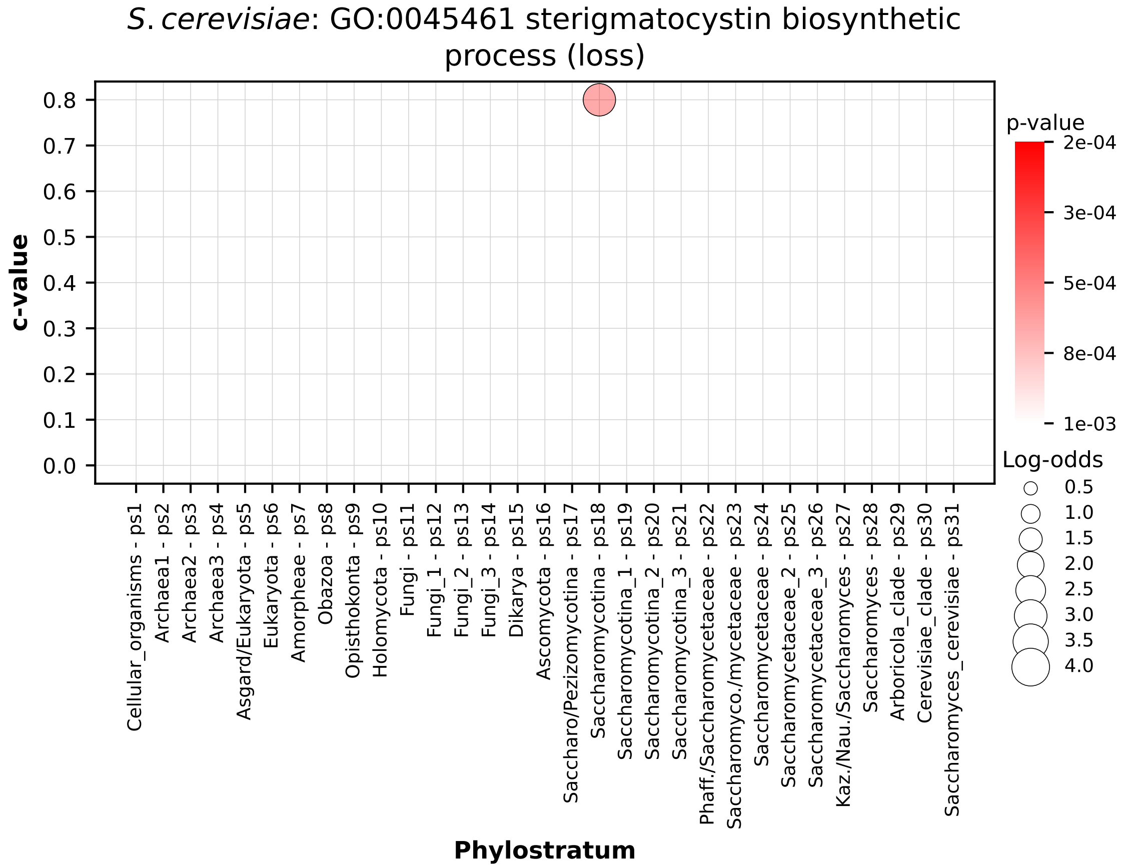

S. cerevisiae: GO:0045461 sterigmatocystin biosynthetic process (loss)
c-value
0.0
0.1
0.2
0.3
0.4
0.5
0.6
0.7
0.8
Phylostratum
Cellular_organisms - ps1
Archaea1 - ps2
Archaea2 - ps3
Archaea3 - ps4
Asgard/Eukaryota - ps5
Eukaryota - ps6
Amorpheae - ps7
Obazoa - ps8
Opisthokonta - ps9
Holomycota - ps10
Fungi - ps11
Fungi_1 - ps12
Fungi_2 - ps13
Fungi_3 - ps14
Dikarya - ps15
Ascomycota - ps16
Saccharo/Pezizomycotina - ps17
Saccharomycotina - ps18
Saccharomycotina_1 - ps19
Saccharomycotina_2 - ps20
Saccharomycotina_3 - ps21
Phaff./Saccharomycetaceae - ps22
Saccharomyco./mycetaceae - ps23
Saccharomycetaceae - ps24
Saccharomycetaceae_2 - ps25
Saccharomycetaceae_3 - ps26
Kaz./Nau./Saccharomyces - ps27
Saccharomyces - ps28
Arboricola_clade - ps29
Cerevisiae_clade - ps30
Saccharomyces_cerevisiae - ps31
p-value
2e-04
3e-04
5e-04
8e-04
1e-03
Log-odds
0.5
1.0
1.5
2.0
2.5
3.0
3.5
4.0

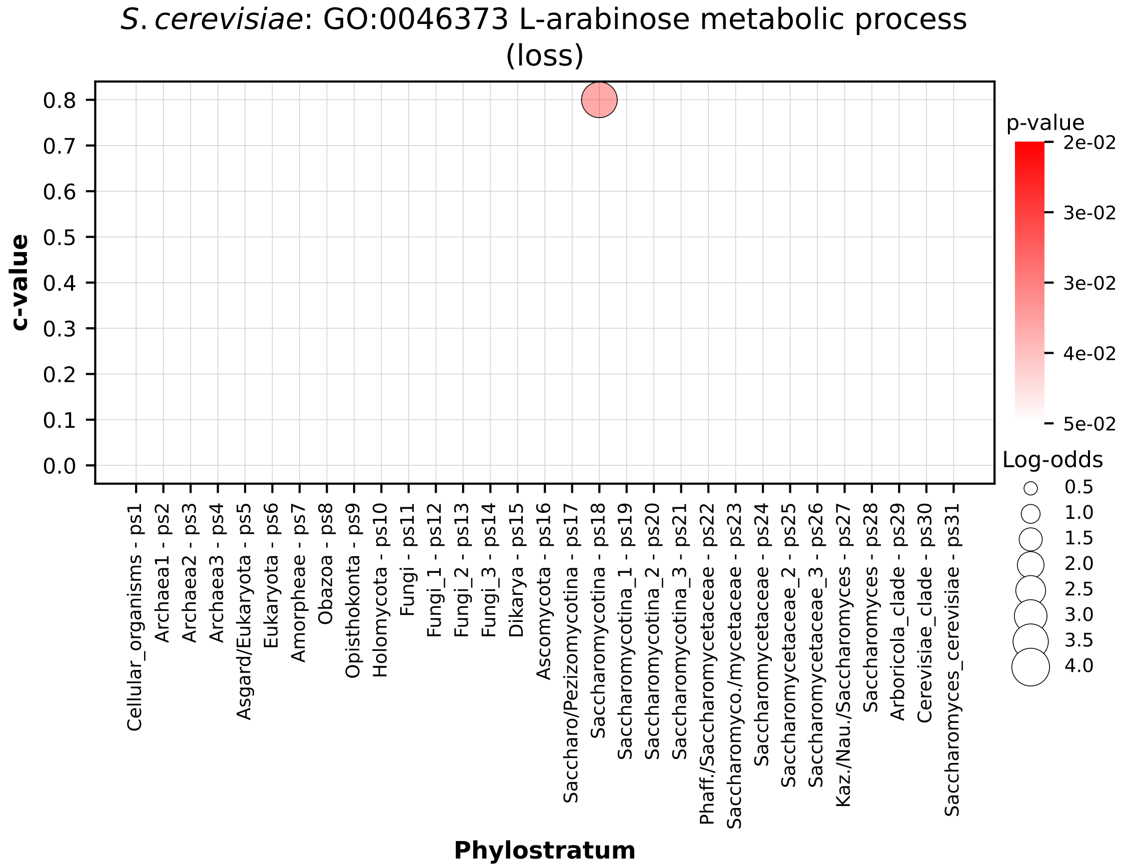
S. cerevisiae: GO:0046373 L-arabinose metabolic process
(loss)
c-value
0.0
0.1
0.2
0.3
0.4
0.5
0.6
0.7
0.8
p-value
2e-02
3e-02
3e-02
4e-02
5e-02
Log-odds
0.5
1.0
1.5
2.0
2.5
3.0
3.5
4.0
Cellular_organisms - ps1
Archaea1 - ps2
Archaea2 - ps3
Archaea3 - ps4
Asgard/Eukaryota - ps5
Eukaryota - ps6
Amorpheae - ps7
Obazoa - ps8
Opisthokonta - ps9
Holomycota - ps10
Fungi - ps11
Fungi_1 - ps12
Fungi_2 - ps13
Fungi_3 - ps14
Dikarya - ps15
Ascomycota - ps16
Saccharo/Pezizomycotina - ps17
Saccharomycotina - ps18
Saccharomycotina_1 - ps19
Saccharomycotina_2 - ps20
Saccharomycotina_3 - ps21
Phaff./Saccharomycetaceae - ps22
Saccharomyco./mycetaceae - ps23
Saccharomycetaceae - ps24
Saccharomycetaceae_2 - ps25
Saccharomycetaceae_3 - ps26
Kaz./Nau./Saccharomyces - ps27
Saccharomyces - ps28
Arboricola_clade - ps29
Cerevisiae_clade - ps30
Saccharomyces_cerevisiae - ps31
Phylostratum

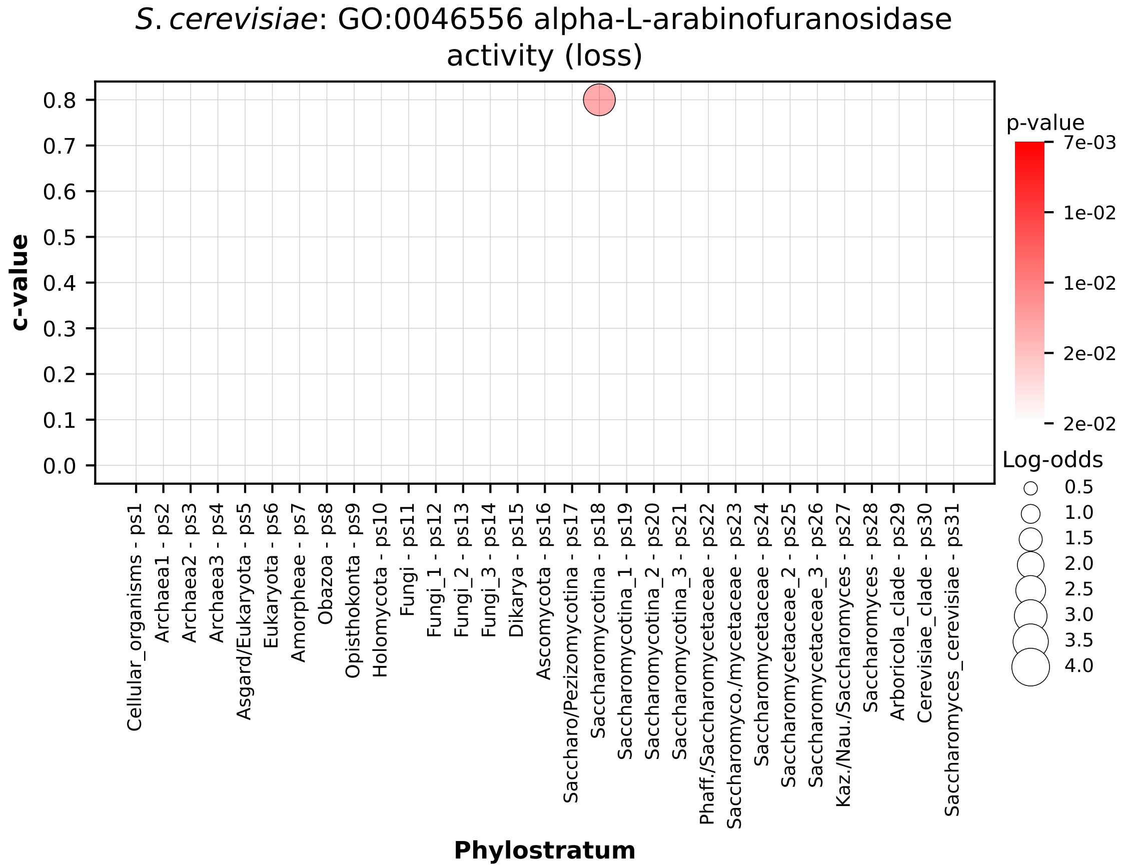

S. cerevisiae: GO:0046556 alpha-L-arabinofuranosidase activity (loss)
c-value
0.8
0.7
0.6
0.5
0.4
0.3
0.2
0.1
0.0
p-value
7e-03
1e-02
1e-02
2e-02
2e-02
Log-odds
0.5
1.0
1.5
2.0
2.5
3.0
3.5
4.0
Cellular_organisms - ps1
Archaea1 - ps2
Archaea2 - ps3
Archaea3 - ps4
Asgard/Eukaryota - ps5
Eukaryota - ps6
Amorpheae - ps7
Obazoa - ps8
Opisthokonta - ps9
Holomycota - ps10
Fungi - ps11
Fungi_1 - ps12
Fungi_2 - ps13
Fungi_3 - ps14
Dikarya - ps15
Ascomycota - ps16
Saccharo/Pezizomycotina - ps17
Saccharomycotina - ps18
Saccharomycotina_1 - ps19
Saccharomycotina_2 - ps20
Saccharomycotina_3 - ps21
Phaff./Saccharomycetaceae - ps22
Saccharomyco./mycetaceae - ps23
Saccharomycetaceae - ps24
Saccharomycetaceae_2 - ps25
Saccharomycetaceae_3 - ps26
Kaz./Nau./Saccharomyces - ps27
Saccharomyces - ps28
Arboricola_clade - ps29
Cerevisiae_clade - ps30
Saccharomyces_cerevisiae - ps31
Phylostratum

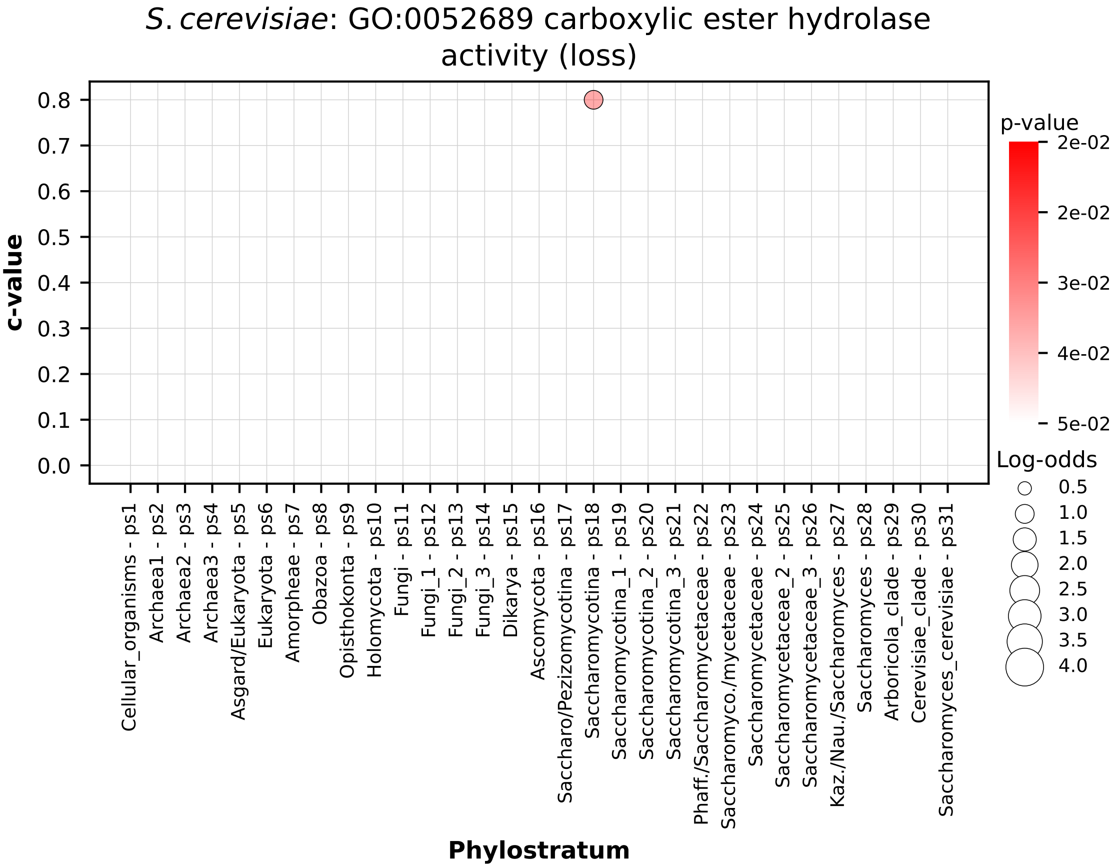

S. cerevisiae: GO:0052689 carboxylic ester hydrolase activity (loss)
c-value
0.8
0.7
0.6
0.5
0.4
0.3
0.2
0.1
0.0
p-value
2e-02
2e-02
3e-02
4e-02
5e-02
Log-odds
0.5
1.0
1.5
2.0
2.5
3.0
3.5
4.0
Cellular_organisms - ps1
Archaea1 - ps2
Archaea2 - ps3
Archaea3 - ps4
Asgard/Eukaryota - ps5
Eukaryota - ps6
Amorpheae - ps7
Obazoa - ps8
Opisthokonta - ps9
Holomycota - ps10
Fungi - ps11
Fungi_1 - ps12
Fungi_2 - ps13
Fungi_3 - ps14
Dikarya - ps15
Ascomycota - ps16
Saccharo/Pezizomycotina - ps17
Saccharomycotina - ps18
Saccharomycotina_1 - ps19
Saccharomycotina_2 - ps20
Saccharomycotina_3 - ps21
Phaff./Saccharomycetaceae - ps22
Saccharomyco./mycetaceae - ps23
Saccharomycetaceae - ps24
Saccharomycetaceae_2 - ps25
Saccharomycetaceae_3 - ps26
Kaz./Nau./Saccharomyces - ps27
Saccharomyces - ps28
Arboricola_clade - ps29
Cerevisiae_clade - ps30
Saccharomyces_cerevisiae - ps31
Phylostratum

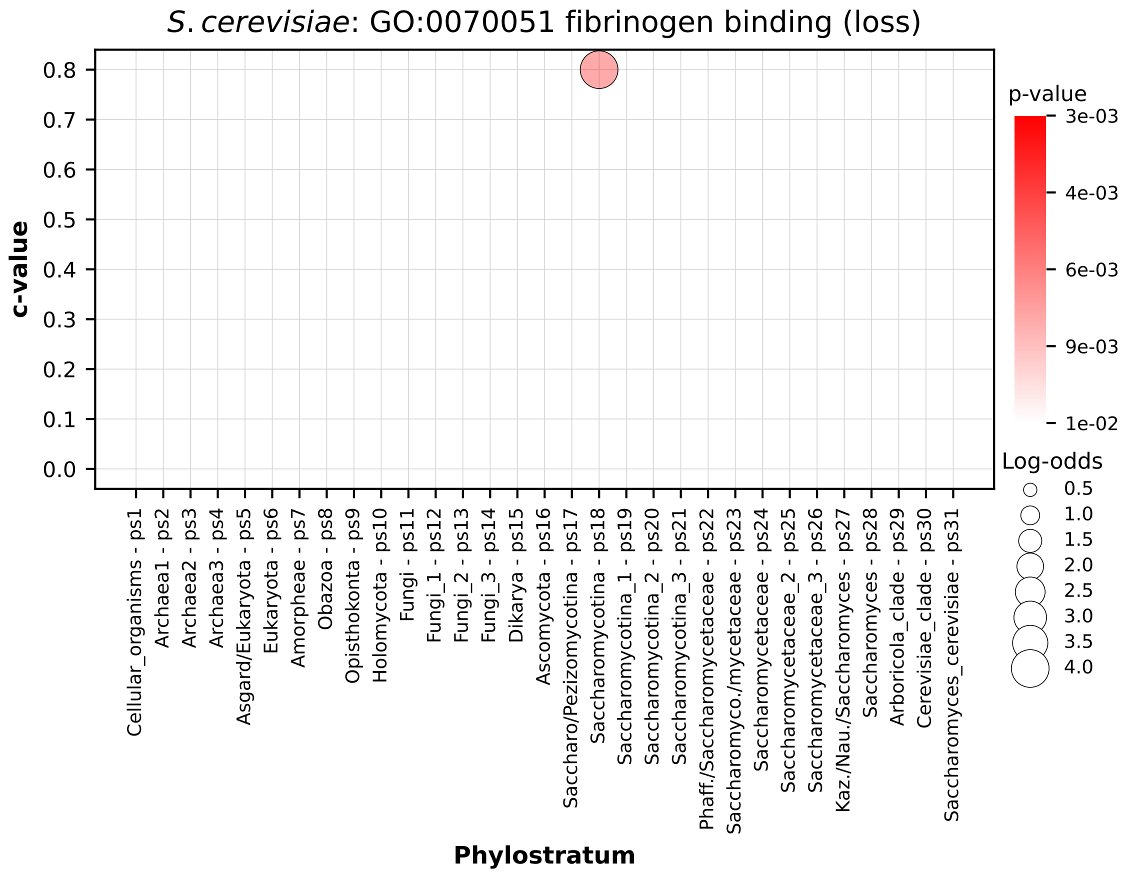

S. cerevisiae: GO:0070051 fibrinogen binding (loss)
c-value
0.0
0.1
0.2
0.3
0.4
0.5
0.6
0.7
0.8
p-value
3e-03
4e-03
6e-03
9e-03
1e-02
Log-odds
0.5
1.0
1.5
2.0
2.5
3.0
3.5
4.0
Cellular_organisms - ps1
Archaea1 - ps2
Archaea2 - ps3
Archaea3 - ps4
Asgard/Eukaryota - ps5
Eukaryota - ps6
Amorpheae - ps7
Obazoa - ps8
Opisthokonta - ps9
Holomycota - ps10
Fungi - ps11
Fungi_1 - ps12
Fungi_2 - ps13
Fungi_3 - ps14
Dikarya - ps15
Ascomycota - ps16
Saccharo/Pezizomycotina - ps17
Saccharomycotina - ps18
Saccharomycotina_1 - ps19
Saccharomycotina_2 - ps20
Saccharomycotina_3 - ps21
Phaff./Saccharomycetaceae - ps22
Saccharomyco./mycetaceae - ps23
Saccharomycetaceae - ps24
Saccharomycetaceae_2 - ps25
Saccharomycetaceae_3 - ps26
Kaz./Nau./Saccharomyces - ps27
Saccharomyces - ps28
Arboricola_clade - ps29
Cerevisiae_clade - ps30
Saccharomyces_cerevisiae - ps31
Phylostratum

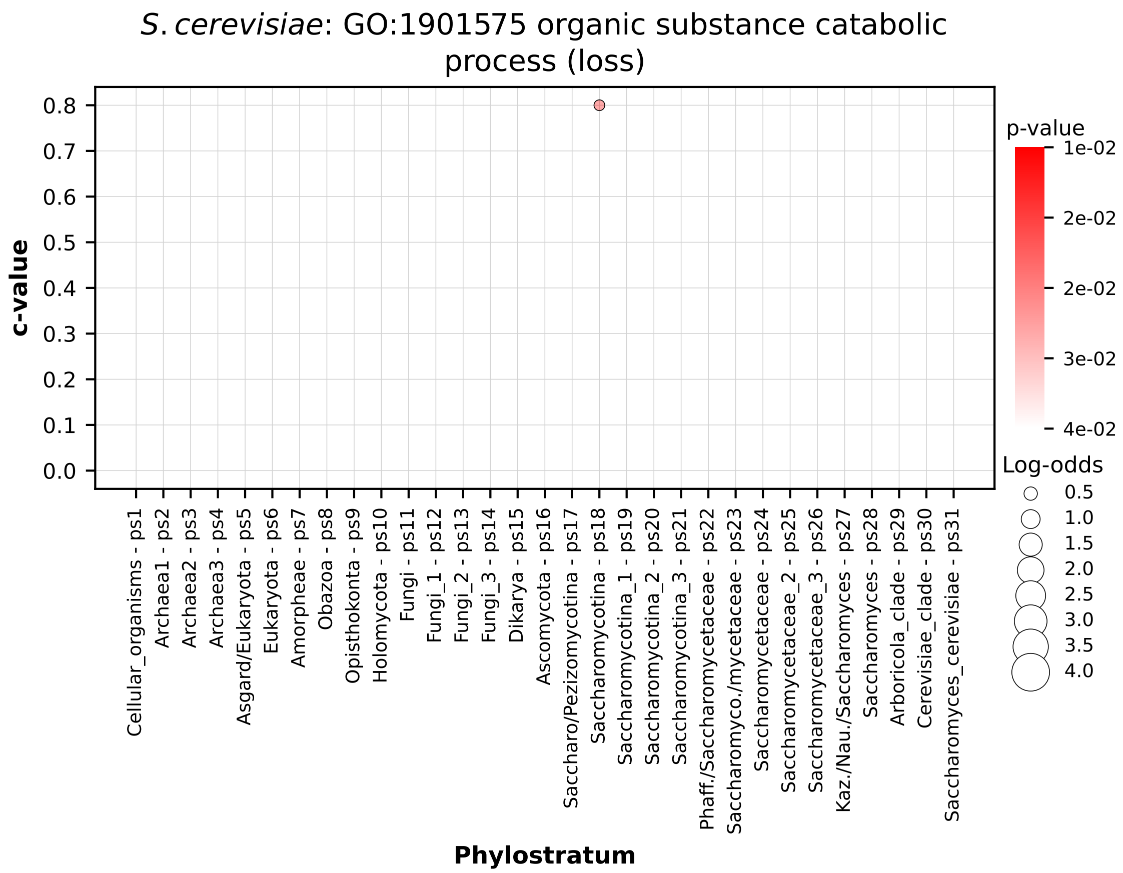
S. cerevisiae: GO:1901575 organic substance catabolic process (loss)
c-value
0.8
0.7
0.6
0.5
0.4
0.3
0.2
0.1
0.0
p-value
1e-02
2e-02
2e-02
3e-02
4e-02
Log-odds
0.5
1.0
1.5
2.0
2.5
3.0
3.5
4.0
Cellular_organisms - ps1
Archaea1 - ps2
Archaea2 - ps3
Archaea3 - ps4
Asgard/Eukaryota - ps5
Eukaryota - ps6
Amorpheae - ps7
Obazoa - ps8
Opisthokonta - ps9
Holomycota - ps10
Fungi - ps11
Fungi_1 - ps12
Fungi_2 - ps13
Fungi_3 - ps14
Dikarya - ps15
Ascomycota - ps16
Saccharo/Pezizomycotina - ps17
Saccharomycotina - ps18
Saccharomycotina_1 - ps19
Saccharomycotina_2 - ps20
Saccharomycotina_3 - ps21
Phaff./Saccharomycetaceae - ps22
Saccharomyco./mycetaceae - ps23
Saccharomycetaceae - ps24
Saccharomycetaceae_2 - ps25
Saccharomycetaceae_3 - ps26
Kaz./Nau./Saccharomyces - ps27
Saccharomyces - ps28
Arboricola_clade - ps29
Cerevisiae_clade - ps30
Saccharomyces_cerevisiae - ps31
Phylostratum

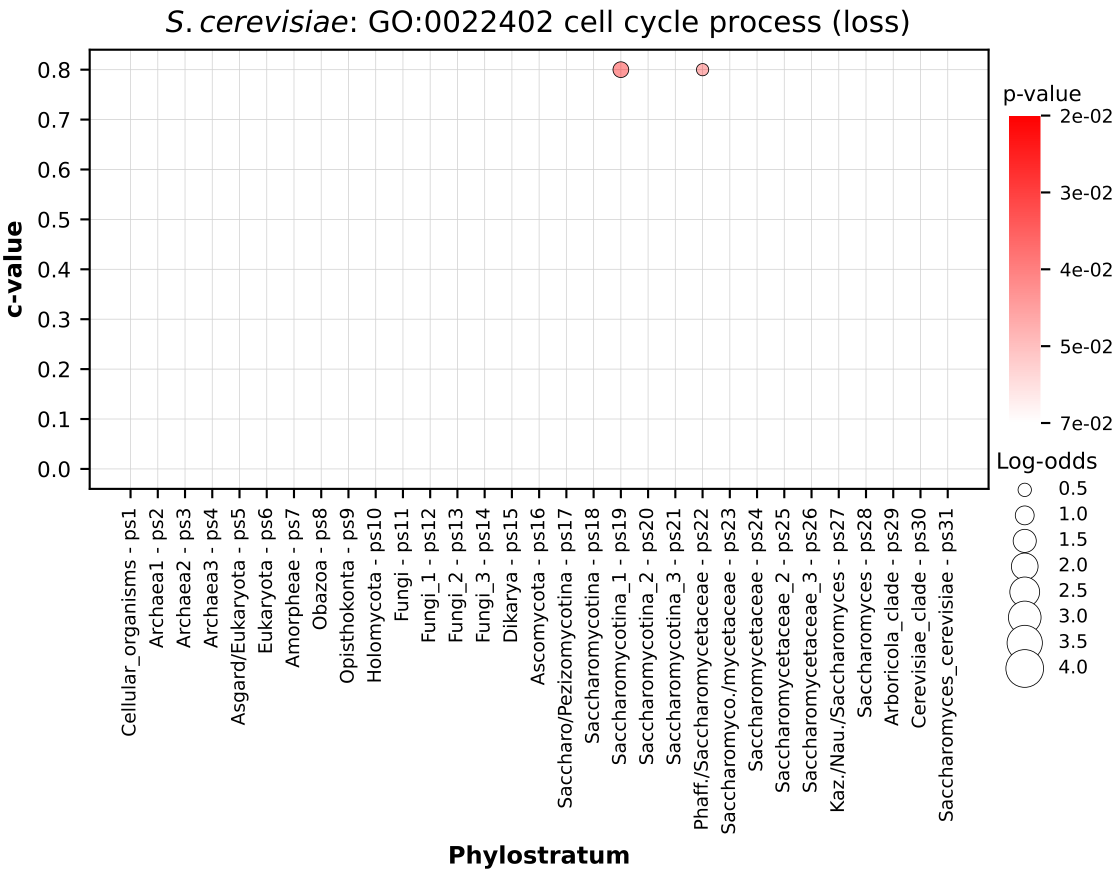

S. cerevisiae: GO:0022402 cell cycle process (loss)
c-value
0.0
0.1
0.2
0.3
0.4
0.5
0.6
0.7
0.8
p-value
2e-02
3e-02
4e-02
5e-02
7e-02
Log-odds
0.5
1.0
1.5
2.0
2.5
3.0
3.5
4.0
Cellular_organisms - ps1
Archaea1 - ps2
Archaea2 - ps3
Archaea3 - ps4
Asgard/Eukaryota - ps5
Eukaryota - ps6
Amorpheae - ps7
Obazoa - ps8
Opisthokonta - ps9
Holomycota - ps10
Fungi - ps11
Fungi_1 - ps12
Fungi_2 - ps13
Fungi_3 - ps14
Dikarya - ps15
Ascomycota - ps16
Saccharo/Pezizomycotina - ps17
Saccharomycotina - ps18
Saccharomycotina_1 - ps19
Saccharomycotina_2 - ps20
Saccharomycotina_3 - ps21
Phaff./Saccharomycetaceae - ps22
Saccharomyco./mycetaceae - ps23
Saccharomycetaceae - ps24
Saccharomycetaceae_2 - ps25
Saccharomycetaceae_3 - ps26
Kaz./Nau./Saccharomyces - ps27
Saccharomyces - ps28
Arboricola_clade - ps29
Cerevisiae_clade - ps30
Saccharomyces_cerevisiae - ps31
Phylostratum

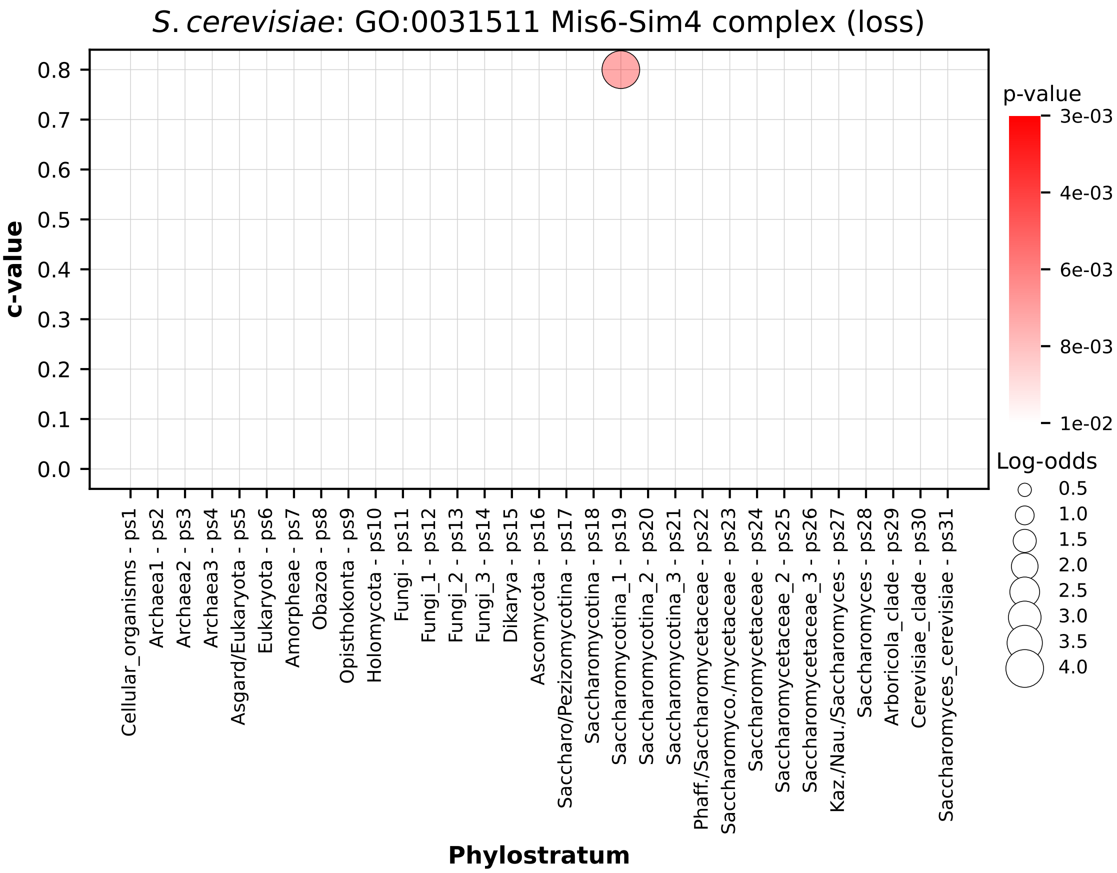
S. cerevisiae: GO:0031511 Mis6-Sim4 complex (loss)
c-value
0.0
0.1
0.2
0.3
0.4
0.5
0.6
0.7
0.8
p-value
3e-03
4e-03
6e-03
8e-03
1e-02
Log-odds
0.5
1.0
1.5
2.0
2.5
3.0
3.5
4.0
Cellular_organisms - ps1
Archaea1 - ps2
Archaea2 - ps3
Archaea3 - ps4
Asgard/Eukaryota - ps5
Eukaryota - ps6
Amorpheae - ps7
Obazoa - ps8
Opisthokonta - ps9
Holomycota - ps10
Fungi - ps11
Fungi_1 - ps12
Fungi_2 - ps13
Fungi_3 - ps14
Dikarya - ps15
Ascomycota - ps16
Saccharo/Pezizomycotina - ps17
Saccharomycotina - ps18
Saccharomycotina_1 - ps19
Saccharomycotina_2 - ps20
Saccharomycotina_3 - ps21
Phaff./Saccharomycetaceae - ps22
Saccharomyco./mycetaceae - ps23
Saccharomycetaceae - ps24
Saccharomycetaceae_2 - ps25
Saccharomycetaceae_3 - ps26
Kaz./Nau./Saccharomyces - ps27
Saccharomyces - ps28
Arboricola_clade - ps29
Cerevisiae_clade - ps30
Saccharomyces_cerevisiae - ps31
Phylostratum

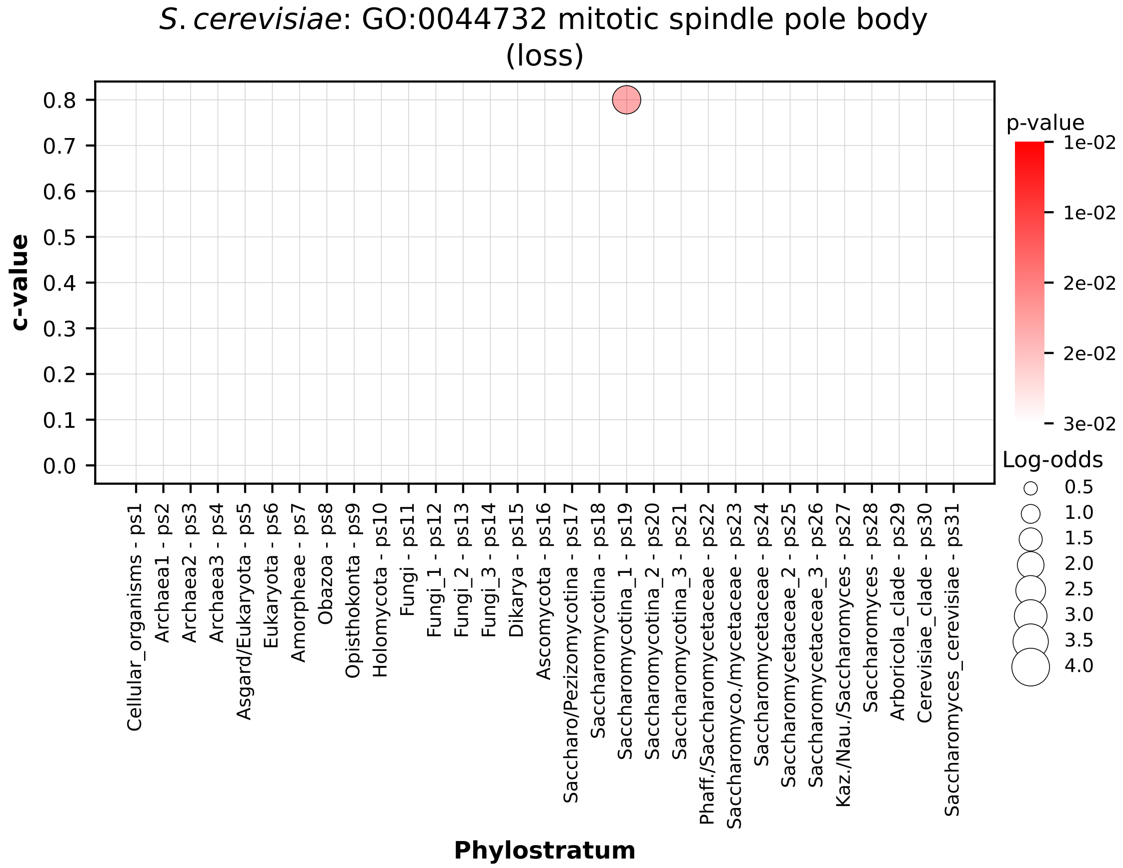
S. cerevisiae: GO:0044732 mitotic spindle pole body
(loss)
c-value
0.8
0.7
0.6
0.5
0.4
0.3
0.2
0.1
0.0
p-value
1e-02
1e-02
2e-02
2e-02
3e-02
Log-odds
0.5
1.0
1.5
2.0
2.5
3.0
3.5
4.0
Cellular_organisms - ps1
Archaea1 - ps2
Archaea2 - ps3
Archaea3 - ps4
Asgard/Eukaryota - ps5
Eukaryota - ps6
Amorpheae - ps7
Obazoa - ps8
Opisthokonta - ps9
Holomycota - ps10
Fungi - ps11
Fungi_1 - ps12
Fungi_2 - ps13
Fungi_3 - ps14
Dikarya - ps15
Ascomycota - ps16
Saccharo/Pezizomycotina - ps17
Saccharomycotina - ps18
Saccharomycotina_1 - ps19
Saccharomycotina_2 - ps20
Saccharomycotina_3 - ps21
Phaff./Saccharomycetaceae - ps22
Saccharomyco./mycetaceae - ps23
Saccharomycetaceae - ps24
Saccharomycetaceae_2 - ps25
Saccharomycetaceae_3 - ps26
Kaz./Nau./Saccharomyces - ps27
Saccharomyces - ps28
Arboricola_clade - ps29
Cerevisiae_clade - ps30
Saccharomyces_cerevisiae - ps31
Phylostratum

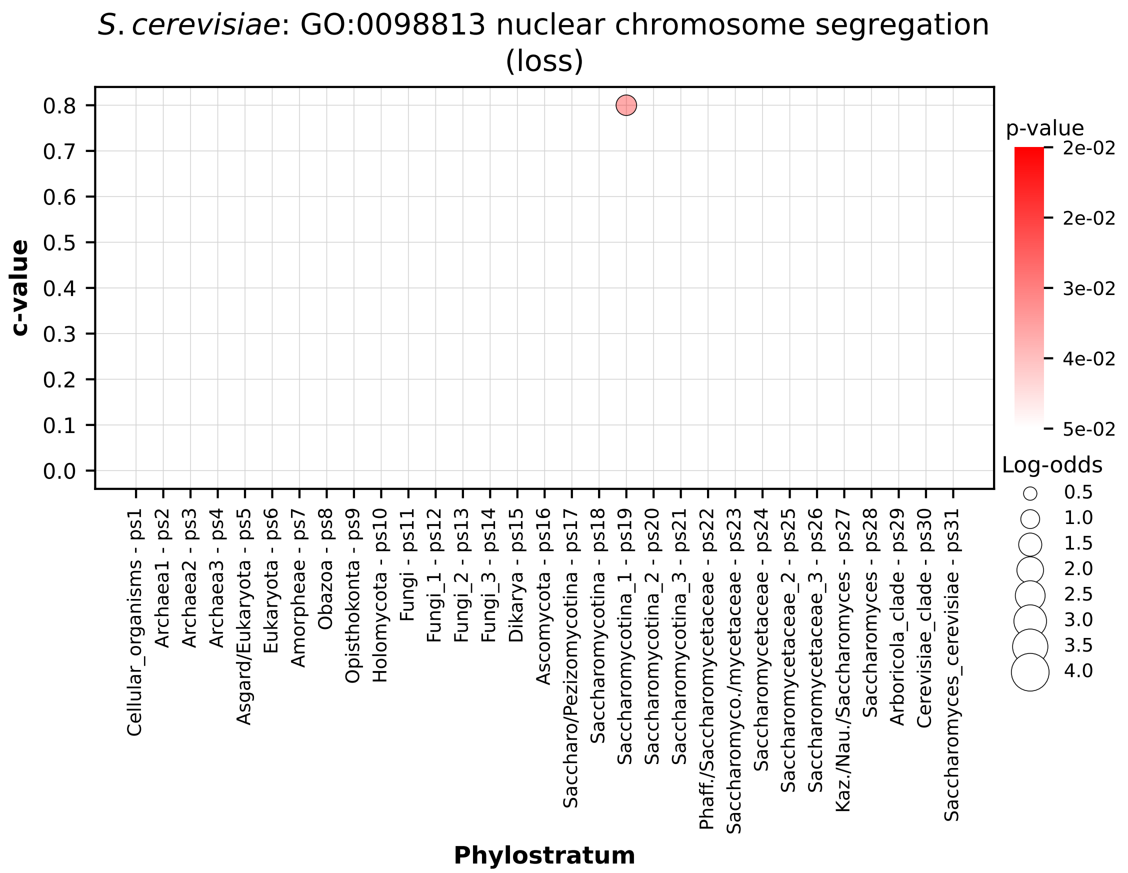

S. cerevisiae: GO:0098813 nuclear chromosome segregation
(loss)
c-value
0.8
0.7
0.6
0.5
0.4
0.3
0.2
0.1
0.0
p-value
2e-02
2e-02
3e-02
4e-02
5e-02
Log-odds
0.5
1.0
1.5
2.0
2.5
3.0
3.5
4.0
Cellular_organisms - ps1
Archaea1 - ps2
Archaea2 - ps3
Archaea3 - ps4
Asgard/Eukaryota - ps5
Eukaryota - ps6
Amorpheae - ps7
Obazoa - ps8
Opisthokonta - ps9
Holomycota - ps10
Fungi - ps11
Fungi_1 - ps12
Fungi_2 - ps13
Fungi_3 - ps14
Dikarya - ps15
Ascomycota - ps16
Saccharo/Pezizomycotina - ps17
Saccharomycotina - ps18
Saccharomycotina_1 - ps19
Saccharomycotina_2 - ps20
Saccharomycotina_3 - ps21
Phaff./Saccharomycetaceae - ps22
Saccharomyco./mycetaceae - ps23
Saccharomycetaceae - ps24
Saccharomycetaceae_2 - ps25
Saccharomycetaceae_3 - ps26
Kaz./Nau./Saccharomyces - ps27
Saccharomyces - ps28
Arboricola_clade - ps29
Cerevisiae_clade - ps30
Saccharomyces_cerevisiae - ps31
Phylostratum

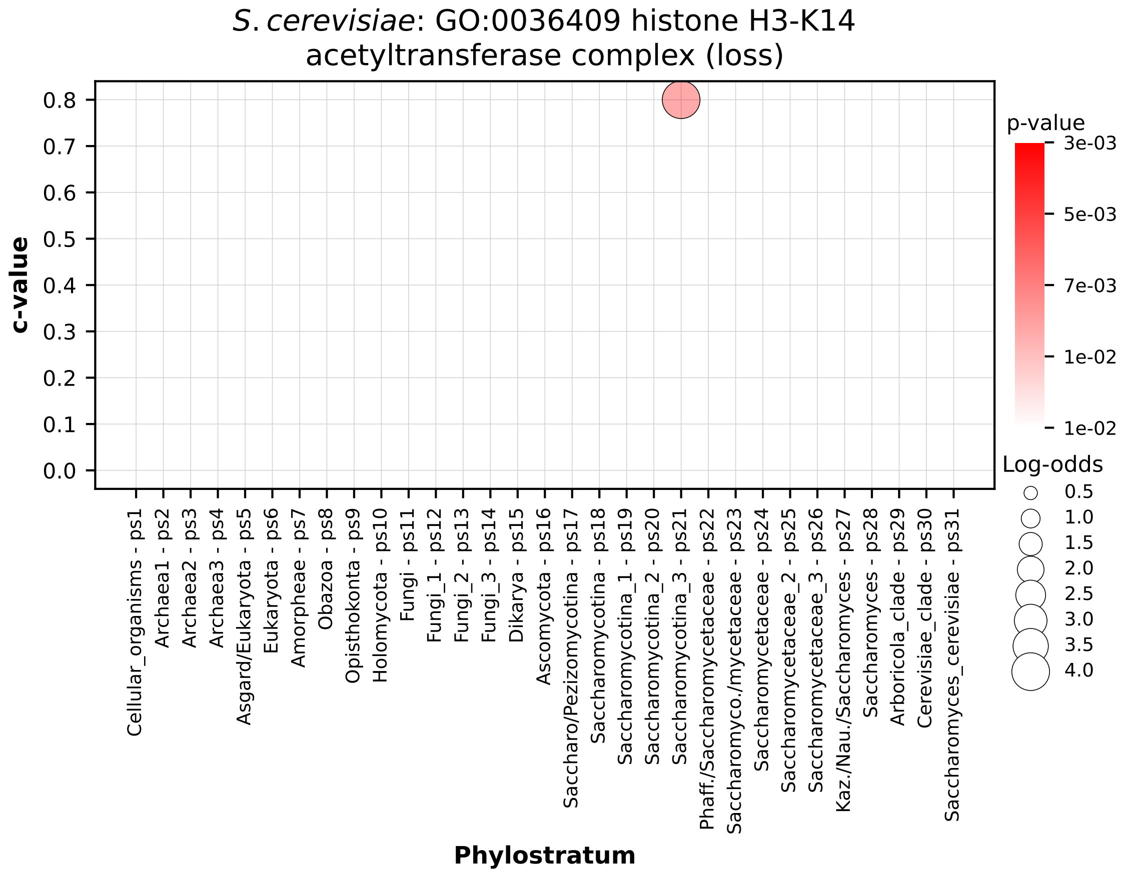

S. cerevisiae: GO:0036409 histone H3-K14 acetyltransferase complex (loss)
c-value
0.8
0.7
0.6
0.5
0.4
0.3
0.2
0.1
0.0
Phylostratum
Cellular_organisms - ps1
Archaea1 - ps2
Archaea2 - ps3
Archaea3 - ps4
Asgard/Eukaryota - ps5
Eukaryota - ps6
Amorpheae - ps7
Obazoa - ps8
Opisthokonta - ps9
Holomycota - ps10
Fungi - ps11
Fungi_1 - ps12
Fungi_2 - ps13
Fungi_3 - ps14
Dikarya - ps15
Ascomycota - ps16
Saccharo/Pezizomycotina - ps17
Saccharomycotina - ps18
Saccharomycotina_1 - ps19
Saccharomycotina_2 - ps20
Saccharomycotina_3 - ps21
Phaff./Saccharomycetaceae - ps22
Saccharomyco./mycetaceae - ps23
Saccharomycetaceae - ps24
Saccharomycetaceae_2 - ps25
Saccharomycetaceae_3 - ps26
Kaz./Nau./Saccharomyces - ps27
Saccharomyces - ps28
Arboricola_clade - ps29
Cerevisiae_clade - ps30
Saccharomyces_cerevisiae - ps31
p-value
3e-03
5e-03
7e-03
1e-02
1e-02
Log-odds
0.5
1.0
1.5
2.0
2.5
3.0
3.5
4.0

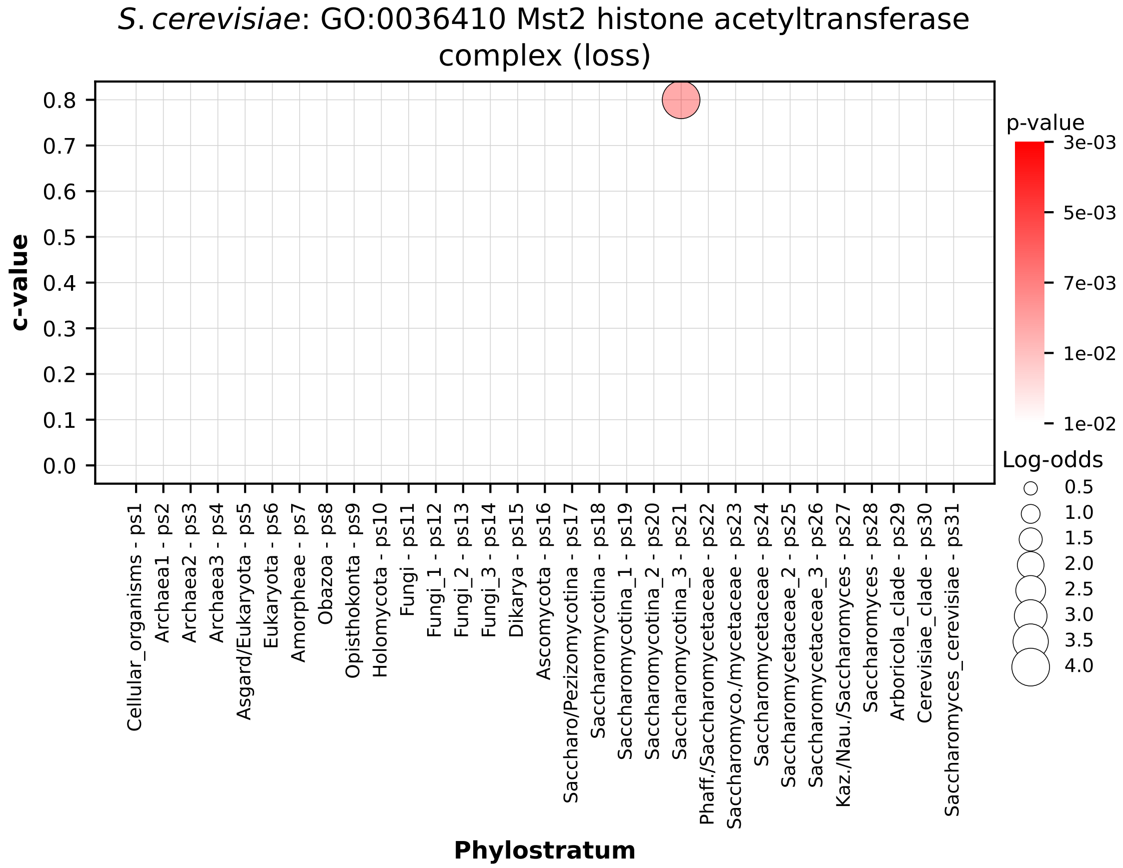

S. cerevisiae: GO:0036410 Mst2 histone acetyltransferase complex (loss)
c-value
0.8
0.7
0.6
0.5
0.4
0.3
0.2
0.1
0.0
p-value
3e-03
5e-03
7e-03
1e-02
1e-02
Log-odds
0.5
1.0
1.5
2.0
2.5
3.0
3.5
4.0
Cellular_organisms - ps1
Archaea1 - ps2
Archaea2 - ps3
Archaea3 - ps4
Asgard/Eukaryota - ps5
Eukaryota - ps6
Amorpheae - ps7
Obazoa - ps8
Opisthokonta - ps9
Holomycota - ps10
Fungi - ps11
Fungi_1 - ps12
Fungi_2 - ps13
Fungi_3 - ps14
Dikarya - ps15
Ascomycota - ps16
Saccharo/Pezizomycotina - ps17
Saccharomycotina - ps18
Saccharomycotina_1 - ps19
Saccharomycotina_2 - ps20
Saccharomycotina_3 - ps21
Phaff./Saccharomycetaceae - ps22
Saccharomyco./mycetaceae - ps23
Saccharomycetaceae - ps24
Saccharomycetaceae_2 - ps25
Saccharomycetaceae_3 - ps26
Kaz./Nau./Saccharomyces - ps27
Saccharomyces - ps28
Arboricola_clade - ps29
Cerevisiae_clade - ps30
Saccharomyces_cerevisiae - ps31
Phylostratum

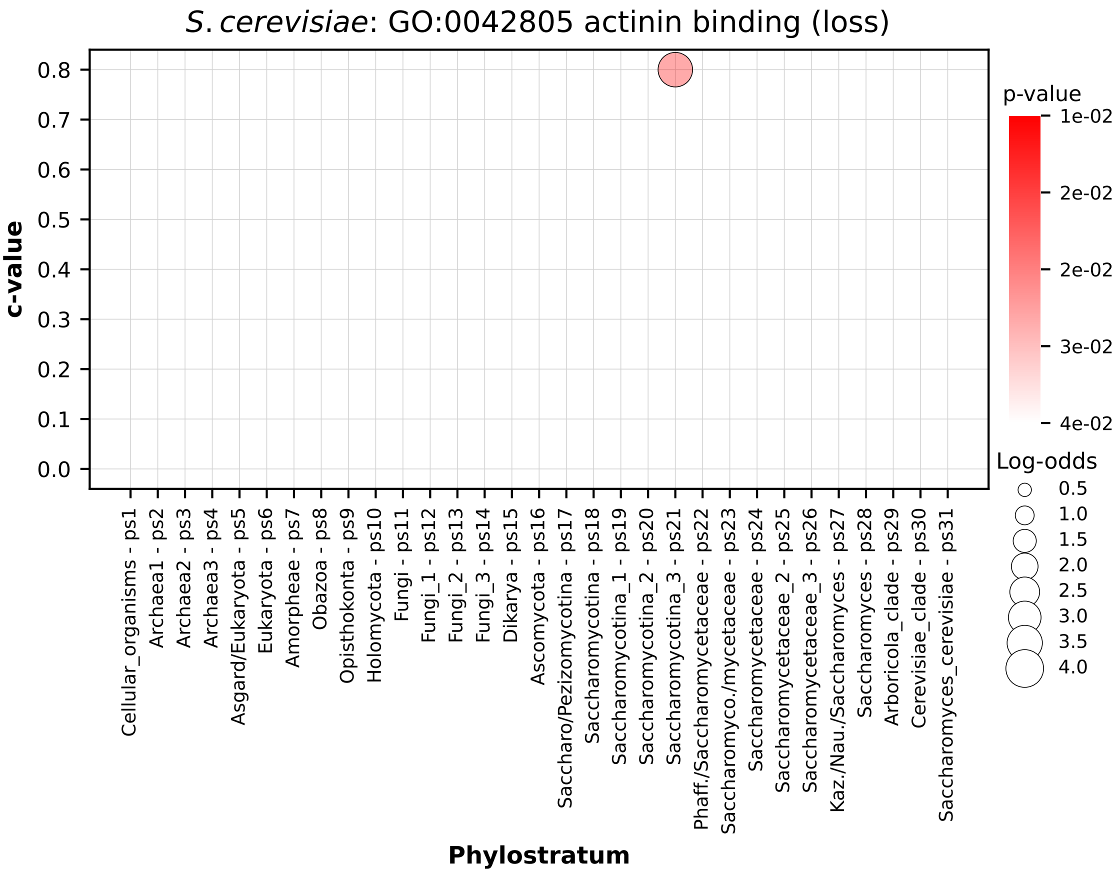

S. cerevisiae: GO:0042805 actinin binding (loss)
c-value
0.0
0.1
0.2
0.3
0.4
0.5
0.6
0.7
0.8
p-value
1e-02
2e-02
2e-02
3e-02
4e-02
Log-odds
0.5
1.0
1.5
2.0
2.5
3.0
3.5
4.0
Cellular_organisms - ps1
Archaea1 - ps2
Archaea2 - ps3
Archaea3 - ps4
Asgard/Eukaryota - ps5
Eukaryota - ps6
Amorpheae - ps7
Obazoa - ps8
Opisthokonta - ps9
Holomycota - ps10
Fungi - ps11
Fungi_1 - ps12
Fungi_2 - ps13
Fungi_3 - ps14
Dikarya - ps15
Ascomycota - ps16
Saccharo/Pezizomycotina - ps17
Saccharomycotina - ps18
Saccharomycotina_1 - ps19
Saccharomycotina_2 - ps20
Saccharomycotina_3 - ps21
Phaff./Saccharomycetaceae - ps22
Saccharomyco./mycetaceae - ps23
Saccharomycetaceae - ps24
Saccharomycetaceae_2 - ps25
Saccharomycetaceae_3 - ps26
Kaz./Nau./Saccharomyces - ps27
Saccharomyces - ps28
Arboricola_clade - ps29
Cerevisiae_clade - ps30
Saccharomyces_cerevisiae - ps31
Phylostratum

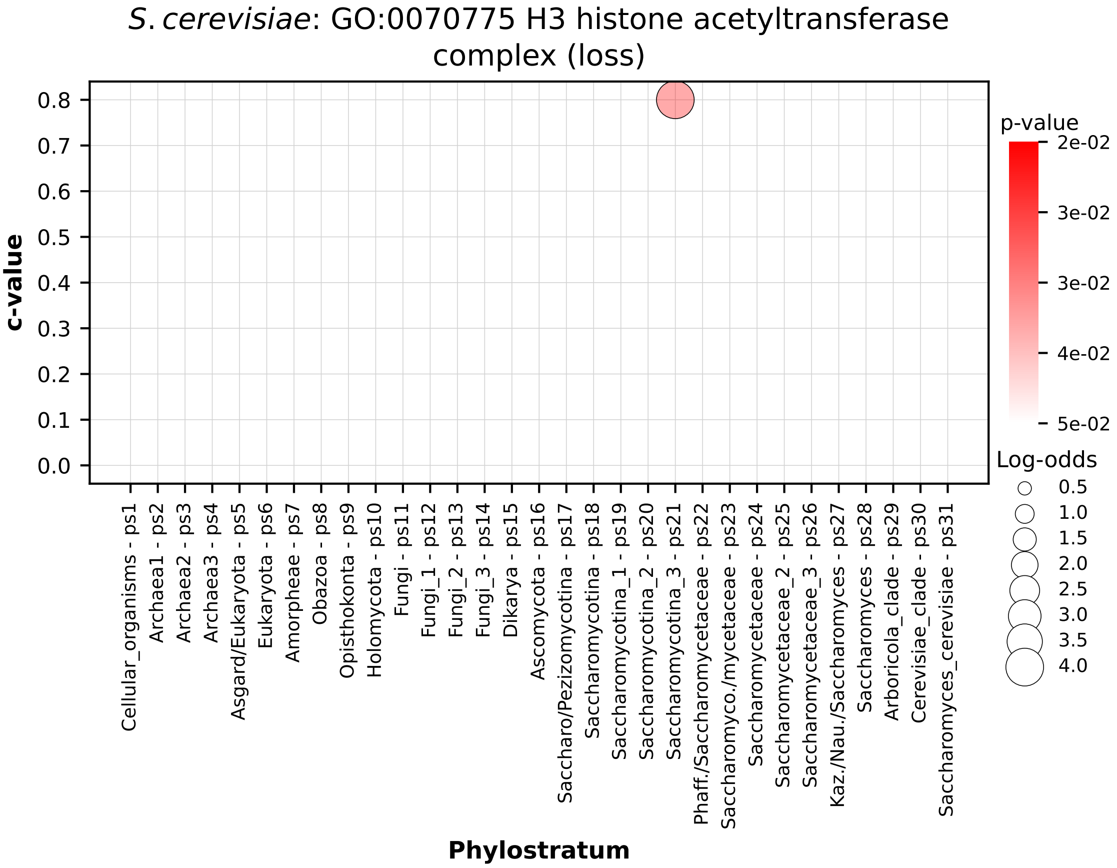

S. cerevisiae: GO:0070775 H3 histone acetyltransferase complex (loss)
c-value
0.8
0.7
0.6
0.5
0.4
0.3
0.2
0.1
0.0
p-value
2e-02
3e-02
3e-02
4e-02
5e-02
Log-odds
0.5
1.0
1.5
2.0
2.5
3.0
3.5
4.0
Cellular_organisms - ps1
Archaea1 - ps2
Archaea2 - ps3
Archaea3 - ps4
Asgard/Eukaryota - ps5
Eukaryota - ps6
Amorpheae - ps7
Obazoa - ps8
Opisthokonta - ps9
Holomycota - ps10
Fungi - ps11
Fungi_1 - ps12
Fungi_2 - ps13
Fungi_3 - ps14
Dikarya - ps15
Ascomycota - ps16
Saccharo/Pezizomycotina - ps17
Saccharomycotina - ps18
Saccharomycotina_1 - ps19
Saccharomycotina_2 - ps20
Saccharomycotina_3 - ps21
Phaff./Saccharomycetaceae - ps22
Saccharomyco./mycetaceae - ps23
Saccharomycetaceae - ps24
Saccharomycetaceae_2 - ps25
Saccharomycetaceae_3 - ps26
Kaz./Nau./Saccharomyces - ps27
Saccharomyces - ps28
Arboricola_clade - ps29
Cerevisiae_clade - ps30
Saccharomyces_cerevisiae - ps31
Phylostratum

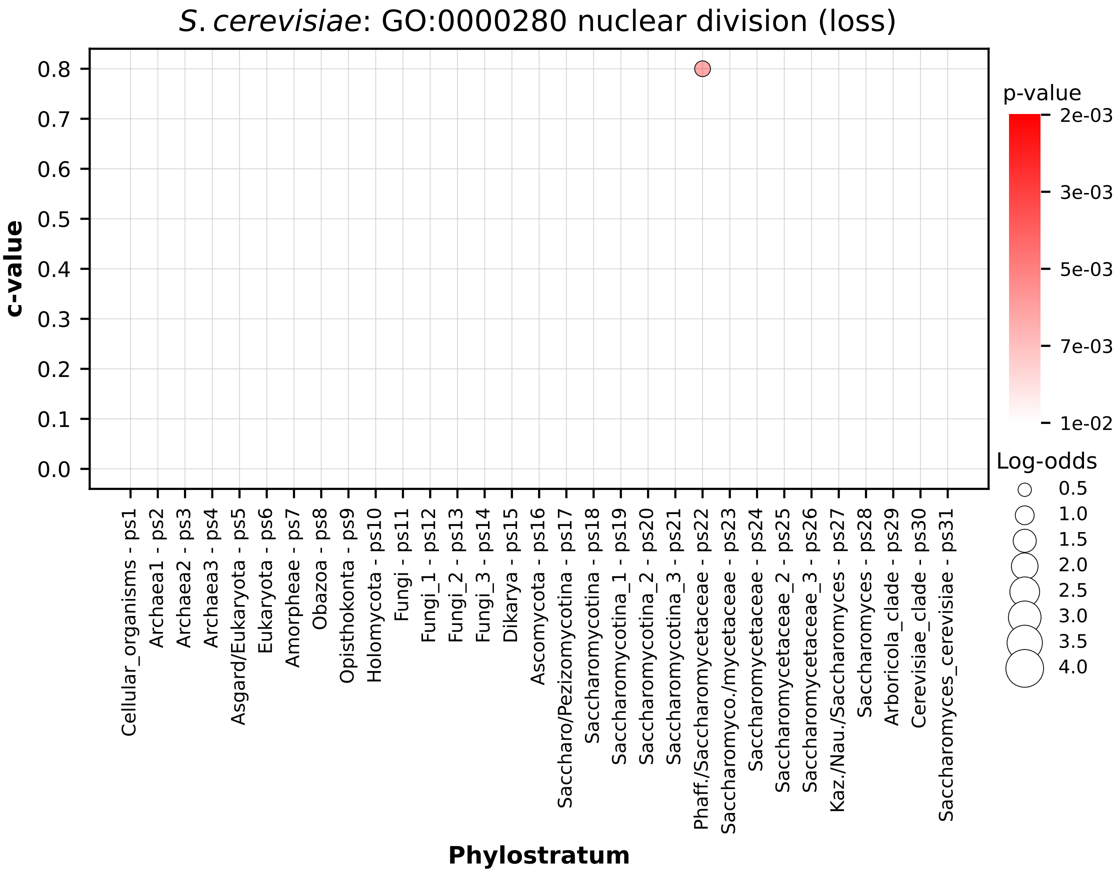

S. cerevisiae: GO:0000280 nuclear division (loss)
c-value
0.8
0.7
0.6
0.5
0.4
0.3
0.2
0.1
0.0
p-value
2e-03
3e-03
5e-03
7e-03
1e-02
Log-odds
0.5
1.0
1.5
2.0
2.5
3.0
3.5
4.0
Cellular_organisms - ps1
Archaea1 - ps2
Archaea2 - ps3
Archaea3 - ps4
Asgard/Eukaryota - ps5
Eukaryota - ps6
Amorpheae - ps7
Obazoa - ps8
Opisthokonta - ps9
Holomycota - ps10
Fungi - ps11
Fungi_1 - ps12
Fungi_2 - ps13
Fungi_3 - ps14
Dikarya - ps15
Ascomycota - ps16
Saccharo/Pezizomycotina - ps17
Saccharomycotina - ps18
Saccharomycotina_1 - ps19
Saccharomycotina_2 - ps20
Saccharomycotina_3 - ps21
Phaff./Saccharomycetaceae - ps22
Saccharomyco./mycetaceae - ps23
Saccharomycetaceae - ps24
Saccharomycetaceae_2 - ps25
Saccharomycetaceae_3 - ps26
Kaz./Nau./Saccharomyces - ps27
Saccharomyces - ps28
Arboricola_clade - ps29
Cerevisiae_clade - ps30
Saccharomyces_cerevisiae - ps31
Phylostratum

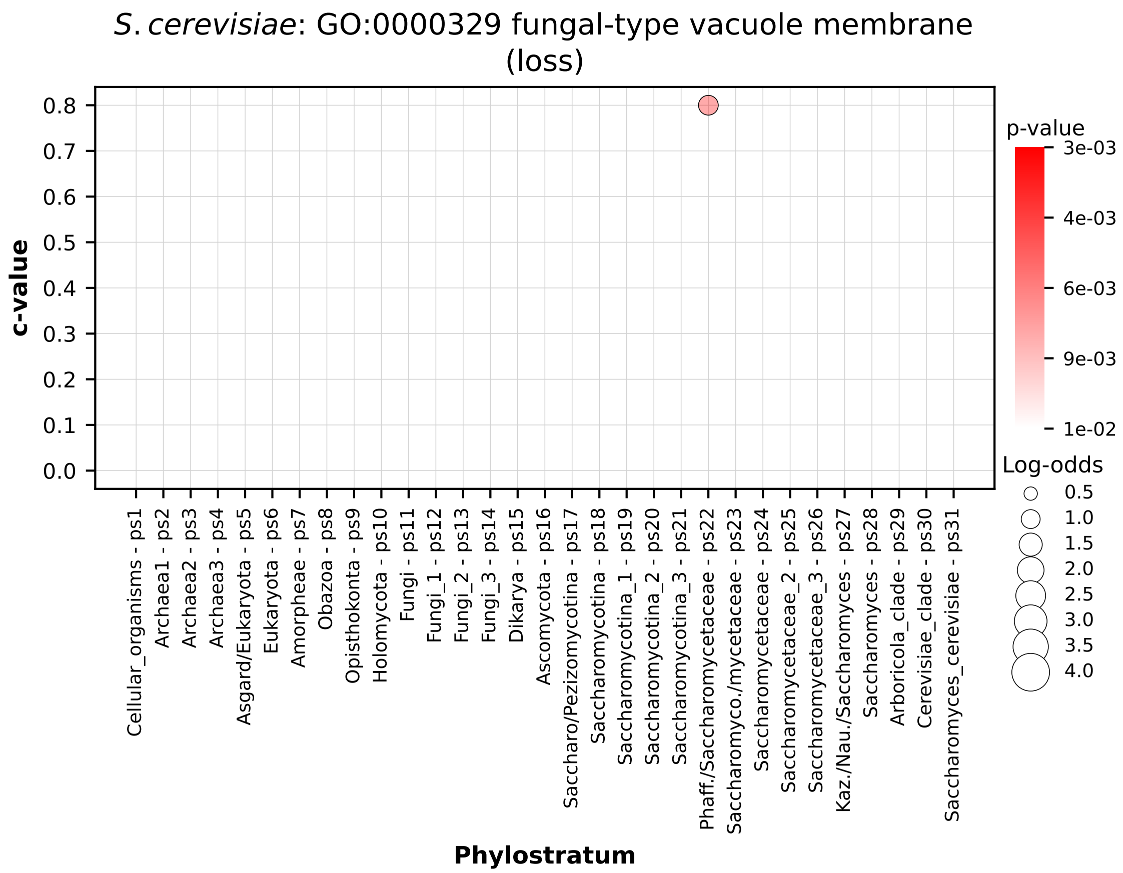

S. cerevisiae: GO:0000329 fungal-type vacuole membrane (loss)
c-value
0.8
0.7
0.6
0.5
0.4
0.3
0.2
0.1
0.0
p-value
3e-03
4e-03
6e-03
9e-03
1e-02
Log-odds
0.5
1.0
1.5
2.0
2.5
3.0
3.5
4.0
Cellular_organisms - ps1
Archaea1 - ps2
Archaea2 - ps3
Archaea3 - ps4
Asgard/Eukaryota - ps5
Eukaryota - ps6
Amorpheae - ps7
Obazoa - ps8
Opisthokonta - ps9
Holomycota - ps10
Fungi - ps11
Fungi_1 - ps12
Fungi_2 - ps13
Fungi_3 - ps14
Dikarya - ps15
Ascomycota - ps16
Saccharo/Pezizomycotina - ps17
Saccharomycotina - ps18
Saccharomycotina_1 - ps19
Saccharomycotina_2 - ps20
Saccharomycotina_3 - ps21
Phaff./Saccharomycetaceae - ps22
Saccharomyco./mycetaceae - ps23
Saccharomycetaceae - ps24
Saccharomycetaceae_2 - ps25
Saccharomycetaceae_3 - ps26
Kaz./Nau./Saccharomyces - ps27
Saccharomyces - ps28
Arboricola_clade - ps29
Cerevisiae_clade - ps30
Saccharomyces_cerevisiae - ps31
Phylostratum

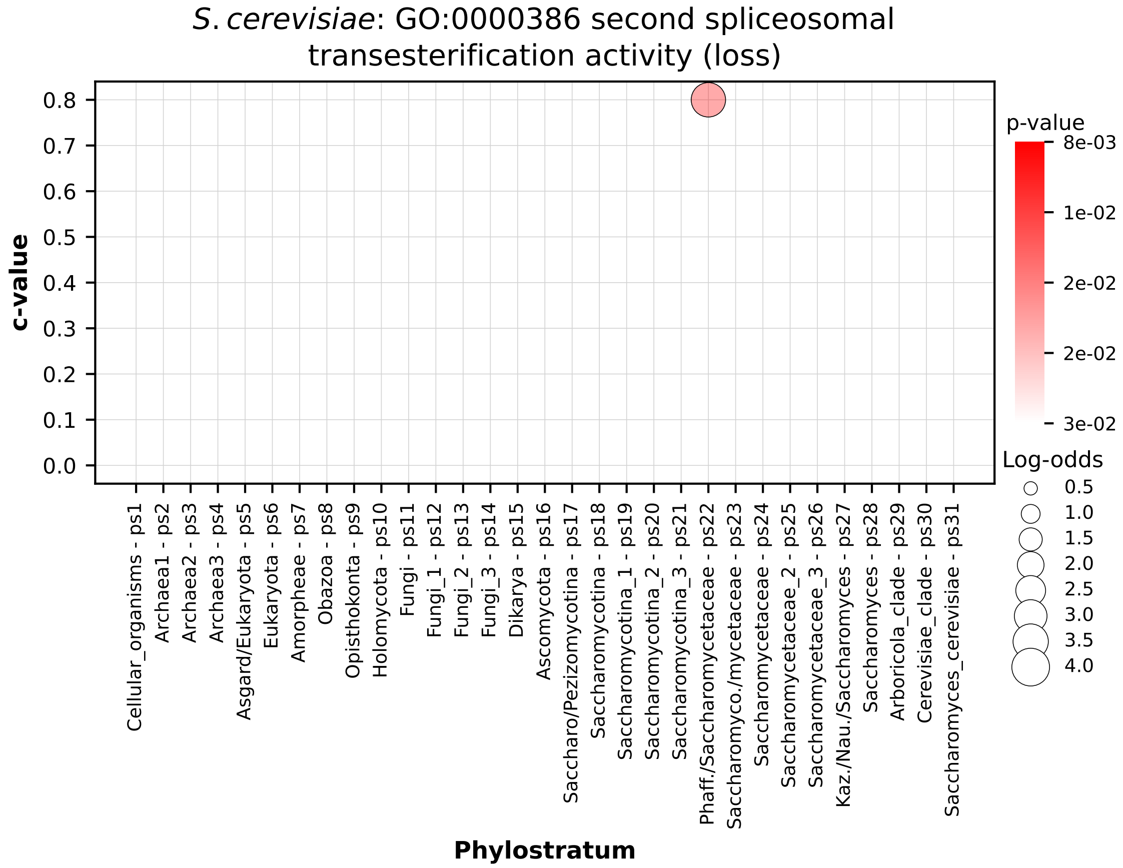

S. cerevisiae: GO:0000386 second spliceosomal transesterification activity (loss)
c-value
0.8
0.7
0.6
0.5
0.4
0.3
0.2
0.1
0.0
Phylostratum
Cellular_organisms - ps1
Archaea1 - ps2
Archaea2 - ps3
Archaea3 - ps4
Asgard/Eukaryota - ps5
Eukaryota - ps6
Amorpheae - ps7
Obazoa - ps8
Opisthokonta - ps9
Holomycota - ps10
Fungi - ps11
Fungi_1 - ps12
Fungi_2 - ps13
Fungi_3 - ps14
Dikarya - ps15
Ascomycota - ps16
Saccharo/Pezizomycotina - ps17
Saccharomycotina - ps18
Saccharomycotina_1 - ps19
Saccharomycotina_2 - ps20
Saccharomycotina_3 - ps21
Phaff./Saccharomycetaceae - ps22
Saccharomyco./mycetaceae - ps23
Saccharomycetaceae - ps24
Saccharomycetaceae_2 - ps25
Saccharomycetaceae_3 - ps26
Kaz./Nau./Saccharomyces - ps27
Saccharomyces - ps28
Arboricola_clade - ps29
Cerevisiae_clade - ps30
Saccharomyces_cerevisiae - ps31
p-value
8e-03
1e-02
2e-02
2e-02
3e-02
Log-odds
0.5
1.0
1.5
2.0
2.5
3.0
3.5
4.0

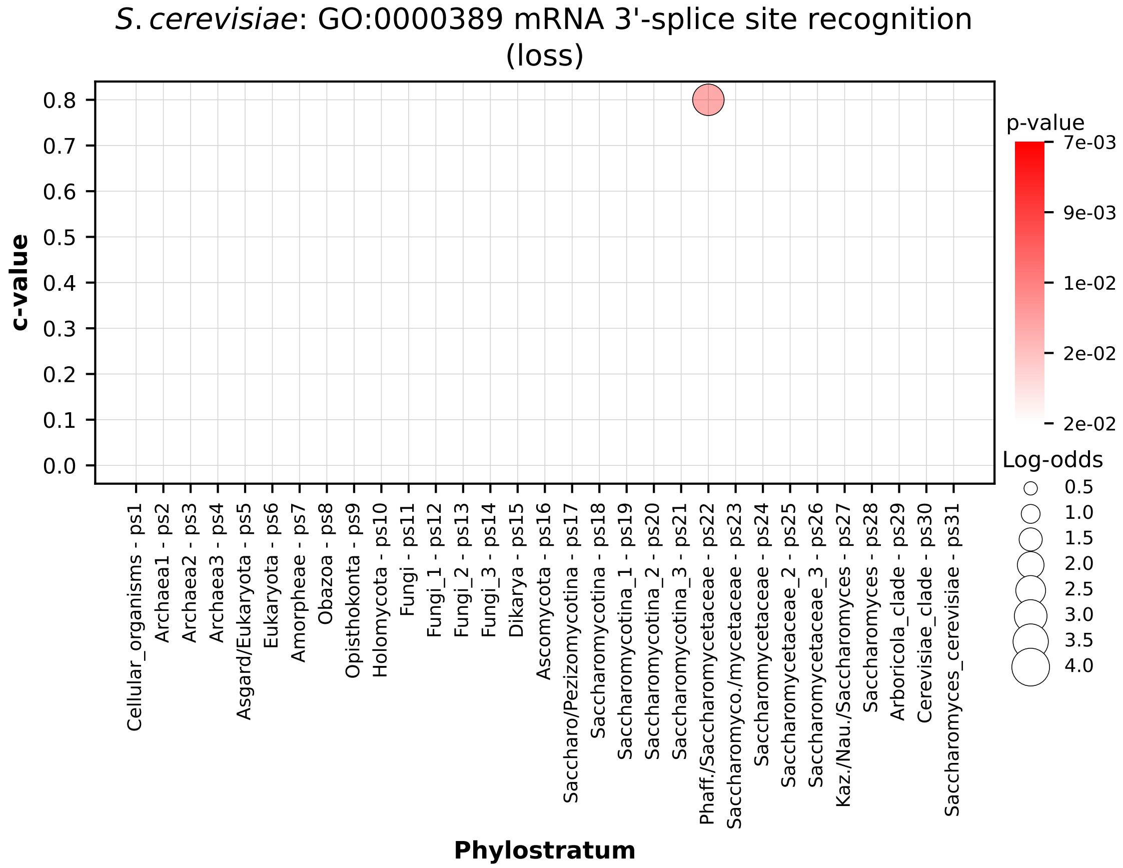

S. cerevisiae: GO:0000389 mRNA 3'-splice site recognition (loss)
c-value
0.0
0.1
0.2
0.3
0.4
0.5
0.6
0.7
0.8
Phylostratum
Cellular_organisms - ps1
Archaea1 - ps2
Archaea2 - ps3
Archaea3 - ps4
Asgard/Eukaryota - ps5
Eukaryota - ps6
Amorpheae - ps7
Obazoa - ps8
Opisthokonta - ps9
Holomycota - ps10
Fungi - ps11
Fungi_1 - ps12
Fungi_2 - ps13
Fungi_3 - ps14
Dikarya - ps15
Ascomycota - ps16
Saccharo/Pezizomycotina - ps17
Saccharomycotina - ps18
Saccharomycotina_1 - ps19
Saccharomycotina_2 - ps20
Saccharomycotina_3 - ps21
Phaff./Saccharomycetaceae - ps22
Saccharomyco./mycetaceae - ps23
Saccharomycetaceae - ps24
Saccharomycetaceae_2 - ps25
Saccharomycetaceae_3 - ps26
Kaz./Nau./Saccharomyces - ps27
Saccharomyces - ps28
Arboricola_clade - ps29
Cerevisiae_clade - ps30
Saccharomyces_cerevisiae - ps31
p-value
7e-03
9e-03
1e-02
2e-02
2e-02
Log-odds
0.5
1.0
1.5
2.0
2.5
3.0
3.5
4.0

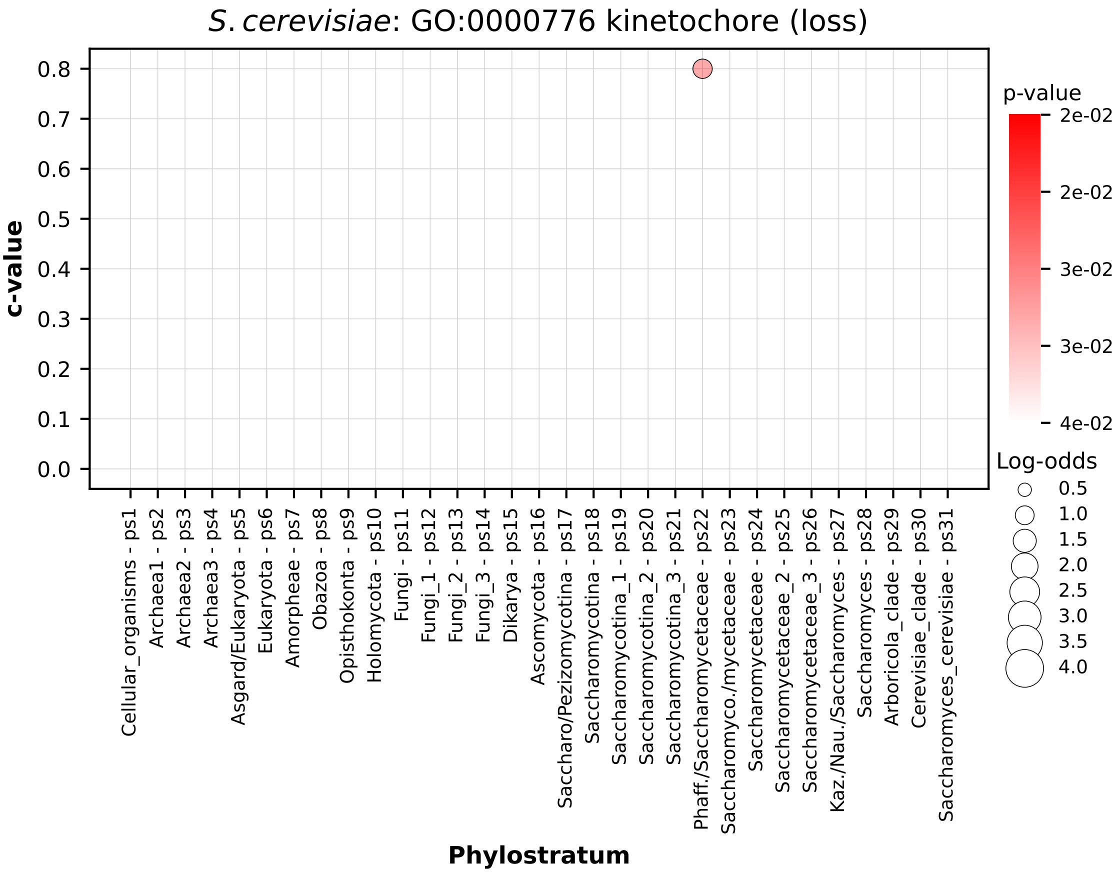
S. cerevisiae: GO:0000776 kinetochore (loss)
c-value
0.8
0.7
0.6
0.5
0.4
0.3
0.2
0.1
0.0
p-value
2e-02
2e-02
3e-02
3e-02
4e-02
Log-odds
0.5
1.0
1.5
2.0
2.5
3.0
3.5
4.0
Cellular_organisms - ps1
Archaea1 - ps2
Archaea2 - ps3
Archaea3 - ps4
Asgard/Eukaryota - ps5
Eukaryota - ps6
Amorpheae - ps7
Obazoa - ps8
Opisthokonta - ps9
Holomycota - ps10
Fungi - ps11
Fungi_1 - ps12
Fungi_2 - ps13
Fungi_3 - ps14
Dikarya - ps15
Ascomycota - ps16
Saccharo/Pezizomycotina - ps17
Saccharomycotina - ps18
Saccharomycotina_1 - ps19
Saccharomycotina_2 - ps20
Saccharomycotina_3 - ps21
Phaff./Saccharomycetaceae - ps22
Saccharomyco./mycetaceae - ps23
Saccharomycetaceae - ps24
Saccharomycetaceae_2 - ps25
Saccharomycetaceae_3 - ps26
Kaz./Nau./Saccharomyces - ps27
Saccharomyces - ps28
Arboricola_clade - ps29
Cerevisiae_clade - ps30
Saccharomyces_cerevisiae - ps31
Phylostratum

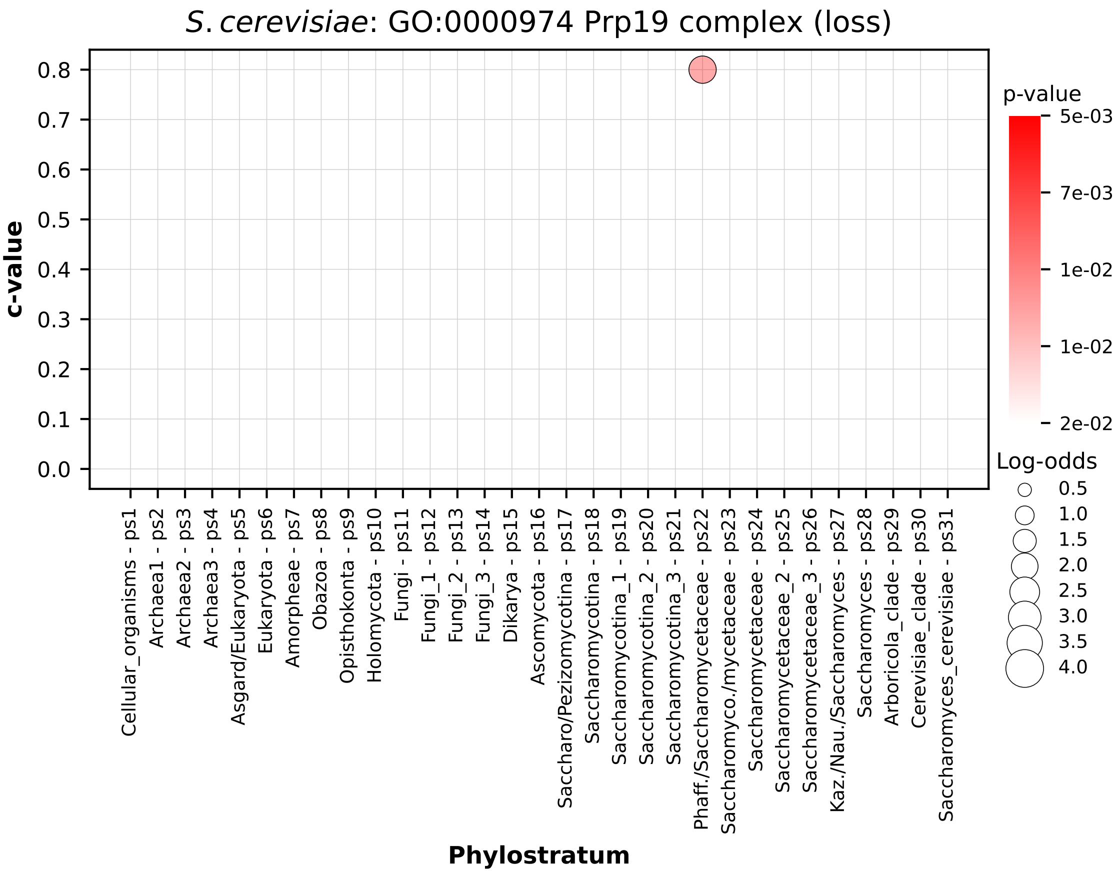

S. cerevisiae: GO:0000974 Prp19 complex (loss)
c-value
0.0
0.1
0.2
0.3
0.4
0.5
0.6
0.7
0.8
p-value
5e-03
7e-03
1e-02
1e-02
2e-02
Log-odds
0.5
1.0
1.5
2.0
2.5
3.0
3.5
4.0
Cellular_organisms - ps1
Archaea1 - ps2
Archaea2 - ps3
Archaea3 - ps4
Asgard/Eukaryota - ps5
Eukaryota - ps6
Amorpheae - ps7
Obazoa - ps8
Opisthokonta - ps9
Holomycota - ps10
Fungi - ps11
Fungi_1 - ps12
Fungi_2 - ps13
Fungi_3 - ps14
Dikarya - ps15
Ascomycota - ps16
Saccharo/Pezizomycotina - ps17
Saccharomycotina - ps18
Saccharomycotina_1 - ps19
Saccharomycotina_2 - ps20
Saccharomycotina_3 - ps21
Phaff./Saccharomycetaceae - ps22
Saccharomyco./mycetaceae - ps23
Saccharomycetaceae - ps24
Saccharomycetaceae_2 - ps25
Saccharomycetaceae_3 - ps26
Kaz./Nau./Saccharomyces - ps27
Saccharomyces - ps28
Arboricola_clade - ps29
Cerevisiae_clade - ps30
Saccharomyces_cerevisiae - ps31
Phylostratum

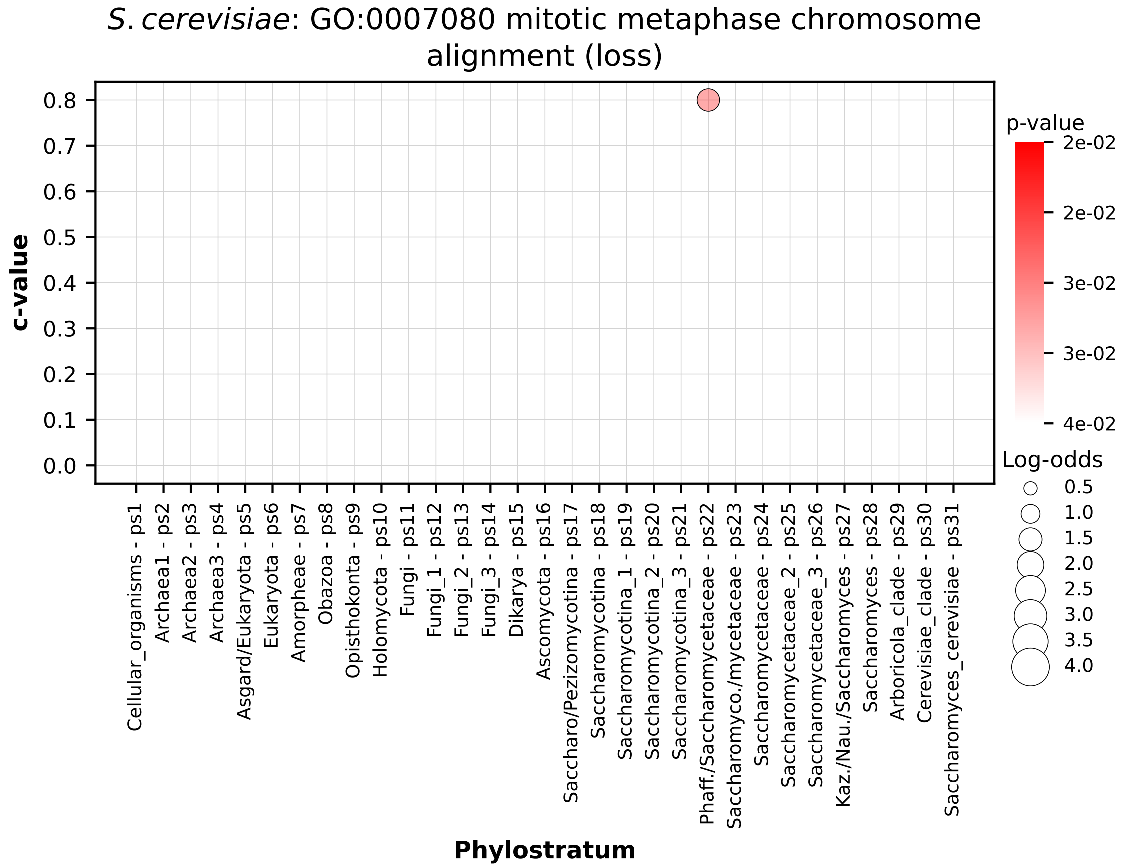

S. cerevisiae: GO:0007080 mitotic metaphase chromosome alignment (loss)
c-value
0.0
0.1
0.2
0.3
0.4
0.5
0.6
0.7
0.8
p-value
2e-02
2e-02
3e-02
3e-02
4e-02
Log-odds
0.5
1.0
1.5
2.0
2.5
3.0
3.5
4.0
Cellular_organisms - ps1
Archaea1 - ps2
Archaea2 - ps3
Archaea3 - ps4
Asgard/Eukaryota - ps5
Eukaryota - ps6
Amorpheae - ps7
Obazoa - ps8
Opisthokonta - ps9
Holomycota - ps10
Fungi - ps11
Fungi_1 - ps12
Fungi_2 - ps13
Fungi_3 - ps14
Dikarya - ps15
Ascomycota - ps16
Saccharo/Pezizomycotina - ps17
Saccharomycotina - ps18
Saccharomycotina_1 - ps19
Saccharomycotina_2 - ps20
Saccharomycotina_3 - ps21
Phaff./Saccharomycetaceae - ps22
Saccharomyco./mycetaceae - ps23
Saccharomycetaceae - ps24
Saccharomycetaceae_2 - ps25
Saccharomycetaceae_3 - ps26
Kaz./Nau./Saccharomyces - ps27
Saccharomyces - ps28
Arboricola_clade - ps29
Cerevisiae_clade - ps30
Saccharomyces_cerevisiae - ps31
Phylostratum

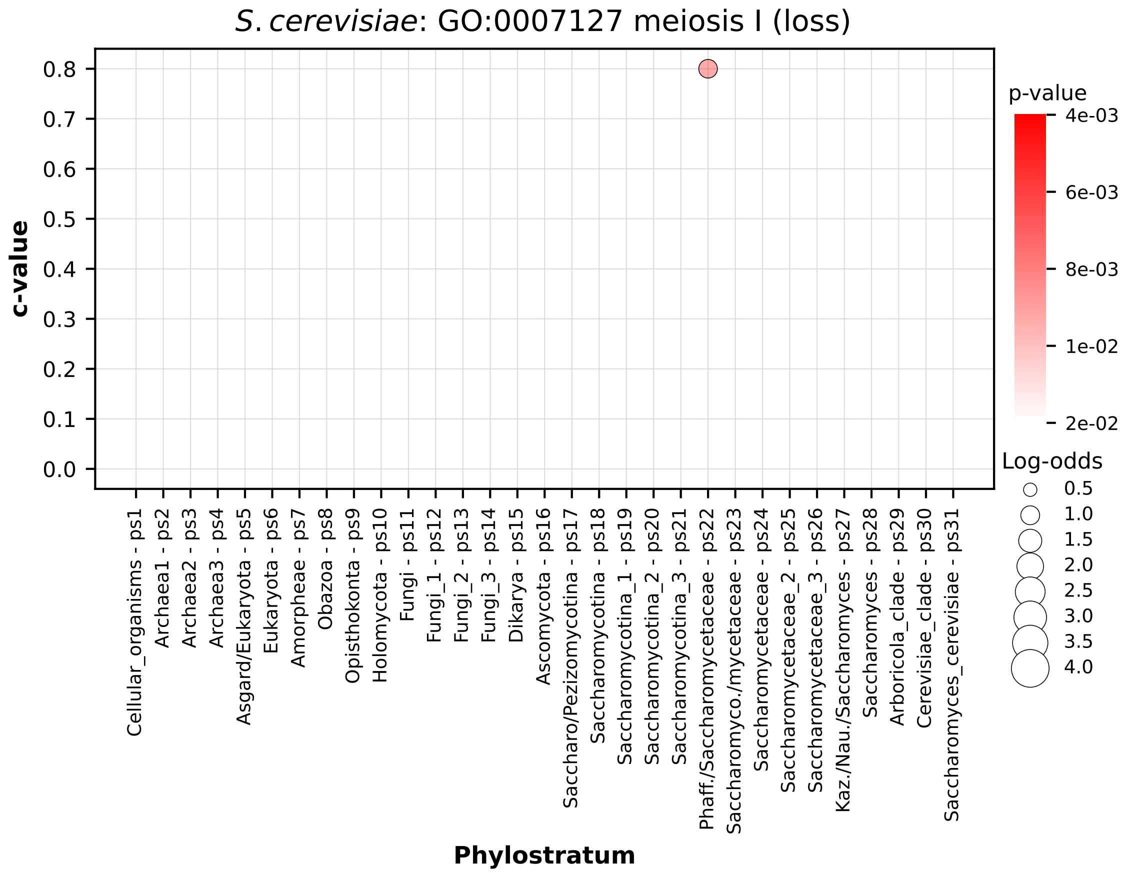
S. cerevisiae: GO:0007127 meiosis I (loss)
c-value
0.0
0.1
0.2
0.3
0.4
0.5
0.6
0.7
0.8
p-value
4e-03
6e-03
8e-03
1e-02
2e-02
Log-odds
0.5
1.0
1.5
2.0
2.5
3.0
3.5
4.0
Cellular_organisms - ps1
Archaea1 - ps2
Archaea2 - ps3
Archaea3 - ps4
Asgard/Eukaryota - ps5
Eukaryota - ps6
Amorpheae - ps7
Obazoa - ps8
Opisthokonta - ps9
Holomycota - ps10
Fungi - ps11
Fungi_1 - ps12
Fungi_2 - ps13
Fungi_3 - ps14
Dikarya - ps15
Ascomycota - ps16
Saccharo/Pezizomycotina - ps17
Saccharomycotina - ps18
Saccharomycotina_1 - ps19
Saccharomycotina_2 - ps20
Saccharomycotina_3 - ps21
Phaff./Saccharomycetaceae - ps22
Saccharomyco./mycetaceae - ps23
Saccharomycetaceae - ps24
Saccharomycetaceae_2 - ps25
Saccharomycetaceae_3 - ps26
Kaz./Nau./Saccharomyces - ps27
Saccharomyces - ps28
Arboricola_clade - ps29
Cerevisiae_clade - ps30
Saccharomyces_cerevisiae - ps31
Phylostratum

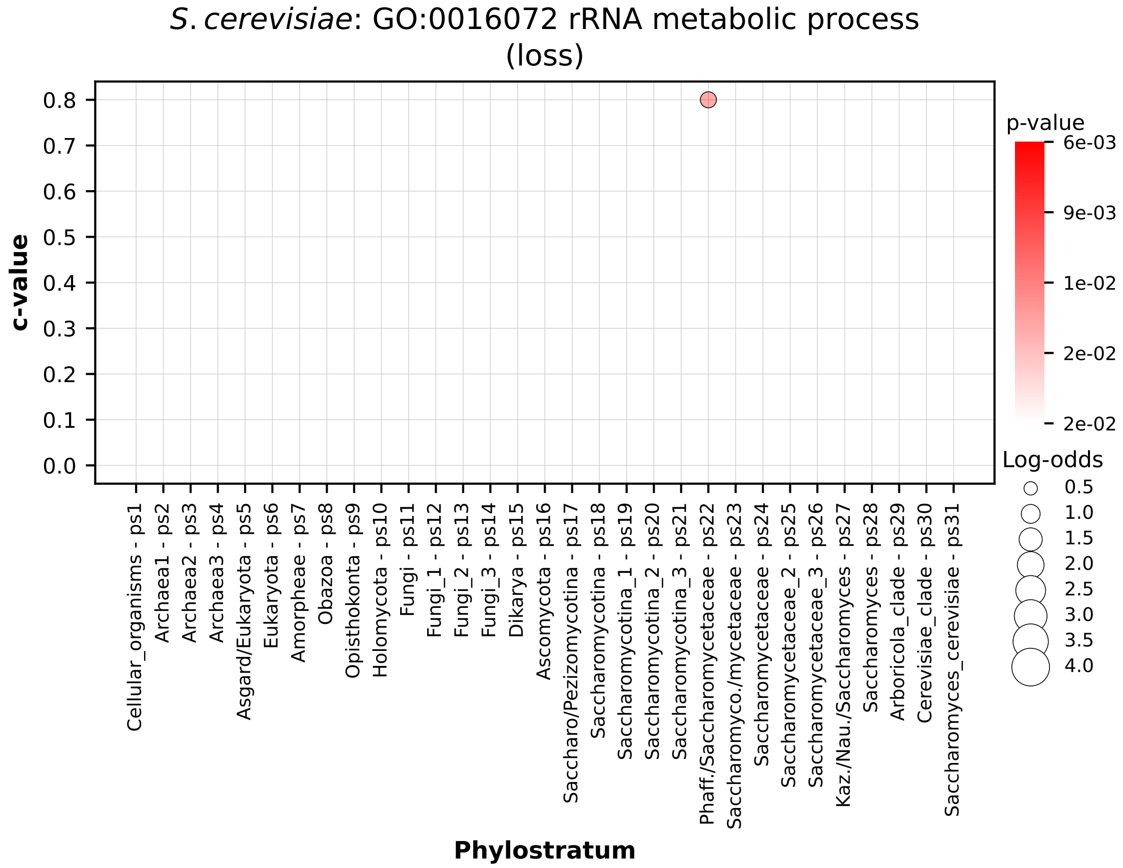

S. cerevisiae: GO:0016072 rRNA metabolic process
(loss)
c-value
0.8
0.7
0.6
0.5
0.4
0.3
0.2
0.1
0.0
p-value
6e-03
9e-03
1e-02
2e-02
2e-02
Log-odds
0.5
1.0
1.5
2.0
2.5
3.0
3.5
4.0
Cellular_organisms - ps1
Archaea1 - ps2
Archaea2 - ps3
Archaea3 - ps4
Asgard/Eukaryota - ps5
Eukaryota - ps6
Amorpheae - ps7
Obazoa - ps8
Opisthokonta - ps9
Holomycota - ps10
Fungi - ps11
Fungi_1 - ps12
Fungi_2 - ps13
Fungi_3 - ps14
Dikarya - ps15
Ascomycota - ps16
Saccharo/Pezizomycotina - ps17
Saccharomycotina - ps18
Saccharomycotina_1 - ps19
Saccharomycotina_2 - ps20
Saccharomycotina_3 - ps21
Phaff./Saccharomycetaceae - ps22
Saccharomyco./mycetaceae - ps23
Saccharomycetaceae - ps24
Saccharomycetaceae_2 - ps25
Saccharomycetaceae_3 - ps26
Kaz./Nau./Saccharomyces - ps27
Saccharomyces - ps28
Arboricola_clade - ps29
Cerevisiae_clade - ps30
Saccharomyces_cerevisiae - ps31
Phylostratum

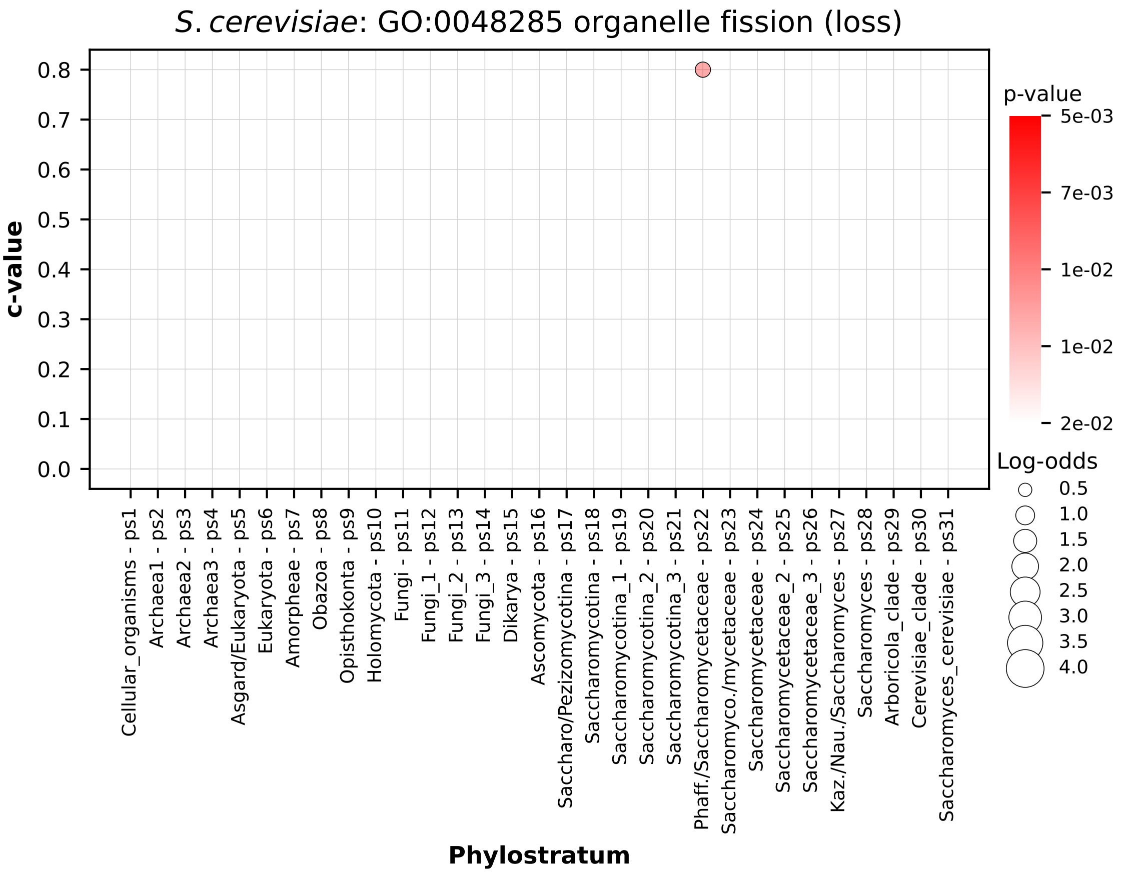

S. cerevisiae: GO:0048285 organelle fission (loss)
c-value
0.8
0.7
0.6
0.5
0.4
0.3
0.2
0.1
0.0
p-value
5e-03
7e-03
1e-02
1e-02
2e-02
Log-odds
0.5
1.0
1.5
2.0
2.5
3.0
3.5
4.0
Cellular_organisms - ps1
Archaea1 - ps2
Archaea2 - ps3
Archaea3 - ps4
Asgard/Eukaryota - ps5
Eukaryota - ps6
Amorpheae - ps7
Obazoa - ps8
Opisthokonta - ps9
Holomycota - ps10
Fungi - ps11
Fungi_1 - ps12
Fungi_2 - ps13
Fungi_3 - ps14
Dikarya - ps15
Ascomycota - ps16
Saccharo/Pezizomycotina - ps17
Saccharomycotina - ps18
Saccharomycotina_1 - ps19
Saccharomycotina_2 - ps20
Saccharomycotina_3 - ps21
Phaff./Saccharomycetaceae - ps22
Saccharomyco./mycetaceae - ps23
Saccharomycetaceae - ps24
Saccharomycetaceae_2 - ps25
Saccharomycetaceae_3 - ps26
Kaz./Nau./Saccharomyces - ps27
Saccharomyces - ps28
Arboricola_clade - ps29
Cerevisiae_clade - ps30
Saccharomyces_cerevisiae - ps31
Phylostratum

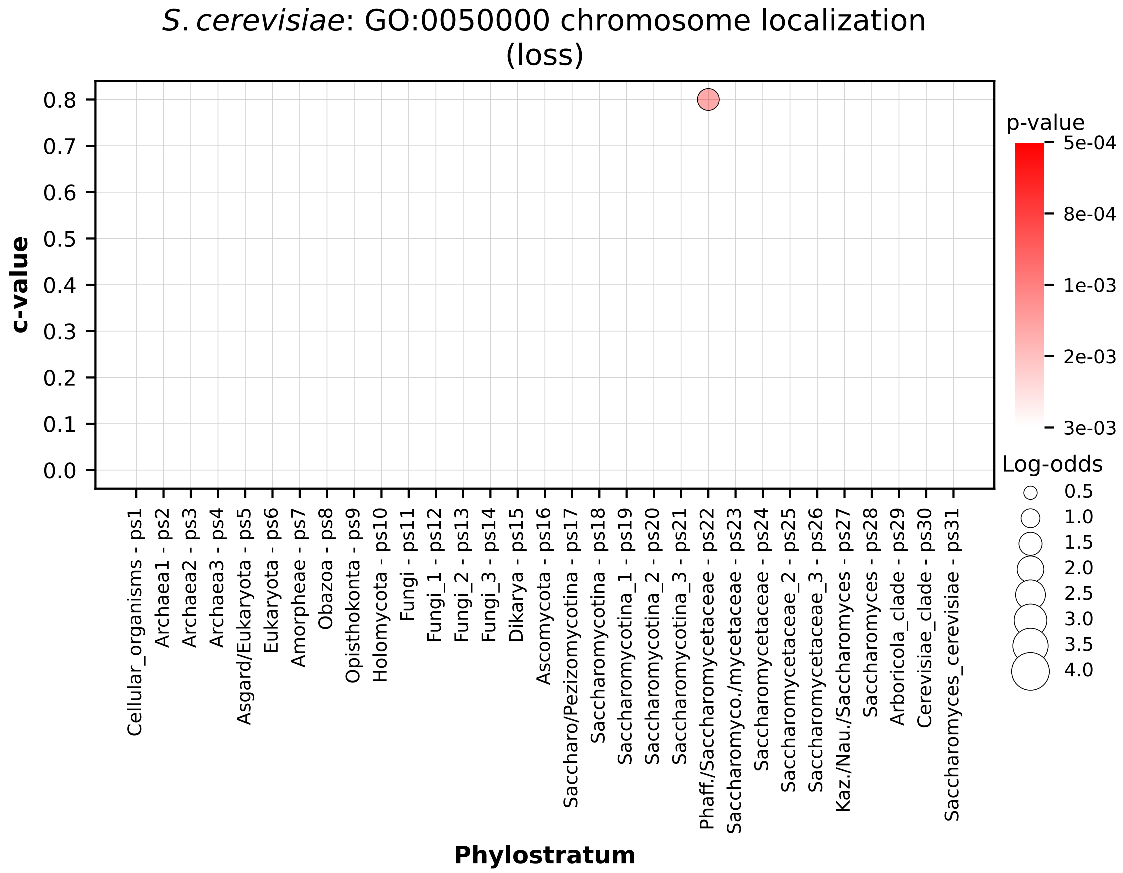

S. cerevisiae: GO:0050000 chromosome localization
(loss)
c-value
0.0
0.1
0.2
0.3
0.4
0.5
0.6
0.7
0.8
p-value
5e-04
8e-04
1e-03
2e-03
3e-03
Log-odds
0.5
1.0
1.5
2.0
2.5
3.0
3.5
4.0
Cellular_organisms - ps1
Archaea1 - ps2
Archaea2 - ps3
Archaea3 - ps4
Asgard/Eukaryota - ps5
Eukaryota - ps6
Amorpheae - ps7
Obazoa - ps8
Opisthokonta - ps9
Holomycota - ps10
Fungi - ps11
Fungi_1 - ps12
Fungi_2 - ps13
Fungi_3 - ps14
Dikarya - ps15
Ascomycota - ps16
Saccharo/Pezizomycotina - ps17
Saccharomycotina - ps18
Saccharomycotina_1 - ps19
Saccharomycotina_2 - ps20
Saccharomycotina_3 - ps21
Phaff./Saccharomycetaceae - ps22
Saccharomyco./mycetaceae - ps23
Saccharomycetaceae - ps24
Saccharomycetaceae_2 - ps25
Saccharomycetaceae_3 - ps26
Kaz./Nau./Saccharomyces - ps27
Saccharomyces - ps28
Arboricola_clade - ps29
Cerevisiae_clade - ps30
Saccharomyces_cerevisiae - ps31
Phylostratum

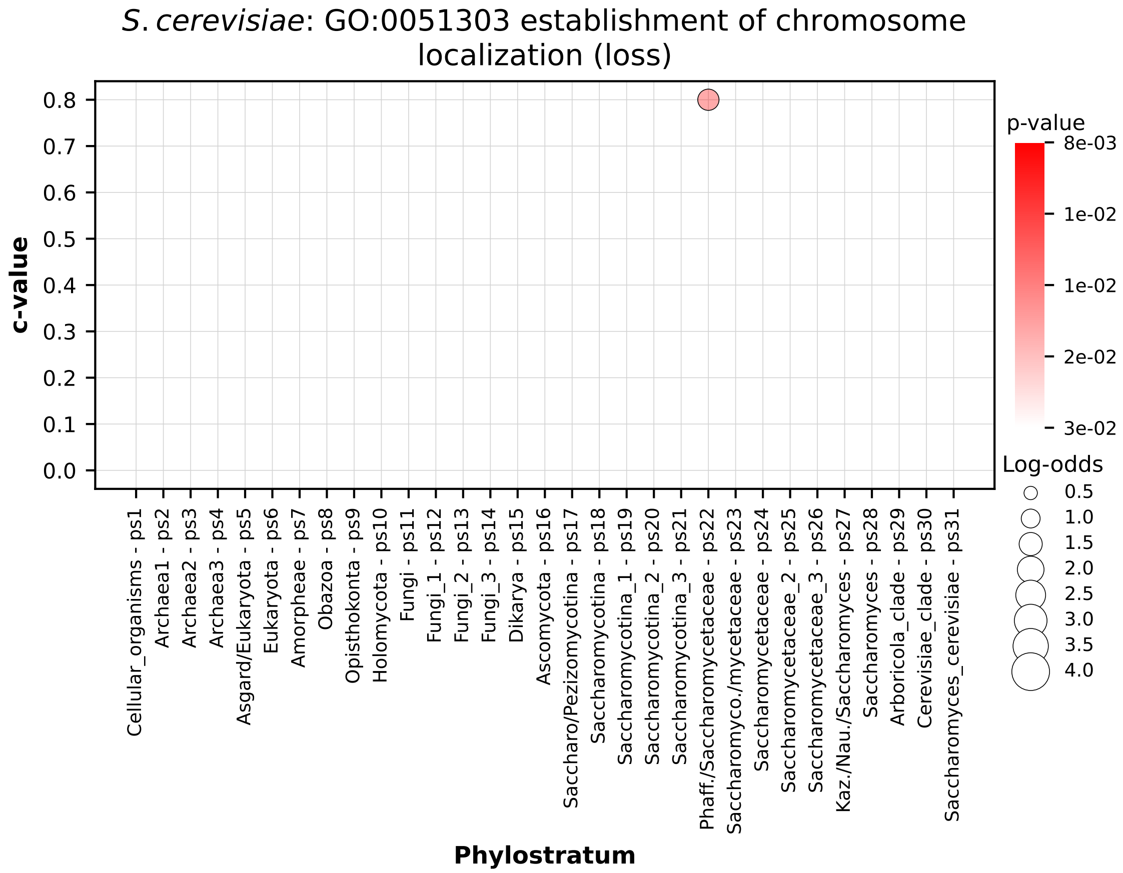

S. cerevisiae: GO:0051303 establishment of chromosome localization (loss)
c-value
0.8
0.7
0.6
0.5
0.4
0.3
0.2
0.1
0.0
p-value
8e-03
1e-02
1e-02
2e-02
3e-02
Log-odds
0.5
1.0
1.5
2.0
2.5
3.0
3.5
4.0
Cellular_organisms - ps1
Archaea1 - ps2
Archaea2 - ps3
Archaea3 - ps4
Asgard/Eukaryota - ps5
Eukaryota - ps6
Amorpheae - ps7
Obazoa - ps8
Opisthokonta - ps9
Holomycota - ps10
Fungi - ps11
Fungi_1 - ps12
Fungi_2 - ps13
Fungi_3 - ps14
Dikarya - ps15
Ascomycota - ps16
Saccharo/Pezizomycotina - ps17
Saccharomycotina - ps18
Saccharomycotina_1 - ps19
Saccharomycotina_2 - ps20
Saccharomycotina_3 - ps21
Phaff./Saccharomycetaceae - ps22
Saccharomyco./mycetaceae - ps23
Saccharomycetaceae - ps24
Saccharomycetaceae_2 - ps25
Saccharomycetaceae_3 - ps26
Kaz./Nau./Saccharomyces - ps27
Saccharomyces - ps28
Arboricola_clade - ps29
Cerevisiae_clade - ps30
Saccharomyces_cerevisiae - ps31
Phylostratum

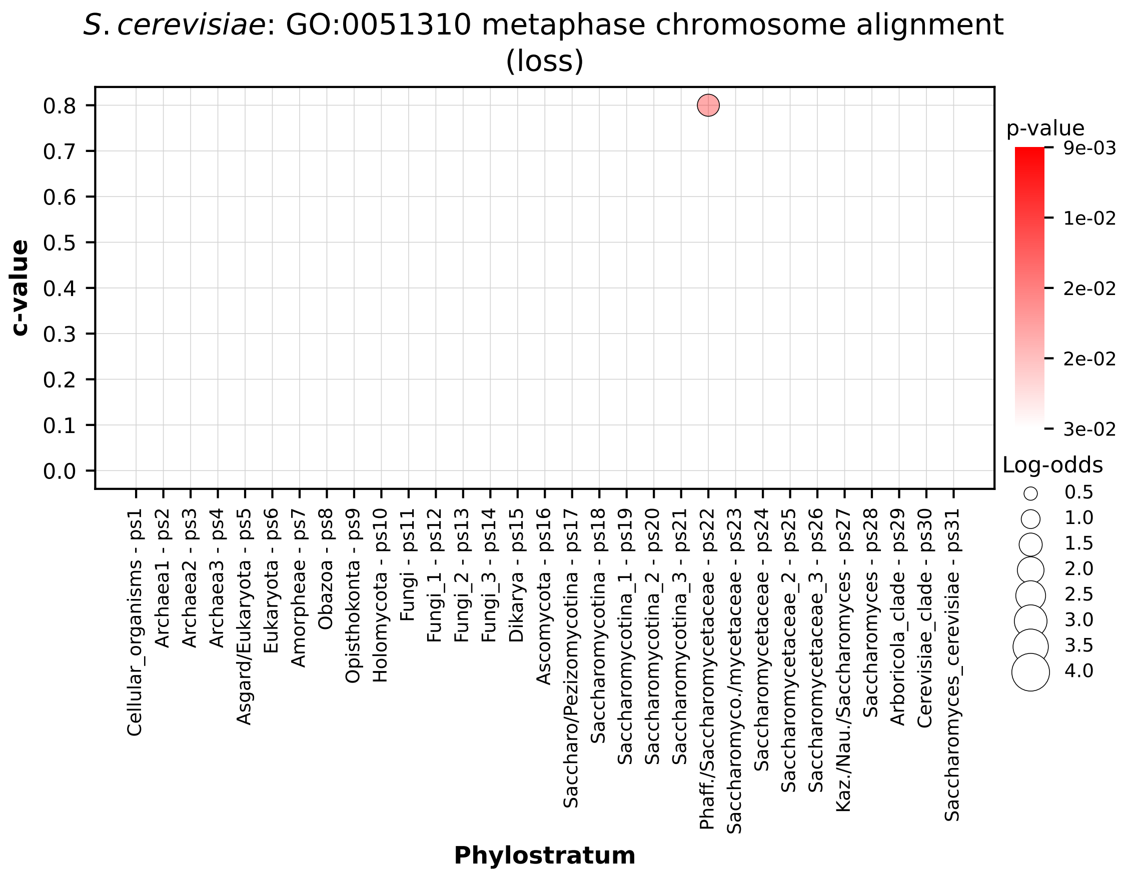

S. cerevisiae: GO:0051310 metaphase chromosome alignment (loss)
c-value
0.0
0.1
0.2
0.3
0.4
0.5
0.6
0.7
0.8
Phylostratum
Cellular_organisms - ps1
Archaea1 - ps2
Archaea2 - ps3
Archaea3 - ps4
Asgard/Eukaryota - ps5
Eukaryota - ps6
Amorpheae - ps7
Obazoa - ps8
Opisthokonta - ps9
Holomycota - ps10
Fungi - ps11
Fungi_1 - ps12
Fungi_2 - ps13
Fungi_3 - ps14
Dikarya - ps15
Ascomycota - ps16
Saccharo/Pezizomycotina - ps17
Saccharomycotina - ps18
Saccharomycotina_1 - ps19
Saccharomycotina_2 - ps20
Saccharomycotina_3 - ps21
Phaff./Saccharomycetaceae - ps22
Saccharomyco./mycetaceae - ps23
Saccharomycetaceae - ps24
Saccharomycetaceae_2 - ps25
Saccharomycetaceae_3 - ps26
Kaz./Nau./Saccharomyces - ps27
Saccharomyces - ps28
Arboricola_clade - ps29
Cerevisiae_clade - ps30
Saccharomyces_cerevisiae - ps31
p-value
9e-03
1e-02
2e-02
2e-02
3e-02
Log-odds
0.5
1.0
1.5
2.0
2.5
3.0
3.5
4.0

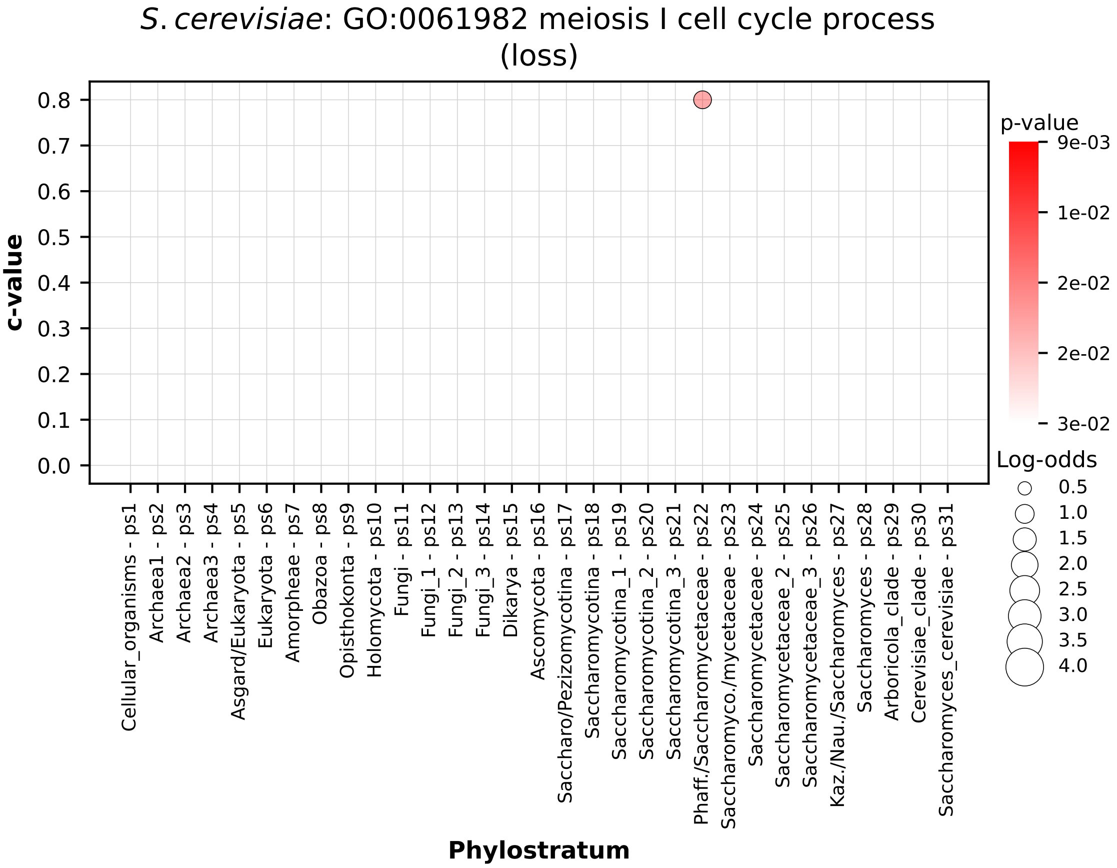
S. cerevisiae: GO:0061982 meiosis I cell cycle process
(loss)
c-value
0.8
0.7
0.6
0.5
0.4
0.3
0.2
0.1
0.0
p-value
9e-03
1e-02
2e-02
2e-02
3e-02
Log-odds
0.5
1.0
1.5
2.0
2.5
3.0
3.5
4.0
Cellular_organisms - ps1
Archaea1 - ps2
Archaea2 - ps3
Archaea3 - ps4
Asgard/Eukaryota - ps5
Eukaryota - ps6
Amorpheae - ps7
Obazoa - ps8
Opisthokonta - ps9
Holomycota - ps10
Fungi - ps11
Fungi_1 - ps12
Fungi_2 - ps13
Fungi_3 - ps14
Dikarya - ps15
Ascomycota - ps16
Saccharo/Pezizomycotina - ps17
Saccharomycotina - ps18
Saccharomycotina_1 - ps19
Saccharomycotina_2 - ps20
Saccharomycotina_3 - ps21
Phaff./Saccharomycetaceae - ps22
Saccharomyco./mycetaceae - ps23
Saccharomycetaceae - ps24
Saccharomycetaceae_2 - ps25
Saccharomycetaceae_3 - ps26
Kaz./Nau./Saccharomyces - ps27
Saccharomyces - ps28
Arboricola_clade - ps29
Cerevisiae_clade - ps30
Saccharomyces_cerevisiae - ps31
Phylostratum

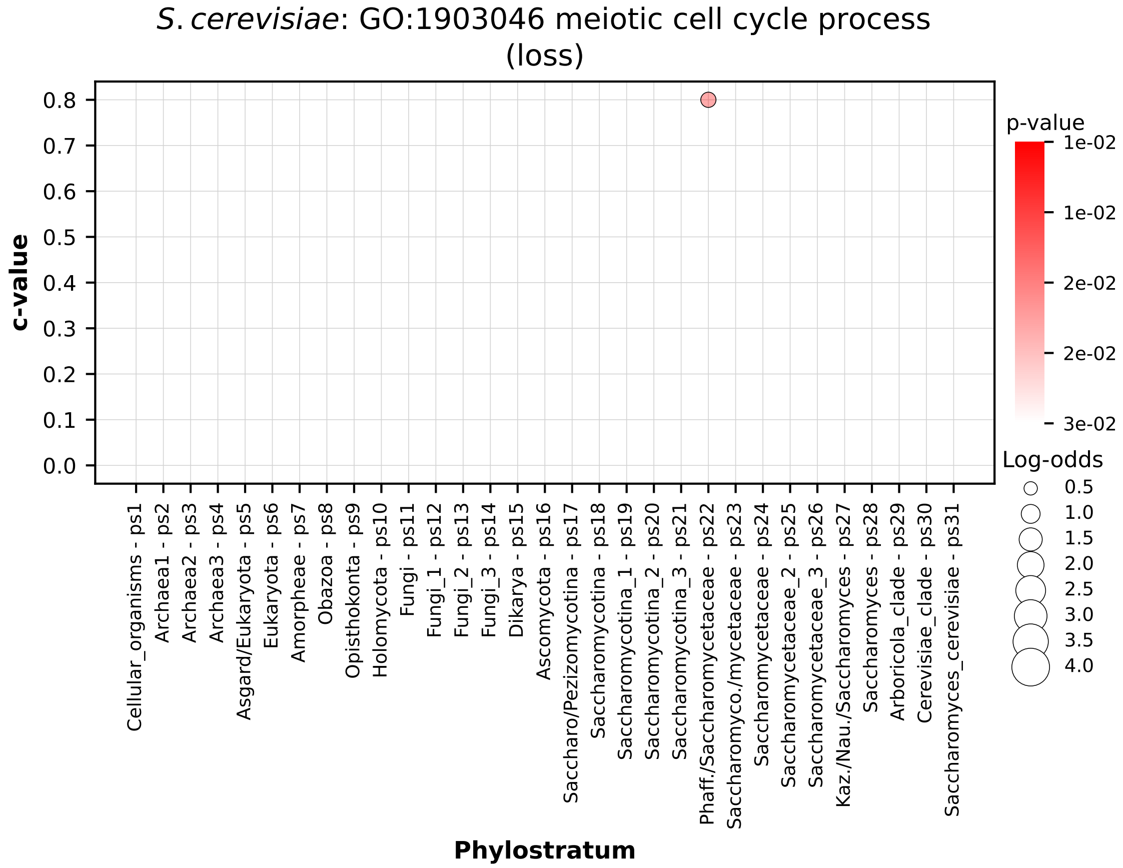
S. cerevisiae: GO:1903046 meiotic cell cycle process
(loss)
c-value
0.8
0.7
0.6
0.5
0.4
0.3
0.2
0.1
0.0
p-value
1e-02
1e-02
2e-02
2e-02
3e-02
Log-odds
0.5
1.0
1.5
2.0
2.5
3.0
3.5
4.0
Cellular_organisms - ps1
Archaea1 - ps2
Archaea2 - ps3
Archaea3 - ps4
Asgard/Eukaryota - ps5
Eukaryota - ps6
Amorpheae - ps7
Obazoa - ps8
Opisthokonta - ps9
Holomycota - ps10
Fungi - ps11
Fungi_1 - ps12
Fungi_2 - ps13
Fungi_3 - ps14
Dikarya - ps15
Ascomycota - ps16
Saccharo/Pezizomycotina - ps17
Saccharomycotina - ps18
Saccharomycotina_1 - ps19
Saccharomycotina_2 - ps20
Saccharomycotina_3 - ps21
Phaff./Saccharomycetaceae - ps22
Saccharomyco./mycetaceae - ps23
Saccharomycetaceae - ps24
Saccharomycetaceae_2 - ps25
Saccharomycetaceae_3 - ps26
Kaz./Nau./Saccharomyces - ps27
Saccharomyces - ps28
Arboricola_clade - ps29
Cerevisiae_clade - ps30
Saccharomyces_cerevisiae - ps31
Phylostratum

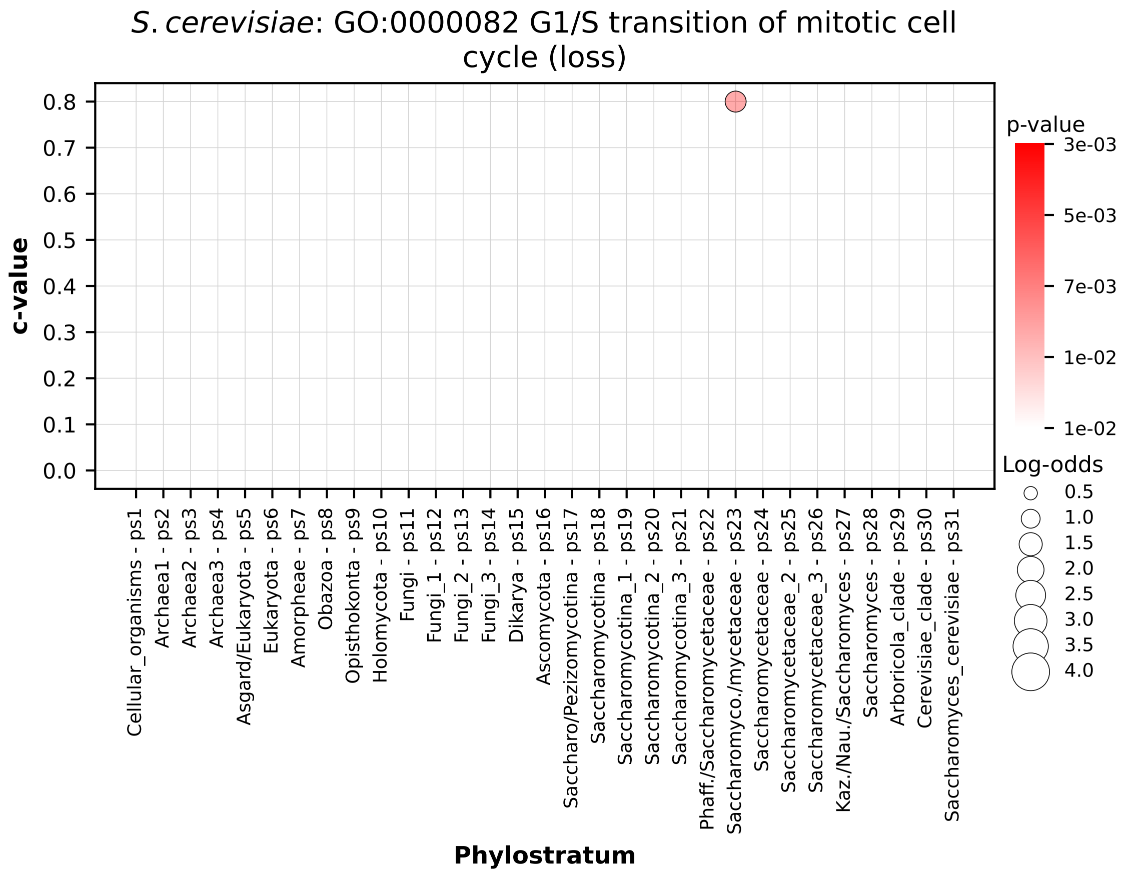

S. cerevisiae: GO:0000082 G1/S transition of mitotic cell cycle (loss)
c-value
0.0
0.1
0.2
0.3
0.4
0.5
0.6
0.7
0.8
p-value
3e-03
5e-03
7e-03
1e-02
1e-02
Log-odds
0.5
1.0
1.5
2.0
2.5
3.0
3.5
4.0
Cellular_organisms - ps1
Archaea1 - ps2
Archaea2 - ps3
Archaea3 - ps4
Asgard/Eukaryota - ps5
Eukaryota - ps6
Amorpheae - ps7
Obazoa - ps8
Opisthokonta - ps9
Holomycota - ps10
Fungi - ps11
Fungi_1 - ps12
Fungi_2 - ps13
Fungi_3 - ps14
Dikarya - ps15
Ascomycota - ps16
Saccharo/Pezizomycotina - ps17
Saccharomycotina - ps18
Saccharomycotina_1 - ps19
Saccharomycotina_2 - ps20
Saccharomycotina_3 - ps21
Phaff./Saccharomycetaceae - ps22
Saccharomyco./mycetaceae - ps23
Saccharomycetaceae - ps24
Saccharomycetaceae_2 - ps25
Saccharomycetaceae_3 - ps26
Kaz./Nau./Saccharomyces - ps27
Saccharomyces - ps28
Arboricola_clade - ps29
Cerevisiae_clade - ps30
Saccharomyces_cerevisiae - ps31
Phylostratum

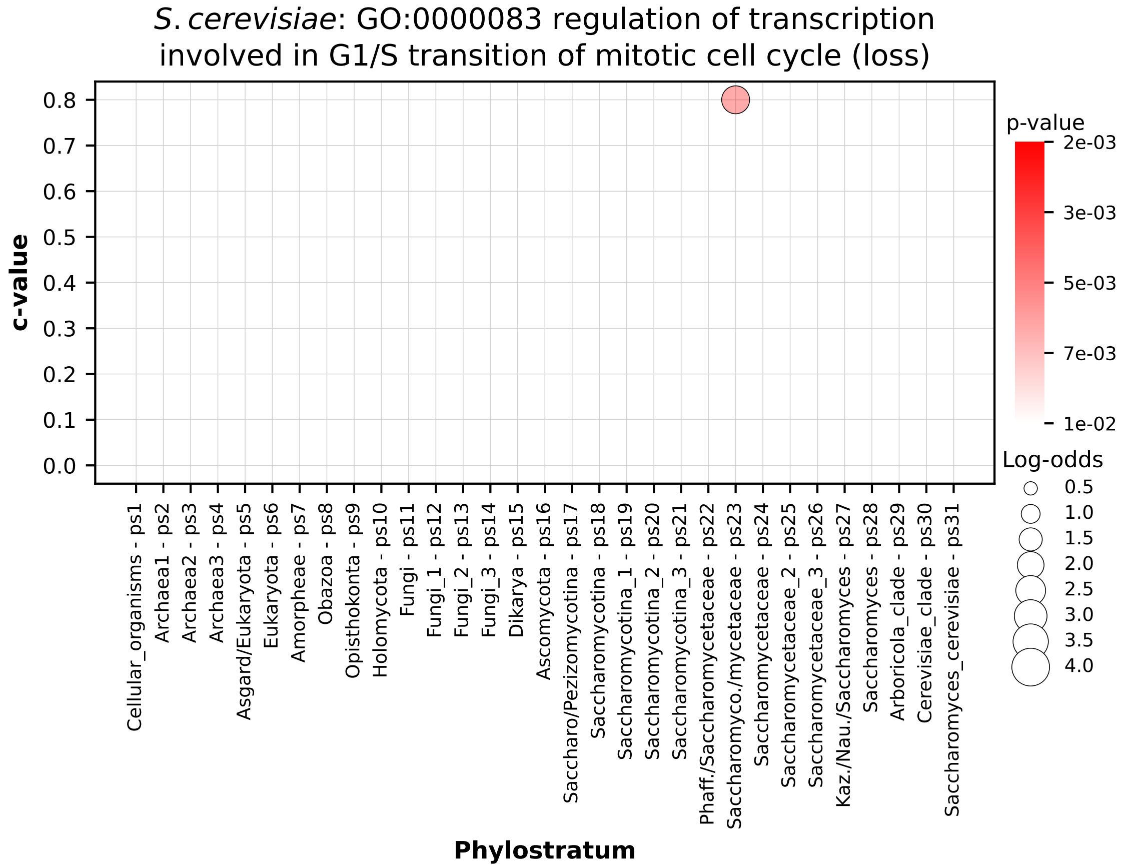

S. cerevisiae: GO:0000083 regulation of transcription involved in G1/S transition of mitotic cell cycle (loss)
c-value
0.0
0.1
0.2
0.3
0.4
0.5
0.6
0.7
0.8
p-value
2e-03
3e-03
5e-03
7e-03
1e-02
Log-odds
0.5
1.0
1.5
2.0
2.5
3.0
3.5
4.0
Cellular_organisms - ps1
Archaea1 - ps2
Archaea2 - ps3
Archaea3 - ps4
Asgard/Eukaryota - ps5
Eukaryota - ps6
Amorpheae - ps7
Obazoa - ps8
Opisthokonta - ps9
Holomycota - ps10
Fungi - ps11
Fungi_1 - ps12
Fungi_2 - ps13
Fungi_3 - ps14
Dikarya - ps15
Ascomycota - ps16
Saccharo/Pezizomycotina - ps17
Saccharomycotina - ps18
Saccharomycotina_1 - ps19
Saccharomycotina_2 - ps20
Saccharomycotina_3 - ps21
Phaff./Saccharomycetaceae - ps22
Saccharomyco./mycetaceae - ps23
Saccharomycetaceae - ps24
Saccharomycetaceae_2 - ps25
Saccharomycetaceae_3 - ps26
Kaz./Nau./Saccharomyces - ps27
Saccharomyces - ps28
Arboricola_clade - ps29
Cerevisiae_clade - ps30
Saccharomyces_cerevisiae - ps31
Phylostratum

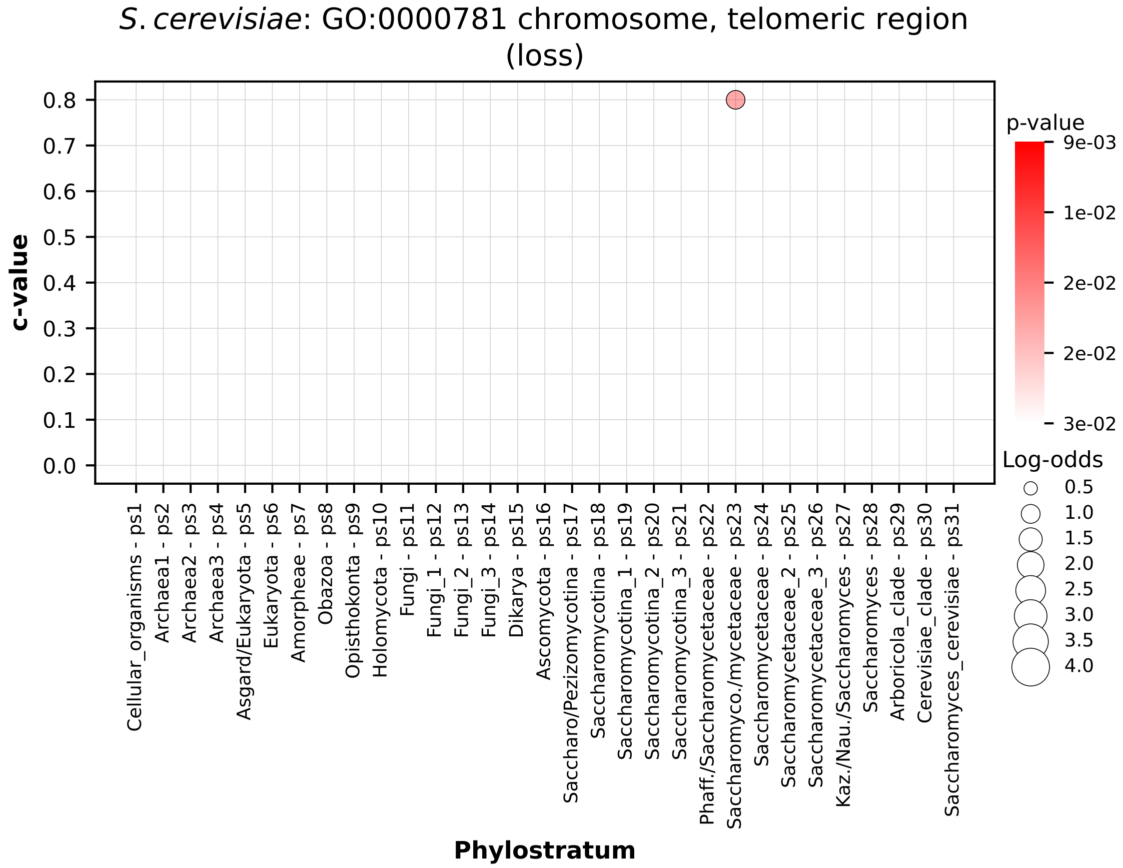

S. cerevisiae: GO:0000781 chromosome, telomeric region (loss)
c-value
0.8
0.7
0.6
0.5
0.4
0.3
0.2
0.1
0.0
p-value
9e-03
1e-02
2e-02
2e-02
3e-02
Log-odds
0.5
1.0
1.5
2.0
2.5
3.0
3.5
4.0
Cellular_organisms - ps1
Archaea1 - ps2
Archaea2 - ps3
Archaea3 - ps4
Asgard/Eukaryota - ps5
Eukaryota - ps6
Amorpheae - ps7
Obazoa - ps8
Opisthokonta - ps9
Holomycota - ps10
Fungi - ps11
Fungi_1 - ps12
Fungi_2 - ps13
Fungi_3 - ps14
Dikarya - ps15
Ascomycota - ps16
Saccharo/Pezizomycotina - ps17
Saccharomycotina - ps18
Saccharomycotina_1 - ps19
Saccharomycotina_2 - ps20
Saccharomycotina_3 - ps21
Phaff./Saccharomycetaceae - ps22
Saccharomyco./mycetaceae - ps23
Saccharomycetaceae - ps24
Saccharomycetaceae_2 - ps25
Saccharomycetaceae_3 - ps26
Kaz./Nau./Saccharomyces - ps27
Saccharomyces - ps28
Arboricola_clade - ps29
Cerevisiae_clade - ps30
Saccharomyces_cerevisiae - ps31
Phylostratum

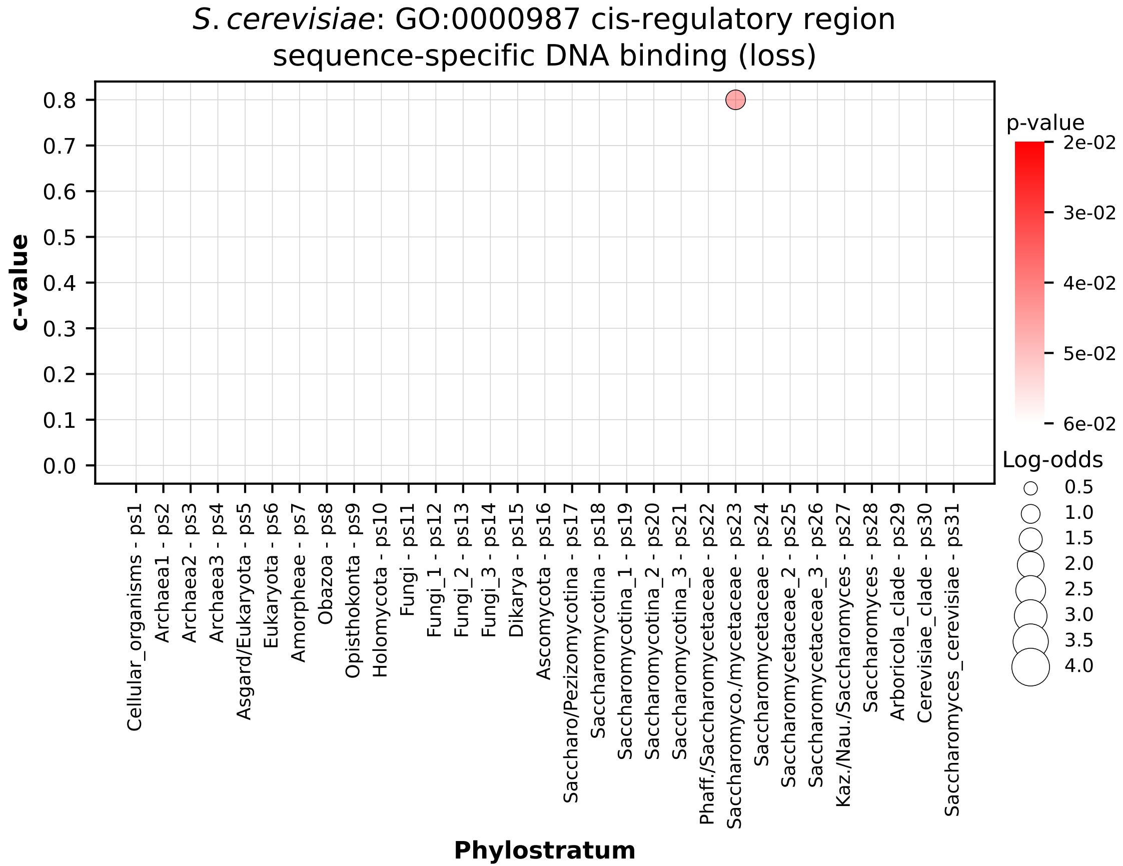

S. cerevisiae: GO:0000987 cis-regulatory region sequence-specific DNA binding (loss)
c-value
0.0
0.1
0.2
0.3
0.4
0.5
0.6
0.7
0.8
Phylostratum
Cellular_organisms - ps1
Archaea1 - ps2
Archaea2 - ps3
Archaea3 - ps4
Asgard/Eukaryota - ps5
Eukaryota - ps6
Amorpheae - ps7
Obazoa - ps8
Opisthokonta - ps9
Holomycota - ps10
Fungi - ps11
Fungi_1 - ps12
Fungi_2 - ps13
Fungi_3 - ps14
Dikarya - ps15
Ascomycota - ps16
Saccharo/Pezizomycotina - ps17
Saccharomycotina - ps18
Saccharomycotina_1 - ps19
Saccharomycotina_2 - ps20
Saccharomycotina_3 - ps21
Phaff./Saccharomycetaceae - ps22
Saccharomyco./mycetaceae - ps23
Saccharomycetaceae - ps24
Saccharomycetaceae_2 - ps25
Saccharomycetaceae_3 - ps26
Kaz./Nau./Saccharomyces - ps27
Saccharomyces - ps28
Arboricola_clade - ps29
Cerevisiae_clade - ps30
Saccharomyces_cerevisiae - ps31
p-value
2e-02
3e-02
4e-02
5e-02
6e-02
Log-odds
0.5
1.0
1.5
2.0
2.5
3.0
3.5
4.0

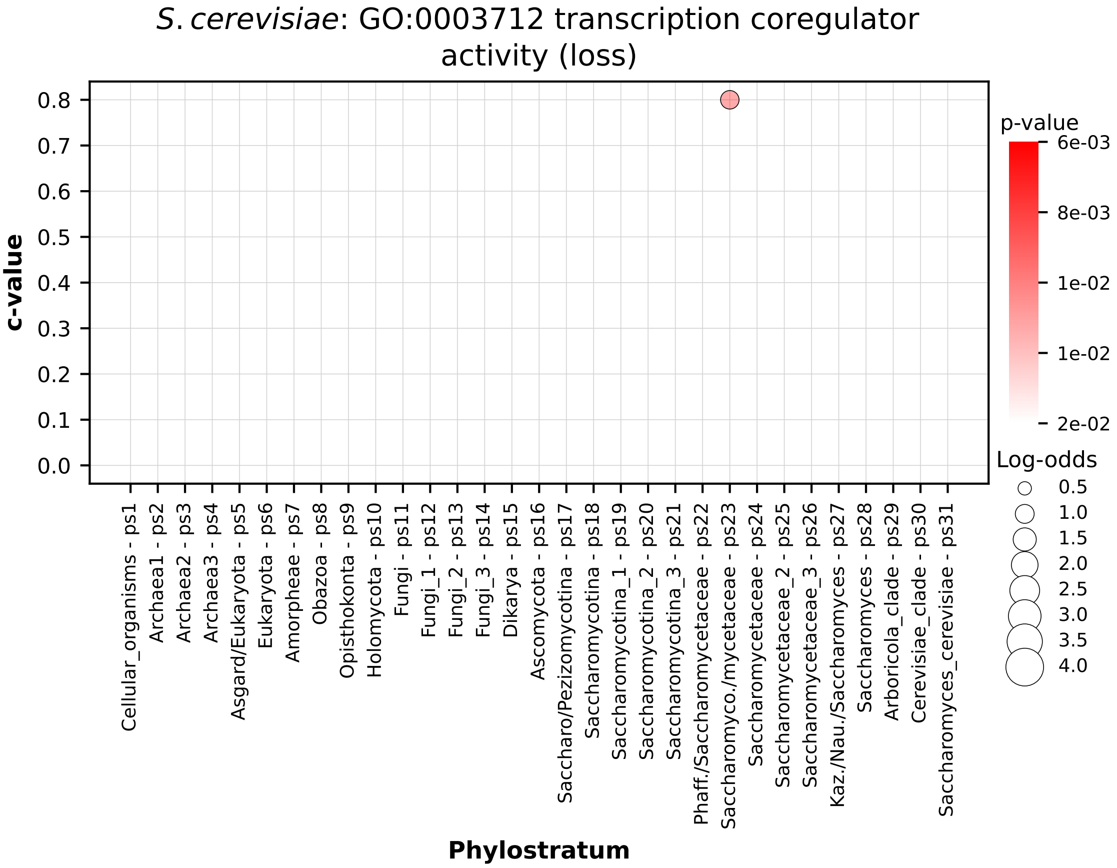

S. cerevisiae: GO:0003712 transcription coregulator activity (loss)
c-value
0.0
0.1
0.2
0.3
0.4
0.5
0.6
0.7
0.8
p-value
6e-03
8e-03
1e-02
1e-02
2e-02
Log-odds
0.5
1.0
1.5
2.0
2.5
3.0
3.5
4.0
Cellular_organisms - ps1
Archaea1 - ps2
Archaea2 - ps3
Archaea3 - ps4
Asgard/Eukaryota - ps5
Eukaryota - ps6
Amorpheae - ps7
Obazoa - ps8
Opisthokonta - ps9
Holomycota - ps10
Fungi - ps11
Fungi_1 - ps12
Fungi_2 - ps13
Fungi_3 - ps14
Dikarya - ps15
Ascomycota - ps16
Saccharo/Pezizomycotina - ps17
Saccharomycotina - ps18
Saccharomycotina_1 - ps19
Saccharomycotina_2 - ps20
Saccharomycotina_3 - ps21
Phaff./Saccharomycetaceae - ps22
Saccharomyco./mycetaceae - ps23
Saccharomycetaceae - ps24
Saccharomycetaceae_2 - ps25
Saccharomycetaceae_3 - ps26
Kaz./Nau./Saccharomyces - ps27
Saccharomyces - ps28
Arboricola_clade - ps29
Cerevisiae_clade - ps30
Saccharomyces_cerevisiae - ps31
Phylostratum

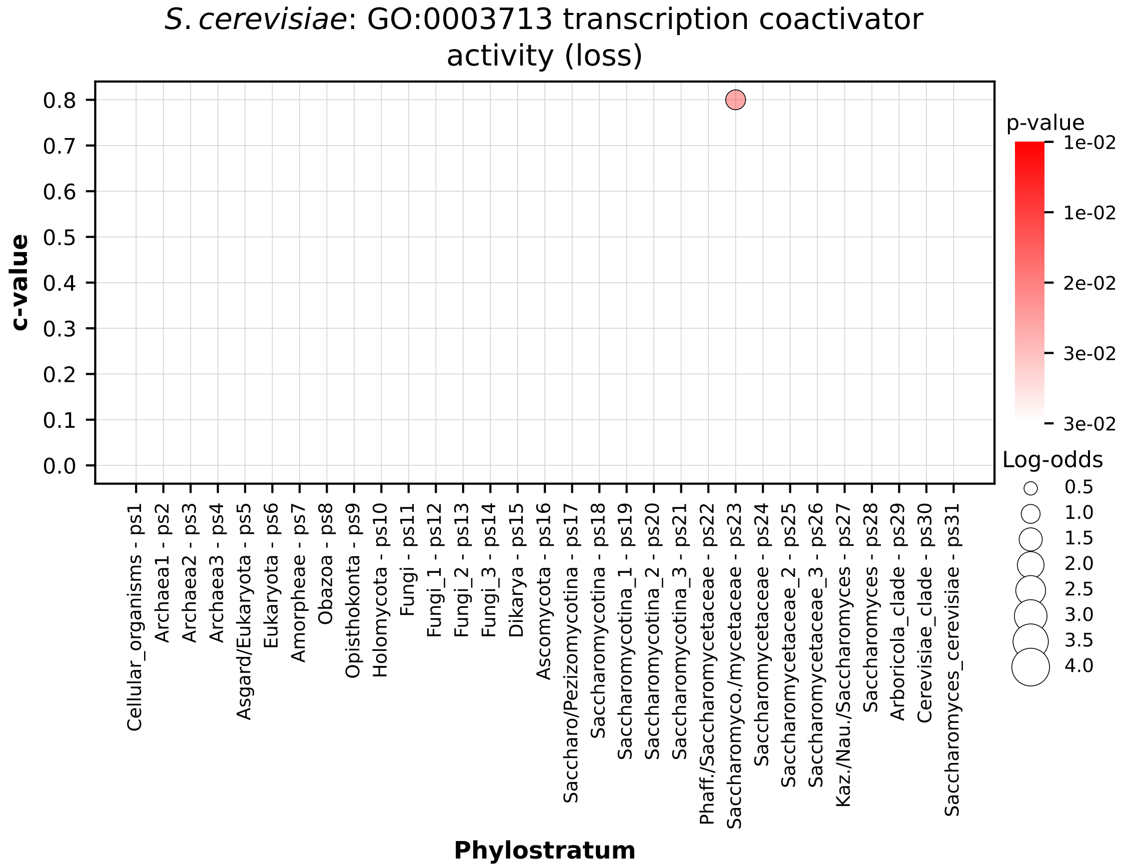

S. cerevisiae: GO:0003713 transcription coactivator activity (loss)
c-value
0.8
0.7
0.6
0.5
0.4
0.3
0.2
0.1
0.0
p-value
1e-02
1e-02
2e-02
3e-02
3e-02
Log-odds
0.5
1.0
1.5
2.0
2.5
3.0
3.5
4.0
Cellular_organisms - ps1
Archaea1 - ps2
Archaea2 - ps3
Archaea3 - ps4
Asgard/Eukaryota - ps5
Eukaryota - ps6
Amorpheae - ps7
Obazoa - ps8
Opisthokonta - ps9
Holomycota - ps10
Fungi - ps11
Fungi_1 - ps12
Fungi_2 - ps13
Fungi_3 - ps14
Dikarya - ps15
Ascomycota - ps16
Saccharo/Pezizomycotina - ps17
Saccharomycotina - ps18
Saccharomycotina_1 - ps19
Saccharomycotina_2 - ps20
Saccharomycotina_3 - ps21
Phaff./Saccharomycetaceae - ps22
Saccharomyco./mycetaceae - ps23
Saccharomycetaceae - ps24
Saccharomycetaceae_2 - ps25
Saccharomycetaceae_3 - ps26
Kaz./Nau./Saccharomyces - ps27
Saccharomyces - ps28
Arboricola_clade - ps29
Cerevisiae_clade - ps30
Saccharomyces_cerevisiae - ps31
Phylostratum

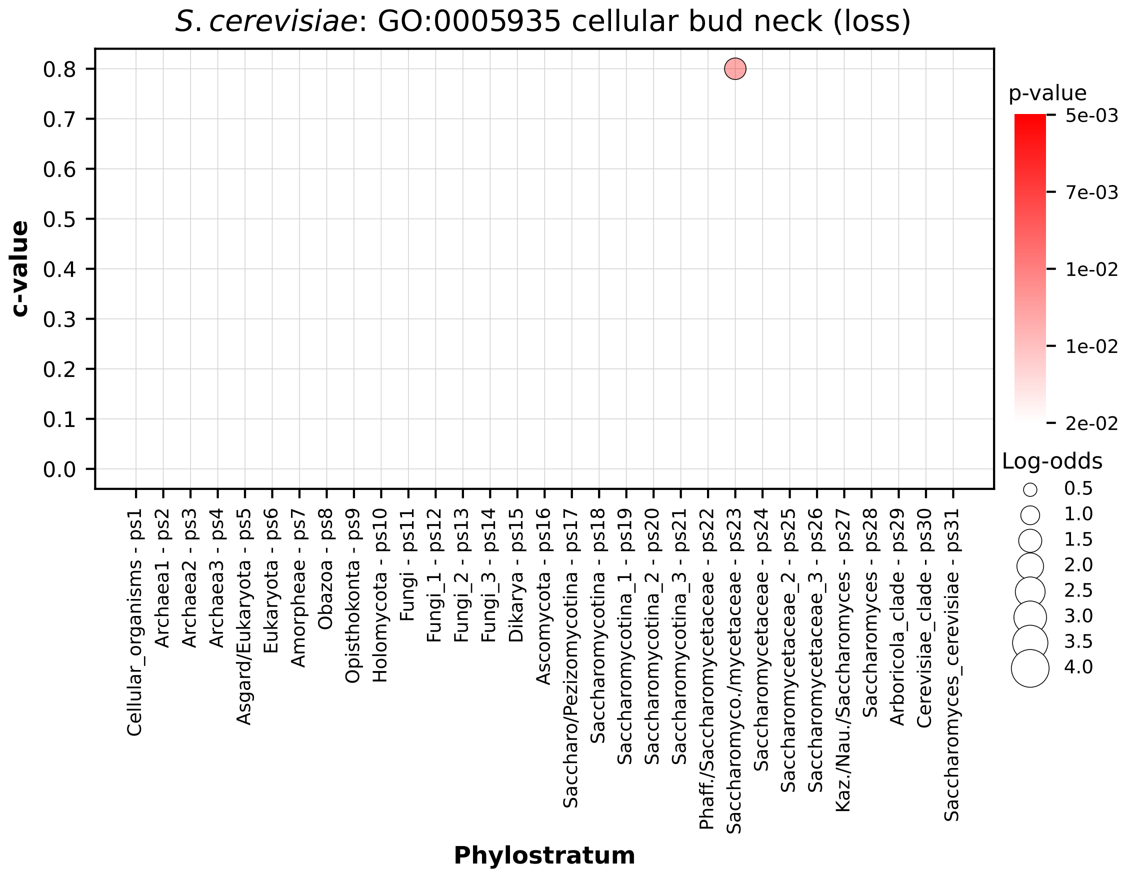
S. cerevisiae: GO:0005935 cellular bud neck (loss)
c-value
0.8
0.7
0.6
0.5
0.4
0.3
0.2
0.1
0.0
p-value
5e-03
7e-03
1e-02
1e-02
2e-02
Log-odds
0.5
1.0
1.5
2.0
2.5
3.0
3.5
4.0
Cellular_organisms - ps1
Archaea1 - ps2
Archaea2 - ps3
Archaea3 - ps4
Asgard/Eukaryota - ps5
Eukaryota - ps6
Amorpheae - ps7
Obazoa - ps8
Opisthokonta - ps9
Holomycota - ps10
Fungi - ps11
Fungi_1 - ps12
Fungi_2 - ps13
Fungi_3 - ps14
Dikarya - ps15
Ascomycota - ps16
Saccharo/Pezizomycotina - ps17
Saccharomycotina - ps18
Saccharomycotina_1 - ps19
Saccharomycotina_2 - ps20
Saccharomycotina_3 - ps21
Phaff./Saccharomycetaceae - ps22
Saccharomyco./mycetaceae - ps23
Saccharomycetaceae - ps24
Saccharomycetaceae_2 - ps25
Saccharomycetaceae_3 - ps26
Kaz./Nau./Saccharomyces - ps27
Saccharomyces - ps28
Arboricola_clade - ps29
Cerevisiae_clade - ps30
Saccharomyces_cerevisiae - ps31
Phylostratum

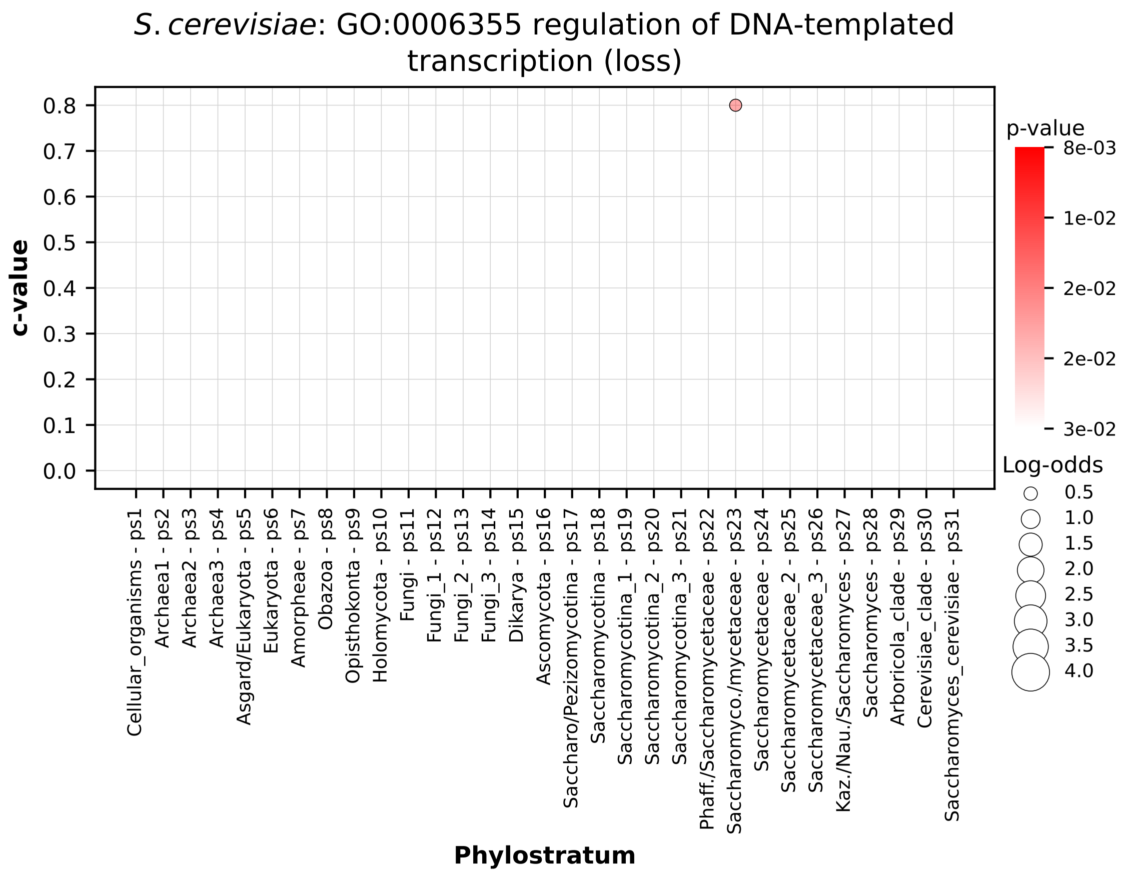

S. cerevisiae: GO:0006355 regulation of DNA-templated transcription (loss)
c-value
0.0
0.1
0.2
0.3
0.4
0.5
0.6
0.7
0.8
Phylostratum
Cellular_organisms - ps1
Archaea1 - ps2
Archaea2 - ps3
Archaea3 - ps4
Asgard/Eukaryota - ps5
Eukaryota - ps6
Amorpheae - ps7
Obazoa - ps8
Opisthokonta - ps9
Holomycota - ps10
Fungi - ps11
Fungi_1 - ps12
Fungi_2 - ps13
Fungi_3 - ps14
Dikarya - ps15
Ascomycota - ps16
Saccharo/Pezizomycotina - ps17
Saccharomycotina - ps18
Saccharomycotina_1 - ps19
Saccharomycotina_2 - ps20
Saccharomycotina_3 - ps21
Phaff./Saccharomycetaceae - ps22
Saccharomyco./mycetaceae - ps23
Saccharomycetaceae - ps24
Saccharomycetaceae_2 - ps25
Saccharomycetaceae_3 - ps26
Kaz./Nau./Saccharomyces - ps27
Saccharomyces - ps28
Arboricola_clade - ps29
Cerevisiae_clade - ps30
Saccharomyces_cerevisiae - ps31
p-value
8e-03
1e-02
2e-02
2e-02
3e-02
Log-odds
0.5
1.0
1.5
2.0
2.5
3.0
3.5
4.0

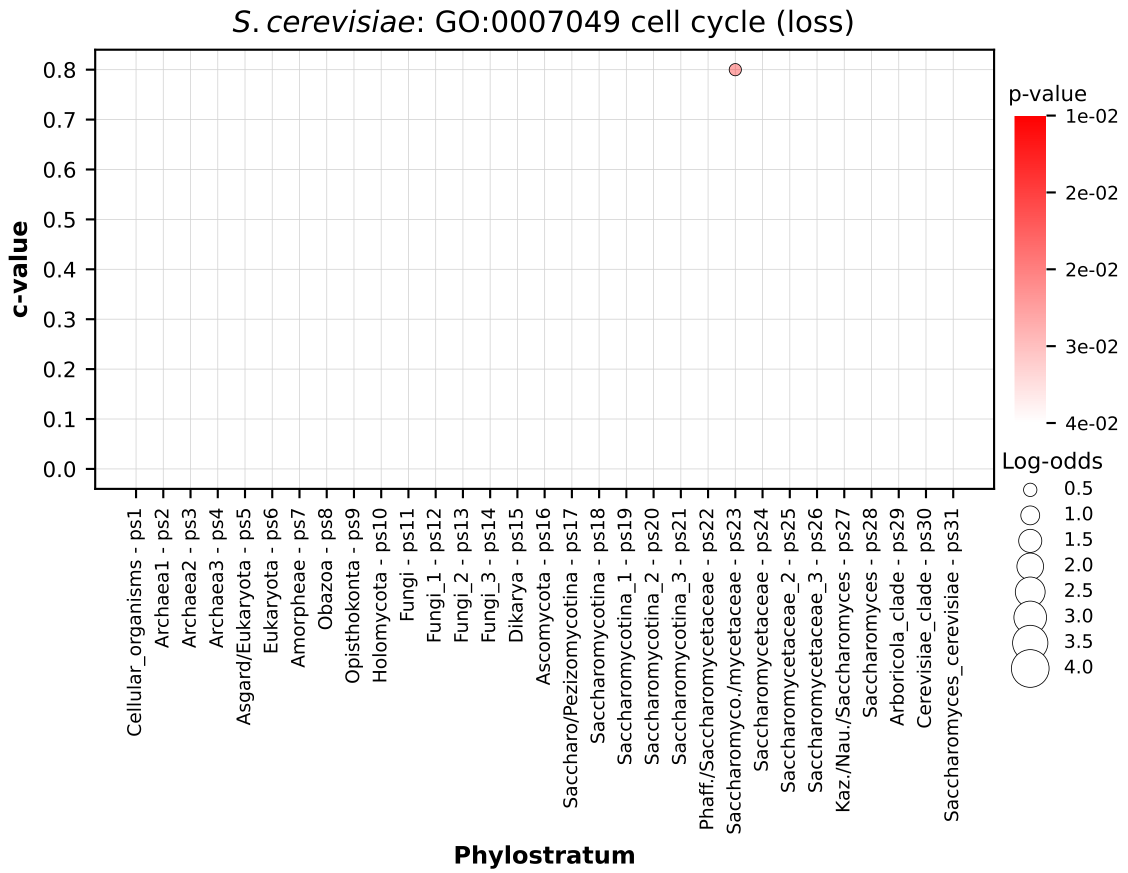
S. cerevisiae: GO:0007049 cell cycle (loss)
c-value
0.0
0.1
0.2
0.3
0.4
0.5
0.6
0.7
0.8
p-value
1e-02
2e-02
2e-02
3e-02
4e-02
Log-odds
0.5
1.0
1.5
2.0
2.5
3.0
3.5
4.0
Cellular_organisms - ps1
Archaea1 - ps2
Archaea2 - ps3
Archaea3 - ps4
Asgard/Eukaryota - ps5
Eukaryota - ps6
Amorpheae - ps7
Obazoa - ps8
Opisthokonta - ps9
Holomycota - ps10
Fungi - ps11
Fungi_1 - ps12
Fungi_2 - ps13
Fungi_3 - ps14
Dikarya - ps15
Ascomycota - ps16
Saccharo/Pezizomycotina - ps17
Saccharomycotina - ps18
Saccharomycotina_1 - ps19
Saccharomycotina_2 - ps20
Saccharomycotina_3 - ps21
Phaff./Saccharomycetaceae - ps22
Saccharomyco./mycetaceae - ps23
Saccharomycetaceae - ps24
Saccharomycetaceae_2 - ps25
Saccharomycetaceae_3 - ps26
Kaz./Nau./Saccharomyces - ps27
Saccharomyces - ps28
Arboricola_clade - ps29
Cerevisiae_clade - ps30
Saccharomyces_cerevisiae - ps31
Phylostratum

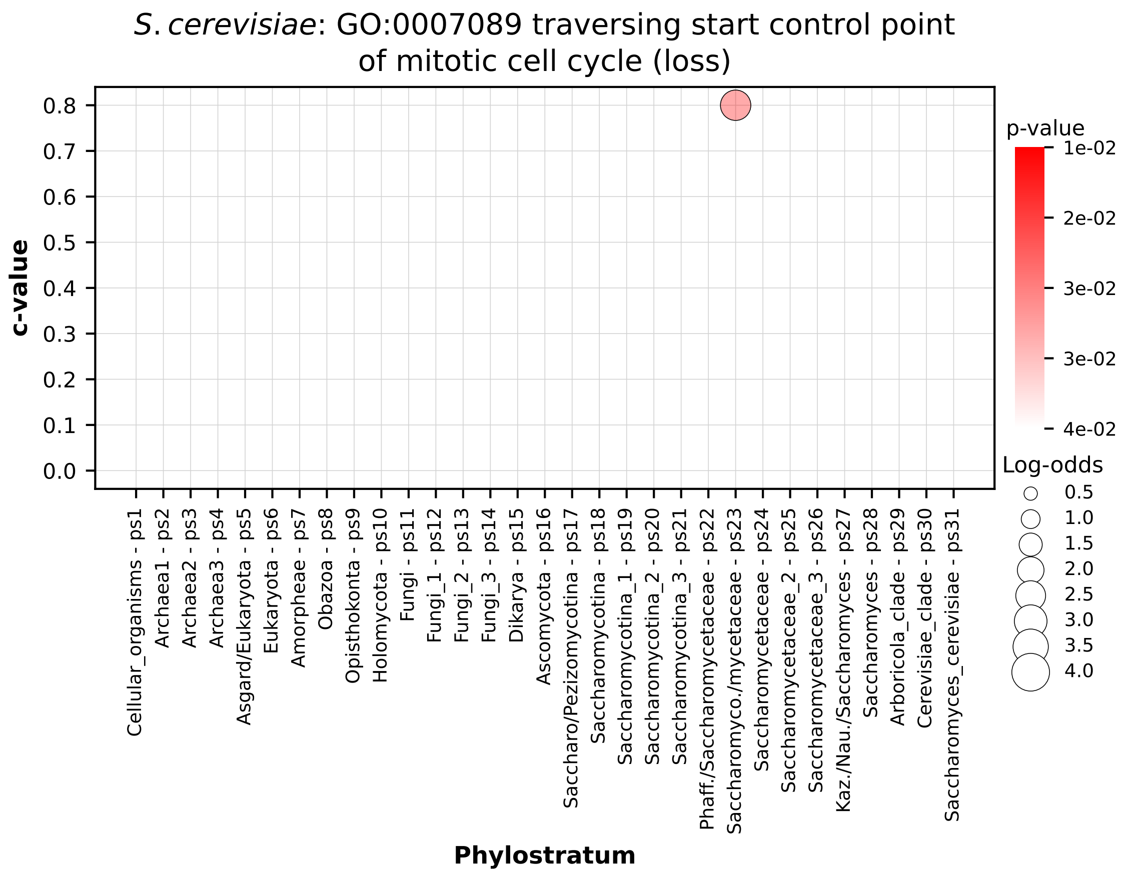
S. cerevisiae: GO:0007089 traversing start control point of mitotic cell cycle (loss)
c-value
0.0
0.1
0.2
0.3
0.4
0.5
0.6
0.7
0.8
p-value
1e-02
2e-02
3e-02
3e-02
4e-02
Log-odds
0.5
1.0
1.5
2.0
2.5
3.0
3.5
4.0
Cellular_organisms - ps1
Archaea1 - ps2
Archaea2 - ps3
Archaea3 - ps4
Asgard/Eukaryota - ps5
Eukaryota - ps6
Amorpheae - ps7
Obazoa - ps8
Opisthokonta - ps9
Holomycota - ps10
Fungi - ps11
Fungi_1 - ps12
Fungi_2 - ps13
Fungi_3 - ps14
Dikarya - ps15
Ascomycota - ps16
Saccharo/Pezizomycotina - ps17
Saccharomycotina - ps18
Saccharomycotina_1 - ps19
Saccharomycotina_2 - ps20
Saccharomycotina_3 - ps21
Phaff./Saccharomycetaceae - ps22
Saccharomyco./mycetaceae - ps23
Saccharomycetaceae - ps24
Saccharomycetaceae_2 - ps25
Saccharomycetaceae_3 - ps26
Kaz./Nau./Saccharomyces - ps27
Saccharomyces - ps28
Arboricola_clade - ps29
Cerevisiae_clade - ps30
Saccharomyces_cerevisiae - ps31
Phylostratum

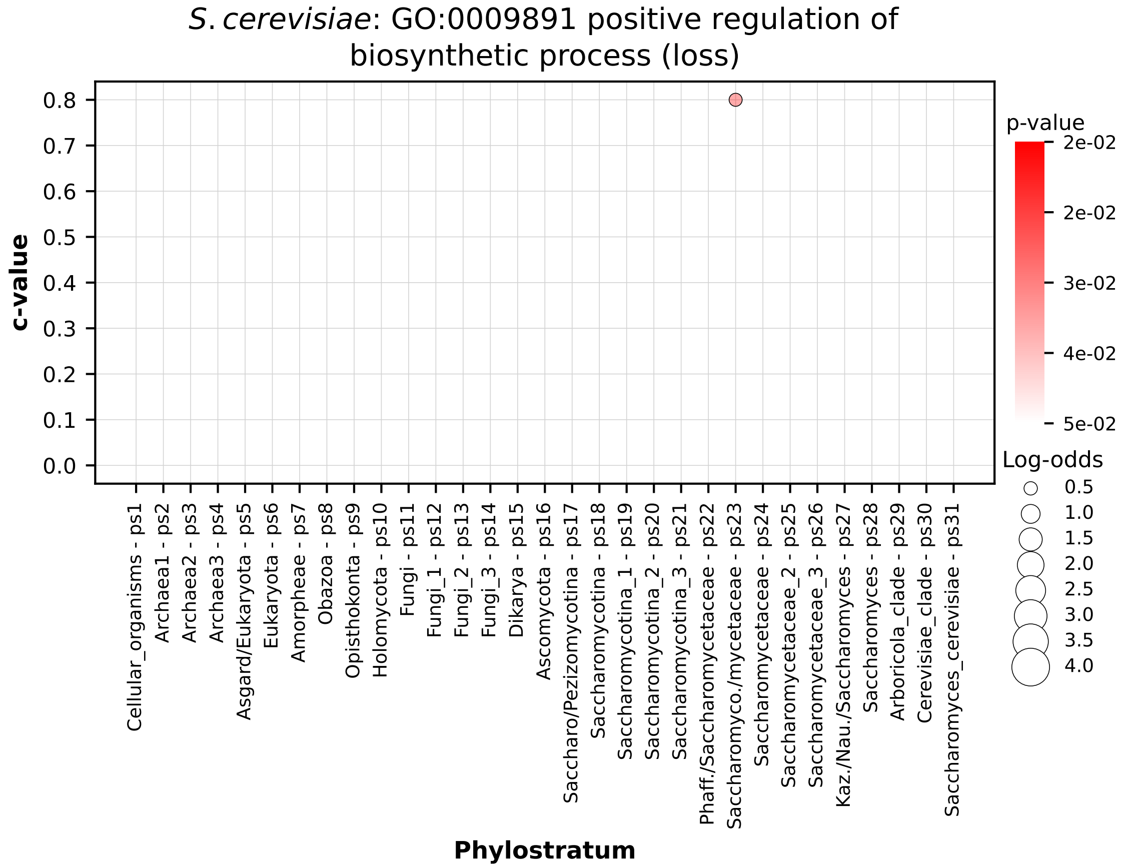

S. cerevisiae: GO:0009891 positive regulation of biosynthetic process (loss)
c-value
0.0
0.1
0.2
0.3
0.4
0.5
0.6
0.7
0.8
Phylostratum
Cellular_organisms - ps1
Archaea1 - ps2
Archaea2 - ps3
Archaea3 - ps4
Asgard/Eukaryota - ps5
Eukaryota - ps6
Amorpheae - ps7
Obazoa - ps8
Opisthokonta - ps9
Holomycota - ps10
Fungi - ps11
Fungi_1 - ps12
Fungi_2 - ps13
Fungi_3 - ps14
Dikarya - ps15
Ascomycota - ps16
Saccharo/Pezizomycotina - ps17
Saccharomycotina - ps18
Saccharomycotina_1 - ps19
Saccharomycotina_2 - ps20
Saccharomycotina_3 - ps21
Phaff./Saccharomycetaceae - ps22
Saccharomyco./mycetaceae - ps23
Saccharomycetaceae - ps24
Saccharomycetaceae_2 - ps25
Saccharomycetaceae_3 - ps26
Kaz./Nau./Saccharomyces - ps27
Saccharomyces - ps28
Arboricola_clade - ps29
Cerevisiae_clade - ps30
Saccharomyces_cerevisiae - ps31
p-value
2e-02
2e-02
3e-02
4e-02
5e-02
Log-odds
0.5
1.0
1.5
2.0
2.5
3.0
3.5
4.0

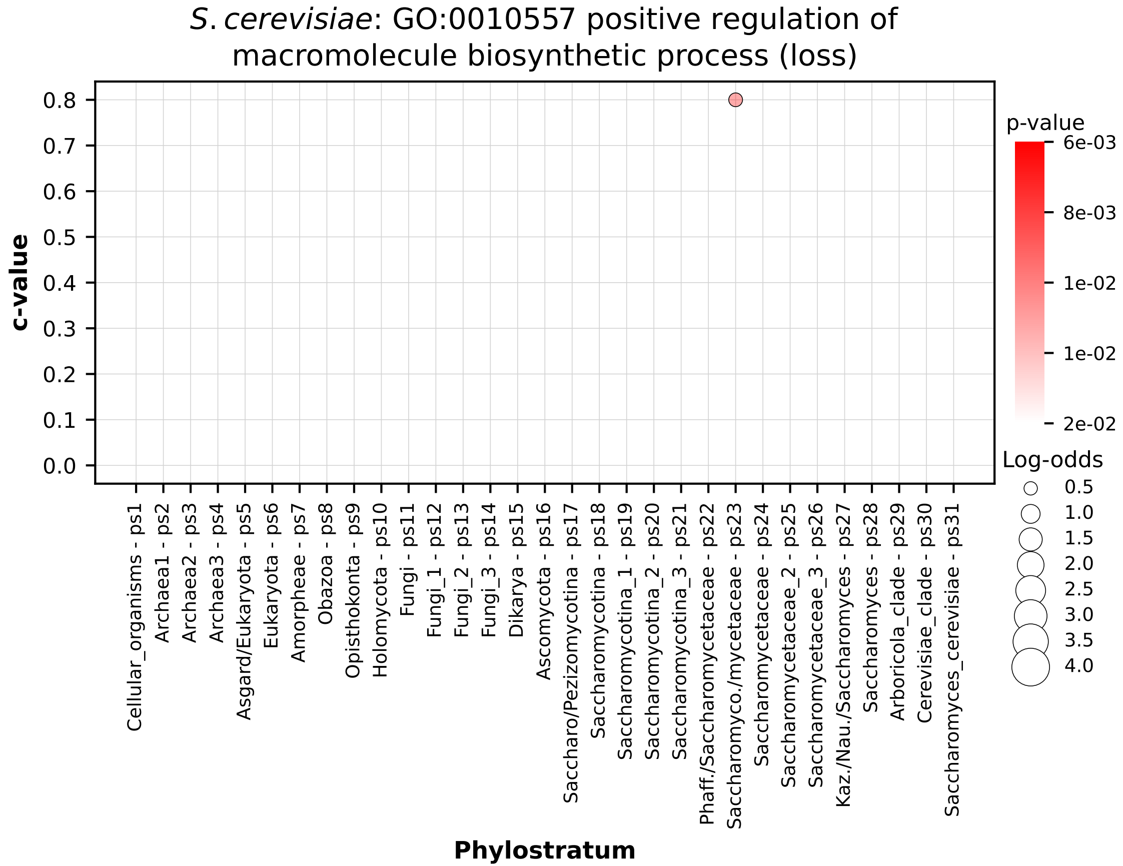
S. cerevisiae: GO:0010557 positive regulation of macromolecule biosynthetic process (loss)
c-value
0.8
0.7
0.6
0.5
0.4
0.3
0.2
0.1
0.0
p-value
6e-03
8e-03
1e-02
1e-02
2e-02
Log-odds
0.5
1.0
1.5
2.0
2.5
3.0
3.5
4.0
Cellular_organisms - ps1
Archaea1 - ps2
Archaea2 - ps3
Archaea3 - ps4
Asgard/Eukaryota - ps5
Eukaryota - ps6
Amorpheae - ps7
Obazoa - ps8
Opisthokonta - ps9
Holomycota - ps10
Fungi - ps11
Fungi_1 - ps12
Fungi_2 - ps13
Fungi_3 - ps14
Dikarya - ps15
Ascomycota - ps16
Saccharo/Pezizomycotina - ps17
Saccharomycotina - ps18
Saccharomycotina_1 - ps19
Saccharomycotina_2 - ps20
Saccharomycotina_3 - ps21
Phaff./Saccharomycetaceae - ps22
Saccharomyco./mycetaceae - ps23
Saccharomycetaceae - ps24
Saccharomycetaceae_2 - ps25
Saccharomycetaceae_3 - ps26
Kaz./Nau./Saccharomyces - ps27
Saccharomyces - ps28
Arboricola_clade - ps29
Cerevisiae_clade - ps30
Saccharomyces_cerevisiae - ps31
Phylostratum

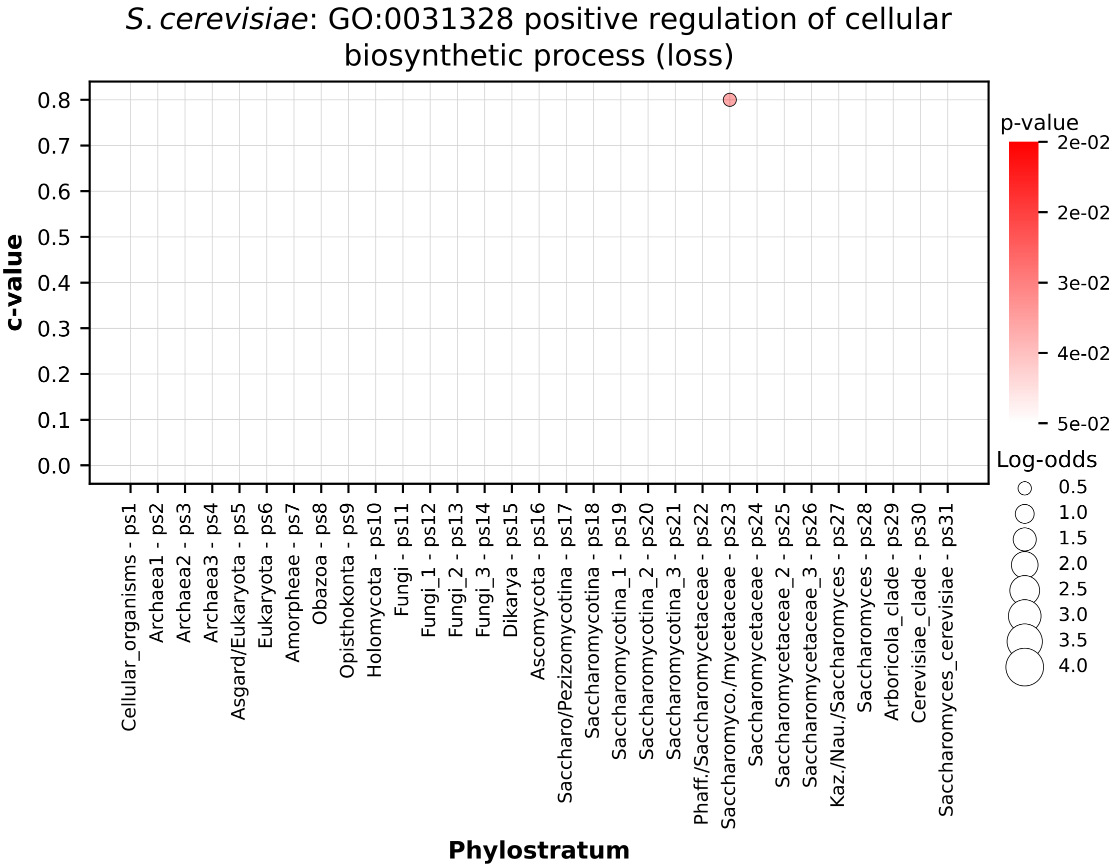

S. cerevisiae: GO:0031328 positive regulation of cellular biosynthetic process (loss)
c-value
0.0
0.1
0.2
0.3
0.4
0.5
0.6
0.7
0.8
p-value
2e-02
2e-02
3e-02
4e-02
5e-02
Log-odds
0.5
1.0
1.5
2.0
2.5
3.0
3.5
4.0
Cellular_organisms - ps1
Archaea1 - ps2
Archaea2 - ps3
Archaea3 - ps4
Asgard/Eukaryota - ps5
Eukaryota - ps6
Amorpheae - ps7
Obazoa - ps8
Opisthokonta - ps9
Holomycota - ps10
Fungi - ps11
Fungi_1 - ps12
Fungi_2 - ps13
Fungi_3 - ps14
Dikarya - ps15
Ascomycota - ps16
Saccharo/Pezizomycotina - ps17
Saccharomycotina - ps18
Saccharomycotina_1 - ps19
Saccharomycotina_2 - ps20
Saccharomycotina_3 - ps21
Phaff./Saccharomycetaceae - ps22
Saccharomyco./mycetaceae - ps23
Saccharomycetaceae - ps24
Saccharomycetaceae_2 - ps25
Saccharomycetaceae_3 - ps26
Kaz./Nau./Saccharomyces - ps27
Saccharomyces - ps28
Arboricola_clade - ps29
Cerevisiae_clade - ps30
Saccharomyces_cerevisiae - ps31
Phylostratum

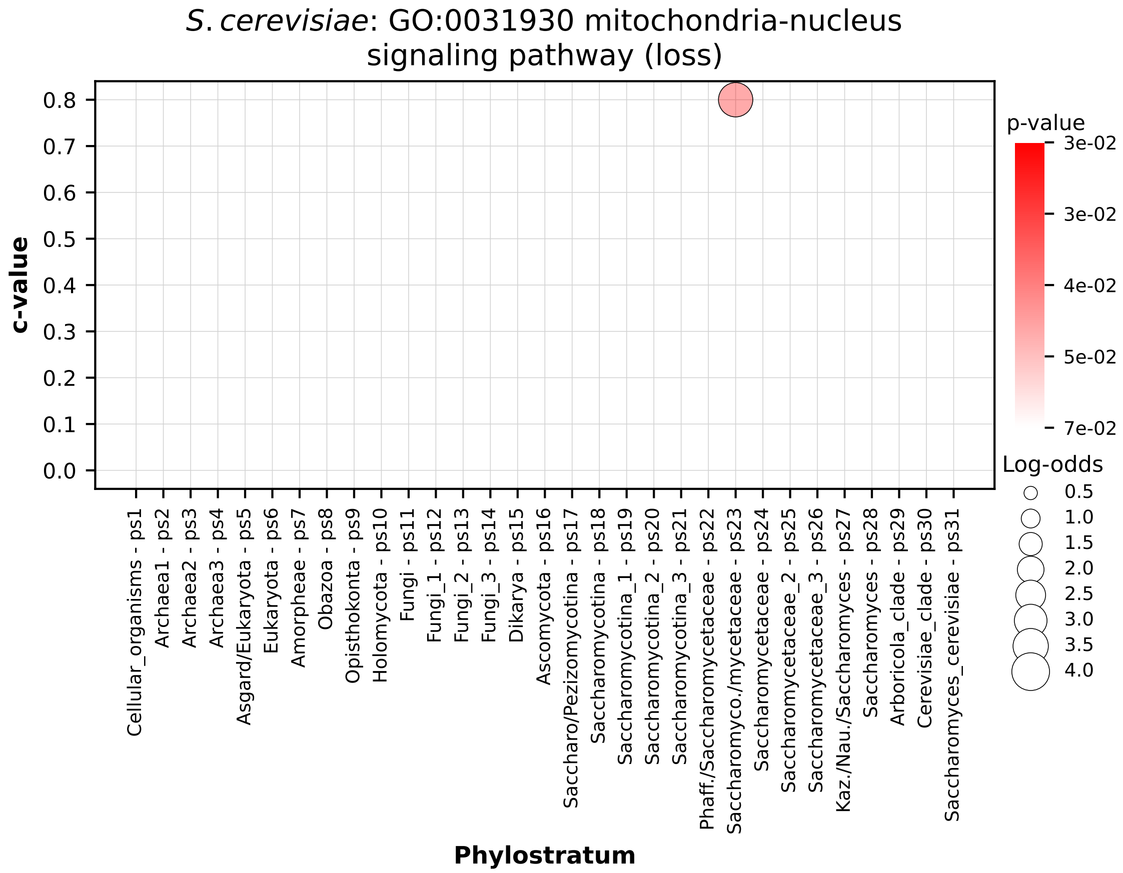

S. cerevisiae: GO:0031930 mitochondria-nucleus signaling pathway (loss)
c-value
0.8
0.7
0.6
0.5
0.4
0.3
0.2
0.1
0.0
p-value
3e-02
3e-02
4e-02
5e-02
7e-02
Log-odds
0.5
1.0
1.5
2.0
2.5
3.0
3.5
4.0
Cellular_organisms - ps1
Archaea1 - ps2
Archaea2 - ps3
Archaea3 - ps4
Asgard/Eukaryota - ps5
Eukaryota - ps6
Amorpheae - ps7
Obazoa - ps8
Opisthokonta - ps9
Holomycota - ps10
Fungi - ps11
Fungi_1 - ps12
Fungi_2 - ps13
Fungi_3 - ps14
Dikarya - ps15
Ascomycota - ps16
Saccharo/Pezizomycotina - ps17
Saccharomycotina - ps18
Saccharomycotina_1 - ps19
Saccharomycotina_2 - ps20
Saccharomycotina_3 - ps21
Phaff./Saccharomycetaceae - ps22
Saccharomyco./mycetaceae - ps23
Saccharomycetaceae - ps24
Saccharomycetaceae_2 - ps25
Saccharomycetaceae_3 - ps26
Kaz./Nau./Saccharomyces - ps27
Saccharomyces - ps28
Arboricola_clade - ps29
Cerevisiae_clade - ps30
Saccharomyces_cerevisiae - ps31
Phylostratum

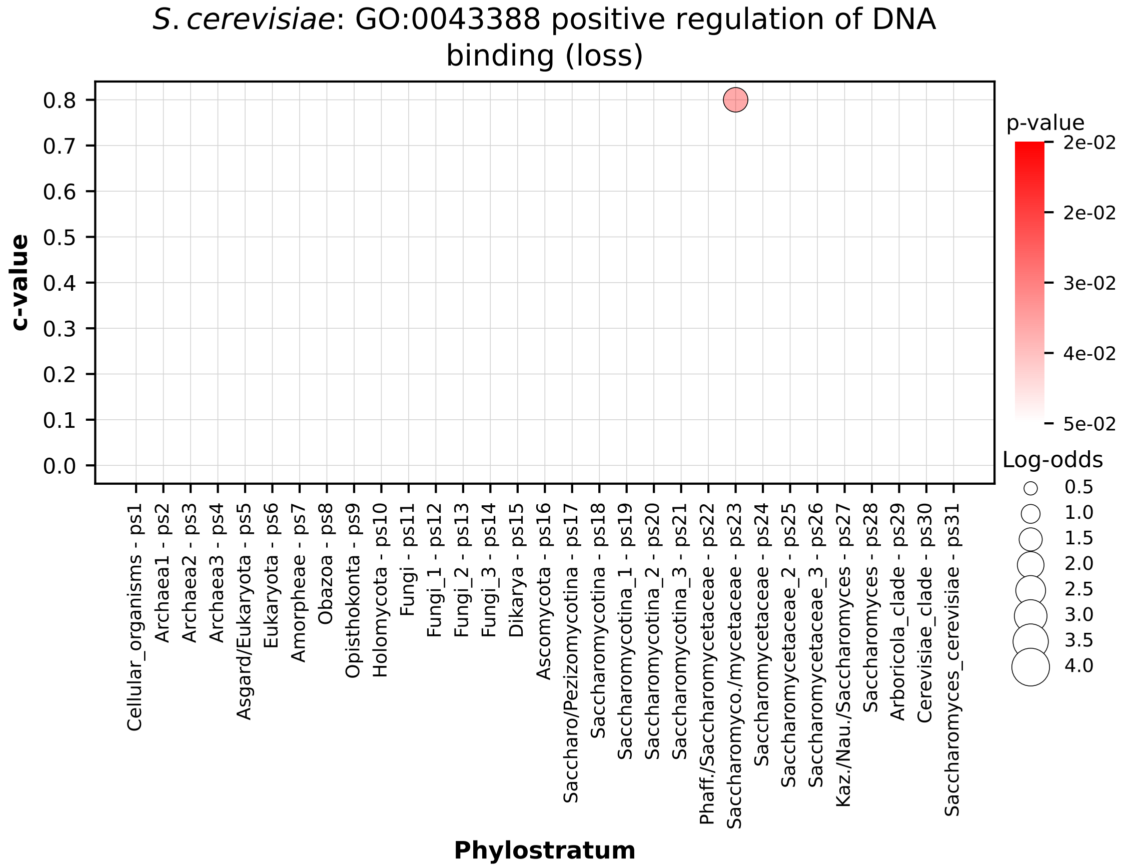

S. cerevisiae: GO:0043388 positive regulation of DNA binding (loss)
c-value
0.8
0.7
0.6
0.5
0.4
0.3
0.2
0.1
0.0
Phylostratum
Cellular_organisms - ps1
Archaea1 - ps2
Archaea2 - ps3
Archaea3 - ps4
Asgard/Eukaryota - ps5
Eukaryota - ps6
Amorpheae - ps7
Obazoa - ps8
Opisthokonta - ps9
Holomycota - ps10
Fungi - ps11
Fungi_1 - ps12
Fungi_2 - ps13
Fungi_3 - ps14
Dikarya - ps15
Ascomycota - ps16
Saccharo/Pezizomycotina - ps17
Saccharomycotina - ps18
Saccharomycotina_1 - ps19
Saccharomycotina_2 - ps20
Saccharomycotina_3 - ps21
Phaff./Saccharomycetaceae - ps22
Saccharomyco./mycetaceae - ps23
Saccharomycetaceae - ps24
Saccharomycetaceae_2 - ps25
Saccharomycetaceae_3 - ps26
Kaz./Nau./Saccharomyces - ps27
Saccharomyces - ps28
Arboricola_clade - ps29
Cerevisiae_clade - ps30
Saccharomyces_cerevisiae - ps31
p-value
2e-02
2e-02
3e-02
4e-02
5e-02
Log-odds
0.5
1.0
1.5
2.0
2.5
3.0
3.5
4.0

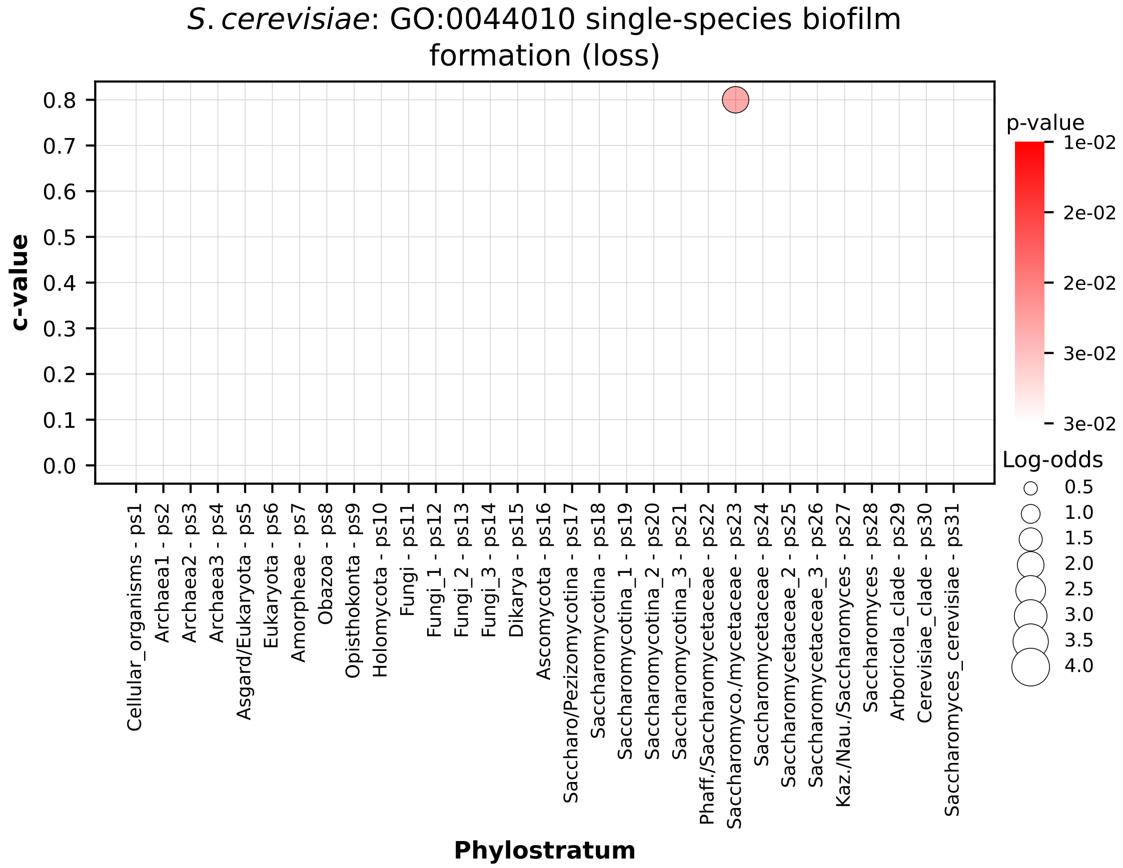

S. cerevisiae: GO:0044010 single-species biofilm formation (loss)
c-value
0.8
0.7
0.6
0.5
0.4
0.3
0.2
0.1
0.0
p-value
1e-02
2e-02
2e-02
3e-02
3e-02
Log-odds
0.5
1.0
1.5
2.0
2.5
3.0
3.5
4.0
Cellular_organisms - ps1
Archaea1 - ps2
Archaea2 - ps3
Archaea3 - ps4
Asgard/Eukaryota - ps5
Eukaryota - ps6
Amorpheae - ps7
Obazoa - ps8
Opisthokonta - ps9
Holomycota - ps10
Fungi - ps11
Fungi_1 - ps12
Fungi_2 - ps13
Fungi_3 - ps14
Dikarya - ps15
Ascomycota - ps16
Saccharo/Pezizomycotina - ps17
Saccharomycotina - ps18
Saccharomycotina_1 - ps19
Saccharomycotina_2 - ps20
Saccharomycotina_3 - ps21
Phaff./Saccharomycetaceae - ps22
Saccharomyco./mycetaceae - ps23
Saccharomycetaceae - ps24
Saccharomycetaceae_2 - ps25
Saccharomycetaceae_3 - ps26
Kaz./Nau./Saccharomyces - ps27
Saccharomyces - ps28
Arboricola_clade - ps29
Cerevisiae_clade - ps30
Saccharomyces_cerevisiae - ps31
Phylostratum

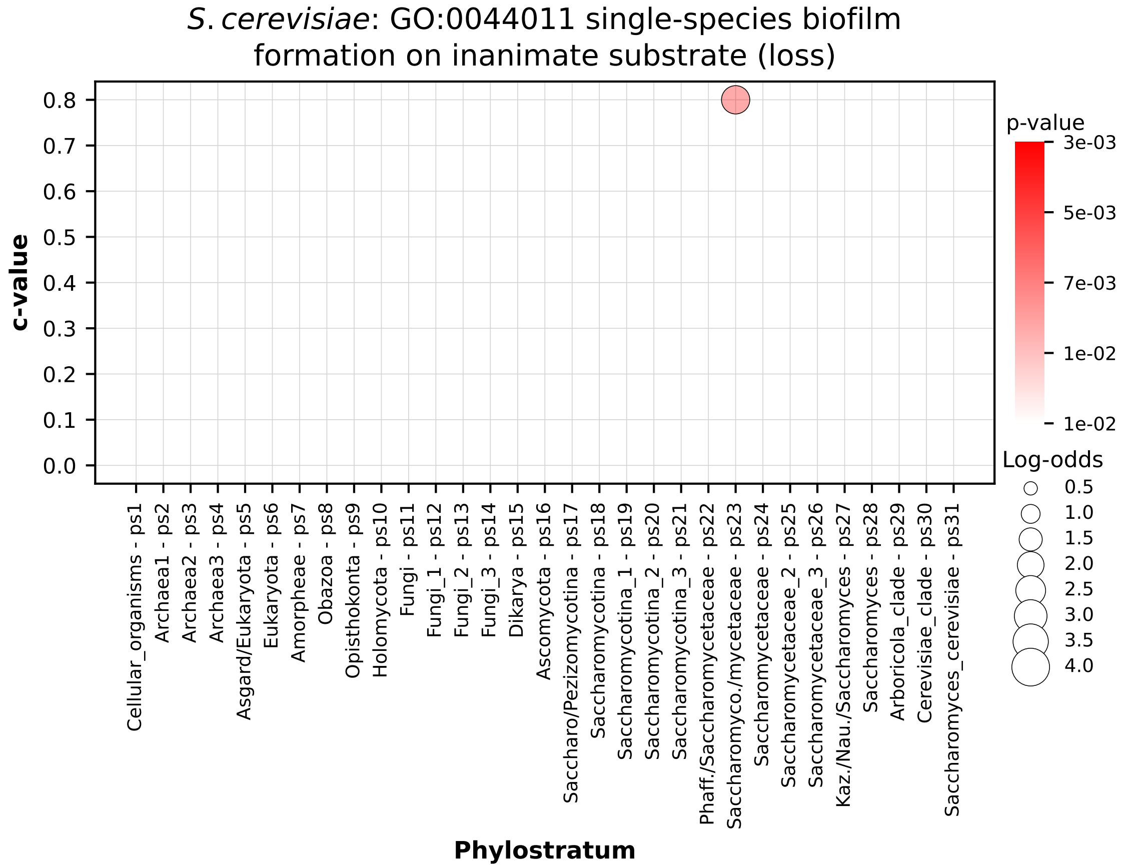

S. cerevisiae: GO:0044011 single-species biofilm formation on inanimate substrate (loss)
c-value
0.8
0.7
0.6
0.5
0.4
0.3
0.2
0.1
0.0
p-value
3e-03
5e-03
7e-03
1e-02
1e-02
Log-odds
0.5
1.0
1.5
2.0
2.5
3.0
3.5
4.0
Cellular_organisms - ps1
Archaea1 - ps2
Archaea2 - ps3
Archaea3 - ps4
Asgard/Eukaryota - ps5
Eukaryota - ps6
Amorpheae - ps7
Obazoa - ps8
Opisthokonta - ps9
Holomycota - ps10
Fungi - ps11
Fungi_1 - ps12
Fungi_2 - ps13
Fungi_3 - ps14
Dikarya - ps15
Ascomycota - ps16
Saccharo/Pezizomycotina - ps17
Saccharomycotina - ps18
Saccharomycotina_1 - ps19
Saccharomycotina_2 - ps20
Saccharomycotina_3 - ps21
Phaff./Saccharomycetaceae - ps22
Saccharomyco./mycetaceae - ps23
Saccharomycetaceae - ps24
Saccharomycetaceae_2 - ps25
Saccharomycetaceae_3 - ps26
Kaz./Nau./Saccharomyces - ps27
Saccharomyces - ps28
Arboricola_clade - ps29
Cerevisiae_clade - ps30
Saccharomyces_cerevisiae - ps31
Phylostratum

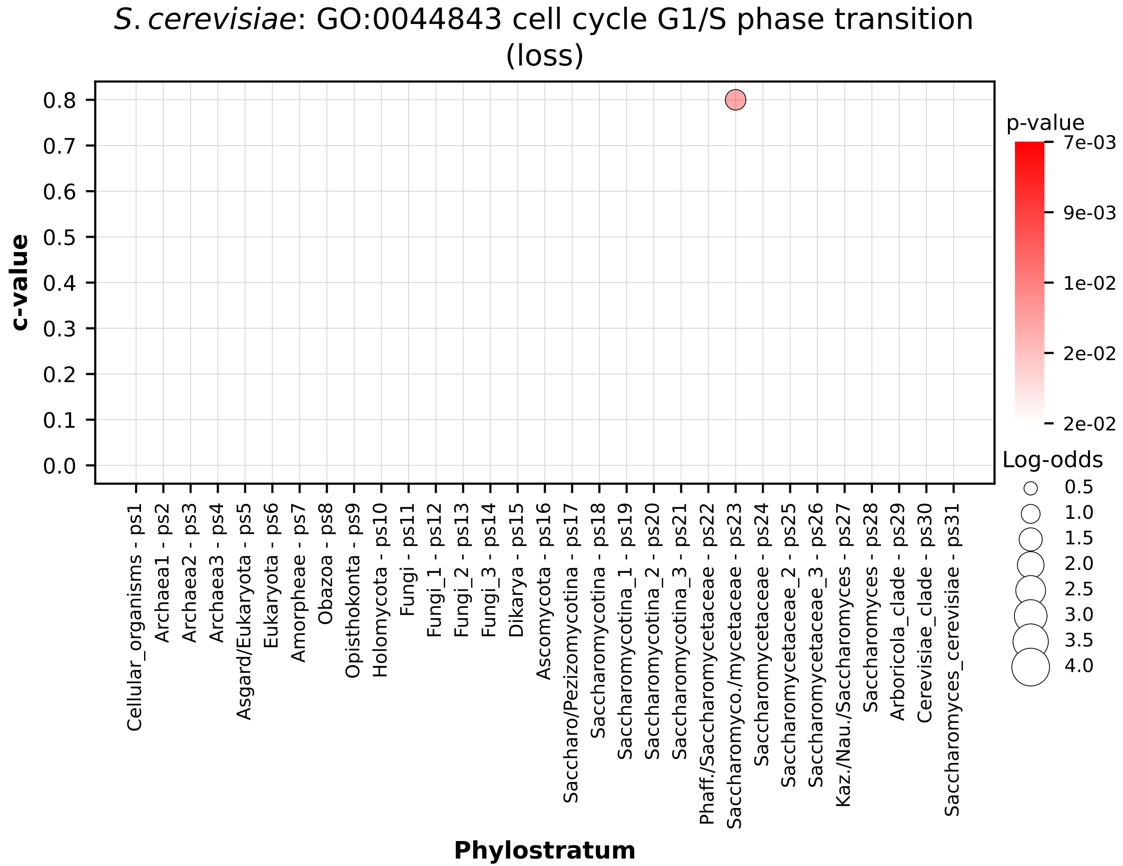
S. cerevisiae: GO:0044843 cell cycle G1/S phase transition (loss)
c-value
0.0
0.1
0.2
0.3
0.4
0.5
0.6
0.7
0.8
Phylostratum
Cellular_organisms - ps1
Archaea1 - ps2
Archaea2 - ps3
Archaea3 - ps4
Asgard/Eukaryota - ps5
Eukaryota - ps6
Amorpheae - ps7
Obazoa - ps8
Opisthokonta - ps9
Holomycota - ps10
Fungi - ps11
Fungi_1 - ps12
Fungi_2 - ps13
Fungi_3 - ps14
Dikarya - ps15
Ascomycota - ps16
Saccharo/Pezizomycotina - ps17
Saccharomycotina - ps18
Saccharomycotina_1 - ps19
Saccharomycotina_2 - ps20
Saccharomycotina_3 - ps21
Phaff./Saccharomycetaceae - ps22
Saccharomyco./mycetaceae - ps23
Saccharomycetaceae - ps24
Saccharomycetaceae_2 - ps25
Saccharomycetaceae_3 - ps26
Kaz./Nau./Saccharomyces - ps27
Saccharomyces - ps28
Arboricola_clade - ps29
Cerevisiae_clade - ps30
Saccharomyces_cerevisiae - ps31
p-value
7e-03
9e-03
1e-02
2e-02
2e-02
Log-odds
0.5
1.0
1.5
2.0
2.5
3.0
3.5
4.0

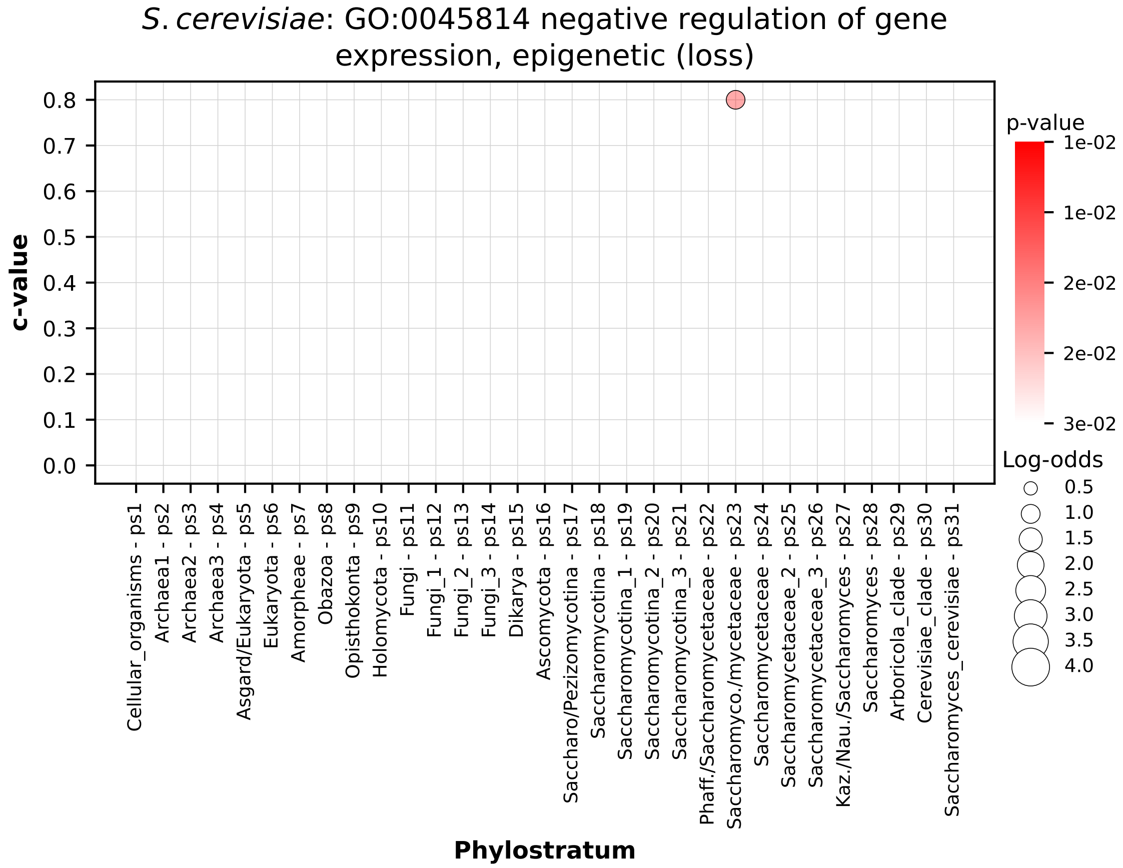

S. cerevisiae: GO:0045814 negative regulation of gene expression, epigenetic (loss)
c-value
0.0
0.1
0.2
0.3
0.4
0.5
0.6
0.7
0.8
p-value
1e-02
1e-02
2e-02
2e-02
3e-02
Log-odds
0.5
1.0
1.5
2.0
2.5
3.0
3.5
4.0
Cellular_organisms - ps1
Archaea1 - ps2
Archaea2 - ps3
Archaea3 - ps4
Asgard/Eukaryota - ps5
Eukaryota - ps6
Amorpheae - ps7
Obazoa - ps8
Opisthokonta - ps9
Holomycota - ps10
Fungi - ps11
Fungi_1 - ps12
Fungi_2 - ps13
Fungi_3 - ps14
Dikarya - ps15
Ascomycota - ps16
Saccharo/Pezizomycotina - ps17
Saccharomycotina - ps18
Saccharomycotina_1 - ps19
Saccharomycotina_2 - ps20
Saccharomycotina_3 - ps21
Phaff./Saccharomycetaceae - ps22
Saccharomyco./mycetaceae - ps23
Saccharomycetaceae - ps24
Saccharomycetaceae_2 - ps25
Saccharomycetaceae_3 - ps26
Kaz./Nau./Saccharomyces - ps27
Saccharomyces - ps28
Arboricola_clade - ps29
Cerevisiae_clade - ps30
Saccharomyces_cerevisiae - ps31
Phylostratum

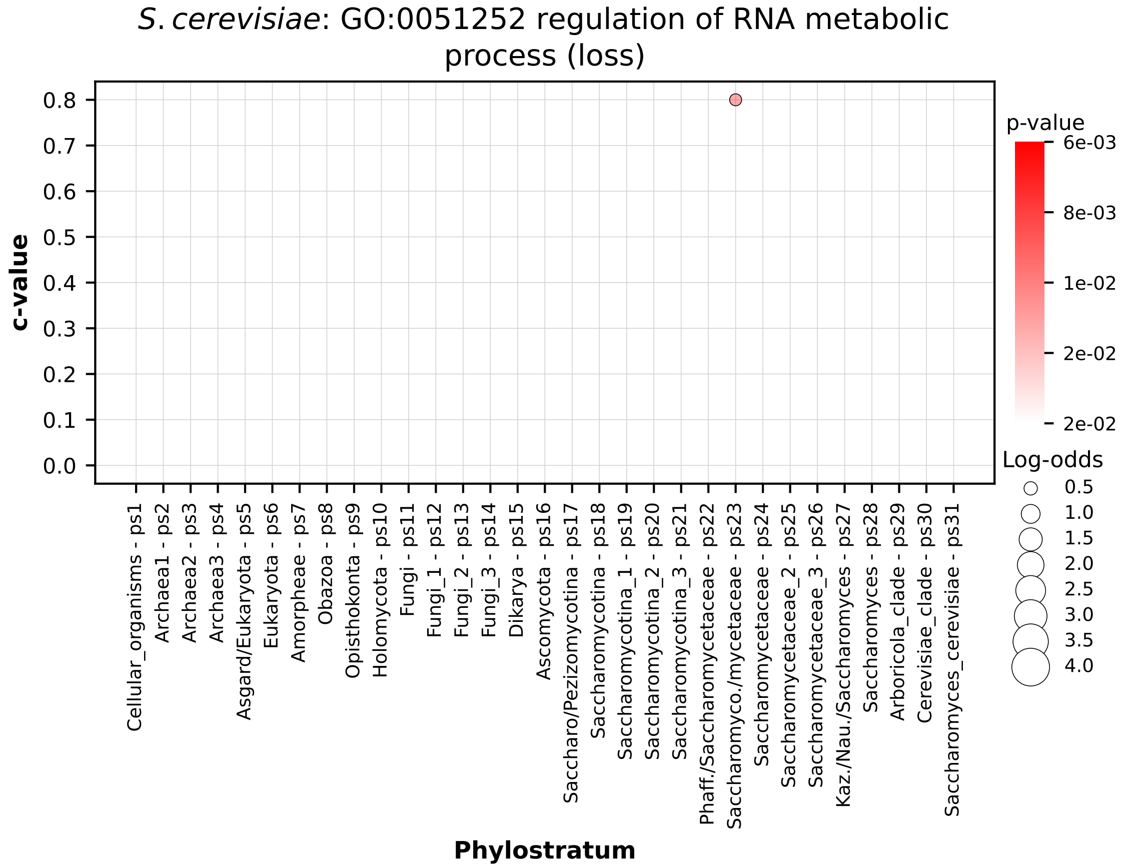
S. cerevisiae: GO:0051252 regulation of RNA metabolic process (loss)
c-value
0.0
0.1
0.2
0.3
0.4
0.5
0.6
0.7
0.8
p-value
6e-03
8e-03
1e-02
2e-02
2e-02
Log-odds
0.5
1.0
1.5
2.0
2.5
3.0
3.5
4.0
Cellular_organisms - ps1
Archaea1 - ps2
Archaea2 - ps3
Archaea3 - ps4
Asgard/Eukaryota - ps5
Eukaryota - ps6
Amorpheae - ps7
Obazoa - ps8
Opisthokonta - ps9
Holomycota - ps10
Fungi - ps11
Fungi_1 - ps12
Fungi_2 - ps13
Fungi_3 - ps14
Dikarya - ps15
Ascomycota - ps16
Saccharo/Pezizomycotina - ps17
Saccharomycotina - ps18
Saccharomycotina_1 - ps19
Saccharomycotina_2 - ps20
Saccharomycotina_3 - ps21
Phaff./Saccharomycetaceae - ps22
Saccharomyco./mycetaceae - ps23
Saccharomycetaceae - ps24
Saccharomycetaceae_2 - ps25
Saccharomycetaceae_3 - ps26
Kaz./Nau./Saccharomyces - ps27
Saccharomyces - ps28
Arboricola_clade - ps29
Cerevisiae_clade - ps30
Saccharomyces_cerevisiae - ps31
Phylostratum

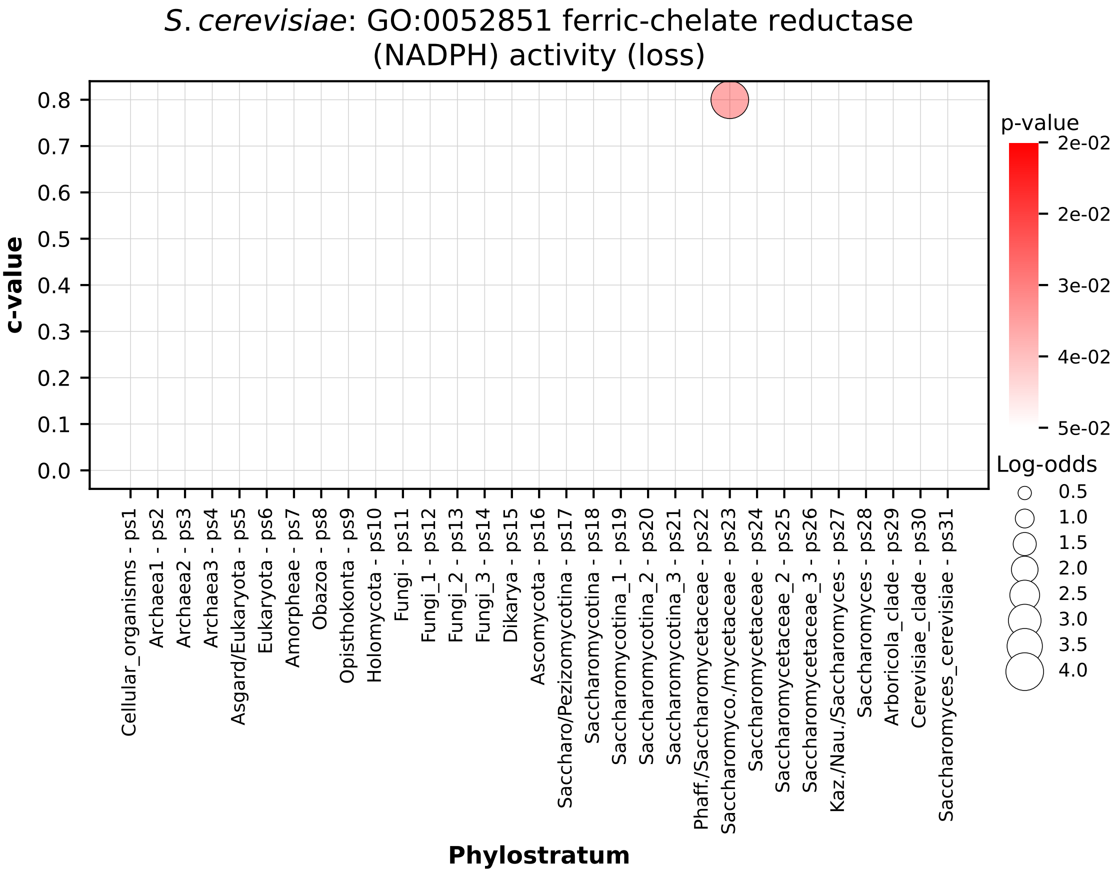
S. cerevisiae: GO:0052851 ferric-chelate reductase (NADPH) activity (loss)
c-value
0.0
0.1
0.2
0.3
0.4
0.5
0.6
0.7
0.8
p-value
2e-02
2e-02
3e-02
4e-02
5e-02
Log-odds
0.5
1.0
1.5
2.0
2.5
3.0
3.5
4.0
Cellular_organisms - ps1
Archaea1 - ps2
Archaea2 - ps3
Archaea3 - ps4
Asgard/Eukaryota - ps5
Eukaryota - ps6
Amorpheae - ps7
Obazoa - ps8
Opisthokonta - ps9
Holomycota - ps10
Fungi - ps11
Fungi_1 - ps12
Fungi_2 - ps13
Fungi_3 - ps14
Dikarya - ps15
Ascomycota - ps16
Saccharo/Pezizomycotina - ps17
Saccharomycotina - ps18
Saccharomycotina_1 - ps19
Saccharomycotina_2 - ps20
Saccharomycotina_3 - ps21
Phaff./Saccharomycetaceae - ps22
Saccharomyco./mycetaceae - ps23
Saccharomycetaceae - ps24
Saccharomycetaceae_2 - ps25
Saccharomycetaceae_3 - ps26
Kaz./Nau./Saccharomyces - ps27
Saccharomyces - ps28
Arboricola_clade - ps29
Cerevisiae_clade - ps30
Saccharomyces_cerevisiae - ps31
Phylostratum

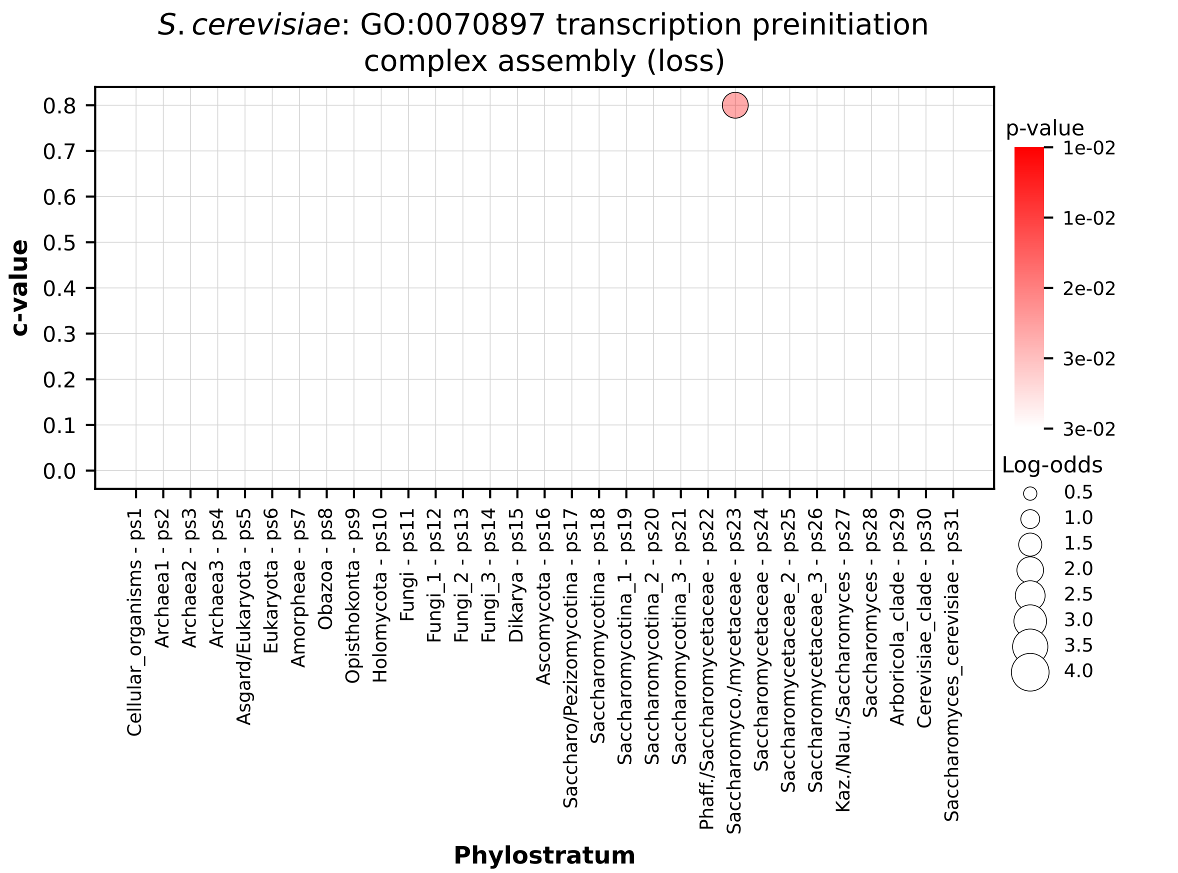

S. cerevisiae: GO:0070897 transcription preinitiation complex assembly (loss)
c-value
0.0
0.1
0.2
0.3
0.4
0.5
0.6
0.7
0.8
p-value
1e-02
1e-02
2e-02
3e-02
3e-02
Log-odds
0.5
1.0
1.5
2.0
2.5
3.0
3.5
4.0
Cellular_organisms - ps1
Archaea1 - ps2
Archaea2 - ps3
Archaea3 - ps4
Asgard/Eukaryota - ps5
Eukaryota - ps6
Amorpheae - ps7
Obazoa - ps8
Opisthokonta - ps9
Holomycota - ps10
Fungi - ps11
Fungi_1 - ps12
Fungi_2 - ps13
Fungi_3 - ps14
Dikarya - ps15
Ascomycota - ps16
Saccharo/Pezizomycotina - ps17
Saccharomycotina - ps18
Saccharomycotina_1 - ps19
Saccharomycotina_2 - ps20
Saccharomycotina_3 - ps21
Phaff./Saccharomycetaceae - ps22
Saccharomyco./mycetaceae - ps23
Saccharomycetaceae - ps24
Saccharomycetaceae_2 - ps25
Saccharomycetaceae_3 - ps26
Kaz./Nau./Saccharomyces - ps27
Saccharomyces - ps28
Arboricola_clade - ps29
Cerevisiae_clade - ps30
Saccharomyces_cerevisiae - ps31
Phylostratum

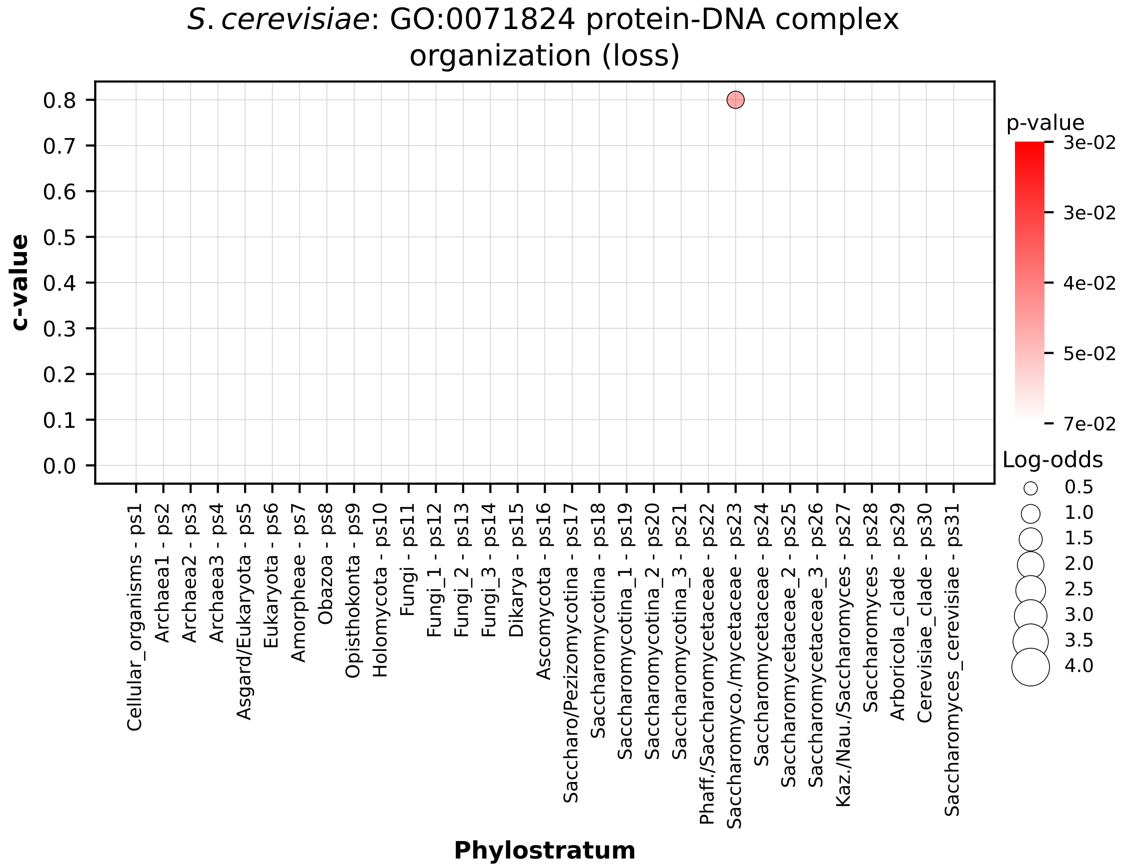

S. cerevisiae: GO:0071824 protein-DNA complex organization (loss)
c-value
0.8
0.7
0.6
0.5
0.4
0.3
0.2
0.1
0.0
p-value
3e-02
3e-02
4e-02
5e-02
7e-02
Log-odds
0.5
1.0
1.5
2.0
2.5
3.0
3.5
4.0
Cellular_organisms - ps1
Archaea1 - ps2
Archaea2 - ps3
Archaea3 - ps4
Asgard/Eukaryota - ps5
Eukaryota - ps6
Amorpheae - ps7
Obazoa - ps8
Opisthokonta - ps9
Holomycota - ps10
Fungi - ps11
Fungi_1 - ps12
Fungi_2 - ps13
Fungi_3 - ps14
Dikarya - ps15
Ascomycota - ps16
Saccharo/Pezizomycotina - ps17
Saccharomycotina - ps18
Saccharomycotina_1 - ps19
Saccharomycotina_2 - ps20
Saccharomycotina_3 - ps21
Phaff./Saccharomycetaceae - ps22
Saccharomyco./mycetaceae - ps23
Saccharomycetaceae - ps24
Saccharomycetaceae_2 - ps25
Saccharomycetaceae_3 - ps26
Kaz./Nau./Saccharomyces - ps27
Saccharomyces - ps28
Arboricola_clade - ps29
Cerevisiae_clade - ps30
Saccharomyces_cerevisiae - ps31
Phylostratum

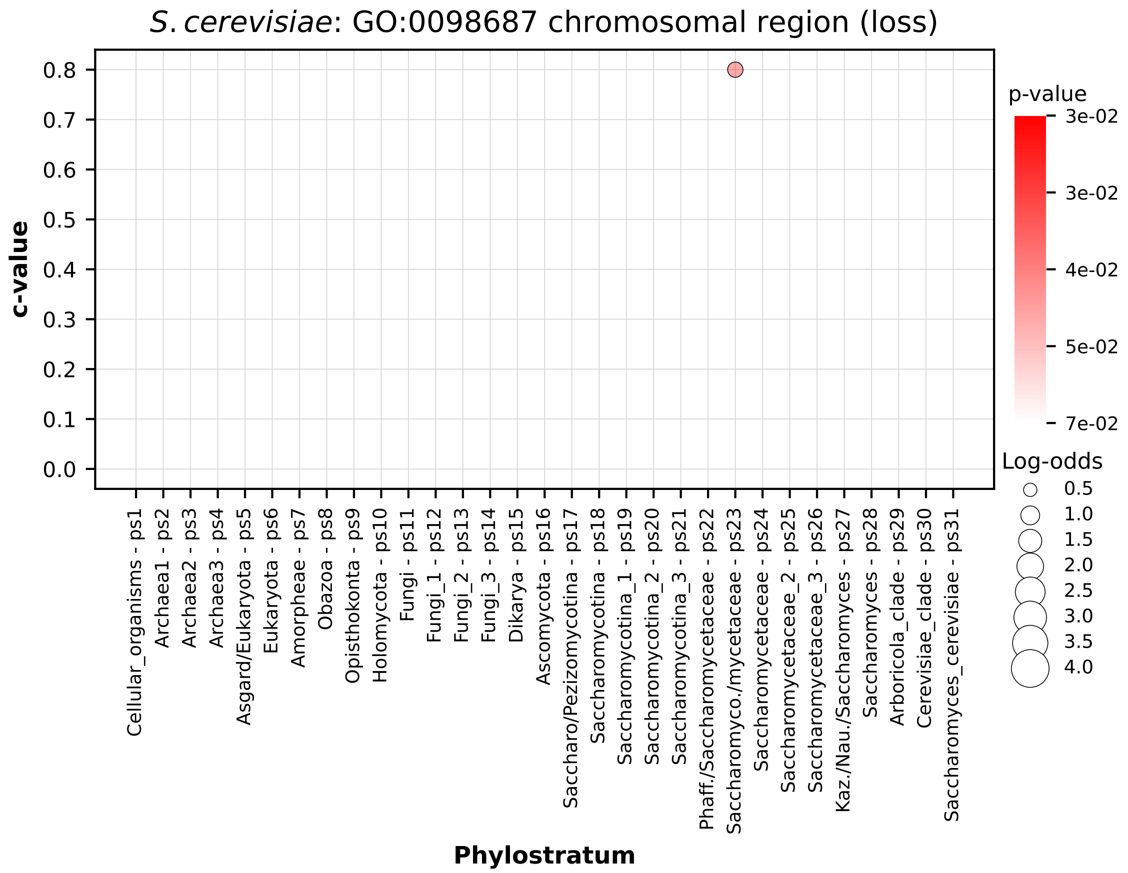

S. cerevisiae: GO:0098687 chromosomal region (loss)
c-value
0.8
0.7
0.6
0.5
0.4
0.3
0.2
0.1
0.0
Phylostratum
Cellular_organisms - ps1
Archaea1 - ps2
Archaea2 - ps3
Archaea3 - ps4
Asgard/Eukaryota - ps5
Eukaryota - ps6
Amorpheae - ps7
Obazoa - ps8
Opisthokonta - ps9
Holomycota - ps10
Fungi - ps11
Fungi_1 - ps12
Fungi_2 - ps13
Fungi_3 - ps14
Dikarya - ps15
Ascomycota - ps16
Saccharo/Pezizomycotina - ps17
Saccharomycotina - ps18
Saccharomycotina_1 - ps19
Saccharomycotina_2 - ps20
Saccharomycotina_3 - ps21
Phaff./Saccharomycetaceae - ps22
Saccharomyco./mycetaceae - ps23
Saccharomycetaceae - ps24
Saccharomycetaceae_2 - ps25
Saccharomycetaceae_3 - ps26
Kaz./Nau./Saccharomyces - ps27
Saccharomyces - ps28
Arboricola_clade - ps29
Cerevisiae_clade - ps30
Saccharomyces_cerevisiae - ps31
p-value
3e-02
3e-02
4e-02
5e-02
7e-02
Log-odds
0.5
1.0
1.5
2.0
2.5
3.0
3.5
4.0

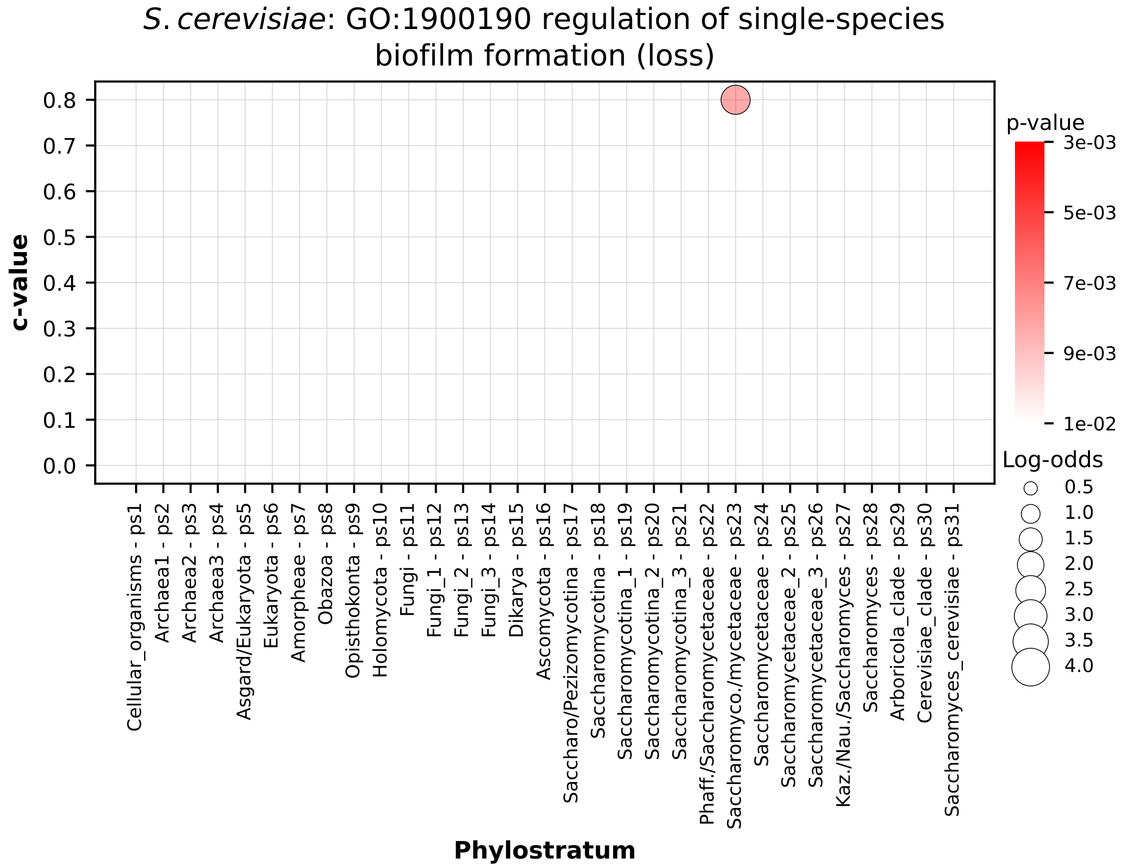

S. cerevisiae: GO:1900190 regulation of single-species biofilm formation (loss)
c-value
0.0
0.1
0.2
0.3
0.4
0.5
0.6
0.7
0.8
p-value
3e-03
5e-03
7e-03
9e-03
1e-02
Log-odds
0.5
1.0
1.5
2.0
2.5
3.0
3.5
4.0
Cellular_organisms - ps1
Archaea1 - ps2
Archaea2 - ps3
Archaea3 - ps4
Asgard/Eukaryota - ps5
Eukaryota - ps6
Amorpheae - ps7
Obazoa - ps8
Opisthokonta - ps9
Holomycota - ps10
Fungi - ps11
Fungi_1 - ps12
Fungi_2 - ps13
Fungi_3 - ps14
Dikarya - ps15
Ascomycota - ps16
Saccharo/Pezizomycotina - ps17
Saccharomycotina - ps18
Saccharomycotina_1 - ps19
Saccharomycotina_2 - ps20
Saccharomycotina_3 - ps21
Phaff./Saccharomycetaceae - ps22
Saccharomyco./mycetaceae - ps23
Saccharomycetaceae - ps24
Saccharomycetaceae_2 - ps25
Saccharomycetaceae_3 - ps26
Kaz./Nau./Saccharomyces - ps27
Saccharomyces - ps28
Arboricola_clade - ps29
Cerevisiae_clade - ps30
Saccharomyces_cerevisiae - ps31
Phylostratum

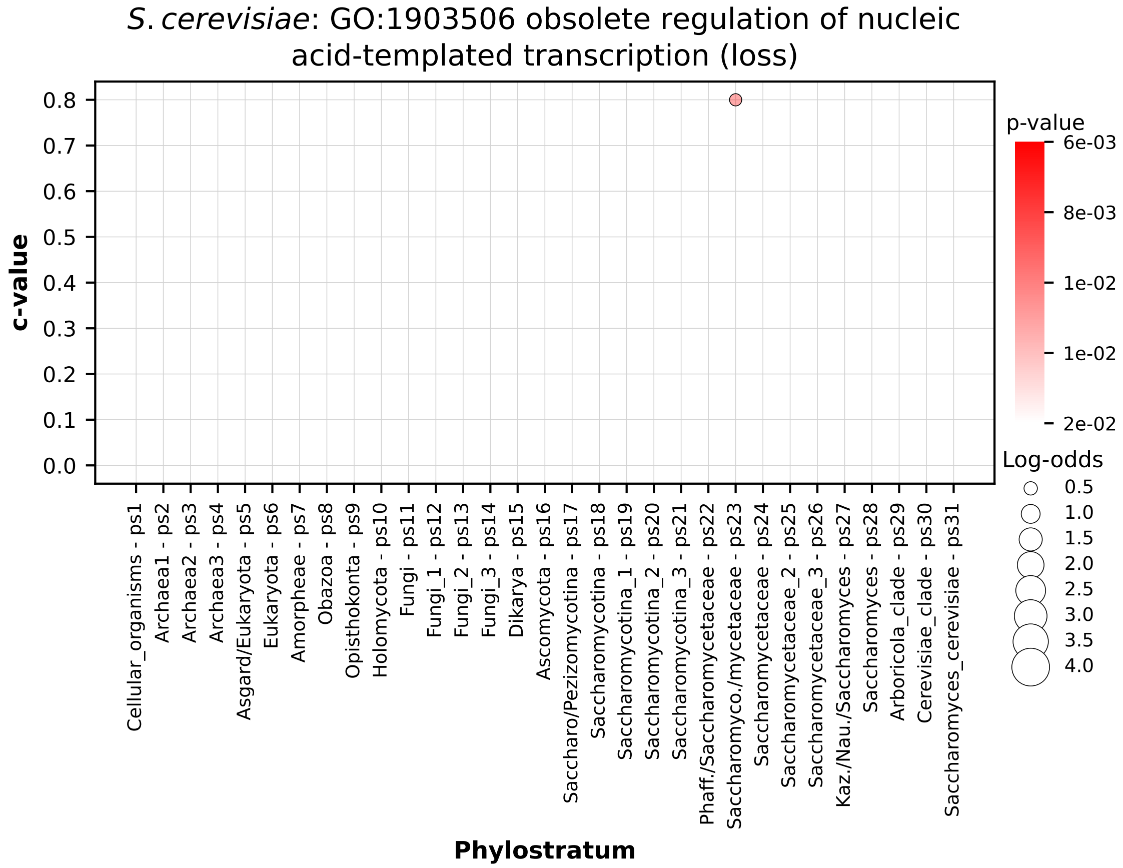
S. cerevisiae: GO:1903506 obsolete regulation of nucleic acid-templated transcription (loss)
c-value
0.8
0.7
0.6
0.5
0.4
0.3
0.2
0.1
0.0
p-value
6e-03
8e-03
1e-02
1e-02
2e-02
Log-odds
0.5
1.0
1.5
2.0
2.5
3.0
3.5
4.0
Cellular_organisms - ps1
Archaea1 - ps2
Archaea2 - ps3
Archaea3 - ps4
Asgard/Eukaryota - ps5
Eukaryota - ps6
Amorpheae - ps7
Obazoa - ps8
Opisthokonta - ps9
Holomycota - ps10
Fungi - ps11
Fungi_1 - ps12
Fungi_2 - ps13
Fungi_3 - ps14
Dikarya - ps15
Ascomycota - ps16
Saccharo/Pezizomycotina - ps17
Saccharomycotina - ps18
Saccharomycotina_1 - ps19
Saccharomycotina_2 - ps20
Saccharomycotina_3 - ps21
Phaff./Saccharomycetaceae - ps22
Saccharomyco./mycetaceae - ps23
Saccharomycetaceae - ps24
Saccharomycetaceae_2 - ps25
Saccharomycetaceae_3 - ps26
Kaz./Nau./Saccharomyces - ps27
Saccharomyces - ps28
Arboricola_clade - ps29
Cerevisiae_clade - ps30
Saccharomyces_cerevisiae - ps31
Phylostratum

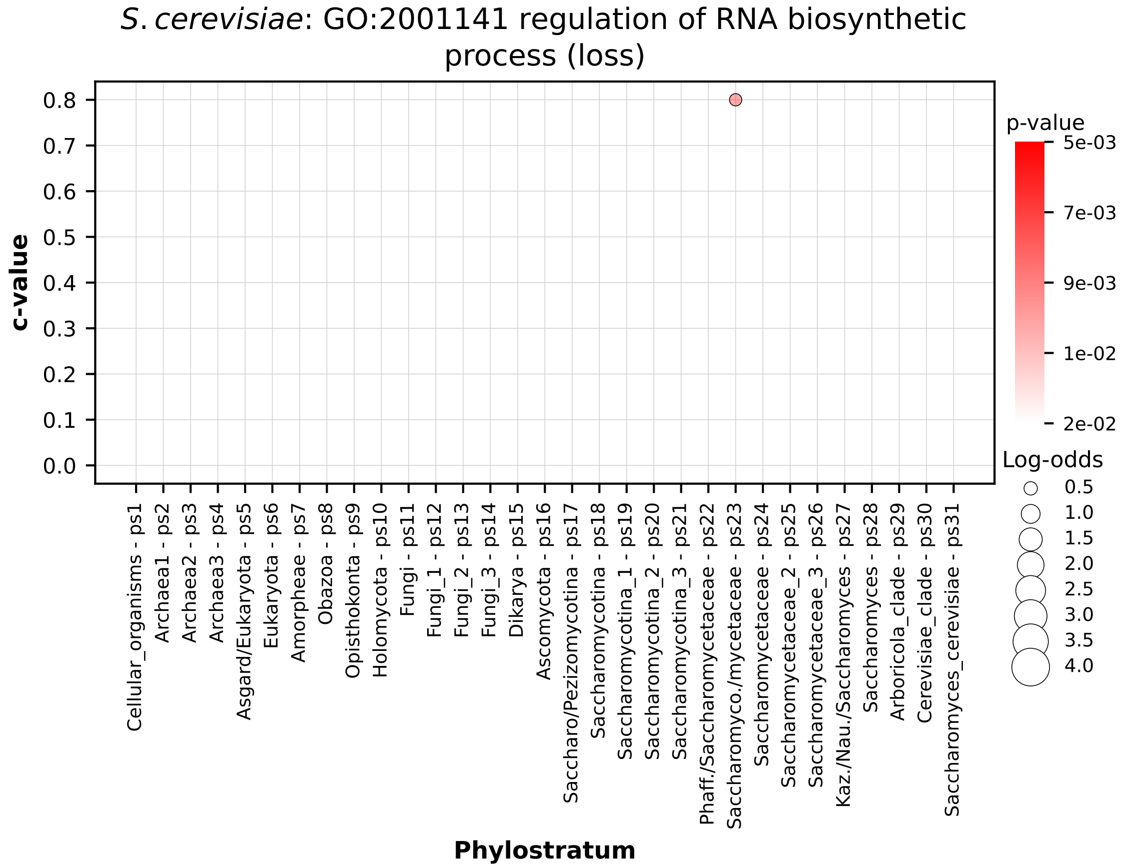
S. cerevisiae: GO:2001141 regulation of RNA biosynthetic process (loss)
c-value
0.8
0.7
0.6
0.5
0.4
0.3
0.2
0.1
0.0
p-value
5e-03
7e-03
9e-03
1e-02
2e-02
Log-odds
0.5
1.0
1.5
2.0
2.5
3.0
3.5
4.0
Cellular_organisms - ps1
Archaea1 - ps2
Archaea2 - ps3
Archaea3 - ps4
Asgard/Eukaryota - ps5
Eukaryota - ps6
Amorpheae - ps7
Obazoa - ps8
Opisthokonta - ps9
Holomycota - ps10
Fungi - ps11
Fungi_1 - ps12
Fungi_2 - ps13
Fungi_3 - ps14
Dikarya - ps15
Ascomycota - ps16
Saccharo/Pezizomycotina - ps17
Saccharomycotina - ps18
Saccharomycotina_1 - ps19
Saccharomycotina_2 - ps20
Saccharomycotina_3 - ps21
Phaff./Saccharomycetaceae - ps22
Saccharomyco./mycetaceae - ps23
Saccharomycetaceae - ps24
Saccharomycetaceae_2 - ps25
Saccharomycetaceae_3 - ps26
Kaz./Nau./Saccharomyces - ps27
Saccharomyces - ps28
Arboricola_clade - ps29
Cerevisiae_clade - ps30
Saccharomyces_cerevisiae - ps31
Phylostratum

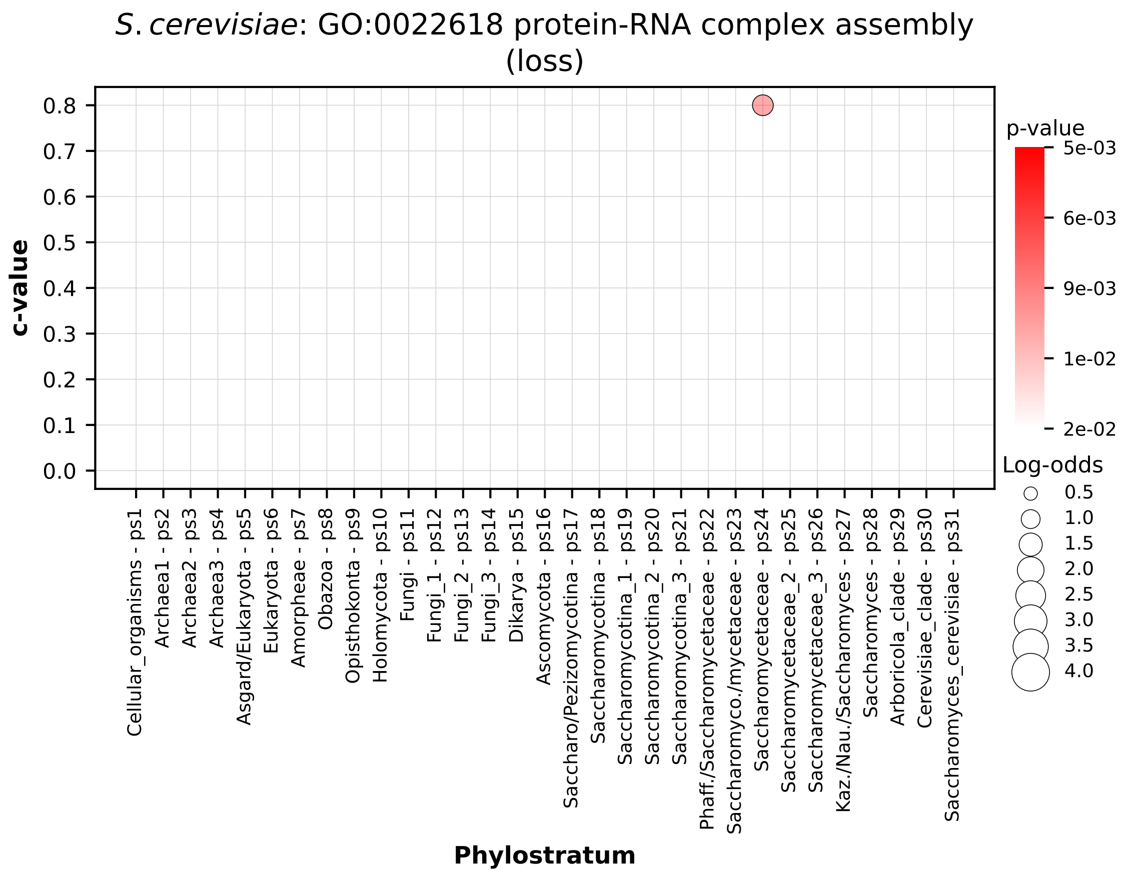
S. cerevisiae: GO:0022618 protein-RNA complex assembly
(loss)
c-value
0.0
0.1
0.2
0.3
0.4
0.5
0.6
0.7
0.8
Phylostratum
Cellular_organisms - ps1
Archaea1 - ps2
Archaea2 - ps3
Archaea3 - ps4
Asgard/Eukaryota - ps5
Eukaryota - ps6
Amorpheae - ps7
Obazoa - ps8
Opisthokonta - ps9
Holomycota - ps10
Fungi - ps11
Fungi_1 - ps12
Fungi_2 - ps13
Fungi_3 - ps14
Dikarya - ps15
Ascomycota - ps16
Saccharo/Pezizomycotina - ps17
Saccharomycotina - ps18
Saccharomycotina_1 - ps19
Saccharomycotina_2 - ps20
Saccharomycotina_3 - ps21
Phaff./Saccharomycetaceae - ps22
Saccharomyco./mycetaceae - ps23
Saccharomycetaceae - ps24
Saccharomycetaceae_2 - ps25
Saccharomycetaceae_3 - ps26
Kaz./Nau./Saccharomyces - ps27
Saccharomyces - ps28
Arboricola_clade - ps29
Cerevisiae_clade - ps30
Saccharomyces_cerevisiae - ps31
p-value
5e-03
6e-03
9e-03
1e-02
2e-02
Log-odds
0.5
1.0
1.5
2.0
2.5
3.0
3.5
4.0

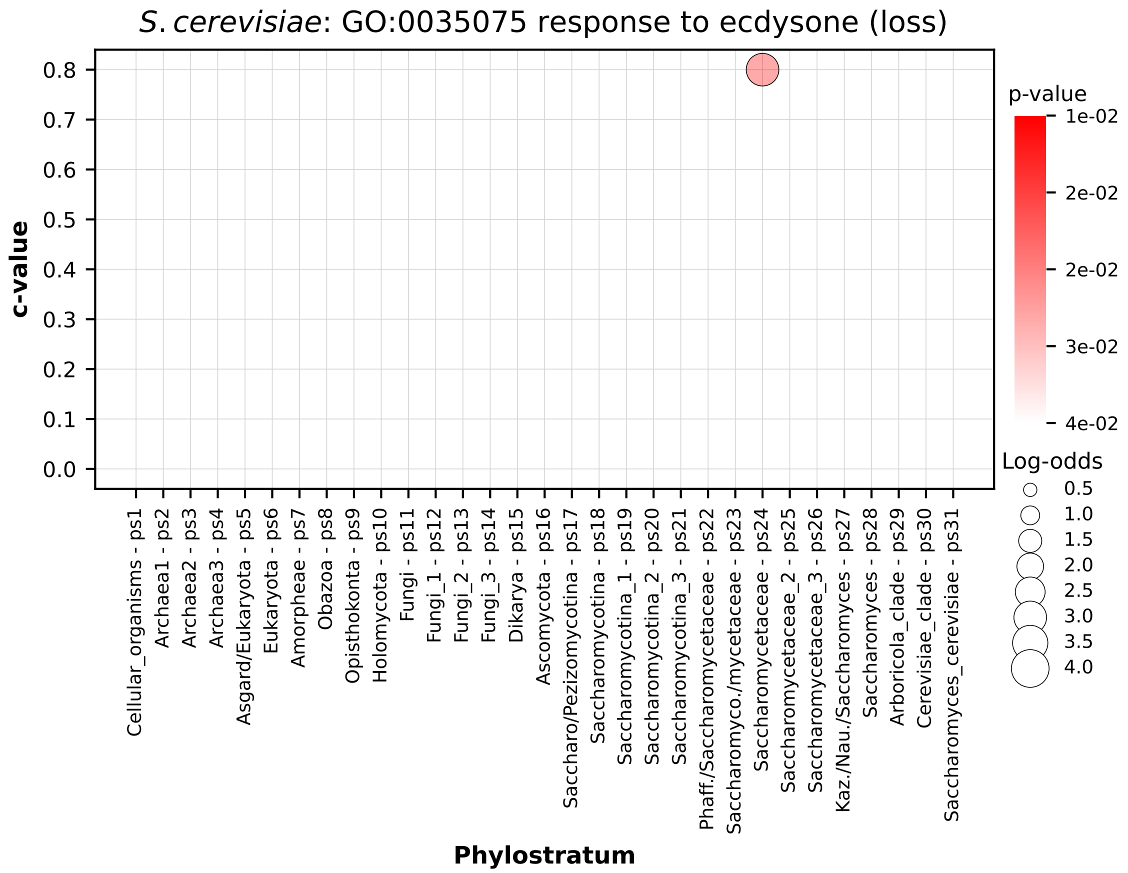

S. cerevisiae: GO:0035075 response to ecdysone (loss)
c-value
0.8
0.7
0.6
0.5
0.4
0.3
0.2
0.1
0.0
p-value
1e-02
2e-02
2e-02
3e-02
4e-02
Log-odds
0.5
1.0
1.5
2.0
2.5
3.0
3.5
4.0
Cellular_organisms - ps1
Archaea1 - ps2
Archaea2 - ps3
Archaea3 - ps4
Asgard/Eukaryota - ps5
Eukaryota - ps6
Amorpheae - ps7
Obazoa - ps8
Opisthokonta - ps9
Holomycota - ps10
Fungi - ps11
Fungi_1 - ps12
Fungi_2 - ps13
Fungi_3 - ps14
Dikarya - ps15
Ascomycota - ps16
Saccharo/Pezizomycotina - ps17
Saccharomycotina - ps18
Saccharomycotina_1 - ps19
Saccharomycotina_2 - ps20
Saccharomycotina_3 - ps21
Phaff./Saccharomycetaceae - ps22
Saccharomyco./mycetaceae - ps23
Saccharomycetaceae - ps24
Saccharomycetaceae_2 - ps25
Saccharomycetaceae_3 - ps26
Kaz./Nau./Saccharomyces - ps27
Saccharomyces - ps28
Arboricola_clade - ps29
Cerevisiae_clade - ps30
Saccharomyces_cerevisiae - ps31
Phylostratum

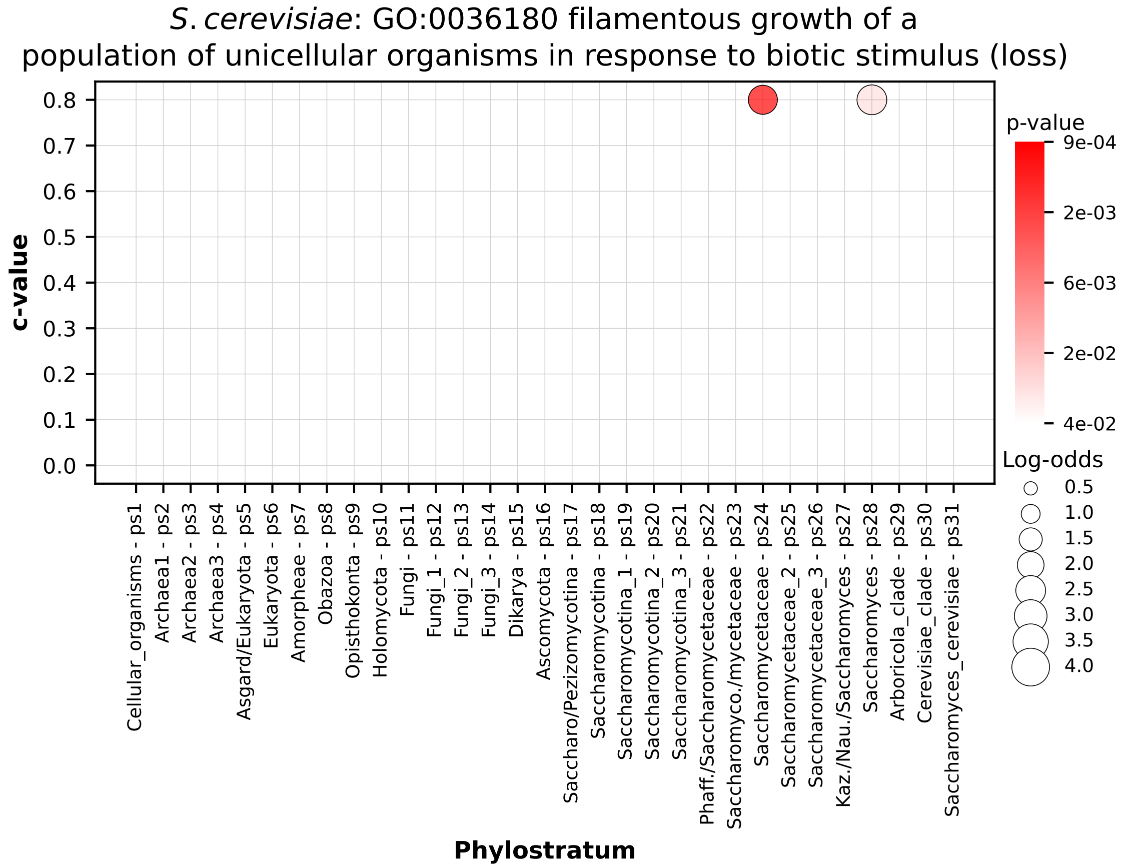

S. cerevisiae: GO:0036180 filamentous growth of a population of unicellular organisms in response to biotic stimulus (loss)
c-value
0.0
0.1
0.2
0.3
0.4
0.5
0.6
0.7
0.8
p-value
9e-04
2e-03
6e-03
2e-02
4e-02
Log-odds
0.5
1.0
1.5
2.0
2.5
3.0
3.5
4.0
Cellular_organisms - ps1
Archaea1 - ps2
Archaea2 - ps3
Archaea3 - ps4
Asgard/Eukaryota - ps5
Eukaryota - ps6
Amorpheae - ps7
Obazoa - ps8
Opisthokonta - ps9
Holomycota - ps10
Fungi - ps11
Fungi_1 - ps12
Fungi_2 - ps13
Fungi_3 - ps14
Dikarya - ps15
Ascomycota - ps16
Saccharo/Pezizomycotina - ps17
Saccharomycotina - ps18
Saccharomycotina_1 - ps19
Saccharomycotina_2 - ps20
Saccharomycotina_3 - ps21
Phaff./Saccharomycetaceae - ps22
Saccharomyco./mycetaceae - ps23
Saccharomycetaceae - ps24
Saccharomycetaceae_2 - ps25
Saccharomycetaceae_3 - ps26
Kaz./Nau./Saccharomyces - ps27
Saccharomyces - ps28
Arboricola_clade - ps29
Cerevisiae_clade - ps30
Saccharomyces_cerevisiae - ps31
Phylostratum

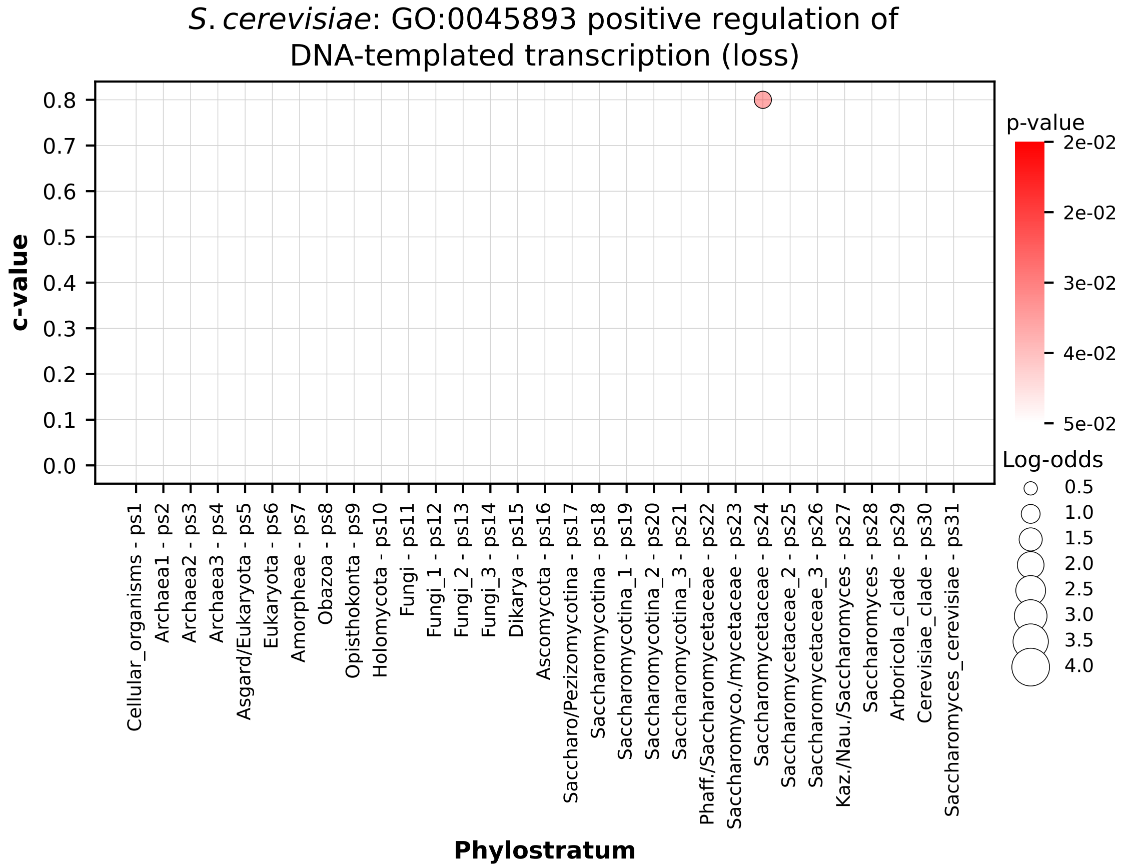

S. cerevisiae: GO:0045893 positive regulation of DNA-templated transcription (loss)
c-value
0.0
0.1
0.2
0.3
0.4
0.5
0.6
0.7
0.8
Phylostratum
Cellular_organisms - ps1
Archaea1 - ps2
Archaea2 - ps3
Archaea3 - ps4
Asgard/Eukaryota - ps5
Eukaryota - ps6
Amorpheae - ps7
Obazoa - ps8
Opisthokonta - ps9
Holomycota - ps10
Fungi - ps11
Fungi_1 - ps12
Fungi_2 - ps13
Fungi_3 - ps14
Dikarya - ps15
Ascomycota - ps16
Saccharo/Pezizomycotina - ps17
Saccharomycotina - ps18
Saccharomycotina_1 - ps19
Saccharomycotina_2 - ps20
Saccharomycotina_3 - ps21
Phaff./Saccharomycetaceae - ps22
Saccharomyco./mycetaceae - ps23
Saccharomycetaceae - ps24
Saccharomycetaceae_2 - ps25
Saccharomycetaceae_3 - ps26
Kaz./Nau./Saccharomyces - ps27
Saccharomyces - ps28
Arboricola_clade - ps29
Cerevisiae_clade - ps30
Saccharomyces_cerevisiae - ps31
p-value
2e-02
2e-02
3e-02
4e-02
5e-02
Log-odds
0.5
1.0
1.5
2.0
2.5
3.0
3.5
4.0

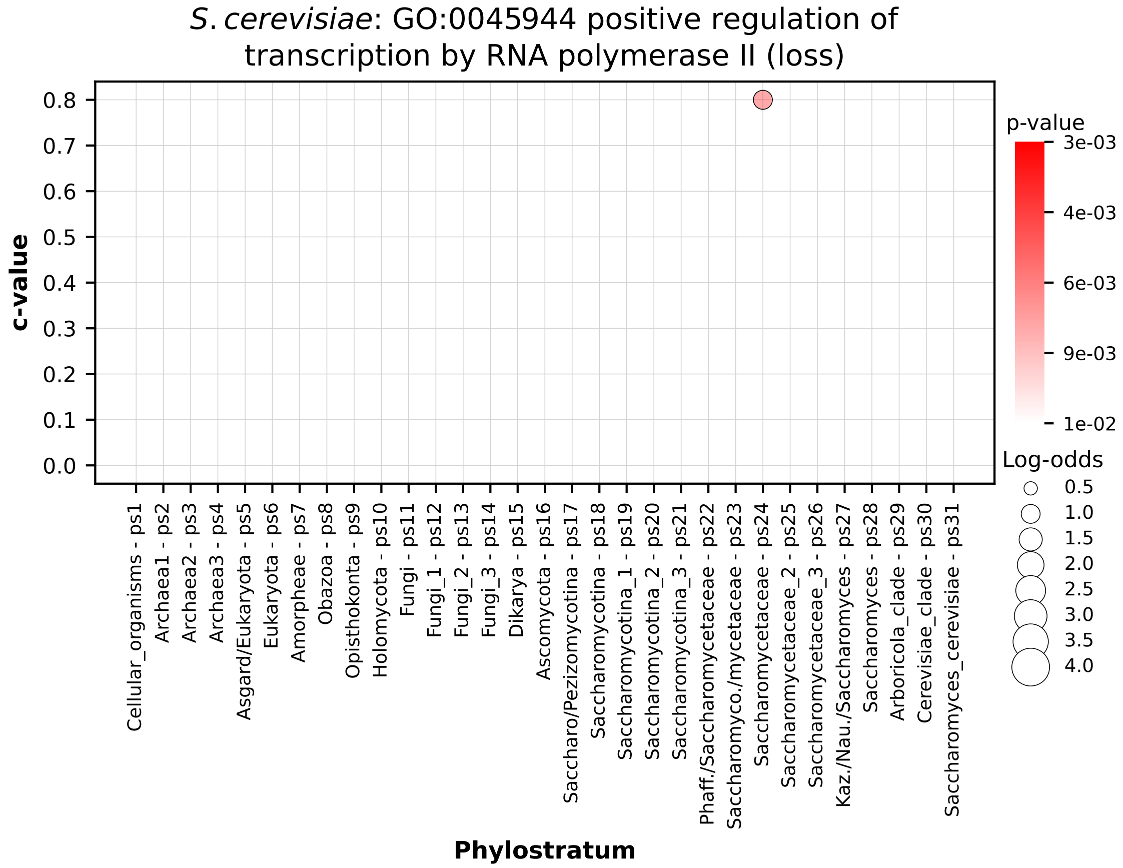
S. cerevisiae: GO:0045944 positive regulation of transcription by RNA polymerase II (loss)
c-value
0.0
0.1
0.2
0.3
0.4
0.5
0.6
0.7
0.8
p-value
3e-03
4e-03
6e-03
9e-03
1e-02
Log-odds
0.5
1.0
1.5
2.0
2.5
3.0
3.5
4.0
Cellular_organisms - ps1
Archaea1 - ps2
Archaea2 - ps3
Archaea3 - ps4
Asgard/Eukaryota - ps5
Eukaryota - ps6
Amorpheae - ps7
Obazoa - ps8
Opisthokonta - ps9
Holomycota - ps10
Fungi - ps11
Fungi_1 - ps12
Fungi_2 - ps13
Fungi_3 - ps14
Dikarya - ps15
Ascomycota - ps16
Saccharo/Pezizomycotina - ps17
Saccharomycotina - ps18
Saccharomycotina_1 - ps19
Saccharomycotina_2 - ps20
Saccharomycotina_3 - ps21
Phaff./Saccharomycetaceae - ps22
Saccharomyco./mycetaceae - ps23
Saccharomycetaceae - ps24
Saccharomycetaceae_2 - ps25
Saccharomycetaceae_3 - ps26
Kaz./Nau./Saccharomyces - ps27
Saccharomyces - ps28
Arboricola_clade - ps29
Cerevisiae_clade - ps30
Saccharomyces_cerevisiae - ps31
Phylostratum

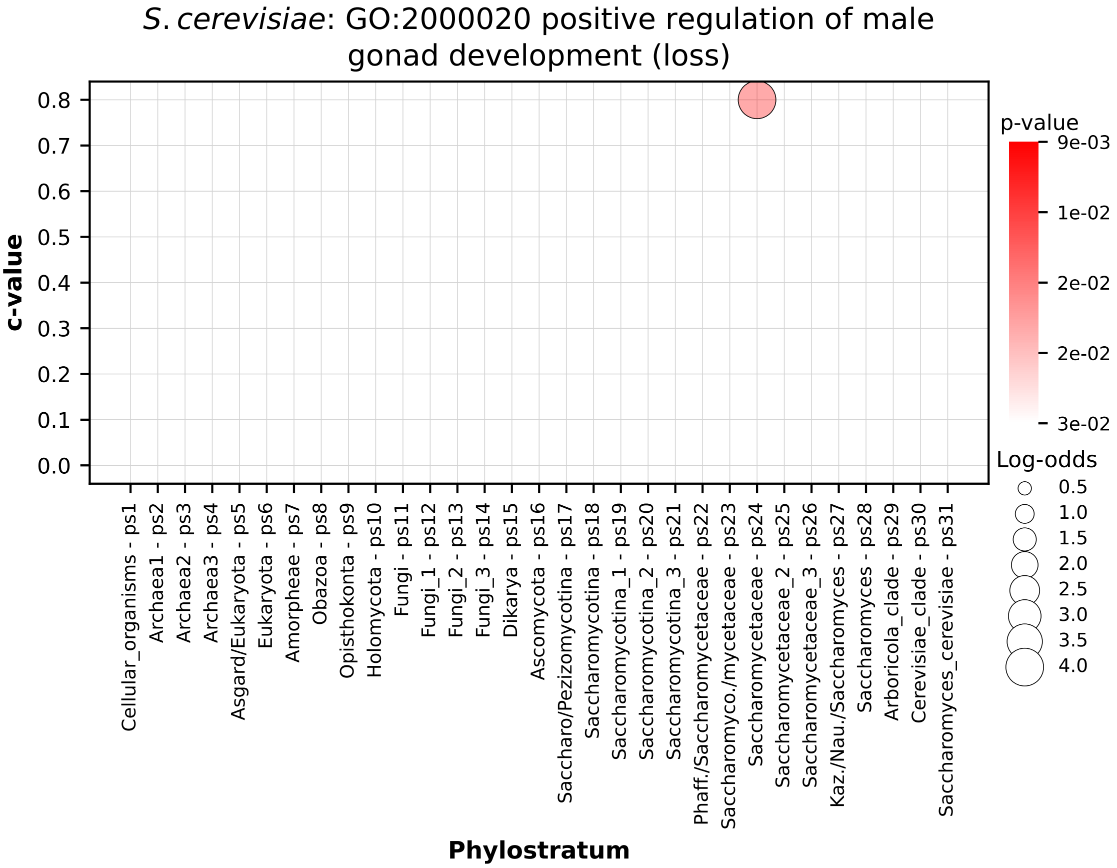

S. cerevisiae: GO:2000020 positive regulation of male gonad development (loss)
c-value
0.0
0.1
0.2
0.3
0.4
0.5
0.6
0.7
0.8
p-value
9e-03
1e-02
2e-02
2e-02
3e-02
Log-odds
0.5
1.0
1.5
2.0
2.5
3.0
3.5
4.0
Cellular_organisms - ps1
Archaea1 - ps2
Archaea2 - ps3
Archaea3 - ps4
Asgard/Eukaryota - ps5
Eukaryota - ps6
Amorpheae - ps7
Obazoa - ps8
Opisthokonta - ps9
Holomycota - ps10
Fungi - ps11
Fungi_1 - ps12
Fungi_2 - ps13
Fungi_3 - ps14
Dikarya - ps15
Ascomycota - ps16
Saccharo/Pezizomycotina - ps17
Saccharomycotina - ps18
Saccharomycotina_1 - ps19
Saccharomycotina_2 - ps20
Saccharomycotina_3 - ps21
Phaff./Saccharomycetaceae - ps22
Saccharomyco./mycetaceae - ps23
Saccharomycetaceae - ps24
Saccharomycetaceae_2 - ps25
Saccharomycetaceae_3 - ps26
Kaz./Nau./Saccharomyces - ps27
Saccharomyces - ps28
Arboricola_clade - ps29
Cerevisiae_clade - ps30
Saccharomyces_cerevisiae - ps31
Phylostratum

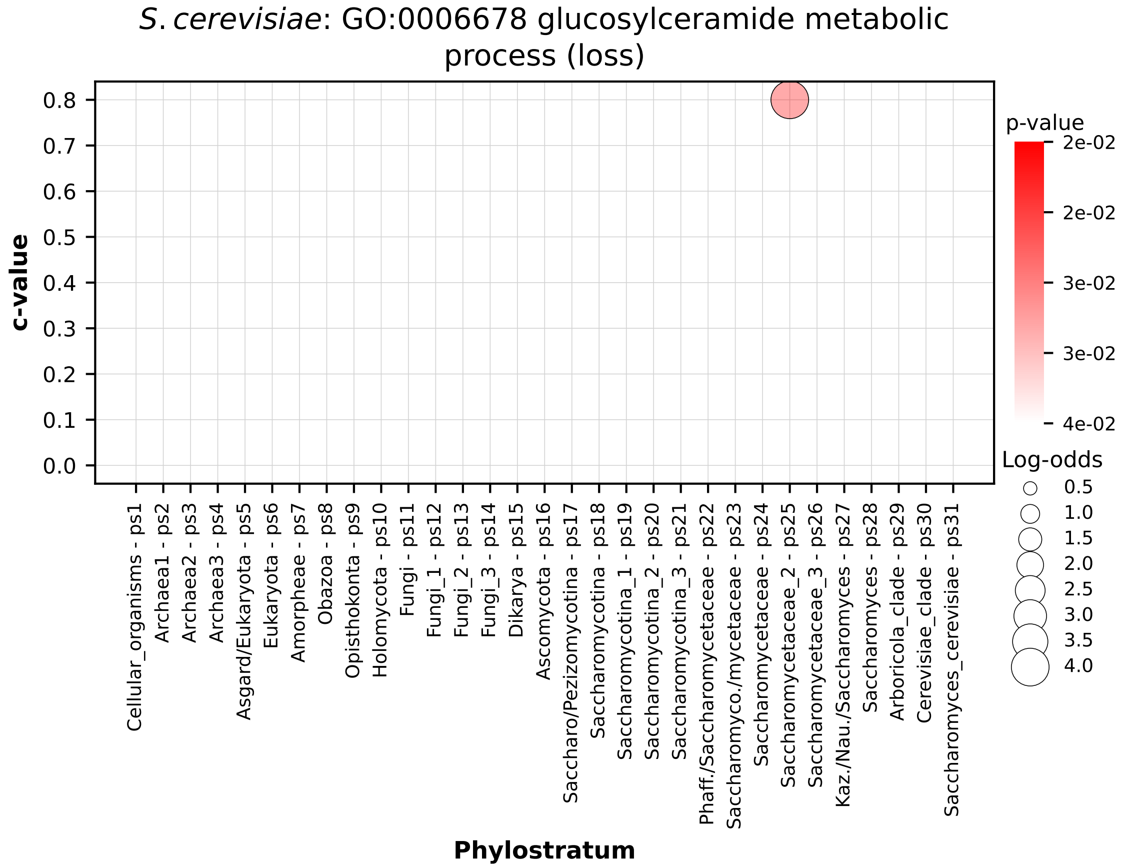
S. cerevisiae: GO:0006678 glucosylceramide metabolic process (loss)
c-value
0.0
0.1
0.2
0.3
0.4
0.5
0.6
0.7
0.8
p-value
2e-02
2e-02
3e-02
3e-02
4e-02
Log-odds
0.5
1.0
1.5
2.0
2.5
3.0
3.5
4.0
Cellular_organisms - ps1
Archaea1 - ps2
Archaea2 - ps3
Archaea3 - ps4
Asgard/Eukaryota - ps5
Eukaryota - ps6
Amorpheae - ps7
Obazoa - ps8
Opisthokonta - ps9
Holomycota - ps10
Fungi - ps11
Fungi_1 - ps12
Fungi_2 - ps13
Fungi_3 - ps14
Dikarya - ps15
Ascomycota - ps16
Saccharo/Pezizomycotina - ps17
Saccharomycotina - ps18
Saccharomycotina_1 - ps19
Saccharomycotina_2 - ps20
Saccharomycotina_3 - ps21
Phaff./Saccharomycetaceae - ps22
Saccharomyco./mycetaceae - ps23
Saccharomycetaceae - ps24
Saccharomycetaceae_2 - ps25
Saccharomycetaceae_3 - ps26
Kaz./Nau./Saccharomyces - ps27
Saccharomyces - ps28
Arboricola_clade - ps29
Cerevisiae_clade - ps30
Saccharomyces_cerevisiae - ps31
Phylostratum

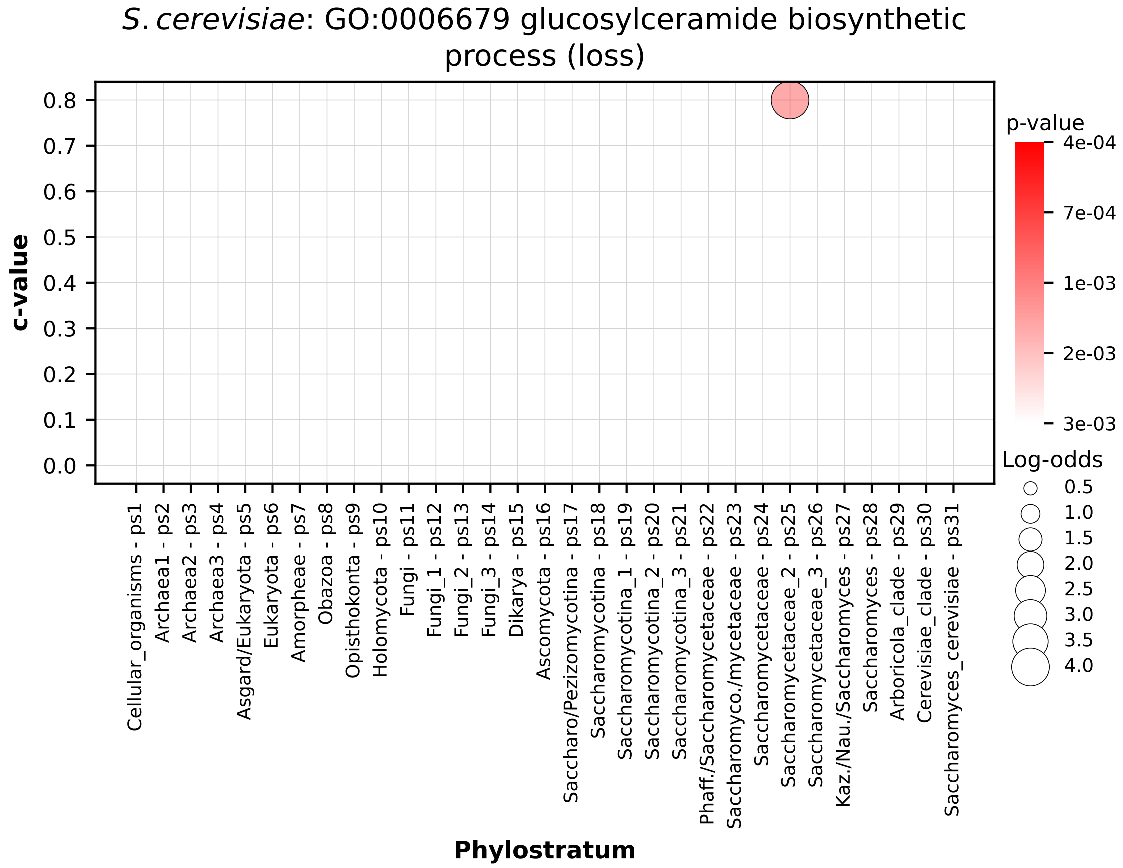

S. cerevisiae: GO:0006679 glucosylceramide biosynthetic process (loss)
c-value
0.0
0.1
0.2
0.3
0.4
0.5
0.6
0.7
0.8
p-value
4e-04
7e-04
1e-03
2e-03
3e-03
Log-odds
0.5
1.0
1.5
2.0
2.5
3.0
3.5
4.0
Cellular_organisms - ps1
Archaea1 - ps2
Archaea2 - ps3
Archaea3 - ps4
Asgard/Eukaryota - ps5
Eukaryota - ps6
Amorpheae - ps7
Obazoa - ps8
Opisthokonta - ps9
Holomycota - ps10
Fungi - ps11
Fungi_1 - ps12
Fungi_2 - ps13
Fungi_3 - ps14
Dikarya - ps15
Ascomycota - ps16
Saccharo/Pezizomycotina - ps17
Saccharomycotina - ps18
Saccharomycotina_1 - ps19
Saccharomycotina_2 - ps20
Saccharomycotina_3 - ps21
Phaff./Saccharomycetaceae - ps22
Saccharomyco./mycetaceae - ps23
Saccharomycetaceae - ps24
Saccharomycetaceae_2 - ps25
Saccharomycetaceae_3 - ps26
Kaz./Nau./Saccharomyces - ps27
Saccharomyces - ps28
Arboricola_clade - ps29
Cerevisiae_clade - ps30
Saccharomyces_cerevisiae - ps31
Phylostratum

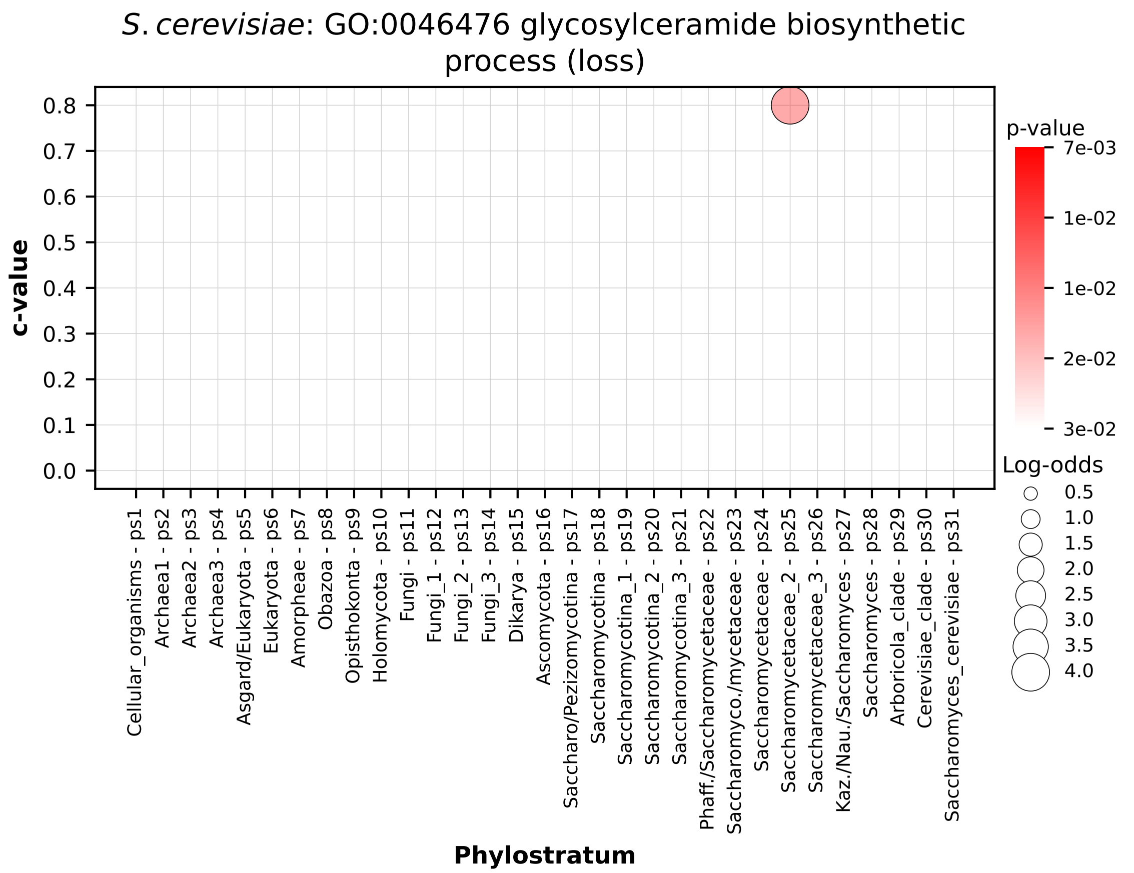

S. cerevisiae: GO:0046476 glycosylceramide biosynthetic process (loss)
c-value
0.8
0.7
0.6
0.5
0.4
0.3
0.2
0.1
0.0
p-value
7e-03
1e-02
1e-02
2e-02
3e-02
Log-odds
0.5
1.0
1.5
2.0
2.5
3.0
3.5
4.0
Cellular_organisms - ps1
Archaea1 - ps2
Archaea2 - ps3
Archaea3 - ps4
Asgard/Eukaryota - ps5
Eukaryota - ps6
Amorpheae - ps7
Obazoa - ps8
Opisthokonta - ps9
Holomycota - ps10
Fungi - ps11
Fungi_1 - ps12
Fungi_2 - ps13
Fungi_3 - ps14
Dikarya - ps15
Ascomycota - ps16
Saccharo/Pezizomycotina - ps17
Saccharomycotina - ps18
Saccharomycotina_1 - ps19
Saccharomycotina_2 - ps20
Saccharomycotina_3 - ps21
Phaff./Saccharomycetaceae - ps22
Saccharomyco./mycetaceae - ps23
Saccharomycetaceae - ps24
Saccharomycetaceae_2 - ps25
Saccharomycetaceae_3 - ps26
Kaz./Nau./Saccharomyces - ps27
Saccharomyces - ps28
Arboricola_clade - ps29
Cerevisiae_clade - ps30
Saccharomyces_cerevisiae - ps31
Phylostratum

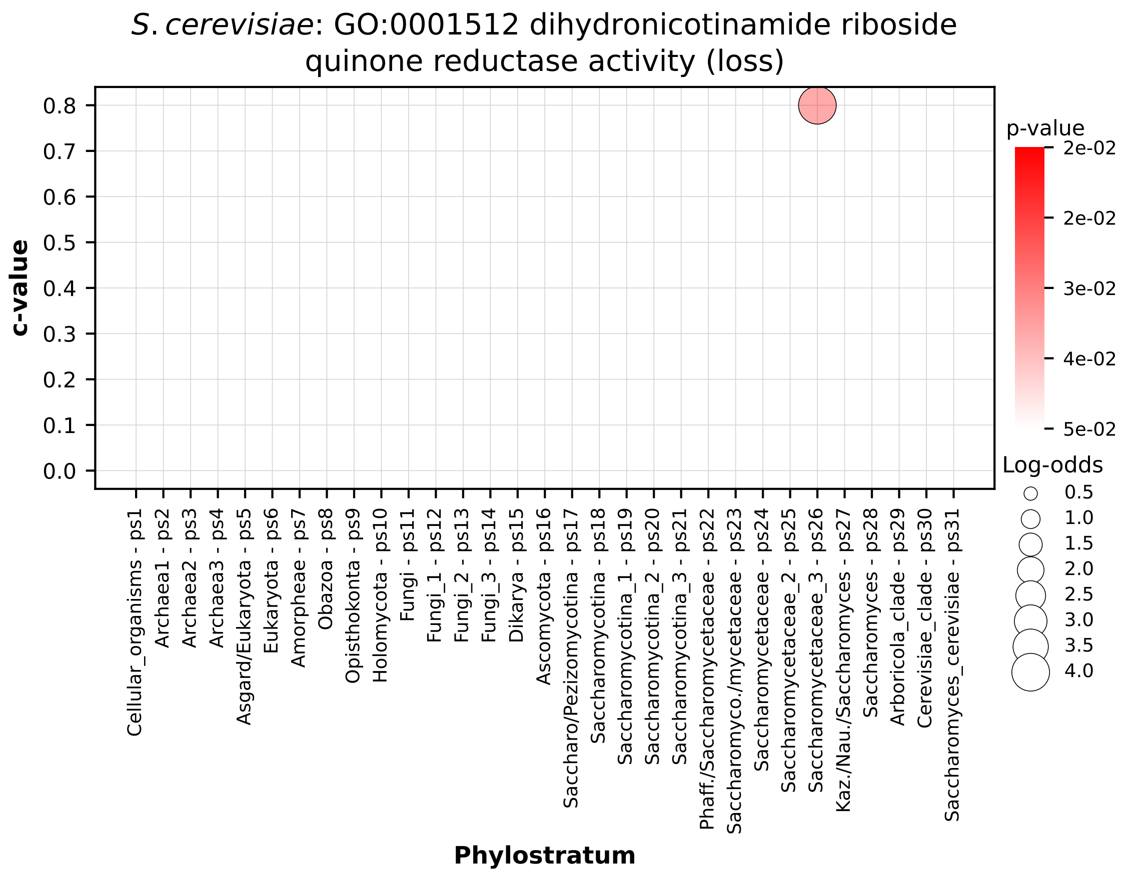

S. cerevisiae: GO:0001512 dihydronicotinamide riboside quinone reductase activity (loss)
c-value
0.0
0.1
0.2
0.3
0.4
0.5
0.6
0.7
0.8
p-value
2e-02
2e-02
3e-02
4e-02
5e-02
Log-odds
0.5
1.0
1.5
2.0
2.5
3.0
3.5
4.0
Cellular_organisms - ps1
Archaea1 - ps2
Archaea2 - ps3
Archaea3 - ps4
Asgard/Eukaryota - ps5
Eukaryota - ps6
Amorpheae - ps7
Obazoa - ps8
Opisthokonta - ps9
Holomycota - ps10
Fungi - ps11
Fungi_1 - ps12
Fungi_2 - ps13
Fungi_3 - ps14
Dikarya - ps15
Ascomycota - ps16
Saccharo/Pezizomycotina - ps17
Saccharomycotina - ps18
Saccharomycotina_1 - ps19
Saccharomycotina_2 - ps20
Saccharomycotina_3 - ps21
Phaff./Saccharomycetaceae - ps22
Saccharomyco./mycetaceae - ps23
Saccharomycetaceae - ps24
Saccharomycetaceae_2 - ps25
Saccharomycetaceae_3 - ps26
Kaz./Nau./Saccharomyces - ps27
Saccharomyces - ps28
Arboricola_clade - ps29
Cerevisiae_clade - ps30
Saccharomyces_cerevisiae - ps31
Phylostratum

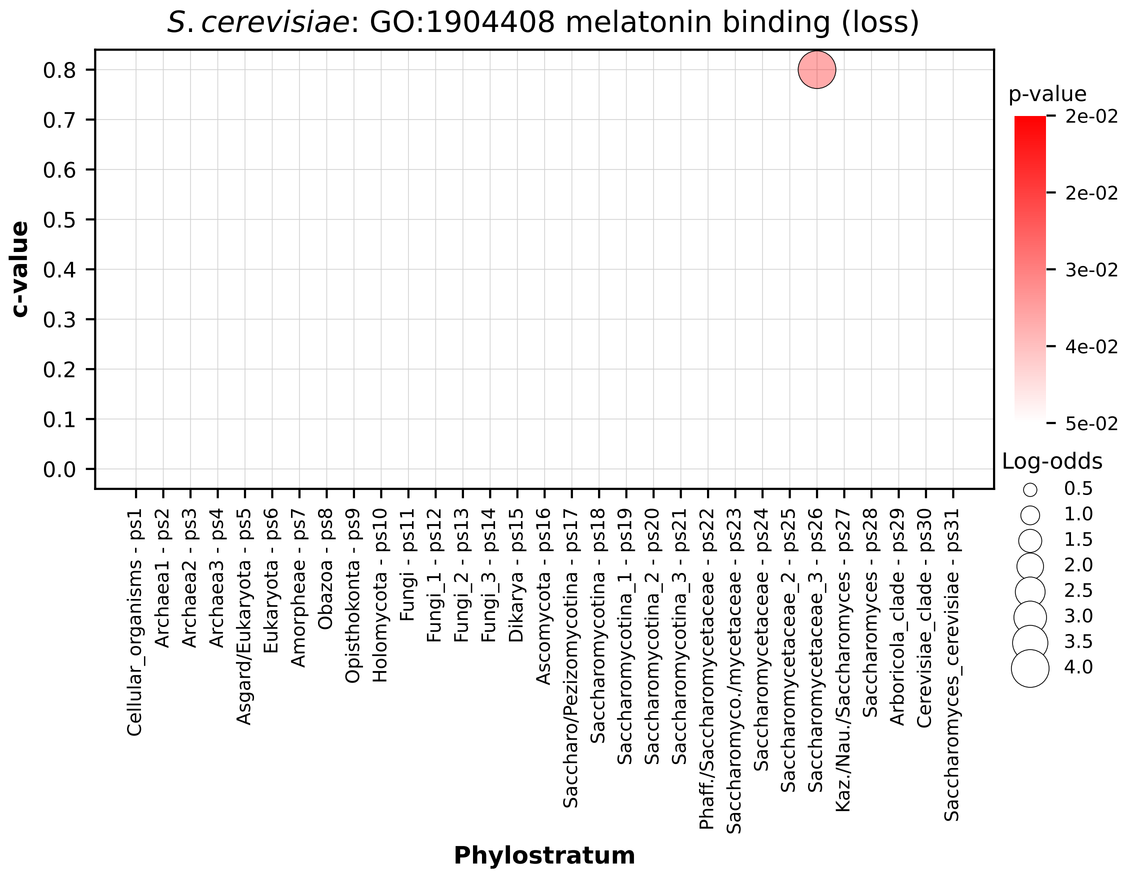

S. cerevisiae: GO:1904408 melatonin binding (loss)
c-value
0.0
0.1
0.2
0.3
0.4
0.5
0.6
0.7
0.8
p-value
2e-02
2e-02
3e-02
4e-02
5e-02
Log-odds
0.5
1.0
1.5
2.0
2.5
3.0
3.5
4.0
Cellular_organisms - ps1
Archaea1 - ps2
Archaea2 - ps3
Archaea3 - ps4
Asgard/Eukaryota - ps5
Eukaryota - ps6
Amorpheae - ps7
Obazoa - ps8
Opisthokonta - ps9
Holomycota - ps10
Fungi - ps11
Fungi_1 - ps12
Fungi_2 - ps13
Fungi_3 - ps14
Dikarya - ps15
Ascomycota - ps16
Saccharo/Pezizomycotina - ps17
Saccharomycotina - ps18
Saccharomycotina_1 - ps19
Saccharomycotina_2 - ps20
Saccharomycotina_3 - ps21
Phaff./Saccharomycetaceae - ps22
Saccharomyco./mycetaceae - ps23
Saccharomycetaceae - ps24
Saccharomycetaceae_2 - ps25
Saccharomycetaceae_3 - ps26
Kaz./Nau./Saccharomyces - ps27
Saccharomyces - ps28
Arboricola_clade - ps29
Cerevisiae_clade - ps30
Saccharomyces_cerevisiae - ps31
Phylostratum

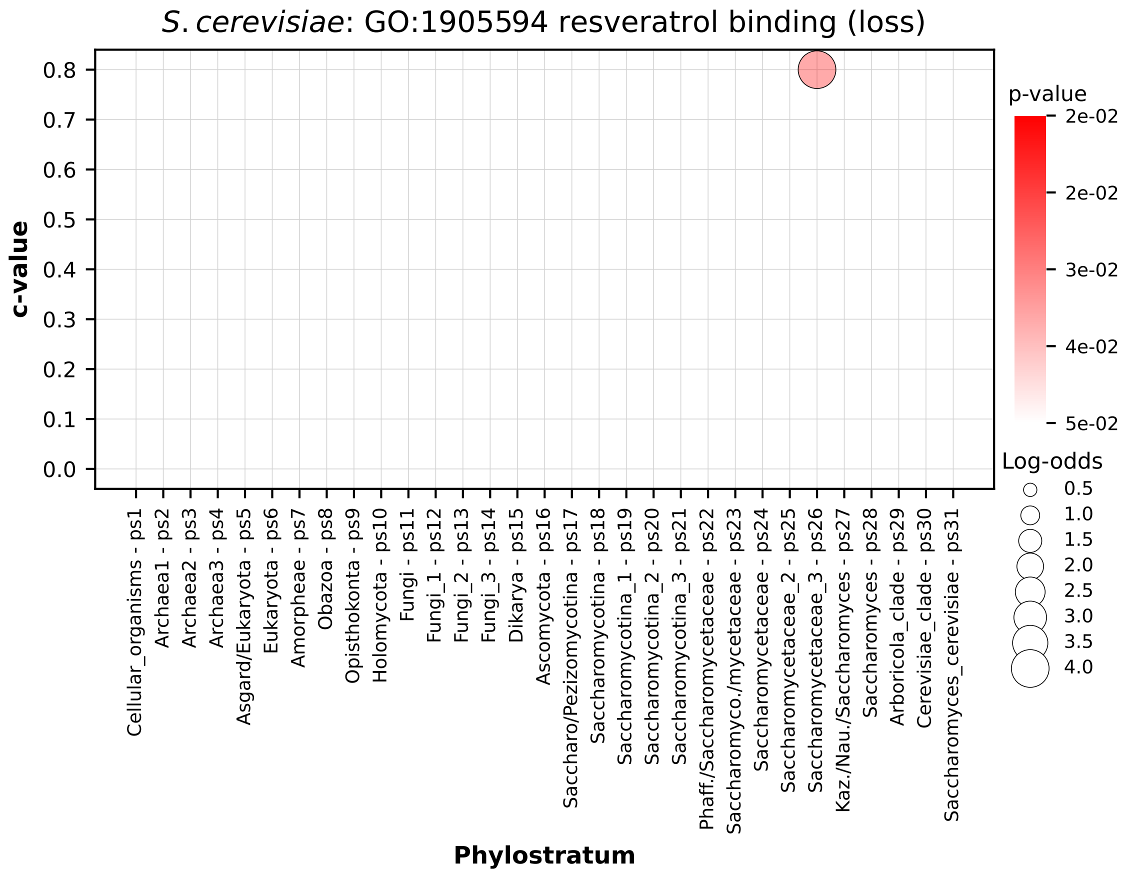

S. cerevisiae: GO:1905594 resveratrol binding (loss)
c-value
0.0
0.1
0.2
0.3
0.4
0.5
0.6
0.7
0.8
p-value
2e-02
2e-02
3e-02
4e-02
5e-02
Log-odds
0.5
1.0
1.5
2.0
2.5
3.0
3.5
4.0
Cellular_organisms - ps1
Archaea1 - ps2
Archaea2 - ps3
Archaea3 - ps4
Asgard/Eukaryota - ps5
Eukaryota - ps6
Amorpheae - ps7
Obazoa - ps8
Opisthokonta - ps9
Holomycota - ps10
Fungi - ps11
Fungi_1 - ps12
Fungi_2 - ps13
Fungi_3 - ps14
Dikarya - ps15
Ascomycota - ps16
Saccharo/Pezizomycotina - ps17
Saccharomycotina - ps18
Saccharomycotina_1 - ps19
Saccharomycotina_2 - ps20
Saccharomycotina_3 - ps21
Phaff./Saccharomycetaceae - ps22
Saccharomyco./mycetaceae - ps23
Saccharomycetaceae - ps24
Saccharomycetaceae_2 - ps25
Saccharomycetaceae_3 - ps26
Kaz./Nau./Saccharomyces - ps27
Saccharomyces - ps28
Arboricola_clade - ps29
Cerevisiae_clade - ps30
Saccharomyces_cerevisiae - ps31
Phylostratum

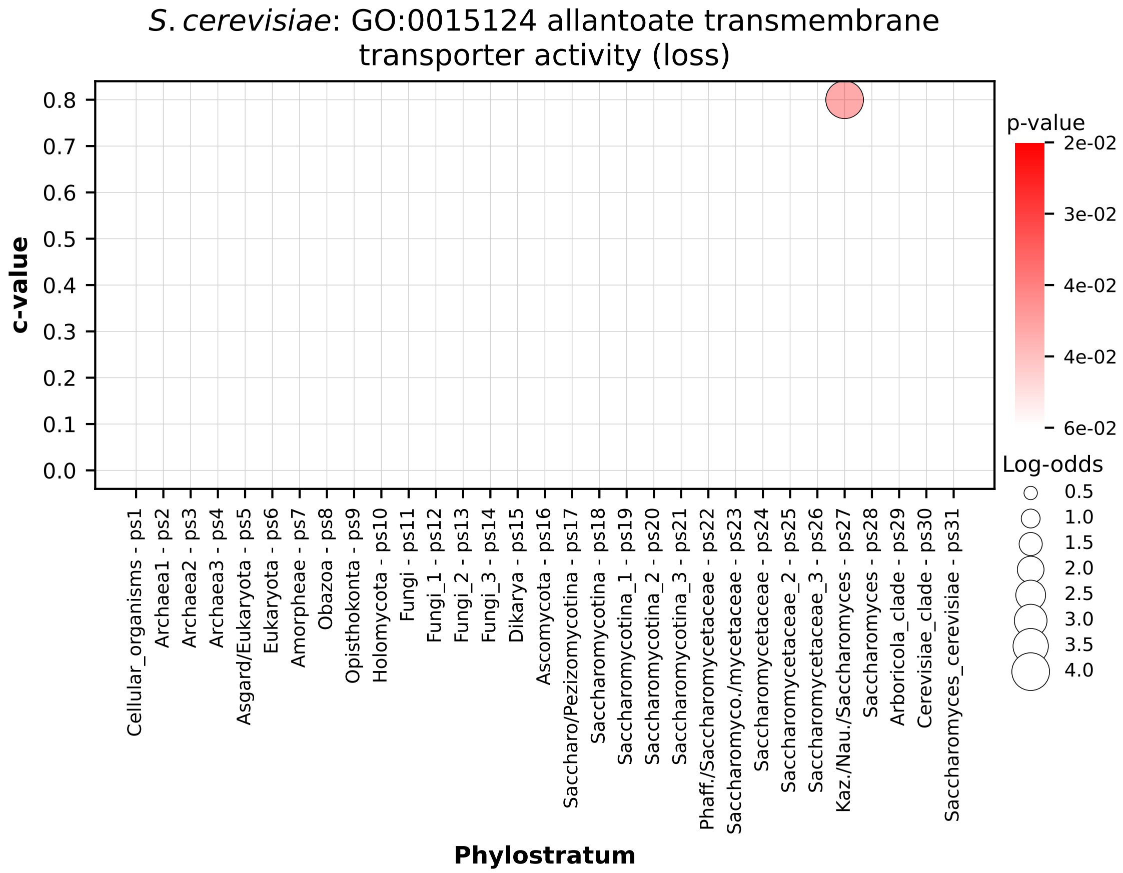

S. cerevisiae: GO:0015124 allantoate transmembrane transporter activity (loss)
c-value
0.0
0.1
0.2
0.3
0.4
0.5
0.6
0.7
0.8
Phylostratum
Cellular_organisms - ps1
Archaea1 - ps2
Archaea2 - ps3
Archaea3 - ps4
Asgard/Eukaryota - ps5
Eukaryota - ps6
Amorpheae - ps7
Obazoa - ps8
Opisthokonta - ps9
Holomycota - ps10
Fungi - ps11
Fungi_1 - ps12
Fungi_2 - ps13
Fungi_3 - ps14
Dikarya - ps15
Ascomycota - ps16
Saccharo/Pezizomycotina - ps17
Saccharomycotina - ps18
Saccharomycotina_1 - ps19
Saccharomycotina_2 - ps20
Saccharomycotina_3 - ps21
Phaff./Saccharomycetaceae - ps22
Saccharomyco./mycetaceae - ps23
Saccharomycetaceae - ps24
Saccharomycetaceae_2 - ps25
Saccharomycetaceae_3 - ps26
Kaz./Nau./Saccharomyces - ps27
Saccharomyces - ps28
Arboricola_clade - ps29
Cerevisiae_clade - ps30
Saccharomyces_cerevisiae - ps31
p-value
2e-02
3e-02
4e-02
4e-02
6e-02
Log-odds
0.5
1.0
1.5
2.0
2.5
3.0
3.5
4.0

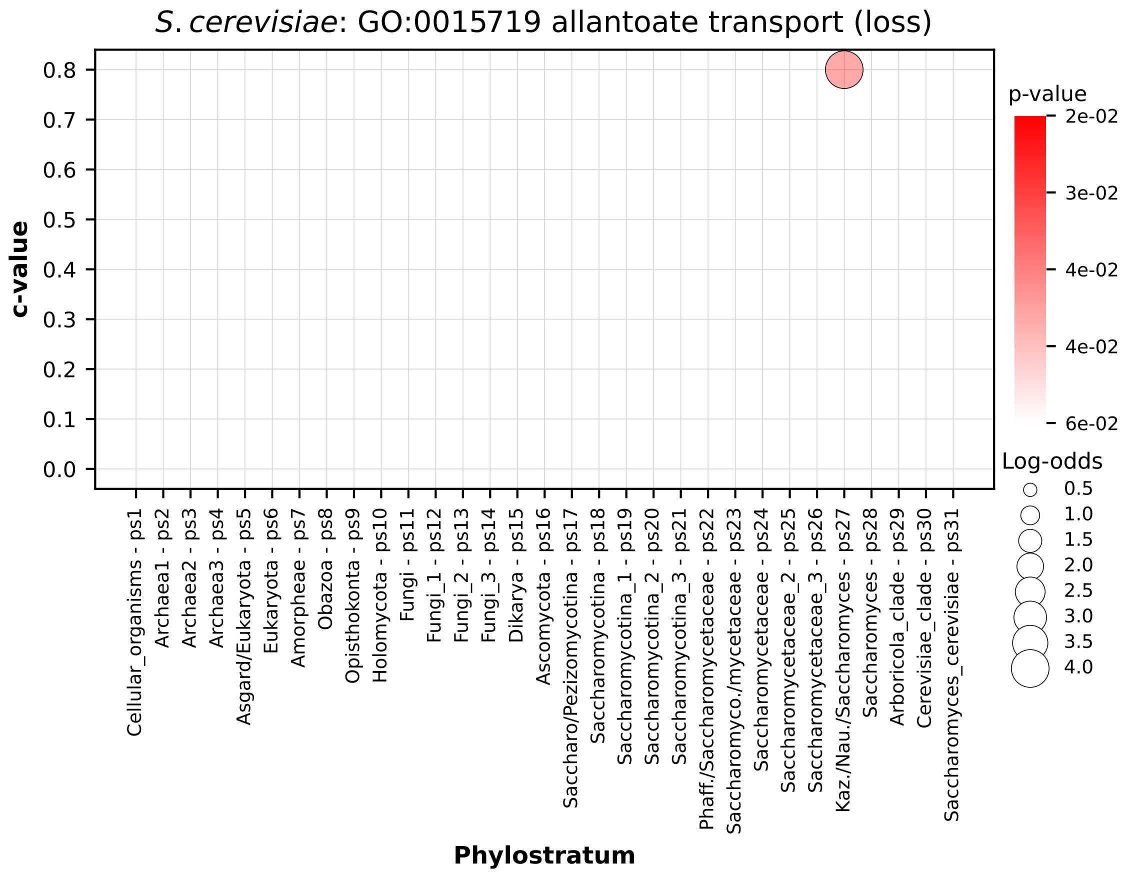
S. cerevisiae: GO:0015719 allantoate transport (loss)
c-value
0.0
0.1
0.2
0.3
0.4
0.5
0.6
0.7
0.8
p-value
2e-02
3e-02
4e-02
4e-02
6e-02
Log-odds
0.5
1.0
1.5
2.0
2.5
3.0
3.5
4.0
Cellular_organisms - ps1
Archaea1 - ps2
Archaea2 - ps3
Archaea3 - ps4
Asgard/Eukaryota - ps5
Eukaryota - ps6
Amorpheae - ps7
Obazoa - ps8
Opisthokonta - ps9
Holomycota - ps10
Fungi - ps11
Fungi_1 - ps12
Fungi_2 - ps13
Fungi_3 - ps14
Dikarya - ps15
Ascomycota - ps16
Saccharo/Pezizomycotina - ps17
Saccharomycotina - ps18
Saccharomycotina_1 - ps19
Saccharomycotina_2 - ps20
Saccharomycotina_3 - ps21
Phaff./Saccharomycetaceae - ps22
Saccharomyco./mycetaceae - ps23
Saccharomycetaceae - ps24
Saccharomycetaceae_2 - ps25
Saccharomycetaceae_3 - ps26
Kaz./Nau./Saccharomyces - ps27
Saccharomyces - ps28
Arboricola_clade - ps29
Cerevisiae_clade - ps30
Saccharomyces_cerevisiae - ps31
Phylostratum

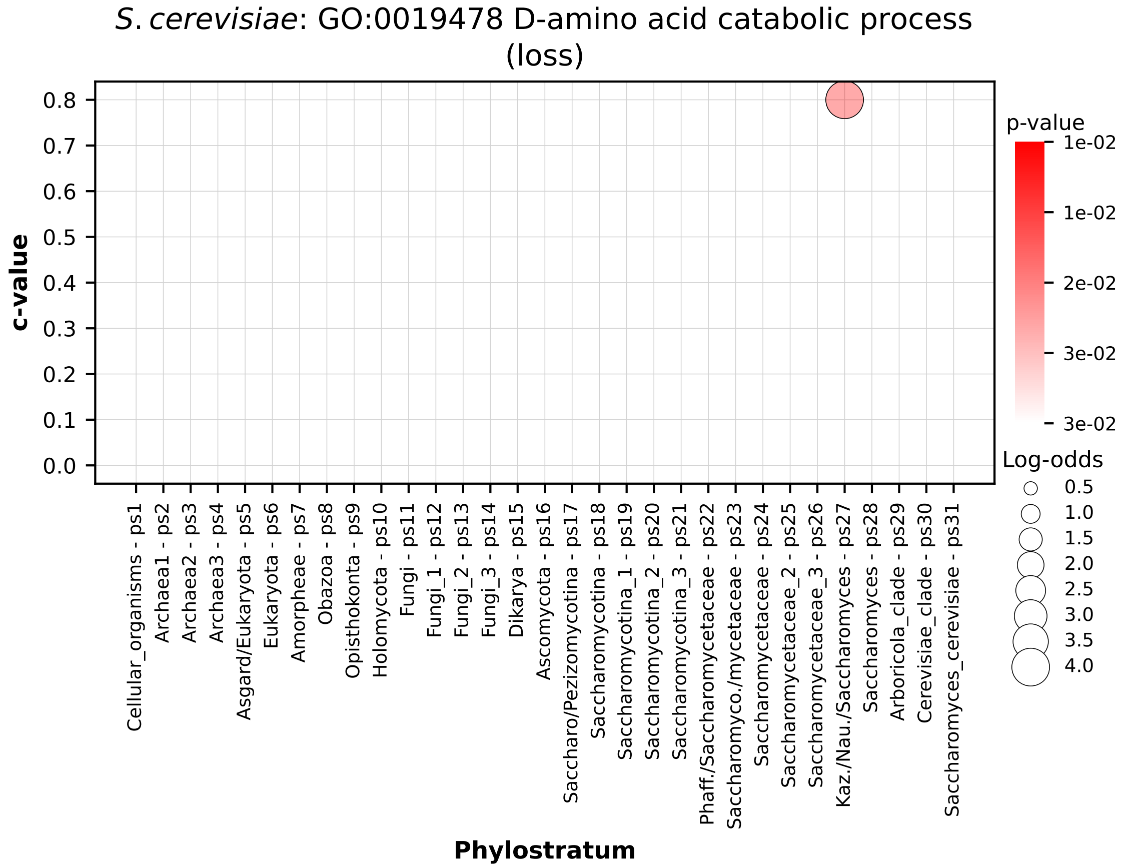

S. cerevisiae: GO:0019478 D-amino acid catabolic process (loss)
c-value
0.8
0.7
0.6
0.5
0.4
0.3
0.2
0.1
0.0
p-value
1e-02
1e-02
2e-02
3e-02
3e-02
Log-odds
0.5
1.0
1.5
2.0
2.5
3.0
3.5
4.0
Cellular_organisms - ps1
Archaea1 - ps2
Archaea2 - ps3
Archaea3 - ps4
Asgard/Eukaryota - ps5
Eukaryota - ps6
Amorpheae - ps7
Obazoa - ps8
Opisthokonta - ps9
Holomycota - ps10
Fungi - ps11
Fungi_1 - ps12
Fungi_2 - ps13
Fungi_3 - ps14
Dikarya - ps15
Ascomycota - ps16
Saccharo/Pezizomycotina - ps17
Saccharomycotina - ps18
Saccharomycotina_1 - ps19
Saccharomycotina_2 - ps20
Saccharomycotina_3 - ps21
Phaff./Saccharomycetaceae - ps22
Saccharomyco./mycetaceae - ps23
Saccharomycetaceae - ps24
Saccharomycetaceae_2 - ps25
Saccharomycetaceae_3 - ps26
Kaz./Nau./Saccharomyces - ps27
Saccharomyces - ps28
Arboricola_clade - ps29
Cerevisiae_clade - ps30
Saccharomyces_cerevisiae - ps31
Phylostratum

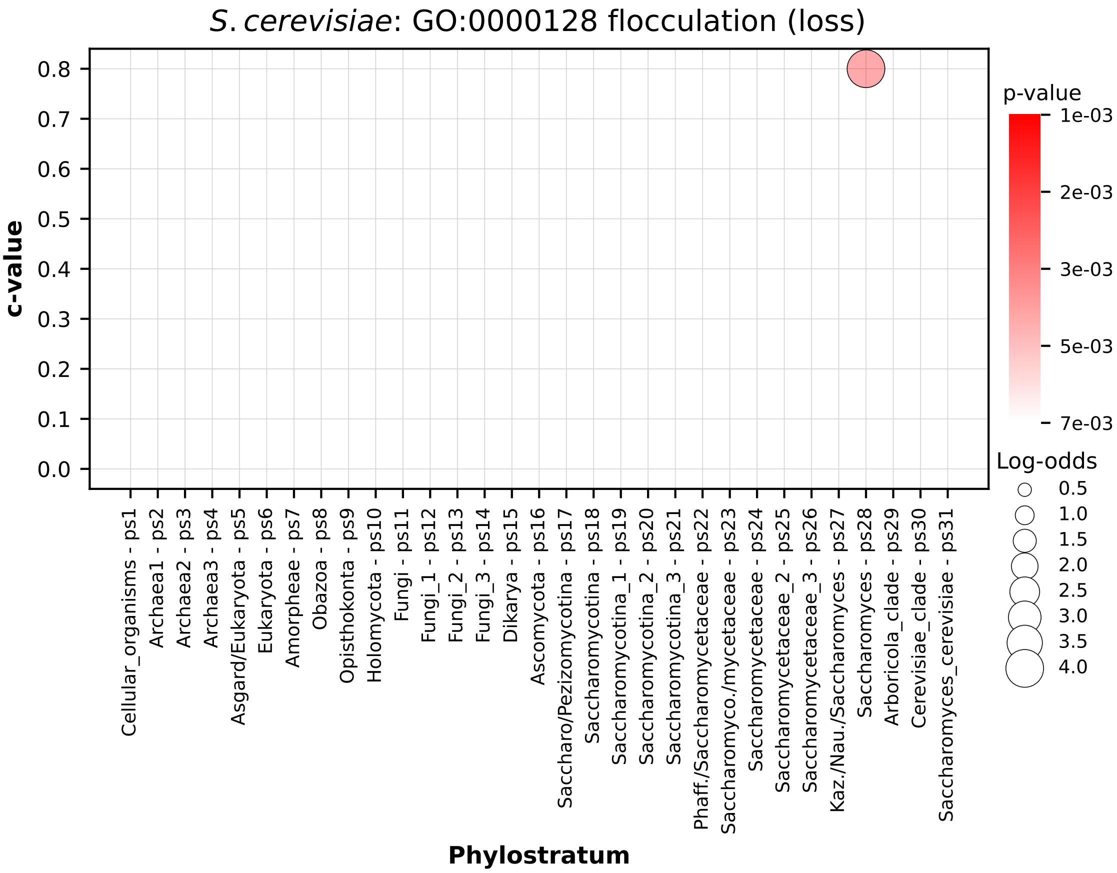

S. cerevisiae: GO:0000128 flocculation (loss)
c-value
0.8
0.7
0.6
0.5
0.4
0.3
0.2
0.1
0.0
p-value
1e-03
2e-03
3e-03
5e-03
7e-03
Log-odds
0.5
1.0
1.5
2.0
2.5
3.0
3.5
4.0
Cellular_organisms - ps1
Archaea1 - ps2
Archaea2 - ps3
Archaea3 - ps4
Asgard/Eukaryota - ps5
Eukaryota - ps6
Amorpheae - ps7
Obazoa - ps8
Opisthokonta - ps9
Holomycota - ps10
Fungi - ps11
Fungi_1 - ps12
Fungi_2 - ps13
Fungi_3 - ps14
Dikarya - ps15
Ascomycota - ps16
Saccharo/Pezizomycotina - ps17
Saccharomycotina - ps18
Saccharomycotina_1 - ps19
Saccharomycotina_2 - ps20
Saccharomycotina_3 - ps21
Phaff./Saccharomycetaceae - ps22
Saccharomyco./mycetaceae - ps23
Saccharomycetaceae - ps24
Saccharomycetaceae_2 - ps25
Saccharomycetaceae_3 - ps26
Kaz./Nau./Saccharomyces - ps27
Saccharomyces - ps28
Arboricola_clade - ps29
Cerevisiae_clade - ps30
Saccharomyces_cerevisiae - ps31
Phylostratum

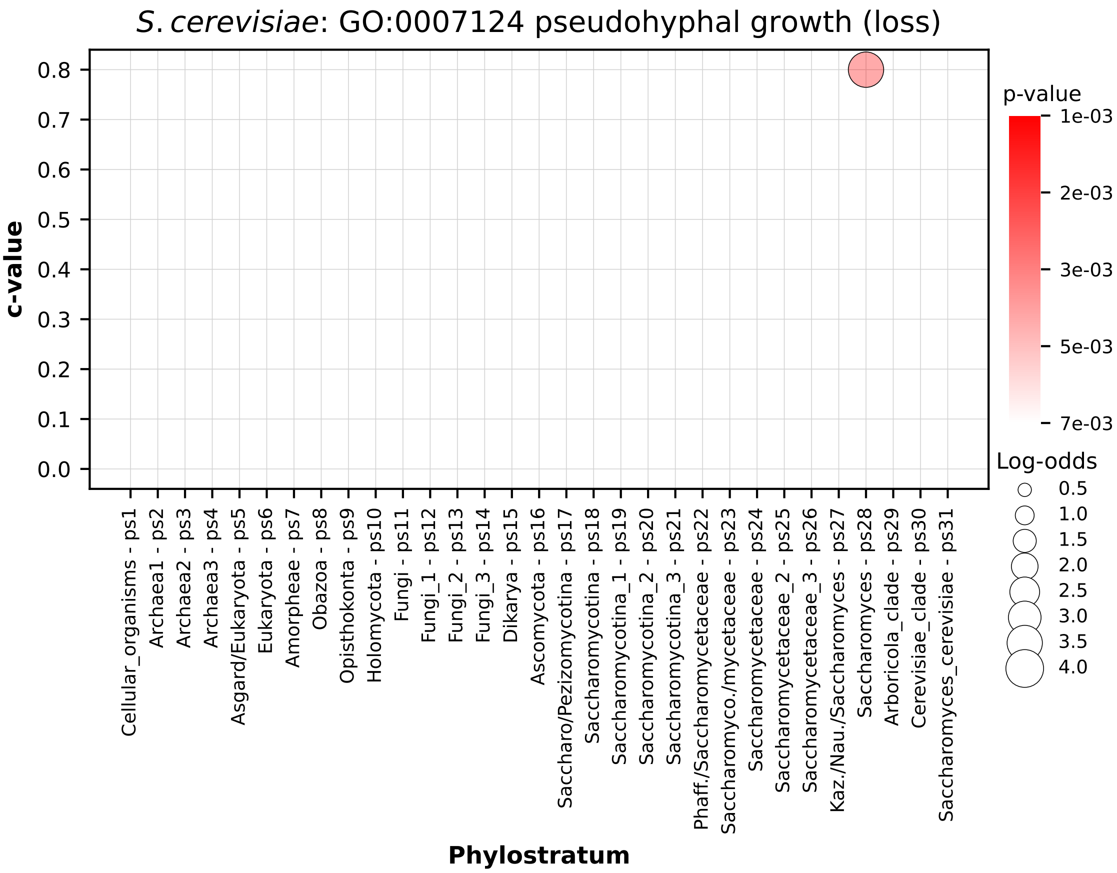

S. cerevisiae: GO:0007124 pseudohyphal growth (loss)
c-value
0.0
0.1
0.2
0.3
0.4
0.5
0.6
0.7
0.8
p-value
1e-03
2e-03
3e-03
5e-03
7e-03
Log-odds
0.5
1.0
1.5
2.0
2.5
3.0
3.5
4.0
Cellular_organisms - ps1
Archaea1 - ps2
Archaea2 - ps3
Archaea3 - ps4
Asgard/Eukaryota - ps5
Eukaryota - ps6
Amorpheae - ps7
Obazoa - ps8
Opisthokonta - ps9
Holomycota - ps10
Fungi - ps11
Fungi_1 - ps12
Fungi_2 - ps13
Fungi_3 - ps14
Dikarya - ps15
Ascomycota - ps16
Saccharo/Pezizomycotina - ps17
Saccharomycotina - ps18
Saccharomycotina_1 - ps19
Saccharomycotina_2 - ps20
Saccharomycotina_3 - ps21
Phaff./Saccharomycetaceae - ps22
Saccharomyco./mycetaceae - ps23
Saccharomycetaceae - ps24
Saccharomycetaceae_2 - ps25
Saccharomycetaceae_3 - ps26
Kaz./Nau./Saccharomyces - ps27
Saccharomyces - ps28
Arboricola_clade - ps29
Cerevisiae_clade - ps30
Saccharomyces_cerevisiae - ps31
Phylostratum

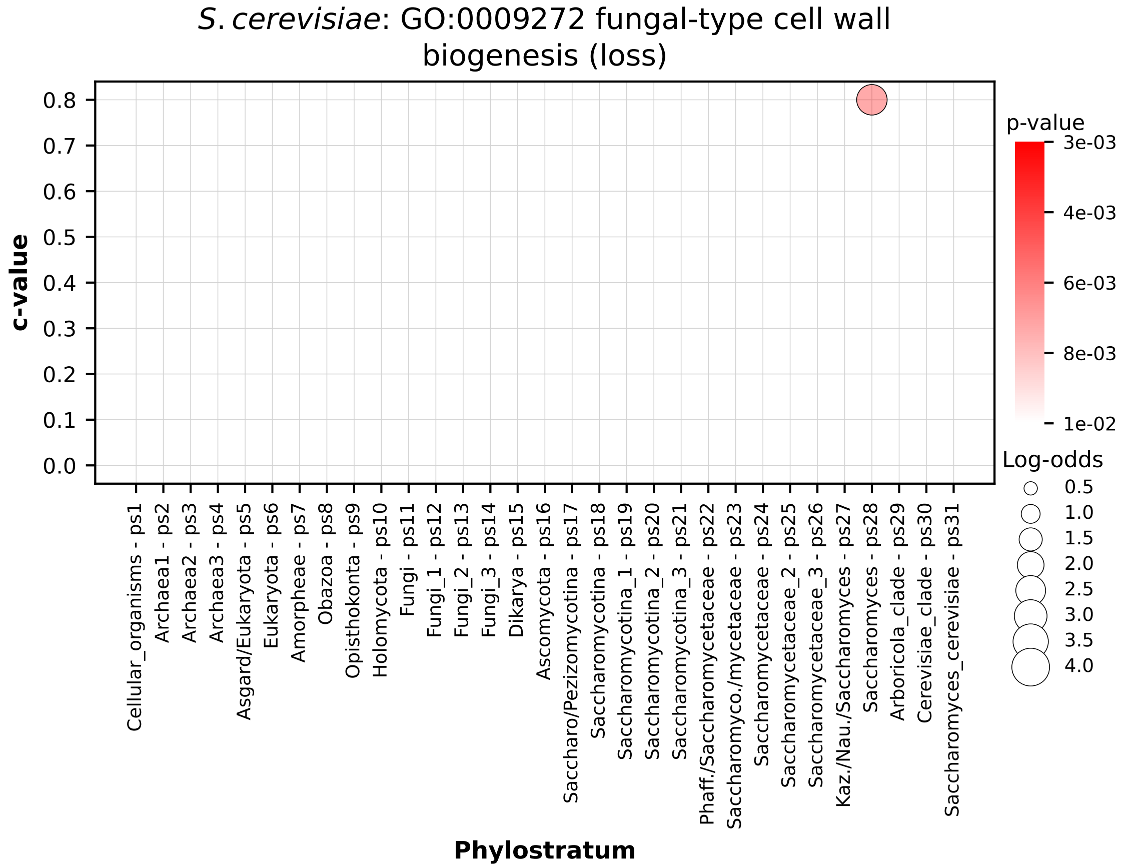

S. cerevisiae: GO:0009272 fungal-type cell wall biogenesis (loss)
c-value
0.0
0.1
0.2
0.3
0.4
0.5
0.6
0.7
0.8
p-value
3e-03
4e-03
6e-03
8e-03
1e-02
Log-odds
0.5
1.0
1.5
2.0
2.5
3.0
3.5
4.0
Cellular_organisms - ps1
Archaea1 - ps2
Archaea2 - ps3
Archaea3 - ps4
Asgard/Eukaryota - ps5
Eukaryota - ps6
Amorpheae - ps7
Obazoa - ps8
Opisthokonta - ps9
Holomycota - ps10
Fungi - ps11
Fungi_1 - ps12
Fungi_2 - ps13
Fungi_3 - ps14
Dikarya - ps15
Ascomycota - ps16
Saccharo/Pezizomycotina - ps17
Saccharomycotina - ps18
Saccharomycotina_1 - ps19
Saccharomycotina_2 - ps20
Saccharomycotina_3 - ps21
Phaff./Saccharomycetaceae - ps22
Saccharomyco./mycetaceae - ps23
Saccharomycetaceae - ps24
Saccharomycetaceae_2 - ps25
Saccharomycetaceae_3 - ps26
Kaz./Nau./Saccharomyces - ps27
Saccharomyces - ps28
Arboricola_clade - ps29
Cerevisiae_clade - ps30
Saccharomyces_cerevisiae - ps31
Phylostratum

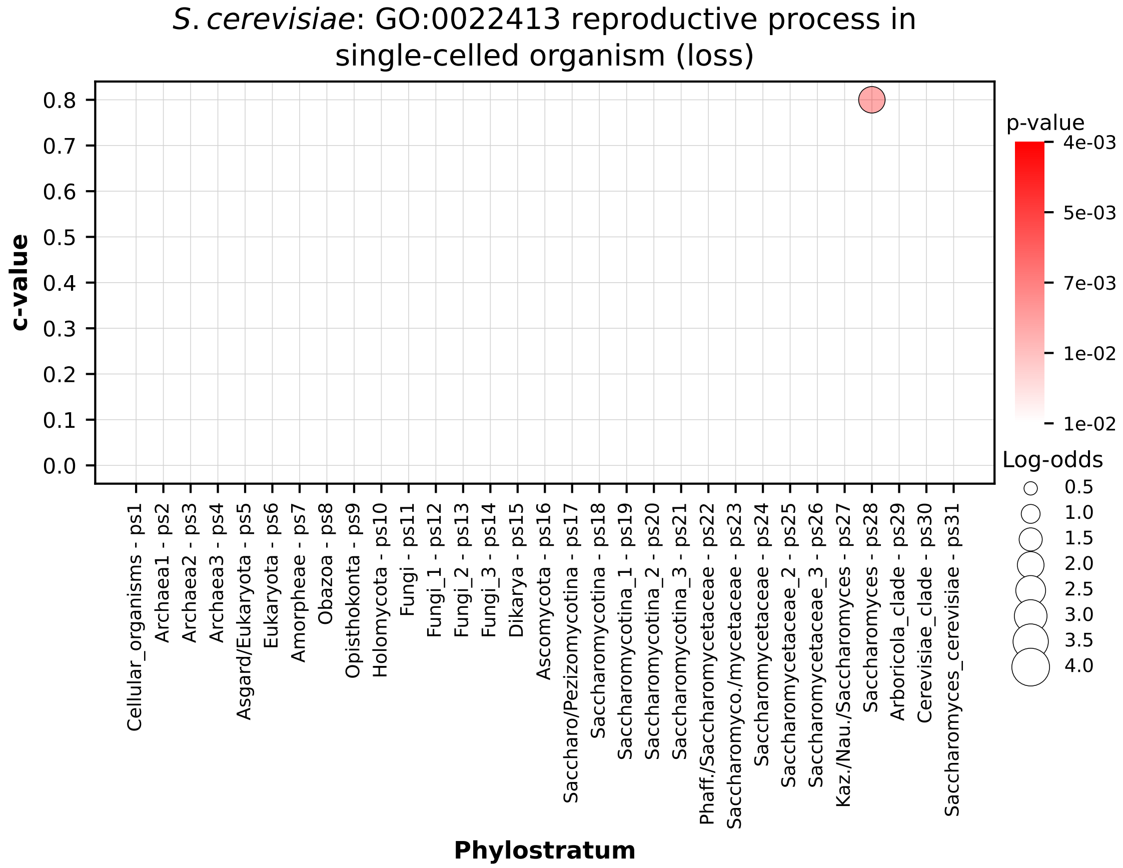

S. cerevisiae: GO:0022413 reproductive process in single-celled organism (loss)
c-value
0.0
0.1
0.2
0.3
0.4
0.5
0.6
0.7
0.8
p-value
4e-03
5e-03
7e-03
1e-02
1e-02
Log-odds
0.5
1.0
1.5
2.0
2.5
3.0
3.5
4.0
Cellular_organisms - ps1
Archaea1 - ps2
Archaea2 - ps3
Archaea3 - ps4
Asgard/Eukaryota - ps5
Eukaryota - ps6
Amorpheae - ps7
Obazoa - ps8
Opisthokonta - ps9
Holomycota - ps10
Fungi - ps11
Fungi_1 - ps12
Fungi_2 - ps13
Fungi_3 - ps14
Dikarya - ps15
Ascomycota - ps16
Saccharo/Pezizomycotina - ps17
Saccharomycotina - ps18
Saccharomycotina_1 - ps19
Saccharomycotina_2 - ps20
Saccharomycotina_3 - ps21
Phaff./Saccharomycetaceae - ps22
Saccharomyco./mycetaceae - ps23
Saccharomycetaceae - ps24
Saccharomycetaceae_2 - ps25
Saccharomycetaceae_3 - ps26
Kaz./Nau./Saccharomyces - ps27
Saccharomyces - ps28
Arboricola_clade - ps29
Cerevisiae_clade - ps30
Saccharomyces_cerevisiae - ps31
Phylostratum

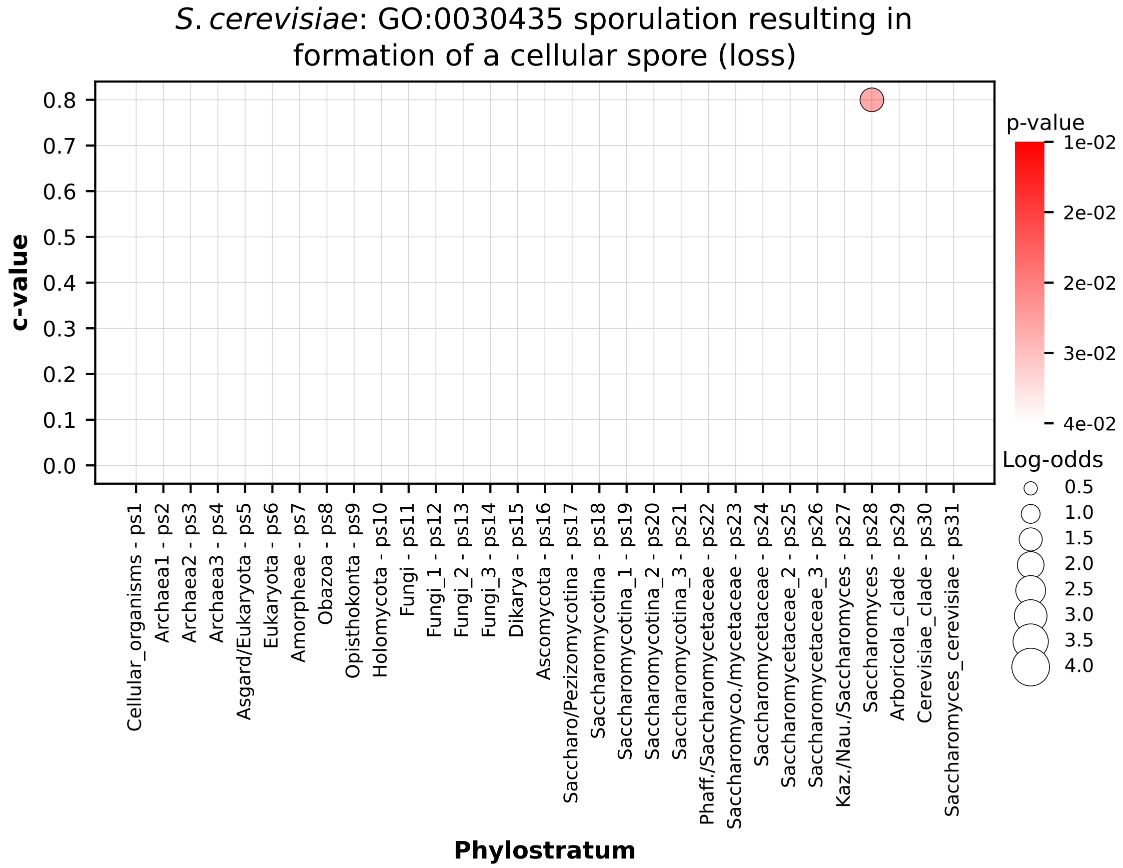

S. cerevisiae: GO:0030435 sporulation resulting in formation of a cellular spore (loss)
c-value
0.0
0.1
0.2
0.3
0.4
0.5
0.6
0.7
0.8
p-value
1e-02
2e-02
2e-02
3e-02
4e-02
Log-odds
0.5
1.0
1.5
2.0
2.5
3.0
3.5
4.0
Cellular_organisms - ps1
Archaea1 - ps2
Archaea2 - ps3
Archaea3 - ps4
Asgard/Eukaryota - ps5
Eukaryota - ps6
Amorpheae - ps7
Obazoa - ps8
Opisthokonta - ps9
Holomycota - ps10
Fungi - ps11
Fungi_1 - ps12
Fungi_2 - ps13
Fungi_3 - ps14
Dikarya - ps15
Ascomycota - ps16
Saccharo/Pezizomycotina - ps17
Saccharomycotina - ps18
Saccharomycotina_1 - ps19
Saccharomycotina_2 - ps20
Saccharomycotina_3 - ps21
Phaff./Saccharomycetaceae - ps22
Saccharomyco./mycetaceae - ps23
Saccharomycetaceae - ps24
Saccharomycetaceae_2 - ps25
Saccharomycetaceae_3 - ps26
Kaz./Nau./Saccharomyces - ps27
Saccharomyces - ps28
Arboricola_clade - ps29
Cerevisiae_clade - ps30
Saccharomyces_cerevisiae - ps31
Phylostratum

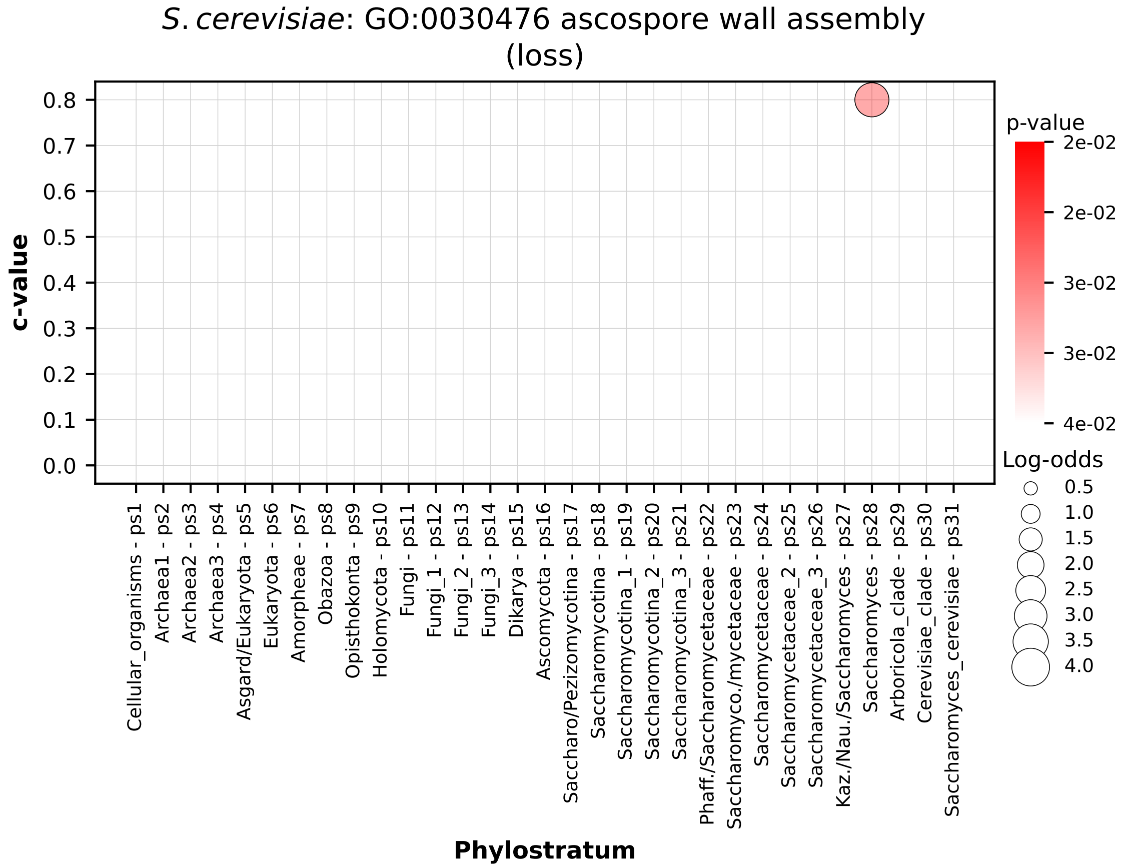

S. cerevisiae: GO:0030476 ascospore wall assembly
(loss)
c-value
0.8
0.7
0.6
0.5
0.4
0.3
0.2
0.1
0.0
p-value
2e-02
2e-02
3e-02
3e-02
4e-02
Log-odds
0.5
1.0
1.5
2.0
2.5
3.0
3.5
4.0
Cellular_organisms - ps1
Archaea1 - ps2
Archaea2 - ps3
Archaea3 - ps4
Asgard/Eukaryota - ps5
Eukaryota - ps6
Amorpheae - ps7
Obazoa - ps8
Opisthokonta - ps9
Holomycota - ps10
Fungi - ps11
Fungi_1 - ps12
Fungi_2 - ps13
Fungi_3 - ps14
Dikarya - ps15
Ascomycota - ps16
Saccharo/Pezizomycotina - ps17
Saccharomycotina - ps18
Saccharomycotina_1 - ps19
Saccharomycotina_2 - ps20
Saccharomycotina_3 - ps21
Phaff./Saccharomycetaceae - ps22
Saccharomyco./mycetaceae - ps23
Saccharomycetaceae - ps24
Saccharomycetaceae_2 - ps25
Saccharomycetaceae_3 - ps26
Kaz./Nau./Saccharomyces - ps27
Saccharomyces - ps28
Arboricola_clade - ps29
Cerevisiae_clade - ps30
Saccharomyces_cerevisiae - ps31
Phylostratum

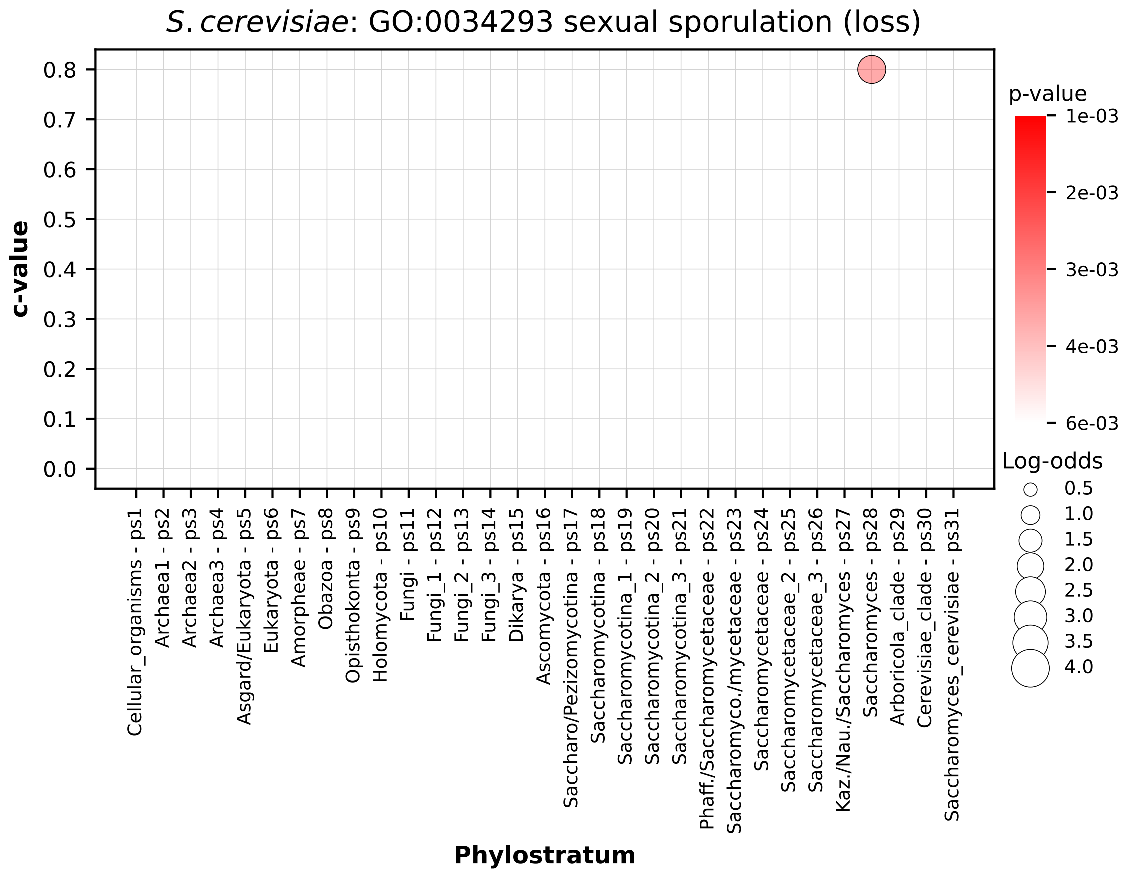
S. cerevisiae: GO:0034293 sexual sporulation (loss)
c-value
0.0
0.1
0.2
0.3
0.4
0.5
0.6
0.7
0.8
p-value
1e-03
2e-03
3e-03
4e-03
6e-03
Log-odds
0.5
1.0
1.5
2.0
2.5
3.0
3.5
4.0
Cellular_organisms - ps1
Archaea1 - ps2
Archaea2 - ps3
Archaea3 - ps4
Asgard/Eukaryota - ps5
Eukaryota - ps6
Amorpheae - ps7
Obazoa - ps8
Opisthokonta - ps9
Holomycota - ps10
Fungi - ps11
Fungi_1 - ps12
Fungi_2 - ps13
Fungi_3 - ps14
Dikarya - ps15
Ascomycota - ps16
Saccharo/Pezizomycotina - ps17
Saccharomycotina - ps18
Saccharomycotina_1 - ps19
Saccharomycotina_2 - ps20
Saccharomycotina_3 - ps21
Phaff./Saccharomycetaceae - ps22
Saccharomyco./mycetaceae - ps23
Saccharomycetaceae - ps24
Saccharomycetaceae_2 - ps25
Saccharomycetaceae_3 - ps26
Kaz./Nau./Saccharomyces - ps27
Saccharomyces - ps28
Arboricola_clade - ps29
Cerevisiae_clade - ps30
Saccharomyces_cerevisiae - ps31
Phylostratum

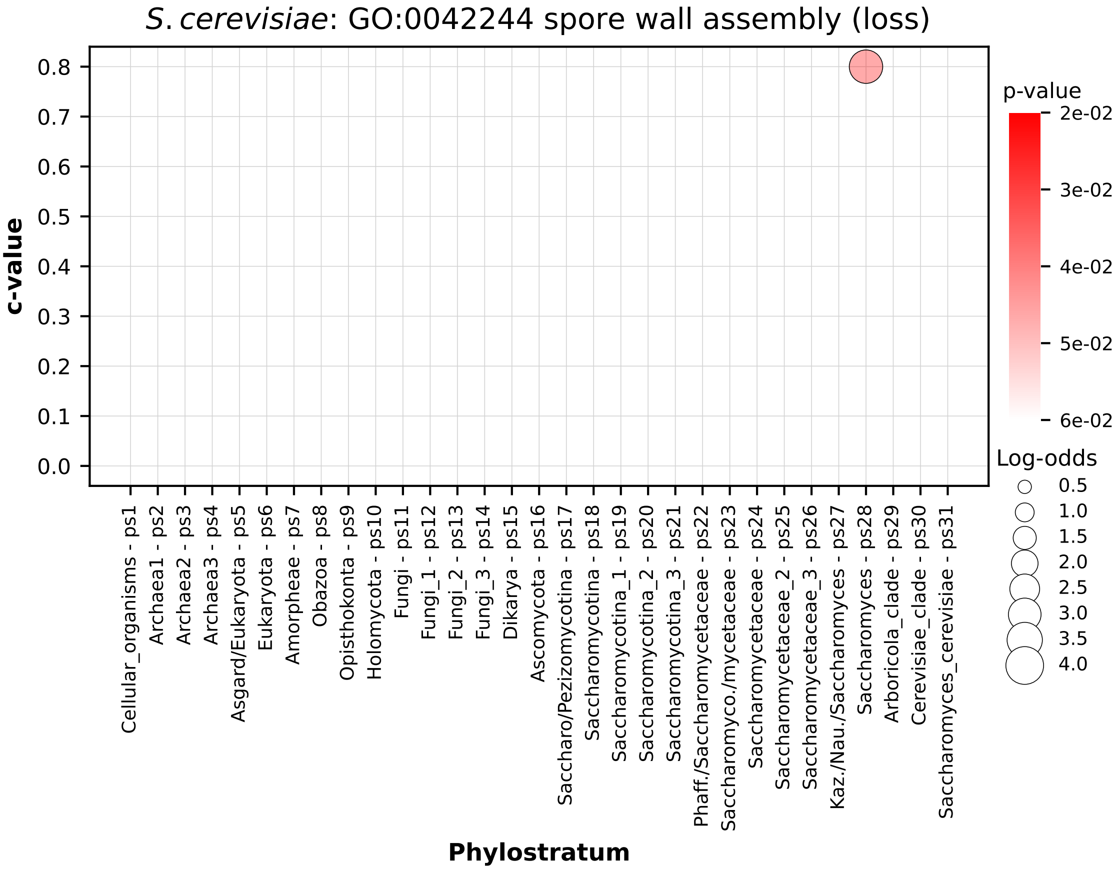
S. cerevisiae: GO:0042244 spore wall assembly (loss)
c-value
0.8
0.7
0.6
0.5
0.4
0.3
0.2
0.1
0.0
p-value
2e-02
3e-02
4e-02
5e-02
6e-02
Log-odds
0.5
1.0
1.5
2.0
2.5
3.0
3.5
4.0
Cellular_organisms - ps1
Archaea1 - ps2
Archaea2 - ps3
Archaea3 - ps4
Asgard/Eukaryota - ps5
Eukaryota - ps6
Amorpheae - ps7
Obazoa - ps8
Opisthokonta - ps9
Holomycota - ps10
Fungi - ps11
Fungi_1 - ps12
Fungi_2 - ps13
Fungi_3 - ps14
Dikarya - ps15
Ascomycota - ps16
Saccharo/Pezizomycotina - ps17
Saccharomycotina - ps18
Saccharomycotina_1 - ps19
Saccharomycotina_2 - ps20
Saccharomycotina_3 - ps21
Phaff./Saccharomycetaceae - ps22
Saccharomyco./mycetaceae - ps23
Saccharomycetaceae - ps24
Saccharomycetaceae_2 - ps25
Saccharomycetaceae_3 - ps26
Kaz./Nau./Saccharomyces - ps27
Saccharomyces - ps28
Arboricola_clade - ps29
Cerevisiae_clade - ps30
Saccharomyces_cerevisiae - ps31
Phylostratum

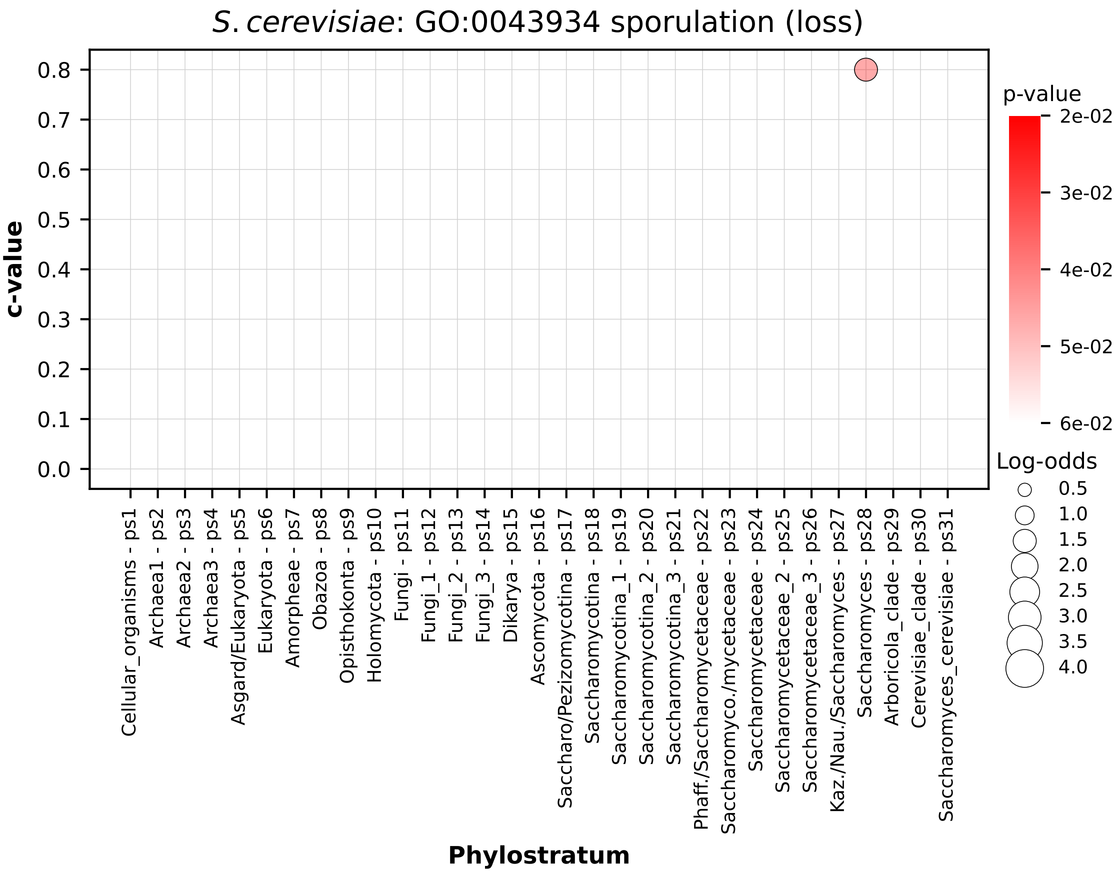

S. cerevisiae: GO:0043934 sporulation (loss)
c-value
0.0
0.1
0.2
0.3
0.4
0.5
0.6
0.7
0.8
Phylostratum
Cellular_organisms - ps1
Archaea1 - ps2
Archaea2 - ps3
Archaea3 - ps4
Asgard/Eukaryota - ps5
Eukaryota - ps6
Amorpheae - ps7
Obazoa - ps8
Opisthokonta - ps9
Holomycota - ps10
Fungi - ps11
Fungi_1 - ps12
Fungi_2 - ps13
Fungi_3 - ps14
Dikarya - ps15
Ascomycota - ps16
Saccharo/Pezizomycotina - ps17
Saccharomycotina - ps18
Saccharomycotina_1 - ps19
Saccharomycotina_2 - ps20
Saccharomycotina_3 - ps21
Phaff./Saccharomycetaceae - ps22
Saccharomyco./mycetaceae - ps23
Saccharomycetaceae - ps24
Saccharomycetaceae_2 - ps25
Saccharomycetaceae_3 - ps26
Kaz./Nau./Saccharomyces - ps27
Saccharomyces - ps28
Arboricola_clade - ps29
Cerevisiae_clade - ps30
Saccharomyces_cerevisiae - ps31
p-value
2e-02
3e-02
4e-02
5e-02
6e-02
Log-odds
0.5
1.0
1.5
2.0
2.5
3.0
3.5
4.0

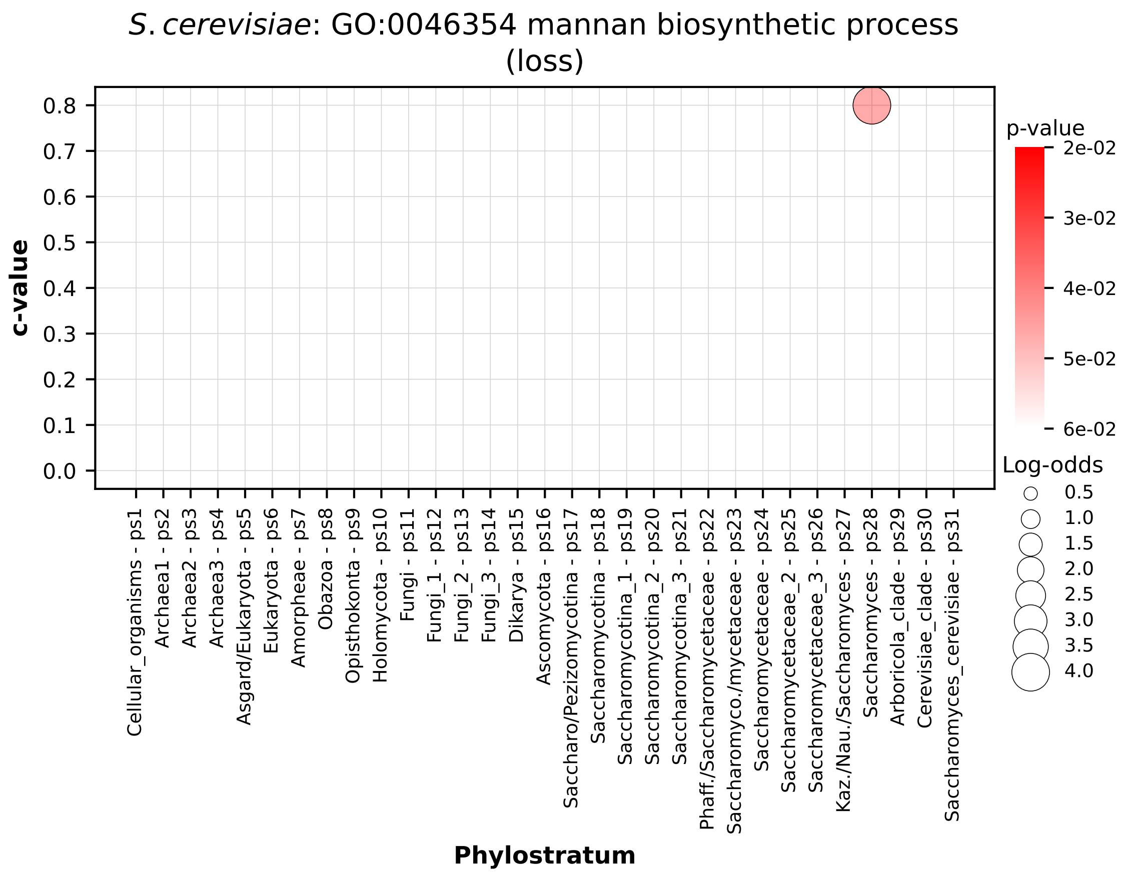

S. cerevisiae: GO:0046354 mannan biosynthetic process
(loss)
c-value
0.0
0.1
0.2
0.3
0.4
0.5
0.6
0.7
0.8
p-value
2e-02
3e-02
4e-02
5e-02
6e-02
Log-odds
0.5
1.0
1.5
2.0
2.5
3.0
3.5
4.0
Cellular_organisms - ps1
Archaea1 - ps2
Archaea2 - ps3
Archaea3 - ps4
Asgard/Eukaryota - ps5
Eukaryota - ps6
Amorpheae - ps7
Obazoa - ps8
Opisthokonta - ps9
Holomycota - ps10
Fungi - ps11
Fungi_1 - ps12
Fungi_2 - ps13
Fungi_3 - ps14
Dikarya - ps15
Ascomycota - ps16
Saccharo/Pezizomycotina - ps17
Saccharomycotina - ps18
Saccharomycotina_1 - ps19
Saccharomycotina_2 - ps20
Saccharomycotina_3 - ps21
Phaff./Saccharomycetaceae - ps22
Saccharomyco./mycetaceae - ps23
Saccharomycetaceae - ps24
Saccharomycetaceae_2 - ps25
Saccharomycetaceae_3 - ps26
Kaz./Nau./Saccharomyces - ps27
Saccharomyces - ps28
Arboricola_clade - ps29
Cerevisiae_clade - ps30
Saccharomyces_cerevisiae - ps31
Phylostratum

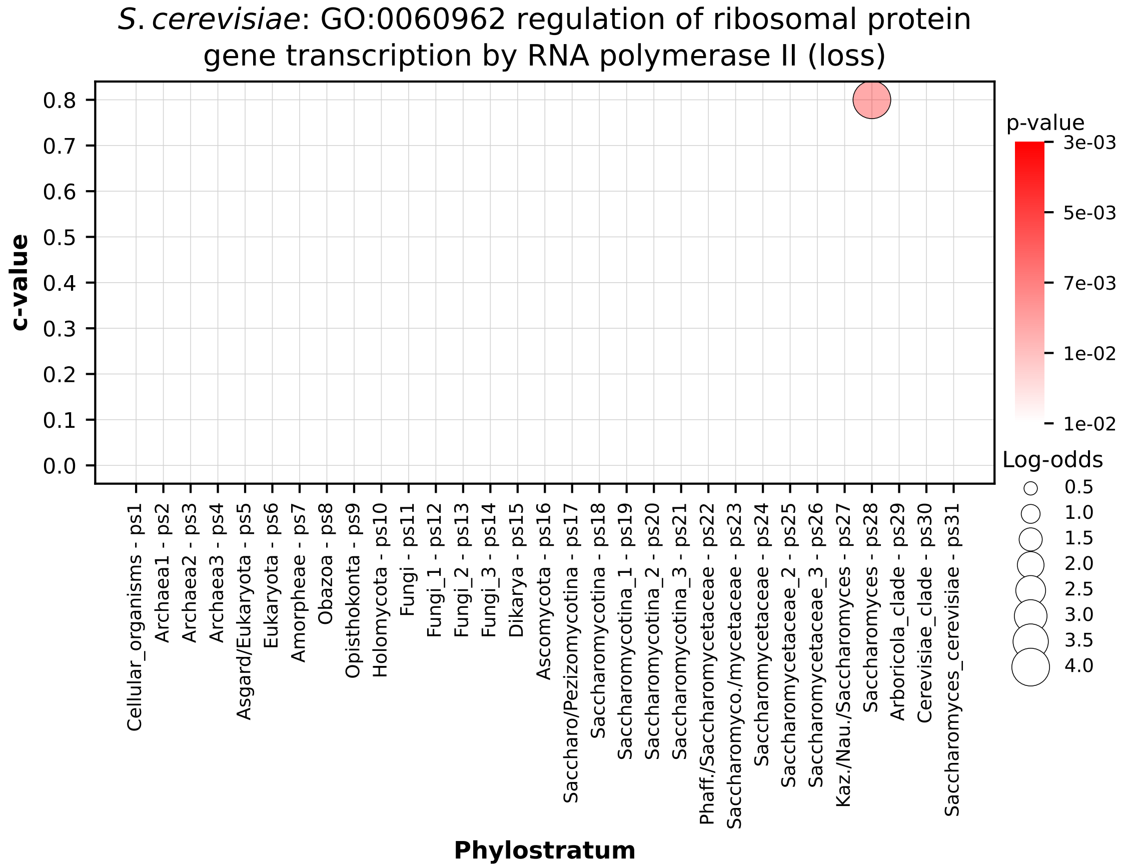
S. cerevisiae: GO:0060962 regulation of ribosomal protein gene transcription by RNA polymerase II (loss)
c-value
0.0
0.1
0.2
0.3
0.4
0.5
0.6
0.7
0.8
p-value
3e-03
5e-03
7e-03
1e-02
1e-02
Log-odds
0.5
1.0
1.5
2.0
2.5
3.0
3.5
4.0
Cellular_organisms - ps1
Archaea1 - ps2
Archaea2 - ps3
Archaea3 - ps4
Asgard/Eukaryota - ps5
Eukaryota - ps6
Amorpheae - ps7
Obazoa - ps8
Opisthokonta - ps9
Holomycota - ps10
Fungi - ps11
Fungi_1 - ps12
Fungi_2 - ps13
Fungi_3 - ps14
Dikarya - ps15
Ascomycota - ps16
Saccharo/Pezizomycotina - ps17
Saccharomycotina - ps18
Saccharomycotina_1 - ps19
Saccharomycotina_2 - ps20
Saccharomycotina_3 - ps21
Phaff./Saccharomycetaceae - ps22
Saccharomyco./mycetaceae - ps23
Saccharomycetaceae - ps24
Saccharomycetaceae_2 - ps25
Saccharomycetaceae_3 - ps26
Kaz./Nau./Saccharomyces - ps27
Saccharomyces - ps28
Arboricola_clade - ps29
Cerevisiae_clade - ps30
Saccharomyces_cerevisiae - ps31
Phylostratum

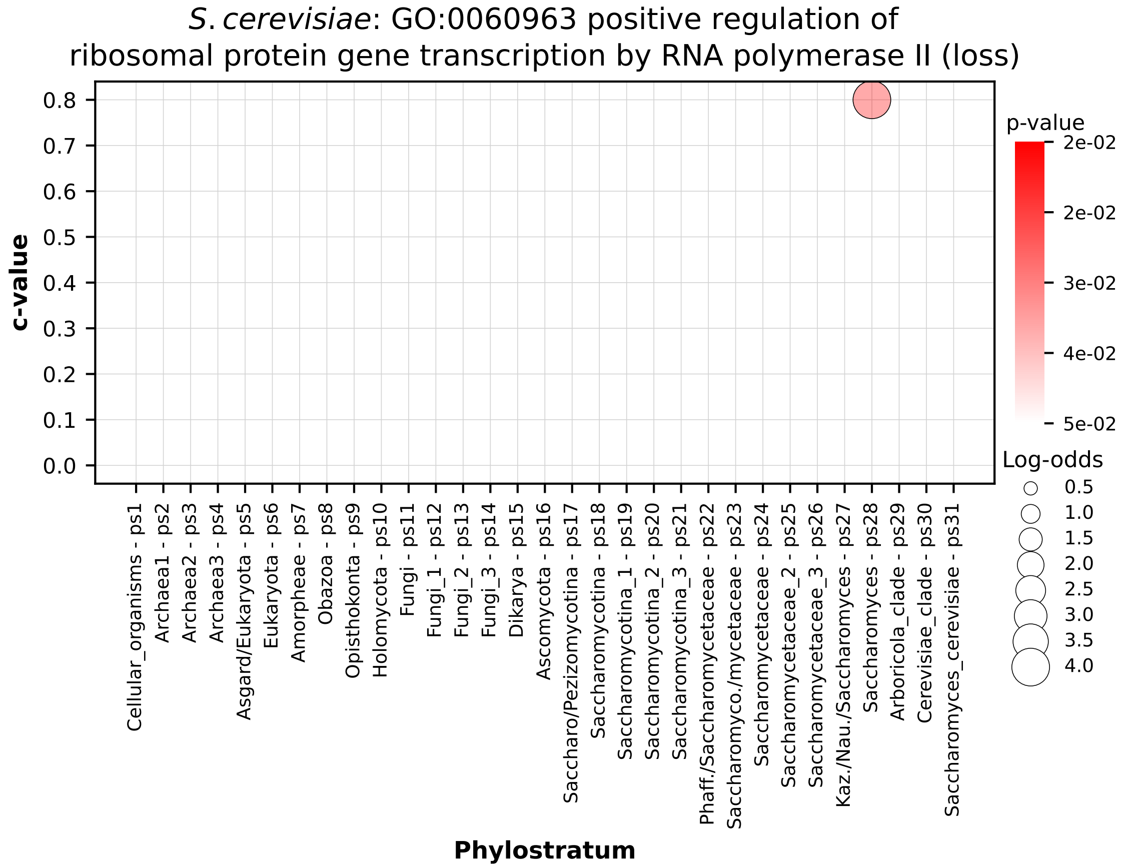

S. cerevisiae: GO:0060963 positive regulation of ribosomal protein gene transcription by RNA polymerase II (loss)
c-value
0.0
0.1
0.2
0.3
0.4
0.5
0.6
0.7
0.8
p-value
2e-02
2e-02
3e-02
4e-02
5e-02
Log-odds
0.5
1.0
1.5
2.0
2.5
3.0
3.5
4.0
Cellular_organisms - ps1
Archaea1 - ps2
Archaea2 - ps3
Archaea3 - ps4
Asgard/Eukaryota - ps5
Eukaryota - ps6
Amorpheae - ps7
Obazoa - ps8
Opisthokonta - ps9
Holomycota - ps10
Fungi - ps11
Fungi_1 - ps12
Fungi_2 - ps13
Fungi_3 - ps14
Dikarya - ps15
Ascomycota - ps16
Saccharo/Pezizomycotina - ps17
Saccharomycotina - ps18
Saccharomycotina_1 - ps19
Saccharomycotina_2 - ps20
Saccharomycotina_3 - ps21
Phaff./Saccharomycetaceae - ps22
Saccharomyco./mycetaceae - ps23
Saccharomycetaceae - ps24
Saccharomycetaceae_2 - ps25
Saccharomycetaceae_3 - ps26
Kaz./Nau./Saccharomyces - ps27
Saccharomyces - ps28
Arboricola_clade - ps29
Cerevisiae_clade - ps30
Saccharomyces_cerevisiae - ps31
Phylostratum

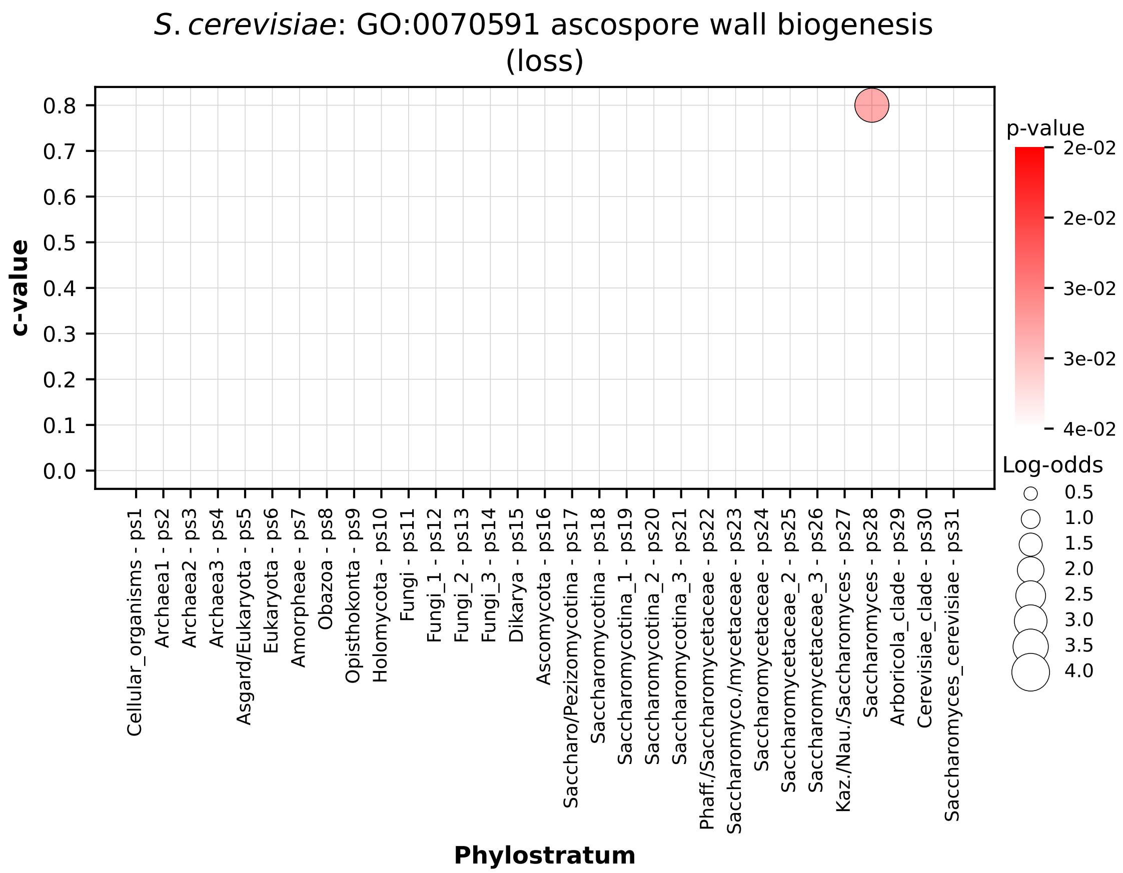

S. cerevisiae: GO:0070591 ascospore wall biogenesis
(loss)
c-value
Phylostratum
p-value
2e-02
2e-02
3e-02
3e-02
4e-02
Log-odds
0.5
1.0
1.5
2.0
2.5
3.0
3.5
4.0
Cellular_organisms - ps1
Archaea1 - ps2
Archaea2 - ps3
Archaea3 - ps4
Asgard/Eukaryota - ps5
Eukaryota - ps6
Amorpheae - ps7
Obazoa - ps8
Opisthokonta - ps9
Holomycota - ps10
Fungi - ps11
Fungi_1 - ps12
Fungi_2 - ps13
Fungi_3 - ps14
Dikarya - ps15
Ascomycota - ps16
Saccharo/Pezizomycotina - ps17
Saccharomycotina - ps18
Saccharomycotina_1 - ps19
Saccharomycotina_2 - ps20
Saccharomycotina_3 - ps21
Phaff./Saccharomycetaceae - ps22
Saccharomyco./mycetaceae - ps23
Saccharomycetaceae - ps24
Saccharomycetaceae_2 - ps25
Saccharomycetaceae_3 - ps26
Kaz./Nau./Saccharomyces - ps27
Saccharomyces - ps28
Arboricola_clade - ps29
Cerevisiae_clade - ps30
Saccharomyces_cerevisiae - ps31

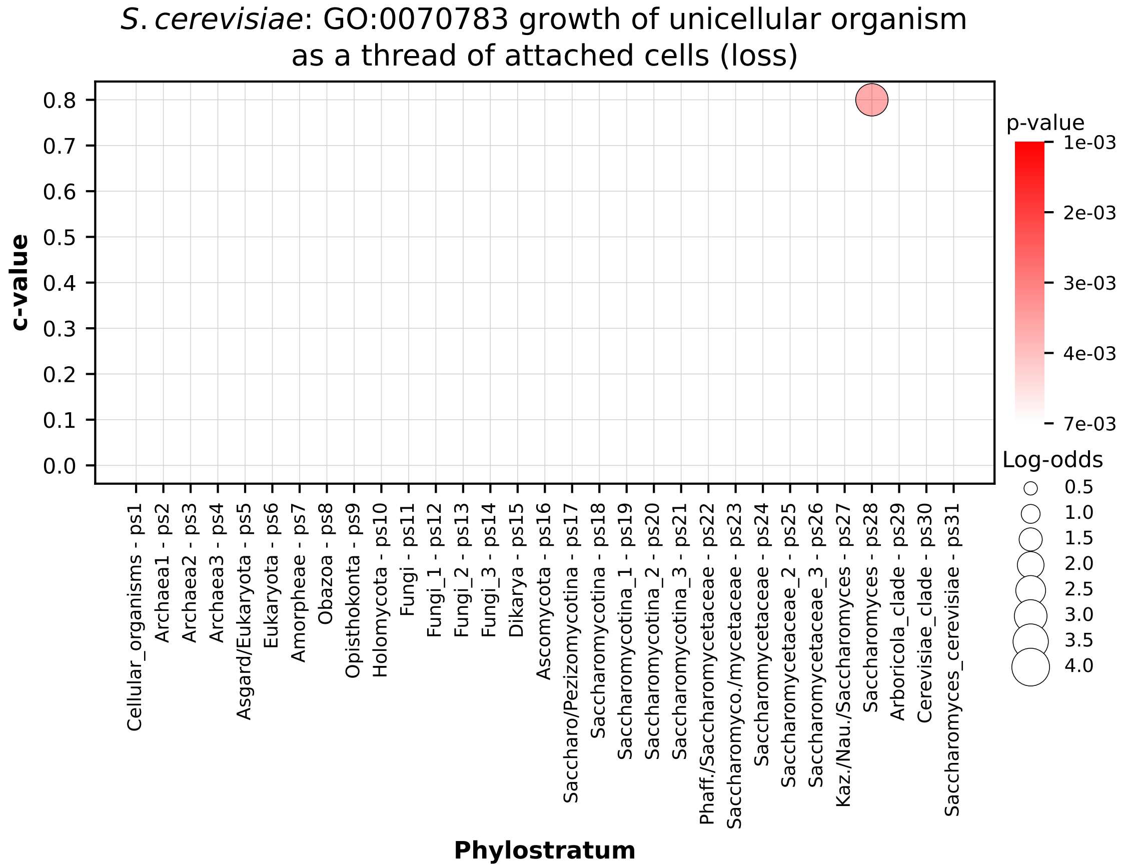

S. cerevisiae: GO:0070783 growth of unicellular organism as a thread of attached cells (loss)
c-value
0.0
0.1
0.2
0.3
0.4
0.5
0.6
0.7
0.8
Phylostratum
Cellular_organisms - ps1
Archaea1 - ps2
Archaea2 - ps3
Archaea3 - ps4
Asgard/Eukaryota - ps5
Eukaryota - ps6
Amorpheae - ps7
Obazoa - ps8
Opisthokonta - ps9
Holomycota - ps10
Fungi - ps11
Fungi_1 - ps12
Fungi_2 - ps13
Fungi_3 - ps14
Dikarya - ps15
Ascomycota - ps16
Saccharo/Pezizomycotina - ps17
Saccharomycotina - ps18
Saccharomycotina_1 - ps19
Saccharomycotina_2 - ps20
Saccharomycotina_3 - ps21
Phaff./Saccharomycetaceae - ps22
Saccharomyco./mycetaceae - ps23
Saccharomycetaceae - ps24
Saccharomycetaceae_2 - ps25
Saccharomycetaceae_3 - ps26
Kaz./Nau./Saccharomyces - ps27
Saccharomyces - ps28
Arboricola_clade - ps29
Cerevisiae_clade - ps30
Saccharomyces_cerevisiae - ps31
p-value
1e-03
2e-03
3e-03
4e-03
7e-03
Log-odds
0.5
1.0
1.5
2.0
2.5
3.0
3.5
4.0

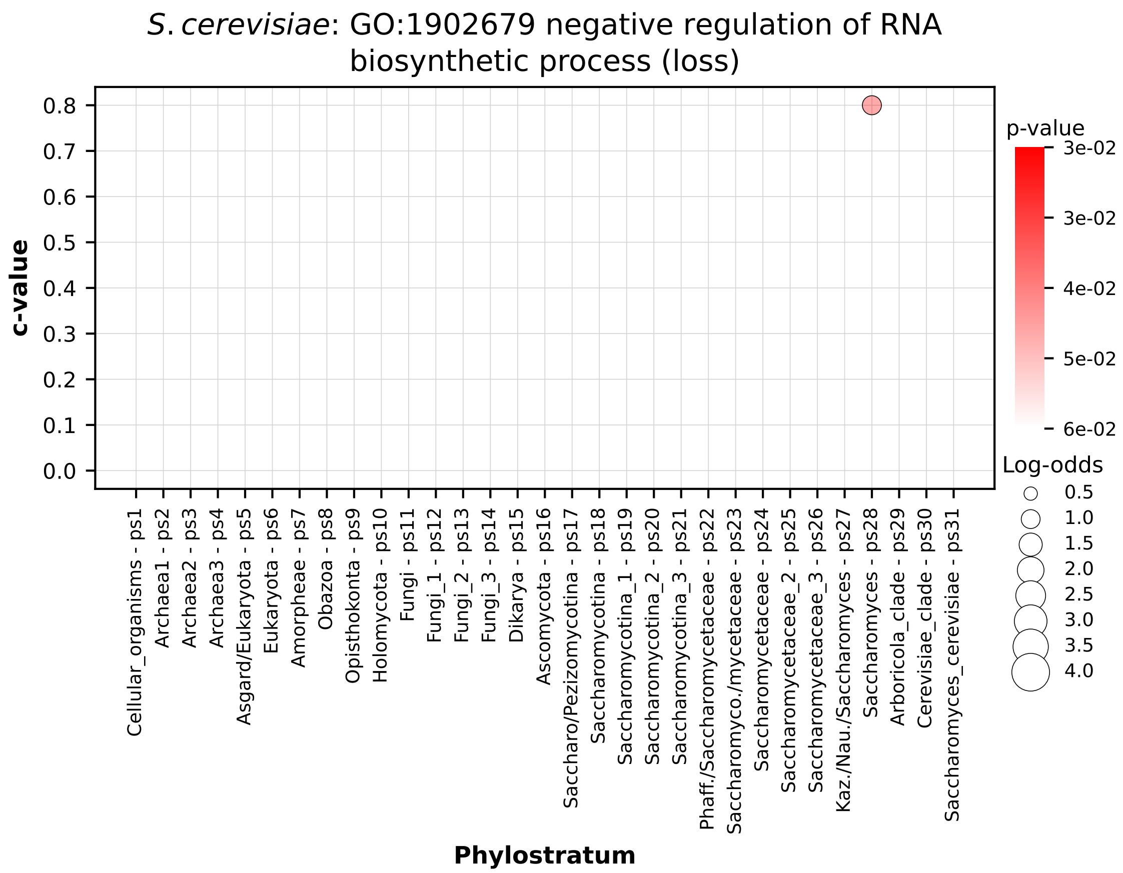
S. cerevisiae: GO:1902679 negative regulation of RNA biosynthetic process (loss)
c-value
0.0
0.1
0.2
0.3
0.4
0.5
0.6
0.7
0.8
Phylostratum
Cellular_organisms - ps1
Archaea1 - ps2
Archaea2 - ps3
Archaea3 - ps4
Asgard/Eukaryota - ps5
Eukaryota - ps6
Amorpheae - ps7
Obazoa - ps8
Opisthokonta - ps9
Holomycota - ps10
Fungi - ps11
Fungi_1 - ps12
Fungi_2 - ps13
Fungi_3 - ps14
Dikarya - ps15
Ascomycota - ps16
Saccharo/Pezizomycotina - ps17
Saccharomycotina - ps18
Saccharomycotina_1 - ps19
Saccharomycotina_2 - ps20
Saccharomycotina_3 - ps21
Phaff./Saccharomycetaceae - ps22
Saccharomyco./mycetaceae - ps23
Saccharomycetaceae - ps24
Saccharomycetaceae_2 - ps25
Saccharomycetaceae_3 - ps26
Kaz./Nau./Saccharomyces - ps27
Saccharomyces - ps28
Arboricola_clade - ps29
Cerevisiae_clade - ps30
Saccharomyces_cerevisiae - ps31
p-value
3e-02
3e-02
4e-02
5e-02
6e-02
Log-odds
0.5
1.0
1.5
2.0
2.5
3.0
3.5
4.0

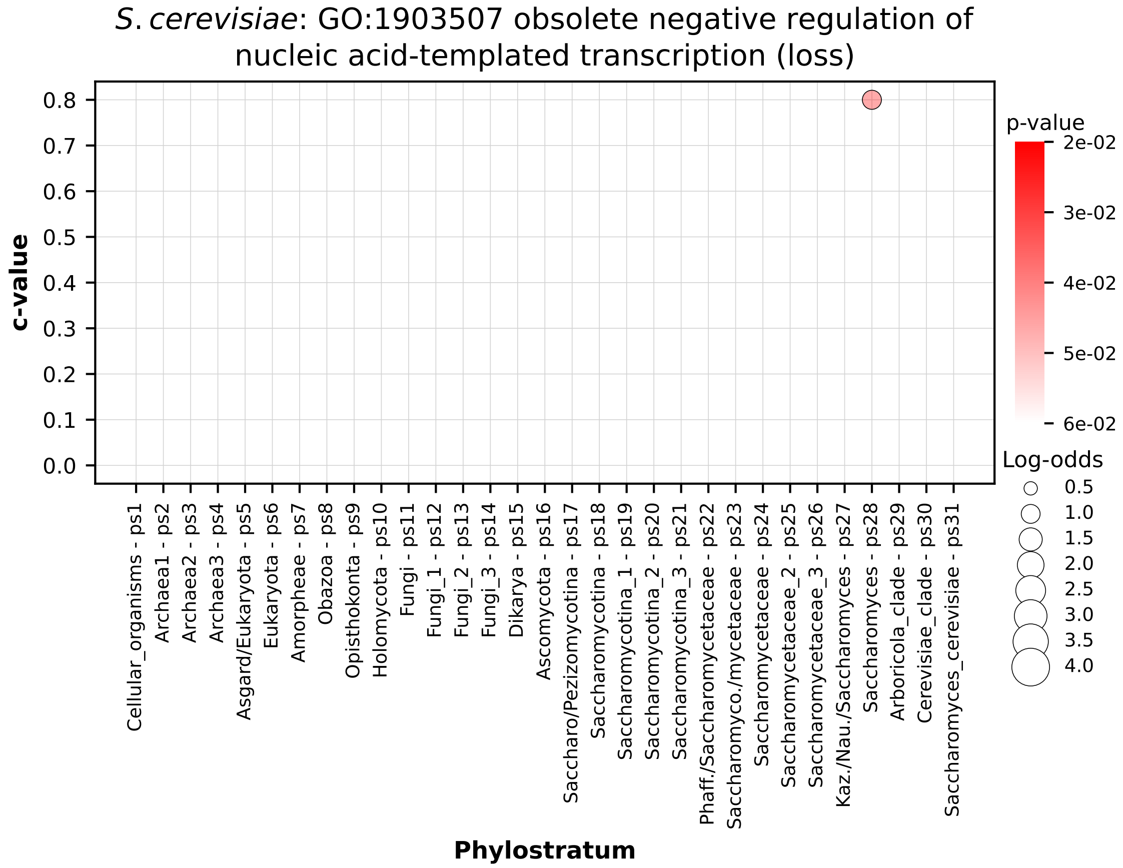

S. cerevisiae: GO:1903507 obsolete negative regulation of nucleic acid-templated transcription (loss)
c-value
0.0
0.1
0.2
0.3
0.4
0.5
0.6
0.7
0.8
p-value
2e-02
3e-02
4e-02
5e-02
6e-02
Log-odds
0.5
1.0
1.5
2.0
2.5
3.0
3.5
4.0
Cellular_organisms - ps1
Archaea1 - ps2
Archaea2 - ps3
Archaea3 - ps4
Asgard/Eukaryota - ps5
Eukaryota - ps6
Amorpheae - ps7
Obazoa - ps8
Opisthokonta - ps9
Holomycota - ps10
Fungi - ps11
Fungi_1 - ps12
Fungi_2 - ps13
Fungi_3 - ps14
Dikarya - ps15
Ascomycota - ps16
Saccharo/Pezizomycotina - ps17
Saccharomycotina - ps18
Saccharomycotina_1 - ps19
Saccharomycotina_2 - ps20
Saccharomycotina_3 - ps21
Phaff./Saccharomycetaceae - ps22
Saccharomyco./mycetaceae - ps23
Saccharomycetaceae - ps24
Saccharomycetaceae_2 - ps25
Saccharomycetaceae_3 - ps26
Kaz./Nau./Saccharomyces - ps27
Saccharomyces - ps28
Arboricola_clade - ps29
Cerevisiae_clade - ps30
Saccharomyces_cerevisiae - ps31
Phylostratum

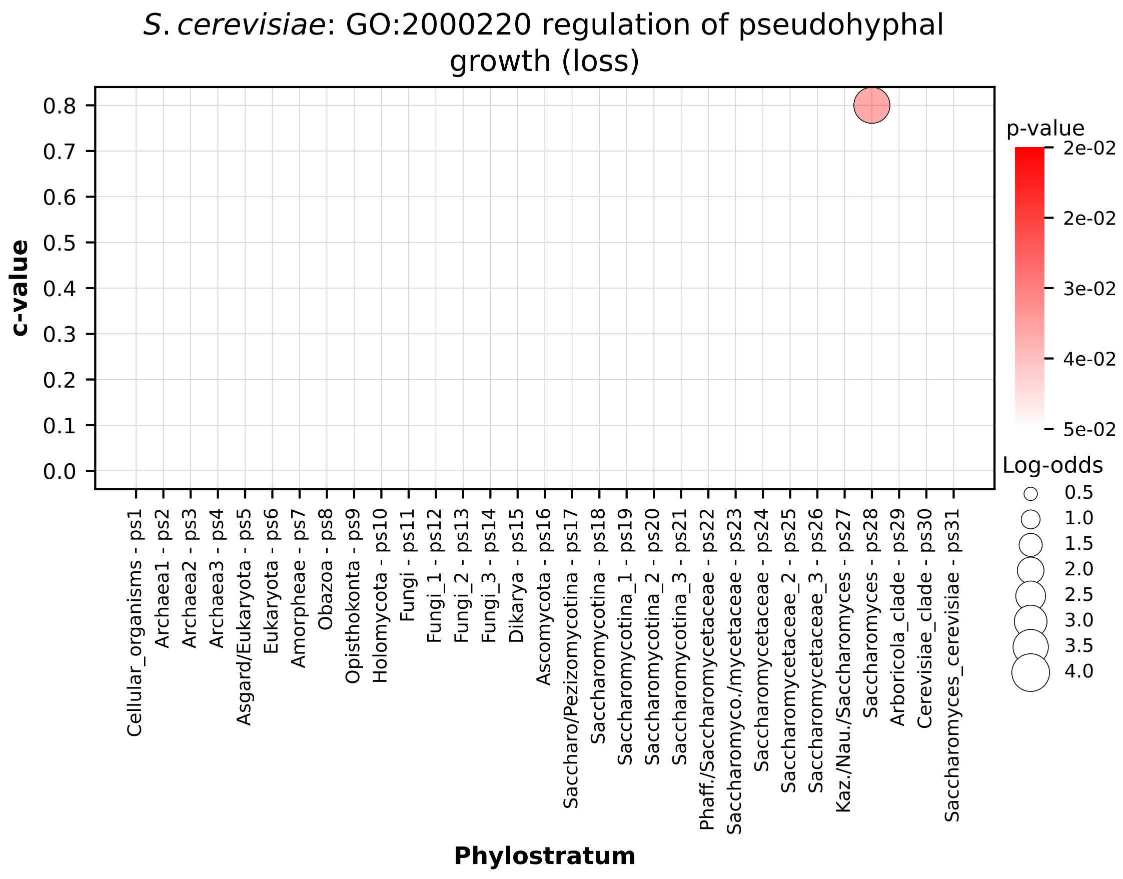

S. cerevisiae: GO:2000220 regulation of pseudohyphal growth (loss)
c-value
0.0
0.1
0.2
0.3
0.4
0.5
0.6
0.7
0.8
Phylostratum
Cellular_organisms - ps1
Archaea1 - ps2
Archaea2 - ps3
Archaea3 - ps4
Asgard/Eukaryota - ps5
Eukaryota - ps6
Amorpheae - ps7
Obazoa - ps8
Opisthokonta - ps9
Holomycota - ps10
Fungi - ps11
Fungi_1 - ps12
Fungi_2 - ps13
Fungi_3 - ps14
Dikarya - ps15
Ascomycota - ps16
Saccharo/Pezizomycotina - ps17
Saccharomycotina - ps18
Saccharomycotina_1 - ps19
Saccharomycotina_2 - ps20
Saccharomycotina_3 - ps21
Phaff./Saccharomycetaceae - ps22
Saccharomyco./mycetaceae - ps23
Saccharomycetaceae - ps24
Saccharomycetaceae_2 - ps25
Saccharomycetaceae_3 - ps26
Kaz./Nau./Saccharomyces - ps27
Saccharomyces - ps28
Arboricola_clade - ps29
Cerevisiae_clade - ps30
Saccharomyces_cerevisiae - ps31
p-value
2e-02
2e-02
3e-02
4e-02
5e-02
Log-odds
0.5
1.0
1.5
2.0
2.5
3.0
3.5
4.0

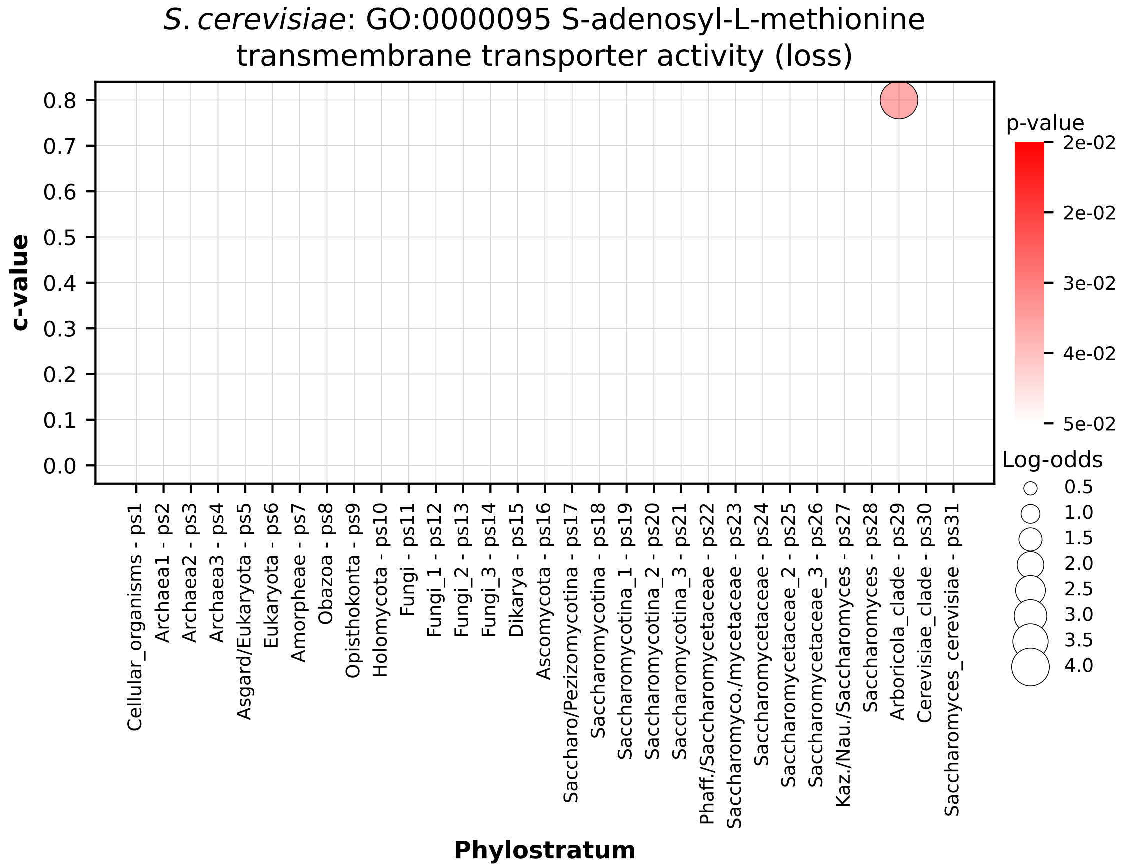

S. cerevisiae: GO:0000095 S-adenosyl-L-methionine transmembrane transporter activity (loss)
c-value
0.8
0.7
0.6
0.5
0.4
0.3
0.2
0.1
0.0
p-value
2e-02
2e-02
3e-02
4e-02
5e-02
Log-odds
0.5
1.0
1.5
2.0
2.5
3.0
3.5
4.0
Cellular_organisms - ps1
Archaea1 - ps2
Archaea2 - ps3
Archaea3 - ps4
Asgard/Eukaryota - ps5
Eukaryota - ps6
Amorpheae - ps7
Obazoa - ps8
Opisthokonta - ps9
Holomycota - ps10
Fungi - ps11
Fungi_1 - ps12
Fungi_2 - ps13
Fungi_3 - ps14
Dikarya - ps15
Ascomycota - ps16
Saccharo/Pezizomycotina - ps17
Saccharomycotina - ps18
Saccharomycotina_1 - ps19
Saccharomycotina_2 - ps20
Saccharomycotina_3 - ps21
Phaff./Saccharomycetaceae - ps22
Saccharomyco./mycetaceae - ps23
Saccharomycetaceae - ps24
Saccharomycetaceae_2 - ps25
Saccharomycetaceae_3 - ps26
Kaz./Nau./Saccharomyces - ps27
Saccharomyces - ps28
Arboricola_clade - ps29
Cerevisiae_clade - ps30
Saccharomyces_cerevisiae - ps31
Phylostratum

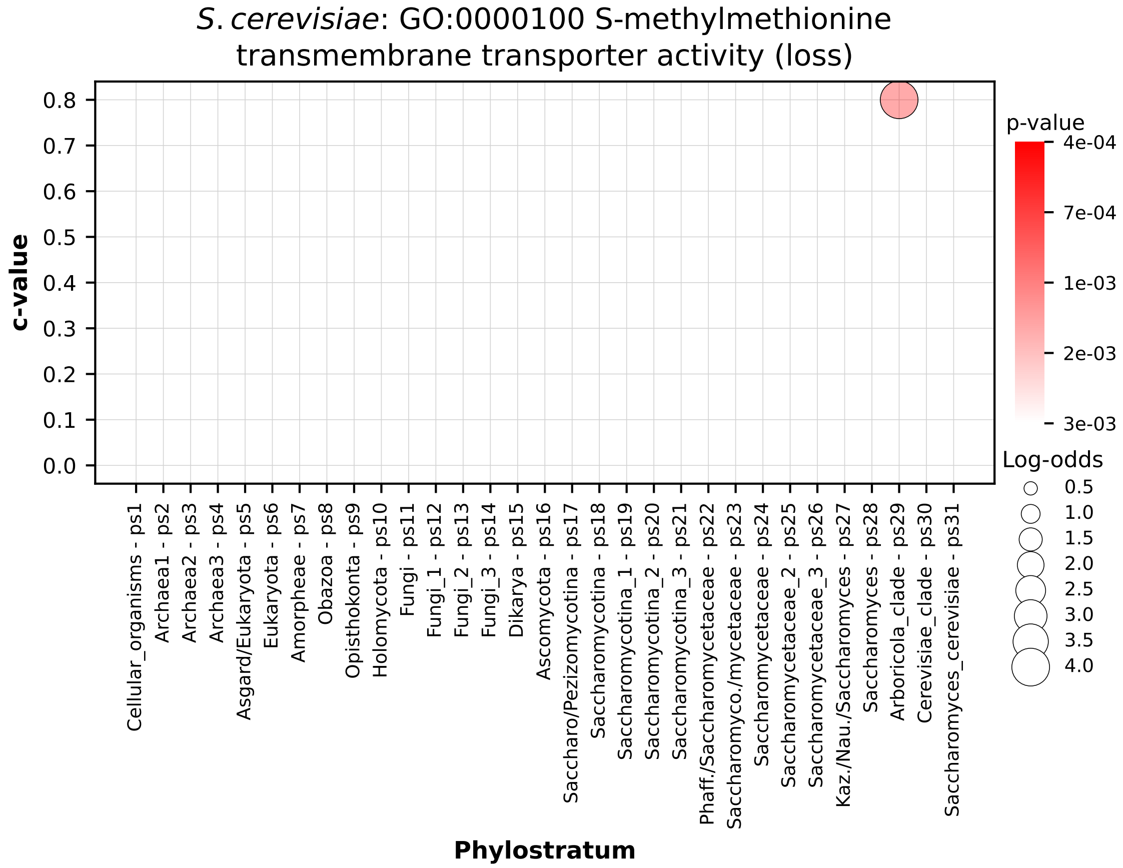

S. cerevisiae: GO:0000100 S-methylmethionine transmembrane transporter activity (loss)
c-value
0.8
0.7
0.6
0.5
0.4
0.3
0.2
0.1
0.0
p-value
4e-04
7e-04
1e-03
2e-03
3e-03
Log-odds
0.5
1.0
1.5
2.0
2.5
3.0
3.5
4.0
Cellular_organisms - ps1
Archaea1 - ps2
Archaea2 - ps3
Archaea3 - ps4
Asgard/Eukaryota - ps5
Eukaryota - ps6
Amorpheae - ps7
Obazoa - ps8
Opisthokonta - ps9
Holomycota - ps10
Fungi - ps11
Fungi_1 - ps12
Fungi_2 - ps13
Fungi_3 - ps14
Dikarya - ps15
Ascomycota - ps16
Saccharo/Pezizomycotina - ps17
Saccharomycotina - ps18
Saccharomycotina_1 - ps19
Saccharomycotina_2 - ps20
Saccharomycotina_3 - ps21
Phaff./Saccharomycetaceae - ps22
Saccharomyco./mycetaceae - ps23
Saccharomycetaceae - ps24
Saccharomycetaceae_2 - ps25
Saccharomycetaceae_3 - ps26
Kaz./Nau./Saccharomyces - ps27
Saccharomyces - ps28
Arboricola_clade - ps29
Cerevisiae_clade - ps30
Saccharomyces_cerevisiae - ps31
Phylostratum

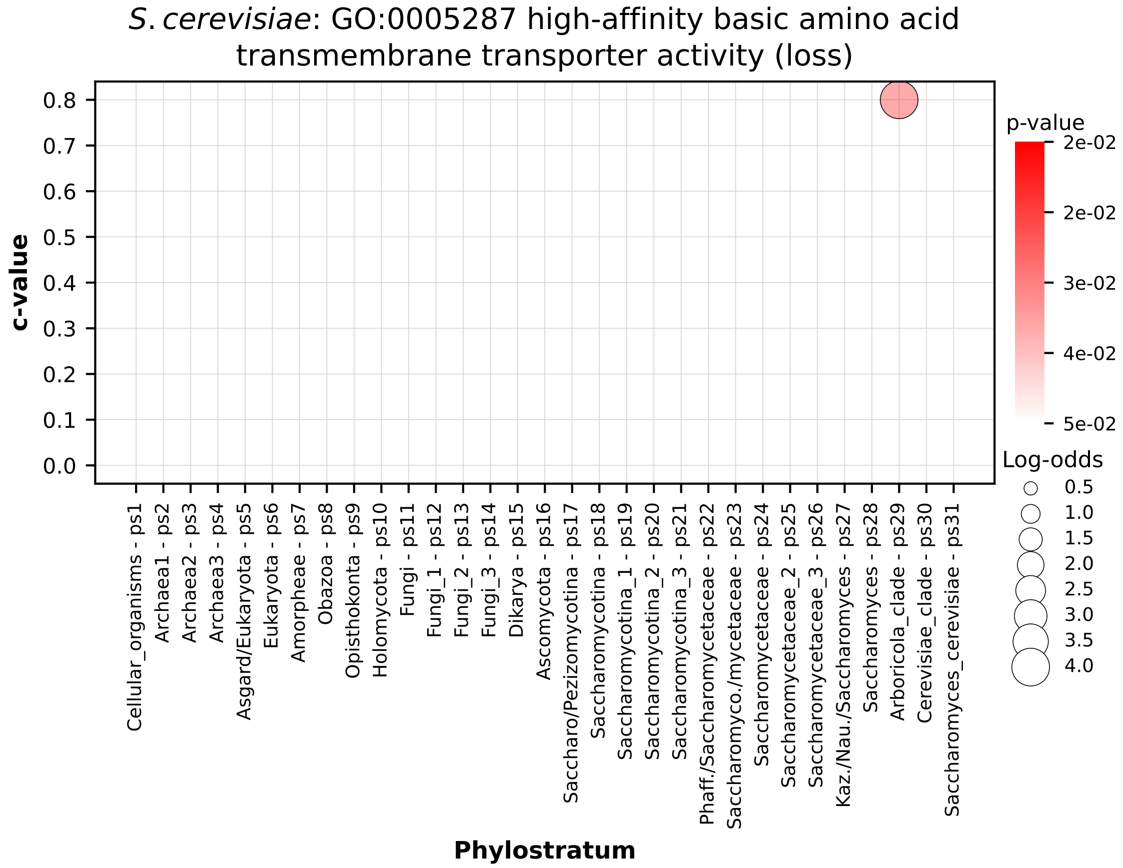
S. cerevisiae: GO:0005287 high-affinity basic amino acid transmembrane transporter activity (loss)
c-value
0.0
0.1
0.2
0.3
0.4
0.5
0.6
0.7
0.8
p-value
2e-02
2e-02
3e-02
4e-02
5e-02
Log-odds
0.5
1.0
1.5
2.0
2.5
3.0
3.5
4.0
Cellular_organisms - ps1
Archaea1 - ps2
Archaea2 - ps3
Archaea3 - ps4
Asgard/Eukaryota - ps5
Eukaryota - ps6
Amorpheae - ps7
Obazoa - ps8
Opisthokonta - ps9
Holomycota - ps10
Fungi - ps11
Fungi_1 - ps12
Fungi_2 - ps13
Fungi_3 - ps14
Dikarya - ps15
Ascomycota - ps16
Saccharo/Pezizomycotina - ps17
Saccharomycotina - ps18
Saccharomycotina_1 - ps19
Saccharomycotina_2 - ps20
Saccharomycotina_3 - ps21
Phaff./Saccharomycetaceae - ps22
Saccharomyco./mycetaceae - ps23
Saccharomycetaceae - ps24
Saccharomycetaceae_2 - ps25
Saccharomycetaceae_3 - ps26
Kaz./Nau./Saccharomyces - ps27
Saccharomyces - ps28
Arboricola_clade - ps29
Cerevisiae_clade - ps30
Saccharomyces_cerevisiae - ps31
Phylostratum

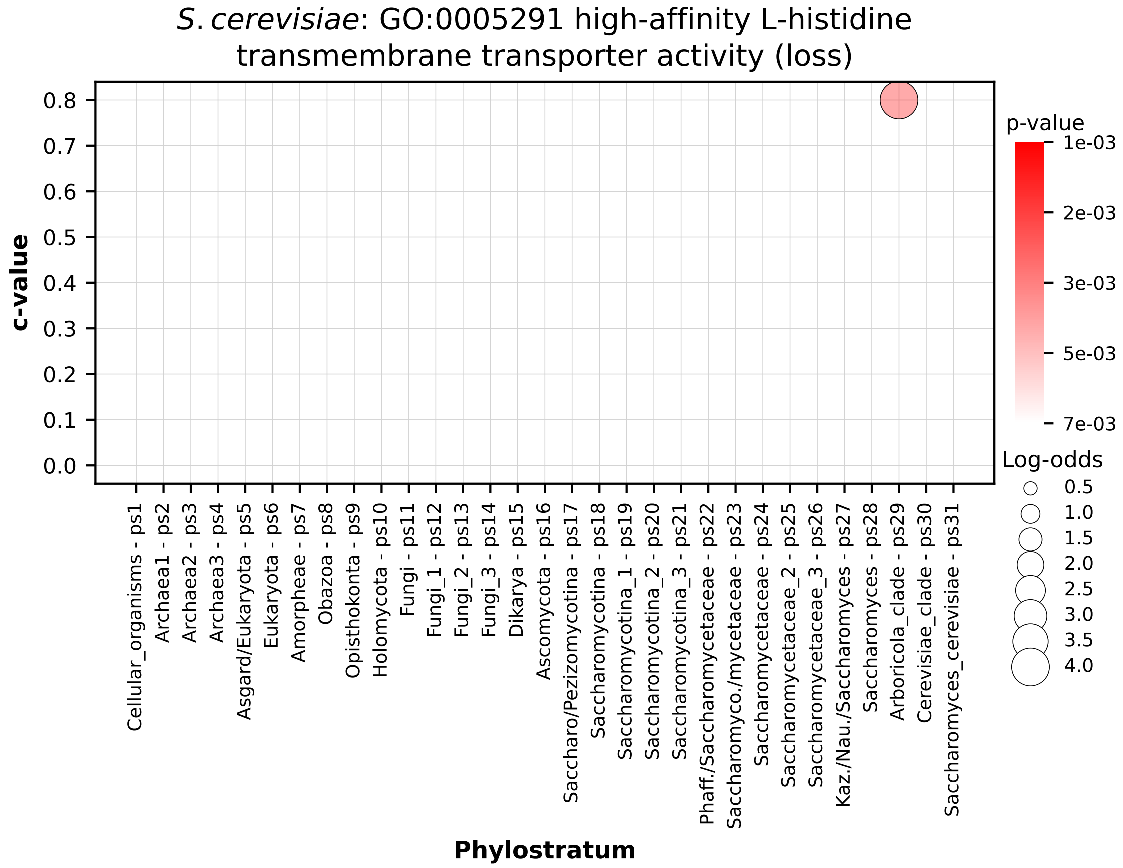

S. cerevisiae: GO:0005291 high-affinity L-histidine transmembrane transporter activity (loss)
c-value
0.0
0.1
0.2
0.3
0.4
0.5
0.6
0.7
0.8
p-value
1e-03
2e-03
3e-03
5e-03
7e-03
Log-odds
0.5
1.0
1.5
2.0
2.5
3.0
3.5
4.0
Cellular_organisms - ps1
Archaea1 - ps2
Archaea2 - ps3
Archaea3 - ps4
Asgard/Eukaryota - ps5
Eukaryota - ps6
Amorpheae - ps7
Obazoa - ps8
Opisthokonta - ps9
Holomycota - ps10
Fungi - ps11
Fungi_1 - ps12
Fungi_2 - ps13
Fungi_3 - ps14
Dikarya - ps15
Ascomycota - ps16
Saccharo/Pezizomycotina - ps17
Saccharomycotina - ps18
Saccharomycotina_1 - ps19
Saccharomycotina_2 - ps20
Saccharomycotina_3 - ps21
Phaff./Saccharomycetaceae - ps22
Saccharomyco./mycetaceae - ps23
Saccharomycetaceae - ps24
Saccharomycetaceae_2 - ps25
Saccharomycetaceae_3 - ps26
Kaz./Nau./Saccharomyces - ps27
Saccharomyces - ps28
Arboricola_clade - ps29
Cerevisiae_clade - ps30
Saccharomyces_cerevisiae - ps31
Phylostratum

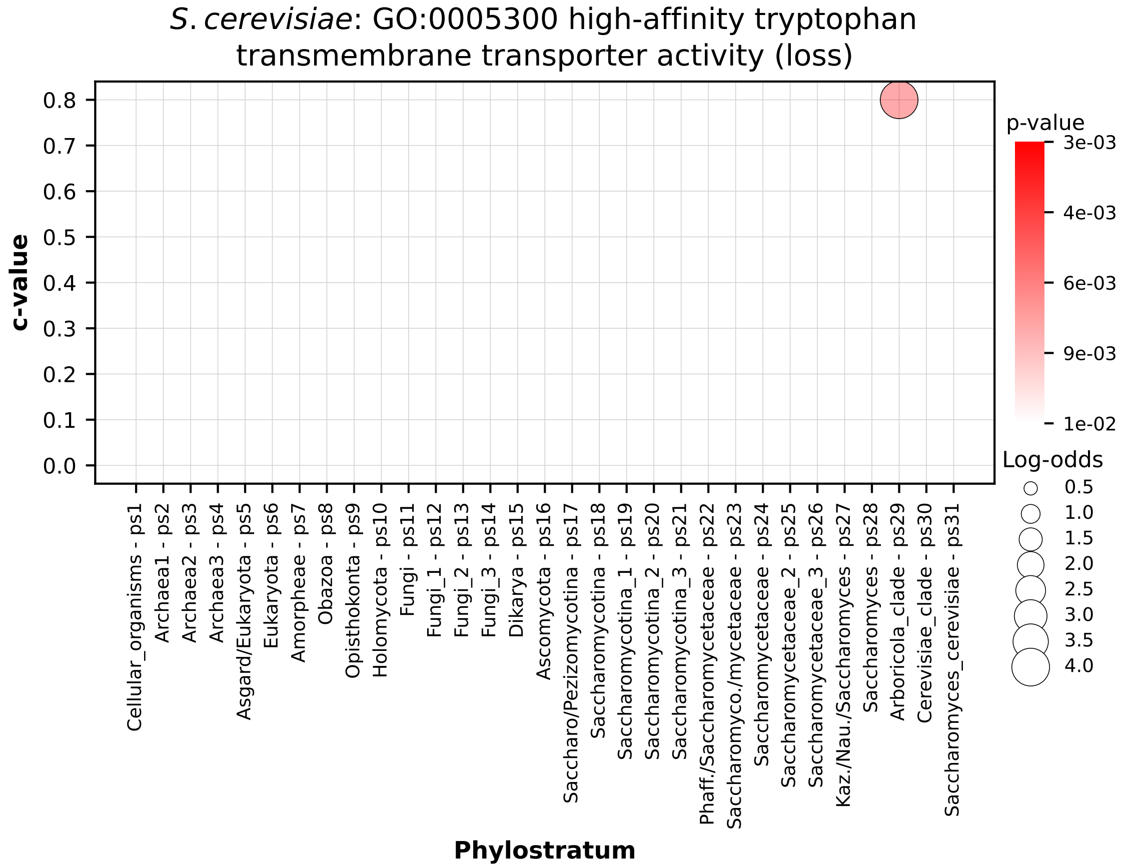

S. cerevisiae: GO:0005300 high-affinity tryptophan transmembrane transporter activity (loss)
c-value
0.8
0.7
0.6
0.5
0.4
0.3
0.2
0.1
0.0
Phylostratum
Cellular_organisms - ps1
Archaea1 - ps2
Archaea2 - ps3
Archaea3 - ps4
Asgard/Eukaryota - ps5
Eukaryota - ps6
Amorpheae - ps7
Obazoa - ps8
Opisthokonta - ps9
Holomycota - ps10
Fungi - ps11
Fungi_1 - ps12
Fungi_2 - ps13
Fungi_3 - ps14
Dikarya - ps15
Ascomycota - ps16
Saccharo/Pezizomycotina - ps17
Saccharomycotina - ps18
Saccharomycotina_1 - ps19
Saccharomycotina_2 - ps20
Saccharomycotina_3 - ps21
Phaff./Saccharomycetaceae - ps22
Saccharomyco./mycetaceae - ps23
Saccharomycetaceae - ps24
Saccharomycetaceae_2 - ps25
Saccharomycetaceae_3 - ps26
Kaz./Nau./Saccharomyces - ps27
Saccharomyces - ps28
Arboricola_clade - ps29
Cerevisiae_clade - ps30
Saccharomyces_cerevisiae - ps31
p-value
3e-03
4e-03
6e-03
9e-03
1e-02
Log-odds
0.5
1.0
1.5
2.0
2.5
3.0
3.5
4.0

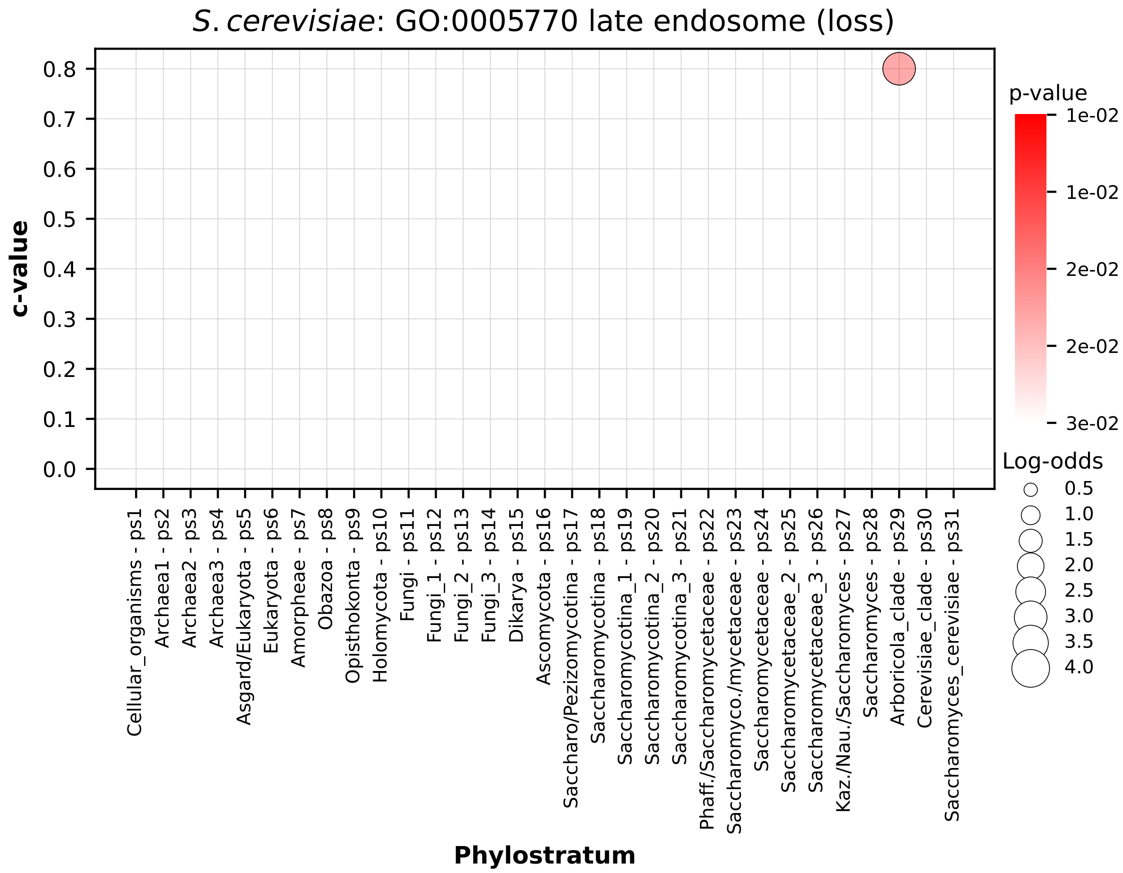
S. cerevisiae: GO:0005770 late endosome (loss)
c-value
0.8
0.7
0.6
0.5
0.4
0.3
0.2
0.1
0.0
p-value
1e-02
1e-02
2e-02
2e-02
3e-02
Log-odds
0.5
1.0
1.5
2.0
2.5
3.0
3.5
4.0
Cellular_organisms - ps1
Archaea1 - ps2
Archaea2 - ps3
Archaea3 - ps4
Asgard/Eukaryota - ps5
Eukaryota - ps6
Amorpheae - ps7
Obazoa - ps8
Opisthokonta - ps9
Holomycota - ps10
Fungi - ps11
Fungi_1 - ps12
Fungi_2 - ps13
Fungi_3 - ps14
Dikarya - ps15
Ascomycota - ps16
Saccharo/Pezizomycotina - ps17
Saccharomycotina - ps18
Saccharomycotina_1 - ps19
Saccharomycotina_2 - ps20
Saccharomycotina_3 - ps21
Phaff./Saccharomycetaceae - ps22
Saccharomyco./mycetaceae - ps23
Saccharomycetaceae - ps24
Saccharomycetaceae_2 - ps25
Saccharomycetaceae_3 - ps26
Kaz./Nau./Saccharomyces - ps27
Saccharomyces - ps28
Arboricola_clade - ps29
Cerevisiae_clade - ps30
Saccharomyces_cerevisiae - ps31
Phylostratum

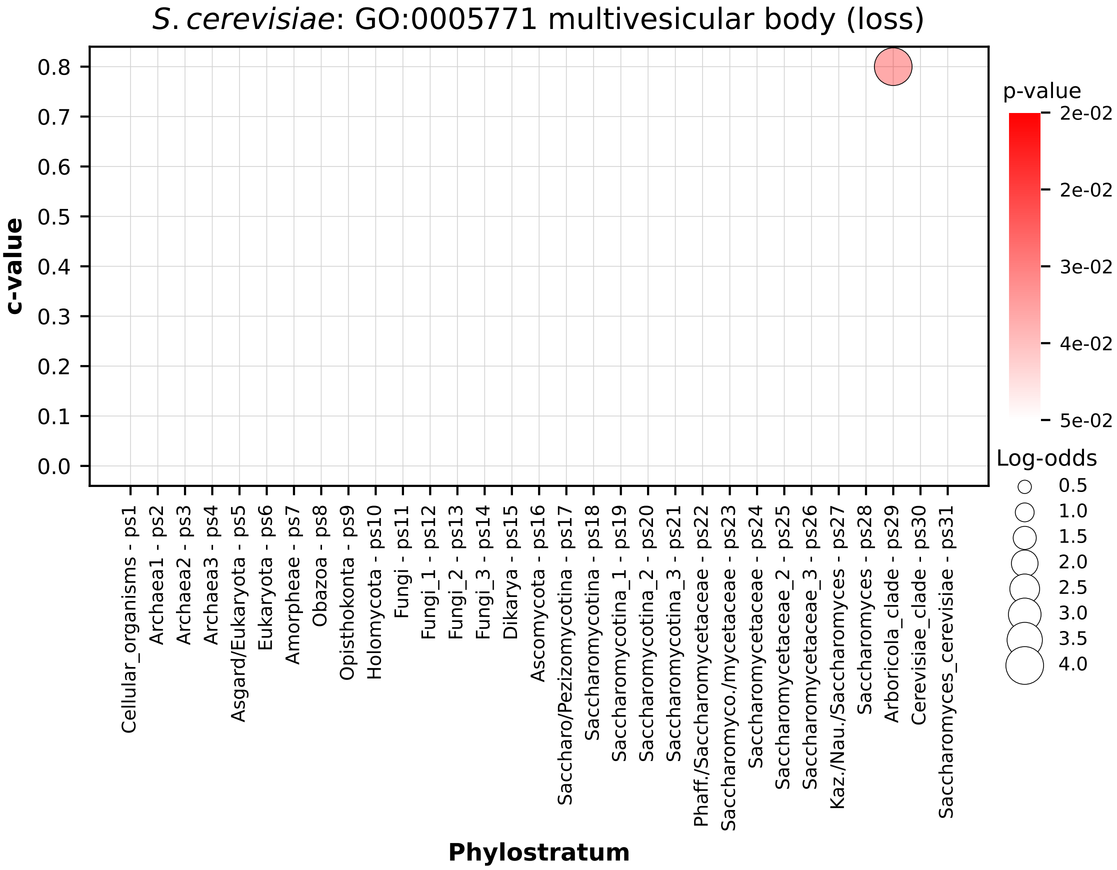
S. cerevisiae: GO:0005771 multivesicular body (loss)
c-value
0.8
0.7
0.6
0.5
0.4
0.3
0.2
0.1
0.0
p-value
2e-02
2e-02
3e-02
4e-02
5e-02
Log-odds
0.5
1.0
1.5
2.0
2.5
3.0
3.5
4.0
Cellular_organisms - ps1
Archaea1 - ps2
Archaea2 - ps3
Archaea3 - ps4
Asgard/Eukaryota - ps5
Eukaryota - ps6
Amorpheae - ps7
Obazoa - ps8
Opisthokonta - ps9
Holomycota - ps10
Fungi - ps11
Fungi_1 - ps12
Fungi_2 - ps13
Fungi_3 - ps14
Dikarya - ps15
Ascomycota - ps16
Saccharo/Pezizomycotina - ps17
Saccharomycotina - ps18
Saccharomycotina_1 - ps19
Saccharomycotina_2 - ps20
Saccharomycotina_3 - ps21
Phaff./Saccharomycetaceae - ps22
Saccharomyco./mycetaceae - ps23
Saccharomycetaceae - ps24
Saccharomycetaceae_2 - ps25
Saccharomycetaceae_3 - ps26
Kaz./Nau./Saccharomyces - ps27
Saccharomyces - ps28
Arboricola_clade - ps29
Cerevisiae_clade - ps30
Saccharomyces_cerevisiae - ps31
Phylostratum

S. cerevisiae: GO:0015174 basic amino acid transmembrane transporter activity (loss)

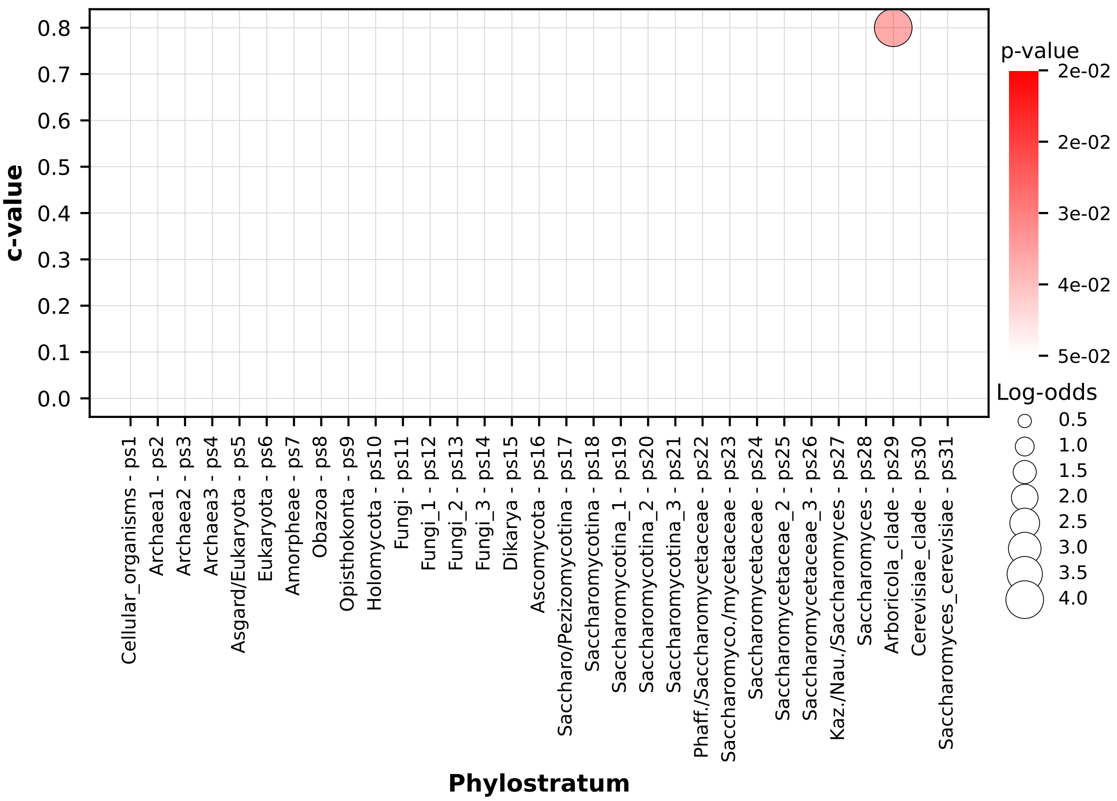

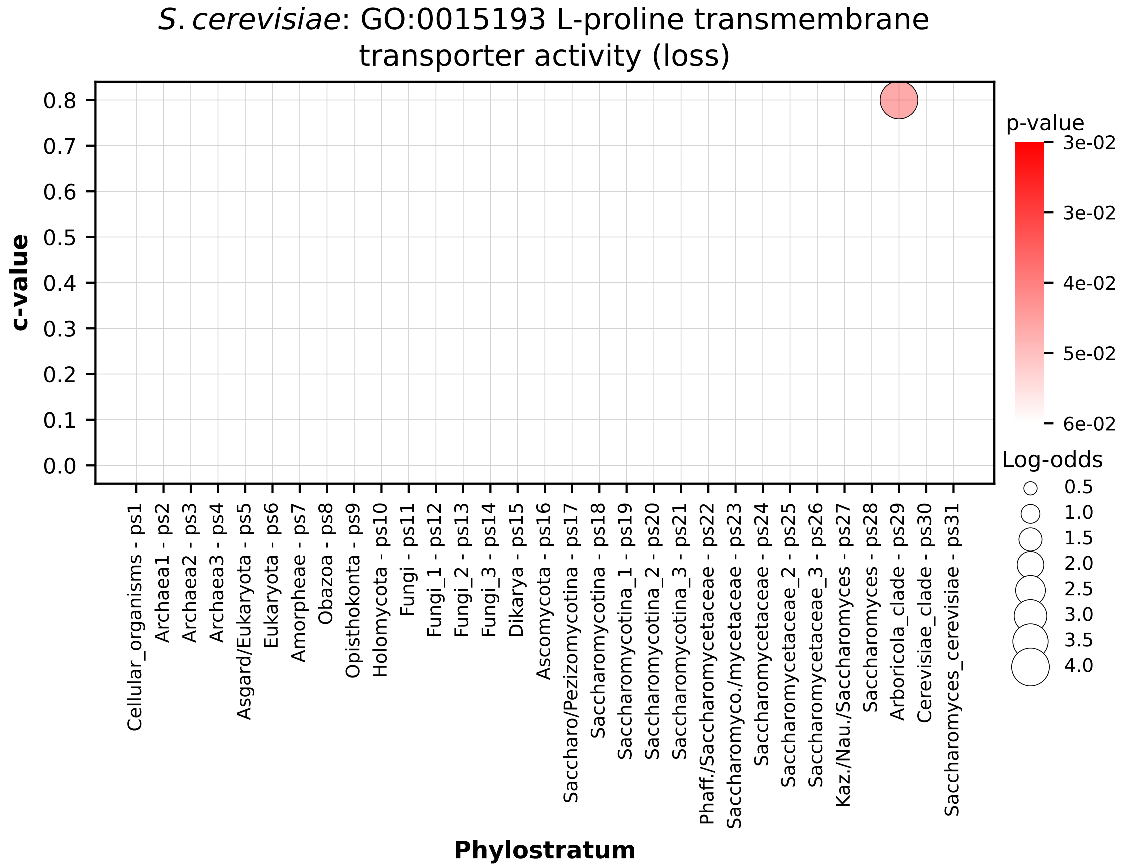

S. cerevisiae: GO:0015193 L-proline transmembrane transporter activity (loss)
c-value
0.0
0.1
0.2
0.3
0.4
0.5
0.6
0.7
0.8
Phylostratum
Cellular_organisms - ps1
Archaea1 - ps2
Archaea2 - ps3
Archaea3 - ps4
Asgard/Eukaryota - ps5
Eukaryota - ps6
Amorpheae - ps7
Obazoa - ps8
Opisthokonta - ps9
Holomycota - ps10
Fungi - ps11
Fungi_1 - ps12
Fungi_2 - ps13
Fungi_3 - ps14
Dikarya - ps15
Ascomycota - ps16
Saccharo/Pezizomycotina - ps17
Saccharomycotina - ps18
Saccharomycotina_1 - ps19
Saccharomycotina_2 - ps20
Saccharomycotina_3 - ps21
Phaff./Saccharomycetaceae - ps22
Saccharomyco./mycetaceae - ps23
Saccharomycetaceae - ps24
Saccharomycetaceae_2 - ps25
Saccharomycetaceae_3 - ps26
Kaz./Nau./Saccharomyces - ps27
Saccharomyces - ps28
Arboricola_clade - ps29
Cerevisiae_clade - ps30
Saccharomyces_cerevisiae - ps31
p-value
3e-02
3e-02
4e-02
5e-02
6e-02
Log-odds
0.5
1.0
1.5
2.0
2.5
3.0
3.5
4.0

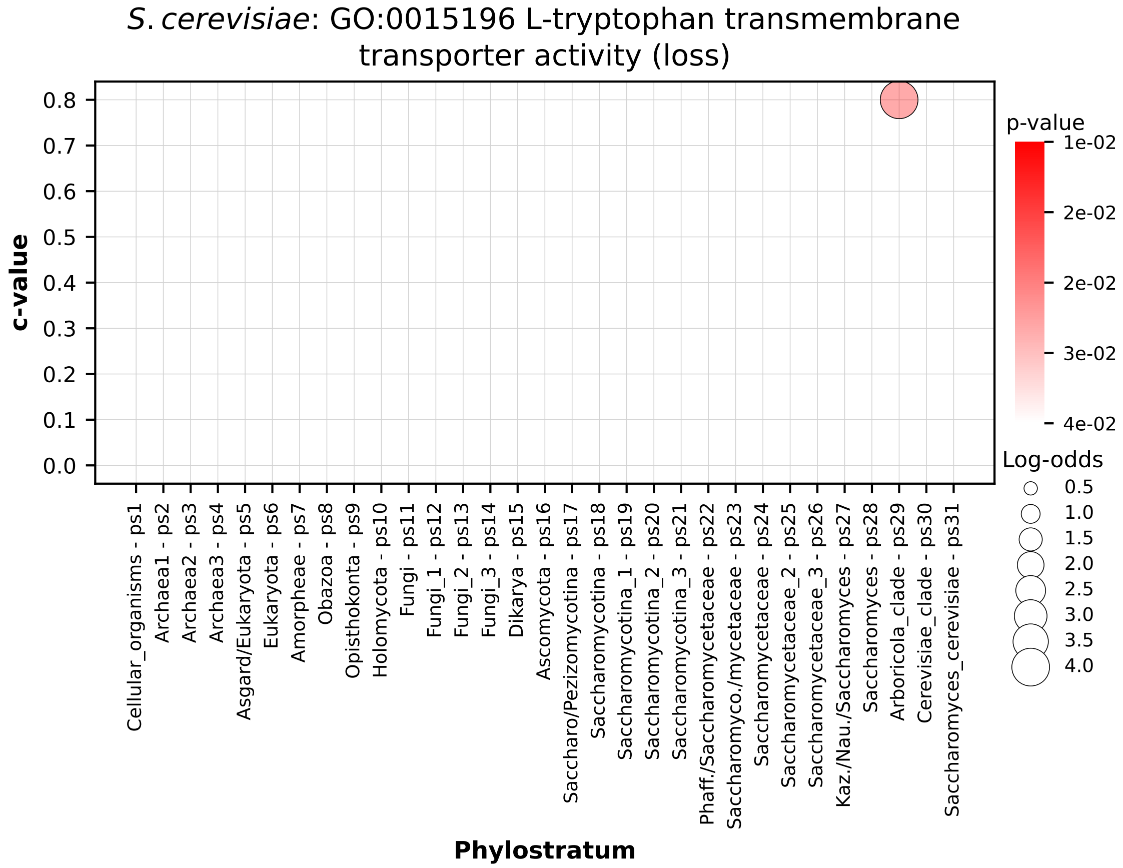
S. cerevisiae: GO:0015196 L-tryptophan transmembrane transporter activity (loss)
c-value
0.8
0.7
0.6
0.5
0.4
0.3
0.2
0.1
0.0
Phylostratum
Cellular_organisms - ps1
Archaea1 - ps2
Archaea2 - ps3
Archaea3 - ps4
Asgard/Eukaryota - ps5
Eukaryota - ps6
Amorpheae - ps7
Obazoa - ps8
Opisthokonta - ps9
Holomycota - ps10
Fungi - ps11
Fungi_1 - ps12
Fungi_2 - ps13
Fungi_3 - ps14
Dikarya - ps15
Ascomycota - ps16
Saccharo/Pezizomycotina - ps17
Saccharomycotina - ps18
Saccharomycotina_1 - ps19
Saccharomycotina_2 - ps20
Saccharomycotina_3 - ps21
Phaff./Saccharomycetaceae - ps22
Saccharomyco./mycetaceae - ps23
Saccharomycetaceae - ps24
Saccharomycetaceae_2 - ps25
Saccharomycetaceae_3 - ps26
Kaz./Nau./Saccharomyces - ps27
Saccharomyces - ps28
Arboricola_clade - ps29
Cerevisiae_clade - ps30
Saccharomyces_cerevisiae - ps31
p-value
1e-02
2e-02
2e-02
3e-02
4e-02
Log-odds
0.5
1.0
1.5
2.0
2.5
3.0
3.5
4.0

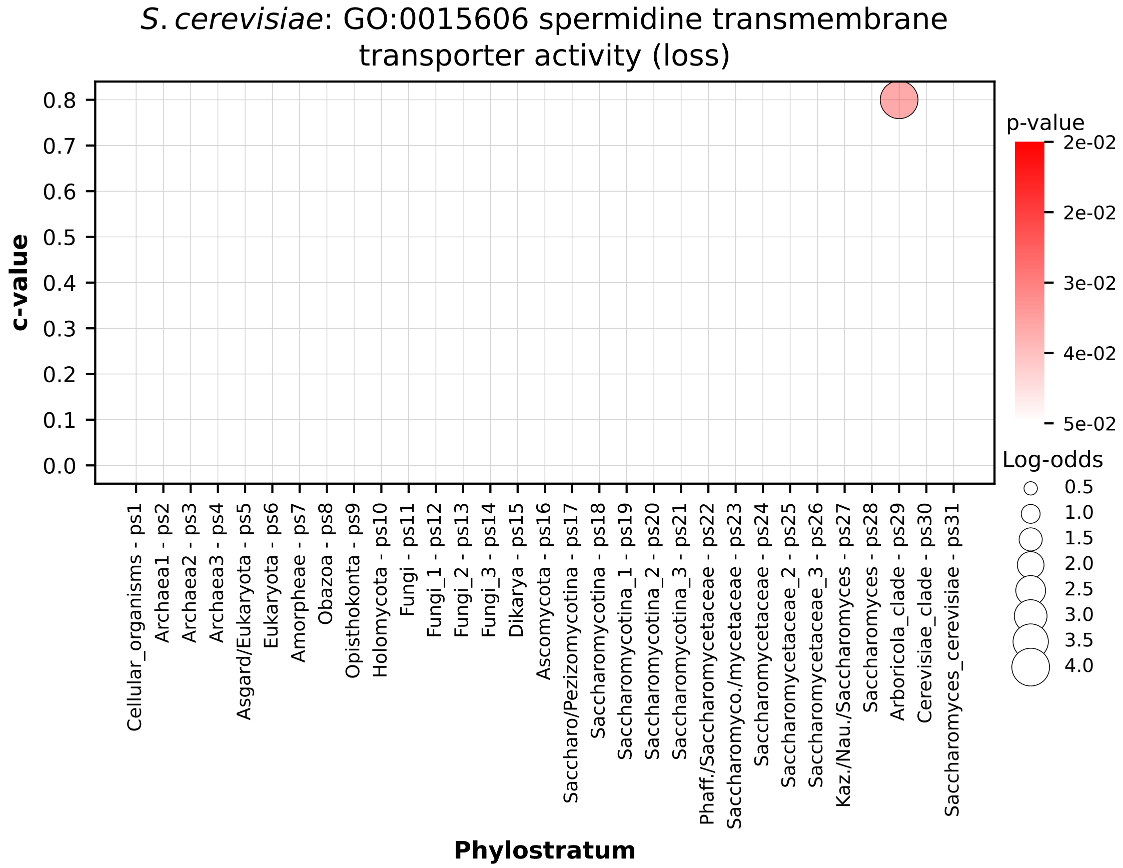

S. cerevisiae: GO:0015606 spermidine transmembrane transporter activity (loss)
c-value
0.8
0.7
0.6
0.5
0.4
0.3
0.2
0.1
0.0
p-value
2e-02
2e-02
3e-02
4e-02
5e-02
Log-odds
0.5
1.0
1.5
2.0
2.5
3.0
3.5
4.0
Cellular_organisms - ps1
Archaea1 - ps2
Archaea2 - ps3
Archaea3 - ps4
Asgard/Eukaryota - ps5
Eukaryota - ps6
Amorpheae - ps7
Obazoa - ps8
Opisthokonta - ps9
Holomycota - ps10
Fungi - ps11
Fungi_1 - ps12
Fungi_2 - ps13
Fungi_3 - ps14
Dikarya - ps15
Ascomycota - ps16
Saccharo/Pezizomycotina - ps17
Saccharomycotina - ps18
Saccharomycotina_1 - ps19
Saccharomycotina_2 - ps20
Saccharomycotina_3 - ps21
Phaff./Saccharomycetaceae - ps22
Saccharomyco./mycetaceae - ps23
Saccharomycetaceae - ps24
Saccharomycetaceae_2 - ps25
Saccharomycetaceae_3 - ps26
Kaz./Nau./Saccharomyces - ps27
Saccharomyces - ps28
Arboricola_clade - ps29
Cerevisiae_clade - ps30
Saccharomyces_cerevisiae - ps31
Phylostratum

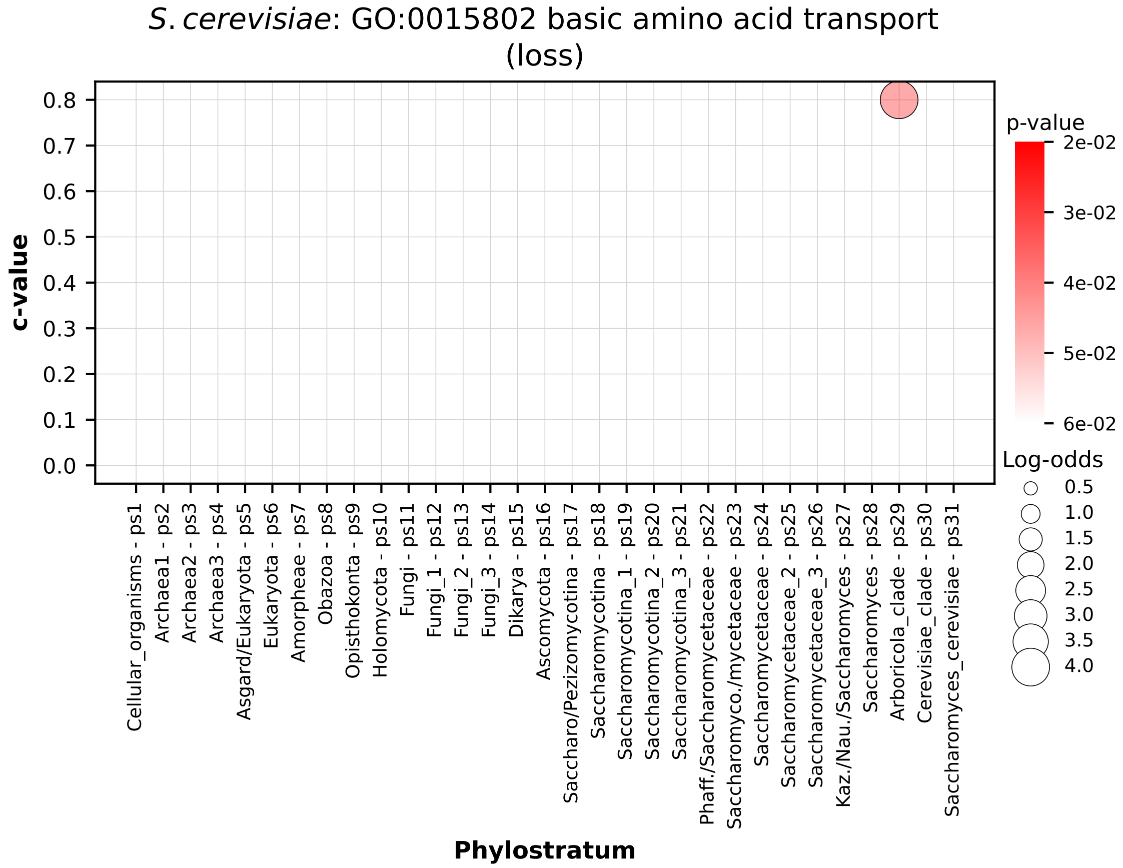

S. cerevisiae: GO:0015802 basic amino acid transport
(loss)
c-value
0.0
0.1
0.2
0.3
0.4
0.5
0.6
0.7
0.8
Phylostratum
Cellular_organisms - ps1
Archaea1 - ps2
Archaea2 - ps3
Archaea3 - ps4
Asgard/Eukaryota - ps5
Eukaryota - ps6
Amorpheae - ps7
Obazoa - ps8
Opisthokonta - ps9
Holomycota - ps10
Fungi - ps11
Fungi_1 - ps12
Fungi_2 - ps13
Fungi_3 - ps14
Dikarya - ps15
Ascomycota - ps16
Saccharo/Pezizomycotina - ps17
Saccharomycotina - ps18
Saccharomycotina_1 - ps19
Saccharomycotina_2 - ps20
Saccharomycotina_3 - ps21
Phaff./Saccharomycetaceae - ps22
Saccharomyco./mycetaceae - ps23
Saccharomycetaceae - ps24
Saccharomycetaceae_2 - ps25
Saccharomycetaceae_3 - ps26
Kaz./Nau./Saccharomyces - ps27
Saccharomyces - ps28
Arboricola_clade - ps29
Cerevisiae_clade - ps30
Saccharomyces_cerevisiae - ps31
p-value
2e-02
3e-02
4e-02
5e-02
6e-02
Log-odds
0.5
1.0
1.5
2.0
2.5
3.0
3.5
4.0

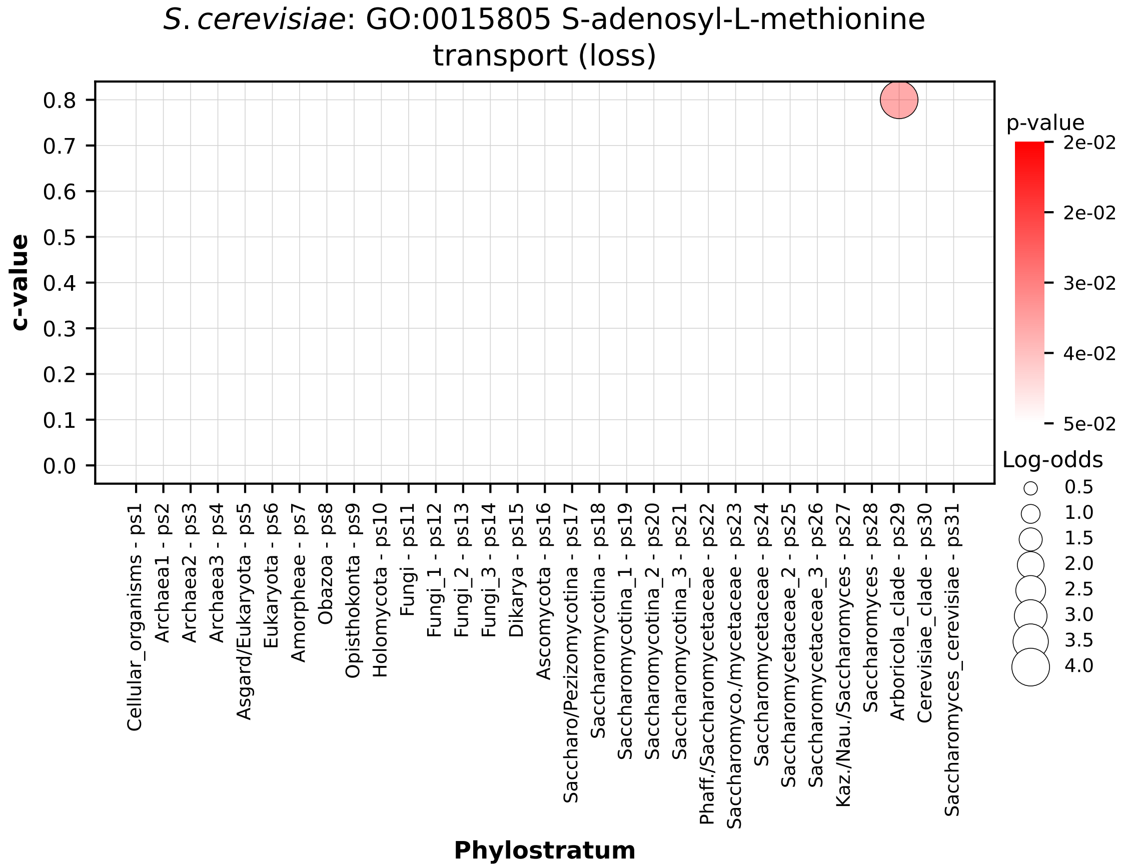
S. cerevisiae: GO:0015805 S-adenosyl-L-methionine transport (loss)
c-value
0.0
0.1
0.2
0.3
0.4
0.5
0.6
0.7
0.8
p-value
2e-02
2e-02
3e-02
4e-02
5e-02
Log-odds
0.5
1.0
1.5
2.0
2.5
3.0
3.5
4.0
Cellular_organisms - ps1
Archaea1 - ps2
Archaea2 - ps3
Archaea3 - ps4
Asgard/Eukaryota - ps5
Eukaryota - ps6
Amorpheae - ps7
Obazoa - ps8
Opisthokonta - ps9
Holomycota - ps10
Fungi - ps11
Fungi_1 - ps12
Fungi_2 - ps13
Fungi_3 - ps14
Dikarya - ps15
Ascomycota - ps16
Saccharo/Pezizomycotina - ps17
Saccharomycotina - ps18
Saccharomycotina_1 - ps19
Saccharomycotina_2 - ps20
Saccharomycotina_3 - ps21
Phaff./Saccharomycetaceae - ps22
Saccharomyco./mycetaceae - ps23
Saccharomycetaceae - ps24
Saccharomycetaceae_2 - ps25
Saccharomycetaceae_3 - ps26
Kaz./Nau./Saccharomyces - ps27
Saccharomyces - ps28
Arboricola_clade - ps29
Cerevisiae_clade - ps30
Saccharomyces_cerevisiae - ps31
Phylostratum

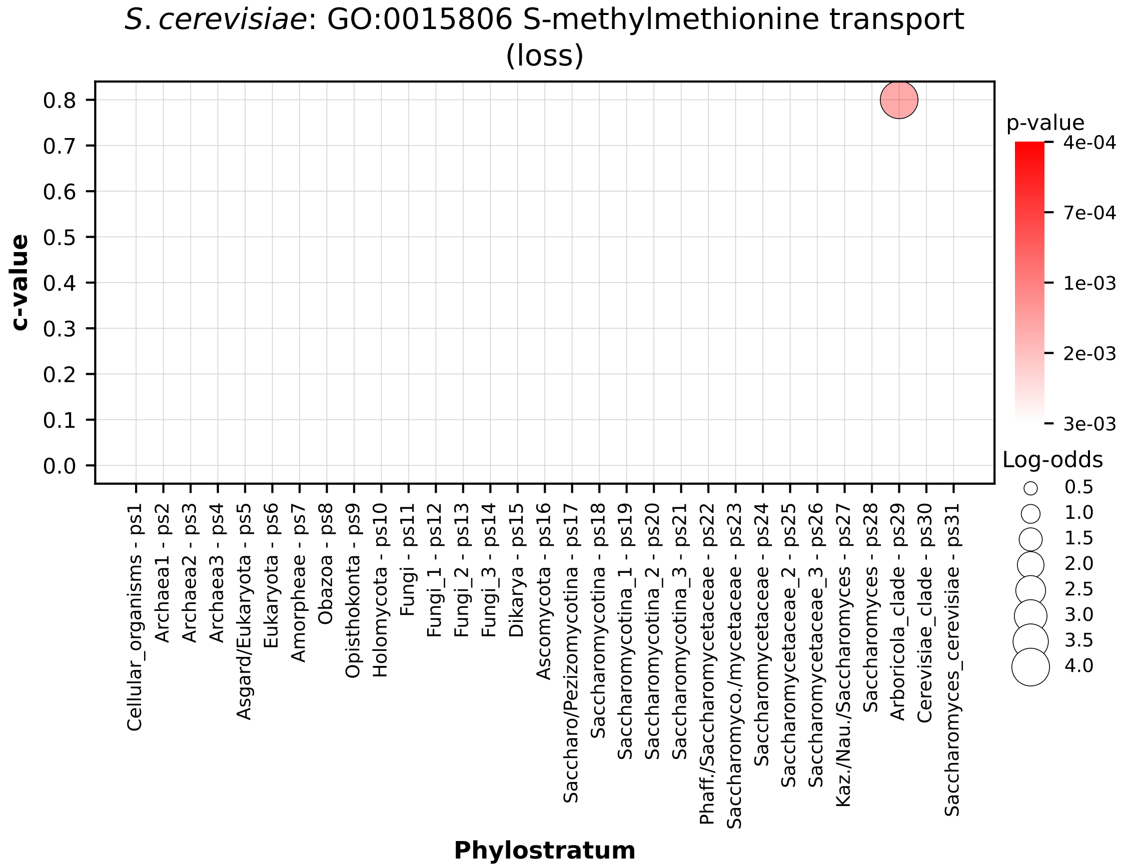
$S. cerevisiae$: GO:0015806 S-methylmethionine transport
(loss)
c-value
0.0
0.1
0.2
0.3
0.4
0.5
0.6
0.7
0.8
p-value
4e-04
7e-04
1e-03
2e-03
3e-03
Log-odds
0.5
1.0
1.5
2.0
2.5
3.0
3.5
4.0
Cellular_organisms - ps1
Archaea1 - ps2
Archaea2 - ps3
Archaea3 - ps4
Asgard/Eukaryota - ps5
Eukaryota - ps6
Amorpheae - ps7
Obazoa - ps8
Opisthokonta - ps9
Holomycota - ps10
Fungi - ps11
Fungi_1 - ps12
Fungi_2 - ps13
Fungi_3 - ps14
Dikarya - ps15
Ascomycota - ps16
Saccharo/Pezizomycotina - ps17
Saccharomycotina - ps18
Saccharomycotina_1 - ps19
Saccharomycotina_2 - ps20
Saccharomycotina_3 - ps21
Phaff./Saccharomycetaceae - ps22
Saccharomyco./mycetaceae - ps23
Saccharomycetaceae - ps24
Saccharomycetaceae_2 - ps25
Saccharomycetaceae_3 - ps26
Kaz./Nau./Saccharomyces - ps27
Saccharomyces - ps28
Arboricola_clade - ps29
Cerevisiae_clade - ps30
Saccharomyces_cerevisiae - ps31
Phylostratum

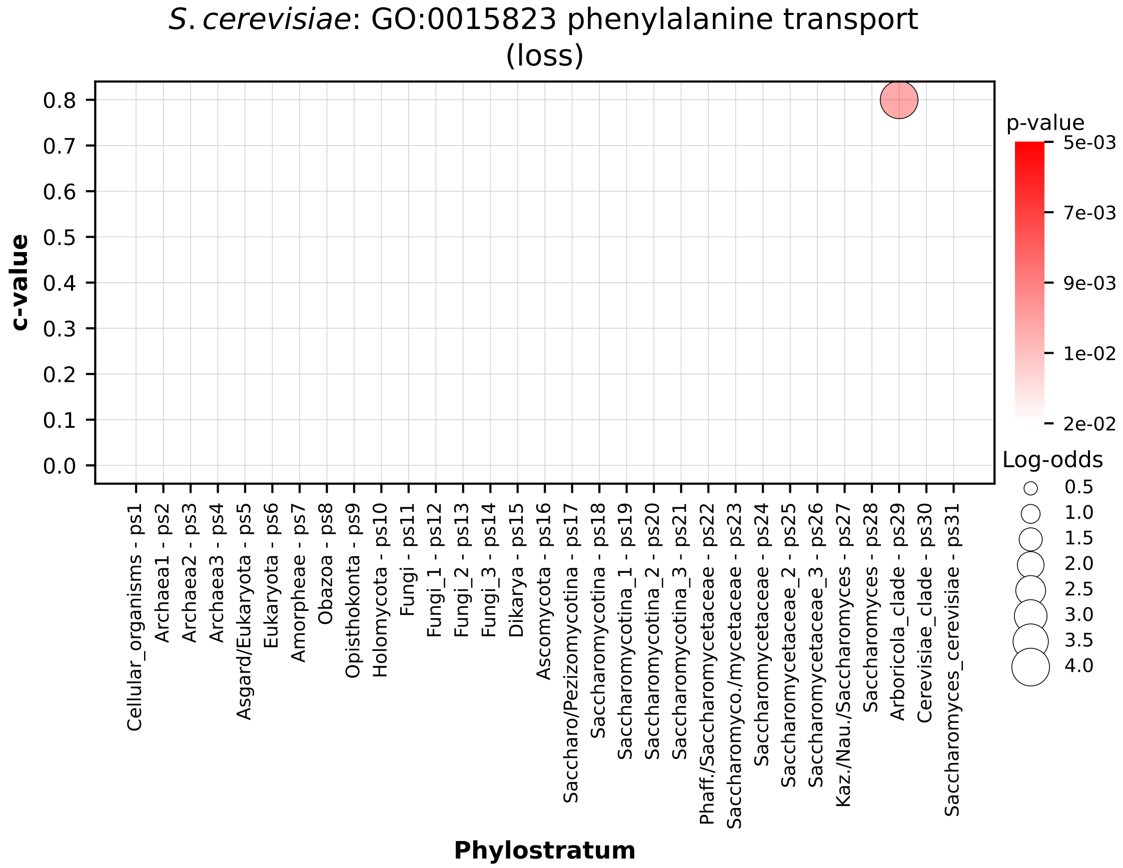
S. cerevisiae: GO:0015823 phenylalanine transport
(loss)
c-value
0.8
0.7
0.6
0.5
0.4
0.3
0.2
0.1
0.0
p-value
5e-03
7e-03
9e-03
1e-02
2e-02
Log-odds
0.5
1.0
1.5
2.0
2.5
3.0
3.5
4.0
Cellular_organisms - ps1
Archaea1 - ps2
Archaea2 - ps3
Archaea3 - ps4
Asgard/Eukaryota - ps5
Eukaryota - ps6
Amorpheae - ps7
Obazoa - ps8
Opisthokonta - ps9
Holomycota - ps10
Fungi - ps11
Fungi_1 - ps12
Fungi_2 - ps13
Fungi_3 - ps14
Dikarya - ps15
Ascomycota - ps16
Saccharo/Pezizomycotina - ps17
Saccharomycotina - ps18
Saccharomycotina_1 - ps19
Saccharomycotina_2 - ps20
Saccharomycotina_3 - ps21
Phaff./Saccharomycetaceae - ps22
Saccharomyco./mycetaceae - ps23
Saccharomycetaceae - ps24
Saccharomycetaceae_2 - ps25
Saccharomycetaceae_3 - ps26
Kaz./Nau./Saccharomyces - ps27
Saccharomyces - ps28
Arboricola_clade - ps29
Cerevisiae_clade - ps30
Saccharomyces_cerevisiae - ps31
Phylostratum

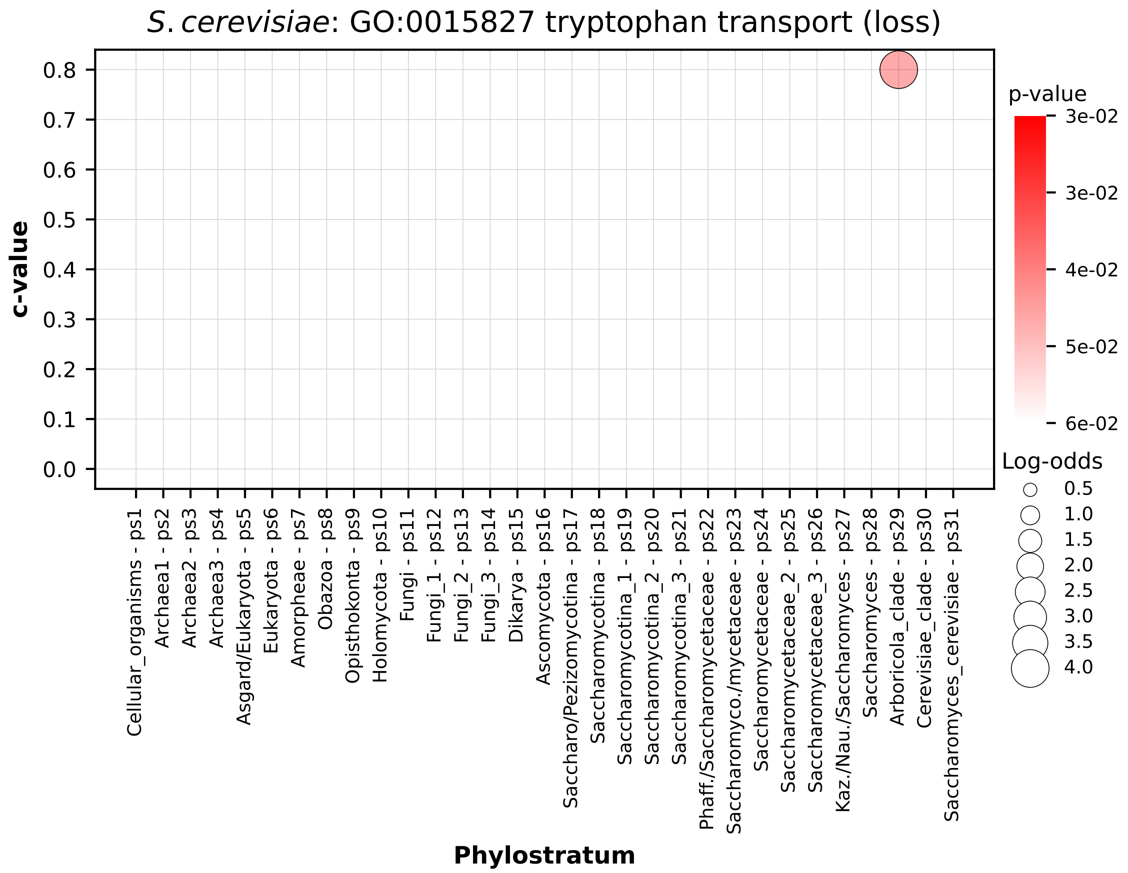
S. cerevisiae: GO:0015827 tryptophan transport (loss)
c-value
0.8
0.7
0.6
0.5
0.4
0.3
0.2
0.1
0.0
p-value
3e-02
3e-02
4e-02
5e-02
6e-02
Log-odds
0.5
1.0
1.5
2.0
2.5
3.0
3.5
4.0
Cellular_organisms - ps1
Archaea1 - ps2
Archaea2 - ps3
Archaea3 - ps4
Asgard/Eukaryota - ps5
Eukaryota - ps6
Amorpheae - ps7
Obazoa - ps8
Opisthokonta - ps9
Holomycota - ps10
Fungi - ps11
Fungi_1 - ps12
Fungi_2 - ps13
Fungi_3 - ps14
Dikarya - ps15
Ascomycota - ps16
Saccharo/Pezizomycotina - ps17
Saccharomycotina - ps18
Saccharomycotina_1 - ps19
Saccharomycotina_2 - ps20
Saccharomycotina_3 - ps21
Phaff./Saccharomycetaceae - ps22
Saccharomyco./mycetaceae - ps23
Saccharomycetaceae - ps24
Saccharomycetaceae_2 - ps25
Saccharomycetaceae_3 - ps26
Kaz./Nau./Saccharomyces - ps27
Saccharomyces - ps28
Arboricola_clade - ps29
Cerevisiae_clade - ps30
Saccharomyces_cerevisiae - ps31
Phylostratum

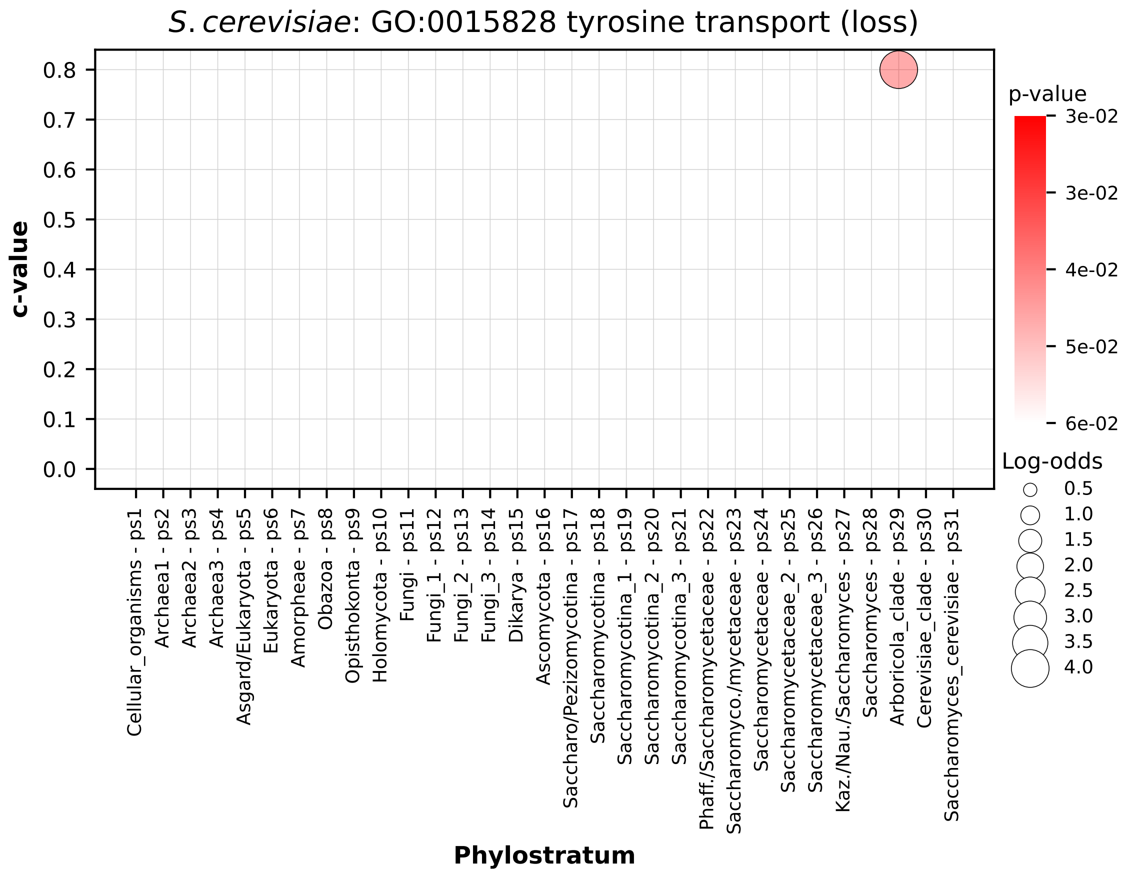

S. cerevisiae: GO:0015828 tyrosine transport (loss)
c-value
0.8
0.7
0.6
0.5
0.4
0.3
0.2
0.1
0.0
p-value
3e-02
3e-02
4e-02
5e-02
6e-02
Log-odds
0.5
1.0
1.5
2.0
2.5
3.0
3.5
4.0
Cellular_organisms - ps1
Archaea1 - ps2
Archaea2 - ps3
Archaea3 - ps4
Asgard/Eukaryota - ps5
Eukaryota - ps6
Amorpheae - ps7
Obazoa - ps8
Opisthokonta - ps9
Holomycota - ps10
Fungi - ps11
Fungi_1 - ps12
Fungi_2 - ps13
Fungi_3 - ps14
Dikarya - ps15
Ascomycota - ps16
Saccharo/Pezizomycotina - ps17
Saccharomycotina - ps18
Saccharomycotina_1 - ps19
Saccharomycotina_2 - ps20
Saccharomycotina_3 - ps21
Phaff./Saccharomycetaceae - ps22
Saccharomyco./mycetaceae - ps23
Saccharomycetaceae - ps24
Saccharomycetaceae_2 - ps25
Saccharomycetaceae_3 - ps26
Kaz./Nau./Saccharomyces - ps27
Saccharomyces - ps28
Arboricola_clade - ps29
Cerevisiae_clade - ps30
Saccharomyces_cerevisiae - ps31
Phylostratum

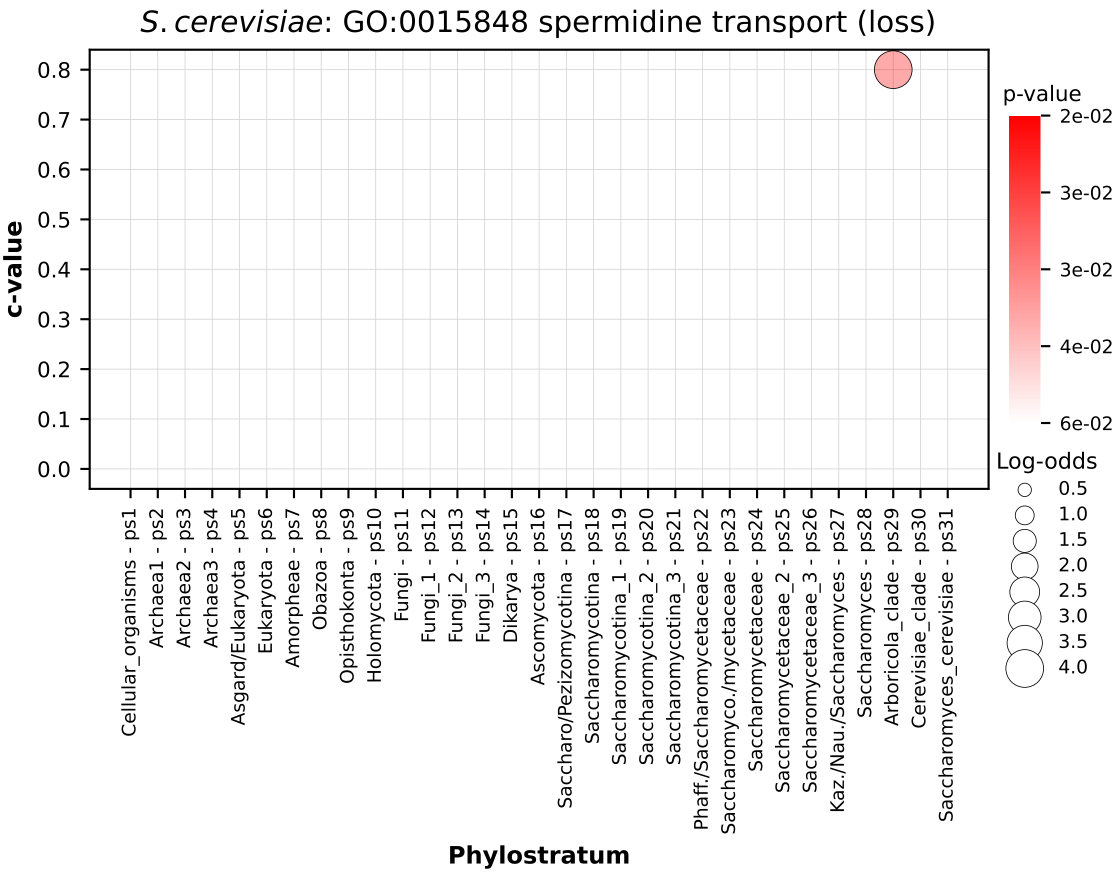
S. cerevisiae: GO:0015848 spermidine transport (loss)
c-value
0.0
0.1
0.2
0.3
0.4
0.5
0.6
0.7
0.8
p-value
2e-02
3e-02
3e-02
4e-02
6e-02
Log-odds
0.5
1.0
1.5
2.0
2.5
3.0
3.5
4.0
Cellular_organisms - ps1
Archaea1 - ps2
Archaea2 - ps3
Archaea3 - ps4
Asgard/Eukaryota - ps5
Eukaryota - ps6
Amorpheae - ps7
Obazoa - ps8
Opisthokonta - ps9
Holomycota - ps10
Fungi - ps11
Fungi_1 - ps12
Fungi_2 - ps13
Fungi_3 - ps14
Dikarya - ps15
Ascomycota - ps16
Saccharo/Pezizomycotina - ps17
Saccharomycotina - ps18
Saccharomycotina_1 - ps19
Saccharomycotina_2 - ps20
Saccharomycotina_3 - ps21
Phaff./Saccharomycetaceae - ps22
Saccharomyco./mycetaceae - ps23
Saccharomycetaceae - ps24
Saccharomycetaceae_2 - ps25
Saccharomycetaceae_3 - ps26
Kaz./Nau./Saccharomyces - ps27
Saccharomyces - ps28
Arboricola_clade - ps29
Cerevisiae_clade - ps30
Saccharomyces_cerevisiae - ps31
Phylostratum

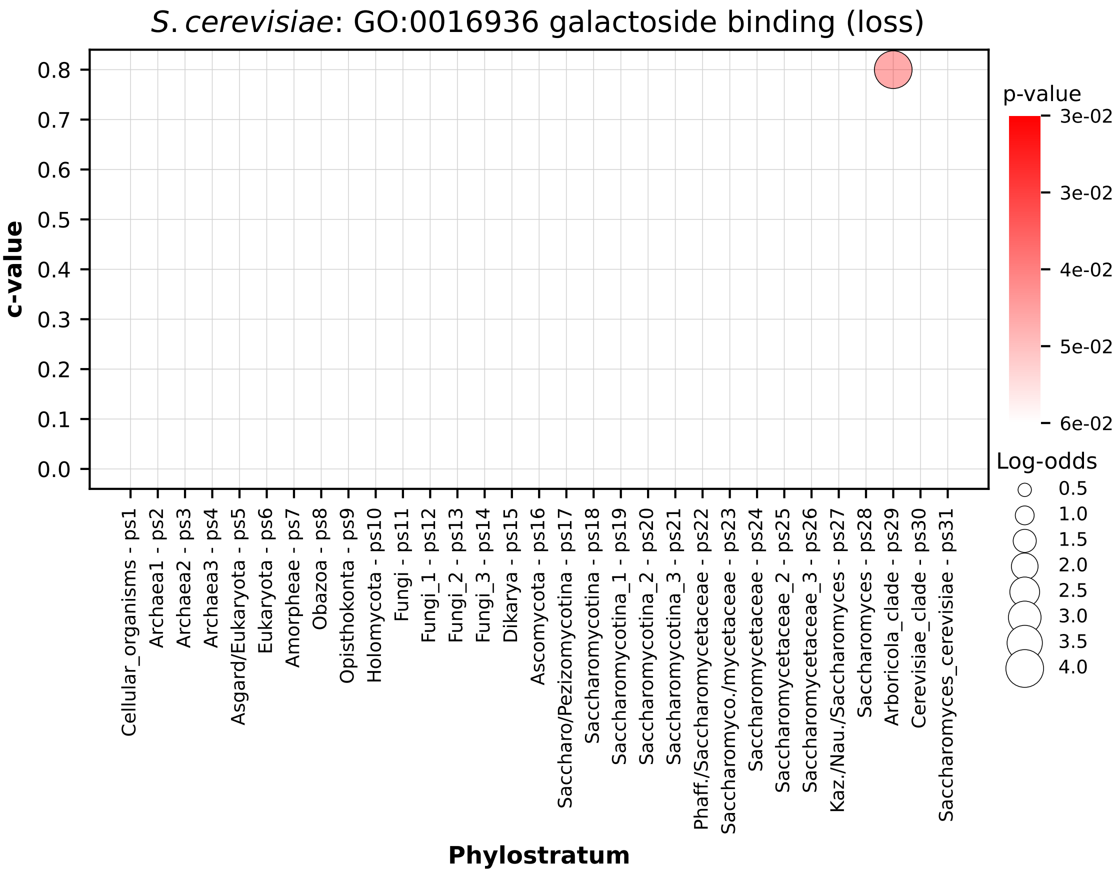
S. cerevisiae: GO:0016936 galactoside binding (loss)
c-value
0.8
0.7
0.6
0.5
0.4
0.3
0.2
0.1
0.0
p-value
3e-02
3e-02
4e-02
5e-02
6e-02
Log-odds
0.5
1.0
1.5
2.0
2.5
3.0
3.5
4.0
Cellular_organisms - ps1
Archaea1 - ps2
Archaea2 - ps3
Archaea3 - ps4
Asgard/Eukaryota - ps5
Eukaryota - ps6
Amorpheae - ps7
Obazoa - ps8
Opisthokonta - ps9
Holomycota - ps10
Fungi - ps11
Fungi_1 - ps12
Fungi_2 - ps13
Fungi_3 - ps14
Dikarya - ps15
Ascomycota - ps16
Saccharo/Pezizomycotina - ps17
Saccharomycotina - ps18
Saccharomycotina_1 - ps19
Saccharomycotina_2 - ps20
Saccharomycotina_3 - ps21
Phaff./Saccharomycetaceae - ps22
Saccharomyco./mycetaceae - ps23
Saccharomycetaceae - ps24
Saccharomycetaceae_2 - ps25
Saccharomycetaceae_3 - ps26
Kaz./Nau./Saccharomyces - ps27
Saccharomyces - ps28
Arboricola_clade - ps29
Cerevisiae_clade - ps30
Saccharomyces_cerevisiae - ps31
Phylostratum

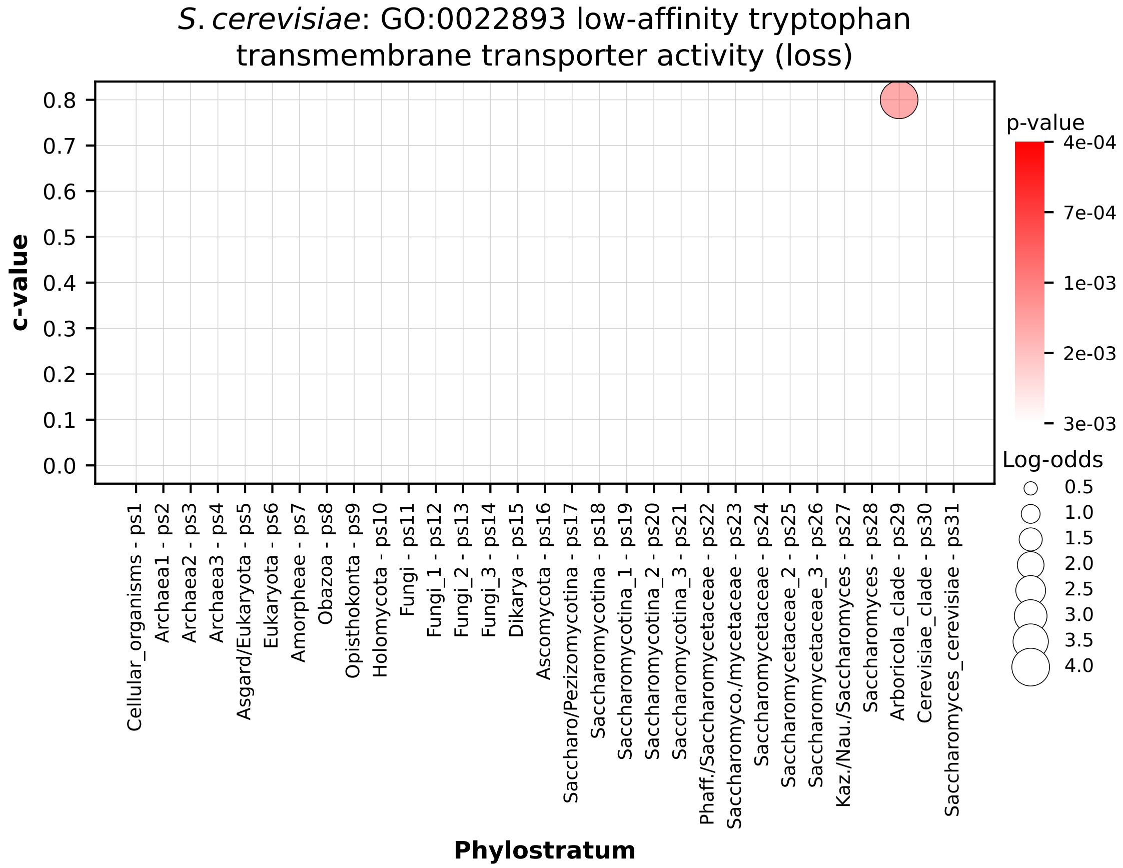

S. cerevisiae: GO:0022893 low-affinity tryptophan transmembrane transporter activity (loss)
c-value
0.8
0.7
0.6
0.5
0.4
0.3
0.2
0.1
0.0
p-value
4e-04
7e-04
1e-03
2e-03
3e-03
Log-odds
0.5
1.0
1.5
2.0
2.5
3.0
3.5
4.0
Cellular_organisms - ps1
Archaea1 - ps2
Archaea2 - ps3
Archaea3 - ps4
Asgard/Eukaryota - ps5
Eukaryota - ps6
Amorpheae - ps7
Obazoa - ps8
Opisthokonta - ps9
Holomycota - ps10
Fungi - ps11
Fungi_1 - ps12
Fungi_2 - ps13
Fungi_3 - ps14
Dikarya - ps15
Ascomycota - ps16
Saccharo/Pezizomycotina - ps17
Saccharomycotina - ps18
Saccharomycotina_1 - ps19
Saccharomycotina_2 - ps20
Saccharomycotina_3 - ps21
Phaff./Saccharomycetaceae - ps22
Saccharomyco./mycetaceae - ps23
Saccharomycetaceae - ps24
Saccharomycetaceae_2 - ps25
Saccharomycetaceae_3 - ps26
Kaz./Nau./Saccharomyces - ps27
Saccharomyces - ps28
Arboricola_clade - ps29
Cerevisiae_clade - ps30
Saccharomyces_cerevisiae - ps31
Phylostratum

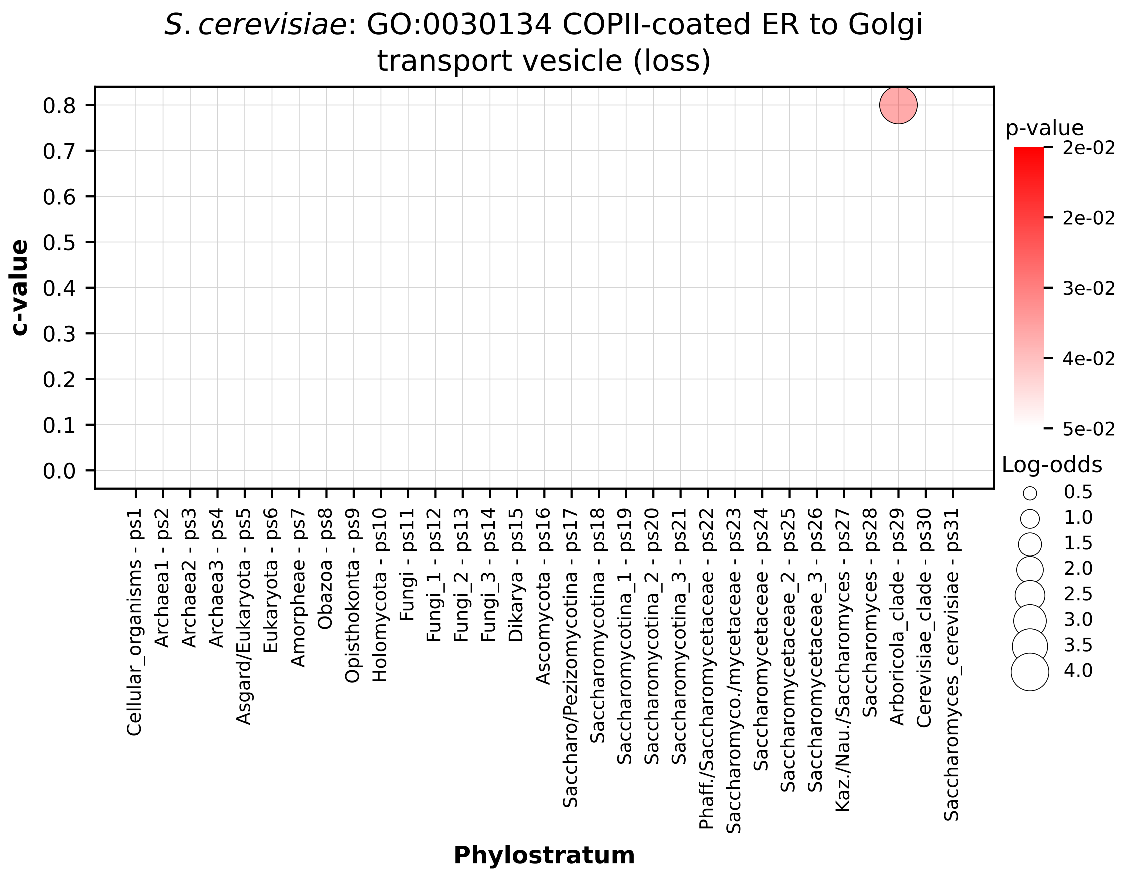
S. cerevisiae: GO:0030134 COPII-coated ER to Golgi transport vesicle (loss)
c-value
0.0
0.1
0.2
0.3
0.4
0.5
0.6
0.7
0.8
Phylostratum
Cellular_organisms - ps1
Archaea1 - ps2
Archaea2 - ps3
Archaea3 - ps4
Asgard/Eukaryota - ps5
Eukaryota - ps6
Amorpheae - ps7
Obazoa - ps8
Opisthokonta - ps9
Holomycota - ps10
Fungi - ps11
Fungi_1 - ps12
Fungi_2 - ps13
Fungi_3 - ps14
Dikarya - ps15
Ascomycota - ps16
Saccharo/Pezizomycotina - ps17
Saccharomycotina - ps18
Saccharomycotina_1 - ps19
Saccharomycotina_2 - ps20
Saccharomycotina_3 - ps21
Phaff./Saccharomycetaceae - ps22
Saccharomyco./mycetaceae - ps23
Saccharomycetaceae - ps24
Saccharomycetaceae_2 - ps25
Saccharomycetaceae_3 - ps26
Kaz./Nau./Saccharomyces - ps27
Saccharomyces - ps28
Arboricola_clade - ps29
Cerevisiae_clade - ps30
Saccharomyces_cerevisiae - ps31
p-value
2e-02
2e-02
3e-02
4e-02
5e-02
Log-odds
0.5
1.0
1.5
2.0
2.5
3.0
3.5
4.0

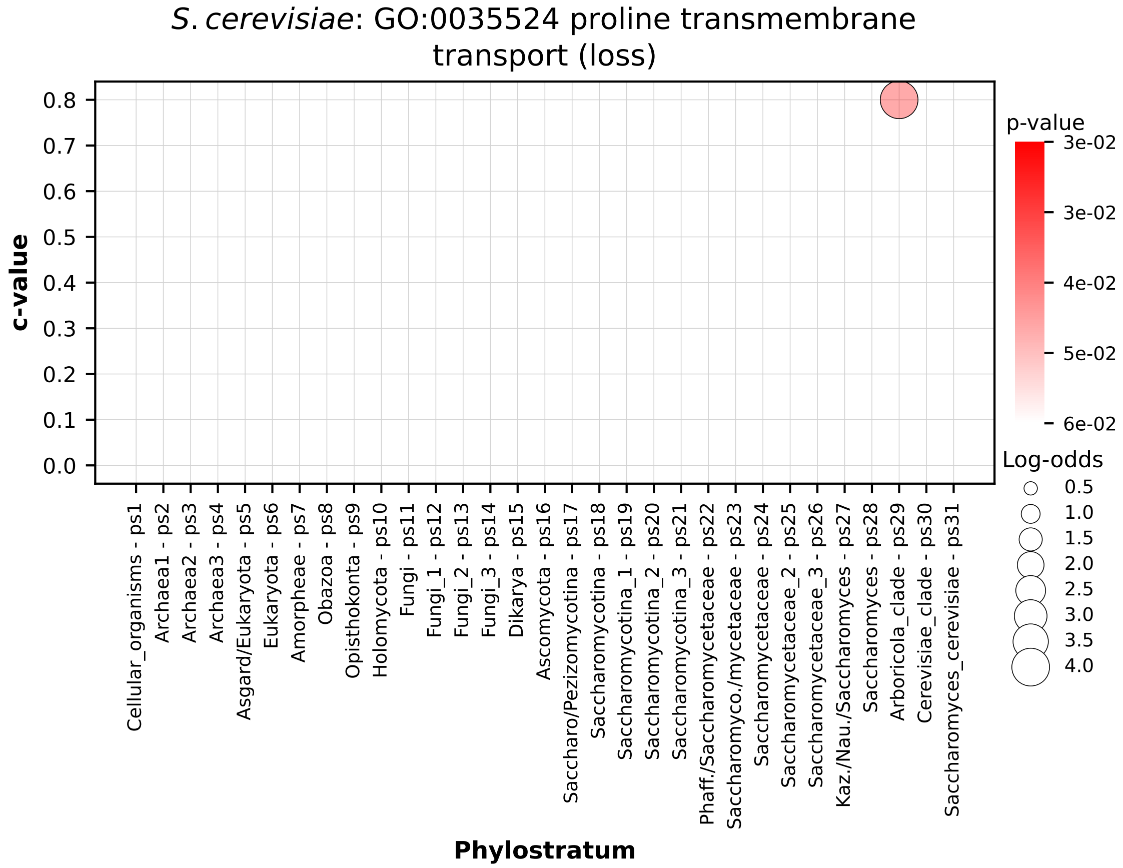

S. cerevisiae: GO:0035524 proline transmembrane transport (loss)
c-value
0.8
0.7
0.6
0.5
0.4
0.3
0.2
0.1
0.0
p-value
3e-02
3e-02
4e-02
5e-02
6e-02
Log-odds
0.5
1.0
1.5
2.0
2.5
3.0
3.5
4.0
Cellular_organisms - ps1
Archaea1 - ps2
Archaea2 - ps3
Archaea3 - ps4
Asgard/Eukaryota - ps5
Eukaryota - ps6
Amorpheae - ps7
Obazoa - ps8
Opisthokonta - ps9
Holomycota - ps10
Fungi - ps11
Fungi_1 - ps12
Fungi_2 - ps13
Fungi_3 - ps14
Dikarya - ps15
Ascomycota - ps16
Saccharo/Pezizomycotina - ps17
Saccharomycotina - ps18
Saccharomycotina_1 - ps19
Saccharomycotina_2 - ps20
Saccharomycotina_3 - ps21
Phaff./Saccharomycetaceae - ps22
Saccharomyco./mycetaceae - ps23
Saccharomycetaceae - ps24
Saccharomycetaceae_2 - ps25
Saccharomycetaceae_3 - ps26
Kaz./Nau./Saccharomyces - ps27
Saccharomyces - ps28
Arboricola_clade - ps29
Cerevisiae_clade - ps30
Saccharomyces_cerevisiae - ps31
Phylostratum

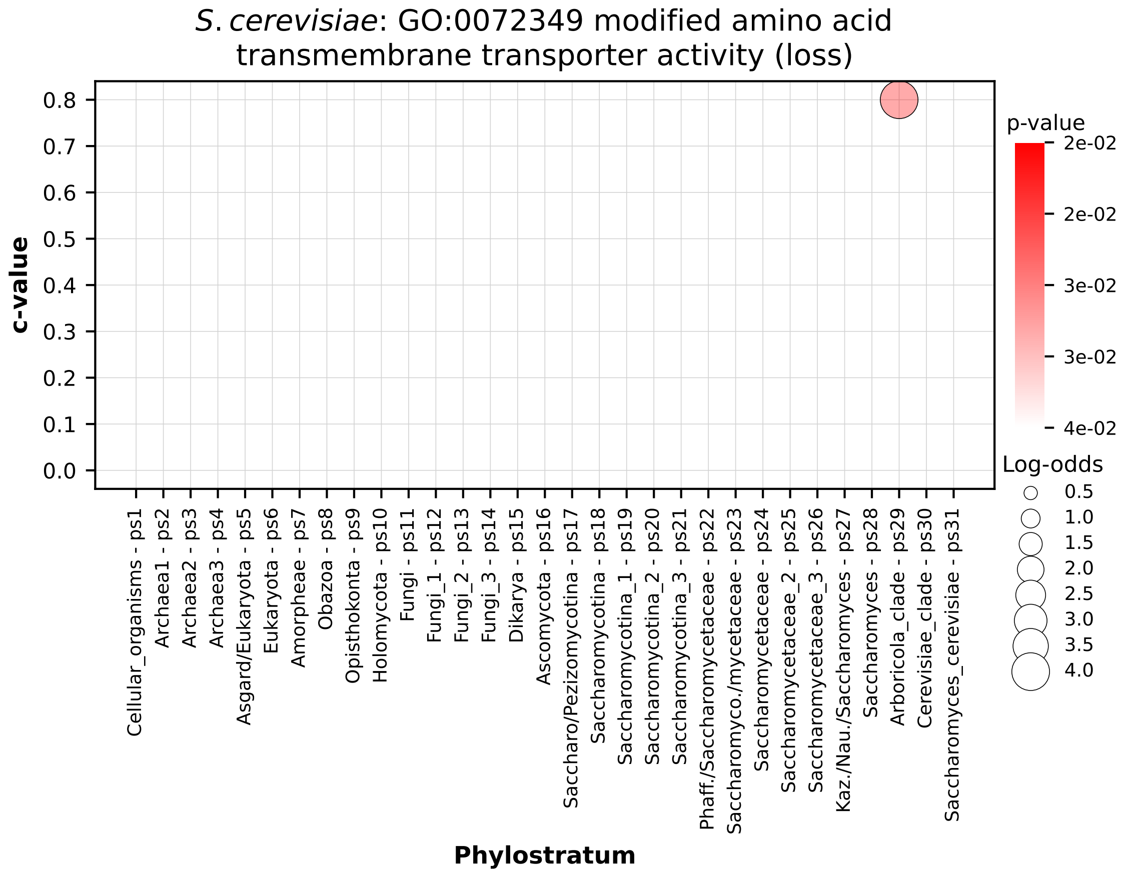

S. cerevisiae: GO:0072349 modified amino acid transmembrane transporter activity (loss)
c-value
0.8
0.7
0.6
0.5
0.4
0.3
0.2
0.1
0.0
p-value
2e-02
2e-02
3e-02
3e-02
4e-02
Log-odds
0.5
1.0
1.5
2.0
2.5
3.0
3.5
4.0
Cellular_organisms - ps1
Archaea1 - ps2
Archaea2 - ps3
Archaea3 - ps4
Asgard/Eukaryota - ps5
Eukaryota - ps6
Amorpheae - ps7
Obazoa - ps8
Opisthokonta - ps9
Holomycota - ps10
Fungi - ps11
Fungi_1 - ps12
Fungi_2 - ps13
Fungi_3 - ps14
Dikarya - ps15
Ascomycota - ps16
Saccharo/Pezizomycotina - ps17
Saccharomycotina - ps18
Saccharomycotina_1 - ps19
Saccharomycotina_2 - ps20
Saccharomycotina_3 - ps21
Phaff./Saccharomycetaceae - ps22
Saccharomyco./mycetaceae - ps23
Saccharomycetaceae - ps24
Saccharomycetaceae_2 - ps25
Saccharomycetaceae_3 - ps26
Kaz./Nau./Saccharomyces - ps27
Saccharomyces - ps28
Arboricola_clade - ps29
Cerevisiae_clade - ps30
Saccharomyces_cerevisiae - ps31
Phylostratum

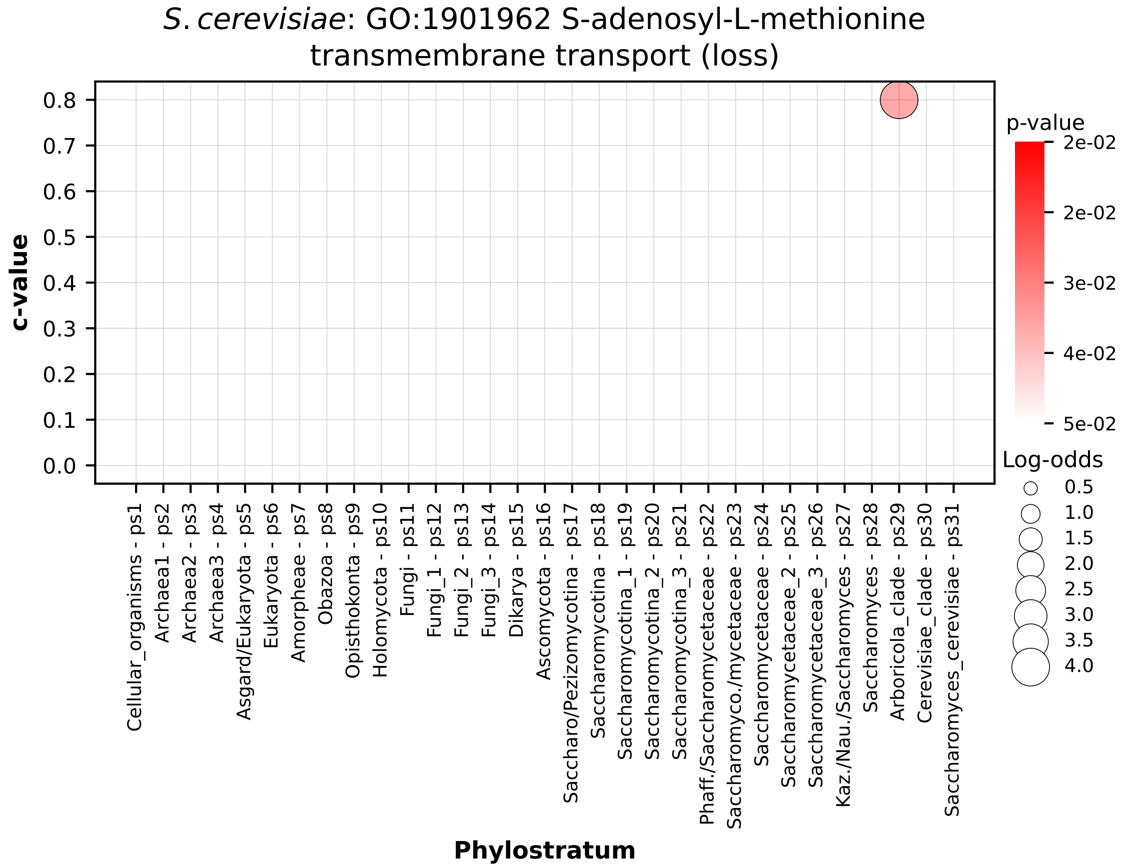
S. cerevisiae: GO:1901962 S-adenosyl-L-methionine transmembrane transport (loss)
c-value
0.0
0.1
0.2
0.3
0.4
0.5
0.6
0.7
0.8
p-value
2e-02
2e-02
3e-02
4e-02
5e-02
Log-odds
0.5
1.0
1.5
2.0
2.5
3.0
3.5
4.0
Cellular_organisms - ps1
Archaea1 - ps2
Archaea2 - ps3
Archaea3 - ps4
Asgard/Eukaryota - ps5
Eukaryota - ps6
Amorpheae - ps7
Obazoa - ps8
Opisthokonta - ps9
Holomycota - ps10
Fungi - ps11
Fungi_1 - ps12
Fungi_2 - ps13
Fungi_3 - ps14
Dikarya - ps15
Ascomycota - ps16
Saccharo/Pezizomycotina - ps17
Saccharomycotina - ps18
Saccharomycotina_1 - ps19
Saccharomycotina_2 - ps20
Saccharomycotina_3 - ps21
Phaff./Saccharomycetaceae - ps22
Saccharomyco./mycetaceae - ps23
Saccharomycetaceae - ps24
Saccharomycetaceae_2 - ps25
Saccharomycetaceae_3 - ps26
Kaz./Nau./Saccharomyces - ps27
Saccharomyces - ps28
Arboricola_clade - ps29
Cerevisiae_clade - ps30
Saccharomyces_cerevisiae - ps31
Phylostratum

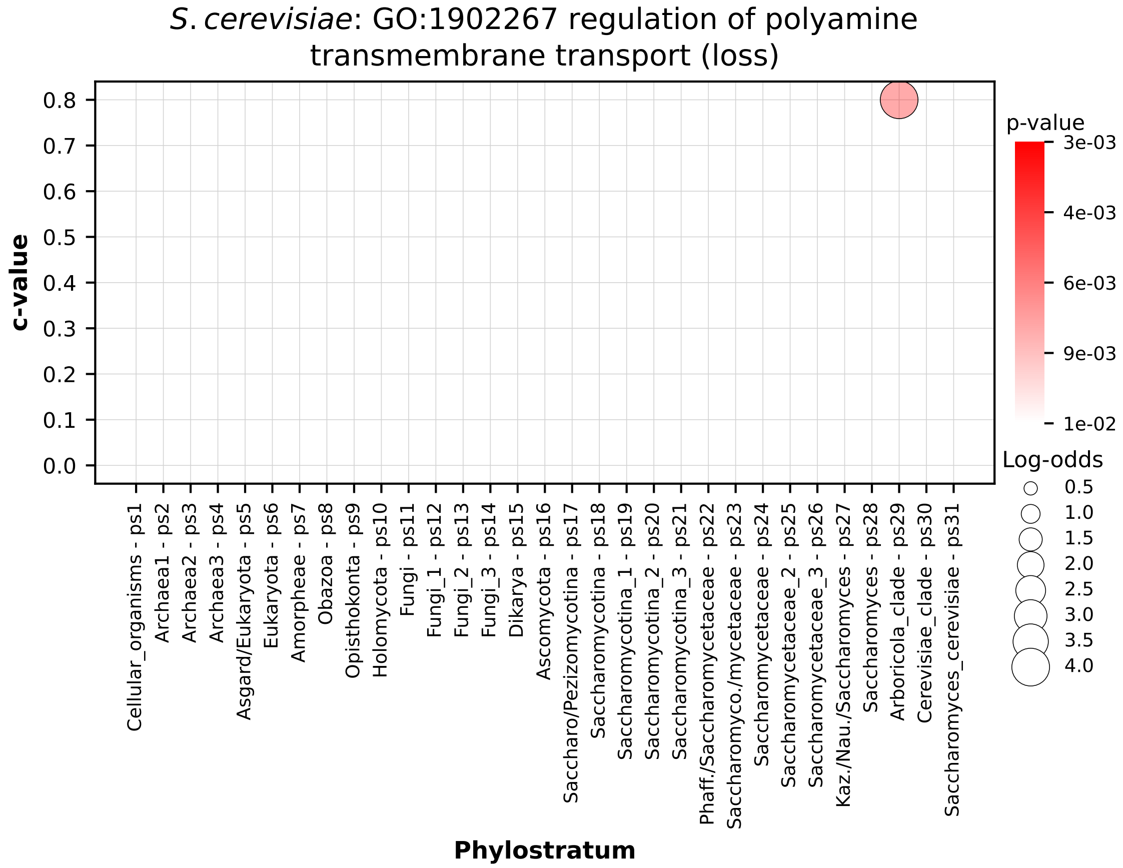
S. cerevisiae: GO:1902267 regulation of polyamine transmembrane transport (loss)
c-value
0.8
0.7
0.6
0.5
0.4
0.3
0.2
0.1
0.0
p-value
3e-03
4e-03
6e-03
9e-03
1e-02
Log-odds
0.5
1.0
1.5
2.0
2.5
3.0
3.5
4.0
Cellular_organisms - ps1
Archaea1 - ps2
Archaea2 - ps3
Archaea3 - ps4
Asgard/Eukaryota - ps5
Eukaryota - ps6
Amorpheae - ps7
Obazoa - ps8
Opisthokonta - ps9
Holomycota - ps10
Fungi - ps11
Fungi_1 - ps12
Fungi_2 - ps13
Fungi_3 - ps14
Dikarya - ps15
Ascomycota - ps16
Saccharo/Pezizomycotina - ps17
Saccharomycotina - ps18
Saccharomycotina_1 - ps19
Saccharomycotina_2 - ps20
Saccharomycotina_3 - ps21
Phaff./Saccharomycetaceae - ps22
Saccharomyco./mycetaceae - ps23
Saccharomycetaceae - ps24
Saccharomycetaceae_2 - ps25
Saccharomycetaceae_3 - ps26
Kaz./Nau./Saccharomyces - ps27
Saccharomyces - ps28
Arboricola_clade - ps29
Cerevisiae_clade - ps30
Saccharomyces_cerevisiae - ps31
Phylostratum

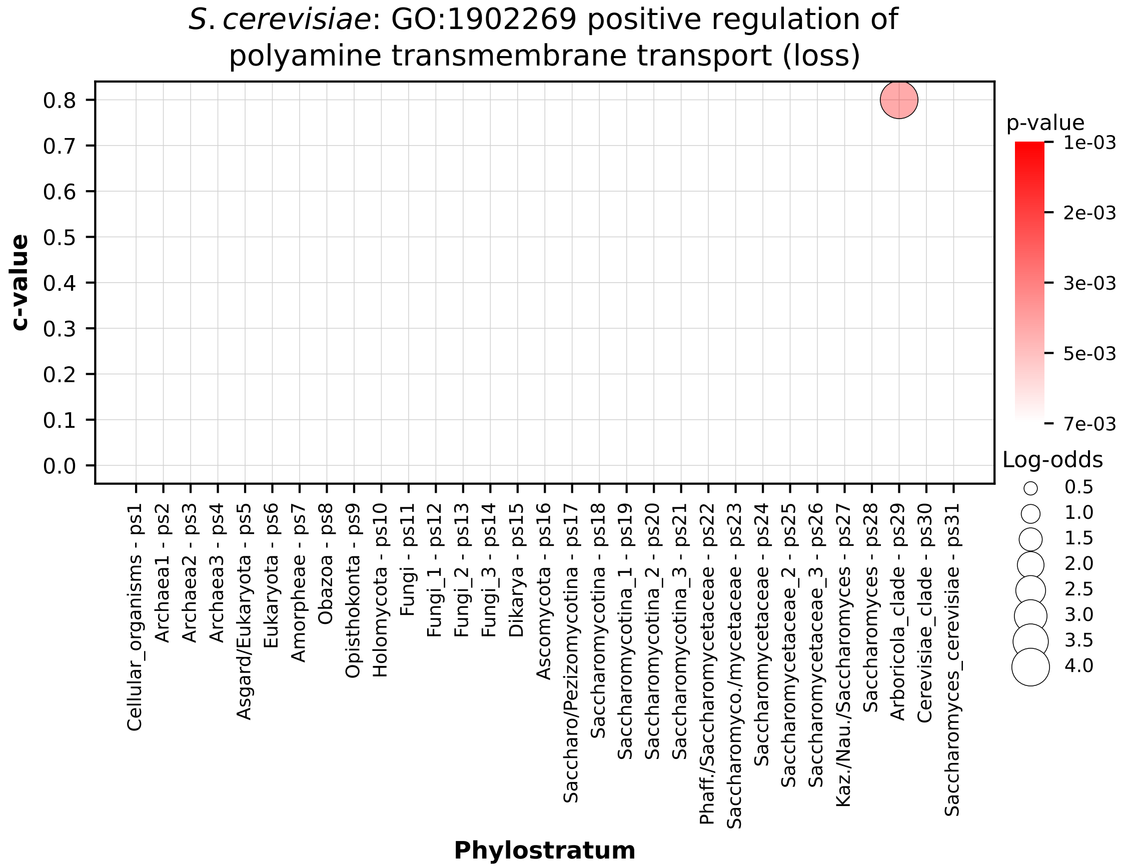
S. cerevisiae: GO:1902269 positive regulation of polyamine transmembrane transport (loss)
c-value
0.8
0.7
0.6
0.5
0.4
0.3
0.2
0.1
0.0
p-value
1e-03
2e-03
3e-03
5e-03
7e-03
Log-odds
0.5
1.0
1.5
2.0
2.5
3.0
3.5
4.0
Cellular_organisms - ps1
Archaea1 - ps2
Archaea2 - ps3
Archaea3 - ps4
Asgard/Eukaryota - ps5
Eukaryota - ps6
Amorpheae - ps7
Obazoa - ps8
Opisthokonta - ps9
Holomycota - ps10
Fungi - ps11
Fungi_1 - ps12
Fungi_2 - ps13
Fungi_3 - ps14
Dikarya - ps15
Ascomycota - ps16
Saccharo/Pezizomycotina - ps17
Saccharomycotina - ps18
Saccharomycotina_1 - ps19
Saccharomycotina_2 - ps20
Saccharomycotina_3 - ps21
Phaff./Saccharomycetaceae - ps22
Saccharomyco./mycetaceae - ps23
Saccharomycetaceae - ps24
Saccharomycetaceae_2 - ps25
Saccharomycetaceae_3 - ps26
Kaz./Nau./Saccharomyces - ps27
Saccharomyces - ps28
Arboricola_clade - ps29
Cerevisiae_clade - ps30
Saccharomyces_cerevisiae - ps31
Phylostratum

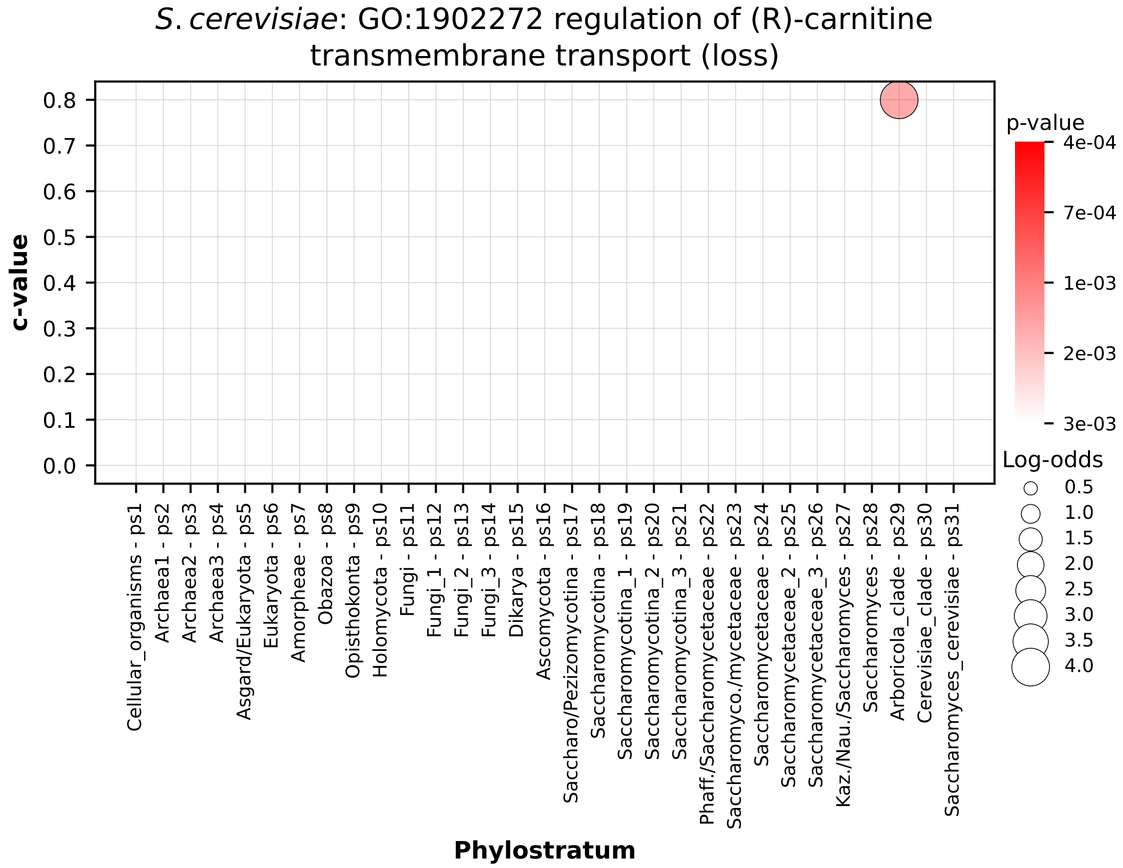
S. cerevisiae: GO:1902272 regulation of (R)-carnitine transmembrane transport (loss)
c-value
Phylostratum
p-value
4e-04
7e-04
1e-03
2e-03
3e-03
Log-odds
0.5
1.0
1.5
2.0
2.5
3.0
3.5
4.0
Cellular_organisms - ps1
Archaea1 - ps2
Archaea2 - ps3
Archaea3 - ps4
Asgard/Eukaryota - ps5
Eukaryota - ps6
Amorpheae - ps7
Obazoa - ps8
Opisthokonta - ps9
Holomycota - ps10
Fungi - ps11
Fungi_1 - ps12
Fungi_2 - ps13
Fungi_3 - ps14
Dikarya - ps15
Ascomycota - ps16
Saccharo/Pezizomycotina - ps17
Saccharomycotina - ps18
Saccharomycotina_1 - ps19
Saccharomycotina_2 - ps20
Saccharomycotina_3 - ps21
Phaff./Saccharomycetaceae - ps22
Saccharomyco./mycetaceae - ps23
Saccharomycetaceae - ps24
Saccharomycetaceae_2 - ps25
Saccharomycetaceae_3 - ps26
Kaz./Nau./Saccharomyces - ps27
Saccharomyces - ps28
Arboricola_clade - ps29
Cerevisiae_clade - ps30
Saccharomyces_cerevisiae - ps31

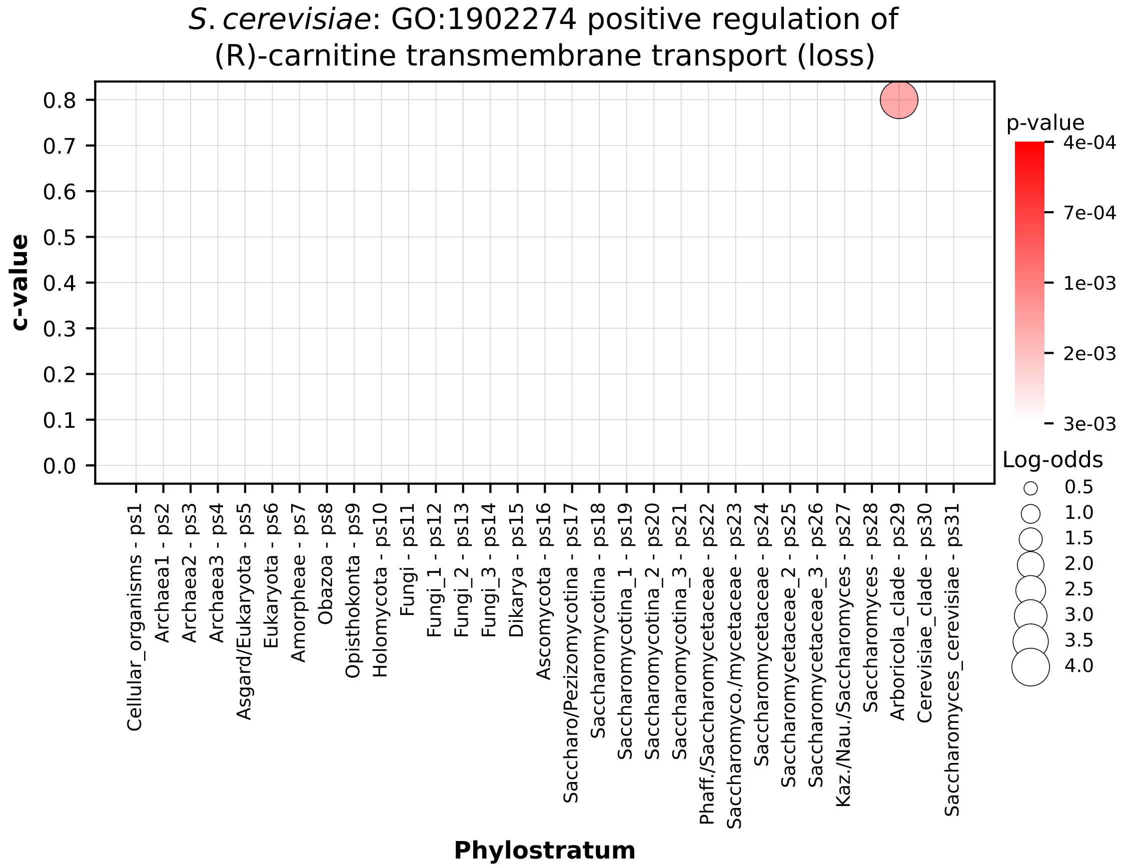
S. cerevisiae: GO:1902274 positive regulation of (R)-carnitine transmembrane transport (loss)
c-value
0.0
0.1
0.2
0.3
0.4
0.5
0.6
0.7
0.8
p-value
4e-04
7e-04
1e-03
2e-03
3e-03
Log-odds
0.5
1.0
1.5
2.0
2.5
3.0
3.5
4.0
Cellular_organisms - ps1
Archaea1 - ps2
Archaea2 - ps3
Archaea3 - ps4
Asgard/Eukaryota - ps5
Eukaryota - ps6
Amorpheae - ps7
Obazoa - ps8
Opisthokonta - ps9
Holomycota - ps10
Fungi - ps11
Fungi_1 - ps12
Fungi_2 - ps13
Fungi_3 - ps14
Dikarya - ps15
Ascomycota - ps16
Saccharo/Pezizomycotina - ps17
Saccharomycotina - ps18
Saccharomycotina_1 - ps19
Saccharomycotina_2 - ps20
Saccharomycotina_3 - ps21
Phaff./Saccharomycetaceae - ps22
Saccharomyco./mycetaceae - ps23
Saccharomycetaceae - ps24
Saccharomycetaceae_2 - ps25
Saccharomycetaceae_3 - ps26
Kaz./Nau./Saccharomyces - ps27
Saccharomyces - ps28
Arboricola_clade - ps29
Cerevisiae_clade - ps30
Saccharomyces_cerevisiae - ps31
Phylostratum

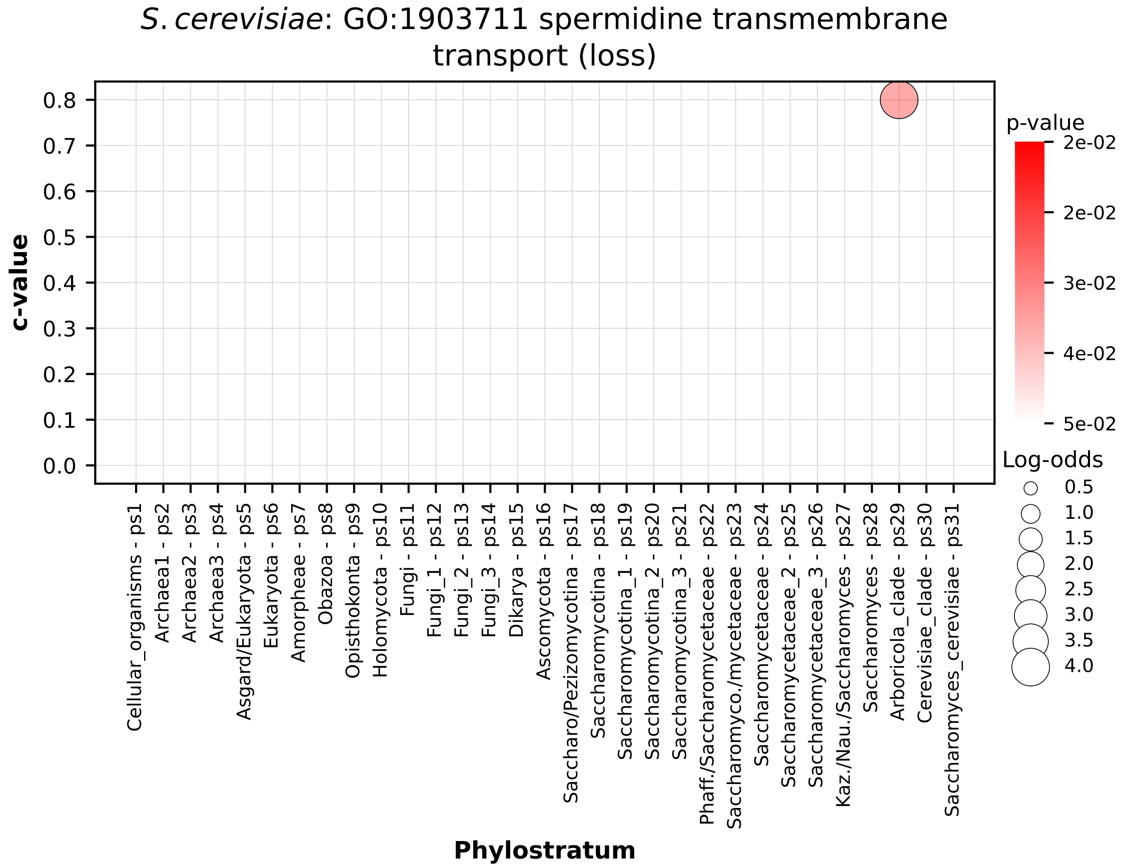

S. cerevisiae: GO:1903711 spermidine transmembrane transport (loss)
c-value
0.8
0.7
0.6
0.5
0.4
0.3
0.2
0.1
0.0
Phylostratum
Cellular_organisms - ps1
Archaea1 - ps2
Archaea2 - ps3
Archaea3 - ps4
Asgard/Eukaryota - ps5
Eukaryota - ps6
Amorpheae - ps7
Obazoa - ps8
Opisthokonta - ps9
Holomycota - ps10
Fungi - ps11
Fungi_1 - ps12
Fungi_2 - ps13
Fungi_3 - ps14
Dikarya - ps15
Ascomycota - ps16
Saccharo/Pezizomycotina - ps17
Saccharomycotina - ps18
Saccharomycotina_1 - ps19
Saccharomycotina_2 - ps20
Saccharomycotina_3 - ps21
Phaff./Saccharomycetaceae - ps22
Saccharomyco./mycetaceae - ps23
Saccharomycetaceae - ps24
Saccharomycetaceae_2 - ps25
Saccharomycetaceae_3 - ps26
Kaz./Nau./Saccharomyces - ps27
Saccharomyces - ps28
Arboricola_clade - ps29
Cerevisiae_clade - ps30
Saccharomyces_cerevisiae - ps31
p-value
2e-02
2e-02
3e-02
4e-02
5e-02
Log-odds
0.5
1.0
1.5
2.0
2.5
3.0
3.5
4.0

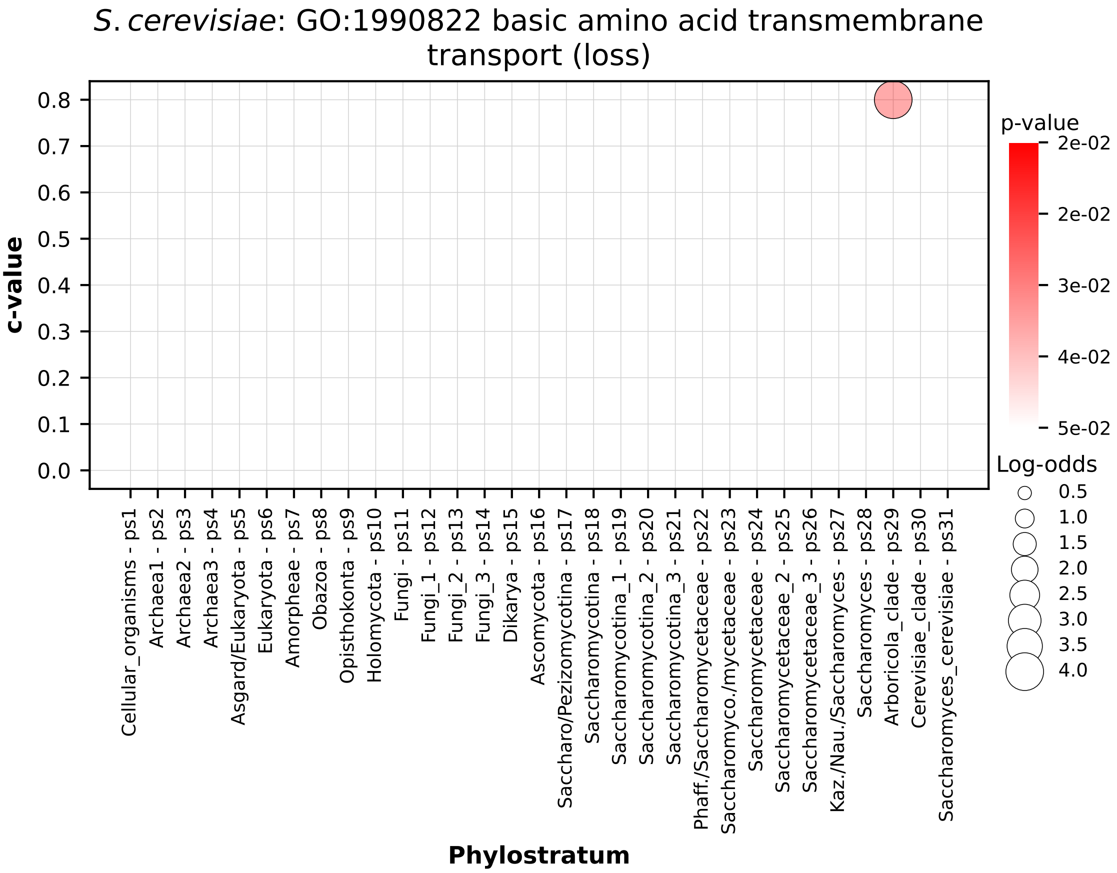

S. cerevisiae: GO:1990822 basic amino acid transmembrane transport (loss)
c-value
0.0
0.1
0.2
0.3
0.4
0.5
0.6
0.7
0.8
p-value
2e-02
2e-02
3e-02
4e-02
5e-02
Log-odds
0.5
1.0
1.5
2.0
2.5
3.0
3.5
4.0
Cellular_organisms - ps1
Archaea1 - ps2
Archaea2 - ps3
Archaea3 - ps4
Asgard/Eukaryota - ps5
Eukaryota - ps6
Amorpheae - ps7
Obazoa - ps8
Opisthokonta - ps9
Holomycota - ps10
Fungi - ps11
Fungi_1 - ps12
Fungi_2 - ps13
Fungi_3 - ps14
Dikarya - ps15
Ascomycota - ps16
Saccharo/Pezizomycotina - ps17
Saccharomycotina - ps18
Saccharomycotina_1 - ps19
Saccharomycotina_2 - ps20
Saccharomycotina_3 - ps21
Phaff./Saccharomycetaceae - ps22
Saccharomyco./mycetaceae - ps23
Saccharomycetaceae - ps24
Saccharomycetaceae_2 - ps25
Saccharomycetaceae_3 - ps26
Kaz./Nau./Saccharomyces - ps27
Saccharomyces - ps28
Arboricola_clade - ps29
Cerevisiae_clade - ps30
Saccharomyces_cerevisiae - ps31
Phylostratum
