# Supplementary material for: Macroevolutionary dynamics of gene family gain and loss along multicellular eukaryotic lineages
Source: Nat Commun. 2024 Mar 26;15:2663. doi: 10.1038/s41467-024-47017-w (PMC10966110; doi:10.1038/s41467-024-47017-w)
Supplement: Supplementary file 18 — Supplementary Dataset 13 [file 41467_2024_47017_MOESM18_ESM.pdf]

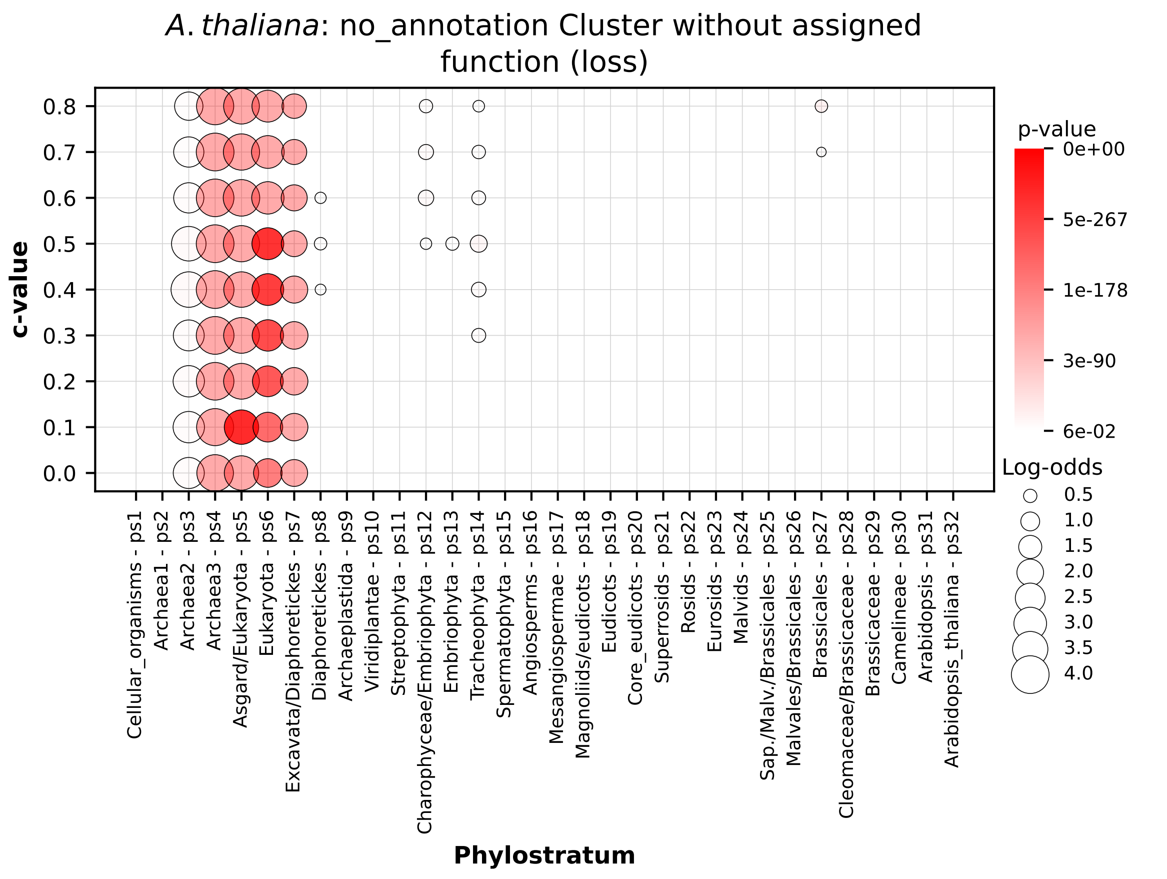

$A. thaliana$: no_annotation Cluster without assigned function (loss)
c-value
0.8
0.7
0.6
0.5
0.4
0.3
0.2
0.1
0.0
Phylostratum
Cellular_organisms - ps1
Archaea1 - ps2
Archaea2 - ps3
Archaea3 - ps4
Asgard/Eukaryota - ps5
Eukaryota - ps6
Excavata/Diaphoretickes - ps7
Diaphoretickes - ps8
Archaeplastida - ps9
Viridiplantae - ps10
Streptophyta - ps11
Charophyceae/Embriophyta - ps12
Embriophyta - ps13
Tracheophyta - ps14
Spermatophyta - ps15
Angiosperms - ps16
Mesangiospermae - ps17
Magnoliids/eudicots - ps18
Eudicots - ps19
Core_eudicots - ps20
Superrosids - ps21
Rosids - ps22
Eurosids - ps23
Malvids - ps24
Sap./Malv./Brassicales - ps25
Malvales/Brassicales - ps26
Brassicales - ps27
Cleomaceae/Brassicaceae - ps28
Brassicaceae - ps29
Camelineae - ps30
Arabidopsis - ps31
Arabidopsis_thaliana - ps32
p-value
0e+00
5e-267
1e-178
3e-90
6e-02
Log-odds
0.5
1.0
1.5
2.0
2.5
3.0
3.5
4.0

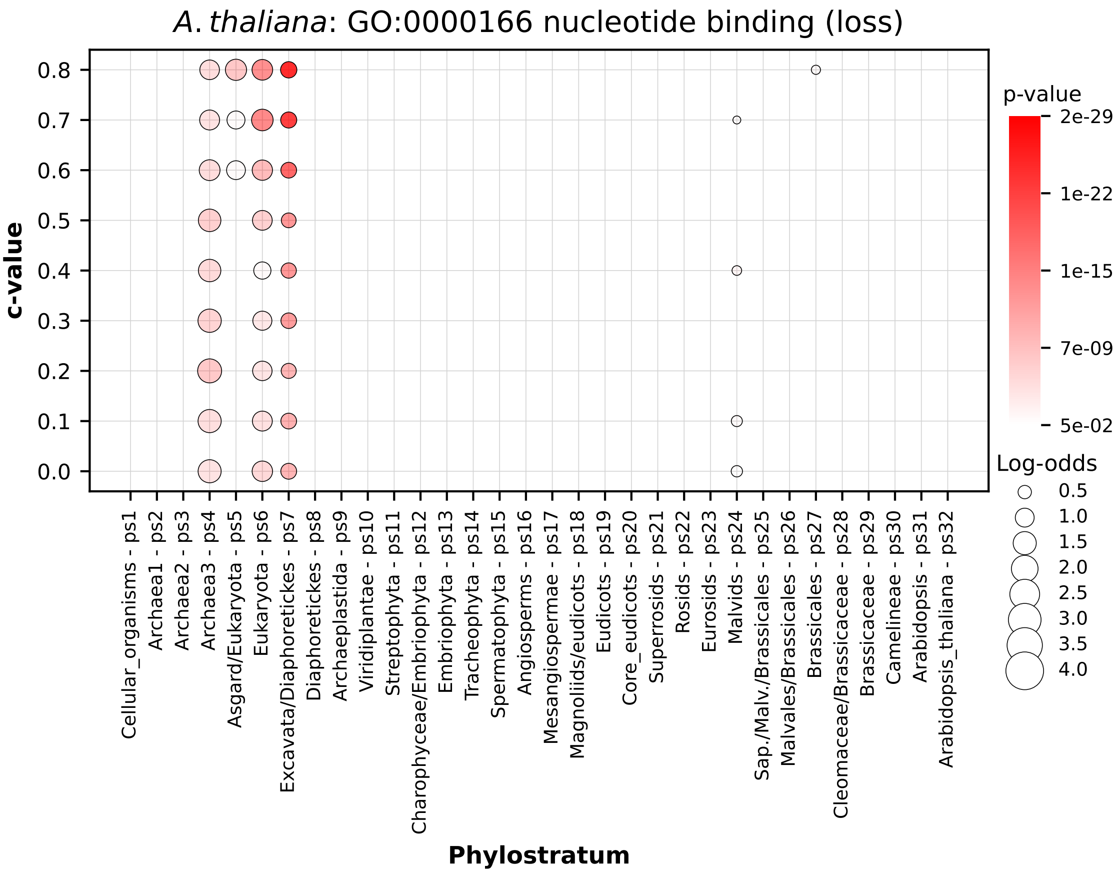

A. thaliana: GO:0000166 nucleotide binding (loss)
c-value
0.8
0.7
0.6
0.5
0.4
0.3
0.2
0.1
0.0
Phylostratum
Cellular_organisms - ps1
Archaea1 - ps2
Archaea2 - ps3
Archaea3 - ps4
Asgard/Eukaryota - ps5
Eukaryota - ps6
Excavata/Diaphoretickes - ps7
Diaphoretickes - ps8
Archaeplastida - ps9
Viridiplantae - ps10
Streptophyta - ps11
Charophyceae/Embriophyta - ps12
Embriophyta - ps13
Tracheophyta - ps14
Spermatophyta - ps15
Angiosperms - ps16
Mesangiospermae - ps17
Magnoliids/eudicots - ps18
Eudicots - ps19
Core_eudicots - ps20
Superrosids - ps21
Rosids - ps22
Eurosids - ps23
Malvids - ps24
Sap./Malv./Brassicales - ps25
Malvales/Brassicales - ps26
Brassicales - ps27
Cleomaceae/Brassicaceae - ps28
Brassicaceae - ps29
Camelineae - ps30
Arabidopsis - ps31
Arabidopsis_thaliana - ps32
p-value
2e-29
1e-22
1e-15
7e-09
5e-02
Log-odds
0.5
1.0
1.5
2.0
2.5
3.0
3.5
4.0

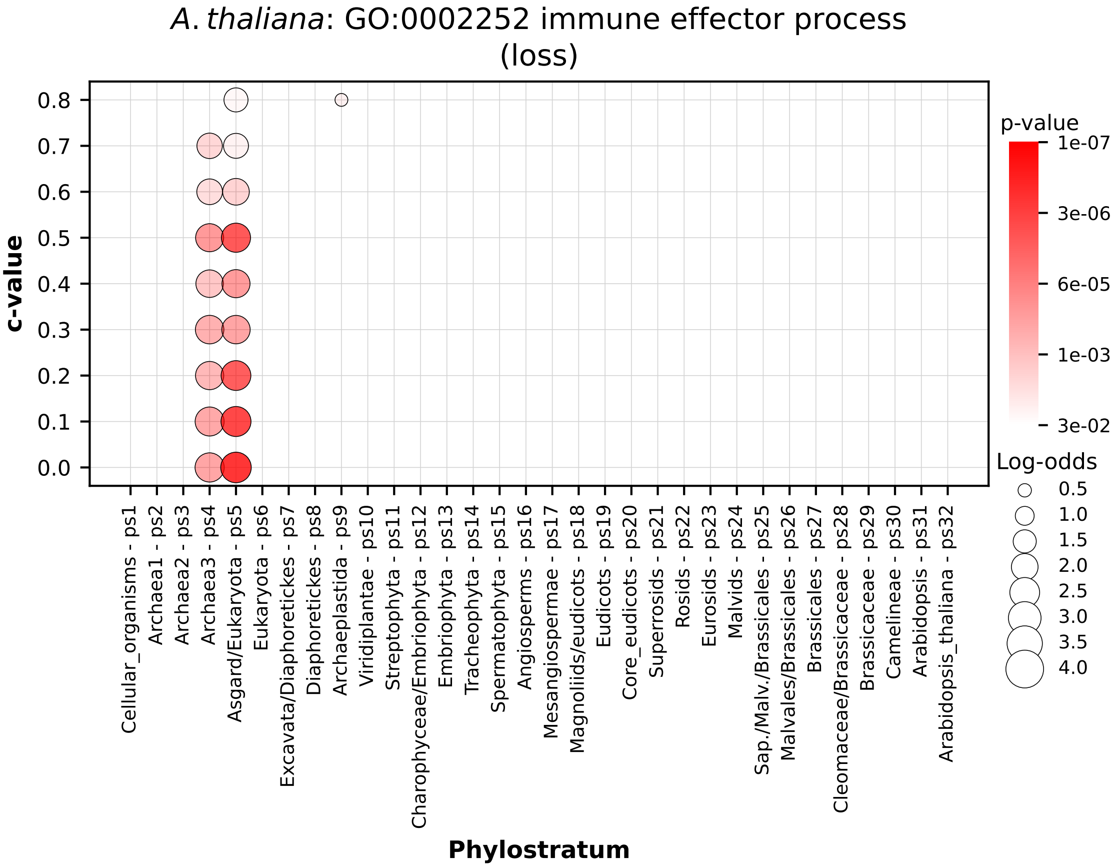
A. thaliana: GO:0002252 immune effector process
(loss)
c-value
0.8
0.7
0.6
0.5
0.4
0.3
0.2
0.1
0.0
p-value
1e-07
3e-06
6e-05
1e-03
3e-02
Log-odds
0.5
1.0
1.5
2.0
2.5
3.0
3.5
4.0
Cellular_organisms - ps1
Archaea1 - ps2
Archaea2 - ps3
Archaea3 - ps4
Asgard/Eukaryota - ps5
Eukaryota - ps6
Excavata/Diaphoretickes - ps7
Diaphoretickes - ps8
Archaeplastida - ps9
Viridiplantae - ps10
Streptophyta - ps11
Charophyceae/Embriophyta - ps12
Embriophyta - ps13
Tracheophyta - ps14
Spermatophyta - ps15
Angiosperms - ps16
Mesangiospermae - ps17
Magnoliids/eudicots - ps18
Eudicots - ps19
Core_eudicots - ps20
Superrosids - ps21
Rosids - ps22
Eurosids - ps23
Malvids - ps24
Sap./Malv./Brassicales - ps25
Malvales/Brassicales - ps26
Brassicales - ps27
Cleomaceae/Brassicaceae - ps28
Brassicaceae - ps29
Camelineae - ps30
Arabidopsis - ps31
Arabidopsis_thaliana - ps32
Phylostratum

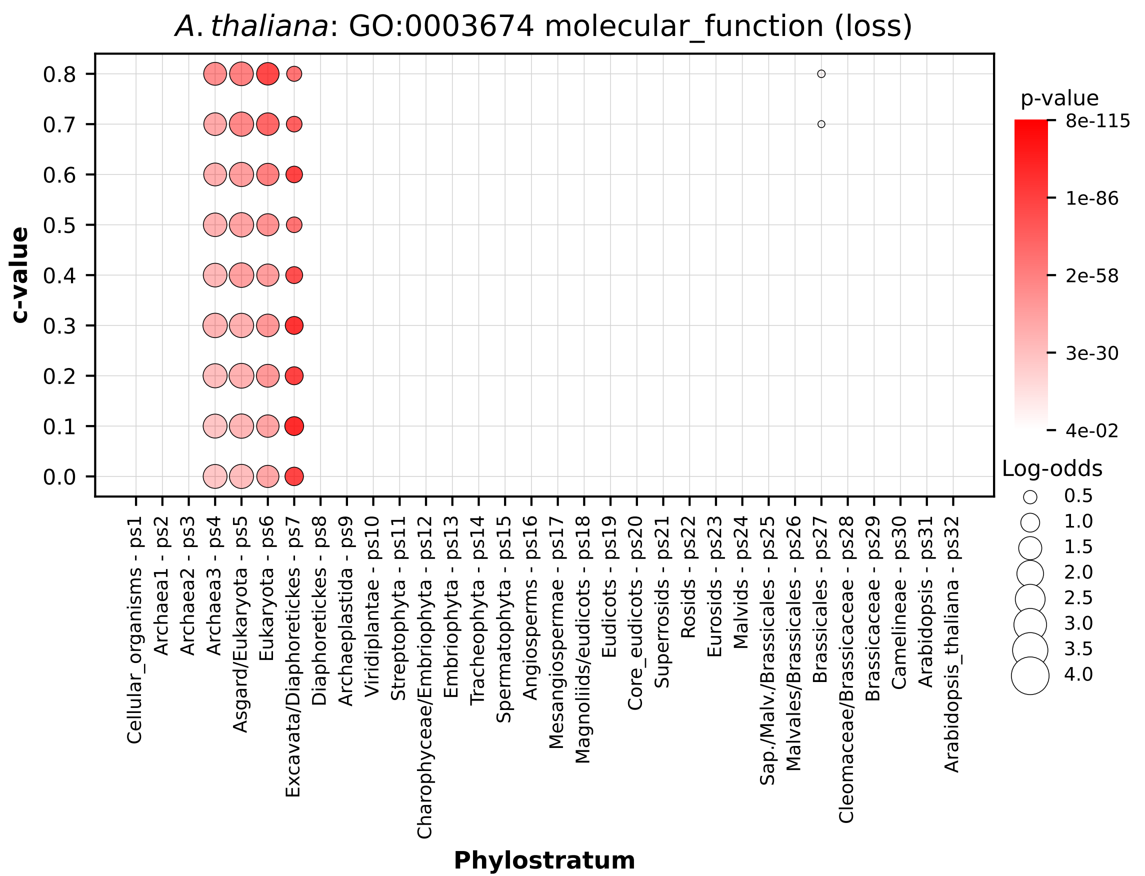

A. thaliana: GO:0003674 molecular_function (loss)
c-value
0.8
0.7
0.6
0.5
0.4
0.3
0.2
0.1
0.0
Phylostratum
Cellular_organisms - ps1
Archaea1 - ps2
Archaea2 - ps3
Archaea3 - ps4
Asgard/Eukaryota - ps5
Eukaryota - ps6
Excavata/Diaphoretickes - ps7
Diaphoretickes - ps8
Archaeplastida - ps9
Viridiplantae - ps10
Streptophyta - ps11
Charophyceae/Embriophyta - ps12
Embriophyta - ps13
Tracheophyta - ps14
Spermatophyta - ps15
Angiosperms - ps16
Mesangiospermae - ps17
Magnoliids/eudicots - ps18
Eudicots - ps19
Core_eudicots - ps20
Superrosids - ps21
Rosids - ps22
Eurosids - ps23
Malvids - ps24
Sap./Malv./Brassicales - ps25
Malvales/Brassicales - ps26
Brassicales - ps27
Cleomaceae/Brassicaceae - ps28
Brassicaceae - ps29
Camelineae - ps30
Arabidopsis - ps31
Arabidopsis_thaliana - ps32
p-value
8e-115
1e-86
2e-58
3e-30
4e-02
Log-odds
0.5
1.0
1.5
2.0
2.5
3.0
3.5
4.0

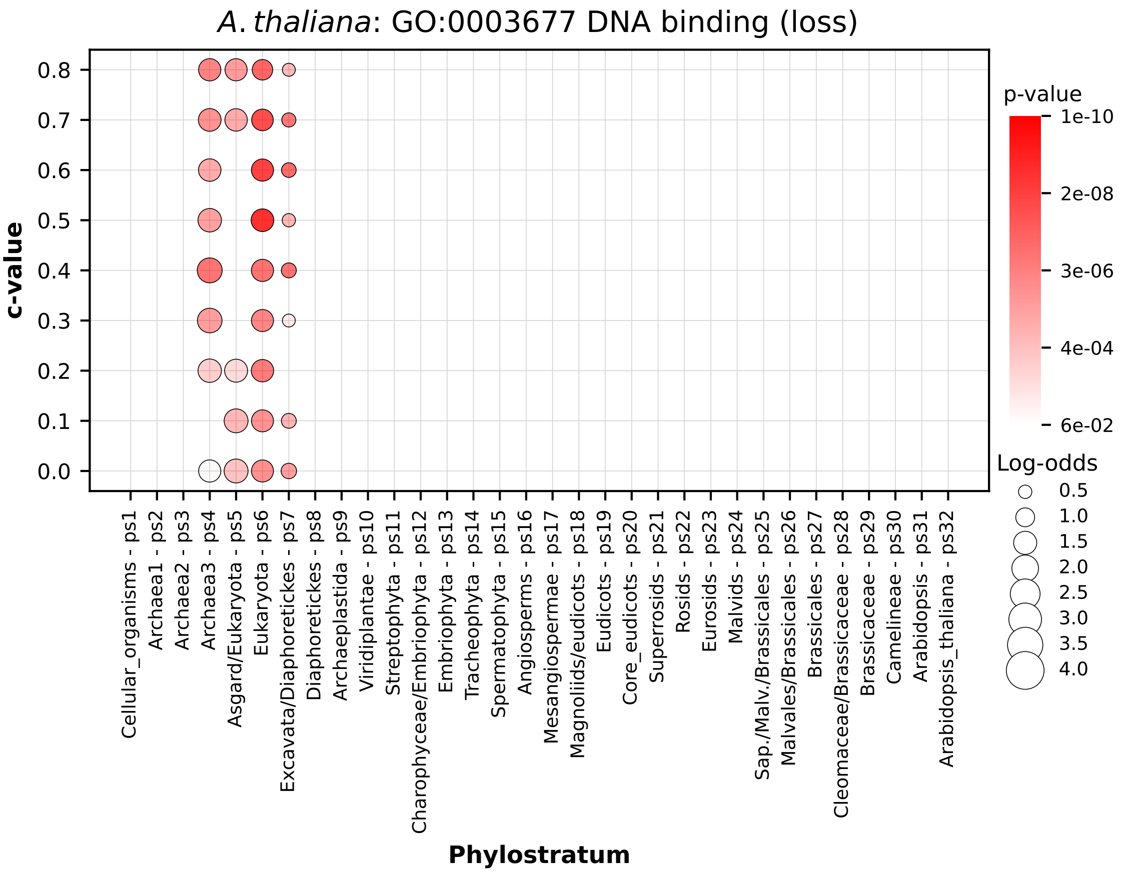
A. thaliana: GO:0003677 DNA binding (loss)
c-value
0.8
0.7
0.6
0.5
0.4
0.3
0.2
0.1
0.0
Phylostratum
Cellular_organisms - ps1
Archaea1 - ps2
Archaea2 - ps3
Archaea3 - ps4
Asgard/Eukaryota - ps5
Eukaryota - ps6
Excavata/Diaphoretickes - ps7
Diaphoretickes - ps8
Archaeplastida - ps9
Viridiplantae - ps10
Streptophyta - ps11
Charophyceae/Embriophyta - ps12
Embriophyta - ps13
Tracheophyta - ps14
Spermatophyta - ps15
Angiosperms - ps16
Mesangiospermae - ps17
Magnoliids/eudicots - ps18
Eudicots - ps19
Core_eudicots - ps20
Superrosids - ps21
Rosids - ps22
Eurosids - ps23
Malvids - ps24
Sap./Malv./Brassicales - ps25
Malvales/Brassicales - ps26
Brassicales - ps27
Cleomaceae/Brassicaceae - ps28
Brassicaceae - ps29
Camelineae - ps30
Arabidopsis - ps31
Arabidopsis_thaliana - ps32
p-value
1e-10
2e-08
3e-06
4e-04
6e-02
Log-odds
0.5
1.0
1.5
2.0
2.5
3.0
3.5
4.0

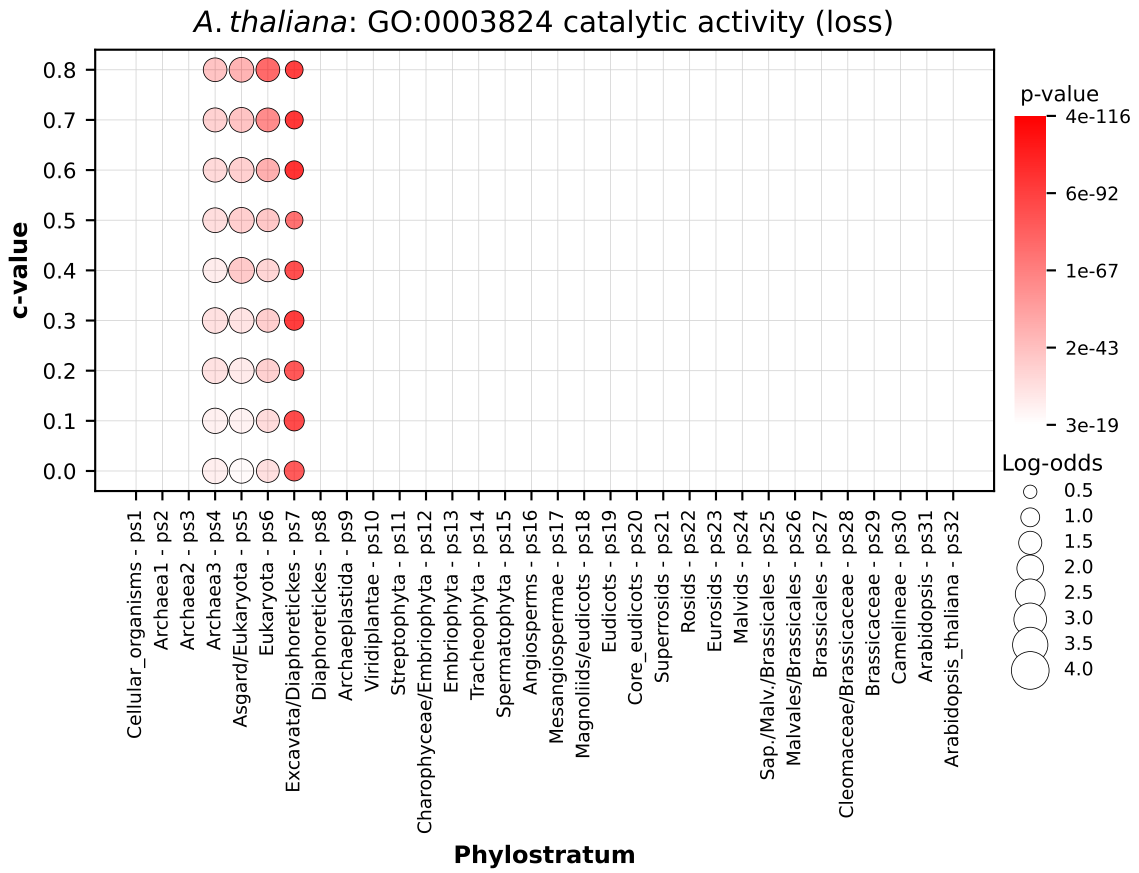

A. thaliana: GO:0003824 catalytic activity (loss)
c-value
0.8
0.7
0.6
0.5
0.4
0.3
0.2
0.1
0.0
Phylostratum
Cellular_organisms - ps1
Archaea1 - ps2
Archaea2 - ps3
Archaea3 - ps4
Asgard/Eukaryota - ps5
Eukaryota - ps6
Excavata/Diaphoretickes - ps7
Diaphoretickes - ps8
Archaeplastida - ps9
Viridiplantae - ps10
Streptophyta - ps11
Charophyceae/Embriophyta - ps12
Embriophyta - ps13
Tracheophyta - ps14
Spermatophyta - ps15
Angiosperms - ps16
Mesangiospermae - ps17
Magnoliids/eudicots - ps18
Eudicots - ps19
Core_eudicots - ps20
Superrosids - ps21
Rosids - ps22
Eurosids - ps23
Malvids - ps24
Sap./Malv./Brassicales - ps25
Malvales/Brassicales - ps26
Brassicales - ps27
Cleomaceae/Brassicaceae - ps28
Brassicaceae - ps29
Camelineae - ps30
Arabidopsis - ps31
Arabidopsis_thaliana - ps32
p-value
4e-116
6e-92
1e-67
2e-43
3e-19
Log-odds
0.5
1.0
1.5
2.0
2.5
3.0
3.5
4.0

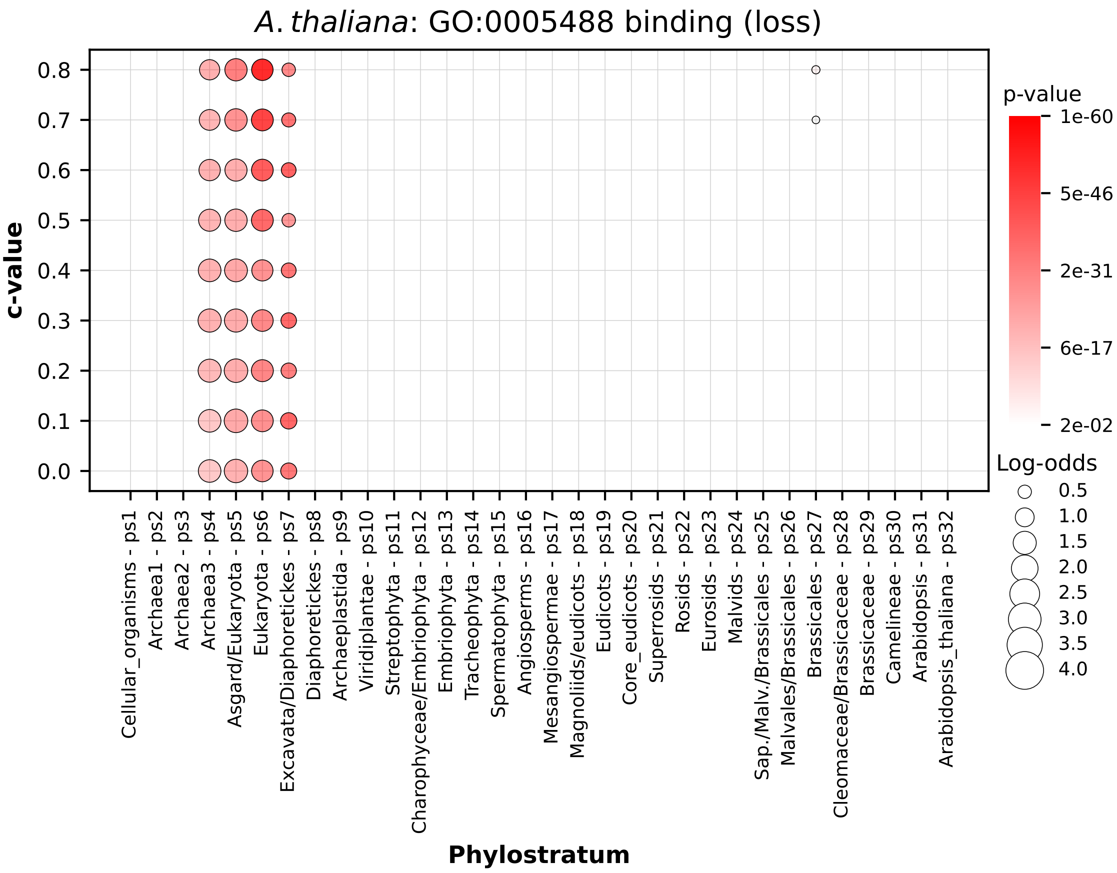
A. thaliana: GO:0005488 binding (loss)
c-value
0.0
0.1
0.2
0.3
0.4
0.5
0.6
0.7
0.8
Phylostratum
Cellular_organisms - ps1
Archaea1 - ps2
Archaea2 - ps3
Archaea3 - ps4
Asgard/Eukaryota - ps5
Eukaryota - ps6
Excavata/Diaphoretickes - ps7
Diaphoretickes - ps8
Archaeplastida - ps9
Viridiplantae - ps10
Streptophyta - ps11
Charophyceae/Embriophyta - ps12
Embriophyta - ps13
Tracheophyta - ps14
Spermatophyta - ps15
Angiosperms - ps16
Mesangiospermae - ps17
Magnoliids/eudicots - ps18
Eudicots - ps19
Core_eudicots - ps20
Superrosids - ps21
Rosids - ps22
Eurosids - ps23
Malvids - ps24
Sap./Malv./Brassicales - ps25
Malvales/Brassicales - ps26
Brassicales - ps27
Cleomaceae/Brassicaceae - ps28
Brassicaceae - ps29
Camelineae - ps30
Arabidopsis - ps31
Arabidopsis_thaliana - ps32
p-value
1e-60
5e-46
2e-31
6e-17
2e-02
Log-odds
0.5
1.0
1.5
2.0
2.5
3.0
3.5
4.0

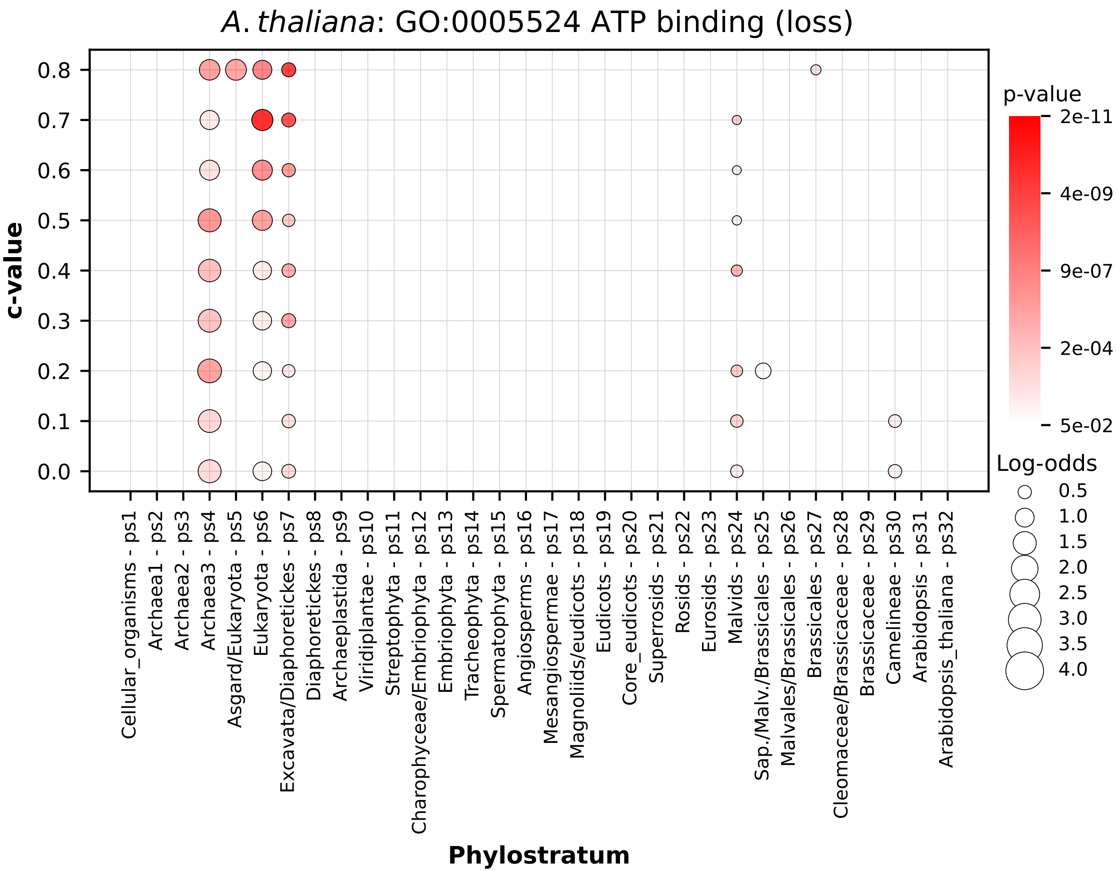

A. thaliana: GO:0005524 ATP binding (loss)
c-value
0.8
0.7
0.6
0.5
0.4
0.3
0.2
0.1
0.0
Phylostratum
Cellular_organisms - ps1
Archaea1 - ps2
Archaea2 - ps3
Archaea3 - ps4
Asgard/Eukaryota - ps5
Eukaryota - ps6
Excavata/Diaphoretickes - ps7
Diaphoretickes - ps8
Archaeplastida - ps9
Viridiplantae - ps10
Streptophyta - ps11
Charophyceae/Embriophyta - ps12
Embriophyta - ps13
Tracheophyta - ps14
Spermatophyta - ps15
Angiosperms - ps16
Mesangiospermae - ps17
Magnoliids/eudicots - ps18
Eudicots - ps19
Core_eudicots - ps20
Superrosids - ps21
Rosids - ps22
Eurosids - ps23
Malvids - ps24
Sap./Malv./Brassicales - ps25
Malvales/Brassicales - ps26
Brassicales - ps27
Cleomaceae/Brassicaceae - ps28
Brassicaceae - ps29
Camelineae - ps30
Arabidopsis - ps31
Arabidopsis_thaliana - ps32
p-value
2e-11
4e-09
9e-07
2e-04
5e-02
Log-odds
0.5
1.0
1.5
2.0
2.5
3.0
3.5
4.0

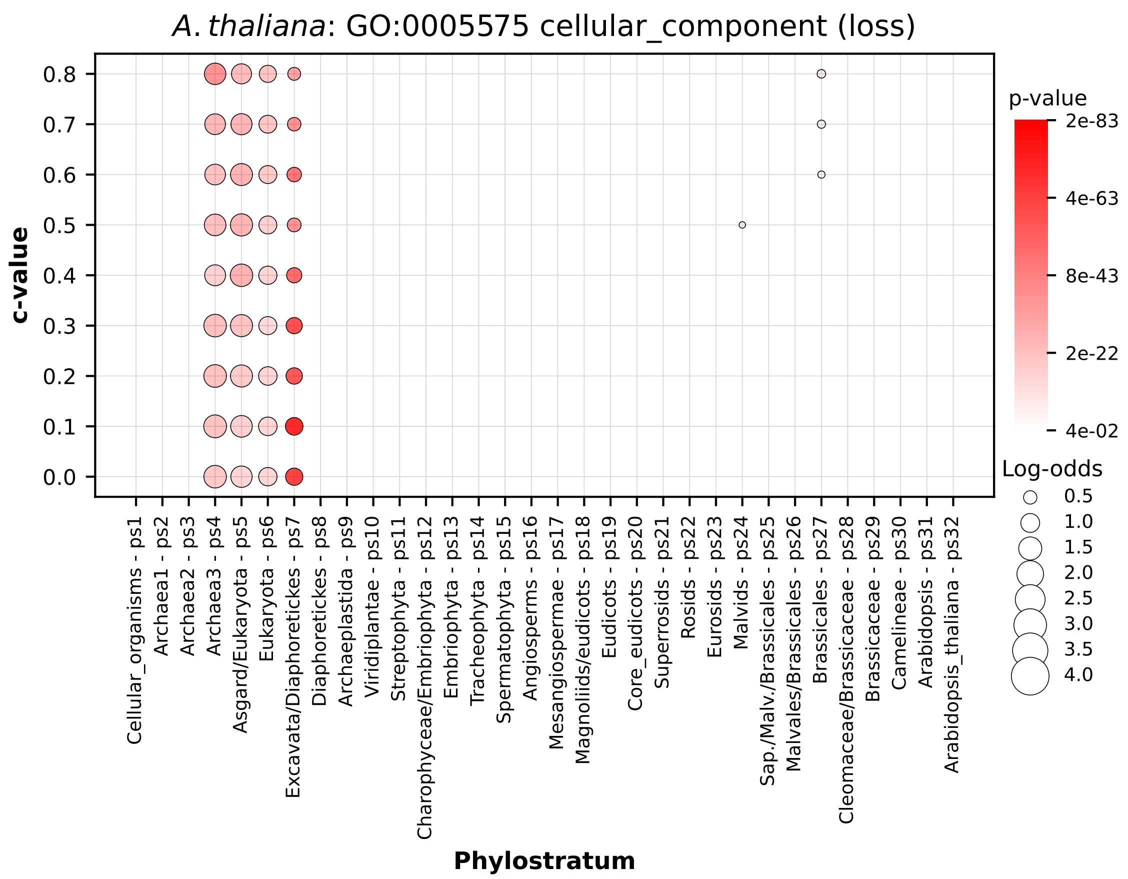
A. thaliana: GO:0005575 cellular_component (loss)
c-value
0.8
0.7
0.6
0.5
0.4
0.3
0.2
0.1
0.0
Phylostratum
Cellular_organisms - ps1
Archaea1 - ps2
Archaea2 - ps3
Archaea3 - ps4
Asgard/Eukaryota - ps5
Eukaryota - ps6
Excavata/Diaphoretickes - ps7
Diaphoretickes - ps8
Archaeplastida - ps9
Viridiplantae - ps10
Streptophyta - ps11
Charophyceae/Embriophyta - ps12
Embriophyta - ps13
Tracheophyta - ps14
Spermatophyta - ps15
Angiosperms - ps16
Mesangiospermae - ps17
Magnoliids/eudicots - ps18
Eudicots - ps19
Core_eudicots - ps20
Superrosids - ps21
Rosids - ps22
Eurosids - ps23
Malvids - ps24
Sap./Malv./Brassicales - ps25
Malvales/Brassicales - ps26
Brassicales - ps27
Cleomaceae/Brassicaceae - ps28
Brassicaceae - ps29
Camelineae - ps30
Arabidopsis - ps31
Arabidopsis_thaliana - ps32
p-value
2e-83
4e-63
8e-43
2e-22
4e-02
Log-odds
0.5
1.0
1.5
2.0
2.5
3.0
3.5
4.0

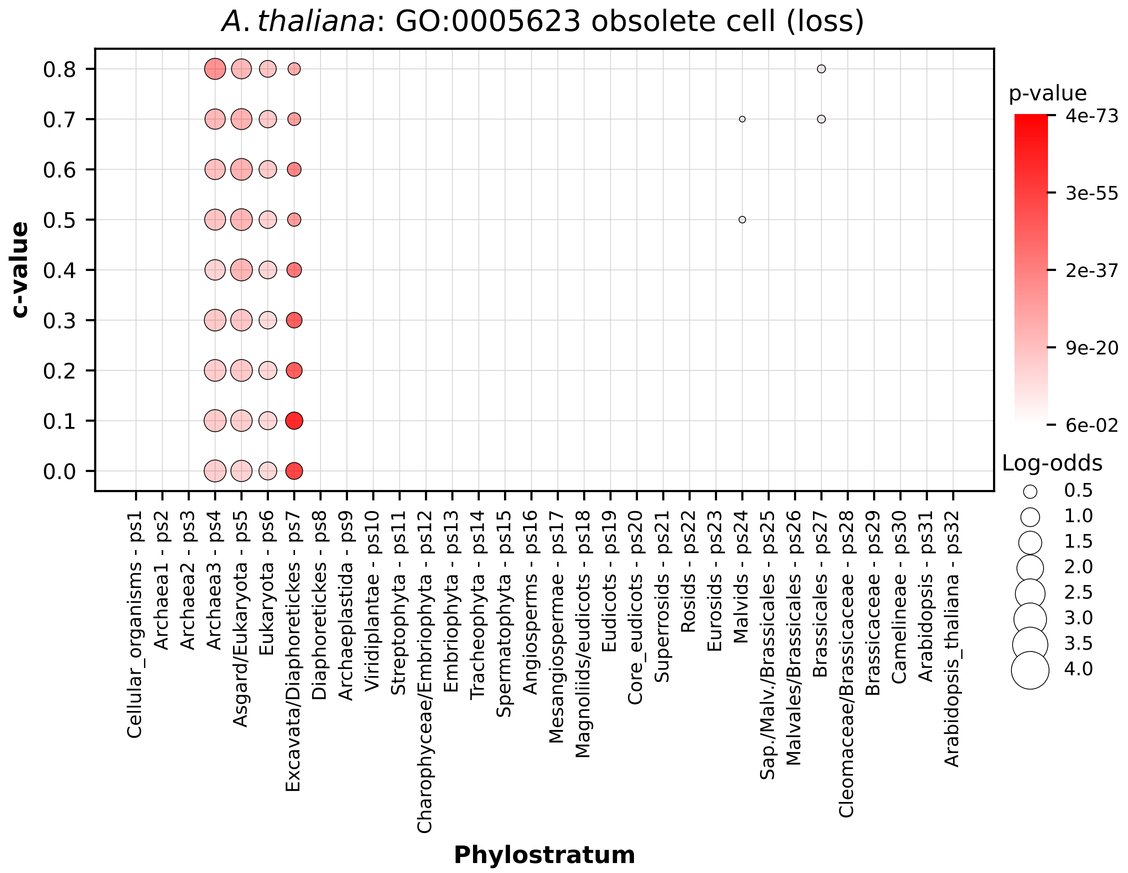

A. thaliana: GO:0005623 obsolete cell (loss)
c-value
0.8
0.7
0.6
0.5
0.4
0.3
0.2
0.1
0.0
Phylostratum
Cellular_organisms - ps1
Archaea1 - ps2
Archaea2 - ps3
Archaea3 - ps4
Asgard/Eukaryota - ps5
Eukaryota - ps6
Excavata/Diaphoretickes - ps7
Diaphoretickes - ps8
Archaeplastida - ps9
Viridiplantae - ps10
Streptophyta - ps11
Charophyceae/Embriophyta - ps12
Embriophyta - ps13
Tracheophyta - ps14
Spermatophyta - ps15
Angiosperms - ps16
Mesangiospermae - ps17
Magnoliids/eudicots - ps18
Eudicots - ps19
Core_eudicots - ps20
Superrosids - ps21
Rosids - ps22
Eurosids - ps23
Malvids - ps24
Sap./Malv./Brassicales - ps25
Malvales/Brassicales - ps26
Brassicales - ps27
Cleomaceae/Brassicaceae - ps28
Brassicaceae - ps29
Camelineae - ps30
Arabidopsis - ps31
Arabidopsis_thaliana - ps32
p-value
4e-73
3e-55
2e-37
9e-20
6e-02
Log-odds
0.5
1.0
1.5
2.0
2.5
3.0
3.5
4.0

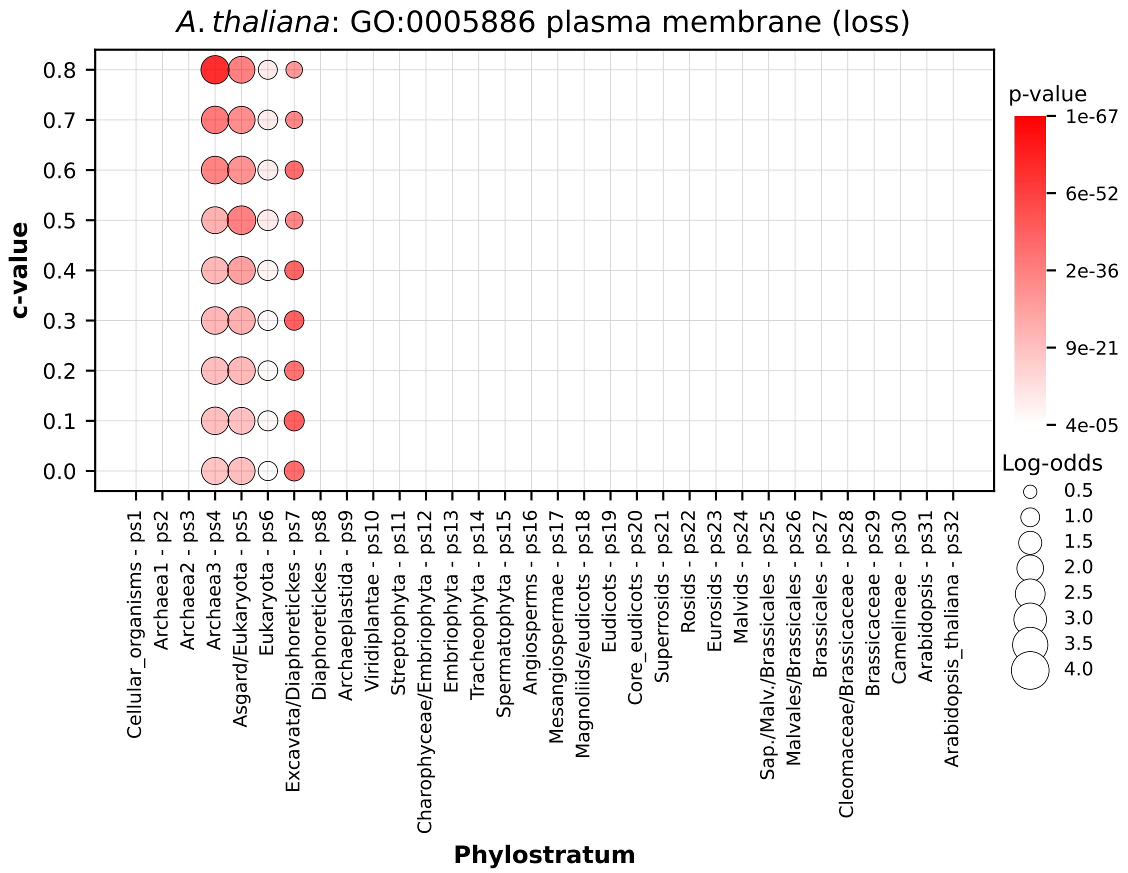

A. thaliana: GO:0005886 plasma membrane (loss)
c-value
0.8
0.7
0.6
0.5
0.4
0.3
0.2
0.1
0.0
p-value
1e-67
6e-52
2e-36
9e-21
4e-05
Log-odds
0.5
1.0
1.5
2.0
2.5
3.0
3.5
4.0
Cellular_organisms - ps1
Archaea1 - ps2
Archaea2 - ps3
Archaea3 - ps4
Asgard/Eukaryota - ps5
Eukaryota - ps6
Excavata/Diaphoretickes - ps7
Diaphoretickes - ps8
Archaeplastida - ps9
Viridiplantae - ps10
Streptophyta - ps11
Charophyceae/Embriophyta - ps12
Embriophyta - ps13
Tracheophyta - ps14
Spermatophyta - ps15
Angiosperms - ps16
Mesangiospermae - ps17
Magnoliids/eudicots - ps18
Eudicots - ps19
Core_eudicots - ps20
Superrosids - ps21
Rosids - ps22
Eurosids - ps23
Malvids - ps24
Sap./Malv./Brassicales - ps25
Malvales/Brassicales - ps26
Brassicales - ps27
Cleomaceae/Brassicaceae - ps28
Brassicaceae - ps29
Camelineae - ps30
Arabidopsis - ps31
Arabidopsis_thaliana - ps32
Phylostratum

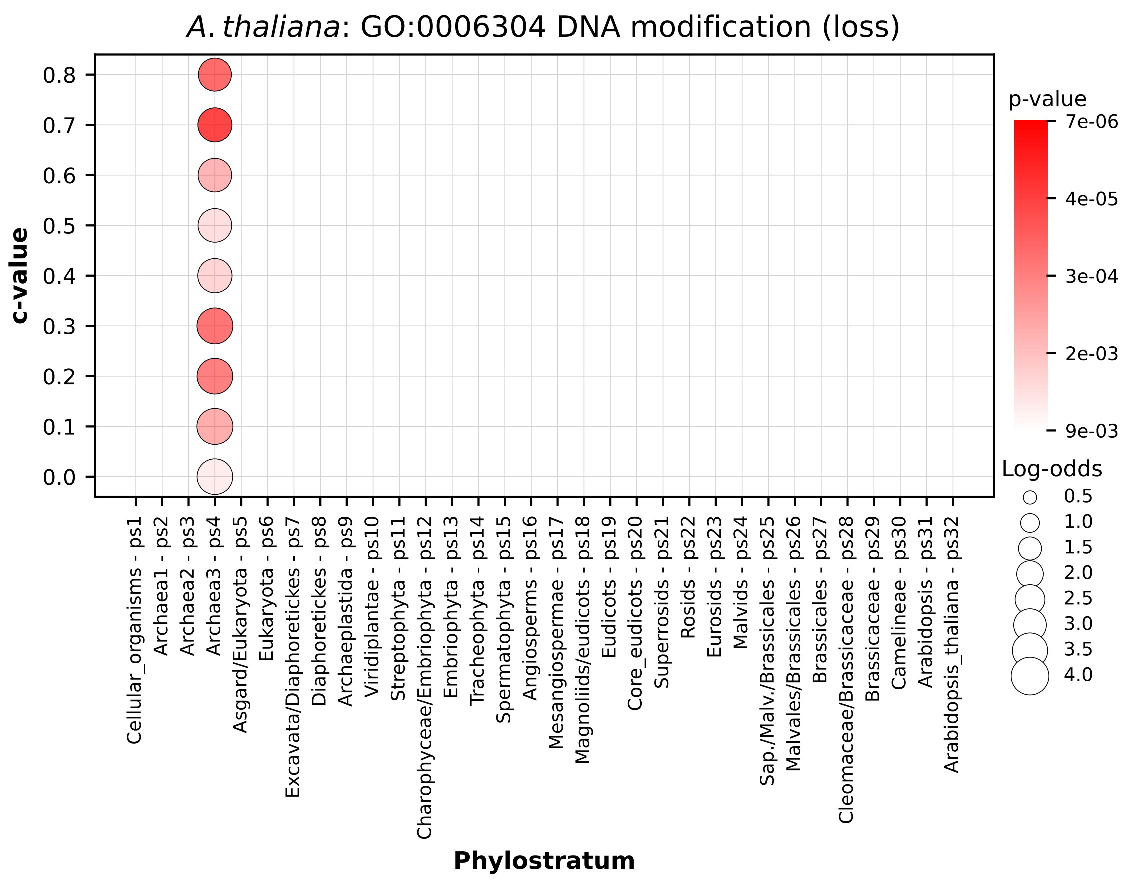

A. thaliana: GO:0006304 DNA modification (loss)
c-value
0.8
0.7
0.6
0.5
0.4
0.3
0.2
0.1
0.0
Phylostratum
Cellular_organisms - ps1
Archaea1 - ps2
Archaea2 - ps3
Archaea3 - ps4
Asgard/Eukaryota - ps5
Eukaryota - ps6
Excavata/Diaphoretickes - ps7
Diaphoretickes - ps8
Archaeplastida - ps9
Viridiplantae - ps10
Streptophyta - ps11
Charophyceae/Embriophyta - ps12
Embriophyta - ps13
Tracheophyta - ps14
Spermatophyta - ps15
Angiosperms - ps16
Mesangiospermae - ps17
Magnoliids/eudicots - ps18
Eudicots - ps19
Core_eudicots - ps20
Superrosids - ps21
Rosids - ps22
Eurosids - ps23
Malvids - ps24
Sap./Malv./Brassicales - ps25
Malvales/Brassicales - ps26
Brassicales - ps27
Cleomaceae/Brassicaceae - ps28
Brassicaceae - ps29
Camelineae - ps30
Arabidopsis - ps31
Arabidopsis_thaliana - ps32
p-value
7e-06
4e-05
3e-04
2e-03
9e-03
Log-odds
0.5
1.0
1.5
2.0
2.5
3.0
3.5
4.0

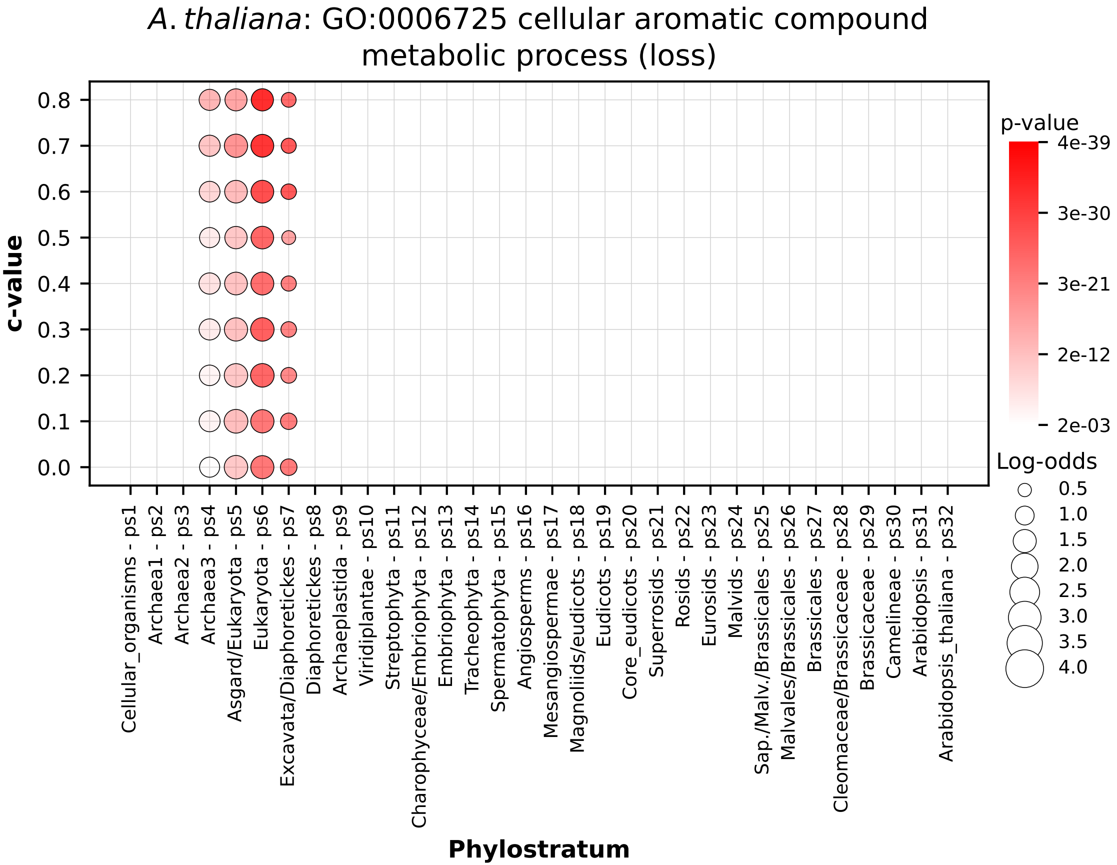
A. thaliana: GO:0006725 cellular aromatic compound metabolic process (loss)
c-value
0.8
0.7
0.6
0.5
0.4
0.3
0.2
0.1
0.0
Phylostratum
Cellular_organisms - ps1
Archaea1 - ps2
Archaea2 - ps3
Archaea3 - ps4
Asgard/Eukaryota - ps5
Eukaryota - ps6
Excavata/Diaphoretickes - ps7
Diaphoretickes - ps8
Archaeplastida - ps9
Viridiplantae - ps10
Streptophyta - ps11
Charophyceae/Embriophyta - ps12
Embriophyta - ps13
Tracheophyta - ps14
Spermatophyta - ps15
Angiosperms - ps16
Mesangiospermae - ps17
Magnoliids/eudicots - ps18
Eudicots - ps19
Core_eudicots - ps20
Superrosids - ps21
Rosids - ps22
Eurosids - ps23
Malvids - ps24
Sap./Malv./Brassicales - ps25
Malvales/Brassicales - ps26
Brassicales - ps27
Cleomaceae/Brassicaceae - ps28
Brassicaceae - ps29
Camelineae - ps30
Arabidopsis - ps31
Arabidopsis_thaliana - ps32
p-value
4e-39
3e-30
3e-21
2e-12
2e-03
Log-odds
0.5
1.0
1.5
2.0
2.5
3.0
3.5
4.0

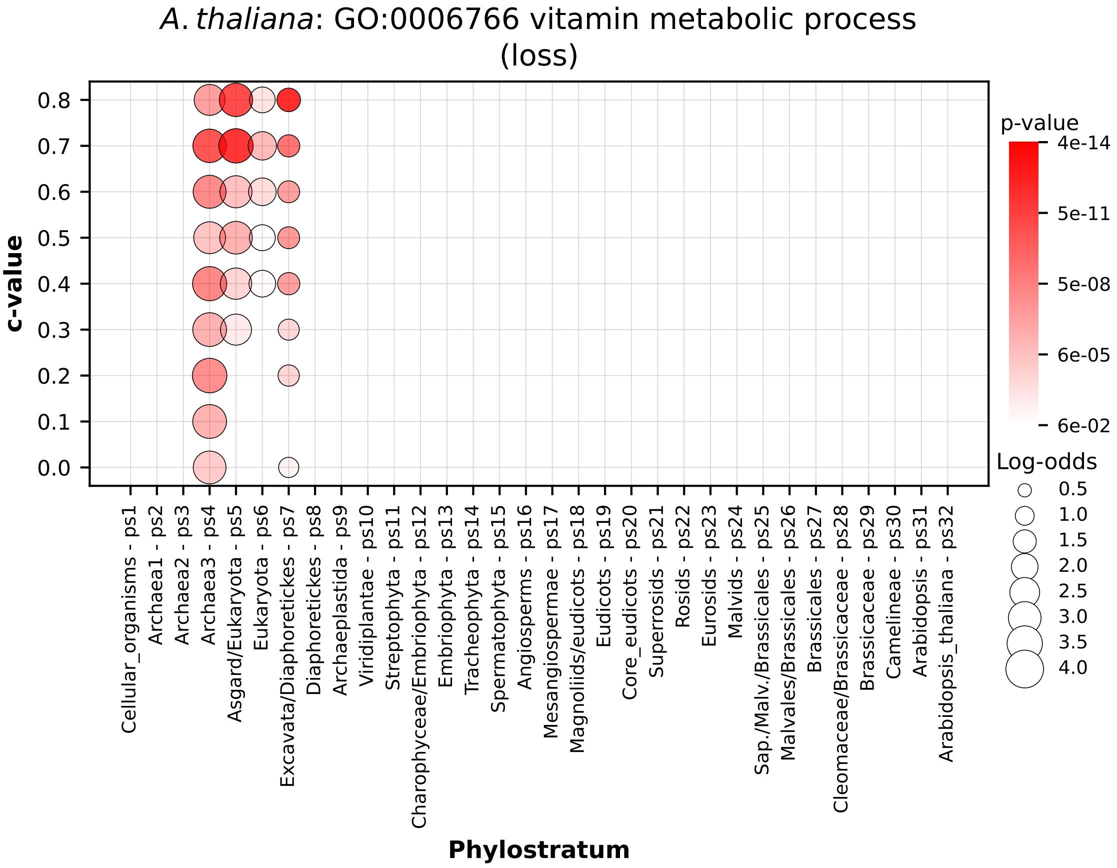

A. thaliana: GO:0006766 vitamin metabolic process (loss)
c-value
0.8
0.7
0.6
0.5
0.4
0.3
0.2
0.1
0.0
p-value
4e-14
5e-11
5e-08
6e-05
6e-02
Log-odds
0.5
1.0
1.5
2.0
2.5
3.0
3.5
4.0
Cellular_organisms - ps1
Archaea1 - ps2
Archaea2 - ps3
Archaea3 - ps4
Asgard/Eukaryota - ps5
Eukaryota - ps6
Excavata/Diaphoretickes - ps7
Diaphoretickes - ps8
Archaeplastida - ps9
Viridiplantae - ps10
Streptophyta - ps11
Charophyceae/Embriophyta - ps12
Embriophyta - ps13
Tracheophyta - ps14
Spermatophyta - ps15
Angiosperms - ps16
Mesangiospermae - ps17
Magnoliids/eudicots - ps18
Eudicots - ps19
Core_eudicots - ps20
Superrosids - ps21
Rosids - ps22
Eurosids - ps23
Malvids - ps24
Sap./Malv./Brassicales - ps25
Malvales/Brassicales - ps26
Brassicales - ps27
Cleomaceae/Brassicaceae - ps28
Brassicaceae - ps29
Camelineae - ps30
Arabidopsis - ps31
Arabidopsis_thaliana - ps32
Phylostratum

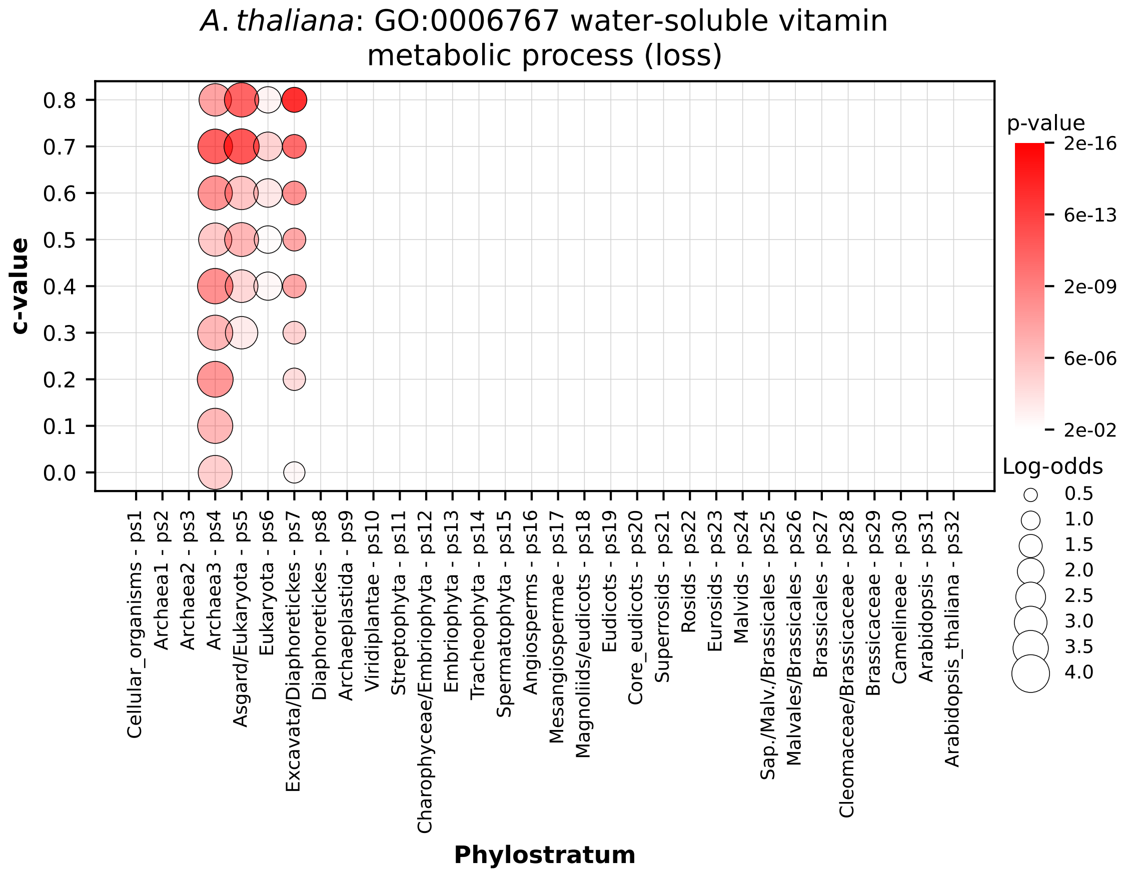

A. thaliana: GO:0006767 water-soluble vitamin metabolic process (loss)
c-value
0.8
0.7
0.6
0.5
0.4
0.3
0.2
0.1
0.0
p-value
2e-16
6e-13
2e-09
6e-06
2e-02
Log-odds
0.5
1.0
1.5
2.0
2.5
3.0
3.5
4.0
Cellular_organisms - ps1
Archaea1 - ps2
Archaea2 - ps3
Archaea3 - ps4
Asgard/Eukaryota - ps5
Eukaryota - ps6
Excavata/Diaphoretickes - ps7
Diaphoretickes - ps8
Archaeplastida - ps9
Viridiplantae - ps10
Streptophyta - ps11
Charophyceae/Embriophyta - ps12
Embriophyta - ps13
Tracheophyta - ps14
Spermatophyta - ps15
Angiosperms - ps16
Mesangiospermae - ps17
Magnoliids/eudicots - ps18
Eudicots - ps19
Core_eudicots - ps20
Superrosids - ps21
Rosids - ps22
Eurosids - ps23
Malvids - ps24
Sap./Malv./Brassicales - ps25
Malvales/Brassicales - ps26
Brassicales - ps27
Cleomaceae/Brassicaceae - ps28
Brassicaceae - ps29
Camelineae - ps30
Arabidopsis - ps31
Arabidopsis_thaliana - ps32
Phylostratum

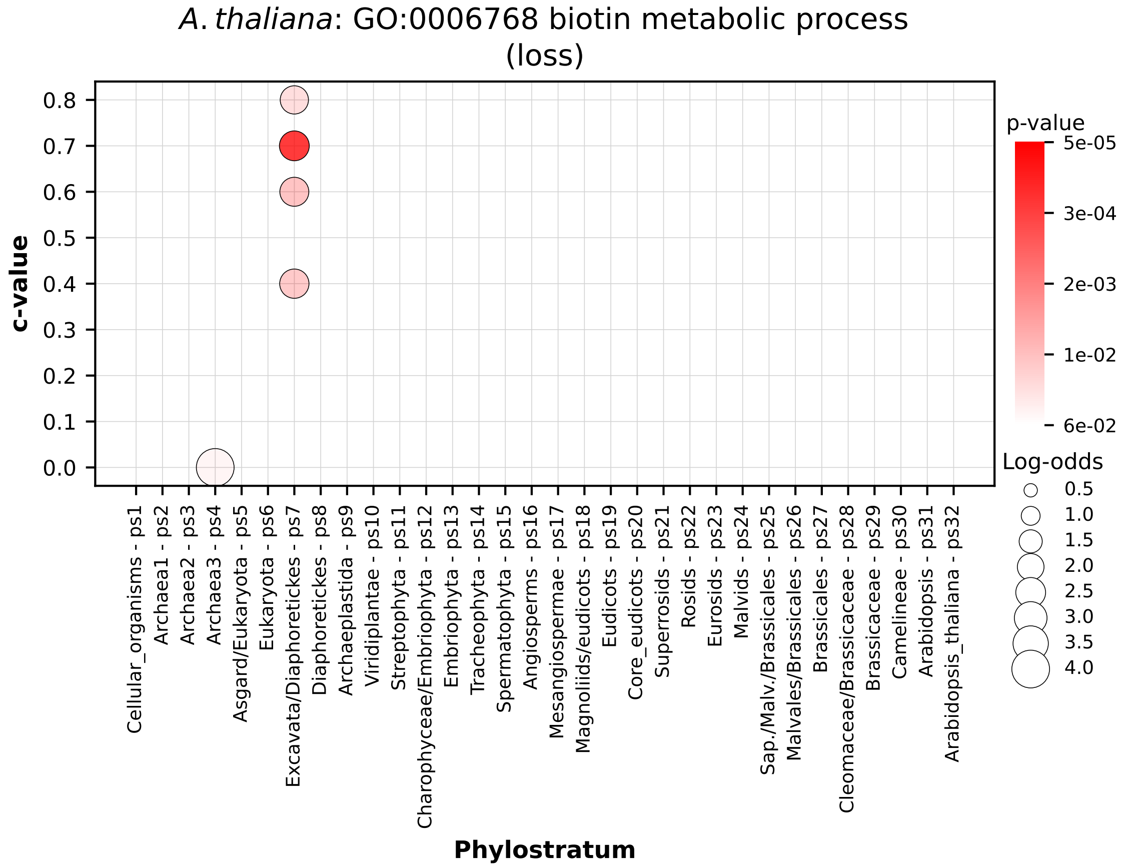

A. thaliana: GO:0006768 biotin metabolic process
(loss)
c-value
0.8
0.7
0.6
0.5
0.4
0.3
0.2
0.1
0.0
p-value
5e-05
3e-04
2e-03
1e-02
6e-02
Log-odds
0.5
1.0
1.5
2.0
2.5
3.0
3.5
4.0
Cellular_organisms - ps1
Archaea1 - ps2
Archaea2 - ps3
Archaea3 - ps4
Asgard/Eukaryota - ps5
Eukaryota - ps6
Excavata/Diaphoretickes - ps7
Diaphoretickes - ps8
Archaeplastida - ps9
Viridiplantae - ps10
Streptophyta - ps11
Charophyceae/Embriophyta - ps12
Embriophyta - ps13
Tracheophyta - ps14
Spermatophyta - ps15
Angiosperms - ps16
Mesangiospermae - ps17
Magnoliids/eudicots - ps18
Eudicots - ps19
Core_eudicots - ps20
Superrosids - ps21
Rosids - ps22
Eurosids - ps23
Malvids - ps24
Sap./Malv./Brassicales - ps25
Malvales/Brassicales - ps26
Brassicales - ps27
Cleomaceae/Brassicaceae - ps28
Brassicaceae - ps29
Camelineae - ps30
Arabidopsis - ps31
Arabidopsis_thaliana - ps32
Phylostratum

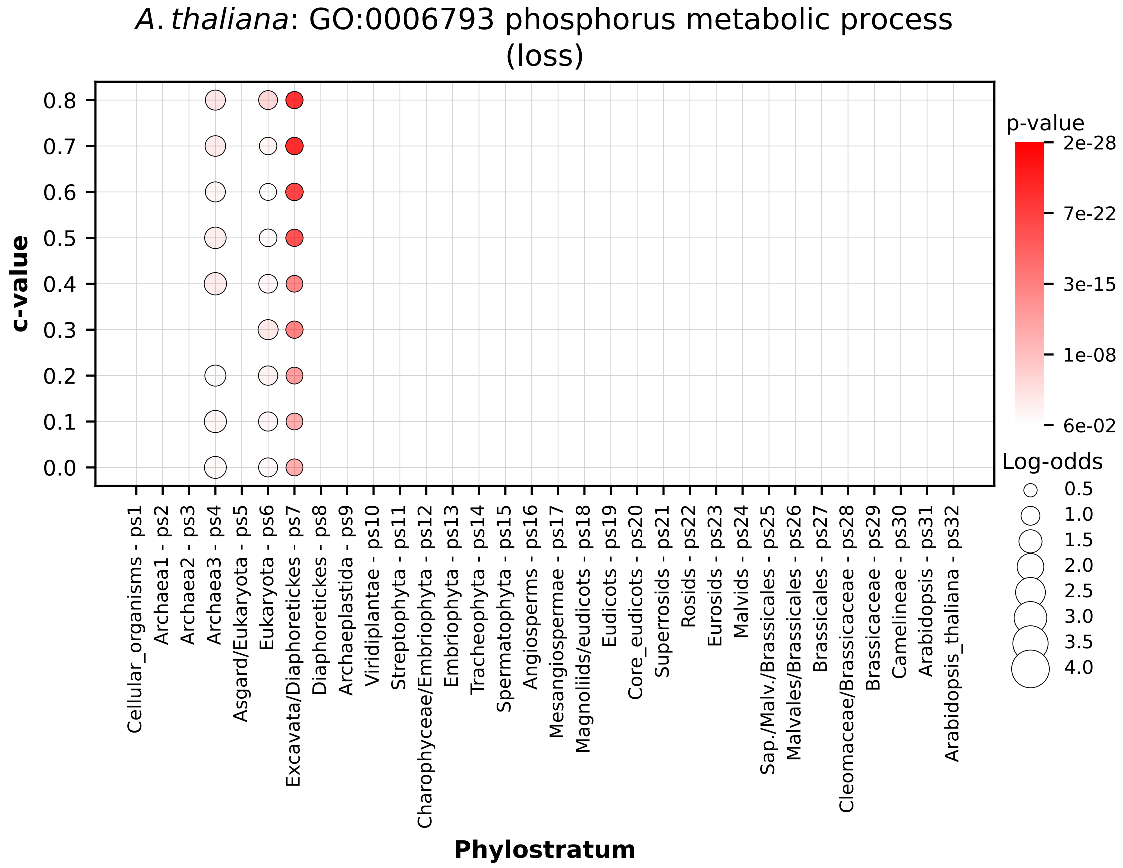

A. thaliana: GO:0006793 phosphorus metabolic process
(loss)
c-value
0.8
0.7
0.6
0.5
0.4
0.3
0.2
0.1
0.0
p-value
2e-28
7e-22
3e-15
1e-08
6e-02
Log-odds
0.5
1.0
1.5
2.0
2.5
3.0
3.5
4.0
Cellular_organisms - ps1
Archaea1 - ps2
Archaea2 - ps3
Archaea3 - ps4
Asgard/Eukaryota - ps5
Eukaryota - ps6
Excavata/Diaphoretickes - ps7
Diaphoretickes - ps8
Archaeplastida - ps9
Viridiplantae - ps10
Streptophyta - ps11
Charophyceae/Embriophyta - ps12
Embriophyta - ps13
Tracheophyta - ps14
Spermatophyta - ps15
Angiosperms - ps16
Mesangiospermae - ps17
Magnoliids/eudicots - ps18
Eudicots - ps19
Core_eudicots - ps20
Superrosids - ps21
Rosids - ps22
Eurosids - ps23
Malvids - ps24
Sap./Malv./Brassicales - ps25
Malvales/Brassicales - ps26
Brassicales - ps27
Cleomaceae/Brassicaceae - ps28
Brassicaceae - ps29
Camelineae - ps30
Arabidopsis - ps31
Arabidopsis_thaliana - ps32
Phylostratum

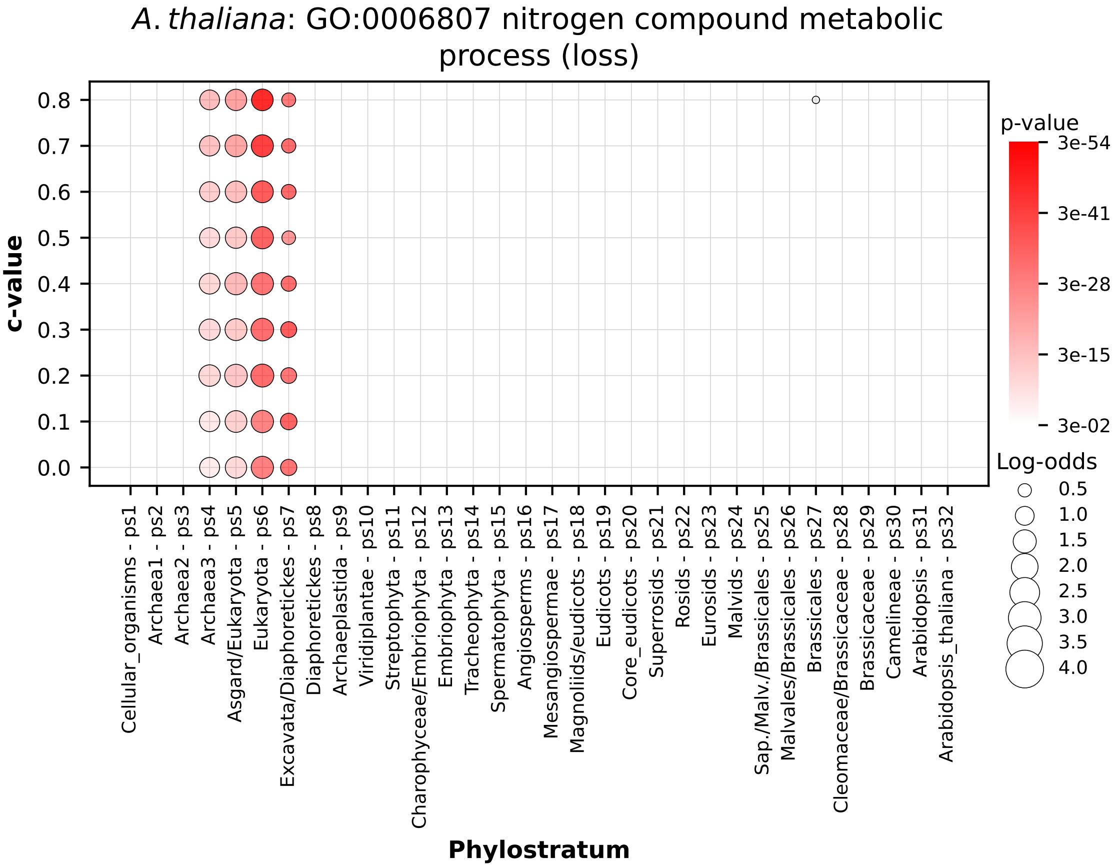

A. thaliana: GO:0006807 nitrogen compound metabolic process (loss)
c-value
0.8
0.7
0.6
0.5
0.4
0.3
0.2
0.1
0.0
p-value
3e-54
3e-41
3e-28
3e-15
3e-02
Log-odds
0.5
1.0
1.5
2.0
2.5
3.0
3.5
4.0
Cellular_organisms - ps1
Archaea1 - ps2
Archaea2 - ps3
Archaea3 - ps4
Asgard/Eukaryota - ps5
Eukaryota - ps6
Excavata/Diaphoretickes - ps7
Diaphoretickes - ps8
Archaeplastida - ps9
Viridiplantae - ps10
Streptophyta - ps11
Charophyceae/Embriophyta - ps12
Embriophyta - ps13
Tracheophyta - ps14
Spermatophyta - ps15
Angiosperms - ps16
Mesangiospermae - ps17
Magnoliids/eudicots - ps18
Eudicots - ps19
Core_eudicots - ps20
Superrosids - ps21
Rosids - ps22
Eurosids - ps23
Malvids - ps24
Sap./Malv./Brassicales - ps25
Malvales/Brassicales - ps26
Brassicales - ps27
Cleomaceae/Brassicaceae - ps28
Brassicaceae - ps29
Camelineae - ps30
Arabidopsis - ps31
Arabidopsis_thaliana - ps32
Phylostratum

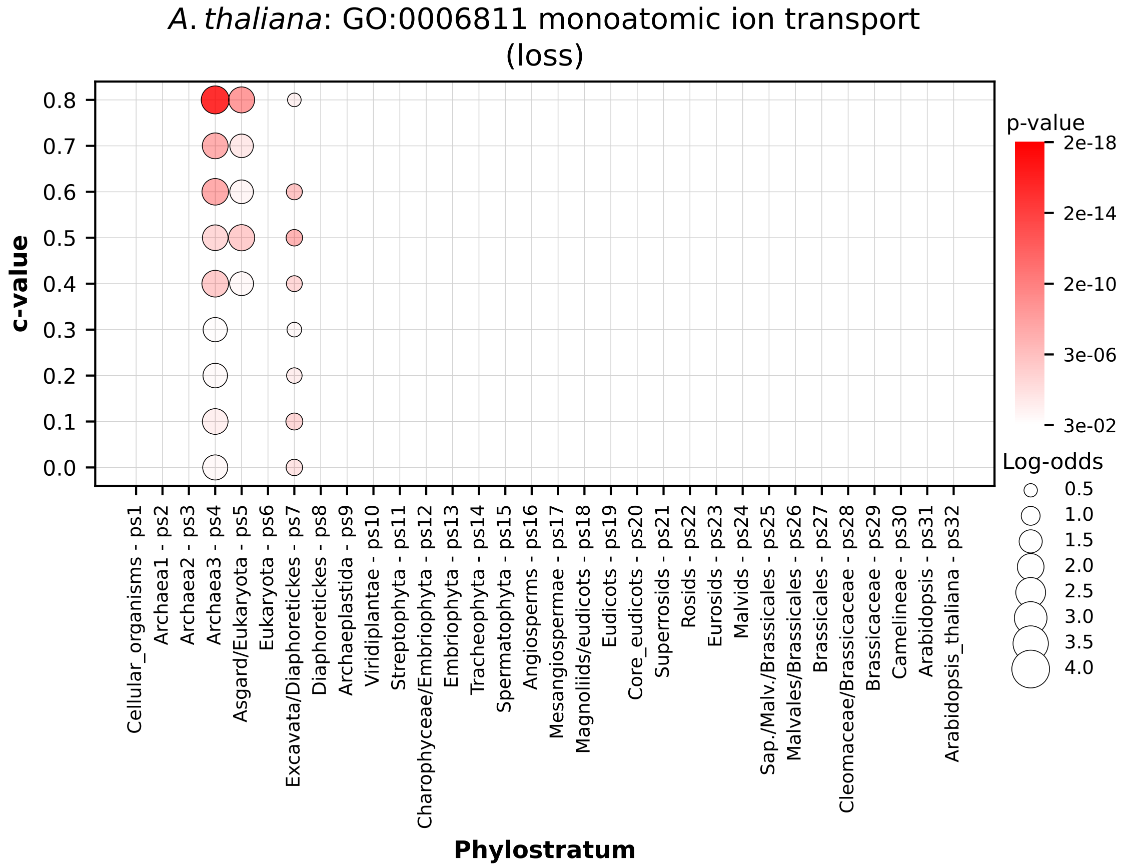
A. thaliana: GO:0006811 monoatomic ion transport
(loss)
c-value
0.8
0.7
0.6
0.5
0.4
0.3
0.2
0.1
0.0
Phylostratum
Cellular_organisms - ps1
Archaea1 - ps2
Archaea2 - ps3
Archaea3 - ps4
Asgard/Eukaryota - ps5
Eukaryota - ps6
Excavata/Diaphoretickes - ps7
Diaphoretickes - ps8
Archaeplastida - ps9
Viridiplantae - ps10
Streptophyta - ps11
Charophyceae/Embriophyta - ps12
Embriophyta - ps13
Tracheophyta - ps14
Spermatophyta - ps15
Angiosperms - ps16
Mesangiospermae - ps17
Magnoliids/eudicots - ps18
Eudicots - ps19
Core_eudicots - ps20
Superrosids - ps21
Rosids - ps22
Eurosids - ps23
Malvids - ps24
Sap./Malv./Brassicales - ps25
Malvales/Brassicales - ps26
Brassicales - ps27
Cleomaceae/Brassicaceae - ps28
Brassicaceae - ps29
Camelineae - ps30
Arabidopsis - ps31
Arabidopsis_thaliana - ps32
p-value
2e-18
2e-14
2e-10
3e-06
3e-02
Log-odds
0.5
1.0
1.5
2.0
2.5
3.0
3.5
4.0

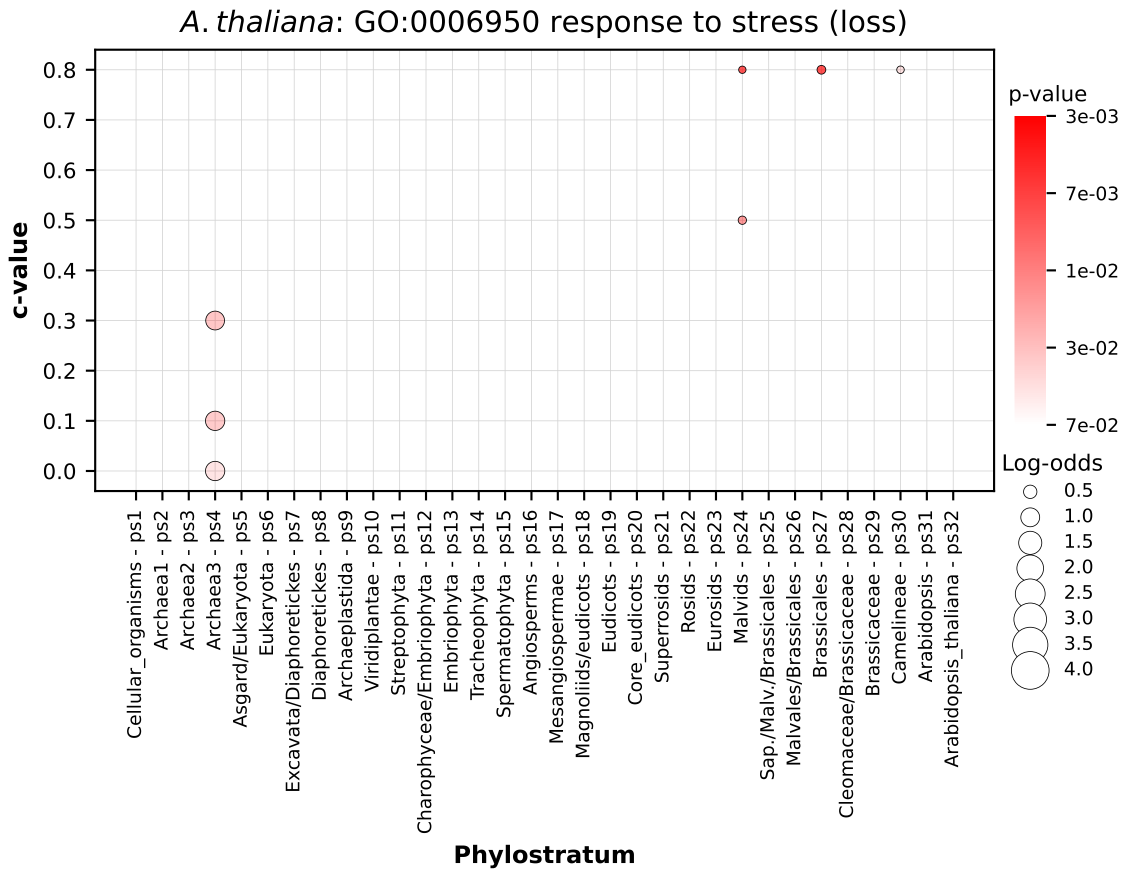
A. thaliana: GO:0006950 response to stress (loss)
c-value
0.8
0.7
0.6
0.5
0.4
0.3
0.2
0.1
0.0
Phylostratum
Cellular_organisms - ps1
Archaea1 - ps2
Archaea2 - ps3
Archaea3 - ps4
Asgard/Eukaryota - ps5
Eukaryota - ps6
Excavata/Diaphoretickes - ps7
Diaphoretickes - ps8
Archaeplastida - ps9
Viridiplantae - ps10
Streptophyta - ps11
Charophyceae/Embriophyta - ps12
Embriophyta - ps13
Tracheophyta - ps14
Spermatophyta - ps15
Angiosperms - ps16
Mesangiospermae - ps17
Magnoliids/eudicots - ps18
Eudicots - ps19
Core_eudicots - ps20
Superrosids - ps21
Rosids - ps22
Eurosids - ps23
Malvids - ps24
Sap./Malv./Brassicales - ps25
Malvales/Brassicales - ps26
Brassicales - ps27
Cleomaceae/Brassicaceae - ps28
Brassicaceae - ps29
Camelineae - ps30
Arabidopsis - ps31
Arabidopsis_thaliana - ps32
p-value
3e-03
7e-03
1e-02
3e-02
7e-02
Log-odds
0.5
1.0
1.5
2.0
2.5
3.0
3.5
4.0

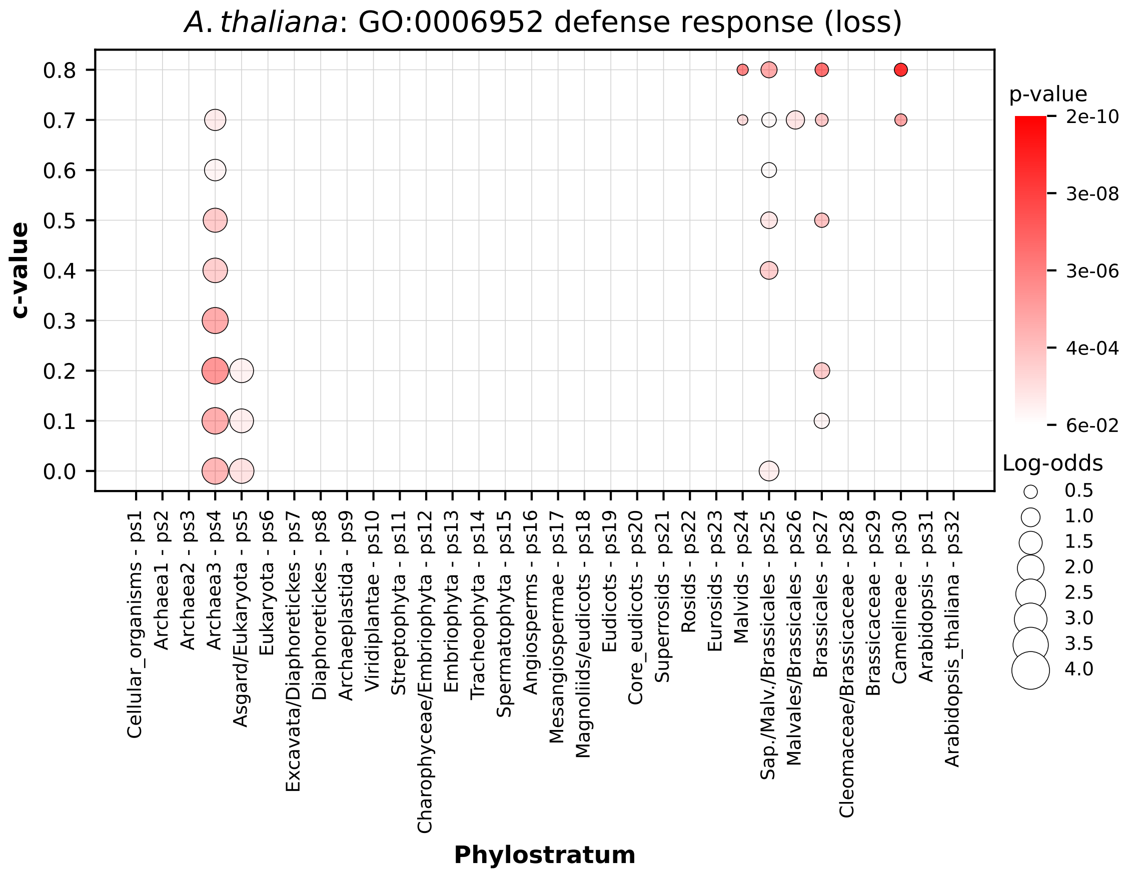

A. thaliana: GO:0006952 defense response (loss)
c-value
0.8
0.7
0.6
0.5
0.4
0.3
0.2
0.1
0.0
Phylostratum
Cellular_organisms - ps1
Archaea1 - ps2
Archaea2 - ps3
Archaea3 - ps4
Asgard/Eukaryota - ps5
Eukaryota - ps6
Excavata/Diaphoretickes - ps7
Diaphoretickes - ps8
Archaeplastida - ps9
Viridiplantae - ps10
Streptophyta - ps11
Charophyceae/Embriophyta - ps12
Embriophyta - ps13
Tracheophyta - ps14
Spermatophyta - ps15
Angiosperms - ps16
Mesangiospermae - ps17
Magnoliids/eudicots - ps18
Eudicots - ps19
Core_eudicots - ps20
Superrosids - ps21
Rosids - ps22
Eurosids - ps23
Malvids - ps24
Sap./Malv./Brassicales - ps25
Malvales/Brassicales - ps26
Brassicales - ps27
Cleomaceae/Brassicaceae - ps28
Brassicaceae - ps29
Camelineae - ps30
Arabidopsis - ps31
Arabidopsis_thaliana - ps32
p-value
2e-10
3e-08
3e-06
4e-04
6e-02
Log-odds
0.5
1.0
1.5
2.0
2.5
3.0
3.5
4.0

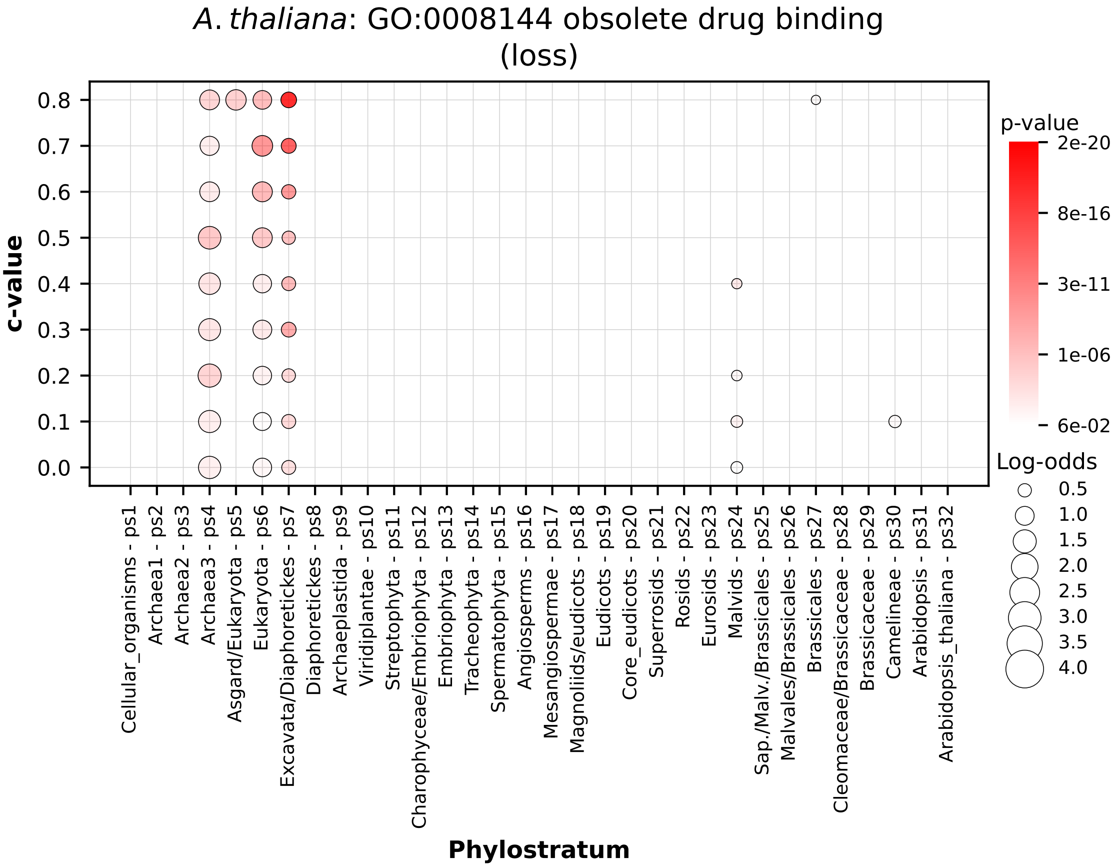

A. thaliana: GO:0008144 obsolete drug binding
(loss)
c-value
0.8
0.7
0.6
0.5
0.4
0.3
0.2
0.1
0.0
Phylostratum
Cellular_organisms - ps1
Archaea1 - ps2
Archaea2 - ps3
Archaea3 - ps4
Asgard/Eukaryota - ps5
Eukaryota - ps6
Excavata/Diaphoretickes - ps7
Diaphoretickes - ps8
Archaeplastida - ps9
Viridiplantae - ps10
Streptophyta - ps11
Charophyceae/Embriophyta - ps12
Embriophyta - ps13
Tracheophyta - ps14
Spermatophyta - ps15
Angiosperms - ps16
Mesangiospermae - ps17
Magnoliids/eudicots - ps18
Eudicots - ps19
Core_eudicots - ps20
Superrosids - ps21
Rosids - ps22
Eurosids - ps23
Malvids - ps24
Sap./Malv./Brassicales - ps25
Malvales/Brassicales - ps26
Brassicales - ps27
Cleomaceae/Brassicaceae - ps28
Brassicaceae - ps29
Camelineae - ps30
Arabidopsis - ps31
Arabidopsis_thaliana - ps32
p-value
2e-20
8e-16
3e-11
1e-06
6e-02
Log-odds
0.5
1.0
1.5
2.0
2.5
3.0
3.5
4.0

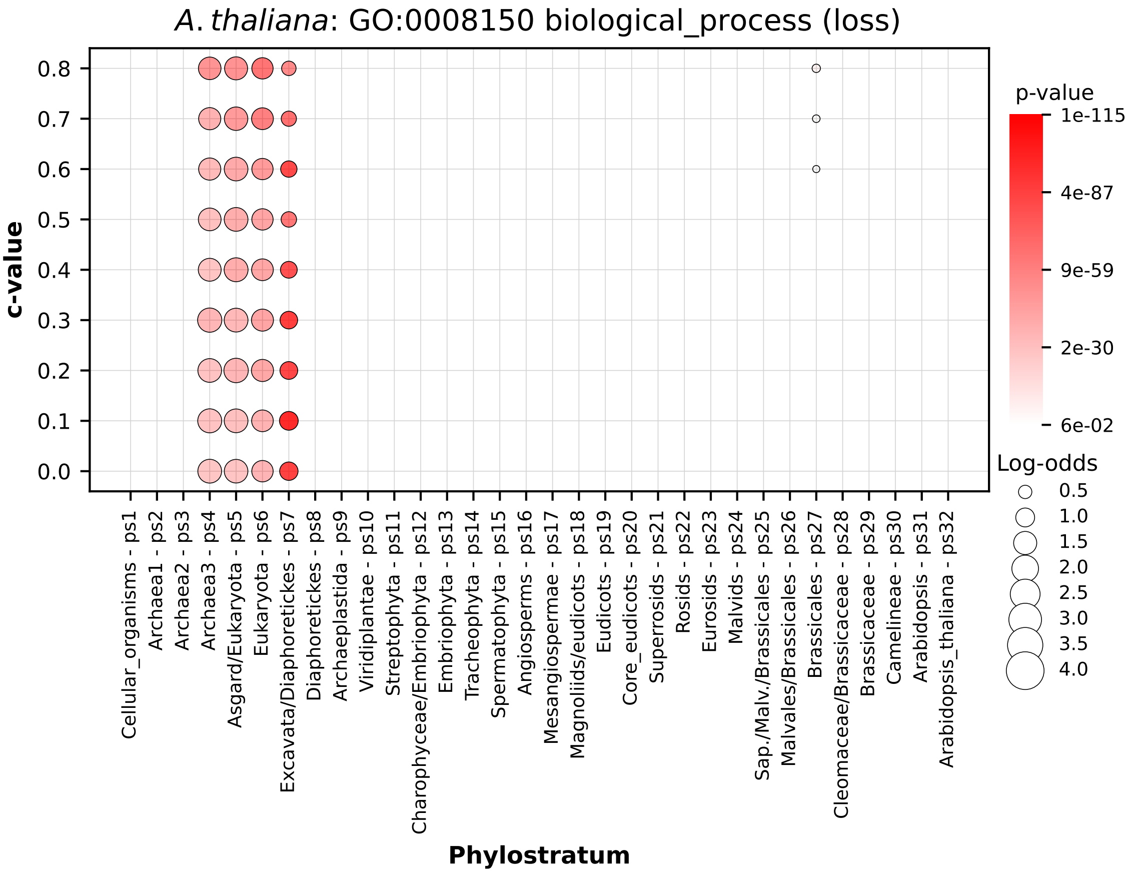

A. thaliana: GO:0008150 biological_process (loss)
c-value
0.8
0.7
0.6
0.5
0.4
0.3
0.2
0.1
0.0
p-value
1e-115
4e-87
9e-59
2e-30
6e-02
Log-odds
0.5
1.0
1.5
2.0
2.5
3.0
3.5
4.0
Cellular_organisms - ps1
Archaea1 - ps2
Archaea2 - ps3
Archaea3 - ps4
Asgard/Eukaryota - ps5
Eukaryota - ps6
Excavata/Diaphoretickes - ps7
Diaphoretickes - ps8
Archaeplastida - ps9
Viridiplantae - ps10
Streptophyta - ps11
Charophyceae/Embriophyta - ps12
Embriophyta - ps13
Tracheophyta - ps14
Spermatophyta - ps15
Angiosperms - ps16
Mesangiospermae - ps17
Magnoliids/eudicots - ps18
Eudicots - ps19
Core_eudicots - ps20
Superrosids - ps21
Rosids - ps22
Eurosids - ps23
Malvids - ps24
Sap./Malv./Brassicales - ps25
Malvales/Brassicales - ps26
Brassicales - ps27
Cleomaceae/Brassicaceae - ps28
Brassicaceae - ps29
Camelineae - ps30
Arabidopsis - ps31
Arabidopsis_thaliana - ps32
Phylostratum

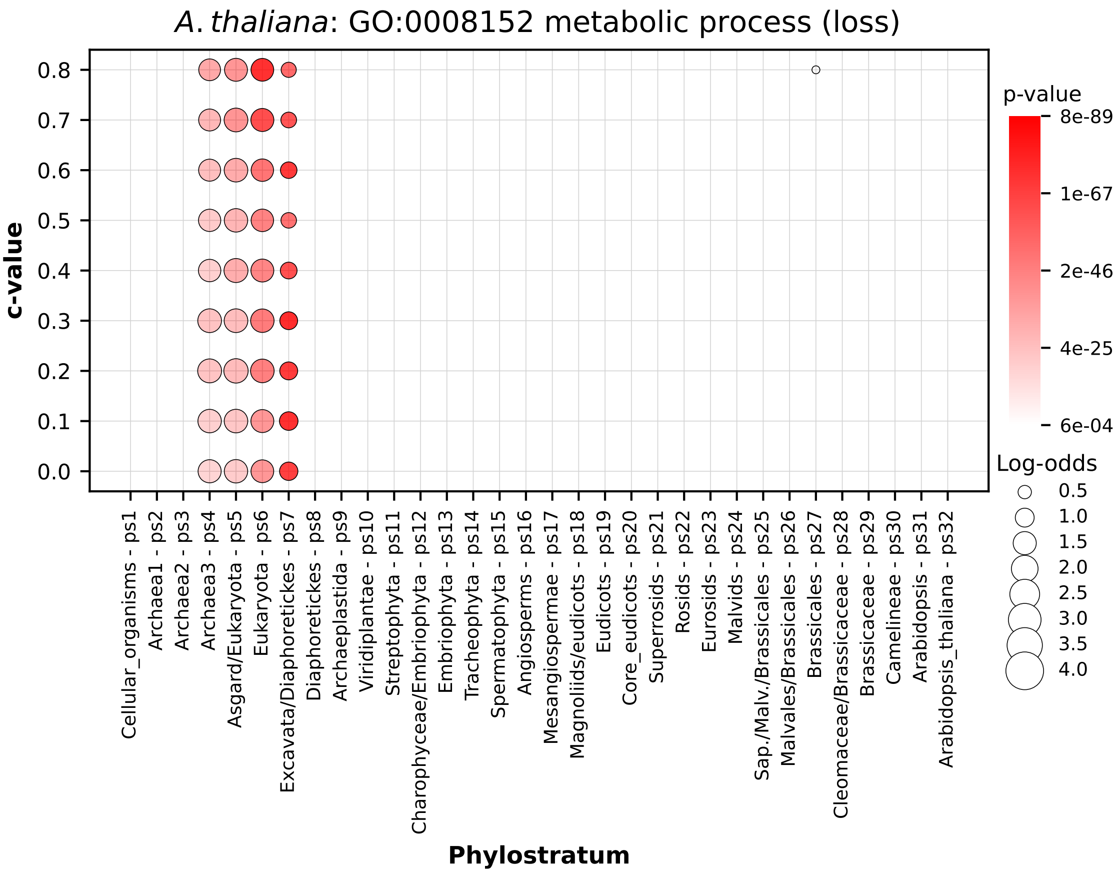
A. thaliana: GO:0008152 metabolic process (loss)
c-value
0.8
0.7
0.6
0.5
0.4
0.3
0.2
0.1
0.0
p-value
8e-89
1e-67
2e-46
4e-25
6e-04
Log-odds
0.5
1.0
1.5
2.0
2.5
3.0
3.5
4.0
Cellular_organisms - ps1
Archaea1 - ps2
Archaea2 - ps3
Archaea3 - ps4
Asgard/Eukaryota - ps5
Eukaryota - ps6
Excavata/Diaphoretickes - ps7
Diaphoretickes - ps8
Archaeplastida - ps9
Viridiplantae - ps10
Streptophyta - ps11
Charophyceae/Embriophyta - ps12
Embriophyta - ps13
Tracheophyta - ps14
Spermatophyta - ps15
Angiosperms - ps16
Mesangiospermae - ps17
Magnoliids/eudicots - ps18
Eudicots - ps19
Core_eudicots - ps20
Superrosids - ps21
Rosids - ps22
Eurosids - ps23
Malvids - ps24
Sap./Malv./Brassicales - ps25
Malvales/Brassicales - ps26
Brassicales - ps27
Cleomaceae/Brassicaceae - ps28
Brassicaceae - ps29
Camelineae - ps30
Arabidopsis - ps31
Arabidopsis_thaliana - ps32
Phylostratum

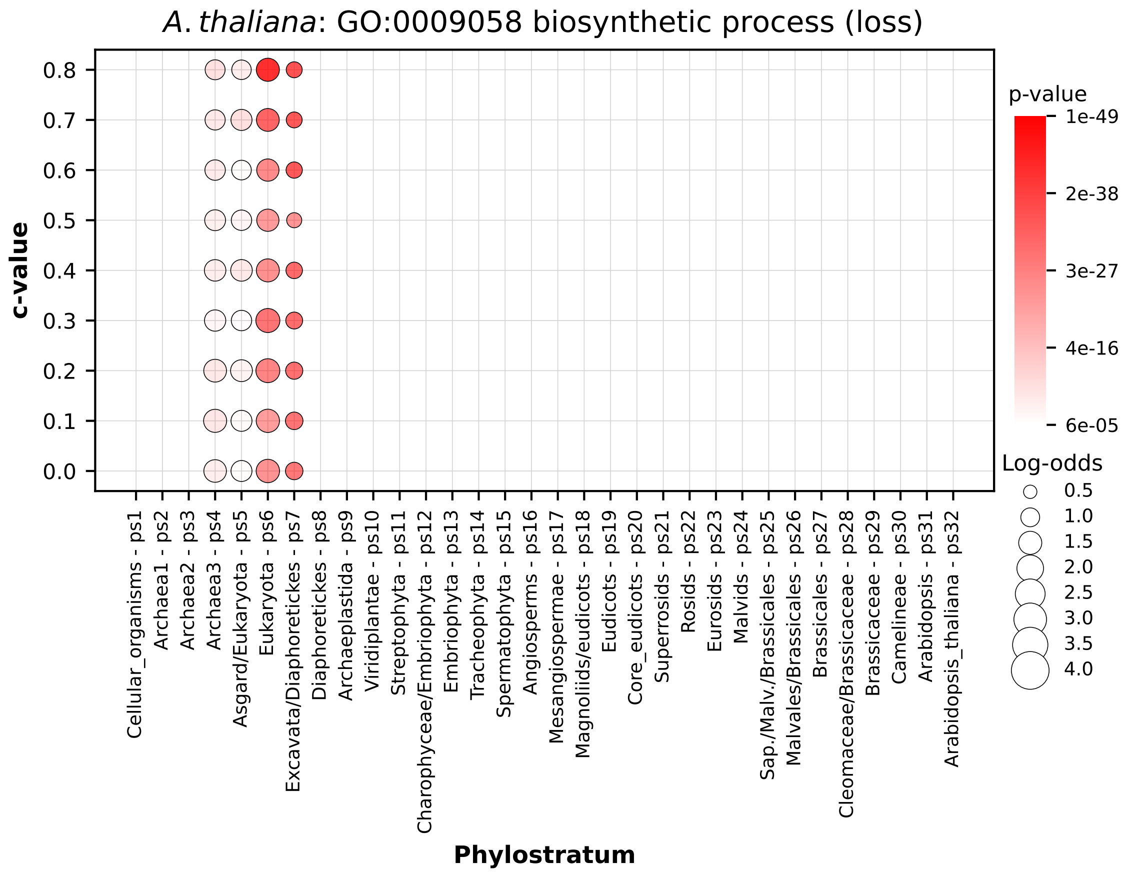
A. thaliana: GO:0009058 biosynthetic process (loss)
c-value
0.0
0.1
0.2
0.3
0.4
0.5
0.6
0.7
0.8
p-value
1e-49
2e-38
3e-27
4e-16
6e-05
Log-odds
0.5
1.0
1.5
2.0
2.5
3.0
3.5
4.0
Cellular_organisms - ps1
Archaea1 - ps2
Archaea2 - ps3
Archaea3 - ps4
Asgard/Eukaryota - ps5
Eukaryota - ps6
Excavata/Diaphoretickes - ps7
Diaphoretickes - ps8
Archaeplastida - ps9
Viridiplantae - ps10
Streptophyta - ps11
Charophyceae/Embriophyta - ps12
Embriophyta - ps13
Tracheophyta - ps14
Spermatophyta - ps15
Angiosperms - ps16
Mesangiospermae - ps17
Magnoliids/eudicots - ps18
Eudicots - ps19
Core_eudicots - ps20
Superrosids - ps21
Rosids - ps22
Eurosids - ps23
Malvids - ps24
Sap./Malv./Brassicales - ps25
Malvales/Brassicales - ps26
Brassicales - ps27
Cleomaceae/Brassicaceae - ps28
Brassicaceae - ps29
Camelineae - ps30
Arabidopsis - ps31
Arabidopsis_thaliana - ps32
Phylostratum

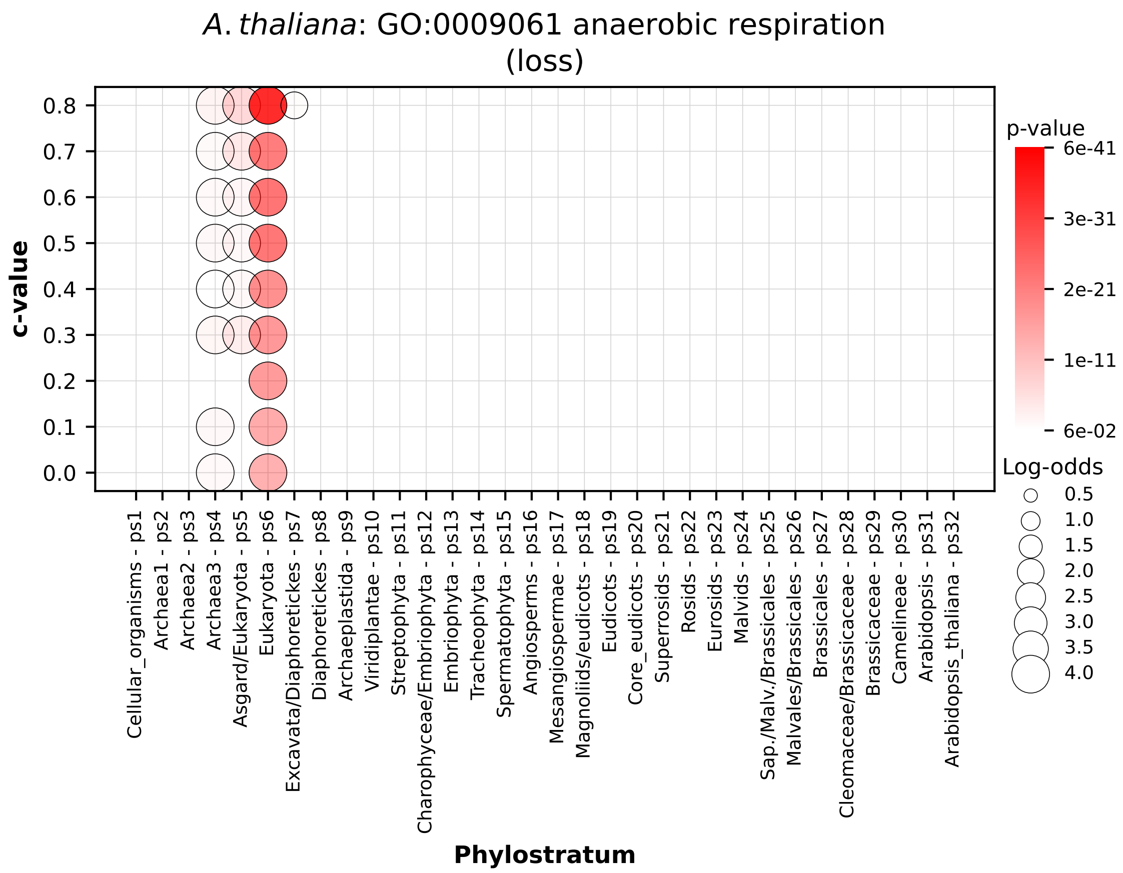
A. thaliana: GO:0009061 anaerobic respiration
(loss)
c-value
0.8
0.7
0.6
0.5
0.4
0.3
0.2
0.1
0.0
p-value
6e-41
3e-31
2e-21
1e-11
6e-02
Log-odds
0.5
1.0
1.5
2.0
2.5
3.0
3.5
4.0
Cellular_organisms - ps1
Archaea1 - ps2
Archaea2 - ps3
Archaea3 - ps4
Asgard/Eukaryota - ps5
Eukaryota - ps6
Excavata/Diaphoretickes - ps7
Diaphoretickes - ps8
Archaeplastida - ps9
Viridiplantae - ps10
Streptophyta - ps11
Charophyceae/Embriophyta - ps12
Embriophyta - ps13
Tracheophyta - ps14
Spermatophyta - ps15
Angiosperms - ps16
Mesangiospermae - ps17
Magnoliids/eudicots - ps18
Eudicots - ps19
Core_eudicots - ps20
Superrosids - ps21
Rosids - ps22
Eurosids - ps23
Malvids - ps24
Sap./Malv./Brassicales - ps25
Malvales/Brassicales - ps26
Brassicales - ps27
Cleomaceae/Brassicaceae - ps28
Brassicaceae - ps29
Camelineae - ps30
Arabidopsis - ps31
Arabidopsis_thaliana - ps32
Phylostratum

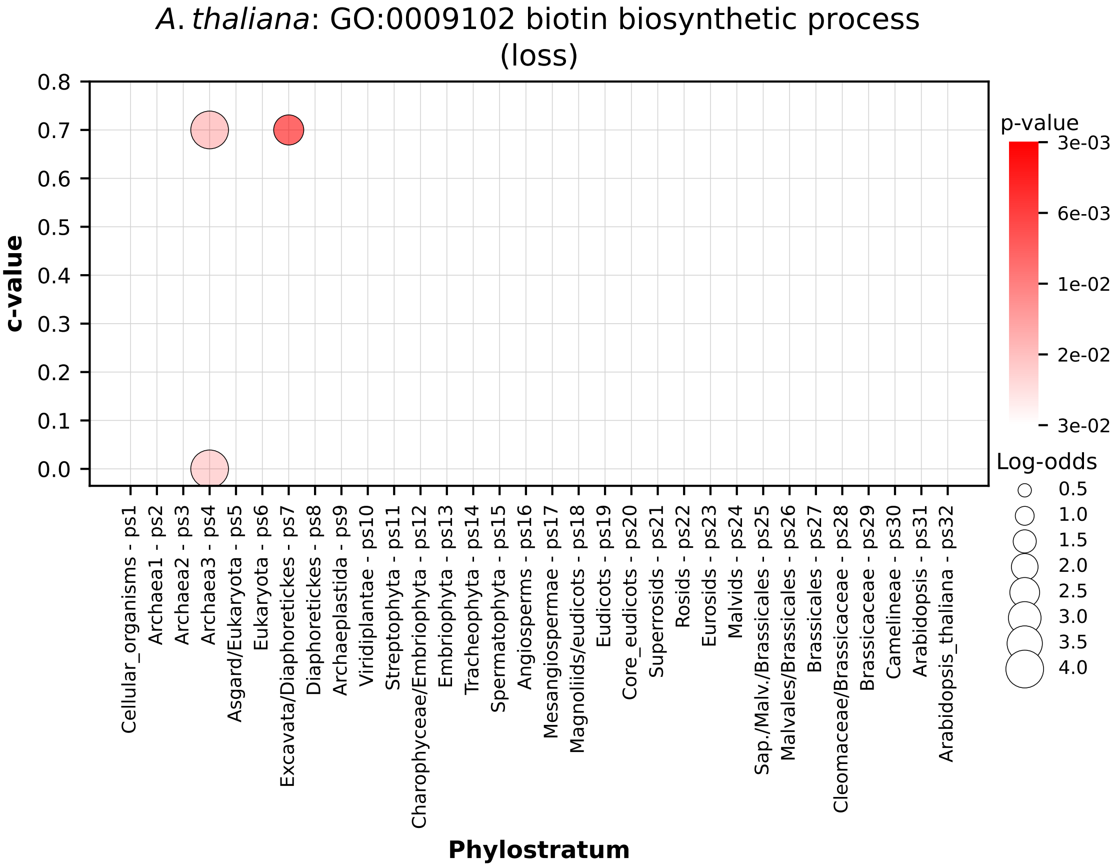

A. thaliana: GO:0009102 biotin biosynthetic process
(loss)
c-value
0.8
0.7
0.6
0.5
0.4
0.3
0.2
0.1
0.0
p-value
3e-03
6e-03
1e-02
2e-02
3e-02
Log-odds
0.5
1.0
1.5
2.0
2.5
3.0
3.5
4.0
Cellular_organisms - ps1
Archaea1 - ps2
Archaea2 - ps3
Archaea3 - ps4
Asgard/Eukaryota - ps5
Eukaryota - ps6
Excavata/Diaphoretickes - ps7
Diaphoretickes - ps8
Archaeplastida - ps9
Viridiplantae - ps10
Streptophyta - ps11
Charophyceae/Embriophyta - ps12
Embriophyta - ps13
Tracheophyta - ps14
Spermatophyta - ps15
Angiosperms - ps16
Mesangiospermae - ps17
Magnoliids/eudicots - ps18
Eudicots - ps19
Core_eudicots - ps20
Superrosids - ps21
Rosids - ps22
Eurosids - ps23
Malvids - ps24
Sap./Malv./Brassicales - ps25
Malvales/Brassicales - ps26
Brassicales - ps27
Cleomaceae/Brassicaceae - ps28
Brassicaceae - ps29
Camelineae - ps30
Arabidopsis - ps31
Arabidopsis_thaliana - ps32
Phylostratum

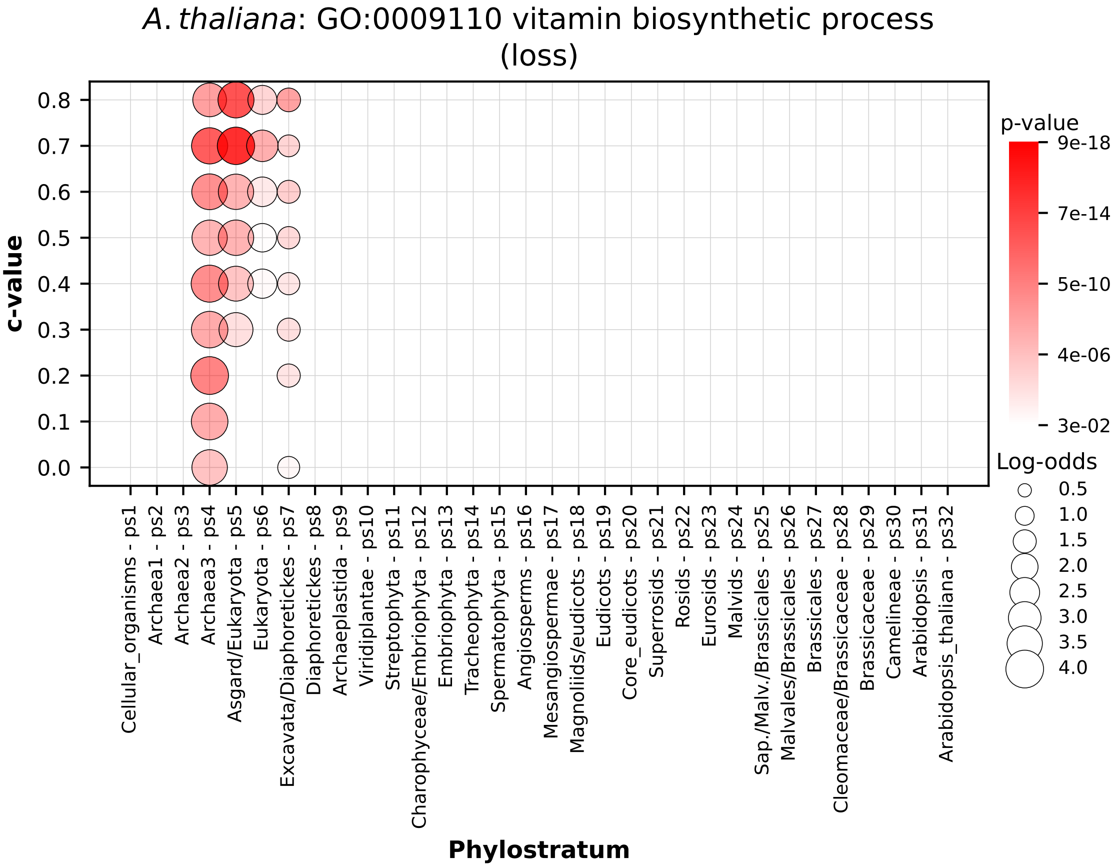
A. thaliana: GO:0009110 vitamin biosynthetic process
(loss)
c-value
0.8
0.7
0.6
0.5
0.4
0.3
0.2
0.1
0.0
Phylostratum
Cellular_organisms - ps1
Archaea1 - ps2
Archaea2 - ps3
Archaea3 - ps4
Asgard/Eukaryota - ps5
Eukaryota - ps6
Excavata/Diaphoretickes - ps7
Diaphoretickes - ps8
Archaeplastida - ps9
Viridiplantae - ps10
Streptophyta - ps11
Charophyceae/Embriophyta - ps12
Embriophyta - ps13
Tracheophyta - ps14
Spermatophyta - ps15
Angiosperms - ps16
Mesangiospermae - ps17
Magnoliids/eudicots - ps18
Eudicots - ps19
Core_eudicots - ps20
Superrosids - ps21
Rosids - ps22
Eurosids - ps23
Malvids - ps24
Sap./Malv./Brassicales - ps25
Malvales/Brassicales - ps26
Brassicales - ps27
Cleomaceae/Brassicaceae - ps28
Brassicaceae - ps29
Camelineae - ps30
Arabidopsis - ps31
Arabidopsis_thaliana - ps32
p-value
9e-18
7e-14
5e-10
4e-06
3e-02
Log-odds
0.5
1.0
1.5
2.0
2.5
3.0
3.5
4.0

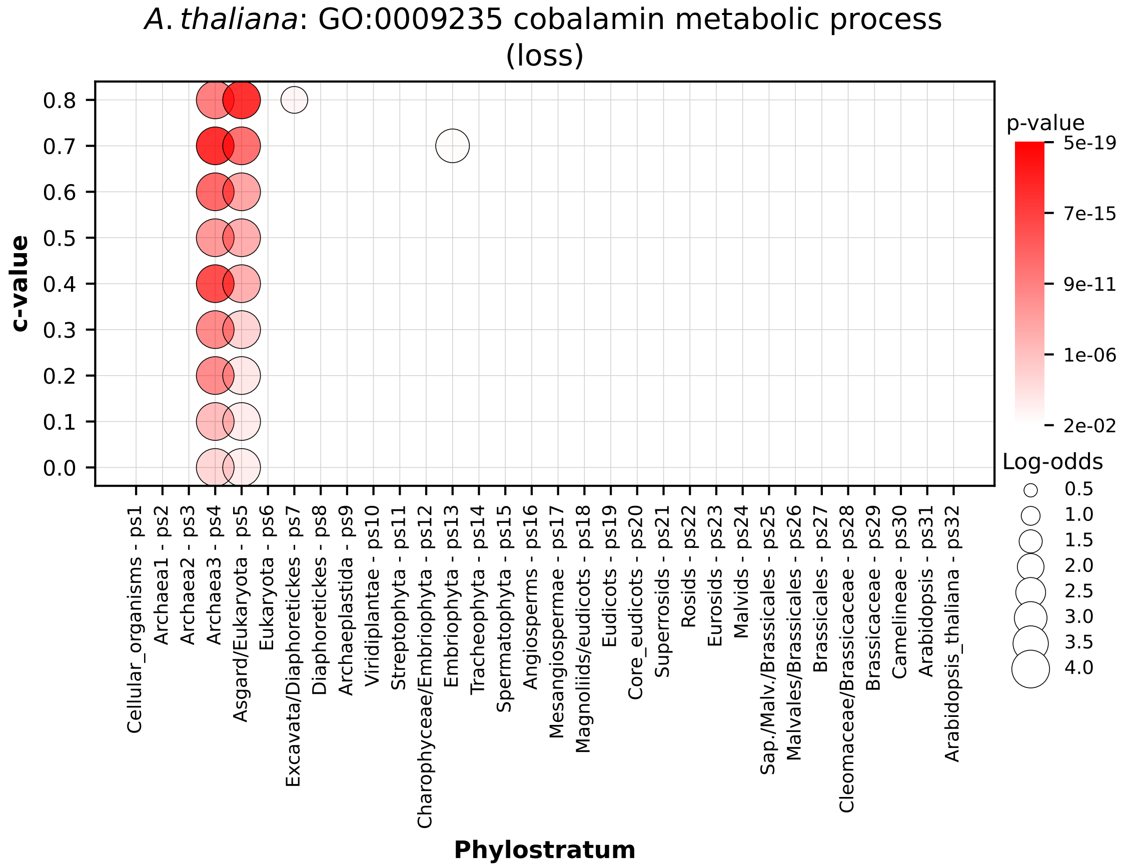
A. thaliana: GO:0009235 cobalamin metabolic process
(loss)
c-value
0.8
0.7
0.6
0.5
0.4
0.3
0.2
0.1
0.0
Phylostratum
Cellular_organisms - ps1
Archaea1 - ps2
Archaea2 - ps3
Archaea3 - ps4
Asgard/Eukaryota - ps5
Eukaryota - ps6
Excavata/Diaphoretickes - ps7
Diaphoretickes - ps8
Archaeplastida - ps9
Viridiplantae - ps10
Streptophyta - ps11
Charophyceae/Embriophyta - ps12
Embriophyta - ps13
Tracheophyta - ps14
Spermatophyta - ps15
Angiosperms - ps16
Mesangiospermae - ps17
Magnoliids/eudicots - ps18
Eudicots - ps19
Core_eudicots - ps20
Superrosids - ps21
Rosids - ps22
Eurosids - ps23
Malvids - ps24
Sap./Malv./Brassicales - ps25
Malvales/Brassicales - ps26
Brassicales - ps27
Cleomaceae/Brassicaceae - ps28
Brassicaceae - ps29
Camelineae - ps30
Arabidopsis - ps31
Arabidopsis_thaliana - ps32
p-value
5e-19
7e-15
9e-11
1e-06
2e-02
Log-odds
0.5
1.0
1.5
2.0
2.5
3.0
3.5
4.0

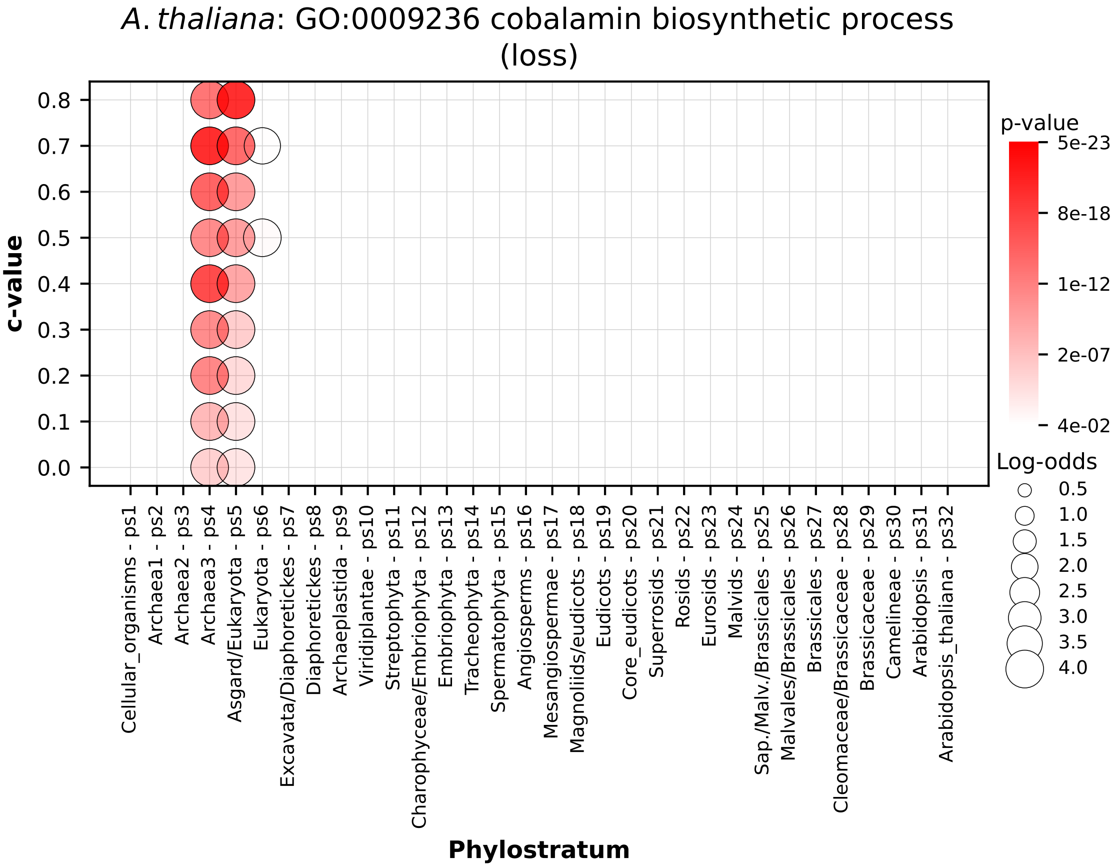
A. thaliana: GO:0009236 cobalamin biosynthetic process
(loss)
c-value
0.8
0.7
0.6
0.5
0.4
0.3
0.2
0.1
0.0
Phylostratum
Cellular_organisms - ps1
Archaea1 - ps2
Archaea2 - ps3
Archaea3 - ps4
Asgard/Eukaryota - ps5
Eukaryota - ps6
Excavata/Diaphoretickes - ps7
Diaphoretickes - ps8
Archaeplastida - ps9
Viridiplantae - ps10
Streptophyta - ps11
Charophyceae/Embriophyta - ps12
Embriophyta - ps13
Tracheophyta - ps14
Spermatophyta - ps15
Angiosperms - ps16
Mesangiospermae - ps17
Magnoliids/eudicots - ps18
Eudicots - ps19
Core_eudicots - ps20
Superrosids - ps21
Rosids - ps22
Eurosids - ps23
Malvids - ps24
Sap./Malv./Brassicales - ps25
Malvales/Brassicales - ps26
Brassicales - ps27
Cleomaceae/Brassicaceae - ps28
Brassicaceae - ps29
Camelineae - ps30
Arabidopsis - ps31
Arabidopsis_thaliana - ps32
p-value
5e-23
8e-18
1e-12
2e-07
4e-02
Log-odds
0.5
1.0
1.5
2.0
2.5
3.0
3.5
4.0

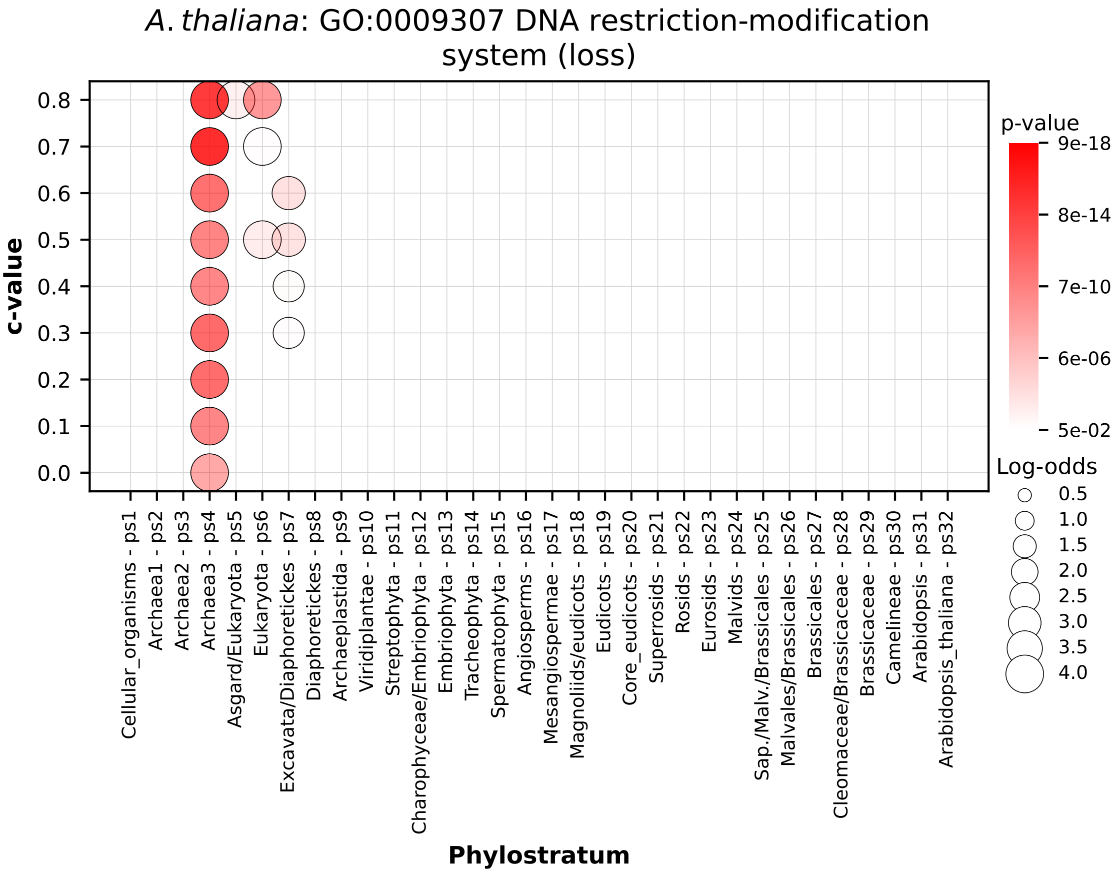

A. thaliana: GO:0009307 DNA restriction-modification system (loss)
c-value
0.8
0.7
0.6
0.5
0.4
0.3
0.2
0.1
0.0
Phylostratum
Cellular_organisms - ps1
Archaea1 - ps2
Archaea2 - ps3
Archaea3 - ps4
Asgard/Eukaryota - ps5
Eukaryota - ps6
Excavata/Diaphoretickes - ps7
Diaphoretickes - ps8
Archaeplastida - ps9
Viridiplantae - ps10
Streptophyta - ps11
Charophyceae/Embriophyta - ps12
Embriophyta - ps13
Tracheophyta - ps14
Spermatophyta - ps15
Angiosperms - ps16
Mesangiospermae - ps17
Magnoliids/eudicots - ps18
Eudicots - ps19
Core_eudicots - ps20
Superrosids - ps21
Rosids - ps22
Eurosids - ps23
Malvids - ps24
Sap./Malv./Brassicales - ps25
Malvales/Brassicales - ps26
Brassicales - ps27
Cleomaceae/Brassicaceae - ps28
Brassicaceae - ps29
Camelineae - ps30
Arabidopsis - ps31
Arabidopsis_thaliana - ps32
p-value
9e-18
8e-14
7e-10
6e-06
5e-02
Log-odds
0.5
1.0
1.5
2.0
2.5
3.0
3.5
4.0

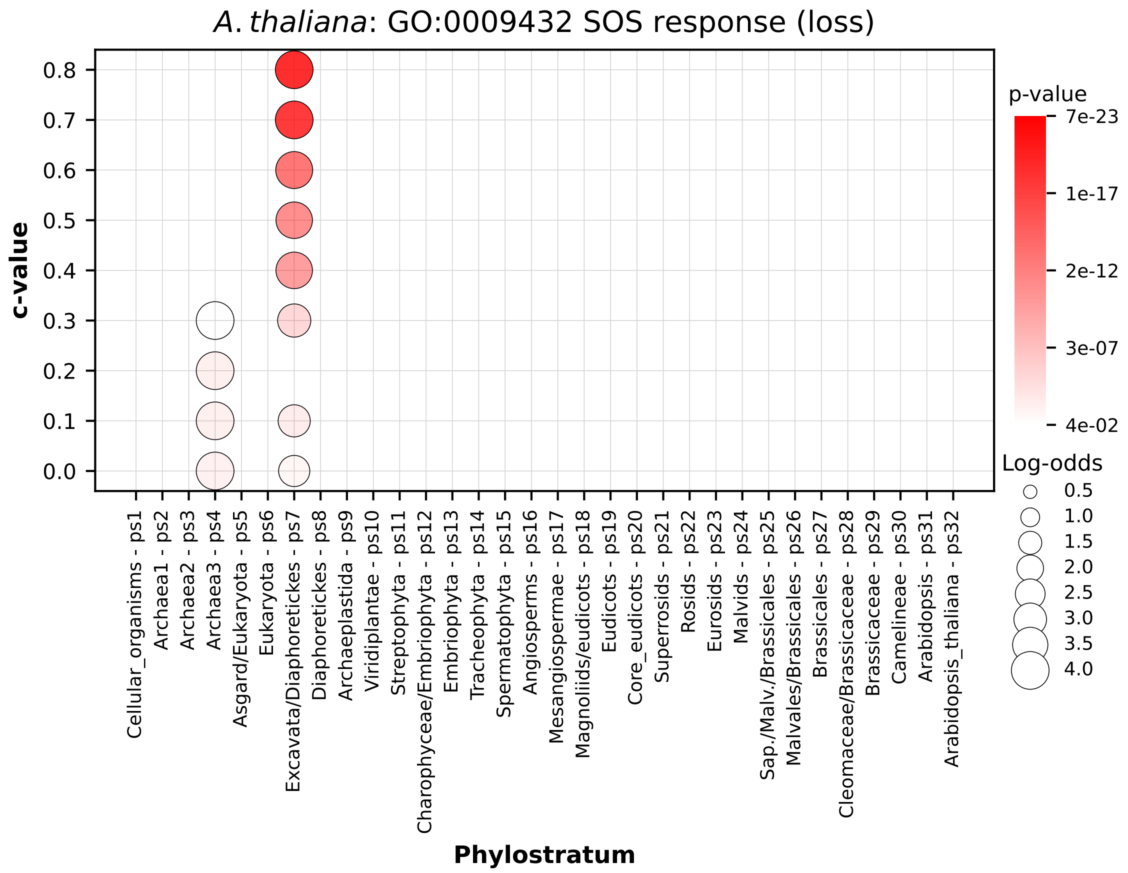
A. thaliana: GO:0009432 SOS response (loss)
c-value
0.8
0.7
0.6
0.5
0.4
0.3
0.2
0.1
0.0
p-value
7e-23
1e-17
2e-12
3e-07
4e-02
Log-odds
0.5
1.0
1.5
2.0
2.5
3.0
3.5
4.0
Cellular_organisms - ps1
Archaea1 - ps2
Archaea2 - ps3
Archaea3 - ps4
Asgard/Eukaryota - ps5
Eukaryota - ps6
Excavata/Diaphoretickes - ps7
Diaphoretickes - ps8
Archaeplastida - ps9
Viridiplantae - ps10
Streptophyta - ps11
Charophyceae/Embriophyta - ps12
Embriophyta - ps13
Tracheophyta - ps14
Spermatophyta - ps15
Angiosperms - ps16
Mesangiospermae - ps17
Magnoliids/eudicots - ps18
Eudicots - ps19
Core_eudicots - ps20
Superrosids - ps21
Rosids - ps22
Eurosids - ps23
Malvids - ps24
Sap./Malv./Brassicales - ps25
Malvales/Brassicales - ps26
Brassicales - ps27
Cleomaceae/Brassicaceae - ps28
Brassicaceae - ps29
Camelineae - ps30
Arabidopsis - ps31
Arabidopsis_thaliana - ps32
Phylostratum

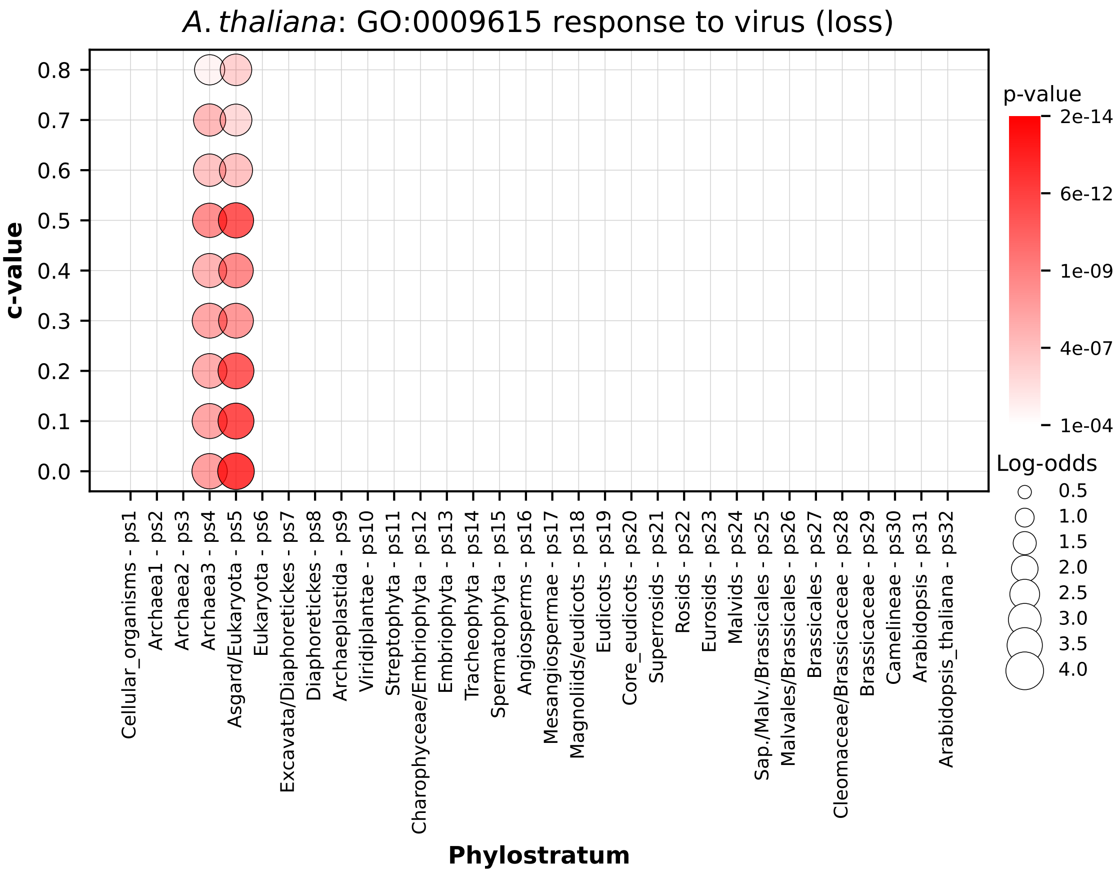
A. thaliana: GO:0009615 response to virus (loss)
c-value
0.8
0.7
0.6
0.5
0.4
0.3
0.2
0.1
0.0
Phylostratum
Cellular_organisms - ps1
Archaea1 - ps2
Archaea2 - ps3
Archaea3 - ps4
Asgard/Eukaryota - ps5
Eukaryota - ps6
Excavata/Diaphoretickes - ps7
Diaphoretickes - ps8
Archaeplastida - ps9
Viridiplantae - ps10
Streptophyta - ps11
Charophyceae/Embriophyta - ps12
Embriophyta - ps13
Tracheophyta - ps14
Spermatophyta - ps15
Angiosperms - ps16
Mesangiospermae - ps17
Magnoliids/eudicots - ps18
Eudicots - ps19
Core_eudicots - ps20
Superrosids - ps21
Rosids - ps22
Eurosids - ps23
Malvids - ps24
Sap./Malv./Brassicales - ps25
Malvales/Brassicales - ps26
Brassicales - ps27
Cleomaceae/Brassicaceae - ps28
Brassicaceae - ps29
Camelineae - ps30
Arabidopsis - ps31
Arabidopsis_thaliana - ps32
p-value
2e-14
6e-12
1e-09
4e-07
1e-04
Log-odds
0.5
1.0
1.5
2.0
2.5
3.0
3.5
4.0

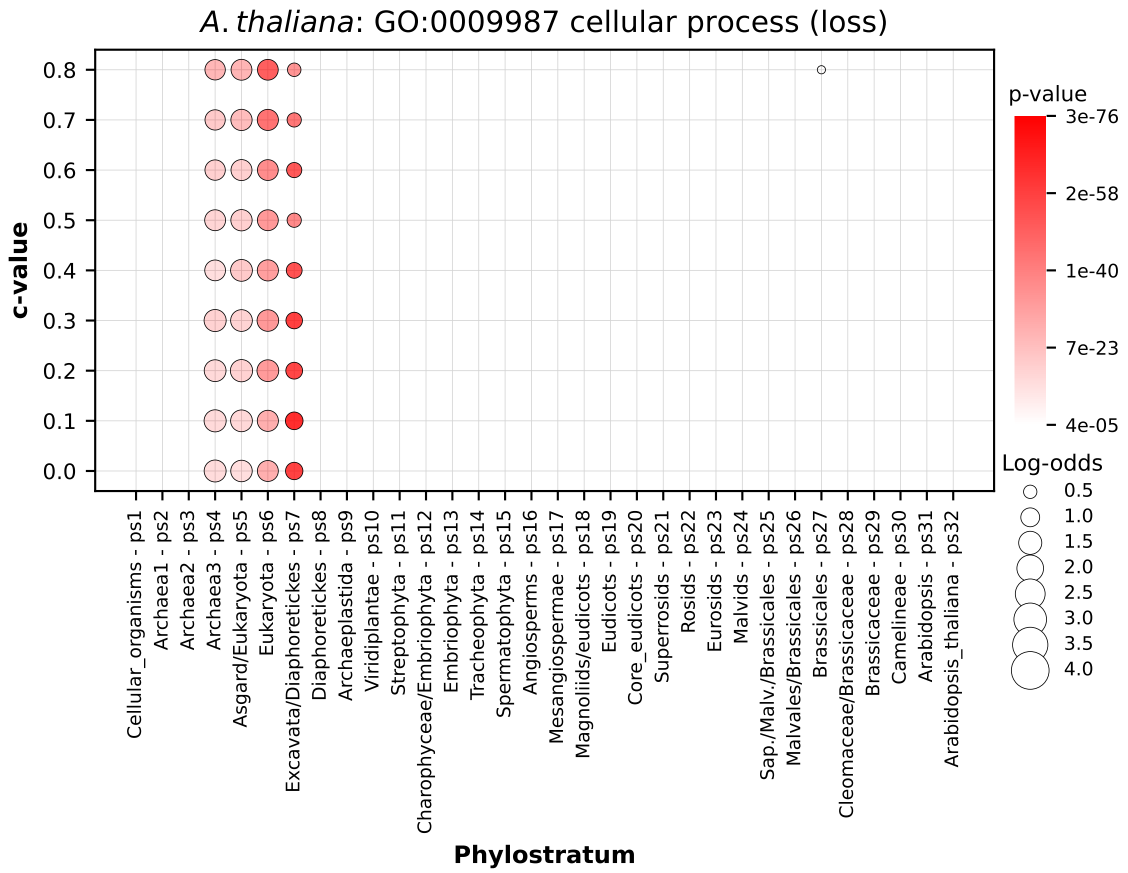

A. thaliana: GO:0009987 cellular process (loss)
c-value
0.0
0.1
0.2
0.3
0.4
0.5
0.6
0.7
0.8
p-value
3e-76
2e-58
1e-40
7e-23
4e-05
Log-odds
0.5
1.0
1.5
2.0
2.5
3.0
3.5
4.0
Cellular_organisms - ps1
Archaea1 - ps2
Archaea2 - ps3
Archaea3 - ps4
Asgard/Eukaryota - ps5
Eukaryota - ps6
Excavata/Diaphoretickes - ps7
Diaphoretickes - ps8
Archaeplastida - ps9
Viridiplantae - ps10
Streptophyta - ps11
Charophyceae/Embriophyta - ps12
Embriophyta - ps13
Tracheophyta - ps14
Spermatophyta - ps15
Angiosperms - ps16
Mesangiospermae - ps17
Magnoliids/eudicots - ps18
Eudicots - ps19
Core_eudicots - ps20
Superrosids - ps21
Rosids - ps22
Eurosids - ps23
Malvids - ps24
Sap./Malv./Brassicales - ps25
Malvales/Brassicales - ps26
Brassicales - ps27
Cleomaceae/Brassicaceae - ps28
Brassicaceae - ps29
Camelineae - ps30
Arabidopsis - ps31
Arabidopsis_thaliana - ps32
Phylostratum

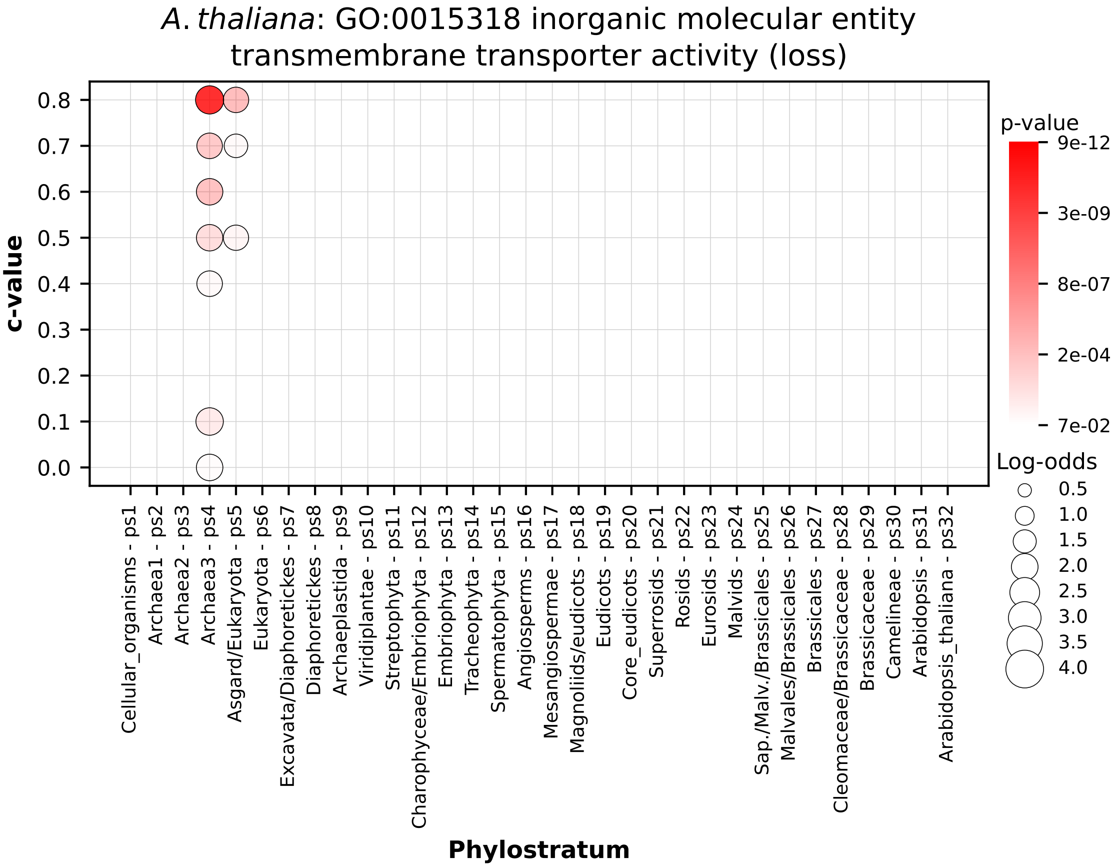
A. thaliana: GO:0015318 inorganic molecular entity transmembrane transporter activity (loss)
c-value
0.8
0.7
0.6
0.5
0.4
0.3
0.2
0.1
0.0
Phylostratum
Cellular_organisms - ps1
Archaea1 - ps2
Archaea2 - ps3
Archaea3 - ps4
Asgard/Eukaryota - ps5
Eukaryota - ps6
Excavata/Diaphoretickes - ps7
Diaphoretickes - ps8
Archaeplastida - ps9
Viridiplantae - ps10
Streptophyta - ps11
Charophyceae/Embriophyta - ps12
Embriophyta - ps13
Tracheophyta - ps14
Spermatophyta - ps15
Angiosperms - ps16
Mesangiospermae - ps17
Magnoliids/eudicots - ps18
Eudicots - ps19
Core_eudicots - ps20
Superrosids - ps21
Rosids - ps22
Eurosids - ps23
Malvids - ps24
Sap./Malv./Brassicales - ps25
Malvales/Brassicales - ps26
Brassicales - ps27
Cleomaceae/Brassicaceae - ps28
Brassicaceae - ps29
Camelineae - ps30
Arabidopsis - ps31
Arabidopsis_thaliana - ps32
p-value
9e-12
3e-09
8e-07
2e-04
7e-02
Log-odds
0.5
1.0
1.5
2.0
2.5
3.0
3.5
4.0

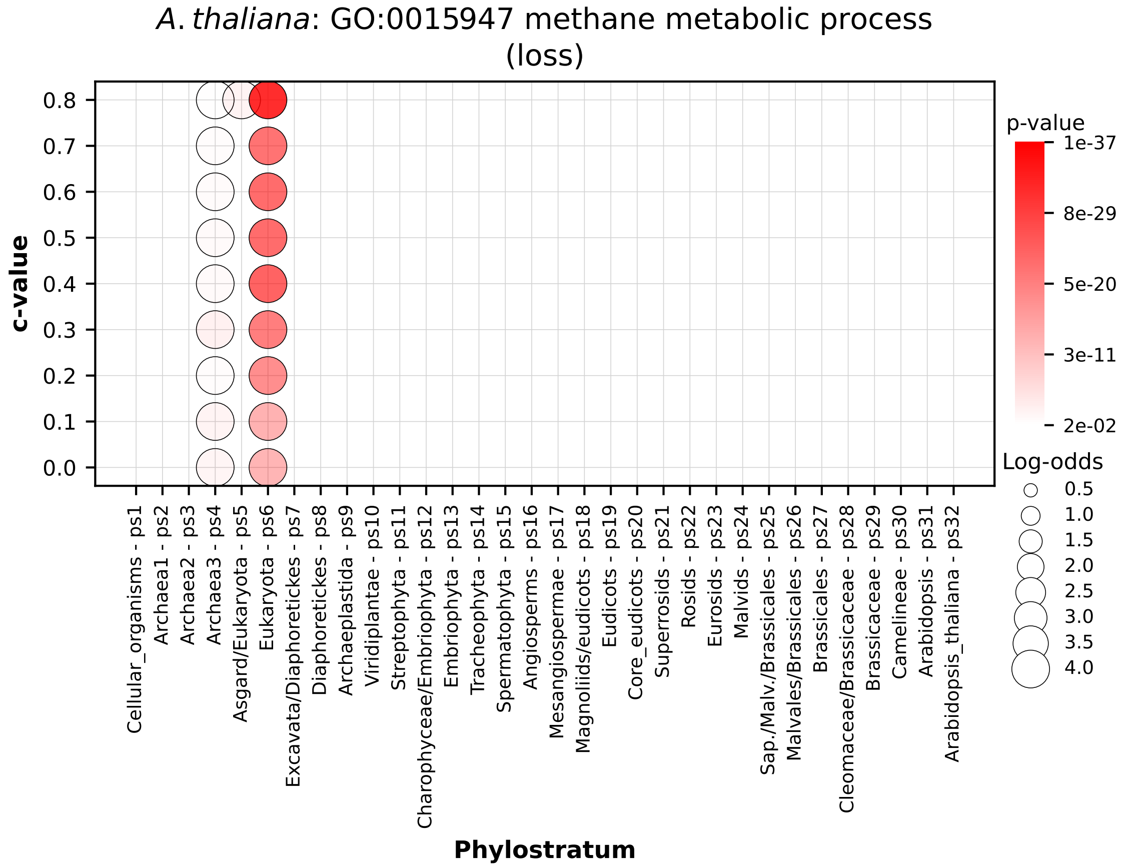

A. thaliana: GO:0015947 methane metabolic process
(loss)
c-value
0.8
0.7
0.6
0.5
0.4
0.3
0.2
0.1
0.0
p-value
1e-37
8e-29
5e-20
3e-11
2e-02
Log-odds
0.5
1.0
1.5
2.0
2.5
3.0
3.5
4.0
Cellular_organisms - ps1
Archaea1 - ps2
Archaea2 - ps3
Archaea3 - ps4
Asgard/Eukaryota - ps5
Eukaryota - ps6
Excavata/Diaphoretickes - ps7
Diaphoretickes - ps8
Archaeplastida - ps9
Viridiplantae - ps10
Streptophyta - ps11
Charophyceae/Embriophyta - ps12
Embriophyta - ps13
Tracheophyta - ps14
Spermatophyta - ps15
Angiosperms - ps16
Mesangiospermae - ps17
Magnoliids/eudicots - ps18
Eudicots - ps19
Core_eudicots - ps20
Superrosids - ps21
Rosids - ps22
Eurosids - ps23
Malvids - ps24
Sap./Malv./Brassicales - ps25
Malvales/Brassicales - ps26
Brassicales - ps27
Cleomaceae/Brassicaceae - ps28
Brassicaceae - ps29
Camelineae - ps30
Arabidopsis - ps31
Arabidopsis_thaliana - ps32
Phylostratum

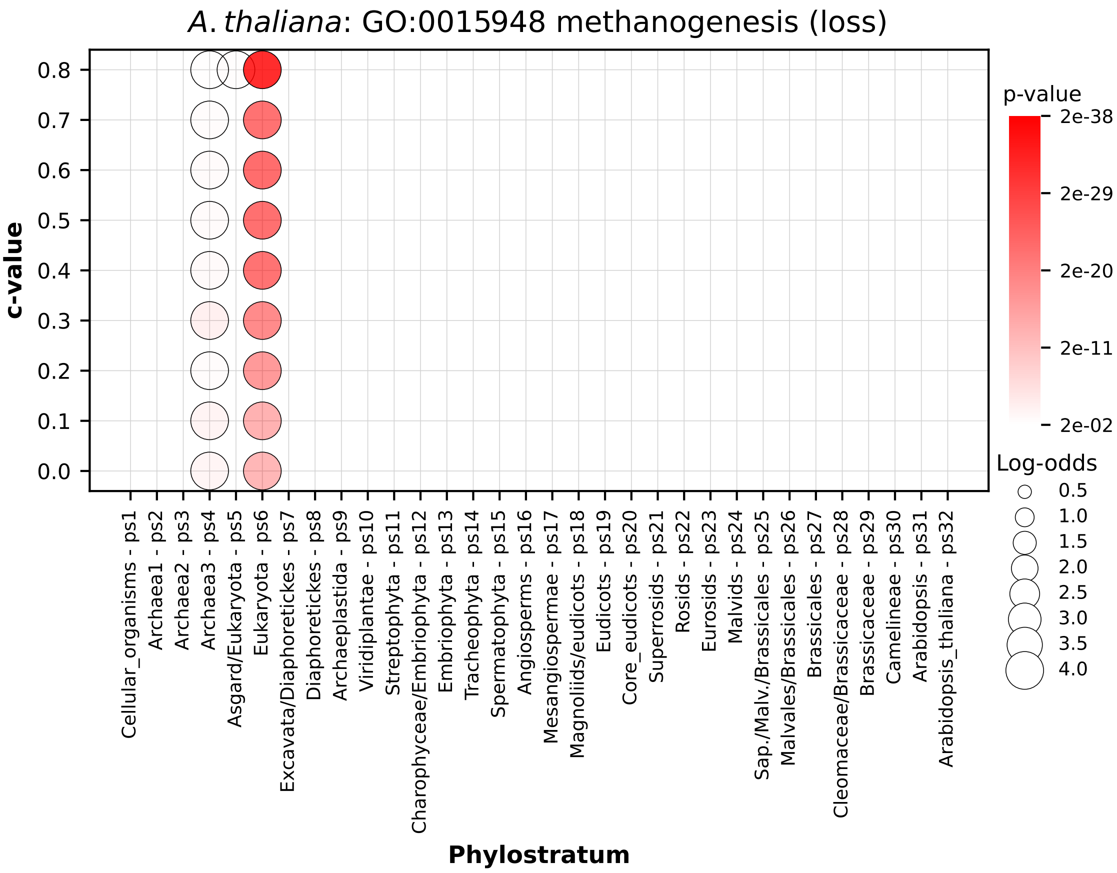
A. thaliana: GO:0015948 methanogenesis (loss)
c-value
0.8
0.7
0.6
0.5
0.4
0.3
0.2
0.1
0.0
p-value
2e-38
2e-29
2e-20
2e-11
2e-02
Log-odds
0.5
1.0
1.5
2.0
2.5
3.0
3.5
4.0
Cellular_organisms - ps1
Archaea1 - ps2
Archaea2 - ps3
Archaea3 - ps4
Asgard/Eukaryota - ps5
Eukaryota - ps6
Excavata/Diaphoretickes - ps7
Diaphoretickes - ps8
Archaeplastida - ps9
Viridiplantae - ps10
Streptophyta - ps11
Charophyceae/Embriophyta - ps12
Embriophyta - ps13
Tracheophyta - ps14
Spermatophyta - ps15
Angiosperms - ps16
Mesangiospermae - ps17
Magnoliids/eudicots - ps18
Eudicots - ps19
Core_eudicots - ps20
Superrosids - ps21
Rosids - ps22
Eurosids - ps23
Malvids - ps24
Sap./Malv./Brassicales - ps25
Malvales/Brassicales - ps26
Brassicales - ps27
Cleomaceae/Brassicaceae - ps28
Brassicaceae - ps29
Camelineae - ps30
Arabidopsis - ps31
Arabidopsis_thaliana - ps32
Phylostratum

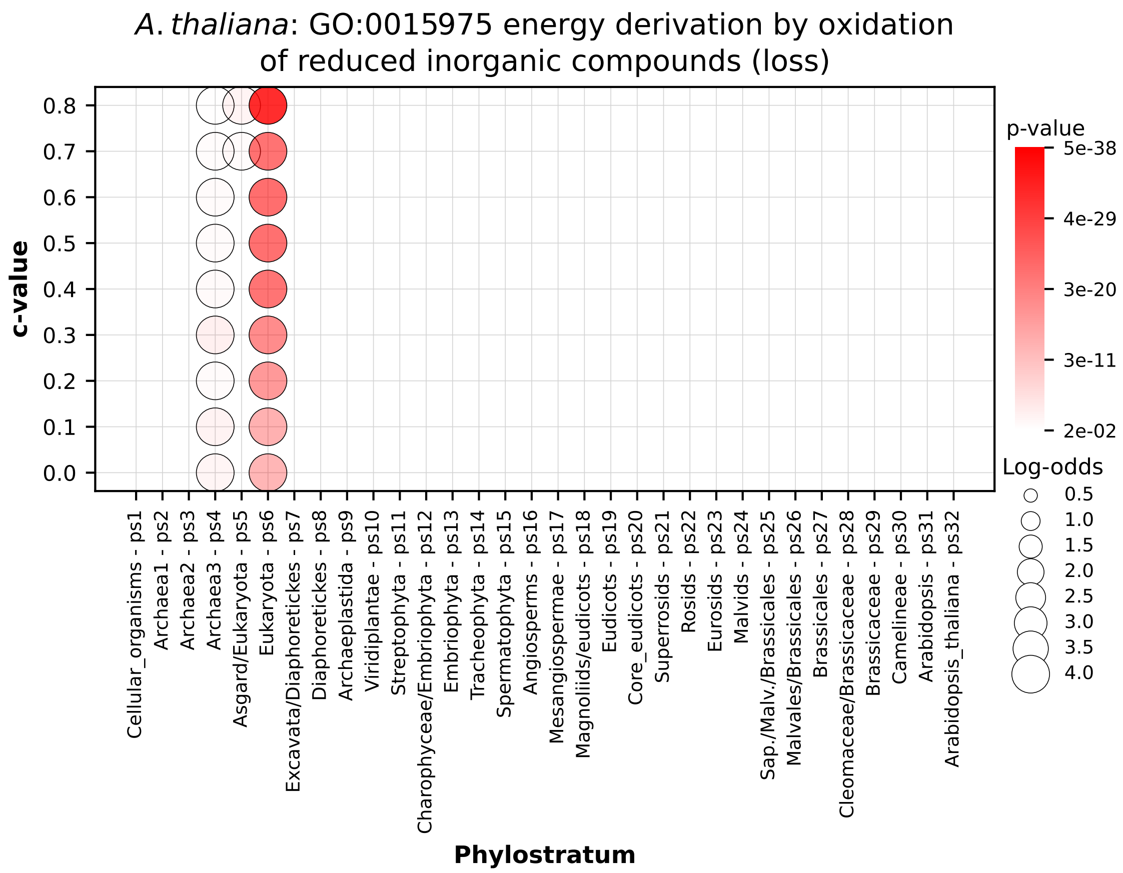
A. thaliana: GO:0015975 energy derivation by oxidation of reduced inorganic compounds (loss)
c-value
0.8
0.7
0.6
0.5
0.4
0.3
0.2
0.1
0.0
p-value
5e-38
4e-29
3e-20
3e-11
2e-02
Log-odds
0.5
1.0
1.5
2.0
2.5
3.0
3.5
4.0
Cellular_organisms - ps1
Archaea1 - ps2
Archaea2 - ps3
Archaea3 - ps4
Asgard/Eukaryota - ps5
Eukaryota - ps6
Excavata/Diaphoretickes - ps7
Diaphoretickes - ps8
Archaeplastida - ps9
Viridiplantae - ps10
Streptophyta - ps11
Charophyceae/Embriophyta - ps12
Embriophyta - ps13
Tracheophyta - ps14
Spermatophyta - ps15
Angiosperms - ps16
Mesangiospermae - ps17
Magnoliids/eudicots - ps18
Eudicots - ps19
Core_eudicots - ps20
Superrosids - ps21
Rosids - ps22
Eurosids - ps23
Malvids - ps24
Sap./Malv./Brassicales - ps25
Malvales/Brassicales - ps26
Brassicales - ps27
Cleomaceae/Brassicaceae - ps28
Brassicaceae - ps29
Camelineae - ps30
Arabidopsis - ps31
Arabidopsis_thaliana - ps32
Phylostratum

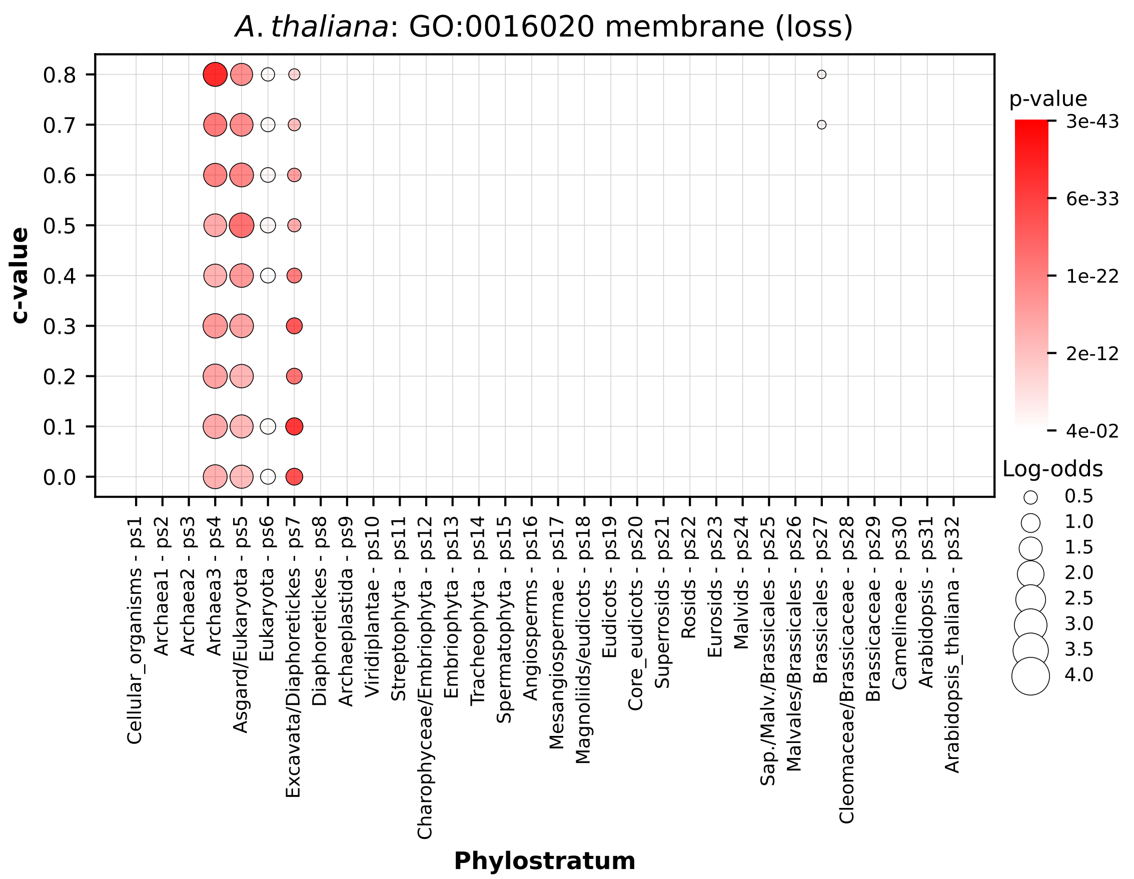

A. thaliana: GO:0016020 membrane (loss)
c-value
0.8
0.7
0.6
0.5
0.4
0.3
0.2
0.1
0.0
Phylostratum
Cellular_organisms - ps1
Archaea1 - ps2
Archaea2 - ps3
Archaea3 - ps4
Asgard/Eukaryota - ps5
Eukaryota - ps6
Excavata/Diaphoretickes - ps7
Diaphoretickes - ps8
Archaeplastida - ps9
Viridiplantae - ps10
Streptophyta - ps11
Charophyceae/Embriophyta - ps12
Embriophyta - ps13
Tracheophyta - ps14
Spermatophyta - ps15
Angiosperms - ps16
Mesangiospermae - ps17
Magnoliids/eudicots - ps18
Eudicots - ps19
Core_eudicots - ps20
Superrosids - ps21
Rosids - ps22
Eurosids - ps23
Malvids - ps24
Sap./Malv./Brassicales - ps25
Malvales/Brassicales - ps26
Brassicales - ps27
Cleomaceae/Brassicaceae - ps28
Brassicaceae - ps29
Camelineae - ps30
Arabidopsis - ps31
Arabidopsis_thaliana - ps32
p-value
3e-43
6e-33
1e-22
2e-12
4e-02
Log-odds
0.5
1.0
1.5
2.0
2.5
3.0
3.5
4.0

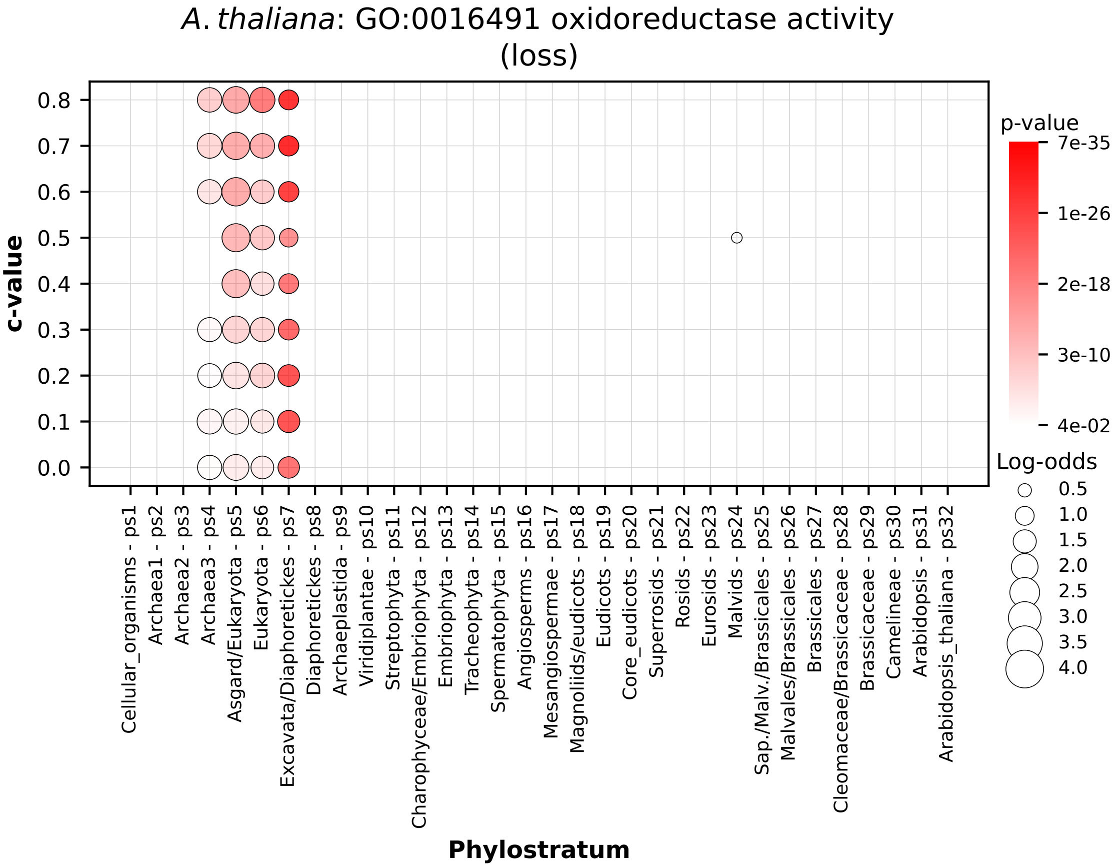

A. thaliana: GO:0016491 oxidoreductase activity
(loss)
c-value
0.8
0.7
0.6
0.5
0.4
0.3
0.2
0.1
0.0
Phylostratum
Cellular_organisms - ps1
Archaea1 - ps2
Archaea2 - ps3
Archaea3 - ps4
Asgard/Eukaryota - ps5
Eukaryota - ps6
Excavata/Diaphoretickes - ps7
Diaphoretickes - ps8
Archaeplastida - ps9
Viridiplantae - ps10
Streptophyta - ps11
Charophyceae/Embriophyta - ps12
Embriophyta - ps13
Tracheophyta - ps14
Spermatophyta - ps15
Angiosperms - ps16
Mesangiospermae - ps17
Magnoliids/eudicots - ps18
Eudicots - ps19
Core_eudicots - ps20
Superrosids - ps21
Rosids - ps22
Eurosids - ps23
Malvids - ps24
Sap./Malv./Brassicales - ps25
Malvales/Brassicales - ps26
Brassicales - ps27
Cleomaceae/Brassicaceae - ps28
Brassicaceae - ps29
Camelineae - ps30
Arabidopsis - ps31
Arabidopsis_thaliana - ps32
p-value
7e-35
1e-26
2e-18
3e-10
4e-02
Log-odds
0.5
1.0
1.5
2.0
2.5
3.0
3.5
4.0

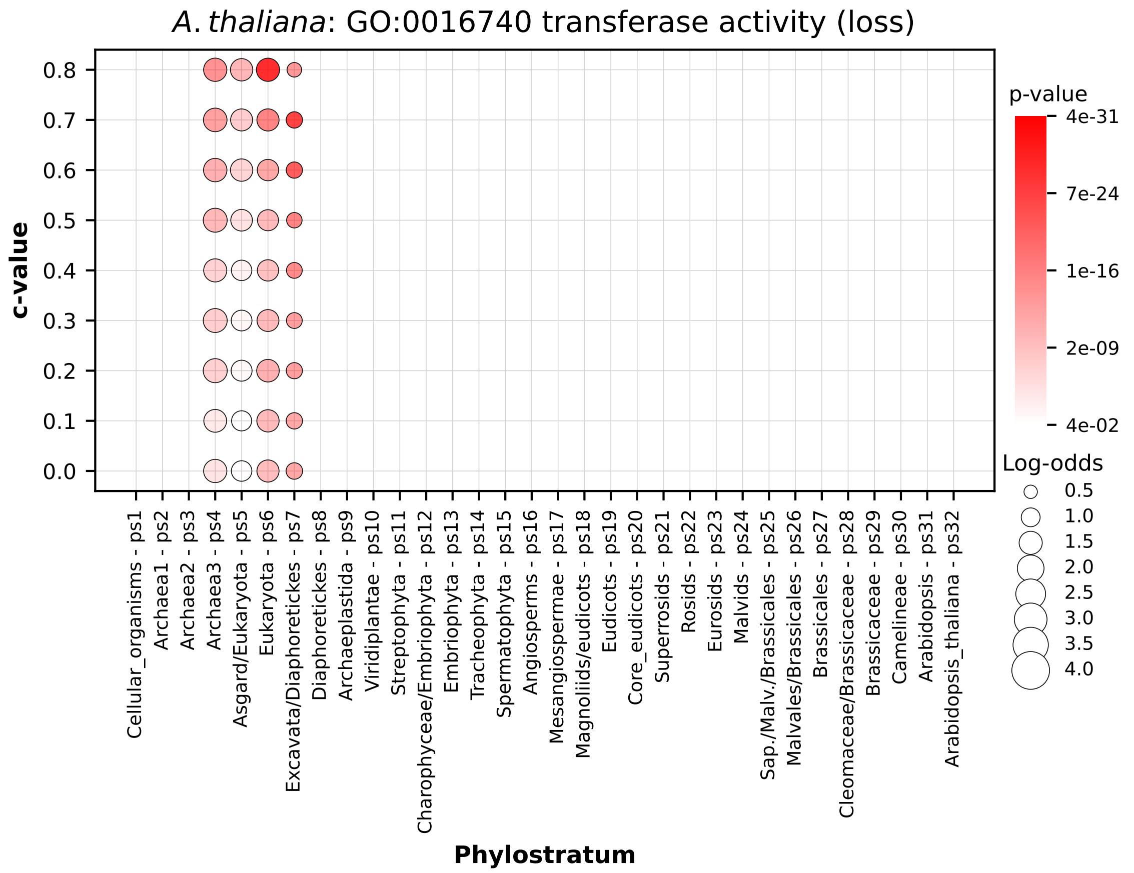

A. thaliana: GO:0016740 transferase activity (loss)
c-value
0.0
0.1
0.2
0.3
0.4
0.5
0.6
0.7
0.8
Phylostratum
Cellular_organisms - ps1
Archaea1 - ps2
Archaea2 - ps3
Archaea3 - ps4
Asgard/Eukaryota - ps5
Eukaryota - ps6
Excavata/Diaphoretickes - ps7
Diaphoretickes - ps8
Archaeplastida - ps9
Viridiplantae - ps10
Streptophyta - ps11
Charophyceae/Embriophyta - ps12
Embriophyta - ps13
Tracheophyta - ps14
Spermatophyta - ps15
Angiosperms - ps16
Mesangiospermae - ps17
Magnoliids/eudicots - ps18
Eudicots - ps19
Core_eudicots - ps20
Superrosids - ps21
Rosids - ps22
Eurosids - ps23
Malvids - ps24
Sap./Malv./Brassicales - ps25
Malvales/Brassicales - ps26
Brassicales - ps27
Cleomaceae/Brassicaceae - ps28
Brassicaceae - ps29
Camelineae - ps30
Arabidopsis - ps31
Arabidopsis_thaliana - ps32
p-value
4e-31
7e-24
1e-16
2e-09
4e-02
Log-odds
0.5
1.0
1.5
2.0
2.5
3.0
3.5
4.0

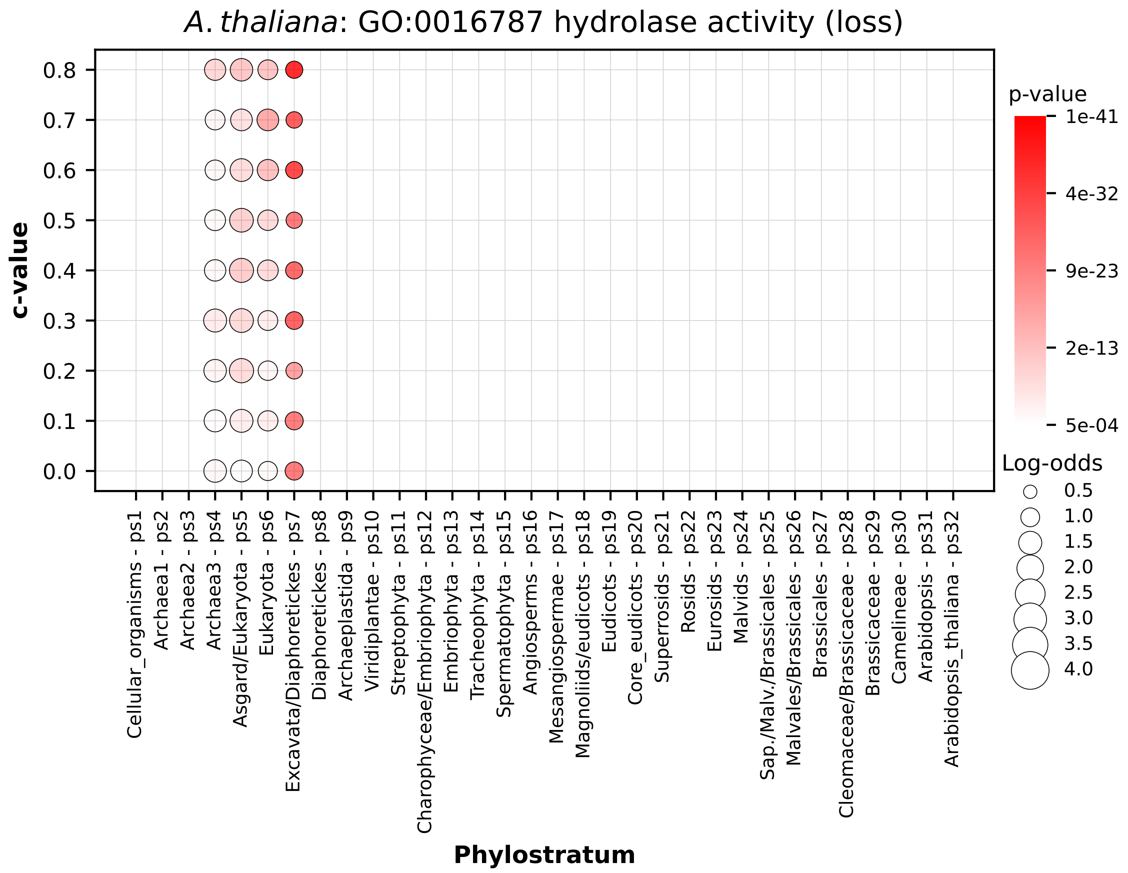

A. thaliana: GO:0016787 hydrolase activity (loss)
c-value
0.8
0.7
0.6
0.5
0.4
0.3
0.2
0.1
0.0
p-value
1e-41
4e-32
9e-23
2e-13
5e-04
Log-odds
0.5
1.0
1.5
2.0
2.5
3.0
3.5
4.0
Cellular_organisms - ps1
Archaea1 - ps2
Archaea2 - ps3
Archaea3 - ps4
Asgard/Eukaryota - ps5
Eukaryota - ps6
Excavata/Diaphoretickes - ps7
Diaphoretickes - ps8
Archaeplastida - ps9
Viridiplantae - ps10
Streptophyta - ps11
Charophyceae/Embriophyta - ps12
Embriophyta - ps13
Tracheophyta - ps14
Spermatophyta - ps15
Angiosperms - ps16
Mesangiospermae - ps17
Magnoliids/eudicots - ps18
Eudicots - ps19
Core_eudicots - ps20
Superrosids - ps21
Rosids - ps22
Eurosids - ps23
Malvids - ps24
Sap./Malv./Brassicales - ps25
Malvales/Brassicales - ps26
Brassicales - ps27
Cleomaceae/Brassicaceae - ps28
Brassicaceae - ps29
Camelineae - ps30
Arabidopsis - ps31
Arabidopsis_thaliana - ps32
Phylostratum

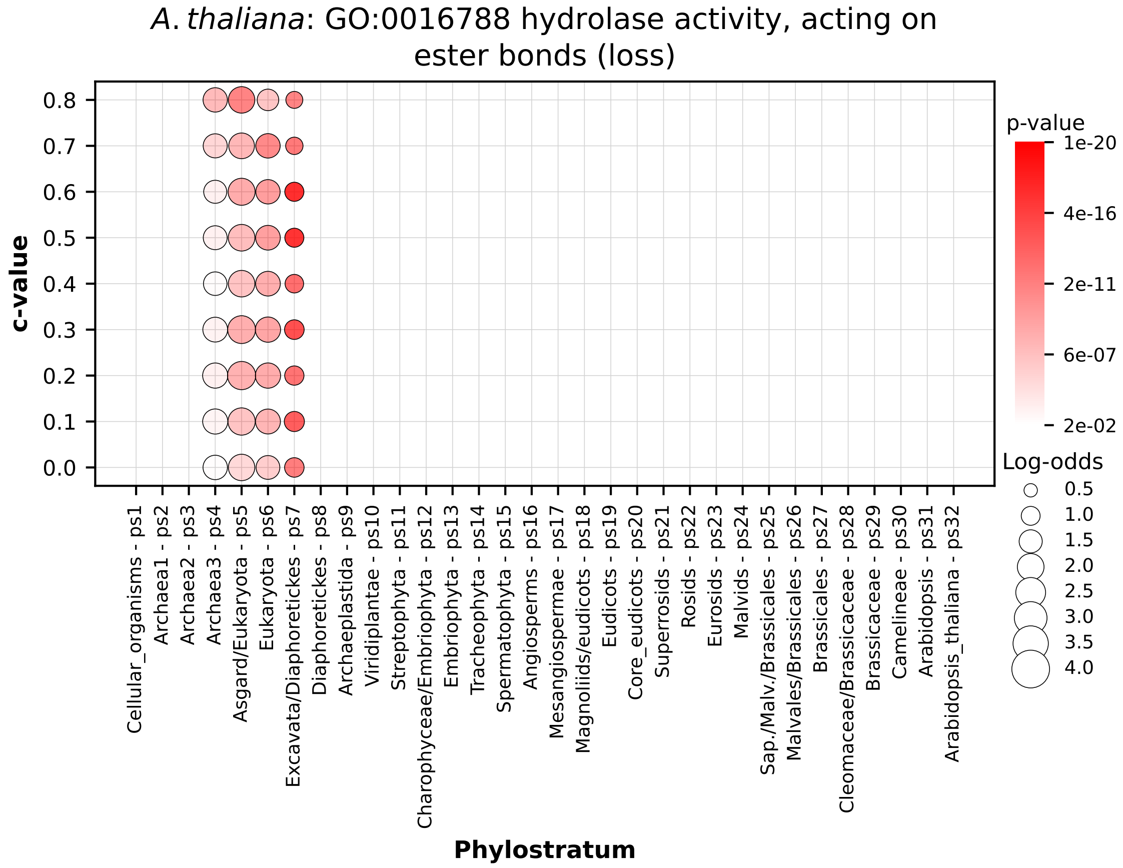

A. thaliana: GO:0016788 hydrolase activity, acting on ester bonds (loss)
c-value
0.8
0.7
0.6
0.5
0.4
0.3
0.2
0.1
0.0
p-value
1e-20
4e-16
2e-11
6e-07
2e-02
Log-odds
0.5
1.0
1.5
2.0
2.5
3.0
3.5
4.0
Cellular_organisms - ps1
Archaea1 - ps2
Archaea2 - ps3
Archaea3 - ps4
Asgard/Eukaryota - ps5
Eukaryota - ps6
Excavata/Diaphoretickes - ps7
Diaphoretickes - ps8
Archaeplastida - ps9
Viridiplantae - ps10
Streptophyta - ps11
Charophyceae/Embriophyta - ps12
Embriophyta - ps13
Tracheophyta - ps14
Spermatophyta - ps15
Angiosperms - ps16
Mesangiospermae - ps17
Magnoliids/eudicots - ps18
Eudicots - ps19
Core_eudicots - ps20
Superrosids - ps21
Rosids - ps22
Eurosids - ps23
Malvids - ps24
Sap./Malv./Brassicales - ps25
Malvales/Brassicales - ps26
Brassicales - ps27
Cleomaceae/Brassicaceae - ps28
Brassicaceae - ps29
Camelineae - ps30
Arabidopsis - ps31
Arabidopsis_thaliana - ps32
Phylostratum

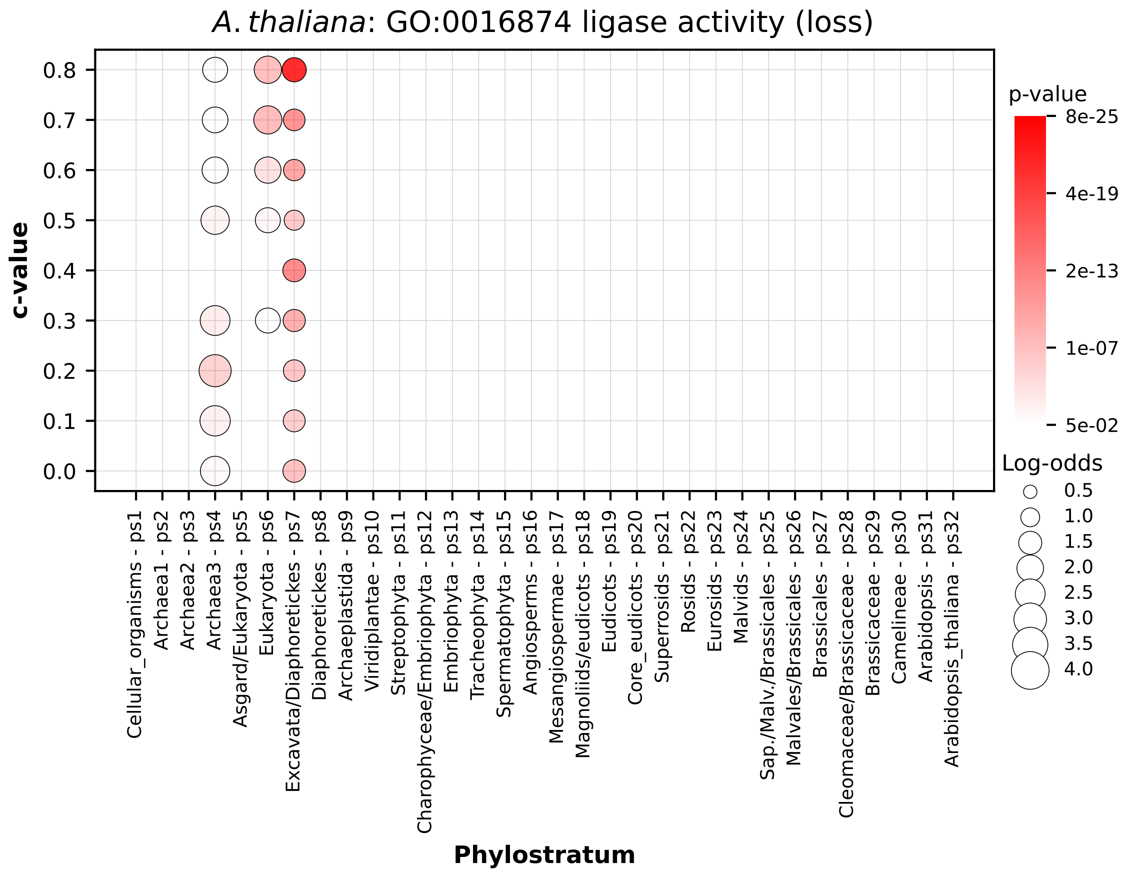
A. thaliana: GO:0016874 ligase activity (loss)
c-value
0.0
0.1
0.2
0.3
0.4
0.5
0.6
0.7
0.8
p-value
8e-25
4e-19
2e-13
1e-07
5e-02
Log-odds
0.5
1.0
1.5
2.0
2.5
3.0
3.5
4.0
Cellular_organisms - ps1
Archaea1 - ps2
Archaea2 - ps3
Archaea3 - ps4
Asgard/Eukaryota - ps5
Eukaryota - ps6
Excavata/Diaphoretickes - ps7
Diaphoretickes - ps8
Archaeplastida - ps9
Viridiplantae - ps10
Streptophyta - ps11
Charophyceae/Embriophyta - ps12
Embriophyta - ps13
Tracheophyta - ps14
Spermatophyta - ps15
Angiosperms - ps16
Mesangiospermae - ps17
Magnoliids/eudicots - ps18
Eudicots - ps19
Core_eudicots - ps20
Superrosids - ps21
Rosids - ps22
Eurosids - ps23
Malvids - ps24
Sap./Malv./Brassicales - ps25
Malvales/Brassicales - ps26
Brassicales - ps27
Cleomaceae/Brassicaceae - ps28
Brassicaceae - ps29
Camelineae - ps30
Arabidopsis - ps31
Arabidopsis_thaliana - ps32
Phylostratum

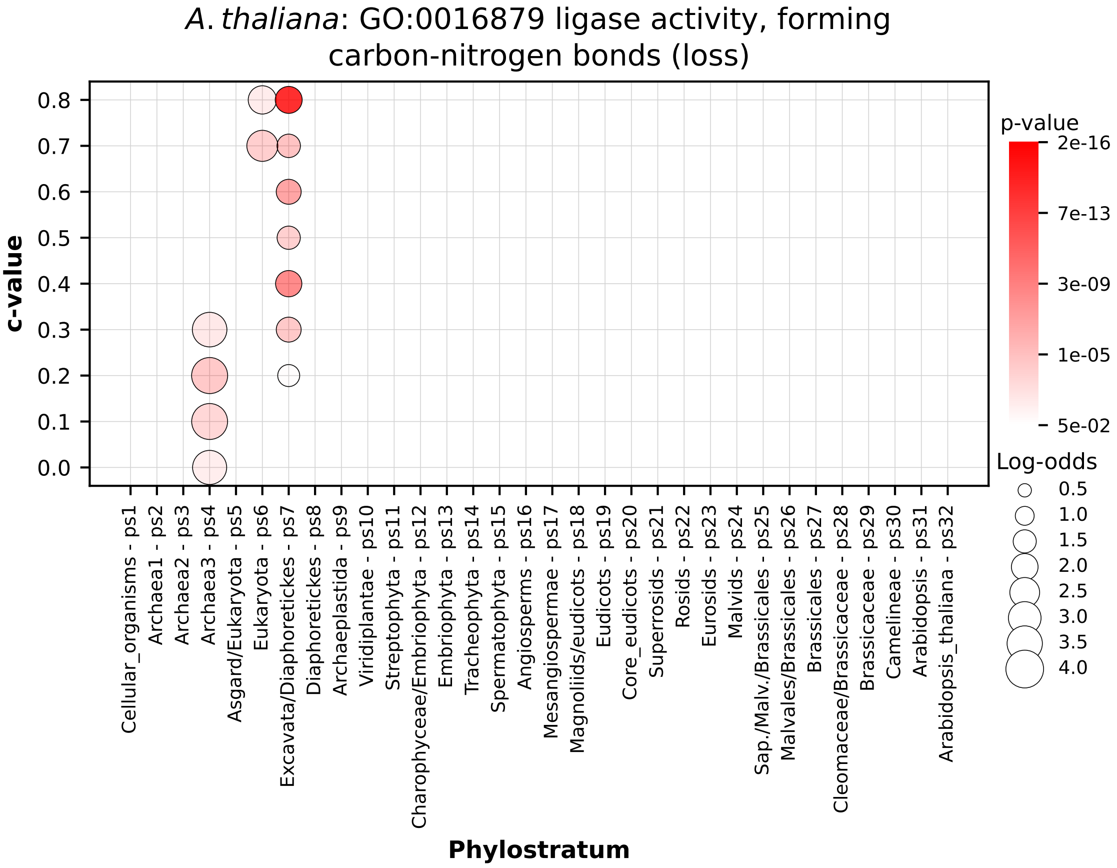
A. thaliana: GO:0016879 ligase activity, forming carbon-nitrogen bonds (loss)
c-value
0.8
0.7
0.6
0.5
0.4
0.3
0.2
0.1
0.0
p-value
2e-16
7e-13
3e-09
1e-05
5e-02
Log-odds
0.5
1.0
1.5
2.0
2.5
3.0
3.5
4.0
Cellular_organisms - ps1
Archaea1 - ps2
Archaea2 - ps3
Archaea3 - ps4
Asgard/Eukaryota - ps5
Eukaryota - ps6
Excavata/Diaphoretickes - ps7
Diaphoretickes - ps8
Archaeplastida - ps9
Viridiplantae - ps10
Streptophyta - ps11
Charophyceae/Embriophyta - ps12
Embriophyta - ps13
Tracheophyta - ps14
Spermatophyta - ps15
Angiosperms - ps16
Mesangiospermae - ps17
Magnoliids/eudicots - ps18
Eudicots - ps19
Core_eudicots - ps20
Superrosids - ps21
Rosids - ps22
Eurosids - ps23
Malvids - ps24
Sap./Malv./Brassicales - ps25
Malvales/Brassicales - ps26
Brassicales - ps27
Cleomaceae/Brassicaceae - ps28
Brassicaceae - ps29
Camelineae - ps30
Arabidopsis - ps31
Arabidopsis_thaliana - ps32
Phylostratum

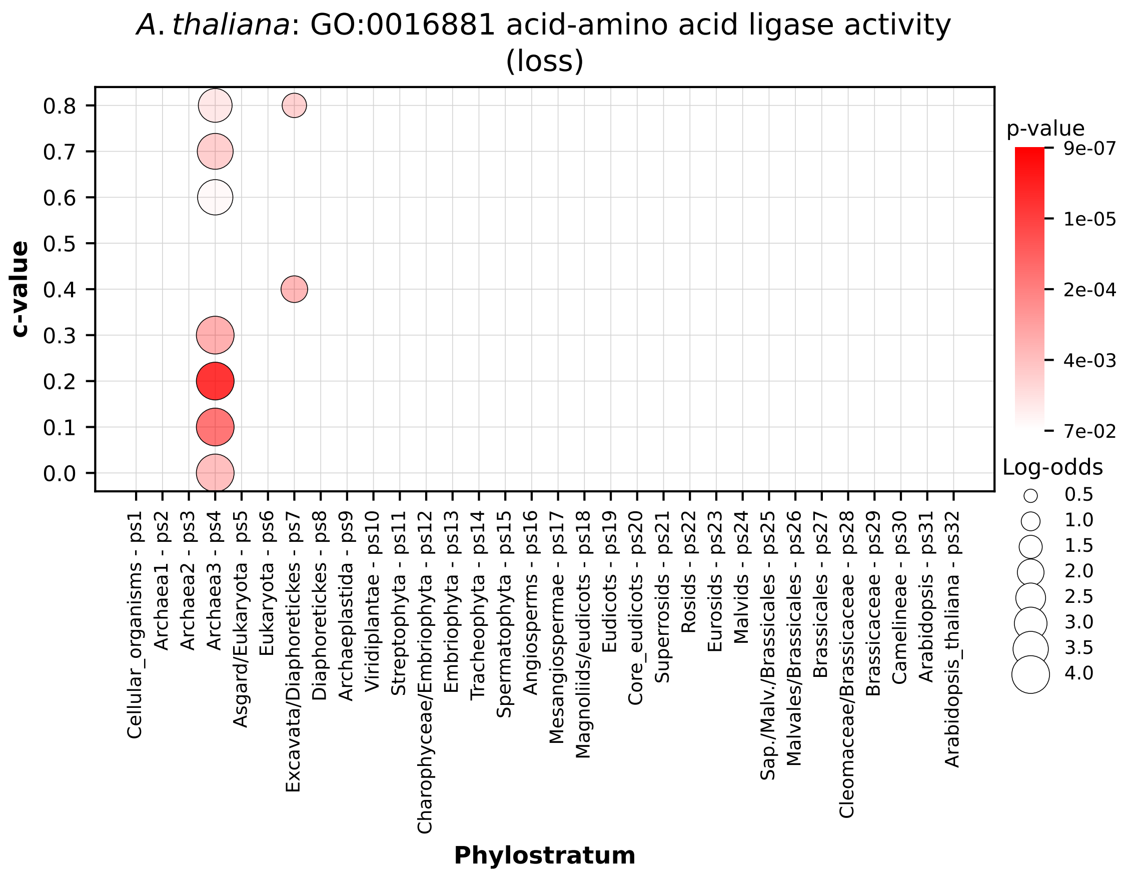
A. thaliana: GO:0016881 acid-amino acid ligase activity (loss)
c-value
0.8
0.7
0.6
0.5
0.4
0.3
0.2
0.1
0.0
Phylostratum
Cellular_organisms - ps1
Archaea1 - ps2
Archaea2 - ps3
Archaea3 - ps4
Asgard/Eukaryota - ps5
Eukaryota - ps6
Excavata/Diaphoretickes - ps7
Diaphoretickes - ps8
Archaeplastida - ps9
Viridiplantae - ps10
Streptophyta - ps11
Charophyceae/Embriophyta - ps12
Embriophyta - ps13
Tracheophyta - ps14
Spermatophyta - ps15
Angiosperms - ps16
Mesangiospermae - ps17
Magnoliids/eudicots - ps18
Eudicots - ps19
Core_eudicots - ps20
Superrosids - ps21
Rosids - ps22
Eurosids - ps23
Malvids - ps24
Sap./Malv./Brassicales - ps25
Malvales/Brassicales - ps26
Brassicales - ps27
Cleomaceae/Brassicaceae - ps28
Brassicaceae - ps29
Camelineae - ps30
Arabidopsis - ps31
Arabidopsis_thaliana - ps32
p-value
9e-07
1e-05
2e-04
4e-03
7e-02
Log-odds
0.5
1.0
1.5
2.0
2.5
3.0
3.5
4.0

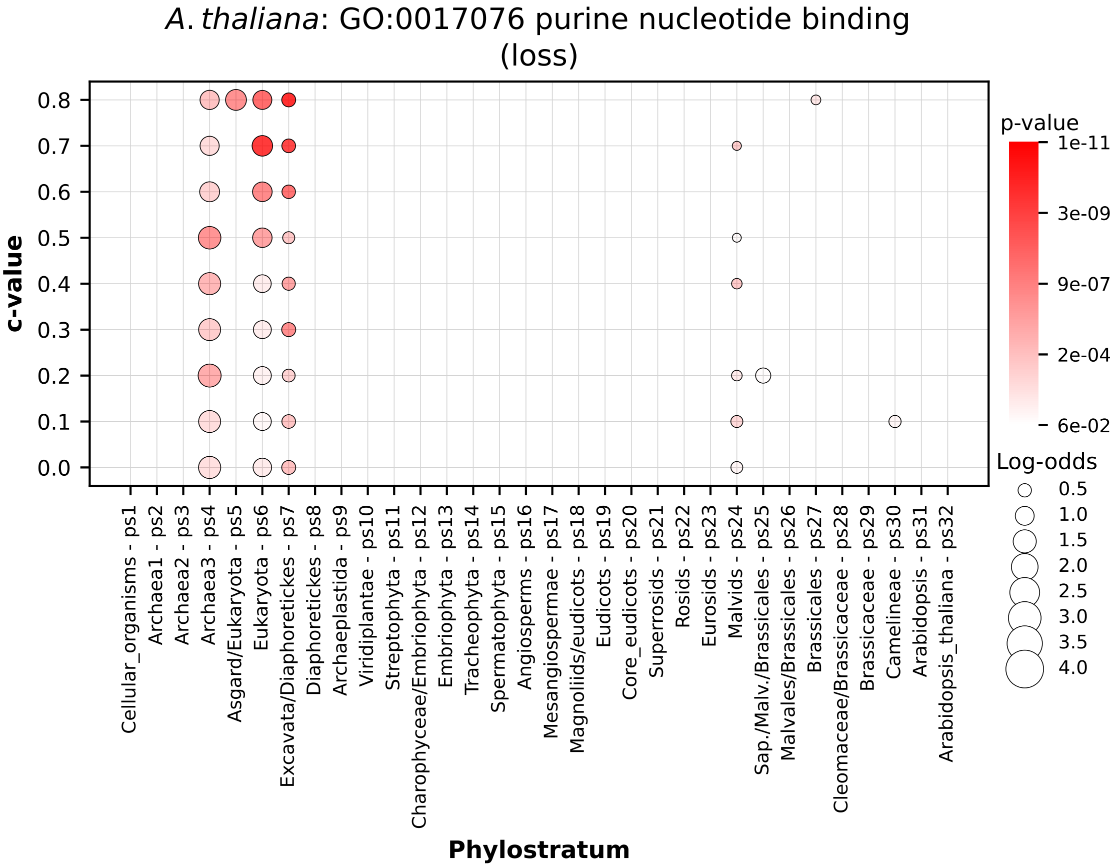

A. thaliana: GO:0017076 purine nucleotide binding (loss)
c-value
0.8
0.7
0.6
0.5
0.4
0.3
0.2
0.1
0.0
Phylostratum
Cellular_organisms - ps1
Archaea1 - ps2
Archaea2 - ps3
Archaea3 - ps4
Asgard/Eukaryota - ps5
Eukaryota - ps6
Excavata/Diaphoretickes - ps7
Diaphoretickes - ps8
Archaeplastida - ps9
Viridiplantae - ps10
Streptophyta - ps11
Charophyceae/Embriophyta - ps12
Embriophyta - ps13
Tracheophyta - ps14
Spermatophyta - ps15
Angiosperms - ps16
Mesangiospermae - ps17
Magnoliids/eudicots - ps18
Eudicots - ps19
Core_eudicots - ps20
Superrosids - ps21
Rosids - ps22
Eurosids - ps23
Malvids - ps24
Sap./Malv./Brassicales - ps25
Malvales/Brassicales - ps26
Brassicales - ps27
Cleomaceae/Brassicaceae - ps28
Brassicaceae - ps29
Camelineae - ps30
Arabidopsis - ps31
Arabidopsis_thaliana - ps32
p-value
1e-11
3e-09
9e-07
2e-04
6e-02
Log-odds
0.5
1.0
1.5
2.0
2.5
3.0
3.5
4.0

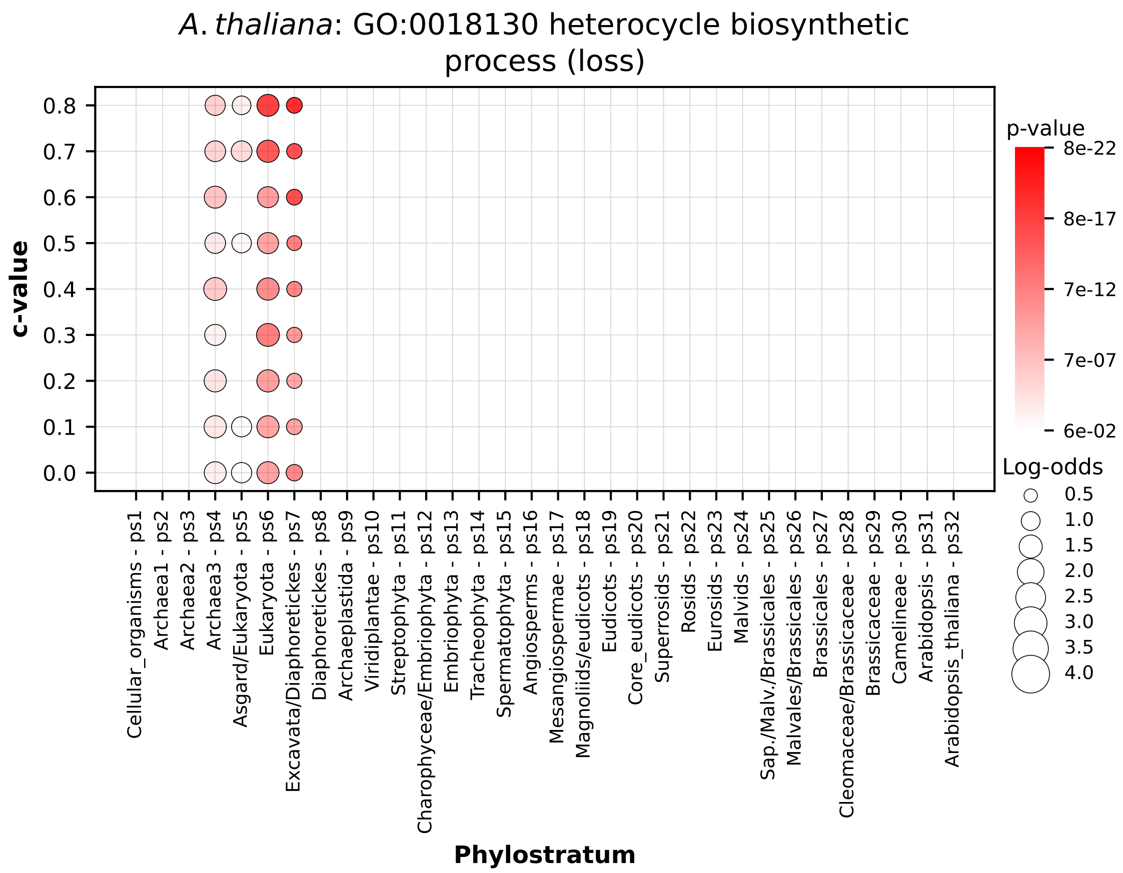

A. thaliana: GO:0018130 heterocycle biosynthetic process (loss)
c-value
0.8
0.7
0.6
0.5
0.4
0.3
0.2
0.1
0.0
Phylostratum
Cellular_organisms - ps1
Archaea1 - ps2
Archaea2 - ps3
Archaea3 - ps4
Asgard/Eukaryota - ps5
Eukaryota - ps6
Excavata/Diaphoretickes - ps7
Diaphoretickes - ps8
Archaeplastida - ps9
Viridiplantae - ps10
Streptophyta - ps11
Charophyceae/Embriophyta - ps12
Embriophyta - ps13
Tracheophyta - ps14
Spermatophyta - ps15
Angiosperms - ps16
Mesangiospermae - ps17
Magnoliids/eudicots - ps18
Eudicots - ps19
Core_eudicots - ps20
Superrosids - ps21
Rosids - ps22
Eurosids - ps23
Malvids - ps24
Sap./Malv./Brassicales - ps25
Malvales/Brassicales - ps26
Brassicales - ps27
Cleomaceae/Brassicaceae - ps28
Brassicaceae - ps29
Camelineae - ps30
Arabidopsis - ps31
Arabidopsis_thaliana - ps32
p-value
8e-22
8e-17
7e-12
7e-07
6e-02
Log-odds
0.5
1.0
1.5
2.0
2.5
3.0
3.5
4.0

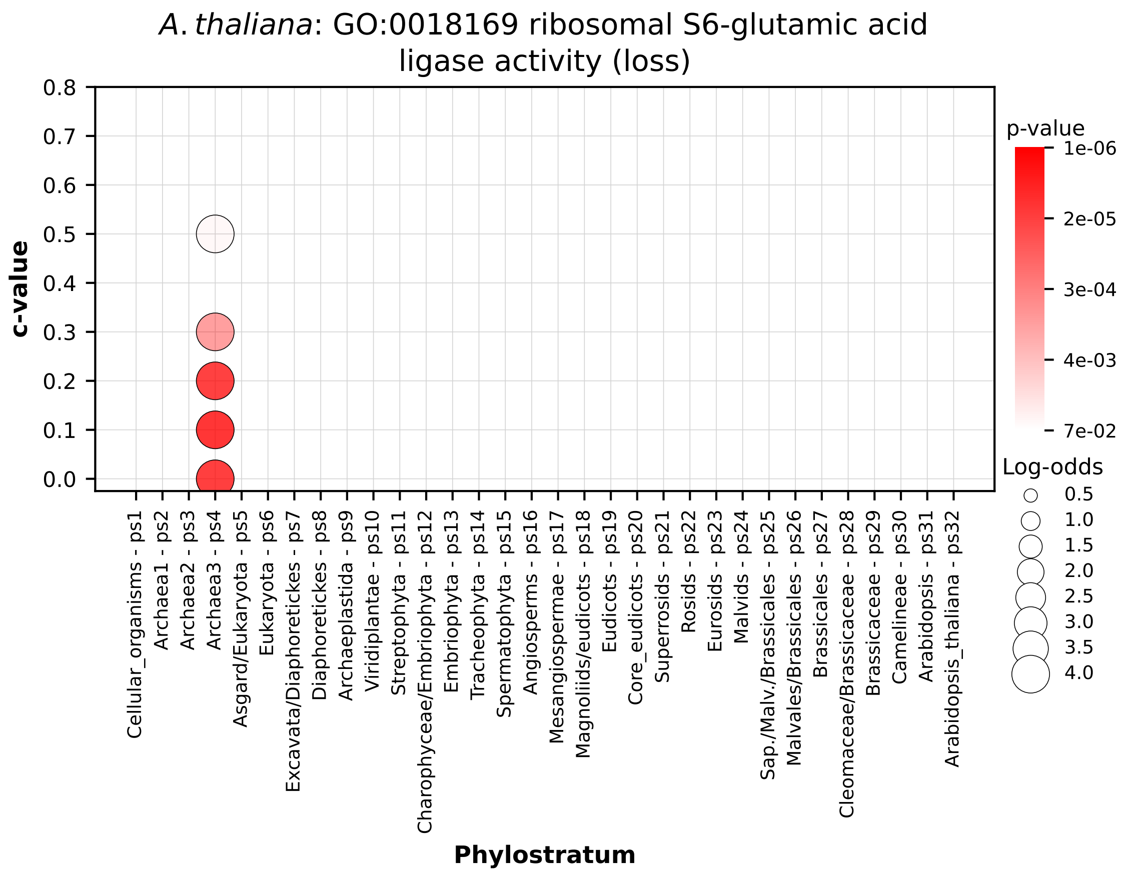

A. thaliana: GO:0018169 ribosomal S6-glutamic acid ligase activity (loss)
c-value
0.8
0.7
0.6
0.5
0.4
0.3
0.2
0.1
0.0
p-value
1e-06
2e-05
3e-04
4e-03
7e-02
Log-odds
0.5
1.0
1.5
2.0
2.5
3.0
3.5
4.0
Cellular_organisms - ps1
Archaea1 - ps2
Archaea2 - ps3
Archaea3 - ps4
Asgard/Eukaryota - ps5
Eukaryota - ps6
Excavata/Diaphoretickes - ps7
Diaphoretickes - ps8
Archaeplastida - ps9
Viridiplantae - ps10
Streptophyta - ps11
Charophyceae/Embriophyta - ps12
Embriophyta - ps13
Tracheophyta - ps14
Spermatophyta - ps15
Angiosperms - ps16
Mesangiospermae - ps17
Magnoliids/eudicots - ps18
Eudicots - ps19
Core_eudicots - ps20
Superrosids - ps21
Rosids - ps22
Eurosids - ps23
Malvids - ps24
Sap./Malv./Brassicales - ps25
Malvales/Brassicales - ps26
Brassicales - ps27
Cleomaceae/Brassicaceae - ps28
Brassicaceae - ps29
Camelineae - ps30
Arabidopsis - ps31
Arabidopsis_thaliana - ps32
Phylostratum

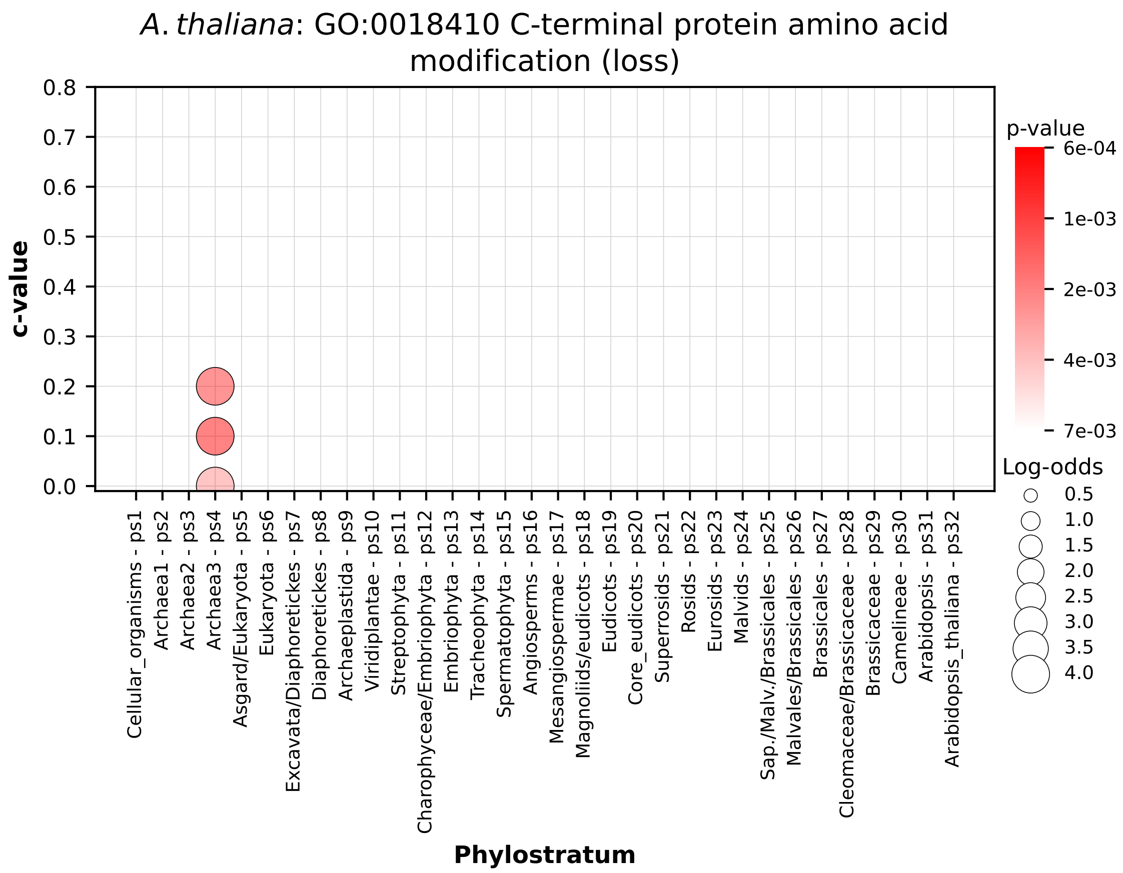

A. thaliana: GO:0018410 C-terminal protein amino acid modification (loss)
c-value
0.0
0.1
0.2
0.3
0.4
0.5
0.6
0.7
0.8
Phylostratum
Cellular_organisms - ps1
Archaea1 - ps2
Archaea2 - ps3
Archaea3 - ps4
Asgard/Eukaryota - ps5
Eukaryota - ps6
Excavata/Diaphoretickes - ps7
Diaphoretickes - ps8
Archaeplastida - ps9
Viridiplantae - ps10
Streptophyta - ps11
Charophyceae/Embriophyta - ps12
Embriophyta - ps13
Tracheophyta - ps14
Spermatophyta - ps15
Angiosperms - ps16
Mesangiospermae - ps17
Magnoliids/eudicots - ps18
Eudicots - ps19
Core_eudicots - ps20
Superrosids - ps21
Rosids - ps22
Eurosids - ps23
Malvids - ps24
Sap./Malv./Brassicales - ps25
Malvales/Brassicales - ps26
Brassicales - ps27
Cleomaceae/Brassicaceae - ps28
Brassicaceae - ps29
Camelineae - ps30
Arabidopsis - ps31
Arabidopsis_thaliana - ps32
p-value
6e-04
1e-03
2e-03
4e-03
7e-03
Log-odds
0.5
1.0
1.5
2.0
2.5
3.0
3.5
4.0

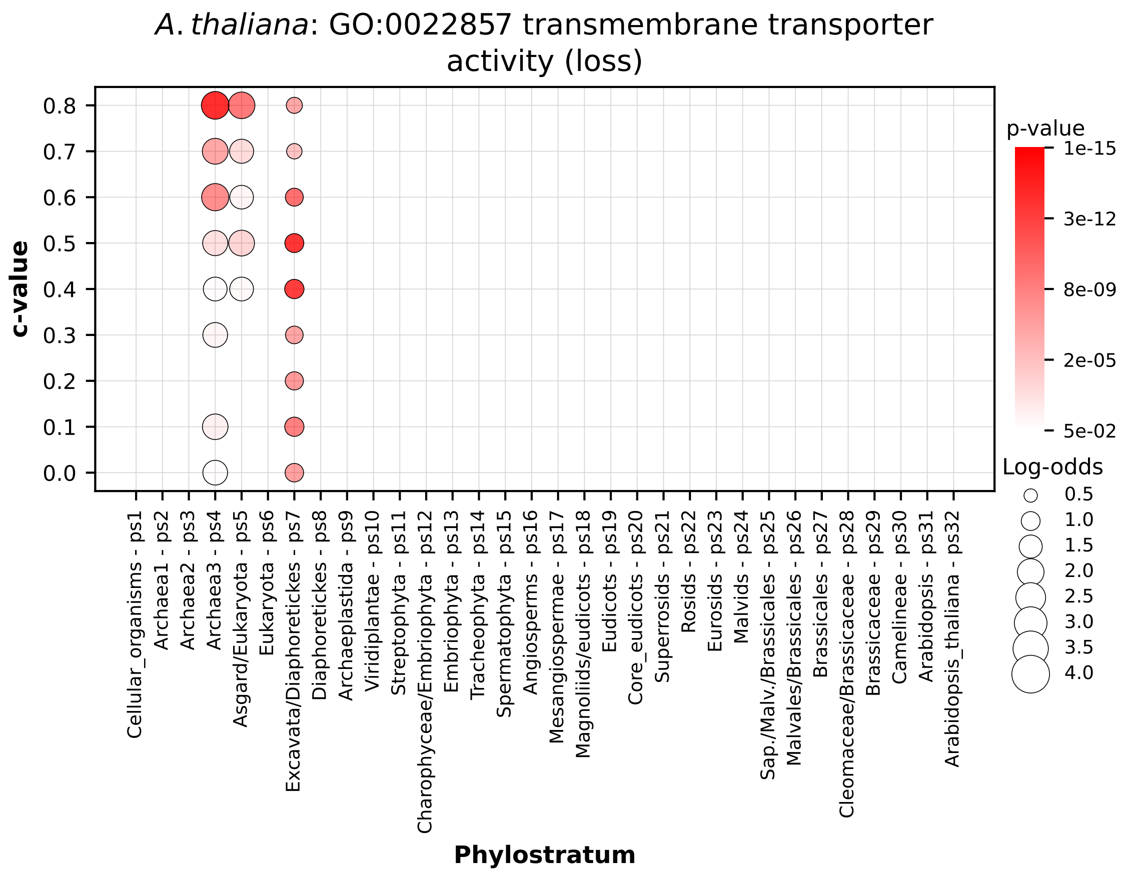
A. thaliana: GO:0022857 transmembrane transporter activity (loss)
c-value
0.8
0.7
0.6
0.5
0.4
0.3
0.2
0.1
0.0
Phylostratum
Cellular_organisms - ps1
Archaea1 - ps2
Archaea2 - ps3
Archaea3 - ps4
Asgard/Eukaryota - ps5
Eukaryota - ps6
Excavata/Diaphoretickes - ps7
Diaphoretickes - ps8
Archaeplastida - ps9
Viridiplantae - ps10
Streptophyta - ps11
Charophyceae/Embriophyta - ps12
Embriophyta - ps13
Tracheophyta - ps14
Spermatophyta - ps15
Angiosperms - ps16
Mesangiospermae - ps17
Magnoliids/eudicots - ps18
Eudicots - ps19
Core_eudicots - ps20
Superrosids - ps21
Rosids - ps22
Eurosids - ps23
Malvids - ps24
Sap./Malv./Brassicales - ps25
Malvales/Brassicales - ps26
Brassicales - ps27
Cleomaceae/Brassicaceae - ps28
Brassicaceae - ps29
Camelineae - ps30
Arabidopsis - ps31
Arabidopsis_thaliana - ps32
p-value
1e-15
3e-12
8e-09
2e-05
5e-02
Log-odds
0.5
1.0
1.5
2.0
2.5
3.0
3.5
4.0

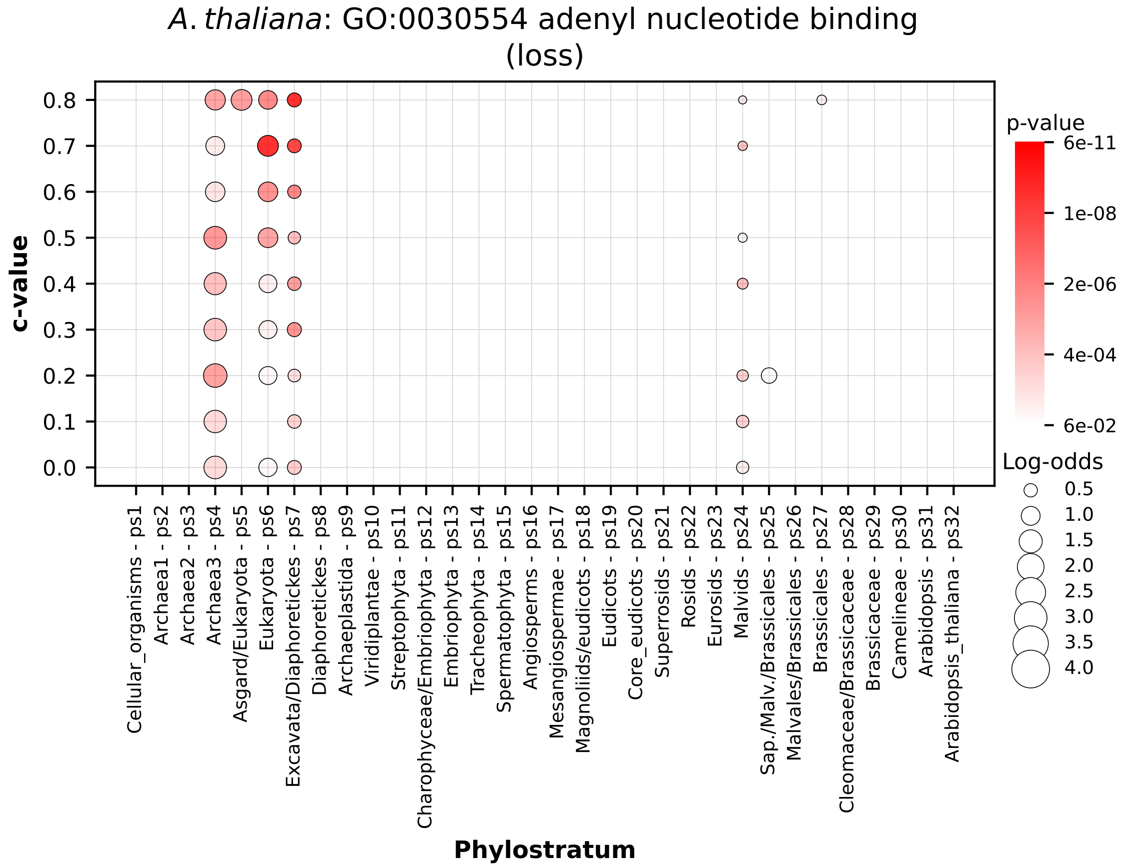

A. thaliana: GO:0030554 adenyl nucleotide binding
(loss)
c-value
0.8
0.7
0.6
0.5
0.4
0.3
0.2
0.1
0.0
p-value
6e-11
1e-08
2e-06
4e-04
6e-02
Log-odds
0.5
1.0
1.5
2.0
2.5
3.0
3.5
4.0
Cellular_organisms - ps1
Archaea1 - ps2
Archaea2 - ps3
Archaea3 - ps4
Asgard/Eukaryota - ps5
Eukaryota - ps6
Excavata/Diaphoretickes - ps7
Diaphoretickes - ps8
Archaeplastida - ps9
Viridiplantae - ps10
Streptophyta - ps11
Charophyceae/Embriophyta - ps12
Embriophyta - ps13
Tracheophyta - ps14
Spermatophyta - ps15
Angiosperms - ps16
Mesangiospermae - ps17
Magnoliids/eudicots - ps18
Eudicots - ps19
Core_eudicots - ps20
Superrosids - ps21
Rosids - ps22
Eurosids - ps23
Malvids - ps24
Sap./Malv./Brassicales - ps25
Malvales/Brassicales - ps26
Brassicales - ps27
Cleomaceae/Brassicaceae - ps28
Brassicaceae - ps29
Camelineae - ps30
Arabidopsis - ps31
Arabidopsis_thaliana - ps32
Phylostratum

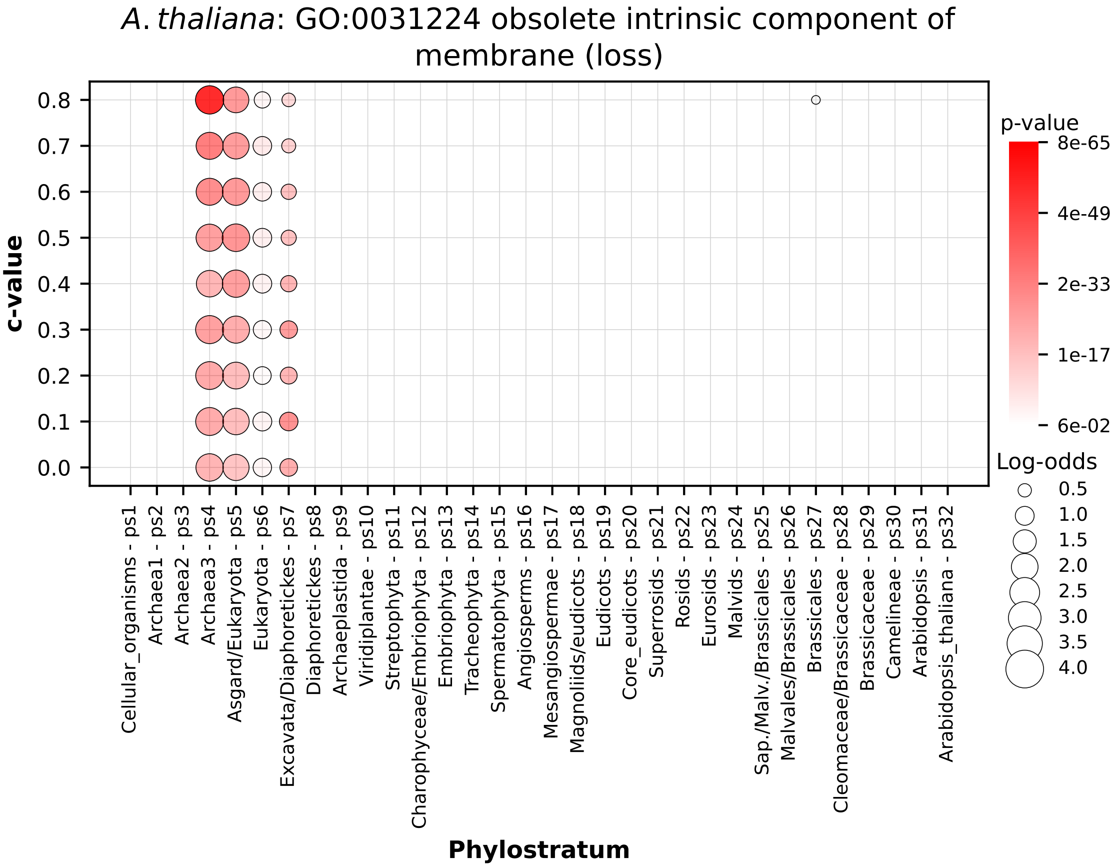

A. thaliana: GO:0031224 obsolete intrinsic component of membrane (loss)
c-value
0.8
0.7
0.6
0.5
0.4
0.3
0.2
0.1
0.0
Phylostratum
Cellular_organisms - ps1
Archaea1 - ps2
Archaea2 - ps3
Archaea3 - ps4
Asgard/Eukaryota - ps5
Eukaryota - ps6
Excavata/Diaphoretickes - ps7
Diaphoretickes - ps8
Archaeplastida - ps9
Viridiplantae - ps10
Streptophyta - ps11
Charophyceae/Embriophyta - ps12
Embriophyta - ps13
Tracheophyta - ps14
Spermatophyta - ps15
Angiosperms - ps16
Mesangiospermae - ps17
Magnoliids/eudicots - ps18
Eudicots - ps19
Core_eudicots - ps20
Superrosids - ps21
Rosids - ps22
Eurosids - ps23
Malvids - ps24
Sap./Malv./Brassicales - ps25
Malvales/Brassicales - ps26
Brassicales - ps27
Cleomaceae/Brassicaceae - ps28
Brassicaceae - ps29
Camelineae - ps30
Arabidopsis - ps31
Arabidopsis_thaliana - ps32
p-value
8e-65
4e-49
2e-33
1e-17
6e-02
Log-odds
0.5
1.0
1.5
2.0
2.5
3.0
3.5
4.0

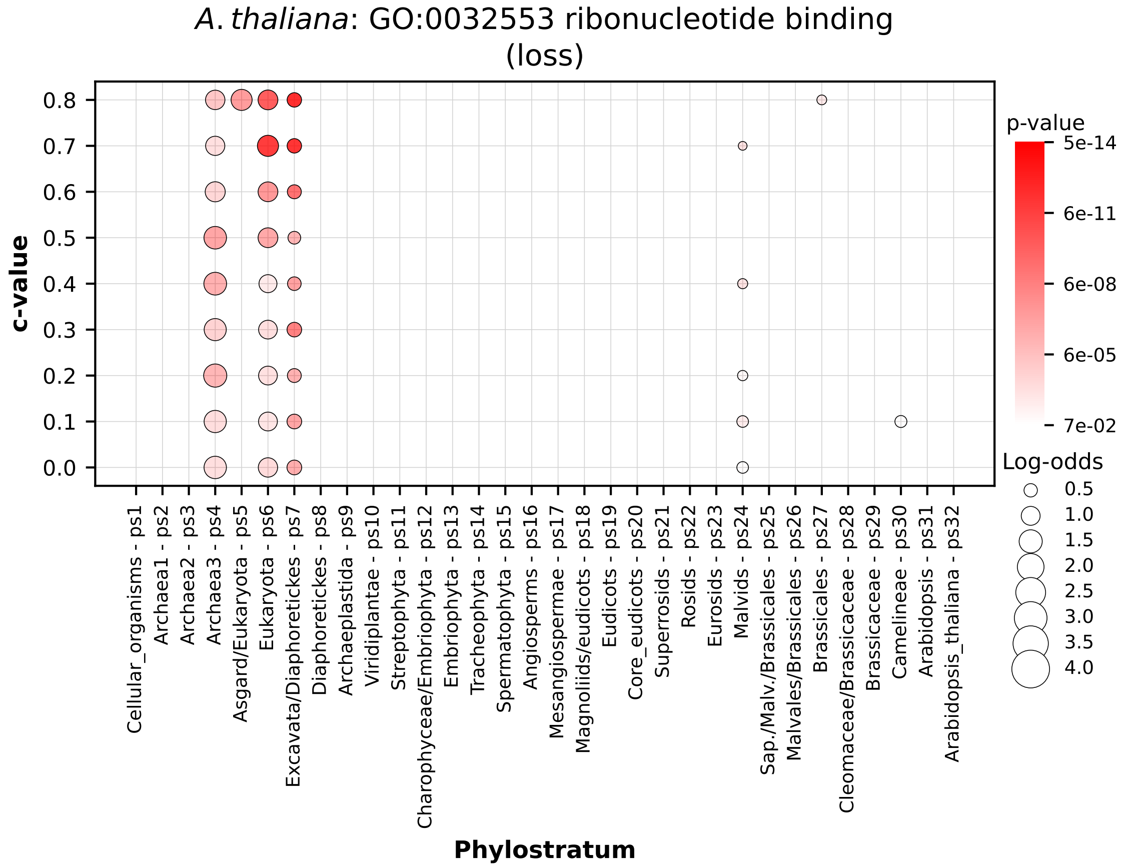

A. thaliana: GO:0032553 ribonucleotide binding (loss)
c-value
0.8
0.7
0.6
0.5
0.4
0.3
0.2
0.1
0.0
p-value
5e-14
6e-11
6e-08
6e-05
7e-02
Log-odds
0.5
1.0
1.5
2.0
2.5
3.0
3.5
4.0
Cellular_organisms - ps1
Archaea1 - ps2
Archaea2 - ps3
Archaea3 - ps4
Asgard/Eukaryota - ps5
Eukaryota - ps6
Excavata/Diaphoretickes - ps7
Diaphoretickes - ps8
Archaeplastida - ps9
Viridiplantae - ps10
Streptophyta - ps11
Charophyceae/Embriophyta - ps12
Embriophyta - ps13
Tracheophyta - ps14
Spermatophyta - ps15
Angiosperms - ps16
Mesangiospermae - ps17
Magnoliids/eudicots - ps18
Eudicots - ps19
Core_eudicots - ps20
Superrosids - ps21
Rosids - ps22
Eurosids - ps23
Malvids - ps24
Sap./Malv./Brassicales - ps25
Malvales/Brassicales - ps26
Brassicales - ps27
Cleomaceae/Brassicaceae - ps28
Brassicaceae - ps29
Camelineae - ps30
Arabidopsis - ps31
Arabidopsis_thaliana - ps32
Phylostratum

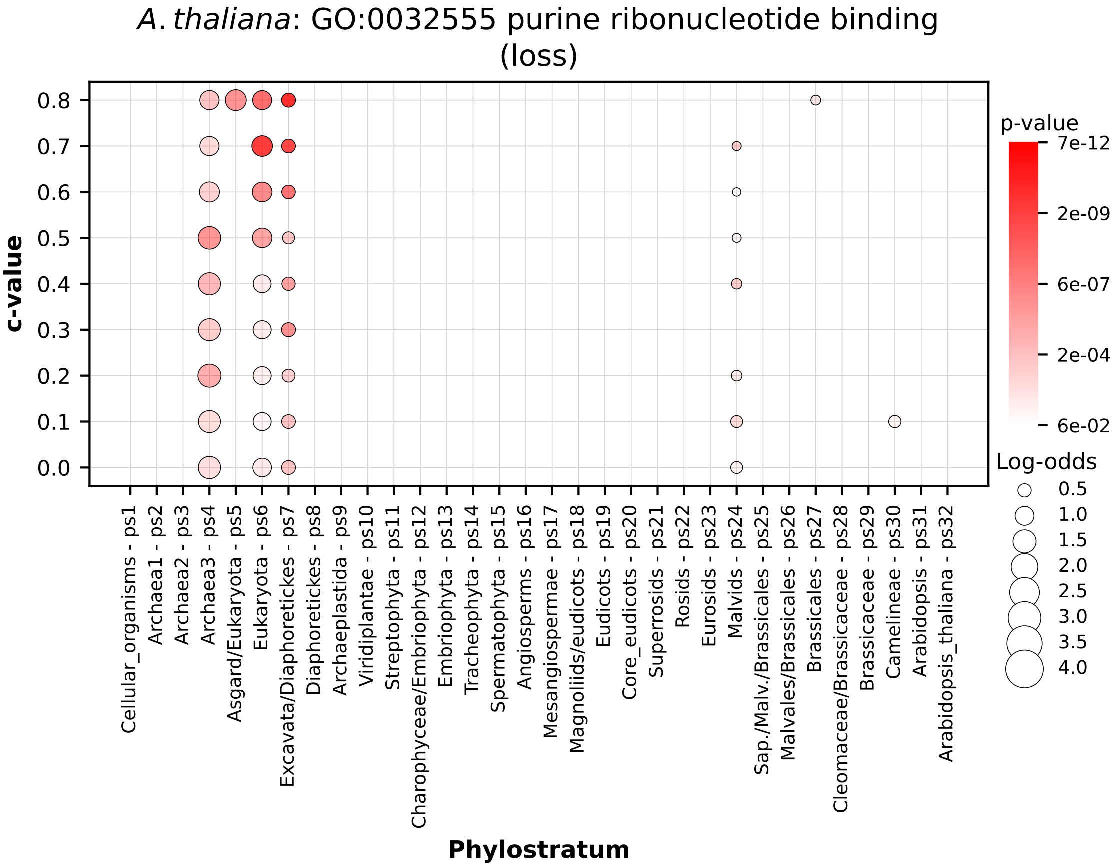

A. thaliana: GO:0032555 purine ribonucleotide binding (loss)
c-value
0.8
0.7
0.6
0.5
0.4
0.3
0.2
0.1
0.0
p-value
7e-12
2e-09
6e-07
2e-04
6e-02
Log-odds
0.5
1.0
1.5
2.0
2.5
3.0
3.5
4.0
Cellular_organisms - ps1
Archaea1 - ps2
Archaea2 - ps3
Archaea3 - ps4
Asgard/Eukaryota - ps5
Eukaryota - ps6
Excavata/Diaphoretickes - ps7
Diaphoretickes - ps8
Archaeplastida - ps9
Viridiplantae - ps10
Streptophyta - ps11
Charophyceae/Embriophyta - ps12
Embriophyta - ps13
Tracheophyta - ps14
Spermatophyta - ps15
Angiosperms - ps16
Mesangiospermae - ps17
Magnoliids/eudicots - ps18
Eudicots - ps19
Core_eudicots - ps20
Superrosids - ps21
Rosids - ps22
Eurosids - ps23
Malvids - ps24
Sap./Malv./Brassicales - ps25
Malvales/Brassicales - ps26
Brassicales - ps27
Cleomaceae/Brassicaceae - ps28
Brassicaceae - ps29
Camelineae - ps30
Arabidopsis - ps31
Arabidopsis_thaliana - ps32
Phylostratum

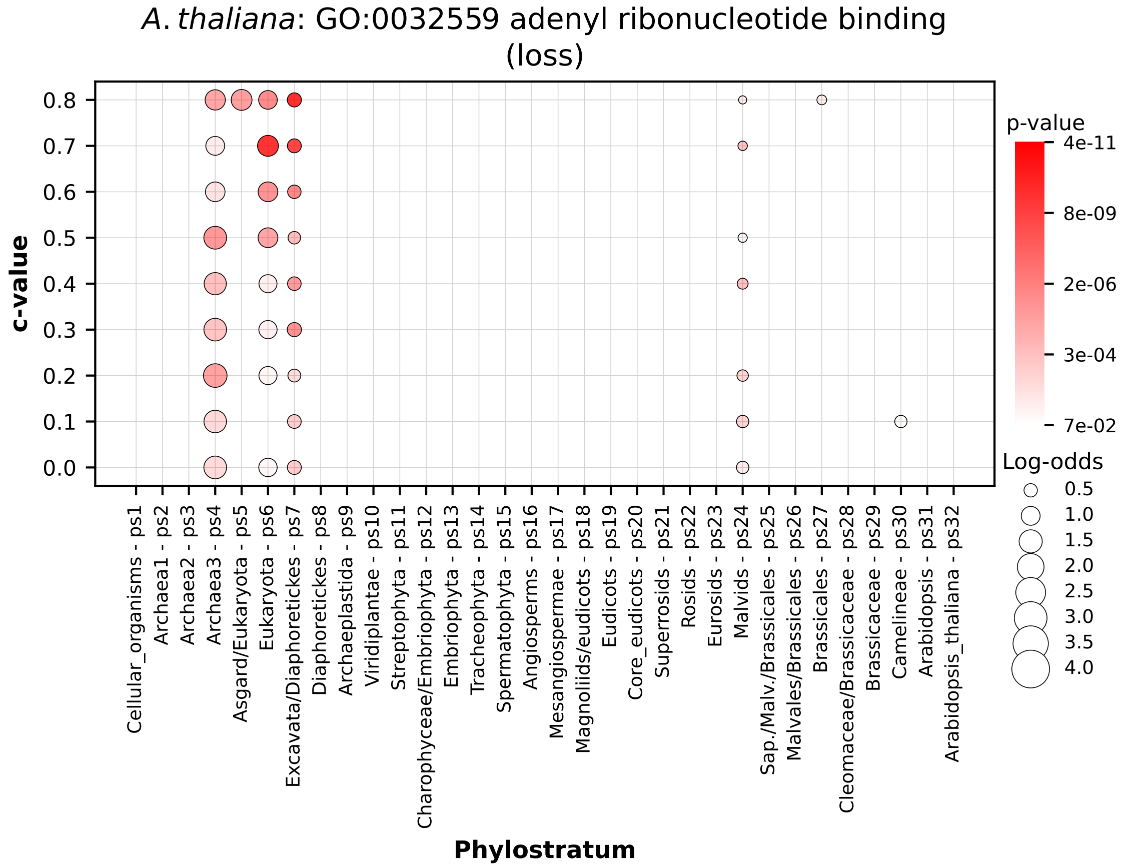

A. thaliana: GO:0032559 adenyl ribonucleotide binding
(loss)
c-value
0.8
0.7
0.6
0.5
0.4
0.3
0.2
0.1
0.0
p-value
4e-11
8e-09
2e-06
3e-04
7e-02
Log-odds
0.5
1.0
1.5
2.0
2.5
3.0
3.5
4.0
Cellular_organisms - ps1
Archaea1 - ps2
Archaea2 - ps3
Archaea3 - ps4
Asgard/Eukaryota - ps5
Eukaryota - ps6
Excavata/Diaphoretickes - ps7
Diaphoretickes - ps8
Archaeplastida - ps9
Viridiplantae - ps10
Streptophyta - ps11
Charophyceae/Embriophyta - ps12
Embriophyta - ps13
Tracheophyta - ps14
Spermatophyta - ps15
Angiosperms - ps16
Mesangiospermae - ps17
Magnoliids/eudicots - ps18
Eudicots - ps19
Core_eudicots - ps20
Superrosids - ps21
Rosids - ps22
Eurosids - ps23
Malvids - ps24
Sap./Malv./Brassicales - ps25
Malvales/Brassicales - ps26
Brassicales - ps27
Cleomaceae/Brassicaceae - ps28
Brassicaceae - ps29
Camelineae - ps30
Arabidopsis - ps31
Arabidopsis_thaliana - ps32
Phylostratum

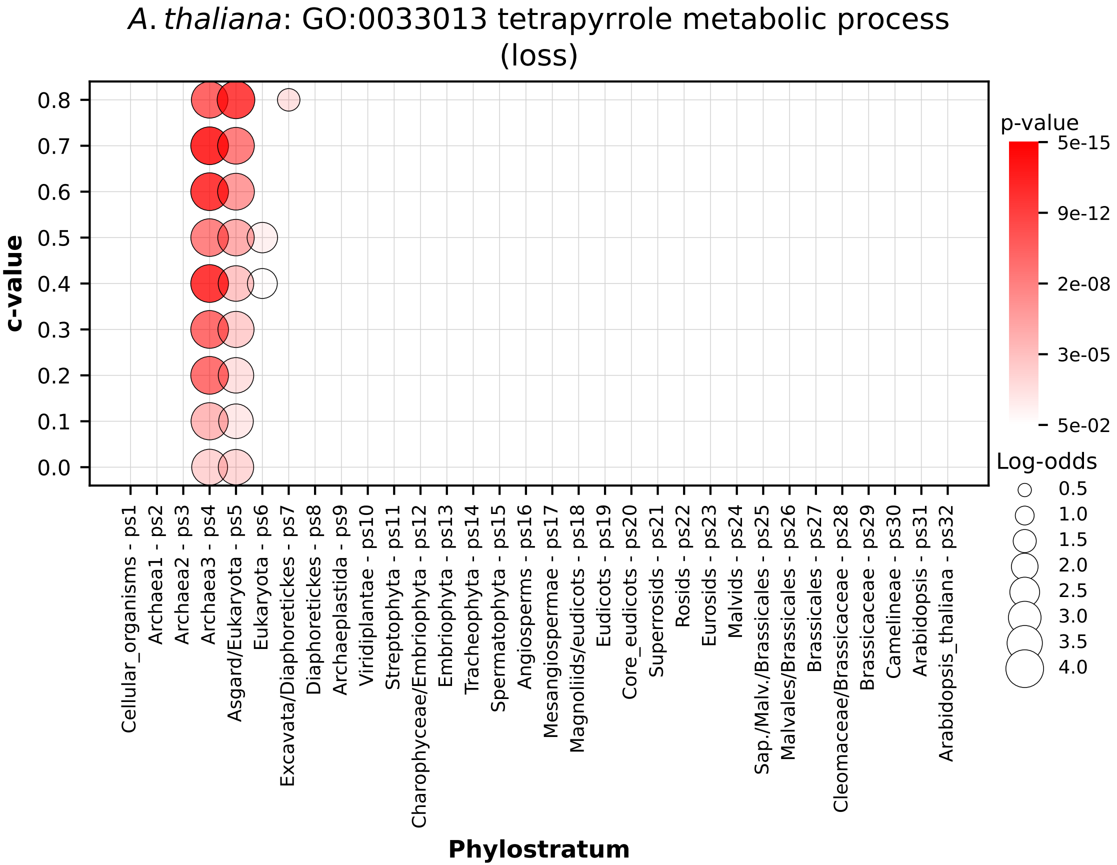
A. thaliana: GO:0033013 tetrapyrrole metabolic process
(loss)
c-value
0.8
0.7
0.6
0.5
0.4
0.3
0.2
0.1
0.0
Phylostratum
Cellular_organisms - ps1
Archaea1 - ps2
Archaea2 - ps3
Archaea3 - ps4
Asgard/Eukaryota - ps5
Eukaryota - ps6
Excavata/Diaphoretickes - ps7
Diaphoretickes - ps8
Archaeplastida - ps9
Viridiplantae - ps10
Streptophyta - ps11
Charophyceae/Embriophyta - ps12
Embriophyta - ps13
Tracheophyta - ps14
Spermatophyta - ps15
Angiosperms - ps16
Mesangiospermae - ps17
Magnoliids/eudicots - ps18
Eudicots - ps19
Core_eudicots - ps20
Superrosids - ps21
Rosids - ps22
Eurosids - ps23
Malvids - ps24
Sap./Malv./Brassicales - ps25
Malvales/Brassicales - ps26
Brassicales - ps27
Cleomaceae/Brassicaceae - ps28
Brassicaceae - ps29
Camelineae - ps30
Arabidopsis - ps31
Arabidopsis_thaliana - ps32
p-value
5e-15
9e-12
2e-08
3e-05
5e-02
Log-odds
0.5
1.0
1.5
2.0
2.5
3.0
3.5
4.0

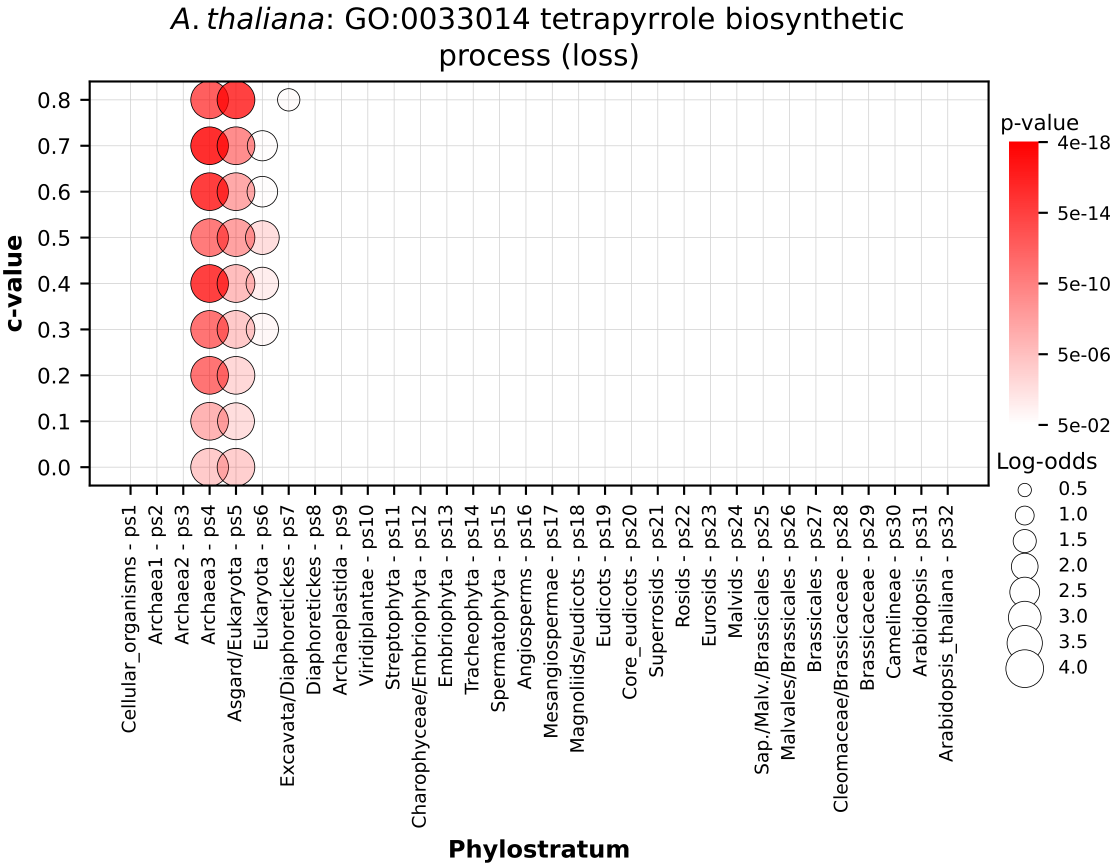
A. thaliana: GO:0033014 tetrapyrrole biosynthetic process (loss)
c-value
0.8
0.7
0.6
0.5
0.4
0.3
0.2
0.1
0.0
p-value
4e-18
5e-14
5e-10
5e-06
5e-02
Log-odds
0.5
1.0
1.5
2.0
2.5
3.0
3.5
4.0
Cellular_organisms - ps1
Archaea1 - ps2
Archaea2 - ps3
Archaea3 - ps4
Asgard/Eukaryota - ps5
Eukaryota - ps6
Excavata/Diaphoretickes - ps7
Diaphoretickes - ps8
Archaeplastida - ps9
Viridiplantae - ps10
Streptophyta - ps11
Charophyceae/Embriophyta - ps12
Embriophyta - ps13
Tracheophyta - ps14
Spermatophyta - ps15
Angiosperms - ps16
Mesangiospermae - ps17
Magnoliids/eudicots - ps18
Eudicots - ps19
Core_eudicots - ps20
Superrosids - ps21
Rosids - ps22
Eurosids - ps23
Malvids - ps24
Sap./Malv./Brassicales - ps25
Malvales/Brassicales - ps26
Brassicales - ps27
Cleomaceae/Brassicaceae - ps28
Brassicaceae - ps29
Camelineae - ps30
Arabidopsis - ps31
Arabidopsis_thaliana - ps32
Phylostratum

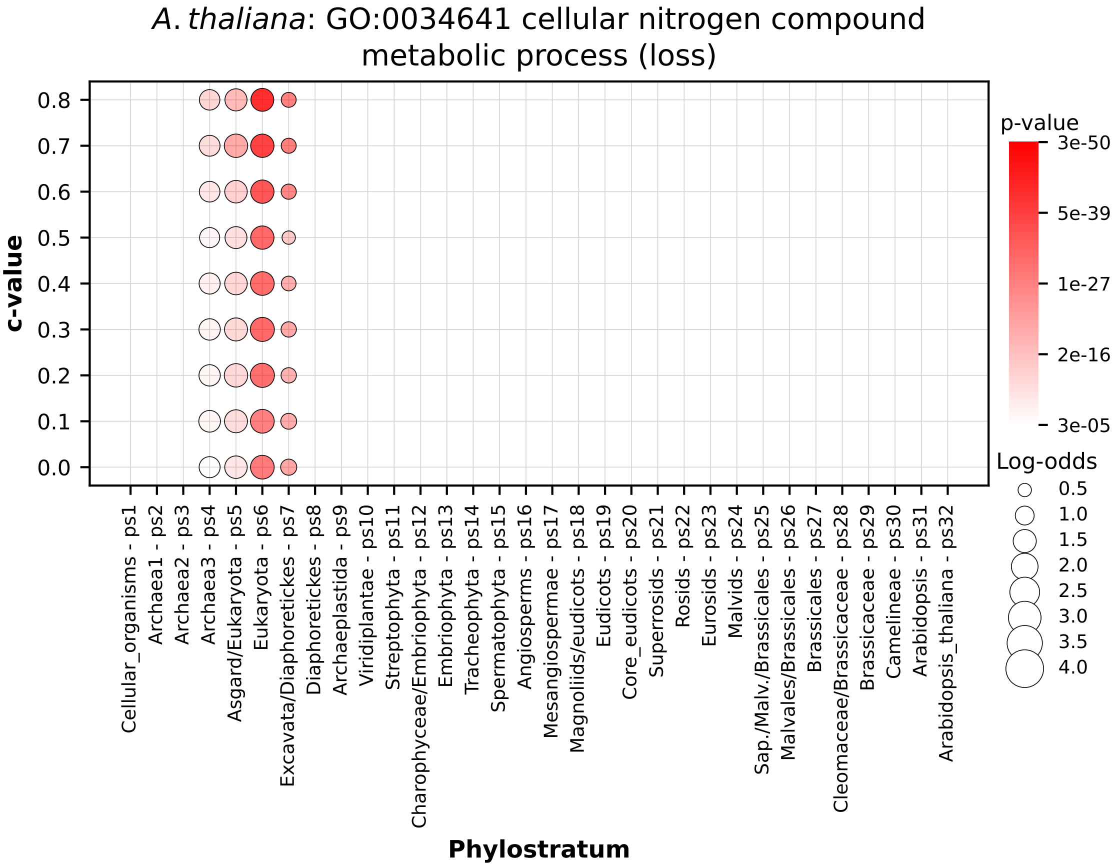

A. thaliana: GO:0034641 cellular nitrogen compound metabolic process (loss)
c-value
0.0
0.1
0.2
0.3
0.4
0.5
0.6
0.7
0.8
Phylostratum
Cellular_organisms - ps1
Archaea1 - ps2
Archaea2 - ps3
Archaea3 - ps4
Asgard/Eukaryota - ps5
Eukaryota - ps6
Excavata/Diaphoretickes - ps7
Diaphoretickes - ps8
Archaeplastida - ps9
Viridiplantae - ps10
Streptophyta - ps11
Charophyceae/Embriophyta - ps12
Embriophyta - ps13
Tracheophyta - ps14
Spermatophyta - ps15
Angiosperms - ps16
Mesangiospermae - ps17
Magnoliids/eudicots - ps18
Eudicots - ps19
Core_eudicots - ps20
Superrosids - ps21
Rosids - ps22
Eurosids - ps23
Malvids - ps24
Sap./Malv./Brassicales - ps25
Malvales/Brassicales - ps26
Brassicales - ps27
Cleomaceae/Brassicaceae - ps28
Brassicaceae - ps29
Camelineae - ps30
Arabidopsis - ps31
Arabidopsis_thaliana - ps32
p-value
3e-50
5e-39
1e-27
2e-16
3e-05
Log-odds
0.5
1.0
1.5
2.0
2.5
3.0
3.5
4.0

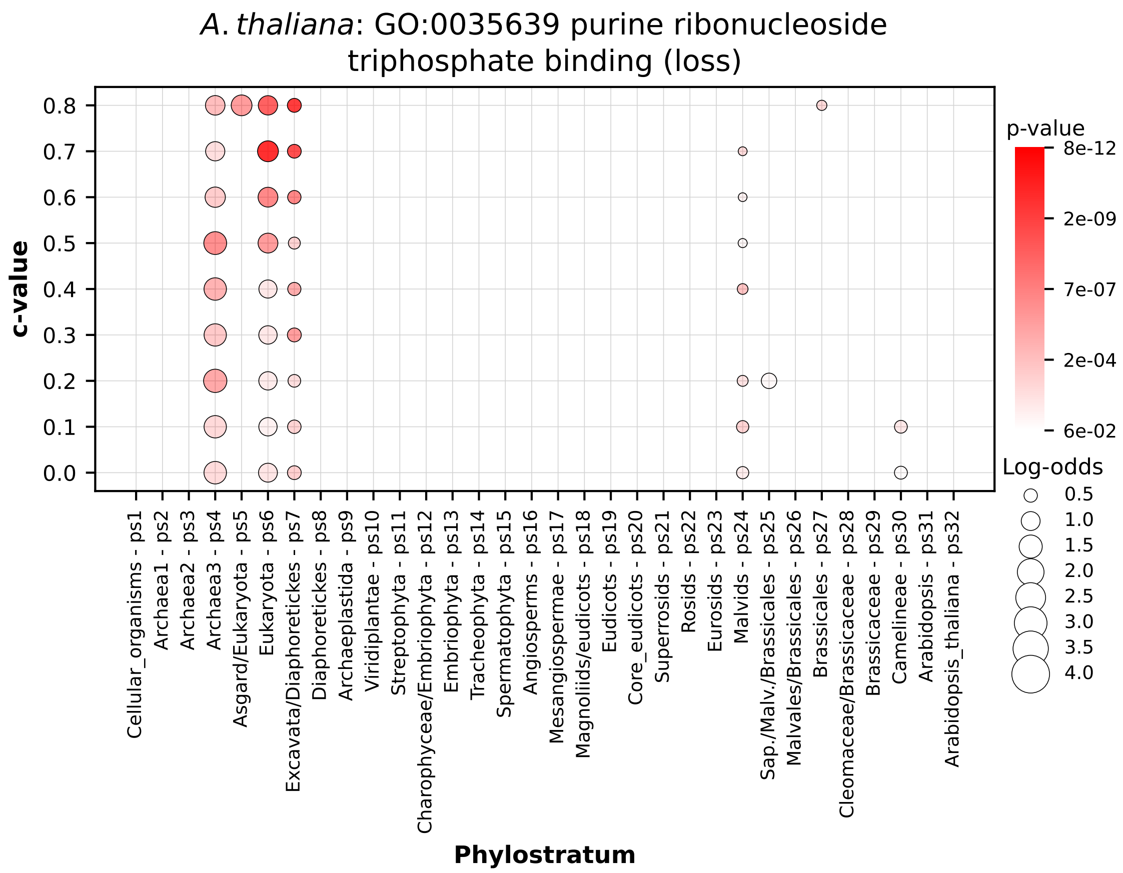
A. thaliana: GO:0035639 purine ribonucleoside triphosphate binding (loss)
c-value
0.8
0.7
0.6
0.5
0.4
0.3
0.2
0.1
0.0
p-value
8e-12
2e-09
7e-07
2e-04
6e-02
Log-odds
0.5
1.0
1.5
2.0
2.5
3.0
3.5
4.0
Cellular_organisms - ps1
Archaea1 - ps2
Archaea2 - ps3
Archaea3 - ps4
Asgard/Eukaryota - ps5
Eukaryota - ps6
Excavata/Diaphoretickes - ps7
Diaphoretickes - ps8
Archaeplastida - ps9
Viridiplantae - ps10
Streptophyta - ps11
Charophyceae/Embriophyta - ps12
Embriophyta - ps13
Tracheophyta - ps14
Spermatophyta - ps15
Angiosperms - ps16
Mesangiospermae - ps17
Magnoliids/eudicots - ps18
Eudicots - ps19
Core_eudicots - ps20
Superrosids - ps21
Rosids - ps22
Eurosids - ps23
Malvids - ps24
Sap./Malv./Brassicales - ps25
Malvales/Brassicales - ps26
Brassicales - ps27
Cleomaceae/Brassicaceae - ps28
Brassicaceae - ps29
Camelineae - ps30
Arabidopsis - ps31
Arabidopsis_thaliana - ps32
Phylostratum

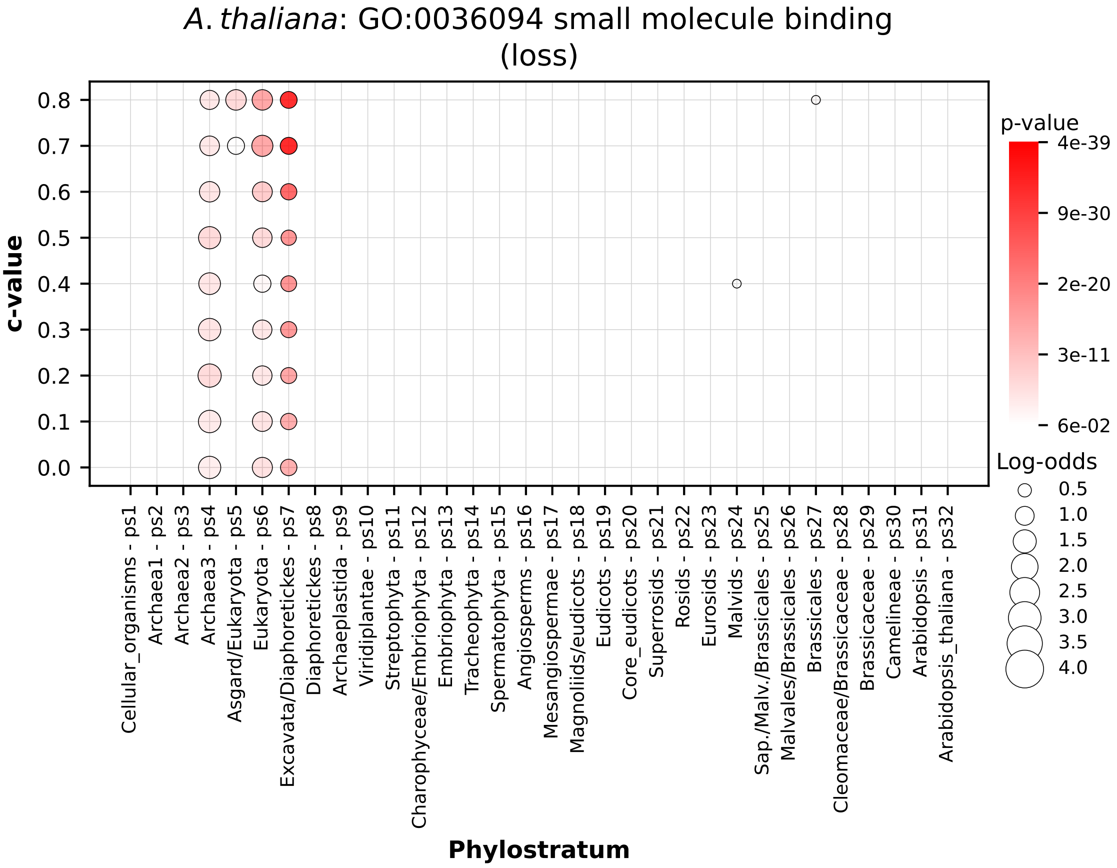

A. thaliana: GO:0036094 small molecule binding
(loss)
c-value
0.8
0.7
0.6
0.5
0.4
0.3
0.2
0.1
0.0
Phylostratum
Cellular_organisms - ps1
Archaea1 - ps2
Archaea2 - ps3
Archaea3 - ps4
Asgard/Eukaryota - ps5
Eukaryota - ps6
Excavata/Diaphoretickes - ps7
Diaphoretickes - ps8
Archaeplastida - ps9
Viridiplantae - ps10
Streptophyta - ps11
Charophyceae/Embriophyta - ps12
Embriophyta - ps13
Tracheophyta - ps14
Spermatophyta - ps15
Angiosperms - ps16
Mesangiospermae - ps17
Magnoliids/eudicots - ps18
Eudicots - ps19
Core_eudicots - ps20
Superrosids - ps21
Rosids - ps22
Eurosids - ps23
Malvids - ps24
Sap./Malv./Brassicales - ps25
Malvales/Brassicales - ps26
Brassicales - ps27
Cleomaceae/Brassicaceae - ps28
Brassicaceae - ps29
Camelineae - ps30
Arabidopsis - ps31
Arabidopsis_thaliana - ps32
p-value
4e-39
9e-30
2e-20
3e-11
6e-02
Log-odds
0.5
1.0
1.5
2.0
2.5
3.0
3.5
4.0

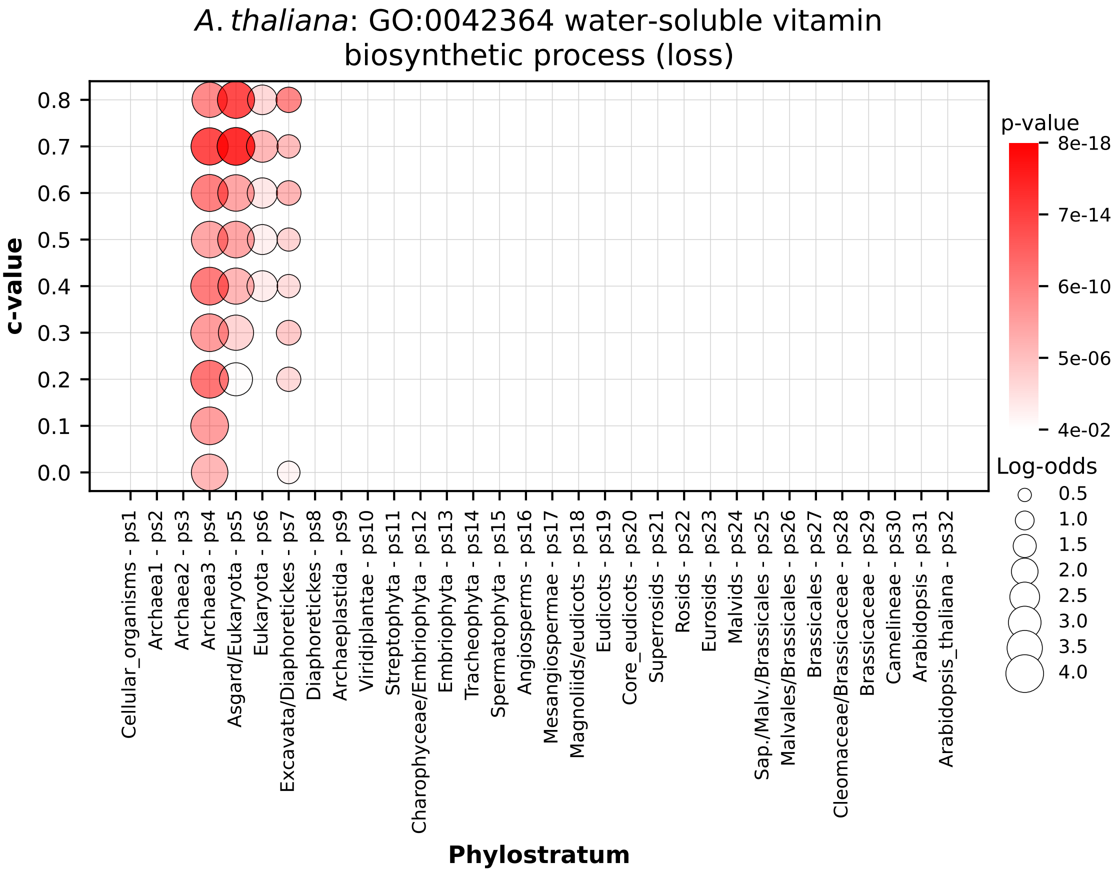

$A. thaliana$: GO:0042364 water-soluble vitamin biosynthetic process (loss)
c-value
0.8
0.7
0.6
0.5
0.4
0.3
0.2
0.1
0.0
p-value
8e-18
7e-14
6e-10
5e-06
4e-02
Log-odds
0.5
1.0
1.5
2.0
2.5
3.0
3.5
4.0
Cellular_organisms - ps1
Archaea1 - ps2
Archaea2 - ps3
Archaea3 - ps4
Asgard/Eukaryota - ps5
Eukaryota - ps6
Excavata/Diaphoretickes - ps7
Diaphoretickes - ps8
Archaeplastida - ps9
Viridiplantae - ps10
Streptophyta - ps11
Charophyceae/Embriophyta - ps12
Embriophyta - ps13
Tracheophyta - ps14
Spermatophyta - ps15
Angiosperms - ps16
Mesangiospermae - ps17
Magnoliids/eudicots - ps18
Eudicots - ps19
Core_eudicots - ps20
Superrosids - ps21
Rosids - ps22
Eurosids - ps23
Malvids - ps24
Sap./Malv./Brassicales - ps25
Malvales/Brassicales - ps26
Brassicales - ps27
Cleomaceae/Brassicaceae - ps28
Brassicaceae - ps29
Camelineae - ps30
Arabidopsis - ps31
Arabidopsis_thaliana - ps32
Phylostratum

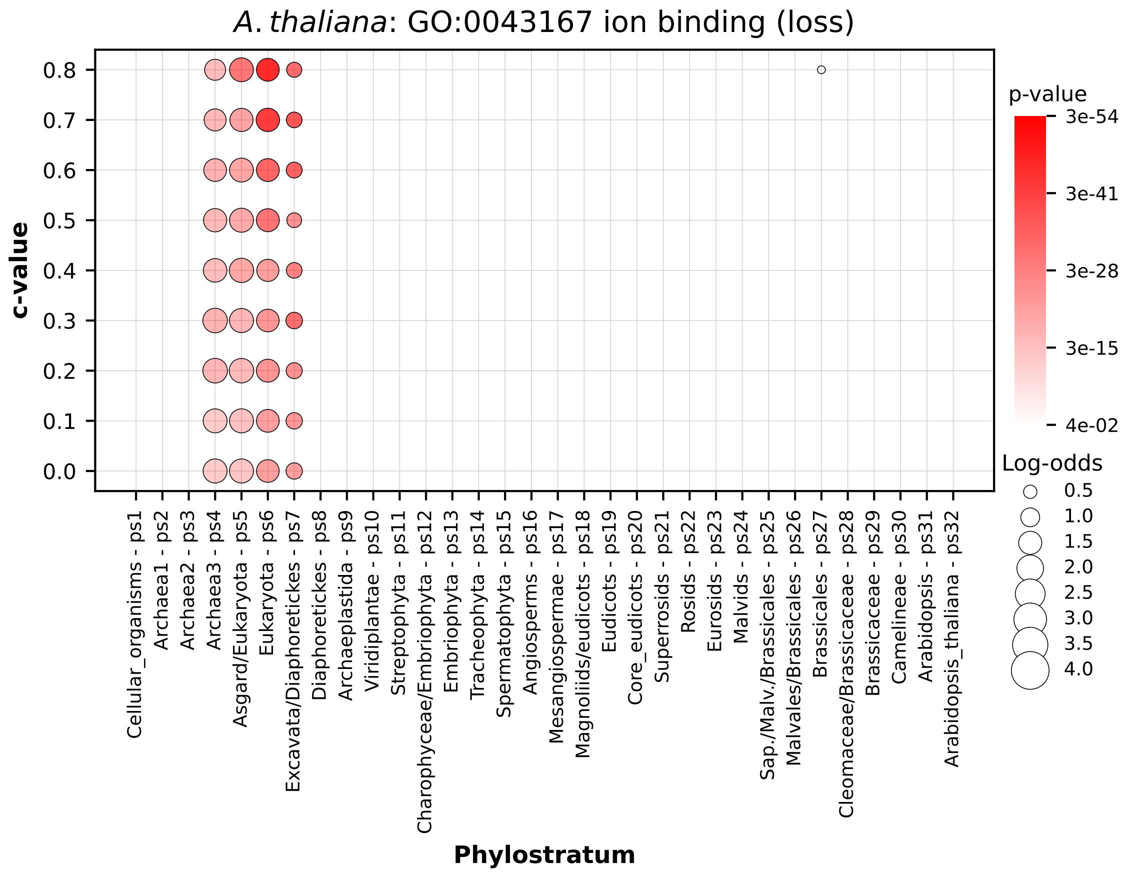

A. thaliana: GO:0043167 ion binding (loss)
c-value
0.8
0.7
0.6
0.5
0.4
0.3
0.2
0.1
0.0
Phylostratum
Cellular_organisms - ps1
Archaea1 - ps2
Archaea2 - ps3
Archaea3 - ps4
Asgard/Eukaryota - ps5
Eukaryota - ps6
Excavata/Diaphoretickes - ps7
Diaphoretickes - ps8
Archaeplastida - ps9
Viridiplantae - ps10
Streptophyta - ps11
Charophyceae/Embriophyta - ps12
Embriophyta - ps13
Tracheophyta - ps14
Spermatophyta - ps15
Angiosperms - ps16
Mesangiospermae - ps17
Magnoliids/eudicots - ps18
Eudicots - ps19
Core_eudicots - ps20
Superrosids - ps21
Rosids - ps22
Eurosids - ps23
Malvids - ps24
Sap./Malv./Brassicales - ps25
Malvales/Brassicales - ps26
Brassicales - ps27
Cleomaceae/Brassicaceae - ps28
Brassicaceae - ps29
Camelineae - ps30
Arabidopsis - ps31
Arabidopsis_thaliana - ps32
p-value
3e-54
3e-41
3e-28
3e-15
4e-02
Log-odds
0.5
1.0
1.5
2.0
2.5
3.0
3.5
4.0

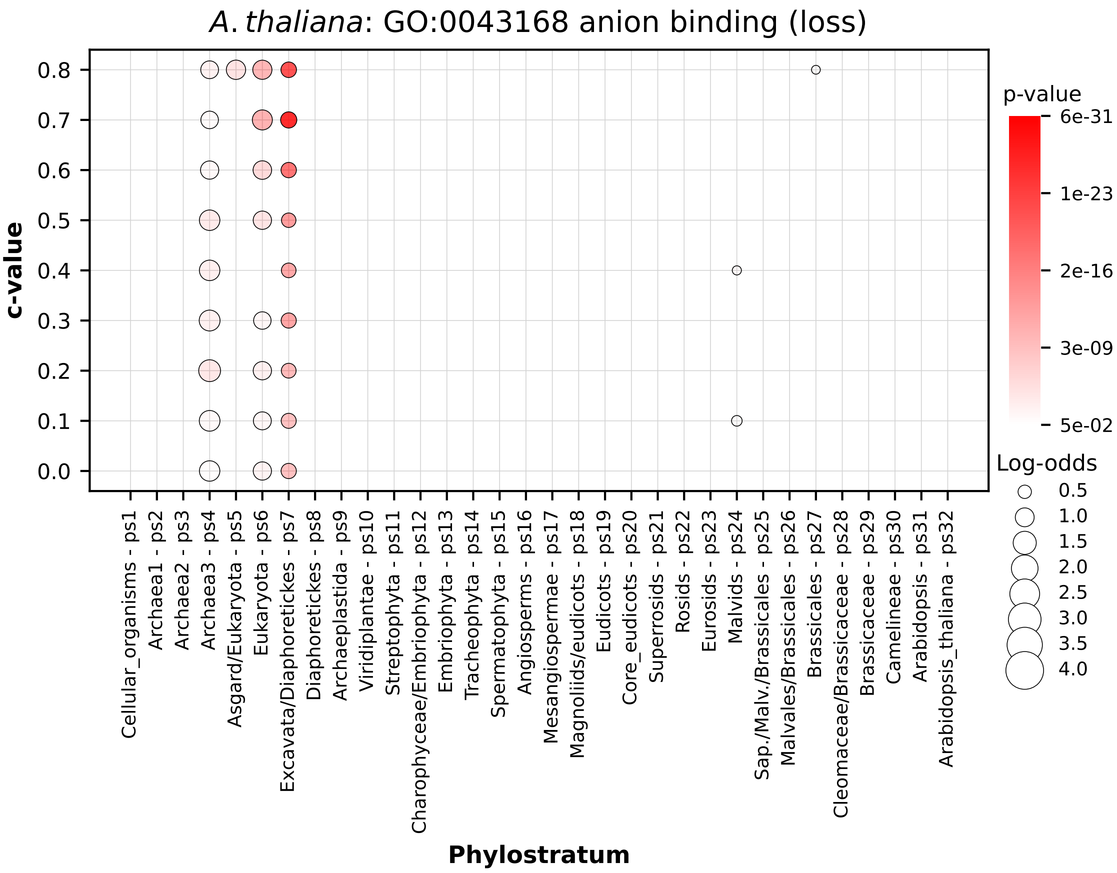
A. thaliana: GO:0043168 anion binding (loss)
c-value
0.0
0.1
0.2
0.3
0.4
0.5
0.6
0.7
0.8
Phylostratum
Cellular_organisms - ps1
Archaea1 - ps2
Archaea2 - ps3
Archaea3 - ps4
Asgard/Eukaryota - ps5
Eukaryota - ps6
Excavata/Diaphoretickes - ps7
Diaphoretickes - ps8
Archaeplastida - ps9
Viridiplantae - ps10
Streptophyta - ps11
Charophyceae/Embriophyta - ps12
Embriophyta - ps13
Tracheophyta - ps14
Spermatophyta - ps15
Angiosperms - ps16
Mesangiospermae - ps17
Magnoliids/eudicots - ps18
Eudicots - ps19
Core_eudicots - ps20
Superrosids - ps21
Rosids - ps22
Eurosids - ps23
Malvids - ps24
Sap./Malv./Brassicales - ps25
Malvales/Brassicales - ps26
Brassicales - ps27
Cleomaceae/Brassicaceae - ps28
Brassicaceae - ps29
Camelineae - ps30
Arabidopsis - ps31
Arabidopsis_thaliana - ps32
p-value
6e-31
1e-23
2e-16
3e-09
5e-02
Log-odds
0.5
1.0
1.5
2.0
2.5
3.0
3.5
4.0

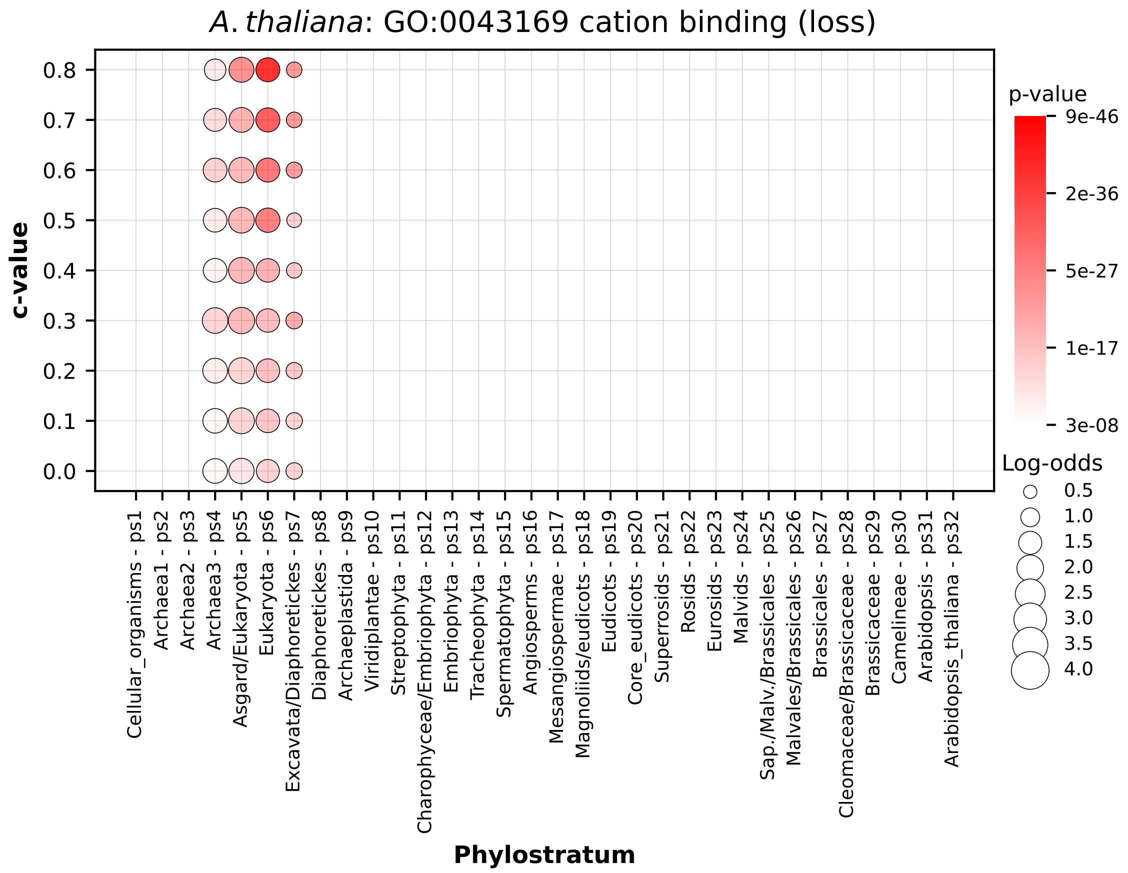

A. thaliana: GO:0043169 cation binding (loss)
c-value
0.8
0.7
0.6
0.5
0.4
0.3
0.2
0.1
0.0
Phylostratum
Cellular_organisms - ps1
Archaea1 - ps2
Archaea2 - ps3
Archaea3 - ps4
Asgard/Eukaryota - ps5
Eukaryota - ps6
Excavata/Diaphoretickes - ps7
Diaphoretickes - ps8
Archaeplastida - ps9
Viridiplantae - ps10
Streptophyta - ps11
Charophyceae/Embriophyta - ps12
Embriophyta - ps13
Tracheophyta - ps14
Spermatophyta - ps15
Angiosperms - ps16
Mesangiospermae - ps17
Magnoliids/eudicots - ps18
Eudicots - ps19
Core_eudicots - ps20
Superrosids - ps21
Rosids - ps22
Eurosids - ps23
Malvids - ps24
Sap./Malv./Brassicales - ps25
Malvales/Brassicales - ps26
Brassicales - ps27
Cleomaceae/Brassicaceae - ps28
Brassicaceae - ps29
Camelineae - ps30
Arabidopsis - ps31
Arabidopsis_thaliana - ps32
p-value
9e-46
2e-36
5e-27
1e-17
3e-08
Log-odds
0.5
1.0
1.5
2.0
2.5
3.0
3.5
4.0

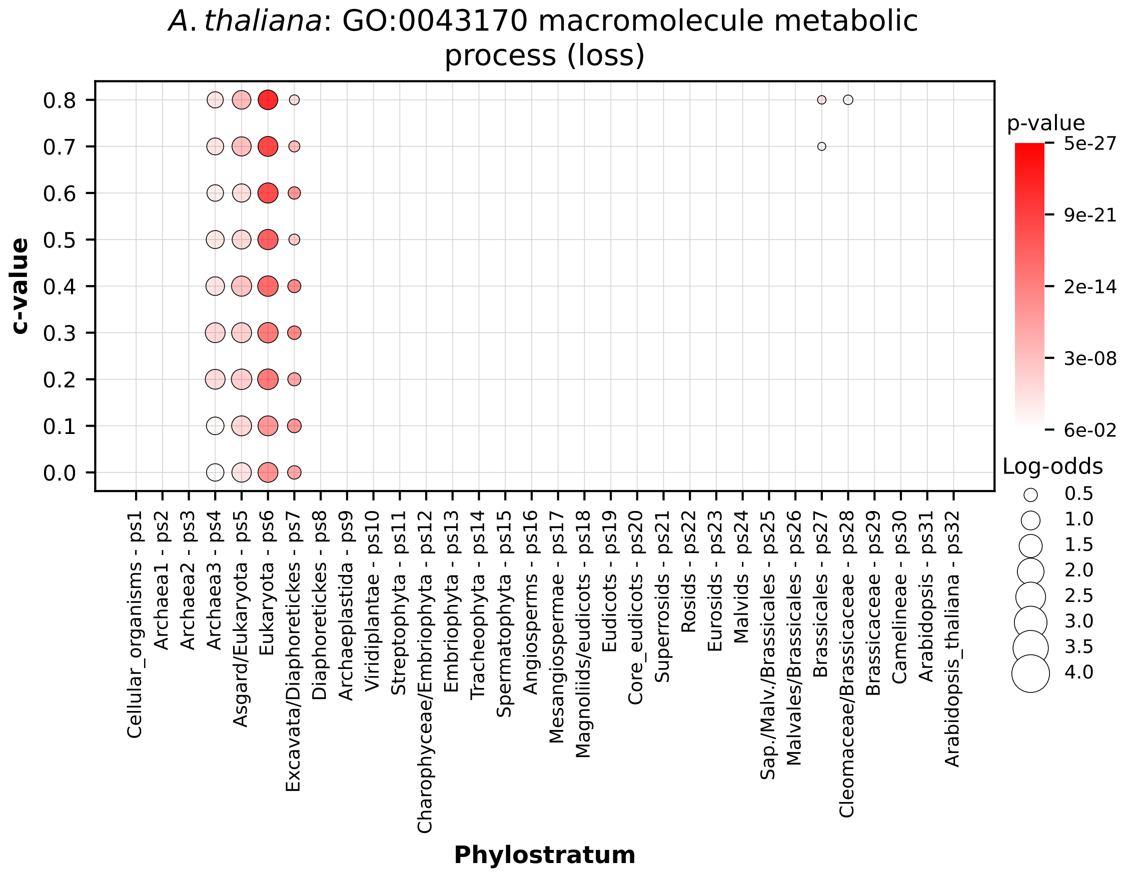

A. thaliana: GO:0043170 macromolecule metabolic process (loss)
c-value
0.8
0.7
0.6
0.5
0.4
0.3
0.2
0.1
0.0
Phylostratum
Cellular_organisms - ps1
Archaea1 - ps2
Archaea2 - ps3
Archaea3 - ps4
Asgard/Eukaryota - ps5
Eukaryota - ps6
Excavata/Diaphoretickes - ps7
Diaphoretickes - ps8
Archaeplastida - ps9
Viridiplantae - ps10
Streptophyta - ps11
Charophyceae/Embriophyta - ps12
Embriophyta - ps13
Tracheophyta - ps14
Spermatophyta - ps15
Angiosperms - ps16
Mesangiospermae - ps17
Magnoliids/eudicots - ps18
Eudicots - ps19
Core_eudicots - ps20
Superrosids - ps21
Rosids - ps22
Eurosids - ps23
Malvids - ps24
Sap./Malv./Brassicales - ps25
Malvales/Brassicales - ps26
Brassicales - ps27
Cleomaceae/Brassicaceae - ps28
Brassicaceae - ps29
Camelineae - ps30
Arabidopsis - ps31
Arabidopsis_thaliana - ps32
p-value
5e-27
9e-21
2e-14
3e-08
6e-02
Log-odds
0.5
1.0
1.5
2.0
2.5
3.0
3.5
4.0

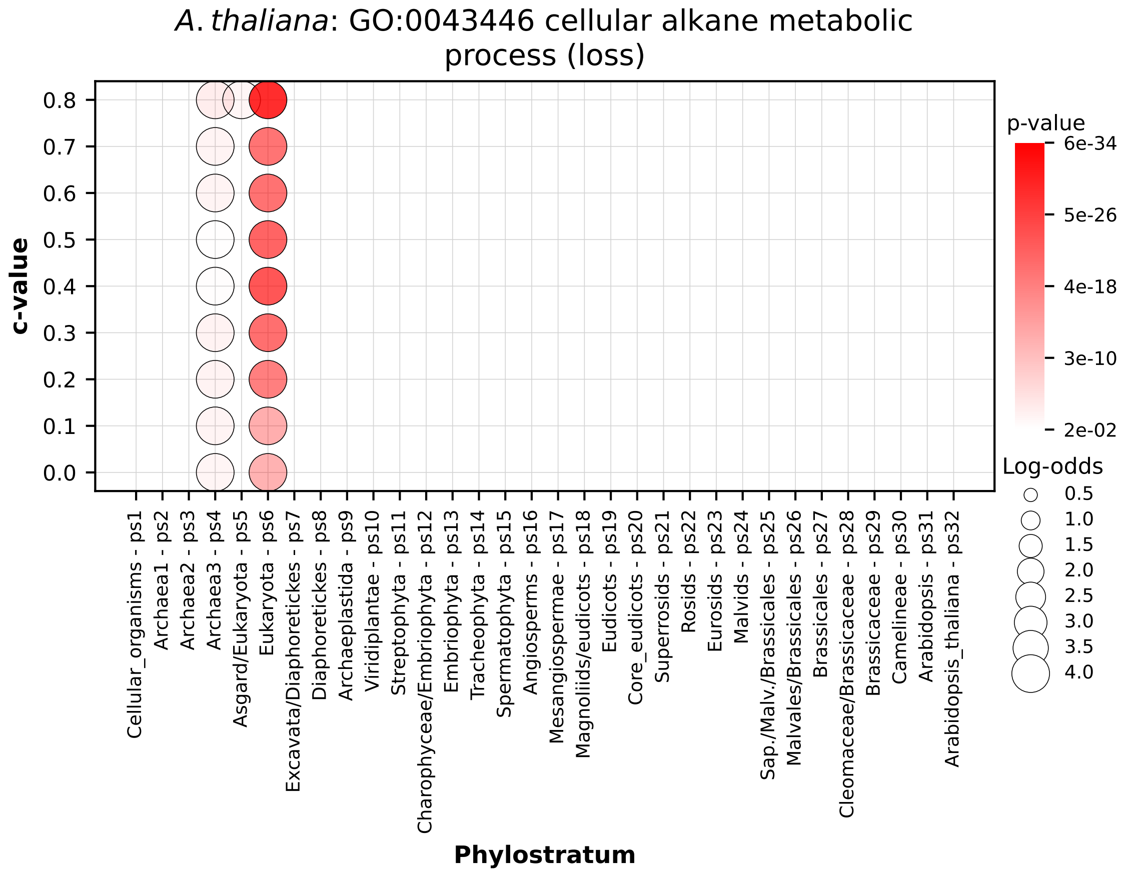

A. thaliana: GO:0043446 cellular alkane metabolic process (loss)
c-value
0.8
0.7
0.6
0.5
0.4
0.3
0.2
0.1
0.0
p-value
6e-34
5e-26
4e-18
3e-10
2e-02
Log-odds
0.5
1.0
1.5
2.0
2.5
3.0
3.5
4.0
Cellular_organisms - ps1
Archaea1 - ps2
Archaea2 - ps3
Archaea3 - ps4
Asgard/Eukaryota - ps5
Eukaryota - ps6
Excavata/Diaphoretickes - ps7
Diaphoretickes - ps8
Archaeplastida - ps9
Viridiplantae - ps10
Streptophyta - ps11
Charophyceae/Embriophyta - ps12
Embriophyta - ps13
Tracheophyta - ps14
Spermatophyta - ps15
Angiosperms - ps16
Mesangiospermae - ps17
Magnoliids/eudicots - ps18
Eudicots - ps19
Core_eudicots - ps20
Superrosids - ps21
Rosids - ps22
Eurosids - ps23
Malvids - ps24
Sap./Malv./Brassicales - ps25
Malvales/Brassicales - ps26
Brassicales - ps27
Cleomaceae/Brassicaceae - ps28
Brassicaceae - ps29
Camelineae - ps30
Arabidopsis - ps31
Arabidopsis_thaliana - ps32
Phylostratum

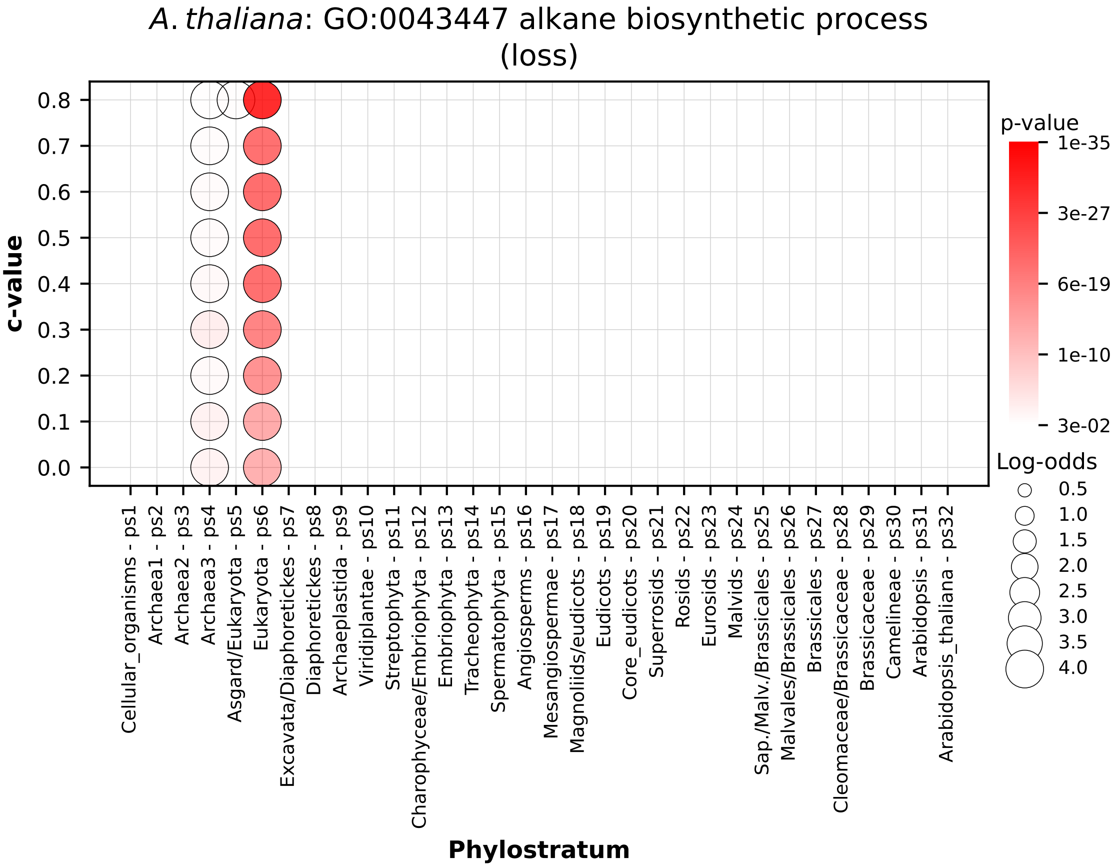

A. thaliana: GO:0043447 alkane biosynthetic process
(loss)
c-value
0.8
0.7
0.6
0.5
0.4
0.3
0.2
0.1
0.0
p-value
1e-35
3e-27
6e-19
1e-10
3e-02
Log-odds
0.5
1.0
1.5
2.0
2.5
3.0
3.5
4.0
Cellular_organisms - ps1
Archaea1 - ps2
Archaea2 - ps3
Archaea3 - ps4
Asgard/Eukaryota - ps5
Eukaryota - ps6
Excavata/Diaphoretickes - ps7
Diaphoretickes - ps8
Archaeplastida - ps9
Viridiplantae - ps10
Streptophyta - ps11
Charophyceae/Embriophyta - ps12
Embriophyta - ps13
Tracheophyta - ps14
Spermatophyta - ps15
Angiosperms - ps16
Mesangiospermae - ps17
Magnoliids/eudicots - ps18
Eudicots - ps19
Core_eudicots - ps20
Superrosids - ps21
Rosids - ps22
Eurosids - ps23
Malvids - ps24
Sap./Malv./Brassicales - ps25
Malvales/Brassicales - ps26
Brassicales - ps27
Cleomaceae/Brassicaceae - ps28
Brassicaceae - ps29
Camelineae - ps30
Arabidopsis - ps31
Arabidopsis_thaliana - ps32
Phylostratum

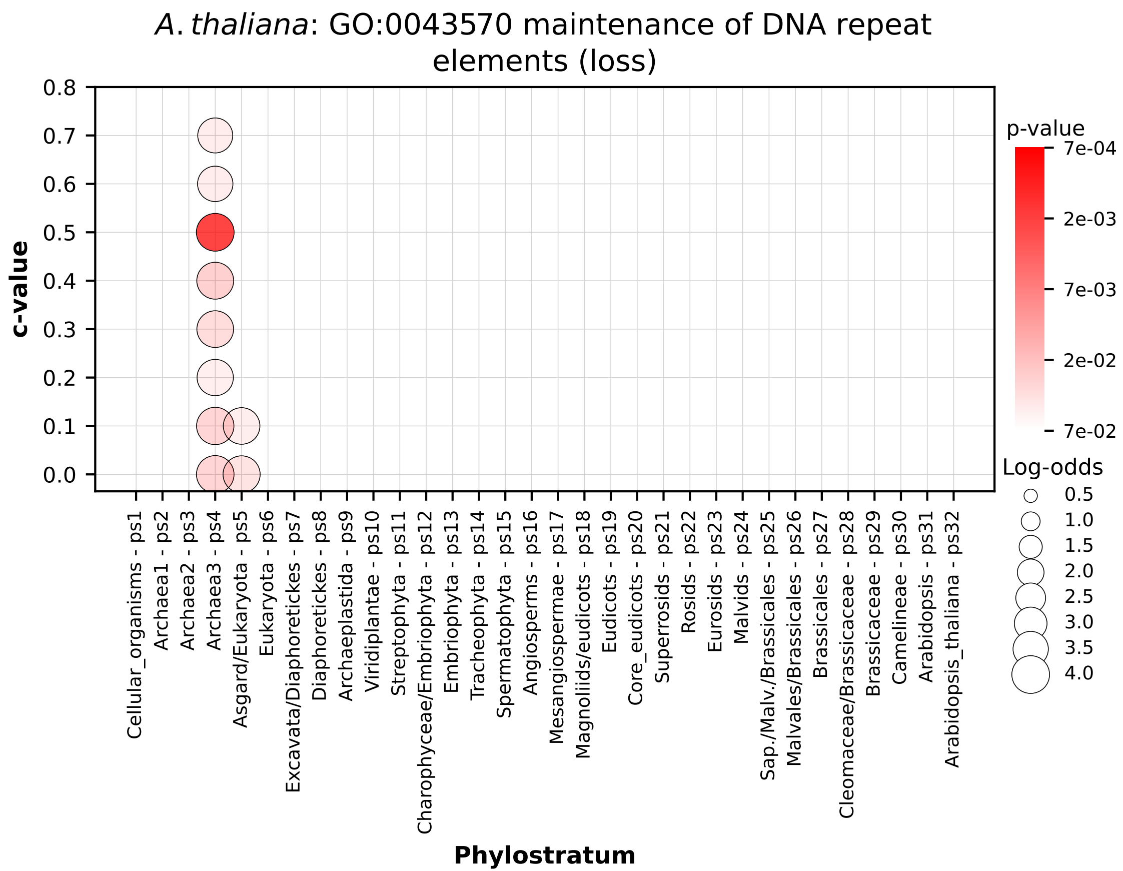

A. thaliana: GO:0043570 maintenance of DNA repeat elements (loss)
c-value
0.8
0.7
0.6
0.5
0.4
0.3
0.2
0.1
0.0
p-value
7e-04
2e-03
7e-03
2e-02
7e-02
Log-odds
0.5
1.0
1.5
2.0
2.5
3.0
3.5
4.0
Cellular_organisms - ps1
Archaea1 - ps2
Archaea2 - ps3
Archaea3 - ps4
Asgard/Eukaryota - ps5
Eukaryota - ps6
Excavata/Diaphoretickes - ps7
Diaphoretickes - ps8
Archaeplastida - ps9
Viridiplantae - ps10
Streptophyta - ps11
Charophyceae/Embriophyta - ps12
Embriophyta - ps13
Tracheophyta - ps14
Spermatophyta - ps15
Angiosperms - ps16
Mesangiospermae - ps17
Magnoliids/eudicots - ps18
Eudicots - ps19
Core_eudicots - ps20
Superrosids - ps21
Rosids - ps22
Eurosids - ps23
Malvids - ps24
Sap./Malv./Brassicales - ps25
Malvales/Brassicales - ps26
Brassicales - ps27
Cleomaceae/Brassicaceae - ps28
Brassicaceae - ps29
Camelineae - ps30
Arabidopsis - ps31
Arabidopsis_thaliana - ps32
Phylostratum

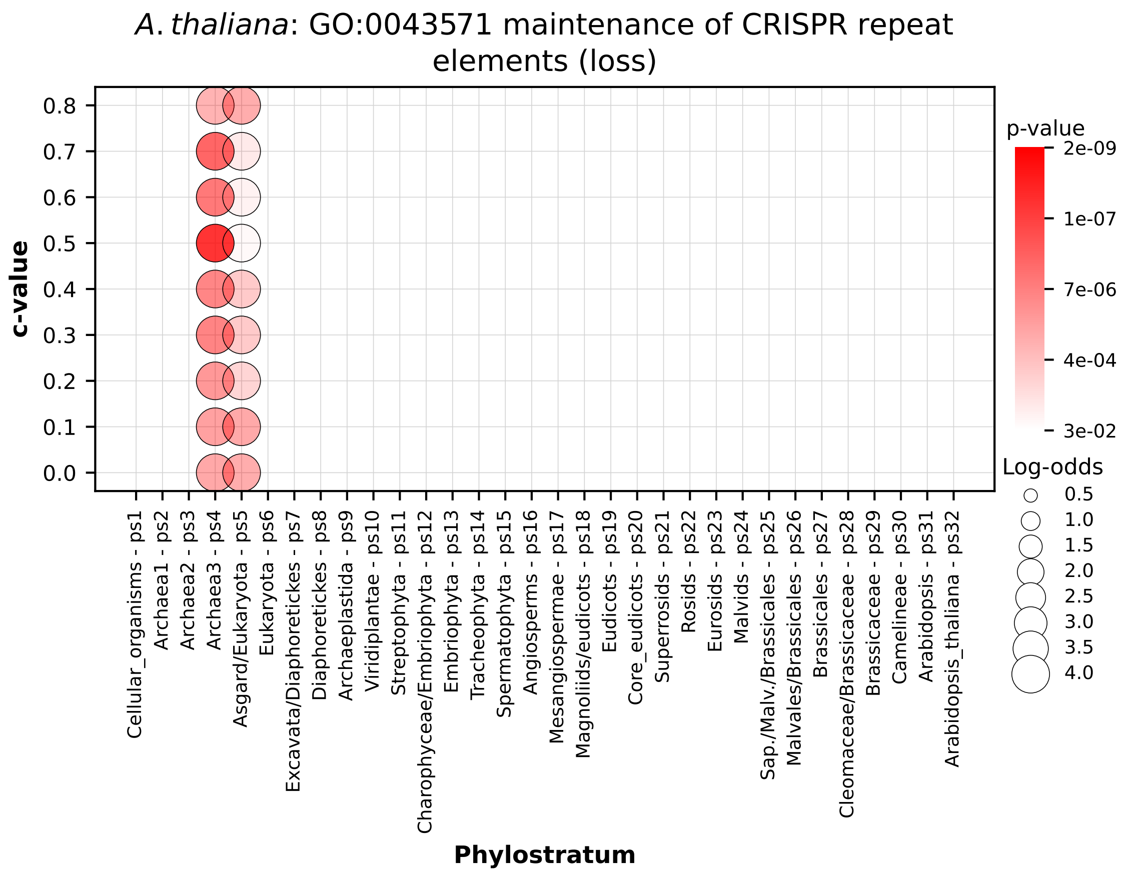

A. thaliana: GO:0043571 maintenance of CRISPR repeat elements (loss)
c-value
0.8
0.7
0.6
0.5
0.4
0.3
0.2
0.1
0.0
p-value
2e-09
1e-07
7e-06
4e-04
3e-02
Log-odds
0.5
1.0
1.5
2.0
2.5
3.0
3.5
4.0
Cellular_organisms - ps1
Archaea1 - ps2
Archaea2 - ps3
Archaea3 - ps4
Asgard/Eukaryota - ps5
Eukaryota - ps6
Excavata/Diaphoretickes - ps7
Diaphoretickes - ps8
Archaeplastida - ps9
Viridiplantae - ps10
Streptophyta - ps11
Charophyceae/Embriophyta - ps12
Embriophyta - ps13
Tracheophyta - ps14
Spermatophyta - ps15
Angiosperms - ps16
Mesangiospermae - ps17
Magnoliids/eudicots - ps18
Eudicots - ps19
Core_eudicots - ps20
Superrosids - ps21
Rosids - ps22
Eurosids - ps23
Malvids - ps24
Sap./Malv./Brassicales - ps25
Malvales/Brassicales - ps26
Brassicales - ps27
Cleomaceae/Brassicaceae - ps28
Brassicaceae - ps29
Camelineae - ps30
Arabidopsis - ps31
Arabidopsis_thaliana - ps32
Phylostratum

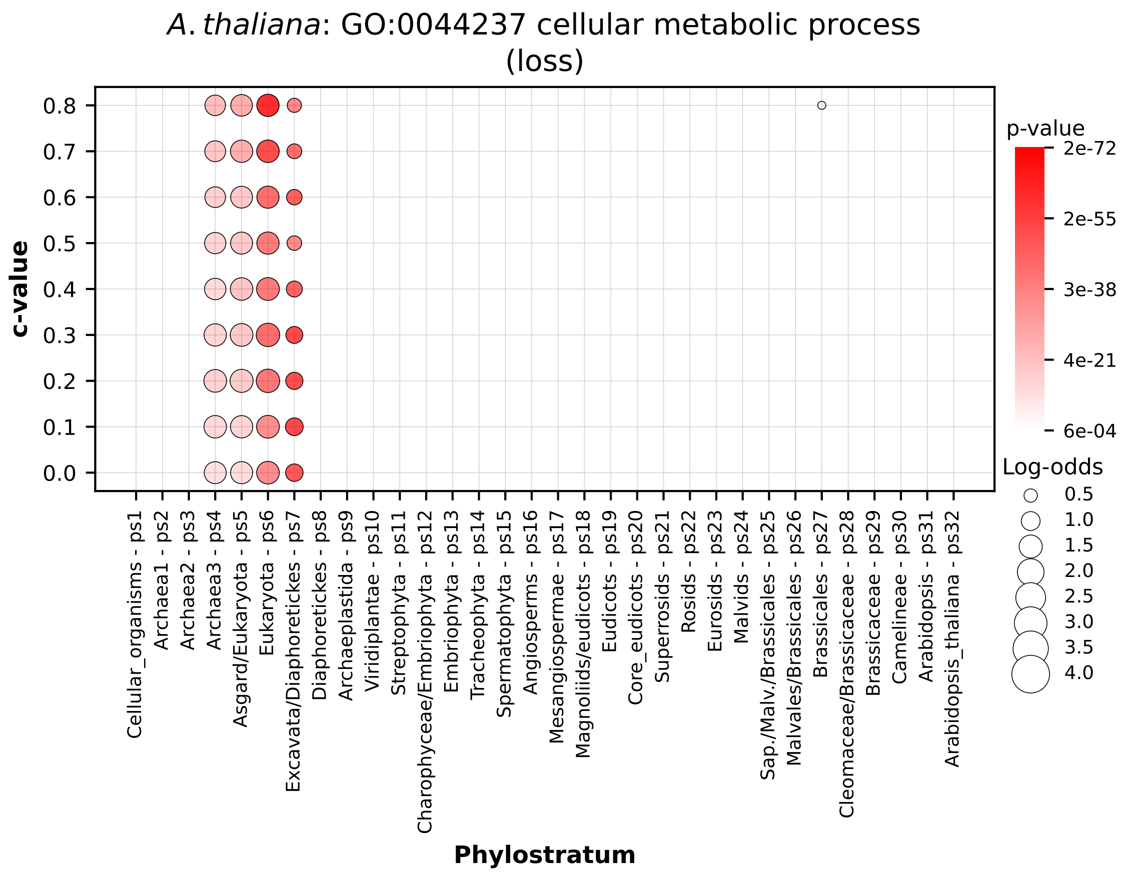

A. thaliana: GO:0044237 cellular metabolic process
(loss)
c-value
0.0
0.1
0.2
0.3
0.4
0.5
0.6
0.7
0.8
Phylostratum
Cellular_organisms - ps1
Archaea1 - ps2
Archaea2 - ps3
Archaea3 - ps4
Asgard/Eukaryota - ps5
Eukaryota - ps6
Excavata/Diaphoretickes - ps7
Diaphoretickes - ps8
Archaeplastida - ps9
Viridiplantae - ps10
Streptophyta - ps11
Charophyceae/Embriophyta - ps12
Embriophyta - ps13
Tracheophyta - ps14
Spermatophyta - ps15
Angiosperms - ps16
Mesangiospermae - ps17
Magnoliids/eudicots - ps18
Eudicots - ps19
Core_eudicots - ps20
Superrosids - ps21
Rosids - ps22
Eurosids - ps23
Malvids - ps24
Sap./Malv./Brassicales - ps25
Malvales/Brassicales - ps26
Brassicales - ps27
Cleomaceae/Brassicaceae - ps28
Brassicaceae - ps29
Camelineae - ps30
Arabidopsis - ps31
Arabidopsis_thaliana - ps32
p-value
2e-72
2e-55
3e-38
4e-21
6e-04
Log-odds
0.5
1.0
1.5
2.0
2.5
3.0
3.5
4.0

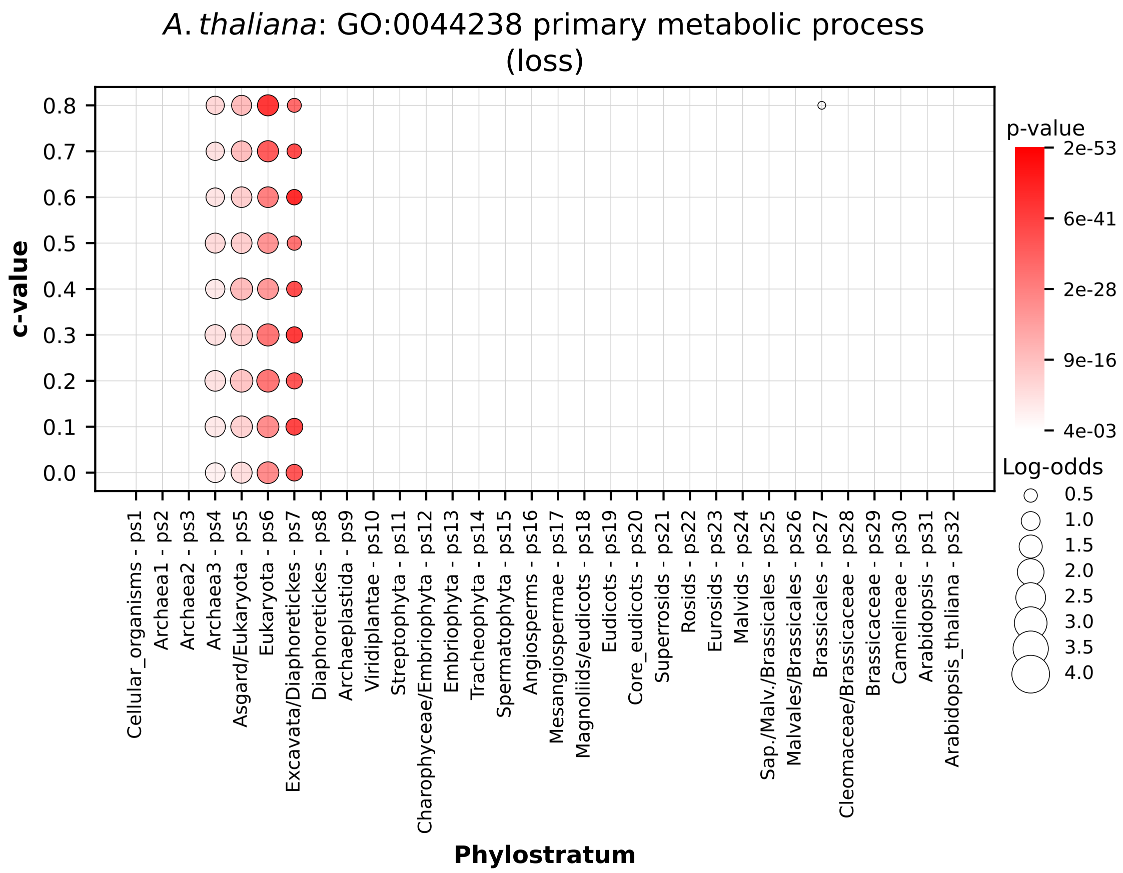

A. thaliana: GO:0044238 primary metabolic process (loss)
c-value
0.8
0.7
0.6
0.5
0.4
0.3
0.2
0.1
0.0
Phylostratum
Cellular_organisms - ps1
Archaea1 - ps2
Archaea2 - ps3
Archaea3 - ps4
Asgard/Eukaryota - ps5
Eukaryota - ps6
Excavata/Diaphoretickes - ps7
Diaphoretickes - ps8
Archaeplastida - ps9
Viridiplantae - ps10
Streptophyta - ps11
Charophyceae/Embriophyta - ps12
Embriophyta - ps13
Tracheophyta - ps14
Spermatophyta - ps15
Angiosperms - ps16
Mesangiospermae - ps17
Magnoliids/eudicots - ps18
Eudicots - ps19
Core_eudicots - ps20
Superrosids - ps21
Rosids - ps22
Eurosids - ps23
Malvids - ps24
Sap./Malv./Brassicales - ps25
Malvales/Brassicales - ps26
Brassicales - ps27
Cleomaceae/Brassicaceae - ps28
Brassicaceae - ps29
Camelineae - ps30
Arabidopsis - ps31
Arabidopsis_thaliana - ps32
p-value
2e-53
6e-41
2e-28
9e-16
4e-03
Log-odds
0.5
1.0
1.5
2.0
2.5
3.0
3.5
4.0

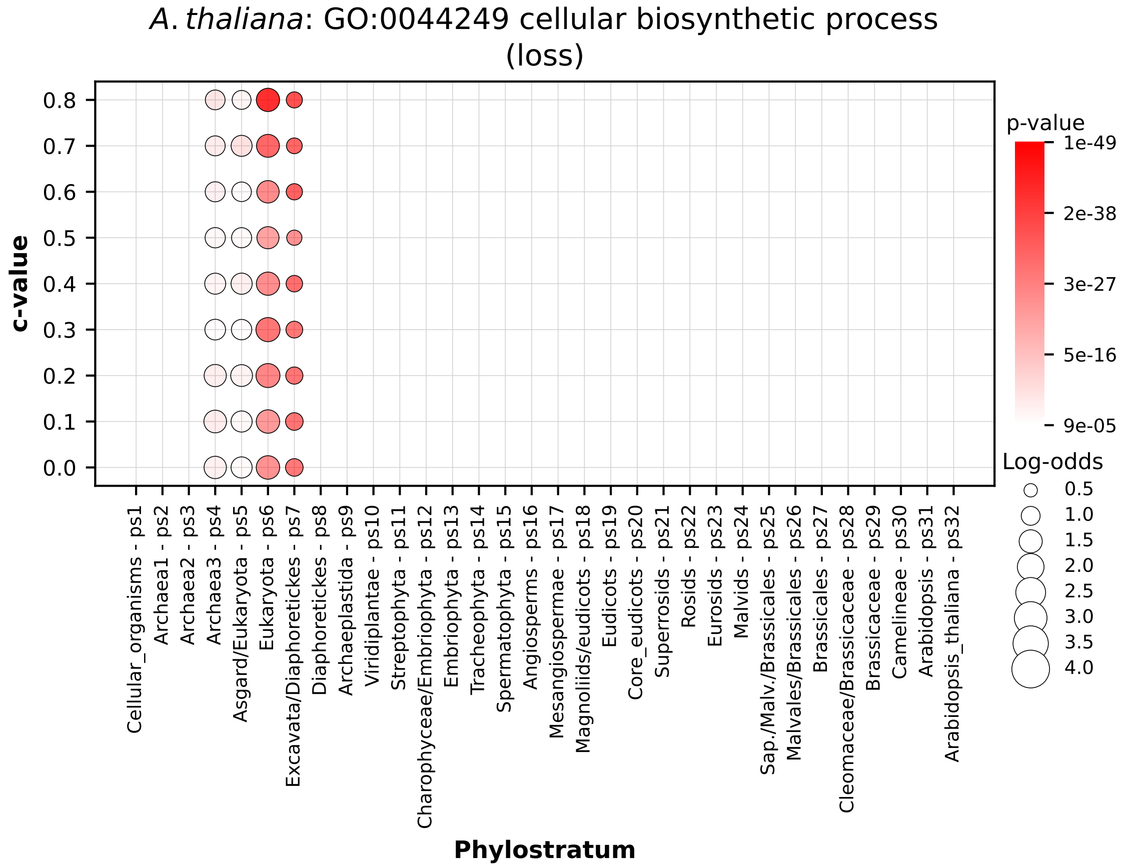

A. thaliana: GO:0044249 cellular biosynthetic process
(loss)
c-value
0.8
0.7
0.6
0.5
0.4
0.3
0.2
0.1
0.0
Phylostratum
Cellular_organisms - ps1
Archaea1 - ps2
Archaea2 - ps3
Archaea3 - ps4
Asgard/Eukaryota - ps5
Eukaryota - ps6
Excavata/Diaphoretickes - ps7
Diaphoretickes - ps8
Archaeplastida - ps9
Viridiplantae - ps10
Streptophyta - ps11
Charophyceae/Embriophyta - ps12
Embriophyta - ps13
Tracheophyta - ps14
Spermatophyta - ps15
Angiosperms - ps16
Mesangiospermae - ps17
Magnoliids/eudicots - ps18
Eudicots - ps19
Core_eudicots - ps20
Superrosids - ps21
Rosids - ps22
Eurosids - ps23
Malvids - ps24
Sap./Malv./Brassicales - ps25
Malvales/Brassicales - ps26
Brassicales - ps27
Cleomaceae/Brassicaceae - ps28
Brassicaceae - ps29
Camelineae - ps30
Arabidopsis - ps31
Arabidopsis_thaliana - ps32
p-value
1e-49
2e-38
3e-27
5e-16
9e-05
Log-odds
0.5
1.0
1.5
2.0
2.5
3.0
3.5
4.0

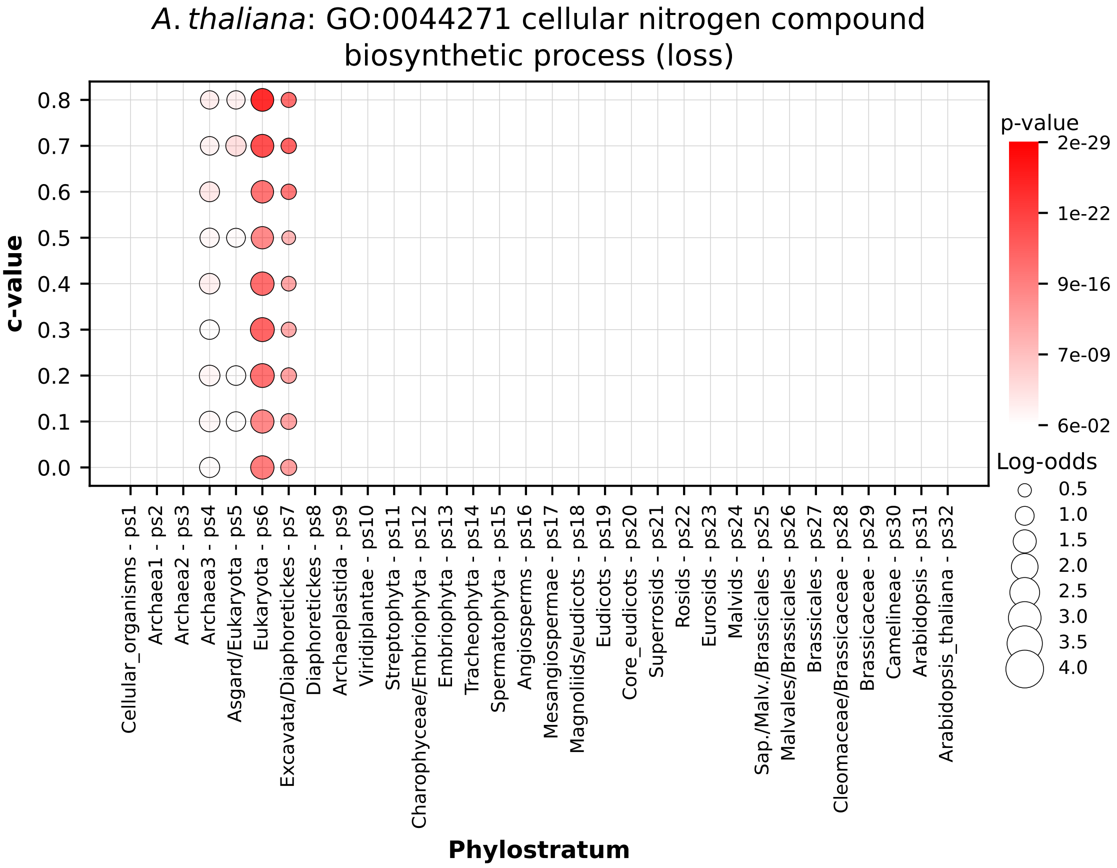

A. thaliana: GO:0044271 cellular nitrogen compound biosynthetic process (loss)
c-value
0.8
0.7
0.6
0.5
0.4
0.3
0.2
0.1
0.0
Phylostratum
Cellular_organisms - ps1
Archaea1 - ps2
Archaea2 - ps3
Archaea3 - ps4
Asgard/Eukaryota - ps5
Eukaryota - ps6
Excavata/Diaphoretickes - ps7
Diaphoretickes - ps8
Archaeplastida - ps9
Viridiplantae - ps10
Streptophyta - ps11
Charophyceae/Embriophyta - ps12
Embriophyta - ps13
Tracheophyta - ps14
Spermatophyta - ps15
Angiosperms - ps16
Mesangiospermae - ps17
Magnoliids/eudicots - ps18
Eudicots - ps19
Core_eudicots - ps20
Superrosids - ps21
Rosids - ps22
Eurosids - ps23
Malvids - ps24
Sap./Malv./Brassicales - ps25
Malvales/Brassicales - ps26
Brassicales - ps27
Cleomaceae/Brassicaceae - ps28
Brassicaceae - ps29
Camelineae - ps30
Arabidopsis - ps31
Arabidopsis_thaliana - ps32
p-value
2e-29
1e-22
9e-16
7e-09
6e-02
Log-odds
0.5
1.0
1.5
2.0
2.5
3.0
3.5
4.0

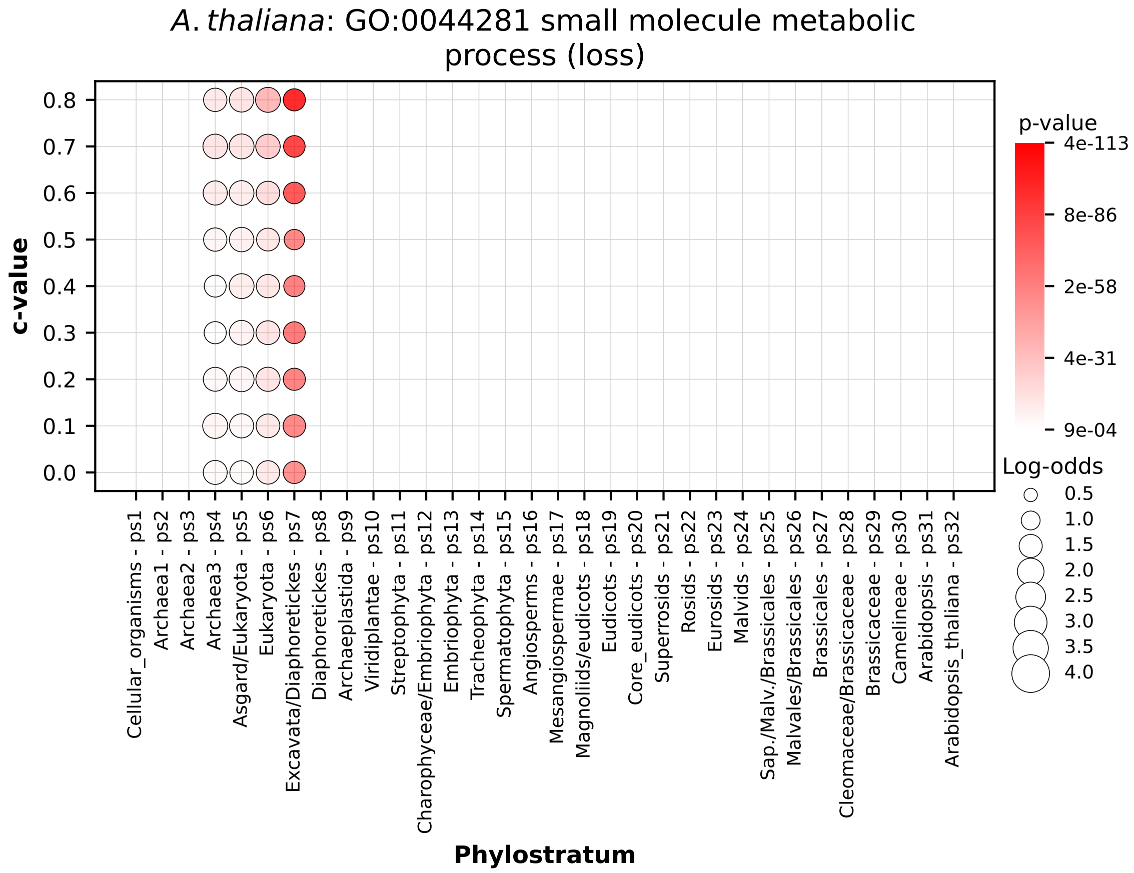
A. thaliana: GO:0044281 small molecule metabolic process (loss)
c-value
0.8
0.7
0.6
0.5
0.4
0.3
0.2
0.1
0.0
p-value
4e-113
8e-86
2e-58
4e-31
9e-04
Log-odds
0.5
1.0
1.5
2.0
2.5
3.0
3.5
4.0
Cellular_organisms - ps1
Archaea1 - ps2
Archaea2 - ps3
Archaea3 - ps4
Asgard/Eukaryota - ps5
Eukaryota - ps6
Excavata/Diaphoretickes - ps7
Diaphoretickes - ps8
Archaeplastida - ps9
Viridiplantae - ps10
Streptophyta - ps11
Charophyceae/Embriophyta - ps12
Embriophyta - ps13
Tracheophyta - ps14
Spermatophyta - ps15
Angiosperms - ps16
Mesangiospermae - ps17
Magnoliids/eudicots - ps18
Eudicots - ps19
Core_eudicots - ps20
Superrosids - ps21
Rosids - ps22
Eurosids - ps23
Malvids - ps24
Sap./Malv./Brassicales - ps25
Malvales/Brassicales - ps26
Brassicales - ps27
Cleomaceae/Brassicaceae - ps28
Brassicaceae - ps29
Camelineae - ps30
Arabidopsis - ps31
Arabidopsis_thaliana - ps32
Phylostratum

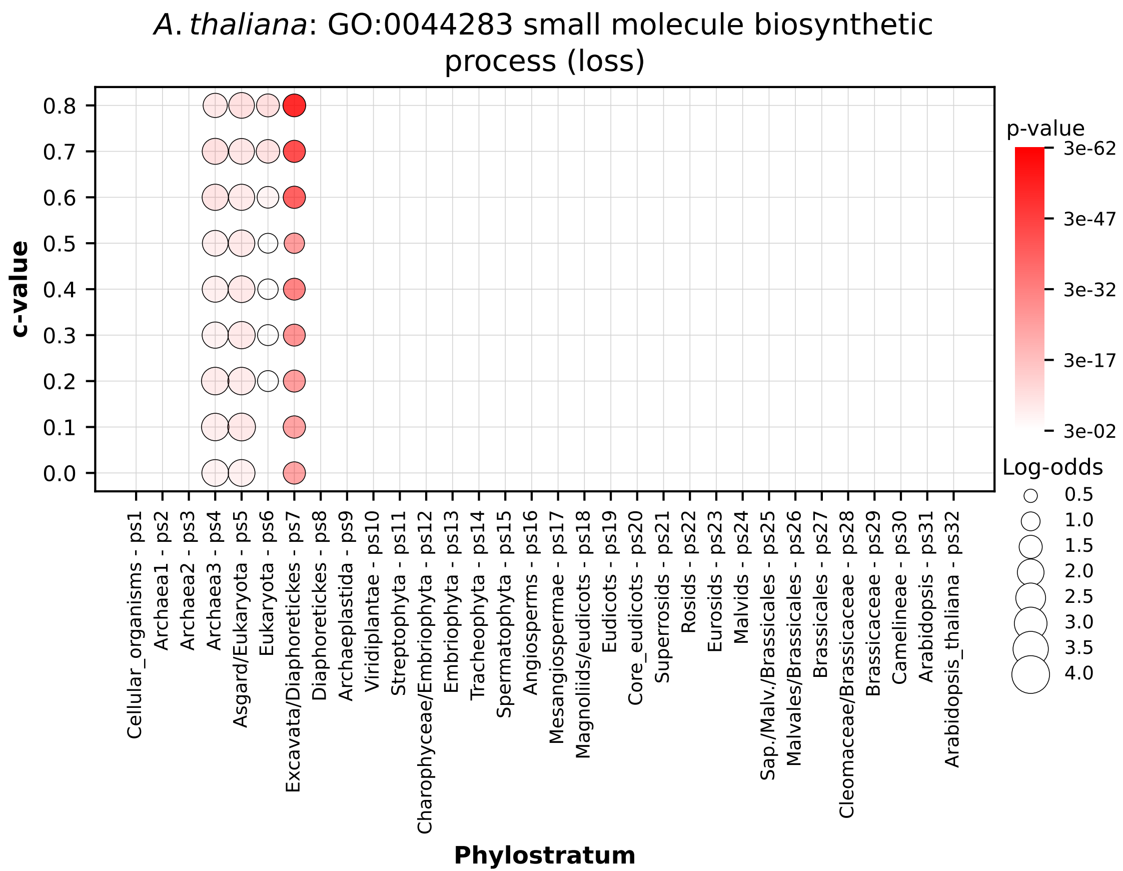

A. thaliana: GO:0044283 small molecule biosynthetic process (loss)
c-value
0.8
0.7
0.6
0.5
0.4
0.3
0.2
0.1
0.0
Phylostratum
Cellular_organisms - ps1
Archaea1 - ps2
Archaea2 - ps3
Archaea3 - ps4
Asgard/Eukaryota - ps5
Eukaryota - ps6
Excavata/Diaphoretickes - ps7
Diaphoretickes - ps8
Archaeplastida - ps9
Viridiplantae - ps10
Streptophyta - ps11
Charophyceae/Embriophyta - ps12
Embriophyta - ps13
Tracheophyta - ps14
Spermatophyta - ps15
Angiosperms - ps16
Mesangiospermae - ps17
Magnoliids/eudicots - ps18
Eudicots - ps19
Core_eudicots - ps20
Superrosids - ps21
Rosids - ps22
Eurosids - ps23
Malvids - ps24
Sap./Malv./Brassicales - ps25
Malvales/Brassicales - ps26
Brassicales - ps27
Cleomaceae/Brassicaceae - ps28
Brassicaceae - ps29
Camelineae - ps30
Arabidopsis - ps31
Arabidopsis_thaliana - ps32
p-value
3e-62
3e-47
3e-32
3e-17
3e-02
Log-odds
0.5
1.0
1.5
2.0
2.5
3.0
3.5
4.0

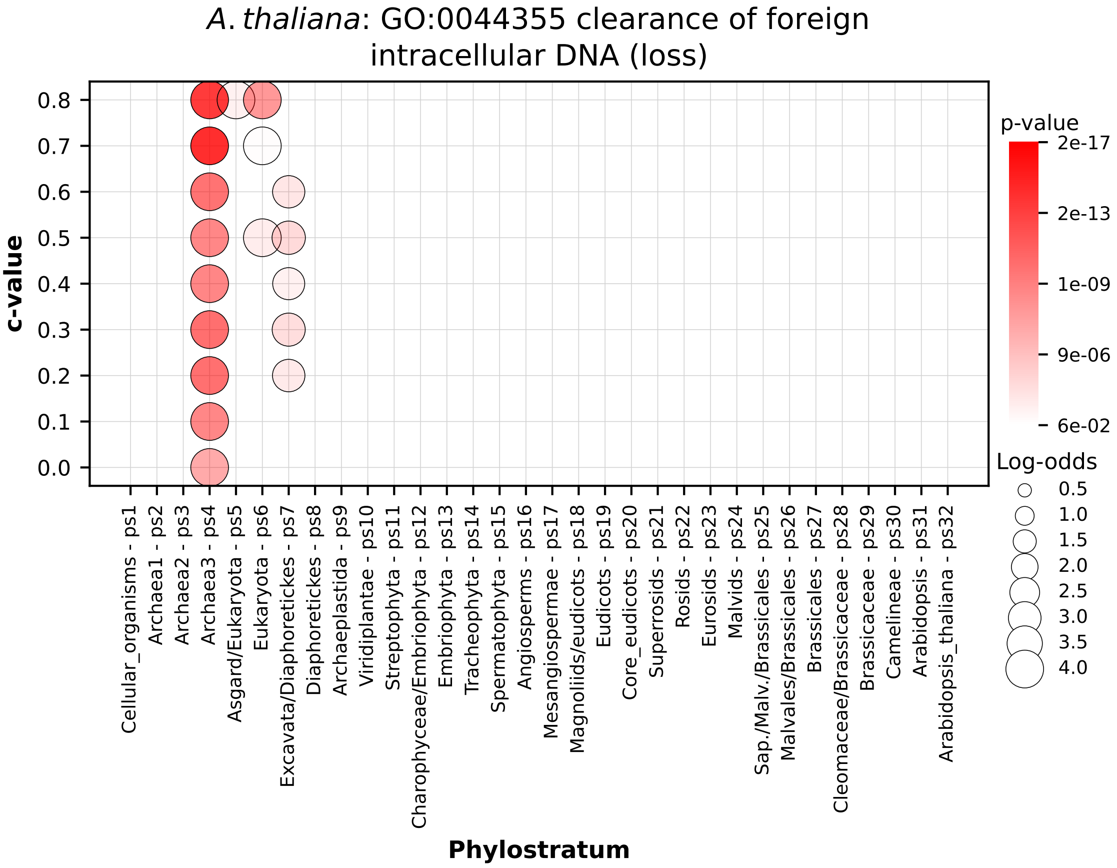

A. thaliana: GO:0044355 clearance of foreign intracellular DNA (loss)
c-value
0.8
0.7
0.6
0.5
0.4
0.3
0.2
0.1
0.0
p-value
2e-17
2e-13
1e-09
9e-06
6e-02
Log-odds
0.5
1.0
1.5
2.0
2.5
3.0
3.5
4.0
Cellular_organisms - ps1
Archaea1 - ps2
Archaea2 - ps3
Archaea3 - ps4
Asgard/Eukaryota - ps5
Eukaryota - ps6
Excavata/Diaphoretickes - ps7
Diaphoretickes - ps8
Archaeplastida - ps9
Viridiplantae - ps10
Streptophyta - ps11
Charophyceae/Embriophyta - ps12
Embriophyta - ps13
Tracheophyta - ps14
Spermatophyta - ps15
Angiosperms - ps16
Mesangiospermae - ps17
Magnoliids/eudicots - ps18
Eudicots - ps19
Core_eudicots - ps20
Superrosids - ps21
Rosids - ps22
Eurosids - ps23
Malvids - ps24
Sap./Malv./Brassicales - ps25
Malvales/Brassicales - ps26
Brassicales - ps27
Cleomaceae/Brassicaceae - ps28
Brassicaceae - ps29
Camelineae - ps30
Arabidopsis - ps31
Arabidopsis_thaliana - ps32
Phylostratum

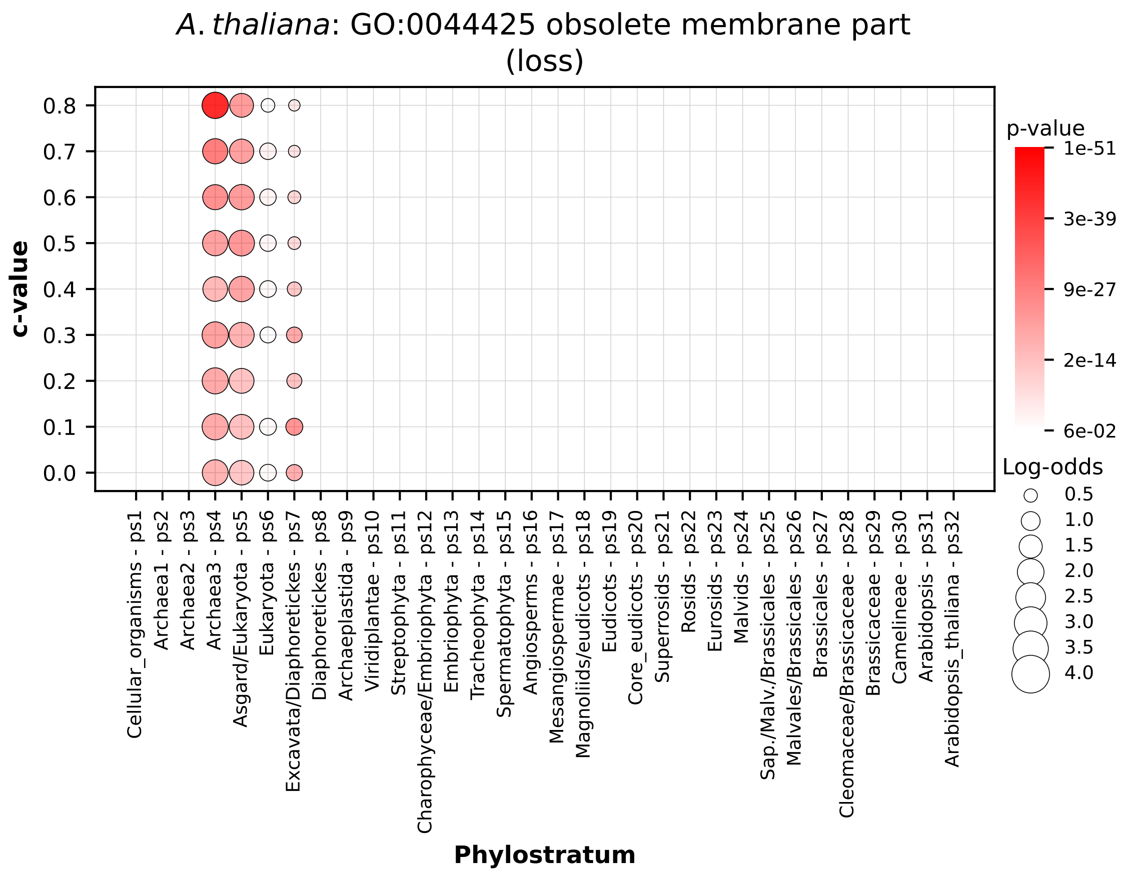

A. thaliana: GO:0044425 obsolete membrane part
(loss)
c-value
0.8
0.7
0.6
0.5
0.4
0.3
0.2
0.1
0.0
p-value
1e-51
3e-39
9e-27
2e-14
6e-02
Log-odds
0.5
1.0
1.5
2.0
2.5
3.0
3.5
4.0
Cellular_organisms - ps1
Archaea1 - ps2
Archaea2 - ps3
Archaea3 - ps4
Asgard/Eukaryota - ps5
Eukaryota - ps6
Excavata/Diaphoretickes - ps7
Diaphoretickes - ps8
Archaeplastida - ps9
Viridiplantae - ps10
Streptophyta - ps11
Charophyceae/Embriophyta - ps12
Embriophyta - ps13
Tracheophyta - ps14
Spermatophyta - ps15
Angiosperms - ps16
Mesangiospermae - ps17
Magnoliids/eudicots - ps18
Eudicots - ps19
Core_eudicots - ps20
Superrosids - ps21
Rosids - ps22
Eurosids - ps23
Malvids - ps24
Sap./Malv./Brassicales - ps25
Malvales/Brassicales - ps26
Brassicales - ps27
Cleomaceae/Brassicaceae - ps28
Brassicaceae - ps29
Camelineae - ps30
Arabidopsis - ps31
Arabidopsis_thaliana - ps32
Phylostratum

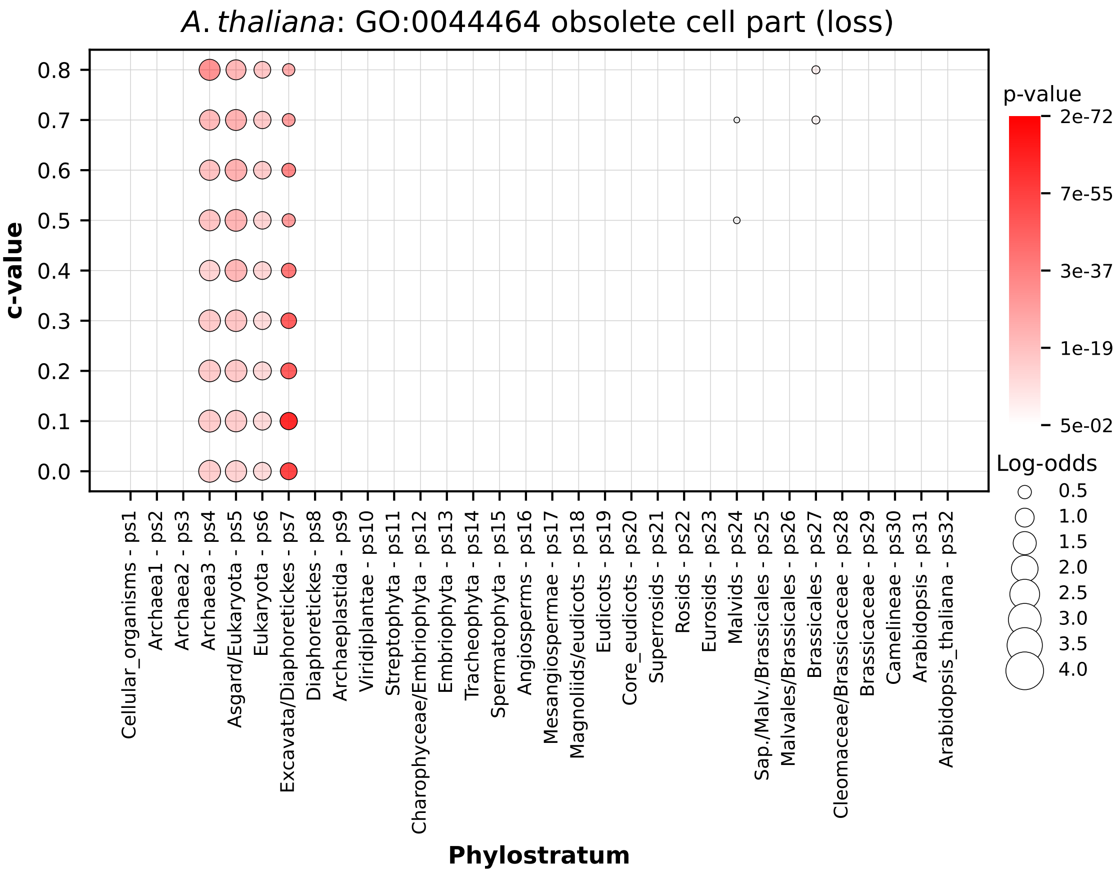

A. thaliana: GO:0044464 obsolete cell part (loss)
c-value
0.8
0.7
0.6
0.5
0.4
0.3
0.2
0.1
0.0
Phylostratum
Cellular_organisms - ps1
Archaea1 - ps2
Archaea2 - ps3
Archaea3 - ps4
Asgard/Eukaryota - ps5
Eukaryota - ps6
Excavata/Diaphoretickes - ps7
Diaphoretickes - ps8
Archaeplastida - ps9
Viridiplantae - ps10
Streptophyta - ps11
Charophyceae/Embriophyta - ps12
Embriophyta - ps13
Tracheophyta - ps14
Spermatophyta - ps15
Angiosperms - ps16
Mesangiospermae - ps17
Magnoliids/eudicots - ps18
Eudicots - ps19
Core_eudicots - ps20
Superrosids - ps21
Rosids - ps22
Eurosids - ps23
Malvids - ps24
Sap./Malv./Brassicales - ps25
Malvales/Brassicales - ps26
Brassicales - ps27
Cleomaceae/Brassicaceae - ps28
Brassicaceae - ps29
Camelineae - ps30
Arabidopsis - ps31
Arabidopsis_thaliana - ps32
p-value
2e-72
7e-55
3e-37
1e-19
5e-02
Log-odds
0.5
1.0
1.5
2.0
2.5
3.0
3.5
4.0

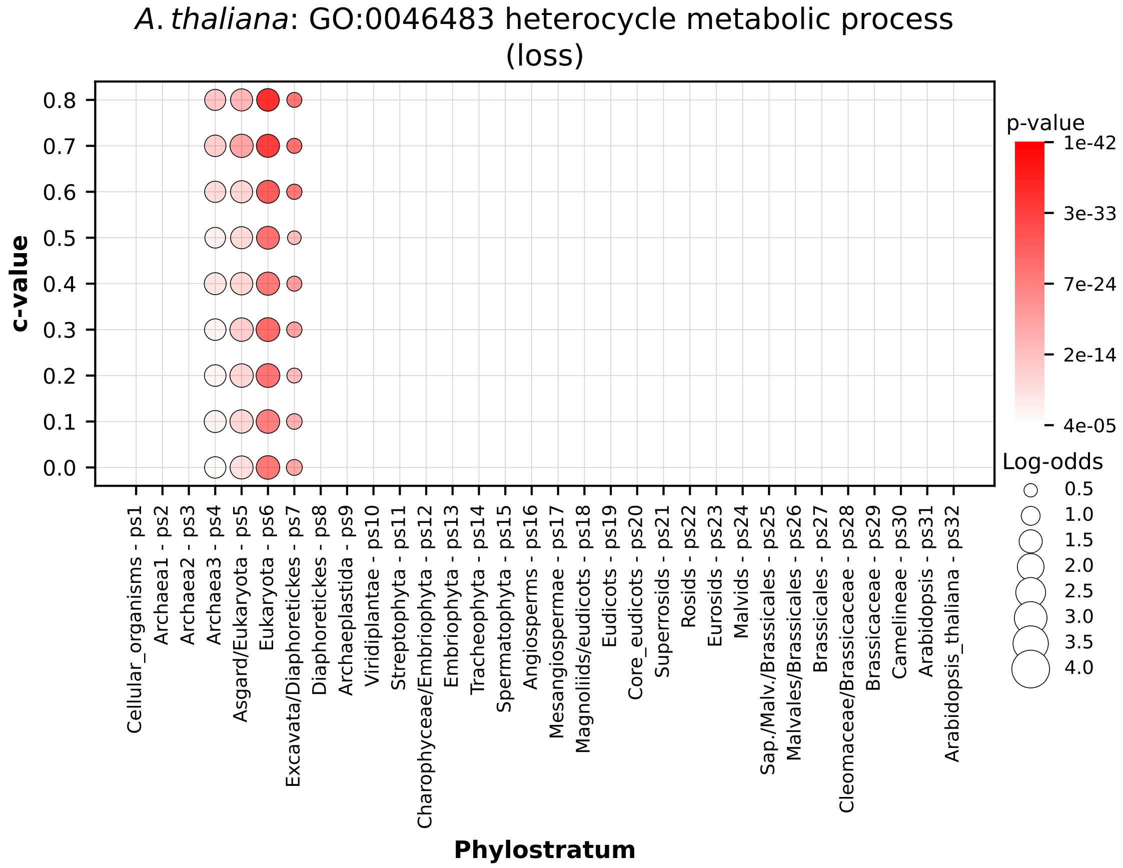

A. thaliana: GO:0046483 heterocycle metabolic process
(loss)
c-value
0.8
0.7
0.6
0.5
0.4
0.3
0.2
0.1
0.0
p-value
1e-42
3e-33
7e-24
2e-14
4e-05
Log-odds
0.5
1.0
1.5
2.0
2.5
3.0
3.5
4.0
Cellular_organisms - ps1
Archaea1 - ps2
Archaea2 - ps3
Archaea3 - ps4
Asgard/Eukaryota - ps5
Eukaryota - ps6
Excavata/Diaphoretickes - ps7
Diaphoretickes - ps8
Archaeplastida - ps9
Viridiplantae - ps10
Streptophyta - ps11
Charophyceae/Embriophyta - ps12
Embriophyta - ps13
Tracheophyta - ps14
Spermatophyta - ps15
Angiosperms - ps16
Mesangiospermae - ps17
Magnoliids/eudicots - ps18
Eudicots - ps19
Core_eudicots - ps20
Superrosids - ps21
Rosids - ps22
Eurosids - ps23
Malvids - ps24
Sap./Malv./Brassicales - ps25
Malvales/Brassicales - ps26
Brassicales - ps27
Cleomaceae/Brassicaceae - ps28
Brassicaceae - ps29
Camelineae - ps30
Arabidopsis - ps31
Arabidopsis_thaliana - ps32
Phylostratum

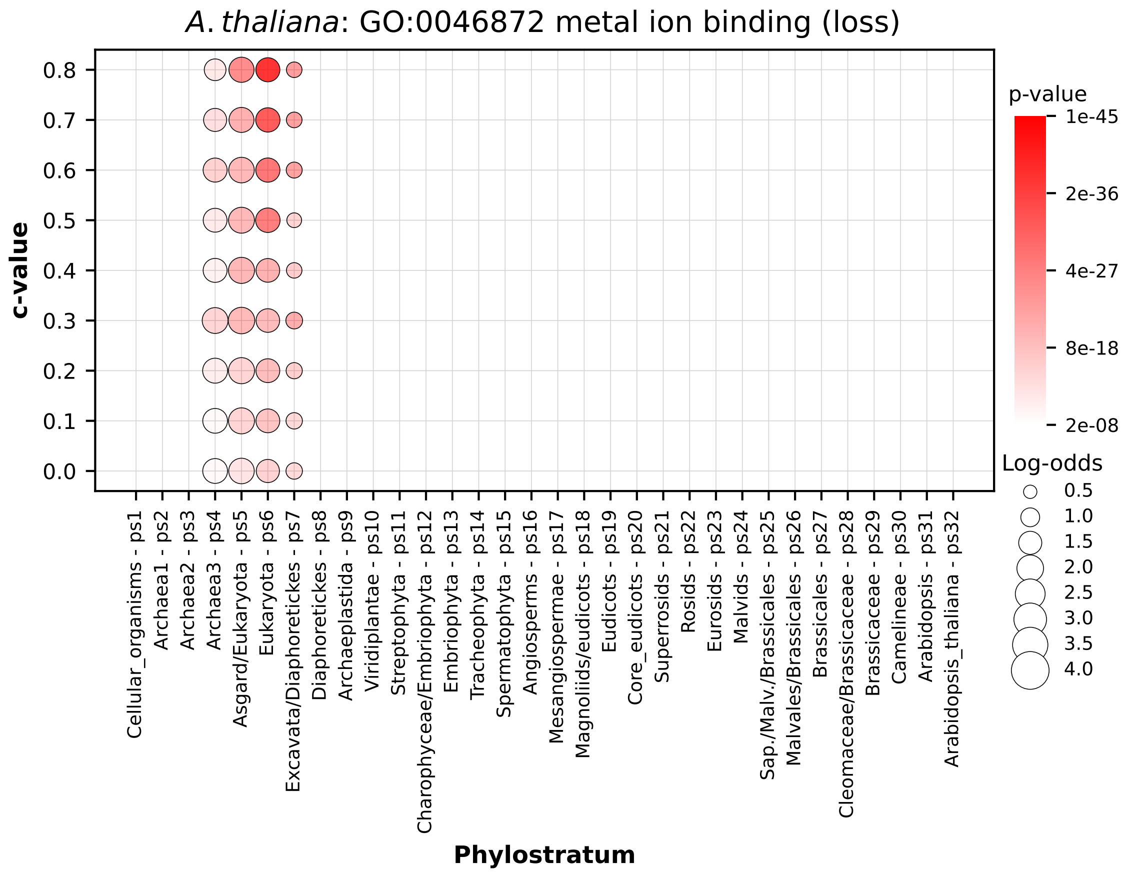

A. thaliana: GO:0046872 metal ion binding (loss)
c-value
0.0
0.1
0.2
0.3
0.4
0.5
0.6
0.7
0.8
Phylostratum
Cellular_organisms - ps1
Archaea1 - ps2
Archaea2 - ps3
Archaea3 - ps4
Asgard/Eukaryota - ps5
Eukaryota - ps6
Excavata/Diaphoretickes - ps7
Diaphoretickes - ps8
Archaeplastida - ps9
Viridiplantae - ps10
Streptophyta - ps11
Charophyceae/Embriophyta - ps12
Embriophyta - ps13
Tracheophyta - ps14
Spermatophyta - ps15
Angiosperms - ps16
Mesangiospermae - ps17
Magnoliids/eudicots - ps18
Eudicots - ps19
Core_eudicots - ps20
Superrosids - ps21
Rosids - ps22
Eurosids - ps23
Malvids - ps24
Sap./Malv./Brassicales - ps25
Malvales/Brassicales - ps26
Brassicales - ps27
Cleomaceae/Brassicaceae - ps28
Brassicaceae - ps29
Camelineae - ps30
Arabidopsis - ps31
Arabidopsis_thaliana - ps32
p-value
1e-45
2e-36
4e-27
8e-18
2e-08
Log-odds
0.5
1.0
1.5
2.0
2.5
3.0
3.5
4.0

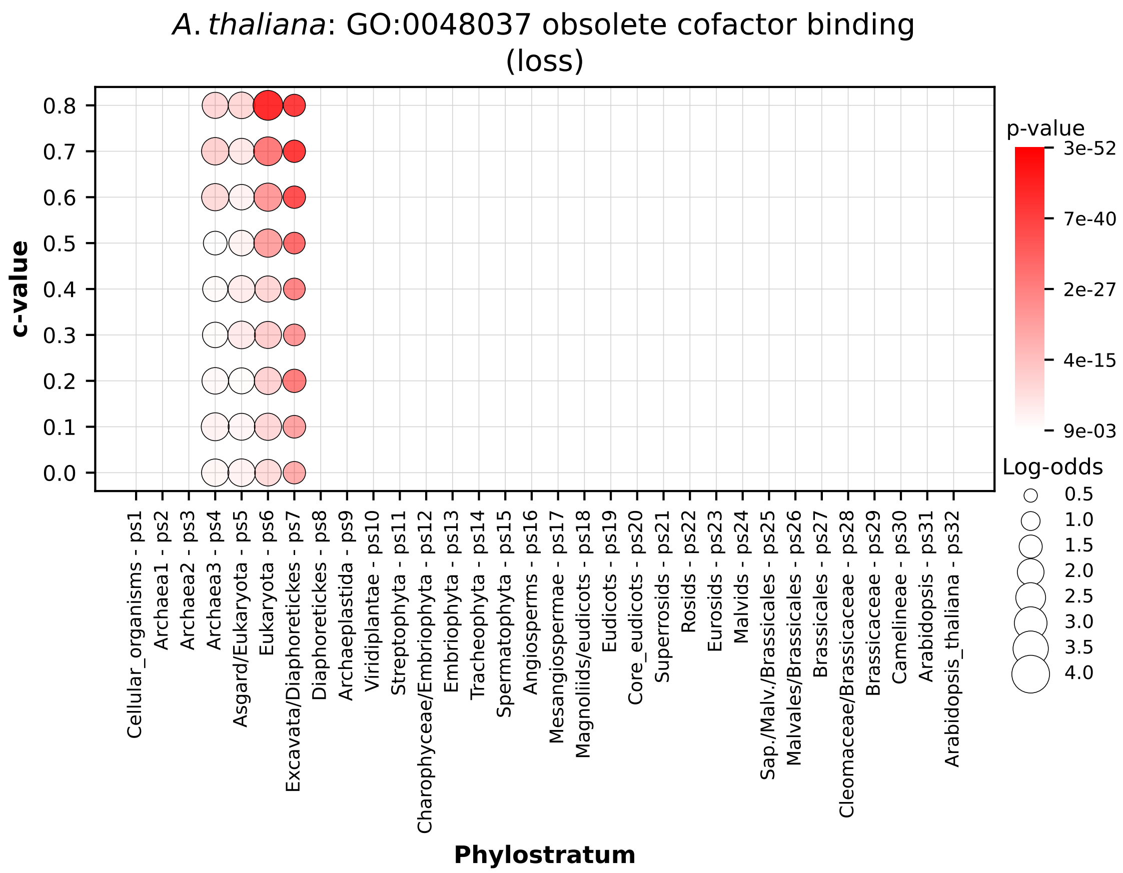

A. thaliana: GO:0048037 obsolete cofactor binding (loss)
c-value
0.8
0.7
0.6
0.5
0.4
0.3
0.2
0.1
0.0
p-value
3e-52
7e-40
2e-27
4e-15
9e-03
Log-odds
0.5
1.0
1.5
2.0
2.5
3.0
3.5
4.0
Cellular_organisms - ps1
Archaea1 - ps2
Archaea2 - ps3
Archaea3 - ps4
Asgard/Eukaryota - ps5
Eukaryota - ps6
Excavata/Diaphoretickes - ps7
Diaphoretickes - ps8
Archaeplastida - ps9
Viridiplantae - ps10
Streptophyta - ps11
Charophyceae/Embriophyta - ps12
Embriophyta - ps13
Tracheophyta - ps14
Spermatophyta - ps15
Angiosperms - ps16
Mesangiospermae - ps17
Magnoliids/eudicots - ps18
Eudicots - ps19
Core_eudicots - ps20
Superrosids - ps21
Rosids - ps22
Eurosids - ps23
Malvids - ps24
Sap./Malv./Brassicales - ps25
Malvales/Brassicales - ps26
Brassicales - ps27
Cleomaceae/Brassicaceae - ps28
Brassicaceae - ps29
Camelineae - ps30
Arabidopsis - ps31
Arabidopsis_thaliana - ps32
Phylostratum

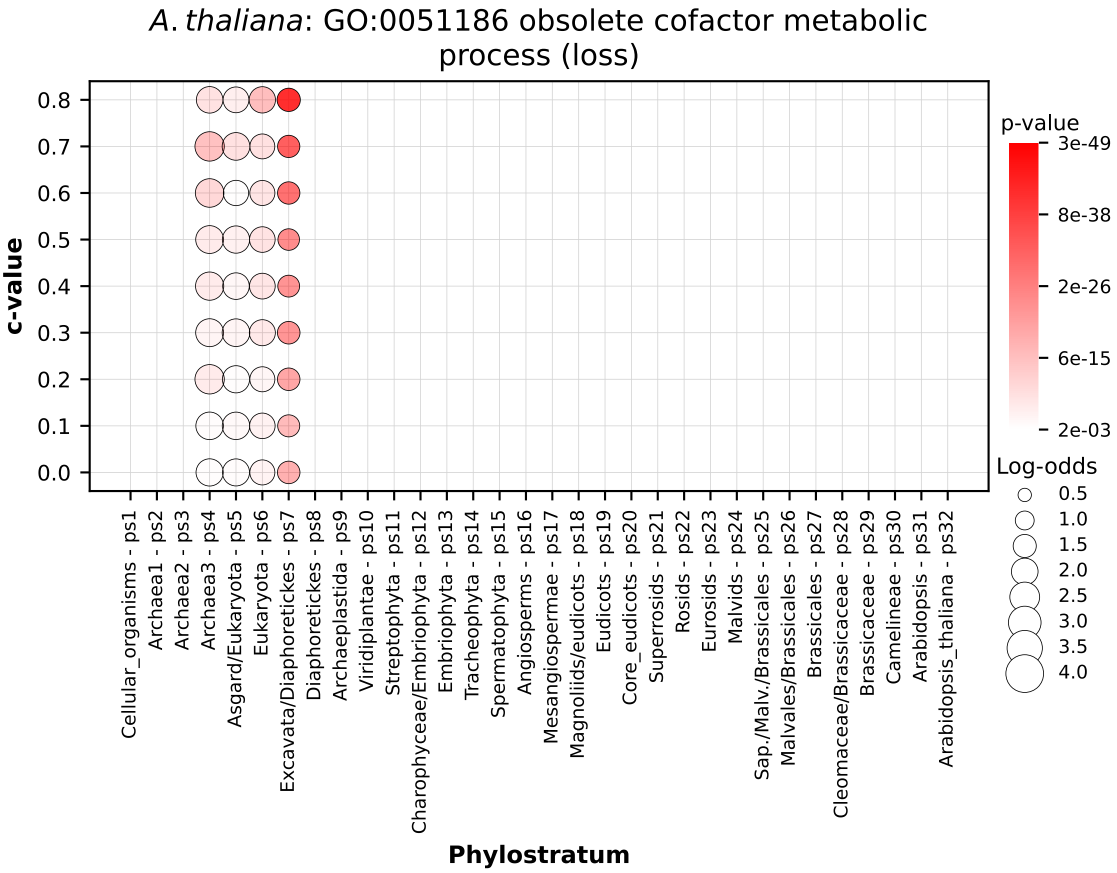
A. thaliana: GO:0051186 obsolete cofactor metabolic process (loss)
c-value
0.8
0.7
0.6
0.5
0.4
0.3
0.2
0.1
0.0
p-value
3e-49
8e-38
2e-26
6e-15
2e-03
Log-odds
0.5
1.0
1.5
2.0
2.5
3.0
3.5
4.0
Cellular_organisms - ps1
Archaea1 - ps2
Archaea2 - ps3
Archaea3 - ps4
Asgard/Eukaryota - ps5
Eukaryota - ps6
Excavata/Diaphoretickes - ps7
Diaphoretickes - ps8
Archaeplastida - ps9
Viridiplantae - ps10
Streptophyta - ps11
Charophyceae/Embriophyta - ps12
Embriophyta - ps13
Tracheophyta - ps14
Spermatophyta - ps15
Angiosperms - ps16
Mesangiospermae - ps17
Magnoliids/eudicots - ps18
Eudicots - ps19
Core_eudicots - ps20
Superrosids - ps21
Rosids - ps22
Eurosids - ps23
Malvids - ps24
Sap./Malv./Brassicales - ps25
Malvales/Brassicales - ps26
Brassicales - ps27
Cleomaceae/Brassicaceae - ps28
Brassicaceae - ps29
Camelineae - ps30
Arabidopsis - ps31
Arabidopsis_thaliana - ps32
Phylostratum

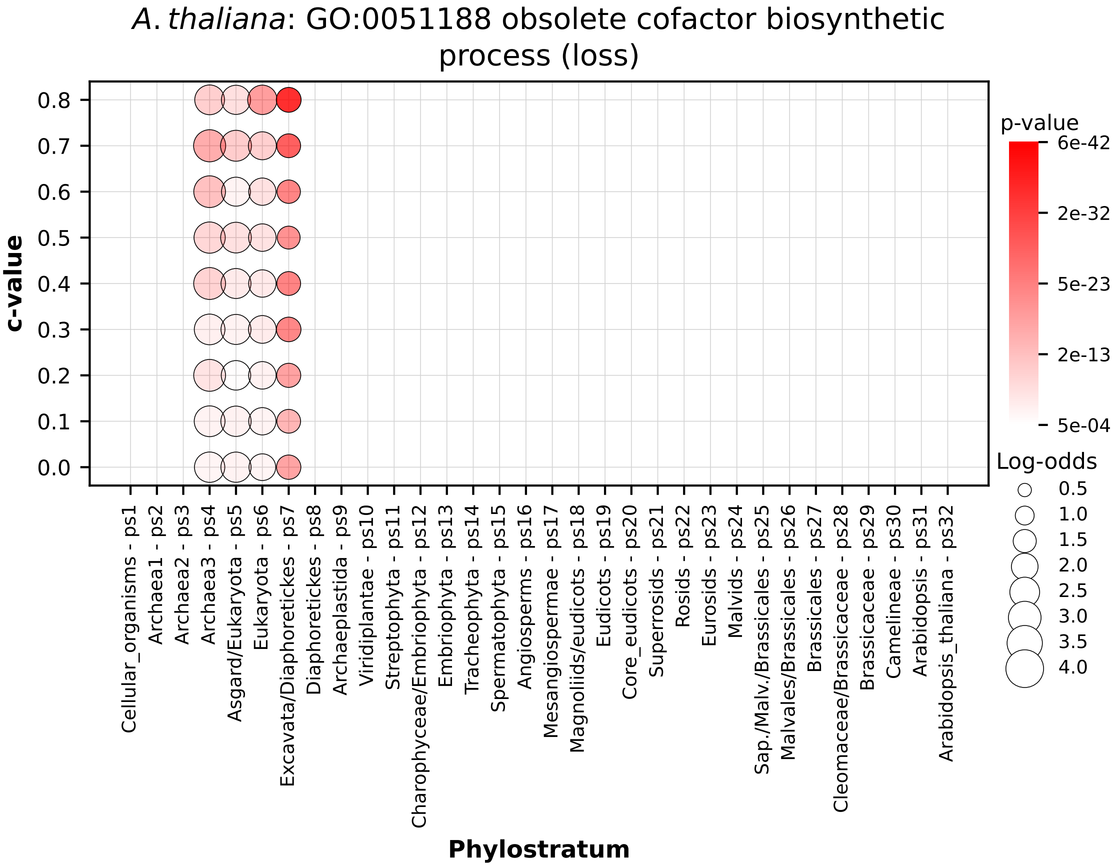

A. thaliana: GO:0051188 obsolete cofactor biosynthetic process (loss)
c-value
0.8
0.7
0.6
0.5
0.4
0.3
0.2
0.1
0.0
Phylostratum
Cellular_organisms - ps1
Archaea1 - ps2
Archaea2 - ps3
Archaea3 - ps4
Asgard/Eukaryota - ps5
Eukaryota - ps6
Excavata/Diaphoretickes - ps7
Diaphoretickes - ps8
Archaeplastida - ps9
Viridiplantae - ps10
Streptophyta - ps11
Charophyceae/Embriophyta - ps12
Embriophyta - ps13
Tracheophyta - ps14
Spermatophyta - ps15
Angiosperms - ps16
Mesangiospermae - ps17
Magnoliids/eudicots - ps18
Eudicots - ps19
Core_eudicots - ps20
Superrosids - ps21
Rosids - ps22
Eurosids - ps23
Malvids - ps24
Sap./Malv./Brassicales - ps25
Malvales/Brassicales - ps26
Brassicales - ps27
Cleomaceae/Brassicaceae - ps28
Brassicaceae - ps29
Camelineae - ps30
Arabidopsis - ps31
Arabidopsis_thaliana - ps32
p-value
6e-42
2e-32
5e-23
2e-13
5e-04
Log-odds
0.5
1.0
1.5
2.0
2.5
3.0
3.5
4.0

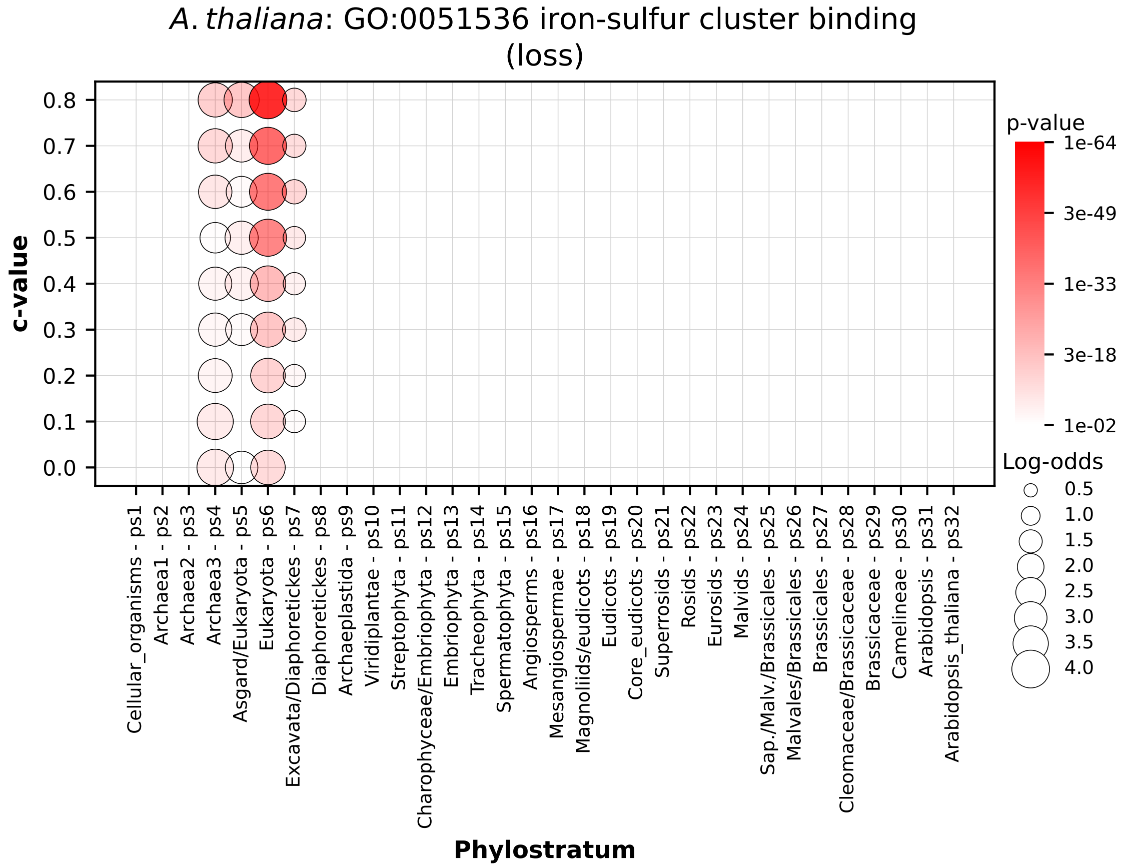

A. thaliana: GO:0051536 iron-sulfur cluster binding
(loss)
c-value
0.8
0.7
0.6
0.5
0.4
0.3
0.2
0.1
0.0
p-value
1e-64
3e-49
1e-33
3e-18
1e-02
Log-odds
0.5
1.0
1.5
2.0
2.5
3.0
3.5
4.0
Cellular_organisms - ps1
Archaea1 - ps2
Archaea2 - ps3
Archaea3 - ps4
Asgard/Eukaryota - ps5
Eukaryota - ps6
Excavata/Diaphoretickes - ps7
Diaphoretickes - ps8
Archaeplastida - ps9
Viridiplantae - ps10
Streptophyta - ps11
Charophyceae/Embriophyta - ps12
Embriophyta - ps13
Tracheophyta - ps14
Spermatophyta - ps15
Angiosperms - ps16
Mesangiospermae - ps17
Magnoliids/eudicots - ps18
Eudicots - ps19
Core_eudicots - ps20
Superrosids - ps21
Rosids - ps22
Eurosids - ps23
Malvids - ps24
Sap./Malv./Brassicales - ps25
Malvales/Brassicales - ps26
Brassicales - ps27
Cleomaceae/Brassicaceae - ps28
Brassicaceae - ps29
Camelineae - ps30
Arabidopsis - ps31
Arabidopsis_thaliana - ps32
Phylostratum

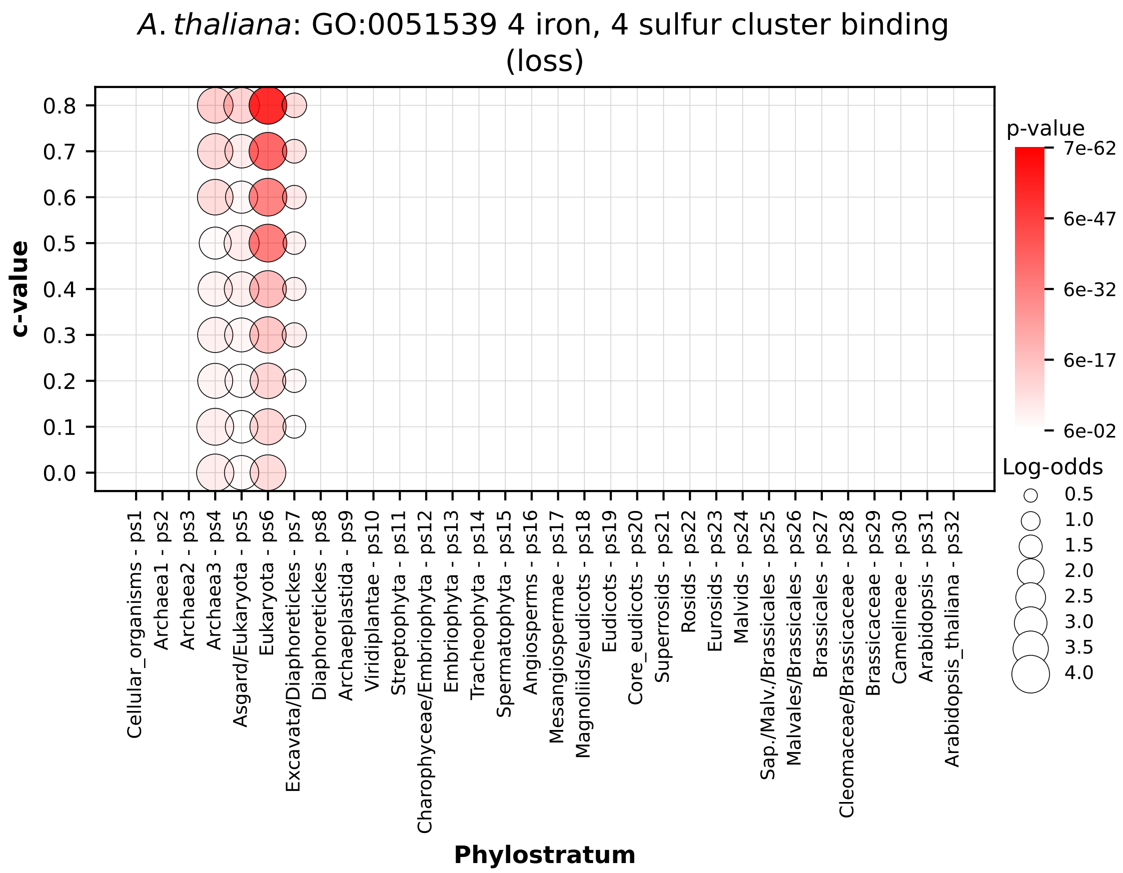

A. thaliana: GO:0051539 4 iron, 4 sulfur cluster binding (loss)
c-value
0.8
0.7
0.6
0.5
0.4
0.3
0.2
0.1
0.0
Phylostratum
Cellular_organisms - ps1
Archaea1 - ps2
Archaea2 - ps3
Archaea3 - ps4
Asgard/Eukaryota - ps5
Eukaryota - ps6
Excavata/Diaphoretickes - ps7
Diaphoretickes - ps8
Archaeplastida - ps9
Viridiplantae - ps10
Streptophyta - ps11
Charophyceae/Embriophyta - ps12
Embriophyta - ps13
Tracheophyta - ps14
Spermatophyta - ps15
Angiosperms - ps16
Mesangiospermae - ps17
Magnoliids/eudicots - ps18
Eudicots - ps19
Core_eudicots - ps20
Superrosids - ps21
Rosids - ps22
Eurosids - ps23
Malvids - ps24
Sap./Malv./Brassicales - ps25
Malvales/Brassicales - ps26
Brassicales - ps27
Cleomaceae/Brassicaceae - ps28
Brassicaceae - ps29
Camelineae - ps30
Arabidopsis - ps31
Arabidopsis_thaliana - ps32
p-value
7e-62
6e-47
6e-32
6e-17
6e-02
Log-odds
0.5
1.0
1.5
2.0
2.5
3.0
3.5
4.0

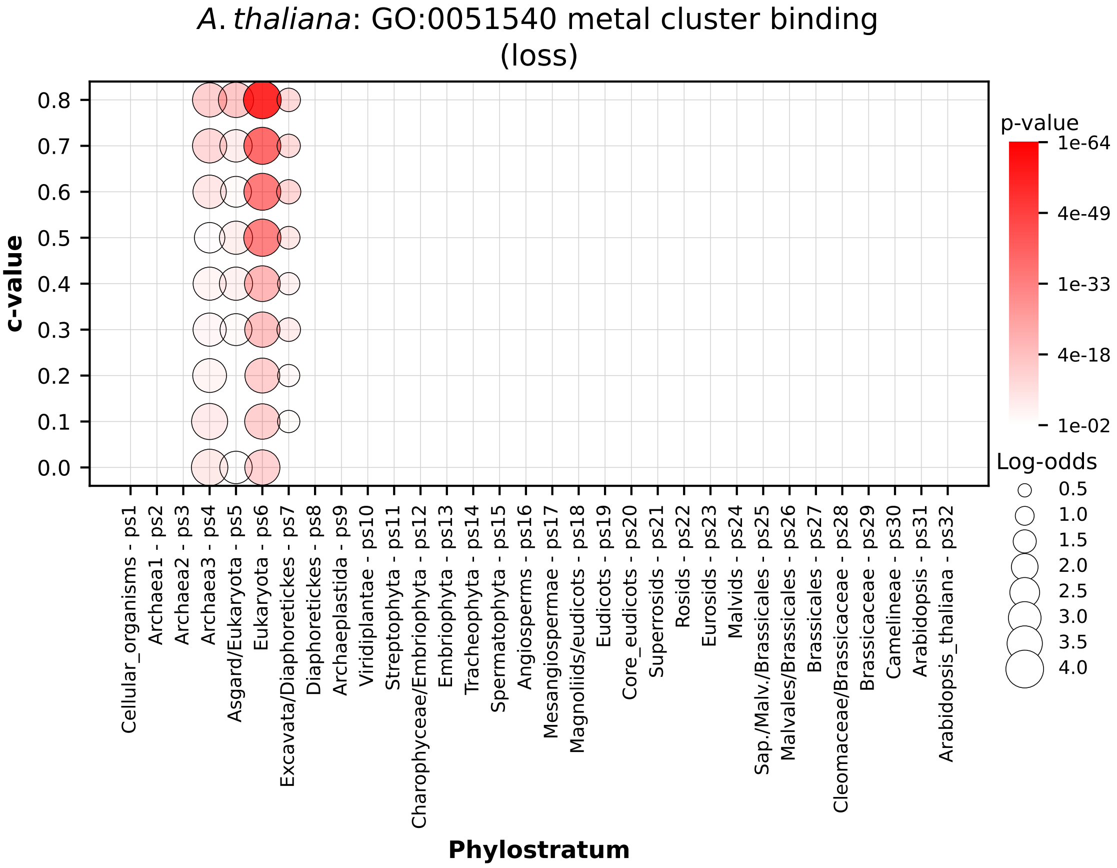

A. thaliana: GO:0051540 metal cluster binding
(loss)
c-value
0.8
0.7
0.6
0.5
0.4
0.3
0.2
0.1
0.0
p-value
1e-64
4e-49
1e-33
4e-18
1e-02
Log-odds
0.5
1.0
1.5
2.0
2.5
3.0
3.5
4.0
Cellular_organisms - ps1
Archaea1 - ps2
Archaea2 - ps3
Archaea3 - ps4
Asgard/Eukaryota - ps5
Eukaryota - ps6
Excavata/Diaphoretickes - ps7
Diaphoretickes - ps8
Archaeplastida - ps9
Viridiplantae - ps10
Streptophyta - ps11
Charophyceae/Embriophyta - ps12
Embriophyta - ps13
Tracheophyta - ps14
Spermatophyta - ps15
Angiosperms - ps16
Mesangiospermae - ps17
Magnoliids/eudicots - ps18
Eudicots - ps19
Core_eudicots - ps20
Superrosids - ps21
Rosids - ps22
Eurosids - ps23
Malvids - ps24
Sap./Malv./Brassicales - ps25
Malvales/Brassicales - ps26
Brassicales - ps27
Cleomaceae/Brassicaceae - ps28
Brassicaceae - ps29
Camelineae - ps30
Arabidopsis - ps31
Arabidopsis_thaliana - ps32
Phylostratum

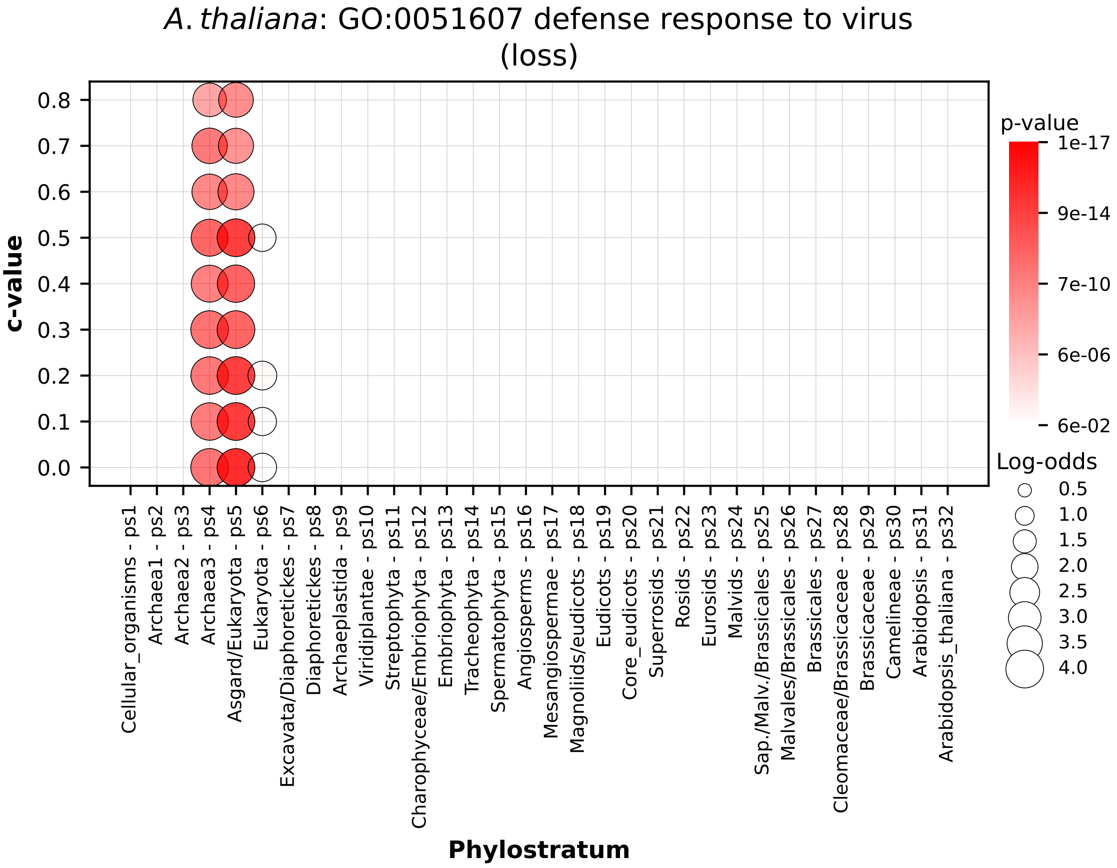

A. thaliana: GO:0051607 defense response to virus
(loss)
c-value
0.8
0.7
0.6
0.5
0.4
0.3
0.2
0.1
0.0
p-value
1e-17
9e-14
7e-10
6e-06
6e-02
Log-odds
0.5
1.0
1.5
2.0
2.5
3.0
3.5
4.0
Cellular_organisms - ps1
Archaea1 - ps2
Archaea2 - ps3
Archaea3 - ps4
Asgard/Eukaryota - ps5
Eukaryota - ps6
Excavata/Diaphoretickes - ps7
Diaphoretickes - ps8
Archaeplastida - ps9
Viridiplantae - ps10
Streptophyta - ps11
Charophyceae/Embriophyta - ps12
Embriophyta - ps13
Tracheophyta - ps14
Spermatophyta - ps15
Angiosperms - ps16
Mesangiospermae - ps17
Magnoliids/eudicots - ps18
Eudicots - ps19
Core_eudicots - ps20
Superrosids - ps21
Rosids - ps22
Eurosids - ps23
Malvids - ps24
Sap./Malv./Brassicales - ps25
Malvales/Brassicales - ps26
Brassicales - ps27
Cleomaceae/Brassicaceae - ps28
Brassicaceae - ps29
Camelineae - ps30
Arabidopsis - ps31
Arabidopsis_thaliana - ps32
Phylostratum

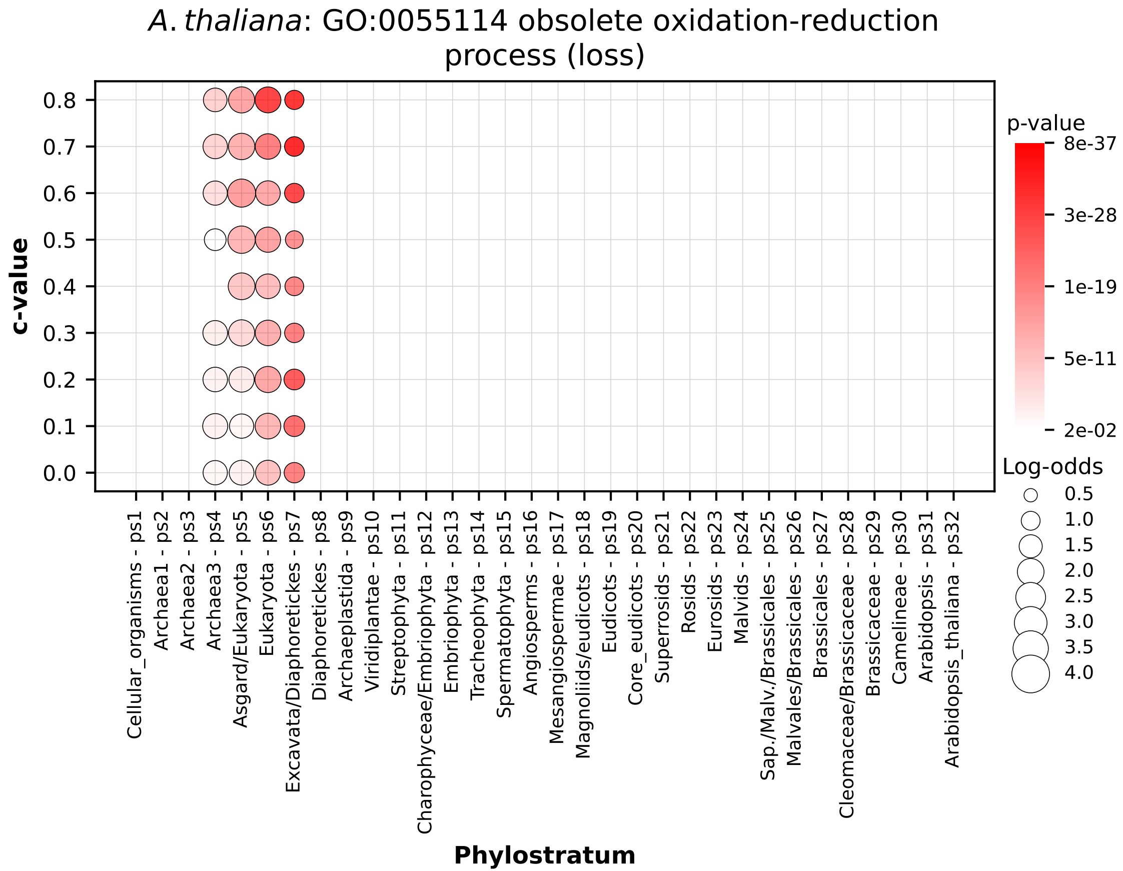

A. thaliana: GO:0055114 obsolete oxidation-reduction process (loss)
c-value
0.8
0.7
0.6
0.5
0.4
0.3
0.2
0.1
0.0
p-value
8e-37
3e-28
1e-19
5e-11
2e-02
Log-odds
0.5
1.0
1.5
2.0
2.5
3.0
3.5
4.0
Cellular_organisms - ps1
Archaea1 - ps2
Archaea2 - ps3
Archaea3 - ps4
Asgard/Eukaryota - ps5
Eukaryota - ps6
Excavata/Diaphoretickes - ps7
Diaphoretickes - ps8
Archaeplastida - ps9
Viridiplantae - ps10
Streptophyta - ps11
Charophyceae/Embriophyta - ps12
Embriophyta - ps13
Tracheophyta - ps14
Spermatophyta - ps15
Angiosperms - ps16
Mesangiospermae - ps17
Magnoliids/eudicots - ps18
Eudicots - ps19
Core_eudicots - ps20
Superrosids - ps21
Rosids - ps22
Eurosids - ps23
Malvids - ps24
Sap./Malv./Brassicales - ps25
Malvales/Brassicales - ps26
Brassicales - ps27
Cleomaceae/Brassicaceae - ps28
Brassicaceae - ps29
Camelineae - ps30
Arabidopsis - ps31
Arabidopsis_thaliana - ps32
Phylostratum

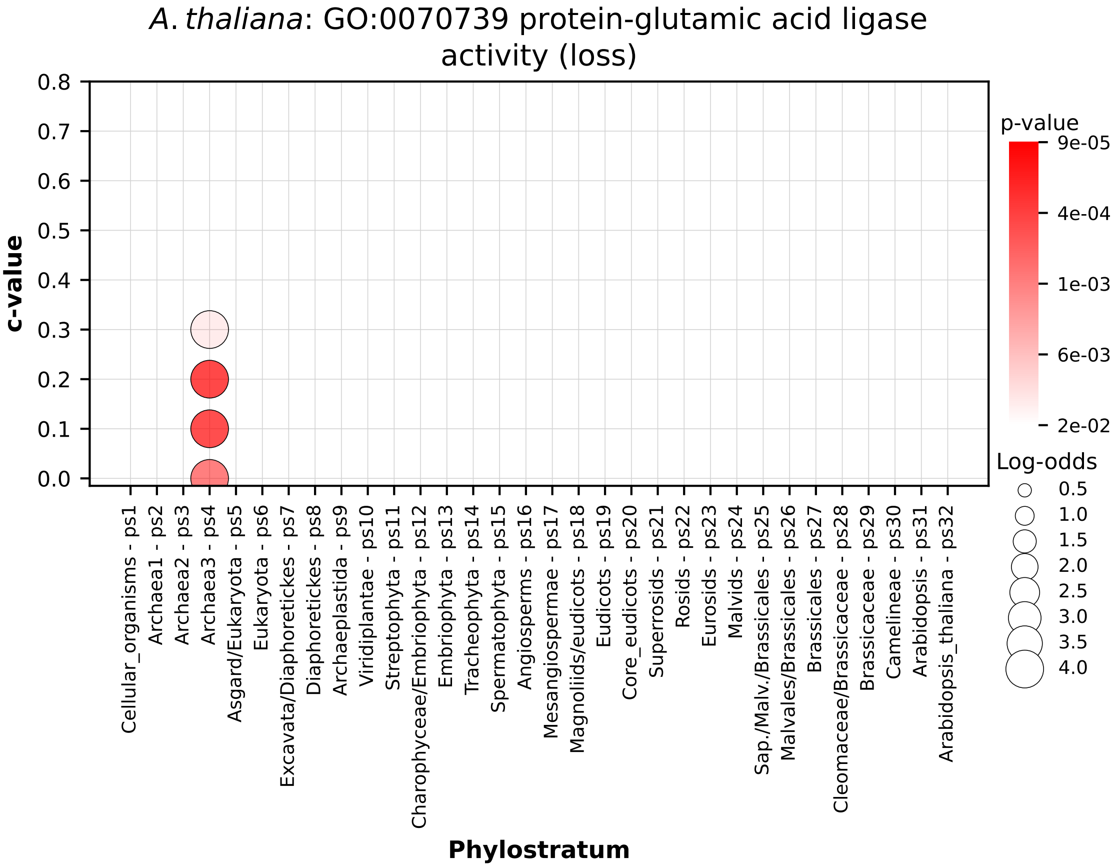
A. thaliana: GO:0070739 protein-glutamic acid ligase activity (loss)
c-value
0.8
0.7
0.6
0.5
0.4
0.3
0.2
0.1
0.0
p-value
9e-05
4e-04
1e-03
6e-03
2e-02
Log-odds
0.5
1.0
1.5
2.0
2.5
3.0
3.5
4.0
Cellular_organisms - ps1
Archaea1 - ps2
Archaea2 - ps3
Archaea3 - ps4
Asgard/Eukaryota - ps5
Eukaryota - ps6
Excavata/Diaphoretickes - ps7
Diaphoretickes - ps8
Archaeplastida - ps9
Viridiplantae - ps10
Streptophyta - ps11
Charophyceae/Embriophyta - ps12
Embriophyta - ps13
Tracheophyta - ps14
Spermatophyta - ps15
Angiosperms - ps16
Mesangiospermae - ps17
Magnoliids/eudicots - ps18
Eudicots - ps19
Core_eudicots - ps20
Superrosids - ps21
Rosids - ps22
Eurosids - ps23
Malvids - ps24
Sap./Malv./Brassicales - ps25
Malvales/Brassicales - ps26
Brassicales - ps27
Cleomaceae/Brassicaceae - ps28
Brassicaceae - ps29
Camelineae - ps30
Arabidopsis - ps31
Arabidopsis_thaliana - ps32
Phylostratum

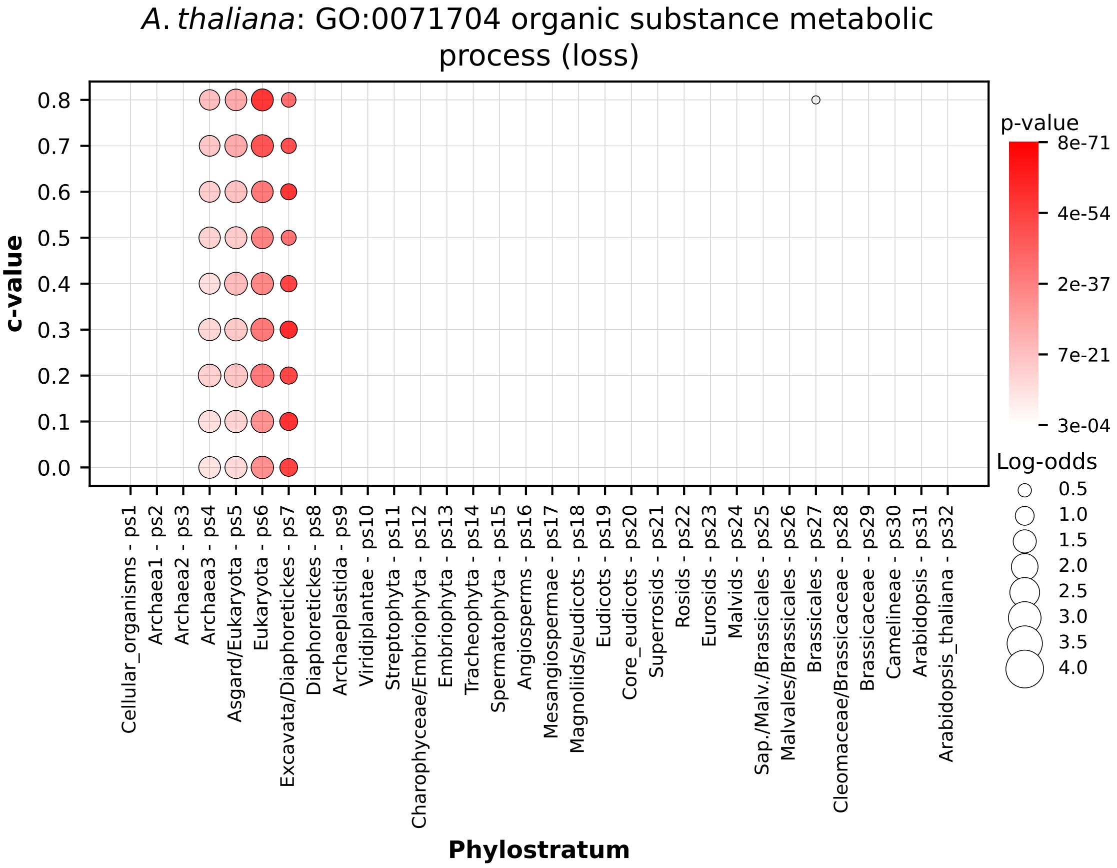

A. thaliana: GO:0071704 organic substance metabolic process (loss)
c-value
0.8
0.7
0.6
0.5
0.4
0.3
0.2
0.1
0.0
Phylostratum
Cellular_organisms - ps1
Archaea1 - ps2
Archaea2 - ps3
Archaea3 - ps4
Asgard/Eukaryota - ps5
Eukaryota - ps6
Excavata/Diaphoretickes - ps7
Diaphoretickes - ps8
Archaeplastida - ps9
Viridiplantae - ps10
Streptophyta - ps11
Charophyceae/Embriophyta - ps12
Embriophyta - ps13
Tracheophyta - ps14
Spermatophyta - ps15
Angiosperms - ps16
Mesangiospermae - ps17
Magnoliids/eudicots - ps18
Eudicots - ps19
Core_eudicots - ps20
Superrosids - ps21
Rosids - ps22
Eurosids - ps23
Malvids - ps24
Sap./Malv./Brassicales - ps25
Malvales/Brassicales - ps26
Brassicales - ps27
Cleomaceae/Brassicaceae - ps28
Brassicaceae - ps29
Camelineae - ps30
Arabidopsis - ps31
Arabidopsis_thaliana - ps32
p-value
8e-71
4e-54
2e-37
7e-21
3e-04
Log-odds
0.5
1.0
1.5
2.0
2.5
3.0
3.5
4.0

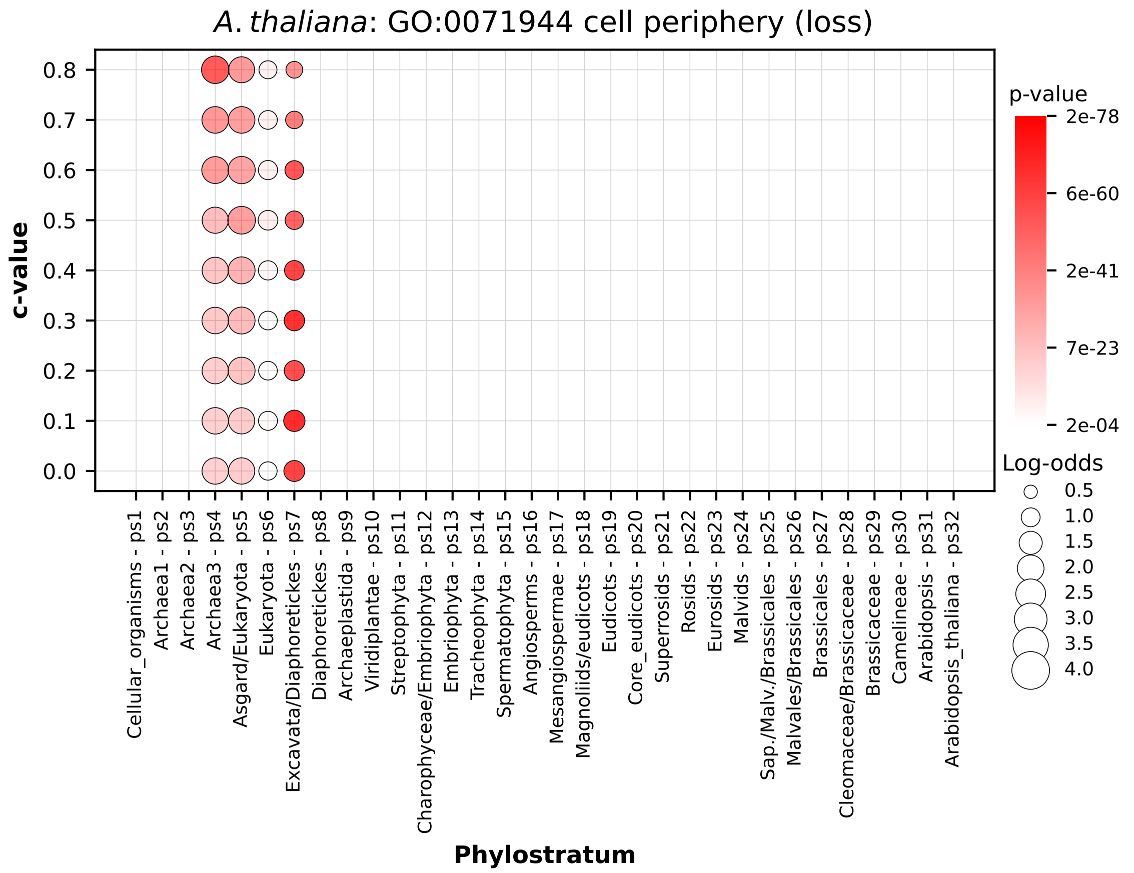

A. thaliana: GO:0071944 cell periphery (loss)
c-value
0.8
0.7
0.6
0.5
0.4
0.3
0.2
0.1
0.0
p-value
2e-78
6e-60
2e-41
7e-23
2e-04
Log-odds
0.5
1.0
1.5
2.0
2.5
3.0
3.5
4.0
Cellular_organisms - ps1
Archaea1 - ps2
Archaea2 - ps3
Archaea3 - ps4
Asgard/Eukaryota - ps5
Eukaryota - ps6
Excavata/Diaphoretickes - ps7
Diaphoretickes - ps8
Archaeplastida - ps9
Viridiplantae - ps10
Streptophyta - ps11
Charophyceae/Embriophyta - ps12
Embriophyta - ps13
Tracheophyta - ps14
Spermatophyta - ps15
Angiosperms - ps16
Mesangiospermae - ps17
Magnoliids/eudicots - ps18
Eudicots - ps19
Core_eudicots - ps20
Superrosids - ps21
Rosids - ps22
Eurosids - ps23
Malvids - ps24
Sap./Malv./Brassicales - ps25
Malvales/Brassicales - ps26
Brassicales - ps27
Cleomaceae/Brassicaceae - ps28
Brassicaceae - ps29
Camelineae - ps30
Arabidopsis - ps31
Arabidopsis_thaliana - ps32
Phylostratum

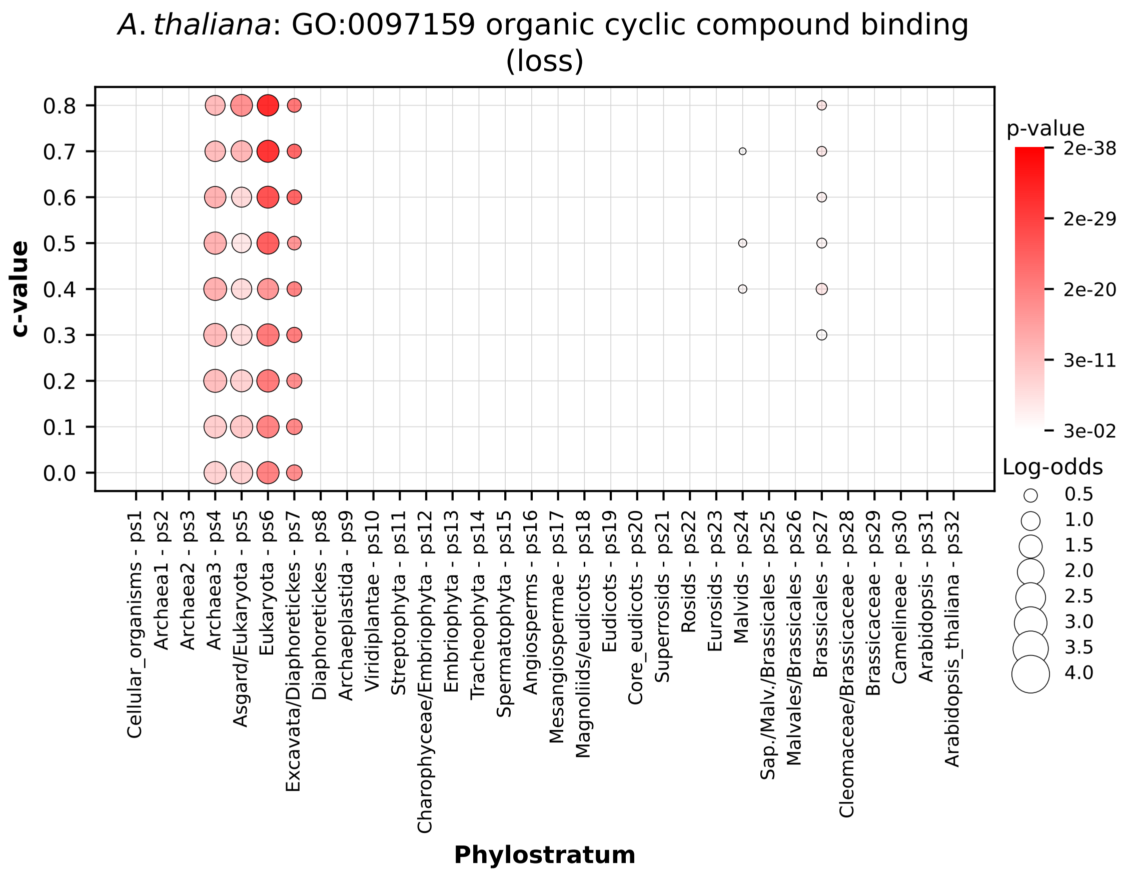
A. thaliana: GO:0097159 organic cyclic compound binding
(loss)
c-value
0.8
0.7
0.6
0.5
0.4
0.3
0.2
0.1
0.0
p-value
2e-38
2e-29
2e-20
3e-11
3e-02
Log-odds
0.5
1.0
1.5
2.0
2.5
3.0
3.5
4.0
Cellular_organisms - ps1
Archaea1 - ps2
Archaea2 - ps3
Archaea3 - ps4
Asgard/Eukaryota - ps5
Eukaryota - ps6
Excavata/Diaphoretickes - ps7
Diaphoretickes - ps8
Archaeplastida - ps9
Viridiplantae - ps10
Streptophyta - ps11
Charophyceae/Embriophyta - ps12
Embriophyta - ps13
Tracheophyta - ps14
Spermatophyta - ps15
Angiosperms - ps16
Mesangiospermae - ps17
Magnoliids/eudicots - ps18
Eudicots - ps19
Core_eudicots - ps20
Superrosids - ps21
Rosids - ps22
Eurosids - ps23
Malvids - ps24
Sap./Malv./Brassicales - ps25
Malvales/Brassicales - ps26
Brassicales - ps27
Cleomaceae/Brassicaceae - ps28
Brassicaceae - ps29
Camelineae - ps30
Arabidopsis - ps31
Arabidopsis_thaliana - ps32
Phylostratum

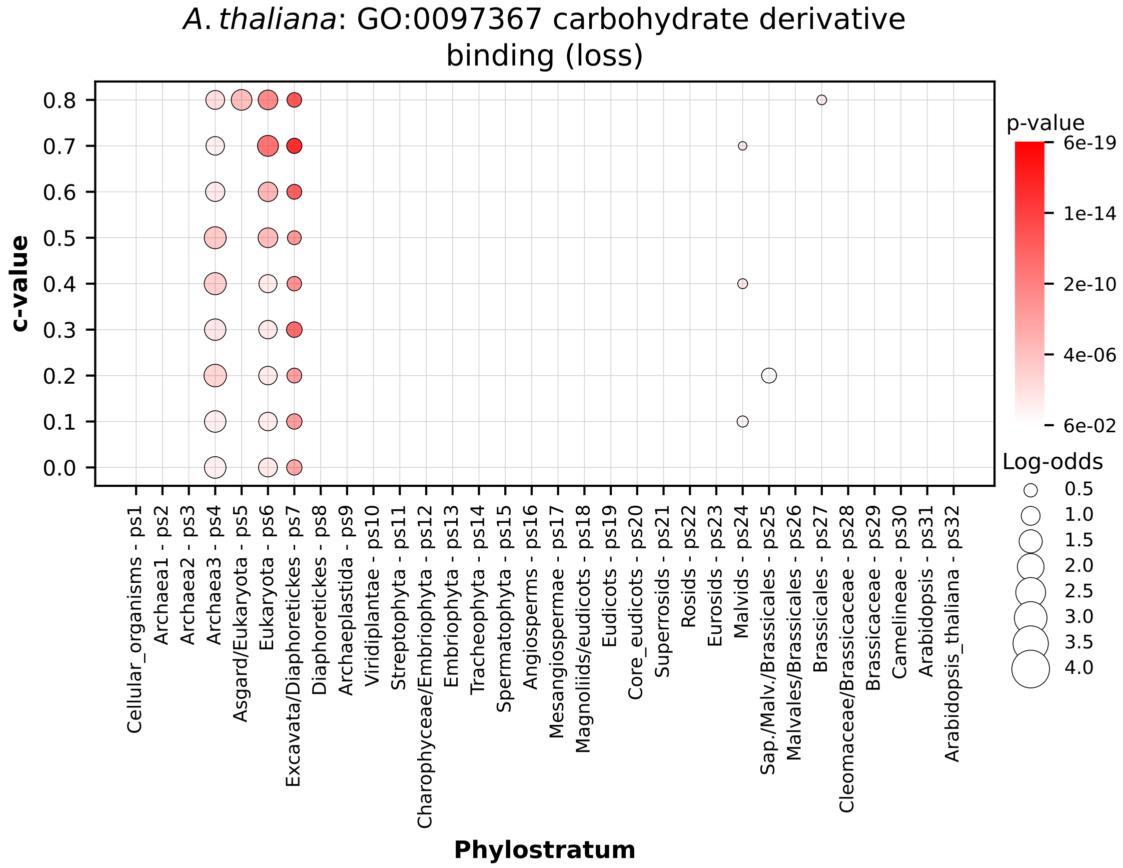

A. thaliana: GO:0097367 carbohydrate derivative binding (loss)
c-value
0.8
0.7
0.6
0.5
0.4
0.3
0.2
0.1
0.0
p-value
6e-19
1e-14
2e-10
4e-06
6e-02
Log-odds
0.5
1.0
1.5
2.0
2.5
3.0
3.5
4.0
Cellular_organisms - ps1
Archaea1 - ps2
Archaea2 - ps3
Archaea3 - ps4
Asgard/Eukaryota - ps5
Eukaryota - ps6
Excavata/Diaphoretickes - ps7
Diaphoretickes - ps8
Archaeplastida - ps9
Viridiplantae - ps10
Streptophyta - ps11
Charophyceae/Embriophyta - ps12
Embriophyta - ps13
Tracheophyta - ps14
Spermatophyta - ps15
Angiosperms - ps16
Mesangiospermae - ps17
Magnoliids/eudicots - ps18
Eudicots - ps19
Core_eudicots - ps20
Superrosids - ps21
Rosids - ps22
Eurosids - ps23
Malvids - ps24
Sap./Malv./Brassicales - ps25
Malvales/Brassicales - ps26
Brassicales - ps27
Cleomaceae/Brassicaceae - ps28
Brassicaceae - ps29
Camelineae - ps30
Arabidopsis - ps31
Arabidopsis_thaliana - ps32
Phylostratum

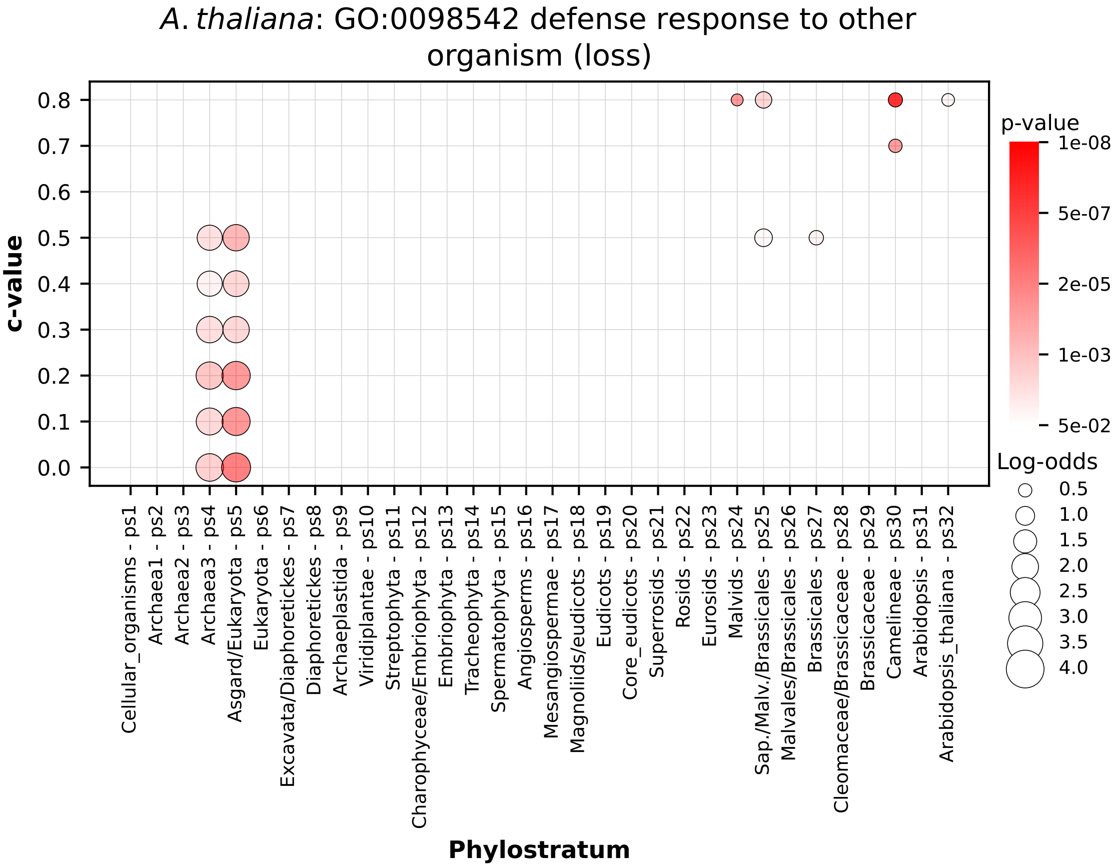

A. thaliana: GO:0098542 defense response to other organism (loss)
c-value
0.0
0.1
0.2
0.3
0.4
0.5
0.6
0.7
0.8
Phylostratum
Cellular_organisms - ps1
Archaea1 - ps2
Archaea2 - ps3
Archaea3 - ps4
Asgard/Eukaryota - ps5
Eukaryota - ps6
Excavata/Diaphoretickes - ps7
Diaphoretickes - ps8
Archaeplastida - ps9
Viridiplantae - ps10
Streptophyta - ps11
Charophyceae/Embriophyta - ps12
Embriophyta - ps13
Tracheophyta - ps14
Spermatophyta - ps15
Angiosperms - ps16
Mesangiospermae - ps17
Magnoliids/eudicots - ps18
Eudicots - ps19
Core_eudicots - ps20
Superrosids - ps21
Rosids - ps22
Eurosids - ps23
Malvids - ps24
Sap./Malv./Brassicales - ps25
Malvales/Brassicales - ps26
Brassicales - ps27
Cleomaceae/Brassicaceae - ps28
Brassicaceae - ps29
Camelineae - ps30
Arabidopsis - ps31
Arabidopsis_thaliana - ps32
p-value
1e-08
5e-07
2e-05
1e-03
5e-02
Log-odds
0.5
1.0
1.5
2.0
2.5
3.0
3.5
4.0

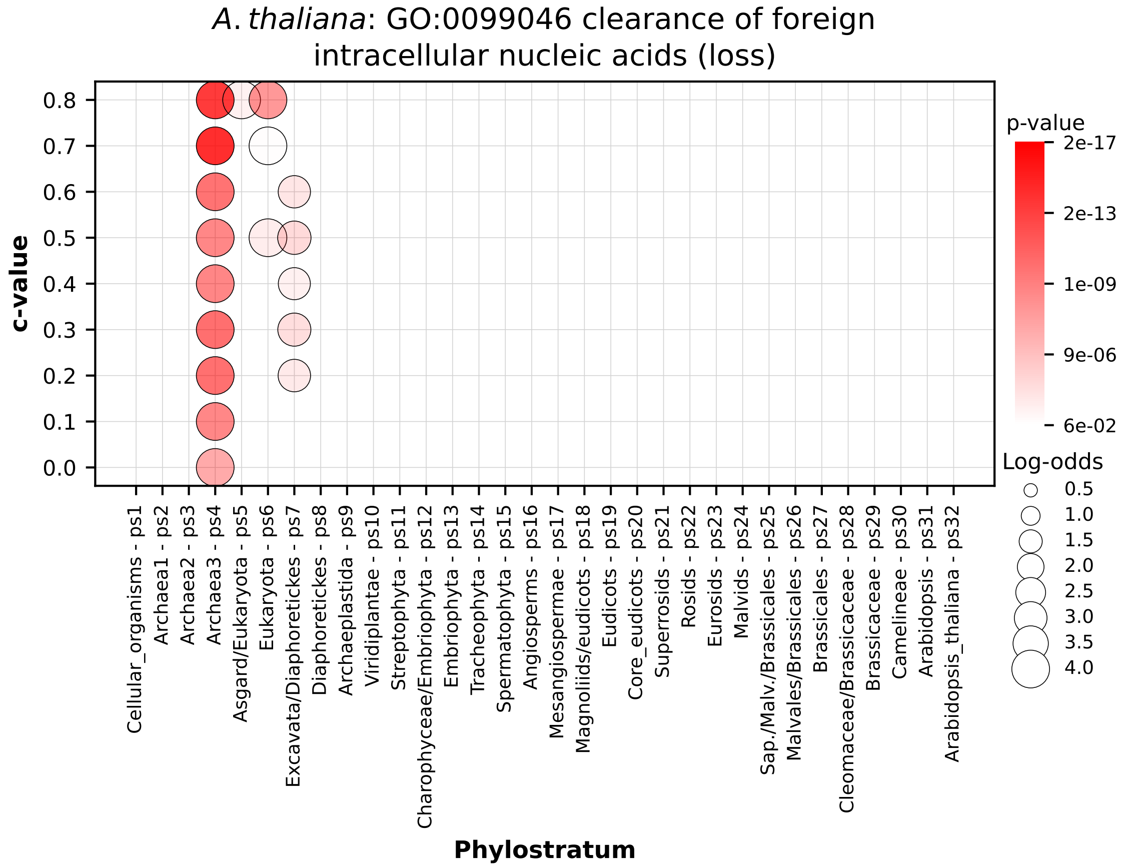

A. thaliana: GO:0099046 clearance of foreign intracellular nucleic acids (loss)
c-value
0.8
0.7
0.6
0.5
0.4
0.3
0.2
0.1
0.0
Phylostratum
Cellular_organisms - ps1
Archaea1 - ps2
Archaea2 - ps3
Archaea3 - ps4
Asgard/Eukaryota - ps5
Eukaryota - ps6
Excavata/Diaphoretickes - ps7
Diaphoretickes - ps8
Archaeplastida - ps9
Viridiplantae - ps10
Streptophyta - ps11
Charophyceae/Embriophyta - ps12
Embriophyta - ps13
Tracheophyta - ps14
Spermatophyta - ps15
Angiosperms - ps16
Mesangiospermae - ps17
Magnoliids/eudicots - ps18
Eudicots - ps19
Core_eudicots - ps20
Superrosids - ps21
Rosids - ps22
Eurosids - ps23
Malvids - ps24
Sap./Malv./Brassicales - ps25
Malvales/Brassicales - ps26
Brassicales - ps27
Cleomaceae/Brassicaceae - ps28
Brassicaceae - ps29
Camelineae - ps30
Arabidopsis - ps31
Arabidopsis_thaliana - ps32
p-value
2e-17
2e-13
1e-09
9e-06
6e-02
Log-odds
0.5
1.0
1.5
2.0
2.5
3.0
3.5
4.0

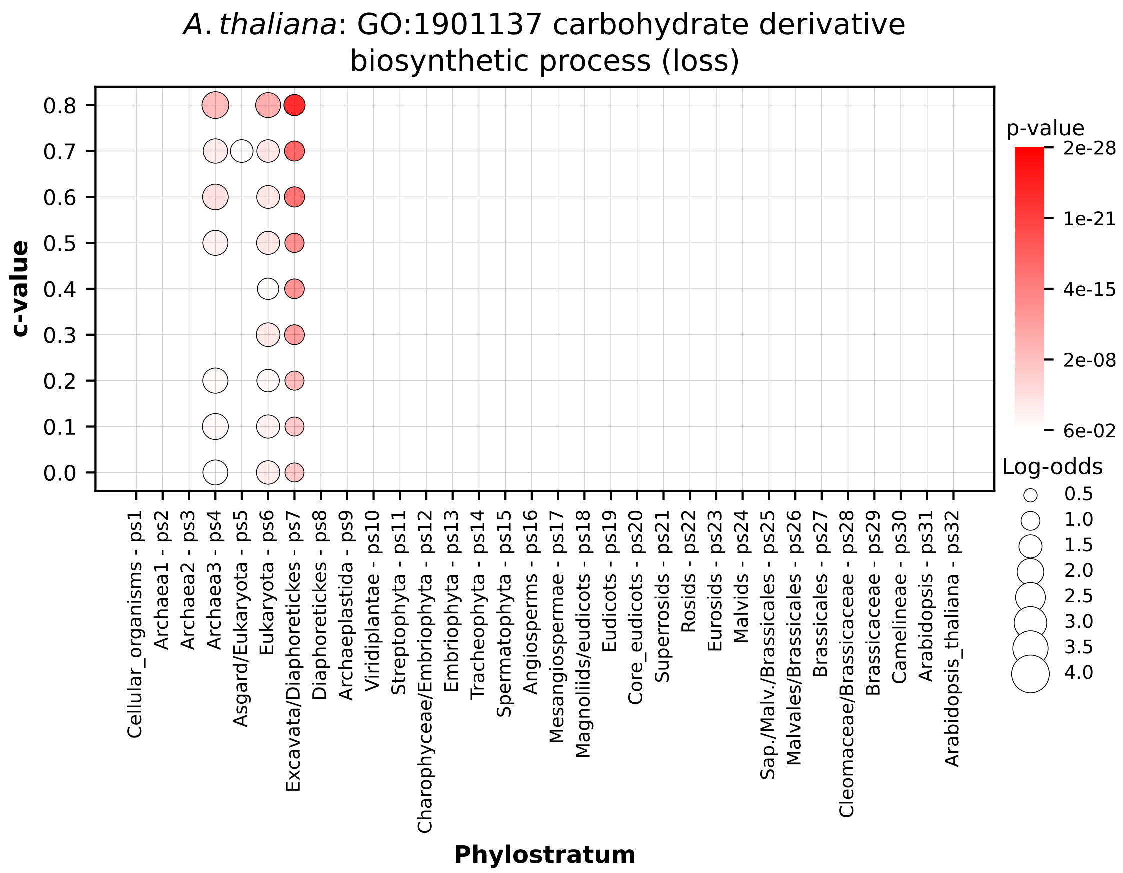

A. thaliana: GO:1901137 carbohydrate derivative biosynthetic process (loss)
c-value
0.8
0.7
0.6
0.5
0.4
0.3
0.2
0.1
0.0
p-value
2e-28
1e-21
4e-15
2e-08
6e-02
Log-odds
0.5
1.0
1.5
2.0
2.5
3.0
3.5
4.0
Cellular_organisms - ps1
Archaea1 - ps2
Archaea2 - ps3
Archaea3 - ps4
Asgard/Eukaryota - ps5
Eukaryota - ps6
Excavata/Diaphoretickes - ps7
Diaphoretickes - ps8
Archaeplastida - ps9
Viridiplantae - ps10
Streptophyta - ps11
Charophyceae/Embriophyta - ps12
Embriophyta - ps13
Tracheophyta - ps14
Spermatophyta - ps15
Angiosperms - ps16
Mesangiospermae - ps17
Magnoliids/eudicots - ps18
Eudicots - ps19
Core_eudicots - ps20
Superrosids - ps21
Rosids - ps22
Eurosids - ps23
Malvids - ps24
Sap./Malv./Brassicales - ps25
Malvales/Brassicales - ps26
Brassicales - ps27
Cleomaceae/Brassicaceae - ps28
Brassicaceae - ps29
Camelineae - ps30
Arabidopsis - ps31
Arabidopsis_thaliana - ps32
Phylostratum

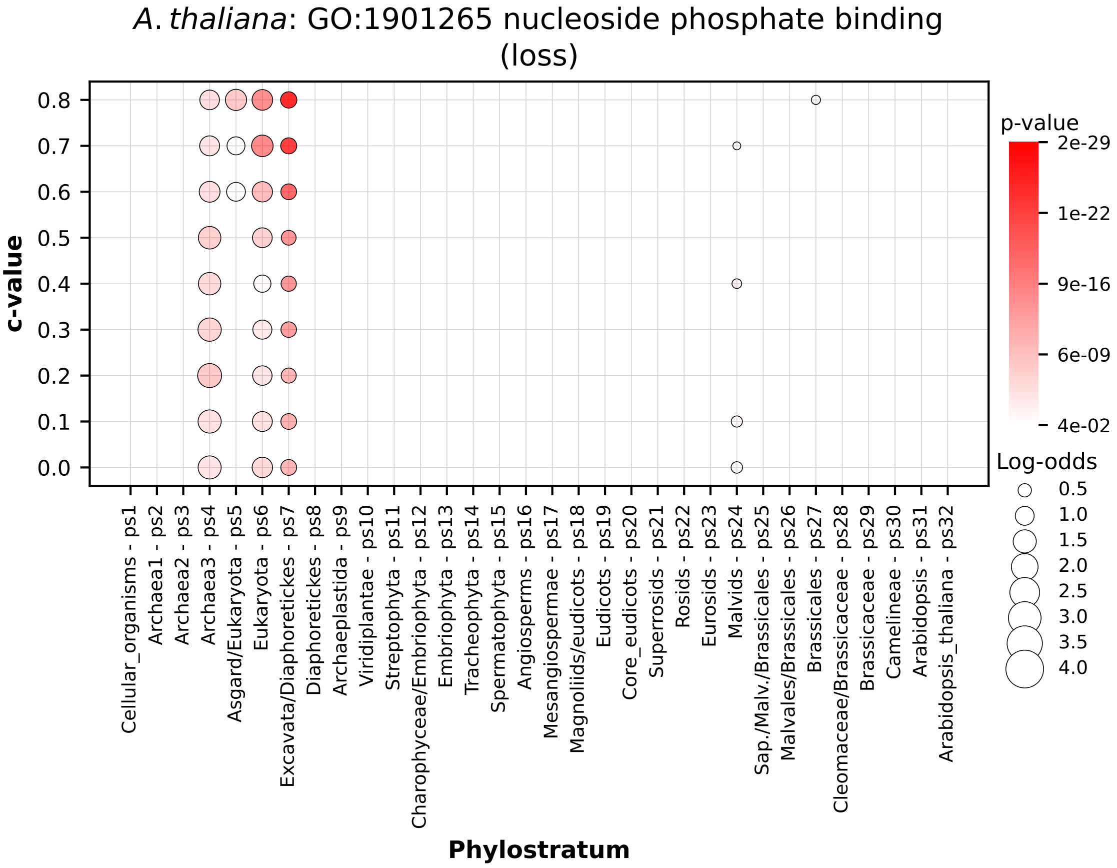

A. thaliana: GO:1901265 nucleoside phosphate binding
(loss)
c-value
0.8
0.7
0.6
0.5
0.4
0.3
0.2
0.1
0.0
Phylostratum
Cellular_organisms - ps1
Archaea1 - ps2
Archaea2 - ps3
Archaea3 - ps4
Asgard/Eukaryota - ps5
Eukaryota - ps6
Excavata/Diaphoretickes - ps7
Diaphoretickes - ps8
Archaeplastida - ps9
Viridiplantae - ps10
Streptophyta - ps11
Charophyceae/Embriophyta - ps12
Embriophyta - ps13
Tracheophyta - ps14
Spermatophyta - ps15
Angiosperms - ps16
Mesangiospermae - ps17
Magnoliids/eudicots - ps18
Eudicots - ps19
Core_eudicots - ps20
Superrosids - ps21
Rosids - ps22
Eurosids - ps23
Malvids - ps24
Sap./Malv./Brassicales - ps25
Malvales/Brassicales - ps26
Brassicales - ps27
Cleomaceae/Brassicaceae - ps28
Brassicaceae - ps29
Camelineae - ps30
Arabidopsis - ps31
Arabidopsis_thaliana - ps32
p-value
2e-29
1e-22
9e-16
6e-09
4e-02
Log-odds
0.5
1.0
1.5
2.0
2.5
3.0
3.5
4.0

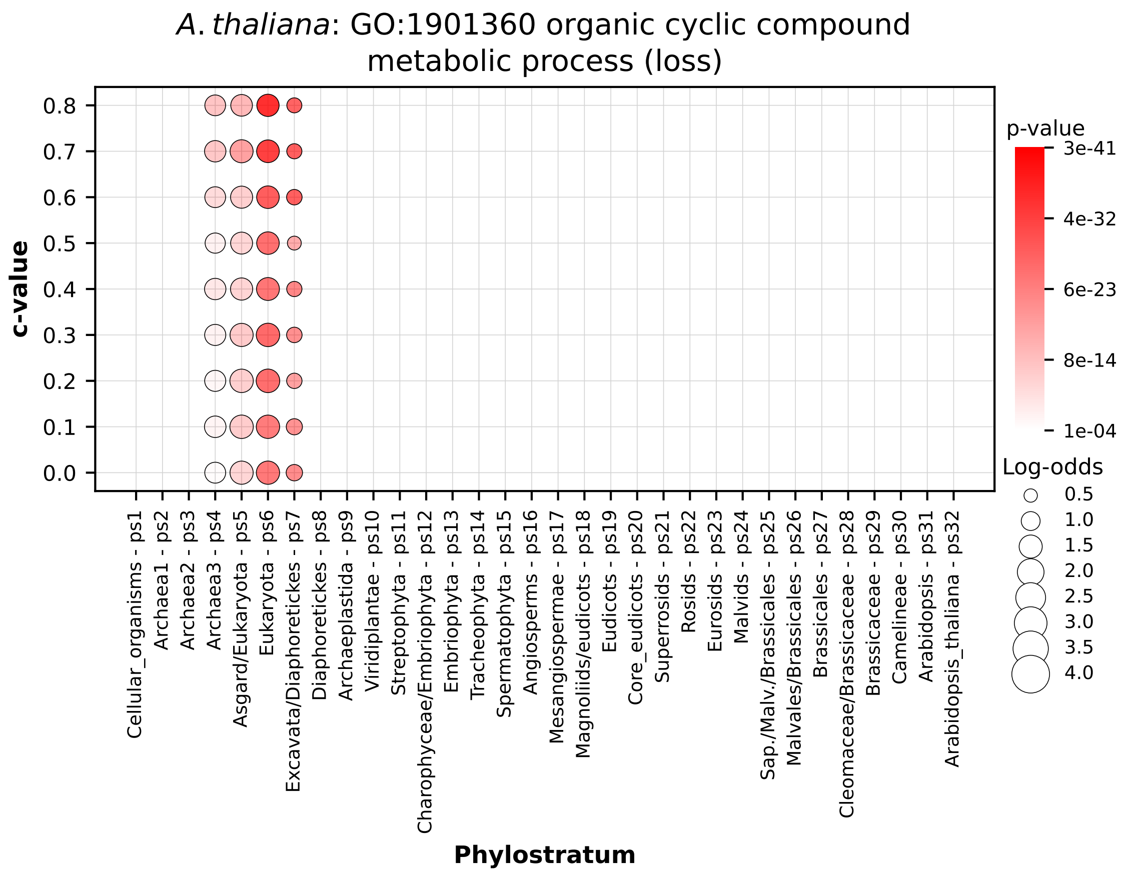

A. thaliana: GO:1901360 organic cyclic compound metabolic process (loss)
c-value
0.8
0.7
0.6
0.5
0.4
0.3
0.2
0.1
0.0
p-value
3e-41
4e-32
6e-23
8e-14
1e-04
Log-odds
0.5
1.0
1.5
2.0
2.5
3.0
3.5
4.0
Cellular_organisms - ps1
Archaea1 - ps2
Archaea2 - ps3
Archaea3 - ps4
Asgard/Eukaryota - ps5
Eukaryota - ps6
Excavata/Diaphoretickes - ps7
Diaphoretickes - ps8
Archaeplastida - ps9
Viridiplantae - ps10
Streptophyta - ps11
Charophyceae/Embriophyta - ps12
Embriophyta - ps13
Tracheophyta - ps14
Spermatophyta - ps15
Angiosperms - ps16
Mesangiospermae - ps17
Magnoliids/eudicots - ps18
Eudicots - ps19
Core_eudicots - ps20
Superrosids - ps21
Rosids - ps22
Eurosids - ps23
Malvids - ps24
Sap./Malv./Brassicales - ps25
Malvales/Brassicales - ps26
Brassicales - ps27
Cleomaceae/Brassicaceae - ps28
Brassicaceae - ps29
Camelineae - ps30
Arabidopsis - ps31
Arabidopsis_thaliana - ps32
Phylostratum

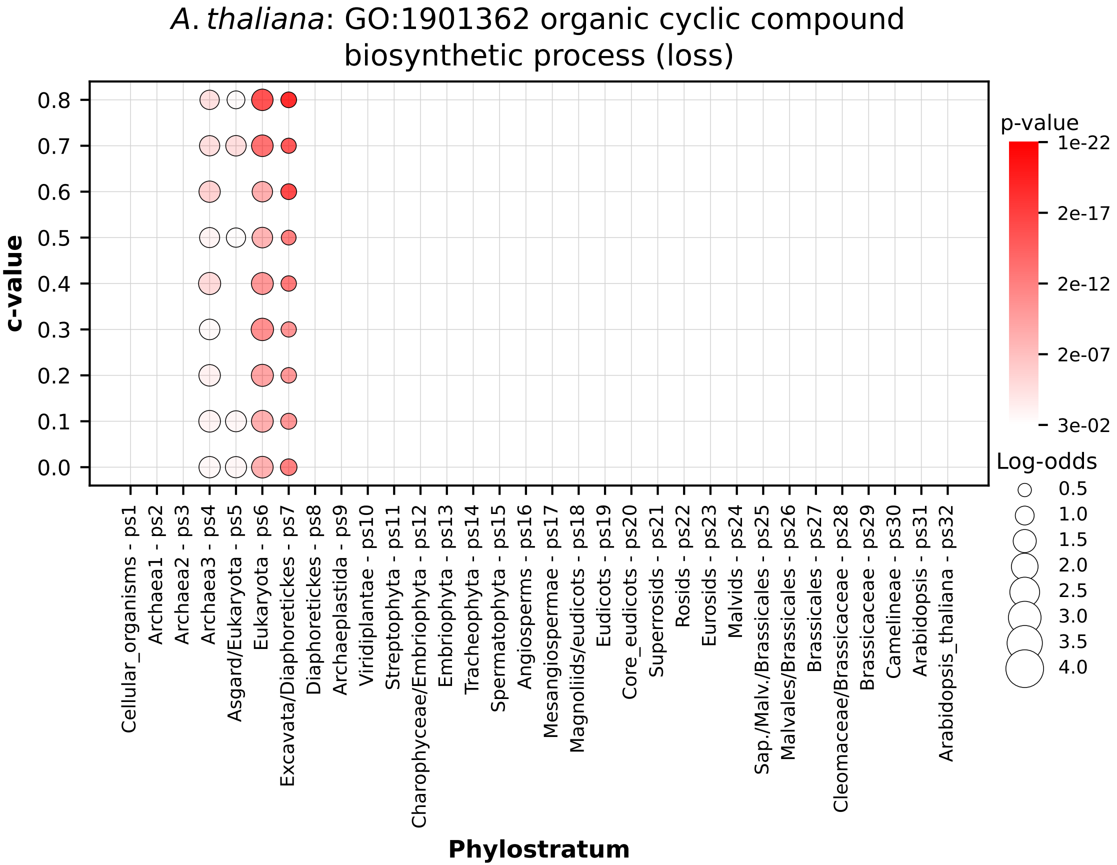

A. thaliana: GO:1901362 organic cyclic compound biosynthetic process (loss)
c-value
0.8
0.7
0.6
0.5
0.4
0.3
0.2
0.1
0.0
p-value
1e-22
2e-17
2e-12
2e-07
3e-02
Log-odds
0.5
1.0
1.5
2.0
2.5
3.0
3.5
4.0
Cellular_organisms - ps1
Archaea1 - ps2
Archaea2 - ps3
Archaea3 - ps4
Asgard/Eukaryota - ps5
Eukaryota - ps6
Excavata/Diaphoretickes - ps7
Diaphoretickes - ps8
Archaeplastida - ps9
Viridiplantae - ps10
Streptophyta - ps11
Charophyceae/Embriophyta - ps12
Embriophyta - ps13
Tracheophyta - ps14
Spermatophyta - ps15
Angiosperms - ps16
Mesangiospermae - ps17
Magnoliids/eudicots - ps18
Eudicots - ps19
Core_eudicots - ps20
Superrosids - ps21
Rosids - ps22
Eurosids - ps23
Malvids - ps24
Sap./Malv./Brassicales - ps25
Malvales/Brassicales - ps26
Brassicales - ps27
Cleomaceae/Brassicaceae - ps28
Brassicaceae - ps29
Camelineae - ps30
Arabidopsis - ps31
Arabidopsis_thaliana - ps32
Phylostratum

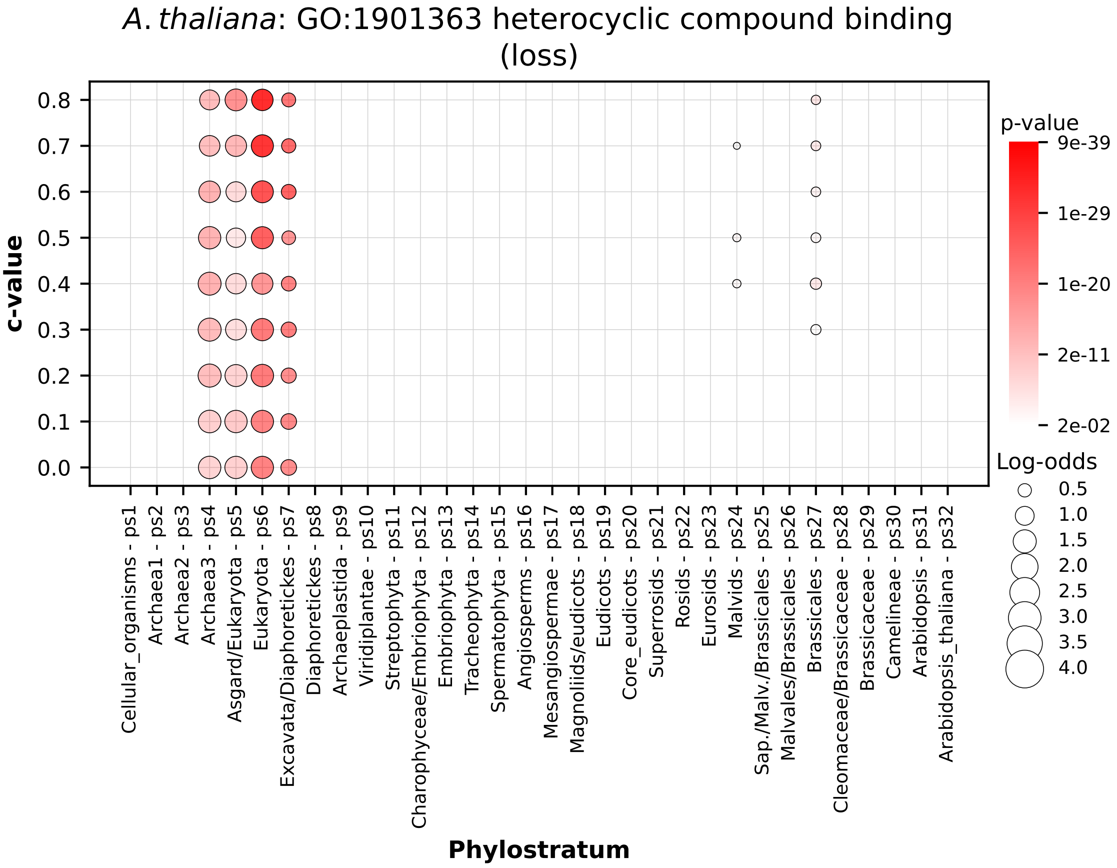

$A. thaliana$: GO:1901363 heterocyclic compound binding
(loss)
c-value
0.8
0.7
0.6
0.5
0.4
0.3
0.2
0.1
0.0
p-value
9e-39
1e-29
1e-20
2e-11
2e-02
Log-odds
0.5
1.0
1.5
2.0
2.5
3.0
3.5
4.0
Cellular_organisms - ps1
Archaea1 - ps2
Archaea2 - ps3
Archaea3 - ps4
Asgard/Eukaryota - ps5
Eukaryota - ps6
Excavata/Diaphoretickes - ps7
Diaphoretickes - ps8
Archaeplastida - ps9
Viridiplantae - ps10
Streptophyta - ps11
Charophyceae/Embriophyta - ps12
Embriophyta - ps13
Tracheophyta - ps14
Spermatophyta - ps15
Angiosperms - ps16
Mesangiospermae - ps17
Magnoliids/eudicots - ps18
Eudicots - ps19
Core_eudicots - ps20
Superrosids - ps21
Rosids - ps22
Eurosids - ps23
Malvids - ps24
Sap./Malv./Brassicales - ps25
Malvales/Brassicales - ps26
Brassicales - ps27
Cleomaceae/Brassicaceae - ps28
Brassicaceae - ps29
Camelineae - ps30
Arabidopsis - ps31
Arabidopsis_thaliana - ps32
Phylostratum

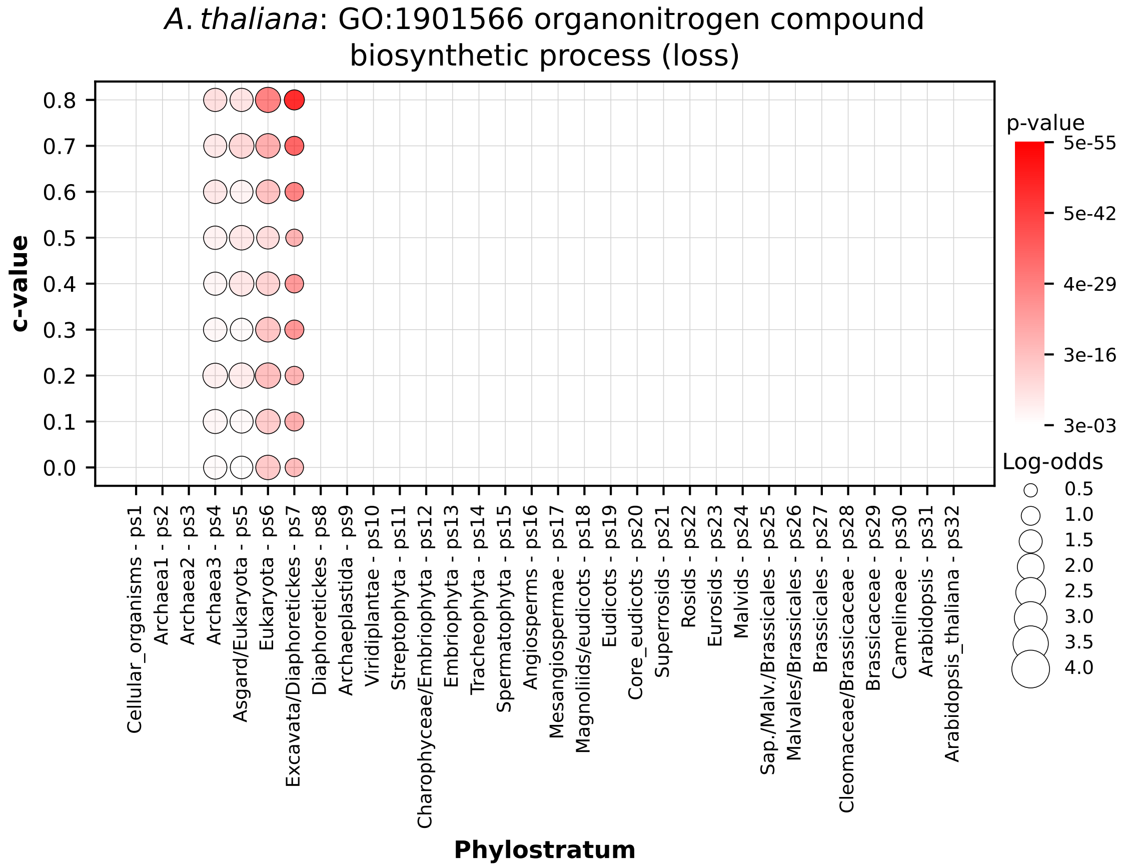

A. thaliana: GO:1901566 organonitrogen compound biosynthetic process (loss)
c-value
0.8
0.7
0.6
0.5
0.4
0.3
0.2
0.1
0.0
Phylostratum
Cellular_organisms - ps1
Archaea1 - ps2
Archaea2 - ps3
Archaea3 - ps4
Asgard/Eukaryota - ps5
Eukaryota - ps6
Excavata/Diaphoretickes - ps7
Diaphoretickes - ps8
Archaeplastida - ps9
Viridiplantae - ps10
Streptophyta - ps11
Charophyceae/Embriophyta - ps12
Embriophyta - ps13
Tracheophyta - ps14
Spermatophyta - ps15
Angiosperms - ps16
Mesangiospermae - ps17
Magnoliids/eudicots - ps18
Eudicots - ps19
Core_eudicots - ps20
Superrosids - ps21
Rosids - ps22
Eurosids - ps23
Malvids - ps24
Sap./Malv./Brassicales - ps25
Malvales/Brassicales - ps26
Brassicales - ps27
Cleomaceae/Brassicaceae - ps28
Brassicaceae - ps29
Camelineae - ps30
Arabidopsis - ps31
Arabidopsis_thaliana - ps32
p-value
5e-55
5e-42
4e-29
3e-16
3e-03
Log-odds
0.5
1.0
1.5
2.0
2.5
3.0
3.5
4.0

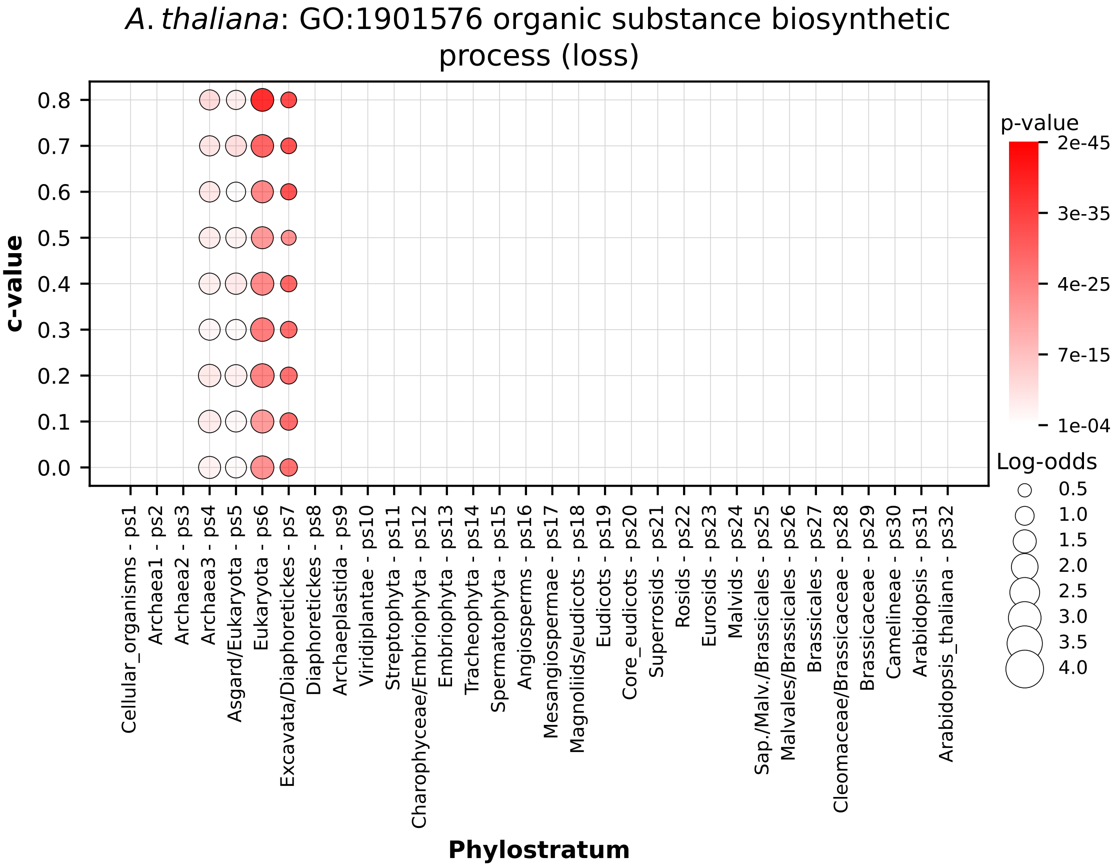
A. thaliana: GO:1901576 organic substance biosynthetic process (loss)
c-value
0.8
0.7
0.6
0.5
0.4
0.3
0.2
0.1
0.0
Phylostratum
Cellular_organisms - ps1
Archaea1 - ps2
Archaea2 - ps3
Archaea3 - ps4
Asgard/Eukaryota - ps5
Eukaryota - ps6
Excavata/Diaphoretickes - ps7
Diaphoretickes - ps8
Archaeplastida - ps9
Viridiplantae - ps10
Streptophyta - ps11
Charophyceae/Embriophyta - ps12
Embriophyta - ps13
Tracheophyta - ps14
Spermatophyta - ps15
Angiosperms - ps16
Mesangiospermae - ps17
Magnoliids/eudicots - ps18
Eudicots - ps19
Core_eudicots - ps20
Superrosids - ps21
Rosids - ps22
Eurosids - ps23
Malvids - ps24
Sap./Malv./Brassicales - ps25
Malvales/Brassicales - ps26
Brassicales - ps27
Cleomaceae/Brassicaceae - ps28
Brassicaceae - ps29
Camelineae - ps30
Arabidopsis - ps31
Arabidopsis_thaliana - ps32
p-value
2e-45
3e-35
4e-25
7e-15
1e-04
Log-odds
0.5
1.0
1.5
2.0
2.5
3.0
3.5
4.0

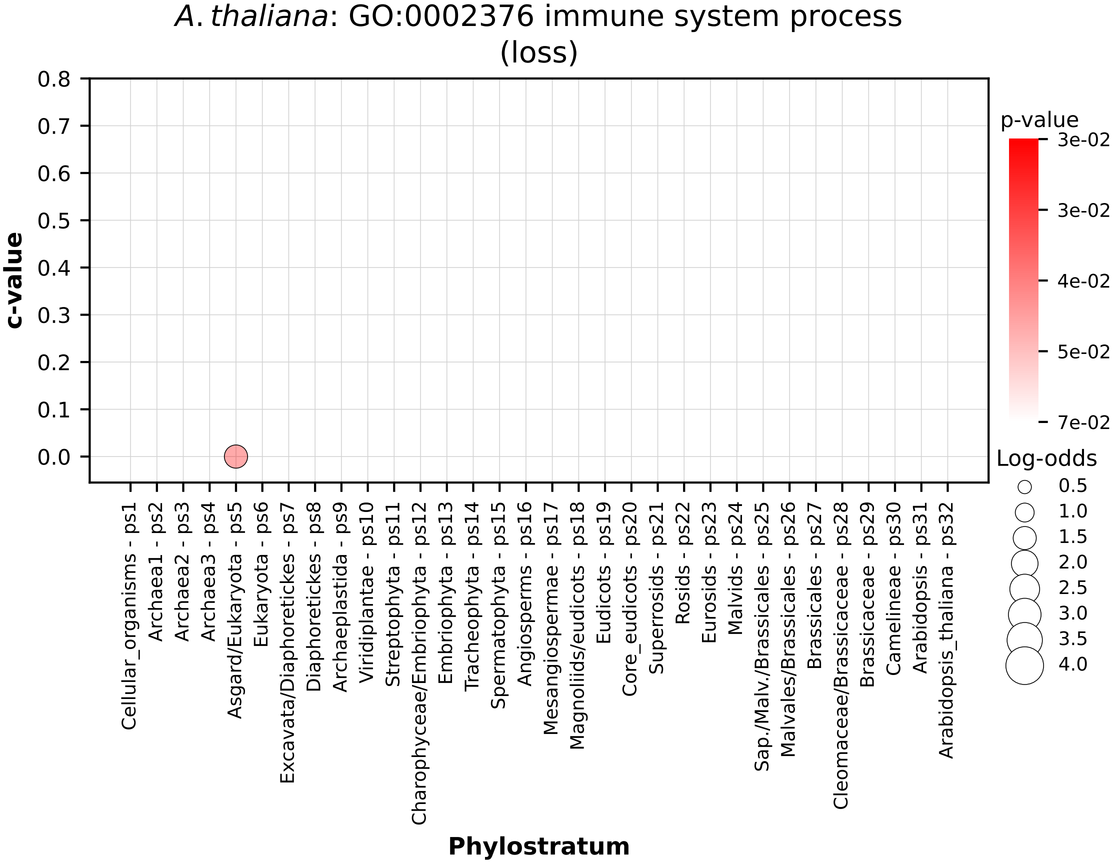
A. thaliana: GO:0002376 immune system process
(loss)
c-value
0.8
0.7
0.6
0.5
0.4
0.3
0.2
0.1
0.0
Phylostratum
Cellular_organisms - ps1
Archaea1 - ps2
Archaea2 - ps3
Archaea3 - ps4
Asgard/Eukaryota - ps5
Eukaryota - ps6
Excavata/Diaphoretickes - ps7
Diaphoretickes - ps8
Archaeplastida - ps9
Viridiplantae - ps10
Streptophyta - ps11
Charophyceae/Embriophyta - ps12
Embriophyta - ps13
Tracheophyta - ps14
Spermatophyta - ps15
Angiosperms - ps16
Mesangiospermae - ps17
Magnoliids/eudicots - ps18
Eudicots - ps19
Core_eudicots - ps20
Superrosids - ps21
Rosids - ps22
Eurosids - ps23
Malvids - ps24
Sap./Malv./Brassicales - ps25
Malvales/Brassicales - ps26
Brassicales - ps27
Cleomaceae/Brassicaceae - ps28
Brassicaceae - ps29
Camelineae - ps30
Arabidopsis - ps31
Arabidopsis_thaliana - ps32
p-value
3e-02
3e-02
4e-02
5e-02
7e-02
Log-odds
0.5
1.0
1.5
2.0
2.5
3.0
3.5
4.0

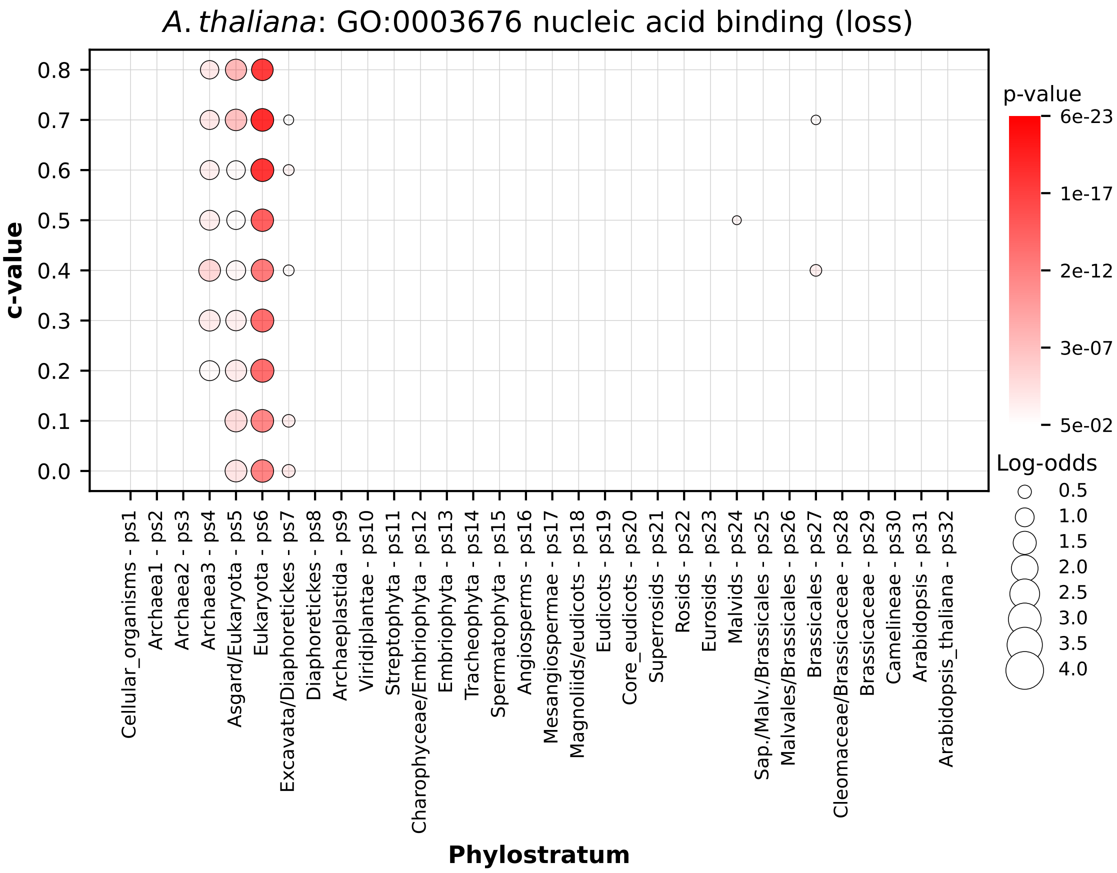

A. thaliana: GO:0003676 nucleic acid binding (loss)
c-value
0.8
0.7
0.6
0.5
0.4
0.3
0.2
0.1
0.0
p-value
6e-23
1e-17
2e-12
3e-07
5e-02
Log-odds
0.5
1.0
1.5
2.0
2.5
3.0
3.5
4.0
Cellular_organisms - ps1
Archaea1 - ps2
Archaea2 - ps3
Archaea3 - ps4
Asgard/Eukaryota - ps5
Eukaryota - ps6
Excavata/Diaphoretickes - ps7
Diaphoretickes - ps8
Archaeplastida - ps9
Viridiplantae - ps10
Streptophyta - ps11
Charophyceae/Embriophyta - ps12
Embriophyta - ps13
Tracheophyta - ps14
Spermatophyta - ps15
Angiosperms - ps16
Mesangiospermae - ps17
Magnoliids/eudicots - ps18
Eudicots - ps19
Core_eudicots - ps20
Superrosids - ps21
Rosids - ps22
Eurosids - ps23
Malvids - ps24
Sap./Malv./Brassicales - ps25
Malvales/Brassicales - ps26
Brassicales - ps27
Cleomaceae/Brassicaceae - ps28
Brassicaceae - ps29
Camelineae - ps30
Arabidopsis - ps31
Arabidopsis_thaliana - ps32
Phylostratum

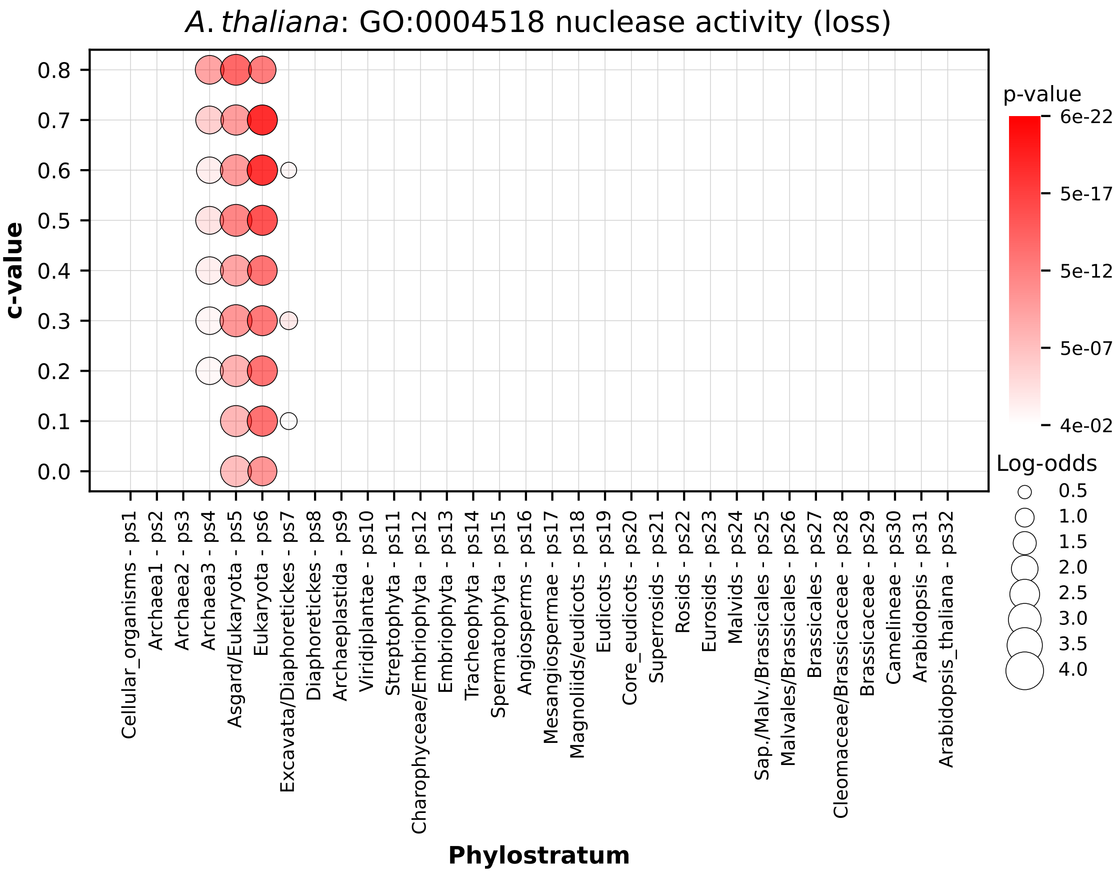

A. thaliana: GO:0004518 nuclease activity (loss)
c-value
0.8
0.7
0.6
0.5
0.4
0.3
0.2
0.1
0.0
Phylostratum
Cellular_organisms - ps1
Archaea1 - ps2
Archaea2 - ps3
Archaea3 - ps4
Asgard/Eukaryota - ps5
Eukaryota - ps6
Excavata/Diaphoretickes - ps7
Diaphoretickes - ps8
Archaeplastida - ps9
Viridiplantae - ps10
Streptophyta - ps11
Charophyceae/Embriophyta - ps12
Embriophyta - ps13
Tracheophyta - ps14
Spermatophyta - ps15
Angiosperms - ps16
Mesangiospermae - ps17
Magnoliids/eudicots - ps18
Eudicots - ps19
Core_eudicots - ps20
Superrosids - ps21
Rosids - ps22
Eurosids - ps23
Malvids - ps24
Sap./Malv./Brassicales - ps25
Malvales/Brassicales - ps26
Brassicales - ps27
Cleomaceae/Brassicaceae - ps28
Brassicaceae - ps29
Camelineae - ps30
Arabidopsis - ps31
Arabidopsis_thaliana - ps32
p-value
6e-22
5e-17
5e-12
5e-07
4e-02
Log-odds
0.5
1.0
1.5
2.0
2.5
3.0
3.5
4.0

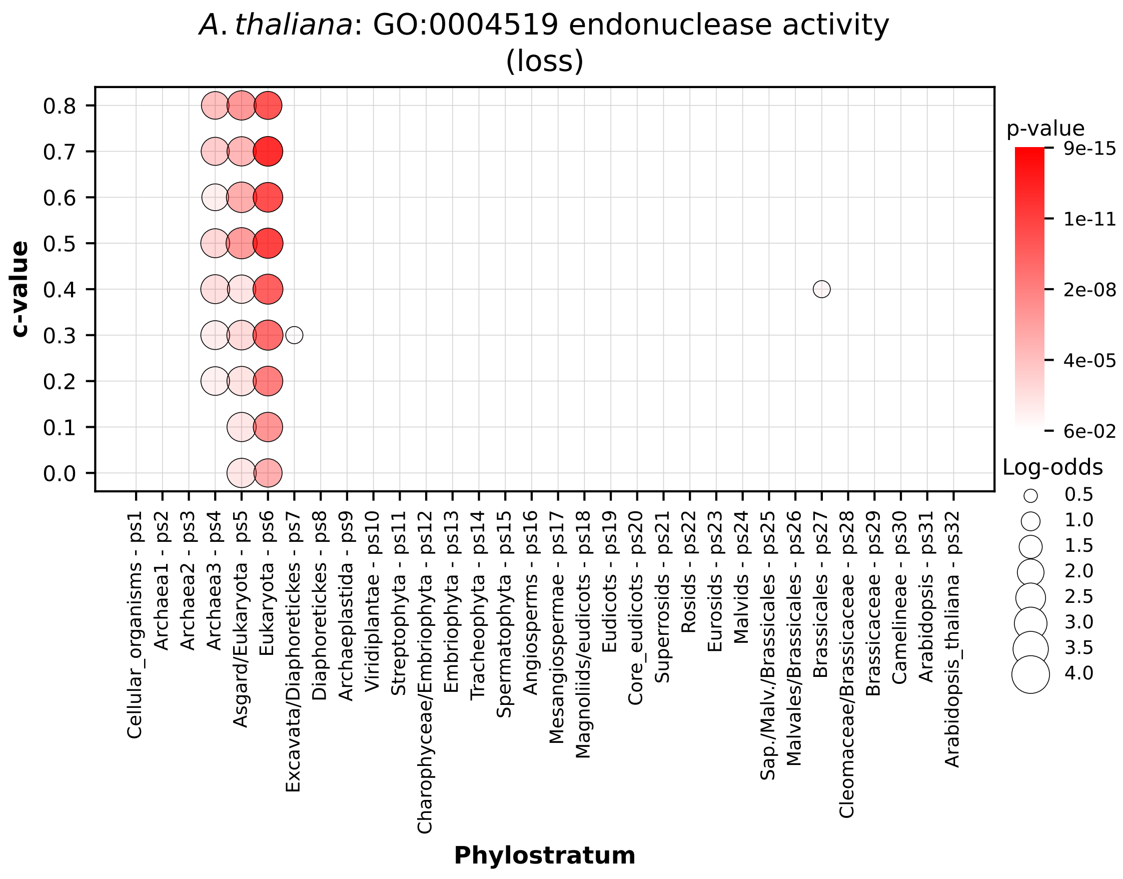
A. thaliana: GO:0004519 endonuclease activity
(loss)
c-value
0.8
0.7
0.6
0.5
0.4
0.3
0.2
0.1
0.0
Phylostratum
Cellular_organisms - ps1
Archaea1 - ps2
Archaea2 - ps3
Archaea3 - ps4
Asgard/Eukaryota - ps5
Eukaryota - ps6
Excavata/Diaphoretickes - ps7
Diaphoretickes - ps8
Archaeplastida - ps9
Viridiplantae - ps10
Streptophyta - ps11
Charophyceae/Embriophyta - ps12
Embriophyta - ps13
Tracheophyta - ps14
Spermatophyta - ps15
Angiosperms - ps16
Mesangiospermae - ps17
Magnoliids/eudicots - ps18
Eudicots - ps19
Core_eudicots - ps20
Superrosids - ps21
Rosids - ps22
Eurosids - ps23
Malvids - ps24
Sap./Malv./Brassicales - ps25
Malvales/Brassicales - ps26
Brassicales - ps27
Cleomaceae/Brassicaceae - ps28
Brassicaceae - ps29
Camelineae - ps30
Arabidopsis - ps31
Arabidopsis_thaliana - ps32
p-value
9e-15
1e-11
2e-08
4e-05
6e-02
Log-odds
0.5
1.0
1.5
2.0
2.5
3.0
3.5
4.0

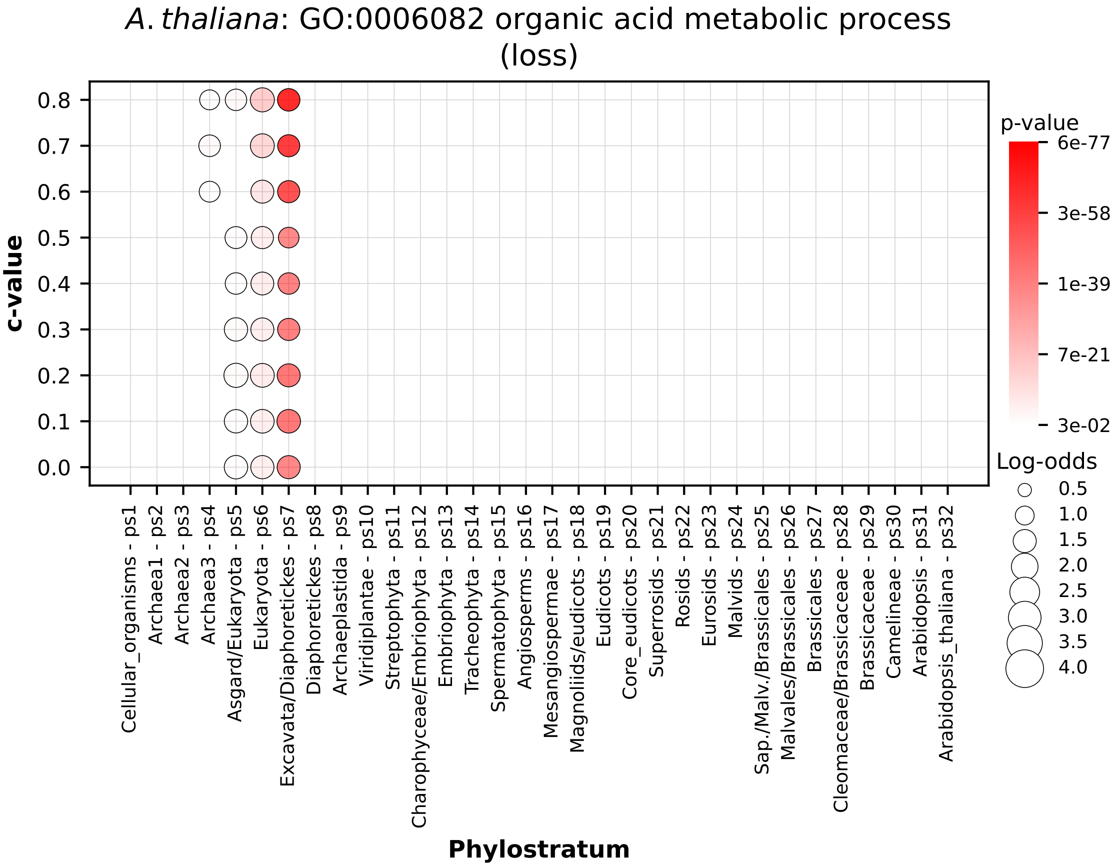

A. thaliana: GO:0006082 organic acid metabolic process
(loss)
c-value
0.8
0.7
0.6
0.5
0.4
0.3
0.2
0.1
0.0
Phylostratum
Cellular_organisms - ps1
Archaea1 - ps2
Archaea2 - ps3
Archaea3 - ps4
Asgard/Eukaryota - ps5
Eukaryota - ps6
Excavata/Diaphoretickes - ps7
Diaphoretickes - ps8
Archaeplastida - ps9
Viridiplantae - ps10
Streptophyta - ps11
Charophyceae/Embriophyta - ps12
Embriophyta - ps13
Tracheophyta - ps14
Spermatophyta - ps15
Angiosperms - ps16
Mesangiospermae - ps17
Magnoliids/eudicots - ps18
Eudicots - ps19
Core_eudicots - ps20
Superrosids - ps21
Rosids - ps22
Eurosids - ps23
Malvids - ps24
Sap./Malv./Brassicales - ps25
Malvales/Brassicales - ps26
Brassicales - ps27
Cleomaceae/Brassicaceae - ps28
Brassicaceae - ps29
Camelineae - ps30
Arabidopsis - ps31
Arabidopsis_thaliana - ps32
p-value
6e-77
3e-58
1e-39
7e-21
3e-02
Log-odds
0.5
1.0
1.5
2.0
2.5
3.0
3.5
4.0

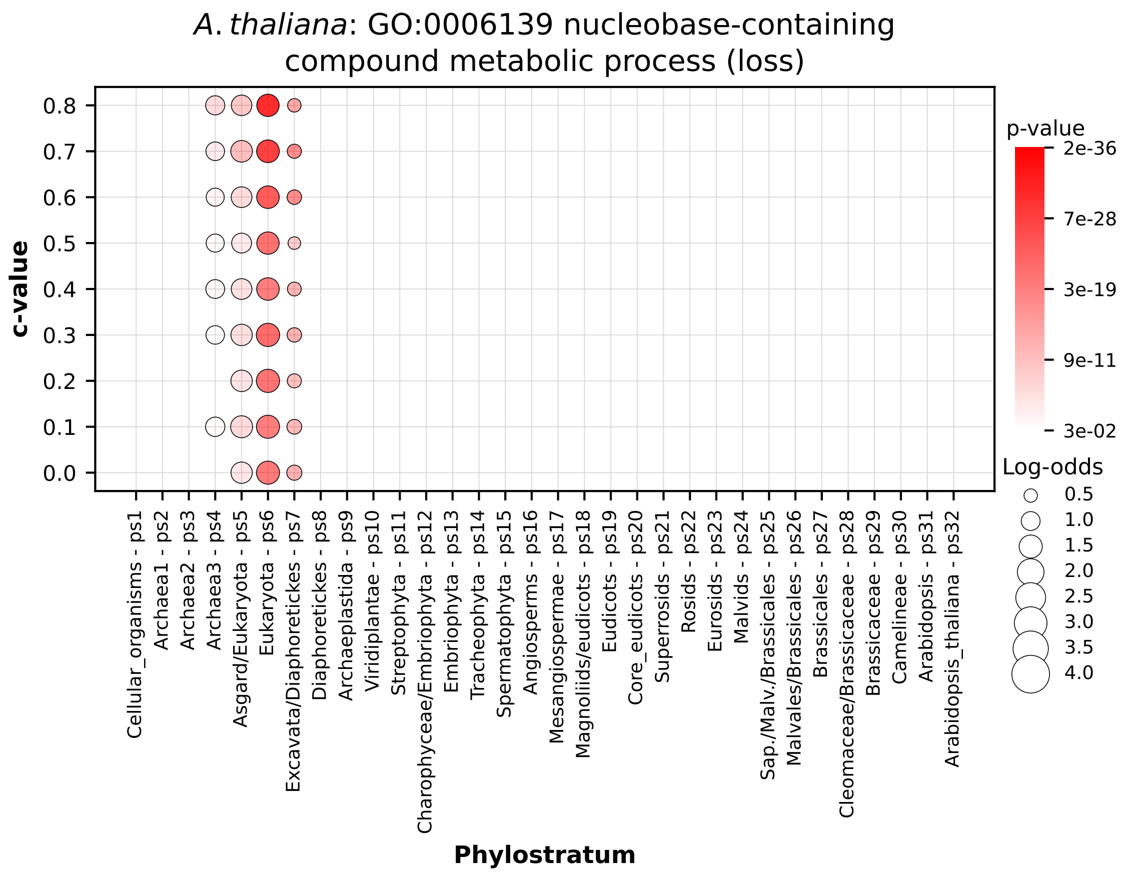

A. thaliana: GO:0006139 nucleobase-containing compound metabolic process (loss)
c-value
0.8
0.7
0.6
0.5
0.4
0.3
0.2
0.1
0.0
Phylostratum
Cellular_organisms - ps1
Archaea1 - ps2
Archaea2 - ps3
Archaea3 - ps4
Asgard/Eukaryota - ps5
Eukaryota - ps6
Excavata/Diaphoretickes - ps7
Diaphoretickes - ps8
Archaeplastida - ps9
Viridiplantae - ps10
Streptophyta - ps11
Charophyceae/Embriophyta - ps12
Embriophyta - ps13
Tracheophyta - ps14
Spermatophyta - ps15
Angiosperms - ps16
Mesangiospermae - ps17
Magnoliids/eudicots - ps18
Eudicots - ps19
Core_eudicots - ps20
Superrosids - ps21
Rosids - ps22
Eurosids - ps23
Malvids - ps24
Sap./Malv./Brassicales - ps25
Malvales/Brassicales - ps26
Brassicales - ps27
Cleomaceae/Brassicaceae - ps28
Brassicaceae - ps29
Camelineae - ps30
Arabidopsis - ps31
Arabidopsis_thaliana - ps32
p-value
2e-36
7e-28
3e-19
9e-11
3e-02
Log-odds
0.5
1.0
1.5
2.0
2.5
3.0
3.5
4.0

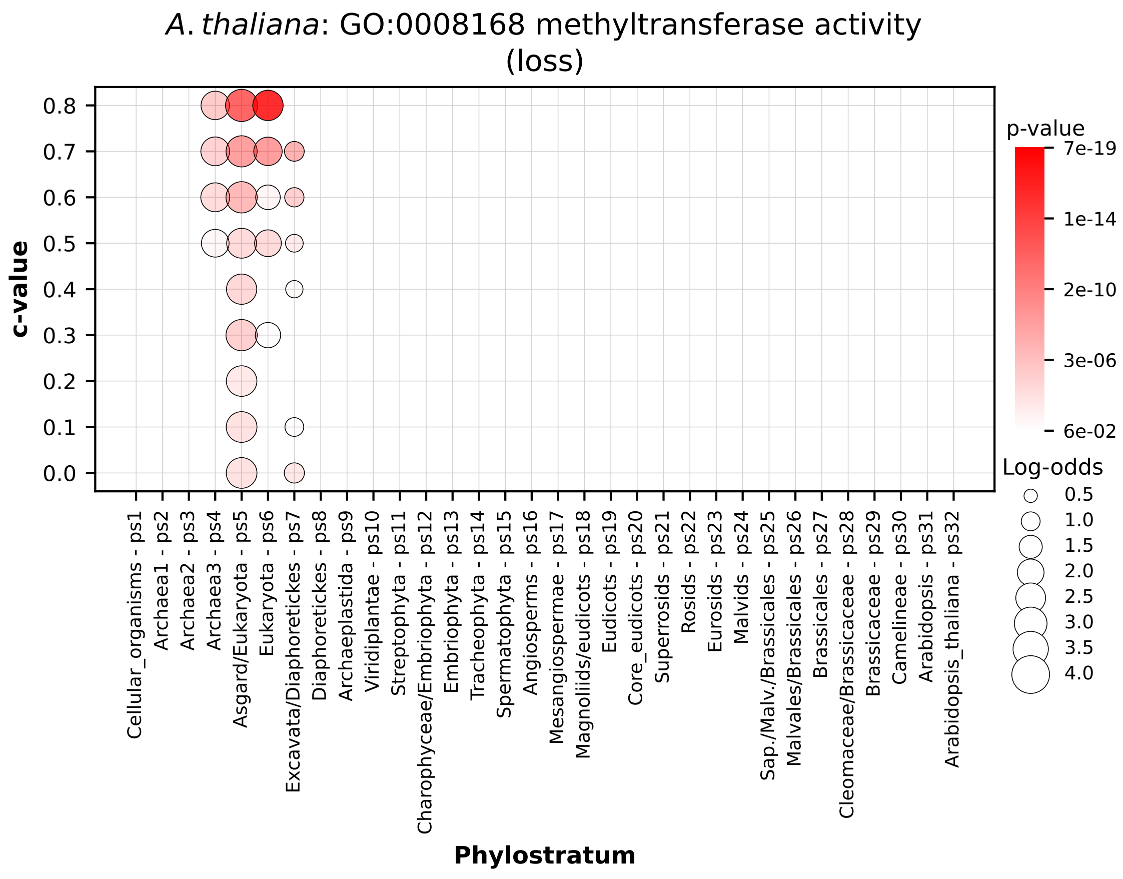

A. thaliana: GO:0008168 methyltransferase activity
(loss)
c-value
0.8
0.7
0.6
0.5
0.4
0.3
0.2
0.1
0.0
Phylostratum
Cellular_organisms - ps1
Archaea1 - ps2
Archaea2 - ps3
Archaea3 - ps4
Asgard/Eukaryota - ps5
Eukaryota - ps6
Excavata/Diaphoretickes - ps7
Diaphoretickes - ps8
Archaeplastida - ps9
Viridiplantae - ps10
Streptophyta - ps11
Charophyceae/Embriophyta - ps12
Embriophyta - ps13
Tracheophyta - ps14
Spermatophyta - ps15
Angiosperms - ps16
Mesangiospermae - ps17
Magnoliids/eudicots - ps18
Eudicots - ps19
Core_eudicots - ps20
Superrosids - ps21
Rosids - ps22
Eurosids - ps23
Malvids - ps24
Sap./Malv./Brassicales - ps25
Malvales/Brassicales - ps26
Brassicales - ps27
Cleomaceae/Brassicaceae - ps28
Brassicaceae - ps29
Camelineae - ps30
Arabidopsis - ps31
Arabidopsis_thaliana - ps32
p-value
7e-19
1e-14
2e-10
3e-06
6e-02
Log-odds
0.5
1.0
1.5
2.0
2.5
3.0
3.5
4.0

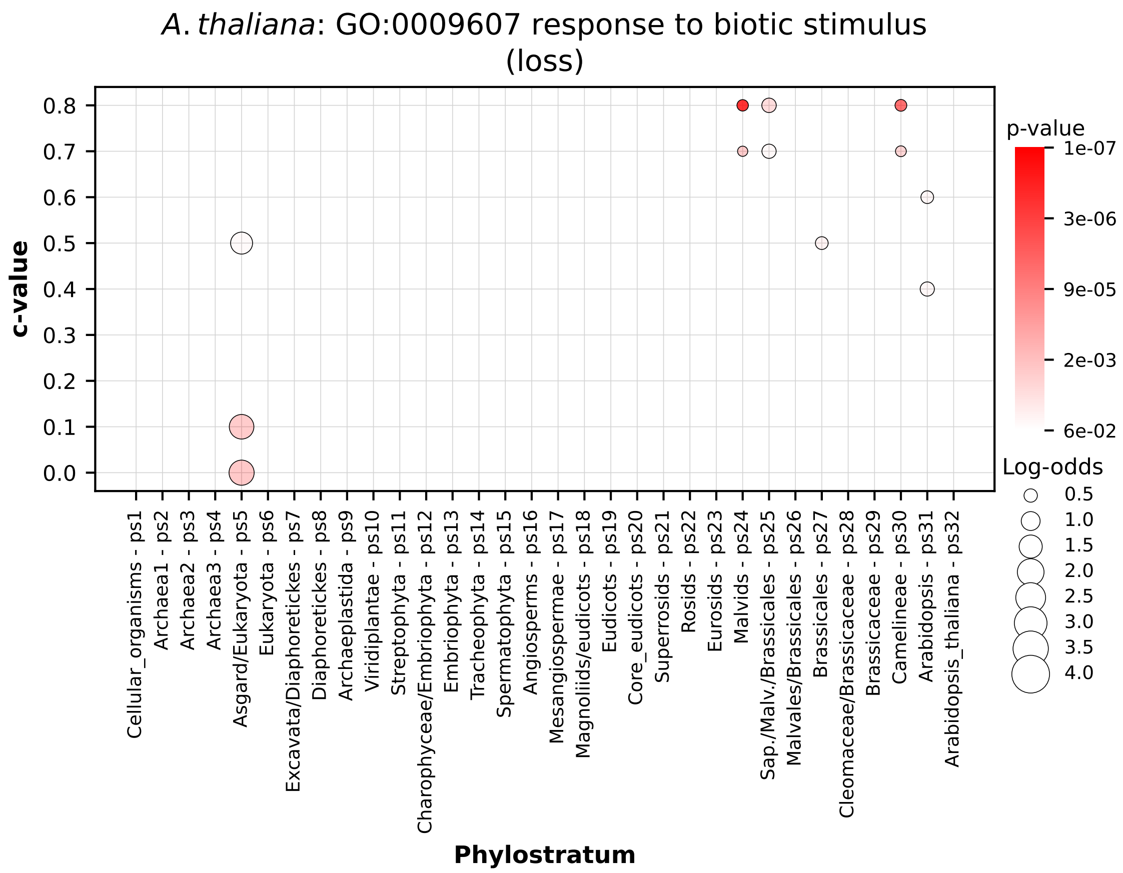
A. thaliana: GO:0009607 response to biotic stimulus
(loss)
c-value
0.8
0.7
0.6
0.5
0.4
0.3
0.2
0.1
0.0
p-value
1e-07
3e-06
9e-05
2e-03
6e-02
Log-odds
0.5
1.0
1.5
2.0
2.5
3.0
3.5
4.0
Cellular_organisms - ps1
Archaea1 - ps2
Archaea2 - ps3
Archaea3 - ps4
Asgard/Eukaryota - ps5
Eukaryota - ps6
Excavata/Diaphoretickes - ps7
Diaphoretickes - ps8
Archaeplastida - ps9
Viridiplantae - ps10
Streptophyta - ps11
Charophyceae/Embriophyta - ps12
Embriophyta - ps13
Tracheophyta - ps14
Spermatophyta - ps15
Angiosperms - ps16
Mesangiospermae - ps17
Magnoliids/eudicots - ps18
Eudicots - ps19
Core_eudicots - ps20
Superrosids - ps21
Rosids - ps22
Eurosids - ps23
Malvids - ps24
Sap./Malv./Brassicales - ps25
Malvales/Brassicales - ps26
Brassicales - ps27
Cleomaceae/Brassicaceae - ps28
Brassicaceae - ps29
Camelineae - ps30
Arabidopsis - ps31
Arabidopsis_thaliana - ps32
Phylostratum

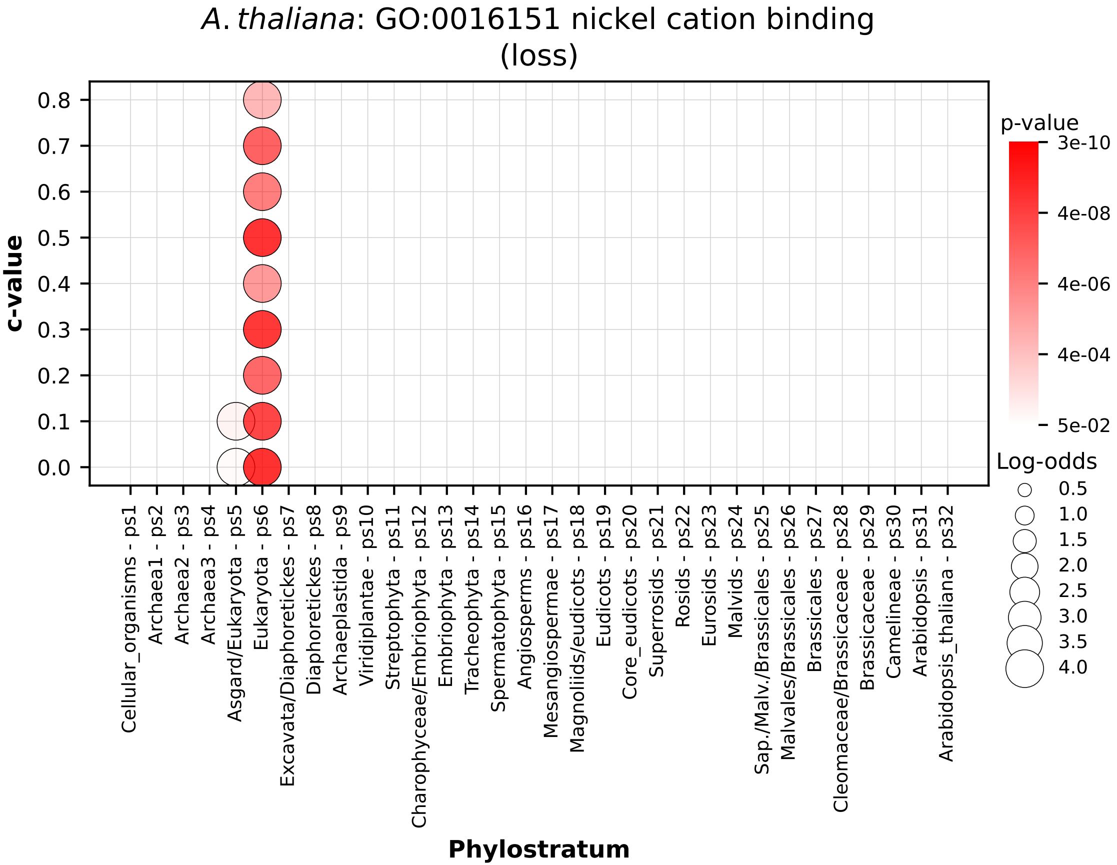
A. thaliana: GO:0016151 nickel cation binding
(loss)
c-value
0.8
0.7
0.6
0.5
0.4
0.3
0.2
0.1
0.0
p-value
3e-10
4e-08
4e-06
4e-04
5e-02
Log-odds
0.5
1.0
1.5
2.0
2.5
3.0
3.5
4.0
Cellular_organisms - ps1
Archaea1 - ps2
Archaea2 - ps3
Archaea3 - ps4
Asgard/Eukaryota - ps5
Eukaryota - ps6
Excavata/Diaphoretickes - ps7
Diaphoretickes - ps8
Archaeplastida - ps9
Viridiplantae - ps10
Streptophyta - ps11
Charophyceae/Embriophyta - ps12
Embriophyta - ps13
Tracheophyta - ps14
Spermatophyta - ps15
Angiosperms - ps16
Mesangiospermae - ps17
Magnoliids/eudicots - ps18
Eudicots - ps19
Core_eudicots - ps20
Superrosids - ps21
Rosids - ps22
Eurosids - ps23
Malvids - ps24
Sap./Malv./Brassicales - ps25
Malvales/Brassicales - ps26
Brassicales - ps27
Cleomaceae/Brassicaceae - ps28
Brassicaceae - ps29
Camelineae - ps30
Arabidopsis - ps31
Arabidopsis_thaliana - ps32
Phylostratum

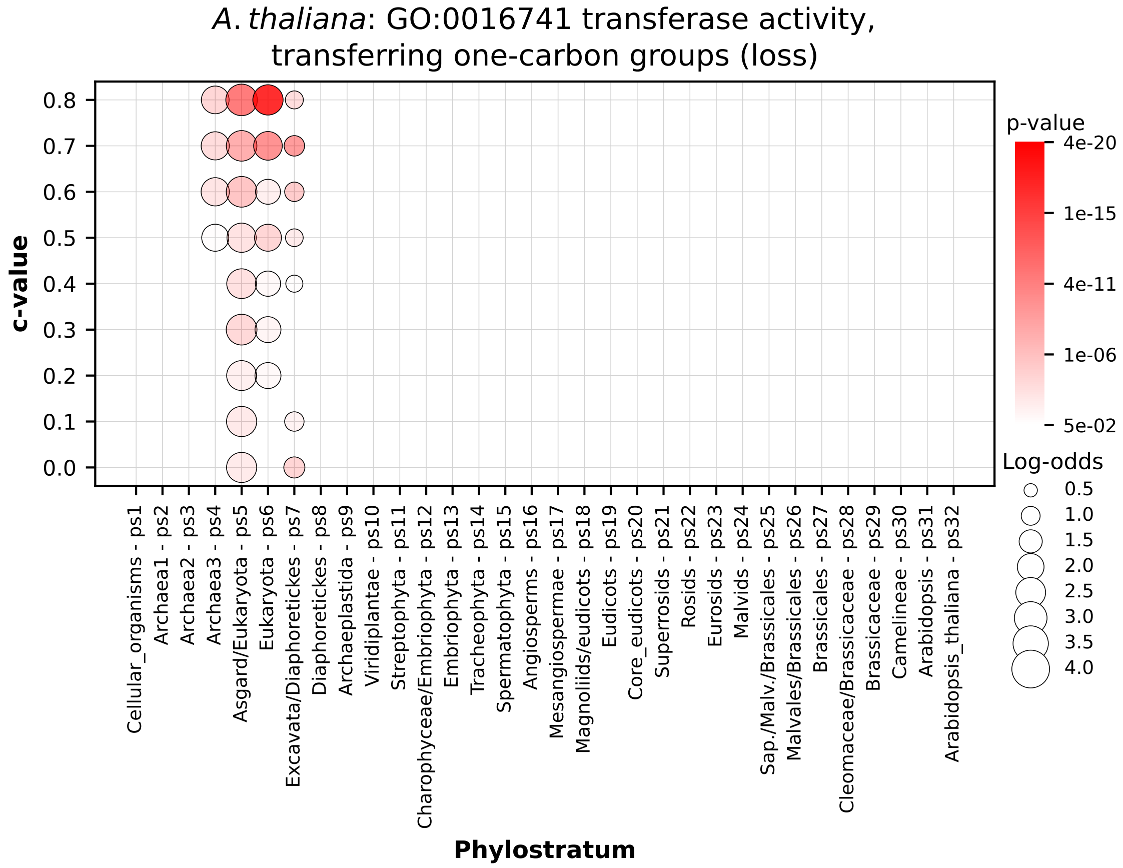

A. thaliana: GO:0016741 transferase activity, transferring one-carbon groups (loss)
c-value
0.8
0.7
0.6
0.5
0.4
0.3
0.2
0.1
0.0
p-value
4e-20
1e-15
4e-11
1e-06
5e-02
Log-odds
0.5
1.0
1.5
2.0
2.5
3.0
3.5
4.0
Cellular_organisms - ps1
Archaea1 - ps2
Archaea2 - ps3
Archaea3 - ps4
Asgard/Eukaryota - ps5
Eukaryota - ps6
Excavata/Diaphoretickes - ps7
Diaphoretickes - ps8
Archaeplastida - ps9
Viridiplantae - ps10
Streptophyta - ps11
Charophyceae/Embriophyta - ps12
Embriophyta - ps13
Tracheophyta - ps14
Spermatophyta - ps15
Angiosperms - ps16
Mesangiospermae - ps17
Magnoliids/eudicots - ps18
Eudicots - ps19
Core_eudicots - ps20
Superrosids - ps21
Rosids - ps22
Eurosids - ps23
Malvids - ps24
Sap./Malv./Brassicales - ps25
Malvales/Brassicales - ps26
Brassicales - ps27
Cleomaceae/Brassicaceae - ps28
Brassicaceae - ps29
Camelineae - ps30
Arabidopsis - ps31
Arabidopsis_thaliana - ps32
Phylostratum

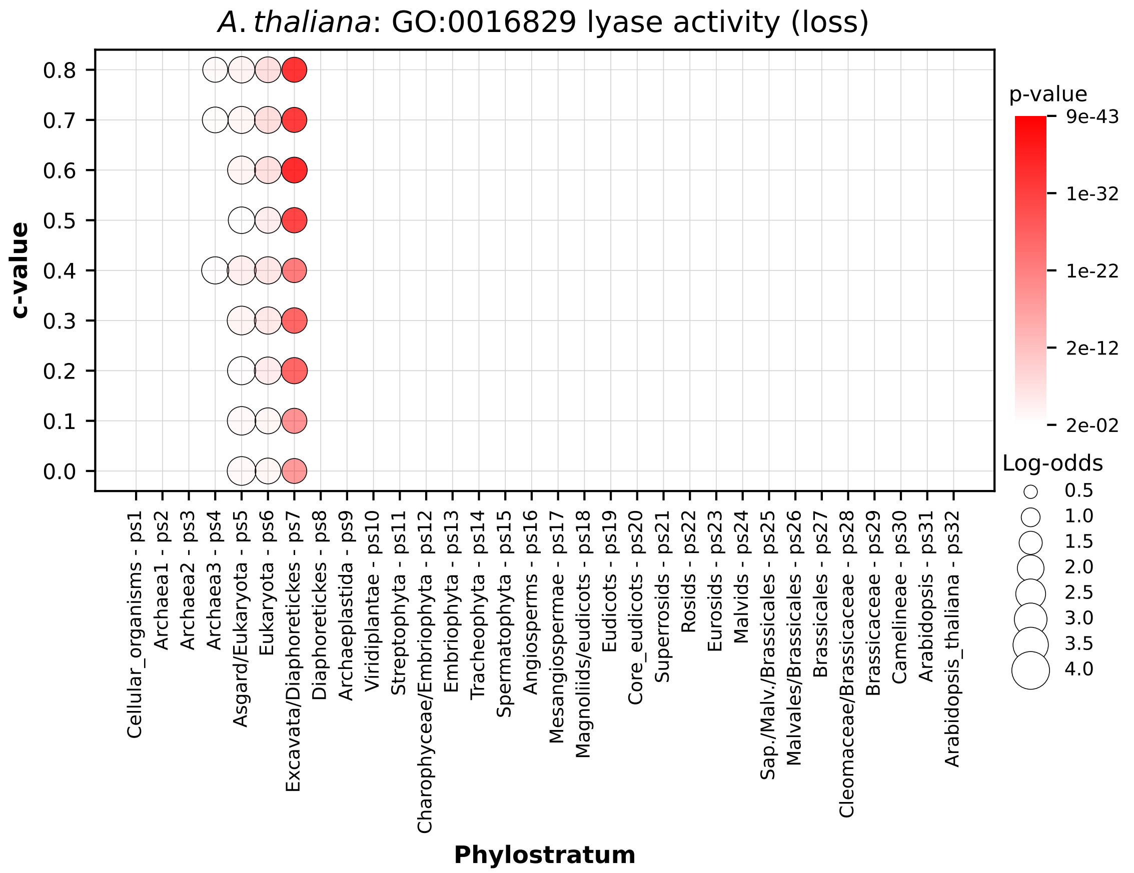
A. thaliana: GO:0016829 lyase activity (loss)
c-value
0.8
0.7
0.6
0.5
0.4
0.3
0.2
0.1
0.0
p-value
9e-43
1e-32
1e-22
2e-12
2e-02
Log-odds
0.5
1.0
1.5
2.0
2.5
3.0
3.5
4.0
Cellular_organisms - ps1
Archaea1 - ps2
Archaea2 - ps3
Archaea3 - ps4
Asgard/Eukaryota - ps5
Eukaryota - ps6
Excavata/Diaphoretickes - ps7
Diaphoretickes - ps8
Archaeplastida - ps9
Viridiplantae - ps10
Streptophyta - ps11
Charophyceae/Embriophyta - ps12
Embriophyta - ps13
Tracheophyta - ps14
Spermatophyta - ps15
Angiosperms - ps16
Mesangiospermae - ps17
Magnoliids/eudicots - ps18
Eudicots - ps19
Core_eudicots - ps20
Superrosids - ps21
Rosids - ps22
Eurosids - ps23
Malvids - ps24
Sap./Malv./Brassicales - ps25
Malvales/Brassicales - ps26
Brassicales - ps27
Cleomaceae/Brassicaceae - ps28
Brassicaceae - ps29
Camelineae - ps30
Arabidopsis - ps31
Arabidopsis_thaliana - ps32
Phylostratum

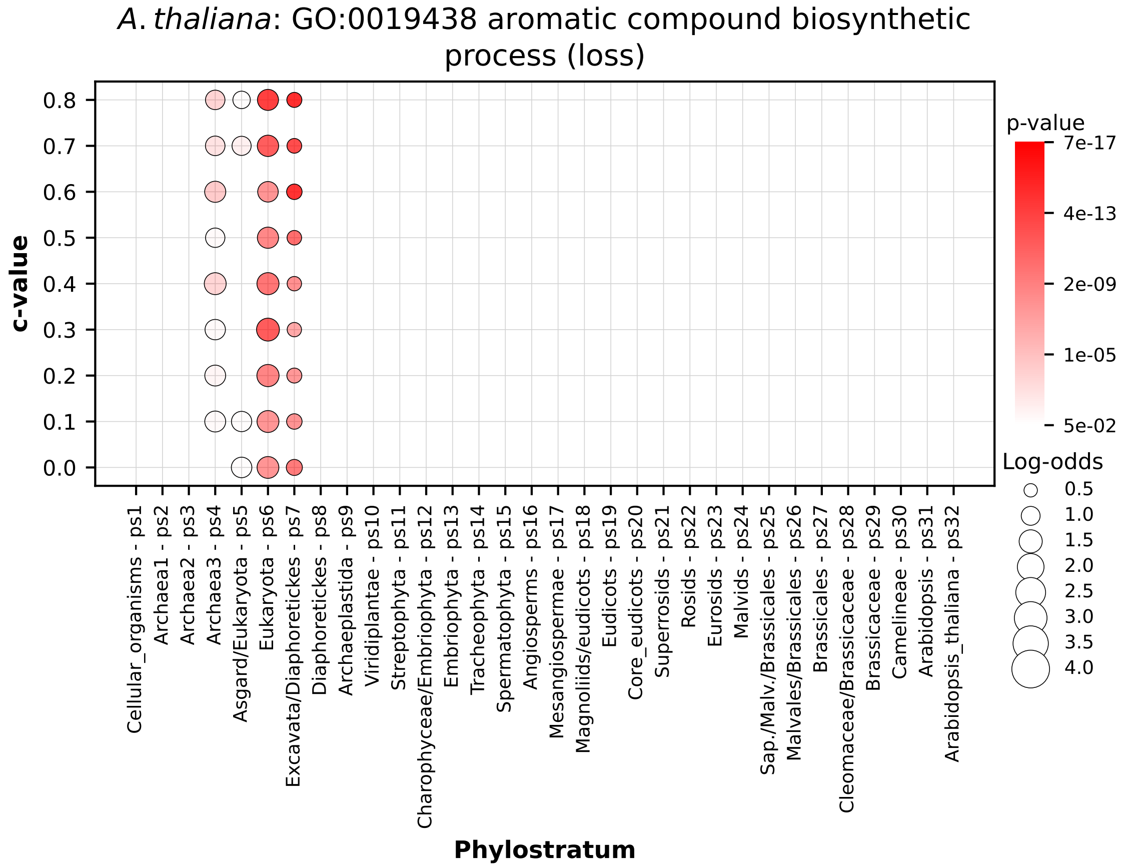

A. thaliana: GO:0019438 aromatic compound biosynthetic process (loss)
c-value
0.8
0.7
0.6
0.5
0.4
0.3
0.2
0.1
0.0
Phylostratum
Cellular_organisms - ps1
Archaea1 - ps2
Archaea2 - ps3
Archaea3 - ps4
Asgard/Eukaryota - ps5
Eukaryota - ps6
Excavata/Diaphoretickes - ps7
Diaphoretickes - ps8
Archaeplastida - ps9
Viridiplantae - ps10
Streptophyta - ps11
Charophyceae/Embriophyta - ps12
Embriophyta - ps13
Tracheophyta - ps14
Spermatophyta - ps15
Angiosperms - ps16
Mesangiospermae - ps17
Magnoliids/eudicots - ps18
Eudicots - ps19
Core_eudicots - ps20
Superrosids - ps21
Rosids - ps22
Eurosids - ps23
Malvids - ps24
Sap./Malv./Brassicales - ps25
Malvales/Brassicales - ps26
Brassicales - ps27
Cleomaceae/Brassicaceae - ps28
Brassicaceae - ps29
Camelineae - ps30
Arabidopsis - ps31
Arabidopsis_thaliana - ps32
p-value
7e-17
4e-13
2e-09
1e-05
5e-02
Log-odds
0.5
1.0
1.5
2.0
2.5
3.0
3.5
4.0

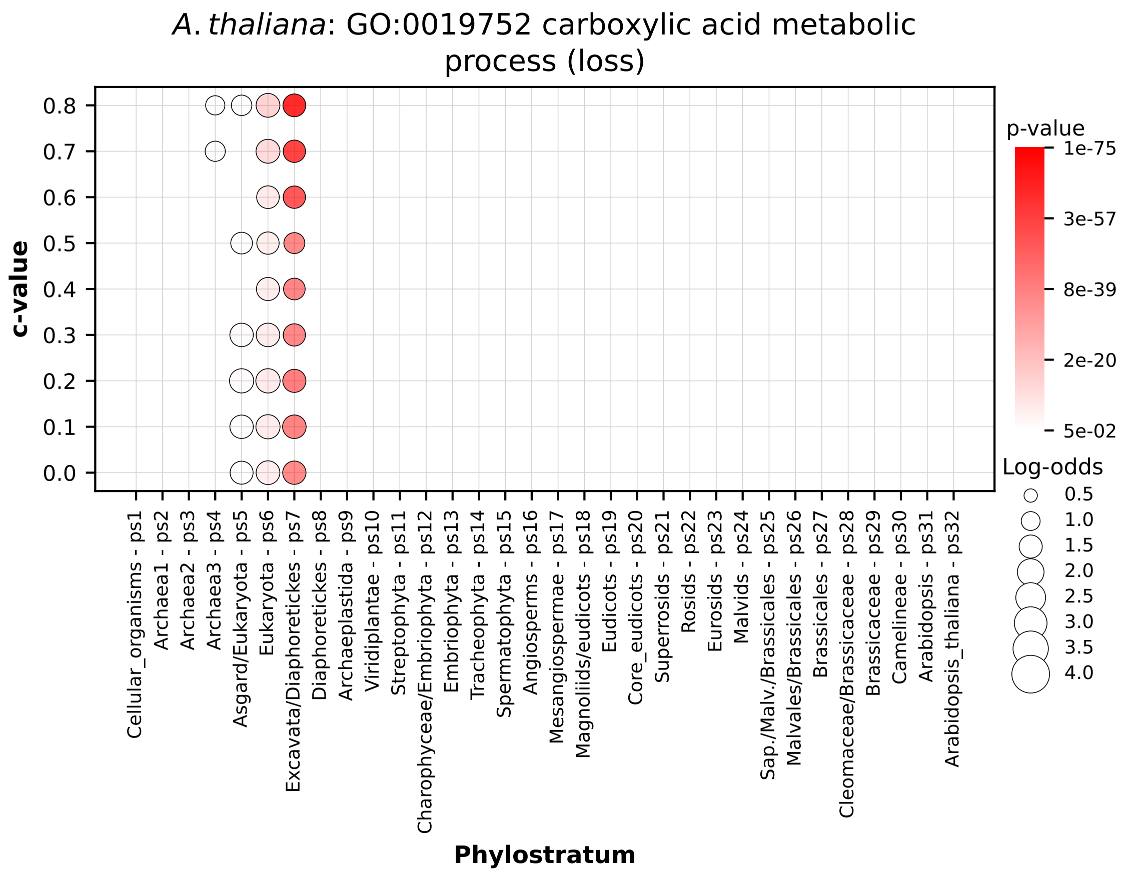

A. thaliana: GO:0019752 carboxylic acid metabolic process (loss)
c-value
0.8
0.7
0.6
0.5
0.4
0.3
0.2
0.1
0.0
p-value
1e-75
3e-57
8e-39
2e-20
5e-02
Log-odds
0.5
1.0
1.5
2.0
2.5
3.0
3.5
4.0
Cellular_organisms - ps1
Archaea1 - ps2
Archaea2 - ps3
Archaea3 - ps4
Asgard/Eukaryota - ps5
Eukaryota - ps6
Excavata/Diaphoretickes - ps7
Diaphoretickes - ps8
Archaeplastida - ps9
Viridiplantae - ps10
Streptophyta - ps11
Charophyceae/Embriophyta - ps12
Embriophyta - ps13
Tracheophyta - ps14
Spermatophyta - ps15
Angiosperms - ps16
Mesangiospermae - ps17
Magnoliids/eudicots - ps18
Eudicots - ps19
Core_eudicots - ps20
Superrosids - ps21
Rosids - ps22
Eurosids - ps23
Malvids - ps24
Sap./Malv./Brassicales - ps25
Malvales/Brassicales - ps26
Brassicales - ps27
Cleomaceae/Brassicaceae - ps28
Brassicaceae - ps29
Camelineae - ps30
Arabidopsis - ps31
Arabidopsis_thaliana - ps32
Phylostratum

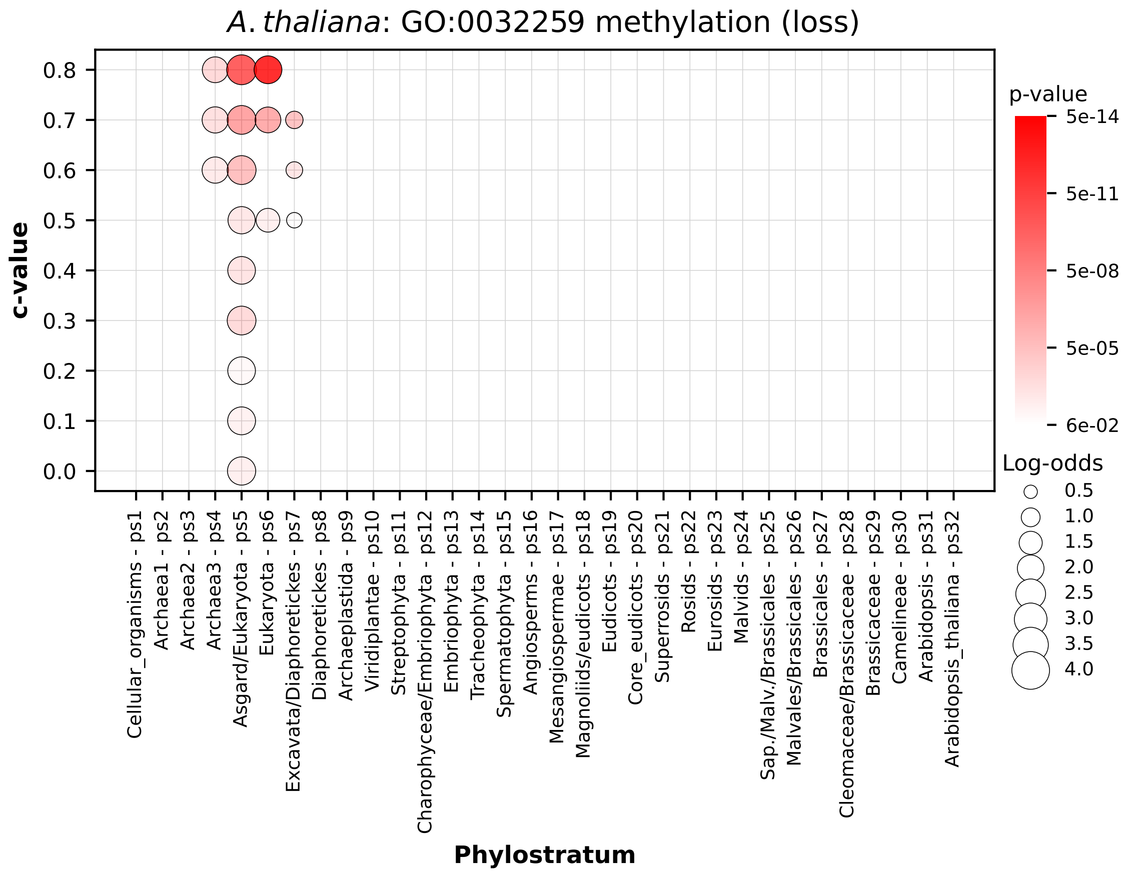

A. thaliana: GO:0032259 methylation (loss)
c-value
0.0
0.1
0.2
0.3
0.4
0.5
0.6
0.7
0.8
Phylostratum
Cellular_organisms - ps1
Archaea1 - ps2
Archaea2 - ps3
Archaea3 - ps4
Asgard/Eukaryota - ps5
Eukaryota - ps6
Excavata/Diaphoretickes - ps7
Diaphoretickes - ps8
Archaeplastida - ps9
Viridiplantae - ps10
Streptophyta - ps11
Charophyceae/Embriophyta - ps12
Embriophyta - ps13
Tracheophyta - ps14
Spermatophyta - ps15
Angiosperms - ps16
Mesangiospermae - ps17
Magnoliids/eudicots - ps18
Eudicots - ps19
Core_eudicots - ps20
Superrosids - ps21
Rosids - ps22
Eurosids - ps23
Malvids - ps24
Sap./Malv./Brassicales - ps25
Malvales/Brassicales - ps26
Brassicales - ps27
Cleomaceae/Brassicaceae - ps28
Brassicaceae - ps29
Camelineae - ps30
Arabidopsis - ps31
Arabidopsis_thaliana - ps32
p-value
5e-14
5e-11
5e-08
5e-05
6e-02
Log-odds
0.5
1.0
1.5
2.0
2.5
3.0
3.5
4.0

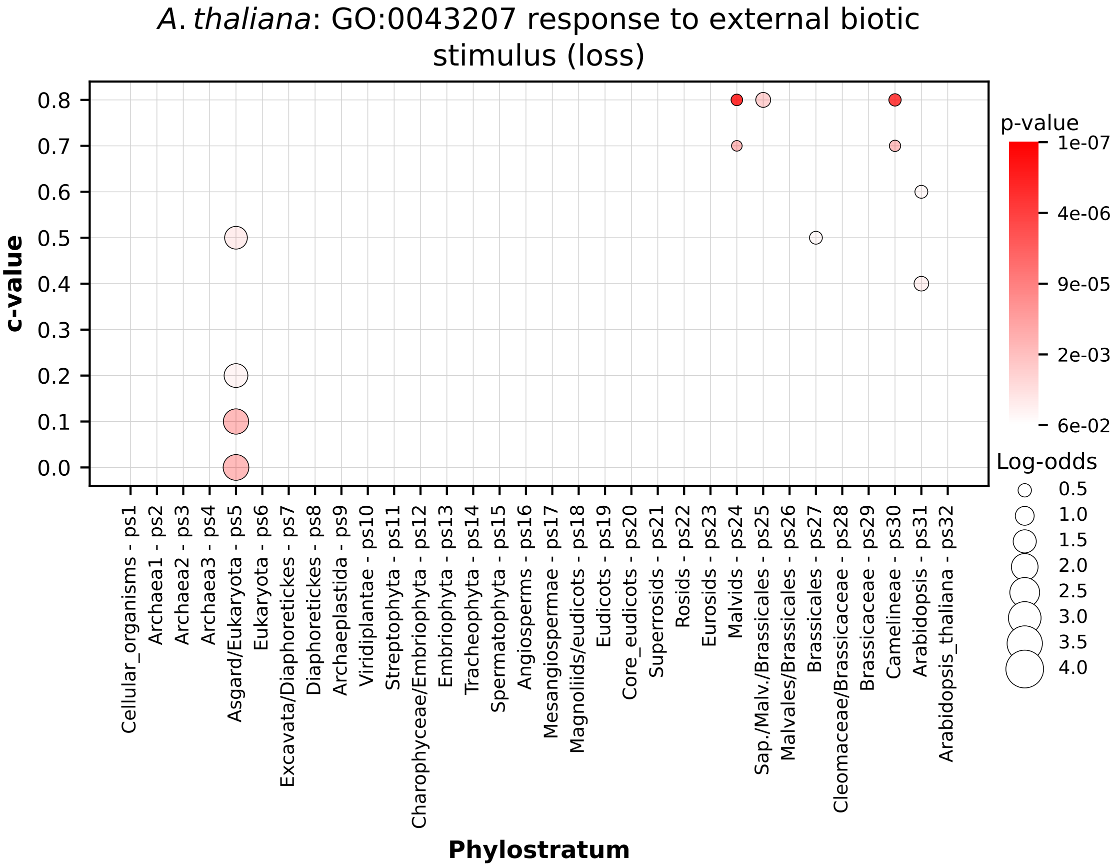

A. thaliana: GO:0043207 response to external biotic stimulus (loss)
c-value
0.8
0.7
0.6
0.5
0.4
0.3
0.2
0.1
0.0
Phylostratum
Cellular_organisms - ps1
Archaea1 - ps2
Archaea2 - ps3
Archaea3 - ps4
Asgard/Eukaryota - ps5
Eukaryota - ps6
Excavata/Diaphoretickes - ps7
Diaphoretickes - ps8
Archaeplastida - ps9
Viridiplantae - ps10
Streptophyta - ps11
Charophyceae/Embriophyta - ps12
Embriophyta - ps13
Tracheophyta - ps14
Spermatophyta - ps15
Angiosperms - ps16
Mesangiospermae - ps17
Magnoliids/eudicots - ps18
Eudicots - ps19
Core_eudicots - ps20
Superrosids - ps21
Rosids - ps22
Eurosids - ps23
Malvids - ps24
Sap./Malv./Brassicales - ps25
Malvales/Brassicales - ps26
Brassicales - ps27
Cleomaceae/Brassicaceae - ps28
Brassicaceae - ps29
Camelineae - ps30
Arabidopsis - ps31
Arabidopsis_thaliana - ps32
p-value
1e-07
4e-06
9e-05
2e-03
6e-02
Log-odds
0.5
1.0
1.5
2.0
2.5
3.0
3.5
4.0

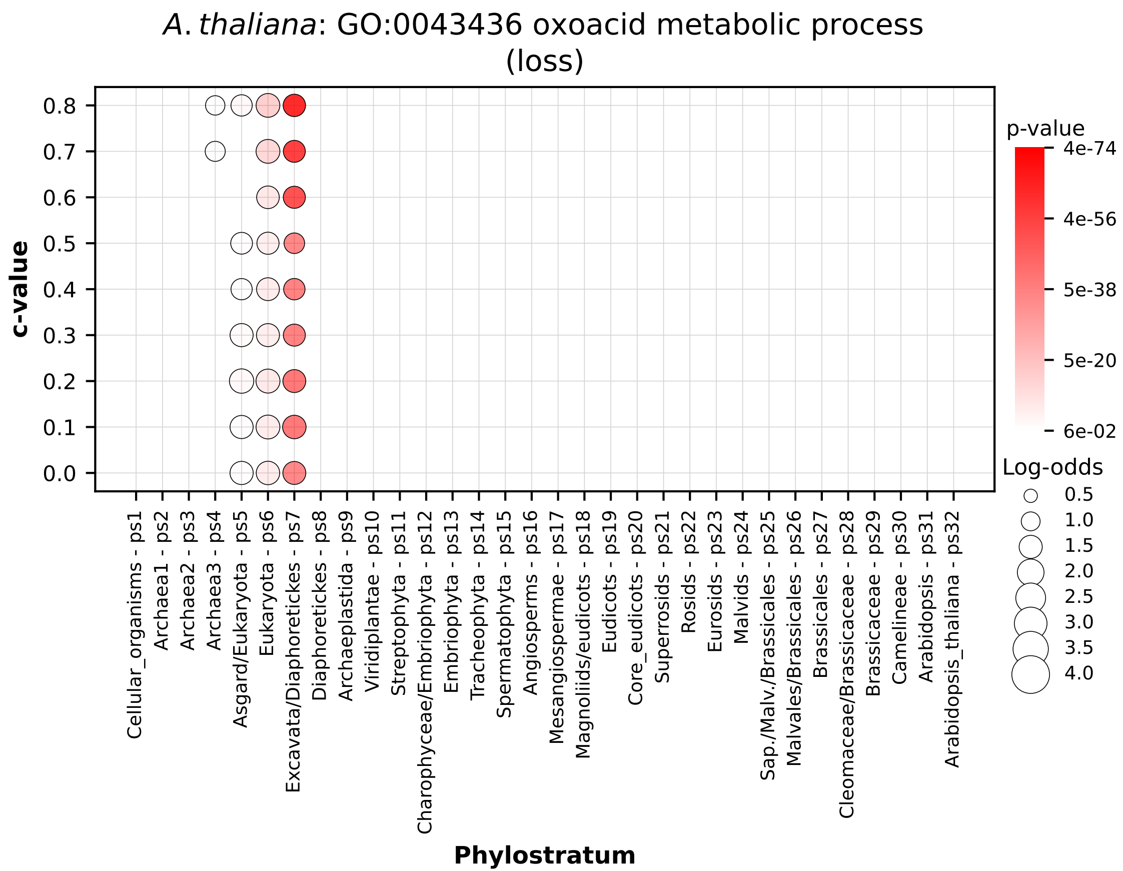
A. thaliana: GO:0043436 oxoacid metabolic process
(loss)
c-value
0.8
0.7
0.6
0.5
0.4
0.3
0.2
0.1
0.0
p-value
4e-74
4e-56
5e-38
5e-20
6e-02
Log-odds
0.5
1.0
1.5
2.0
2.5
3.0
3.5
4.0
Cellular_organisms - ps1
Archaea1 - ps2
Archaea2 - ps3
Archaea3 - ps4
Asgard/Eukaryota - ps5
Eukaryota - ps6
Excavata/Diaphoretickes - ps7
Diaphoretickes - ps8
Archaeplastida - ps9
Viridiplantae - ps10
Streptophyta - ps11
Charophyceae/Embriophyta - ps12
Embriophyta - ps13
Tracheophyta - ps14
Spermatophyta - ps15
Angiosperms - ps16
Mesangiospermae - ps17
Magnoliids/eudicots - ps18
Eudicots - ps19
Core_eudicots - ps20
Superrosids - ps21
Rosids - ps22
Eurosids - ps23
Malvids - ps24
Sap./Malv./Brassicales - ps25
Malvales/Brassicales - ps26
Brassicales - ps27
Cleomaceae/Brassicaceae - ps28
Brassicaceae - ps29
Camelineae - ps30
Arabidopsis - ps31
Arabidopsis_thaliana - ps32
Phylostratum

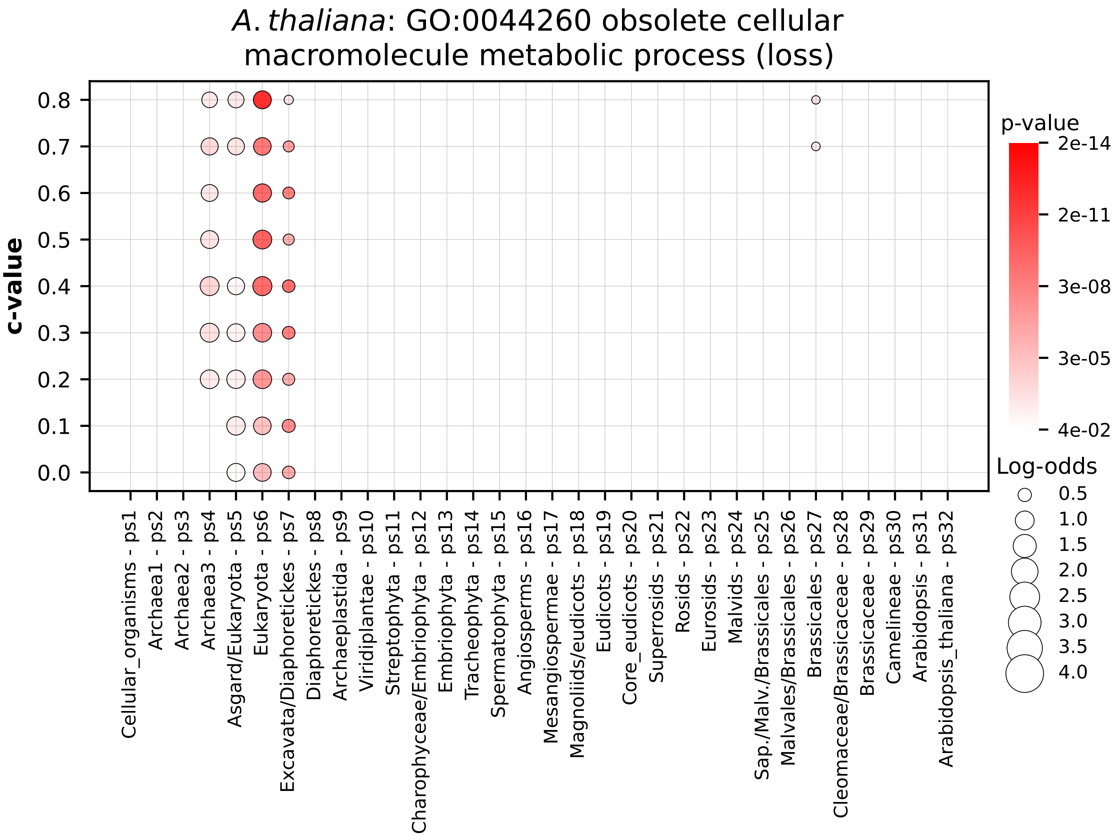

A. thaliana: GO:0044260 obsolete cellular macromolecule metabolic process (loss)
c-value
0.8
0.7
0.6
0.5
0.4
0.3
0.2
0.1
0.0
p-value
2e-14
2e-11
3e-08
3e-05
4e-02
Log-odds
0.5
1.0
1.5
2.0
2.5
3.0
3.5
4.0
Cellular_organisms - ps1
Archaea1 - ps2
Archaea2 - ps3
Archaea3 - ps4
Asgard/Eukaryota - ps5
Eukaryota - ps6
Excavata/Diaphoretickes - ps7
Diaphoretickes - ps8
Archaeplastida - ps9
Viridiplantae - ps10
Streptophyta - ps11
Charophyceae/Embriophyta - ps12
Embriophyta - ps13
Tracheophyta - ps14
Spermatophyta - ps15
Angiosperms - ps16
Mesangiospermae - ps17
Magnoliids/eudicots - ps18
Eudicots - ps19
Core_eudicots - ps20
Superrosids - ps21
Rosids - ps22
Eurosids - ps23
Malvids - ps24
Sap./Malv./Brassicales - ps25
Malvales/Brassicales - ps26
Brassicales - ps27
Cleomaceae/Brassicaceae - ps28
Brassicaceae - ps29
Camelineae - ps30
Arabidopsis - ps31
Arabidopsis_thaliana - ps32
Phylostratum

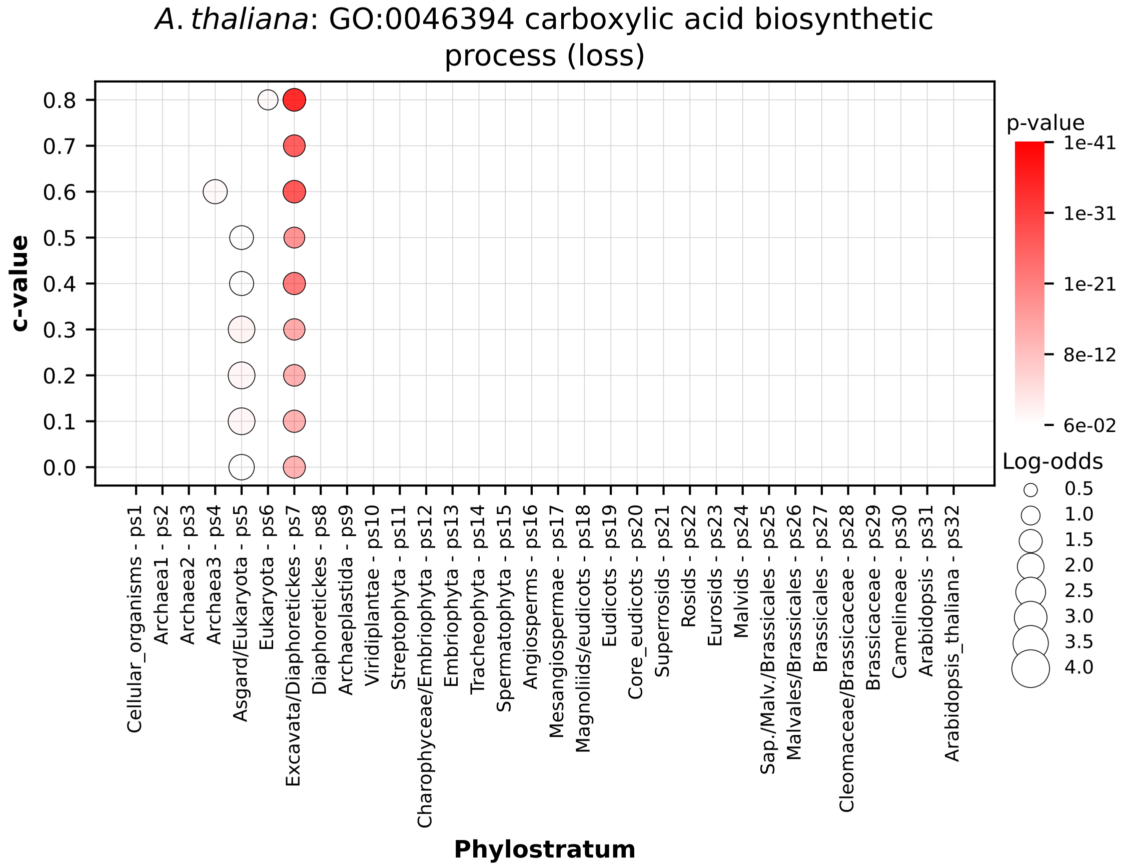

A. thaliana: GO:0046394 carboxylic acid biosynthetic process (loss)
c-value
0.8
0.7
0.6
0.5
0.4
0.3
0.2
0.1
0.0
p-value
1e-41
1e-31
1e-21
8e-12
6e-02
Log-odds
0.5
1.0
1.5
2.0
2.5
3.0
3.5
4.0
Cellular_organisms - ps1
Archaea1 - ps2
Archaea2 - ps3
Archaea3 - ps4
Asgard/Eukaryota - ps5
Eukaryota - ps6
Excavata/Diaphoretickes - ps7
Diaphoretickes - ps8
Archaeplastida - ps9
Viridiplantae - ps10
Streptophyta - ps11
Charophyceae/Embriophyta - ps12
Embriophyta - ps13
Tracheophyta - ps14
Spermatophyta - ps15
Angiosperms - ps16
Mesangiospermae - ps17
Magnoliids/eudicots - ps18
Eudicots - ps19
Core_eudicots - ps20
Superrosids - ps21
Rosids - ps22
Eurosids - ps23
Malvids - ps24
Sap./Malv./Brassicales - ps25
Malvales/Brassicales - ps26
Brassicales - ps27
Cleomaceae/Brassicaceae - ps28
Brassicaceae - ps29
Camelineae - ps30
Arabidopsis - ps31
Arabidopsis_thaliana - ps32
Phylostratum

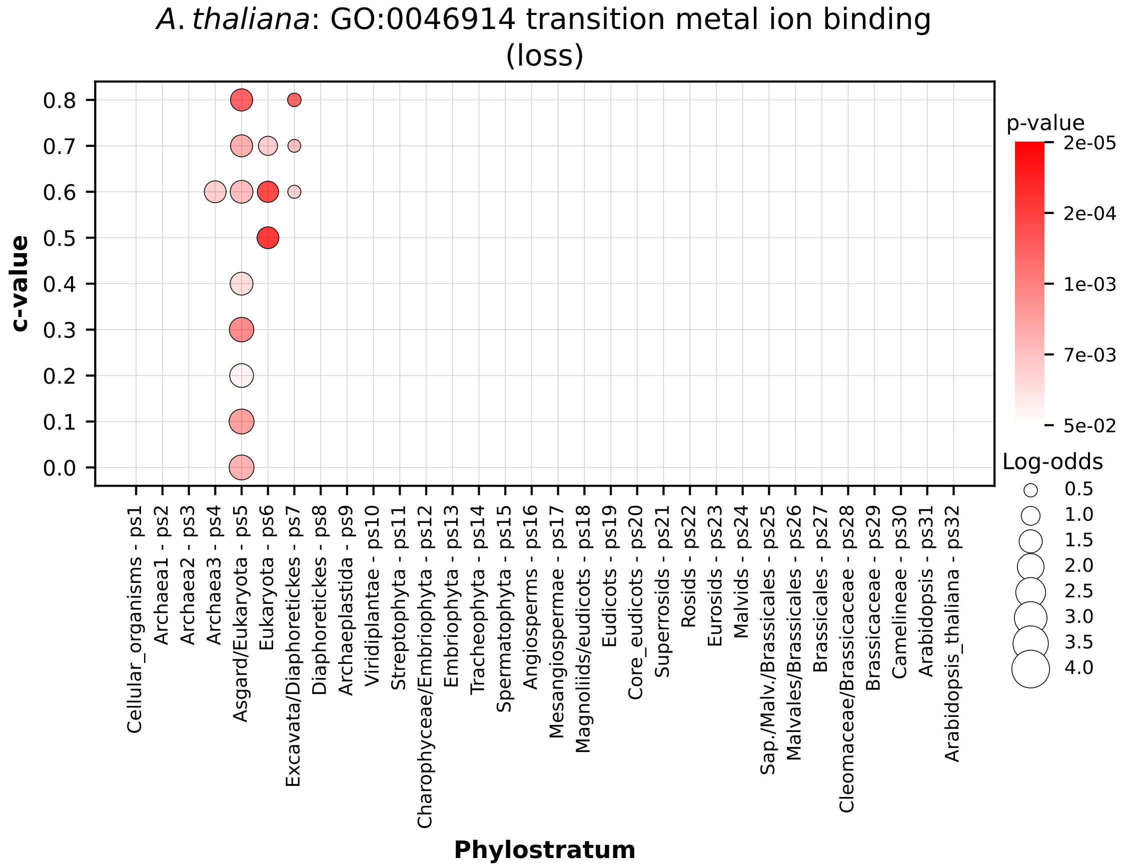

A. thaliana: GO:0046914 transition metal ion binding
(loss)
c-value
0.0
0.1
0.2
0.3
0.4
0.5
0.6
0.7
0.8
Phylostratum
Cellular_organisms - ps1
Archaea1 - ps2
Archaea2 - ps3
Archaea3 - ps4
Asgard/Eukaryota - ps5
Eukaryota - ps6
Excavata/Diaphoretickes - ps7
Diaphoretickes - ps8
Archaeplastida - ps9
Viridiplantae - ps10
Streptophyta - ps11
Charophyceae/Embriophyta - ps12
Embriophyta - ps13
Tracheophyta - ps14
Spermatophyta - ps15
Angiosperms - ps16
Mesangiospermae - ps17
Magnoliids/eudicots - ps18
Eudicots - ps19
Core_eudicots - ps20
Superrosids - ps21
Rosids - ps22
Eurosids - ps23
Malvids - ps24
Sap./Malv./Brassicales - ps25
Malvales/Brassicales - ps26
Brassicales - ps27
Cleomaceae/Brassicaceae - ps28
Brassicaceae - ps29
Camelineae - ps30
Arabidopsis - ps31
Arabidopsis_thaliana - ps32
p-value
2e-05
2e-04
1e-03
7e-03
5e-02
Log-odds
0.5
1.0
1.5
2.0
2.5
3.0
3.5
4.0

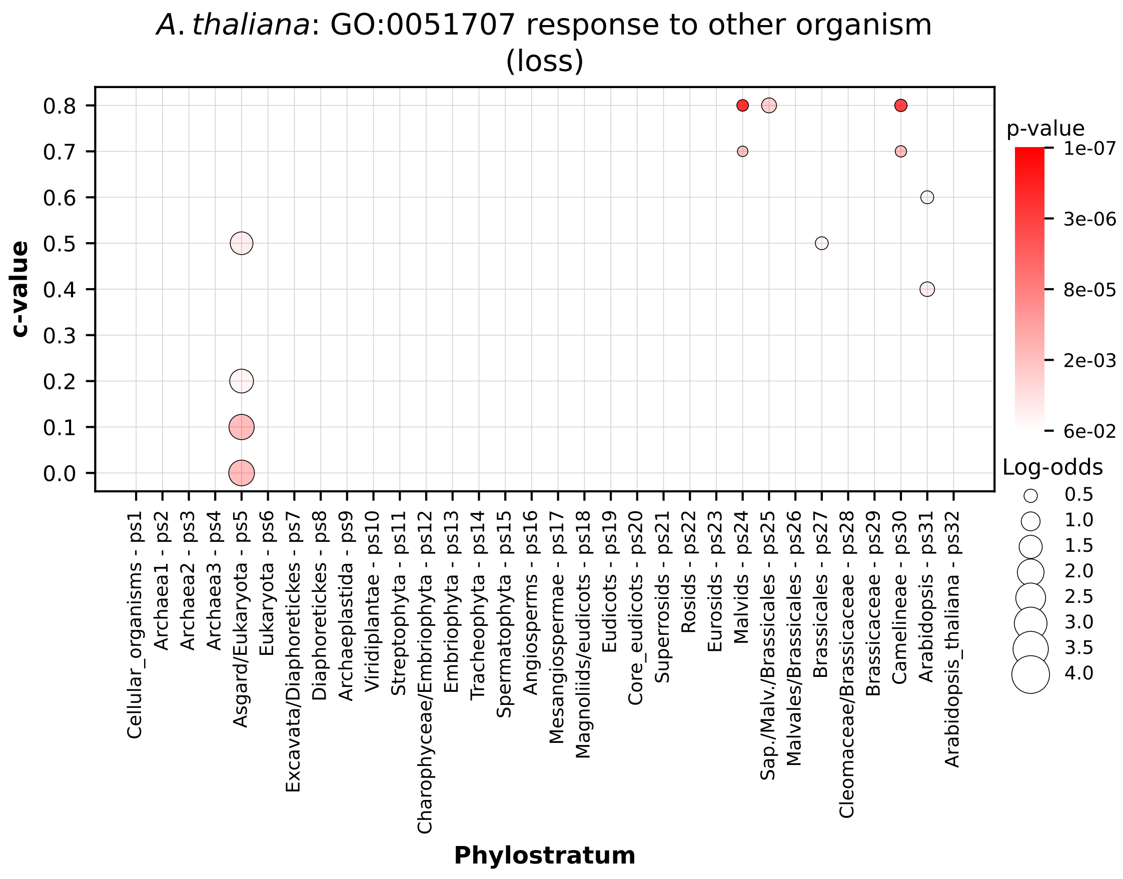
A. thaliana: GO:0051707 response to other organism
(loss)
c-value
0.8
0.7
0.6
0.5
0.4
0.3
0.2
0.1
0.0
p-value
1e-07
3e-06
8e-05
2e-03
6e-02
Log-odds
0.5
1.0
1.5
2.0
2.5
3.0
3.5
4.0
Cellular_organisms - ps1
Archaea1 - ps2
Archaea2 - ps3
Archaea3 - ps4
Asgard/Eukaryota - ps5
Eukaryota - ps6
Excavata/Diaphoretickes - ps7
Diaphoretickes - ps8
Archaeplastida - ps9
Viridiplantae - ps10
Streptophyta - ps11
Charophyceae/Embriophyta - ps12
Embriophyta - ps13
Tracheophyta - ps14
Spermatophyta - ps15
Angiosperms - ps16
Mesangiospermae - ps17
Magnoliids/eudicots - ps18
Eudicots - ps19
Core_eudicots - ps20
Superrosids - ps21
Rosids - ps22
Eurosids - ps23
Malvids - ps24
Sap./Malv./Brassicales - ps25
Malvales/Brassicales - ps26
Brassicales - ps27
Cleomaceae/Brassicaceae - ps28
Brassicaceae - ps29
Camelineae - ps30
Arabidopsis - ps31
Arabidopsis_thaliana - ps32
Phylostratum

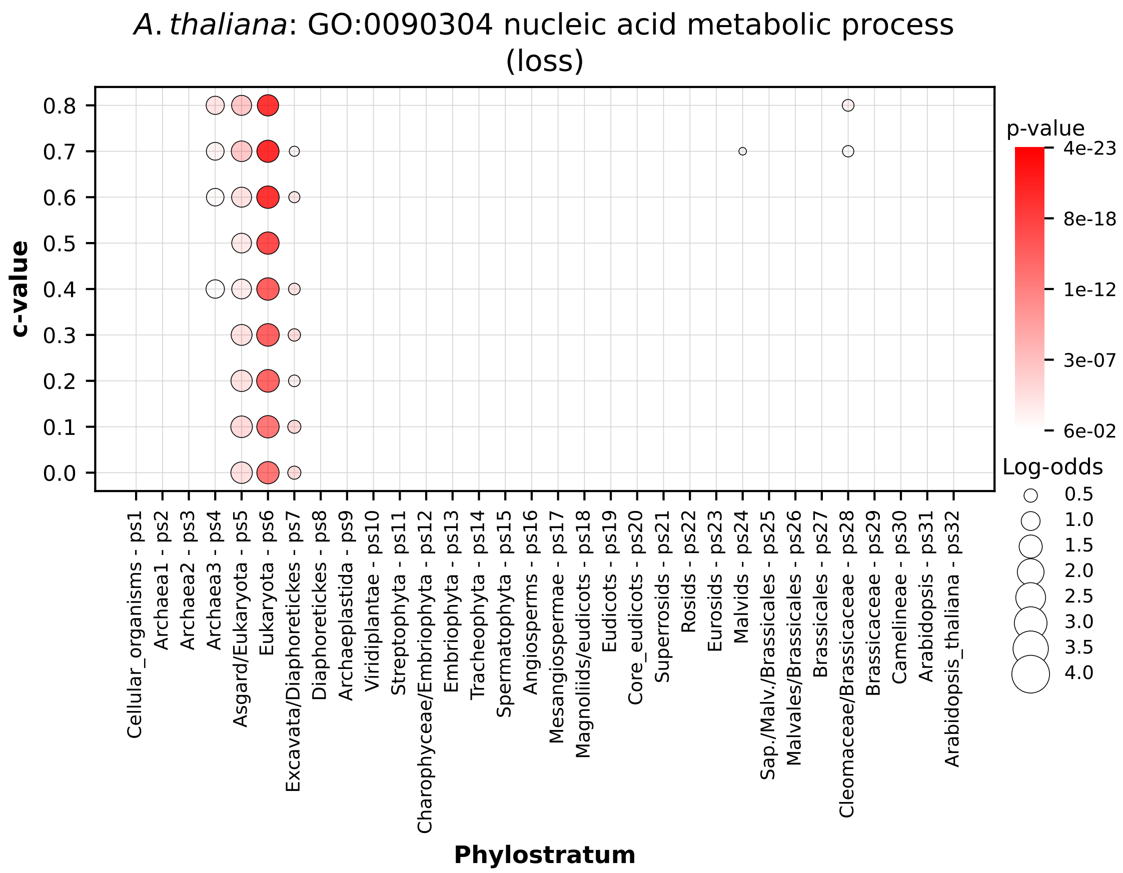

A. thaliana: GO:0090304 nucleic acid metabolic process (loss)
c-value
0.8
0.7
0.6
0.5
0.4
0.3
0.2
0.1
0.0
p-value
4e-23
8e-18
1e-12
3e-07
6e-02
Log-odds
0.5
1.0
1.5
2.0
2.5
3.0
3.5
4.0
Cellular_organisms - ps1
Archaea1 - ps2
Archaea2 - ps3
Archaea3 - ps4
Asgard/Eukaryota - ps5
Eukaryota - ps6
Excavata/Diaphoretickes - ps7
Diaphoretickes - ps8
Archaeplastida - ps9
Viridiplantae - ps10
Streptophyta - ps11
Charophyceae/Embriophyta - ps12
Embriophyta - ps13
Tracheophyta - ps14
Spermatophyta - ps15
Angiosperms - ps16
Mesangiospermae - ps17
Magnoliids/eudicots - ps18
Eudicots - ps19
Core_eudicots - ps20
Superrosids - ps21
Rosids - ps22
Eurosids - ps23
Malvids - ps24
Sap./Malv./Brassicales - ps25
Malvales/Brassicales - ps26
Brassicales - ps27
Cleomaceae/Brassicaceae - ps28
Brassicaceae - ps29
Camelineae - ps30
Arabidopsis - ps31
Arabidopsis_thaliana - ps32
Phylostratum

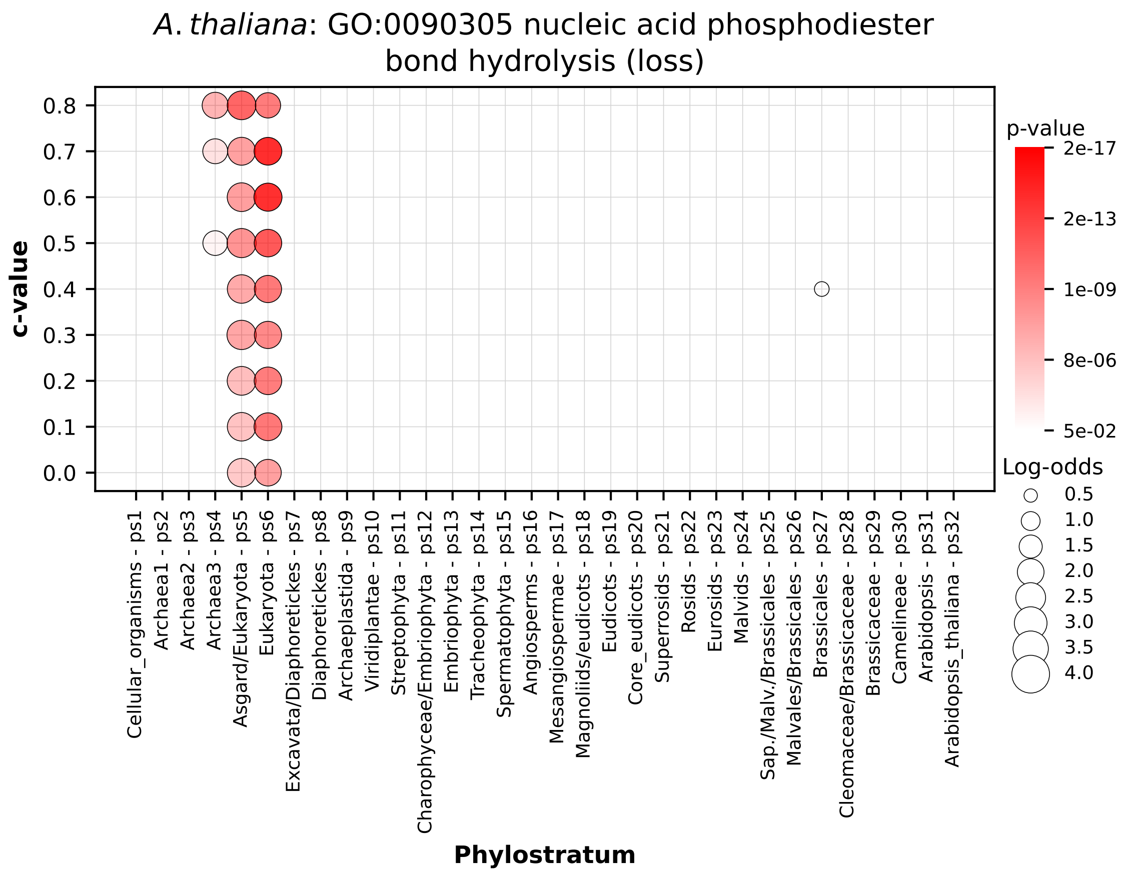

A. thaliana: GO:0090305 nucleic acid phosphodiester bond hydrolysis (loss)
c-value
0.8
0.7
0.6
0.5
0.4
0.3
0.2
0.1
0.0
p-value
2e-17
2e-13
1e-09
8e-06
5e-02
Log-odds
0.5
1.0
1.5
2.0
2.5
3.0
3.5
4.0
Cellular_organisms - ps1
Archaea1 - ps2
Archaea2 - ps3
Archaea3 - ps4
Asgard/Eukaryota - ps5
Eukaryota - ps6
Excavata/Diaphoretickes - ps7
Diaphoretickes - ps8
Archaeplastida - ps9
Viridiplantae - ps10
Streptophyta - ps11
Charophyceae/Embriophyta - ps12
Embriophyta - ps13
Tracheophyta - ps14
Spermatophyta - ps15
Angiosperms - ps16
Mesangiospermae - ps17
Magnoliids/eudicots - ps18
Eudicots - ps19
Core_eudicots - ps20
Superrosids - ps21
Rosids - ps22
Eurosids - ps23
Malvids - ps24
Sap./Malv./Brassicales - ps25
Malvales/Brassicales - ps26
Brassicales - ps27
Cleomaceae/Brassicaceae - ps28
Brassicaceae - ps29
Camelineae - ps30
Arabidopsis - ps31
Arabidopsis_thaliana - ps32
Phylostratum

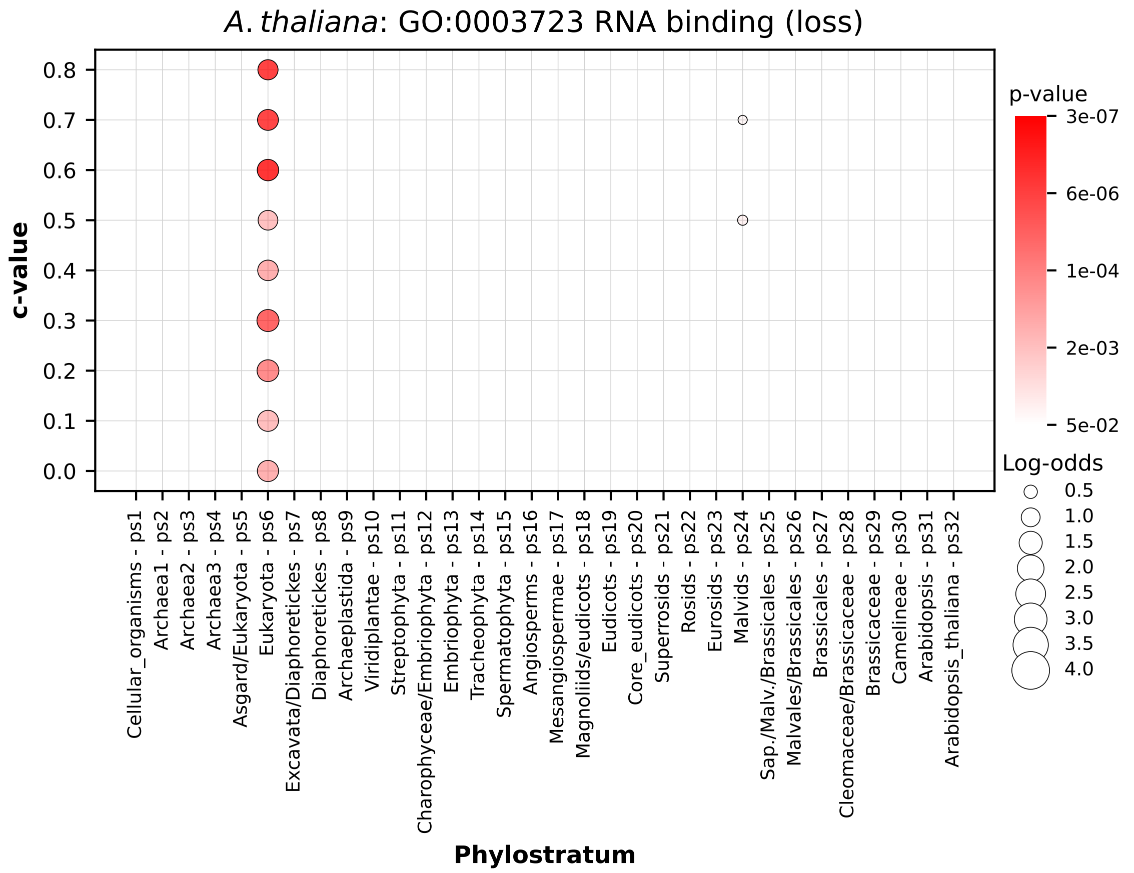

A. thaliana: GO:0003723 RNA binding (loss)
c-value
0.0
0.1
0.2
0.3
0.4
0.5
0.6
0.7
0.8
p-value
3e-07
6e-06
1e-04
2e-03
5e-02
Log-odds
0.5
1.0
1.5
2.0
2.5
3.0
3.5
4.0
Cellular_organisms - ps1
Archaea1 - ps2
Archaea2 - ps3
Archaea3 - ps4
Asgard/Eukaryota - ps5
Eukaryota - ps6
Excavata/Diaphoretickes - ps7
Diaphoretickes - ps8
Archaeplastida - ps9
Viridiplantae - ps10
Streptophyta - ps11
Charophyceae/Embriophyta - ps12
Embriophyta - ps13
Tracheophyta - ps14
Spermatophyta - ps15
Angiosperms - ps16
Mesangiospermae - ps17
Magnoliids/eudicots - ps18
Eudicots - ps19
Core_eudicots - ps20
Superrosids - ps21
Rosids - ps22
Eurosids - ps23
Malvids - ps24
Sap./Malv./Brassicales - ps25
Malvales/Brassicales - ps26
Brassicales - ps27
Cleomaceae/Brassicaceae - ps28
Brassicaceae - ps29
Camelineae - ps30
Arabidopsis - ps31
Arabidopsis_thaliana - ps32
Phylostratum

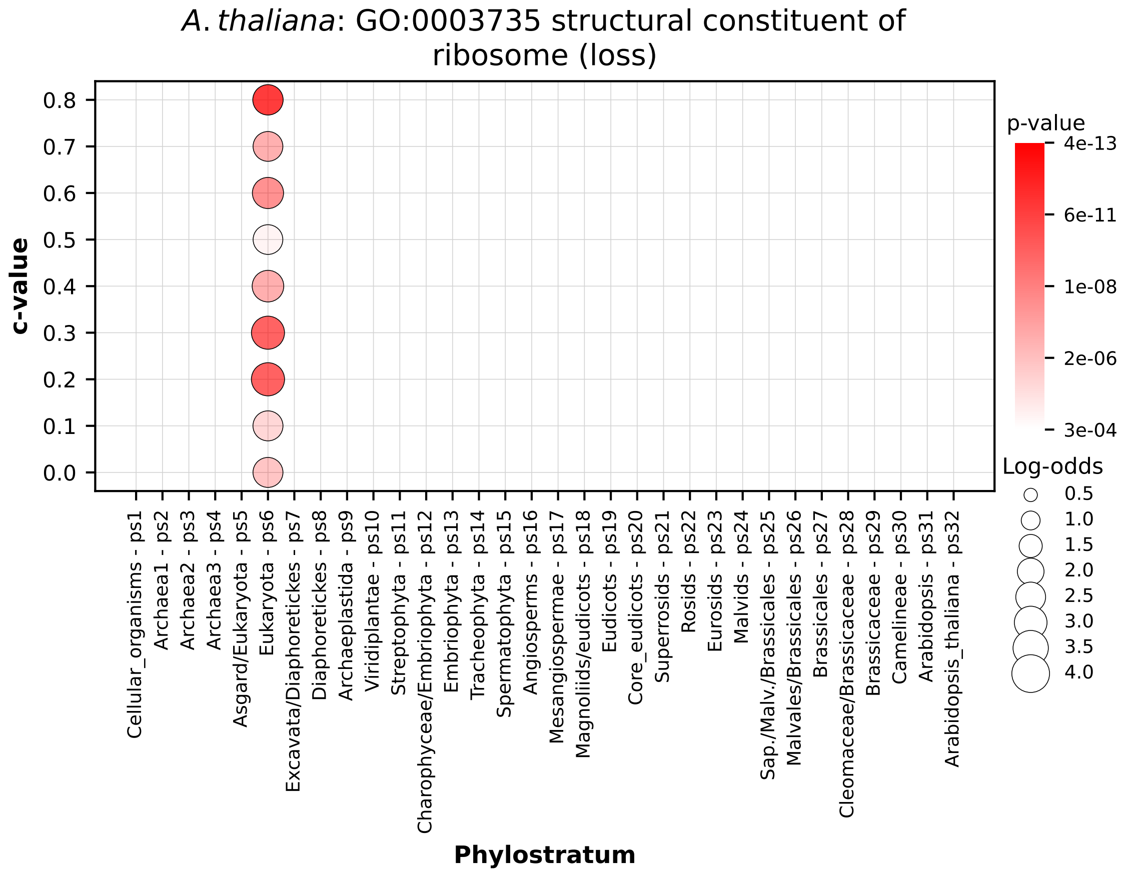

A. thaliana: GO:0003735 structural constituent of ribosome (loss)
c-value
0.0
0.1
0.2
0.3
0.4
0.5
0.6
0.7
0.8
Phylostratum
Cellular_organisms - ps1
Archaea1 - ps2
Archaea2 - ps3
Archaea3 - ps4
Asgard/Eukaryota - ps5
Eukaryota - ps6
Excavata/Diaphoretickes - ps7
Diaphoretickes - ps8
Archaeplastida - ps9
Viridiplantae - ps10
Streptophyta - ps11
Charophyceae/Embriophyta - ps12
Embriophyta - ps13
Tracheophyta - ps14
Spermatophyta - ps15
Angiosperms - ps16
Mesangiospermae - ps17
Magnoliids/eudicots - ps18
Eudicots - ps19
Core_eudicots - ps20
Superrosids - ps21
Rosids - ps22
Eurosids - ps23
Malvids - ps24
Sap./Malv./Brassicales - ps25
Malvales/Brassicales - ps26
Brassicales - ps27
Cleomaceae/Brassicaceae - ps28
Brassicaceae - ps29
Camelineae - ps30
Arabidopsis - ps31
Arabidopsis_thaliana - ps32
p-value
4e-13
6e-11
1e-08
2e-06
3e-04
Log-odds
0.5
1.0
1.5
2.0
2.5
3.0
3.5
4.0

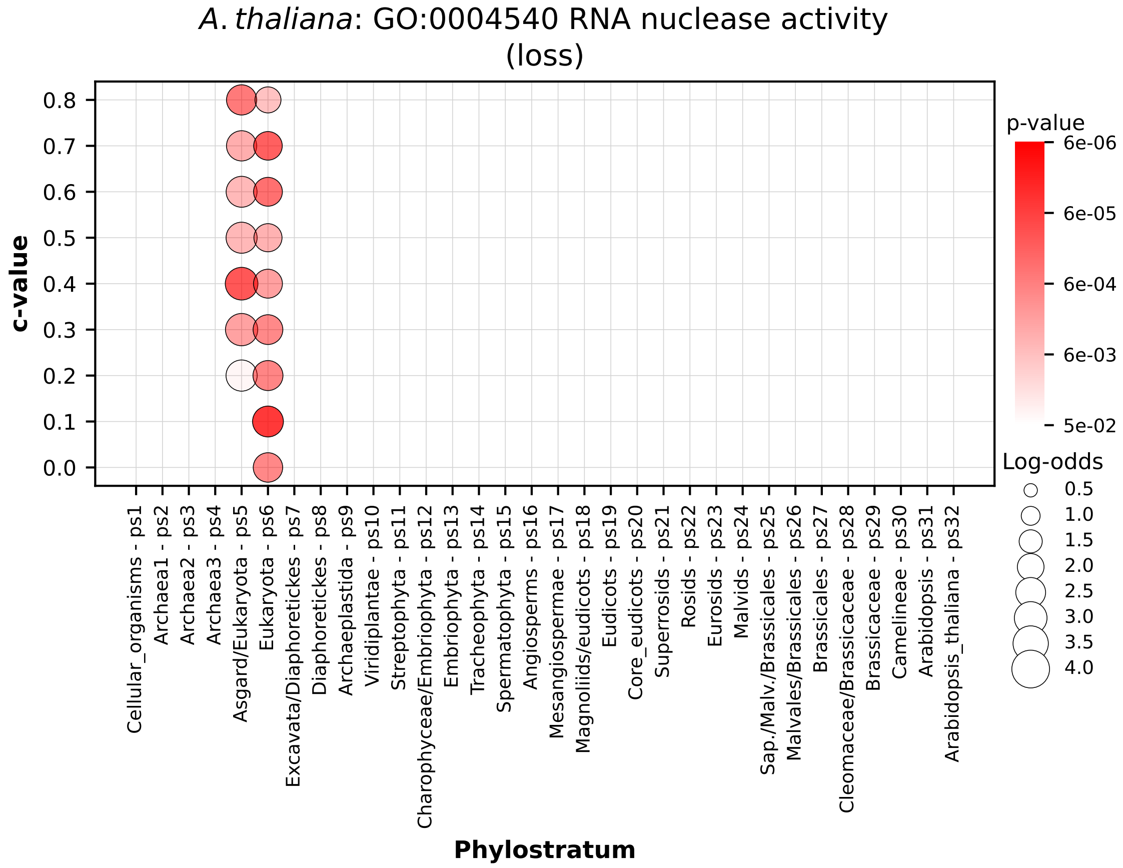

A. thaliana: GO:0004540 RNA nuclease activity
(loss)
c-value
0.8
0.7
0.6
0.5
0.4
0.3
0.2
0.1
0.0
Phylostratum
Cellular_organisms - ps1
Archaea1 - ps2
Archaea2 - ps3
Archaea3 - ps4
Asgard/Eukaryota - ps5
Eukaryota - ps6
Excavata/Diaphoretickes - ps7
Diaphoretickes - ps8
Archaeplastida - ps9
Viridiplantae - ps10
Streptophyta - ps11
Charophyceae/Embriophyta - ps12
Embriophyta - ps13
Tracheophyta - ps14
Spermatophyta - ps15
Angiosperms - ps16
Mesangiospermae - ps17
Magnoliids/eudicots - ps18
Eudicots - ps19
Core_eudicots - ps20
Superrosids - ps21
Rosids - ps22
Eurosids - ps23
Malvids - ps24
Sap./Malv./Brassicales - ps25
Malvales/Brassicales - ps26
Brassicales - ps27
Cleomaceae/Brassicaceae - ps28
Brassicaceae - ps29
Camelineae - ps30
Arabidopsis - ps31
Arabidopsis_thaliana - ps32
p-value
6e-06
6e-05
6e-04
6e-03
5e-02
Log-odds
0.5
1.0
1.5
2.0
2.5
3.0
3.5
4.0

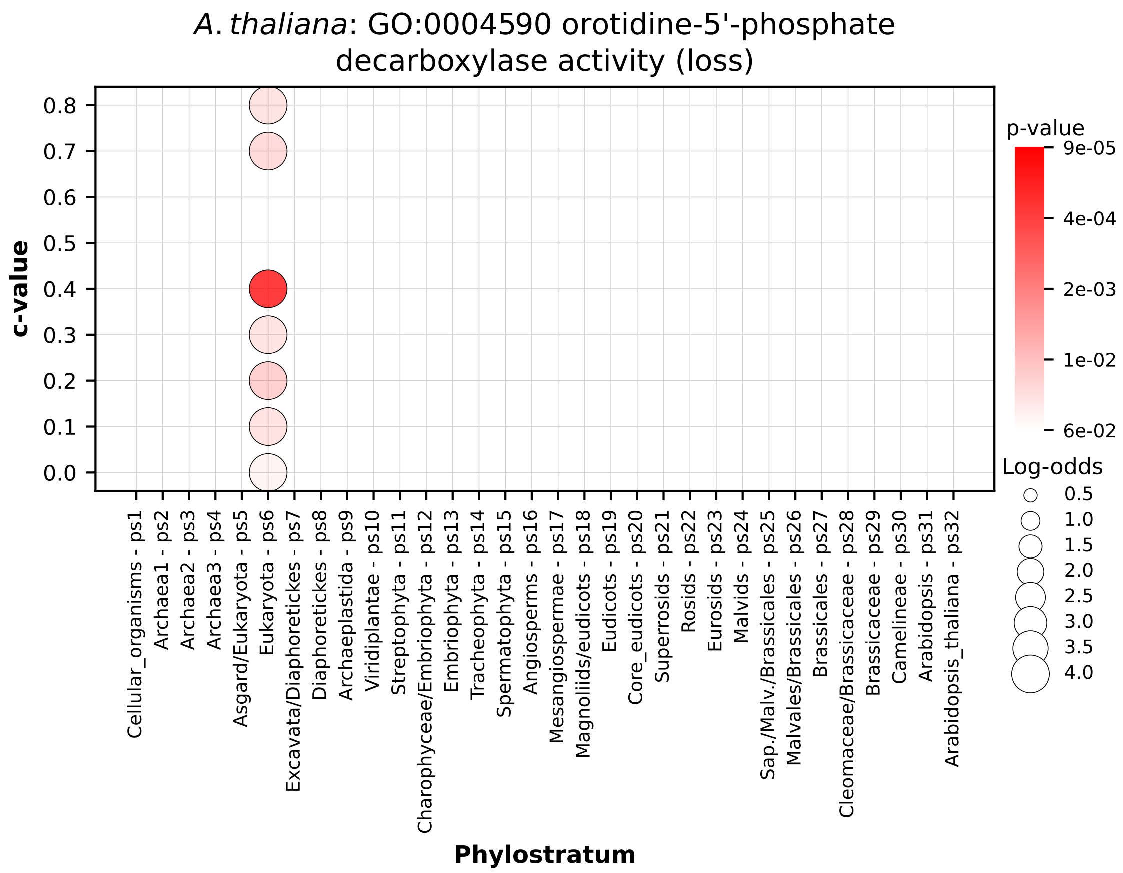

A. thaliana: GO:0004590 orotidine-5'-phosphate decarboxylase activity (loss)
c-value
0.8
0.7
0.6
0.5
0.4
0.3
0.2
0.1
0.0
p-value
9e-05
4e-04
2e-03
1e-02
6e-02
Log-odds
0.5
1.0
1.5
2.0
2.5
3.0
3.5
4.0
Cellular_organisms - ps1
Archaea1 - ps2
Archaea2 - ps3
Archaea3 - ps4
Asgard/Eukaryota - ps5
Eukaryota - ps6
Excavata/Diaphoretickes - ps7
Diaphoretickes - ps8
Archaeplastida - ps9
Viridiplantae - ps10
Streptophyta - ps11
Charophyceae/Embriophyta - ps12
Embriophyta - ps13
Tracheophyta - ps14
Spermatophyta - ps15
Angiosperms - ps16
Mesangiospermae - ps17
Magnoliids/eudicots - ps18
Eudicots - ps19
Core_eudicots - ps20
Superrosids - ps21
Rosids - ps22
Eurosids - ps23
Malvids - ps24
Sap./Malv./Brassicales - ps25
Malvales/Brassicales - ps26
Brassicales - ps27
Cleomaceae/Brassicaceae - ps28
Brassicaceae - ps29
Camelineae - ps30
Arabidopsis - ps31
Arabidopsis_thaliana - ps32
Phylostratum

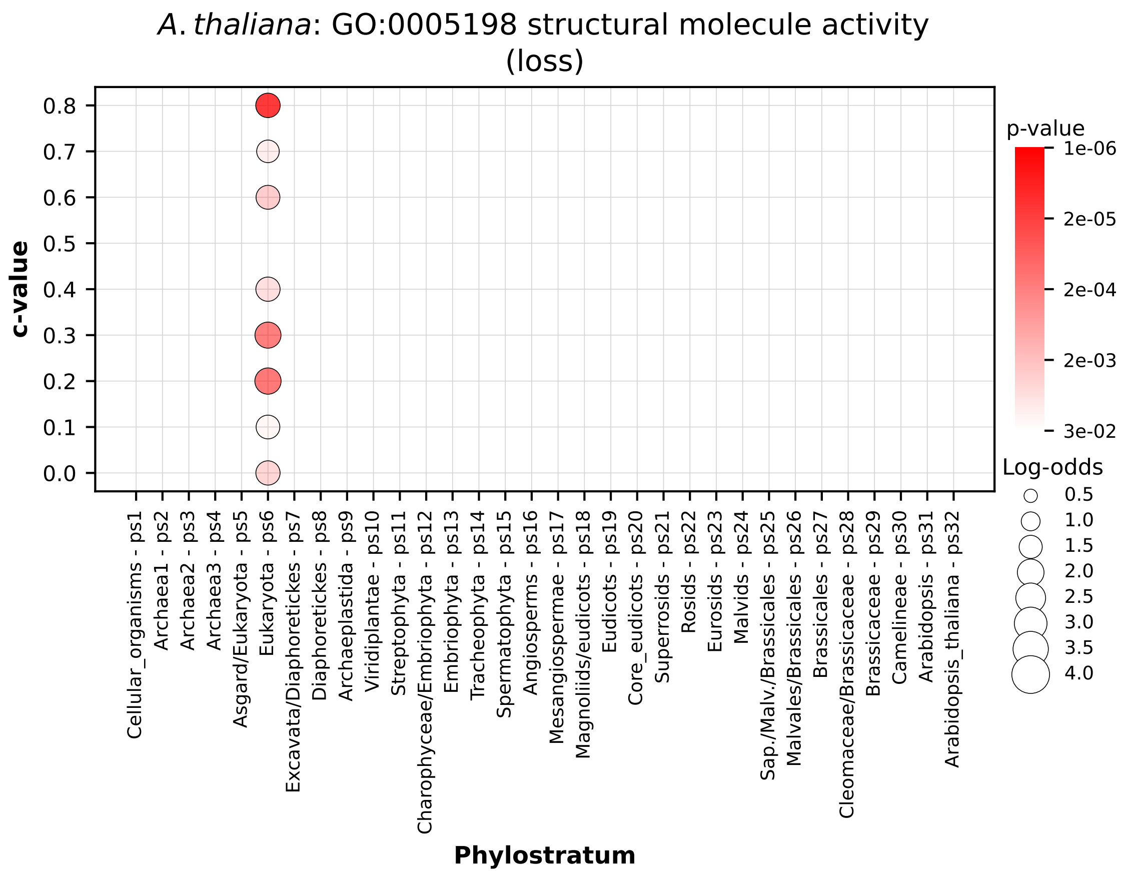

A. thaliana: GO:0005198 structural molecule activity (loss)
c-value
0.8
0.7
0.6
0.5
0.4
0.3
0.2
0.1
0.0
Phylostratum
Cellular_organisms - ps1
Archaea1 - ps2
Archaea2 - ps3
Archaea3 - ps4
Asgard/Eukaryota - ps5
Eukaryota - ps6
Excavata/Diaphoretickes - ps7
Diaphoretickes - ps8
Archaeplastida - ps9
Viridiplantae - ps10
Streptophyta - ps11
Charophyceae/Embriophyta - ps12
Embriophyta - ps13
Tracheophyta - ps14
Spermatophyta - ps15
Angiosperms - ps16
Mesangiospermae - ps17
Magnoliids/eudicots - ps18
Eudicots - ps19
Core_eudicots - ps20
Superrosids - ps21
Rosids - ps22
Eurosids - ps23
Malvids - ps24
Sap./Malv./Brassicales - ps25
Malvales/Brassicales - ps26
Brassicales - ps27
Cleomaceae/Brassicaceae - ps28
Brassicaceae - ps29
Camelineae - ps30
Arabidopsis - ps31
Arabidopsis_thaliana - ps32
p-value
1e-06
2e-05
2e-04
2e-03
3e-02
Log-odds
0.5
1.0
1.5
2.0
2.5
3.0
3.5
4.0

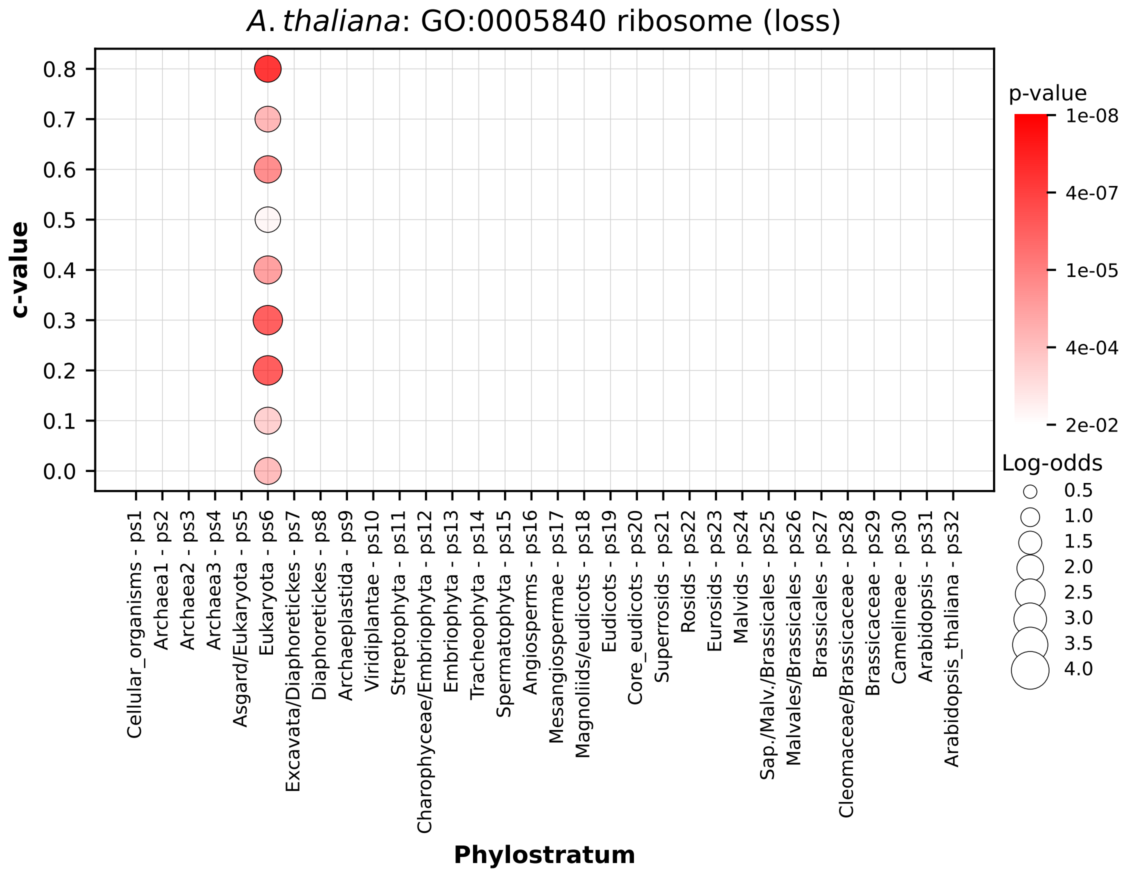

A. thaliana: GO:0005840 ribosome (loss)
c-value
0.8
0.7
0.6
0.5
0.4
0.3
0.2
0.1
0.0
p-value
1e-08
4e-07
1e-05
4e-04
2e-02
Log-odds
0.5
1.0
1.5
2.0
2.5
3.0
3.5
4.0
Cellular_organisms - ps1
Archaea1 - ps2
Archaea2 - ps3
Archaea3 - ps4
Asgard/Eukaryota - ps5
Eukaryota - ps6
Excavata/Diaphoretickes - ps7
Diaphoretickes - ps8
Archaeplastida - ps9
Viridiplantae - ps10
Streptophyta - ps11
Charophyceae/Embriophyta - ps12
Embriophyta - ps13
Tracheophyta - ps14
Spermatophyta - ps15
Angiosperms - ps16
Mesangiospermae - ps17
Magnoliids/eudicots - ps18
Eudicots - ps19
Core_eudicots - ps20
Superrosids - ps21
Rosids - ps22
Eurosids - ps23
Malvids - ps24
Sap./Malv./Brassicales - ps25
Malvales/Brassicales - ps26
Brassicales - ps27
Cleomaceae/Brassicaceae - ps28
Brassicaceae - ps29
Camelineae - ps30
Arabidopsis - ps31
Arabidopsis_thaliana - ps32
Phylostratum

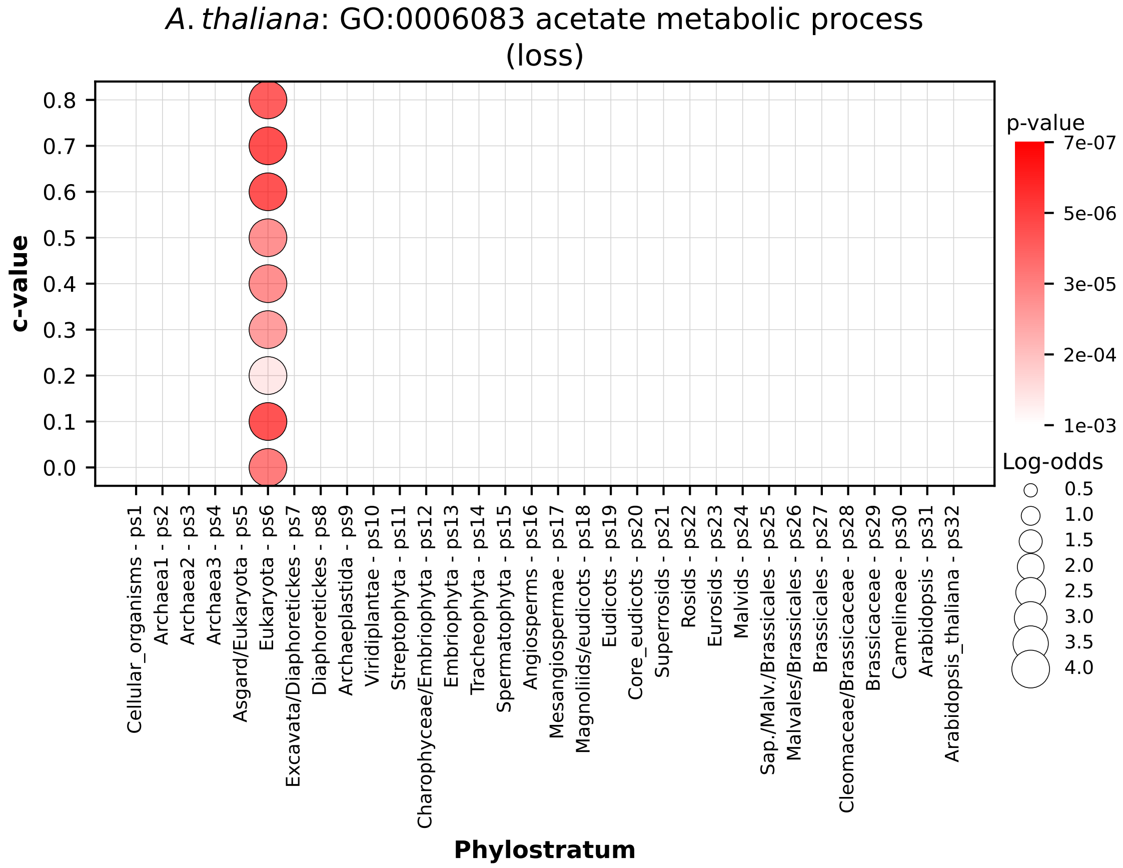

A. thaliana: GO:0006083 acetate metabolic process
(loss)
c-value
0.8
0.7
0.6
0.5
0.4
0.3
0.2
0.1
0.0
p-value
7e-07
5e-06
3e-05
2e-04
1e-03
Log-odds
0.5
1.0
1.5
2.0
2.5
3.0
3.5
4.0
Cellular_organisms - ps1
Archaea1 - ps2
Archaea2 - ps3
Archaea3 - ps4
Asgard/Eukaryota - ps5
Eukaryota - ps6
Excavata/Diaphoretickes - ps7
Diaphoretickes - ps8
Archaeplastida - ps9
Viridiplantae - ps10
Streptophyta - ps11
Charophyceae/Embriophyta - ps12
Embriophyta - ps13
Tracheophyta - ps14
Spermatophyta - ps15
Angiosperms - ps16
Mesangiospermae - ps17
Magnoliids/eudicots - ps18
Eudicots - ps19
Core_eudicots - ps20
Superrosids - ps21
Rosids - ps22
Eurosids - ps23
Malvids - ps24
Sap./Malv./Brassicales - ps25
Malvales/Brassicales - ps26
Brassicales - ps27
Cleomaceae/Brassicaceae - ps28
Brassicaceae - ps29
Camelineae - ps30
Arabidopsis - ps31
Arabidopsis_thaliana - ps32
Phylostratum

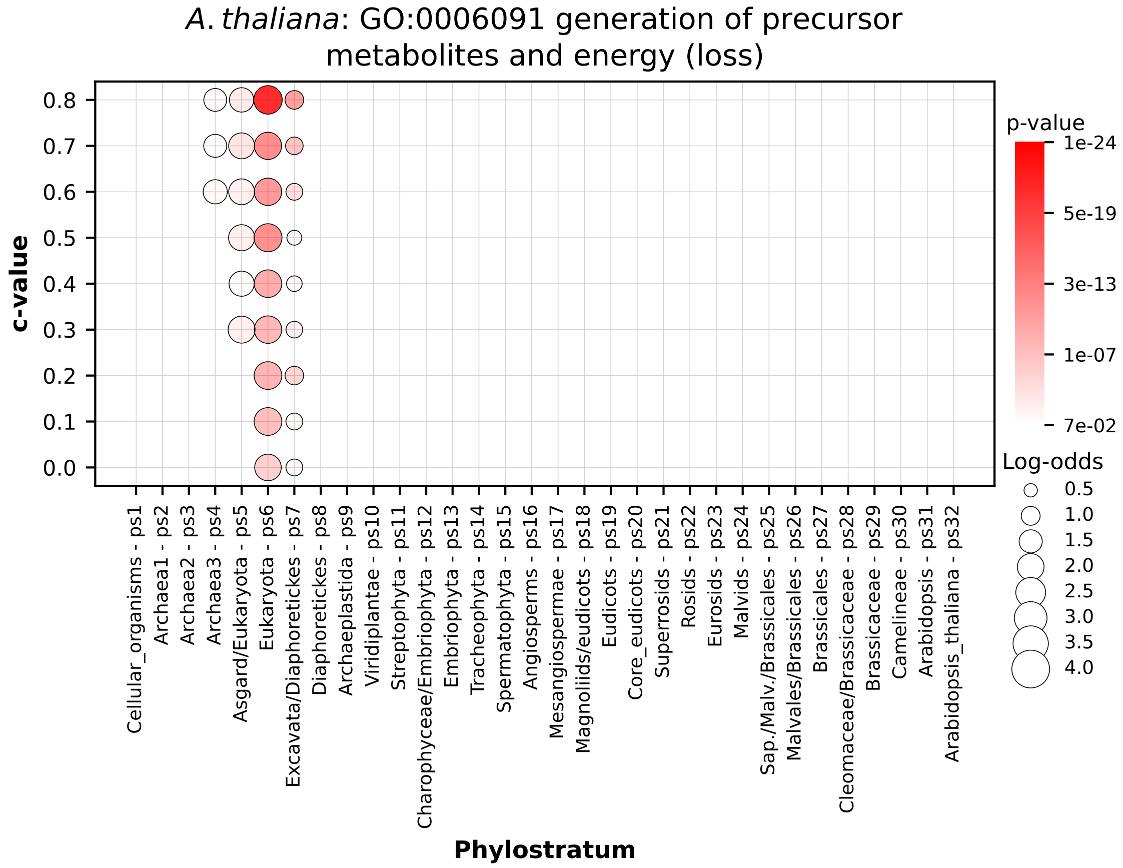
A. thaliana: GO:0006091 generation of precursor metabolites and energy (loss)
c-value
0.8
0.7
0.6
0.5
0.4
0.3
0.2
0.1
0.0
Phylostratum
Cellular_organisms - ps1
Archaea1 - ps2
Archaea2 - ps3
Archaea3 - ps4
Asgard/Eukaryota - ps5
Eukaryota - ps6
Excavata/Diaphoretickes - ps7
Diaphoretickes - ps8
Archaeplastida - ps9
Viridiplantae - ps10
Streptophyta - ps11
Charophyceae/Embriophyta - ps12
Embriophyta - ps13
Tracheophyta - ps14
Spermatophyta - ps15
Angiosperms - ps16
Mesangiospermae - ps17
Magnoliids/eudicots - ps18
Eudicots - ps19
Core_eudicots - ps20
Superrosids - ps21
Rosids - ps22
Eurosids - ps23
Malvids - ps24
Sap./Malv./Brassicales - ps25
Malvales/Brassicales - ps26
Brassicales - ps27
Cleomaceae/Brassicaceae - ps28
Brassicaceae - ps29
Camelineae - ps30
Arabidopsis - ps31
Arabidopsis_thaliana - ps32
p-value
1e-24
5e-19
3e-13
1e-07
7e-02
Log-odds
0.5
1.0
1.5
2.0
2.5
3.0
3.5
4.0

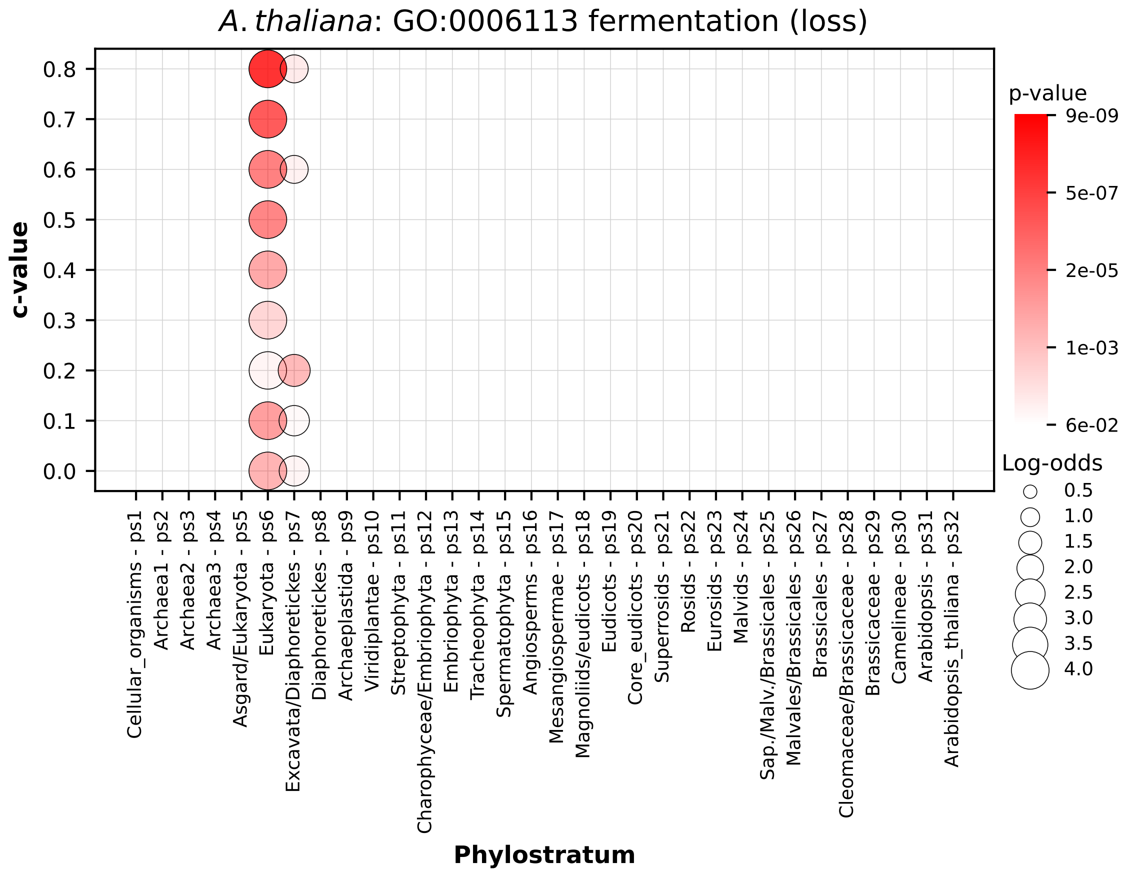

A. thaliana: GO:0006113 fermentation (loss)
c-value
0.8
0.7
0.6
0.5
0.4
0.3
0.2
0.1
0.0
Phylostratum
Cellular_organisms - ps1
Archaea1 - ps2
Archaea2 - ps3
Archaea3 - ps4
Asgard/Eukaryota - ps5
Eukaryota - ps6
Excavata/Diaphoretickes - ps7
Diaphoretickes - ps8
Archaeplastida - ps9
Viridiplantae - ps10
Streptophyta - ps11
Charophyceae/Embriophyta - ps12
Embriophyta - ps13
Tracheophyta - ps14
Spermatophyta - ps15
Angiosperms - ps16
Mesangiospermae - ps17
Magnoliids/eudicots - ps18
Eudicots - ps19
Core_eudicots - ps20
Superrosids - ps21
Rosids - ps22
Eurosids - ps23
Malvids - ps24
Sap./Malv./Brassicales - ps25
Malvales/Brassicales - ps26
Brassicales - ps27
Cleomaceae/Brassicaceae - ps28
Brassicaceae - ps29
Camelineae - ps30
Arabidopsis - ps31
Arabidopsis_thaliana - ps32
p-value
9e-09
5e-07
2e-05
1e-03
6e-02
Log-odds
0.5
1.0
1.5
2.0
2.5
3.0
3.5
4.0

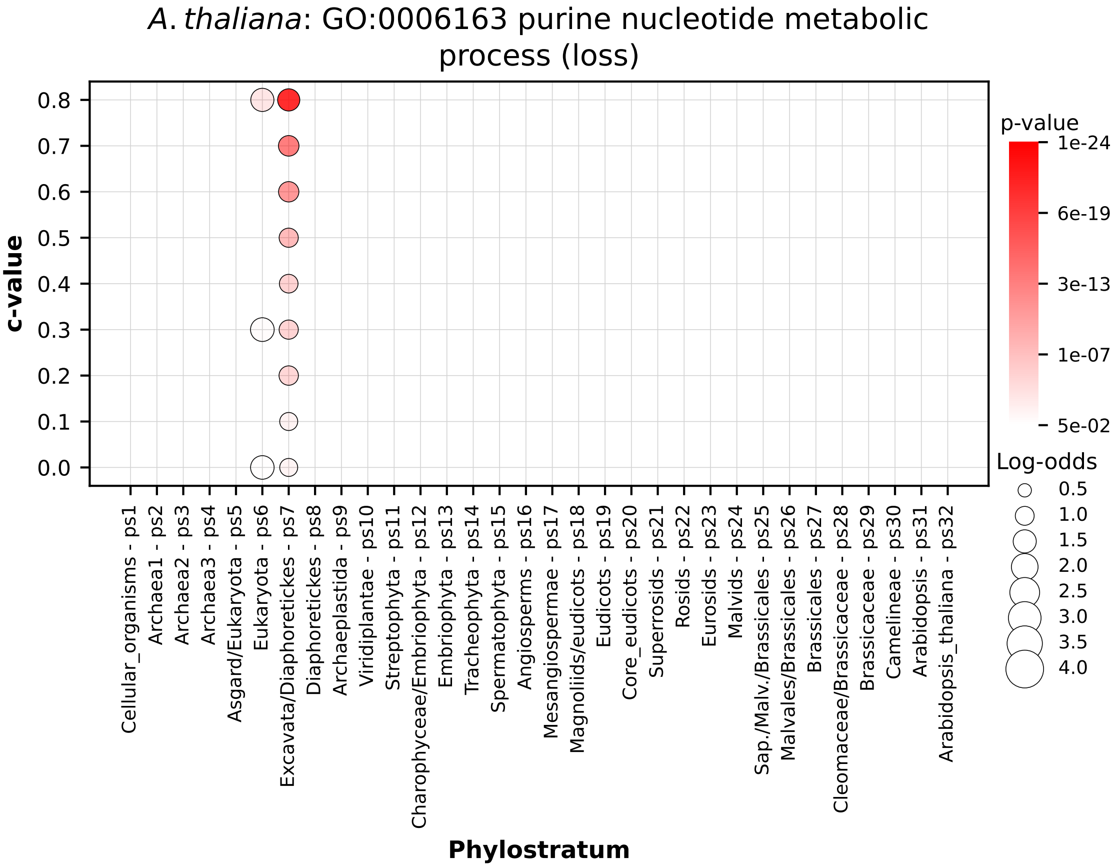

A. thaliana: GO:0006163 purine nucleotide metabolic process (loss)
c-value
0.8
0.7
0.6
0.5
0.4
0.3
0.2
0.1
0.0
p-value
1e-24
6e-19
3e-13
1e-07
5e-02
Log-odds
0.5
1.0
1.5
2.0
2.5
3.0
3.5
4.0
Cellular_organisms - ps1
Archaea1 - ps2
Archaea2 - ps3
Archaea3 - ps4
Asgard/Eukaryota - ps5
Eukaryota - ps6
Excavata/Diaphoretickes - ps7
Diaphoretickes - ps8
Archaeplastida - ps9
Viridiplantae - ps10
Streptophyta - ps11
Charophyceae/Embriophyta - ps12
Embriophyta - ps13
Tracheophyta - ps14
Spermatophyta - ps15
Angiosperms - ps16
Mesangiospermae - ps17
Magnoliids/eudicots - ps18
Eudicots - ps19
Core_eudicots - ps20
Superrosids - ps21
Rosids - ps22
Eurosids - ps23
Malvids - ps24
Sap./Malv./Brassicales - ps25
Malvales/Brassicales - ps26
Brassicales - ps27
Cleomaceae/Brassicaceae - ps28
Brassicaceae - ps29
Camelineae - ps30
Arabidopsis - ps31
Arabidopsis_thaliana - ps32
Phylostratum

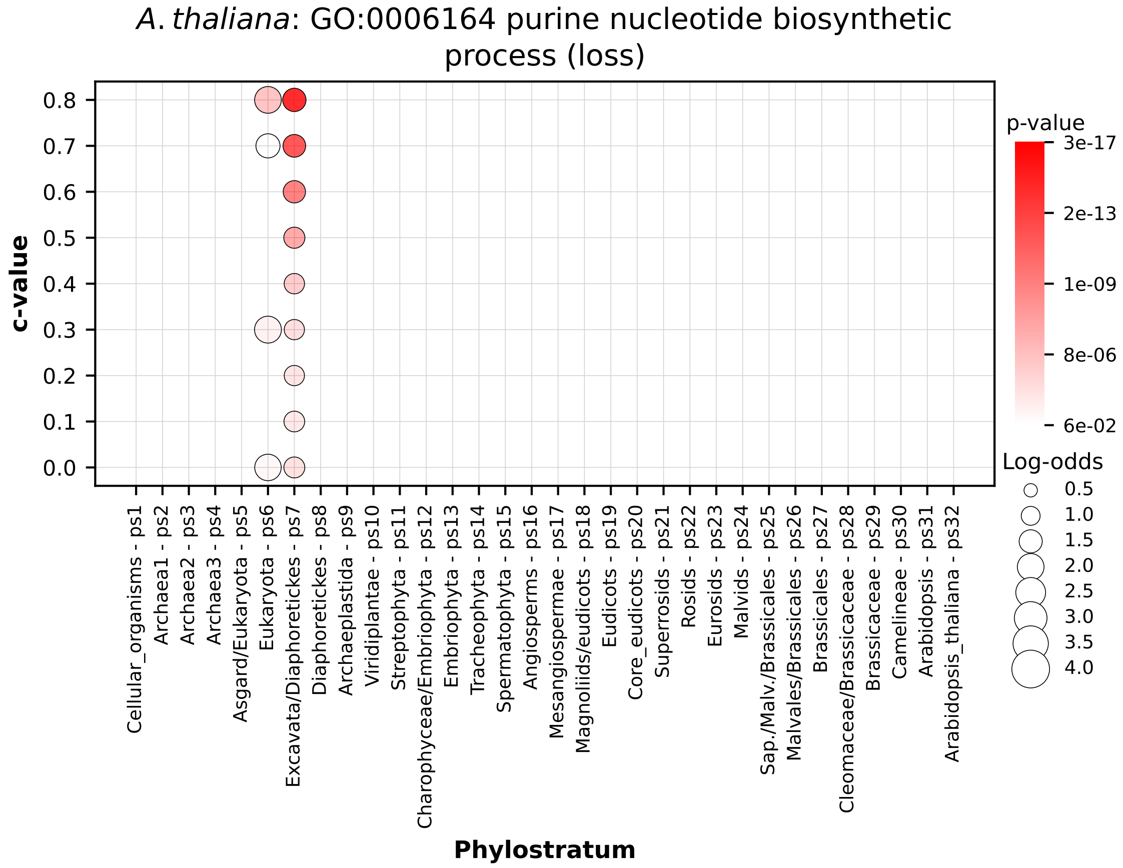
A. thaliana: GO:0006164 purine nucleotide biosynthetic process (loss)
c-value
0.8
0.7
0.6
0.5
0.4
0.3
0.2
0.1
0.0
Phylostratum
Cellular_organisms - ps1
Archaea1 - ps2
Archaea2 - ps3
Archaea3 - ps4
Asgard/Eukaryota - ps5
Eukaryota - ps6
Excavata/Diaphoretickes - ps7
Diaphoretickes - ps8
Archaeplastida - ps9
Viridiplantae - ps10
Streptophyta - ps11
Charophyceae/Embriophyta - ps12
Embriophyta - ps13
Tracheophyta - ps14
Spermatophyta - ps15
Angiosperms - ps16
Mesangiospermae - ps17
Magnoliids/eudicots - ps18
Eudicots - ps19
Core_eudicots - ps20
Superrosids - ps21
Rosids - ps22
Eurosids - ps23
Malvids - ps24
Sap./Malv./Brassicales - ps25
Malvales/Brassicales - ps26
Brassicales - ps27
Cleomaceae/Brassicaceae - ps28
Brassicaceae - ps29
Camelineae - ps30
Arabidopsis - ps31
Arabidopsis_thaliana - ps32
p-value
3e-17
2e-13
1e-09
8e-06
6e-02
Log-odds
0.5
1.0
1.5
2.0
2.5
3.0
3.5
4.0

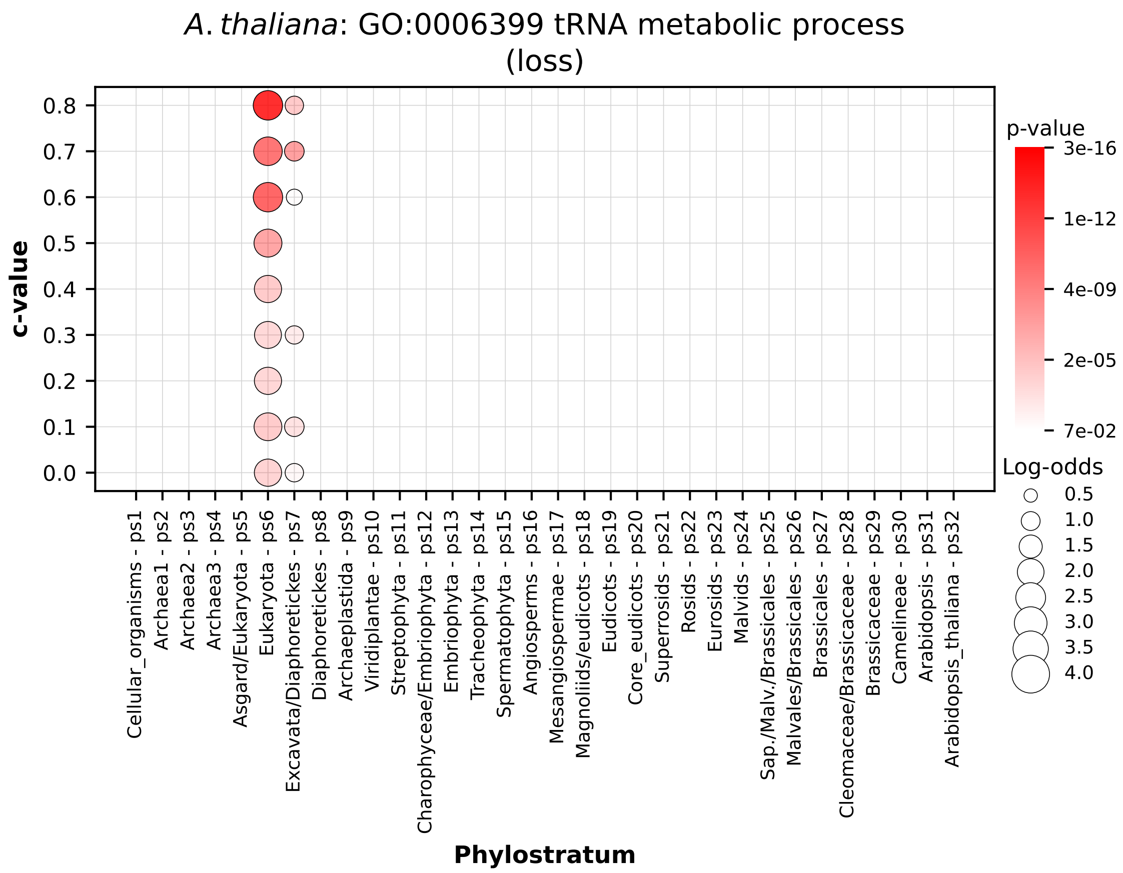

A. thaliana: GO:0006399 tRNA metabolic process
(loss)
c-value
0.8
0.7
0.6
0.5
0.4
0.3
0.2
0.1
0.0
Phylostratum
Cellular_organisms - ps1
Archaea1 - ps2
Archaea2 - ps3
Archaea3 - ps4
Asgard/Eukaryota - ps5
Eukaryota - ps6
Excavata/Diaphoretickes - ps7
Diaphoretickes - ps8
Archaeplastida - ps9
Viridiplantae - ps10
Streptophyta - ps11
Charophyceae/Embriophyta - ps12
Embriophyta - ps13
Tracheophyta - ps14
Spermatophyta - ps15
Angiosperms - ps16
Mesangiospermae - ps17
Magnoliids/eudicots - ps18
Eudicots - ps19
Core_eudicots - ps20
Superrosids - ps21
Rosids - ps22
Eurosids - ps23
Malvids - ps24
Sap./Malv./Brassicales - ps25
Malvales/Brassicales - ps26
Brassicales - ps27
Cleomaceae/Brassicaceae - ps28
Brassicaceae - ps29
Camelineae - ps30
Arabidopsis - ps31
Arabidopsis_thaliana - ps32
p-value
3e-16
1e-12
4e-09
2e-05
7e-02
Log-odds
0.5
1.0
1.5
2.0
2.5
3.0
3.5
4.0

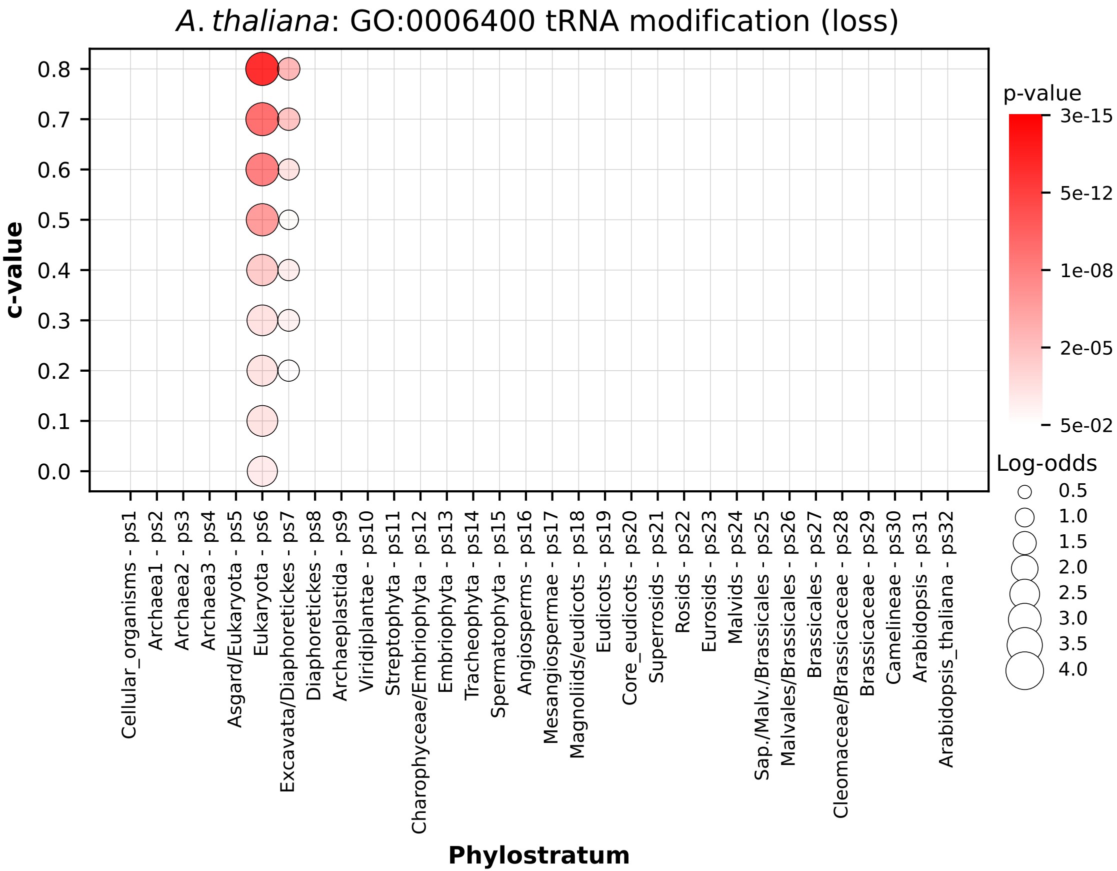

A. thaliana: GO:0006400 tRNA modification (loss)
c-value
0.8
0.7
0.6
0.5
0.4
0.3
0.2
0.1
0.0
p-value
3e-15
5e-12
1e-08
2e-05
5e-02
Log-odds
0.5
1.0
1.5
2.0
2.5
3.0
3.5
4.0
Cellular_organisms - ps1
Archaea1 - ps2
Archaea2 - ps3
Archaea3 - ps4
Asgard/Eukaryota - ps5
Eukaryota - ps6
Excavata/Diaphoretickes - ps7
Diaphoretickes - ps8
Archaeplastida - ps9
Viridiplantae - ps10
Streptophyta - ps11
Charophyceae/Embriophyta - ps12
Embriophyta - ps13
Tracheophyta - ps14
Spermatophyta - ps15
Angiosperms - ps16
Mesangiospermae - ps17
Magnoliids/eudicots - ps18
Eudicots - ps19
Core_eudicots - ps20
Superrosids - ps21
Rosids - ps22
Eurosids - ps23
Malvids - ps24
Sap./Malv./Brassicales - ps25
Malvales/Brassicales - ps26
Brassicales - ps27
Cleomaceae/Brassicaceae - ps28
Brassicaceae - ps29
Camelineae - ps30
Arabidopsis - ps31
Arabidopsis_thaliana - ps32
Phylostratum

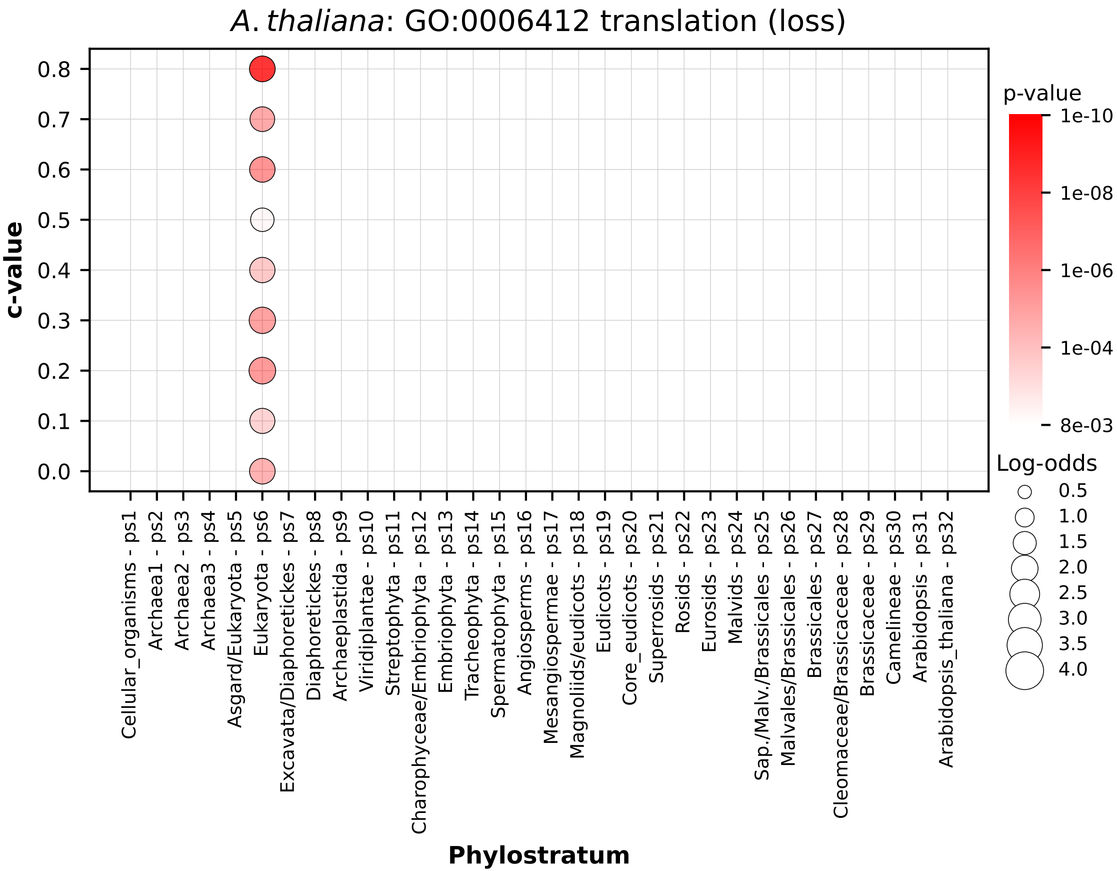

A. thaliana: GO:0006412 translation (loss)
c-value
0.8
0.7
0.6
0.5
0.4
0.3
0.2
0.1
0.0
p-value
1e-10
1e-08
1e-06
1e-04
8e-03
Log-odds
0.5
1.0
1.5
2.0
2.5
3.0
3.5
4.0
Cellular_organisms - ps1
Archaea1 - ps2
Archaea2 - ps3
Archaea3 - ps4
Asgard/Eukaryota - ps5
Eukaryota - ps6
Excavata/Diaphoretickes - ps7
Diaphoretickes - ps8
Archaeplastida - ps9
Viridiplantae - ps10
Streptophyta - ps11
Charophyceae/Embriophyta - ps12
Embriophyta - ps13
Tracheophyta - ps14
Spermatophyta - ps15
Angiosperms - ps16
Mesangiospermae - ps17
Magnoliids/eudicots - ps18
Eudicots - ps19
Core_eudicots - ps20
Superrosids - ps21
Rosids - ps22
Eurosids - ps23
Malvids - ps24
Sap./Malv./Brassicales - ps25
Malvales/Brassicales - ps26
Brassicales - ps27
Cleomaceae/Brassicaceae - ps28
Brassicaceae - ps29
Camelineae - ps30
Arabidopsis - ps31
Arabidopsis_thaliana - ps32
Phylostratum

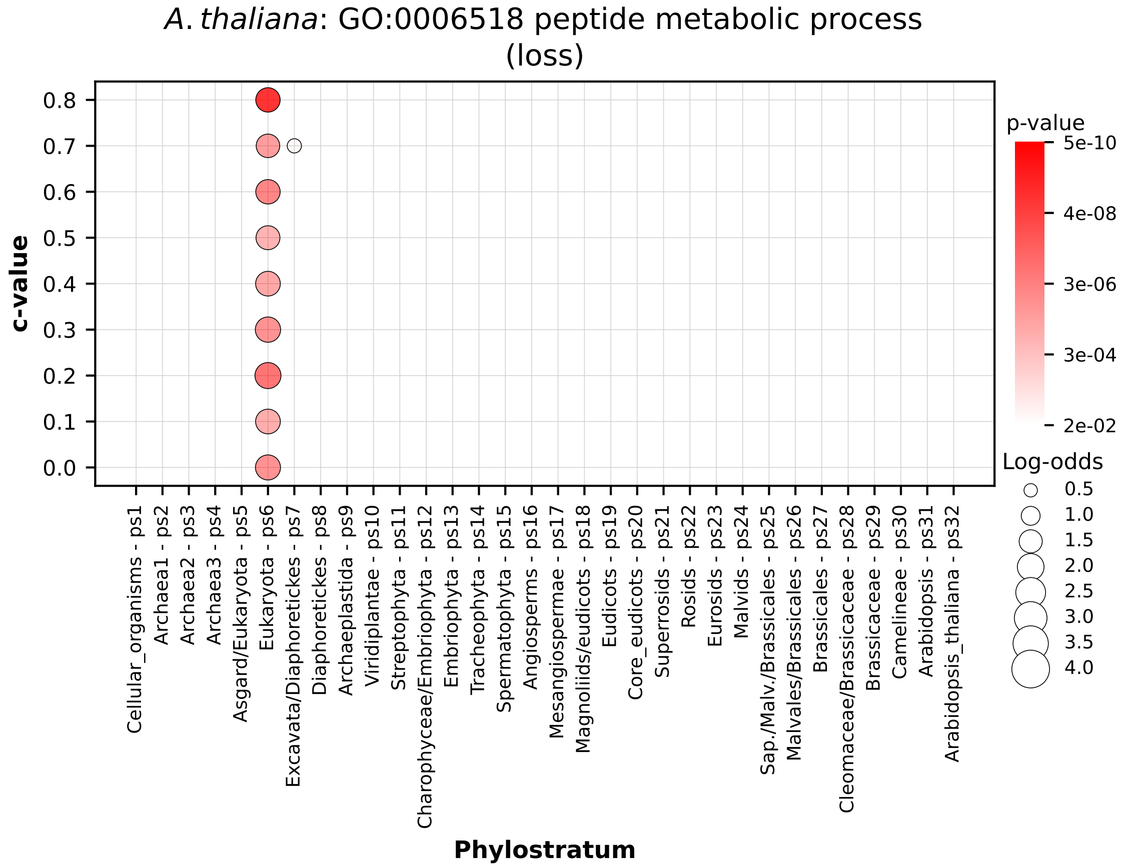

A. thaliana: GO:0006518 peptide metabolic process (loss)
c-value
0.0
0.1
0.2
0.3
0.4
0.5
0.6
0.7
0.8
Phylostratum
Cellular_organisms - ps1
Archaea1 - ps2
Archaea2 - ps3
Archaea3 - ps4
Asgard/Eukaryota - ps5
Eukaryota - ps6
Excavata/Diaphoretickes - ps7
Diaphoretickes - ps8
Archaeplastida - ps9
Viridiplantae - ps10
Streptophyta - ps11
Charophyceae/Embriophyta - ps12
Embriophyta - ps13
Tracheophyta - ps14
Spermatophyta - ps15
Angiosperms - ps16
Mesangiospermae - ps17
Magnoliids/eudicots - ps18
Eudicots - ps19
Core_eudicots - ps20
Superrosids - ps21
Rosids - ps22
Eurosids - ps23
Malvids - ps24
Sap./Malv./Brassicales - ps25
Malvales/Brassicales - ps26
Brassicales - ps27
Cleomaceae/Brassicaceae - ps28
Brassicaceae - ps29
Camelineae - ps30
Arabidopsis - ps31
Arabidopsis_thaliana - ps32
p-value
5e-10
4e-08
3e-06
3e-04
2e-02
Log-odds
0.5
1.0
1.5
2.0
2.5
3.0
3.5
4.0

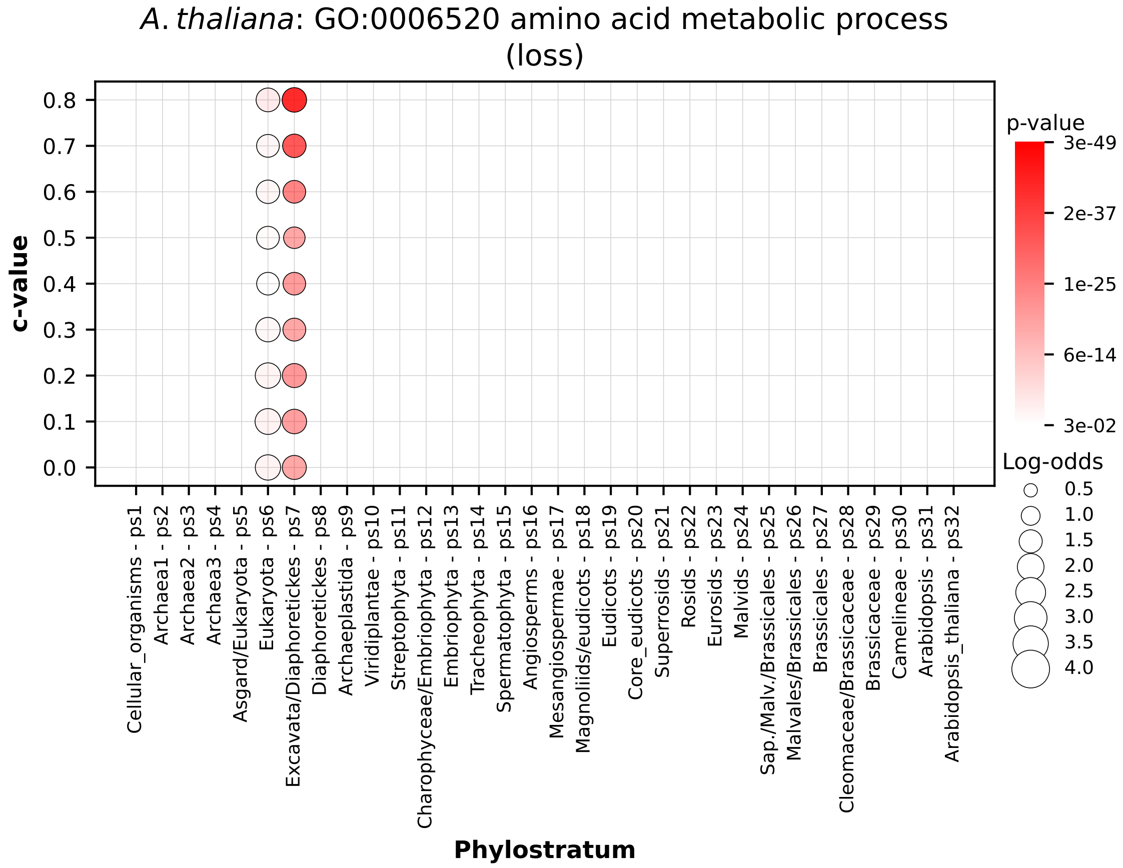
A. thaliana: GO:0006520 amino acid metabolic process
(loss)
c-value
0.8
0.7
0.6
0.5
0.4
0.3
0.2
0.1
0.0
p-value
3e-49
2e-37
1e-25
6e-14
3e-02
Log-odds
0.5
1.0
1.5
2.0
2.5
3.0
3.5
4.0
Cellular_organisms - ps1
Archaea1 - ps2
Archaea2 - ps3
Archaea3 - ps4
Asgard/Eukaryota - ps5
Eukaryota - ps6
Excavata/Diaphoretickes - ps7
Diaphoretickes - ps8
Archaeplastida - ps9
Viridiplantae - ps10
Streptophyta - ps11
Charophyceae/Embriophyta - ps12
Embriophyta - ps13
Tracheophyta - ps14
Spermatophyta - ps15
Angiosperms - ps16
Mesangiospermae - ps17
Magnoliids/eudicots - ps18
Eudicots - ps19
Core_eudicots - ps20
Superrosids - ps21
Rosids - ps22
Eurosids - ps23
Malvids - ps24
Sap./Malv./Brassicales - ps25
Malvales/Brassicales - ps26
Brassicales - ps27
Cleomaceae/Brassicaceae - ps28
Brassicaceae - ps29
Camelineae - ps30
Arabidopsis - ps31
Arabidopsis_thaliana - ps32
Phylostratum

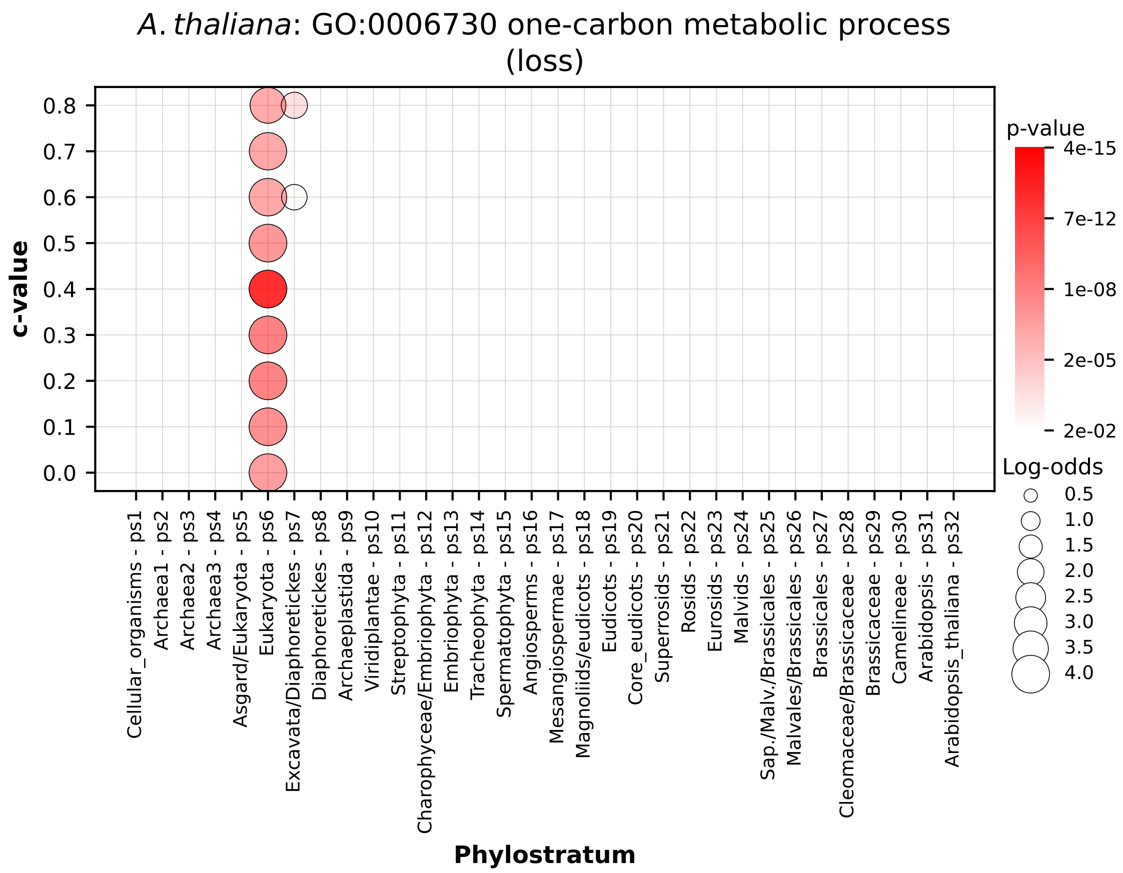

A. thaliana: GO:0006730 one-carbon metabolic process
(loss)
c-value
0.8
0.7
0.6
0.5
0.4
0.3
0.2
0.1
0.0
Phylostratum
Cellular_organisms - ps1
Archaea1 - ps2
Archaea2 - ps3
Archaea3 - ps4
Asgard/Eukaryota - ps5
Eukaryota - ps6
Excavata/Diaphoretickes - ps7
Diaphoretickes - ps8
Archaeplastida - ps9
Viridiplantae - ps10
Streptophyta - ps11
Charophyceae/Embriophyta - ps12
Embriophyta - ps13
Tracheophyta - ps14
Spermatophyta - ps15
Angiosperms - ps16
Mesangiospermae - ps17
Magnoliids/eudicots - ps18
Eudicots - ps19
Core_eudicots - ps20
Superrosids - ps21
Rosids - ps22
Eurosids - ps23
Malvids - ps24
Sap./Malv./Brassicales - ps25
Malvales/Brassicales - ps26
Brassicales - ps27
Cleomaceae/Brassicaceae - ps28
Brassicaceae - ps29
Camelineae - ps30
Arabidopsis - ps31
Arabidopsis_thaliana - ps32
p-value
4e-15
7e-12
1e-08
2e-05
2e-02
Log-odds
0.5
1.0
1.5
2.0
2.5
3.0
3.5
4.0

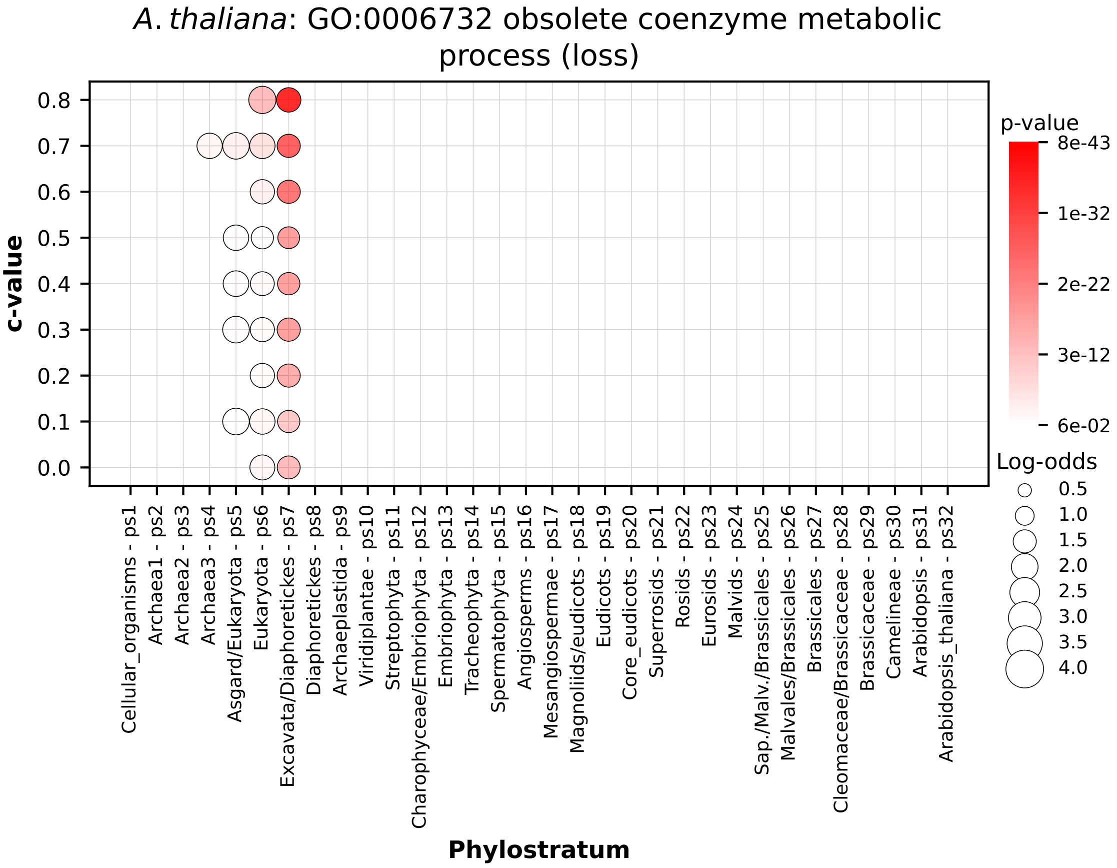

A. thaliana: GO:0006732 obsolete coenzyme metabolic process (loss)
c-value
0.8
0.7
0.6
0.5
0.4
0.3
0.2
0.1
0.0
p-value
8e-43
1e-32
2e-22
3e-12
6e-02
Log-odds
0.5
1.0
1.5
2.0
2.5
3.0
3.5
4.0
Cellular_organisms - ps1
Archaea1 - ps2
Archaea2 - ps3
Archaea3 - ps4
Asgard/Eukaryota - ps5
Eukaryota - ps6
Excavata/Diaphoretickes - ps7
Diaphoretickes - ps8
Archaeplastida - ps9
Viridiplantae - ps10
Streptophyta - ps11
Charophyceae/Embriophyta - ps12
Embriophyta - ps13
Tracheophyta - ps14
Spermatophyta - ps15
Angiosperms - ps16
Mesangiospermae - ps17
Magnoliids/eudicots - ps18
Eudicots - ps19
Core_eudicots - ps20
Superrosids - ps21
Rosids - ps22
Eurosids - ps23
Malvids - ps24
Sap./Malv./Brassicales - ps25
Malvales/Brassicales - ps26
Brassicales - ps27
Cleomaceae/Brassicaceae - ps28
Brassicaceae - ps29
Camelineae - ps30
Arabidopsis - ps31
Arabidopsis_thaliana - ps32
Phylostratum

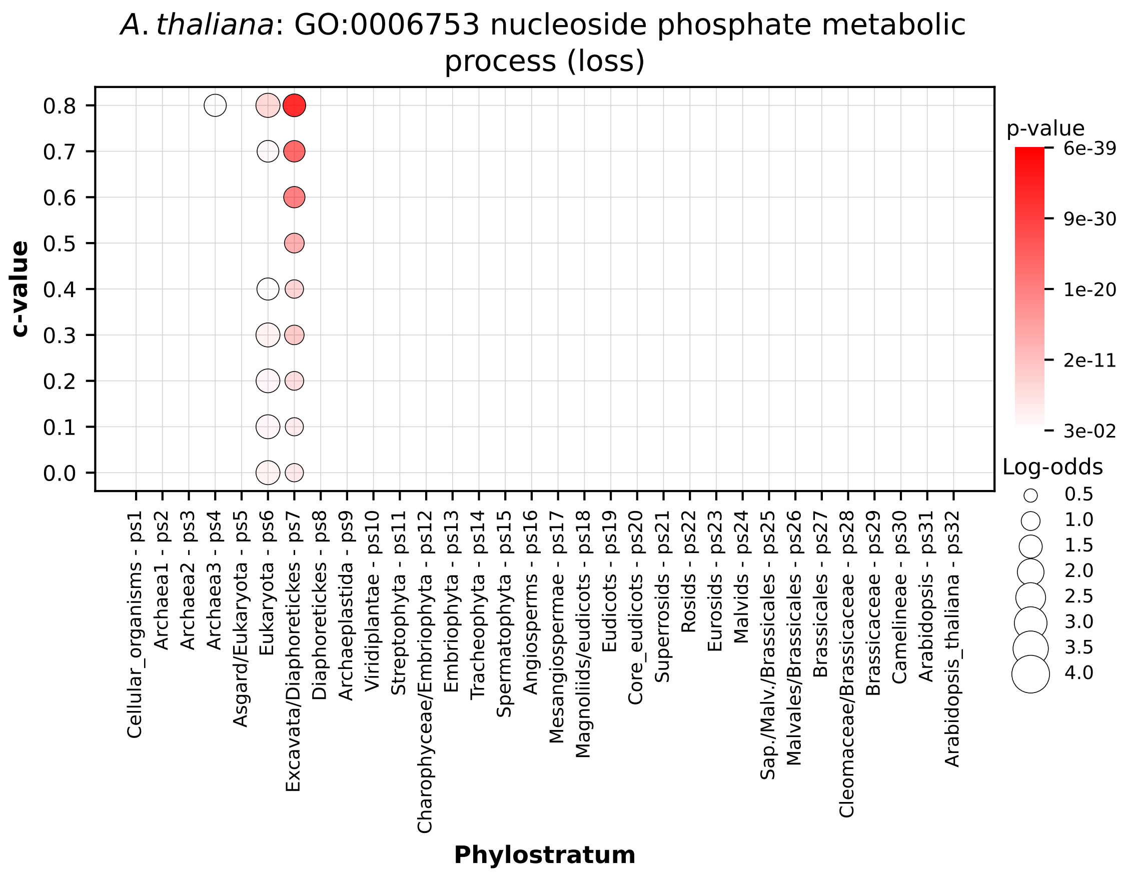

A. thaliana: GO:0006753 nucleoside phosphate metabolic process (loss)
c-value
0.8
0.7
0.6
0.5
0.4
0.3
0.2
0.1
0.0
Phylostratum
Cellular_organisms - ps1
Archaea1 - ps2
Archaea2 - ps3
Archaea3 - ps4
Asgard/Eukaryota - ps5
Eukaryota - ps6
Excavata/Diaphoretickes - ps7
Diaphoretickes - ps8
Archaeplastida - ps9
Viridiplantae - ps10
Streptophyta - ps11
Charophyceae/Embriophyta - ps12
Embriophyta - ps13
Tracheophyta - ps14
Spermatophyta - ps15
Angiosperms - ps16
Mesangiospermae - ps17
Magnoliids/eudicots - ps18
Eudicots - ps19
Core_eudicots - ps20
Superrosids - ps21
Rosids - ps22
Eurosids - ps23
Malvids - ps24
Sap./Malv./Brassicales - ps25
Malvales/Brassicales - ps26
Brassicales - ps27
Cleomaceae/Brassicaceae - ps28
Brassicaceae - ps29
Camelineae - ps30
Arabidopsis - ps31
Arabidopsis_thaliana - ps32
p-value
6e-39
9e-30
1e-20
2e-11
3e-02
Log-odds
0.5
1.0
1.5
2.0
2.5
3.0
3.5
4.0

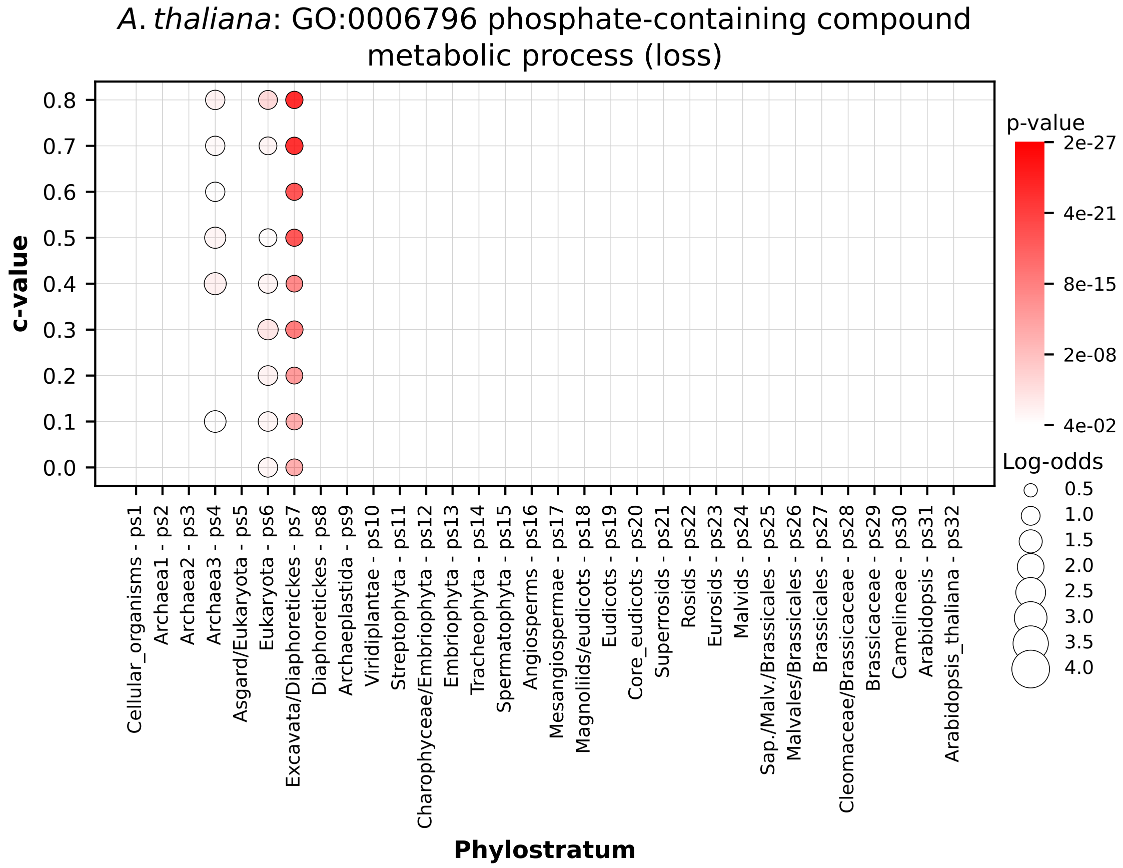
A. thaliana: GO:0006796 phosphate-containing compound metabolic process (loss)
c-value
0.8
0.7
0.6
0.5
0.4
0.3
0.2
0.1
0.0
p-value
2e-27
4e-21
8e-15
2e-08
4e-02
Log-odds
0.5
1.0
1.5
2.0
2.5
3.0
3.5
4.0
Cellular_organisms - ps1
Archaea1 - ps2
Archaea2 - ps3
Archaea3 - ps4
Asgard/Eukaryota - ps5
Eukaryota - ps6
Excavata/Diaphoretickes - ps7
Diaphoretickes - ps8
Archaeplastida - ps9
Viridiplantae - ps10
Streptophyta - ps11
Charophyceae/Embriophyta - ps12
Embriophyta - ps13
Tracheophyta - ps14
Spermatophyta - ps15
Angiosperms - ps16
Mesangiospermae - ps17
Magnoliids/eudicots - ps18
Eudicots - ps19
Core_eudicots - ps20
Superrosids - ps21
Rosids - ps22
Eurosids - ps23
Malvids - ps24
Sap./Malv./Brassicales - ps25
Malvales/Brassicales - ps26
Brassicales - ps27
Cleomaceae/Brassicaceae - ps28
Brassicaceae - ps29
Camelineae - ps30
Arabidopsis - ps31
Arabidopsis_thaliana - ps32
Phylostratum

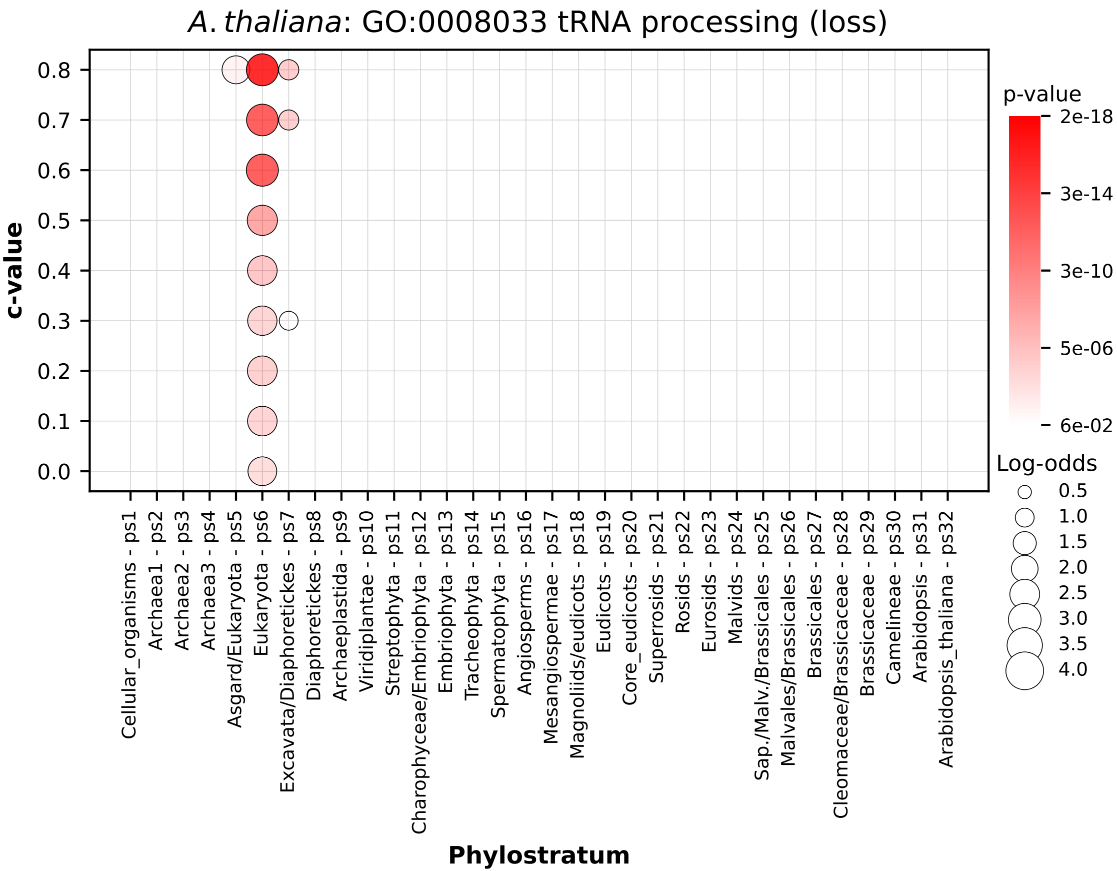

A. thaliana: GO:0008033 tRNA processing (loss)
c-value
0.0
0.1
0.2
0.3
0.4
0.5
0.6
0.7
0.8
Phylostratum
Cellular_organisms - ps1
Archaea1 - ps2
Archaea2 - ps3
Archaea3 - ps4
Asgard/Eukaryota - ps5
Eukaryota - ps6
Excavata/Diaphoretickes - ps7
Diaphoretickes - ps8
Archaeplastida - ps9
Viridiplantae - ps10
Streptophyta - ps11
Charophyceae/Embriophyta - ps12
Embriophyta - ps13
Tracheophyta - ps14
Spermatophyta - ps15
Angiosperms - ps16
Mesangiospermae - ps17
Magnoliids/eudicots - ps18
Eudicots - ps19
Core_eudicots - ps20
Superrosids - ps21
Rosids - ps22
Eurosids - ps23
Malvids - ps24
Sap./Malv./Brassicales - ps25
Malvales/Brassicales - ps26
Brassicales - ps27
Cleomaceae/Brassicaceae - ps28
Brassicaceae - ps29
Camelineae - ps30
Arabidopsis - ps31
Arabidopsis_thaliana - ps32
p-value
2e-18
3e-14
3e-10
5e-06
6e-02
Log-odds
0.5
1.0
1.5
2.0
2.5
3.0
3.5
4.0

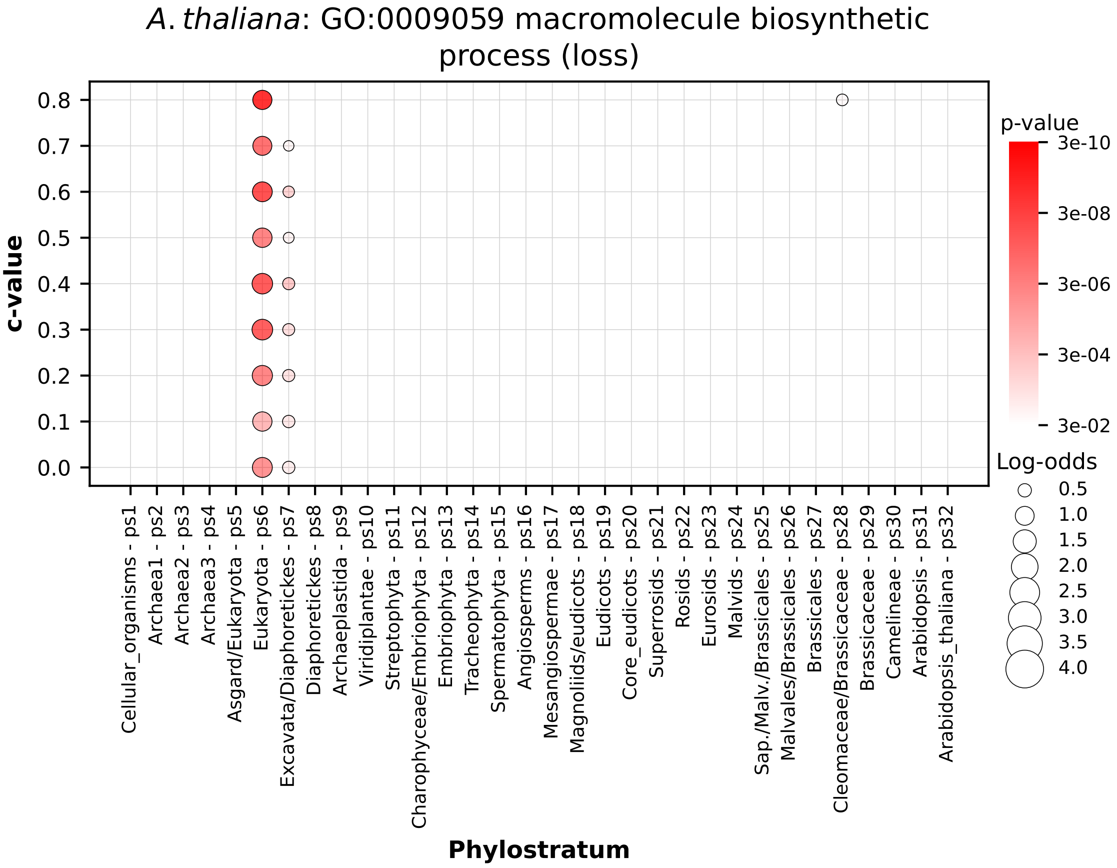
A. thaliana: GO:0009059 macromolecule biosynthetic process (loss)
c-value
0.8
0.7
0.6
0.5
0.4
0.3
0.2
0.1
0.0
p-value
3e-10
3e-08
3e-06
3e-04
3e-02
Log-odds
0.5
1.0
1.5
2.0
2.5
3.0
3.5
4.0
Cellular_organisms - ps1
Archaea1 - ps2
Archaea2 - ps3
Archaea3 - ps4
Asgard/Eukaryota - ps5
Eukaryota - ps6
Excavata/Diaphoretickes - ps7
Diaphoretickes - ps8
Archaeplastida - ps9
Viridiplantae - ps10
Streptophyta - ps11
Charophyceae/Embriophyta - ps12
Embriophyta - ps13
Tracheophyta - ps14
Spermatophyta - ps15
Angiosperms - ps16
Mesangiospermae - ps17
Magnoliids/eudicots - ps18
Eudicots - ps19
Core_eudicots - ps20
Superrosids - ps21
Rosids - ps22
Eurosids - ps23
Malvids - ps24
Sap./Malv./Brassicales - ps25
Malvales/Brassicales - ps26
Brassicales - ps27
Cleomaceae/Brassicaceae - ps28
Brassicaceae - ps29
Camelineae - ps30
Arabidopsis - ps31
Arabidopsis_thaliana - ps32
Phylostratum

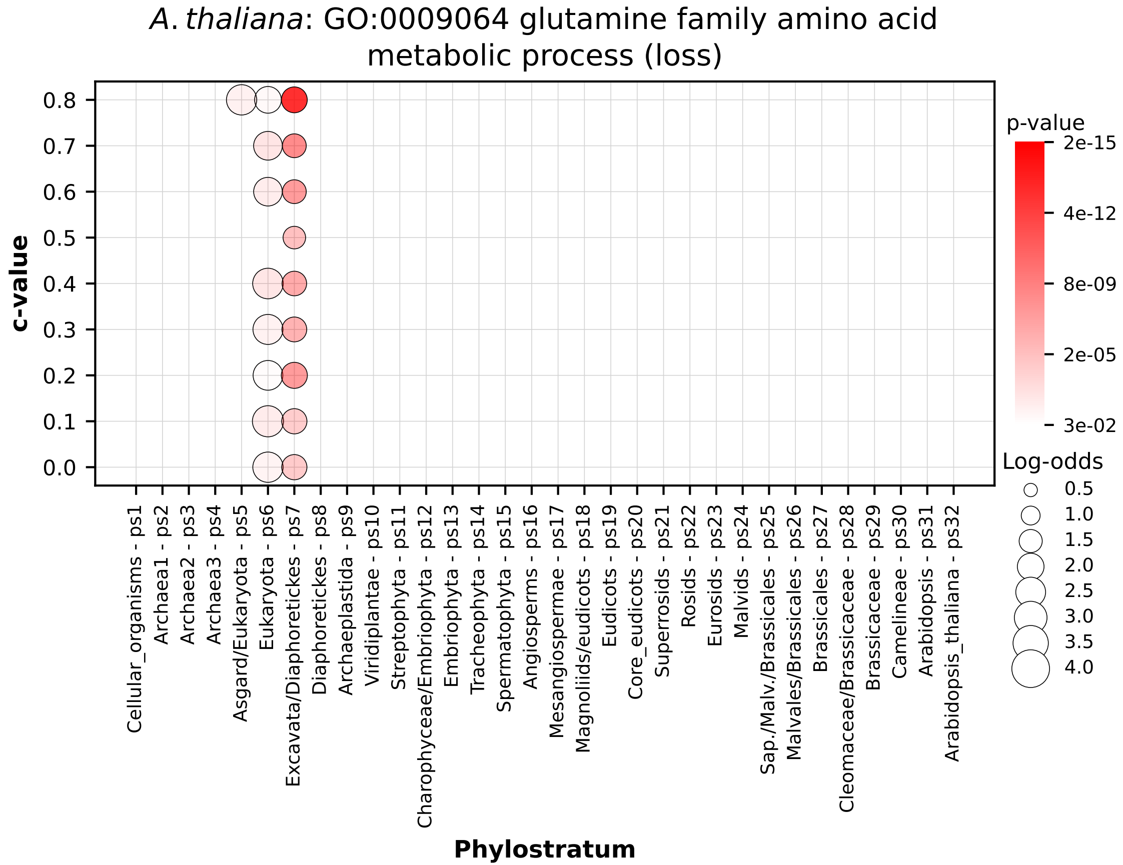

$A. thaliana$: GO:0009064 glutamine family amino acid metabolic process (loss)
c-value
0.8
0.7
0.6
0.5
0.4
0.3
0.2
0.1
0.0
p-value
2e-15
4e-12
8e-09
2e-05
3e-02
Log-odds
0.5
1.0
1.5
2.0
2.5
3.0
3.5
4.0
Cellular_organisms - ps1
Archaea1 - ps2
Archaea2 - ps3
Archaea3 - ps4
Asgard/Eukaryota - ps5
Eukaryota - ps6
Excavata/Diaphoretickes - ps7
Diaphoretickes - ps8
Archaeplastida - ps9
Viridiplantae - ps10
Streptophyta - ps11
Charophyceae/Embriophyta - ps12
Embriophyta - ps13
Tracheophyta - ps14
Spermatophyta - ps15
Angiosperms - ps16
Mesangiospermae - ps17
Magnoliids/eudicots - ps18
Eudicots - ps19
Core_eudicots - ps20
Superrosids - ps21
Rosids - ps22
Eurosids - ps23
Malvids - ps24
Sap./Malv./Brassicales - ps25
Malvales/Brassicales - ps26
Brassicales - ps27
Cleomaceae/Brassicaceae - ps28
Brassicaceae - ps29
Camelineae - ps30
Arabidopsis - ps31
Arabidopsis_thaliana - ps32
Phylostratum

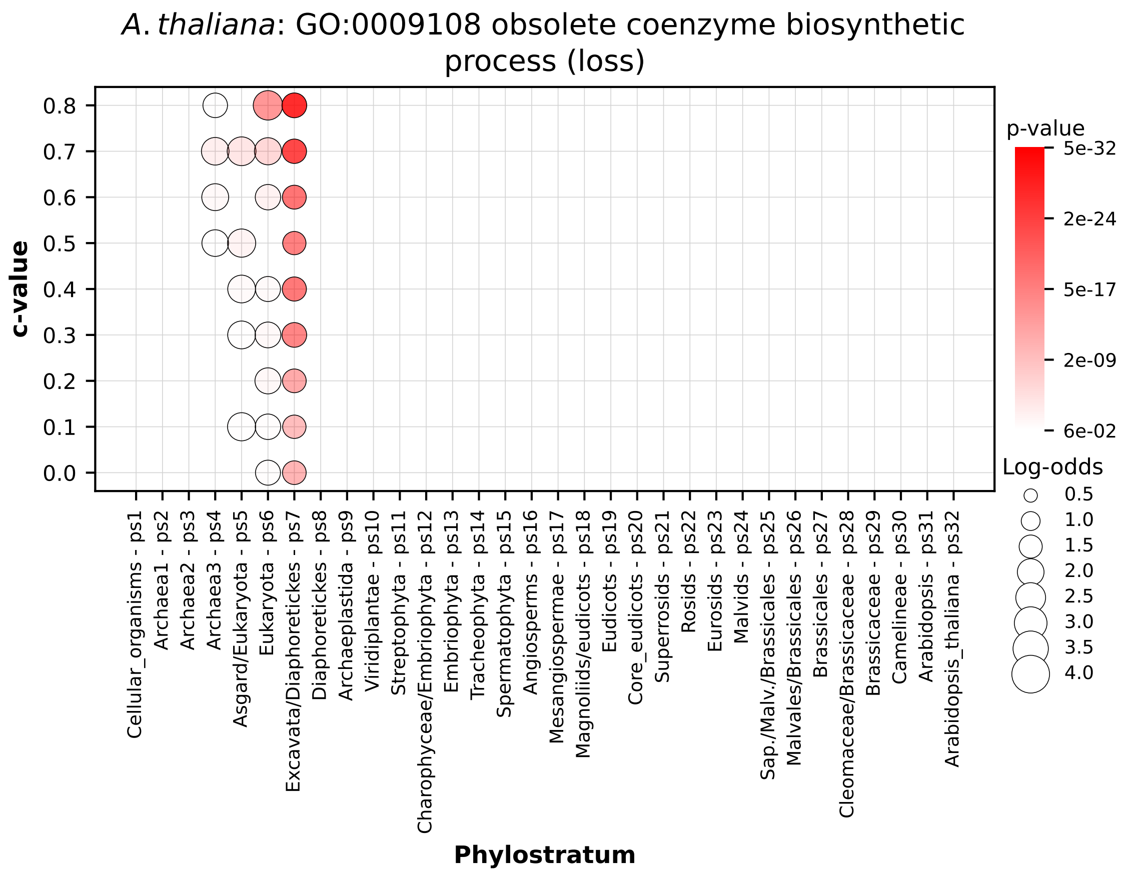
A. thaliana: GO:0009108 obsolete coenzyme biosynthetic process (loss)
c-value
0.8
0.7
0.6
0.5
0.4
0.3
0.2
0.1
0.0
Phylostratum
Cellular_organisms - ps1
Archaea1 - ps2
Archaea2 - ps3
Archaea3 - ps4
Asgard/Eukaryota - ps5
Eukaryota - ps6
Excavata/Diaphoretickes - ps7
Diaphoretickes - ps8
Archaeplastida - ps9
Viridiplantae - ps10
Streptophyta - ps11
Charophyceae/Embriophyta - ps12
Embriophyta - ps13
Tracheophyta - ps14
Spermatophyta - ps15
Angiosperms - ps16
Mesangiospermae - ps17
Magnoliids/eudicots - ps18
Eudicots - ps19
Core_eudicots - ps20
Superrosids - ps21
Rosids - ps22
Eurosids - ps23
Malvids - ps24
Sap./Malv./Brassicales - ps25
Malvales/Brassicales - ps26
Brassicales - ps27
Cleomaceae/Brassicaceae - ps28
Brassicaceae - ps29
Camelineae - ps30
Arabidopsis - ps31
Arabidopsis_thaliana - ps32
p-value
5e-32
2e-24
5e-17
2e-09
6e-02
Log-odds
0.5
1.0
1.5
2.0
2.5
3.0
3.5
4.0

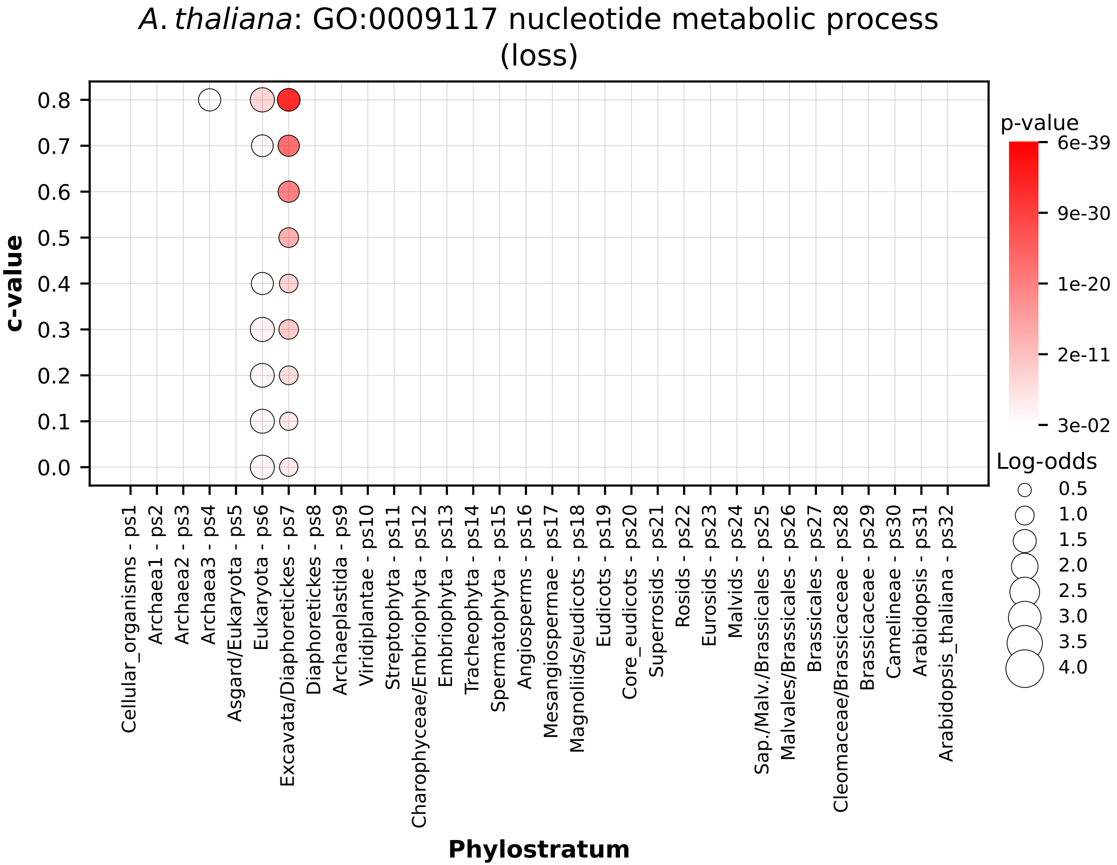

A. thaliana: GO:0009117 nucleotide metabolic process
(loss)
c-value
0.8
0.7
0.6
0.5
0.4
0.3
0.2
0.1
0.0
p-value
6e-39
9e-30
1e-20
2e-11
3e-02
Log-odds
0.5
1.0
1.5
2.0
2.5
3.0
3.5
4.0
Cellular_organisms - ps1
Archaea1 - ps2
Archaea2 - ps3
Archaea3 - ps4
Asgard/Eukaryota - ps5
Eukaryota - ps6
Excavata/Diaphoretickes - ps7
Diaphoretickes - ps8
Archaeplastida - ps9
Viridiplantae - ps10
Streptophyta - ps11
Charophyceae/Embriophyta - ps12
Embriophyta - ps13
Tracheophyta - ps14
Spermatophyta - ps15
Angiosperms - ps16
Mesangiospermae - ps17
Magnoliids/eudicots - ps18
Eudicots - ps19
Core_eudicots - ps20
Superrosids - ps21
Rosids - ps22
Eurosids - ps23
Malvids - ps24
Sap./Malv./Brassicales - ps25
Malvales/Brassicales - ps26
Brassicales - ps27
Cleomaceae/Brassicaceae - ps28
Brassicaceae - ps29
Camelineae - ps30
Arabidopsis - ps31
Arabidopsis_thaliana - ps32
Phylostratum

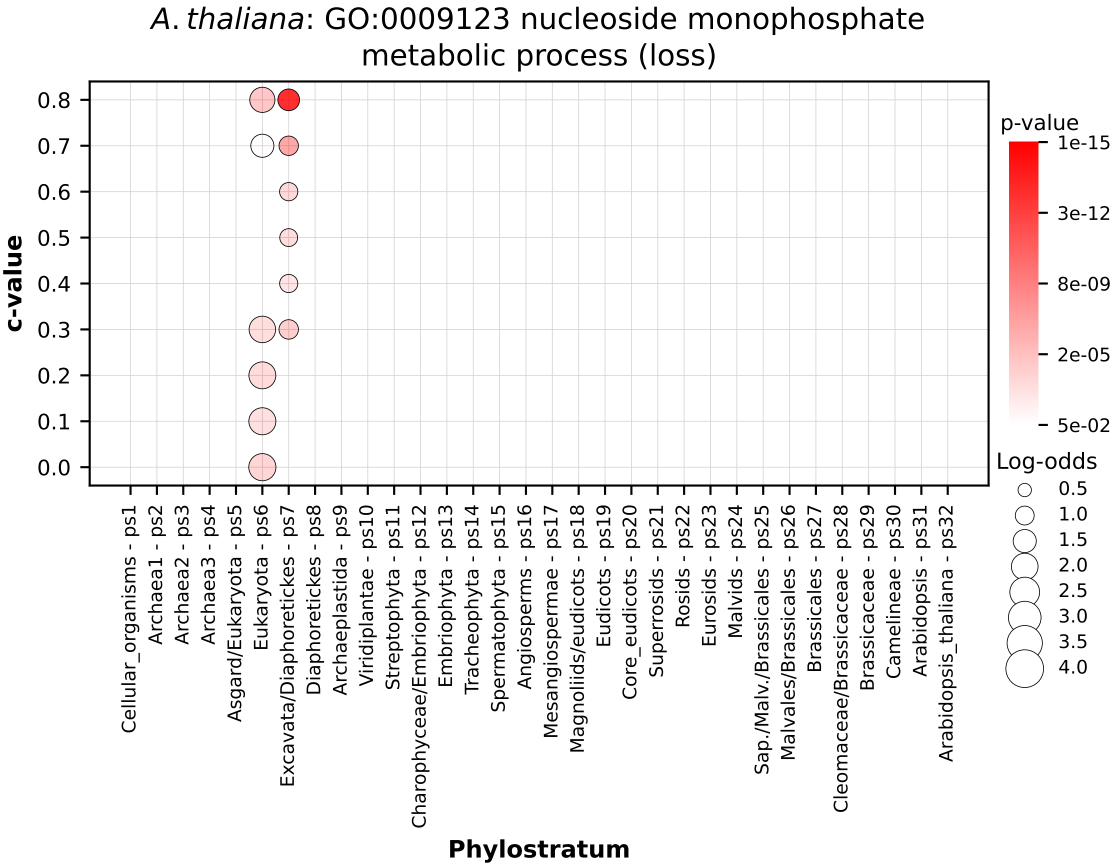
A. thaliana: GO:0009123 nucleoside monophosphate metabolic process (loss)
c-value
0.8
0.7
0.6
0.5
0.4
0.3
0.2
0.1
0.0
Phylostratum
Cellular_organisms - ps1
Archaea1 - ps2
Archaea2 - ps3
Archaea3 - ps4
Asgard/Eukaryota - ps5
Eukaryota - ps6
Excavata/Diaphoretickes - ps7
Diaphoretickes - ps8
Archaeplastida - ps9
Viridiplantae - ps10
Streptophyta - ps11
Charophyceae/Embriophyta - ps12
Embriophyta - ps13
Tracheophyta - ps14
Spermatophyta - ps15
Angiosperms - ps16
Mesangiospermae - ps17
Magnoliids/eudicots - ps18
Eudicots - ps19
Core_eudicots - ps20
Superrosids - ps21
Rosids - ps22
Eurosids - ps23
Malvids - ps24
Sap./Malv./Brassicales - ps25
Malvales/Brassicales - ps26
Brassicales - ps27
Cleomaceae/Brassicaceae - ps28
Brassicaceae - ps29
Camelineae - ps30
Arabidopsis - ps31
Arabidopsis_thaliana - ps32
p-value
1e-15
3e-12
8e-09
2e-05
5e-02
Log-odds
0.5
1.0
1.5
2.0
2.5
3.0
3.5
4.0

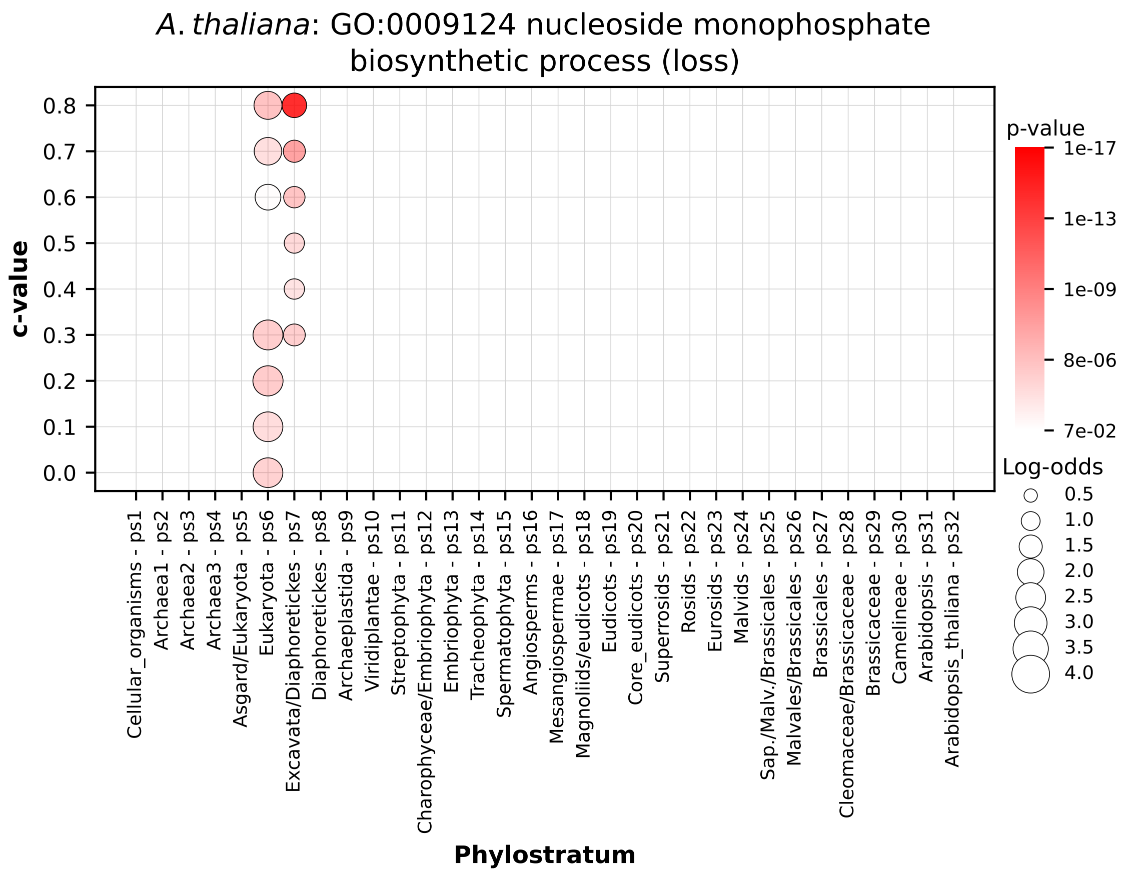

A. thaliana: GO:0009124 nucleoside monophosphate biosynthetic process (loss)
c-value
0.8
0.7
0.6
0.5
0.4
0.3
0.2
0.1
0.0
p-value
1e-17
1e-13
1e-09
8e-06
7e-02
Log-odds
0.5
1.0
1.5
2.0
2.5
3.0
3.5
4.0
Cellular_organisms - ps1
Archaea1 - ps2
Archaea2 - ps3
Archaea3 - ps4
Asgard/Eukaryota - ps5
Eukaryota - ps6
Excavata/Diaphoretickes - ps7
Diaphoretickes - ps8
Archaeplastida - ps9
Viridiplantae - ps10
Streptophyta - ps11
Charophyceae/Embriophyta - ps12
Embriophyta - ps13
Tracheophyta - ps14
Spermatophyta - ps15
Angiosperms - ps16
Mesangiospermae - ps17
Magnoliids/eudicots - ps18
Eudicots - ps19
Core_eudicots - ps20
Superrosids - ps21
Rosids - ps22
Eurosids - ps23
Malvids - ps24
Sap./Malv./Brassicales - ps25
Malvales/Brassicales - ps26
Brassicales - ps27
Cleomaceae/Brassicaceae - ps28
Brassicaceae - ps29
Camelineae - ps30
Arabidopsis - ps31
Arabidopsis_thaliana - ps32
Phylostratum

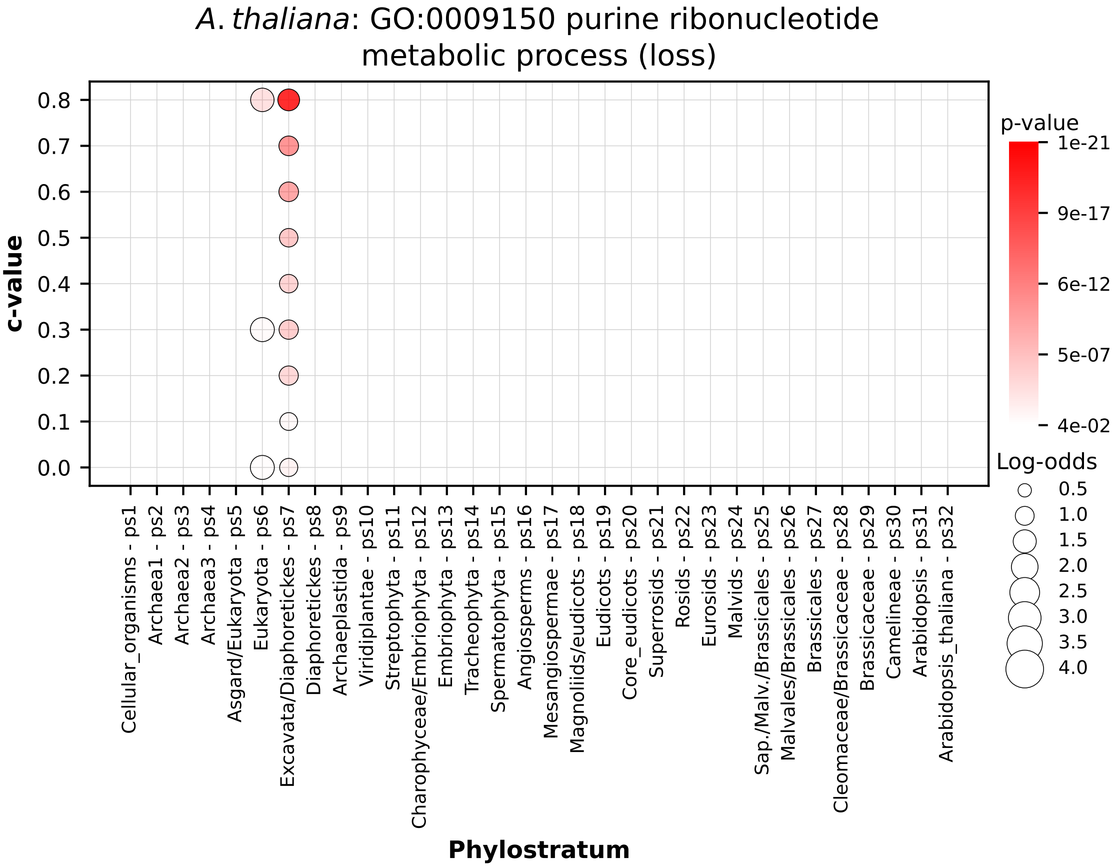

A. thaliana: GO:0009150 purine ribonucleotide metabolic process (loss)
c-value
0.8
0.7
0.6
0.5
0.4
0.3
0.2
0.1
0.0
p-value
1e-21
9e-17
6e-12
5e-07
4e-02
Log-odds
0.5
1.0
1.5
2.0
2.5
3.0
3.5
4.0
Cellular_organisms - ps1
Archaea1 - ps2
Archaea2 - ps3
Archaea3 - ps4
Asgard/Eukaryota - ps5
Eukaryota - ps6
Excavata/Diaphoretickes - ps7
Diaphoretickes - ps8
Archaeplastida - ps9
Viridiplantae - ps10
Streptophyta - ps11
Charophyceae/Embriophyta - ps12
Embriophyta - ps13
Tracheophyta - ps14
Spermatophyta - ps15
Angiosperms - ps16
Mesangiospermae - ps17
Magnoliids/eudicots - ps18
Eudicots - ps19
Core_eudicots - ps20
Superrosids - ps21
Rosids - ps22
Eurosids - ps23
Malvids - ps24
Sap./Malv./Brassicales - ps25
Malvales/Brassicales - ps26
Brassicales - ps27
Cleomaceae/Brassicaceae - ps28
Brassicaceae - ps29
Camelineae - ps30
Arabidopsis - ps31
Arabidopsis_thaliana - ps32
Phylostratum

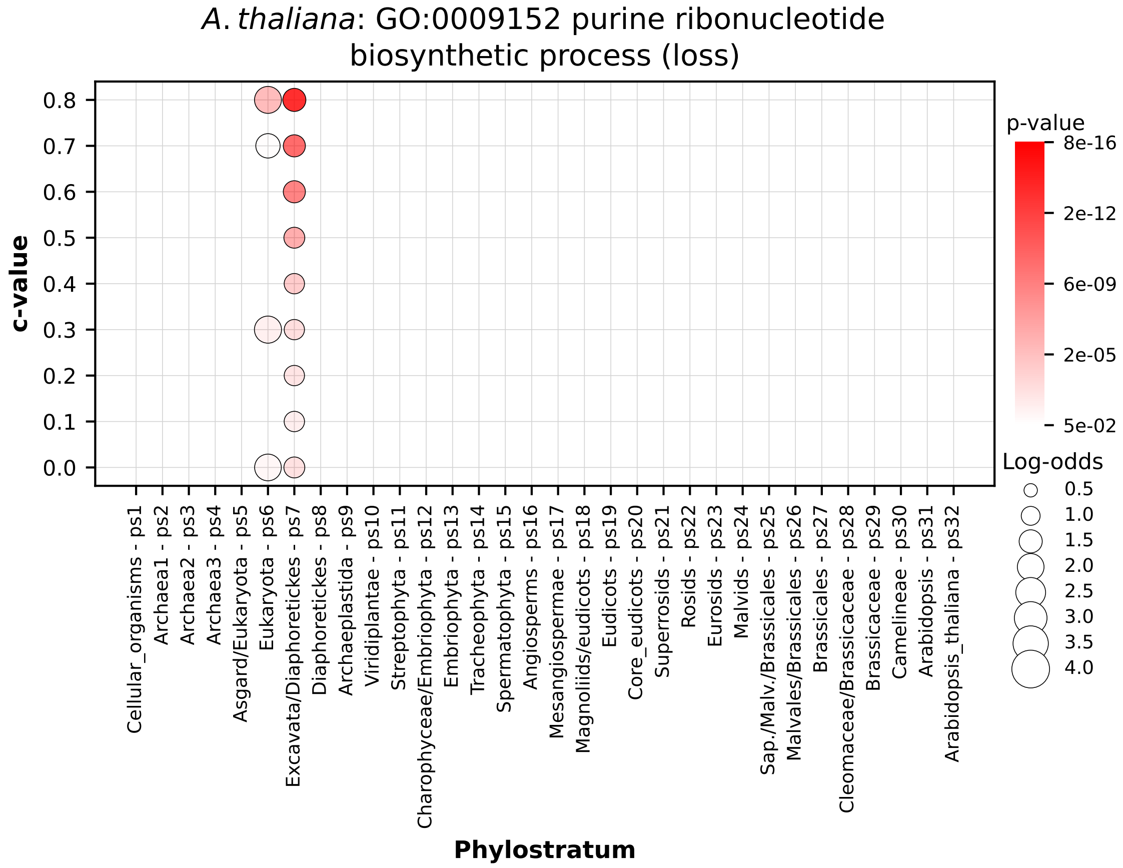

A. thaliana: GO:0009152 purine ribonucleotide biosynthetic process (loss)
c-value
0.8
0.7
0.6
0.5
0.4
0.3
0.2
0.1
0.0
p-value
8e-16
2e-12
6e-09
2e-05
5e-02
Log-odds
0.5
1.0
1.5
2.0
2.5
3.0
3.5
4.0
Cellular_organisms - ps1
Archaea1 - ps2
Archaea2 - ps3
Archaea3 - ps4
Asgard/Eukaryota - ps5
Eukaryota - ps6
Excavata/Diaphoretickes - ps7
Diaphoretickes - ps8
Archaeplastida - ps9
Viridiplantae - ps10
Streptophyta - ps11
Charophyceae/Embriophyta - ps12
Embriophyta - ps13
Tracheophyta - ps14
Spermatophyta - ps15
Angiosperms - ps16
Mesangiospermae - ps17
Magnoliids/eudicots - ps18
Eudicots - ps19
Core_eudicots - ps20
Superrosids - ps21
Rosids - ps22
Eurosids - ps23
Malvids - ps24
Sap./Malv./Brassicales - ps25
Malvales/Brassicales - ps26
Brassicales - ps27
Cleomaceae/Brassicaceae - ps28
Brassicaceae - ps29
Camelineae - ps30
Arabidopsis - ps31
Arabidopsis_thaliana - ps32
Phylostratum

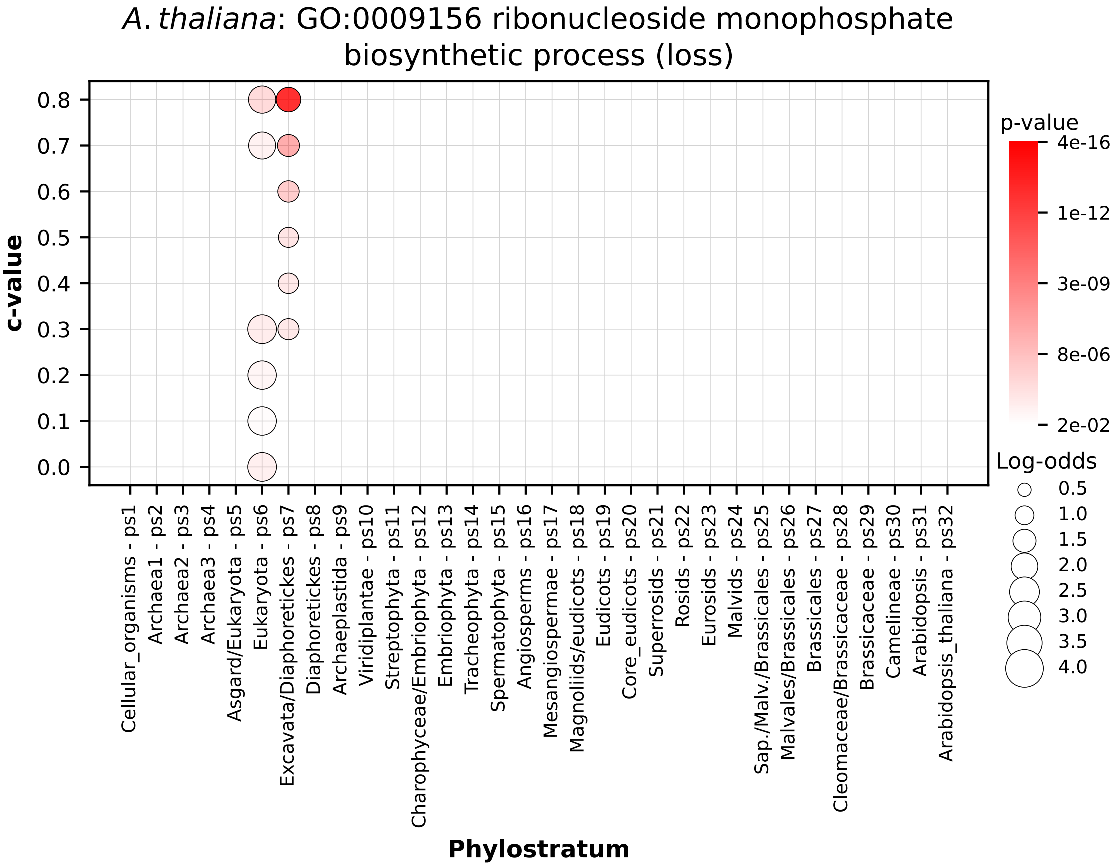

A. thaliana: GO:0009156 ribonucleoside monophosphate biosynthetic process (loss)
c-value
0.8
0.7
0.6
0.5
0.4
0.3
0.2
0.1
0.0
Phylostratum
Cellular_organisms - ps1
Archaea1 - ps2
Archaea2 - ps3
Archaea3 - ps4
Asgard/Eukaryota - ps5
Eukaryota - ps6
Excavata/Diaphoretickes - ps7
Diaphoretickes - ps8
Archaeplastida - ps9
Viridiplantae - ps10
Streptophyta - ps11
Charophyceae/Embriophyta - ps12
Embriophyta - ps13
Tracheophyta - ps14
Spermatophyta - ps15
Angiosperms - ps16
Mesangiospermae - ps17
Magnoliids/eudicots - ps18
Eudicots - ps19
Core_eudicots - ps20
Superrosids - ps21
Rosids - ps22
Eurosids - ps23
Malvids - ps24
Sap./Malv./Brassicales - ps25
Malvales/Brassicales - ps26
Brassicales - ps27
Cleomaceae/Brassicaceae - ps28
Brassicaceae - ps29
Camelineae - ps30
Arabidopsis - ps31
Arabidopsis_thaliana - ps32
p-value
4e-16
1e-12
3e-09
8e-06
2e-02
Log-odds
0.5
1.0
1.5
2.0
2.5
3.0
3.5
4.0

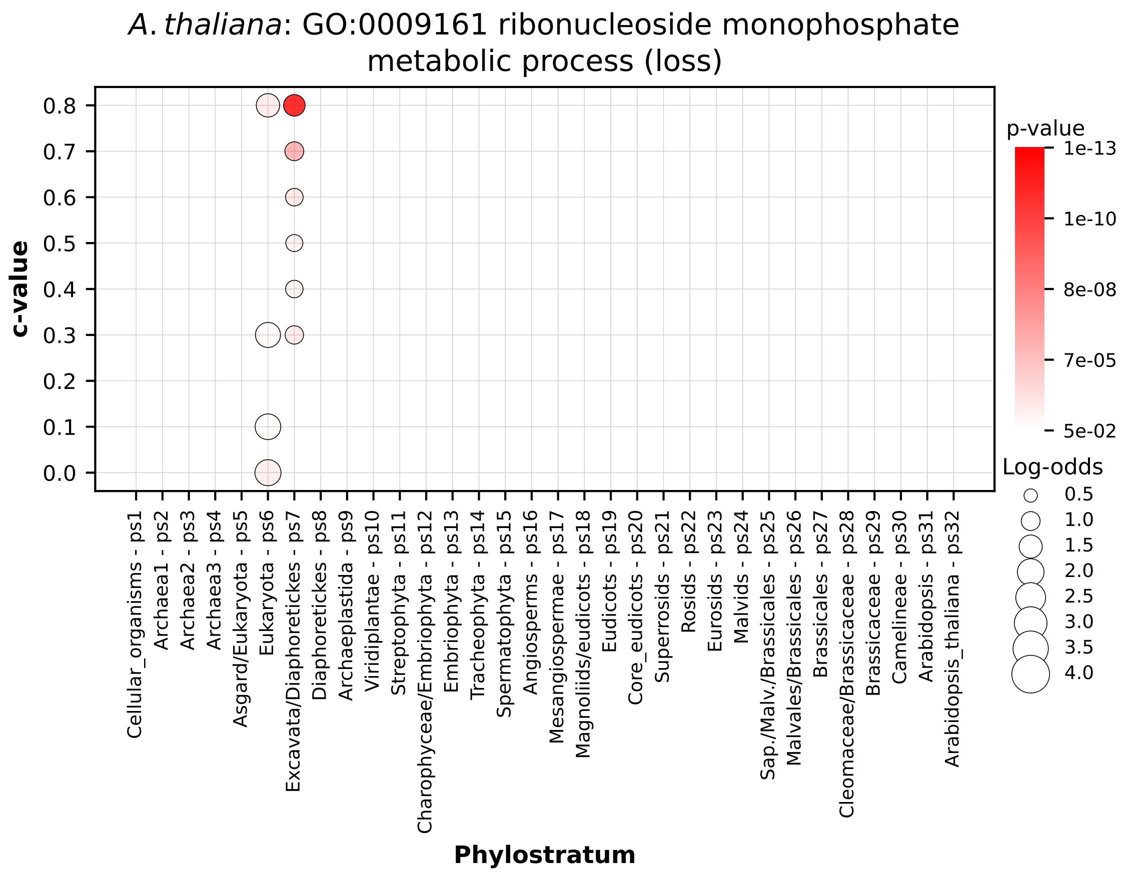

A. thaliana: GO:0009161 ribonucleoside monophosphate metabolic process (loss)
c-value
0.8
0.7
0.6
0.5
0.4
0.3
0.2
0.1
0.0
p-value
1e-13
1e-10
8e-08
7e-05
5e-02
Log-odds
0.5
1.0
1.5
2.0
2.5
3.0
3.5
4.0
Cellular_organisms - ps1
Archaea1 - ps2
Archaea2 - ps3
Archaea3 - ps4
Asgard/Eukaryota - ps5
Eukaryota - ps6
Excavata/Diaphoretickes - ps7
Diaphoretickes - ps8
Archaeplastida - ps9
Viridiplantae - ps10
Streptophyta - ps11
Charophyceae/Embriophyta - ps12
Embriophyta - ps13
Tracheophyta - ps14
Spermatophyta - ps15
Angiosperms - ps16
Mesangiospermae - ps17
Magnoliids/eudicots - ps18
Eudicots - ps19
Core_eudicots - ps20
Superrosids - ps21
Rosids - ps22
Eurosids - ps23
Malvids - ps24
Sap./Malv./Brassicales - ps25
Malvales/Brassicales - ps26
Brassicales - ps27
Cleomaceae/Brassicaceae - ps28
Brassicaceae - ps29
Camelineae - ps30
Arabidopsis - ps31
Arabidopsis_thaliana - ps32
Phylostratum

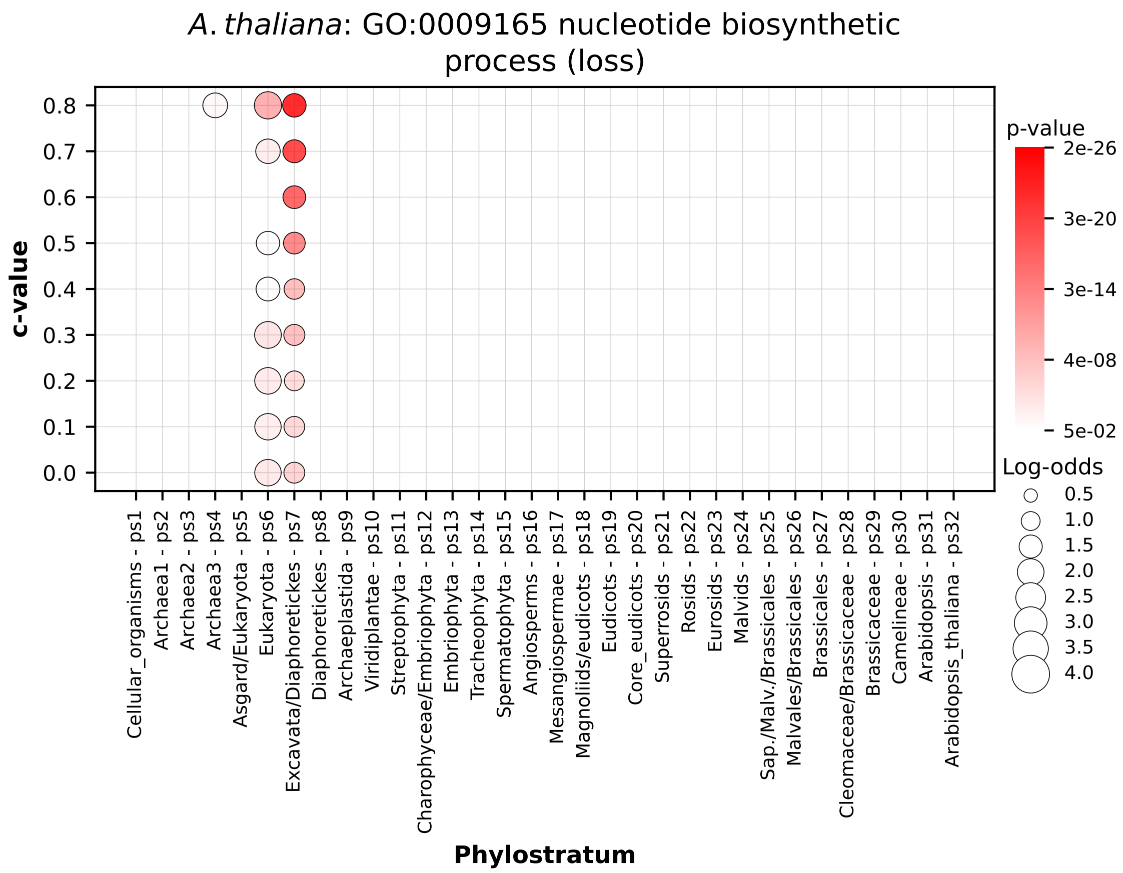

A. thaliana: GO:0009165 nucleotide biosynthetic process (loss)
c-value
0.8
0.7
0.6
0.5
0.4
0.3
0.2
0.1
0.0
p-value
2e-26
3e-20
3e-14
4e-08
5e-02
Log-odds
0.5
1.0
1.5
2.0
2.5
3.0
3.5
4.0
Cellular_organisms - ps1
Archaea1 - ps2
Archaea2 - ps3
Archaea3 - ps4
Asgard/Eukaryota - ps5
Eukaryota - ps6
Excavata/Diaphoretickes - ps7
Diaphoretickes - ps8
Archaeplastida - ps9
Viridiplantae - ps10
Streptophyta - ps11
Charophyceae/Embriophyta - ps12
Embriophyta - ps13
Tracheophyta - ps14
Spermatophyta - ps15
Angiosperms - ps16
Mesangiospermae - ps17
Magnoliids/eudicots - ps18
Eudicots - ps19
Core_eudicots - ps20
Superrosids - ps21
Rosids - ps22
Eurosids - ps23
Malvids - ps24
Sap./Malv./Brassicales - ps25
Malvales/Brassicales - ps26
Brassicales - ps27
Cleomaceae/Brassicaceae - ps28
Brassicaceae - ps29
Camelineae - ps30
Arabidopsis - ps31
Arabidopsis_thaliana - ps32
Phylostratum

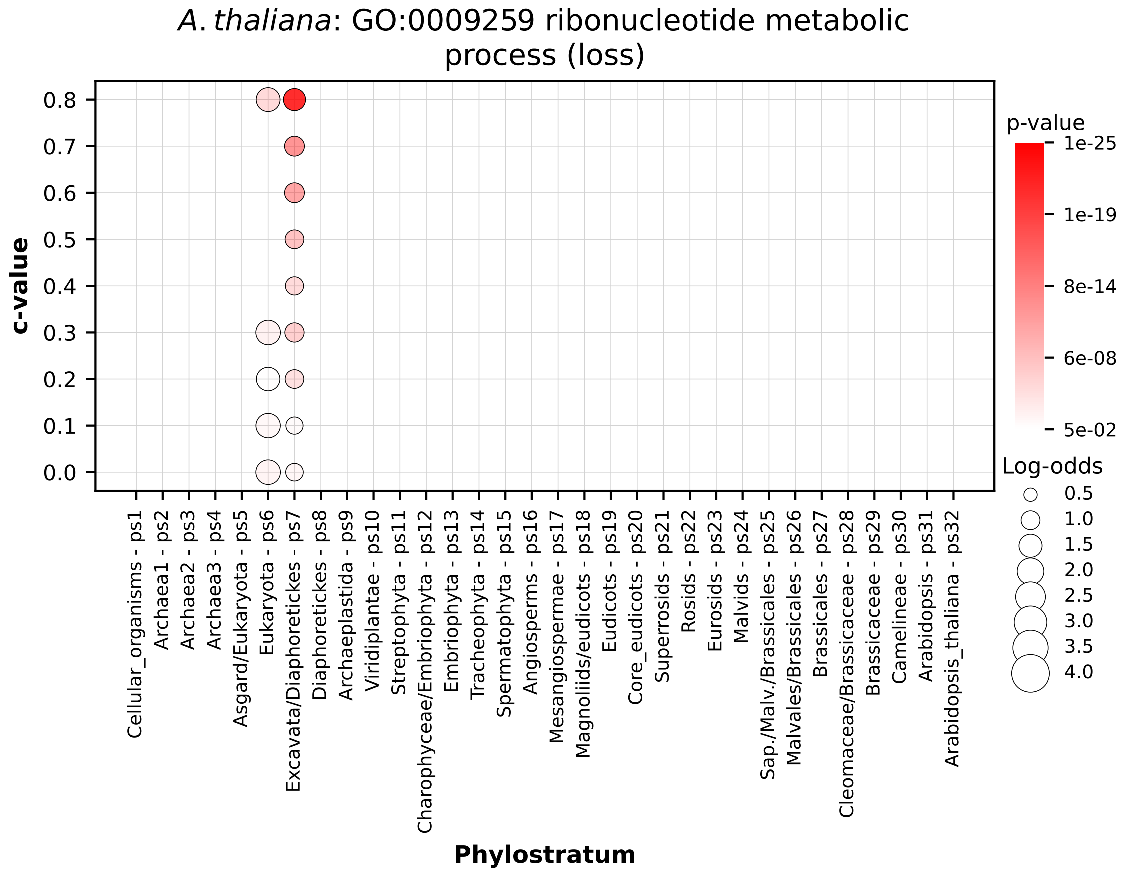

A. thaliana: GO:0009259 ribonucleotide metabolic process (loss)
c-value
0.8
0.7
0.6
0.5
0.4
0.3
0.2
0.1
0.0
p-value
1e-25
1e-19
8e-14
6e-08
5e-02
Log-odds
0.5
1.0
1.5
2.0
2.5
3.0
3.5
4.0
Cellular_organisms - ps1
Archaea1 - ps2
Archaea2 - ps3
Archaea3 - ps4
Asgard/Eukaryota - ps5
Eukaryota - ps6
Excavata/Diaphoretickes - ps7
Diaphoretickes - ps8
Archaeplastida - ps9
Viridiplantae - ps10
Streptophyta - ps11
Charophyceae/Embriophyta - ps12
Embriophyta - ps13
Tracheophyta - ps14
Spermatophyta - ps15
Angiosperms - ps16
Mesangiospermae - ps17
Magnoliids/eudicots - ps18
Eudicots - ps19
Core_eudicots - ps20
Superrosids - ps21
Rosids - ps22
Eurosids - ps23
Malvids - ps24
Sap./Malv./Brassicales - ps25
Malvales/Brassicales - ps26
Brassicales - ps27
Cleomaceae/Brassicaceae - ps28
Brassicaceae - ps29
Camelineae - ps30
Arabidopsis - ps31
Arabidopsis_thaliana - ps32
Phylostratum

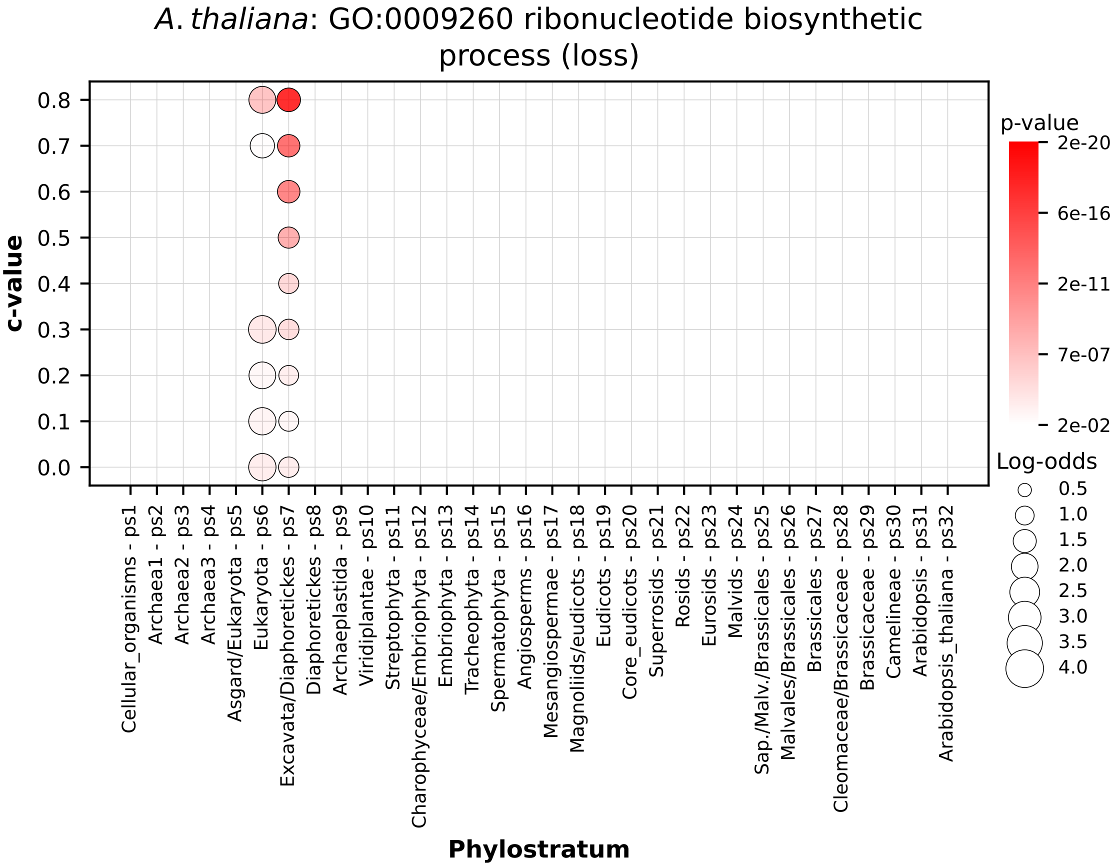

A. thaliana: GO:0009260 ribonucleotide biosynthetic process (loss)
c-value
0.8
0.7
0.6
0.5
0.4
0.3
0.2
0.1
0.0
p-value
2e-20
6e-16
2e-11
7e-07
2e-02
Log-odds
0.5
1.0
1.5
2.0
2.5
3.0
3.5
4.0
Cellular_organisms - ps1
Archaea1 - ps2
Archaea2 - ps3
Archaea3 - ps4
Asgard/Eukaryota - ps5
Eukaryota - ps6
Excavata/Diaphoretickes - ps7
Diaphoretickes - ps8
Archaeplastida - ps9
Viridiplantae - ps10
Streptophyta - ps11
Charophyceae/Embriophyta - ps12
Embriophyta - ps13
Tracheophyta - ps14
Spermatophyta - ps15
Angiosperms - ps16
Mesangiospermae - ps17
Magnoliids/eudicots - ps18
Eudicots - ps19
Core_eudicots - ps20
Superrosids - ps21
Rosids - ps22
Eurosids - ps23
Malvids - ps24
Sap./Malv./Brassicales - ps25
Malvales/Brassicales - ps26
Brassicales - ps27
Cleomaceae/Brassicaceae - ps28
Brassicaceae - ps29
Camelineae - ps30
Arabidopsis - ps31
Arabidopsis_thaliana - ps32
Phylostratum

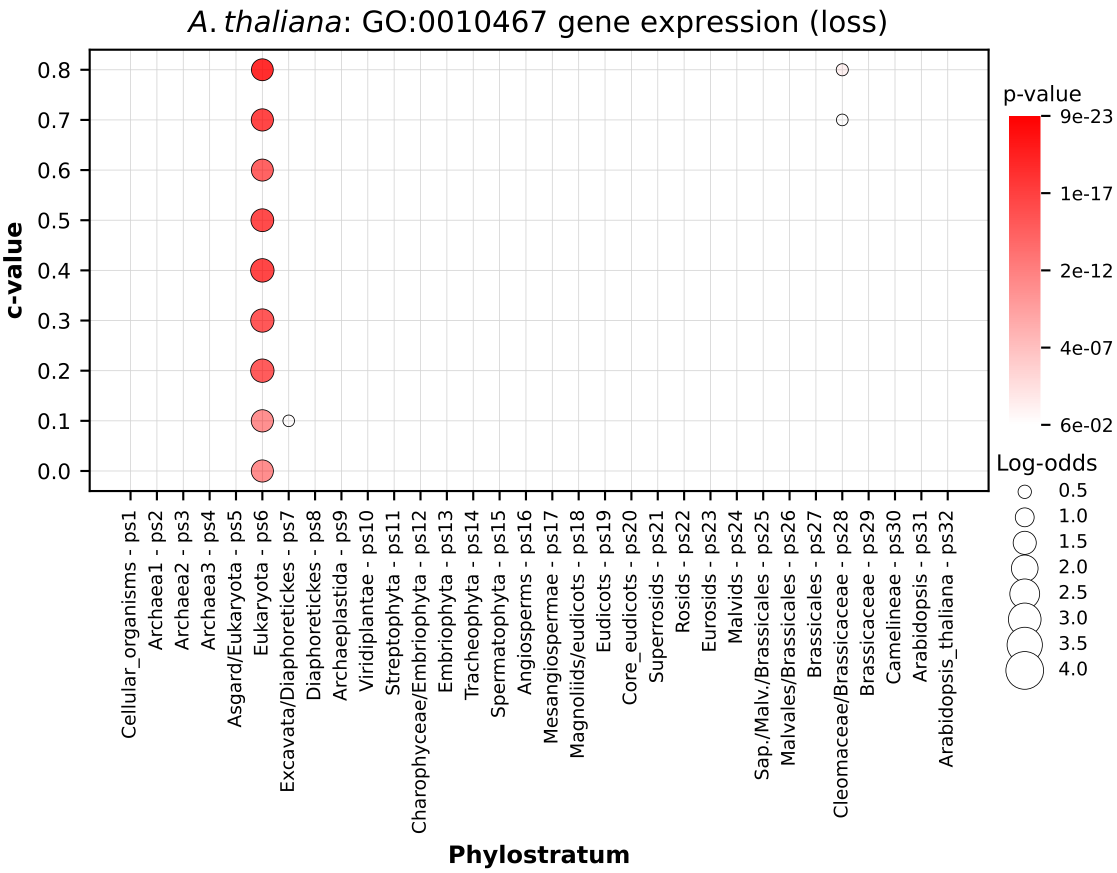

A. thaliana: GO:0010467 gene expression (loss)
c-value
0.8
0.7
0.6
0.5
0.4
0.3
0.2
0.1
0.0
p-value
9e-23
1e-17
2e-12
4e-07
6e-02
Log-odds
0.5
1.0
1.5
2.0
2.5
3.0
3.5
4.0
Cellular_organisms - ps1
Archaea1 - ps2
Archaea2 - ps3
Archaea3 - ps4
Asgard/Eukaryota - ps5
Eukaryota - ps6
Excavata/Diaphoretickes - ps7
Diaphoretickes - ps8
Archaeplastida - ps9
Viridiplantae - ps10
Streptophyta - ps11
Charophyceae/Embriophyta - ps12
Embriophyta - ps13
Tracheophyta - ps14
Spermatophyta - ps15
Angiosperms - ps16
Mesangiospermae - ps17
Magnoliids/eudicots - ps18
Eudicots - ps19
Core_eudicots - ps20
Superrosids - ps21
Rosids - ps22
Eurosids - ps23
Malvids - ps24
Sap./Malv./Brassicales - ps25
Malvales/Brassicales - ps26
Brassicales - ps27
Cleomaceae/Brassicaceae - ps28
Brassicaceae - ps29
Camelineae - ps30
Arabidopsis - ps31
Arabidopsis_thaliana - ps32
Phylostratum

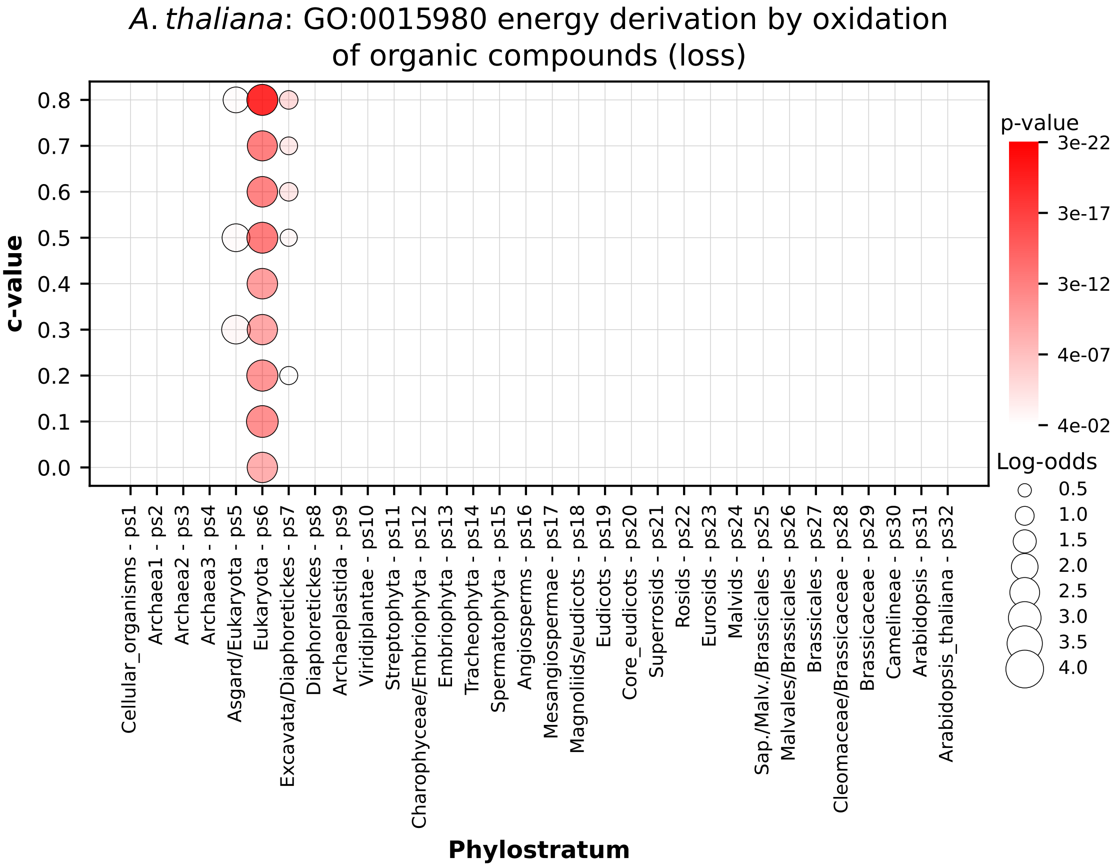

A. thaliana: GO:0015980 energy derivation by oxidation of organic compounds (loss)
c-value
0.8
0.7
0.6
0.5
0.4
0.3
0.2
0.1
0.0
p-value
3e-22
3e-17
3e-12
4e-07
4e-02
Log-odds
0.5
1.0
1.5
2.0
2.5
3.0
3.5
4.0
Cellular_organisms - ps1
Archaea1 - ps2
Archaea2 - ps3
Archaea3 - ps4
Asgard/Eukaryota - ps5
Eukaryota - ps6
Excavata/Diaphoretickes - ps7
Diaphoretickes - ps8
Archaeplastida - ps9
Viridiplantae - ps10
Streptophyta - ps11
Charophyceae/Embriophyta - ps12
Embriophyta - ps13
Tracheophyta - ps14
Spermatophyta - ps15
Angiosperms - ps16
Mesangiospermae - ps17
Magnoliids/eudicots - ps18
Eudicots - ps19
Core_eudicots - ps20
Superrosids - ps21
Rosids - ps22
Eurosids - ps23
Malvids - ps24
Sap./Malv./Brassicales - ps25
Malvales/Brassicales - ps26
Brassicales - ps27
Cleomaceae/Brassicaceae - ps28
Brassicaceae - ps29
Camelineae - ps30
Arabidopsis - ps31
Arabidopsis_thaliana - ps32
Phylostratum

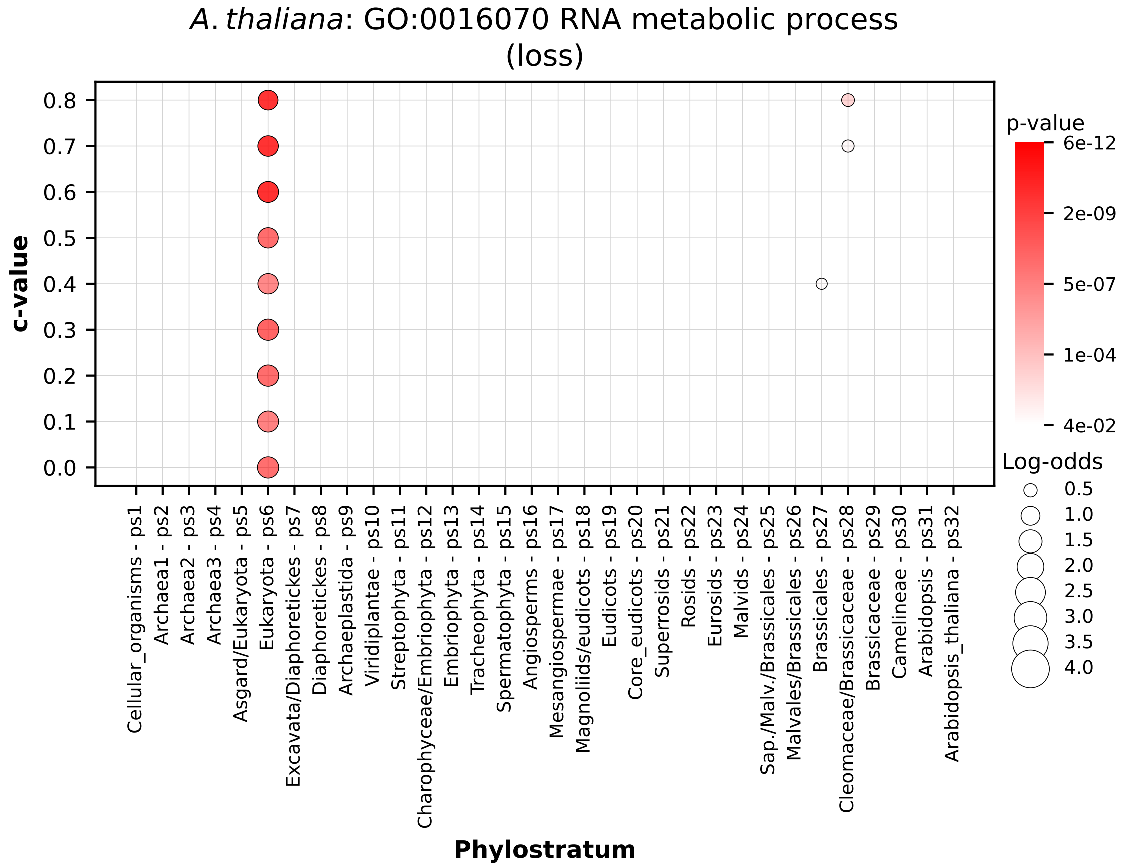

A. thaliana: GO:0016070 RNA metabolic process
(loss)
c-value
0.8
0.7
0.6
0.5
0.4
0.3
0.2
0.1
0.0
Phylostratum
Cellular_organisms - ps1
Archaea1 - ps2
Archaea2 - ps3
Archaea3 - ps4
Asgard/Eukaryota - ps5
Eukaryota - ps6
Excavata/Diaphoretickes - ps7
Diaphoretickes - ps8
Archaeplastida - ps9
Viridiplantae - ps10
Streptophyta - ps11
Charophyceae/Embriophyta - ps12
Embriophyta - ps13
Tracheophyta - ps14
Spermatophyta - ps15
Angiosperms - ps16
Mesangiospermae - ps17
Magnoliids/eudicots - ps18
Eudicots - ps19
Core_eudicots - ps20
Superrosids - ps21
Rosids - ps22
Eurosids - ps23
Malvids - ps24
Sap./Malv./Brassicales - ps25
Malvales/Brassicales - ps26
Brassicales - ps27
Cleomaceae/Brassicaceae - ps28
Brassicaceae - ps29
Camelineae - ps30
Arabidopsis - ps31
Arabidopsis_thaliana - ps32
p-value
6e-12
2e-09
5e-07
1e-04
4e-02
Log-odds
0.5
1.0
1.5
2.0
2.5
3.0
3.5
4.0

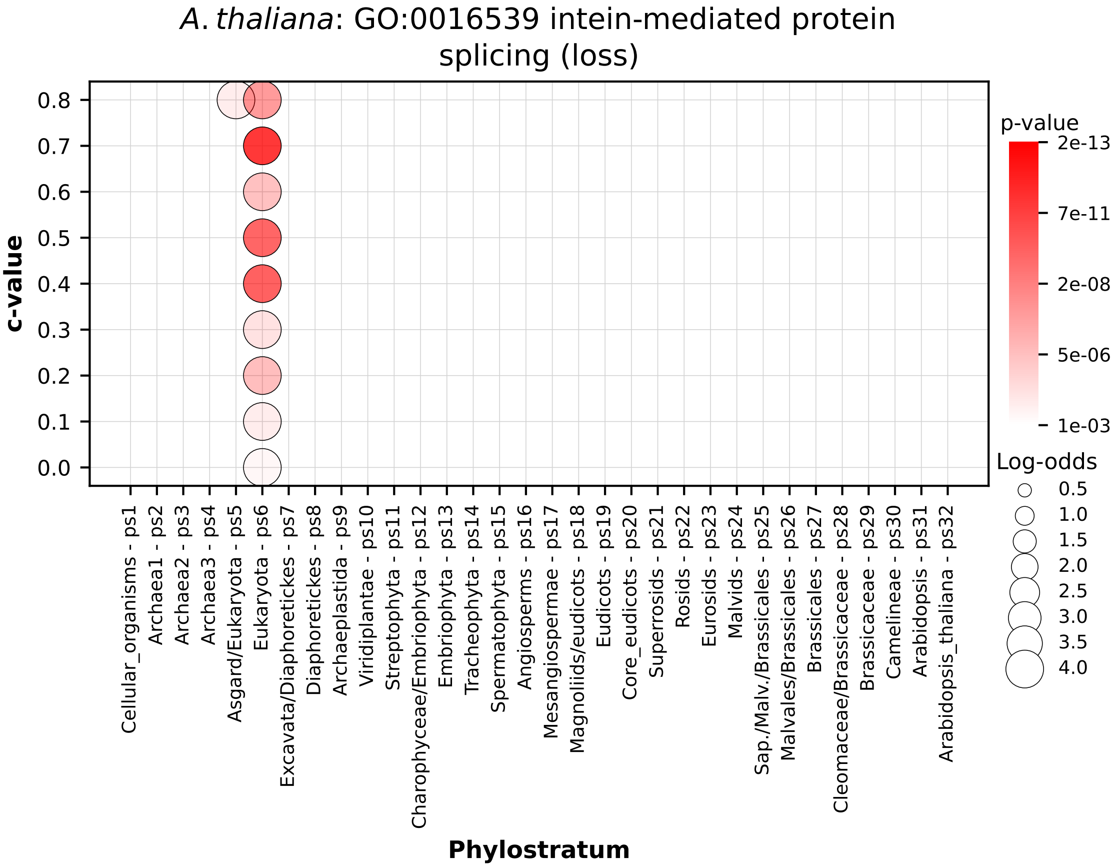

A. thaliana: GO:0016539 intein-mediated protein splicing (loss)
c-value
0.8
0.7
0.6
0.5
0.4
0.3
0.2
0.1
0.0
p-value
2e-13
7e-11
2e-08
5e-06
1e-03
Log-odds
0.5
1.0
1.5
2.0
2.5
3.0
3.5
4.0
Cellular_organisms - ps1
Archaea1 - ps2
Archaea2 - ps3
Archaea3 - ps4
Asgard/Eukaryota - ps5
Eukaryota - ps6
Excavata/Diaphoretickes - ps7
Diaphoretickes - ps8
Archaeplastida - ps9
Viridiplantae - ps10
Streptophyta - ps11
Charophyceae/Embriophyta - ps12
Embriophyta - ps13
Tracheophyta - ps14
Spermatophyta - ps15
Angiosperms - ps16
Mesangiospermae - ps17
Magnoliids/eudicots - ps18
Eudicots - ps19
Core_eudicots - ps20
Superrosids - ps21
Rosids - ps22
Eurosids - ps23
Malvids - ps24
Sap./Malv./Brassicales - ps25
Malvales/Brassicales - ps26
Brassicales - ps27
Cleomaceae/Brassicaceae - ps28
Brassicaceae - ps29
Camelineae - ps30
Arabidopsis - ps31
Arabidopsis_thaliana - ps32
Phylostratum

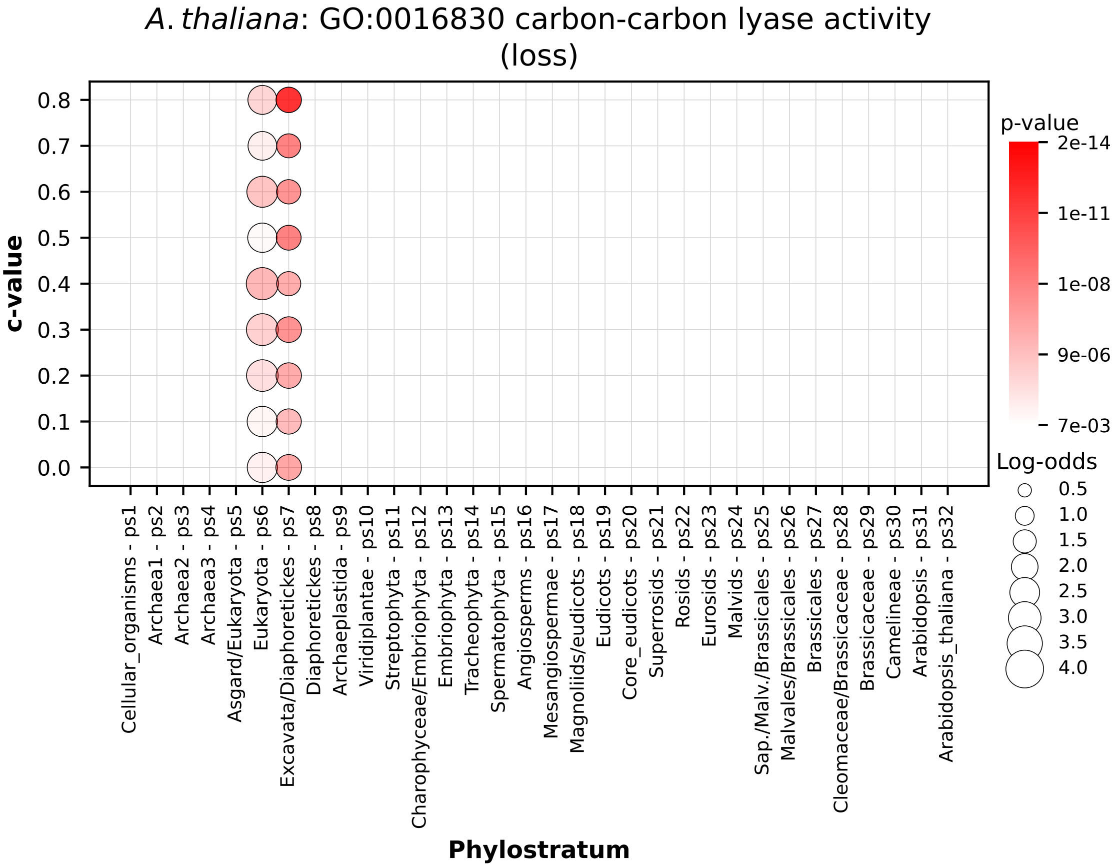
A. thaliana: GO:0016830 carbon-carbon lyase activity
(loss)
c-value
0.8
0.7
0.6
0.5
0.4
0.3
0.2
0.1
0.0
p-value
2e-14
1e-11
1e-08
9e-06
7e-03
Log-odds
0.5
1.0
1.5
2.0
2.5
3.0
3.5
4.0
Cellular_organisms - ps1
Archaea1 - ps2
Archaea2 - ps3
Archaea3 - ps4
Asgard/Eukaryota - ps5
Eukaryota - ps6
Excavata/Diaphoretickes - ps7
Diaphoretickes - ps8
Archaeplastida - ps9
Viridiplantae - ps10
Streptophyta - ps11
Charophyceae/Embriophyta - ps12
Embriophyta - ps13
Tracheophyta - ps14
Spermatophyta - ps15
Angiosperms - ps16
Mesangiospermae - ps17
Magnoliids/eudicots - ps18
Eudicots - ps19
Core_eudicots - ps20
Superrosids - ps21
Rosids - ps22
Eurosids - ps23
Malvids - ps24
Sap./Malv./Brassicales - ps25
Malvales/Brassicales - ps26
Brassicales - ps27
Cleomaceae/Brassicaceae - ps28
Brassicaceae - ps29
Camelineae - ps30
Arabidopsis - ps31
Arabidopsis_thaliana - ps32
Phylostratum

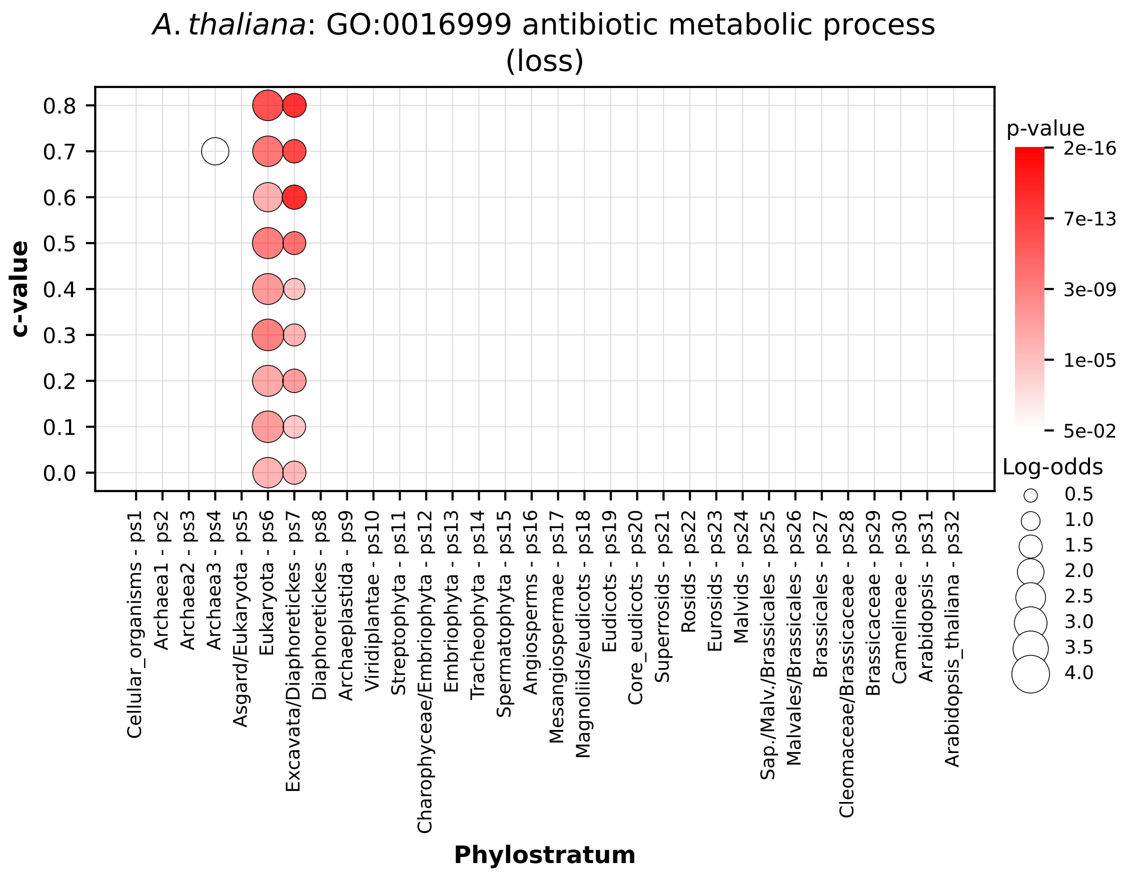

A. thaliana: GO:0016999 antibiotic metabolic process
(loss)
c-value
0.8
0.7
0.6
0.5
0.4
0.3
0.2
0.1
0.0
p-value
2e-16
7e-13
3e-09
1e-05
5e-02
Log-odds
0.5
1.0
1.5
2.0
2.5
3.0
3.5
4.0
Cellular_organisms - ps1
Archaea1 - ps2
Archaea2 - ps3
Archaea3 - ps4
Asgard/Eukaryota - ps5
Eukaryota - ps6
Excavata/Diaphoretickes - ps7
Diaphoretickes - ps8
Archaeplastida - ps9
Viridiplantae - ps10
Streptophyta - ps11
Charophyceae/Embriophyta - ps12
Embriophyta - ps13
Tracheophyta - ps14
Spermatophyta - ps15
Angiosperms - ps16
Mesangiospermae - ps17
Magnoliids/eudicots - ps18
Eudicots - ps19
Core_eudicots - ps20
Superrosids - ps21
Rosids - ps22
Eurosids - ps23
Malvids - ps24
Sap./Malv./Brassicales - ps25
Malvales/Brassicales - ps26
Brassicales - ps27
Cleomaceae/Brassicaceae - ps28
Brassicaceae - ps29
Camelineae - ps30
Arabidopsis - ps31
Arabidopsis_thaliana - ps32
Phylostratum

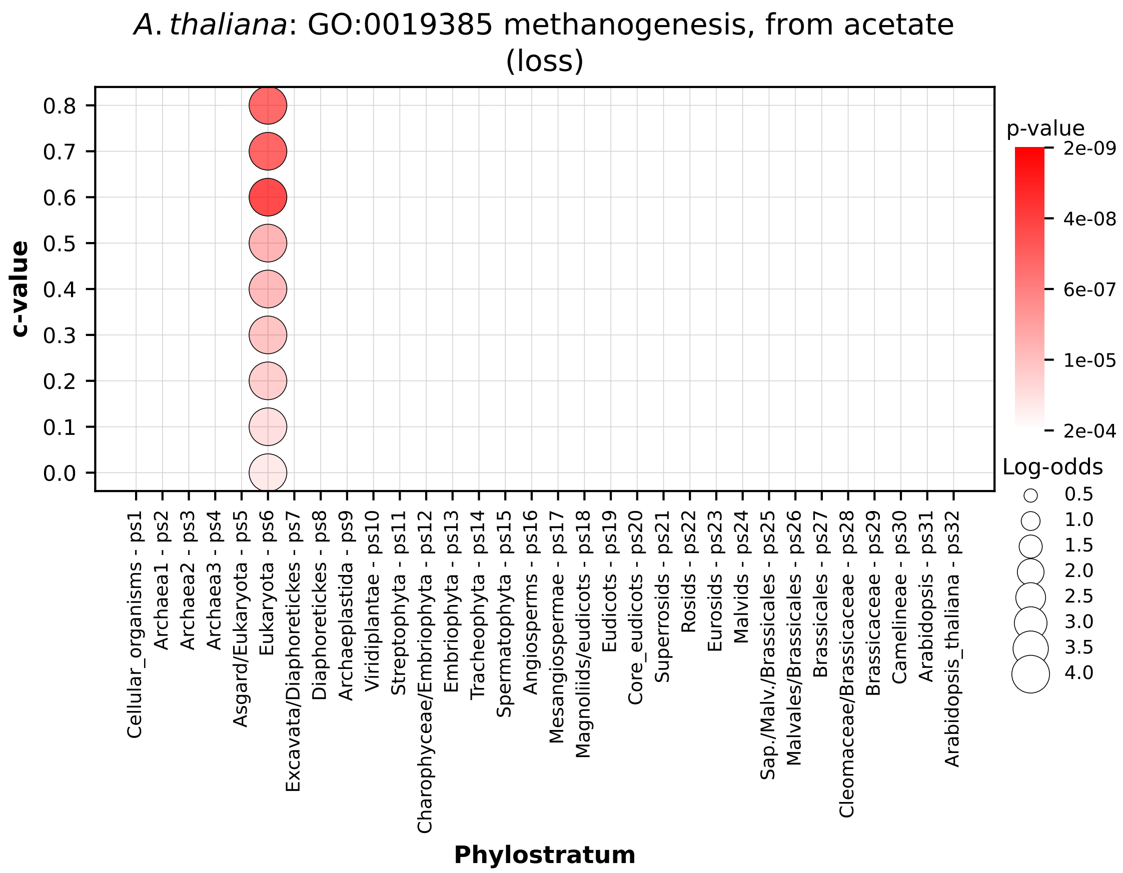

A. thaliana: GO:0019385 methanogenesis, from acetate (loss)
c-value
0.0
0.1
0.2
0.3
0.4
0.5
0.6
0.7
0.8
p-value
2e-09
4e-08
6e-07
1e-05
2e-04
Log-odds
0.5
1.0
1.5
2.0
2.5
3.0
3.5
4.0
Cellular_organisms - ps1
Archaea1 - ps2
Archaea2 - ps3
Archaea3 - ps4
Asgard/Eukaryota - ps5
Eukaryota - ps6
Excavata/Diaphoretickes - ps7
Diaphoretickes - ps8
Archaeplastida - ps9
Viridiplantae - ps10
Streptophyta - ps11
Charophyceae/Embriophyta - ps12
Embriophyta - ps13
Tracheophyta - ps14
Spermatophyta - ps15
Angiosperms - ps16
Mesangiospermae - ps17
Magnoliids/eudicots - ps18
Eudicots - ps19
Core_eudicots - ps20
Superrosids - ps21
Rosids - ps22
Eurosids - ps23
Malvids - ps24
Sap./Malv./Brassicales - ps25
Malvales/Brassicales - ps26
Brassicales - ps27
Cleomaceae/Brassicaceae - ps28
Brassicaceae - ps29
Camelineae - ps30
Arabidopsis - ps31
Arabidopsis_thaliana - ps32
Phylostratum

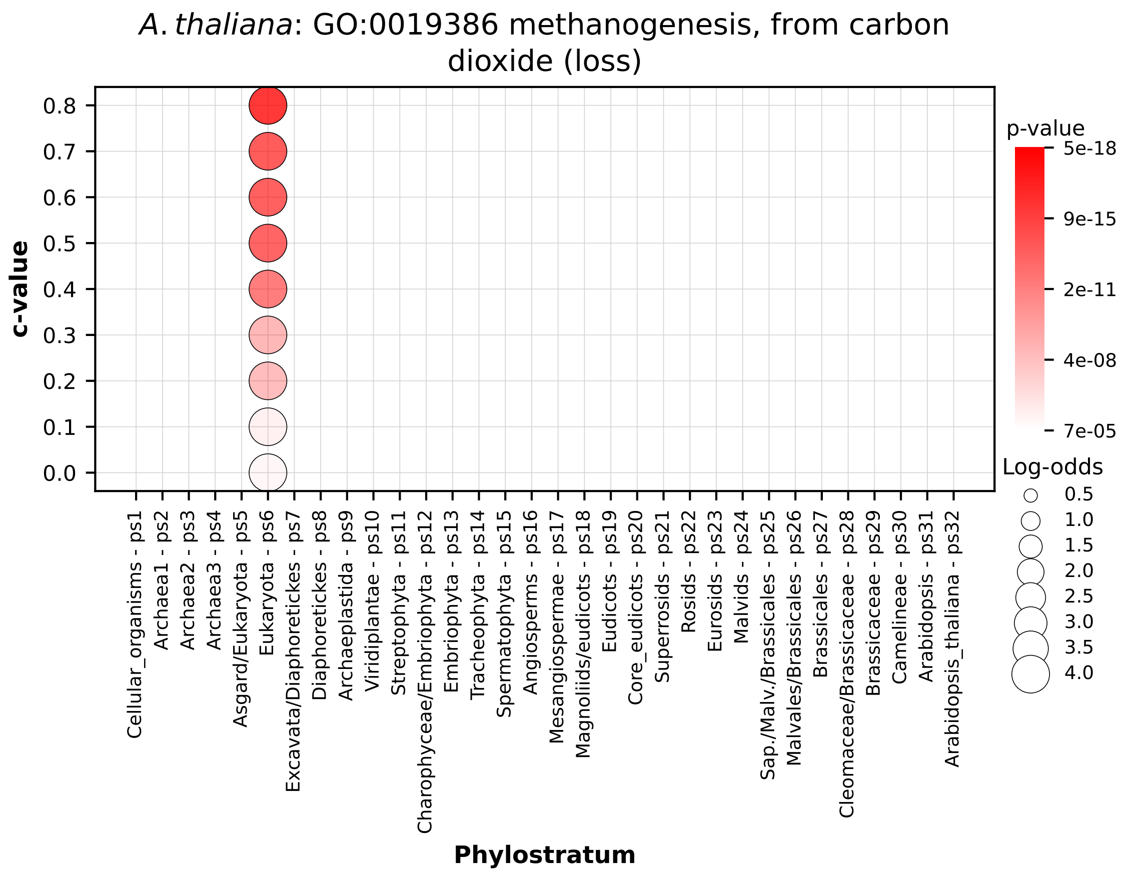

A. thaliana: GO:0019386 methanogenesis, from carbon dioxide (loss)
c-value
0.8
0.7
0.6
0.5
0.4
0.3
0.2
0.1
0.0
p-value
5e-18
9e-15
2e-11
4e-08
7e-05
Log-odds
0.5
1.0
1.5
2.0
2.5
3.0
3.5
4.0
Cellular_organisms - ps1
Archaea1 - ps2
Archaea2 - ps3
Archaea3 - ps4
Asgard/Eukaryota - ps5
Eukaryota - ps6
Excavata/Diaphoretickes - ps7
Diaphoretickes - ps8
Archaeplastida - ps9
Viridiplantae - ps10
Streptophyta - ps11
Charophyceae/Embriophyta - ps12
Embriophyta - ps13
Tracheophyta - ps14
Spermatophyta - ps15
Angiosperms - ps16
Mesangiospermae - ps17
Magnoliids/eudicots - ps18
Eudicots - ps19
Core_eudicots - ps20
Superrosids - ps21
Rosids - ps22
Eurosids - ps23
Malvids - ps24
Sap./Malv./Brassicales - ps25
Malvales/Brassicales - ps26
Brassicales - ps27
Cleomaceae/Brassicaceae - ps28
Brassicaceae - ps29
Camelineae - ps30
Arabidopsis - ps31
Arabidopsis_thaliana - ps32
Phylostratum

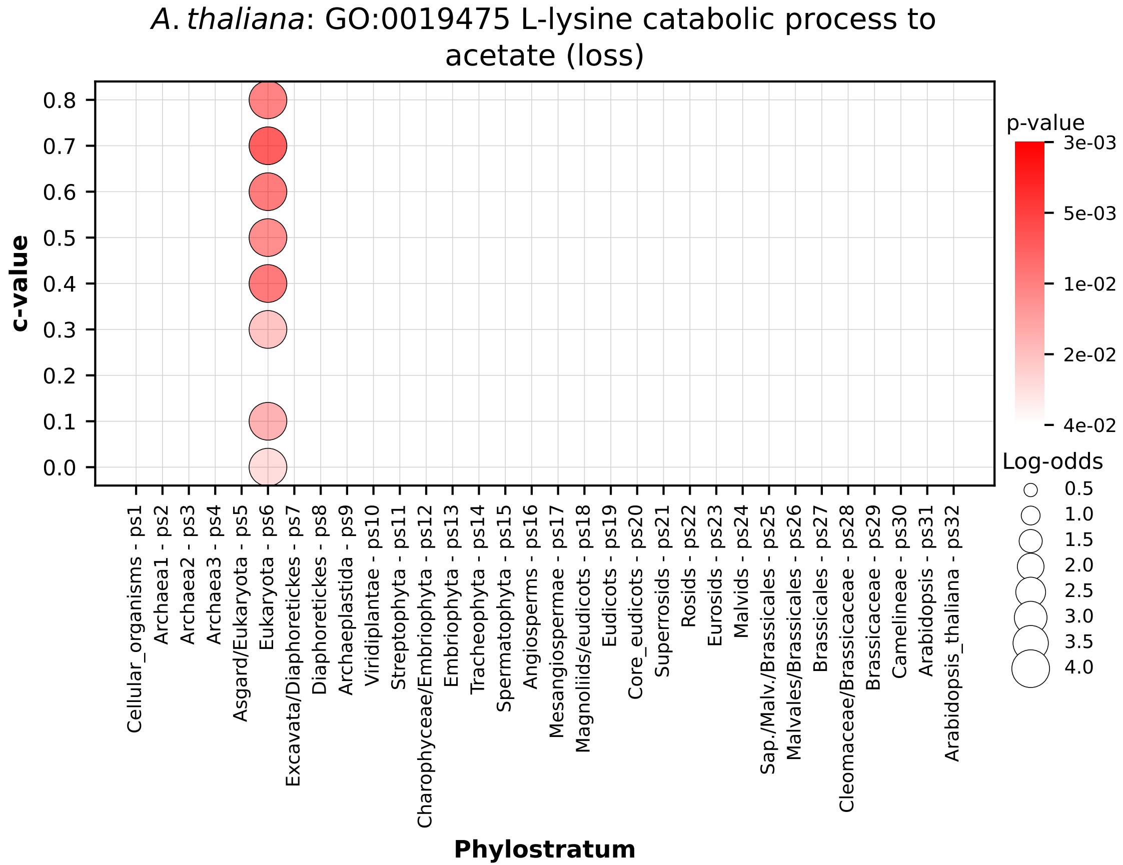

A. thaliana: GO:0019475 L-lysine catabolic process to acetate (loss)
c-value
0.8
0.7
0.6
0.5
0.4
0.3
0.2
0.1
0.0
p-value
3e-03
5e-03
1e-02
2e-02
4e-02
Log-odds
0.5
1.0
1.5
2.0
2.5
3.0
3.5
4.0
Cellular_organisms - ps1
Archaea1 - ps2
Archaea2 - ps3
Archaea3 - ps4
Asgard/Eukaryota - ps5
Eukaryota - ps6
Excavata/Diaphoretickes - ps7
Diaphoretickes - ps8
Archaeplastida - ps9
Viridiplantae - ps10
Streptophyta - ps11
Charophyceae/Embriophyta - ps12
Embriophyta - ps13
Tracheophyta - ps14
Spermatophyta - ps15
Angiosperms - ps16
Mesangiospermae - ps17
Magnoliids/eudicots - ps18
Eudicots - ps19
Core_eudicots - ps20
Superrosids - ps21
Rosids - ps22
Eurosids - ps23
Malvids - ps24
Sap./Malv./Brassicales - ps25
Malvales/Brassicales - ps26
Brassicales - ps27
Cleomaceae/Brassicaceae - ps28
Brassicaceae - ps29
Camelineae - ps30
Arabidopsis - ps31
Arabidopsis_thaliana - ps32
Phylostratum

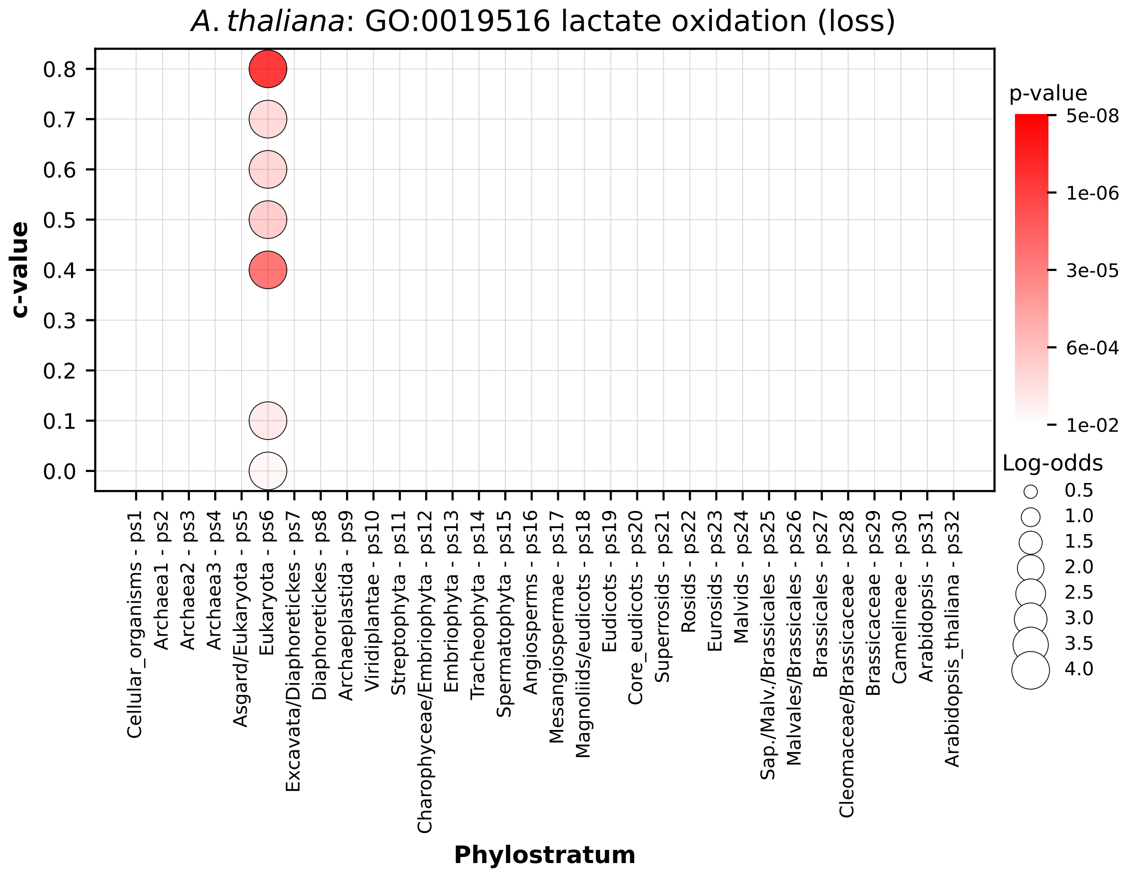

A. thaliana: GO:0019516 lactate oxidation (loss)
c-value
0.0
0.1
0.2
0.3
0.4
0.5
0.6
0.7
0.8
Phylostratum
Cellular_organisms - ps1
Archaea1 - ps2
Archaea2 - ps3
Archaea3 - ps4
Asgard/Eukaryota - ps5
Eukaryota - ps6
Excavata/Diaphoretickes - ps7
Diaphoretickes - ps8
Archaeplastida - ps9
Viridiplantae - ps10
Streptophyta - ps11
Charophyceae/Embriophyta - ps12
Embriophyta - ps13
Tracheophyta - ps14
Spermatophyta - ps15
Angiosperms - ps16
Mesangiospermae - ps17
Magnoliids/eudicots - ps18
Eudicots - ps19
Core_eudicots - ps20
Superrosids - ps21
Rosids - ps22
Eurosids - ps23
Malvids - ps24
Sap./Malv./Brassicales - ps25
Malvales/Brassicales - ps26
Brassicales - ps27
Cleomaceae/Brassicaceae - ps28
Brassicaceae - ps29
Camelineae - ps30
Arabidopsis - ps31
Arabidopsis_thaliana - ps32
p-value
5e-08
1e-06
3e-05
6e-04
1e-02
Log-odds
0.5
1.0
1.5
2.0
2.5
3.0
3.5
4.0

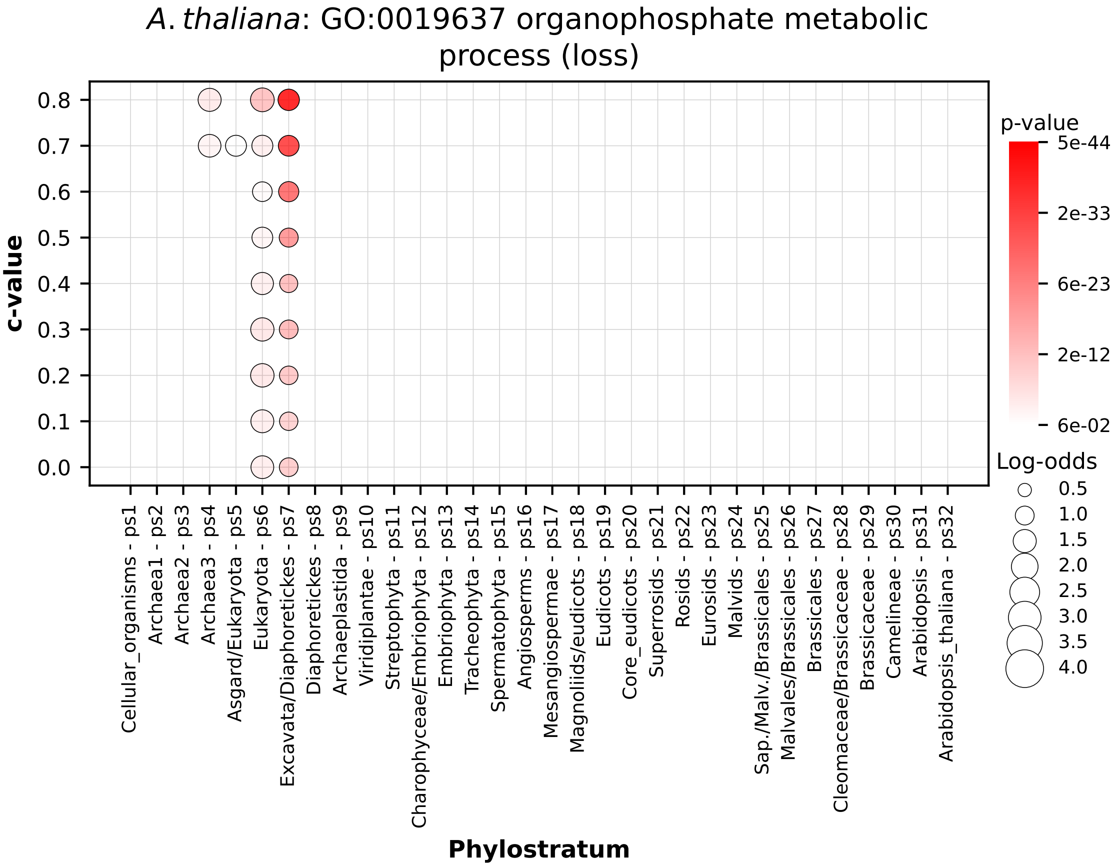

A. thaliana: GO:0019637 organophosphate metabolic process (loss)
c-value
0.8
0.7
0.6
0.5
0.4
0.3
0.2
0.1
0.0
Phylostratum
Cellular_organisms - ps1
Archaea1 - ps2
Archaea2 - ps3
Archaea3 - ps4
Asgard/Eukaryota - ps5
Eukaryota - ps6
Excavata/Diaphoretickes - ps7
Diaphoretickes - ps8
Archaeplastida - ps9
Viridiplantae - ps10
Streptophyta - ps11
Charophyceae/Embriophyta - ps12
Embriophyta - ps13
Tracheophyta - ps14
Spermatophyta - ps15
Angiosperms - ps16
Mesangiospermae - ps17
Magnoliids/eudicots - ps18
Eudicots - ps19
Core_eudicots - ps20
Superrosids - ps21
Rosids - ps22
Eurosids - ps23
Malvids - ps24
Sap./Malv./Brassicales - ps25
Malvales/Brassicales - ps26
Brassicales - ps27
Cleomaceae/Brassicaceae - ps28
Brassicaceae - ps29
Camelineae - ps30
Arabidopsis - ps31
Arabidopsis_thaliana - ps32
p-value
5e-44
2e-33
6e-23
2e-12
6e-02
Log-odds
0.5
1.0
1.5
2.0
2.5
3.0
3.5
4.0

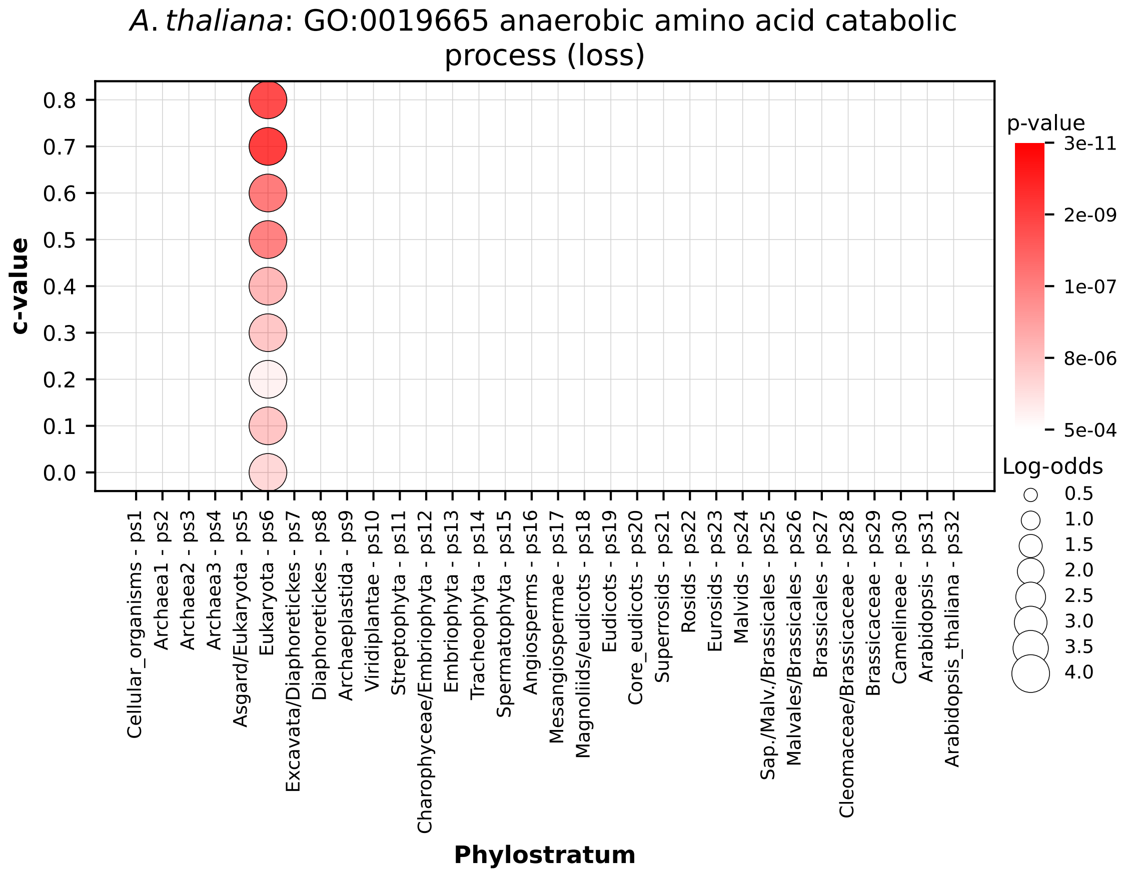
A. thaliana: GO:0019665 anaerobic amino acid catabolic process (loss)
c-value
0.8
0.7
0.6
0.5
0.4
0.3
0.2
0.1
0.0
p-value
3e-11
2e-09
1e-07
8e-06
5e-04
Log-odds
0.5
1.0
1.5
2.0
2.5
3.0
3.5
4.0
Cellular_organisms - ps1
Archaea1 - ps2
Archaea2 - ps3
Archaea3 - ps4
Asgard/Eukaryota - ps5
Eukaryota - ps6
Excavata/Diaphoretickes - ps7
Diaphoretickes - ps8
Archaeplastida - ps9
Viridiplantae - ps10
Streptophyta - ps11
Charophyceae/Embriophyta - ps12
Embriophyta - ps13
Tracheophyta - ps14
Spermatophyta - ps15
Angiosperms - ps16
Mesangiospermae - ps17
Magnoliids/eudicots - ps18
Eudicots - ps19
Core_eudicots - ps20
Superrosids - ps21
Rosids - ps22
Eurosids - ps23
Malvids - ps24
Sap./Malv./Brassicales - ps25
Malvales/Brassicales - ps26
Brassicales - ps27
Cleomaceae/Brassicaceae - ps28
Brassicaceae - ps29
Camelineae - ps30
Arabidopsis - ps31
Arabidopsis_thaliana - ps32
Phylostratum

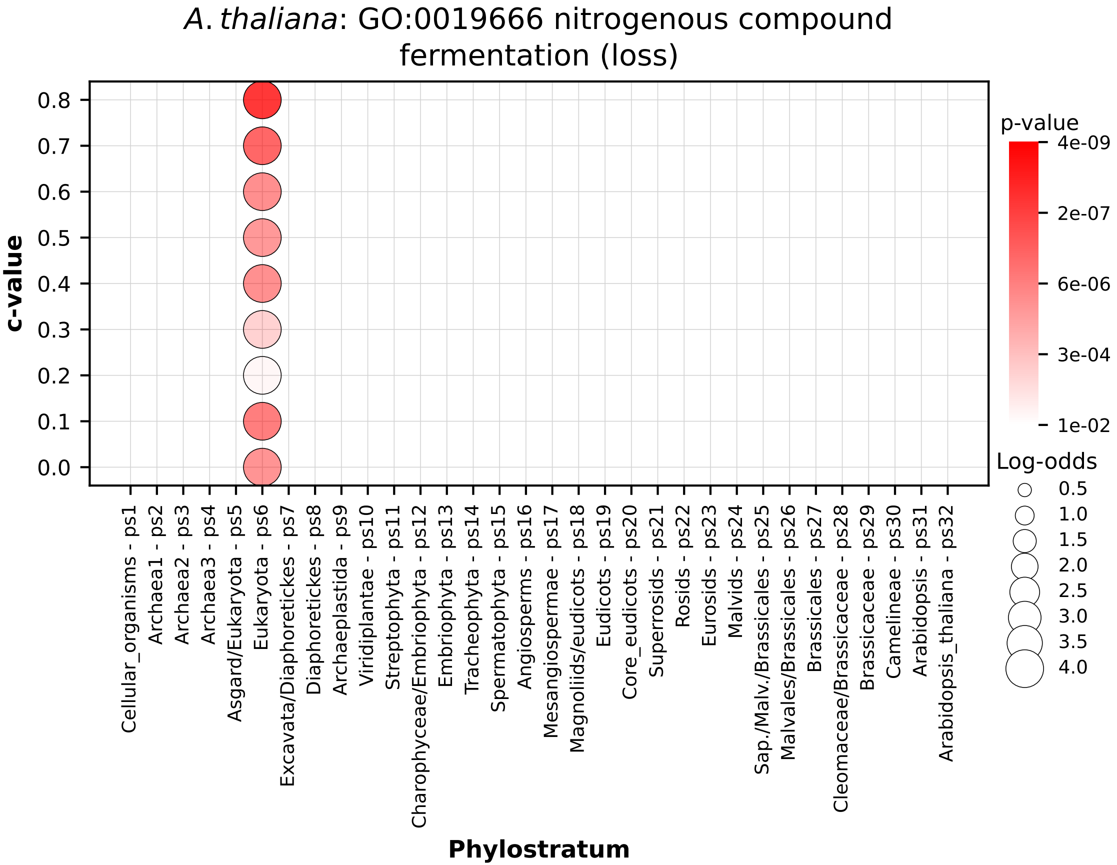

A. thaliana: GO:0019666 nitrogenous compound fermentation (loss)
c-value
0.8
0.7
0.6
0.5
0.4
0.3
0.2
0.1
0.0
p-value
4e-09
2e-07
6e-06
3e-04
1e-02
Log-odds
0.5
1.0
1.5
2.0
2.5
3.0
3.5
4.0
Cellular_organisms - ps1
Archaea1 - ps2
Archaea2 - ps3
Archaea3 - ps4
Asgard/Eukaryota - ps5
Eukaryota - ps6
Excavata/Diaphoretickes - ps7
Diaphoretickes - ps8
Archaeplastida - ps9
Viridiplantae - ps10
Streptophyta - ps11
Charophyceae/Embriophyta - ps12
Embriophyta - ps13
Tracheophyta - ps14
Spermatophyta - ps15
Angiosperms - ps16
Mesangiospermae - ps17
Magnoliids/eudicots - ps18
Eudicots - ps19
Core_eudicots - ps20
Superrosids - ps21
Rosids - ps22
Eurosids - ps23
Malvids - ps24
Sap./Malv./Brassicales - ps25
Malvales/Brassicales - ps26
Brassicales - ps27
Cleomaceae/Brassicaceae - ps28
Brassicaceae - ps29
Camelineae - ps30
Arabidopsis - ps31
Arabidopsis_thaliana - ps32
Phylostratum

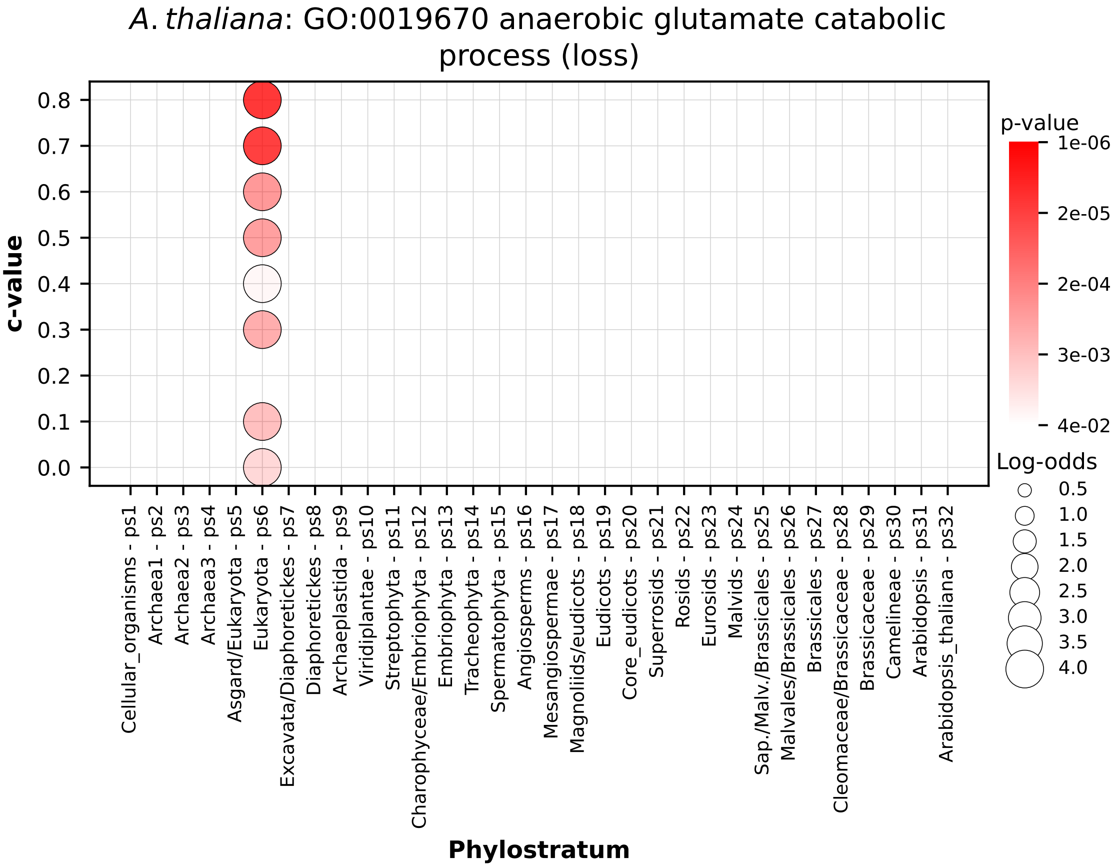
A. thaliana: GO:0019670 anaerobic glutamate catabolic process (loss)
c-value
0.0
0.1
0.2
0.3
0.4
0.5
0.6
0.7
0.8
p-value
1e-06
2e-05
2e-04
3e-03
4e-02
Log-odds
0.5
1.0
1.5
2.0
2.5
3.0
3.5
4.0
Cellular_organisms - ps1
Archaea1 - ps2
Archaea2 - ps3
Archaea3 - ps4
Asgard/Eukaryota - ps5
Eukaryota - ps6
Excavata/Diaphoretickes - ps7
Diaphoretickes - ps8
Archaeplastida - ps9
Viridiplantae - ps10
Streptophyta - ps11
Charophyceae/Embriophyta - ps12
Embriophyta - ps13
Tracheophyta - ps14
Spermatophyta - ps15
Angiosperms - ps16
Mesangiospermae - ps17
Magnoliids/eudicots - ps18
Eudicots - ps19
Core_eudicots - ps20
Superrosids - ps21
Rosids - ps22
Eurosids - ps23
Malvids - ps24
Sap./Malv./Brassicales - ps25
Malvales/Brassicales - ps26
Brassicales - ps27
Cleomaceae/Brassicaceae - ps28
Brassicaceae - ps29
Camelineae - ps30
Arabidopsis - ps31
Arabidopsis_thaliana - ps32
Phylostratum

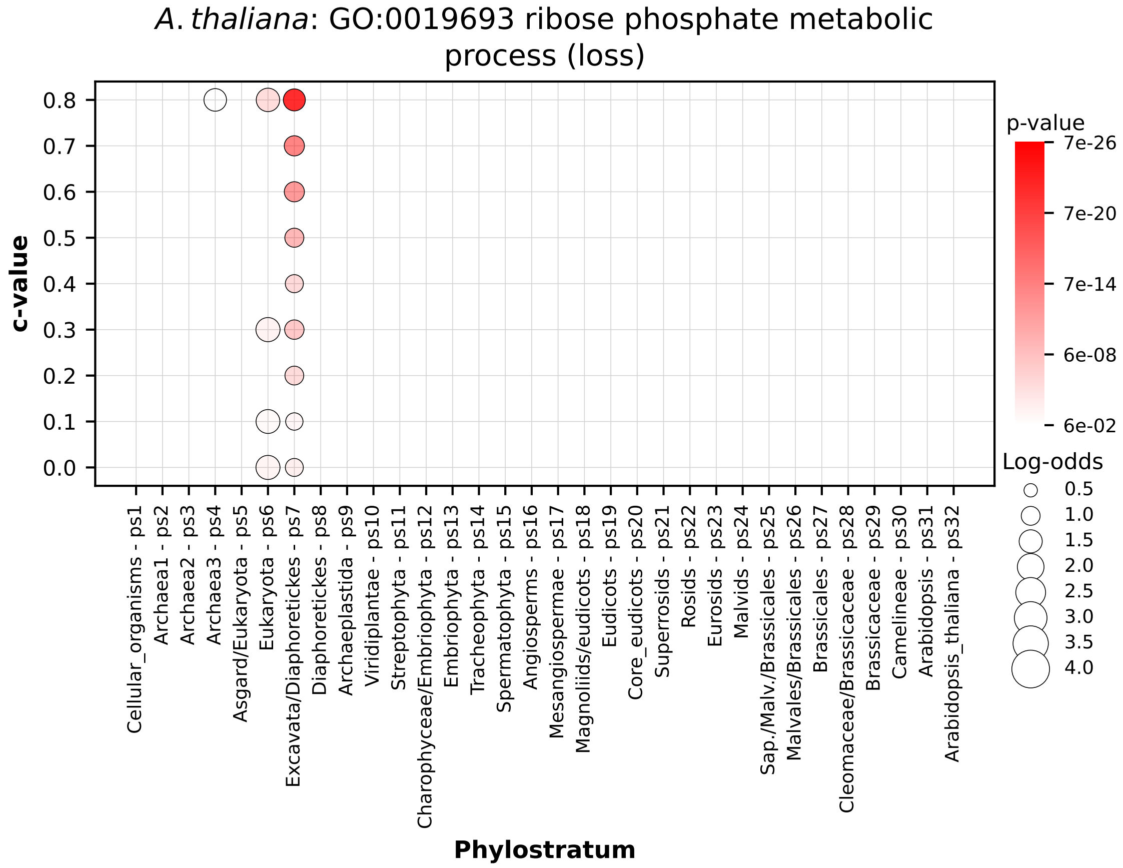

A. thaliana: GO:0019693 ribose phosphate metabolic process (loss)
c-value
0.8
0.7
0.6
0.5
0.4
0.3
0.2
0.1
0.0
p-value
7e-26
7e-20
7e-14
6e-08
6e-02
Log-odds
0.5
1.0
1.5
2.0
2.5
3.0
3.5
4.0
Cellular_organisms - ps1
Archaea1 - ps2
Archaea2 - ps3
Archaea3 - ps4
Asgard/Eukaryota - ps5
Eukaryota - ps6
Excavata/Diaphoretickes - ps7
Diaphoretickes - ps8
Archaeplastida - ps9
Viridiplantae - ps10
Streptophyta - ps11
Charophyceae/Embriophyta - ps12
Embriophyta - ps13
Tracheophyta - ps14
Spermatophyta - ps15
Angiosperms - ps16
Mesangiospermae - ps17
Magnoliids/eudicots - ps18
Eudicots - ps19
Core_eudicots - ps20
Superrosids - ps21
Rosids - ps22
Eurosids - ps23
Malvids - ps24
Sap./Malv./Brassicales - ps25
Malvales/Brassicales - ps26
Brassicales - ps27
Cleomaceae/Brassicaceae - ps28
Brassicaceae - ps29
Camelineae - ps30
Arabidopsis - ps31
Arabidopsis_thaliana - ps32
Phylostratum

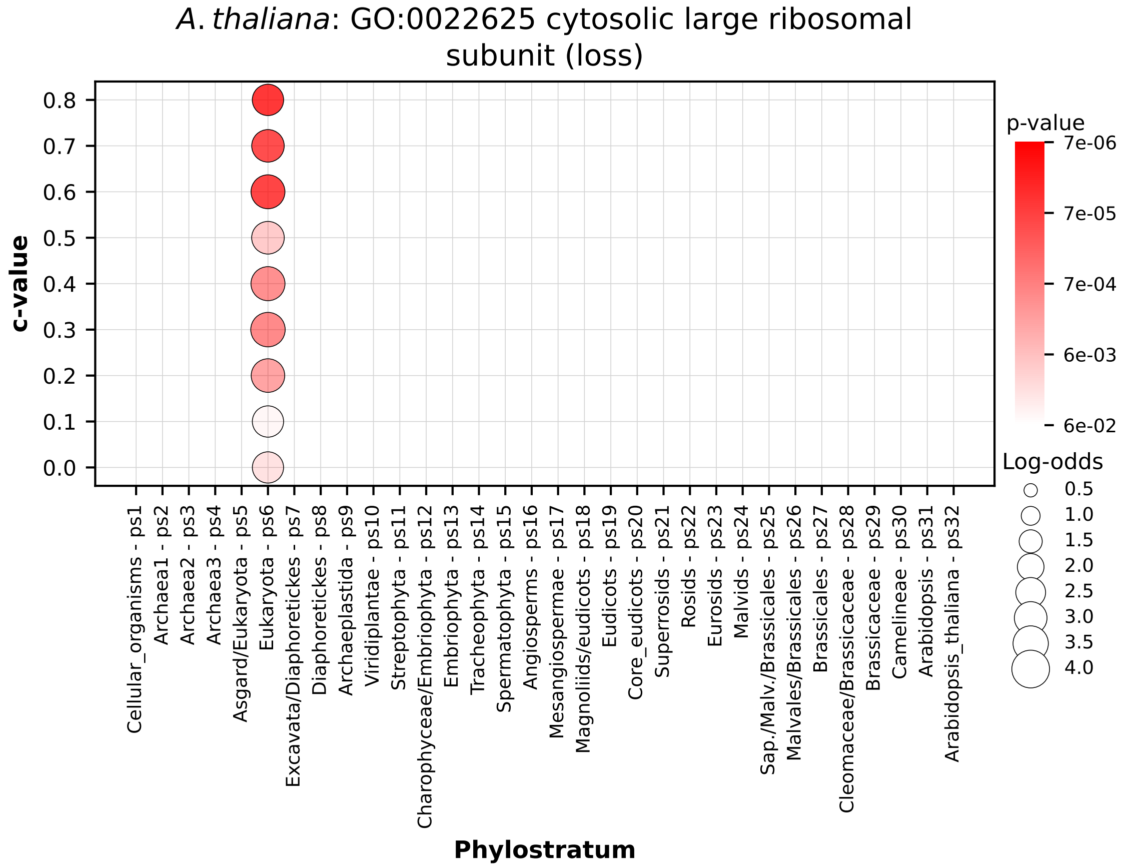

A. thaliana: GO:0022625 cytosolic large ribosomal subunit (loss)
c-value
0.8
0.7
0.6
0.5
0.4
0.3
0.2
0.1
0.0
Phylostratum
Cellular_organisms - ps1
Archaea1 - ps2
Archaea2 - ps3
Archaea3 - ps4
Asgard/Eukaryota - ps5
Eukaryota - ps6
Excavata/Diaphoretickes - ps7
Diaphoretickes - ps8
Archaeplastida - ps9
Viridiplantae - ps10
Streptophyta - ps11
Charophyceae/Embriophyta - ps12
Embriophyta - ps13
Tracheophyta - ps14
Spermatophyta - ps15
Angiosperms - ps16
Mesangiospermae - ps17
Magnoliids/eudicots - ps18
Eudicots - ps19
Core_eudicots - ps20
Superrosids - ps21
Rosids - ps22
Eurosids - ps23
Malvids - ps24
Sap./Malv./Brassicales - ps25
Malvales/Brassicales - ps26
Brassicales - ps27
Cleomaceae/Brassicaceae - ps28
Brassicaceae - ps29
Camelineae - ps30
Arabidopsis - ps31
Arabidopsis_thaliana - ps32
p-value
7e-06
7e-05
7e-04
6e-03
6e-02
Log-odds
0.5
1.0
1.5
2.0
2.5
3.0
3.5
4.0

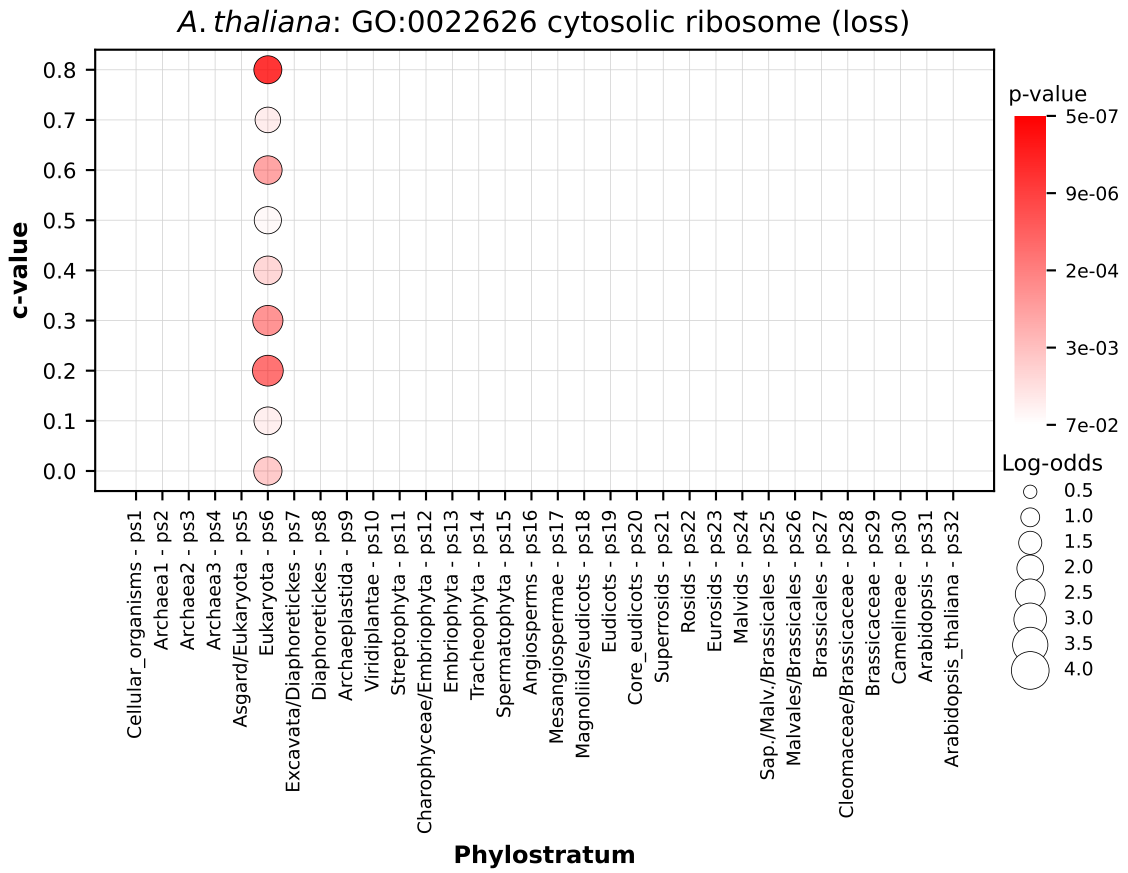
A. thaliana: GO:0022626 cytosolic ribosome (loss)
c-value
0.8
0.7
0.6
0.5
0.4
0.3
0.2
0.1
0.0
p-value
5e-07
9e-06
2e-04
3e-03
7e-02
Log-odds
0.5
1.0
1.5
2.0
2.5
3.0
3.5
4.0
Cellular_organisms - ps1
Archaea1 - ps2
Archaea2 - ps3
Archaea3 - ps4
Asgard/Eukaryota - ps5
Eukaryota - ps6
Excavata/Diaphoretickes - ps7
Diaphoretickes - ps8
Archaeplastida - ps9
Viridiplantae - ps10
Streptophyta - ps11
Charophyceae/Embriophyta - ps12
Embriophyta - ps13
Tracheophyta - ps14
Spermatophyta - ps15
Angiosperms - ps16
Mesangiospermae - ps17
Magnoliids/eudicots - ps18
Eudicots - ps19
Core_eudicots - ps20
Superrosids - ps21
Rosids - ps22
Eurosids - ps23
Malvids - ps24
Sap./Malv./Brassicales - ps25
Malvales/Brassicales - ps26
Brassicales - ps27
Cleomaceae/Brassicaceae - ps28
Brassicaceae - ps29
Camelineae - ps30
Arabidopsis - ps31
Arabidopsis_thaliana - ps32
Phylostratum

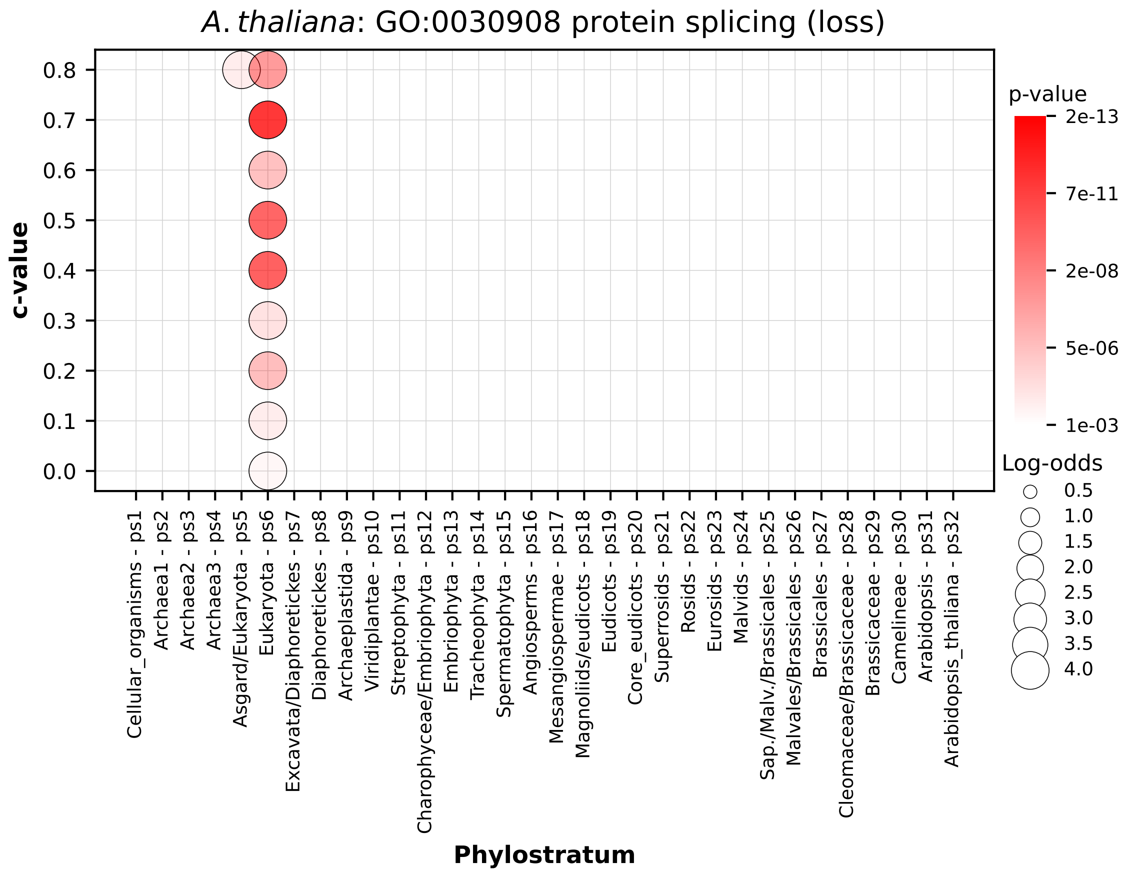

A. thaliana: GO:0030908 protein splicing (loss)
c-value
0.0
0.1
0.2
0.3
0.4
0.5
0.6
0.7
0.8
Phylostratum
Cellular_organisms - ps1
Archaea1 - ps2
Archaea2 - ps3
Archaea3 - ps4
Asgard/Eukaryota - ps5
Eukaryota - ps6
Excavata/Diaphoretickes - ps7
Diaphoretickes - ps8
Archaeplastida - ps9
Viridiplantae - ps10
Streptophyta - ps11
Charophyceae/Embriophyta - ps12
Embriophyta - ps13
Tracheophyta - ps14
Spermatophyta - ps15
Angiosperms - ps16
Mesangiospermae - ps17
Magnoliids/eudicots - ps18
Eudicots - ps19
Core_eudicots - ps20
Superrosids - ps21
Rosids - ps22
Eurosids - ps23
Malvids - ps24
Sap./Malv./Brassicales - ps25
Malvales/Brassicales - ps26
Brassicales - ps27
Cleomaceae/Brassicaceae - ps28
Brassicaceae - ps29
Camelineae - ps30
Arabidopsis - ps31
Arabidopsis_thaliana - ps32
p-value
2e-13
7e-11
2e-08
5e-06
1e-03
Log-odds
0.5
1.0
1.5
2.0
2.5
3.0
3.5
4.0

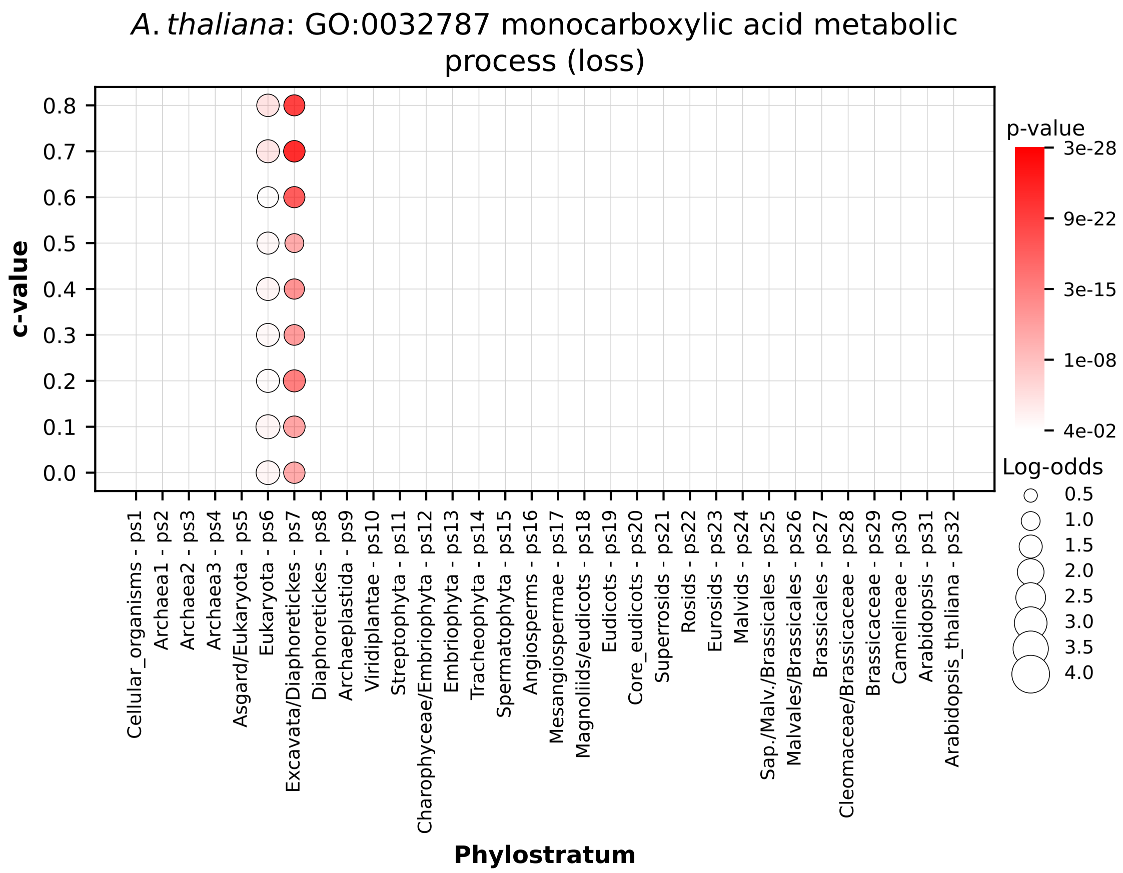

A. thaliana: GO:0032787 monocarboxylic acid metabolic process (loss)
c-value
0.8
0.7
0.6
0.5
0.4
0.3
0.2
0.1
0.0
p-value
3e-28
9e-22
3e-15
1e-08
4e-02
Log-odds
0.5
1.0
1.5
2.0
2.5
3.0
3.5
4.0
Cellular_organisms - ps1
Archaea1 - ps2
Archaea2 - ps3
Archaea3 - ps4
Asgard/Eukaryota - ps5
Eukaryota - ps6
Excavata/Diaphoretickes - ps7
Diaphoretickes - ps8
Archaeplastida - ps9
Viridiplantae - ps10
Streptophyta - ps11
Charophyceae/Embriophyta - ps12
Embriophyta - ps13
Tracheophyta - ps14
Spermatophyta - ps15
Angiosperms - ps16
Mesangiospermae - ps17
Magnoliids/eudicots - ps18
Eudicots - ps19
Core_eudicots - ps20
Superrosids - ps21
Rosids - ps22
Eurosids - ps23
Malvids - ps24
Sap./Malv./Brassicales - ps25
Malvales/Brassicales - ps26
Brassicales - ps27
Cleomaceae/Brassicaceae - ps28
Brassicaceae - ps29
Camelineae - ps30
Arabidopsis - ps31
Arabidopsis_thaliana - ps32
Phylostratum

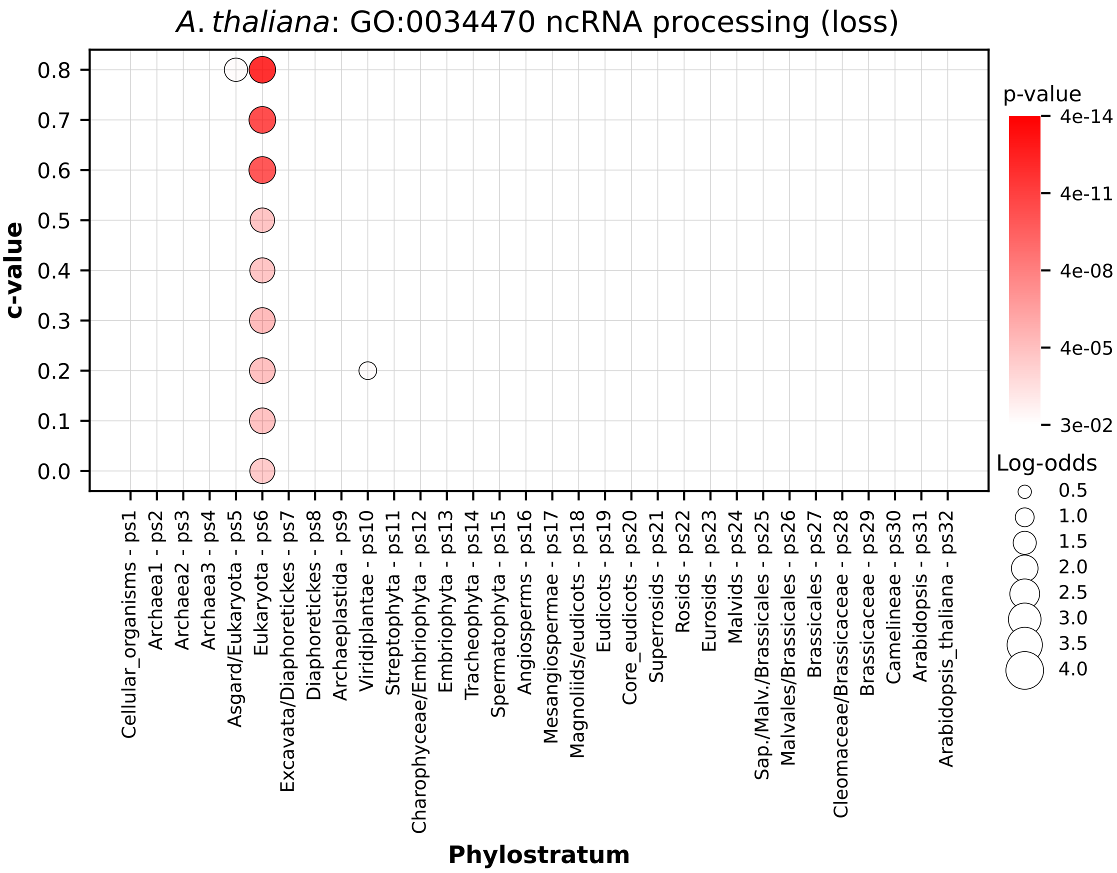
A. thaliana: GO:0034470 ncRNA processing (loss)
c-value
0.8
0.7
0.6
0.5
0.4
0.3
0.2
0.1
0.0
p-value
4e-14
4e-11
4e-08
4e-05
3e-02
Log-odds
0.5
1.0
1.5
2.0
2.5
3.0
3.5
4.0
Cellular_organisms - ps1
Archaea1 - ps2
Archaea2 - ps3
Archaea3 - ps4
Asgard/Eukaryota - ps5
Eukaryota - ps6
Excavata/Diaphoretickes - ps7
Diaphoretickes - ps8
Archaeplastida - ps9
Viridiplantae - ps10
Streptophyta - ps11
Charophyceae/Embriophyta - ps12
Embriophyta - ps13
Tracheophyta - ps14
Spermatophyta - ps15
Angiosperms - ps16
Mesangiospermae - ps17
Magnoliids/eudicots - ps18
Eudicots - ps19
Core_eudicots - ps20
Superrosids - ps21
Rosids - ps22
Eurosids - ps23
Malvids - ps24
Sap./Malv./Brassicales - ps25
Malvales/Brassicales - ps26
Brassicales - ps27
Cleomaceae/Brassicaceae - ps28
Brassicaceae - ps29
Camelineae - ps30
Arabidopsis - ps31
Arabidopsis_thaliana - ps32
Phylostratum

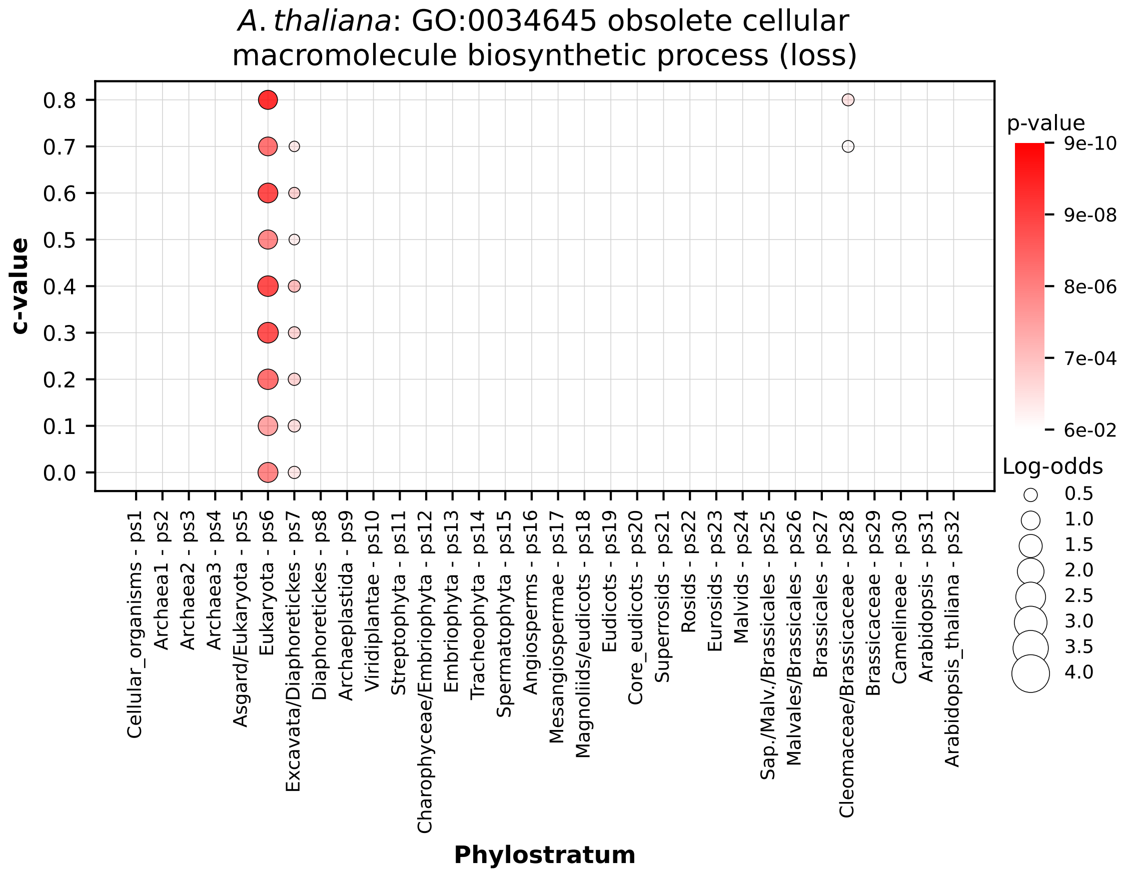

A. thaliana: GO:0034645 obsolete cellular macromolecule biosynthetic process (loss)
c-value
0.8
0.7
0.6
0.5
0.4
0.3
0.2
0.1
0.0
p-value
9e-10
9e-08
8e-06
7e-04
6e-02
Log-odds
0.5
1.0
1.5
2.0
2.5
3.0
3.5
4.0
Cellular_organisms - ps1
Archaea1 - ps2
Archaea2 - ps3
Archaea3 - ps4
Asgard/Eukaryota - ps5
Eukaryota - ps6
Excavata/Diaphoretickes - ps7
Diaphoretickes - ps8
Archaeplastida - ps9
Viridiplantae - ps10
Streptophyta - ps11
Charophyceae/Embriophyta - ps12
Embriophyta - ps13
Tracheophyta - ps14
Spermatophyta - ps15
Angiosperms - ps16
Mesangiospermae - ps17
Magnoliids/eudicots - ps18
Eudicots - ps19
Core_eudicots - ps20
Superrosids - ps21
Rosids - ps22
Eurosids - ps23
Malvids - ps24
Sap./Malv./Brassicales - ps25
Malvales/Brassicales - ps26
Brassicales - ps27
Cleomaceae/Brassicaceae - ps28
Brassicaceae - ps29
Camelineae - ps30
Arabidopsis - ps31
Arabidopsis_thaliana - ps32
Phylostratum

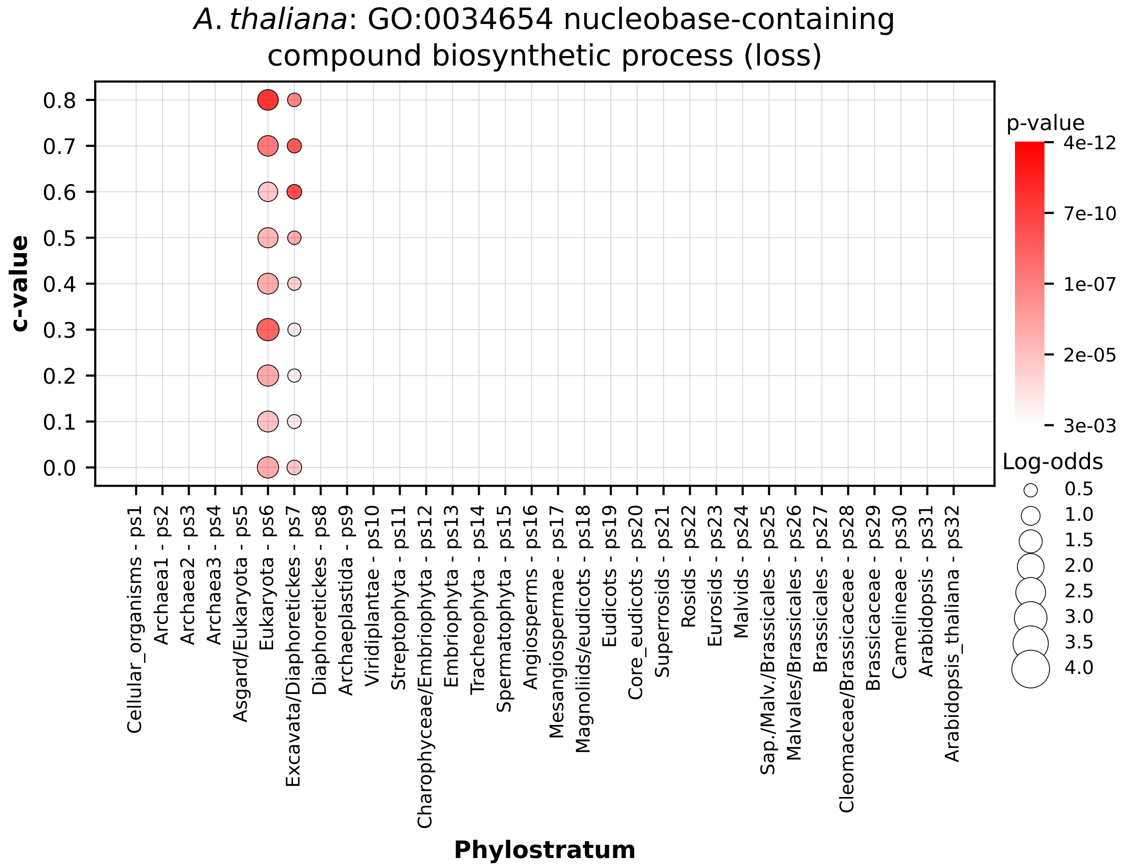
A. thaliana: GO:0034654 nucleobase-containing compound biosynthetic process (loss)
c-value
0.8
0.7
0.6
0.5
0.4
0.3
0.2
0.1
0.0
Phylostratum
Cellular_organisms - ps1
Archaea1 - ps2
Archaea2 - ps3
Archaea3 - ps4
Asgard/Eukaryota - ps5
Eukaryota - ps6
Excavata/Diaphoretickes - ps7
Diaphoretickes - ps8
Archaeplastida - ps9
Viridiplantae - ps10
Streptophyta - ps11
Charophyceae/Embriophyta - ps12
Embriophyta - ps13
Tracheophyta - ps14
Spermatophyta - ps15
Angiosperms - ps16
Mesangiospermae - ps17
Magnoliids/eudicots - ps18
Eudicots - ps19
Core_eudicots - ps20
Superrosids - ps21
Rosids - ps22
Eurosids - ps23
Malvids - ps24
Sap./Malv./Brassicales - ps25
Malvales/Brassicales - ps26
Brassicales - ps27
Cleomaceae/Brassicaceae - ps28
Brassicaceae - ps29
Camelineae - ps30
Arabidopsis - ps31
Arabidopsis_thaliana - ps32
p-value
4e-12
7e-10
1e-07
2e-05
3e-03
Log-odds
0.5
1.0
1.5
2.0
2.5
3.0
3.5
4.0

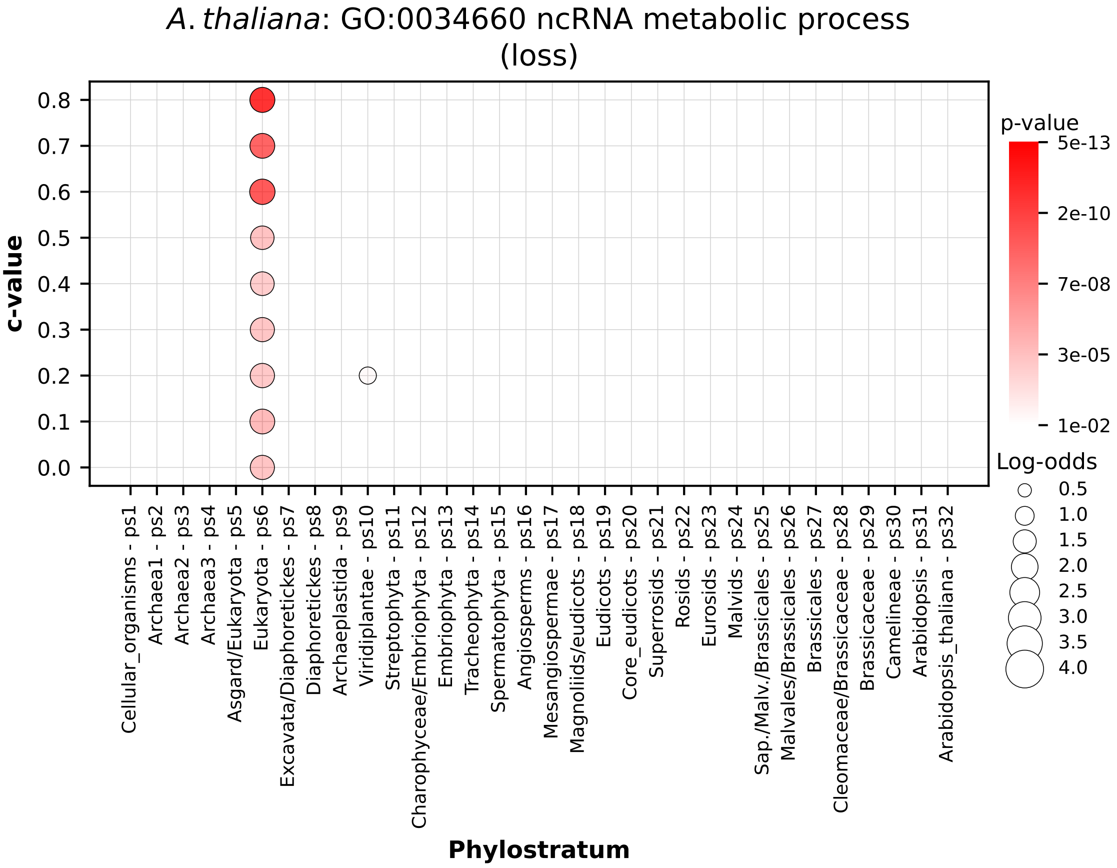
A. thaliana: GO:0034660 ncRNA metabolic process
(loss)
c-value
0.8
0.7
0.6
0.5
0.4
0.3
0.2
0.1
0.0
p-value
5e-13
2e-10
7e-08
3e-05
1e-02
Log-odds
0.5
1.0
1.5
2.0
2.5
3.0
3.5
4.0
Cellular_organisms - ps1
Archaea1 - ps2
Archaea2 - ps3
Archaea3 - ps4
Asgard/Eukaryota - ps5
Eukaryota - ps6
Excavata/Diaphoretickes - ps7
Diaphoretickes - ps8
Archaeplastida - ps9
Viridiplantae - ps10
Streptophyta - ps11
Charophyceae/Embriophyta - ps12
Embriophyta - ps13
Tracheophyta - ps14
Spermatophyta - ps15
Angiosperms - ps16
Mesangiospermae - ps17
Magnoliids/eudicots - ps18
Eudicots - ps19
Core_eudicots - ps20
Superrosids - ps21
Rosids - ps22
Eurosids - ps23
Malvids - ps24
Sap./Malv./Brassicales - ps25
Malvales/Brassicales - ps26
Brassicales - ps27
Cleomaceae/Brassicaceae - ps28
Brassicaceae - ps29
Camelineae - ps30
Arabidopsis - ps31
Arabidopsis_thaliana - ps32
Phylostratum

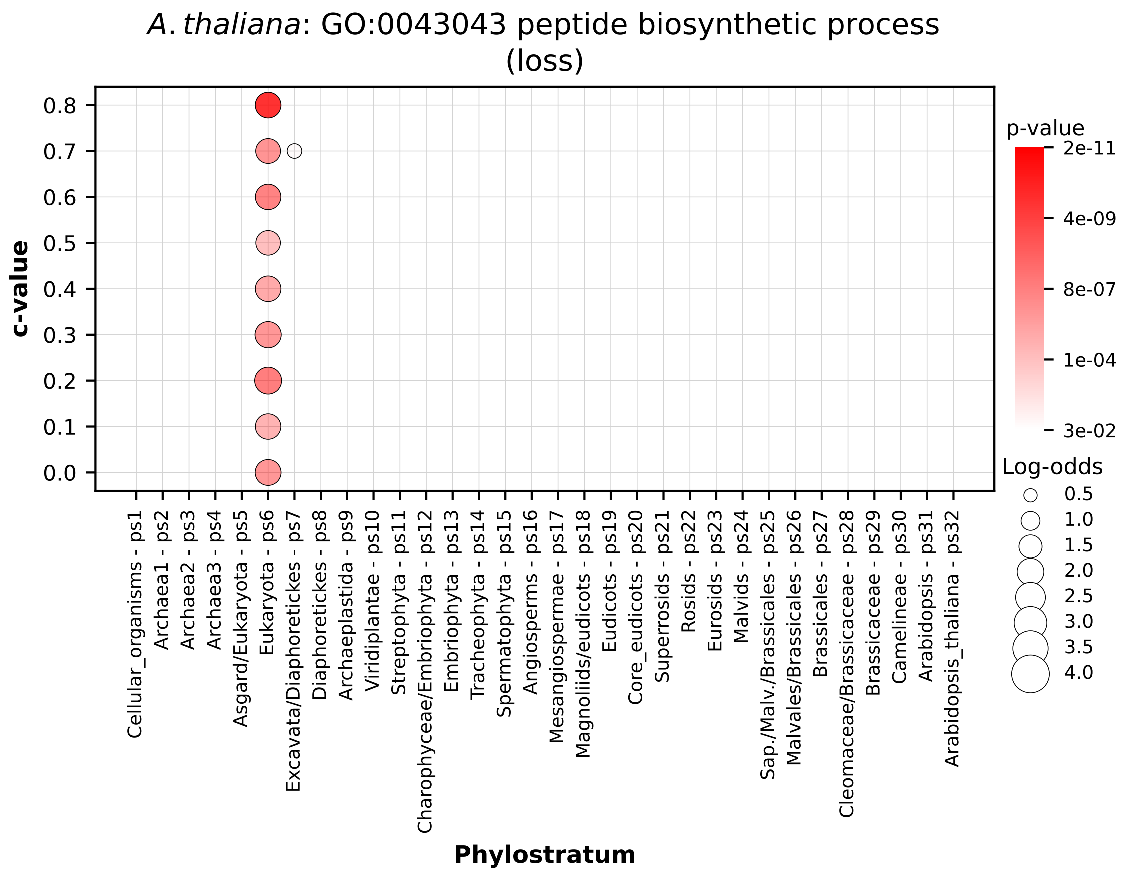

A. thaliana: GO:0043043 peptide biosynthetic process
(loss)
c-value
0.8
0.7
0.6
0.5
0.4
0.3
0.2
0.1
0.0
p-value
2e-11
4e-09
8e-07
1e-04
3e-02
Log-odds
0.5
1.0
1.5
2.0
2.5
3.0
3.5
4.0
Cellular_organisms - ps1
Archaea1 - ps2
Archaea2 - ps3
Archaea3 - ps4
Asgard/Eukaryota - ps5
Eukaryota - ps6
Excavata/Diaphoretickes - ps7
Diaphoretickes - ps8
Archaeplastida - ps9
Viridiplantae - ps10
Streptophyta - ps11
Charophyceae/Embriophyta - ps12
Embriophyta - ps13
Tracheophyta - ps14
Spermatophyta - ps15
Angiosperms - ps16
Mesangiospermae - ps17
Magnoliids/eudicots - ps18
Eudicots - ps19
Core_eudicots - ps20
Superrosids - ps21
Rosids - ps22
Eurosids - ps23
Malvids - ps24
Sap./Malv./Brassicales - ps25
Malvales/Brassicales - ps26
Brassicales - ps27
Cleomaceae/Brassicaceae - ps28
Brassicaceae - ps29
Camelineae - ps30
Arabidopsis - ps31
Arabidopsis_thaliana - ps32
Phylostratum

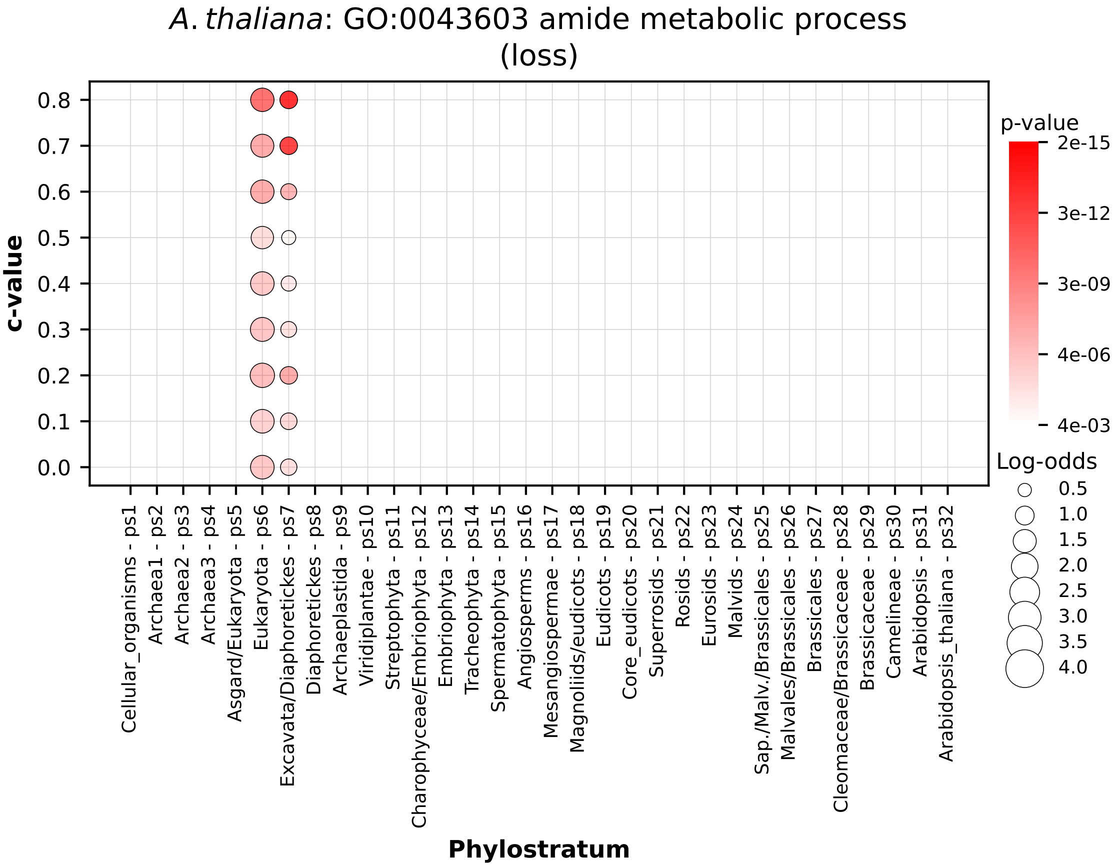

A. thaliana: GO:0043603 amide metabolic process
(loss)
c-value
0.8
0.7
0.6
0.5
0.4
0.3
0.2
0.1
0.0
p-value
2e-15
3e-12
3e-09
4e-06
4e-03
Log-odds
0.5
1.0
1.5
2.0
2.5
3.0
3.5
4.0
Cellular_organisms - ps1
Archaea1 - ps2
Archaea2 - ps3
Archaea3 - ps4
Asgard/Eukaryota - ps5
Eukaryota - ps6
Excavata/Diaphoretickes - ps7
Diaphoretickes - ps8
Archaeplastida - ps9
Viridiplantae - ps10
Streptophyta - ps11
Charophyceae/Embriophyta - ps12
Embriophyta - ps13
Tracheophyta - ps14
Spermatophyta - ps15
Angiosperms - ps16
Mesangiospermae - ps17
Magnoliids/eudicots - ps18
Eudicots - ps19
Core_eudicots - ps20
Superrosids - ps21
Rosids - ps22
Eurosids - ps23
Malvids - ps24
Sap./Malv./Brassicales - ps25
Malvales/Brassicales - ps26
Brassicales - ps27
Cleomaceae/Brassicaceae - ps28
Brassicaceae - ps29
Camelineae - ps30
Arabidopsis - ps31
Arabidopsis_thaliana - ps32
Phylostratum

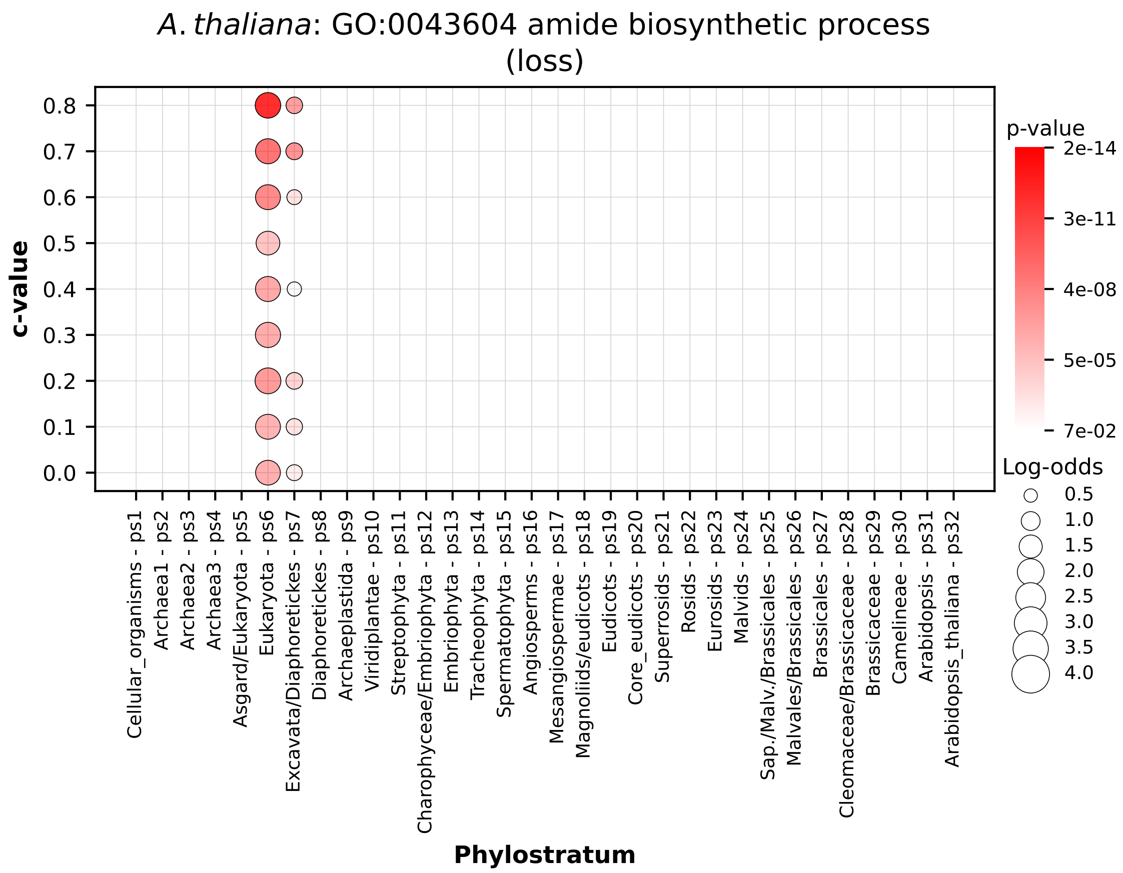

A. thaliana: GO:0043604 amide biosynthetic process
(loss)
c-value
0.8
0.7
0.6
0.5
0.4
0.3
0.2
0.1
0.0
Phylostratum
Cellular_organisms - ps1
Archaea1 - ps2
Archaea2 - ps3
Archaea3 - ps4
Asgard/Eukaryota - ps5
Eukaryota - ps6
Excavata/Diaphoretickes - ps7
Diaphoretickes - ps8
Archaeplastida - ps9
Viridiplantae - ps10
Streptophyta - ps11
Charophyceae/Embriophyta - ps12
Embriophyta - ps13
Tracheophyta - ps14
Spermatophyta - ps15
Angiosperms - ps16
Mesangiospermae - ps17
Magnoliids/eudicots - ps18
Eudicots - ps19
Core_eudicots - ps20
Superrosids - ps21
Rosids - ps22
Eurosids - ps23
Malvids - ps24
Sap./Malv./Brassicales - ps25
Malvales/Brassicales - ps26
Brassicales - ps27
Cleomaceae/Brassicaceae - ps28
Brassicaceae - ps29
Camelineae - ps30
Arabidopsis - ps31
Arabidopsis_thaliana - ps32
p-value
2e-14
3e-11
4e-08
5e-05
7e-02
Log-odds
0.5
1.0
1.5
2.0
2.5
3.0
3.5
4.0

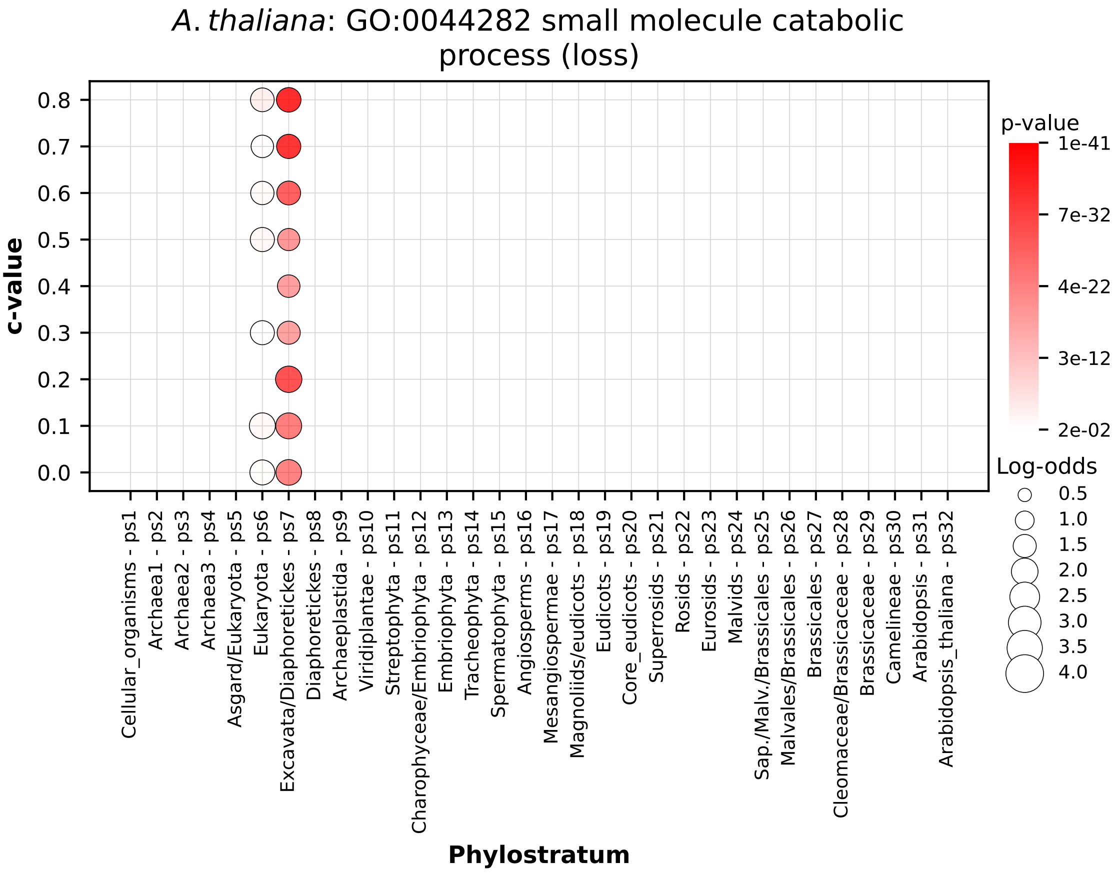

A. thaliana: GO:0044282 small molecule catabolic process (loss)
c-value
0.8
0.7
0.6
0.5
0.4
0.3
0.2
0.1
0.0
p-value
1e-41
7e-32
4e-22
3e-12
2e-02
Log-odds
0.5
1.0
1.5
2.0
2.5
3.0
3.5
4.0
Cellular_organisms - ps1
Archaea1 - ps2
Archaea2 - ps3
Archaea3 - ps4
Asgard/Eukaryota - ps5
Eukaryota - ps6
Excavata/Diaphoretickes - ps7
Diaphoretickes - ps8
Archaeplastida - ps9
Viridiplantae - ps10
Streptophyta - ps11
Charophyceae/Embriophyta - ps12
Embriophyta - ps13
Tracheophyta - ps14
Spermatophyta - ps15
Angiosperms - ps16
Mesangiospermae - ps17
Magnoliids/eudicots - ps18
Eudicots - ps19
Core_eudicots - ps20
Superrosids - ps21
Rosids - ps22
Eurosids - ps23
Malvids - ps24
Sap./Malv./Brassicales - ps25
Malvales/Brassicales - ps26
Brassicales - ps27
Cleomaceae/Brassicaceae - ps28
Brassicaceae - ps29
Camelineae - ps30
Arabidopsis - ps31
Arabidopsis_thaliana - ps32
Phylostratum

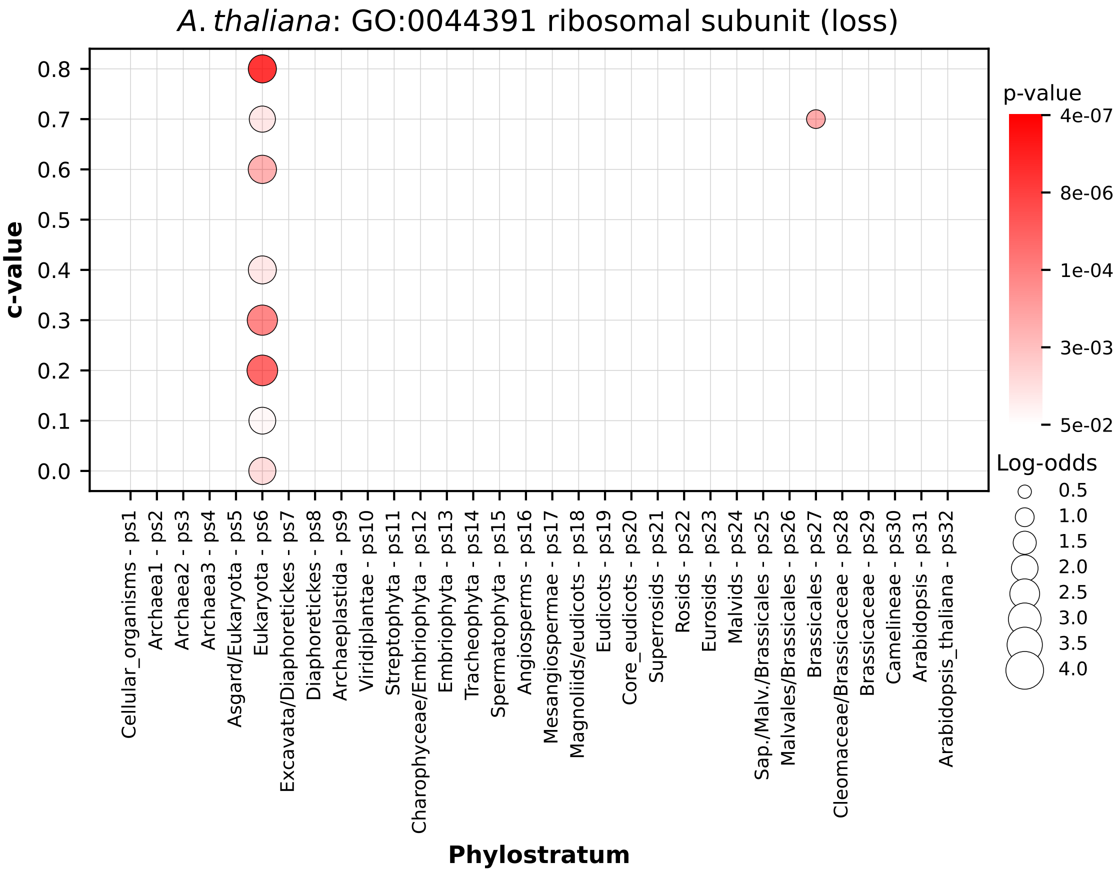

A. thaliana: GO:0044391 ribosomal subunit (loss)
c-value
0.0
0.1
0.2
0.3
0.4
0.5
0.6
0.7
0.8
p-value
4e-07
8e-06
1e-04
3e-03
5e-02
Log-odds
0.5
1.0
1.5
2.0
2.5
3.0
3.5
4.0
Cellular_organisms - ps1
Archaea1 - ps2
Archaea2 - ps3
Archaea3 - ps4
Asgard/Eukaryota - ps5
Eukaryota - ps6
Excavata/Diaphoretickes - ps7
Diaphoretickes - ps8
Archaeplastida - ps9
Viridiplantae - ps10
Streptophyta - ps11
Charophyceae/Embriophyta - ps12
Embriophyta - ps13
Tracheophyta - ps14
Spermatophyta - ps15
Angiosperms - ps16
Mesangiospermae - ps17
Magnoliids/eudicots - ps18
Eudicots - ps19
Core_eudicots - ps20
Superrosids - ps21
Rosids - ps22
Eurosids - ps23
Malvids - ps24
Sap./Malv./Brassicales - ps25
Malvales/Brassicales - ps26
Brassicales - ps27
Cleomaceae/Brassicaceae - ps28
Brassicaceae - ps29
Camelineae - ps30
Arabidopsis - ps31
Arabidopsis_thaliana - ps32
Phylostratum

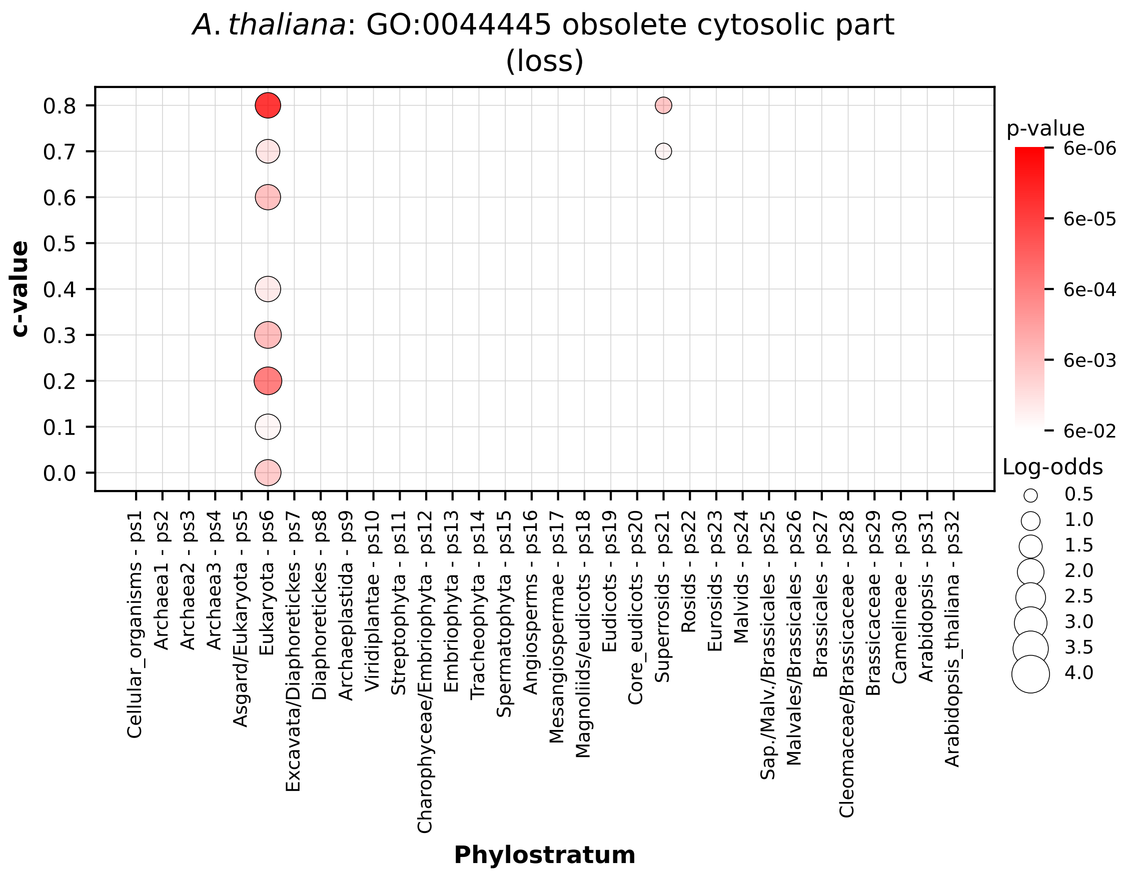

A. thaliana: GO:0044445 obsolete cytosolic part
(loss)
c-value
0.8
0.7
0.6
0.5
0.4
0.3
0.2
0.1
0.0
p-value
6e-06
6e-05
6e-04
6e-03
6e-02
Log-odds
0.5
1.0
1.5
2.0
2.5
3.0
3.5
4.0
Cellular_organisms - ps1
Archaea1 - ps2
Archaea2 - ps3
Archaea3 - ps4
Asgard/Eukaryota - ps5
Eukaryota - ps6
Excavata/Diaphoretickes - ps7
Diaphoretickes - ps8
Archaeplastida - ps9
Viridiplantae - ps10
Streptophyta - ps11
Charophyceae/Embriophyta - ps12
Embriophyta - ps13
Tracheophyta - ps14
Spermatophyta - ps15
Angiosperms - ps16
Mesangiospermae - ps17
Magnoliids/eudicots - ps18
Eudicots - ps19
Core_eudicots - ps20
Superrosids - ps21
Rosids - ps22
Eurosids - ps23
Malvids - ps24
Sap./Malv./Brassicales - ps25
Malvales/Brassicales - ps26
Brassicales - ps27
Cleomaceae/Brassicaceae - ps28
Brassicaceae - ps29
Camelineae - ps30
Arabidopsis - ps31
Arabidopsis_thaliana - ps32
Phylostratum

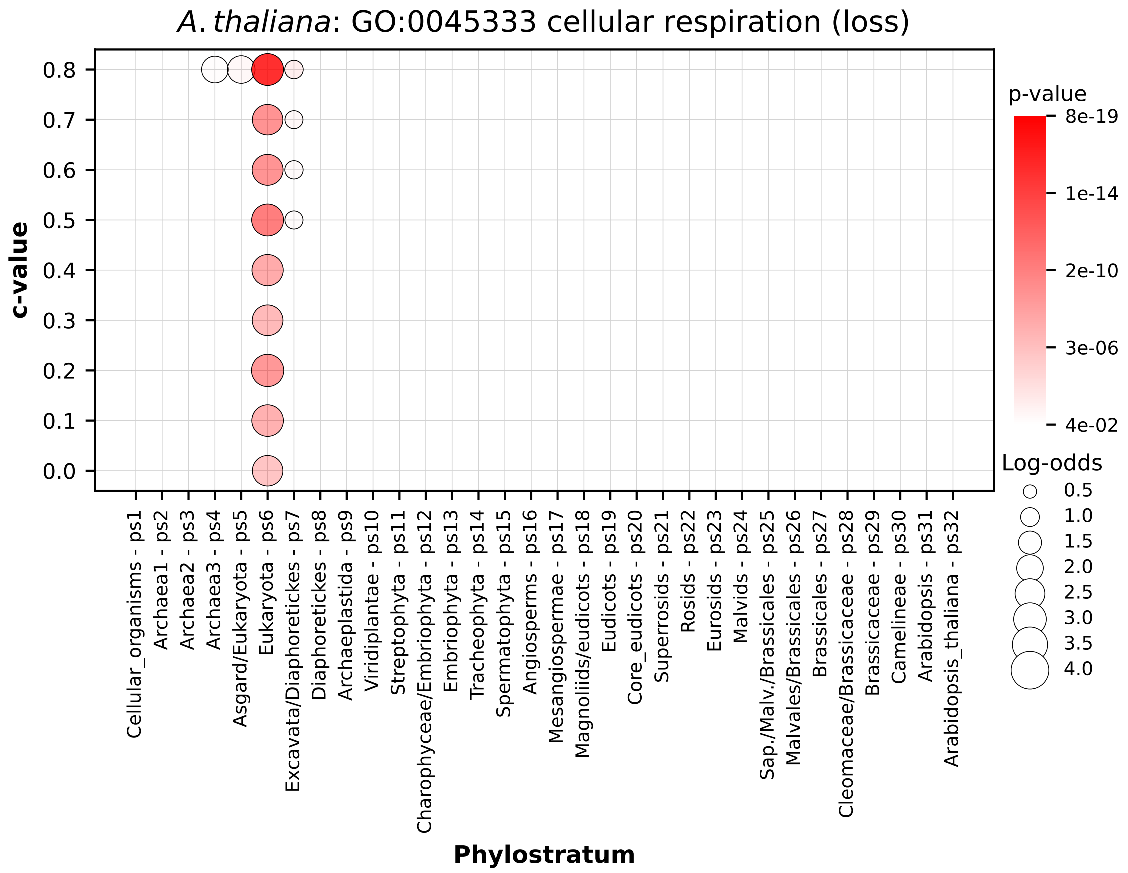

A. thaliana: GO:0045333 cellular respiration (loss)
c-value
0.0
0.1
0.2
0.3
0.4
0.5
0.6
0.7
0.8
p-value
8e-19
1e-14
2e-10
3e-06
4e-02
Log-odds
0.5
1.0
1.5
2.0
2.5
3.0
3.5
4.0
Cellular_organisms - ps1
Archaea1 - ps2
Archaea2 - ps3
Archaea3 - ps4
Asgard/Eukaryota - ps5
Eukaryota - ps6
Excavata/Diaphoretickes - ps7
Diaphoretickes - ps8
Archaeplastida - ps9
Viridiplantae - ps10
Streptophyta - ps11
Charophyceae/Embriophyta - ps12
Embriophyta - ps13
Tracheophyta - ps14
Spermatophyta - ps15
Angiosperms - ps16
Mesangiospermae - ps17
Magnoliids/eudicots - ps18
Eudicots - ps19
Core_eudicots - ps20
Superrosids - ps21
Rosids - ps22
Eurosids - ps23
Malvids - ps24
Sap./Malv./Brassicales - ps25
Malvales/Brassicales - ps26
Brassicales - ps27
Cleomaceae/Brassicaceae - ps28
Brassicaceae - ps29
Camelineae - ps30
Arabidopsis - ps31
Arabidopsis_thaliana - ps32
Phylostratum

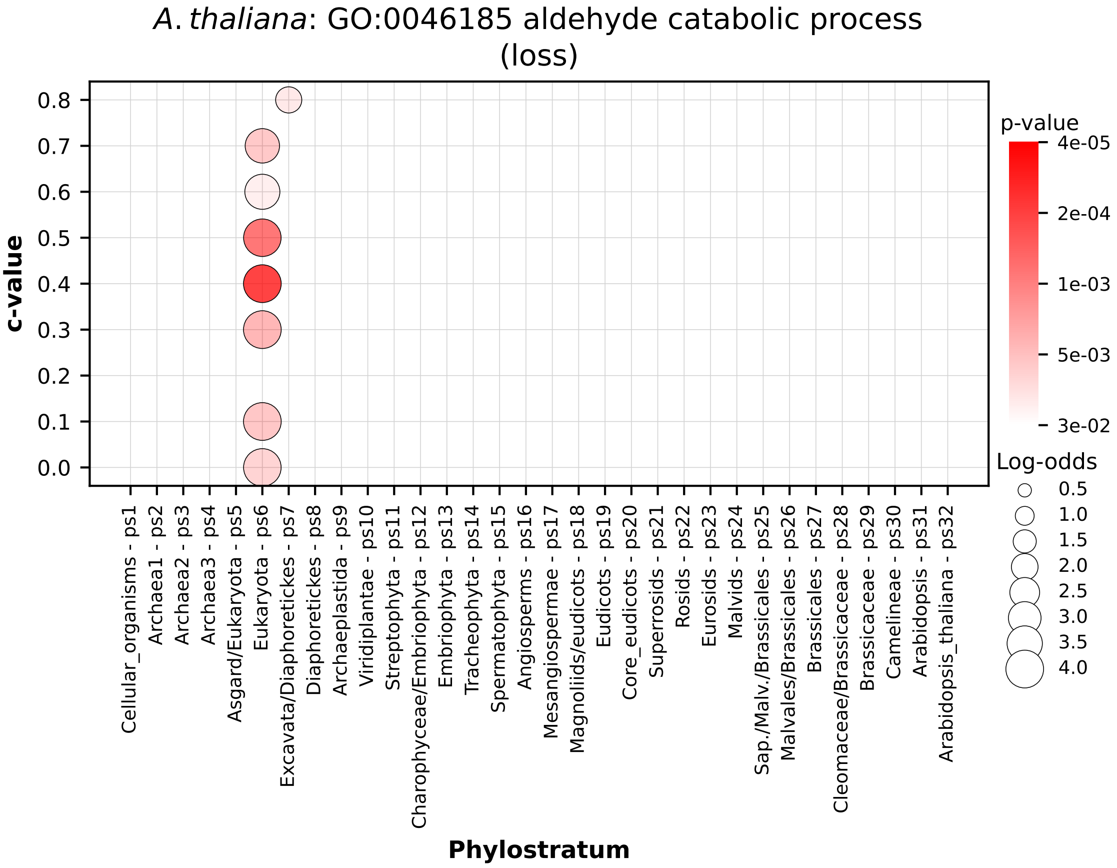

A. thaliana: GO:0046185 aldehyde catabolic process
(loss)
c-value
0.8
0.7
0.6
0.5
0.4
0.3
0.2
0.1
0.0
p-value
4e-05
2e-04
1e-03
5e-03
3e-02
Log-odds
0.5
1.0
1.5
2.0
2.5
3.0
3.5
4.0
Cellular_organisms - ps1
Archaea1 - ps2
Archaea2 - ps3
Archaea3 - ps4
Asgard/Eukaryota - ps5
Eukaryota - ps6
Excavata/Diaphoretickes - ps7
Diaphoretickes - ps8
Archaeplastida - ps9
Viridiplantae - ps10
Streptophyta - ps11
Charophyceae/Embriophyta - ps12
Embriophyta - ps13
Tracheophyta - ps14
Spermatophyta - ps15
Angiosperms - ps16
Mesangiospermae - ps17
Magnoliids/eudicots - ps18
Eudicots - ps19
Core_eudicots - ps20
Superrosids - ps21
Rosids - ps22
Eurosids - ps23
Malvids - ps24
Sap./Malv./Brassicales - ps25
Malvales/Brassicales - ps26
Brassicales - ps27
Cleomaceae/Brassicaceae - ps28
Brassicaceae - ps29
Camelineae - ps30
Arabidopsis - ps31
Arabidopsis_thaliana - ps32
Phylostratum

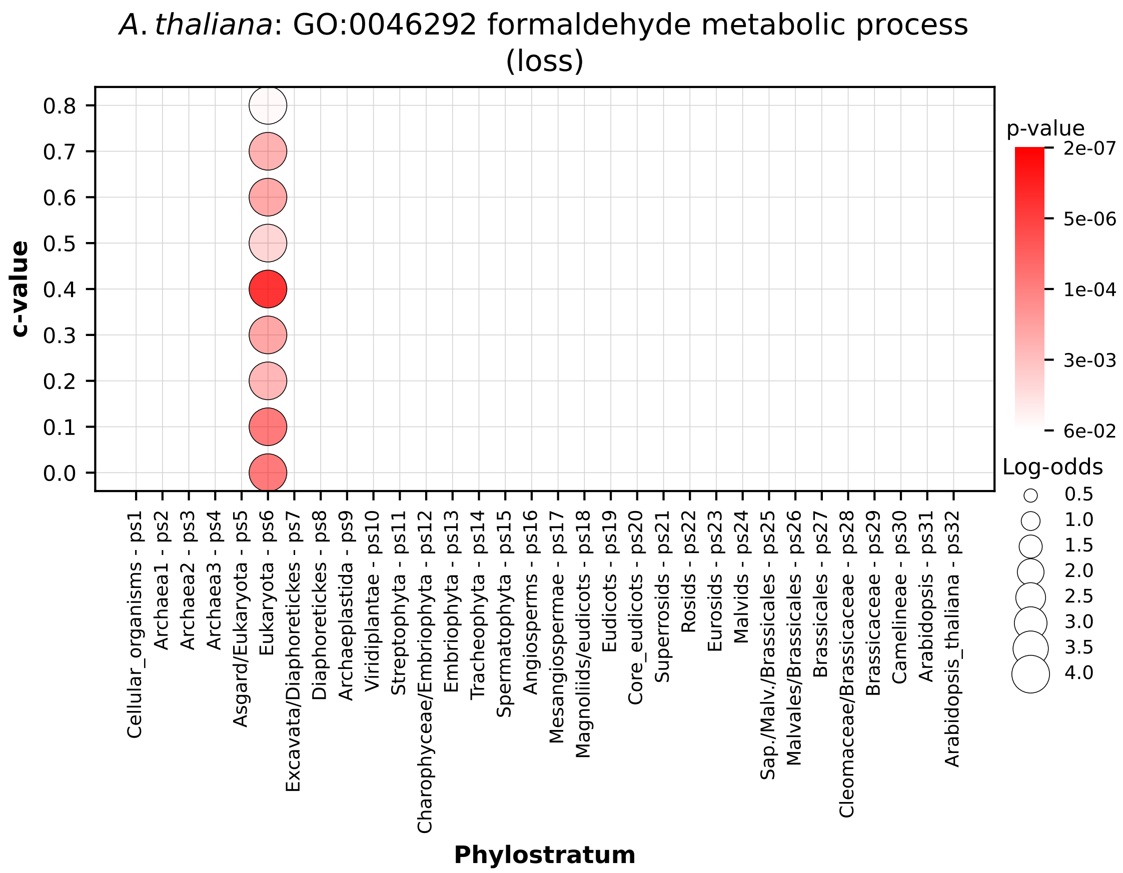
A. thaliana: GO:0046292 formaldehyde metabolic process
(loss)
c-value
0.8
0.7
0.6
0.5
0.4
0.3
0.2
0.1
0.0
Phylostratum
Cellular_organisms - ps1
Archaea1 - ps2
Archaea2 - ps3
Archaea3 - ps4
Asgard/Eukaryota - ps5
Eukaryota - ps6
Excavata/Diaphoretickes - ps7
Diaphoretickes - ps8
Archaeplastida - ps9
Viridiplantae - ps10
Streptophyta - ps11
Charophyceae/Embriophyta - ps12
Embriophyta - ps13
Tracheophyta - ps14
Spermatophyta - ps15
Angiosperms - ps16
Mesangiospermae - ps17
Magnoliids/eudicots - ps18
Eudicots - ps19
Core_eudicots - ps20
Superrosids - ps21
Rosids - ps22
Eurosids - ps23
Malvids - ps24
Sap./Malv./Brassicales - ps25
Malvales/Brassicales - ps26
Brassicales - ps27
Cleomaceae/Brassicaceae - ps28
Brassicaceae - ps29
Camelineae - ps30
Arabidopsis - ps31
Arabidopsis_thaliana - ps32
p-value
2e-07
5e-06
1e-04
3e-03
6e-02
Log-odds
0.5
1.0
1.5
2.0
2.5
3.0
3.5
4.0

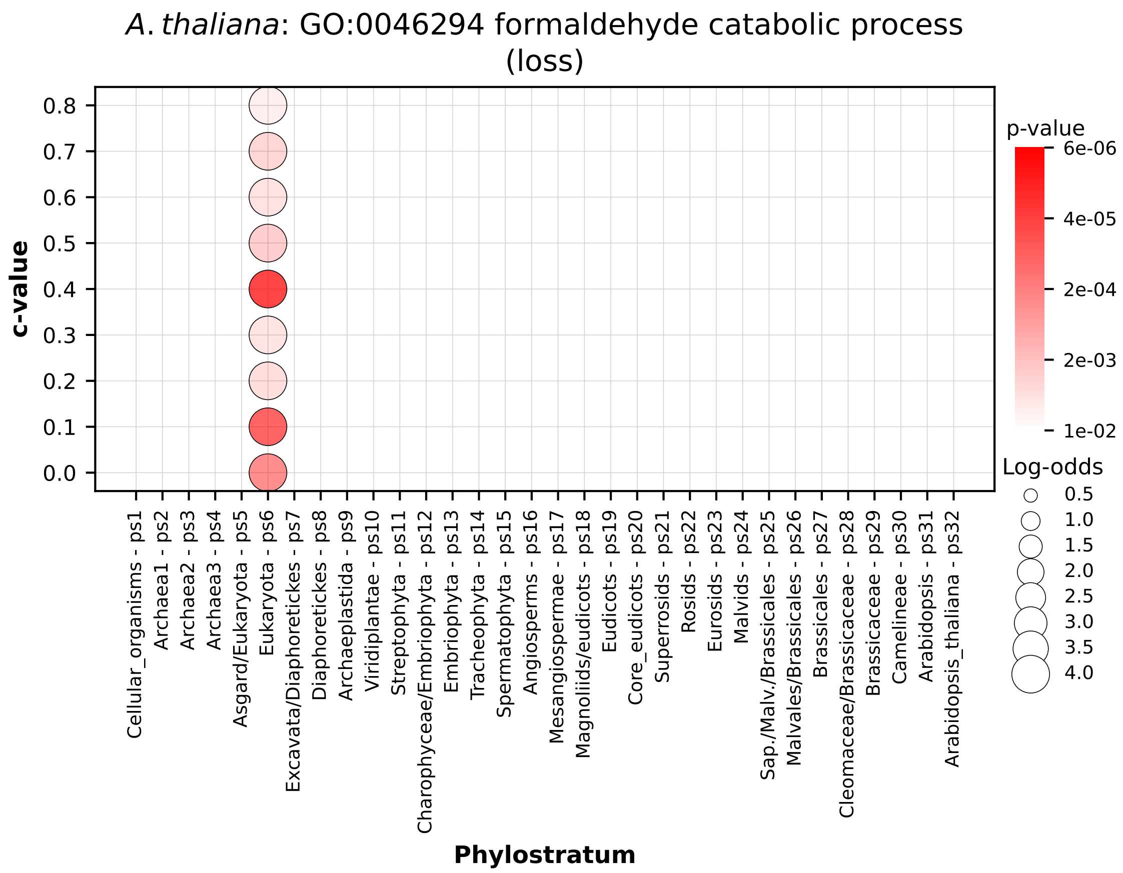
A. thaliana: GO:0046294 formaldehyde catabolic process
(loss)
c-value
0.8
0.7
0.6
0.5
0.4
0.3
0.2
0.1
0.0
p-value
6e-06
4e-05
2e-04
2e-03
1e-02
Log-odds
0.5
1.0
1.5
2.0
2.5
3.0
3.5
4.0
Cellular_organisms - ps1
Archaea1 - ps2
Archaea2 - ps3
Archaea3 - ps4
Asgard/Eukaryota - ps5
Eukaryota - ps6
Excavata/Diaphoretickes - ps7
Diaphoretickes - ps8
Archaeplastida - ps9
Viridiplantae - ps10
Streptophyta - ps11
Charophyceae/Embriophyta - ps12
Embriophyta - ps13
Tracheophyta - ps14
Spermatophyta - ps15
Angiosperms - ps16
Mesangiospermae - ps17
Magnoliids/eudicots - ps18
Eudicots - ps19
Core_eudicots - ps20
Superrosids - ps21
Rosids - ps22
Eurosids - ps23
Malvids - ps24
Sap./Malv./Brassicales - ps25
Malvales/Brassicales - ps26
Brassicales - ps27
Cleomaceae/Brassicaceae - ps28
Brassicaceae - ps29
Camelineae - ps30
Arabidopsis - ps31
Arabidopsis_thaliana - ps32
Phylostratum

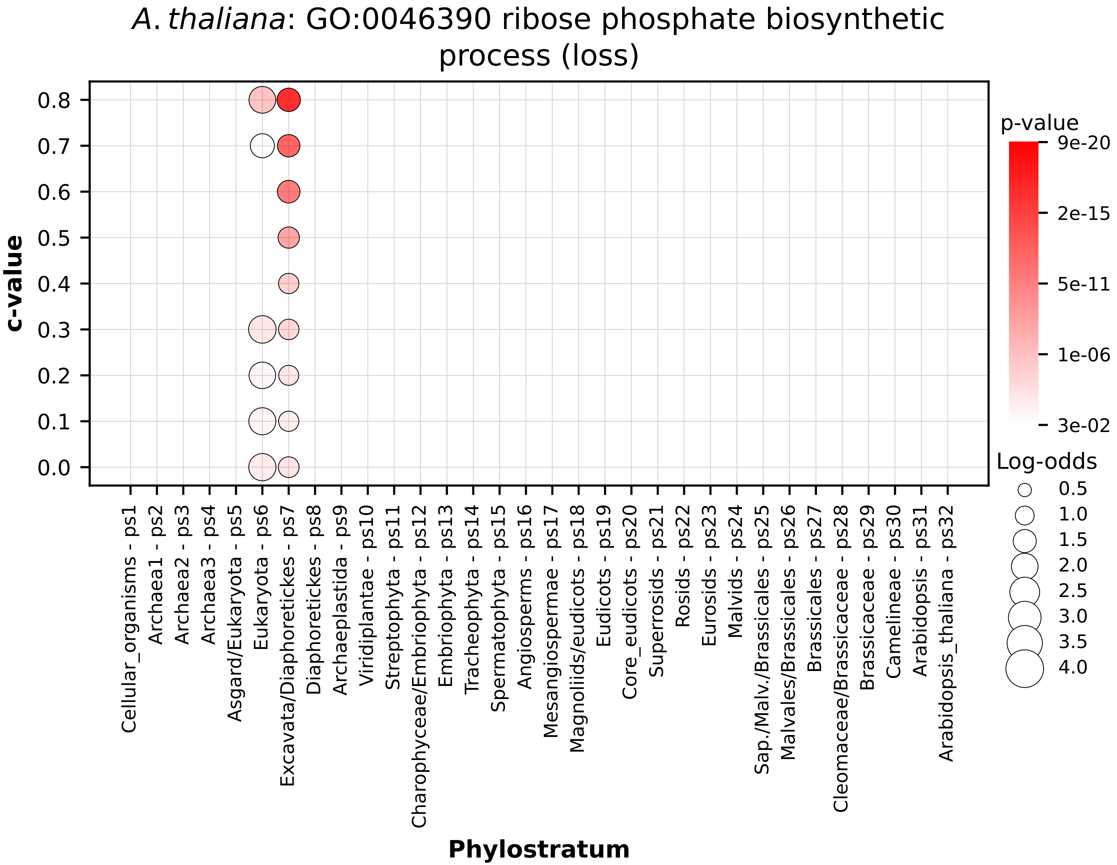

A. thaliana: GO:0046390 ribose phosphate biosynthetic process (loss)
c-value
0.8
0.7
0.6
0.5
0.4
0.3
0.2
0.1
0.0
p-value
9e-20
2e-15
5e-11
1e-06
3e-02
Log-odds
0.5
1.0
1.5
2.0
2.5
3.0
3.5
4.0
Cellular_organisms - ps1
Archaea1 - ps2
Archaea2 - ps3
Archaea3 - ps4
Asgard/Eukaryota - ps5
Eukaryota - ps6
Excavata/Diaphoretickes - ps7
Diaphoretickes - ps8
Archaeplastida - ps9
Viridiplantae - ps10
Streptophyta - ps11
Charophyceae/Embriophyta - ps12
Embriophyta - ps13
Tracheophyta - ps14
Spermatophyta - ps15
Angiosperms - ps16
Mesangiospermae - ps17
Magnoliids/eudicots - ps18
Eudicots - ps19
Core_eudicots - ps20
Superrosids - ps21
Rosids - ps22
Eurosids - ps23
Malvids - ps24
Sap./Malv./Brassicales - ps25
Malvales/Brassicales - ps26
Brassicales - ps27
Cleomaceae/Brassicaceae - ps28
Brassicaceae - ps29
Camelineae - ps30
Arabidopsis - ps31
Arabidopsis_thaliana - ps32
Phylostratum

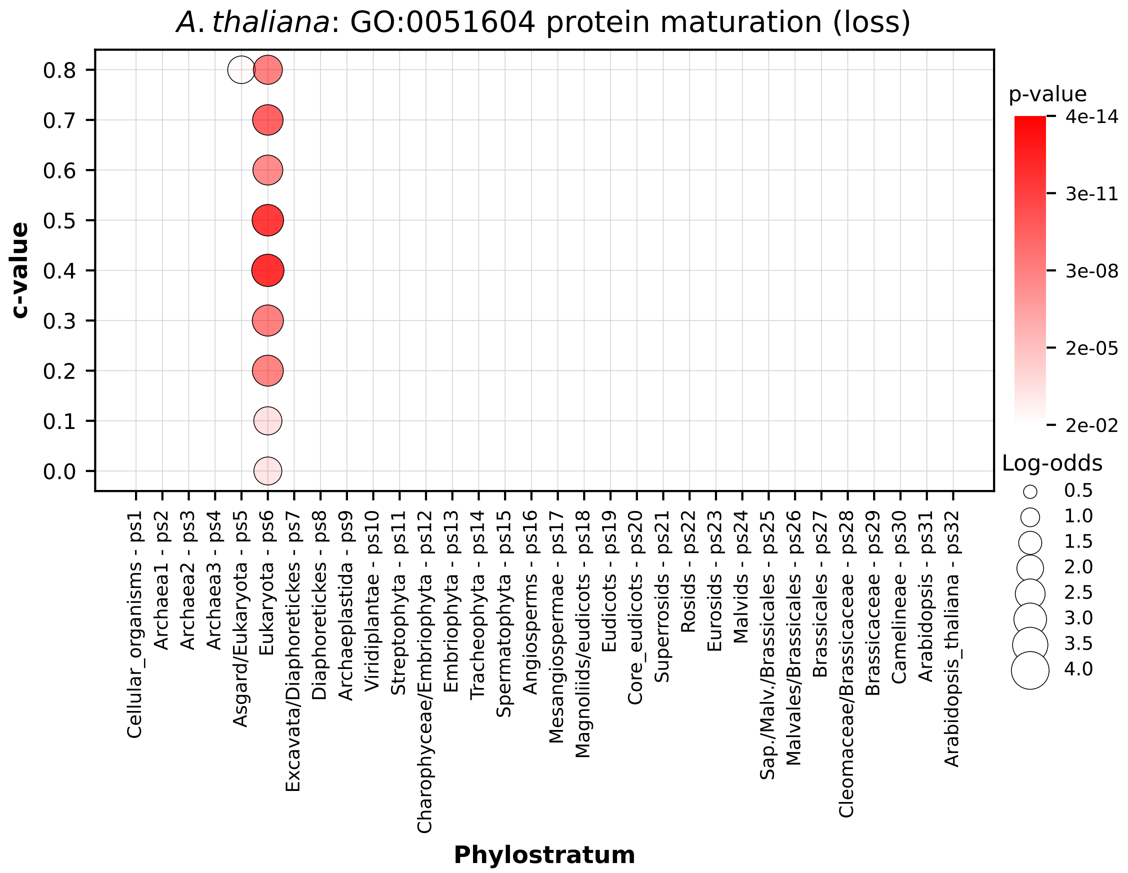

A. thaliana: GO:0051604 protein maturation (loss)
c-value
0.8
0.7
0.6
0.5
0.4
0.3
0.2
0.1
0.0
p-value
4e-14
3e-11
3e-08
2e-05
2e-02
Log-odds
0.5
1.0
1.5
2.0
2.5
3.0
3.5
4.0
Cellular_organisms - ps1
Archaea1 - ps2
Archaea2 - ps3
Archaea3 - ps4
Asgard/Eukaryota - ps5
Eukaryota - ps6
Excavata/Diaphoretickes - ps7
Diaphoretickes - ps8
Archaeplastida - ps9
Viridiplantae - ps10
Streptophyta - ps11
Charophyceae/Embriophyta - ps12
Embriophyta - ps13
Tracheophyta - ps14
Spermatophyta - ps15
Angiosperms - ps16
Mesangiospermae - ps17
Magnoliids/eudicots - ps18
Eudicots - ps19
Core_eudicots - ps20
Superrosids - ps21
Rosids - ps22
Eurosids - ps23
Malvids - ps24
Sap./Malv./Brassicales - ps25
Malvales/Brassicales - ps26
Brassicales - ps27
Cleomaceae/Brassicaceae - ps28
Brassicaceae - ps29
Camelineae - ps30
Arabidopsis - ps31
Arabidopsis_thaliana - ps32
Phylostratum

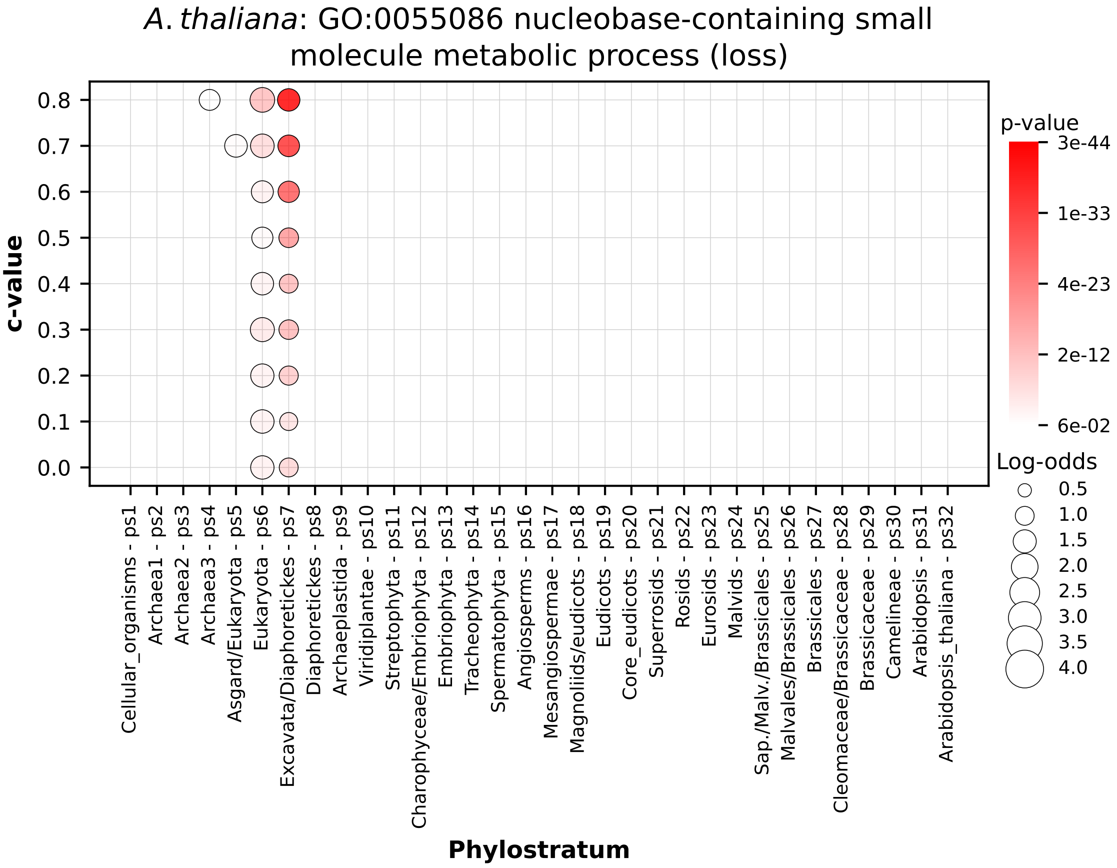

A. thaliana: GO:0055086 nucleobase-containing small molecule metabolic process (loss)
c-value
0.8
0.7
0.6
0.5
0.4
0.3
0.2
0.1
0.0
p-value
3e-44
1e-33
4e-23
2e-12
6e-02
Log-odds
0.5
1.0
1.5
2.0
2.5
3.0
3.5
4.0
Cellular_organisms - ps1
Archaea1 - ps2
Archaea2 - ps3
Archaea3 - ps4
Asgard/Eukaryota - ps5
Eukaryota - ps6
Excavata/Diaphoretickes - ps7
Diaphoretickes - ps8
Archaeplastida - ps9
Viridiplantae - ps10
Streptophyta - ps11
Charophyceae/Embriophyta - ps12
Embriophyta - ps13
Tracheophyta - ps14
Spermatophyta - ps15
Angiosperms - ps16
Mesangiospermae - ps17
Magnoliids/eudicots - ps18
Eudicots - ps19
Core_eudicots - ps20
Superrosids - ps21
Rosids - ps22
Eurosids - ps23
Malvids - ps24
Sap./Malv./Brassicales - ps25
Malvales/Brassicales - ps26
Brassicales - ps27
Cleomaceae/Brassicaceae - ps28
Brassicaceae - ps29
Camelineae - ps30
Arabidopsis - ps31
Arabidopsis_thaliana - ps32
Phylostratum

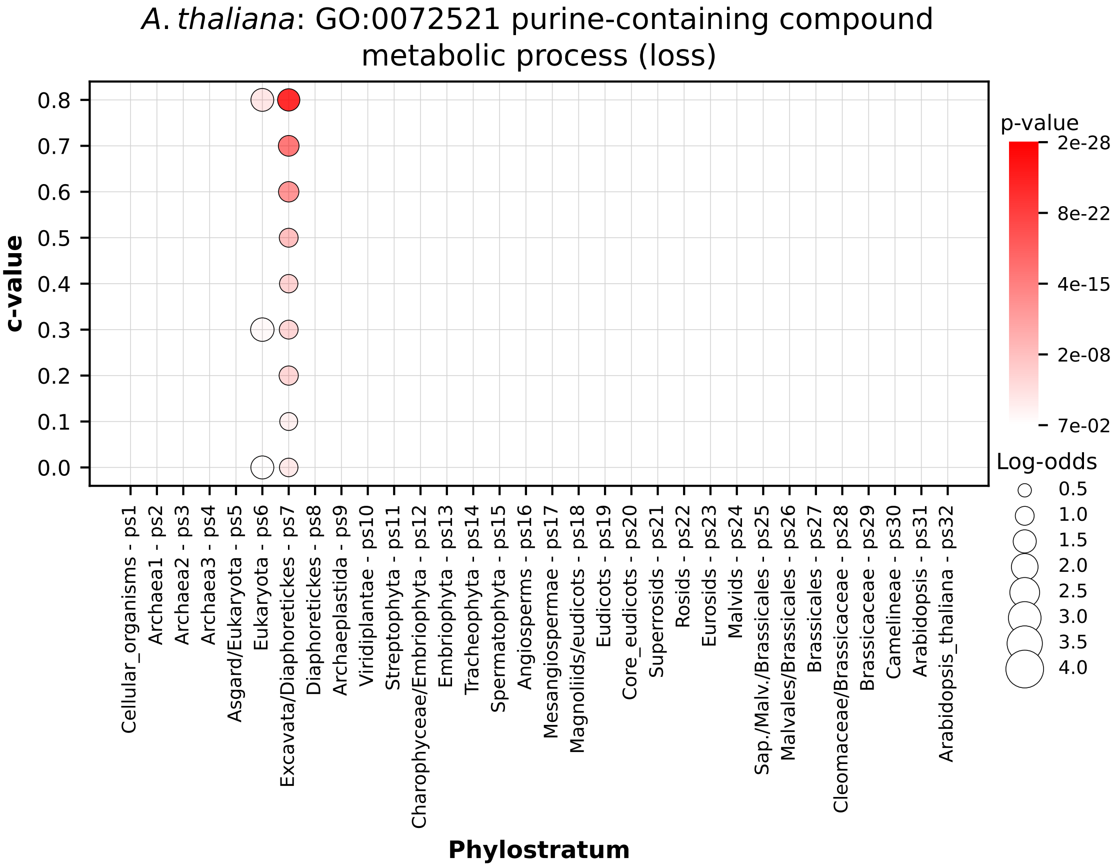

A. thaliana: GO:0072521 purine-containing compound metabolic process (loss)
c-value
0.0
0.1
0.2
0.3
0.4
0.5
0.6
0.7
0.8
Phylostratum
Cellular_organisms - ps1
Archaea1 - ps2
Archaea2 - ps3
Archaea3 - ps4
Asgard/Eukaryota - ps5
Eukaryota - ps6
Excavata/Diaphoretickes - ps7
Diaphoretickes - ps8
Archaeplastida - ps9
Viridiplantae - ps10
Streptophyta - ps11
Charophyceae/Embriophyta - ps12
Embriophyta - ps13
Tracheophyta - ps14
Spermatophyta - ps15
Angiosperms - ps16
Mesangiospermae - ps17
Magnoliids/eudicots - ps18
Eudicots - ps19
Core_eudicots - ps20
Superrosids - ps21
Rosids - ps22
Eurosids - ps23
Malvids - ps24
Sap./Malv./Brassicales - ps25
Malvales/Brassicales - ps26
Brassicales - ps27
Cleomaceae/Brassicaceae - ps28
Brassicaceae - ps29
Camelineae - ps30
Arabidopsis - ps31
Arabidopsis_thaliana - ps32
p-value
2e-28
8e-22
4e-15
2e-08
7e-02
Log-odds
0.5
1.0
1.5
2.0
2.5
3.0
3.5
4.0

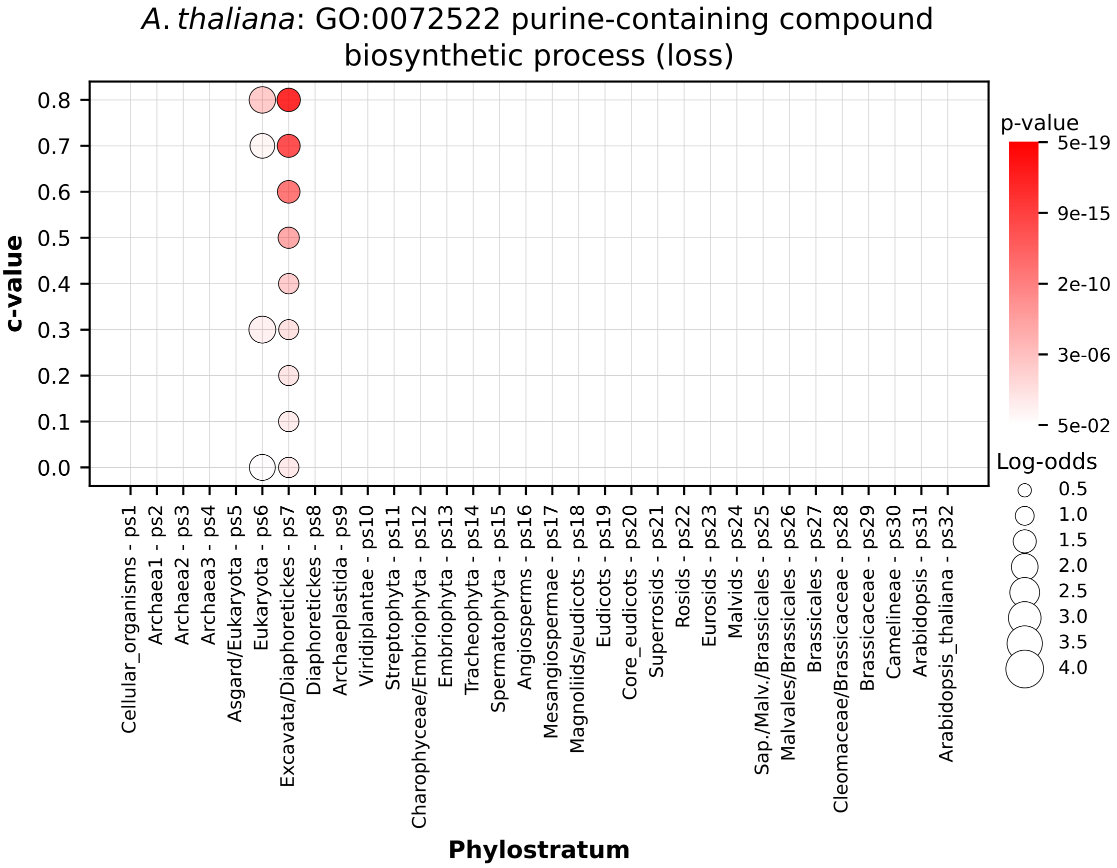
A. thaliana: GO:0072522 purine-containing compound biosynthetic process (loss)
c-value
0.8
0.7
0.6
0.5
0.4
0.3
0.2
0.1
0.0
p-value
5e-19
9e-15
2e-10
3e-06
5e-02
Log-odds
0.5
1.0
1.5
2.0
2.5
3.0
3.5
4.0
Cellular_organisms - ps1
Archaea1 - ps2
Archaea2 - ps3
Archaea3 - ps4
Asgard/Eukaryota - ps5
Eukaryota - ps6
Excavata/Diaphoretickes - ps7
Diaphoretickes - ps8
Archaeplastida - ps9
Viridiplantae - ps10
Streptophyta - ps11
Charophyceae/Embriophyta - ps12
Embriophyta - ps13
Tracheophyta - ps14
Spermatophyta - ps15
Angiosperms - ps16
Mesangiospermae - ps17
Magnoliids/eudicots - ps18
Eudicots - ps19
Core_eudicots - ps20
Superrosids - ps21
Rosids - ps22
Eurosids - ps23
Malvids - ps24
Sap./Malv./Brassicales - ps25
Malvales/Brassicales - ps26
Brassicales - ps27
Cleomaceae/Brassicaceae - ps28
Brassicaceae - ps29
Camelineae - ps30
Arabidopsis - ps31
Arabidopsis_thaliana - ps32
Phylostratum

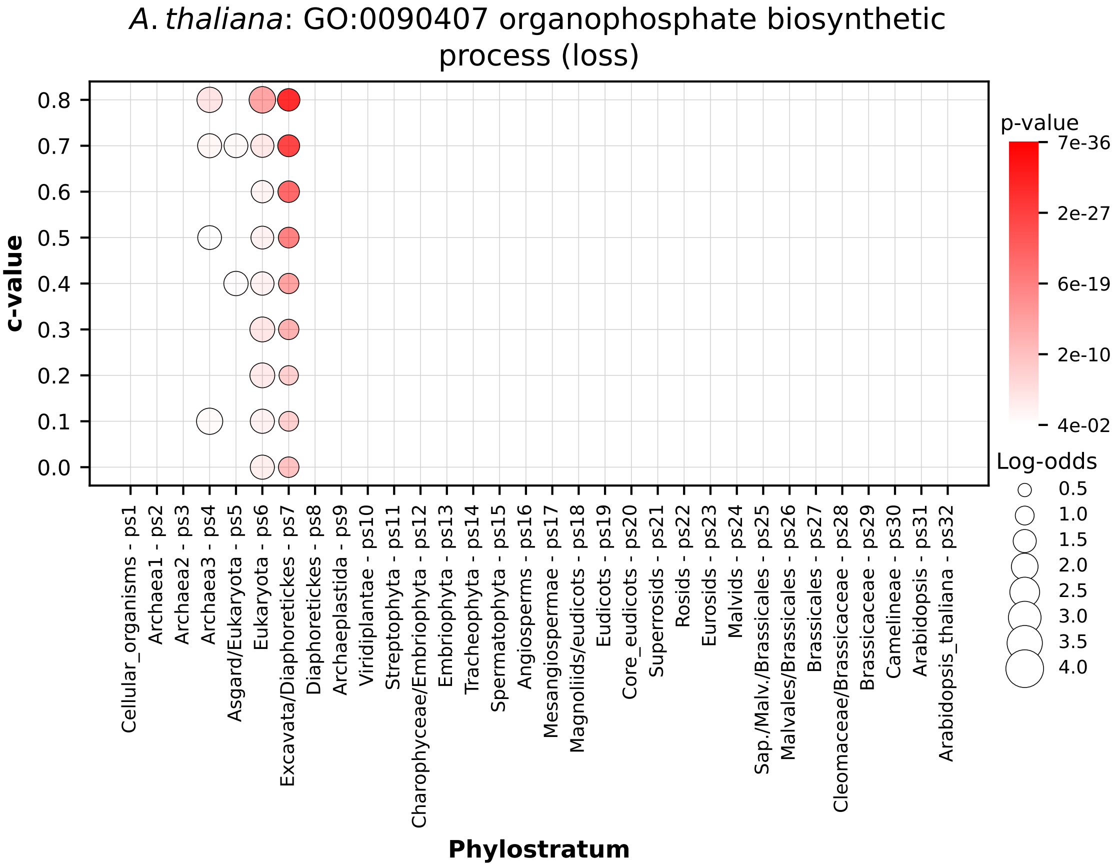

A. thaliana: GO:0090407 organophosphate biosynthetic process (loss)
c-value
0.8
0.7
0.6
0.5
0.4
0.3
0.2
0.1
0.0
p-value
7e-36
2e-27
6e-19
2e-10
4e-02
Log-odds
0.5
1.0
1.5
2.0
2.5
3.0
3.5
4.0
Cellular_organisms - ps1
Archaea1 - ps2
Archaea2 - ps3
Archaea3 - ps4
Asgard/Eukaryota - ps5
Eukaryota - ps6
Excavata/Diaphoretickes - ps7
Diaphoretickes - ps8
Archaeplastida - ps9
Viridiplantae - ps10
Streptophyta - ps11
Charophyceae/Embriophyta - ps12
Embriophyta - ps13
Tracheophyta - ps14
Spermatophyta - ps15
Angiosperms - ps16
Mesangiospermae - ps17
Magnoliids/eudicots - ps18
Eudicots - ps19
Core_eudicots - ps20
Superrosids - ps21
Rosids - ps22
Eurosids - ps23
Malvids - ps24
Sap./Malv./Brassicales - ps25
Malvales/Brassicales - ps26
Brassicales - ps27
Cleomaceae/Brassicaceae - ps28
Brassicaceae - ps29
Camelineae - ps30
Arabidopsis - ps31
Arabidopsis_thaliana - ps32
Phylostratum

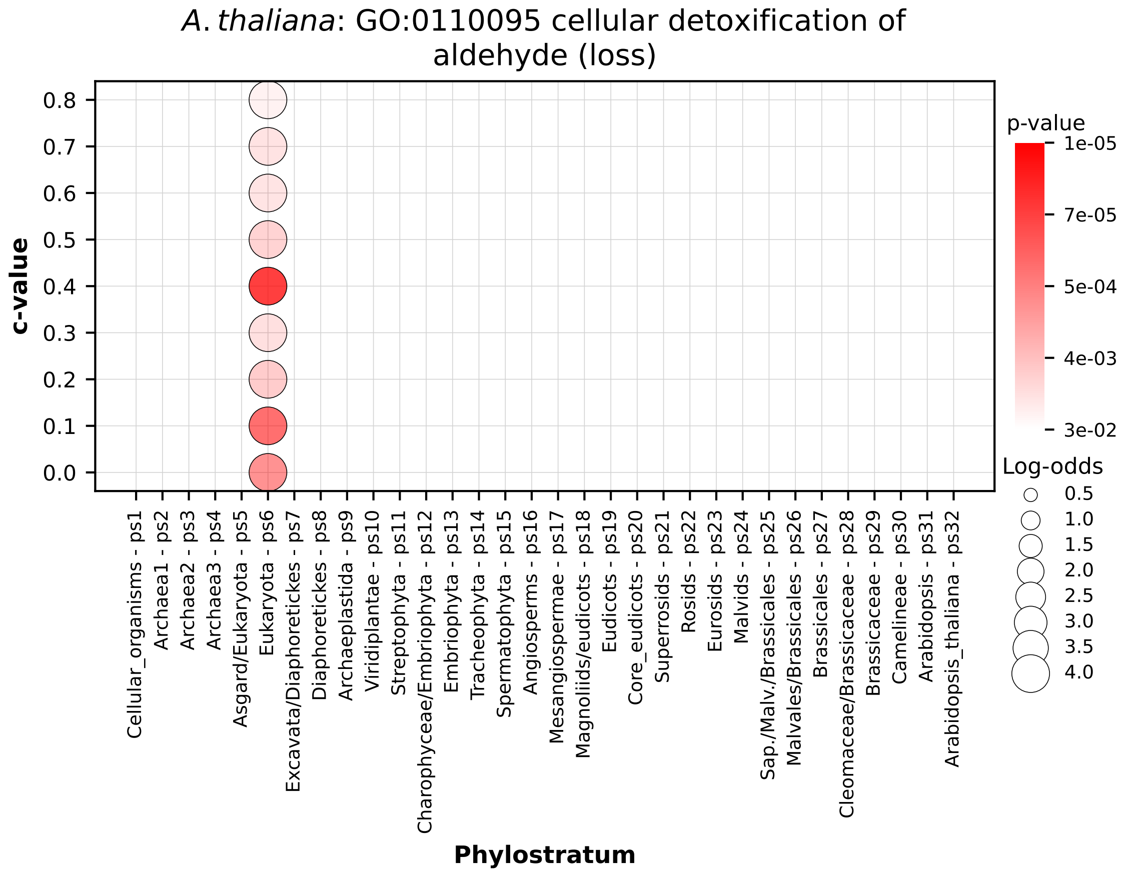

A. thaliana: GO:0110095 cellular detoxification of aldehyde (loss)
c-value
0.8
0.7
0.6
0.5
0.4
0.3
0.2
0.1
0.0
p-value
1e-05
7e-05
5e-04
4e-03
3e-02
Log-odds
0.5
1.0
1.5
2.0
2.5
3.0
3.5
4.0
Cellular_organisms - ps1
Archaea1 - ps2
Archaea2 - ps3
Archaea3 - ps4
Asgard/Eukaryota - ps5
Eukaryota - ps6
Excavata/Diaphoretickes - ps7
Diaphoretickes - ps8
Archaeplastida - ps9
Viridiplantae - ps10
Streptophyta - ps11
Charophyceae/Embriophyta - ps12
Embriophyta - ps13
Tracheophyta - ps14
Spermatophyta - ps15
Angiosperms - ps16
Mesangiospermae - ps17
Magnoliids/eudicots - ps18
Eudicots - ps19
Core_eudicots - ps20
Superrosids - ps21
Rosids - ps22
Eurosids - ps23
Malvids - ps24
Sap./Malv./Brassicales - ps25
Malvales/Brassicales - ps26
Brassicales - ps27
Cleomaceae/Brassicaceae - ps28
Brassicaceae - ps29
Camelineae - ps30
Arabidopsis - ps31
Arabidopsis_thaliana - ps32
Phylostratum

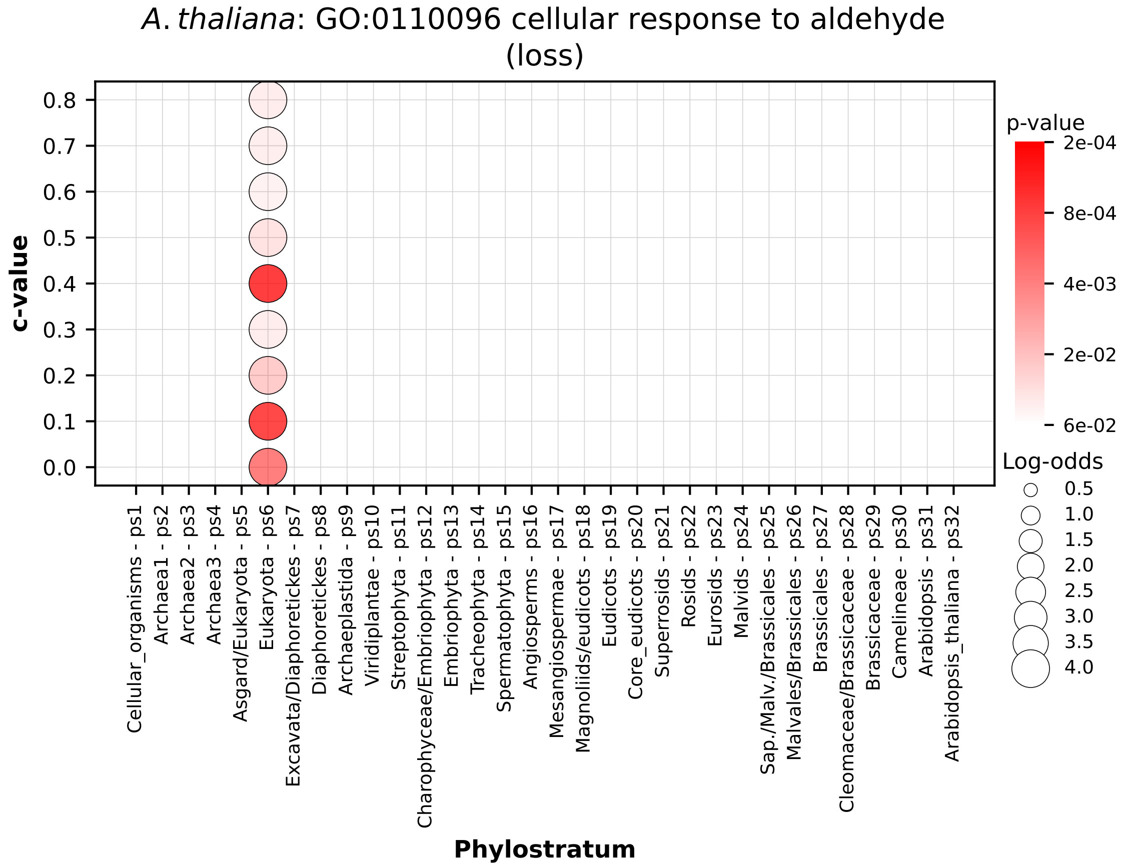

A. thaliana: GO:0110096 cellular response to aldehyde (loss)
c-value
0.0
0.1
0.2
0.3
0.4
0.5
0.6
0.7
0.8
Phylostratum
Cellular_organisms - ps1
Archaea1 - ps2
Archaea2 - ps3
Archaea3 - ps4
Asgard/Eukaryota - ps5
Eukaryota - ps6
Excavata/Diaphoretickes - ps7
Diaphoretickes - ps8
Archaeplastida - ps9
Viridiplantae - ps10
Streptophyta - ps11
Charophyceae/Embriophyta - ps12
Embriophyta - ps13
Tracheophyta - ps14
Spermatophyta - ps15
Angiosperms - ps16
Mesangiospermae - ps17
Magnoliids/eudicots - ps18
Eudicots - ps19
Core_eudicots - ps20
Superrosids - ps21
Rosids - ps22
Eurosids - ps23
Malvids - ps24
Sap./Malv./Brassicales - ps25
Malvales/Brassicales - ps26
Brassicales - ps27
Cleomaceae/Brassicaceae - ps28
Brassicaceae - ps29
Camelineae - ps30
Arabidopsis - ps31
Arabidopsis_thaliana - ps32
p-value
2e-04
8e-04
4e-03
2e-02
6e-02
Log-odds
0.5
1.0
1.5
2.0
2.5
3.0
3.5
4.0

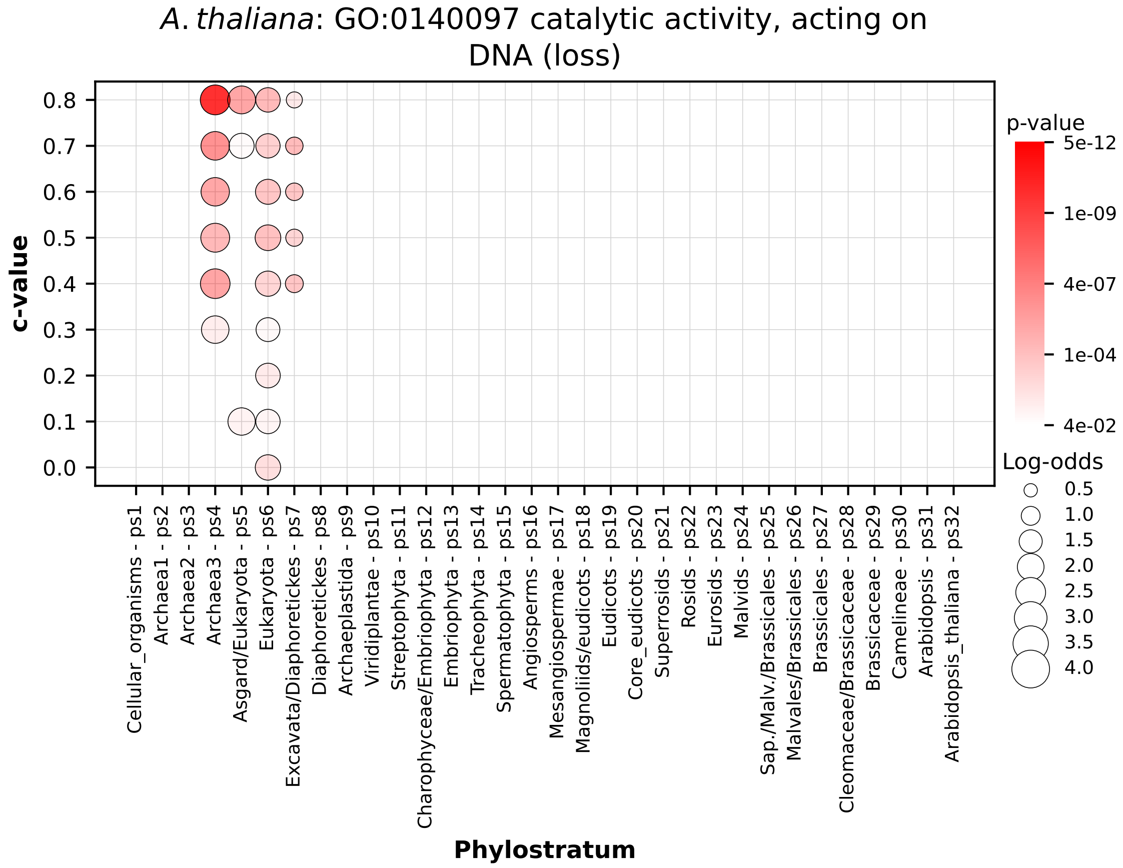

A. thaliana: GO:0140097 catalytic activity, acting on DNA (loss)
c-value
Phylostratum
p-value
5e-12
1e-09
4e-07
1e-04
4e-02
Log-odds
0.5
1.0
1.5
2.0
2.5
3.0
3.5
4.0
Cellular_organisms - ps1
Archaea1 - ps2
Archaea2 - ps3
Archaea3 - ps4
Asgard/Eukaryota - ps5
Eukaryota - ps6
Excavata/Diaphoretickes - ps7
Diaphoretickes - ps8
Archaeplastida - ps9
Viridiplantae - ps10
Streptophyta - ps11
Charophyceae/Embriophyta - ps12
Embriophyta - ps13
Tracheophyta - ps14
Spermatophyta - ps15
Angiosperms - ps16
Mesangiospermae - ps17
Magnoliids/eudicots - ps18
Eudicots - ps19
Core_eudicots - ps20
Superrosids - ps21
Rosids - ps22
Eurosids - ps23
Malvids - ps24
Sap./Malv./Brassicales - ps25
Malvales/Brassicales - ps26
Brassicales - ps27
Cleomaceae/Brassicaceae - ps28
Brassicaceae - ps29
Camelineae - ps30
Arabidopsis - ps31
Arabidopsis_thaliana - ps32

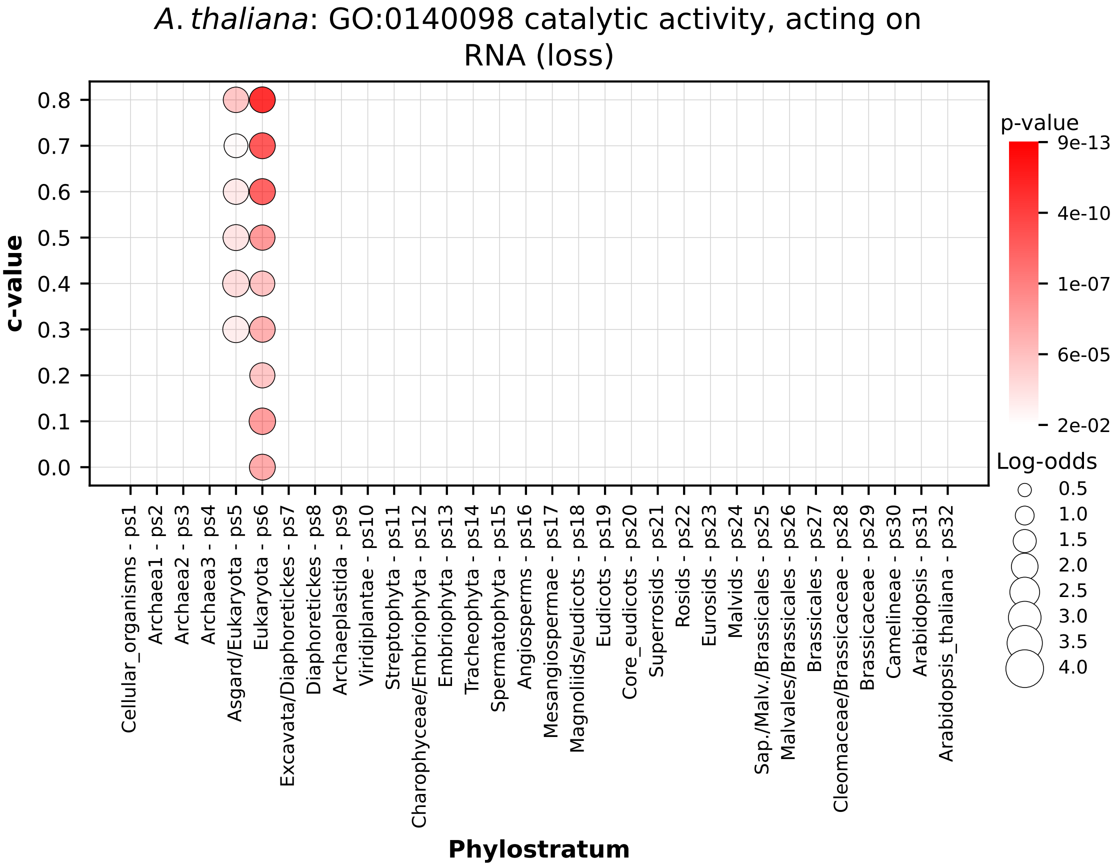
A. thaliana: GO:0140098 catalytic activity, acting on RNA (loss)
c-value
0.8
0.7
0.6
0.5
0.4
0.3
0.2
0.1
0.0
Phylostratum
Cellular_organisms - ps1
Archaea1 - ps2
Archaea2 - ps3
Archaea3 - ps4
Asgard/Eukaryota - ps5
Eukaryota - ps6
Excavata/Diaphoretickes - ps7
Diaphoretickes - ps8
Archaeplastida - ps9
Viridiplantae - ps10
Streptophyta - ps11
Charophyceae/Embriophyta - ps12
Embriophyta - ps13
Tracheophyta - ps14
Spermatophyta - ps15
Angiosperms - ps16
Mesangiospermae - ps17
Magnoliids/eudicots - ps18
Eudicots - ps19
Core_eudicots - ps20
Superrosids - ps21
Rosids - ps22
Eurosids - ps23
Malvids - ps24
Sap./Malv./Brassicales - ps25
Malvales/Brassicales - ps26
Brassicales - ps27
Cleomaceae/Brassicaceae - ps28
Brassicaceae - ps29
Camelineae - ps30
Arabidopsis - ps31
Arabidopsis_thaliana - ps32
p-value
9e-13
4e-10
1e-07
6e-05
2e-02
Log-odds
0.5
1.0
1.5
2.0
2.5
3.0
3.5
4.0

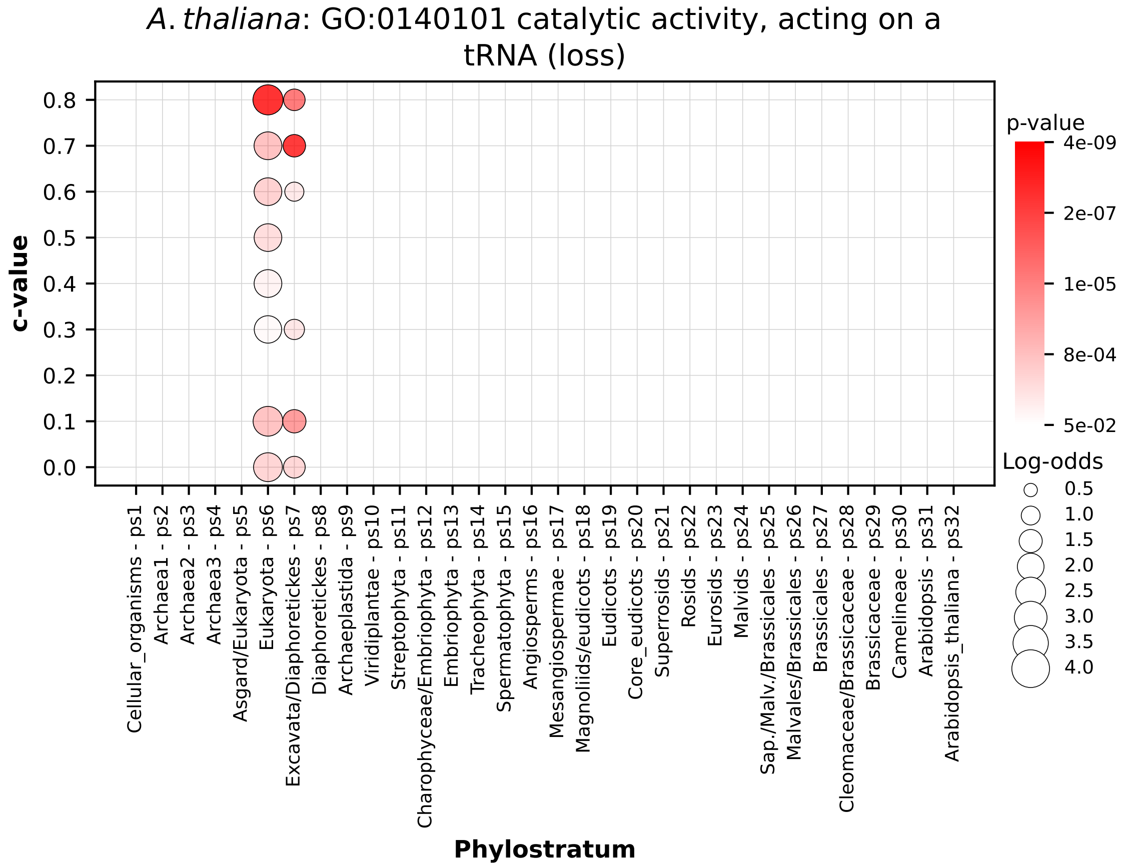
A. thaliana: GO:0140101 catalytic activity, acting on a tRNA (loss)
c-value
0.8
0.7
0.6
0.5
0.4
0.3
0.2
0.1
0.0
Phylostratum
Cellular_organisms - ps1
Archaea1 - ps2
Archaea2 - ps3
Archaea3 - ps4
Asgard/Eukaryota - ps5
Eukaryota - ps6
Excavata/Diaphoretickes - ps7
Diaphoretickes - ps8
Archaeplastida - ps9
Viridiplantae - ps10
Streptophyta - ps11
Charophyceae/Embriophyta - ps12
Embriophyta - ps13
Tracheophyta - ps14
Spermatophyta - ps15
Angiosperms - ps16
Mesangiospermae - ps17
Magnoliids/eudicots - ps18
Eudicots - ps19
Core_eudicots - ps20
Superrosids - ps21
Rosids - ps22
Eurosids - ps23
Malvids - ps24
Sap./Malv./Brassicales - ps25
Malvales/Brassicales - ps26
Brassicales - ps27
Cleomaceae/Brassicaceae - ps28
Brassicaceae - ps29
Camelineae - ps30
Arabidopsis - ps31
Arabidopsis_thaliana - ps32
p-value
4e-09
2e-07
1e-05
8e-04
5e-02
Log-odds
0.5
1.0
1.5
2.0
2.5
3.0
3.5
4.0

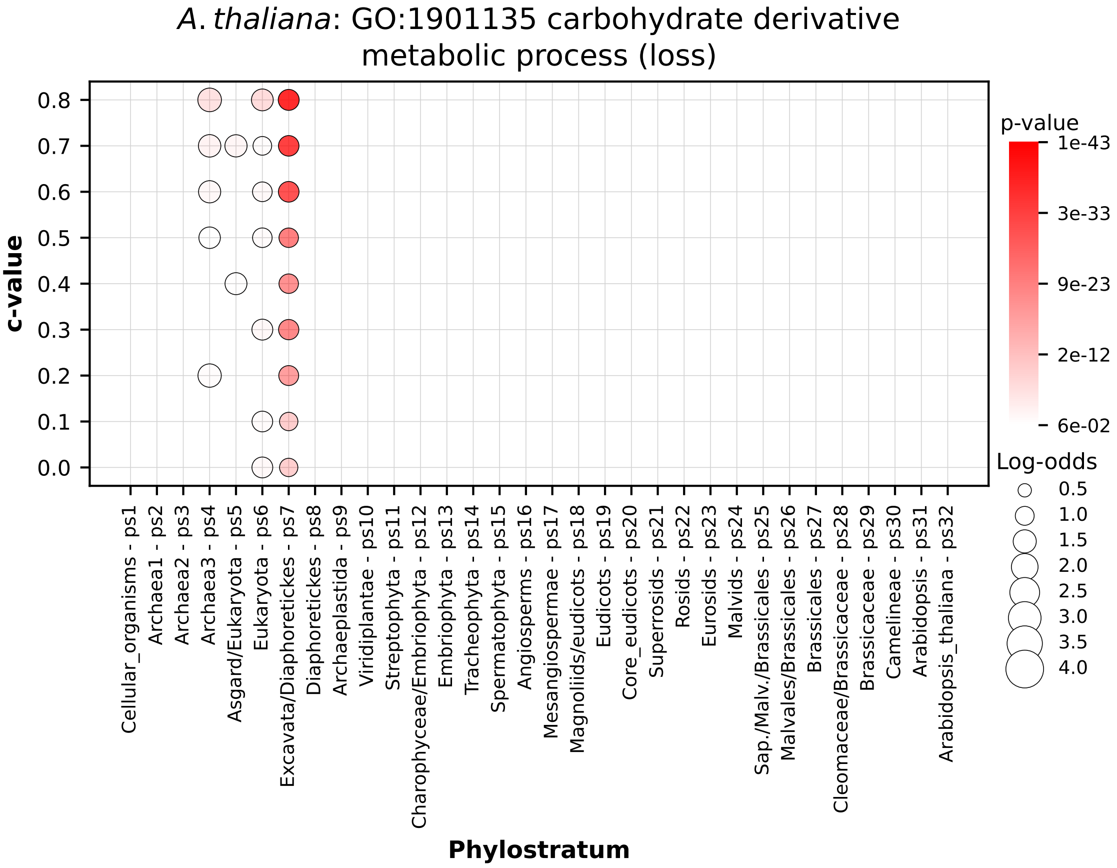

A. thaliana: GO:1901135 carbohydrate derivative metabolic process (loss)
c-value
0.8
0.7
0.6
0.5
0.4
0.3
0.2
0.1
0.0
p-value
1e-43
3e-33
9e-23
2e-12
6e-02
Log-odds
0.5
1.0
1.5
2.0
2.5
3.0
3.5
4.0
Cellular_organisms - ps1
Archaea1 - ps2
Archaea2 - ps3
Archaea3 - ps4
Asgard/Eukaryota - ps5
Eukaryota - ps6
Excavata/Diaphoretickes - ps7
Diaphoretickes - ps8
Archaeplastida - ps9
Viridiplantae - ps10
Streptophyta - ps11
Charophyceae/Embriophyta - ps12
Embriophyta - ps13
Tracheophyta - ps14
Spermatophyta - ps15
Angiosperms - ps16
Mesangiospermae - ps17
Magnoliids/eudicots - ps18
Eudicots - ps19
Core_eudicots - ps20
Superrosids - ps21
Rosids - ps22
Eurosids - ps23
Malvids - ps24
Sap./Malv./Brassicales - ps25
Malvales/Brassicales - ps26
Brassicales - ps27
Cleomaceae/Brassicaceae - ps28
Brassicaceae - ps29
Camelineae - ps30
Arabidopsis - ps31
Arabidopsis_thaliana - ps32
Phylostratum

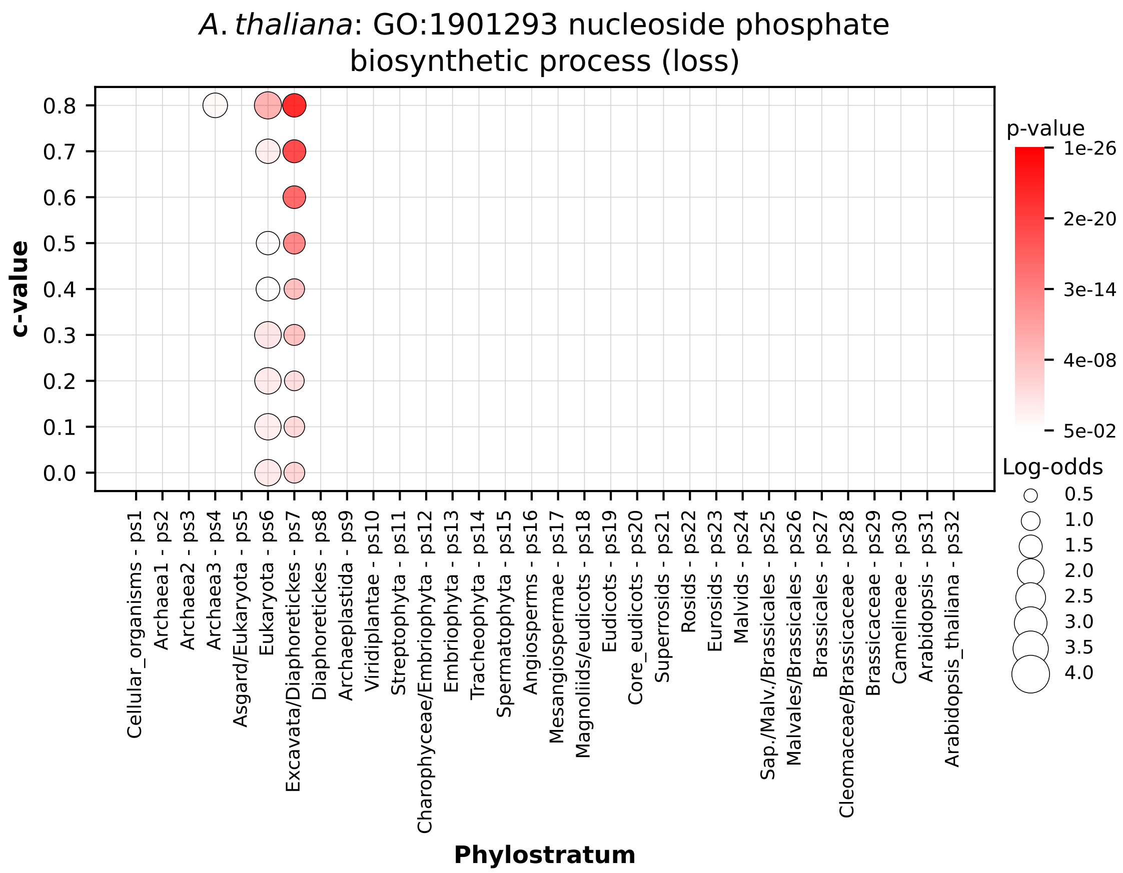

A. thaliana: GO:1901293 nucleoside phosphate biosynthetic process (loss)
c-value
0.8
0.7
0.6
0.5
0.4
0.3
0.2
0.1
0.0
Phylostratum
Cellular_organisms - ps1
Archaea1 - ps2
Archaea2 - ps3
Archaea3 - ps4
Asgard/Eukaryota - ps5
Eukaryota - ps6
Excavata/Diaphoretickes - ps7
Diaphoretickes - ps8
Archaeplastida - ps9
Viridiplantae - ps10
Streptophyta - ps11
Charophyceae/Embriophyta - ps12
Embriophyta - ps13
Tracheophyta - ps14
Spermatophyta - ps15
Angiosperms - ps16
Mesangiospermae - ps17
Magnoliids/eudicots - ps18
Eudicots - ps19
Core_eudicots - ps20
Superrosids - ps21
Rosids - ps22
Eurosids - ps23
Malvids - ps24
Sap./Malv./Brassicales - ps25
Malvales/Brassicales - ps26
Brassicales - ps27
Cleomaceae/Brassicaceae - ps28
Brassicaceae - ps29
Camelineae - ps30
Arabidopsis - ps31
Arabidopsis_thaliana - ps32
p-value
1e-26
2e-20
3e-14
4e-08
5e-02
Log-odds
0.5
1.0
1.5
2.0
2.5
3.0
3.5
4.0

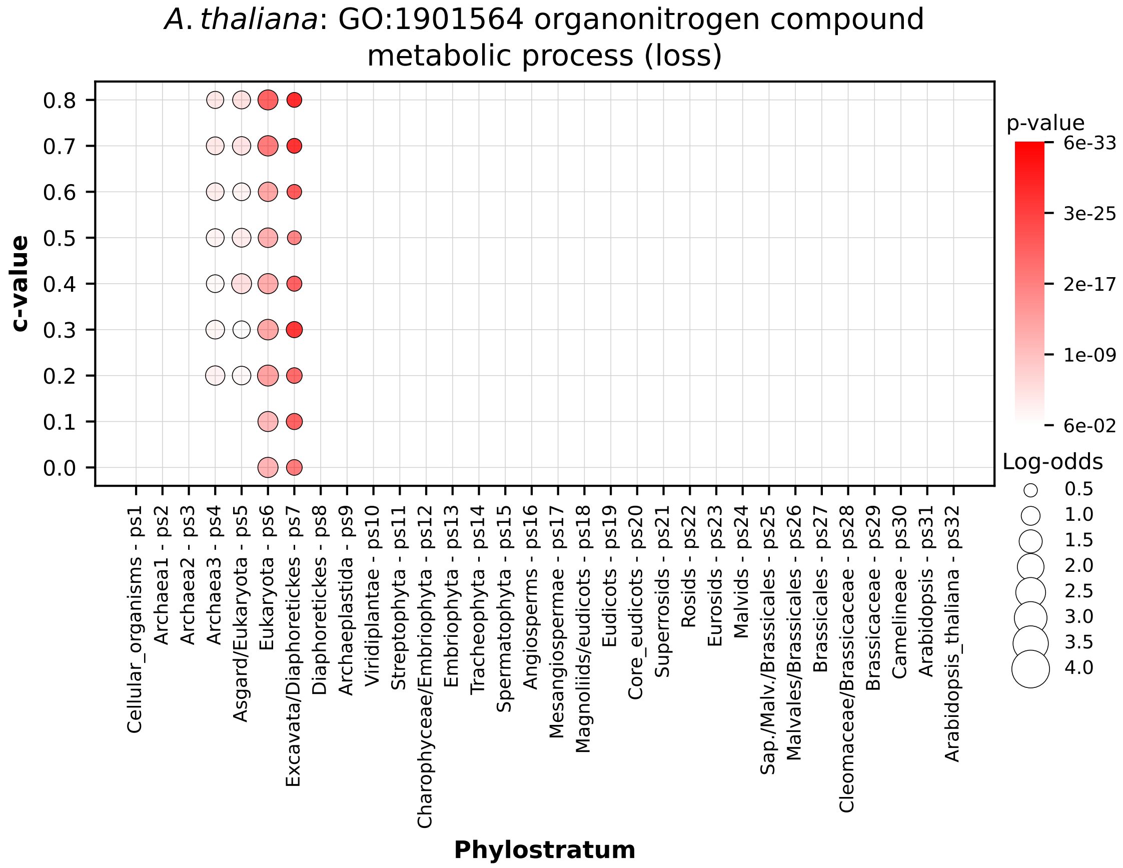
A. thaliana: GO:1901564 organonitrogen compound metabolic process (loss)
c-value
0.8
0.7
0.6
0.5
0.4
0.3
0.2
0.1
0.0
Phylostratum
Cellular_organisms - ps1
Archaea1 - ps2
Archaea2 - ps3
Archaea3 - ps4
Asgard/Eukaryota - ps5
Eukaryota - ps6
Excavata/Diaphoretickes - ps7
Diaphoretickes - ps8
Archaeplastida - ps9
Viridiplantae - ps10
Streptophyta - ps11
Charophyceae/Embriophyta - ps12
Embriophyta - ps13
Tracheophyta - ps14
Spermatophyta - ps15
Angiosperms - ps16
Mesangiospermae - ps17
Magnoliids/eudicots - ps18
Eudicots - ps19
Core_eudicots - ps20
Superrosids - ps21
Rosids - ps22
Eurosids - ps23
Malvids - ps24
Sap./Malv./Brassicales - ps25
Malvales/Brassicales - ps26
Brassicales - ps27
Cleomaceae/Brassicaceae - ps28
Brassicaceae - ps29
Camelineae - ps30
Arabidopsis - ps31
Arabidopsis_thaliana - ps32
p-value
6e-33
3e-25
2e-17
1e-09
6e-02
Log-odds
0.5
1.0
1.5
2.0
2.5
3.0
3.5
4.0

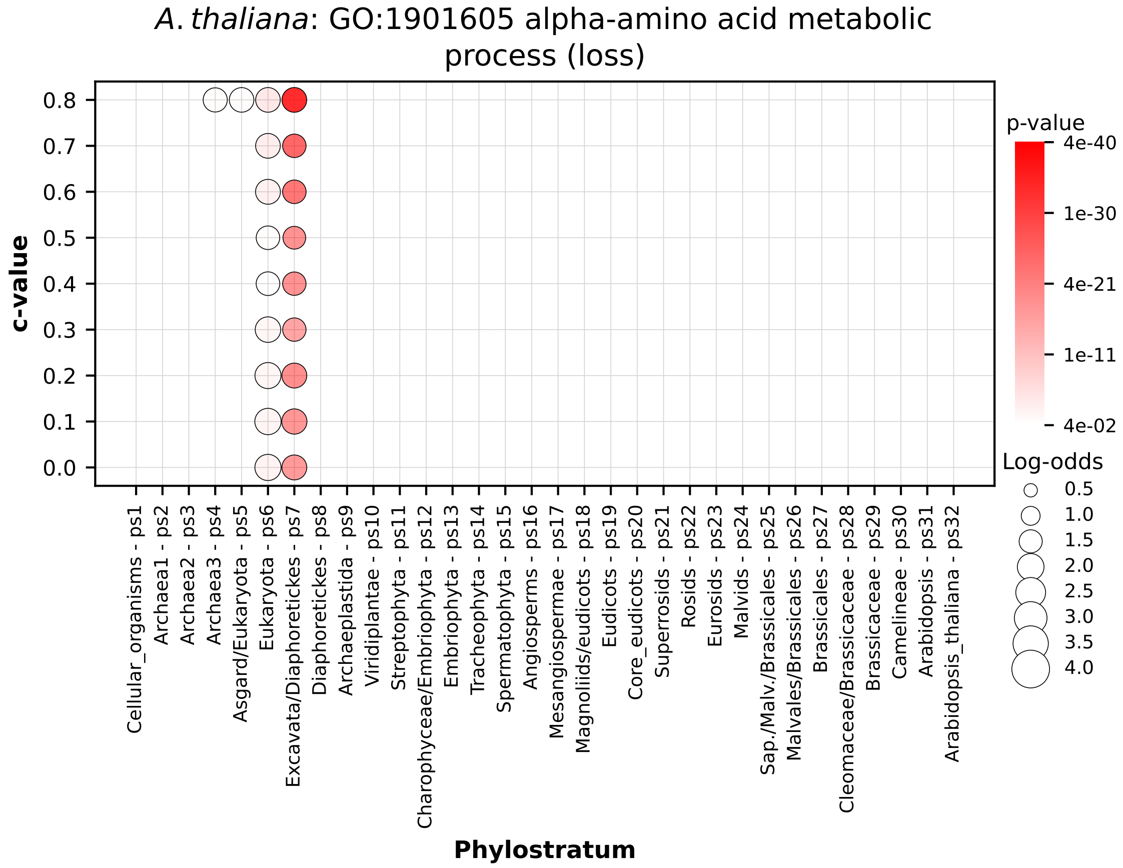
A. thaliana: GO:1901605 alpha-amino acid metabolic process (loss)
c-value
0.8
0.7
0.6
0.5
0.4
0.3
0.2
0.1
0.0
Phylostratum
Cellular_organisms - ps1
Archaea1 - ps2
Archaea2 - ps3
Archaea3 - ps4
Asgard/Eukaryota - ps5
Eukaryota - ps6
Excavata/Diaphoretickes - ps7
Diaphoretickes - ps8
Archaeplastida - ps9
Viridiplantae - ps10
Streptophyta - ps11
Charophyceae/Embriophyta - ps12
Embriophyta - ps13
Tracheophyta - ps14
Spermatophyta - ps15
Angiosperms - ps16
Mesangiospermae - ps17
Magnoliids/eudicots - ps18
Eudicots - ps19
Core_eudicots - ps20
Superrosids - ps21
Rosids - ps22
Eurosids - ps23
Malvids - ps24
Sap./Malv./Brassicales - ps25
Malvales/Brassicales - ps26
Brassicales - ps27
Cleomaceae/Brassicaceae - ps28
Brassicaceae - ps29
Camelineae - ps30
Arabidopsis - ps31
Arabidopsis_thaliana - ps32
p-value
4e-40
1e-30
4e-21
1e-11
4e-02
Log-odds
0.5
1.0
1.5
2.0
2.5
3.0
3.5
4.0

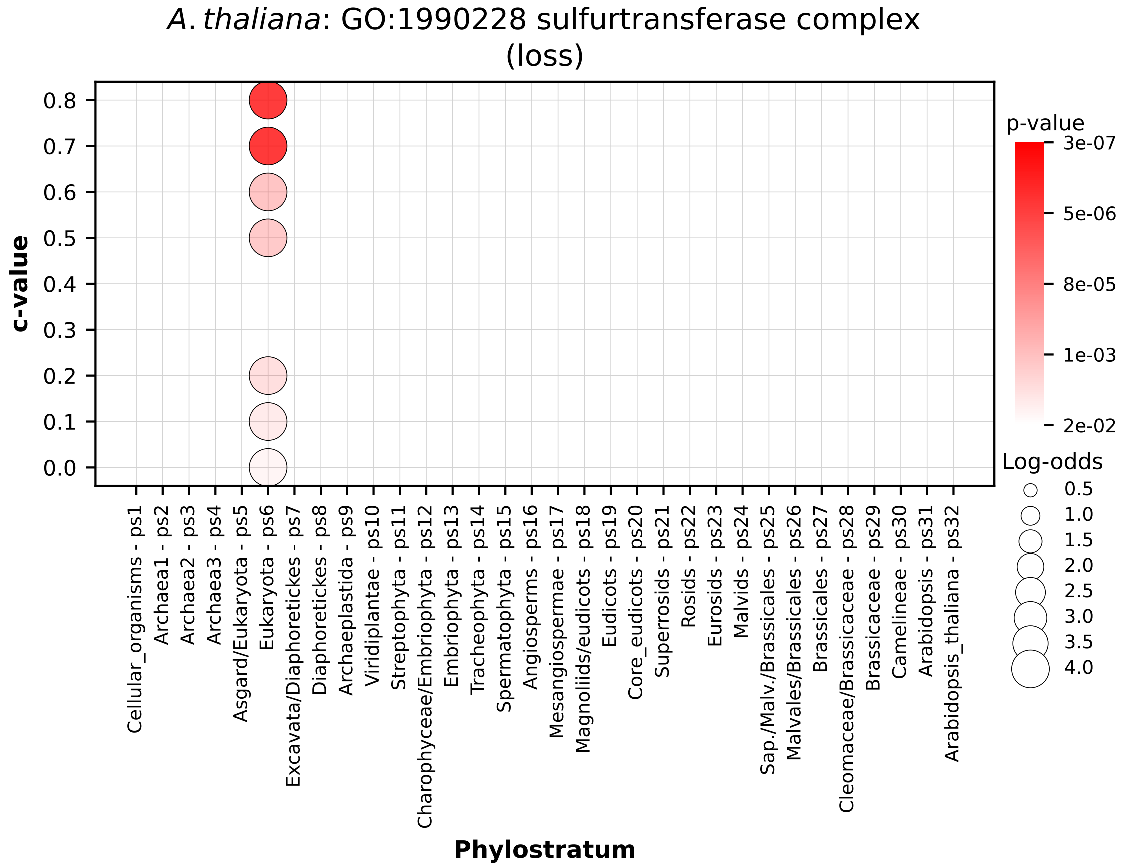

A. thaliana: GO:1990228 sulfurtransferase complex
(loss)
c-value
0.0
0.1
0.2
0.3
0.4
0.5
0.6
0.7
0.8
p-value
3e-07
5e-06
8e-05
1e-03
2e-02
Log-odds
0.5
1.0
1.5
2.0
2.5
3.0
3.5
4.0
Cellular_organisms - ps1
Archaea1 - ps2
Archaea2 - ps3
Archaea3 - ps4
Asgard/Eukaryota - ps5
Eukaryota - ps6
Excavata/Diaphoretickes - ps7
Diaphoretickes - ps8
Archaeplastida - ps9
Viridiplantae - ps10
Streptophyta - ps11
Charophyceae/Embriophyta - ps12
Embriophyta - ps13
Tracheophyta - ps14
Spermatophyta - ps15
Angiosperms - ps16
Mesangiospermae - ps17
Magnoliids/eudicots - ps18
Eudicots - ps19
Core_eudicots - ps20
Superrosids - ps21
Rosids - ps22
Eurosids - ps23
Malvids - ps24
Sap./Malv./Brassicales - ps25
Malvales/Brassicales - ps26
Brassicales - ps27
Cleomaceae/Brassicaceae - ps28
Brassicaceae - ps29
Camelineae - ps30
Arabidopsis - ps31
Arabidopsis_thaliana - ps32
Phylostratum

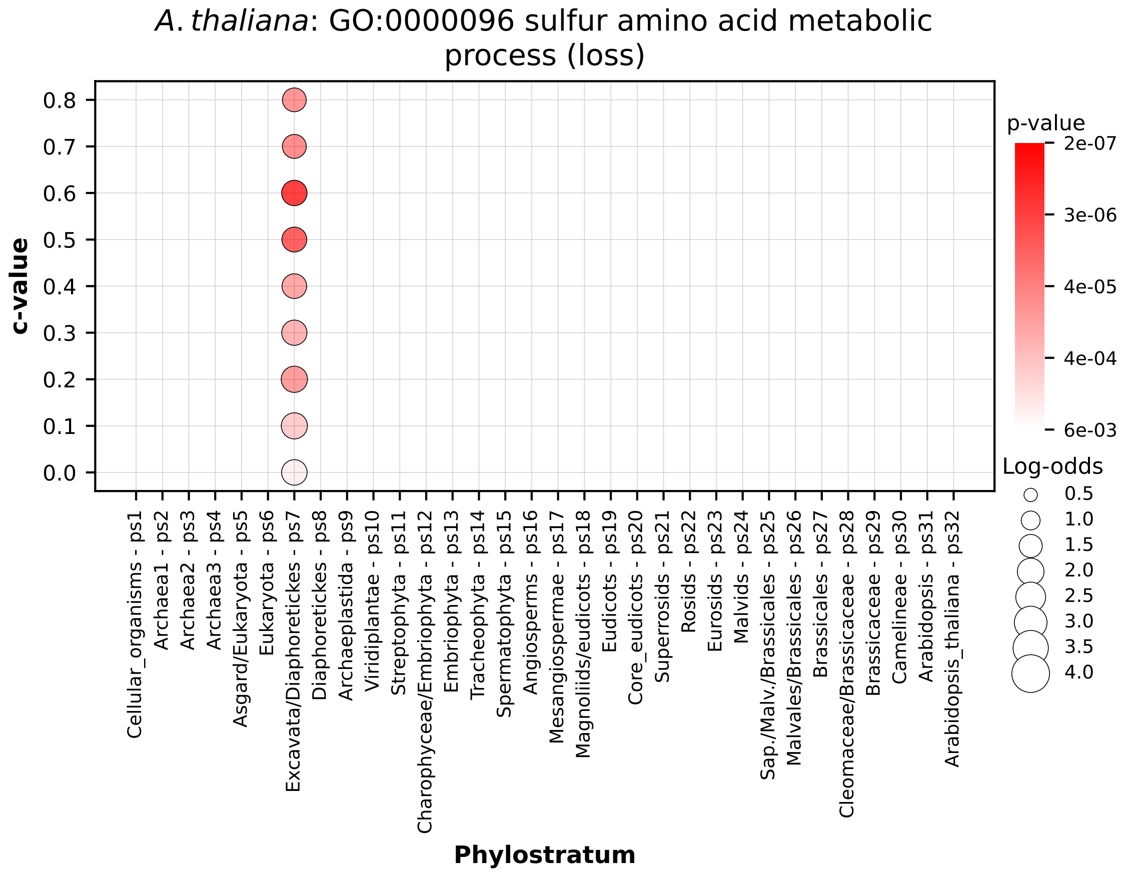

A. thaliana: GO:0000096 sulfur amino acid metabolic process (loss)
c-value
0.8
0.7
0.6
0.5
0.4
0.3
0.2
0.1
0.0
p-value
2e-07
3e-06
4e-05
4e-04
6e-03
Log-odds
0.5
1.0
1.5
2.0
2.5
3.0
3.5
4.0
Cellular_organisms - ps1
Archaea1 - ps2
Archaea2 - ps3
Archaea3 - ps4
Asgard/Eukaryota - ps5
Eukaryota - ps6
Excavata/Diaphoretickes - ps7
Diaphoretickes - ps8
Archaeplastida - ps9
Viridiplantae - ps10
Streptophyta - ps11
Charophyceae/Embriophyta - ps12
Embriophyta - ps13
Tracheophyta - ps14
Spermatophyta - ps15
Angiosperms - ps16
Mesangiospermae - ps17
Magnoliids/eudicots - ps18
Eudicots - ps19
Core_eudicots - ps20
Superrosids - ps21
Rosids - ps22
Eurosids - ps23
Malvids - ps24
Sap./Malv./Brassicales - ps25
Malvales/Brassicales - ps26
Brassicales - ps27
Cleomaceae/Brassicaceae - ps28
Brassicaceae - ps29
Camelineae - ps30
Arabidopsis - ps31
Arabidopsis_thaliana - ps32
Phylostratum

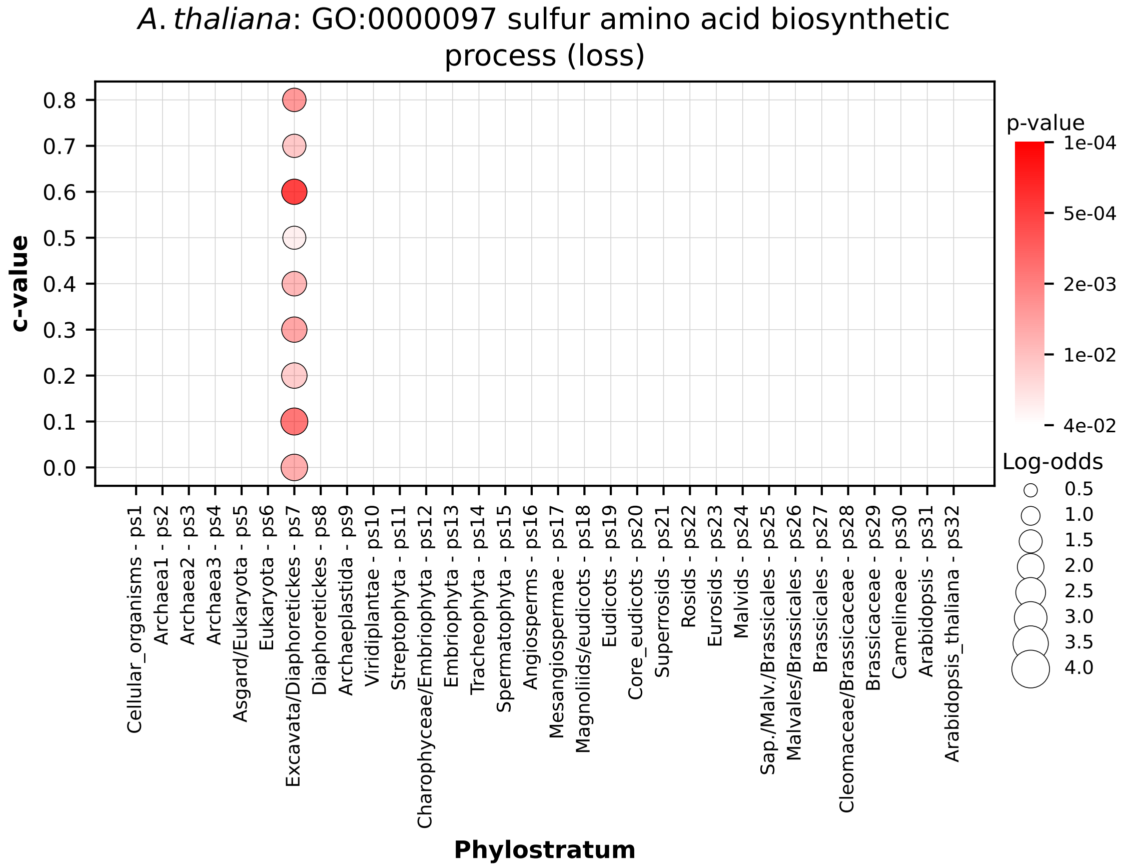
A. thaliana: GO:0000097 sulfur amino acid biosynthetic process (loss)
c-value
0.8
0.7
0.6
0.5
0.4
0.3
0.2
0.1
0.0
Phylostratum
Cellular_organisms - ps1
Archaea1 - ps2
Archaea2 - ps3
Archaea3 - ps4
Asgard/Eukaryota - ps5
Eukaryota - ps6
Excavata/Diaphoretickes - ps7
Diaphoretickes - ps8
Archaeplastida - ps9
Viridiplantae - ps10
Streptophyta - ps11
Charophyceae/Embriophyta - ps12
Embriophyta - ps13
Tracheophyta - ps14
Spermatophyta - ps15
Angiosperms - ps16
Mesangiospermae - ps17
Magnoliids/eudicots - ps18
Eudicots - ps19
Core_eudicots - ps20
Superrosids - ps21
Rosids - ps22
Eurosids - ps23
Malvids - ps24
Sap./Malv./Brassicales - ps25
Malvales/Brassicales - ps26
Brassicales - ps27
Cleomaceae/Brassicaceae - ps28
Brassicaceae - ps29
Camelineae - ps30
Arabidopsis - ps31
Arabidopsis_thaliana - ps32
p-value
1e-04
5e-04
2e-03
1e-02
4e-02
Log-odds
0.5
1.0
1.5
2.0
2.5
3.0
3.5
4.0

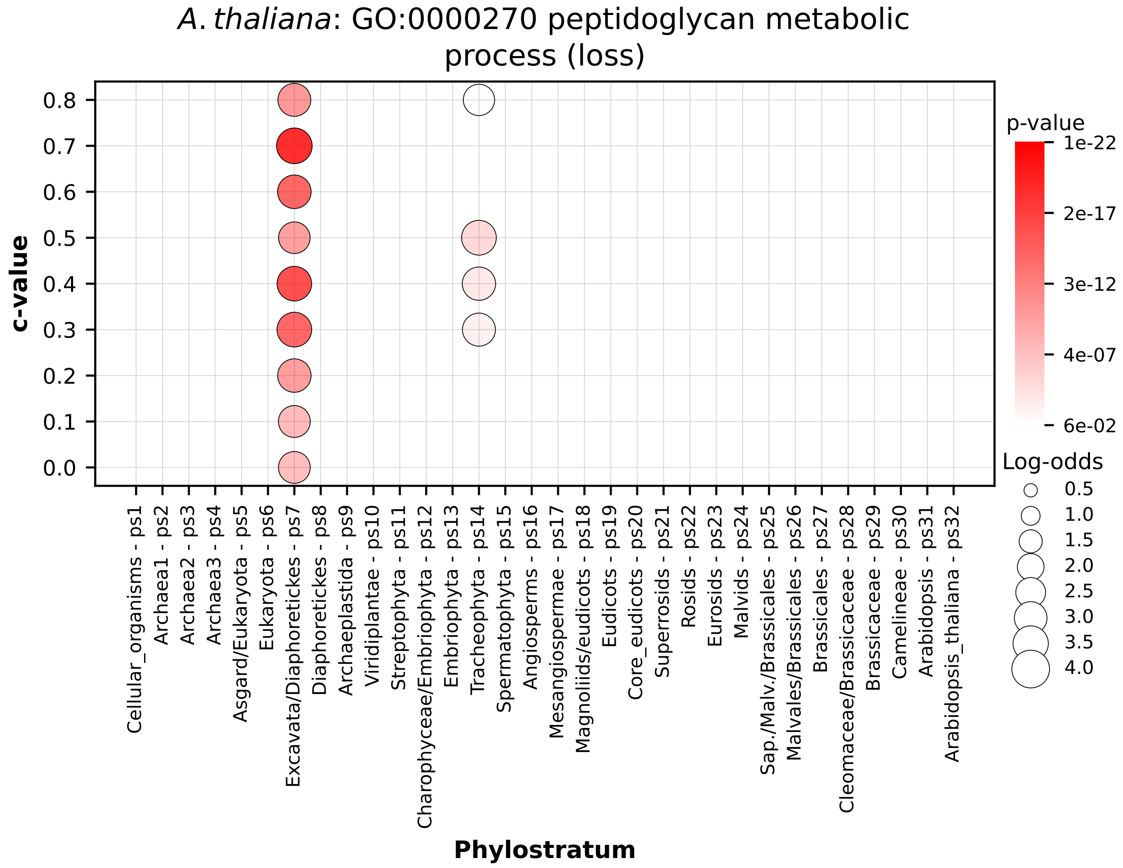

A. thaliana: GO:0000270 peptidoglycan metabolic process (loss)
c-value
0.8
0.7
0.6
0.5
0.4
0.3
0.2
0.1
0.0
p-value
1e-22
2e-17
3e-12
4e-07
6e-02
Log-odds
0.5
1.0
1.5
2.0
2.5
3.0
3.5
4.0
Cellular_organisms - ps1
Archaea1 - ps2
Archaea2 - ps3
Archaea3 - ps4
Asgard/Eukaryota - ps5
Eukaryota - ps6
Excavata/Diaphoretickes - ps7
Diaphoretickes - ps8
Archaeplastida - ps9
Viridiplantae - ps10
Streptophyta - ps11
Charophyceae/Embriophyta - ps12
Embriophyta - ps13
Tracheophyta - ps14
Spermatophyta - ps15
Angiosperms - ps16
Mesangiospermae - ps17
Magnoliids/eudicots - ps18
Eudicots - ps19
Core_eudicots - ps20
Superrosids - ps21
Rosids - ps22
Eurosids - ps23
Malvids - ps24
Sap./Malv./Brassicales - ps25
Malvales/Brassicales - ps26
Brassicales - ps27
Cleomaceae/Brassicaceae - ps28
Brassicaceae - ps29
Camelineae - ps30
Arabidopsis - ps31
Arabidopsis_thaliana - ps32
Phylostratum

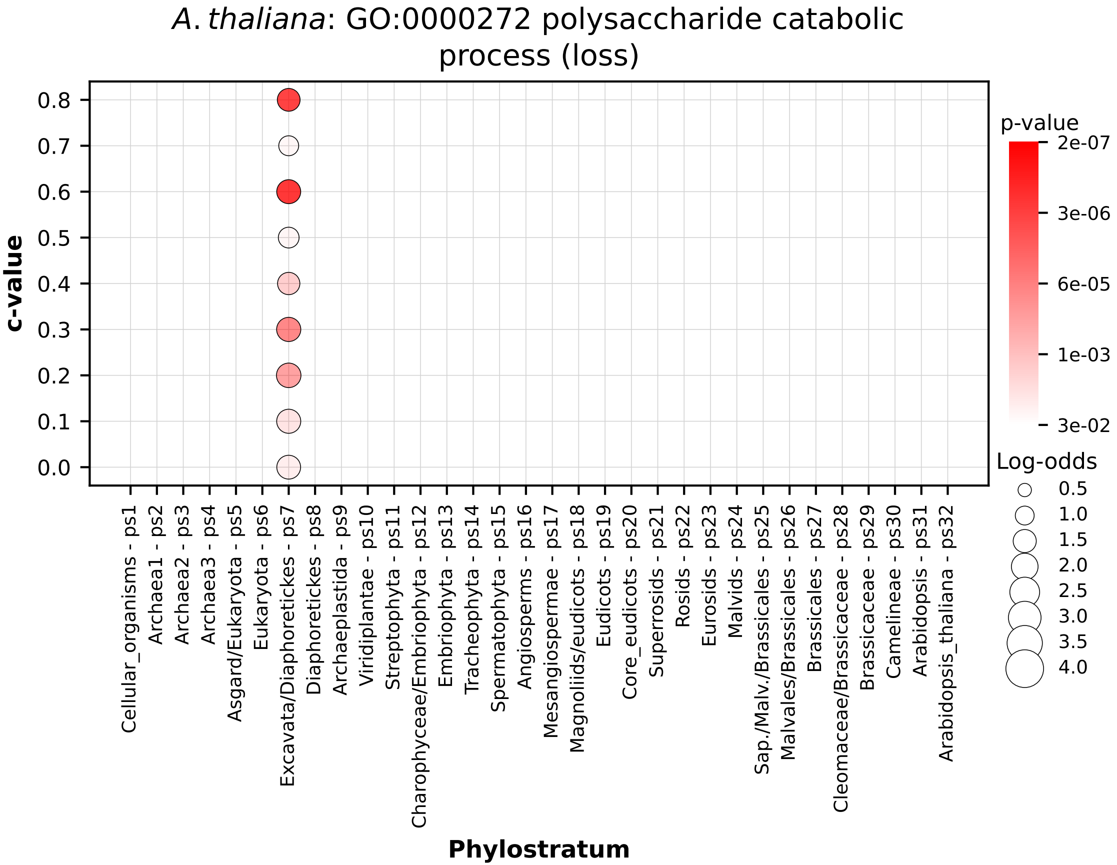
A. thaliana: GO:0000272 polysaccharide catabolic process (loss)
c-value
0.8
0.7
0.6
0.5
0.4
0.3
0.2
0.1
0.0
p-value
2e-07
3e-06
6e-05
1e-03
3e-02
Log-odds
0.5
1.0
1.5
2.0
2.5
3.0
3.5
4.0
Cellular_organisms - ps1
Archaea1 - ps2
Archaea2 - ps3
Archaea3 - ps4
Asgard/Eukaryota - ps5
Eukaryota - ps6
Excavata/Diaphoretickes - ps7
Diaphoretickes - ps8
Archaeplastida - ps9
Viridiplantae - ps10
Streptophyta - ps11
Charophyceae/Embriophyta - ps12
Embriophyta - ps13
Tracheophyta - ps14
Spermatophyta - ps15
Angiosperms - ps16
Mesangiospermae - ps17
Magnoliids/eudicots - ps18
Eudicots - ps19
Core_eudicots - ps20
Superrosids - ps21
Rosids - ps22
Eurosids - ps23
Malvids - ps24
Sap./Malv./Brassicales - ps25
Malvales/Brassicales - ps26
Brassicales - ps27
Cleomaceae/Brassicaceae - ps28
Brassicaceae - ps29
Camelineae - ps30
Arabidopsis - ps31
Arabidopsis_thaliana - ps32
Phylostratum

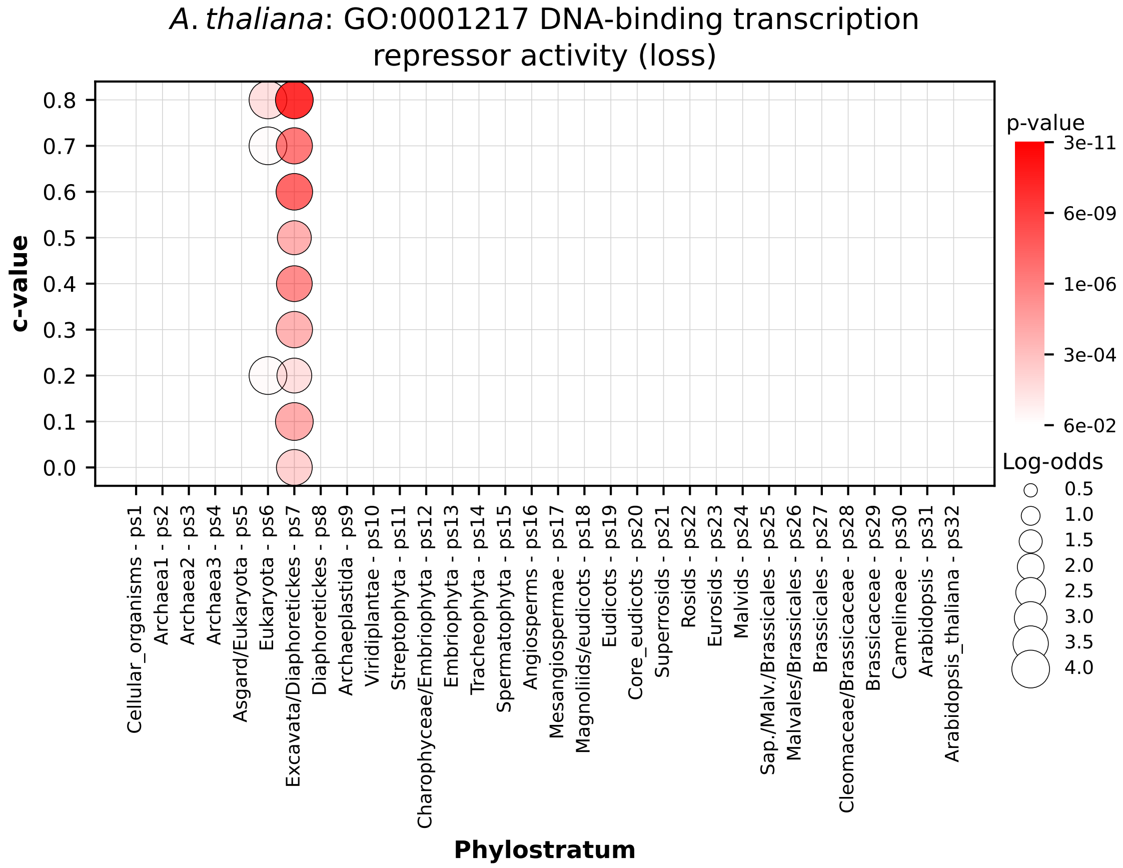

A. thaliana: GO:0001217 DNA-binding transcription repressor activity (loss)
c-value
0.8
0.7
0.6
0.5
0.4
0.3
0.2
0.1
0.0
Phylostratum
Cellular_organisms - ps1
Archaea1 - ps2
Archaea2 - ps3
Archaea3 - ps4
Asgard/Eukaryota - ps5
Eukaryota - ps6
Excavata/Diaphoretickes - ps7
Diaphoretickes - ps8
Archaeplastida - ps9
Viridiplantae - ps10
Streptophyta - ps11
Charophyceae/Embriophyta - ps12
Embriophyta - ps13
Tracheophyta - ps14
Spermatophyta - ps15
Angiosperms - ps16
Mesangiospermae - ps17
Magnoliids/eudicots - ps18
Eudicots - ps19
Core_eudicots - ps20
Superrosids - ps21
Rosids - ps22
Eurosids - ps23
Malvids - ps24
Sap./Malv./Brassicales - ps25
Malvales/Brassicales - ps26
Brassicales - ps27
Cleomaceae/Brassicaceae - ps28
Brassicaceae - ps29
Camelineae - ps30
Arabidopsis - ps31
Arabidopsis_thaliana - ps32
p-value
3e-11
6e-09
1e-06
3e-04
6e-02
Log-odds
0.5
1.0
1.5
2.0
2.5
3.0
3.5
4.0

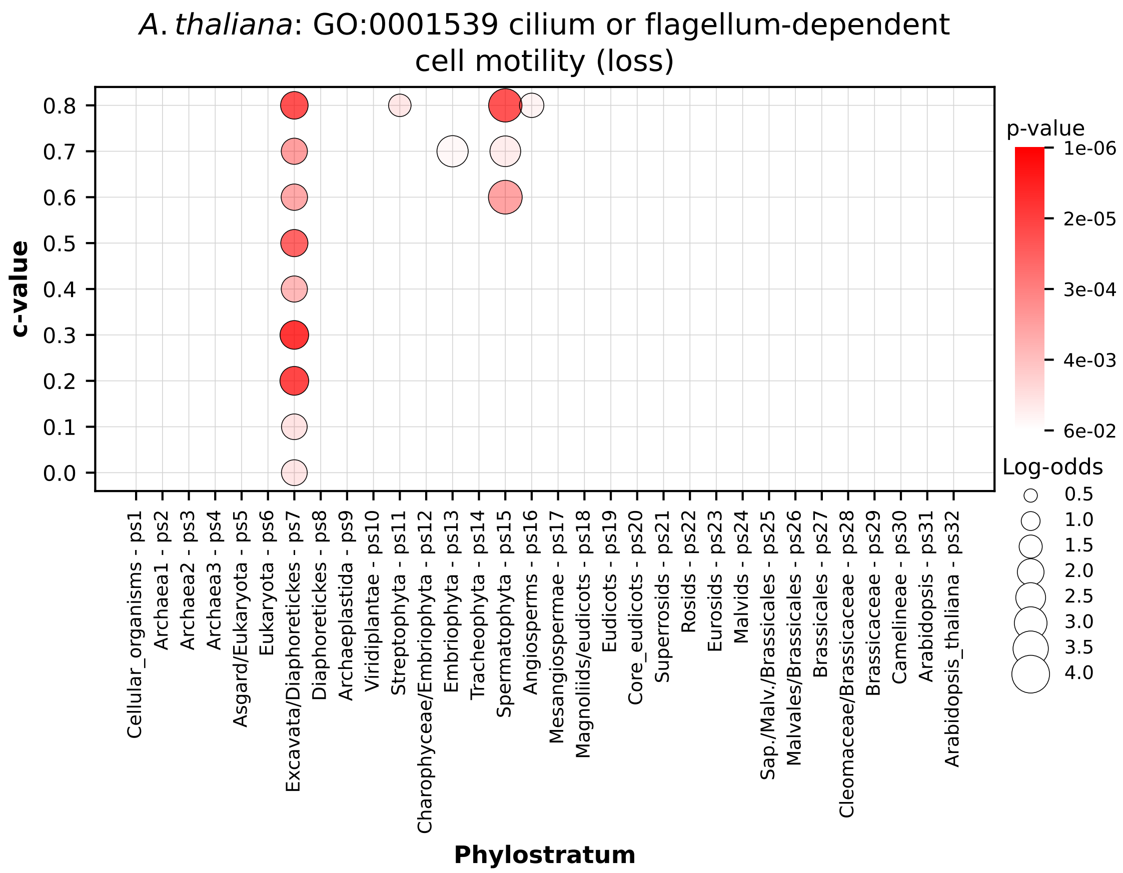

A. thaliana: GO:0001539 cilium or flagellum-dependent cell motility (loss)
c-value
0.8
0.7
0.6
0.5
0.4
0.3
0.2
0.1
0.0
Phylostratum
Cellular_organisms - ps1
Archaea1 - ps2
Archaea2 - ps3
Archaea3 - ps4
Asgard/Eukaryota - ps5
Eukaryota - ps6
Excavata/Diaphoretickes - ps7
Diaphoretickes - ps8
Archaeplastida - ps9
Viridiplantae - ps10
Streptophyta - ps11
Charophyceae/Embriophyta - ps12
Embriophyta - ps13
Tracheophyta - ps14
Spermatophyta - ps15
Angiosperms - ps16
Mesangiospermae - ps17
Magnoliids/eudicots - ps18
Eudicots - ps19
Core_eudicots - ps20
Superrosids - ps21
Rosids - ps22
Eurosids - ps23
Malvids - ps24
Sap./Malv./Brassicales - ps25
Malvales/Brassicales - ps26
Brassicales - ps27
Cleomaceae/Brassicaceae - ps28
Brassicaceae - ps29
Camelineae - ps30
Arabidopsis - ps31
Arabidopsis_thaliana - ps32
p-value
1e-06
2e-05
3e-04
4e-03
6e-02
Log-odds
0.5
1.0
1.5
2.0
2.5
3.0
3.5
4.0

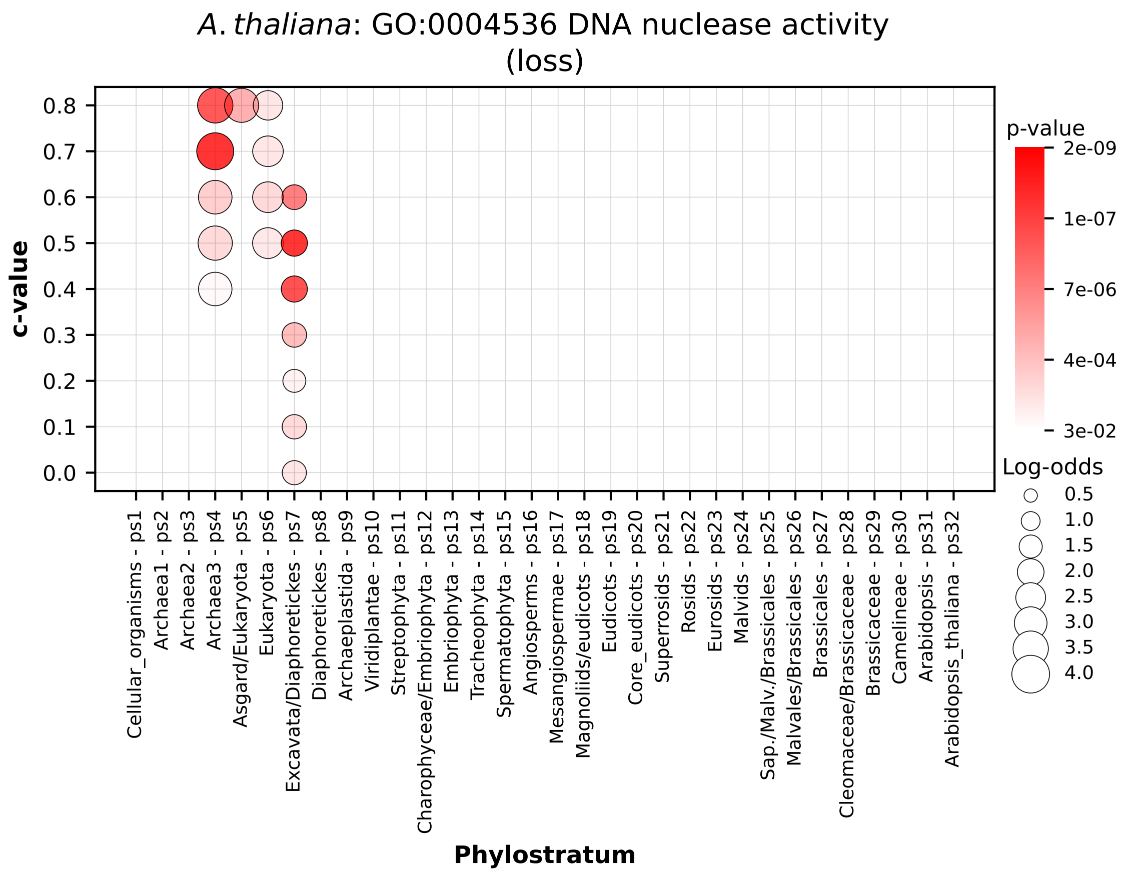

A. thaliana: GO:0004536 DNA nuclease activity
(loss)
c-value
0.8
0.7
0.6
0.5
0.4
0.3
0.2
0.1
0.0
p-value
2e-09
1e-07
7e-06
4e-04
3e-02
Log-odds
0.5
1.0
1.5
2.0
2.5
3.0
3.5
4.0
Cellular_organisms - ps1
Archaea1 - ps2
Archaea2 - ps3
Archaea3 - ps4
Asgard/Eukaryota - ps5
Eukaryota - ps6
Excavata/Diaphoretickes - ps7
Diaphoretickes - ps8
Archaeplastida - ps9
Viridiplantae - ps10
Streptophyta - ps11
Charophyceae/Embriophyta - ps12
Embriophyta - ps13
Tracheophyta - ps14
Spermatophyta - ps15
Angiosperms - ps16
Mesangiospermae - ps17
Magnoliids/eudicots - ps18
Eudicots - ps19
Core_eudicots - ps20
Superrosids - ps21
Rosids - ps22
Eurosids - ps23
Malvids - ps24
Sap./Malv./Brassicales - ps25
Malvales/Brassicales - ps26
Brassicales - ps27
Cleomaceae/Brassicaceae - ps28
Brassicaceae - ps29
Camelineae - ps30
Arabidopsis - ps31
Arabidopsis_thaliana - ps32
Phylostratum

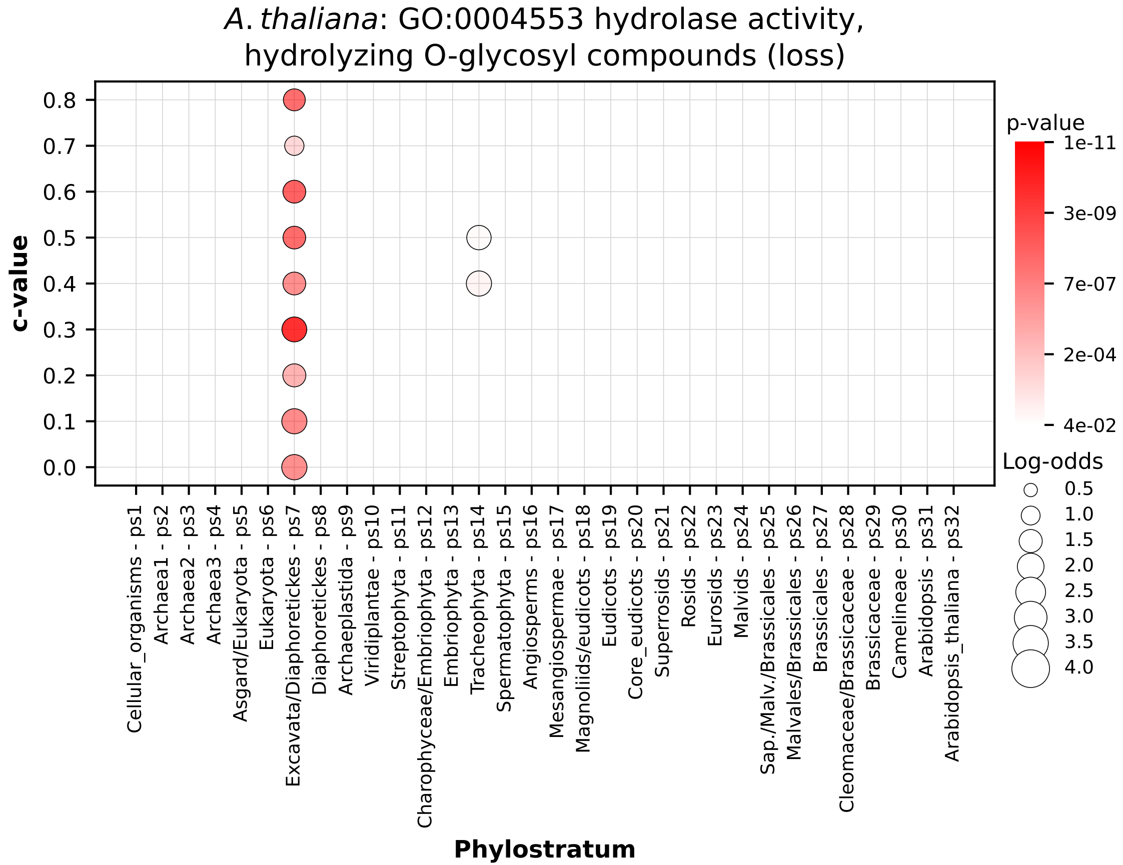

A. thaliana: GO:0004553 hydrolase activity, hydrolyzing O-glycosyl compounds (loss)
c-value
0.8
0.7
0.6
0.5
0.4
0.3
0.2
0.1
0.0
p-value
1e-11
3e-09
7e-07
2e-04
4e-02
Log-odds
0.5
1.0
1.5
2.0
2.5
3.0
3.5
4.0
Cellular_organisms - ps1
Archaea1 - ps2
Archaea2 - ps3
Archaea3 - ps4
Asgard/Eukaryota - ps5
Eukaryota - ps6
Excavata/Diaphoretickes - ps7
Diaphoretickes - ps8
Archaeplastida - ps9
Viridiplantae - ps10
Streptophyta - ps11
Charophyceae/Embriophyta - ps12
Embriophyta - ps13
Tracheophyta - ps14
Spermatophyta - ps15
Angiosperms - ps16
Mesangiospermae - ps17
Magnoliids/eudicots - ps18
Eudicots - ps19
Core_eudicots - ps20
Superrosids - ps21
Rosids - ps22
Eurosids - ps23
Malvids - ps24
Sap./Malv./Brassicales - ps25
Malvales/Brassicales - ps26
Brassicales - ps27
Cleomaceae/Brassicaceae - ps28
Brassicaceae - ps29
Camelineae - ps30
Arabidopsis - ps31
Arabidopsis_thaliana - ps32
Phylostratum

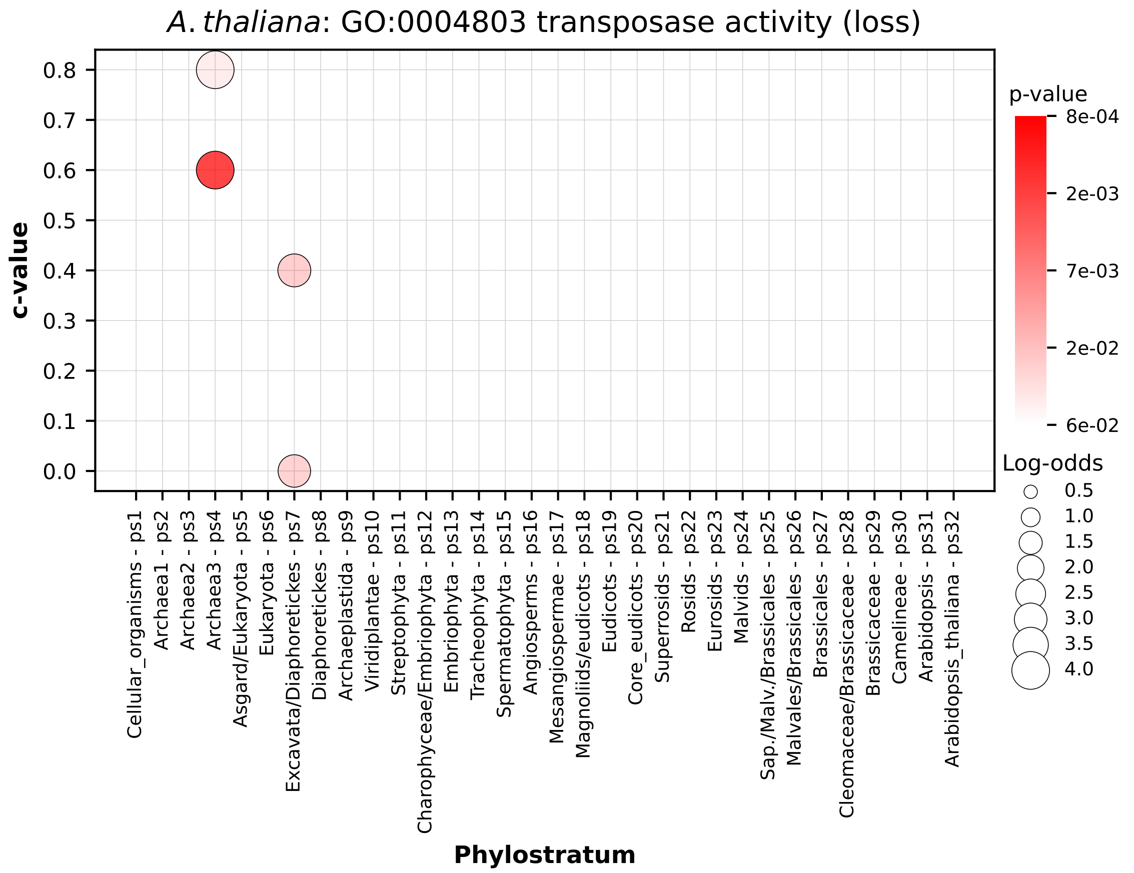
A. thaliana: GO:0004803 transposase activity (loss)
c-value
0.8
0.7
0.6
0.5
0.4
0.3
0.2
0.1
0.0
Phylostratum
Cellular_organisms - ps1
Archaea1 - ps2
Archaea2 - ps3
Archaea3 - ps4
Asgard/Eukaryota - ps5
Eukaryota - ps6
Excavata/Diaphoretickes - ps7
Diaphoretickes - ps8
Archaeplastida - ps9
Viridiplantae - ps10
Streptophyta - ps11
Charophyceae/Embriophyta - ps12
Embriophyta - ps13
Tracheophyta - ps14
Spermatophyta - ps15
Angiosperms - ps16
Mesangiospermae - ps17
Magnoliids/eudicots - ps18
Eudicots - ps19
Core_eudicots - ps20
Superrosids - ps21
Rosids - ps22
Eurosids - ps23
Malvids - ps24
Sap./Malv./Brassicales - ps25
Malvales/Brassicales - ps26
Brassicales - ps27
Cleomaceae/Brassicaceae - ps28
Brassicaceae - ps29
Camelineae - ps30
Arabidopsis - ps31
Arabidopsis_thaliana - ps32
p-value
8e-04
2e-03
7e-03
2e-02
6e-02
Log-odds
0.5
1.0
1.5
2.0
2.5
3.0
3.5
4.0

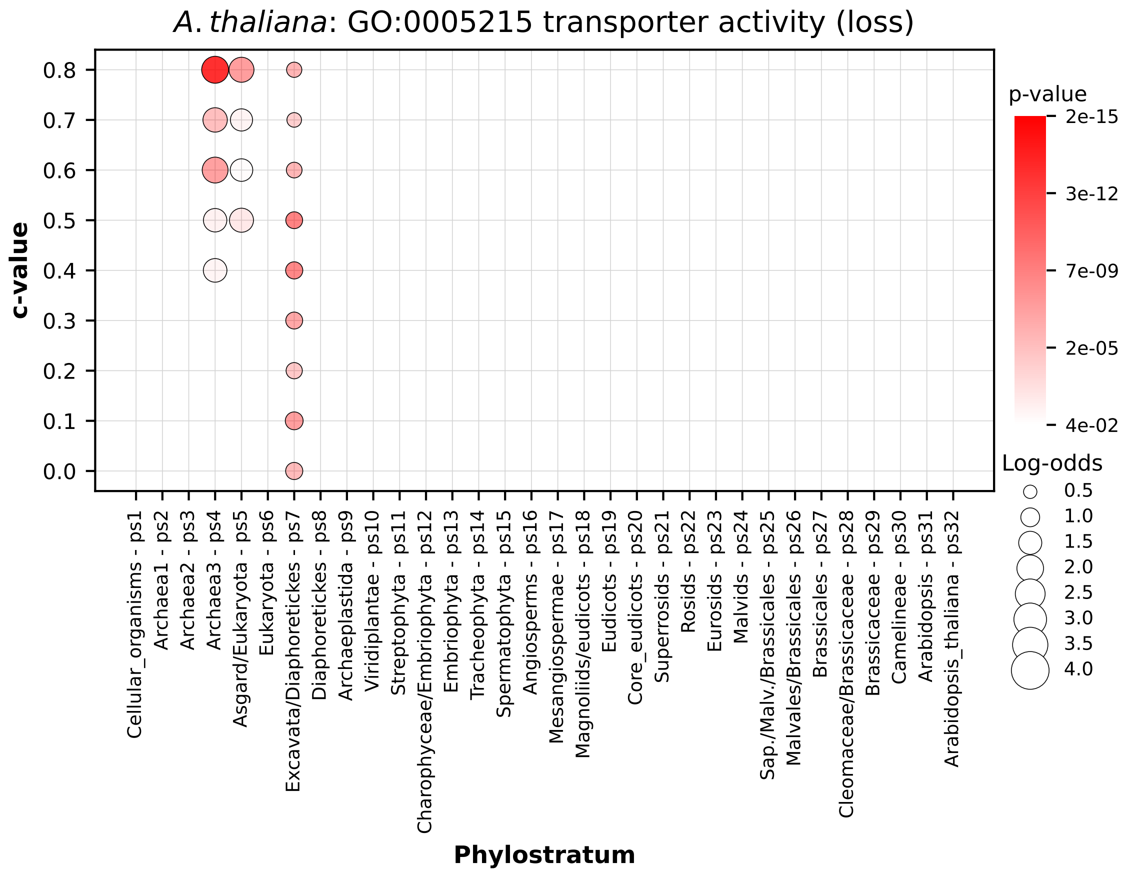

A. thaliana: GO:0005215 transporter activity (loss)
c-value
0.8
0.7
0.6
0.5
0.4
0.3
0.2
0.1
0.0
Phylostratum
Cellular_organisms - ps1
Archaea1 - ps2
Archaea2 - ps3
Archaea3 - ps4
Asgard/Eukaryota - ps5
Eukaryota - ps6
Excavata/Diaphoretickes - ps7
Diaphoretickes - ps8
Archaeplastida - ps9
Viridiplantae - ps10
Streptophyta - ps11
Charophyceae/Embriophyta - ps12
Embriophyta - ps13
Tracheophyta - ps14
Spermatophyta - ps15
Angiosperms - ps16
Mesangiospermae - ps17
Magnoliids/eudicots - ps18
Eudicots - ps19
Core_eudicots - ps20
Superrosids - ps21
Rosids - ps22
Eurosids - ps23
Malvids - ps24
Sap./Malv./Brassicales - ps25
Malvales/Brassicales - ps26
Brassicales - ps27
Cleomaceae/Brassicaceae - ps28
Brassicaceae - ps29
Camelineae - ps30
Arabidopsis - ps31
Arabidopsis_thaliana - ps32
p-value
2e-15
3e-12
7e-09
2e-05
4e-02
Log-odds
0.5
1.0
1.5
2.0
2.5
3.0
3.5
4.0

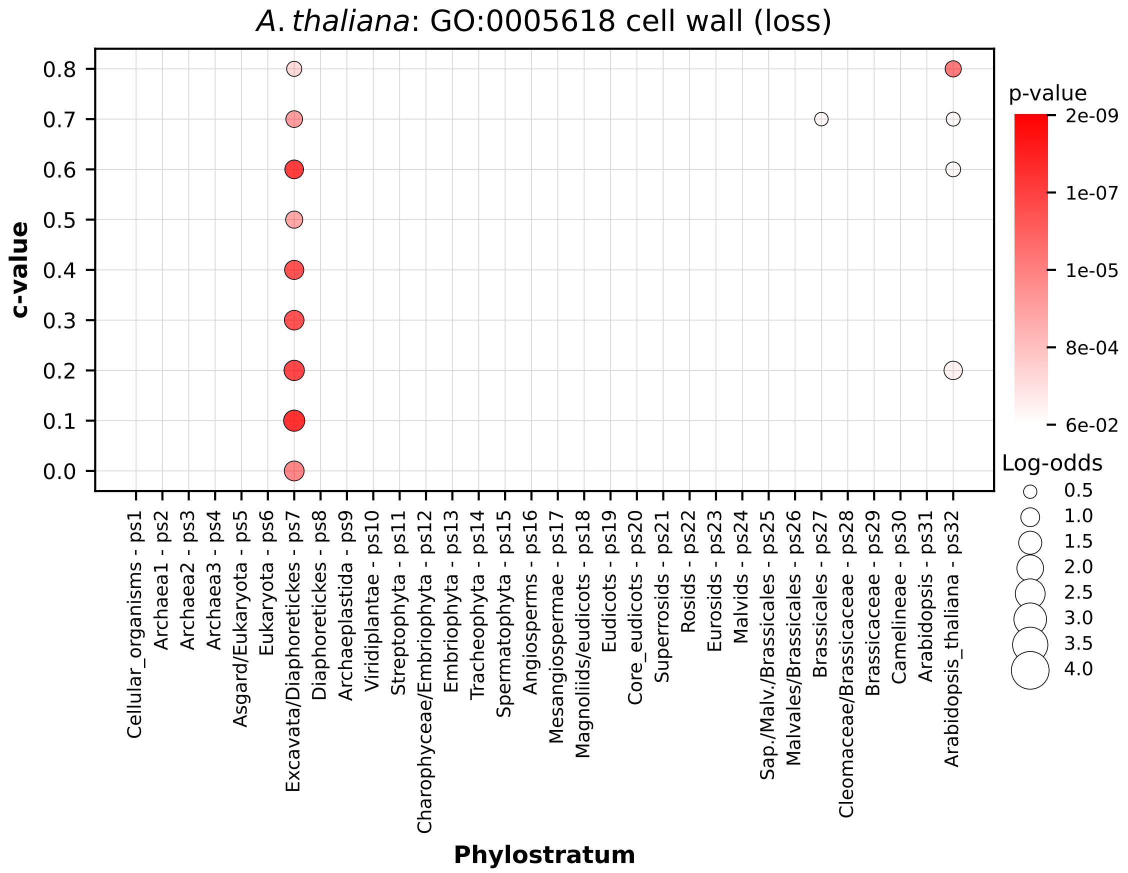

A. thaliana: GO:0005618 cell wall (loss)
c-value
0.0
0.1
0.2
0.3
0.4
0.5
0.6
0.7
0.8
p-value
2e-09
1e-07
1e-05
8e-04
6e-02
Log-odds
0.5
1.0
1.5
2.0
2.5
3.0
3.5
4.0
Cellular_organisms - ps1
Archaea1 - ps2
Archaea2 - ps3
Archaea3 - ps4
Asgard/Eukaryota - ps5
Eukaryota - ps6
Excavata/Diaphoretickes - ps7
Diaphoretickes - ps8
Archaeplastida - ps9
Viridiplantae - ps10
Streptophyta - ps11
Charophyceae/Embriophyta - ps12
Embriophyta - ps13
Tracheophyta - ps14
Spermatophyta - ps15
Angiosperms - ps16
Mesangiospermae - ps17
Magnoliids/eudicots - ps18
Eudicots - ps19
Core_eudicots - ps20
Superrosids - ps21
Rosids - ps22
Eurosids - ps23
Malvids - ps24
Sap./Malv./Brassicales - ps25
Malvales/Brassicales - ps26
Brassicales - ps27
Cleomaceae/Brassicaceae - ps28
Brassicaceae - ps29
Camelineae - ps30
Arabidopsis - ps31
Arabidopsis_thaliana - ps32
Phylostratum

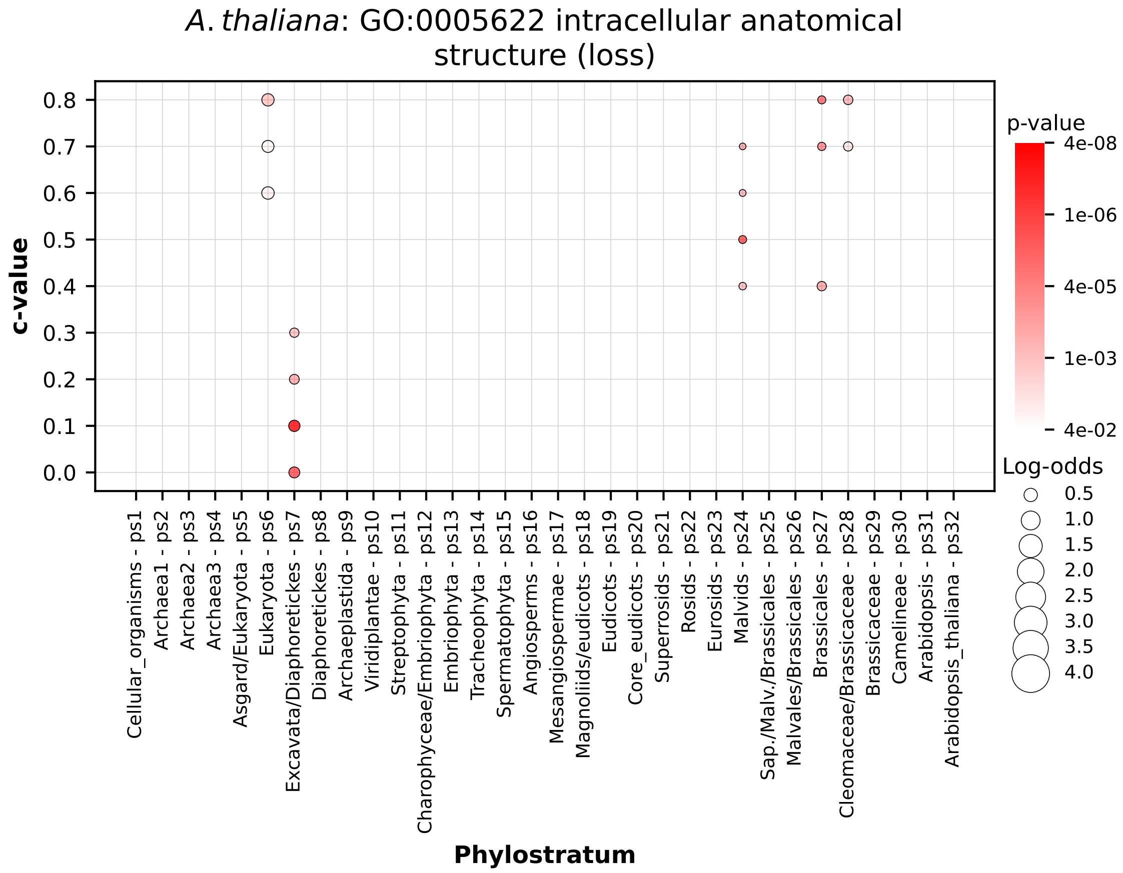

A. thaliana: GO:0005622 intracellular anatomical structure (loss)
c-value
0.8
0.7
0.6
0.5
0.4
0.3
0.2
0.1
0.0
p-value
4e-08
1e-06
4e-05
1e-03
4e-02
Log-odds
0.5
1.0
1.5
2.0
2.5
3.0
3.5
4.0
Cellular_organisms - ps1
Archaea1 - ps2
Archaea2 - ps3
Archaea3 - ps4
Asgard/Eukaryota - ps5
Eukaryota - ps6
Excavata/Diaphoretickes - ps7
Diaphoretickes - ps8
Archaeplastida - ps9
Viridiplantae - ps10
Streptophyta - ps11
Charophyceae/Embriophyta - ps12
Embriophyta - ps13
Tracheophyta - ps14
Spermatophyta - ps15
Angiosperms - ps16
Mesangiospermae - ps17
Magnoliids/eudicots - ps18
Eudicots - ps19
Core_eudicots - ps20
Superrosids - ps21
Rosids - ps22
Eurosids - ps23
Malvids - ps24
Sap./Malv./Brassicales - ps25
Malvales/Brassicales - ps26
Brassicales - ps27
Cleomaceae/Brassicaceae - ps28
Brassicaceae - ps29
Camelineae - ps30
Arabidopsis - ps31
Arabidopsis_thaliana - ps32
Phylostratum

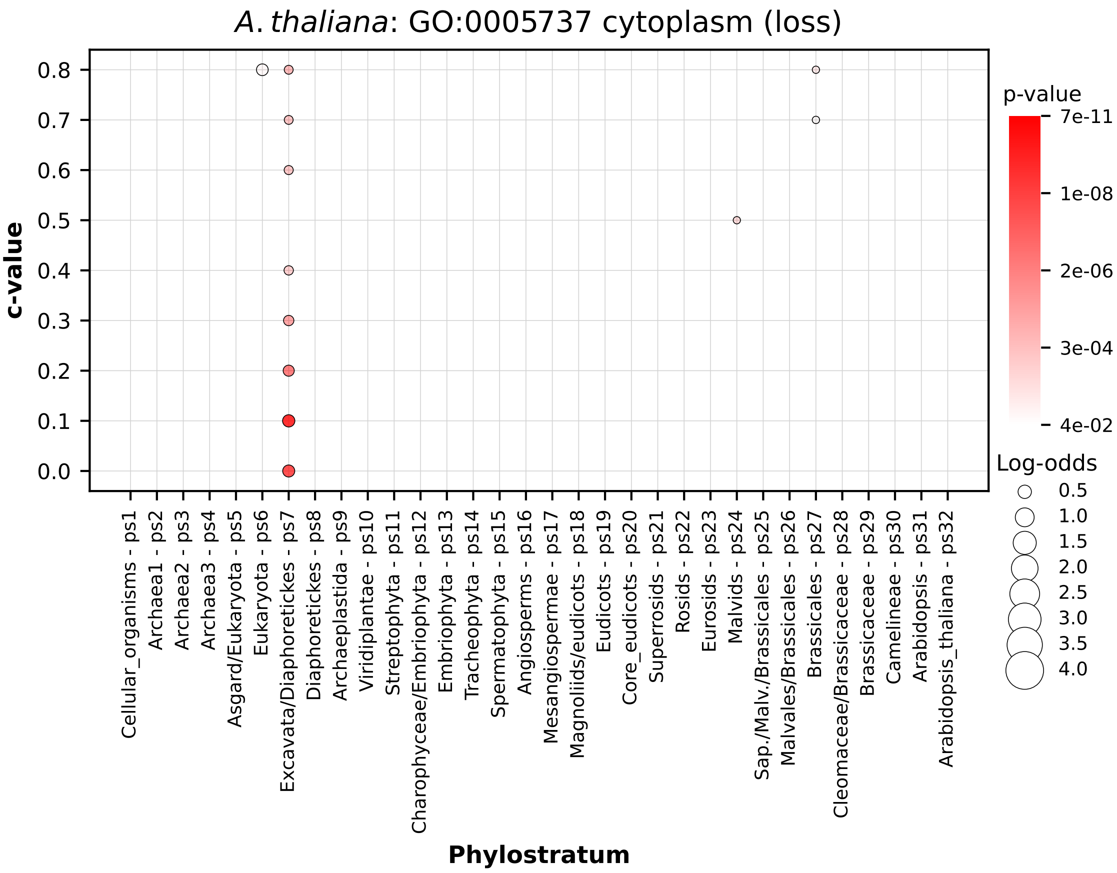
A. thaliana: GO:0005737 cytoplasm (loss)
c-value
0.8
0.7
0.6
0.5
0.4
0.3
0.2
0.1
0.0
Phylostratum
Cellular_organisms - ps1
Archaea1 - ps2
Archaea2 - ps3
Archaea3 - ps4
Asgard/Eukaryota - ps5
Eukaryota - ps6
Excavata/Diaphoretickes - ps7
Diaphoretickes - ps8
Archaeplastida - ps9
Viridiplantae - ps10
Streptophyta - ps11
Charophyceae/Embriophyta - ps12
Embriophyta - ps13
Tracheophyta - ps14
Spermatophyta - ps15
Angiosperms - ps16
Mesangiospermae - ps17
Magnoliids/eudicots - ps18
Eudicots - ps19
Core_eudicots - ps20
Superrosids - ps21
Rosids - ps22
Eurosids - ps23
Malvids - ps24
Sap./Malv./Brassicales - ps25
Malvales/Brassicales - ps26
Brassicales - ps27
Cleomaceae/Brassicaceae - ps28
Brassicaceae - ps29
Camelineae - ps30
Arabidopsis - ps31
Arabidopsis_thaliana - ps32
p-value
7e-11
1e-08
2e-06
3e-04
4e-02
Log-odds
0.5
1.0
1.5
2.0
2.5
3.0
3.5
4.0

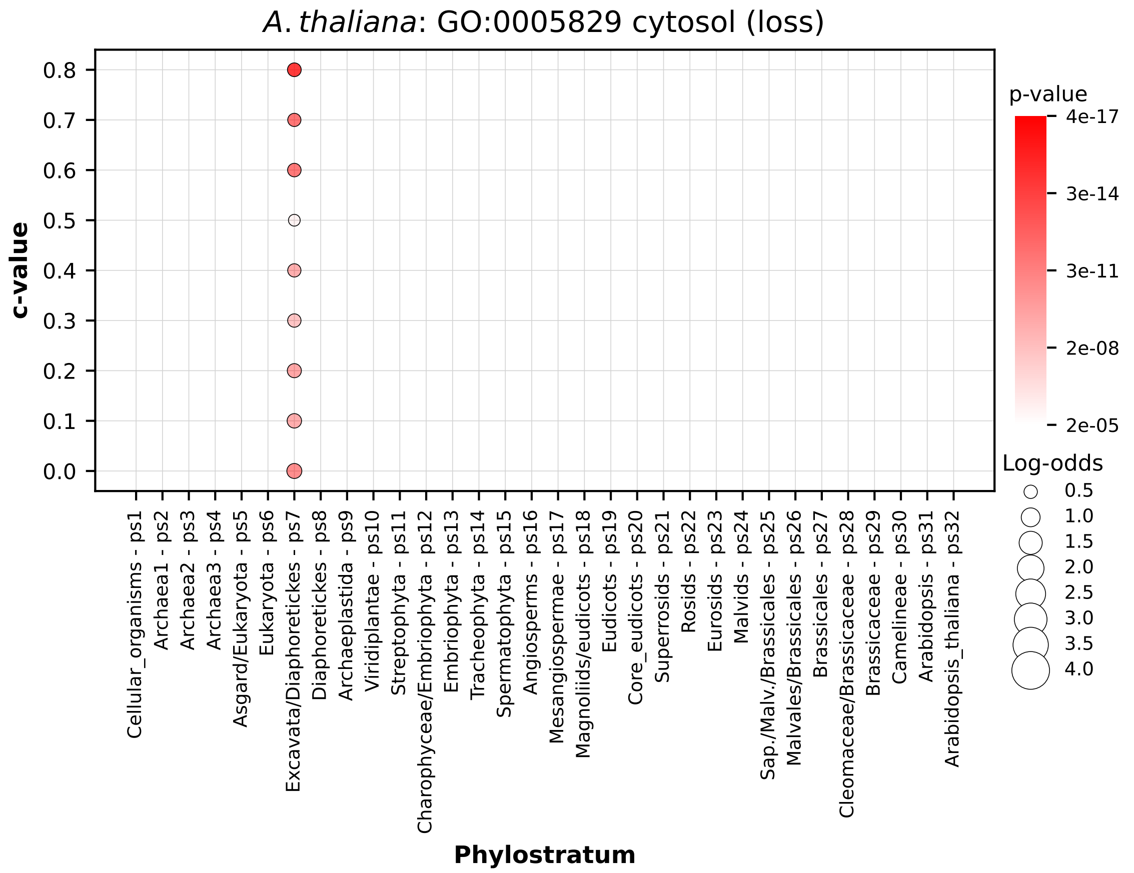
A. thaliana: GO:0005829 cytosol (loss)
c-value
0.8
0.7
0.6
0.5
0.4
0.3
0.2
0.1
0.0
Phylostratum
Cellular_organisms - ps1
Archaea1 - ps2
Archaea2 - ps3
Archaea3 - ps4
Asgard/Eukaryota - ps5
Eukaryota - ps6
Excavata/Diaphoretickes - ps7
Diaphoretickes - ps8
Archaeplastida - ps9
Viridiplantae - ps10
Streptophyta - ps11
Charophyceae/Embriophyta - ps12
Embriophyta - ps13
Tracheophyta - ps14
Spermatophyta - ps15
Angiosperms - ps16
Mesangiospermae - ps17
Magnoliids/eudicots - ps18
Eudicots - ps19
Core_eudicots - ps20
Superrosids - ps21
Rosids - ps22
Eurosids - ps23
Malvids - ps24
Sap./Malv./Brassicales - ps25
Malvales/Brassicales - ps26
Brassicales - ps27
Cleomaceae/Brassicaceae - ps28
Brassicaceae - ps29
Camelineae - ps30
Arabidopsis - ps31
Arabidopsis_thaliana - ps32
p-value
4e-17
3e-14
3e-11
2e-08
2e-05
Log-odds
0.5
1.0
1.5
2.0
2.5
3.0
3.5
4.0

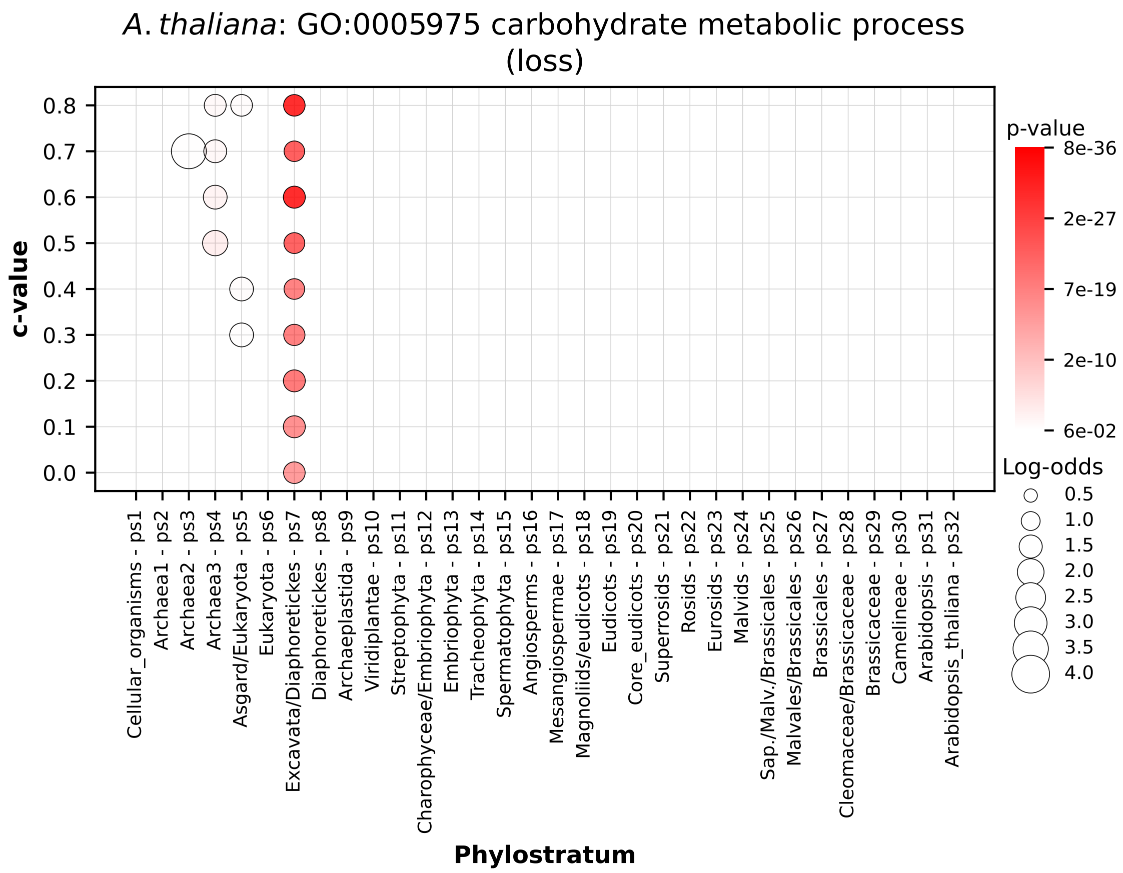

A. thaliana: GO:0005975 carbohydrate metabolic process (loss)
c-value
0.8
0.7
0.6
0.5
0.4
0.3
0.2
0.1
0.0
Phylostratum
Cellular_organisms - ps1
Archaea1 - ps2
Archaea2 - ps3
Archaea3 - ps4
Asgard/Eukaryota - ps5
Eukaryota - ps6
Excavata/Diaphoretickes - ps7
Diaphoretickes - ps8
Archaeplastida - ps9
Viridiplantae - ps10
Streptophyta - ps11
Charophyceae/Embriophyta - ps12
Embriophyta - ps13
Tracheophyta - ps14
Spermatophyta - ps15
Angiosperms - ps16
Mesangiospermae - ps17
Magnoliids/eudicots - ps18
Eudicots - ps19
Core_eudicots - ps20
Superrosids - ps21
Rosids - ps22
Eurosids - ps23
Malvids - ps24
Sap./Malv./Brassicales - ps25
Malvales/Brassicales - ps26
Brassicales - ps27
Cleomaceae/Brassicaceae - ps28
Brassicaceae - ps29
Camelineae - ps30
Arabidopsis - ps31
Arabidopsis_thaliana - ps32
p-value
8e-36
2e-27
7e-19
2e-10
6e-02
Log-odds
0.5
1.0
1.5
2.0
2.5
3.0
3.5
4.0

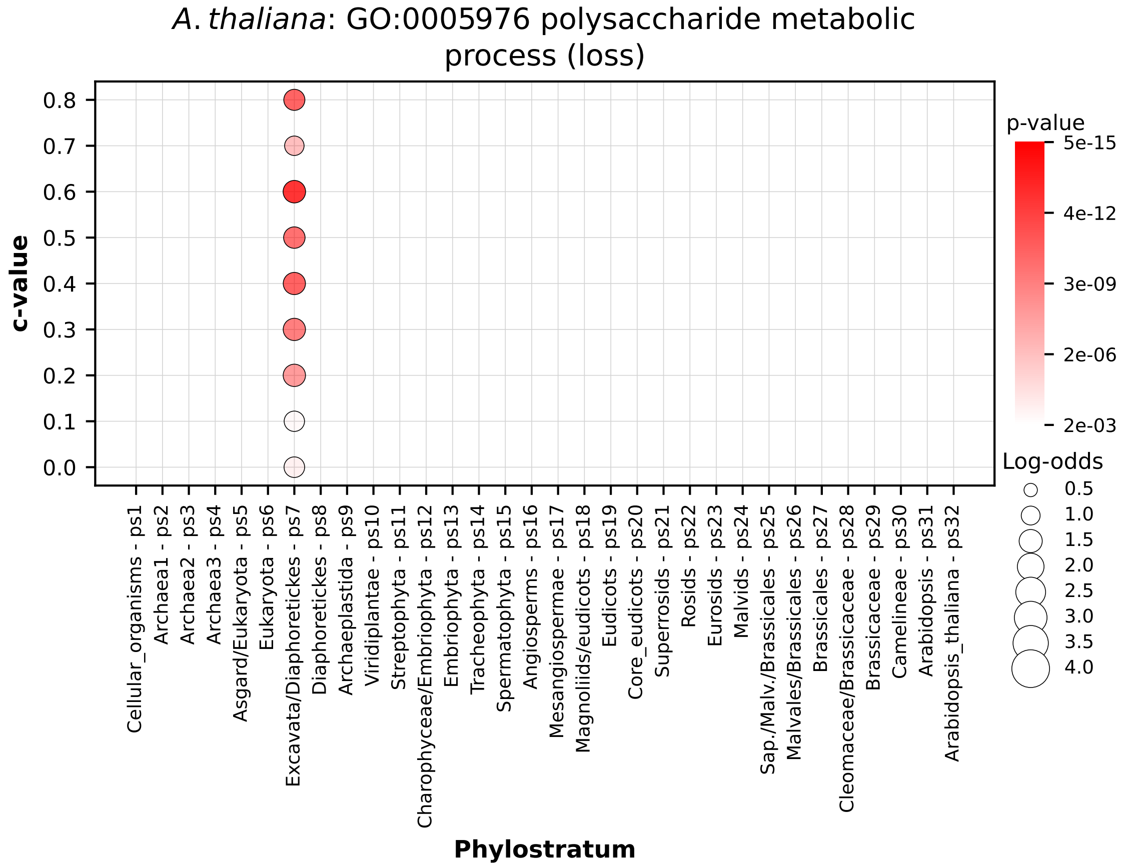
A. thaliana: GO:0005976 polysaccharide metabolic process (loss)
c-value
0.8
0.7
0.6
0.5
0.4
0.3
0.2
0.1
0.0
p-value
5e-15
4e-12
3e-09
2e-06
2e-03
Log-odds
0.5
1.0
1.5
2.0
2.5
3.0
3.5
4.0
Cellular_organisms - ps1
Archaea1 - ps2
Archaea2 - ps3
Archaea3 - ps4
Asgard/Eukaryota - ps5
Eukaryota - ps6
Excavata/Diaphoretickes - ps7
Diaphoretickes - ps8
Archaeplastida - ps9
Viridiplantae - ps10
Streptophyta - ps11
Charophyceae/Embriophyta - ps12
Embriophyta - ps13
Tracheophyta - ps14
Spermatophyta - ps15
Angiosperms - ps16
Mesangiospermae - ps17
Magnoliids/eudicots - ps18
Eudicots - ps19
Core_eudicots - ps20
Superrosids - ps21
Rosids - ps22
Eurosids - ps23
Malvids - ps24
Sap./Malv./Brassicales - ps25
Malvales/Brassicales - ps26
Brassicales - ps27
Cleomaceae/Brassicaceae - ps28
Brassicaceae - ps29
Camelineae - ps30
Arabidopsis - ps31
Arabidopsis_thaliana - ps32
Phylostratum

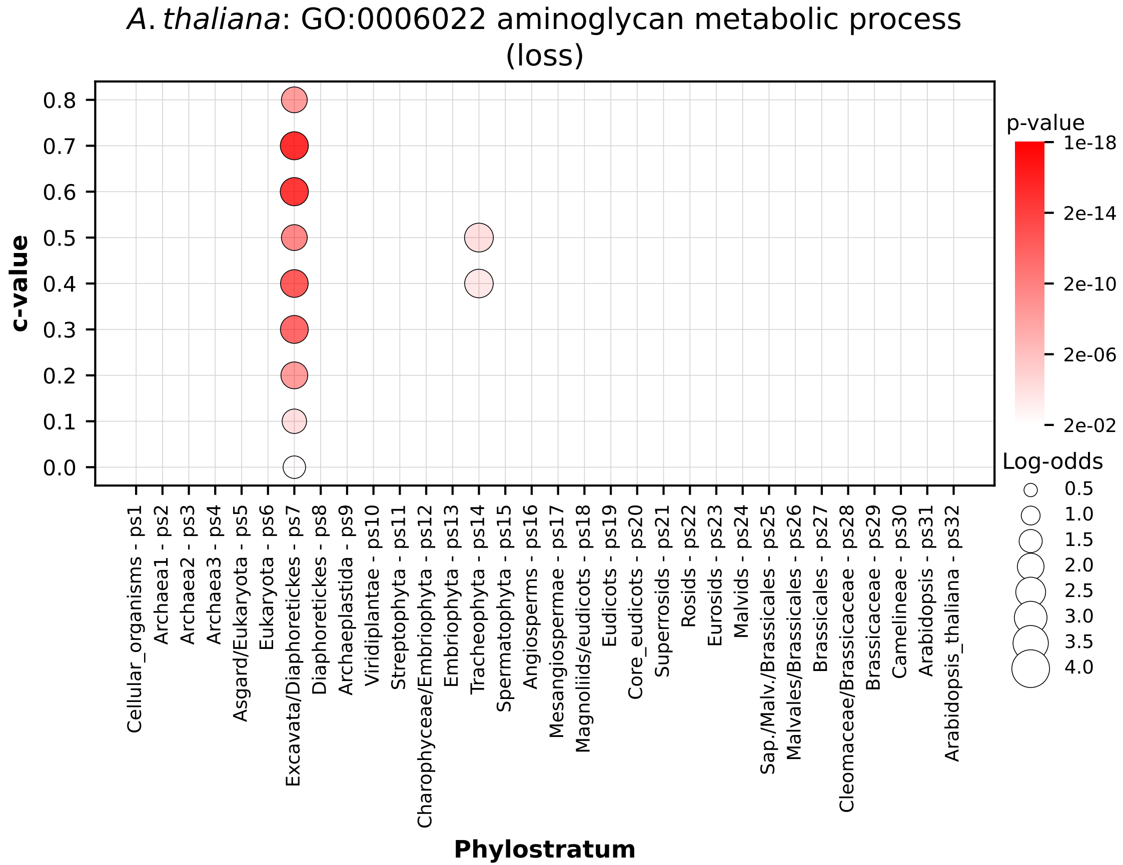

A. thaliana: GO:0006022 aminoglycan metabolic process
(loss)
c-value
0.8
0.7
0.6
0.5
0.4
0.3
0.2
0.1
0.0
p-value
1e-18
2e-14
2e-10
2e-06
2e-02
Log-odds
0.5
1.0
1.5
2.0
2.5
3.0
3.5
4.0
Cellular_organisms - ps1
Archaea1 - ps2
Archaea2 - ps3
Archaea3 - ps4
Asgard/Eukaryota - ps5
Eukaryota - ps6
Excavata/Diaphoretickes - ps7
Diaphoretickes - ps8
Archaeplastida - ps9
Viridiplantae - ps10
Streptophyta - ps11
Charophyceae/Embriophyta - ps12
Embriophyta - ps13
Tracheophyta - ps14
Spermatophyta - ps15
Angiosperms - ps16
Mesangiospermae - ps17
Magnoliids/eudicots - ps18
Eudicots - ps19
Core_eudicots - ps20
Superrosids - ps21
Rosids - ps22
Eurosids - ps23
Malvids - ps24
Sap./Malv./Brassicales - ps25
Malvales/Brassicales - ps26
Brassicales - ps27
Cleomaceae/Brassicaceae - ps28
Brassicaceae - ps29
Camelineae - ps30
Arabidopsis - ps31
Arabidopsis_thaliana - ps32
Phylostratum

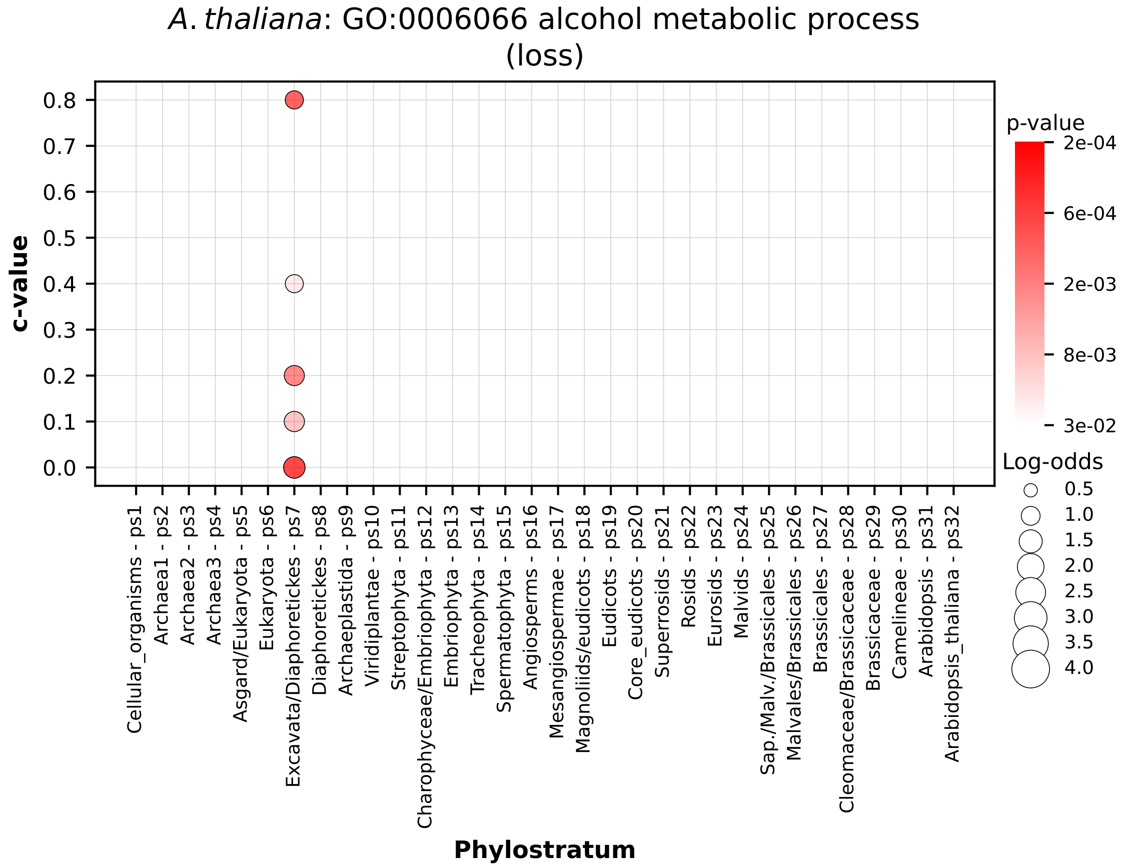

A. thaliana: GO:0006066 alcohol metabolic process
(loss)
c-value
0.8
0.7
0.6
0.5
0.4
0.3
0.2
0.1
0.0
p-value
2e-04
6e-04
2e-03
8e-03
3e-02
Log-odds
0.5
1.0
1.5
2.0
2.5
3.0
3.5
4.0
Cellular_organisms - ps1
Archaea1 - ps2
Archaea2 - ps3
Archaea3 - ps4
Asgard/Eukaryota - ps5
Eukaryota - ps6
Excavata/Diaphoretickes - ps7
Diaphoretickes - ps8
Archaeplastida - ps9
Viridiplantae - ps10
Streptophyta - ps11
Charophyceae/Embriophyta - ps12
Embriophyta - ps13
Tracheophyta - ps14
Spermatophyta - ps15
Angiosperms - ps16
Mesangiospermae - ps17
Magnoliids/eudicots - ps18
Eudicots - ps19
Core_eudicots - ps20
Superrosids - ps21
Rosids - ps22
Eurosids - ps23
Malvids - ps24
Sap./Malv./Brassicales - ps25
Malvales/Brassicales - ps26
Brassicales - ps27
Cleomaceae/Brassicaceae - ps28
Brassicaceae - ps29
Camelineae - ps30
Arabidopsis - ps31
Arabidopsis_thaliana - ps32
Phylostratum

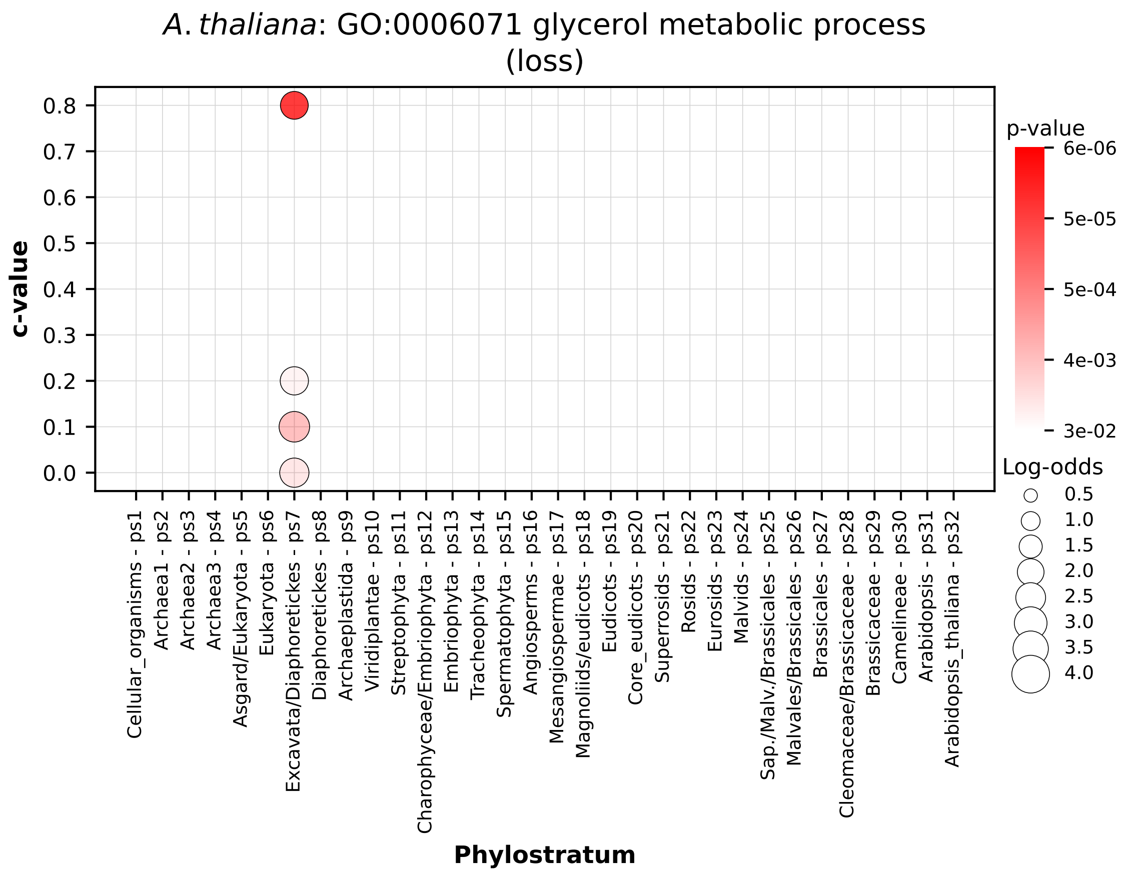
A. thaliana: GO:0006071 glycerol metabolic process
(loss)
c-value
0.8
0.7
0.6
0.5
0.4
0.3
0.2
0.1
0.0
Phylostratum
Cellular_organisms - ps1
Archaea1 - ps2
Archaea2 - ps3
Archaea3 - ps4
Asgard/Eukaryota - ps5
Eukaryota - ps6
Excavata/Diaphoretickes - ps7
Diaphoretickes - ps8
Archaeplastida - ps9
Viridiplantae - ps10
Streptophyta - ps11
Charophyceae/Embriophyta - ps12
Embriophyta - ps13
Tracheophyta - ps14
Spermatophyta - ps15
Angiosperms - ps16
Mesangiospermae - ps17
Magnoliids/eudicots - ps18
Eudicots - ps19
Core_eudicots - ps20
Superrosids - ps21
Rosids - ps22
Eurosids - ps23
Malvids - ps24
Sap./Malv./Brassicales - ps25
Malvales/Brassicales - ps26
Brassicales - ps27
Cleomaceae/Brassicaceae - ps28
Brassicaceae - ps29
Camelineae - ps30
Arabidopsis - ps31
Arabidopsis_thaliana - ps32
p-value
6e-06
5e-05
5e-04
4e-03
3e-02
Log-odds
0.5
1.0
1.5
2.0
2.5
3.0
3.5
4.0

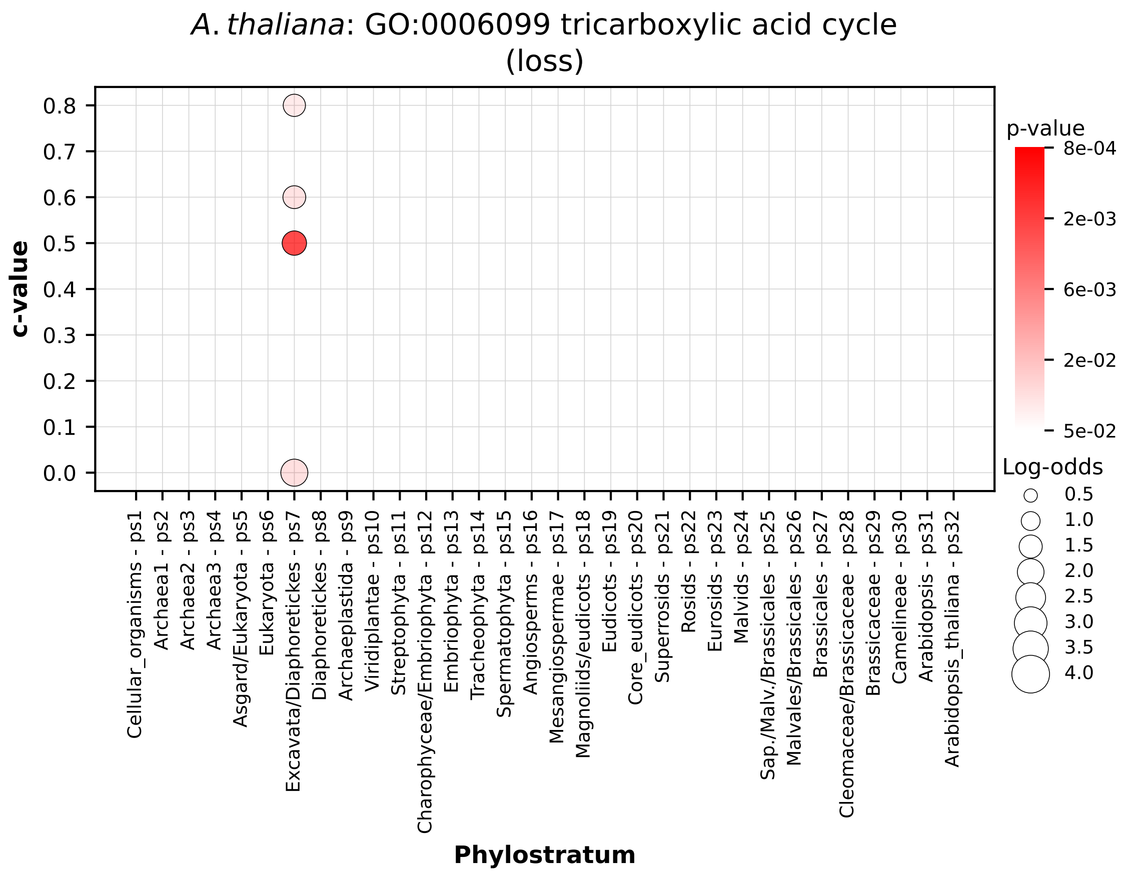

A. thaliana: GO:0006099 tricarboxylic acid cycle
(loss)
c-value
0.8
0.7
0.6
0.5
0.4
0.3
0.2
0.1
0.0
p-value
8e-04
2e-03
6e-03
2e-02
5e-02
Log-odds
0.5
1.0
1.5
2.0
2.5
3.0
3.5
4.0
Cellular_organisms - ps1
Archaea1 - ps2
Archaea2 - ps3
Archaea3 - ps4
Asgard/Eukaryota - ps5
Eukaryota - ps6
Excavata/Diaphoretickes - ps7
Diaphoretickes - ps8
Archaeplastida - ps9
Viridiplantae - ps10
Streptophyta - ps11
Charophyceae/Embriophyta - ps12
Embriophyta - ps13
Tracheophyta - ps14
Spermatophyta - ps15
Angiosperms - ps16
Mesangiospermae - ps17
Magnoliids/eudicots - ps18
Eudicots - ps19
Core_eudicots - ps20
Superrosids - ps21
Rosids - ps22
Eurosids - ps23
Malvids - ps24
Sap./Malv./Brassicales - ps25
Malvales/Brassicales - ps26
Brassicales - ps27
Cleomaceae/Brassicaceae - ps28
Brassicaceae - ps29
Camelineae - ps30
Arabidopsis - ps31
Arabidopsis_thaliana - ps32
Phylostratum

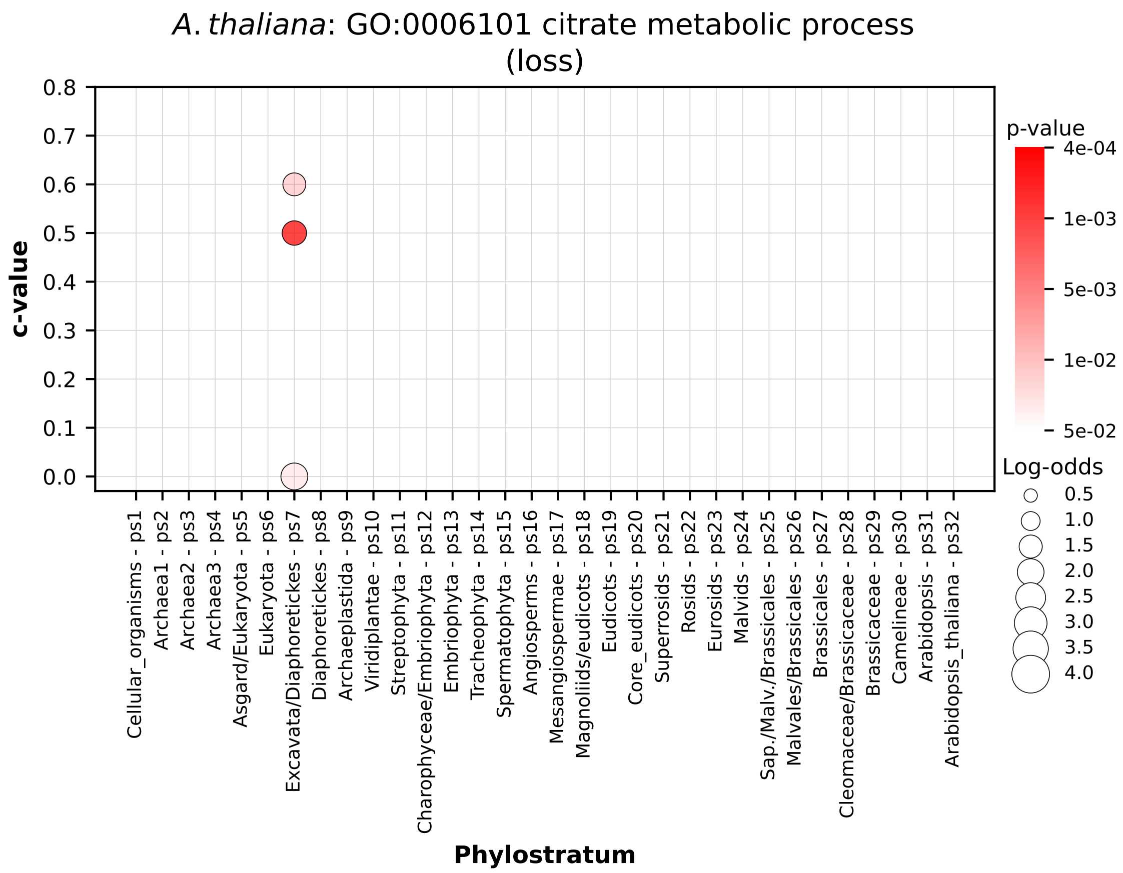

A. thaliana: GO:0006101 citrate metabolic process
(loss)
c-value
0.8
0.7
0.6
0.5
0.4
0.3
0.2
0.1
0.0
p-value
4e-04
1e-03
5e-03
1e-02
5e-02
Log-odds
0.5
1.0
1.5
2.0
2.5
3.0
3.5
4.0
Cellular_organisms - ps1
Archaea1 - ps2
Archaea2 - ps3
Archaea3 - ps4
Asgard/Eukaryota - ps5
Eukaryota - ps6
Excavata/Diaphoretickes - ps7
Diaphoretickes - ps8
Archaeplastida - ps9
Viridiplantae - ps10
Streptophyta - ps11
Charophyceae/Embriophyta - ps12
Embriophyta - ps13
Tracheophyta - ps14
Spermatophyta - ps15
Angiosperms - ps16
Mesangiospermae - ps17
Magnoliids/eudicots - ps18
Eudicots - ps19
Core_eudicots - ps20
Superrosids - ps21
Rosids - ps22
Eurosids - ps23
Malvids - ps24
Sap./Malv./Brassicales - ps25
Malvales/Brassicales - ps26
Brassicales - ps27
Cleomaceae/Brassicaceae - ps28
Brassicaceae - ps29
Camelineae - ps30
Arabidopsis - ps31
Arabidopsis_thaliana - ps32
Phylostratum

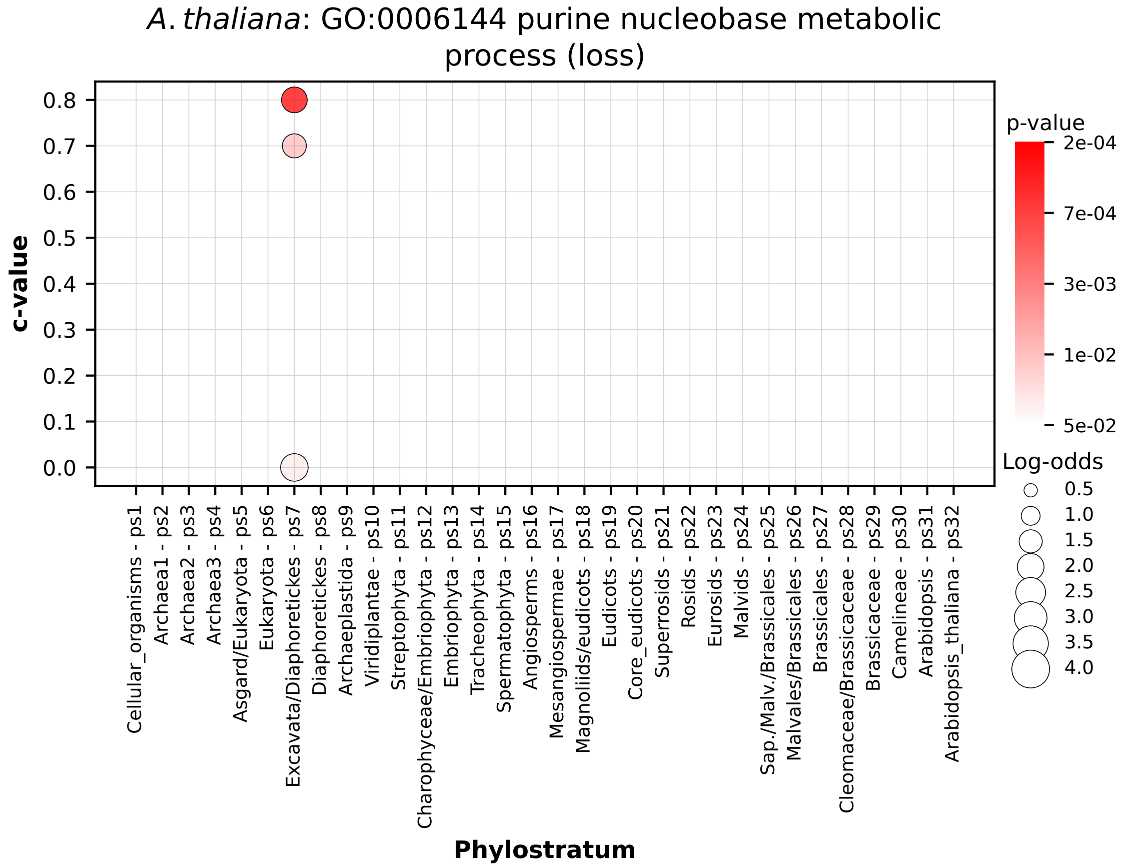
A. thaliana: GO:0006144 purine nucleobase metabolic process (loss)
c-value
0.8
0.7
0.6
0.5
0.4
0.3
0.2
0.1
0.0
p-value
2e-04
7e-04
3e-03
1e-02
5e-02
Log-odds
0.5
1.0
1.5
2.0
2.5
3.0
3.5
4.0
Cellular_organisms - ps1
Archaea1 - ps2
Archaea2 - ps3
Archaea3 - ps4
Asgard/Eukaryota - ps5
Eukaryota - ps6
Excavata/Diaphoretickes - ps7
Diaphoretickes - ps8
Archaeplastida - ps9
Viridiplantae - ps10
Streptophyta - ps11
Charophyceae/Embriophyta - ps12
Embriophyta - ps13
Tracheophyta - ps14
Spermatophyta - ps15
Angiosperms - ps16
Mesangiospermae - ps17
Magnoliids/eudicots - ps18
Eudicots - ps19
Core_eudicots - ps20
Superrosids - ps21
Rosids - ps22
Eurosids - ps23
Malvids - ps24
Sap./Malv./Brassicales - ps25
Malvales/Brassicales - ps26
Brassicales - ps27
Cleomaceae/Brassicaceae - ps28
Brassicaceae - ps29
Camelineae - ps30
Arabidopsis - ps31
Arabidopsis_thaliana - ps32
Phylostratum

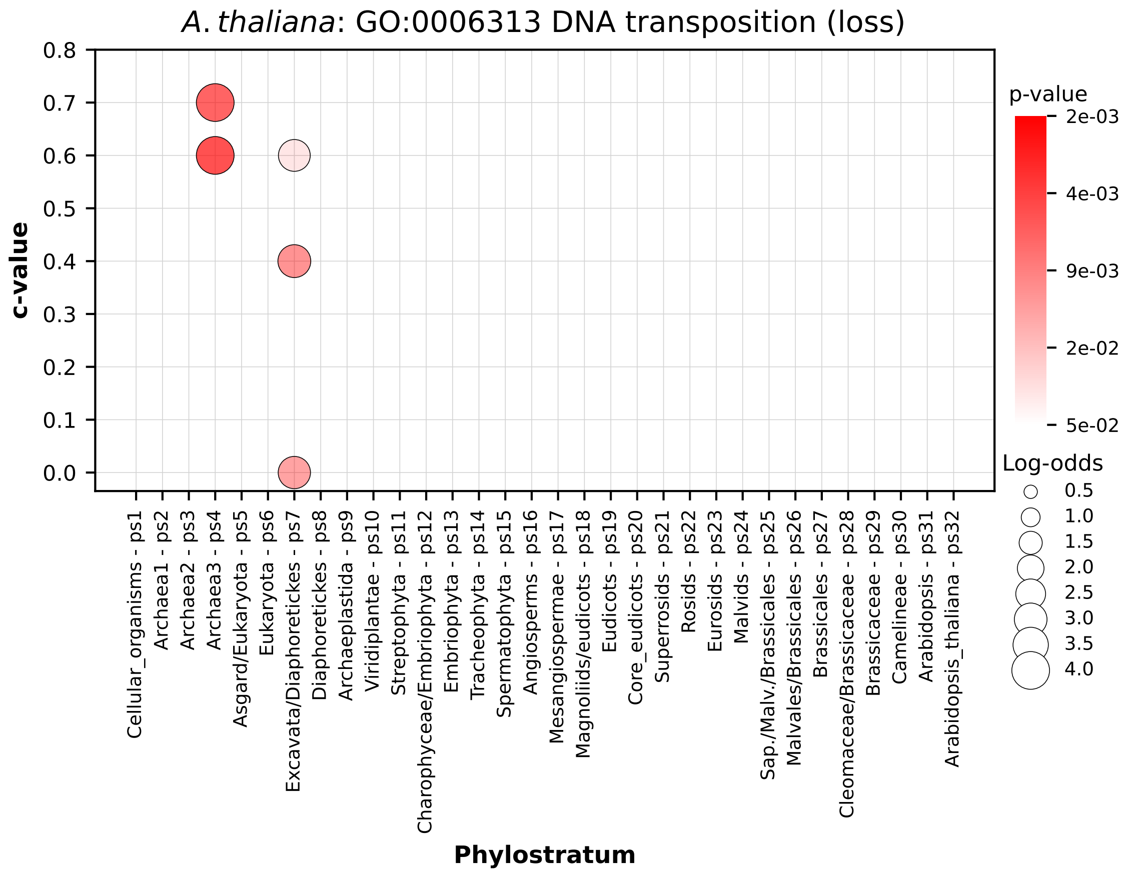

A. thaliana: GO:0006313 DNA transposition (loss)
c-value
0.8
0.7
0.6
0.5
0.4
0.3
0.2
0.1
0.0
p-value
2e-03
4e-03
9e-03
2e-02
5e-02
Log-odds
0.5
1.0
1.5
2.0
2.5
3.0
3.5
4.0
Cellular_organisms - ps1
Archaea1 - ps2
Archaea2 - ps3
Archaea3 - ps4
Asgard/Eukaryota - ps5
Eukaryota - ps6
Excavata/Diaphoretickes - ps7
Diaphoretickes - ps8
Archaeplastida - ps9
Viridiplantae - ps10
Streptophyta - ps11
Charophyceae/Embriophyta - ps12
Embriophyta - ps13
Tracheophyta - ps14
Spermatophyta - ps15
Angiosperms - ps16
Mesangiospermae - ps17
Magnoliids/eudicots - ps18
Eudicots - ps19
Core_eudicots - ps20
Superrosids - ps21
Rosids - ps22
Eurosids - ps23
Malvids - ps24
Sap./Malv./Brassicales - ps25
Malvales/Brassicales - ps26
Brassicales - ps27
Cleomaceae/Brassicaceae - ps28
Brassicaceae - ps29
Camelineae - ps30
Arabidopsis - ps31
Arabidopsis_thaliana - ps32
Phylostratum

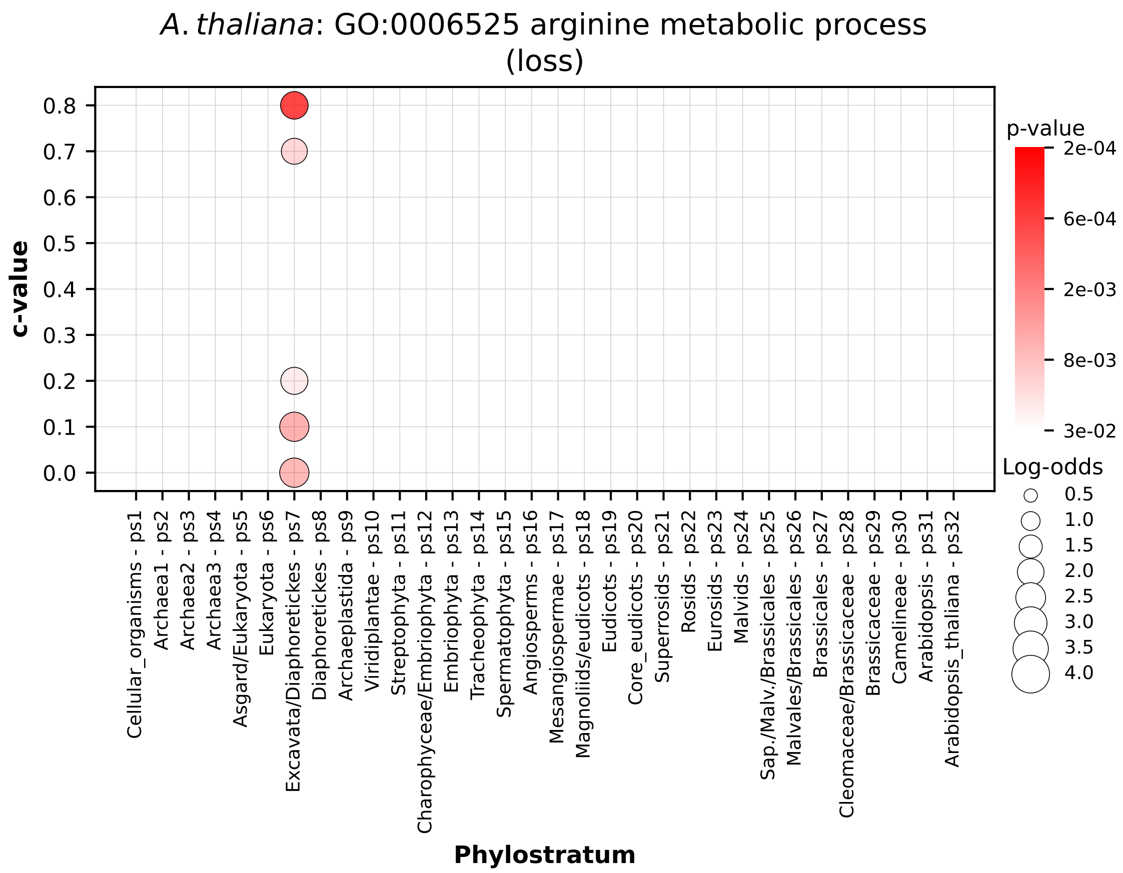

A. thaliana: GO:0006525 arginine metabolic process
(loss)
c-value
0.8
0.7
0.6
0.5
0.4
0.3
0.2
0.1
0.0
Phylostratum
Cellular_organisms - ps1
Archaea1 - ps2
Archaea2 - ps3
Archaea3 - ps4
Asgard/Eukaryota - ps5
Eukaryota - ps6
Excavata/Diaphoretickes - ps7
Diaphoretickes - ps8
Archaeplastida - ps9
Viridiplantae - ps10
Streptophyta - ps11
Charophyceae/Embriophyta - ps12
Embriophyta - ps13
Tracheophyta - ps14
Spermatophyta - ps15
Angiosperms - ps16
Mesangiospermae - ps17
Magnoliids/eudicots - ps18
Eudicots - ps19
Core_eudicots - ps20
Superrosids - ps21
Rosids - ps22
Eurosids - ps23
Malvids - ps24
Sap./Malv./Brassicales - ps25
Malvales/Brassicales - ps26
Brassicales - ps27
Cleomaceae/Brassicaceae - ps28
Brassicaceae - ps29
Camelineae - ps30
Arabidopsis - ps31
Arabidopsis_thaliana - ps32
p-value
2e-04
6e-04
2e-03
8e-03
3e-02
Log-odds
0.5
1.0
1.5
2.0
2.5
3.0
3.5
4.0

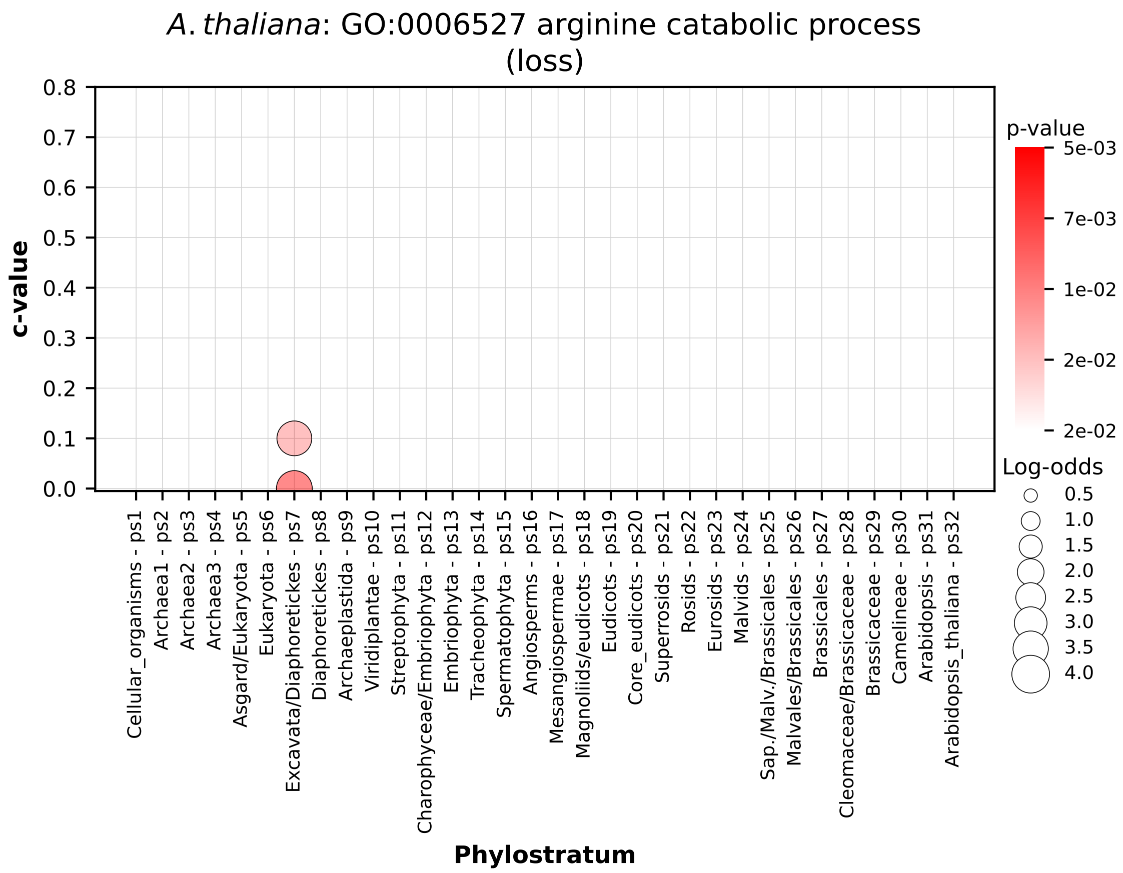
A. thaliana: GO:0006527 arginine catabolic process
(loss)
c-value
0.8
0.7
0.6
0.5
0.4
0.3
0.2
0.1
0.0
p-value
5e-03
7e-03
1e-02
2e-02
2e-02
Log-odds
0.5
1.0
1.5
2.0
2.5
3.0
3.5
4.0
Cellular_organisms - ps1
Archaea1 - ps2
Archaea2 - ps3
Archaea3 - ps4
Asgard/Eukaryota - ps5
Eukaryota - ps6
Excavata/Diaphoretickes - ps7
Diaphoretickes - ps8
Archaeplastida - ps9
Viridiplantae - ps10
Streptophyta - ps11
Charophyceae/Embriophyta - ps12
Embriophyta - ps13
Tracheophyta - ps14
Spermatophyta - ps15
Angiosperms - ps16
Mesangiospermae - ps17
Magnoliids/eudicots - ps18
Eudicots - ps19
Core_eudicots - ps20
Superrosids - ps21
Rosids - ps22
Eurosids - ps23
Malvids - ps24
Sap./Malv./Brassicales - ps25
Malvales/Brassicales - ps26
Brassicales - ps27
Cleomaceae/Brassicaceae - ps28
Brassicaceae - ps29
Camelineae - ps30
Arabidopsis - ps31
Arabidopsis_thaliana - ps32
Phylostratum

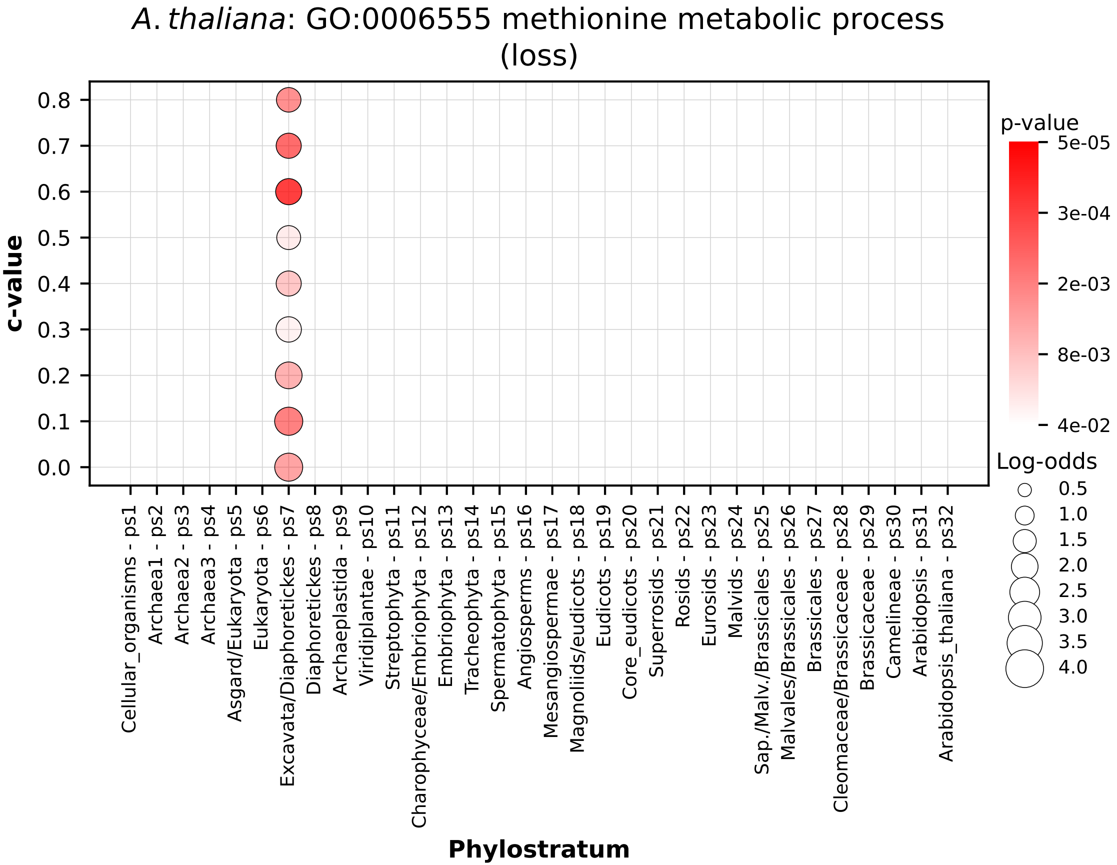
A. thaliana: GO:0006555 methionine metabolic process
(loss)
c-value
0.8
0.7
0.6
0.5
0.4
0.3
0.2
0.1
0.0
p-value
5e-05
3e-04
2e-03
8e-03
4e-02
Log-odds
0.5
1.0
1.5
2.0
2.5
3.0
3.5
4.0
Cellular_organisms - ps1
Archaea1 - ps2
Archaea2 - ps3
Archaea3 - ps4
Asgard/Eukaryota - ps5
Eukaryota - ps6
Excavata/Diaphoretickes - ps7
Diaphoretickes - ps8
Archaeplastida - ps9
Viridiplantae - ps10
Streptophyta - ps11
Charophyceae/Embriophyta - ps12
Embriophyta - ps13
Tracheophyta - ps14
Spermatophyta - ps15
Angiosperms - ps16
Mesangiospermae - ps17
Magnoliids/eudicots - ps18
Eudicots - ps19
Core_eudicots - ps20
Superrosids - ps21
Rosids - ps22
Eurosids - ps23
Malvids - ps24
Sap./Malv./Brassicales - ps25
Malvales/Brassicales - ps26
Brassicales - ps27
Cleomaceae/Brassicaceae - ps28
Brassicaceae - ps29
Camelineae - ps30
Arabidopsis - ps31
Arabidopsis_thaliana - ps32
Phylostratum

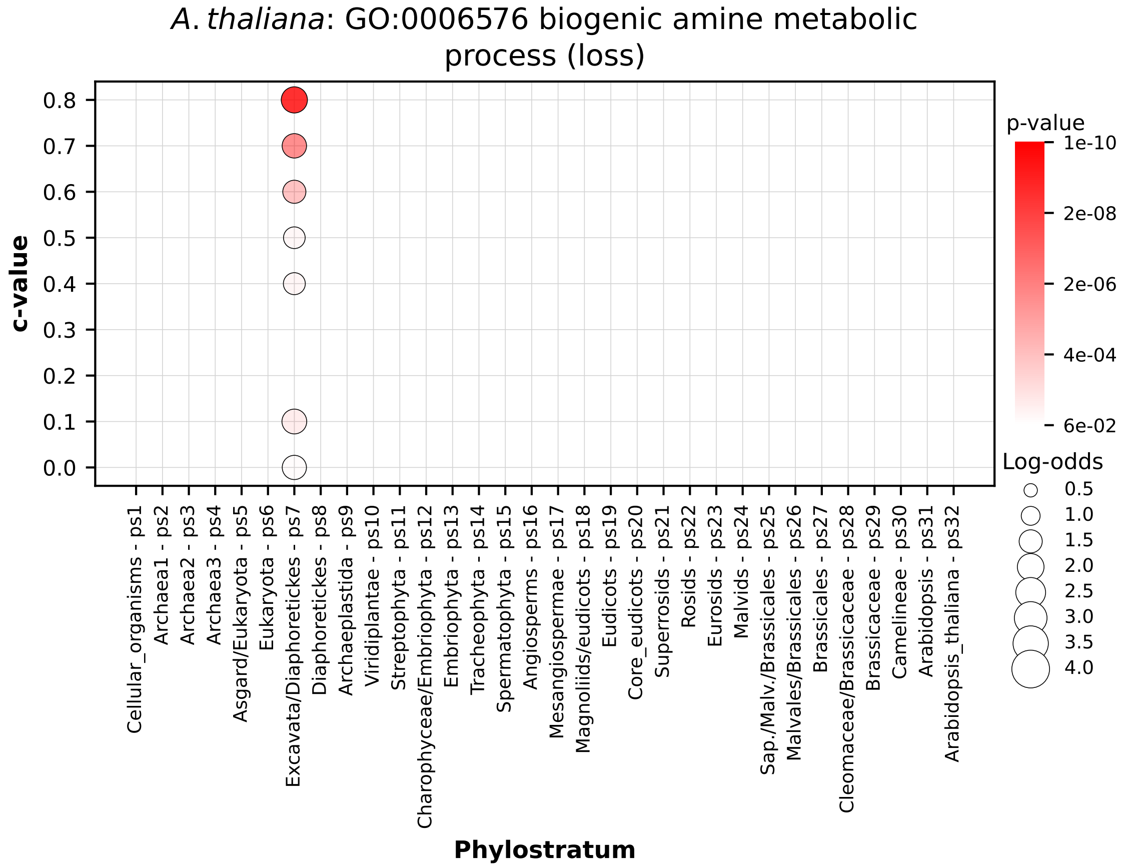

A. thaliana: GO:0006576 biogenic amine metabolic process (loss)
c-value
0.8
0.7
0.6
0.5
0.4
0.3
0.2
0.1
0.0
Phylostratum
Cellular_organisms - ps1
Archaea1 - ps2
Archaea2 - ps3
Archaea3 - ps4
Asgard/Eukaryota - ps5
Eukaryota - ps6
Excavata/Diaphoretickes - ps7
Diaphoretickes - ps8
Archaeplastida - ps9
Viridiplantae - ps10
Streptophyta - ps11
Charophyceae/Embriophyta - ps12
Embriophyta - ps13
Tracheophyta - ps14
Spermatophyta - ps15
Angiosperms - ps16
Mesangiospermae - ps17
Magnoliids/eudicots - ps18
Eudicots - ps19
Core_eudicots - ps20
Superrosids - ps21
Rosids - ps22
Eurosids - ps23
Malvids - ps24
Sap./Malv./Brassicales - ps25
Malvales/Brassicales - ps26
Brassicales - ps27
Cleomaceae/Brassicaceae - ps28
Brassicaceae - ps29
Camelineae - ps30
Arabidopsis - ps31
Arabidopsis_thaliana - ps32
p-value
1e-10
2e-08
2e-06
4e-04
6e-02
Log-odds
0.5
1.0
1.5
2.0
2.5
3.0
3.5
4.0

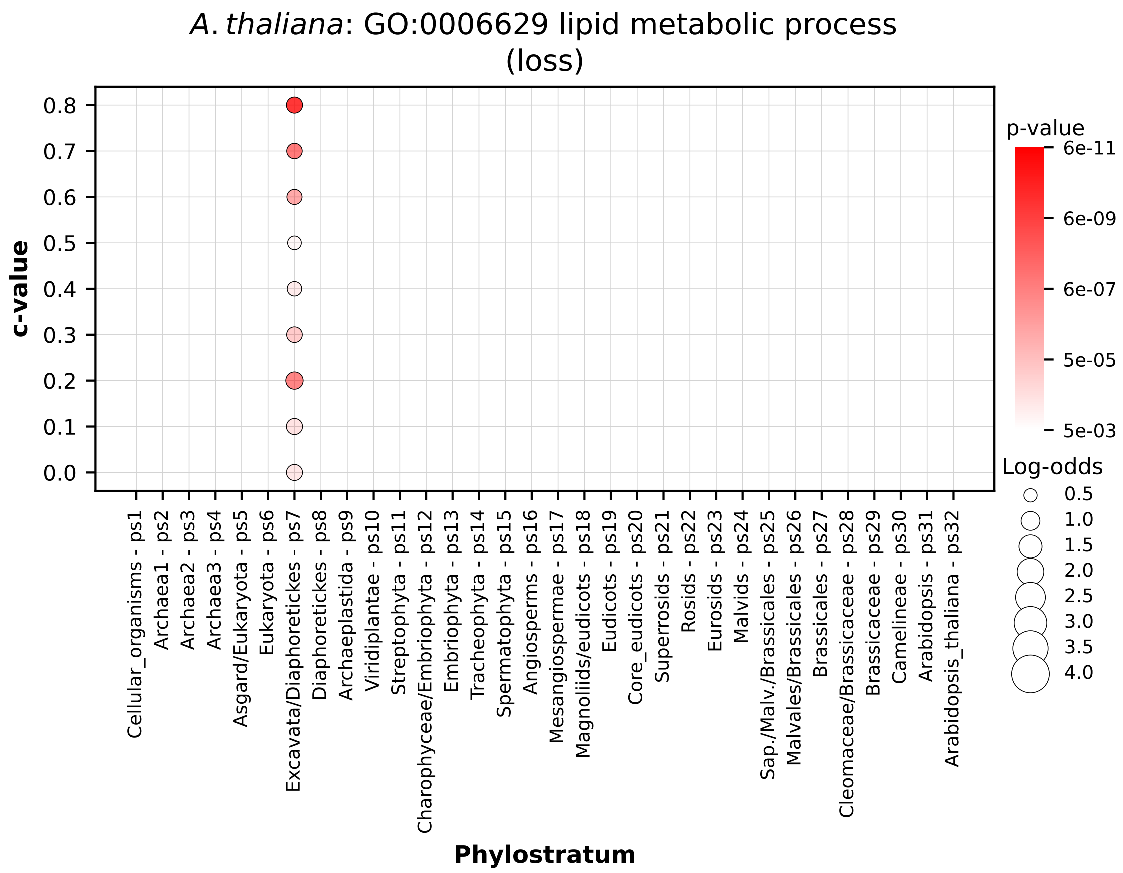

A. thaliana: GO:0006629 lipid metabolic process
(loss)
c-value
0.0
0.1
0.2
0.3
0.4
0.5
0.6
0.7
0.8
Phylostratum
Cellular_organisms - ps1
Archaea1 - ps2
Archaea2 - ps3
Archaea3 - ps4
Asgard/Eukaryota - ps5
Eukaryota - ps6
Excavata/Diaphoretickes - ps7
Diaphoretickes - ps8
Archaeplastida - ps9
Viridiplantae - ps10
Streptophyta - ps11
Charophyceae/Embriophyta - ps12
Embriophyta - ps13
Tracheophyta - ps14
Spermatophyta - ps15
Angiosperms - ps16
Mesangiospermae - ps17
Magnoliids/eudicots - ps18
Eudicots - ps19
Core_eudicots - ps20
Superrosids - ps21
Rosids - ps22
Eurosids - ps23
Malvids - ps24
Sap./Malv./Brassicales - ps25
Malvales/Brassicales - ps26
Brassicales - ps27
Cleomaceae/Brassicaceae - ps28
Brassicaceae - ps29
Camelineae - ps30
Arabidopsis - ps31
Arabidopsis_thaliana - ps32
p-value
6e-11
6e-09
6e-07
5e-05
5e-03
Log-odds
0.5
1.0
1.5
2.0
2.5
3.0
3.5
4.0

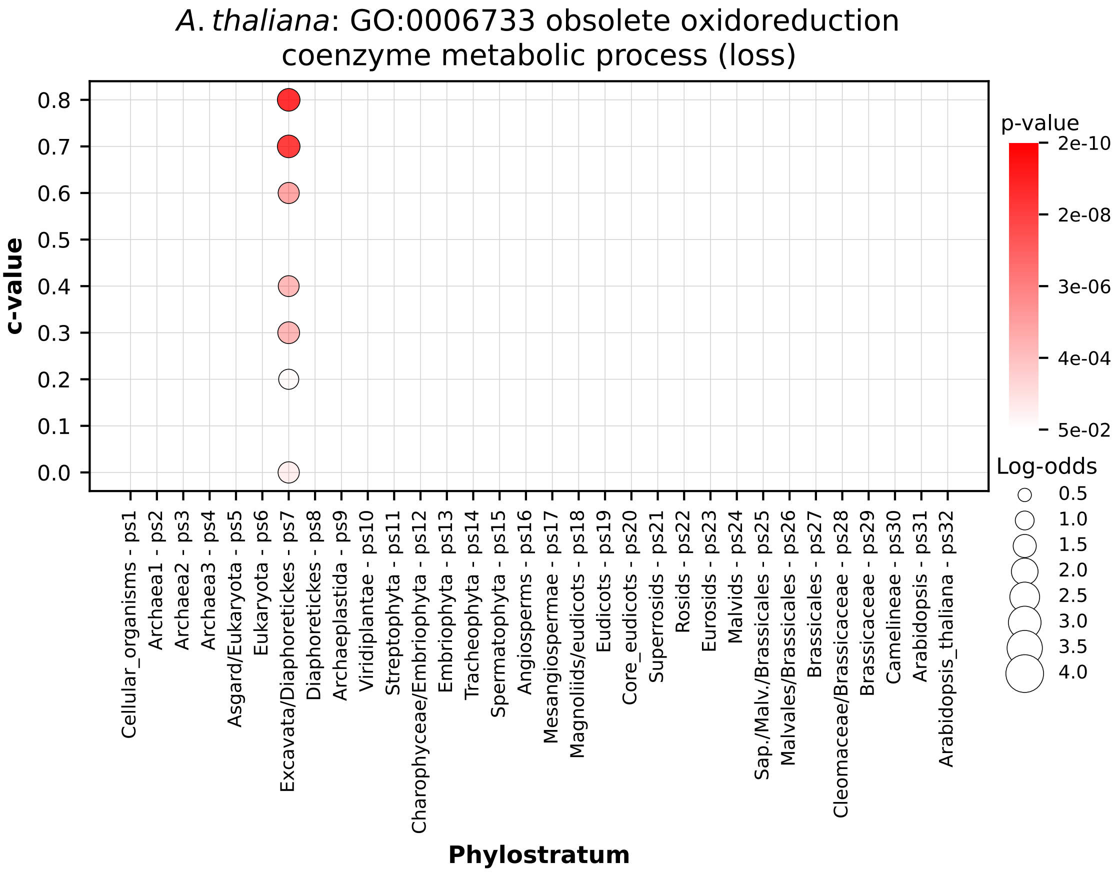

$A. thaliana$: GO:0006733 obsolete oxidoreduction coenzyme metabolic process (loss)
c-value
0.8
0.7
0.6
0.5
0.4
0.3
0.2
0.1
0.0
p-value
2e-10
2e-08
3e-06
4e-04
5e-02
Log-odds
0.5
1.0
1.5
2.0
2.5
3.0
3.5
4.0
Cellular_organisms - ps1
Archaea1 - ps2
Archaea2 - ps3
Archaea3 - ps4
Asgard/Eukaryota - ps5
Eukaryota - ps6
Excavata/Diaphoretickes - ps7
Diaphoretickes - ps8
Archaeplastida - ps9
Viridiplantae - ps10
Streptophyta - ps11
Charophyceae/Embriophyta - ps12
Embriophyta - ps13
Tracheophyta - ps14
Spermatophyta - ps15
Angiosperms - ps16
Mesangiospermae - ps17
Magnoliids/eudicots - ps18
Eudicots - ps19
Core_eudicots - ps20
Superrosids - ps21
Rosids - ps22
Eurosids - ps23
Malvids - ps24
Sap./Malv./Brassicales - ps25
Malvales/Brassicales - ps26
Brassicales - ps27
Cleomaceae/Brassicaceae - ps28
Brassicaceae - ps29
Camelineae - ps30
Arabidopsis - ps31
Arabidopsis_thaliana - ps32
Phylostratum

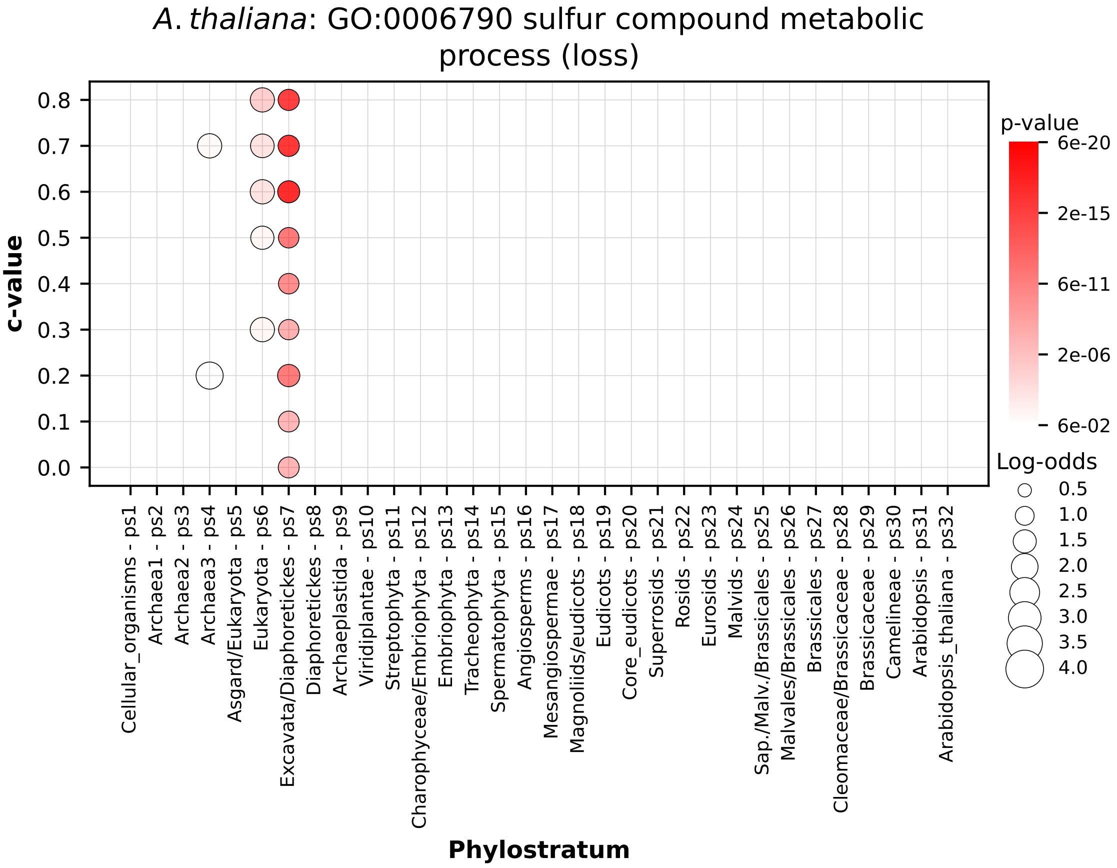
A. thaliana: GO:0006790 sulfur compound metabolic process (loss)
c-value
0.8
0.7
0.6
0.5
0.4
0.3
0.2
0.1
0.0
p-value
6e-20
2e-15
6e-11
2e-06
6e-02
Log-odds
0.5
1.0
1.5
2.0
2.5
3.0
3.5
4.0
Cellular_organisms - ps1
Archaea1 - ps2
Archaea2 - ps3
Archaea3 - ps4
Asgard/Eukaryota - ps5
Eukaryota - ps6
Excavata/Diaphoretickes - ps7
Diaphoretickes - ps8
Archaeplastida - ps9
Viridiplantae - ps10
Streptophyta - ps11
Charophyceae/Embriophyta - ps12
Embriophyta - ps13
Tracheophyta - ps14
Spermatophyta - ps15
Angiosperms - ps16
Mesangiospermae - ps17
Magnoliids/eudicots - ps18
Eudicots - ps19
Core_eudicots - ps20
Superrosids - ps21
Rosids - ps22
Eurosids - ps23
Malvids - ps24
Sap./Malv./Brassicales - ps25
Malvales/Brassicales - ps26
Brassicales - ps27
Cleomaceae/Brassicaceae - ps28
Brassicaceae - ps29
Camelineae - ps30
Arabidopsis - ps31
Arabidopsis_thaliana - ps32
Phylostratum

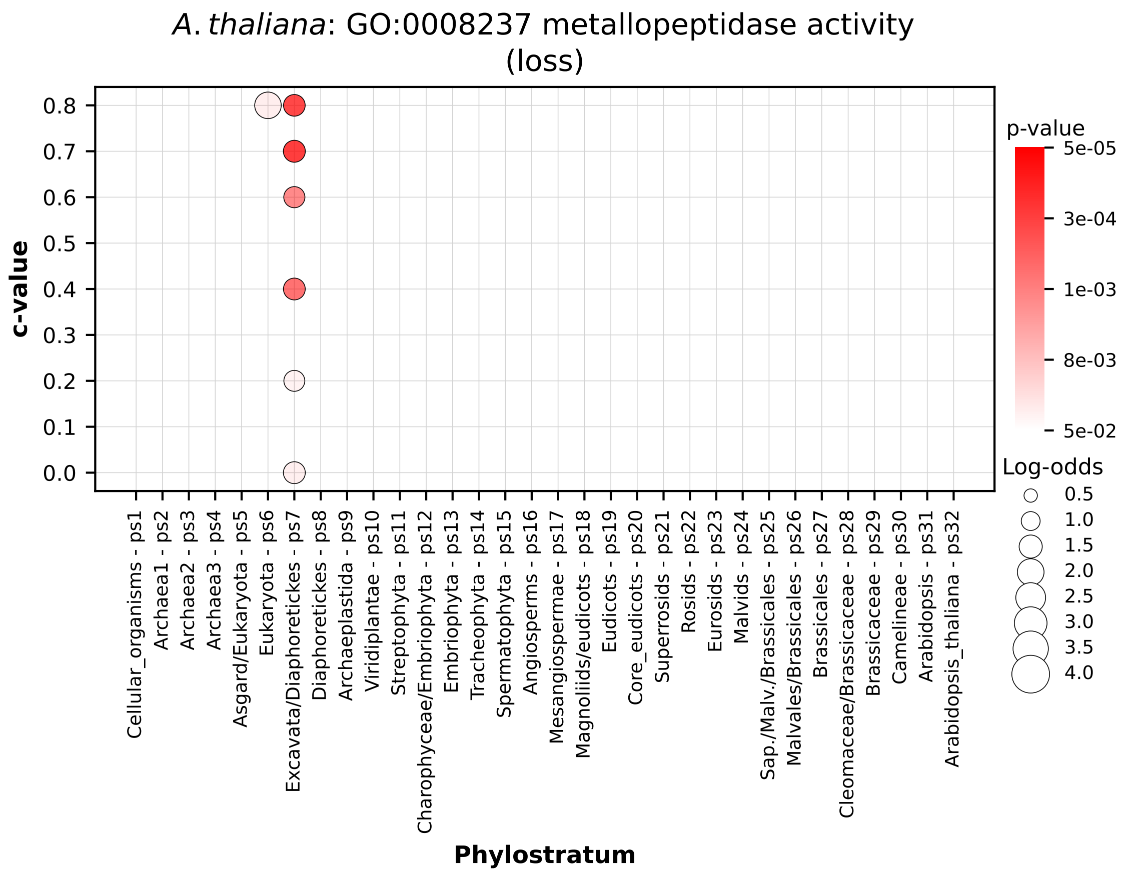

A. thaliana: GO:0008237 metallopeptidase activity (loss)
c-value
0.8
0.7
0.6
0.5
0.4
0.3
0.2
0.1
0.0
p-value
5e-05
3e-04
1e-03
8e-03
5e-02
Log-odds
0.5
1.0
1.5
2.0
2.5
3.0
3.5
4.0
Cellular_organisms - ps1
Archaea1 - ps2
Archaea2 - ps3
Archaea3 - ps4
Asgard/Eukaryota - ps5
Eukaryota - ps6
Excavata/Diaphoretickes - ps7
Diaphoretickes - ps8
Archaeplastida - ps9
Viridiplantae - ps10
Streptophyta - ps11
Charophyceae/Embriophyta - ps12
Embriophyta - ps13
Tracheophyta - ps14
Spermatophyta - ps15
Angiosperms - ps16
Mesangiospermae - ps17
Magnoliids/eudicots - ps18
Eudicots - ps19
Core_eudicots - ps20
Superrosids - ps21
Rosids - ps22
Eurosids - ps23
Malvids - ps24
Sap./Malv./Brassicales - ps25
Malvales/Brassicales - ps26
Brassicales - ps27
Cleomaceae/Brassicaceae - ps28
Brassicaceae - ps29
Camelineae - ps30
Arabidopsis - ps31
Arabidopsis_thaliana - ps32
Phylostratum

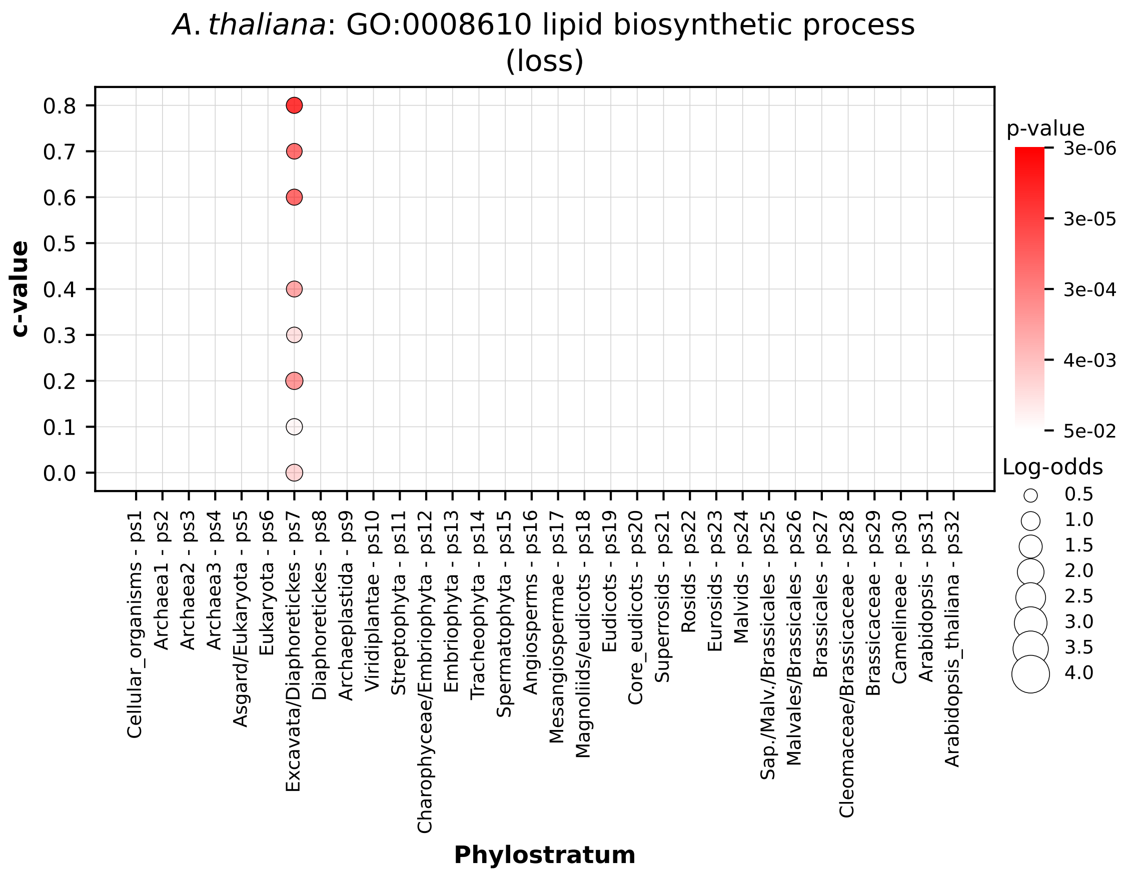

A. thaliana: GO:0008610 lipid biosynthetic process
(loss)
c-value
0.8
0.7
0.6
0.5
0.4
0.3
0.2
0.1
0.0
p-value
3e-06
3e-05
3e-04
4e-03
5e-02
Log-odds
0.5
1.0
1.5
2.0
2.5
3.0
3.5
4.0
Cellular_organisms - ps1
Archaea1 - ps2
Archaea2 - ps3
Archaea3 - ps4
Asgard/Eukaryota - ps5
Eukaryota - ps6
Excavata/Diaphoretickes - ps7
Diaphoretickes - ps8
Archaeplastida - ps9
Viridiplantae - ps10
Streptophyta - ps11
Charophyceae/Embriophyta - ps12
Embriophyta - ps13
Tracheophyta - ps14
Spermatophyta - ps15
Angiosperms - ps16
Mesangiospermae - ps17
Magnoliids/eudicots - ps18
Eudicots - ps19
Core_eudicots - ps20
Superrosids - ps21
Rosids - ps22
Eurosids - ps23
Malvids - ps24
Sap./Malv./Brassicales - ps25
Malvales/Brassicales - ps26
Brassicales - ps27
Cleomaceae/Brassicaceae - ps28
Brassicaceae - ps29
Camelineae - ps30
Arabidopsis - ps31
Arabidopsis_thaliana - ps32
Phylostratum

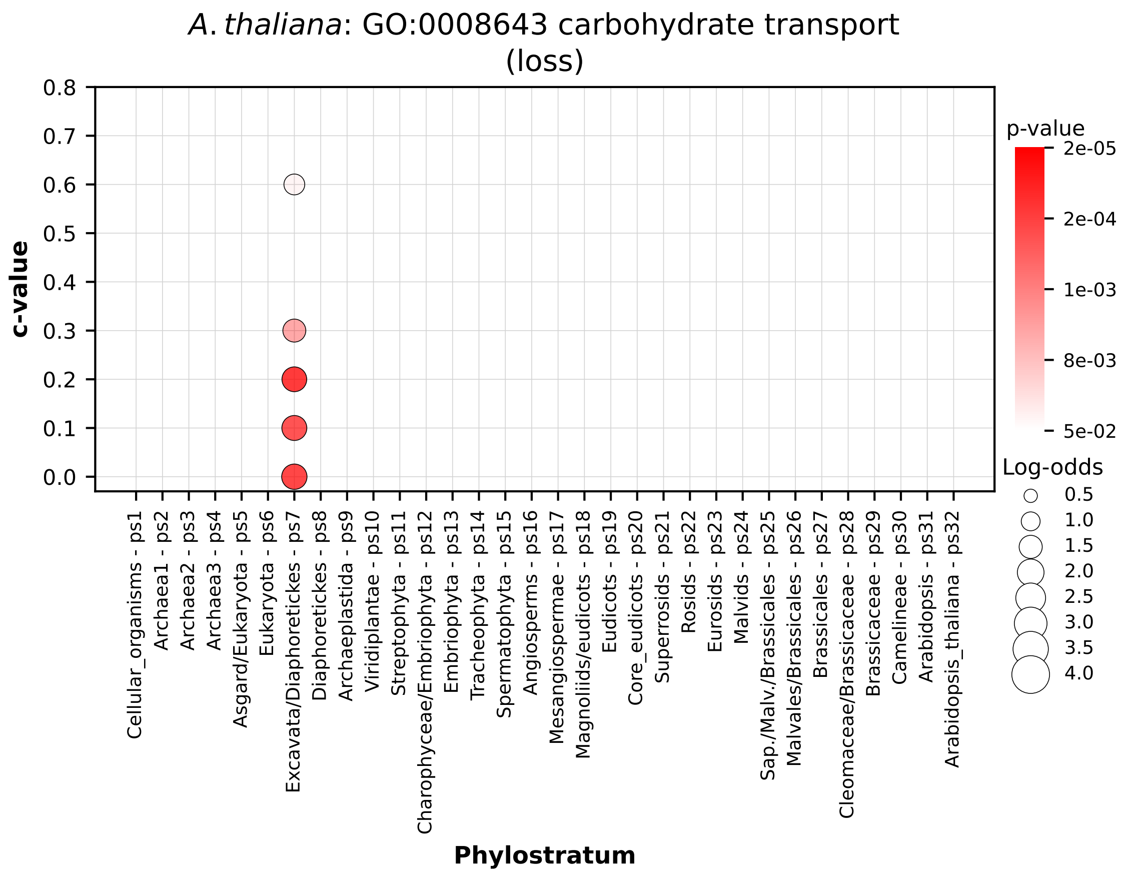
A. thaliana: GO:0008643 carbohydrate transport
(loss)
c-value
0.8
0.7
0.6
0.5
0.4
0.3
0.2
0.1
0.0
p-value
2e-05
2e-04
1e-03
8e-03
5e-02
Log-odds
0.5
1.0
1.5
2.0
2.5
3.0
3.5
4.0
Cellular_organisms - ps1
Archaea1 - ps2
Archaea2 - ps3
Archaea3 - ps4
Asgard/Eukaryota - ps5
Eukaryota - ps6
Excavata/Diaphoretickes - ps7
Diaphoretickes - ps8
Archaeplastida - ps9
Viridiplantae - ps10
Streptophyta - ps11
Charophyceae/Embriophyta - ps12
Embriophyta - ps13
Tracheophyta - ps14
Spermatophyta - ps15
Angiosperms - ps16
Mesangiospermae - ps17
Magnoliids/eudicots - ps18
Eudicots - ps19
Core_eudicots - ps20
Superrosids - ps21
Rosids - ps22
Eurosids - ps23
Malvids - ps24
Sap./Malv./Brassicales - ps25
Malvales/Brassicales - ps26
Brassicales - ps27
Cleomaceae/Brassicaceae - ps28
Brassicaceae - ps29
Camelineae - ps30
Arabidopsis - ps31
Arabidopsis_thaliana - ps32
Phylostratum

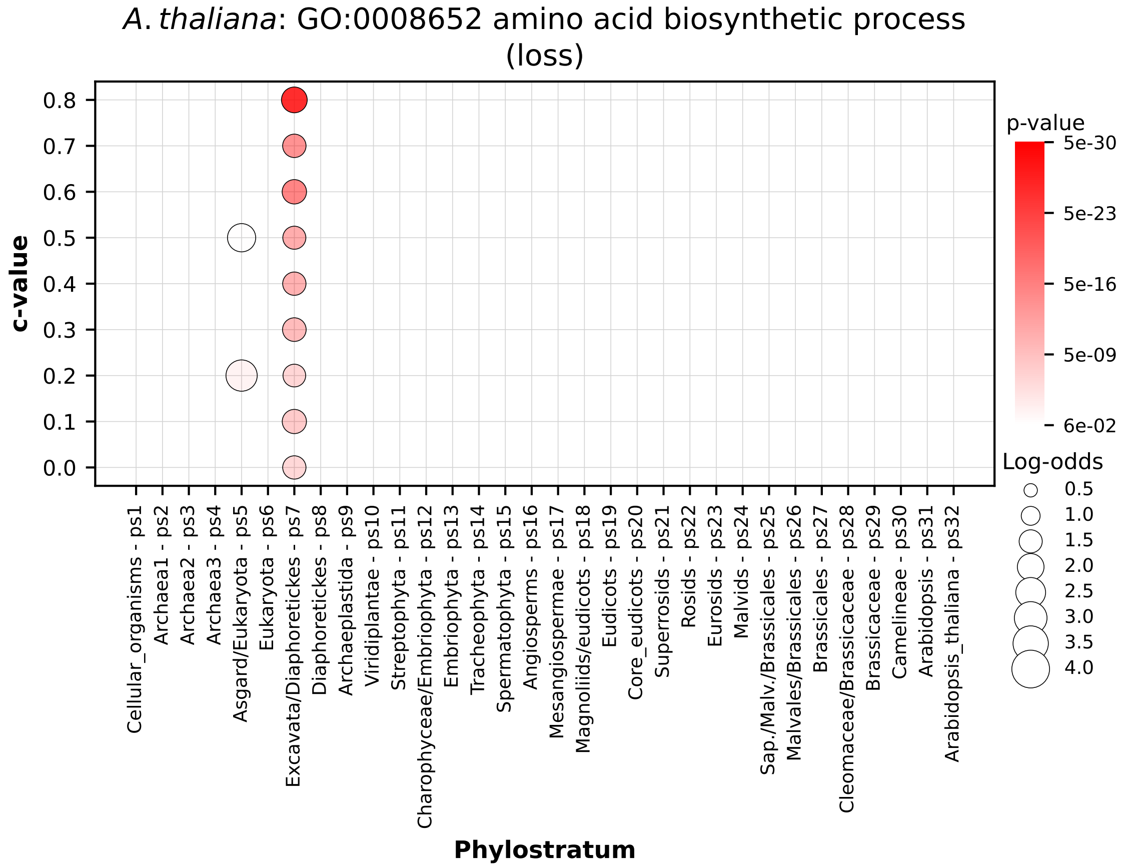

A. thaliana: GO:0008652 amino acid biosynthetic process
(loss)
c-value
0.8
0.7
0.6
0.5
0.4
0.3
0.2
0.1
0.0
p-value
5e-30
5e-23
5e-16
5e-09
6e-02
Log-odds
0.5
1.0
1.5
2.0
2.5
3.0
3.5
4.0
Cellular_organisms - ps1
Archaea1 - ps2
Archaea2 - ps3
Archaea3 - ps4
Asgard/Eukaryota - ps5
Eukaryota - ps6
Excavata/Diaphoretickes - ps7
Diaphoretickes - ps8
Archaeplastida - ps9
Viridiplantae - ps10
Streptophyta - ps11
Charophyceae/Embriophyta - ps12
Embriophyta - ps13
Tracheophyta - ps14
Spermatophyta - ps15
Angiosperms - ps16
Mesangiospermae - ps17
Magnoliids/eudicots - ps18
Eudicots - ps19
Core_eudicots - ps20
Superrosids - ps21
Rosids - ps22
Eurosids - ps23
Malvids - ps24
Sap./Malv./Brassicales - ps25
Malvales/Brassicales - ps26
Brassicales - ps27
Cleomaceae/Brassicaceae - ps28
Brassicaceae - ps29
Camelineae - ps30
Arabidopsis - ps31
Arabidopsis_thaliana - ps32
Phylostratum

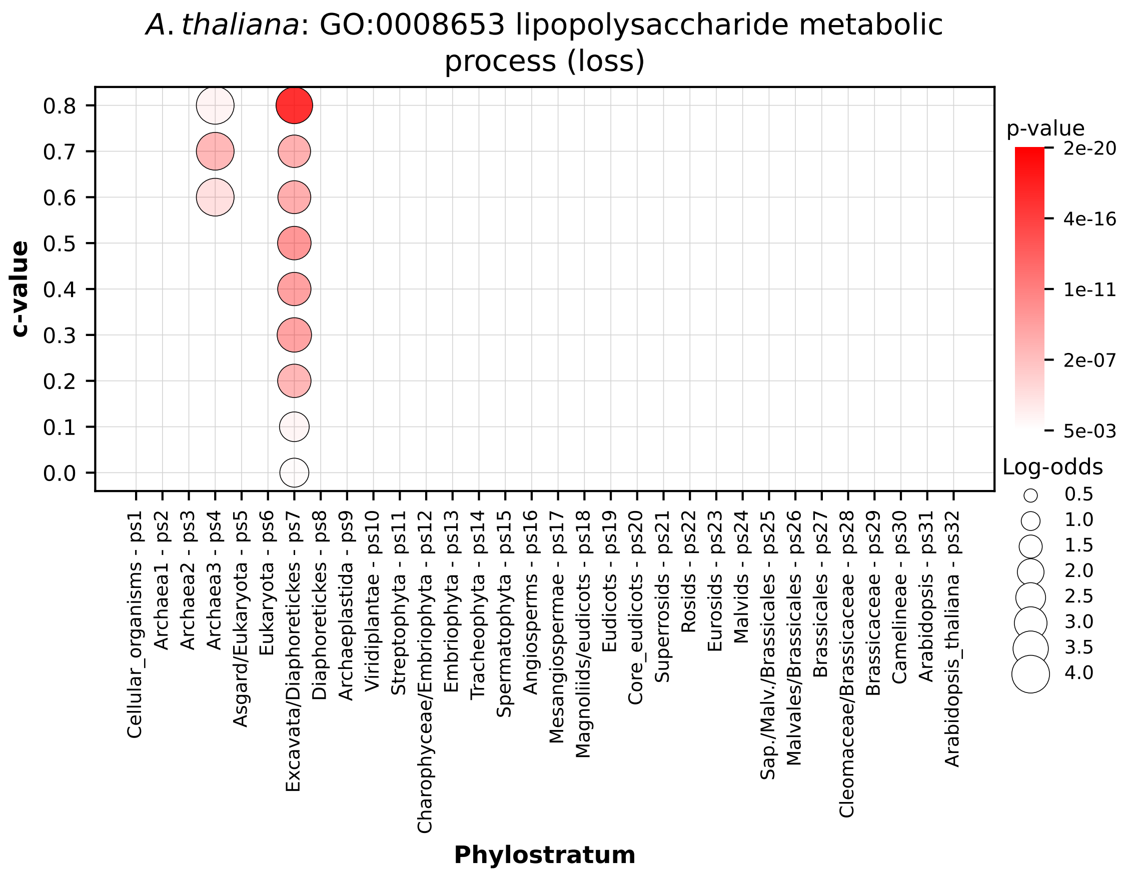

A. thaliana: GO:0008653 lipopolysaccharide metabolic process (loss)
c-value
0.0
0.1
0.2
0.3
0.4
0.5
0.6
0.7
0.8
p-value
2e-20
4e-16
1e-11
2e-07
5e-03
Log-odds
0.5
1.0
1.5
2.0
2.5
3.0
3.5
4.0
Cellular_organisms - ps1
Archaea1 - ps2
Archaea2 - ps3
Archaea3 - ps4
Asgard/Eukaryota - ps5
Eukaryota - ps6
Excavata/Diaphoretickes - ps7
Diaphoretickes - ps8
Archaeplastida - ps9
Viridiplantae - ps10
Streptophyta - ps11
Charophyceae/Embriophyta - ps12
Embriophyta - ps13
Tracheophyta - ps14
Spermatophyta - ps15
Angiosperms - ps16
Mesangiospermae - ps17
Magnoliids/eudicots - ps18
Eudicots - ps19
Core_eudicots - ps20
Superrosids - ps21
Rosids - ps22
Eurosids - ps23
Malvids - ps24
Sap./Malv./Brassicales - ps25
Malvales/Brassicales - ps26
Brassicales - ps27
Cleomaceae/Brassicaceae - ps28
Brassicaceae - ps29
Camelineae - ps30
Arabidopsis - ps31
Arabidopsis_thaliana - ps32
Phylostratum

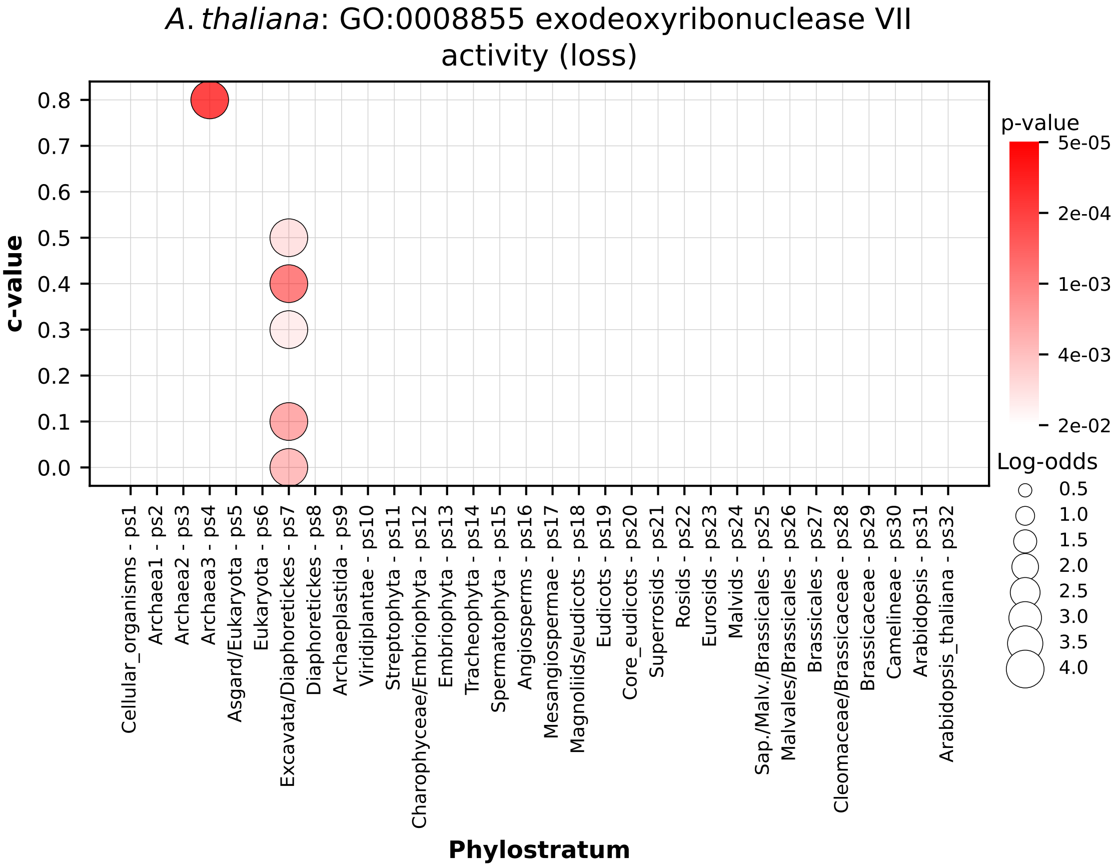
A. thaliana: GO:0008855 exodeoxyribonuclease VII activity (loss)
c-value
0.8
0.7
0.6
0.5
0.4
0.3
0.2
0.1
0.0
p-value
5e-05
2e-04
1e-03
4e-03
2e-02
Log-odds
0.5
1.0
1.5
2.0
2.5
3.0
3.5
4.0
Cellular_organisms - ps1
Archaea1 - ps2
Archaea2 - ps3
Archaea3 - ps4
Asgard/Eukaryota - ps5
Eukaryota - ps6
Excavata/Diaphoretickes - ps7
Diaphoretickes - ps8
Archaeplastida - ps9
Viridiplantae - ps10
Streptophyta - ps11
Charophyceae/Embriophyta - ps12
Embriophyta - ps13
Tracheophyta - ps14
Spermatophyta - ps15
Angiosperms - ps16
Mesangiospermae - ps17
Magnoliids/eudicots - ps18
Eudicots - ps19
Core_eudicots - ps20
Superrosids - ps21
Rosids - ps22
Eurosids - ps23
Malvids - ps24
Sap./Malv./Brassicales - ps25
Malvales/Brassicales - ps26
Brassicales - ps27
Cleomaceae/Brassicaceae - ps28
Brassicaceae - ps29
Camelineae - ps30
Arabidopsis - ps31
Arabidopsis_thaliana - ps32
Phylostratum

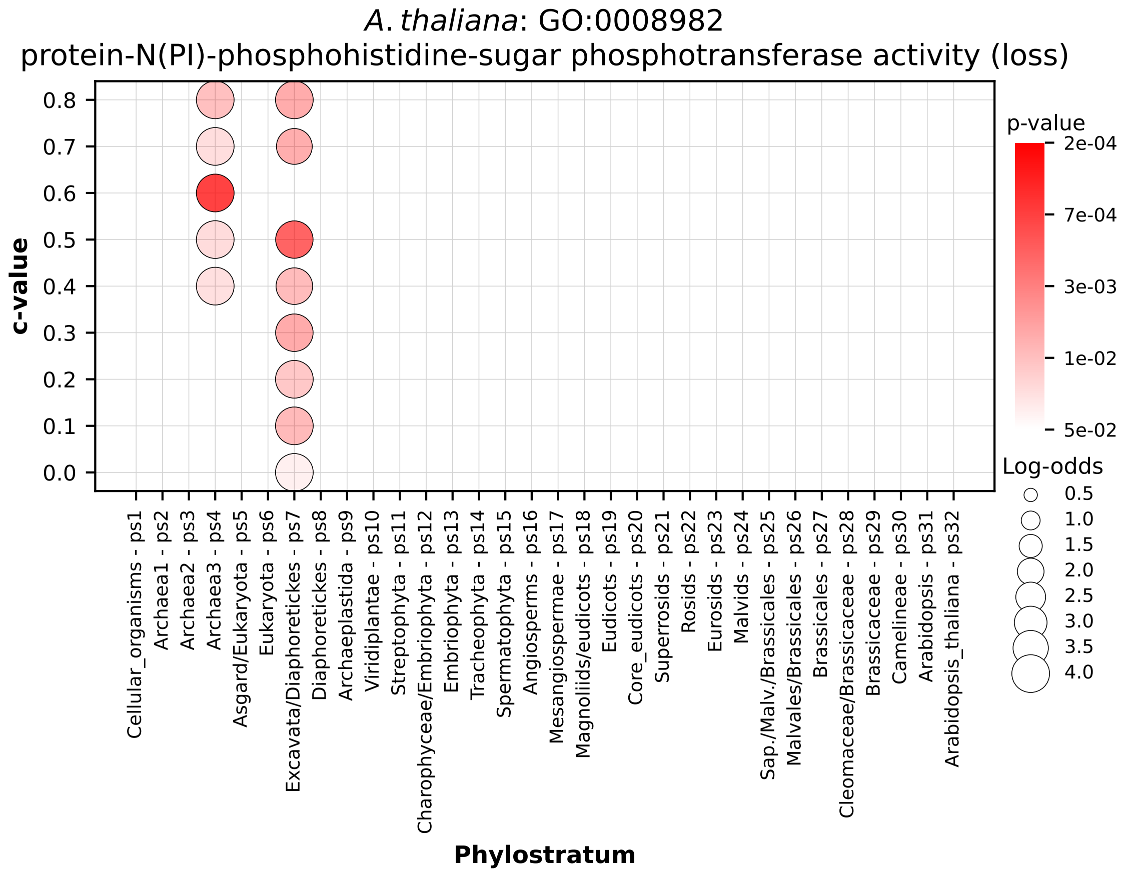

A. thaliana: GO:0008982
protein-N(PI)-phosphohistidine-sugar phosphotransferase activity (loss)
c-value
0.8
0.7
0.6
0.5
0.4
0.3
0.2
0.1
0.0
p-value
2e-04
7e-04
3e-03
1e-02
5e-02
Log-odds
0.5
1.0
1.5
2.0
2.5
3.0
3.5
4.0
Cellular_organisms - ps1
Archaea1 - ps2
Archaea2 - ps3
Archaea3 - ps4
Asgard/Eukaryota - ps5
Eukaryota - ps6
Excavata/Diaphoretickes - ps7
Diaphoretickes - ps8
Archaeplastida - ps9
Viridiplantae - ps10
Streptophyta - ps11
Charophyceae/Embriophyta - ps12
Embriophyta - ps13
Tracheophyta - ps14
Spermatophyta - ps15
Angiosperms - ps16
Mesangiospermae - ps17
Magnoliids/eudicots - ps18
Eudicots - ps19
Core_eudicots - ps20
Superrosids - ps21
Rosids - ps22
Eurosids - ps23
Malvids - ps24
Sap./Malv./Brassicales - ps25
Malvales/Brassicales - ps26
Brassicales - ps27
Cleomaceae/Brassicaceae - ps28
Brassicaceae - ps29
Camelineae - ps30
Arabidopsis - ps31
Arabidopsis_thaliana - ps32
Phylostratum

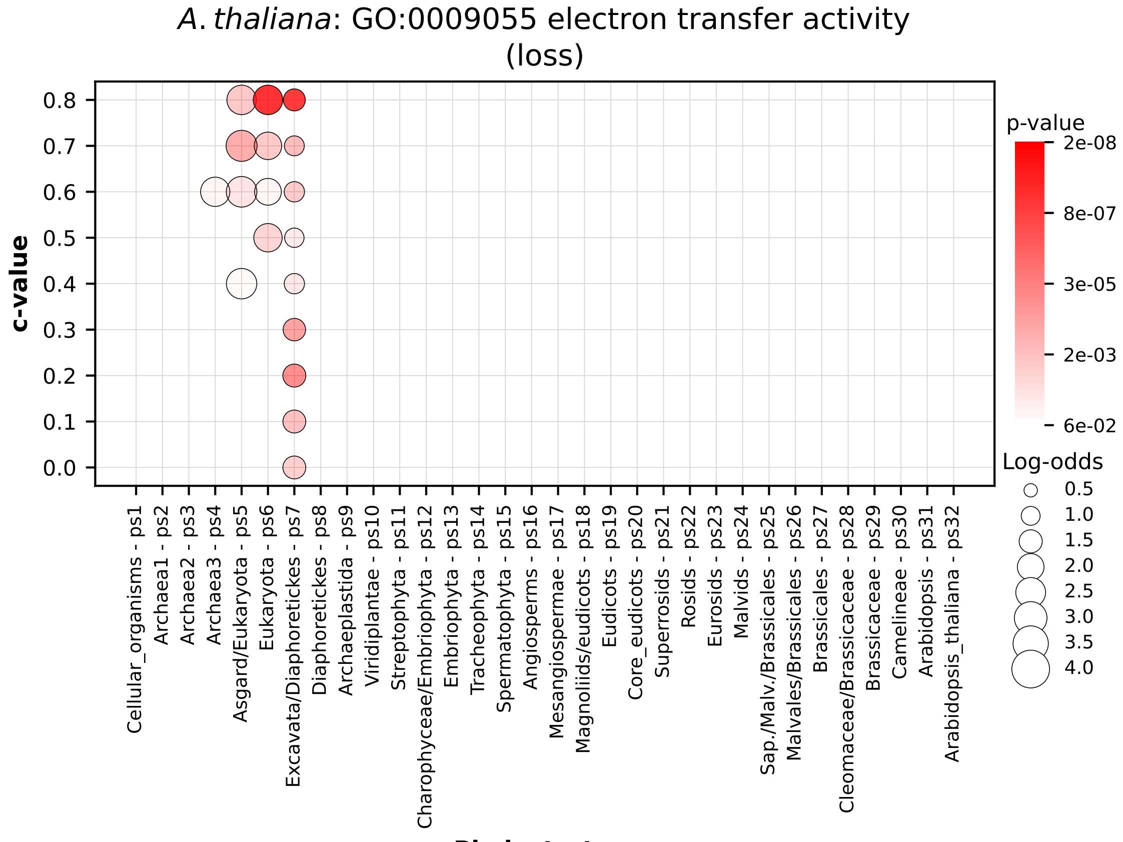

A. thaliana: GO:0009055 electron transfer activity
(loss)
c-value
0.8
0.7
0.6
0.5
0.4
0.3
0.2
0.1
0.0
p-value
2e-08
8e-07
3e-05
2e-03
6e-02
Log-odds
0.5
1.0
1.5
2.0
2.5
3.0
3.5
4.0
Cellular_organisms - ps1
Archaea1 - ps2
Archaea2 - ps3
Archaea3 - ps4
Asgard/Eukaryota - ps5
Eukaryota - ps6
Excavata/Diaphoretickes - ps7
Diaphoretickes - ps8
Archaeplastida - ps9
Viridiplantae - ps10
Streptophyta - ps11
Charophyceae/Embriophyta - ps12
Embriophyta - ps13
Tracheophyta - ps14
Spermatophyta - ps15
Angiosperms - ps16
Mesangiospermae - ps17
Magnoliids/eudicots - ps18
Eudicots - ps19
Core_eudicots - ps20
Superrosids - ps21
Rosids - ps22
Eurosids - ps23
Malvids - ps24
Sap./Malv./Brassicales - ps25
Malvales/Brassicales - ps26
Brassicales - ps27
Cleomaceae/Brassicaceae - ps28
Brassicaceae - ps29
Camelineae - ps30
Arabidopsis - ps31
Arabidopsis_thaliana - ps32
Phylostratum

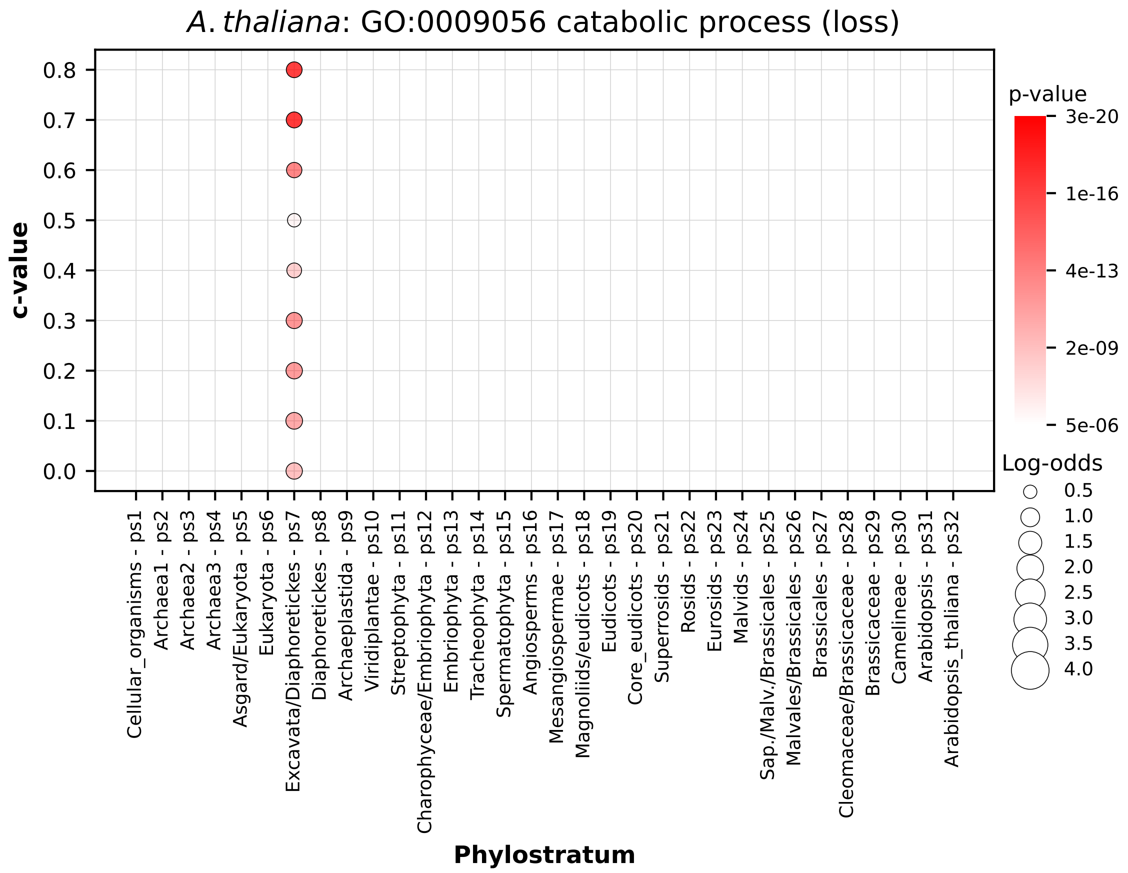
A. thaliana: GO:0009056 catabolic process (loss)
c-value
0.0
0.1
0.2
0.3
0.4
0.5
0.6
0.7
0.8
Phylostratum
Cellular_organisms - ps1
Archaea1 - ps2
Archaea2 - ps3
Archaea3 - ps4
Asgard/Eukaryota - ps5
Eukaryota - ps6
Excavata/Diaphoretickes - ps7
Diaphoretickes - ps8
Archaeplastida - ps9
Viridiplantae - ps10
Streptophyta - ps11
Charophyceae/Embriophyta - ps12
Embriophyta - ps13
Tracheophyta - ps14
Spermatophyta - ps15
Angiosperms - ps16
Mesangiospermae - ps17
Magnoliids/eudicots - ps18
Eudicots - ps19
Core_eudicots - ps20
Superrosids - ps21
Rosids - ps22
Eurosids - ps23
Malvids - ps24
Sap./Malv./Brassicales - ps25
Malvales/Brassicales - ps26
Brassicales - ps27
Cleomaceae/Brassicaceae - ps28
Brassicaceae - ps29
Camelineae - ps30
Arabidopsis - ps31
Arabidopsis_thaliana - ps32
p-value
3e-20
1e-16
4e-13
2e-09
5e-06
Log-odds
0.5
1.0
1.5
2.0
2.5
3.0
3.5
4.0

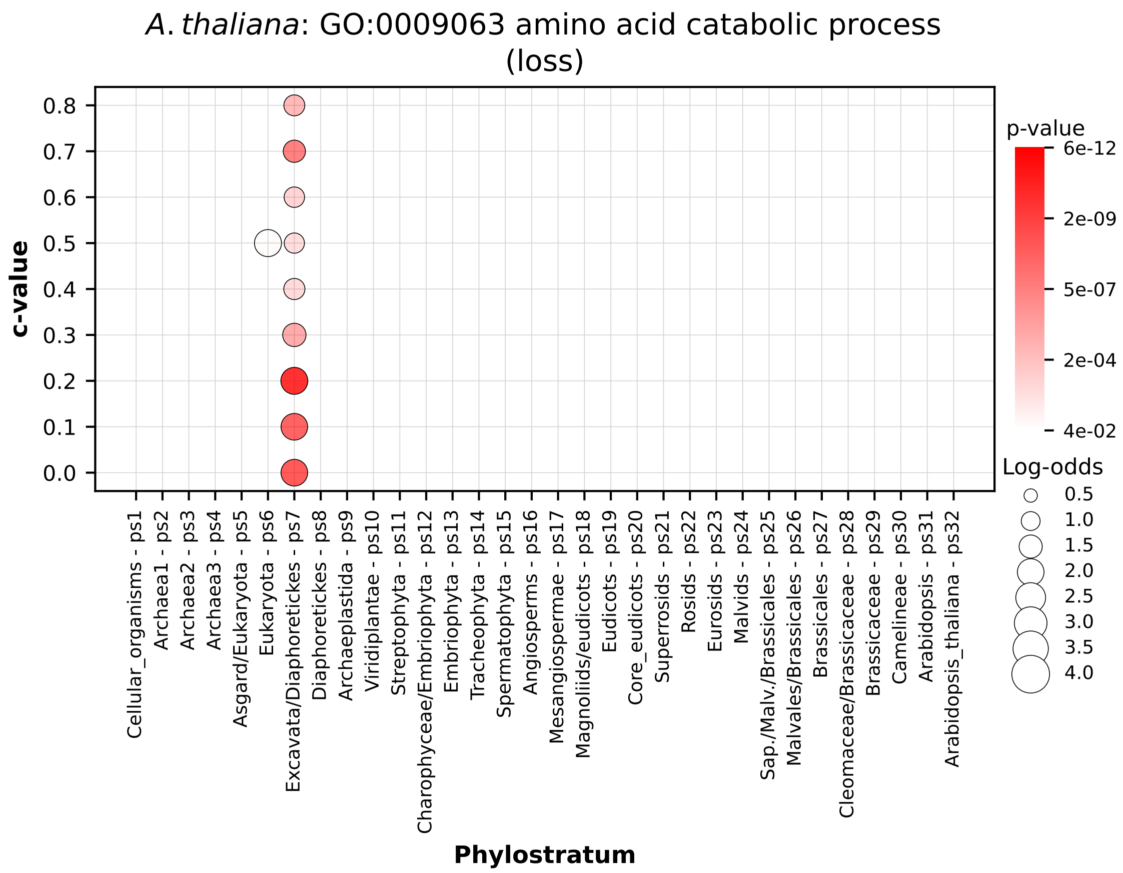

A. thaliana: GO:0009063 amino acid catabolic process
(loss)
c-value
0.8
0.7
0.6
0.5
0.4
0.3
0.2
0.1
0.0
p-value
6e-12
2e-09
5e-07
2e-04
4e-02
Log-odds
0.5
1.0
1.5
2.0
2.5
3.0
3.5
4.0
Cellular_organisms - ps1
Archaea1 - ps2
Archaea2 - ps3
Archaea3 - ps4
Asgard/Eukaryota - ps5
Eukaryota - ps6
Excavata/Diaphoretickes - ps7
Diaphoretickes - ps8
Archaeplastida - ps9
Viridiplantae - ps10
Streptophyta - ps11
Charophyceae/Embriophyta - ps12
Embriophyta - ps13
Tracheophyta - ps14
Spermatophyta - ps15
Angiosperms - ps16
Mesangiospermae - ps17
Magnoliids/eudicots - ps18
Eudicots - ps19
Core_eudicots - ps20
Superrosids - ps21
Rosids - ps22
Eurosids - ps23
Malvids - ps24
Sap./Malv./Brassicales - ps25
Malvales/Brassicales - ps26
Brassicales - ps27
Cleomaceae/Brassicaceae - ps28
Brassicaceae - ps29
Camelineae - ps30
Arabidopsis - ps31
Arabidopsis_thaliana - ps32
Phylostratum

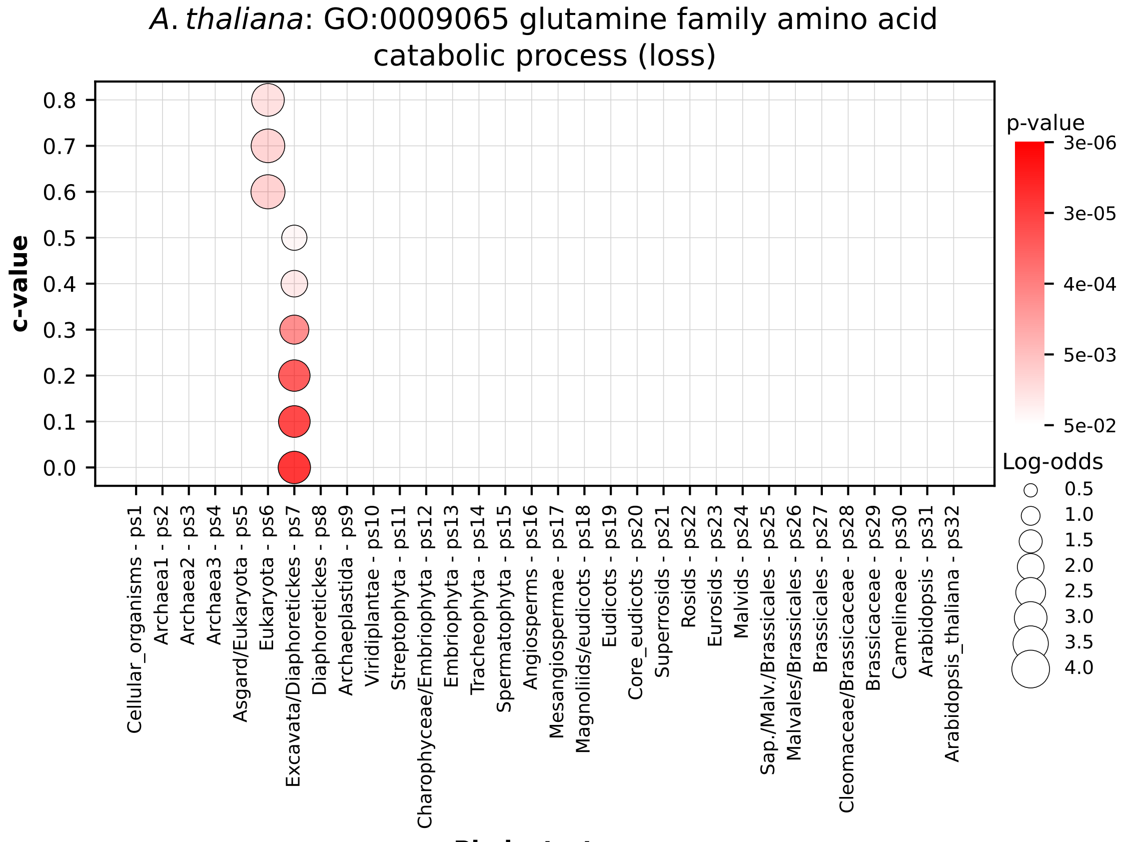

A. thaliana: GO:0009065 glutamine family amino acid catabolic process (loss)
c-value
0.8
0.7
0.6
0.5
0.4
0.3
0.2
0.1
0.0
p-value
3e-06
3e-05
4e-04
5e-03
5e-02
Log-odds
0.5
1.0
1.5
2.0
2.5
3.0
3.5
4.0
Cellular_organisms - ps1
Archaea1 - ps2
Archaea2 - ps3
Archaea3 - ps4
Asgard/Eukaryota - ps5
Eukaryota - ps6
Excavata/Diaphoretickes - ps7
Diaphoretickes - ps8
Archaeplastida - ps9
Viridiplantae - ps10
Streptophyta - ps11
Charophyceae/Embriophyta - ps12
Embriophyta - ps13
Tracheophyta - ps14
Spermatophyta - ps15
Angiosperms - ps16
Mesangiospermae - ps17
Magnoliids/eudicots - ps18
Eudicots - ps19
Core_eudicots - ps20
Superrosids - ps21
Rosids - ps22
Eurosids - ps23
Malvids - ps24
Sap./Malv./Brassicales - ps25
Malvales/Brassicales - ps26
Brassicales - ps27
Cleomaceae/Brassicaceae - ps28
Brassicaceae - ps29
Camelineae - ps30
Arabidopsis - ps31
Arabidopsis_thaliana - ps32
Phylostratum

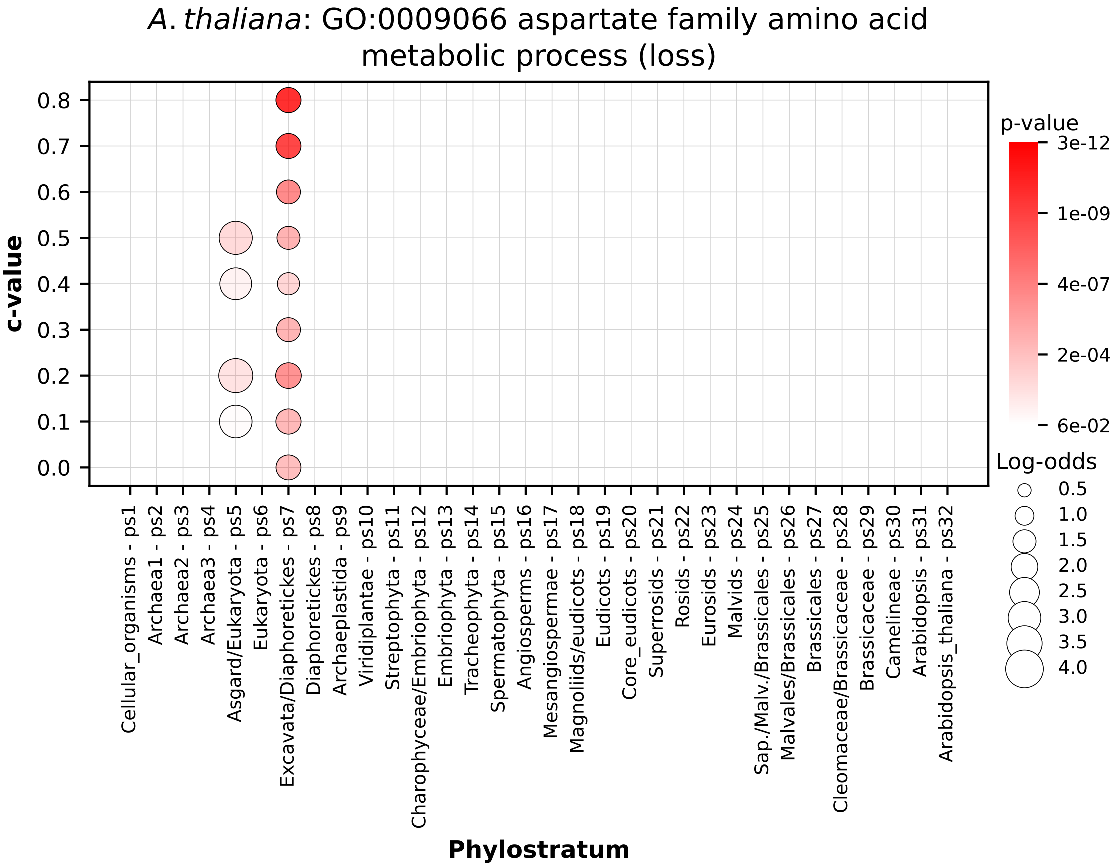
A. thaliana: GO:0009066 aspartate family amino acid metabolic process (loss)
c-value
0.8
0.7
0.6
0.5
0.4
0.3
0.2
0.1
0.0
p-value
3e-12
1e-09
4e-07
2e-04
6e-02
Log-odds
0.5
1.0
1.5
2.0
2.5
3.0
3.5
4.0
Cellular_organisms - ps1
Archaea1 - ps2
Archaea2 - ps3
Archaea3 - ps4
Asgard/Eukaryota - ps5
Eukaryota - ps6
Excavata/Diaphoretickes - ps7
Diaphoretickes - ps8
Archaeplastida - ps9
Viridiplantae - ps10
Streptophyta - ps11
Charophyceae/Embriophyta - ps12
Embriophyta - ps13
Tracheophyta - ps14
Spermatophyta - ps15
Angiosperms - ps16
Mesangiospermae - ps17
Magnoliids/eudicots - ps18
Eudicots - ps19
Core_eudicots - ps20
Superrosids - ps21
Rosids - ps22
Eurosids - ps23
Malvids - ps24
Sap./Malv./Brassicales - ps25
Malvales/Brassicales - ps26
Brassicales - ps27
Cleomaceae/Brassicaceae - ps28
Brassicaceae - ps29
Camelineae - ps30
Arabidopsis - ps31
Arabidopsis_thaliana - ps32
Phylostratum

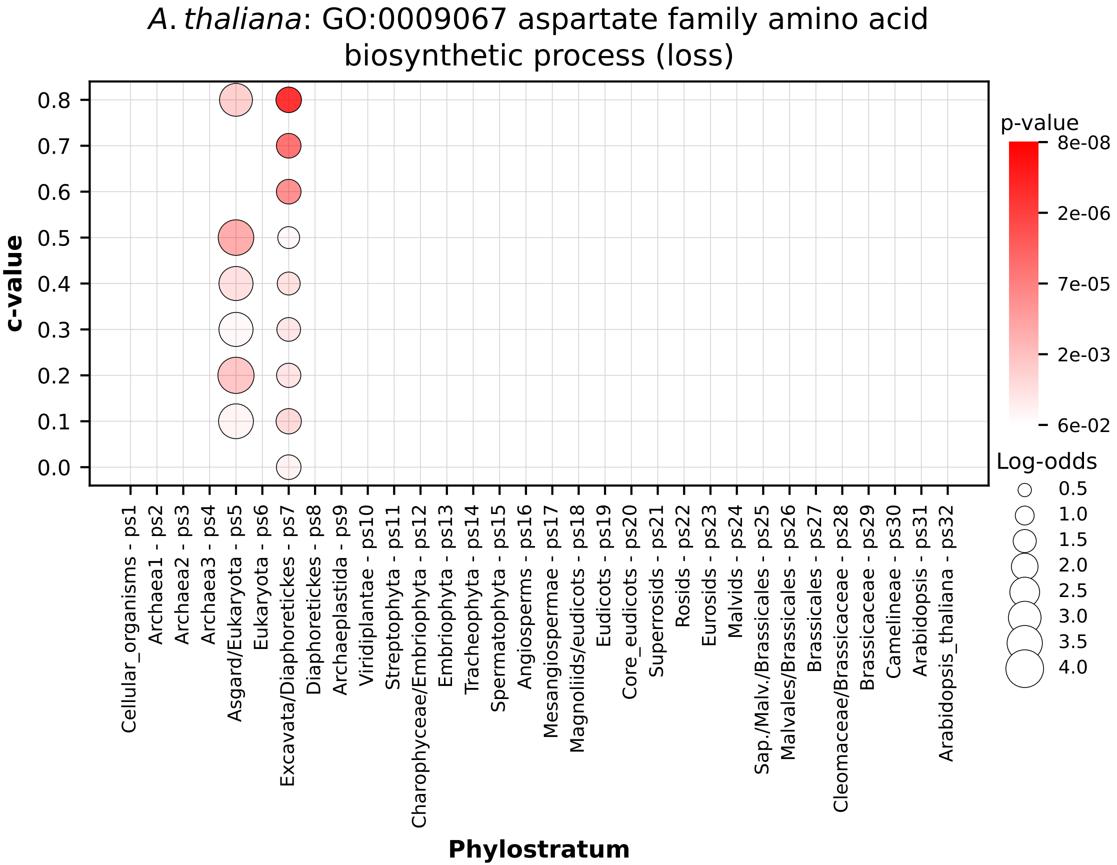

A. thaliana: GO:0009067 aspartate family amino acid biosynthetic process (loss)
c-value
0.8
0.7
0.6
0.5
0.4
0.3
0.2
0.1
0.0
Phylostratum
Cellular_organisms - ps1
Archaea1 - ps2
Archaea2 - ps3
Archaea3 - ps4
Asgard/Eukaryota - ps5
Eukaryota - ps6
Excavata/Diaphoretickes - ps7
Diaphoretickes - ps8
Archaeplastida - ps9
Viridiplantae - ps10
Streptophyta - ps11
Charophyceae/Embriophyta - ps12
Embriophyta - ps13
Tracheophyta - ps14
Spermatophyta - ps15
Angiosperms - ps16
Mesangiospermae - ps17
Magnoliids/eudicots - ps18
Eudicots - ps19
Core_eudicots - ps20
Superrosids - ps21
Rosids - ps22
Eurosids - ps23
Malvids - ps24
Sap./Malv./Brassicales - ps25
Malvales/Brassicales - ps26
Brassicales - ps27
Cleomaceae/Brassicaceae - ps28
Brassicaceae - ps29
Camelineae - ps30
Arabidopsis - ps31
Arabidopsis_thaliana - ps32
p-value
8e-08
2e-06
7e-05
2e-03
6e-02
Log-odds
0.5
1.0
1.5
2.0
2.5
3.0
3.5
4.0

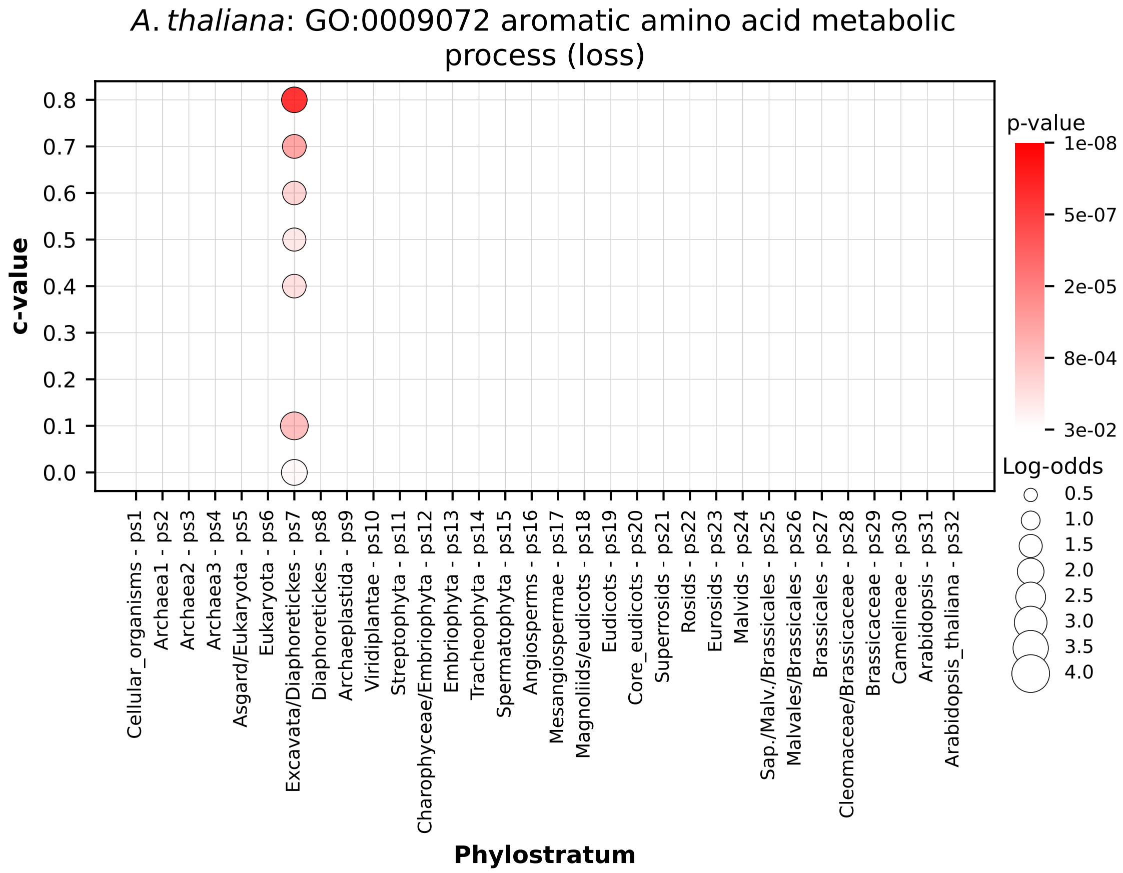

A. thaliana: GO:0009072 aromatic amino acid metabolic process (loss)
c-value
0.8
0.7
0.6
0.5
0.4
0.3
0.2
0.1
0.0
p-value
1e-08
5e-07
2e-05
8e-04
3e-02
Log-odds
0.5
1.0
1.5
2.0
2.5
3.0
3.5
4.0
Cellular_organisms - ps1
Archaea1 - ps2
Archaea2 - ps3
Archaea3 - ps4
Asgard/Eukaryota - ps5
Eukaryota - ps6
Excavata/Diaphoretickes - ps7
Diaphoretickes - ps8
Archaeplastida - ps9
Viridiplantae - ps10
Streptophyta - ps11
Charophyceae/Embriophyta - ps12
Embriophyta - ps13
Tracheophyta - ps14
Spermatophyta - ps15
Angiosperms - ps16
Mesangiospermae - ps17
Magnoliids/eudicots - ps18
Eudicots - ps19
Core_eudicots - ps20
Superrosids - ps21
Rosids - ps22
Eurosids - ps23
Malvids - ps24
Sap./Malv./Brassicales - ps25
Malvales/Brassicales - ps26
Brassicales - ps27
Cleomaceae/Brassicaceae - ps28
Brassicaceae - ps29
Camelineae - ps30
Arabidopsis - ps31
Arabidopsis_thaliana - ps32
Phylostratum

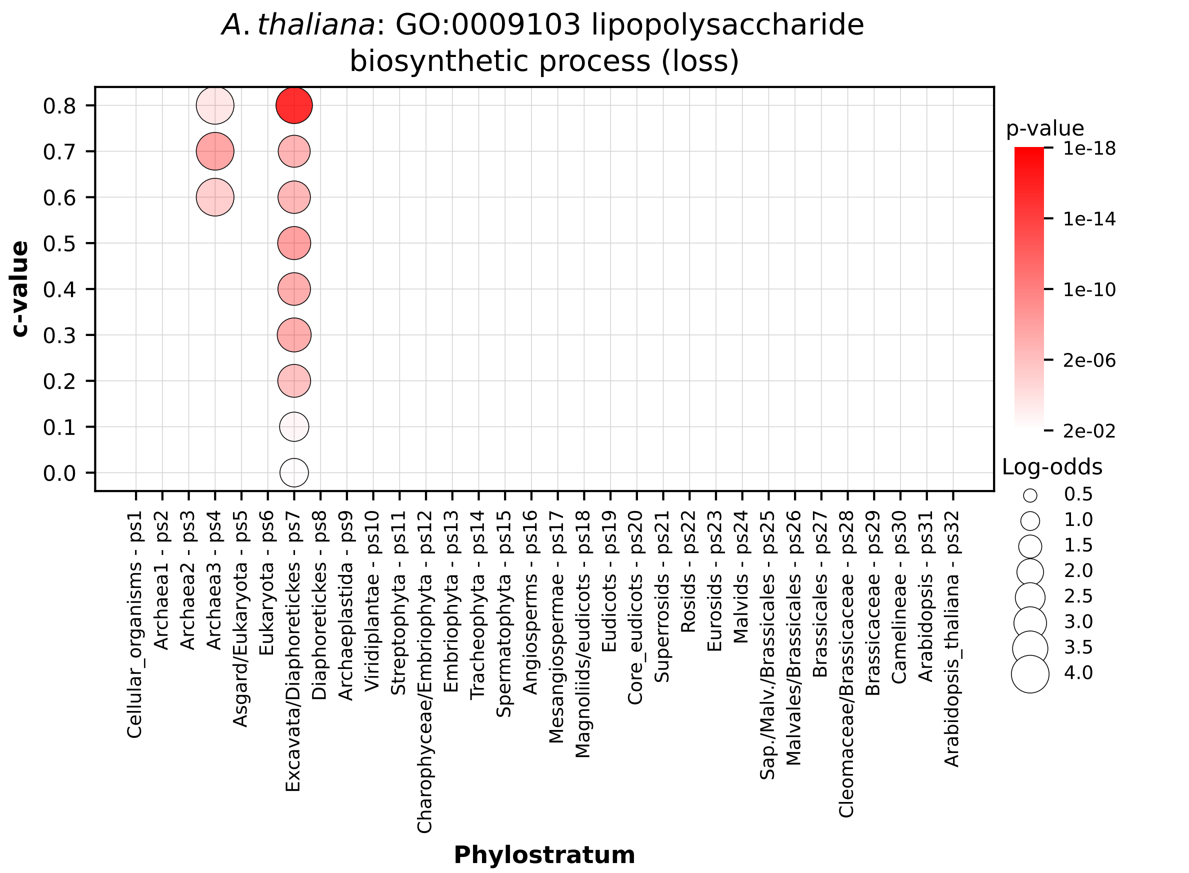

A. thaliana: GO:0009103 lipopolysaccharide biosynthetic process (loss)
c-value
0.8
0.7
0.6
0.5
0.4
0.3
0.2
0.1
0.0
p-value
1e-18
1e-14
1e-10
2e-06
2e-02
Log-odds
0.5
1.0
1.5
2.0
2.5
3.0
3.5
4.0
Cellular_organisms - ps1
Archaea1 - ps2
Archaea2 - ps3
Archaea3 - ps4
Asgard/Eukaryota - ps5
Eukaryota - ps6
Excavata/Diaphoretickes - ps7
Diaphoretickes - ps8
Archaeplastida - ps9
Viridiplantae - ps10
Streptophyta - ps11
Charophyceae/Embriophyta - ps12
Embriophyta - ps13
Tracheophyta - ps14
Spermatophyta - ps15
Angiosperms - ps16
Mesangiospermae - ps17
Magnoliids/eudicots - ps18
Eudicots - ps19
Core_eudicots - ps20
Superrosids - ps21
Rosids - ps22
Eurosids - ps23
Malvids - ps24
Sap./Malv./Brassicales - ps25
Malvales/Brassicales - ps26
Brassicales - ps27
Cleomaceae/Brassicaceae - ps28
Brassicaceae - ps29
Camelineae - ps30
Arabidopsis - ps31
Arabidopsis_thaliana - ps32
Phylostratum

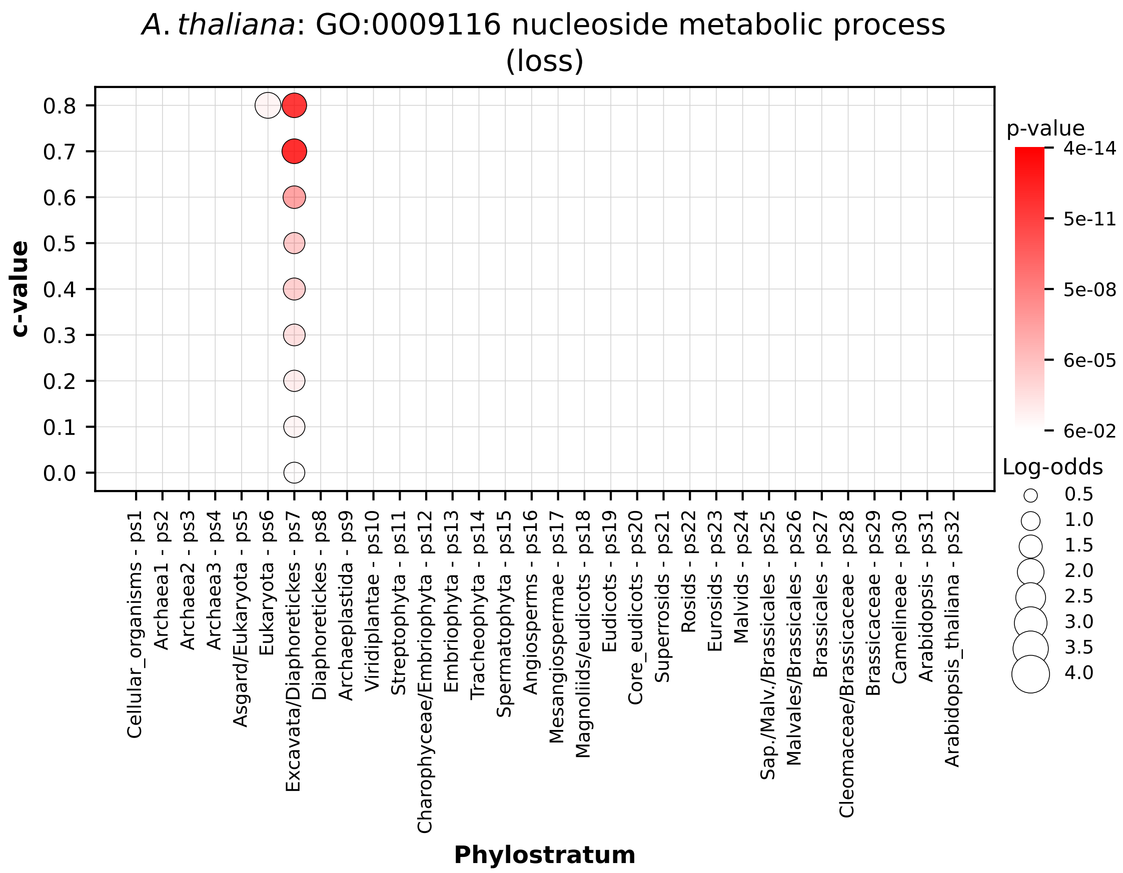

A. thaliana: GO:0009116 nucleoside metabolic process
(loss)
c-value
0.8
0.7
0.6
0.5
0.4
0.3
0.2
0.1
0.0
p-value
4e-14
5e-11
5e-08
6e-05
6e-02
Log-odds
0.5
1.0
1.5
2.0
2.5
3.0
3.5
4.0
Cellular_organisms - ps1
Archaea1 - ps2
Archaea2 - ps3
Archaea3 - ps4
Asgard/Eukaryota - ps5
Eukaryota - ps6
Excavata/Diaphoretickes - ps7
Diaphoretickes - ps8
Archaeplastida - ps9
Viridiplantae - ps10
Streptophyta - ps11
Charophyceae/Embriophyta - ps12
Embriophyta - ps13
Tracheophyta - ps14
Spermatophyta - ps15
Angiosperms - ps16
Mesangiospermae - ps17
Magnoliids/eudicots - ps18
Eudicots - ps19
Core_eudicots - ps20
Superrosids - ps21
Rosids - ps22
Eurosids - ps23
Malvids - ps24
Sap./Malv./Brassicales - ps25
Malvales/Brassicales - ps26
Brassicales - ps27
Cleomaceae/Brassicaceae - ps28
Brassicaceae - ps29
Camelineae - ps30
Arabidopsis - ps31
Arabidopsis_thaliana - ps32
Phylostratum

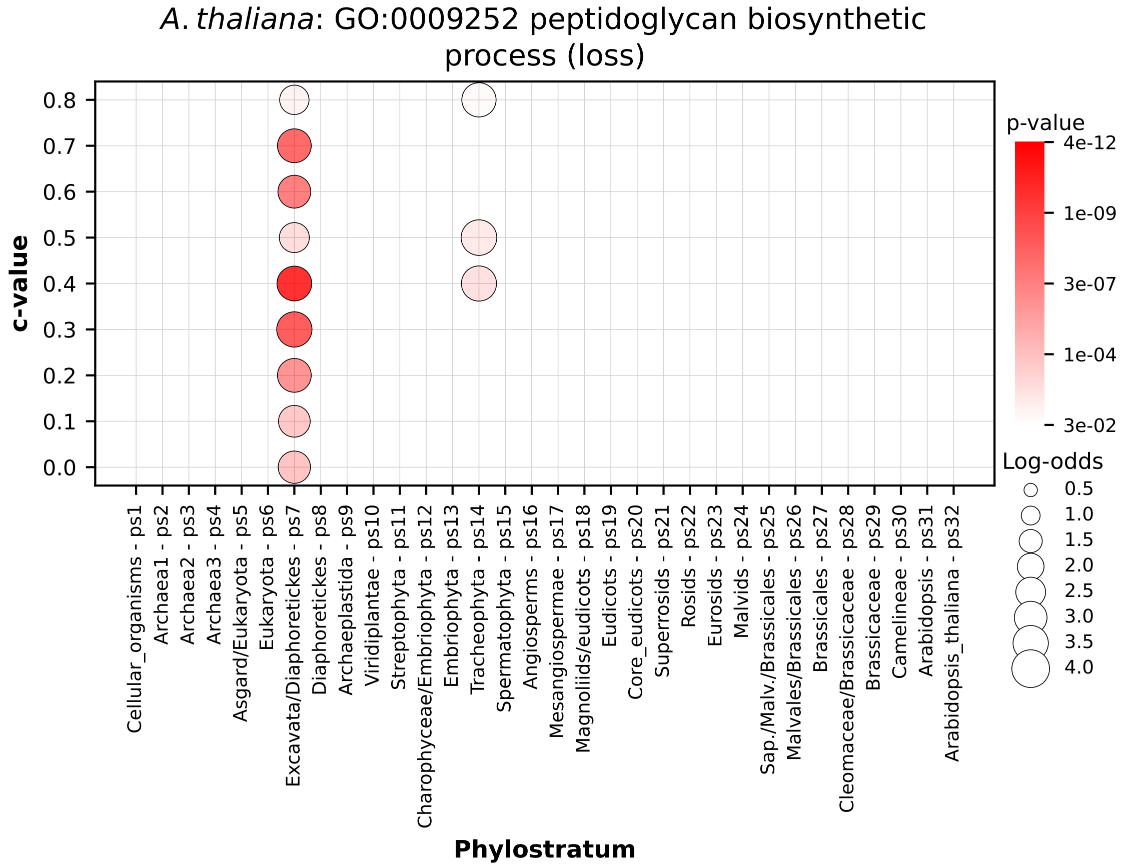

A. thaliana: GO:0009252 peptidoglycan biosynthetic process (loss)
c-value
0.8
0.7
0.6
0.5
0.4
0.3
0.2
0.1
0.0
p-value
4e-12
1e-09
3e-07
1e-04
3e-02
Log-odds
0.5
1.0
1.5
2.0
2.5
3.0
3.5
4.0
Cellular_organisms - ps1
Archaea1 - ps2
Archaea2 - ps3
Archaea3 - ps4
Asgard/Eukaryota - ps5
Eukaryota - ps6
Excavata/Diaphoretickes - ps7
Diaphoretickes - ps8
Archaeplastida - ps9
Viridiplantae - ps10
Streptophyta - ps11
Charophyceae/Embriophyta - ps12
Embriophyta - ps13
Tracheophyta - ps14
Spermatophyta - ps15
Angiosperms - ps16
Mesangiospermae - ps17
Magnoliids/eudicots - ps18
Eudicots - ps19
Core_eudicots - ps20
Superrosids - ps21
Rosids - ps22
Eurosids - ps23
Malvids - ps24
Sap./Malv./Brassicales - ps25
Malvales/Brassicales - ps26
Brassicales - ps27
Cleomaceae/Brassicaceae - ps28
Brassicaceae - ps29
Camelineae - ps30
Arabidopsis - ps31
Arabidopsis_thaliana - ps32
Phylostratum

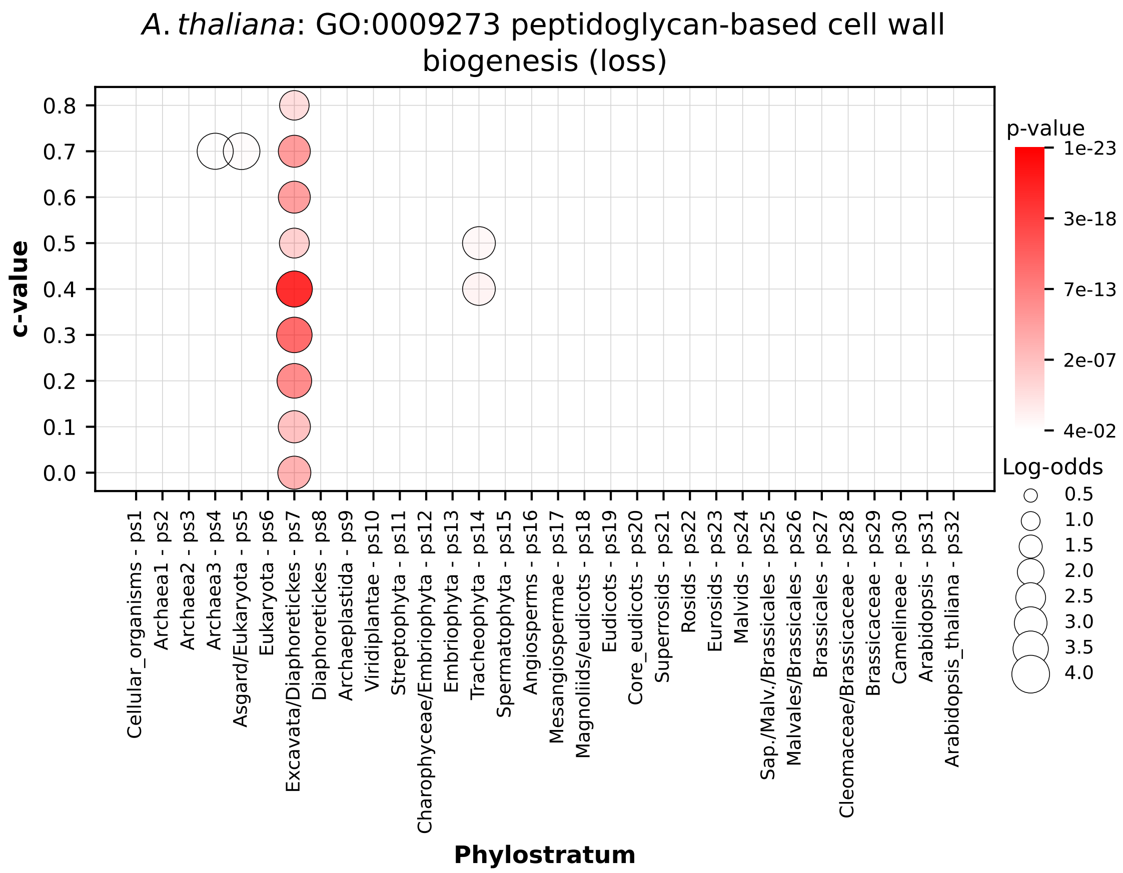

A. thaliana: GO:0009273 peptidoglycan-based cell wall biogenesis (loss)
c-value
0.8
0.7
0.6
0.5
0.4
0.3
0.2
0.1
0.0
Phylostratum
Cellular_organisms - ps1
Archaea1 - ps2
Archaea2 - ps3
Archaea3 - ps4
Asgard/Eukaryota - ps5
Eukaryota - ps6
Excavata/Diaphoretickes - ps7
Diaphoretickes - ps8
Archaeplastida - ps9
Viridiplantae - ps10
Streptophyta - ps11
Charophyceae/Embriophyta - ps12
Embriophyta - ps13
Tracheophyta - ps14
Spermatophyta - ps15
Angiosperms - ps16
Mesangiospermae - ps17
Magnoliids/eudicots - ps18
Eudicots - ps19
Core_eudicots - ps20
Superrosids - ps21
Rosids - ps22
Eurosids - ps23
Malvids - ps24
Sap./Malv./Brassicales - ps25
Malvales/Brassicales - ps26
Brassicales - ps27
Cleomaceae/Brassicaceae - ps28
Brassicaceae - ps29
Camelineae - ps30
Arabidopsis - ps31
Arabidopsis_thaliana - ps32
p-value
1e-23
3e-18
7e-13
2e-07
4e-02
Log-odds
0.5
1.0
1.5
2.0
2.5
3.0
3.5
4.0

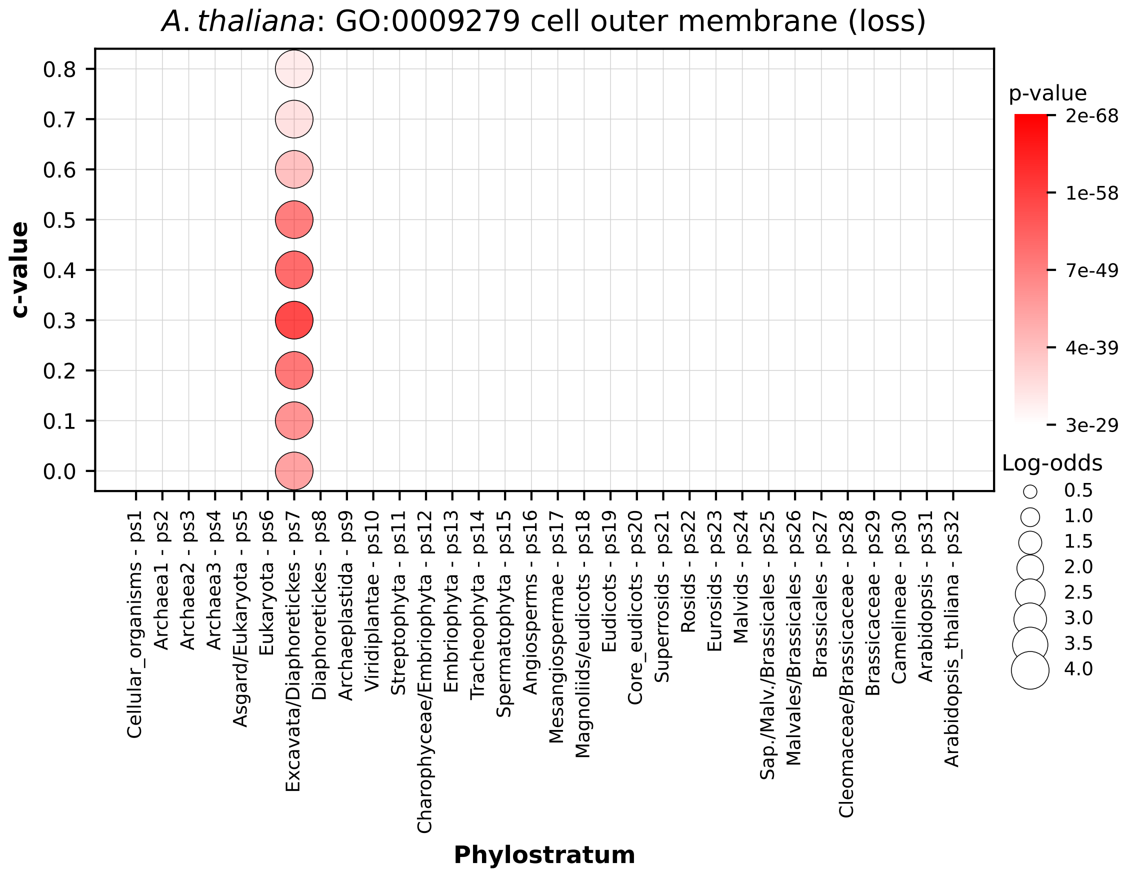
A. thaliana: GO:0009279 cell outer membrane (loss)
c-value
0.8
0.7
0.6
0.5
0.4
0.3
0.2
0.1
0.0
p-value
2e-68
1e-58
7e-49
4e-39
3e-29
Log-odds
0.5
1.0
1.5
2.0
2.5
3.0
3.5
4.0
Cellular_organisms - ps1
Archaea1 - ps2
Archaea2 - ps3
Archaea3 - ps4
Asgard/Eukaryota - ps5
Eukaryota - ps6
Excavata/Diaphoretickes - ps7
Diaphoretickes - ps8
Archaeplastida - ps9
Viridiplantae - ps10
Streptophyta - ps11
Charophyceae/Embriophyta - ps12
Embriophyta - ps13
Tracheophyta - ps14
Spermatophyta - ps15
Angiosperms - ps16
Mesangiospermae - ps17
Magnoliids/eudicots - ps18
Eudicots - ps19
Core_eudicots - ps20
Superrosids - ps21
Rosids - ps22
Eurosids - ps23
Malvids - ps24
Sap./Malv./Brassicales - ps25
Malvales/Brassicales - ps26
Brassicales - ps27
Cleomaceae/Brassicaceae - ps28
Brassicaceae - ps29
Camelineae - ps30
Arabidopsis - ps31
Arabidopsis_thaliana - ps32
Phylostratum

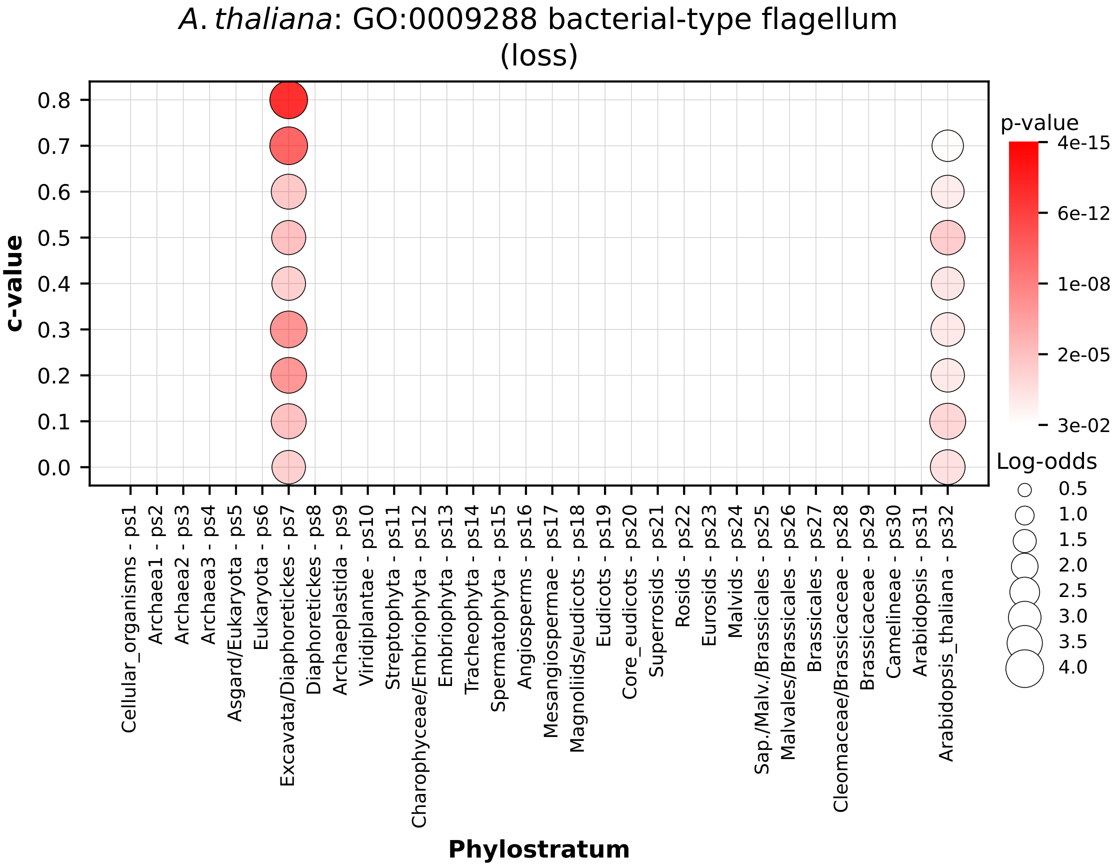

A. thaliana: GO:0009288 bacterial-type flagellum
(loss)
c-value
0.8
0.7
0.6
0.5
0.4
0.3
0.2
0.1
0.0
p-value
4e-15
6e-12
1e-08
2e-05
3e-02
Log-odds
0.5
1.0
1.5
2.0
2.5
3.0
3.5
4.0
Cellular_organisms - ps1
Archaea1 - ps2
Archaea2 - ps3
Archaea3 - ps4
Asgard/Eukaryota - ps5
Eukaryota - ps6
Excavata/Diaphoretickes - ps7
Diaphoretickes - ps8
Archaeplastida - ps9
Viridiplantae - ps10
Streptophyta - ps11
Charophyceae/Embriophyta - ps12
Embriophyta - ps13
Tracheophyta - ps14
Spermatophyta - ps15
Angiosperms - ps16
Mesangiospermae - ps17
Magnoliids/eudicots - ps18
Eudicots - ps19
Core_eudicots - ps20
Superrosids - ps21
Rosids - ps22
Eurosids - ps23
Malvids - ps24
Sap./Malv./Brassicales - ps25
Malvales/Brassicales - ps26
Brassicales - ps27
Cleomaceae/Brassicaceae - ps28
Brassicaceae - ps29
Camelineae - ps30
Arabidopsis - ps31
Arabidopsis_thaliana - ps32
Phylostratum

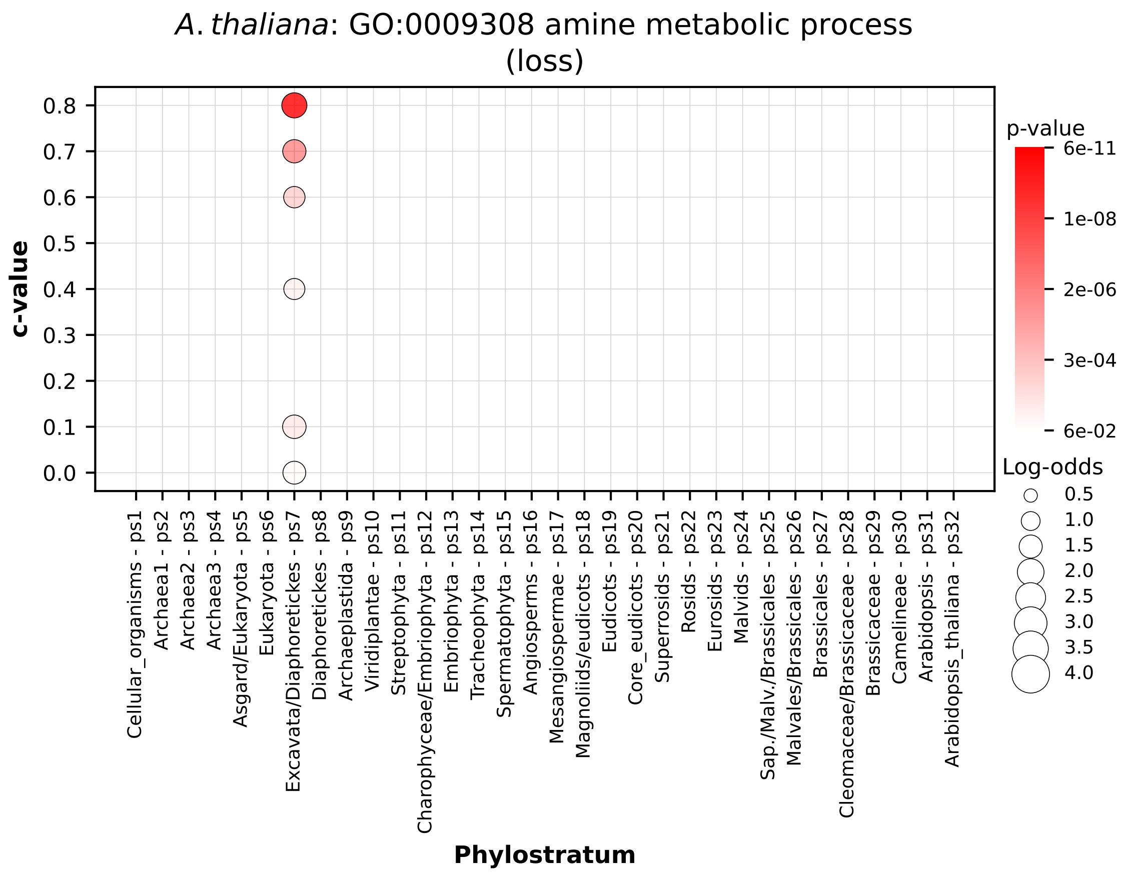
A. thaliana: GO:0009308 amine metabolic process
(loss)
c-value
0.8
0.7
0.6
0.5
0.4
0.3
0.2
0.1
0.0
p-value
6e-11
1e-08
2e-06
3e-04
6e-02
Log-odds
0.5
1.0
1.5
2.0
2.5
3.0
3.5
4.0
Cellular_organisms - ps1
Archaea1 - ps2
Archaea2 - ps3
Archaea3 - ps4
Asgard/Eukaryota - ps5
Eukaryota - ps6
Excavata/Diaphoretickes - ps7
Diaphoretickes - ps8
Archaeplastida - ps9
Viridiplantae - ps10
Streptophyta - ps11
Charophyceae/Embriophyta - ps12
Embriophyta - ps13
Tracheophyta - ps14
Spermatophyta - ps15
Angiosperms - ps16
Mesangiospermae - ps17
Magnoliids/eudicots - ps18
Eudicots - ps19
Core_eudicots - ps20
Superrosids - ps21
Rosids - ps22
Eurosids - ps23
Malvids - ps24
Sap./Malv./Brassicales - ps25
Malvales/Brassicales - ps26
Brassicales - ps27
Cleomaceae/Brassicaceae - ps28
Brassicaceae - ps29
Camelineae - ps30
Arabidopsis - ps31
Arabidopsis_thaliana - ps32
Phylostratum

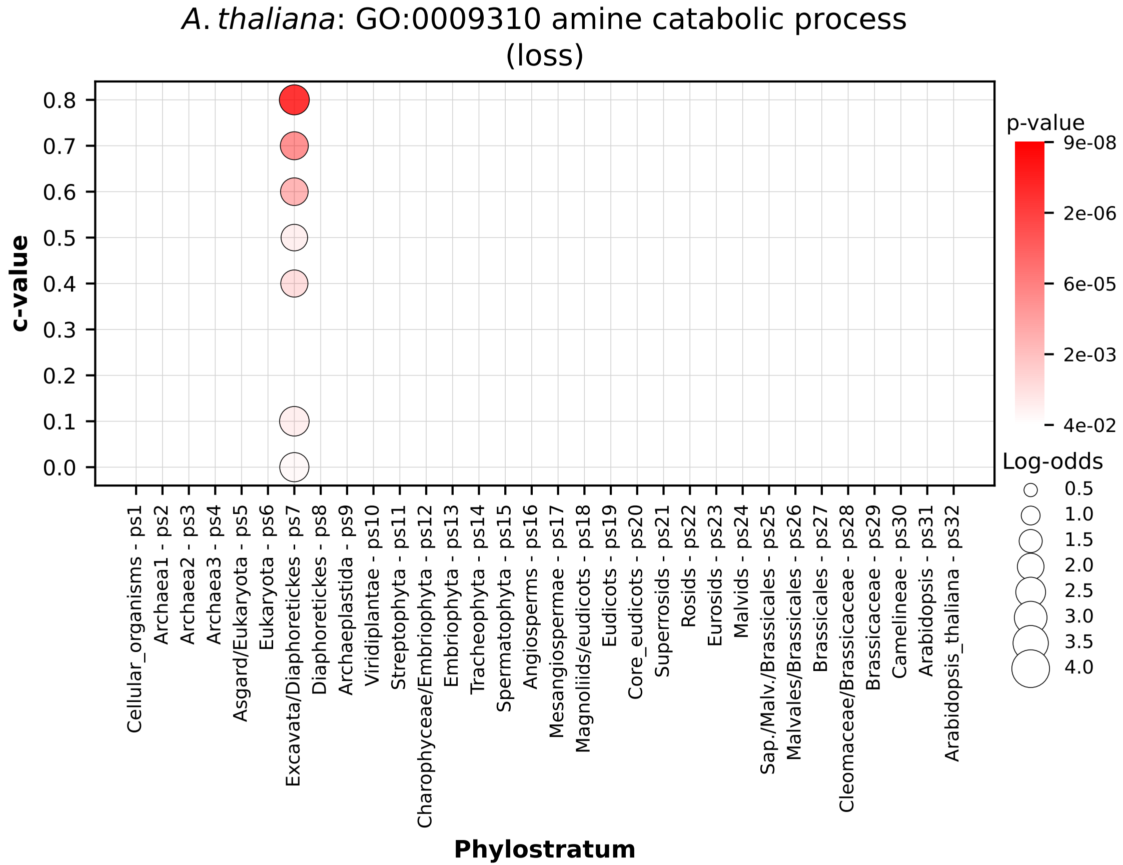

A. thaliana: GO:0009310 amine catabolic process
(loss)
c-value
0.8
0.7
0.6
0.5
0.4
0.3
0.2
0.1
0.0
p-value
9e-08
2e-06
6e-05
2e-03
4e-02
Log-odds
0.5
1.0
1.5
2.0
2.5
3.0
3.5
4.0
Cellular_organisms - ps1
Archaea1 - ps2
Archaea2 - ps3
Archaea3 - ps4
Asgard/Eukaryota - ps5
Eukaryota - ps6
Excavata/Diaphoretickes - ps7
Diaphoretickes - ps8
Archaeplastida - ps9
Viridiplantae - ps10
Streptophyta - ps11
Charophyceae/Embriophyta - ps12
Embriophyta - ps13
Tracheophyta - ps14
Spermatophyta - ps15
Angiosperms - ps16
Mesangiospermae - ps17
Magnoliids/eudicots - ps18
Eudicots - ps19
Core_eudicots - ps20
Superrosids - ps21
Rosids - ps22
Eurosids - ps23
Malvids - ps24
Sap./Malv./Brassicales - ps25
Malvales/Brassicales - ps26
Brassicales - ps27
Cleomaceae/Brassicaceae - ps28
Brassicaceae - ps29
Camelineae - ps30
Arabidopsis - ps31
Arabidopsis_thaliana - ps32
Phylostratum

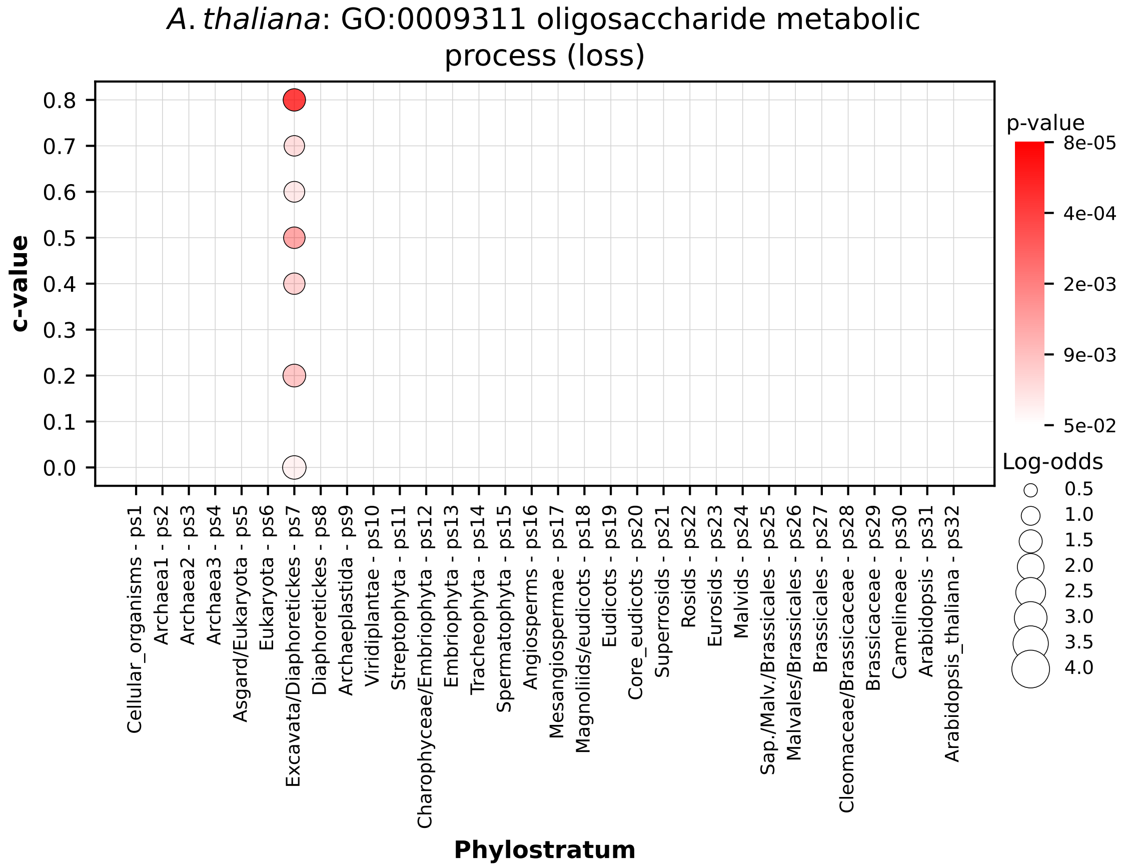

A. thaliana: GO:0009311 oligosaccharide metabolic process (loss)
c-value
0.8
0.7
0.6
0.5
0.4
0.3
0.2
0.1
0.0
p-value
8e-05
4e-04
2e-03
9e-03
5e-02
Log-odds
0.5
1.0
1.5
2.0
2.5
3.0
3.5
4.0
Cellular_organisms - ps1
Archaea1 - ps2
Archaea2 - ps3
Archaea3 - ps4
Asgard/Eukaryota - ps5
Eukaryota - ps6
Excavata/Diaphoretickes - ps7
Diaphoretickes - ps8
Archaeplastida - ps9
Viridiplantae - ps10
Streptophyta - ps11
Charophyceae/Embriophyta - ps12
Embriophyta - ps13
Tracheophyta - ps14
Spermatophyta - ps15
Angiosperms - ps16
Mesangiospermae - ps17
Magnoliids/eudicots - ps18
Eudicots - ps19
Core_eudicots - ps20
Superrosids - ps21
Rosids - ps22
Eurosids - ps23
Malvids - ps24
Sap./Malv./Brassicales - ps25
Malvales/Brassicales - ps26
Brassicales - ps27
Cleomaceae/Brassicaceae - ps28
Brassicaceae - ps29
Camelineae - ps30
Arabidopsis - ps31
Arabidopsis_thaliana - ps32
Phylostratum

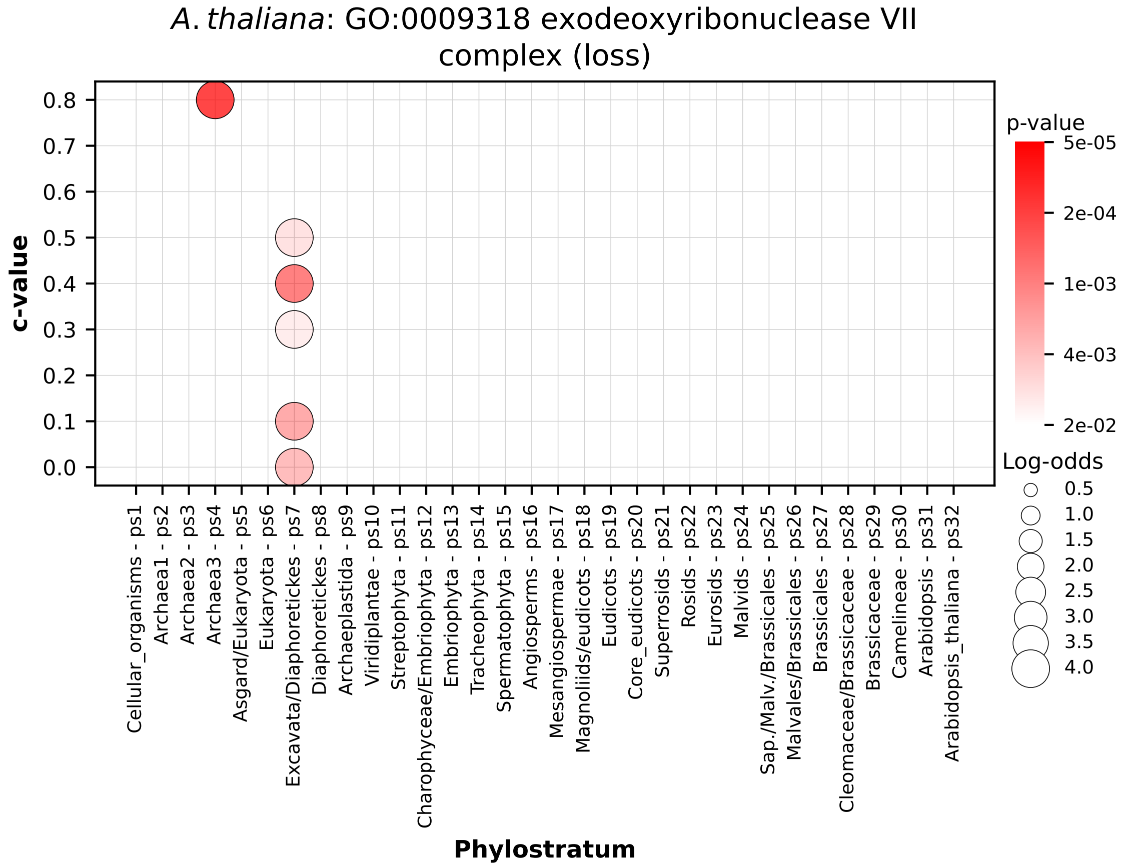
A. thaliana: GO:0009318 exodeoxyribonuclease VII complex (loss)
c-value
0.0
0.1
0.2
0.3
0.4
0.5
0.6
0.7
0.8
p-value
5e-05
2e-04
1e-03
4e-03
2e-02
Log-odds
0.5
1.0
1.5
2.0
2.5
3.0
3.5
4.0
Cellular_organisms - ps1
Archaea1 - ps2
Archaea2 - ps3
Archaea3 - ps4
Asgard/Eukaryota - ps5
Eukaryota - ps6
Excavata/Diaphoretickes - ps7
Diaphoretickes - ps8
Archaeplastida - ps9
Viridiplantae - ps10
Streptophyta - ps11
Charophyceae/Embriophyta - ps12
Embriophyta - ps13
Tracheophyta - ps14
Spermatophyta - ps15
Angiosperms - ps16
Mesangiospermae - ps17
Magnoliids/eudicots - ps18
Eudicots - ps19
Core_eudicots - ps20
Superrosids - ps21
Rosids - ps22
Eurosids - ps23
Malvids - ps24
Sap./Malv./Brassicales - ps25
Malvales/Brassicales - ps26
Brassicales - ps27
Cleomaceae/Brassicaceae - ps28
Brassicaceae - ps29
Camelineae - ps30
Arabidopsis - ps31
Arabidopsis_thaliana - ps32
Phylostratum

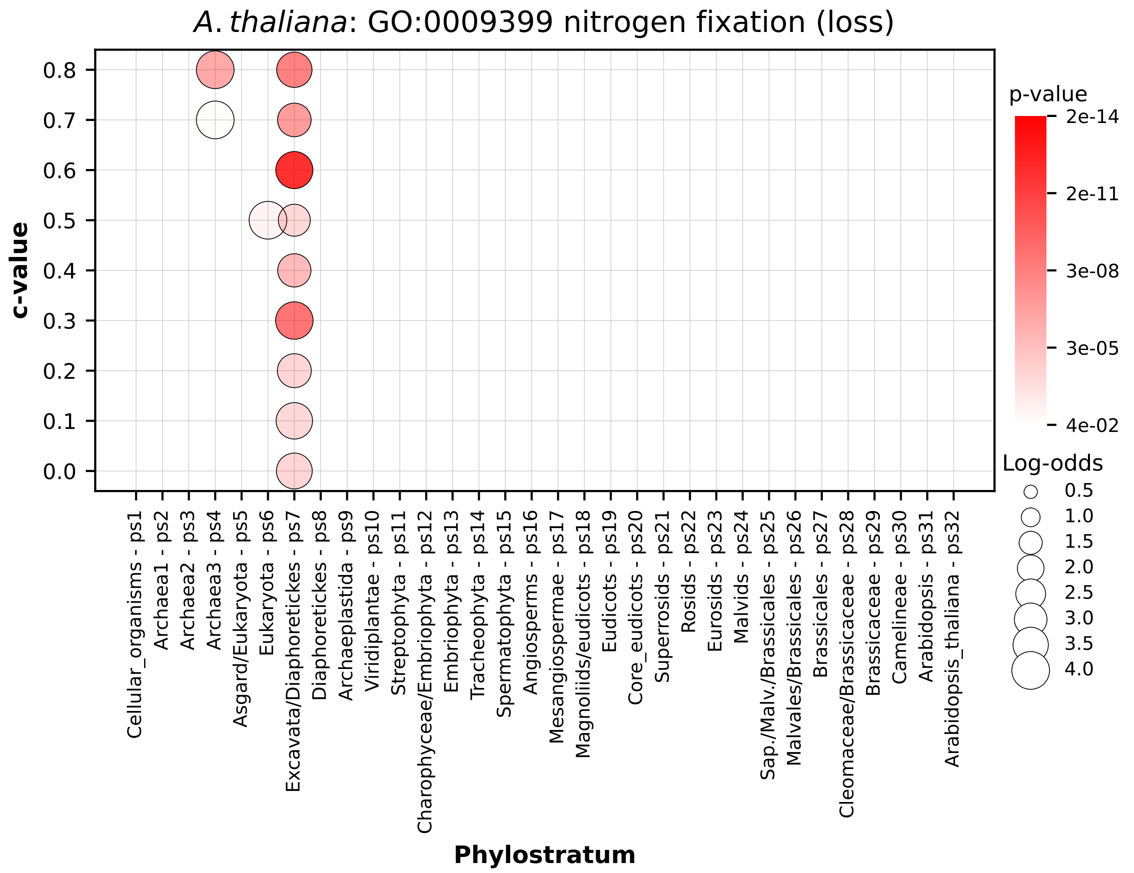
A. thaliana: GO:0009399 nitrogen fixation (loss)
c-value
0.8
0.7
0.6
0.5
0.4
0.3
0.2
0.1
0.0
Phylostratum
Cellular_organisms - ps1
Archaea1 - ps2
Archaea2 - ps3
Archaea3 - ps4
Asgard/Eukaryota - ps5
Eukaryota - ps6
Excavata/Diaphoretickes - ps7
Diaphoretickes - ps8
Archaeplastida - ps9
Viridiplantae - ps10
Streptophyta - ps11
Charophyceae/Embriophyta - ps12
Embriophyta - ps13
Tracheophyta - ps14
Spermatophyta - ps15
Angiosperms - ps16
Mesangiospermae - ps17
Magnoliids/eudicots - ps18
Eudicots - ps19
Core_eudicots - ps20
Superrosids - ps21
Rosids - ps22
Eurosids - ps23
Malvids - ps24
Sap./Malv./Brassicales - ps25
Malvales/Brassicales - ps26
Brassicales - ps27
Cleomaceae/Brassicaceae - ps28
Brassicaceae - ps29
Camelineae - ps30
Arabidopsis - ps31
Arabidopsis_thaliana - ps32
p-value
2e-14
2e-11
3e-08
3e-05
4e-02
Log-odds
0.5
1.0
1.5
2.0
2.5
3.0
3.5
4.0

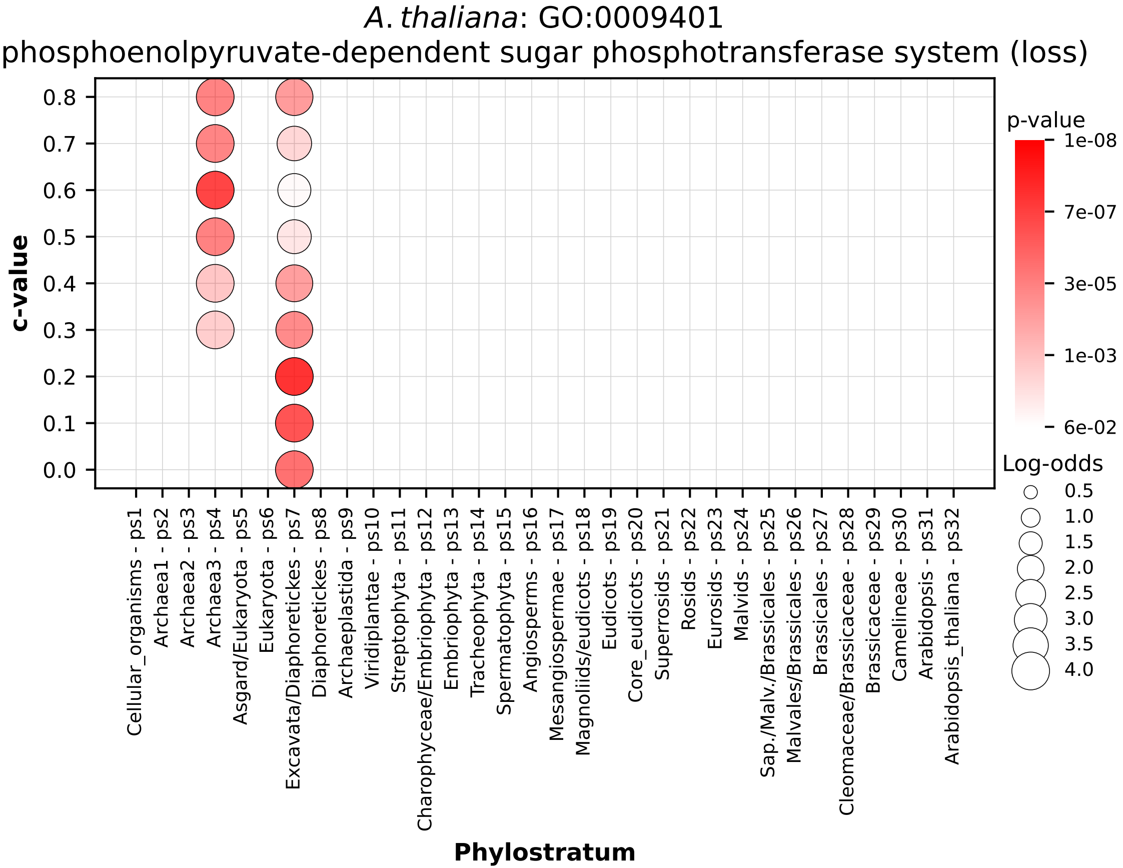

A. thaliana: GO:0009401
phosphoenolpyruvate-dependent sugar phosphotransferase system (loss)
c-value
0.8
0.7
0.6
0.5
0.4
0.3
0.2
0.1
0.0
p-value
1e-08
7e-07
3e-05
1e-03
6e-02
Log-odds
0.5
1.0
1.5
2.0
2.5
3.0
3.5
4.0
Cellular_organisms - ps1
Archaea1 - ps2
Archaea2 - ps3
Archaea3 - ps4
Asgard/Eukaryota - ps5
Eukaryota - ps6
Excavata/Diaphoretickes - ps7
Diaphoretickes - ps8
Archaeplastida - ps9
Viridiplantae - ps10
Streptophyta - ps11
Charophyceae/Embriophyta - ps12
Embriophyta - ps13
Tracheophyta - ps14
Spermatophyta - ps15
Angiosperms - ps16
Mesangiospermae - ps17
Magnoliids/eudicots - ps18
Eudicots - ps19
Core_eudicots - ps20
Superrosids - ps21
Rosids - ps22
Eurosids - ps23
Malvids - ps24
Sap./Malv./Brassicales - ps25
Malvales/Brassicales - ps26
Brassicales - ps27
Cleomaceae/Brassicaceae - ps28
Brassicaceae - ps29
Camelineae - ps30
Arabidopsis - ps31
Arabidopsis_thaliana - ps32
Phylostratum

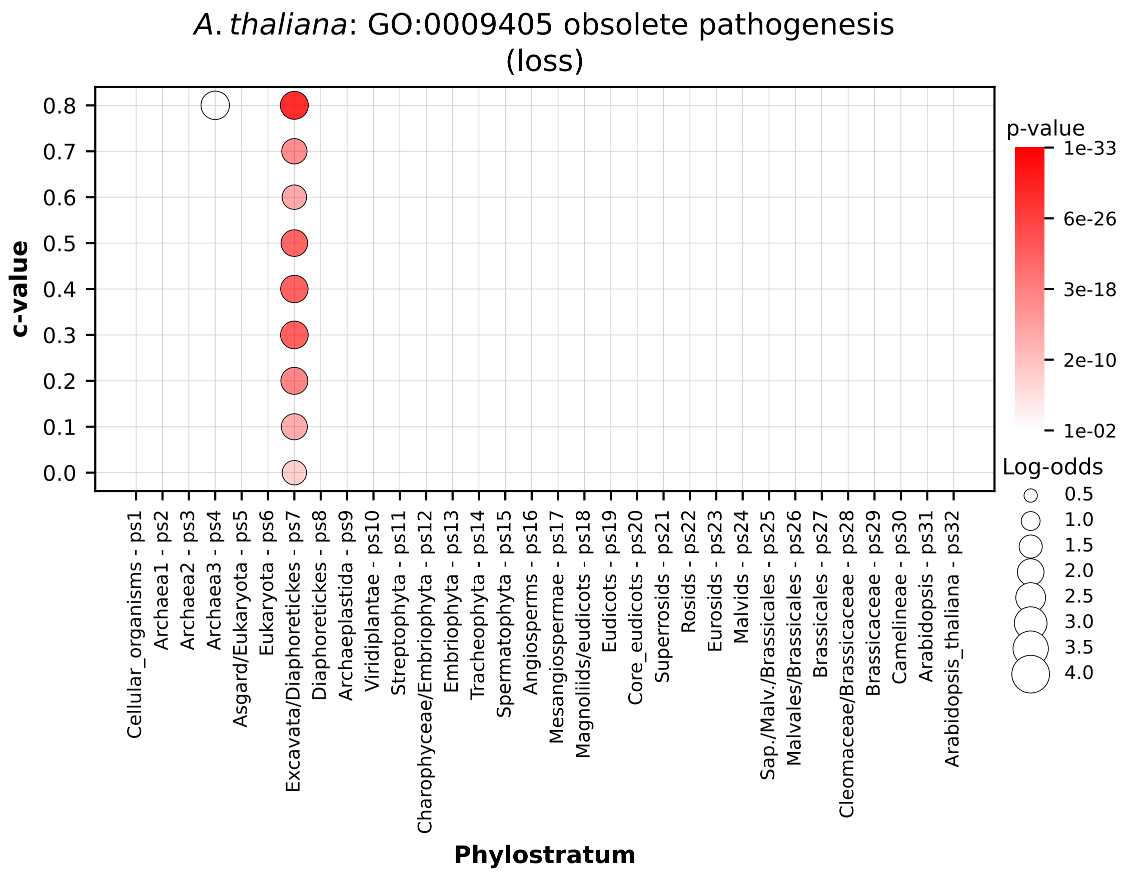

A. thaliana: GO:0009405 obsolete pathogenesis (loss)
c-value
0.8
0.7
0.6
0.5
0.4
0.3
0.2
0.1
0.0
p-value
1e-33
6e-26
3e-18
2e-10
1e-02
Log-odds
0.5
1.0
1.5
2.0
2.5
3.0
3.5
4.0
Cellular_organisms - ps1
Archaea1 - ps2
Archaea2 - ps3
Archaea3 - ps4
Asgard/Eukaryota - ps5
Eukaryota - ps6
Excavata/Diaphoretickes - ps7
Diaphoretickes - ps8
Archaeplastida - ps9
Viridiplantae - ps10
Streptophyta - ps11
Charophyceae/Embriophyta - ps12
Embriophyta - ps13
Tracheophyta - ps14
Spermatophyta - ps15
Angiosperms - ps16
Mesangiospermae - ps17
Magnoliids/eudicots - ps18
Eudicots - ps19
Core_eudicots - ps20
Superrosids - ps21
Rosids - ps22
Eurosids - ps23
Malvids - ps24
Sap./Malv./Brassicales - ps25
Malvales/Brassicales - ps26
Brassicales - ps27
Cleomaceae/Brassicaceae - ps28
Brassicaceae - ps29
Camelineae - ps30
Arabidopsis - ps31
Arabidopsis_thaliana - ps32
Phylostratum

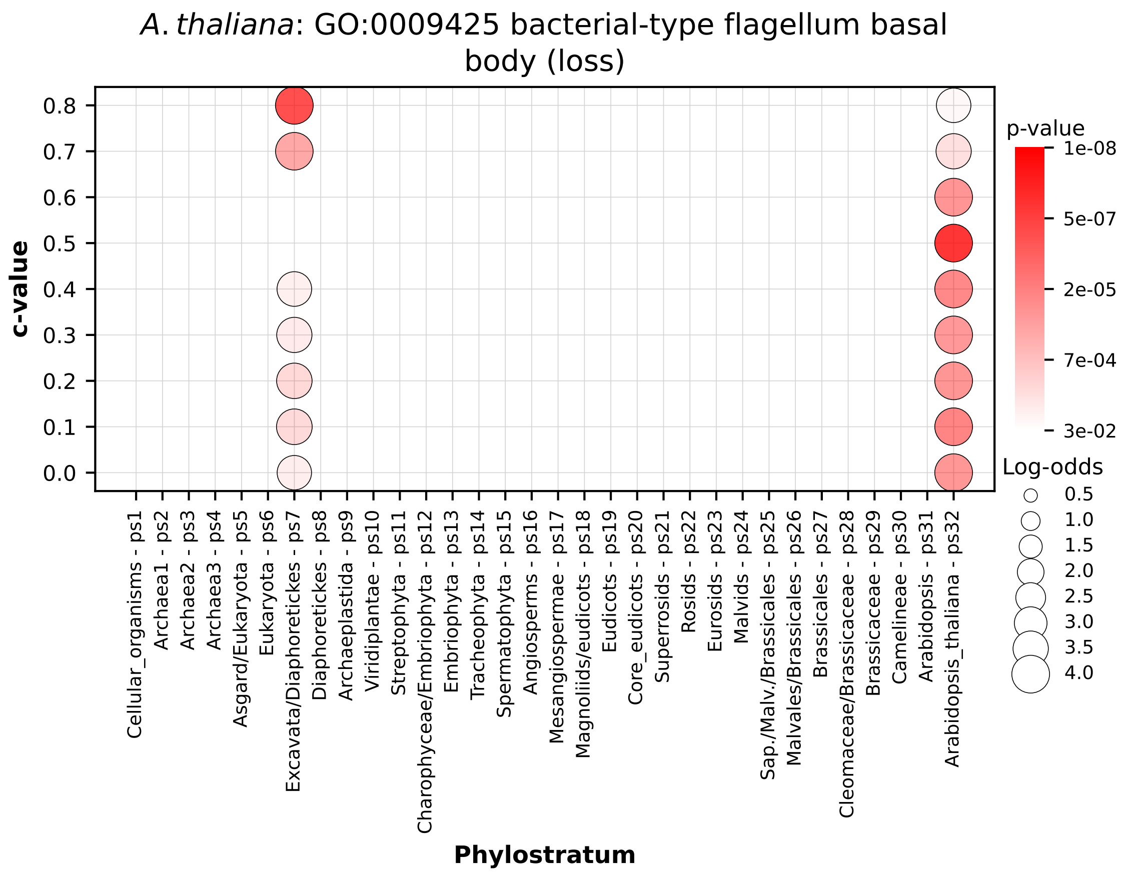

A. thaliana: GO:0009425 bacterial-type flagellum basal body (loss)
c-value
0.8
0.7
0.6
0.5
0.4
0.3
0.2
0.1
0.0
p-value
1e-08
5e-07
2e-05
7e-04
3e-02
Log-odds
0.5
1.0
1.5
2.0
2.5
3.0
3.5
4.0
Cellular_organisms - ps1
Archaea1 - ps2
Archaea2 - ps3
Archaea3 - ps4
Asgard/Eukaryota - ps5
Eukaryota - ps6
Excavata/Diaphoretickes - ps7
Diaphoretickes - ps8
Archaeplastida - ps9
Viridiplantae - ps10
Streptophyta - ps11
Charophyceae/Embriophyta - ps12
Embriophyta - ps13
Tracheophyta - ps14
Spermatophyta - ps15
Angiosperms - ps16
Mesangiospermae - ps17
Magnoliids/eudicots - ps18
Eudicots - ps19
Core_eudicots - ps20
Superrosids - ps21
Rosids - ps22
Eurosids - ps23
Malvids - ps24
Sap./Malv./Brassicales - ps25
Malvales/Brassicales - ps26
Brassicales - ps27
Cleomaceae/Brassicaceae - ps28
Brassicaceae - ps29
Camelineae - ps30
Arabidopsis - ps31
Arabidopsis_thaliana - ps32
Phylostratum

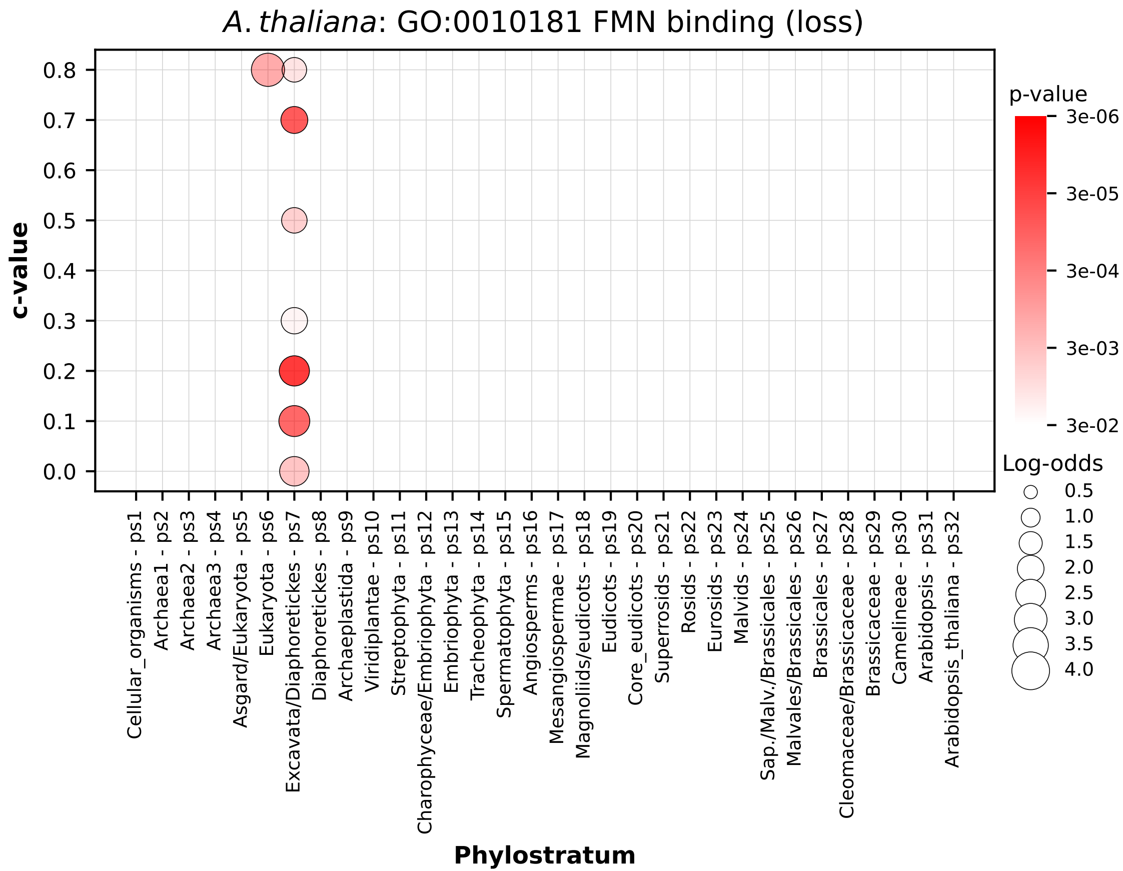

A. thaliana: GO:0010181 FMN binding (loss)
c-value
0.8
0.7
0.6
0.5
0.4
0.3
0.2
0.1
0.0
p-value
3e-06
3e-05
3e-04
3e-03
3e-02
Log-odds
0.5
1.0
1.5
2.0
2.5
3.0
3.5
4.0
Cellular_organisms - ps1
Archaea1 - ps2
Archaea2 - ps3
Archaea3 - ps4
Asgard/Eukaryota - ps5
Eukaryota - ps6
Excavata/Diaphoretickes - ps7
Diaphoretickes - ps8
Archaeplastida - ps9
Viridiplantae - ps10
Streptophyta - ps11
Charophyceae/Embriophyta - ps12
Embriophyta - ps13
Tracheophyta - ps14
Spermatophyta - ps15
Angiosperms - ps16
Mesangiospermae - ps17
Magnoliids/eudicots - ps18
Eudicots - ps19
Core_eudicots - ps20
Superrosids - ps21
Rosids - ps22
Eurosids - ps23
Malvids - ps24
Sap./Malv./Brassicales - ps25
Malvales/Brassicales - ps26
Brassicales - ps27
Cleomaceae/Brassicaceae - ps28
Brassicaceae - ps29
Camelineae - ps30
Arabidopsis - ps31
Arabidopsis_thaliana - ps32
Phylostratum

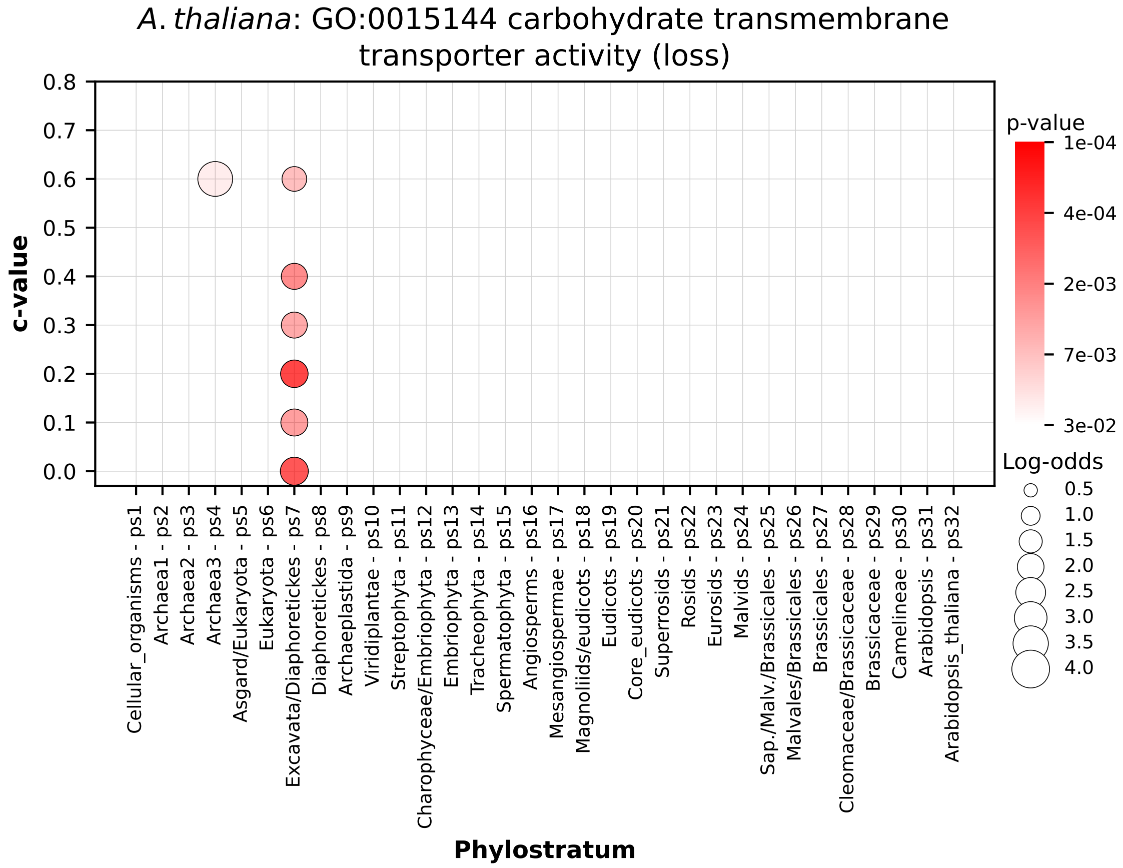

A. thaliana: GO:0015144 carbohydrate transmembrane transporter activity (loss)
c-value
0.8
0.7
0.6
0.5
0.4
0.3
0.2
0.1
0.0
Phylostratum
Cellular_organisms - ps1
Archaea1 - ps2
Archaea2 - ps3
Archaea3 - ps4
Asgard/Eukaryota - ps5
Eukaryota - ps6
Excavata/Diaphoretickes - ps7
Diaphoretickes - ps8
Archaeplastida - ps9
Viridiplantae - ps10
Streptophyta - ps11
Charophyceae/Embriophyta - ps12
Embriophyta - ps13
Tracheophyta - ps14
Spermatophyta - ps15
Angiosperms - ps16
Mesangiospermae - ps17
Magnoliids/eudicots - ps18
Eudicots - ps19
Core_eudicots - ps20
Superrosids - ps21
Rosids - ps22
Eurosids - ps23
Malvids - ps24
Sap./Malv./Brassicales - ps25
Malvales/Brassicales - ps26
Brassicales - ps27
Cleomaceae/Brassicaceae - ps28
Brassicaceae - ps29
Camelineae - ps30
Arabidopsis - ps31
Arabidopsis_thaliana - ps32
p-value
1e-04
4e-04
2e-03
7e-03
3e-02
Log-odds
0.5
1.0
1.5
2.0
2.5
3.0
3.5
4.0

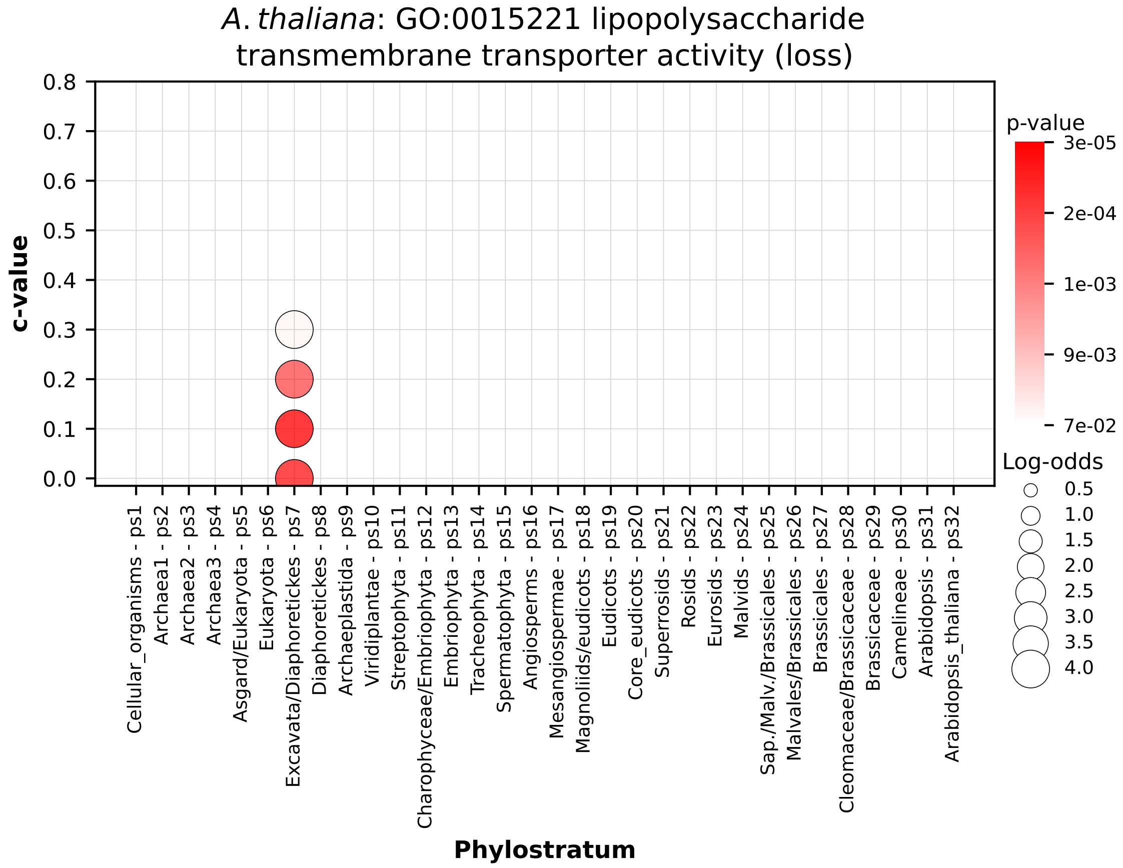

A. thaliana: GO:0015221 lipopolysaccharide transmembrane transporter activity (loss)
c-value
0.8
0.7
0.6
0.5
0.4
0.3
0.2
0.1
0.0
Phylostratum
Cellular_organisms - ps1
Archaea1 - ps2
Archaea2 - ps3
Archaea3 - ps4
Asgard/Eukaryota - ps5
Eukaryota - ps6
Excavata/Diaphoretickes - ps7
Diaphoretickes - ps8
Archaeplastida - ps9
Viridiplantae - ps10
Streptophyta - ps11
Charophyceae/Embriophyta - ps12
Embriophyta - ps13
Tracheophyta - ps14
Spermatophyta - ps15
Angiosperms - ps16
Mesangiospermae - ps17
Magnoliids/eudicots - ps18
Eudicots - ps19
Core_eudicots - ps20
Superrosids - ps21
Rosids - ps22
Eurosids - ps23
Malvids - ps24
Sap./Malv./Brassicales - ps25
Malvales/Brassicales - ps26
Brassicales - ps27
Cleomaceae/Brassicaceae - ps28
Brassicaceae - ps29
Camelineae - ps30
Arabidopsis - ps31
Arabidopsis_thaliana - ps32
p-value
3e-05
2e-04
1e-03
9e-03
7e-02
Log-odds
0.5
1.0
1.5
2.0
2.5
3.0
3.5
4.0

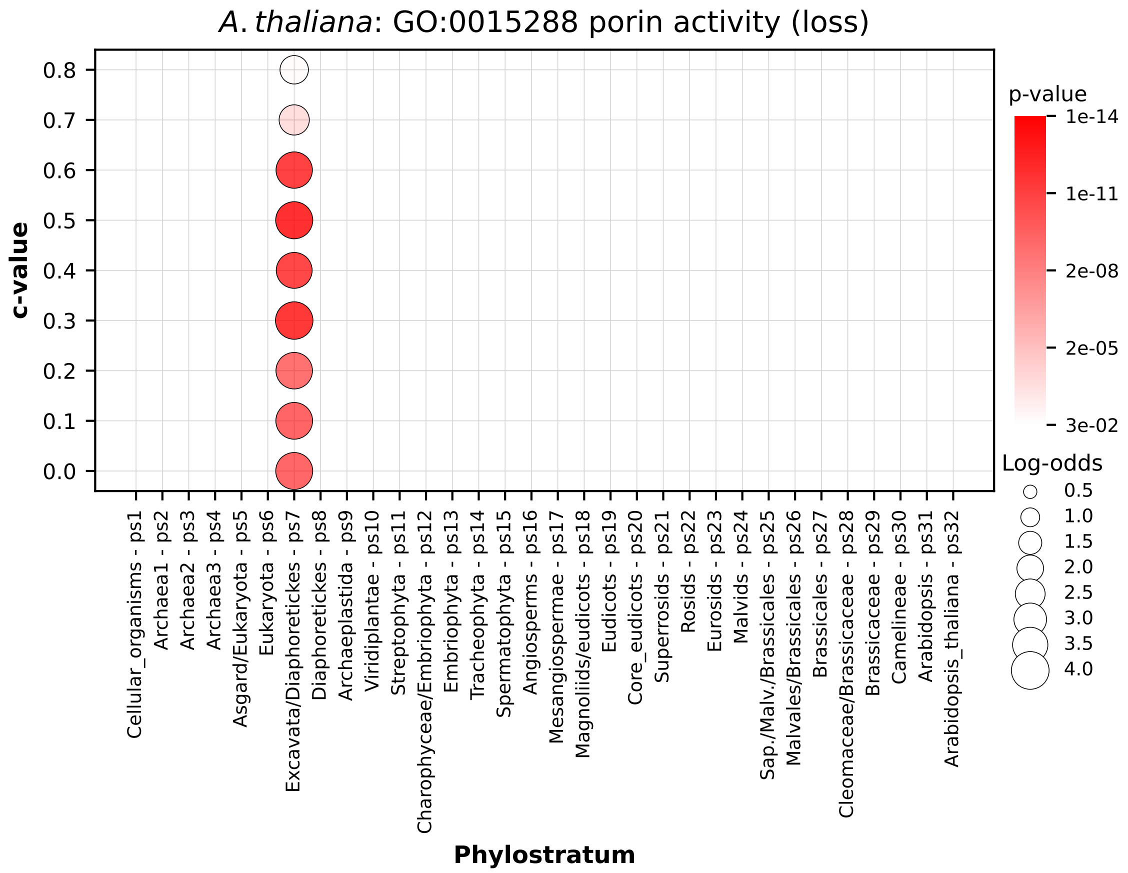

A. thaliana: GO:0015288 porin activity (loss)
c-value
0.8
0.7
0.6
0.5
0.4
0.3
0.2
0.1
0.0
Phylostratum
Cellular_organisms - ps1
Archaea1 - ps2
Archaea2 - ps3
Archaea3 - ps4
Asgard/Eukaryota - ps5
Eukaryota - ps6
Excavata/Diaphoretickes - ps7
Diaphoretickes - ps8
Archaeplastida - ps9
Viridiplantae - ps10
Streptophyta - ps11
Charophyceae/Embriophyta - ps12
Embriophyta - ps13
Tracheophyta - ps14
Spermatophyta - ps15
Angiosperms - ps16
Mesangiospermae - ps17
Magnoliids/eudicots - ps18
Eudicots - ps19
Core_eudicots - ps20
Superrosids - ps21
Rosids - ps22
Eurosids - ps23
Malvids - ps24
Sap./Malv./Brassicales - ps25
Malvales/Brassicales - ps26
Brassicales - ps27
Cleomaceae/Brassicaceae - ps28
Brassicaceae - ps29
Camelineae - ps30
Arabidopsis - ps31
Arabidopsis_thaliana - ps32
p-value
1e-14
1e-11
2e-08
2e-05
3e-02
Log-odds
0.5
1.0
1.5
2.0
2.5
3.0
3.5
4.0

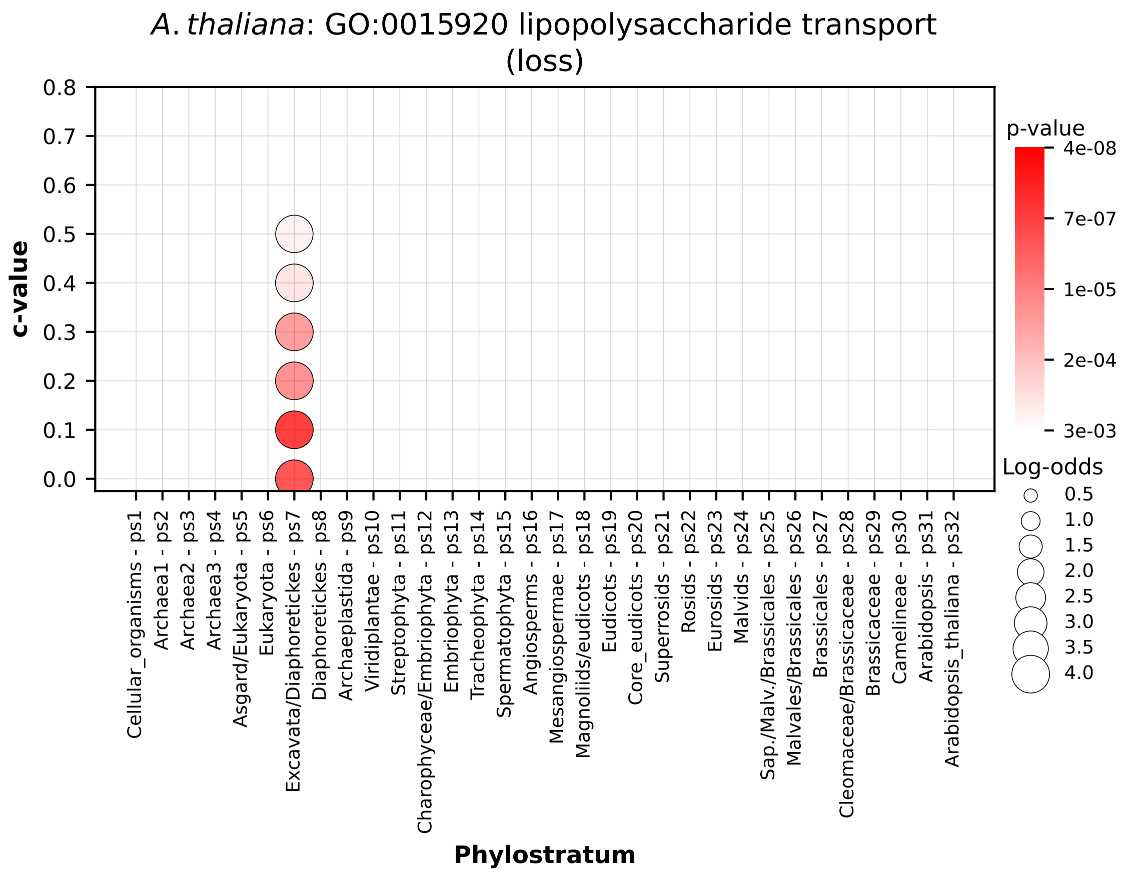

A. thaliana: GO:0015920 lipopolysaccharide transport
(loss)
c-value
0.8
0.7
0.6
0.5
0.4
0.3
0.2
0.1
0.0
p-value
4e-08
7e-07
1e-05
2e-04
3e-03
Log-odds
0.5
1.0
1.5
2.0
2.5
3.0
3.5
4.0
Cellular_organisms - ps1
Archaea1 - ps2
Archaea2 - ps3
Archaea3 - ps4
Asgard/Eukaryota - ps5
Eukaryota - ps6
Excavata/Diaphoretickes - ps7
Diaphoretickes - ps8
Archaeplastida - ps9
Viridiplantae - ps10
Streptophyta - ps11
Charophyceae/Embriophyta - ps12
Embriophyta - ps13
Tracheophyta - ps14
Spermatophyta - ps15
Angiosperms - ps16
Mesangiospermae - ps17
Magnoliids/eudicots - ps18
Eudicots - ps19
Core_eudicots - ps20
Superrosids - ps21
Rosids - ps22
Eurosids - ps23
Malvids - ps24
Sap./Malv./Brassicales - ps25
Malvales/Brassicales - ps26
Brassicales - ps27
Cleomaceae/Brassicaceae - ps28
Brassicaceae - ps29
Camelineae - ps30
Arabidopsis - ps31
Arabidopsis_thaliana - ps32
Phylostratum

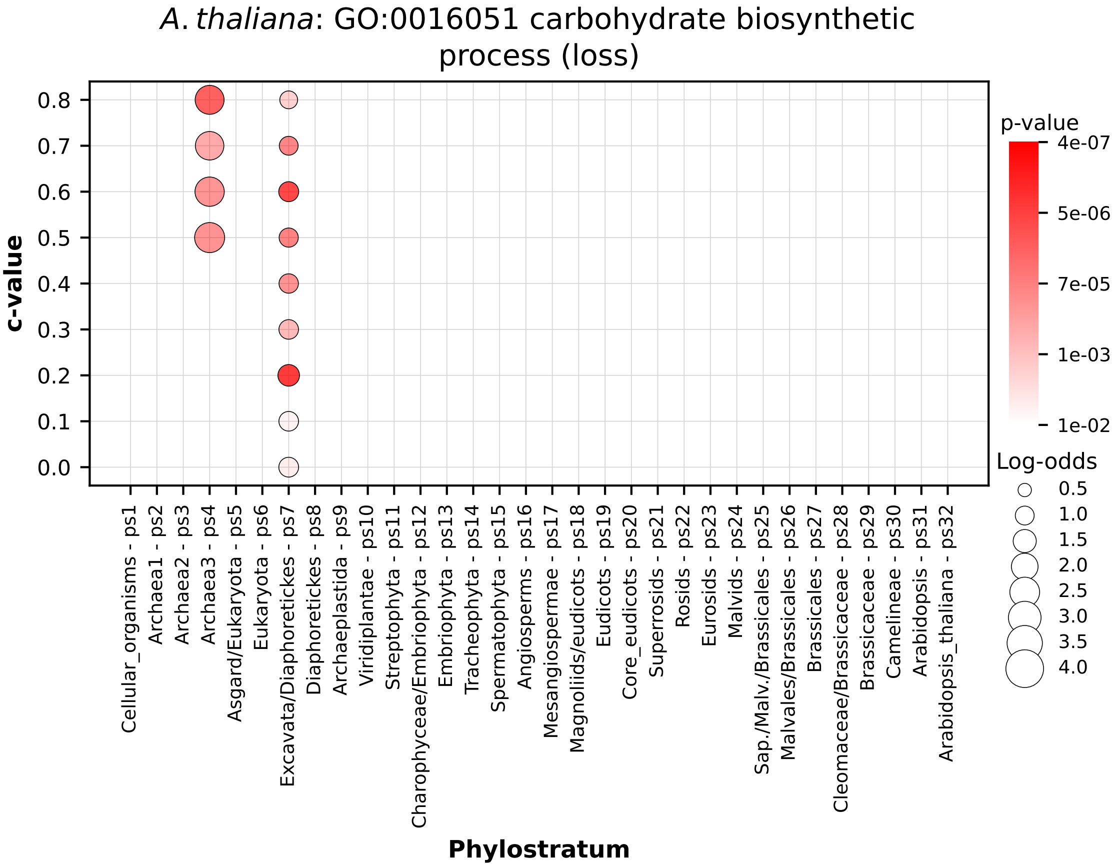

A. thaliana: GO:0016051 carbohydrate biosynthetic process (loss)
c-value
0.8
0.7
0.6
0.5
0.4
0.3
0.2
0.1
0.0
p-value
4e-07
5e-06
7e-05
1e-03
1e-02
Log-odds
0.5
1.0
1.5
2.0
2.5
3.0
3.5
4.0
Cellular_organisms - ps1
Archaea1 - ps2
Archaea2 - ps3
Archaea3 - ps4
Asgard/Eukaryota - ps5
Eukaryota - ps6
Excavata/Diaphoretickes - ps7
Diaphoretickes - ps8
Archaeplastida - ps9
Viridiplantae - ps10
Streptophyta - ps11
Charophyceae/Embriophyta - ps12
Embriophyta - ps13
Tracheophyta - ps14
Spermatophyta - ps15
Angiosperms - ps16
Mesangiospermae - ps17
Magnoliids/eudicots - ps18
Eudicots - ps19
Core_eudicots - ps20
Superrosids - ps21
Rosids - ps22
Eurosids - ps23
Malvids - ps24
Sap./Malv./Brassicales - ps25
Malvales/Brassicales - ps26
Brassicales - ps27
Cleomaceae/Brassicaceae - ps28
Brassicaceae - ps29
Camelineae - ps30
Arabidopsis - ps31
Arabidopsis_thaliana - ps32
Phylostratum

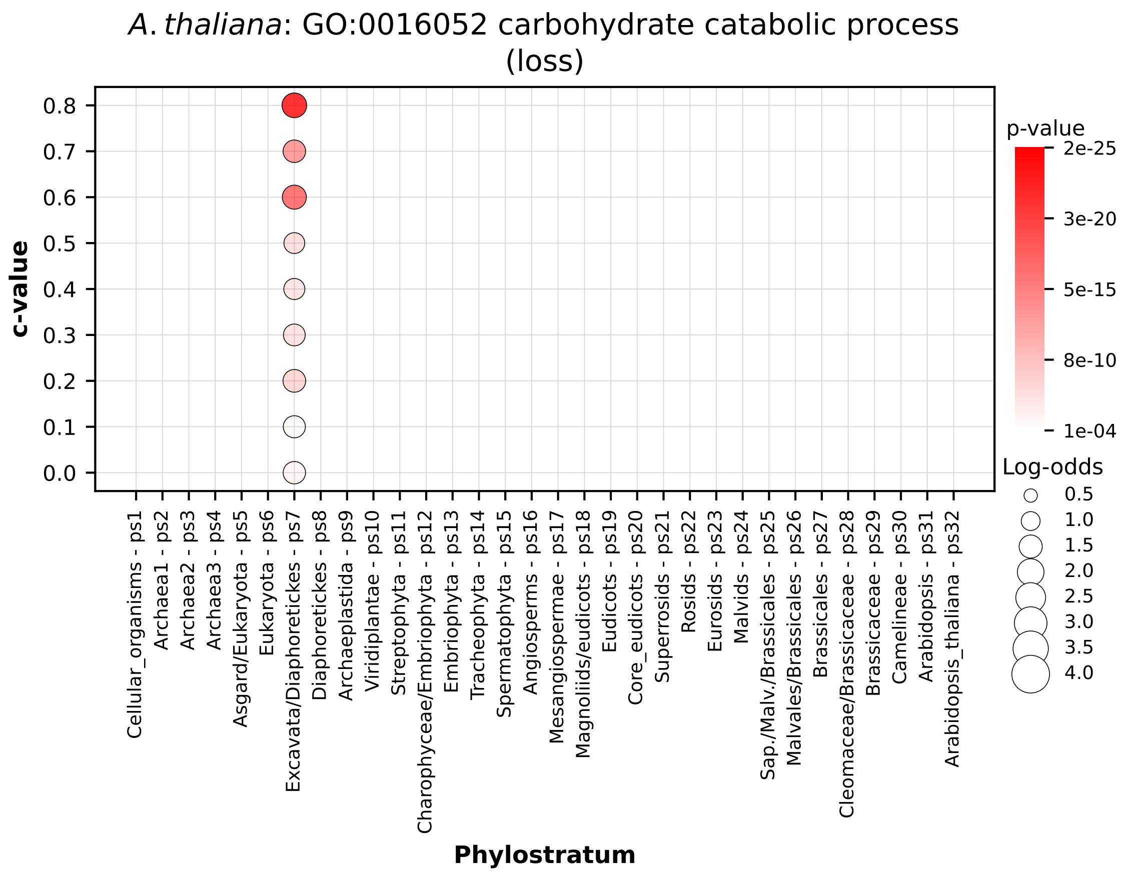
A. thaliana: GO:0016052 carbohydrate catabolic process
(loss)
c-value
0.8
0.7
0.6
0.5
0.4
0.3
0.2
0.1
0.0
p-value
2e-25
3e-20
5e-15
8e-10
1e-04
Log-odds
0.5
1.0
1.5
2.0
2.5
3.0
3.5
4.0
Cellular_organisms - ps1
Archaea1 - ps2
Archaea2 - ps3
Archaea3 - ps4
Asgard/Eukaryota - ps5
Eukaryota - ps6
Excavata/Diaphoretickes - ps7
Diaphoretickes - ps8
Archaeplastida - ps9
Viridiplantae - ps10
Streptophyta - ps11
Charophyceae/Embriophyta - ps12
Embriophyta - ps13
Tracheophyta - ps14
Spermatophyta - ps15
Angiosperms - ps16
Mesangiospermae - ps17
Magnoliids/eudicots - ps18
Eudicots - ps19
Core_eudicots - ps20
Superrosids - ps21
Rosids - ps22
Eurosids - ps23
Malvids - ps24
Sap./Malv./Brassicales - ps25
Malvales/Brassicales - ps26
Brassicales - ps27
Cleomaceae/Brassicaceae - ps28
Brassicaceae - ps29
Camelineae - ps30
Arabidopsis - ps31
Arabidopsis_thaliana - ps32
Phylostratum

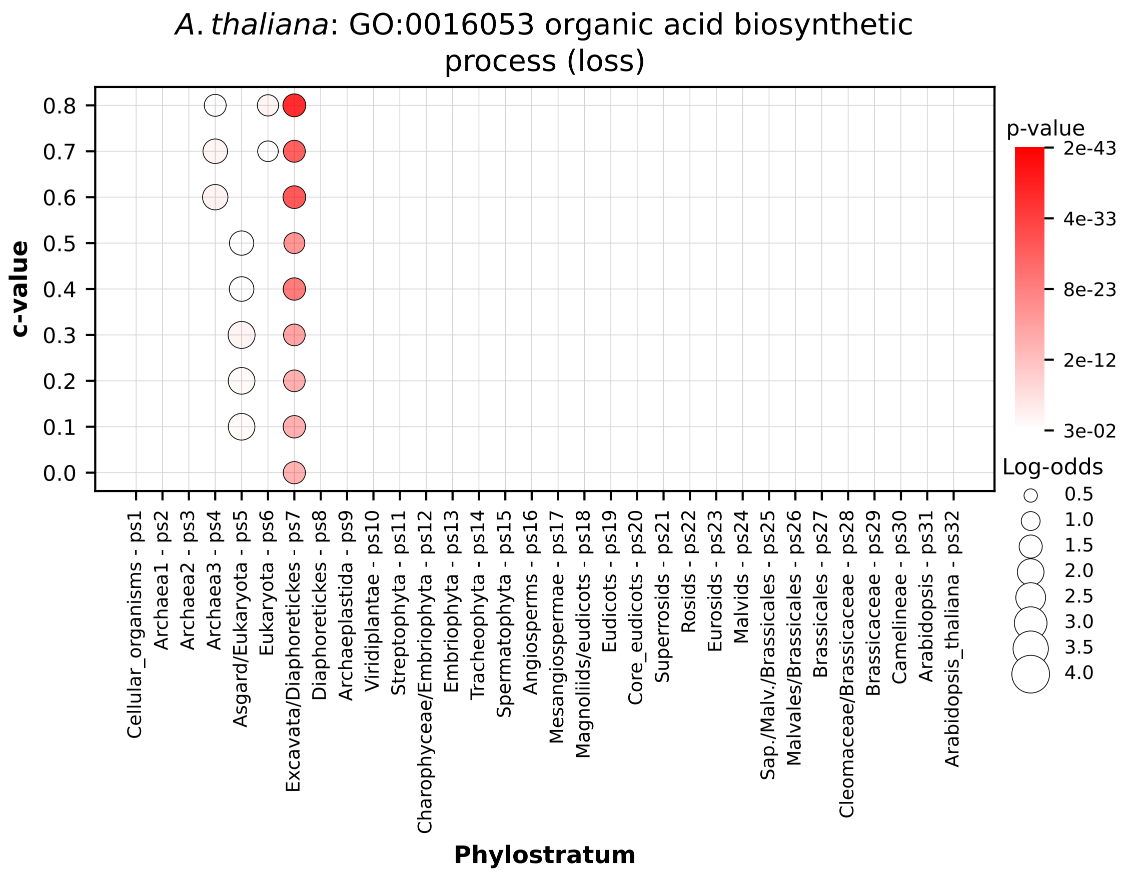

A. thaliana: GO:0016053 organic acid biosynthetic process (loss)
c-value
0.8
0.7
0.6
0.5
0.4
0.3
0.2
0.1
0.0
p-value
2e-43
4e-33
8e-23
2e-12
3e-02
Log-odds
0.5
1.0
1.5
2.0
2.5
3.0
3.5
4.0
Cellular_organisms - ps1
Archaea1 - ps2
Archaea2 - ps3
Archaea3 - ps4
Asgard/Eukaryota - ps5
Eukaryota - ps6
Excavata/Diaphoretickes - ps7
Diaphoretickes - ps8
Archaeplastida - ps9
Viridiplantae - ps10
Streptophyta - ps11
Charophyceae/Embriophyta - ps12
Embriophyta - ps13
Tracheophyta - ps14
Spermatophyta - ps15
Angiosperms - ps16
Mesangiospermae - ps17
Magnoliids/eudicots - ps18
Eudicots - ps19
Core_eudicots - ps20
Superrosids - ps21
Rosids - ps22
Eurosids - ps23
Malvids - ps24
Sap./Malv./Brassicales - ps25
Malvales/Brassicales - ps26
Brassicales - ps27
Cleomaceae/Brassicaceae - ps28
Brassicaceae - ps29
Camelineae - ps30
Arabidopsis - ps31
Arabidopsis_thaliana - ps32
Phylostratum

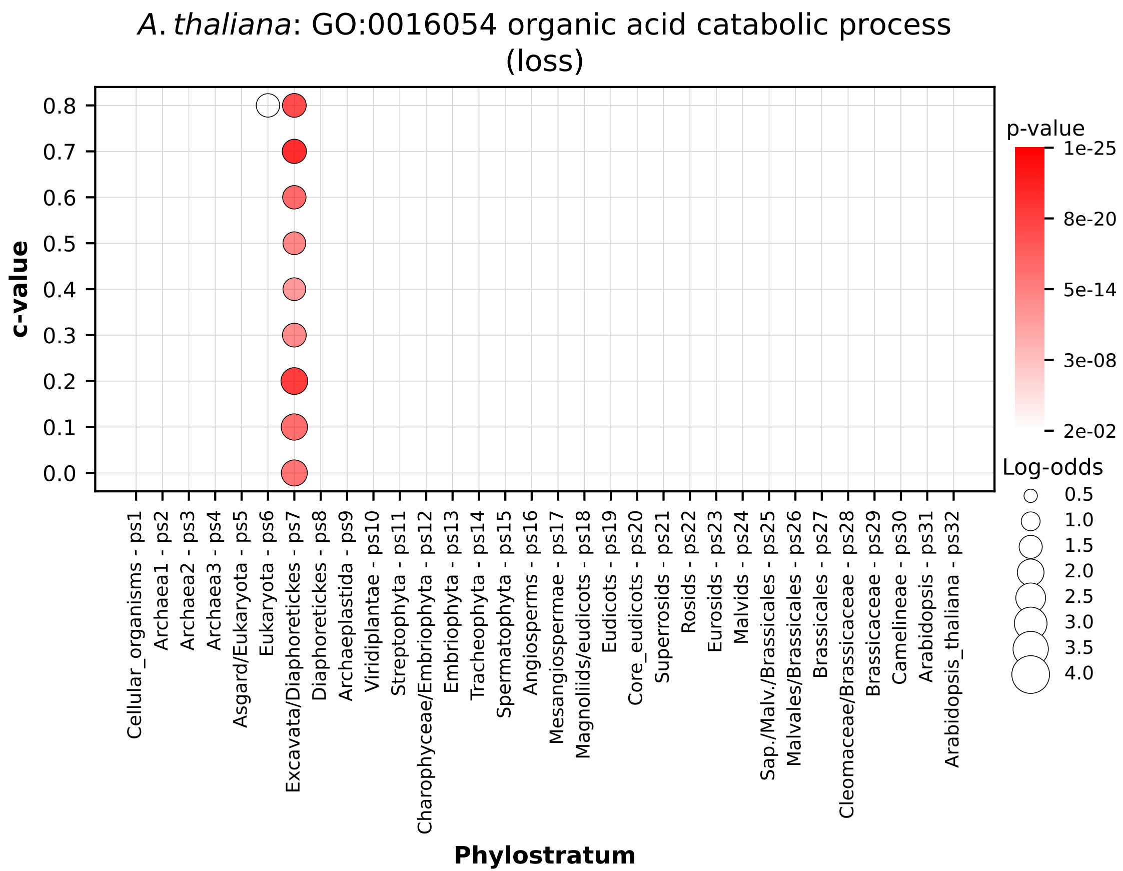
A. thaliana: GO:0016054 organic acid catabolic process
(loss)
c-value
0.8
0.7
0.6
0.5
0.4
0.3
0.2
0.1
0.0
Phylostratum
Cellular_organisms - ps1
Archaea1 - ps2
Archaea2 - ps3
Archaea3 - ps4
Asgard/Eukaryota - ps5
Eukaryota - ps6
Excavata/Diaphoretickes - ps7
Diaphoretickes - ps8
Archaeplastida - ps9
Viridiplantae - ps10
Streptophyta - ps11
Charophyceae/Embriophyta - ps12
Embriophyta - ps13
Tracheophyta - ps14
Spermatophyta - ps15
Angiosperms - ps16
Mesangiospermae - ps17
Magnoliids/eudicots - ps18
Eudicots - ps19
Core_eudicots - ps20
Superrosids - ps21
Rosids - ps22
Eurosids - ps23
Malvids - ps24
Sap./Malv./Brassicales - ps25
Malvales/Brassicales - ps26
Brassicales - ps27
Cleomaceae/Brassicaceae - ps28
Brassicaceae - ps29
Camelineae - ps30
Arabidopsis - ps31
Arabidopsis_thaliana - ps32
p-value
1e-25
8e-20
5e-14
3e-08
2e-02
Log-odds
0.5
1.0
1.5
2.0
2.5
3.0
3.5
4.0

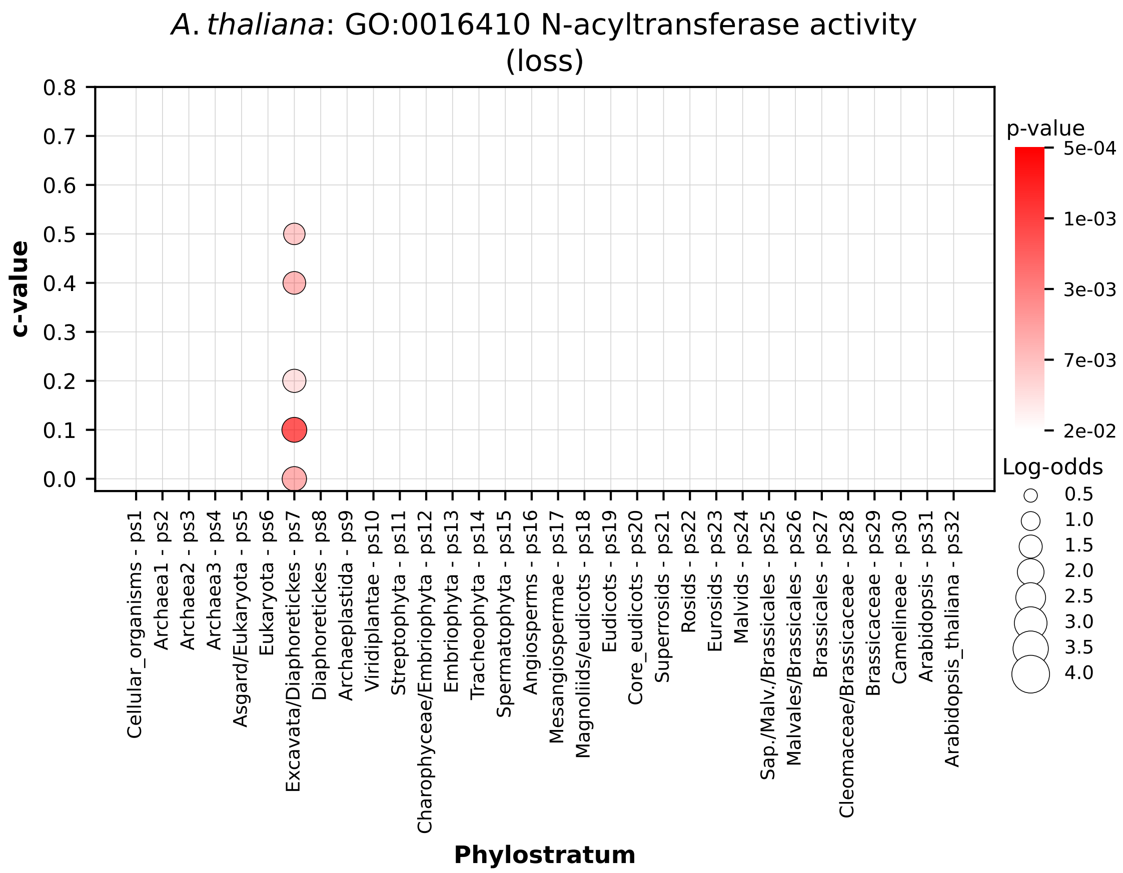

A. thaliana: GO:0016410 N-acyltransferase activity
(loss)
c-value
0.8
0.7
0.6
0.5
0.4
0.3
0.2
0.1
0.0
p-value
5e-04
1e-03
3e-03
7e-03
2e-02
Log-odds
0.5
1.0
1.5
2.0
2.5
3.0
3.5
4.0
Cellular_organisms - ps1
Archaea1 - ps2
Archaea2 - ps3
Archaea3 - ps4
Asgard/Eukaryota - ps5
Eukaryota - ps6
Excavata/Diaphoretickes - ps7
Diaphoretickes - ps8
Archaeplastida - ps9
Viridiplantae - ps10
Streptophyta - ps11
Charophyceae/Embriophyta - ps12
Embriophyta - ps13
Tracheophyta - ps14
Spermatophyta - ps15
Angiosperms - ps16
Mesangiospermae - ps17
Magnoliids/eudicots - ps18
Eudicots - ps19
Core_eudicots - ps20
Superrosids - ps21
Rosids - ps22
Eurosids - ps23
Malvids - ps24
Sap./Malv./Brassicales - ps25
Malvales/Brassicales - ps26
Brassicales - ps27
Cleomaceae/Brassicaceae - ps28
Brassicaceae - ps29
Camelineae - ps30
Arabidopsis - ps31
Arabidopsis_thaliana - ps32
Phylostratum

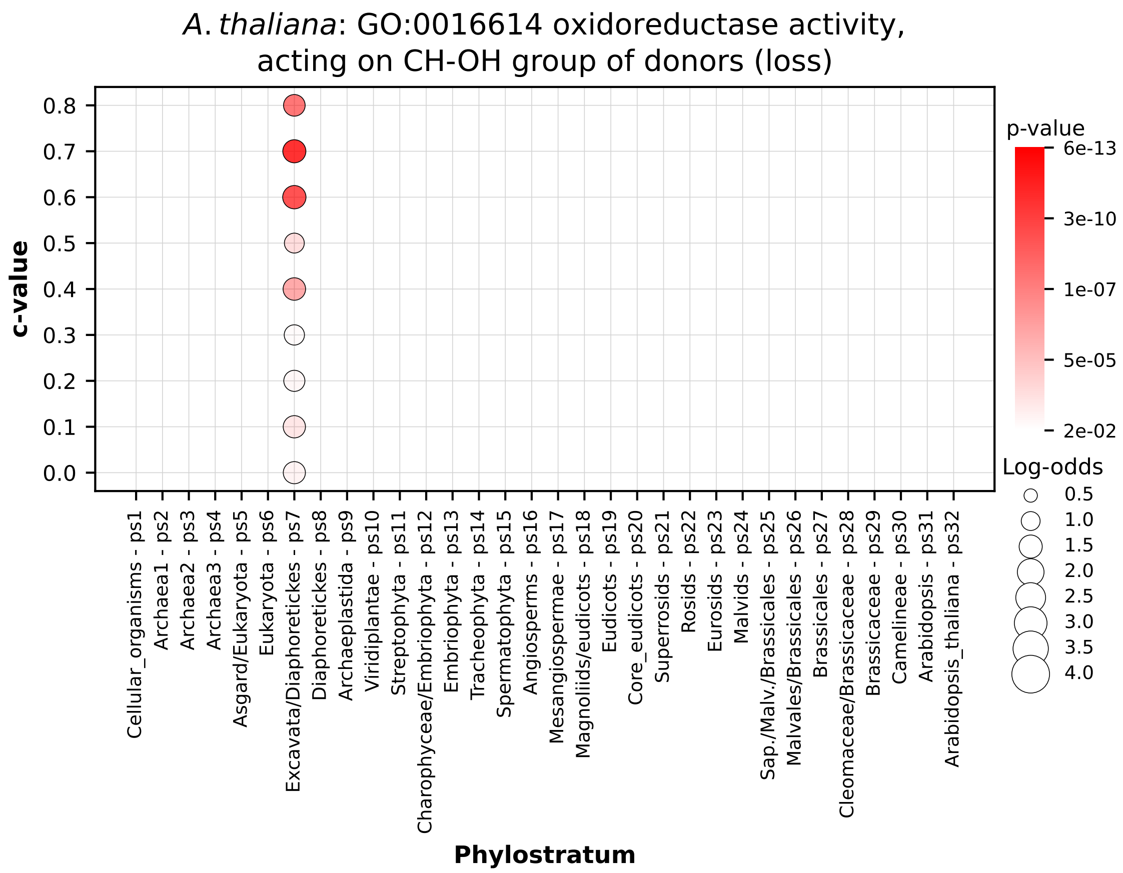

A. thaliana: GO:0016614 oxidoreductase activity, acting on CH-OH group of donors (loss)
c-value
0.0
0.1
0.2
0.3
0.4
0.5
0.6
0.7
0.8
p-value
6e-13
3e-10
1e-07
5e-05
2e-02
Log-odds
0.5
1.0
1.5
2.0
2.5
3.0
3.5
4.0
Cellular_organisms - ps1
Archaea1 - ps2
Archaea2 - ps3
Archaea3 - ps4
Asgard/Eukaryota - ps5
Eukaryota - ps6
Excavata/Diaphoretickes - ps7
Diaphoretickes - ps8
Archaeplastida - ps9
Viridiplantae - ps10
Streptophyta - ps11
Charophyceae/Embriophyta - ps12
Embriophyta - ps13
Tracheophyta - ps14
Spermatophyta - ps15
Angiosperms - ps16
Mesangiospermae - ps17
Magnoliids/eudicots - ps18
Eudicots - ps19
Core_eudicots - ps20
Superrosids - ps21
Rosids - ps22
Eurosids - ps23
Malvids - ps24
Sap./Malv./Brassicales - ps25
Malvales/Brassicales - ps26
Brassicales - ps27
Cleomaceae/Brassicaceae - ps28
Brassicaceae - ps29
Camelineae - ps30
Arabidopsis - ps31
Arabidopsis_thaliana - ps32
Phylostratum

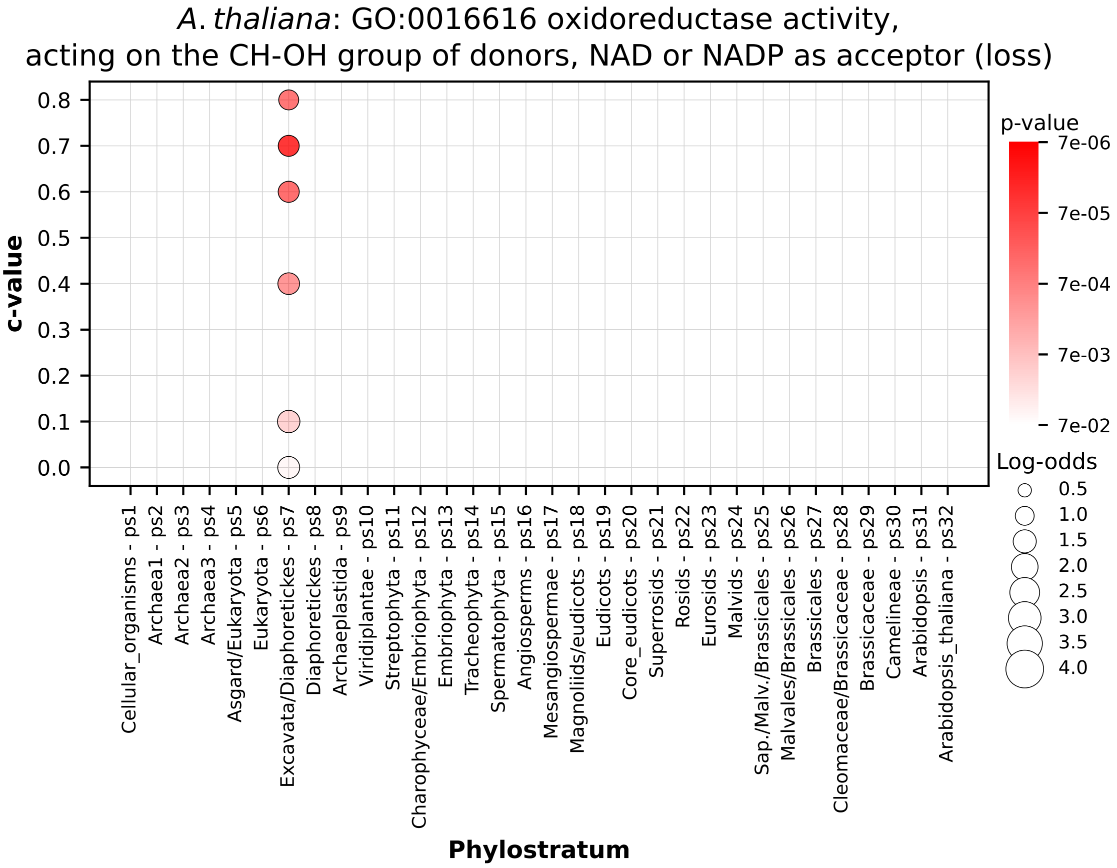
A. thaliana: GO:0016616 oxidoreductase activity,
acting on the CH-OH group of donors, NAD or NADP as acceptor (loss)
c-value
0.8
0.7
0.6
0.5
0.4
0.3
0.2
0.1
0.0
p-value
7e-06
7e-05
7e-04
7e-03
7e-02
Log-odds
0.5
1.0
1.5
2.0
2.5
3.0
3.5
4.0
Cellular_organisms - ps1
Archaea1 - ps2
Archaea2 - ps3
Archaea3 - ps4
Asgard/Eukaryota - ps5
Eukaryota - ps6
Excavata/Diaphoretickes - ps7
Diaphoretickes - ps8
Archaeplastida - ps9
Viridiplantae - ps10
Streptophyta - ps11
Charophyceae/Embriophyta - ps12
Embriophyta - ps13
Tracheophyta - ps14
Spermatophyta - ps15
Angiosperms - ps16
Mesangiospermae - ps17
Magnoliids/eudicots - ps18
Eudicots - ps19
Core_eudicots - ps20
Superrosids - ps21
Rosids - ps22
Eurosids - ps23
Malvids - ps24
Sap./Malv./Brassicales - ps25
Malvales/Brassicales - ps26
Brassicales - ps27
Cleomaceae/Brassicaceae - ps28
Brassicaceae - ps29
Camelineae - ps30
Arabidopsis - ps31
Arabidopsis_thaliana - ps32
Phylostratum

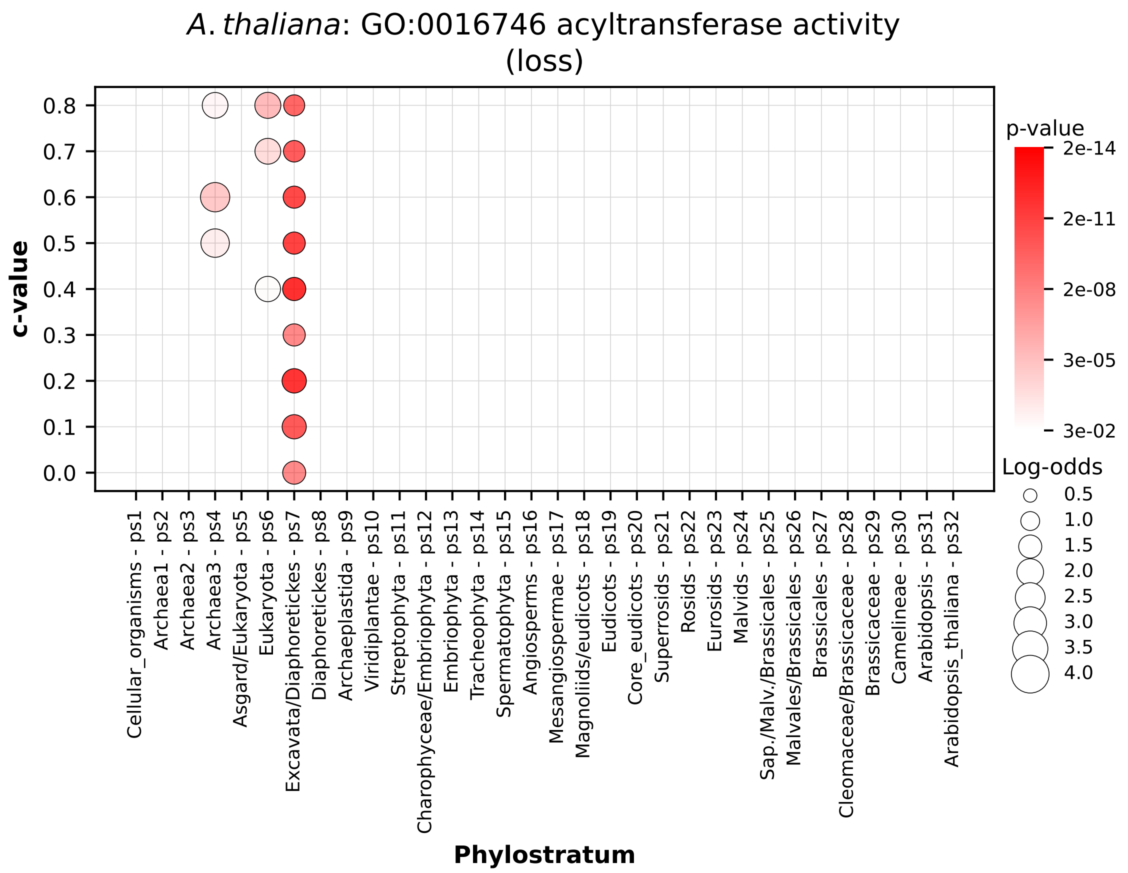

$A. thaliana$: GO:0016746 acyltransferase activity
(loss)
c-value
0.8
0.7
0.6
0.5
0.4
0.3
0.2
0.1
0.0
Phylostratum
Cellular_organisms - ps1
Archaea1 - ps2
Archaea2 - ps3
Archaea3 - ps4
Asgard/Eukaryota - ps5
Eukaryota - ps6
Excavata/Diaphoretickes - ps7
Diaphoretickes - ps8
Archaeplastida - ps9
Viridiplantae - ps10
Streptophyta - ps11
Charophyceae/Embriophyta - ps12
Embriophyta - ps13
Tracheophyta - ps14
Spermatophyta - ps15
Angiosperms - ps16
Mesangiospermae - ps17
Magnoliids/eudicots - ps18
Eudicots - ps19
Core_eudicots - ps20
Superrosids - ps21
Rosids - ps22
Eurosids - ps23
Malvids - ps24
Sap./Malv./Brassicales - ps25
Malvales/Brassicales - ps26
Brassicales - ps27
Cleomaceae/Brassicaceae - ps28
Brassicaceae - ps29
Camelineae - ps30
Arabidopsis - ps31
Arabidopsis_thaliana - ps32
p-value
2e-14
2e-11
2e-08
3e-05
3e-02
Log-odds
0.5
1.0
1.5
2.0
2.5
3.0
3.5
4.0

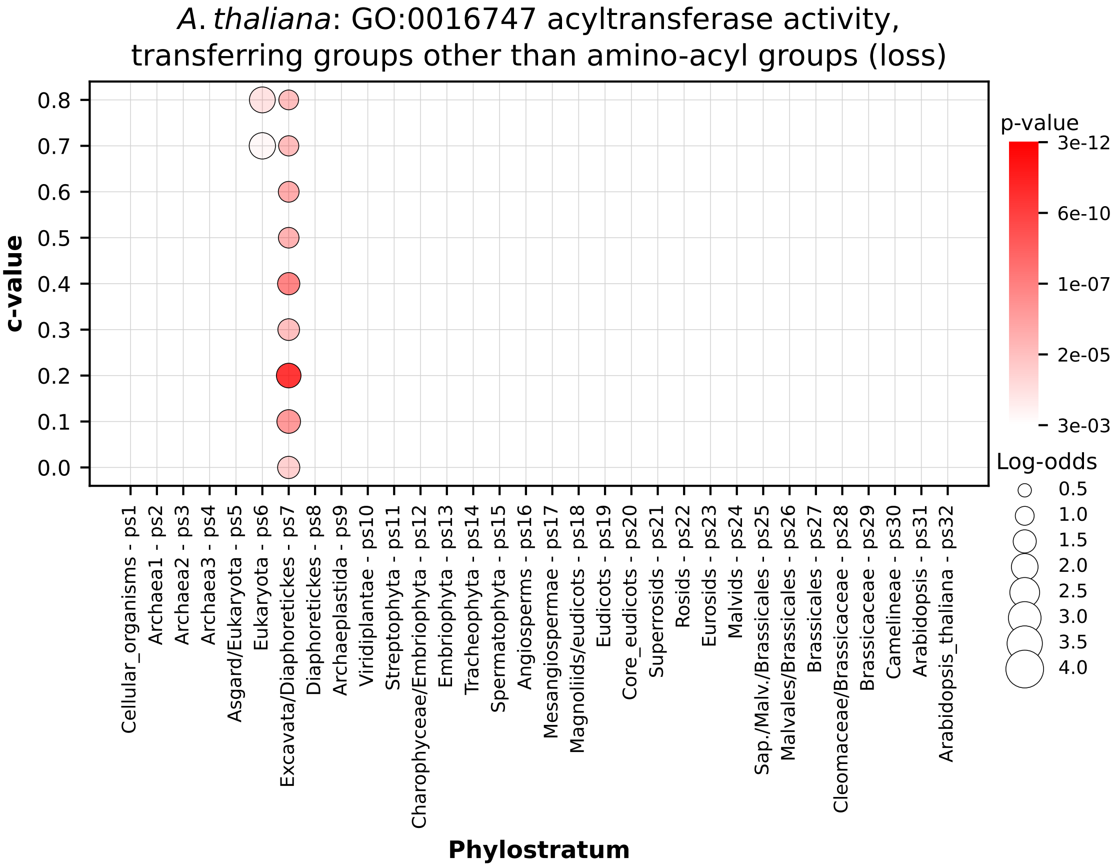
A. thaliana: GO:0016747 acyltransferase activity, transferring groups other than amino-acyl groups (loss)
c-value
0.8
0.7
0.6
0.5
0.4
0.3
0.2
0.1
0.0
Phylostratum
Cellular_organisms - ps1
Archaea1 - ps2
Archaea2 - ps3
Archaea3 - ps4
Asgard/Eukaryota - ps5
Eukaryota - ps6
Excavata/Diaphoretickes - ps7
Diaphoretickes - ps8
Archaeplastida - ps9
Viridiplantae - ps10
Streptophyta - ps11
Charophyceae/Embriophyta - ps12
Embriophyta - ps13
Tracheophyta - ps14
Spermatophyta - ps15
Angiosperms - ps16
Mesangiospermae - ps17
Magnoliids/eudicots - ps18
Eudicots - ps19
Core_eudicots - ps20
Superrosids - ps21
Rosids - ps22
Eurosids - ps23
Malvids - ps24
Sap./Malv./Brassicales - ps25
Malvales/Brassicales - ps26
Brassicales - ps27
Cleomaceae/Brassicaceae - ps28
Brassicaceae - ps29
Camelineae - ps30
Arabidopsis - ps31
Arabidopsis_thaliana - ps32
p-value
3e-12
6e-10
1e-07
2e-05
3e-03
Log-odds
0.5
1.0
1.5
2.0
2.5
3.0
3.5
4.0

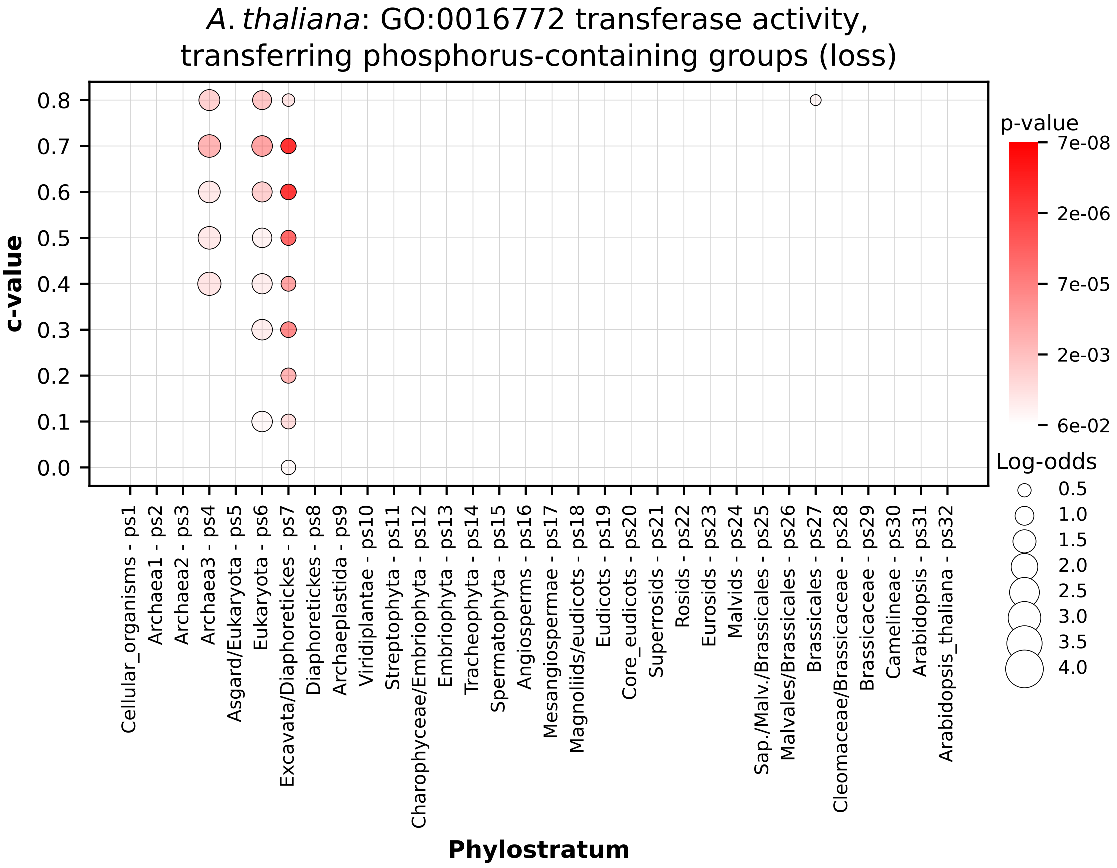
A. thaliana: GO:0016772 transferase activity, transferring phosphorus-containing groups (loss)
c-value
0.8
0.7
0.6
0.5
0.4
0.3
0.2
0.1
0.0
p-value
7e-08
2e-06
7e-05
2e-03
6e-02
Log-odds
0.5
1.0
1.5
2.0
2.5
3.0
3.5
4.0
Cellular_organisms - ps1
Archaea1 - ps2
Archaea2 - ps3
Archaea3 - ps4
Asgard/Eukaryota - ps5
Eukaryota - ps6
Excavata/Diaphoretickes - ps7
Diaphoretickes - ps8
Archaeplastida - ps9
Viridiplantae - ps10
Streptophyta - ps11
Charophyceae/Embriophyta - ps12
Embriophyta - ps13
Tracheophyta - ps14
Spermatophyta - ps15
Angiosperms - ps16
Mesangiospermae - ps17
Magnoliids/eudicots - ps18
Eudicots - ps19
Core_eudicots - ps20
Superrosids - ps21
Rosids - ps22
Eurosids - ps23
Malvids - ps24
Sap./Malv./Brassicales - ps25
Malvales/Brassicales - ps26
Brassicales - ps27
Cleomaceae/Brassicaceae - ps28
Brassicaceae - ps29
Camelineae - ps30
Arabidopsis - ps31
Arabidopsis_thaliana - ps32
Phylostratum

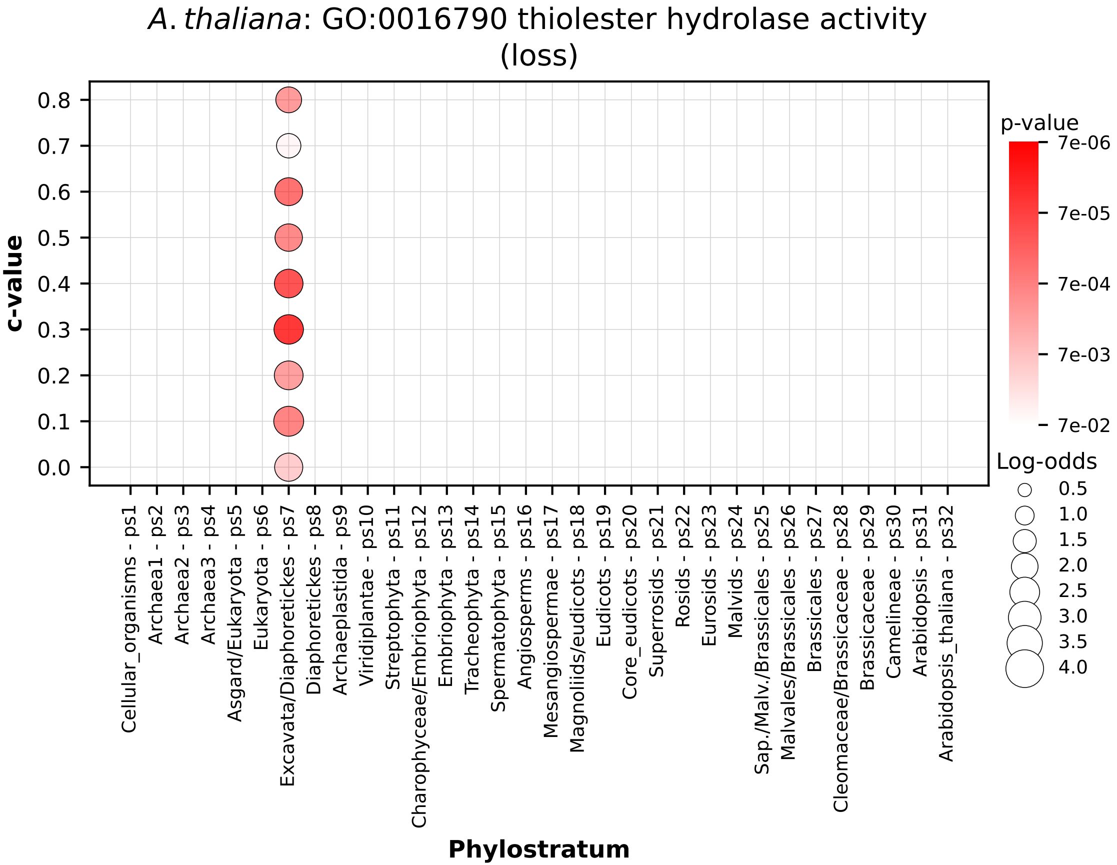

A. thaliana: GO:0016790 thiolester hydrolase activity
(loss)
c-value
0.8
0.7
0.6
0.5
0.4
0.3
0.2
0.1
0.0
p-value
7e-06
7e-05
7e-04
7e-03
7e-02
Log-odds
0.5
1.0
1.5
2.0
2.5
3.0
3.5
4.0
Cellular_organisms - ps1
Archaea1 - ps2
Archaea2 - ps3
Archaea3 - ps4
Asgard/Eukaryota - ps5
Eukaryota - ps6
Excavata/Diaphoretickes - ps7
Diaphoretickes - ps8
Archaeplastida - ps9
Viridiplantae - ps10
Streptophyta - ps11
Charophyceae/Embriophyta - ps12
Embriophyta - ps13
Tracheophyta - ps14
Spermatophyta - ps15
Angiosperms - ps16
Mesangiospermae - ps17
Magnoliids/eudicots - ps18
Eudicots - ps19
Core_eudicots - ps20
Superrosids - ps21
Rosids - ps22
Eurosids - ps23
Malvids - ps24
Sap./Malv./Brassicales - ps25
Malvales/Brassicales - ps26
Brassicales - ps27
Cleomaceae/Brassicaceae - ps28
Brassicaceae - ps29
Camelineae - ps30
Arabidopsis - ps31
Arabidopsis_thaliana - ps32
Phylostratum

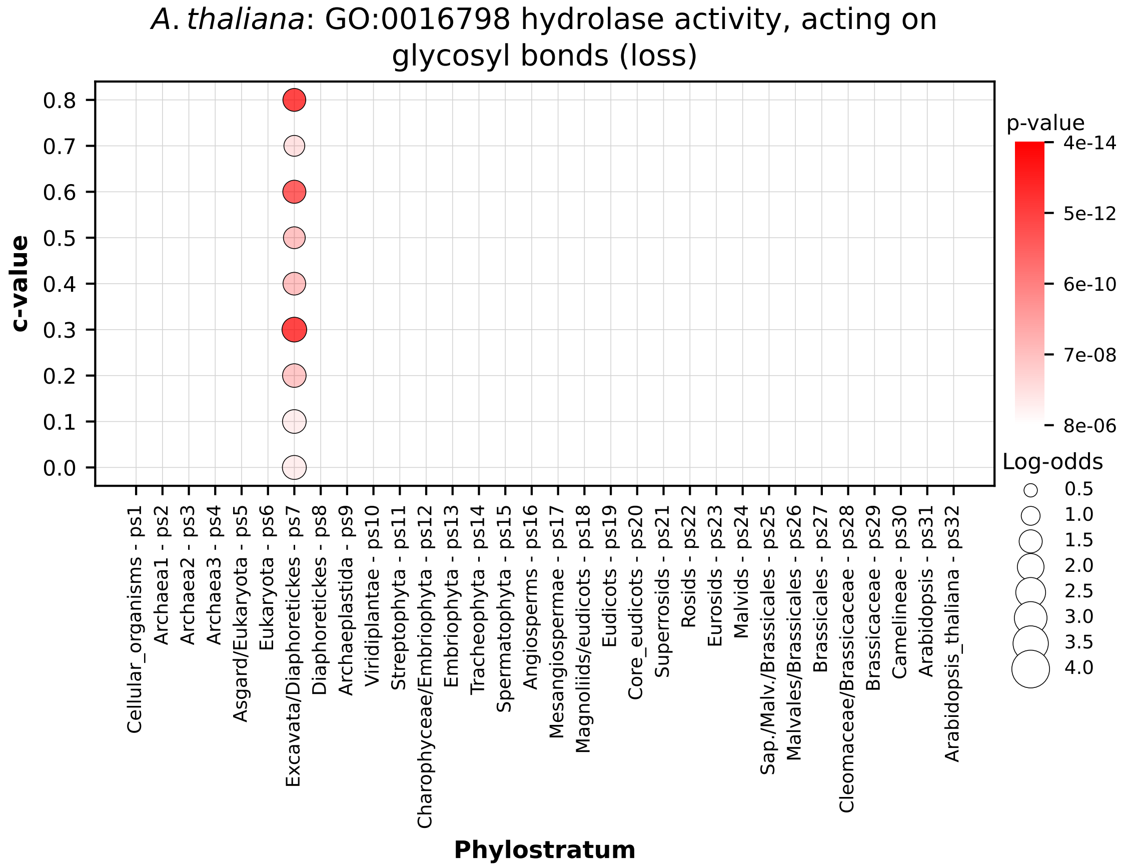

A. thaliana: GO:0016798 hydrolase activity, acting on glycosyl bonds (loss)
c-value
0.0
0.1
0.2
0.3
0.4
0.5
0.6
0.7
0.8
Phylostratum
Cellular_organisms - ps1
Archaea1 - ps2
Archaea2 - ps3
Archaea3 - ps4
Asgard/Eukaryota - ps5
Eukaryota - ps6
Excavata/Diaphoretickes - ps7
Diaphoretickes - ps8
Archaeplastida - ps9
Viridiplantae - ps10
Streptophyta - ps11
Charophyceae/Embriophyta - ps12
Embriophyta - ps13
Tracheophyta - ps14
Spermatophyta - ps15
Angiosperms - ps16
Mesangiospermae - ps17
Magnoliids/eudicots - ps18
Eudicots - ps19
Core_eudicots - ps20
Superrosids - ps21
Rosids - ps22
Eurosids - ps23
Malvids - ps24
Sap./Malv./Brassicales - ps25
Malvales/Brassicales - ps26
Brassicales - ps27
Cleomaceae/Brassicaceae - ps28
Brassicaceae - ps29
Camelineae - ps30
Arabidopsis - ps31
Arabidopsis_thaliana - ps32
p-value
4e-14
5e-12
6e-10
7e-08
8e-06
Log-odds
0.5
1.0
1.5
2.0
2.5
3.0
3.5
4.0

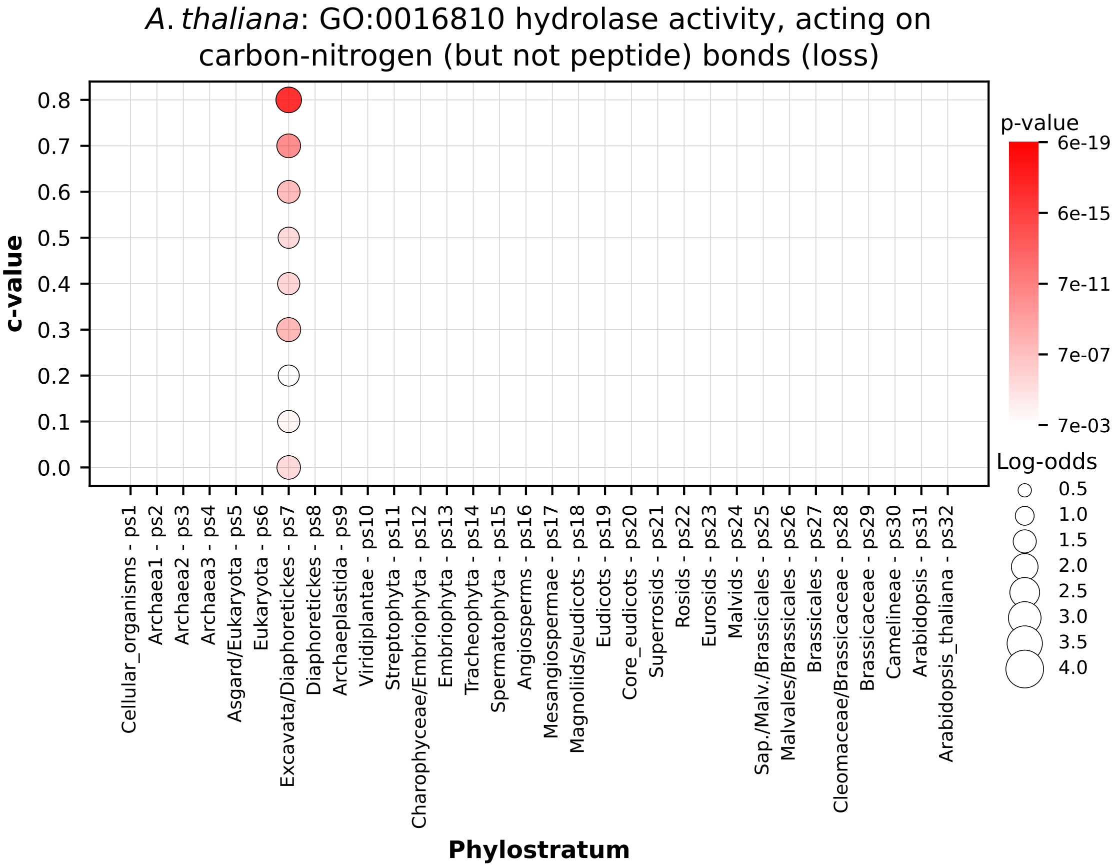

A. thaliana: GO:0016810 hydrolase activity, acting on carbon-nitrogen (but not peptide) bonds (loss)
c-value
0.8
0.7
0.6
0.5
0.4
0.3
0.2
0.1
0.0
p-value
6e-19
6e-15
7e-11
7e-07
7e-03
Log-odds
0.5
1.0
1.5
2.0
2.5
3.0
3.5
4.0
Cellular_organisms - ps1
Archaea1 - ps2
Archaea2 - ps3
Archaea3 - ps4
Asgard/Eukaryota - ps5
Eukaryota - ps6
Excavata/Diaphoretickes - ps7
Diaphoretickes - ps8
Archaeplastida - ps9
Viridiplantae - ps10
Streptophyta - ps11
Charophyceae/Embriophyta - ps12
Embriophyta - ps13
Tracheophyta - ps14
Spermatophyta - ps15
Angiosperms - ps16
Mesangiospermae - ps17
Magnoliids/eudicots - ps18
Eudicots - ps19
Core_eudicots - ps20
Superrosids - ps21
Rosids - ps22
Eurosids - ps23
Malvids - ps24
Sap./Malv./Brassicales - ps25
Malvales/Brassicales - ps26
Brassicales - ps27
Cleomaceae/Brassicaceae - ps28
Brassicaceae - ps29
Camelineae - ps30
Arabidopsis - ps31
Arabidopsis_thaliana - ps32
Phylostratum

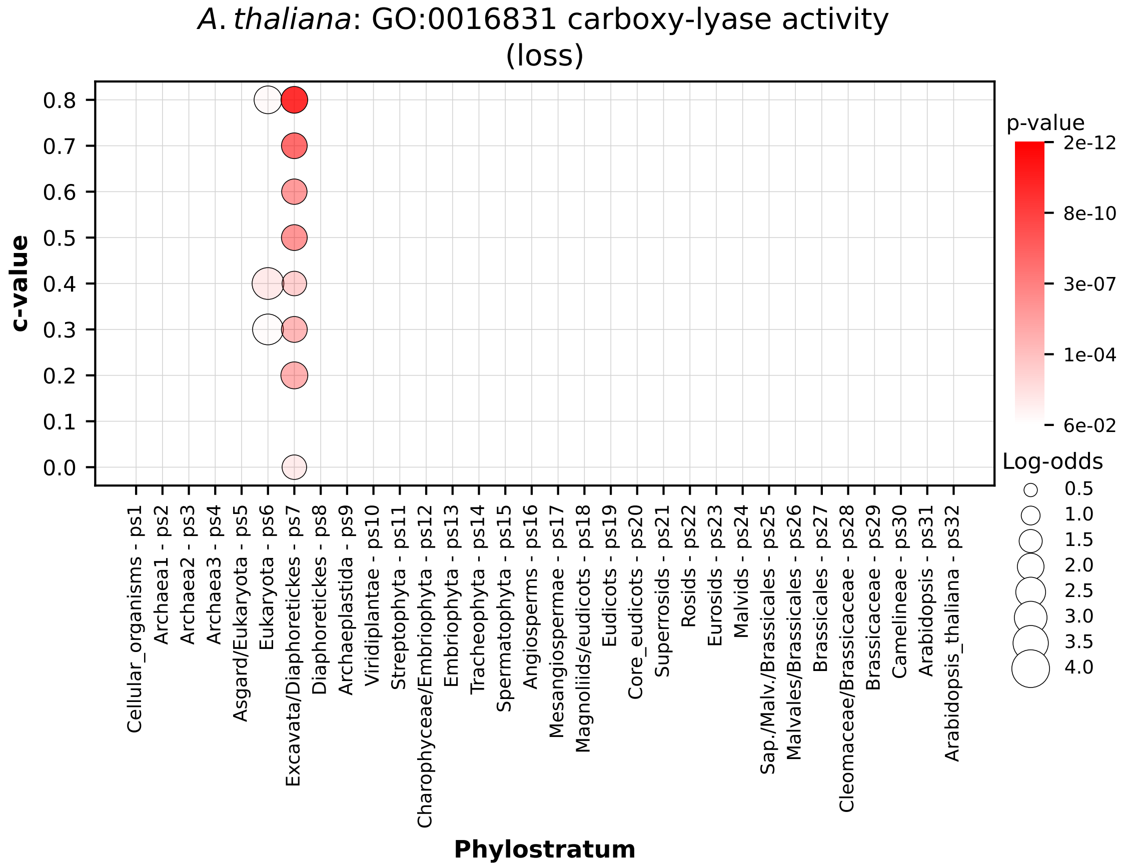

A. thaliana: GO:0016831 carboxy-lyase activity
(loss)
c-value
0.8
0.7
0.6
0.5
0.4
0.3
0.2
0.1
0.0
p-value
2e-12
8e-10
3e-07
1e-04
6e-02
Log-odds
0.5
1.0
1.5
2.0
2.5
3.0
3.5
4.0
Cellular_organisms - ps1
Archaea1 - ps2
Archaea2 - ps3
Archaea3 - ps4
Asgard/Eukaryota - ps5
Eukaryota - ps6
Excavata/Diaphoretickes - ps7
Diaphoretickes - ps8
Archaeplastida - ps9
Viridiplantae - ps10
Streptophyta - ps11
Charophyceae/Embriophyta - ps12
Embriophyta - ps13
Tracheophyta - ps14
Spermatophyta - ps15
Angiosperms - ps16
Mesangiospermae - ps17
Magnoliids/eudicots - ps18
Eudicots - ps19
Core_eudicots - ps20
Superrosids - ps21
Rosids - ps22
Eurosids - ps23
Malvids - ps24
Sap./Malv./Brassicales - ps25
Malvales/Brassicales - ps26
Brassicales - ps27
Cleomaceae/Brassicaceae - ps28
Brassicaceae - ps29
Camelineae - ps30
Arabidopsis - ps31
Arabidopsis_thaliana - ps32
Phylostratum

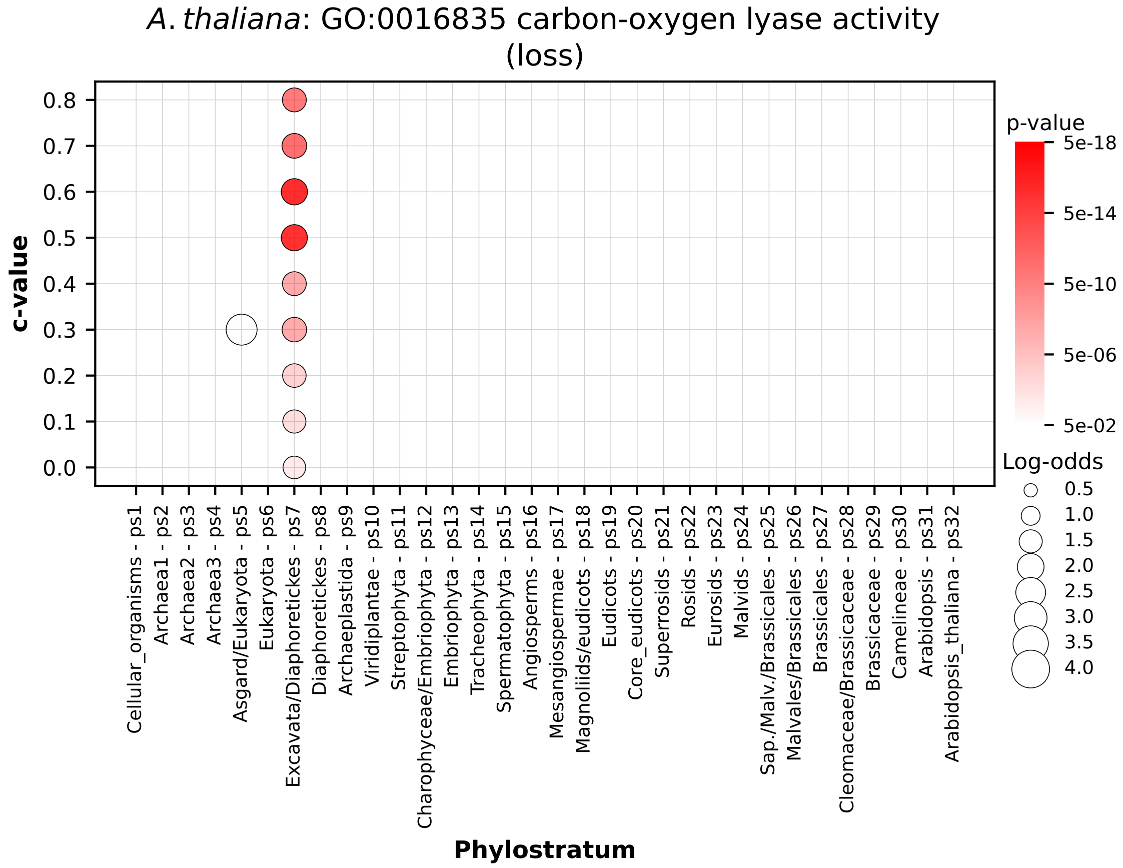

A. thaliana: GO:0016835 carbon-oxygen lyase activity (loss)
c-value
0.8
0.7
0.6
0.5
0.4
0.3
0.2
0.1
0.0
p-value
5e-18
5e-14
5e-10
5e-06
5e-02
Log-odds
0.5
1.0
1.5
2.0
2.5
3.0
3.5
4.0
Cellular_organisms - ps1
Archaea1 - ps2
Archaea2 - ps3
Archaea3 - ps4
Asgard/Eukaryota - ps5
Eukaryota - ps6
Excavata/Diaphoretickes - ps7
Diaphoretickes - ps8
Archaeplastida - ps9
Viridiplantae - ps10
Streptophyta - ps11
Charophyceae/Embriophyta - ps12
Embriophyta - ps13
Tracheophyta - ps14
Spermatophyta - ps15
Angiosperms - ps16
Mesangiospermae - ps17
Magnoliids/eudicots - ps18
Eudicots - ps19
Core_eudicots - ps20
Superrosids - ps21
Rosids - ps22
Eurosids - ps23
Malvids - ps24
Sap./Malv./Brassicales - ps25
Malvales/Brassicales - ps26
Brassicales - ps27
Cleomaceae/Brassicaceae - ps28
Brassicaceae - ps29
Camelineae - ps30
Arabidopsis - ps31
Arabidopsis_thaliana - ps32
Phylostratum

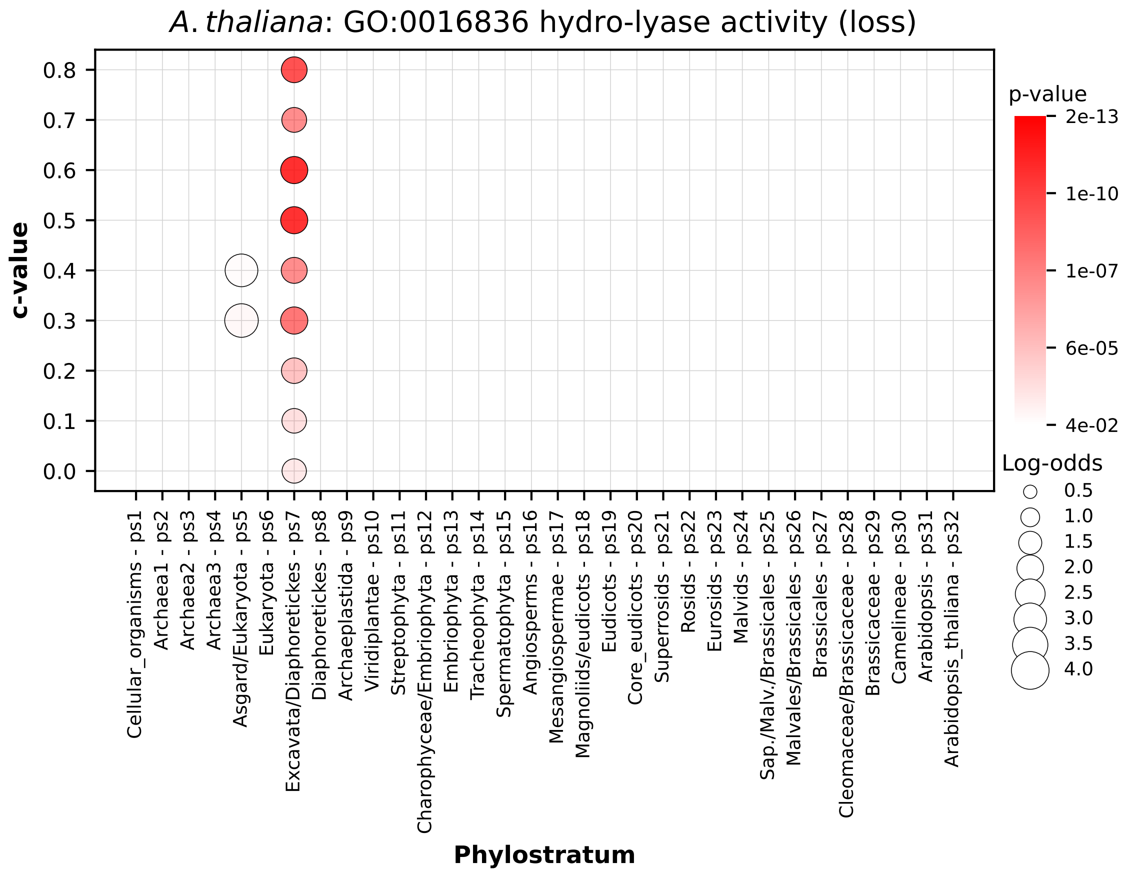

A. thaliana: GO:0016836 hydro-lyase activity (loss)
c-value
0.8
0.7
0.6
0.5
0.4
0.3
0.2
0.1
0.0
p-value
2e-13
1e-10
1e-07
6e-05
4e-02
Log-odds
0.5
1.0
1.5
2.0
2.5
3.0
3.5
4.0
Cellular_organisms - ps1
Archaea1 - ps2
Archaea2 - ps3
Archaea3 - ps4
Asgard/Eukaryota - ps5
Eukaryota - ps6
Excavata/Diaphoretickes - ps7
Diaphoretickes - ps8
Archaeplastida - ps9
Viridiplantae - ps10
Streptophyta - ps11
Charophyceae/Embriophyta - ps12
Embriophyta - ps13
Tracheophyta - ps14
Spermatophyta - ps15
Angiosperms - ps16
Mesangiospermae - ps17
Magnoliids/eudicots - ps18
Eudicots - ps19
Core_eudicots - ps20
Superrosids - ps21
Rosids - ps22
Eurosids - ps23
Malvids - ps24
Sap./Malv./Brassicales - ps25
Malvales/Brassicales - ps26
Brassicales - ps27
Cleomaceae/Brassicaceae - ps28
Brassicaceae - ps29
Camelineae - ps30
Arabidopsis - ps31
Arabidopsis_thaliana - ps32
Phylostratum

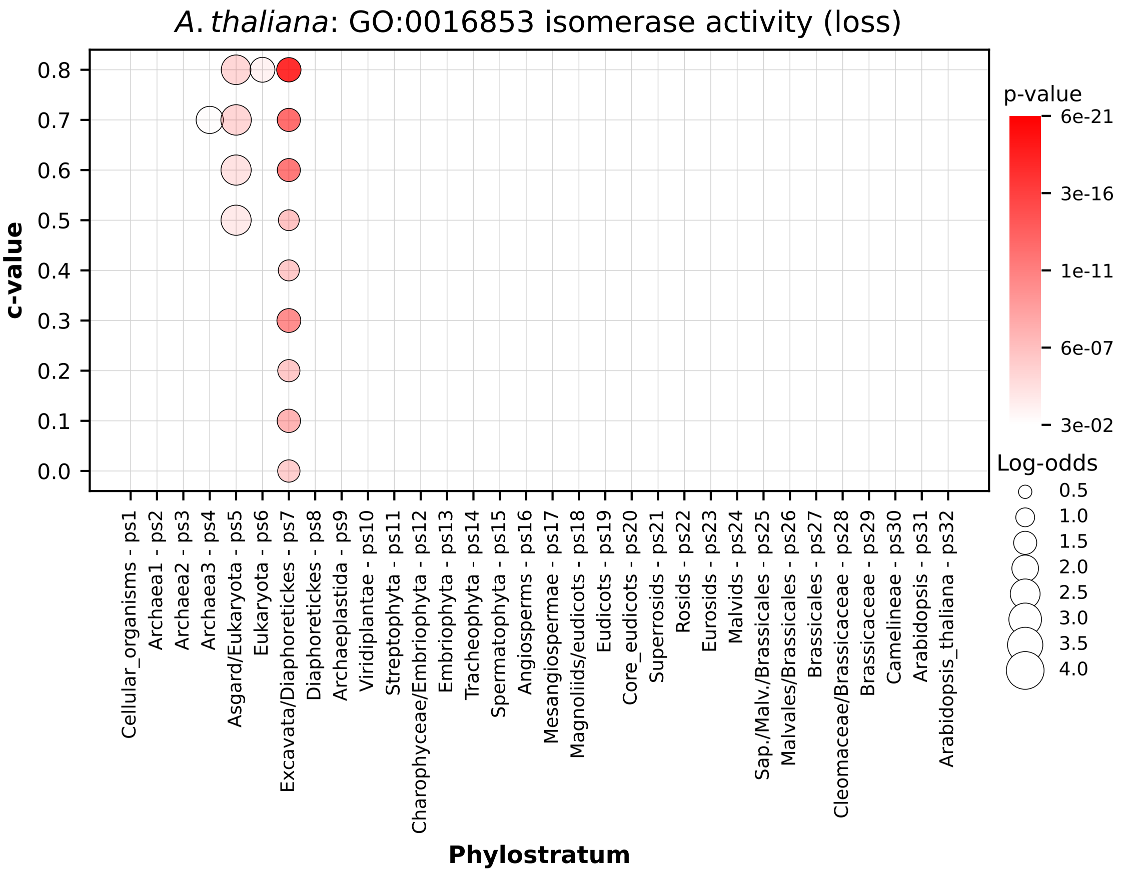

A. thaliana: GO:0016853 isomerase activity (loss)
c-value
0.0
0.1
0.2
0.3
0.4
0.5
0.6
0.7
0.8
Phylostratum
Cellular_organisms - ps1
Archaea1 - ps2
Archaea2 - ps3
Archaea3 - ps4
Asgard/Eukaryota - ps5
Eukaryota - ps6
Excavata/Diaphoretickes - ps7
Diaphoretickes - ps8
Archaeplastida - ps9
Viridiplantae - ps10
Streptophyta - ps11
Charophyceae/Embriophyta - ps12
Embriophyta - ps13
Tracheophyta - ps14
Spermatophyta - ps15
Angiosperms - ps16
Mesangiospermae - ps17
Magnoliids/eudicots - ps18
Eudicots - ps19
Core_eudicots - ps20
Superrosids - ps21
Rosids - ps22
Eurosids - ps23
Malvids - ps24
Sap./Malv./Brassicales - ps25
Malvales/Brassicales - ps26
Brassicales - ps27
Cleomaceae/Brassicaceae - ps28
Brassicaceae - ps29
Camelineae - ps30
Arabidopsis - ps31
Arabidopsis_thaliana - ps32
p-value
6e-21
3e-16
1e-11
6e-07
3e-02
Log-odds
0.5
1.0
1.5
2.0
2.5
3.0
3.5
4.0

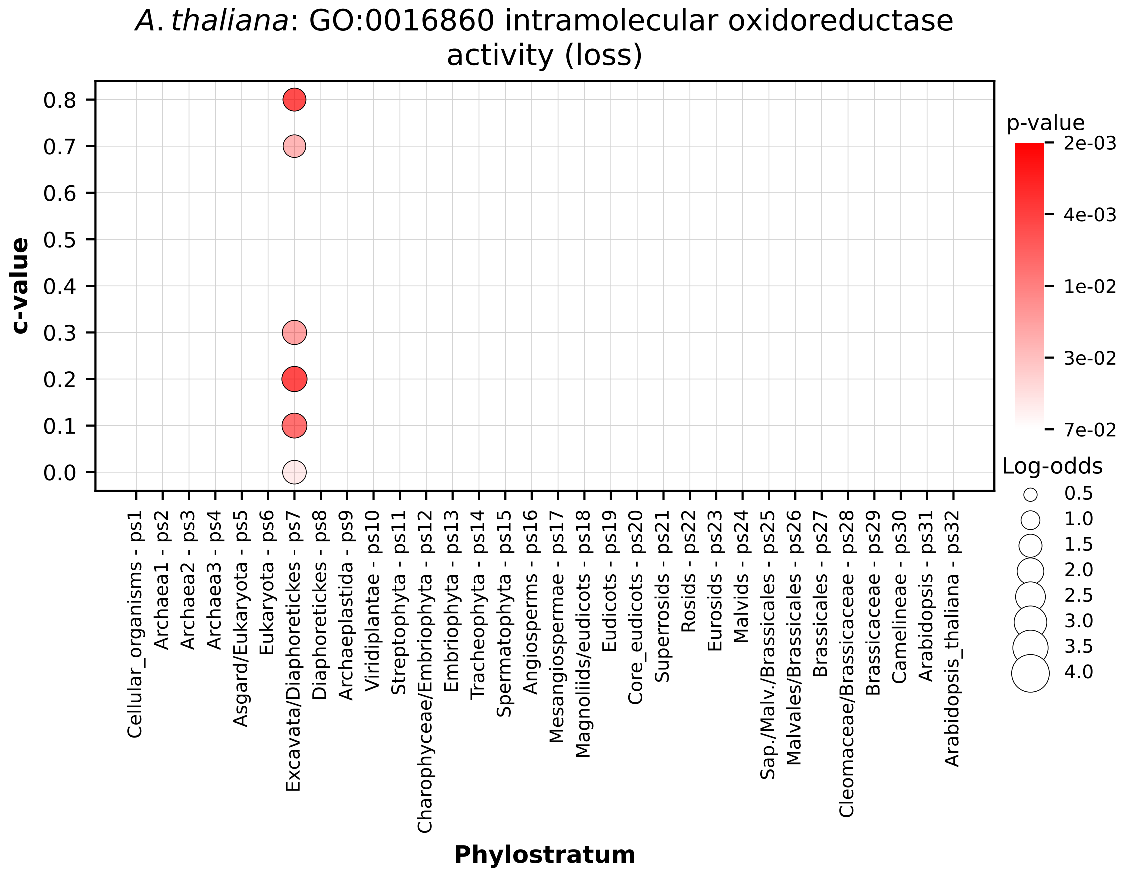

A. thaliana: GO:0016860 intramolecular oxidoreductase activity (loss)
c-value
0.8
0.7
0.6
0.5
0.4
0.3
0.2
0.1
0.0
Phylostratum
Cellular_organisms - ps1
Archaea1 - ps2
Archaea2 - ps3
Archaea3 - ps4
Asgard/Eukaryota - ps5
Eukaryota - ps6
Excavata/Diaphoretickes - ps7
Diaphoretickes - ps8
Archaeplastida - ps9
Viridiplantae - ps10
Streptophyta - ps11
Charophyceae/Embriophyta - ps12
Embriophyta - ps13
Tracheophyta - ps14
Spermatophyta - ps15
Angiosperms - ps16
Mesangiospermae - ps17
Magnoliids/eudicots - ps18
Eudicots - ps19
Core_eudicots - ps20
Superrosids - ps21
Rosids - ps22
Eurosids - ps23
Malvids - ps24
Sap./Malv./Brassicales - ps25
Malvales/Brassicales - ps26
Brassicales - ps27
Cleomaceae/Brassicaceae - ps28
Brassicaceae - ps29
Camelineae - ps30
Arabidopsis - ps31
Arabidopsis_thaliana - ps32
p-value
2e-03
4e-03
1e-02
3e-02
7e-02
Log-odds
0.5
1.0
1.5
2.0
2.5
3.0
3.5
4.0

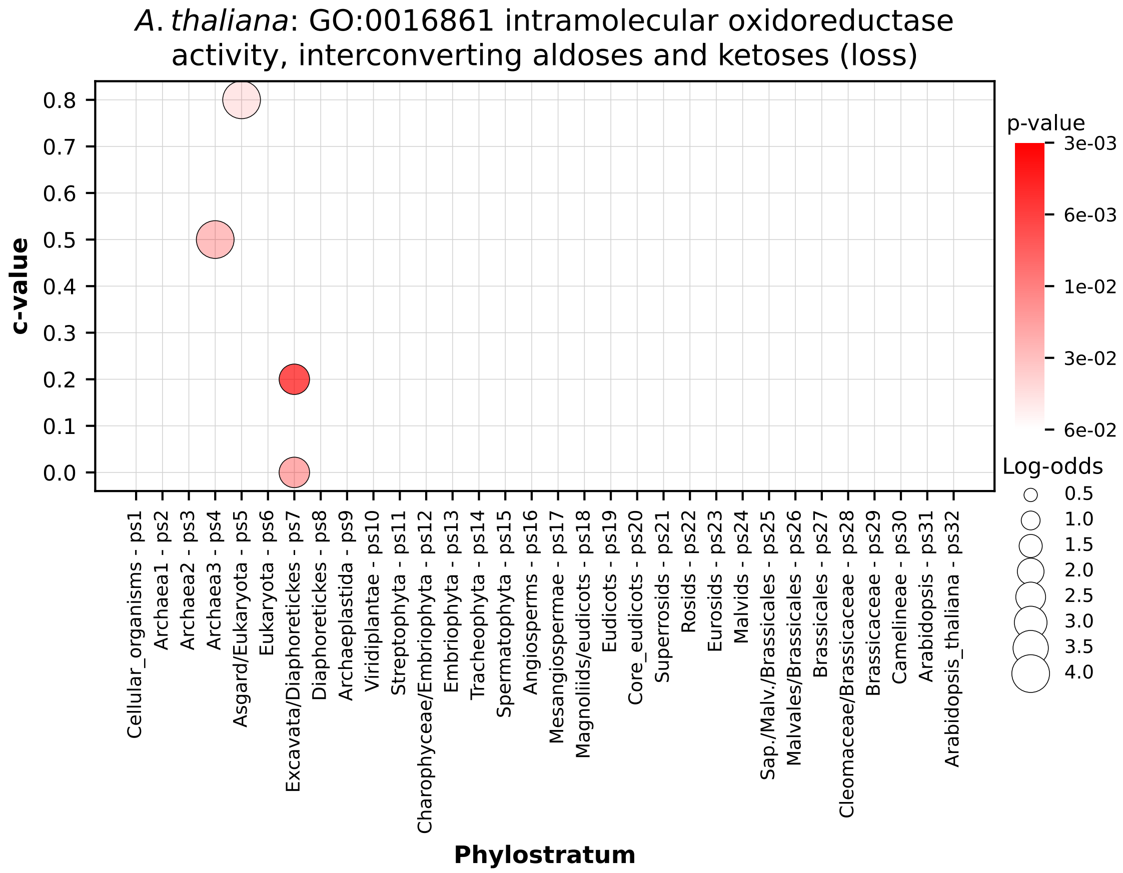

A. thaliana: GO:0016861 intramolecular oxidoreductase activity, interconverting aldoses and ketoses (loss)
c-value
0.8
0.7
0.6
0.5
0.4
0.3
0.2
0.1
0.0
p-value
3e-03
6e-03
1e-02
3e-02
6e-02
Log-odds
0.5
1.0
1.5
2.0
2.5
3.0
3.5
4.0
Cellular_organisms - ps1
Archaea1 - ps2
Archaea2 - ps3
Archaea3 - ps4
Asgard/Eukaryota - ps5
Eukaryota - ps6
Excavata/Diaphoretickes - ps7
Diaphoretickes - ps8
Archaeplastida - ps9
Viridiplantae - ps10
Streptophyta - ps11
Charophyceae/Embriophyta - ps12
Embriophyta - ps13
Tracheophyta - ps14
Spermatophyta - ps15
Angiosperms - ps16
Mesangiospermae - ps17
Magnoliids/eudicots - ps18
Eudicots - ps19
Core_eudicots - ps20
Superrosids - ps21
Rosids - ps22
Eurosids - ps23
Malvids - ps24
Sap./Malv./Brassicales - ps25
Malvales/Brassicales - ps26
Brassicales - ps27
Cleomaceae/Brassicaceae - ps28
Brassicaceae - ps29
Camelineae - ps30
Arabidopsis - ps31
Arabidopsis_thaliana - ps32
Phylostratum

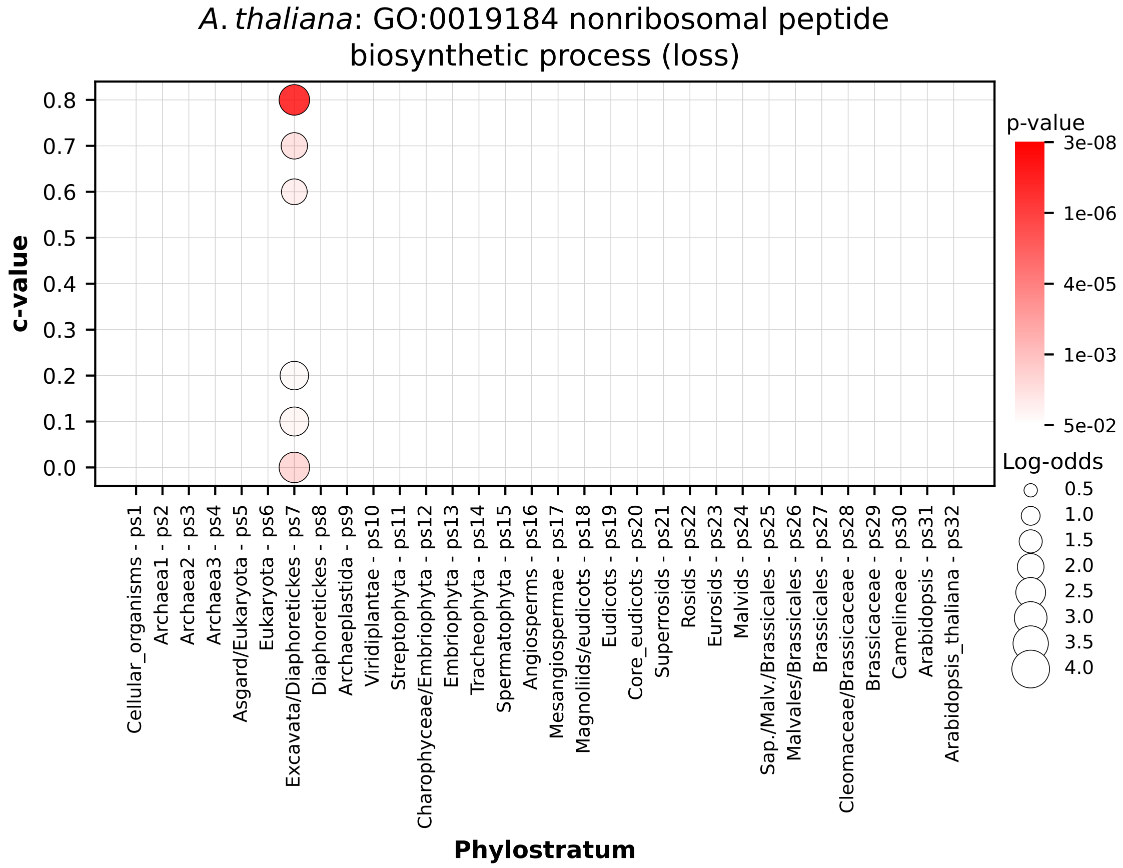

A. thaliana: GO:0019184 nonribosomal peptide biosynthetic process (loss)
c-value
0.0
0.1
0.2
0.3
0.4
0.5
0.6
0.7
0.8
Phylostratum
Cellular_organisms - ps1
Archaea1 - ps2
Archaea2 - ps3
Archaea3 - ps4
Asgard/Eukaryota - ps5
Eukaryota - ps6
Excavata/Diaphoretickes - ps7
Diaphoretickes - ps8
Archaeplastida - ps9
Viridiplantae - ps10
Streptophyta - ps11
Charophyceae/Embriophyta - ps12
Embriophyta - ps13
Tracheophyta - ps14
Spermatophyta - ps15
Angiosperms - ps16
Mesangiospermae - ps17
Magnoliids/eudicots - ps18
Eudicots - ps19
Core_eudicots - ps20
Superrosids - ps21
Rosids - ps22
Eurosids - ps23
Malvids - ps24
Sap./Malv./Brassicales - ps25
Malvales/Brassicales - ps26
Brassicales - ps27
Cleomaceae/Brassicaceae - ps28
Brassicaceae - ps29
Camelineae - ps30
Arabidopsis - ps31
Arabidopsis_thaliana - ps32
p-value
3e-08
1e-06
4e-05
1e-03
5e-02
Log-odds
0.5
1.0
1.5
2.0
2.5
3.0
3.5
4.0

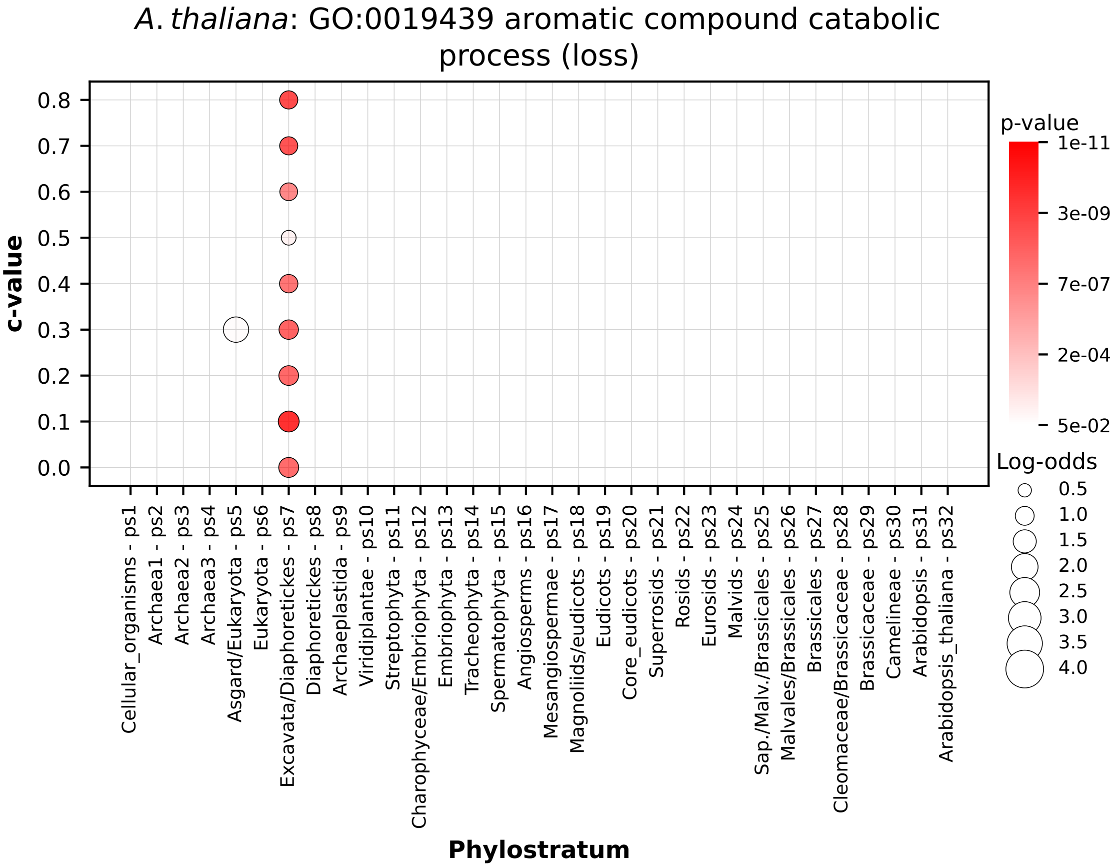
A. thaliana: GO:0019439 aromatic compound catabolic process (loss)
c-value
0.8
0.7
0.6
0.5
0.4
0.3
0.2
0.1
0.0
Phylostratum
Cellular_organisms - ps1
Archaea1 - ps2
Archaea2 - ps3
Archaea3 - ps4
Asgard/Eukaryota - ps5
Eukaryota - ps6
Excavata/Diaphoretickes - ps7
Diaphoretickes - ps8
Archaeplastida - ps9
Viridiplantae - ps10
Streptophyta - ps11
Charophyceae/Embriophyta - ps12
Embriophyta - ps13
Tracheophyta - ps14
Spermatophyta - ps15
Angiosperms - ps16
Mesangiospermae - ps17
Magnoliids/eudicots - ps18
Eudicots - ps19
Core_eudicots - ps20
Superrosids - ps21
Rosids - ps22
Eurosids - ps23
Malvids - ps24
Sap./Malv./Brassicales - ps25
Malvales/Brassicales - ps26
Brassicales - ps27
Cleomaceae/Brassicaceae - ps28
Brassicaceae - ps29
Camelineae - ps30
Arabidopsis - ps31
Arabidopsis_thaliana - ps32
p-value
1e-11
3e-09
7e-07
2e-04
5e-02
Log-odds
0.5
1.0
1.5
2.0
2.5
3.0
3.5
4.0

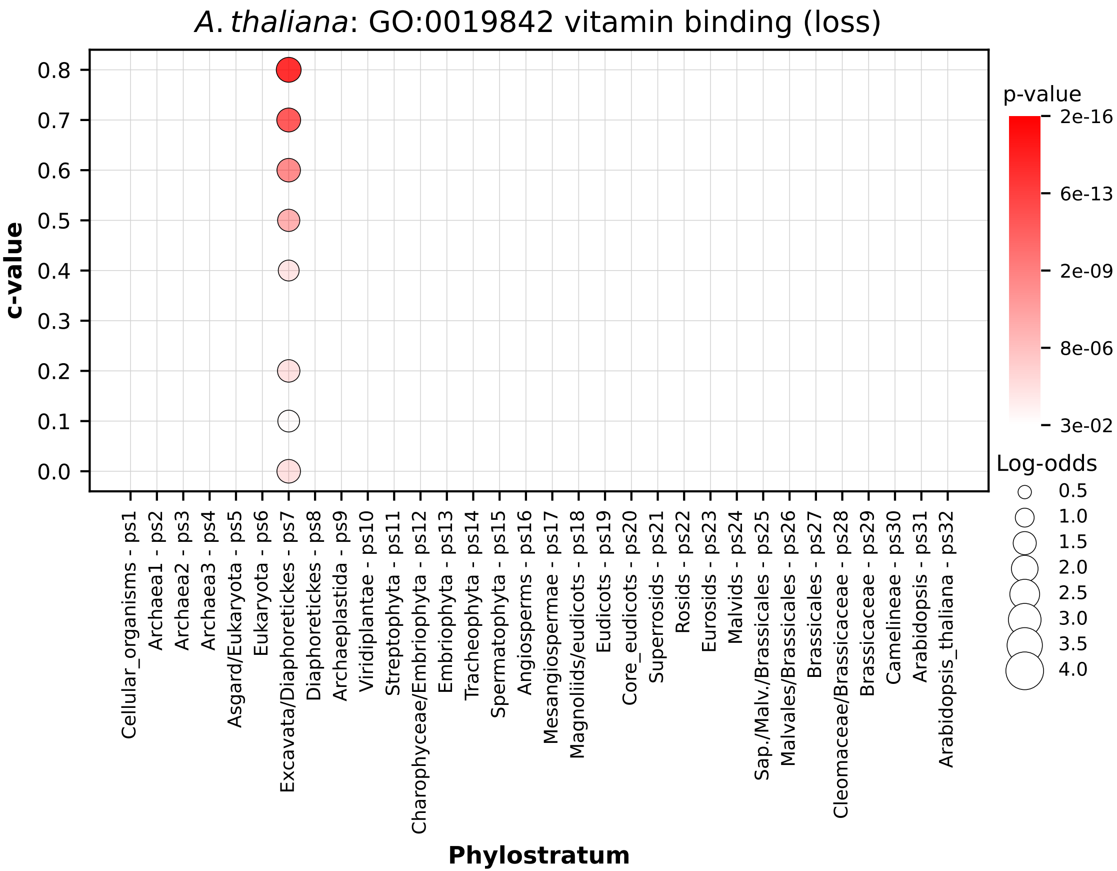

A. thaliana: GO:0019842 vitamin binding (loss)
c-value
0.8
0.7
0.6
0.5
0.4
0.3
0.2
0.1
0.0
p-value
2e-16
6e-13
2e-09
8e-06
3e-02
Log-odds
0.5
1.0
1.5
2.0
2.5
3.0
3.5
4.0
Cellular_organisms - ps1
Archaea1 - ps2
Archaea2 - ps3
Archaea3 - ps4
Asgard/Eukaryota - ps5
Eukaryota - ps6
Excavata/Diaphoretickes - ps7
Diaphoretickes - ps8
Archaeplastida - ps9
Viridiplantae - ps10
Streptophyta - ps11
Charophyceae/Embriophyta - ps12
Embriophyta - ps13
Tracheophyta - ps14
Spermatophyta - ps15
Angiosperms - ps16
Mesangiospermae - ps17
Magnoliids/eudicots - ps18
Eudicots - ps19
Core_eudicots - ps20
Superrosids - ps21
Rosids - ps22
Eurosids - ps23
Malvids - ps24
Sap./Malv./Brassicales - ps25
Malvales/Brassicales - ps26
Brassicales - ps27
Cleomaceae/Brassicaceae - ps28
Brassicaceae - ps29
Camelineae - ps30
Arabidopsis - ps31
Arabidopsis_thaliana - ps32
Phylostratum

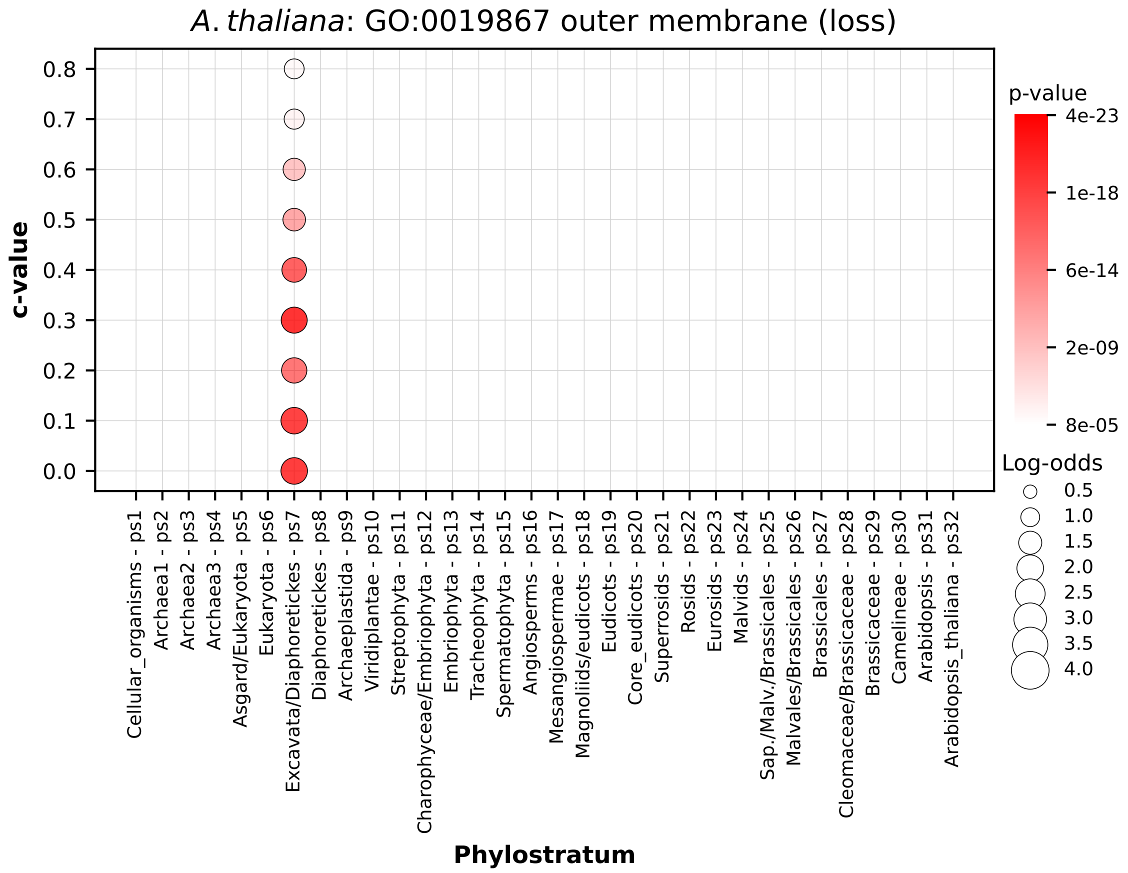
A. thaliana: GO:0019867 outer membrane (loss)
c-value
0.8
0.7
0.6
0.5
0.4
0.3
0.2
0.1
0.0
p-value
4e-23
1e-18
6e-14
2e-09
8e-05
Log-odds
0.5
1.0
1.5
2.0
2.5
3.0
3.5
4.0
Cellular_organisms - ps1
Archaea1 - ps2
Archaea2 - ps3
Archaea3 - ps4
Asgard/Eukaryota - ps5
Eukaryota - ps6
Excavata/Diaphoretickes - ps7
Diaphoretickes - ps8
Archaeplastida - ps9
Viridiplantae - ps10
Streptophyta - ps11
Charophyceae/Embriophyta - ps12
Embriophyta - ps13
Tracheophyta - ps14
Spermatophyta - ps15
Angiosperms - ps16
Mesangiospermae - ps17
Magnoliids/eudicots - ps18
Eudicots - ps19
Core_eudicots - ps20
Superrosids - ps21
Rosids - ps22
Eurosids - ps23
Malvids - ps24
Sap./Malv./Brassicales - ps25
Malvales/Brassicales - ps26
Brassicales - ps27
Cleomaceae/Brassicaceae - ps28
Brassicaceae - ps29
Camelineae - ps30
Arabidopsis - ps31
Arabidopsis_thaliana - ps32
Phylostratum

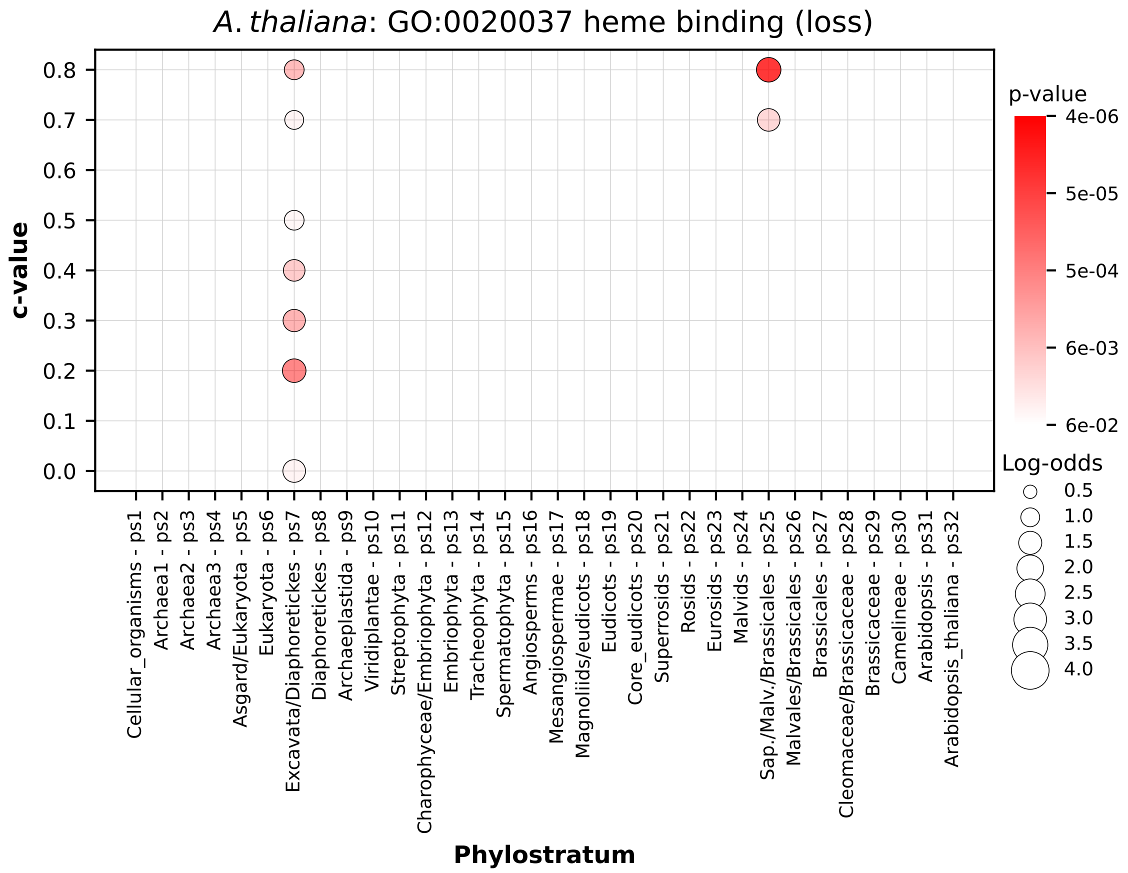

A. thaliana: GO:0020037 heme binding (loss)
c-value
0.8
0.7
0.6
0.5
0.4
0.3
0.2
0.1
0.0
p-value
4e-06
5e-05
5e-04
6e-03
6e-02
Log-odds
0.5
1.0
1.5
2.0
2.5
3.0
3.5
4.0
Cellular_organisms - ps1
Archaea1 - ps2
Archaea2 - ps3
Archaea3 - ps4
Asgard/Eukaryota - ps5
Eukaryota - ps6
Excavata/Diaphoretickes - ps7
Diaphoretickes - ps8
Archaeplastida - ps9
Viridiplantae - ps10
Streptophyta - ps11
Charophyceae/Embriophyta - ps12
Embriophyta - ps13
Tracheophyta - ps14
Spermatophyta - ps15
Angiosperms - ps16
Mesangiospermae - ps17
Magnoliids/eudicots - ps18
Eudicots - ps19
Core_eudicots - ps20
Superrosids - ps21
Rosids - ps22
Eurosids - ps23
Malvids - ps24
Sap./Malv./Brassicales - ps25
Malvales/Brassicales - ps26
Brassicales - ps27
Cleomaceae/Brassicaceae - ps28
Brassicaceae - ps29
Camelineae - ps30
Arabidopsis - ps31
Arabidopsis_thaliana - ps32
Phylostratum

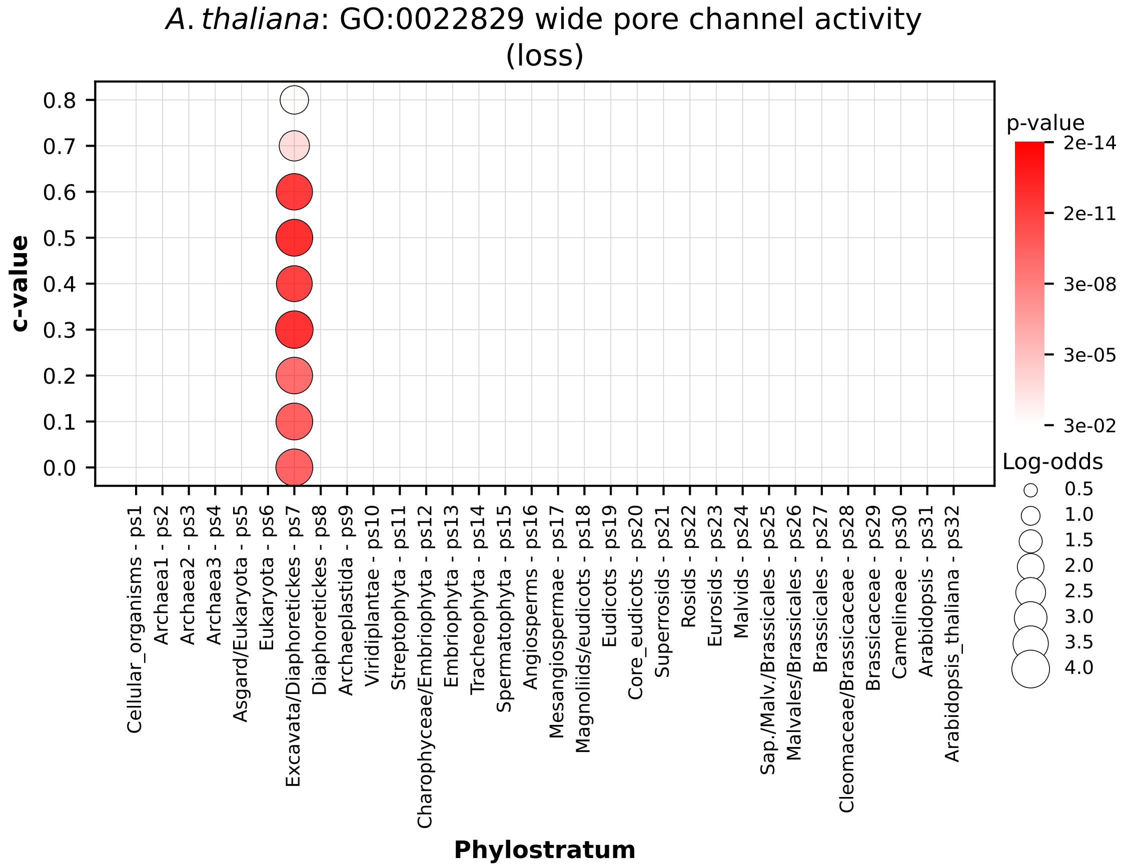
A. thaliana: GO:0022829 wide pore channel activity
(loss)
c-value
0.8
0.7
0.6
0.5
0.4
0.3
0.2
0.1
0.0
p-value
2e-14
2e-11
3e-08
3e-05
3e-02
Log-odds
0.5
1.0
1.5
2.0
2.5
3.0
3.5
4.0
Cellular_organisms - ps1
Archaea1 - ps2
Archaea2 - ps3
Archaea3 - ps4
Asgard/Eukaryota - ps5
Eukaryota - ps6
Excavata/Diaphoretickes - ps7
Diaphoretickes - ps8
Archaeplastida - ps9
Viridiplantae - ps10
Streptophyta - ps11
Charophyceae/Embriophyta - ps12
Embriophyta - ps13
Tracheophyta - ps14
Spermatophyta - ps15
Angiosperms - ps16
Mesangiospermae - ps17
Magnoliids/eudicots - ps18
Eudicots - ps19
Core_eudicots - ps20
Superrosids - ps21
Rosids - ps22
Eurosids - ps23
Malvids - ps24
Sap./Malv./Brassicales - ps25
Malvales/Brassicales - ps26
Brassicales - ps27
Cleomaceae/Brassicaceae - ps28
Brassicaceae - ps29
Camelineae - ps30
Arabidopsis - ps31
Arabidopsis_thaliana - ps32
Phylostratum

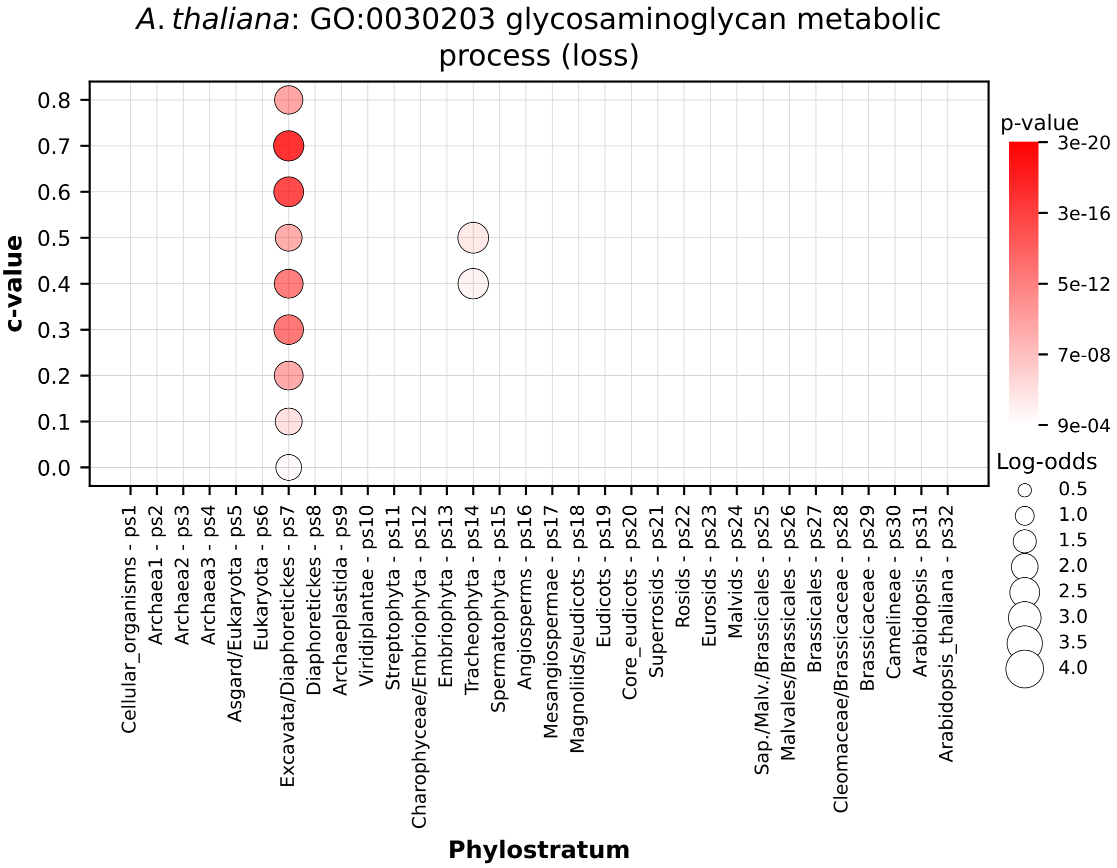

A. thaliana: GO:0030203 glycosaminoglycan metabolic process (loss)
c-value
0.8
0.7
0.6
0.5
0.4
0.3
0.2
0.1
0.0
p-value
3e-20
3e-16
5e-12
7e-08
9e-04
Log-odds
0.5
1.0
1.5
2.0
2.5
3.0
3.5
4.0
Cellular_organisms - ps1
Archaea1 - ps2
Archaea2 - ps3
Archaea3 - ps4
Asgard/Eukaryota - ps5
Eukaryota - ps6
Excavata/Diaphoretickes - ps7
Diaphoretickes - ps8
Archaeplastida - ps9
Viridiplantae - ps10
Streptophyta - ps11
Charophyceae/Embriophyta - ps12
Embriophyta - ps13
Tracheophyta - ps14
Spermatophyta - ps15
Angiosperms - ps16
Mesangiospermae - ps17
Magnoliids/eudicots - ps18
Eudicots - ps19
Core_eudicots - ps20
Superrosids - ps21
Rosids - ps22
Eurosids - ps23
Malvids - ps24
Sap./Malv./Brassicales - ps25
Malvales/Brassicales - ps26
Brassicales - ps27
Cleomaceae/Brassicaceae - ps28
Brassicaceae - ps29
Camelineae - ps30
Arabidopsis - ps31
Arabidopsis_thaliana - ps32
Phylostratum

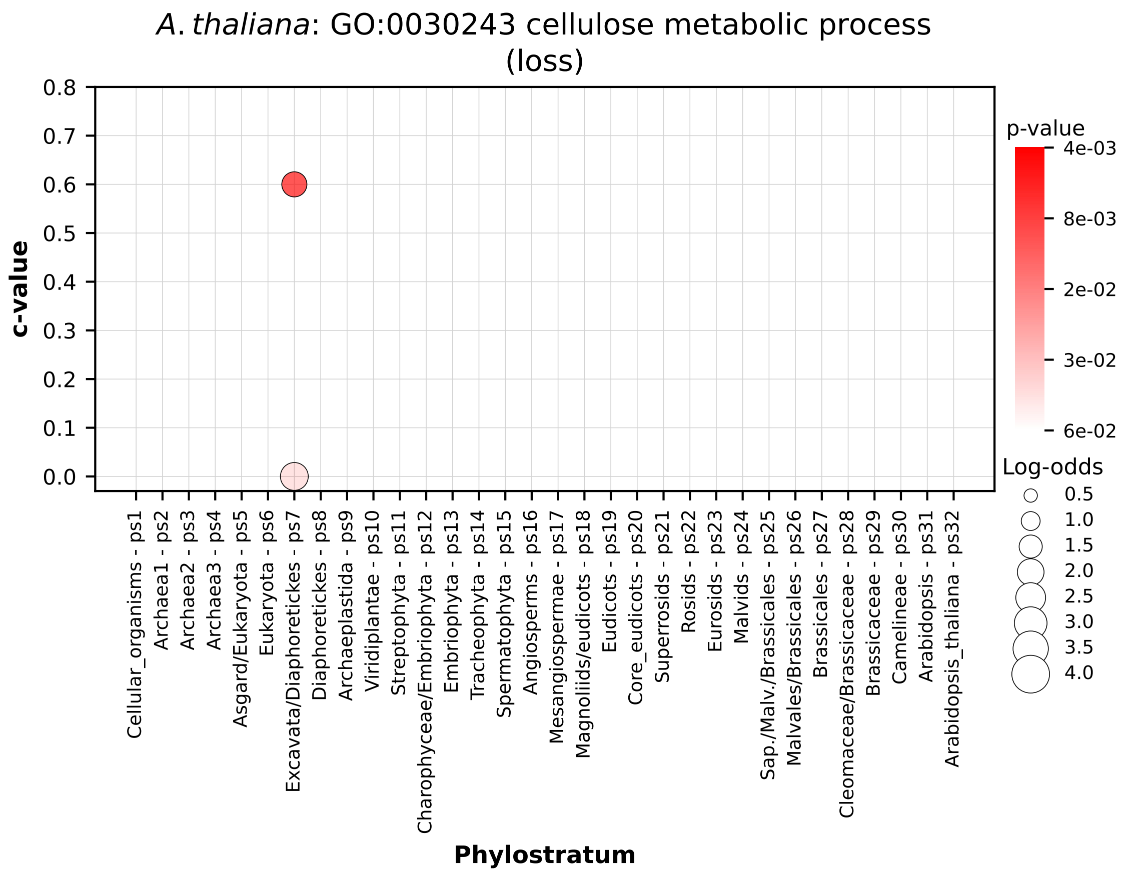

A. thaliana: GO:0030243 cellulose metabolic process
(loss)
c-value
0.8
0.7
0.6
0.5
0.4
0.3
0.2
0.1
0.0
p-value
4e-03
8e-03
2e-02
3e-02
6e-02
Log-odds
0.5
1.0
1.5
2.0
2.5
3.0
3.5
4.0
Cellular_organisms - ps1
Archaea1 - ps2
Archaea2 - ps3
Archaea3 - ps4
Asgard/Eukaryota - ps5
Eukaryota - ps6
Excavata/Diaphoretickes - ps7
Diaphoretickes - ps8
Archaeplastida - ps9
Viridiplantae - ps10
Streptophyta - ps11
Charophyceae/Embriophyta - ps12
Embriophyta - ps13
Tracheophyta - ps14
Spermatophyta - ps15
Angiosperms - ps16
Mesangiospermae - ps17
Magnoliids/eudicots - ps18
Eudicots - ps19
Core_eudicots - ps20
Superrosids - ps21
Rosids - ps22
Eurosids - ps23
Malvids - ps24
Sap./Malv./Brassicales - ps25
Malvales/Brassicales - ps26
Brassicales - ps27
Cleomaceae/Brassicaceae - ps28
Brassicaceae - ps29
Camelineae - ps30
Arabidopsis - ps31
Arabidopsis_thaliana - ps32
Phylostratum

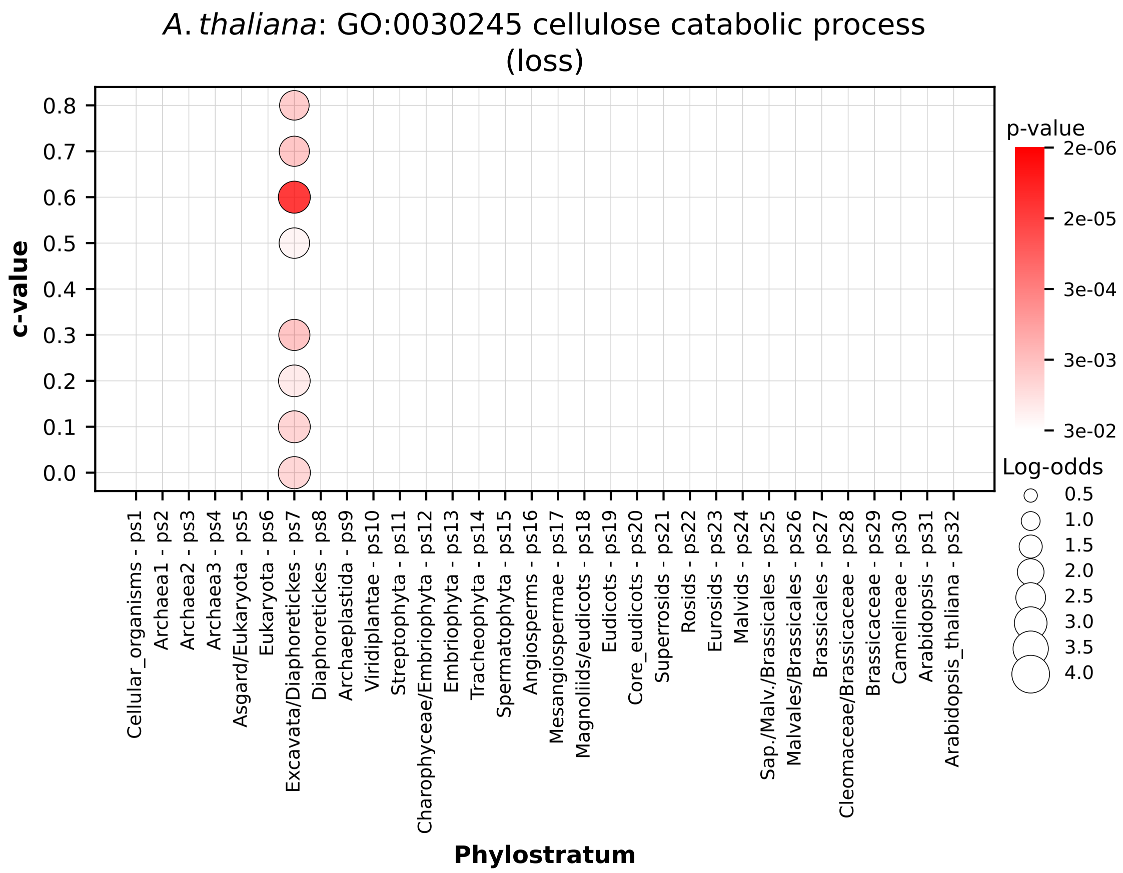
A. thaliana: GO:0030245 cellulose catabolic process (loss)
c-value
0.8
0.7
0.6
0.5
0.4
0.3
0.2
0.1
0.0
Phylostratum
Cellular_organisms - ps1
Archaea1 - ps2
Archaea2 - ps3
Archaea3 - ps4
Asgard/Eukaryota - ps5
Eukaryota - ps6
Excavata/Diaphoretickes - ps7
Diaphoretickes - ps8
Archaeplastida - ps9
Viridiplantae - ps10
Streptophyta - ps11
Charophyceae/Embriophyta - ps12
Embriophyta - ps13
Tracheophyta - ps14
Spermatophyta - ps15
Angiosperms - ps16
Mesangiospermae - ps17
Magnoliids/eudicots - ps18
Eudicots - ps19
Core_eudicots - ps20
Superrosids - ps21
Rosids - ps22
Eurosids - ps23
Malvids - ps24
Sap./Malv./Brassicales - ps25
Malvales/Brassicales - ps26
Brassicales - ps27
Cleomaceae/Brassicaceae - ps28
Brassicaceae - ps29
Camelineae - ps30
Arabidopsis - ps31
Arabidopsis_thaliana - ps32
p-value
2e-06
2e-05
3e-04
3e-03
3e-02
Log-odds
0.5
1.0
1.5
2.0
2.5
3.0
3.5
4.0

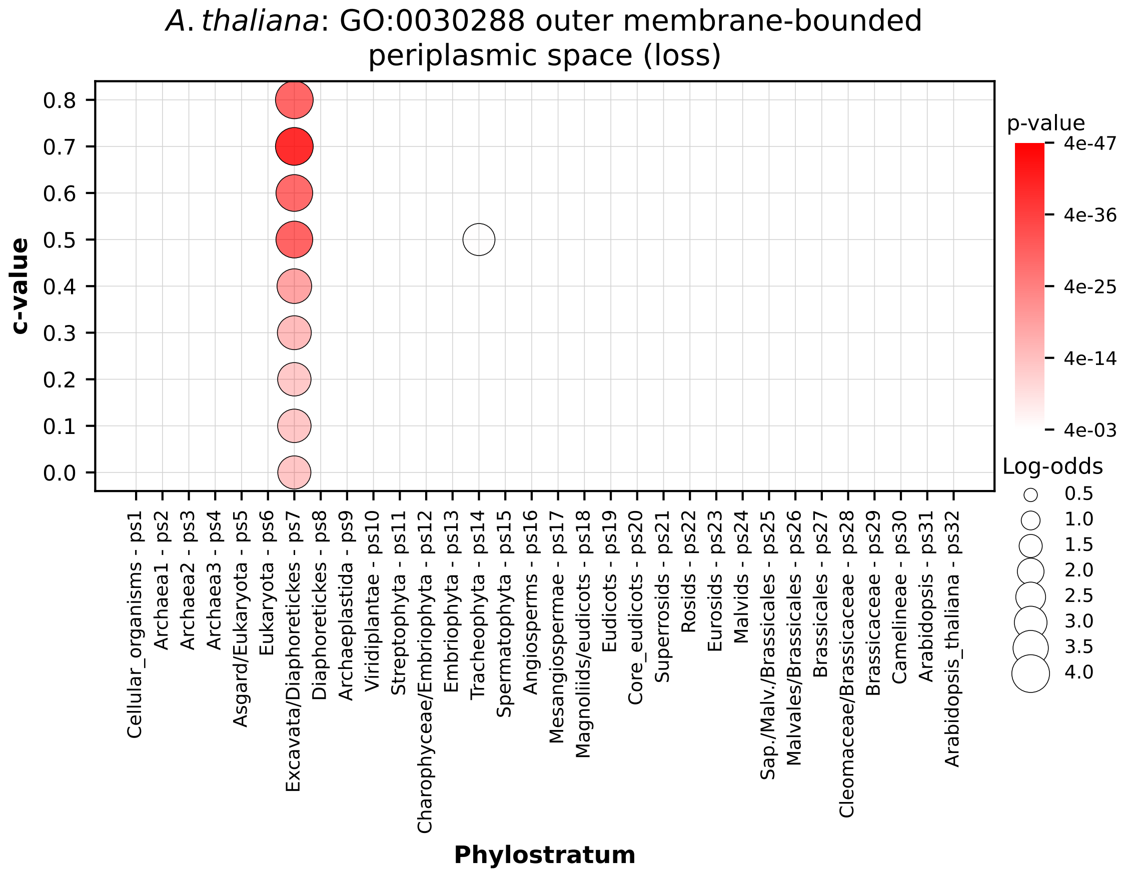

A. thaliana: GO:0030288 outer membrane-bounded periplasmic space (loss)
c-value
0.8
0.7
0.6
0.5
0.4
0.3
0.2
0.1
0.0
p-value
4e-47
4e-36
4e-25
4e-14
4e-03
Log-odds
0.5
1.0
1.5
2.0
2.5
3.0
3.5
4.0
Cellular_organisms - ps1
Archaea1 - ps2
Archaea2 - ps3
Archaea3 - ps4
Asgard/Eukaryota - ps5
Eukaryota - ps6
Excavata/Diaphoretickes - ps7
Diaphoretickes - ps8
Archaeplastida - ps9
Viridiplantae - ps10
Streptophyta - ps11
Charophyceae/Embriophyta - ps12
Embriophyta - ps13
Tracheophyta - ps14
Spermatophyta - ps15
Angiosperms - ps16
Mesangiospermae - ps17
Magnoliids/eudicots - ps18
Eudicots - ps19
Core_eudicots - ps20
Superrosids - ps21
Rosids - ps22
Eurosids - ps23
Malvids - ps24
Sap./Malv./Brassicales - ps25
Malvales/Brassicales - ps26
Brassicales - ps27
Cleomaceae/Brassicaceae - ps28
Brassicaceae - ps29
Camelineae - ps30
Arabidopsis - ps31
Arabidopsis_thaliana - ps32
Phylostratum

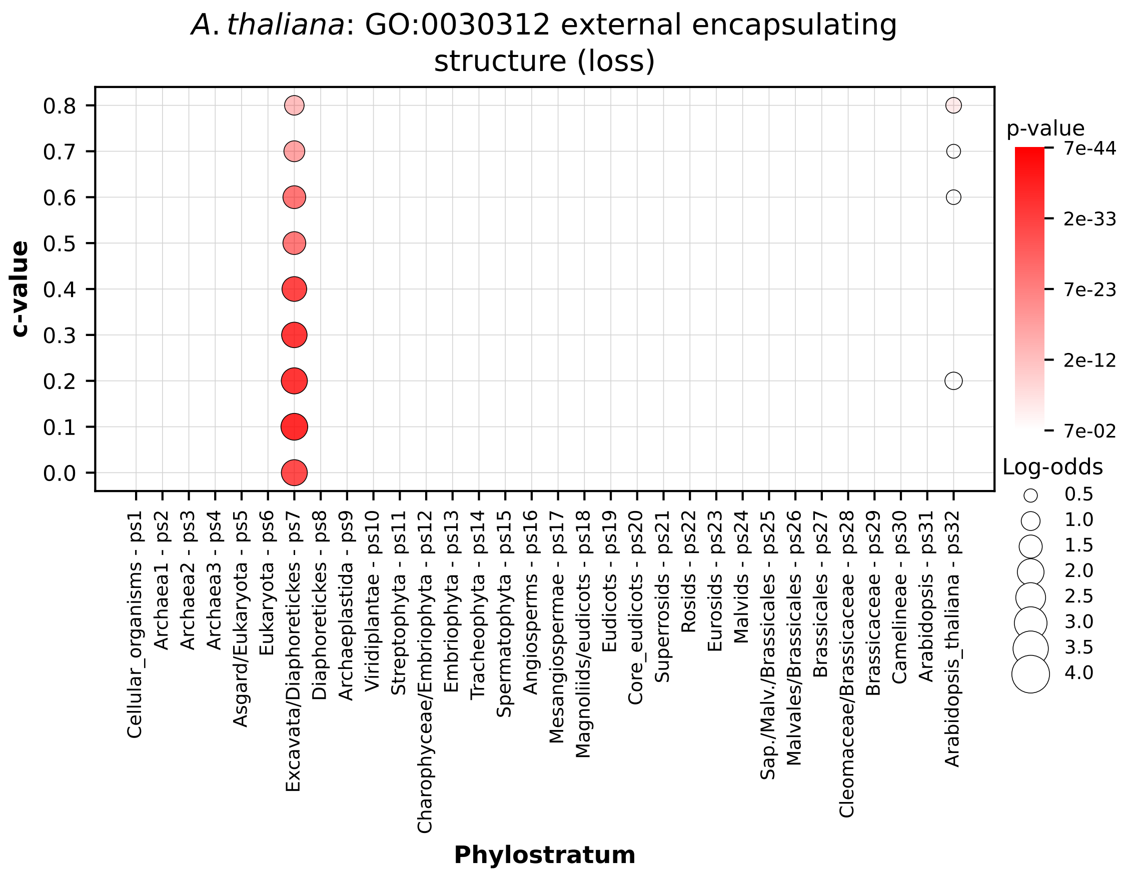

A. thaliana: GO:0030312 external encapsulating structure (loss)
c-value
0.0
0.1
0.2
0.3
0.4
0.5
0.6
0.7
0.8
Phylostratum
Cellular_organisms - ps1
Archaea1 - ps2
Archaea2 - ps3
Archaea3 - ps4
Asgard/Eukaryota - ps5
Eukaryota - ps6
Excavata/Diaphoretickes - ps7
Diaphoretickes - ps8
Archaeplastida - ps9
Viridiplantae - ps10
Streptophyta - ps11
Charophyceae/Embriophyta - ps12
Embriophyta - ps13
Tracheophyta - ps14
Spermatophyta - ps15
Angiosperms - ps16
Mesangiospermae - ps17
Magnoliids/eudicots - ps18
Eudicots - ps19
Core_eudicots - ps20
Superrosids - ps21
Rosids - ps22
Eurosids - ps23
Malvids - ps24
Sap./Malv./Brassicales - ps25
Malvales/Brassicales - ps26
Brassicales - ps27
Cleomaceae/Brassicaceae - ps28
Brassicaceae - ps29
Camelineae - ps30
Arabidopsis - ps31
Arabidopsis_thaliana - ps32
p-value
7e-44
2e-33
7e-23
2e-12
7e-02
Log-odds
0.5
1.0
1.5
2.0
2.5
3.0
3.5
4.0

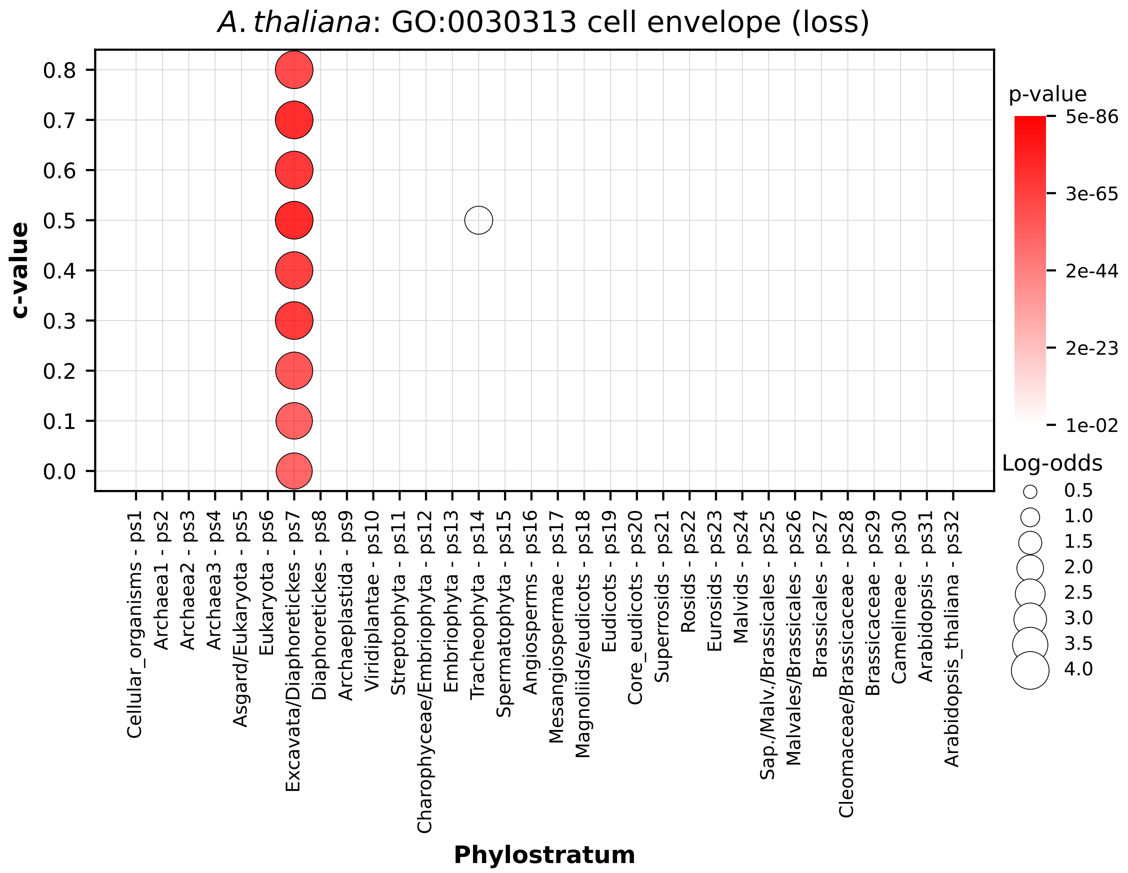

A. thaliana: GO:0030313 cell envelope (loss)
c-value
0.0
0.1
0.2
0.3
0.4
0.5
0.6
0.7
0.8
Phylostratum
Cellular_organisms - ps1
Archaea1 - ps2
Archaea2 - ps3
Archaea3 - ps4
Asgard/Eukaryota - ps5
Eukaryota - ps6
Excavata/Diaphoretickes - ps7
Diaphoretickes - ps8
Archaeplastida - ps9
Viridiplantae - ps10
Streptophyta - ps11
Charophyceae/Embriophyta - ps12
Embriophyta - ps13
Tracheophyta - ps14
Spermatophyta - ps15
Angiosperms - ps16
Mesangiospermae - ps17
Magnoliids/eudicots - ps18
Eudicots - ps19
Core_eudicots - ps20
Superrosids - ps21
Rosids - ps22
Eurosids - ps23
Malvids - ps24
Sap./Malv./Brassicales - ps25
Malvales/Brassicales - ps26
Brassicales - ps27
Cleomaceae/Brassicaceae - ps28
Brassicaceae - ps29
Camelineae - ps30
Arabidopsis - ps31
Arabidopsis_thaliana - ps32
p-value
5e-86
3e-65
2e-44
2e-23
1e-02
Log-odds
0.5
1.0
1.5
2.0
2.5
3.0
3.5
4.0

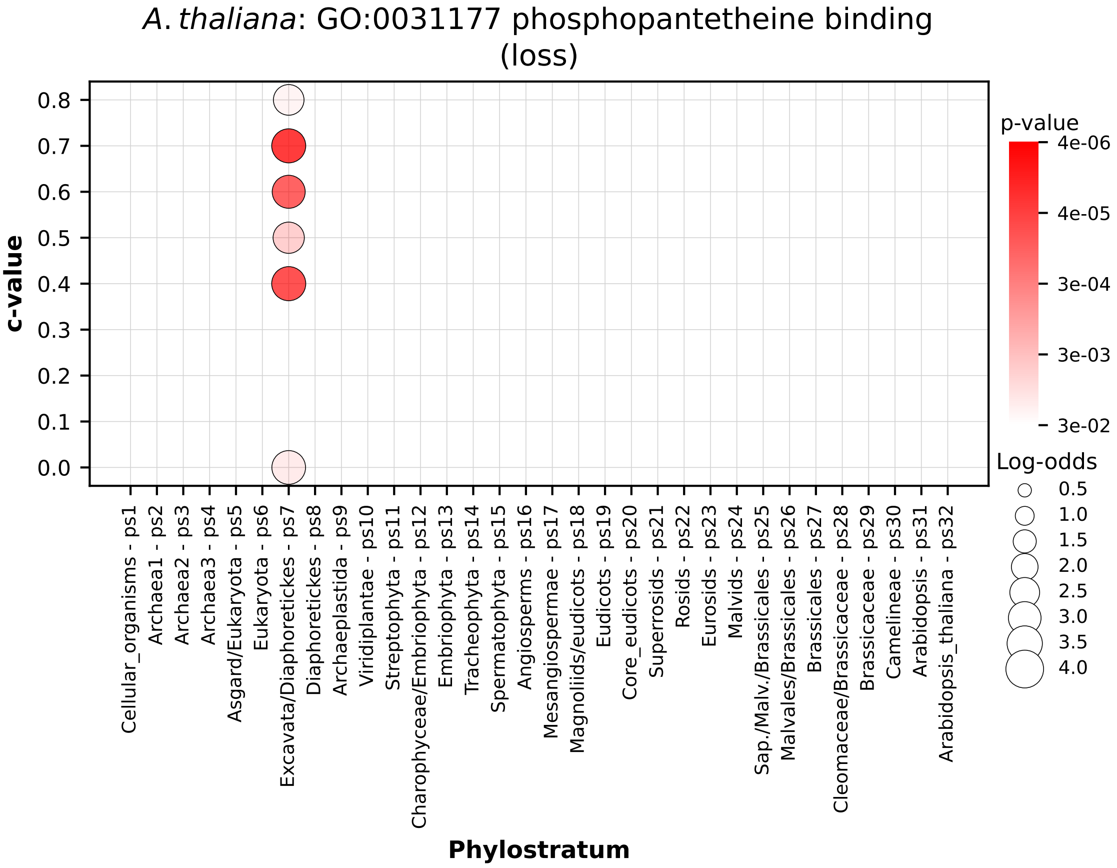

A. thaliana: GO:0031177 phosphopantetheine binding
(loss)
c-value
0.8
0.7
0.6
0.5
0.4
0.3
0.2
0.1
0.0
p-value
4e-06
4e-05
3e-04
3e-03
3e-02
Log-odds
0.5
1.0
1.5
2.0
2.5
3.0
3.5
4.0
Cellular_organisms - ps1
Archaea1 - ps2
Archaea2 - ps3
Archaea3 - ps4
Asgard/Eukaryota - ps5
Eukaryota - ps6
Excavata/Diaphoretickes - ps7
Diaphoretickes - ps8
Archaeplastida - ps9
Viridiplantae - ps10
Streptophyta - ps11
Charophyceae/Embriophyta - ps12
Embriophyta - ps13
Tracheophyta - ps14
Spermatophyta - ps15
Angiosperms - ps16
Mesangiospermae - ps17
Magnoliids/eudicots - ps18
Eudicots - ps19
Core_eudicots - ps20
Superrosids - ps21
Rosids - ps22
Eurosids - ps23
Malvids - ps24
Sap./Malv./Brassicales - ps25
Malvales/Brassicales - ps26
Brassicales - ps27
Cleomaceae/Brassicaceae - ps28
Brassicaceae - ps29
Camelineae - ps30
Arabidopsis - ps31
Arabidopsis_thaliana - ps32
Phylostratum

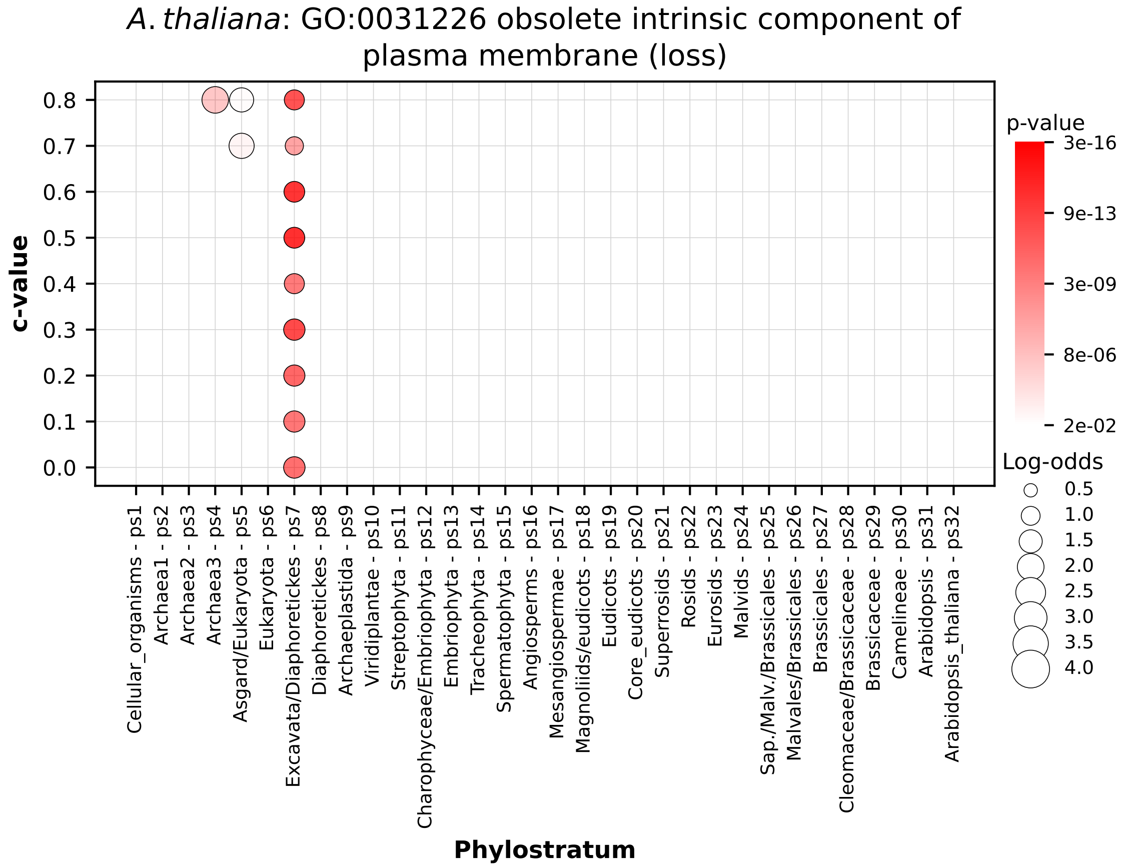

A. thaliana: GO:0031226 obsolete intrinsic component of plasma membrane (loss)
c-value
0.8
0.7
0.6
0.5
0.4
0.3
0.2
0.1
0.0
p-value
3e-16
9e-13
3e-09
8e-06
2e-02
Log-odds
0.5
1.0
1.5
2.0
2.5
3.0
3.5
4.0
Cellular_organisms - ps1
Archaea1 - ps2
Archaea2 - ps3
Archaea3 - ps4
Asgard/Eukaryota - ps5
Eukaryota - ps6
Excavata/Diaphoretickes - ps7
Diaphoretickes - ps8
Archaeplastida - ps9
Viridiplantae - ps10
Streptophyta - ps11
Charophyceae/Embriophyta - ps12
Embriophyta - ps13
Tracheophyta - ps14
Spermatophyta - ps15
Angiosperms - ps16
Mesangiospermae - ps17
Magnoliids/eudicots - ps18
Eudicots - ps19
Core_eudicots - ps20
Superrosids - ps21
Rosids - ps22
Eurosids - ps23
Malvids - ps24
Sap./Malv./Brassicales - ps25
Malvales/Brassicales - ps26
Brassicales - ps27
Cleomaceae/Brassicaceae - ps28
Brassicaceae - ps29
Camelineae - ps30
Arabidopsis - ps31
Arabidopsis_thaliana - ps32
Phylostratum

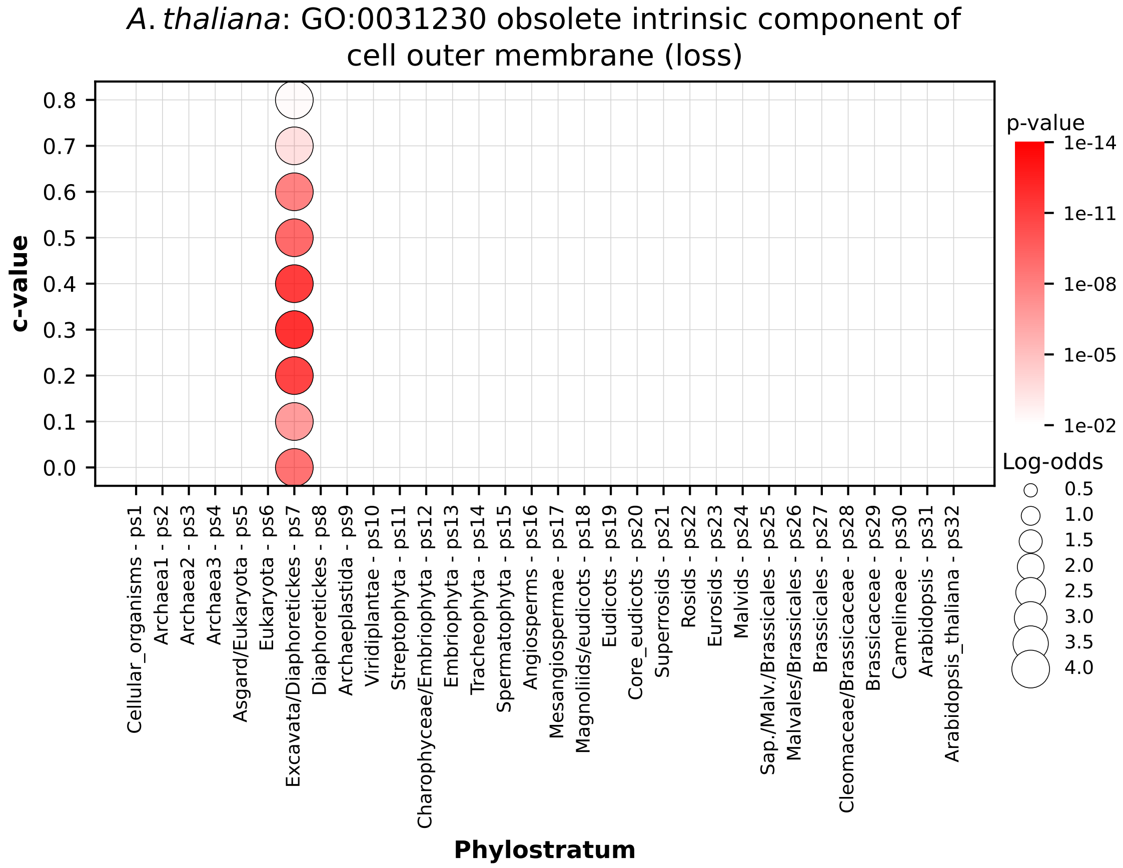

A. thaliana: GO:0031230 obsolete intrinsic component of cell outer membrane (loss)
c-value
0.8
0.7
0.6
0.5
0.4
0.3
0.2
0.1
0.0
p-value
1e-14
1e-11
1e-08
1e-05
1e-02
Log-odds
0.5
1.0
1.5
2.0
2.5
3.0
3.5
4.0
Cellular_organisms - ps1
Archaea1 - ps2
Archaea2 - ps3
Archaea3 - ps4
Asgard/Eukaryota - ps5
Eukaryota - ps6
Excavata/Diaphoretickes - ps7
Diaphoretickes - ps8
Archaeplastida - ps9
Viridiplantae - ps10
Streptophyta - ps11
Charophyceae/Embriophyta - ps12
Embriophyta - ps13
Tracheophyta - ps14
Spermatophyta - ps15
Angiosperms - ps16
Mesangiospermae - ps17
Magnoliids/eudicots - ps18
Eudicots - ps19
Core_eudicots - ps20
Superrosids - ps21
Rosids - ps22
Eurosids - ps23
Malvids - ps24
Sap./Malv./Brassicales - ps25
Malvales/Brassicales - ps26
Brassicales - ps27
Cleomaceae/Brassicaceae - ps28
Brassicaceae - ps29
Camelineae - ps30
Arabidopsis - ps31
Arabidopsis_thaliana - ps32
Phylostratum

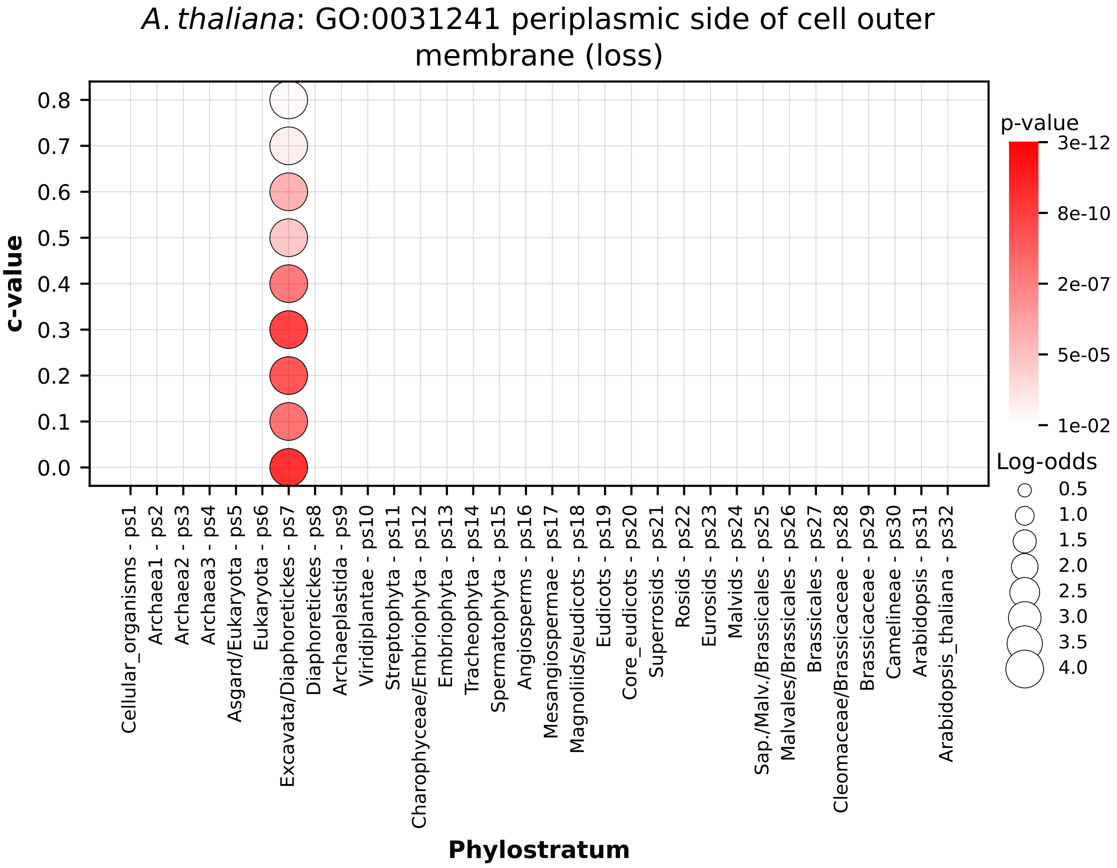

A. thaliana: GO:0031241 periplasmic side of cell outer membrane (loss)
c-value
0.8
0.7
0.6
0.5
0.4
0.3
0.2
0.1
0.0
p-value
3e-12
8e-10
2e-07
5e-05
1e-02
Log-odds
0.5
1.0
1.5
2.0
2.5
3.0
3.5
4.0
Cellular_organisms - ps1
Archaea1 - ps2
Archaea2 - ps3
Archaea3 - ps4
Asgard/Eukaryota - ps5
Eukaryota - ps6
Excavata/Diaphoretickes - ps7
Diaphoretickes - ps8
Archaeplastida - ps9
Viridiplantae - ps10
Streptophyta - ps11
Charophyceae/Embriophyta - ps12
Embriophyta - ps13
Tracheophyta - ps14
Spermatophyta - ps15
Angiosperms - ps16
Mesangiospermae - ps17
Magnoliids/eudicots - ps18
Eudicots - ps19
Core_eudicots - ps20
Superrosids - ps21
Rosids - ps22
Eurosids - ps23
Malvids - ps24
Sap./Malv./Brassicales - ps25
Malvales/Brassicales - ps26
Brassicales - ps27
Cleomaceae/Brassicaceae - ps28
Brassicaceae - ps29
Camelineae - ps30
Arabidopsis - ps31
Arabidopsis_thaliana - ps32
Phylostratum

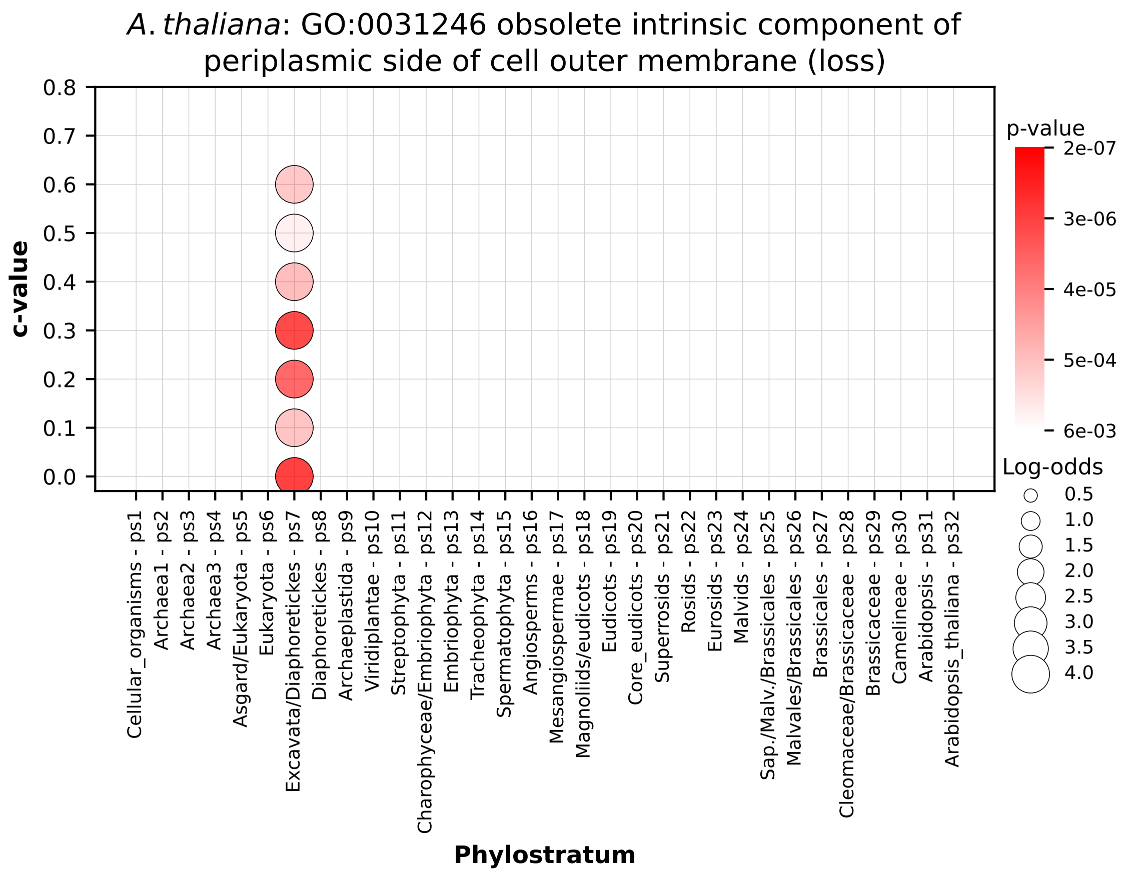

A. thaliana: GO:0031246 obsolete intrinsic component of periplasmic side of cell outer membrane (loss)
c-value
0.8
0.7
0.6
0.5
0.4
0.3
0.2
0.1
0.0
p-value
2e-07
3e-06
4e-05
5e-04
6e-03
Log-odds
0.5
1.0
1.5
2.0
2.5
3.0
3.5
4.0
Cellular_organisms - ps1
Archaea1 - ps2
Archaea2 - ps3
Archaea3 - ps4
Asgard/Eukaryota - ps5
Eukaryota - ps6
Excavata/Diaphoretickes - ps7
Diaphoretickes - ps8
Archaeplastida - ps9
Viridiplantae - ps10
Streptophyta - ps11
Charophyceae/Embriophyta - ps12
Embriophyta - ps13
Tracheophyta - ps14
Spermatophyta - ps15
Angiosperms - ps16
Mesangiospermae - ps17
Magnoliids/eudicots - ps18
Eudicots - ps19
Core_eudicots - ps20
Superrosids - ps21
Rosids - ps22
Eurosids - ps23
Malvids - ps24
Sap./Malv./Brassicales - ps25
Malvales/Brassicales - ps26
Brassicales - ps27
Cleomaceae/Brassicaceae - ps28
Brassicaceae - ps29
Camelineae - ps30
Arabidopsis - ps31
Arabidopsis_thaliana - ps32
Phylostratum

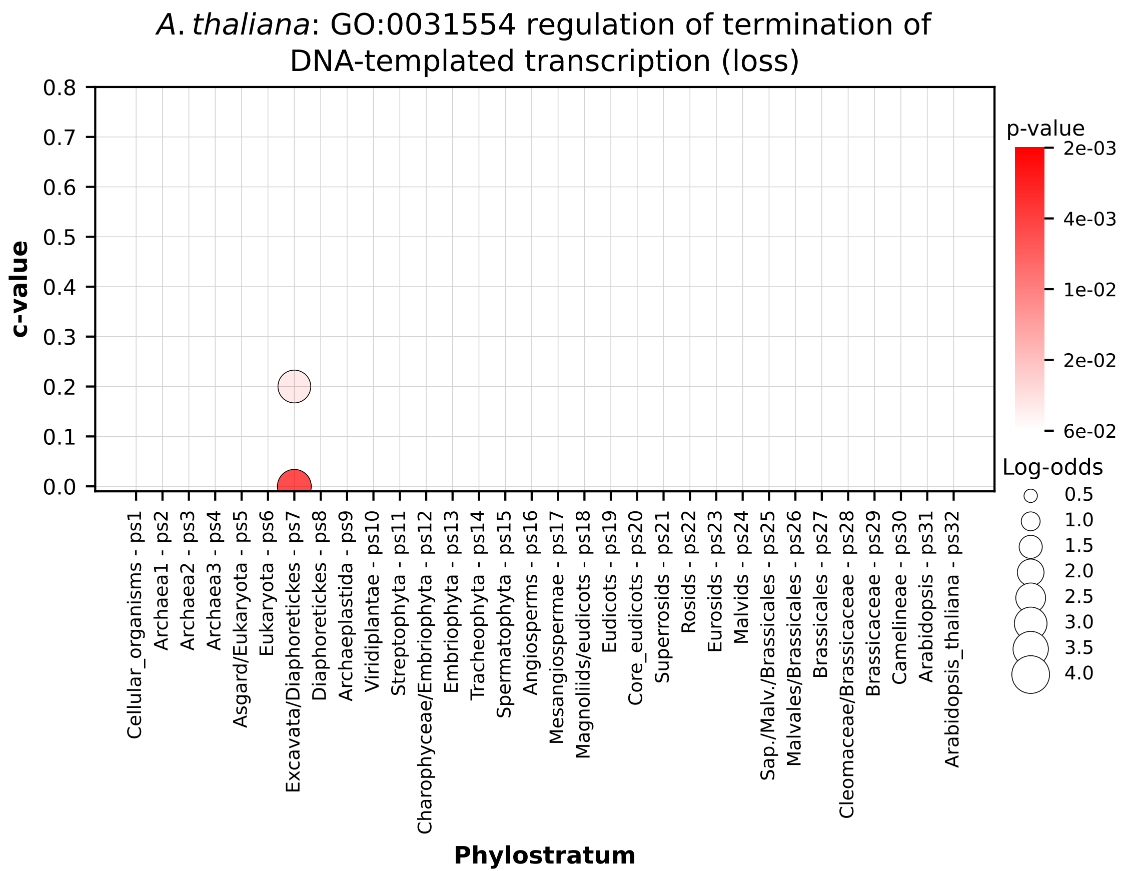

A. thaliana: GO:0031554 regulation of termination of DNA-templated transcription (loss)
c-value
0.0
0.1
0.2
0.3
0.4
0.5
0.6
0.7
0.8
p-value
2e-03
4e-03
1e-02
2e-02
6e-02
Log-odds
0.5
1.0
1.5
2.0
2.5
3.0
3.5
4.0
Cellular_organisms - ps1
Archaea1 - ps2
Archaea2 - ps3
Archaea3 - ps4
Asgard/Eukaryota - ps5
Eukaryota - ps6
Excavata/Diaphoretickes - ps7
Diaphoretickes - ps8
Archaeplastida - ps9
Viridiplantae - ps10
Streptophyta - ps11
Charophyceae/Embriophyta - ps12
Embriophyta - ps13
Tracheophyta - ps14
Spermatophyta - ps15
Angiosperms - ps16
Mesangiospermae - ps17
Magnoliids/eudicots - ps18
Eudicots - ps19
Core_eudicots - ps20
Superrosids - ps21
Rosids - ps22
Eurosids - ps23
Malvids - ps24
Sap./Malv./Brassicales - ps25
Malvales/Brassicales - ps26
Brassicales - ps27
Cleomaceae/Brassicaceae - ps28
Brassicaceae - ps29
Camelineae - ps30
Arabidopsis - ps31
Arabidopsis_thaliana - ps32
Phylostratum

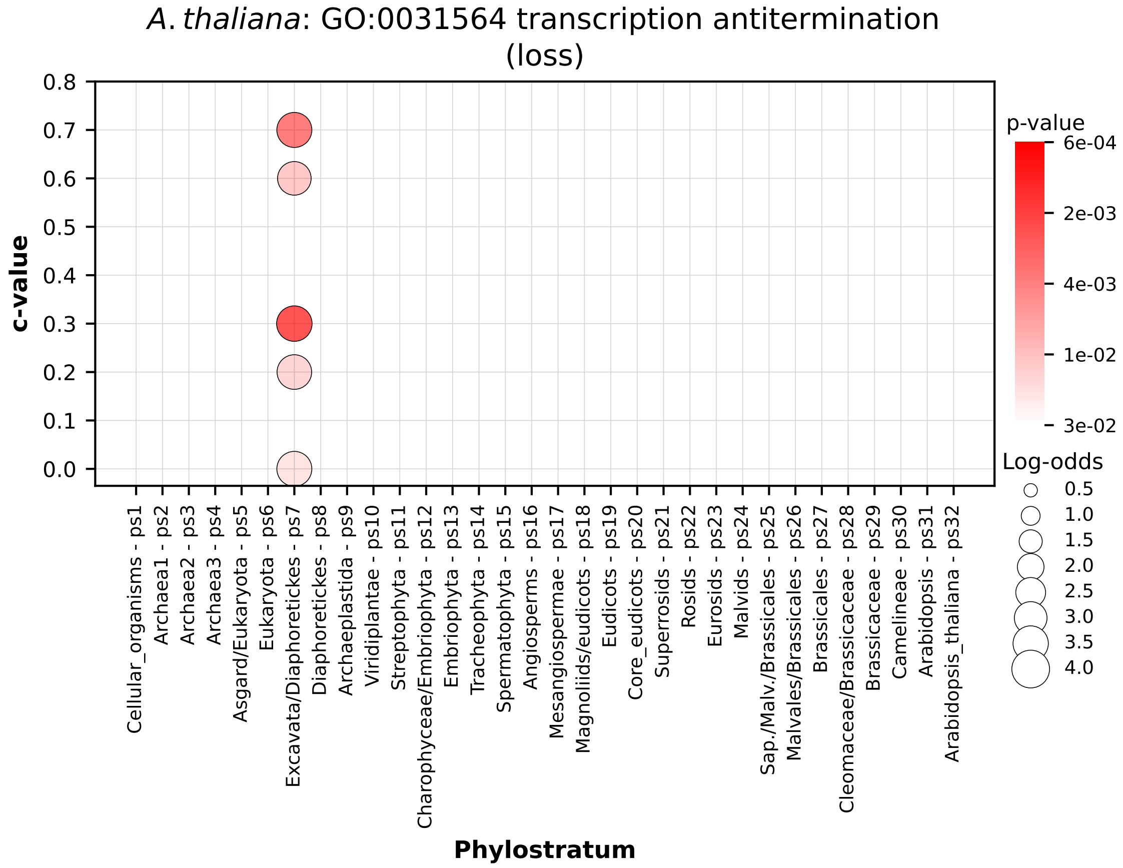

A. thaliana: GO:0031564 transcription antitermination (loss)
c-value
0.8
0.7
0.6
0.5
0.4
0.3
0.2
0.1
0.0
p-value
6e-04
2e-03
4e-03
1e-02
3e-02
Log-odds
0.5
1.0
1.5
2.0
2.5
3.0
3.5
4.0
Cellular_organisms - ps1
Archaea1 - ps2
Archaea2 - ps3
Archaea3 - ps4
Asgard/Eukaryota - ps5
Eukaryota - ps6
Excavata/Diaphoretickes - ps7
Diaphoretickes - ps8
Archaeplastida - ps9
Viridiplantae - ps10
Streptophyta - ps11
Charophyceae/Embriophyta - ps12
Embriophyta - ps13
Tracheophyta - ps14
Spermatophyta - ps15
Angiosperms - ps16
Mesangiospermae - ps17
Magnoliids/eudicots - ps18
Eudicots - ps19
Core_eudicots - ps20
Superrosids - ps21
Rosids - ps22
Eurosids - ps23
Malvids - ps24
Sap./Malv./Brassicales - ps25
Malvales/Brassicales - ps26
Brassicales - ps27
Cleomaceae/Brassicaceae - ps28
Brassicaceae - ps29
Camelineae - ps30
Arabidopsis - ps31
Arabidopsis_thaliana - ps32
Phylostratum

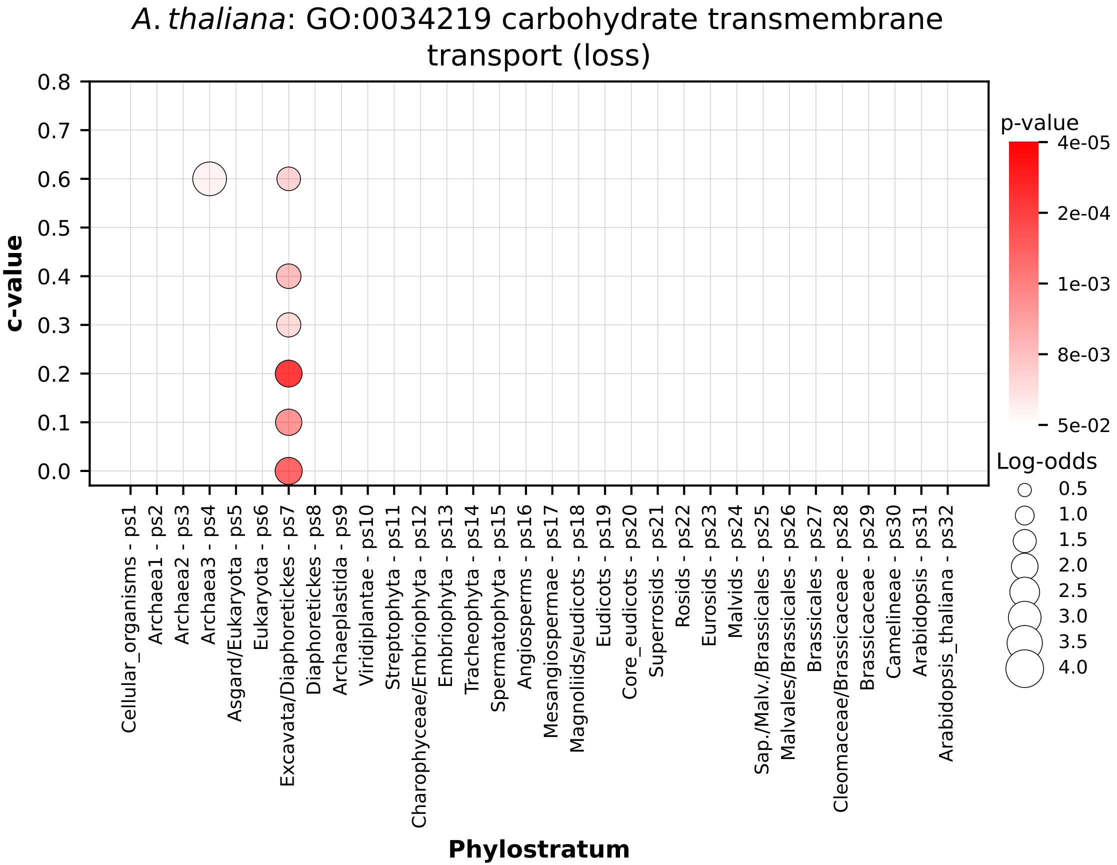

A. thaliana: GO:0034219 carbohydrate transmembrane transport (loss)
c-value
0.8
0.7
0.6
0.5
0.4
0.3
0.2
0.1
0.0
Phylostratum
Cellular_organisms - ps1
Archaea1 - ps2
Archaea2 - ps3
Archaea3 - ps4
Asgard/Eukaryota - ps5
Eukaryota - ps6
Excavata/Diaphoretickes - ps7
Diaphoretickes - ps8
Archaeplastida - ps9
Viridiplantae - ps10
Streptophyta - ps11
Charophyceae/Embriophyta - ps12
Embriophyta - ps13
Tracheophyta - ps14
Spermatophyta - ps15
Angiosperms - ps16
Mesangiospermae - ps17
Magnoliids/eudicots - ps18
Eudicots - ps19
Core_eudicots - ps20
Superrosids - ps21
Rosids - ps22
Eurosids - ps23
Malvids - ps24
Sap./Malv./Brassicales - ps25
Malvales/Brassicales - ps26
Brassicales - ps27
Cleomaceae/Brassicaceae - ps28
Brassicaceae - ps29
Camelineae - ps30
Arabidopsis - ps31
Arabidopsis_thaliana - ps32
p-value
4e-05
2e-04
1e-03
8e-03
5e-02
Log-odds
0.5
1.0
1.5
2.0
2.5
3.0
3.5
4.0

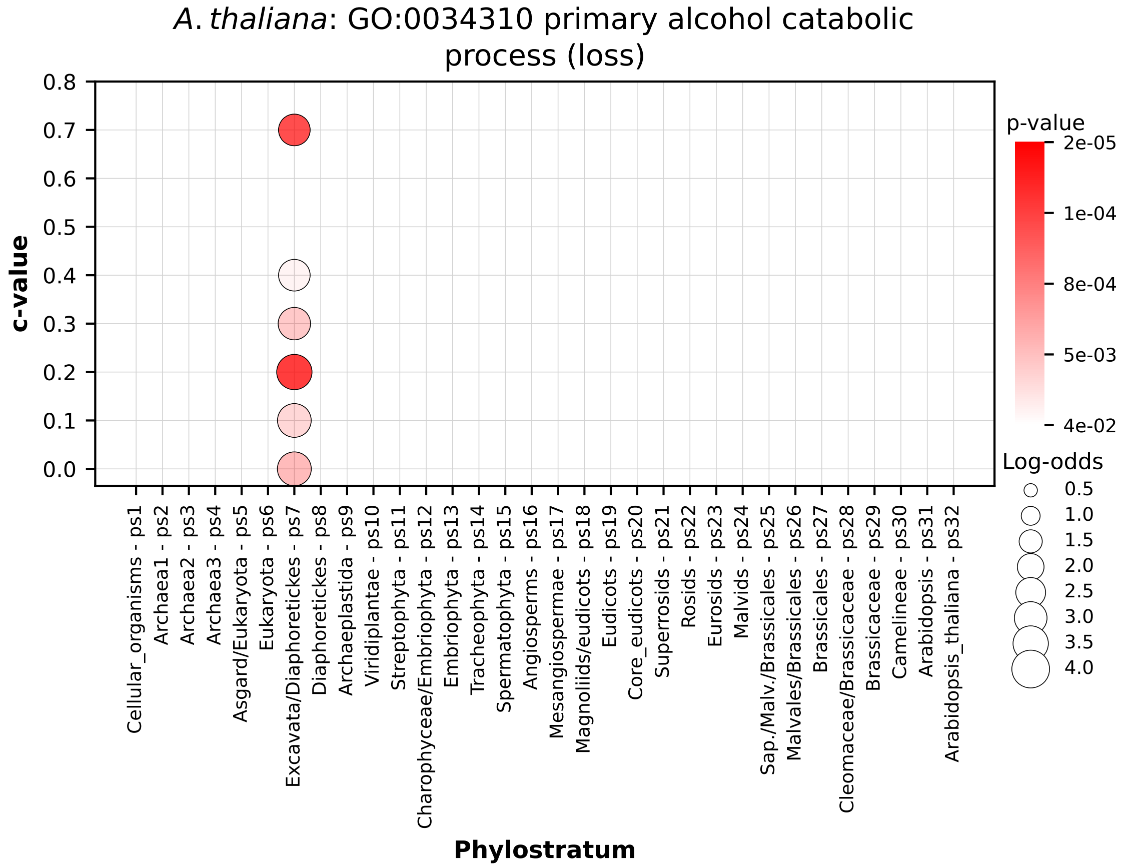
A. thaliana: GO:0034310 primary alcohol catabolic process (loss)
c-value
0.8
0.7
0.6
0.5
0.4
0.3
0.2
0.1
0.0
Phylostratum
Cellular_organisms - ps1
Archaea1 - ps2
Archaea2 - ps3
Archaea3 - ps4
Asgard/Eukaryota - ps5
Eukaryota - ps6
Excavata/Diaphoretickes - ps7
Diaphoretickes - ps8
Archaeplastida - ps9
Viridiplantae - ps10
Streptophyta - ps11
Charophyceae/Embriophyta - ps12
Embriophyta - ps13
Tracheophyta - ps14
Spermatophyta - ps15
Angiosperms - ps16
Mesangiospermae - ps17
Magnoliids/eudicots - ps18
Eudicots - ps19
Core_eudicots - ps20
Superrosids - ps21
Rosids - ps22
Eurosids - ps23
Malvids - ps24
Sap./Malv./Brassicales - ps25
Malvales/Brassicales - ps26
Brassicales - ps27
Cleomaceae/Brassicaceae - ps28
Brassicaceae - ps29
Camelineae - ps30
Arabidopsis - ps31
Arabidopsis_thaliana - ps32
p-value
2e-05
1e-04
8e-04
5e-03
4e-02
Log-odds
0.5
1.0
1.5
2.0
2.5
3.0
3.5
4.0

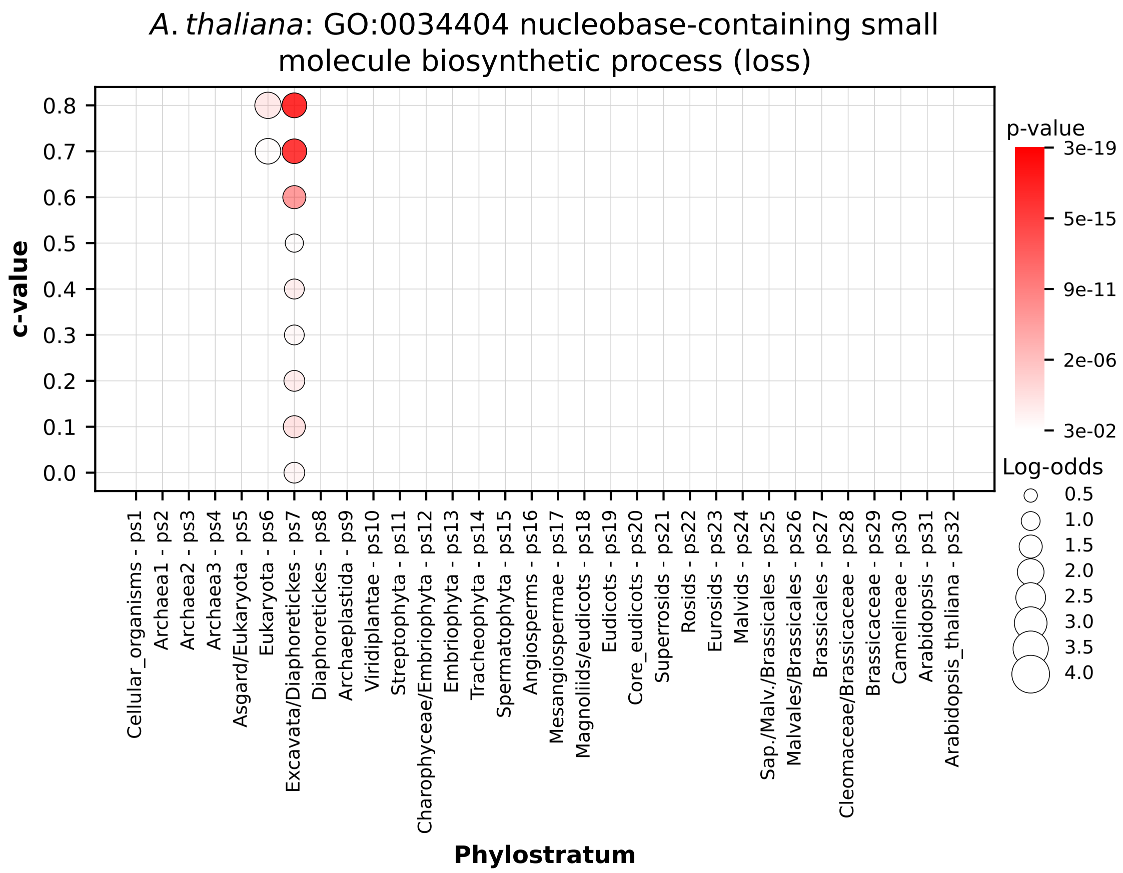
A. thaliana: GO:0034404 nucleobase-containing small molecule biosynthetic process (loss)
c-value
0.8
0.7
0.6
0.5
0.4
0.3
0.2
0.1
0.0
Phylostratum
Cellular_organisms - ps1
Archaea1 - ps2
Archaea2 - ps3
Archaea3 - ps4
Asgard/Eukaryota - ps5
Eukaryota - ps6
Excavata/Diaphoretickes - ps7
Diaphoretickes - ps8
Archaeplastida - ps9
Viridiplantae - ps10
Streptophyta - ps11
Charophyceae/Embriophyta - ps12
Embriophyta - ps13
Tracheophyta - ps14
Spermatophyta - ps15
Angiosperms - ps16
Mesangiospermae - ps17
Magnoliids/eudicots - ps18
Eudicots - ps19
Core_eudicots - ps20
Superrosids - ps21
Rosids - ps22
Eurosids - ps23
Malvids - ps24
Sap./Malv./Brassicales - ps25
Malvales/Brassicales - ps26
Brassicales - ps27
Cleomaceae/Brassicaceae - ps28
Brassicaceae - ps29
Camelineae - ps30
Arabidopsis - ps31
Arabidopsis_thaliana - ps32
p-value
3e-19
5e-15
9e-11
2e-06
3e-02
Log-odds
0.5
1.0
1.5
2.0
2.5
3.0
3.5
4.0

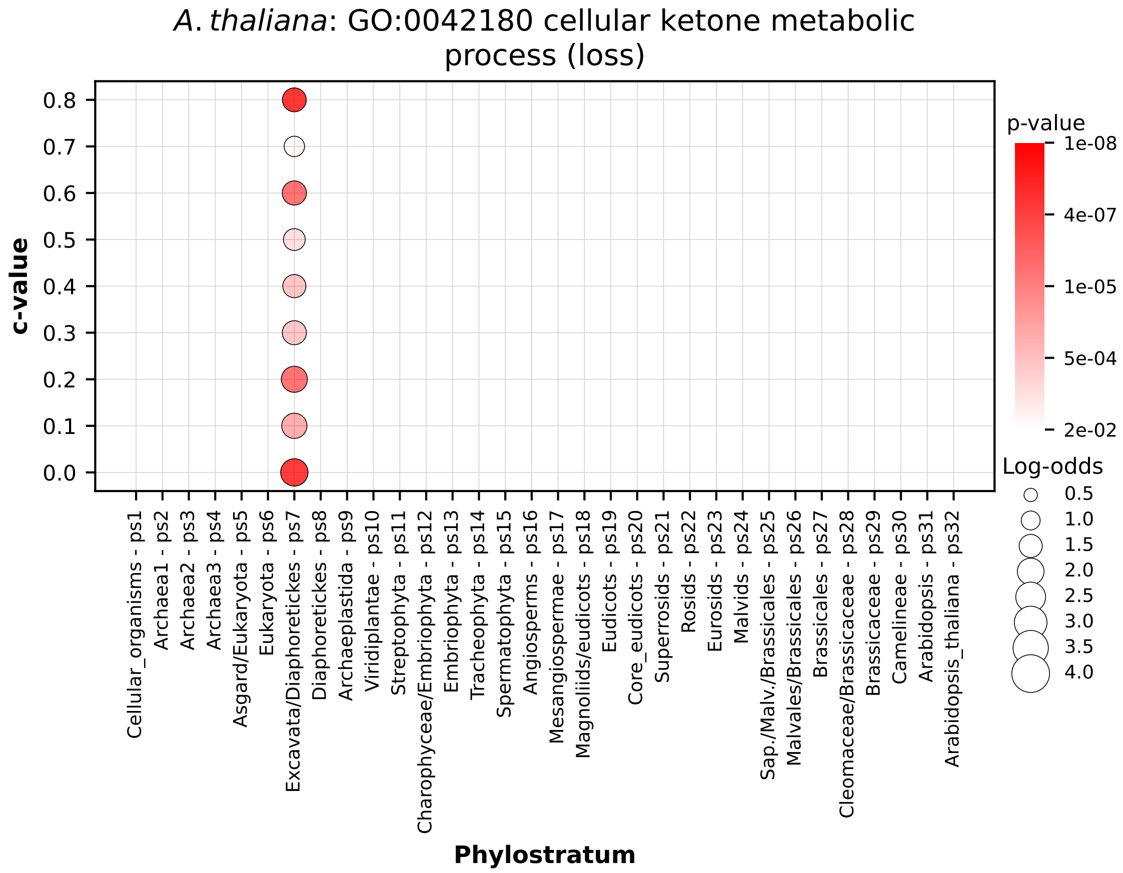

A. thaliana: GO:0042180 cellular ketone metabolic process (loss)
c-value
0.8
0.7
0.6
0.5
0.4
0.3
0.2
0.1
0.0
p-value
1e-08
4e-07
1e-05
5e-04
2e-02
Log-odds
0.5
1.0
1.5
2.0
2.5
3.0
3.5
4.0
Cellular_organisms - ps1
Archaea1 - ps2
Archaea2 - ps3
Archaea3 - ps4
Asgard/Eukaryota - ps5
Eukaryota - ps6
Excavata/Diaphoretickes - ps7
Diaphoretickes - ps8
Archaeplastida - ps9
Viridiplantae - ps10
Streptophyta - ps11
Charophyceae/Embriophyta - ps12
Embriophyta - ps13
Tracheophyta - ps14
Spermatophyta - ps15
Angiosperms - ps16
Mesangiospermae - ps17
Magnoliids/eudicots - ps18
Eudicots - ps19
Core_eudicots - ps20
Superrosids - ps21
Rosids - ps22
Eurosids - ps23
Malvids - ps24
Sap./Malv./Brassicales - ps25
Malvales/Brassicales - ps26
Brassicales - ps27
Cleomaceae/Brassicaceae - ps28
Brassicaceae - ps29
Camelineae - ps30
Arabidopsis - ps31
Arabidopsis_thaliana - ps32
Phylostratum

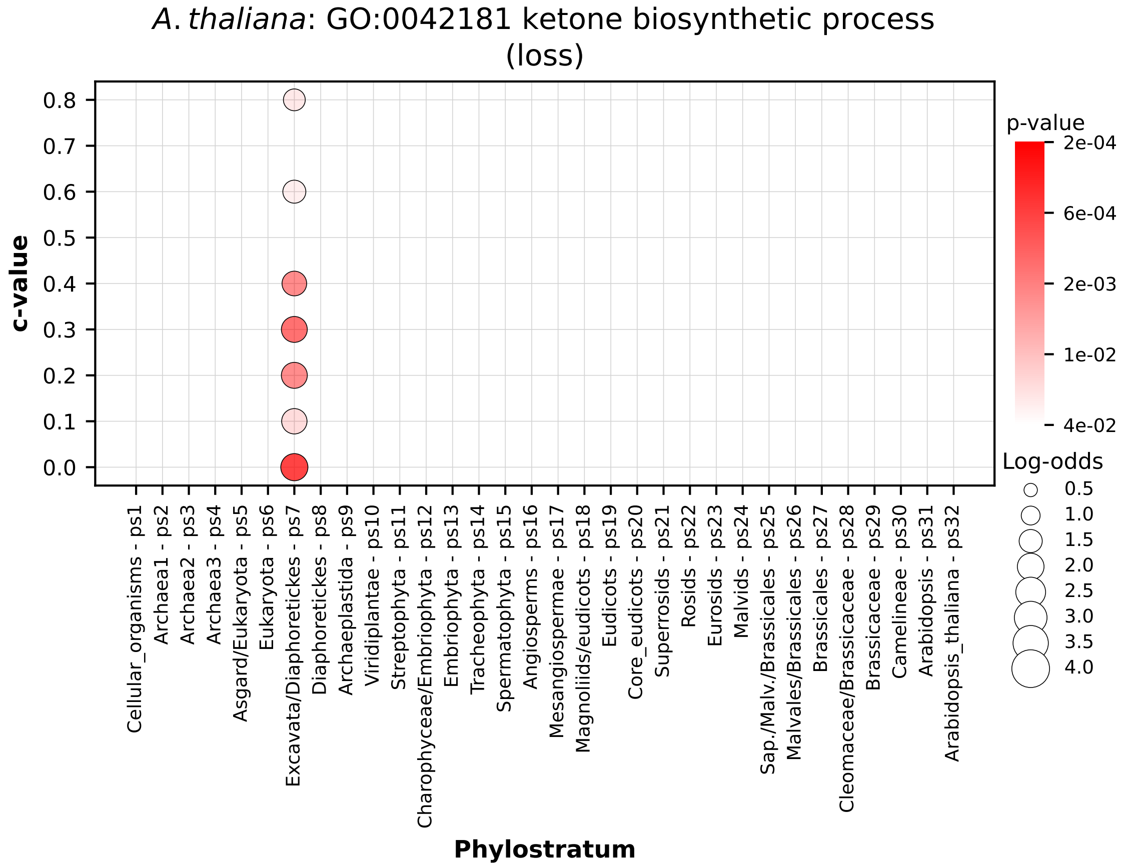

A. thaliana: GO:0042181 ketone biosynthetic process
(loss)
c-value
0.8
0.7
0.6
0.5
0.4
0.3
0.2
0.1
0.0
p-value
2e-04
6e-04
2e-03
1e-02
4e-02
Log-odds
0.5
1.0
1.5
2.0
2.5
3.0
3.5
4.0
Cellular_organisms - ps1
Archaea1 - ps2
Archaea2 - ps3
Archaea3 - ps4
Asgard/Eukaryota - ps5
Eukaryota - ps6
Excavata/Diaphoretickes - ps7
Diaphoretickes - ps8
Archaeplastida - ps9
Viridiplantae - ps10
Streptophyta - ps11
Charophyceae/Embriophyta - ps12
Embriophyta - ps13
Tracheophyta - ps14
Spermatophyta - ps15
Angiosperms - ps16
Mesangiospermae - ps17
Magnoliids/eudicots - ps18
Eudicots - ps19
Core_eudicots - ps20
Superrosids - ps21
Rosids - ps22
Eurosids - ps23
Malvids - ps24
Sap./Malv./Brassicales - ps25
Malvales/Brassicales - ps26
Brassicales - ps27
Cleomaceae/Brassicaceae - ps28
Brassicaceae - ps29
Camelineae - ps30
Arabidopsis - ps31
Arabidopsis_thaliana - ps32
Phylostratum

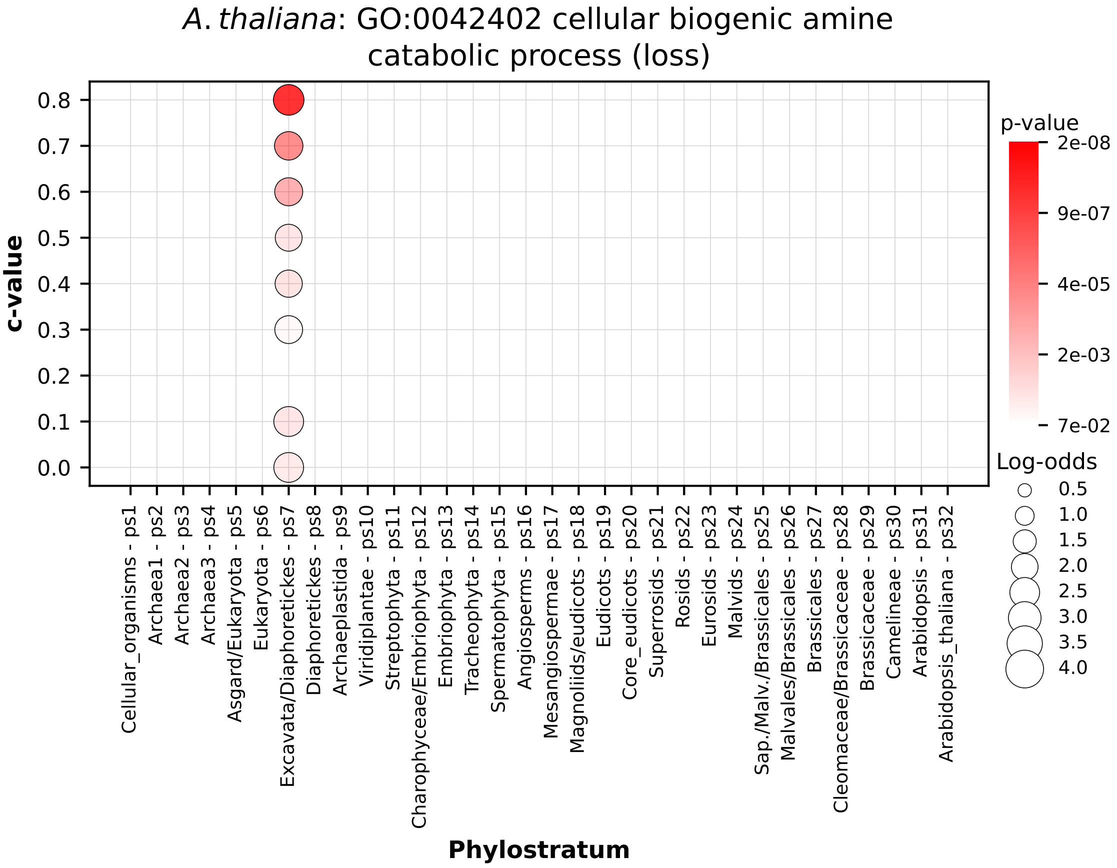

A. thaliana: GO:0042402 cellular biogenic amine catabolic process (loss)
c-value
0.8
0.7
0.6
0.5
0.4
0.3
0.2
0.1
0.0
Phylostratum
Cellular_organisms - ps1
Archaea1 - ps2
Archaea2 - ps3
Archaea3 - ps4
Asgard/Eukaryota - ps5
Eukaryota - ps6
Excavata/Diaphoretickes - ps7
Diaphoretickes - ps8
Archaeplastida - ps9
Viridiplantae - ps10
Streptophyta - ps11
Charophyceae/Embriophyta - ps12
Embriophyta - ps13
Tracheophyta - ps14
Spermatophyta - ps15
Angiosperms - ps16
Mesangiospermae - ps17
Magnoliids/eudicots - ps18
Eudicots - ps19
Core_eudicots - ps20
Superrosids - ps21
Rosids - ps22
Eurosids - ps23
Malvids - ps24
Sap./Malv./Brassicales - ps25
Malvales/Brassicales - ps26
Brassicales - ps27
Cleomaceae/Brassicaceae - ps28
Brassicaceae - ps29
Camelineae - ps30
Arabidopsis - ps31
Arabidopsis_thaliana - ps32
p-value
2e-08
9e-07
4e-05
2e-03
7e-02
Log-odds
0.5
1.0
1.5
2.0
2.5
3.0
3.5
4.0

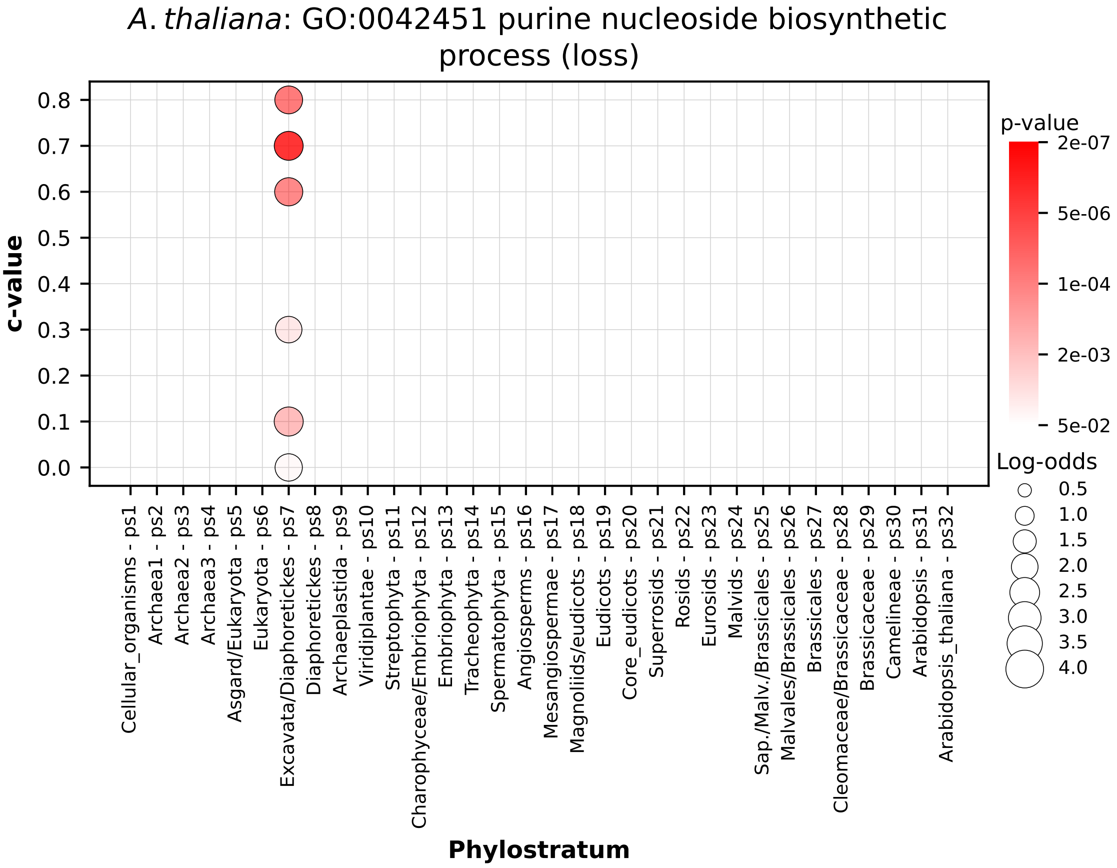
A. thaliana: GO:0042451 purine nucleoside biosynthetic process (loss)
c-value
0.8
0.7
0.6
0.5
0.4
0.3
0.2
0.1
0.0
Phylostratum
Cellular_organisms - ps1
Archaea1 - ps2
Archaea2 - ps3
Archaea3 - ps4
Asgard/Eukaryota - ps5
Eukaryota - ps6
Excavata/Diaphoretickes - ps7
Diaphoretickes - ps8
Archaeplastida - ps9
Viridiplantae - ps10
Streptophyta - ps11
Charophyceae/Embriophyta - ps12
Embriophyta - ps13
Tracheophyta - ps14
Spermatophyta - ps15
Angiosperms - ps16
Mesangiospermae - ps17
Magnoliids/eudicots - ps18
Eudicots - ps19
Core_eudicots - ps20
Superrosids - ps21
Rosids - ps22
Eurosids - ps23
Malvids - ps24
Sap./Malv./Brassicales - ps25
Malvales/Brassicales - ps26
Brassicales - ps27
Cleomaceae/Brassicaceae - ps28
Brassicaceae - ps29
Camelineae - ps30
Arabidopsis - ps31
Arabidopsis_thaliana - ps32
p-value
2e-07
5e-06
1e-04
2e-03
5e-02
Log-odds
0.5
1.0
1.5
2.0
2.5
3.0
3.5
4.0

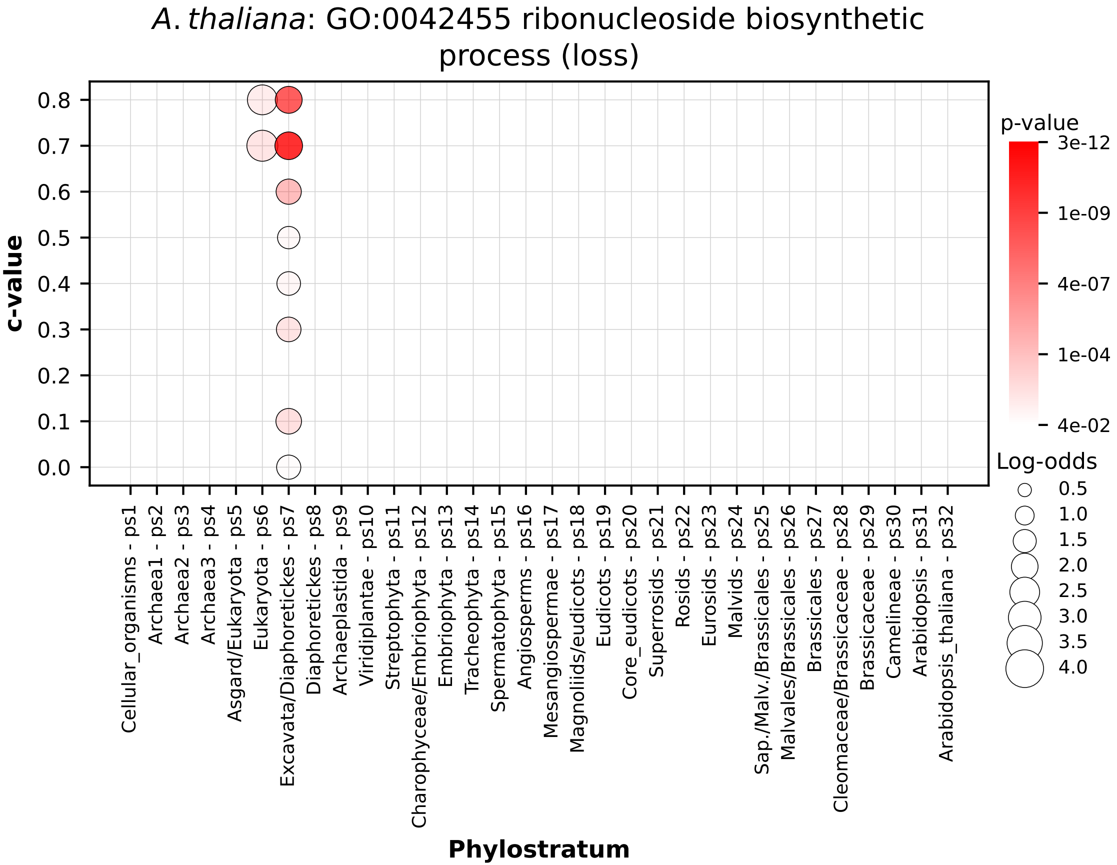

A. thaliana: GO:0042455 ribonucleoside biosynthetic process (loss)
c-value
0.8
0.7
0.6
0.5
0.4
0.3
0.2
0.1
0.0
Phylostratum
Cellular_organisms - ps1
Archaea1 - ps2
Archaea2 - ps3
Archaea3 - ps4
Asgard/Eukaryota - ps5
Eukaryota - ps6
Excavata/Diaphoretickes - ps7
Diaphoretickes - ps8
Archaeplastida - ps9
Viridiplantae - ps10
Streptophyta - ps11
Charophyceae/Embriophyta - ps12
Embriophyta - ps13
Tracheophyta - ps14
Spermatophyta - ps15
Angiosperms - ps16
Mesangiospermae - ps17
Magnoliids/eudicots - ps18
Eudicots - ps19
Core_eudicots - ps20
Superrosids - ps21
Rosids - ps22
Eurosids - ps23
Malvids - ps24
Sap./Malv./Brassicales - ps25
Malvales/Brassicales - ps26
Brassicales - ps27
Cleomaceae/Brassicaceae - ps28
Brassicaceae - ps29
Camelineae - ps30
Arabidopsis - ps31
Arabidopsis_thaliana - ps32
p-value
3e-12
1e-09
4e-07
1e-04
4e-02
Log-odds
0.5
1.0
1.5
2.0
2.5
3.0
3.5
4.0

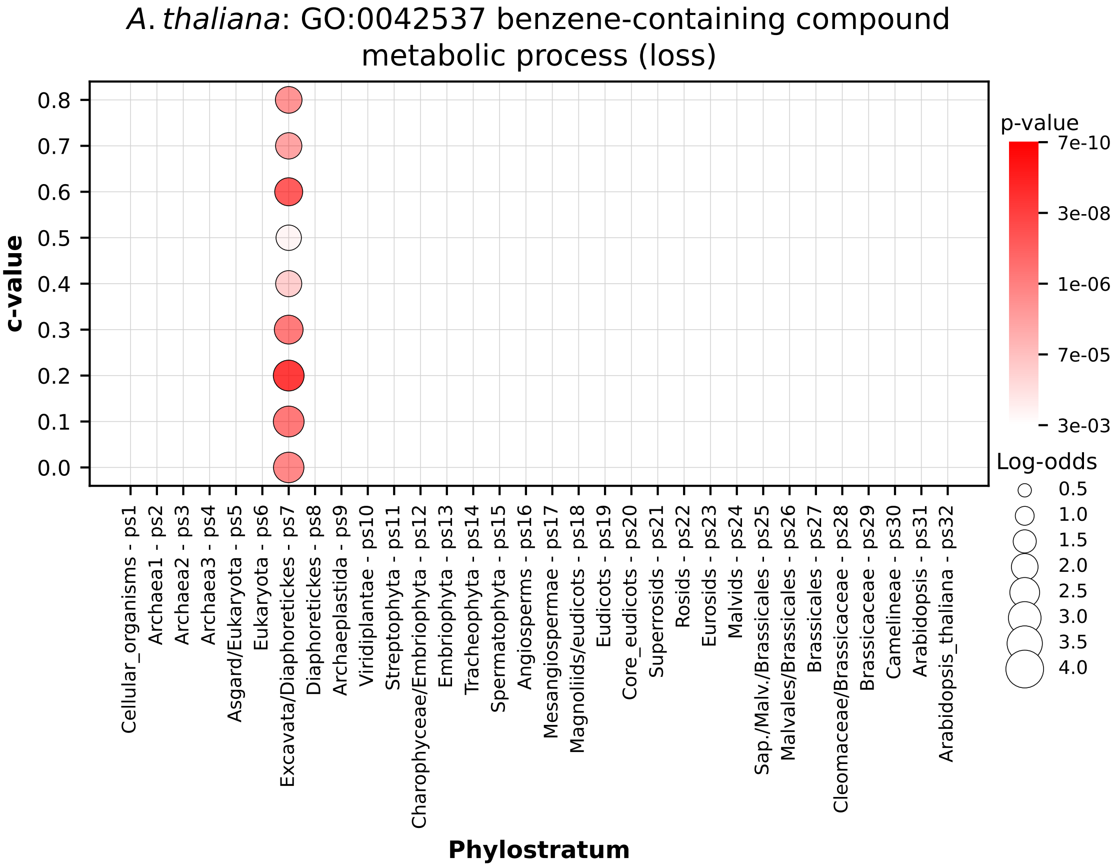

A. thaliana: GO:0042537 benzene-containing compound metabolic process (loss)
c-value
0.8
0.7
0.6
0.5
0.4
0.3
0.2
0.1
0.0
p-value
7e-10
3e-08
1e-06
7e-05
3e-03
Log-odds
0.5
1.0
1.5
2.0
2.5
3.0
3.5
4.0
Cellular_organisms - ps1
Archaea1 - ps2
Archaea2 - ps3
Archaea3 - ps4
Asgard/Eukaryota - ps5
Eukaryota - ps6
Excavata/Diaphoretickes - ps7
Diaphoretickes - ps8
Archaeplastida - ps9
Viridiplantae - ps10
Streptophyta - ps11
Charophyceae/Embriophyta - ps12
Embriophyta - ps13
Tracheophyta - ps14
Spermatophyta - ps15
Angiosperms - ps16
Mesangiospermae - ps17
Magnoliids/eudicots - ps18
Eudicots - ps19
Core_eudicots - ps20
Superrosids - ps21
Rosids - ps22
Eurosids - ps23
Malvids - ps24
Sap./Malv./Brassicales - ps25
Malvales/Brassicales - ps26
Brassicales - ps27
Cleomaceae/Brassicaceae - ps28
Brassicaceae - ps29
Camelineae - ps30
Arabidopsis - ps31
Arabidopsis_thaliana - ps32
Phylostratum

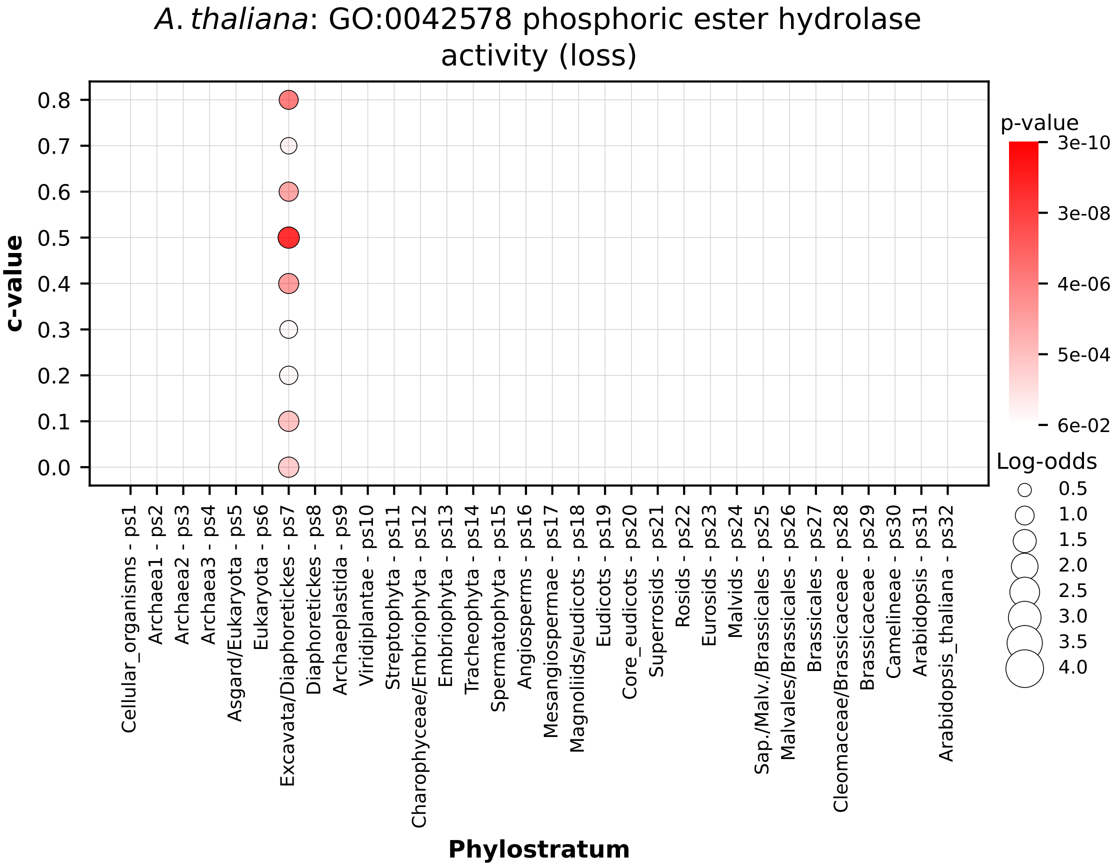

A. thaliana: GO:0042578 phosphoric ester hydrolase activity (loss)
c-value
0.8
0.7
0.6
0.5
0.4
0.3
0.2
0.1
0.0
p-value
3e-10
3e-08
4e-06
5e-04
6e-02
Log-odds
0.5
1.0
1.5
2.0
2.5
3.0
3.5
4.0
Cellular_organisms - ps1
Archaea1 - ps2
Archaea2 - ps3
Archaea3 - ps4
Asgard/Eukaryota - ps5
Eukaryota - ps6
Excavata/Diaphoretickes - ps7
Diaphoretickes - ps8
Archaeplastida - ps9
Viridiplantae - ps10
Streptophyta - ps11
Charophyceae/Embriophyta - ps12
Embriophyta - ps13
Tracheophyta - ps14
Spermatophyta - ps15
Angiosperms - ps16
Mesangiospermae - ps17
Magnoliids/eudicots - ps18
Eudicots - ps19
Core_eudicots - ps20
Superrosids - ps21
Rosids - ps22
Eurosids - ps23
Malvids - ps24
Sap./Malv./Brassicales - ps25
Malvales/Brassicales - ps26
Brassicales - ps27
Cleomaceae/Brassicaceae - ps28
Brassicaceae - ps29
Camelineae - ps30
Arabidopsis - ps31
Arabidopsis_thaliana - ps32
Phylostratum

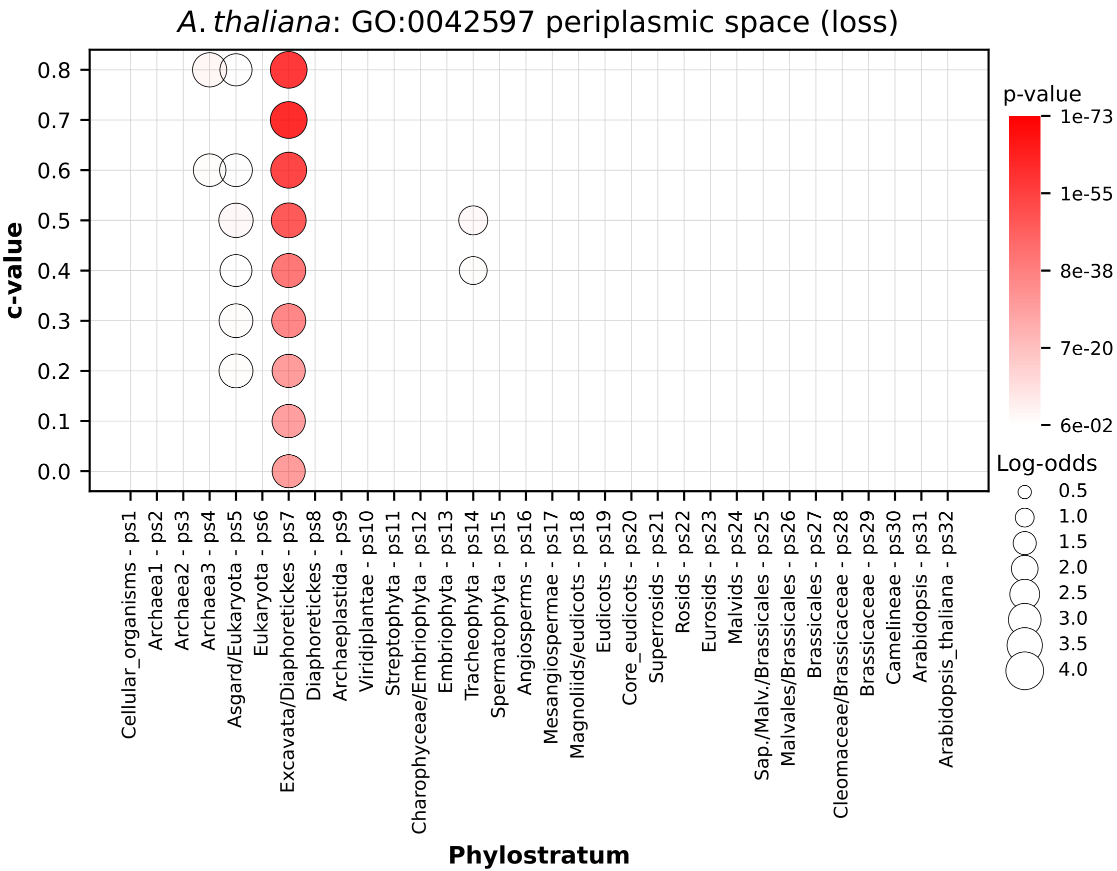

A. thaliana: GO:0042597 periplasmic space (loss)
c-value
0.8
0.7
0.6
0.5
0.4
0.3
0.2
0.1
0.0
p-value
1e-73
1e-55
8e-38
7e-20
6e-02
Log-odds
0.5
1.0
1.5
2.0
2.5
3.0
3.5
4.0
Cellular_organisms - ps1
Archaea1 - ps2
Archaea2 - ps3
Archaea3 - ps4
Asgard/Eukaryota - ps5
Eukaryota - ps6
Excavata/Diaphoretickes - ps7
Diaphoretickes - ps8
Archaeplastida - ps9
Viridiplantae - ps10
Streptophyta - ps11
Charophyceae/Embriophyta - ps12
Embriophyta - ps13
Tracheophyta - ps14
Spermatophyta - ps15
Angiosperms - ps16
Mesangiospermae - ps17
Magnoliids/eudicots - ps18
Eudicots - ps19
Core_eudicots - ps20
Superrosids - ps21
Rosids - ps22
Eurosids - ps23
Malvids - ps24
Sap./Malv./Brassicales - ps25
Malvales/Brassicales - ps26
Brassicales - ps27
Cleomaceae/Brassicaceae - ps28
Brassicaceae - ps29
Camelineae - ps30
Arabidopsis - ps31
Arabidopsis_thaliana - ps32
Phylostratum

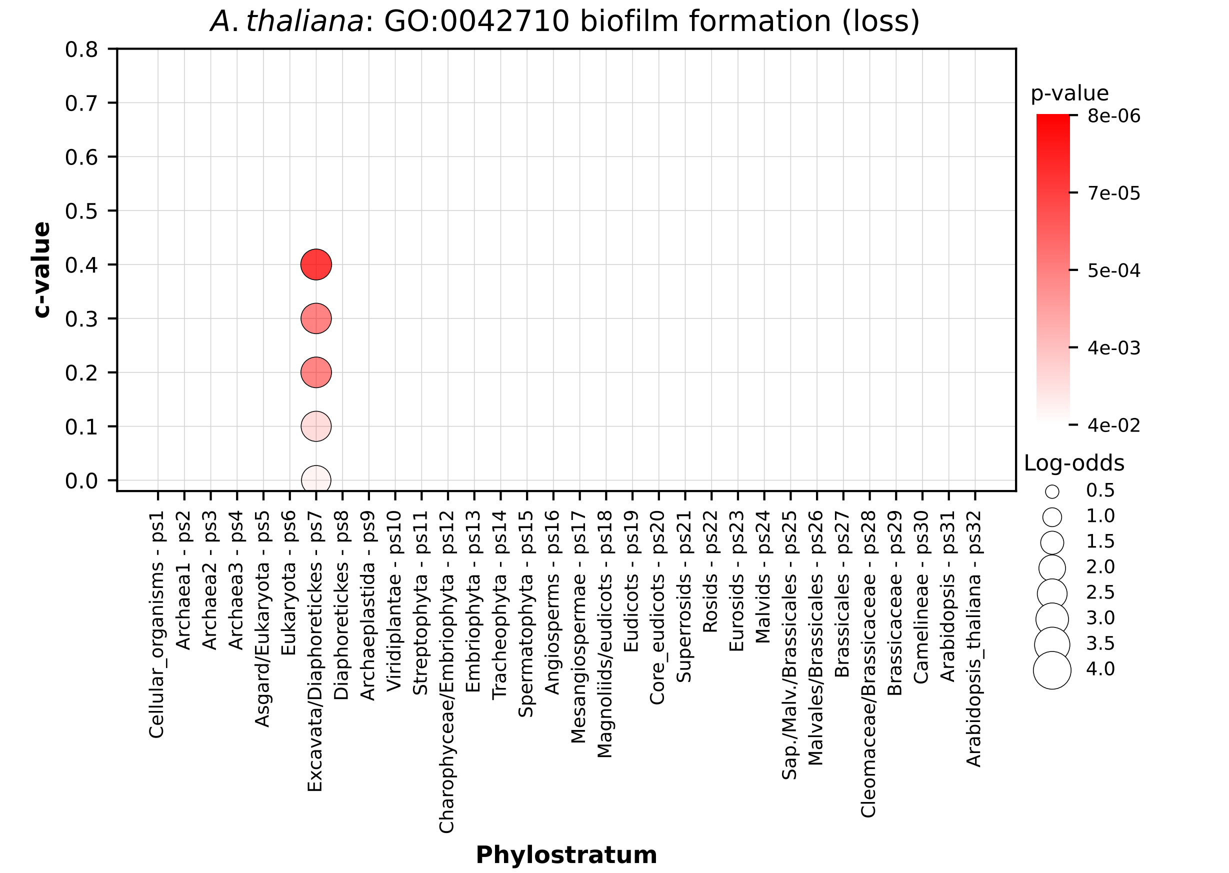

A. thaliana: GO:0042710 biofilm formation (loss)
c-value
0.0
0.1
0.2
0.3
0.4
0.5
0.6
0.7
0.8
Phylostratum
Cellular_organisms - ps1
Archaea1 - ps2
Archaea2 - ps3
Archaea3 - ps4
Asgard/Eukaryota - ps5
Eukaryota - ps6
Excavata/Diaphoretickes - ps7
Diaphoretickes - ps8
Archaeplastida - ps9
Viridiplantae - ps10
Streptophyta - ps11
Charophyceae/Embriophyta - ps12
Embriophyta - ps13
Tracheophyta - ps14
Spermatophyta - ps15
Angiosperms - ps16
Mesangiospermae - ps17
Magnoliids/eudicots - ps18
Eudicots - ps19
Core_eudicots - ps20
Superrosids - ps21
Rosids - ps22
Eurosids - ps23
Malvids - ps24
Sap./Malv./Brassicales - ps25
Malvales/Brassicales - ps26
Brassicales - ps27
Cleomaceae/Brassicaceae - ps28
Brassicaceae - ps29
Camelineae - ps30
Arabidopsis - ps31
Arabidopsis_thaliana - ps32
p-value
8e-06
7e-05
5e-04
4e-03
4e-02
Log-odds
0.5
1.0
1.5
2.0
2.5
3.0
3.5
4.0

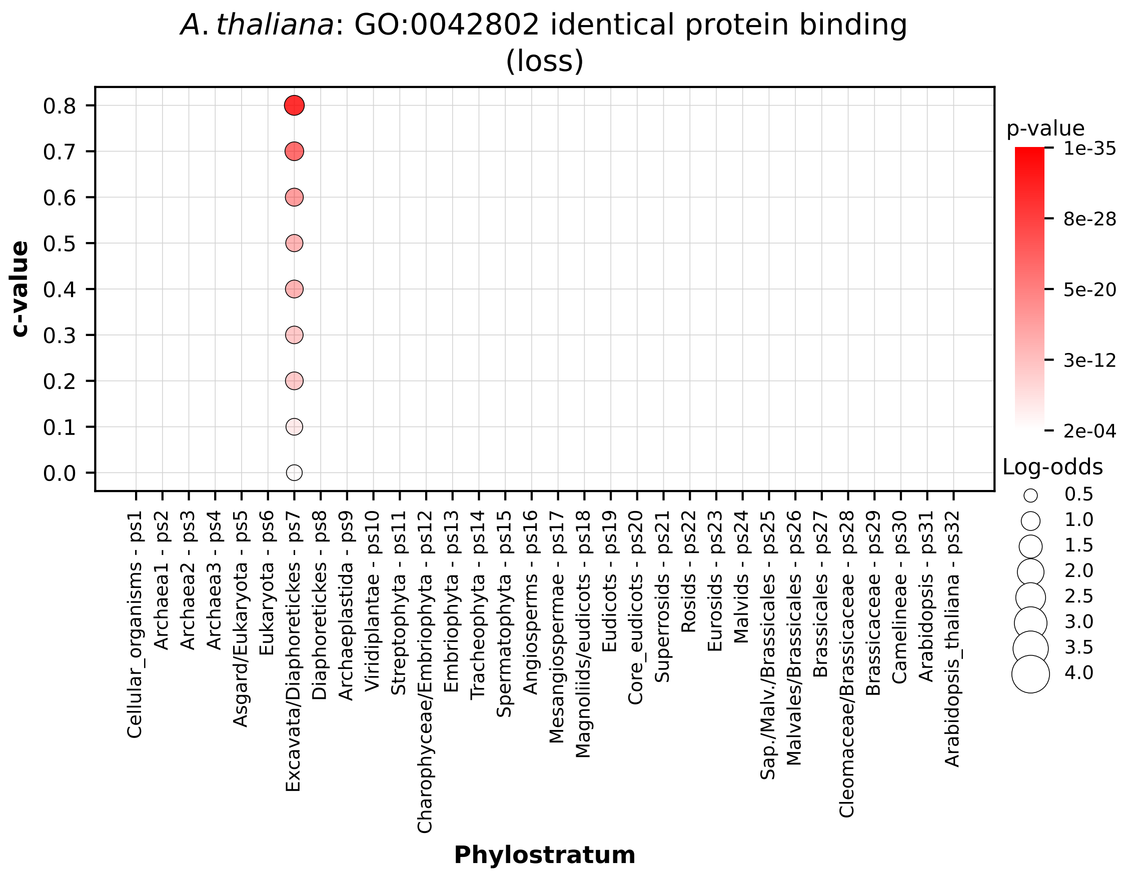

A. thaliana: GO:0042802 identical protein binding (loss)
c-value
0.0
0.1
0.2
0.3
0.4
0.5
0.6
0.7
0.8
Phylostratum
Cellular_organisms - ps1
Archaea1 - ps2
Archaea2 - ps3
Archaea3 - ps4
Asgard/Eukaryota - ps5
Eukaryota - ps6
Excavata/Diaphoretickes - ps7
Diaphoretickes - ps8
Archaeplastida - ps9
Viridiplantae - ps10
Streptophyta - ps11
Charophyceae/Embriophyta - ps12
Embriophyta - ps13
Tracheophyta - ps14
Spermatophyta - ps15
Angiosperms - ps16
Mesangiospermae - ps17
Magnoliids/eudicots - ps18
Eudicots - ps19
Core_eudicots - ps20
Superrosids - ps21
Rosids - ps22
Eurosids - ps23
Malvids - ps24
Sap./Malv./Brassicales - ps25
Malvales/Brassicales - ps26
Brassicales - ps27
Cleomaceae/Brassicaceae - ps28
Brassicaceae - ps29
Camelineae - ps30
Arabidopsis - ps31
Arabidopsis_thaliana - ps32
p-value
1e-35
8e-28
5e-20
3e-12
2e-04
Log-odds
0.5
1.0
1.5
2.0
2.5
3.0
3.5
4.0

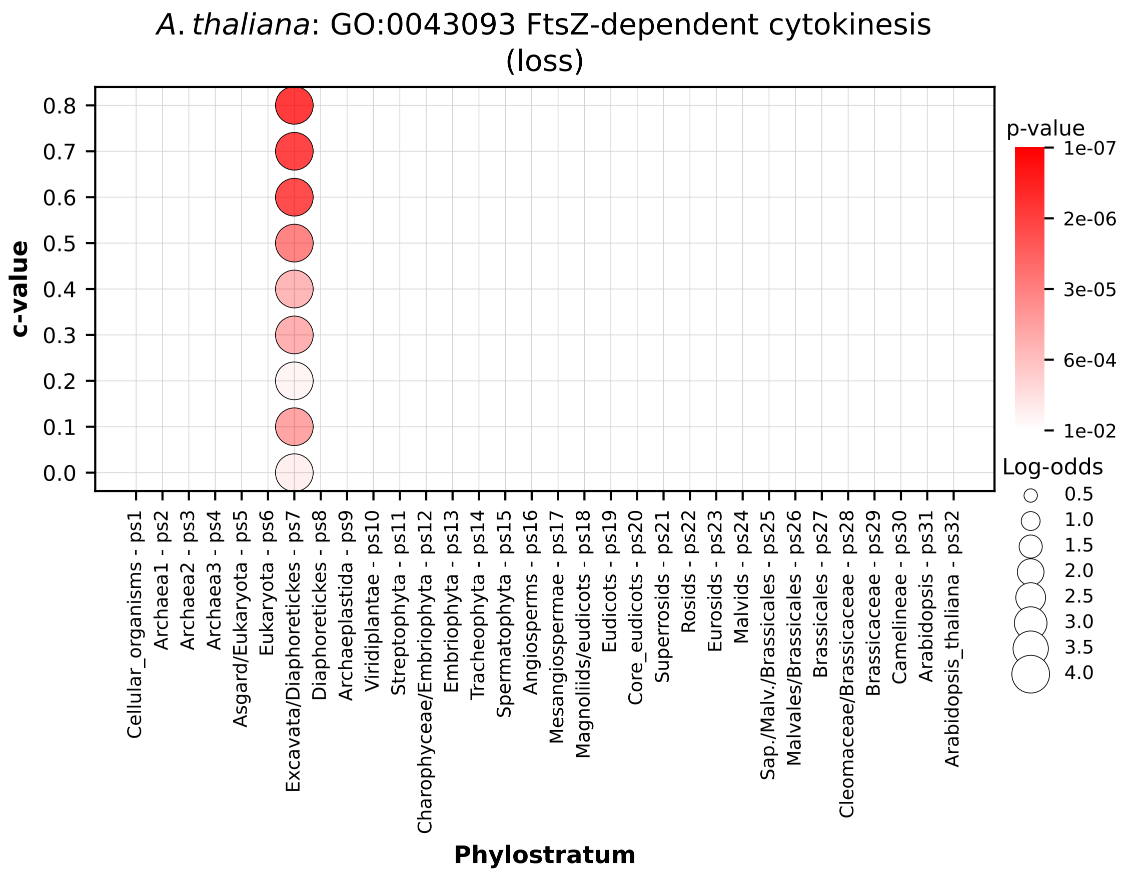

A. thaliana: GO:0043093 FtsZ-dependent cytokinesis
(loss)
c-value
0.8
0.7
0.6
0.5
0.4
0.3
0.2
0.1
0.0
Phylostratum
Cellular_organisms - ps1
Archaea1 - ps2
Archaea2 - ps3
Archaea3 - ps4
Asgard/Eukaryota - ps5
Eukaryota - ps6
Excavata/Diaphoretickes - ps7
Diaphoretickes - ps8
Archaeplastida - ps9
Viridiplantae - ps10
Streptophyta - ps11
Charophyceae/Embriophyta - ps12
Embriophyta - ps13
Tracheophyta - ps14
Spermatophyta - ps15
Angiosperms - ps16
Mesangiospermae - ps17
Magnoliids/eudicots - ps18
Eudicots - ps19
Core_eudicots - ps20
Superrosids - ps21
Rosids - ps22
Eurosids - ps23
Malvids - ps24
Sap./Malv./Brassicales - ps25
Malvales/Brassicales - ps26
Brassicales - ps27
Cleomaceae/Brassicaceae - ps28
Brassicaceae - ps29
Camelineae - ps30
Arabidopsis - ps31
Arabidopsis_thaliana - ps32
p-value
1e-07
2e-06
3e-05
6e-04
1e-02
Log-odds
0.5
1.0
1.5
2.0
2.5
3.0
3.5
4.0

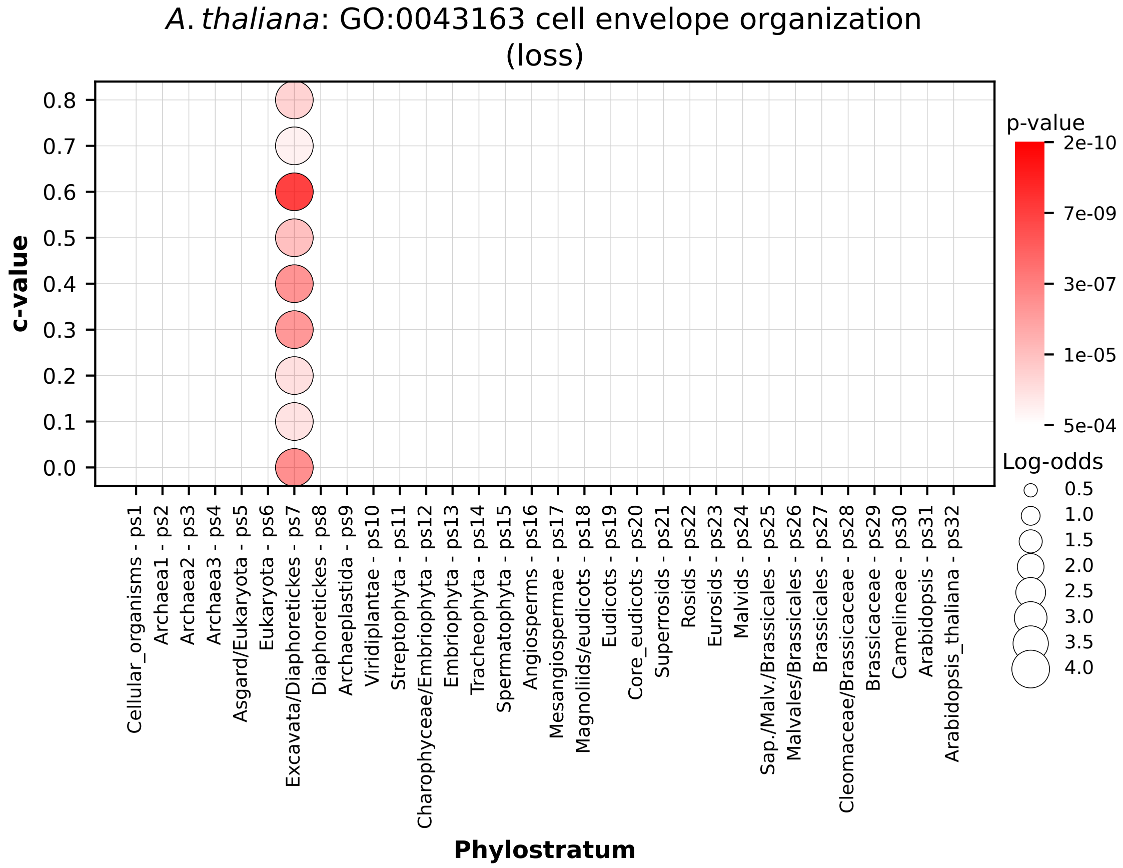

A. thaliana: GO:0043163 cell envelope organization
(loss)
c-value
0.0
0.1
0.2
0.3
0.4
0.5
0.6
0.7
0.8
Phylostratum
Cellular_organisms - ps1
Archaea1 - ps2
Archaea2 - ps3
Archaea3 - ps4
Asgard/Eukaryota - ps5
Eukaryota - ps6
Excavata/Diaphoretickes - ps7
Diaphoretickes - ps8
Archaeplastida - ps9
Viridiplantae - ps10
Streptophyta - ps11
Charophyceae/Embriophyta - ps12
Embriophyta - ps13
Tracheophyta - ps14
Spermatophyta - ps15
Angiosperms - ps16
Mesangiospermae - ps17
Magnoliids/eudicots - ps18
Eudicots - ps19
Core_eudicots - ps20
Superrosids - ps21
Rosids - ps22
Eurosids - ps23
Malvids - ps24
Sap./Malv./Brassicales - ps25
Malvales/Brassicales - ps26
Brassicales - ps27
Cleomaceae/Brassicaceae - ps28
Brassicaceae - ps29
Camelineae - ps30
Arabidopsis - ps31
Arabidopsis_thaliana - ps32
p-value
2e-10
7e-09
3e-07
1e-05
5e-04
Log-odds
0.5
1.0
1.5
2.0
2.5
3.0
3.5
4.0

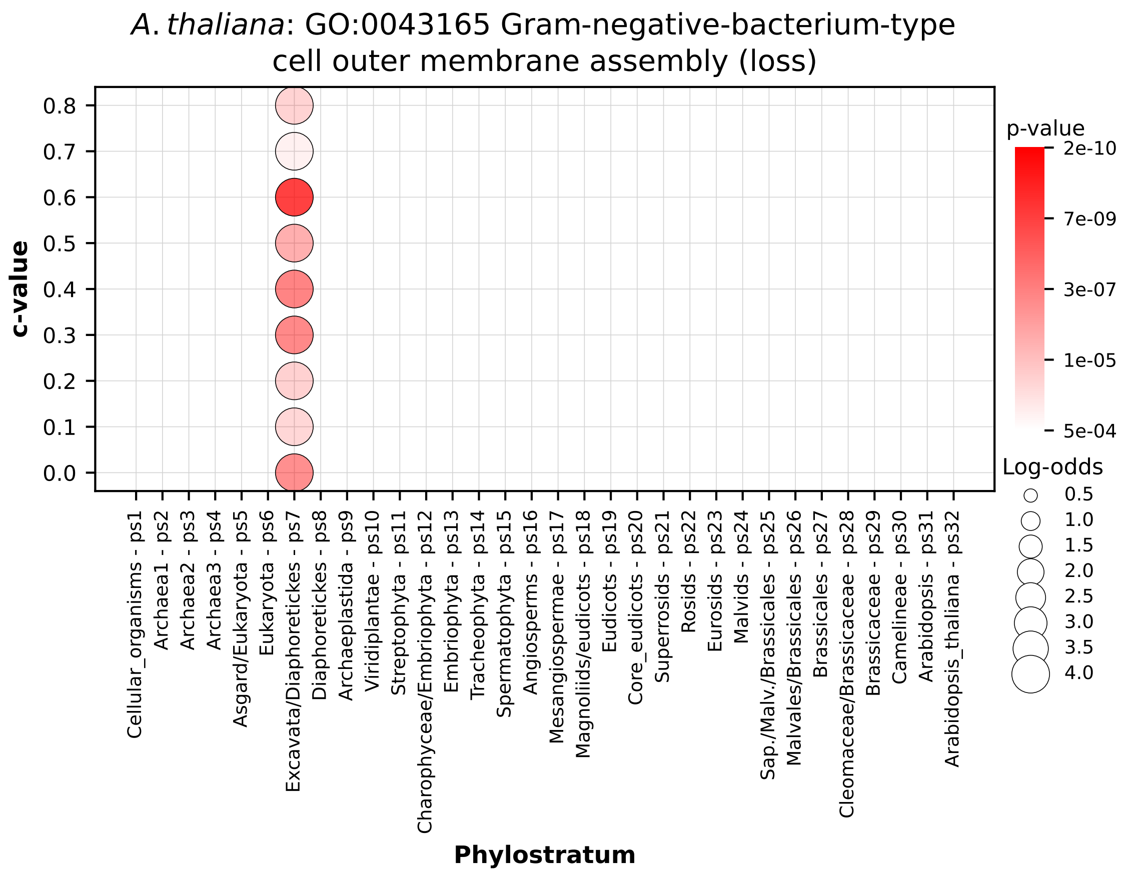

A. thaliana: GO:0043165 Gram-negative-bacterium-type cell outer membrane assembly (loss)
c-value
0.8
0.7
0.6
0.5
0.4
0.3
0.2
0.1
0.0
Phylostratum
Cellular_organisms - ps1
Archaea1 - ps2
Archaea2 - ps3
Archaea3 - ps4
Asgard/Eukaryota - ps5
Eukaryota - ps6
Excavata/Diaphoretickes - ps7
Diaphoretickes - ps8
Archaeplastida - ps9
Viridiplantae - ps10
Streptophyta - ps11
Charophyceae/Embriophyta - ps12
Embriophyta - ps13
Tracheophyta - ps14
Spermatophyta - ps15
Angiosperms - ps16
Mesangiospermae - ps17
Magnoliids/eudicots - ps18
Eudicots - ps19
Core_eudicots - ps20
Superrosids - ps21
Rosids - ps22
Eurosids - ps23
Malvids - ps24
Sap./Malv./Brassicales - ps25
Malvales/Brassicales - ps26
Brassicales - ps27
Cleomaceae/Brassicaceae - ps28
Brassicaceae - ps29
Camelineae - ps30
Arabidopsis - ps31
Arabidopsis_thaliana - ps32
p-value
2e-10
7e-09
3e-07
1e-05
5e-04
Log-odds
0.5
1.0
1.5
2.0
2.5
3.0
3.5
4.0

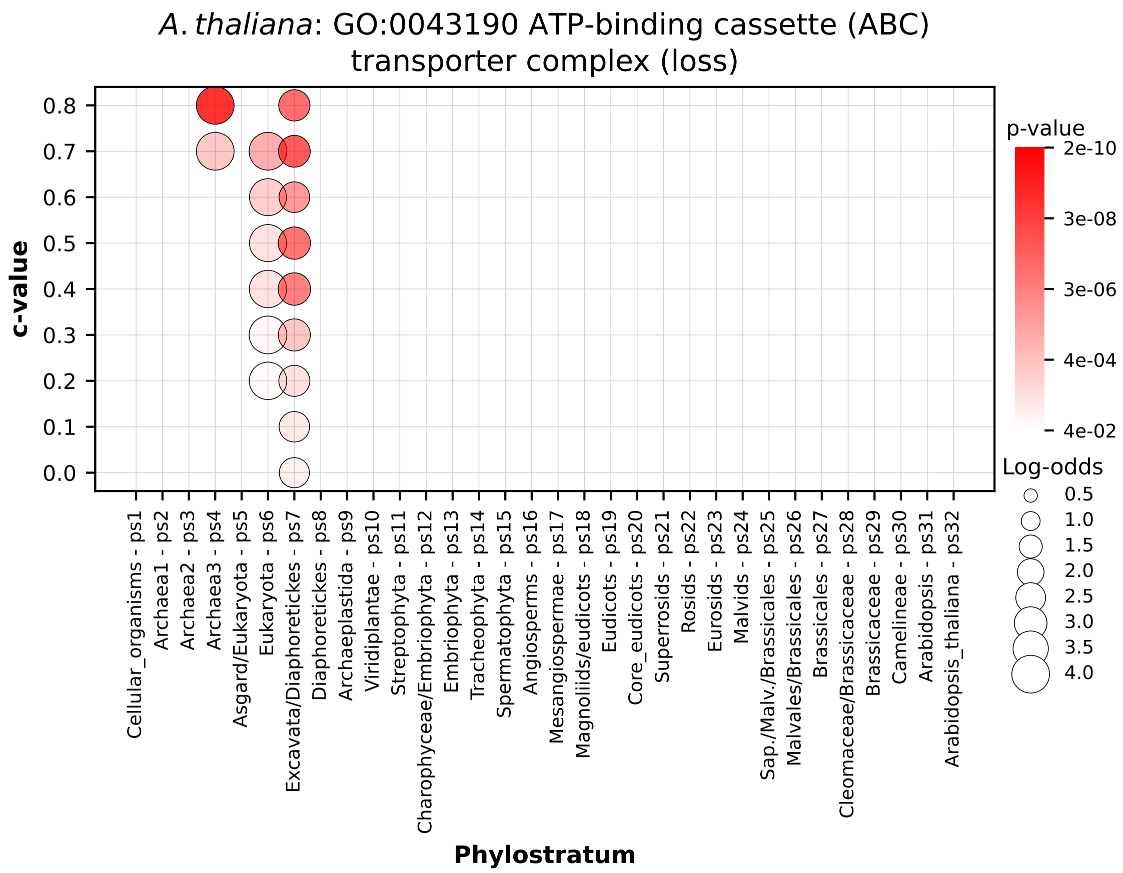

A. thaliana: GO:0043190 ATP-binding cassette (ABC) transporter complex (loss)
c-value
0.8
0.7
0.6
0.5
0.4
0.3
0.2
0.1
0.0
p-value
2e-10
3e-08
3e-06
4e-04
4e-02
Log-odds
0.5
1.0
1.5
2.0
2.5
3.0
3.5
4.0
Cellular_organisms - ps1
Archaea1 - ps2
Archaea2 - ps3
Archaea3 - ps4
Asgard/Eukaryota - ps5
Eukaryota - ps6
Excavata/Diaphoretickes - ps7
Diaphoretickes - ps8
Archaeplastida - ps9
Viridiplantae - ps10
Streptophyta - ps11
Charophyceae/Embriophyta - ps12
Embriophyta - ps13
Tracheophyta - ps14
Spermatophyta - ps15
Angiosperms - ps16
Mesangiospermae - ps17
Magnoliids/eudicots - ps18
Eudicots - ps19
Core_eudicots - ps20
Superrosids - ps21
Rosids - ps22
Eurosids - ps23
Malvids - ps24
Sap./Malv./Brassicales - ps25
Malvales/Brassicales - ps26
Brassicales - ps27
Cleomaceae/Brassicaceae - ps28
Brassicaceae - ps29
Camelineae - ps30
Arabidopsis - ps31
Arabidopsis_thaliana - ps32
Phylostratum

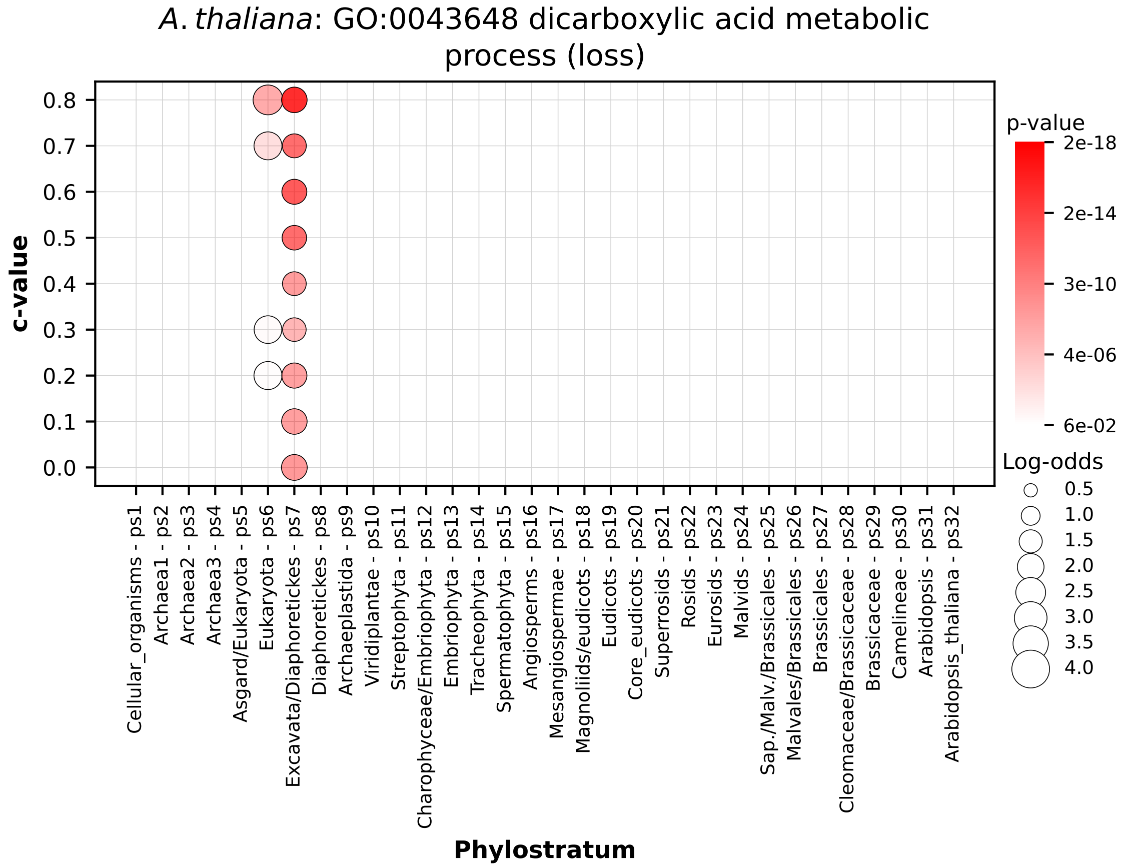

A. thaliana: GO:0043648 dicarboxylic acid metabolic process (loss)
c-value
0.8
0.7
0.6
0.5
0.4
0.3
0.2
0.1
0.0
p-value
2e-18
2e-14
3e-10
4e-06
6e-02
Log-odds
0.5
1.0
1.5
2.0
2.5
3.0
3.5
4.0
Cellular_organisms - ps1
Archaea1 - ps2
Archaea2 - ps3
Archaea3 - ps4
Asgard/Eukaryota - ps5
Eukaryota - ps6
Excavata/Diaphoretickes - ps7
Diaphoretickes - ps8
Archaeplastida - ps9
Viridiplantae - ps10
Streptophyta - ps11
Charophyceae/Embriophyta - ps12
Embriophyta - ps13
Tracheophyta - ps14
Spermatophyta - ps15
Angiosperms - ps16
Mesangiospermae - ps17
Magnoliids/eudicots - ps18
Eudicots - ps19
Core_eudicots - ps20
Superrosids - ps21
Rosids - ps22
Eurosids - ps23
Malvids - ps24
Sap./Malv./Brassicales - ps25
Malvales/Brassicales - ps26
Brassicales - ps27
Cleomaceae/Brassicaceae - ps28
Brassicaceae - ps29
Camelineae - ps30
Arabidopsis - ps31
Arabidopsis_thaliana - ps32
Phylostratum

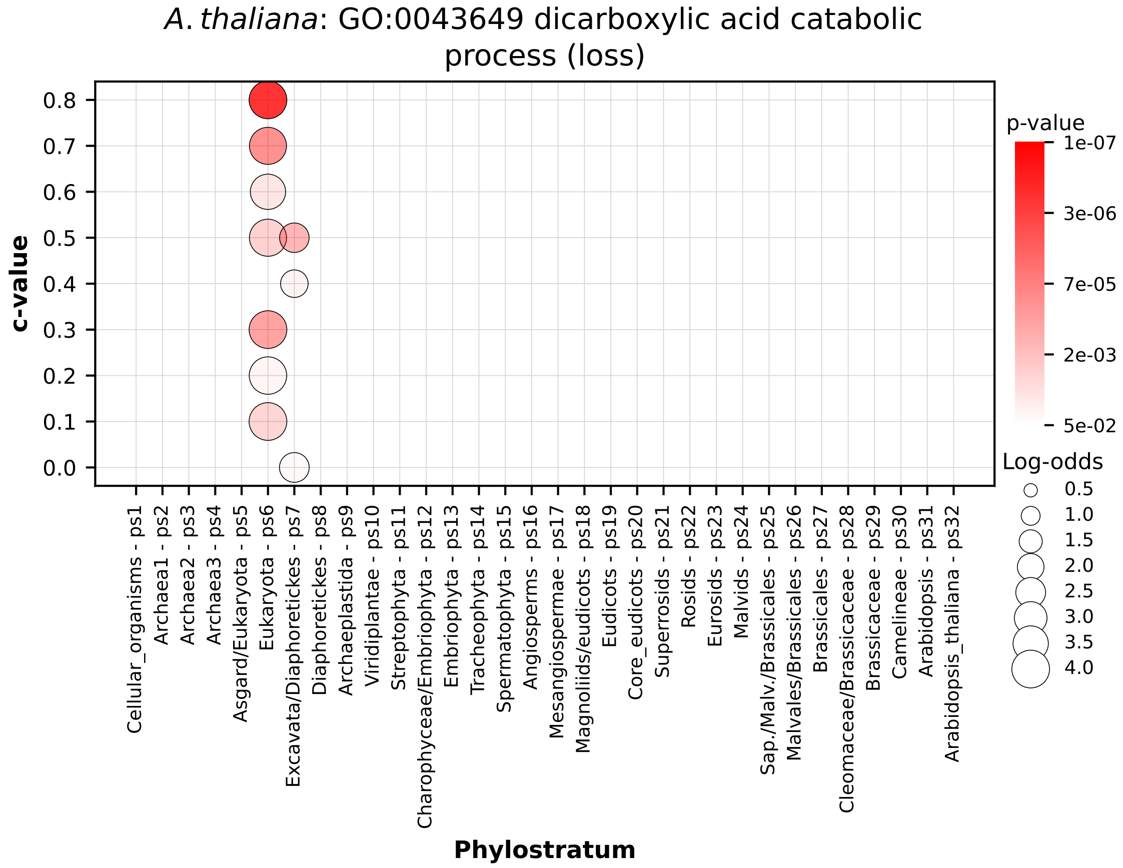

A. thaliana: GO:0043649 dicarboxylic acid catabolic process (loss)
c-value
0.8
0.7
0.6
0.5
0.4
0.3
0.2
0.1
0.0
p-value
1e-07
3e-06
7e-05
2e-03
5e-02
Log-odds
0.5
1.0
1.5
2.0
2.5
3.0
3.5
4.0
Cellular_organisms - ps1
Archaea1 - ps2
Archaea2 - ps3
Archaea3 - ps4
Asgard/Eukaryota - ps5
Eukaryota - ps6
Excavata/Diaphoretickes - ps7
Diaphoretickes - ps8
Archaeplastida - ps9
Viridiplantae - ps10
Streptophyta - ps11
Charophyceae/Embriophyta - ps12
Embriophyta - ps13
Tracheophyta - ps14
Spermatophyta - ps15
Angiosperms - ps16
Mesangiospermae - ps17
Magnoliids/eudicots - ps18
Eudicots - ps19
Core_eudicots - ps20
Superrosids - ps21
Rosids - ps22
Eurosids - ps23
Malvids - ps24
Sap./Malv./Brassicales - ps25
Malvales/Brassicales - ps26
Brassicales - ps27
Cleomaceae/Brassicaceae - ps28
Brassicaceae - ps29
Camelineae - ps30
Arabidopsis - ps31
Arabidopsis_thaliana - ps32
Phylostratum

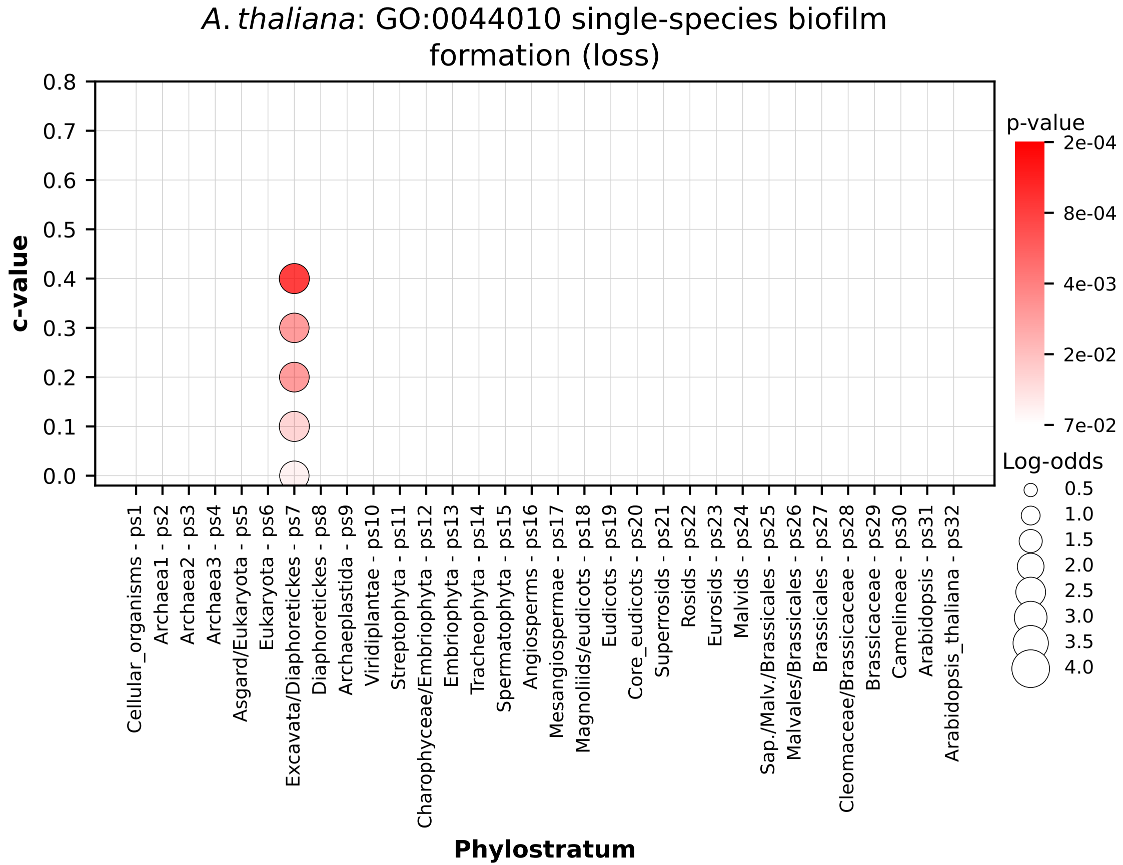
A. thaliana: GO:0044010 single-species biofilm formation (loss)
c-value
0.8
0.7
0.6
0.5
0.4
0.3
0.2
0.1
0.0
Phylostratum
Cellular_organisms - ps1
Archaea1 - ps2
Archaea2 - ps3
Archaea3 - ps4
Asgard/Eukaryota - ps5
Eukaryota - ps6
Excavata/Diaphoretickes - ps7
Diaphoretickes - ps8
Archaeplastida - ps9
Viridiplantae - ps10
Streptophyta - ps11
Charophyceae/Embriophyta - ps12
Embriophyta - ps13
Tracheophyta - ps14
Spermatophyta - ps15
Angiosperms - ps16
Mesangiospermae - ps17
Magnoliids/eudicots - ps18
Eudicots - ps19
Core_eudicots - ps20
Superrosids - ps21
Rosids - ps22
Eurosids - ps23
Malvids - ps24
Sap./Malv./Brassicales - ps25
Malvales/Brassicales - ps26
Brassicales - ps27
Cleomaceae/Brassicaceae - ps28
Brassicaceae - ps29
Camelineae - ps30
Arabidopsis - ps31
Arabidopsis_thaliana - ps32
p-value
2e-04
8e-04
4e-03
2e-02
7e-02
Log-odds
0.5
1.0
1.5
2.0
2.5
3.0
3.5
4.0

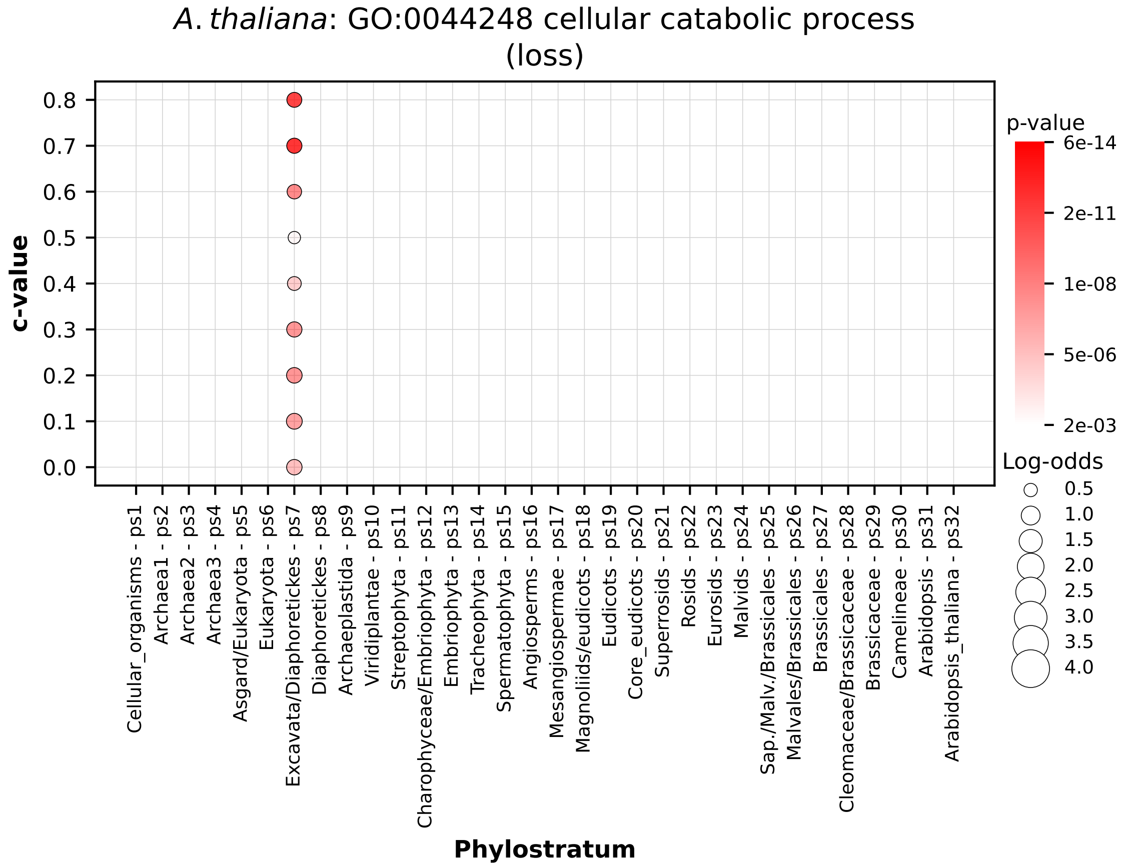

A. thaliana: GO:0044248 cellular catabolic process
(loss)
c-value
0.8
0.7
0.6
0.5
0.4
0.3
0.2
0.1
0.0
Phylostratum
Cellular_organisms - ps1
Archaea1 - ps2
Archaea2 - ps3
Archaea3 - ps4
Asgard/Eukaryota - ps5
Eukaryota - ps6
Excavata/Diaphoretickes - ps7
Diaphoretickes - ps8
Archaeplastida - ps9
Viridiplantae - ps10
Streptophyta - ps11
Charophyceae/Embriophyta - ps12
Embriophyta - ps13
Tracheophyta - ps14
Spermatophyta - ps15
Angiosperms - ps16
Mesangiospermae - ps17
Magnoliids/eudicots - ps18
Eudicots - ps19
Core_eudicots - ps20
Superrosids - ps21
Rosids - ps22
Eurosids - ps23
Malvids - ps24
Sap./Malv./Brassicales - ps25
Malvales/Brassicales - ps26
Brassicales - ps27
Cleomaceae/Brassicaceae - ps28
Brassicaceae - ps29
Camelineae - ps30
Arabidopsis - ps31
Arabidopsis_thaliana - ps32
p-value
6e-14
2e-11
1e-08
5e-06
2e-03
Log-odds
0.5
1.0
1.5
2.0
2.5
3.0
3.5
4.0

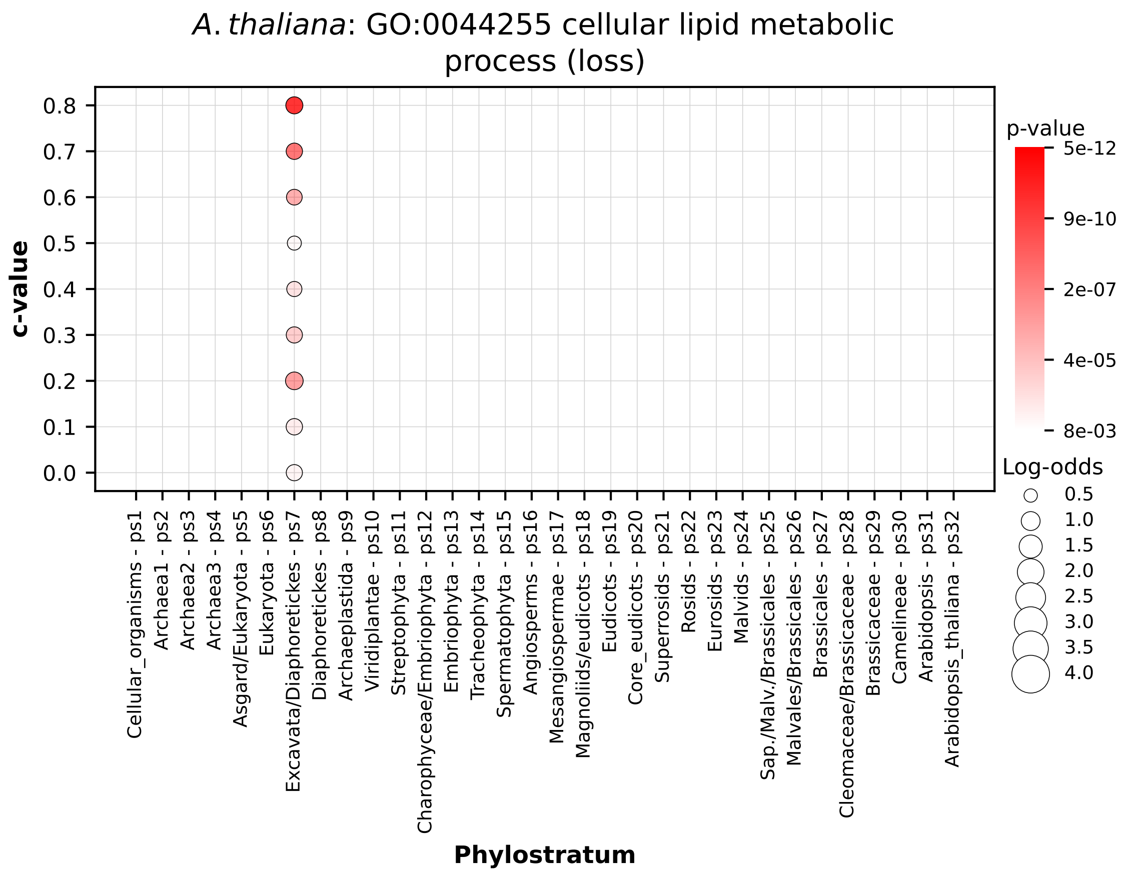
A. thaliana: GO:0044255 cellular lipid metabolic process (loss)
c-value
0.8
0.7
0.6
0.5
0.4
0.3
0.2
0.1
0.0
p-value
5e-12
9e-10
2e-07
4e-05
8e-03
Log-odds
0.5
1.0
1.5
2.0
2.5
3.0
3.5
4.0
Cellular_organisms - ps1
Archaea1 - ps2
Archaea2 - ps3
Archaea3 - ps4
Asgard/Eukaryota - ps5
Eukaryota - ps6
Excavata/Diaphoretickes - ps7
Diaphoretickes - ps8
Archaeplastida - ps9
Viridiplantae - ps10
Streptophyta - ps11
Charophyceae/Embriophyta - ps12
Embriophyta - ps13
Tracheophyta - ps14
Spermatophyta - ps15
Angiosperms - ps16
Mesangiospermae - ps17
Magnoliids/eudicots - ps18
Eudicots - ps19
Core_eudicots - ps20
Superrosids - ps21
Rosids - ps22
Eurosids - ps23
Malvids - ps24
Sap./Malv./Brassicales - ps25
Malvales/Brassicales - ps26
Brassicales - ps27
Cleomaceae/Brassicaceae - ps28
Brassicaceae - ps29
Camelineae - ps30
Arabidopsis - ps31
Arabidopsis_thaliana - ps32
Phylostratum

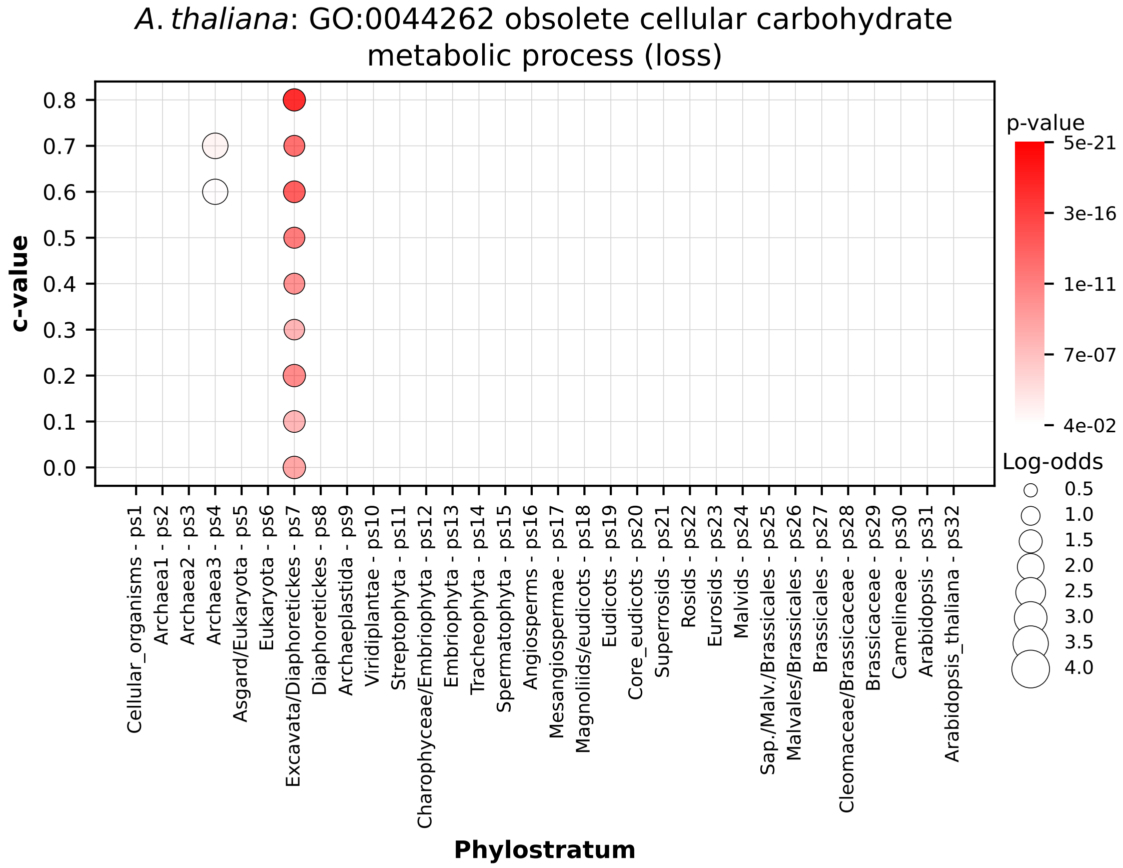

A. thaliana: GO:0044262 obsolete cellular carbohydrate metabolic process (loss)
c-value
0.8
0.7
0.6
0.5
0.4
0.3
0.2
0.1
0.0
p-value
5e-21
3e-16
1e-11
7e-07
4e-02
Log-odds
0.5
1.0
1.5
2.0
2.5
3.0
3.5
4.0
Cellular_organisms - ps1
Archaea1 - ps2
Archaea2 - ps3
Archaea3 - ps4
Asgard/Eukaryota - ps5
Eukaryota - ps6
Excavata/Diaphoretickes - ps7
Diaphoretickes - ps8
Archaeplastida - ps9
Viridiplantae - ps10
Streptophyta - ps11
Charophyceae/Embriophyta - ps12
Embriophyta - ps13
Tracheophyta - ps14
Spermatophyta - ps15
Angiosperms - ps16
Mesangiospermae - ps17
Magnoliids/eudicots - ps18
Eudicots - ps19
Core_eudicots - ps20
Superrosids - ps21
Rosids - ps22
Eurosids - ps23
Malvids - ps24
Sap./Malv./Brassicales - ps25
Malvales/Brassicales - ps26
Brassicales - ps27
Cleomaceae/Brassicaceae - ps28
Brassicaceae - ps29
Camelineae - ps30
Arabidopsis - ps31
Arabidopsis_thaliana - ps32
Phylostratum

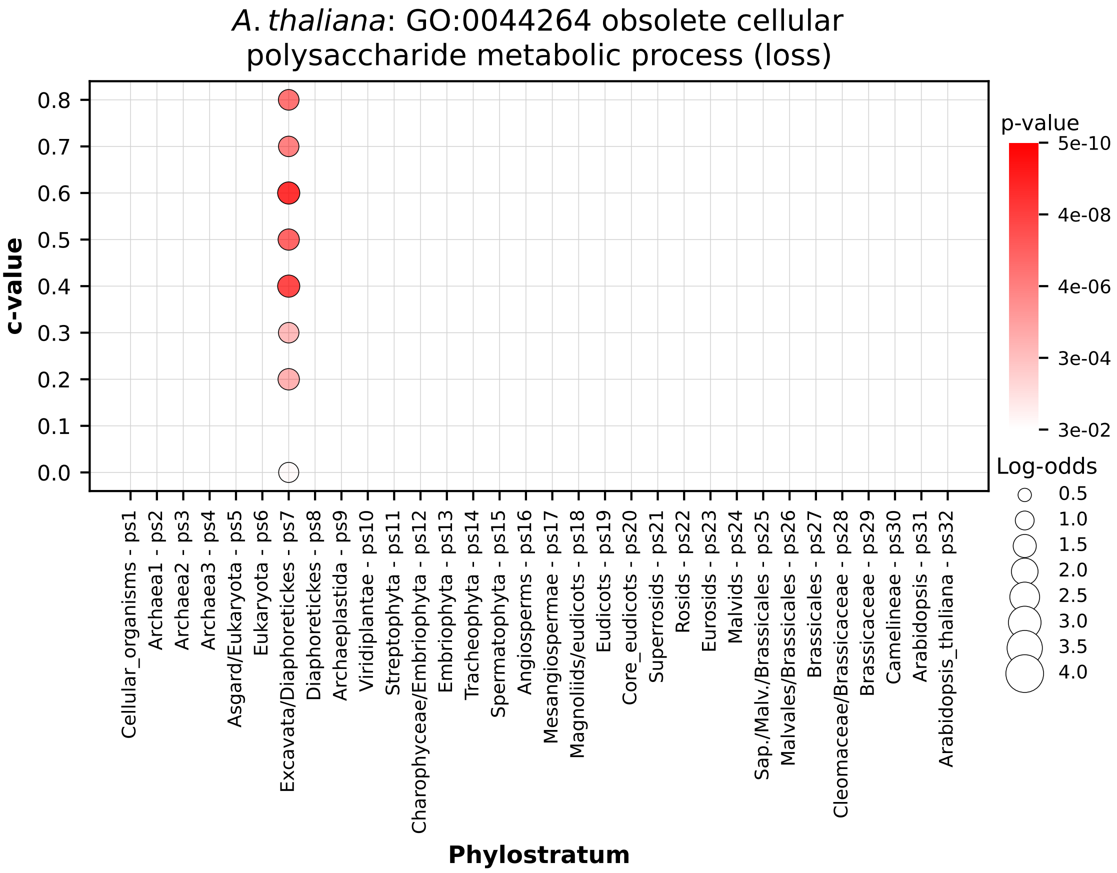
A. thaliana: GO:0044264 obsolete cellular polysaccharide metabolic process (loss)
c-value
0.8
0.7
0.6
0.5
0.4
0.3
0.2
0.1
0.0
p-value
5e-10
4e-08
4e-06
3e-04
3e-02
Log-odds
0.5
1.0
1.5
2.0
2.5
3.0
3.5
4.0
Cellular_organisms - ps1
Archaea1 - ps2
Archaea2 - ps3
Archaea3 - ps4
Asgard/Eukaryota - ps5
Eukaryota - ps6
Excavata/Diaphoretickes - ps7
Diaphoretickes - ps8
Archaeplastida - ps9
Viridiplantae - ps10
Streptophyta - ps11
Charophyceae/Embriophyta - ps12
Embriophyta - ps13
Tracheophyta - ps14
Spermatophyta - ps15
Angiosperms - ps16
Mesangiospermae - ps17
Magnoliids/eudicots - ps18
Eudicots - ps19
Core_eudicots - ps20
Superrosids - ps21
Rosids - ps22
Eurosids - ps23
Malvids - ps24
Sap./Malv./Brassicales - ps25
Malvales/Brassicales - ps26
Brassicales - ps27
Cleomaceae/Brassicaceae - ps28
Brassicaceae - ps29
Camelineae - ps30
Arabidopsis - ps31
Arabidopsis_thaliana - ps32
Phylostratum

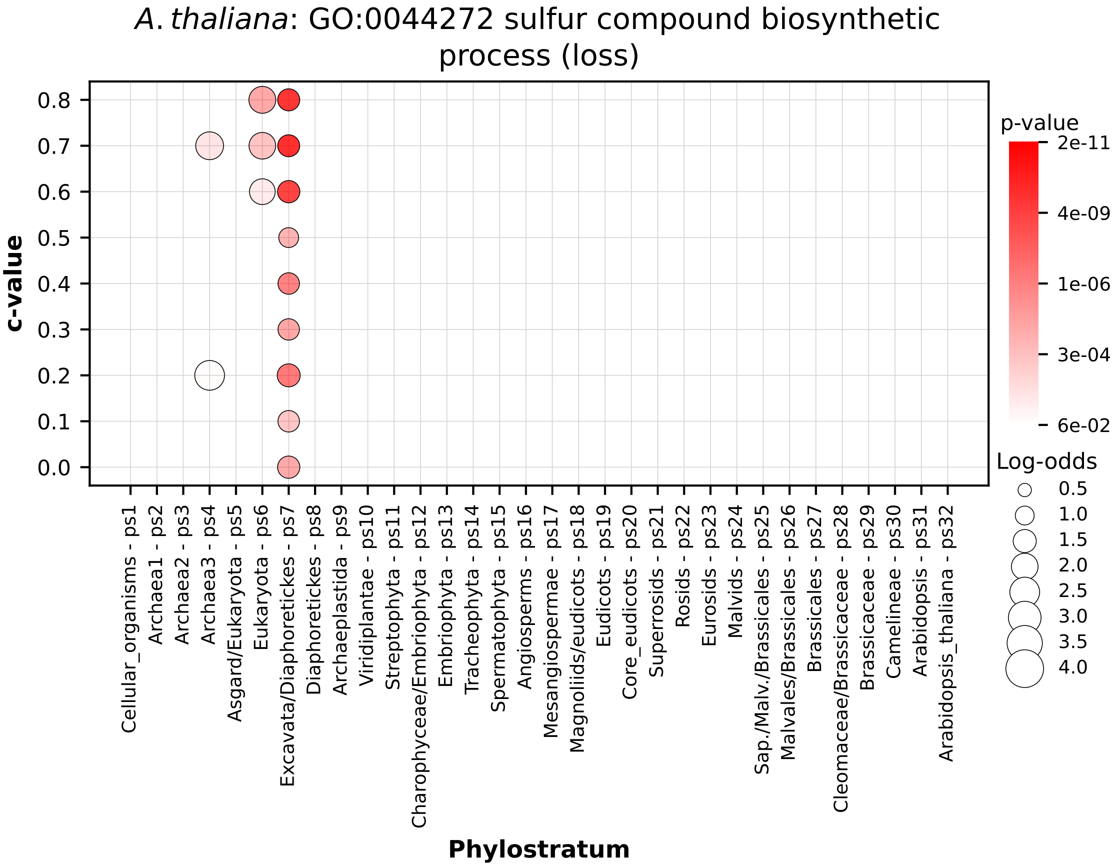

A. thaliana: GO:0044272 sulfur compound biosynthetic process (loss)
c-value
0.8
0.7
0.6
0.5
0.4
0.3
0.2
0.1
0.0
p-value
2e-11
4e-09
1e-06
3e-04
6e-02
Log-odds
0.5
1.0
1.5
2.0
2.5
3.0
3.5
4.0
Cellular_organisms - ps1
Archaea1 - ps2
Archaea2 - ps3
Archaea3 - ps4
Asgard/Eukaryota - ps5
Eukaryota - ps6
Excavata/Diaphoretickes - ps7
Diaphoretickes - ps8
Archaeplastida - ps9
Viridiplantae - ps10
Streptophyta - ps11
Charophyceae/Embriophyta - ps12
Embriophyta - ps13
Tracheophyta - ps14
Spermatophyta - ps15
Angiosperms - ps16
Mesangiospermae - ps17
Magnoliids/eudicots - ps18
Eudicots - ps19
Core_eudicots - ps20
Superrosids - ps21
Rosids - ps22
Eurosids - ps23
Malvids - ps24
Sap./Malv./Brassicales - ps25
Malvales/Brassicales - ps26
Brassicales - ps27
Cleomaceae/Brassicaceae - ps28
Brassicaceae - ps29
Camelineae - ps30
Arabidopsis - ps31
Arabidopsis_thaliana - ps32
Phylostratum

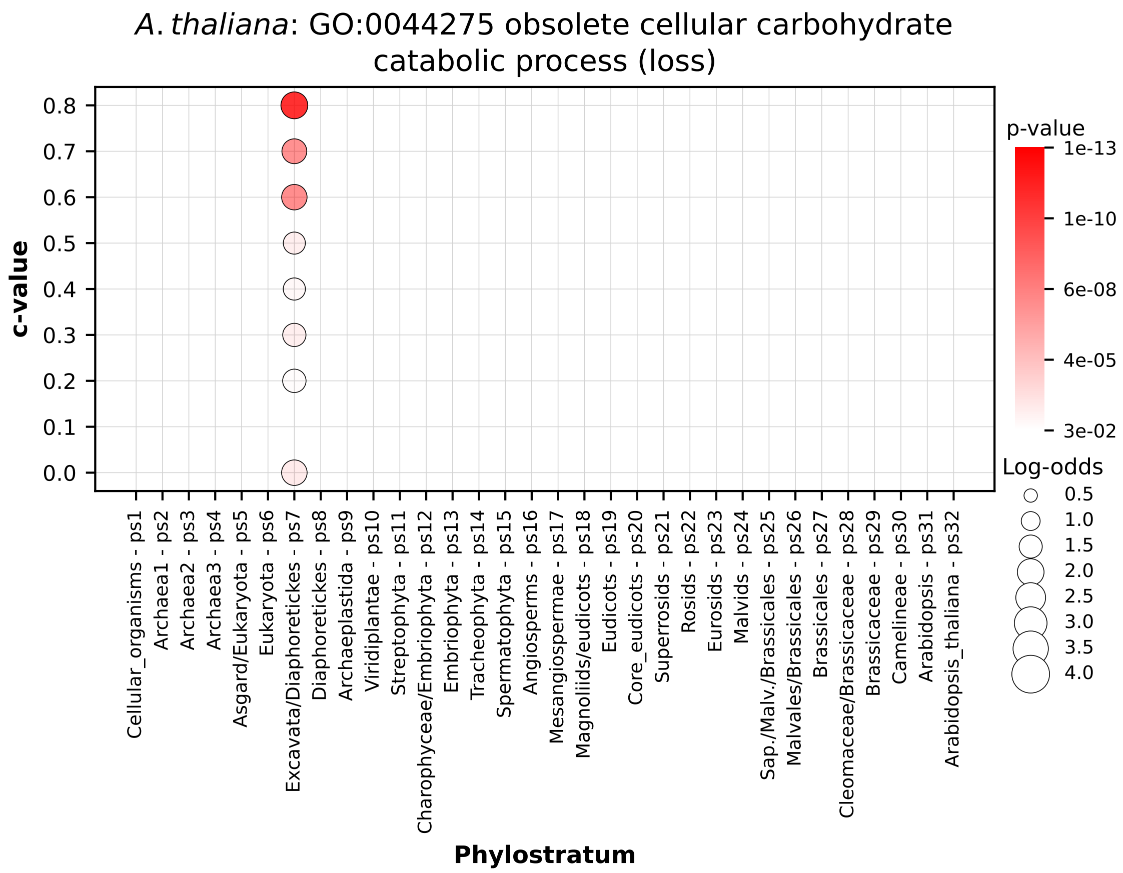

A. thaliana: GO:0044275 obsolete cellular carbohydrate catabolic process (loss)
c-value
0.8
0.7
0.6
0.5
0.4
0.3
0.2
0.1
0.0
p-value
1e-13
1e-10
6e-08
4e-05
3e-02
Log-odds
0.5
1.0
1.5
2.0
2.5
3.0
3.5
4.0
Cellular_organisms - ps1
Archaea1 - ps2
Archaea2 - ps3
Archaea3 - ps4
Asgard/Eukaryota - ps5
Eukaryota - ps6
Excavata/Diaphoretickes - ps7
Diaphoretickes - ps8
Archaeplastida - ps9
Viridiplantae - ps10
Streptophyta - ps11
Charophyceae/Embriophyta - ps12
Embriophyta - ps13
Tracheophyta - ps14
Spermatophyta - ps15
Angiosperms - ps16
Mesangiospermae - ps17
Magnoliids/eudicots - ps18
Eudicots - ps19
Core_eudicots - ps20
Superrosids - ps21
Rosids - ps22
Eurosids - ps23
Malvids - ps24
Sap./Malv./Brassicales - ps25
Malvales/Brassicales - ps26
Brassicales - ps27
Cleomaceae/Brassicaceae - ps28
Brassicaceae - ps29
Camelineae - ps30
Arabidopsis - ps31
Arabidopsis_thaliana - ps32
Phylostratum

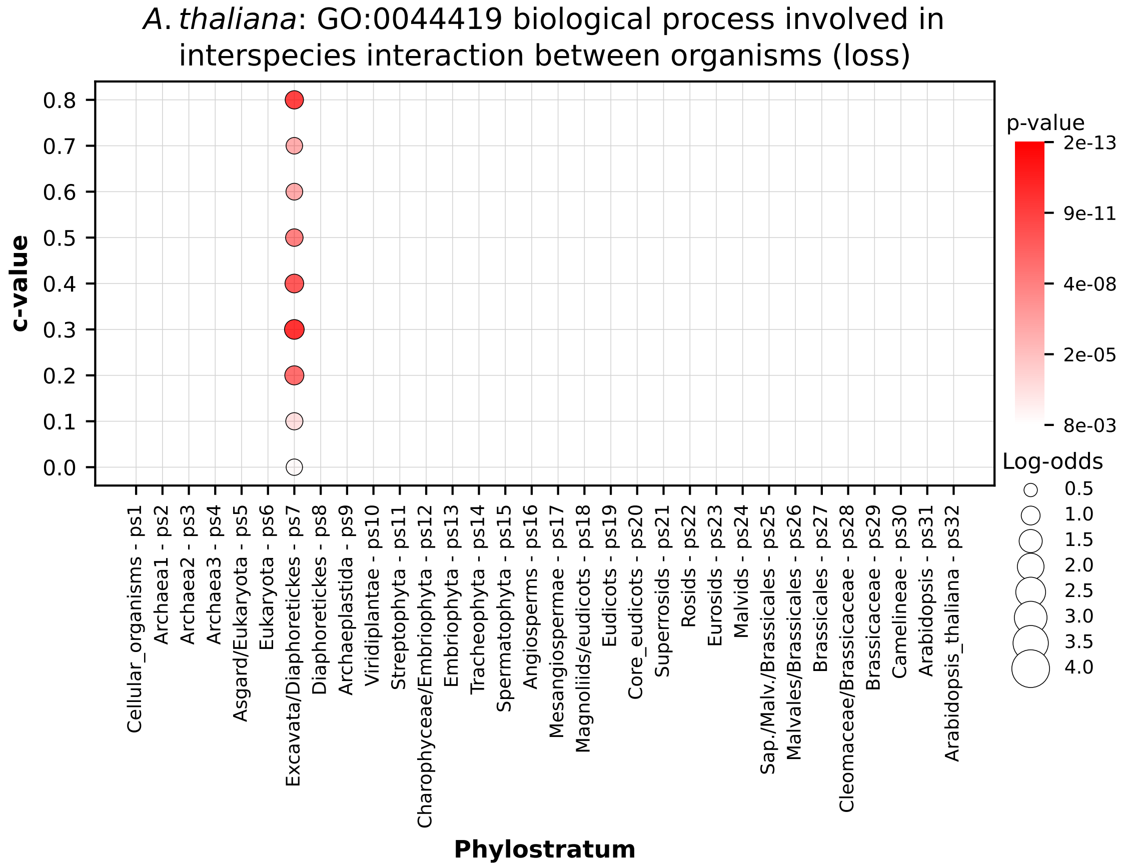

A. thaliana: GO:0044419 biological process involved in interspecies interaction between organisms (loss)
c-value
0.8
0.7
0.6
0.5
0.4
0.3
0.2
0.1
0.0
p-value
2e-13
9e-11
4e-08
2e-05
8e-03
Log-odds
0.5
1.0
1.5
2.0
2.5
3.0
3.5
4.0
Cellular_organisms - ps1
Archaea1 - ps2
Archaea2 - ps3
Archaea3 - ps4
Asgard/Eukaryota - ps5
Eukaryota - ps6
Excavata/Diaphoretickes - ps7
Diaphoretickes - ps8
Archaeplastida - ps9
Viridiplantae - ps10
Streptophyta - ps11
Charophyceae/Embriophyta - ps12
Embriophyta - ps13
Tracheophyta - ps14
Spermatophyta - ps15
Angiosperms - ps16
Mesangiospermae - ps17
Magnoliids/eudicots - ps18
Eudicots - ps19
Core_eudicots - ps20
Superrosids - ps21
Rosids - ps22
Eurosids - ps23
Malvids - ps24
Sap./Malv./Brassicales - ps25
Malvales/Brassicales - ps26
Brassicales - ps27
Cleomaceae/Brassicaceae - ps28
Brassicaceae - ps29
Camelineae - ps30
Arabidopsis - ps31
Arabidopsis_thaliana - ps32
Phylostratum

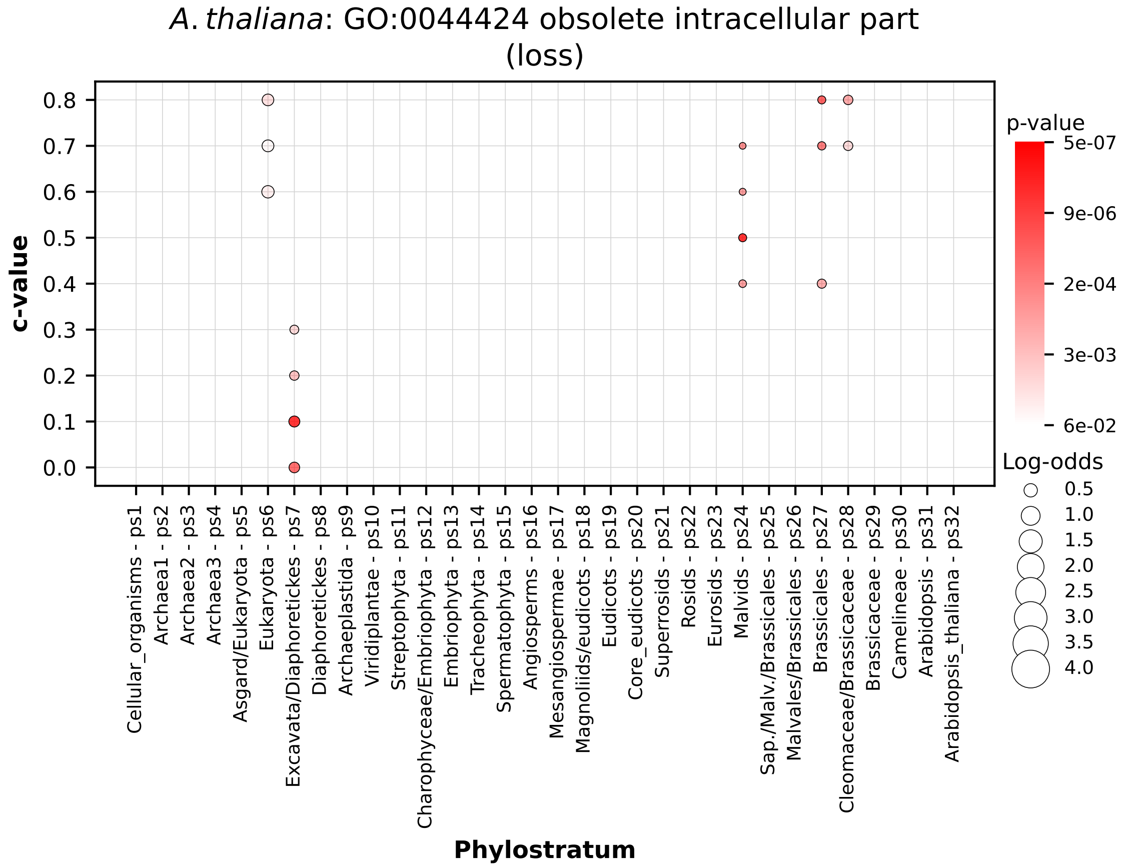
A. thaliana: GO:0044424 obsolete intracellular part
(loss)
c-value
0.8
0.7
0.6
0.5
0.4
0.3
0.2
0.1
0.0
p-value
5e-07
9e-06
2e-04
3e-03
6e-02
Log-odds
0.5
1.0
1.5
2.0
2.5
3.0
3.5
4.0
Cellular_organisms - ps1
Archaea1 - ps2
Archaea2 - ps3
Archaea3 - ps4
Asgard/Eukaryota - ps5
Eukaryota - ps6
Excavata/Diaphoretickes - ps7
Diaphoretickes - ps8
Archaeplastida - ps9
Viridiplantae - ps10
Streptophyta - ps11
Charophyceae/Embriophyta - ps12
Embriophyta - ps13
Tracheophyta - ps14
Spermatophyta - ps15
Angiosperms - ps16
Mesangiospermae - ps17
Magnoliids/eudicots - ps18
Eudicots - ps19
Core_eudicots - ps20
Superrosids - ps21
Rosids - ps22
Eurosids - ps23
Malvids - ps24
Sap./Malv./Brassicales - ps25
Malvales/Brassicales - ps26
Brassicales - ps27
Cleomaceae/Brassicaceae - ps28
Brassicaceae - ps29
Camelineae - ps30
Arabidopsis - ps31
Arabidopsis_thaliana - ps32
Phylostratum

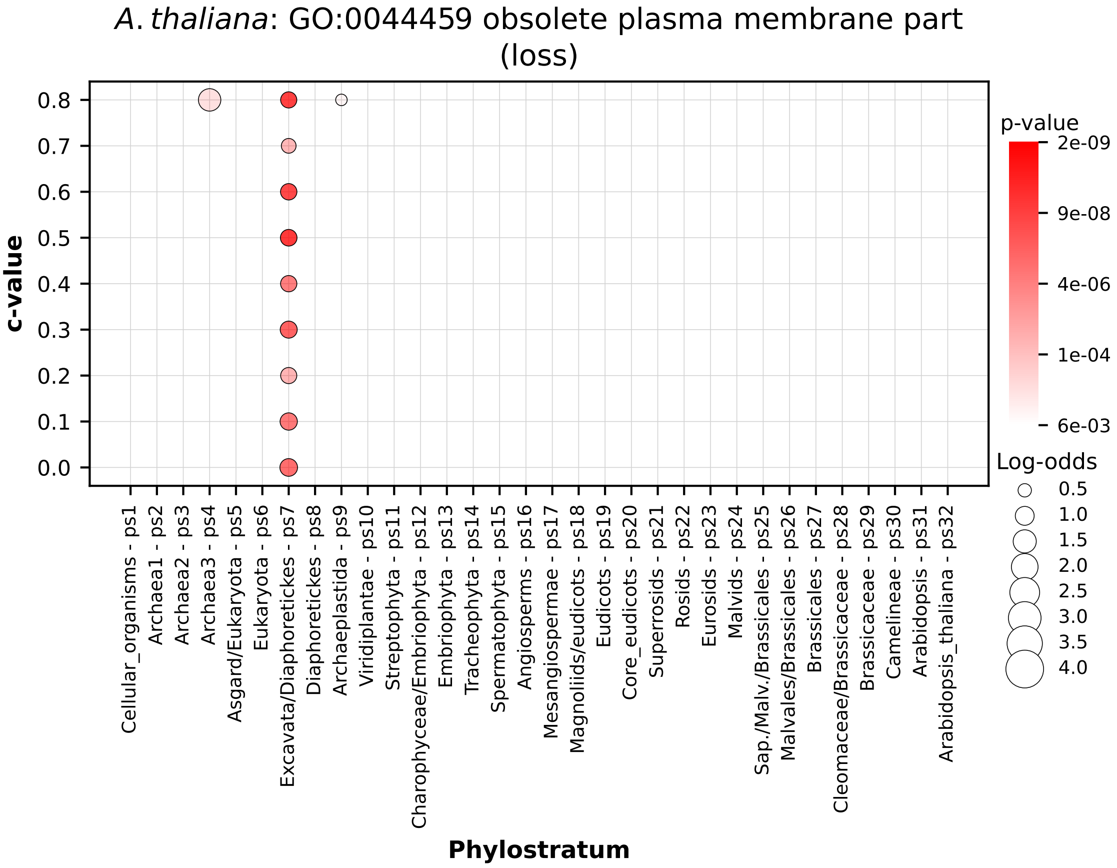

A. thaliana: GO:0044459 obsolete plasma membrane part (loss)
c-value
0.8
0.7
0.6
0.5
0.4
0.3
0.2
0.1
0.0
p-value
2e-09
9e-08
4e-06
1e-04
6e-03
Log-odds
0.5
1.0
1.5
2.0
2.5
3.0
3.5
4.0
Cellular_organisms - ps1
Archaea1 - ps2
Archaea2 - ps3
Archaea3 - ps4
Asgard/Eukaryota - ps5
Eukaryota - ps6
Excavata/Diaphoretickes - ps7
Diaphoretickes - ps8
Archaeplastida - ps9
Viridiplantae - ps10
Streptophyta - ps11
Charophyceae/Embriophyta - ps12
Embriophyta - ps13
Tracheophyta - ps14
Spermatophyta - ps15
Angiosperms - ps16
Mesangiospermae - ps17
Magnoliids/eudicots - ps18
Eudicots - ps19
Core_eudicots - ps20
Superrosids - ps21
Rosids - ps22
Eurosids - ps23
Malvids - ps24
Sap./Malv./Brassicales - ps25
Malvales/Brassicales - ps26
Brassicales - ps27
Cleomaceae/Brassicaceae - ps28
Brassicaceae - ps29
Camelineae - ps30
Arabidopsis - ps31
Arabidopsis_thaliana - ps32
Phylostratum

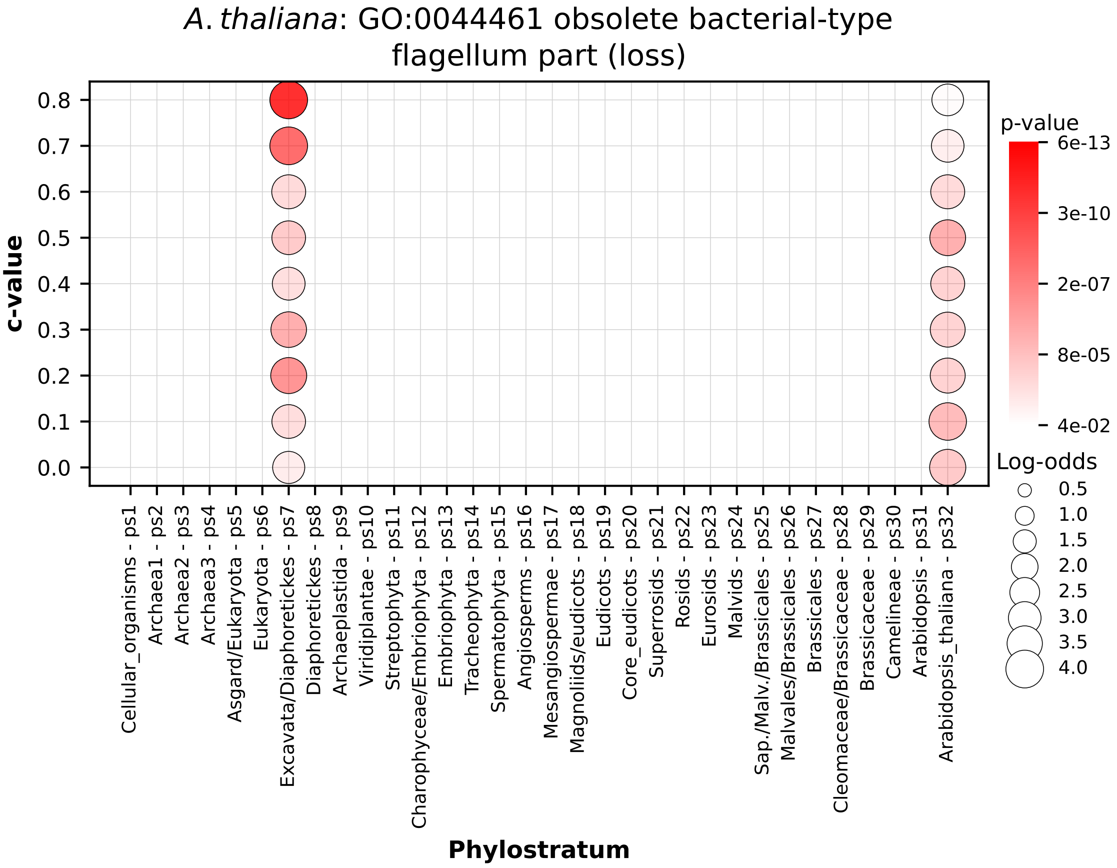

A. thaliana: GO:0044461 obsolete bacterial-type flagellum part (loss)
c-value
0.8
0.7
0.6
0.5
0.4
0.3
0.2
0.1
0.0
p-value
6e-13
3e-10
2e-07
8e-05
4e-02
Log-odds
0.5
1.0
1.5
2.0
2.5
3.0
3.5
4.0
Cellular_organisms - ps1
Archaea1 - ps2
Archaea2 - ps3
Archaea3 - ps4
Asgard/Eukaryota - ps5
Eukaryota - ps6
Excavata/Diaphoretickes - ps7
Diaphoretickes - ps8
Archaeplastida - ps9
Viridiplantae - ps10
Streptophyta - ps11
Charophyceae/Embriophyta - ps12
Embriophyta - ps13
Tracheophyta - ps14
Spermatophyta - ps15
Angiosperms - ps16
Mesangiospermae - ps17
Magnoliids/eudicots - ps18
Eudicots - ps19
Core_eudicots - ps20
Superrosids - ps21
Rosids - ps22
Eurosids - ps23
Malvids - ps24
Sap./Malv./Brassicales - ps25
Malvales/Brassicales - ps26
Brassicales - ps27
Cleomaceae/Brassicaceae - ps28
Brassicaceae - ps29
Camelineae - ps30
Arabidopsis - ps31
Arabidopsis_thaliana - ps32
Phylostratum

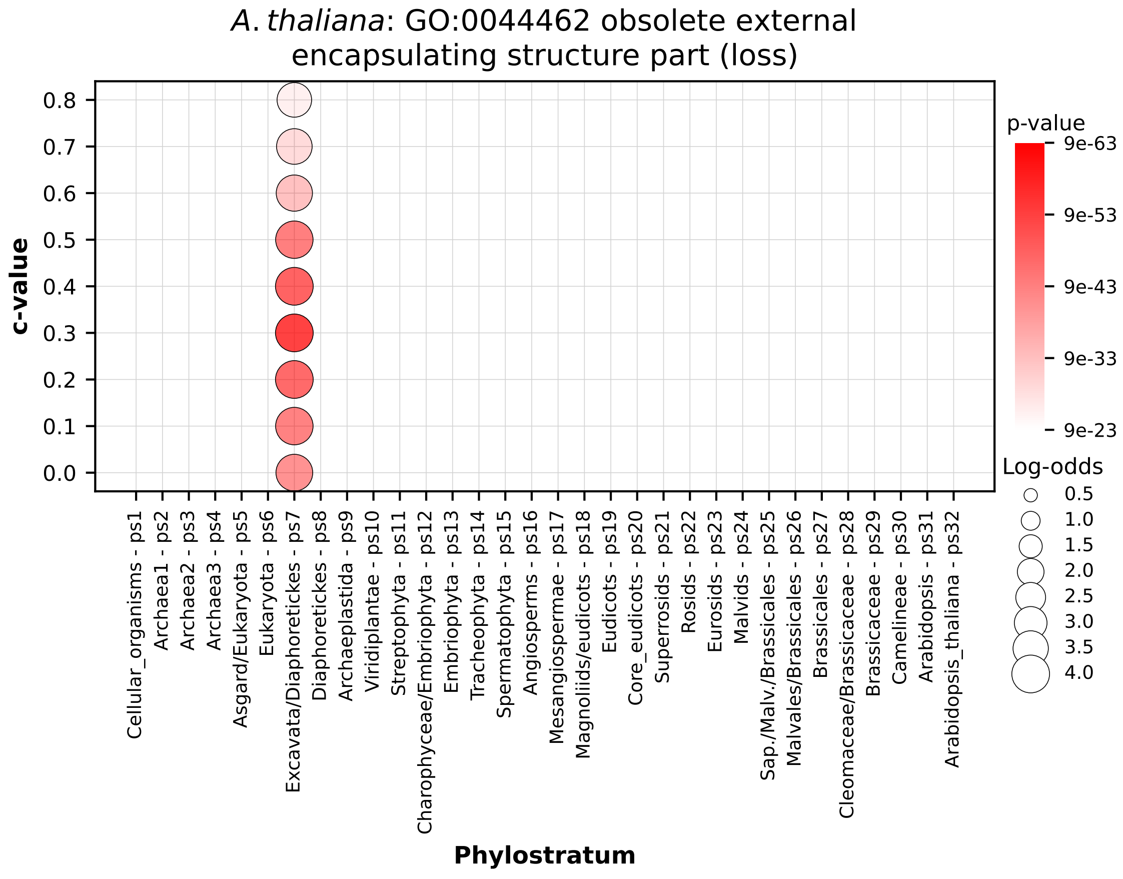

A. thaliana: GO:0044462 obsolete external encapsulating structure part (loss)
c-value
0.8
0.7
0.6
0.5
0.4
0.3
0.2
0.1
0.0
Phylostratum
Cellular_organisms - ps1
Archaea1 - ps2
Archaea2 - ps3
Archaea3 - ps4
Asgard/Eukaryota - ps5
Eukaryota - ps6
Excavata/Diaphoretickes - ps7
Diaphoretickes - ps8
Archaeplastida - ps9
Viridiplantae - ps10
Streptophyta - ps11
Charophyceae/Embriophyta - ps12
Embriophyta - ps13
Tracheophyta - ps14
Spermatophyta - ps15
Angiosperms - ps16
Mesangiospermae - ps17
Magnoliids/eudicots - ps18
Eudicots - ps19
Core_eudicots - ps20
Superrosids - ps21
Rosids - ps22
Eurosids - ps23
Malvids - ps24
Sap./Malv./Brassicales - ps25
Malvales/Brassicales - ps26
Brassicales - ps27
Cleomaceae/Brassicaceae - ps28
Brassicaceae - ps29
Camelineae - ps30
Arabidopsis - ps31
Arabidopsis_thaliana - ps32
p-value
9e-63
9e-53
9e-43
9e-33
9e-23
Log-odds
0.5
1.0
1.5
2.0
2.5
3.0
3.5
4.0

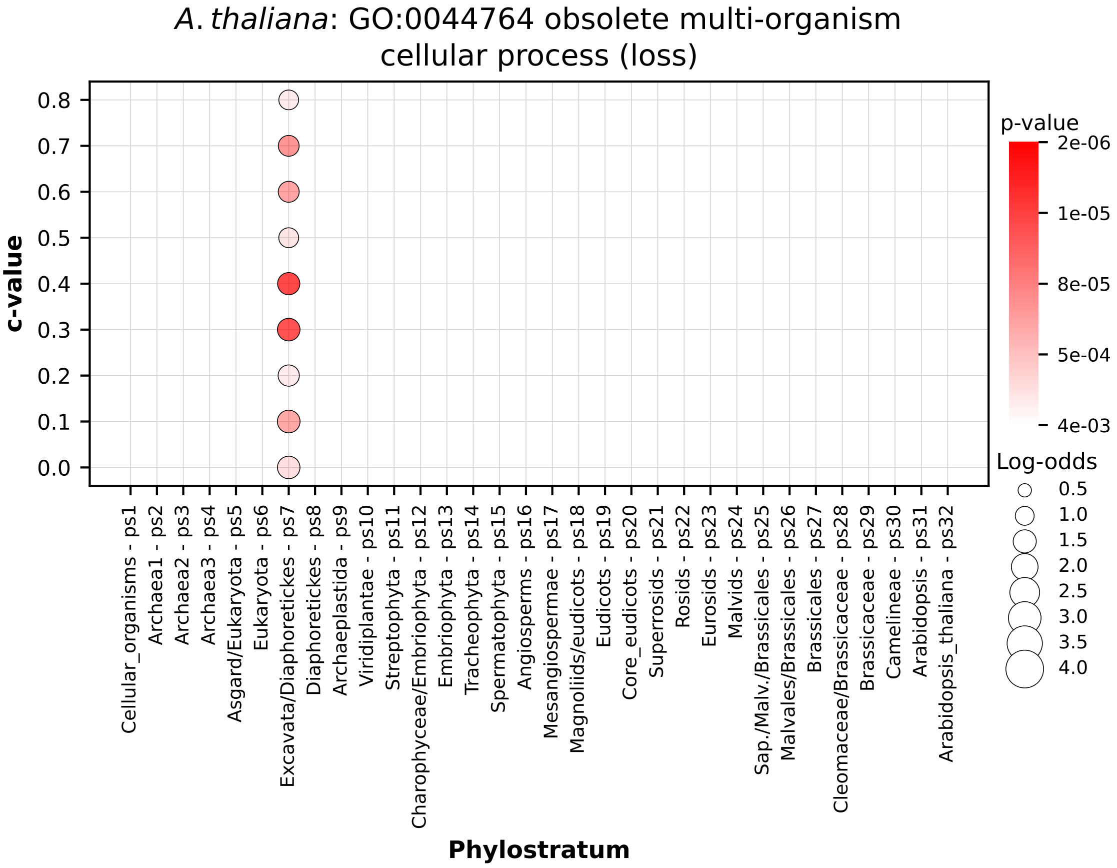

A. thaliana: GO:0044764 obsolete multi-organism cellular process (loss)
c-value
0.8
0.7
0.6
0.5
0.4
0.3
0.2
0.1
0.0
p-value
2e-06
1e-05
8e-05
5e-04
4e-03
Log-odds
0.5
1.0
1.5
2.0
2.5
3.0
3.5
4.0
Cellular_organisms - ps1
Archaea1 - ps2
Archaea2 - ps3
Archaea3 - ps4
Asgard/Eukaryota - ps5
Eukaryota - ps6
Excavata/Diaphoretickes - ps7
Diaphoretickes - ps8
Archaeplastida - ps9
Viridiplantae - ps10
Streptophyta - ps11
Charophyceae/Embriophyta - ps12
Embriophyta - ps13
Tracheophyta - ps14
Spermatophyta - ps15
Angiosperms - ps16
Mesangiospermae - ps17
Magnoliids/eudicots - ps18
Eudicots - ps19
Core_eudicots - ps20
Superrosids - ps21
Rosids - ps22
Eurosids - ps23
Malvids - ps24
Sap./Malv./Brassicales - ps25
Malvales/Brassicales - ps26
Brassicales - ps27
Cleomaceae/Brassicaceae - ps28
Brassicaceae - ps29
Camelineae - ps30
Arabidopsis - ps31
Arabidopsis_thaliana - ps32
Phylostratum

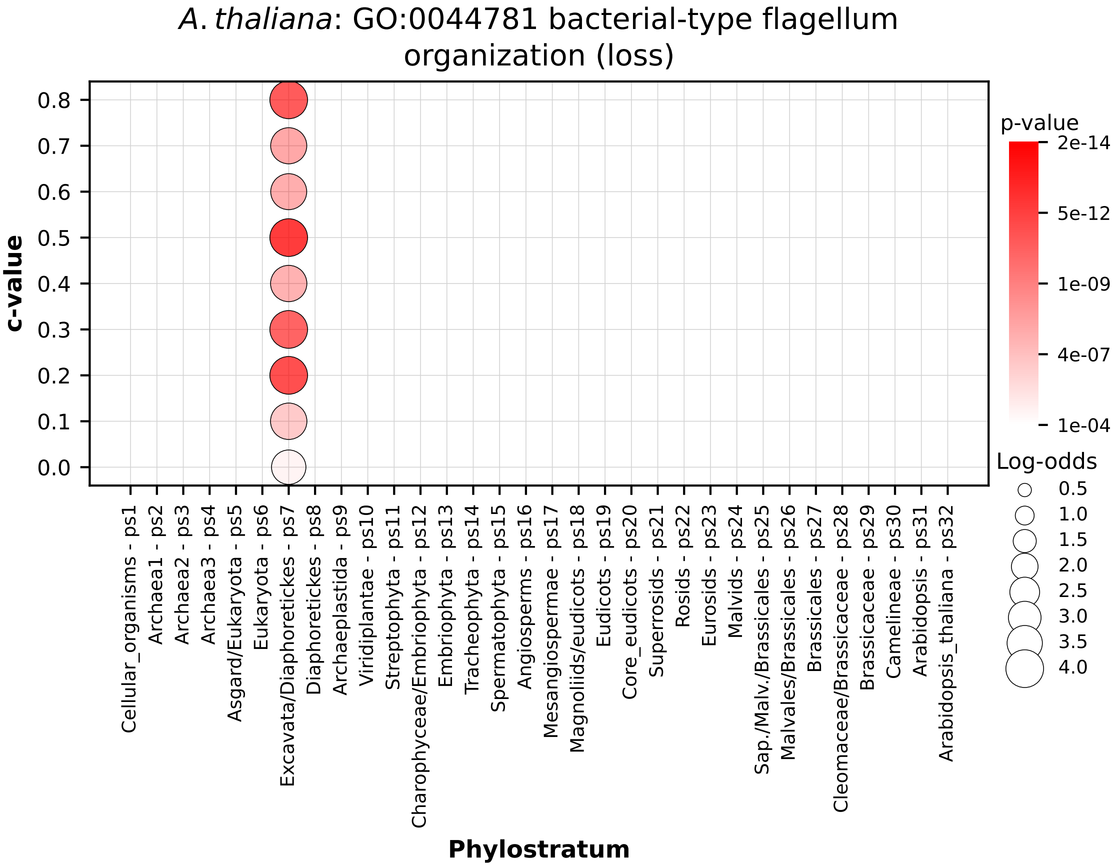
A. thaliana: GO:0044781 bacterial-type flagellum organization (loss)
c-value
0.8
0.7
0.6
0.5
0.4
0.3
0.2
0.1
0.0
p-value
2e-14
5e-12
1e-09
4e-07
1e-04
Log-odds
0.5
1.0
1.5
2.0
2.5
3.0
3.5
4.0
Cellular_organisms - ps1
Archaea1 - ps2
Archaea2 - ps3
Archaea3 - ps4
Asgard/Eukaryota - ps5
Eukaryota - ps6
Excavata/Diaphoretickes - ps7
Diaphoretickes - ps8
Archaeplastida - ps9
Viridiplantae - ps10
Streptophyta - ps11
Charophyceae/Embriophyta - ps12
Embriophyta - ps13
Tracheophyta - ps14
Spermatophyta - ps15
Angiosperms - ps16
Mesangiospermae - ps17
Magnoliids/eudicots - ps18
Eudicots - ps19
Core_eudicots - ps20
Superrosids - ps21
Rosids - ps22
Eurosids - ps23
Malvids - ps24
Sap./Malv./Brassicales - ps25
Malvales/Brassicales - ps26
Brassicales - ps27
Cleomaceae/Brassicaceae - ps28
Brassicaceae - ps29
Camelineae - ps30
Arabidopsis - ps31
Arabidopsis_thaliana - ps32
Phylostratum

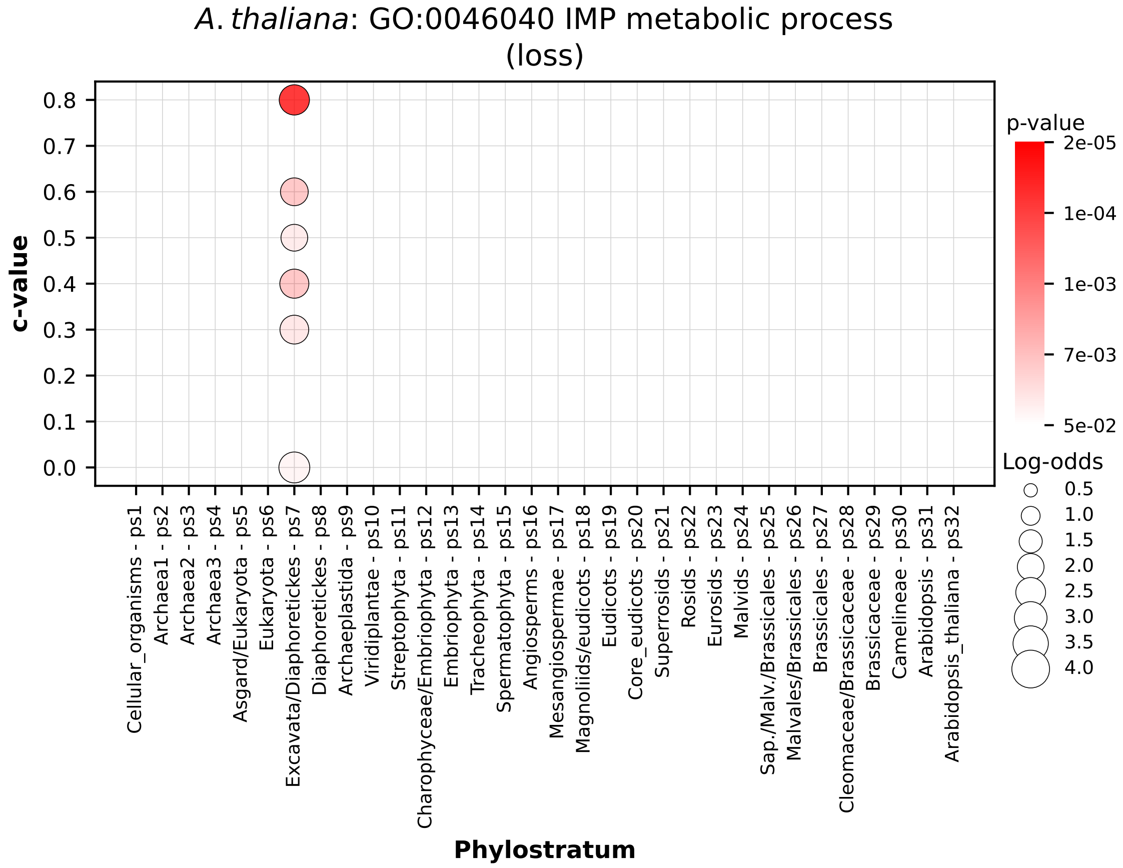
A. thaliana: GO:0046040 IMP metabolic process
(loss)
c-value
0.8
0.7
0.6
0.5
0.4
0.3
0.2
0.1
0.0
p-value
2e-05
1e-04
1e-03
7e-03
5e-02
Log-odds
0.5
1.0
1.5
2.0
2.5
3.0
3.5
4.0
Cellular_organisms - ps1
Archaea1 - ps2
Archaea2 - ps3
Archaea3 - ps4
Asgard/Eukaryota - ps5
Eukaryota - ps6
Excavata/Diaphoretickes - ps7
Diaphoretickes - ps8
Archaeplastida - ps9
Viridiplantae - ps10
Streptophyta - ps11
Charophyceae/Embriophyta - ps12
Embriophyta - ps13
Tracheophyta - ps14
Spermatophyta - ps15
Angiosperms - ps16
Mesangiospermae - ps17
Magnoliids/eudicots - ps18
Eudicots - ps19
Core_eudicots - ps20
Superrosids - ps21
Rosids - ps22
Eurosids - ps23
Malvids - ps24
Sap./Malv./Brassicales - ps25
Malvales/Brassicales - ps26
Brassicales - ps27
Cleomaceae/Brassicaceae - ps28
Brassicaceae - ps29
Camelineae - ps30
Arabidopsis - ps31
Arabidopsis_thaliana - ps32
Phylostratum

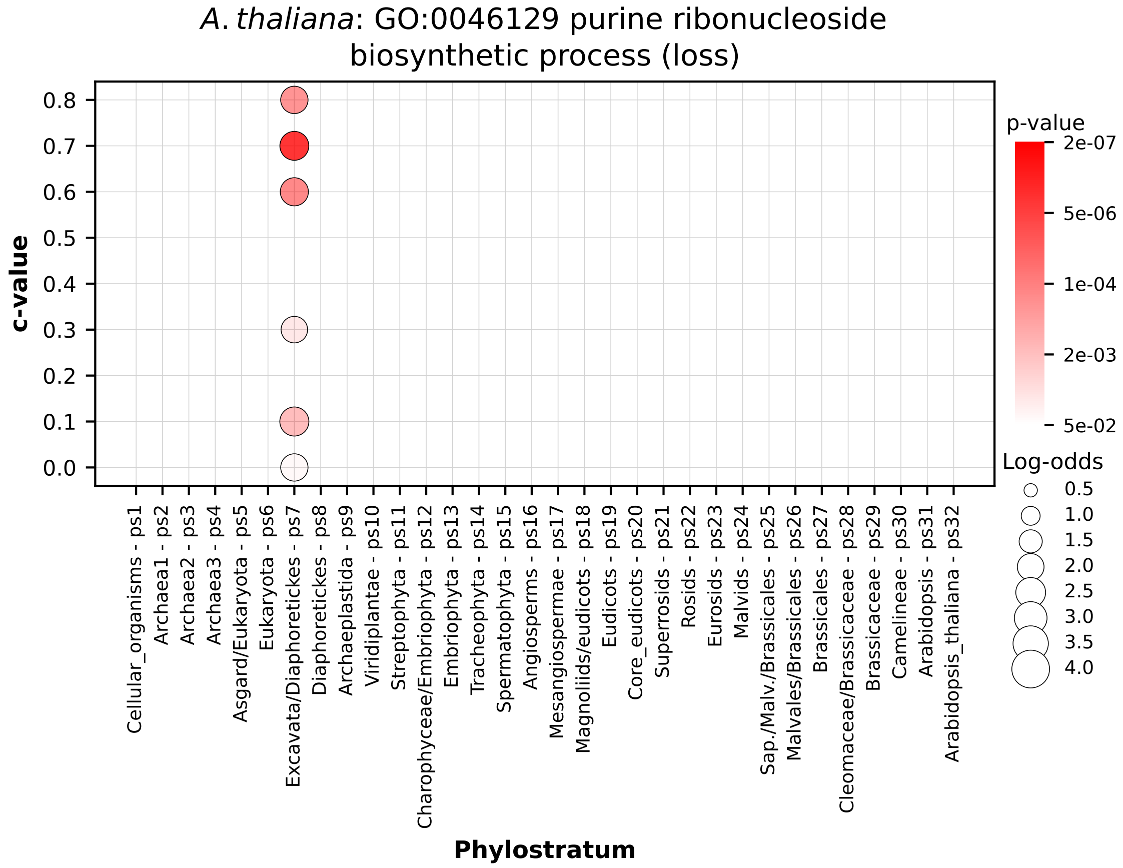

A. thaliana: GO:0046129 purine ribonucleoside biosynthetic process (loss)
c-value
0.8
0.7
0.6
0.5
0.4
0.3
0.2
0.1
0.0
p-value
2e-07
5e-06
1e-04
2e-03
5e-02
Log-odds
0.5
1.0
1.5
2.0
2.5
3.0
3.5
4.0
Cellular_organisms - ps1
Archaea1 - ps2
Archaea2 - ps3
Archaea3 - ps4
Asgard/Eukaryota - ps5
Eukaryota - ps6
Excavata/Diaphoretickes - ps7
Diaphoretickes - ps8
Archaeplastida - ps9
Viridiplantae - ps10
Streptophyta - ps11
Charophyceae/Embriophyta - ps12
Embriophyta - ps13
Tracheophyta - ps14
Spermatophyta - ps15
Angiosperms - ps16
Mesangiospermae - ps17
Magnoliids/eudicots - ps18
Eudicots - ps19
Core_eudicots - ps20
Superrosids - ps21
Rosids - ps22
Eurosids - ps23
Malvids - ps24
Sap./Malv./Brassicales - ps25
Malvales/Brassicales - ps26
Brassicales - ps27
Cleomaceae/Brassicaceae - ps28
Brassicaceae - ps29
Camelineae - ps30
Arabidopsis - ps31
Arabidopsis_thaliana - ps32
Phylostratum

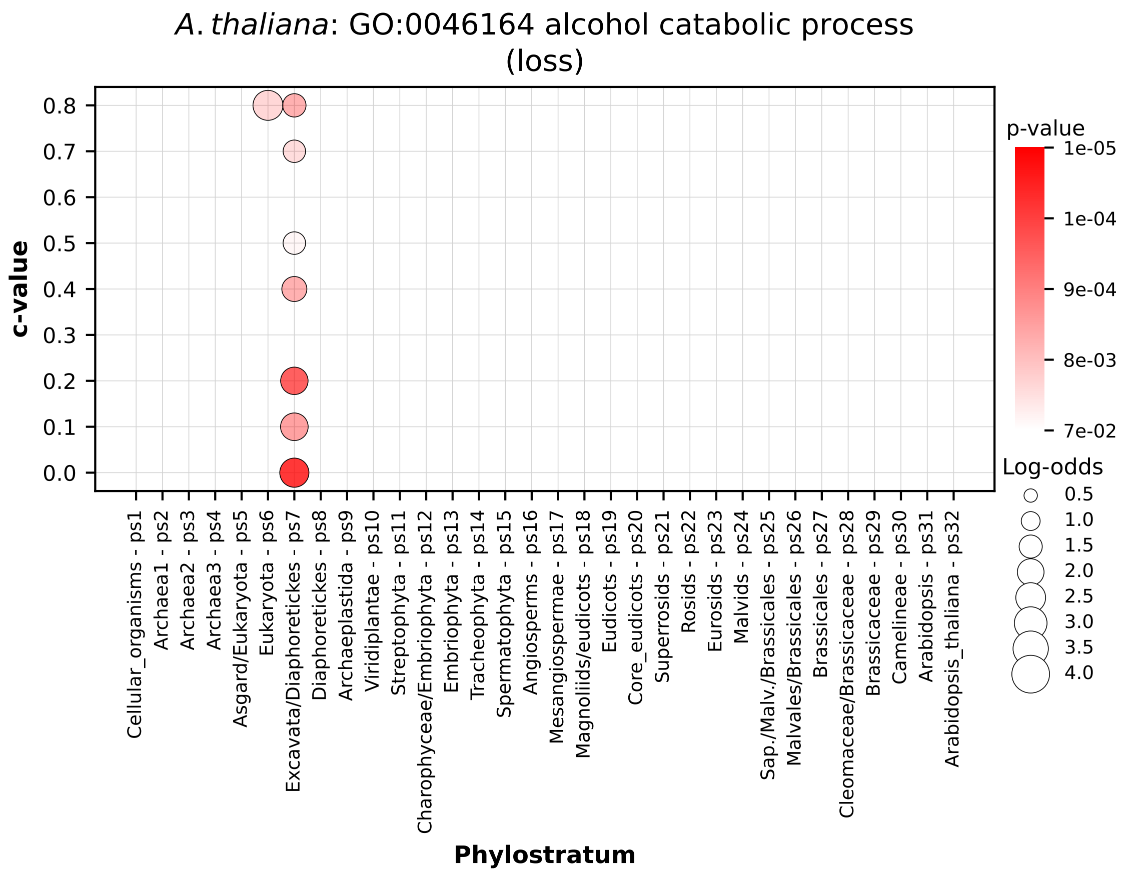

A. thaliana: GO:0046164 alcohol catabolic process
(loss)
c-value
0.8
0.7
0.6
0.5
0.4
0.3
0.2
0.1
0.0
p-value
1e-05
1e-04
9e-04
8e-03
7e-02
Log-odds
0.5
1.0
1.5
2.0
2.5
3.0
3.5
4.0
Cellular_organisms - ps1
Archaea1 - ps2
Archaea2 - ps3
Archaea3 - ps4
Asgard/Eukaryota - ps5
Eukaryota - ps6
Excavata/Diaphoretickes - ps7
Diaphoretickes - ps8
Archaeplastida - ps9
Viridiplantae - ps10
Streptophyta - ps11
Charophyceae/Embriophyta - ps12
Embriophyta - ps13
Tracheophyta - ps14
Spermatophyta - ps15
Angiosperms - ps16
Mesangiospermae - ps17
Magnoliids/eudicots - ps18
Eudicots - ps19
Core_eudicots - ps20
Superrosids - ps21
Rosids - ps22
Eurosids - ps23
Malvids - ps24
Sap./Malv./Brassicales - ps25
Malvales/Brassicales - ps26
Brassicales - ps27
Cleomaceae/Brassicaceae - ps28
Brassicaceae - ps29
Camelineae - ps30
Arabidopsis - ps31
Arabidopsis_thaliana - ps32
Phylostratum

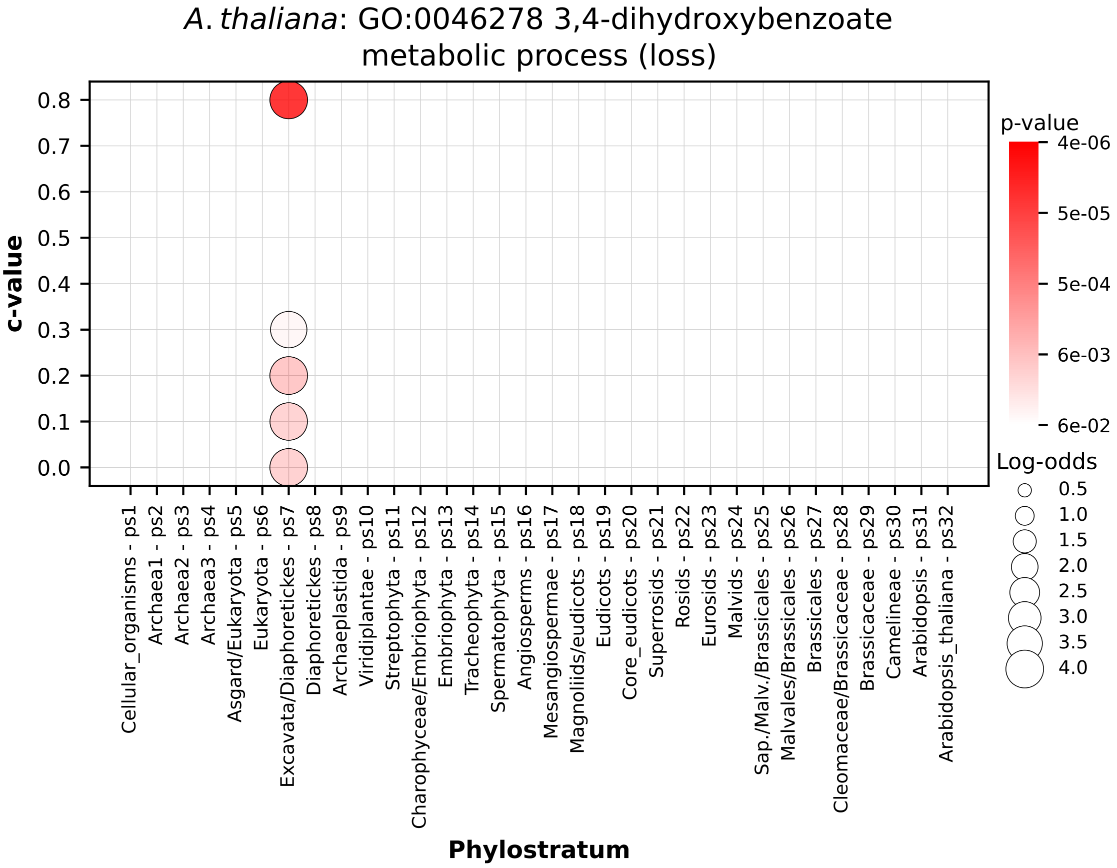

A. thaliana: GO:0046278 3,4-dihydroxybenzoate metabolic process (loss)
c-value
0.8
0.7
0.6
0.5
0.4
0.3
0.2
0.1
0.0
p-value
4e-06
5e-05
5e-04
6e-03
6e-02
Log-odds
0.5
1.0
1.5
2.0
2.5
3.0
3.5
4.0
Cellular_organisms - ps1
Archaea1 - ps2
Archaea2 - ps3
Archaea3 - ps4
Asgard/Eukaryota - ps5
Eukaryota - ps6
Excavata/Diaphoretickes - ps7
Diaphoretickes - ps8
Archaeplastida - ps9
Viridiplantae - ps10
Streptophyta - ps11
Charophyceae/Embriophyta - ps12
Embriophyta - ps13
Tracheophyta - ps14
Spermatophyta - ps15
Angiosperms - ps16
Mesangiospermae - ps17
Magnoliids/eudicots - ps18
Eudicots - ps19
Core_eudicots - ps20
Superrosids - ps21
Rosids - ps22
Eurosids - ps23
Malvids - ps24
Sap./Malv./Brassicales - ps25
Malvales/Brassicales - ps26
Brassicales - ps27
Cleomaceae/Brassicaceae - ps28
Brassicaceae - ps29
Camelineae - ps30
Arabidopsis - ps31
Arabidopsis_thaliana - ps32
Phylostratum

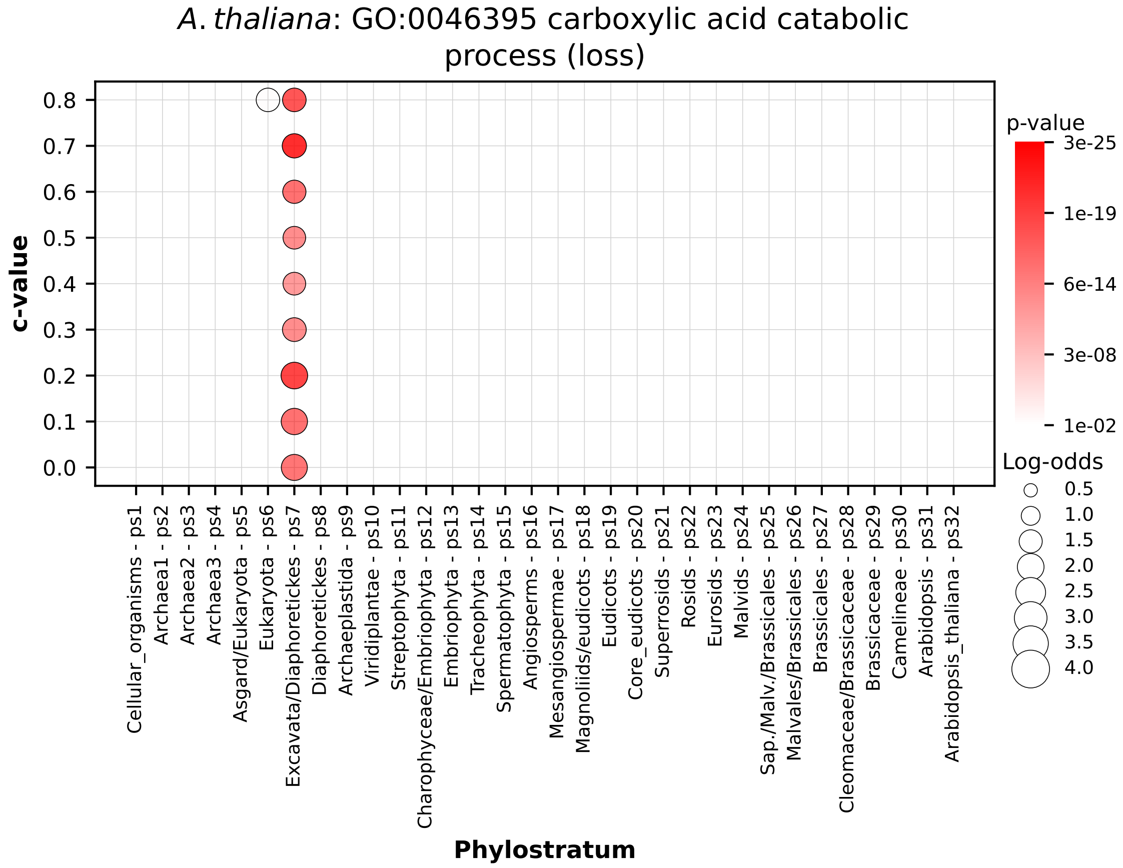
A. thaliana: GO:0046395 carboxylic acid catabolic process (loss)
c-value
0.8
0.7
0.6
0.5
0.4
0.3
0.2
0.1
0.0
p-value
3e-25
1e-19
6e-14
3e-08
1e-02
Log-odds
0.5
1.0
1.5
2.0
2.5
3.0
3.5
4.0
Cellular_organisms - ps1
Archaea1 - ps2
Archaea2 - ps3
Archaea3 - ps4
Asgard/Eukaryota - ps5
Eukaryota - ps6
Excavata/Diaphoretickes - ps7
Diaphoretickes - ps8
Archaeplastida - ps9
Viridiplantae - ps10
Streptophyta - ps11
Charophyceae/Embriophyta - ps12
Embriophyta - ps13
Tracheophyta - ps14
Spermatophyta - ps15
Angiosperms - ps16
Mesangiospermae - ps17
Magnoliids/eudicots - ps18
Eudicots - ps19
Core_eudicots - ps20
Superrosids - ps21
Rosids - ps22
Eurosids - ps23
Malvids - ps24
Sap./Malv./Brassicales - ps25
Malvales/Brassicales - ps26
Brassicales - ps27
Cleomaceae/Brassicaceae - ps28
Brassicaceae - ps29
Camelineae - ps30
Arabidopsis - ps31
Arabidopsis_thaliana - ps32
Phylostratum

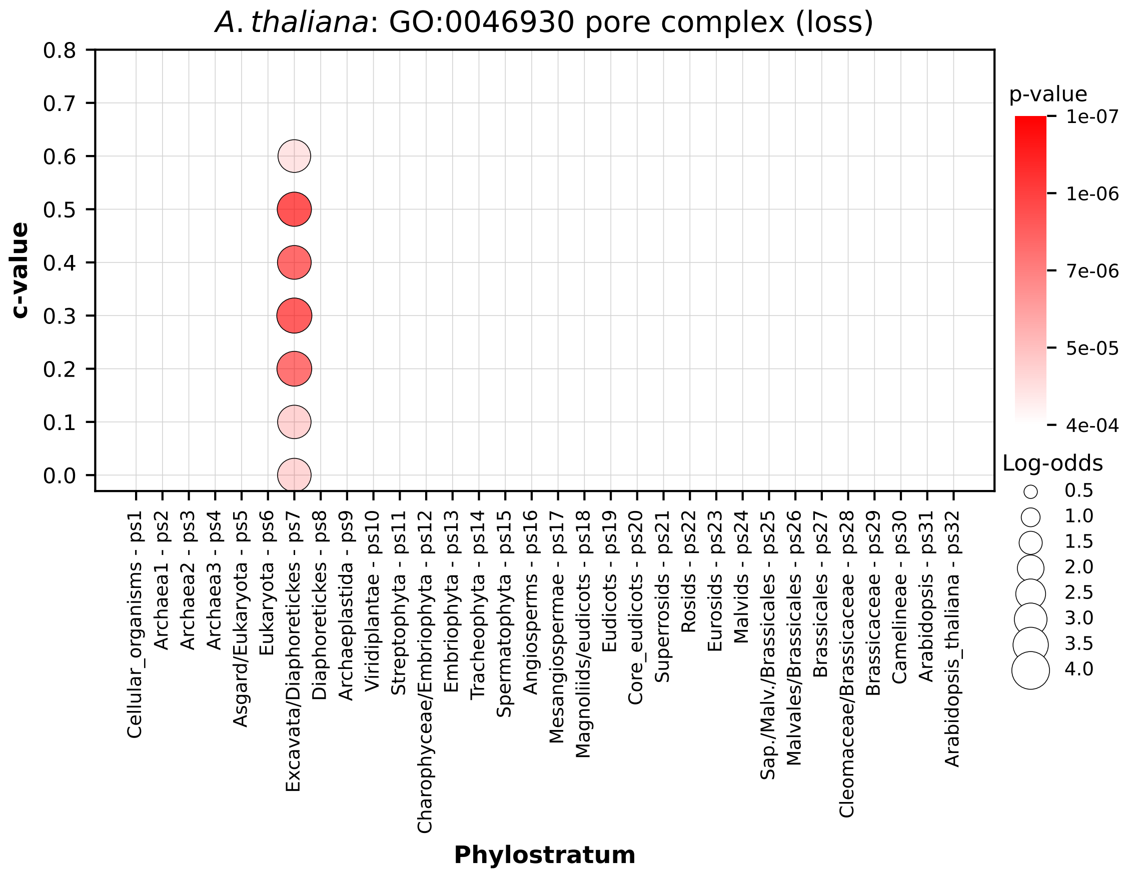

A. thaliana: GO:0046930 pore complex (loss)
c-value
0.0
0.1
0.2
0.3
0.4
0.5
0.6
0.7
0.8
Phylostratum
Cellular_organisms - ps1
Archaea1 - ps2
Archaea2 - ps3
Archaea3 - ps4
Asgard/Eukaryota - ps5
Eukaryota - ps6
Excavata/Diaphoretickes - ps7
Diaphoretickes - ps8
Archaeplastida - ps9
Viridiplantae - ps10
Streptophyta - ps11
Charophyceae/Embriophyta - ps12
Embriophyta - ps13
Tracheophyta - ps14
Spermatophyta - ps15
Angiosperms - ps16
Mesangiospermae - ps17
Magnoliids/eudicots - ps18
Eudicots - ps19
Core_eudicots - ps20
Superrosids - ps21
Rosids - ps22
Eurosids - ps23
Malvids - ps24
Sap./Malv./Brassicales - ps25
Malvales/Brassicales - ps26
Brassicales - ps27
Cleomaceae/Brassicaceae - ps28
Brassicaceae - ps29
Camelineae - ps30
Arabidopsis - ps31
Arabidopsis_thaliana - ps32
p-value
1e-07
1e-06
7e-06
5e-05
4e-04
Log-odds
0.5
1.0
1.5
2.0
2.5
3.0
3.5
4.0

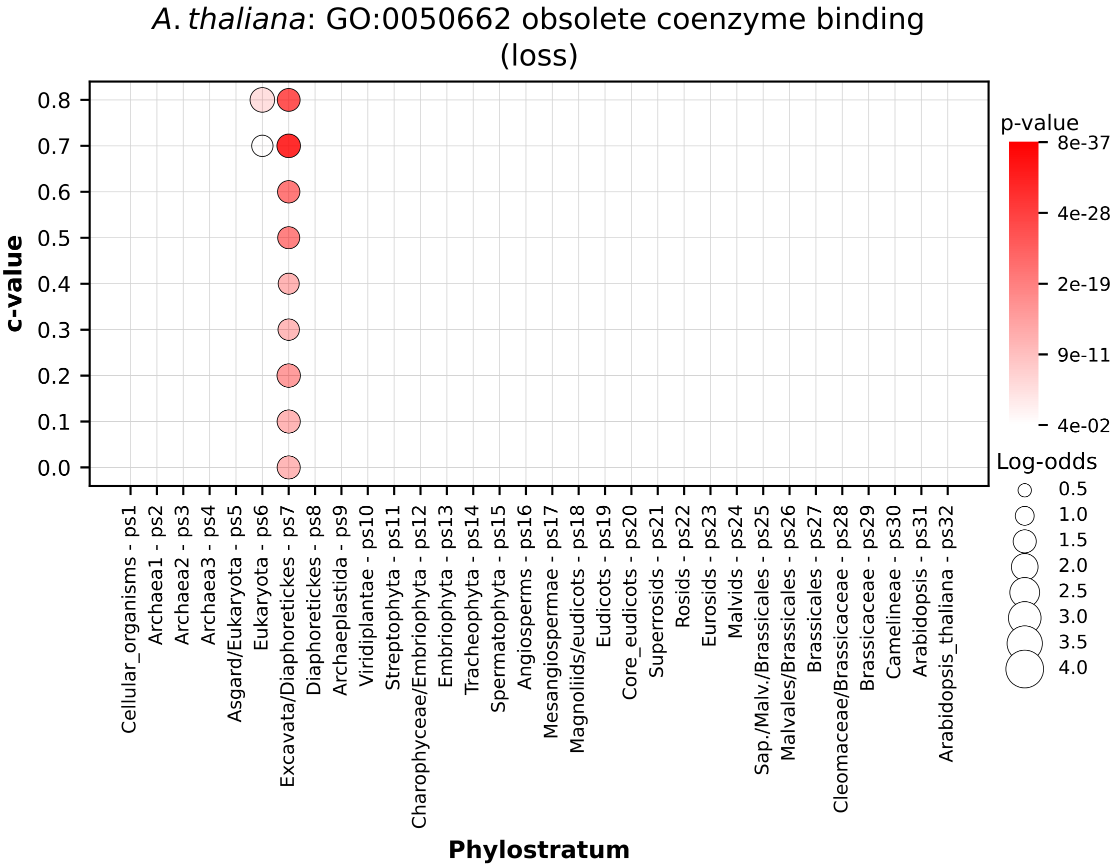

A. thaliana: GO:0050662 obsolete coenzyme binding
(loss)
c-value
0.8
0.7
0.6
0.5
0.4
0.3
0.2
0.1
0.0
p-value
8e-37
4e-28
2e-19
9e-11
4e-02
Log-odds
0.5
1.0
1.5
2.0
2.5
3.0
3.5
4.0
Cellular_organisms - ps1
Archaea1 - ps2
Archaea2 - ps3
Archaea3 - ps4
Asgard/Eukaryota - ps5
Eukaryota - ps6
Excavata/Diaphoretickes - ps7
Diaphoretickes - ps8
Archaeplastida - ps9
Viridiplantae - ps10
Streptophyta - ps11
Charophyceae/Embriophyta - ps12
Embriophyta - ps13
Tracheophyta - ps14
Spermatophyta - ps15
Angiosperms - ps16
Mesangiospermae - ps17
Magnoliids/eudicots - ps18
Eudicots - ps19
Core_eudicots - ps20
Superrosids - ps21
Rosids - ps22
Eurosids - ps23
Malvids - ps24
Sap./Malv./Brassicales - ps25
Malvales/Brassicales - ps26
Brassicales - ps27
Cleomaceae/Brassicaceae - ps28
Brassicaceae - ps29
Camelineae - ps30
Arabidopsis - ps31
Arabidopsis_thaliana - ps32
Phylostratum

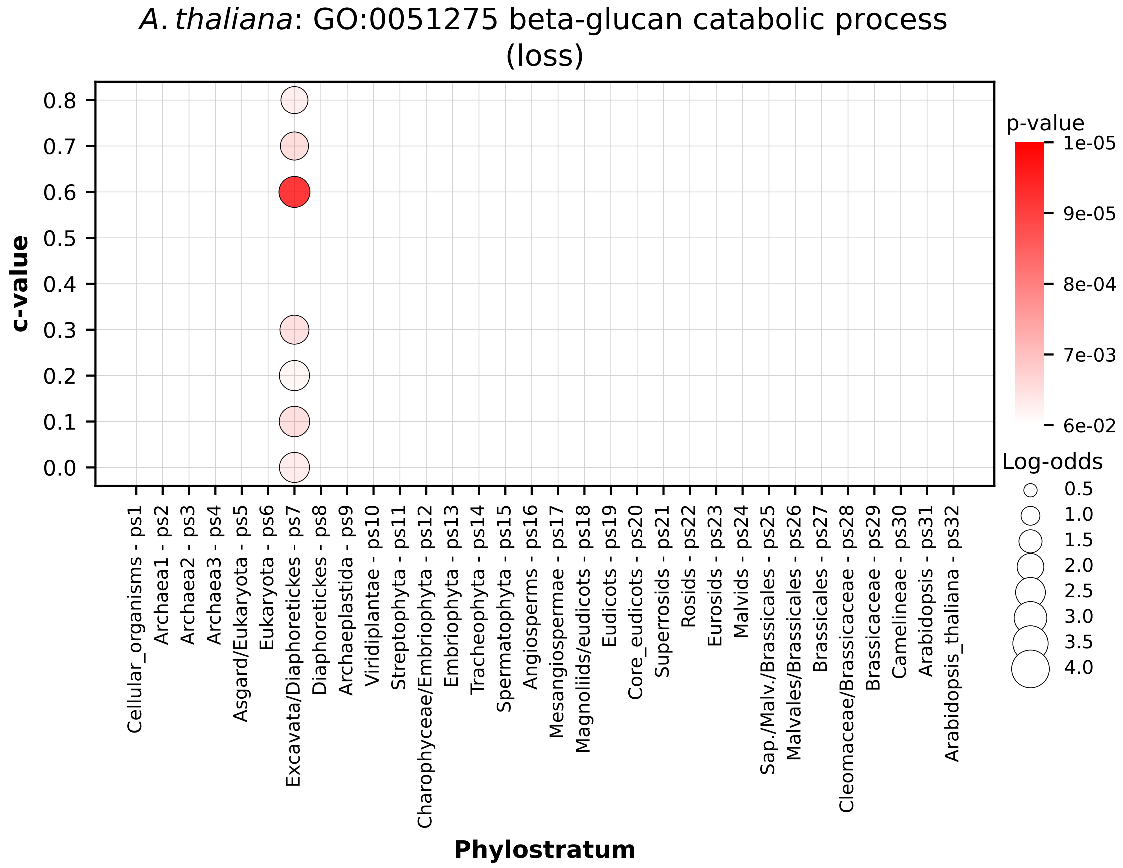

A. thaliana: GO:0051275 beta-glucan catabolic process
(loss)
c-value
0.8
0.7
0.6
0.5
0.4
0.3
0.2
0.1
0.0
p-value
1e-05
9e-05
8e-04
7e-03
6e-02
Log-odds
0.5
1.0
1.5
2.0
2.5
3.0
3.5
4.0
Cellular_organisms - ps1
Archaea1 - ps2
Archaea2 - ps3
Archaea3 - ps4
Asgard/Eukaryota - ps5
Eukaryota - ps6
Excavata/Diaphoretickes - ps7
Diaphoretickes - ps8
Archaeplastida - ps9
Viridiplantae - ps10
Streptophyta - ps11
Charophyceae/Embriophyta - ps12
Embriophyta - ps13
Tracheophyta - ps14
Spermatophyta - ps15
Angiosperms - ps16
Mesangiospermae - ps17
Magnoliids/eudicots - ps18
Eudicots - ps19
Core_eudicots - ps20
Superrosids - ps21
Rosids - ps22
Eurosids - ps23
Malvids - ps24
Sap./Malv./Brassicales - ps25
Malvales/Brassicales - ps26
Brassicales - ps27
Cleomaceae/Brassicaceae - ps28
Brassicaceae - ps29
Camelineae - ps30
Arabidopsis - ps31
Arabidopsis_thaliana - ps32
Phylostratum

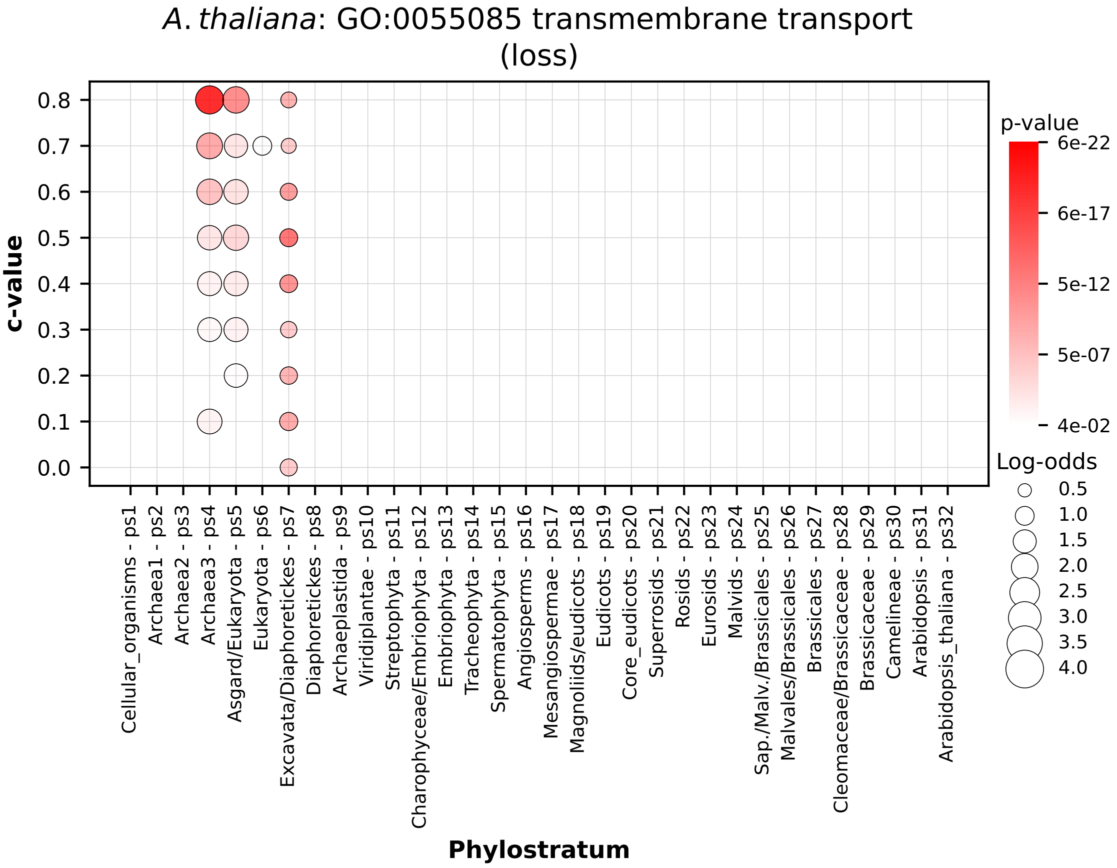

A. thaliana: GO:0055085 transmembrane transport
(loss)
c-value
0.8
0.7
0.6
0.5
0.4
0.3
0.2
0.1
0.0
Phylostratum
Cellular_organisms - ps1
Archaea1 - ps2
Archaea2 - ps3
Archaea3 - ps4
Asgard/Eukaryota - ps5
Eukaryota - ps6
Excavata/Diaphoretickes - ps7
Diaphoretickes - ps8
Archaeplastida - ps9
Viridiplantae - ps10
Streptophyta - ps11
Charophyceae/Embriophyta - ps12
Embriophyta - ps13
Tracheophyta - ps14
Spermatophyta - ps15
Angiosperms - ps16
Mesangiospermae - ps17
Magnoliids/eudicots - ps18
Eudicots - ps19
Core_eudicots - ps20
Superrosids - ps21
Rosids - ps22
Eurosids - ps23
Malvids - ps24
Sap./Malv./Brassicales - ps25
Malvales/Brassicales - ps26
Brassicales - ps27
Cleomaceae/Brassicaceae - ps28
Brassicaceae - ps29
Camelineae - ps30
Arabidopsis - ps31
Arabidopsis_thaliana - ps32
p-value
6e-22
6e-17
5e-12
5e-07
4e-02
Log-odds
0.5
1.0
1.5
2.0
2.5
3.0
3.5
4.0

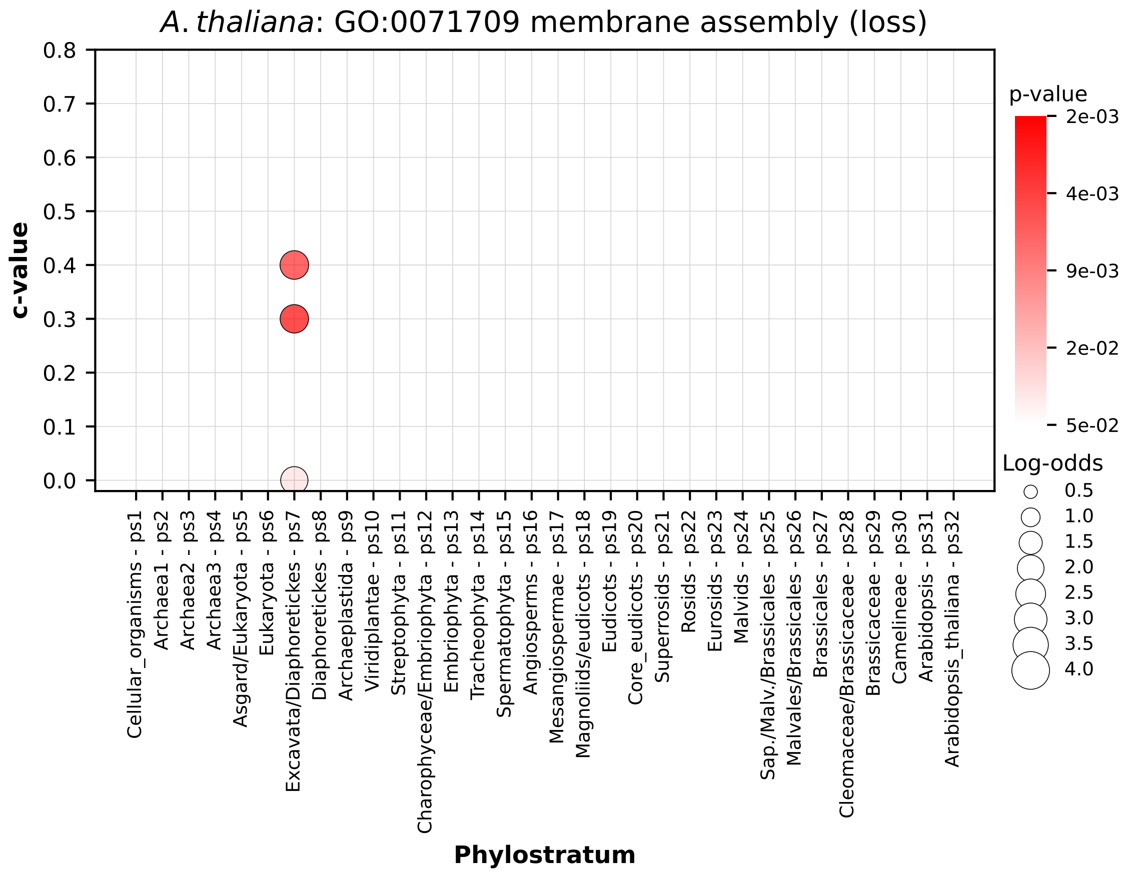
A. thaliana: GO:0071709 membrane assembly (loss)
c-value
0.8
0.7
0.6
0.5
0.4
0.3
0.2
0.1
0.0
Phylostratum
Cellular_organisms - ps1
Archaea1 - ps2
Archaea2 - ps3
Archaea3 - ps4
Asgard/Eukaryota - ps5
Eukaryota - ps6
Excavata/Diaphoretickes - ps7
Diaphoretickes - ps8
Archaeplastida - ps9
Viridiplantae - ps10
Streptophyta - ps11
Charophyceae/Embriophyta - ps12
Embriophyta - ps13
Tracheophyta - ps14
Spermatophyta - ps15
Angiosperms - ps16
Mesangiospermae - ps17
Magnoliids/eudicots - ps18
Eudicots - ps19
Core_eudicots - ps20
Superrosids - ps21
Rosids - ps22
Eurosids - ps23
Malvids - ps24
Sap./Malv./Brassicales - ps25
Malvales/Brassicales - ps26
Brassicales - ps27
Cleomaceae/Brassicaceae - ps28
Brassicaceae - ps29
Camelineae - ps30
Arabidopsis - ps31
Arabidopsis_thaliana - ps32
p-value
2e-03
4e-03
9e-03
2e-02
5e-02
Log-odds
0.5
1.0
1.5
2.0
2.5
3.0
3.5
4.0

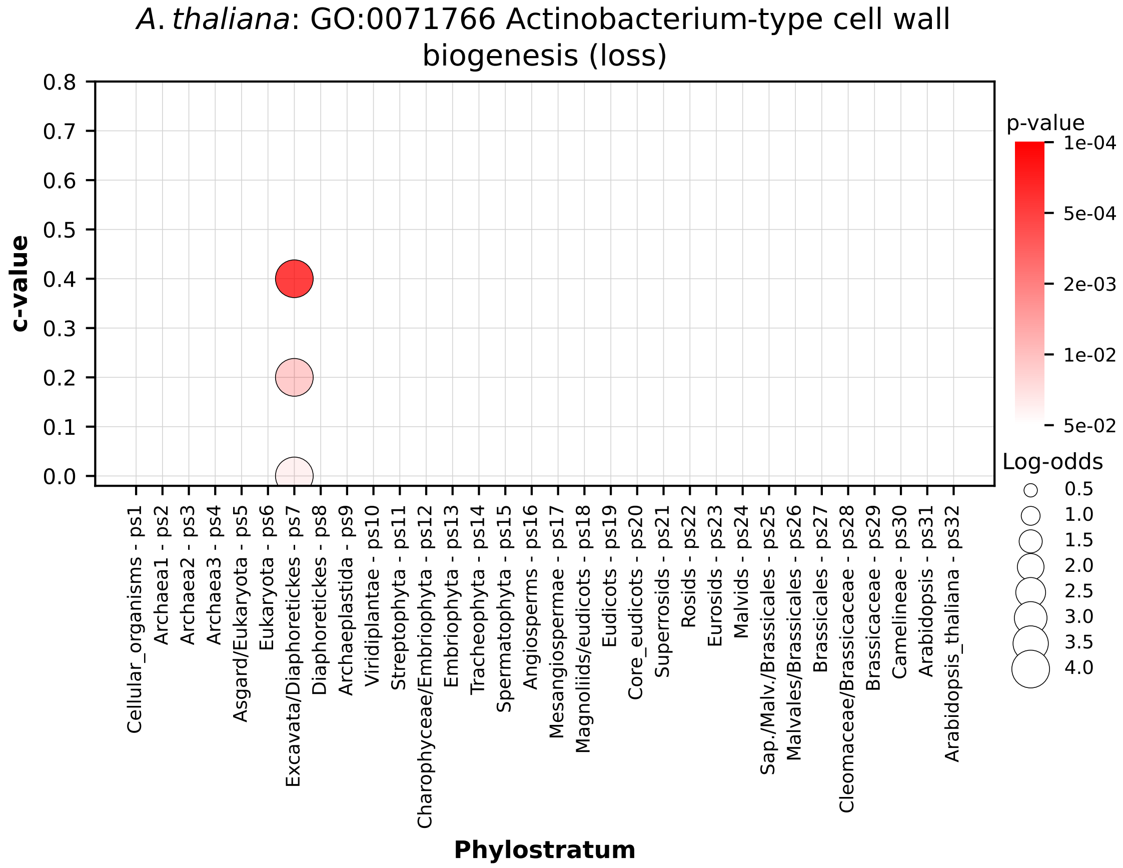

A. thaliana: GO:0071766 Actinobacterium-type cell wall biogenesis (loss)
c-value
0.0
0.1
0.2
0.3
0.4
0.5
0.6
0.7
0.8
Phylostratum
Cellular_organisms - ps1
Archaea1 - ps2
Archaea2 - ps3
Archaea3 - ps4
Asgard/Eukaryota - ps5
Eukaryota - ps6
Excavata/Diaphoretickes - ps7
Diaphoretickes - ps8
Archaeplastida - ps9
Viridiplantae - ps10
Streptophyta - ps11
Charophyceae/Embriophyta - ps12
Embriophyta - ps13
Tracheophyta - ps14
Spermatophyta - ps15
Angiosperms - ps16
Mesangiospermae - ps17
Magnoliids/eudicots - ps18
Eudicots - ps19
Core_eudicots - ps20
Superrosids - ps21
Rosids - ps22
Eurosids - ps23
Malvids - ps24
Sap./Malv./Brassicales - ps25
Malvales/Brassicales - ps26
Brassicales - ps27
Cleomaceae/Brassicaceae - ps28
Brassicaceae - ps29
Camelineae - ps30
Arabidopsis - ps31
Arabidopsis_thaliana - ps32
p-value
1e-04
5e-04
2e-03
1e-02
5e-02
Log-odds
0.5
1.0
1.5
2.0
2.5
3.0
3.5
4.0

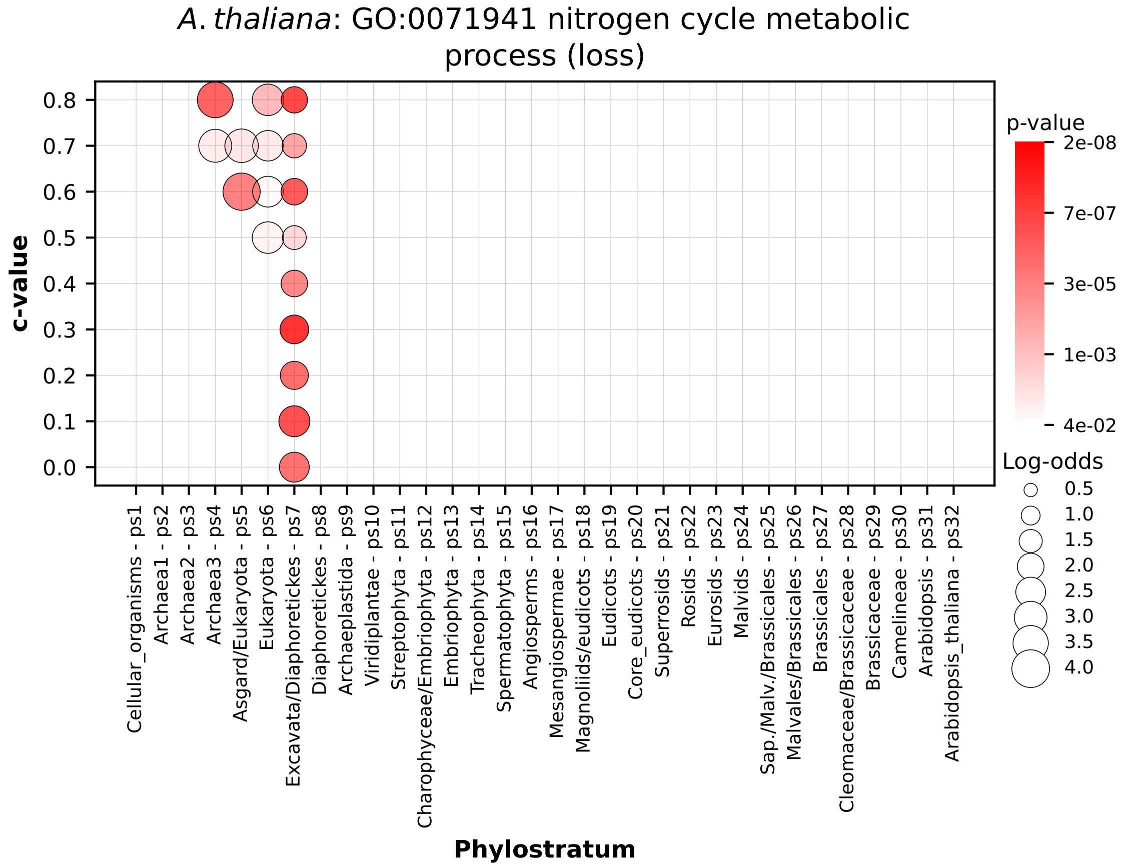
A. thaliana: GO:0071941 nitrogen cycle metabolic process (loss)
c-value
0.8
0.7
0.6
0.5
0.4
0.3
0.2
0.1
0.0
p-value
2e-08
7e-07
3e-05
1e-03
4e-02
Log-odds
0.5
1.0
1.5
2.0
2.5
3.0
3.5
4.0
Cellular_organisms - ps1
Archaea1 - ps2
Archaea2 - ps3
Archaea3 - ps4
Asgard/Eukaryota - ps5
Eukaryota - ps6
Excavata/Diaphoretickes - ps7
Diaphoretickes - ps8
Archaeplastida - ps9
Viridiplantae - ps10
Streptophyta - ps11
Charophyceae/Embriophyta - ps12
Embriophyta - ps13
Tracheophyta - ps14
Spermatophyta - ps15
Angiosperms - ps16
Mesangiospermae - ps17
Magnoliids/eudicots - ps18
Eudicots - ps19
Core_eudicots - ps20
Superrosids - ps21
Rosids - ps22
Eurosids - ps23
Malvids - ps24
Sap./Malv./Brassicales - ps25
Malvales/Brassicales - ps26
Brassicales - ps27
Cleomaceae/Brassicaceae - ps28
Brassicaceae - ps29
Camelineae - ps30
Arabidopsis - ps31
Arabidopsis_thaliana - ps32
Phylostratum

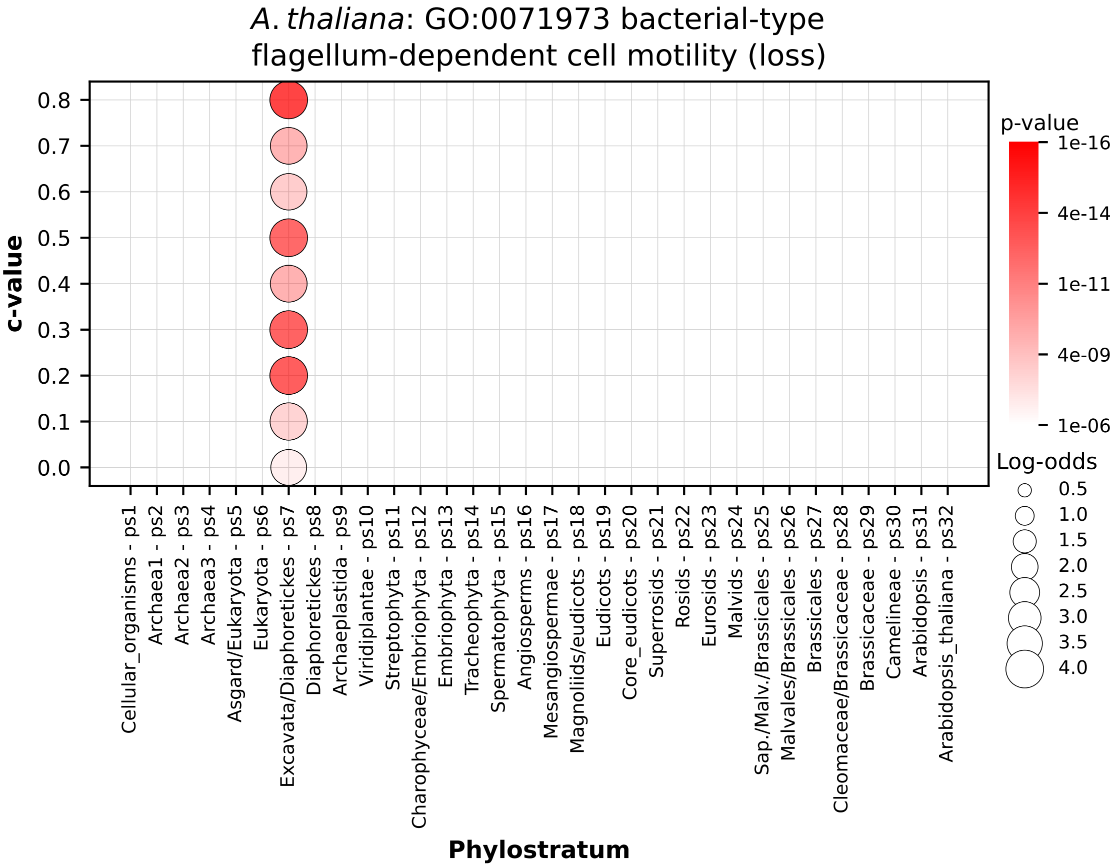
A. thaliana: GO:0071973 bacterial-type flagellum-dependent cell motility (loss)
c-value
0.8
0.7
0.6
0.5
0.4
0.3
0.2
0.1
0.0
Phylostratum
Cellular_organisms - ps1
Archaea1 - ps2
Archaea2 - ps3
Archaea3 - ps4
Asgard/Eukaryota - ps5
Eukaryota - ps6
Excavata/Diaphoretickes - ps7
Diaphoretickes - ps8
Archaeplastida - ps9
Viridiplantae - ps10
Streptophyta - ps11
Charophyceae/Embriophyta - ps12
Embriophyta - ps13
Tracheophyta - ps14
Spermatophyta - ps15
Angiosperms - ps16
Mesangiospermae - ps17
Magnoliids/eudicots - ps18
Eudicots - ps19
Core_eudicots - ps20
Superrosids - ps21
Rosids - ps22
Eurosids - ps23
Malvids - ps24
Sap./Malv./Brassicales - ps25
Malvales/Brassicales - ps26
Brassicales - ps27
Cleomaceae/Brassicaceae - ps28
Brassicaceae - ps29
Camelineae - ps30
Arabidopsis - ps31
Arabidopsis_thaliana - ps32
p-value
1e-16
4e-14
1e-11
4e-09
1e-06
Log-odds
0.5
1.0
1.5
2.0
2.5
3.0
3.5
4.0

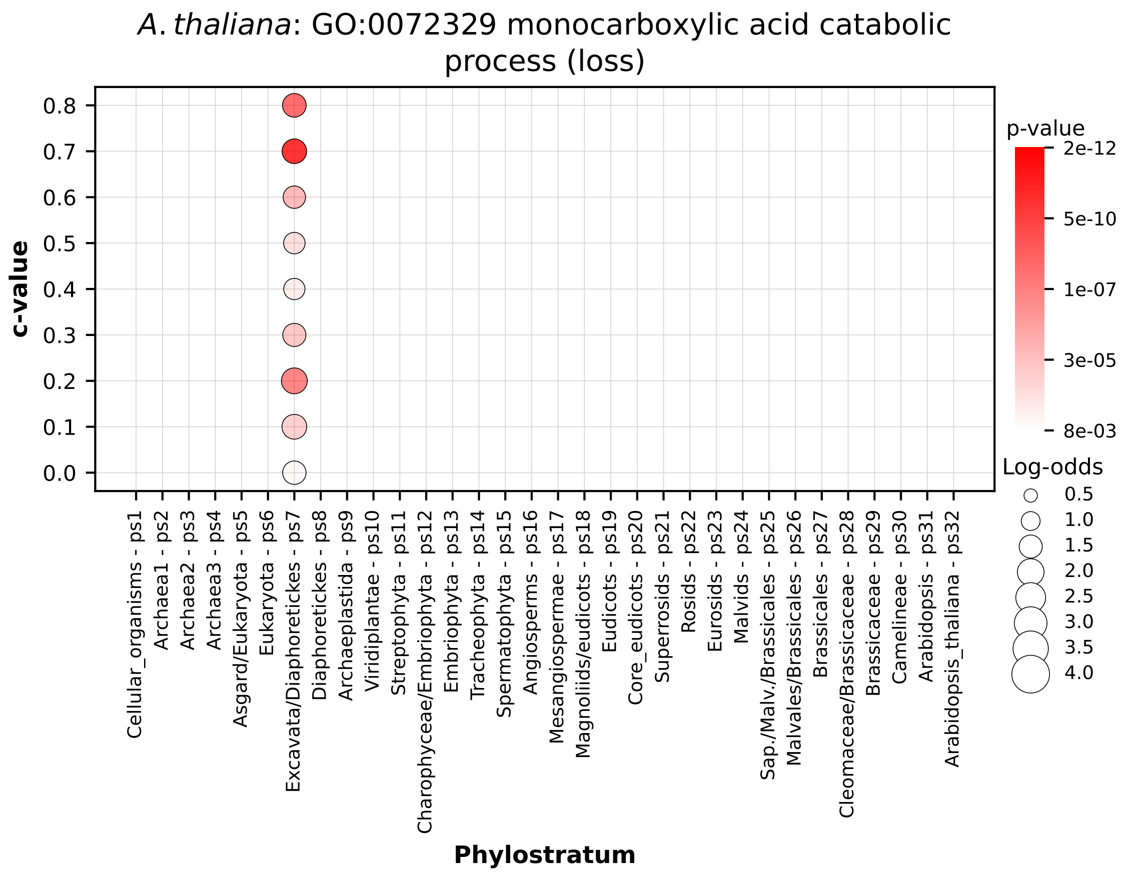

A. thaliana: GO:0072329 monocarboxylic acid catabolic process (loss)
c-value
0.8
0.7
0.6
0.5
0.4
0.3
0.2
0.1
0.0
p-value
2e-12
5e-10
1e-07
3e-05
8e-03
Log-odds
0.5
1.0
1.5
2.0
2.5
3.0
3.5
4.0
Cellular_organisms - ps1
Archaea1 - ps2
Archaea2 - ps3
Archaea3 - ps4
Asgard/Eukaryota - ps5
Eukaryota - ps6
Excavata/Diaphoretickes - ps7
Diaphoretickes - ps8
Archaeplastida - ps9
Viridiplantae - ps10
Streptophyta - ps11
Charophyceae/Embriophyta - ps12
Embriophyta - ps13
Tracheophyta - ps14
Spermatophyta - ps15
Angiosperms - ps16
Mesangiospermae - ps17
Magnoliids/eudicots - ps18
Eudicots - ps19
Core_eudicots - ps20
Superrosids - ps21
Rosids - ps22
Eurosids - ps23
Malvids - ps24
Sap./Malv./Brassicales - ps25
Malvales/Brassicales - ps26
Brassicales - ps27
Cleomaceae/Brassicaceae - ps28
Brassicaceae - ps29
Camelineae - ps30
Arabidopsis - ps31
Arabidopsis_thaliana - ps32
Phylostratum

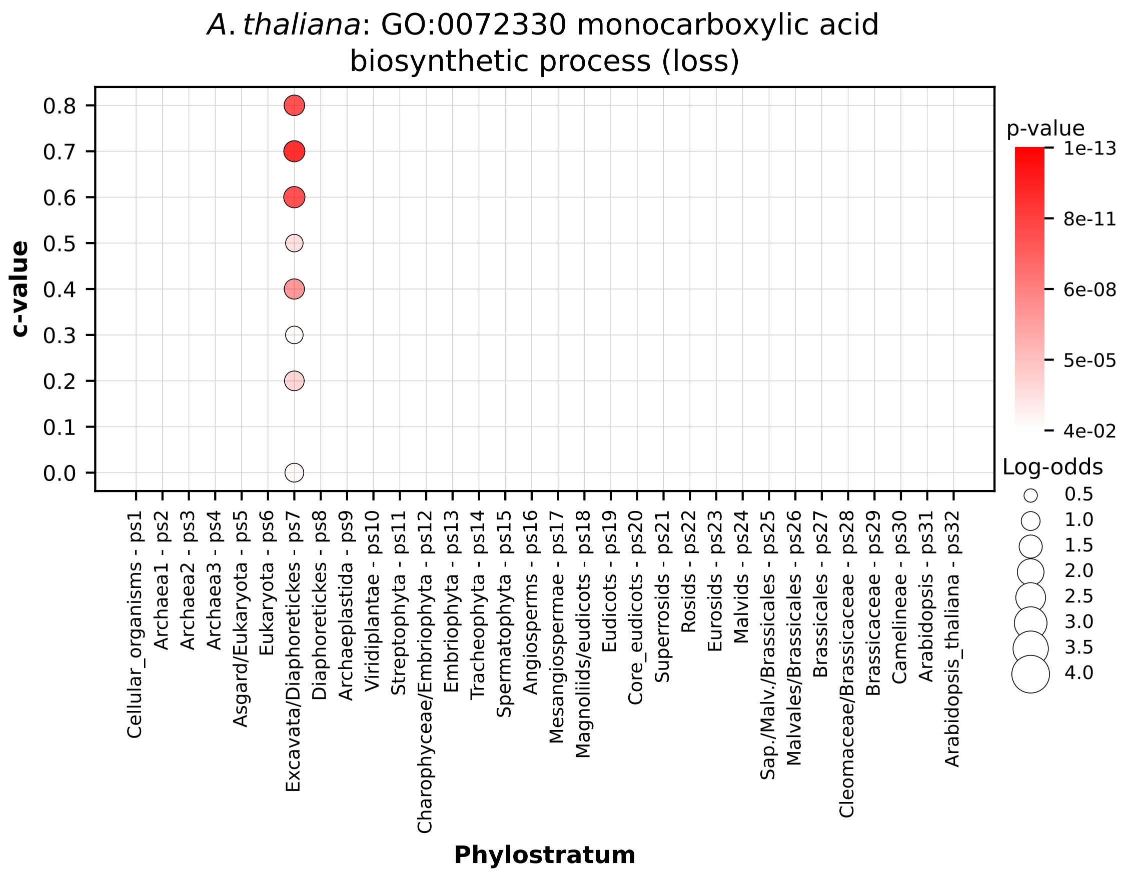

A. thaliana: GO:0072330 monocarboxylic acid biosynthetic process (loss)
c-value
0.8
0.7
0.6
0.5
0.4
0.3
0.2
0.1
0.0
p-value
1e-13
8e-11
6e-08
5e-05
4e-02
Log-odds
0.5
1.0
1.5
2.0
2.5
3.0
3.5
4.0
Cellular_organisms - ps1
Archaea1 - ps2
Archaea2 - ps3
Archaea3 - ps4
Asgard/Eukaryota - ps5
Eukaryota - ps6
Excavata/Diaphoretickes - ps7
Diaphoretickes - ps8
Archaeplastida - ps9
Viridiplantae - ps10
Streptophyta - ps11
Charophyceae/Embriophyta - ps12
Embriophyta - ps13
Tracheophyta - ps14
Spermatophyta - ps15
Angiosperms - ps16
Mesangiospermae - ps17
Magnoliids/eudicots - ps18
Eudicots - ps19
Core_eudicots - ps20
Superrosids - ps21
Rosids - ps22
Eurosids - ps23
Malvids - ps24
Sap./Malv./Brassicales - ps25
Malvales/Brassicales - ps26
Brassicales - ps27
Cleomaceae/Brassicaceae - ps28
Brassicaceae - ps29
Camelineae - ps30
Arabidopsis - ps31
Arabidopsis_thaliana - ps32
Phylostratum

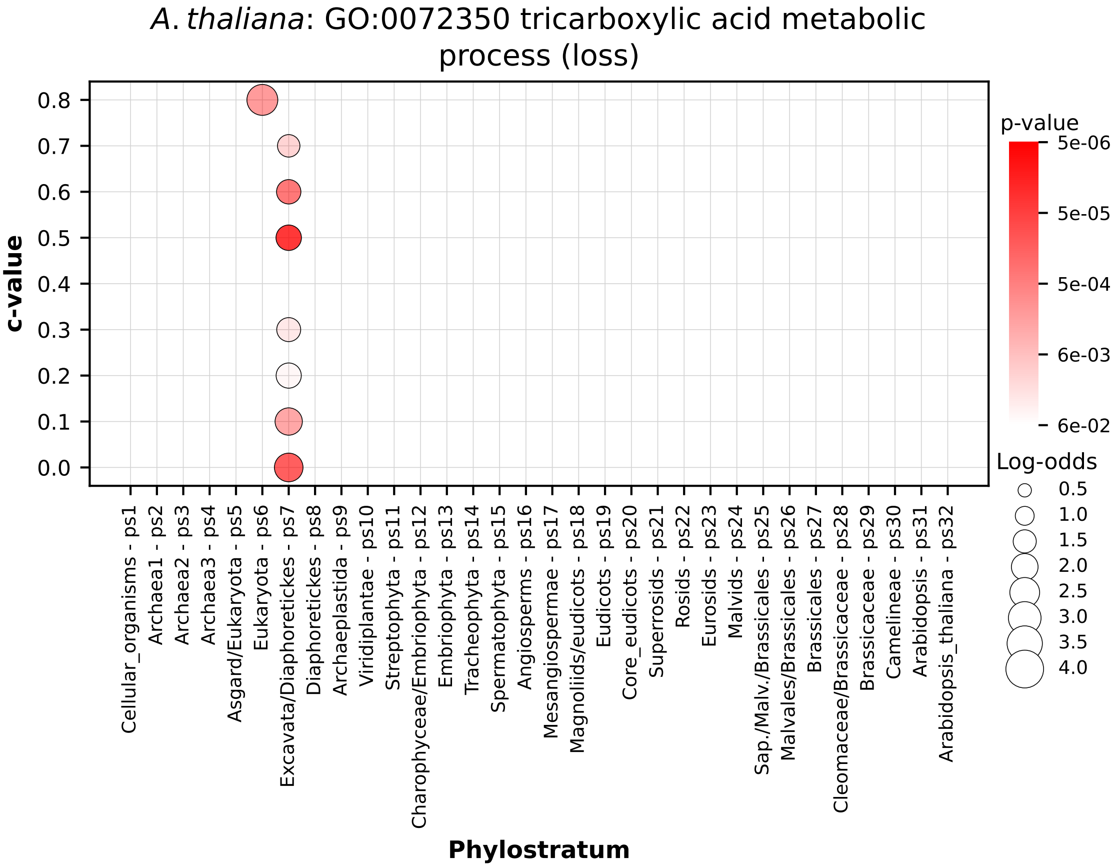

A. thaliana: GO:0072350 tricarboxylic acid metabolic process (loss)
c-value
0.8
0.7
0.6
0.5
0.4
0.3
0.2
0.1
0.0
p-value
5e-06
5e-05
5e-04
6e-03
6e-02
Log-odds
0.5
1.0
1.5
2.0
2.5
3.0
3.5
4.0
Cellular_organisms - ps1
Archaea1 - ps2
Archaea2 - ps3
Archaea3 - ps4
Asgard/Eukaryota - ps5
Eukaryota - ps6
Excavata/Diaphoretickes - ps7
Diaphoretickes - ps8
Archaeplastida - ps9
Viridiplantae - ps10
Streptophyta - ps11
Charophyceae/Embriophyta - ps12
Embriophyta - ps13
Tracheophyta - ps14
Spermatophyta - ps15
Angiosperms - ps16
Mesangiospermae - ps17
Magnoliids/eudicots - ps18
Eudicots - ps19
Core_eudicots - ps20
Superrosids - ps21
Rosids - ps22
Eurosids - ps23
Malvids - ps24
Sap./Malv./Brassicales - ps25
Malvales/Brassicales - ps26
Brassicales - ps27
Cleomaceae/Brassicaceae - ps28
Brassicaceae - ps29
Camelineae - ps30
Arabidopsis - ps31
Arabidopsis_thaliana - ps32
Phylostratum

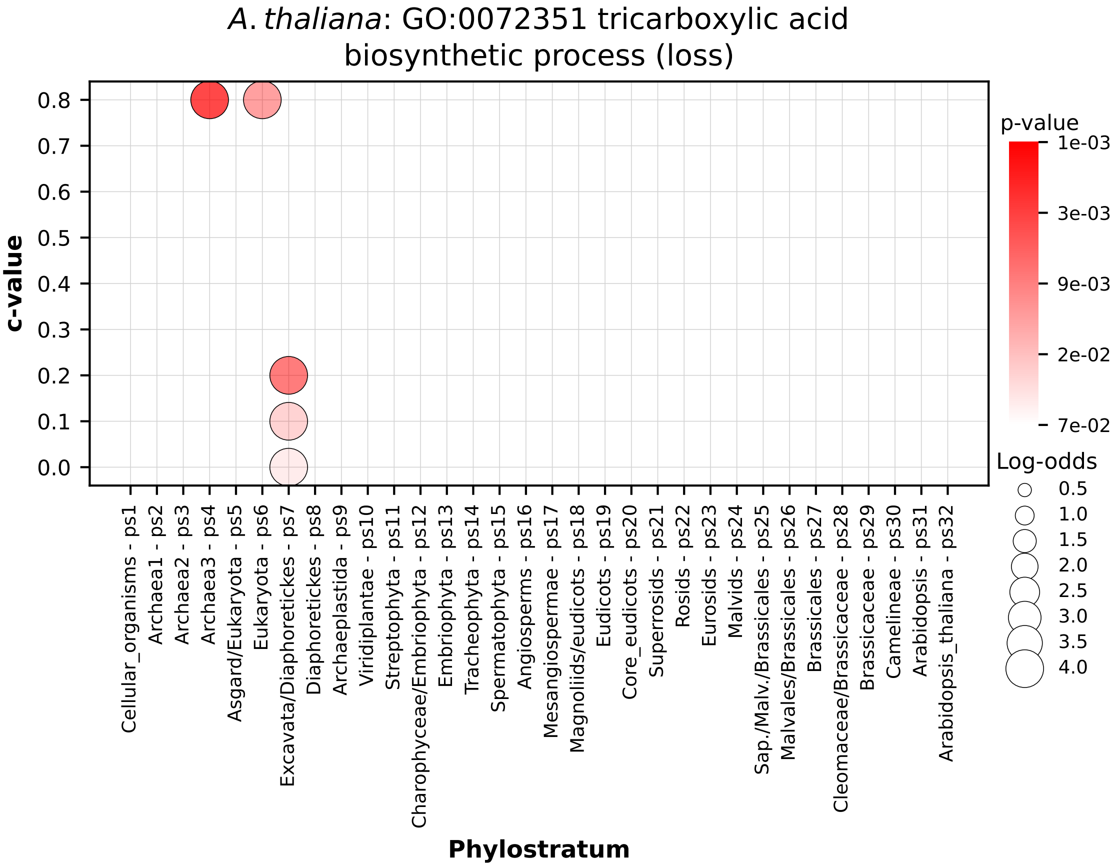
A. thaliana: GO:0072351 tricarboxylic acid biosynthetic process (loss)
c-value
0.8
0.7
0.6
0.5
0.4
0.3
0.2
0.1
0.0
Phylostratum
Cellular_organisms - ps1
Archaea1 - ps2
Archaea2 - ps3
Archaea3 - ps4
Asgard/Eukaryota - ps5
Eukaryota - ps6
Excavata/Diaphoretickes - ps7
Diaphoretickes - ps8
Archaeplastida - ps9
Viridiplantae - ps10
Streptophyta - ps11
Charophyceae/Embriophyta - ps12
Embriophyta - ps13
Tracheophyta - ps14
Spermatophyta - ps15
Angiosperms - ps16
Mesangiospermae - ps17
Magnoliids/eudicots - ps18
Eudicots - ps19
Core_eudicots - ps20
Superrosids - ps21
Rosids - ps22
Eurosids - ps23
Malvids - ps24
Sap./Malv./Brassicales - ps25
Malvales/Brassicales - ps26
Brassicales - ps27
Cleomaceae/Brassicaceae - ps28
Brassicaceae - ps29
Camelineae - ps30
Arabidopsis - ps31
Arabidopsis_thaliana - ps32
p-value
1e-03
3e-03
9e-03
2e-02
7e-02
Log-odds
0.5
1.0
1.5
2.0
2.5
3.0
3.5
4.0

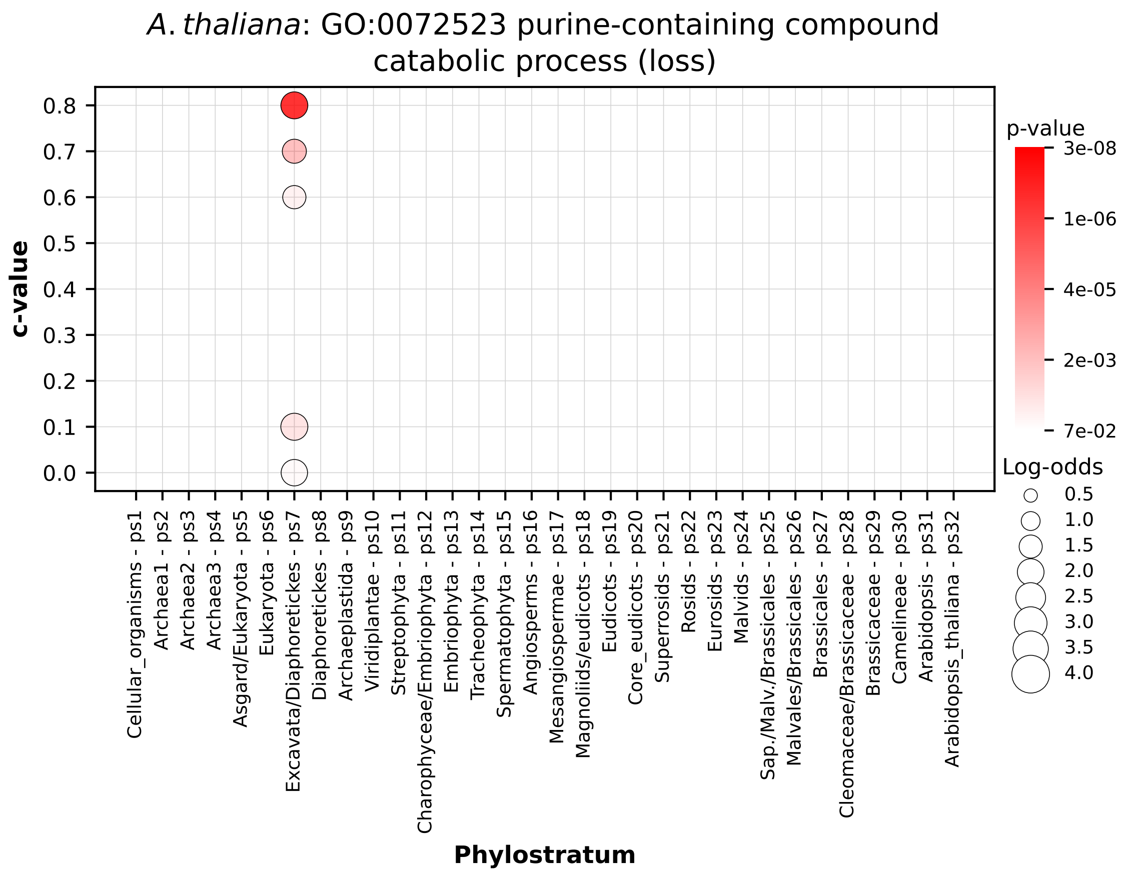
A. thaliana: GO:0072523 purine-containing compound catabolic process (loss)
c-value
0.8
0.7
0.6
0.5
0.4
0.3
0.2
0.1
0.0
p-value
3e-08
1e-06
4e-05
2e-03
7e-02
Log-odds
0.5
1.0
1.5
2.0
2.5
3.0
3.5
4.0
Cellular_organisms - ps1
Archaea1 - ps2
Archaea2 - ps3
Archaea3 - ps4
Asgard/Eukaryota - ps5
Eukaryota - ps6
Excavata/Diaphoretickes - ps7
Diaphoretickes - ps8
Archaeplastida - ps9
Viridiplantae - ps10
Streptophyta - ps11
Charophyceae/Embriophyta - ps12
Embriophyta - ps13
Tracheophyta - ps14
Spermatophyta - ps15
Angiosperms - ps16
Mesangiospermae - ps17
Magnoliids/eudicots - ps18
Eudicots - ps19
Core_eudicots - ps20
Superrosids - ps21
Rosids - ps22
Eurosids - ps23
Malvids - ps24
Sap./Malv./Brassicales - ps25
Malvales/Brassicales - ps26
Brassicales - ps27
Cleomaceae/Brassicaceae - ps28
Brassicaceae - ps29
Camelineae - ps30
Arabidopsis - ps31
Arabidopsis_thaliana - ps32
Phylostratum

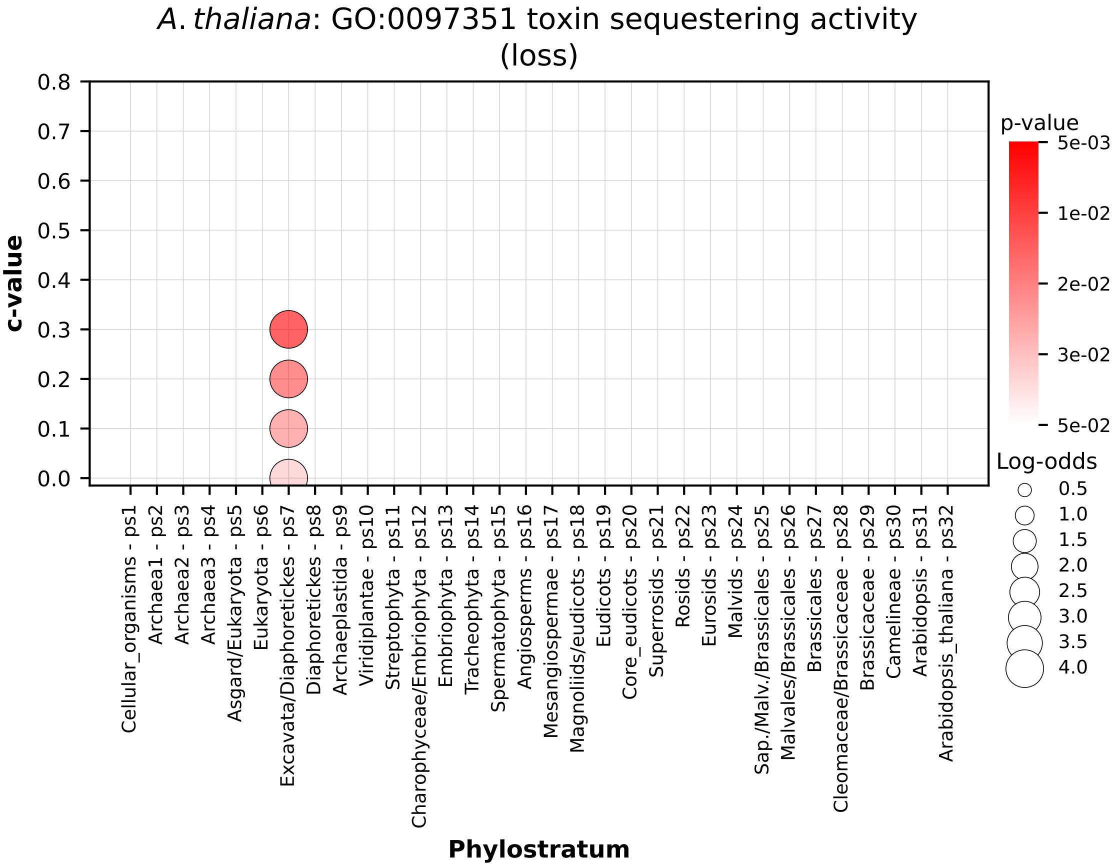

A. thaliana: GO:0097351 toxin sequestering activity (loss)
c-value
0.8
0.7
0.6
0.5
0.4
0.3
0.2
0.1
0.0
Phylostratum
Cellular_organisms - ps1
Archaea1 - ps2
Archaea2 - ps3
Archaea3 - ps4
Asgard/Eukaryota - ps5
Eukaryota - ps6
Excavata/Diaphoretickes - ps7
Diaphoretickes - ps8
Archaeplastida - ps9
Viridiplantae - ps10
Streptophyta - ps11
Charophyceae/Embriophyta - ps12
Embriophyta - ps13
Tracheophyta - ps14
Spermatophyta - ps15
Angiosperms - ps16
Mesangiospermae - ps17
Magnoliids/eudicots - ps18
Eudicots - ps19
Core_eudicots - ps20
Superrosids - ps21
Rosids - ps22
Eurosids - ps23
Malvids - ps24
Sap./Malv./Brassicales - ps25
Malvales/Brassicales - ps26
Brassicales - ps27
Cleomaceae/Brassicaceae - ps28
Brassicaceae - ps29
Camelineae - ps30
Arabidopsis - ps31
Arabidopsis_thaliana - ps32
p-value
5e-03
1e-02
2e-02
3e-02
5e-02
Log-odds
0.5
1.0
1.5
2.0
2.5
3.0
3.5
4.0

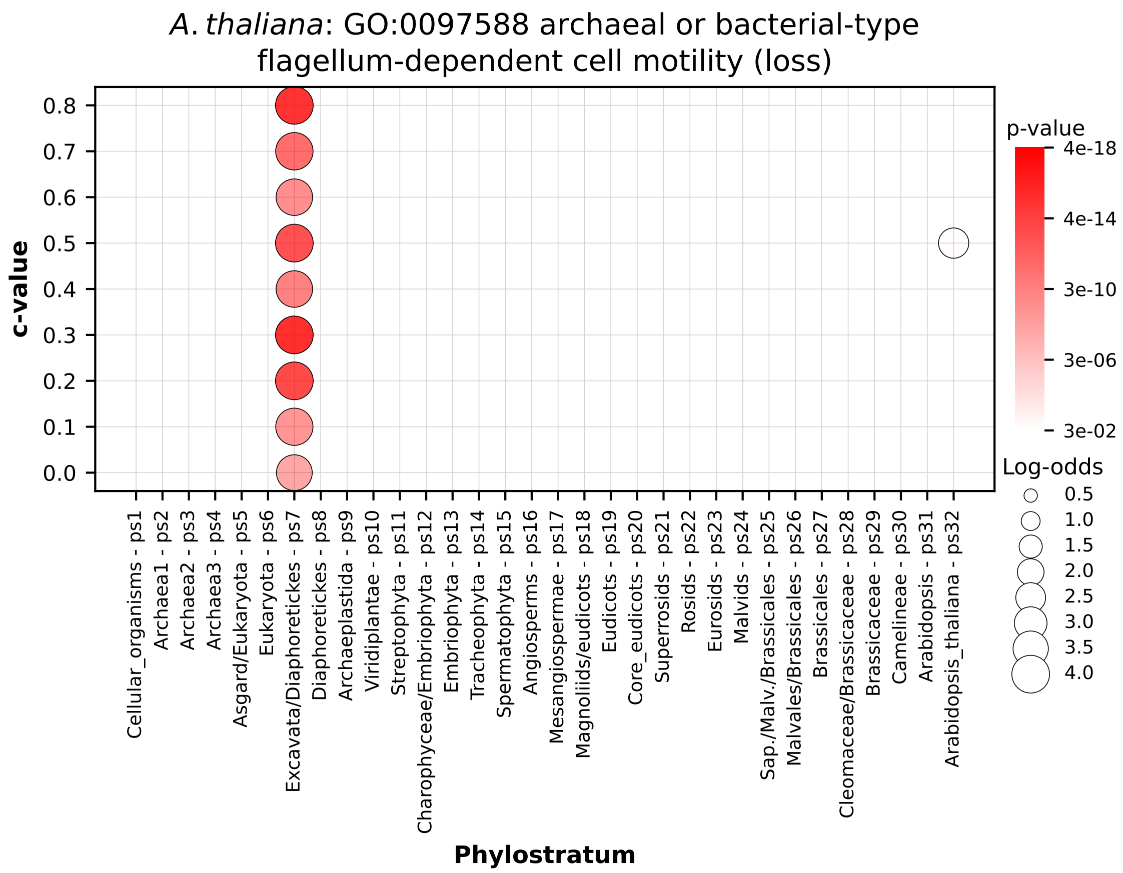

A. thaliana: GO:0097588 archaeal or bacterial-type flagellum-dependent cell motility (loss)
c-value
0.8
0.7
0.6
0.5
0.4
0.3
0.2
0.1
0.0
p-value
4e-18
4e-14
3e-10
3e-06
3e-02
Log-odds
0.5
1.0
1.5
2.0
2.5
3.0
3.5
4.0
Cellular_organisms - ps1
Archaea1 - ps2
Archaea2 - ps3
Archaea3 - ps4
Asgard/Eukaryota - ps5
Eukaryota - ps6
Excavata/Diaphoretickes - ps7
Diaphoretickes - ps8
Archaeplastida - ps9
Viridiplantae - ps10
Streptophyta - ps11
Charophyceae/Embriophyta - ps12
Embriophyta - ps13
Tracheophyta - ps14
Spermatophyta - ps15
Angiosperms - ps16
Mesangiospermae - ps17
Magnoliids/eudicots - ps18
Eudicots - ps19
Core_eudicots - ps20
Superrosids - ps21
Rosids - ps22
Eurosids - ps23
Malvids - ps24
Sap./Malv./Brassicales - ps25
Malvales/Brassicales - ps26
Brassicales - ps27
Cleomaceae/Brassicaceae - ps28
Brassicaceae - ps29
Camelineae - ps30
Arabidopsis - ps31
Arabidopsis_thaliana - ps32
Phylostratum

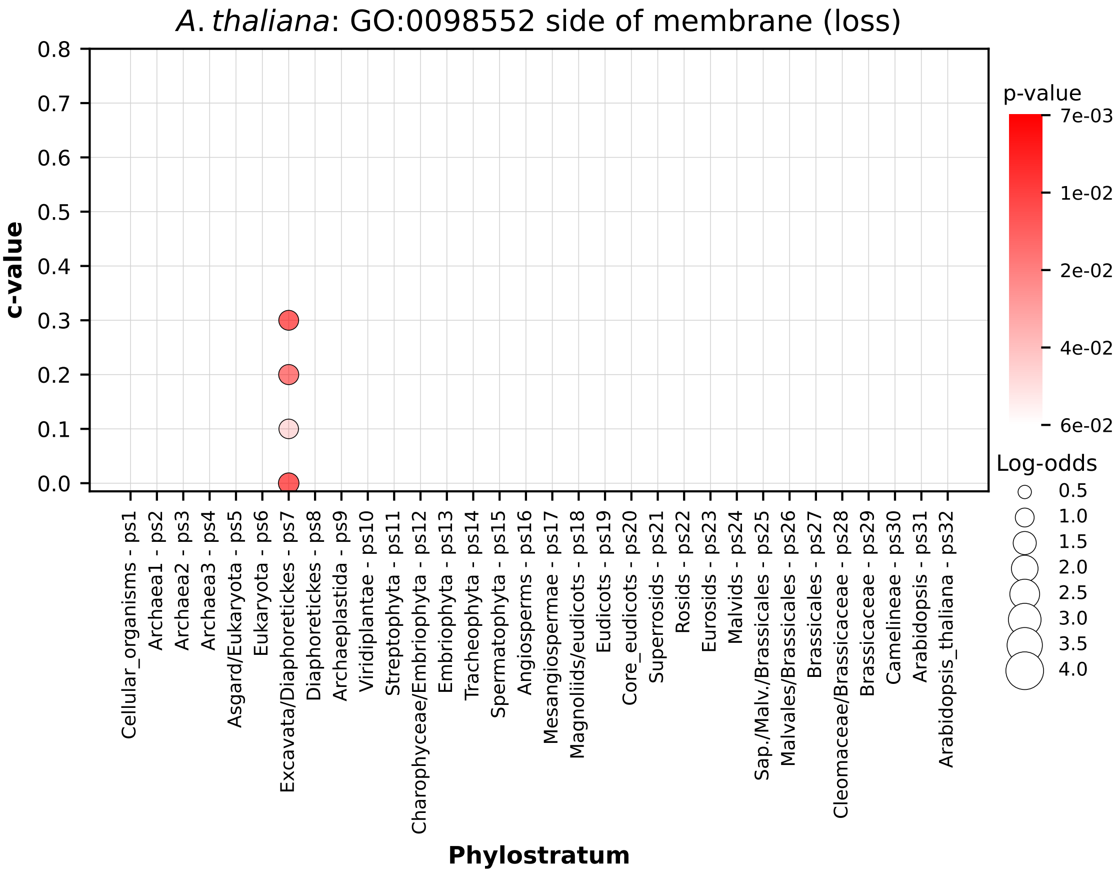

A. thaliana: GO:0098552 side of membrane (loss)
c-value
0.0
0.1
0.2
0.3
0.4
0.5
0.6
0.7
0.8
p-value
7e-03
1e-02
2e-02
4e-02
6e-02
Log-odds
0.5
1.0
1.5
2.0
2.5
3.0
3.5
4.0
Cellular_organisms - ps1
Archaea1 - ps2
Archaea2 - ps3
Archaea3 - ps4
Asgard/Eukaryota - ps5
Eukaryota - ps6
Excavata/Diaphoretickes - ps7
Diaphoretickes - ps8
Archaeplastida - ps9
Viridiplantae - ps10
Streptophyta - ps11
Charophyceae/Embriophyta - ps12
Embriophyta - ps13
Tracheophyta - ps14
Spermatophyta - ps15
Angiosperms - ps16
Mesangiospermae - ps17
Magnoliids/eudicots - ps18
Eudicots - ps19
Core_eudicots - ps20
Superrosids - ps21
Rosids - ps22
Eurosids - ps23
Malvids - ps24
Sap./Malv./Brassicales - ps25
Malvales/Brassicales - ps26
Brassicales - ps27
Cleomaceae/Brassicaceae - ps28
Brassicaceae - ps29
Camelineae - ps30
Arabidopsis - ps31
Arabidopsis_thaliana - ps32
Phylostratum

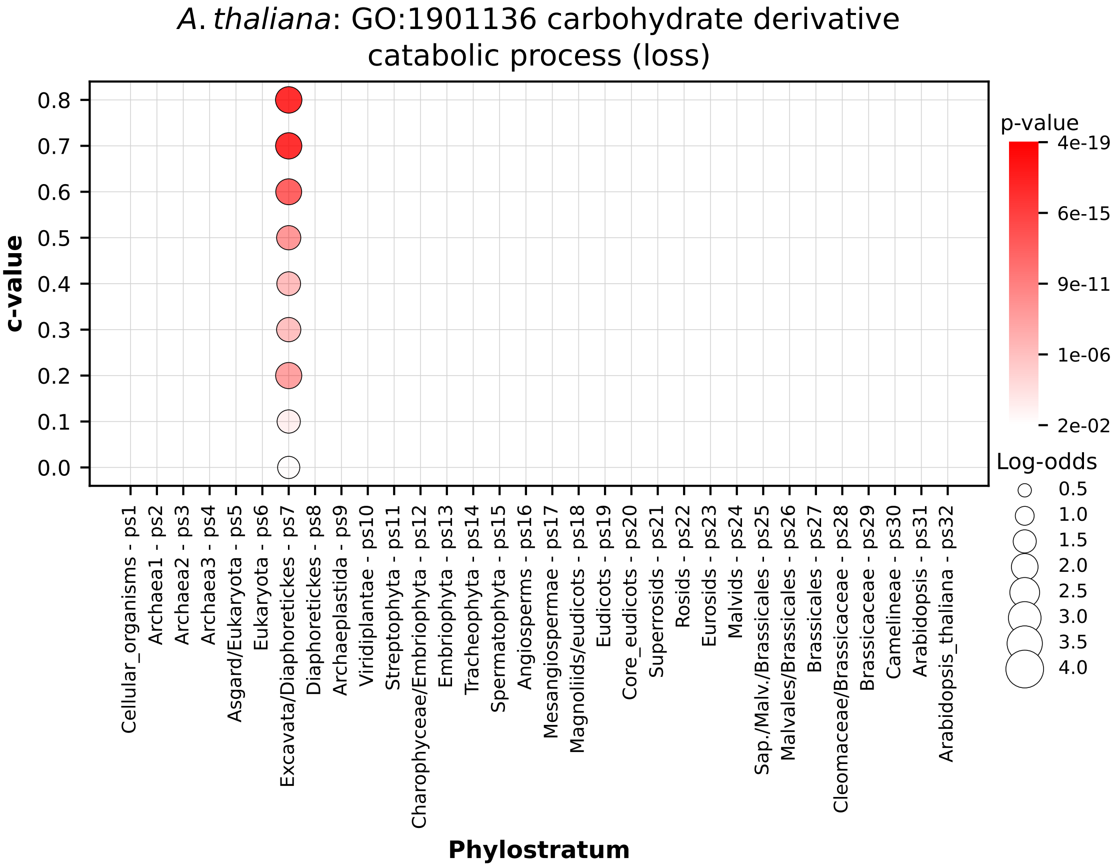
A. thaliana: GO:1901136 carbohydrate derivative catabolic process (loss)
c-value
0.8
0.7
0.6
0.5
0.4
0.3
0.2
0.1
0.0
Phylostratum
Cellular_organisms - ps1
Archaea1 - ps2
Archaea2 - ps3
Archaea3 - ps4
Asgard/Eukaryota - ps5
Eukaryota - ps6
Excavata/Diaphoretickes - ps7
Diaphoretickes - ps8
Archaeplastida - ps9
Viridiplantae - ps10
Streptophyta - ps11
Charophyceae/Embriophyta - ps12
Embriophyta - ps13
Tracheophyta - ps14
Spermatophyta - ps15
Angiosperms - ps16
Mesangiospermae - ps17
Magnoliids/eudicots - ps18
Eudicots - ps19
Core_eudicots - ps20
Superrosids - ps21
Rosids - ps22
Eurosids - ps23
Malvids - ps24
Sap./Malv./Brassicales - ps25
Malvales/Brassicales - ps26
Brassicales - ps27
Cleomaceae/Brassicaceae - ps28
Brassicaceae - ps29
Camelineae - ps30
Arabidopsis - ps31
Arabidopsis_thaliana - ps32
p-value
4e-19
6e-15
9e-11
1e-06
2e-02
Log-odds
0.5
1.0
1.5
2.0
2.5
3.0
3.5
4.0

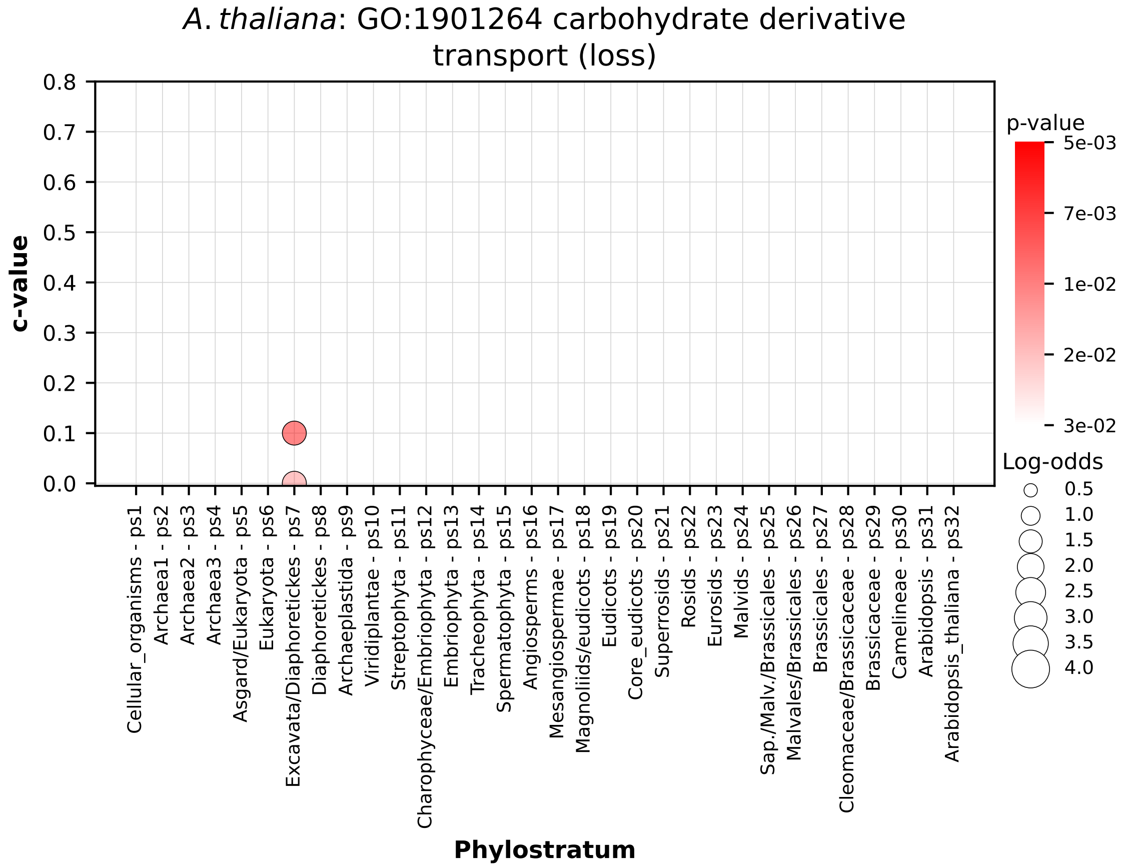
A. thaliana: GO:1901264 carbohydrate derivative transport (loss)
c-value
0.0
0.1
0.2
0.3
0.4
0.5
0.6
0.7
0.8
Phylostratum
Cellular_organisms - ps1
Archaea1 - ps2
Archaea2 - ps3
Archaea3 - ps4
Asgard/Eukaryota - ps5
Eukaryota - ps6
Excavata/Diaphoretickes - ps7
Diaphoretickes - ps8
Archaeplastida - ps9
Viridiplantae - ps10
Streptophyta - ps11
Charophyceae/Embriophyta - ps12
Embriophyta - ps13
Tracheophyta - ps14
Spermatophyta - ps15
Angiosperms - ps16
Mesangiospermae - ps17
Magnoliids/eudicots - ps18
Eudicots - ps19
Core_eudicots - ps20
Superrosids - ps21
Rosids - ps22
Eurosids - ps23
Malvids - ps24
Sap./Malv./Brassicales - ps25
Malvales/Brassicales - ps26
Brassicales - ps27
Cleomaceae/Brassicaceae - ps28
Brassicaceae - ps29
Camelineae - ps30
Arabidopsis - ps31
Arabidopsis_thaliana - ps32
p-value
5e-03
7e-03
1e-02
2e-02
3e-02
Log-odds
0.5
1.0
1.5
2.0
2.5
3.0
3.5
4.0

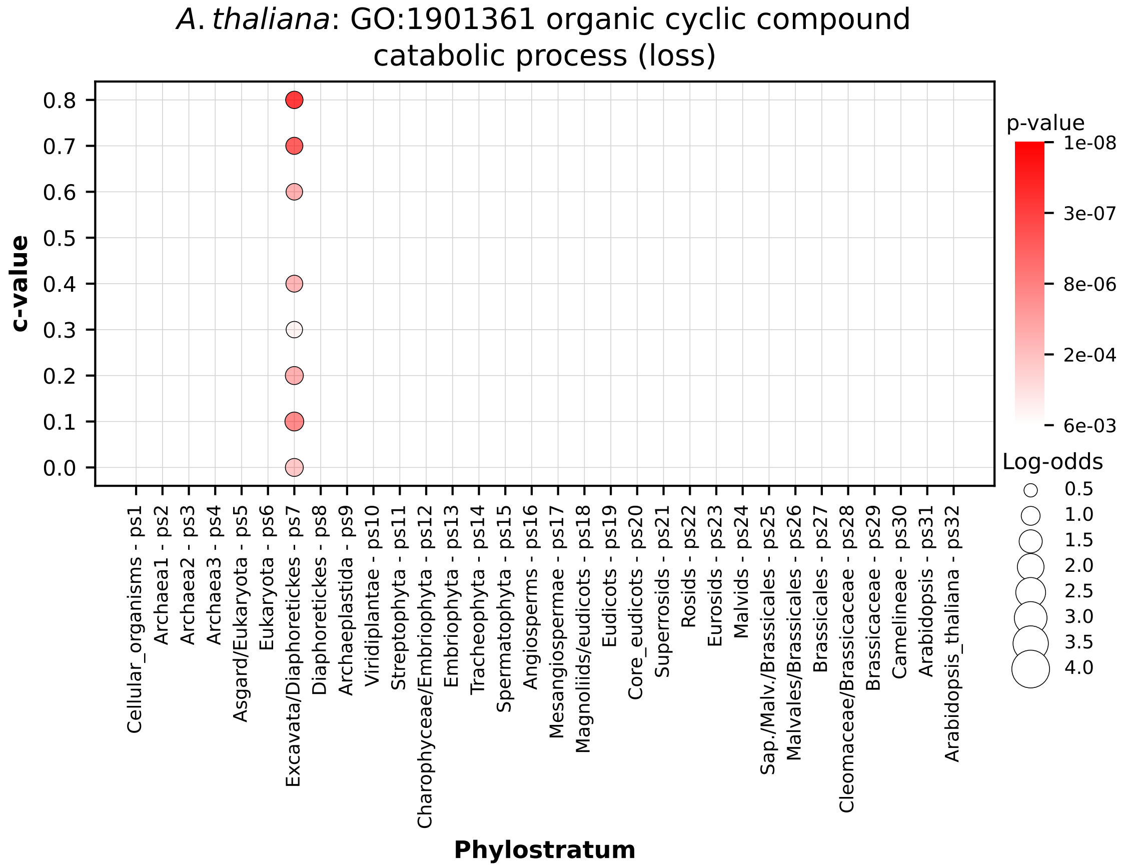
A. thaliana: GO:1901361 organic cyclic compound catabolic process (loss)
c-value
0.8
0.7
0.6
0.5
0.4
0.3
0.2
0.1
0.0
p-value
1e-08
3e-07
8e-06
2e-04
6e-03
Log-odds
0.5
1.0
1.5
2.0
2.5
3.0
3.5
4.0
Cellular_organisms - ps1
Archaea1 - ps2
Archaea2 - ps3
Archaea3 - ps4
Asgard/Eukaryota - ps5
Eukaryota - ps6
Excavata/Diaphoretickes - ps7
Diaphoretickes - ps8
Archaeplastida - ps9
Viridiplantae - ps10
Streptophyta - ps11
Charophyceae/Embriophyta - ps12
Embriophyta - ps13
Tracheophyta - ps14
Spermatophyta - ps15
Angiosperms - ps16
Mesangiospermae - ps17
Magnoliids/eudicots - ps18
Eudicots - ps19
Core_eudicots - ps20
Superrosids - ps21
Rosids - ps22
Eurosids - ps23
Malvids - ps24
Sap./Malv./Brassicales - ps25
Malvales/Brassicales - ps26
Brassicales - ps27
Cleomaceae/Brassicaceae - ps28
Brassicaceae - ps29
Camelineae - ps30
Arabidopsis - ps31
Arabidopsis_thaliana - ps32
Phylostratum

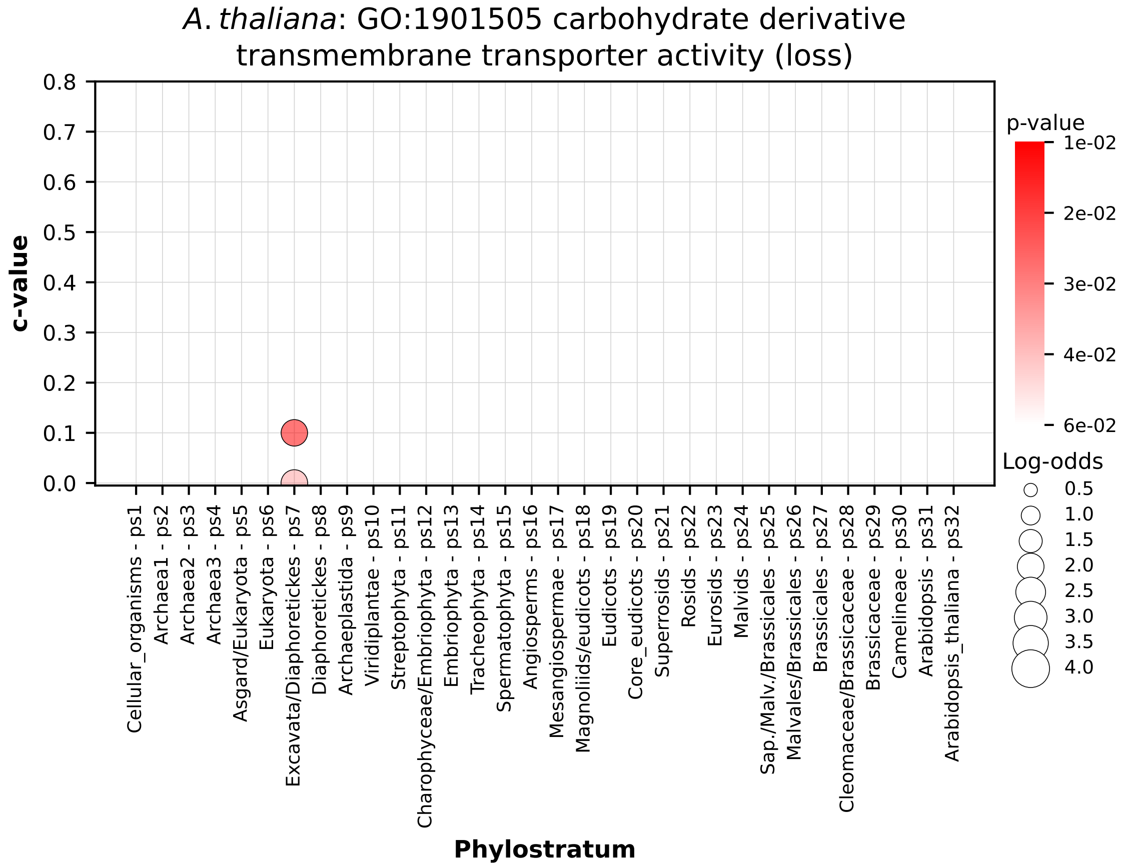

A. thaliana: GO:1901505 carbohydrate derivative transmembrane transporter activity (loss)
c-value
0.8
0.7
0.6
0.5
0.4
0.3
0.2
0.1
0.0
p-value
1e-02
2e-02
3e-02
4e-02
6e-02
Log-odds
0.5
1.0
1.5
2.0
2.5
3.0
3.5
4.0
Cellular_organisms - ps1
Archaea1 - ps2
Archaea2 - ps3
Archaea3 - ps4
Asgard/Eukaryota - ps5
Eukaryota - ps6
Excavata/Diaphoretickes - ps7
Diaphoretickes - ps8
Archaeplastida - ps9
Viridiplantae - ps10
Streptophyta - ps11
Charophyceae/Embriophyta - ps12
Embriophyta - ps13
Tracheophyta - ps14
Spermatophyta - ps15
Angiosperms - ps16
Mesangiospermae - ps17
Magnoliids/eudicots - ps18
Eudicots - ps19
Core_eudicots - ps20
Superrosids - ps21
Rosids - ps22
Eurosids - ps23
Malvids - ps24
Sap./Malv./Brassicales - ps25
Malvales/Brassicales - ps26
Brassicales - ps27
Cleomaceae/Brassicaceae - ps28
Brassicaceae - ps29
Camelineae - ps30
Arabidopsis - ps31
Arabidopsis_thaliana - ps32
Phylostratum

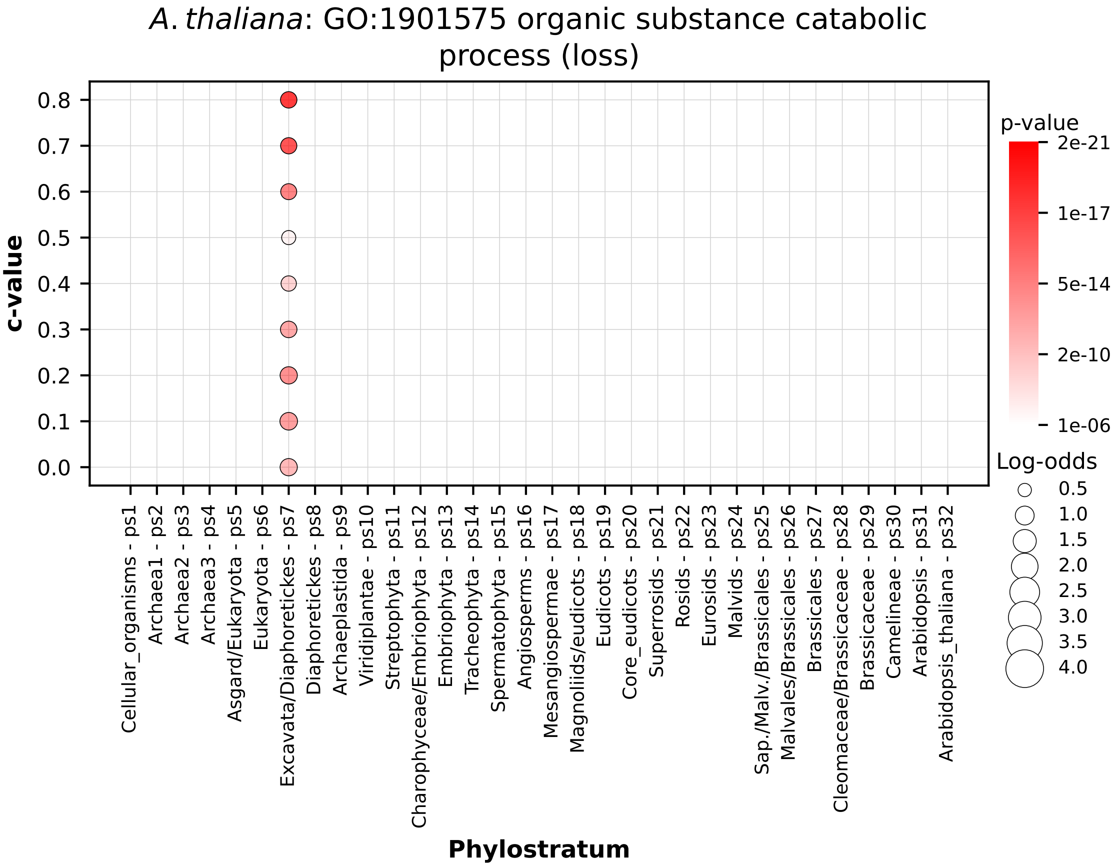

A. thaliana: GO:1901575 organic substance catabolic process (loss)
c-value
0.8
0.7
0.6
0.5
0.4
0.3
0.2
0.1
0.0
p-value
2e-21
1e-17
5e-14
2e-10
1e-06
Log-odds
0.5
1.0
1.5
2.0
2.5
3.0
3.5
4.0
Cellular_organisms - ps1
Archaea1 - ps2
Archaea2 - ps3
Archaea3 - ps4
Asgard/Eukaryota - ps5
Eukaryota - ps6
Excavata/Diaphoretickes - ps7
Diaphoretickes - ps8
Archaeplastida - ps9
Viridiplantae - ps10
Streptophyta - ps11
Charophyceae/Embriophyta - ps12
Embriophyta - ps13
Tracheophyta - ps14
Spermatophyta - ps15
Angiosperms - ps16
Mesangiospermae - ps17
Magnoliids/eudicots - ps18
Eudicots - ps19
Core_eudicots - ps20
Superrosids - ps21
Rosids - ps22
Eurosids - ps23
Malvids - ps24
Sap./Malv./Brassicales - ps25
Malvales/Brassicales - ps26
Brassicales - ps27
Cleomaceae/Brassicaceae - ps28
Brassicaceae - ps29
Camelineae - ps30
Arabidopsis - ps31
Arabidopsis_thaliana - ps32
Phylostratum

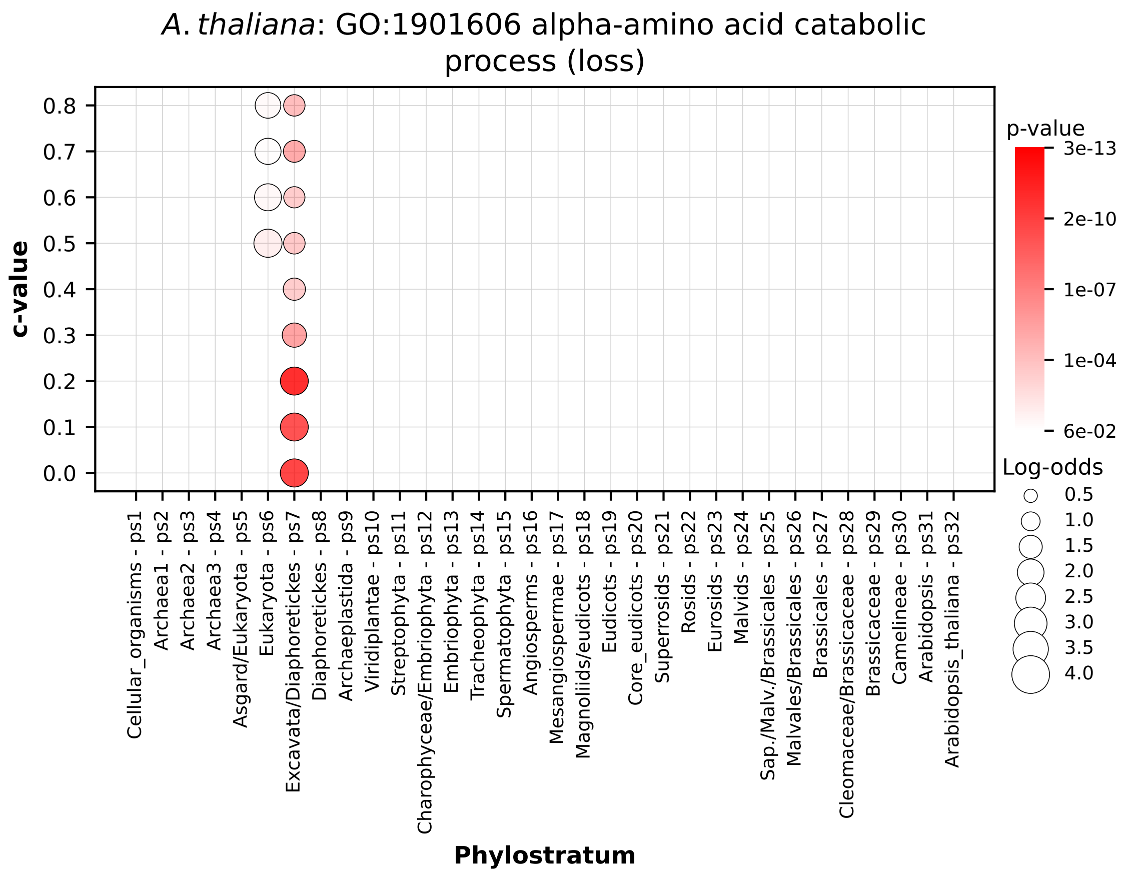

A. thaliana: GO:1901606 alpha-amino acid catabolic process (loss)
c-value
0.8
0.7
0.6
0.5
0.4
0.3
0.2
0.1
0.0
p-value
3e-13
2e-10
1e-07
1e-04
6e-02
Log-odds
0.5
1.0
1.5
2.0
2.5
3.0
3.5
4.0
Cellular_organisms - ps1
Archaea1 - ps2
Archaea2 - ps3
Archaea3 - ps4
Asgard/Eukaryota - ps5
Eukaryota - ps6
Excavata/Diaphoretickes - ps7
Diaphoretickes - ps8
Archaeplastida - ps9
Viridiplantae - ps10
Streptophyta - ps11
Charophyceae/Embriophyta - ps12
Embriophyta - ps13
Tracheophyta - ps14
Spermatophyta - ps15
Angiosperms - ps16
Mesangiospermae - ps17
Magnoliids/eudicots - ps18
Eudicots - ps19
Core_eudicots - ps20
Superrosids - ps21
Rosids - ps22
Eurosids - ps23
Malvids - ps24
Sap./Malv./Brassicales - ps25
Malvales/Brassicales - ps26
Brassicales - ps27
Cleomaceae/Brassicaceae - ps28
Brassicaceae - ps29
Camelineae - ps30
Arabidopsis - ps31
Arabidopsis_thaliana - ps32
Phylostratum

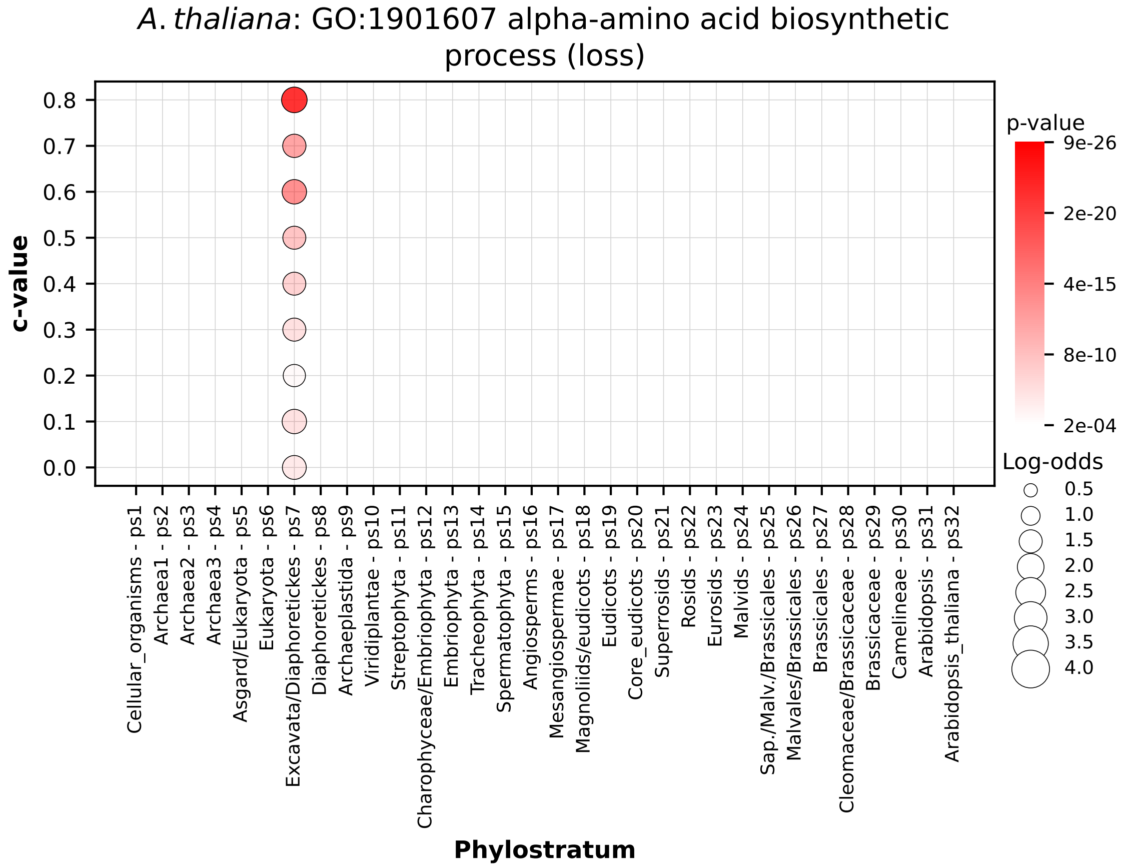
A. thaliana: GO:1901607 alpha-amino acid biosynthetic process (loss)
c-value
0.8
0.7
0.6
0.5
0.4
0.3
0.2
0.1
0.0
p-value
9e-26
2e-20
4e-15
8e-10
2e-04
Log-odds
0.5
1.0
1.5
2.0
2.5
3.0
3.5
4.0
Cellular_organisms - ps1
Archaea1 - ps2
Archaea2 - ps3
Archaea3 - ps4
Asgard/Eukaryota - ps5
Eukaryota - ps6
Excavata/Diaphoretickes - ps7
Diaphoretickes - ps8
Archaeplastida - ps9
Viridiplantae - ps10
Streptophyta - ps11
Charophyceae/Embriophyta - ps12
Embriophyta - ps13
Tracheophyta - ps14
Spermatophyta - ps15
Angiosperms - ps16
Mesangiospermae - ps17
Magnoliids/eudicots - ps18
Eudicots - ps19
Core_eudicots - ps20
Superrosids - ps21
Rosids - ps22
Eurosids - ps23
Malvids - ps24
Sap./Malv./Brassicales - ps25
Malvales/Brassicales - ps26
Brassicales - ps27
Cleomaceae/Brassicaceae - ps28
Brassicaceae - ps29
Camelineae - ps30
Arabidopsis - ps31
Arabidopsis_thaliana - ps32
Phylostratum

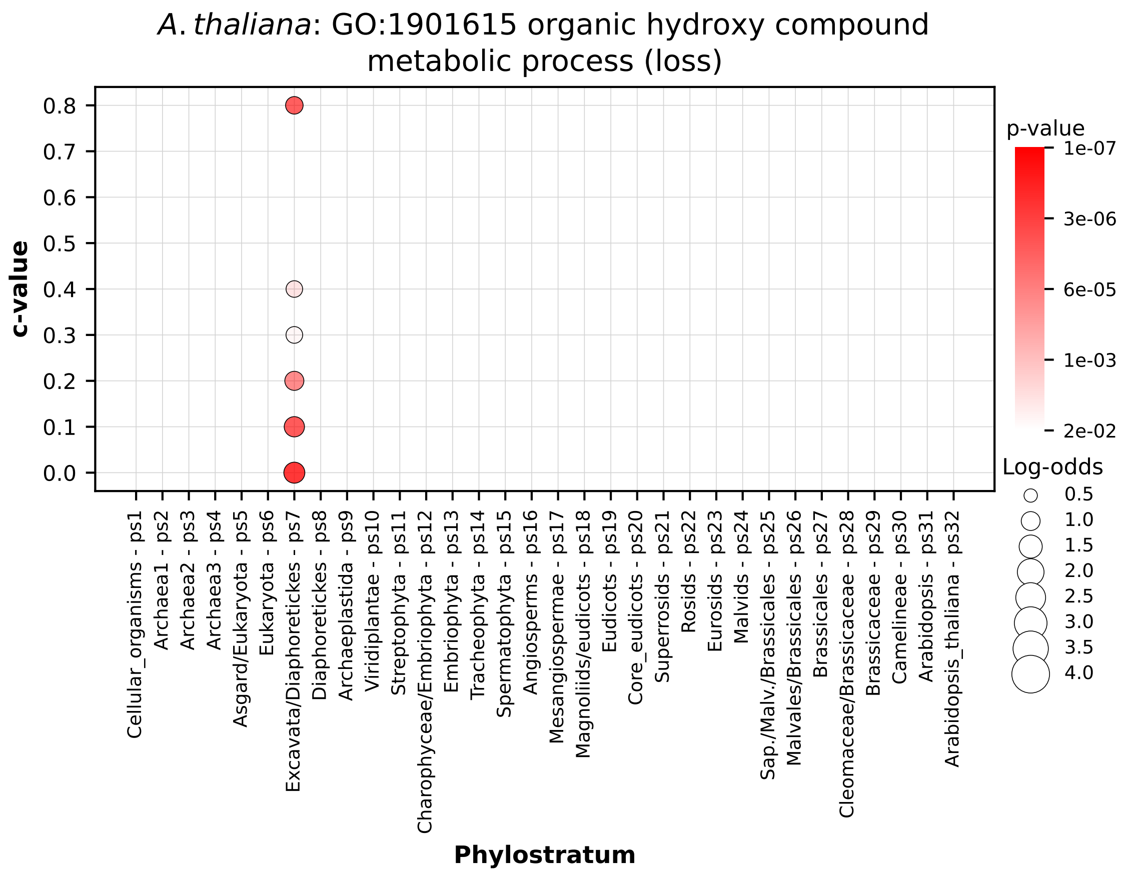

A. thaliana: GO:1901615 organic hydroxy compound metabolic process (loss)
c-value
0.8
0.7
0.6
0.5
0.4
0.3
0.2
0.1
0.0
Phylostratum
Cellular_organisms - ps1
Archaea1 - ps2
Archaea2 - ps3
Archaea3 - ps4
Asgard/Eukaryota - ps5
Eukaryota - ps6
Excavata/Diaphoretickes - ps7
Diaphoretickes - ps8
Archaeplastida - ps9
Viridiplantae - ps10
Streptophyta - ps11
Charophyceae/Embriophyta - ps12
Embriophyta - ps13
Tracheophyta - ps14
Spermatophyta - ps15
Angiosperms - ps16
Mesangiospermae - ps17
Magnoliids/eudicots - ps18
Eudicots - ps19
Core_eudicots - ps20
Superrosids - ps21
Rosids - ps22
Eurosids - ps23
Malvids - ps24
Sap./Malv./Brassicales - ps25
Malvales/Brassicales - ps26
Brassicales - ps27
Cleomaceae/Brassicaceae - ps28
Brassicaceae - ps29
Camelineae - ps30
Arabidopsis - ps31
Arabidopsis_thaliana - ps32
p-value
1e-07
3e-06
6e-05
1e-03
2e-02
Log-odds
0.5
1.0
1.5
2.0
2.5
3.0
3.5
4.0

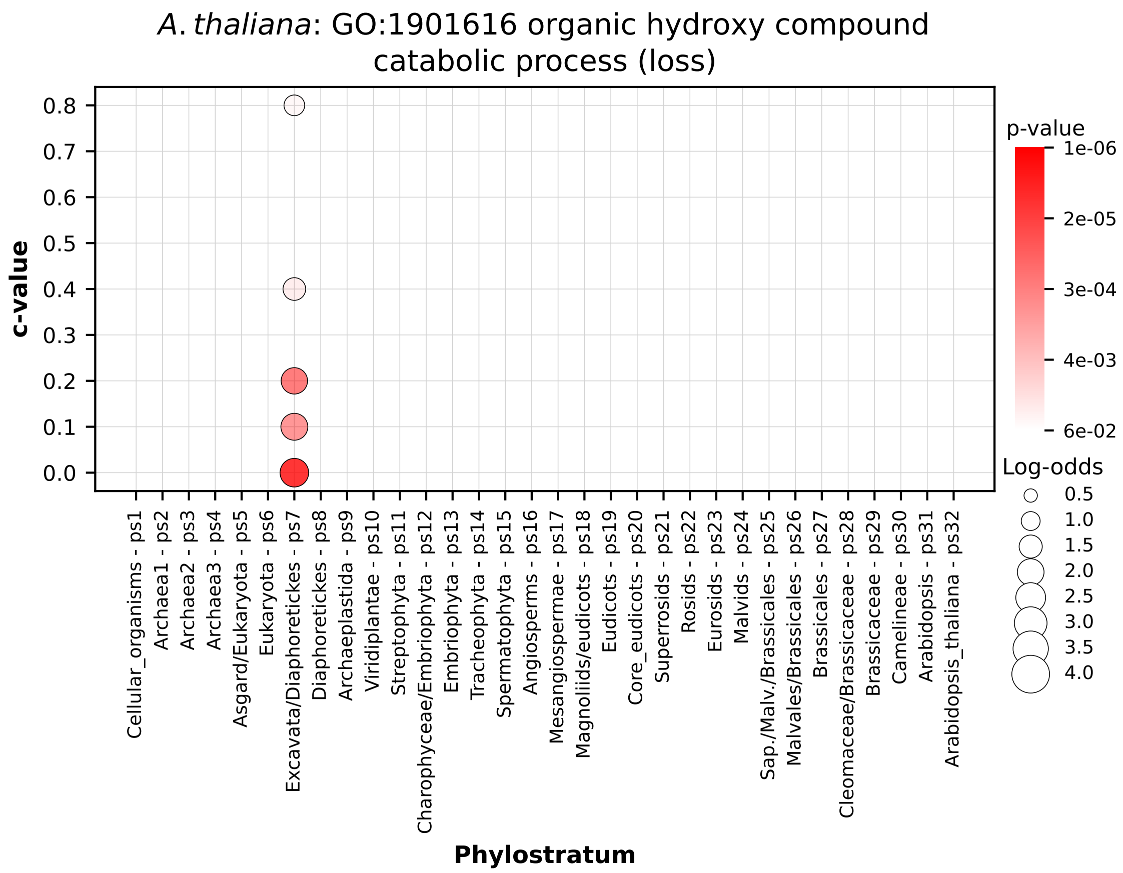

A. thaliana: GO:1901616 organic hydroxy compound catabolic process (loss)
c-value
0.8
0.7
0.6
0.5
0.4
0.3
0.2
0.1
0.0
Phylostratum
Cellular_organisms - ps1
Archaea1 - ps2
Archaea2 - ps3
Archaea3 - ps4
Asgard/Eukaryota - ps5
Eukaryota - ps6
Excavata/Diaphoretickes - ps7
Diaphoretickes - ps8
Archaeplastida - ps9
Viridiplantae - ps10
Streptophyta - ps11
Charophyceae/Embriophyta - ps12
Embriophyta - ps13
Tracheophyta - ps14
Spermatophyta - ps15
Angiosperms - ps16
Mesangiospermae - ps17
Magnoliids/eudicots - ps18
Eudicots - ps19
Core_eudicots - ps20
Superrosids - ps21
Rosids - ps22
Eurosids - ps23
Malvids - ps24
Sap./Malv./Brassicales - ps25
Malvales/Brassicales - ps26
Brassicales - ps27
Cleomaceae/Brassicaceae - ps28
Brassicaceae - ps29
Camelineae - ps30
Arabidopsis - ps31
Arabidopsis_thaliana - ps32
p-value
1e-06
2e-05
3e-04
4e-03
6e-02
Log-odds
0.5
1.0
1.5
2.0
2.5
3.0
3.5
4.0

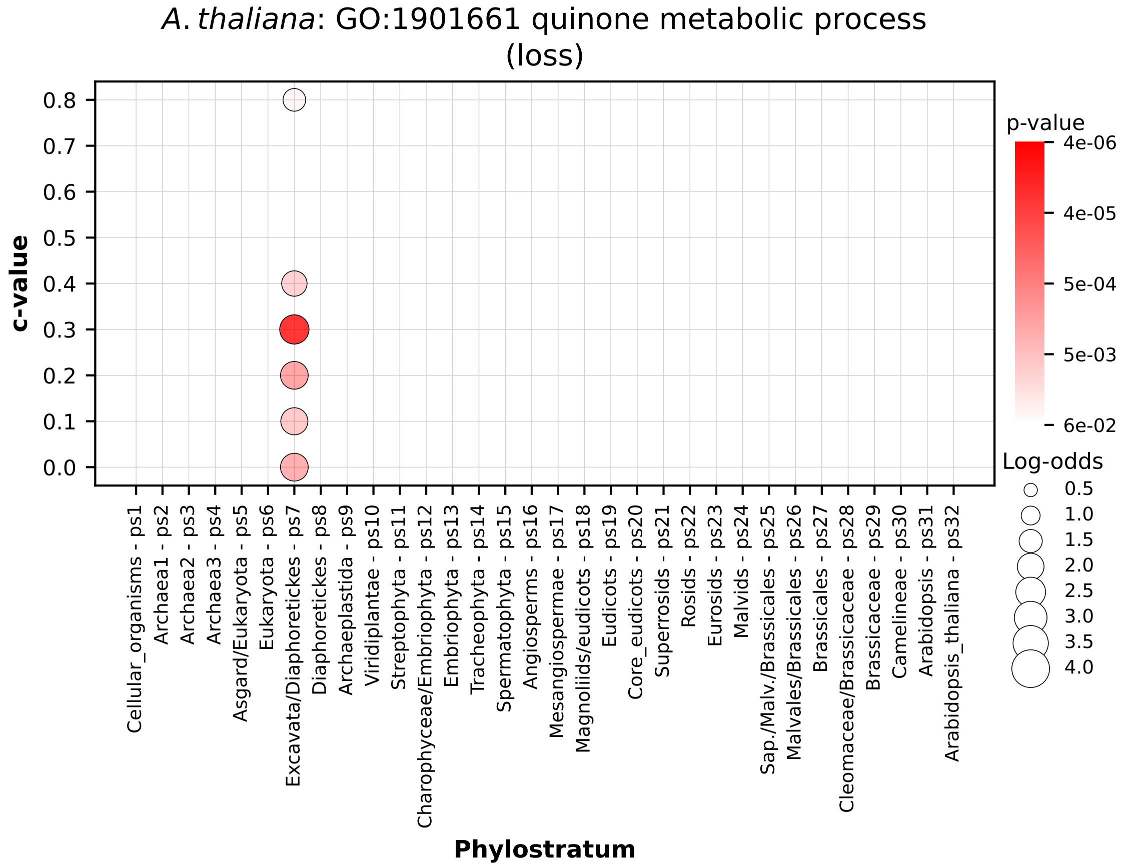

A. thaliana: GO:1901661 quinone metabolic process (loss)
c-value
0.8
0.7
0.6
0.5
0.4
0.3
0.2
0.1
0.0
p-value
4e-06
4e-05
5e-04
5e-03
6e-02
Log-odds
0.5
1.0
1.5
2.0
2.5
3.0
3.5
4.0
Cellular_organisms - ps1
Archaea1 - ps2
Archaea2 - ps3
Archaea3 - ps4
Asgard/Eukaryota - ps5
Eukaryota - ps6
Excavata/Diaphoretickes - ps7
Diaphoretickes - ps8
Archaeplastida - ps9
Viridiplantae - ps10
Streptophyta - ps11
Charophyceae/Embriophyta - ps12
Embriophyta - ps13
Tracheophyta - ps14
Spermatophyta - ps15
Angiosperms - ps16
Mesangiospermae - ps17
Magnoliids/eudicots - ps18
Eudicots - ps19
Core_eudicots - ps20
Superrosids - ps21
Rosids - ps22
Eurosids - ps23
Malvids - ps24
Sap./Malv./Brassicales - ps25
Malvales/Brassicales - ps26
Brassicales - ps27
Cleomaceae/Brassicaceae - ps28
Brassicaceae - ps29
Camelineae - ps30
Arabidopsis - ps31
Arabidopsis_thaliana - ps32
Phylostratum

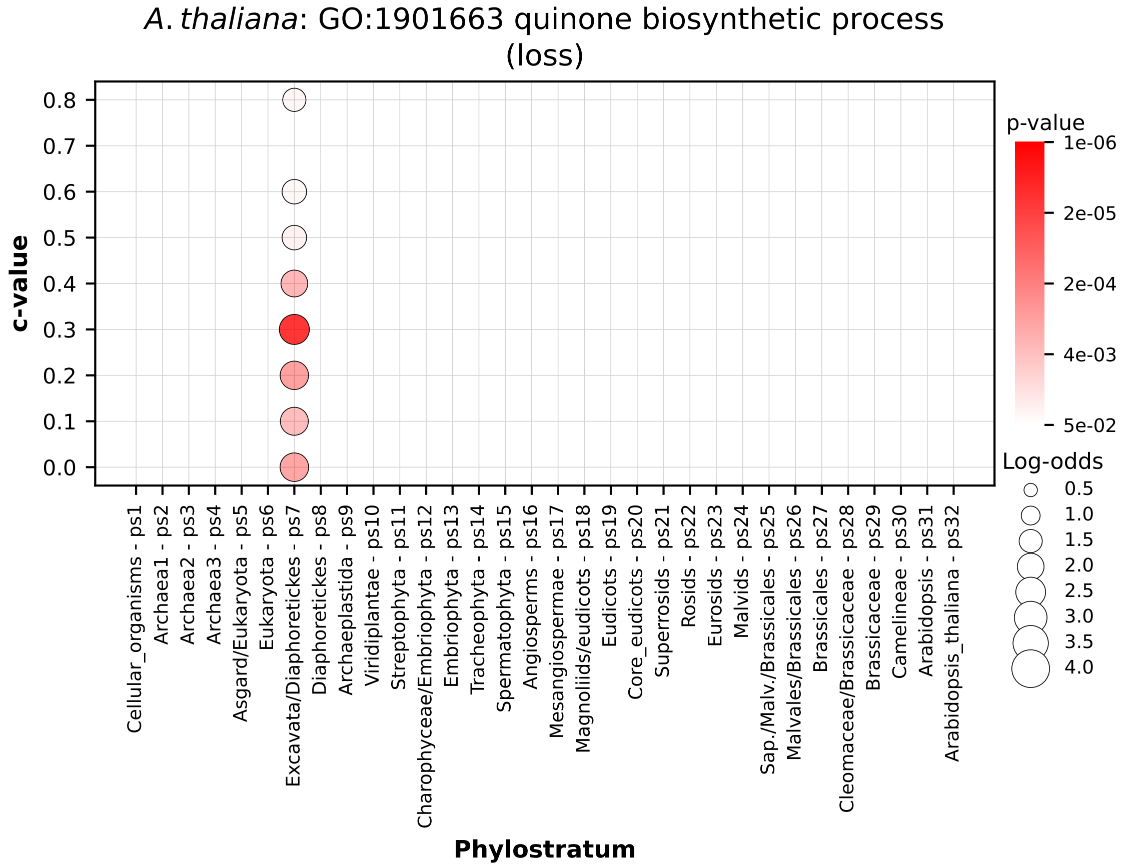

A. thaliana: GO:1901663 quinone biosynthetic process
(loss)
c-value
0.8
0.7
0.6
0.5
0.4
0.3
0.2
0.1
0.0
Phylostratum
Cellular_organisms - ps1
Archaea1 - ps2
Archaea2 - ps3
Archaea3 - ps4
Asgard/Eukaryota - ps5
Eukaryota - ps6
Excavata/Diaphoretickes - ps7
Diaphoretickes - ps8
Archaeplastida - ps9
Viridiplantae - ps10
Streptophyta - ps11
Charophyceae/Embriophyta - ps12
Embriophyta - ps13
Tracheophyta - ps14
Spermatophyta - ps15
Angiosperms - ps16
Mesangiospermae - ps17
Magnoliids/eudicots - ps18
Eudicots - ps19
Core_eudicots - ps20
Superrosids - ps21
Rosids - ps22
Eurosids - ps23
Malvids - ps24
Sap./Malv./Brassicales - ps25
Malvales/Brassicales - ps26
Brassicales - ps27
Cleomaceae/Brassicaceae - ps28
Brassicaceae - ps29
Camelineae - ps30
Arabidopsis - ps31
Arabidopsis_thaliana - ps32
p-value
1e-06
2e-05
2e-04
4e-03
5e-02
Log-odds
0.5
1.0
1.5
2.0
2.5
3.0
3.5
4.0

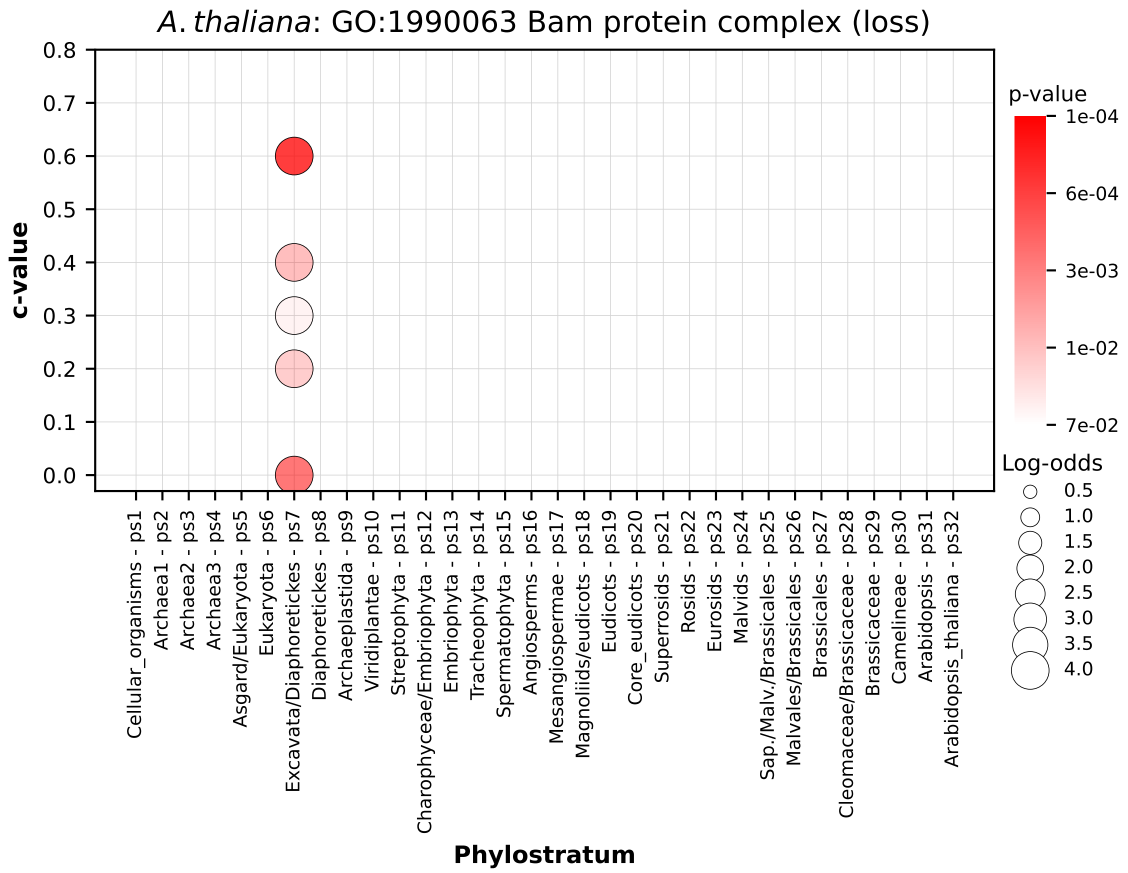

A. thaliana: GO:1990063 Bam protein complex (loss)
c-value
0.8
0.7
0.6
0.5
0.4
0.3
0.2
0.1
0.0
p-value
1e-04
6e-04
3e-03
1e-02
7e-02
Log-odds
0.5
1.0
1.5
2.0
2.5
3.0
3.5
4.0
Cellular_organisms - ps1
Archaea1 - ps2
Archaea2 - ps3
Archaea3 - ps4
Asgard/Eukaryota - ps5
Eukaryota - ps6
Excavata/Diaphoretickes - ps7
Diaphoretickes - ps8
Archaeplastida - ps9
Viridiplantae - ps10
Streptophyta - ps11
Charophyceae/Embriophyta - ps12
Embriophyta - ps13
Tracheophyta - ps14
Spermatophyta - ps15
Angiosperms - ps16
Mesangiospermae - ps17
Magnoliids/eudicots - ps18
Eudicots - ps19
Core_eudicots - ps20
Superrosids - ps21
Rosids - ps22
Eurosids - ps23
Malvids - ps24
Sap./Malv./Brassicales - ps25
Malvales/Brassicales - ps26
Brassicales - ps27
Cleomaceae/Brassicaceae - ps28
Brassicaceae - ps29
Camelineae - ps30
Arabidopsis - ps31
Arabidopsis_thaliana - ps32
Phylostratum

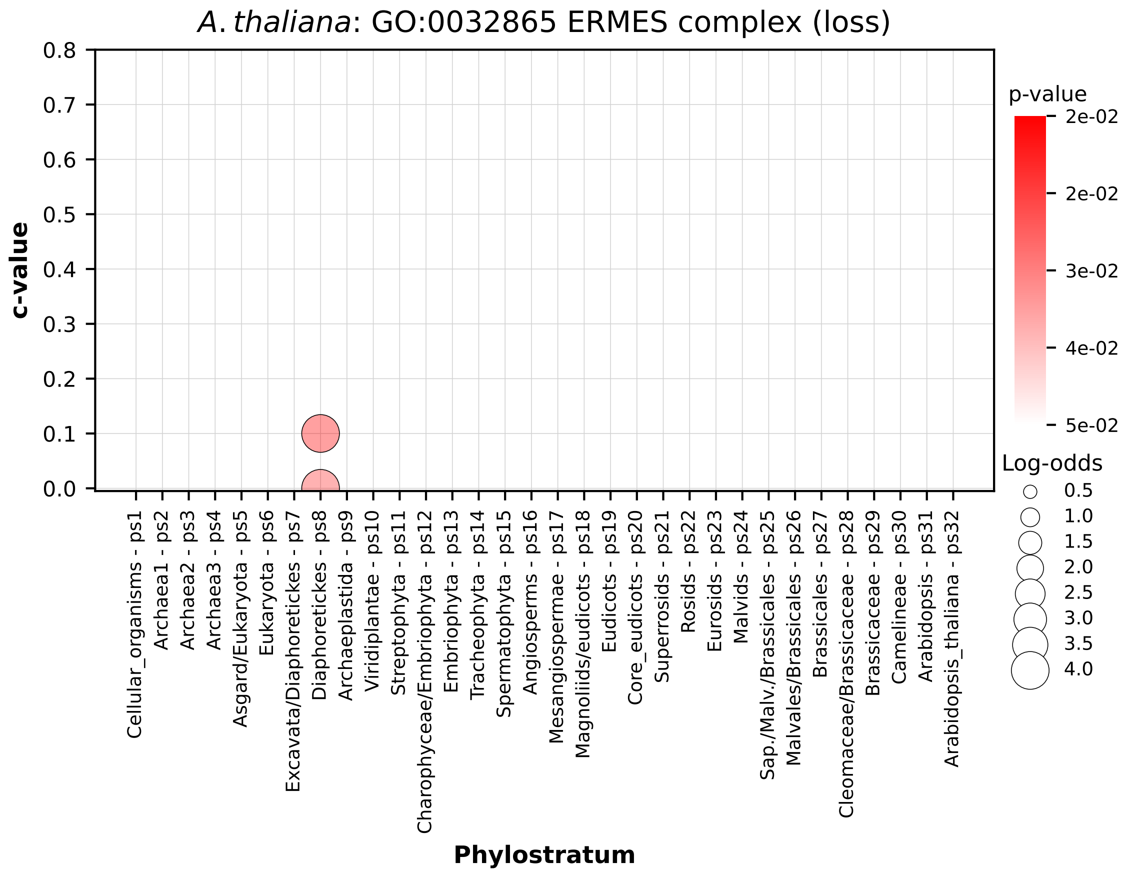
A. thaliana: GO:0032865 ERMES complex (loss)
c-value
0.8
0.7
0.6
0.5
0.4
0.3
0.2
0.1
0.0
p-value
2e-02
2e-02
3e-02
4e-02
5e-02
Log-odds
0.5
1.0
1.5
2.0
2.5
3.0
3.5
4.0
Cellular_organisms - ps1
Archaea1 - ps2
Archaea2 - ps3
Archaea3 - ps4
Asgard/Eukaryota - ps5
Eukaryota - ps6
Excavata/Diaphoretickes - ps7
Diaphoretickes - ps8
Archaeplastida - ps9
Viridiplantae - ps10
Streptophyta - ps11
Charophyceae/Embriophyta - ps12
Embriophyta - ps13
Tracheophyta - ps14
Spermatophyta - ps15
Angiosperms - ps16
Mesangiospermae - ps17
Magnoliids/eudicots - ps18
Eudicots - ps19
Core_eudicots - ps20
Superrosids - ps21
Rosids - ps22
Eurosids - ps23
Malvids - ps24
Sap./Malv./Brassicales - ps25
Malvales/Brassicales - ps26
Brassicales - ps27
Cleomaceae/Brassicaceae - ps28
Brassicaceae - ps29
Camelineae - ps30
Arabidopsis - ps31
Arabidopsis_thaliana - ps32
Phylostratum

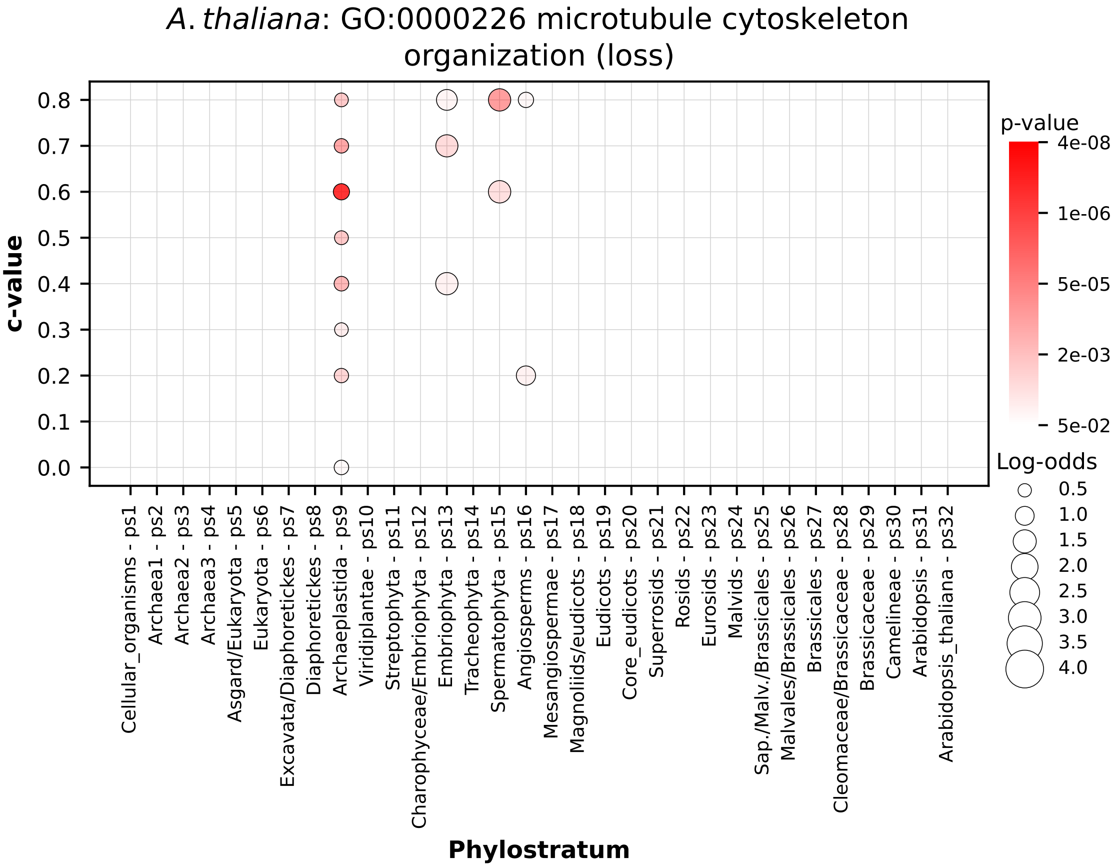
A. thaliana: GO:0000226 microtubule cytoskeleton organization (loss)
c-value
0.8
0.7
0.6
0.5
0.4
0.3
0.2
0.1
0.0
p-value
4e-08
1e-06
5e-05
2e-03
5e-02
Log-odds
0.5
1.0
1.5
2.0
2.5
3.0
3.5
4.0
Cellular_organisms - ps1
Archaea1 - ps2
Archaea2 - ps3
Archaea3 - ps4
Asgard/Eukaryota - ps5
Eukaryota - ps6
Excavata/Diaphoretickes - ps7
Diaphoretickes - ps8
Archaeplastida - ps9
Viridiplantae - ps10
Streptophyta - ps11
Charophyceae/Embriophyta - ps12
Embriophyta - ps13
Tracheophyta - ps14
Spermatophyta - ps15
Angiosperms - ps16
Mesangiospermae - ps17
Magnoliids/eudicots - ps18
Eudicots - ps19
Core_eudicots - ps20
Superrosids - ps21
Rosids - ps22
Eurosids - ps23
Malvids - ps24
Sap./Malv./Brassicales - ps25
Malvales/Brassicales - ps26
Brassicales - ps27
Cleomaceae/Brassicaceae - ps28
Brassicaceae - ps29
Camelineae - ps30
Arabidopsis - ps31
Arabidopsis_thaliana - ps32
Phylostratum

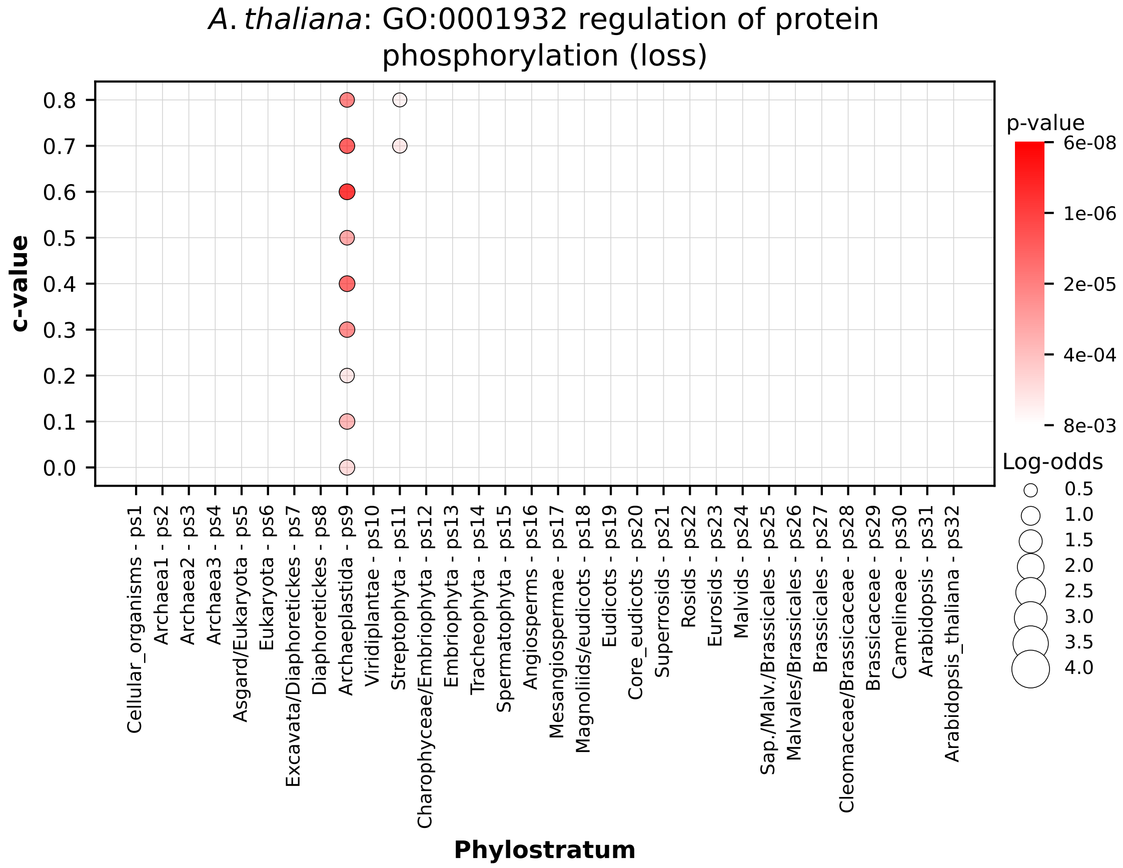

A. thaliana: GO:0001932 regulation of protein phosphorylation (loss)
c-value
0.8
0.7
0.6
0.5
0.4
0.3
0.2
0.1
0.0
Phylostratum
Cellular_organisms - ps1
Archaea1 - ps2
Archaea2 - ps3
Archaea3 - ps4
Asgard/Eukaryota - ps5
Eukaryota - ps6
Excavata/Diaphoretickes - ps7
Diaphoretickes - ps8
Archaeplastida - ps9
Viridiplantae - ps10
Streptophyta - ps11
Charophyceae/Embriophyta - ps12
Embriophyta - ps13
Tracheophyta - ps14
Spermatophyta - ps15
Angiosperms - ps16
Mesangiospermae - ps17
Magnoliids/eudicots - ps18
Eudicots - ps19
Core_eudicots - ps20
Superrosids - ps21
Rosids - ps22
Eurosids - ps23
Malvids - ps24
Sap./Malv./Brassicales - ps25
Malvales/Brassicales - ps26
Brassicales - ps27
Cleomaceae/Brassicaceae - ps28
Brassicaceae - ps29
Camelineae - ps30
Arabidopsis - ps31
Arabidopsis_thaliana - ps32
p-value
6e-08
1e-06
2e-05
4e-04
8e-03
Log-odds
0.5
1.0
1.5
2.0
2.5
3.0
3.5
4.0

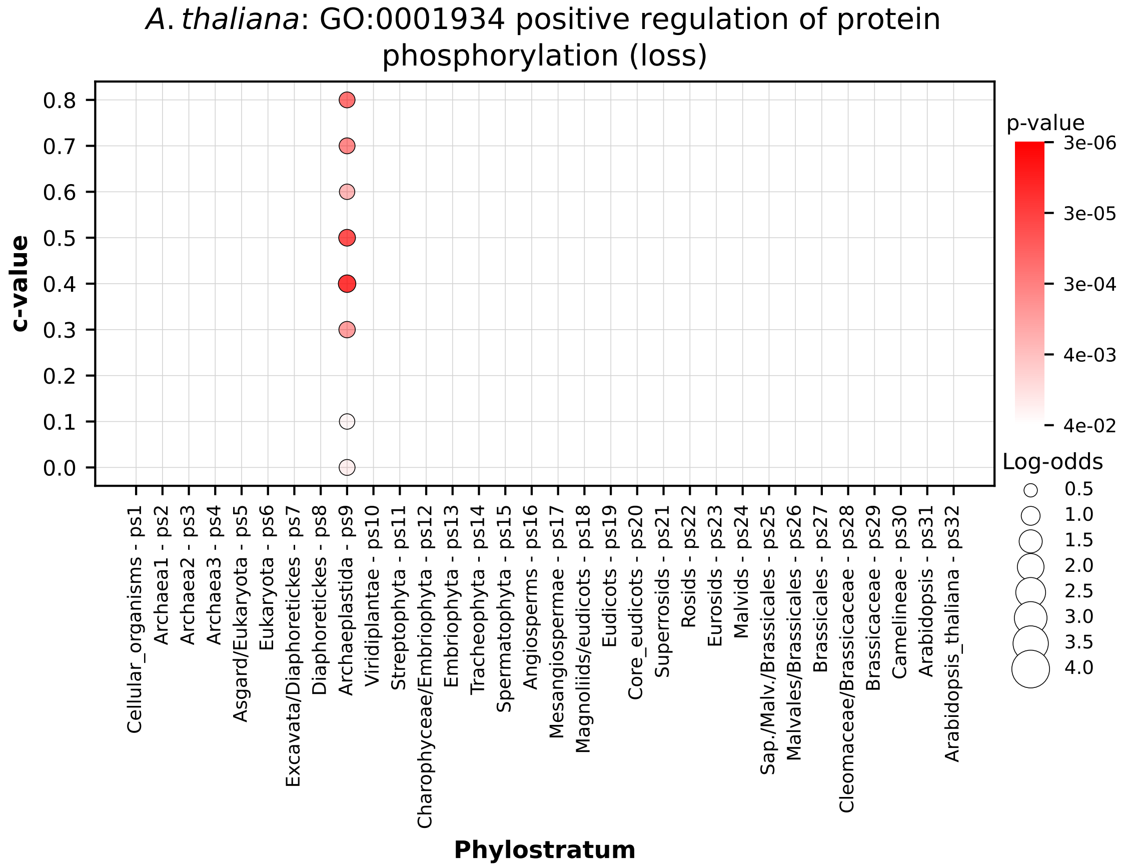

A. thaliana: GO:0001934 positive regulation of protein phosphorylation (loss)
c-value
0.8
0.7
0.6
0.5
0.4
0.3
0.2
0.1
0.0
p-value
3e-06
3e-05
3e-04
4e-03
4e-02
Log-odds
0.5
1.0
1.5
2.0
2.5
3.0
3.5
4.0
Cellular_organisms - ps1
Archaea1 - ps2
Archaea2 - ps3
Archaea3 - ps4
Asgard/Eukaryota - ps5
Eukaryota - ps6
Excavata/Diaphoretickes - ps7
Diaphoretickes - ps8
Archaeplastida - ps9
Viridiplantae - ps10
Streptophyta - ps11
Charophyceae/Embriophyta - ps12
Embriophyta - ps13
Tracheophyta - ps14
Spermatophyta - ps15
Angiosperms - ps16
Mesangiospermae - ps17
Magnoliids/eudicots - ps18
Eudicots - ps19
Core_eudicots - ps20
Superrosids - ps21
Rosids - ps22
Eurosids - ps23
Malvids - ps24
Sap./Malv./Brassicales - ps25
Malvales/Brassicales - ps26
Brassicales - ps27
Cleomaceae/Brassicaceae - ps28
Brassicaceae - ps29
Camelineae - ps30
Arabidopsis - ps31
Arabidopsis_thaliana - ps32
Phylostratum

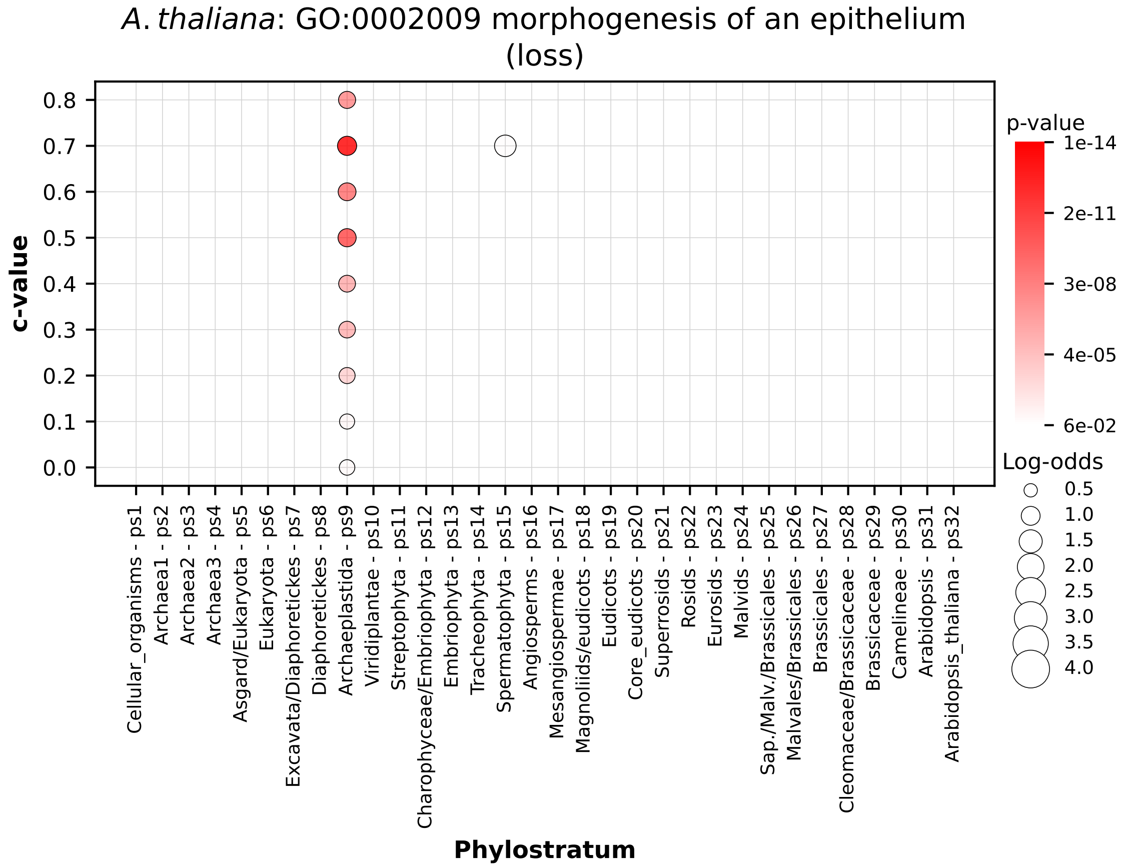

A. thaliana: GO:0002009 morphogenesis of an epithelium (loss)
c-value
0.8
0.7
0.6
0.5
0.4
0.3
0.2
0.1
0.0
p-value
1e-14
2e-11
3e-08
4e-05
6e-02
Log-odds
0.5
1.0
1.5
2.0
2.5
3.0
3.5
4.0
Cellular_organisms - ps1
Archaea1 - ps2
Archaea2 - ps3
Archaea3 - ps4
Asgard/Eukaryota - ps5
Eukaryota - ps6
Excavata/Diaphoretickes - ps7
Diaphoretickes - ps8
Archaeplastida - ps9
Viridiplantae - ps10
Streptophyta - ps11
Charophyceae/Embriophyta - ps12
Embriophyta - ps13
Tracheophyta - ps14
Spermatophyta - ps15
Angiosperms - ps16
Mesangiospermae - ps17
Magnoliids/eudicots - ps18
Eudicots - ps19
Core_eudicots - ps20
Superrosids - ps21
Rosids - ps22
Eurosids - ps23
Malvids - ps24
Sap./Malv./Brassicales - ps25
Malvales/Brassicales - ps26
Brassicales - ps27
Cleomaceae/Brassicaceae - ps28
Brassicaceae - ps29
Camelineae - ps30
Arabidopsis - ps31
Arabidopsis_thaliana - ps32
Phylostratum

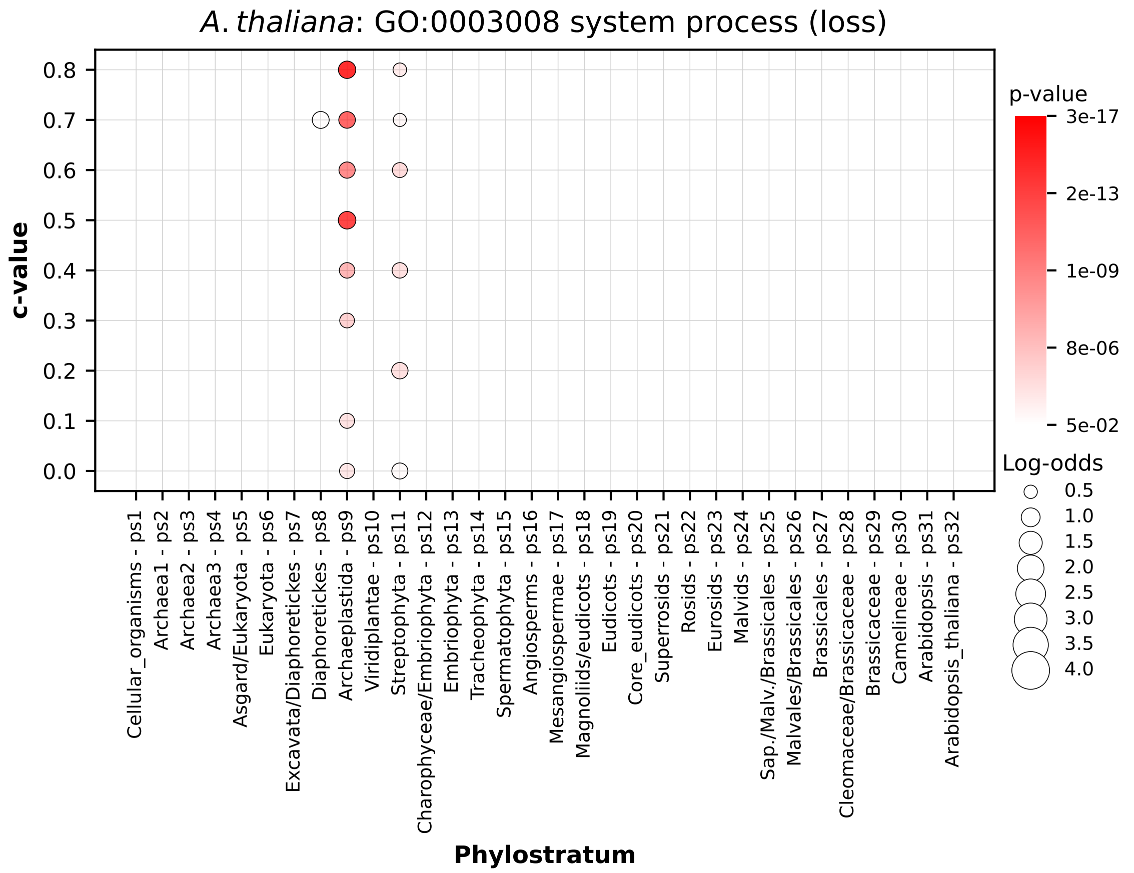

A. thaliana: GO:0003008 system process (loss)
c-value
0.8
0.7
0.6
0.5
0.4
0.3
0.2
0.1
0.0
p-value
3e-17
2e-13
1e-09
8e-06
5e-02
Log-odds
0.5
1.0
1.5
2.0
2.5
3.0
3.5
4.0
Cellular_organisms - ps1
Archaea1 - ps2
Archaea2 - ps3
Archaea3 - ps4
Asgard/Eukaryota - ps5
Eukaryota - ps6
Excavata/Diaphoretickes - ps7
Diaphoretickes - ps8
Archaeplastida - ps9
Viridiplantae - ps10
Streptophyta - ps11
Charophyceae/Embriophyta - ps12
Embriophyta - ps13
Tracheophyta - ps14
Spermatophyta - ps15
Angiosperms - ps16
Mesangiospermae - ps17
Magnoliids/eudicots - ps18
Eudicots - ps19
Core_eudicots - ps20
Superrosids - ps21
Rosids - ps22
Eurosids - ps23
Malvids - ps24
Sap./Malv./Brassicales - ps25
Malvales/Brassicales - ps26
Brassicales - ps27
Cleomaceae/Brassicaceae - ps28
Brassicaceae - ps29
Camelineae - ps30
Arabidopsis - ps31
Arabidopsis_thaliana - ps32
Phylostratum

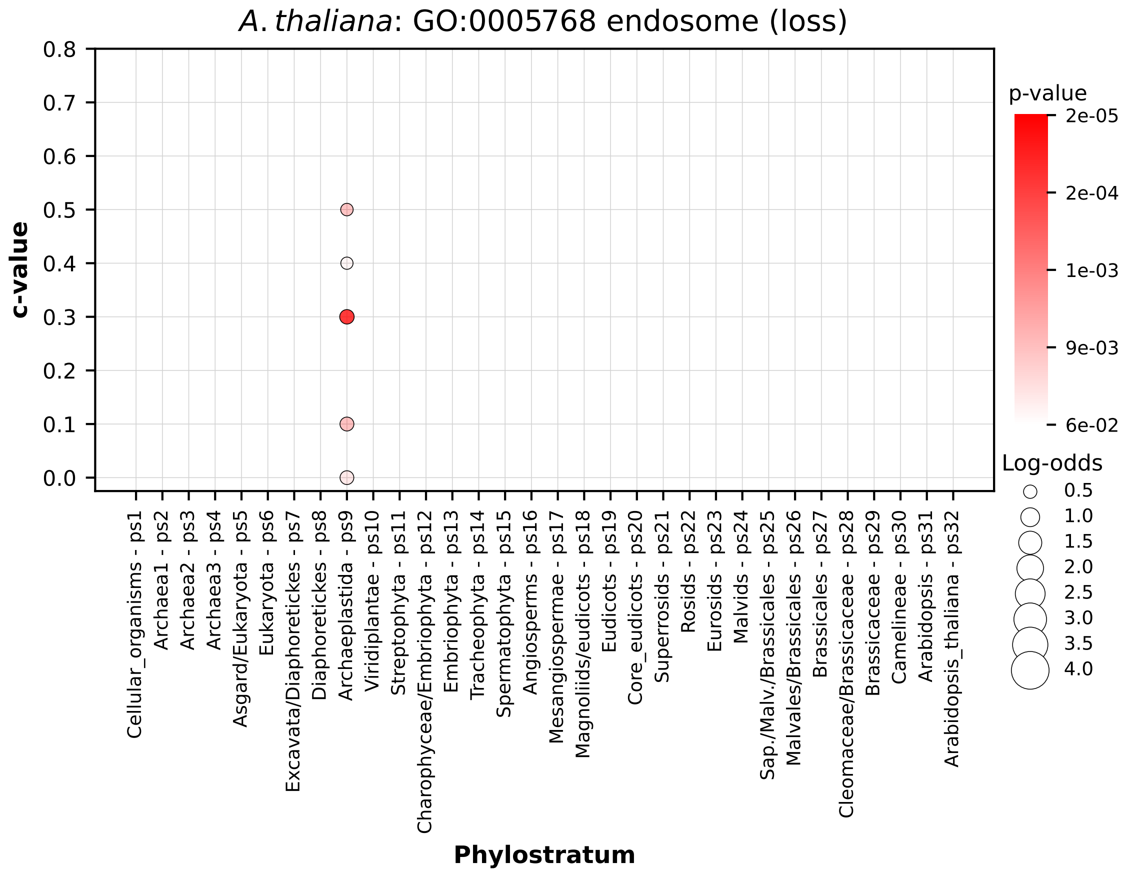

A. thaliana: GO:0005768 endosome (loss)
c-value
0.8
0.7
0.6
0.5
0.4
0.3
0.2
0.1
0.0
p-value
2e-05
2e-04
1e-03
9e-03
6e-02
Log-odds
0.5
1.0
1.5
2.0
2.5
3.0
3.5
4.0
Cellular_organisms - ps1
Archaea1 - ps2
Archaea2 - ps3
Archaea3 - ps4
Asgard/Eukaryota - ps5
Eukaryota - ps6
Excavata/Diaphoretickes - ps7
Diaphoretickes - ps8
Archaeplastida - ps9
Viridiplantae - ps10
Streptophyta - ps11
Charophyceae/Embriophyta - ps12
Embriophyta - ps13
Tracheophyta - ps14
Spermatophyta - ps15
Angiosperms - ps16
Mesangiospermae - ps17
Magnoliids/eudicots - ps18
Eudicots - ps19
Core_eudicots - ps20
Superrosids - ps21
Rosids - ps22
Eurosids - ps23
Malvids - ps24
Sap./Malv./Brassicales - ps25
Malvales/Brassicales - ps26
Brassicales - ps27
Cleomaceae/Brassicaceae - ps28
Brassicaceae - ps29
Camelineae - ps30
Arabidopsis - ps31
Arabidopsis_thaliana - ps32
Phylostratum

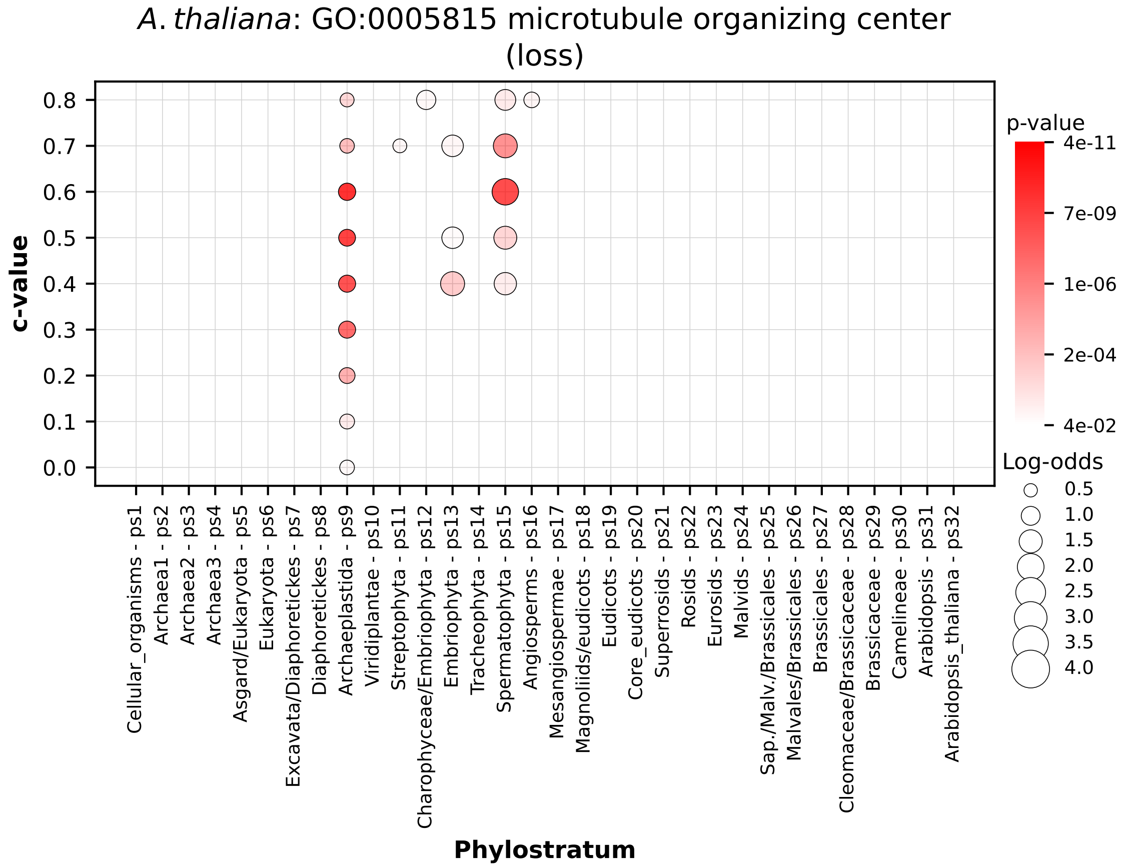
A. thaliana: GO:0005815 microtubule organizing center
(loss)
c-value
0.8
0.7
0.6
0.5
0.4
0.3
0.2
0.1
0.0
Phylostratum
Cellular_organisms - ps1
Archaea1 - ps2
Archaea2 - ps3
Archaea3 - ps4
Asgard/Eukaryota - ps5
Eukaryota - ps6
Excavata/Diaphoretickes - ps7
Diaphoretickes - ps8
Archaeplastida - ps9
Viridiplantae - ps10
Streptophyta - ps11
Charophyceae/Embriophyta - ps12
Embriophyta - ps13
Tracheophyta - ps14
Spermatophyta - ps15
Angiosperms - ps16
Mesangiospermae - ps17
Magnoliids/eudicots - ps18
Eudicots - ps19
Core_eudicots - ps20
Superrosids - ps21
Rosids - ps22
Eurosids - ps23
Malvids - ps24
Sap./Malv./Brassicales - ps25
Malvales/Brassicales - ps26
Brassicales - ps27
Cleomaceae/Brassicaceae - ps28
Brassicaceae - ps29
Camelineae - ps30
Arabidopsis - ps31
Arabidopsis_thaliana - ps32
p-value
4e-11
7e-09
1e-06
2e-04
4e-02
Log-odds
0.5
1.0
1.5
2.0
2.5
3.0
3.5
4.0

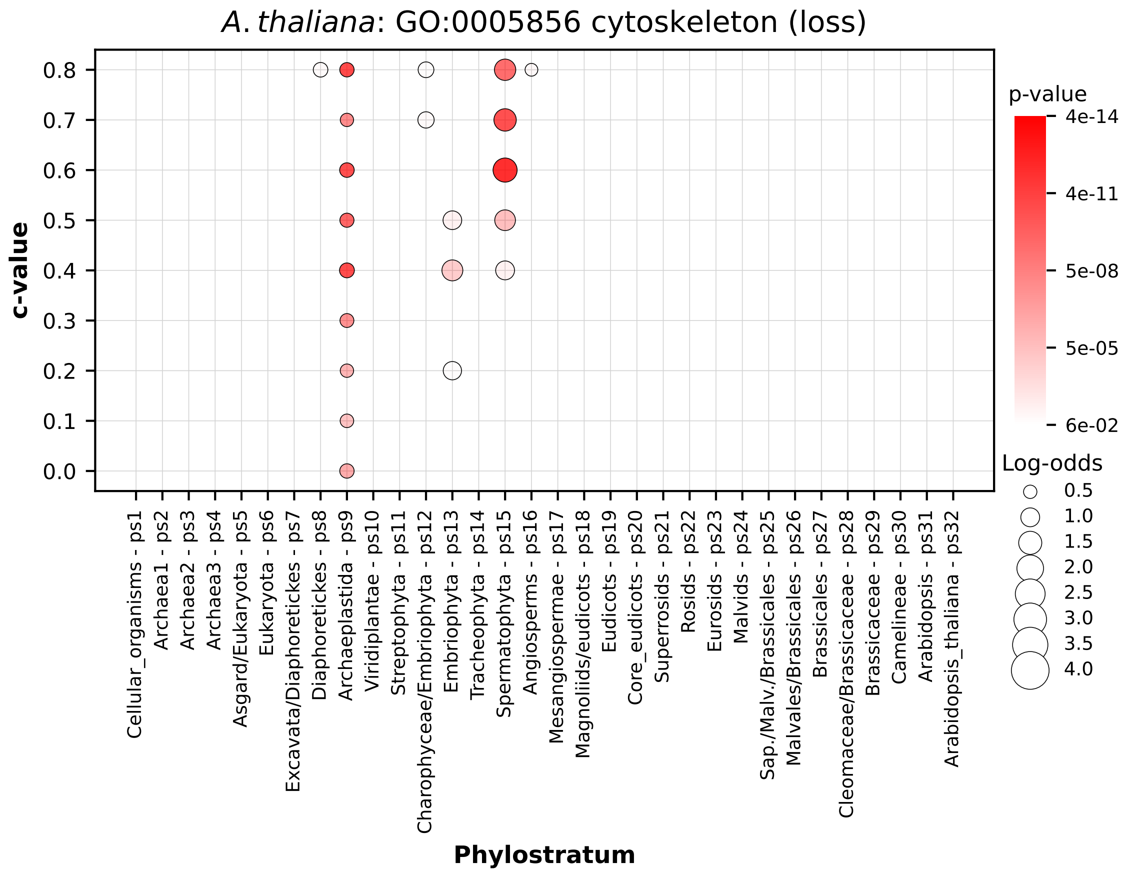

A. thaliana: GO:0005856 cytoskeleton (loss)
c-value
0.8
0.7
0.6
0.5
0.4
0.3
0.2
0.1
0.0
Phylostratum
Cellular_organisms - ps1
Archaea1 - ps2
Archaea2 - ps3
Archaea3 - ps4
Asgard/Eukaryota - ps5
Eukaryota - ps6
Excavata/Diaphoretickes - ps7
Diaphoretickes - ps8
Archaeplastida - ps9
Viridiplantae - ps10
Streptophyta - ps11
Charophyceae/Embriophyta - ps12
Embriophyta - ps13
Tracheophyta - ps14
Spermatophyta - ps15
Angiosperms - ps16
Mesangiospermae - ps17
Magnoliids/eudicots - ps18
Eudicots - ps19
Core_eudicots - ps20
Superrosids - ps21
Rosids - ps22
Eurosids - ps23
Malvids - ps24
Sap./Malv./Brassicales - ps25
Malvales/Brassicales - ps26
Brassicales - ps27
Cleomaceae/Brassicaceae - ps28
Brassicaceae - ps29
Camelineae - ps30
Arabidopsis - ps31
Arabidopsis_thaliana - ps32
p-value
4e-14
4e-11
5e-08
5e-05
6e-02
Log-odds
0.5
1.0
1.5
2.0
2.5
3.0
3.5
4.0

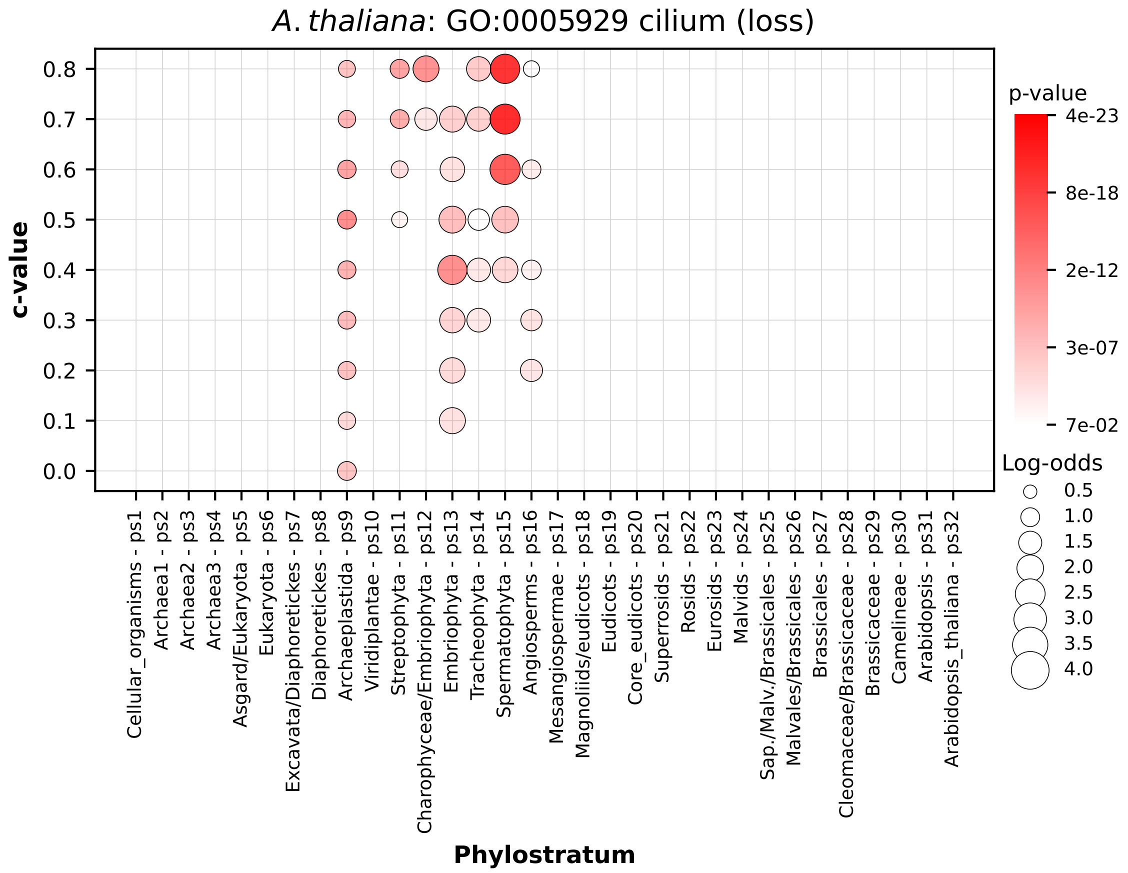

A. thaliana: GO:0005929 cilium (loss)
c-value
0.8
0.7
0.6
0.5
0.4
0.3
0.2
0.1
0.0
p-value
4e-23
8e-18
2e-12
3e-07
7e-02
Log-odds
0.5
1.0
1.5
2.0
2.5
3.0
3.5
4.0
Cellular_organisms - ps1
Archaea1 - ps2
Archaea2 - ps3
Archaea3 - ps4
Asgard/Eukaryota - ps5
Eukaryota - ps6
Excavata/Diaphoretickes - ps7
Diaphoretickes - ps8
Archaeplastida - ps9
Viridiplantae - ps10
Streptophyta - ps11
Charophyceae/Embriophyta - ps12
Embriophyta - ps13
Tracheophyta - ps14
Spermatophyta - ps15
Angiosperms - ps16
Mesangiospermae - ps17
Magnoliids/eudicots - ps18
Eudicots - ps19
Core_eudicots - ps20
Superrosids - ps21
Rosids - ps22
Eurosids - ps23
Malvids - ps24
Sap./Malv./Brassicales - ps25
Malvales/Brassicales - ps26
Brassicales - ps27
Cleomaceae/Brassicaceae - ps28
Brassicaceae - ps29
Camelineae - ps30
Arabidopsis - ps31
Arabidopsis_thaliana - ps32
Phylostratum

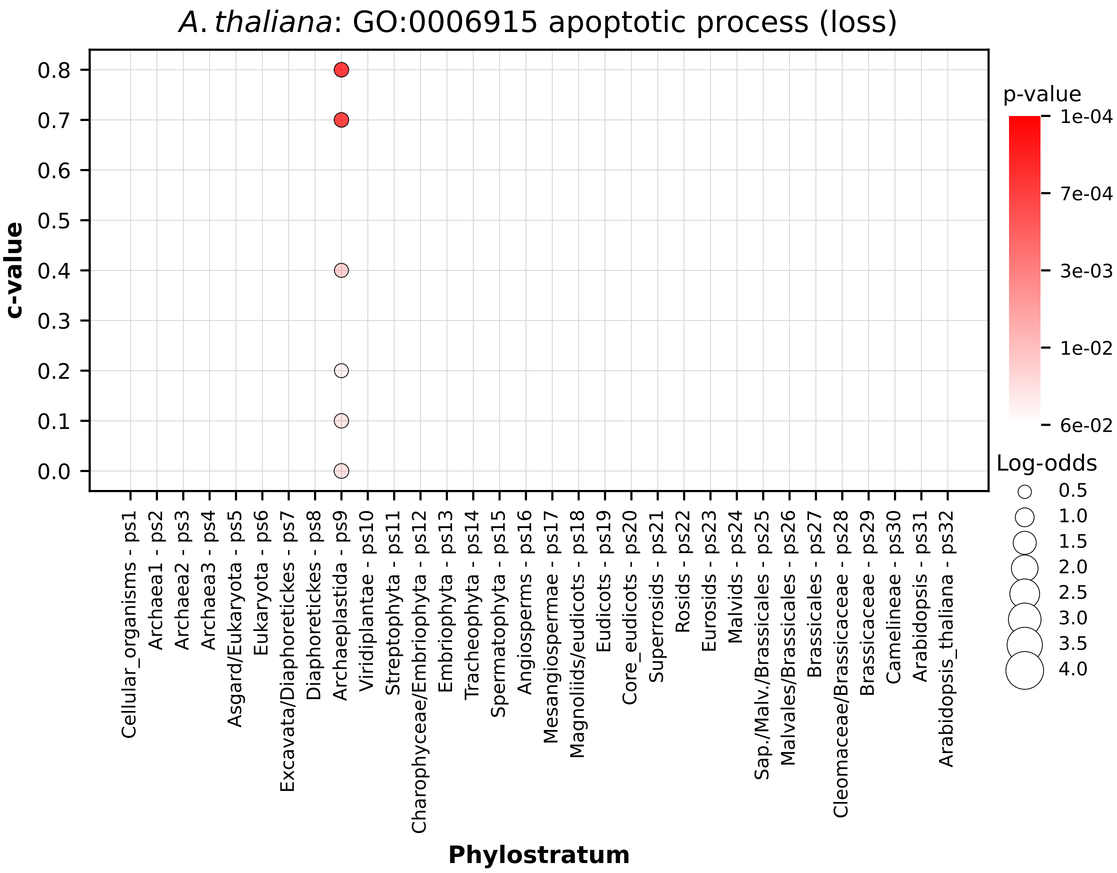
A. thaliana: GO:0006915 apoptotic process (loss)
c-value
0.8
0.7
0.6
0.5
0.4
0.3
0.2
0.1
0.0
p-value
1e-04
7e-04
3e-03
1e-02
6e-02
Log-odds
0.5
1.0
1.5
2.0
2.5
3.0
3.5
4.0
Cellular_organisms - ps1
Archaea1 - ps2
Archaea2 - ps3
Archaea3 - ps4
Asgard/Eukaryota - ps5
Eukaryota - ps6
Excavata/Diaphoretickes - ps7
Diaphoretickes - ps8
Archaeplastida - ps9
Viridiplantae - ps10
Streptophyta - ps11
Charophyceae/Embriophyta - ps12
Embriophyta - ps13
Tracheophyta - ps14
Spermatophyta - ps15
Angiosperms - ps16
Mesangiospermae - ps17
Magnoliids/eudicots - ps18
Eudicots - ps19
Core_eudicots - ps20
Superrosids - ps21
Rosids - ps22
Eurosids - ps23
Malvids - ps24
Sap./Malv./Brassicales - ps25
Malvales/Brassicales - ps26
Brassicales - ps27
Cleomaceae/Brassicaceae - ps28
Brassicaceae - ps29
Camelineae - ps30
Arabidopsis - ps31
Arabidopsis_thaliana - ps32
Phylostratum

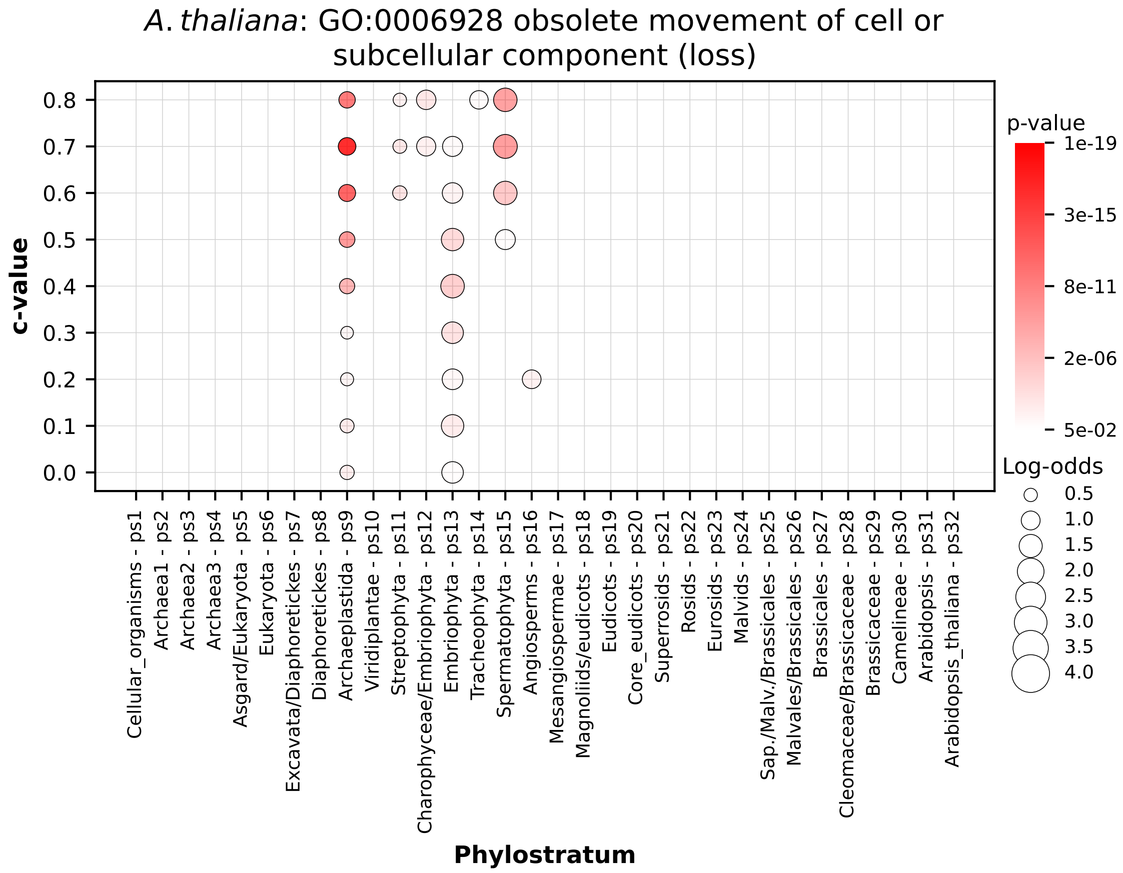

A. thaliana: GO:0006928 obsolete movement of cell or subcellular component (loss)
c-value
0.8
0.7
0.6
0.5
0.4
0.3
0.2
0.1
0.0
p-value
1e-19
3e-15
8e-11
2e-06
5e-02
Log-odds
0.5
1.0
1.5
2.0
2.5
3.0
3.5
4.0
Cellular_organisms - ps1
Archaea1 - ps2
Archaea2 - ps3
Archaea3 - ps4
Asgard/Eukaryota - ps5
Eukaryota - ps6
Excavata/Diaphoretickes - ps7
Diaphoretickes - ps8
Archaeplastida - ps9
Viridiplantae - ps10
Streptophyta - ps11
Charophyceae/Embriophyta - ps12
Embriophyta - ps13
Tracheophyta - ps14
Spermatophyta - ps15
Angiosperms - ps16
Mesangiospermae - ps17
Magnoliids/eudicots - ps18
Eudicots - ps19
Core_eudicots - ps20
Superrosids - ps21
Rosids - ps22
Eurosids - ps23
Malvids - ps24
Sap./Malv./Brassicales - ps25
Malvales/Brassicales - ps26
Brassicales - ps27
Cleomaceae/Brassicaceae - ps28
Brassicaceae - ps29
Camelineae - ps30
Arabidopsis - ps31
Arabidopsis_thaliana - ps32
Phylostratum

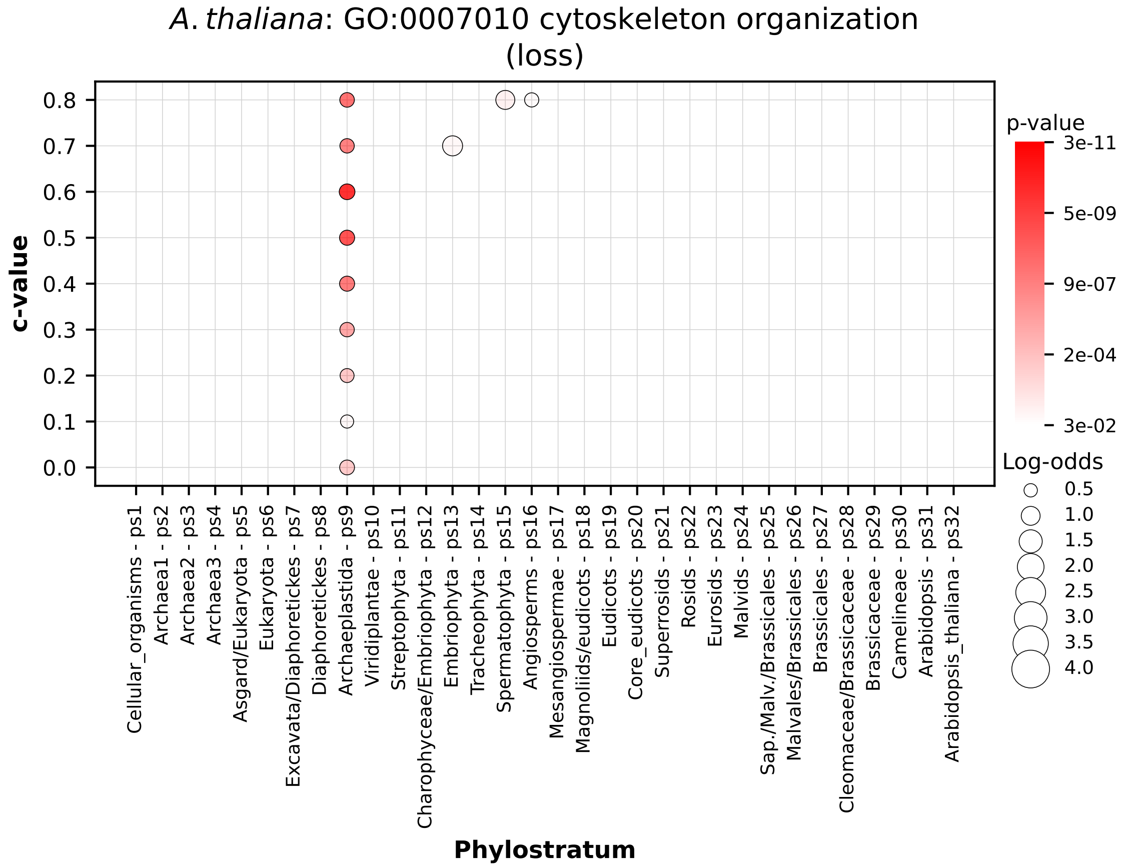
A. thaliana: GO:0007010 cytoskeleton organization (loss)
c-value
0.8
0.7
0.6
0.5
0.4
0.3
0.2
0.1
0.0
Phylostratum
Cellular_organisms - ps1
Archaea1 - ps2
Archaea2 - ps3
Archaea3 - ps4
Asgard/Eukaryota - ps5
Eukaryota - ps6
Excavata/Diaphoretickes - ps7
Diaphoretickes - ps8
Archaeplastida - ps9
Viridiplantae - ps10
Streptophyta - ps11
Charophyceae/Embriophyta - ps12
Embriophyta - ps13
Tracheophyta - ps14
Spermatophyta - ps15
Angiosperms - ps16
Mesangiospermae - ps17
Magnoliids/eudicots - ps18
Eudicots - ps19
Core_eudicots - ps20
Superrosids - ps21
Rosids - ps22
Eurosids - ps23
Malvids - ps24
Sap./Malv./Brassicales - ps25
Malvales/Brassicales - ps26
Brassicales - ps27
Cleomaceae/Brassicaceae - ps28
Brassicaceae - ps29
Camelineae - ps30
Arabidopsis - ps31
Arabidopsis_thaliana - ps32
p-value
3e-11
5e-09
9e-07
2e-04
3e-02
Log-odds
0.5
1.0
1.5
2.0
2.5
3.0
3.5
4.0

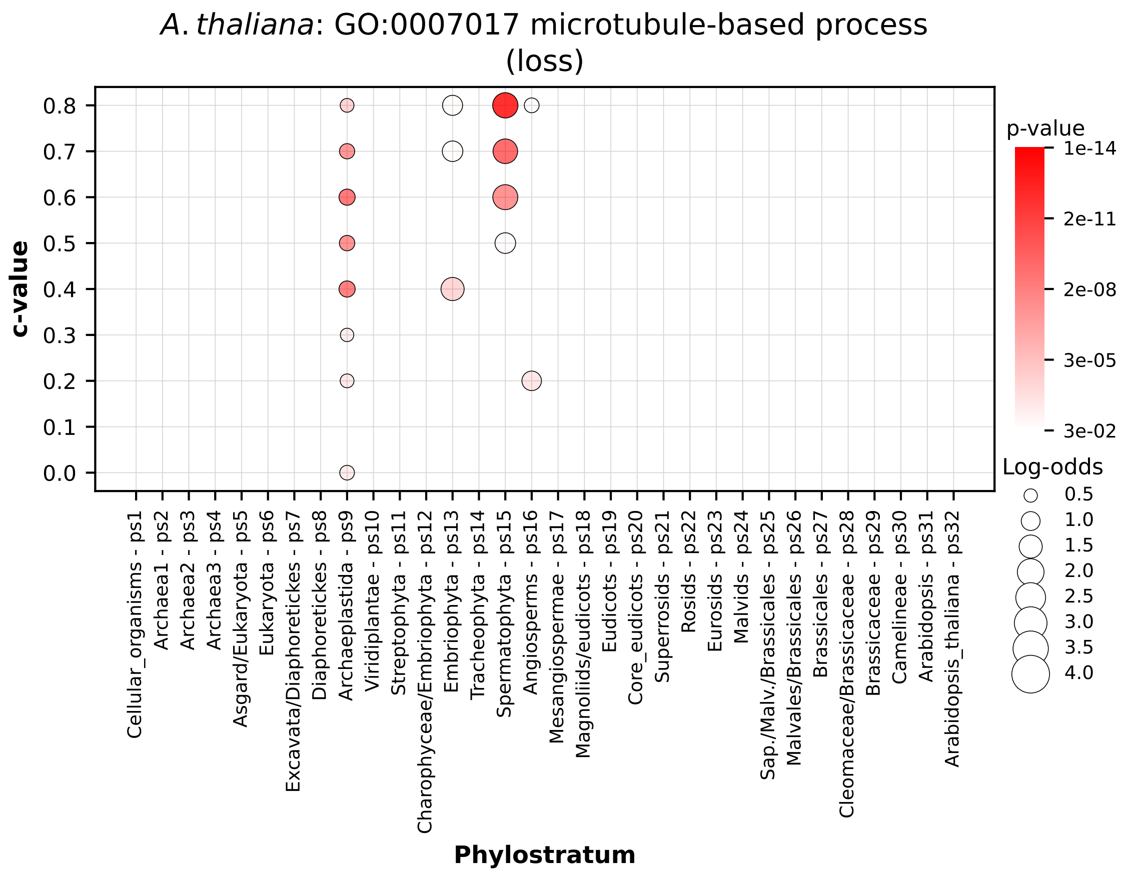

A. thaliana: GO:0007017 microtubule-based process
(loss)
c-value
0.8
0.7
0.6
0.5
0.4
0.3
0.2
0.1
0.0
Phylostratum
Cellular_organisms - ps1
Archaea1 - ps2
Archaea2 - ps3
Archaea3 - ps4
Asgard/Eukaryota - ps5
Eukaryota - ps6
Excavata/Diaphoretickes - ps7
Diaphoretickes - ps8
Archaeplastida - ps9
Viridiplantae - ps10
Streptophyta - ps11
Charophyceae/Embriophyta - ps12
Embriophyta - ps13
Tracheophyta - ps14
Spermatophyta - ps15
Angiosperms - ps16
Mesangiospermae - ps17
Magnoliids/eudicots - ps18
Eudicots - ps19
Core_eudicots - ps20
Superrosids - ps21
Rosids - ps22
Eurosids - ps23
Malvids - ps24
Sap./Malv./Brassicales - ps25
Malvales/Brassicales - ps26
Brassicales - ps27
Cleomaceae/Brassicaceae - ps28
Brassicaceae - ps29
Camelineae - ps30
Arabidopsis - ps31
Arabidopsis_thaliana - ps32
p-value
1e-14
2e-11
2e-08
3e-05
3e-02
Log-odds
0.5
1.0
1.5
2.0
2.5
3.0
3.5
4.0

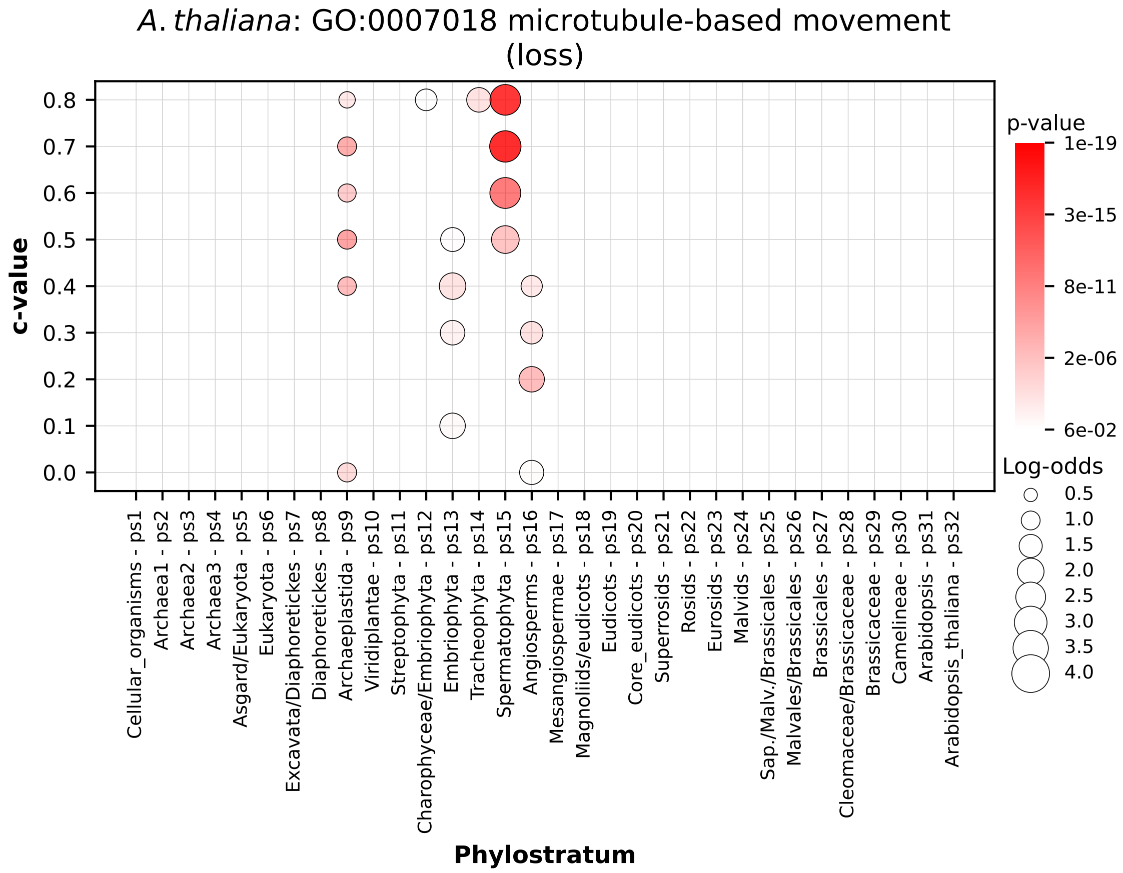

A. thaliana: GO:0007018 microtubule-based movement
(loss)
c-value
0.8
0.7
0.6
0.5
0.4
0.3
0.2
0.1
0.0
Phylostratum
Cellular_organisms - ps1
Archaea1 - ps2
Archaea2 - ps3
Archaea3 - ps4
Asgard/Eukaryota - ps5
Eukaryota - ps6
Excavata/Diaphoretickes - ps7
Diaphoretickes - ps8
Archaeplastida - ps9
Viridiplantae - ps10
Streptophyta - ps11
Charophyceae/Embriophyta - ps12
Embriophyta - ps13
Tracheophyta - ps14
Spermatophyta - ps15
Angiosperms - ps16
Mesangiospermae - ps17
Magnoliids/eudicots - ps18
Eudicots - ps19
Core_eudicots - ps20
Superrosids - ps21
Rosids - ps22
Eurosids - ps23
Malvids - ps24
Sap./Malv./Brassicales - ps25
Malvales/Brassicales - ps26
Brassicales - ps27
Cleomaceae/Brassicaceae - ps28
Brassicaceae - ps29
Camelineae - ps30
Arabidopsis - ps31
Arabidopsis_thaliana - ps32
p-value
1e-19
3e-15
8e-11
2e-06
6e-02
Log-odds
0.5
1.0
1.5
2.0
2.5
3.0
3.5
4.0

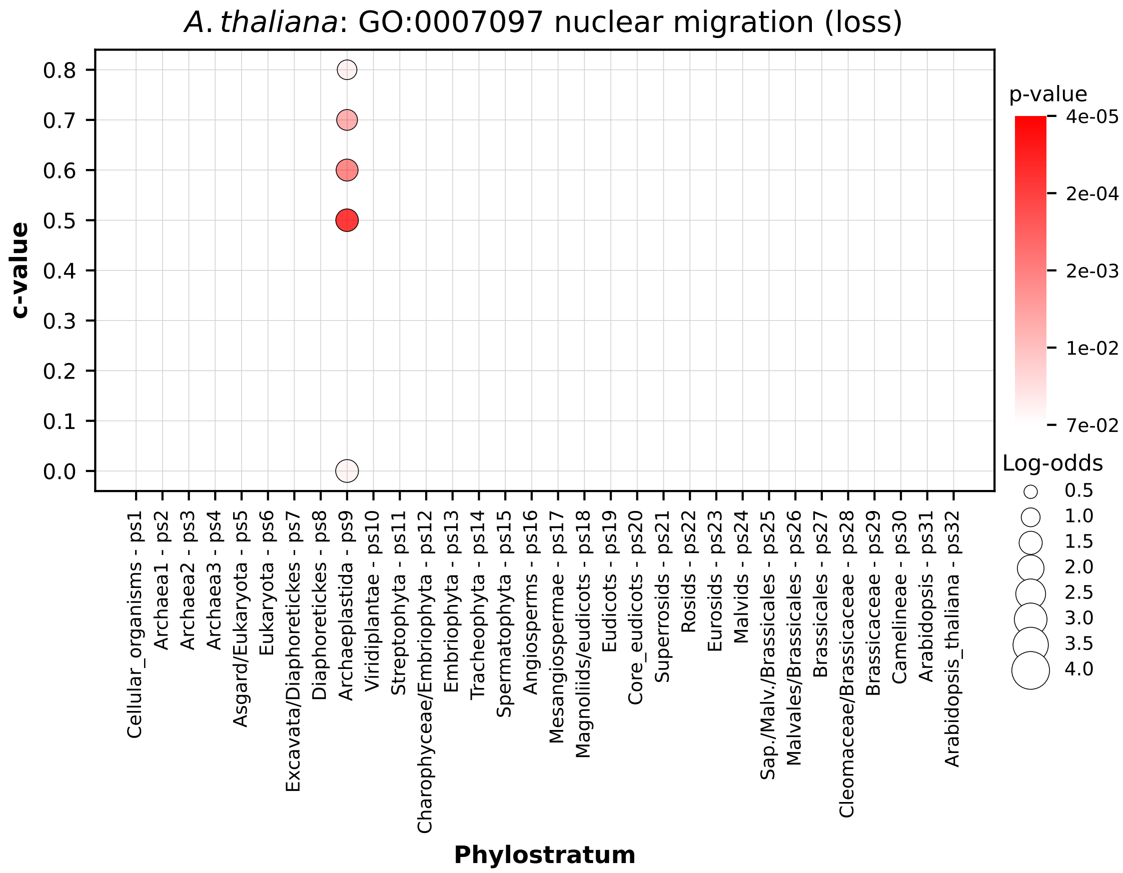

A. thaliana: GO:0007097 nuclear migration (loss)
c-value
0.8
0.7
0.6
0.5
0.4
0.3
0.2
0.1
0.0
Phylostratum
Cellular_organisms - ps1
Archaea1 - ps2
Archaea2 - ps3
Archaea3 - ps4
Asgard/Eukaryota - ps5
Eukaryota - ps6
Excavata/Diaphoretickes - ps7
Diaphoretickes - ps8
Archaeplastida - ps9
Viridiplantae - ps10
Streptophyta - ps11
Charophyceae/Embriophyta - ps12
Embriophyta - ps13
Tracheophyta - ps14
Spermatophyta - ps15
Angiosperms - ps16
Mesangiospermae - ps17
Magnoliids/eudicots - ps18
Eudicots - ps19
Core_eudicots - ps20
Superrosids - ps21
Rosids - ps22
Eurosids - ps23
Malvids - ps24
Sap./Malv./Brassicales - ps25
Malvales/Brassicales - ps26
Brassicales - ps27
Cleomaceae/Brassicaceae - ps28
Brassicaceae - ps29
Camelineae - ps30
Arabidopsis - ps31
Arabidopsis_thaliana - ps32
p-value
4e-05
2e-04
2e-03
1e-02
7e-02
Log-odds
0.5
1.0
1.5
2.0
2.5
3.0
3.5
4.0

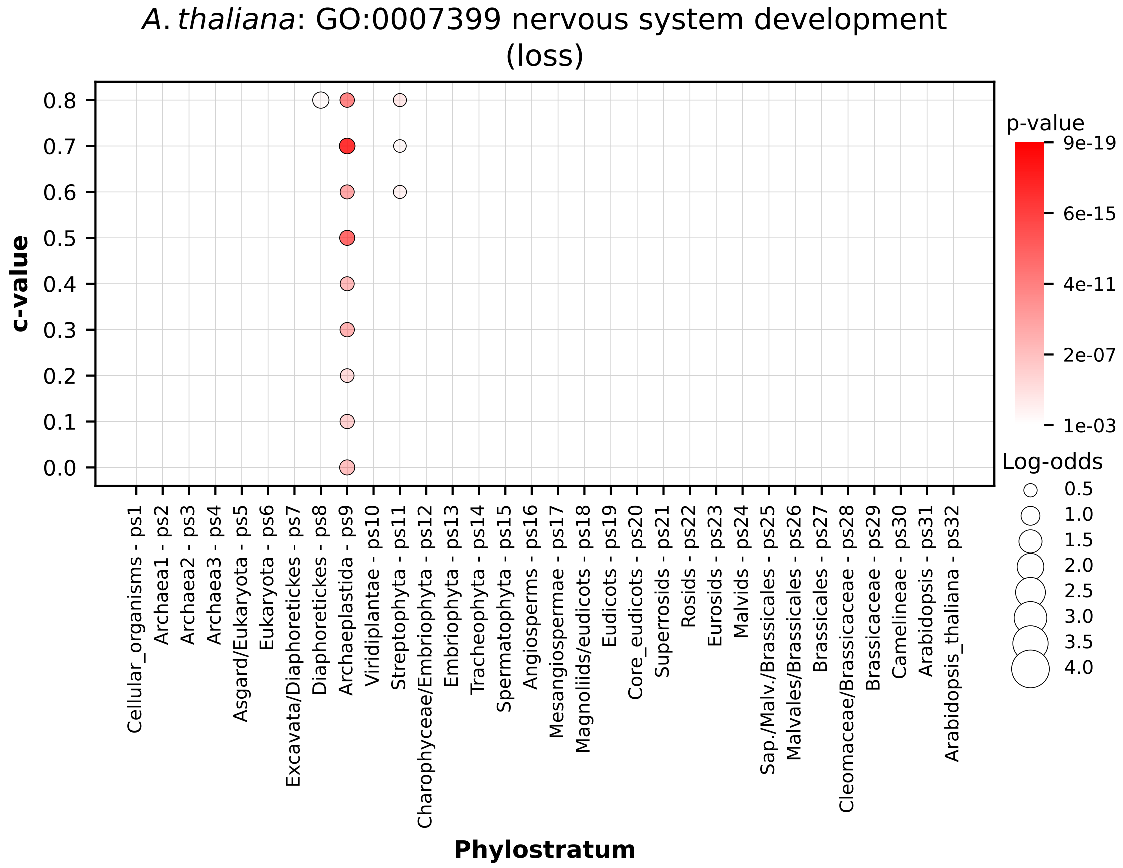

$A. thaliana$: GO:0007399 nervous system development (loss)
c-value
0.8
0.7
0.6
0.5
0.4
0.3
0.2
0.1
0.0
Phylostratum
Cellular_organisms - ps1
Archaea1 - ps2
Archaea2 - ps3
Archaea3 - ps4
Asgard/Eukaryota - ps5
Eukaryota - ps6
Excavata/Diaphoretickes - ps7
Diaphoretickes - ps8
Archaeplastida - ps9
Viridiplantae - ps10
Streptophyta - ps11
Charophyceae/Embriophyta - ps12
Embriophyta - ps13
Tracheophyta - ps14
Spermatophyta - ps15
Angiosperms - ps16
Mesangiospermae - ps17
Magnoliids/eudicots - ps18
Eudicots - ps19
Core_eudicots - ps20
Superrosids - ps21
Rosids - ps22
Eurosids - ps23
Malvids - ps24
Sap./Malv./Brassicales - ps25
Malvales/Brassicales - ps26
Brassicales - ps27
Cleomaceae/Brassicaceae - ps28
Brassicaceae - ps29
Camelineae - ps30
Arabidopsis - ps31
Arabidopsis_thaliana - ps32
p-value
9e-19
6e-15
4e-11
2e-07
1e-03
Log-odds
0.5
1.0
1.5
2.0
2.5
3.0
3.5
4.0

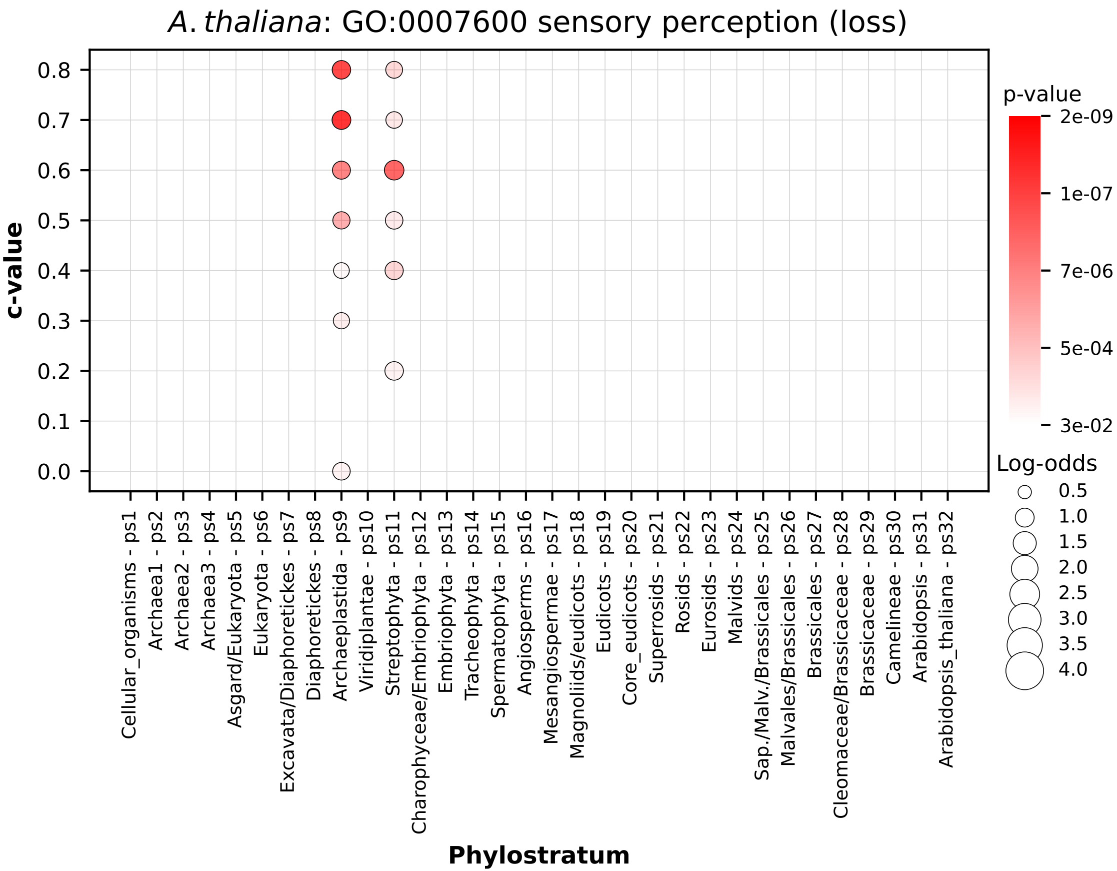

A. thaliana: GO:0007600 sensory perception (loss)
c-value
0.8
0.7
0.6
0.5
0.4
0.3
0.2
0.1
0.0
p-value
2e-09
1e-07
7e-06
5e-04
3e-02
Log-odds
0.5
1.0
1.5
2.0
2.5
3.0
3.5
4.0
Cellular_organisms - ps1
Archaea1 - ps2
Archaea2 - ps3
Archaea3 - ps4
Asgard/Eukaryota - ps5
Eukaryota - ps6
Excavata/Diaphoretickes - ps7
Diaphoretickes - ps8
Archaeplastida - ps9
Viridiplantae - ps10
Streptophyta - ps11
Charophyceae/Embriophyta - ps12
Embriophyta - ps13
Tracheophyta - ps14
Spermatophyta - ps15
Angiosperms - ps16
Mesangiospermae - ps17
Magnoliids/eudicots - ps18
Eudicots - ps19
Core_eudicots - ps20
Superrosids - ps21
Rosids - ps22
Eurosids - ps23
Malvids - ps24
Sap./Malv./Brassicales - ps25
Malvales/Brassicales - ps26
Brassicales - ps27
Cleomaceae/Brassicaceae - ps28
Brassicaceae - ps29
Camelineae - ps30
Arabidopsis - ps31
Arabidopsis_thaliana - ps32
Phylostratum

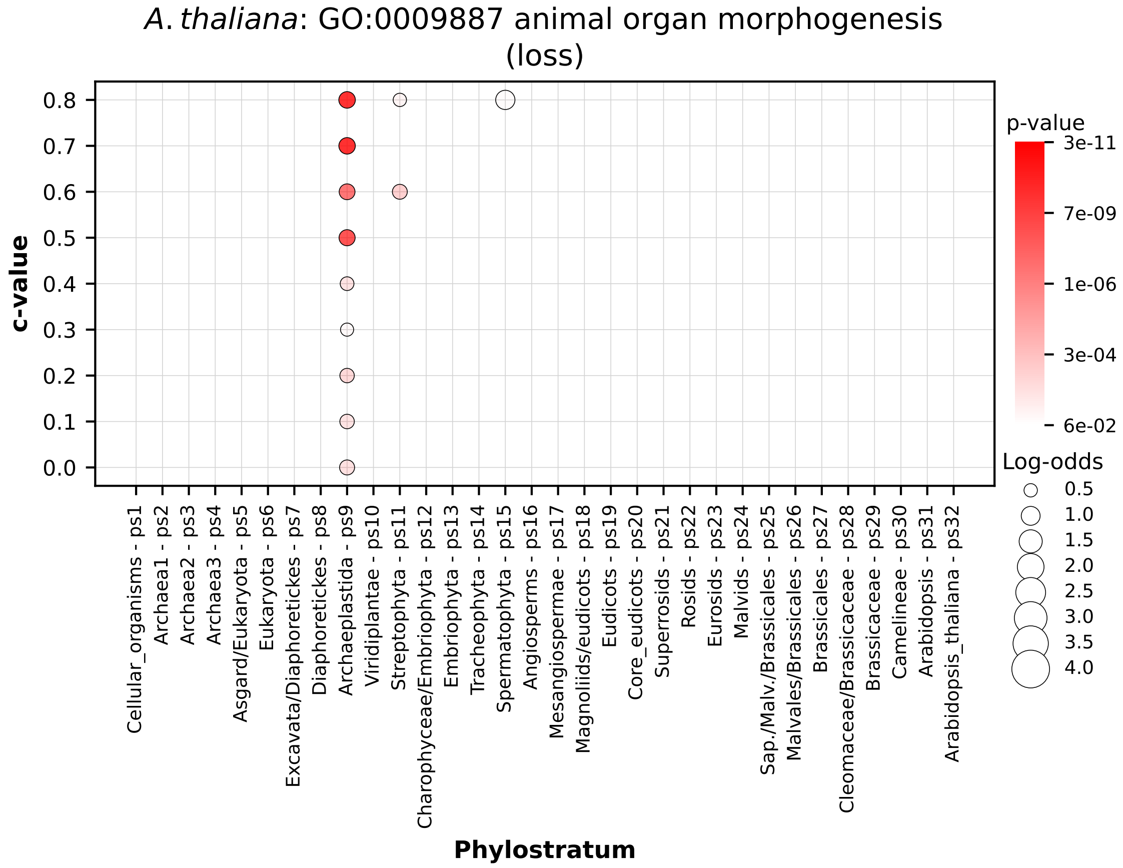

A. thaliana: GO:0009887 animal organ morphogenesis
(loss)
c-value
0.0
0.1
0.2
0.3
0.4
0.5
0.6
0.7
0.8
p-value
3e-11
7e-09
1e-06
3e-04
6e-02
Log-odds
0.5
1.0
1.5
2.0
2.5
3.0
3.5
4.0
Cellular_organisms - ps1
Archaea1 - ps2
Archaea2 - ps3
Archaea3 - ps4
Asgard/Eukaryota - ps5
Eukaryota - ps6
Excavata/Diaphoretickes - ps7
Diaphoretickes - ps8
Archaeplastida - ps9
Viridiplantae - ps10
Streptophyta - ps11
Charophyceae/Embriophyta - ps12
Embriophyta - ps13
Tracheophyta - ps14
Spermatophyta - ps15
Angiosperms - ps16
Mesangiospermae - ps17
Magnoliids/eudicots - ps18
Eudicots - ps19
Core_eudicots - ps20
Superrosids - ps21
Rosids - ps22
Eurosids - ps23
Malvids - ps24
Sap./Malv./Brassicales - ps25
Malvales/Brassicales - ps26
Brassicales - ps27
Cleomaceae/Brassicaceae - ps28
Brassicaceae - ps29
Camelineae - ps30
Arabidopsis - ps31
Arabidopsis_thaliana - ps32
Phylostratum

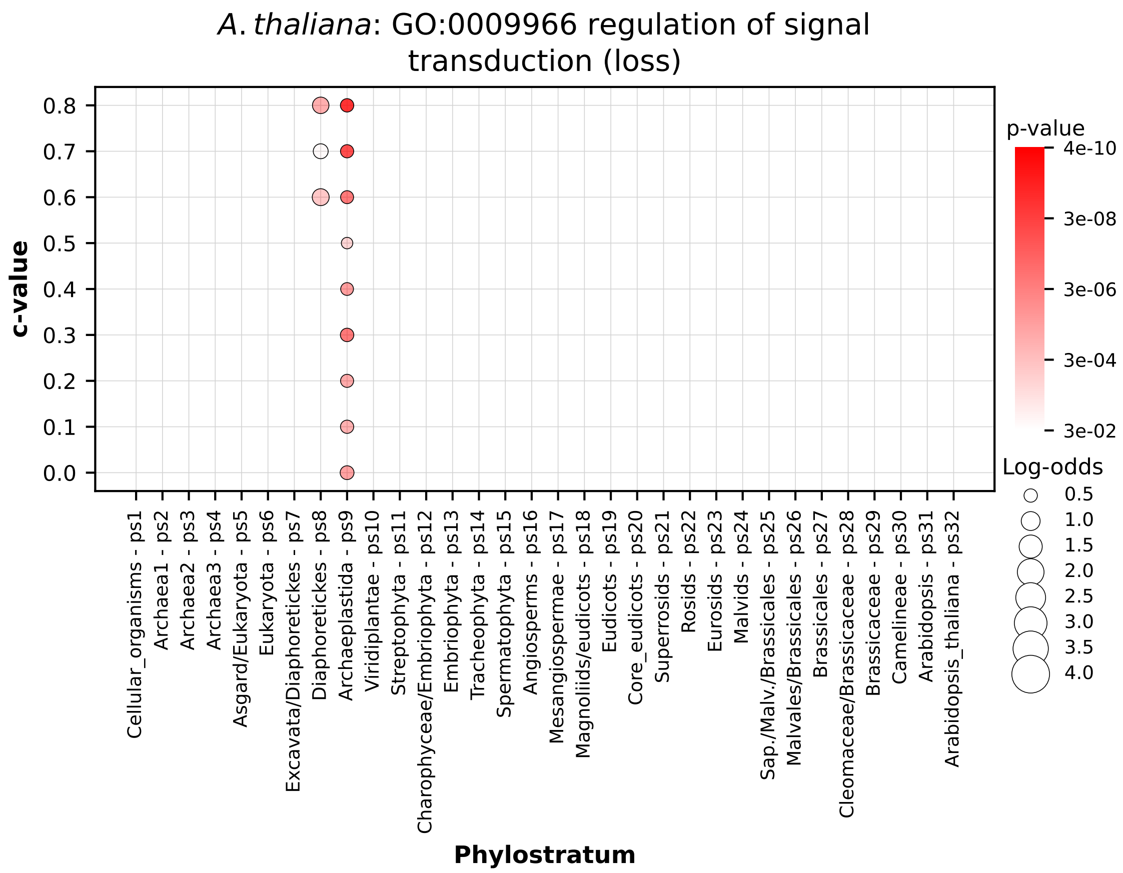

A. thaliana: GO:0009966 regulation of signal transduction (loss)
c-value
0.8
0.7
0.6
0.5
0.4
0.3
0.2
0.1
0.0
p-value
4e-10
3e-08
3e-06
3e-04
3e-02
Log-odds
0.5
1.0
1.5
2.0
2.5
3.0
3.5
4.0
Cellular_organisms - ps1
Archaea1 - ps2
Archaea2 - ps3
Archaea3 - ps4
Asgard/Eukaryota - ps5
Eukaryota - ps6
Excavata/Diaphoretickes - ps7
Diaphoretickes - ps8
Archaeplastida - ps9
Viridiplantae - ps10
Streptophyta - ps11
Charophyceae/Embriophyta - ps12
Embriophyta - ps13
Tracheophyta - ps14
Spermatophyta - ps15
Angiosperms - ps16
Mesangiospermae - ps17
Magnoliids/eudicots - ps18
Eudicots - ps19
Core_eudicots - ps20
Superrosids - ps21
Rosids - ps22
Eurosids - ps23
Malvids - ps24
Sap./Malv./Brassicales - ps25
Malvales/Brassicales - ps26
Brassicales - ps27
Cleomaceae/Brassicaceae - ps28
Brassicaceae - ps29
Camelineae - ps30
Arabidopsis - ps31
Arabidopsis_thaliana - ps32
Phylostratum

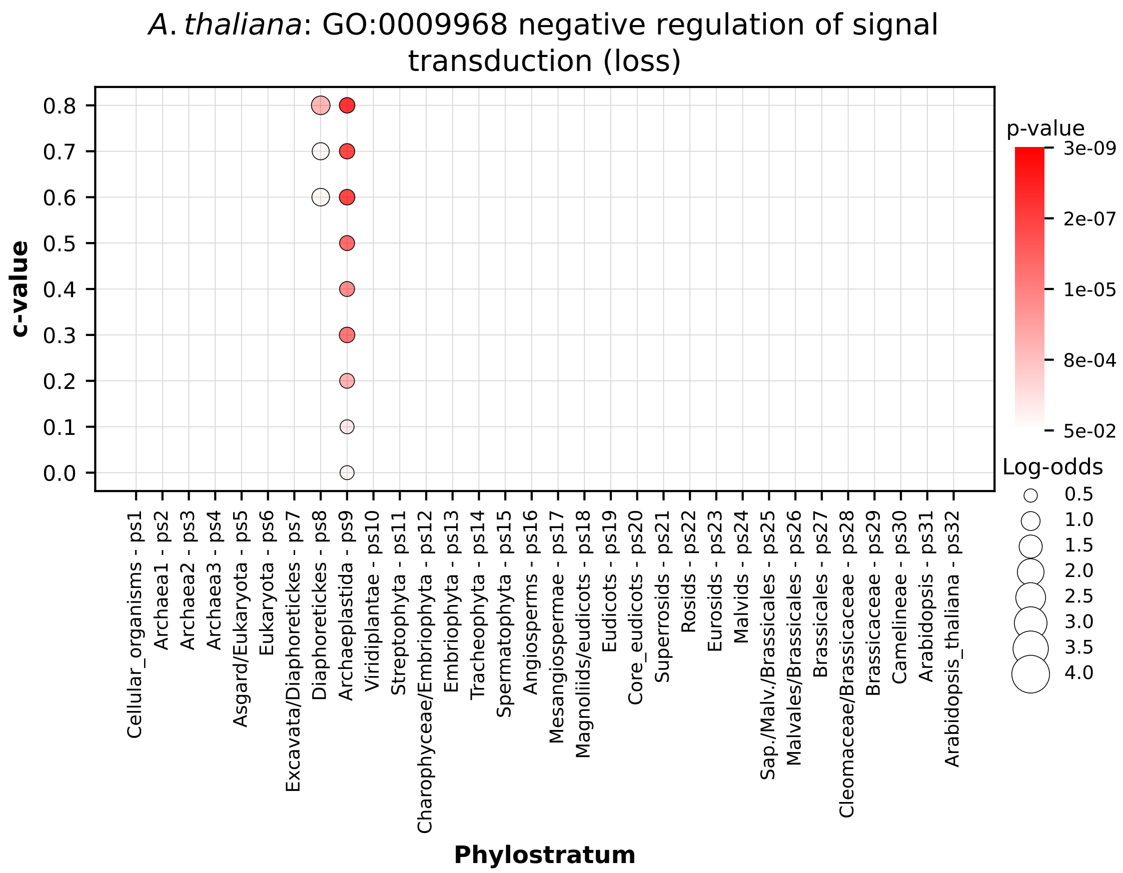

A. thaliana: GO:0009968 negative regulation of signal transduction (loss)
c-value
0.8
0.7
0.6
0.5
0.4
0.3
0.2
0.1
0.0
p-value
3e-09
2e-07
1e-05
8e-04
5e-02
Log-odds
0.5
1.0
1.5
2.0
2.5
3.0
3.5
4.0
Cellular_organisms - ps1
Archaea1 - ps2
Archaea2 - ps3
Archaea3 - ps4
Asgard/Eukaryota - ps5
Eukaryota - ps6
Excavata/Diaphoretickes - ps7
Diaphoretickes - ps8
Archaeplastida - ps9
Viridiplantae - ps10
Streptophyta - ps11
Charophyceae/Embriophyta - ps12
Embriophyta - ps13
Tracheophyta - ps14
Spermatophyta - ps15
Angiosperms - ps16
Mesangiospermae - ps17
Magnoliids/eudicots - ps18
Eudicots - ps19
Core_eudicots - ps20
Superrosids - ps21
Rosids - ps22
Eurosids - ps23
Malvids - ps24
Sap./Malv./Brassicales - ps25
Malvales/Brassicales - ps26
Brassicales - ps27
Cleomaceae/Brassicaceae - ps28
Brassicaceae - ps29
Camelineae - ps30
Arabidopsis - ps31
Arabidopsis_thaliana - ps32
Phylostratum

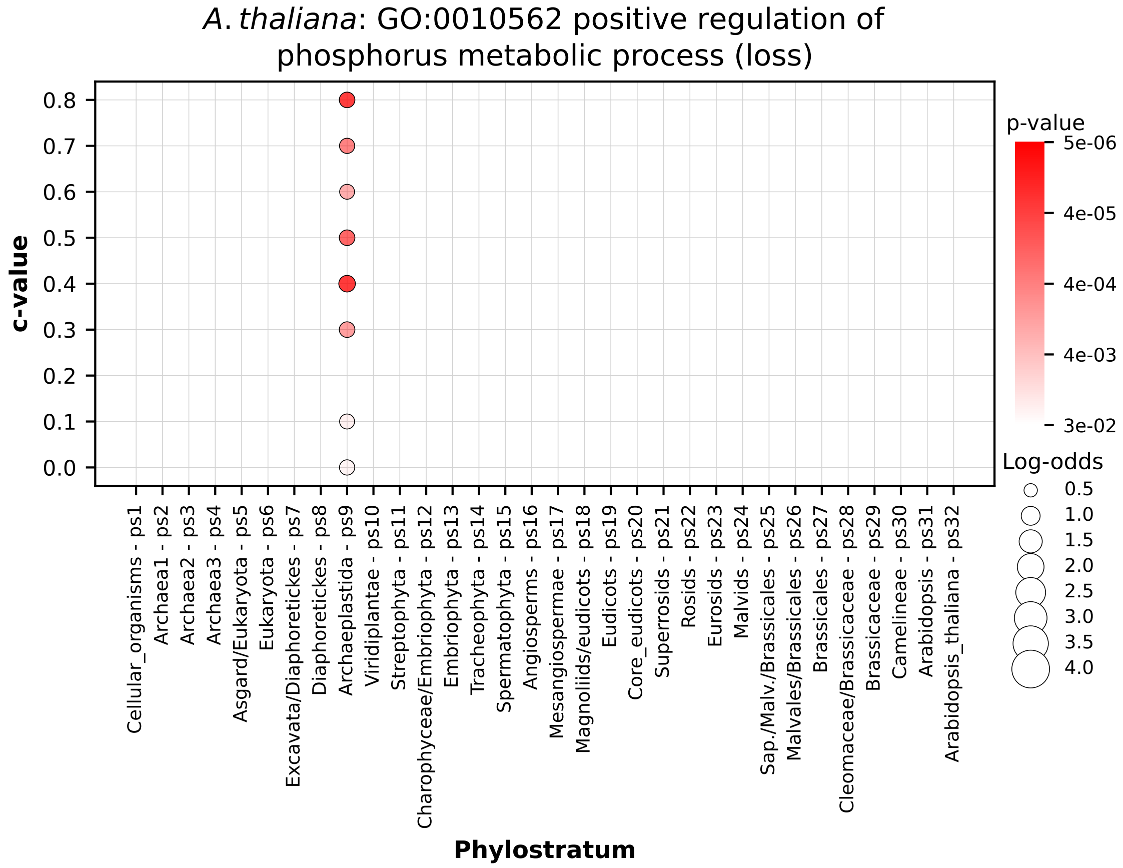

A. thaliana: GO:0010562 positive regulation of phosphorus metabolic process (loss)
c-value
0.8
0.7
0.6
0.5
0.4
0.3
0.2
0.1
0.0
p-value
5e-06
4e-05
4e-04
4e-03
3e-02
Log-odds
0.5
1.0
1.5
2.0
2.5
3.0
3.5
4.0
Cellular_organisms - ps1
Archaea1 - ps2
Archaea2 - ps3
Archaea3 - ps4
Asgard/Eukaryota - ps5
Eukaryota - ps6
Excavata/Diaphoretickes - ps7
Diaphoretickes - ps8
Archaeplastida - ps9
Viridiplantae - ps10
Streptophyta - ps11
Charophyceae/Embriophyta - ps12
Embriophyta - ps13
Tracheophyta - ps14
Spermatophyta - ps15
Angiosperms - ps16
Mesangiospermae - ps17
Magnoliids/eudicots - ps18
Eudicots - ps19
Core_eudicots - ps20
Superrosids - ps21
Rosids - ps22
Eurosids - ps23
Malvids - ps24
Sap./Malv./Brassicales - ps25
Malvales/Brassicales - ps26
Brassicales - ps27
Cleomaceae/Brassicaceae - ps28
Brassicaceae - ps29
Camelineae - ps30
Arabidopsis - ps31
Arabidopsis_thaliana - ps32
Phylostratum

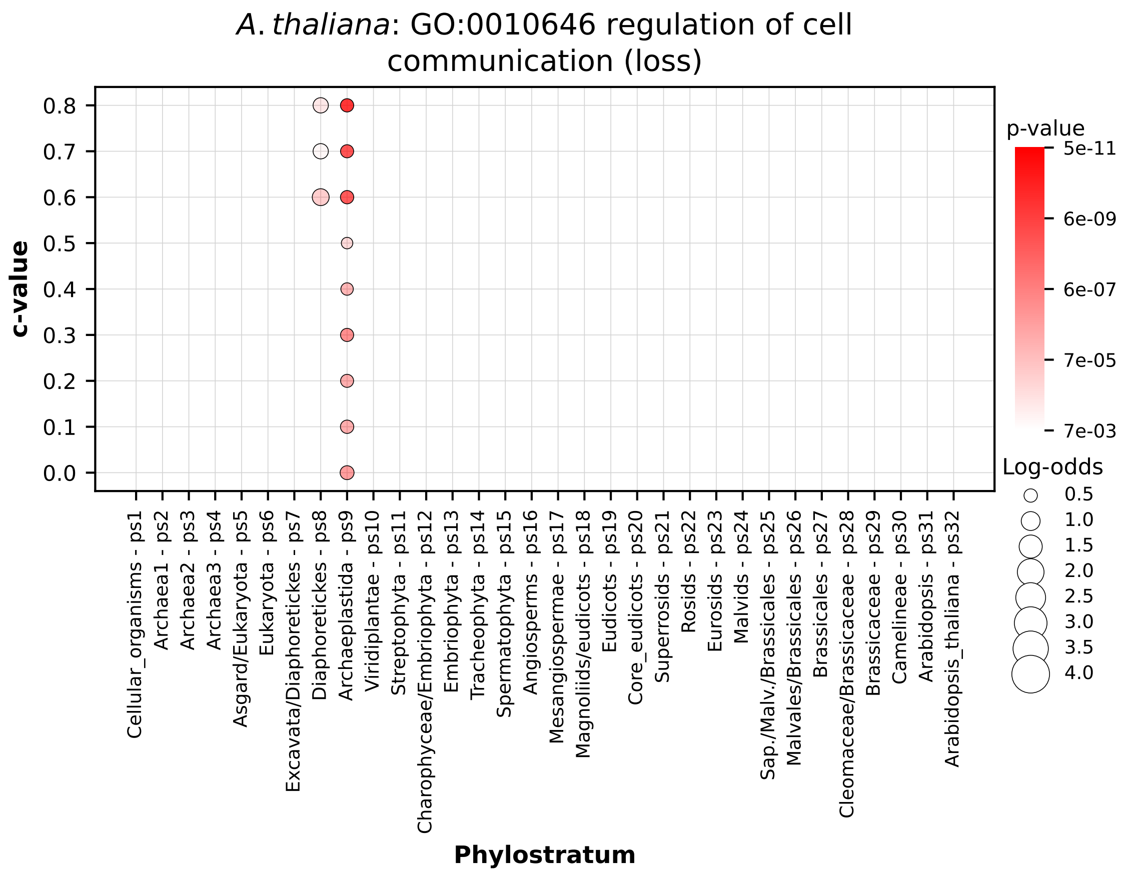

A. thaliana: GO:0010646 regulation of cell communication (loss)
c-value
0.8
0.7
0.6
0.5
0.4
0.3
0.2
0.1
0.0
p-value
5e-11
6e-09
6e-07
7e-05
7e-03
Log-odds
0.5
1.0
1.5
2.0
2.5
3.0
3.5
4.0
Cellular_organisms - ps1
Archaea1 - ps2
Archaea2 - ps3
Archaea3 - ps4
Asgard/Eukaryota - ps5
Eukaryota - ps6
Excavata/Diaphoretickes - ps7
Diaphoretickes - ps8
Archaeplastida - ps9
Viridiplantae - ps10
Streptophyta - ps11
Charophyceae/Embriophyta - ps12
Embriophyta - ps13
Tracheophyta - ps14
Spermatophyta - ps15
Angiosperms - ps16
Mesangiospermae - ps17
Magnoliids/eudicots - ps18
Eudicots - ps19
Core_eudicots - ps20
Superrosids - ps21
Rosids - ps22
Eurosids - ps23
Malvids - ps24
Sap./Malv./Brassicales - ps25
Malvales/Brassicales - ps26
Brassicales - ps27
Cleomaceae/Brassicaceae - ps28
Brassicaceae - ps29
Camelineae - ps30
Arabidopsis - ps31
Arabidopsis_thaliana - ps32
Phylostratum

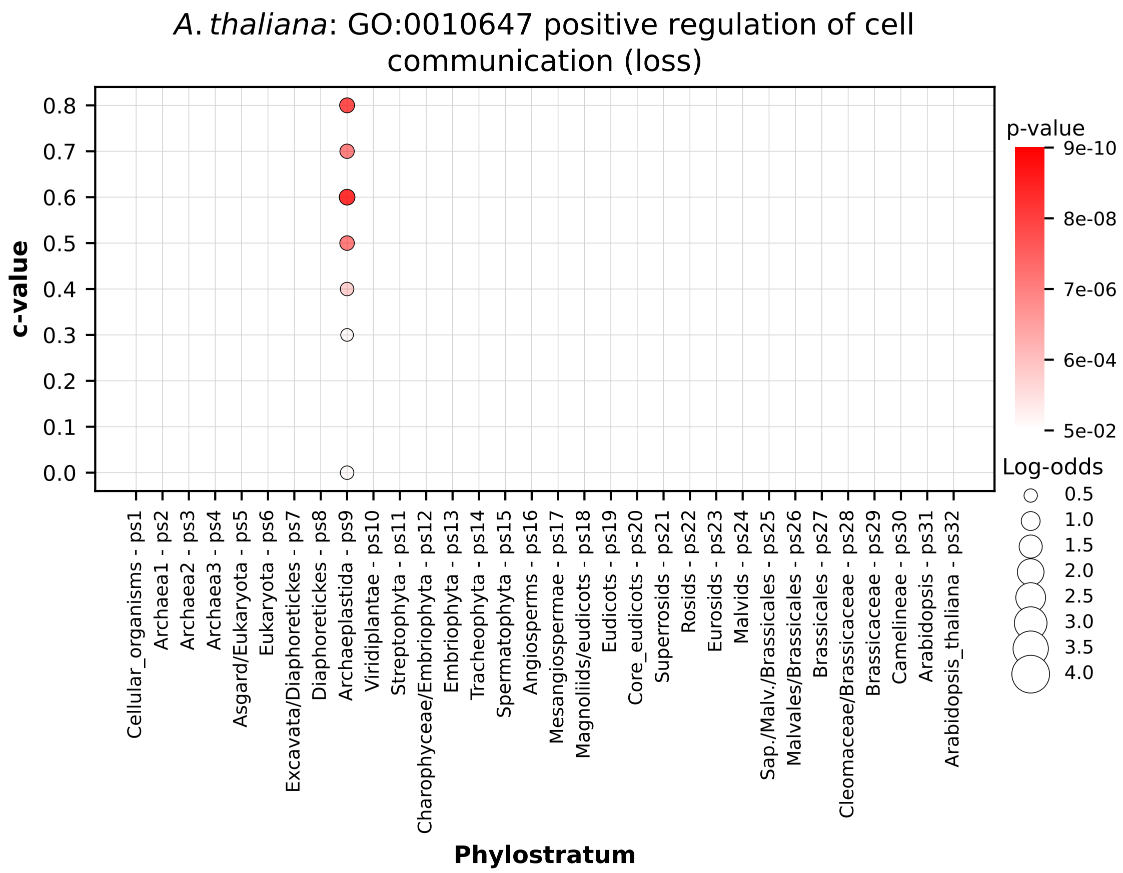
A. thaliana: GO:0010647 positive regulation of cell communication (loss)
c-value
0.8
0.7
0.6
0.5
0.4
0.3
0.2
0.1
0.0
Phylostratum
Cellular_organisms - ps1
Archaea1 - ps2
Archaea2 - ps3
Archaea3 - ps4
Asgard/Eukaryota - ps5
Eukaryota - ps6
Excavata/Diaphoretickes - ps7
Diaphoretickes - ps8
Archaeplastida - ps9
Viridiplantae - ps10
Streptophyta - ps11
Charophyceae/Embriophyta - ps12
Embriophyta - ps13
Tracheophyta - ps14
Spermatophyta - ps15
Angiosperms - ps16
Mesangiospermae - ps17
Magnoliids/eudicots - ps18
Eudicots - ps19
Core_eudicots - ps20
Superrosids - ps21
Rosids - ps22
Eurosids - ps23
Malvids - ps24
Sap./Malv./Brassicales - ps25
Malvales/Brassicales - ps26
Brassicales - ps27
Cleomaceae/Brassicaceae - ps28
Brassicaceae - ps29
Camelineae - ps30
Arabidopsis - ps31
Arabidopsis_thaliana - ps32
p-value
9e-10
8e-08
7e-06
6e-04
5e-02
Log-odds
0.5
1.0
1.5
2.0
2.5
3.0
3.5
4.0

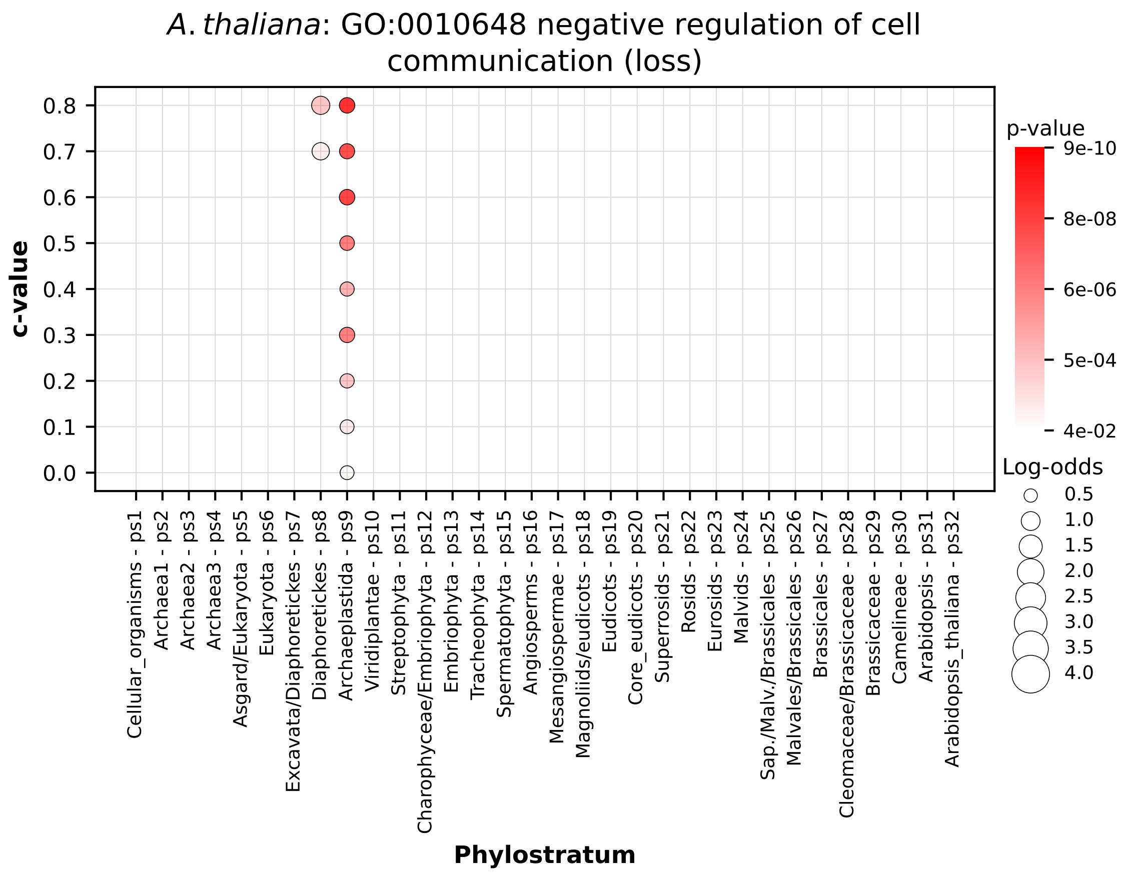
A. thaliana: GO:0010648 negative regulation of cell communication (loss)
c-value
0.8
0.7
0.6
0.5
0.4
0.3
0.2
0.1
0.0
p-value
9e-10
8e-08
6e-06
5e-04
4e-02
Log-odds
0.5
1.0
1.5
2.0
2.5
3.0
3.5
4.0
Cellular_organisms - ps1
Archaea1 - ps2
Archaea2 - ps3
Archaea3 - ps4
Asgard/Eukaryota - ps5
Eukaryota - ps6
Excavata/Diaphoretickes - ps7
Diaphoretickes - ps8
Archaeplastida - ps9
Viridiplantae - ps10
Streptophyta - ps11
Charophyceae/Embriophyta - ps12
Embriophyta - ps13
Tracheophyta - ps14
Spermatophyta - ps15
Angiosperms - ps16
Mesangiospermae - ps17
Magnoliids/eudicots - ps18
Eudicots - ps19
Core_eudicots - ps20
Superrosids - ps21
Rosids - ps22
Eurosids - ps23
Malvids - ps24
Sap./Malv./Brassicales - ps25
Malvales/Brassicales - ps26
Brassicales - ps27
Cleomaceae/Brassicaceae - ps28
Brassicaceae - ps29
Camelineae - ps30
Arabidopsis - ps31
Arabidopsis_thaliana - ps32
Phylostratum

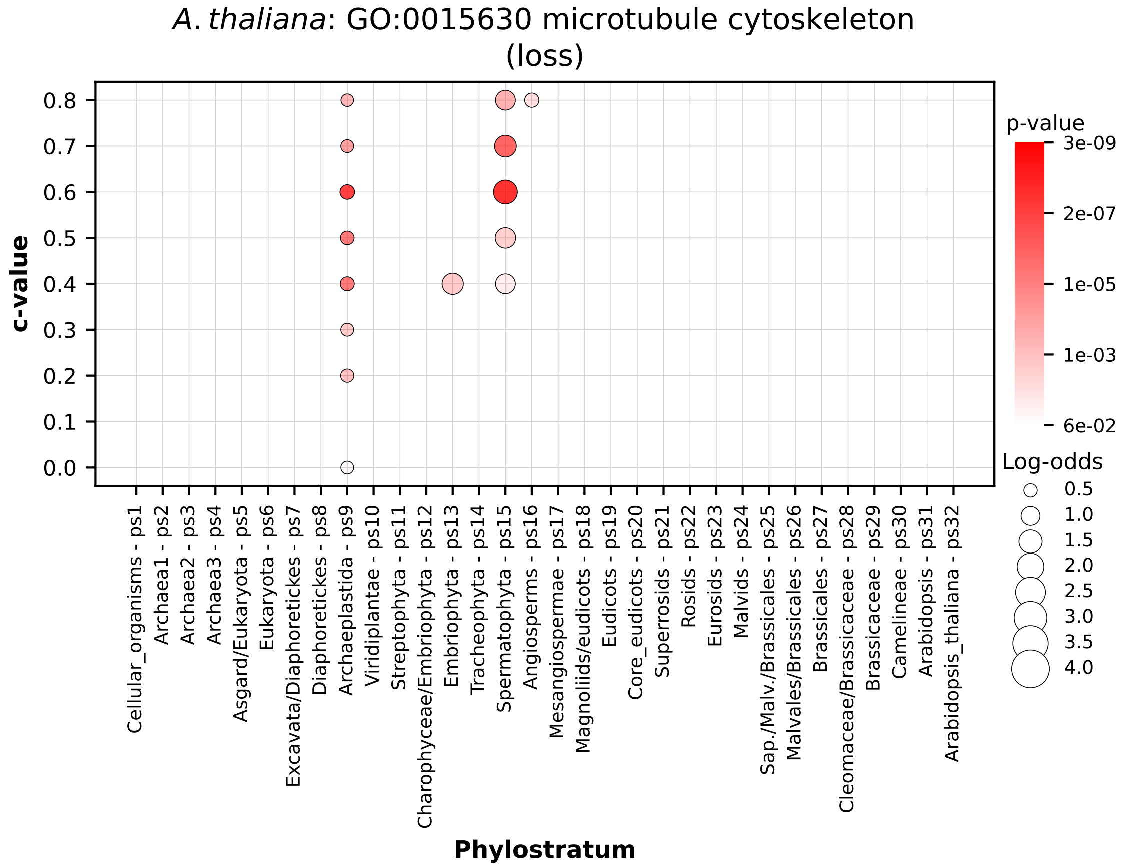

A. thaliana: GO:0015630 microtubule cytoskeleton
(loss)
c-value
0.8
0.7
0.6
0.5
0.4
0.3
0.2
0.1
0.0
p-value
3e-09
2e-07
1e-05
1e-03
6e-02
Log-odds
0.5
1.0
1.5
2.0
2.5
3.0
3.5
4.0
Cellular_organisms - ps1
Archaea1 - ps2
Archaea2 - ps3
Archaea3 - ps4
Asgard/Eukaryota - ps5
Eukaryota - ps6
Excavata/Diaphoretickes - ps7
Diaphoretickes - ps8
Archaeplastida - ps9
Viridiplantae - ps10
Streptophyta - ps11
Charophyceae/Embriophyta - ps12
Embriophyta - ps13
Tracheophyta - ps14
Spermatophyta - ps15
Angiosperms - ps16
Mesangiospermae - ps17
Magnoliids/eudicots - ps18
Eudicots - ps19
Core_eudicots - ps20
Superrosids - ps21
Rosids - ps22
Eurosids - ps23
Malvids - ps24
Sap./Malv./Brassicales - ps25
Malvales/Brassicales - ps26
Brassicales - ps27
Cleomaceae/Brassicaceae - ps28
Brassicaceae - ps29
Camelineae - ps30
Arabidopsis - ps31
Arabidopsis_thaliana - ps32
Phylostratum

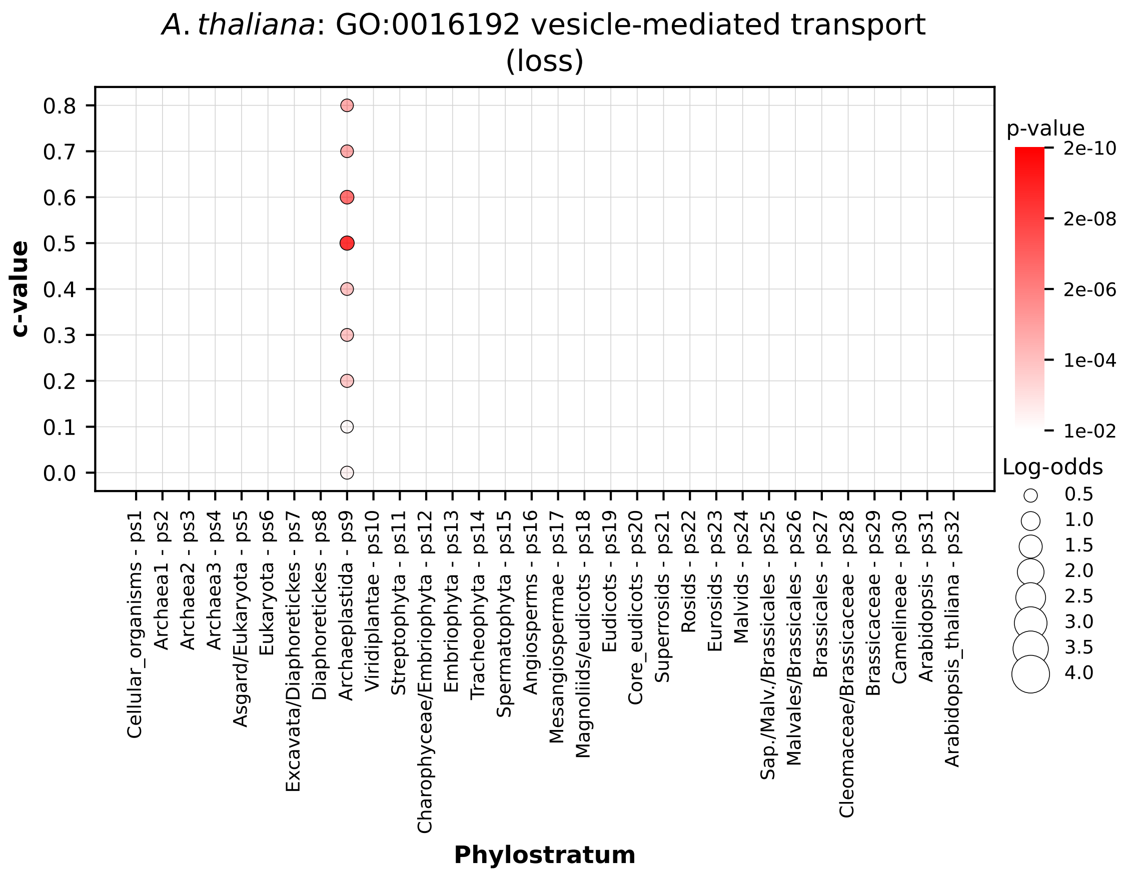

A. thaliana: GO:0016192 vesicle-mediated transport
(loss)
c-value
0.8
0.7
0.6
0.5
0.4
0.3
0.2
0.1
0.0
p-value
2e-10
2e-08
2e-06
1e-04
1e-02
Log-odds
0.5
1.0
1.5
2.0
2.5
3.0
3.5
4.0
Cellular_organisms - ps1
Archaea1 - ps2
Archaea2 - ps3
Archaea3 - ps4
Asgard/Eukaryota - ps5
Eukaryota - ps6
Excavata/Diaphoretickes - ps7
Diaphoretickes - ps8
Archaeplastida - ps9
Viridiplantae - ps10
Streptophyta - ps11
Charophyceae/Embriophyta - ps12
Embriophyta - ps13
Tracheophyta - ps14
Spermatophyta - ps15
Angiosperms - ps16
Mesangiospermae - ps17
Magnoliids/eudicots - ps18
Eudicots - ps19
Core_eudicots - ps20
Superrosids - ps21
Rosids - ps22
Eurosids - ps23
Malvids - ps24
Sap./Malv./Brassicales - ps25
Malvales/Brassicales - ps26
Brassicales - ps27
Cleomaceae/Brassicaceae - ps28
Brassicaceae - ps29
Camelineae - ps30
Arabidopsis - ps31
Arabidopsis_thaliana - ps32
Phylostratum

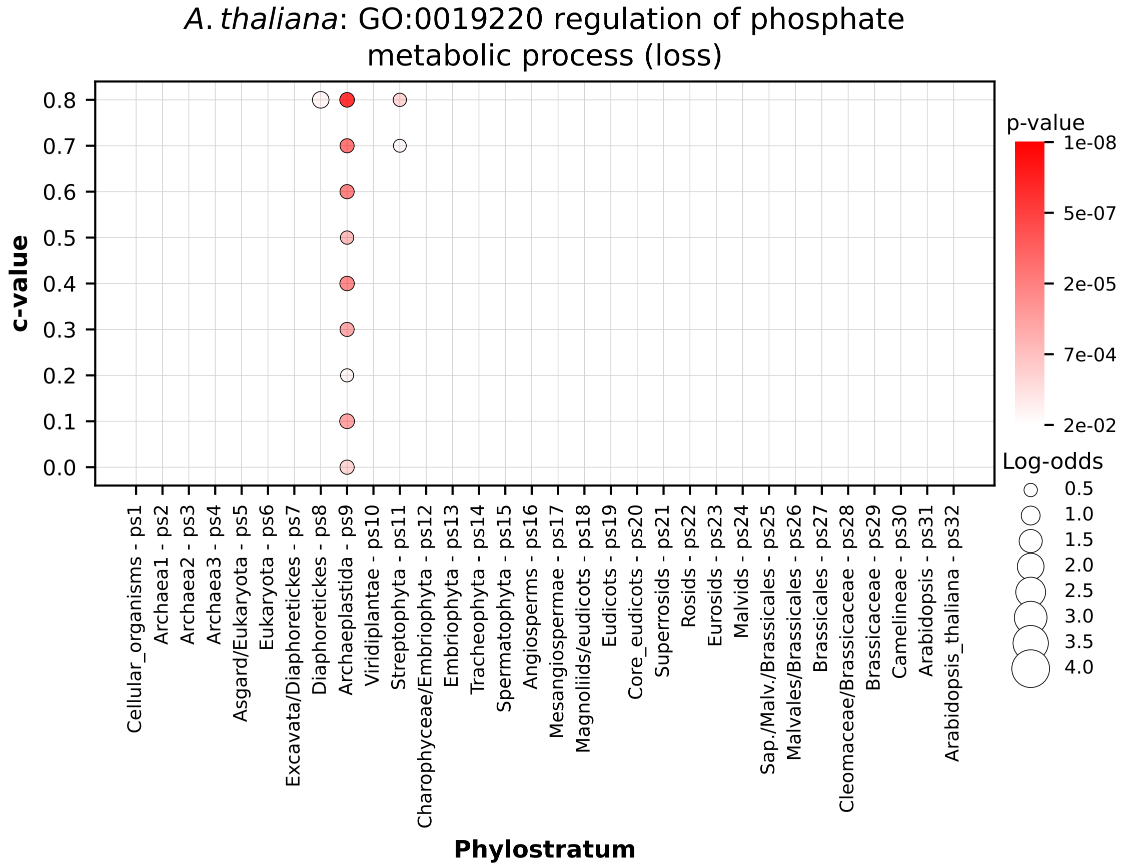
A. thaliana: GO:0019220 regulation of phosphate metabolic process (loss)
c-value
0.8
0.7
0.6
0.5
0.4
0.3
0.2
0.1
0.0
p-value
1e-08
5e-07
2e-05
7e-04
2e-02
Log-odds
0.5
1.0
1.5
2.0
2.5
3.0
3.5
4.0
Cellular_organisms - ps1
Archaea1 - ps2
Archaea2 - ps3
Archaea3 - ps4
Asgard/Eukaryota - ps5
Eukaryota - ps6
Excavata/Diaphoretickes - ps7
Diaphoretickes - ps8
Archaeplastida - ps9
Viridiplantae - ps10
Streptophyta - ps11
Charophyceae/Embriophyta - ps12
Embriophyta - ps13
Tracheophyta - ps14
Spermatophyta - ps15
Angiosperms - ps16
Mesangiospermae - ps17
Magnoliids/eudicots - ps18
Eudicots - ps19
Core_eudicots - ps20
Superrosids - ps21
Rosids - ps22
Eurosids - ps23
Malvids - ps24
Sap./Malv./Brassicales - ps25
Malvales/Brassicales - ps26
Brassicales - ps27
Cleomaceae/Brassicaceae - ps28
Brassicaceae - ps29
Camelineae - ps30
Arabidopsis - ps31
Arabidopsis_thaliana - ps32
Phylostratum

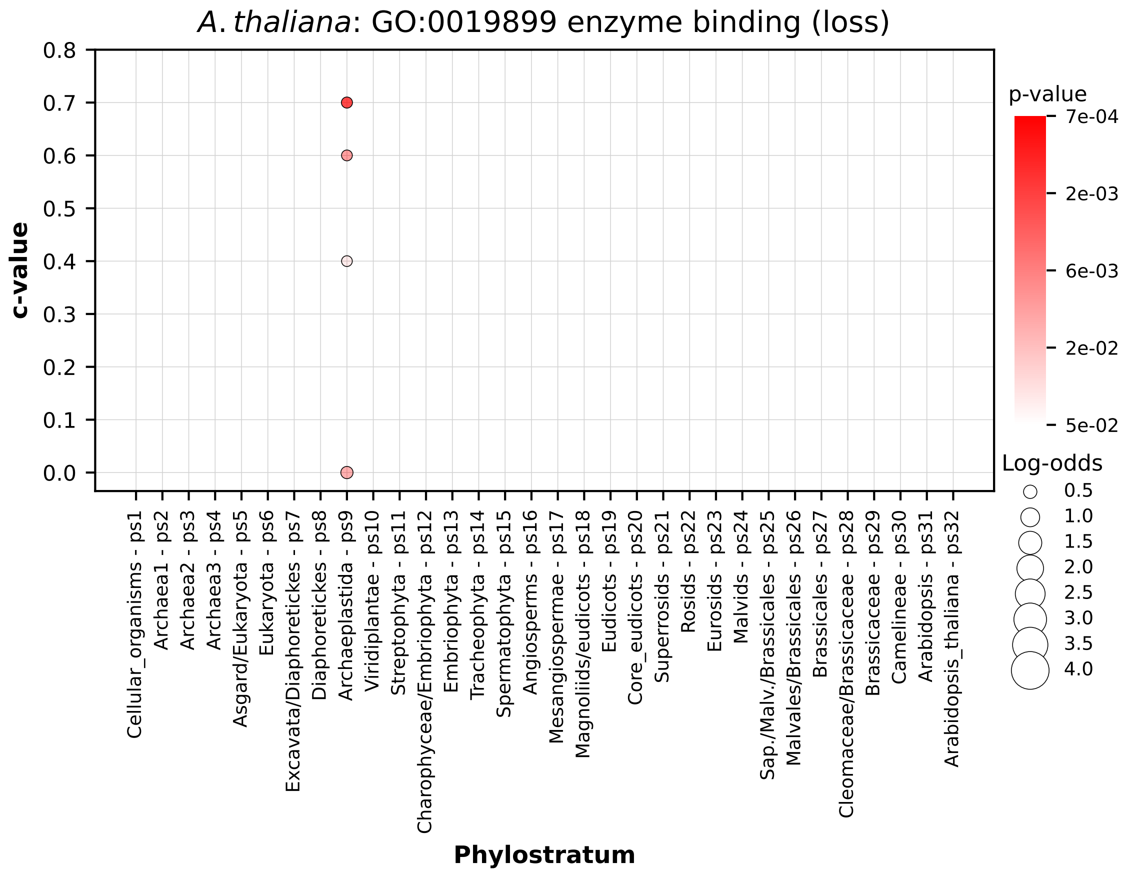

A. thaliana: GO:0019899 enzyme binding (loss)
c-value
0.0
0.1
0.2
0.3
0.4
0.5
0.6
0.7
0.8
Phylostratum
Cellular_organisms - ps1
Archaea1 - ps2
Archaea2 - ps3
Archaea3 - ps4
Asgard/Eukaryota - ps5
Eukaryota - ps6
Excavata/Diaphoretickes - ps7
Diaphoretickes - ps8
Archaeplastida - ps9
Viridiplantae - ps10
Streptophyta - ps11
Charophyceae/Embriophyta - ps12
Embriophyta - ps13
Tracheophyta - ps14
Spermatophyta - ps15
Angiosperms - ps16
Mesangiospermae - ps17
Magnoliids/eudicots - ps18
Eudicots - ps19
Core_eudicots - ps20
Superrosids - ps21
Rosids - ps22
Eurosids - ps23
Malvids - ps24
Sap./Malv./Brassicales - ps25
Malvales/Brassicales - ps26
Brassicales - ps27
Cleomaceae/Brassicaceae - ps28
Brassicaceae - ps29
Camelineae - ps30
Arabidopsis - ps31
Arabidopsis_thaliana - ps32
p-value
7e-04
2e-03
6e-03
2e-02
5e-02
Log-odds
0.5
1.0
1.5
2.0
2.5
3.0
3.5
4.0

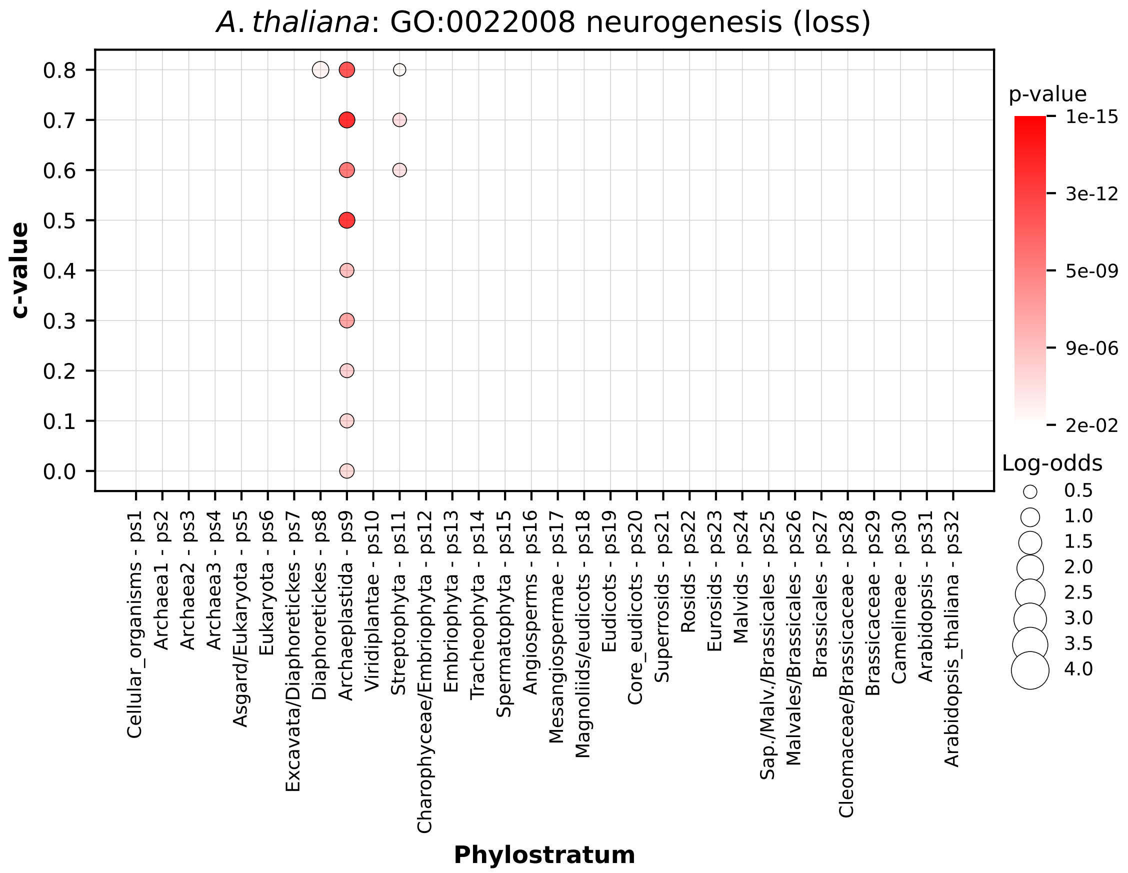

A. thaliana: GO:0022008 neurogenesis (loss)
c-value
0.8
0.7
0.6
0.5
0.4
0.3
0.2
0.1
0.0
p-value
1e-15
3e-12
5e-09
9e-06
2e-02
Log-odds
0.5
1.0
1.5
2.0
2.5
3.0
3.5
4.0
Cellular_organisms - ps1
Archaea1 - ps2
Archaea2 - ps3
Archaea3 - ps4
Asgard/Eukaryota - ps5
Eukaryota - ps6
Excavata/Diaphoretickes - ps7
Diaphoretickes - ps8
Archaeplastida - ps9
Viridiplantae - ps10
Streptophyta - ps11
Charophyceae/Embriophyta - ps12
Embriophyta - ps13
Tracheophyta - ps14
Spermatophyta - ps15
Angiosperms - ps16
Mesangiospermae - ps17
Magnoliids/eudicots - ps18
Eudicots - ps19
Core_eudicots - ps20
Superrosids - ps21
Rosids - ps22
Eurosids - ps23
Malvids - ps24
Sap./Malv./Brassicales - ps25
Malvales/Brassicales - ps26
Brassicales - ps27
Cleomaceae/Brassicaceae - ps28
Brassicaceae - ps29
Camelineae - ps30
Arabidopsis - ps31
Arabidopsis_thaliana - ps32
Phylostratum

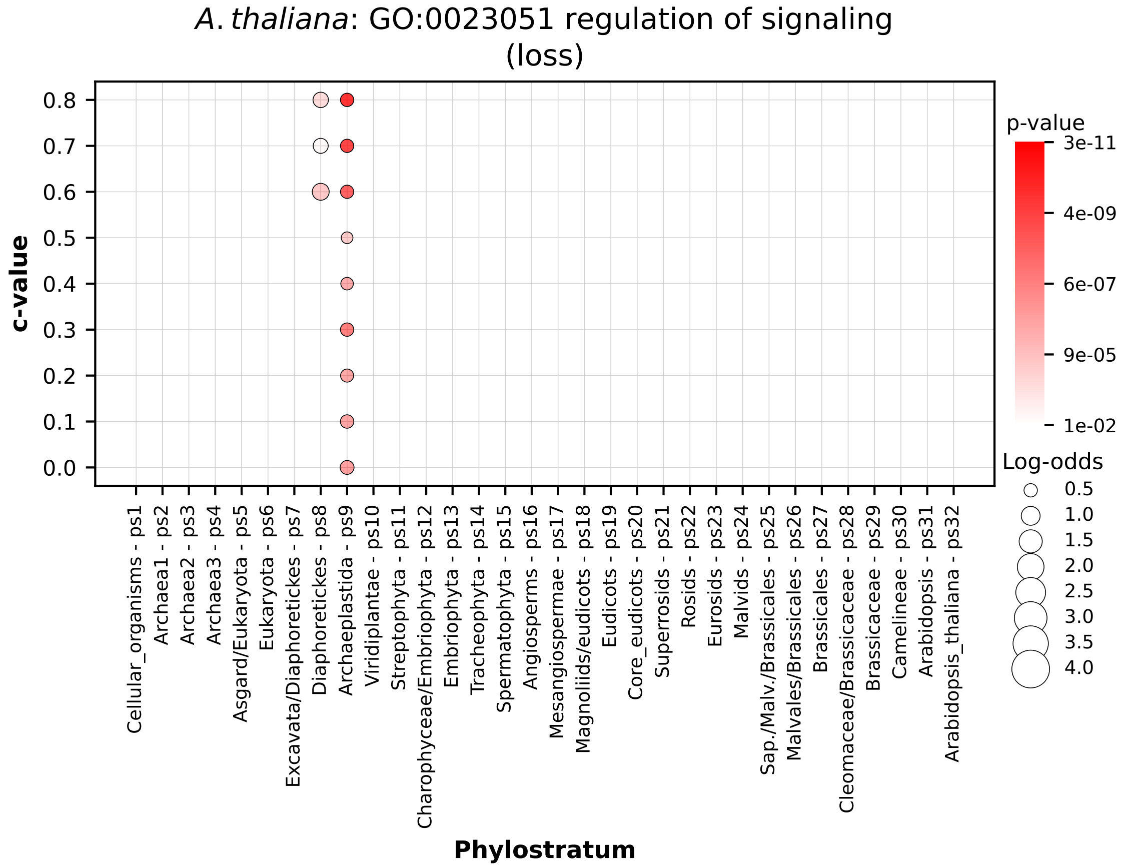

A. thaliana: GO:0023051 regulation of signaling
(loss)
c-value
0.8
0.7
0.6
0.5
0.4
0.3
0.2
0.1
0.0
p-value
3e-11
4e-09
6e-07
9e-05
1e-02
Log-odds
0.5
1.0
1.5
2.0
2.5
3.0
3.5
4.0
Cellular_organisms - ps1
Archaea1 - ps2
Archaea2 - ps3
Archaea3 - ps4
Asgard/Eukaryota - ps5
Eukaryota - ps6
Excavata/Diaphoretickes - ps7
Diaphoretickes - ps8
Archaeplastida - ps9
Viridiplantae - ps10
Streptophyta - ps11
Charophyceae/Embriophyta - ps12
Embriophyta - ps13
Tracheophyta - ps14
Spermatophyta - ps15
Angiosperms - ps16
Mesangiospermae - ps17
Magnoliids/eudicots - ps18
Eudicots - ps19
Core_eudicots - ps20
Superrosids - ps21
Rosids - ps22
Eurosids - ps23
Malvids - ps24
Sap./Malv./Brassicales - ps25
Malvales/Brassicales - ps26
Brassicales - ps27
Cleomaceae/Brassicaceae - ps28
Brassicaceae - ps29
Camelineae - ps30
Arabidopsis - ps31
Arabidopsis_thaliana - ps32
Phylostratum

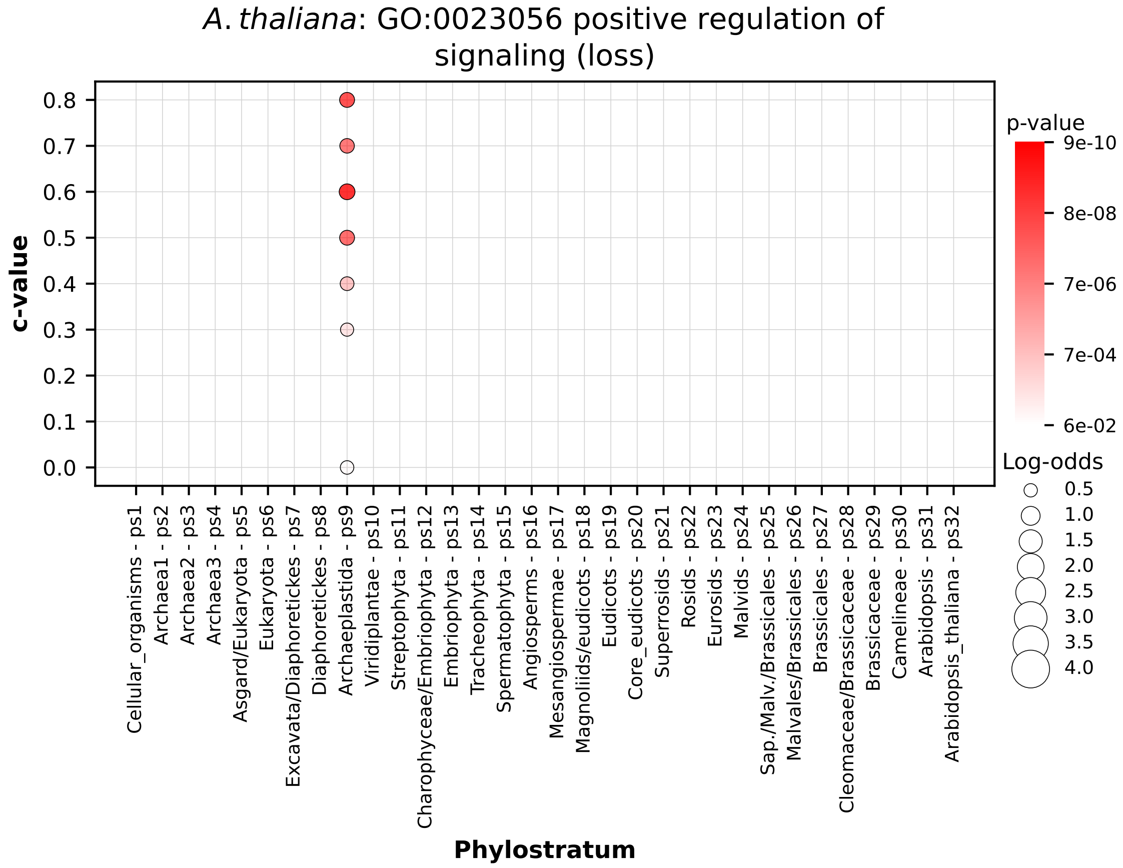
A. thaliana: GO:0023056 positive regulation of signaling (loss)
c-value
0.8
0.7
0.6
0.5
0.4
0.3
0.2
0.1
0.0
p-value
9e-10
8e-08
7e-06
7e-04
6e-02
Log-odds
0.5
1.0
1.5
2.0
2.5
3.0
3.5
4.0
Cellular_organisms - ps1
Archaea1 - ps2
Archaea2 - ps3
Archaea3 - ps4
Asgard/Eukaryota - ps5
Eukaryota - ps6
Excavata/Diaphoretickes - ps7
Diaphoretickes - ps8
Archaeplastida - ps9
Viridiplantae - ps10
Streptophyta - ps11
Charophyceae/Embriophyta - ps12
Embriophyta - ps13
Tracheophyta - ps14
Spermatophyta - ps15
Angiosperms - ps16
Mesangiospermae - ps17
Magnoliids/eudicots - ps18
Eudicots - ps19
Core_eudicots - ps20
Superrosids - ps21
Rosids - ps22
Eurosids - ps23
Malvids - ps24
Sap./Malv./Brassicales - ps25
Malvales/Brassicales - ps26
Brassicales - ps27
Cleomaceae/Brassicaceae - ps28
Brassicaceae - ps29
Camelineae - ps30
Arabidopsis - ps31
Arabidopsis_thaliana - ps32
Phylostratum

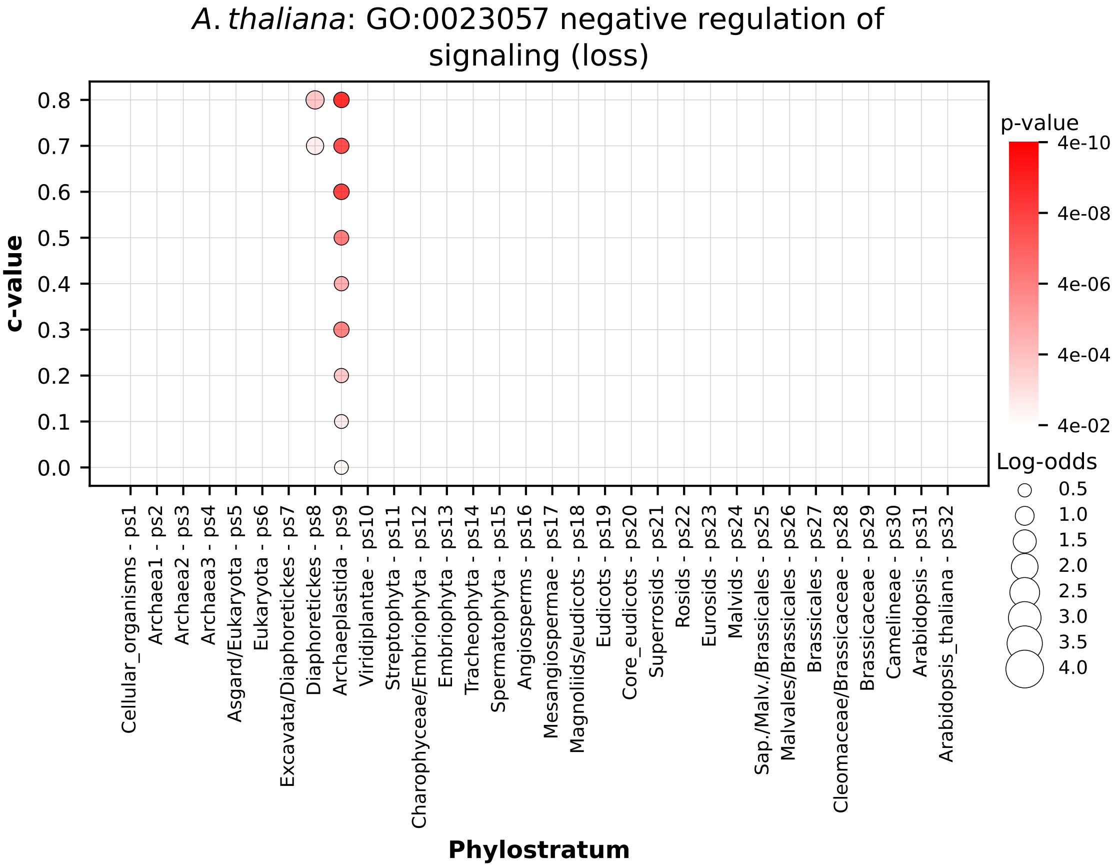
A. thaliana: GO:0023057 negative regulation of signaling (loss)
c-value
0.8
0.7
0.6
0.5
0.4
0.3
0.2
0.1
0.0
p-value
4e-10
4e-08
4e-06
4e-04
4e-02
Log-odds
0.5
1.0
1.5
2.0
2.5
3.0
3.5
4.0
Cellular_organisms - ps1
Archaea1 - ps2
Archaea2 - ps3
Archaea3 - ps4
Asgard/Eukaryota - ps5
Eukaryota - ps6
Excavata/Diaphoretickes - ps7
Diaphoretickes - ps8
Archaeplastida - ps9
Viridiplantae - ps10
Streptophyta - ps11
Charophyceae/Embriophyta - ps12
Embriophyta - ps13
Tracheophyta - ps14
Spermatophyta - ps15
Angiosperms - ps16
Mesangiospermae - ps17
Magnoliids/eudicots - ps18
Eudicots - ps19
Core_eudicots - ps20
Superrosids - ps21
Rosids - ps22
Eurosids - ps23
Malvids - ps24
Sap./Malv./Brassicales - ps25
Malvales/Brassicales - ps26
Brassicales - ps27
Cleomaceae/Brassicaceae - ps28
Brassicaceae - ps29
Camelineae - ps30
Arabidopsis - ps31
Arabidopsis_thaliana - ps32
Phylostratum

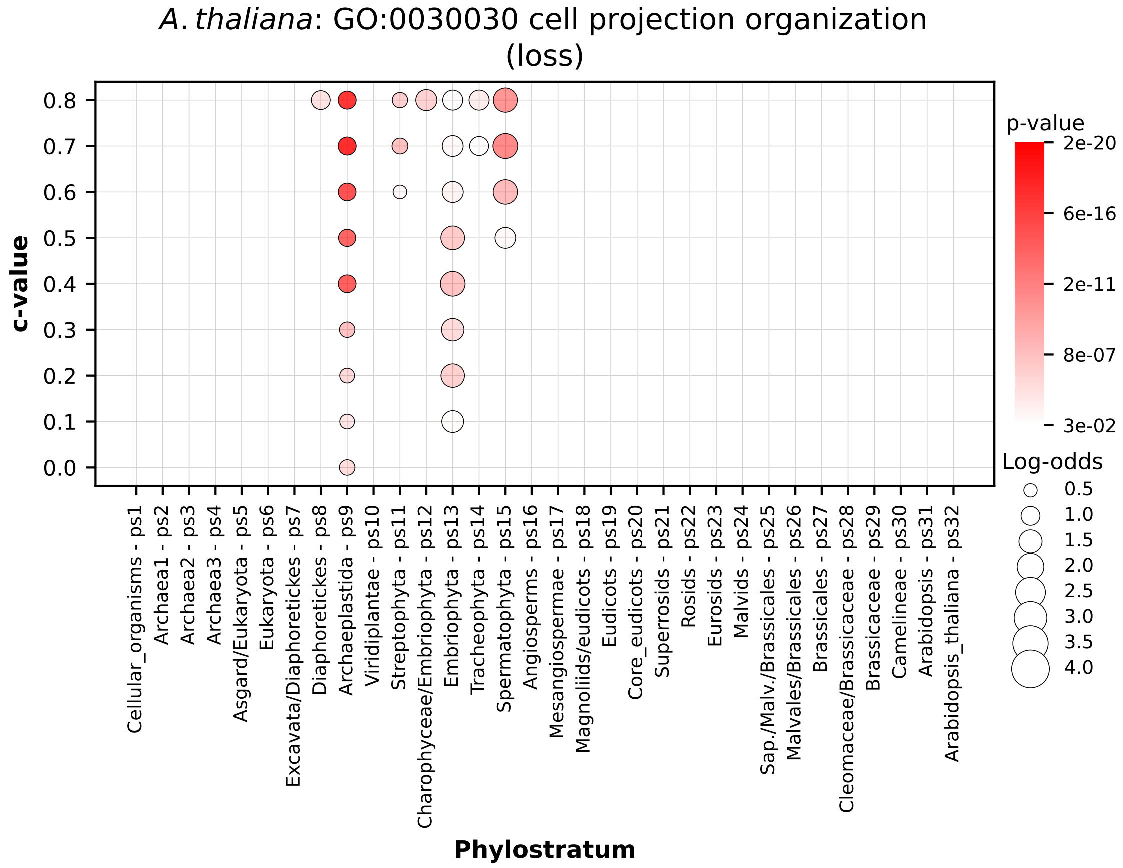

A. thaliana: GO:0030030 cell projection organization
(loss)
c-value
0.8
0.7
0.6
0.5
0.4
0.3
0.2
0.1
0.0
p-value
2e-20
6e-16
2e-11
8e-07
3e-02
Log-odds
0.5
1.0
1.5
2.0
2.5
3.0
3.5
4.0
Cellular_organisms - ps1
Archaea1 - ps2
Archaea2 - ps3
Archaea3 - ps4
Asgard/Eukaryota - ps5
Eukaryota - ps6
Excavata/Diaphoretickes - ps7
Diaphoretickes - ps8
Archaeplastida - ps9
Viridiplantae - ps10
Streptophyta - ps11
Charophyceae/Embriophyta - ps12
Embriophyta - ps13
Tracheophyta - ps14
Spermatophyta - ps15
Angiosperms - ps16
Mesangiospermae - ps17
Magnoliids/eudicots - ps18
Eudicots - ps19
Core_eudicots - ps20
Superrosids - ps21
Rosids - ps22
Eurosids - ps23
Malvids - ps24
Sap./Malv./Brassicales - ps25
Malvales/Brassicales - ps26
Brassicales - ps27
Cleomaceae/Brassicaceae - ps28
Brassicaceae - ps29
Camelineae - ps30
Arabidopsis - ps31
Arabidopsis_thaliana - ps32
Phylostratum

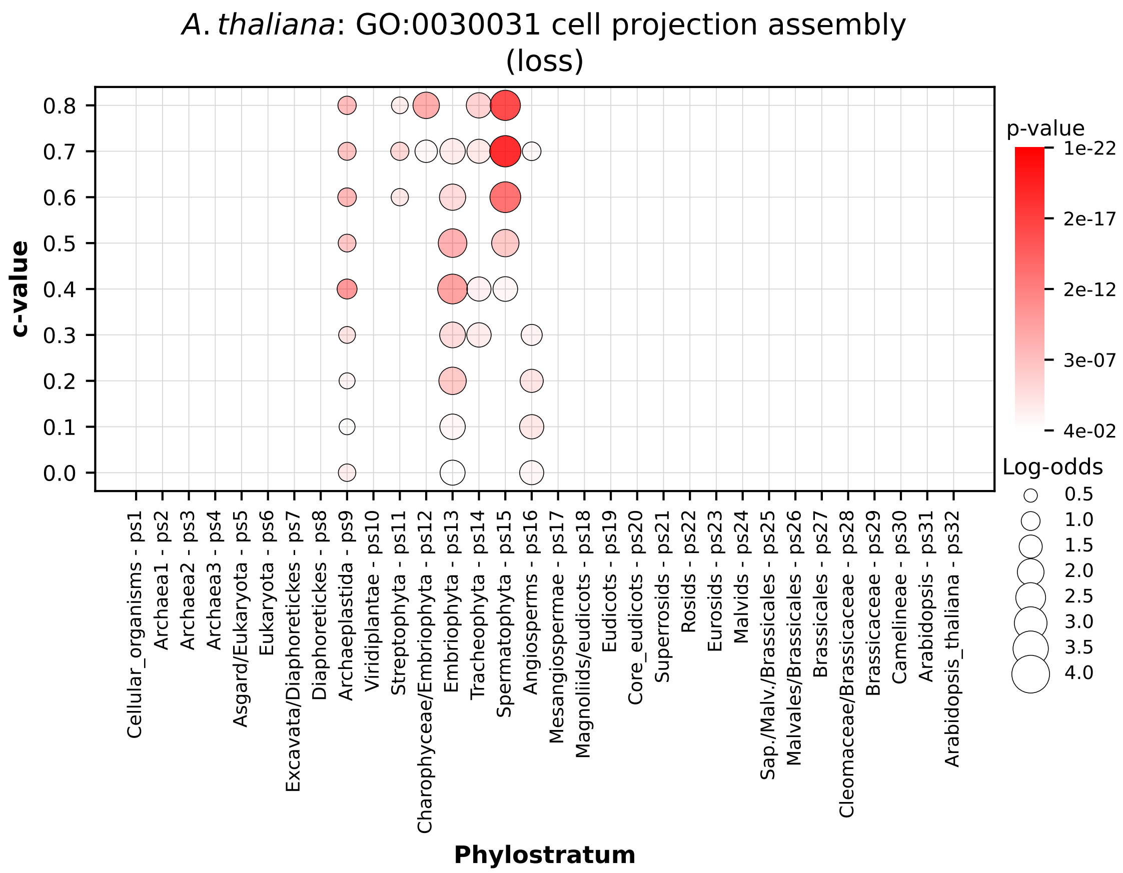

A. thaliana: GO:0030031 cell projection assembly
(loss)
c-value
0.8
0.7
0.6
0.5
0.4
0.3
0.2
0.1
0.0
Phylostratum
Cellular_organisms - ps1
Archaea1 - ps2
Archaea2 - ps3
Archaea3 - ps4
Asgard/Eukaryota - ps5
Eukaryota - ps6
Excavata/Diaphoretickes - ps7
Diaphoretickes - ps8
Archaeplastida - ps9
Viridiplantae - ps10
Streptophyta - ps11
Charophyceae/Embriophyta - ps12
Embriophyta - ps13
Tracheophyta - ps14
Spermatophyta - ps15
Angiosperms - ps16
Mesangiospermae - ps17
Magnoliids/eudicots - ps18
Eudicots - ps19
Core_eudicots - ps20
Superrosids - ps21
Rosids - ps22
Eurosids - ps23
Malvids - ps24
Sap./Malv./Brassicales - ps25
Malvales/Brassicales - ps26
Brassicales - ps27
Cleomaceae/Brassicaceae - ps28
Brassicaceae - ps29
Camelineae - ps30
Arabidopsis - ps31
Arabidopsis_thaliana - ps32
p-value
1e-22
2e-17
2e-12
3e-07
4e-02
Log-odds
0.5
1.0
1.5
2.0
2.5
3.0
3.5
4.0

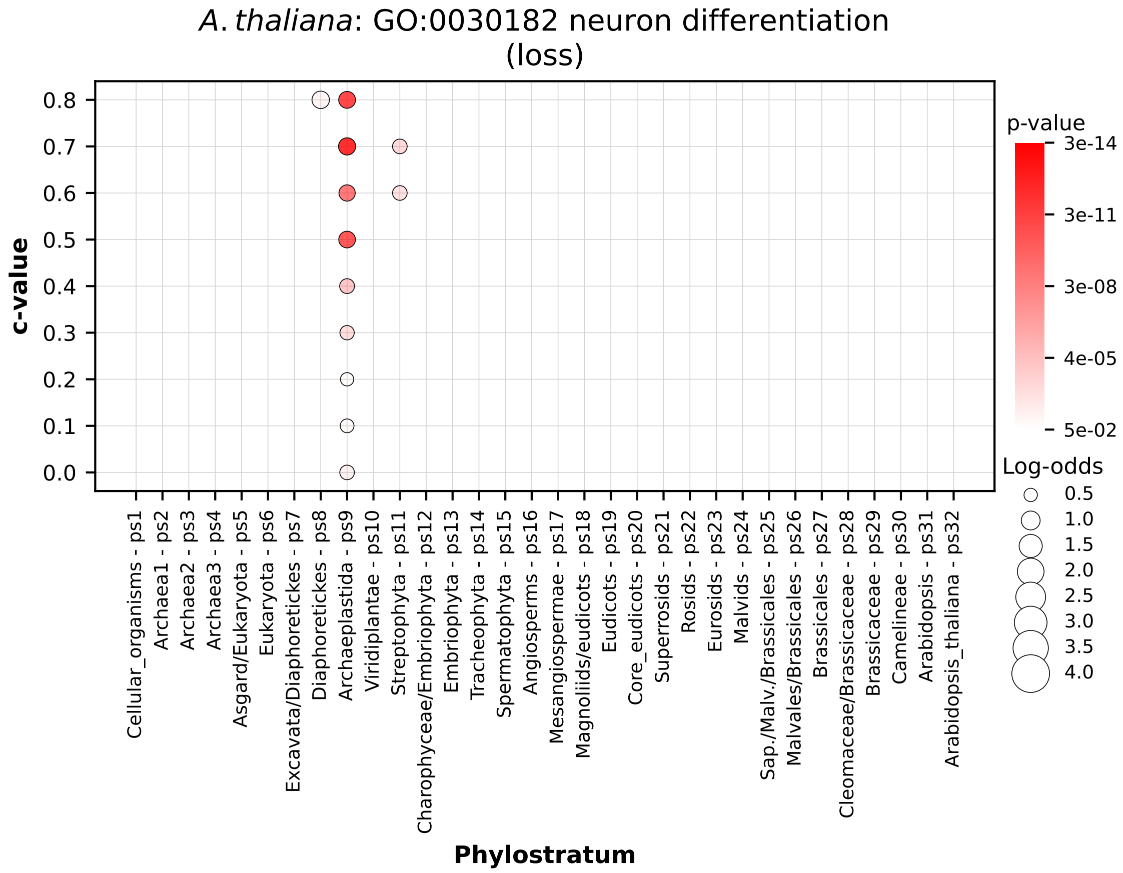

A. thaliana: GO:0030182 neuron differentiation
(loss)
c-value
0.8
0.7
0.6
0.5
0.4
0.3
0.2
0.1
0.0
Phylostratum
Cellular_organisms - ps1
Archaea1 - ps2
Archaea2 - ps3
Archaea3 - ps4
Asgard/Eukaryota - ps5
Eukaryota - ps6
Excavata/Diaphoretickes - ps7
Diaphoretickes - ps8
Archaeplastida - ps9
Viridiplantae - ps10
Streptophyta - ps11
Charophyceae/Embriophyta - ps12
Embriophyta - ps13
Tracheophyta - ps14
Spermatophyta - ps15
Angiosperms - ps16
Mesangiospermae - ps17
Magnoliids/eudicots - ps18
Eudicots - ps19
Core_eudicots - ps20
Superrosids - ps21
Rosids - ps22
Eurosids - ps23
Malvids - ps24
Sap./Malv./Brassicales - ps25
Malvales/Brassicales - ps26
Brassicales - ps27
Cleomaceae/Brassicaceae - ps28
Brassicaceae - ps29
Camelineae - ps30
Arabidopsis - ps31
Arabidopsis_thaliana - ps32
p-value
3e-14
3e-11
3e-08
4e-05
5e-02
Log-odds
0.5
1.0
1.5
2.0
2.5
3.0
3.5
4.0

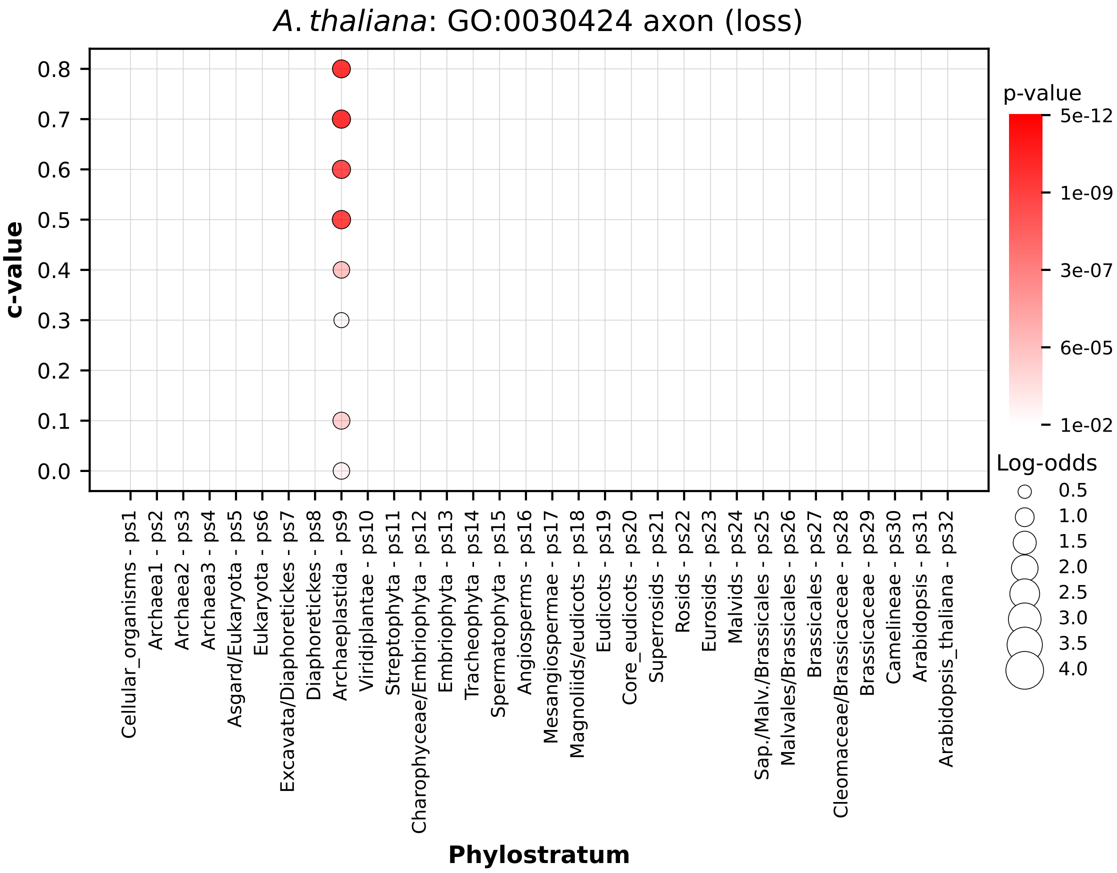

A. thaliana: GO:0030424 axon (loss)
c-value
0.8
0.7
0.6
0.5
0.4
0.3
0.2
0.1
0.0
Phylostratum
Cellular_organisms - ps1
Archaea1 - ps2
Archaea2 - ps3
Archaea3 - ps4
Asgard/Eukaryota - ps5
Eukaryota - ps6
Excavata/Diaphoretickes - ps7
Diaphoretickes - ps8
Archaeplastida - ps9
Viridiplantae - ps10
Streptophyta - ps11
Charophyceae/Embriophyta - ps12
Embriophyta - ps13
Tracheophyta - ps14
Spermatophyta - ps15
Angiosperms - ps16
Mesangiospermae - ps17
Magnoliids/eudicots - ps18
Eudicots - ps19
Core_eudicots - ps20
Superrosids - ps21
Rosids - ps22
Eurosids - ps23
Malvids - ps24
Sap./Malv./Brassicales - ps25
Malvales/Brassicales - ps26
Brassicales - ps27
Cleomaceae/Brassicaceae - ps28
Brassicaceae - ps29
Camelineae - ps30
Arabidopsis - ps31
Arabidopsis_thaliana - ps32
p-value
5e-12
1e-09
3e-07
6e-05
1e-02
Log-odds
0.5
1.0
1.5
2.0
2.5
3.0
3.5
4.0

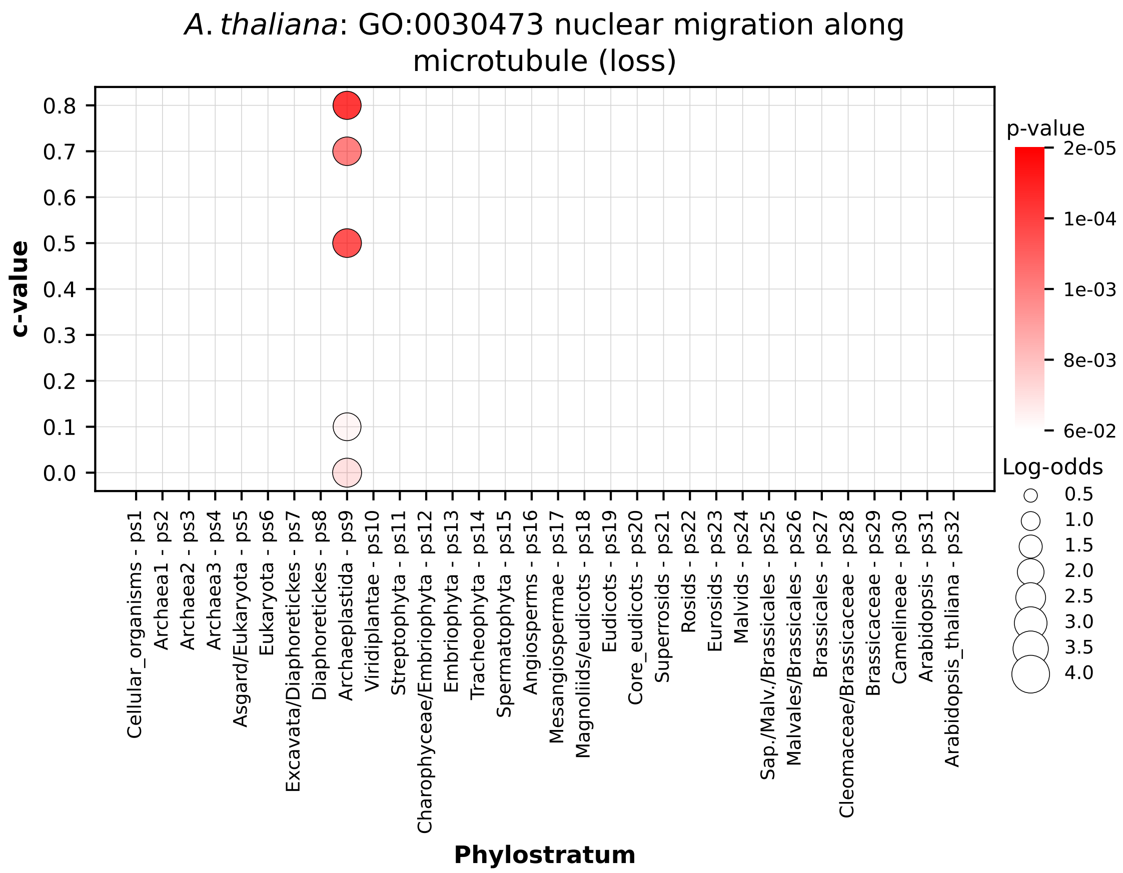

A. thaliana: GO:0030473 nuclear migration along microtubule (loss)
c-value
0.8
0.7
0.6
0.5
0.4
0.3
0.2
0.1
0.0
p-value
2e-05
1e-04
1e-03
8e-03
6e-02
Log-odds
0.5
1.0
1.5
2.0
2.5
3.0
3.5
4.0
Cellular_organisms - ps1
Archaea1 - ps2
Archaea2 - ps3
Archaea3 - ps4
Asgard/Eukaryota - ps5
Eukaryota - ps6
Excavata/Diaphoretickes - ps7
Diaphoretickes - ps8
Archaeplastida - ps9
Viridiplantae - ps10
Streptophyta - ps11
Charophyceae/Embriophyta - ps12
Embriophyta - ps13
Tracheophyta - ps14
Spermatophyta - ps15
Angiosperms - ps16
Mesangiospermae - ps17
Magnoliids/eudicots - ps18
Eudicots - ps19
Core_eudicots - ps20
Superrosids - ps21
Rosids - ps22
Eurosids - ps23
Malvids - ps24
Sap./Malv./Brassicales - ps25
Malvales/Brassicales - ps26
Brassicales - ps27
Cleomaceae/Brassicaceae - ps28
Brassicaceae - ps29
Camelineae - ps30
Arabidopsis - ps31
Arabidopsis_thaliana - ps32
Phylostratum

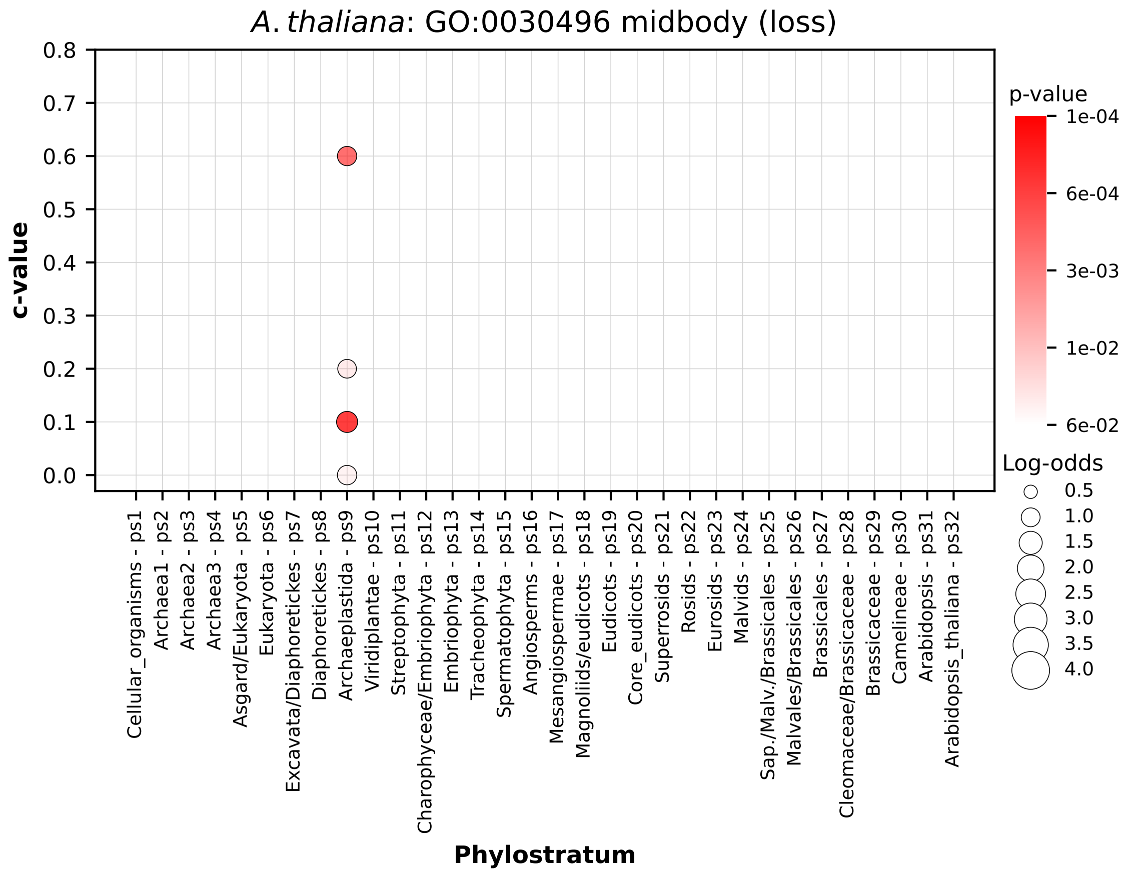

A. thaliana: GO:0030496 midbody (loss)
c-value
0.8
0.7
0.6
0.5
0.4
0.3
0.2
0.1
0.0
Phylostratum
Cellular_organisms - ps1
Archaea1 - ps2
Archaea2 - ps3
Archaea3 - ps4
Asgard/Eukaryota - ps5
Eukaryota - ps6
Excavata/Diaphoretickes - ps7
Diaphoretickes - ps8
Archaeplastida - ps9
Viridiplantae - ps10
Streptophyta - ps11
Charophyceae/Embriophyta - ps12
Embriophyta - ps13
Tracheophyta - ps14
Spermatophyta - ps15
Angiosperms - ps16
Mesangiospermae - ps17
Magnoliids/eudicots - ps18
Eudicots - ps19
Core_eudicots - ps20
Superrosids - ps21
Rosids - ps22
Eurosids - ps23
Malvids - ps24
Sap./Malv./Brassicales - ps25
Malvales/Brassicales - ps26
Brassicales - ps27
Cleomaceae/Brassicaceae - ps28
Brassicaceae - ps29
Camelineae - ps30
Arabidopsis - ps31
Arabidopsis_thaliana - ps32
p-value
1e-04
6e-04
3e-03
1e-02
6e-02
Log-odds
0.5
1.0
1.5
2.0
2.5
3.0
3.5
4.0

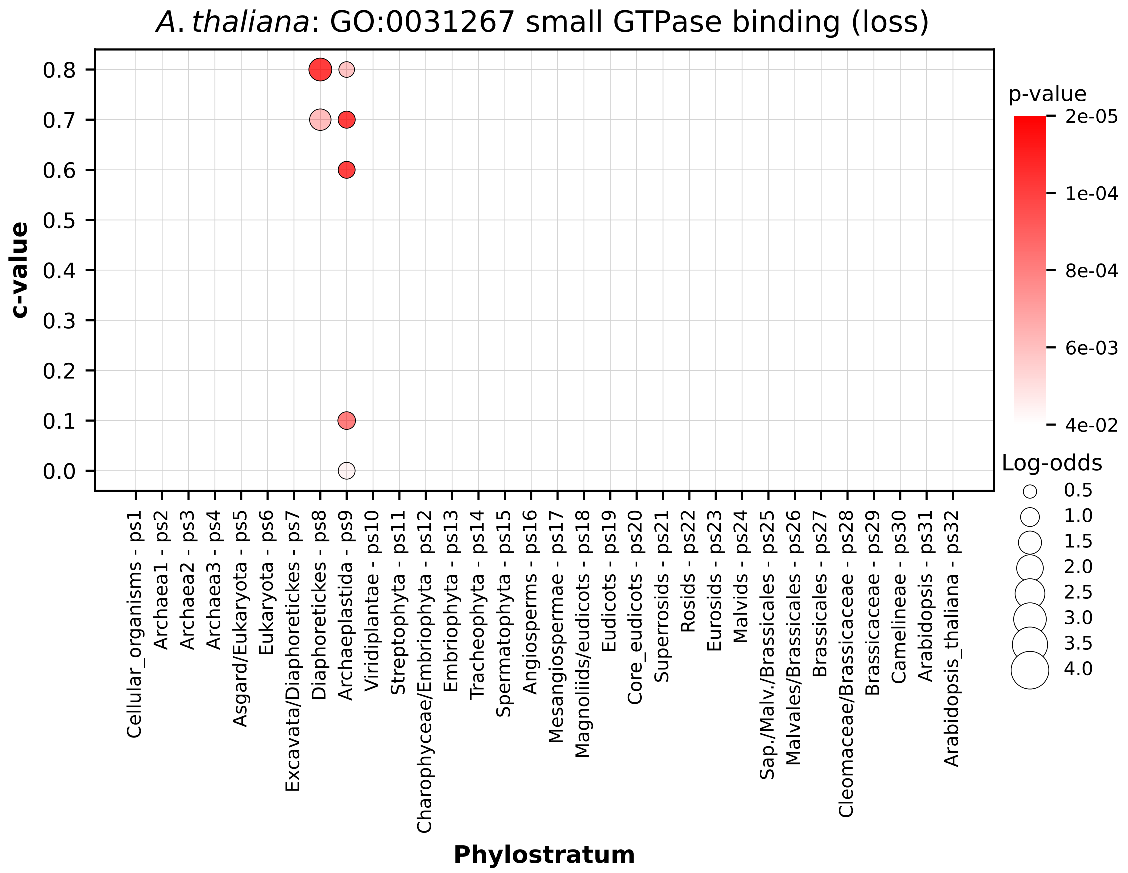

A. thaliana: GO:0031267 small GTPase binding (loss)
c-value
0.8
0.7
0.6
0.5
0.4
0.3
0.2
0.1
0.0
p-value
2e-05
1e-04
8e-04
6e-03
4e-02
Log-odds
0.5
1.0
1.5
2.0
2.5
3.0
3.5
4.0
Cellular_organisms - ps1
Archaea1 - ps2
Archaea2 - ps3
Archaea3 - ps4
Asgard/Eukaryota - ps5
Eukaryota - ps6
Excavata/Diaphoretickes - ps7
Diaphoretickes - ps8
Archaeplastida - ps9
Viridiplantae - ps10
Streptophyta - ps11
Charophyceae/Embriophyta - ps12
Embriophyta - ps13
Tracheophyta - ps14
Spermatophyta - ps15
Angiosperms - ps16
Mesangiospermae - ps17
Magnoliids/eudicots - ps18
Eudicots - ps19
Core_eudicots - ps20
Superrosids - ps21
Rosids - ps22
Eurosids - ps23
Malvids - ps24
Sap./Malv./Brassicales - ps25
Malvales/Brassicales - ps26
Brassicales - ps27
Cleomaceae/Brassicaceae - ps28
Brassicaceae - ps29
Camelineae - ps30
Arabidopsis - ps31
Arabidopsis_thaliana - ps32
Phylostratum

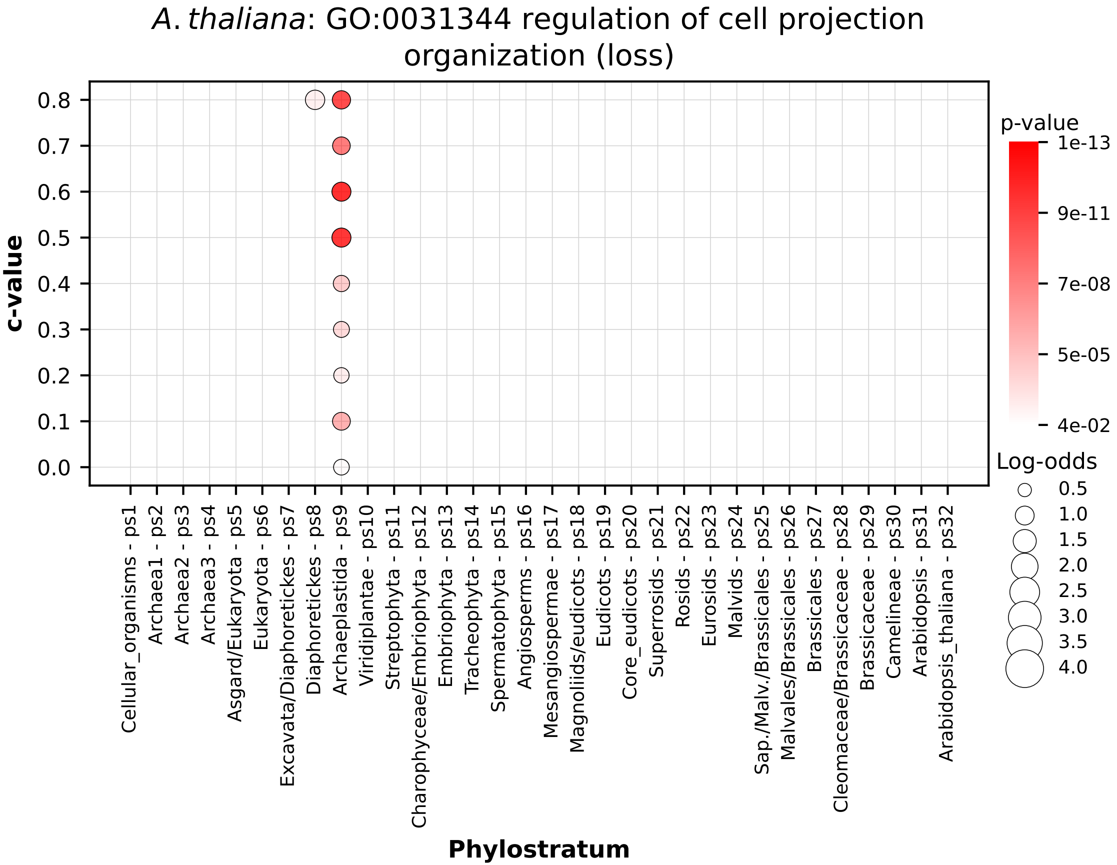

A. thaliana: GO:0031344 regulation of cell projection organization (loss)
c-value
0.8
0.7
0.6
0.5
0.4
0.3
0.2
0.1
0.0
p-value
1e-13
9e-11
7e-08
5e-05
4e-02
Log-odds
0.5
1.0
1.5
2.0
2.5
3.0
3.5
4.0
Cellular_organisms - ps1
Archaea1 - ps2
Archaea2 - ps3
Archaea3 - ps4
Asgard/Eukaryota - ps5
Eukaryota - ps6
Excavata/Diaphoretickes - ps7
Diaphoretickes - ps8
Archaeplastida - ps9
Viridiplantae - ps10
Streptophyta - ps11
Charophyceae/Embriophyta - ps12
Embriophyta - ps13
Tracheophyta - ps14
Spermatophyta - ps15
Angiosperms - ps16
Mesangiospermae - ps17
Magnoliids/eudicots - ps18
Eudicots - ps19
Core_eudicots - ps20
Superrosids - ps21
Rosids - ps22
Eurosids - ps23
Malvids - ps24
Sap./Malv./Brassicales - ps25
Malvales/Brassicales - ps26
Brassicales - ps27
Cleomaceae/Brassicaceae - ps28
Brassicaceae - ps29
Camelineae - ps30
Arabidopsis - ps31
Arabidopsis_thaliana - ps32
Phylostratum

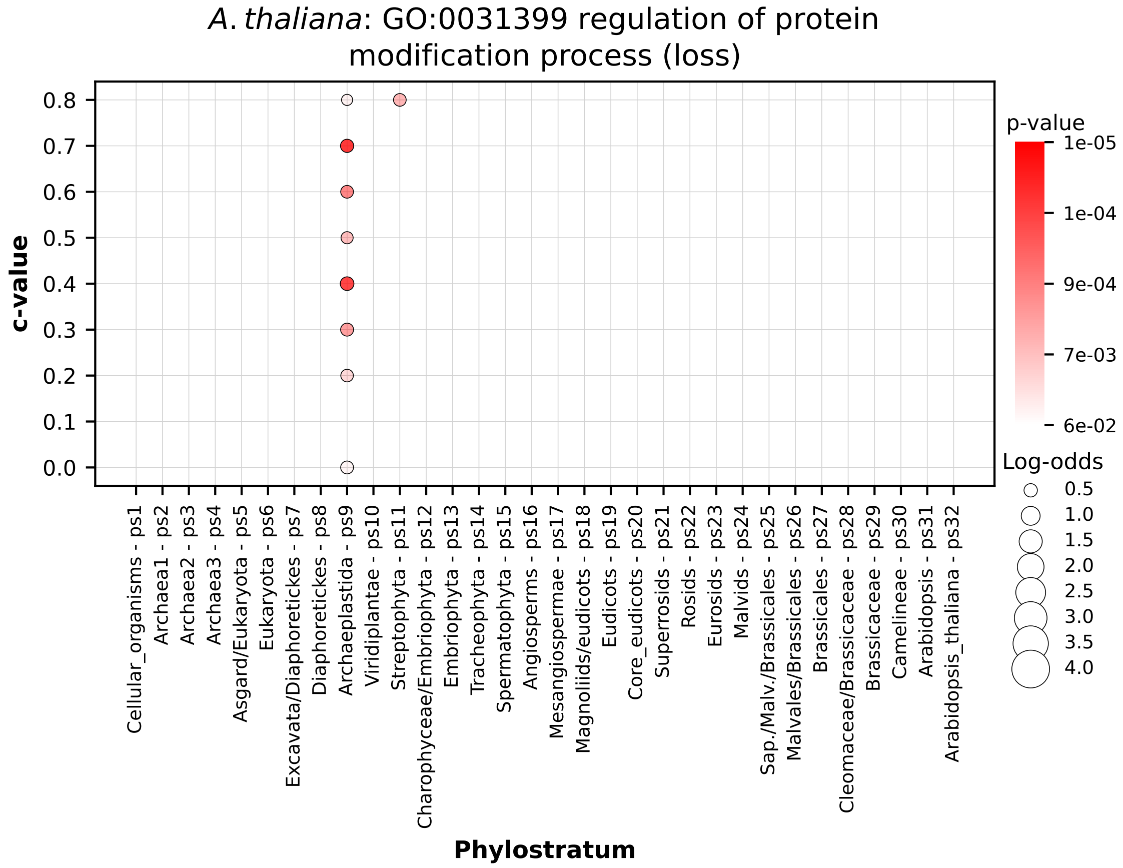
A. thaliana: GO:0031399 regulation of protein modification process (loss)
c-value
0.8
0.7
0.6
0.5
0.4
0.3
0.2
0.1
0.0
p-value
1e-05
1e-04
9e-04
7e-03
6e-02
Log-odds
0.5
1.0
1.5
2.0
2.5
3.0
3.5
4.0
Cellular_organisms - ps1
Archaea1 - ps2
Archaea2 - ps3
Archaea3 - ps4
Asgard/Eukaryota - ps5
Eukaryota - ps6
Excavata/Diaphoretickes - ps7
Diaphoretickes - ps8
Archaeplastida - ps9
Viridiplantae - ps10
Streptophyta - ps11
Charophyceae/Embriophyta - ps12
Embriophyta - ps13
Tracheophyta - ps14
Spermatophyta - ps15
Angiosperms - ps16
Mesangiospermae - ps17
Magnoliids/eudicots - ps18
Eudicots - ps19
Core_eudicots - ps20
Superrosids - ps21
Rosids - ps22
Eurosids - ps23
Malvids - ps24
Sap./Malv./Brassicales - ps25
Malvales/Brassicales - ps26
Brassicales - ps27
Cleomaceae/Brassicaceae - ps28
Brassicaceae - ps29
Camelineae - ps30
Arabidopsis - ps31
Arabidopsis_thaliana - ps32
Phylostratum

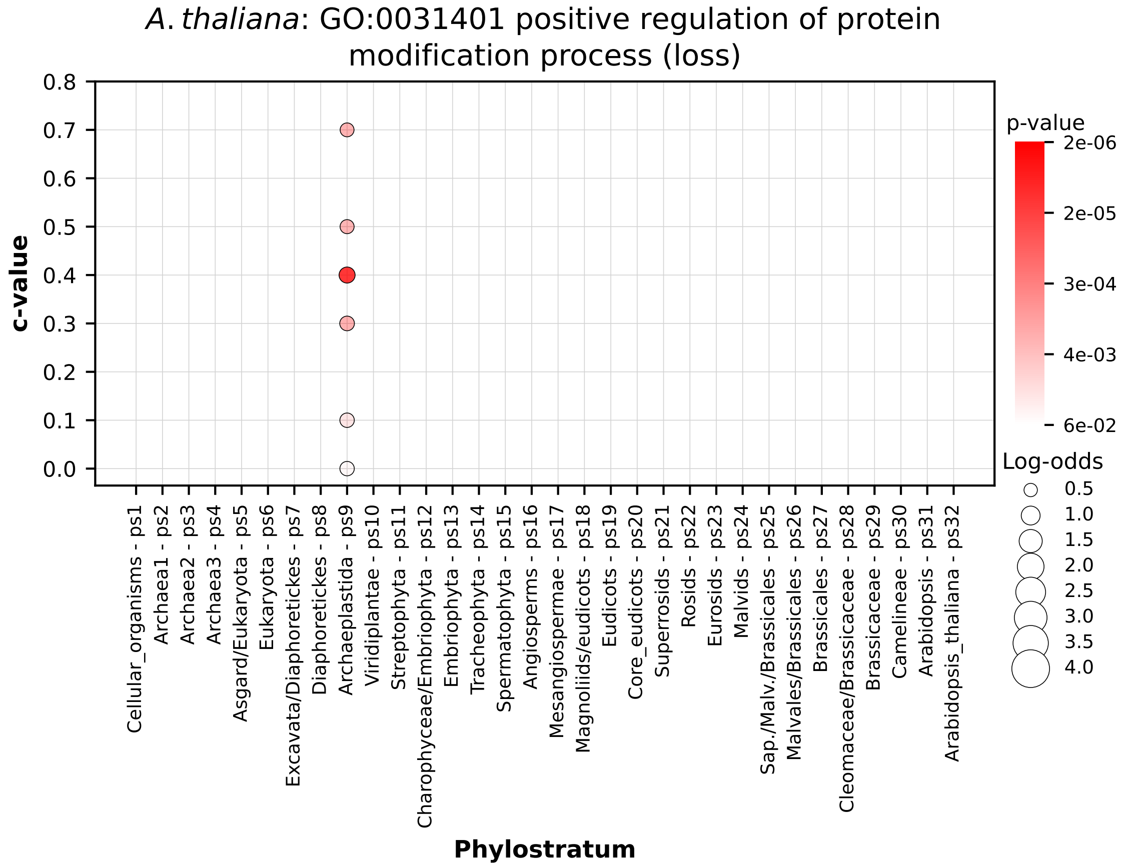

$A. thaliana$: GO:0031401 positive regulation of protein modification process (loss)
c-value
0.8
0.7
0.6
0.5
0.4
0.3
0.2
0.1
0.0
Phylostratum
Cellular_organisms - ps1
Archaea1 - ps2
Archaea2 - ps3
Archaea3 - ps4
Asgard/Eukaryota - ps5
Eukaryota - ps6
Excavata/Diaphoretickes - ps7
Diaphoretickes - ps8
Archaeplastida - ps9
Viridiplantae - ps10
Streptophyta - ps11
Charophyceae/Embriophyta - ps12
Embriophyta - ps13
Tracheophyta - ps14
Spermatophyta - ps15
Angiosperms - ps16
Mesangiospermae - ps17
Magnoliids/eudicots - ps18
Eudicots - ps19
Core_eudicots - ps20
Superrosids - ps21
Rosids - ps22
Eurosids - ps23
Malvids - ps24
Sap./Malv./Brassicales - ps25
Malvales/Brassicales - ps26
Brassicales - ps27
Cleomaceae/Brassicaceae - ps28
Brassicaceae - ps29
Camelineae - ps30
Arabidopsis - ps31
Arabidopsis_thaliana - ps32
p-value
2e-06
2e-05
3e-04
4e-03
6e-02
Log-odds
0.5
1.0
1.5
2.0
2.5
3.0
3.5
4.0

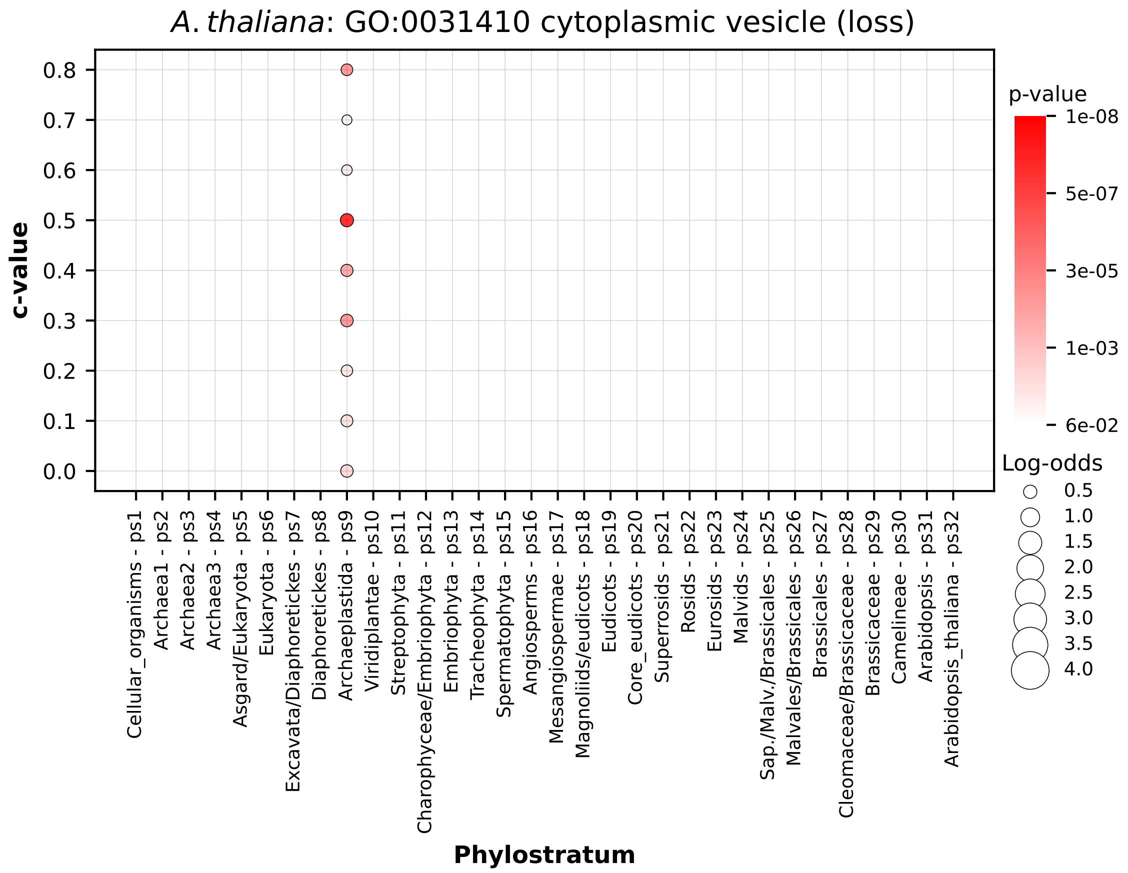

A. thaliana: GO:0031410 cytoplasmic vesicle (loss)
c-value
0.8
0.7
0.6
0.5
0.4
0.3
0.2
0.1
0.0
Phylostratum
Cellular_organisms - ps1
Archaea1 - ps2
Archaea2 - ps3
Archaea3 - ps4
Asgard/Eukaryota - ps5
Eukaryota - ps6
Excavata/Diaphoretickes - ps7
Diaphoretickes - ps8
Archaeplastida - ps9
Viridiplantae - ps10
Streptophyta - ps11
Charophyceae/Embriophyta - ps12
Embriophyta - ps13
Tracheophyta - ps14
Spermatophyta - ps15
Angiosperms - ps16
Mesangiospermae - ps17
Magnoliids/eudicots - ps18
Eudicots - ps19
Core_eudicots - ps20
Superrosids - ps21
Rosids - ps22
Eurosids - ps23
Malvids - ps24
Sap./Malv./Brassicales - ps25
Malvales/Brassicales - ps26
Brassicales - ps27
Cleomaceae/Brassicaceae - ps28
Brassicaceae - ps29
Camelineae - ps30
Arabidopsis - ps31
Arabidopsis_thaliana - ps32
p-value
1e-08
5e-07
3e-05
1e-03
6e-02
Log-odds
0.5
1.0
1.5
2.0
2.5
3.0
3.5
4.0

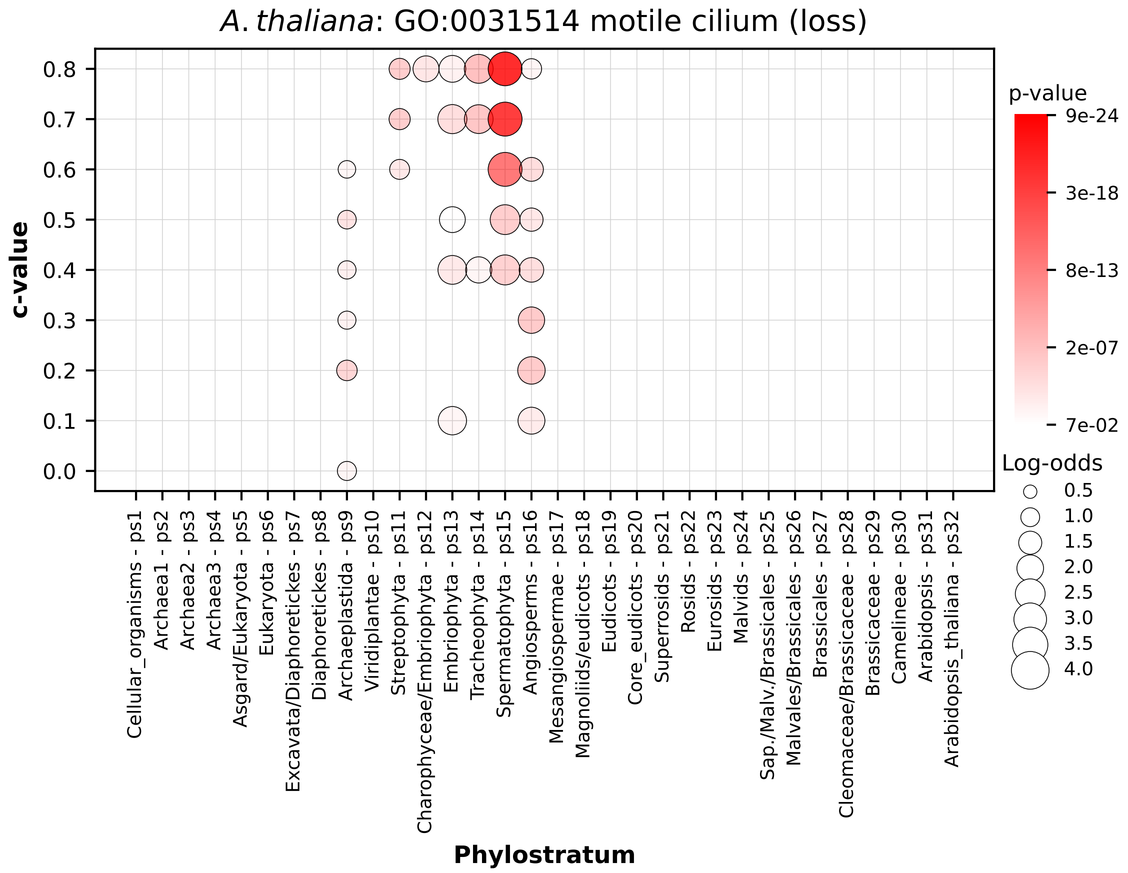

A. thaliana: GO:0031514 motile cilium (loss)
c-value
0.8
0.7
0.6
0.5
0.4
0.3
0.2
0.1
0.0
Phylostratum
Cellular_organisms - ps1
Archaea1 - ps2
Archaea2 - ps3
Archaea3 - ps4
Asgard/Eukaryota - ps5
Eukaryota - ps6
Excavata/Diaphoretickes - ps7
Diaphoretickes - ps8
Archaeplastida - ps9
Viridiplantae - ps10
Streptophyta - ps11
Charophyceae/Embriophyta - ps12
Embriophyta - ps13
Tracheophyta - ps14
Spermatophyta - ps15
Angiosperms - ps16
Mesangiospermae - ps17
Magnoliids/eudicots - ps18
Eudicots - ps19
Core_eudicots - ps20
Superrosids - ps21
Rosids - ps22
Eurosids - ps23
Malvids - ps24
Sap./Malv./Brassicales - ps25
Malvales/Brassicales - ps26
Brassicales - ps27
Cleomaceae/Brassicaceae - ps28
Brassicaceae - ps29
Camelineae - ps30
Arabidopsis - ps31
Arabidopsis_thaliana - ps32
p-value
9e-24
3e-18
8e-13
2e-07
7e-02
Log-odds
0.5
1.0
1.5
2.0
2.5
3.0
3.5
4.0

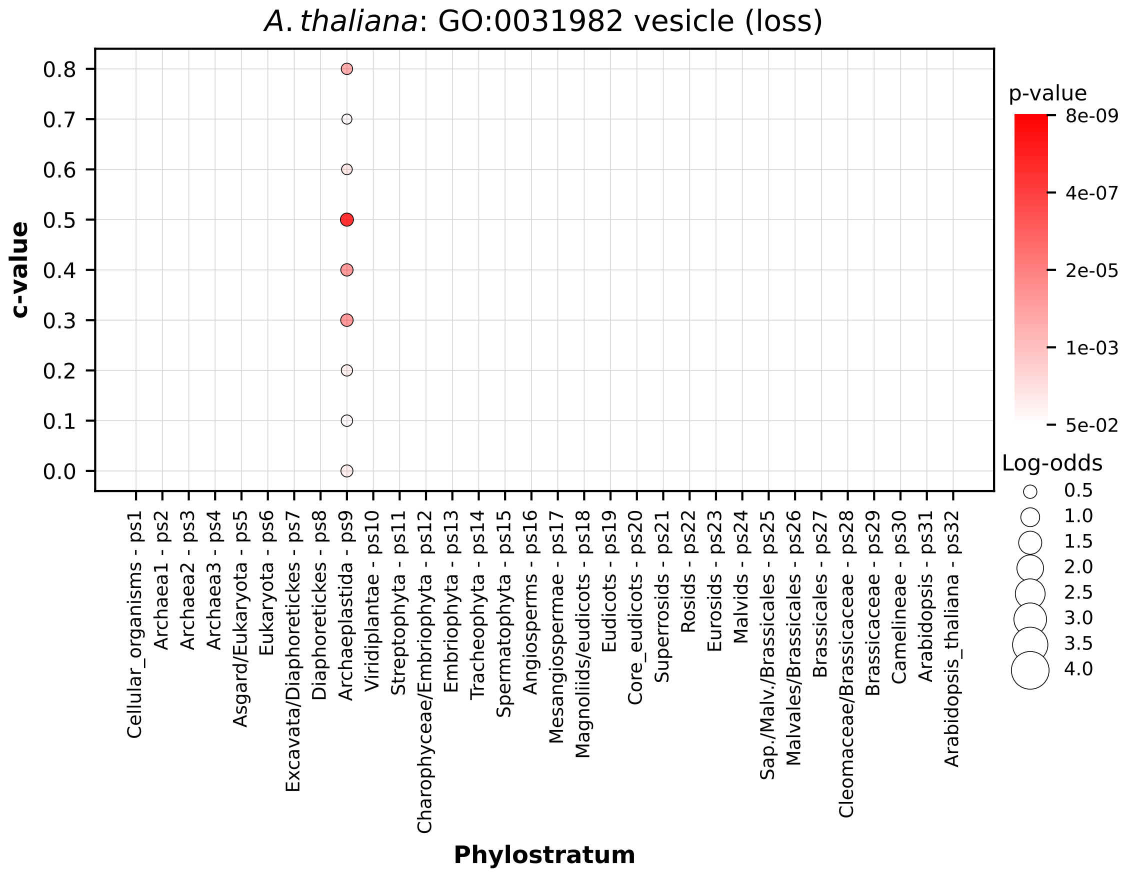
A. thaliana: GO:0031982 vesicle (loss)
c-value
0.0
0.1
0.2
0.3
0.4
0.5
0.6
0.7
0.8
Phylostratum
Cellular_organisms - ps1
Archaea1 - ps2
Archaea2 - ps3
Archaea3 - ps4
Asgard/Eukaryota - ps5
Eukaryota - ps6
Excavata/Diaphoretickes - ps7
Diaphoretickes - ps8
Archaeplastida - ps9
Viridiplantae - ps10
Streptophyta - ps11
Charophyceae/Embriophyta - ps12
Embriophyta - ps13
Tracheophyta - ps14
Spermatophyta - ps15
Angiosperms - ps16
Mesangiospermae - ps17
Magnoliids/eudicots - ps18
Eudicots - ps19
Core_eudicots - ps20
Superrosids - ps21
Rosids - ps22
Eurosids - ps23
Malvids - ps24
Sap./Malv./Brassicales - ps25
Malvales/Brassicales - ps26
Brassicales - ps27
Cleomaceae/Brassicaceae - ps28
Brassicaceae - ps29
Camelineae - ps30
Arabidopsis - ps31
Arabidopsis_thaliana - ps32
p-value
8e-09
4e-07
2e-05
1e-03
5e-02
Log-odds
0.5
1.0
1.5
2.0
2.5
3.0
3.5
4.0

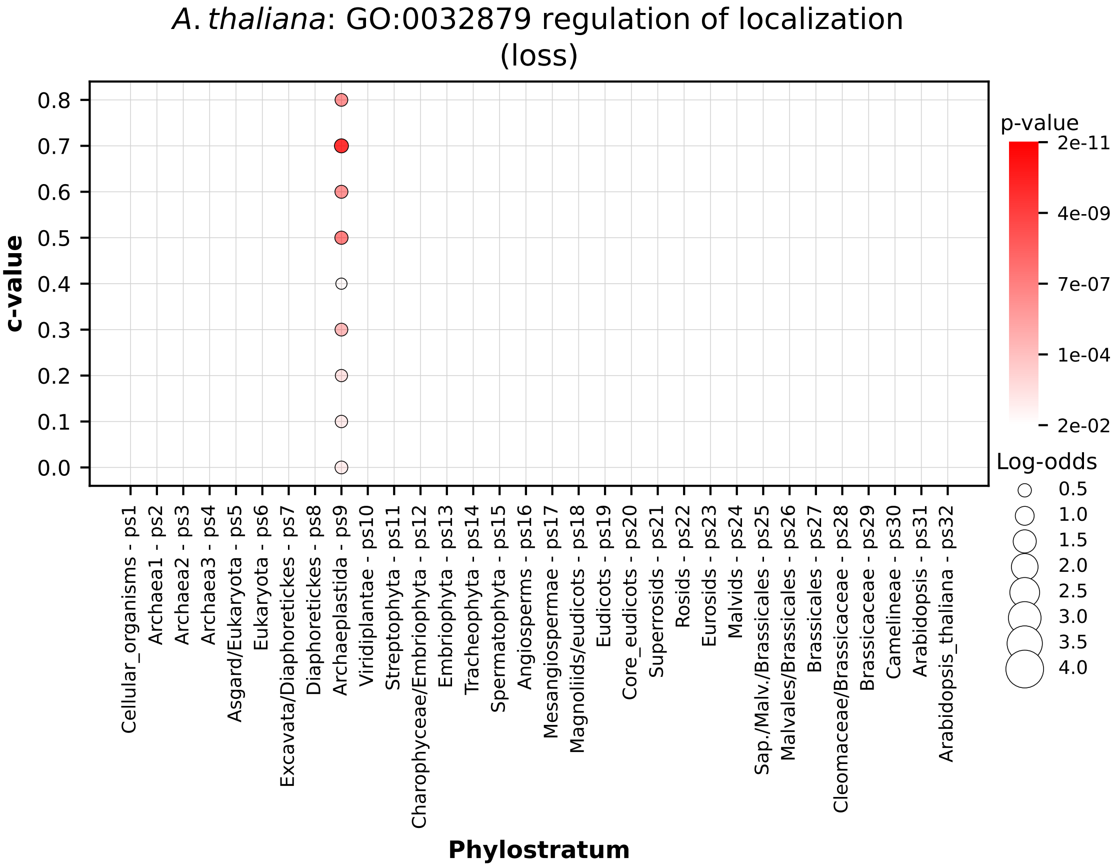

A. thaliana: GO:0032879 regulation of localization
(loss)
c-value
0.0
0.1
0.2
0.3
0.4
0.5
0.6
0.7
0.8
Phylostratum
Cellular_organisms - ps1
Archaea1 - ps2
Archaea2 - ps3
Archaea3 - ps4
Asgard/Eukaryota - ps5
Eukaryota - ps6
Excavata/Diaphoretickes - ps7
Diaphoretickes - ps8
Archaeplastida - ps9
Viridiplantae - ps10
Streptophyta - ps11
Charophyceae/Embriophyta - ps12
Embriophyta - ps13
Tracheophyta - ps14
Spermatophyta - ps15
Angiosperms - ps16
Mesangiospermae - ps17
Magnoliids/eudicots - ps18
Eudicots - ps19
Core_eudicots - ps20
Superrosids - ps21
Rosids - ps22
Eurosids - ps23
Malvids - ps24
Sap./Malv./Brassicales - ps25
Malvales/Brassicales - ps26
Brassicales - ps27
Cleomaceae/Brassicaceae - ps28
Brassicaceae - ps29
Camelineae - ps30
Arabidopsis - ps31
Arabidopsis_thaliana - ps32
p-value
2e-11
4e-09
7e-07
1e-04
2e-02
Log-odds
0.5
1.0
1.5
2.0
2.5
3.0
3.5
4.0

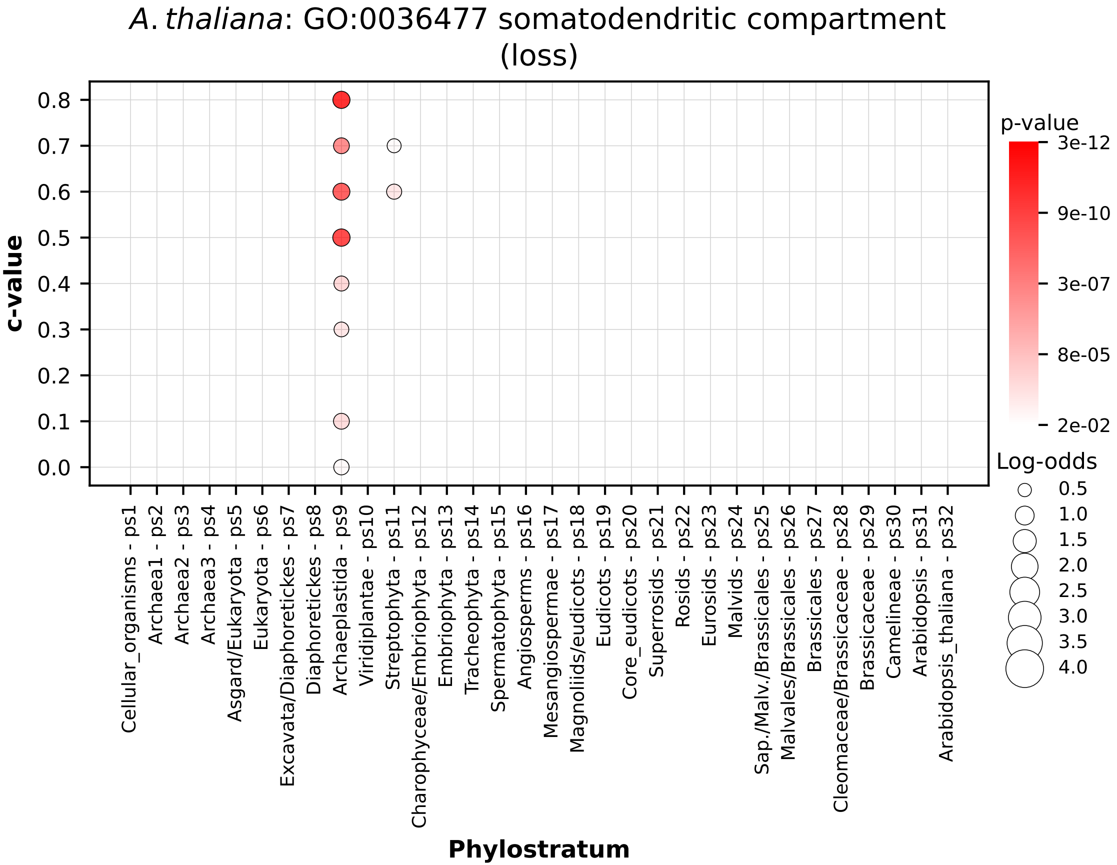

A. thaliana: GO:0036477 somatodendritic compartment (loss)
c-value
0.8
0.7
0.6
0.5
0.4
0.3
0.2
0.1
0.0
p-value
3e-12
9e-10
3e-07
8e-05
2e-02
Log-odds
0.5
1.0
1.5
2.0
2.5
3.0
3.5
4.0
Cellular_organisms - ps1
Archaea1 - ps2
Archaea2 - ps3
Archaea3 - ps4
Asgard/Eukaryota - ps5
Eukaryota - ps6
Excavata/Diaphoretickes - ps7
Diaphoretickes - ps8
Archaeplastida - ps9
Viridiplantae - ps10
Streptophyta - ps11
Charophyceae/Embriophyta - ps12
Embriophyta - ps13
Tracheophyta - ps14
Spermatophyta - ps15
Angiosperms - ps16
Mesangiospermae - ps17
Magnoliids/eudicots - ps18
Eudicots - ps19
Core_eudicots - ps20
Superrosids - ps21
Rosids - ps22
Eurosids - ps23
Malvids - ps24
Sap./Malv./Brassicales - ps25
Malvales/Brassicales - ps26
Brassicales - ps27
Cleomaceae/Brassicaceae - ps28
Brassicaceae - ps29
Camelineae - ps30
Arabidopsis - ps31
Arabidopsis_thaliana - ps32
Phylostratum

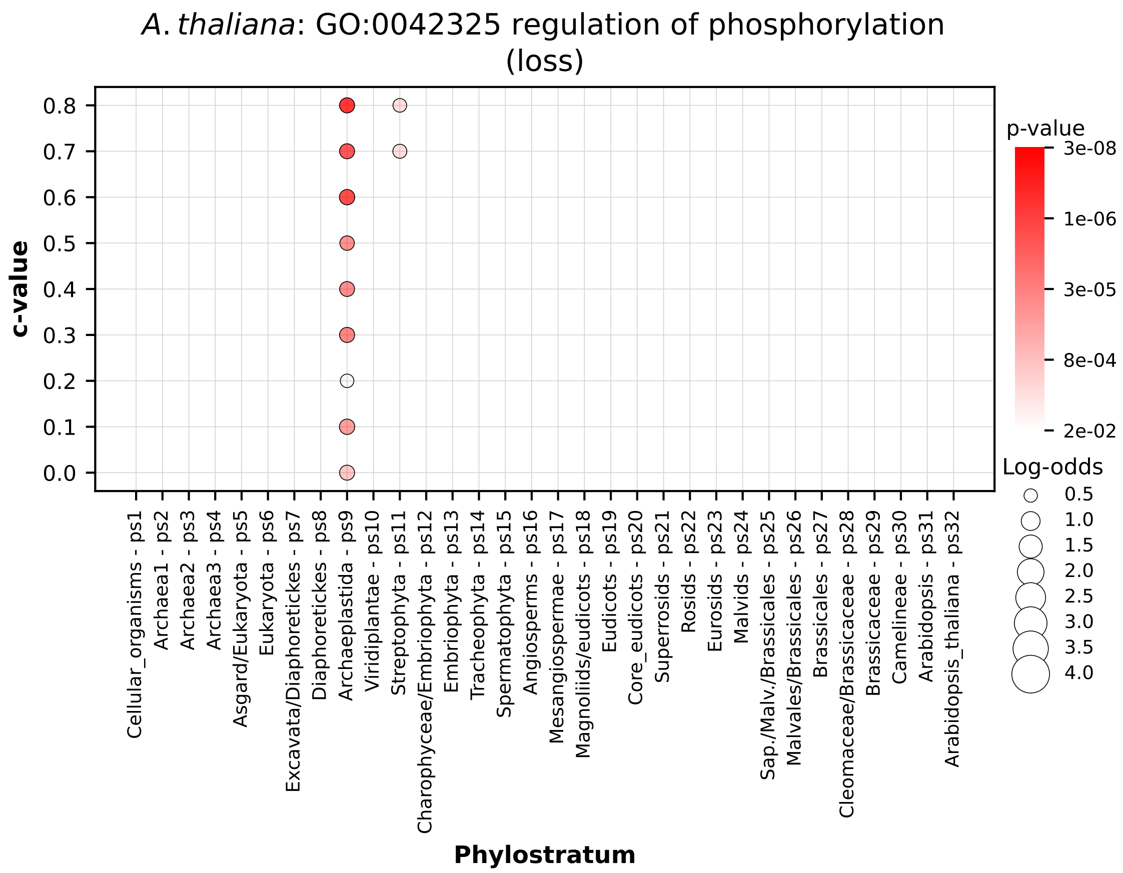

A. thaliana: GO:0042325 regulation of phosphorylation (loss)
c-value
0.8
0.7
0.6
0.5
0.4
0.3
0.2
0.1
0.0
p-value
3e-08
1e-06
3e-05
8e-04
2e-02
Log-odds
0.5
1.0
1.5
2.0
2.5
3.0
3.5
4.0
Cellular_organisms - ps1
Archaea1 - ps2
Archaea2 - ps3
Archaea3 - ps4
Asgard/Eukaryota - ps5
Eukaryota - ps6
Excavata/Diaphoretickes - ps7
Diaphoretickes - ps8
Archaeplastida - ps9
Viridiplantae - ps10
Streptophyta - ps11
Charophyceae/Embriophyta - ps12
Embriophyta - ps13
Tracheophyta - ps14
Spermatophyta - ps15
Angiosperms - ps16
Mesangiospermae - ps17
Magnoliids/eudicots - ps18
Eudicots - ps19
Core_eudicots - ps20
Superrosids - ps21
Rosids - ps22
Eurosids - ps23
Malvids - ps24
Sap./Malv./Brassicales - ps25
Malvales/Brassicales - ps26
Brassicales - ps27
Cleomaceae/Brassicaceae - ps28
Brassicaceae - ps29
Camelineae - ps30
Arabidopsis - ps31
Arabidopsis_thaliana - ps32
Phylostratum

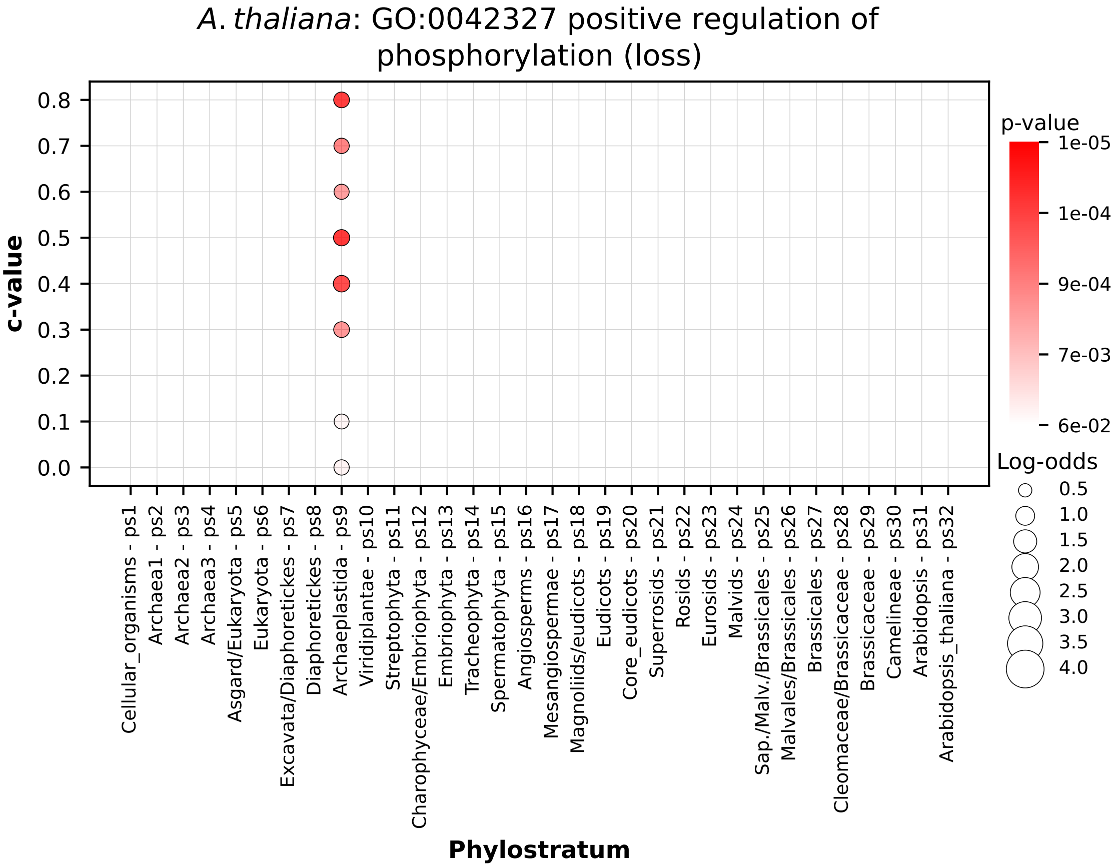

A. thaliana: GO:0042327 positive regulation of phosphorylation (loss)
c-value
0.8
0.7
0.6
0.5
0.4
0.3
0.2
0.1
0.0
p-value
1e-05
1e-04
9e-04
7e-03
6e-02
Log-odds
0.5
1.0
1.5
2.0
2.5
3.0
3.5
4.0
Cellular_organisms - ps1
Archaea1 - ps2
Archaea2 - ps3
Archaea3 - ps4
Asgard/Eukaryota - ps5
Eukaryota - ps6
Excavata/Diaphoretickes - ps7
Diaphoretickes - ps8
Archaeplastida - ps9
Viridiplantae - ps10
Streptophyta - ps11
Charophyceae/Embriophyta - ps12
Embriophyta - ps13
Tracheophyta - ps14
Spermatophyta - ps15
Angiosperms - ps16
Mesangiospermae - ps17
Magnoliids/eudicots - ps18
Eudicots - ps19
Core_eudicots - ps20
Superrosids - ps21
Rosids - ps22
Eurosids - ps23
Malvids - ps24
Sap./Malv./Brassicales - ps25
Malvales/Brassicales - ps26
Brassicales - ps27
Cleomaceae/Brassicaceae - ps28
Brassicaceae - ps29
Camelineae - ps30
Arabidopsis - ps31
Arabidopsis_thaliana - ps32
Phylostratum

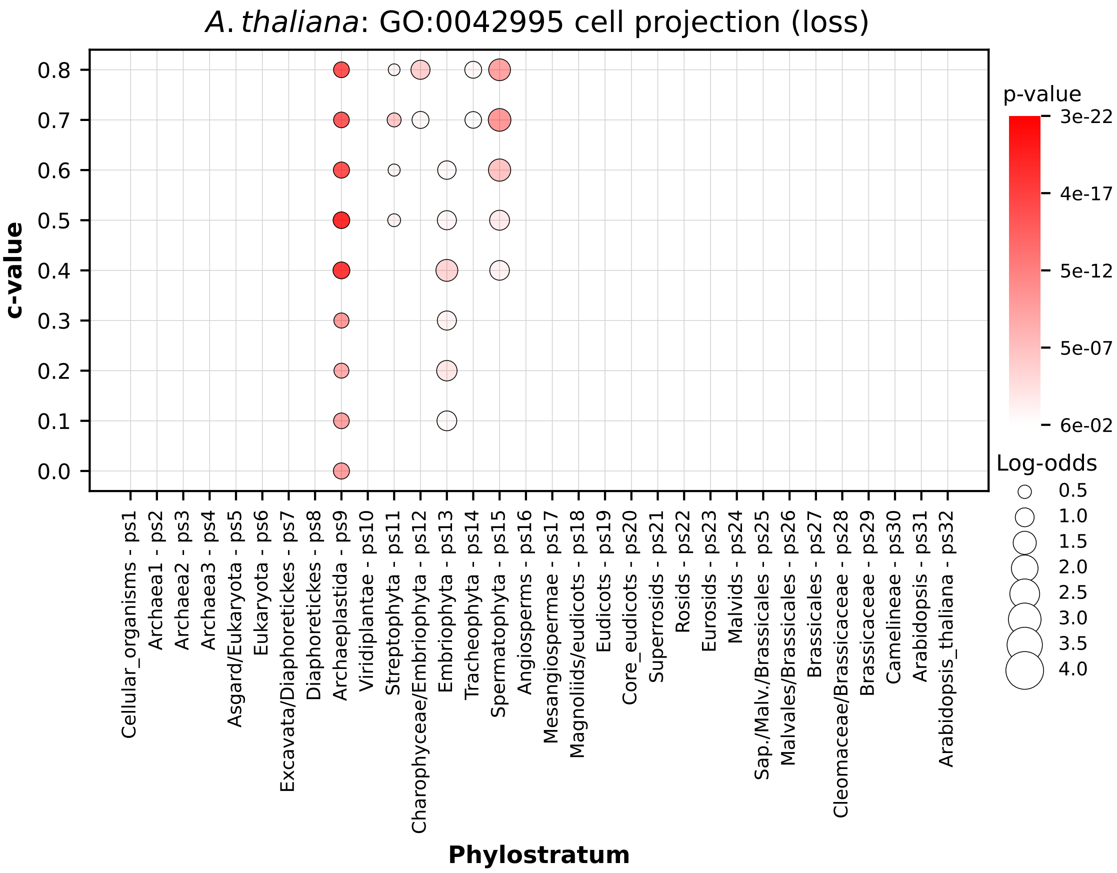
A. thaliana: GO:0042995 cell projection (loss)
c-value
0.8
0.7
0.6
0.5
0.4
0.3
0.2
0.1
0.0
Phylostratum
Cellular_organisms - ps1
Archaea1 - ps2
Archaea2 - ps3
Archaea3 - ps4
Asgard/Eukaryota - ps5
Eukaryota - ps6
Excavata/Diaphoretickes - ps7
Diaphoretickes - ps8
Archaeplastida - ps9
Viridiplantae - ps10
Streptophyta - ps11
Charophyceae/Embriophyta - ps12
Embriophyta - ps13
Tracheophyta - ps14
Spermatophyta - ps15
Angiosperms - ps16
Mesangiospermae - ps17
Magnoliids/eudicots - ps18
Eudicots - ps19
Core_eudicots - ps20
Superrosids - ps21
Rosids - ps22
Eurosids - ps23
Malvids - ps24
Sap./Malv./Brassicales - ps25
Malvales/Brassicales - ps26
Brassicales - ps27
Cleomaceae/Brassicaceae - ps28
Brassicaceae - ps29
Camelineae - ps30
Arabidopsis - ps31
Arabidopsis_thaliana - ps32
p-value
3e-22
4e-17
5e-12
5e-07
6e-02
Log-odds
0.5
1.0
1.5
2.0
2.5
3.0
3.5
4.0

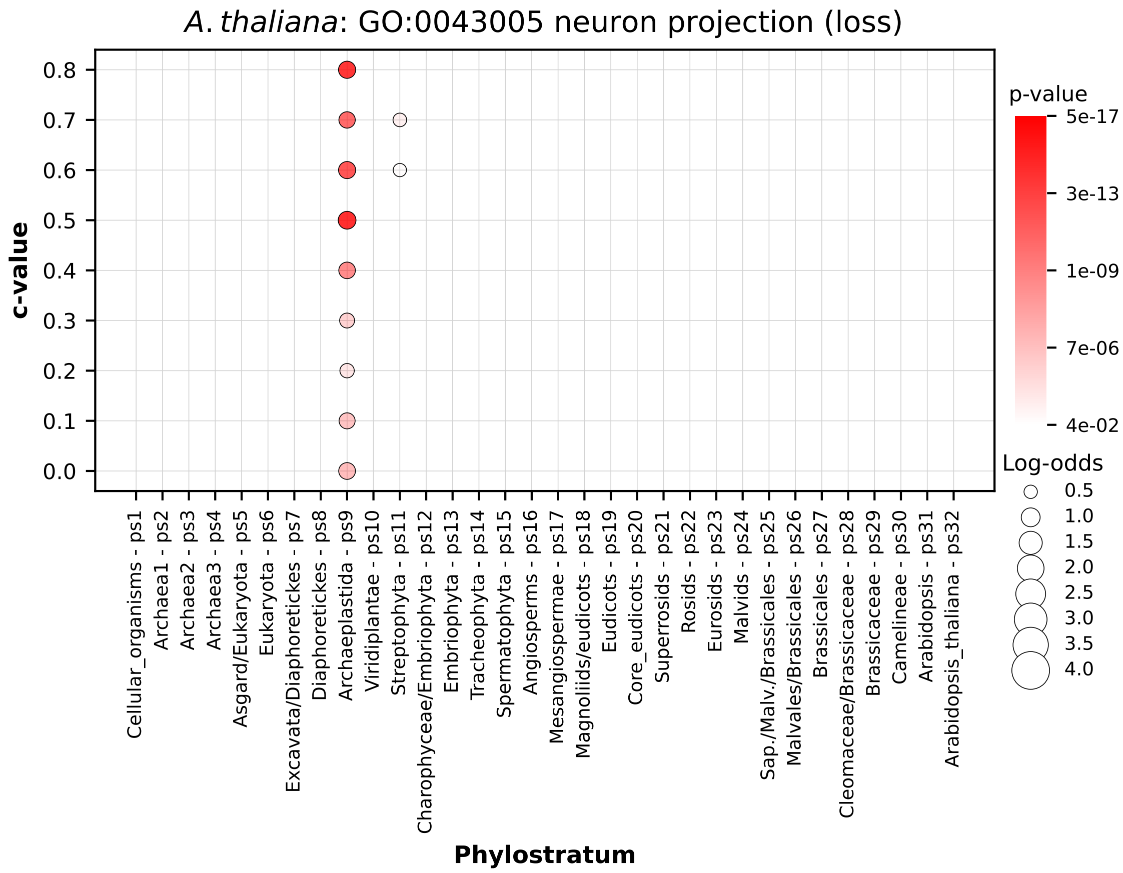

A. thaliana: GO:0043005 neuron projection (loss)
c-value
0.0
0.1
0.2
0.3
0.4
0.5
0.6
0.7
0.8
p-value
5e-17
3e-13
1e-09
7e-06
4e-02
Log-odds
0.5
1.0
1.5
2.0
2.5
3.0
3.5
4.0
Cellular_organisms - ps1
Archaea1 - ps2
Archaea2 - ps3
Archaea3 - ps4
Asgard/Eukaryota - ps5
Eukaryota - ps6
Excavata/Diaphoretickes - ps7
Diaphoretickes - ps8
Archaeplastida - ps9
Viridiplantae - ps10
Streptophyta - ps11
Charophyceae/Embriophyta - ps12
Embriophyta - ps13
Tracheophyta - ps14
Spermatophyta - ps15
Angiosperms - ps16
Mesangiospermae - ps17
Magnoliids/eudicots - ps18
Eudicots - ps19
Core_eudicots - ps20
Superrosids - ps21
Rosids - ps22
Eurosids - ps23
Malvids - ps24
Sap./Malv./Brassicales - ps25
Malvales/Brassicales - ps26
Brassicales - ps27
Cleomaceae/Brassicaceae - ps28
Brassicaceae - ps29
Camelineae - ps30
Arabidopsis - ps31
Arabidopsis_thaliana - ps32
Phylostratum

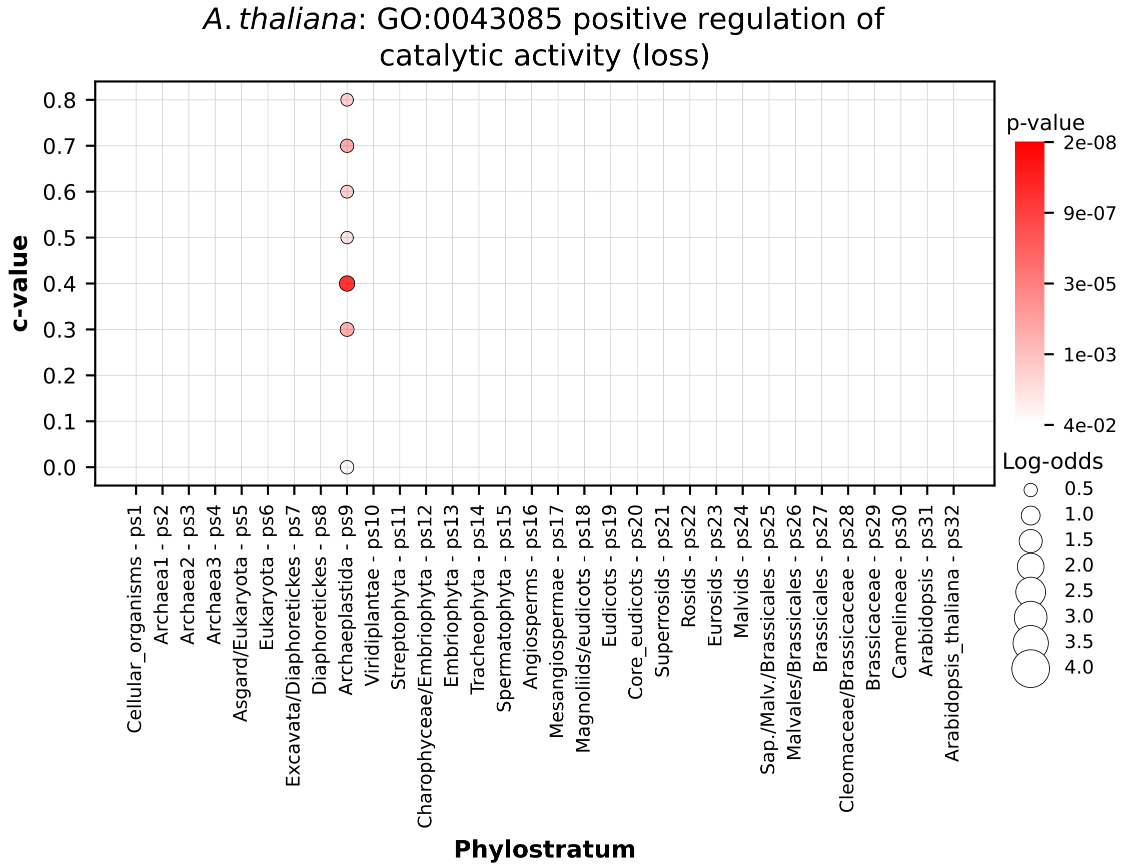

A. thaliana: GO:0043085 positive regulation of catalytic activity (loss)
c-value
0.8
0.7
0.6
0.5
0.4
0.3
0.2
0.1
0.0
p-value
2e-08
9e-07
3e-05
1e-03
4e-02
Log-odds
0.5
1.0
1.5
2.0
2.5
3.0
3.5
4.0
Cellular_organisms - ps1
Archaea1 - ps2
Archaea2 - ps3
Archaea3 - ps4
Asgard/Eukaryota - ps5
Eukaryota - ps6
Excavata/Diaphoretickes - ps7
Diaphoretickes - ps8
Archaeplastida - ps9
Viridiplantae - ps10
Streptophyta - ps11
Charophyceae/Embriophyta - ps12
Embriophyta - ps13
Tracheophyta - ps14
Spermatophyta - ps15
Angiosperms - ps16
Mesangiospermae - ps17
Magnoliids/eudicots - ps18
Eudicots - ps19
Core_eudicots - ps20
Superrosids - ps21
Rosids - ps22
Eurosids - ps23
Malvids - ps24
Sap./Malv./Brassicales - ps25
Malvales/Brassicales - ps26
Brassicales - ps27
Cleomaceae/Brassicaceae - ps28
Brassicaceae - ps29
Camelineae - ps30
Arabidopsis - ps31
Arabidopsis_thaliana - ps32
Phylostratum

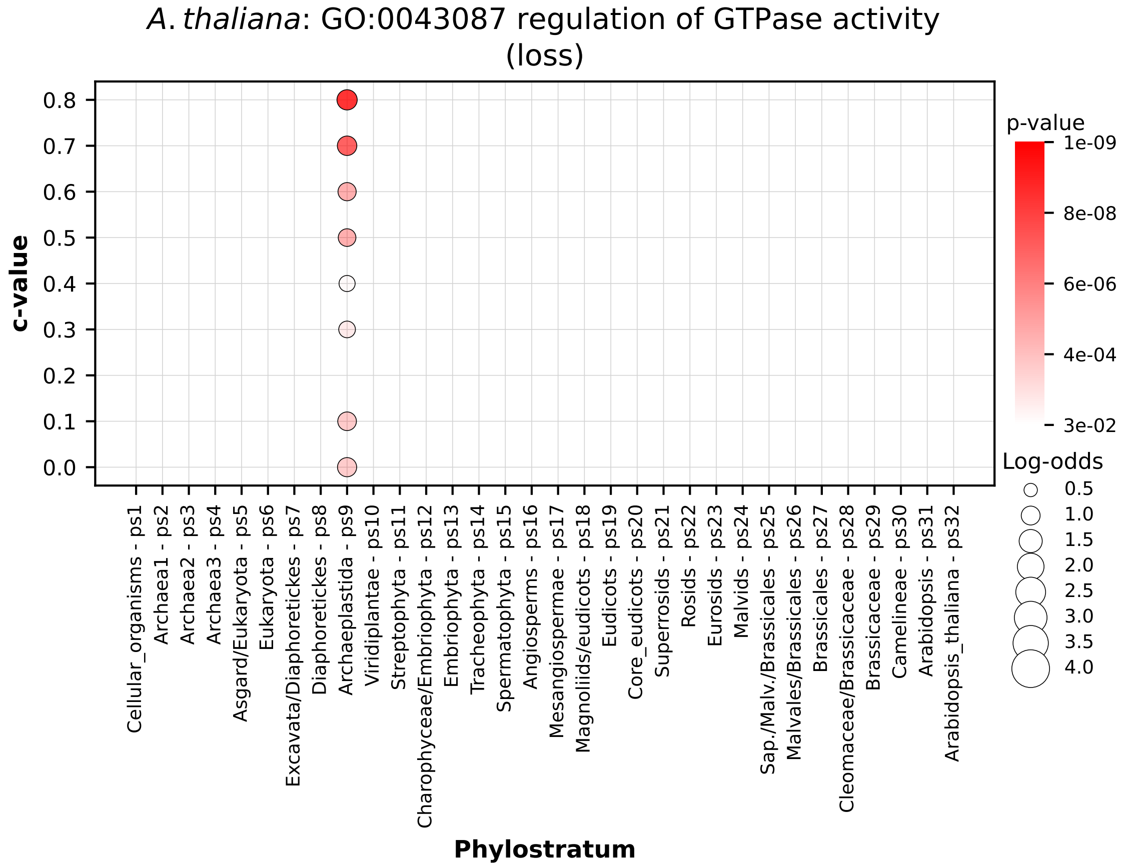

A. thaliana: GO:0043087 regulation of GTPase activity
(loss)
c-value
0.8
0.7
0.6
0.5
0.4
0.3
0.2
0.1
0.0
p-value
1e-09
8e-08
6e-06
4e-04
3e-02
Log-odds
0.5
1.0
1.5
2.0
2.5
3.0
3.5
4.0
Cellular_organisms - ps1
Archaea1 - ps2
Archaea2 - ps3
Archaea3 - ps4
Asgard/Eukaryota - ps5
Eukaryota - ps6
Excavata/Diaphoretickes - ps7
Diaphoretickes - ps8
Archaeplastida - ps9
Viridiplantae - ps10
Streptophyta - ps11
Charophyceae/Embriophyta - ps12
Embriophyta - ps13
Tracheophyta - ps14
Spermatophyta - ps15
Angiosperms - ps16
Mesangiospermae - ps17
Magnoliids/eudicots - ps18
Eudicots - ps19
Core_eudicots - ps20
Superrosids - ps21
Rosids - ps22
Eurosids - ps23
Malvids - ps24
Sap./Malv./Brassicales - ps25
Malvales/Brassicales - ps26
Brassicales - ps27
Cleomaceae/Brassicaceae - ps28
Brassicaceae - ps29
Camelineae - ps30
Arabidopsis - ps31
Arabidopsis_thaliana - ps32
Phylostratum

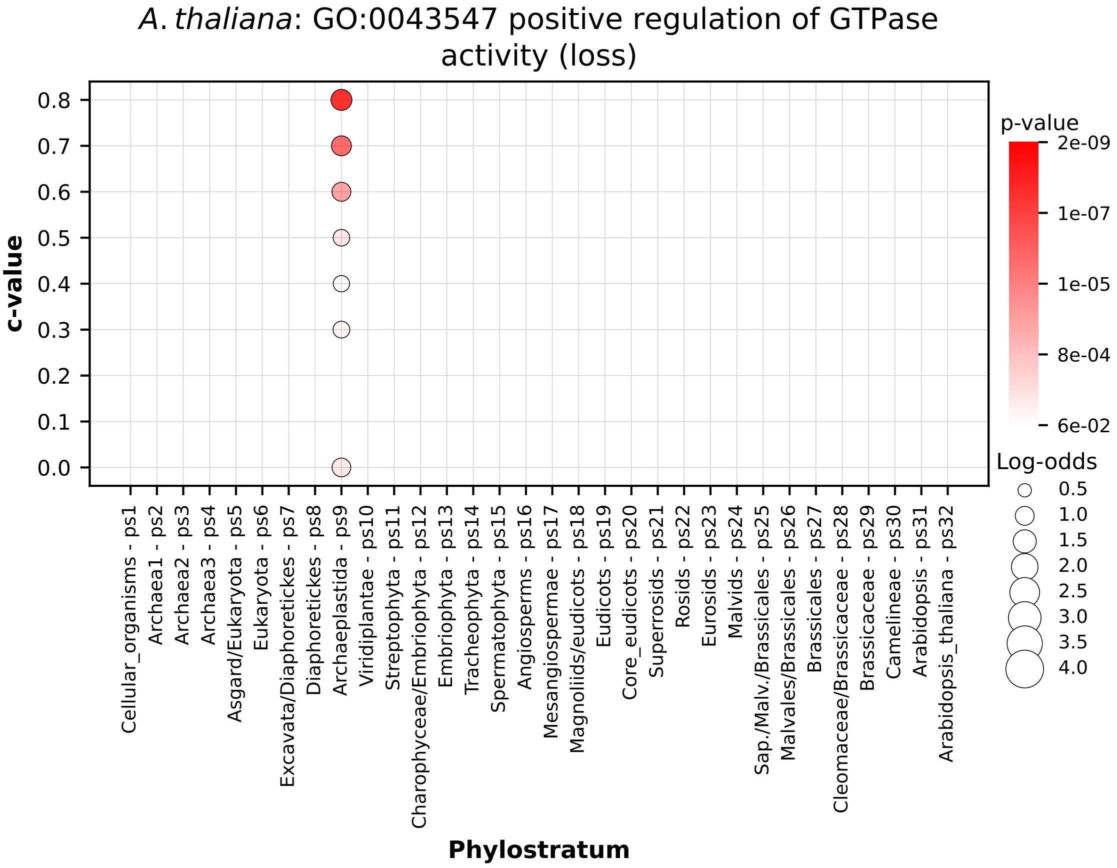

A. thaliana: GO:0043547 positive regulation of GTPase activity (loss)
c-value
0.8
0.7
0.6
0.5
0.4
0.3
0.2
0.1
0.0
p-value
2e-09
1e-07
1e-05
8e-04
6e-02
Log-odds
0.5
1.0
1.5
2.0
2.5
3.0
3.5
4.0
Cellular_organisms - ps1
Archaea1 - ps2
Archaea2 - ps3
Archaea3 - ps4
Asgard/Eukaryota - ps5
Eukaryota - ps6
Excavata/Diaphoretickes - ps7
Diaphoretickes - ps8
Archaeplastida - ps9
Viridiplantae - ps10
Streptophyta - ps11
Charophyceae/Embriophyta - ps12
Embriophyta - ps13
Tracheophyta - ps14
Spermatophyta - ps15
Angiosperms - ps16
Mesangiospermae - ps17
Magnoliids/eudicots - ps18
Eudicots - ps19
Core_eudicots - ps20
Superrosids - ps21
Rosids - ps22
Eurosids - ps23
Malvids - ps24
Sap./Malv./Brassicales - ps25
Malvales/Brassicales - ps26
Brassicales - ps27
Cleomaceae/Brassicaceae - ps28
Brassicaceae - ps29
Camelineae - ps30
Arabidopsis - ps31
Arabidopsis_thaliana - ps32
Phylostratum

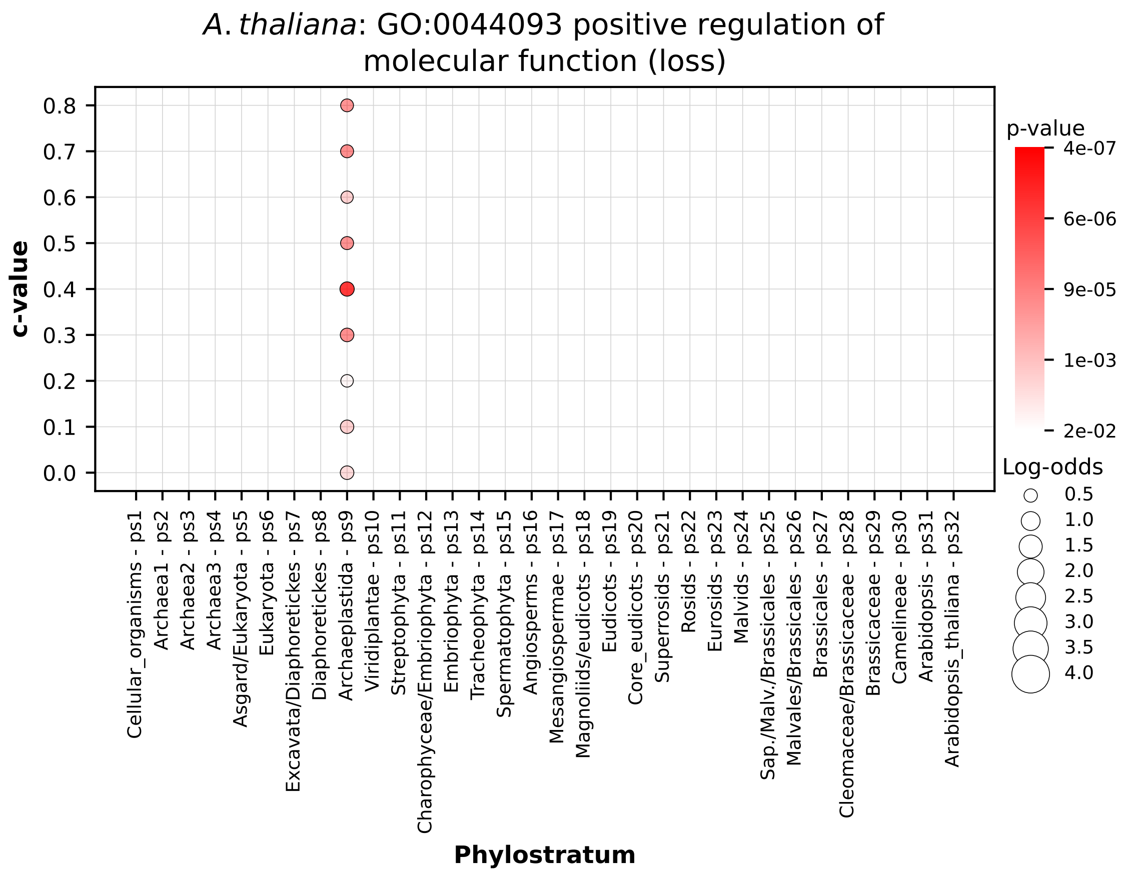
A. thaliana: GO:0044093 positive regulation of molecular function (loss)
c-value
0.8
0.7
0.6
0.5
0.4
0.3
0.2
0.1
0.0
p-value
4e-07
6e-06
9e-05
1e-03
2e-02
Log-odds
0.5
1.0
1.5
2.0
2.5
3.0
3.5
4.0
Cellular_organisms - ps1
Archaea1 - ps2
Archaea2 - ps3
Archaea3 - ps4
Asgard/Eukaryota - ps5
Eukaryota - ps6
Excavata/Diaphoretickes - ps7
Diaphoretickes - ps8
Archaeplastida - ps9
Viridiplantae - ps10
Streptophyta - ps11
Charophyceae/Embriophyta - ps12
Embriophyta - ps13
Tracheophyta - ps14
Spermatophyta - ps15
Angiosperms - ps16
Mesangiospermae - ps17
Magnoliids/eudicots - ps18
Eudicots - ps19
Core_eudicots - ps20
Superrosids - ps21
Rosids - ps22
Eurosids - ps23
Malvids - ps24
Sap./Malv./Brassicales - ps25
Malvales/Brassicales - ps26
Brassicales - ps27
Cleomaceae/Brassicaceae - ps28
Brassicaceae - ps29
Camelineae - ps30
Arabidopsis - ps31
Arabidopsis_thaliana - ps32
Phylostratum

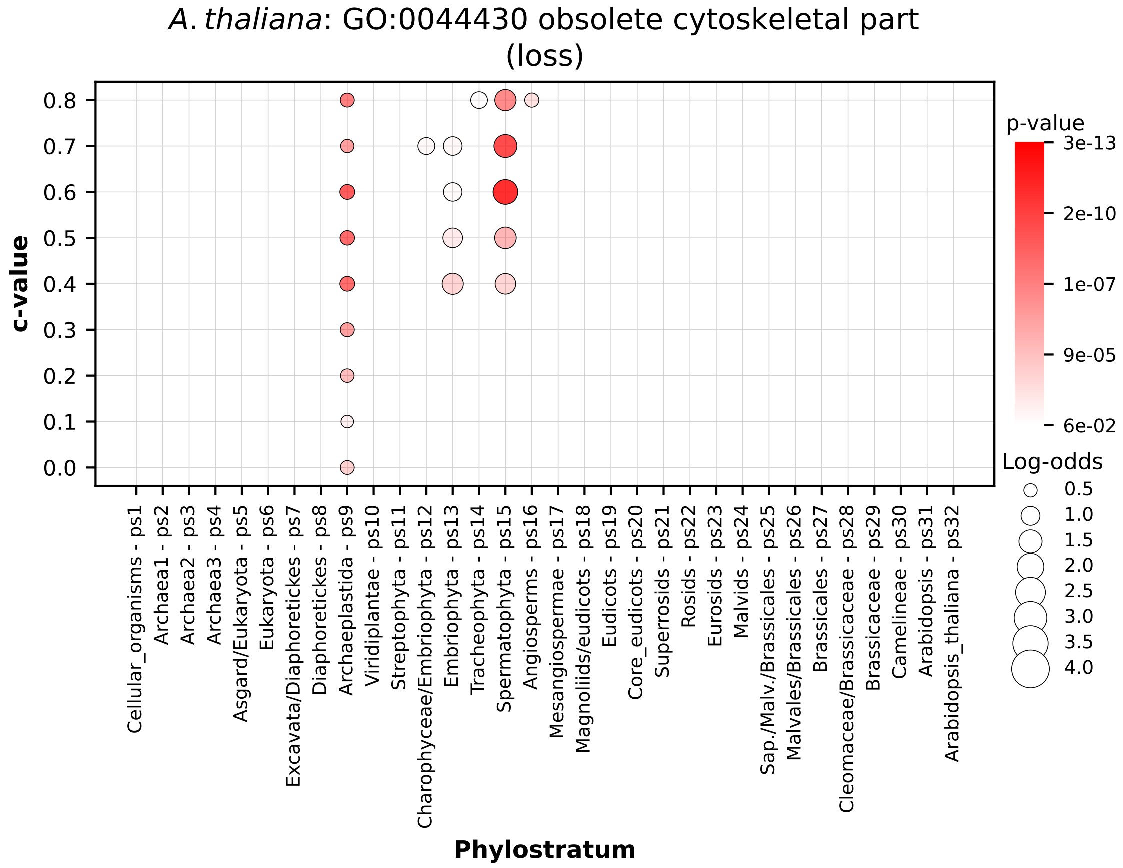

A. thaliana: GO:0044430 obsolete cytoskeletal part
(loss)
c-value
0.8
0.7
0.6
0.5
0.4
0.3
0.2
0.1
0.0
Phylostratum
Cellular_organisms - ps1
Archaea1 - ps2
Archaea2 - ps3
Archaea3 - ps4
Asgard/Eukaryota - ps5
Eukaryota - ps6
Excavata/Diaphoretickes - ps7
Diaphoretickes - ps8
Archaeplastida - ps9
Viridiplantae - ps10
Streptophyta - ps11
Charophyceae/Embriophyta - ps12
Embriophyta - ps13
Tracheophyta - ps14
Spermatophyta - ps15
Angiosperms - ps16
Mesangiospermae - ps17
Magnoliids/eudicots - ps18
Eudicots - ps19
Core_eudicots - ps20
Superrosids - ps21
Rosids - ps22
Eurosids - ps23
Malvids - ps24
Sap./Malv./Brassicales - ps25
Malvales/Brassicales - ps26
Brassicales - ps27
Cleomaceae/Brassicaceae - ps28
Brassicaceae - ps29
Camelineae - ps30
Arabidopsis - ps31
Arabidopsis_thaliana - ps32
p-value
3e-13
2e-10
1e-07
9e-05
6e-02
Log-odds
0.5
1.0
1.5
2.0
2.5
3.0
3.5
4.0

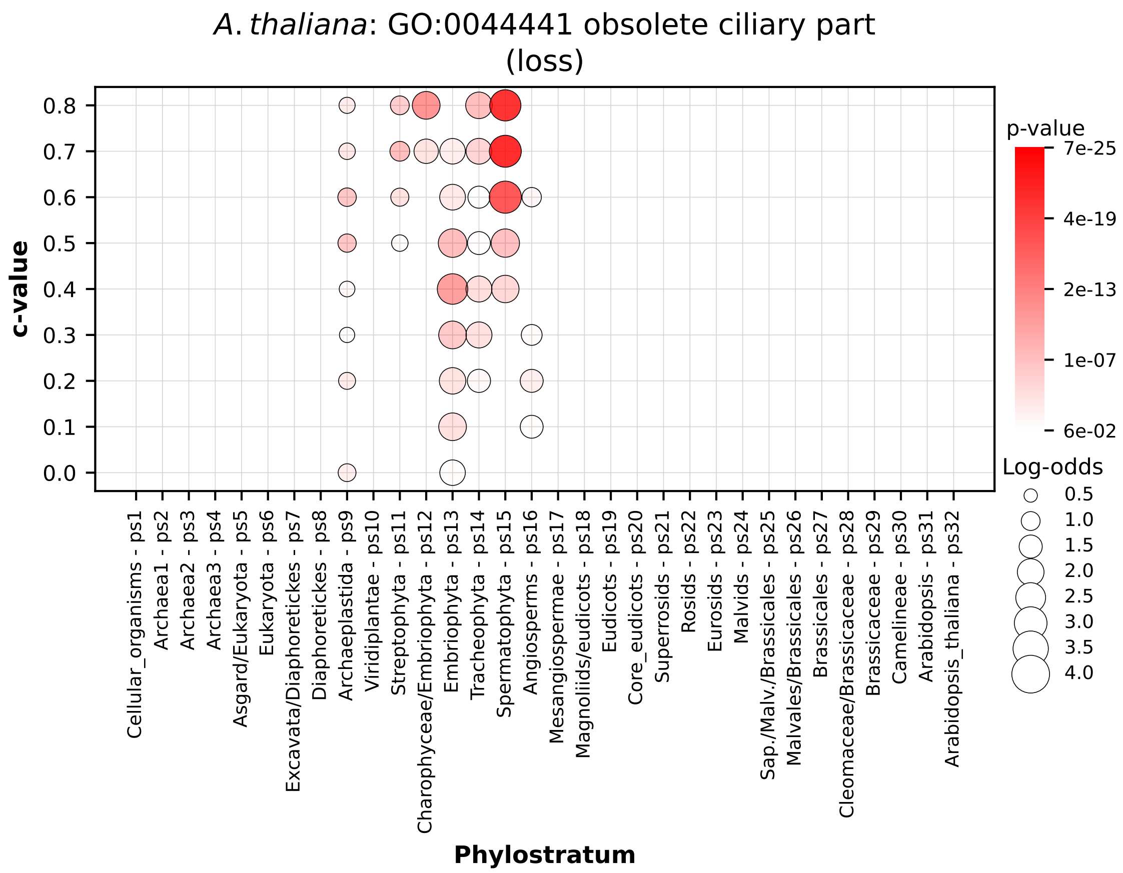

A. thaliana: GO:0044441 obsolete ciliary part
(loss)
c-value
0.8
0.7
0.6
0.5
0.4
0.3
0.2
0.1
0.0
p-value
7e-25
4e-19
2e-13
1e-07
6e-02
Log-odds
0.5
1.0
1.5
2.0
2.5
3.0
3.5
4.0
Cellular_organisms - ps1
Archaea1 - ps2
Archaea2 - ps3
Archaea3 - ps4
Asgard/Eukaryota - ps5
Eukaryota - ps6
Excavata/Diaphoretickes - ps7
Diaphoretickes - ps8
Archaeplastida - ps9
Viridiplantae - ps10
Streptophyta - ps11
Charophyceae/Embriophyta - ps12
Embriophyta - ps13
Tracheophyta - ps14
Spermatophyta - ps15
Angiosperms - ps16
Mesangiospermae - ps17
Magnoliids/eudicots - ps18
Eudicots - ps19
Core_eudicots - ps20
Superrosids - ps21
Rosids - ps22
Eurosids - ps23
Malvids - ps24
Sap./Malv./Brassicales - ps25
Malvales/Brassicales - ps26
Brassicales - ps27
Cleomaceae/Brassicaceae - ps28
Brassicaceae - ps29
Camelineae - ps30
Arabidopsis - ps31
Arabidopsis_thaliana - ps32
Phylostratum

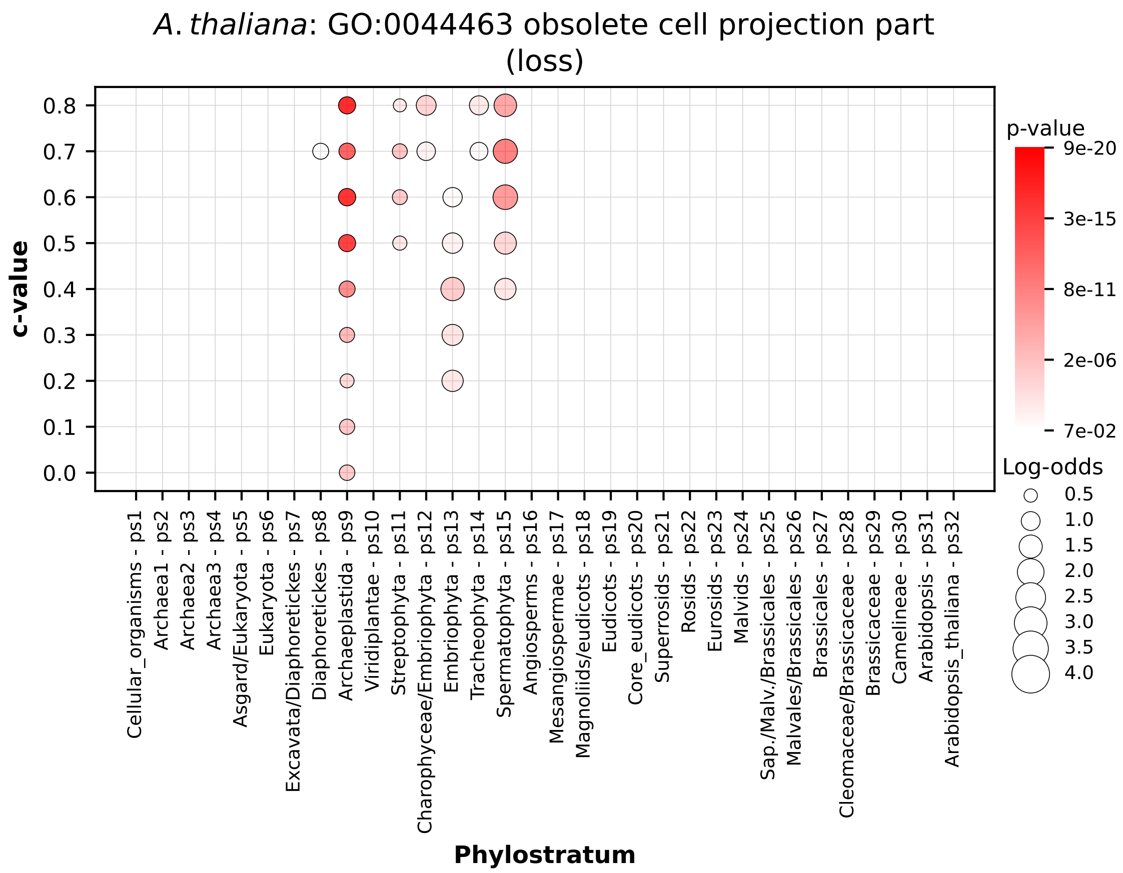

A. thaliana: GO:0044463 obsolete cell projection part
(loss)
c-value
0.8
0.7
0.6
0.5
0.4
0.3
0.2
0.1
0.0
p-value
9e-20
3e-15
8e-11
2e-06
7e-02
Log-odds
0.5
1.0
1.5
2.0
2.5
3.0
3.5
4.0
Cellular_organisms - ps1
Archaea1 - ps2
Archaea2 - ps3
Archaea3 - ps4
Asgard/Eukaryota - ps5
Eukaryota - ps6
Excavata/Diaphoretickes - ps7
Diaphoretickes - ps8
Archaeplastida - ps9
Viridiplantae - ps10
Streptophyta - ps11
Charophyceae/Embriophyta - ps12
Embriophyta - ps13
Tracheophyta - ps14
Spermatophyta - ps15
Angiosperms - ps16
Mesangiospermae - ps17
Magnoliids/eudicots - ps18
Eudicots - ps19
Core_eudicots - ps20
Superrosids - ps21
Rosids - ps22
Eurosids - ps23
Malvids - ps24
Sap./Malv./Brassicales - ps25
Malvales/Brassicales - ps26
Brassicales - ps27
Cleomaceae/Brassicaceae - ps28
Brassicaceae - ps29
Camelineae - ps30
Arabidopsis - ps31
Arabidopsis_thaliana - ps32
Phylostratum

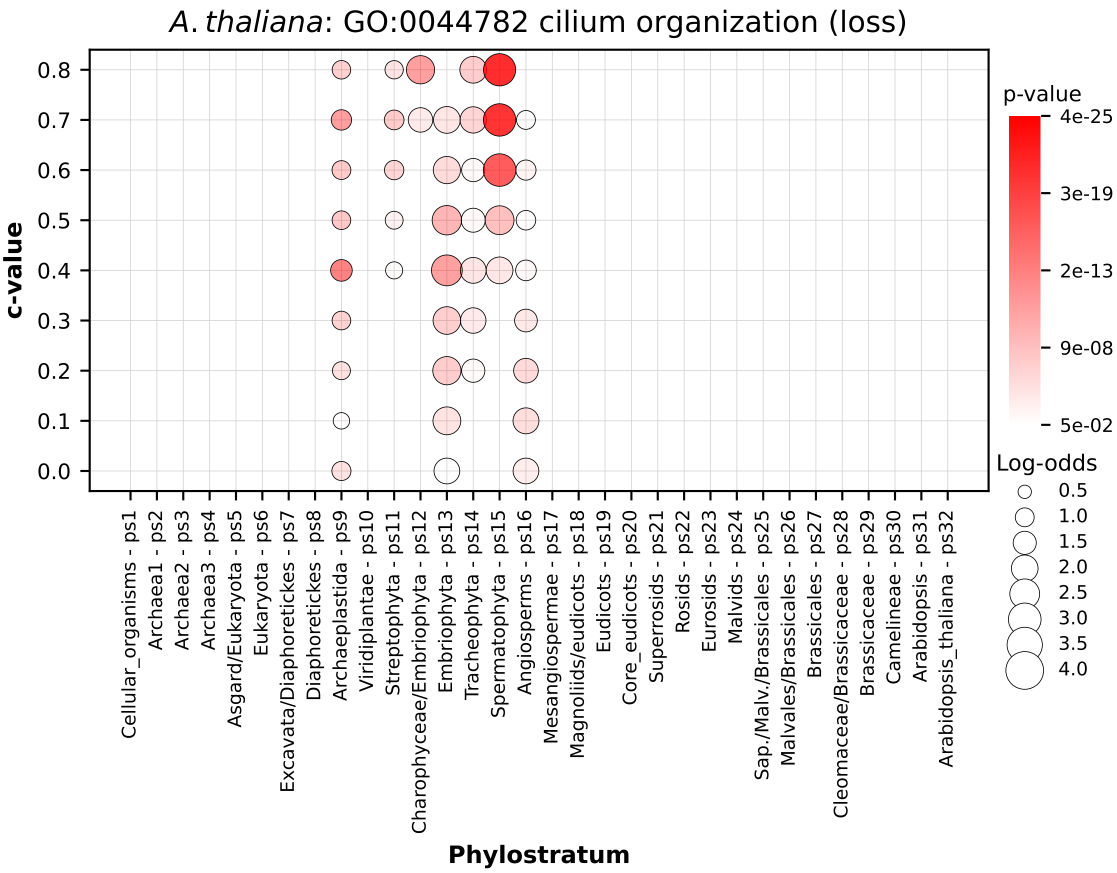

A. thaliana: GO:0044782 cilium organization (loss)
c-value
0.8
0.7
0.6
0.5
0.4
0.3
0.2
0.1
0.0
p-value
4e-25
3e-19
2e-13
9e-08
5e-02
Log-odds
0.5
1.0
1.5
2.0
2.5
3.0
3.5
4.0
Cellular_organisms - ps1
Archaea1 - ps2
Archaea2 - ps3
Archaea3 - ps4
Asgard/Eukaryota - ps5
Eukaryota - ps6
Excavata/Diaphoretickes - ps7
Diaphoretickes - ps8
Archaeplastida - ps9
Viridiplantae - ps10
Streptophyta - ps11
Charophyceae/Embriophyta - ps12
Embriophyta - ps13
Tracheophyta - ps14
Spermatophyta - ps15
Angiosperms - ps16
Mesangiospermae - ps17
Magnoliids/eudicots - ps18
Eudicots - ps19
Core_eudicots - ps20
Superrosids - ps21
Rosids - ps22
Eurosids - ps23
Malvids - ps24
Sap./Malv./Brassicales - ps25
Malvales/Brassicales - ps26
Brassicales - ps27
Cleomaceae/Brassicaceae - ps28
Brassicaceae - ps29
Camelineae - ps30
Arabidopsis - ps31
Arabidopsis_thaliana - ps32
Phylostratum

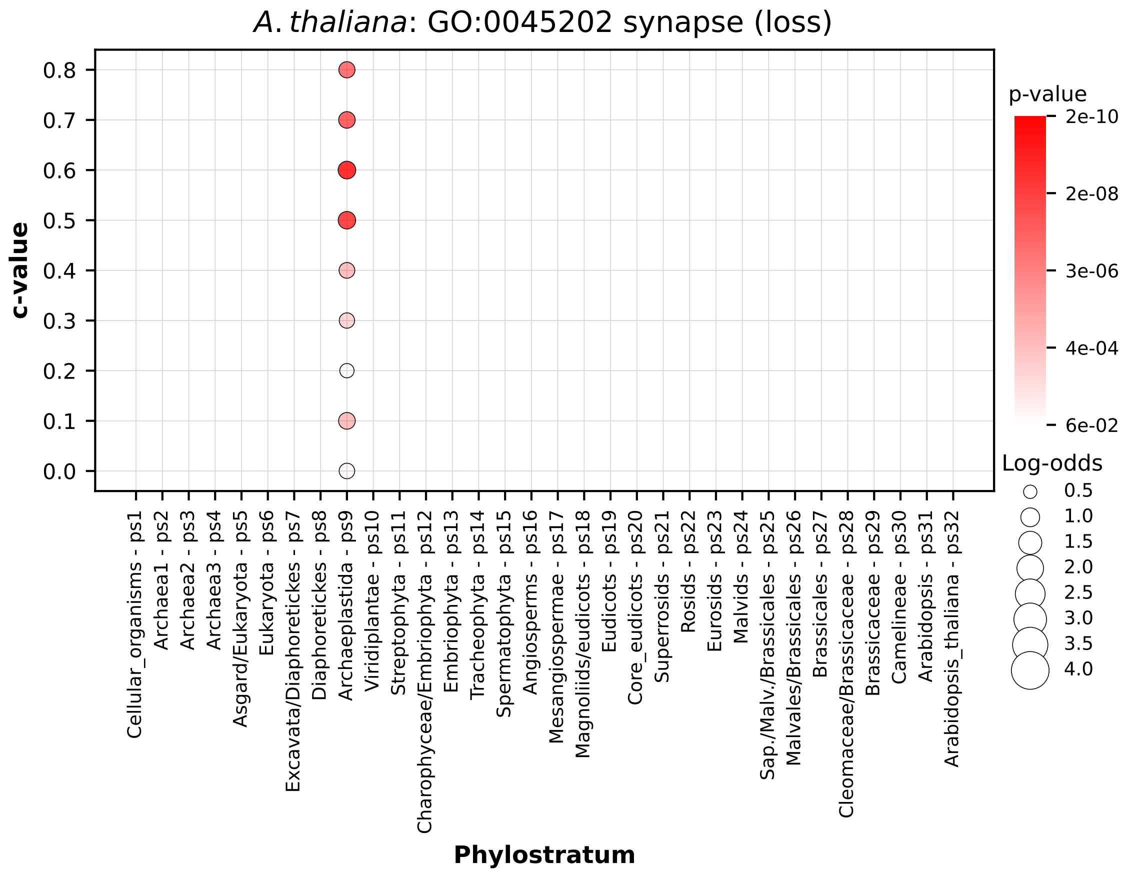
A. thaliana: GO:0045202 synapse (loss)
c-value
0.0
0.1
0.2
0.3
0.4
0.5
0.6
0.7
0.8
Phylostratum
Cellular_organisms - ps1
Archaea1 - ps2
Archaea2 - ps3
Archaea3 - ps4
Asgard/Eukaryota - ps5
Eukaryota - ps6
Excavata/Diaphoretickes - ps7
Diaphoretickes - ps8
Archaeplastida - ps9
Viridiplantae - ps10
Streptophyta - ps11
Charophyceae/Embriophyta - ps12
Embriophyta - ps13
Tracheophyta - ps14
Spermatophyta - ps15
Angiosperms - ps16
Mesangiospermae - ps17
Magnoliids/eudicots - ps18
Eudicots - ps19
Core_eudicots - ps20
Superrosids - ps21
Rosids - ps22
Eurosids - ps23
Malvids - ps24
Sap./Malv./Brassicales - ps25
Malvales/Brassicales - ps26
Brassicales - ps27
Cleomaceae/Brassicaceae - ps28
Brassicaceae - ps29
Camelineae - ps30
Arabidopsis - ps31
Arabidopsis_thaliana - ps32
p-value
2e-10
2e-08
3e-06
4e-04
6e-02
Log-odds
0.5
1.0
1.5
2.0
2.5
3.0
3.5
4.0

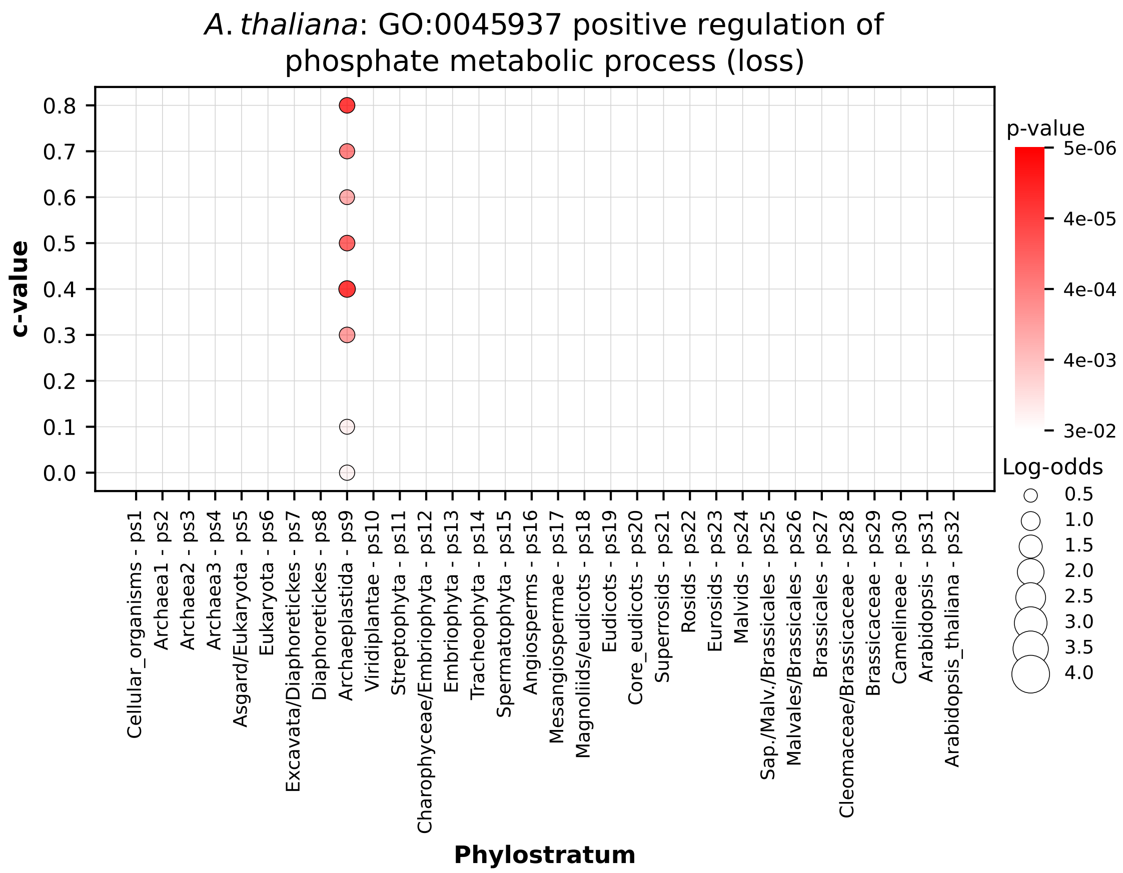
A. thaliana: GO:0045937 positive regulation of phosphate metabolic process (loss)
c-value
0.8
0.7
0.6
0.5
0.4
0.3
0.2
0.1
0.0
p-value
5e-06
4e-05
4e-04
4e-03
3e-02
Log-odds
0.5
1.0
1.5
2.0
2.5
3.0
3.5
4.0
Cellular_organisms - ps1
Archaea1 - ps2
Archaea2 - ps3
Archaea3 - ps4
Asgard/Eukaryota - ps5
Eukaryota - ps6
Excavata/Diaphoretickes - ps7
Diaphoretickes - ps8
Archaeplastida - ps9
Viridiplantae - ps10
Streptophyta - ps11
Charophyceae/Embriophyta - ps12
Embriophyta - ps13
Tracheophyta - ps14
Spermatophyta - ps15
Angiosperms - ps16
Mesangiospermae - ps17
Magnoliids/eudicots - ps18
Eudicots - ps19
Core_eudicots - ps20
Superrosids - ps21
Rosids - ps22
Eurosids - ps23
Malvids - ps24
Sap./Malv./Brassicales - ps25
Malvales/Brassicales - ps26
Brassicales - ps27
Cleomaceae/Brassicaceae - ps28
Brassicaceae - ps29
Camelineae - ps30
Arabidopsis - ps31
Arabidopsis_thaliana - ps32
Phylostratum

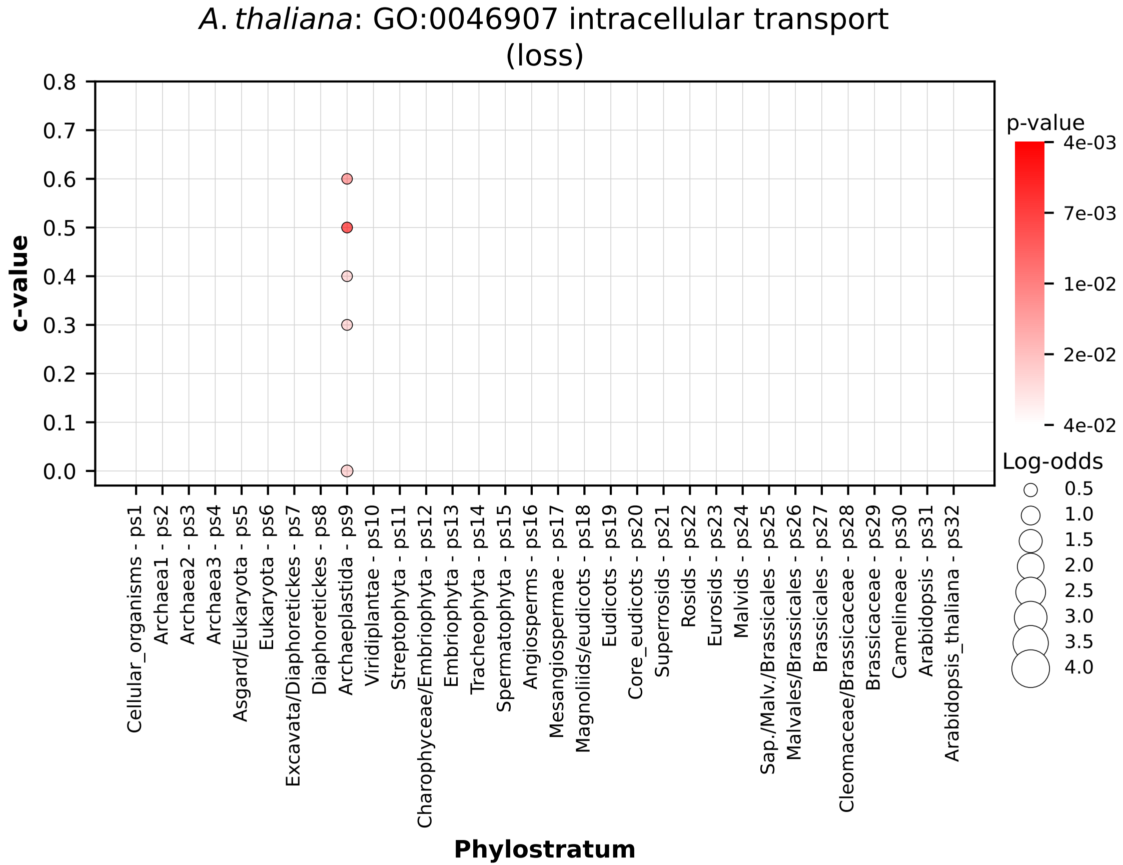

A. thaliana: GO:0046907 intracellular transport
(loss)
c-value
0.8
0.7
0.6
0.5
0.4
0.3
0.2
0.1
0.0
Phylostratum
Cellular_organisms - ps1
Archaea1 - ps2
Archaea2 - ps3
Archaea3 - ps4
Asgard/Eukaryota - ps5
Eukaryota - ps6
Excavata/Diaphoretickes - ps7
Diaphoretickes - ps8
Archaeplastida - ps9
Viridiplantae - ps10
Streptophyta - ps11
Charophyceae/Embriophyta - ps12
Embriophyta - ps13
Tracheophyta - ps14
Spermatophyta - ps15
Angiosperms - ps16
Mesangiospermae - ps17
Magnoliids/eudicots - ps18
Eudicots - ps19
Core_eudicots - ps20
Superrosids - ps21
Rosids - ps22
Eurosids - ps23
Malvids - ps24
Sap./Malv./Brassicales - ps25
Malvales/Brassicales - ps26
Brassicales - ps27
Cleomaceae/Brassicaceae - ps28
Brassicaceae - ps29
Camelineae - ps30
Arabidopsis - ps31
Arabidopsis_thaliana - ps32
p-value
4e-03
7e-03
1e-02
2e-02
4e-02
Log-odds
0.5
1.0
1.5
2.0
2.5
3.0
3.5
4.0

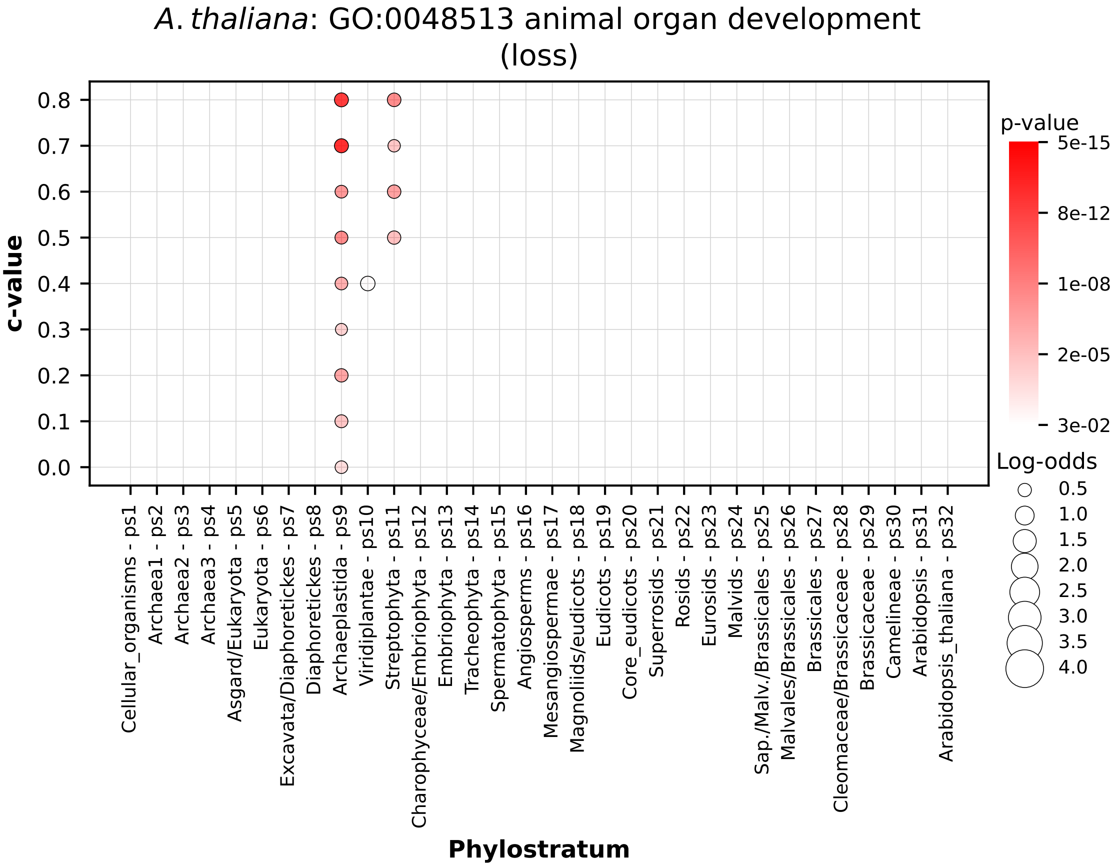
A. thaliana: GO:0048513 animal organ development
(loss)
c-value
0.8
0.7
0.6
0.5
0.4
0.3
0.2
0.1
0.0
Phylostratum
Cellular_organisms - ps1
Archaea1 - ps2
Archaea2 - ps3
Archaea3 - ps4
Asgard/Eukaryota - ps5
Eukaryota - ps6
Excavata/Diaphoretickes - ps7
Diaphoretickes - ps8
Archaeplastida - ps9
Viridiplantae - ps10
Streptophyta - ps11
Charophyceae/Embriophyta - ps12
Embriophyta - ps13
Tracheophyta - ps14
Spermatophyta - ps15
Angiosperms - ps16
Mesangiospermae - ps17
Magnoliids/eudicots - ps18
Eudicots - ps19
Core_eudicots - ps20
Superrosids - ps21
Rosids - ps22
Eurosids - ps23
Malvids - ps24
Sap./Malv./Brassicales - ps25
Malvales/Brassicales - ps26
Brassicales - ps27
Cleomaceae/Brassicaceae - ps28
Brassicaceae - ps29
Camelineae - ps30
Arabidopsis - ps31
Arabidopsis_thaliana - ps32
p-value
5e-15
8e-12
1e-08
2e-05
3e-02
Log-odds
0.5
1.0
1.5
2.0
2.5
3.0
3.5
4.0

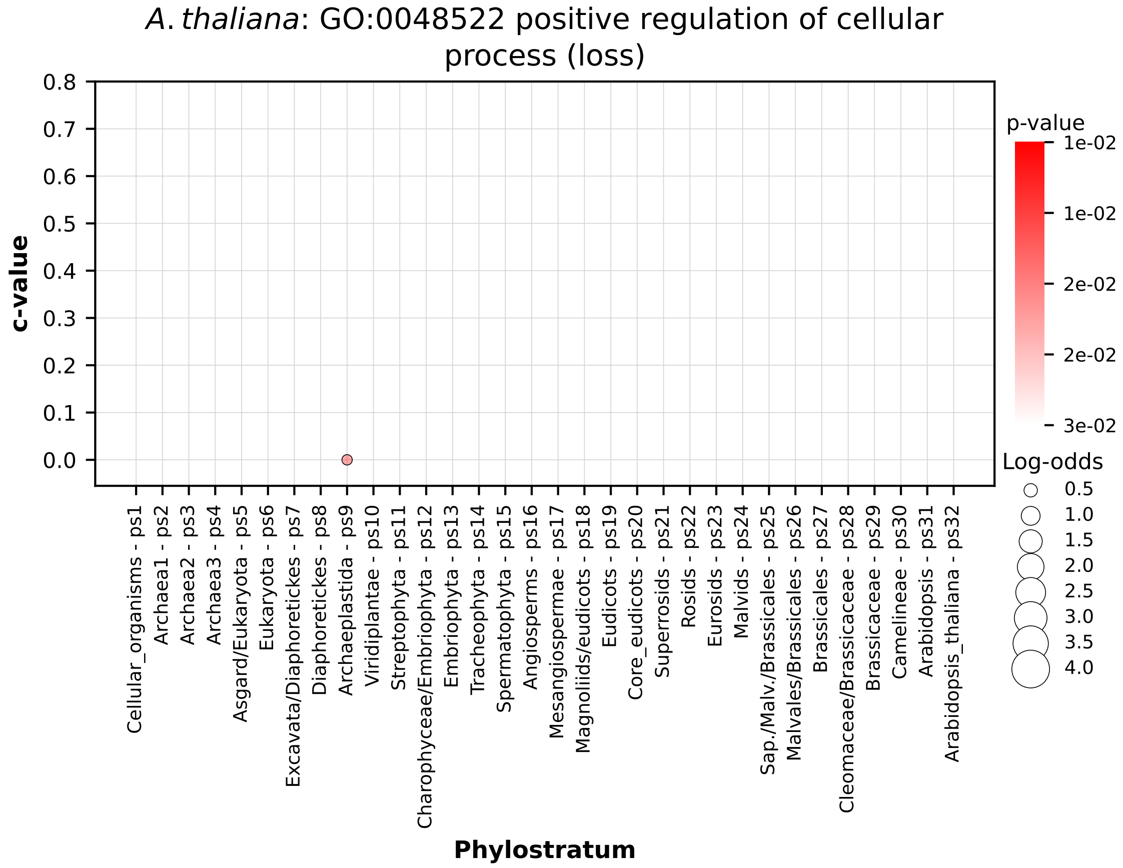

A. thaliana: GO:0048522 positive regulation of cellular process (loss)
c-value
0.8
0.7
0.6
0.5
0.4
0.3
0.2
0.1
0.0
Phylostratum
Cellular_organisms - ps1
Archaea1 - ps2
Archaea2 - ps3
Archaea3 - ps4
Asgard/Eukaryota - ps5
Eukaryota - ps6
Excavata/Diaphoretickes - ps7
Diaphoretickes - ps8
Archaeplastida - ps9
Viridiplantae - ps10
Streptophyta - ps11
Charophyceae/Embriophyta - ps12
Embriophyta - ps13
Tracheophyta - ps14
Spermatophyta - ps15
Angiosperms - ps16
Mesangiospermae - ps17
Magnoliids/eudicots - ps18
Eudicots - ps19
Core_eudicots - ps20
Superrosids - ps21
Rosids - ps22
Eurosids - ps23
Malvids - ps24
Sap./Malv./Brassicales - ps25
Malvales/Brassicales - ps26
Brassicales - ps27
Cleomaceae/Brassicaceae - ps28
Brassicaceae - ps29
Camelineae - ps30
Arabidopsis - ps31
Arabidopsis_thaliana - ps32
p-value
1e-02
1e-02
2e-02
2e-02
3e-02
Log-odds
0.5
1.0
1.5
2.0
2.5
3.0
3.5
4.0

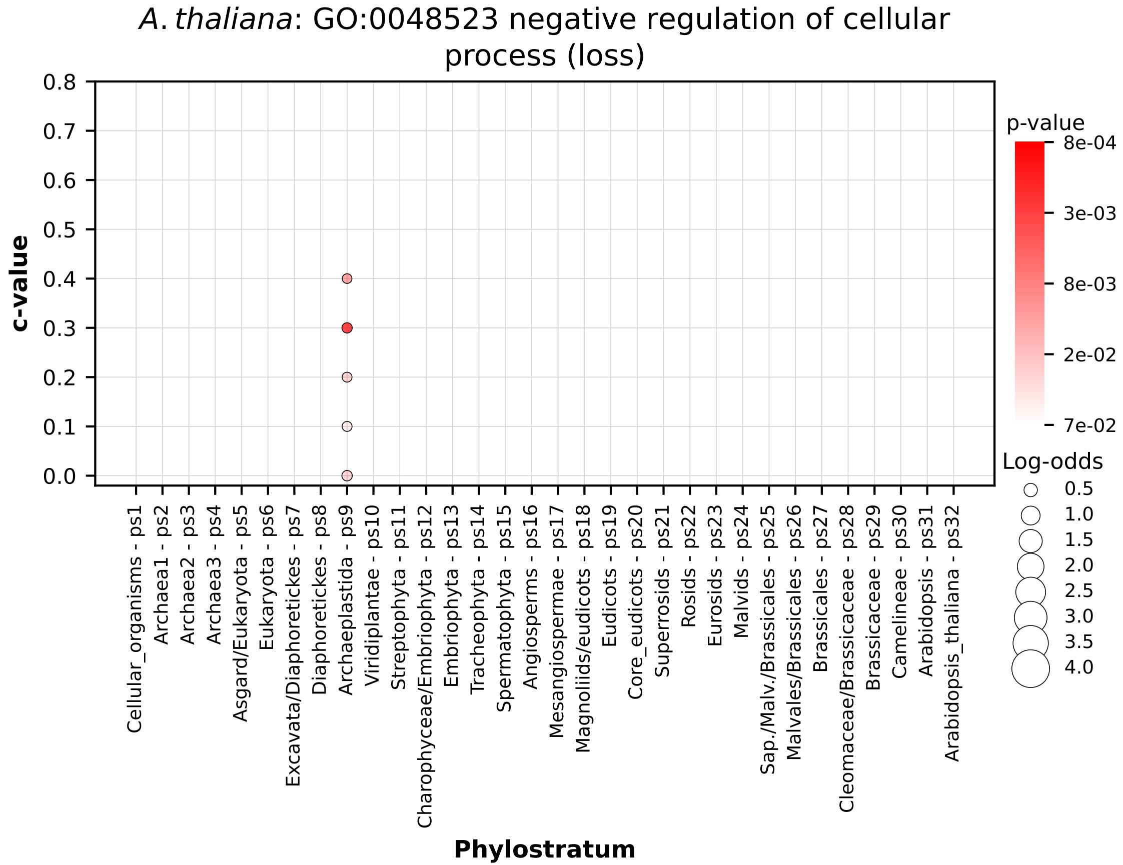

A. thaliana: GO:0048523 negative regulation of cellular process (loss)
c-value
0.8
0.7
0.6
0.5
0.4
0.3
0.2
0.1
0.0
p-value
8e-04
3e-03
8e-03
2e-02
7e-02
Log-odds
0.5
1.0
1.5
2.0
2.5
3.0
3.5
4.0
Cellular_organisms - ps1
Archaea1 - ps2
Archaea2 - ps3
Archaea3 - ps4
Asgard/Eukaryota - ps5
Eukaryota - ps6
Excavata/Diaphoretickes - ps7
Diaphoretickes - ps8
Archaeplastida - ps9
Viridiplantae - ps10
Streptophyta - ps11
Charophyceae/Embriophyta - ps12
Embriophyta - ps13
Tracheophyta - ps14
Spermatophyta - ps15
Angiosperms - ps16
Mesangiospermae - ps17
Magnoliids/eudicots - ps18
Eudicots - ps19
Core_eudicots - ps20
Superrosids - ps21
Rosids - ps22
Eurosids - ps23
Malvids - ps24
Sap./Malv./Brassicales - ps25
Malvales/Brassicales - ps26
Brassicales - ps27
Cleomaceae/Brassicaceae - ps28
Brassicaceae - ps29
Camelineae - ps30
Arabidopsis - ps31
Arabidopsis_thaliana - ps32
Phylostratum

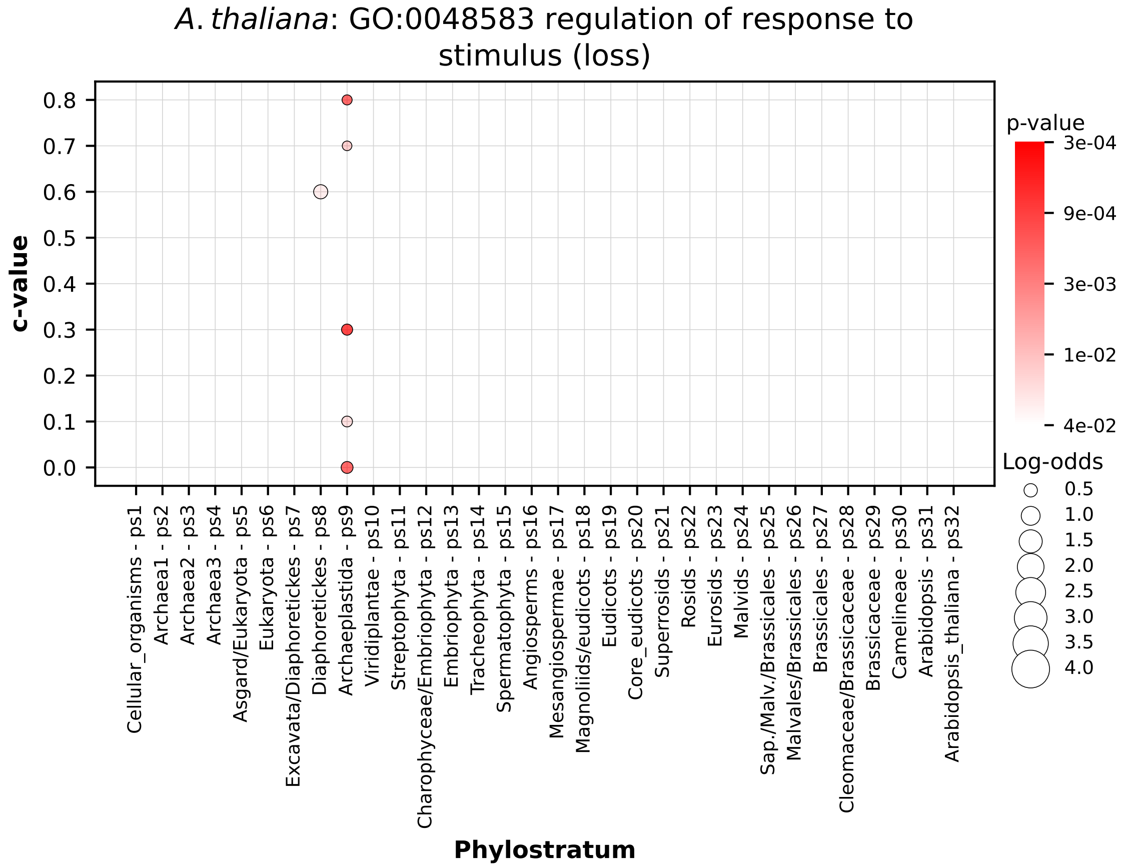
A. thaliana: GO:0048583 regulation of response to stimulus (loss)
c-value
0.8
0.7
0.6
0.5
0.4
0.3
0.2
0.1
0.0
p-value
3e-04
9e-04
3e-03
1e-02
4e-02
Log-odds
0.5
1.0
1.5
2.0
2.5
3.0
3.5
4.0
Cellular_organisms - ps1
Archaea1 - ps2
Archaea2 - ps3
Archaea3 - ps4
Asgard/Eukaryota - ps5
Eukaryota - ps6
Excavata/Diaphoretickes - ps7
Diaphoretickes - ps8
Archaeplastida - ps9
Viridiplantae - ps10
Streptophyta - ps11
Charophyceae/Embriophyta - ps12
Embriophyta - ps13
Tracheophyta - ps14
Spermatophyta - ps15
Angiosperms - ps16
Mesangiospermae - ps17
Magnoliids/eudicots - ps18
Eudicots - ps19
Core_eudicots - ps20
Superrosids - ps21
Rosids - ps22
Eurosids - ps23
Malvids - ps24
Sap./Malv./Brassicales - ps25
Malvales/Brassicales - ps26
Brassicales - ps27
Cleomaceae/Brassicaceae - ps28
Brassicaceae - ps29
Camelineae - ps30
Arabidopsis - ps31
Arabidopsis_thaliana - ps32
Phylostratum

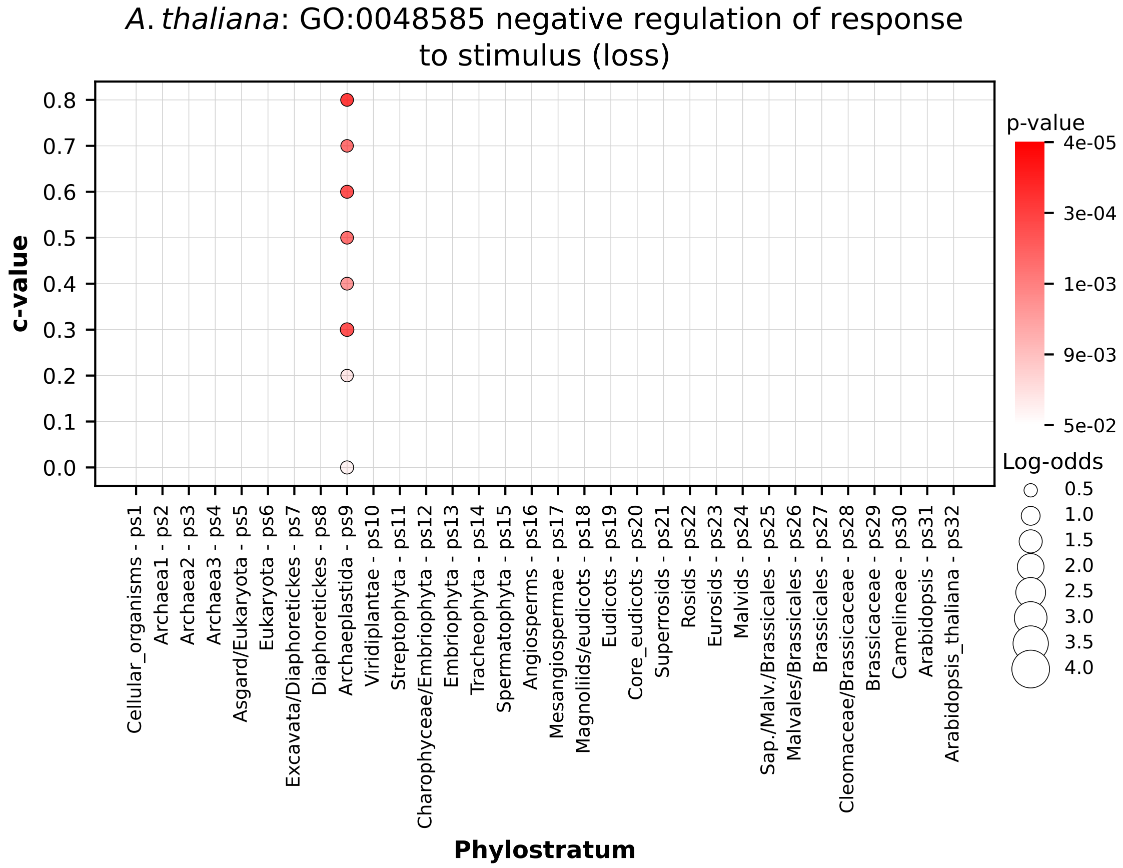

A. thaliana: GO:0048585 negative regulation of response to stimulus (loss)
c-value
0.8
0.7
0.6
0.5
0.4
0.3
0.2
0.1
0.0
Phylostratum
Cellular_organisms - ps1
Archaea1 - ps2
Archaea2 - ps3
Archaea3 - ps4
Asgard/Eukaryota - ps5
Eukaryota - ps6
Excavata/Diaphoretickes - ps7
Diaphoretickes - ps8
Archaeplastida - ps9
Viridiplantae - ps10
Streptophyta - ps11
Charophyceae/Embriophyta - ps12
Embriophyta - ps13
Tracheophyta - ps14
Spermatophyta - ps15
Angiosperms - ps16
Mesangiospermae - ps17
Magnoliids/eudicots - ps18
Eudicots - ps19
Core_eudicots - ps20
Superrosids - ps21
Rosids - ps22
Eurosids - ps23
Malvids - ps24
Sap./Malv./Brassicales - ps25
Malvales/Brassicales - ps26
Brassicales - ps27
Cleomaceae/Brassicaceae - ps28
Brassicaceae - ps29
Camelineae - ps30
Arabidopsis - ps31
Arabidopsis_thaliana - ps32
p-value
4e-05
3e-04
1e-03
9e-03
5e-02
Log-odds
0.5
1.0
1.5
2.0
2.5
3.0
3.5
4.0

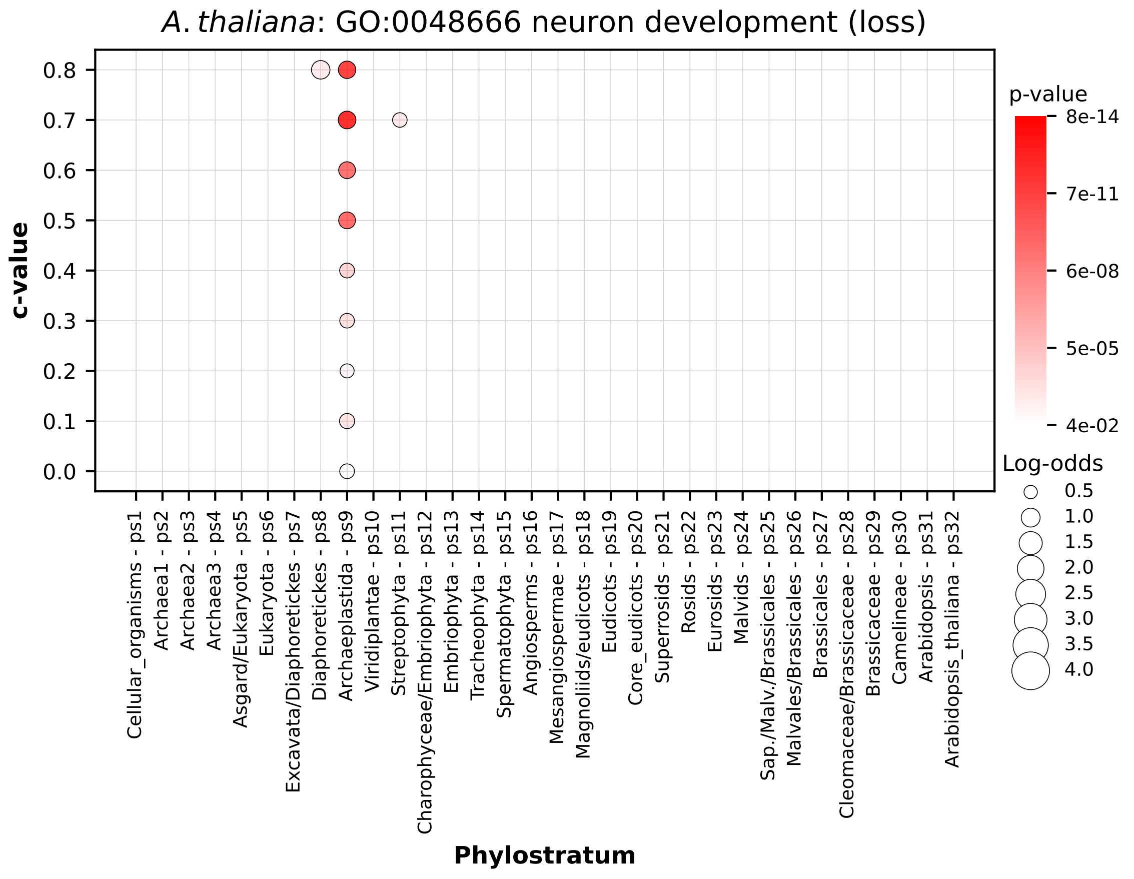
A. thaliana: GO:0048666 neuron development (loss)
c-value
0.8
0.7
0.6
0.5
0.4
0.3
0.2
0.1
0.0
p-value
8e-14
7e-11
6e-08
5e-05
4e-02
Log-odds
0.5
1.0
1.5
2.0
2.5
3.0
3.5
4.0
Cellular_organisms - ps1
Archaea1 - ps2
Archaea2 - ps3
Archaea3 - ps4
Asgard/Eukaryota - ps5
Eukaryota - ps6
Excavata/Diaphoretickes - ps7
Diaphoretickes - ps8
Archaeplastida - ps9
Viridiplantae - ps10
Streptophyta - ps11
Charophyceae/Embriophyta - ps12
Embriophyta - ps13
Tracheophyta - ps14
Spermatophyta - ps15
Angiosperms - ps16
Mesangiospermae - ps17
Magnoliids/eudicots - ps18
Eudicots - ps19
Core_eudicots - ps20
Superrosids - ps21
Rosids - ps22
Eurosids - ps23
Malvids - ps24
Sap./Malv./Brassicales - ps25
Malvales/Brassicales - ps26
Brassicales - ps27
Cleomaceae/Brassicaceae - ps28
Brassicaceae - ps29
Camelineae - ps30
Arabidopsis - ps31
Arabidopsis_thaliana - ps32
Phylostratum

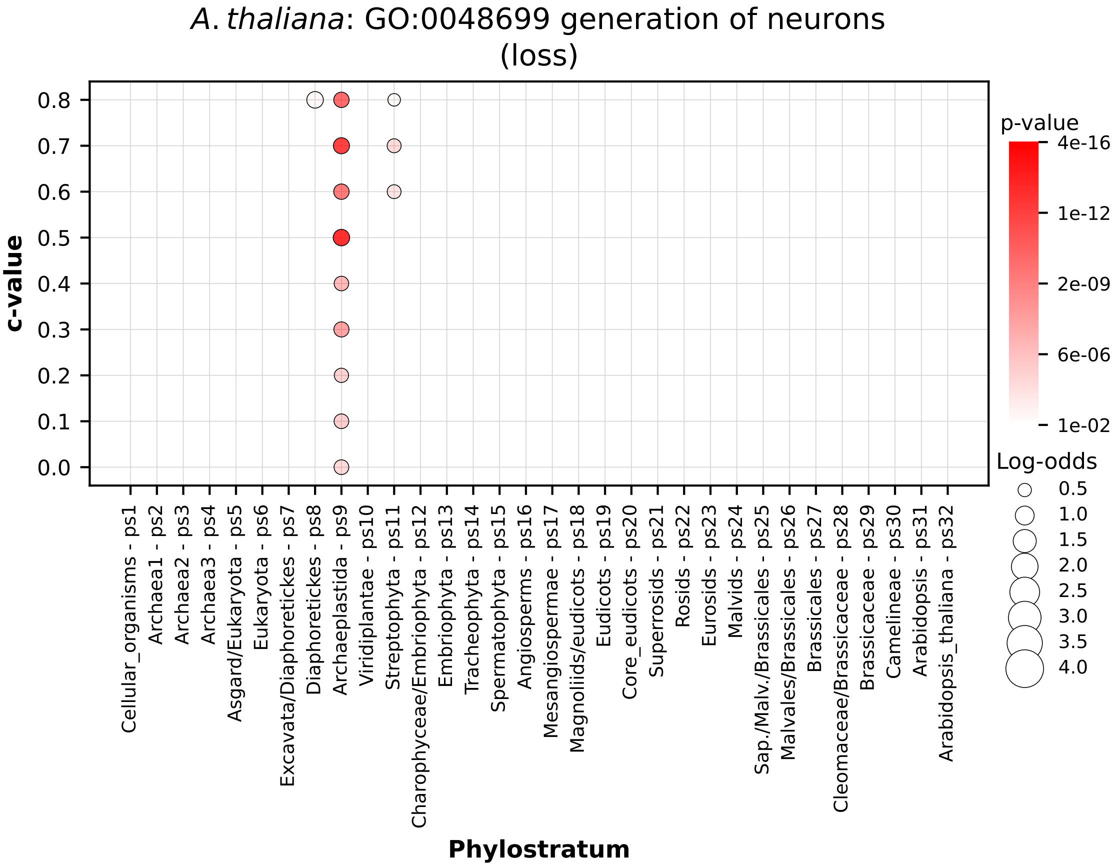

A. thaliana: GO:0048699 generation of neurons
(loss)
c-value
0.8
0.7
0.6
0.5
0.4
0.3
0.2
0.1
0.0
p-value
4e-16
1e-12
2e-09
6e-06
1e-02
Log-odds
0.5
1.0
1.5
2.0
2.5
3.0
3.5
4.0
Cellular_organisms - ps1
Archaea1 - ps2
Archaea2 - ps3
Archaea3 - ps4
Asgard/Eukaryota - ps5
Eukaryota - ps6
Excavata/Diaphoretickes - ps7
Diaphoretickes - ps8
Archaeplastida - ps9
Viridiplantae - ps10
Streptophyta - ps11
Charophyceae/Embriophyta - ps12
Embriophyta - ps13
Tracheophyta - ps14
Spermatophyta - ps15
Angiosperms - ps16
Mesangiospermae - ps17
Magnoliids/eudicots - ps18
Eudicots - ps19
Core_eudicots - ps20
Superrosids - ps21
Rosids - ps22
Eurosids - ps23
Malvids - ps24
Sap./Malv./Brassicales - ps25
Malvales/Brassicales - ps26
Brassicales - ps27
Cleomaceae/Brassicaceae - ps28
Brassicaceae - ps29
Camelineae - ps30
Arabidopsis - ps31
Arabidopsis_thaliana - ps32
Phylostratum

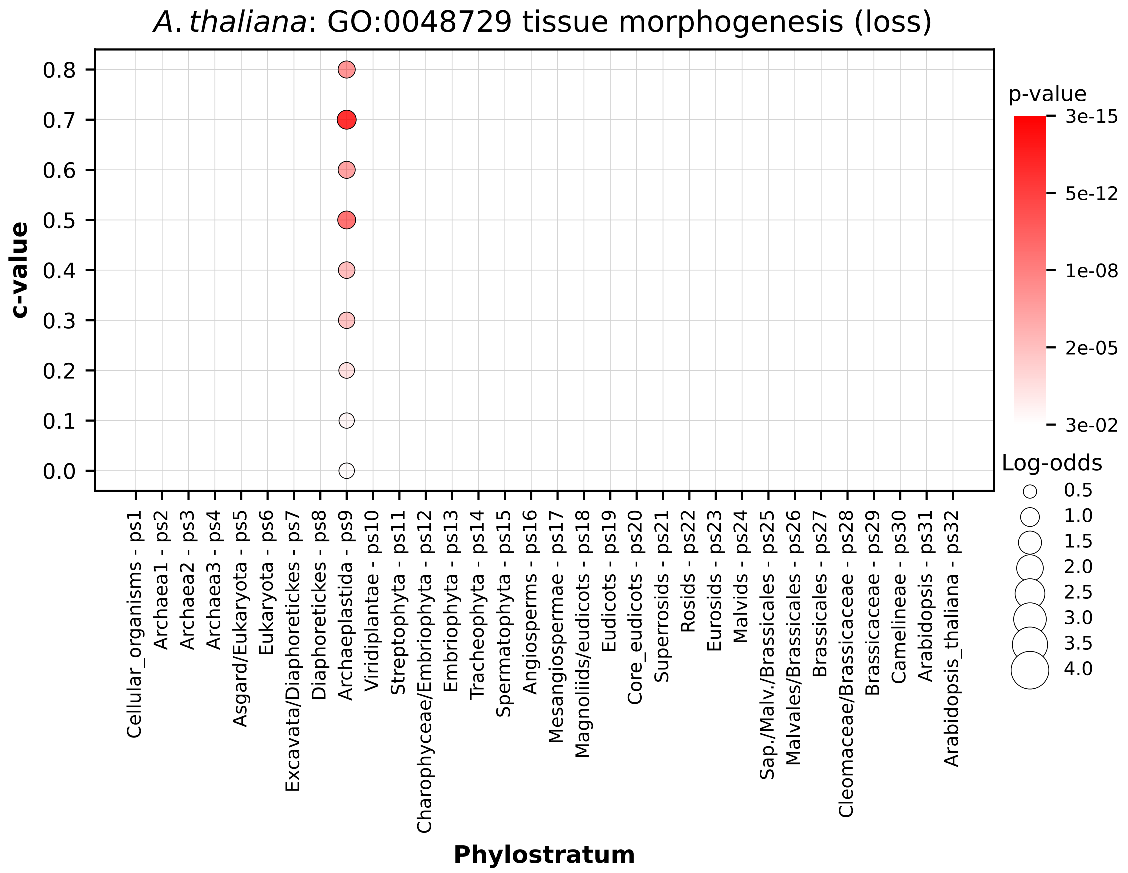

A. thaliana: GO:0048729 tissue morphogenesis (loss)
c-value
0.8
0.7
0.6
0.5
0.4
0.3
0.2
0.1
0.0
p-value
3e-15
5e-12
1e-08
2e-05
3e-02
Log-odds
0.5
1.0
1.5
2.0
2.5
3.0
3.5
4.0
Cellular_organisms - ps1
Archaea1 - ps2
Archaea2 - ps3
Archaea3 - ps4
Asgard/Eukaryota - ps5
Eukaryota - ps6
Excavata/Diaphoretickes - ps7
Diaphoretickes - ps8
Archaeplastida - ps9
Viridiplantae - ps10
Streptophyta - ps11
Charophyceae/Embriophyta - ps12
Embriophyta - ps13
Tracheophyta - ps14
Spermatophyta - ps15
Angiosperms - ps16
Mesangiospermae - ps17
Magnoliids/eudicots - ps18
Eudicots - ps19
Core_eudicots - ps20
Superrosids - ps21
Rosids - ps22
Eurosids - ps23
Malvids - ps24
Sap./Malv./Brassicales - ps25
Malvales/Brassicales - ps26
Brassicales - ps27
Cleomaceae/Brassicaceae - ps28
Brassicaceae - ps29
Camelineae - ps30
Arabidopsis - ps31
Arabidopsis_thaliana - ps32
Phylostratum

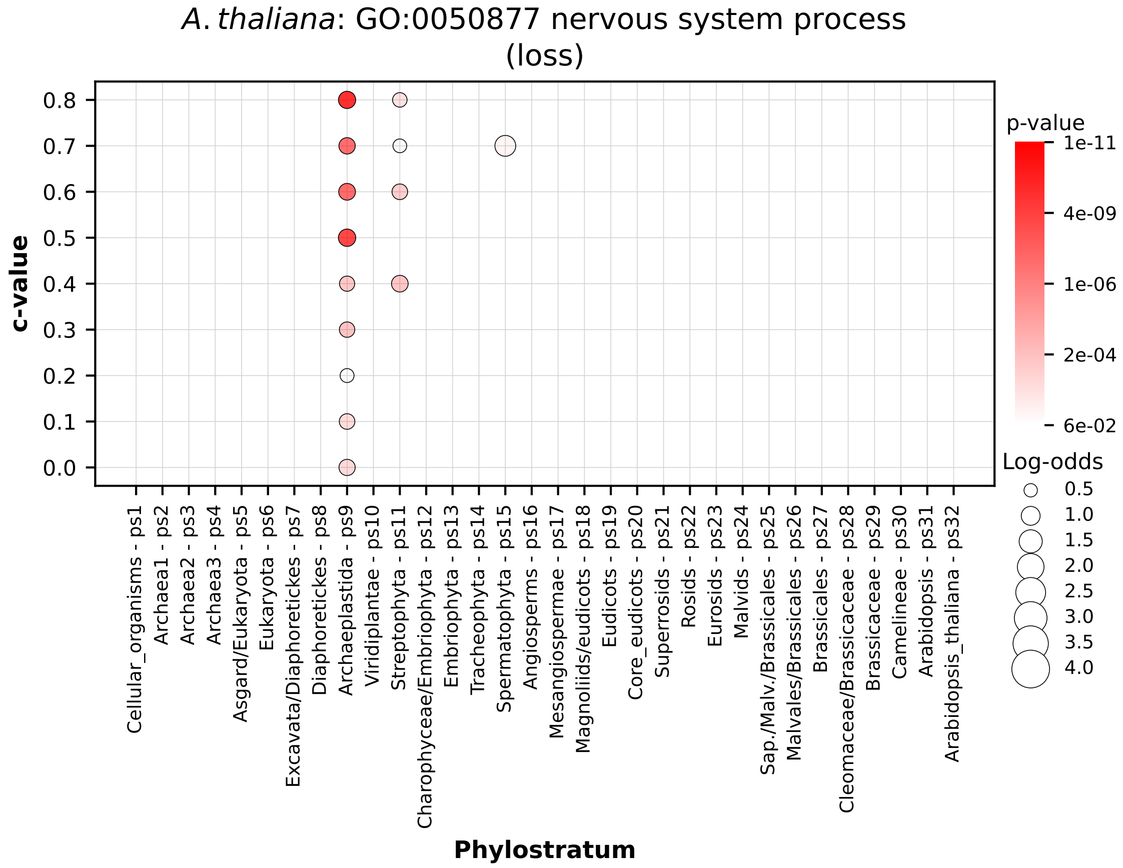

A. thaliana: GO:0050877 nervous system process
(loss)
c-value
0.8
0.7
0.6
0.5
0.4
0.3
0.2
0.1
0.0
p-value
1e-11
4e-09
1e-06
2e-04
6e-02
Log-odds
0.5
1.0
1.5
2.0
2.5
3.0
3.5
4.0
Cellular_organisms - ps1
Archaea1 - ps2
Archaea2 - ps3
Archaea3 - ps4
Asgard/Eukaryota - ps5
Eukaryota - ps6
Excavata/Diaphoretickes - ps7
Diaphoretickes - ps8
Archaeplastida - ps9
Viridiplantae - ps10
Streptophyta - ps11
Charophyceae/Embriophyta - ps12
Embriophyta - ps13
Tracheophyta - ps14
Spermatophyta - ps15
Angiosperms - ps16
Mesangiospermae - ps17
Magnoliids/eudicots - ps18
Eudicots - ps19
Core_eudicots - ps20
Superrosids - ps21
Rosids - ps22
Eurosids - ps23
Malvids - ps24
Sap./Malv./Brassicales - ps25
Malvales/Brassicales - ps26
Brassicales - ps27
Cleomaceae/Brassicaceae - ps28
Brassicaceae - ps29
Camelineae - ps30
Arabidopsis - ps31
Arabidopsis_thaliana - ps32
Phylostratum

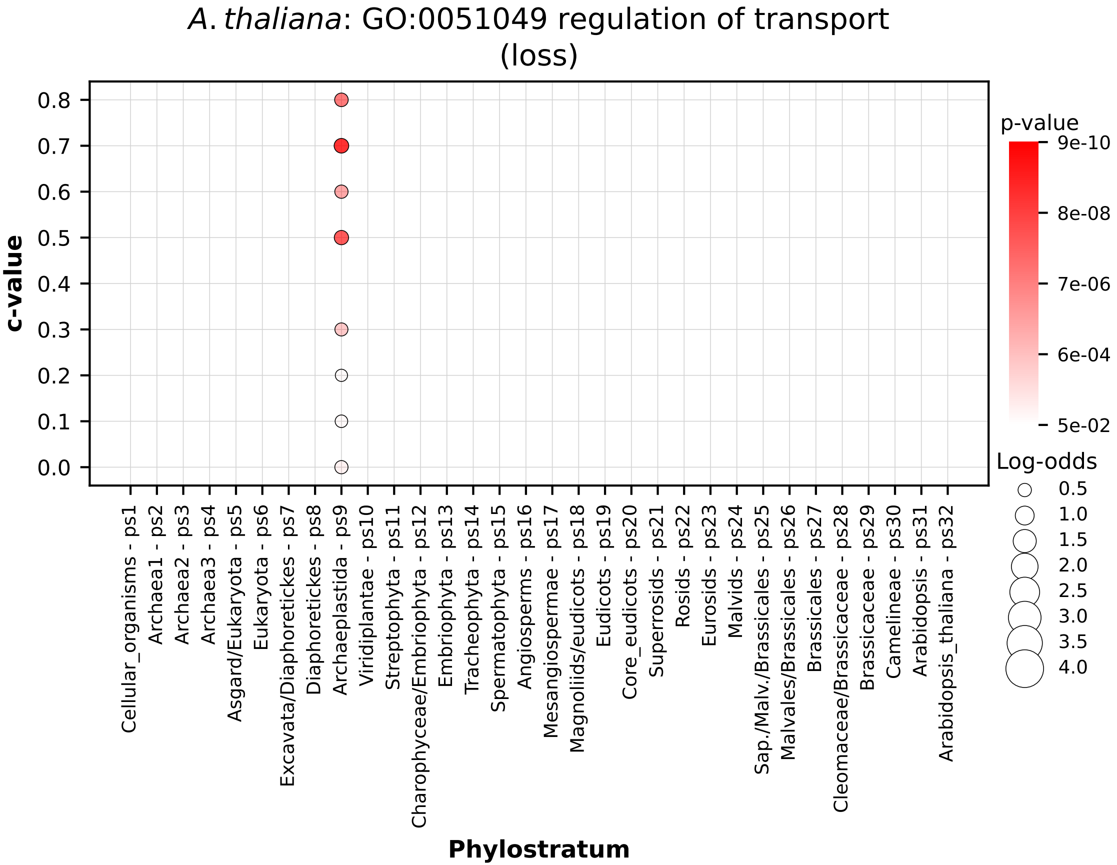
A. thaliana: GO:0051049 regulation of transport
(loss)
c-value
0.8
0.7
0.6
0.5
0.4
0.3
0.2
0.1
0.0
p-value
9e-10
8e-08
7e-06
6e-04
5e-02
Log-odds
0.5
1.0
1.5
2.0
2.5
3.0
3.5
4.0
Cellular_organisms - ps1
Archaea1 - ps2
Archaea2 - ps3
Archaea3 - ps4
Asgard/Eukaryota - ps5
Eukaryota - ps6
Excavata/Diaphoretickes - ps7
Diaphoretickes - ps8
Archaeplastida - ps9
Viridiplantae - ps10
Streptophyta - ps11
Charophyceae/Embriophyta - ps12
Embriophyta - ps13
Tracheophyta - ps14
Spermatophyta - ps15
Angiosperms - ps16
Mesangiospermae - ps17
Magnoliids/eudicots - ps18
Eudicots - ps19
Core_eudicots - ps20
Superrosids - ps21
Rosids - ps22
Eurosids - ps23
Malvids - ps24
Sap./Malv./Brassicales - ps25
Malvales/Brassicales - ps26
Brassicales - ps27
Cleomaceae/Brassicaceae - ps28
Brassicaceae - ps29
Camelineae - ps30
Arabidopsis - ps31
Arabidopsis_thaliana - ps32
Phylostratum

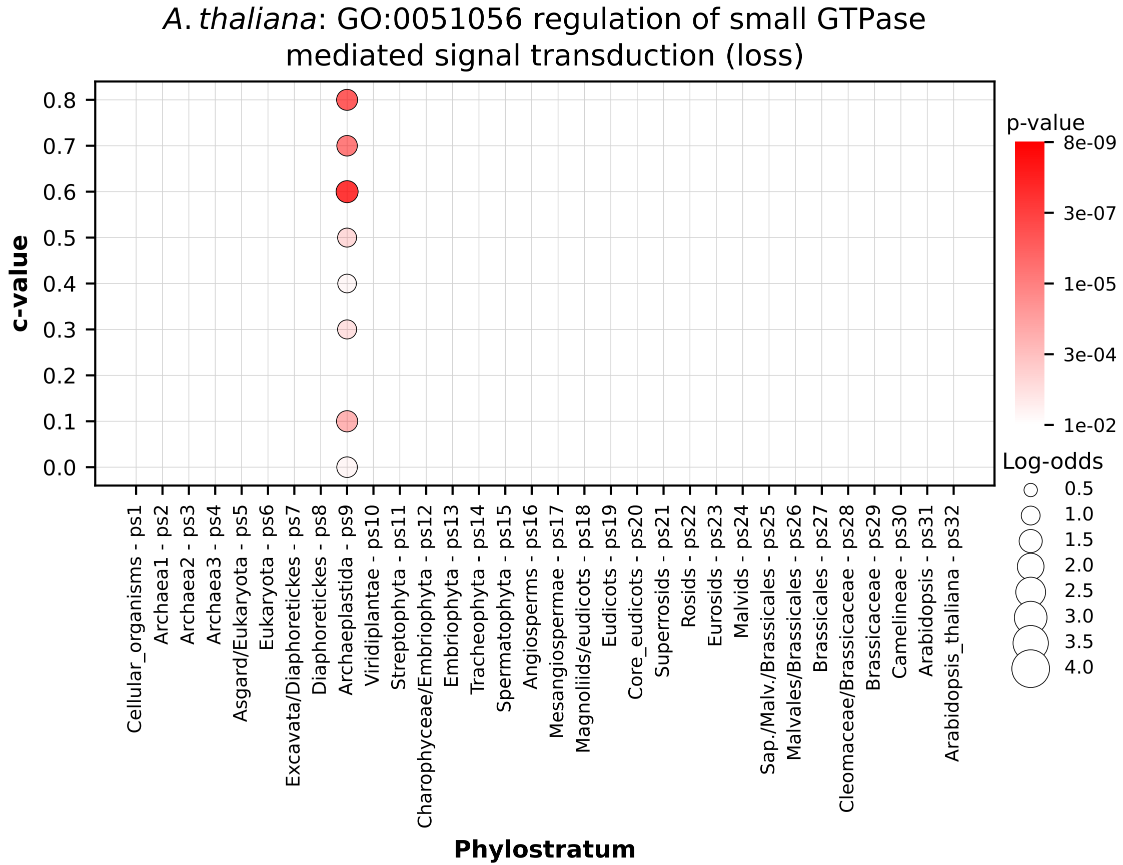
A. thaliana: GO:0051056 regulation of small GTPase mediated signal transduction (loss)
c-value
0.8
0.7
0.6
0.5
0.4
0.3
0.2
0.1
0.0
p-value
8e-09
3e-07
1e-05
3e-04
1e-02
Log-odds
0.5
1.0
1.5
2.0
2.5
3.0
3.5
4.0
Cellular_organisms - ps1
Archaea1 - ps2
Archaea2 - ps3
Archaea3 - ps4
Asgard/Eukaryota - ps5
Eukaryota - ps6
Excavata/Diaphoretickes - ps7
Diaphoretickes - ps8
Archaeplastida - ps9
Viridiplantae - ps10
Streptophyta - ps11
Charophyceae/Embriophyta - ps12
Embriophyta - ps13
Tracheophyta - ps14
Spermatophyta - ps15
Angiosperms - ps16
Mesangiospermae - ps17
Magnoliids/eudicots - ps18
Eudicots - ps19
Core_eudicots - ps20
Superrosids - ps21
Rosids - ps22
Eurosids - ps23
Malvids - ps24
Sap./Malv./Brassicales - ps25
Malvales/Brassicales - ps26
Brassicales - ps27
Cleomaceae/Brassicaceae - ps28
Brassicaceae - ps29
Camelineae - ps30
Arabidopsis - ps31
Arabidopsis_thaliana - ps32
Phylostratum

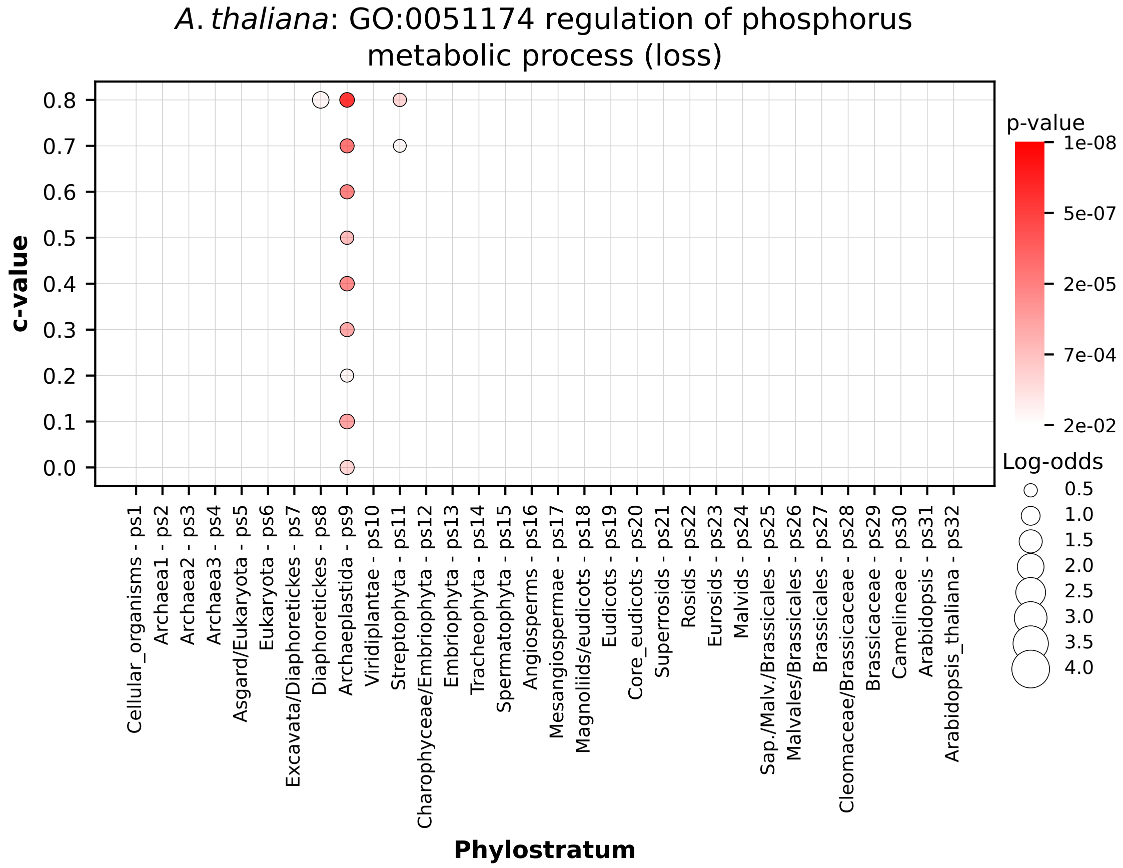

A. thaliana: GO:0051174 regulation of phosphorus metabolic process (loss)
c-value
Phylostratum
p-value
1e-08
5e-07
2e-05
7e-04
2e-02
Log-odds
0.5
1.0
1.5
2.0
2.5
3.0
3.5
4.0
Cellular_organisms - ps1
Archaea1 - ps2
Archaea2 - ps3
Archaea3 - ps4
Asgard/Eukaryota - ps5
Eukaryota - ps6
Excavata/Diaphoretickes - ps7
Diaphoretickes - ps8
Archaeplastida - ps9
Viridiplantae - ps10
Streptophyta - ps11
Charophyceae/Embriophyta - ps12
Embriophyta - ps13
Tracheophyta - ps14
Spermatophyta - ps15
Angiosperms - ps16
Mesangiospermae - ps17
Magnoliids/eudicots - ps18
Eudicots - ps19
Core_eudicots - ps20
Superrosids - ps21
Rosids - ps22
Eurosids - ps23
Malvids - ps24
Sap./Malv./Brassicales - ps25
Malvales/Brassicales - ps26
Brassicales - ps27
Cleomaceae/Brassicaceae - ps28
Brassicaceae - ps29
Camelineae - ps30
Arabidopsis - ps31
Arabidopsis_thaliana - ps32

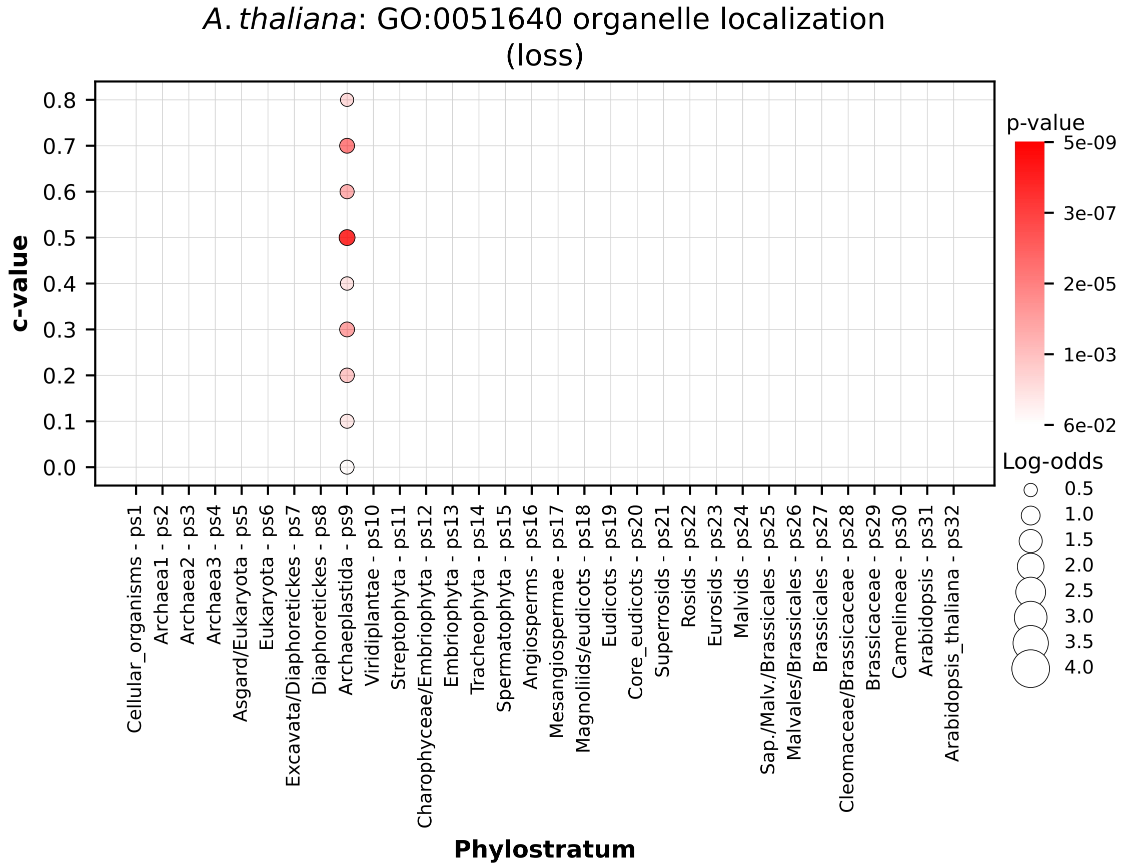

A. thaliana: GO:0051640 organelle localization
(loss)
c-value
0.8
0.7
0.6
0.5
0.4
0.3
0.2
0.1
0.0
Phylostratum
Cellular_organisms - ps1
Archaea1 - ps2
Archaea2 - ps3
Archaea3 - ps4
Asgard/Eukaryota - ps5
Eukaryota - ps6
Excavata/Diaphoretickes - ps7
Diaphoretickes - ps8
Archaeplastida - ps9
Viridiplantae - ps10
Streptophyta - ps11
Charophyceae/Embriophyta - ps12
Embriophyta - ps13
Tracheophyta - ps14
Spermatophyta - ps15
Angiosperms - ps16
Mesangiospermae - ps17
Magnoliids/eudicots - ps18
Eudicots - ps19
Core_eudicots - ps20
Superrosids - ps21
Rosids - ps22
Eurosids - ps23
Malvids - ps24
Sap./Malv./Brassicales - ps25
Malvales/Brassicales - ps26
Brassicales - ps27
Cleomaceae/Brassicaceae - ps28
Brassicaceae - ps29
Camelineae - ps30
Arabidopsis - ps31
Arabidopsis_thaliana - ps32
p-value
5e-09
3e-07
2e-05
1e-03
6e-02
Log-odds
0.5
1.0
1.5
2.0
2.5
3.0
3.5
4.0

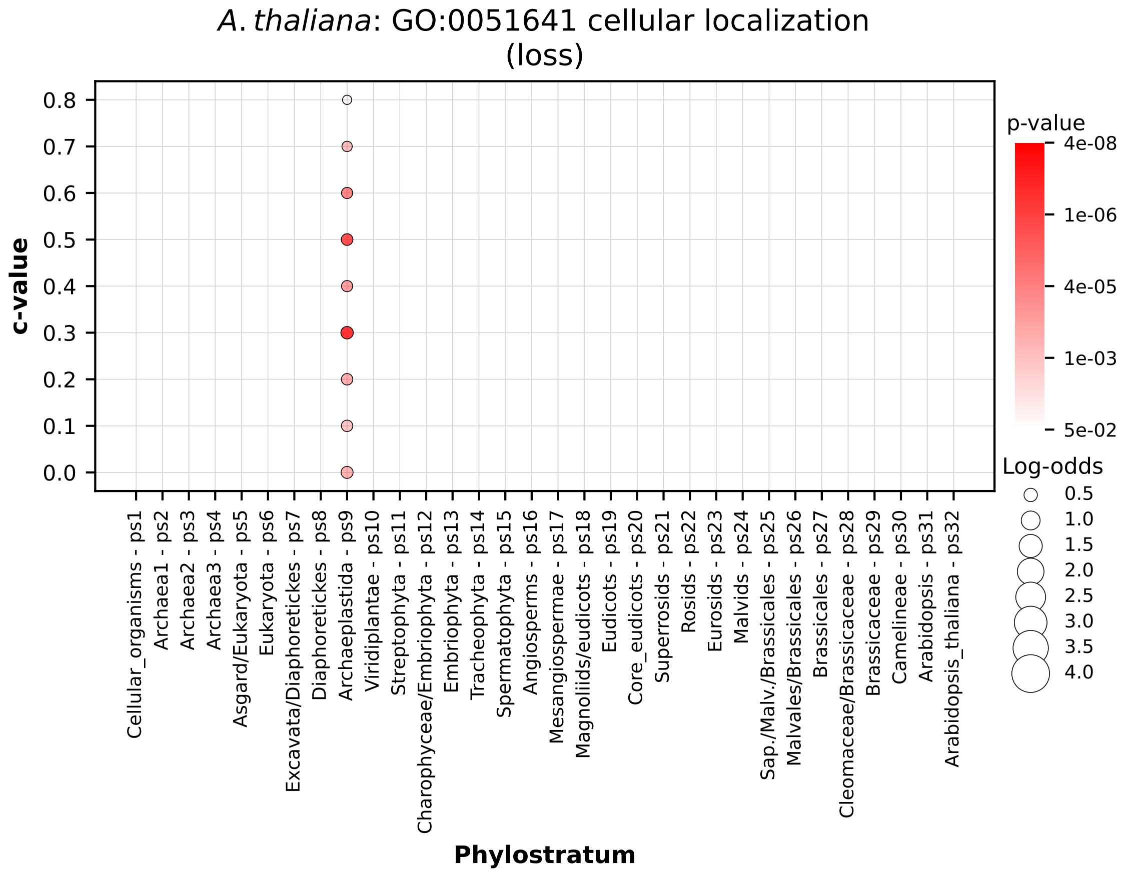

A. thaliana: GO:0051641 cellular localization
(loss)
c-value
0.8
0.7
0.6
0.5
0.4
0.3
0.2
0.1
0.0
p-value
4e-08
1e-06
4e-05
1e-03
5e-02
Log-odds
0.5
1.0
1.5
2.0
2.5
3.0
3.5
4.0
Cellular_organisms - ps1
Archaea1 - ps2
Archaea2 - ps3
Archaea3 - ps4
Asgard/Eukaryota - ps5
Eukaryota - ps6
Excavata/Diaphoretickes - ps7
Diaphoretickes - ps8
Archaeplastida - ps9
Viridiplantae - ps10
Streptophyta - ps11
Charophyceae/Embriophyta - ps12
Embriophyta - ps13
Tracheophyta - ps14
Spermatophyta - ps15
Angiosperms - ps16
Mesangiospermae - ps17
Magnoliids/eudicots - ps18
Eudicots - ps19
Core_eudicots - ps20
Superrosids - ps21
Rosids - ps22
Eurosids - ps23
Malvids - ps24
Sap./Malv./Brassicales - ps25
Malvales/Brassicales - ps26
Brassicales - ps27
Cleomaceae/Brassicaceae - ps28
Brassicaceae - ps29
Camelineae - ps30
Arabidopsis - ps31
Arabidopsis_thaliana - ps32
Phylostratum

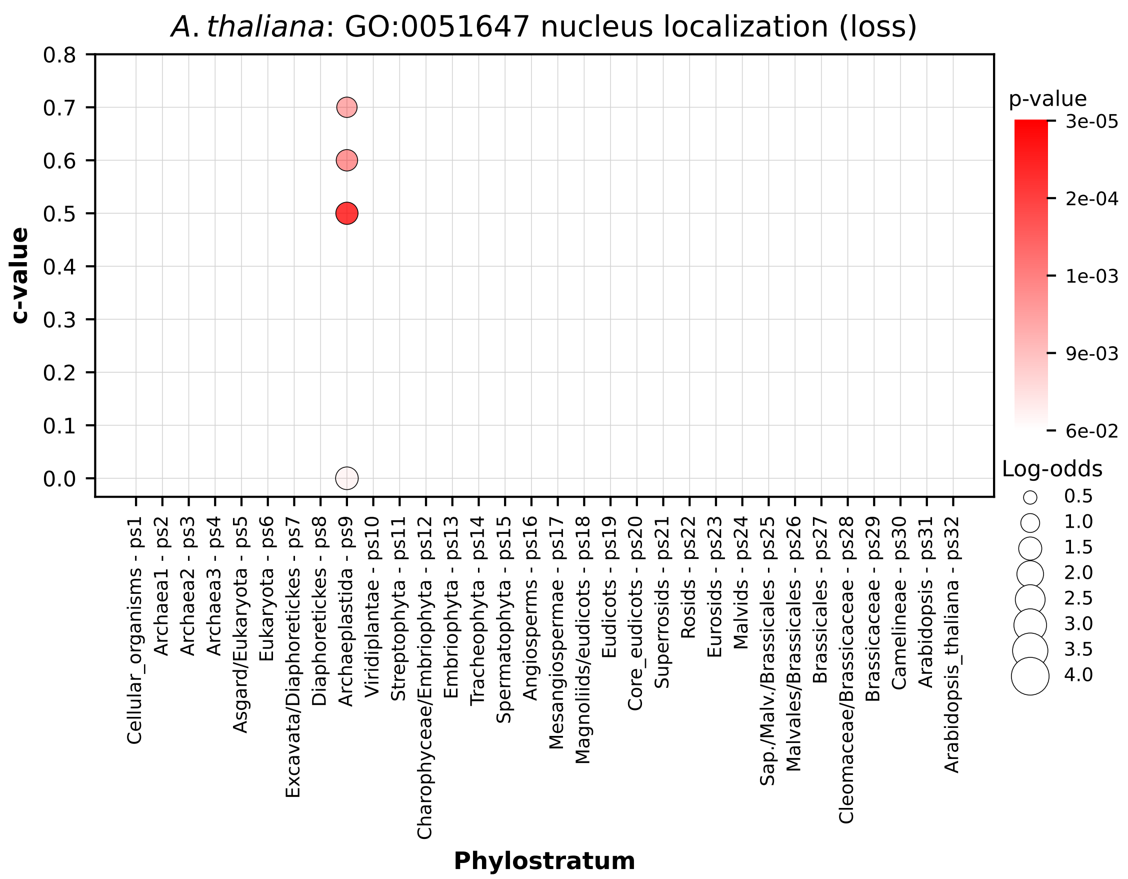

A. thaliana: GO:0051647 nucleus localization (loss)
c-value
0.8
0.7
0.6
0.5
0.4
0.3
0.2
0.1
0.0
Phylostratum
Cellular_organisms - ps1
Archaea1 - ps2
Archaea2 - ps3
Archaea3 - ps4
Asgard/Eukaryota - ps5
Eukaryota - ps6
Excavata/Diaphoretickes - ps7
Diaphoretickes - ps8
Archaeplastida - ps9
Viridiplantae - ps10
Streptophyta - ps11
Charophyceae/Embriophyta - ps12
Embriophyta - ps13
Tracheophyta - ps14
Spermatophyta - ps15
Angiosperms - ps16
Mesangiospermae - ps17
Magnoliids/eudicots - ps18
Eudicots - ps19
Core_eudicots - ps20
Superrosids - ps21
Rosids - ps22
Eurosids - ps23
Malvids - ps24
Sap./Malv./Brassicales - ps25
Malvales/Brassicales - ps26
Brassicales - ps27
Cleomaceae/Brassicaceae - ps28
Brassicaceae - ps29
Camelineae - ps30
Arabidopsis - ps31
Arabidopsis_thaliana - ps32
p-value
3e-05
2e-04
1e-03
9e-03
6e-02
Log-odds
0.5
1.0
1.5
2.0
2.5
3.0
3.5
4.0

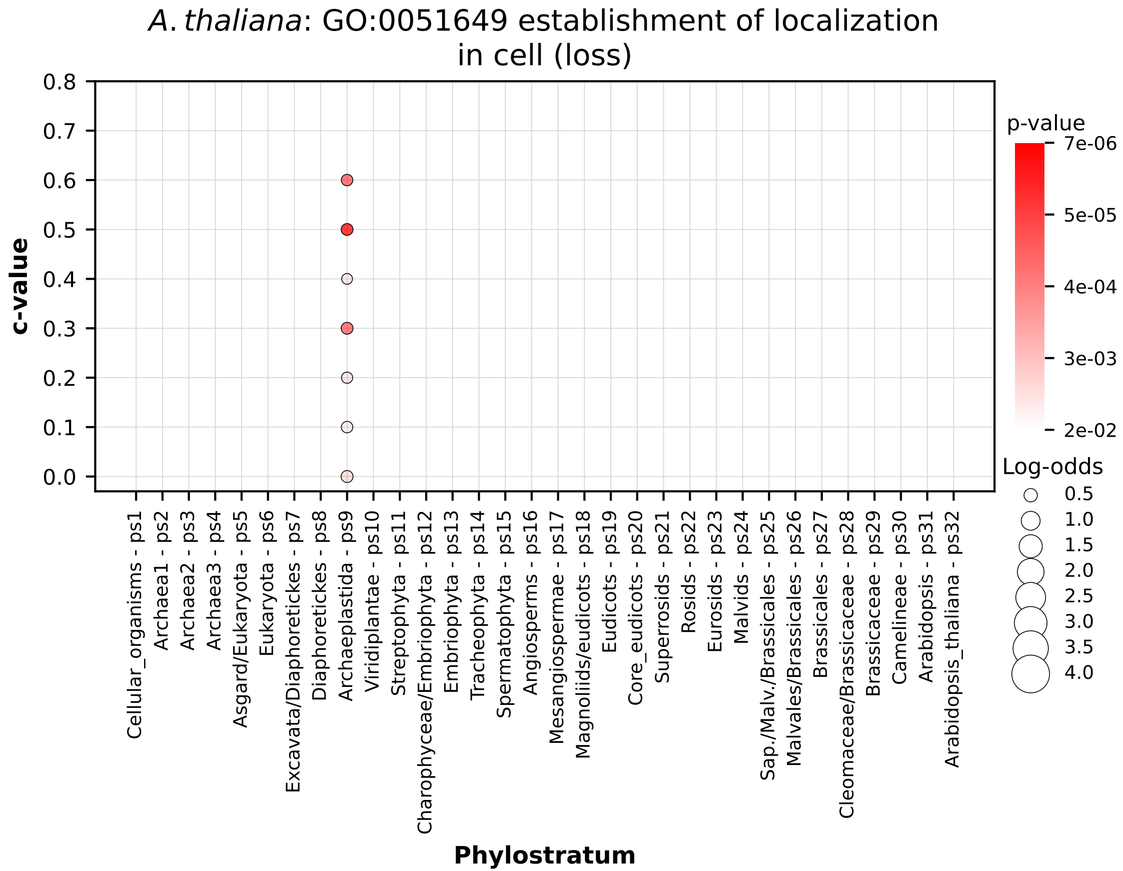

A. thaliana: GO:0051649 establishment of localization in cell (loss)
c-value
0.8
0.7
0.6
0.5
0.4
0.3
0.2
0.1
0.0
p-value
7e-06
5e-05
4e-04
3e-03
2e-02
Log-odds
0.5
1.0
1.5
2.0
2.5
3.0
3.5
4.0
Cellular_organisms - ps1
Archaea1 - ps2
Archaea2 - ps3
Archaea3 - ps4
Asgard/Eukaryota - ps5
Eukaryota - ps6
Excavata/Diaphoretickes - ps7
Diaphoretickes - ps8
Archaeplastida - ps9
Viridiplantae - ps10
Streptophyta - ps11
Charophyceae/Embriophyta - ps12
Embriophyta - ps13
Tracheophyta - ps14
Spermatophyta - ps15
Angiosperms - ps16
Mesangiospermae - ps17
Magnoliids/eudicots - ps18
Eudicots - ps19
Core_eudicots - ps20
Superrosids - ps21
Rosids - ps22
Eurosids - ps23
Malvids - ps24
Sap./Malv./Brassicales - ps25
Malvales/Brassicales - ps26
Brassicales - ps27
Cleomaceae/Brassicaceae - ps28
Brassicaceae - ps29
Camelineae - ps30
Arabidopsis - ps31
Arabidopsis_thaliana - ps32
Phylostratum

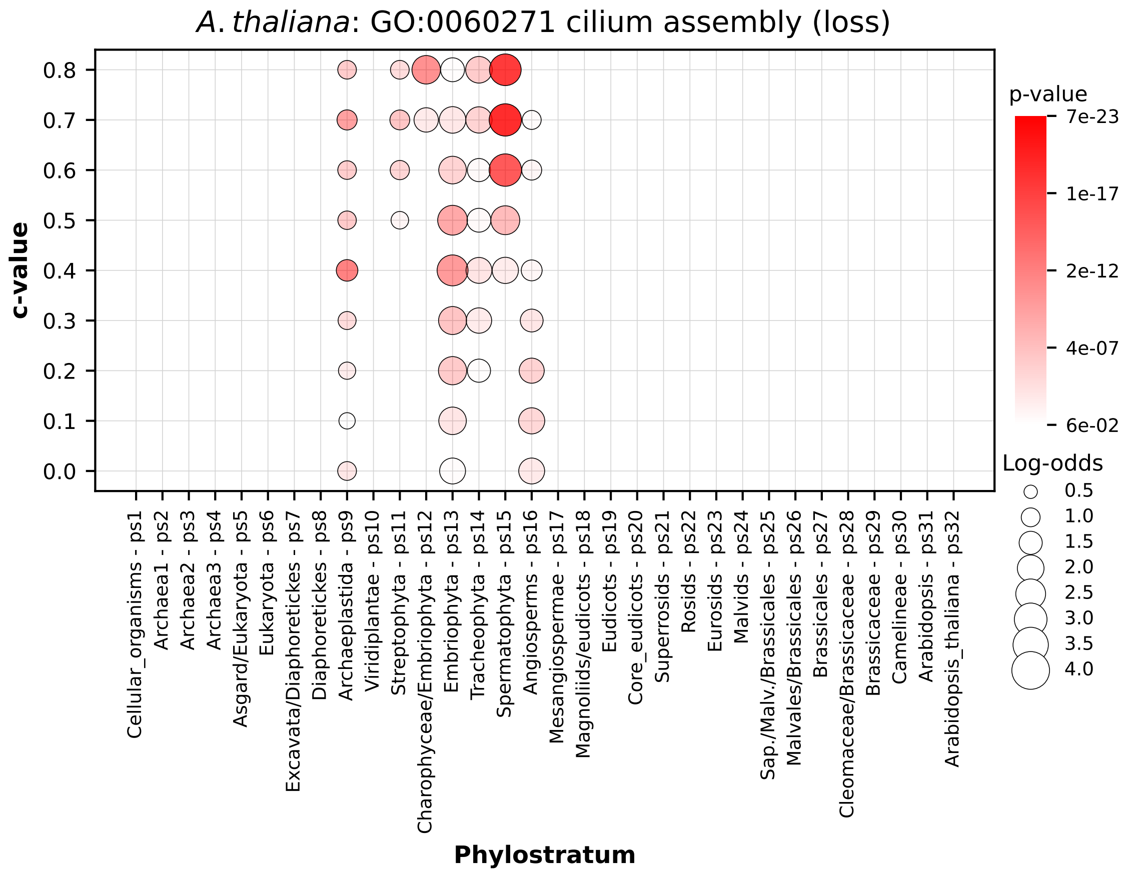

A. thaliana: GO:0060271 cilium assembly (loss)
c-value
0.8
0.7
0.6
0.5
0.4
0.3
0.2
0.1
0.0
p-value
7e-23
1e-17
2e-12
4e-07
6e-02
Log-odds
0.5
1.0
1.5
2.0
2.5
3.0
3.5
4.0
Cellular_organisms - ps1
Archaea1 - ps2
Archaea2 - ps3
Archaea3 - ps4
Asgard/Eukaryota - ps5
Eukaryota - ps6
Excavata/Diaphoretickes - ps7
Diaphoretickes - ps8
Archaeplastida - ps9
Viridiplantae - ps10
Streptophyta - ps11
Charophyceae/Embriophyta - ps12
Embriophyta - ps13
Tracheophyta - ps14
Spermatophyta - ps15
Angiosperms - ps16
Mesangiospermae - ps17
Magnoliids/eudicots - ps18
Eudicots - ps19
Core_eudicots - ps20
Superrosids - ps21
Rosids - ps22
Eurosids - ps23
Malvids - ps24
Sap./Malv./Brassicales - ps25
Malvales/Brassicales - ps26
Brassicales - ps27
Cleomaceae/Brassicaceae - ps28
Brassicaceae - ps29
Camelineae - ps30
Arabidopsis - ps31
Arabidopsis_thaliana - ps32
Phylostratum

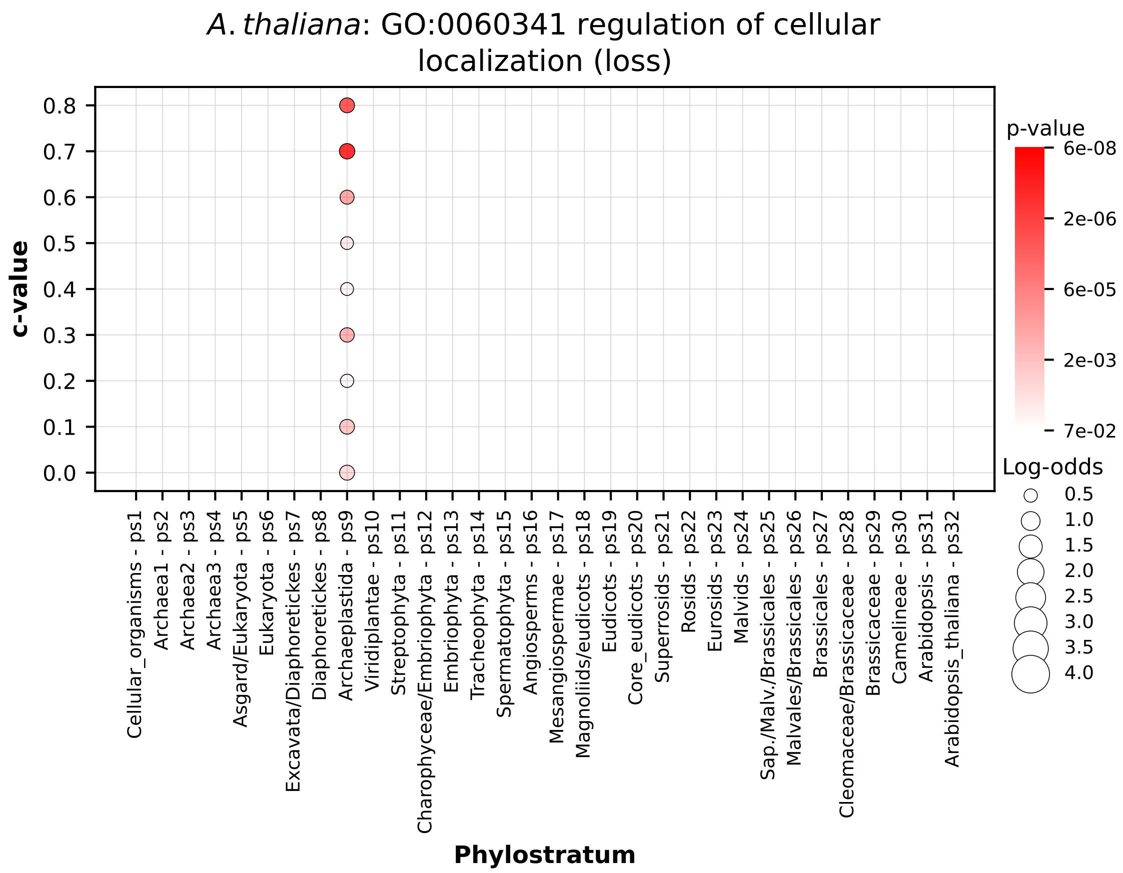

A. thaliana: GO:0060341 regulation of cellular localization (loss)
c-value
0.8
0.7
0.6
0.5
0.4
0.3
0.2
0.1
0.0
Phylostratum
Cellular_organisms - ps1
Archaea1 - ps2
Archaea2 - ps3
Archaea3 - ps4
Asgard/Eukaryota - ps5
Eukaryota - ps6
Excavata/Diaphoretickes - ps7
Diaphoretickes - ps8
Archaeplastida - ps9
Viridiplantae - ps10
Streptophyta - ps11
Charophyceae/Embriophyta - ps12
Embriophyta - ps13
Tracheophyta - ps14
Spermatophyta - ps15
Angiosperms - ps16
Mesangiospermae - ps17
Magnoliids/eudicots - ps18
Eudicots - ps19
Core_eudicots - ps20
Superrosids - ps21
Rosids - ps22
Eurosids - ps23
Malvids - ps24
Sap./Malv./Brassicales - ps25
Malvales/Brassicales - ps26
Brassicales - ps27
Cleomaceae/Brassicaceae - ps28
Brassicaceae - ps29
Camelineae - ps30
Arabidopsis - ps31
Arabidopsis_thaliana - ps32
p-value
6e-08
2e-06
6e-05
2e-03
7e-02
Log-odds
0.5
1.0
1.5
2.0
2.5
3.0
3.5
4.0

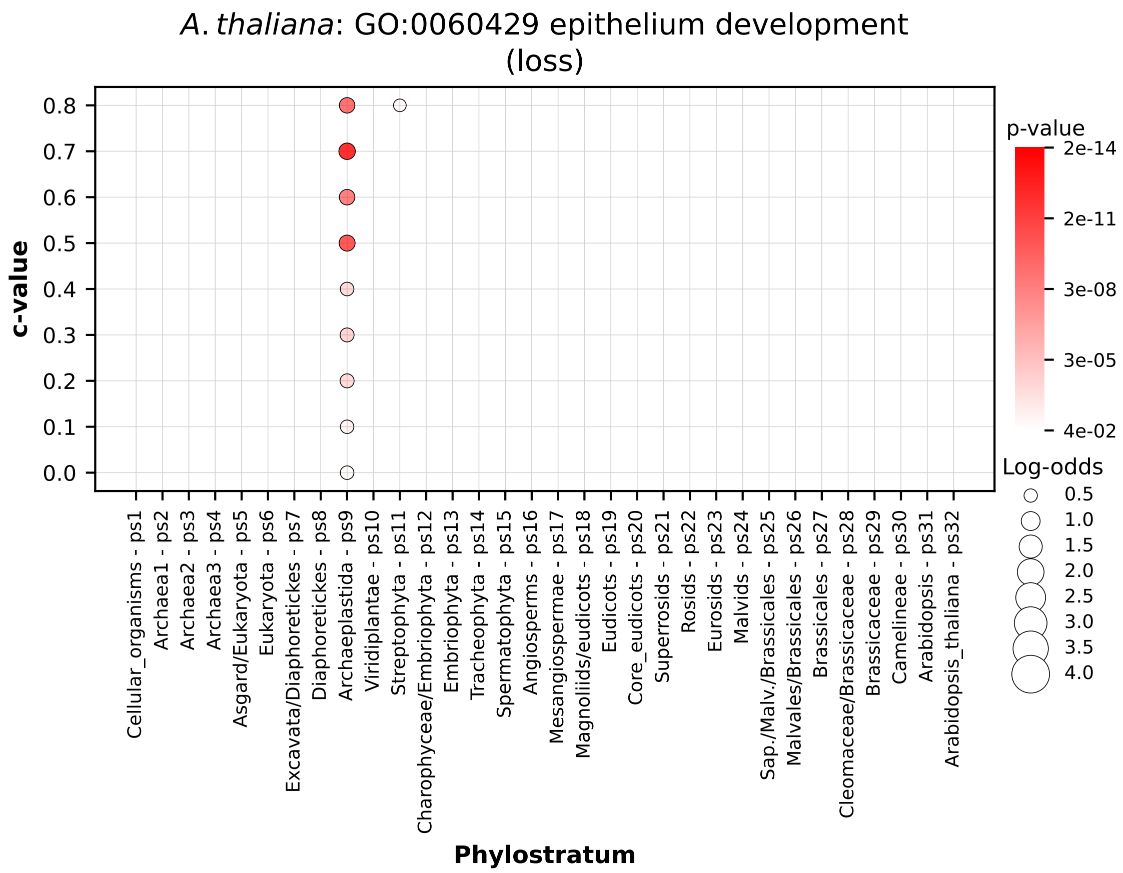

A. thaliana: GO:0060429 epithelium development
(loss)
c-value
0.8
0.7
0.6
0.5
0.4
0.3
0.2
0.1
0.0
p-value
2e-14
2e-11
3e-08
3e-05
4e-02
Log-odds
0.5
1.0
1.5
2.0
2.5
3.0
3.5
4.0
Cellular_organisms - ps1
Archaea1 - ps2
Archaea2 - ps3
Archaea3 - ps4
Asgard/Eukaryota - ps5
Eukaryota - ps6
Excavata/Diaphoretickes - ps7
Diaphoretickes - ps8
Archaeplastida - ps9
Viridiplantae - ps10
Streptophyta - ps11
Charophyceae/Embriophyta - ps12
Embriophyta - ps13
Tracheophyta - ps14
Spermatophyta - ps15
Angiosperms - ps16
Mesangiospermae - ps17
Magnoliids/eudicots - ps18
Eudicots - ps19
Core_eudicots - ps20
Superrosids - ps21
Rosids - ps22
Eurosids - ps23
Malvids - ps24
Sap./Malv./Brassicales - ps25
Malvales/Brassicales - ps26
Brassicales - ps27
Cleomaceae/Brassicaceae - ps28
Brassicaceae - ps29
Camelineae - ps30
Arabidopsis - ps31
Arabidopsis_thaliana - ps32
Phylostratum

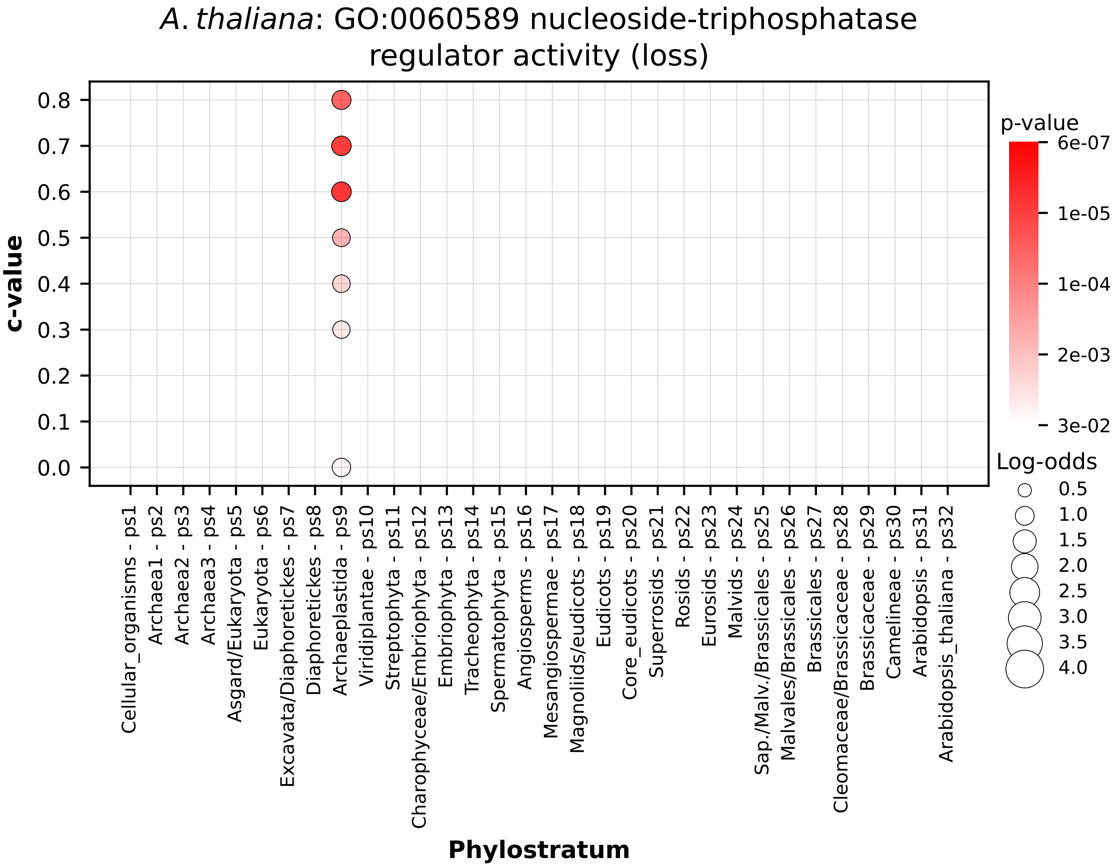
A. thaliana: GO:0060589 nucleoside-triphosphatase regulator activity (loss)
c-value
0.8
0.7
0.6
0.5
0.4
0.3
0.2
0.1
0.0
Phylostratum
Cellular_organisms - ps1
Archaea1 - ps2
Archaea2 - ps3
Archaea3 - ps4
Asgard/Eukaryota - ps5
Eukaryota - ps6
Excavata/Diaphoretickes - ps7
Diaphoretickes - ps8
Archaeplastida - ps9
Viridiplantae - ps10
Streptophyta - ps11
Charophyceae/Embriophyta - ps12
Embriophyta - ps13
Tracheophyta - ps14
Spermatophyta - ps15
Angiosperms - ps16
Mesangiospermae - ps17
Magnoliids/eudicots - ps18
Eudicots - ps19
Core_eudicots - ps20
Superrosids - ps21
Rosids - ps22
Eurosids - ps23
Malvids - ps24
Sap./Malv./Brassicales - ps25
Malvales/Brassicales - ps26
Brassicales - ps27
Cleomaceae/Brassicaceae - ps28
Brassicaceae - ps29
Camelineae - ps30
Arabidopsis - ps31
Arabidopsis_thaliana - ps32
p-value
6e-07
1e-05
1e-04
2e-03
3e-02
Log-odds
0.5
1.0
1.5
2.0
2.5
3.0
3.5
4.0

A. thaliana: GO:0065009 regulation of molecular function (loss)

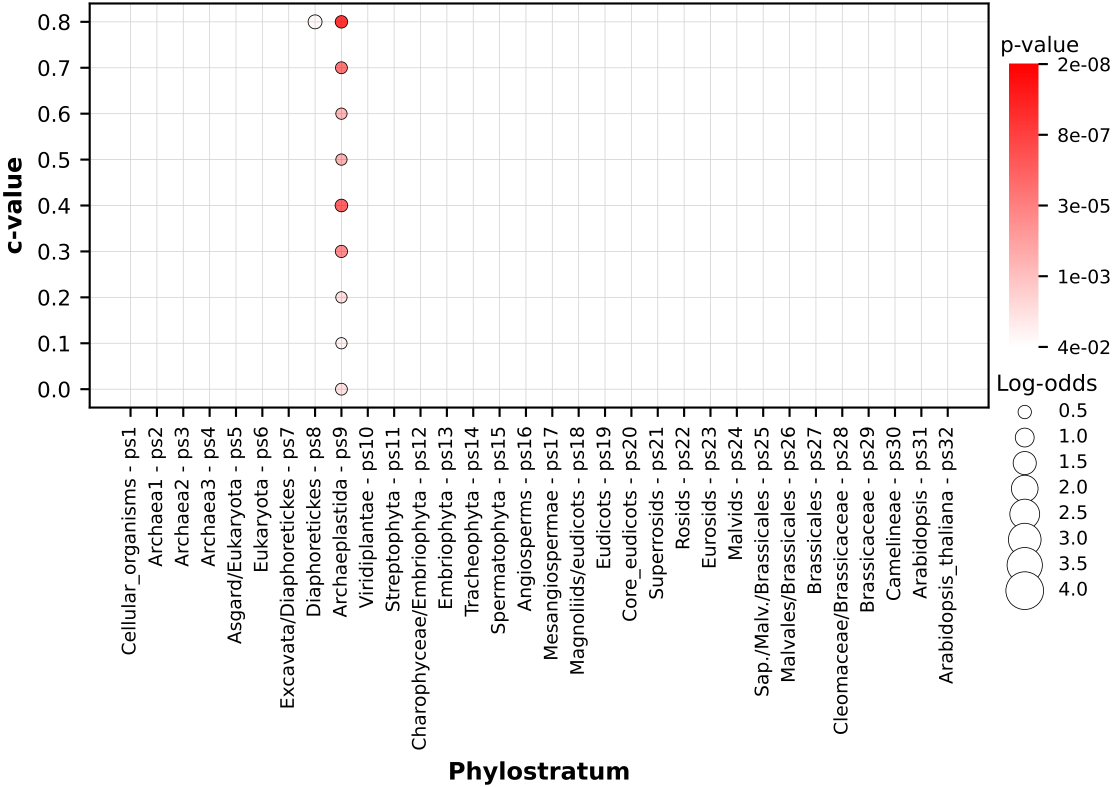

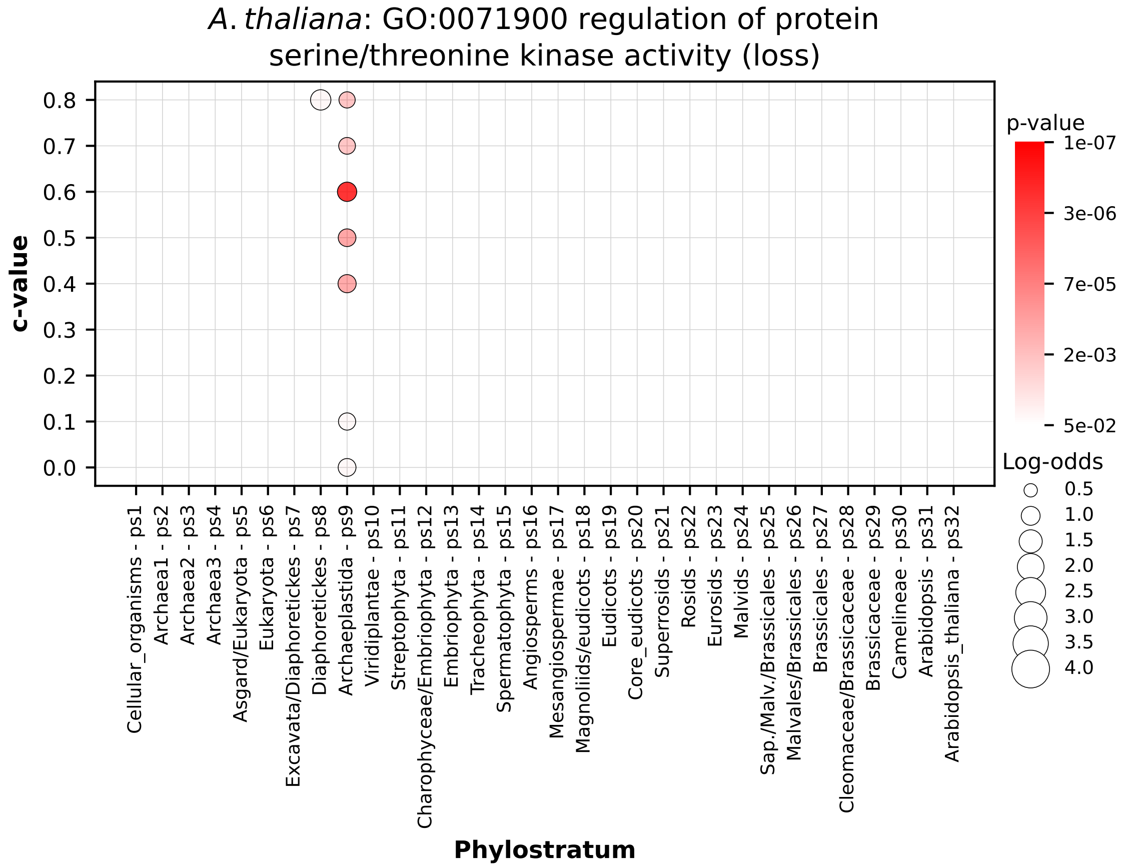

A. thaliana: GO:0071900 regulation of protein serine/threonine kinase activity (loss)
c-value
0.8
0.7
0.6
0.5
0.4
0.3
0.2
0.1
0.0
p-value
1e-07
3e-06
7e-05
2e-03
5e-02
Log-odds
0.5
1.0
1.5
2.0
2.5
3.0
3.5
4.0
Cellular_organisms - ps1
Archaea1 - ps2
Archaea2 - ps3
Archaea3 - ps4
Asgard/Eukaryota - ps5
Eukaryota - ps6
Excavata/Diaphoretickes - ps7
Diaphoretickes - ps8
Archaeplastida - ps9
Viridiplantae - ps10
Streptophyta - ps11
Charophyceae/Embriophyta - ps12
Embriophyta - ps13
Tracheophyta - ps14
Spermatophyta - ps15
Angiosperms - ps16
Mesangiospermae - ps17
Magnoliids/eudicots - ps18
Eudicots - ps19
Core_eudicots - ps20
Superrosids - ps21
Rosids - ps22
Eurosids - ps23
Malvids - ps24
Sap./Malv./Brassicales - ps25
Malvales/Brassicales - ps26
Brassicales - ps27
Cleomaceae/Brassicaceae - ps28
Brassicaceae - ps29
Camelineae - ps30
Arabidopsis - ps31
Arabidopsis_thaliana - ps32
Phylostratum

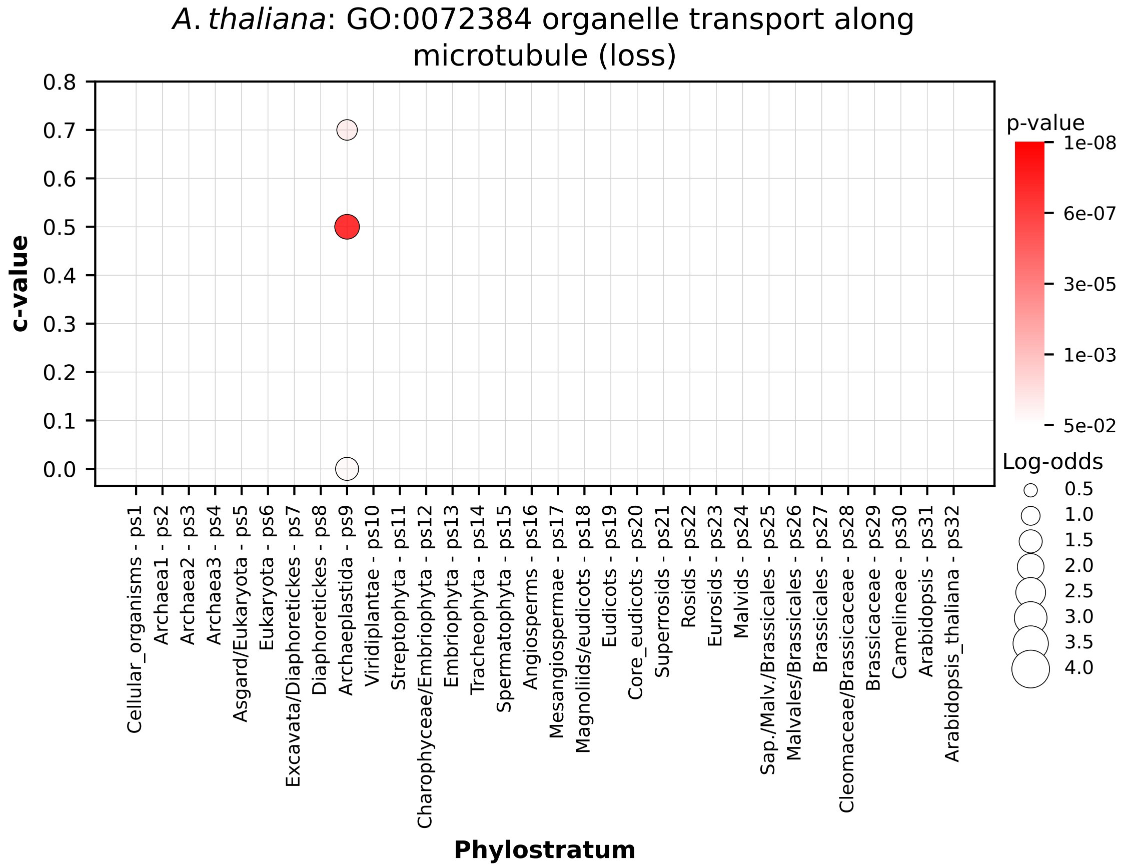

A. thaliana: GO:0072384 organelle transport along microtubule (loss)
c-value
0.8
0.7
0.6
0.5
0.4
0.3
0.2
0.1
0.0
p-value
1e-08
6e-07
3e-05
1e-03
5e-02
Log-odds
0.5
1.0
1.5
2.0
2.5
3.0
3.5
4.0
Cellular_organisms - ps1
Archaea1 - ps2
Archaea2 - ps3
Archaea3 - ps4
Asgard/Eukaryota - ps5
Eukaryota - ps6
Excavata/Diaphoretickes - ps7
Diaphoretickes - ps8
Archaeplastida - ps9
Viridiplantae - ps10
Streptophyta - ps11
Charophyceae/Embriophyta - ps12
Embriophyta - ps13
Tracheophyta - ps14
Spermatophyta - ps15
Angiosperms - ps16
Mesangiospermae - ps17
Magnoliids/eudicots - ps18
Eudicots - ps19
Core_eudicots - ps20
Superrosids - ps21
Rosids - ps22
Eurosids - ps23
Malvids - ps24
Sap./Malv./Brassicales - ps25
Malvales/Brassicales - ps26
Brassicales - ps27
Cleomaceae/Brassicaceae - ps28
Brassicaceae - ps29
Camelineae - ps30
Arabidopsis - ps31
Arabidopsis_thaliana - ps32
Phylostratum

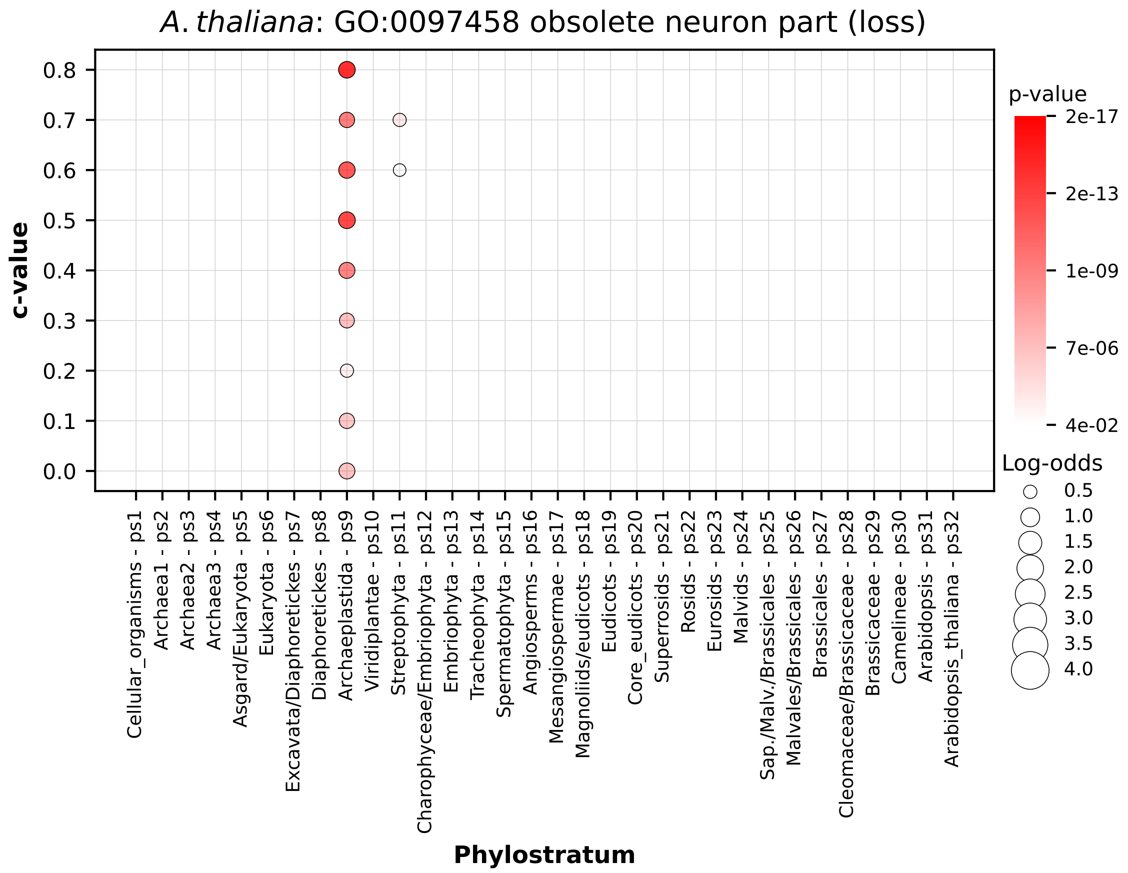

A. thaliana: GO:0097458 obsolete neuron part (loss)
c-value
0.8
0.7
0.6
0.5
0.4
0.3
0.2
0.1
0.0
p-value
2e-17
2e-13
1e-09
7e-06
4e-02
Log-odds
0.5
1.0
1.5
2.0
2.5
3.0
3.5
4.0
Cellular_organisms - ps1
Archaea1 - ps2
Archaea2 - ps3
Archaea3 - ps4
Asgard/Eukaryota - ps5
Eukaryota - ps6
Excavata/Diaphoretickes - ps7
Diaphoretickes - ps8
Archaeplastida - ps9
Viridiplantae - ps10
Streptophyta - ps11
Charophyceae/Embriophyta - ps12
Embriophyta - ps13
Tracheophyta - ps14
Spermatophyta - ps15
Angiosperms - ps16
Mesangiospermae - ps17
Magnoliids/eudicots - ps18
Eudicots - ps19
Core_eudicots - ps20
Superrosids - ps21
Rosids - ps22
Eurosids - ps23
Malvids - ps24
Sap./Malv./Brassicales - ps25
Malvales/Brassicales - ps26
Brassicales - ps27
Cleomaceae/Brassicaceae - ps28
Brassicaceae - ps29
Camelineae - ps30
Arabidopsis - ps31
Arabidopsis_thaliana - ps32
Phylostratum

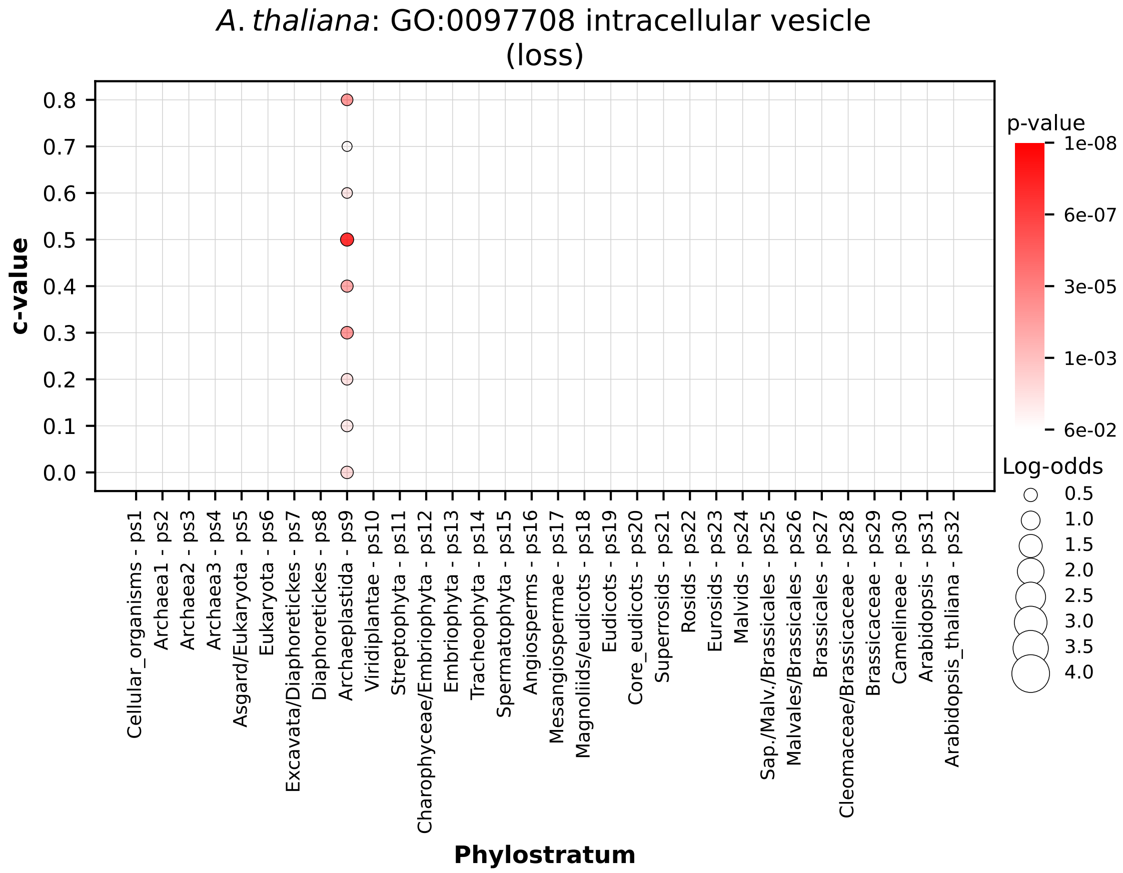

A. thaliana: GO:0097708 intracellular vesicle
(loss)
c-value
0.8
0.7
0.6
0.5
0.4
0.3
0.2
0.1
0.0
p-value
1e-08
6e-07
3e-05
1e-03
6e-02
Log-odds
0.5
1.0
1.5
2.0
2.5
3.0
3.5
4.0
Cellular_organisms - ps1
Archaea1 - ps2
Archaea2 - ps3
Archaea3 - ps4
Asgard/Eukaryota - ps5
Eukaryota - ps6
Excavata/Diaphoretickes - ps7
Diaphoretickes - ps8
Archaeplastida - ps9
Viridiplantae - ps10
Streptophyta - ps11
Charophyceae/Embriophyta - ps12
Embriophyta - ps13
Tracheophyta - ps14
Spermatophyta - ps15
Angiosperms - ps16
Mesangiospermae - ps17
Magnoliids/eudicots - ps18
Eudicots - ps19
Core_eudicots - ps20
Superrosids - ps21
Rosids - ps22
Eurosids - ps23
Malvids - ps24
Sap./Malv./Brassicales - ps25
Malvales/Brassicales - ps26
Brassicales - ps27
Cleomaceae/Brassicaceae - ps28
Brassicaceae - ps29
Camelineae - ps30
Arabidopsis - ps31
Arabidopsis_thaliana - ps32
Phylostratum

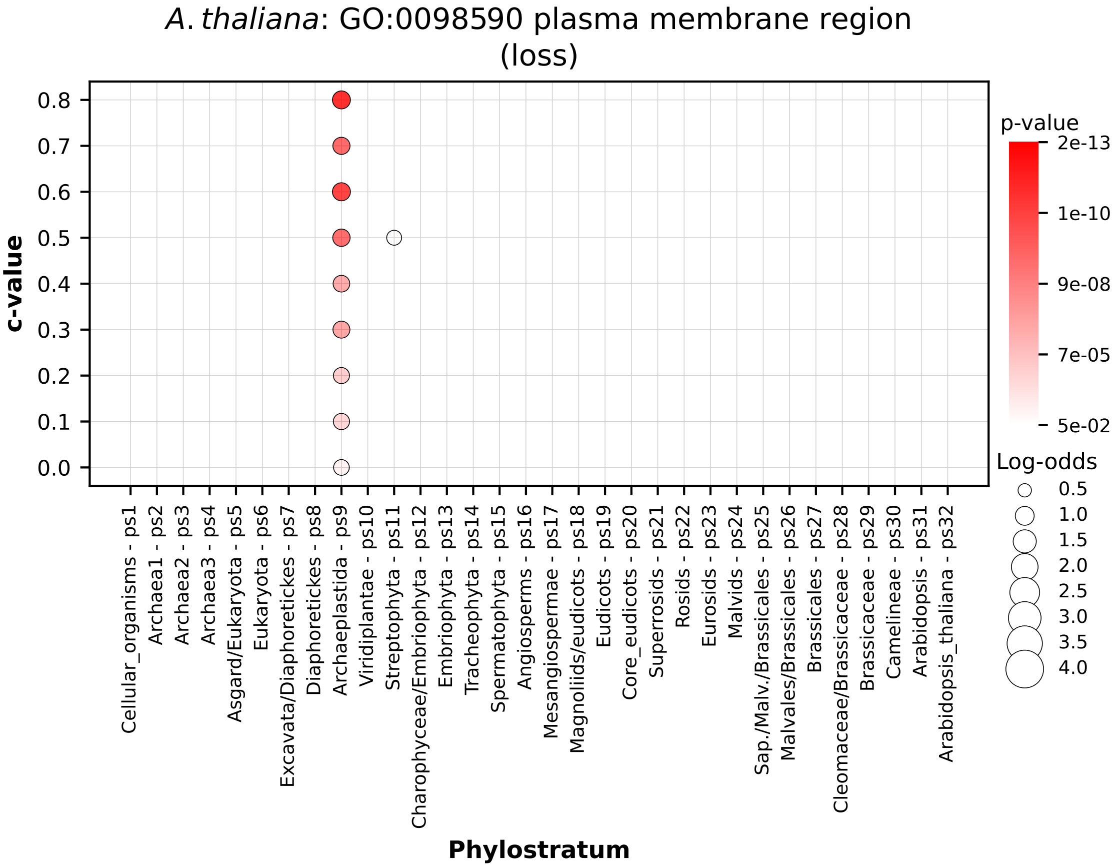

A. thaliana: GO:0098590 plasma membrane region
(loss)
c-value
0.8
0.7
0.6
0.5
0.4
0.3
0.2
0.1
0.0
p-value
2e-13
1e-10
9e-08
7e-05
5e-02
Log-odds
0.5
1.0
1.5
2.0
2.5
3.0
3.5
4.0
Cellular_organisms - ps1
Archaea1 - ps2
Archaea2 - ps3
Archaea3 - ps4
Asgard/Eukaryota - ps5
Eukaryota - ps6
Excavata/Diaphoretickes - ps7
Diaphoretickes - ps8
Archaeplastida - ps9
Viridiplantae - ps10
Streptophyta - ps11
Charophyceae/Embriophyta - ps12
Embriophyta - ps13
Tracheophyta - ps14
Spermatophyta - ps15
Angiosperms - ps16
Mesangiospermae - ps17
Magnoliids/eudicots - ps18
Eudicots - ps19
Core_eudicots - ps20
Superrosids - ps21
Rosids - ps22
Eurosids - ps23
Malvids - ps24
Sap./Malv./Brassicales - ps25
Malvales/Brassicales - ps26
Brassicales - ps27
Cleomaceae/Brassicaceae - ps28
Brassicaceae - ps29
Camelineae - ps30
Arabidopsis - ps31
Arabidopsis_thaliana - ps32
Phylostratum

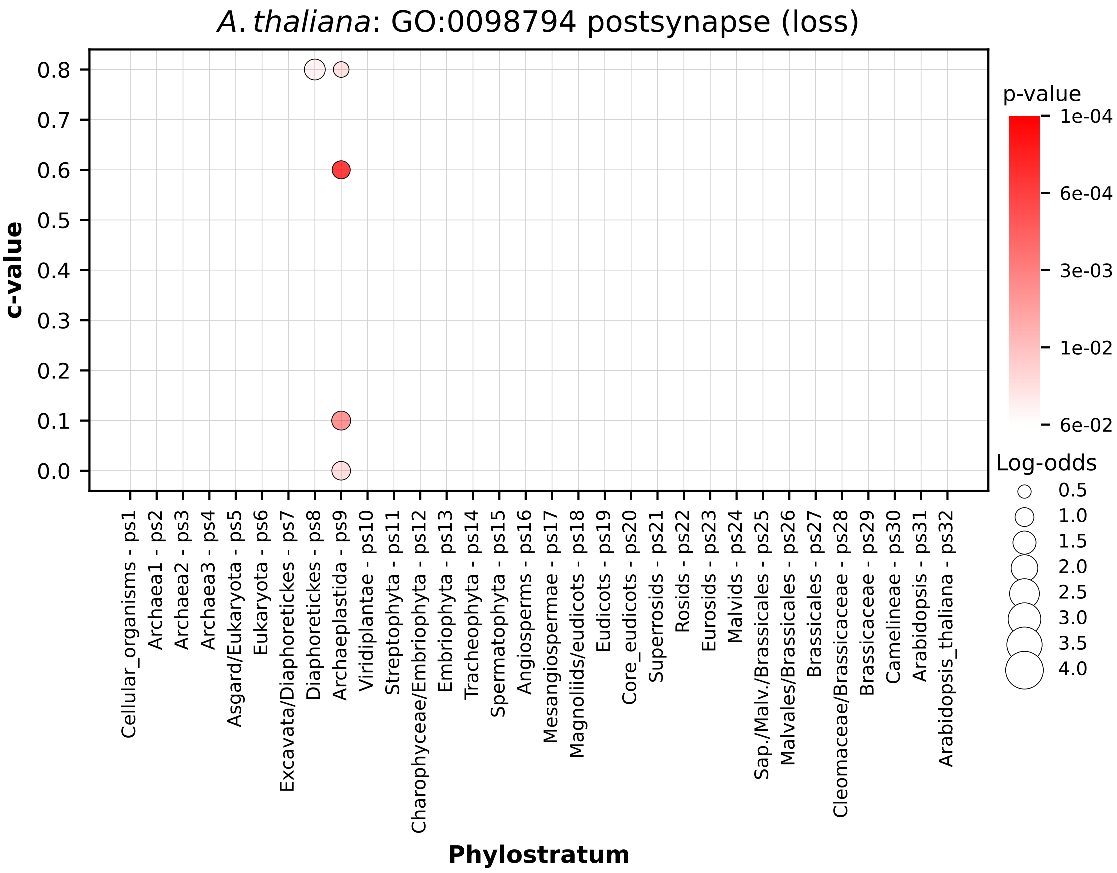

A. thaliana: GO:0098794 postsynapse (loss)
c-value
0.8
0.7
0.6
0.5
0.4
0.3
0.2
0.1
0.0
p-value
1e-04
6e-04
3e-03
1e-02
6e-02
Log-odds
0.5
1.0
1.5
2.0
2.5
3.0
3.5
4.0
Cellular_organisms - ps1
Archaea1 - ps2
Archaea2 - ps3
Archaea3 - ps4
Asgard/Eukaryota - ps5
Eukaryota - ps6
Excavata/Diaphoretickes - ps7
Diaphoretickes - ps8
Archaeplastida - ps9
Viridiplantae - ps10
Streptophyta - ps11
Charophyceae/Embriophyta - ps12
Embriophyta - ps13
Tracheophyta - ps14
Spermatophyta - ps15
Angiosperms - ps16
Mesangiospermae - ps17
Magnoliids/eudicots - ps18
Eudicots - ps19
Core_eudicots - ps20
Superrosids - ps21
Rosids - ps22
Eurosids - ps23
Malvids - ps24
Sap./Malv./Brassicales - ps25
Malvales/Brassicales - ps26
Brassicales - ps27
Cleomaceae/Brassicaceae - ps28
Brassicaceae - ps29
Camelineae - ps30
Arabidopsis - ps31
Arabidopsis_thaliana - ps32
Phylostratum

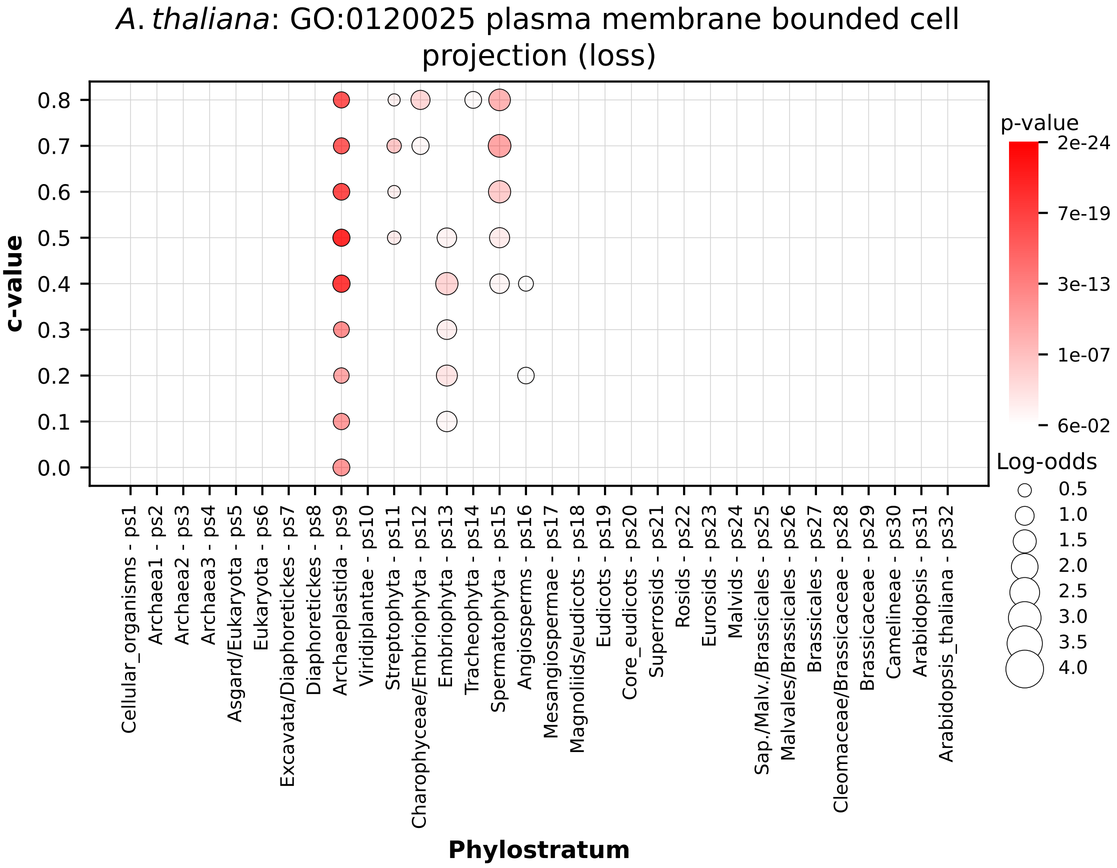
A. thaliana: GO:0120025 plasma membrane bounded cell projection (loss)
c-value
0.8
0.7
0.6
0.5
0.4
0.3
0.2
0.1
0.0
p-value
2e-24
7e-19
3e-13
1e-07
6e-02
Log-odds
0.5
1.0
1.5
2.0
2.5
3.0
3.5
4.0
Cellular_organisms - ps1
Archaea1 - ps2
Archaea2 - ps3
Archaea3 - ps4
Asgard/Eukaryota - ps5
Eukaryota - ps6
Excavata/Diaphoretickes - ps7
Diaphoretickes - ps8
Archaeplastida - ps9
Viridiplantae - ps10
Streptophyta - ps11
Charophyceae/Embriophyta - ps12
Embriophyta - ps13
Tracheophyta - ps14
Spermatophyta - ps15
Angiosperms - ps16
Mesangiospermae - ps17
Magnoliids/eudicots - ps18
Eudicots - ps19
Core_eudicots - ps20
Superrosids - ps21
Rosids - ps22
Eurosids - ps23
Malvids - ps24
Sap./Malv./Brassicales - ps25
Malvales/Brassicales - ps26
Brassicales - ps27
Cleomaceae/Brassicaceae - ps28
Brassicaceae - ps29
Camelineae - ps30
Arabidopsis - ps31
Arabidopsis_thaliana - ps32
Phylostratum

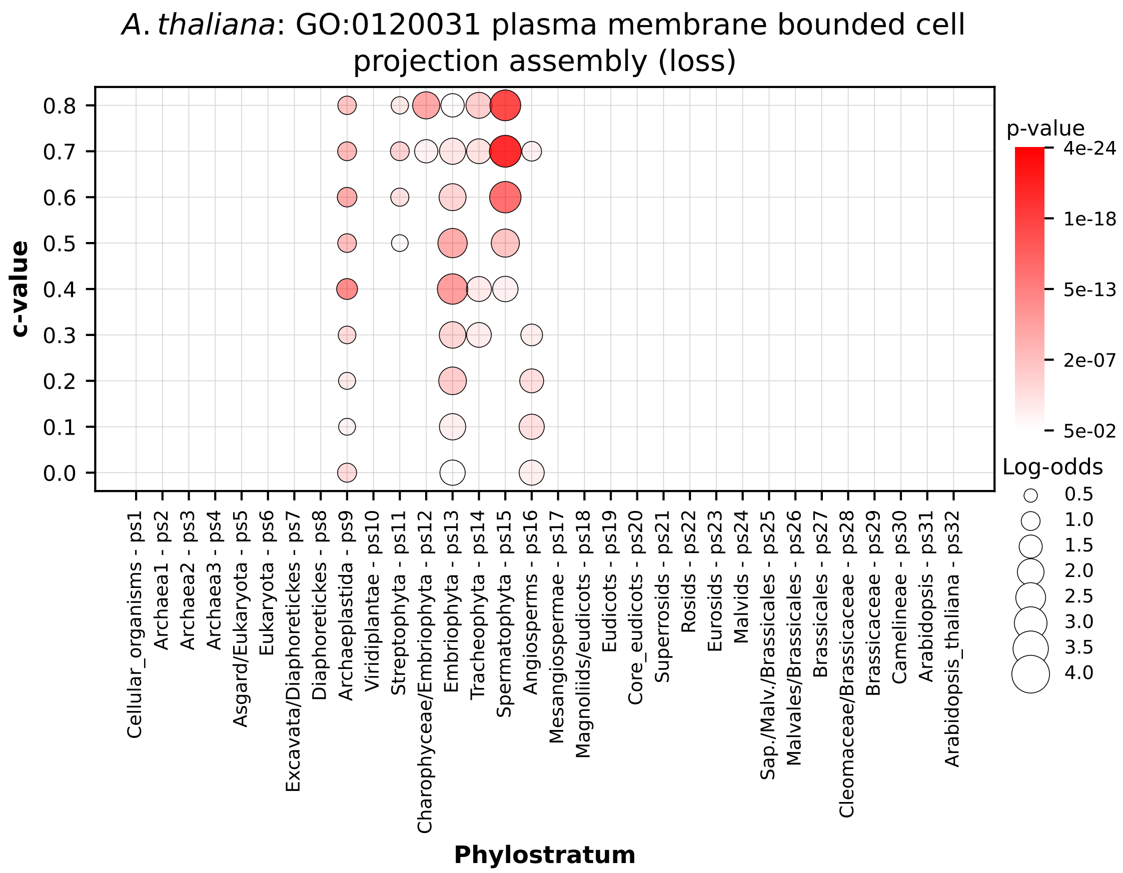
A. thaliana: GO:0120031 plasma membrane bounded cell projection assembly (loss)
c-value
0.8
0.7
0.6
0.5
0.4
0.3
0.2
0.1
0.0
p-value
4e-24
1e-18
5e-13
2e-07
5e-02
Log-odds
0.5
1.0
1.5
2.0
2.5
3.0
3.5
4.0
Cellular_organisms - ps1
Archaea1 - ps2
Archaea2 - ps3
Archaea3 - ps4
Asgard/Eukaryota - ps5
Eukaryota - ps6
Excavata/Diaphoretickes - ps7
Diaphoretickes - ps8
Archaeplastida - ps9
Viridiplantae - ps10
Streptophyta - ps11
Charophyceae/Embriophyta - ps12
Embriophyta - ps13
Tracheophyta - ps14
Spermatophyta - ps15
Angiosperms - ps16
Mesangiospermae - ps17
Magnoliids/eudicots - ps18
Eudicots - ps19
Core_eudicots - ps20
Superrosids - ps21
Rosids - ps22
Eurosids - ps23
Malvids - ps24
Sap./Malv./Brassicales - ps25
Malvales/Brassicales - ps26
Brassicales - ps27
Cleomaceae/Brassicaceae - ps28
Brassicaceae - ps29
Camelineae - ps30
Arabidopsis - ps31
Arabidopsis_thaliana - ps32
Phylostratum

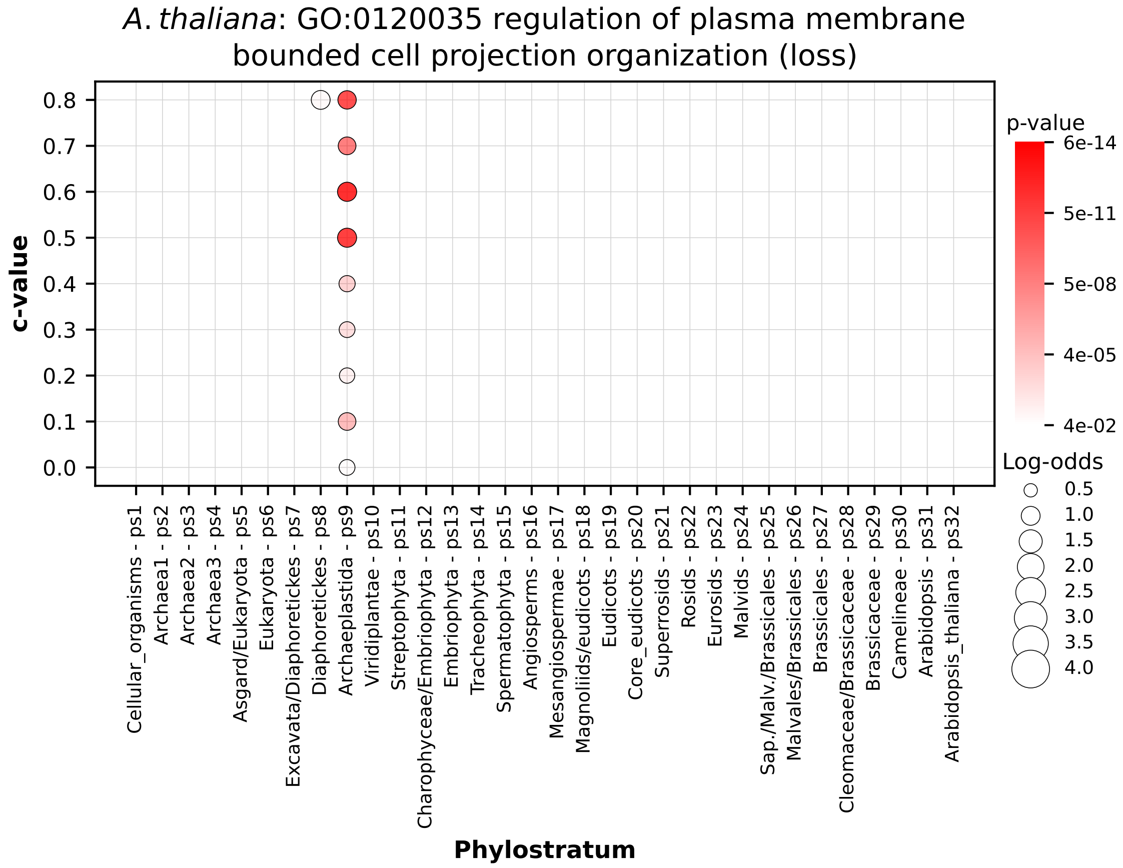

A. thaliana: GO:0120035 regulation of plasma membrane bounded cell projection organization (loss)
c-value
0.8
0.7
0.6
0.5
0.4
0.3
0.2
0.1
0.0
p-value
6e-14
5e-11
5e-08
4e-05
4e-02
Log-odds
0.5
1.0
1.5
2.0
2.5
3.0
3.5
4.0
Cellular_organisms - ps1
Archaea1 - ps2
Archaea2 - ps3
Archaea3 - ps4
Asgard/Eukaryota - ps5
Eukaryota - ps6
Excavata/Diaphoretickes - ps7
Diaphoretickes - ps8
Archaeplastida - ps9
Viridiplantae - ps10
Streptophyta - ps11
Charophyceae/Embriophyta - ps12
Embriophyta - ps13
Tracheophyta - ps14
Spermatophyta - ps15
Angiosperms - ps16
Mesangiospermae - ps17
Magnoliids/eudicots - ps18
Eudicots - ps19
Core_eudicots - ps20
Superrosids - ps21
Rosids - ps22
Eurosids - ps23
Malvids - ps24
Sap./Malv./Brassicales - ps25
Malvales/Brassicales - ps26
Brassicales - ps27
Cleomaceae/Brassicaceae - ps28
Brassicaceae - ps29
Camelineae - ps30
Arabidopsis - ps31
Arabidopsis_thaliana - ps32
Phylostratum

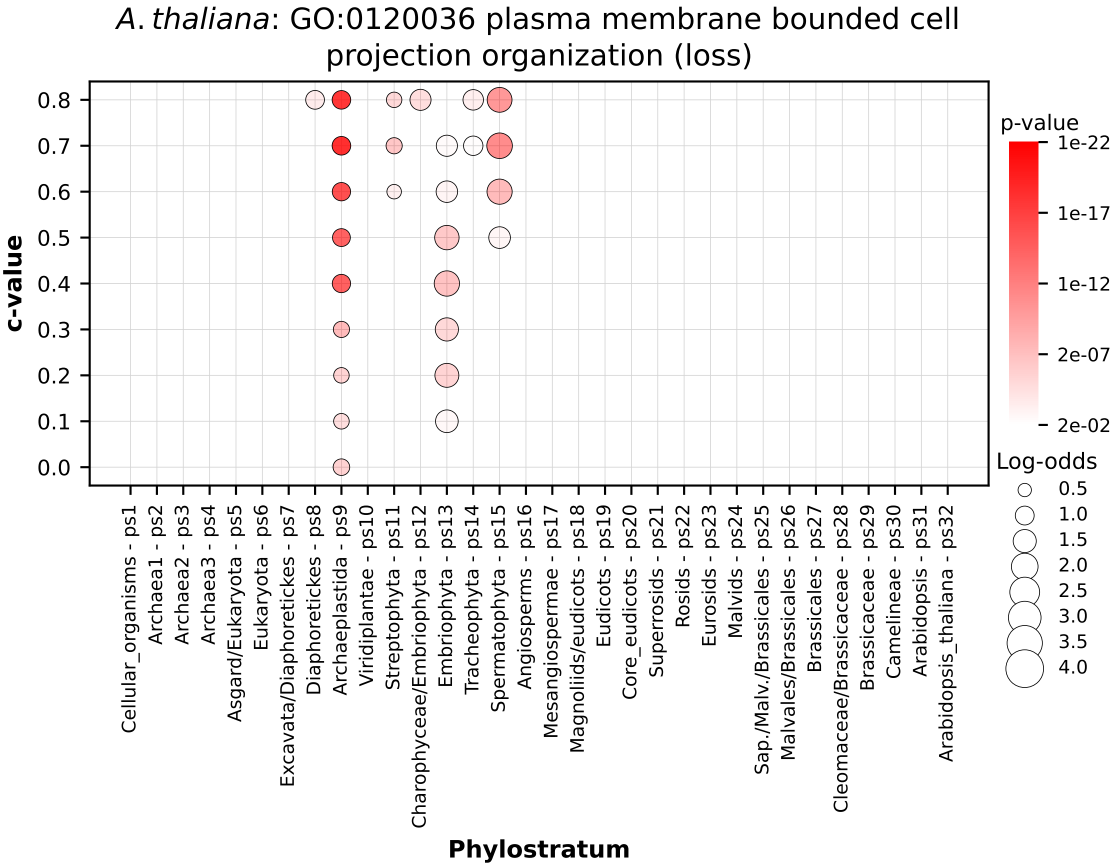
A. thaliana: GO:0120036 plasma membrane bounded cell projection organization (loss)
c-value
0.8
0.7
0.6
0.5
0.4
0.3
0.2
0.1
0.0
p-value
1e-22
1e-17
1e-12
2e-07
2e-02
Log-odds
0.5
1.0
1.5
2.0
2.5
3.0
3.5
4.0
Cellular_organisms - ps1
Archaea1 - ps2
Archaea2 - ps3
Archaea3 - ps4
Asgard/Eukaryota - ps5
Eukaryota - ps6
Excavata/Diaphoretickes - ps7
Diaphoretickes - ps8
Archaeplastida - ps9
Viridiplantae - ps10
Streptophyta - ps11
Charophyceae/Embriophyta - ps12
Embriophyta - ps13
Tracheophyta - ps14
Spermatophyta - ps15
Angiosperms - ps16
Mesangiospermae - ps17
Magnoliids/eudicots - ps18
Eudicots - ps19
Core_eudicots - ps20
Superrosids - ps21
Rosids - ps22
Eurosids - ps23
Malvids - ps24
Sap./Malv./Brassicales - ps25
Malvales/Brassicales - ps26
Brassicales - ps27
Cleomaceae/Brassicaceae - ps28
Brassicaceae - ps29
Camelineae - ps30
Arabidopsis - ps31
Arabidopsis_thaliana - ps32
Phylostratum

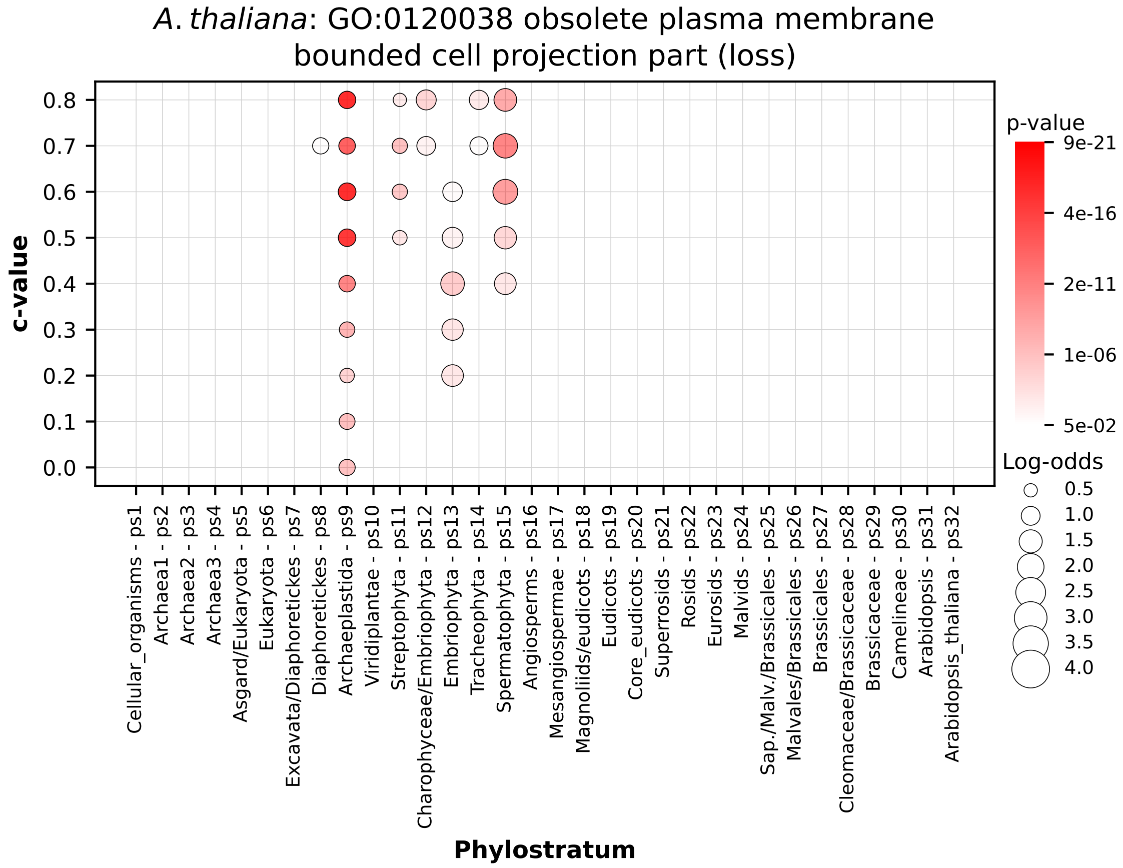
A. thaliana: GO:0120038 obsolete plasma membrane bounded cell projection part (loss)
c-value
0.8
0.7
0.6
0.5
0.4
0.3
0.2
0.1
0.0
p-value
9e-21
4e-16
2e-11
1e-06
5e-02
Log-odds
0.5
1.0
1.5
2.0
2.5
3.0
3.5
4.0
Cellular_organisms - ps1
Archaea1 - ps2
Archaea2 - ps3
Archaea3 - ps4
Asgard/Eukaryota - ps5
Eukaryota - ps6
Excavata/Diaphoretickes - ps7
Diaphoretickes - ps8
Archaeplastida - ps9
Viridiplantae - ps10
Streptophyta - ps11
Charophyceae/Embriophyta - ps12
Embriophyta - ps13
Tracheophyta - ps14
Spermatophyta - ps15
Angiosperms - ps16
Mesangiospermae - ps17
Magnoliids/eudicots - ps18
Eudicots - ps19
Core_eudicots - ps20
Superrosids - ps21
Rosids - ps22
Eurosids - ps23
Malvids - ps24
Sap./Malv./Brassicales - ps25
Malvales/Brassicales - ps26
Brassicales - ps27
Cleomaceae/Brassicaceae - ps28
Brassicaceae - ps29
Camelineae - ps30
Arabidopsis - ps31
Arabidopsis_thaliana - ps32
Phylostratum

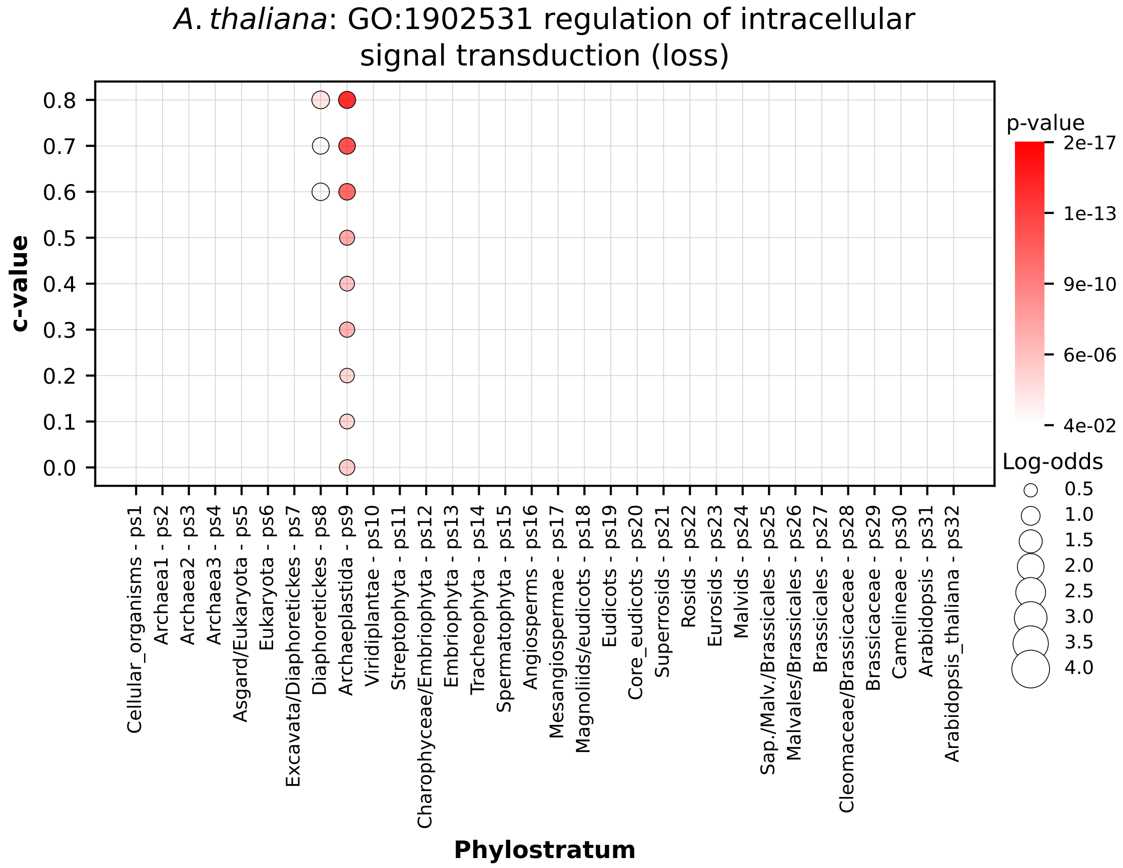

A. thaliana: GO:1902531 regulation of intracellular signal transduction (loss)
c-value
0.8
0.7
0.6
0.5
0.4
0.3
0.2
0.1
0.0
p-value
2e-17
1e-13
9e-10
6e-06
4e-02
Log-odds
0.5
1.0
1.5
2.0
2.5
3.0
3.5
4.0
Cellular_organisms - ps1
Archaea1 - ps2
Archaea2 - ps3
Archaea3 - ps4
Asgard/Eukaryota - ps5
Eukaryota - ps6
Excavata/Diaphoretickes - ps7
Diaphoretickes - ps8
Archaeplastida - ps9
Viridiplantae - ps10
Streptophyta - ps11
Charophyceae/Embriophyta - ps12
Embriophyta - ps13
Tracheophyta - ps14
Spermatophyta - ps15
Angiosperms - ps16
Mesangiospermae - ps17
Magnoliids/eudicots - ps18
Eudicots - ps19
Core_eudicots - ps20
Superrosids - ps21
Rosids - ps22
Eurosids - ps23
Malvids - ps24
Sap./Malv./Brassicales - ps25
Malvales/Brassicales - ps26
Brassicales - ps27
Cleomaceae/Brassicaceae - ps28
Brassicaceae - ps29
Camelineae - ps30
Arabidopsis - ps31
Arabidopsis_thaliana - ps32
Phylostratum

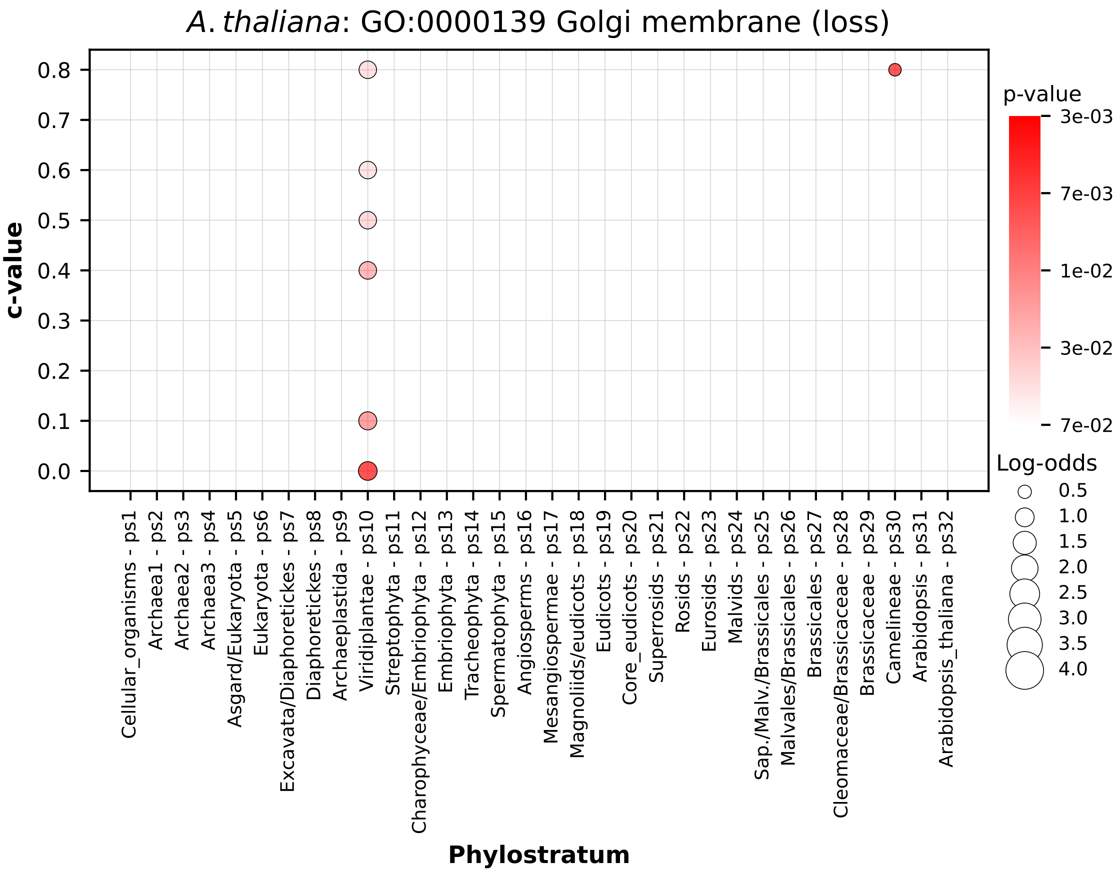

A. thaliana: GO:0000139 Golgi membrane (loss)
c-value
0.8
0.7
0.6
0.5
0.4
0.3
0.2
0.1
0.0
p-value
3e-03
7e-03
1e-02
3e-02
7e-02
Log-odds
0.5
1.0
1.5
2.0
2.5
3.0
3.5
4.0
Cellular_organisms - ps1
Archaea1 - ps2
Archaea2 - ps3
Archaea3 - ps4
Asgard/Eukaryota - ps5
Eukaryota - ps6
Excavata/Diaphoretickes - ps7
Diaphoretickes - ps8
Archaeplastida - ps9
Viridiplantae - ps10
Streptophyta - ps11
Charophyceae/Embriophyta - ps12
Embriophyta - ps13
Tracheophyta - ps14
Spermatophyta - ps15
Angiosperms - ps16
Mesangiospermae - ps17
Magnoliids/eudicots - ps18
Eudicots - ps19
Core_eudicots - ps20
Superrosids - ps21
Rosids - ps22
Eurosids - ps23
Malvids - ps24
Sap./Malv./Brassicales - ps25
Malvales/Brassicales - ps26
Brassicales - ps27
Cleomaceae/Brassicaceae - ps28
Brassicaceae - ps29
Camelineae - ps30
Arabidopsis - ps31
Arabidopsis_thaliana - ps32
Phylostratum

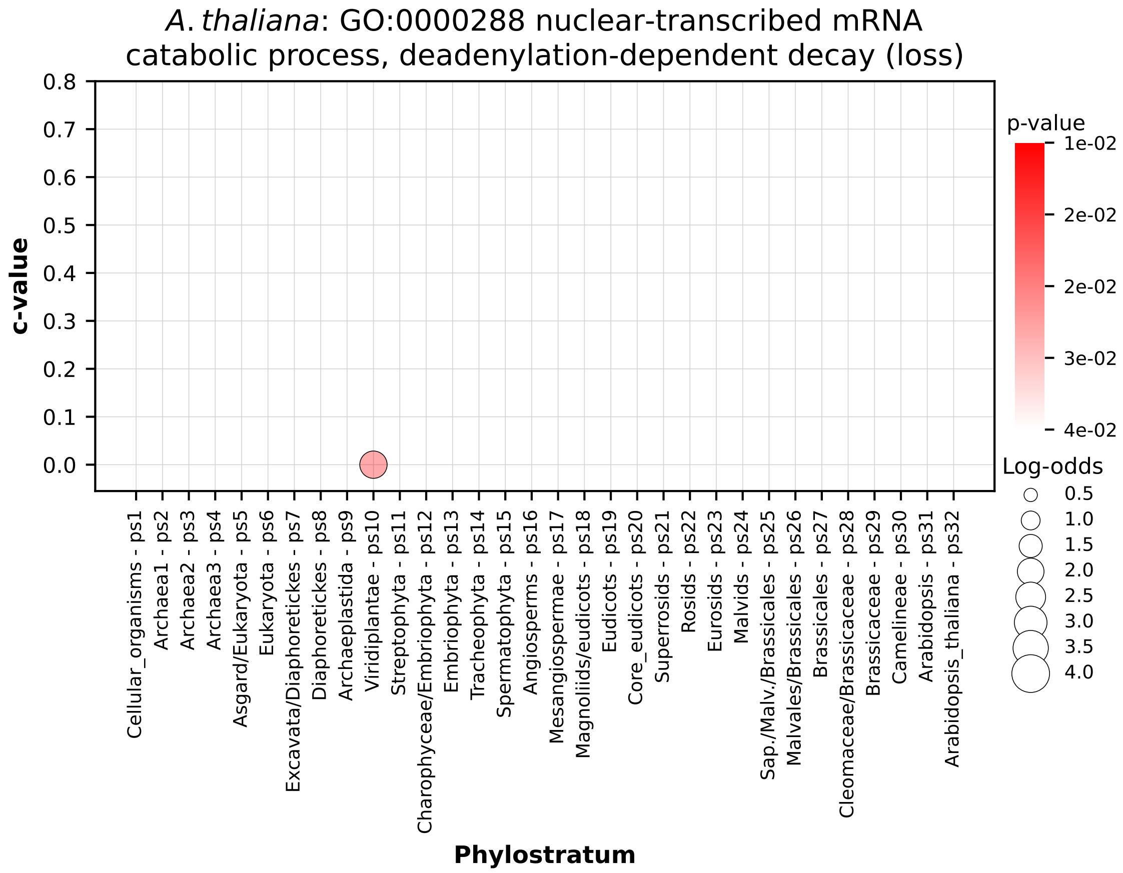

A. thaliana: GO:0000288 nuclear-transcribed mRNA catabolic process, deadenylation-dependent decay (loss)
c-value
0.8
0.7
0.6
0.5
0.4
0.3
0.2
0.1
0.0
p-value
1e-02
2e-02
2e-02
3e-02
4e-02
Log-odds
0.5
1.0
1.5
2.0
2.5
3.0
3.5
4.0
Cellular_organisms - ps1
Archaea1 - ps2
Archaea2 - ps3
Archaea3 - ps4
Asgard/Eukaryota - ps5
Eukaryota - ps6
Excavata/Diaphoretickes - ps7
Diaphoretickes - ps8
Archaeplastida - ps9
Viridiplantae - ps10
Streptophyta - ps11
Charophyceae/Embriophyta - ps12
Embriophyta - ps13
Tracheophyta - ps14
Spermatophyta - ps15
Angiosperms - ps16
Mesangiospermae - ps17
Magnoliids/eudicots - ps18
Eudicots - ps19
Core_eudicots - ps20
Superrosids - ps21
Rosids - ps22
Eurosids - ps23
Malvids - ps24
Sap./Malv./Brassicales - ps25
Malvales/Brassicales - ps26
Brassicales - ps27
Cleomaceae/Brassicaceae - ps28
Brassicaceae - ps29
Camelineae - ps30
Arabidopsis - ps31
Arabidopsis_thaliana - ps32
Phylostratum

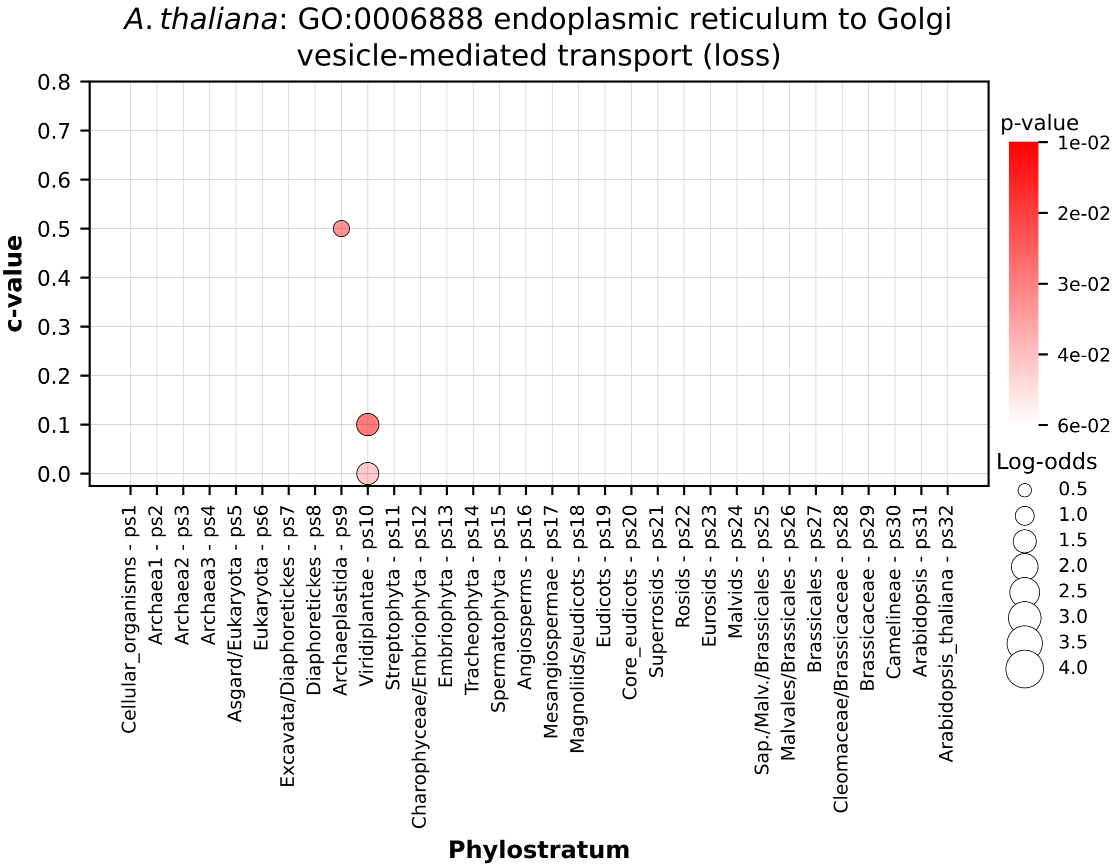
A. thaliana: GO:0006888 endoplasmic reticulum to Golgi vesicle-mediated transport (loss)
c-value
0.8
0.7
0.6
0.5
0.4
0.3
0.2
0.1
0.0
p-value
1e-02
2e-02
3e-02
4e-02
6e-02
Log-odds
0.5
1.0
1.5
2.0
2.5
3.0
3.5
4.0
Cellular_organisms - ps1
Archaea1 - ps2
Archaea2 - ps3
Archaea3 - ps4
Asgard/Eukaryota - ps5
Eukaryota - ps6
Excavata/Diaphoretickes - ps7
Diaphoretickes - ps8
Archaeplastida - ps9
Viridiplantae - ps10
Streptophyta - ps11
Charophyceae/Embriophyta - ps12
Embriophyta - ps13
Tracheophyta - ps14
Spermatophyta - ps15
Angiosperms - ps16
Mesangiospermae - ps17
Magnoliids/eudicots - ps18
Eudicots - ps19
Core_eudicots - ps20
Superrosids - ps21
Rosids - ps22
Eurosids - ps23
Malvids - ps24
Sap./Malv./Brassicales - ps25
Malvales/Brassicales - ps26
Brassicales - ps27
Cleomaceae/Brassicaceae - ps28
Brassicaceae - ps29
Camelineae - ps30
Arabidopsis - ps31
Arabidopsis_thaliana - ps32
Phylostratum

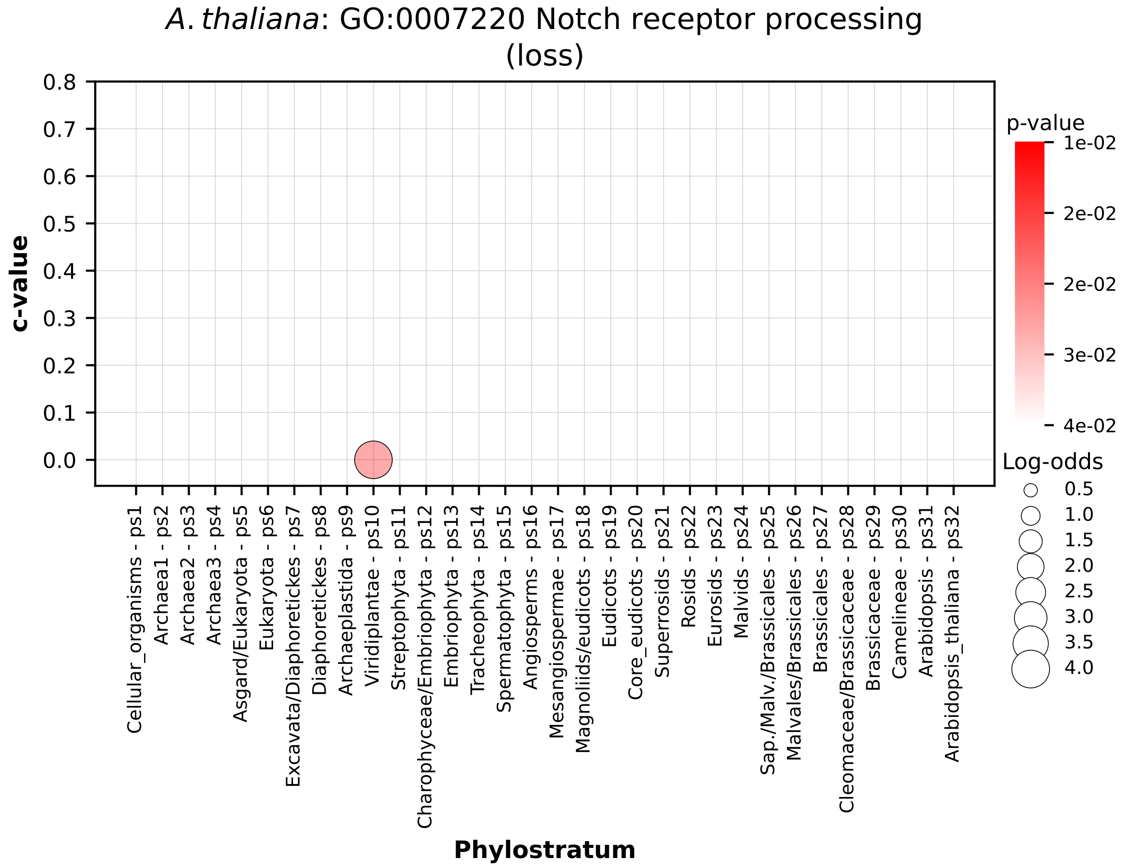

A. thaliana: GO:0007220 Notch receptor processing
(loss)
c-value
0.8
0.7
0.6
0.5
0.4
0.3
0.2
0.1
0.0
Phylostratum
Cellular_organisms - ps1
Archaea1 - ps2
Archaea2 - ps3
Archaea3 - ps4
Asgard/Eukaryota - ps5
Eukaryota - ps6
Excavata/Diaphoretickes - ps7
Diaphoretickes - ps8
Archaeplastida - ps9
Viridiplantae - ps10
Streptophyta - ps11
Charophyceae/Embriophyta - ps12
Embriophyta - ps13
Tracheophyta - ps14
Spermatophyta - ps15
Angiosperms - ps16
Mesangiospermae - ps17
Magnoliids/eudicots - ps18
Eudicots - ps19
Core_eudicots - ps20
Superrosids - ps21
Rosids - ps22
Eurosids - ps23
Malvids - ps24
Sap./Malv./Brassicales - ps25
Malvales/Brassicales - ps26
Brassicales - ps27
Cleomaceae/Brassicaceae - ps28
Brassicaceae - ps29
Camelineae - ps30
Arabidopsis - ps31
Arabidopsis_thaliana - ps32
p-value
1e-02
2e-02
2e-02
3e-02
4e-02
Log-odds
0.5
1.0
1.5
2.0
2.5
3.0
3.5
4.0

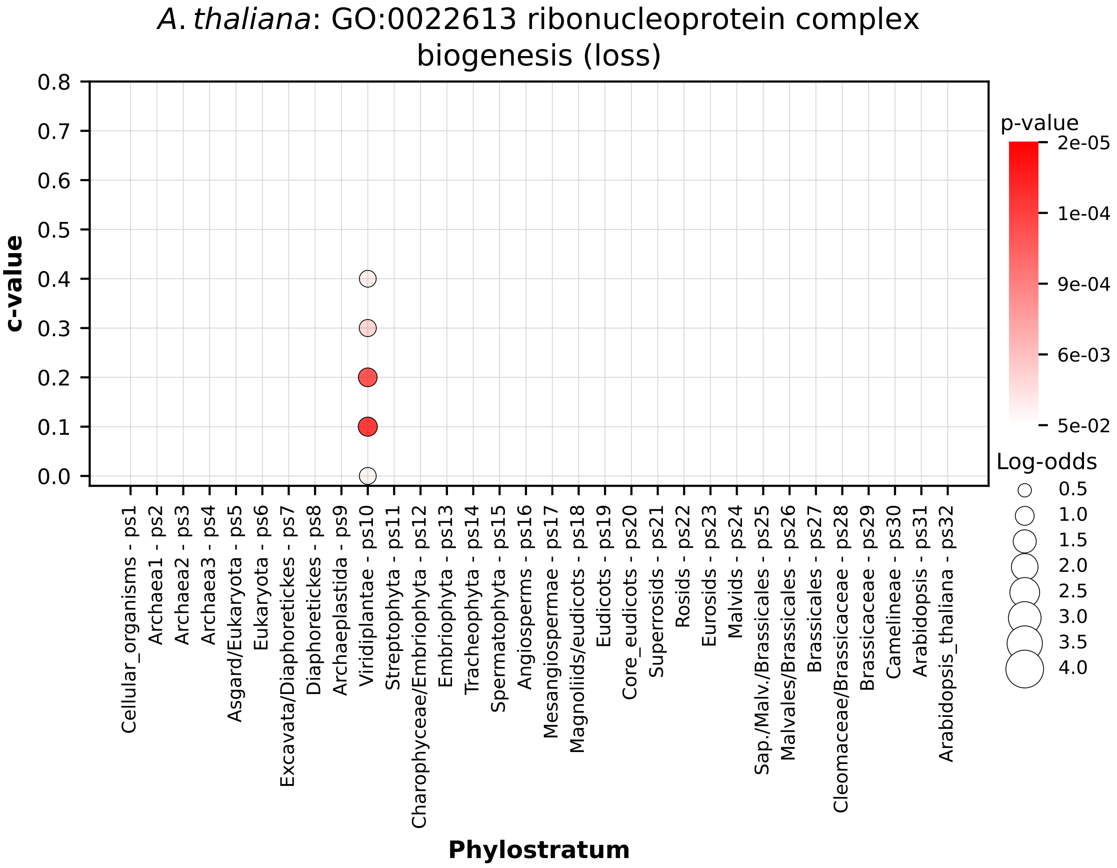

A. thaliana: GO:0022613 ribonucleoprotein complex biogenesis (loss)
c-value
0.8
0.7
0.6
0.5
0.4
0.3
0.2
0.1
0.0
Phylostratum
Cellular_organisms - ps1
Archaea1 - ps2
Archaea2 - ps3
Archaea3 - ps4
Asgard/Eukaryota - ps5
Eukaryota - ps6
Excavata/Diaphoretickes - ps7
Diaphoretickes - ps8
Archaeplastida - ps9
Viridiplantae - ps10
Streptophyta - ps11
Charophyceae/Embriophyta - ps12
Embriophyta - ps13
Tracheophyta - ps14
Spermatophyta - ps15
Angiosperms - ps16
Mesangiospermae - ps17
Magnoliids/eudicots - ps18
Eudicots - ps19
Core_eudicots - ps20
Superrosids - ps21
Rosids - ps22
Eurosids - ps23
Malvids - ps24
Sap./Malv./Brassicales - ps25
Malvales/Brassicales - ps26
Brassicales - ps27
Cleomaceae/Brassicaceae - ps28
Brassicaceae - ps29
Camelineae - ps30
Arabidopsis - ps31
Arabidopsis_thaliana - ps32
p-value
2e-05
1e-04
9e-04
6e-03
5e-02
Log-odds
0.5
1.0
1.5
2.0
2.5
3.0
3.5
4.0

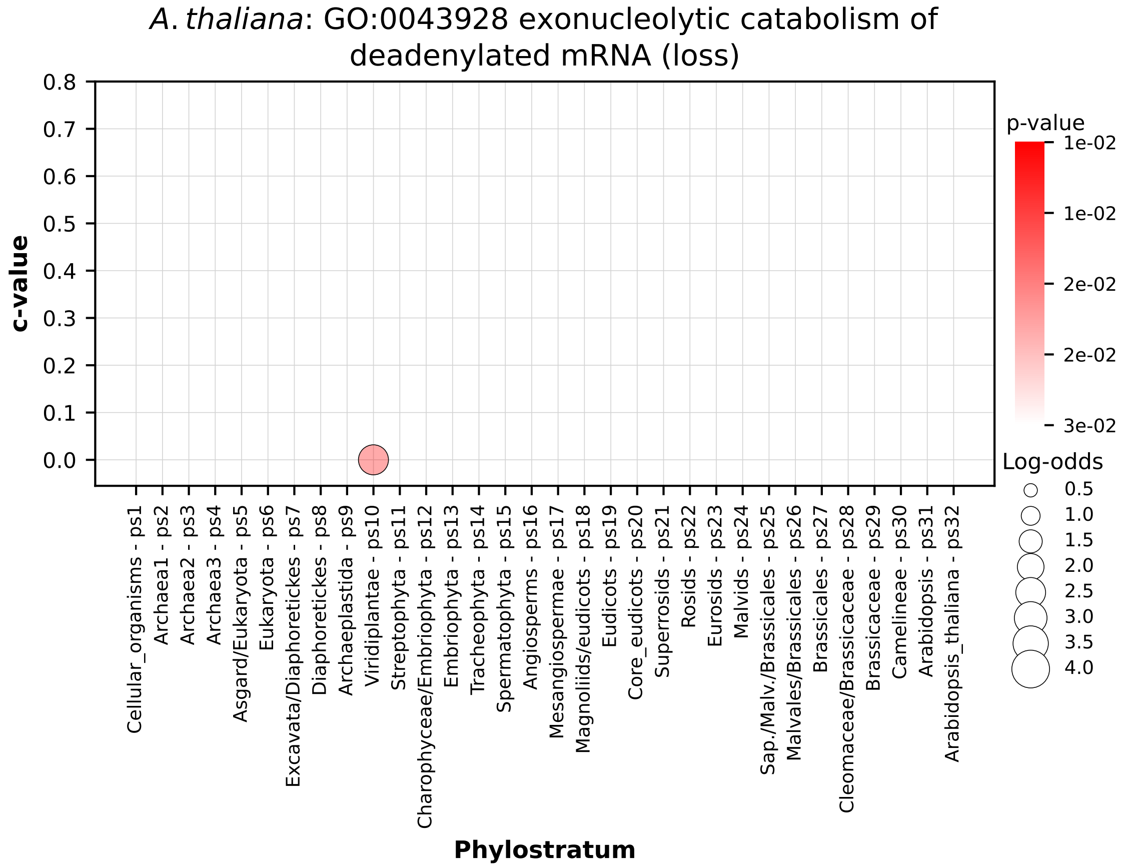

A. thaliana: GO:0043928 exonucleolytic catabolism of deadenylated mRNA (loss)
c-value
0.8
0.7
0.6
0.5
0.4
0.3
0.2
0.1
0.0
p-value
1e-02
1e-02
2e-02
2e-02
3e-02
Log-odds
0.5
1.0
1.5
2.0
2.5
3.0
3.5
4.0
Cellular_organisms - ps1
Archaea1 - ps2
Archaea2 - ps3
Archaea3 - ps4
Asgard/Eukaryota - ps5
Eukaryota - ps6
Excavata/Diaphoretickes - ps7
Diaphoretickes - ps8
Archaeplastida - ps9
Viridiplantae - ps10
Streptophyta - ps11
Charophyceae/Embriophyta - ps12
Embriophyta - ps13
Tracheophyta - ps14
Spermatophyta - ps15
Angiosperms - ps16
Mesangiospermae - ps17
Magnoliids/eudicots - ps18
Eudicots - ps19
Core_eudicots - ps20
Superrosids - ps21
Rosids - ps22
Eurosids - ps23
Malvids - ps24
Sap./Malv./Brassicales - ps25
Malvales/Brassicales - ps26
Brassicales - ps27
Cleomaceae/Brassicaceae - ps28
Brassicaceae - ps29
Camelineae - ps30
Arabidopsis - ps31
Arabidopsis_thaliana - ps32
Phylostratum

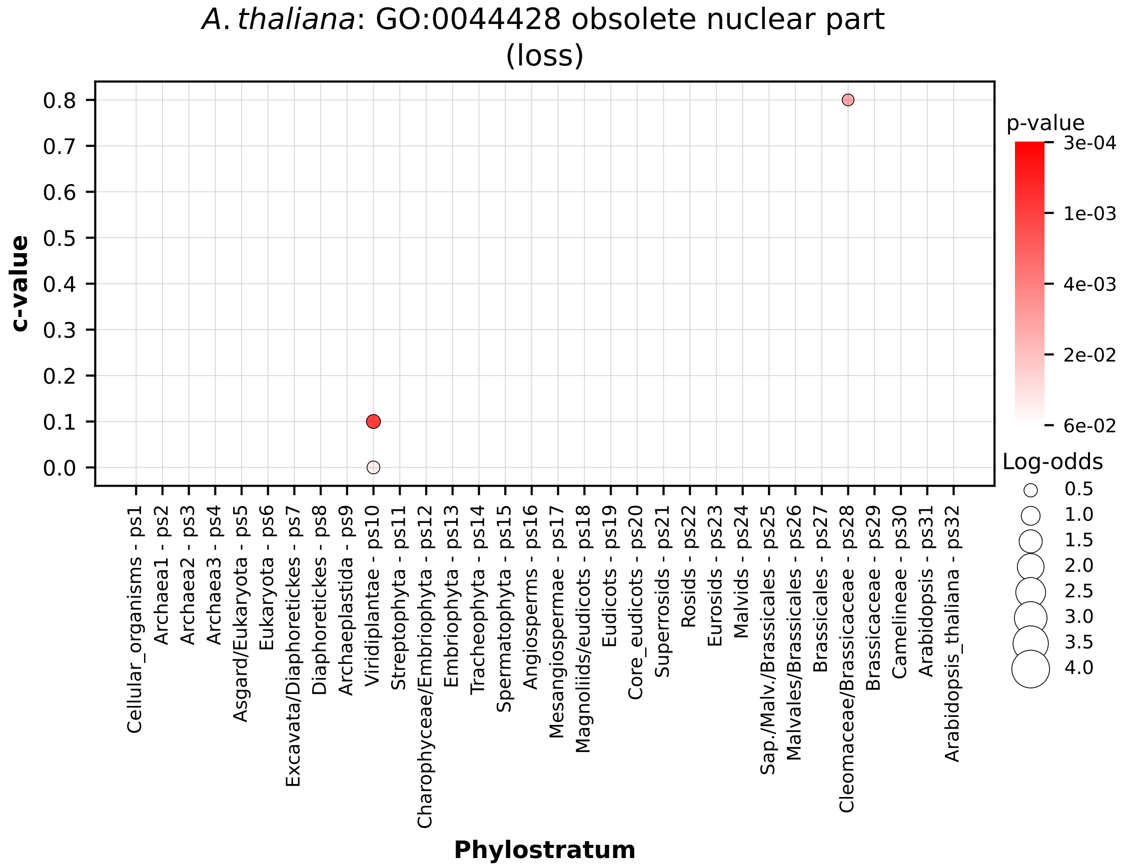

A. thaliana: GO:0044428 obsolete nuclear part
(loss)
c-value
0.8
0.7
0.6
0.5
0.4
0.3
0.2
0.1
0.0
p-value
3e-04
1e-03
4e-03
2e-02
6e-02
Log-odds
0.5
1.0
1.5
2.0
2.5
3.0
3.5
4.0
Cellular_organisms - ps1
Archaea1 - ps2
Archaea2 - ps3
Archaea3 - ps4
Asgard/Eukaryota - ps5
Eukaryota - ps6
Excavata/Diaphoretickes - ps7
Diaphoretickes - ps8
Archaeplastida - ps9
Viridiplantae - ps10
Streptophyta - ps11
Charophyceae/Embriophyta - ps12
Embriophyta - ps13
Tracheophyta - ps14
Spermatophyta - ps15
Angiosperms - ps16
Mesangiospermae - ps17
Magnoliids/eudicots - ps18
Eudicots - ps19
Core_eudicots - ps20
Superrosids - ps21
Rosids - ps22
Eurosids - ps23
Malvids - ps24
Sap./Malv./Brassicales - ps25
Malvales/Brassicales - ps26
Brassicales - ps27
Cleomaceae/Brassicaceae - ps28
Brassicaceae - ps29
Camelineae - ps30
Arabidopsis - ps31
Arabidopsis_thaliana - ps32
Phylostratum

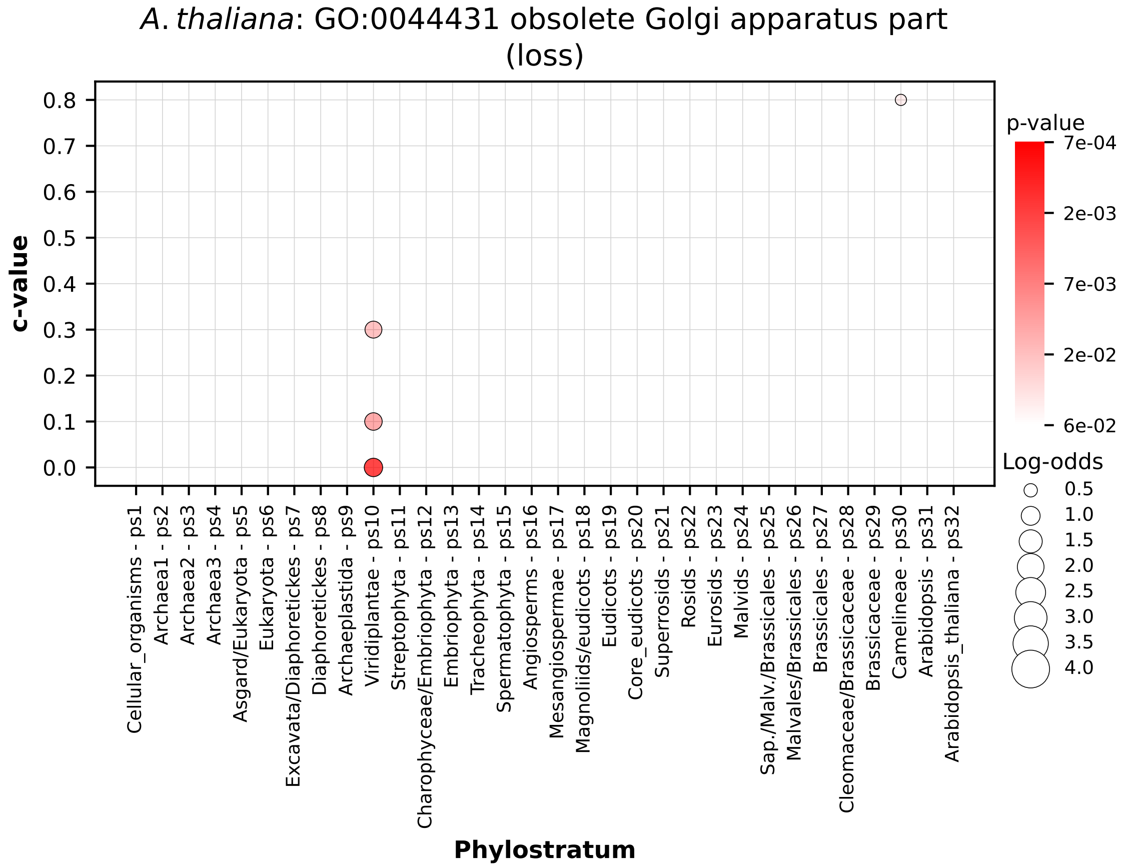

A. thaliana: GO:0044431 obsolete Golgi apparatus part
(loss)
c-value
0.8
0.7
0.6
0.5
0.4
0.3
0.2
0.1
0.0
p-value
7e-04
2e-03
7e-03
2e-02
6e-02
Log-odds
0.5
1.0
1.5
2.0
2.5
3.0
3.5
4.0
Cellular_organisms - ps1
Archaea1 - ps2
Archaea2 - ps3
Archaea3 - ps4
Asgard/Eukaryota - ps5
Eukaryota - ps6
Excavata/Diaphoretickes - ps7
Diaphoretickes - ps8
Archaeplastida - ps9
Viridiplantae - ps10
Streptophyta - ps11
Charophyceae/Embriophyta - ps12
Embriophyta - ps13
Tracheophyta - ps14
Spermatophyta - ps15
Angiosperms - ps16
Mesangiospermae - ps17
Magnoliids/eudicots - ps18
Eudicots - ps19
Core_eudicots - ps20
Superrosids - ps21
Rosids - ps22
Eurosids - ps23
Malvids - ps24
Sap./Malv./Brassicales - ps25
Malvales/Brassicales - ps26
Brassicales - ps27
Cleomaceae/Brassicaceae - ps28
Brassicaceae - ps29
Camelineae - ps30
Arabidopsis - ps31
Arabidopsis_thaliana - ps32
Phylostratum

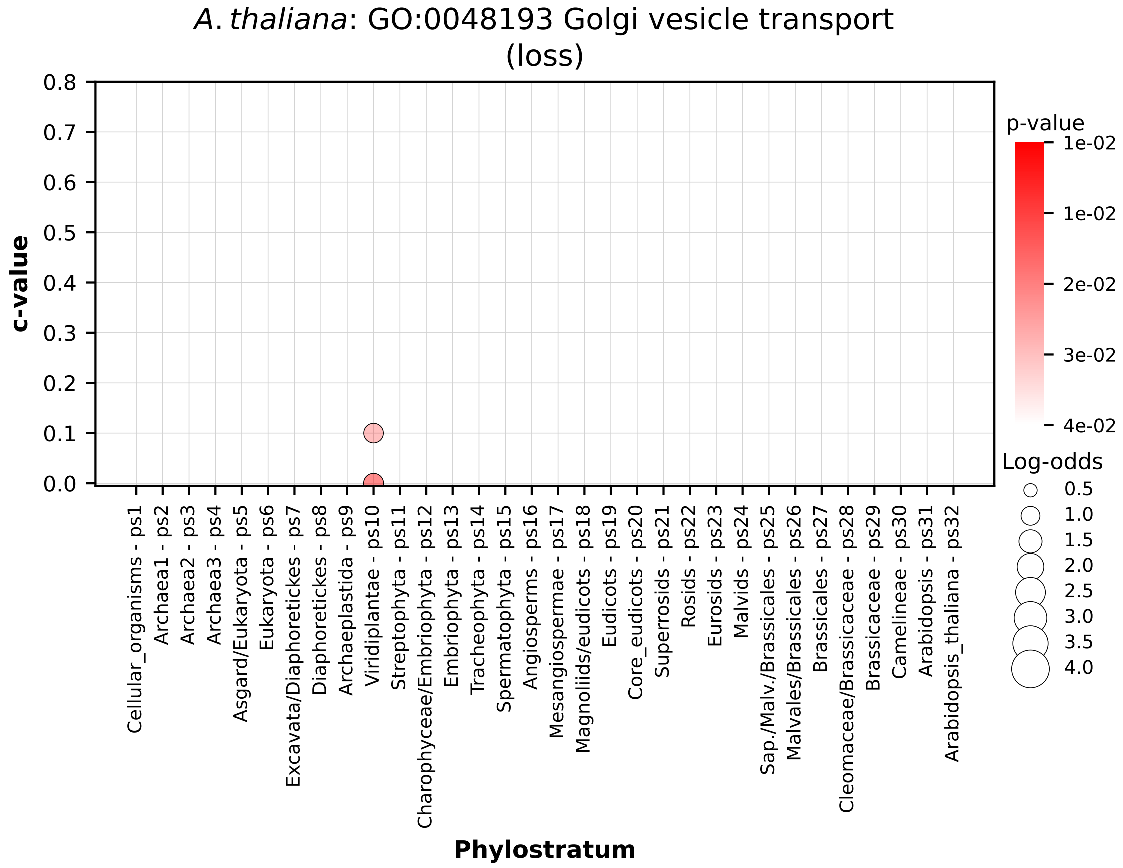

A. thaliana: GO:0048193 Golgi vesicle transport
(loss)
c-value
0.8
0.7
0.6
0.5
0.4
0.3
0.2
0.1
0.0
Phylostratum
Cellular_organisms - ps1
Archaea1 - ps2
Archaea2 - ps3
Archaea3 - ps4
Asgard/Eukaryota - ps5
Eukaryota - ps6
Excavata/Diaphoretickes - ps7
Diaphoretickes - ps8
Archaeplastida - ps9
Viridiplantae - ps10
Streptophyta - ps11
Charophyceae/Embriophyta - ps12
Embriophyta - ps13
Tracheophyta - ps14
Spermatophyta - ps15
Angiosperms - ps16
Mesangiospermae - ps17
Magnoliids/eudicots - ps18
Eudicots - ps19
Core_eudicots - ps20
Superrosids - ps21
Rosids - ps22
Eurosids - ps23
Malvids - ps24
Sap./Malv./Brassicales - ps25
Malvales/Brassicales - ps26
Brassicales - ps27
Cleomaceae/Brassicaceae - ps28
Brassicaceae - ps29
Camelineae - ps30
Arabidopsis - ps31
Arabidopsis_thaliana - ps32
p-value
1e-02
1e-02
2e-02
3e-02
4e-02
Log-odds
0.5
1.0
1.5
2.0
2.5
3.0
3.5
4.0

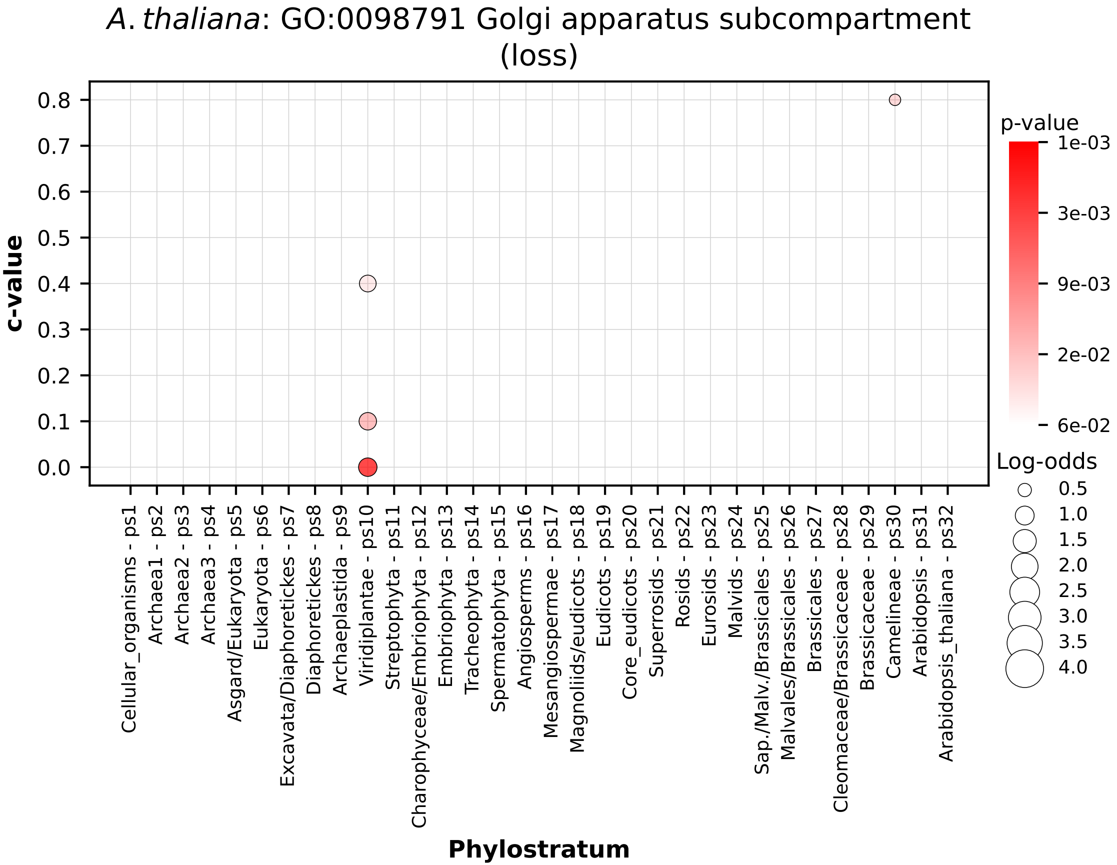
A. thaliana: GO:0098791 Golgi apparatus subcompartment
(loss)
c-value
0.0
0.1
0.2
0.3
0.4
0.5
0.6
0.7
0.8
p-value
1e-03
3e-03
9e-03
2e-02
6e-02
Log-odds
0.5
1.0
1.5
2.0
2.5
3.0
3.5
4.0
Cellular_organisms - ps1
Archaea1 - ps2
Archaea2 - ps3
Archaea3 - ps4
Asgard/Eukaryota - ps5
Eukaryota - ps6
Excavata/Diaphoretickes - ps7
Diaphoretickes - ps8
Archaeplastida - ps9
Viridiplantae - ps10
Streptophyta - ps11
Charophyceae/Embriophyta - ps12
Embriophyta - ps13
Tracheophyta - ps14
Spermatophyta - ps15
Angiosperms - ps16
Mesangiospermae - ps17
Magnoliids/eudicots - ps18
Eudicots - ps19
Core_eudicots - ps20
Superrosids - ps21
Rosids - ps22
Eurosids - ps23
Malvids - ps24
Sap./Malv./Brassicales - ps25
Malvales/Brassicales - ps26
Brassicales - ps27
Cleomaceae/Brassicaceae - ps28
Brassicaceae - ps29
Camelineae - ps30
Arabidopsis - ps31
Arabidopsis_thaliana - ps32
Phylostratum

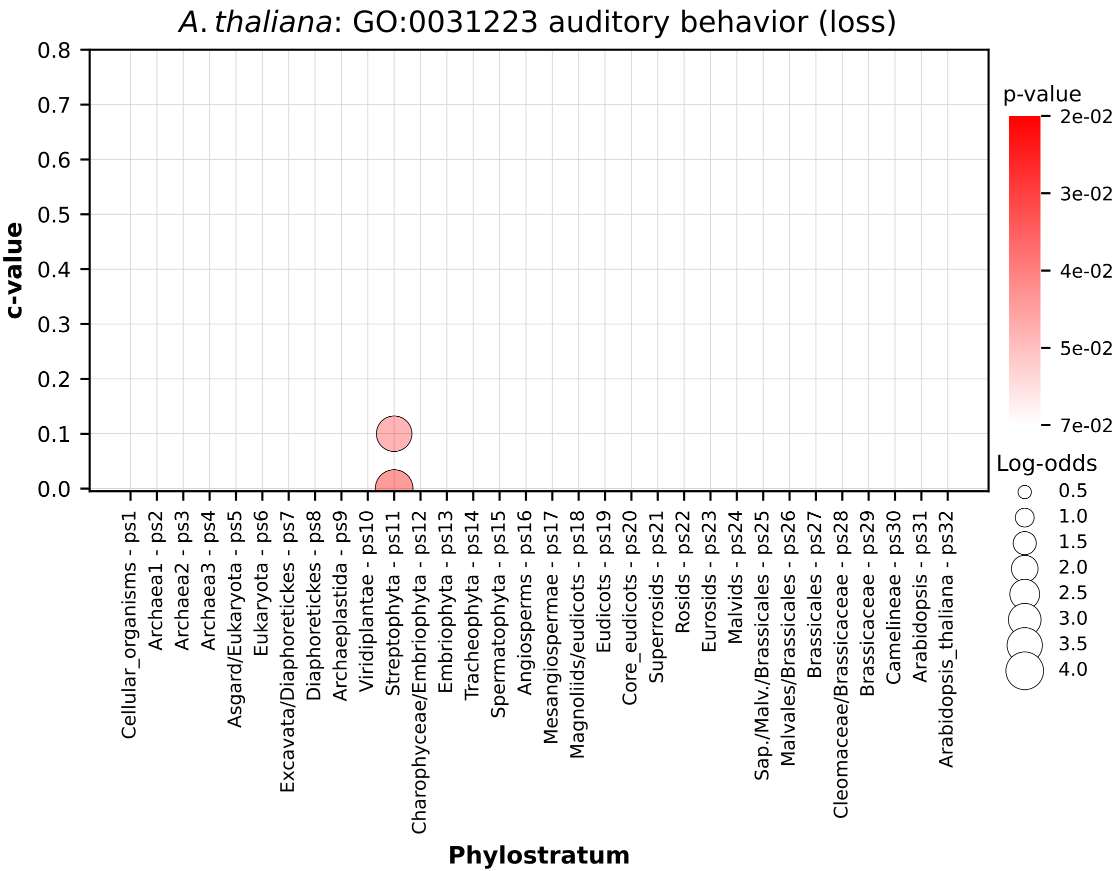

A. thaliana: GO:0031223 auditory behavior (loss)
c-value
0.8
0.7
0.6
0.5
0.4
0.3
0.2
0.1
0.0
Phylostratum
Cellular_organisms - ps1
Archaea1 - ps2
Archaea2 - ps3
Archaea3 - ps4
Asgard/Eukaryota - ps5
Eukaryota - ps6
Excavata/Diaphoretickes - ps7
Diaphoretickes - ps8
Archaeplastida - ps9
Viridiplantae - ps10
Streptophyta - ps11
Charophyceae/Embriophyta - ps12
Embriophyta - ps13
Tracheophyta - ps14
Spermatophyta - ps15
Angiosperms - ps16
Mesangiospermae - ps17
Magnoliids/eudicots - ps18
Eudicots - ps19
Core_eudicots - ps20
Superrosids - ps21
Rosids - ps22
Eurosids - ps23
Malvids - ps24
Sap./Malv./Brassicales - ps25
Malvales/Brassicales - ps26
Brassicales - ps27
Cleomaceae/Brassicaceae - ps28
Brassicaceae - ps29
Camelineae - ps30
Arabidopsis - ps31
Arabidopsis_thaliana - ps32
p-value
2e-02
3e-02
4e-02
5e-02
7e-02
Log-odds
0.5
1.0
1.5
2.0
2.5
3.0
3.5
4.0

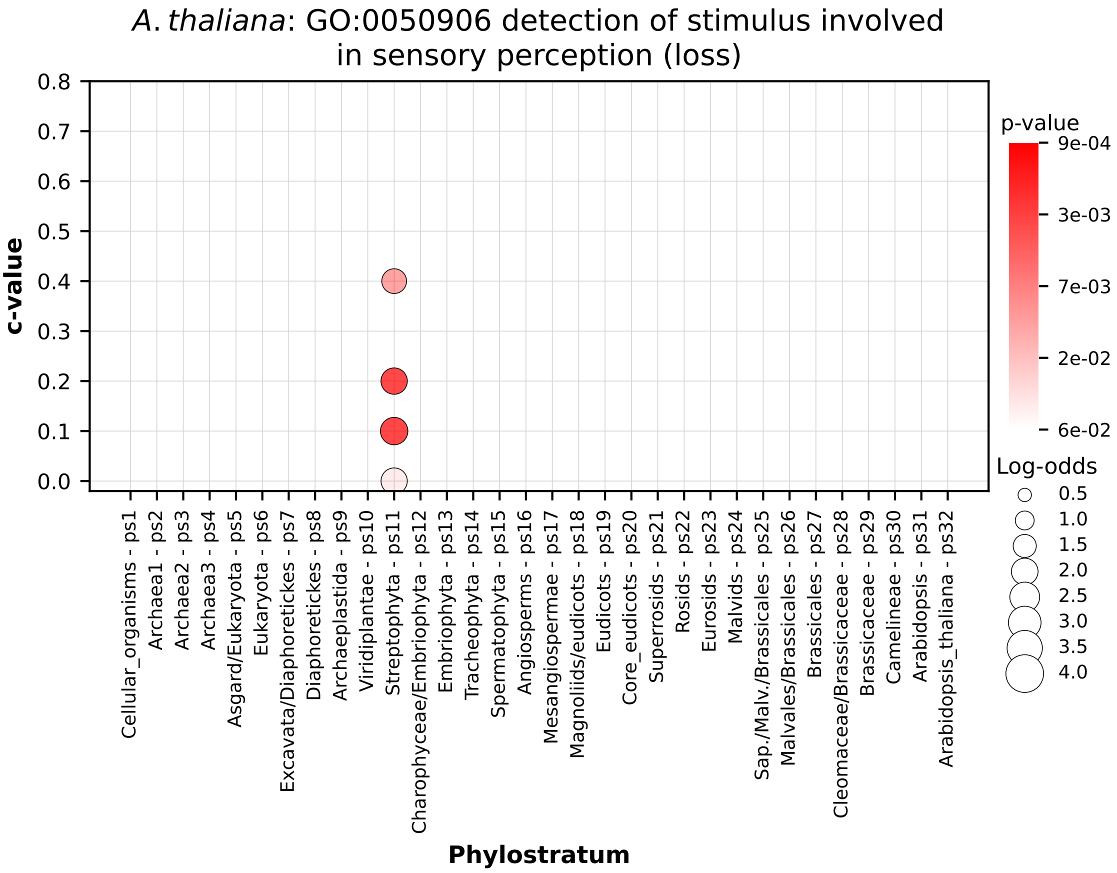

A. thaliana: GO:0050906 detection of stimulus involved in sensory perception (loss)
c-value
0.8
0.7
0.6
0.5
0.4
0.3
0.2
0.1
0.0
p-value
9e-04
3e-03
7e-03
2e-02
6e-02
Log-odds
0.5
1.0
1.5
2.0
2.5
3.0
3.5
4.0
Cellular_organisms - ps1
Archaea1 - ps2
Archaea2 - ps3
Archaea3 - ps4
Asgard/Eukaryota - ps5
Eukaryota - ps6
Excavata/Diaphoretickes - ps7
Diaphoretickes - ps8
Archaeplastida - ps9
Viridiplantae - ps10
Streptophyta - ps11
Charophyceae/Embriophyta - ps12
Embriophyta - ps13
Tracheophyta - ps14
Spermatophyta - ps15
Angiosperms - ps16
Mesangiospermae - ps17
Magnoliids/eudicots - ps18
Eudicots - ps19
Core_eudicots - ps20
Superrosids - ps21
Rosids - ps22
Eurosids - ps23
Malvids - ps24
Sap./Malv./Brassicales - ps25
Malvales/Brassicales - ps26
Brassicales - ps27
Cleomaceae/Brassicaceae - ps28
Brassicaceae - ps29
Camelineae - ps30
Arabidopsis - ps31
Arabidopsis_thaliana - ps32
Phylostratum

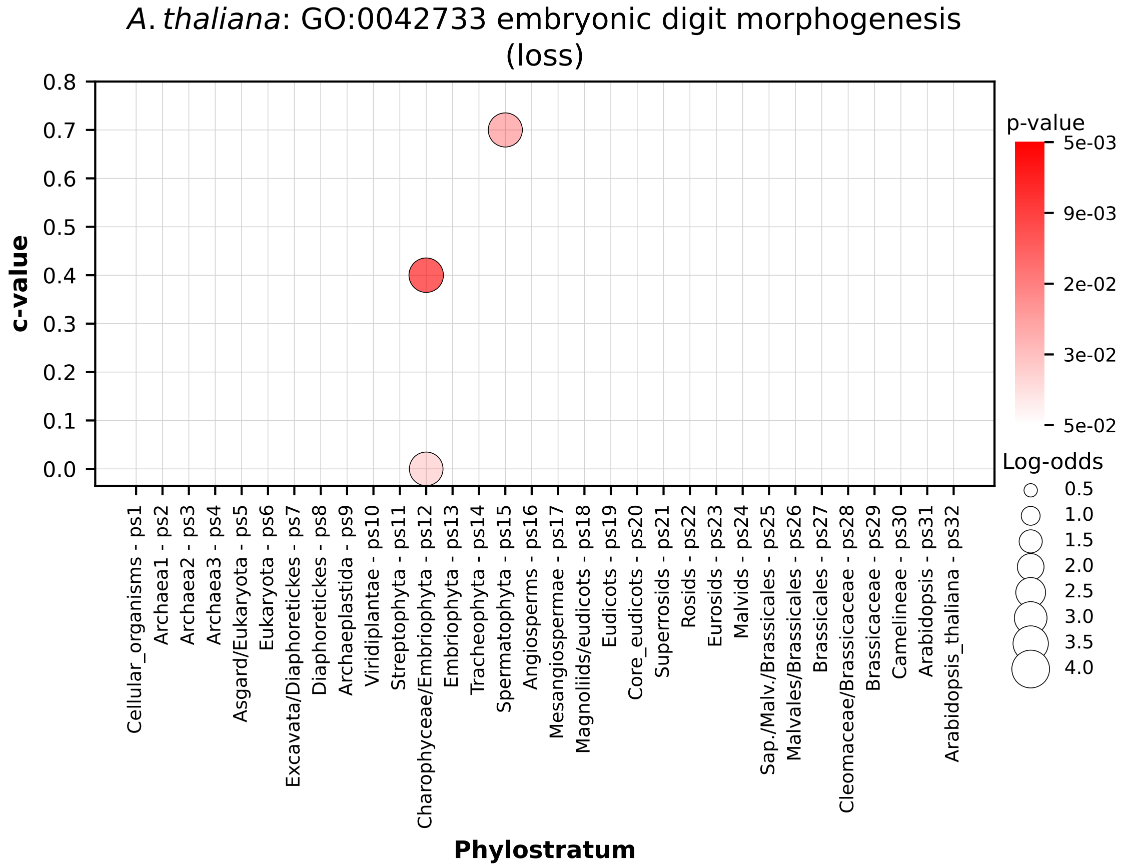
A. thaliana: GO:0042733 embryonic digit morphogenesis (loss)
c-value
0.8
0.7
0.6
0.5
0.4
0.3
0.2
0.1
0.0
p-value
5e-03
9e-03
2e-02
3e-02
5e-02
Log-odds
0.5
1.0
1.5
2.0
2.5
3.0
3.5
4.0
Cellular_organisms - ps1
Archaea1 - ps2
Archaea2 - ps3
Archaea3 - ps4
Asgard/Eukaryota - ps5
Eukaryota - ps6
Excavata/Diaphoretickes - ps7
Diaphoretickes - ps8
Archaeplastida - ps9
Viridiplantae - ps10
Streptophyta - ps11
Charophyceae/Embriophyta - ps12
Embriophyta - ps13
Tracheophyta - ps14
Spermatophyta - ps15
Angiosperms - ps16
Mesangiospermae - ps17
Magnoliids/eudicots - ps18
Eudicots - ps19
Core_eudicots - ps20
Superrosids - ps21
Rosids - ps22
Eurosids - ps23
Malvids - ps24
Sap./Malv./Brassicales - ps25
Malvales/Brassicales - ps26
Brassicales - ps27
Cleomaceae/Brassicaceae - ps28
Brassicaceae - ps29
Camelineae - ps30
Arabidopsis - ps31
Arabidopsis_thaliana - ps32
Phylostratum

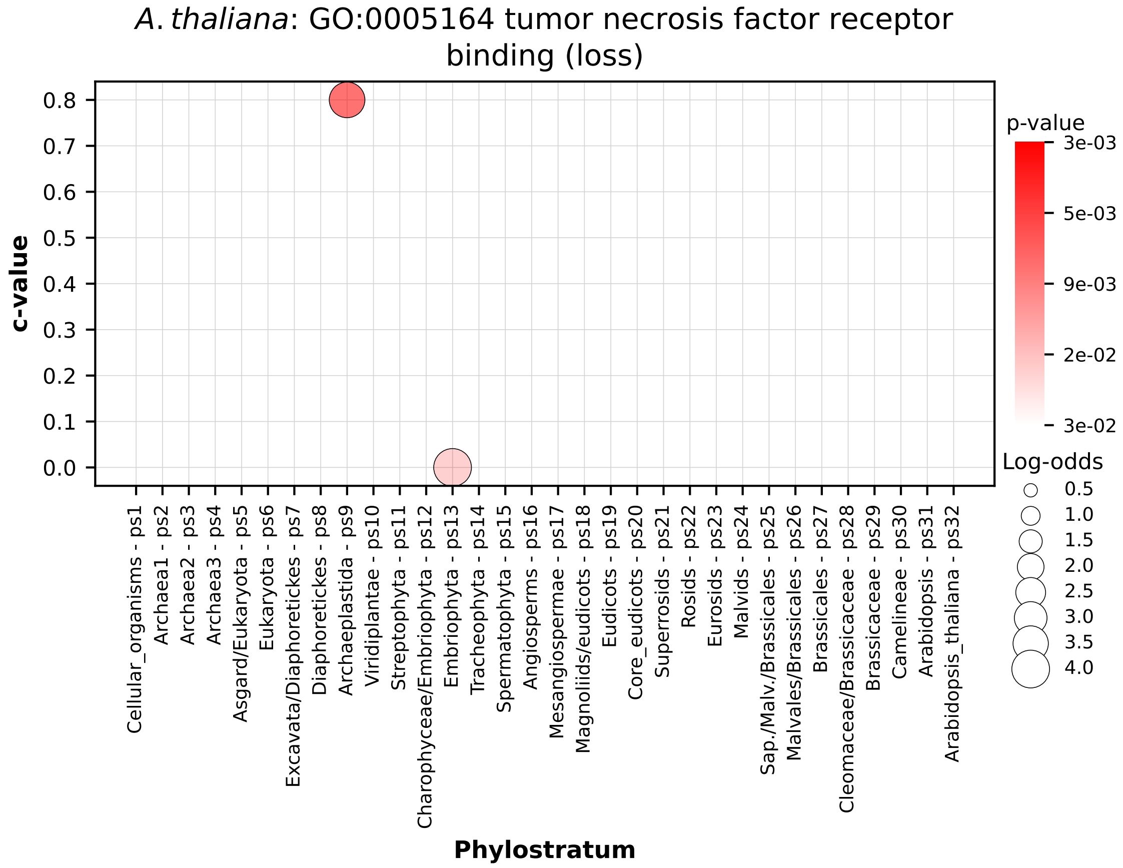
A. thaliana: GO:0005164 tumor necrosis factor receptor binding (loss)
c-value
0.8
0.7
0.6
0.5
0.4
0.3
0.2
0.1
0.0
Phylostratum
Cellular_organisms - ps1
Archaea1 - ps2
Archaea2 - ps3
Archaea3 - ps4
Asgard/Eukaryota - ps5
Eukaryota - ps6
Excavata/Diaphoretickes - ps7
Diaphoretickes - ps8
Archaeplastida - ps9
Viridiplantae - ps10
Streptophyta - ps11
Charophyceae/Embriophyta - ps12
Embriophyta - ps13
Tracheophyta - ps14
Spermatophyta - ps15
Angiosperms - ps16
Mesangiospermae - ps17
Magnoliids/eudicots - ps18
Eudicots - ps19
Core_eudicots - ps20
Superrosids - ps21
Rosids - ps22
Eurosids - ps23
Malvids - ps24
Sap./Malv./Brassicales - ps25
Malvales/Brassicales - ps26
Brassicales - ps27
Cleomaceae/Brassicaceae - ps28
Brassicaceae - ps29
Camelineae - ps30
Arabidopsis - ps31
Arabidopsis_thaliana - ps32
p-value
3e-03
5e-03
9e-03
2e-02
3e-02
Log-odds
0.5
1.0
1.5
2.0
2.5
3.0
3.5
4.0

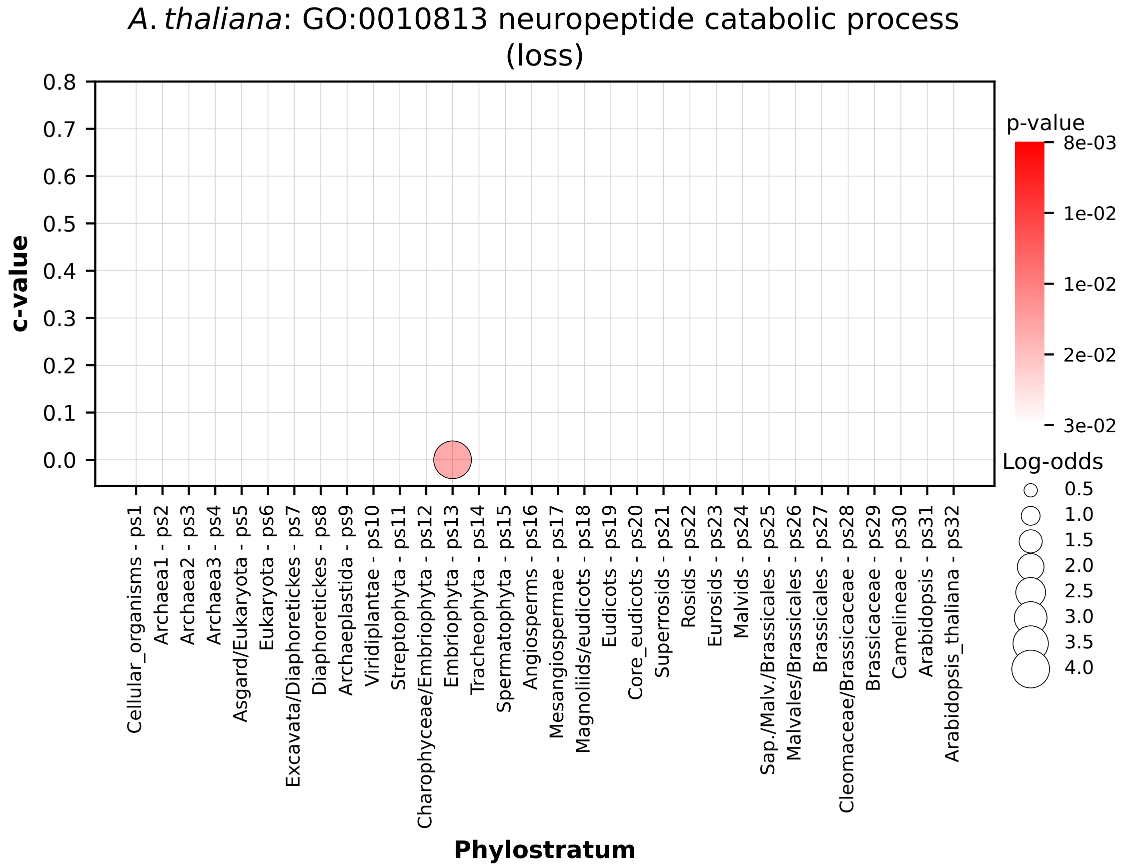

A. thaliana: GO:0010813 neuropeptide catabolic process
(loss)
c-value
0.8
0.7
0.6
0.5
0.4
0.3
0.2
0.1
0.0
p-value
8e-03
1e-02
1e-02
2e-02
3e-02
Log-odds
0.5
1.0
1.5
2.0
2.5
3.0
3.5
4.0
Cellular_organisms - ps1
Archaea1 - ps2
Archaea2 - ps3
Archaea3 - ps4
Asgard/Eukaryota - ps5
Eukaryota - ps6
Excavata/Diaphoretickes - ps7
Diaphoretickes - ps8
Archaeplastida - ps9
Viridiplantae - ps10
Streptophyta - ps11
Charophyceae/Embriophyta - ps12
Embriophyta - ps13
Tracheophyta - ps14
Spermatophyta - ps15
Angiosperms - ps16
Mesangiospermae - ps17
Magnoliids/eudicots - ps18
Eudicots - ps19
Core_eudicots - ps20
Superrosids - ps21
Rosids - ps22
Eurosids - ps23
Malvids - ps24
Sap./Malv./Brassicales - ps25
Malvales/Brassicales - ps26
Brassicales - ps27
Cleomaceae/Brassicaceae - ps28
Brassicaceae - ps29
Camelineae - ps30
Arabidopsis - ps31
Arabidopsis_thaliana - ps32
Phylostratum

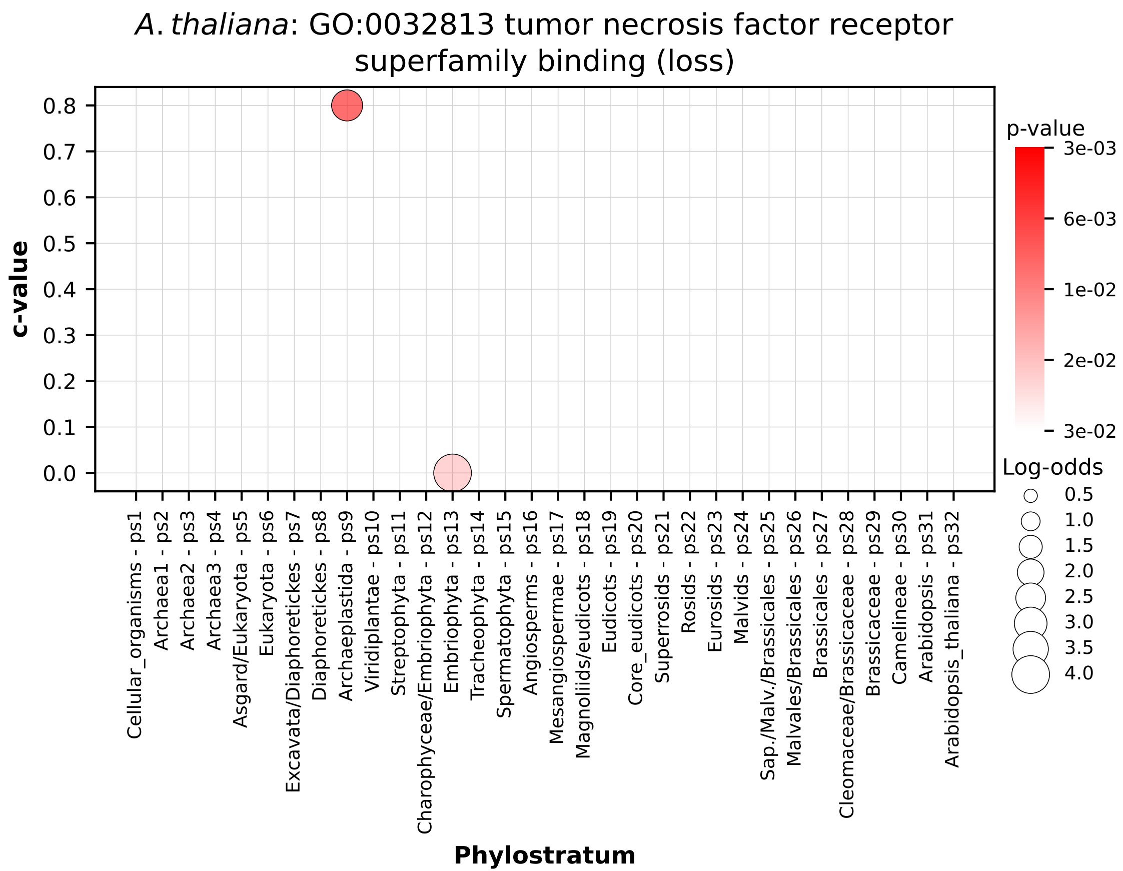

A. thaliana: GO:0032813 tumor necrosis factor receptor superfamily binding (loss)
c-value
0.8
0.7
0.6
0.5
0.4
0.3
0.2
0.1
0.0
p-value
3e-03
6e-03
1e-02
2e-02
3e-02
Log-odds
0.5
1.0
1.5
2.0
2.5
3.0
3.5
4.0
Cellular_organisms - ps1
Archaea1 - ps2
Archaea2 - ps3
Archaea3 - ps4
Asgard/Eukaryota - ps5
Eukaryota - ps6
Excavata/Diaphoretickes - ps7
Diaphoretickes - ps8
Archaeplastida - ps9
Viridiplantae - ps10
Streptophyta - ps11
Charophyceae/Embriophyta - ps12
Embriophyta - ps13
Tracheophyta - ps14
Spermatophyta - ps15
Angiosperms - ps16
Mesangiospermae - ps17
Magnoliids/eudicots - ps18
Eudicots - ps19
Core_eudicots - ps20
Superrosids - ps21
Rosids - ps22
Eurosids - ps23
Malvids - ps24
Sap./Malv./Brassicales - ps25
Malvales/Brassicales - ps26
Brassicales - ps27
Cleomaceae/Brassicaceae - ps28
Brassicaceae - ps29
Camelineae - ps30
Arabidopsis - ps31
Arabidopsis_thaliana - ps32
Phylostratum

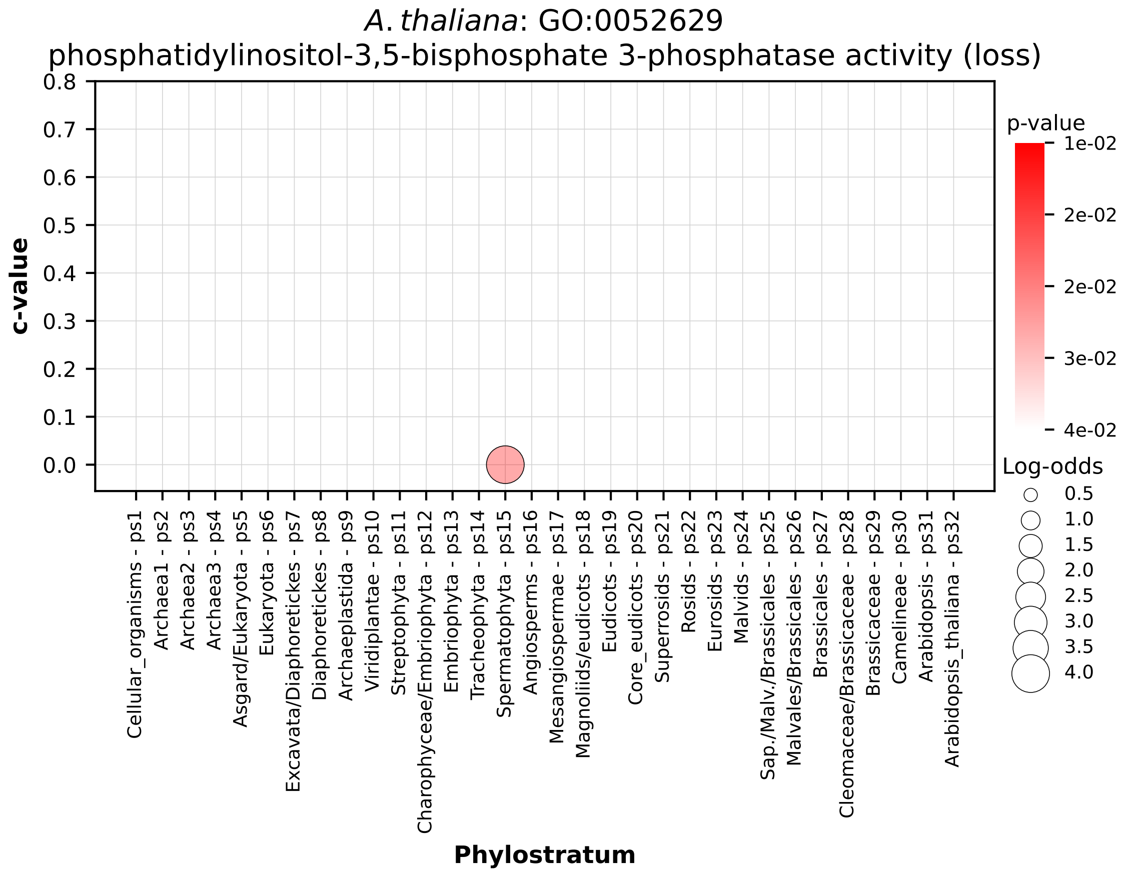

A. thaliana: GO:0052629
phosphatidylinositol-3,5-bisphosphate 3-phosphatase activity (loss)
c-value
0.8
0.7
0.6
0.5
0.4
0.3
0.2
0.1
0.0
p-value
1e-02
2e-02
2e-02
3e-02
4e-02
Log-odds
0.5
1.0
1.5
2.0
2.5
3.0
3.5
4.0
Cellular_organisms - ps1
Archaea1 - ps2
Archaea2 - ps3
Archaea3 - ps4
Asgard/Eukaryota - ps5
Eukaryota - ps6
Excavata/Diaphoretickes - ps7
Diaphoretickes - ps8
Archaeplastida - ps9
Viridiplantae - ps10
Streptophyta - ps11
Charophyceae/Embriophyta - ps12
Embriophyta - ps13
Tracheophyta - ps14
Spermatophyta - ps15
Angiosperms - ps16
Mesangiospermae - ps17
Magnoliids/eudicots - ps18
Eudicots - ps19
Core_eudicots - ps20
Superrosids - ps21
Rosids - ps22
Eurosids - ps23
Malvids - ps24
Sap./Malv./Brassicales - ps25
Malvales/Brassicales - ps26
Brassicales - ps27
Cleomaceae/Brassicaceae - ps28
Brassicaceae - ps29
Camelineae - ps30
Arabidopsis - ps31
Arabidopsis_thaliana - ps32
Phylostratum

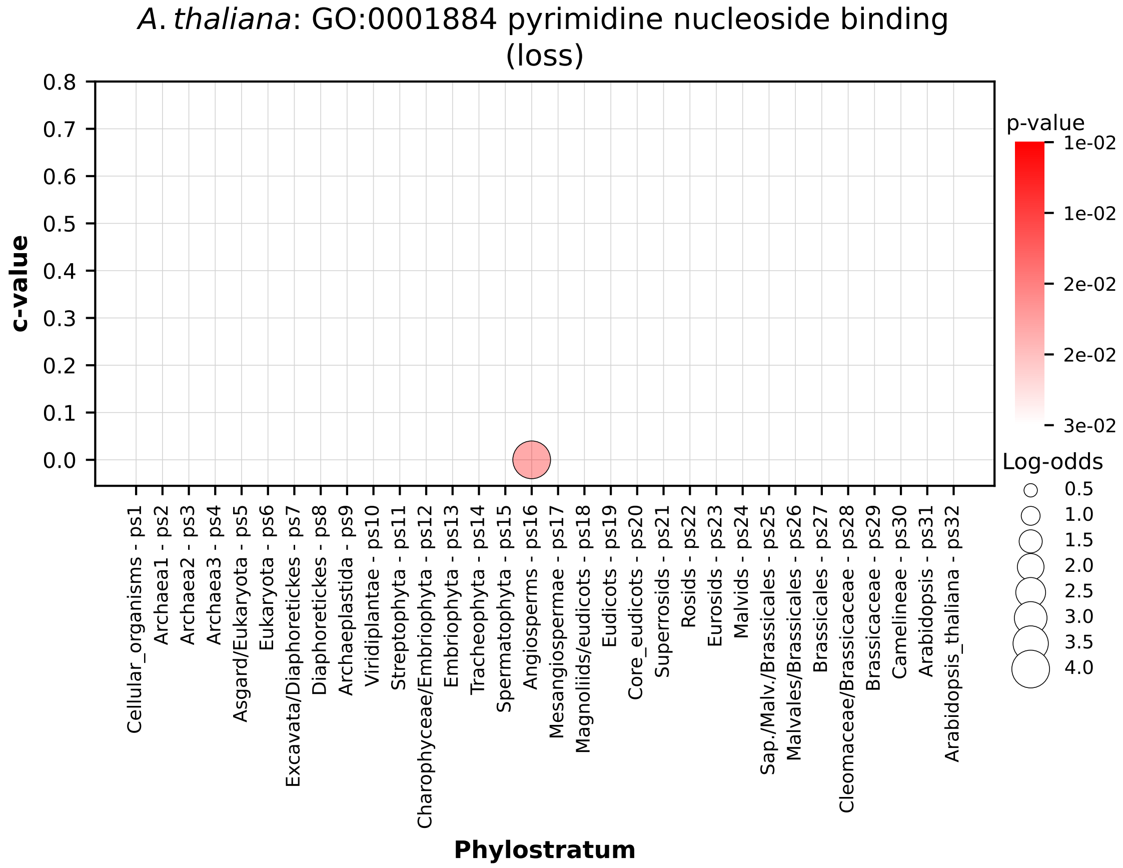

A. thaliana: GO:0001884 pyrimidine nucleoside binding
(loss)
c-value
0.8
0.7
0.6
0.5
0.4
0.3
0.2
0.1
0.0
p-value
1e-02
1e-02
2e-02
2e-02
3e-02
Log-odds
0.5
1.0
1.5
2.0
2.5
3.0
3.5
4.0
Cellular_organisms - ps1
Archaea1 - ps2
Archaea2 - ps3
Archaea3 - ps4
Asgard/Eukaryota - ps5
Eukaryota - ps6
Excavata/Diaphoretickes - ps7
Diaphoretickes - ps8
Archaeplastida - ps9
Viridiplantae - ps10
Streptophyta - ps11
Charophyceae/Embriophyta - ps12
Embriophyta - ps13
Tracheophyta - ps14
Spermatophyta - ps15
Angiosperms - ps16
Mesangiospermae - ps17
Magnoliids/eudicots - ps18
Eudicots - ps19
Core_eudicots - ps20
Superrosids - ps21
Rosids - ps22
Eurosids - ps23
Malvids - ps24
Sap./Malv./Brassicales - ps25
Malvales/Brassicales - ps26
Brassicales - ps27
Cleomaceae/Brassicaceae - ps28
Brassicaceae - ps29
Camelineae - ps30
Arabidopsis - ps31
Arabidopsis_thaliana - ps32
Phylostratum

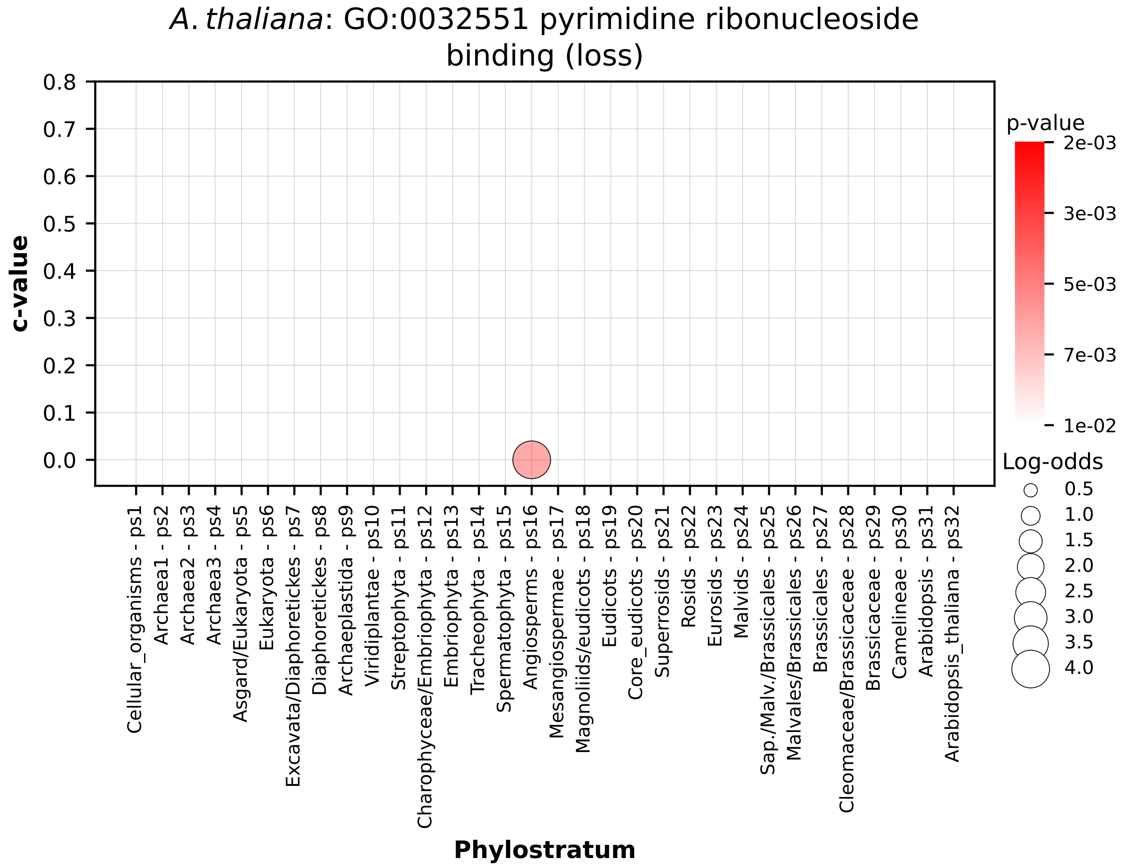

A. thaliana: GO:0032551 pyrimidine ribonucleoside binding (loss)
c-value
0.8
0.7
0.6
0.5
0.4
0.3
0.2
0.1
0.0
p-value
2e-03
3e-03
5e-03
7e-03
1e-02
Log-odds
0.5
1.0
1.5
2.0
2.5
3.0
3.5
4.0
Cellular_organisms - ps1
Archaea1 - ps2
Archaea2 - ps3
Archaea3 - ps4
Asgard/Eukaryota - ps5
Eukaryota - ps6
Excavata/Diaphoretickes - ps7
Diaphoretickes - ps8
Archaeplastida - ps9
Viridiplantae - ps10
Streptophyta - ps11
Charophyceae/Embriophyta - ps12
Embriophyta - ps13
Tracheophyta - ps14
Spermatophyta - ps15
Angiosperms - ps16
Mesangiospermae - ps17
Magnoliids/eudicots - ps18
Eudicots - ps19
Core_eudicots - ps20
Superrosids - ps21
Rosids - ps22
Eurosids - ps23
Malvids - ps24
Sap./Malv./Brassicales - ps25
Malvales/Brassicales - ps26
Brassicales - ps27
Cleomaceae/Brassicaceae - ps28
Brassicaceae - ps29
Camelineae - ps30
Arabidopsis - ps31
Arabidopsis_thaliana - ps32
Phylostratum

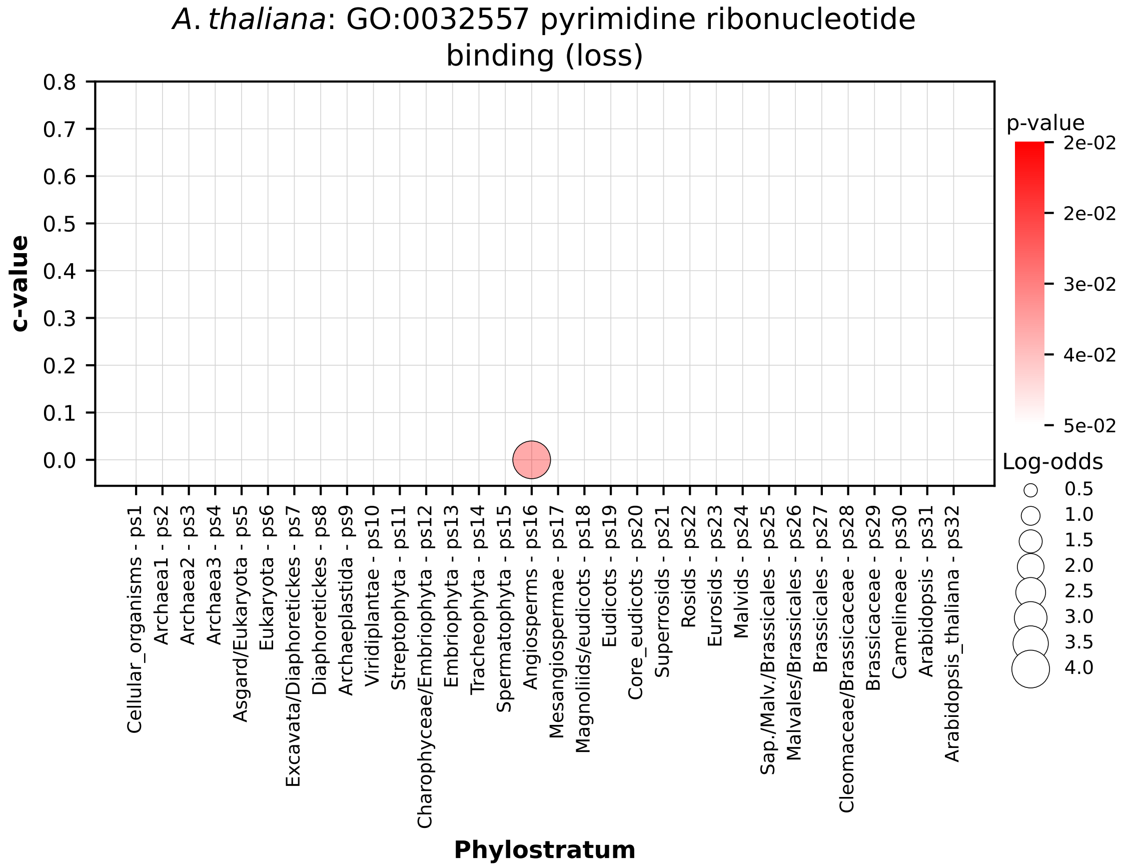
A. thaliana: GO:0032557 pyrimidine ribonucleotide binding (loss)
c-value
0.8
0.7
0.6
0.5
0.4
0.3
0.2
0.1
0.0
p-value
2e-02
2e-02
3e-02
4e-02
5e-02
Log-odds
0.5
1.0
1.5
2.0
2.5
3.0
3.5
4.0
Cellular_organisms - ps1
Archaea1 - ps2
Archaea2 - ps3
Archaea3 - ps4
Asgard/Eukaryota - ps5
Eukaryota - ps6
Excavata/Diaphoretickes - ps7
Diaphoretickes - ps8
Archaeplastida - ps9
Viridiplantae - ps10
Streptophyta - ps11
Charophyceae/Embriophyta - ps12
Embriophyta - ps13
Tracheophyta - ps14
Spermatophyta - ps15
Angiosperms - ps16
Mesangiospermae - ps17
Magnoliids/eudicots - ps18
Eudicots - ps19
Core_eudicots - ps20
Superrosids - ps21
Rosids - ps22
Eurosids - ps23
Malvids - ps24
Sap./Malv./Brassicales - ps25
Malvales/Brassicales - ps26
Brassicales - ps27
Cleomaceae/Brassicaceae - ps28
Brassicaceae - ps29
Camelineae - ps30
Arabidopsis - ps31
Arabidopsis_thaliana - ps32
Phylostratum

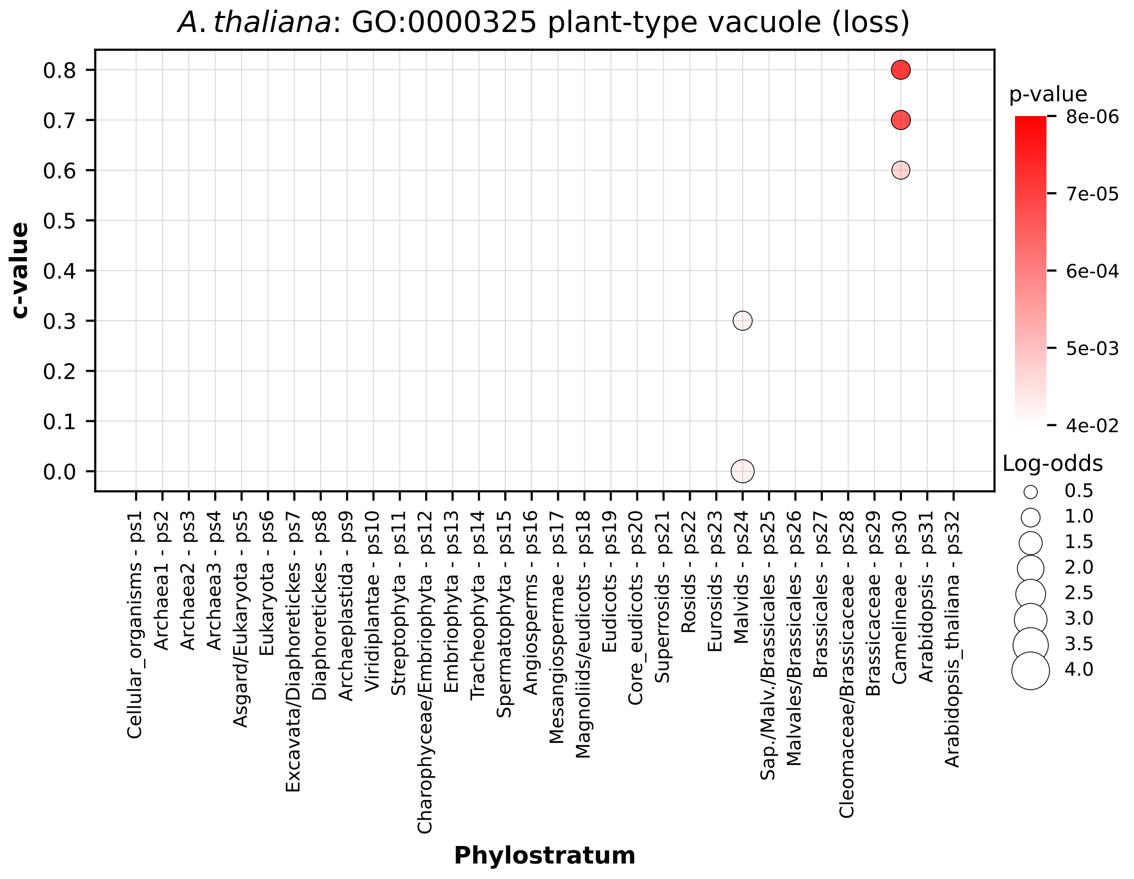

A. thaliana: GO:0000325 plant-type vacuole (loss)
c-value
0.0
0.1
0.2
0.3
0.4
0.5
0.6
0.7
0.8
p-value
8e-06
7e-05
6e-04
5e-03
4e-02
Log-odds
0.5
1.0
1.5
2.0
2.5
3.0
3.5
4.0
Cellular_organisms - ps1
Archaea1 - ps2
Archaea2 - ps3
Archaea3 - ps4
Asgard/Eukaryota - ps5
Eukaryota - ps6
Excavata/Diaphoretickes - ps7
Diaphoretickes - ps8
Archaeplastida - ps9
Viridiplantae - ps10
Streptophyta - ps11
Charophyceae/Embriophyta - ps12
Embriophyta - ps13
Tracheophyta - ps14
Spermatophyta - ps15
Angiosperms - ps16
Mesangiospermae - ps17
Magnoliids/eudicots - ps18
Eudicots - ps19
Core_eudicots - ps20
Superrosids - ps21
Rosids - ps22
Eurosids - ps23
Malvids - ps24
Sap./Malv./Brassicales - ps25
Malvales/Brassicales - ps26
Brassicales - ps27
Cleomaceae/Brassicaceae - ps28
Brassicaceae - ps29
Camelineae - ps30
Arabidopsis - ps31
Arabidopsis_thaliana - ps32
Phylostratum

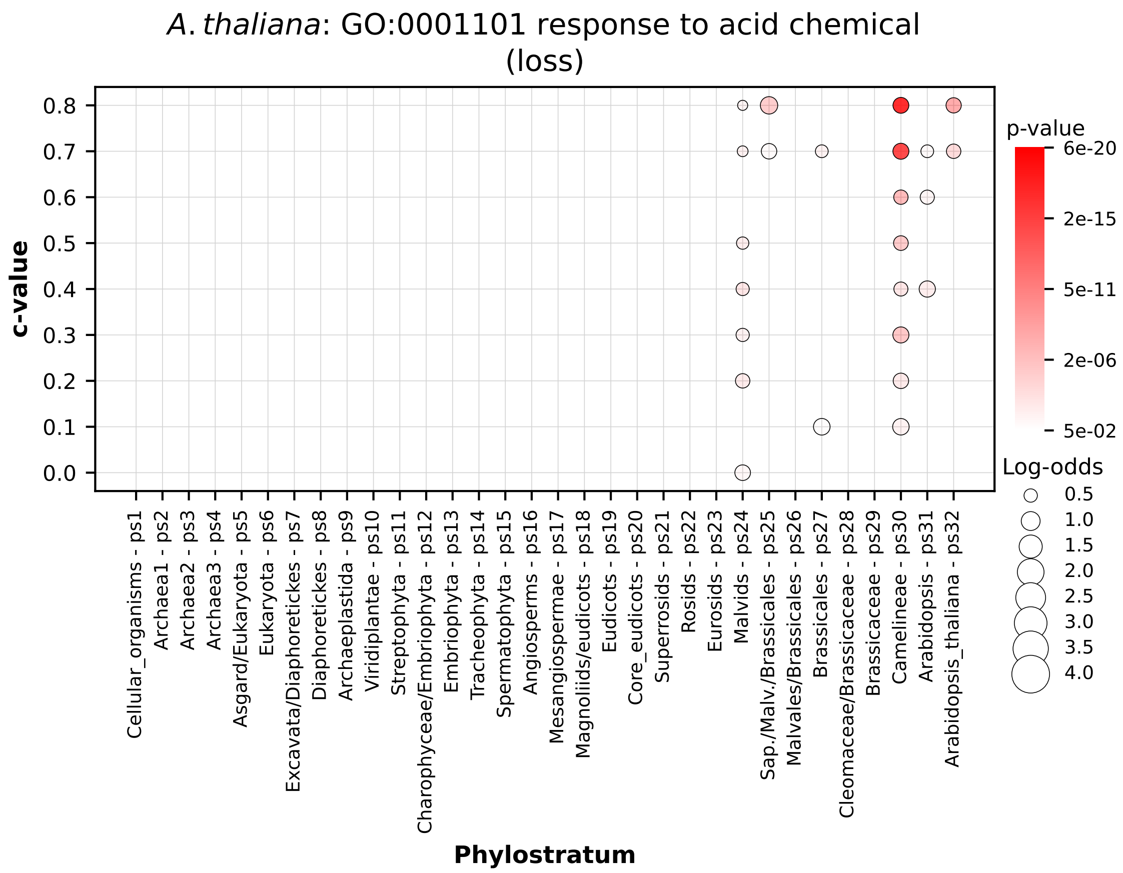
A. thaliana: GO:0001101 response to acid chemical
(loss)
c-value
0.8
0.7
0.6
0.5
0.4
0.3
0.2
0.1
0.0
p-value
6e-20
2e-15
5e-11
2e-06
5e-02
Log-odds
0.5
1.0
1.5
2.0
2.5
3.0
3.5
4.0
Cellular_organisms - ps1
Archaea1 - ps2
Archaea2 - ps3
Archaea3 - ps4
Asgard/Eukaryota - ps5
Eukaryota - ps6
Excavata/Diaphoretickes - ps7
Diaphoretickes - ps8
Archaeplastida - ps9
Viridiplantae - ps10
Streptophyta - ps11
Charophyceae/Embriophyta - ps12
Embriophyta - ps13
Tracheophyta - ps14
Spermatophyta - ps15
Angiosperms - ps16
Mesangiospermae - ps17
Magnoliids/eudicots - ps18
Eudicots - ps19
Core_eudicots - ps20
Superrosids - ps21
Rosids - ps22
Eurosids - ps23
Malvids - ps24
Sap./Malv./Brassicales - ps25
Malvales/Brassicales - ps26
Brassicales - ps27
Cleomaceae/Brassicaceae - ps28
Brassicaceae - ps29
Camelineae - ps30
Arabidopsis - ps31
Arabidopsis_thaliana - ps32
Phylostratum

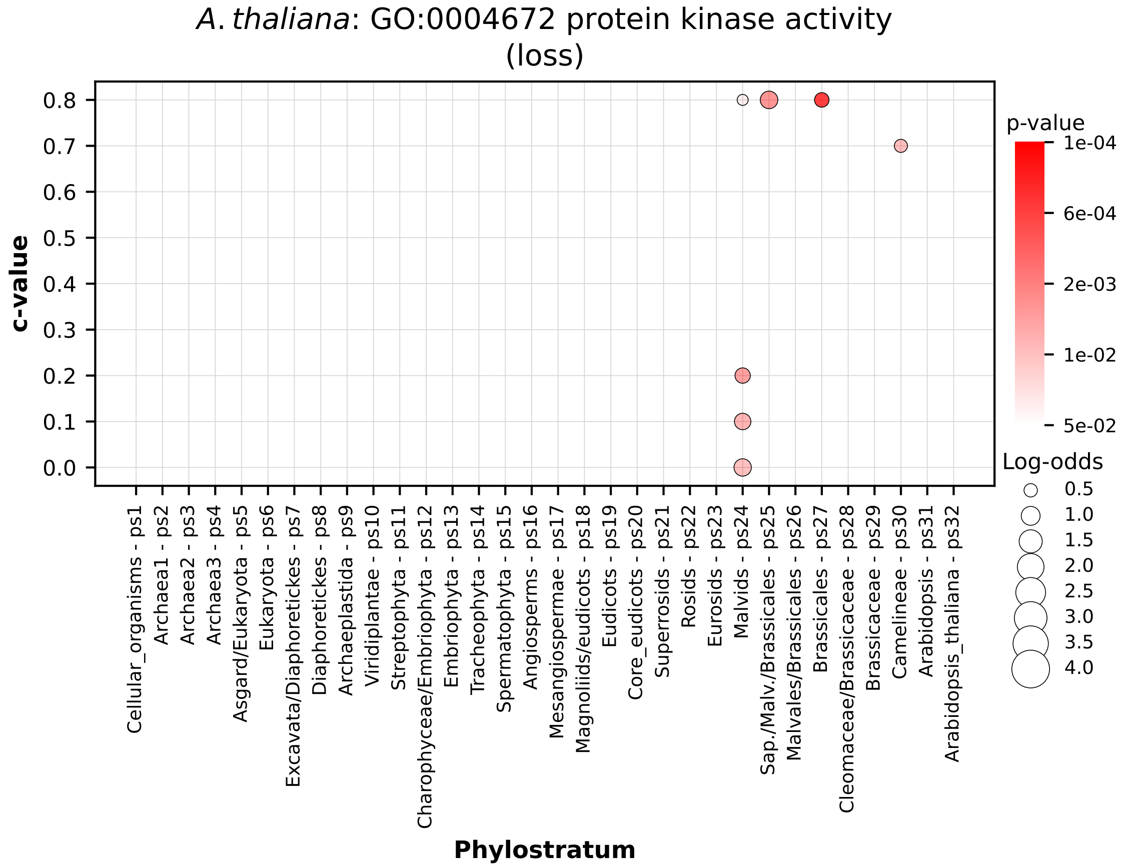

A. thaliana: GO:0004672 protein kinase activity (loss)
c-value
0.8
0.7
0.6
0.5
0.4
0.3
0.2
0.1
0.0
p-value
1e-04
6e-04
2e-03
1e-02
5e-02
Log-odds
0.5
1.0
1.5
2.0
2.5
3.0
3.5
4.0
Cellular_organisms - ps1
Archaea1 - ps2
Archaea2 - ps3
Archaea3 - ps4
Asgard/Eukaryota - ps5
Eukaryota - ps6
Excavata/Diaphoretickes - ps7
Diaphoretickes - ps8
Archaeplastida - ps9
Viridiplantae - ps10
Streptophyta - ps11
Charophyceae/Embriophyta - ps12
Embriophyta - ps13
Tracheophyta - ps14
Spermatophyta - ps15
Angiosperms - ps16
Mesangiospermae - ps17
Magnoliids/eudicots - ps18
Eudicots - ps19
Core_eudicots - ps20
Superrosids - ps21
Rosids - ps22
Eurosids - ps23
Malvids - ps24
Sap./Malv./Brassicales - ps25
Malvales/Brassicales - ps26
Brassicales - ps27
Cleomaceae/Brassicaceae - ps28
Brassicaceae - ps29
Camelineae - ps30
Arabidopsis - ps31
Arabidopsis_thaliana - ps32
Phylostratum

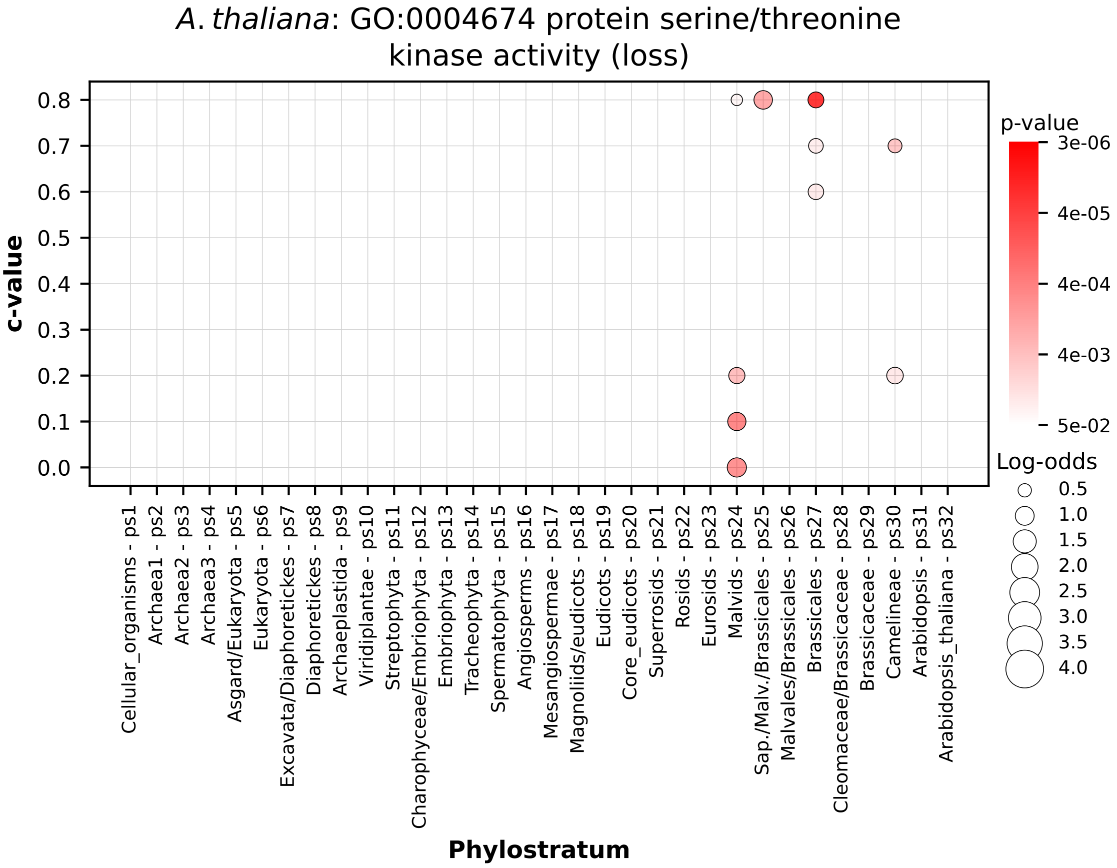

A. thaliana: GO:0004674 protein serine/threonine kinase activity (loss)
c-value
0.8
0.7
0.6
0.5
0.4
0.3
0.2
0.1
0.0
p-value
3e-06
4e-05
4e-04
4e-03
5e-02
Log-odds
0.5
1.0
1.5
2.0
2.5
3.0
3.5
4.0
Cellular_organisms - ps1
Archaea1 - ps2
Archaea2 - ps3
Archaea3 - ps4
Asgard/Eukaryota - ps5
Eukaryota - ps6
Excavata/Diaphoretickes - ps7
Diaphoretickes - ps8
Archaeplastida - ps9
Viridiplantae - ps10
Streptophyta - ps11
Charophyceae/Embriophyta - ps12
Embriophyta - ps13
Tracheophyta - ps14
Spermatophyta - ps15
Angiosperms - ps16
Mesangiospermae - ps17
Magnoliids/eudicots - ps18
Eudicots - ps19
Core_eudicots - ps20
Superrosids - ps21
Rosids - ps22
Eurosids - ps23
Malvids - ps24
Sap./Malv./Brassicales - ps25
Malvales/Brassicales - ps26
Brassicales - ps27
Cleomaceae/Brassicaceae - ps28
Brassicaceae - ps29
Camelineae - ps30
Arabidopsis - ps31
Arabidopsis_thaliana - ps32
Phylostratum

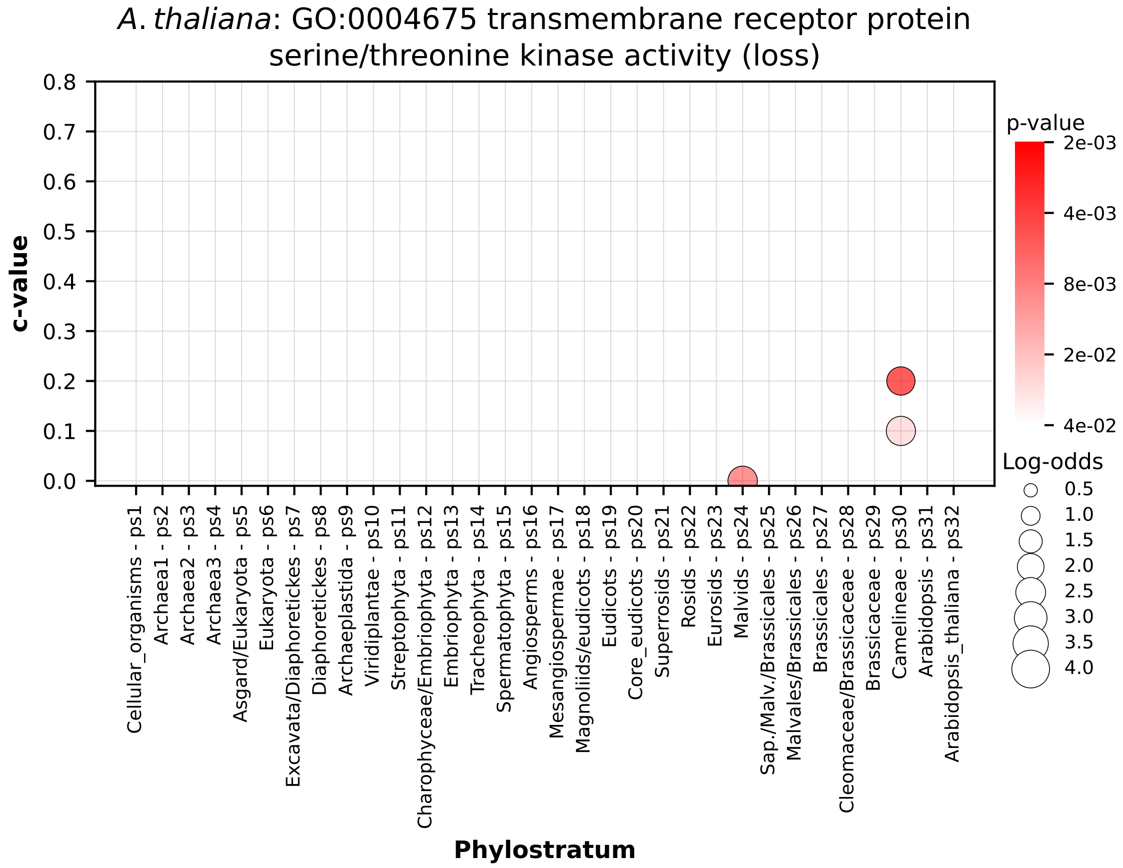

A. thaliana: GO:0004675 transmembrane receptor protein serine/threonine kinase activity (loss)
c-value
0.0
0.1
0.2
0.3
0.4
0.5
0.6
0.7
0.8
Phylostratum
Cellular_organisms - ps1
Archaea1 - ps2
Archaea2 - ps3
Archaea3 - ps4
Asgard/Eukaryota - ps5
Eukaryota - ps6
Excavata/Diaphoretickes - ps7
Diaphoretickes - ps8
Archaeplastida - ps9
Viridiplantae - ps10
Streptophyta - ps11
Charophyceae/Embriophyta - ps12
Embriophyta - ps13
Tracheophyta - ps14
Spermatophyta - ps15
Angiosperms - ps16
Mesangiospermae - ps17
Magnoliids/eudicots - ps18
Eudicots - ps19
Core_eudicots - ps20
Superrosids - ps21
Rosids - ps22
Eurosids - ps23
Malvids - ps24
Sap./Malv./Brassicales - ps25
Malvales/Brassicales - ps26
Brassicales - ps27
Cleomaceae/Brassicaceae - ps28
Brassicaceae - ps29
Camelineae - ps30
Arabidopsis - ps31
Arabidopsis_thaliana - ps32
p-value
2e-03
4e-03
8e-03
2e-02
4e-02
Log-odds
0.5
1.0
1.5
2.0
2.5
3.0
3.5
4.0

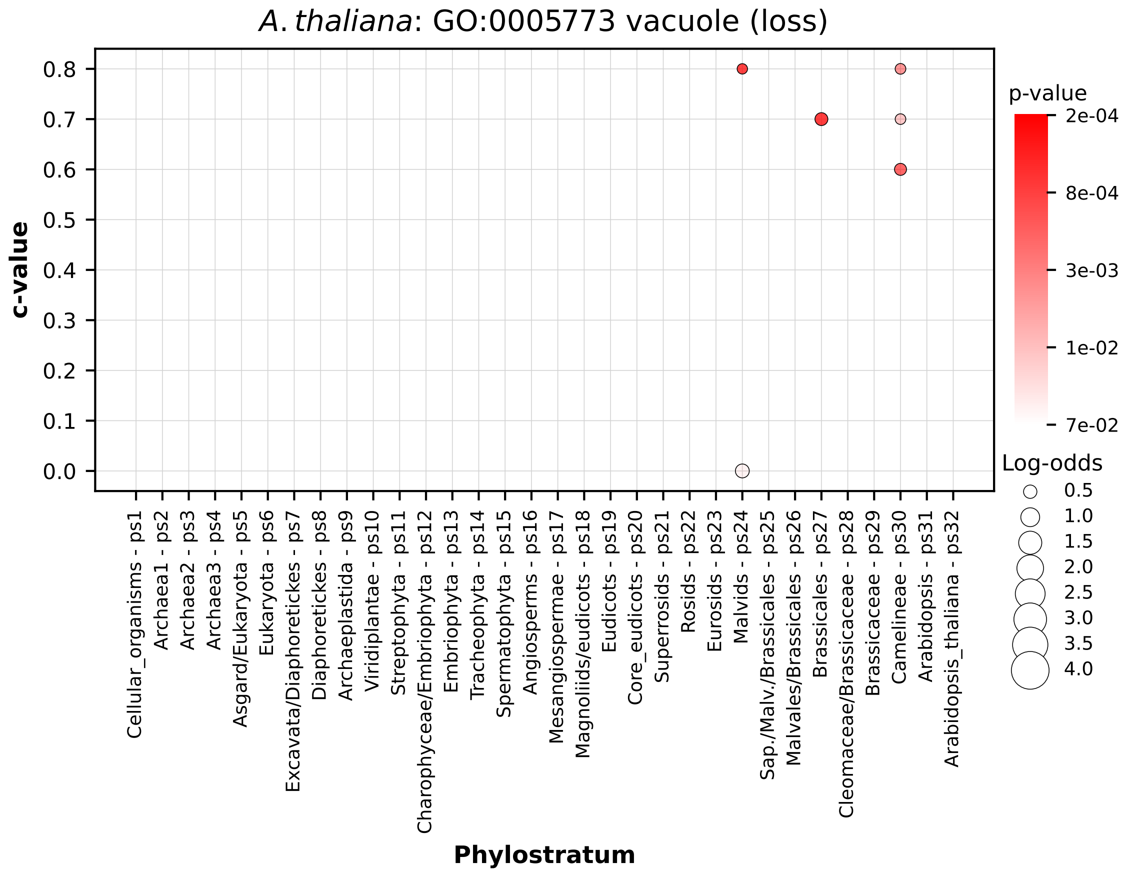

A. thaliana: GO:0005773 vacuole (loss)
c-value
0.0
0.1
0.2
0.3
0.4
0.5
0.6
0.7
0.8
p-value
2e-04
8e-04
3e-03
1e-02
7e-02
Log-odds
0.5
1.0
1.5
2.0
2.5
3.0
3.5
4.0
Cellular_organisms - ps1
Archaea1 - ps2
Archaea2 - ps3
Archaea3 - ps4
Asgard/Eukaryota - ps5
Eukaryota - ps6
Excavata/Diaphoretickes - ps7
Diaphoretickes - ps8
Archaeplastida - ps9
Viridiplantae - ps10
Streptophyta - ps11
Charophyceae/Embriophyta - ps12
Embriophyta - ps13
Tracheophyta - ps14
Spermatophyta - ps15
Angiosperms - ps16
Mesangiospermae - ps17
Magnoliids/eudicots - ps18
Eudicots - ps19
Core_eudicots - ps20
Superrosids - ps21
Rosids - ps22
Eurosids - ps23
Malvids - ps24
Sap./Malv./Brassicales - ps25
Malvales/Brassicales - ps26
Brassicales - ps27
Cleomaceae/Brassicaceae - ps28
Brassicaceae - ps29
Camelineae - ps30
Arabidopsis - ps31
Arabidopsis_thaliana - ps32
Phylostratum

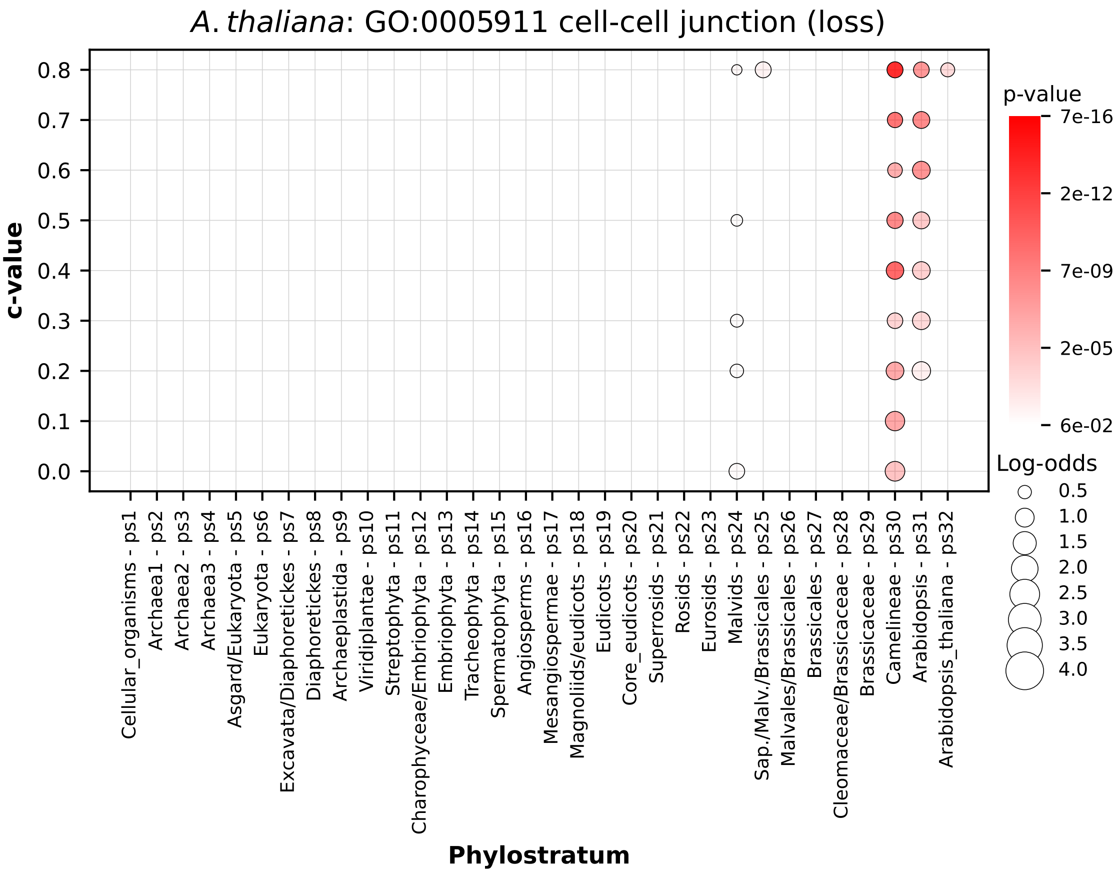

A. thaliana: GO:0005911 cell-cell junction (loss)
c-value
0.0
0.1
0.2
0.3
0.4
0.5
0.6
0.7
0.8
p-value
7e-16
2e-12
7e-09
2e-05
6e-02
Log-odds
0.5
1.0
1.5
2.0
2.5
3.0
3.5
4.0
Cellular_organisms - ps1
Archaea1 - ps2
Archaea2 - ps3
Archaea3 - ps4
Asgard/Eukaryota - ps5
Eukaryota - ps6
Excavata/Diaphoretickes - ps7
Diaphoretickes - ps8
Archaeplastida - ps9
Viridiplantae - ps10
Streptophyta - ps11
Charophyceae/Embriophyta - ps12
Embriophyta - ps13
Tracheophyta - ps14
Spermatophyta - ps15
Angiosperms - ps16
Mesangiospermae - ps17
Magnoliids/eudicots - ps18
Eudicots - ps19
Core_eudicots - ps20
Superrosids - ps21
Rosids - ps22
Eurosids - ps23
Malvids - ps24
Sap./Malv./Brassicales - ps25
Malvales/Brassicales - ps26
Brassicales - ps27
Cleomaceae/Brassicaceae - ps28
Brassicaceae - ps29
Camelineae - ps30
Arabidopsis - ps31
Arabidopsis_thaliana - ps32
Phylostratum

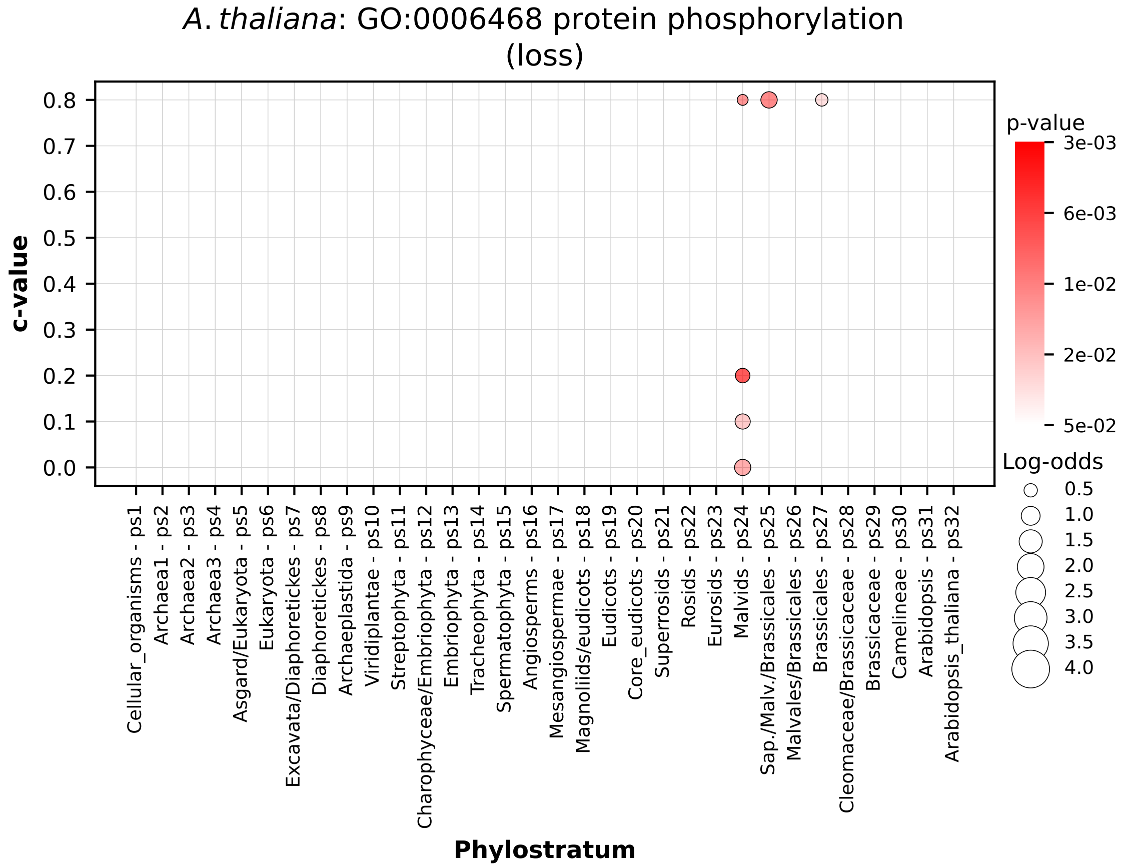

A. thaliana: GO:0006468 protein phosphorylation
(loss)
c-value
0.8
0.7
0.6
0.5
0.4
0.3
0.2
0.1
0.0
p-value
3e-03
6e-03
1e-02
2e-02
5e-02
Log-odds
0.5
1.0
1.5
2.0
2.5
3.0
3.5
4.0
Cellular_organisms - ps1
Archaea1 - ps2
Archaea2 - ps3
Archaea3 - ps4
Asgard/Eukaryota - ps5
Eukaryota - ps6
Excavata/Diaphoretickes - ps7
Diaphoretickes - ps8
Archaeplastida - ps9
Viridiplantae - ps10
Streptophyta - ps11
Charophyceae/Embriophyta - ps12
Embriophyta - ps13
Tracheophyta - ps14
Spermatophyta - ps15
Angiosperms - ps16
Mesangiospermae - ps17
Magnoliids/eudicots - ps18
Eudicots - ps19
Core_eudicots - ps20
Superrosids - ps21
Rosids - ps22
Eurosids - ps23
Malvids - ps24
Sap./Malv./Brassicales - ps25
Malvales/Brassicales - ps26
Brassicales - ps27
Cleomaceae/Brassicaceae - ps28
Brassicaceae - ps29
Camelineae - ps30
Arabidopsis - ps31
Arabidopsis_thaliana - ps32
Phylostratum

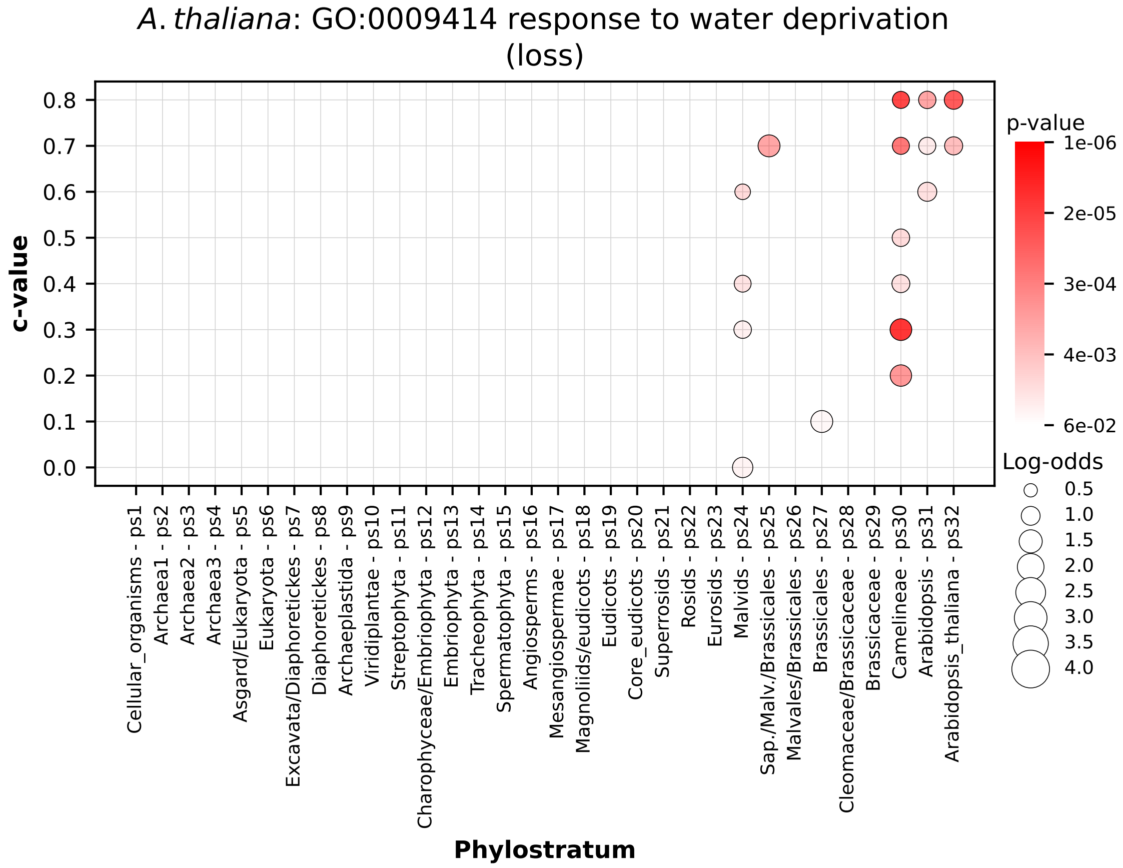
A. thaliana: GO:0009414 response to water deprivation
(loss)
c-value
0.0
0.1
0.2
0.3
0.4
0.5
0.6
0.7
0.8
Phylostratum
Cellular_organisms - ps1
Archaea1 - ps2
Archaea2 - ps3
Archaea3 - ps4
Asgard/Eukaryota - ps5
Eukaryota - ps6
Excavata/Diaphoretickes - ps7
Diaphoretickes - ps8
Archaeplastida - ps9
Viridiplantae - ps10
Streptophyta - ps11
Charophyceae/Embriophyta - ps12
Embriophyta - ps13
Tracheophyta - ps14
Spermatophyta - ps15
Angiosperms - ps16
Mesangiospermae - ps17
Magnoliids/eudicots - ps18
Eudicots - ps19
Core_eudicots - ps20
Superrosids - ps21
Rosids - ps22
Eurosids - ps23
Malvids - ps24
Sap./Malv./Brassicales - ps25
Malvales/Brassicales - ps26
Brassicales - ps27
Cleomaceae/Brassicaceae - ps28
Brassicaceae - ps29
Camelineae - ps30
Arabidopsis - ps31
Arabidopsis_thaliana - ps32
p-value
1e-06
2e-05
3e-04
4e-03
6e-02
Log-odds
0.5
1.0
1.5
2.0
2.5
3.0
3.5
4.0

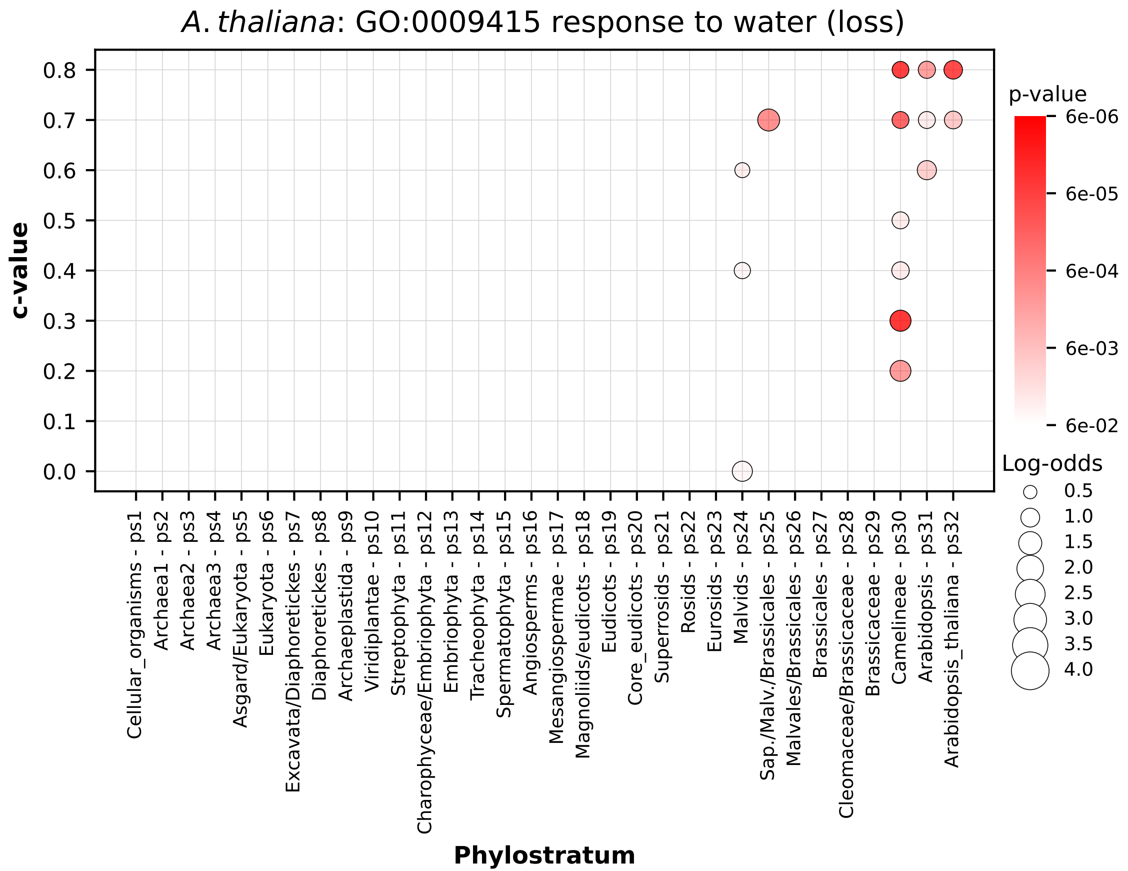
A. thaliana: GO:0009415 response to water (loss)
c-value
0.8
0.7
0.6
0.5
0.4
0.3
0.2
0.1
0.0
Phylostratum
Cellular_organisms - ps1
Archaea1 - ps2
Archaea2 - ps3
Archaea3 - ps4
Asgard/Eukaryota - ps5
Eukaryota - ps6
Excavata/Diaphoretickes - ps7
Diaphoretickes - ps8
Archaeplastida - ps9
Viridiplantae - ps10
Streptophyta - ps11
Charophyceae/Embriophyta - ps12
Embriophyta - ps13
Tracheophyta - ps14
Spermatophyta - ps15
Angiosperms - ps16
Mesangiospermae - ps17
Magnoliids/eudicots - ps18
Eudicots - ps19
Core_eudicots - ps20
Superrosids - ps21
Rosids - ps22
Eurosids - ps23
Malvids - ps24
Sap./Malv./Brassicales - ps25
Malvales/Brassicales - ps26
Brassicales - ps27
Cleomaceae/Brassicaceae - ps28
Brassicaceae - ps29
Camelineae - ps30
Arabidopsis - ps31
Arabidopsis_thaliana - ps32
p-value
6e-06
6e-05
6e-04
6e-03
6e-02
Log-odds
0.5
1.0
1.5
2.0
2.5
3.0
3.5
4.0

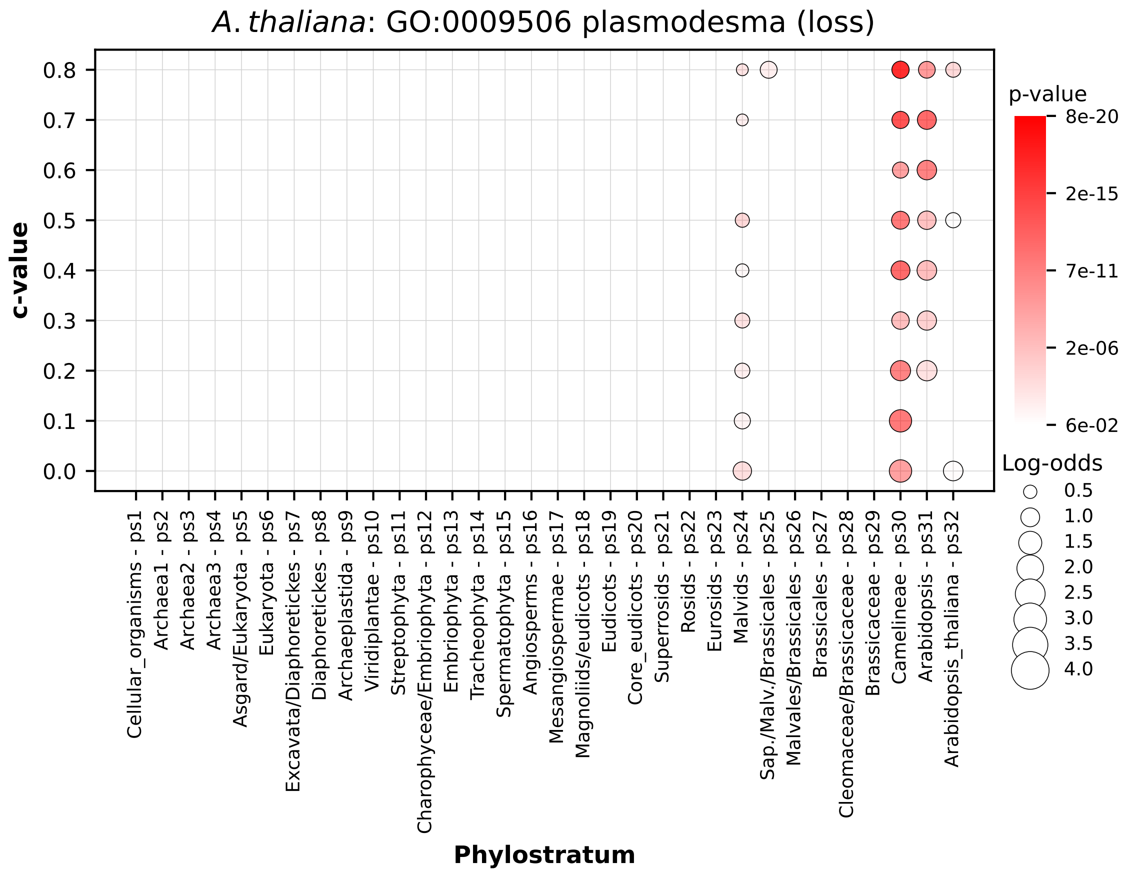
A. thaliana: GO:0009506 plasmodesma (loss)
c-value
0.8
0.7
0.6
0.5
0.4
0.3
0.2
0.1
0.0
p-value
8e-20
2e-15
7e-11
2e-06
6e-02
Log-odds
0.5
1.0
1.5
2.0
2.5
3.0
3.5
4.0
Cellular_organisms - ps1
Archaea1 - ps2
Archaea2 - ps3
Archaea3 - ps4
Asgard/Eukaryota - ps5
Eukaryota - ps6
Excavata/Diaphoretickes - ps7
Diaphoretickes - ps8
Archaeplastida - ps9
Viridiplantae - ps10
Streptophyta - ps11
Charophyceae/Embriophyta - ps12
Embriophyta - ps13
Tracheophyta - ps14
Spermatophyta - ps15
Angiosperms - ps16
Mesangiospermae - ps17
Magnoliids/eudicots - ps18
Eudicots - ps19
Core_eudicots - ps20
Superrosids - ps21
Rosids - ps22
Eurosids - ps23
Malvids - ps24
Sap./Malv./Brassicales - ps25
Malvales/Brassicales - ps26
Brassicales - ps27
Cleomaceae/Brassicaceae - ps28
Brassicaceae - ps29
Camelineae - ps30
Arabidopsis - ps31
Arabidopsis_thaliana - ps32
Phylostratum

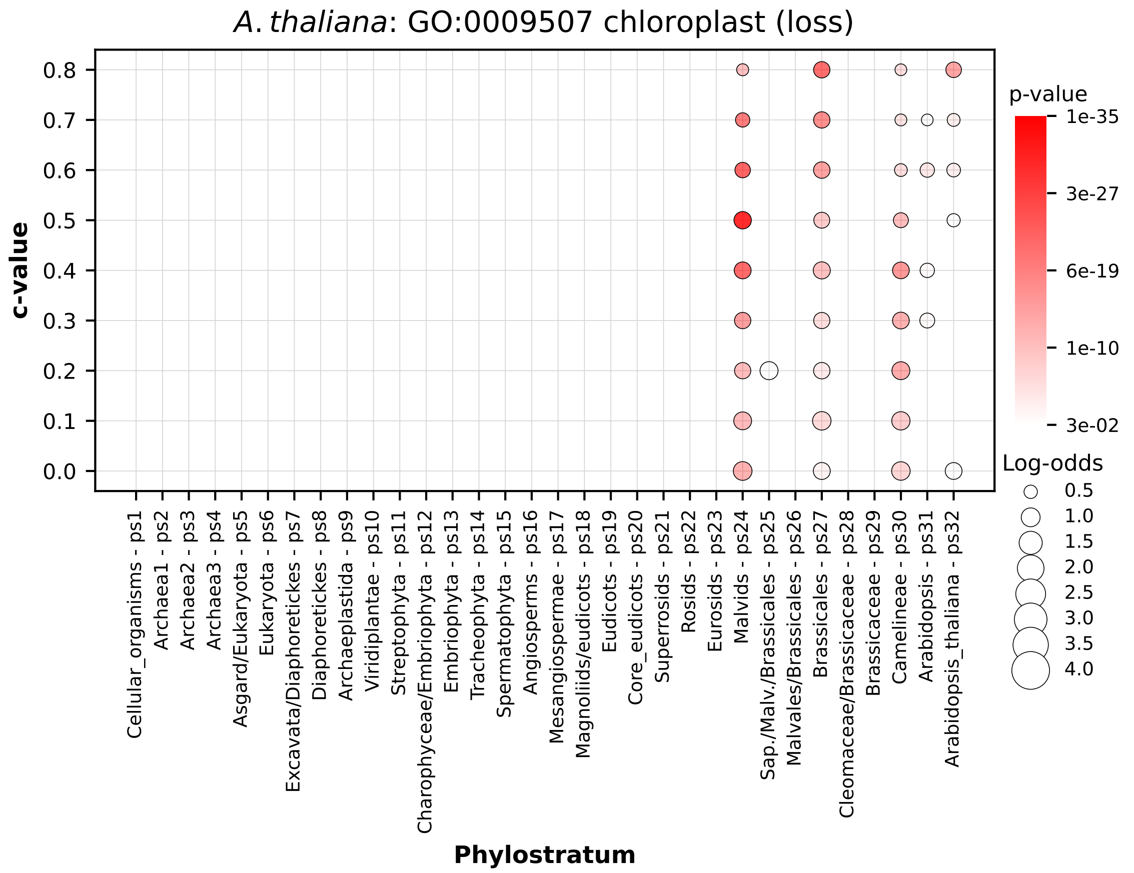

A. thaliana: GO:0009507 chloroplast (loss)
c-value
0.8
0.7
0.6
0.5
0.4
0.3
0.2
0.1
0.0
Phylostratum
Cellular_organisms - ps1
Archaea1 - ps2
Archaea2 - ps3
Archaea3 - ps4
Asgard/Eukaryota - ps5
Eukaryota - ps6
Excavata/Diaphoretickes - ps7
Diaphoretickes - ps8
Archaeplastida - ps9
Viridiplantae - ps10
Streptophyta - ps11
Charophyceae/Embriophyta - ps12
Embriophyta - ps13
Tracheophyta - ps14
Spermatophyta - ps15
Angiosperms - ps16
Mesangiospermae - ps17
Magnoliids/eudicots - ps18
Eudicots - ps19
Core_eudicots - ps20
Superrosids - ps21
Rosids - ps22
Eurosids - ps23
Malvids - ps24
Sap./Malv./Brassicales - ps25
Malvales/Brassicales - ps26
Brassicales - ps27
Cleomaceae/Brassicaceae - ps28
Brassicaceae - ps29
Camelineae - ps30
Arabidopsis - ps31
Arabidopsis_thaliana - ps32
p-value
1e-35
3e-27
6e-19
1e-10
3e-02
Log-odds
0.5
1.0
1.5
2.0
2.5
3.0
3.5
4.0

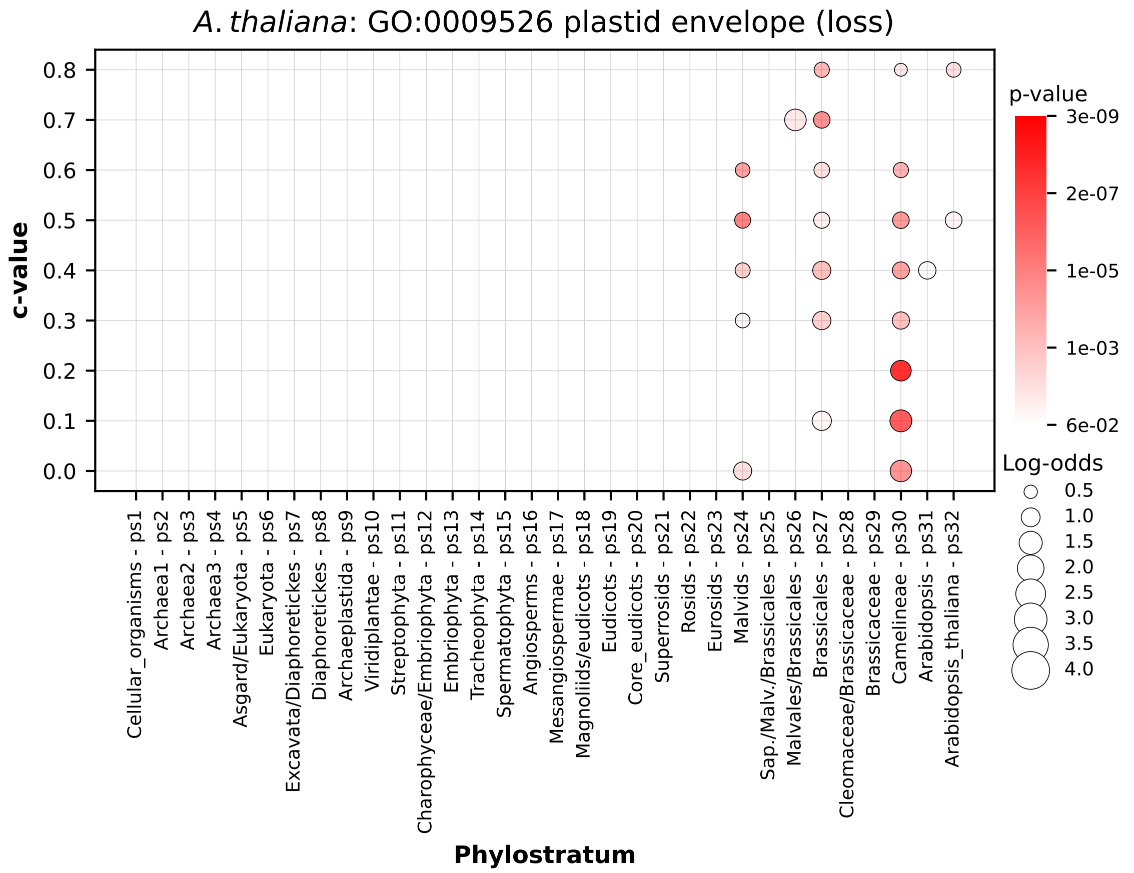
A. thaliana: GO:0009526 plastid envelope (loss)
c-value
0.8
0.7
0.6
0.5
0.4
0.3
0.2
0.1
0.0
Phylostratum
Cellular_organisms - ps1
Archaea1 - ps2
Archaea2 - ps3
Archaea3 - ps4
Asgard/Eukaryota - ps5
Eukaryota - ps6
Excavata/Diaphoretickes - ps7
Diaphoretickes - ps8
Archaeplastida - ps9
Viridiplantae - ps10
Streptophyta - ps11
Charophyceae/Embriophyta - ps12
Embriophyta - ps13
Tracheophyta - ps14
Spermatophyta - ps15
Angiosperms - ps16
Mesangiospermae - ps17
Magnoliids/eudicots - ps18
Eudicots - ps19
Core_eudicots - ps20
Superrosids - ps21
Rosids - ps22
Eurosids - ps23
Malvids - ps24
Sap./Malv./Brassicales - ps25
Malvales/Brassicales - ps26
Brassicales - ps27
Cleomaceae/Brassicaceae - ps28
Brassicaceae - ps29
Camelineae - ps30
Arabidopsis - ps31
Arabidopsis_thaliana - ps32
p-value
3e-09
2e-07
1e-05
1e-03
6e-02
Log-odds
0.5
1.0
1.5
2.0
2.5
3.0
3.5
4.0

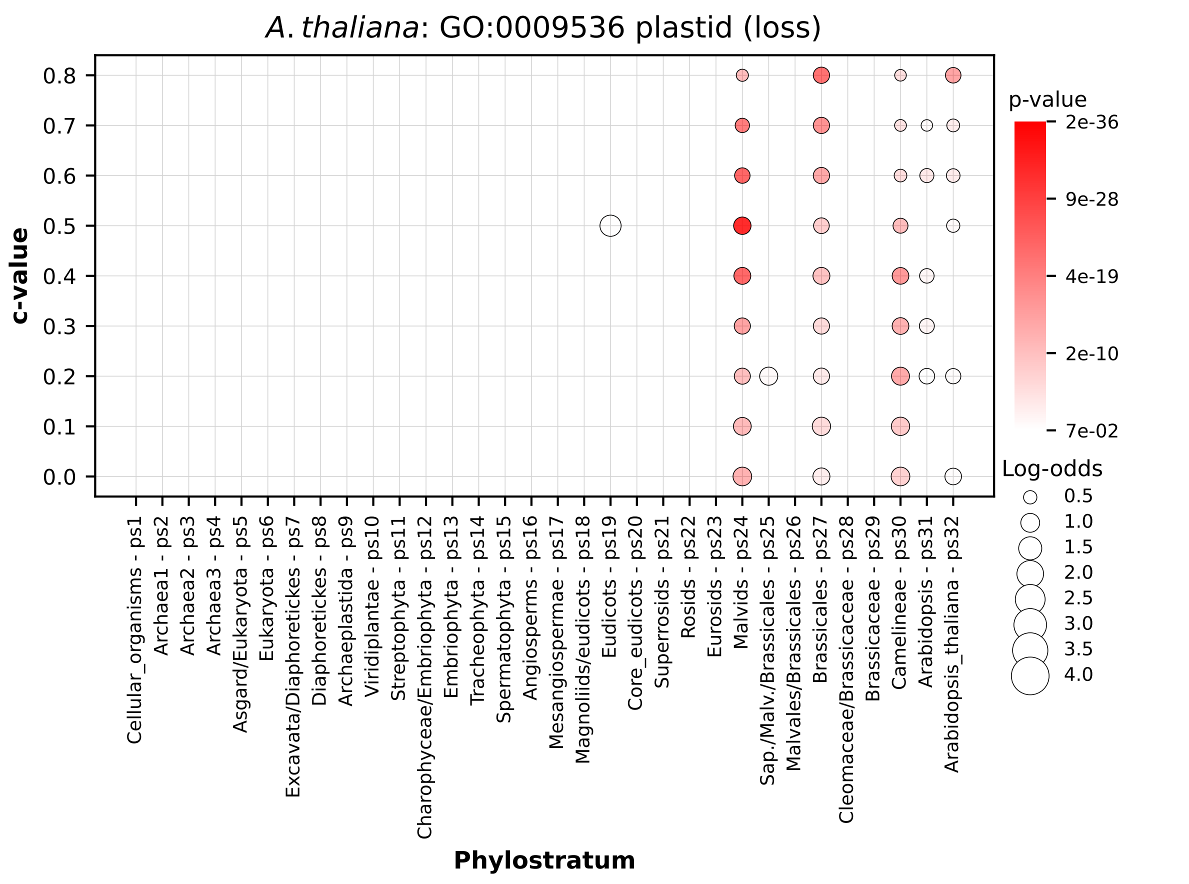

A. thaliana: GO:0009536 plastid (loss)
c-value
0.8
0.7
0.6
0.5
0.4
0.3
0.2
0.1
0.0
p-value
2e-36
9e-28
4e-19
2e-10
7e-02
Log-odds
0.5
1.0
1.5
2.0
2.5
3.0
3.5
4.0
Cellular_organisms - ps1
Archaea1 - ps2
Archaea2 - ps3
Archaea3 - ps4
Asgard/Eukaryota - ps5
Eukaryota - ps6
Excavata/Diaphoretickes - ps7
Diaphoretickes - ps8
Archaeplastida - ps9
Viridiplantae - ps10
Streptophyta - ps11
Charophyceae/Embriophyta - ps12
Embriophyta - ps13
Tracheophyta - ps14
Spermatophyta - ps15
Angiosperms - ps16
Mesangiospermae - ps17
Magnoliids/eudicots - ps18
Eudicots - ps19
Core_eudicots - ps20
Superrosids - ps21
Rosids - ps22
Eurosids - ps23
Malvids - ps24
Sap./Malv./Brassicales - ps25
Malvales/Brassicales - ps26
Brassicales - ps27
Cleomaceae/Brassicaceae - ps28
Brassicaceae - ps29
Camelineae - ps30
Arabidopsis - ps31
Arabidopsis_thaliana - ps32
Phylostratum

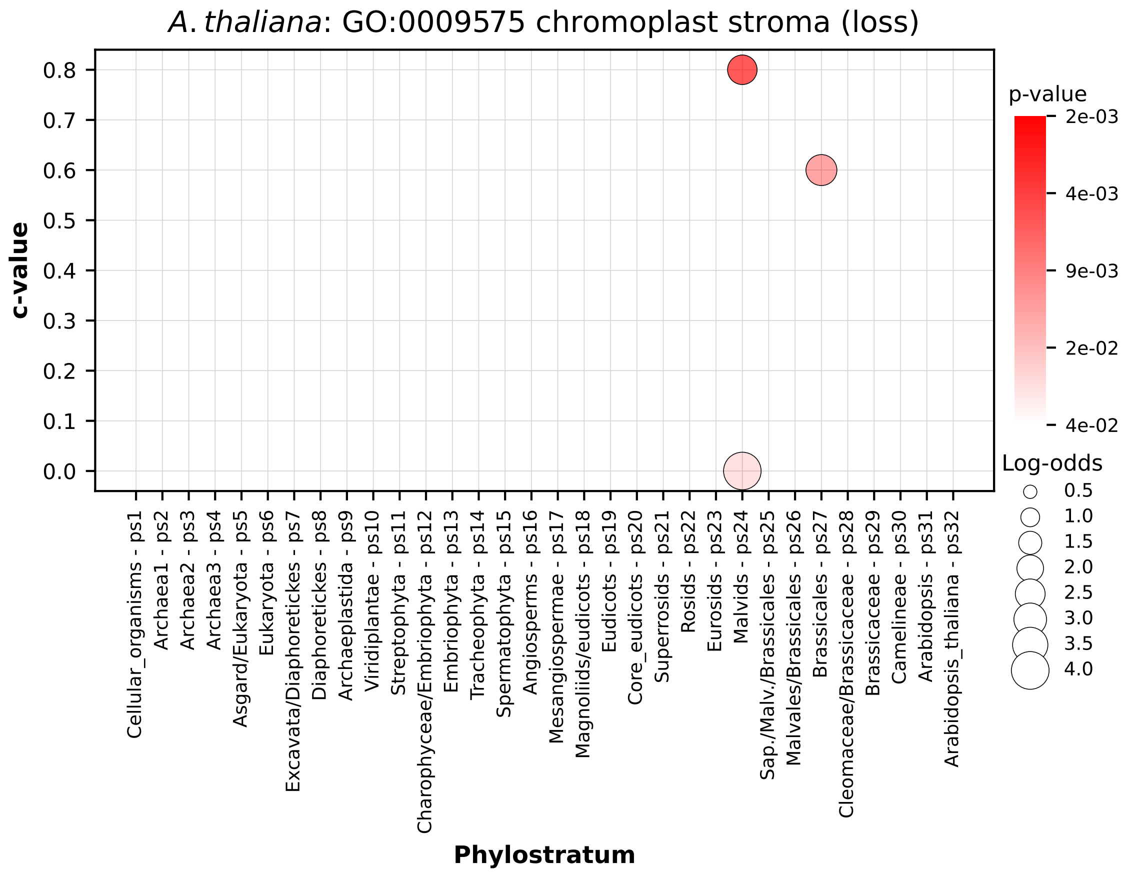

A. thaliana: GO:0009575 chromoplast stroma (loss)
c-value
0.8
0.7
0.6
0.5
0.4
0.3
0.2
0.1
0.0
p-value
2e-03
4e-03
9e-03
2e-02
4e-02
Log-odds
0.5
1.0
1.5
2.0
2.5
3.0
3.5
4.0
Cellular_organisms - ps1
Archaea1 - ps2
Archaea2 - ps3
Archaea3 - ps4
Asgard/Eukaryota - ps5
Eukaryota - ps6
Excavata/Diaphoretickes - ps7
Diaphoretickes - ps8
Archaeplastida - ps9
Viridiplantae - ps10
Streptophyta - ps11
Charophyceae/Embriophyta - ps12
Embriophyta - ps13
Tracheophyta - ps14
Spermatophyta - ps15
Angiosperms - ps16
Mesangiospermae - ps17
Magnoliids/eudicots - ps18
Eudicots - ps19
Core_eudicots - ps20
Superrosids - ps21
Rosids - ps22
Eurosids - ps23
Malvids - ps24
Sap./Malv./Brassicales - ps25
Malvales/Brassicales - ps26
Brassicales - ps27
Cleomaceae/Brassicaceae - ps28
Brassicaceae - ps29
Camelineae - ps30
Arabidopsis - ps31
Arabidopsis_thaliana - ps32
Phylostratum

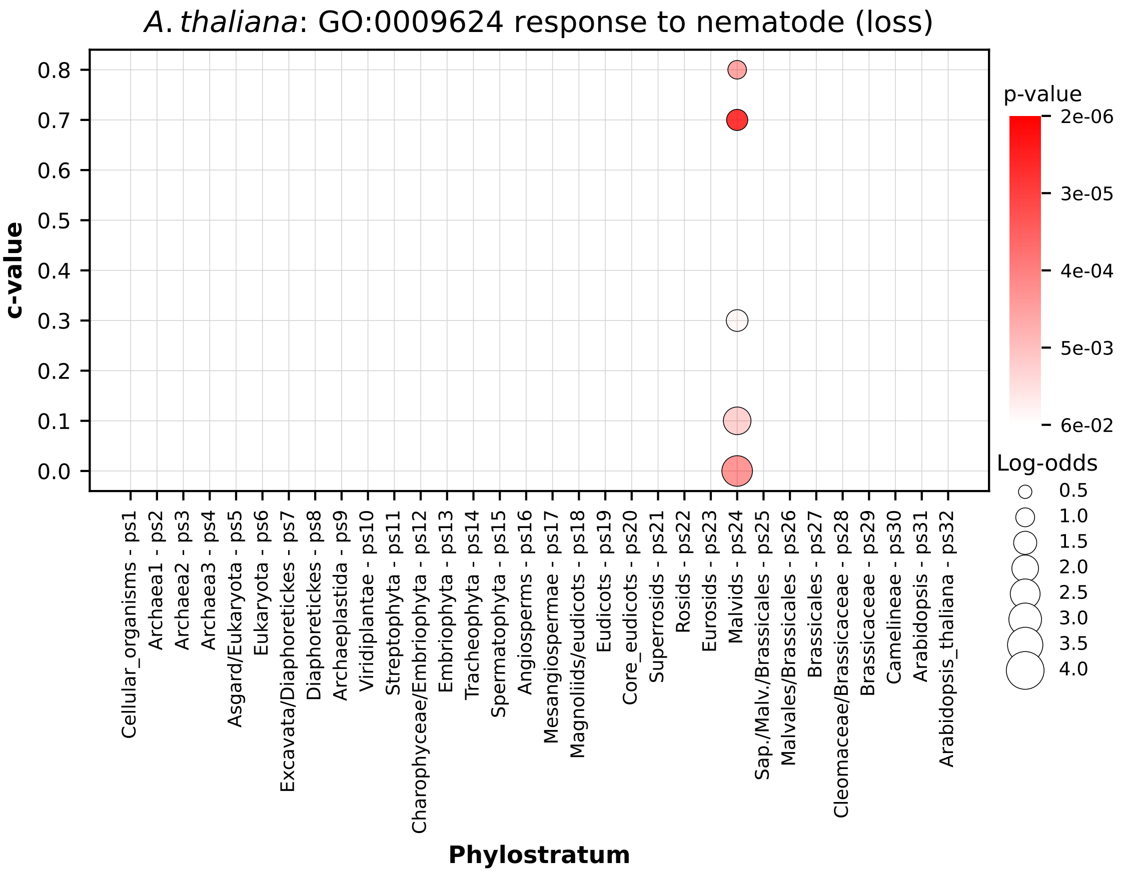

A. thaliana: GO:0009624 response to nematode (loss)
c-value
0.8
0.7
0.6
0.5
0.4
0.3
0.2
0.1
0.0
p-value
2e-06
3e-05
4e-04
5e-03
6e-02
Log-odds
0.5
1.0
1.5
2.0
2.5
3.0
3.5
4.0
Cellular_organisms - ps1
Archaea1 - ps2
Archaea2 - ps3
Archaea3 - ps4
Asgard/Eukaryota - ps5
Eukaryota - ps6
Excavata/Diaphoretickes - ps7
Diaphoretickes - ps8
Archaeplastida - ps9
Viridiplantae - ps10
Streptophyta - ps11
Charophyceae/Embriophyta - ps12
Embriophyta - ps13
Tracheophyta - ps14
Spermatophyta - ps15
Angiosperms - ps16
Mesangiospermae - ps17
Magnoliids/eudicots - ps18
Eudicots - ps19
Core_eudicots - ps20
Superrosids - ps21
Rosids - ps22
Eurosids - ps23
Malvids - ps24
Sap./Malv./Brassicales - ps25
Malvales/Brassicales - ps26
Brassicales - ps27
Cleomaceae/Brassicaceae - ps28
Brassicaceae - ps29
Camelineae - ps30
Arabidopsis - ps31
Arabidopsis_thaliana - ps32
Phylostratum

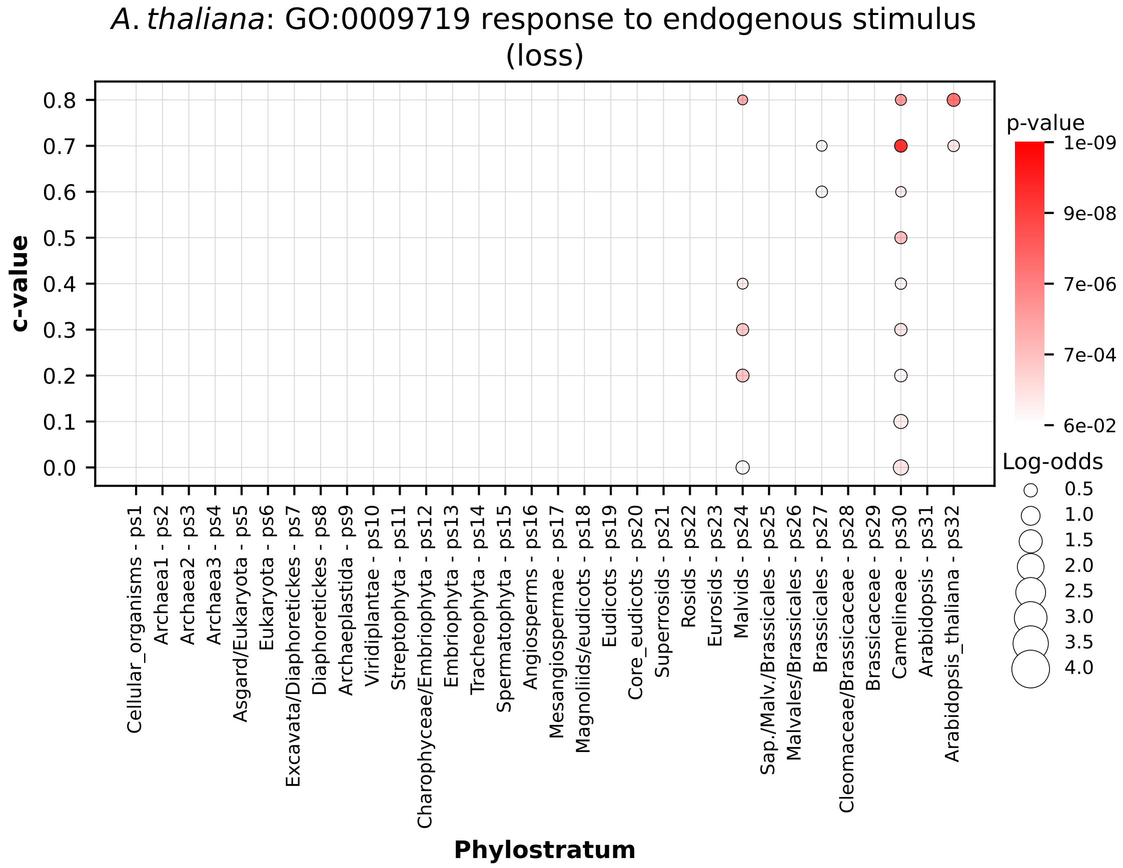

A. thaliana: GO:0009719 response to endogenous stimulus (loss)
c-value
0.8
0.7
0.6
0.5
0.4
0.3
0.2
0.1
0.0
p-value
1e-09
9e-08
7e-06
7e-04
6e-02
Log-odds
0.5
1.0
1.5
2.0
2.5
3.0
3.5
4.0
Cellular_organisms - ps1
Archaea1 - ps2
Archaea2 - ps3
Archaea3 - ps4
Asgard/Eukaryota - ps5
Eukaryota - ps6
Excavata/Diaphoretickes - ps7
Diaphoretickes - ps8
Archaeplastida - ps9
Viridiplantae - ps10
Streptophyta - ps11
Charophyceae/Embriophyta - ps12
Embriophyta - ps13
Tracheophyta - ps14
Spermatophyta - ps15
Angiosperms - ps16
Mesangiospermae - ps17
Magnoliids/eudicots - ps18
Eudicots - ps19
Core_eudicots - ps20
Superrosids - ps21
Rosids - ps22
Eurosids - ps23
Malvids - ps24
Sap./Malv./Brassicales - ps25
Malvales/Brassicales - ps26
Brassicales - ps27
Cleomaceae/Brassicaceae - ps28
Brassicaceae - ps29
Camelineae - ps30
Arabidopsis - ps31
Arabidopsis_thaliana - ps32
Phylostratum

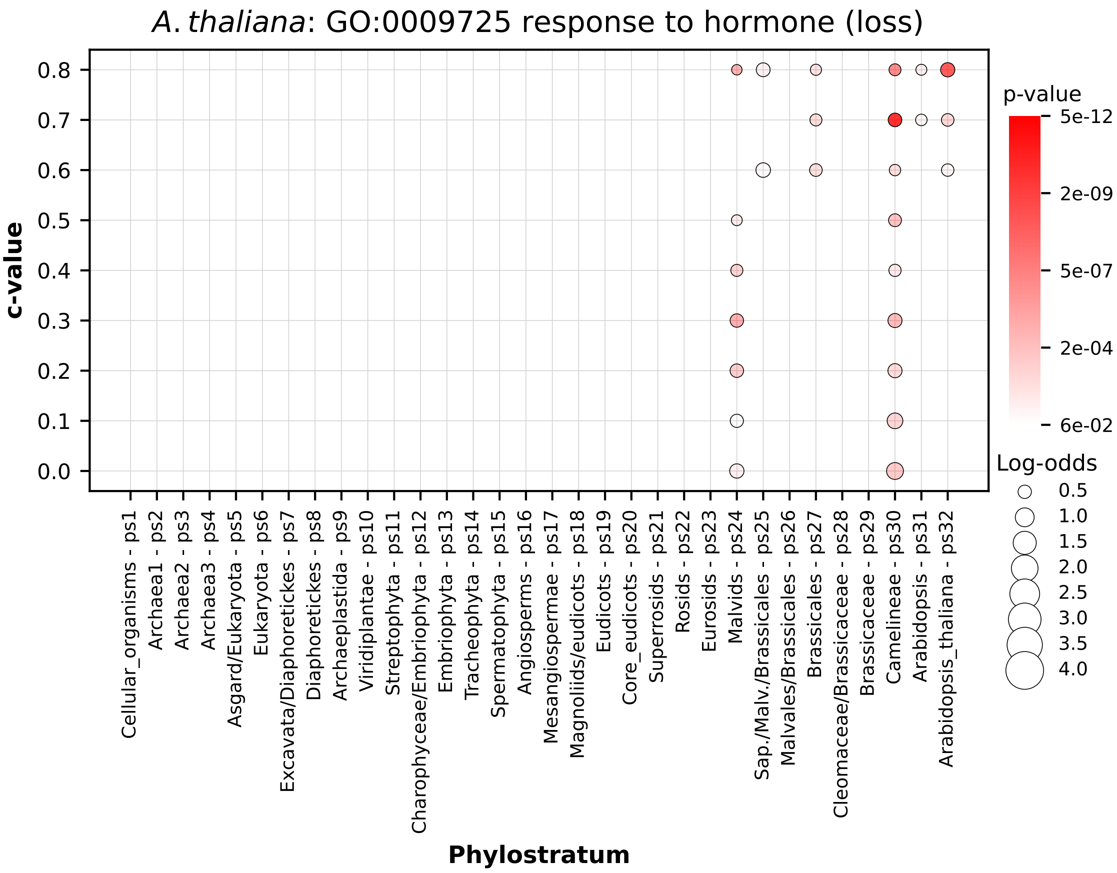

A. thaliana: GO:0009725 response to hormone (loss)
c-value
0.8
0.7
0.6
0.5
0.4
0.3
0.2
0.1
0.0
Phylostratum
Cellular_organisms - ps1
Archaea1 - ps2
Archaea2 - ps3
Archaea3 - ps4
Asgard/Eukaryota - ps5
Eukaryota - ps6
Excavata/Diaphoretickes - ps7
Diaphoretickes - ps8
Archaeplastida - ps9
Viridiplantae - ps10
Streptophyta - ps11
Charophyceae/Embriophyta - ps12
Embriophyta - ps13
Tracheophyta - ps14
Spermatophyta - ps15
Angiosperms - ps16
Mesangiospermae - ps17
Magnoliids/eudicots - ps18
Eudicots - ps19
Core_eudicots - ps20
Superrosids - ps21
Rosids - ps22
Eurosids - ps23
Malvids - ps24
Sap./Malv./Brassicales - ps25
Malvales/Brassicales - ps26
Brassicales - ps27
Cleomaceae/Brassicaceae - ps28
Brassicaceae - ps29
Camelineae - ps30
Arabidopsis - ps31
Arabidopsis_thaliana - ps32
p-value
5e-12
2e-09
5e-07
2e-04
6e-02
Log-odds
0.5
1.0
1.5
2.0
2.5
3.0
3.5
4.0

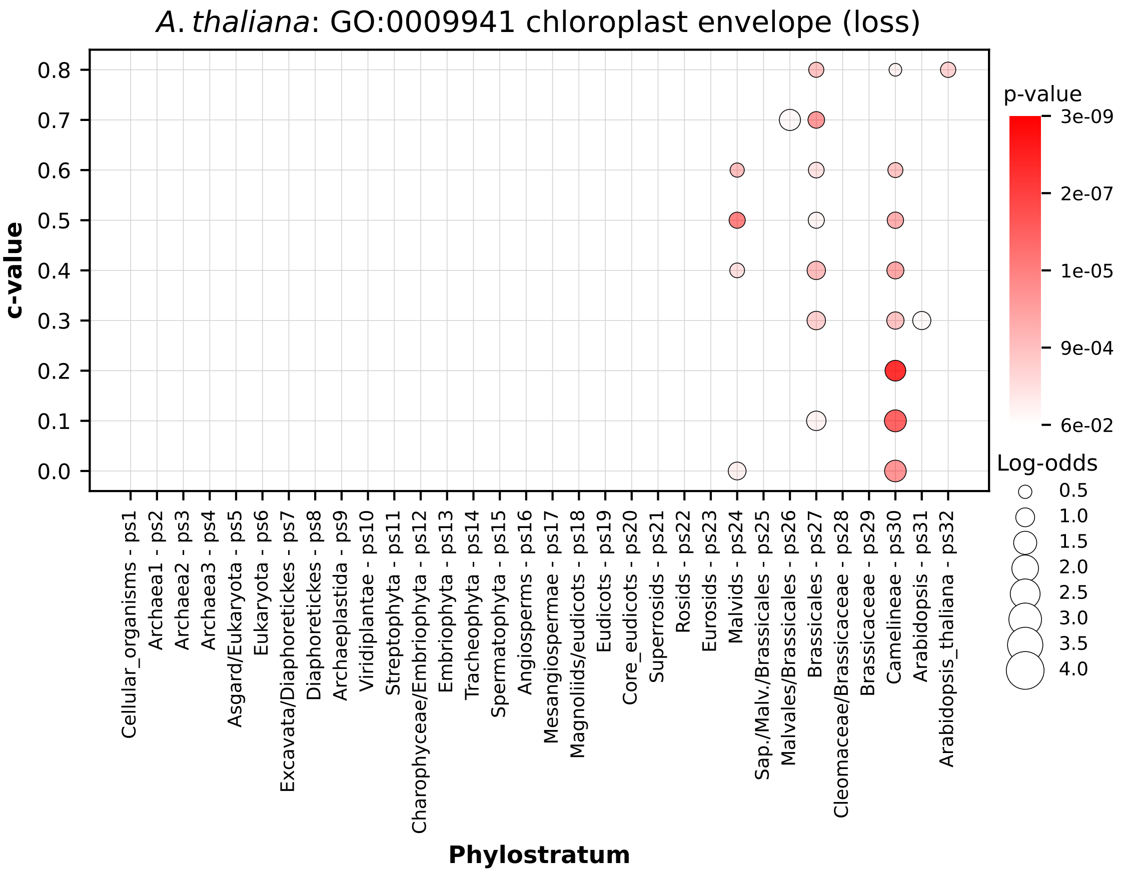

A. thaliana: GO:0009941 chloroplast envelope (loss)
c-value
0.0
0.1
0.2
0.3
0.4
0.5
0.6
0.7
0.8
Phylostratum
Cellular_organisms - ps1
Archaea1 - ps2
Archaea2 - ps3
Archaea3 - ps4
Asgard/Eukaryota - ps5
Eukaryota - ps6
Excavata/Diaphoretickes - ps7
Diaphoretickes - ps8
Archaeplastida - ps9
Viridiplantae - ps10
Streptophyta - ps11
Charophyceae/Embriophyta - ps12
Embriophyta - ps13
Tracheophyta - ps14
Spermatophyta - ps15
Angiosperms - ps16
Mesangiospermae - ps17
Magnoliids/eudicots - ps18
Eudicots - ps19
Core_eudicots - ps20
Superrosids - ps21
Rosids - ps22
Eurosids - ps23
Malvids - ps24
Sap./Malv./Brassicales - ps25
Malvales/Brassicales - ps26
Brassicales - ps27
Cleomaceae/Brassicaceae - ps28
Brassicaceae - ps29
Camelineae - ps30
Arabidopsis - ps31
Arabidopsis_thaliana - ps32
p-value
3e-09
2e-07
1e-05
9e-04
6e-02
Log-odds
0.5
1.0
1.5
2.0
2.5
3.0
3.5
4.0

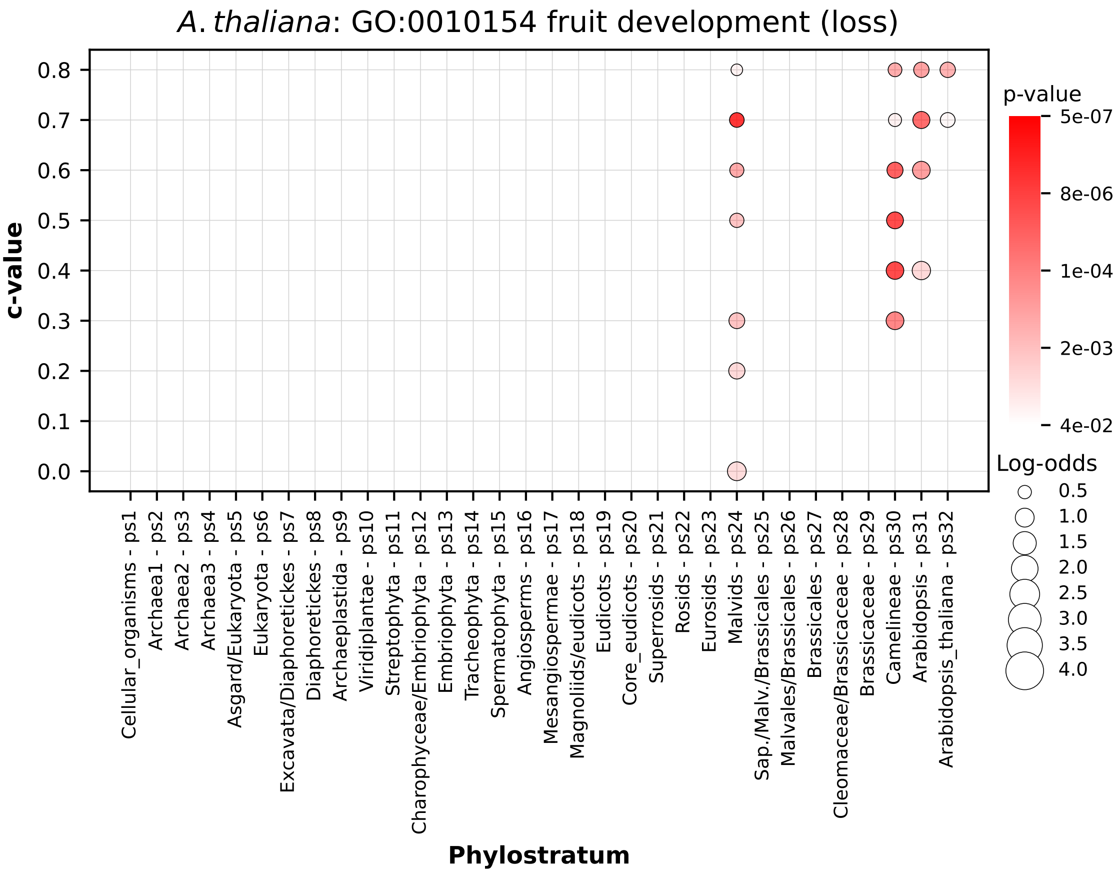
A. thaliana: GO:0010154 fruit development (loss)
c-value
0.8
0.7
0.6
0.5
0.4
0.3
0.2
0.1
0.0
Phylostratum
Cellular_organisms - ps1
Archaea1 - ps2
Archaea2 - ps3
Archaea3 - ps4
Asgard/Eukaryota - ps5
Eukaryota - ps6
Excavata/Diaphoretickes - ps7
Diaphoretickes - ps8
Archaeplastida - ps9
Viridiplantae - ps10
Streptophyta - ps11
Charophyceae/Embriophyta - ps12
Embriophyta - ps13
Tracheophyta - ps14
Spermatophyta - ps15
Angiosperms - ps16
Mesangiospermae - ps17
Magnoliids/eudicots - ps18
Eudicots - ps19
Core_eudicots - ps20
Superrosids - ps21
Rosids - ps22
Eurosids - ps23
Malvids - ps24
Sap./Malv./Brassicales - ps25
Malvales/Brassicales - ps26
Brassicales - ps27
Cleomaceae/Brassicaceae - ps28
Brassicaceae - ps29
Camelineae - ps30
Arabidopsis - ps31
Arabidopsis_thaliana - ps32
p-value
5e-07
8e-06
1e-04
2e-03
4e-02
Log-odds
0.5
1.0
1.5
2.0
2.5
3.0
3.5
4.0

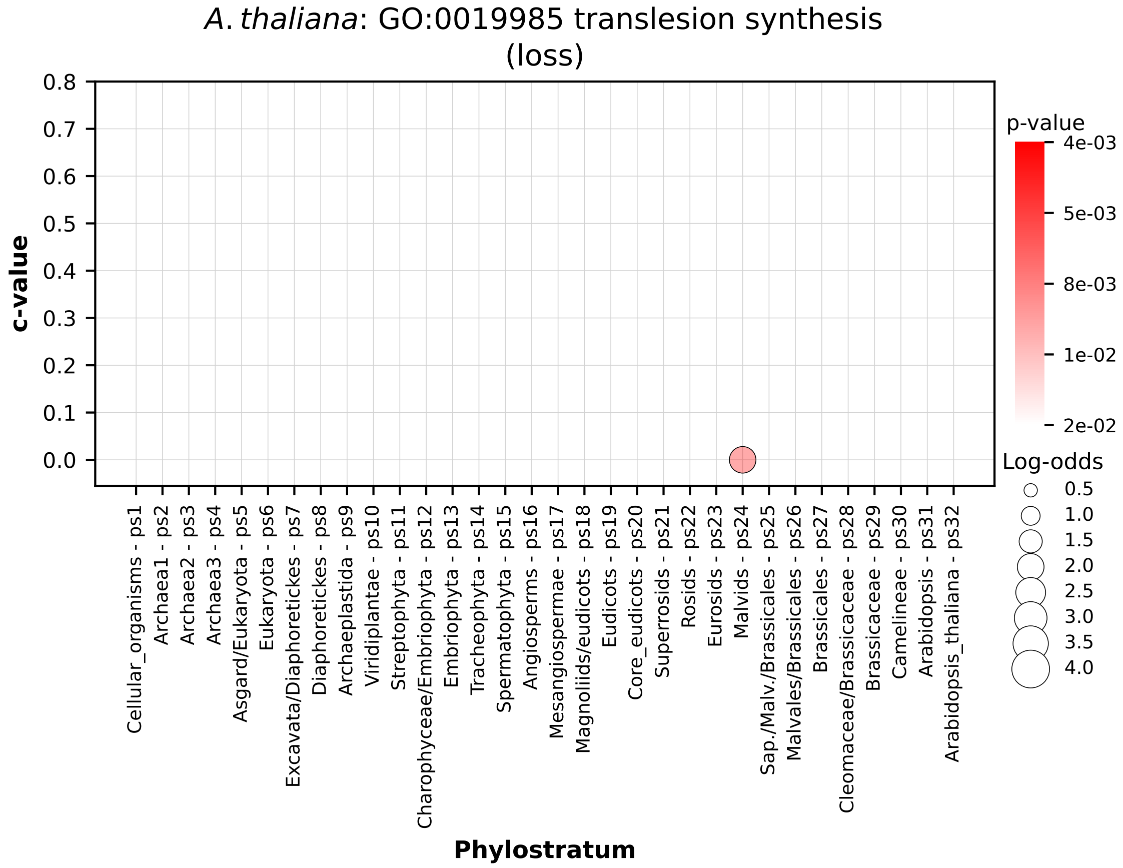

A. thaliana: GO:0019985 translesion synthesis
(loss)
c-value
0.8
0.7
0.6
0.5
0.4
0.3
0.2
0.1
0.0
Phylostratum
Cellular_organisms - ps1
Archaea1 - ps2
Archaea2 - ps3
Archaea3 - ps4
Asgard/Eukaryota - ps5
Eukaryota - ps6
Excavata/Diaphoretickes - ps7
Diaphoretickes - ps8
Archaeplastida - ps9
Viridiplantae - ps10
Streptophyta - ps11
Charophyceae/Embriophyta - ps12
Embriophyta - ps13
Tracheophyta - ps14
Spermatophyta - ps15
Angiosperms - ps16
Mesangiospermae - ps17
Magnoliids/eudicots - ps18
Eudicots - ps19
Core_eudicots - ps20
Superrosids - ps21
Rosids - ps22
Eurosids - ps23
Malvids - ps24
Sap./Malv./Brassicales - ps25
Malvales/Brassicales - ps26
Brassicales - ps27
Cleomaceae/Brassicaceae - ps28
Brassicaceae - ps29
Camelineae - ps30
Arabidopsis - ps31
Arabidopsis_thaliana - ps32
p-value
4e-03
5e-03
8e-03
1e-02
2e-02
Log-odds
0.5
1.0
1.5
2.0
2.5
3.0
3.5
4.0

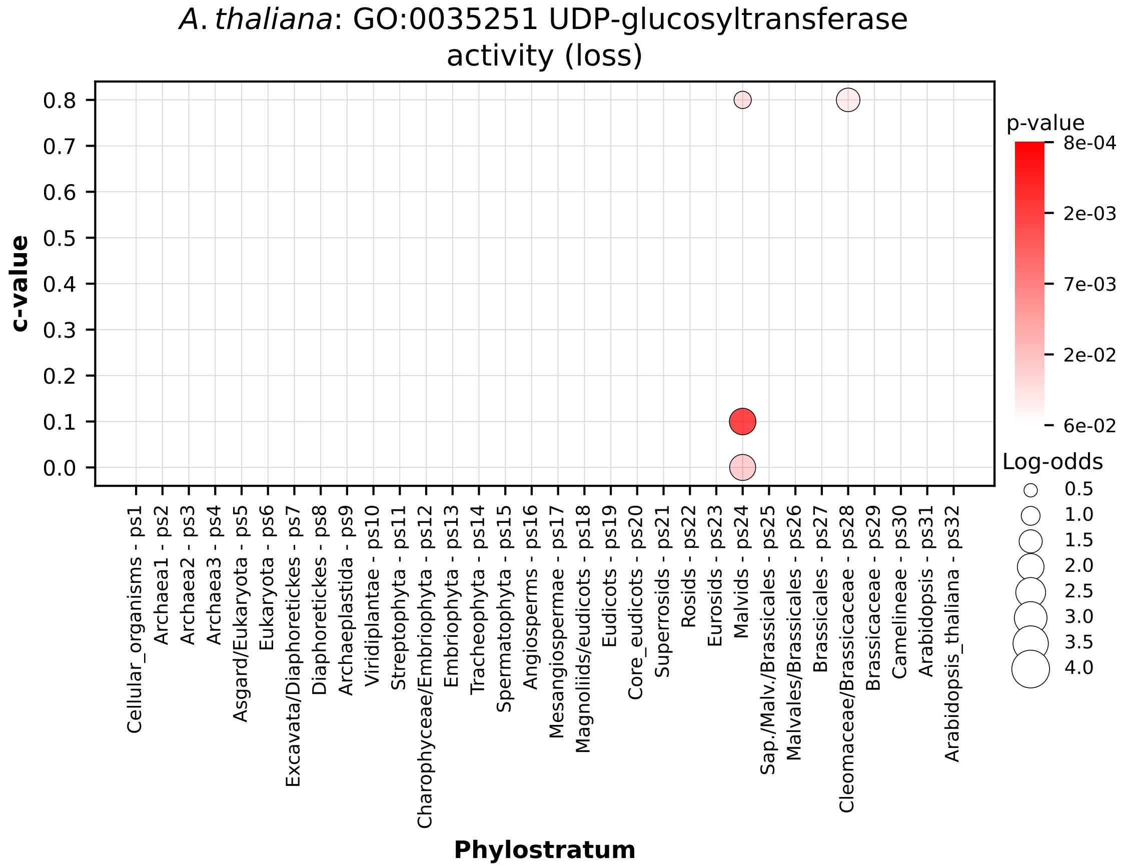

A. thaliana: GO:0035251 UDP-glucosyltransferase activity (loss)
c-value
0.8
0.7
0.6
0.5
0.4
0.3
0.2
0.1
0.0
p-value
8e-04
2e-03
7e-03
2e-02
6e-02
Log-odds
0.5
1.0
1.5
2.0
2.5
3.0
3.5
4.0
Cellular_organisms - ps1
Archaea1 - ps2
Archaea2 - ps3
Archaea3 - ps4
Asgard/Eukaryota - ps5
Eukaryota - ps6
Excavata/Diaphoretickes - ps7
Diaphoretickes - ps8
Archaeplastida - ps9
Viridiplantae - ps10
Streptophyta - ps11
Charophyceae/Embriophyta - ps12
Embriophyta - ps13
Tracheophyta - ps14
Spermatophyta - ps15
Angiosperms - ps16
Mesangiospermae - ps17
Magnoliids/eudicots - ps18
Eudicots - ps19
Core_eudicots - ps20
Superrosids - ps21
Rosids - ps22
Eurosids - ps23
Malvids - ps24
Sap./Malv./Brassicales - ps25
Malvales/Brassicales - ps26
Brassicales - ps27
Cleomaceae/Brassicaceae - ps28
Brassicaceae - ps29
Camelineae - ps30
Arabidopsis - ps31
Arabidopsis_thaliana - ps32
Phylostratum

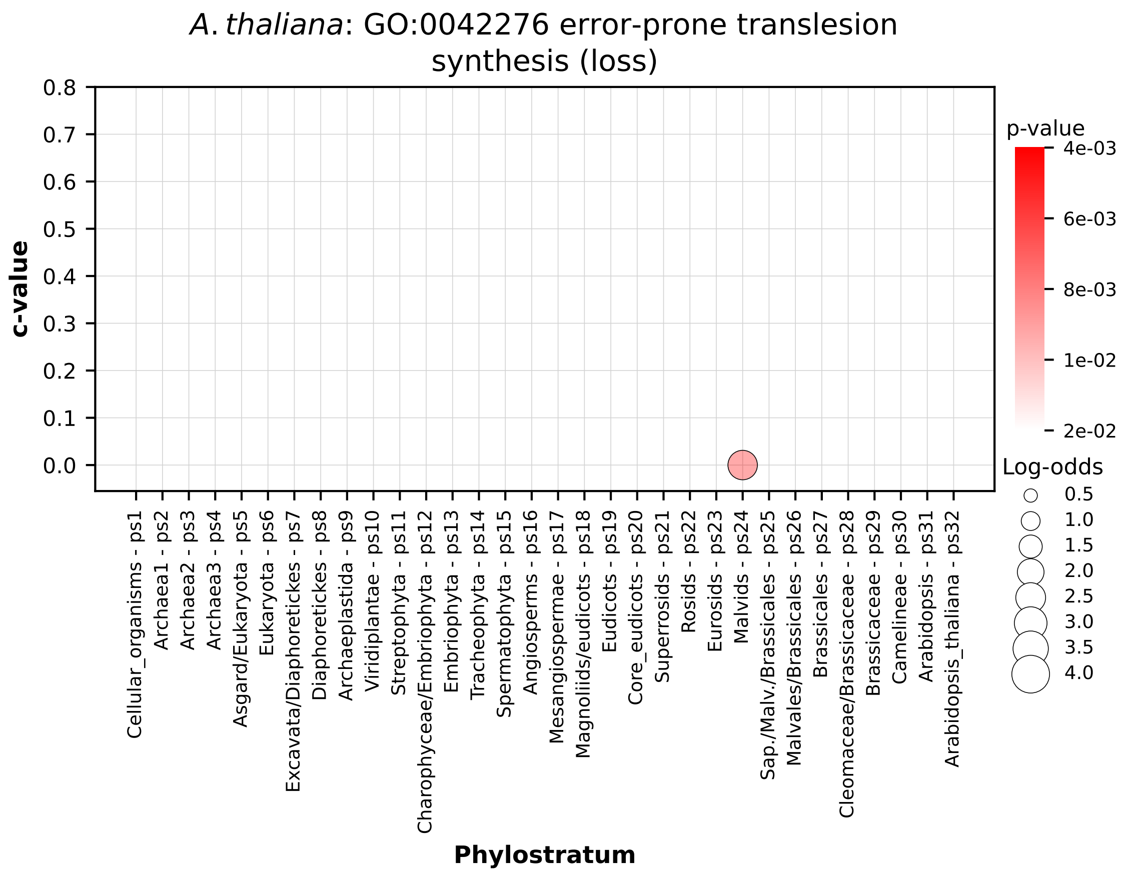

A. thaliana: GO:0042276 error-prone translesion synthesis (loss)
c-value
0.8
0.7
0.6
0.5
0.4
0.3
0.2
0.1
0.0
p-value
4e-03
6e-03
8e-03
1e-02
2e-02
Log-odds
0.5
1.0
1.5
2.0
2.5
3.0
3.5
4.0
Cellular_organisms - ps1
Archaea1 - ps2
Archaea2 - ps3
Archaea3 - ps4
Asgard/Eukaryota - ps5
Eukaryota - ps6
Excavata/Diaphoretickes - ps7
Diaphoretickes - ps8
Archaeplastida - ps9
Viridiplantae - ps10
Streptophyta - ps11
Charophyceae/Embriophyta - ps12
Embriophyta - ps13
Tracheophyta - ps14
Spermatophyta - ps15
Angiosperms - ps16
Mesangiospermae - ps17
Magnoliids/eudicots - ps18
Eudicots - ps19
Core_eudicots - ps20
Superrosids - ps21
Rosids - ps22
Eurosids - ps23
Malvids - ps24
Sap./Malv./Brassicales - ps25
Malvales/Brassicales - ps26
Brassicales - ps27
Cleomaceae/Brassicaceae - ps28
Brassicaceae - ps29
Camelineae - ps30
Arabidopsis - ps31
Arabidopsis_thaliana - ps32
Phylostratum

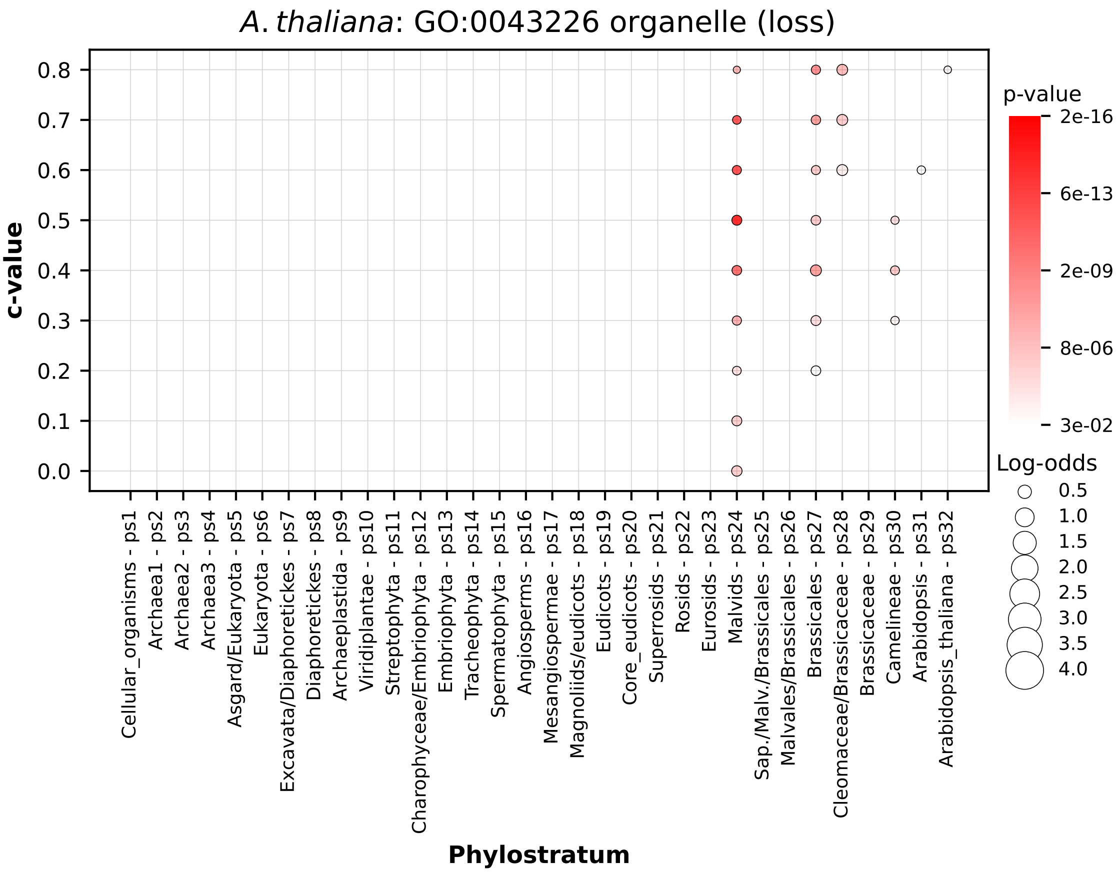

A. thaliana: GO:0043226 organelle (loss)
c-value
0.8
0.7
0.6
0.5
0.4
0.3
0.2
0.1
0.0
p-value
2e-16
6e-13
2e-09
8e-06
3e-02
Log-odds
0.5
1.0
1.5
2.0
2.5
3.0
3.5
4.0
Cellular_organisms - ps1
Archaea1 - ps2
Archaea2 - ps3
Archaea3 - ps4
Asgard/Eukaryota - ps5
Eukaryota - ps6
Excavata/Diaphoretickes - ps7
Diaphoretickes - ps8
Archaeplastida - ps9
Viridiplantae - ps10
Streptophyta - ps11
Charophyceae/Embriophyta - ps12
Embriophyta - ps13
Tracheophyta - ps14
Spermatophyta - ps15
Angiosperms - ps16
Mesangiospermae - ps17
Magnoliids/eudicots - ps18
Eudicots - ps19
Core_eudicots - ps20
Superrosids - ps21
Rosids - ps22
Eurosids - ps23
Malvids - ps24
Sap./Malv./Brassicales - ps25
Malvales/Brassicales - ps26
Brassicales - ps27
Cleomaceae/Brassicaceae - ps28
Brassicaceae - ps29
Camelineae - ps30
Arabidopsis - ps31
Arabidopsis_thaliana - ps32
Phylostratum

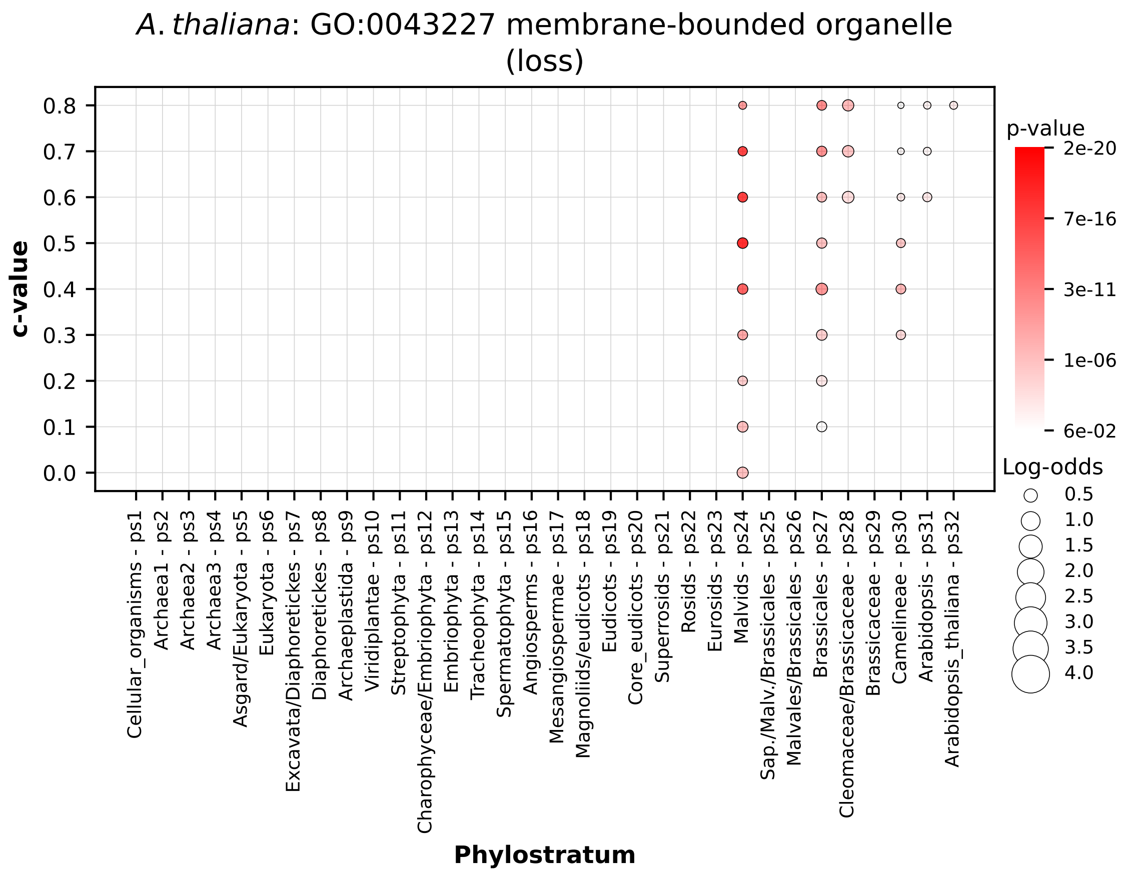

A. thaliana: GO:0043227 membrane-bounded organelle
(loss)
c-value
0.8
0.7
0.6
0.5
0.4
0.3
0.2
0.1
0.0
Phylostratum
Cellular_organisms - ps1
Archaea1 - ps2
Archaea2 - ps3
Archaea3 - ps4
Asgard/Eukaryota - ps5
Eukaryota - ps6
Excavata/Diaphoretickes - ps7
Diaphoretickes - ps8
Archaeplastida - ps9
Viridiplantae - ps10
Streptophyta - ps11
Charophyceae/Embriophyta - ps12
Embriophyta - ps13
Tracheophyta - ps14
Spermatophyta - ps15
Angiosperms - ps16
Mesangiospermae - ps17
Magnoliids/eudicots - ps18
Eudicots - ps19
Core_eudicots - ps20
Superrosids - ps21
Rosids - ps22
Eurosids - ps23
Malvids - ps24
Sap./Malv./Brassicales - ps25
Malvales/Brassicales - ps26
Brassicales - ps27
Cleomaceae/Brassicaceae - ps28
Brassicaceae - ps29
Camelineae - ps30
Arabidopsis - ps31
Arabidopsis_thaliana - ps32
p-value
2e-20
7e-16
3e-11
1e-06
6e-02
Log-odds
0.5
1.0
1.5
2.0
2.5
3.0
3.5
4.0

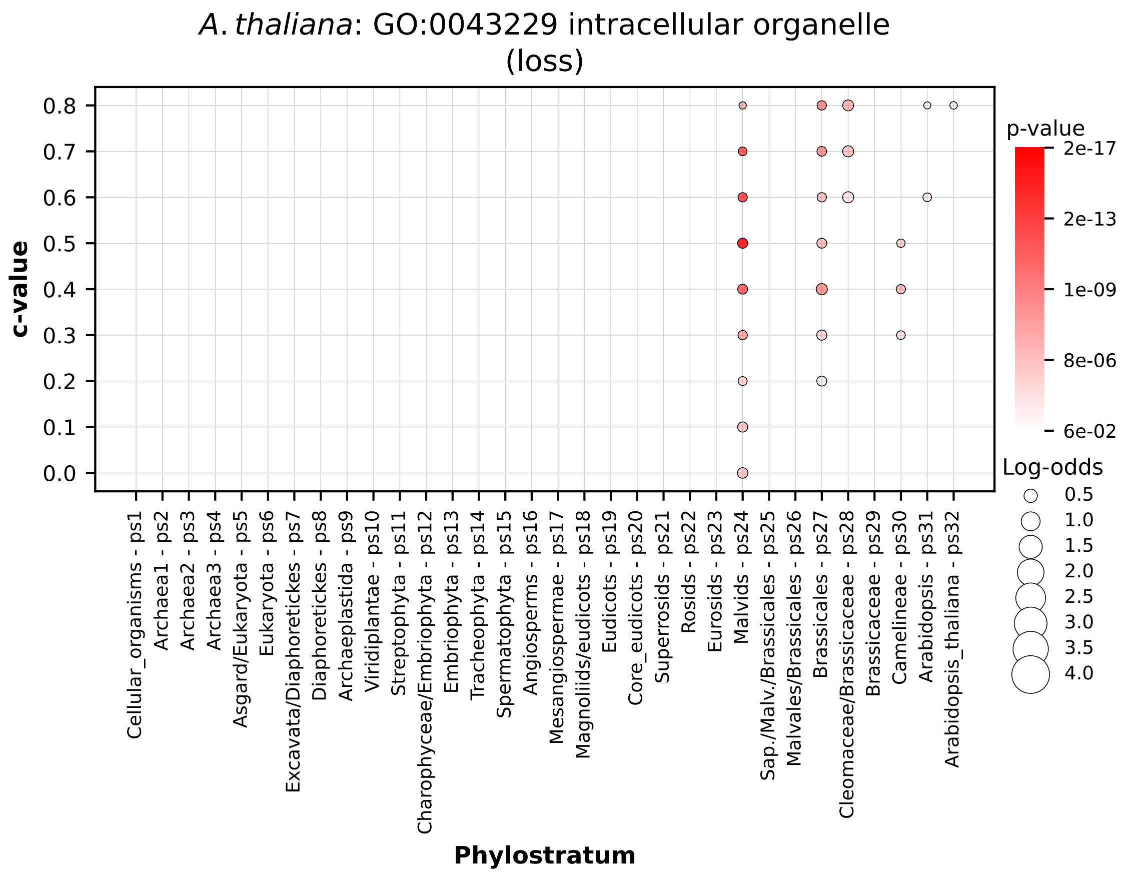
A. thaliana: GO:0043229 intracellular organelle
(loss)
c-value
0.8
0.7
0.6
0.5
0.4
0.3
0.2
0.1
0.0
p-value
2e-17
2e-13
1e-09
8e-06
6e-02
Log-odds
0.5
1.0
1.5
2.0
2.5
3.0
3.5
4.0
Cellular_organisms - ps1
Archaea1 - ps2
Archaea2 - ps3
Archaea3 - ps4
Asgard/Eukaryota - ps5
Eukaryota - ps6
Excavata/Diaphoretickes - ps7
Diaphoretickes - ps8
Archaeplastida - ps9
Viridiplantae - ps10
Streptophyta - ps11
Charophyceae/Embriophyta - ps12
Embriophyta - ps13
Tracheophyta - ps14
Spermatophyta - ps15
Angiosperms - ps16
Mesangiospermae - ps17
Magnoliids/eudicots - ps18
Eudicots - ps19
Core_eudicots - ps20
Superrosids - ps21
Rosids - ps22
Eurosids - ps23
Malvids - ps24
Sap./Malv./Brassicales - ps25
Malvales/Brassicales - ps26
Brassicales - ps27
Cleomaceae/Brassicaceae - ps28
Brassicaceae - ps29
Camelineae - ps30
Arabidopsis - ps31
Arabidopsis_thaliana - ps32
Phylostratum

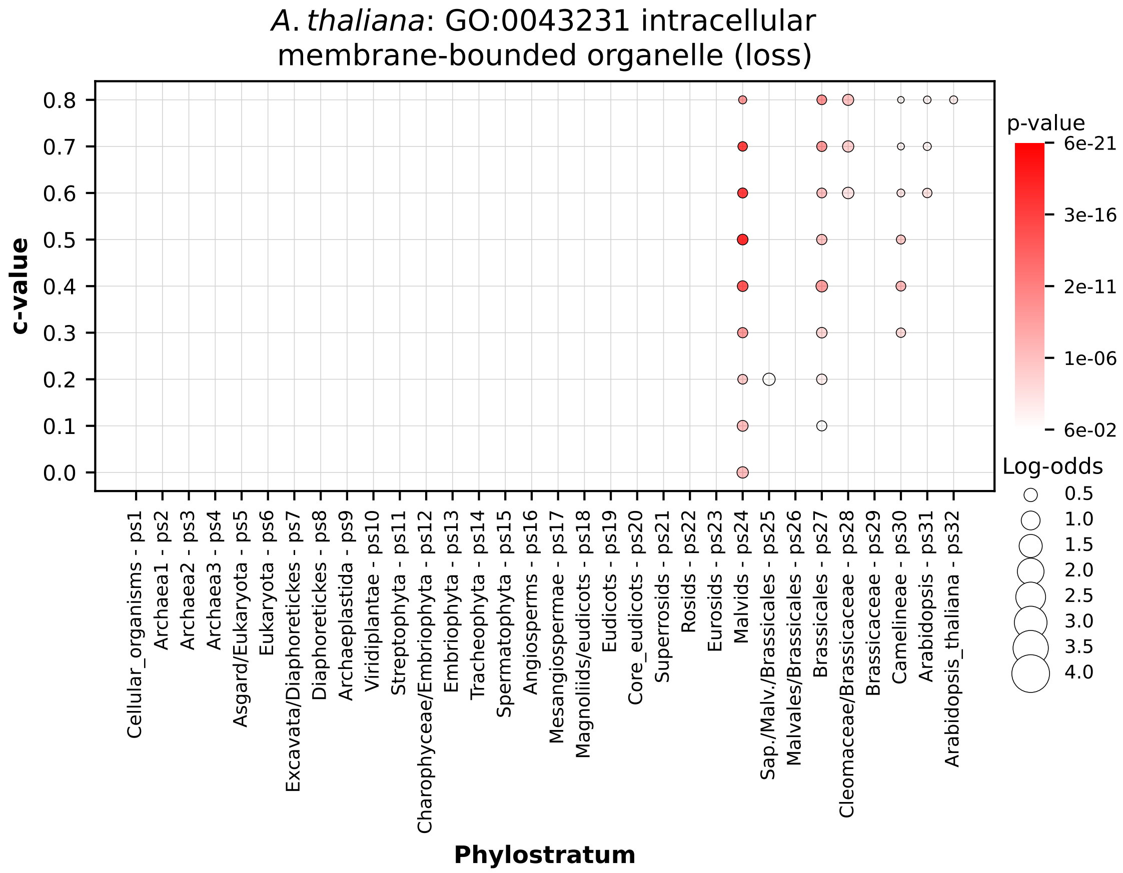

A. thaliana: GO:0043231 intracellular membrane-bounded organelle (loss)
c-value
0.8
0.7
0.6
0.5
0.4
0.3
0.2
0.1
0.0
p-value
6e-21
3e-16
2e-11
1e-06
6e-02
Log-odds
0.5
1.0
1.5
2.0
2.5
3.0
3.5
4.0
Cellular_organisms - ps1
Archaea1 - ps2
Archaea2 - ps3
Archaea3 - ps4
Asgard/Eukaryota - ps5
Eukaryota - ps6
Excavata/Diaphoretickes - ps7
Diaphoretickes - ps8
Archaeplastida - ps9
Viridiplantae - ps10
Streptophyta - ps11
Charophyceae/Embriophyta - ps12
Embriophyta - ps13
Tracheophyta - ps14
Spermatophyta - ps15
Angiosperms - ps16
Mesangiospermae - ps17
Magnoliids/eudicots - ps18
Eudicots - ps19
Core_eudicots - ps20
Superrosids - ps21
Rosids - ps22
Eurosids - ps23
Malvids - ps24
Sap./Malv./Brassicales - ps25
Malvales/Brassicales - ps26
Brassicales - ps27
Cleomaceae/Brassicaceae - ps28
Brassicaceae - ps29
Camelineae - ps30
Arabidopsis - ps31
Arabidopsis_thaliana - ps32
Phylostratum

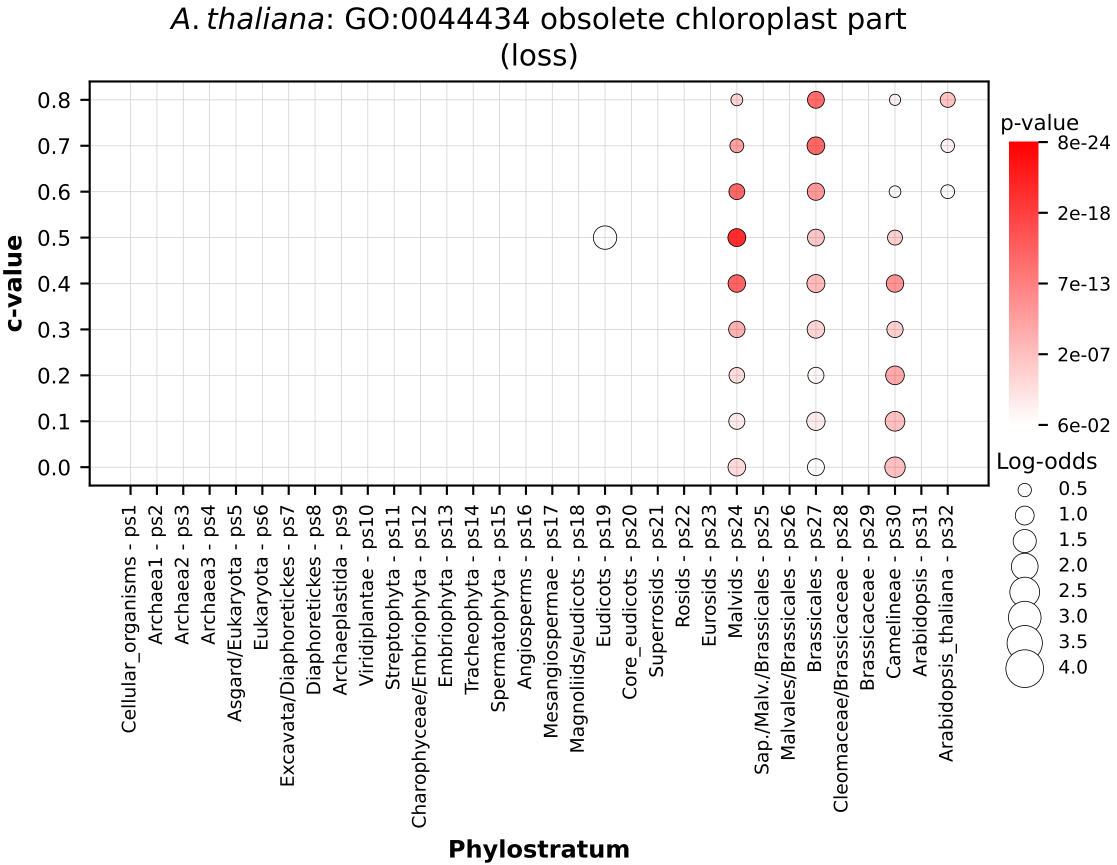
A. thaliana: GO:0044434 obsolete chloroplast part
(loss)
c-value
0.8
0.7
0.6
0.5
0.4
0.3
0.2
0.1
0.0
p-value
8e-24
2e-18
7e-13
2e-07
6e-02
Log-odds
0.5
1.0
1.5
2.0
2.5
3.0
3.5
4.0
Cellular_organisms - ps1
Archaea1 - ps2
Archaea2 - ps3
Archaea3 - ps4
Asgard/Eukaryota - ps5
Eukaryota - ps6
Excavata/Diaphoretickes - ps7
Diaphoretickes - ps8
Archaeplastida - ps9
Viridiplantae - ps10
Streptophyta - ps11
Charophyceae/Embriophyta - ps12
Embriophyta - ps13
Tracheophyta - ps14
Spermatophyta - ps15
Angiosperms - ps16
Mesangiospermae - ps17
Magnoliids/eudicots - ps18
Eudicots - ps19
Core_eudicots - ps20
Superrosids - ps21
Rosids - ps22
Eurosids - ps23
Malvids - ps24
Sap./Malv./Brassicales - ps25
Malvales/Brassicales - ps26
Brassicales - ps27
Cleomaceae/Brassicaceae - ps28
Brassicaceae - ps29
Camelineae - ps30
Arabidopsis - ps31
Arabidopsis_thaliana - ps32
Phylostratum

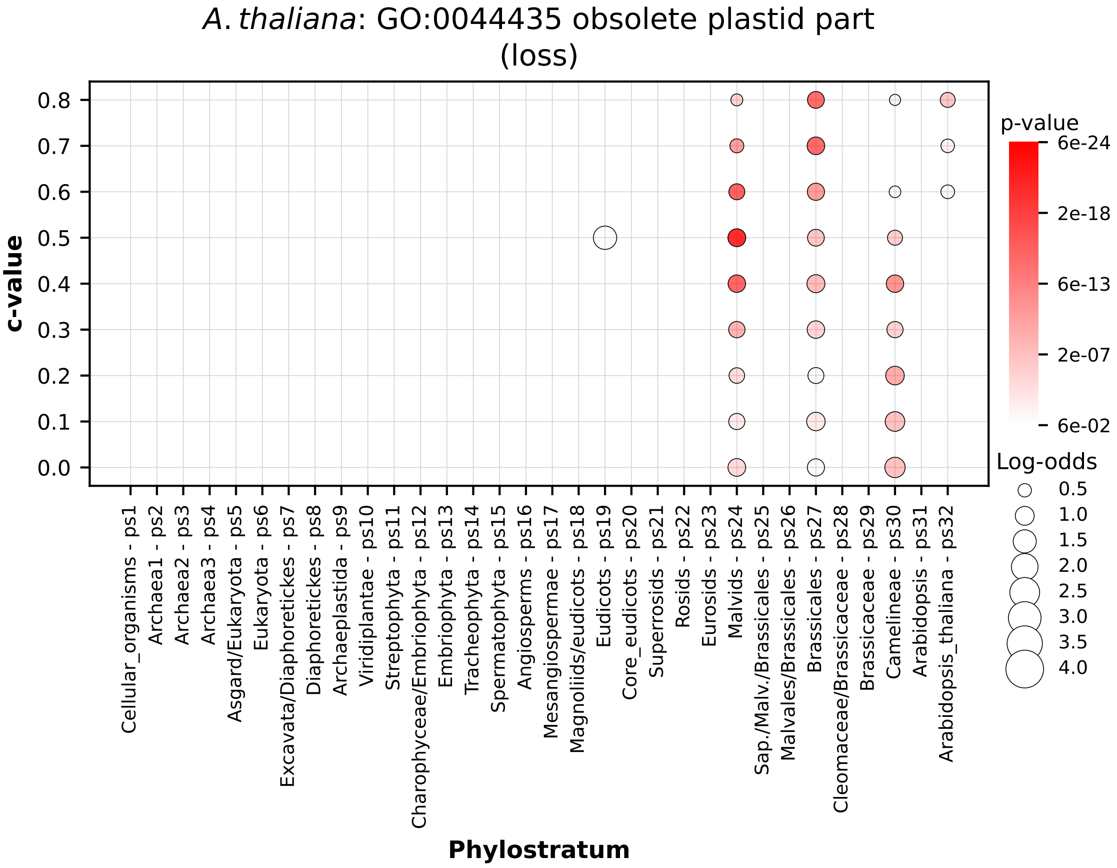

A. thaliana: GO:0044435 obsolete plastid part
(loss)
c-value
0.8
0.7
0.6
0.5
0.4
0.3
0.2
0.1
0.0
p-value
6e-24
2e-18
6e-13
2e-07
6e-02
Log-odds
0.5
1.0
1.5
2.0
2.5
3.0
3.5
4.0
Cellular_organisms - ps1
Archaea1 - ps2
Archaea2 - ps3
Archaea3 - ps4
Asgard/Eukaryota - ps5
Eukaryota - ps6
Excavata/Diaphoretickes - ps7
Diaphoretickes - ps8
Archaeplastida - ps9
Viridiplantae - ps10
Streptophyta - ps11
Charophyceae/Embriophyta - ps12
Embriophyta - ps13
Tracheophyta - ps14
Spermatophyta - ps15
Angiosperms - ps16
Mesangiospermae - ps17
Magnoliids/eudicots - ps18
Eudicots - ps19
Core_eudicots - ps20
Superrosids - ps21
Rosids - ps22
Eurosids - ps23
Malvids - ps24
Sap./Malv./Brassicales - ps25
Malvales/Brassicales - ps26
Brassicales - ps27
Cleomaceae/Brassicaceae - ps28
Brassicaceae - ps29
Camelineae - ps30
Arabidopsis - ps31
Arabidopsis_thaliana - ps32
Phylostratum

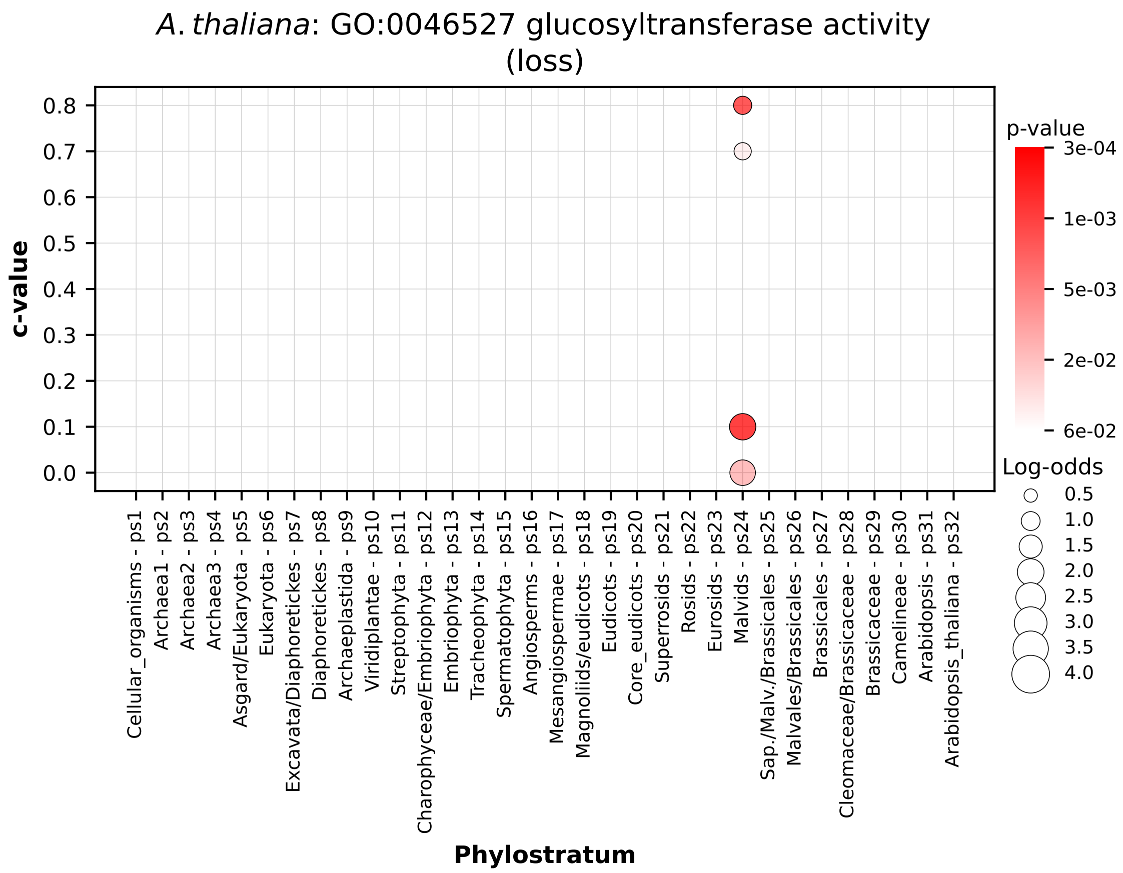

A. thaliana: GO:0046527 glucosyltransferase activity
(loss)
c-value
0.8
0.7
0.6
0.5
0.4
0.3
0.2
0.1
0.0
Phylostratum
Cellular_organisms - ps1
Archaea1 - ps2
Archaea2 - ps3
Archaea3 - ps4
Asgard/Eukaryota - ps5
Eukaryota - ps6
Excavata/Diaphoretickes - ps7
Diaphoretickes - ps8
Archaeplastida - ps9
Viridiplantae - ps10
Streptophyta - ps11
Charophyceae/Embriophyta - ps12
Embriophyta - ps13
Tracheophyta - ps14
Spermatophyta - ps15
Angiosperms - ps16
Mesangiospermae - ps17
Magnoliids/eudicots - ps18
Eudicots - ps19
Core_eudicots - ps20
Superrosids - ps21
Rosids - ps22
Eurosids - ps23
Malvids - ps24
Sap./Malv./Brassicales - ps25
Malvales/Brassicales - ps26
Brassicales - ps27
Cleomaceae/Brassicaceae - ps28
Brassicaceae - ps29
Camelineae - ps30
Arabidopsis - ps31
Arabidopsis_thaliana - ps32
p-value
3e-04
1e-03
5e-03
2e-02
6e-02
Log-odds
0.5
1.0
1.5
2.0
2.5
3.0
3.5
4.0

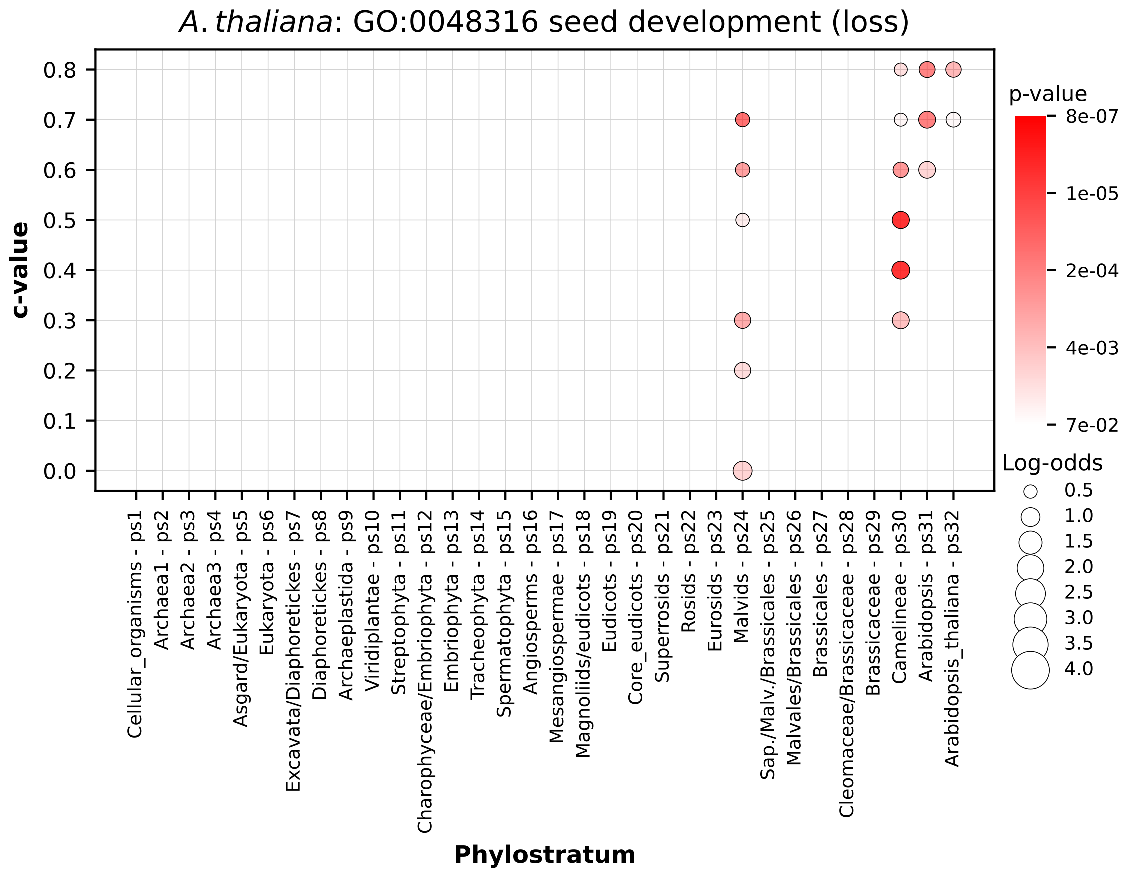

A. thaliana: GO:0048316 seed development (loss)
c-value
0.0
0.1
0.2
0.3
0.4
0.5
0.6
0.7
0.8
p-value
8e-07
1e-05
2e-04
4e-03
7e-02
Log-odds
0.5
1.0
1.5
2.0
2.5
3.0
3.5
4.0
Cellular_organisms - ps1
Archaea1 - ps2
Archaea2 - ps3
Archaea3 - ps4
Asgard/Eukaryota - ps5
Eukaryota - ps6
Excavata/Diaphoretickes - ps7
Diaphoretickes - ps8
Archaeplastida - ps9
Viridiplantae - ps10
Streptophyta - ps11
Charophyceae/Embriophyta - ps12
Embriophyta - ps13
Tracheophyta - ps14
Spermatophyta - ps15
Angiosperms - ps16
Mesangiospermae - ps17
Magnoliids/eudicots - ps18
Eudicots - ps19
Core_eudicots - ps20
Superrosids - ps21
Rosids - ps22
Eurosids - ps23
Malvids - ps24
Sap./Malv./Brassicales - ps25
Malvales/Brassicales - ps26
Brassicales - ps27
Cleomaceae/Brassicaceae - ps28
Brassicaceae - ps29
Camelineae - ps30
Arabidopsis - ps31
Arabidopsis_thaliana - ps32
Phylostratum

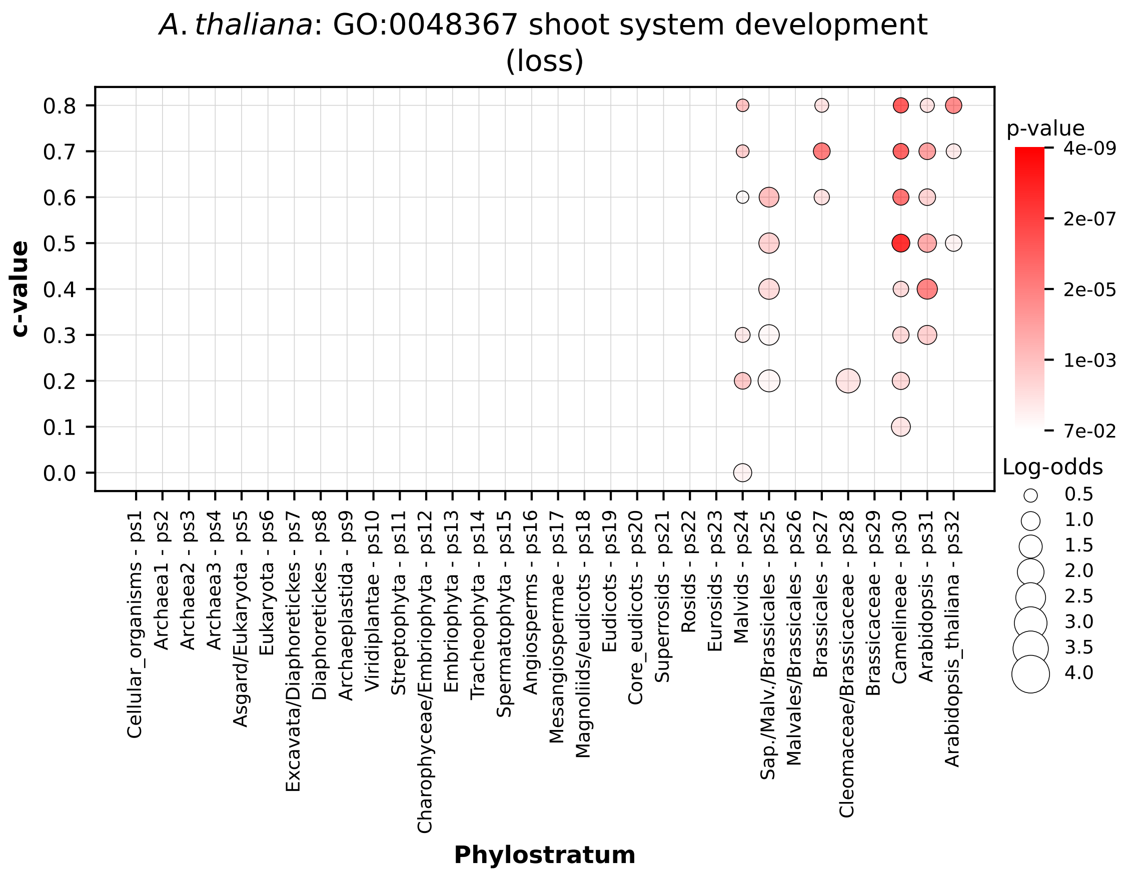

A. thaliana: GO:0048367 shoot system development
(loss)
c-value
0.8
0.7
0.6
0.5
0.4
0.3
0.2
0.1
0.0
Phylostratum
Cellular_organisms - ps1
Archaea1 - ps2
Archaea2 - ps3
Archaea3 - ps4
Asgard/Eukaryota - ps5
Eukaryota - ps6
Excavata/Diaphoretickes - ps7
Diaphoretickes - ps8
Archaeplastida - ps9
Viridiplantae - ps10
Streptophyta - ps11
Charophyceae/Embriophyta - ps12
Embriophyta - ps13
Tracheophyta - ps14
Spermatophyta - ps15
Angiosperms - ps16
Mesangiospermae - ps17
Magnoliids/eudicots - ps18
Eudicots - ps19
Core_eudicots - ps20
Superrosids - ps21
Rosids - ps22
Eurosids - ps23
Malvids - ps24
Sap./Malv./Brassicales - ps25
Malvales/Brassicales - ps26
Brassicales - ps27
Cleomaceae/Brassicaceae - ps28
Brassicaceae - ps29
Camelineae - ps30
Arabidopsis - ps31
Arabidopsis_thaliana - ps32
p-value
4e-09
2e-07
2e-05
1e-03
7e-02
Log-odds
0.5
1.0
1.5
2.0
2.5
3.0
3.5
4.0

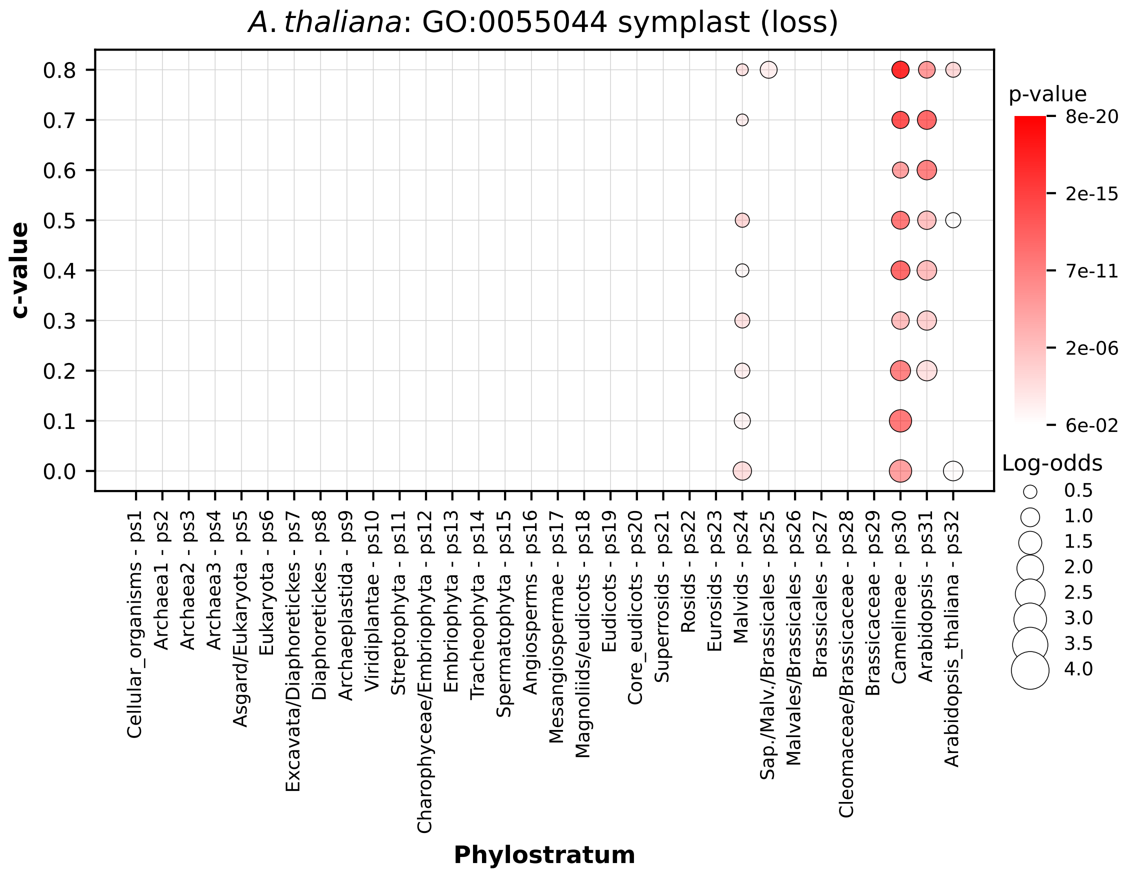
A. thaliana: GO:0055044 symplast (loss)
c-value
0.8
0.7
0.6
0.5
0.4
0.3
0.2
0.1
0.0
p-value
8e-20
2e-15
7e-11
2e-06
6e-02
Log-odds
0.5
1.0
1.5
2.0
2.5
3.0
3.5
4.0
Cellular_organisms - ps1
Archaea1 - ps2
Archaea2 - ps3
Archaea3 - ps4
Asgard/Eukaryota - ps5
Eukaryota - ps6
Excavata/Diaphoretickes - ps7
Diaphoretickes - ps8
Archaeplastida - ps9
Viridiplantae - ps10
Streptophyta - ps11
Charophyceae/Embriophyta - ps12
Embriophyta - ps13
Tracheophyta - ps14
Spermatophyta - ps15
Angiosperms - ps16
Mesangiospermae - ps17
Magnoliids/eudicots - ps18
Eudicots - ps19
Core_eudicots - ps20
Superrosids - ps21
Rosids - ps22
Eurosids - ps23
Malvids - ps24
Sap./Malv./Brassicales - ps25
Malvales/Brassicales - ps26
Brassicales - ps27
Cleomaceae/Brassicaceae - ps28
Brassicaceae - ps29
Camelineae - ps30
Arabidopsis - ps31
Arabidopsis_thaliana - ps32
Phylostratum

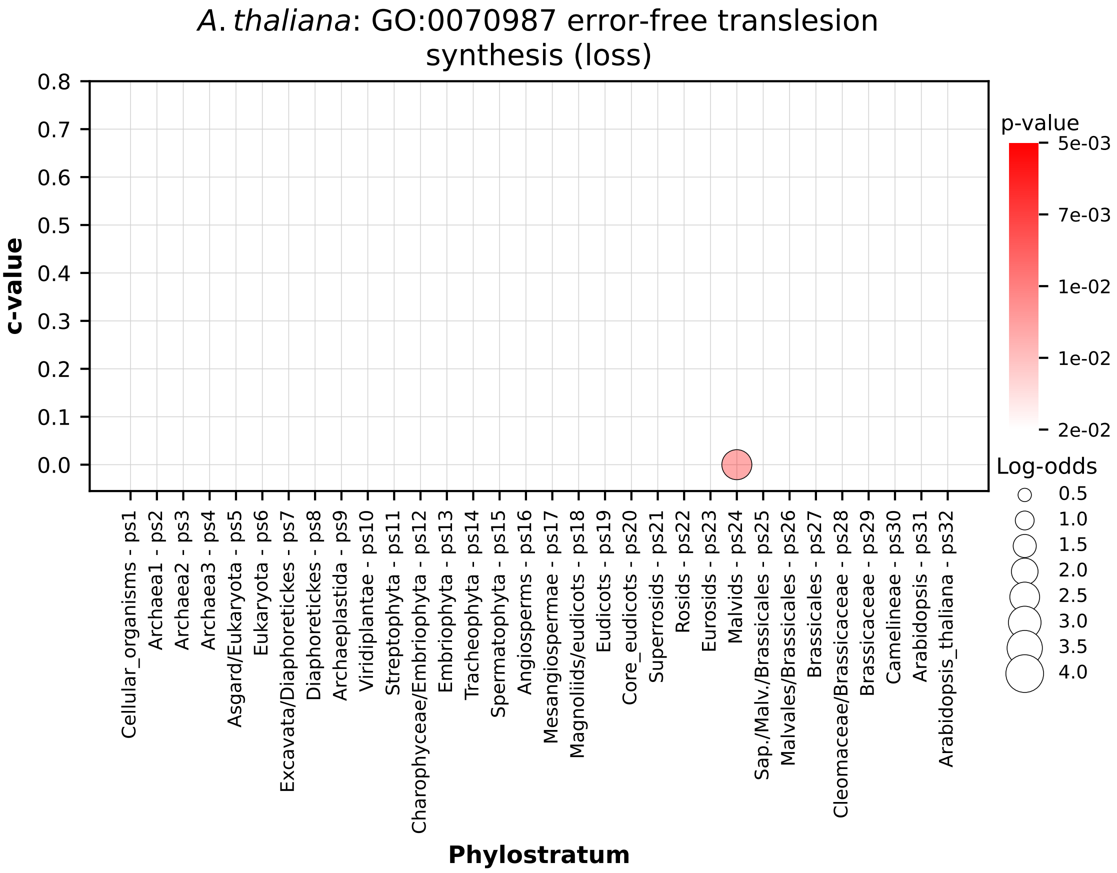

A. thaliana: GO:0070987 error-free translesion synthesis (loss)
c-value
0.8
0.7
0.6
0.5
0.4
0.3
0.2
0.1
0.0
p-value
5e-03
7e-03
1e-02
1e-02
2e-02
Log-odds
0.5
1.0
1.5
2.0
2.5
3.0
3.5
4.0
Cellular_organisms - ps1
Archaea1 - ps2
Archaea2 - ps3
Archaea3 - ps4
Asgard/Eukaryota - ps5
Eukaryota - ps6
Excavata/Diaphoretickes - ps7
Diaphoretickes - ps8
Archaeplastida - ps9
Viridiplantae - ps10
Streptophyta - ps11
Charophyceae/Embriophyta - ps12
Embriophyta - ps13
Tracheophyta - ps14
Spermatophyta - ps15
Angiosperms - ps16
Mesangiospermae - ps17
Magnoliids/eudicots - ps18
Eudicots - ps19
Core_eudicots - ps20
Superrosids - ps21
Rosids - ps22
Eurosids - ps23
Malvids - ps24
Sap./Malv./Brassicales - ps25
Malvales/Brassicales - ps26
Brassicales - ps27
Cleomaceae/Brassicaceae - ps28
Brassicaceae - ps29
Camelineae - ps30
Arabidopsis - ps31
Arabidopsis_thaliana - ps32
Phylostratum

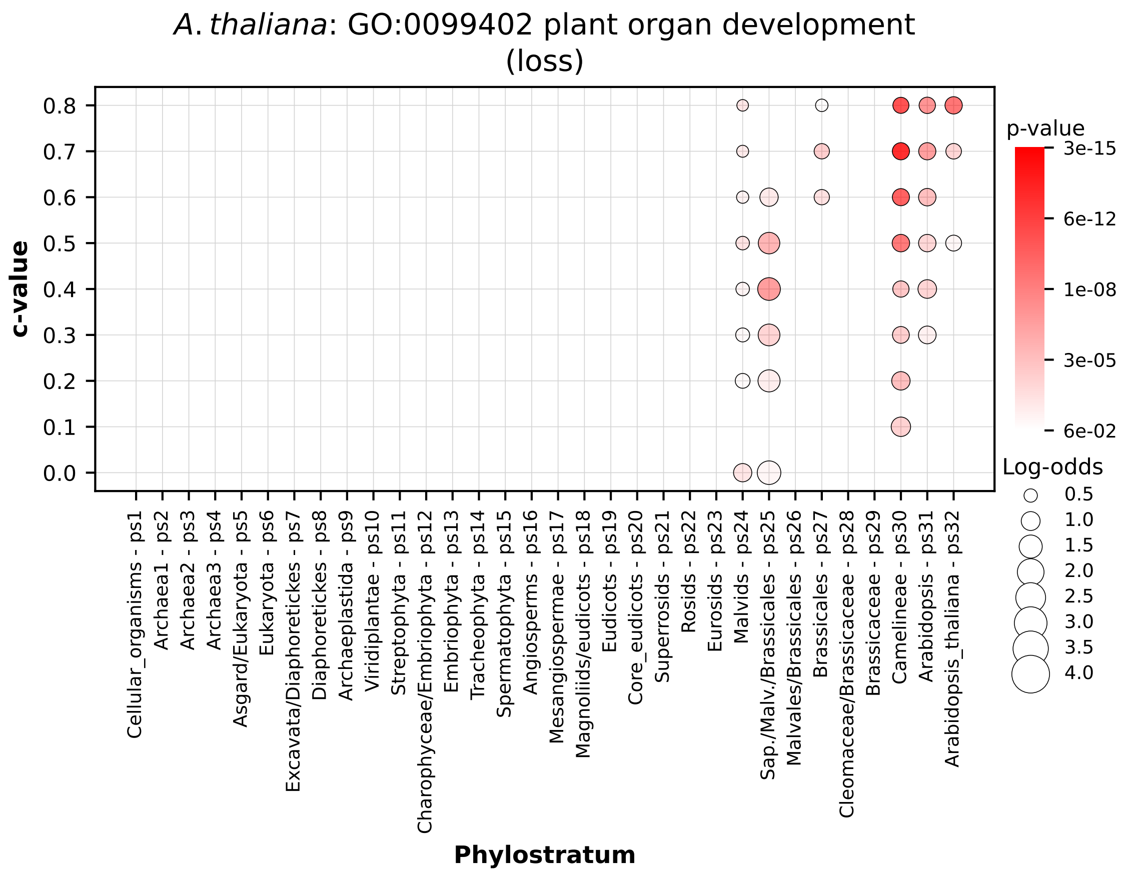

A. thaliana: GO:0099402 plant organ development
(loss)
c-value
0.8
0.7
0.6
0.5
0.4
0.3
0.2
0.1
0.0
p-value
3e-15
6e-12
1e-08
3e-05
6e-02
Log-odds
0.5
1.0
1.5
2.0
2.5
3.0
3.5
4.0
Cellular_organisms - ps1
Archaea1 - ps2
Archaea2 - ps3
Archaea3 - ps4
Asgard/Eukaryota - ps5
Eukaryota - ps6
Excavata/Diaphoretickes - ps7
Diaphoretickes - ps8
Archaeplastida - ps9
Viridiplantae - ps10
Streptophyta - ps11
Charophyceae/Embriophyta - ps12
Embriophyta - ps13
Tracheophyta - ps14
Spermatophyta - ps15
Angiosperms - ps16
Mesangiospermae - ps17
Magnoliids/eudicots - ps18
Eudicots - ps19
Core_eudicots - ps20
Superrosids - ps21
Rosids - ps22
Eurosids - ps23
Malvids - ps24
Sap./Malv./Brassicales - ps25
Malvales/Brassicales - ps26
Brassicales - ps27
Cleomaceae/Brassicaceae - ps28
Brassicaceae - ps29
Camelineae - ps30
Arabidopsis - ps31
Arabidopsis_thaliana - ps32
Phylostratum

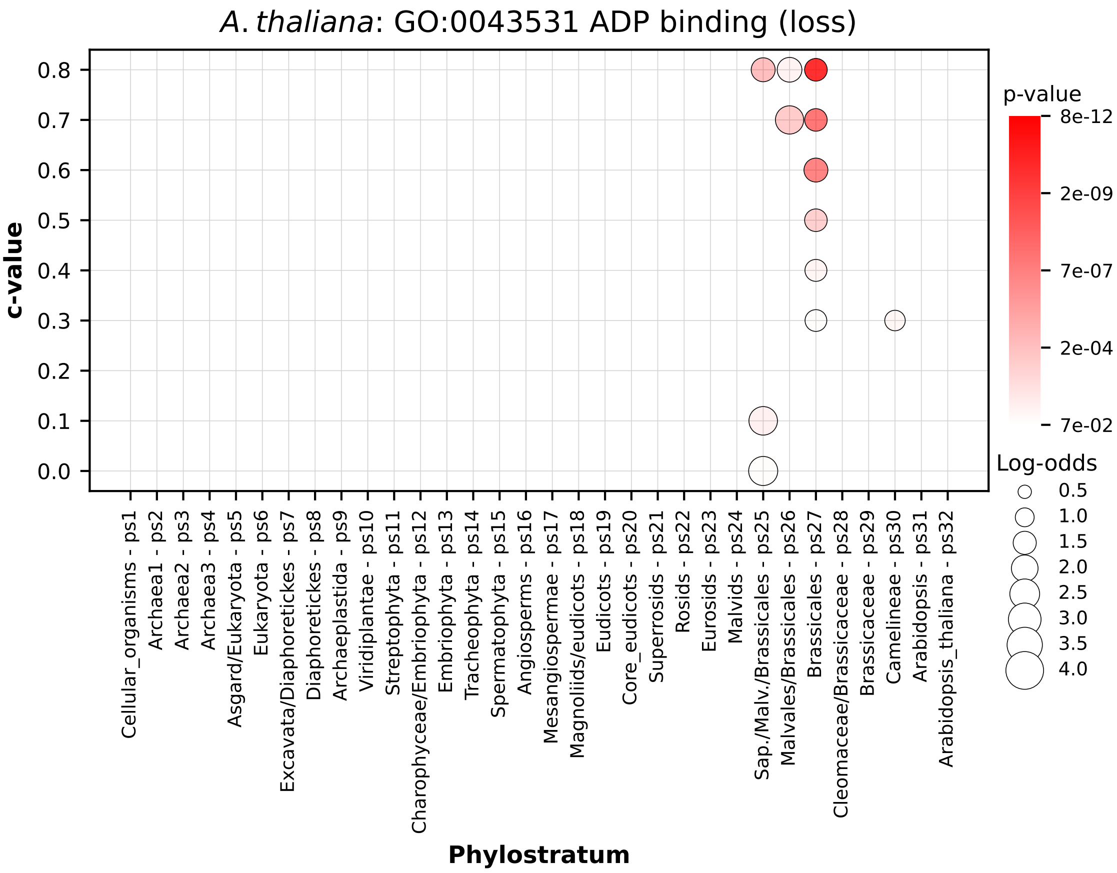

A. thaliana: GO:0043531 ADP binding (loss)
c-value
0.8
0.7
0.6
0.5
0.4
0.3
0.2
0.1
0.0
Phylostratum
Cellular_organisms - ps1
Archaea1 - ps2
Archaea2 - ps3
Archaea3 - ps4
Asgard/Eukaryota - ps5
Eukaryota - ps6
Excavata/Diaphoretickes - ps7
Diaphoretickes - ps8
Archaeplastida - ps9
Viridiplantae - ps10
Streptophyta - ps11
Charophyceae/Embriophyta - ps12
Embriophyta - ps13
Tracheophyta - ps14
Spermatophyta - ps15
Angiosperms - ps16
Mesangiospermae - ps17
Magnoliids/eudicots - ps18
Eudicots - ps19
Core_eudicots - ps20
Superrosids - ps21
Rosids - ps22
Eurosids - ps23
Malvids - ps24
Sap./Malv./Brassicales - ps25
Malvales/Brassicales - ps26
Brassicales - ps27
Cleomaceae/Brassicaceae - ps28
Brassicaceae - ps29
Camelineae - ps30
Arabidopsis - ps31
Arabidopsis_thaliana - ps32
p-value
8e-12
2e-09
7e-07
2e-04
7e-02
Log-odds
0.5
1.0
1.5
2.0
2.5
3.0
3.5
4.0

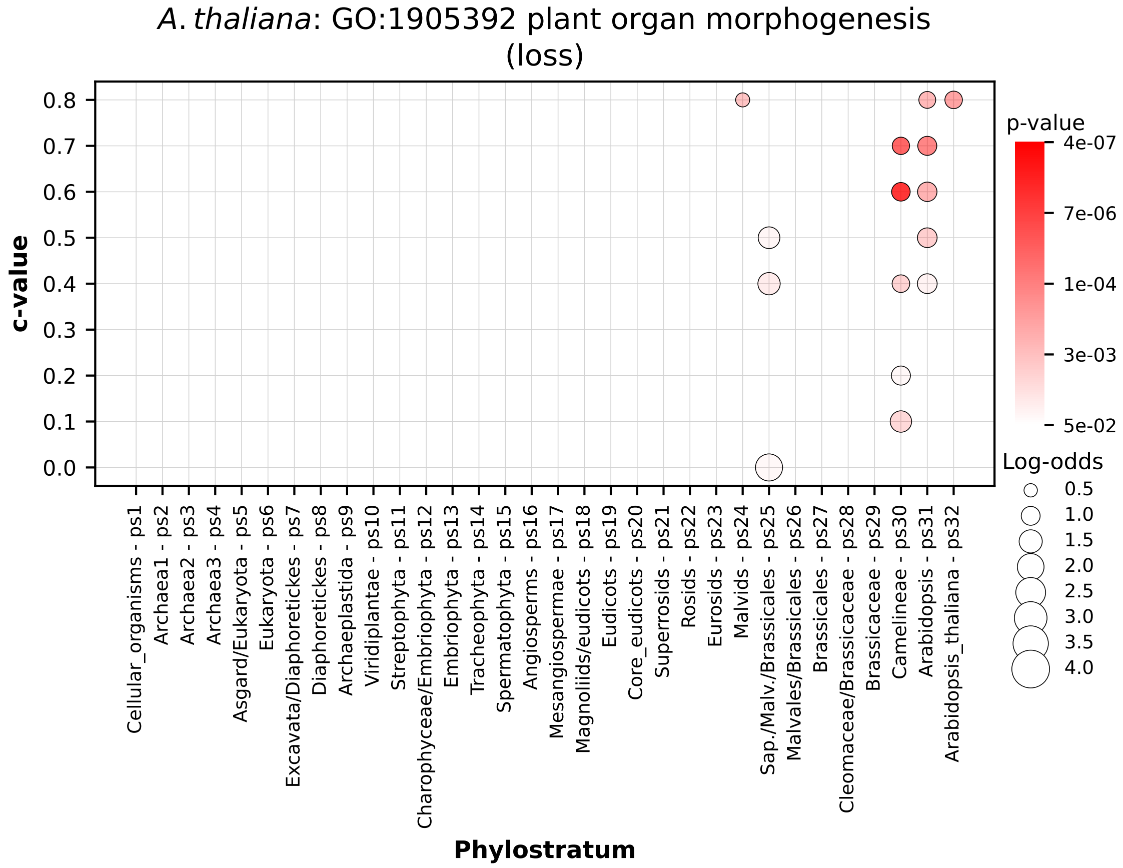

A. thaliana: GO:1905392 plant organ morphogenesis
(loss)
c-value
0.8
0.7
0.6
0.5
0.4
0.3
0.2
0.1
0.0
Phylostratum
Cellular_organisms - ps1
Archaea1 - ps2
Archaea2 - ps3
Archaea3 - ps4
Asgard/Eukaryota - ps5
Eukaryota - ps6
Excavata/Diaphoretickes - ps7
Diaphoretickes - ps8
Archaeplastida - ps9
Viridiplantae - ps10
Streptophyta - ps11
Charophyceae/Embriophyta - ps12
Embriophyta - ps13
Tracheophyta - ps14
Spermatophyta - ps15
Angiosperms - ps16
Mesangiospermae - ps17
Magnoliids/eudicots - ps18
Eudicots - ps19
Core_eudicots - ps20
Superrosids - ps21
Rosids - ps22
Eurosids - ps23
Malvids - ps24
Sap./Malv./Brassicales - ps25
Malvales/Brassicales - ps26
Brassicales - ps27
Cleomaceae/Brassicaceae - ps28
Brassicaceae - ps29
Camelineae - ps30
Arabidopsis - ps31
Arabidopsis_thaliana - ps32
p-value
4e-07
7e-06
1e-04
3e-03
5e-02
Log-odds
0.5
1.0
1.5
2.0
2.5
3.0
3.5
4.0

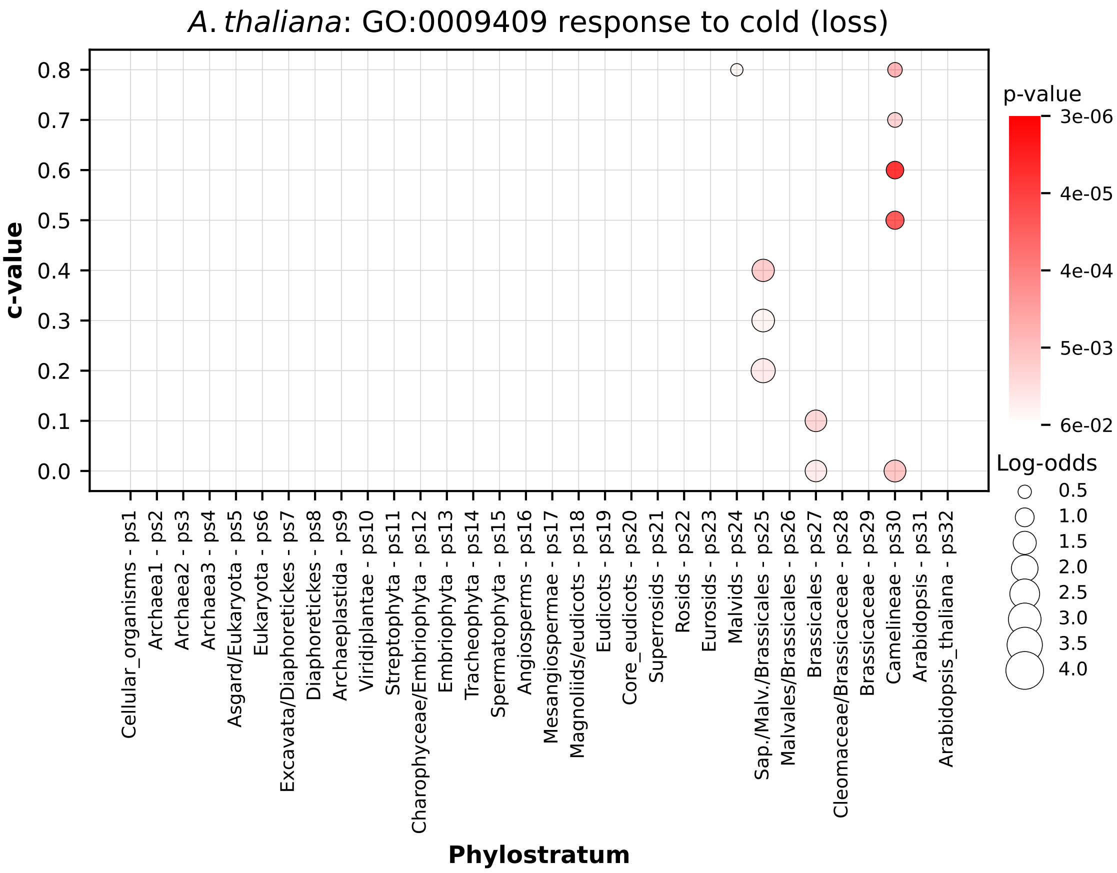

A. thaliana: GO:0009409 response to cold (loss)
c-value
0.8
0.7
0.6
0.5
0.4
0.3
0.2
0.1
0.0
p-value
3e-06
4e-05
4e-04
5e-03
6e-02
Log-odds
0.5
1.0
1.5
2.0
2.5
3.0
3.5
4.0
Cellular_organisms - ps1
Archaea1 - ps2
Archaea2 - ps3
Archaea3 - ps4
Asgard/Eukaryota - ps5
Eukaryota - ps6
Excavata/Diaphoretickes - ps7
Diaphoretickes - ps8
Archaeplastida - ps9
Viridiplantae - ps10
Streptophyta - ps11
Charophyceae/Embriophyta - ps12
Embriophyta - ps13
Tracheophyta - ps14
Spermatophyta - ps15
Angiosperms - ps16
Mesangiospermae - ps17
Magnoliids/eudicots - ps18
Eudicots - ps19
Core_eudicots - ps20
Superrosids - ps21
Rosids - ps22
Eurosids - ps23
Malvids - ps24
Sap./Malv./Brassicales - ps25
Malvales/Brassicales - ps26
Brassicales - ps27
Cleomaceae/Brassicaceae - ps28
Brassicaceae - ps29
Camelineae - ps30
Arabidopsis - ps31
Arabidopsis_thaliana - ps32
Phylostratum

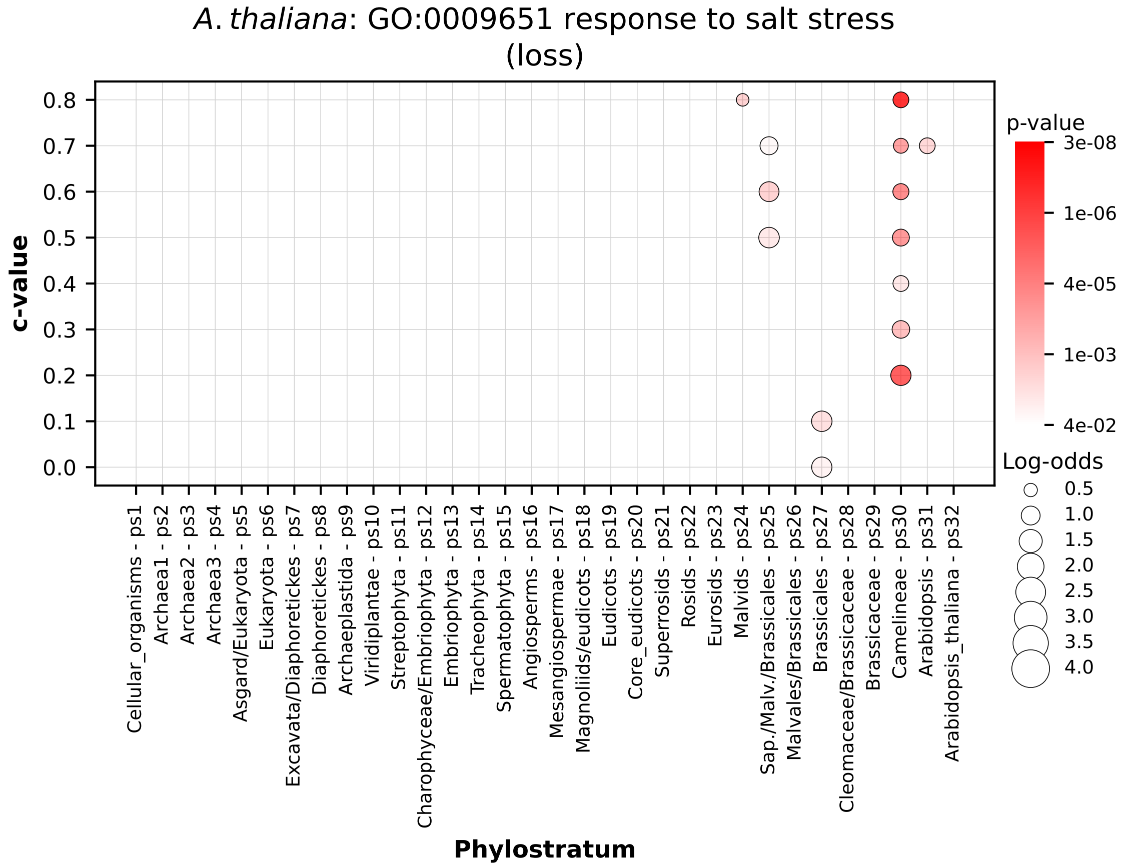
A. thaliana: GO:0009651 response to salt stress
(loss)
c-value
0.8
0.7
0.6
0.5
0.4
0.3
0.2
0.1
0.0
Phylostratum
Cellular_organisms - ps1
Archaea1 - ps2
Archaea2 - ps3
Archaea3 - ps4
Asgard/Eukaryota - ps5
Eukaryota - ps6
Excavata/Diaphoretickes - ps7
Diaphoretickes - ps8
Archaeplastida - ps9
Viridiplantae - ps10
Streptophyta - ps11
Charophyceae/Embriophyta - ps12
Embriophyta - ps13
Tracheophyta - ps14
Spermatophyta - ps15
Angiosperms - ps16
Mesangiospermae - ps17
Magnoliids/eudicots - ps18
Eudicots - ps19
Core_eudicots - ps20
Superrosids - ps21
Rosids - ps22
Eurosids - ps23
Malvids - ps24
Sap./Malv./Brassicales - ps25
Malvales/Brassicales - ps26
Brassicales - ps27
Cleomaceae/Brassicaceae - ps28
Brassicaceae - ps29
Camelineae - ps30
Arabidopsis - ps31
Arabidopsis_thaliana - ps32
p-value
3e-08
1e-06
4e-05
1e-03
4e-02
Log-odds
0.5
1.0
1.5
2.0
2.5
3.0
3.5
4.0

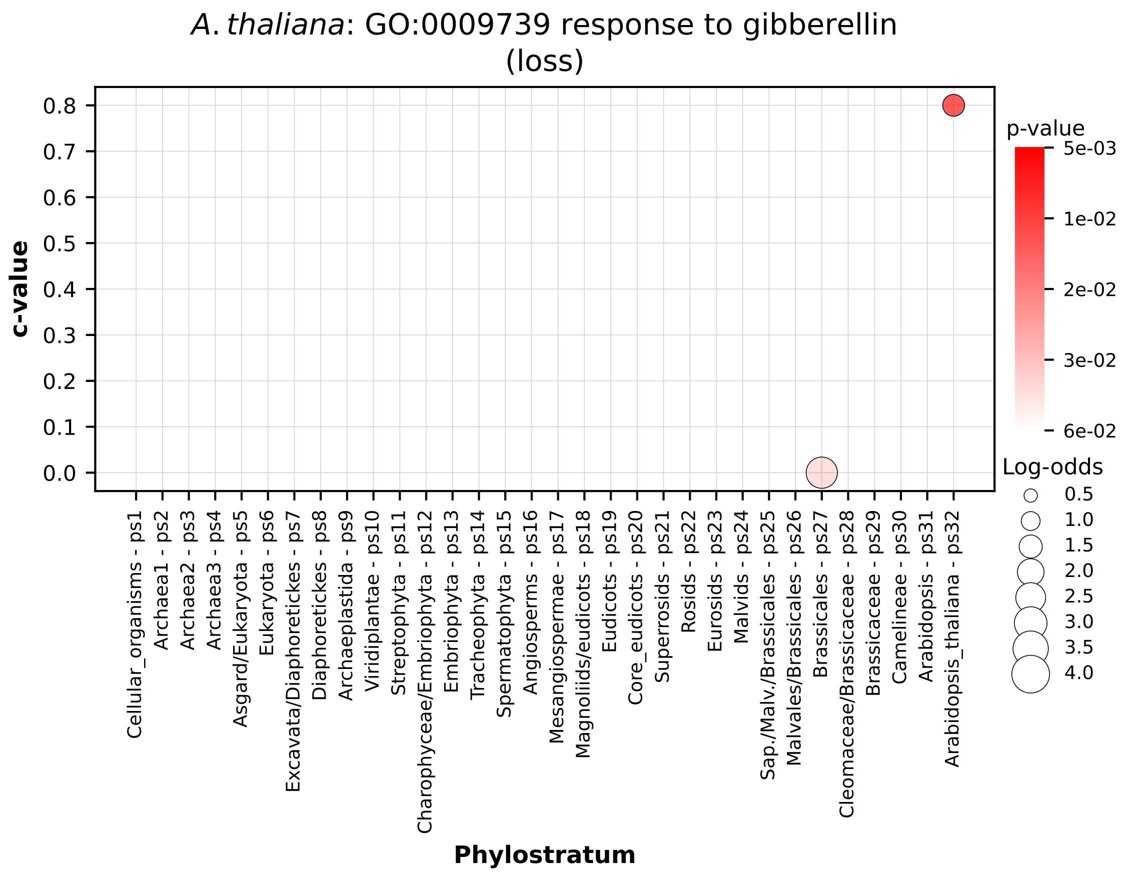

A. thaliana: GO:0009739 response to gibberellin
(loss)
c-value
0.8
0.7
0.6
0.5
0.4
0.3
0.2
0.1
0.0
p-value
5e-03
1e-02
2e-02
3e-02
6e-02
Log-odds
0.5
1.0
1.5
2.0
2.5
3.0
3.5
4.0
Cellular_organisms - ps1
Archaea1 - ps2
Archaea2 - ps3
Archaea3 - ps4
Asgard/Eukaryota - ps5
Eukaryota - ps6
Excavata/Diaphoretickes - ps7
Diaphoretickes - ps8
Archaeplastida - ps9
Viridiplantae - ps10
Streptophyta - ps11
Charophyceae/Embriophyta - ps12
Embriophyta - ps13
Tracheophyta - ps14
Spermatophyta - ps15
Angiosperms - ps16
Mesangiospermae - ps17
Magnoliids/eudicots - ps18
Eudicots - ps19
Core_eudicots - ps20
Superrosids - ps21
Rosids - ps22
Eurosids - ps23
Malvids - ps24
Sap./Malv./Brassicales - ps25
Malvales/Brassicales - ps26
Brassicales - ps27
Cleomaceae/Brassicaceae - ps28
Brassicaceae - ps29
Camelineae - ps30
Arabidopsis - ps31
Arabidopsis_thaliana - ps32
Phylostratum

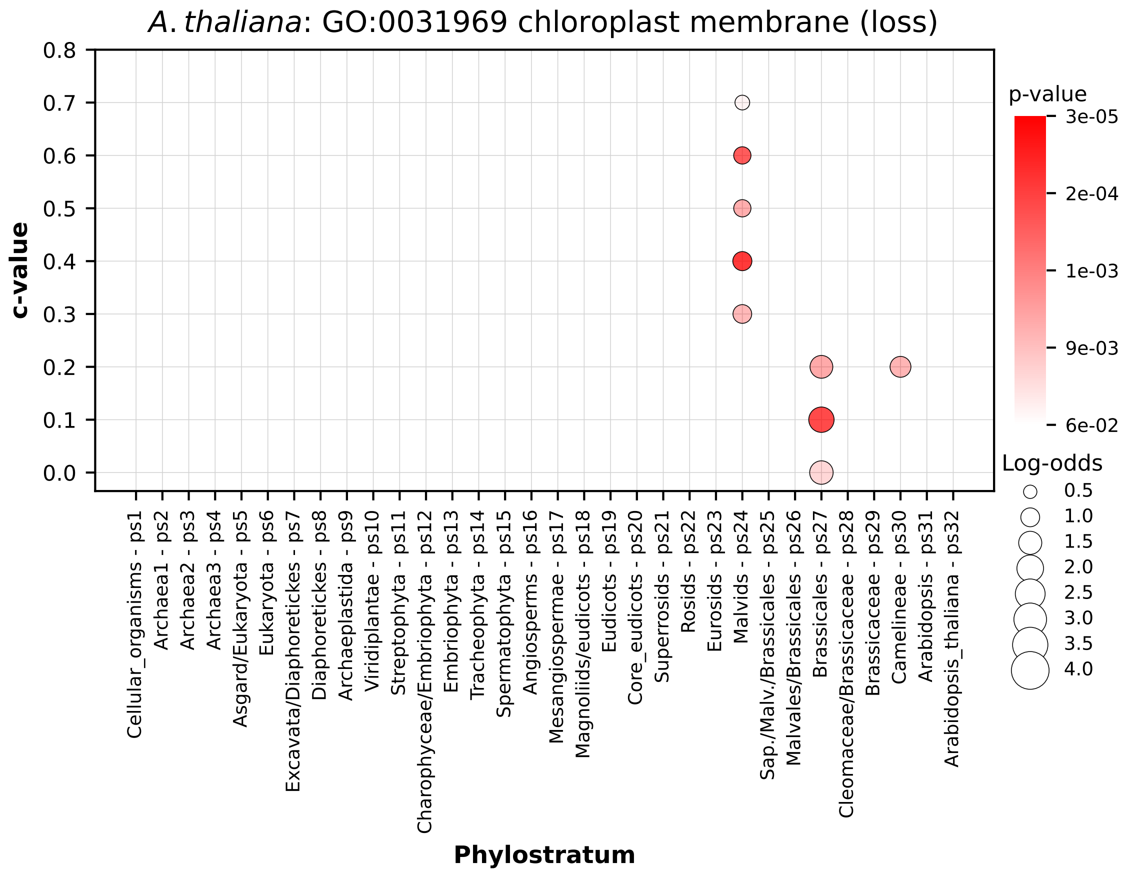

A. thaliana: GO:0031969 chloroplast membrane (loss)
c-value
0.8
0.7
0.6
0.5
0.4
0.3
0.2
0.1
0.0
Phylostratum
Cellular_organisms - ps1
Archaea1 - ps2
Archaea2 - ps3
Archaea3 - ps4
Asgard/Eukaryota - ps5
Eukaryota - ps6
Excavata/Diaphoretickes - ps7
Diaphoretickes - ps8
Archaeplastida - ps9
Viridiplantae - ps10
Streptophyta - ps11
Charophyceae/Embriophyta - ps12
Embriophyta - ps13
Tracheophyta - ps14
Spermatophyta - ps15
Angiosperms - ps16
Mesangiospermae - ps17
Magnoliids/eudicots - ps18
Eudicots - ps19
Core_eudicots - ps20
Superrosids - ps21
Rosids - ps22
Eurosids - ps23
Malvids - ps24
Sap./Malv./Brassicales - ps25
Malvales/Brassicales - ps26
Brassicales - ps27
Cleomaceae/Brassicaceae - ps28
Brassicaceae - ps29
Camelineae - ps30
Arabidopsis - ps31
Arabidopsis_thaliana - ps32
p-value
3e-05
2e-04
1e-03
9e-03
6e-02
Log-odds
0.5
1.0
1.5
2.0
2.5
3.0
3.5
4.0

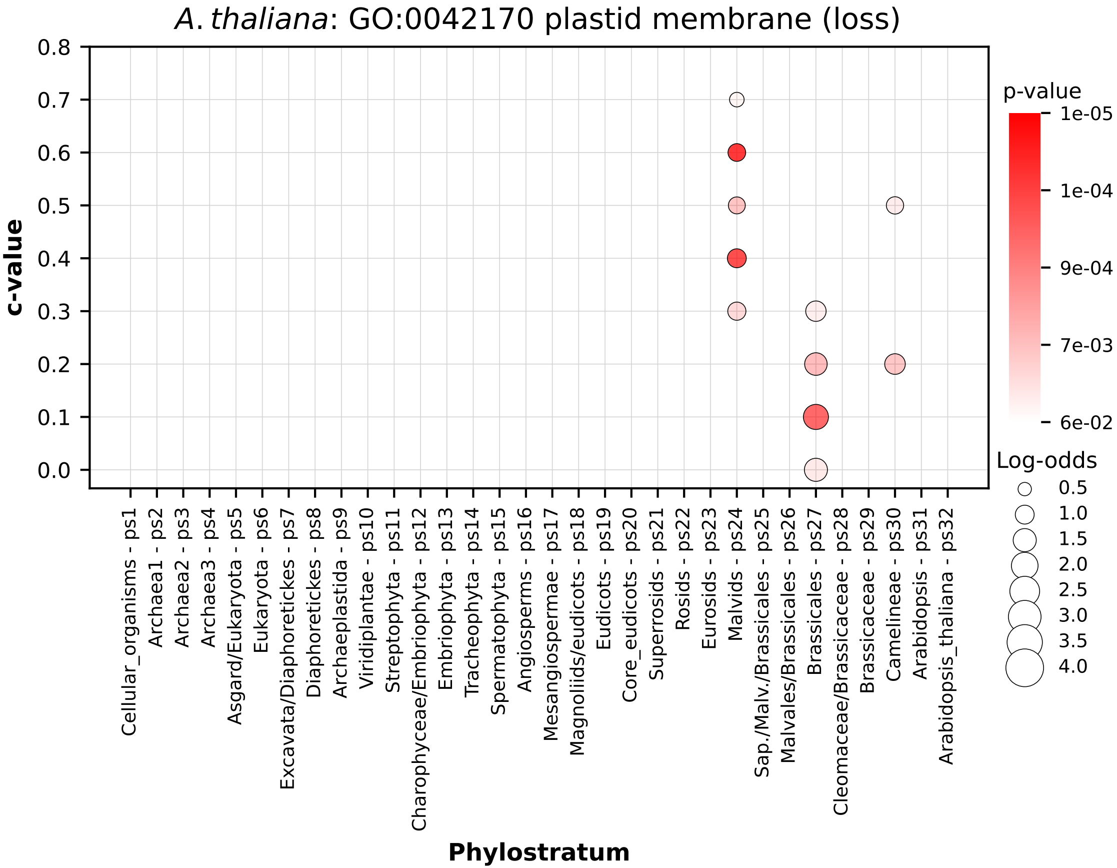

A. thaliana: GO:0042170 plastid membrane (loss)
c-value
0.8
0.7
0.6
0.5
0.4
0.3
0.2
0.1
0.0
p-value
1e-05
1e-04
9e-04
7e-03
6e-02
Log-odds
0.5
1.0
1.5
2.0
2.5
3.0
3.5
4.0
Cellular_organisms - ps1
Archaea1 - ps2
Archaea2 - ps3
Archaea3 - ps4
Asgard/Eukaryota - ps5
Eukaryota - ps6
Excavata/Diaphoretickes - ps7
Diaphoretickes - ps8
Archaeplastida - ps9
Viridiplantae - ps10
Streptophyta - ps11
Charophyceae/Embriophyta - ps12
Embriophyta - ps13
Tracheophyta - ps14
Spermatophyta - ps15
Angiosperms - ps16
Mesangiospermae - ps17
Magnoliids/eudicots - ps18
Eudicots - ps19
Core_eudicots - ps20
Superrosids - ps21
Rosids - ps22
Eurosids - ps23
Malvids - ps24
Sap./Malv./Brassicales - ps25
Malvales/Brassicales - ps26
Brassicales - ps27
Cleomaceae/Brassicaceae - ps28
Brassicaceae - ps29
Camelineae - ps30
Arabidopsis - ps31
Arabidopsis_thaliana - ps32
Phylostratum

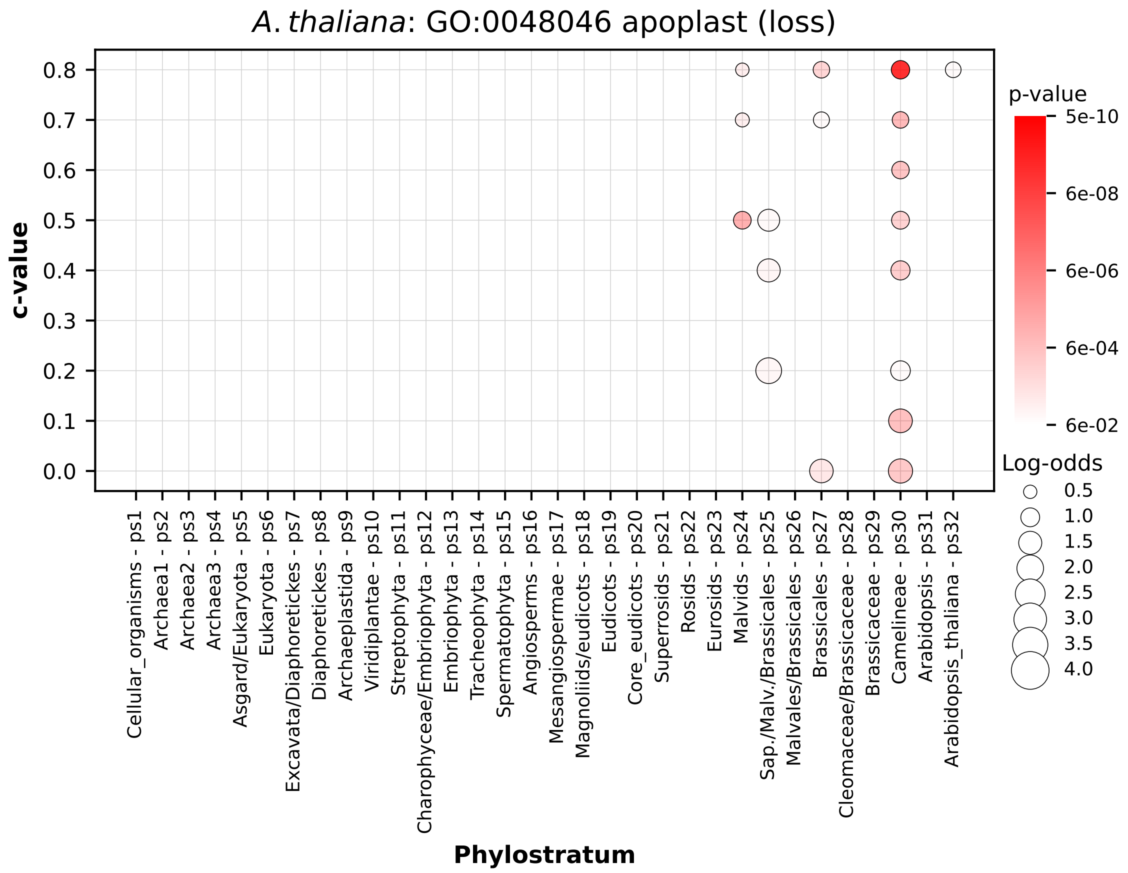

A. thaliana: GO:0048046 apoplast (loss)
c-value
0.8
0.7
0.6
0.5
0.4
0.3
0.2
0.1
0.0
Phylostratum
Cellular_organisms - ps1
Archaea1 - ps2
Archaea2 - ps3
Archaea3 - ps4
Asgard/Eukaryota - ps5
Eukaryota - ps6
Excavata/Diaphoretickes - ps7
Diaphoretickes - ps8
Archaeplastida - ps9
Viridiplantae - ps10
Streptophyta - ps11
Charophyceae/Embriophyta - ps12
Embriophyta - ps13
Tracheophyta - ps14
Spermatophyta - ps15
Angiosperms - ps16
Mesangiospermae - ps17
Magnoliids/eudicots - ps18
Eudicots - ps19
Core_eudicots - ps20
Superrosids - ps21
Rosids - ps22
Eurosids - ps23
Malvids - ps24
Sap./Malv./Brassicales - ps25
Malvales/Brassicales - ps26
Brassicales - ps27
Cleomaceae/Brassicaceae - ps28
Brassicaceae - ps29
Camelineae - ps30
Arabidopsis - ps31
Arabidopsis_thaliana - ps32
p-value
5e-10
6e-08
6e-06
6e-04
6e-02
Log-odds
0.5
1.0
1.5
2.0
2.5
3.0
3.5
4.0

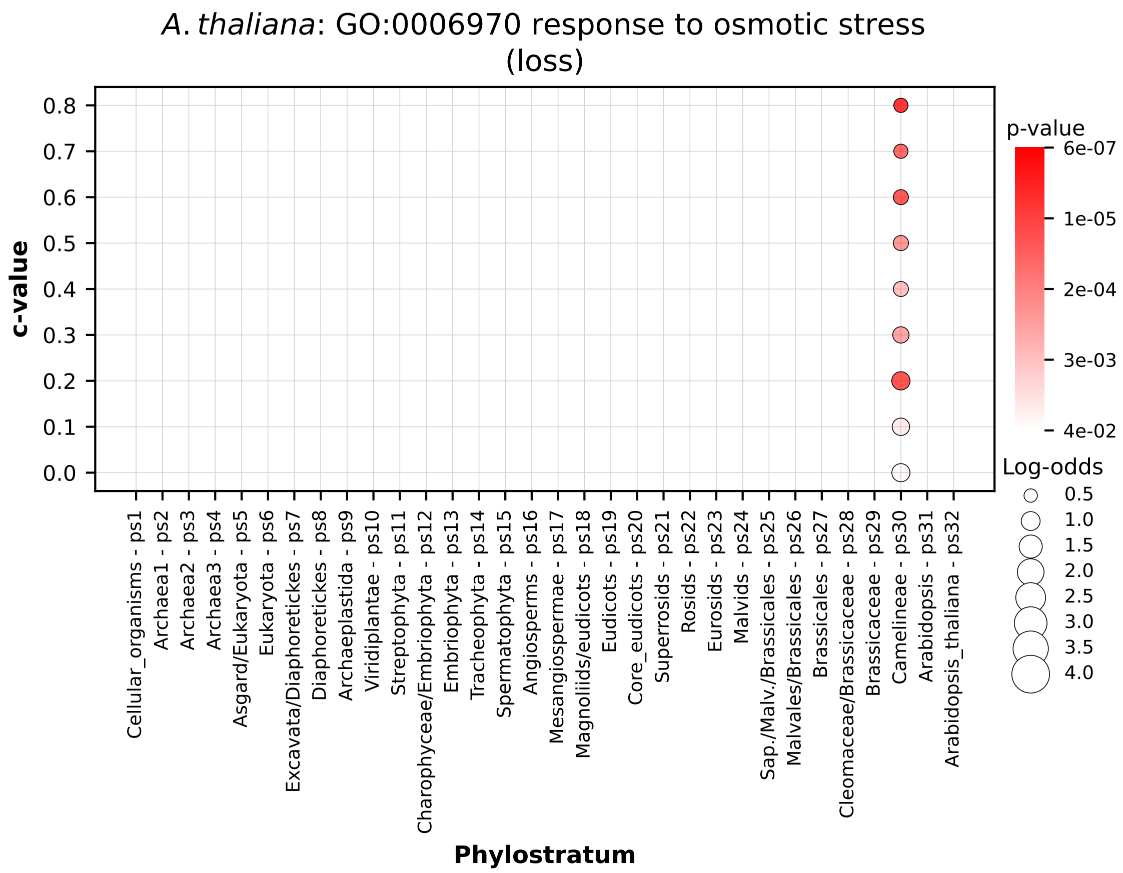

A. thaliana: GO:0006970 response to osmotic stress
(loss)
c-value
0.8
0.7
0.6
0.5
0.4
0.3
0.2
0.1
0.0
p-value
6e-07
1e-05
2e-04
3e-03
4e-02
Log-odds
0.5
1.0
1.5
2.0
2.5
3.0
3.5
4.0
Cellular_organisms - ps1
Archaea1 - ps2
Archaea2 - ps3
Archaea3 - ps4
Asgard/Eukaryota - ps5
Eukaryota - ps6
Excavata/Diaphoretickes - ps7
Diaphoretickes - ps8
Archaeplastida - ps9
Viridiplantae - ps10
Streptophyta - ps11
Charophyceae/Embriophyta - ps12
Embriophyta - ps13
Tracheophyta - ps14
Spermatophyta - ps15
Angiosperms - ps16
Mesangiospermae - ps17
Magnoliids/eudicots - ps18
Eudicots - ps19
Core_eudicots - ps20
Superrosids - ps21
Rosids - ps22
Eurosids - ps23
Malvids - ps24
Sap./Malv./Brassicales - ps25
Malvales/Brassicales - ps26
Brassicales - ps27
Cleomaceae/Brassicaceae - ps28
Brassicaceae - ps29
Camelineae - ps30
Arabidopsis - ps31
Arabidopsis_thaliana - ps32
Phylostratum

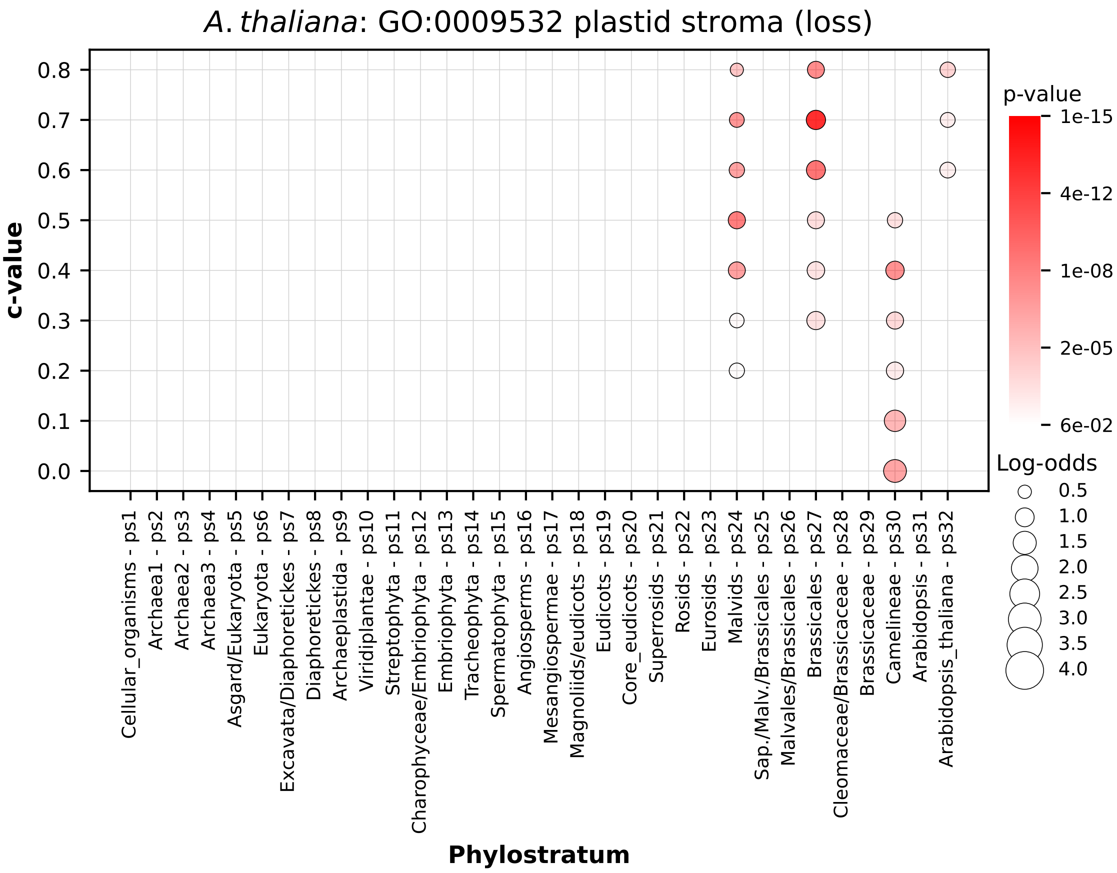

A. thaliana: GO:0009532 plastid stroma (loss)
c-value
0.8
0.7
0.6
0.5
0.4
0.3
0.2
0.1
0.0
Phylostratum
Cellular_organisms - ps1
Archaea1 - ps2
Archaea2 - ps3
Archaea3 - ps4
Asgard/Eukaryota - ps5
Eukaryota - ps6
Excavata/Diaphoretickes - ps7
Diaphoretickes - ps8
Archaeplastida - ps9
Viridiplantae - ps10
Streptophyta - ps11
Charophyceae/Embriophyta - ps12
Embriophyta - ps13
Tracheophyta - ps14
Spermatophyta - ps15
Angiosperms - ps16
Mesangiospermae - ps17
Magnoliids/eudicots - ps18
Eudicots - ps19
Core_eudicots - ps20
Superrosids - ps21
Rosids - ps22
Eurosids - ps23
Malvids - ps24
Sap./Malv./Brassicales - ps25
Malvales/Brassicales - ps26
Brassicales - ps27
Cleomaceae/Brassicaceae - ps28
Brassicaceae - ps29
Camelineae - ps30
Arabidopsis - ps31
Arabidopsis_thaliana - ps32
p-value
1e-15
4e-12
1e-08
2e-05
6e-02
Log-odds
0.5
1.0
1.5
2.0
2.5
3.0
3.5
4.0

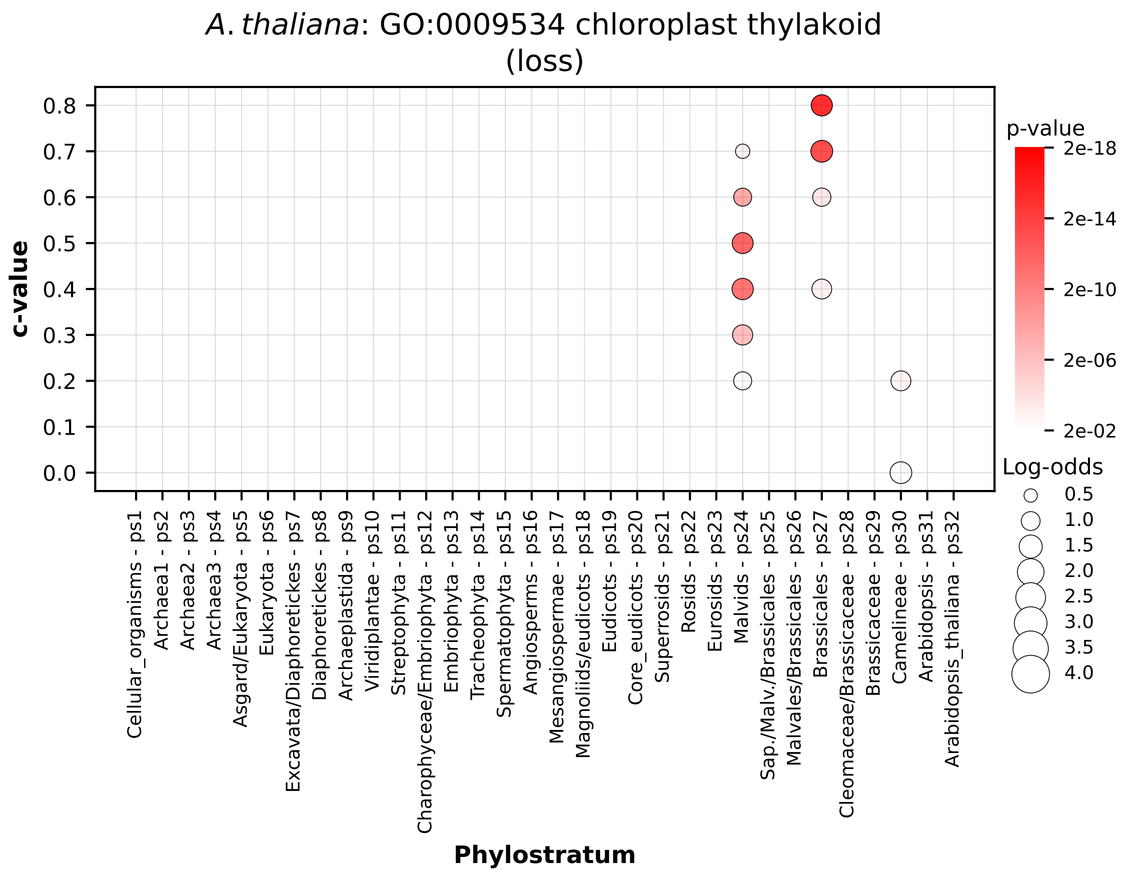

A. thaliana: GO:0009534 chloroplast thylakoid
(loss)
c-value
0.8
0.7
0.6
0.5
0.4
0.3
0.2
0.1
0.0
p-value
2e-18
2e-14
2e-10
2e-06
2e-02
Log-odds
0.5
1.0
1.5
2.0
2.5
3.0
3.5
4.0
Cellular_organisms - ps1
Archaea1 - ps2
Archaea2 - ps3
Archaea3 - ps4
Asgard/Eukaryota - ps5
Eukaryota - ps6
Excavata/Diaphoretickes - ps7
Diaphoretickes - ps8
Archaeplastida - ps9
Viridiplantae - ps10
Streptophyta - ps11
Charophyceae/Embriophyta - ps12
Embriophyta - ps13
Tracheophyta - ps14
Spermatophyta - ps15
Angiosperms - ps16
Mesangiospermae - ps17
Magnoliids/eudicots - ps18
Eudicots - ps19
Core_eudicots - ps20
Superrosids - ps21
Rosids - ps22
Eurosids - ps23
Malvids - ps24
Sap./Malv./Brassicales - ps25
Malvales/Brassicales - ps26
Brassicales - ps27
Cleomaceae/Brassicaceae - ps28
Brassicaceae - ps29
Camelineae - ps30
Arabidopsis - ps31
Arabidopsis_thaliana - ps32
Phylostratum

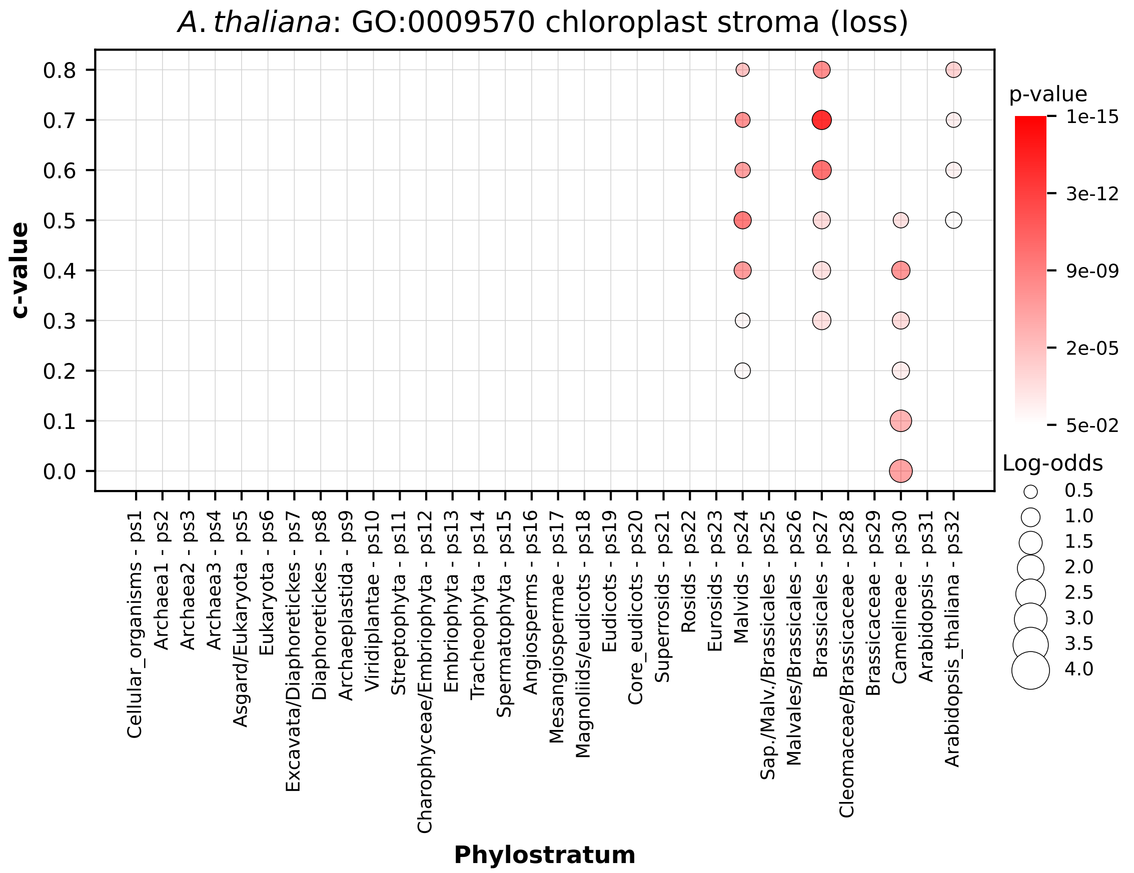

A. thaliana: GO:0009570 chloroplast stroma (loss)
c-value
0.8
0.7
0.6
0.5
0.4
0.3
0.2
0.1
0.0
p-value
1e-15
3e-12
9e-09
2e-05
5e-02
Log-odds
0.5
1.0
1.5
2.0
2.5
3.0
3.5
4.0
Cellular_organisms - ps1
Archaea1 - ps2
Archaea2 - ps3
Archaea3 - ps4
Asgard/Eukaryota - ps5
Eukaryota - ps6
Excavata/Diaphoretickes - ps7
Diaphoretickes - ps8
Archaeplastida - ps9
Viridiplantae - ps10
Streptophyta - ps11
Charophyceae/Embriophyta - ps12
Embriophyta - ps13
Tracheophyta - ps14
Spermatophyta - ps15
Angiosperms - ps16
Mesangiospermae - ps17
Magnoliids/eudicots - ps18
Eudicots - ps19
Core_eudicots - ps20
Superrosids - ps21
Rosids - ps22
Eurosids - ps23
Malvids - ps24
Sap./Malv./Brassicales - ps25
Malvales/Brassicales - ps26
Brassicales - ps27
Cleomaceae/Brassicaceae - ps28
Brassicaceae - ps29
Camelineae - ps30
Arabidopsis - ps31
Arabidopsis_thaliana - ps32
Phylostratum

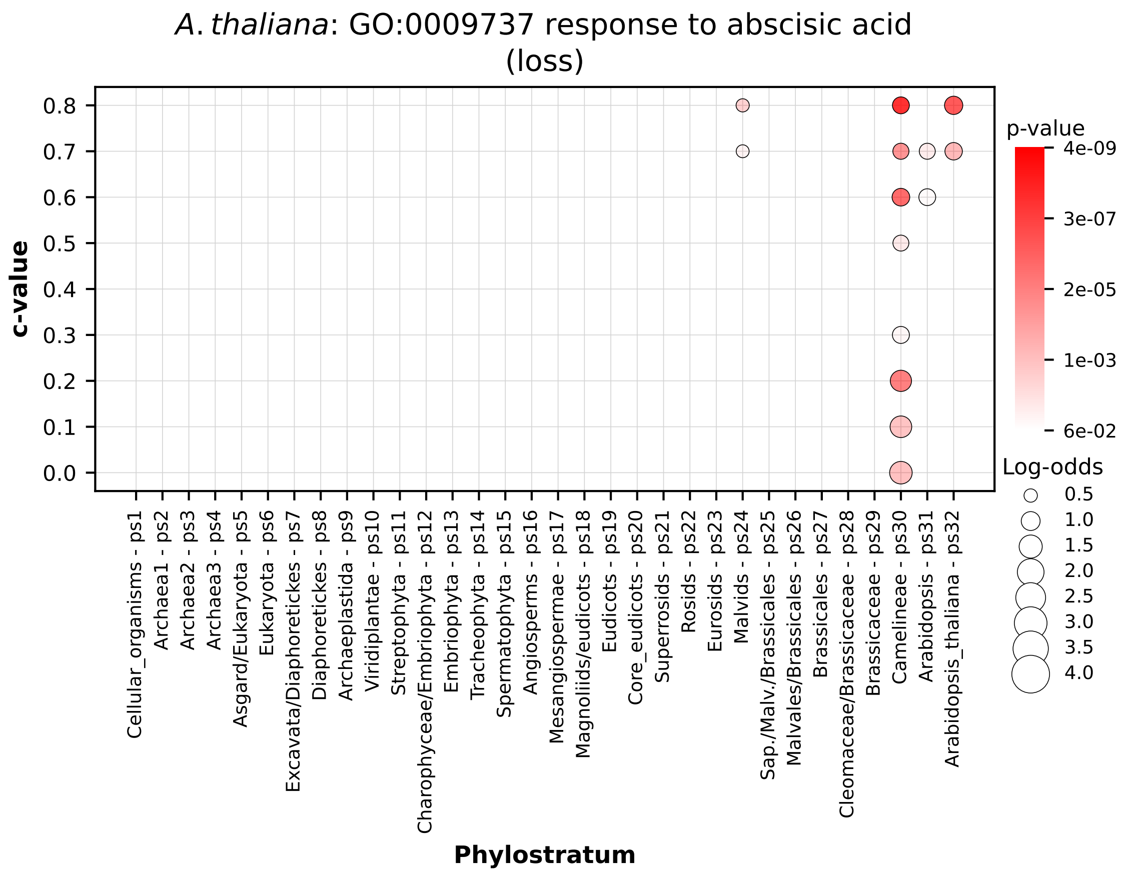

A. thaliana: GO:0009737 response to abscisic acid
(loss)
c-value
0.8
0.7
0.6
0.5
0.4
0.3
0.2
0.1
0.0
p-value
4e-09
3e-07
2e-05
1e-03
6e-02
Log-odds
0.5
1.0
1.5
2.0
2.5
3.0
3.5
4.0
Cellular_organisms - ps1
Archaea1 - ps2
Archaea2 - ps3
Archaea3 - ps4
Asgard/Eukaryota - ps5
Eukaryota - ps6
Excavata/Diaphoretickes - ps7
Diaphoretickes - ps8
Archaeplastida - ps9
Viridiplantae - ps10
Streptophyta - ps11
Charophyceae/Embriophyta - ps12
Embriophyta - ps13
Tracheophyta - ps14
Spermatophyta - ps15
Angiosperms - ps16
Mesangiospermae - ps17
Magnoliids/eudicots - ps18
Eudicots - ps19
Core_eudicots - ps20
Superrosids - ps21
Rosids - ps22
Eurosids - ps23
Malvids - ps24
Sap./Malv./Brassicales - ps25
Malvales/Brassicales - ps26
Brassicales - ps27
Cleomaceae/Brassicaceae - ps28
Brassicaceae - ps29
Camelineae - ps30
Arabidopsis - ps31
Arabidopsis_thaliana - ps32
Phylostratum

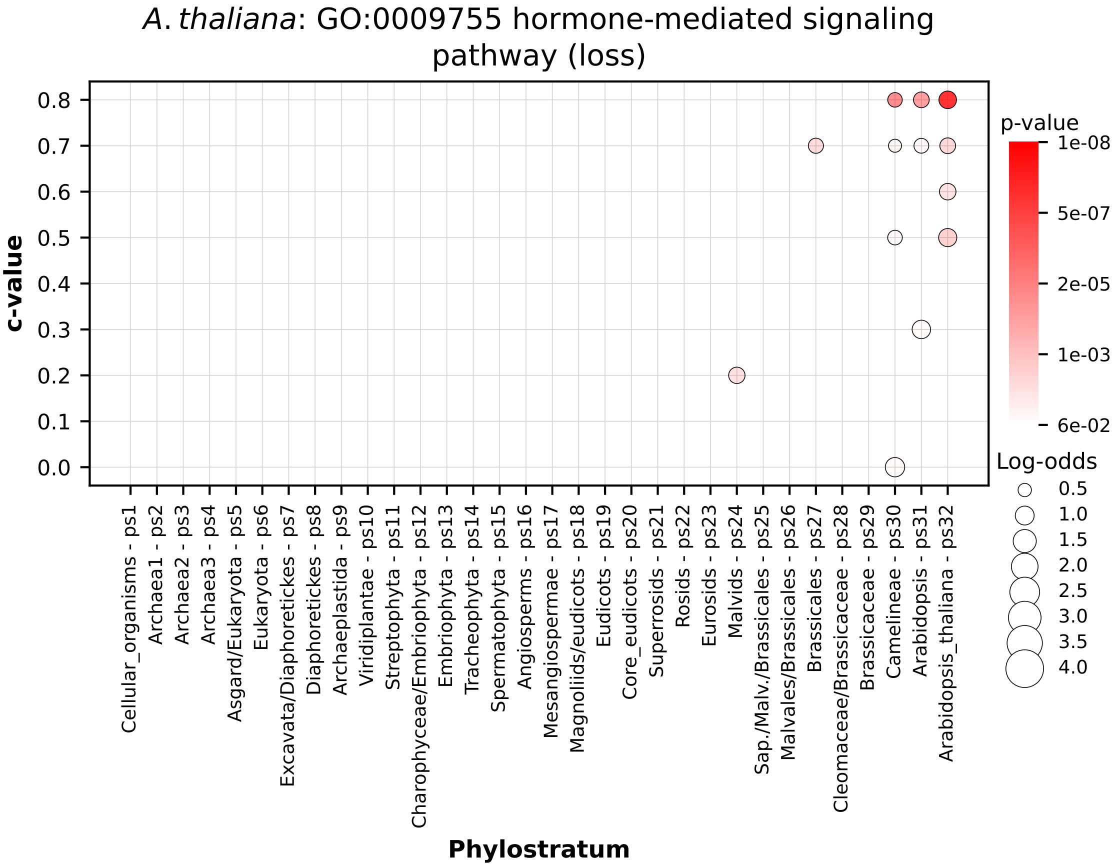

A. thaliana: GO:0009755 hormone-mediated signaling pathway (loss)
c-value
0.8
0.7
0.6
0.5
0.4
0.3
0.2
0.1
0.0
p-value
1e-08
5e-07
2e-05
1e-03
6e-02
Log-odds
0.5
1.0
1.5
2.0
2.5
3.0
3.5
4.0
Cellular_organisms - ps1
Archaea1 - ps2
Archaea2 - ps3
Archaea3 - ps4
Asgard/Eukaryota - ps5
Eukaryota - ps6
Excavata/Diaphoretickes - ps7
Diaphoretickes - ps8
Archaeplastida - ps9
Viridiplantae - ps10
Streptophyta - ps11
Charophyceae/Embriophyta - ps12
Embriophyta - ps13
Tracheophyta - ps14
Spermatophyta - ps15
Angiosperms - ps16
Mesangiospermae - ps17
Magnoliids/eudicots - ps18
Eudicots - ps19
Core_eudicots - ps20
Superrosids - ps21
Rosids - ps22
Eurosids - ps23
Malvids - ps24
Sap./Malv./Brassicales - ps25
Malvales/Brassicales - ps26
Brassicales - ps27
Cleomaceae/Brassicaceae - ps28
Brassicaceae - ps29
Camelineae - ps30
Arabidopsis - ps31
Arabidopsis_thaliana - ps32
Phylostratum

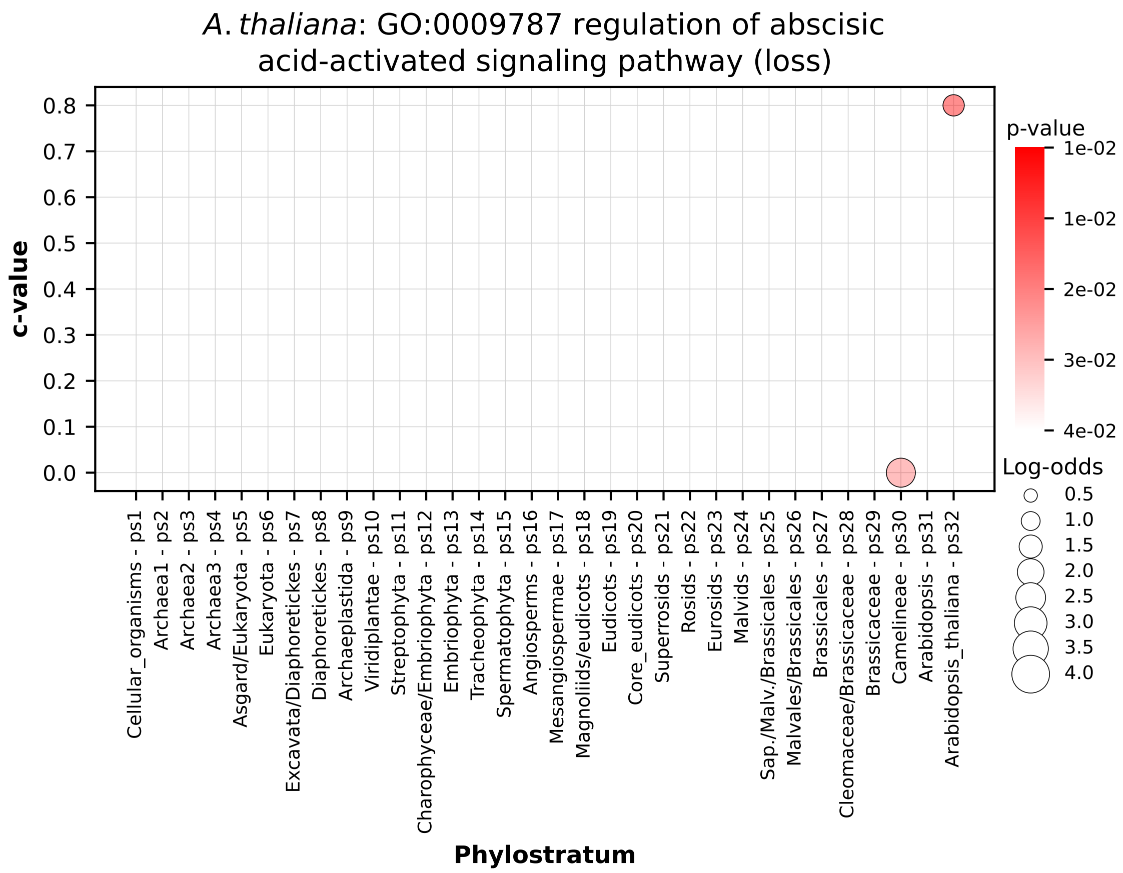
A. thaliana: GO:0009787 regulation of abscisic acid-activated signaling pathway (loss)
c-value
0.8
0.7
0.6
0.5
0.4
0.3
0.2
0.1
0.0
p-value
1e-02
1e-02
2e-02
3e-02
4e-02
Log-odds
0.5
1.0
1.5
2.0
2.5
3.0
3.5
4.0
Cellular_organisms - ps1
Archaea1 - ps2
Archaea2 - ps3
Archaea3 - ps4
Asgard/Eukaryota - ps5
Eukaryota - ps6
Excavata/Diaphoretickes - ps7
Diaphoretickes - ps8
Archaeplastida - ps9
Viridiplantae - ps10
Streptophyta - ps11
Charophyceae/Embriophyta - ps12
Embriophyta - ps13
Tracheophyta - ps14
Spermatophyta - ps15
Angiosperms - ps16
Mesangiospermae - ps17
Magnoliids/eudicots - ps18
Eudicots - ps19
Core_eudicots - ps20
Superrosids - ps21
Rosids - ps22
Eurosids - ps23
Malvids - ps24
Sap./Malv./Brassicales - ps25
Malvales/Brassicales - ps26
Brassicales - ps27
Cleomaceae/Brassicaceae - ps28
Brassicaceae - ps29
Camelineae - ps30
Arabidopsis - ps31
Arabidopsis_thaliana - ps32
Phylostratum

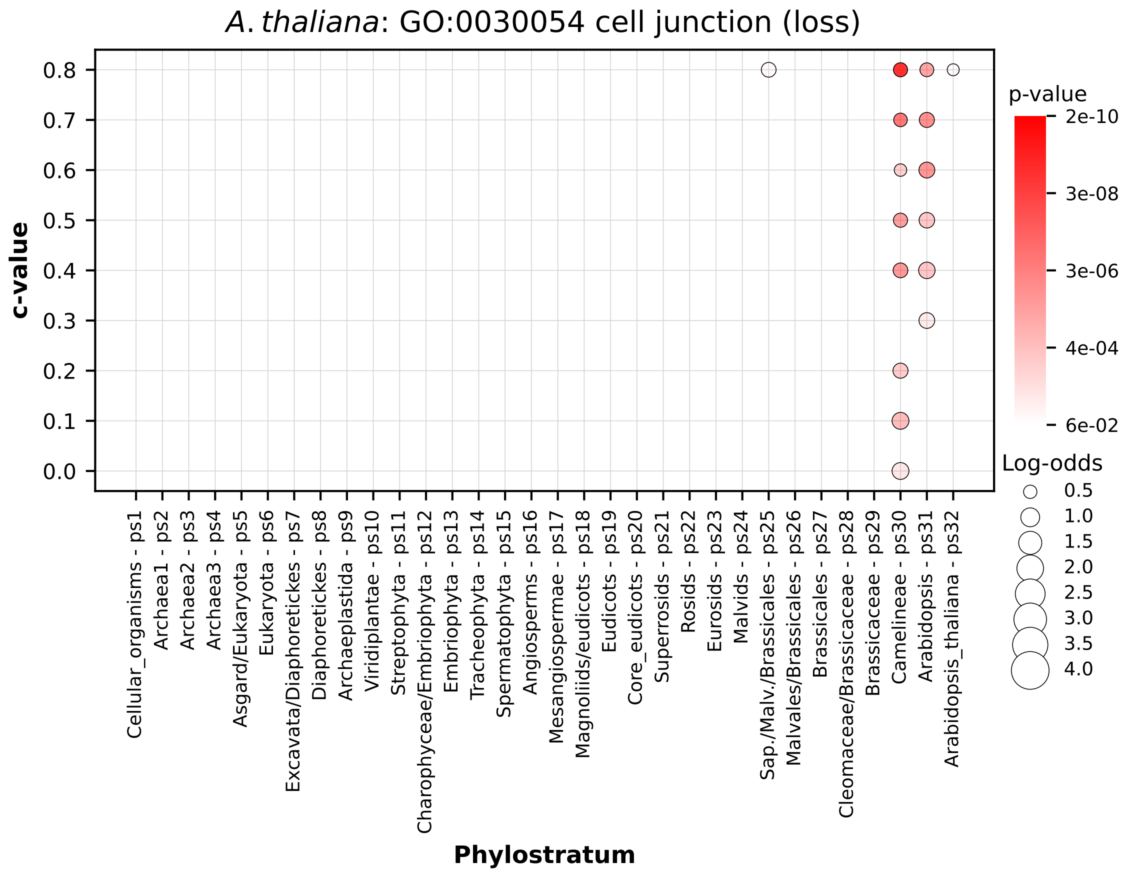
A. thaliana: GO:0030054 cell junction (loss)
c-value
0.8
0.7
0.6
0.5
0.4
0.3
0.2
0.1
0.0
p-value
2e-10
3e-08
3e-06
4e-04
6e-02
Log-odds
0.5
1.0
1.5
2.0
2.5
3.0
3.5
4.0
Cellular_organisms - ps1
Archaea1 - ps2
Archaea2 - ps3
Archaea3 - ps4
Asgard/Eukaryota - ps5
Eukaryota - ps6
Excavata/Diaphoretickes - ps7
Diaphoretickes - ps8
Archaeplastida - ps9
Viridiplantae - ps10
Streptophyta - ps11
Charophyceae/Embriophyta - ps12
Embriophyta - ps13
Tracheophyta - ps14
Spermatophyta - ps15
Angiosperms - ps16
Mesangiospermae - ps17
Magnoliids/eudicots - ps18
Eudicots - ps19
Core_eudicots - ps20
Superrosids - ps21
Rosids - ps22
Eurosids - ps23
Malvids - ps24
Sap./Malv./Brassicales - ps25
Malvales/Brassicales - ps26
Brassicales - ps27
Cleomaceae/Brassicaceae - ps28
Brassicaceae - ps29
Camelineae - ps30
Arabidopsis - ps31
Arabidopsis_thaliana - ps32
Phylostratum

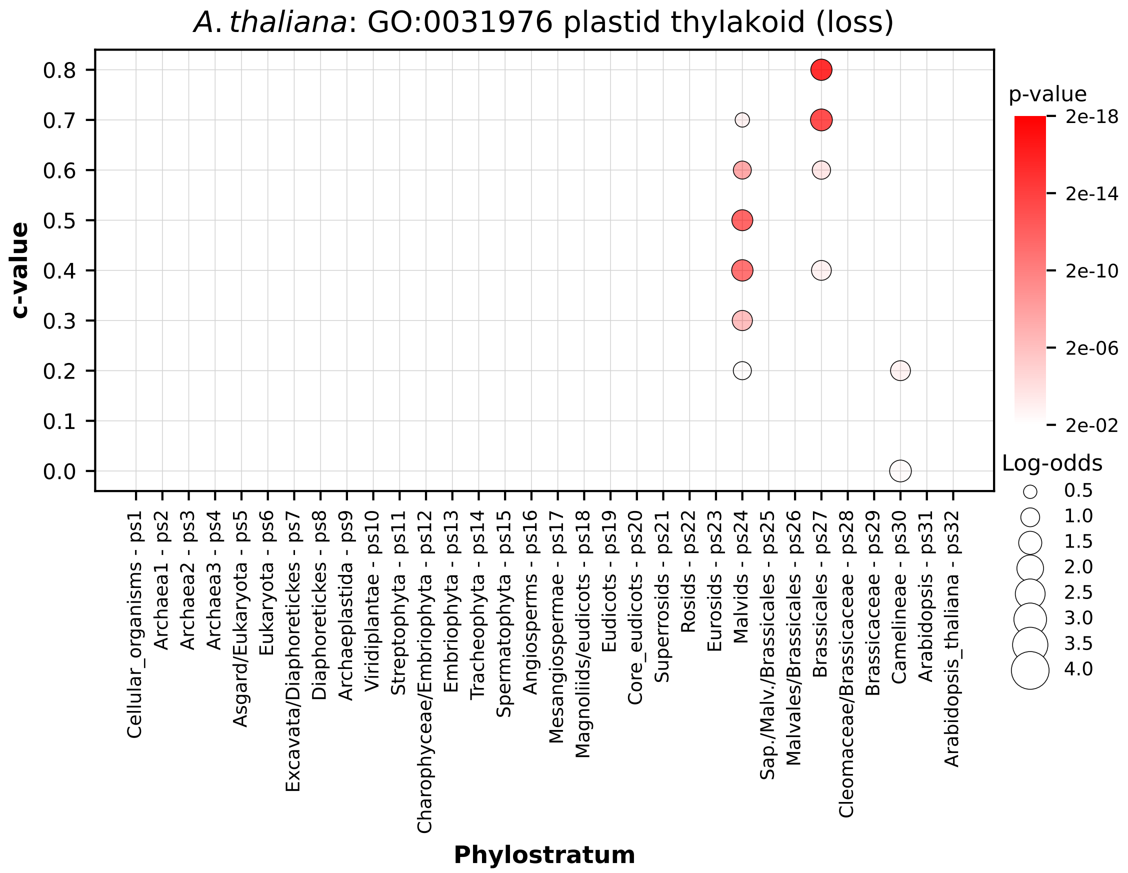
A. thaliana: GO:0031976 plastid thylakoid (loss)
c-value
0.8
0.7
0.6
0.5
0.4
0.3
0.2
0.1
0.0
p-value
2e-18
2e-14
2e-10
2e-06
2e-02
Log-odds
0.5
1.0
1.5
2.0
2.5
3.0
3.5
4.0
Cellular_organisms - ps1
Archaea1 - ps2
Archaea2 - ps3
Archaea3 - ps4
Asgard/Eukaryota - ps5
Eukaryota - ps6
Excavata/Diaphoretickes - ps7
Diaphoretickes - ps8
Archaeplastida - ps9
Viridiplantae - ps10
Streptophyta - ps11
Charophyceae/Embriophyta - ps12
Embriophyta - ps13
Tracheophyta - ps14
Spermatophyta - ps15
Angiosperms - ps16
Mesangiospermae - ps17
Magnoliids/eudicots - ps18
Eudicots - ps19
Core_eudicots - ps20
Superrosids - ps21
Rosids - ps22
Eurosids - ps23
Malvids - ps24
Sap./Malv./Brassicales - ps25
Malvales/Brassicales - ps26
Brassicales - ps27
Cleomaceae/Brassicaceae - ps28
Brassicaceae - ps29
Camelineae - ps30
Arabidopsis - ps31
Arabidopsis_thaliana - ps32
Phylostratum

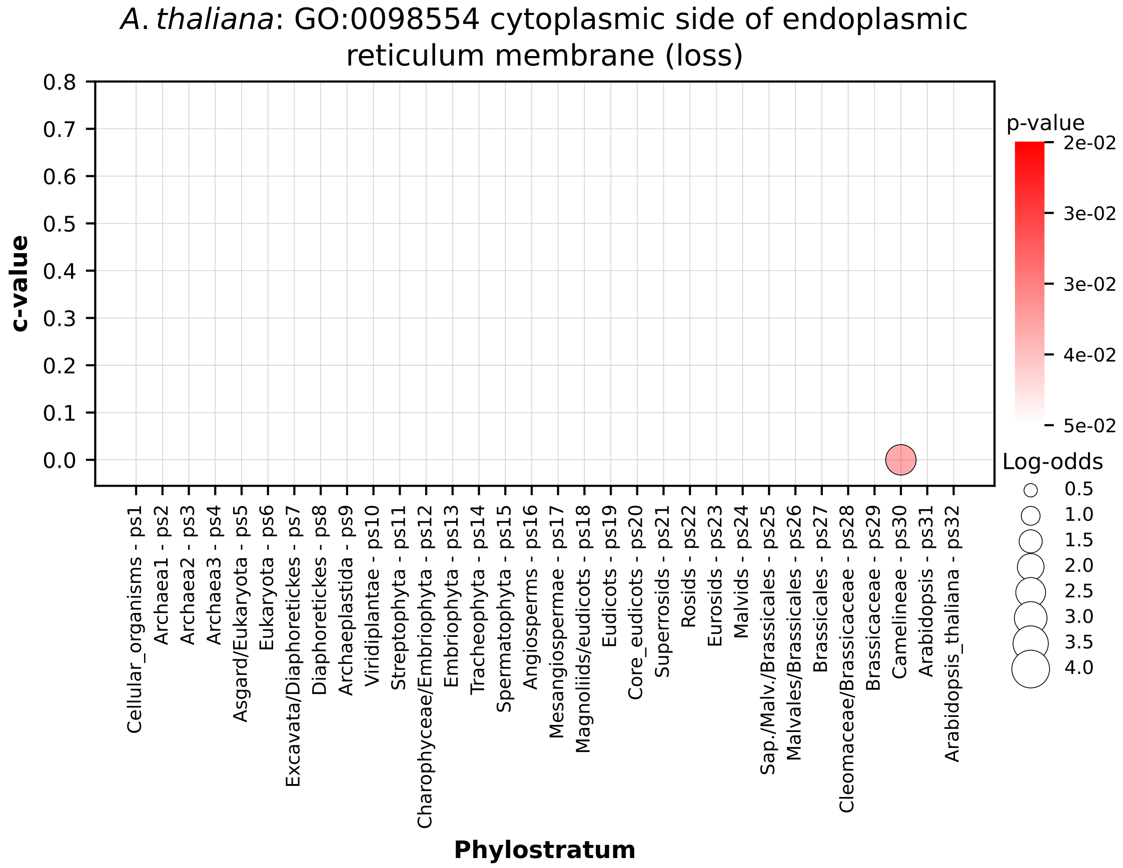

A. thaliana: GO:0098554 cytoplasmic side of endoplasmic reticulum membrane (loss)
c-value
0.8
0.7
0.6
0.5
0.4
0.3
0.2
0.1
0.0
Phylostratum
Cellular_organisms - ps1
Archaea1 - ps2
Archaea2 - ps3
Archaea3 - ps4
Asgard/Eukaryota - ps5
Eukaryota - ps6
Excavata/Diaphoretickes - ps7
Diaphoretickes - ps8
Archaeplastida - ps9
Viridiplantae - ps10
Streptophyta - ps11
Charophyceae/Embriophyta - ps12
Embriophyta - ps13
Tracheophyta - ps14
Spermatophyta - ps15
Angiosperms - ps16
Mesangiospermae - ps17
Magnoliids/eudicots - ps18
Eudicots - ps19
Core_eudicots - ps20
Superrosids - ps21
Rosids - ps22
Eurosids - ps23
Malvids - ps24
Sap./Malv./Brassicales - ps25
Malvales/Brassicales - ps26
Brassicales - ps27
Cleomaceae/Brassicaceae - ps28
Brassicaceae - ps29
Camelineae - ps30
Arabidopsis - ps31
Arabidopsis_thaliana - ps32
p-value
2e-02
3e-02
3e-02
4e-02
5e-02
Log-odds
0.5
1.0
1.5
2.0
2.5
3.0
3.5
4.0

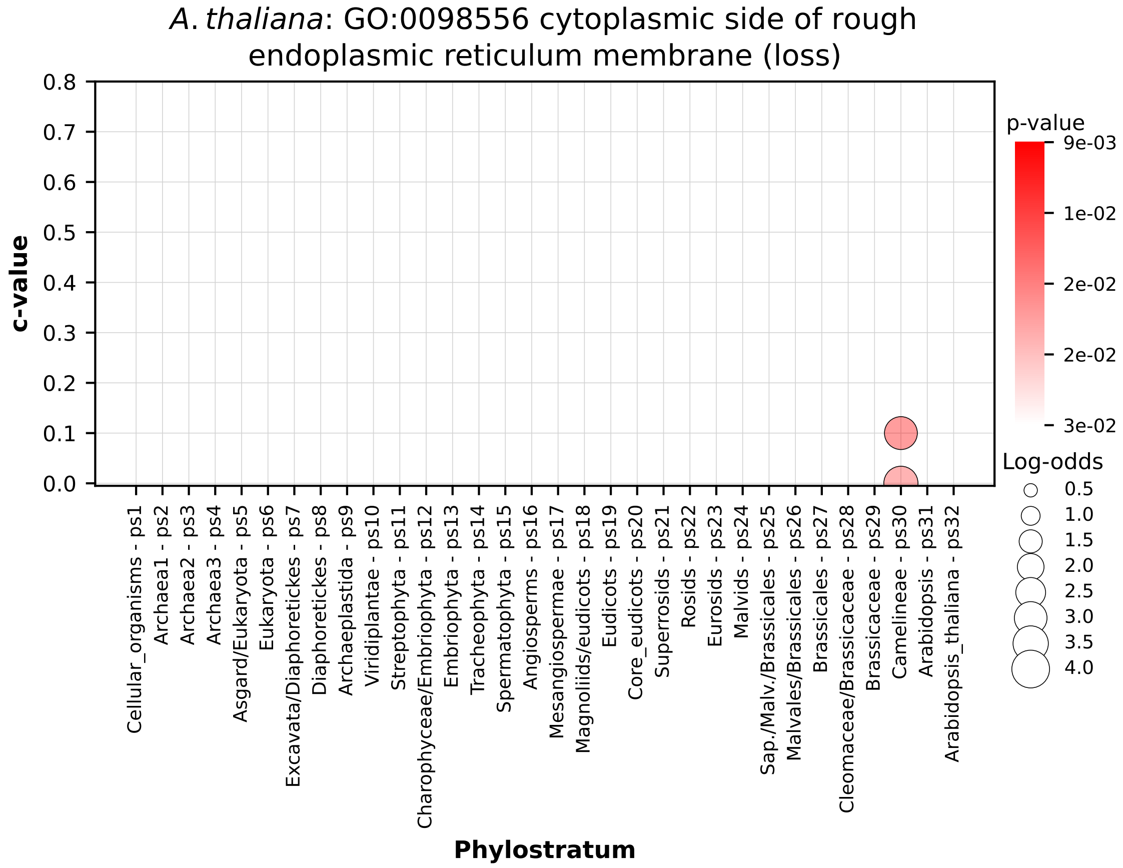

A. thaliana: GO:0098556 cytoplasmic side of rough endoplasmic reticulum membrane (loss)
c-value
0.8
0.7
0.6
0.5
0.4
0.3
0.2
0.1
0.0
p-value
9e-03
1e-02
2e-02
2e-02
3e-02
Log-odds
0.5
1.0
1.5
2.0
2.5
3.0
3.5
4.0
Cellular_organisms - ps1
Archaea1 - ps2
Archaea2 - ps3
Archaea3 - ps4
Asgard/Eukaryota - ps5
Eukaryota - ps6
Excavata/Diaphoretickes - ps7
Diaphoretickes - ps8
Archaeplastida - ps9
Viridiplantae - ps10
Streptophyta - ps11
Charophyceae/Embriophyta - ps12
Embriophyta - ps13
Tracheophyta - ps14
Spermatophyta - ps15
Angiosperms - ps16
Mesangiospermae - ps17
Magnoliids/eudicots - ps18
Eudicots - ps19
Core_eudicots - ps20
Superrosids - ps21
Rosids - ps22
Eurosids - ps23
Malvids - ps24
Sap./Malv./Brassicales - ps25
Malvales/Brassicales - ps26
Brassicales - ps27
Cleomaceae/Brassicaceae - ps28
Brassicaceae - ps29
Camelineae - ps30
Arabidopsis - ps31
Arabidopsis_thaliana - ps32
Phylostratum

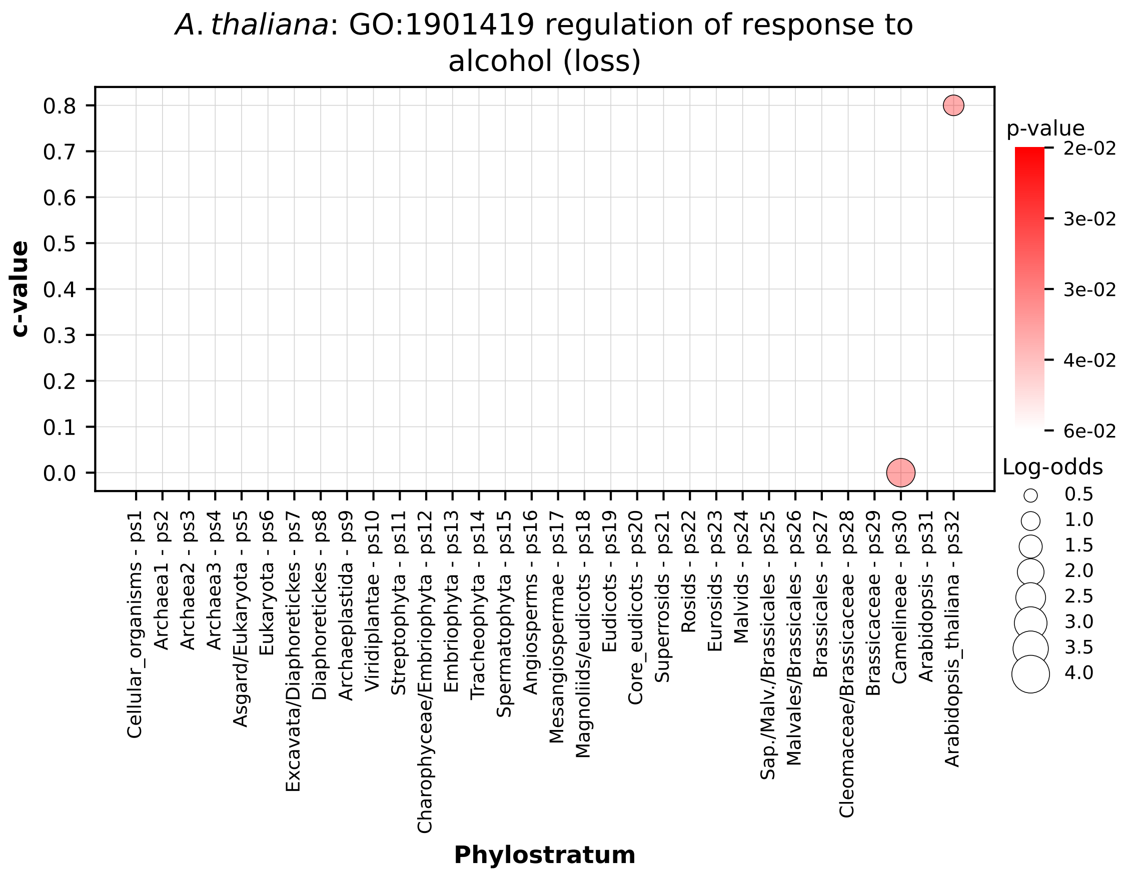

A. thaliana: GO:1901419 regulation of response to alcohol (loss)
c-value
0.8
0.7
0.6
0.5
0.4
0.3
0.2
0.1
0.0
p-value
2e-02
3e-02
3e-02
4e-02
6e-02
Log-odds
0.5
1.0
1.5
2.0
2.5
3.0
3.5
4.0
Cellular_organisms - ps1
Archaea1 - ps2
Archaea2 - ps3
Archaea3 - ps4
Asgard/Eukaryota - ps5
Eukaryota - ps6
Excavata/Diaphoretickes - ps7
Diaphoretickes - ps8
Archaeplastida - ps9
Viridiplantae - ps10
Streptophyta - ps11
Charophyceae/Embriophyta - ps12
Embriophyta - ps13
Tracheophyta - ps14
Spermatophyta - ps15
Angiosperms - ps16
Mesangiospermae - ps17
Magnoliids/eudicots - ps18
Eudicots - ps19
Core_eudicots - ps20
Superrosids - ps21
Rosids - ps22
Eurosids - ps23
Malvids - ps24
Sap./Malv./Brassicales - ps25
Malvales/Brassicales - ps26
Brassicales - ps27
Cleomaceae/Brassicaceae - ps28
Brassicaceae - ps29
Camelineae - ps30
Arabidopsis - ps31
Arabidopsis_thaliana - ps32
Phylostratum

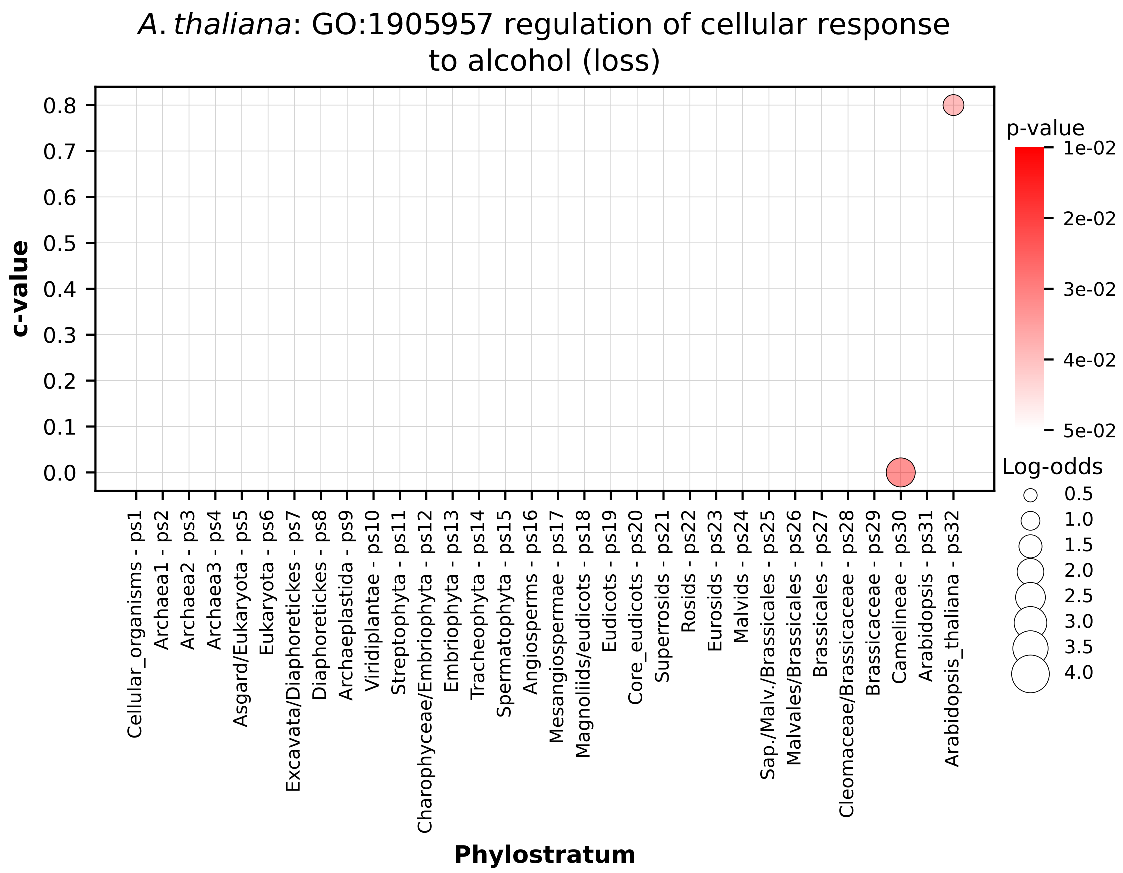

A. thaliana: GO:1905957 regulation of cellular response to alcohol (loss)
c-value
0.8
0.7
0.6
0.5
0.4
0.3
0.2
0.1
0.0
p-value
1e-02
2e-02
3e-02
4e-02
5e-02
Log-odds
0.5
1.0
1.5
2.0
2.5
3.0
3.5
4.0
Cellular_organisms - ps1
Archaea1 - ps2
Archaea2 - ps3
Archaea3 - ps4
Asgard/Eukaryota - ps5
Eukaryota - ps6
Excavata/Diaphoretickes - ps7
Diaphoretickes - ps8
Archaeplastida - ps9
Viridiplantae - ps10
Streptophyta - ps11
Charophyceae/Embriophyta - ps12
Embriophyta - ps13
Tracheophyta - ps14
Spermatophyta - ps15
Angiosperms - ps16
Mesangiospermae - ps17
Magnoliids/eudicots - ps18
Eudicots - ps19
Core_eudicots - ps20
Superrosids - ps21
Rosids - ps22
Eurosids - ps23
Malvids - ps24
Sap./Malv./Brassicales - ps25
Malvales/Brassicales - ps26
Brassicales - ps27
Cleomaceae/Brassicaceae - ps28
Brassicaceae - ps29
Camelineae - ps30
Arabidopsis - ps31
Arabidopsis_thaliana - ps32
Phylostratum

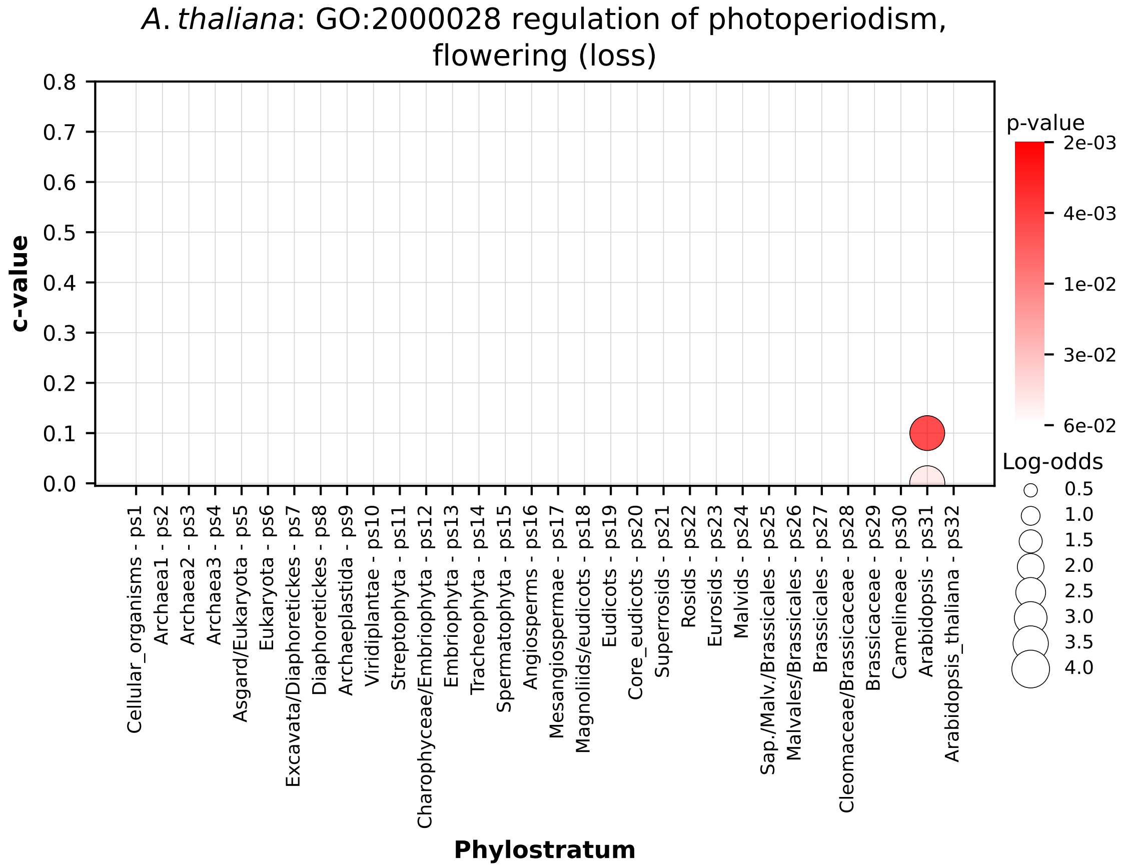

A. thaliana: GO:2000028 regulation of photoperiodism, flowering (loss)
c-value
0.8
0.7
0.6
0.5
0.4
0.3
0.2
0.1
0.0
Cellular_organisms - ps1
Archaea1 - ps2
Archaea2 - ps3
Archaea3 - ps4
Asgard/Eukaryota - ps5
Eukaryota - ps6
Excavata/Diaphoretickes - ps7
Diaphoretickes - ps8
Archaeplastida - ps9
Viridiplantae - ps10
Streptophyta - ps11
Charophyceae/Embriophyta - ps12
Embriophyta - ps13
Tracheophyta - ps14
Spermatophyta - ps15
Angiosperms - ps16
Mesangiospermae - ps17
Magnoliids/eudicots - ps18
Eudicots - ps19
Core_eudicots - ps20
Superrosids - ps21
Rosids - ps22
Eurosids - ps23
Malvids - ps24
Sap./Malv./Brassicales - ps25
Malvales/Brassicales - ps26
Brassicales - ps27
Cleomaceae/Brassicaceae - ps28
Brassicaceae - ps29
Camelineae - ps30
Arabidopsis - ps31
Arabidopsis_thaliana - ps32
Phylostratum
p-value
2e-03
4e-03
1e-02
3e-02
6e-02
Log-odds
0.5
1.0
1.5
2.0
2.5
3.0
3.5
4.0

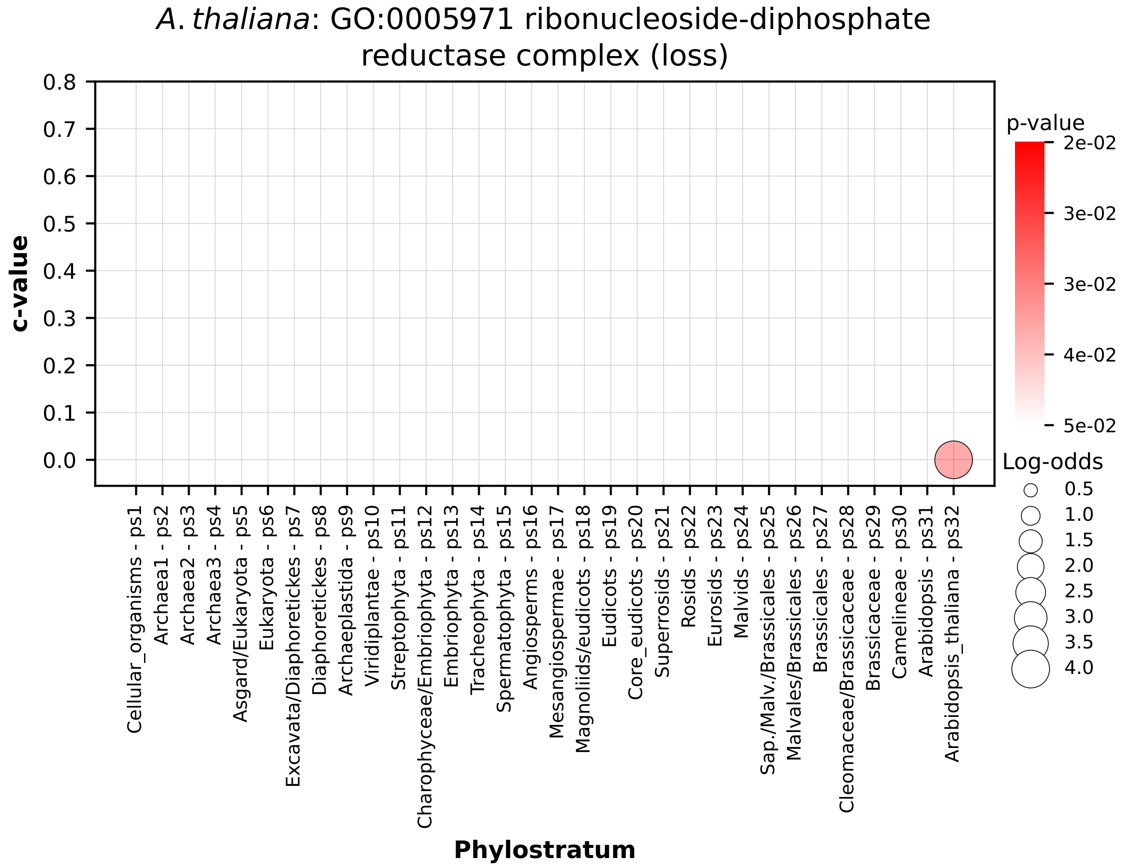

A. thaliana: GO:0005971 ribonucleoside-diphosphate reductase complex (loss)
c-value
0.8
0.7
0.6
0.5
0.4
0.3
0.2
0.1
0.0
Cellular_organisms - ps1
Archaea1 - ps2
Archaea2 - ps3
Archaea3 - ps4
Asgard/Eukaryota - ps5
Eukaryota - ps6
Excavata/Diaphoretickes - ps7
Diaphoretickes - ps8
Archaeplastida - ps9
Viridiplantae - ps10
Streptophyta - ps11
Charophyceae/Embriophyta - ps12
Embriophyta - ps13
Tracheophyta - ps14
Spermatophyta - ps15
Angiosperms - ps16
Mesangiospermae - ps17
Magnoliids/eudicots - ps18
Eudicots - ps19
Core_eudicots - ps20
Superrosids - ps21
Rosids - ps22
Eurosids - ps23
Malvids - ps24
Sap./Malv./Brassicales - ps25
Malvales/Brassicales - ps26
Brassicales - ps27
Cleomaceae/Brassicaceae - ps28
Brassicaceae - ps29
Camelineae - ps30
Arabidopsis - ps31
Arabidopsis_thaliana - ps32
Phylostratum
p-value
2e-02
3e-02
3e-02
4e-02
5e-02
Log-odds
0.5
1.0
1.5
2.0
2.5
3.0
3.5
4.0

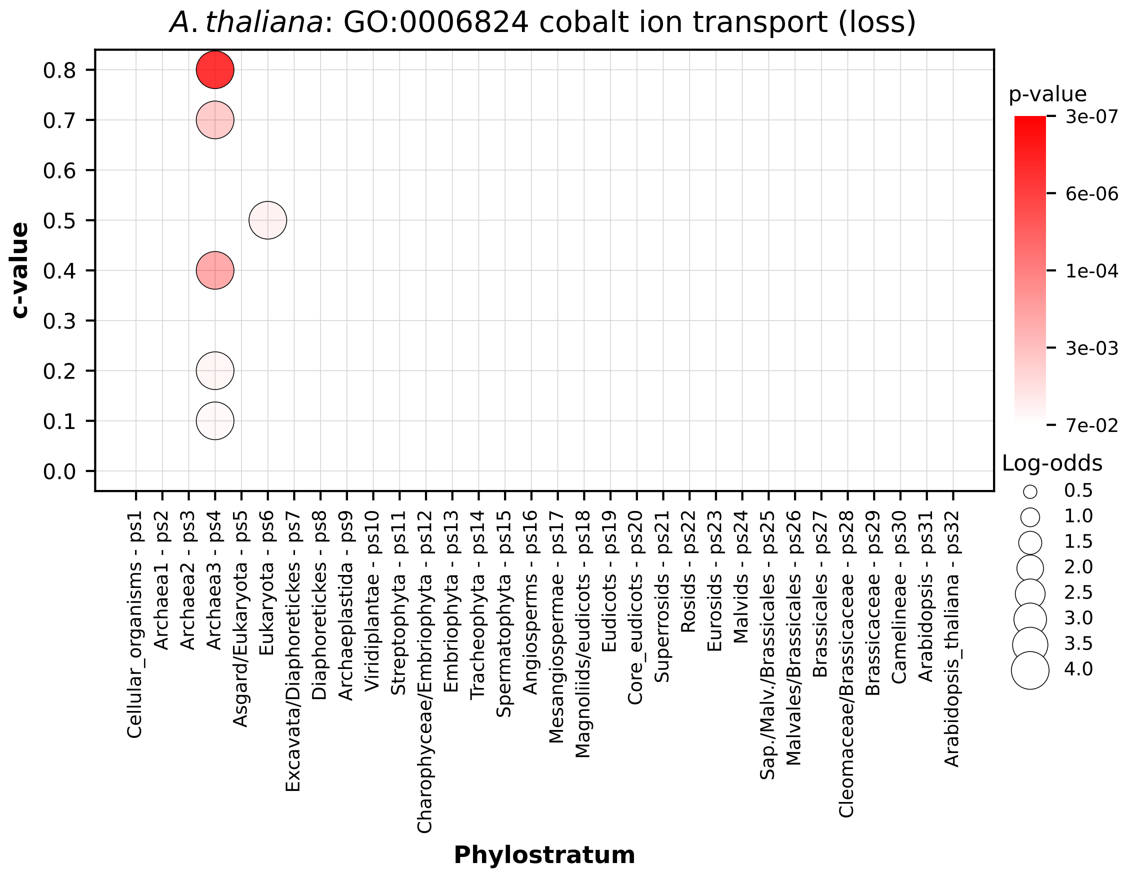

A. thaliana: GO:0006824 cobalt ion transport (loss)
c-value
0.0
0.1
0.2
0.3
0.4
0.5
0.6
0.7
0.8
p-value
3e-07
6e-06
1e-04
3e-03
7e-02
Log-odds
0.5
1.0
1.5
2.0
2.5
3.0
3.5
4.0
Cellular_organisms - ps1
Archaea1 - ps2
Archaea2 - ps3
Archaea3 - ps4
Asgard/Eukaryota - ps5
Eukaryota - ps6
Excavata/Diaphoretickes - ps7
Diaphoretickes - ps8
Archaeplastida - ps9
Viridiplantae - ps10
Streptophyta - ps11
Charophyceae/Embriophyta - ps12
Embriophyta - ps13
Tracheophyta - ps14
Spermatophyta - ps15
Angiosperms - ps16
Mesangiospermae - ps17
Magnoliids/eudicots - ps18
Eudicots - ps19
Core_eudicots - ps20
Superrosids - ps21
Rosids - ps22
Eurosids - ps23
Malvids - ps24
Sap./Malv./Brassicales - ps25
Malvales/Brassicales - ps26
Brassicales - ps27
Cleomaceae/Brassicaceae - ps28
Brassicaceae - ps29
Camelineae - ps30
Arabidopsis - ps31
Arabidopsis_thaliana - ps32
Phylostratum

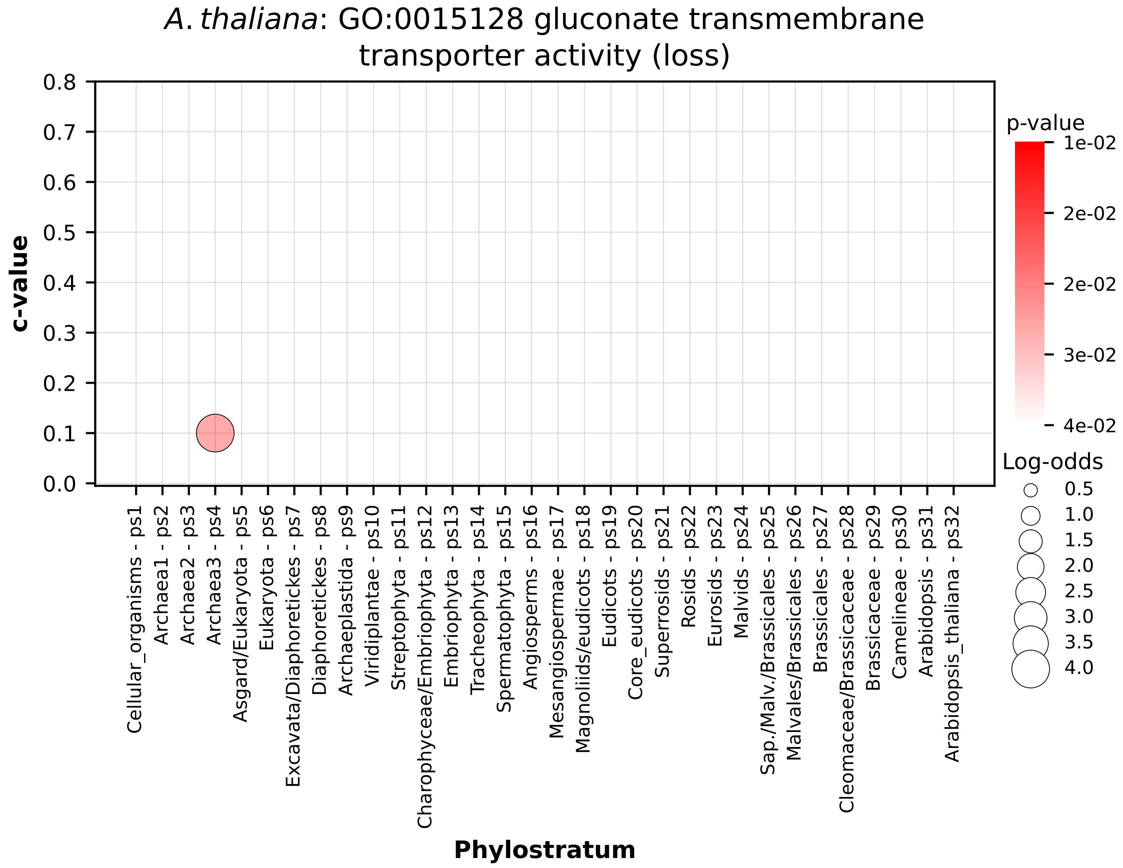

A. thaliana: GO:0015128 gluconate transmembrane transporter activity (loss)
c-value
0.0
0.1
0.2
0.3
0.4
0.5
0.6
0.7
0.8
Phylostratum
Cellular_organisms - ps1
Archaea1 - ps2
Archaea2 - ps3
Archaea3 - ps4
Asgard/Eukaryota - ps5
Eukaryota - ps6
Excavata/Diaphoretickes - ps7
Diaphoretickes - ps8
Archaeplastida - ps9
Viridiplantae - ps10
Streptophyta - ps11
Charophyceae/Embriophyta - ps12
Embriophyta - ps13
Tracheophyta - ps14
Spermatophyta - ps15
Angiosperms - ps16
Mesangiospermae - ps17
Magnoliids/eudicots - ps18
Eudicots - ps19
Core_eudicots - ps20
Superrosids - ps21
Rosids - ps22
Eurosids - ps23
Malvids - ps24
Sap./Malv./Brassicales - ps25
Malvales/Brassicales - ps26
Brassicales - ps27
Cleomaceae/Brassicaceae - ps28
Brassicaceae - ps29
Camelineae - ps30
Arabidopsis - ps31
Arabidopsis_thaliana - ps32
p-value
1e-02
2e-02
2e-02
3e-02
4e-02
Log-odds
0.5
1.0
1.5
2.0
2.5
3.0
3.5
4.0

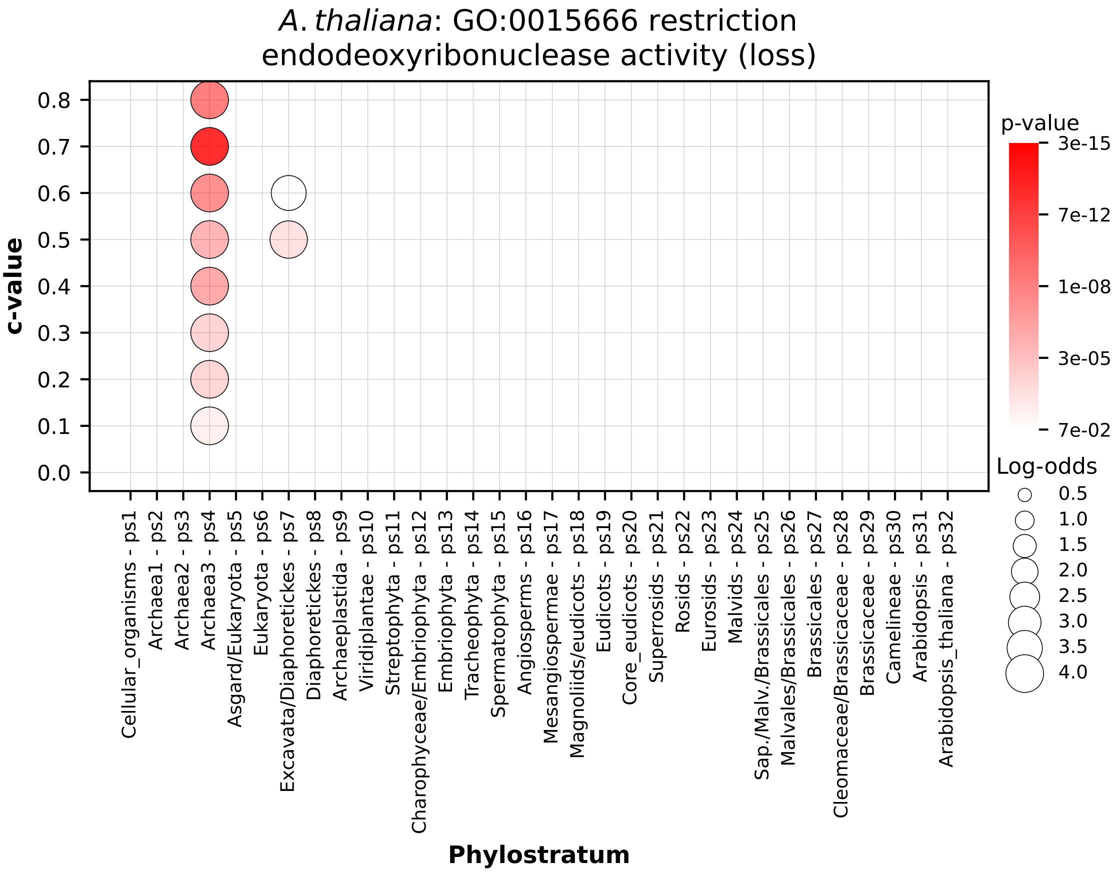
A. thaliana: GO:0015666 restriction endodeoxyribonuclease activity (loss)
c-value
0.0
0.1
0.2
0.3
0.4
0.5
0.6
0.7
0.8
Phylostratum
Cellular_organisms - ps1
Archaea1 - ps2
Archaea2 - ps3
Archaea3 - ps4
Asgard/Eukaryota - ps5
Eukaryota - ps6
Excavata/Diaphoretickes - ps7
Diaphoretickes - ps8
Archaeplastida - ps9
Viridiplantae - ps10
Streptophyta - ps11
Charophyceae/Embriophyta - ps12
Embriophyta - ps13
Tracheophyta - ps14
Spermatophyta - ps15
Angiosperms - ps16
Mesangiospermae - ps17
Magnoliids/eudicots - ps18
Eudicots - ps19
Core_eudicots - ps20
Superrosids - ps21
Rosids - ps22
Eurosids - ps23
Malvids - ps24
Sap./Malv./Brassicales - ps25
Malvales/Brassicales - ps26
Brassicales - ps27
Cleomaceae/Brassicaceae - ps28
Brassicaceae - ps29
Camelineae - ps30
Arabidopsis - ps31
Arabidopsis_thaliana - ps32
p-value
3e-15
7e-12
1e-08
3e-05
7e-02
Log-odds
0.5
1.0
1.5
2.0
2.5
3.0
3.5
4.0

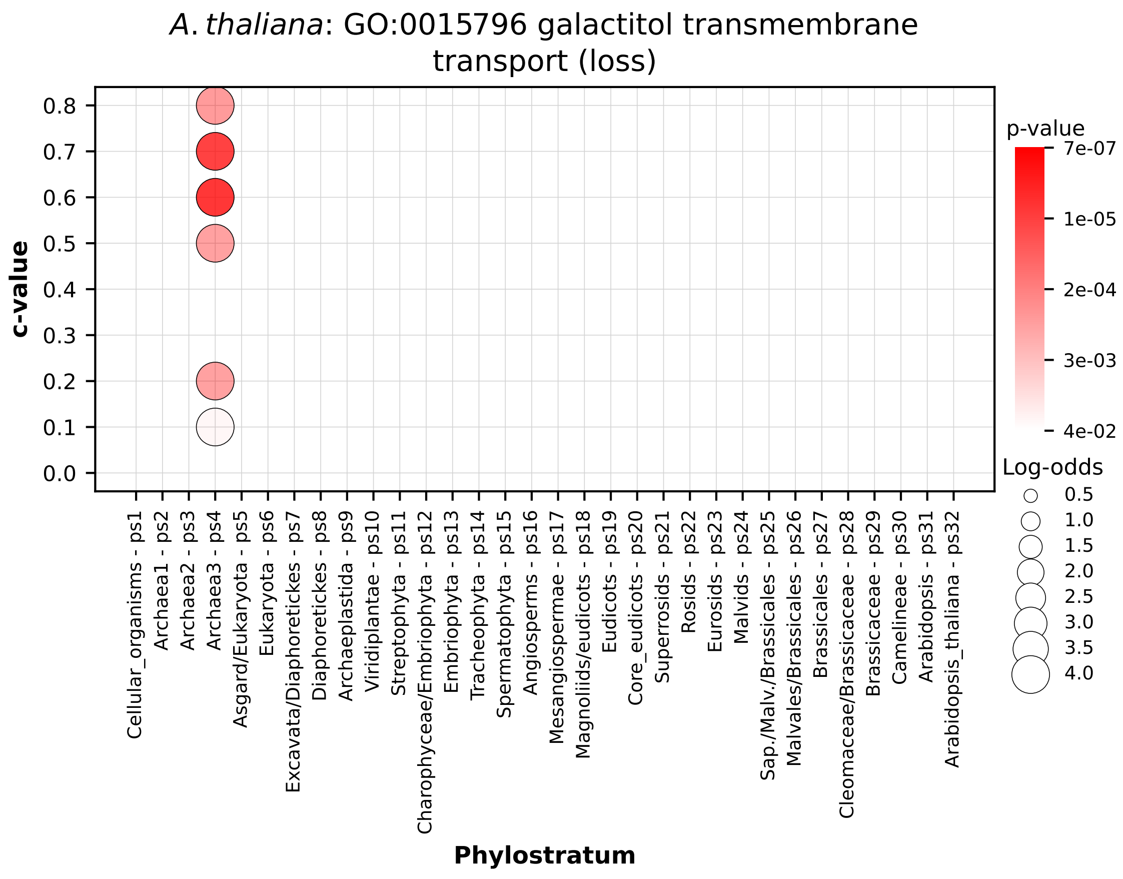

A. thaliana: GO:0015796 galactitol transmembrane transport (loss)
c-value
0.0
0.1
0.2
0.3
0.4
0.5
0.6
0.7
0.8
Phylostratum
Cellular_organisms - ps1
Archaea1 - ps2
Archaea2 - ps3
Archaea3 - ps4
Asgard/Eukaryota - ps5
Eukaryota - ps6
Excavata/Diaphoretickes - ps7
Diaphoretickes - ps8
Archaeplastida - ps9
Viridiplantae - ps10
Streptophyta - ps11
Charophyceae/Embriophyta - ps12
Embriophyta - ps13
Tracheophyta - ps14
Spermatophyta - ps15
Angiosperms - ps16
Mesangiospermae - ps17
Magnoliids/eudicots - ps18
Eudicots - ps19
Core_eudicots - ps20
Superrosids - ps21
Rosids - ps22
Eurosids - ps23
Malvids - ps24
Sap./Malv./Brassicales - ps25
Malvales/Brassicales - ps26
Brassicales - ps27
Cleomaceae/Brassicaceae - ps28
Brassicaceae - ps29
Camelineae - ps30
Arabidopsis - ps31
Arabidopsis_thaliana - ps32
p-value
7e-07
1e-05
2e-04
3e-03
4e-02
Log-odds
0.5
1.0
1.5
2.0
2.5
3.0
3.5
4.0

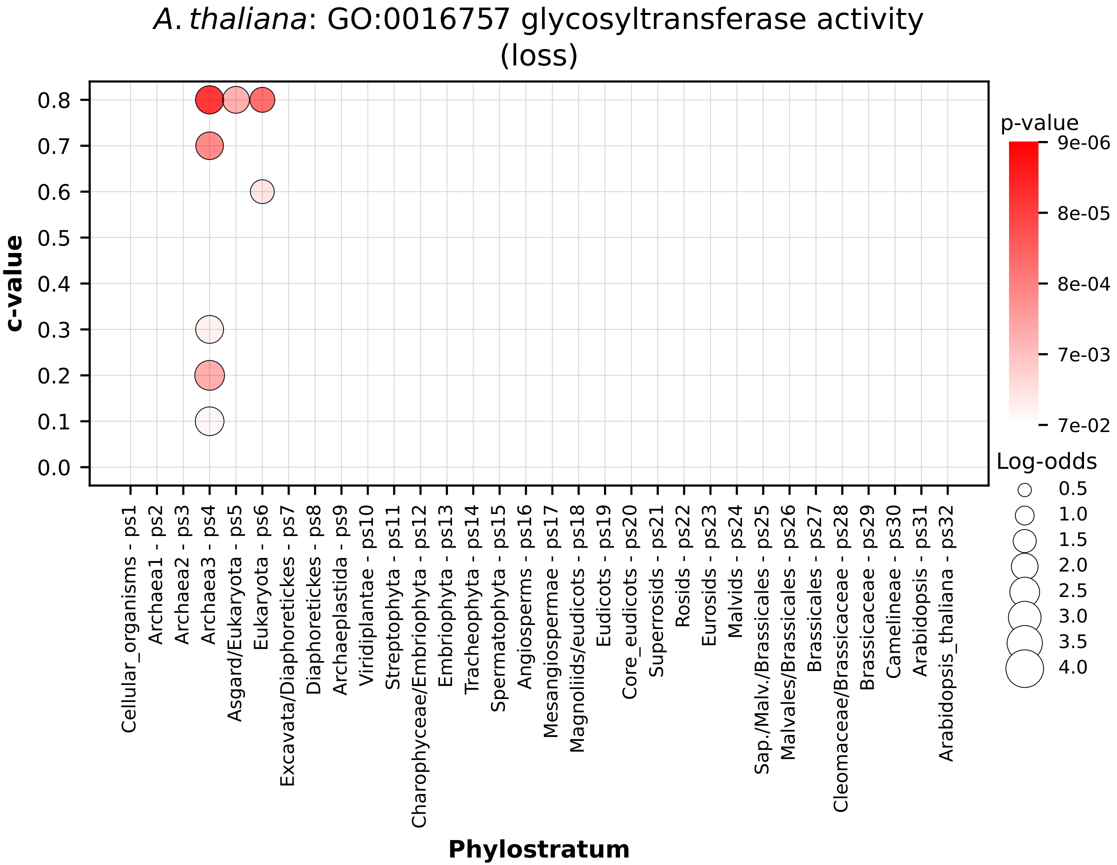

A. thaliana: GO:0016757 glycosyltransferase activity
(loss)
c-value
0.8
0.7
0.6
0.5
0.4
0.3
0.2
0.1
0.0
p-value
9e-06
8e-05
8e-04
7e-03
7e-02
Log-odds
0.5
1.0
1.5
2.0
2.5
3.0
3.5
4.0
Cellular_organisms - ps1
Archaea1 - ps2
Archaea2 - ps3
Archaea3 - ps4
Asgard/Eukaryota - ps5
Eukaryota - ps6
Excavata/Diaphoretickes - ps7
Diaphoretickes - ps8
Archaeplastida - ps9
Viridiplantae - ps10
Streptophyta - ps11
Charophyceae/Embriophyta - ps12
Embriophyta - ps13
Tracheophyta - ps14
Spermatophyta - ps15
Angiosperms - ps16
Mesangiospermae - ps17
Magnoliids/eudicots - ps18
Eudicots - ps19
Core_eudicots - ps20
Superrosids - ps21
Rosids - ps22
Eurosids - ps23
Malvids - ps24
Sap./Malv./Brassicales - ps25
Malvales/Brassicales - ps26
Brassicales - ps27
Cleomaceae/Brassicaceae - ps28
Brassicaceae - ps29
Camelineae - ps30
Arabidopsis - ps31
Arabidopsis_thaliana - ps32
Phylostratum

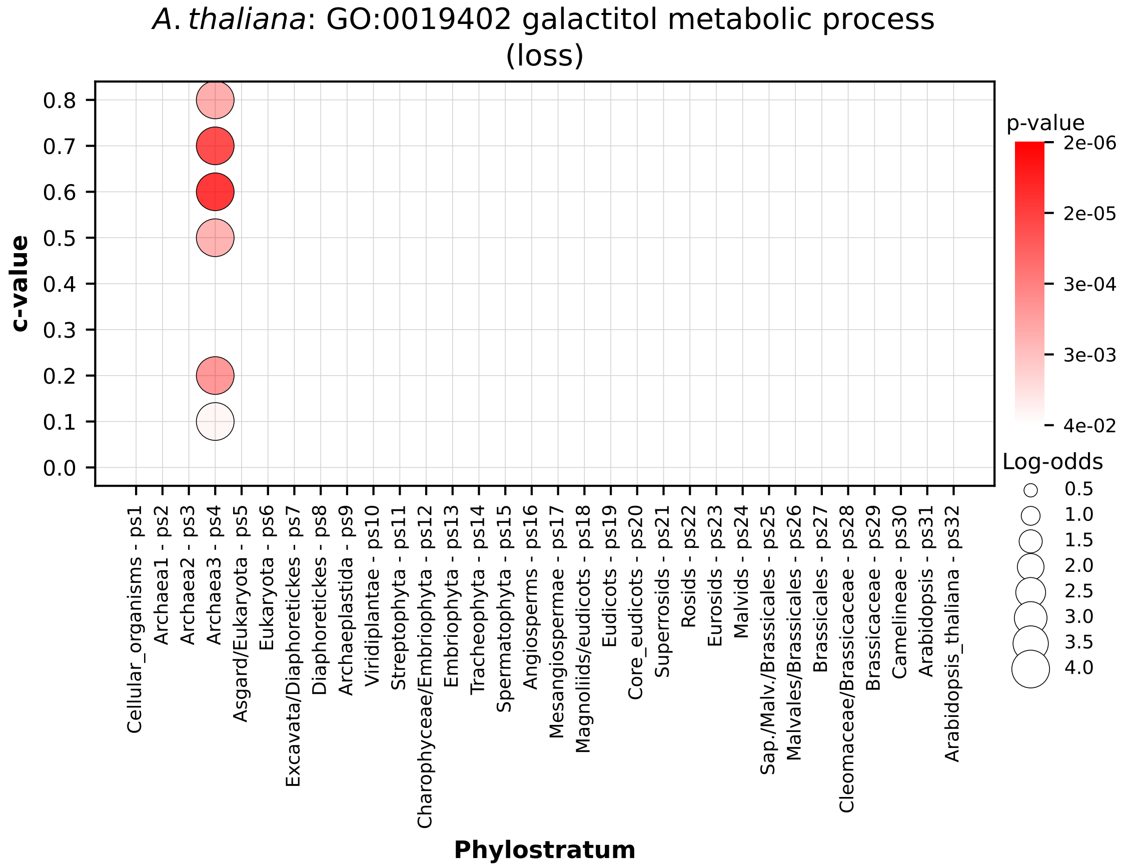

A. thaliana: GO:0019402 galactitol metabolic process
(loss)
c-value
0.8
0.7
0.6
0.5
0.4
0.3
0.2
0.1
0.0
p-value
2e-06
2e-05
3e-04
3e-03
4e-02
Log-odds
0.5
1.0
1.5
2.0
2.5
3.0
3.5
4.0
Cellular_organisms - ps1
Archaea1 - ps2
Archaea2 - ps3
Archaea3 - ps4
Asgard/Eukaryota - ps5
Eukaryota - ps6
Excavata/Diaphoretickes - ps7
Diaphoretickes - ps8
Archaeplastida - ps9
Viridiplantae - ps10
Streptophyta - ps11
Charophyceae/Embriophyta - ps12
Embriophyta - ps13
Tracheophyta - ps14
Spermatophyta - ps15
Angiosperms - ps16
Mesangiospermae - ps17
Magnoliids/eudicots - ps18
Eudicots - ps19
Core_eudicots - ps20
Superrosids - ps21
Rosids - ps22
Eurosids - ps23
Malvids - ps24
Sap./Malv./Brassicales - ps25
Malvales/Brassicales - ps26
Brassicales - ps27
Cleomaceae/Brassicaceae - ps28
Brassicaceae - ps29
Camelineae - ps30
Arabidopsis - ps31
Arabidopsis_thaliana - ps32
Phylostratum

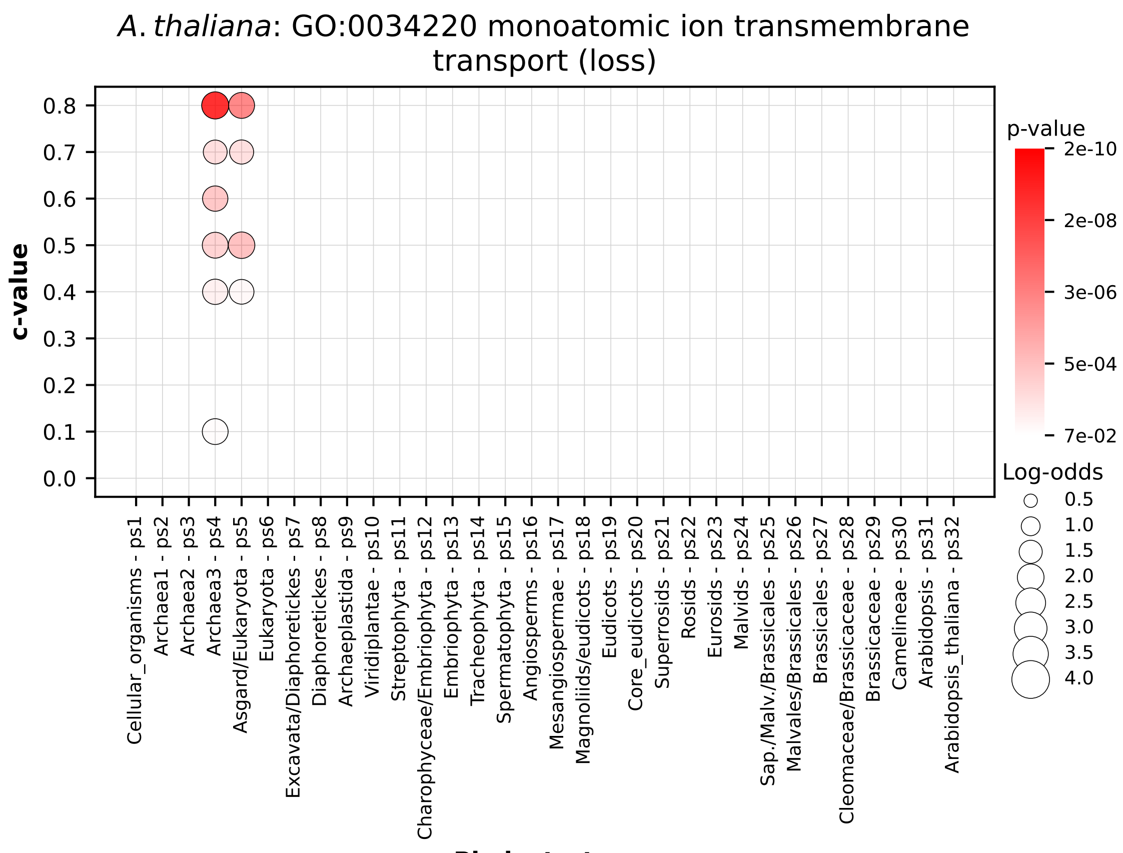
A. thaliana: GO:0034220 monoatomic ion transmembrane transport (loss)
c-value
0.8
0.7
0.6
0.5
0.4
0.3
0.2
0.1
0.0
p-value
2e-10
2e-08
3e-06
5e-04
7e-02
Log-odds
0.5
1.0
1.5
2.0
2.5
3.0
3.5
4.0
Cellular_organisms - ps1
Archaea1 - ps2
Archaea2 - ps3
Archaea3 - ps4
Asgard/Eukaryota - ps5
Eukaryota - ps6
Excavata/Diaphoretickes - ps7
Diaphoretickes - ps8
Archaeplastida - ps9
Viridiplantae - ps10
Streptophyta - ps11
Charophyceae/Embriophyta - ps12
Embriophyta - ps13
Tracheophyta - ps14
Spermatophyta - ps15
Angiosperms - ps16
Mesangiospermae - ps17
Magnoliids/eudicots - ps18
Eudicots - ps19
Core_eudicots - ps20
Superrosids - ps21
Rosids - ps22
Eurosids - ps23
Malvids - ps24
Sap./Malv./Brassicales - ps25
Malvales/Brassicales - ps26
Brassicales - ps27
Cleomaceae/Brassicaceae - ps28
Brassicaceae - ps29
Camelineae - ps30
Arabidopsis - ps31
Arabidopsis_thaliana - ps32
Phylostratum

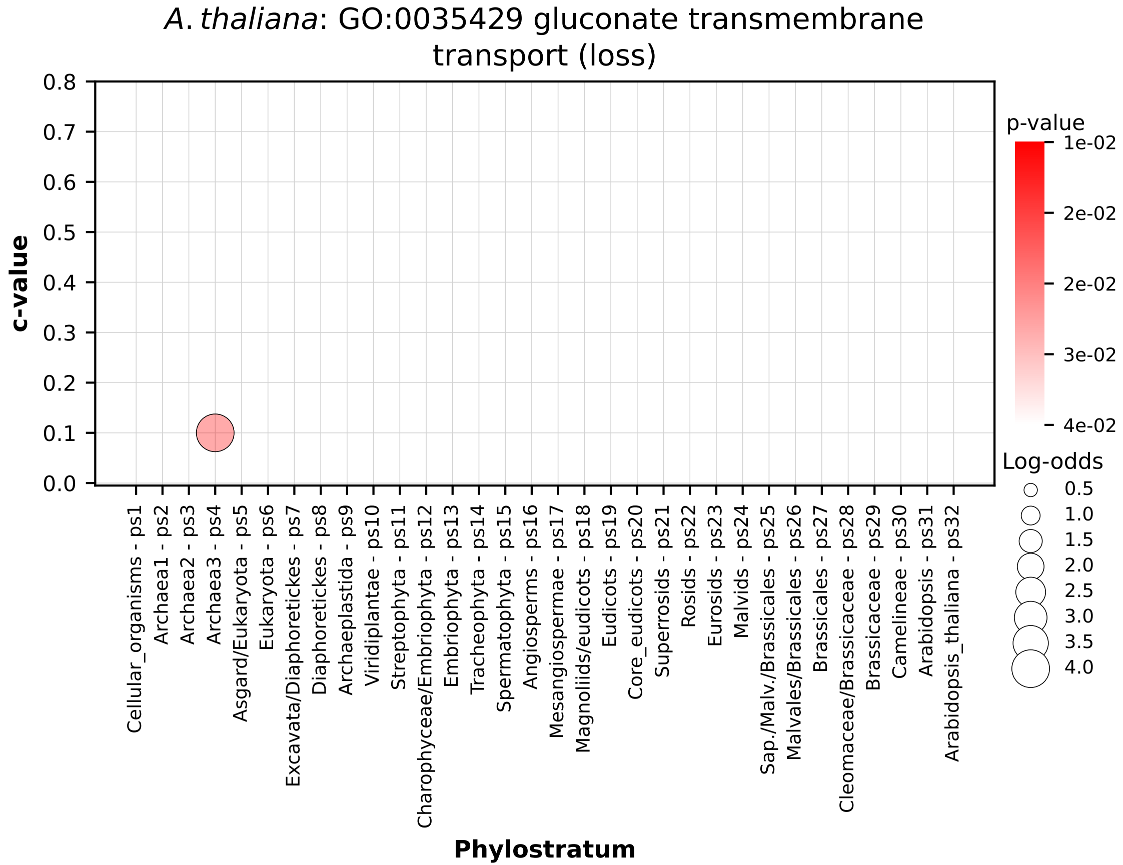
A. thaliana: GO:0035429 gluconate transmembrane transport (loss)
c-value
0.0
0.1
0.2
0.3
0.4
0.5
0.6
0.7
0.8
Phylostratum
Cellular_organisms - ps1
Archaea1 - ps2
Archaea2 - ps3
Archaea3 - ps4
Asgard/Eukaryota - ps5
Eukaryota - ps6
Excavata/Diaphoretickes - ps7
Diaphoretickes - ps8
Archaeplastida - ps9
Viridiplantae - ps10
Streptophyta - ps11
Charophyceae/Embriophyta - ps12
Embriophyta - ps13
Tracheophyta - ps14
Spermatophyta - ps15
Angiosperms - ps16
Mesangiospermae - ps17
Magnoliids/eudicots - ps18
Eudicots - ps19
Core_eudicots - ps20
Superrosids - ps21
Rosids - ps22
Eurosids - ps23
Malvids - ps24
Sap./Malv./Brassicales - ps25
Malvales/Brassicales - ps26
Brassicales - ps27
Cleomaceae/Brassicaceae - ps28
Brassicaceae - ps29
Camelineae - ps30
Arabidopsis - ps31
Arabidopsis_thaliana - ps32
p-value
1e-02
2e-02
2e-02
3e-02
4e-02
Log-odds
0.5
1.0
1.5
2.0
2.5
3.0
3.5
4.0

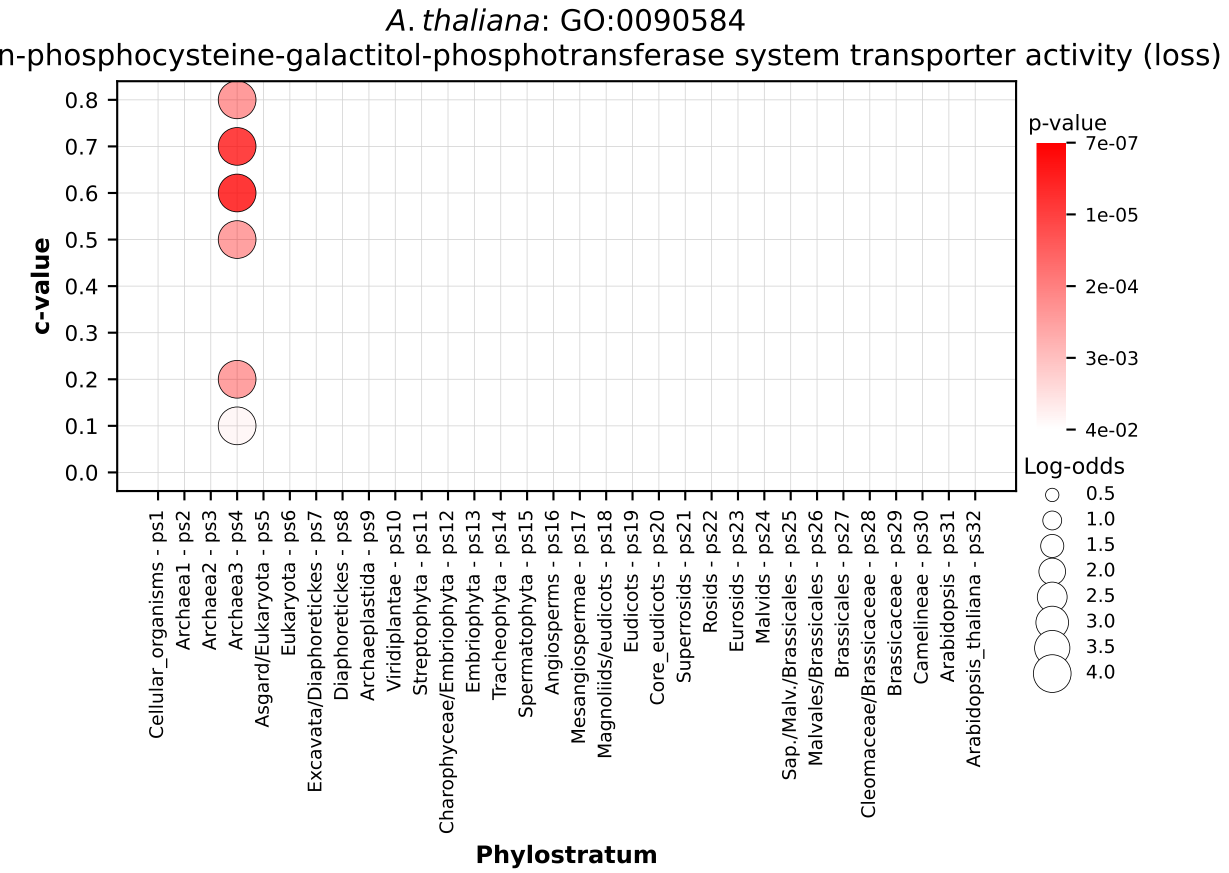

A. thaliana: GO:0090584
n-phosphocysteine-galactitol-phosphotransferase system transporter activity (loss)
c-value
0.8
0.7
0.6
0.5
0.4
0.3
0.2
0.1
0.0
p-value
7e-07
1e-05
2e-04
3e-03
4e-02
Log-odds
0.5
1.0
1.5
2.0
2.5
3.0
3.5
4.0
Cellular_organisms - ps1
Archaea1 - ps2
Archaea2 - ps3
Archaea3 - ps4
Asgard/Eukaryota - ps5
Eukaryota - ps6
Excavata/Diaphoretickes - ps7
Diaphoretickes - ps8
Archaeplastida - ps9
Viridiplantae - ps10
Streptophyta - ps11
Charophyceae/Embriophyta - ps12
Embriophyta - ps13
Tracheophyta - ps14
Spermatophyta - ps15
Angiosperms - ps16
Mesangiospermae - ps17
Magnoliids/eudicots - ps18
Eudicots - ps19
Core_eudicots - ps20
Superrosids - ps21
Rosids - ps22
Eurosids - ps23
Malvids - ps24
Sap./Malv./Brassicales - ps25
Malvales/Brassicales - ps26
Brassicales - ps27
Cleomaceae/Brassicaceae - ps28
Brassicaceae - ps29
Camelineae - ps30
Arabidopsis - ps31
Arabidopsis_thaliana - ps32
Phylostratum

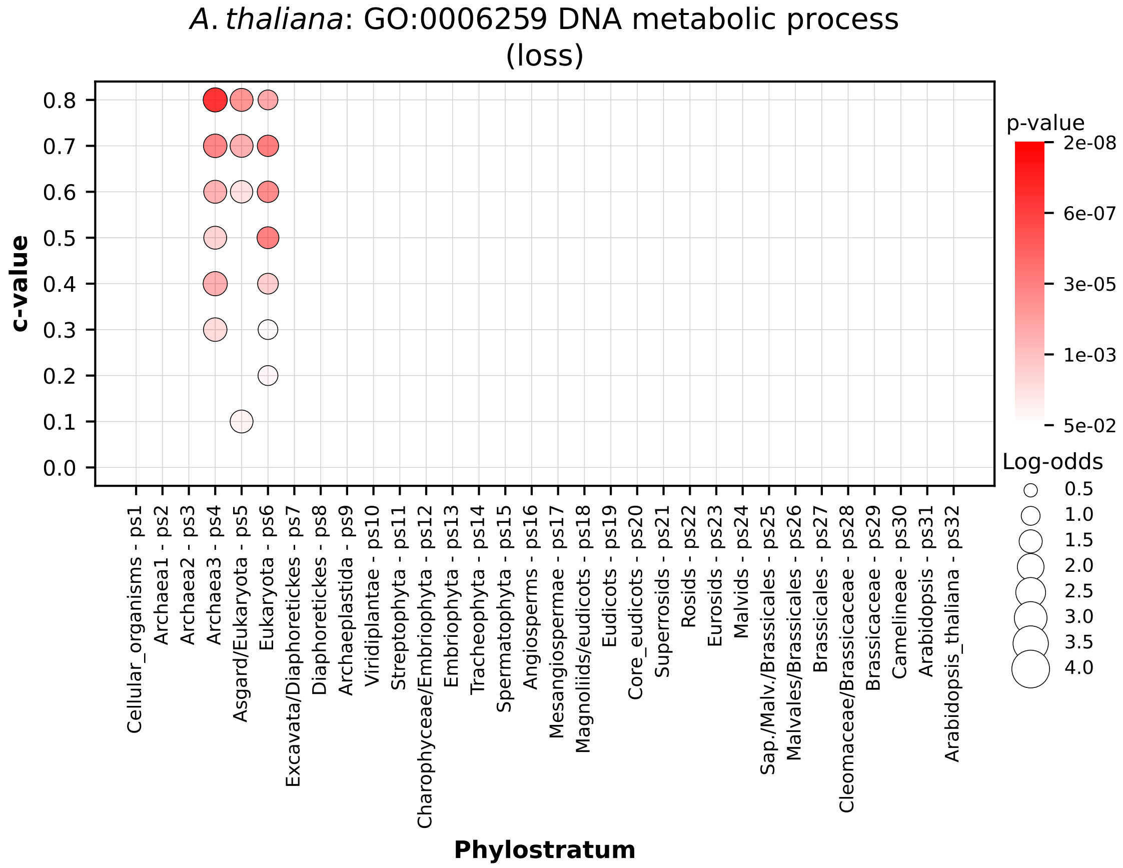

A. thaliana: GO:0006259 DNA metabolic process
(loss)
c-value
0.8
0.7
0.6
0.5
0.4
0.3
0.2
0.1
0.0
Phylostratum
Cellular_organisms - ps1
Archaea1 - ps2
Archaea2 - ps3
Archaea3 - ps4
Asgard/Eukaryota - ps5
Eukaryota - ps6
Excavata/Diaphoretickes - ps7
Diaphoretickes - ps8
Archaeplastida - ps9
Viridiplantae - ps10
Streptophyta - ps11
Charophyceae/Embriophyta - ps12
Embriophyta - ps13
Tracheophyta - ps14
Spermatophyta - ps15
Angiosperms - ps16
Mesangiospermae - ps17
Magnoliids/eudicots - ps18
Eudicots - ps19
Core_eudicots - ps20
Superrosids - ps21
Rosids - ps22
Eurosids - ps23
Malvids - ps24
Sap./Malv./Brassicales - ps25
Malvales/Brassicales - ps26
Brassicales - ps27
Cleomaceae/Brassicaceae - ps28
Brassicaceae - ps29
Camelineae - ps30
Arabidopsis - ps31
Arabidopsis_thaliana - ps32
p-value
2e-08
6e-07
3e-05
1e-03
5e-02
Log-odds
0.5
1.0
1.5
2.0
2.5
3.0
3.5
4.0

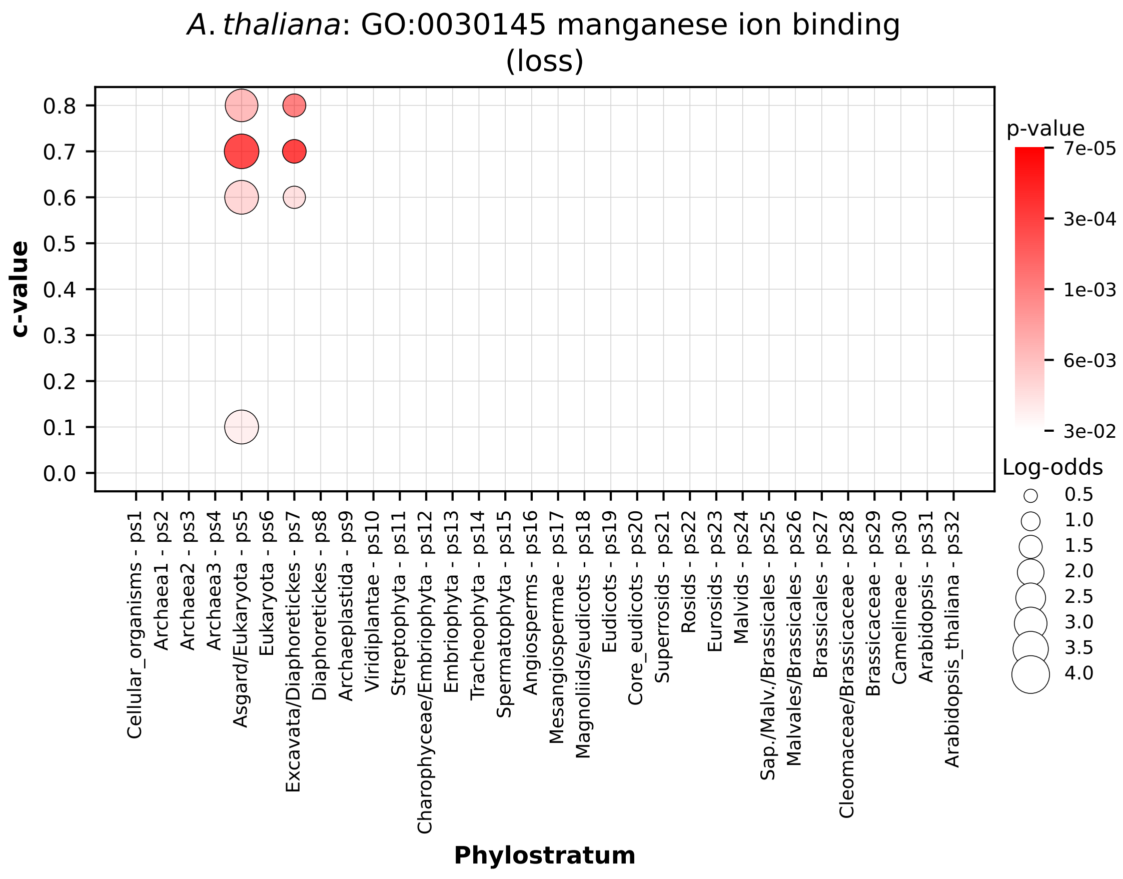

A. thaliana: GO:0030145 manganese ion binding
(loss)
c-value
0.8
0.7
0.6
0.5
0.4
0.3
0.2
0.1
0.0
Phylostratum
Cellular_organisms - ps1
Archaea1 - ps2
Archaea2 - ps3
Archaea3 - ps4
Asgard/Eukaryota - ps5
Eukaryota - ps6
Excavata/Diaphoretickes - ps7
Diaphoretickes - ps8
Archaeplastida - ps9
Viridiplantae - ps10
Streptophyta - ps11
Charophyceae/Embriophyta - ps12
Embriophyta - ps13
Tracheophyta - ps14
Spermatophyta - ps15
Angiosperms - ps16
Mesangiospermae - ps17
Magnoliids/eudicots - ps18
Eudicots - ps19
Core_eudicots - ps20
Superrosids - ps21
Rosids - ps22
Eurosids - ps23
Malvids - ps24
Sap./Malv./Brassicales - ps25
Malvales/Brassicales - ps26
Brassicales - ps27
Cleomaceae/Brassicaceae - ps28
Brassicaceae - ps29
Camelineae - ps30
Arabidopsis - ps31
Arabidopsis_thaliana - ps32
p-value
7e-05
3e-04
1e-03
6e-03
3e-02
Log-odds
0.5
1.0
1.5
2.0
2.5
3.0
3.5
4.0

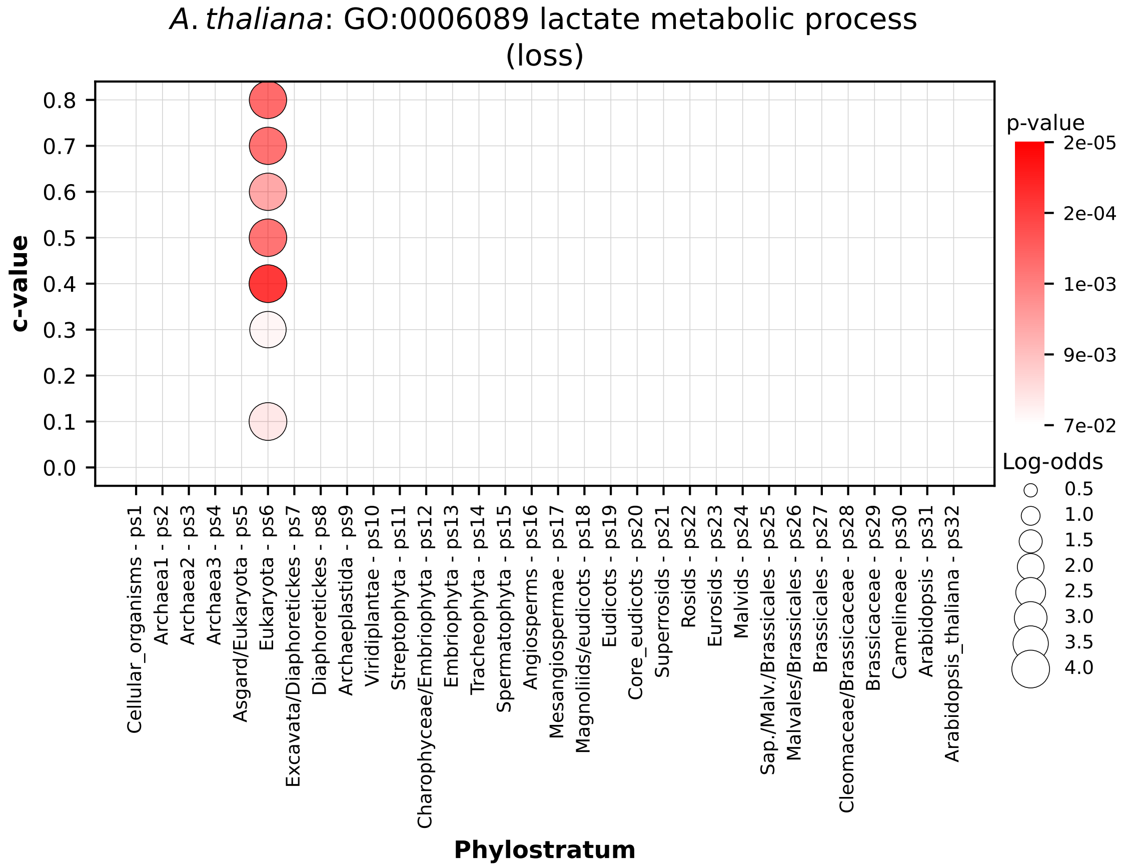

A. thaliana: GO:0006089 lactate metabolic process
(loss)
c-value
0.0
0.1
0.2
0.3
0.4
0.5
0.6
0.7
0.8
Phylostratum
Cellular_organisms - ps1
Archaea1 - ps2
Archaea2 - ps3
Archaea3 - ps4
Asgard/Eukaryota - ps5
Eukaryota - ps6
Excavata/Diaphoretickes - ps7
Diaphoretickes - ps8
Archaeplastida - ps9
Viridiplantae - ps10
Streptophyta - ps11
Charophyceae/Embriophyta - ps12
Embriophyta - ps13
Tracheophyta - ps14
Spermatophyta - ps15
Angiosperms - ps16
Mesangiospermae - ps17
Magnoliids/eudicots - ps18
Eudicots - ps19
Core_eudicots - ps20
Superrosids - ps21
Rosids - ps22
Eurosids - ps23
Malvids - ps24
Sap./Malv./Brassicales - ps25
Malvales/Brassicales - ps26
Brassicales - ps27
Cleomaceae/Brassicaceae - ps28
Brassicaceae - ps29
Camelineae - ps30
Arabidopsis - ps31
Arabidopsis_thaliana - ps32
p-value
2e-05
2e-04
1e-03
9e-03
7e-02
Log-odds
0.5
1.0
1.5
2.0
2.5
3.0
3.5
4.0

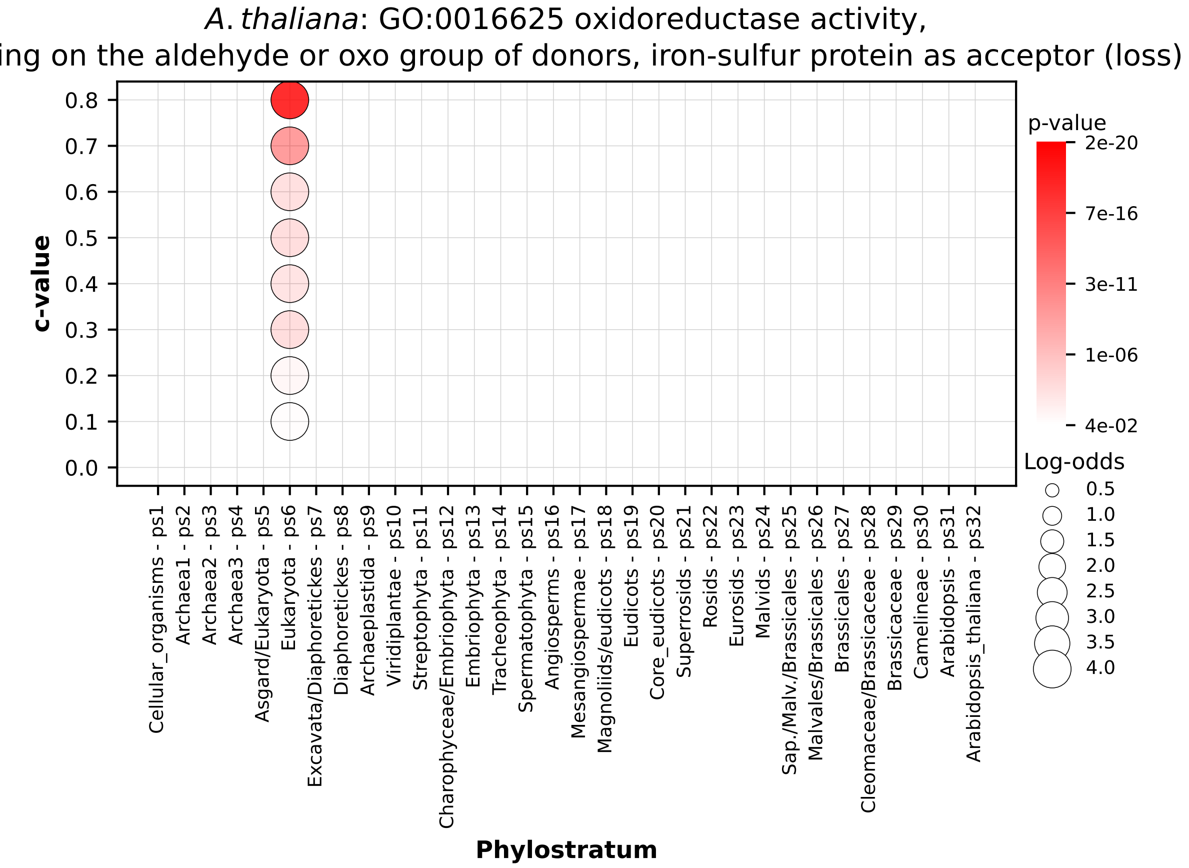

A. thaliana: GO:0016625 oxidoreductase activity,
ing on the aldehyde or oxo group of donors, iron-sulfur protein as acceptor (loss)
c-value
0.0
0.1
0.2
0.3
0.4
0.5
0.6
0.7
0.8
p-value
2e-20
7e-16
3e-11
1e-06
4e-02
Log-odds
0.5
1.0
1.5
2.0
2.5
3.0
3.5
4.0
Cellular_organisms - ps1
Archaea1 - ps2
Archaea2 - ps3
Archaea3 - ps4
Asgard/Eukaryota - ps5
Eukaryota - ps6
Excavata/Diaphoretickes - ps7
Diaphoretickes - ps8
Archaeplastida - ps9
Viridiplantae - ps10
Streptophyta - ps11
Charophyceae/Embriophyta - ps12
Embriophyta - ps13
Tracheophyta - ps14
Spermatophyta - ps15
Angiosperms - ps16
Mesangiospermae - ps17
Magnoliids/eudicots - ps18
Eudicots - ps19
Core_eudicots - ps20
Superrosids - ps21
Rosids - ps22
Eurosids - ps23
Malvids - ps24
Sap./Malv./Brassicales - ps25
Malvales/Brassicales - ps26
Brassicales - ps27
Cleomaceae/Brassicaceae - ps28
Brassicaceae - ps29
Camelineae - ps30
Arabidopsis - ps31
Arabidopsis_thaliana - ps32
Phylostratum

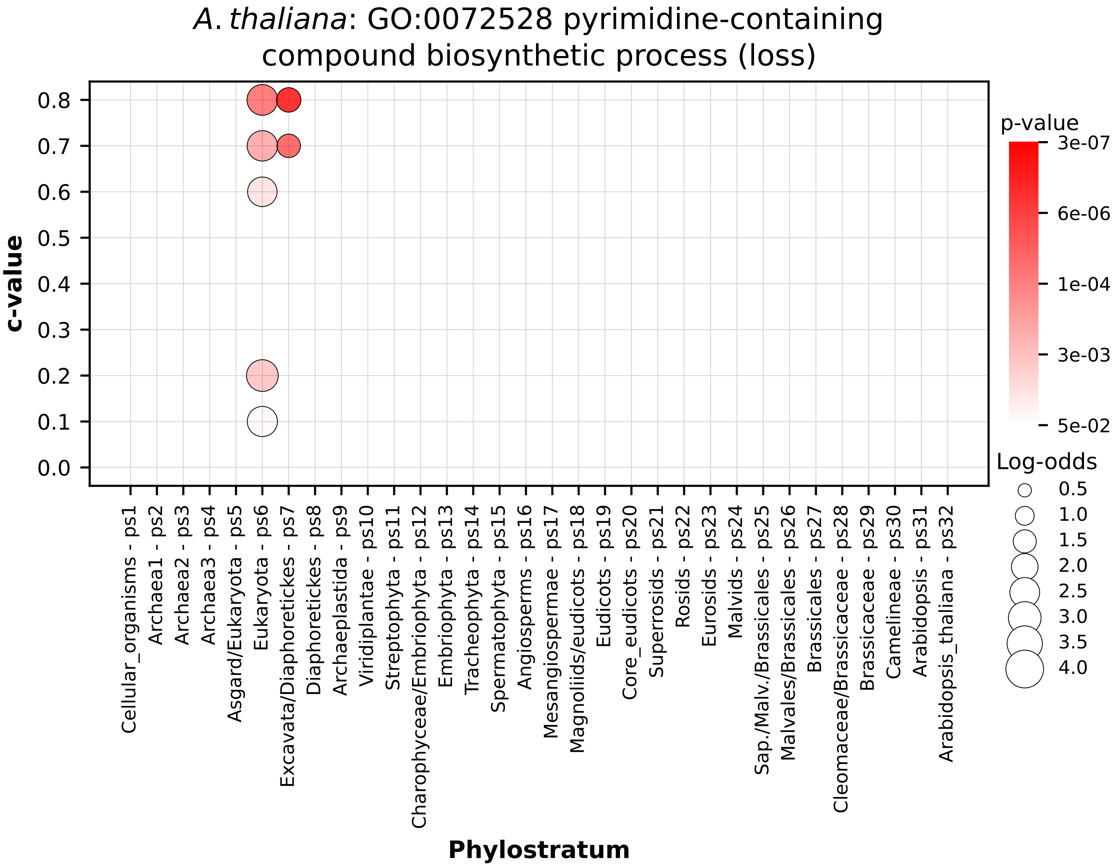
A. thaliana: GO:0072528 pyrimidine-containing compound biosynthetic process (loss)
c-value
0.0
0.1
0.2
0.3
0.4
0.5
0.6
0.7
0.8
Phylostratum
Cellular_organisms - ps1
Archaea1 - ps2
Archaea2 - ps3
Archaea3 - ps4
Asgard/Eukaryota - ps5
Eukaryota - ps6
Excavata/Diaphoretickes - ps7
Diaphoretickes - ps8
Archaeplastida - ps9
Viridiplantae - ps10
Streptophyta - ps11
Charophyceae/Embriophyta - ps12
Embriophyta - ps13
Tracheophyta - ps14
Spermatophyta - ps15
Angiosperms - ps16
Mesangiospermae - ps17
Magnoliids/eudicots - ps18
Eudicots - ps19
Core_eudicots - ps20
Superrosids - ps21
Rosids - ps22
Eurosids - ps23
Malvids - ps24
Sap./Malv./Brassicales - ps25
Malvales/Brassicales - ps26
Brassicales - ps27
Cleomaceae/Brassicaceae - ps28
Brassicaceae - ps29
Camelineae - ps30
Arabidopsis - ps31
Arabidopsis_thaliana - ps32
p-value
3e-07
6e-06
1e-04
3e-03
5e-02
Log-odds
0.5
1.0
1.5
2.0
2.5
3.0
3.5
4.0

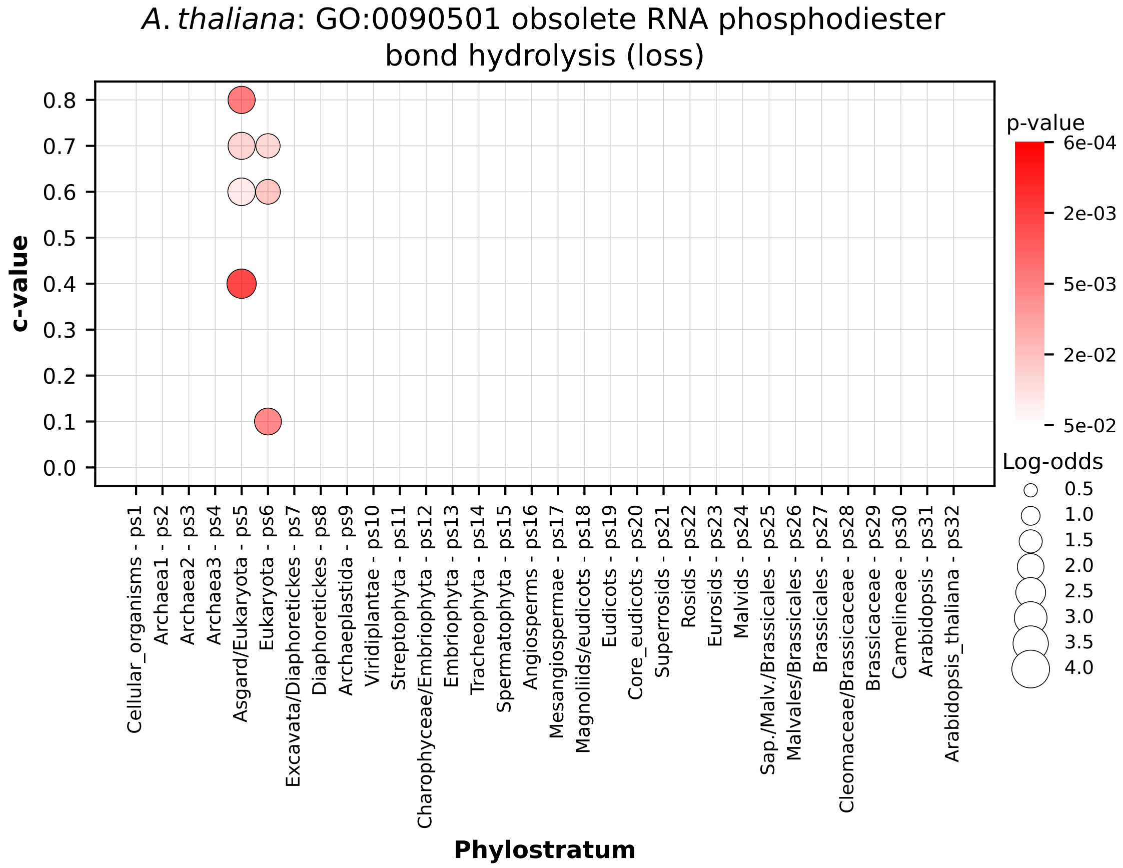

A. thaliana: GO:0090501 obsolete RNA phosphodiester bond hydrolysis (loss)
c-value
0.0
0.1
0.2
0.3
0.4
0.5
0.6
0.7
0.8
p-value
6e-04
2e-03
5e-03
2e-02
5e-02
Log-odds
0.5
1.0
1.5
2.0
2.5
3.0
3.5
4.0
Cellular_organisms - ps1
Archaea1 - ps2
Archaea2 - ps3
Archaea3 - ps4
Asgard/Eukaryota - ps5
Eukaryota - ps6
Excavata/Diaphoretickes - ps7
Diaphoretickes - ps8
Archaeplastida - ps9
Viridiplantae - ps10
Streptophyta - ps11
Charophyceae/Embriophyta - ps12
Embriophyta - ps13
Tracheophyta - ps14
Spermatophyta - ps15
Angiosperms - ps16
Mesangiospermae - ps17
Magnoliids/eudicots - ps18
Eudicots - ps19
Core_eudicots - ps20
Superrosids - ps21
Rosids - ps22
Eurosids - ps23
Malvids - ps24
Sap./Malv./Brassicales - ps25
Malvales/Brassicales - ps26
Brassicales - ps27
Cleomaceae/Brassicaceae - ps28
Brassicaceae - ps29
Camelineae - ps30
Arabidopsis - ps31
Arabidopsis_thaliana - ps32
Phylostratum

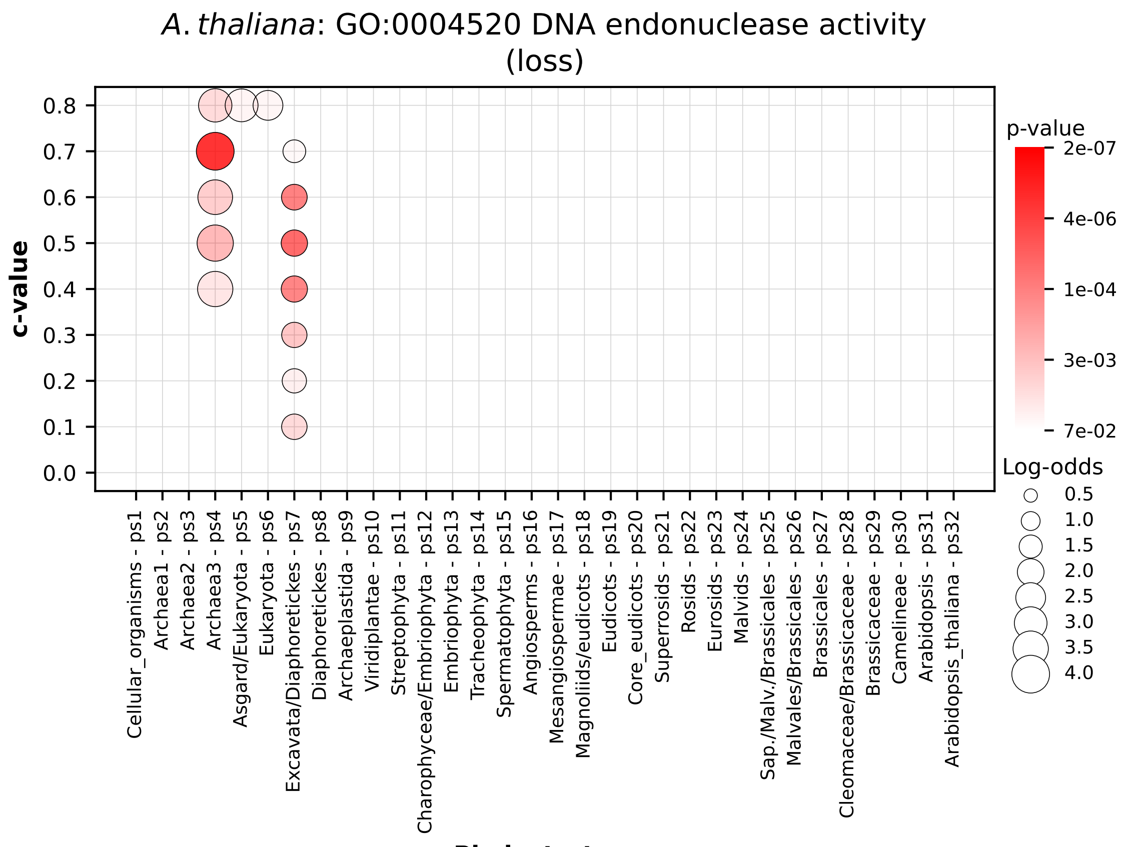
A. thaliana: GO:0004520 DNA endonuclease activity
(loss)
c-value
0.8
0.7
0.6
0.5
0.4
0.3
0.2
0.1
0.0
p-value
2e-07
4e-06
1e-04
3e-03
7e-02
Log-odds
0.5
1.0
1.5
2.0
2.5
3.0
3.5
4.0
Cellular_organisms - ps1
Archaea1 - ps2
Archaea2 - ps3
Archaea3 - ps4
Asgard/Eukaryota - ps5
Eukaryota - ps6
Excavata/Diaphoretickes - ps7
Diaphoretickes - ps8
Archaeplastida - ps9
Viridiplantae - ps10
Streptophyta - ps11
Charophyceae/Embriophyta - ps12
Embriophyta - ps13
Tracheophyta - ps14
Spermatophyta - ps15
Angiosperms - ps16
Mesangiospermae - ps17
Magnoliids/eudicots - ps18
Eudicots - ps19
Core_eudicots - ps20
Superrosids - ps21
Rosids - ps22
Eurosids - ps23
Malvids - ps24
Sap./Malv./Brassicales - ps25
Malvales/Brassicales - ps26
Brassicales - ps27
Cleomaceae/Brassicaceae - ps28
Brassicaceae - ps29
Camelineae - ps30
Arabidopsis - ps31
Arabidopsis_thaliana - ps32
Phylostratum

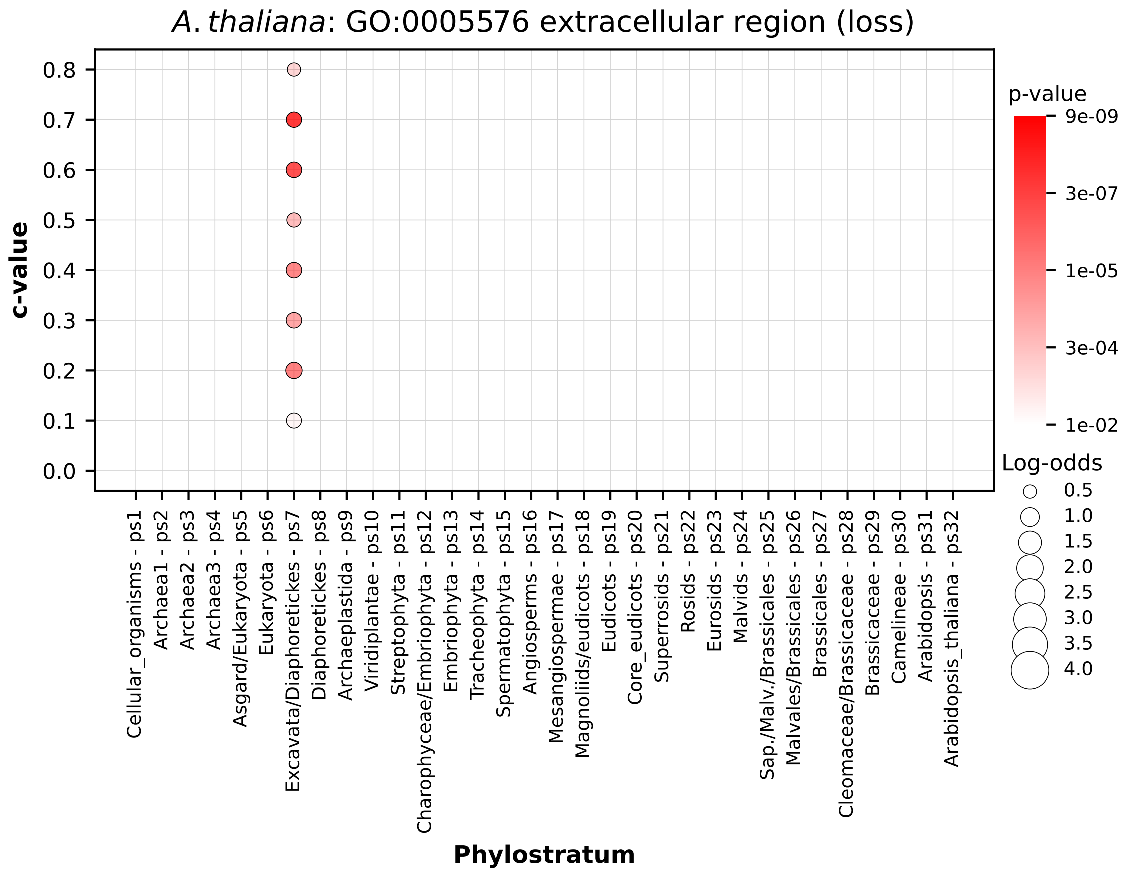

A. thaliana: GO:0005576 extracellular region (loss)
c-value
0.8
0.7
0.6
0.5
0.4
0.3
0.2
0.1
0.0
p-value
9e-09
3e-07
1e-05
3e-04
1e-02
Log-odds
0.5
1.0
1.5
2.0
2.5
3.0
3.5
4.0
Cellular_organisms - ps1
Archaea1 - ps2
Archaea2 - ps3
Archaea3 - ps4
Asgard/Eukaryota - ps5
Eukaryota - ps6
Excavata/Diaphoretickes - ps7
Diaphoretickes - ps8
Archaeplastida - ps9
Viridiplantae - ps10
Streptophyta - ps11
Charophyceae/Embriophyta - ps12
Embriophyta - ps13
Tracheophyta - ps14
Spermatophyta - ps15
Angiosperms - ps16
Mesangiospermae - ps17
Magnoliids/eudicots - ps18
Eudicots - ps19
Core_eudicots - ps20
Superrosids - ps21
Rosids - ps22
Eurosids - ps23
Malvids - ps24
Sap./Malv./Brassicales - ps25
Malvales/Brassicales - ps26
Brassicales - ps27
Cleomaceae/Brassicaceae - ps28
Brassicaceae - ps29
Camelineae - ps30
Arabidopsis - ps31
Arabidopsis_thaliana - ps32
Phylostratum

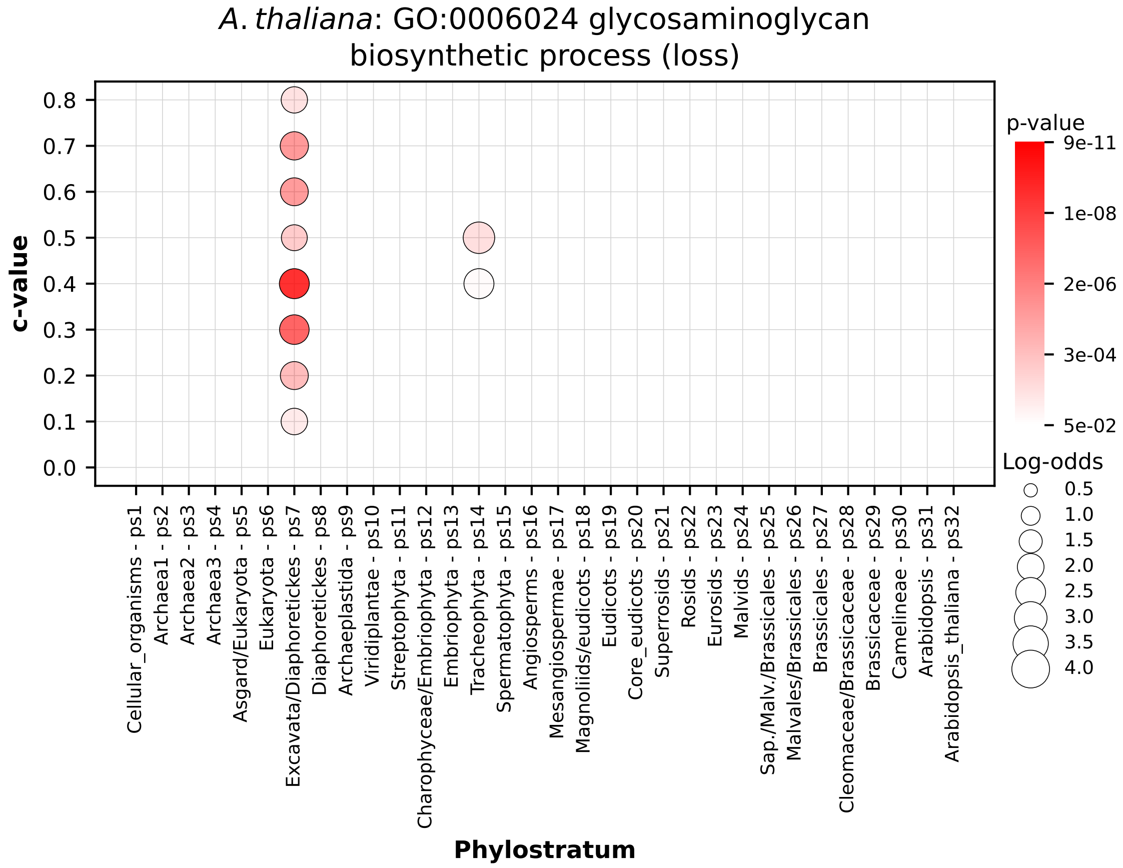

A. thaliana: GO:0006024 glycosaminoglycan biosynthetic process (loss)
c-value
0.0
0.1
0.2
0.3
0.4
0.5
0.6
0.7
0.8
Phylostratum
Cellular_organisms - ps1
Archaea1 - ps2
Archaea2 - ps3
Archaea3 - ps4
Asgard/Eukaryota - ps5
Eukaryota - ps6
Excavata/Diaphoretickes - ps7
Diaphoretickes - ps8
Archaeplastida - ps9
Viridiplantae - ps10
Streptophyta - ps11
Charophyceae/Embriophyta - ps12
Embriophyta - ps13
Tracheophyta - ps14
Spermatophyta - ps15
Angiosperms - ps16
Mesangiospermae - ps17
Magnoliids/eudicots - ps18
Eudicots - ps19
Core_eudicots - ps20
Superrosids - ps21
Rosids - ps22
Eurosids - ps23
Malvids - ps24
Sap./Malv./Brassicales - ps25
Malvales/Brassicales - ps26
Brassicales - ps27
Cleomaceae/Brassicaceae - ps28
Brassicaceae - ps29
Camelineae - ps30
Arabidopsis - ps31
Arabidopsis_thaliana - ps32
p-value
9e-11
1e-08
2e-06
3e-04
5e-02
Log-odds
0.5
1.0
1.5
2.0
2.5
3.0
3.5
4.0

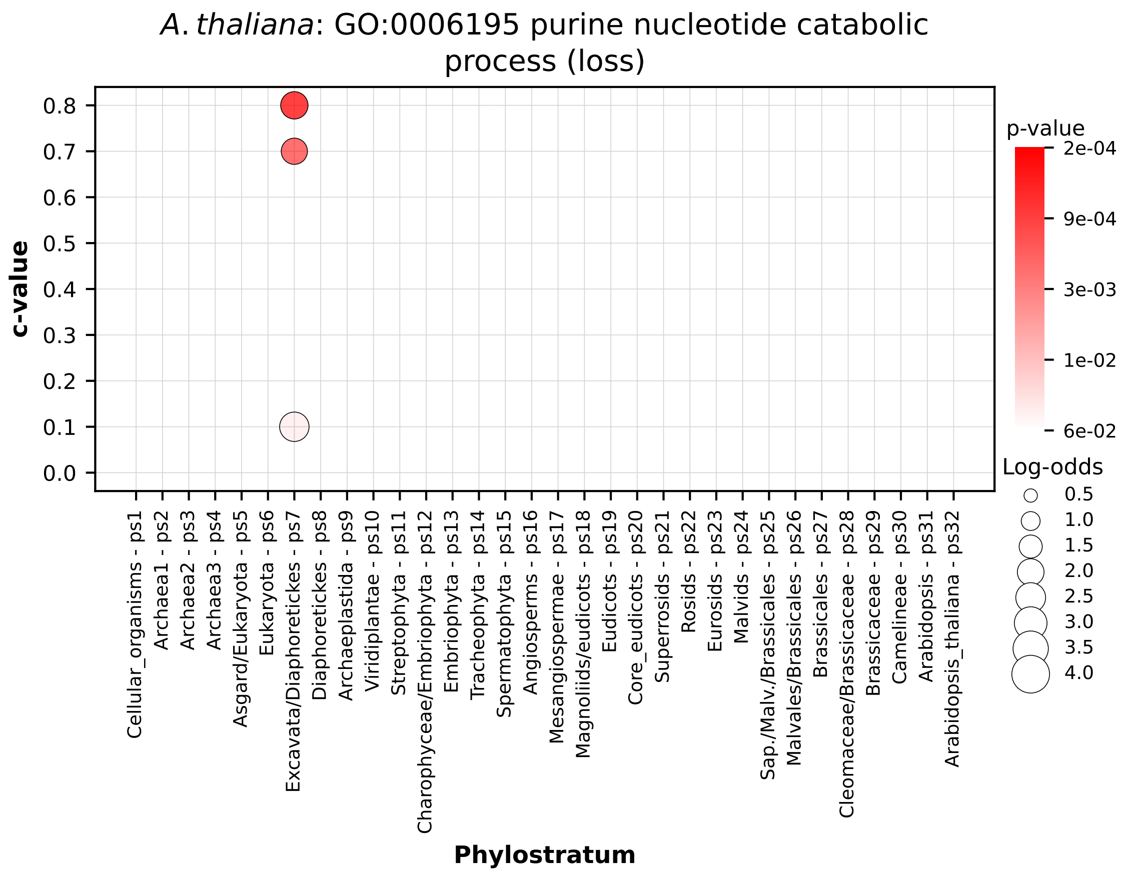
A. thaliana: GO:0006195 purine nucleotide catabolic process (loss)
c-value
0.8
0.7
0.6
0.5
0.4
0.3
0.2
0.1
0.0
Phylostratum
Cellular_organisms - ps1
Archaea1 - ps2
Archaea2 - ps3
Archaea3 - ps4
Asgard/Eukaryota - ps5
Eukaryota - ps6
Excavata/Diaphoretickes - ps7
Diaphoretickes - ps8
Archaeplastida - ps9
Viridiplantae - ps10
Streptophyta - ps11
Charophyceae/Embriophyta - ps12
Embriophyta - ps13
Tracheophyta - ps14
Spermatophyta - ps15
Angiosperms - ps16
Mesangiospermae - ps17
Magnoliids/eudicots - ps18
Eudicots - ps19
Core_eudicots - ps20
Superrosids - ps21
Rosids - ps22
Eurosids - ps23
Malvids - ps24
Sap./Malv./Brassicales - ps25
Malvales/Brassicales - ps26
Brassicales - ps27
Cleomaceae/Brassicaceae - ps28
Brassicaceae - ps29
Camelineae - ps30
Arabidopsis - ps31
Arabidopsis_thaliana - ps32
p-value
2e-04
9e-04
3e-03
1e-02
6e-02
Log-odds
0.5
1.0
1.5
2.0
2.5
3.0
3.5
4.0

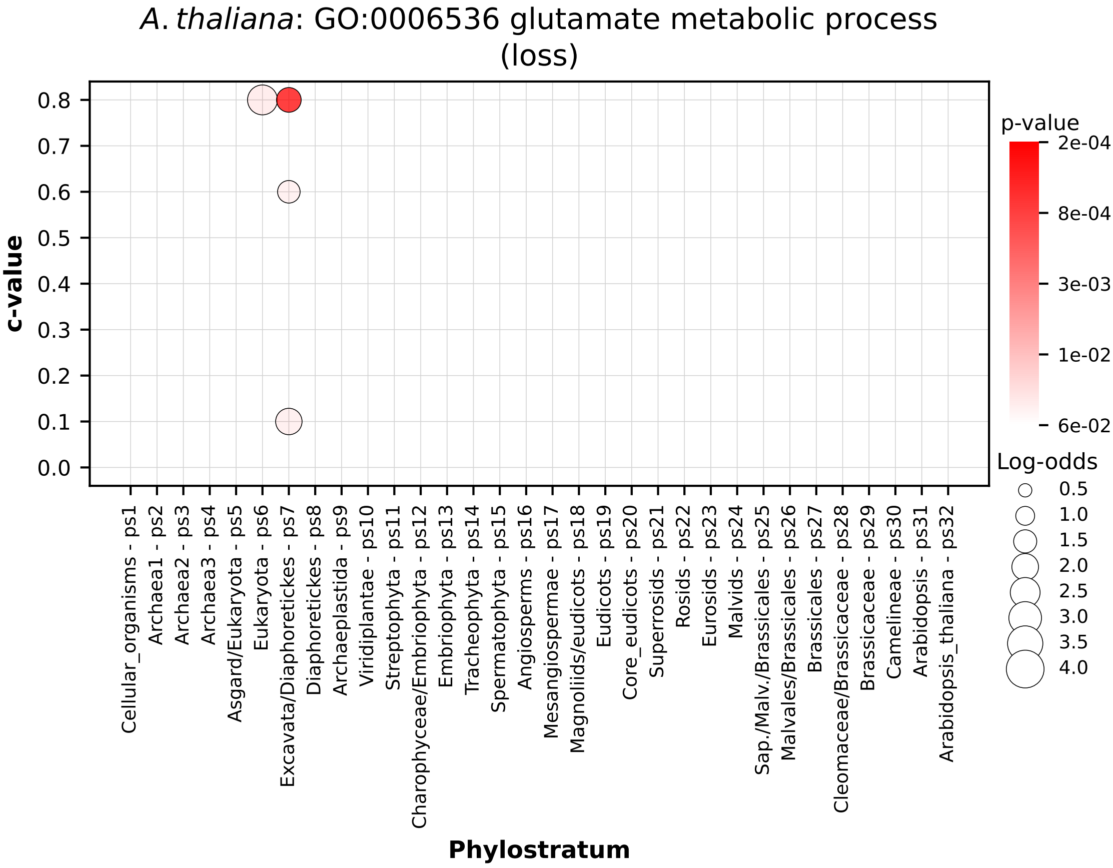
A. thaliana: GO:0006536 glutamate metabolic process
(loss)
c-value
0.0
0.1
0.2
0.3
0.4
0.5
0.6
0.7
0.8
Phylostratum
Cellular_organisms - ps1
Archaea1 - ps2
Archaea2 - ps3
Archaea3 - ps4
Asgard/Eukaryota - ps5
Eukaryota - ps6
Excavata/Diaphoretickes - ps7
Diaphoretickes - ps8
Archaeplastida - ps9
Viridiplantae - ps10
Streptophyta - ps11
Charophyceae/Embriophyta - ps12
Embriophyta - ps13
Tracheophyta - ps14
Spermatophyta - ps15
Angiosperms - ps16
Mesangiospermae - ps17
Magnoliids/eudicots - ps18
Eudicots - ps19
Core_eudicots - ps20
Superrosids - ps21
Rosids - ps22
Eurosids - ps23
Malvids - ps24
Sap./Malv./Brassicales - ps25
Malvales/Brassicales - ps26
Brassicales - ps27
Cleomaceae/Brassicaceae - ps28
Brassicaceae - ps29
Camelineae - ps30
Arabidopsis - ps31
Arabidopsis_thaliana - ps32
p-value
2e-04
8e-04
3e-03
1e-02
6e-02
Log-odds
0.5
1.0
1.5
2.0
2.5
3.0
3.5
4.0

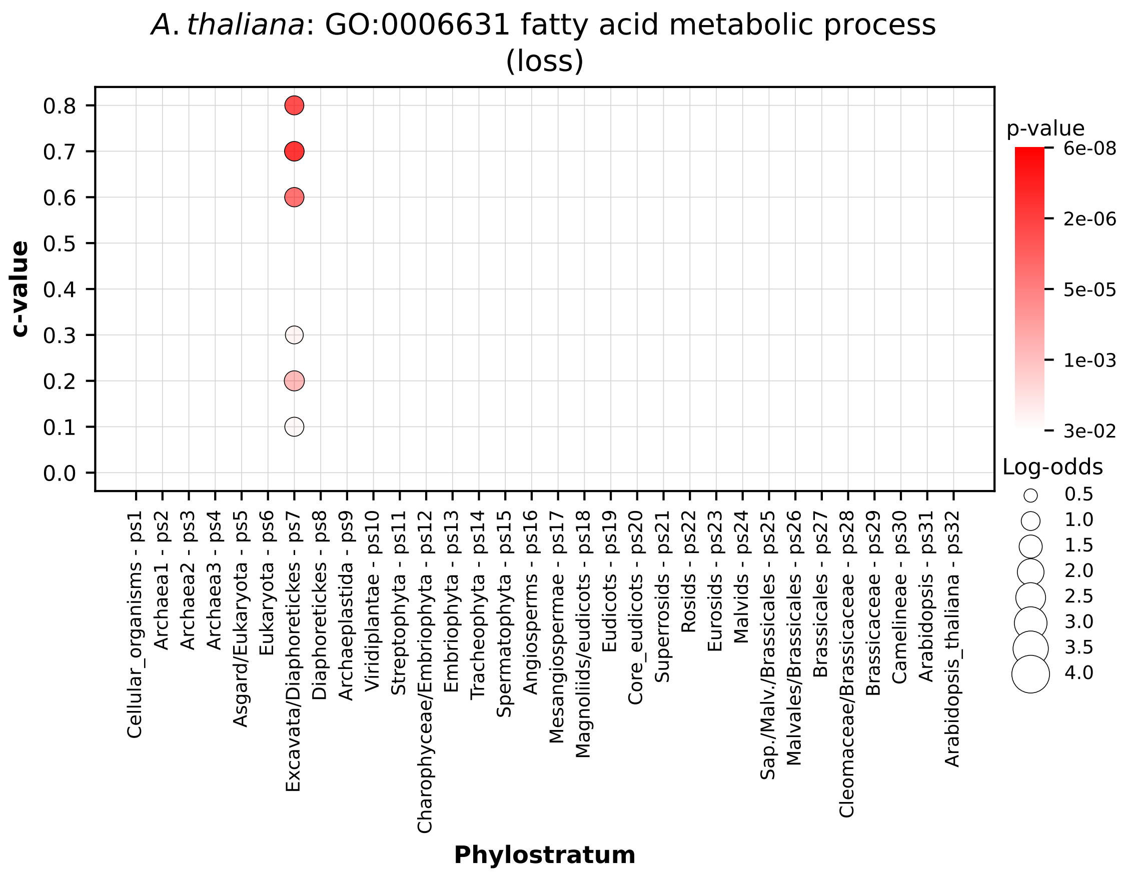
A. thaliana: GO:0006631 fatty acid metabolic process
(loss)
c-value
0.8
0.7
0.6
0.5
0.4
0.3
0.2
0.1
0.0
Phylostratum
Cellular_organisms - ps1
Archaea1 - ps2
Archaea2 - ps3
Archaea3 - ps4
Asgard/Eukaryota - ps5
Eukaryota - ps6
Excavata/Diaphoretickes - ps7
Diaphoretickes - ps8
Archaeplastida - ps9
Viridiplantae - ps10
Streptophyta - ps11
Charophyceae/Embriophyta - ps12
Embriophyta - ps13
Tracheophyta - ps14
Spermatophyta - ps15
Angiosperms - ps16
Mesangiospermae - ps17
Magnoliids/eudicots - ps18
Eudicots - ps19
Core_eudicots - ps20
Superrosids - ps21
Rosids - ps22
Eurosids - ps23
Malvids - ps24
Sap./Malv./Brassicales - ps25
Malvales/Brassicales - ps26
Brassicales - ps27
Cleomaceae/Brassicaceae - ps28
Brassicaceae - ps29
Camelineae - ps30
Arabidopsis - ps31
Arabidopsis_thaliana - ps32
p-value
6e-08
2e-06
5e-05
1e-03
3e-02
Log-odds
0.5
1.0
1.5
2.0
2.5
3.0
3.5
4.0

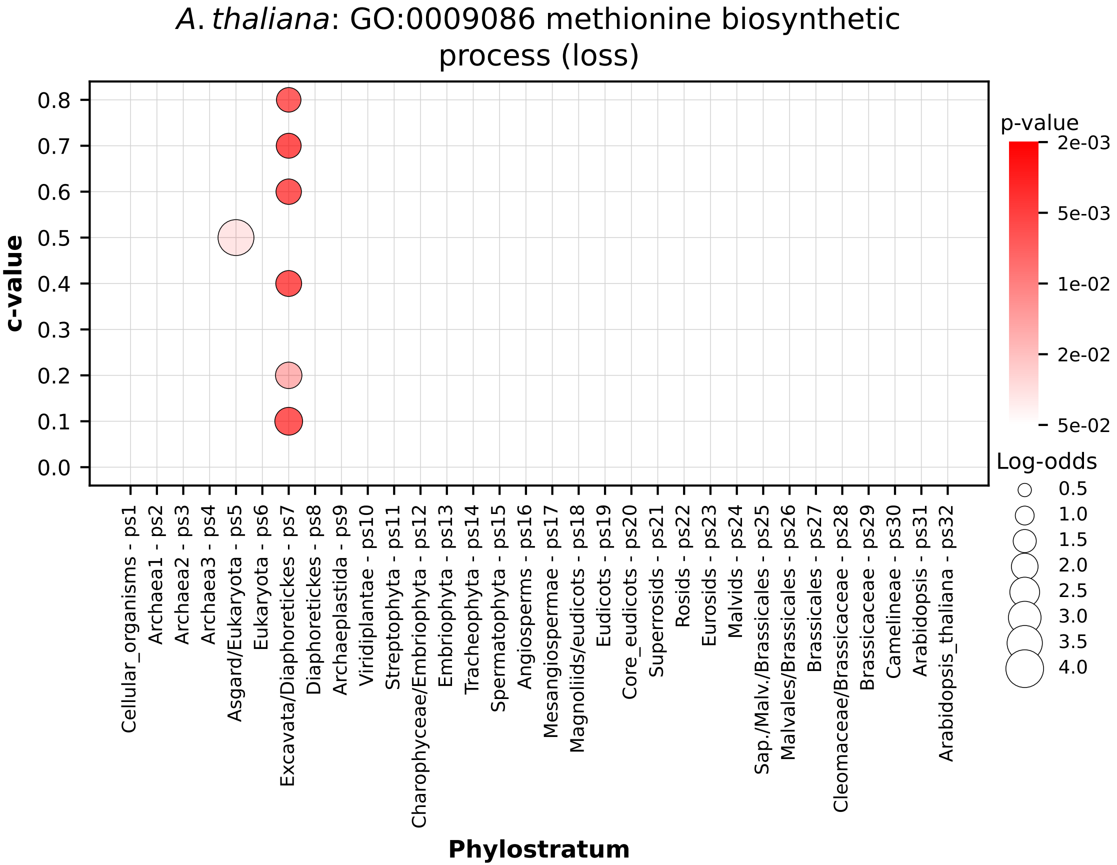

A. thaliana: GO:0009086 methionine biosynthetic process (loss)
c-value
0.8
0.7
0.6
0.5
0.4
0.3
0.2
0.1
0.0
Phylostratum
Cellular_organisms - ps1
Archaea1 - ps2
Archaea2 - ps3
Archaea3 - ps4
Asgard/Eukaryota - ps5
Eukaryota - ps6
Excavata/Diaphoretickes - ps7
Diaphoretickes - ps8
Archaeplastida - ps9
Viridiplantae - ps10
Streptophyta - ps11
Charophyceae/Embriophyta - ps12
Embriophyta - ps13
Tracheophyta - ps14
Spermatophyta - ps15
Angiosperms - ps16
Mesangiospermae - ps17
Magnoliids/eudicots - ps18
Eudicots - ps19
Core_eudicots - ps20
Superrosids - ps21
Rosids - ps22
Eurosids - ps23
Malvids - ps24
Sap./Malv./Brassicales - ps25
Malvales/Brassicales - ps26
Brassicales - ps27
Cleomaceae/Brassicaceae - ps28
Brassicaceae - ps29
Camelineae - ps30
Arabidopsis - ps31
Arabidopsis_thaliana - ps32
p-value
2e-03
5e-03
1e-02
2e-02
5e-02
Log-odds
0.5
1.0
1.5
2.0
2.5
3.0
3.5
4.0

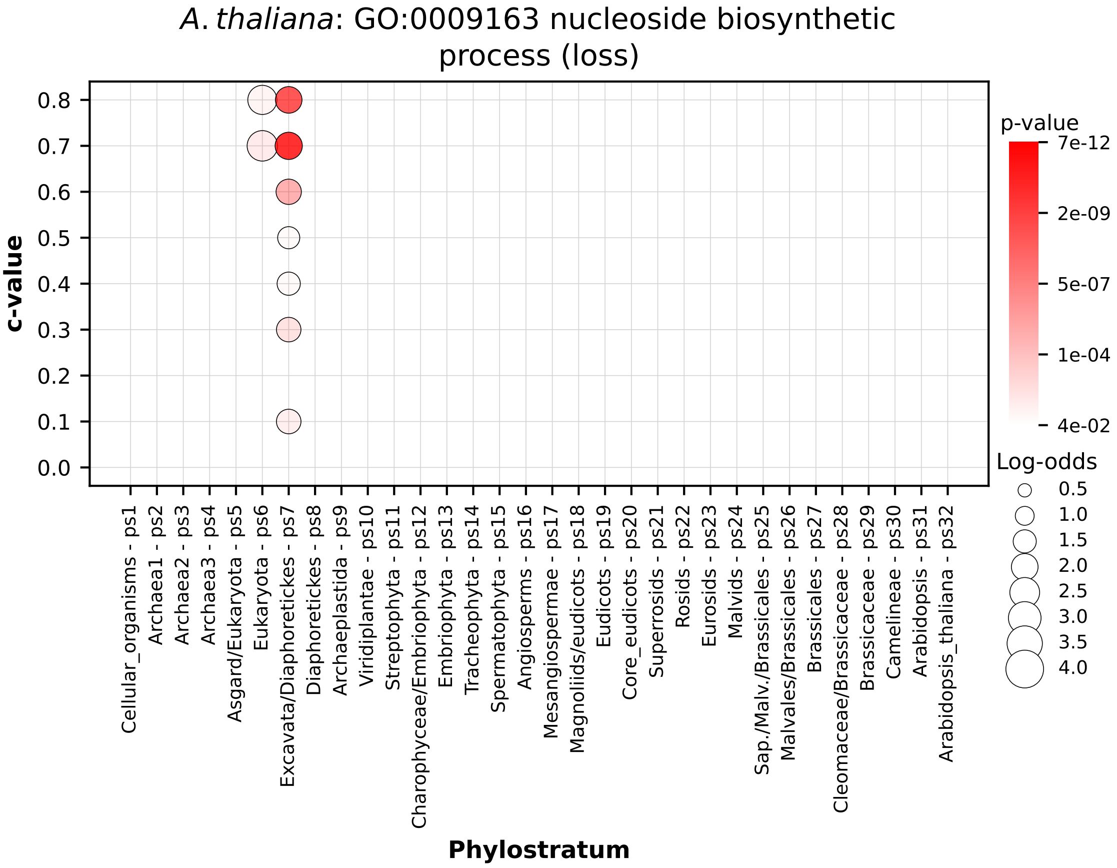

A. thaliana: GO:0009163 nucleoside biosynthetic process (loss)
c-value
0.8
0.7
0.6
0.5
0.4
0.3
0.2
0.1
0.0
Phylostratum
Cellular_organisms - ps1
Archaea1 - ps2
Archaea2 - ps3
Archaea3 - ps4
Asgard/Eukaryota - ps5
Eukaryota - ps6
Excavata/Diaphoretickes - ps7
Diaphoretickes - ps8
Archaeplastida - ps9
Viridiplantae - ps10
Streptophyta - ps11
Charophyceae/Embriophyta - ps12
Embriophyta - ps13
Tracheophyta - ps14
Spermatophyta - ps15
Angiosperms - ps16
Mesangiospermae - ps17
Magnoliids/eudicots - ps18
Eudicots - ps19
Core_eudicots - ps20
Superrosids - ps21
Rosids - ps22
Eurosids - ps23
Malvids - ps24
Sap./Malv./Brassicales - ps25
Malvales/Brassicales - ps26
Brassicales - ps27
Cleomaceae/Brassicaceae - ps28
Brassicaceae - ps29
Camelineae - ps30
Arabidopsis - ps31
Arabidopsis_thaliana - ps32
p-value
7e-12
2e-09
5e-07
1e-04
4e-02
Log-odds
0.5
1.0
1.5
2.0
2.5
3.0
3.5
4.0

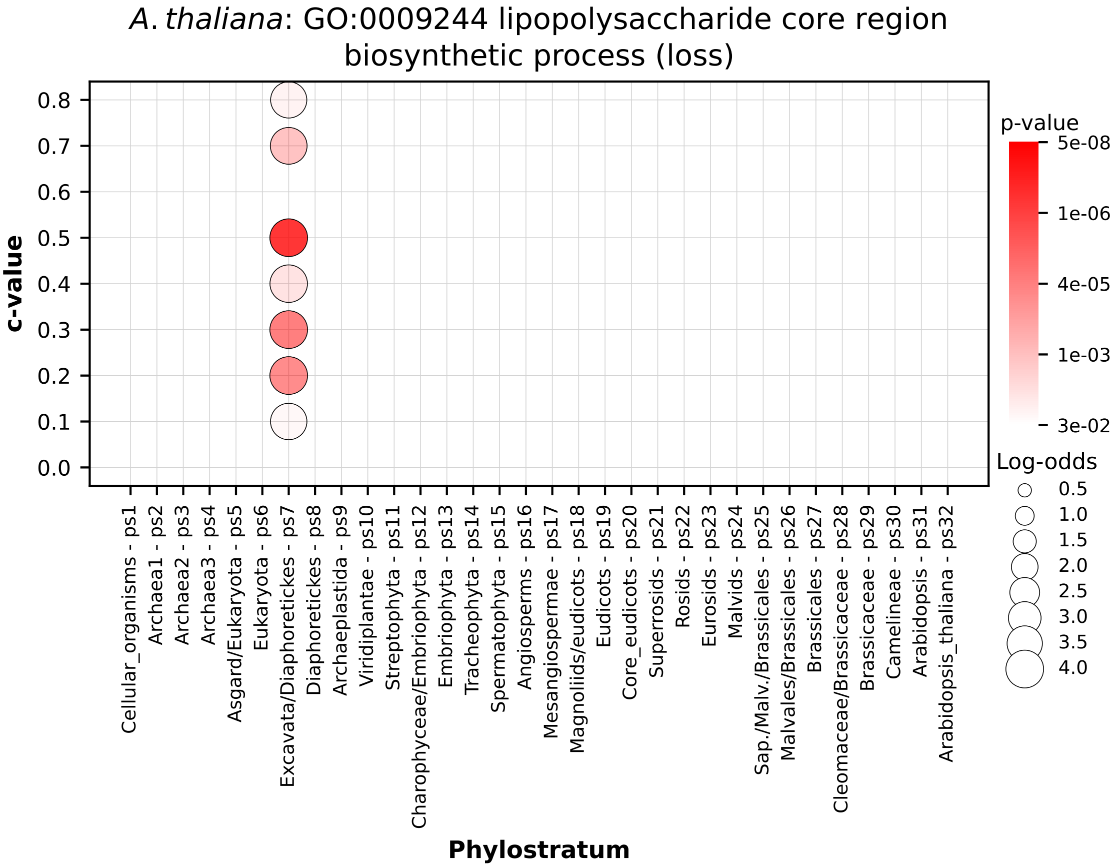

A. thaliana: GO:0009244 lipopolysaccharide core region biosynthetic process (loss)
c-value
0.8
0.7
0.6
0.5
0.4
0.3
0.2
0.1
0.0
p-value
5e-08
1e-06
4e-05
1e-03
3e-02
Log-odds
0.5
1.0
1.5
2.0
2.5
3.0
3.5
4.0
Cellular_organisms - ps1
Archaea1 - ps2
Archaea2 - ps3
Archaea3 - ps4
Asgard/Eukaryota - ps5
Eukaryota - ps6
Excavata/Diaphoretickes - ps7
Diaphoretickes - ps8
Archaeplastida - ps9
Viridiplantae - ps10
Streptophyta - ps11
Charophyceae/Embriophyta - ps12
Embriophyta - ps13
Tracheophyta - ps14
Spermatophyta - ps15
Angiosperms - ps16
Mesangiospermae - ps17
Magnoliids/eudicots - ps18
Eudicots - ps19
Core_eudicots - ps20
Superrosids - ps21
Rosids - ps22
Eurosids - ps23
Malvids - ps24
Sap./Malv./Brassicales - ps25
Malvales/Brassicales - ps26
Brassicales - ps27
Cleomaceae/Brassicaceae - ps28
Brassicaceae - ps29
Camelineae - ps30
Arabidopsis - ps31
Arabidopsis_thaliana - ps32
Phylostratum

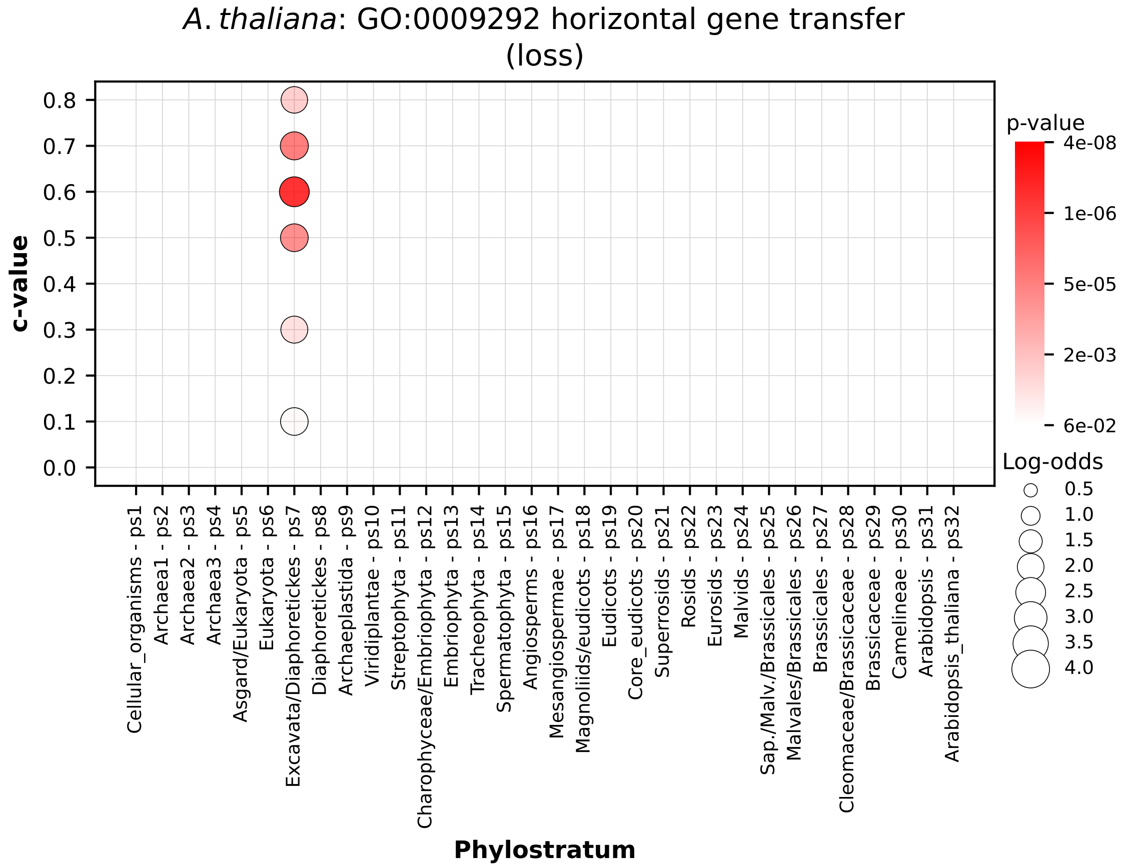

A. thaliana: GO:0009292 horizontal gene transfer
(loss)
c-value
0.8
0.7
0.6
0.5
0.4
0.3
0.2
0.1
0.0
p-value
4e-08
1e-06
5e-05
2e-03
6e-02
Log-odds
0.5
1.0
1.5
2.0
2.5
3.0
3.5
4.0
Cellular_organisms - ps1
Archaea1 - ps2
Archaea2 - ps3
Archaea3 - ps4
Asgard/Eukaryota - ps5
Eukaryota - ps6
Excavata/Diaphoretickes - ps7
Diaphoretickes - ps8
Archaeplastida - ps9
Viridiplantae - ps10
Streptophyta - ps11
Charophyceae/Embriophyta - ps12
Embriophyta - ps13
Tracheophyta - ps14
Spermatophyta - ps15
Angiosperms - ps16
Mesangiospermae - ps17
Magnoliids/eudicots - ps18
Eudicots - ps19
Core_eudicots - ps20
Superrosids - ps21
Rosids - ps22
Eurosids - ps23
Malvids - ps24
Sap./Malv./Brassicales - ps25
Malvales/Brassicales - ps26
Brassicales - ps27
Cleomaceae/Brassicaceae - ps28
Brassicaceae - ps29
Camelineae - ps30
Arabidopsis - ps31
Arabidopsis_thaliana - ps32
Phylostratum

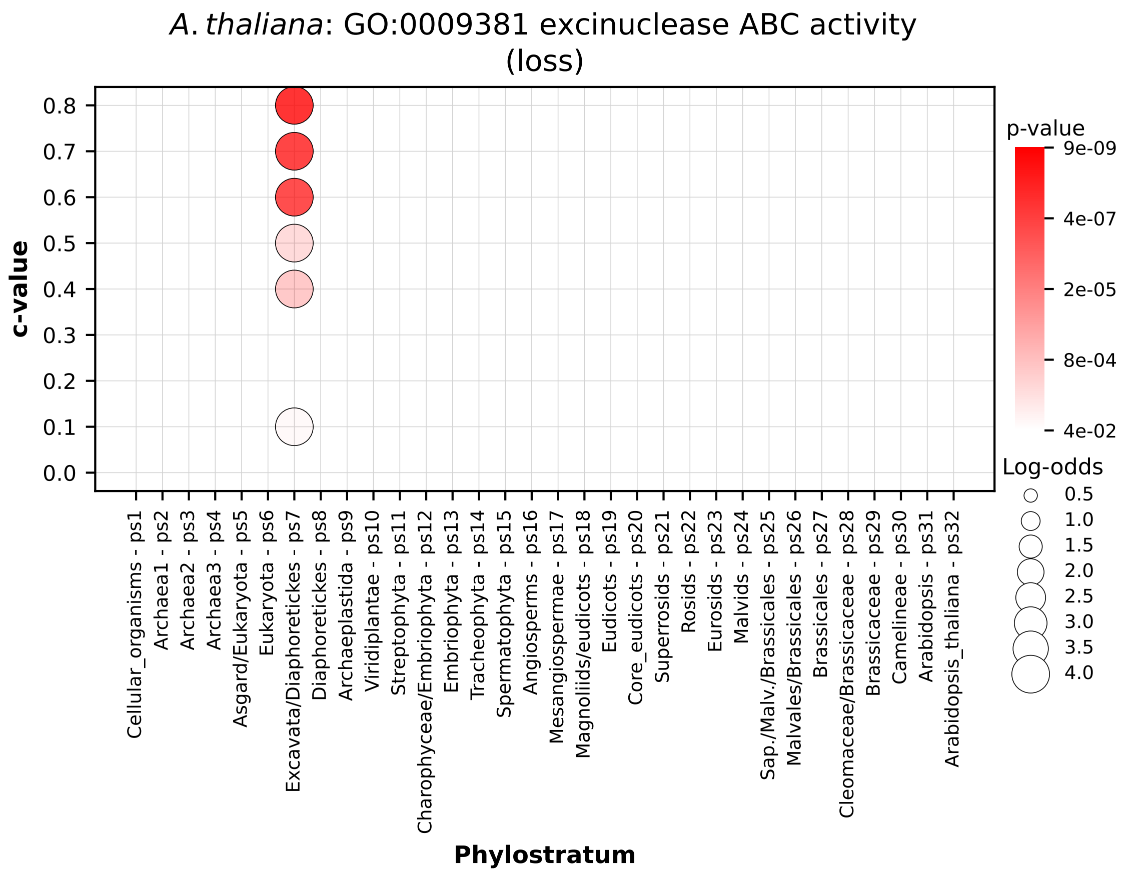

A. thaliana: GO:0009381 excinuclease ABC activity
(loss)
c-value
0.0
0.1
0.2
0.3
0.4
0.5
0.6
0.7
0.8
Phylostratum
Cellular_organisms - ps1
Archaea1 - ps2
Archaea2 - ps3
Archaea3 - ps4
Asgard/Eukaryota - ps5
Eukaryota - ps6
Excavata/Diaphoretickes - ps7
Diaphoretickes - ps8
Archaeplastida - ps9
Viridiplantae - ps10
Streptophyta - ps11
Charophyceae/Embriophyta - ps12
Embriophyta - ps13
Tracheophyta - ps14
Spermatophyta - ps15
Angiosperms - ps16
Mesangiospermae - ps17
Magnoliids/eudicots - ps18
Eudicots - ps19
Core_eudicots - ps20
Superrosids - ps21
Rosids - ps22
Eurosids - ps23
Malvids - ps24
Sap./Malv./Brassicales - ps25
Malvales/Brassicales - ps26
Brassicales - ps27
Cleomaceae/Brassicaceae - ps28
Brassicaceae - ps29
Camelineae - ps30
Arabidopsis - ps31
Arabidopsis_thaliana - ps32
p-value
9e-09
4e-07
2e-05
8e-04
4e-02
Log-odds
0.5
1.0
1.5
2.0
2.5
3.0
3.5
4.0

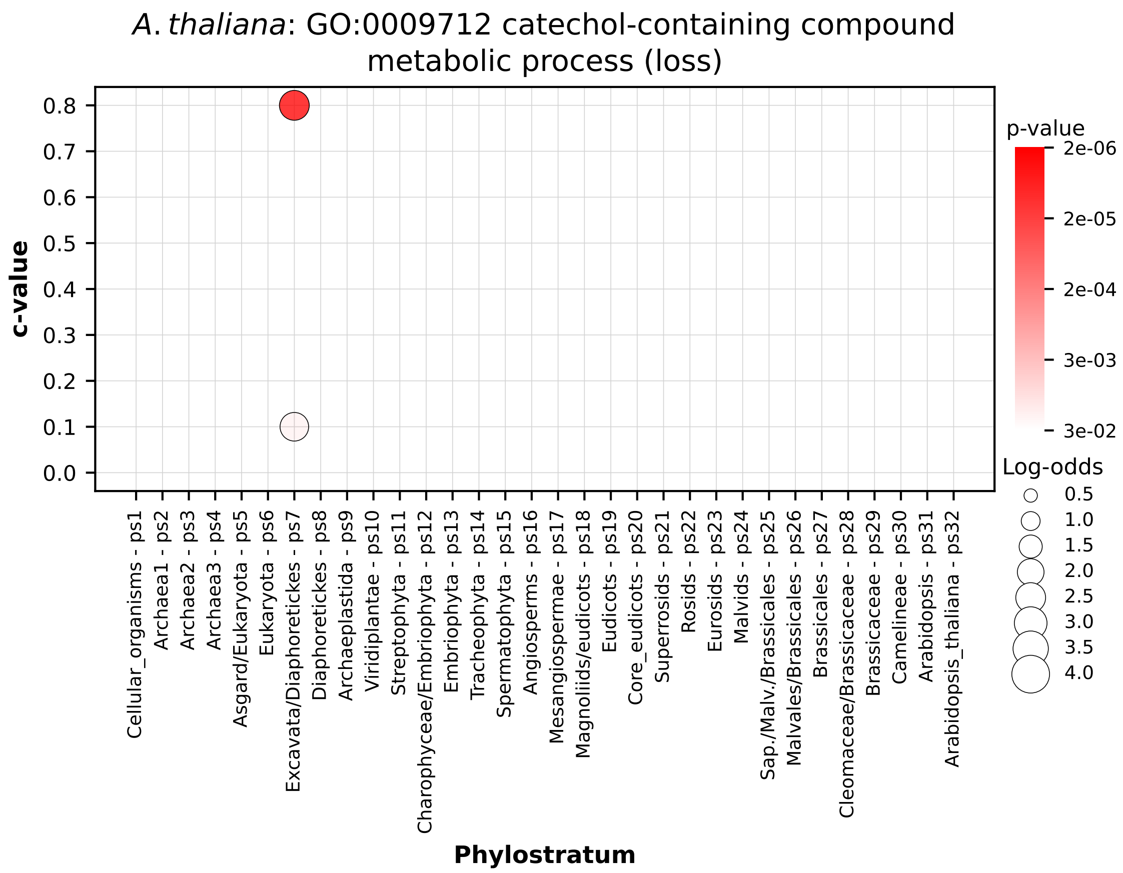

A. thaliana: GO:0009712 catechol-containing compound metabolic process (loss)
c-value
0.0
0.1
0.2
0.3
0.4
0.5
0.6
0.7
0.8
Phylostratum
Cellular_organisms - ps1
Archaea1 - ps2
Archaea2 - ps3
Archaea3 - ps4
Asgard/Eukaryota - ps5
Eukaryota - ps6
Excavata/Diaphoretickes - ps7
Diaphoretickes - ps8
Archaeplastida - ps9
Viridiplantae - ps10
Streptophyta - ps11
Charophyceae/Embriophyta - ps12
Embriophyta - ps13
Tracheophyta - ps14
Spermatophyta - ps15
Angiosperms - ps16
Mesangiospermae - ps17
Magnoliids/eudicots - ps18
Eudicots - ps19
Core_eudicots - ps20
Superrosids - ps21
Rosids - ps22
Eurosids - ps23
Malvids - ps24
Sap./Malv./Brassicales - ps25
Malvales/Brassicales - ps26
Brassicales - ps27
Cleomaceae/Brassicaceae - ps28
Brassicaceae - ps29
Camelineae - ps30
Arabidopsis - ps31
Arabidopsis_thaliana - ps32
p-value
2e-06
2e-05
2e-04
3e-03
3e-02
Log-odds
0.5
1.0
1.5
2.0
2.5
3.0
3.5
4.0

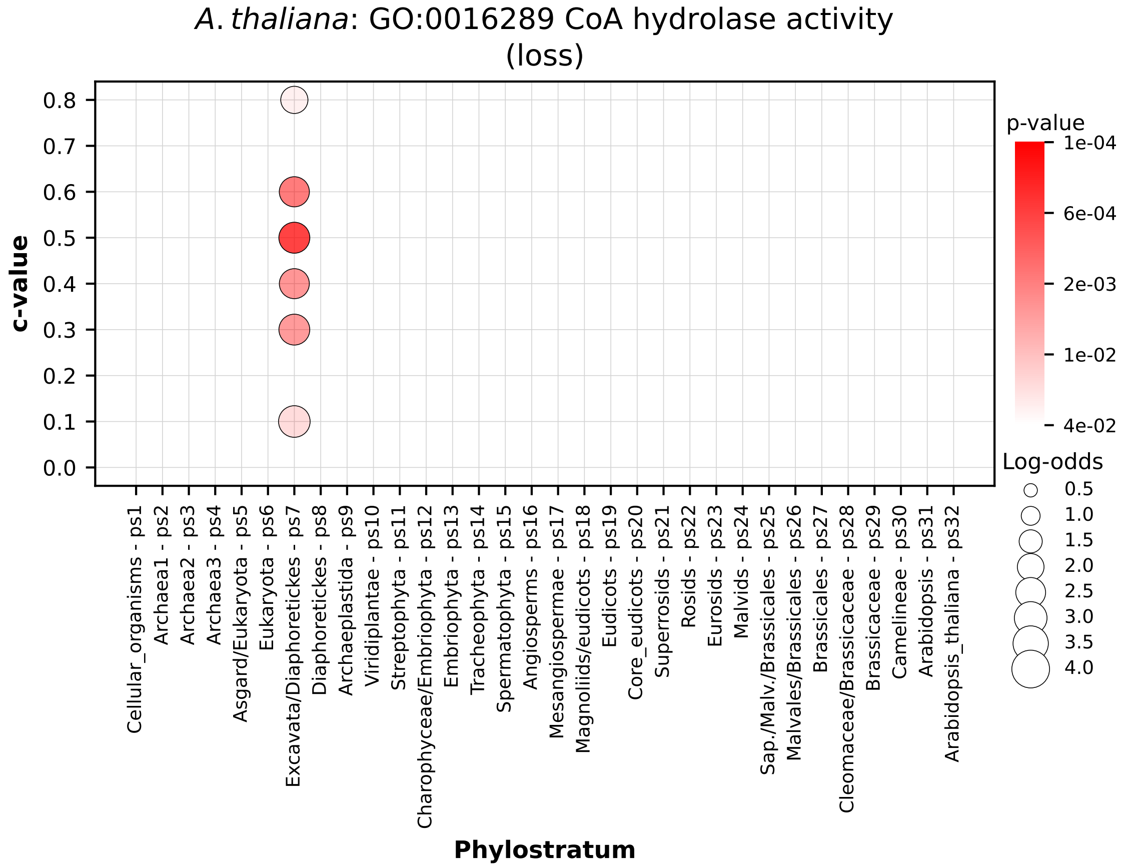
A. thaliana: GO:0016289 CoA hydrolase activity
(loss)
c-value
0.8
0.7
0.6
0.5
0.4
0.3
0.2
0.1
0.0
Phylostratum
Cellular_organisms - ps1
Archaea1 - ps2
Archaea2 - ps3
Archaea3 - ps4
Asgard/Eukaryota - ps5
Eukaryota - ps6
Excavata/Diaphoretickes - ps7
Diaphoretickes - ps8
Archaeplastida - ps9
Viridiplantae - ps10
Streptophyta - ps11
Charophyceae/Embriophyta - ps12
Embriophyta - ps13
Tracheophyta - ps14
Spermatophyta - ps15
Angiosperms - ps16
Mesangiospermae - ps17
Magnoliids/eudicots - ps18
Eudicots - ps19
Core_eudicots - ps20
Superrosids - ps21
Rosids - ps22
Eurosids - ps23
Malvids - ps24
Sap./Malv./Brassicales - ps25
Malvales/Brassicales - ps26
Brassicales - ps27
Cleomaceae/Brassicaceae - ps28
Brassicaceae - ps29
Camelineae - ps30
Arabidopsis - ps31
Arabidopsis_thaliana - ps32
p-value
1e-04
6e-04
2e-03
1e-02
4e-02
Log-odds
0.5
1.0
1.5
2.0
2.5
3.0
3.5
4.0

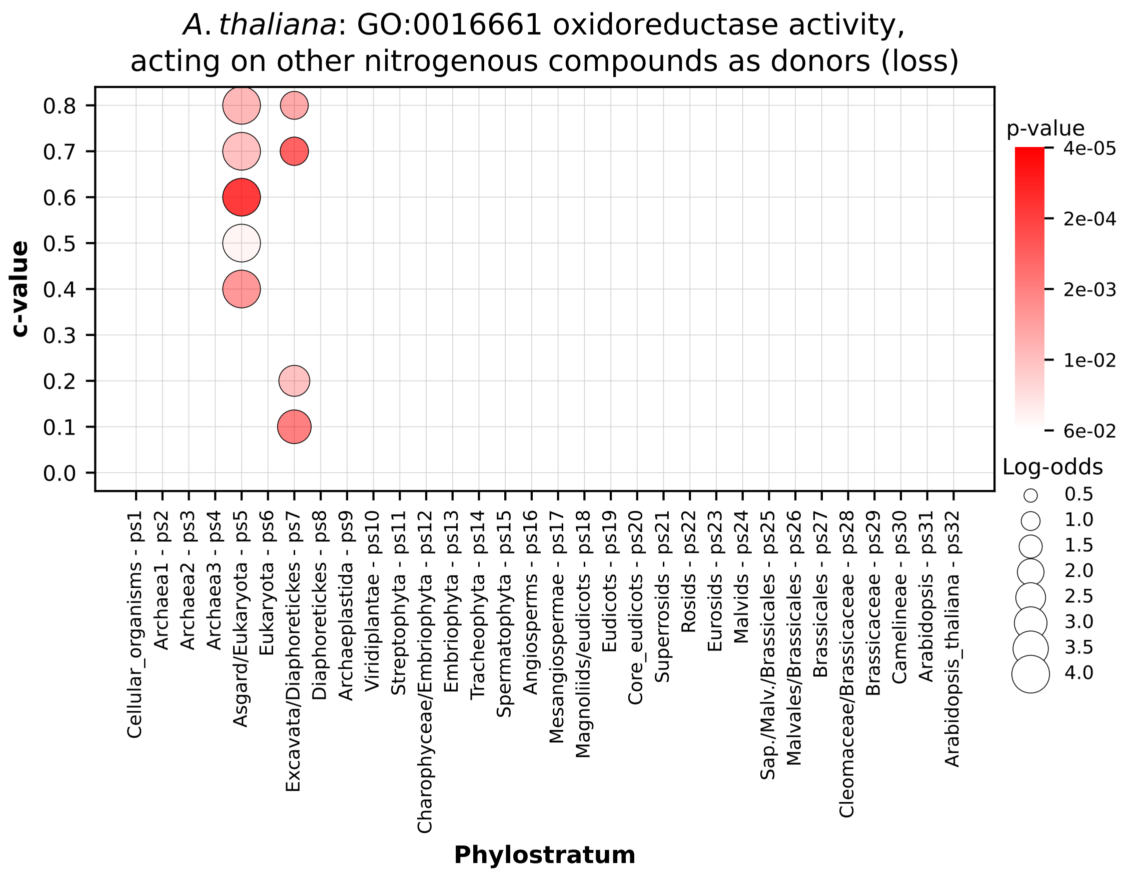
A. thaliana: GO:0016661 oxidoreductase activity,
acting on other nitrogenous compounds as donors (loss)
c-value
0.8
0.7
0.6
0.5
0.4
0.3
0.2
0.1
0.0
p-value
4e-05
2e-04
2e-03
1e-02
6e-02
Log-odds
0.5
1.0
1.5
2.0
2.5
3.0
3.5
4.0
Cellular_organisms - ps1
Archaea1 - ps2
Archaea2 - ps3
Archaea3 - ps4
Asgard/Eukaryota - ps5
Eukaryota - ps6
Excavata/Diaphoretickes - ps7
Diaphoretickes - ps8
Archaeplastida - ps9
Viridiplantae - ps10
Streptophyta - ps11
Charophyceae/Embriophyta - ps12
Embriophyta - ps13
Tracheophyta - ps14
Spermatophyta - ps15
Angiosperms - ps16
Mesangiospermae - ps17
Magnoliids/eudicots - ps18
Eudicots - ps19
Core_eudicots - ps20
Superrosids - ps21
Rosids - ps22
Eurosids - ps23
Malvids - ps24
Sap./Malv./Brassicales - ps25
Malvales/Brassicales - ps26
Brassicales - ps27
Cleomaceae/Brassicaceae - ps28
Brassicaceae - ps29
Camelineae - ps30
Arabidopsis - ps31
Arabidopsis_thaliana - ps32
Phylostratum

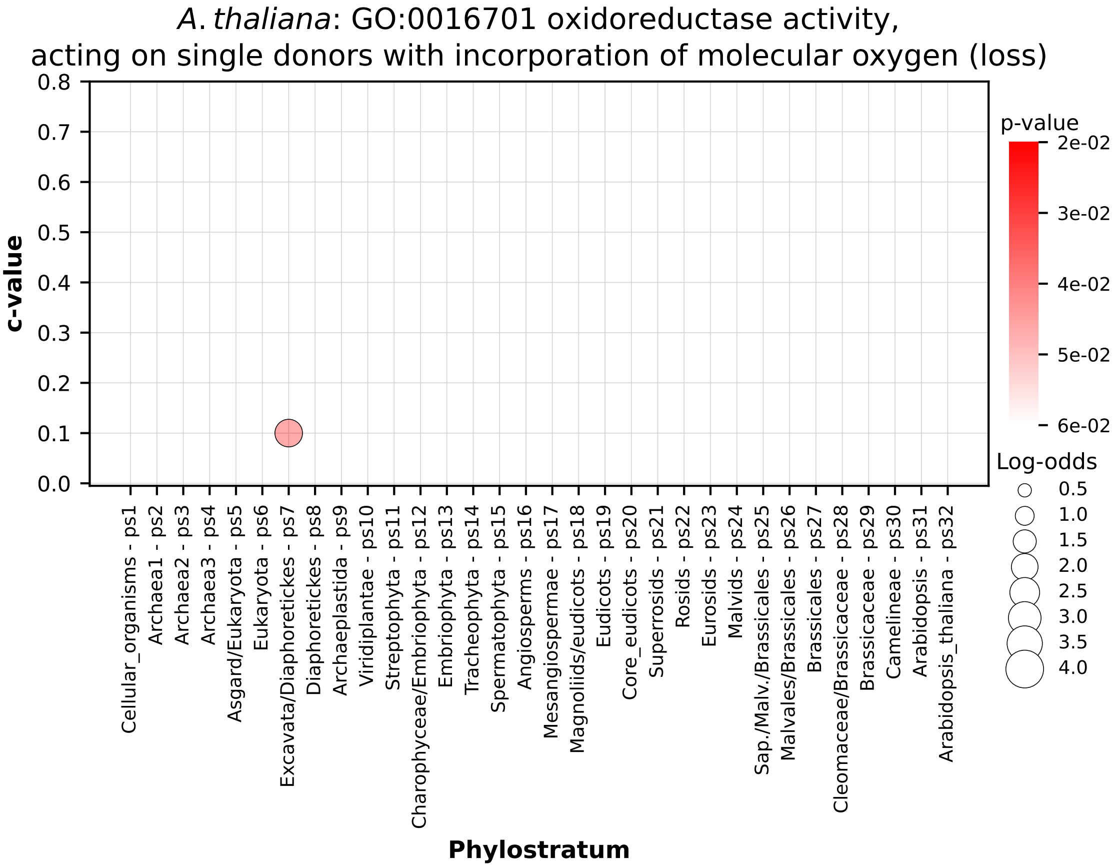

A. thaliana: GO:0016701 oxidoreductase activity,
acting on single donors with incorporation of molecular oxygen (loss)
c-value
0.8
0.7
0.6
0.5
0.4
0.3
0.2
0.1
0.0
Phylostratum
Cellular_organisms - ps1
Archaea1 - ps2
Archaea2 - ps3
Archaea3 - ps4
Asgard/Eukaryota - ps5
Eukaryota - ps6
Excavata/Diaphoretickes - ps7
Diaphoretickes - ps8
Archaeplastida - ps9
Viridiplantae - ps10
Streptophyta - ps11
Charophyceae/Embriophyta - ps12
Embriophyta - ps13
Tracheophyta - ps14
Spermatophyta - ps15
Angiosperms - ps16
Mesangiospermae - ps17
Magnoliids/eudicots - ps18
Eudicots - ps19
Core_eudicots - ps20
Superrosids - ps21
Rosids - ps22
Eurosids - ps23
Malvids - ps24
Sap./Malv./Brassicales - ps25
Malvales/Brassicales - ps26
Brassicales - ps27
Cleomaceae/Brassicaceae - ps28
Brassicaceae - ps29
Camelineae - ps30
Arabidopsis - ps31
Arabidopsis_thaliana - ps32
p-value
2e-02
3e-02
4e-02
5e-02
6e-02
Log-odds
0.5
1.0
1.5
2.0
2.5
3.0
3.5
4.0

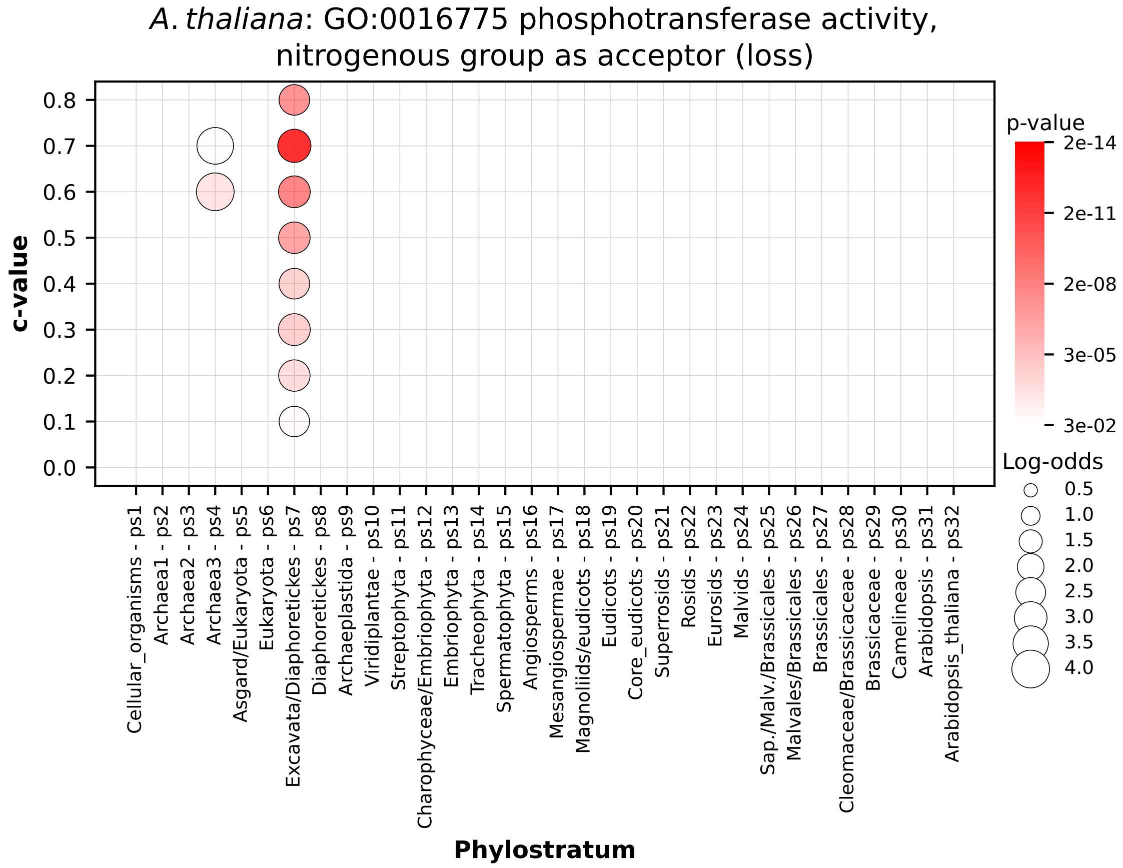

A. thaliana: GO:0016775 phosphotransferase activity, nitrogenous group as acceptor (loss)
c-value
0.0
0.1
0.2
0.3
0.4
0.5
0.6
0.7
0.8
Phylostratum
Cellular_organisms - ps1
Archaea1 - ps2
Archaea2 - ps3
Archaea3 - ps4
Asgard/Eukaryota - ps5
Eukaryota - ps6
Excavata/Diaphoretickes - ps7
Diaphoretickes - ps8
Archaeplastida - ps9
Viridiplantae - ps10
Streptophyta - ps11
Charophyceae/Embriophyta - ps12
Embriophyta - ps13
Tracheophyta - ps14
Spermatophyta - ps15
Angiosperms - ps16
Mesangiospermae - ps17
Magnoliids/eudicots - ps18
Eudicots - ps19
Core_eudicots - ps20
Superrosids - ps21
Rosids - ps22
Eurosids - ps23
Malvids - ps24
Sap./Malv./Brassicales - ps25
Malvales/Brassicales - ps26
Brassicales - ps27
Cleomaceae/Brassicaceae - ps28
Brassicaceae - ps29
Camelineae - ps30
Arabidopsis - ps31
Arabidopsis_thaliana - ps32
p-value
2e-14
2e-11
2e-08
3e-05
3e-02
Log-odds
0.5
1.0
1.5
2.0
2.5
3.0
3.5
4.0

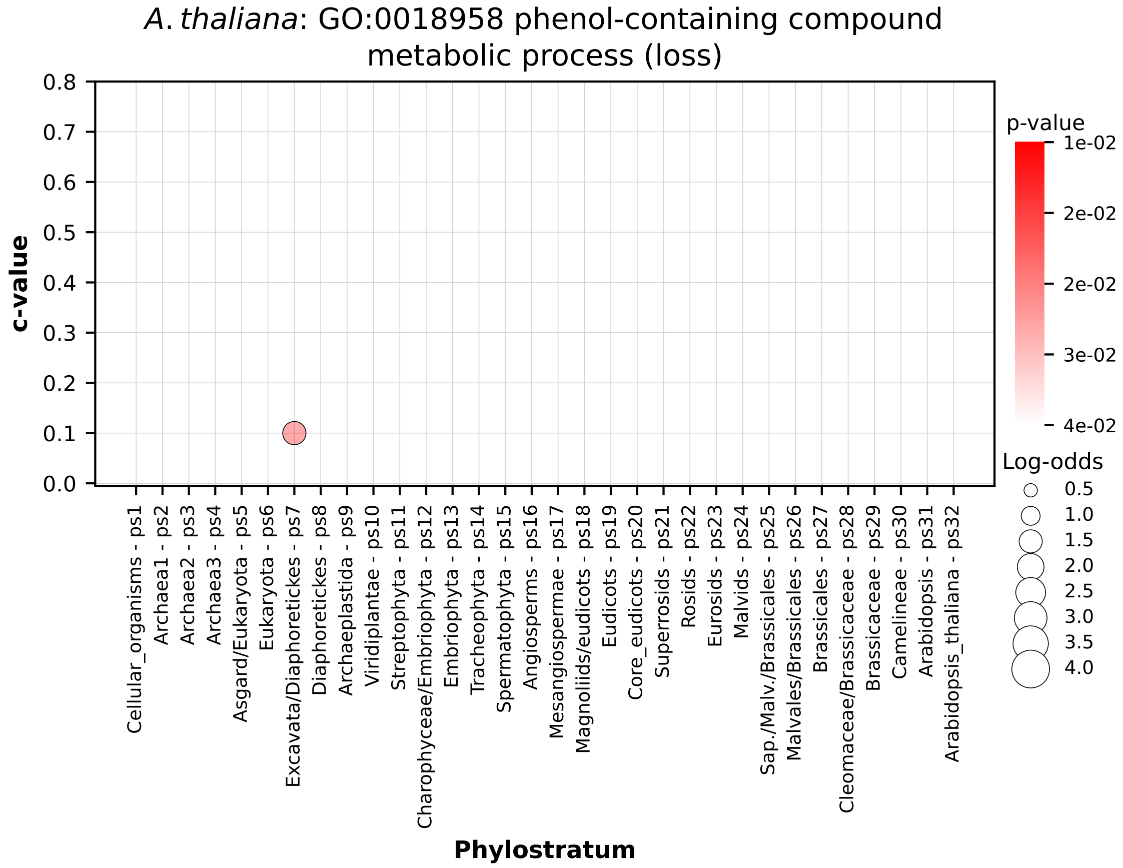
A. thaliana: GO:0018958 phenol-containing compound metabolic process (loss)
c-value
0.8
0.7
0.6
0.5
0.4
0.3
0.2
0.1
0.0
p-value
1e-02
2e-02
2e-02
3e-02
4e-02
Log-odds
0.5
1.0
1.5
2.0
2.5
3.0
3.5
4.0
Cellular_organisms - ps1
Archaea1 - ps2
Archaea2 - ps3
Archaea3 - ps4
Asgard/Eukaryota - ps5
Eukaryota - ps6
Excavata/Diaphoretickes - ps7
Diaphoretickes - ps8
Archaeplastida - ps9
Viridiplantae - ps10
Streptophyta - ps11
Charophyceae/Embriophyta - ps12
Embriophyta - ps13
Tracheophyta - ps14
Spermatophyta - ps15
Angiosperms - ps16
Mesangiospermae - ps17
Magnoliids/eudicots - ps18
Eudicots - ps19
Core_eudicots - ps20
Superrosids - ps21
Rosids - ps22
Eurosids - ps23
Malvids - ps24
Sap./Malv./Brassicales - ps25
Malvales/Brassicales - ps26
Brassicales - ps27
Cleomaceae/Brassicaceae - ps28
Brassicaceae - ps29
Camelineae - ps30
Arabidopsis - ps31
Arabidopsis_thaliana - ps32
Phylostratum

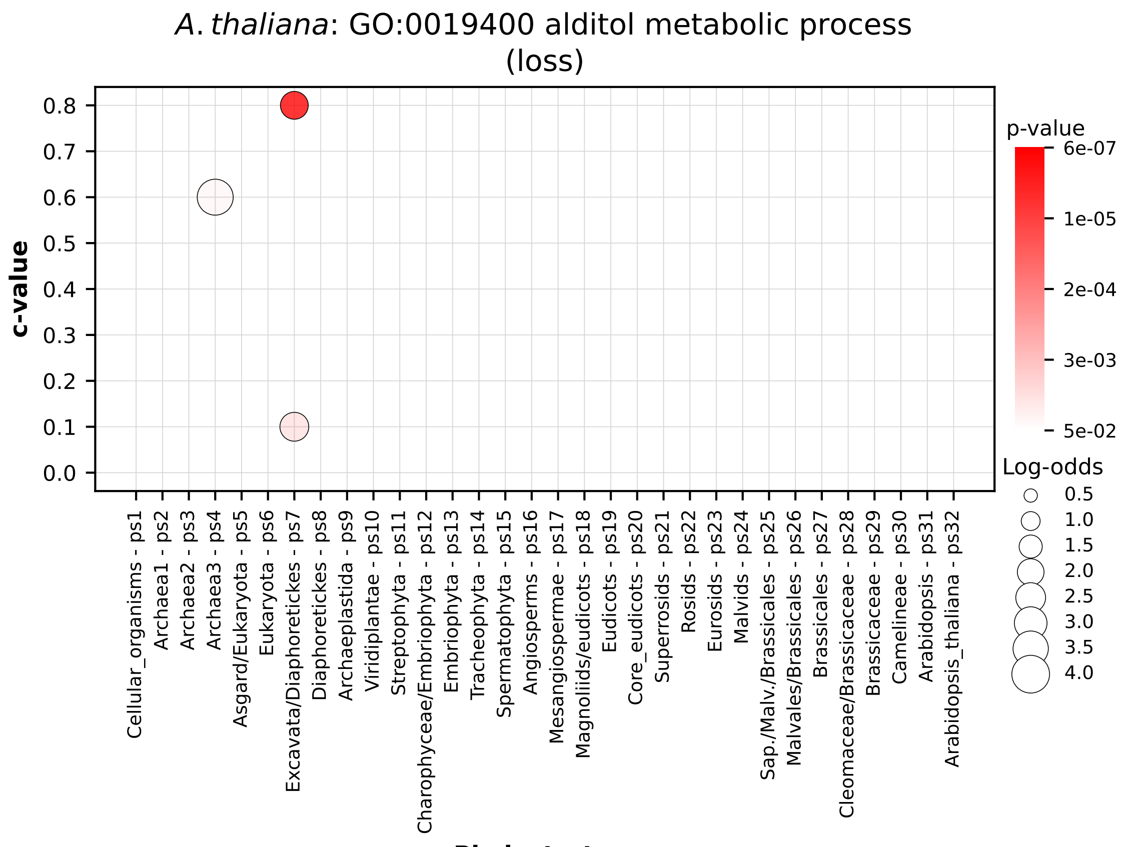

A. thaliana: GO:0019400 alditol metabolic process
(loss)
c-value
0.8
0.7
0.6
0.5
0.4
0.3
0.2
0.1
0.0
p-value
6e-07
1e-05
2e-04
3e-03
5e-02
Log-odds
0.5
1.0
1.5
2.0
2.5
3.0
3.5
4.0
Cellular_organisms - ps1
Archaea1 - ps2
Archaea2 - ps3
Archaea3 - ps4
Asgard/Eukaryota - ps5
Eukaryota - ps6
Excavata/Diaphoretickes - ps7
Diaphoretickes - ps8
Archaeplastida - ps9
Viridiplantae - ps10
Streptophyta - ps11
Charophyceae/Embriophyta - ps12
Embriophyta - ps13
Tracheophyta - ps14
Spermatophyta - ps15
Angiosperms - ps16
Mesangiospermae - ps17
Magnoliids/eudicots - ps18
Eudicots - ps19
Core_eudicots - ps20
Superrosids - ps21
Rosids - ps22
Eurosids - ps23
Malvids - ps24
Sap./Malv./Brassicales - ps25
Malvales/Brassicales - ps26
Brassicales - ps27
Cleomaceae/Brassicaceae - ps28
Brassicaceae - ps29
Camelineae - ps30
Arabidopsis - ps31
Arabidopsis_thaliana - ps32
Phylostratum

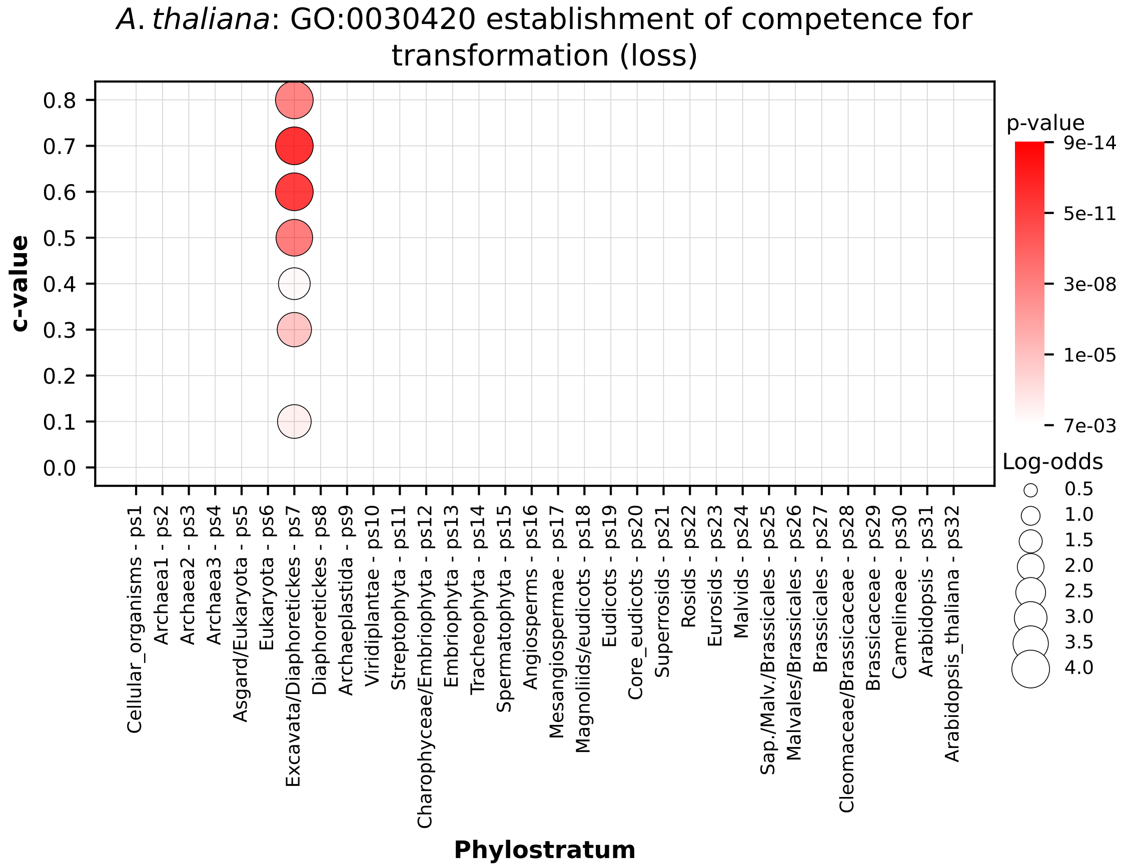
A. thaliana: GO:0030420 establishment of competence for transformation (loss)
c-value
0.8
0.7
0.6
0.5
0.4
0.3
0.2
0.1
0.0
p-value
9e-14
5e-11
3e-08
1e-05
7e-03
Log-odds
0.5
1.0
1.5
2.0
2.5
3.0
3.5
4.0
Cellular_organisms - ps1
Archaea1 - ps2
Archaea2 - ps3
Archaea3 - ps4
Asgard/Eukaryota - ps5
Eukaryota - ps6
Excavata/Diaphoretickes - ps7
Diaphoretickes - ps8
Archaeplastida - ps9
Viridiplantae - ps10
Streptophyta - ps11
Charophyceae/Embriophyta - ps12
Embriophyta - ps13
Tracheophyta - ps14
Spermatophyta - ps15
Angiosperms - ps16
Mesangiospermae - ps17
Magnoliids/eudicots - ps18
Eudicots - ps19
Core_eudicots - ps20
Superrosids - ps21
Rosids - ps22
Eurosids - ps23
Malvids - ps24
Sap./Malv./Brassicales - ps25
Malvales/Brassicales - ps26
Brassicales - ps27
Cleomaceae/Brassicaceae - ps28
Brassicaceae - ps29
Camelineae - ps30
Arabidopsis - ps31
Arabidopsis_thaliana - ps32
Phylostratum

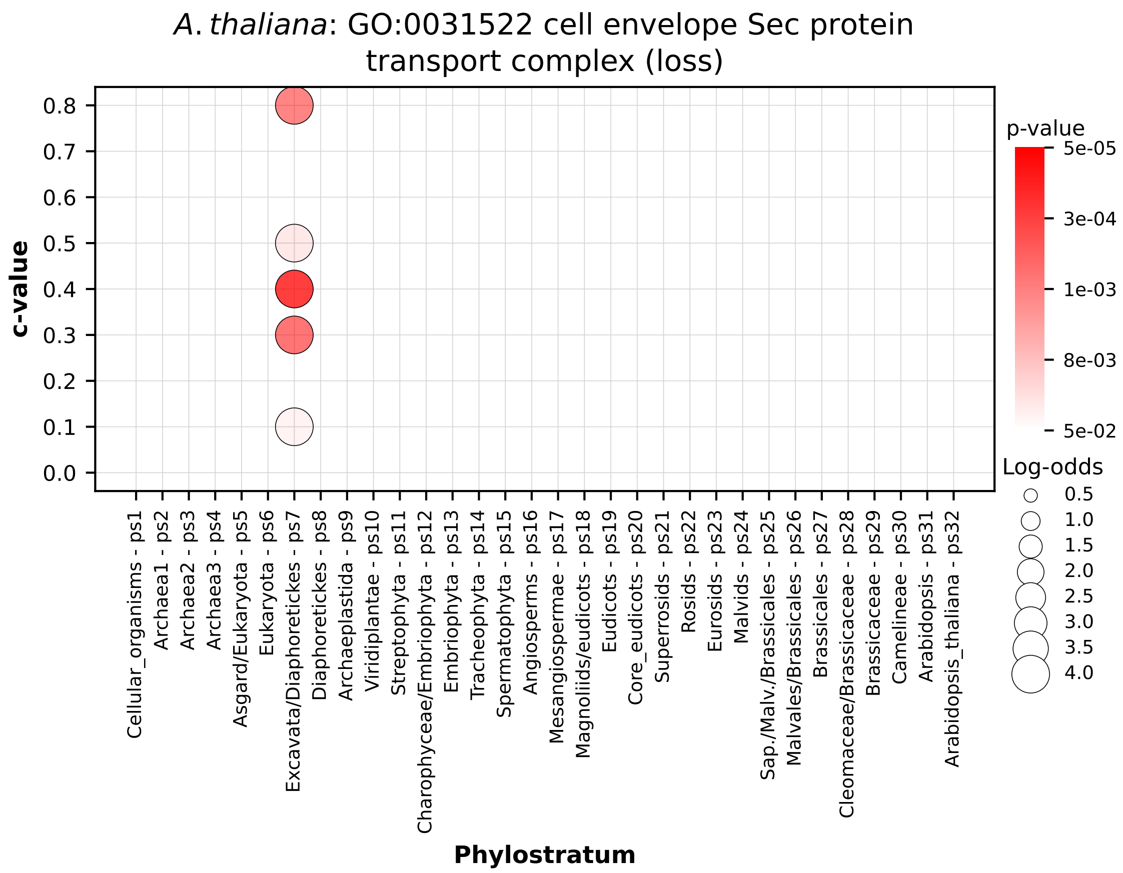

A. thaliana: GO:0031522 cell envelope Sec protein transport complex (loss)
c-value
0.8
0.7
0.6
0.5
0.4
0.3
0.2
0.1
0.0
p-value
5e-05
3e-04
1e-03
8e-03
5e-02
Log-odds
0.5
1.0
1.5
2.0
2.5
3.0
3.5
4.0
Cellular_organisms - ps1
Archaea1 - ps2
Archaea2 - ps3
Archaea3 - ps4
Asgard/Eukaryota - ps5
Eukaryota - ps6
Excavata/Diaphoretickes - ps7
Diaphoretickes - ps8
Archaeplastida - ps9
Viridiplantae - ps10
Streptophyta - ps11
Charophyceae/Embriophyta - ps12
Embriophyta - ps13
Tracheophyta - ps14
Spermatophyta - ps15
Angiosperms - ps16
Mesangiospermae - ps17
Magnoliids/eudicots - ps18
Eudicots - ps19
Core_eudicots - ps20
Superrosids - ps21
Rosids - ps22
Eurosids - ps23
Malvids - ps24
Sap./Malv./Brassicales - ps25
Malvales/Brassicales - ps26
Brassicales - ps27
Cleomaceae/Brassicaceae - ps28
Brassicaceae - ps29
Camelineae - ps30
Arabidopsis - ps31
Arabidopsis_thaliana - ps32
Phylostratum

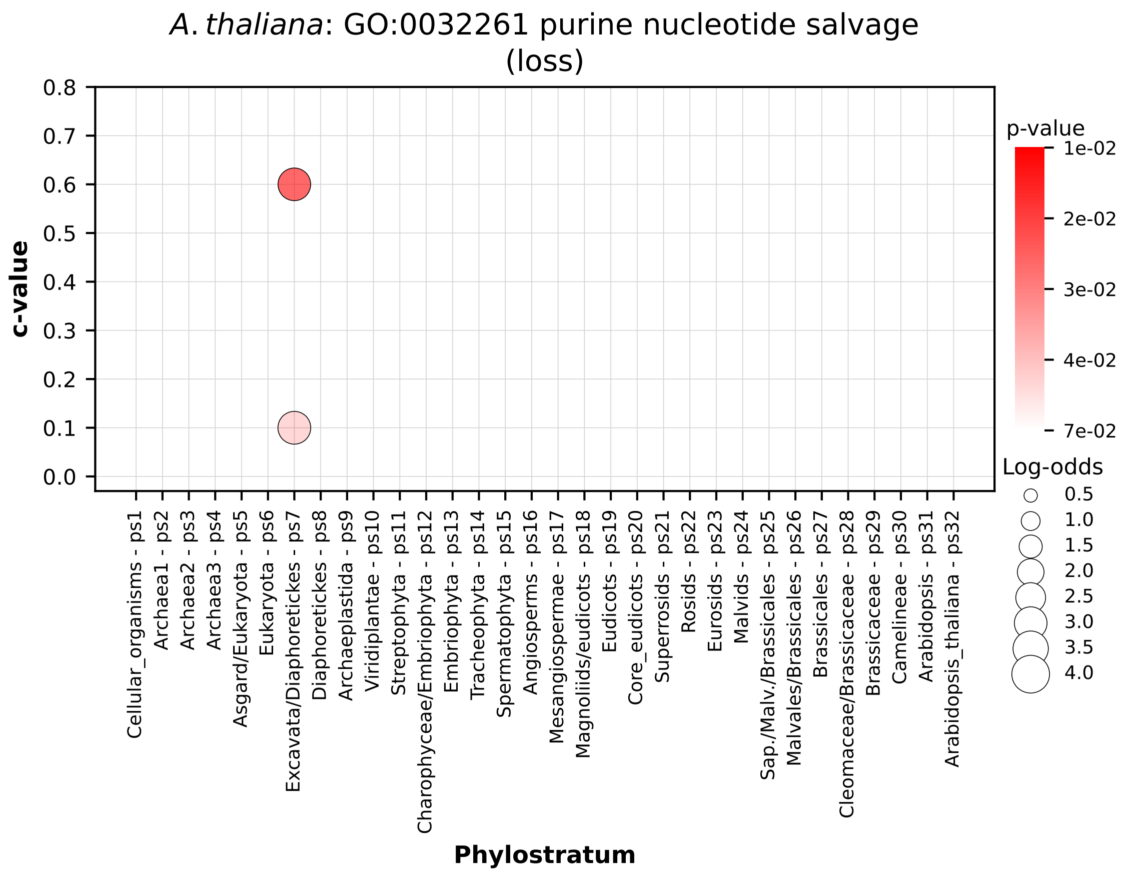

A. thaliana: GO:0032261 purine nucleotide salvage
(loss)
c-value
0.8
0.7
0.6
0.5
0.4
0.3
0.2
0.1
0.0
p-value
1e-02
2e-02
3e-02
4e-02
7e-02
Log-odds
0.5
1.0
1.5
2.0
2.5
3.0
3.5
4.0
Cellular_organisms - ps1
Archaea1 - ps2
Archaea2 - ps3
Archaea3 - ps4
Asgard/Eukaryota - ps5
Eukaryota - ps6
Excavata/Diaphoretickes - ps7
Diaphoretickes - ps8
Archaeplastida - ps9
Viridiplantae - ps10
Streptophyta - ps11
Charophyceae/Embriophyta - ps12
Embriophyta - ps13
Tracheophyta - ps14
Spermatophyta - ps15
Angiosperms - ps16
Mesangiospermae - ps17
Magnoliids/eudicots - ps18
Eudicots - ps19
Core_eudicots - ps20
Superrosids - ps21
Rosids - ps22
Eurosids - ps23
Malvids - ps24
Sap./Malv./Brassicales - ps25
Malvales/Brassicales - ps26
Brassicales - ps27
Cleomaceae/Brassicaceae - ps28
Brassicaceae - ps29
Camelineae - ps30
Arabidopsis - ps31
Arabidopsis_thaliana - ps32
Phylostratum

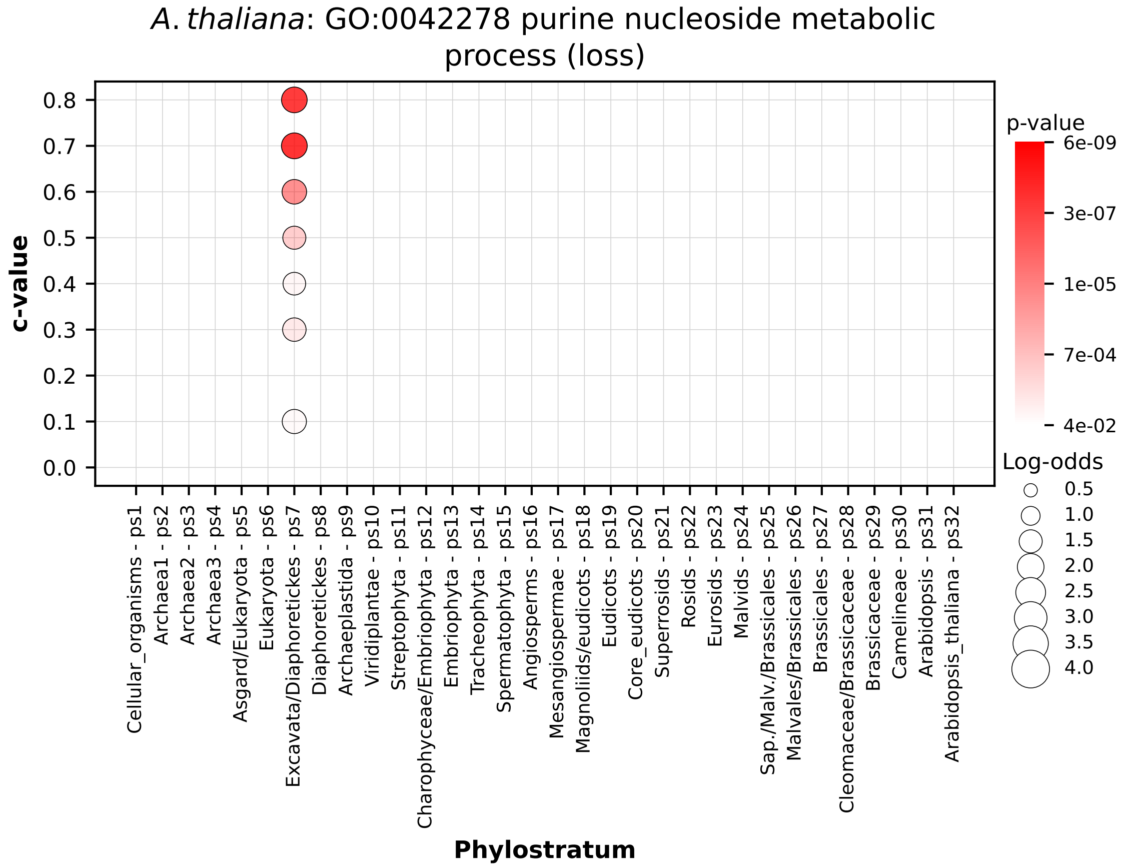

A. thaliana: GO:0042278 purine nucleoside metabolic process (loss)
c-value
0.0
0.1
0.2
0.3
0.4
0.5
0.6
0.7
0.8
p-value
6e-09
3e-07
1e-05
7e-04
4e-02
Log-odds
0.5
1.0
1.5
2.0
2.5
3.0
3.5
4.0
Cellular_organisms - ps1
Archaea1 - ps2
Archaea2 - ps3
Archaea3 - ps4
Asgard/Eukaryota - ps5
Eukaryota - ps6
Excavata/Diaphoretickes - ps7
Diaphoretickes - ps8
Archaeplastida - ps9
Viridiplantae - ps10
Streptophyta - ps11
Charophyceae/Embriophyta - ps12
Embriophyta - ps13
Tracheophyta - ps14
Spermatophyta - ps15
Angiosperms - ps16
Mesangiospermae - ps17
Magnoliids/eudicots - ps18
Eudicots - ps19
Core_eudicots - ps20
Superrosids - ps21
Rosids - ps22
Eurosids - ps23
Malvids - ps24
Sap./Malv./Brassicales - ps25
Malvales/Brassicales - ps26
Brassicales - ps27
Cleomaceae/Brassicaceae - ps28
Brassicaceae - ps29
Camelineae - ps30
Arabidopsis - ps31
Arabidopsis_thaliana - ps32
Phylostratum

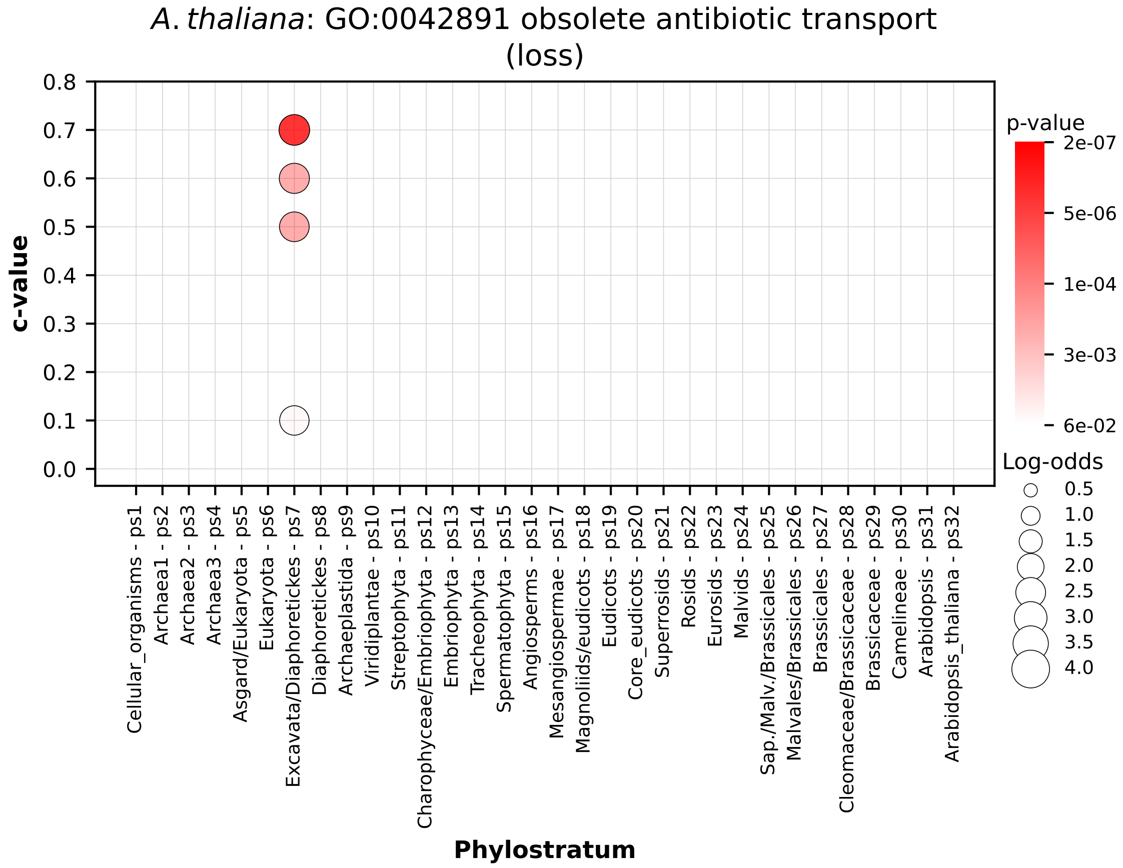
A. thaliana: GO:0042891 obsolete antibiotic transport (loss)
c-value
0.8
0.7
0.6
0.5
0.4
0.3
0.2
0.1
0.0
p-value
2e-07
5e-06
1e-04
3e-03
6e-02
Log-odds
0.5
1.0
1.5
2.0
2.5
3.0
3.5
4.0
Cellular_organisms - ps1
Archaea1 - ps2
Archaea2 - ps3
Archaea3 - ps4
Asgard/Eukaryota - ps5
Eukaryota - ps6
Excavata/Diaphoretickes - ps7
Diaphoretickes - ps8
Archaeplastida - ps9
Viridiplantae - ps10
Streptophyta - ps11
Charophyceae/Embriophyta - ps12
Embriophyta - ps13
Tracheophyta - ps14
Spermatophyta - ps15
Angiosperms - ps16
Mesangiospermae - ps17
Magnoliids/eudicots - ps18
Eudicots - ps19
Core_eudicots - ps20
Superrosids - ps21
Rosids - ps22
Eurosids - ps23
Malvids - ps24
Sap./Malv./Brassicales - ps25
Malvales/Brassicales - ps26
Brassicales - ps27
Cleomaceae/Brassicaceae - ps28
Brassicaceae - ps29
Camelineae - ps30
Arabidopsis - ps31
Arabidopsis_thaliana - ps32
Phylostratum

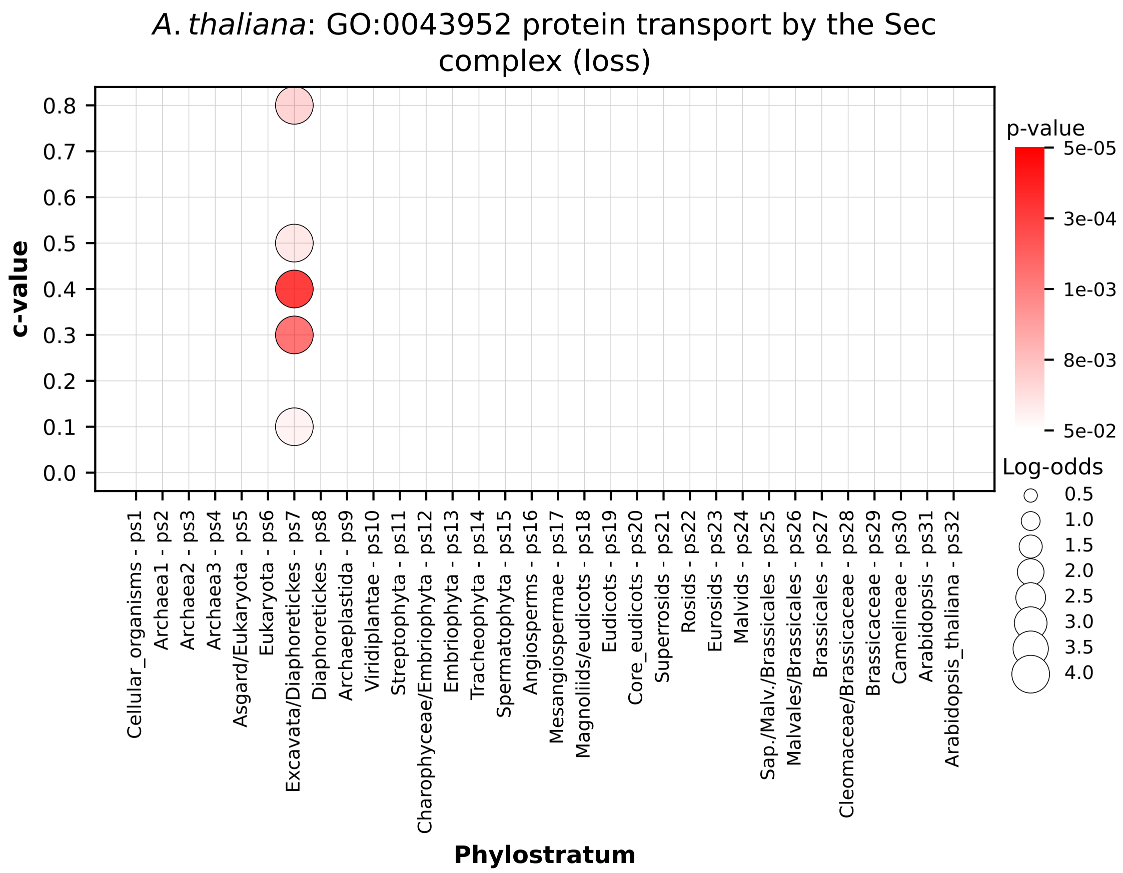
A. thaliana: GO:0043952 protein transport by the Sec complex (loss)
c-value
0.0
0.1
0.2
0.3
0.4
0.5
0.6
0.7
0.8
p-value
5e-05
3e-04
1e-03
8e-03
5e-02
Log-odds
0.5
1.0
1.5
2.0
2.5
3.0
3.5
4.0
Cellular_organisms - ps1
Archaea1 - ps2
Archaea2 - ps3
Archaea3 - ps4
Asgard/Eukaryota - ps5
Eukaryota - ps6
Excavata/Diaphoretickes - ps7
Diaphoretickes - ps8
Archaeplastida - ps9
Viridiplantae - ps10
Streptophyta - ps11
Charophyceae/Embriophyta - ps12
Embriophyta - ps13
Tracheophyta - ps14
Spermatophyta - ps15
Angiosperms - ps16
Mesangiospermae - ps17
Magnoliids/eudicots - ps18
Eudicots - ps19
Core_eudicots - ps20
Superrosids - ps21
Rosids - ps22
Eurosids - ps23
Malvids - ps24
Sap./Malv./Brassicales - ps25
Malvales/Brassicales - ps26
Brassicales - ps27
Cleomaceae/Brassicaceae - ps28
Brassicaceae - ps29
Camelineae - ps30
Arabidopsis - ps31
Arabidopsis_thaliana - ps32
Phylostratum

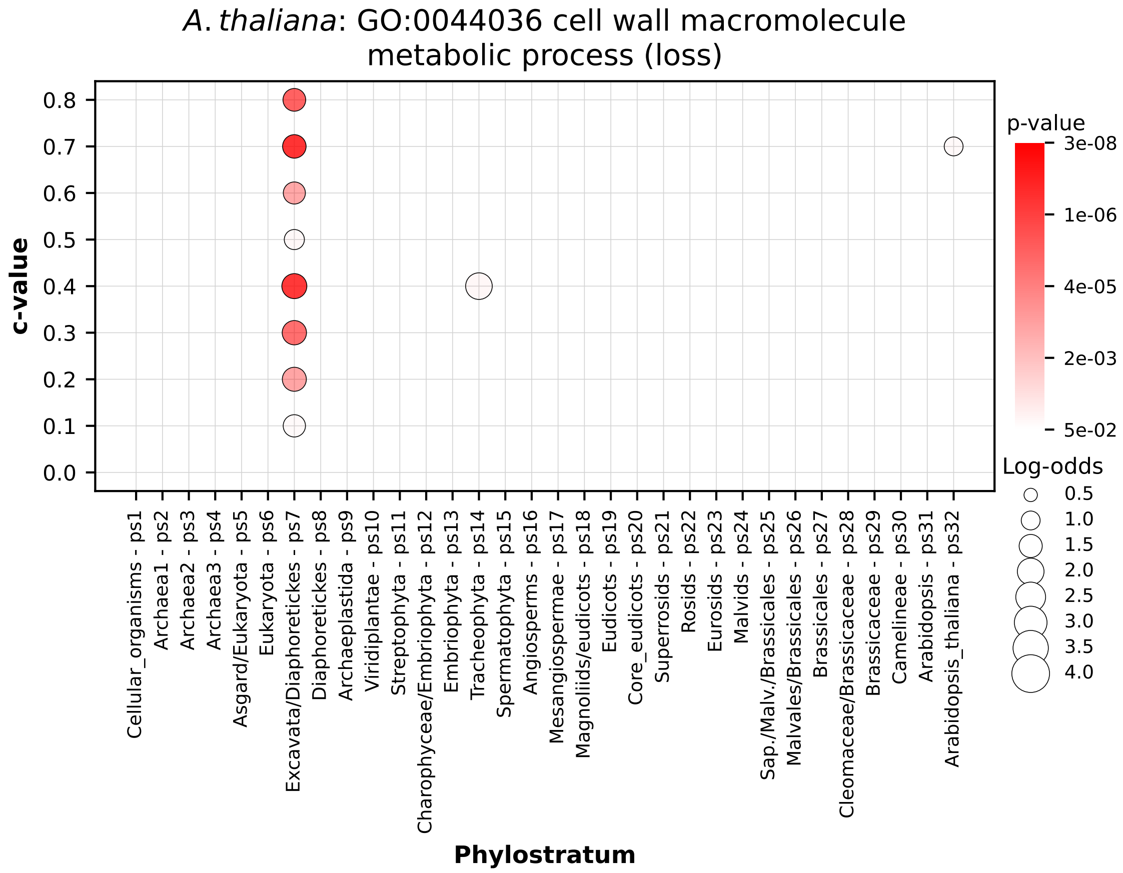
A. thaliana: GO:0044036 cell wall macromolecule metabolic process (loss)
c-value
0.8
0.7
0.6
0.5
0.4
0.3
0.2
0.1
0.0
p-value
3e-08
1e-06
4e-05
2e-03
5e-02
Log-odds
0.5
1.0
1.5
2.0
2.5
3.0
3.5
4.0
Cellular_organisms - ps1
Archaea1 - ps2
Archaea2 - ps3
Archaea3 - ps4
Asgard/Eukaryota - ps5
Eukaryota - ps6
Excavata/Diaphoretickes - ps7
Diaphoretickes - ps8
Archaeplastida - ps9
Viridiplantae - ps10
Streptophyta - ps11
Charophyceae/Embriophyta - ps12
Embriophyta - ps13
Tracheophyta - ps14
Spermatophyta - ps15
Angiosperms - ps16
Mesangiospermae - ps17
Magnoliids/eudicots - ps18
Eudicots - ps19
Core_eudicots - ps20
Superrosids - ps21
Rosids - ps22
Eurosids - ps23
Malvids - ps24
Sap./Malv./Brassicales - ps25
Malvales/Brassicales - ps26
Brassicales - ps27
Cleomaceae/Brassicaceae - ps28
Brassicaceae - ps29
Camelineae - ps30
Arabidopsis - ps31
Arabidopsis_thaliana - ps32
Phylostratum

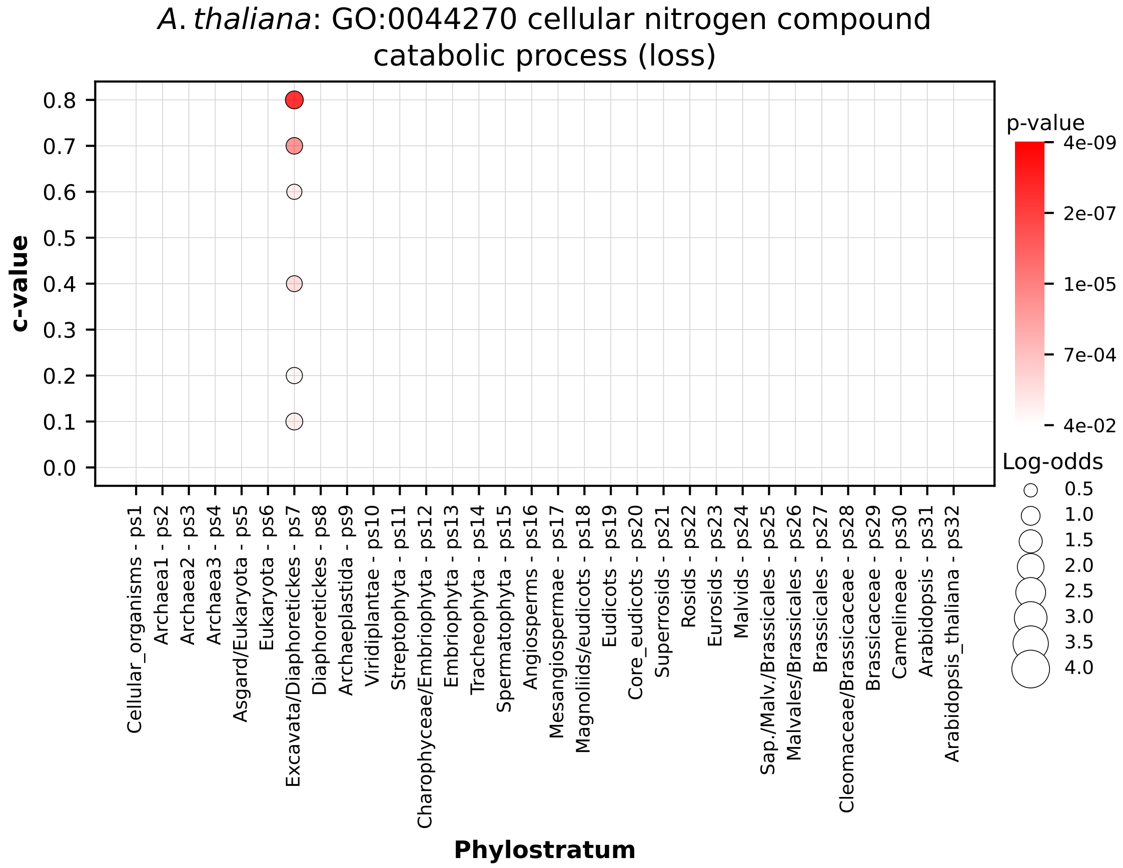

A. thaliana: GO:0044270 cellular nitrogen compound catabolic process (loss)
c-value
0.0
0.1
0.2
0.3
0.4
0.5
0.6
0.7
0.8
Phylostratum
Cellular_organisms - ps1
Archaea1 - ps2
Archaea2 - ps3
Archaea3 - ps4
Asgard/Eukaryota - ps5
Eukaryota - ps6
Excavata/Diaphoretickes - ps7
Diaphoretickes - ps8
Archaeplastida - ps9
Viridiplantae - ps10
Streptophyta - ps11
Charophyceae/Embriophyta - ps12
Embriophyta - ps13
Tracheophyta - ps14
Spermatophyta - ps15
Angiosperms - ps16
Mesangiospermae - ps17
Magnoliids/eudicots - ps18
Eudicots - ps19
Core_eudicots - ps20
Superrosids - ps21
Rosids - ps22
Eurosids - ps23
Malvids - ps24
Sap./Malv./Brassicales - ps25
Malvales/Brassicales - ps26
Brassicales - ps27
Cleomaceae/Brassicaceae - ps28
Brassicaceae - ps29
Camelineae - ps30
Arabidopsis - ps31
Arabidopsis_thaliana - ps32
p-value
4e-09
2e-07
1e-05
7e-04
4e-02
Log-odds
0.5
1.0
1.5
2.0
2.5
3.0
3.5
4.0

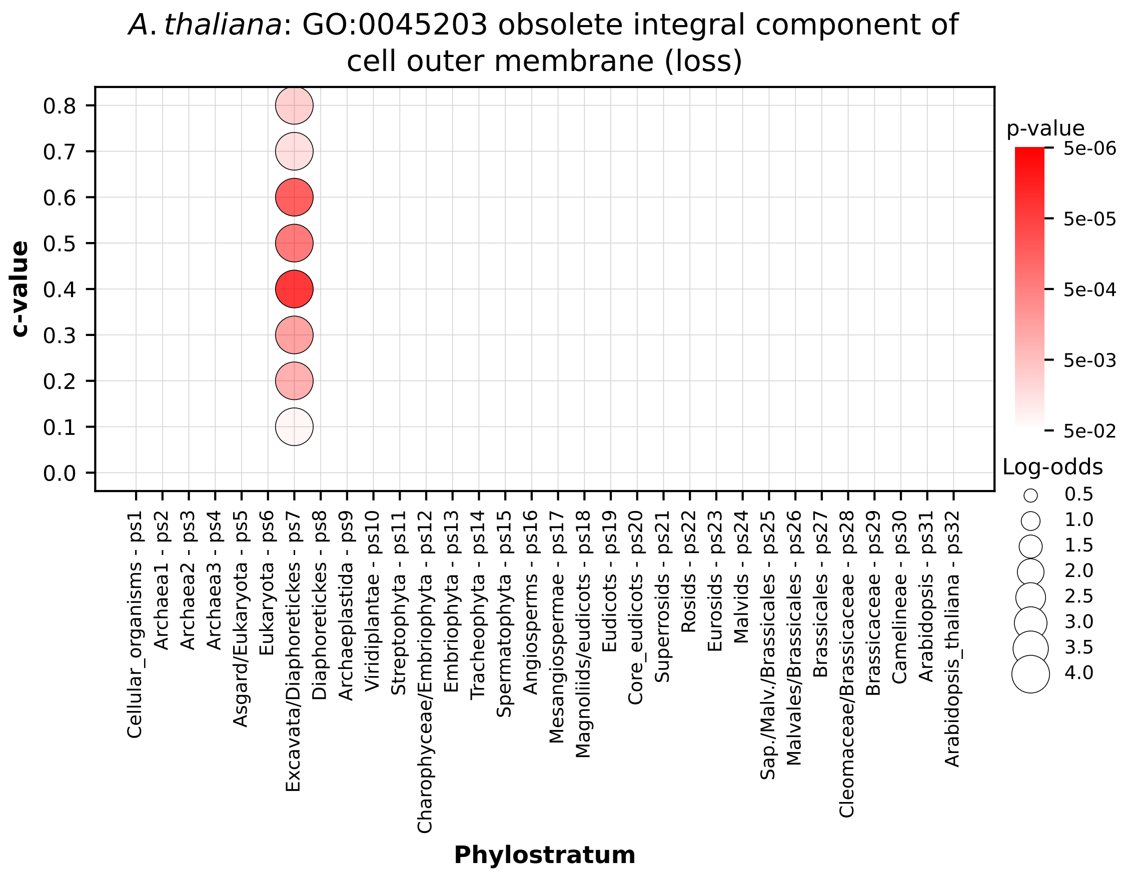

A. thaliana: GO:0045203 obsolete integral component of cell outer membrane (loss)
c-value
0.0
0.1
0.2
0.3
0.4
0.5
0.6
0.7
0.8
p-value
5e-06
5e-05
5e-04
5e-03
5e-02
Log-odds
0.5
1.0
1.5
2.0
2.5
3.0
3.5
4.0
Cellular_organisms - ps1
Archaea1 - ps2
Archaea2 - ps3
Archaea3 - ps4
Asgard/Eukaryota - ps5
Eukaryota - ps6
Excavata/Diaphoretickes - ps7
Diaphoretickes - ps8
Archaeplastida - ps9
Viridiplantae - ps10
Streptophyta - ps11
Charophyceae/Embriophyta - ps12
Embriophyta - ps13
Tracheophyta - ps14
Spermatophyta - ps15
Angiosperms - ps16
Mesangiospermae - ps17
Magnoliids/eudicots - ps18
Eudicots - ps19
Core_eudicots - ps20
Superrosids - ps21
Rosids - ps22
Eurosids - ps23
Malvids - ps24
Sap./Malv./Brassicales - ps25
Malvales/Brassicales - ps26
Brassicales - ps27
Cleomaceae/Brassicaceae - ps28
Brassicaceae - ps29
Camelineae - ps30
Arabidopsis - ps31
Arabidopsis_thaliana - ps32
Phylostratum

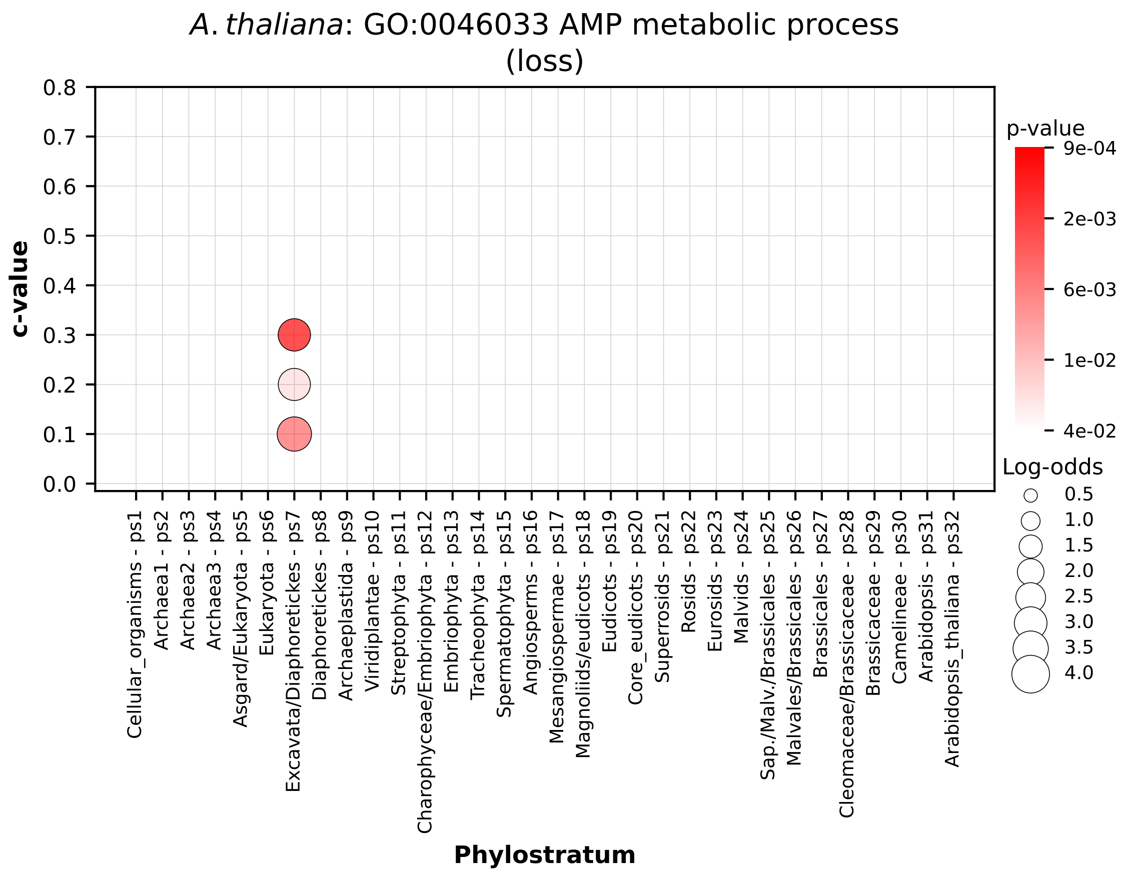

A. thaliana: GO:0046033 AMP metabolic process
(loss)
c-value
0.8
0.7
0.6
0.5
0.4
0.3
0.2
0.1
0.0
p-value
9e-04
2e-03
6e-03
1e-02
4e-02
Log-odds
0.5
1.0
1.5
2.0
2.5
3.0
3.5
4.0
Cellular_organisms - ps1
Archaea1 - ps2
Archaea2 - ps3
Archaea3 - ps4
Asgard/Eukaryota - ps5
Eukaryota - ps6
Excavata/Diaphoretickes - ps7
Diaphoretickes - ps8
Archaeplastida - ps9
Viridiplantae - ps10
Streptophyta - ps11
Charophyceae/Embriophyta - ps12
Embriophyta - ps13
Tracheophyta - ps14
Spermatophyta - ps15
Angiosperms - ps16
Mesangiospermae - ps17
Magnoliids/eudicots - ps18
Eudicots - ps19
Core_eudicots - ps20
Superrosids - ps21
Rosids - ps22
Eurosids - ps23
Malvids - ps24
Sap./Malv./Brassicales - ps25
Malvales/Brassicales - ps26
Brassicales - ps27
Cleomaceae/Brassicaceae - ps28
Brassicaceae - ps29
Camelineae - ps30
Arabidopsis - ps31
Arabidopsis_thaliana - ps32
Phylostratum

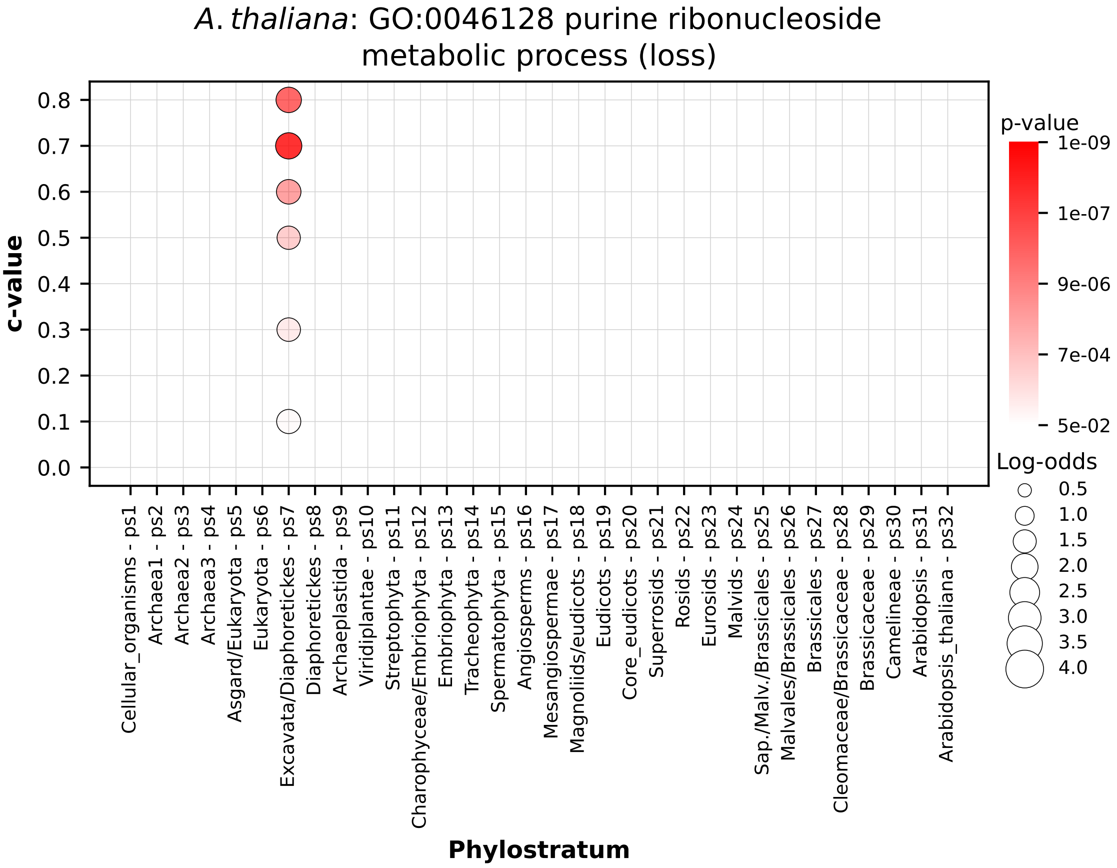

A. thaliana: GO:0046128 purine ribonucleoside metabolic process (loss)
c-value
0.8
0.7
0.6
0.5
0.4
0.3
0.2
0.1
0.0
p-value
1e-09
1e-07
9e-06
7e-04
5e-02
Log-odds
0.5
1.0
1.5
2.0
2.5
3.0
3.5
4.0
Cellular_organisms - ps1
Archaea1 - ps2
Archaea2 - ps3
Archaea3 - ps4
Asgard/Eukaryota - ps5
Eukaryota - ps6
Excavata/Diaphoretickes - ps7
Diaphoretickes - ps8
Archaeplastida - ps9
Viridiplantae - ps10
Streptophyta - ps11
Charophyceae/Embriophyta - ps12
Embriophyta - ps13
Tracheophyta - ps14
Spermatophyta - ps15
Angiosperms - ps16
Mesangiospermae - ps17
Magnoliids/eudicots - ps18
Eudicots - ps19
Core_eudicots - ps20
Superrosids - ps21
Rosids - ps22
Eurosids - ps23
Malvids - ps24
Sap./Malv./Brassicales - ps25
Malvales/Brassicales - ps26
Brassicales - ps27
Cleomaceae/Brassicaceae - ps28
Brassicaceae - ps29
Camelineae - ps30
Arabidopsis - ps31
Arabidopsis_thaliana - ps32
Phylostratum

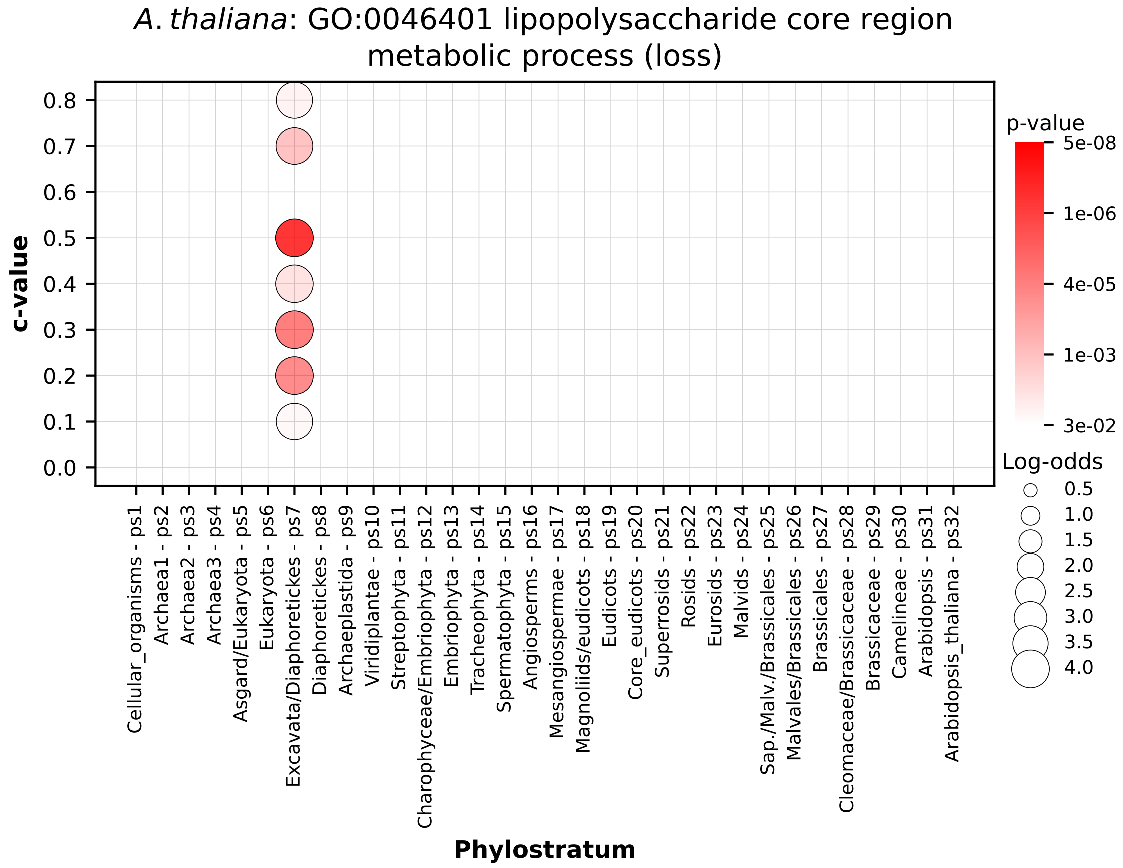

A. thaliana: GO:0046401 lipopolysaccharide core region metabolic process (loss)
c-value
0.8
0.7
0.6
0.5
0.4
0.3
0.2
0.1
0.0
p-value
5e-08
1e-06
4e-05
1e-03
3e-02
Log-odds
0.5
1.0
1.5
2.0
2.5
3.0
3.5
4.0
Cellular_organisms - ps1
Archaea1 - ps2
Archaea2 - ps3
Archaea3 - ps4
Asgard/Eukaryota - ps5
Eukaryota - ps6
Excavata/Diaphoretickes - ps7
Diaphoretickes - ps8
Archaeplastida - ps9
Viridiplantae - ps10
Streptophyta - ps11
Charophyceae/Embriophyta - ps12
Embriophyta - ps13
Tracheophyta - ps14
Spermatophyta - ps15
Angiosperms - ps16
Mesangiospermae - ps17
Magnoliids/eudicots - ps18
Eudicots - ps19
Core_eudicots - ps20
Superrosids - ps21
Rosids - ps22
Eurosids - ps23
Malvids - ps24
Sap./Malv./Brassicales - ps25
Malvales/Brassicales - ps26
Brassicales - ps27
Cleomaceae/Brassicaceae - ps28
Brassicaceae - ps29
Camelineae - ps30
Arabidopsis - ps31
Arabidopsis_thaliana - ps32
Phylostratum

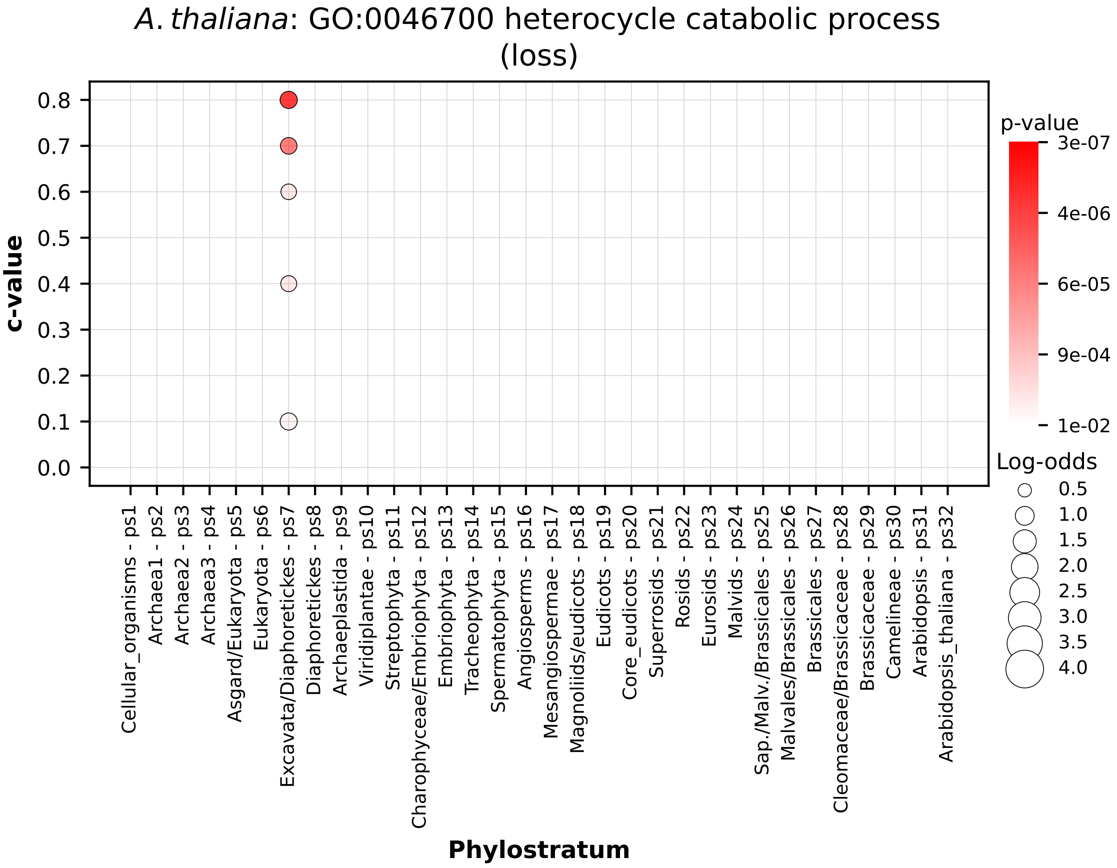

A. thaliana: GO:0046700 heterocycle catabolic process
(loss)
c-value
0.0
0.1
0.2
0.3
0.4
0.5
0.6
0.7
0.8
Phylostratum
Cellular_organisms - ps1
Archaea1 - ps2
Archaea2 - ps3
Archaea3 - ps4
Asgard/Eukaryota - ps5
Eukaryota - ps6
Excavata/Diaphoretickes - ps7
Diaphoretickes - ps8
Archaeplastida - ps9
Viridiplantae - ps10
Streptophyta - ps11
Charophyceae/Embriophyta - ps12
Embriophyta - ps13
Tracheophyta - ps14
Spermatophyta - ps15
Angiosperms - ps16
Mesangiospermae - ps17
Magnoliids/eudicots - ps18
Eudicots - ps19
Core_eudicots - ps20
Superrosids - ps21
Rosids - ps22
Eurosids - ps23
Malvids - ps24
Sap./Malv./Brassicales - ps25
Malvales/Brassicales - ps26
Brassicales - ps27
Cleomaceae/Brassicaceae - ps28
Brassicaceae - ps29
Camelineae - ps30
Arabidopsis - ps31
Arabidopsis_thaliana - ps32
p-value
3e-07
4e-06
6e-05
9e-04
1e-02
Log-odds
0.5
1.0
1.5
2.0
2.5
3.0
3.5
4.0

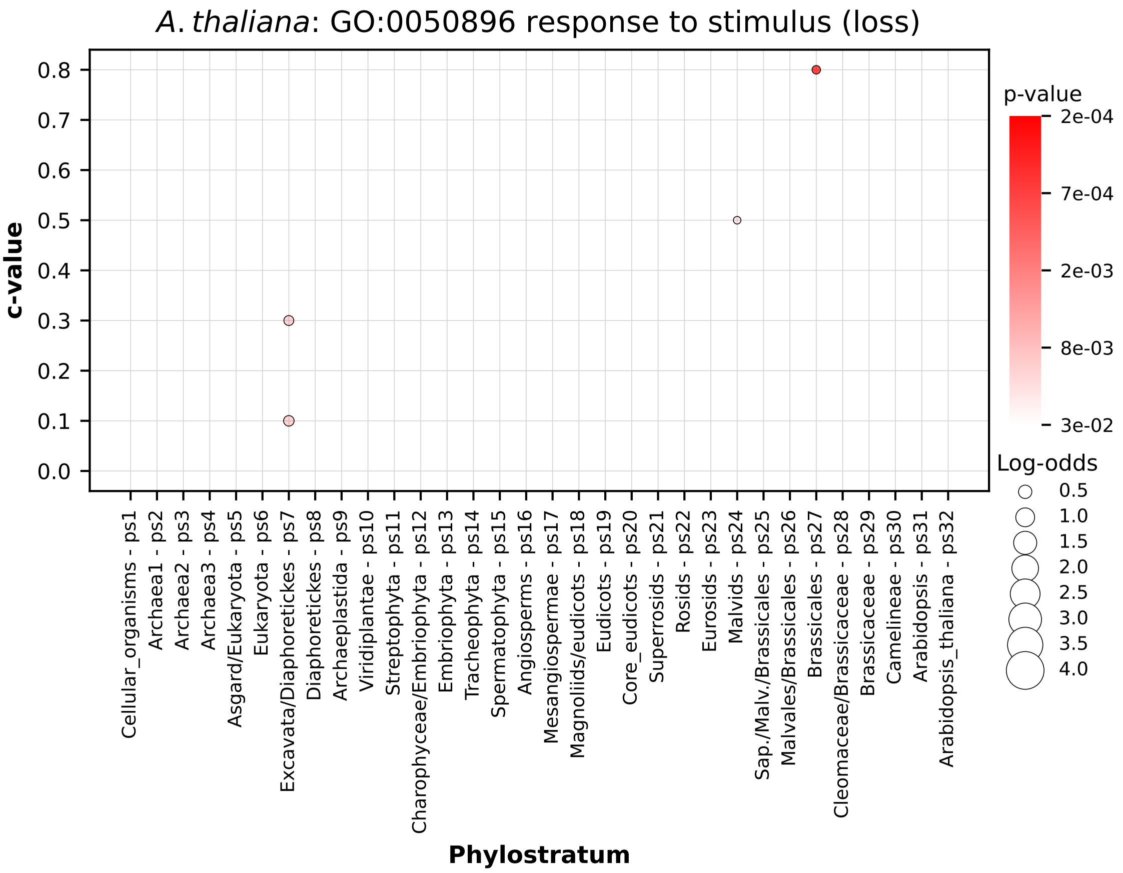

A. thaliana: GO:0050896 response to stimulus (loss)
c-value
0.0
0.1
0.2
0.3
0.4
0.5
0.6
0.7
0.8
p-value
2e-04
7e-04
2e-03
8e-03
3e-02
Log-odds
0.5
1.0
1.5
2.0
2.5
3.0
3.5
4.0
Cellular_organisms - ps1
Archaea1 - ps2
Archaea2 - ps3
Archaea3 - ps4
Asgard/Eukaryota - ps5
Eukaryota - ps6
Excavata/Diaphoretickes - ps7
Diaphoretickes - ps8
Archaeplastida - ps9
Viridiplantae - ps10
Streptophyta - ps11
Charophyceae/Embriophyta - ps12
Embriophyta - ps13
Tracheophyta - ps14
Spermatophyta - ps15
Angiosperms - ps16
Mesangiospermae - ps17
Magnoliids/eudicots - ps18
Eudicots - ps19
Core_eudicots - ps20
Superrosids - ps21
Rosids - ps22
Eurosids - ps23
Malvids - ps24
Sap./Malv./Brassicales - ps25
Malvales/Brassicales - ps26
Brassicales - ps27
Cleomaceae/Brassicaceae - ps28
Brassicaceae - ps29
Camelineae - ps30
Arabidopsis - ps31
Arabidopsis_thaliana - ps32
Phylostratum

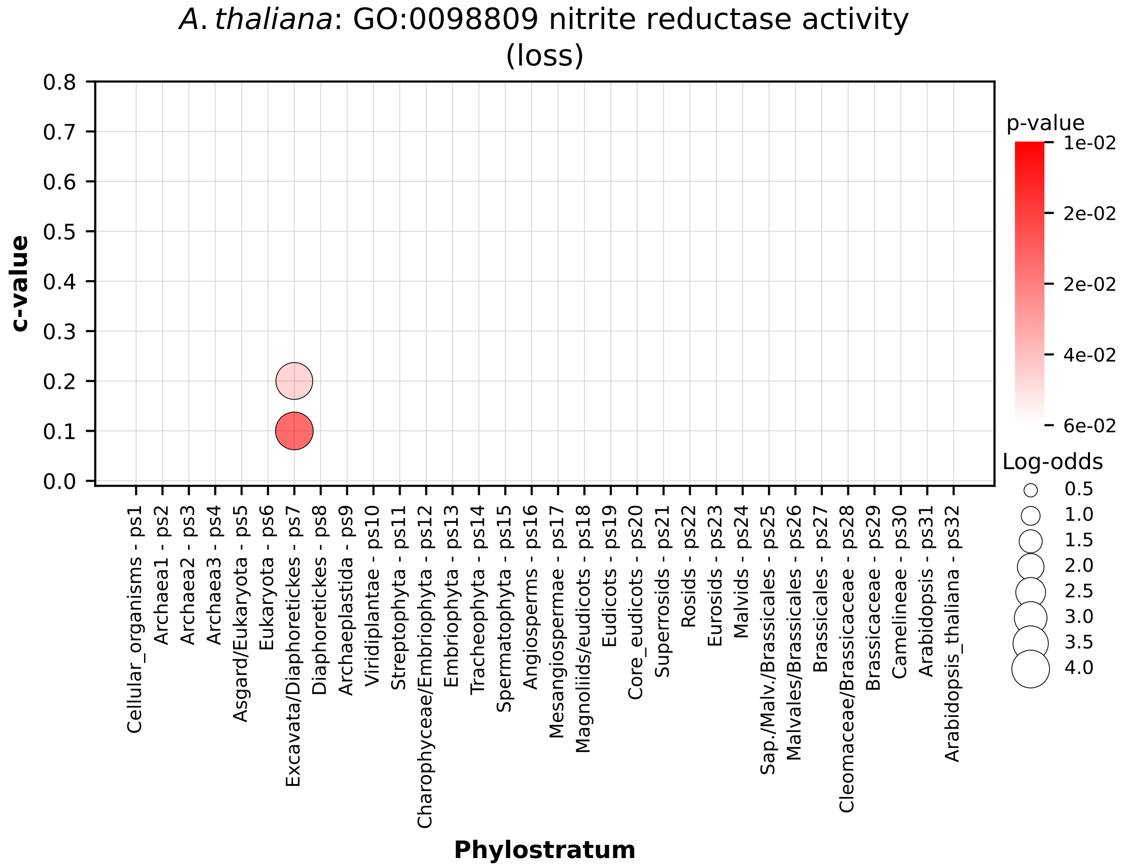
A. thaliana: GO:0098809 nitrite reductase activity
(loss)
c-value
0.8
0.7
0.6
0.5
0.4
0.3
0.2
0.1
0.0
Phylostratum
Cellular_organisms - ps1
Archaea1 - ps2
Archaea2 - ps3
Archaea3 - ps4
Asgard/Eukaryota - ps5
Eukaryota - ps6
Excavata/Diaphoretickes - ps7
Diaphoretickes - ps8
Archaeplastida - ps9
Viridiplantae - ps10
Streptophyta - ps11
Charophyceae/Embriophyta - ps12
Embriophyta - ps13
Tracheophyta - ps14
Spermatophyta - ps15
Angiosperms - ps16
Mesangiospermae - ps17
Magnoliids/eudicots - ps18
Eudicots - ps19
Core_eudicots - ps20
Superrosids - ps21
Rosids - ps22
Eurosids - ps23
Malvids - ps24
Sap./Malv./Brassicales - ps25
Malvales/Brassicales - ps26
Brassicales - ps27
Cleomaceae/Brassicaceae - ps28
Brassicaceae - ps29
Camelineae - ps30
Arabidopsis - ps31
Arabidopsis_thaliana - ps32
p-value
1e-02
2e-02
2e-02
4e-02
6e-02
Log-odds
0.5
1.0
1.5
2.0
2.5
3.0
3.5
4.0

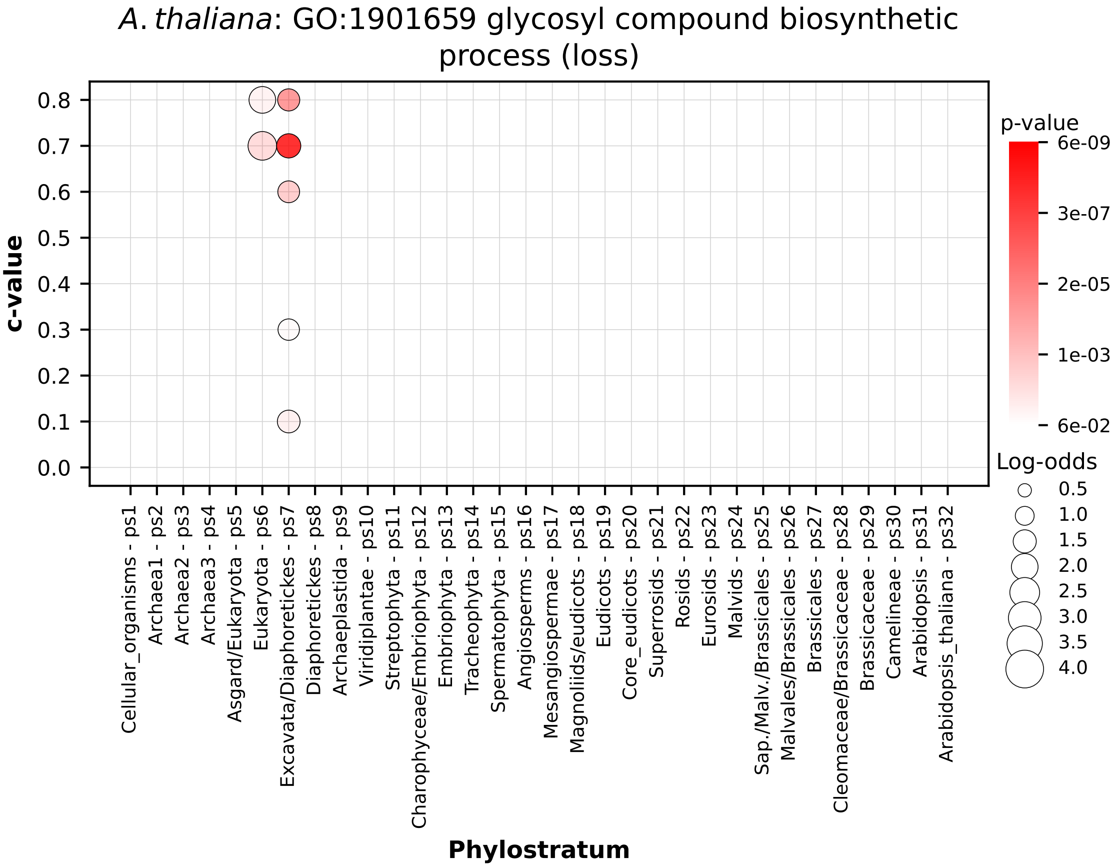

A. thaliana: GO:1901659 glycosyl compound biosynthetic process (loss)
c-value
0.0
0.1
0.2
0.3
0.4
0.5
0.6
0.7
0.8
Phylostratum
Cellular_organisms - ps1
Archaea1 - ps2
Archaea2 - ps3
Archaea3 - ps4
Asgard/Eukaryota - ps5
Eukaryota - ps6
Excavata/Diaphoretickes - ps7
Diaphoretickes - ps8
Archaeplastida - ps9
Viridiplantae - ps10
Streptophyta - ps11
Charophyceae/Embriophyta - ps12
Embriophyta - ps13
Tracheophyta - ps14
Spermatophyta - ps15
Angiosperms - ps16
Mesangiospermae - ps17
Magnoliids/eudicots - ps18
Eudicots - ps19
Core_eudicots - ps20
Superrosids - ps21
Rosids - ps22
Eurosids - ps23
Malvids - ps24
Sap./Malv./Brassicales - ps25
Malvales/Brassicales - ps26
Brassicales - ps27
Cleomaceae/Brassicaceae - ps28
Brassicaceae - ps29
Camelineae - ps30
Arabidopsis - ps31
Arabidopsis_thaliana - ps32
p-value
6e-09
3e-07
2e-05
1e-03
6e-02
Log-odds
0.5
1.0
1.5
2.0
2.5
3.0
3.5
4.0

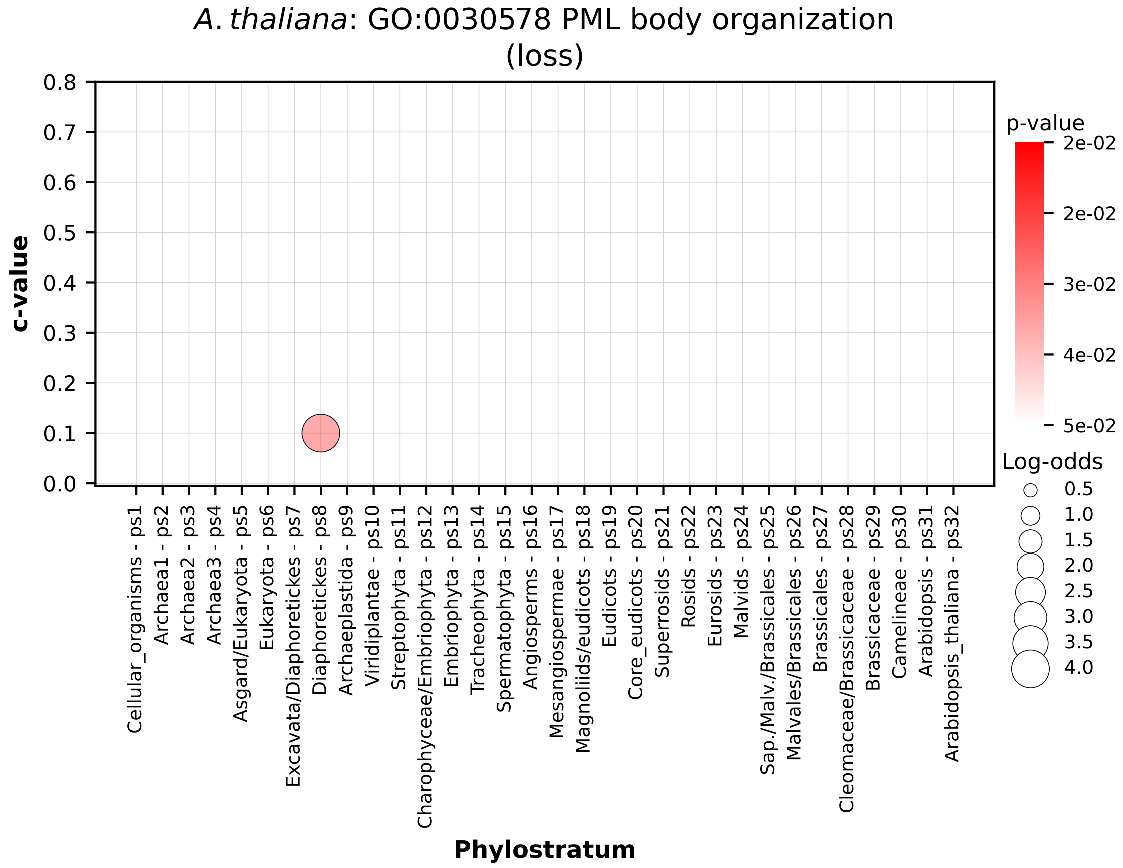

A. thaliana: GO:0030578 PML body organization
(loss)
c-value
0.8
0.7
0.6
0.5
0.4
0.3
0.2
0.1
0.0
p-value
2e-02
2e-02
3e-02
4e-02
5e-02
Log-odds
0.5
1.0
1.5
2.0
2.5
3.0
3.5
4.0
Cellular_organisms - ps1
Archaea1 - ps2
Archaea2 - ps3
Archaea3 - ps4
Asgard/Eukaryota - ps5
Eukaryota - ps6
Excavata/Diaphoretickes - ps7
Diaphoretickes - ps8
Archaeplastida - ps9
Viridiplantae - ps10
Streptophyta - ps11
Charophyceae/Embriophyta - ps12
Embriophyta - ps13
Tracheophyta - ps14
Spermatophyta - ps15
Angiosperms - ps16
Mesangiospermae - ps17
Magnoliids/eudicots - ps18
Eudicots - ps19
Core_eudicots - ps20
Superrosids - ps21
Rosids - ps22
Eurosids - ps23
Malvids - ps24
Sap./Malv./Brassicales - ps25
Malvales/Brassicales - ps26
Brassicales - ps27
Cleomaceae/Brassicaceae - ps28
Brassicaceae - ps29
Camelineae - ps30
Arabidopsis - ps31
Arabidopsis_thaliana - ps32
Phylostratum

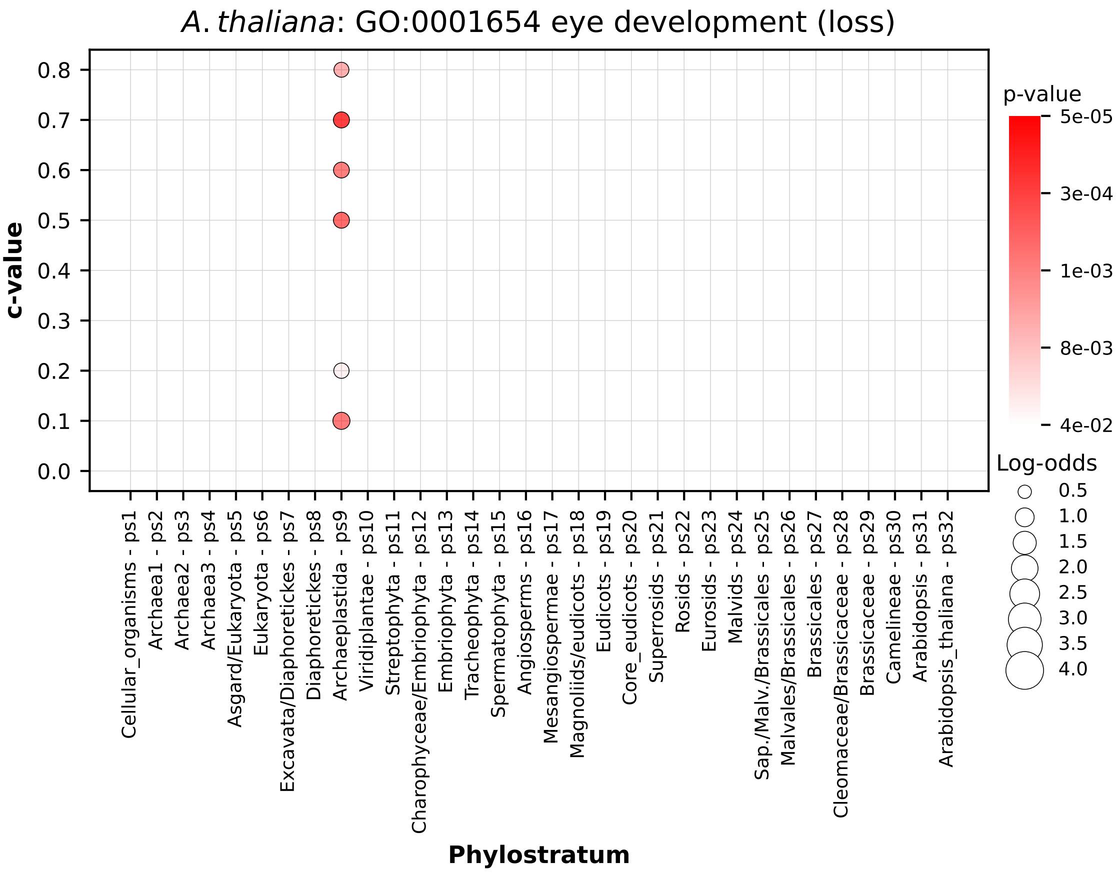
A. thaliana: GO:0001654 eye development (loss)
c-value
0.0
0.1
0.2
0.3
0.4
0.5
0.6
0.7
0.8
Phylostratum
Cellular_organisms - ps1
Archaea1 - ps2
Archaea2 - ps3
Archaea3 - ps4
Asgard/Eukaryota - ps5
Eukaryota - ps6
Excavata/Diaphoretickes - ps7
Diaphoretickes - ps8
Archaeplastida - ps9
Viridiplantae - ps10
Streptophyta - ps11
Charophyceae/Embriophyta - ps12
Embriophyta - ps13
Tracheophyta - ps14
Spermatophyta - ps15
Angiosperms - ps16
Mesangiospermae - ps17
Magnoliids/eudicots - ps18
Eudicots - ps19
Core_eudicots - ps20
Superrosids - ps21
Rosids - ps22
Eurosids - ps23
Malvids - ps24
Sap./Malv./Brassicales - ps25
Malvales/Brassicales - ps26
Brassicales - ps27
Cleomaceae/Brassicaceae - ps28
Brassicaceae - ps29
Camelineae - ps30
Arabidopsis - ps31
Arabidopsis_thaliana - ps32
p-value
5e-05
3e-04
1e-03
8e-03
4e-02
Log-odds
0.5
1.0
1.5
2.0
2.5
3.0
3.5
4.0

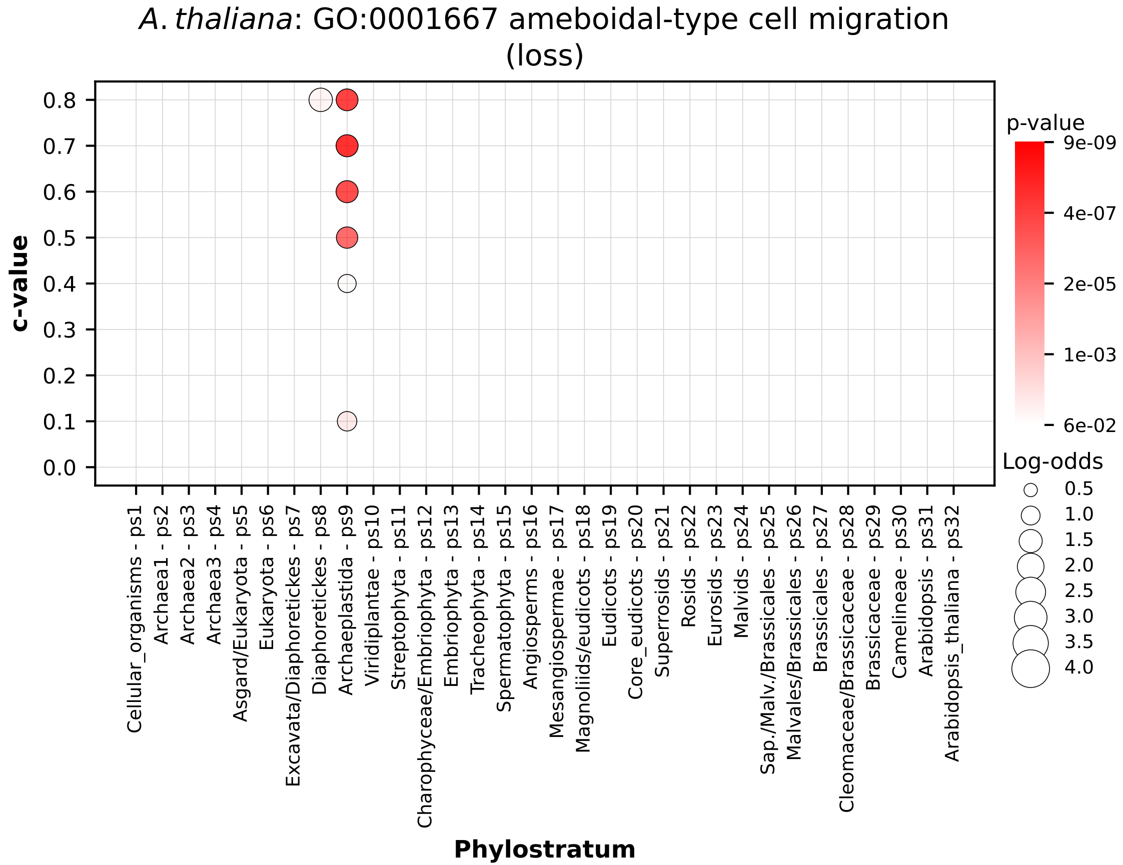

A. thaliana: GO:0001667 ameboidal-type cell migration
(loss)
c-value
0.8
0.7
0.6
0.5
0.4
0.3
0.2
0.1
0.0
p-value
9e-09
4e-07
2e-05
1e-03
6e-02
Log-odds
0.5
1.0
1.5
2.0
2.5
3.0
3.5
4.0
Cellular_organisms - ps1
Archaea1 - ps2
Archaea2 - ps3
Archaea3 - ps4
Asgard/Eukaryota - ps5
Eukaryota - ps6
Excavata/Diaphoretickes - ps7
Diaphoretickes - ps8
Archaeplastida - ps9
Viridiplantae - ps10
Streptophyta - ps11
Charophyceae/Embriophyta - ps12
Embriophyta - ps13
Tracheophyta - ps14
Spermatophyta - ps15
Angiosperms - ps16
Mesangiospermae - ps17
Magnoliids/eudicots - ps18
Eudicots - ps19
Core_eudicots - ps20
Superrosids - ps21
Rosids - ps22
Eurosids - ps23
Malvids - ps24
Sap./Malv./Brassicales - ps25
Malvales/Brassicales - ps26
Brassicales - ps27
Cleomaceae/Brassicaceae - ps28
Brassicaceae - ps29
Camelineae - ps30
Arabidopsis - ps31
Arabidopsis_thaliana - ps32
Phylostratum

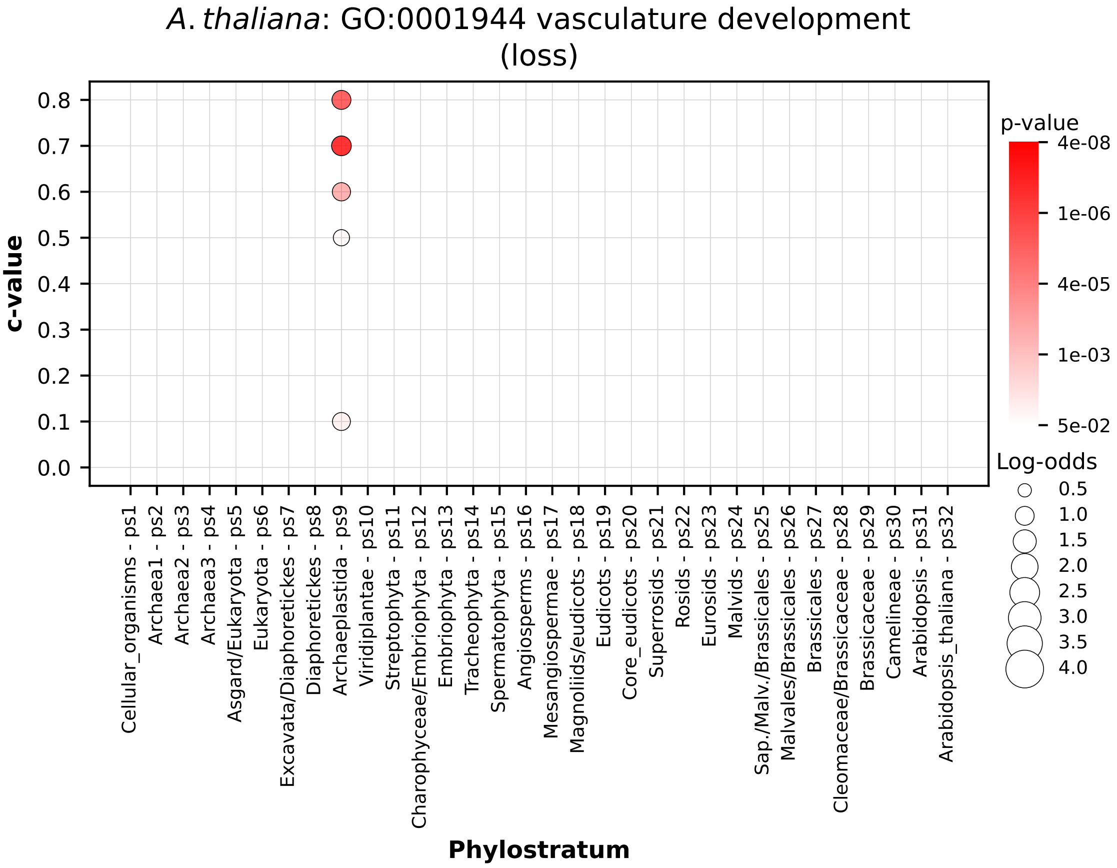

A. thaliana: GO:0001944 vasculature development
(loss)
c-value
0.8
0.7
0.6
0.5
0.4
0.3
0.2
0.1
0.0
Phylostratum
Cellular_organisms - ps1
Archaea1 - ps2
Archaea2 - ps3
Archaea3 - ps4
Asgard/Eukaryota - ps5
Eukaryota - ps6
Excavata/Diaphoretickes - ps7
Diaphoretickes - ps8
Archaeplastida - ps9
Viridiplantae - ps10
Streptophyta - ps11
Charophyceae/Embriophyta - ps12
Embriophyta - ps13
Tracheophyta - ps14
Spermatophyta - ps15
Angiosperms - ps16
Mesangiospermae - ps17
Magnoliids/eudicots - ps18
Eudicots - ps19
Core_eudicots - ps20
Superrosids - ps21
Rosids - ps22
Eurosids - ps23
Malvids - ps24
Sap./Malv./Brassicales - ps25
Malvales/Brassicales - ps26
Brassicales - ps27
Cleomaceae/Brassicaceae - ps28
Brassicaceae - ps29
Camelineae - ps30
Arabidopsis - ps31
Arabidopsis_thaliana - ps32
p-value
4e-08
1e-06
4e-05
1e-03
5e-02
Log-odds
0.5
1.0
1.5
2.0
2.5
3.0
3.5
4.0

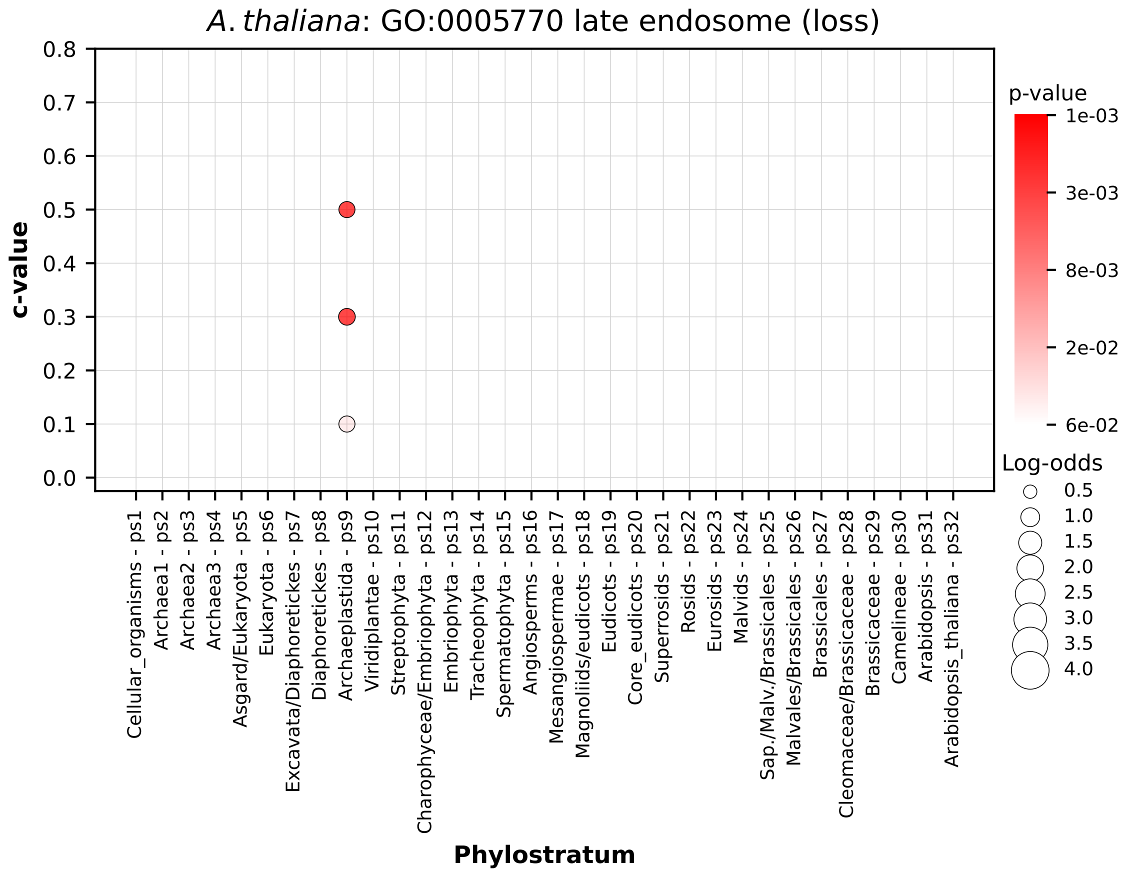

A. thaliana: GO:0005770 late endosome (loss)
c-value
0.8
0.7
0.6
0.5
0.4
0.3
0.2
0.1
0.0
p-value
1e-03
3e-03
8e-03
2e-02
6e-02
Log-odds
0.5
1.0
1.5
2.0
2.5
3.0
3.5
4.0
Cellular_organisms - ps1
Archaea1 - ps2
Archaea2 - ps3
Archaea3 - ps4
Asgard/Eukaryota - ps5
Eukaryota - ps6
Excavata/Diaphoretickes - ps7
Diaphoretickes - ps8
Archaeplastida - ps9
Viridiplantae - ps10
Streptophyta - ps11
Charophyceae/Embriophyta - ps12
Embriophyta - ps13
Tracheophyta - ps14
Spermatophyta - ps15
Angiosperms - ps16
Mesangiospermae - ps17
Magnoliids/eudicots - ps18
Eudicots - ps19
Core_eudicots - ps20
Superrosids - ps21
Rosids - ps22
Eurosids - ps23
Malvids - ps24
Sap./Malv./Brassicales - ps25
Malvales/Brassicales - ps26
Brassicales - ps27
Cleomaceae/Brassicaceae - ps28
Brassicaceae - ps29
Camelineae - ps30
Arabidopsis - ps31
Arabidopsis_thaliana - ps32
Phylostratum

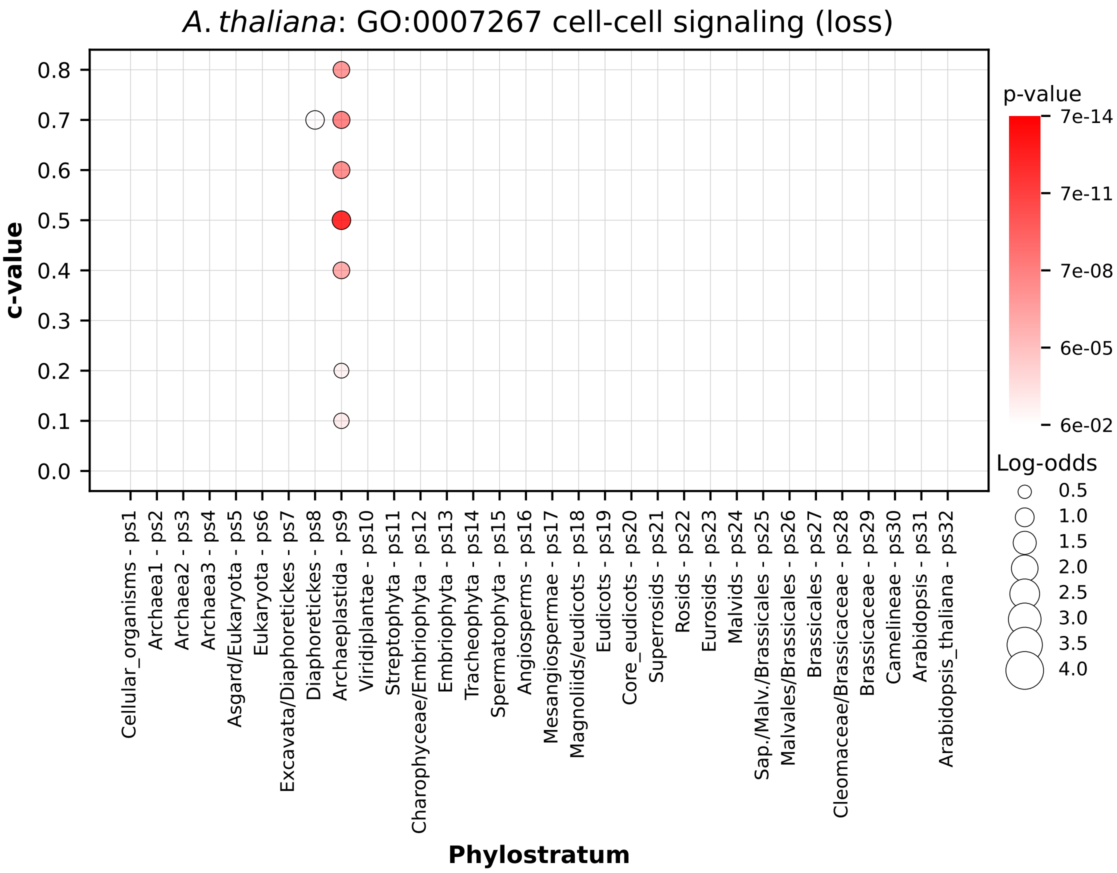

A. thaliana: GO:0007267 cell-cell signaling (loss)
c-value
0.8
0.7
0.6
0.5
0.4
0.3
0.2
0.1
0.0
p-value
7e-14
7e-11
7e-08
6e-05
6e-02
Log-odds
0.5
1.0
1.5
2.0
2.5
3.0
3.5
4.0
Cellular_organisms - ps1
Archaea1 - ps2
Archaea2 - ps3
Archaea3 - ps4
Asgard/Eukaryota - ps5
Eukaryota - ps6
Excavata/Diaphoretickes - ps7
Diaphoretickes - ps8
Archaeplastida - ps9
Viridiplantae - ps10
Streptophyta - ps11
Charophyceae/Embriophyta - ps12
Embriophyta - ps13
Tracheophyta - ps14
Spermatophyta - ps15
Angiosperms - ps16
Mesangiospermae - ps17
Magnoliids/eudicots - ps18
Eudicots - ps19
Core_eudicots - ps20
Superrosids - ps21
Rosids - ps22
Eurosids - ps23
Malvids - ps24
Sap./Malv./Brassicales - ps25
Malvales/Brassicales - ps26
Brassicales - ps27
Cleomaceae/Brassicaceae - ps28
Brassicaceae - ps29
Camelineae - ps30
Arabidopsis - ps31
Arabidopsis_thaliana - ps32
Phylostratum

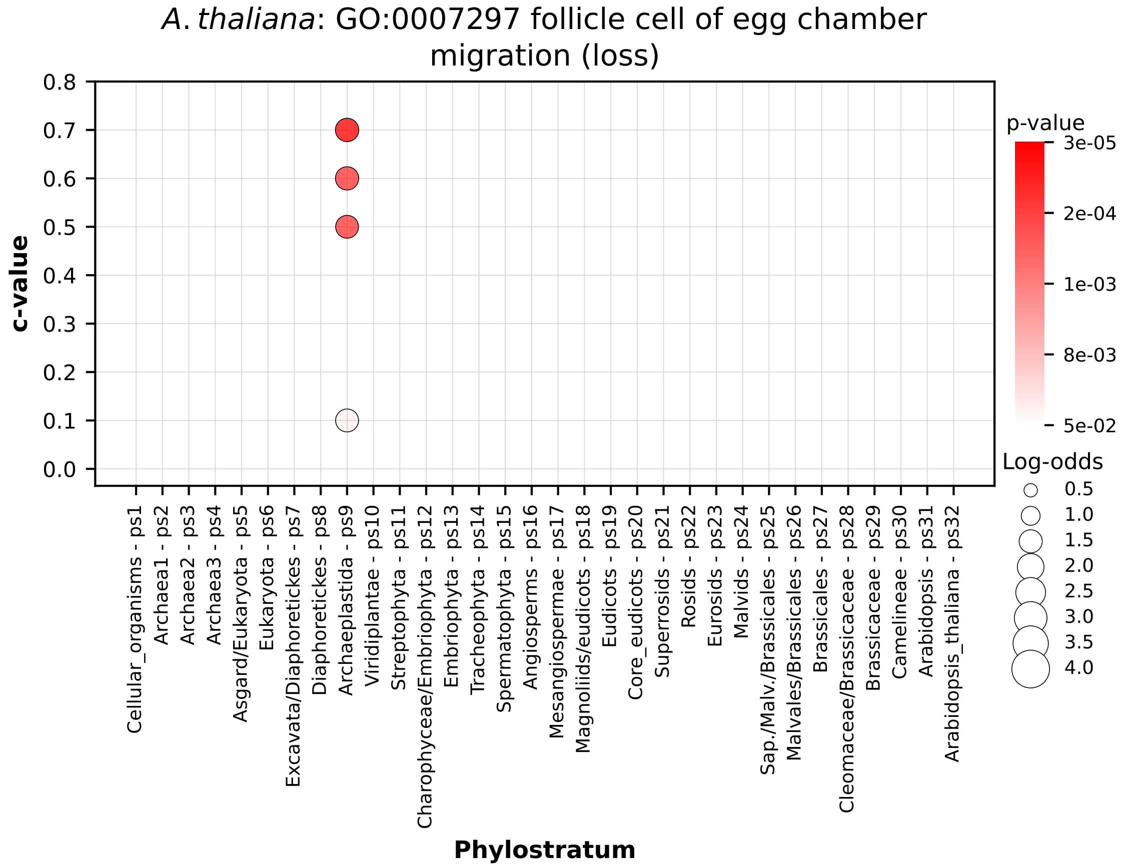

A. thaliana: GO:0007297 follicle cell of egg chamber migration (loss)
c-value
0.8
0.7
0.6
0.5
0.4
0.3
0.2
0.1
0.0
Phylostratum
Cellular_organisms - ps1
Archaea1 - ps2
Archaea2 - ps3
Archaea3 - ps4
Asgard/Eukaryota - ps5
Eukaryota - ps6
Excavata/Diaphoretickes - ps7
Diaphoretickes - ps8
Archaeplastida - ps9
Viridiplantae - ps10
Streptophyta - ps11
Charophyceae/Embriophyta - ps12
Embriophyta - ps13
Tracheophyta - ps14
Spermatophyta - ps15
Angiosperms - ps16
Mesangiospermae - ps17
Magnoliids/eudicots - ps18
Eudicots - ps19
Core_eudicots - ps20
Superrosids - ps21
Rosids - ps22
Eurosids - ps23
Malvids - ps24
Sap./Malv./Brassicales - ps25
Malvales/Brassicales - ps26
Brassicales - ps27
Cleomaceae/Brassicaceae - ps28
Brassicaceae - ps29
Camelineae - ps30
Arabidopsis - ps31
Arabidopsis_thaliana - ps32
p-value
3e-05
2e-04
1e-03
8e-03
5e-02
Log-odds
0.5
1.0
1.5
2.0
2.5
3.0
3.5
4.0

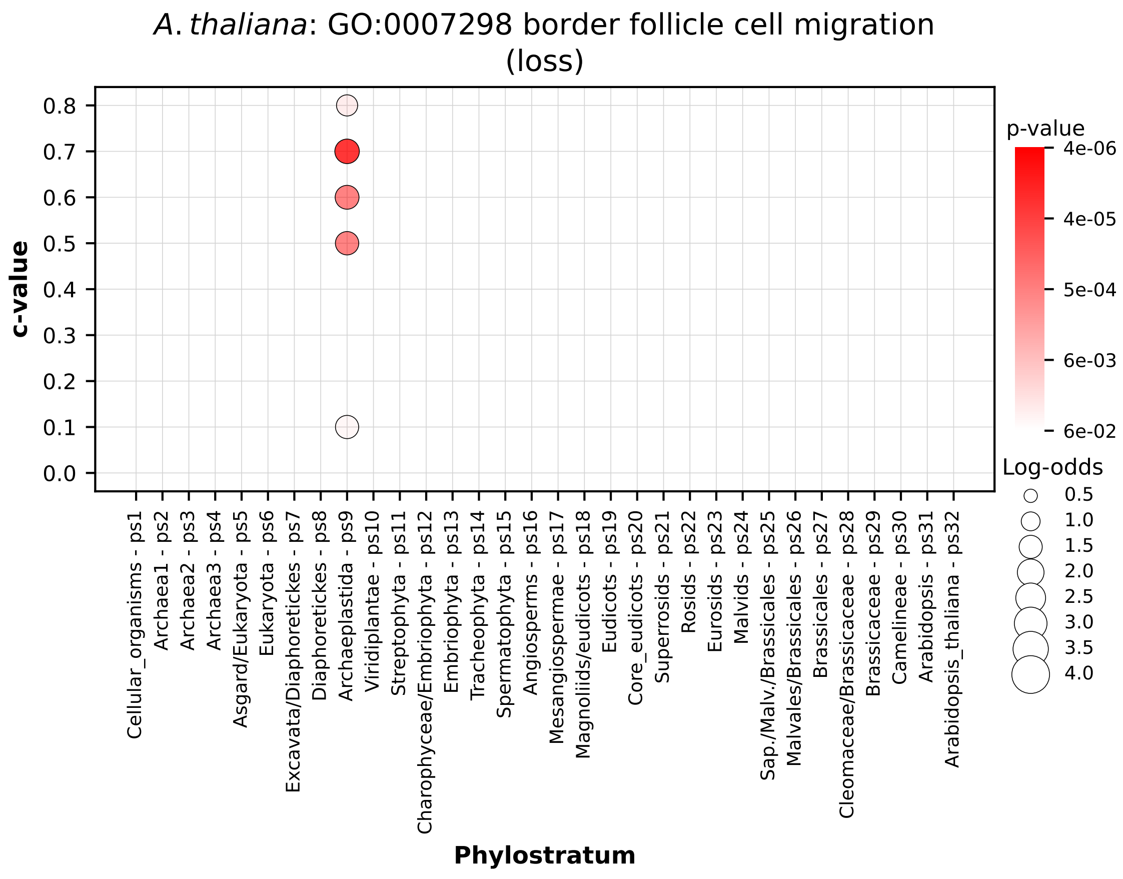

A. thaliana: GO:0007298 border follicle cell migration
(loss)
c-value
0.0
0.1
0.2
0.3
0.4
0.5
0.6
0.7
0.8
Phylostratum
Cellular_organisms - ps1
Archaea1 - ps2
Archaea2 - ps3
Archaea3 - ps4
Asgard/Eukaryota - ps5
Eukaryota - ps6
Excavata/Diaphoretickes - ps7
Diaphoretickes - ps8
Archaeplastida - ps9
Viridiplantae - ps10
Streptophyta - ps11
Charophyceae/Embriophyta - ps12
Embriophyta - ps13
Tracheophyta - ps14
Spermatophyta - ps15
Angiosperms - ps16
Mesangiospermae - ps17
Magnoliids/eudicots - ps18
Eudicots - ps19
Core_eudicots - ps20
Superrosids - ps21
Rosids - ps22
Eurosids - ps23
Malvids - ps24
Sap./Malv./Brassicales - ps25
Malvales/Brassicales - ps26
Brassicales - ps27
Cleomaceae/Brassicaceae - ps28
Brassicaceae - ps29
Camelineae - ps30
Arabidopsis - ps31
Arabidopsis_thaliana - ps32
p-value
4e-06
4e-05
5e-04
6e-03
6e-02
Log-odds
0.5
1.0
1.5
2.0
2.5
3.0
3.5
4.0

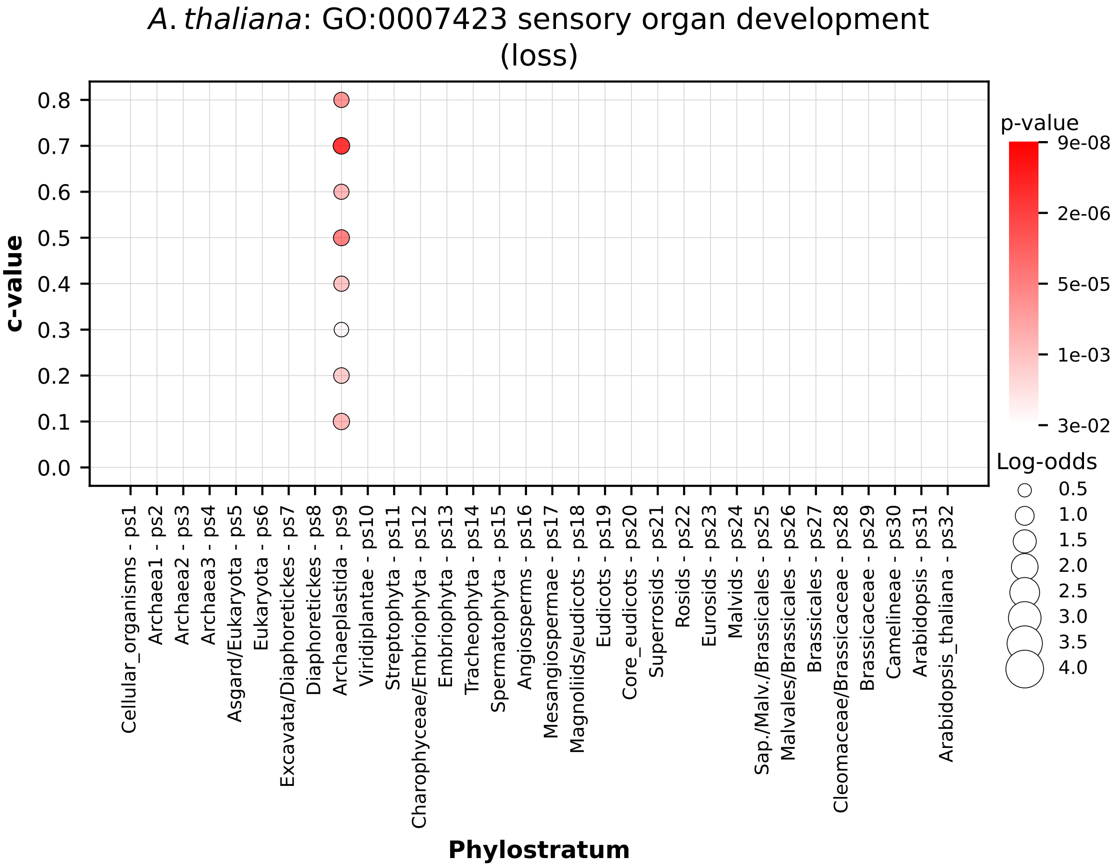
A. thaliana: GO:0007423 sensory organ development
(loss)
c-value
0.0
0.1
0.2
0.3
0.4
0.5
0.6
0.7
0.8
Phylostratum
Cellular_organisms - ps1
Archaea1 - ps2
Archaea2 - ps3
Archaea3 - ps4
Asgard/Eukaryota - ps5
Eukaryota - ps6
Excavata/Diaphoretickes - ps7
Diaphoretickes - ps8
Archaeplastida - ps9
Viridiplantae - ps10
Streptophyta - ps11
Charophyceae/Embriophyta - ps12
Embriophyta - ps13
Tracheophyta - ps14
Spermatophyta - ps15
Angiosperms - ps16
Mesangiospermae - ps17
Magnoliids/eudicots - ps18
Eudicots - ps19
Core_eudicots - ps20
Superrosids - ps21
Rosids - ps22
Eurosids - ps23
Malvids - ps24
Sap./Malv./Brassicales - ps25
Malvales/Brassicales - ps26
Brassicales - ps27
Cleomaceae/Brassicaceae - ps28
Brassicaceae - ps29
Camelineae - ps30
Arabidopsis - ps31
Arabidopsis_thaliana - ps32
p-value
9e-08
2e-06
5e-05
1e-03
3e-02
Log-odds
0.5
1.0
1.5
2.0
2.5
3.0
3.5
4.0

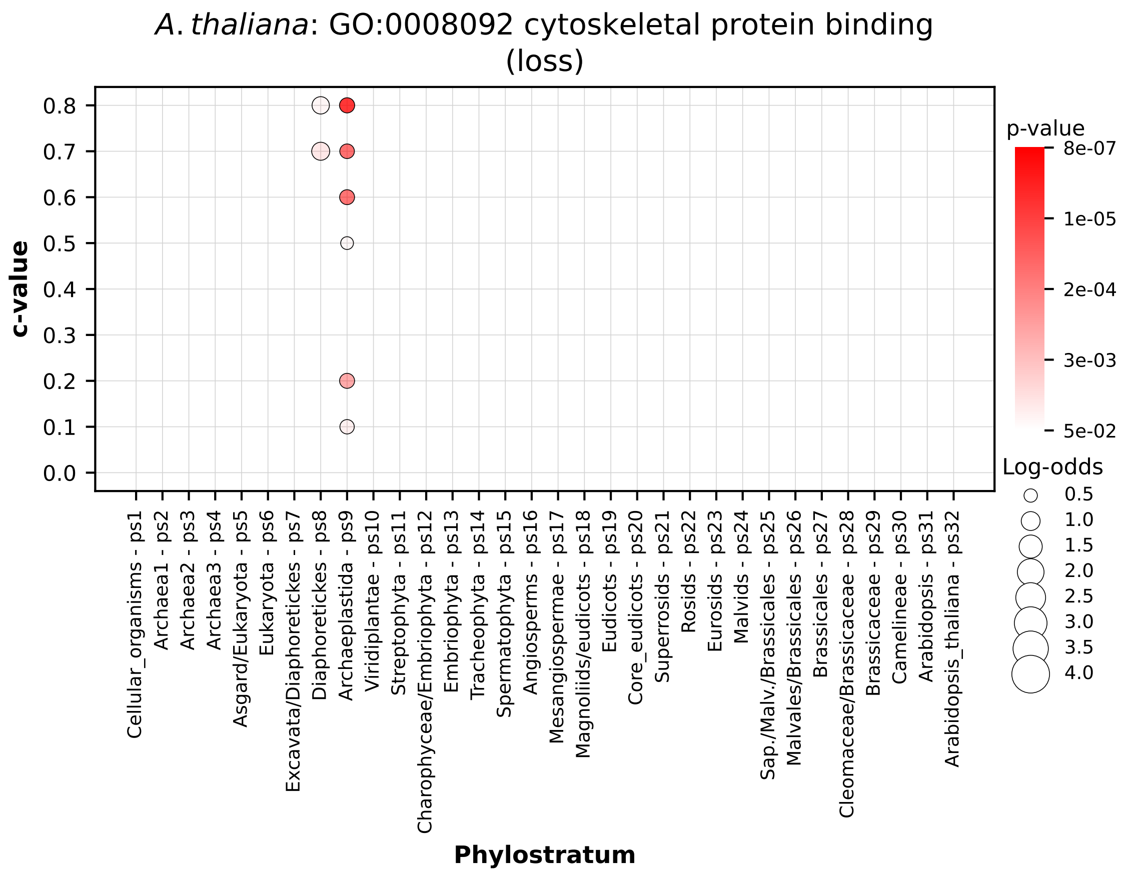

A. thaliana: GO:0008092 cytoskeletal protein binding
(loss)
c-value
0.0
0.1
0.2
0.3
0.4
0.5
0.6
0.7
0.8
p-value
8e-07
1e-05
2e-04
3e-03
5e-02
Log-odds
0.5
1.0
1.5
2.0
2.5
3.0
3.5
4.0
Cellular_organisms - ps1
Archaea1 - ps2
Archaea2 - ps3
Archaea3 - ps4
Asgard/Eukaryota - ps5
Eukaryota - ps6
Excavata/Diaphoretickes - ps7
Diaphoretickes - ps8
Archaeplastida - ps9
Viridiplantae - ps10
Streptophyta - ps11
Charophyceae/Embriophyta - ps12
Embriophyta - ps13
Tracheophyta - ps14
Spermatophyta - ps15
Angiosperms - ps16
Mesangiospermae - ps17
Magnoliids/eudicots - ps18
Eudicots - ps19
Core_eudicots - ps20
Superrosids - ps21
Rosids - ps22
Eurosids - ps23
Malvids - ps24
Sap./Malv./Brassicales - ps25
Malvales/Brassicales - ps26
Brassicales - ps27
Cleomaceae/Brassicaceae - ps28
Brassicaceae - ps29
Camelineae - ps30
Arabidopsis - ps31
Arabidopsis_thaliana - ps32
Phylostratum

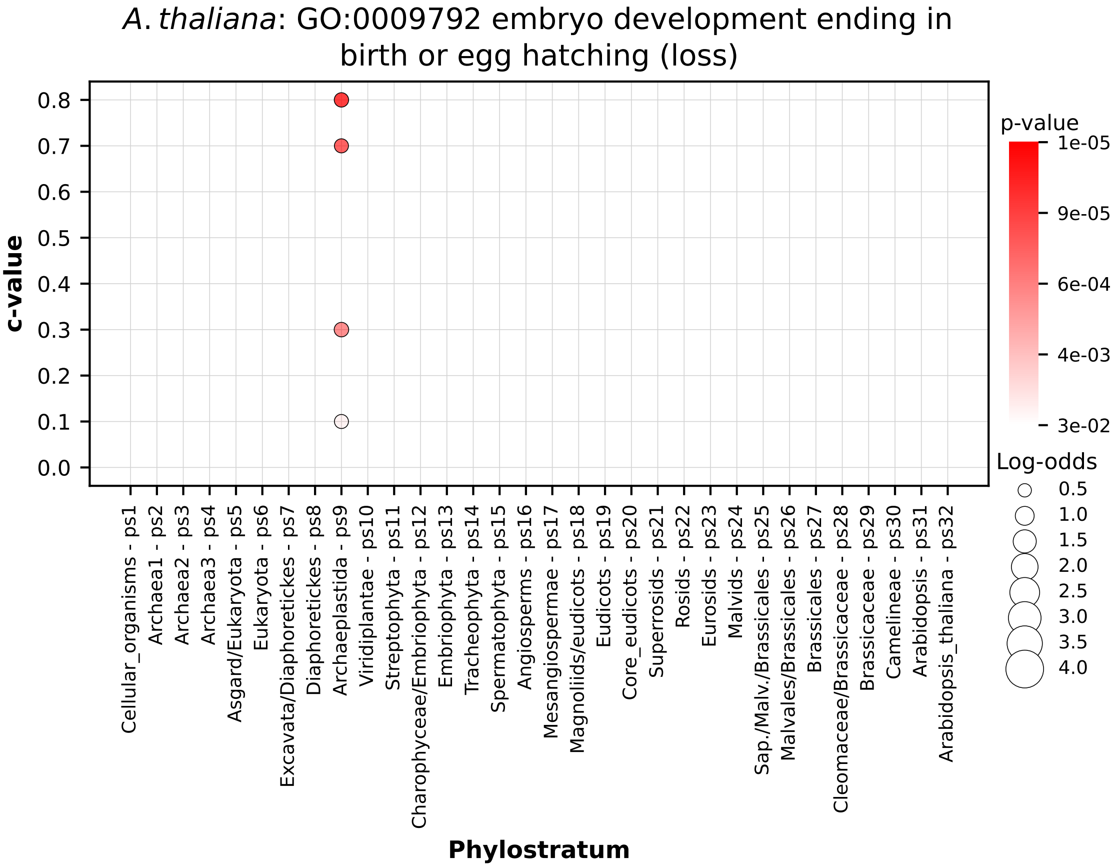
A. thaliana: GO:0009792 embryo development ending in birth or egg hatching (loss)
c-value
0.8
0.7
0.6
0.5
0.4
0.3
0.2
0.1
0.0
Phylostratum
Cellular_organisms - ps1
Archaea1 - ps2
Archaea2 - ps3
Archaea3 - ps4
Asgard/Eukaryota - ps5
Eukaryota - ps6
Excavata/Diaphoretickes - ps7
Diaphoretickes - ps8
Archaeplastida - ps9
Viridiplantae - ps10
Streptophyta - ps11
Charophyceae/Embriophyta - ps12
Embriophyta - ps13
Tracheophyta - ps14
Spermatophyta - ps15
Angiosperms - ps16
Mesangiospermae - ps17
Magnoliids/eudicots - ps18
Eudicots - ps19
Core_eudicots - ps20
Superrosids - ps21
Rosids - ps22
Eurosids - ps23
Malvids - ps24
Sap./Malv./Brassicales - ps25
Malvales/Brassicales - ps26
Brassicales - ps27
Cleomaceae/Brassicaceae - ps28
Brassicaceae - ps29
Camelineae - ps30
Arabidopsis - ps31
Arabidopsis_thaliana - ps32
p-value
1e-05
9e-05
6e-04
4e-03
3e-02
Log-odds
0.5
1.0
1.5
2.0
2.5
3.0
3.5
4.0

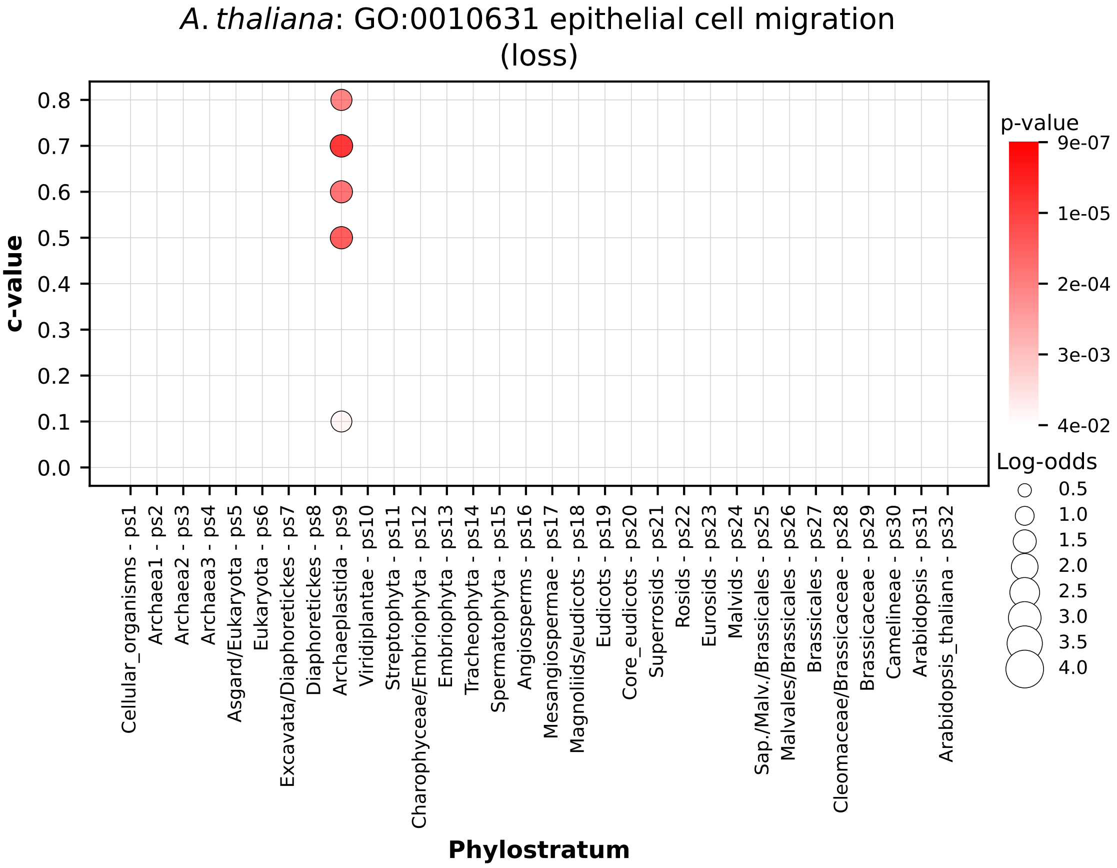

A. thaliana: GO:0010631 epithelial cell migration
(loss)
c-value
0.8
0.7
0.6
0.5
0.4
0.3
0.2
0.1
0.0
p-value
9e-07
1e-05
2e-04
3e-03
4e-02
Log-odds
0.5
1.0
1.5
2.0
2.5
3.0
3.5
4.0
Cellular_organisms - ps1
Archaea1 - ps2
Archaea2 - ps3
Archaea3 - ps4
Asgard/Eukaryota - ps5
Eukaryota - ps6
Excavata/Diaphoretickes - ps7
Diaphoretickes - ps8
Archaeplastida - ps9
Viridiplantae - ps10
Streptophyta - ps11
Charophyceae/Embriophyta - ps12
Embriophyta - ps13
Tracheophyta - ps14
Spermatophyta - ps15
Angiosperms - ps16
Mesangiospermae - ps17
Magnoliids/eudicots - ps18
Eudicots - ps19
Core_eudicots - ps20
Superrosids - ps21
Rosids - ps22
Eurosids - ps23
Malvids - ps24
Sap./Malv./Brassicales - ps25
Malvales/Brassicales - ps26
Brassicales - ps27
Cleomaceae/Brassicaceae - ps28
Brassicaceae - ps29
Camelineae - ps30
Arabidopsis - ps31
Arabidopsis_thaliana - ps32
Phylostratum

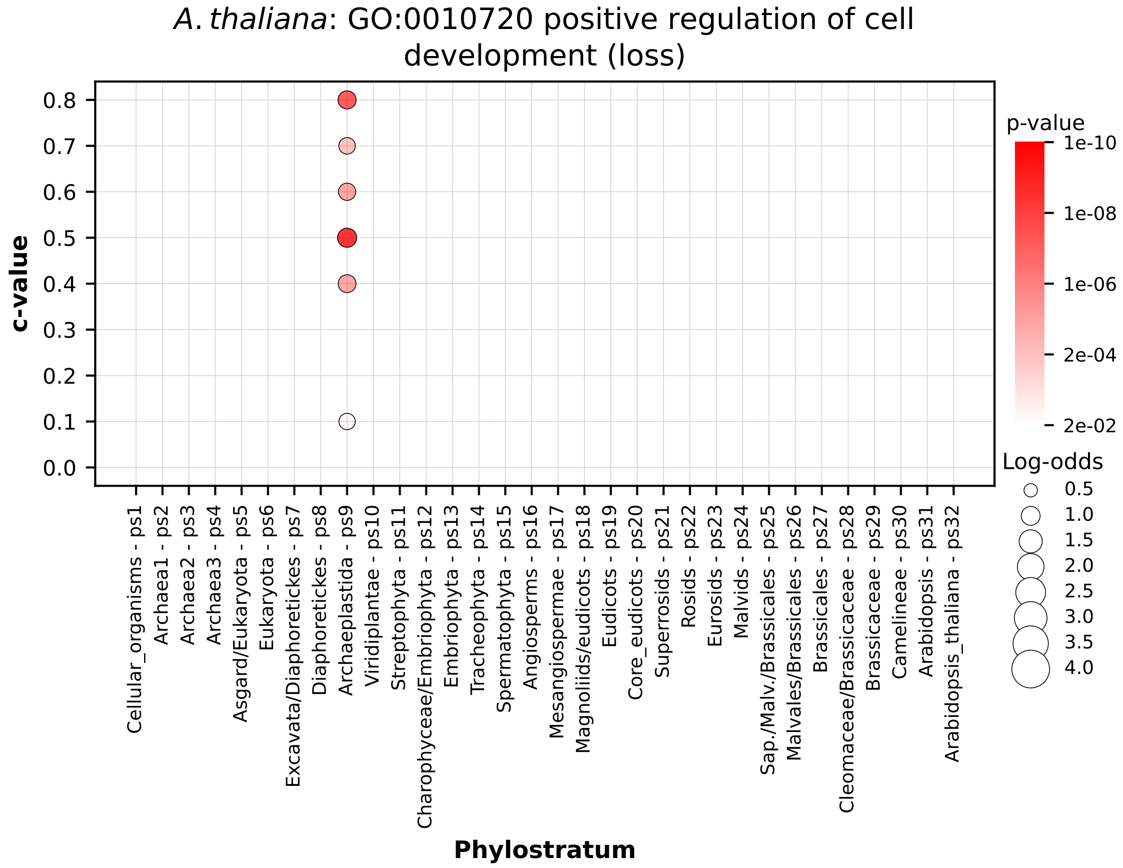

A. thaliana: GO:0010720 positive regulation of cell development (loss)
c-value
0.8
0.7
0.6
0.5
0.4
0.3
0.2
0.1
0.0
p-value
1e-10
1e-08
1e-06
2e-04
2e-02
Log-odds
0.5
1.0
1.5
2.0
2.5
3.0
3.5
4.0
Cellular_organisms - ps1
Archaea1 - ps2
Archaea2 - ps3
Archaea3 - ps4
Asgard/Eukaryota - ps5
Eukaryota - ps6
Excavata/Diaphoretickes - ps7
Diaphoretickes - ps8
Archaeplastida - ps9
Viridiplantae - ps10
Streptophyta - ps11
Charophyceae/Embriophyta - ps12
Embriophyta - ps13
Tracheophyta - ps14
Spermatophyta - ps15
Angiosperms - ps16
Mesangiospermae - ps17
Magnoliids/eudicots - ps18
Eudicots - ps19
Core_eudicots - ps20
Superrosids - ps21
Rosids - ps22
Eurosids - ps23
Malvids - ps24
Sap./Malv./Brassicales - ps25
Malvales/Brassicales - ps26
Brassicales - ps27
Cleomaceae/Brassicaceae - ps28
Brassicaceae - ps29
Camelineae - ps30
Arabidopsis - ps31
Arabidopsis_thaliana - ps32
Phylostratum

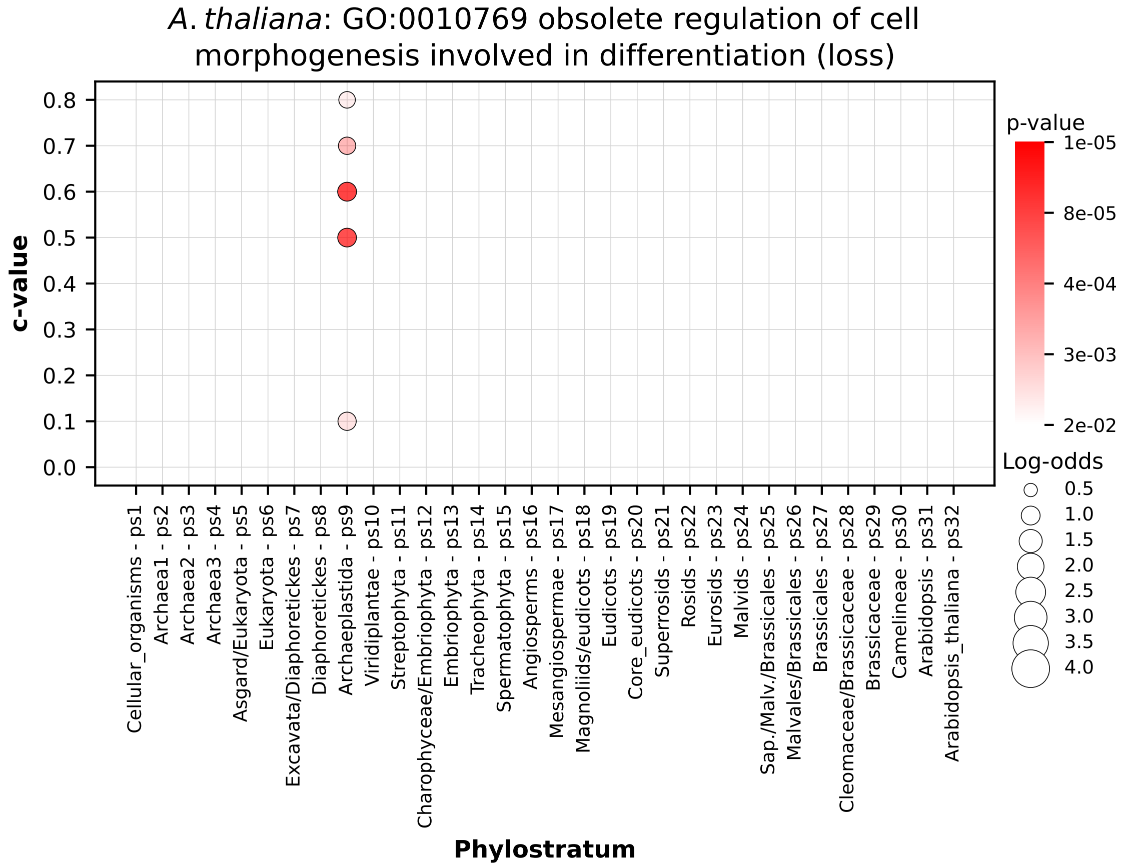

A. thaliana: GO:0010769 obsolete regulation of cell morphogenesis involved in differentiation (loss)
c-value
0.0
0.1
0.2
0.3
0.4
0.5
0.6
0.7
0.8
Phylostratum
Cellular_organisms - ps1
Archaea1 - ps2
Archaea2 - ps3
Archaea3 - ps4
Asgard/Eukaryota - ps5
Eukaryota - ps6
Excavata/Diaphoretickes - ps7
Diaphoretickes - ps8
Archaeplastida - ps9
Viridiplantae - ps10
Streptophyta - ps11
Charophyceae/Embriophyta - ps12
Embriophyta - ps13
Tracheophyta - ps14
Spermatophyta - ps15
Angiosperms - ps16
Mesangiospermae - ps17
Magnoliids/eudicots - ps18
Eudicots - ps19
Core_eudicots - ps20
Superrosids - ps21
Rosids - ps22
Eurosids - ps23
Malvids - ps24
Sap./Malv./Brassicales - ps25
Malvales/Brassicales - ps26
Brassicales - ps27
Cleomaceae/Brassicaceae - ps28
Brassicaceae - ps29
Camelineae - ps30
Arabidopsis - ps31
Arabidopsis_thaliana - ps32
p-value
1e-05
8e-05
4e-04
3e-03
2e-02
Log-odds
0.5
1.0
1.5
2.0
2.5
3.0
3.5
4.0

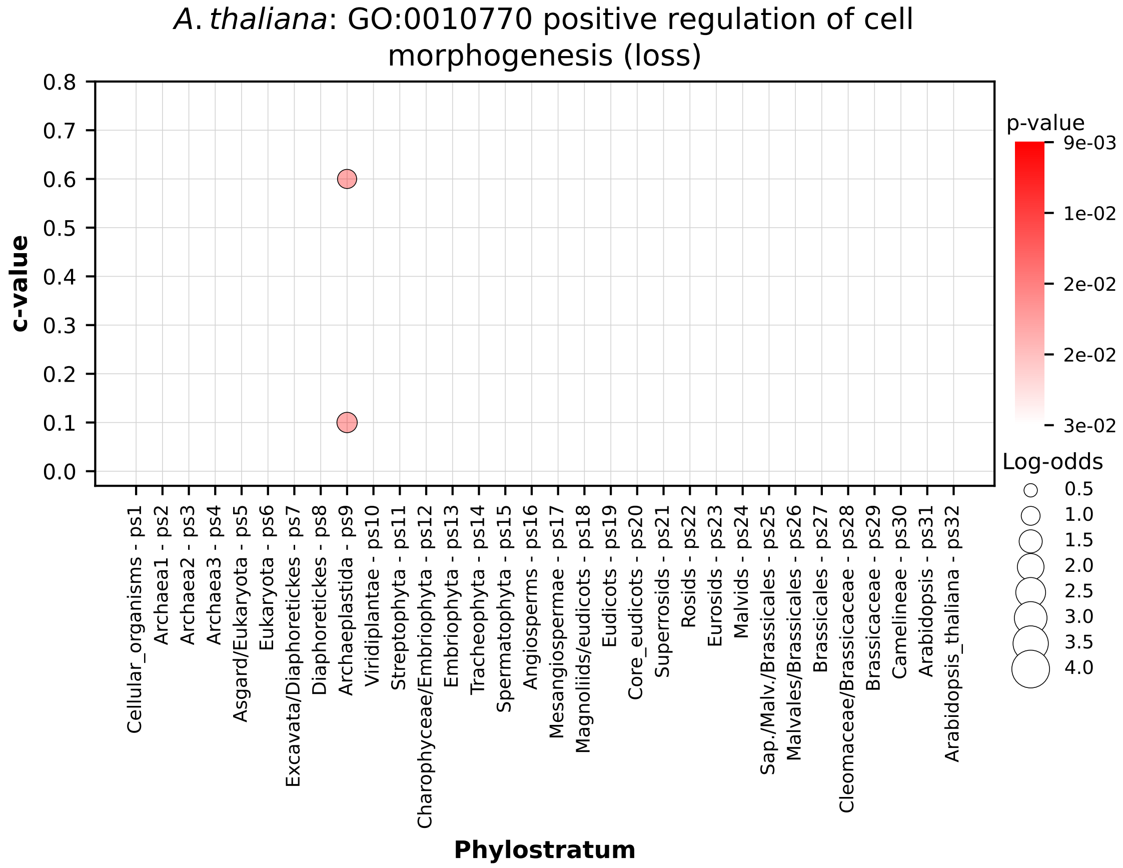

A. thaliana: GO:0010770 positive regulation of cell morphogenesis (loss)
c-value
0.8
0.7
0.6
0.5
0.4
0.3
0.2
0.1
0.0
Phylostratum
Cellular_organisms - ps1
Archaea1 - ps2
Archaea2 - ps3
Archaea3 - ps4
Asgard/Eukaryota - ps5
Eukaryota - ps6
Excavata/Diaphoretickes - ps7
Diaphoretickes - ps8
Archaeplastida - ps9
Viridiplantae - ps10
Streptophyta - ps11
Charophyceae/Embriophyta - ps12
Embriophyta - ps13
Tracheophyta - ps14
Spermatophyta - ps15
Angiosperms - ps16
Mesangiospermae - ps17
Magnoliids/eudicots - ps18
Eudicots - ps19
Core_eudicots - ps20
Superrosids - ps21
Rosids - ps22
Eurosids - ps23
Malvids - ps24
Sap./Malv./Brassicales - ps25
Malvales/Brassicales - ps26
Brassicales - ps27
Cleomaceae/Brassicaceae - ps28
Brassicaceae - ps29
Camelineae - ps30
Arabidopsis - ps31
Arabidopsis_thaliana - ps32
p-value
9e-03
1e-02
2e-02
2e-02
3e-02
Log-odds
0.5
1.0
1.5
2.0
2.5
3.0
3.5
4.0

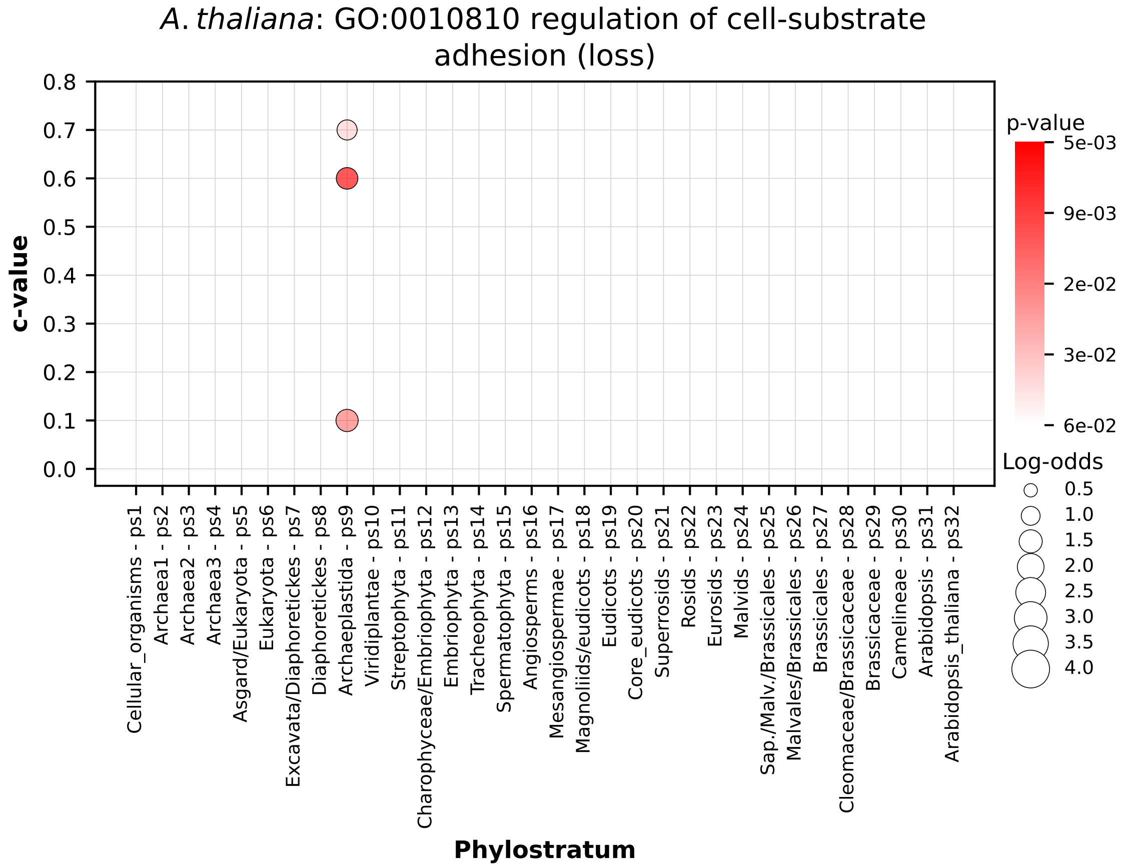

A. thaliana: GO:0010810 regulation of cell-substrate adhesion (loss)
c-value
0.8
0.7
0.6
0.5
0.4
0.3
0.2
0.1
0.0
p-value
5e-03
9e-03
2e-02
3e-02
6e-02
Log-odds
0.5
1.0
1.5
2.0
2.5
3.0
3.5
4.0
Cellular_organisms - ps1
Archaea1 - ps2
Archaea2 - ps3
Archaea3 - ps4
Asgard/Eukaryota - ps5
Eukaryota - ps6
Excavata/Diaphoretickes - ps7
Diaphoretickes - ps8
Archaeplastida - ps9
Viridiplantae - ps10
Streptophyta - ps11
Charophyceae/Embriophyta - ps12
Embriophyta - ps13
Tracheophyta - ps14
Spermatophyta - ps15
Angiosperms - ps16
Mesangiospermae - ps17
Magnoliids/eudicots - ps18
Eudicots - ps19
Core_eudicots - ps20
Superrosids - ps21
Rosids - ps22
Eurosids - ps23
Malvids - ps24
Sap./Malv./Brassicales - ps25
Malvales/Brassicales - ps26
Brassicales - ps27
Cleomaceae/Brassicaceae - ps28
Brassicaceae - ps29
Camelineae - ps30
Arabidopsis - ps31
Arabidopsis_thaliana - ps32
Phylostratum

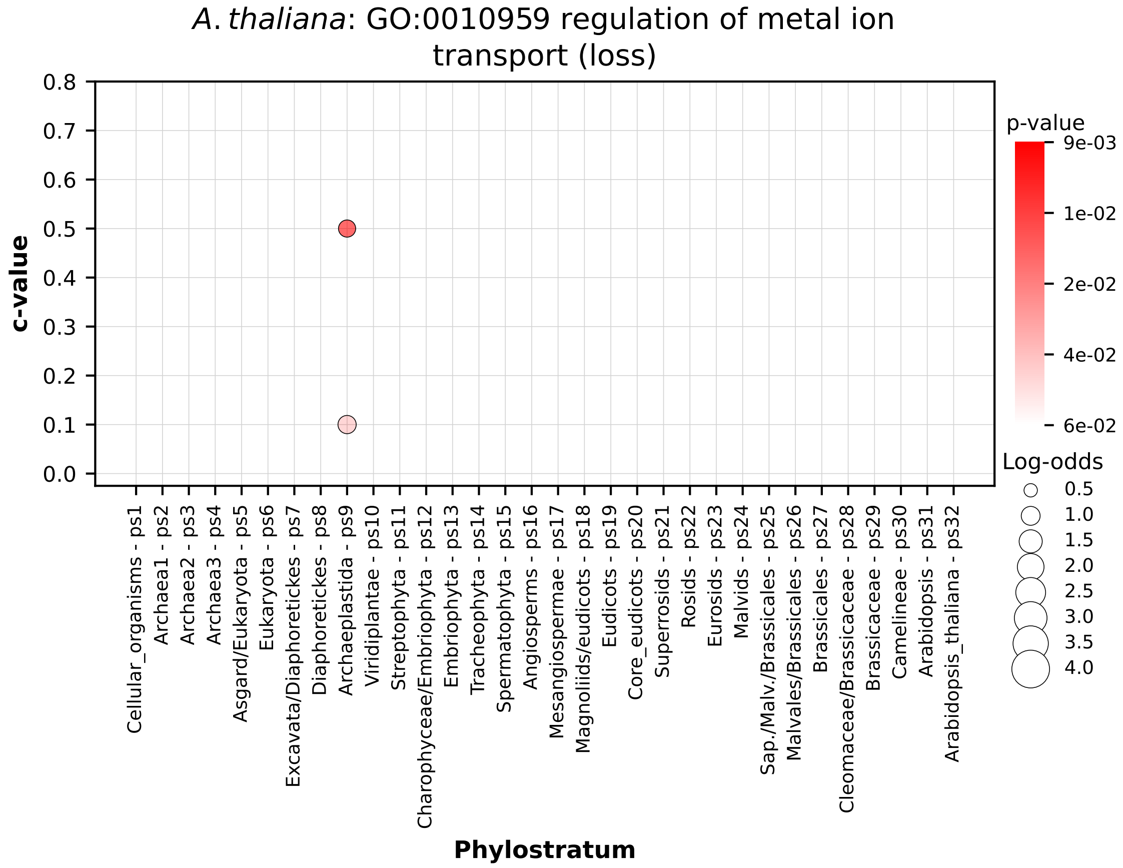

A. thaliana: GO:0010959 regulation of metal ion transport (loss)
c-value
0.8
0.7
0.6
0.5
0.4
0.3
0.2
0.1
0.0
p-value
9e-03
1e-02
2e-02
4e-02
6e-02
Log-odds
0.5
1.0
1.5
2.0
2.5
3.0
3.5
4.0
Cellular_organisms - ps1
Archaea1 - ps2
Archaea2 - ps3
Archaea3 - ps4
Asgard/Eukaryota - ps5
Eukaryota - ps6
Excavata/Diaphoretickes - ps7
Diaphoretickes - ps8
Archaeplastida - ps9
Viridiplantae - ps10
Streptophyta - ps11
Charophyceae/Embriophyta - ps12
Embriophyta - ps13
Tracheophyta - ps14
Spermatophyta - ps15
Angiosperms - ps16
Mesangiospermae - ps17
Magnoliids/eudicots - ps18
Eudicots - ps19
Core_eudicots - ps20
Superrosids - ps21
Rosids - ps22
Eurosids - ps23
Malvids - ps24
Sap./Malv./Brassicales - ps25
Malvales/Brassicales - ps26
Brassicales - ps27
Cleomaceae/Brassicaceae - ps28
Brassicaceae - ps29
Camelineae - ps30
Arabidopsis - ps31
Arabidopsis_thaliana - ps32
Phylostratum

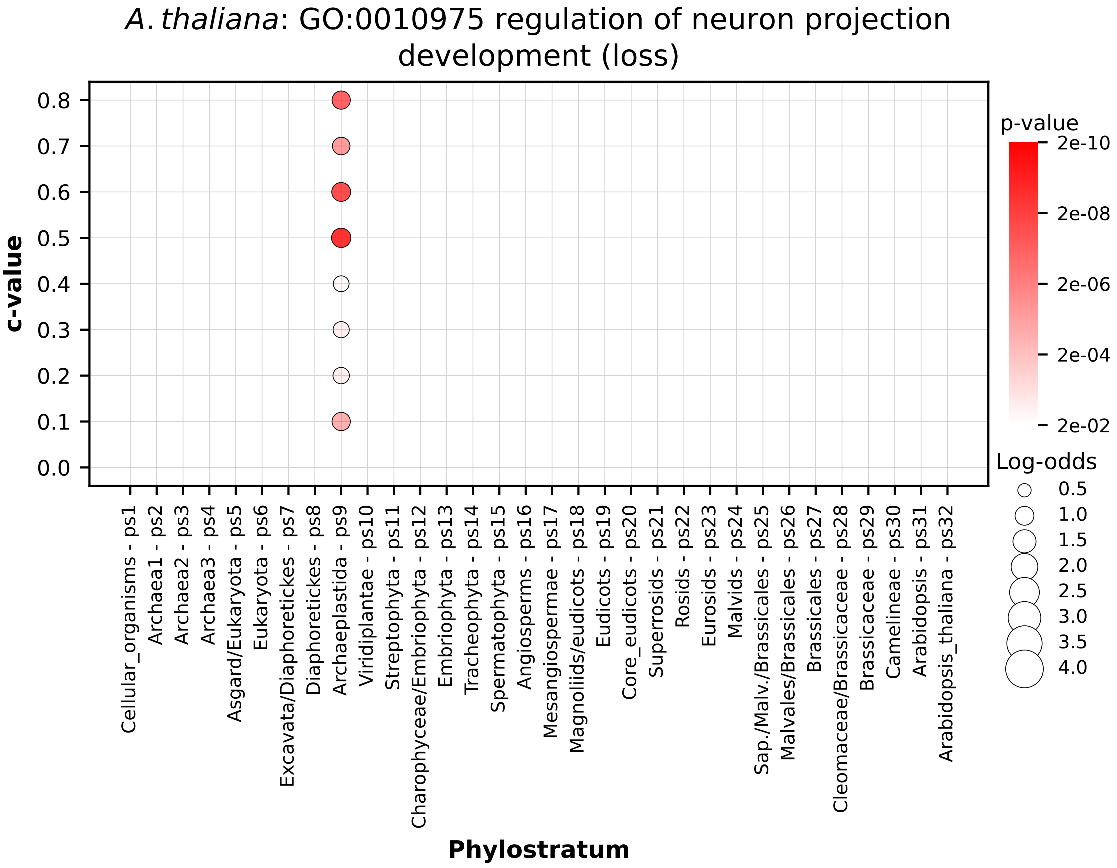

A. thaliana: GO:0010975 regulation of neuron projection development (loss)
c-value
0.8
0.7
0.6
0.5
0.4
0.3
0.2
0.1
0.0
Phylostratum
Cellular_organisms - ps1
Archaea1 - ps2
Archaea2 - ps3
Archaea3 - ps4
Asgard/Eukaryota - ps5
Eukaryota - ps6
Excavata/Diaphoretickes - ps7
Diaphoretickes - ps8
Archaeplastida - ps9
Viridiplantae - ps10
Streptophyta - ps11
Charophyceae/Embriophyta - ps12
Embriophyta - ps13
Tracheophyta - ps14
Spermatophyta - ps15
Angiosperms - ps16
Mesangiospermae - ps17
Magnoliids/eudicots - ps18
Eudicots - ps19
Core_eudicots - ps20
Superrosids - ps21
Rosids - ps22
Eurosids - ps23
Malvids - ps24
Sap./Malv./Brassicales - ps25
Malvales/Brassicales - ps26
Brassicales - ps27
Cleomaceae/Brassicaceae - ps28
Brassicaceae - ps29
Camelineae - ps30
Arabidopsis - ps31
Arabidopsis_thaliana - ps32
p-value
2e-10
2e-08
2e-06
2e-04
2e-02
Log-odds
0.5
1.0
1.5
2.0
2.5
3.0
3.5
4.0

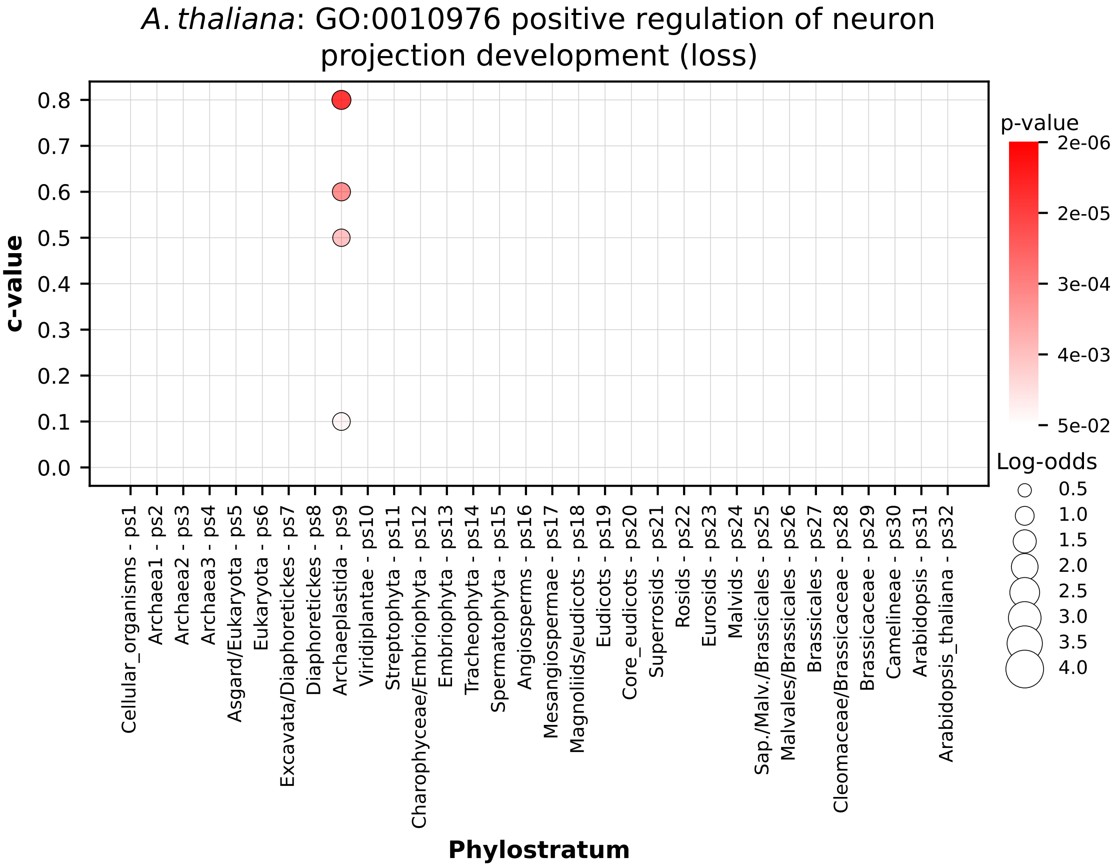
A. thaliana: GO:0010976 positive regulation of neuron projection development (loss)
c-value
0.0
0.1
0.2
0.3
0.4
0.5
0.6
0.7
0.8
Phylostratum
Cellular_organisms - ps1
Archaea1 - ps2
Archaea2 - ps3
Archaea3 - ps4
Asgard/Eukaryota - ps5
Eukaryota - ps6
Excavata/Diaphoretickes - ps7
Diaphoretickes - ps8
Archaeplastida - ps9
Viridiplantae - ps10
Streptophyta - ps11
Charophyceae/Embriophyta - ps12
Embriophyta - ps13
Tracheophyta - ps14
Spermatophyta - ps15
Angiosperms - ps16
Mesangiospermae - ps17
Magnoliids/eudicots - ps18
Eudicots - ps19
Core_eudicots - ps20
Superrosids - ps21
Rosids - ps22
Eurosids - ps23
Malvids - ps24
Sap./Malv./Brassicales - ps25
Malvales/Brassicales - ps26
Brassicales - ps27
Cleomaceae/Brassicaceae - ps28
Brassicaceae - ps29
Camelineae - ps30
Arabidopsis - ps31
Arabidopsis_thaliana - ps32
p-value
2e-06
2e-05
3e-04
4e-03
5e-02
Log-odds
0.5
1.0
1.5
2.0
2.5
3.0
3.5
4.0

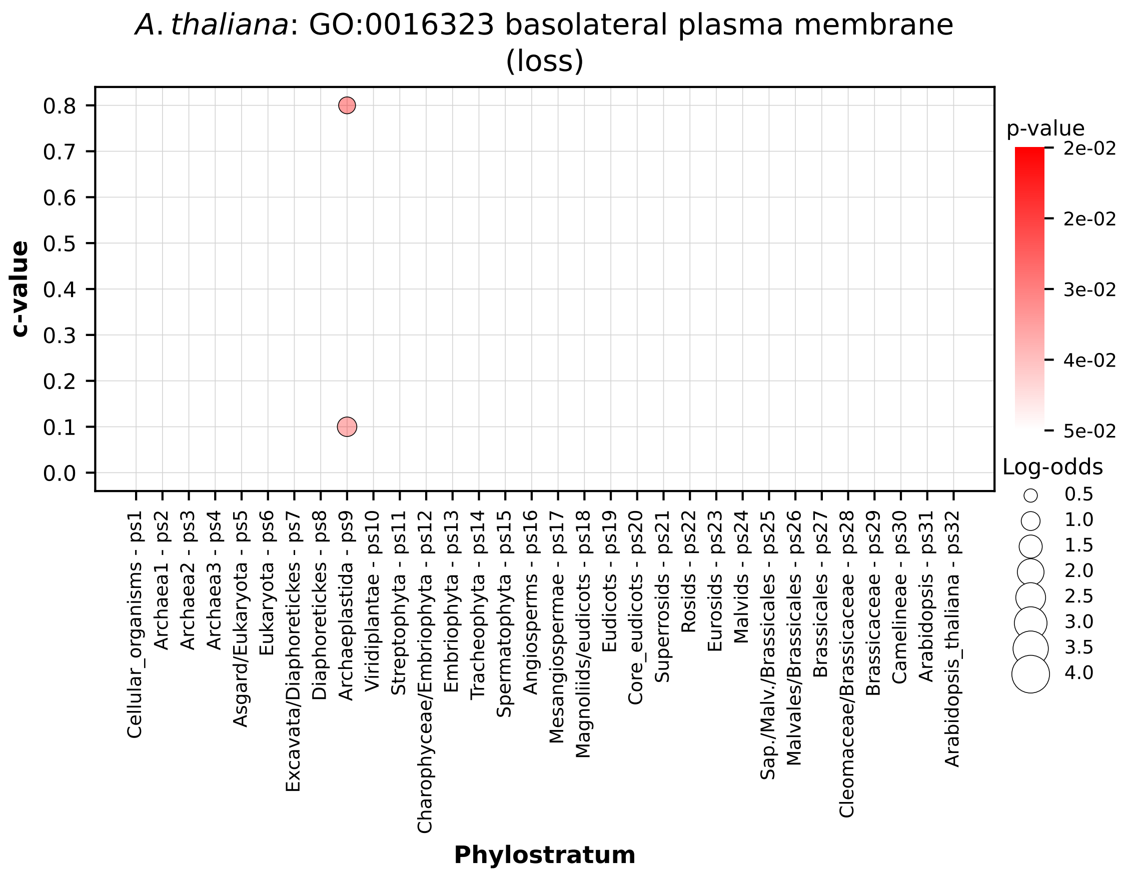

A. thaliana: GO:0016323 basolateral plasma membrane
(loss)
c-value
0.8
0.7
0.6
0.5
0.4
0.3
0.2
0.1
0.0
Phylostratum
Cellular_organisms - ps1
Archaea1 - ps2
Archaea2 - ps3
Archaea3 - ps4
Asgard/Eukaryota - ps5
Eukaryota - ps6
Excavata/Diaphoretickes - ps7
Diaphoretickes - ps8
Archaeplastida - ps9
Viridiplantae - ps10
Streptophyta - ps11
Charophyceae/Embriophyta - ps12
Embriophyta - ps13
Tracheophyta - ps14
Spermatophyta - ps15
Angiosperms - ps16
Mesangiospermae - ps17
Magnoliids/eudicots - ps18
Eudicots - ps19
Core_eudicots - ps20
Superrosids - ps21
Rosids - ps22
Eurosids - ps23
Malvids - ps24
Sap./Malv./Brassicales - ps25
Malvales/Brassicales - ps26
Brassicales - ps27
Cleomaceae/Brassicaceae - ps28
Brassicaceae - ps29
Camelineae - ps30
Arabidopsis - ps31
Arabidopsis_thaliana - ps32
p-value
2e-02
2e-02
3e-02
4e-02
5e-02
Log-odds
0.5
1.0
1.5
2.0
2.5
3.0
3.5
4.0

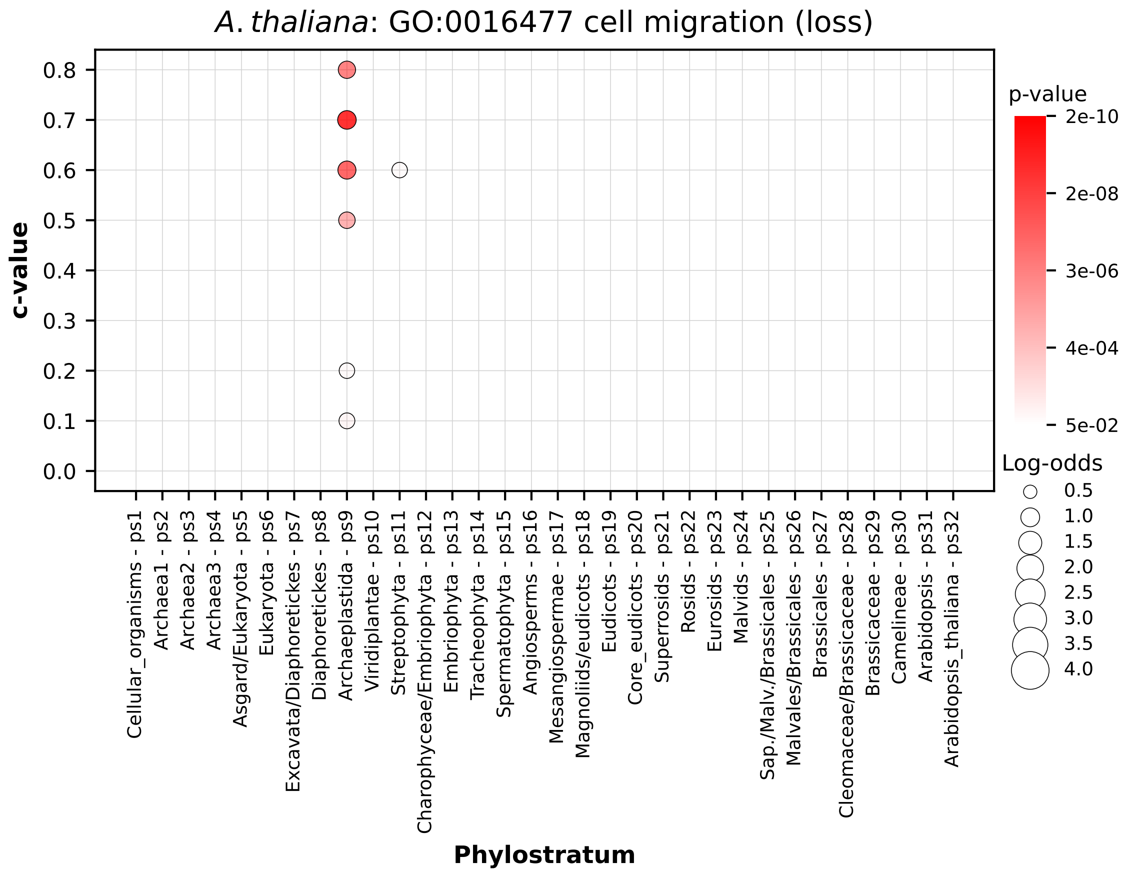
A. thaliana: GO:0016477 cell migration (loss)
c-value
0.0
0.1
0.2
0.3
0.4
0.5
0.6
0.7
0.8
Phylostratum
Cellular_organisms - ps1
Archaea1 - ps2
Archaea2 - ps3
Archaea3 - ps4
Asgard/Eukaryota - ps5
Eukaryota - ps6
Excavata/Diaphoretickes - ps7
Diaphoretickes - ps8
Archaeplastida - ps9
Viridiplantae - ps10
Streptophyta - ps11
Charophyceae/Embriophyta - ps12
Embriophyta - ps13
Tracheophyta - ps14
Spermatophyta - ps15
Angiosperms - ps16
Mesangiospermae - ps17
Magnoliids/eudicots - ps18
Eudicots - ps19
Core_eudicots - ps20
Superrosids - ps21
Rosids - ps22
Eurosids - ps23
Malvids - ps24
Sap./Malv./Brassicales - ps25
Malvales/Brassicales - ps26
Brassicales - ps27
Cleomaceae/Brassicaceae - ps28
Brassicaceae - ps29
Camelineae - ps30
Arabidopsis - ps31
Arabidopsis_thaliana - ps32
p-value
2e-10
2e-08
3e-06
4e-04
5e-02
Log-odds
0.5
1.0
1.5
2.0
2.5
3.0
3.5
4.0

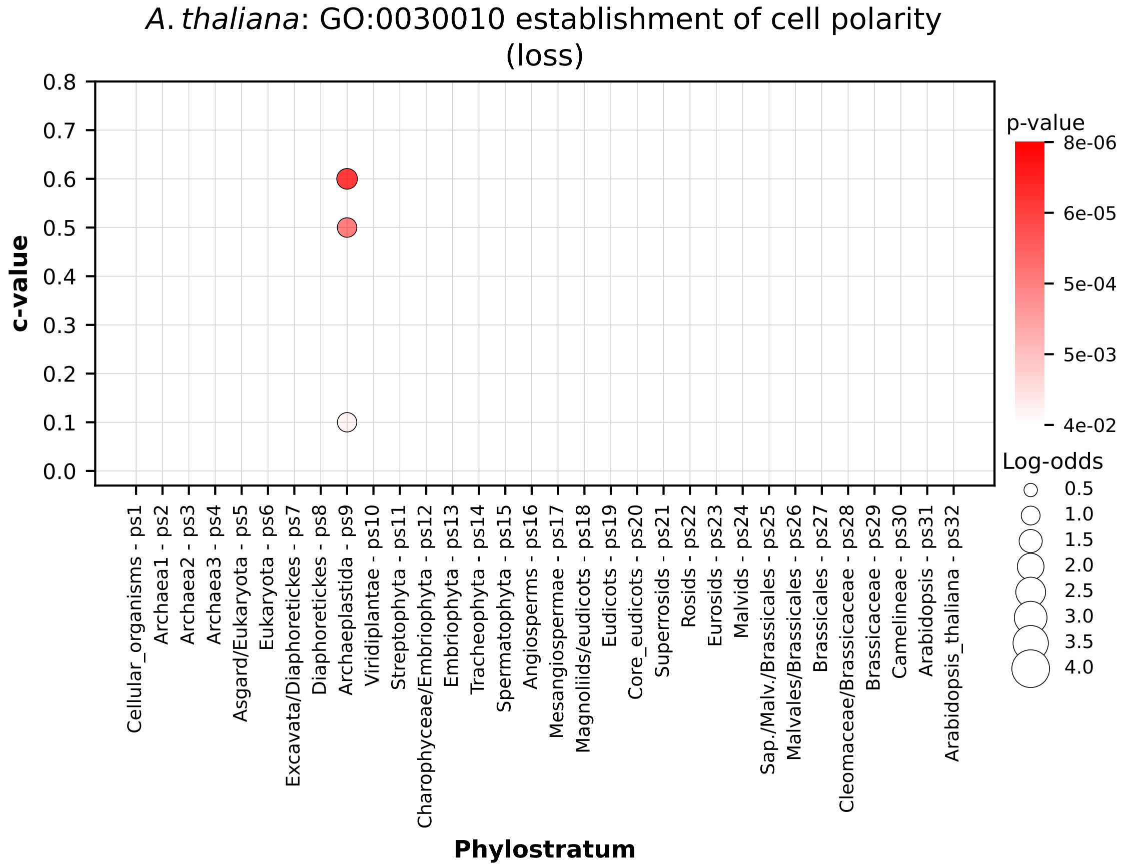
A. thaliana: GO:0030010 establishment of cell polarity (loss)
c-value
0.8
0.7
0.6
0.5
0.4
0.3
0.2
0.1
0.0
p-value
8e-06
6e-05
5e-04
5e-03
4e-02
Log-odds
0.5
1.0
1.5
2.0
2.5
3.0
3.5
4.0
Cellular_organisms - ps1
Archaea1 - ps2
Archaea2 - ps3
Archaea3 - ps4
Asgard/Eukaryota - ps5
Eukaryota - ps6
Excavata/Diaphoretickes - ps7
Diaphoretickes - ps8
Archaeplastida - ps9
Viridiplantae - ps10
Streptophyta - ps11
Charophyceae/Embriophyta - ps12
Embriophyta - ps13
Tracheophyta - ps14
Spermatophyta - ps15
Angiosperms - ps16
Mesangiospermae - ps17
Magnoliids/eudicots - ps18
Eudicots - ps19
Core_eudicots - ps20
Superrosids - ps21
Rosids - ps22
Eurosids - ps23
Malvids - ps24
Sap./Malv./Brassicales - ps25
Malvales/Brassicales - ps26
Brassicales - ps27
Cleomaceae/Brassicaceae - ps28
Brassicaceae - ps29
Camelineae - ps30
Arabidopsis - ps31
Arabidopsis_thaliana - ps32
Phylostratum

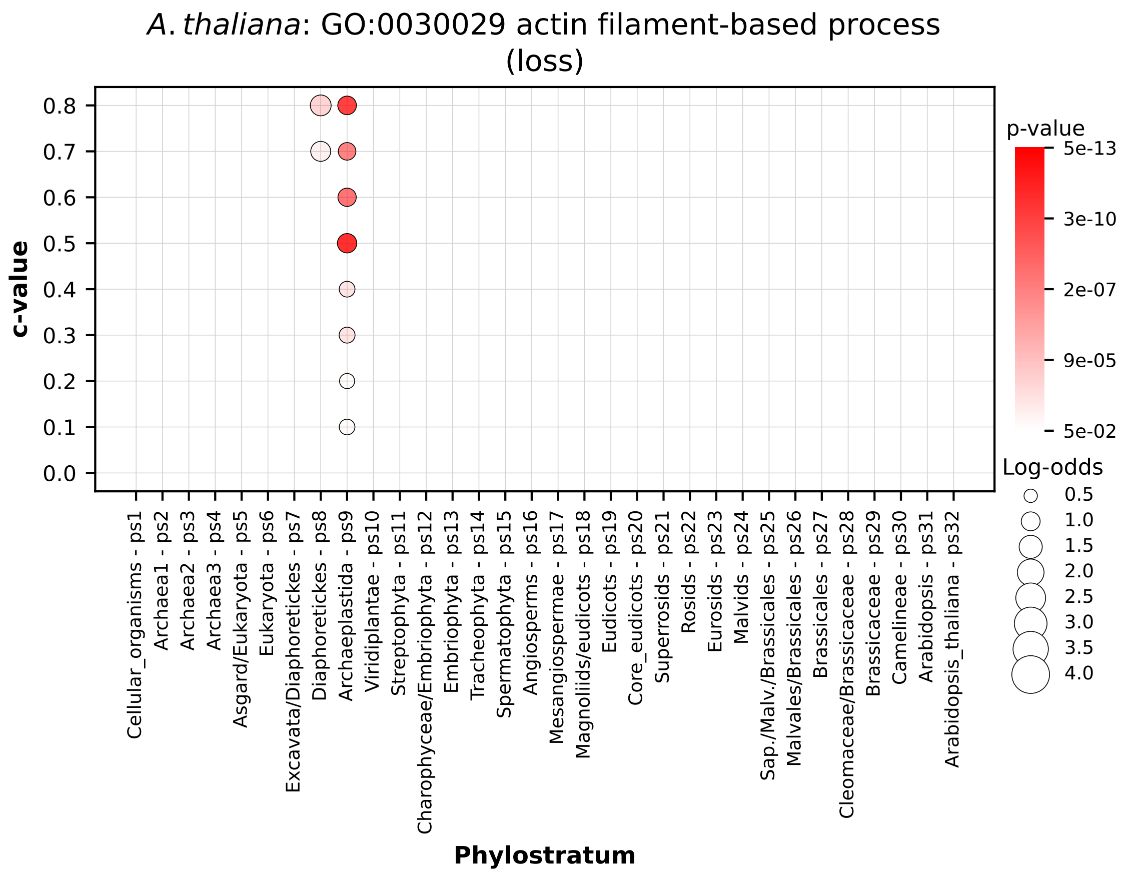

A. thaliana: GO:0030029 actin filament-based process
(loss)
c-value
0.8
0.7
0.6
0.5
0.4
0.3
0.2
0.1
0.0
p-value
5e-13
3e-10
2e-07
9e-05
5e-02
Log-odds
0.5
1.0
1.5
2.0
2.5
3.0
3.5
4.0
Cellular_organisms - ps1
Archaea1 - ps2
Archaea2 - ps3
Archaea3 - ps4
Asgard/Eukaryota - ps5
Eukaryota - ps6
Excavata/Diaphoretickes - ps7
Diaphoretickes - ps8
Archaeplastida - ps9
Viridiplantae - ps10
Streptophyta - ps11
Charophyceae/Embriophyta - ps12
Embriophyta - ps13
Tracheophyta - ps14
Spermatophyta - ps15
Angiosperms - ps16
Mesangiospermae - ps17
Magnoliids/eudicots - ps18
Eudicots - ps19
Core_eudicots - ps20
Superrosids - ps21
Rosids - ps22
Eurosids - ps23
Malvids - ps24
Sap./Malv./Brassicales - ps25
Malvales/Brassicales - ps26
Brassicales - ps27
Cleomaceae/Brassicaceae - ps28
Brassicaceae - ps29
Camelineae - ps30
Arabidopsis - ps31
Arabidopsis_thaliana - ps32
Phylostratum

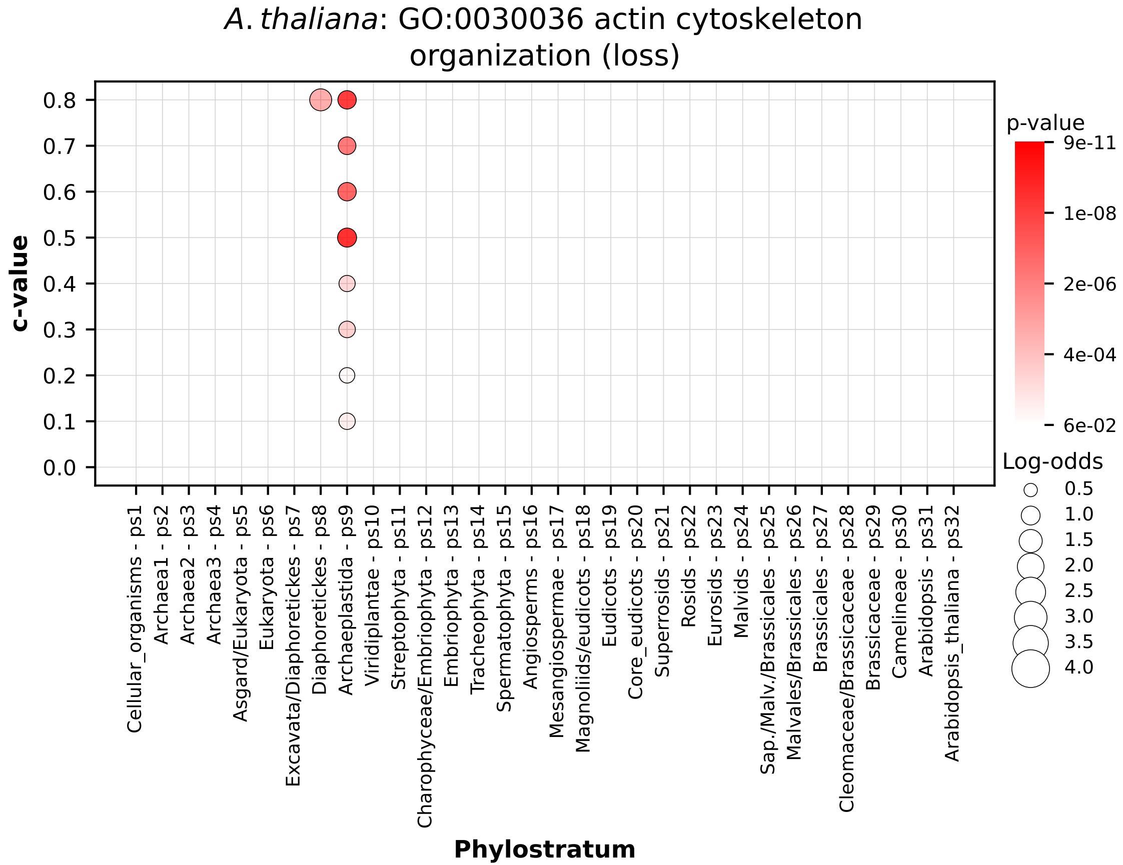

A. thaliana: GO:0030036 actin cytoskeleton organization (loss)
c-value
0.8
0.7
0.6
0.5
0.4
0.3
0.2
0.1
0.0
p-value
9e-11
1e-08
2e-06
4e-04
6e-02
Log-odds
0.5
1.0
1.5
2.0
2.5
3.0
3.5
4.0
Cellular_organisms - ps1
Archaea1 - ps2
Archaea2 - ps3
Archaea3 - ps4
Asgard/Eukaryota - ps5
Eukaryota - ps6
Excavata/Diaphoretickes - ps7
Diaphoretickes - ps8
Archaeplastida - ps9
Viridiplantae - ps10
Streptophyta - ps11
Charophyceae/Embriophyta - ps12
Embriophyta - ps13
Tracheophyta - ps14
Spermatophyta - ps15
Angiosperms - ps16
Mesangiospermae - ps17
Magnoliids/eudicots - ps18
Eudicots - ps19
Core_eudicots - ps20
Superrosids - ps21
Rosids - ps22
Eurosids - ps23
Malvids - ps24
Sap./Malv./Brassicales - ps25
Malvales/Brassicales - ps26
Brassicales - ps27
Cleomaceae/Brassicaceae - ps28
Brassicaceae - ps29
Camelineae - ps30
Arabidopsis - ps31
Arabidopsis_thaliana - ps32
Phylostratum

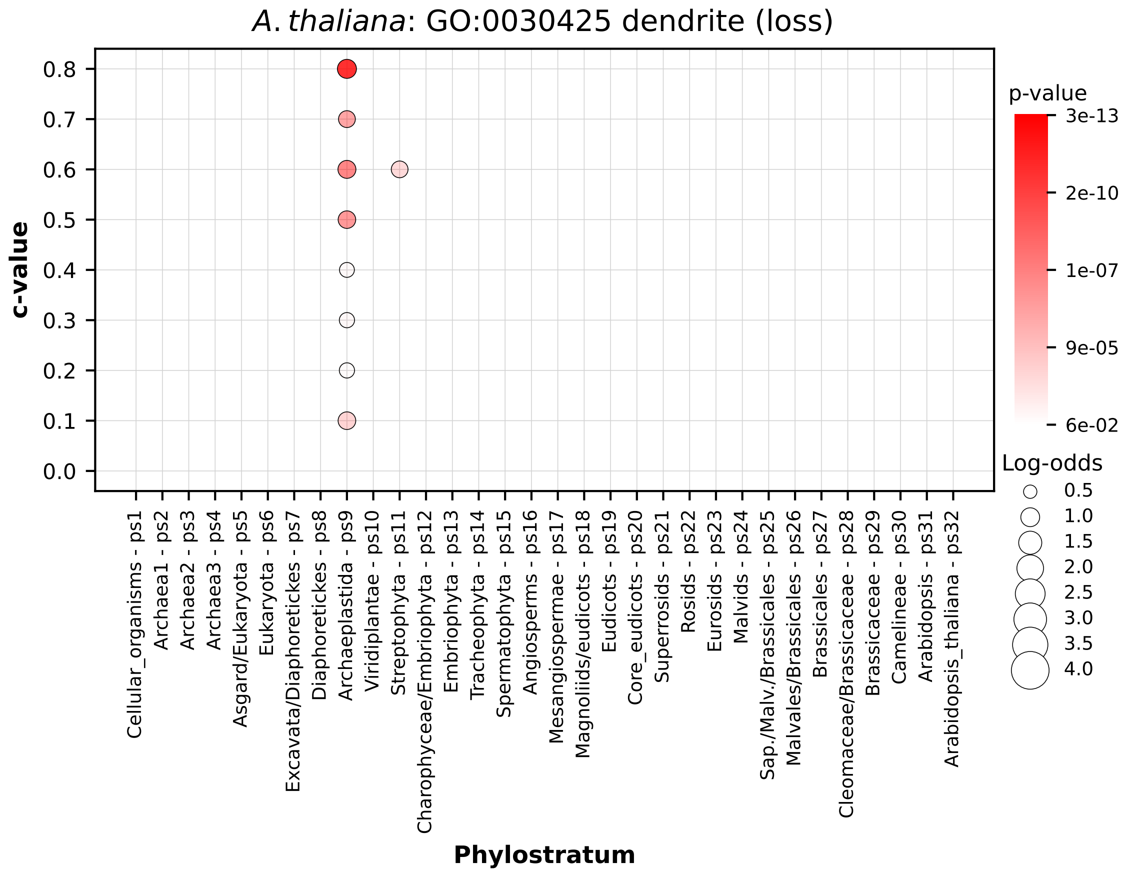

A. thaliana: GO:0030425 dendrite (loss)
c-value
0.0
0.1
0.2
0.3
0.4
0.5
0.6
0.7
0.8
Phylostratum
Cellular_organisms - ps1
Archaea1 - ps2
Archaea2 - ps3
Archaea3 - ps4
Asgard/Eukaryota - ps5
Eukaryota - ps6
Excavata/Diaphoretickes - ps7
Diaphoretickes - ps8
Archaeplastida - ps9
Viridiplantae - ps10
Streptophyta - ps11
Charophyceae/Embriophyta - ps12
Embriophyta - ps13
Tracheophyta - ps14
Spermatophyta - ps15
Angiosperms - ps16
Mesangiospermae - ps17
Magnoliids/eudicots - ps18
Eudicots - ps19
Core_eudicots - ps20
Superrosids - ps21
Rosids - ps22
Eurosids - ps23
Malvids - ps24
Sap./Malv./Brassicales - ps25
Malvales/Brassicales - ps26
Brassicales - ps27
Cleomaceae/Brassicaceae - ps28
Brassicaceae - ps29
Camelineae - ps30
Arabidopsis - ps31
Arabidopsis_thaliana - ps32
p-value
3e-13
2e-10
1e-07
9e-05
6e-02
Log-odds
0.5
1.0
1.5
2.0
2.5
3.0
3.5
4.0

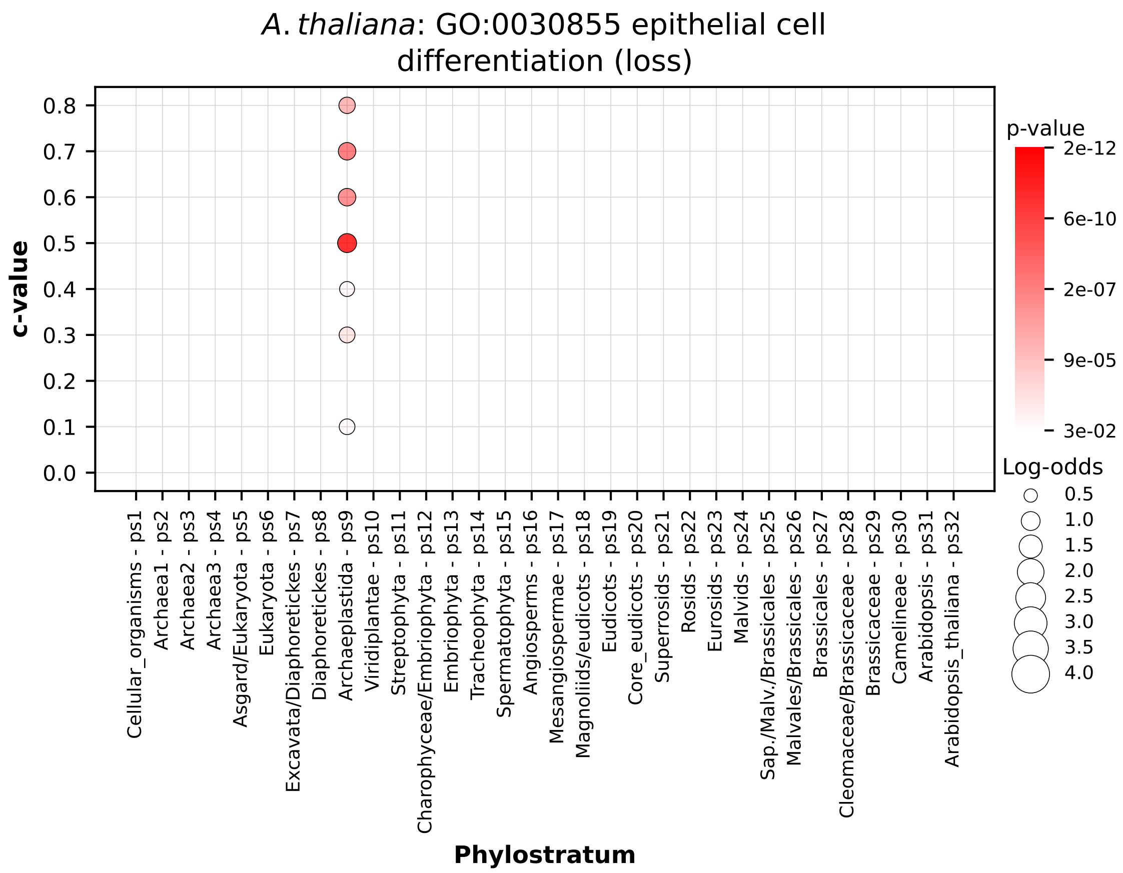

A. thaliana: GO:0030855 epithelial cell differentiation (loss)
c-value
0.8
0.7
0.6
0.5
0.4
0.3
0.2
0.1
0.0
Phylostratum
Cellular_organisms - ps1
Archaea1 - ps2
Archaea2 - ps3
Archaea3 - ps4
Asgard/Eukaryota - ps5
Eukaryota - ps6
Excavata/Diaphoretickes - ps7
Diaphoretickes - ps8
Archaeplastida - ps9
Viridiplantae - ps10
Streptophyta - ps11
Charophyceae/Embriophyta - ps12
Embriophyta - ps13
Tracheophyta - ps14
Spermatophyta - ps15
Angiosperms - ps16
Mesangiospermae - ps17
Magnoliids/eudicots - ps18
Eudicots - ps19
Core_eudicots - ps20
Superrosids - ps21
Rosids - ps22
Eurosids - ps23
Malvids - ps24
Sap./Malv./Brassicales - ps25
Malvales/Brassicales - ps26
Brassicales - ps27
Cleomaceae/Brassicaceae - ps28
Brassicaceae - ps29
Camelineae - ps30
Arabidopsis - ps31
Arabidopsis_thaliana - ps32
p-value
2e-12
6e-10
2e-07
9e-05
3e-02
Log-odds
0.5
1.0
1.5
2.0
2.5
3.0
3.5
4.0

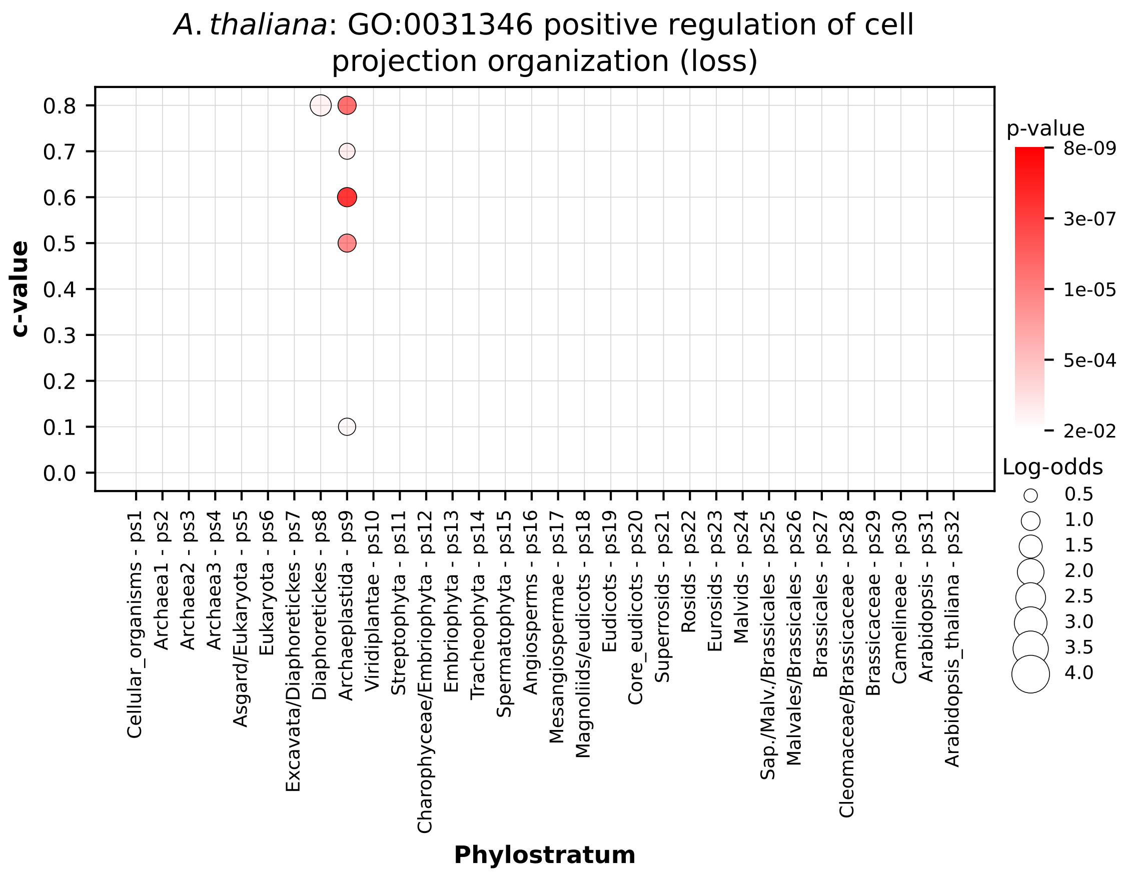

A. thaliana: GO:0031346 positive regulation of cell projection organization (loss)
c-value
0.8
0.7
0.6
0.5
0.4
0.3
0.2
0.1
0.0
p-value
8e-09
3e-07
1e-05
5e-04
2e-02
Log-odds
0.5
1.0
1.5
2.0
2.5
3.0
3.5
4.0
Cellular_organisms - ps1
Archaea1 - ps2
Archaea2 - ps3
Archaea3 - ps4
Asgard/Eukaryota - ps5
Eukaryota - ps6
Excavata/Diaphoretickes - ps7
Diaphoretickes - ps8
Archaeplastida - ps9
Viridiplantae - ps10
Streptophyta - ps11
Charophyceae/Embriophyta - ps12
Embriophyta - ps13
Tracheophyta - ps14
Spermatophyta - ps15
Angiosperms - ps16
Mesangiospermae - ps17
Magnoliids/eudicots - ps18
Eudicots - ps19
Core_eudicots - ps20
Superrosids - ps21
Rosids - ps22
Eurosids - ps23
Malvids - ps24
Sap./Malv./Brassicales - ps25
Malvales/Brassicales - ps26
Brassicales - ps27
Cleomaceae/Brassicaceae - ps28
Brassicaceae - ps29
Camelineae - ps30
Arabidopsis - ps31
Arabidopsis_thaliana - ps32
Phylostratum

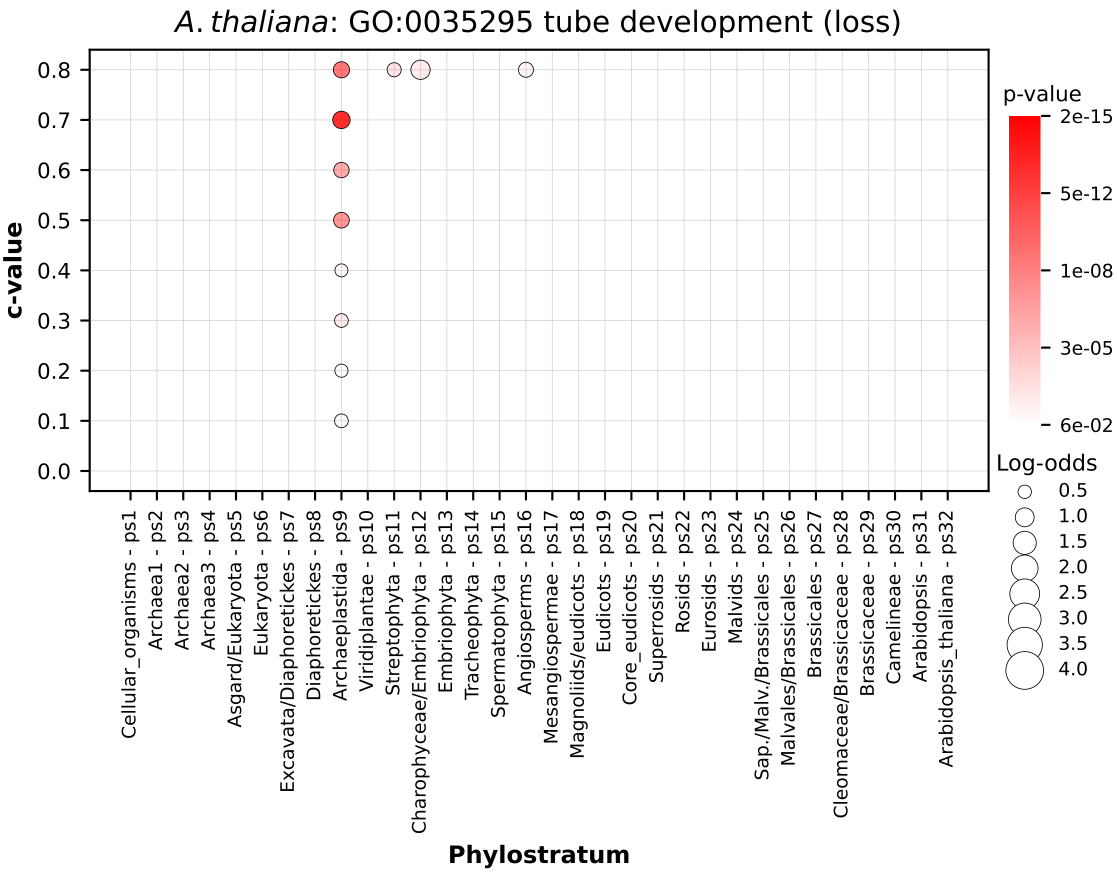

A. thaliana: GO:0035295 tube development (loss)
c-value
0.0
0.1
0.2
0.3
0.4
0.5
0.6
0.7
0.8
Phylostratum
Cellular_organisms - ps1
Archaea1 - ps2
Archaea2 - ps3
Archaea3 - ps4
Asgard/Eukaryota - ps5
Eukaryota - ps6
Excavata/Diaphoretickes - ps7
Diaphoretickes - ps8
Archaeplastida - ps9
Viridiplantae - ps10
Streptophyta - ps11
Charophyceae/Embriophyta - ps12
Embriophyta - ps13
Tracheophyta - ps14
Spermatophyta - ps15
Angiosperms - ps16
Mesangiospermae - ps17
Magnoliids/eudicots - ps18
Eudicots - ps19
Core_eudicots - ps20
Superrosids - ps21
Rosids - ps22
Eurosids - ps23
Malvids - ps24
Sap./Malv./Brassicales - ps25
Malvales/Brassicales - ps26
Brassicales - ps27
Cleomaceae/Brassicaceae - ps28
Brassicaceae - ps29
Camelineae - ps30
Arabidopsis - ps31
Arabidopsis_thaliana - ps32
p-value
2e-15
5e-12
1e-08
3e-05
6e-02
Log-odds
0.5
1.0
1.5
2.0
2.5
3.0
3.5
4.0

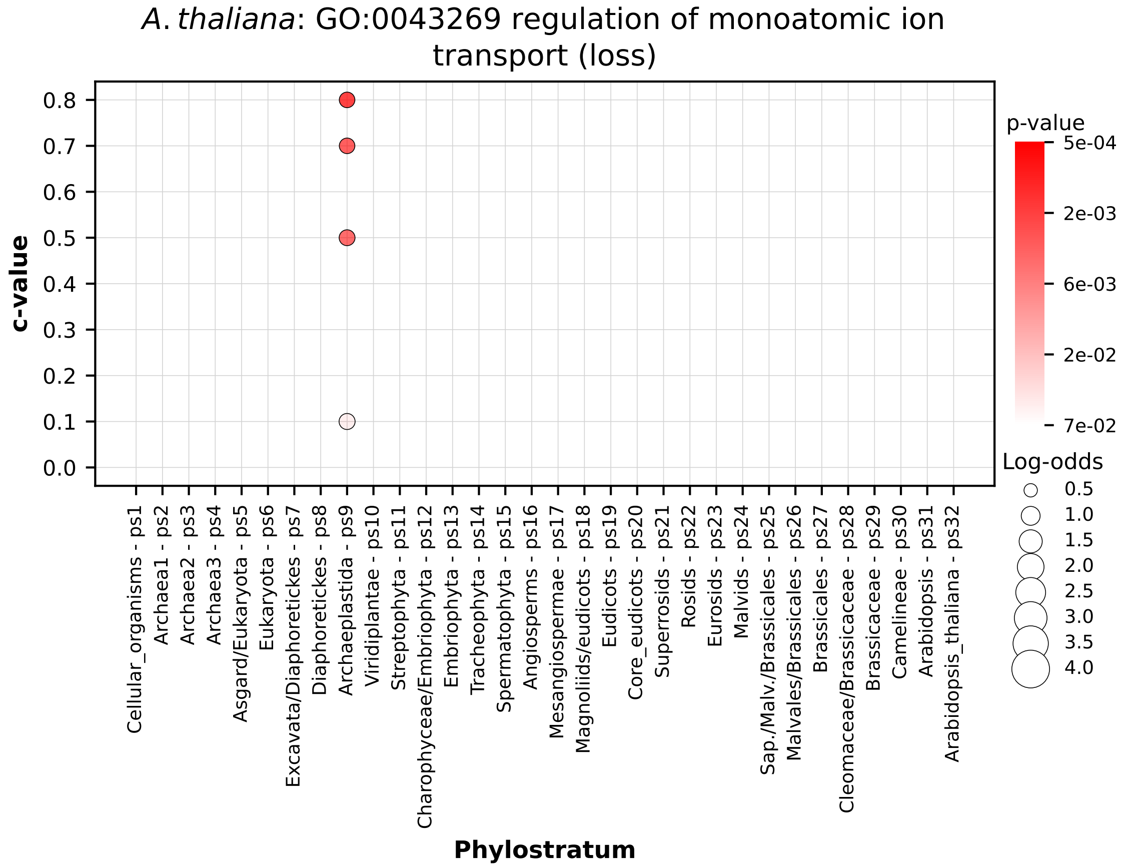
A. thaliana: GO:0043269 regulation of monoatomic ion transport (loss)
c-value
0.0
0.1
0.2
0.3
0.4
0.5
0.6
0.7
0.8
Phylostratum
Cellular_organisms - ps1
Archaea1 - ps2
Archaea2 - ps3
Archaea3 - ps4
Asgard/Eukaryota - ps5
Eukaryota - ps6
Excavata/Diaphoretickes - ps7
Diaphoretickes - ps8
Archaeplastida - ps9
Viridiplantae - ps10
Streptophyta - ps11
Charophyceae/Embriophyta - ps12
Embriophyta - ps13
Tracheophyta - ps14
Spermatophyta - ps15
Angiosperms - ps16
Mesangiospermae - ps17
Magnoliids/eudicots - ps18
Eudicots - ps19
Core_eudicots - ps20
Superrosids - ps21
Rosids - ps22
Eurosids - ps23
Malvids - ps24
Sap./Malv./Brassicales - ps25
Malvales/Brassicales - ps26
Brassicales - ps27
Cleomaceae/Brassicaceae - ps28
Brassicaceae - ps29
Camelineae - ps30
Arabidopsis - ps31
Arabidopsis_thaliana - ps32
p-value
5e-04
2e-03
6e-03
2e-02
7e-02
Log-odds
0.5
1.0
1.5
2.0
2.5
3.0
3.5
4.0

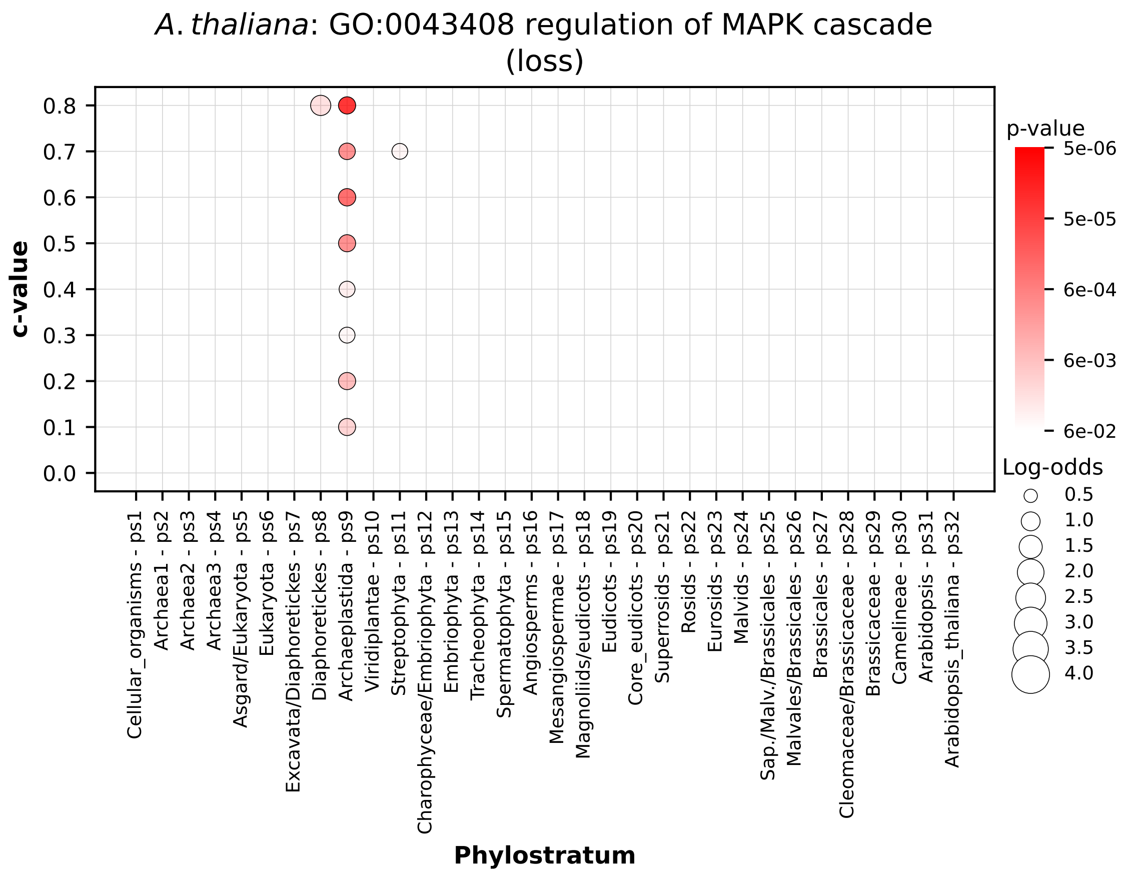

A. thaliana: GO:0043408 regulation of MAPK cascade
(loss)
c-value
0.8
0.7
0.6
0.5
0.4
0.3
0.2
0.1
0.0
Phylostratum
Cellular_organisms - ps1
Archaea1 - ps2
Archaea2 - ps3
Archaea3 - ps4
Asgard/Eukaryota - ps5
Eukaryota - ps6
Excavata/Diaphoretickes - ps7
Diaphoretickes - ps8
Archaeplastida - ps9
Viridiplantae - ps10
Streptophyta - ps11
Charophyceae/Embriophyta - ps12
Embriophyta - ps13
Tracheophyta - ps14
Spermatophyta - ps15
Angiosperms - ps16
Mesangiospermae - ps17
Magnoliids/eudicots - ps18
Eudicots - ps19
Core_eudicots - ps20
Superrosids - ps21
Rosids - ps22
Eurosids - ps23
Malvids - ps24
Sap./Malv./Brassicales - ps25
Malvales/Brassicales - ps26
Brassicales - ps27
Cleomaceae/Brassicaceae - ps28
Brassicaceae - ps29
Camelineae - ps30
Arabidopsis - ps31
Arabidopsis_thaliana - ps32
p-value
5e-06
5e-05
6e-04
6e-03
6e-02
Log-odds
0.5
1.0
1.5
2.0
2.5
3.0
3.5
4.0

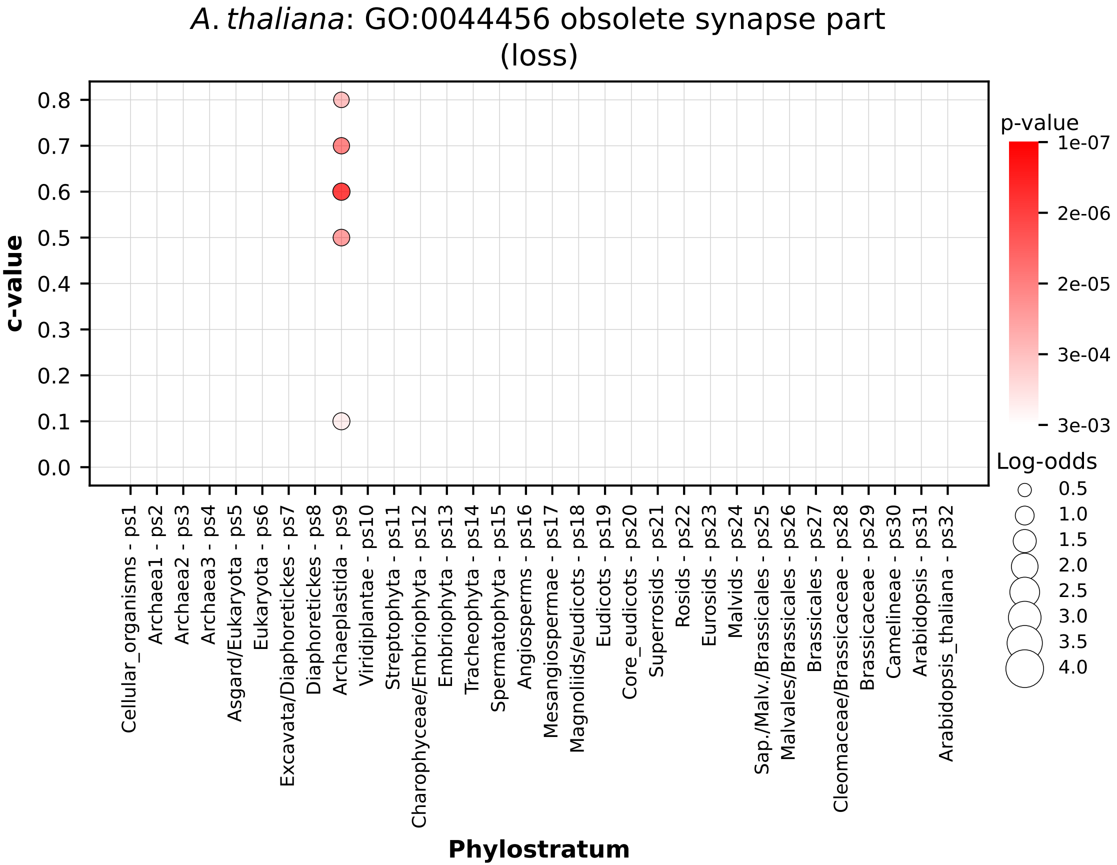

A. thaliana: GO:0044456 obsolete synapse part
(loss)
c-value
0.8
0.7
0.6
0.5
0.4
0.3
0.2
0.1
0.0
p-value
1e-07
2e-06
2e-05
3e-04
3e-03
Log-odds
0.5
1.0
1.5
2.0
2.5
3.0
3.5
4.0
Cellular_organisms - ps1
Archaea1 - ps2
Archaea2 - ps3
Archaea3 - ps4
Asgard/Eukaryota - ps5
Eukaryota - ps6
Excavata/Diaphoretickes - ps7
Diaphoretickes - ps8
Archaeplastida - ps9
Viridiplantae - ps10
Streptophyta - ps11
Charophyceae/Embriophyta - ps12
Embriophyta - ps13
Tracheophyta - ps14
Spermatophyta - ps15
Angiosperms - ps16
Mesangiospermae - ps17
Magnoliids/eudicots - ps18
Eudicots - ps19
Core_eudicots - ps20
Superrosids - ps21
Rosids - ps22
Eurosids - ps23
Malvids - ps24
Sap./Malv./Brassicales - ps25
Malvales/Brassicales - ps26
Brassicales - ps27
Cleomaceae/Brassicaceae - ps28
Brassicaceae - ps29
Camelineae - ps30
Arabidopsis - ps31
Arabidopsis_thaliana - ps32
Phylostratum

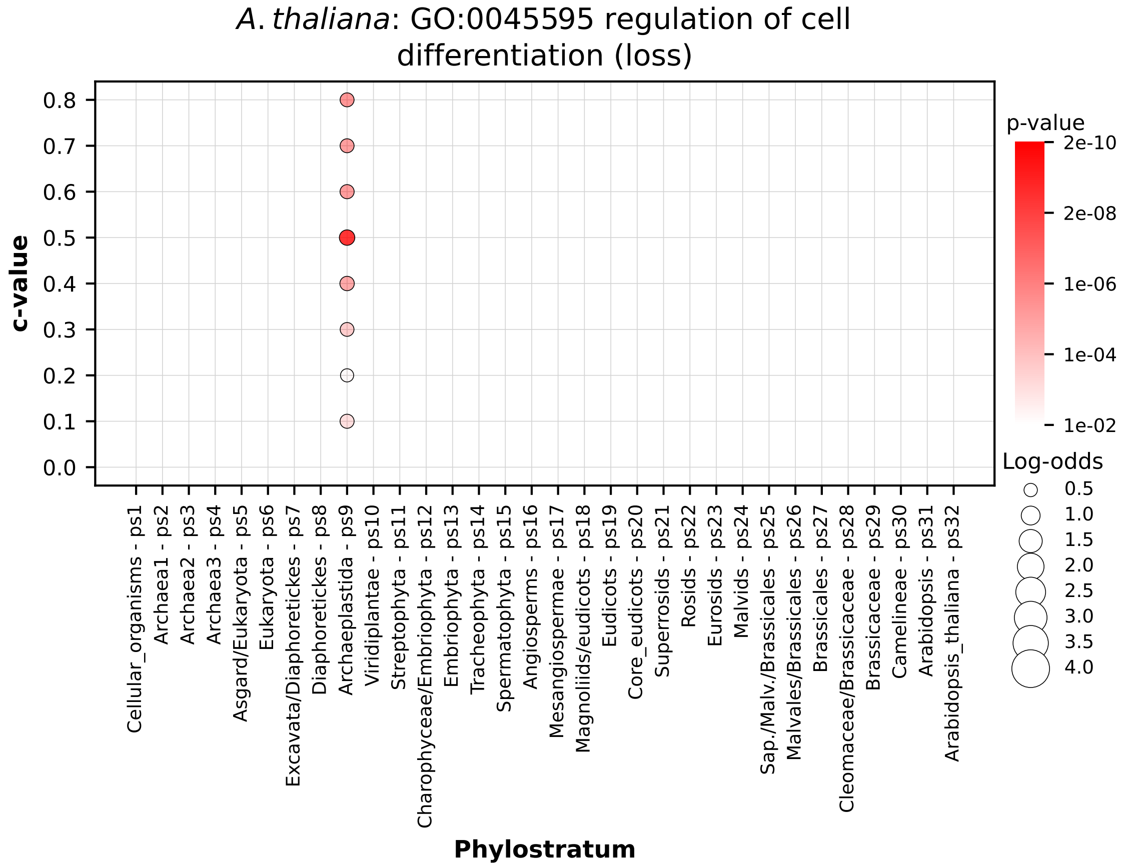

A. thaliana: GO:0045595 regulation of cell differentiation (loss)
c-value
0.8
0.7
0.6
0.5
0.4
0.3
0.2
0.1
0.0
p-value
2e-10
2e-08
1e-06
1e-04
1e-02
Log-odds
0.5
1.0
1.5
2.0
2.5
3.0
3.5
4.0
Cellular_organisms - ps1
Archaea1 - ps2
Archaea2 - ps3
Archaea3 - ps4
Asgard/Eukaryota - ps5
Eukaryota - ps6
Excavata/Diaphoretickes - ps7
Diaphoretickes - ps8
Archaeplastida - ps9
Viridiplantae - ps10
Streptophyta - ps11
Charophyceae/Embriophyta - ps12
Embriophyta - ps13
Tracheophyta - ps14
Spermatophyta - ps15
Angiosperms - ps16
Mesangiospermae - ps17
Magnoliids/eudicots - ps18
Eudicots - ps19
Core_eudicots - ps20
Superrosids - ps21
Rosids - ps22
Eurosids - ps23
Malvids - ps24
Sap./Malv./Brassicales - ps25
Malvales/Brassicales - ps26
Brassicales - ps27
Cleomaceae/Brassicaceae - ps28
Brassicaceae - ps29
Camelineae - ps30
Arabidopsis - ps31
Arabidopsis_thaliana - ps32
Phylostratum

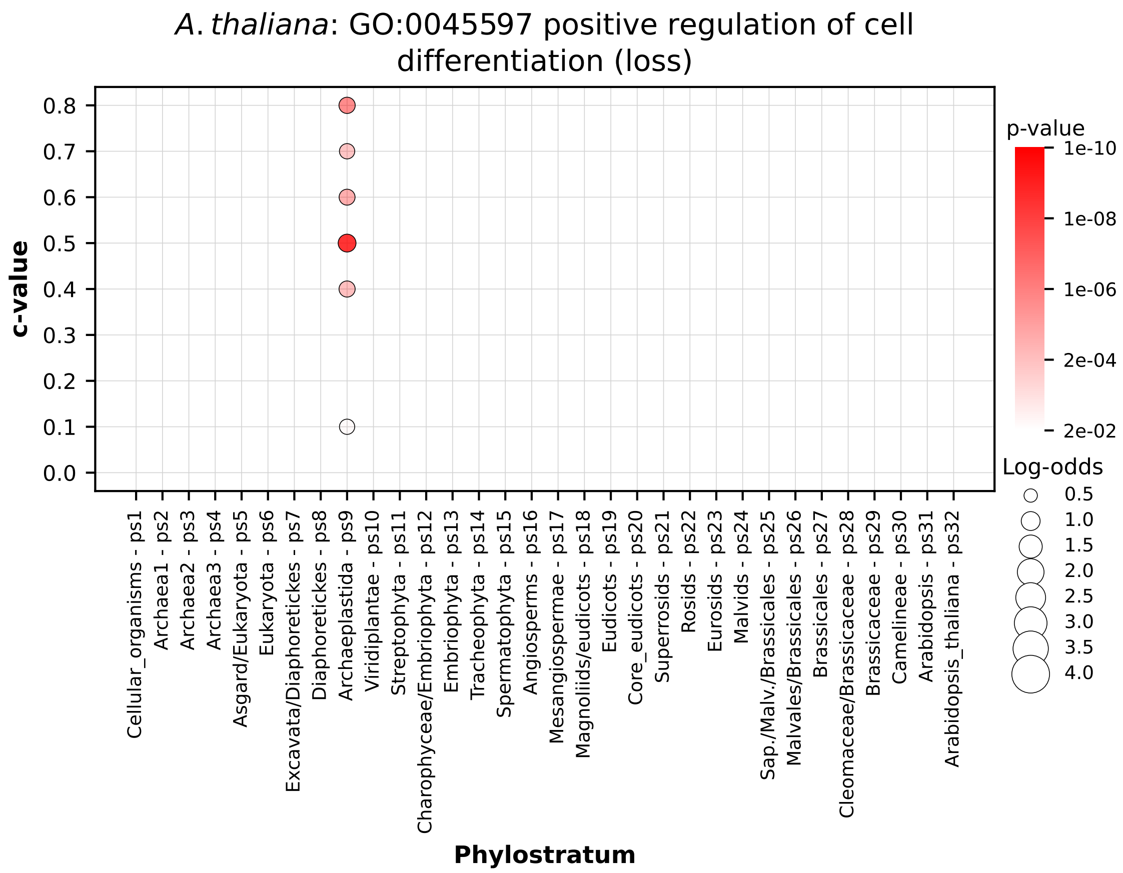

A. thaliana: GO:0045597 positive regulation of cell differentiation (loss)
c-value
0.8
0.7
0.6
0.5
0.4
0.3
0.2
0.1
0.0
Phylostratum
Cellular_organisms - ps1
Archaea1 - ps2
Archaea2 - ps3
Archaea3 - ps4
Asgard/Eukaryota - ps5
Eukaryota - ps6
Excavata/Diaphoretickes - ps7
Diaphoretickes - ps8
Archaeplastida - ps9
Viridiplantae - ps10
Streptophyta - ps11
Charophyceae/Embriophyta - ps12
Embriophyta - ps13
Tracheophyta - ps14
Spermatophyta - ps15
Angiosperms - ps16
Mesangiospermae - ps17
Magnoliids/eudicots - ps18
Eudicots - ps19
Core_eudicots - ps20
Superrosids - ps21
Rosids - ps22
Eurosids - ps23
Malvids - ps24
Sap./Malv./Brassicales - ps25
Malvales/Brassicales - ps26
Brassicales - ps27
Cleomaceae/Brassicaceae - ps28
Brassicaceae - ps29
Camelineae - ps30
Arabidopsis - ps31
Arabidopsis_thaliana - ps32
p-value
1e-10
1e-08
1e-06
2e-04
2e-02
Log-odds
0.5
1.0
1.5
2.0
2.5
3.0
3.5
4.0

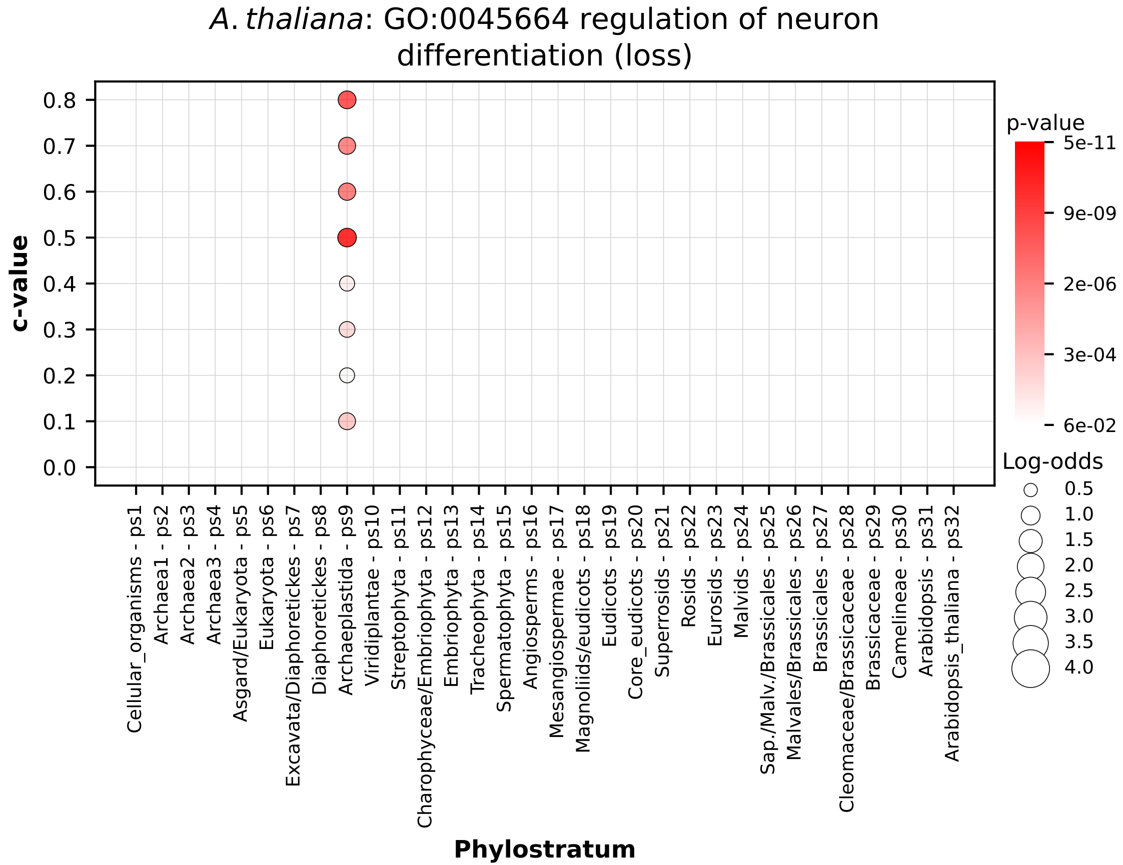

A. thaliana: GO:0045664 regulation of neuron differentiation (loss)
c-value
0.8
0.7
0.6
0.5
0.4
0.3
0.2
0.1
0.0
Phylostratum
Cellular_organisms - ps1
Archaea1 - ps2
Archaea2 - ps3
Archaea3 - ps4
Asgard/Eukaryota - ps5
Eukaryota - ps6
Excavata/Diaphoretickes - ps7
Diaphoretickes - ps8
Archaeplastida - ps9
Viridiplantae - ps10
Streptophyta - ps11
Charophyceae/Embriophyta - ps12
Embriophyta - ps13
Tracheophyta - ps14
Spermatophyta - ps15
Angiosperms - ps16
Mesangiospermae - ps17
Magnoliids/eudicots - ps18
Eudicots - ps19
Core_eudicots - ps20
Superrosids - ps21
Rosids - ps22
Eurosids - ps23
Malvids - ps24
Sap./Malv./Brassicales - ps25
Malvales/Brassicales - ps26
Brassicales - ps27
Cleomaceae/Brassicaceae - ps28
Brassicaceae - ps29
Camelineae - ps30
Arabidopsis - ps31
Arabidopsis_thaliana - ps32
p-value
5e-11
9e-09
2e-06
3e-04
6e-02
Log-odds
0.5
1.0
1.5
2.0
2.5
3.0
3.5
4.0

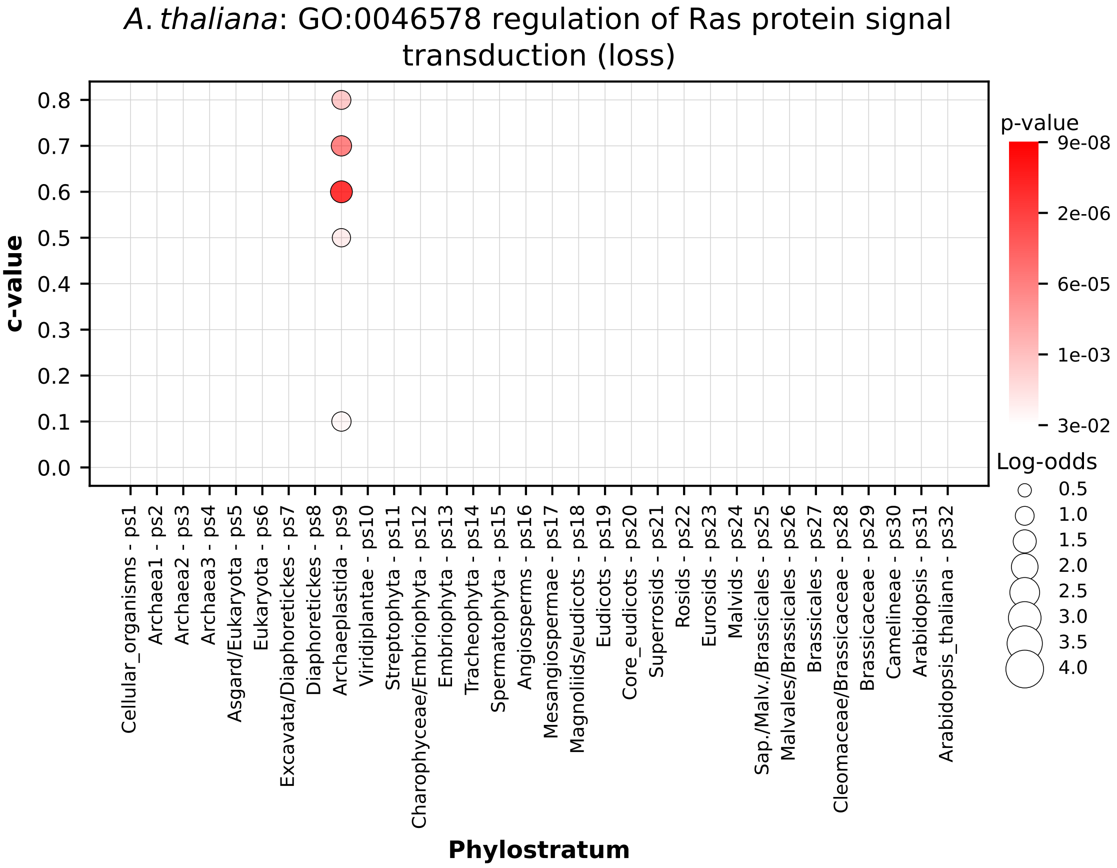

A. thaliana: GO:0046578 regulation of Ras protein signal transduction (loss)
c-value
0.8
0.7
0.6
0.5
0.4
0.3
0.2
0.1
0.0
p-value
9e-08
2e-06
6e-05
1e-03
3e-02
Log-odds
0.5
1.0
1.5
2.0
2.5
3.0
3.5
4.0
Cellular_organisms - ps1
Archaea1 - ps2
Archaea2 - ps3
Archaea3 - ps4
Asgard/Eukaryota - ps5
Eukaryota - ps6
Excavata/Diaphoretickes - ps7
Diaphoretickes - ps8
Archaeplastida - ps9
Viridiplantae - ps10
Streptophyta - ps11
Charophyceae/Embriophyta - ps12
Embriophyta - ps13
Tracheophyta - ps14
Spermatophyta - ps15
Angiosperms - ps16
Mesangiospermae - ps17
Magnoliids/eudicots - ps18
Eudicots - ps19
Core_eudicots - ps20
Superrosids - ps21
Rosids - ps22
Eurosids - ps23
Malvids - ps24
Sap./Malv./Brassicales - ps25
Malvales/Brassicales - ps26
Brassicales - ps27
Cleomaceae/Brassicaceae - ps28
Brassicaceae - ps29
Camelineae - ps30
Arabidopsis - ps31
Arabidopsis_thaliana - ps32
Phylostratum

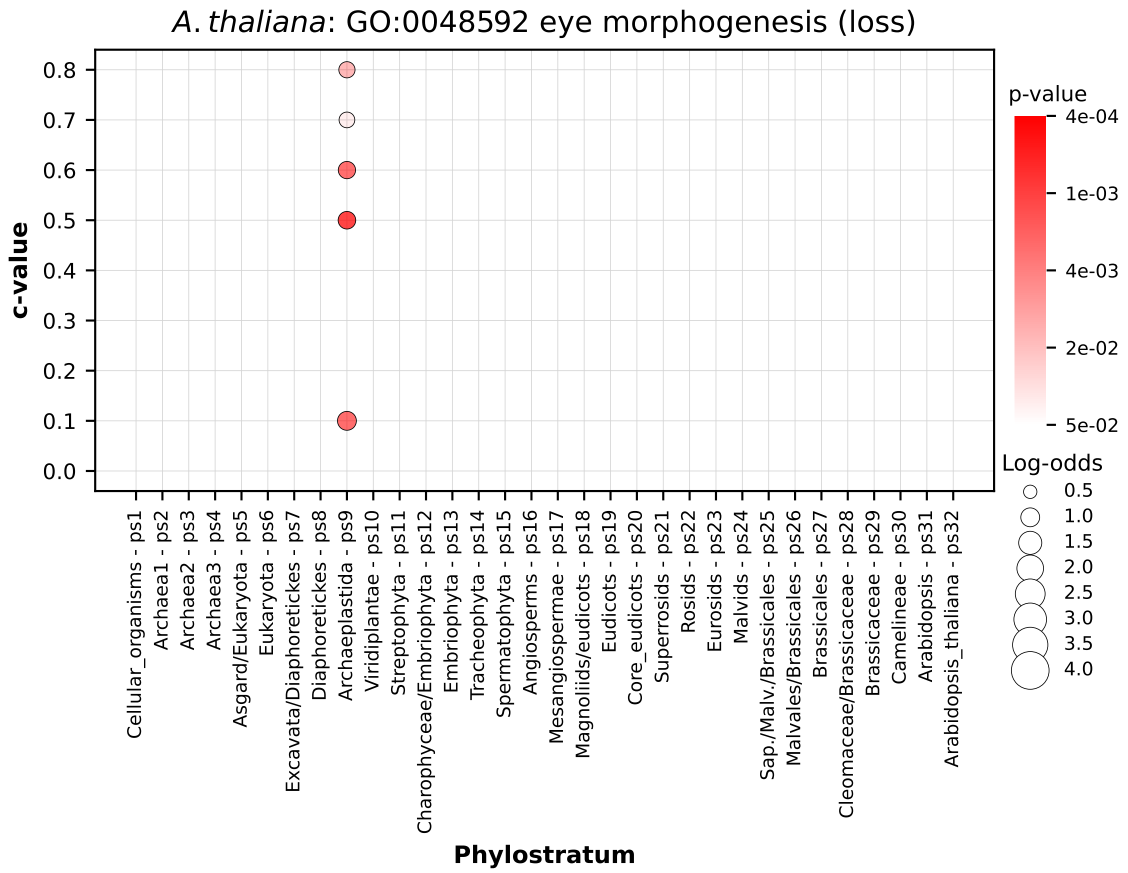
A. thaliana: GO:0048592 eye morphogenesis (loss)
c-value
0.0
0.1
0.2
0.3
0.4
0.5
0.6
0.7
0.8
Phylostratum
Cellular_organisms - ps1
Archaea1 - ps2
Archaea2 - ps3
Archaea3 - ps4
Asgard/Eukaryota - ps5
Eukaryota - ps6
Excavata/Diaphoretickes - ps7
Diaphoretickes - ps8
Archaeplastida - ps9
Viridiplantae - ps10
Streptophyta - ps11
Charophyceae/Embriophyta - ps12
Embriophyta - ps13
Tracheophyta - ps14
Spermatophyta - ps15
Angiosperms - ps16
Mesangiospermae - ps17
Magnoliids/eudicots - ps18
Eudicots - ps19
Core_eudicots - ps20
Superrosids - ps21
Rosids - ps22
Eurosids - ps23
Malvids - ps24
Sap./Malv./Brassicales - ps25
Malvales/Brassicales - ps26
Brassicales - ps27
Cleomaceae/Brassicaceae - ps28
Brassicaceae - ps29
Camelineae - ps30
Arabidopsis - ps31
Arabidopsis_thaliana - ps32
p-value
4e-04
1e-03
4e-03
2e-02
5e-02
Log-odds
0.5
1.0
1.5
2.0
2.5
3.0
3.5
4.0

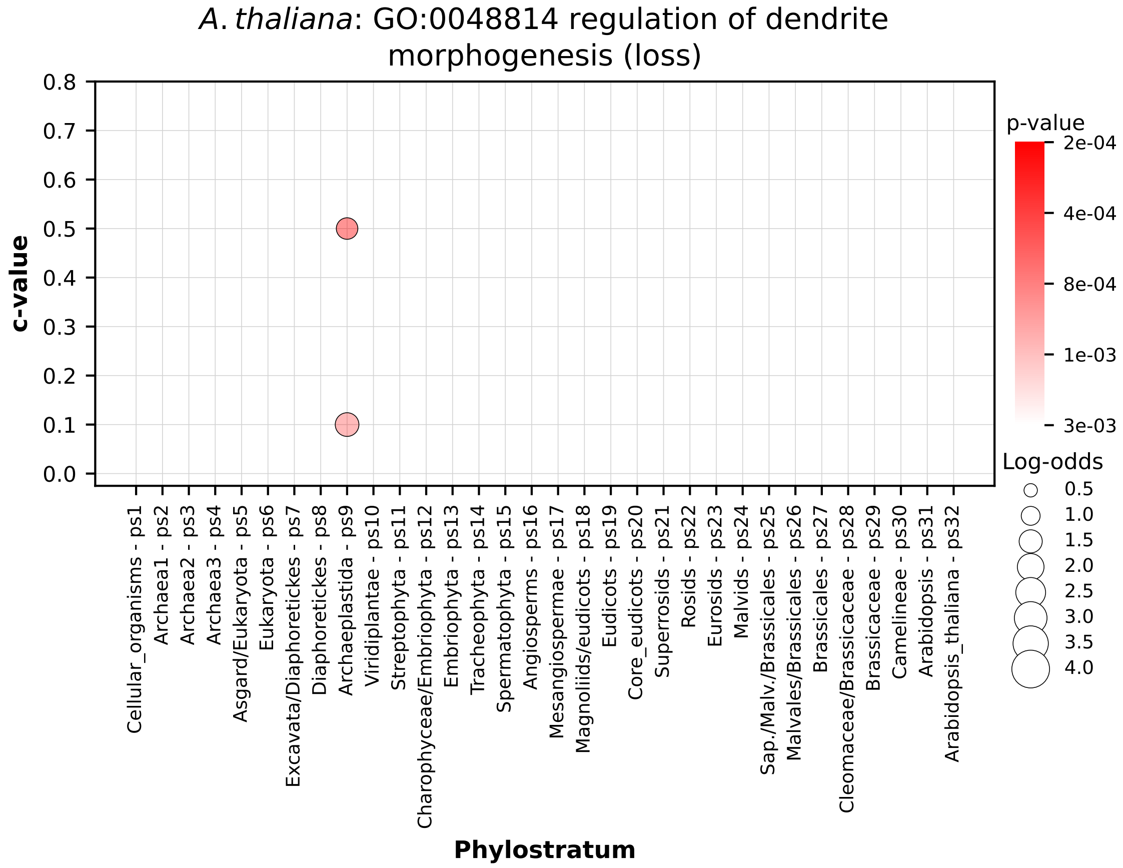
A. thaliana: GO:0048814 regulation of dendrite morphogenesis (loss)
c-value
0.0
0.1
0.2
0.3
0.4
0.5
0.6
0.7
0.8
Phylostratum
Cellular_organisms - ps1
Archaea1 - ps2
Archaea2 - ps3
Archaea3 - ps4
Asgard/Eukaryota - ps5
Eukaryota - ps6
Excavata/Diaphoretickes - ps7
Diaphoretickes - ps8
Archaeplastida - ps9
Viridiplantae - ps10
Streptophyta - ps11
Charophyceae/Embriophyta - ps12
Embriophyta - ps13
Tracheophyta - ps14
Spermatophyta - ps15
Angiosperms - ps16
Mesangiospermae - ps17
Magnoliids/eudicots - ps18
Eudicots - ps19
Core_eudicots - ps20
Superrosids - ps21
Rosids - ps22
Eurosids - ps23
Malvids - ps24
Sap./Malv./Brassicales - ps25
Malvales/Brassicales - ps26
Brassicales - ps27
Cleomaceae/Brassicaceae - ps28
Brassicaceae - ps29
Camelineae - ps30
Arabidopsis - ps31
Arabidopsis_thaliana - ps32
p-value
2e-04
4e-04
8e-04
1e-03
3e-03
Log-odds
0.5
1.0
1.5
2.0
2.5
3.0
3.5
4.0

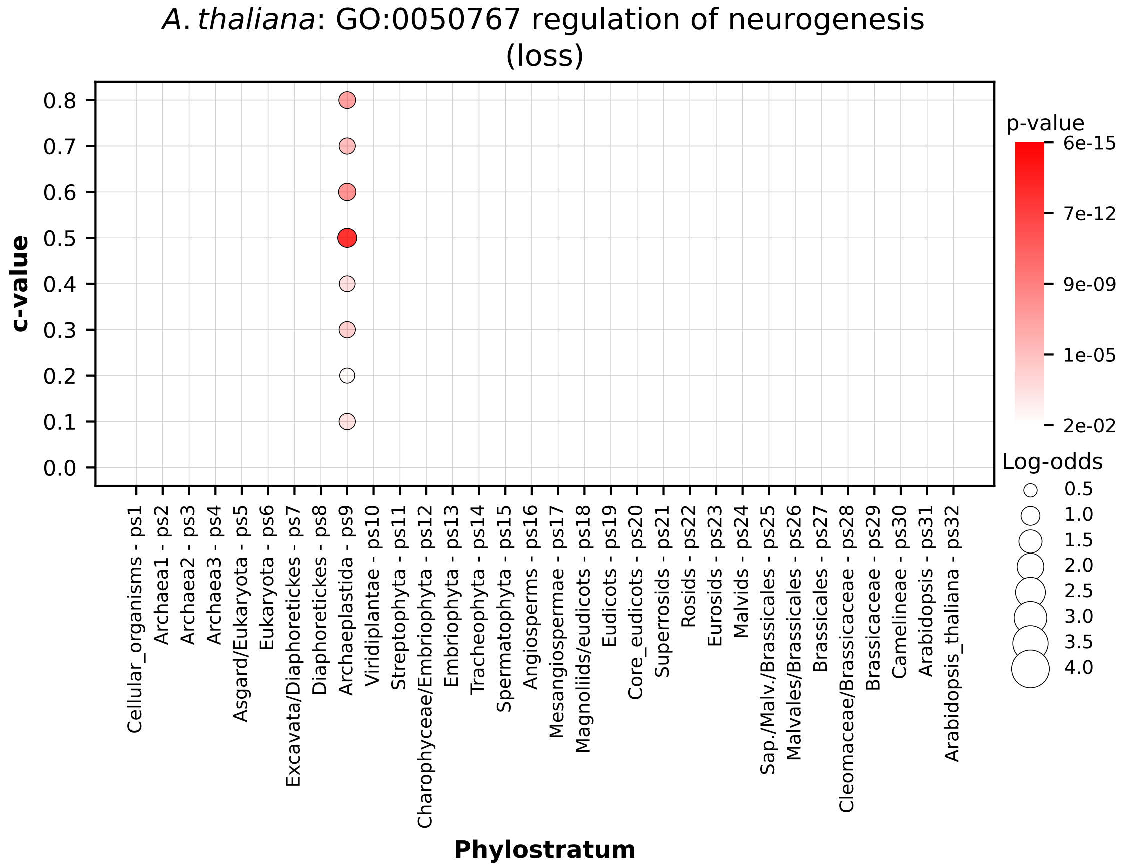

$A. thaliana$: GO:0050767 regulation of neurogenesis
(loss)
c-value
0.8
0.7
0.6
0.5
0.4
0.3
0.2
0.1
0.0
Phylostratum
Cellular_organisms - ps1
Archaea1 - ps2
Archaea2 - ps3
Archaea3 - ps4
Asgard/Eukaryota - ps5
Eukaryota - ps6
Excavata/Diaphoretickes - ps7
Diaphoretickes - ps8
Archaeplastida - ps9
Viridiplantae - ps10
Streptophyta - ps11
Charophyceae/Embriophyta - ps12
Embriophyta - ps13
Tracheophyta - ps14
Spermatophyta - ps15
Angiosperms - ps16
Mesangiospermae - ps17
Magnoliids/eudicots - ps18
Eudicots - ps19
Core_eudicots - ps20
Superrosids - ps21
Rosids - ps22
Eurosids - ps23
Malvids - ps24
Sap./Malv./Brassicales - ps25
Malvales/Brassicales - ps26
Brassicales - ps27
Cleomaceae/Brassicaceae - ps28
Brassicaceae - ps29
Camelineae - ps30
Arabidopsis - ps31
Arabidopsis_thaliana - ps32
p-value
6e-15
7e-12
9e-09
1e-05
2e-02
Log-odds
0.5
1.0
1.5
2.0
2.5
3.0
3.5
4.0

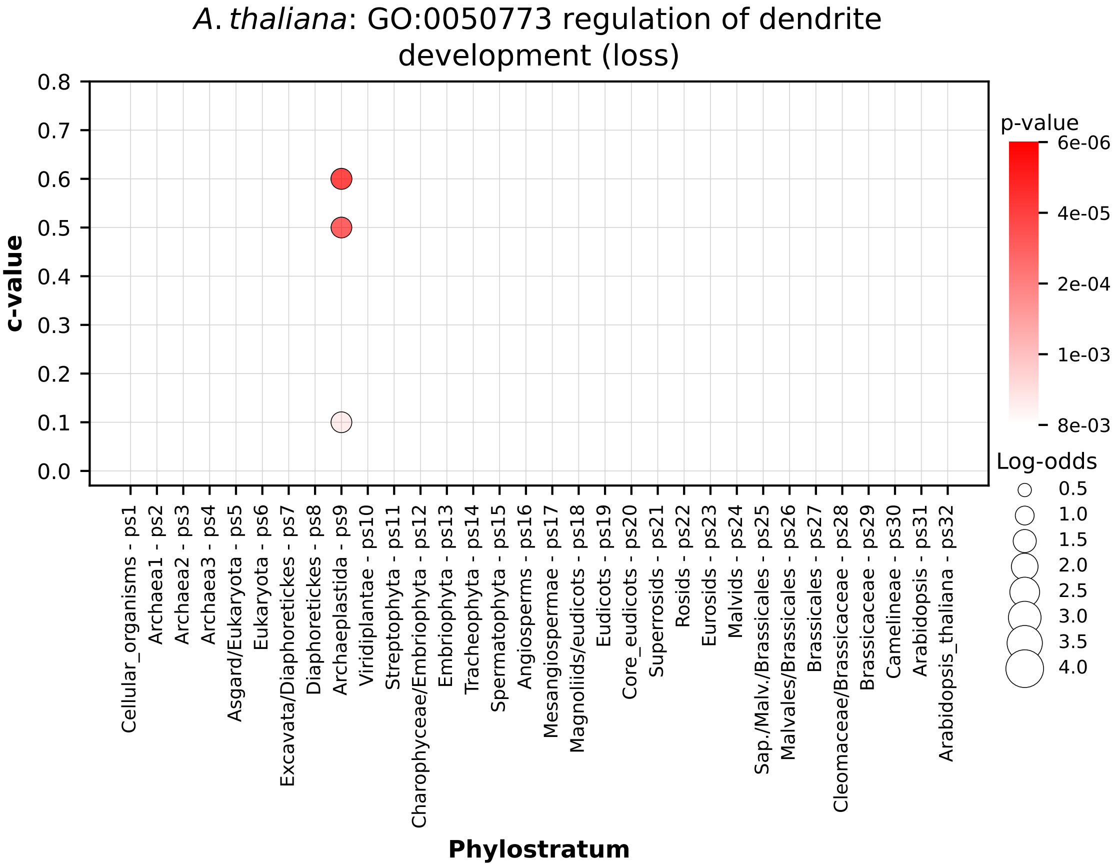

A. thaliana: GO:0050773 regulation of dendrite development (loss)
c-value
0.8
0.7
0.6
0.5
0.4
0.3
0.2
0.1
0.0
Phylostratum
Cellular_organisms - ps1
Archaea1 - ps2
Archaea2 - ps3
Archaea3 - ps4
Asgard/Eukaryota - ps5
Eukaryota - ps6
Excavata/Diaphoretickes - ps7
Diaphoretickes - ps8
Archaeplastida - ps9
Viridiplantae - ps10
Streptophyta - ps11
Charophyceae/Embriophyta - ps12
Embriophyta - ps13
Tracheophyta - ps14
Spermatophyta - ps15
Angiosperms - ps16
Mesangiospermae - ps17
Magnoliids/eudicots - ps18
Eudicots - ps19
Core_eudicots - ps20
Superrosids - ps21
Rosids - ps22
Eurosids - ps23
Malvids - ps24
Sap./Malv./Brassicales - ps25
Malvales/Brassicales - ps26
Brassicales - ps27
Cleomaceae/Brassicaceae - ps28
Brassicaceae - ps29
Camelineae - ps30
Arabidopsis - ps31
Arabidopsis_thaliana - ps32
p-value
6e-06
4e-05
2e-04
1e-03
8e-03
Log-odds
0.5
1.0
1.5
2.0
2.5
3.0
3.5
4.0

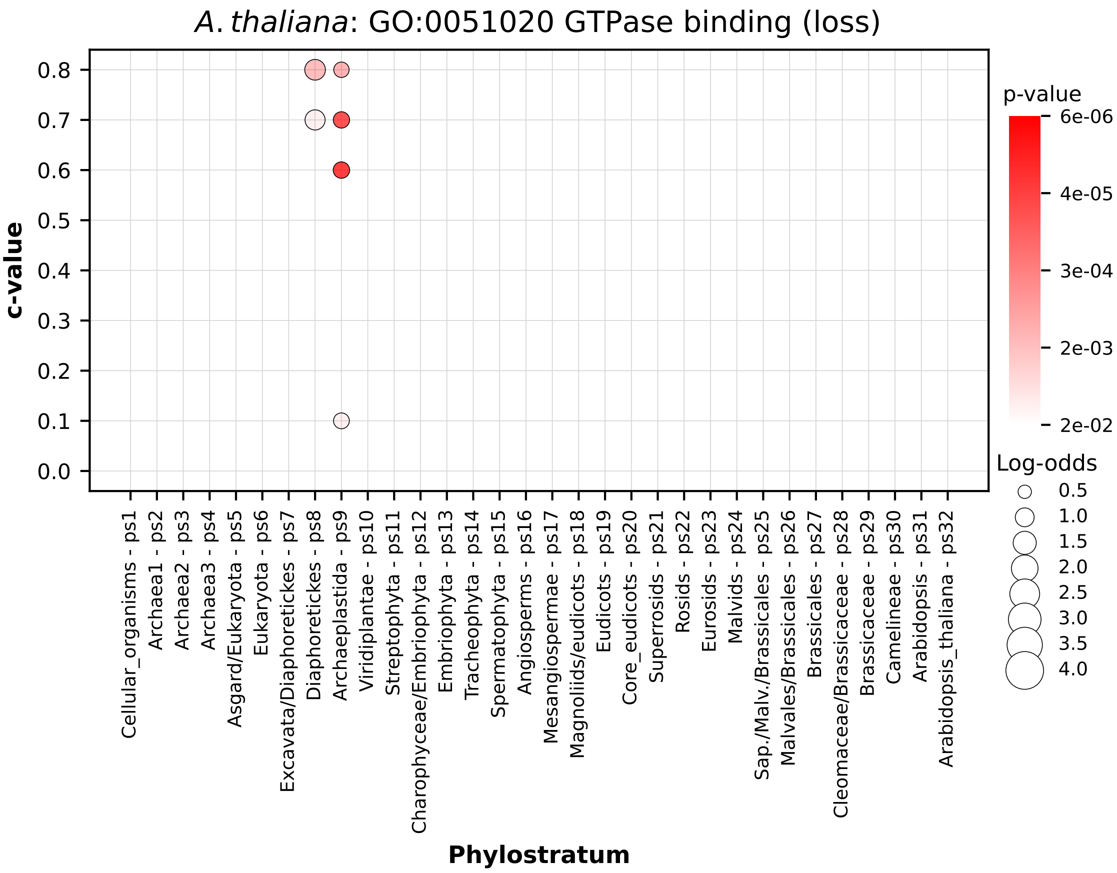

A. thaliana: GO:0051020 GTPase binding (loss)
c-value
0.0
0.1
0.2
0.3
0.4
0.5
0.6
0.7
0.8
Phylostratum
Cellular_organisms - ps1
Archaea1 - ps2
Archaea2 - ps3
Archaea3 - ps4
Asgard/Eukaryota - ps5
Eukaryota - ps6
Excavata/Diaphoretickes - ps7
Diaphoretickes - ps8
Archaeplastida - ps9
Viridiplantae - ps10
Streptophyta - ps11
Charophyceae/Embriophyta - ps12
Embriophyta - ps13
Tracheophyta - ps14
Spermatophyta - ps15
Angiosperms - ps16
Mesangiospermae - ps17
Magnoliids/eudicots - ps18
Eudicots - ps19
Core_eudicots - ps20
Superrosids - ps21
Rosids - ps22
Eurosids - ps23
Malvids - ps24
Sap./Malv./Brassicales - ps25
Malvales/Brassicales - ps26
Brassicales - ps27
Cleomaceae/Brassicaceae - ps28
Brassicaceae - ps29
Camelineae - ps30
Arabidopsis - ps31
Arabidopsis_thaliana - ps32
p-value
6e-06
4e-05
3e-04
2e-03
2e-02
Log-odds
0.5
1.0
1.5
2.0
2.5
3.0
3.5
4.0

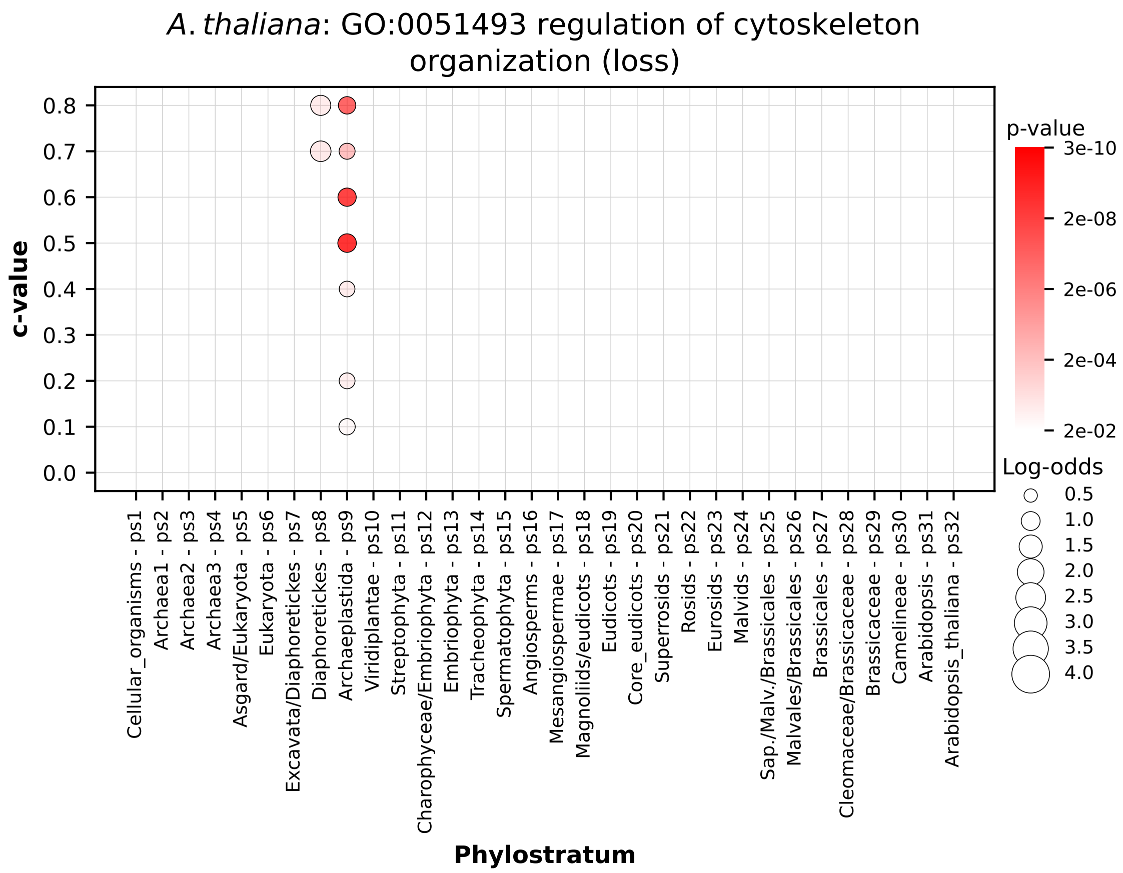

A. thaliana: GO:0051493 regulation of cytoskeleton organization (loss)
c-value
0.8
0.7
0.6
0.5
0.4
0.3
0.2
0.1
0.0
p-value
3e-10
2e-08
2e-06
2e-04
2e-02
Log-odds
0.5
1.0
1.5
2.0
2.5
3.0
3.5
4.0
Cellular_organisms - ps1
Archaea1 - ps2
Archaea2 - ps3
Archaea3 - ps4
Asgard/Eukaryota - ps5
Eukaryota - ps6
Excavata/Diaphoretickes - ps7
Diaphoretickes - ps8
Archaeplastida - ps9
Viridiplantae - ps10
Streptophyta - ps11
Charophyceae/Embriophyta - ps12
Embriophyta - ps13
Tracheophyta - ps14
Spermatophyta - ps15
Angiosperms - ps16
Mesangiospermae - ps17
Magnoliids/eudicots - ps18
Eudicots - ps19
Core_eudicots - ps20
Superrosids - ps21
Rosids - ps22
Eurosids - ps23
Malvids - ps24
Sap./Malv./Brassicales - ps25
Malvales/Brassicales - ps26
Brassicales - ps27
Cleomaceae/Brassicaceae - ps28
Brassicaceae - ps29
Camelineae - ps30
Arabidopsis - ps31
Arabidopsis_thaliana - ps32
Phylostratum

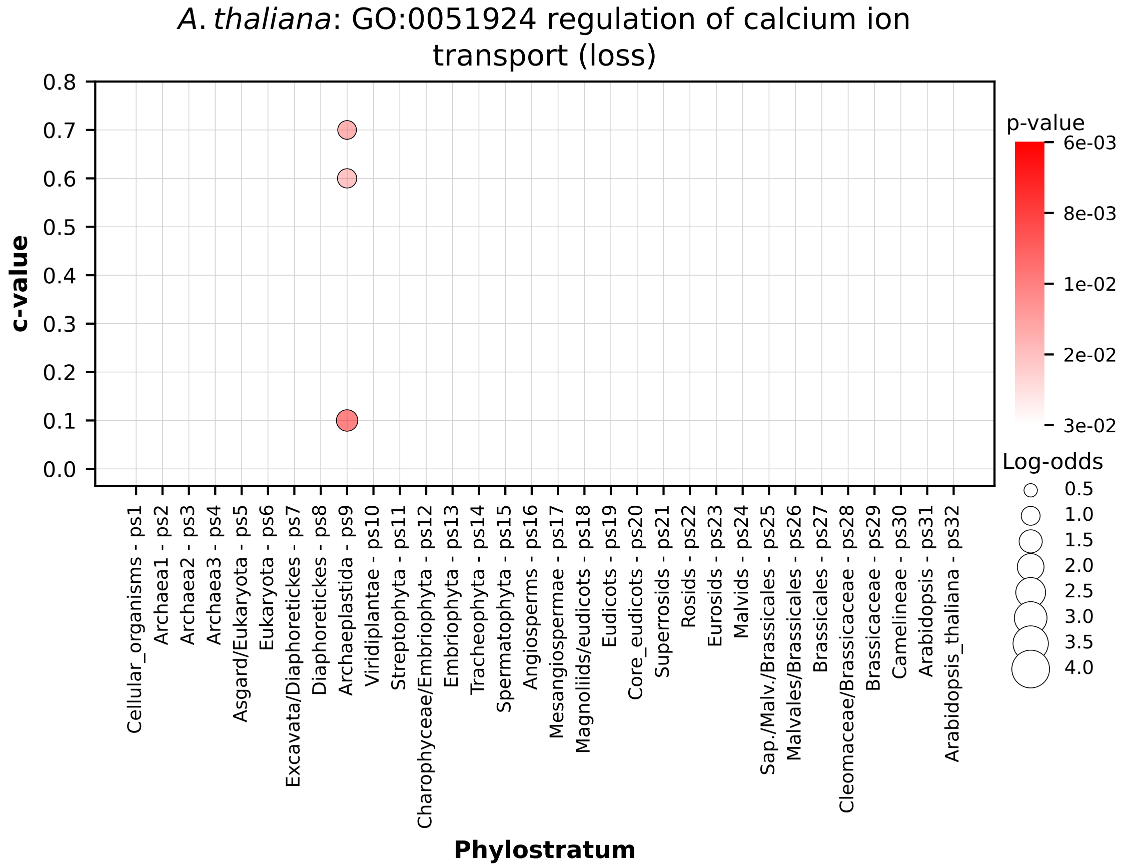

A. thaliana: GO:0051924 regulation of calcium ion transport (loss)
c-value
0.8
0.7
0.6
0.5
0.4
0.3
0.2
0.1
0.0
p-value
6e-03
8e-03
1e-02
2e-02
3e-02
Log-odds
0.5
1.0
1.5
2.0
2.5
3.0
3.5
4.0
Cellular_organisms - ps1
Archaea1 - ps2
Archaea2 - ps3
Archaea3 - ps4
Asgard/Eukaryota - ps5
Eukaryota - ps6
Excavata/Diaphoretickes - ps7
Diaphoretickes - ps8
Archaeplastida - ps9
Viridiplantae - ps10
Streptophyta - ps11
Charophyceae/Embriophyta - ps12
Embriophyta - ps13
Tracheophyta - ps14
Spermatophyta - ps15
Angiosperms - ps16
Mesangiospermae - ps17
Magnoliids/eudicots - ps18
Eudicots - ps19
Core_eudicots - ps20
Superrosids - ps21
Rosids - ps22
Eurosids - ps23
Malvids - ps24
Sap./Malv./Brassicales - ps25
Malvales/Brassicales - ps26
Brassicales - ps27
Cleomaceae/Brassicaceae - ps28
Brassicaceae - ps29
Camelineae - ps30
Arabidopsis - ps31
Arabidopsis_thaliana - ps32
Phylostratum

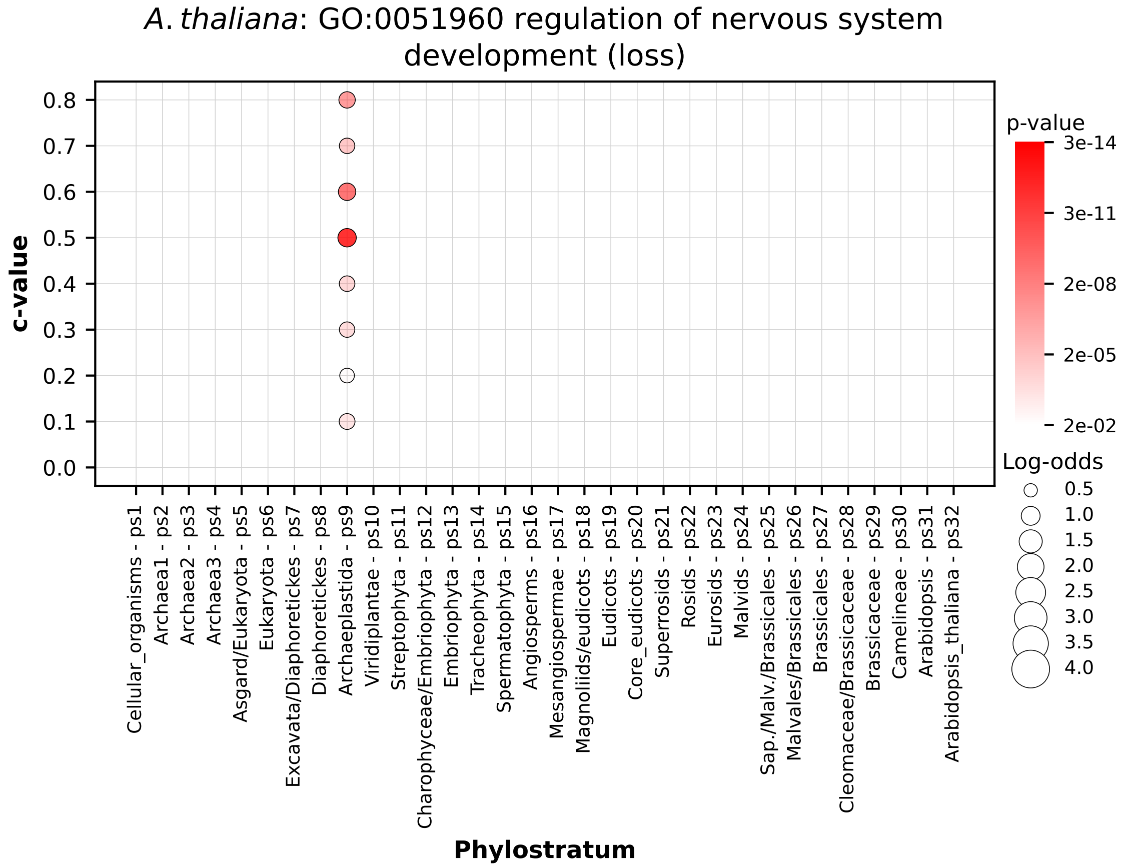

A. thaliana: GO:0051960 regulation of nervous system development (loss)
c-value
0.8
0.7
0.6
0.5
0.4
0.3
0.2
0.1
0.0
p-value
3e-14
3e-11
2e-08
2e-05
2e-02
Log-odds
0.5
1.0
1.5
2.0
2.5
3.0
3.5
4.0
Cellular_organisms - ps1
Archaea1 - ps2
Archaea2 - ps3
Archaea3 - ps4
Asgard/Eukaryota - ps5
Eukaryota - ps6
Excavata/Diaphoretickes - ps7
Diaphoretickes - ps8
Archaeplastida - ps9
Viridiplantae - ps10
Streptophyta - ps11
Charophyceae/Embriophyta - ps12
Embriophyta - ps13
Tracheophyta - ps14
Spermatophyta - ps15
Angiosperms - ps16
Mesangiospermae - ps17
Magnoliids/eudicots - ps18
Eudicots - ps19
Core_eudicots - ps20
Superrosids - ps21
Rosids - ps22
Eurosids - ps23
Malvids - ps24
Sap./Malv./Brassicales - ps25
Malvales/Brassicales - ps26
Brassicales - ps27
Cleomaceae/Brassicaceae - ps28
Brassicaceae - ps29
Camelineae - ps30
Arabidopsis - ps31
Arabidopsis_thaliana - ps32
Phylostratum

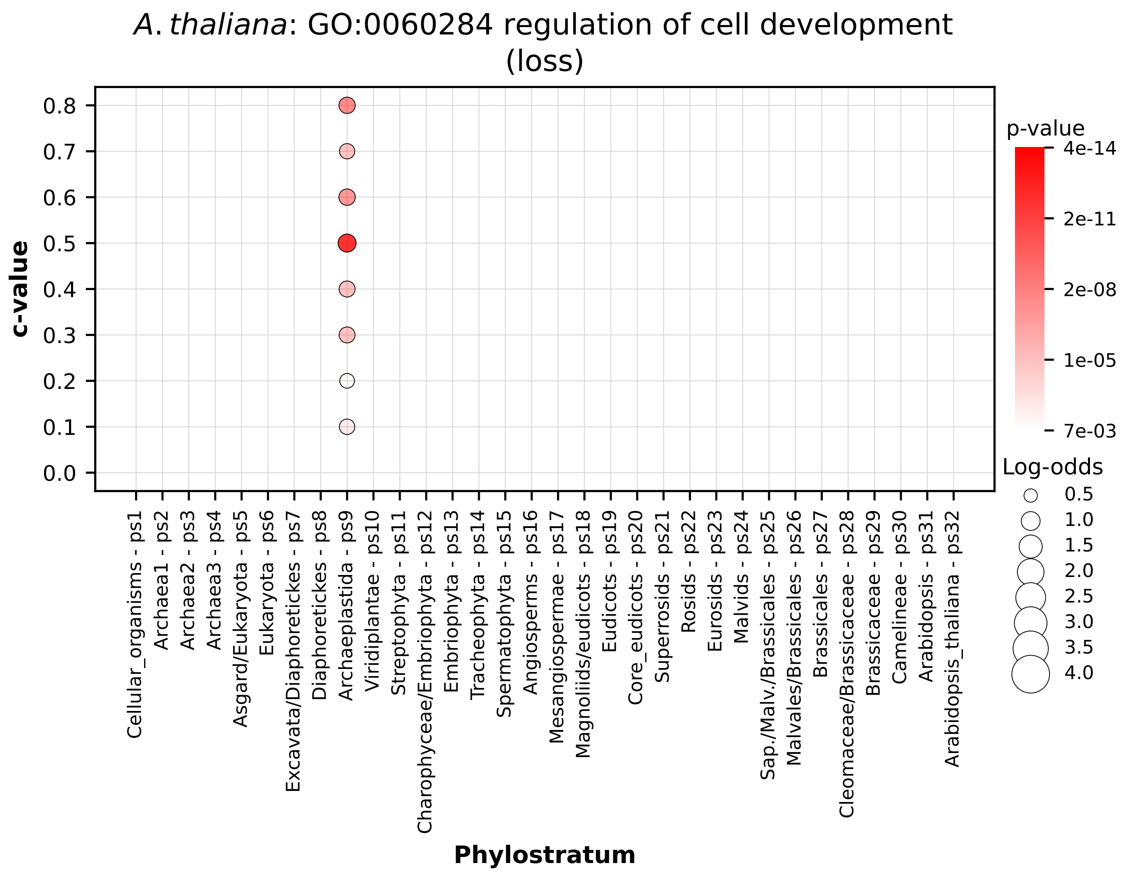

A. thaliana: GO:0060284 regulation of cell development
(loss)
c-value
0.8
0.7
0.6
0.5
0.4
0.3
0.2
0.1
0.0
p-value
4e-14
2e-11
2e-08
1e-05
7e-03
Log-odds
0.5
1.0
1.5
2.0
2.5
3.0
3.5
4.0
Cellular_organisms - ps1
Archaea1 - ps2
Archaea2 - ps3
Archaea3 - ps4
Asgard/Eukaryota - ps5
Eukaryota - ps6
Excavata/Diaphoretickes - ps7
Diaphoretickes - ps8
Archaeplastida - ps9
Viridiplantae - ps10
Streptophyta - ps11
Charophyceae/Embriophyta - ps12
Embriophyta - ps13
Tracheophyta - ps14
Spermatophyta - ps15
Angiosperms - ps16
Mesangiospermae - ps17
Magnoliids/eudicots - ps18
Eudicots - ps19
Core_eudicots - ps20
Superrosids - ps21
Rosids - ps22
Eurosids - ps23
Malvids - ps24
Sap./Malv./Brassicales - ps25
Malvales/Brassicales - ps26
Brassicales - ps27
Cleomaceae/Brassicaceae - ps28
Brassicaceae - ps29
Camelineae - ps30
Arabidopsis - ps31
Arabidopsis_thaliana - ps32
Phylostratum

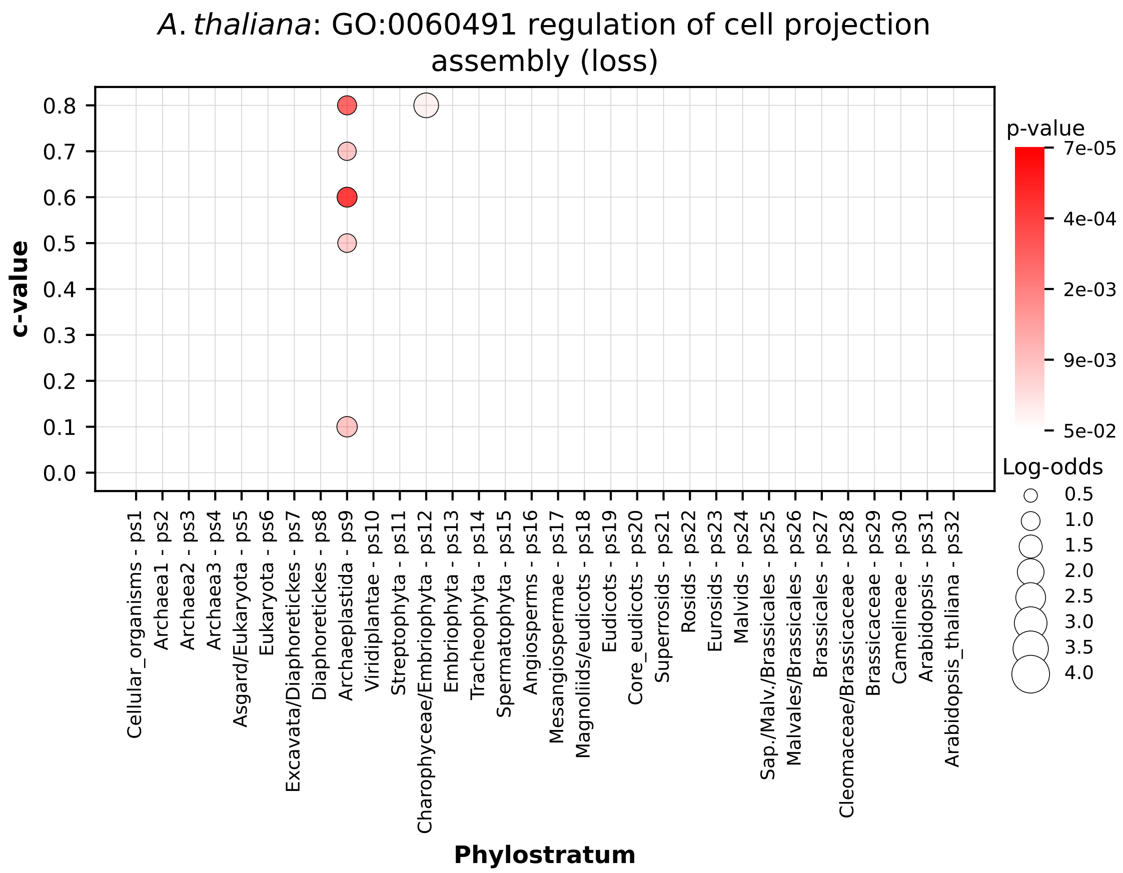
A. thaliana: GO:0060491 regulation of cell projection assembly (loss)
c-value
0.0
0.1
0.2
0.3
0.4
0.5
0.6
0.7
0.8
Phylostratum
Cellular_organisms - ps1
Archaea1 - ps2
Archaea2 - ps3
Archaea3 - ps4
Asgard/Eukaryota - ps5
Eukaryota - ps6
Excavata/Diaphoretickes - ps7
Diaphoretickes - ps8
Archaeplastida - ps9
Viridiplantae - ps10
Streptophyta - ps11
Charophyceae/Embriophyta - ps12
Embriophyta - ps13
Tracheophyta - ps14
Spermatophyta - ps15
Angiosperms - ps16
Mesangiospermae - ps17
Magnoliids/eudicots - ps18
Eudicots - ps19
Core_eudicots - ps20
Superrosids - ps21
Rosids - ps22
Eurosids - ps23
Malvids - ps24
Sap./Malv./Brassicales - ps25
Malvales/Brassicales - ps26
Brassicales - ps27
Cleomaceae/Brassicaceae - ps28
Brassicaceae - ps29
Camelineae - ps30
Arabidopsis - ps31
Arabidopsis_thaliana - ps32
p-value
7e-05
4e-04
2e-03
9e-03
5e-02
Log-odds
0.5
1.0
1.5
2.0
2.5
3.0
3.5
4.0

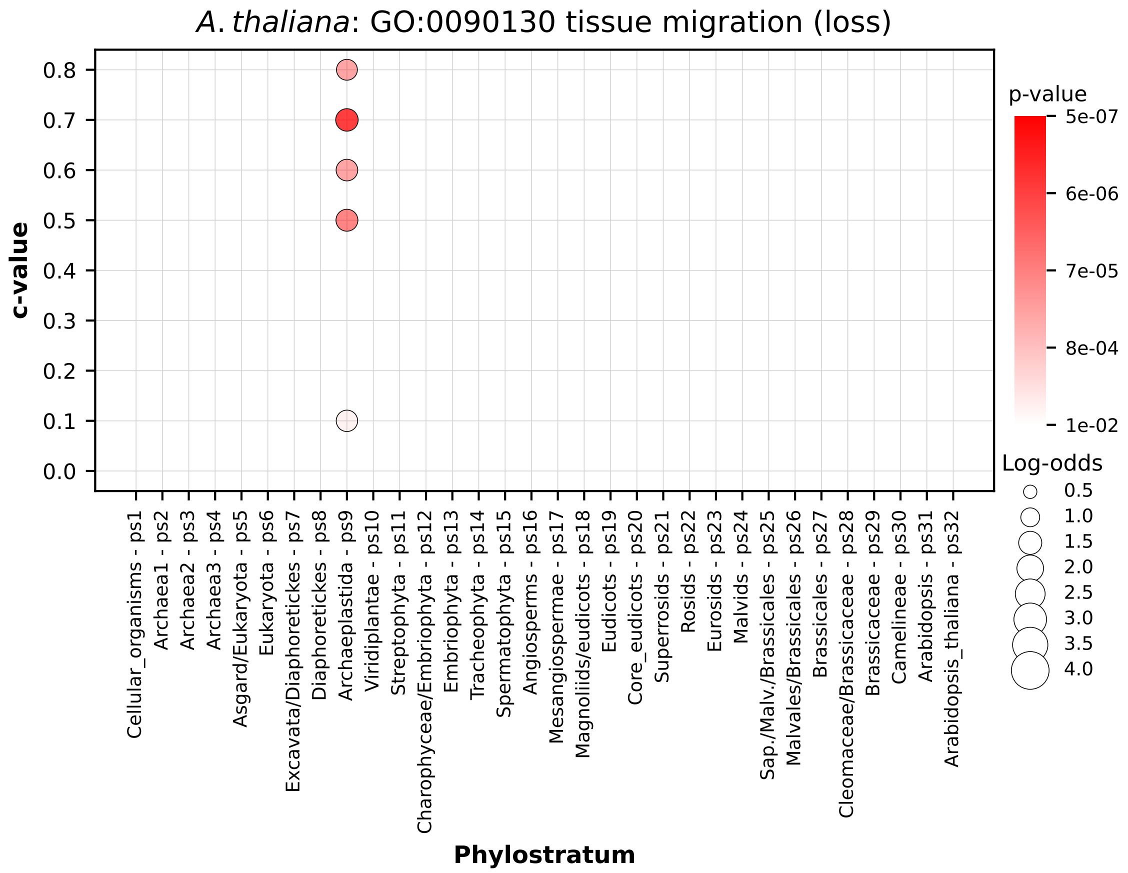

A. thaliana: GO:0090130 tissue migration (loss)
c-value
0.8
0.7
0.6
0.5
0.4
0.3
0.2
0.1
0.0
p-value
5e-07
6e-06
7e-05
8e-04
1e-02
Log-odds
0.5
1.0
1.5
2.0
2.5
3.0
3.5
4.0
Cellular_organisms - ps1
Archaea1 - ps2
Archaea2 - ps3
Archaea3 - ps4
Asgard/Eukaryota - ps5
Eukaryota - ps6
Excavata/Diaphoretickes - ps7
Diaphoretickes - ps8
Archaeplastida - ps9
Viridiplantae - ps10
Streptophyta - ps11
Charophyceae/Embriophyta - ps12
Embriophyta - ps13
Tracheophyta - ps14
Spermatophyta - ps15
Angiosperms - ps16
Mesangiospermae - ps17
Magnoliids/eudicots - ps18
Eudicots - ps19
Core_eudicots - ps20
Superrosids - ps21
Rosids - ps22
Eurosids - ps23
Malvids - ps24
Sap./Malv./Brassicales - ps25
Malvales/Brassicales - ps26
Brassicales - ps27
Cleomaceae/Brassicaceae - ps28
Brassicaceae - ps29
Camelineae - ps30
Arabidopsis - ps31
Arabidopsis_thaliana - ps32
Phylostratum

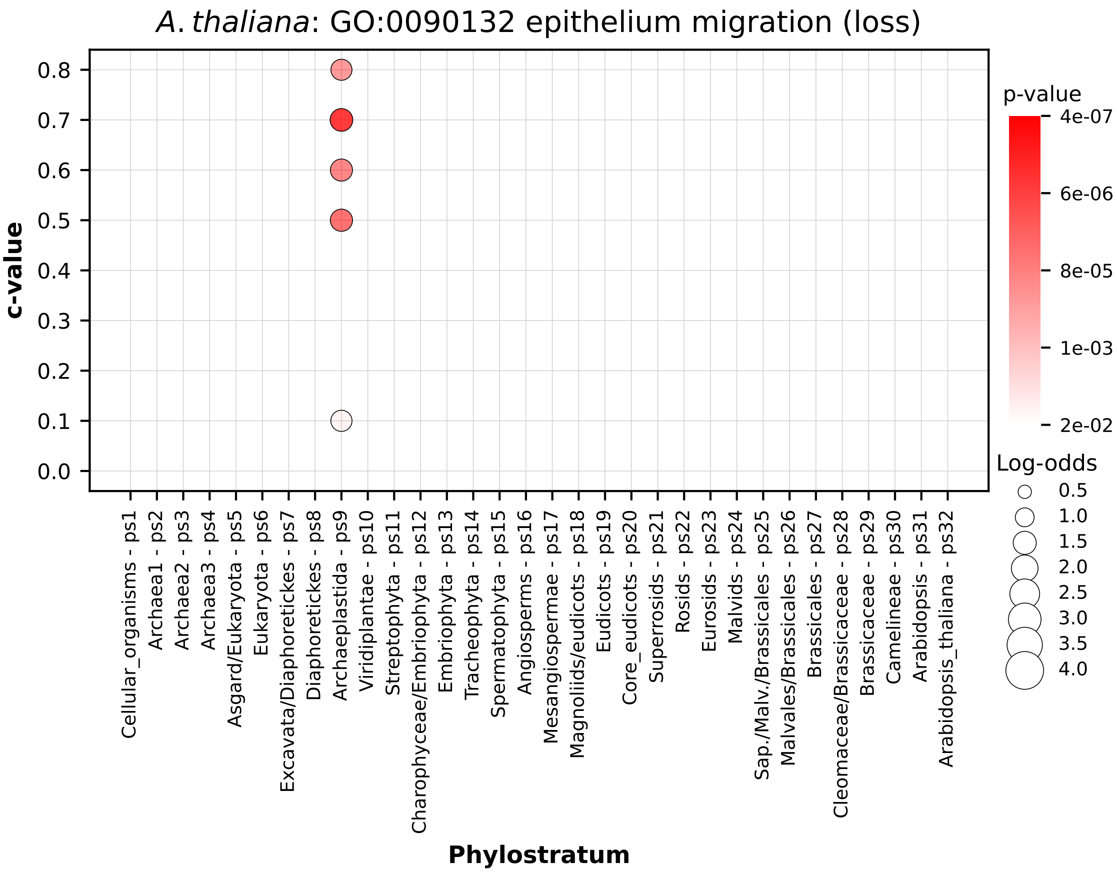

A. thaliana: GO:0090132 epithelium migration (loss)
c-value
0.0
0.1
0.2
0.3
0.4
0.5
0.6
0.7
0.8
p-value
4e-07
6e-06
8e-05
1e-03
2e-02
Log-odds
0.5
1.0
1.5
2.0
2.5
3.0
3.5
4.0
Cellular_organisms - ps1
Archaea1 - ps2
Archaea2 - ps3
Archaea3 - ps4
Asgard/Eukaryota - ps5
Eukaryota - ps6
Excavata/Diaphoretickes - ps7
Diaphoretickes - ps8
Archaeplastida - ps9
Viridiplantae - ps10
Streptophyta - ps11
Charophyceae/Embriophyta - ps12
Embriophyta - ps13
Tracheophyta - ps14
Spermatophyta - ps15
Angiosperms - ps16
Mesangiospermae - ps17
Magnoliids/eudicots - ps18
Eudicots - ps19
Core_eudicots - ps20
Superrosids - ps21
Rosids - ps22
Eurosids - ps23
Malvids - ps24
Sap./Malv./Brassicales - ps25
Malvales/Brassicales - ps26
Brassicales - ps27
Cleomaceae/Brassicaceae - ps28
Brassicaceae - ps29
Camelineae - ps30
Arabidopsis - ps31
Arabidopsis_thaliana - ps32
Phylostratum

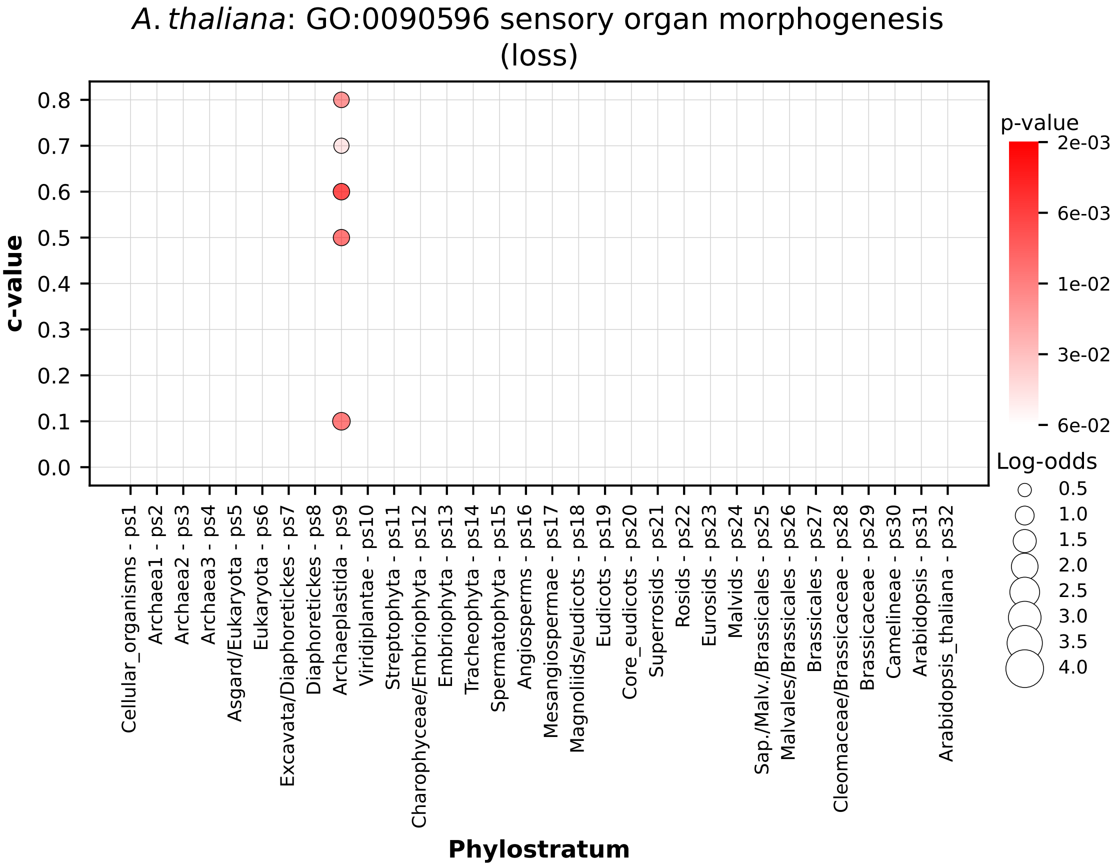

A. thaliana: GO:0090596 sensory organ morphogenesis
(loss)
c-value
0.8
0.7
0.6
0.5
0.4
0.3
0.2
0.1
0.0
Phylostratum
Cellular_organisms - ps1
Archaea1 - ps2
Archaea2 - ps3
Archaea3 - ps4
Asgard/Eukaryota - ps5
Eukaryota - ps6
Excavata/Diaphoretickes - ps7
Diaphoretickes - ps8
Archaeplastida - ps9
Viridiplantae - ps10
Streptophyta - ps11
Charophyceae/Embriophyta - ps12
Embriophyta - ps13
Tracheophyta - ps14
Spermatophyta - ps15
Angiosperms - ps16
Mesangiospermae - ps17
Magnoliids/eudicots - ps18
Eudicots - ps19
Core_eudicots - ps20
Superrosids - ps21
Rosids - ps22
Eurosids - ps23
Malvids - ps24
Sap./Malv./Brassicales - ps25
Malvales/Brassicales - ps26
Brassicales - ps27
Cleomaceae/Brassicaceae - ps28
Brassicaceae - ps29
Camelineae - ps30
Arabidopsis - ps31
Arabidopsis_thaliana - ps32
p-value
2e-03
6e-03
1e-02
3e-02
6e-02
Log-odds
0.5
1.0
1.5
2.0
2.5
3.0
3.5
4.0

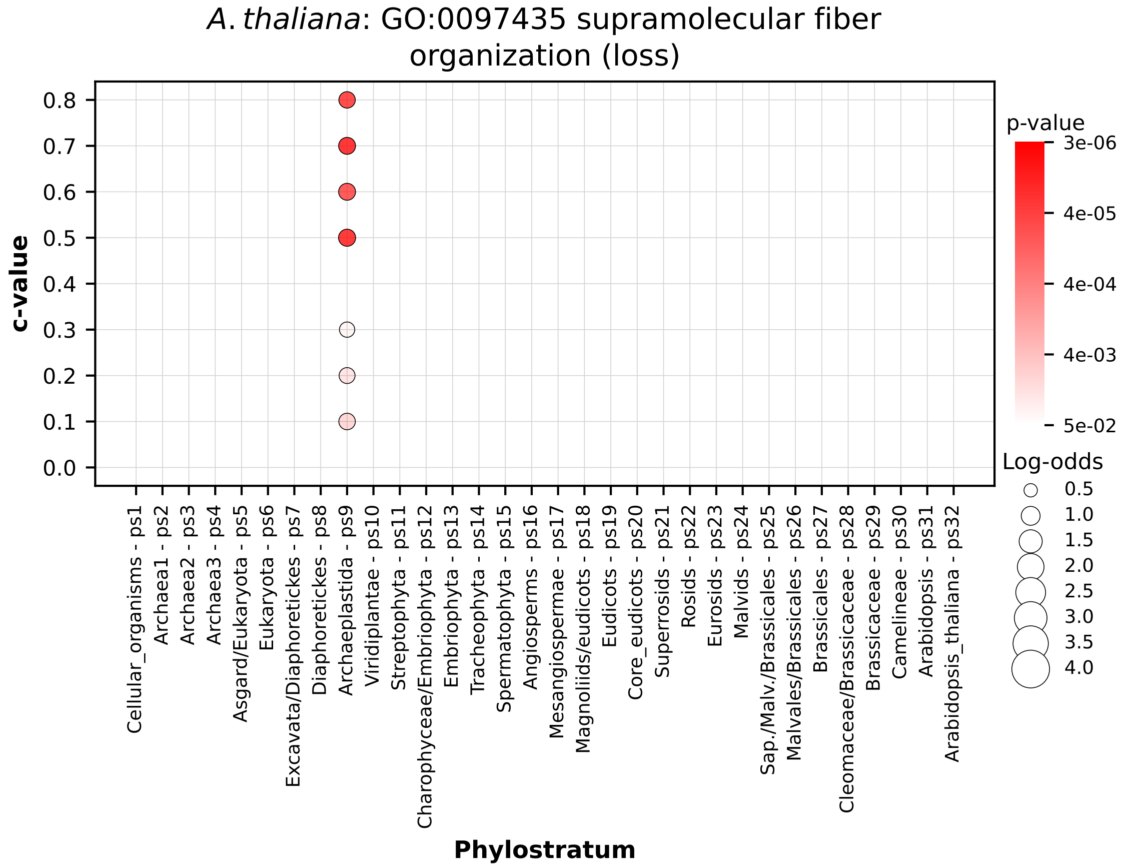

A. thaliana: GO:0097435 supramolecular fiber organization (loss)
c-value
0.8
0.7
0.6
0.5
0.4
0.3
0.2
0.1
0.0
p-value
3e-06
4e-05
4e-04
4e-03
5e-02
Log-odds
0.5
1.0
1.5
2.0
2.5
3.0
3.5
4.0
Cellular_organisms - ps1
Archaea1 - ps2
Archaea2 - ps3
Archaea3 - ps4
Asgard/Eukaryota - ps5
Eukaryota - ps6
Excavata/Diaphoretickes - ps7
Diaphoretickes - ps8
Archaeplastida - ps9
Viridiplantae - ps10
Streptophyta - ps11
Charophyceae/Embriophyta - ps12
Embriophyta - ps13
Tracheophyta - ps14
Spermatophyta - ps15
Angiosperms - ps16
Mesangiospermae - ps17
Magnoliids/eudicots - ps18
Eudicots - ps19
Core_eudicots - ps20
Superrosids - ps21
Rosids - ps22
Eurosids - ps23
Malvids - ps24
Sap./Malv./Brassicales - ps25
Malvales/Brassicales - ps26
Brassicales - ps27
Cleomaceae/Brassicaceae - ps28
Brassicaceae - ps29
Camelineae - ps30
Arabidopsis - ps31
Arabidopsis_thaliana - ps32
Phylostratum

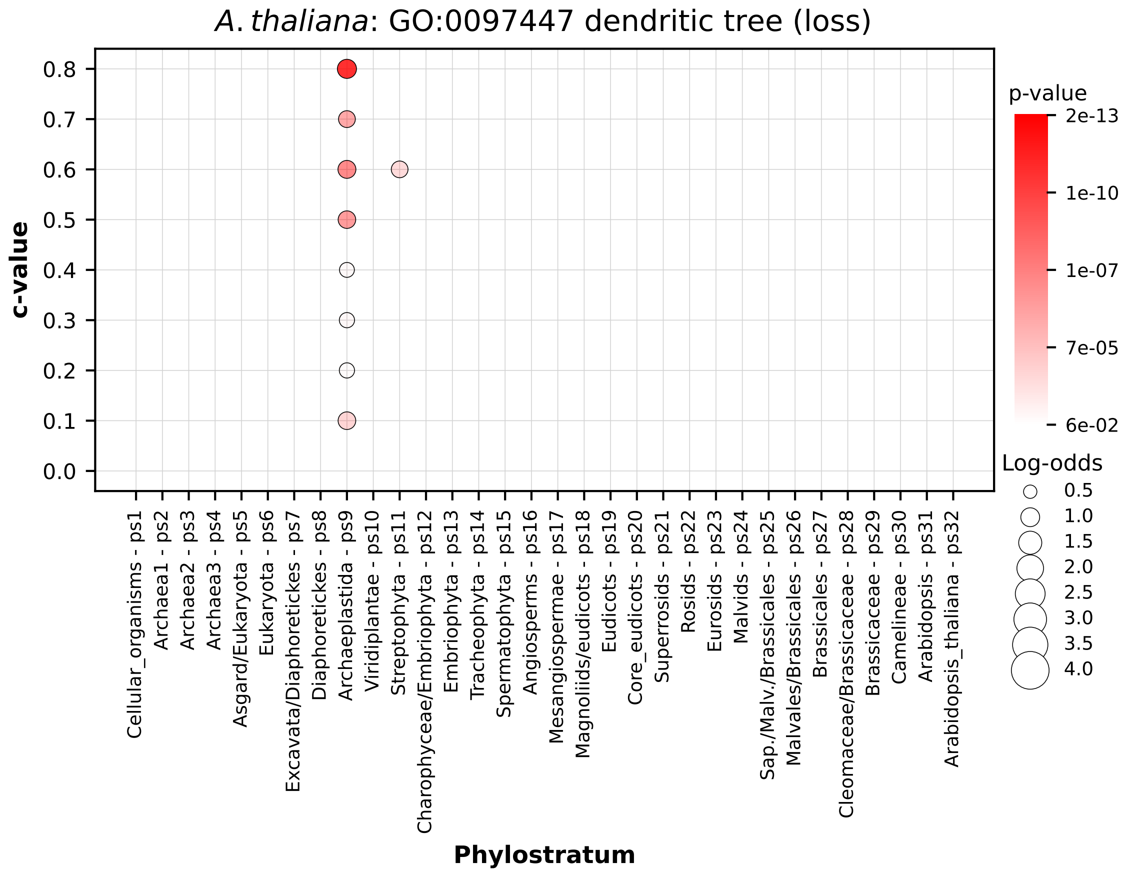

A. thaliana: GO:0097447 dendritic tree (loss)
c-value
0.0
0.1
0.2
0.3
0.4
0.5
0.6
0.7
0.8
p-value
2e-13
1e-10
1e-07
7e-05
6e-02
Log-odds
0.5
1.0
1.5
2.0
2.5
3.0
3.5
4.0
Cellular_organisms - ps1
Archaea1 - ps2
Archaea2 - ps3
Archaea3 - ps4
Asgard/Eukaryota - ps5
Eukaryota - ps6
Excavata/Diaphoretickes - ps7
Diaphoretickes - ps8
Archaeplastida - ps9
Viridiplantae - ps10
Streptophyta - ps11
Charophyceae/Embriophyta - ps12
Embriophyta - ps13
Tracheophyta - ps14
Spermatophyta - ps15
Angiosperms - ps16
Mesangiospermae - ps17
Magnoliids/eudicots - ps18
Eudicots - ps19
Core_eudicots - ps20
Superrosids - ps21
Rosids - ps22
Eurosids - ps23
Malvids - ps24
Sap./Malv./Brassicales - ps25
Malvales/Brassicales - ps26
Brassicales - ps27
Cleomaceae/Brassicaceae - ps28
Brassicaceae - ps29
Camelineae - ps30
Arabidopsis - ps31
Arabidopsis_thaliana - ps32
Phylostratum

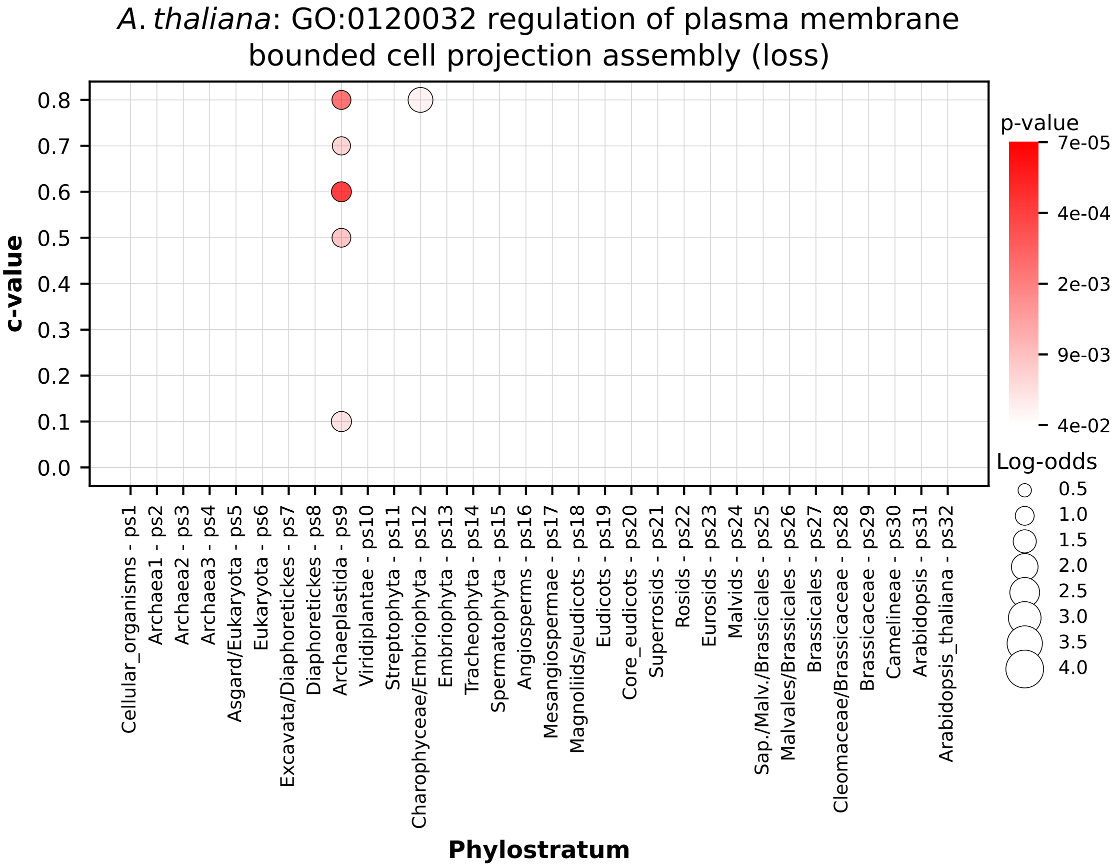
A. thaliana: GO:0120032 regulation of plasma membrane bounded cell projection assembly (loss)
c-value
0.0
0.1
0.2
0.3
0.4
0.5
0.6
0.7
0.8
Phylostratum
Cellular_organisms - ps1
Archaea1 - ps2
Archaea2 - ps3
Archaea3 - ps4
Asgard/Eukaryota - ps5
Eukaryota - ps6
Excavata/Diaphoretickes - ps7
Diaphoretickes - ps8
Archaeplastida - ps9
Viridiplantae - ps10
Streptophyta - ps11
Charophyceae/Embriophyta - ps12
Embriophyta - ps13
Tracheophyta - ps14
Spermatophyta - ps15
Angiosperms - ps16
Mesangiospermae - ps17
Magnoliids/eudicots - ps18
Eudicots - ps19
Core_eudicots - ps20
Superrosids - ps21
Rosids - ps22
Eurosids - ps23
Malvids - ps24
Sap./Malv./Brassicales - ps25
Malvales/Brassicales - ps26
Brassicales - ps27
Cleomaceae/Brassicaceae - ps28
Brassicaceae - ps29
Camelineae - ps30
Arabidopsis - ps31
Arabidopsis_thaliana - ps32
p-value
7e-05
4e-04
2e-03
9e-03
4e-02
Log-odds
0.5
1.0
1.5
2.0
2.5
3.0
3.5
4.0

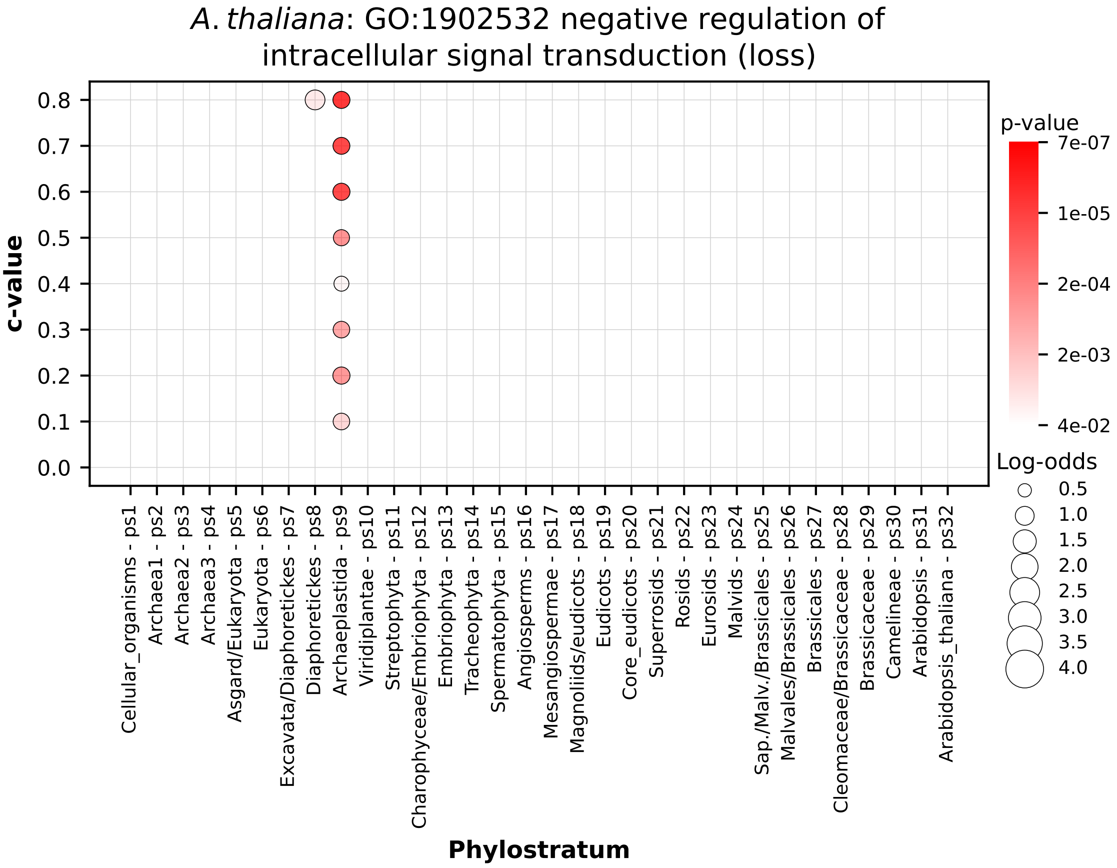

A. thaliana: GO:1902532 negative regulation of intracellular signal transduction (loss)
c-value
0.8
0.7
0.6
0.5
0.4
0.3
0.2
0.1
0.0
Phylostratum
Cellular_organisms - ps1
Archaea1 - ps2
Archaea2 - ps3
Archaea3 - ps4
Asgard/Eukaryota - ps5
Eukaryota - ps6
Excavata/Diaphoretickes - ps7
Diaphoretickes - ps8
Archaeplastida - ps9
Viridiplantae - ps10
Streptophyta - ps11
Charophyceae/Embriophyta - ps12
Embriophyta - ps13
Tracheophyta - ps14
Spermatophyta - ps15
Angiosperms - ps16
Mesangiospermae - ps17
Magnoliids/eudicots - ps18
Eudicots - ps19
Core_eudicots - ps20
Superrosids - ps21
Rosids - ps22
Eurosids - ps23
Malvids - ps24
Sap./Malv./Brassicales - ps25
Malvales/Brassicales - ps26
Brassicales - ps27
Cleomaceae/Brassicaceae - ps28
Brassicaceae - ps29
Camelineae - ps30
Arabidopsis - ps31
Arabidopsis_thaliana - ps32
p-value
7e-07
1e-05
2e-04
2e-03
4e-02
Log-odds
0.5
1.0
1.5
2.0
2.5
3.0
3.5
4.0

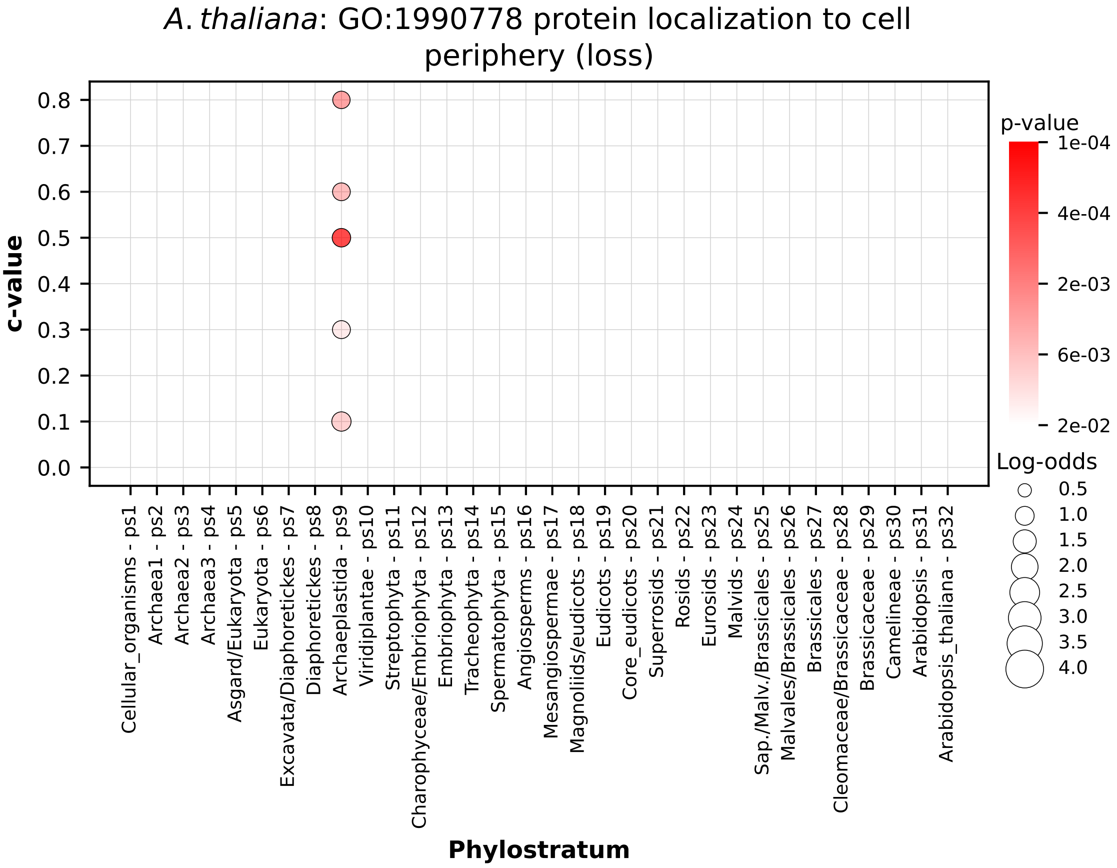

A. thaliana: GO:1990778 protein localization to cell periphery (loss)
c-value
0.8
0.7
0.6
0.5
0.4
0.3
0.2
0.1
0.0
p-value
1e-04
4e-04
2e-03
6e-03
2e-02
Log-odds
0.5
1.0
1.5
2.0
2.5
3.0
3.5
4.0
Cellular_organisms - ps1
Archaea1 - ps2
Archaea2 - ps3
Archaea3 - ps4
Asgard/Eukaryota - ps5
Eukaryota - ps6
Excavata/Diaphoretickes - ps7
Diaphoretickes - ps8
Archaeplastida - ps9
Viridiplantae - ps10
Streptophyta - ps11
Charophyceae/Embriophyta - ps12
Embriophyta - ps13
Tracheophyta - ps14
Spermatophyta - ps15
Angiosperms - ps16
Mesangiospermae - ps17
Magnoliids/eudicots - ps18
Eudicots - ps19
Core_eudicots - ps20
Superrosids - ps21
Rosids - ps22
Eurosids - ps23
Malvids - ps24
Sap./Malv./Brassicales - ps25
Malvales/Brassicales - ps26
Brassicales - ps27
Cleomaceae/Brassicaceae - ps28
Brassicaceae - ps29
Camelineae - ps30
Arabidopsis - ps31
Arabidopsis_thaliana - ps32
Phylostratum

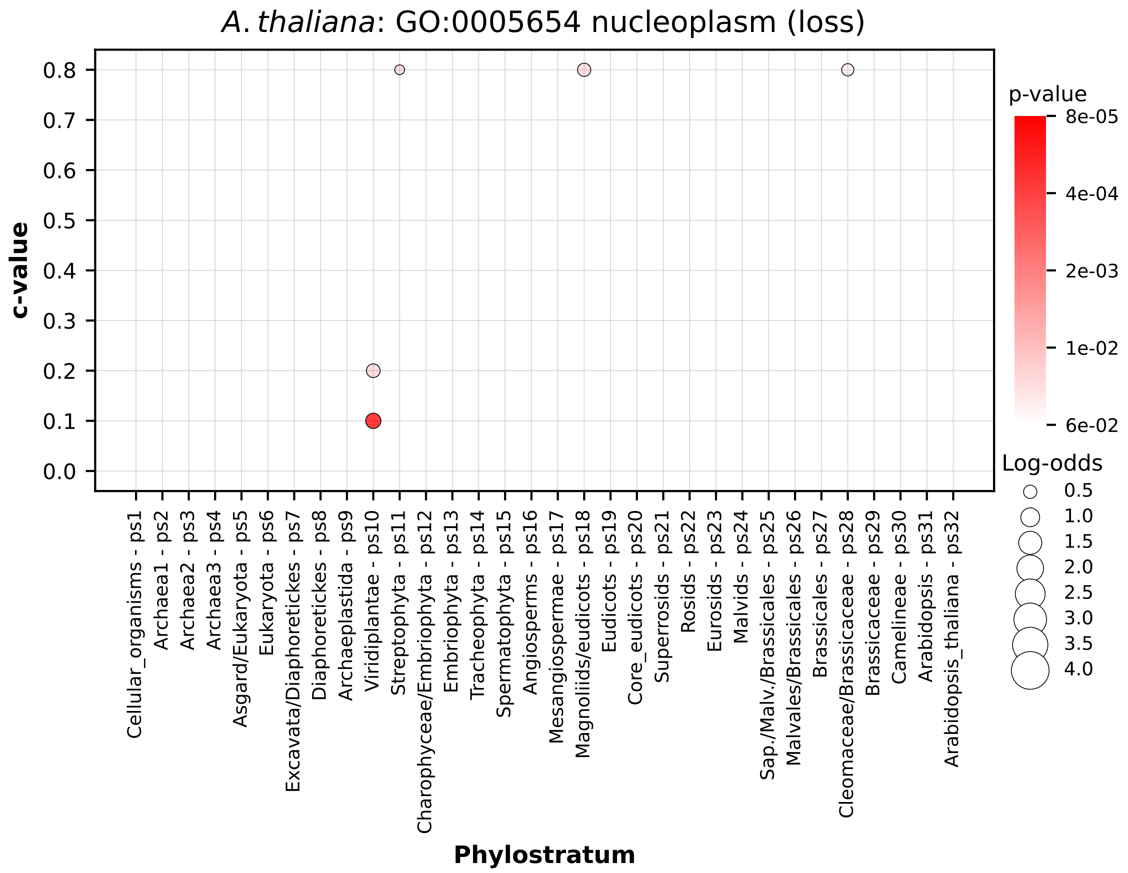

A. thaliana: GO:0005654 nucleoplasm (loss)
c-value
0.0
0.1
0.2
0.3
0.4
0.5
0.6
0.7
0.8
p-value
8e-05
4e-04
2e-03
1e-02
6e-02
Log-odds
0.5
1.0
1.5
2.0
2.5
3.0
3.5
4.0
Cellular_organisms - ps1
Archaea1 - ps2
Archaea2 - ps3
Archaea3 - ps4
Asgard/Eukaryota - ps5
Eukaryota - ps6
Excavata/Diaphoretickes - ps7
Diaphoretickes - ps8
Archaeplastida - ps9
Viridiplantae - ps10
Streptophyta - ps11
Charophyceae/Embriophyta - ps12
Embriophyta - ps13
Tracheophyta - ps14
Spermatophyta - ps15
Angiosperms - ps16
Mesangiospermae - ps17
Magnoliids/eudicots - ps18
Eudicots - ps19
Core_eudicots - ps20
Superrosids - ps21
Rosids - ps22
Eurosids - ps23
Malvids - ps24
Sap./Malv./Brassicales - ps25
Malvales/Brassicales - ps26
Brassicales - ps27
Cleomaceae/Brassicaceae - ps28
Brassicaceae - ps29
Camelineae - ps30
Arabidopsis - ps31
Arabidopsis_thaliana - ps32
Phylostratum

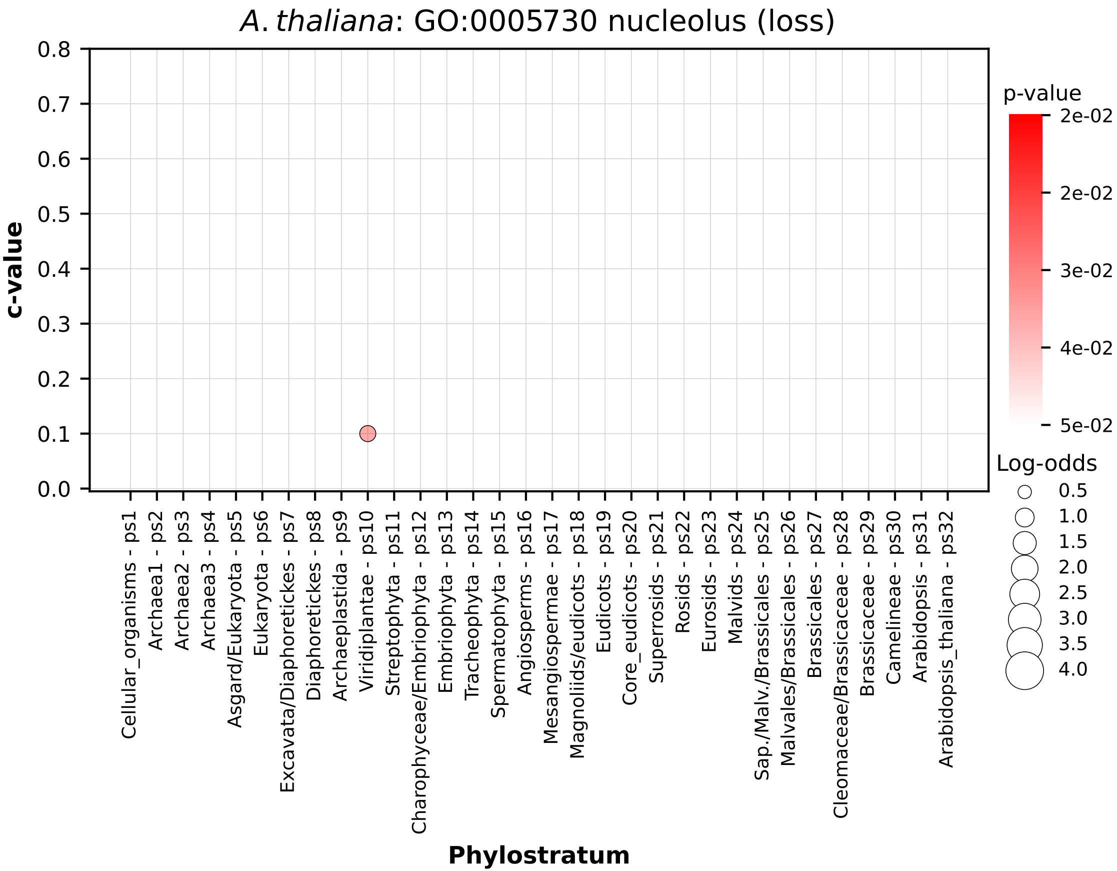

A. thaliana: GO:0005730 nucleolus (loss)
c-value
0.0
0.1
0.2
0.3
0.4
0.5
0.6
0.7
0.8
Phylostratum
Cellular_organisms - ps1
Archaea1 - ps2
Archaea2 - ps3
Archaea3 - ps4
Asgard/Eukaryota - ps5
Eukaryota - ps6
Excavata/Diaphoretickes - ps7
Diaphoretickes - ps8
Archaeplastida - ps9
Viridiplantae - ps10
Streptophyta - ps11
Charophyceae/Embriophyta - ps12
Embriophyta - ps13
Tracheophyta - ps14
Spermatophyta - ps15
Angiosperms - ps16
Mesangiospermae - ps17
Magnoliids/eudicots - ps18
Eudicots - ps19
Core_eudicots - ps20
Superrosids - ps21
Rosids - ps22
Eurosids - ps23
Malvids - ps24
Sap./Malv./Brassicales - ps25
Malvales/Brassicales - ps26
Brassicales - ps27
Cleomaceae/Brassicaceae - ps28
Brassicaceae - ps29
Camelineae - ps30
Arabidopsis - ps31
Arabidopsis_thaliana - ps32
p-value
2e-02
2e-02
3e-02
4e-02
5e-02
Log-odds
0.5
1.0
1.5
2.0
2.5
3.0
3.5
4.0

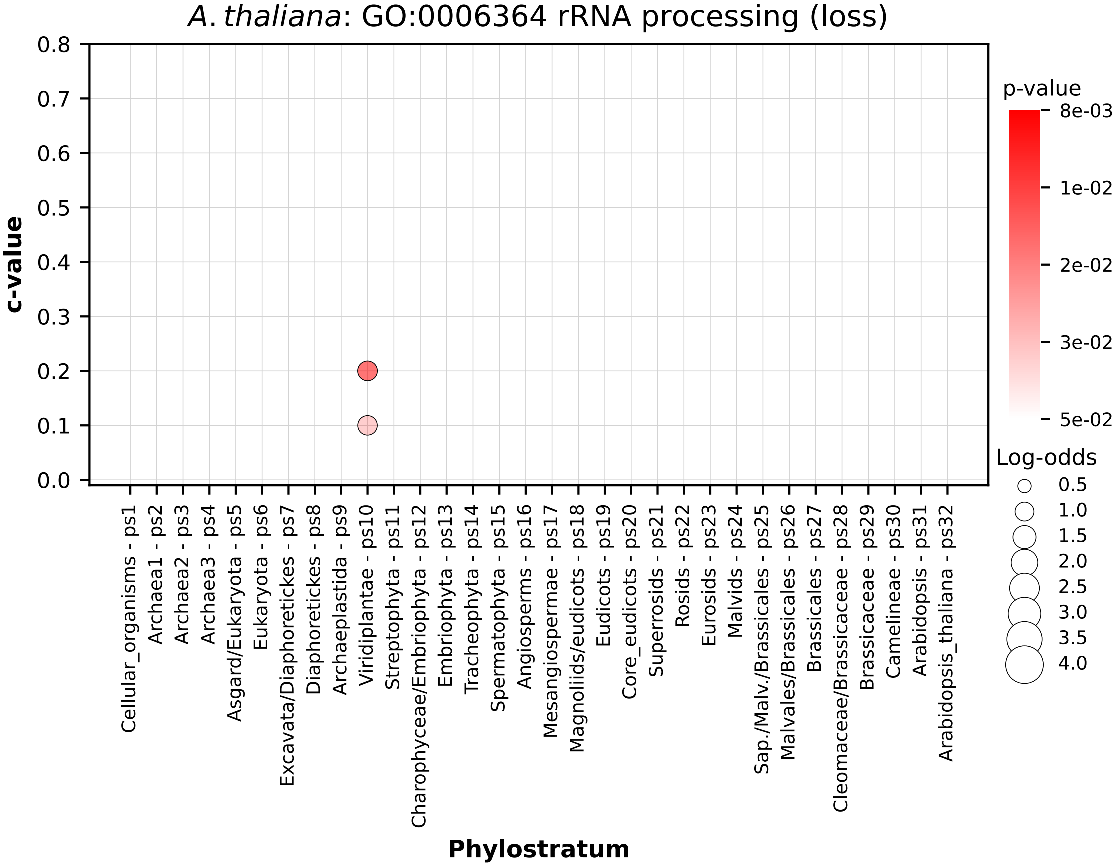

A. thaliana: GO:0006364 rRNA processing (loss)
c-value
0.0
0.1
0.2
0.3
0.4
0.5
0.6
0.7
0.8
Phylostratum
Cellular_organisms - ps1
Archaea1 - ps2
Archaea2 - ps3
Archaea3 - ps4
Asgard/Eukaryota - ps5
Eukaryota - ps6
Excavata/Diaphoretickes - ps7
Diaphoretickes - ps8
Archaeplastida - ps9
Viridiplantae - ps10
Streptophyta - ps11
Charophyceae/Embriophyta - ps12
Embriophyta - ps13
Tracheophyta - ps14
Spermatophyta - ps15
Angiosperms - ps16
Mesangiospermae - ps17
Magnoliids/eudicots - ps18
Eudicots - ps19
Core_eudicots - ps20
Superrosids - ps21
Rosids - ps22
Eurosids - ps23
Malvids - ps24
Sap./Malv./Brassicales - ps25
Malvales/Brassicales - ps26
Brassicales - ps27
Cleomaceae/Brassicaceae - ps28
Brassicaceae - ps29
Camelineae - ps30
Arabidopsis - ps31
Arabidopsis_thaliana - ps32
p-value
8e-03
1e-02
2e-02
3e-02
5e-02
Log-odds
0.5
1.0
1.5
2.0
2.5
3.0
3.5
4.0

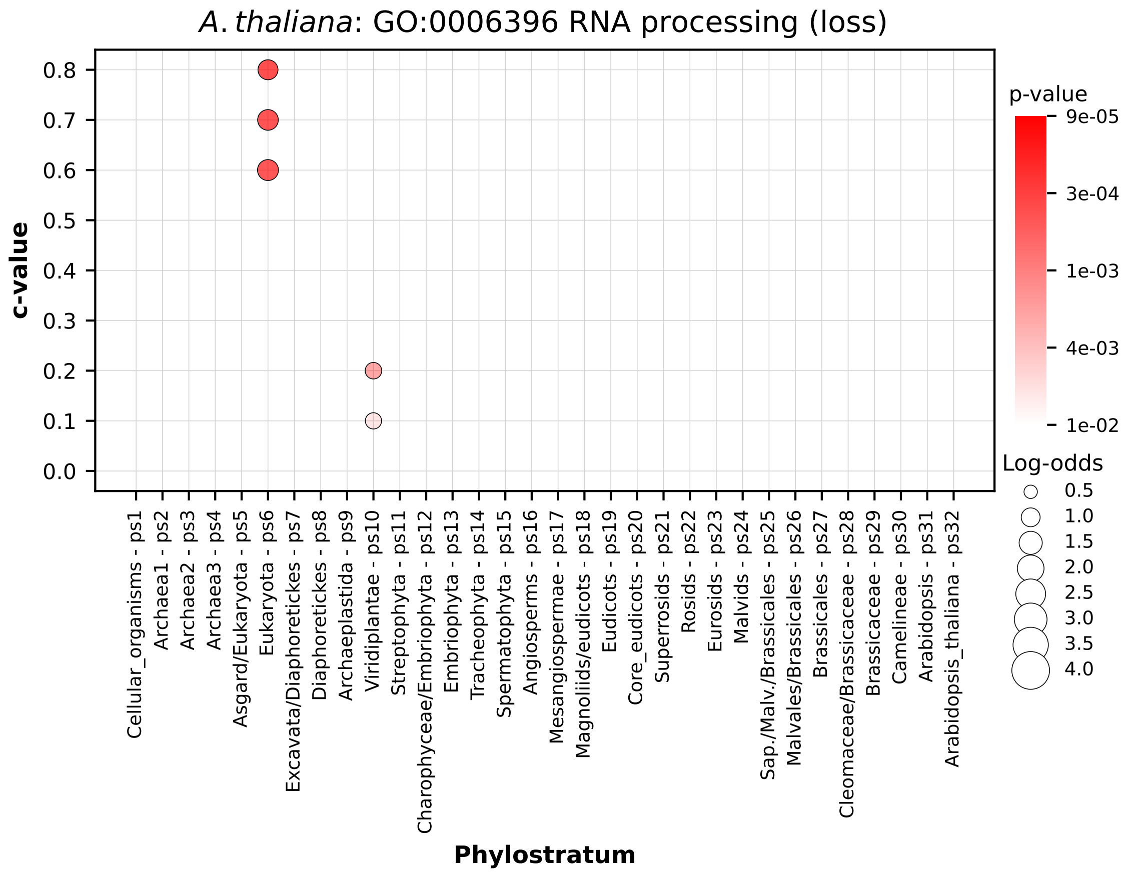

A. thaliana: GO:0006396 RNA processing (loss)
c-value
0.0
0.1
0.2
0.3
0.4
0.5
0.6
0.7
0.8
Phylostratum
Cellular_organisms - ps1
Archaea1 - ps2
Archaea2 - ps3
Archaea3 - ps4
Asgard/Eukaryota - ps5
Eukaryota - ps6
Excavata/Diaphoretickes - ps7
Diaphoretickes - ps8
Archaeplastida - ps9
Viridiplantae - ps10
Streptophyta - ps11
Charophyceae/Embriophyta - ps12
Embriophyta - ps13
Tracheophyta - ps14
Spermatophyta - ps15
Angiosperms - ps16
Mesangiospermae - ps17
Magnoliids/eudicots - ps18
Eudicots - ps19
Core_eudicots - ps20
Superrosids - ps21
Rosids - ps22
Eurosids - ps23
Malvids - ps24
Sap./Malv./Brassicales - ps25
Malvales/Brassicales - ps26
Brassicales - ps27
Cleomaceae/Brassicaceae - ps28
Brassicaceae - ps29
Camelineae - ps30
Arabidopsis - ps31
Arabidopsis_thaliana - ps32
p-value
9e-05
3e-04
1e-03
4e-03
1e-02
Log-odds
0.5
1.0
1.5
2.0
2.5
3.0
3.5
4.0

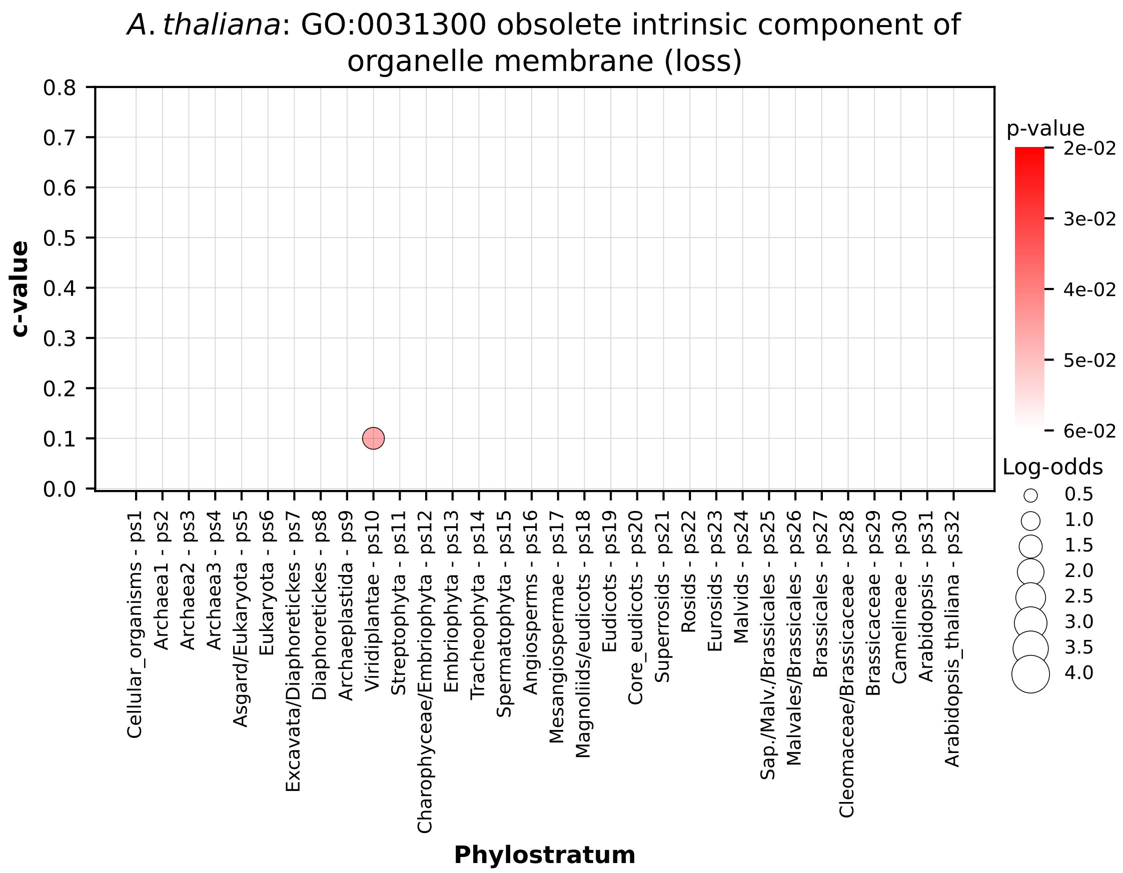
A. thaliana: GO:0031300 obsolete intrinsic component of organelle membrane (loss)
c-value
0.8
0.7
0.6
0.5
0.4
0.3
0.2
0.1
0.0
Phylostratum
Cellular_organisms - ps1
Archaea1 - ps2
Archaea2 - ps3
Archaea3 - ps4
Asgard/Eukaryota - ps5
Eukaryota - ps6
Excavata/Diaphoretickes - ps7
Diaphoretickes - ps8
Archaeplastida - ps9
Viridiplantae - ps10
Streptophyta - ps11
Charophyceae/Embriophyta - ps12
Embriophyta - ps13
Tracheophyta - ps14
Spermatophyta - ps15
Angiosperms - ps16
Mesangiospermae - ps17
Magnoliids/eudicots - ps18
Eudicots - ps19
Core_eudicots - ps20
Superrosids - ps21
Rosids - ps22
Eurosids - ps23
Malvids - ps24
Sap./Malv./Brassicales - ps25
Malvales/Brassicales - ps26
Brassicales - ps27
Cleomaceae/Brassicaceae - ps28
Brassicaceae - ps29
Camelineae - ps30
Arabidopsis - ps31
Arabidopsis_thaliana - ps32
p-value
2e-02
3e-02
4e-02
5e-02
6e-02
Log-odds
0.5
1.0
1.5
2.0
2.5
3.0
3.5
4.0

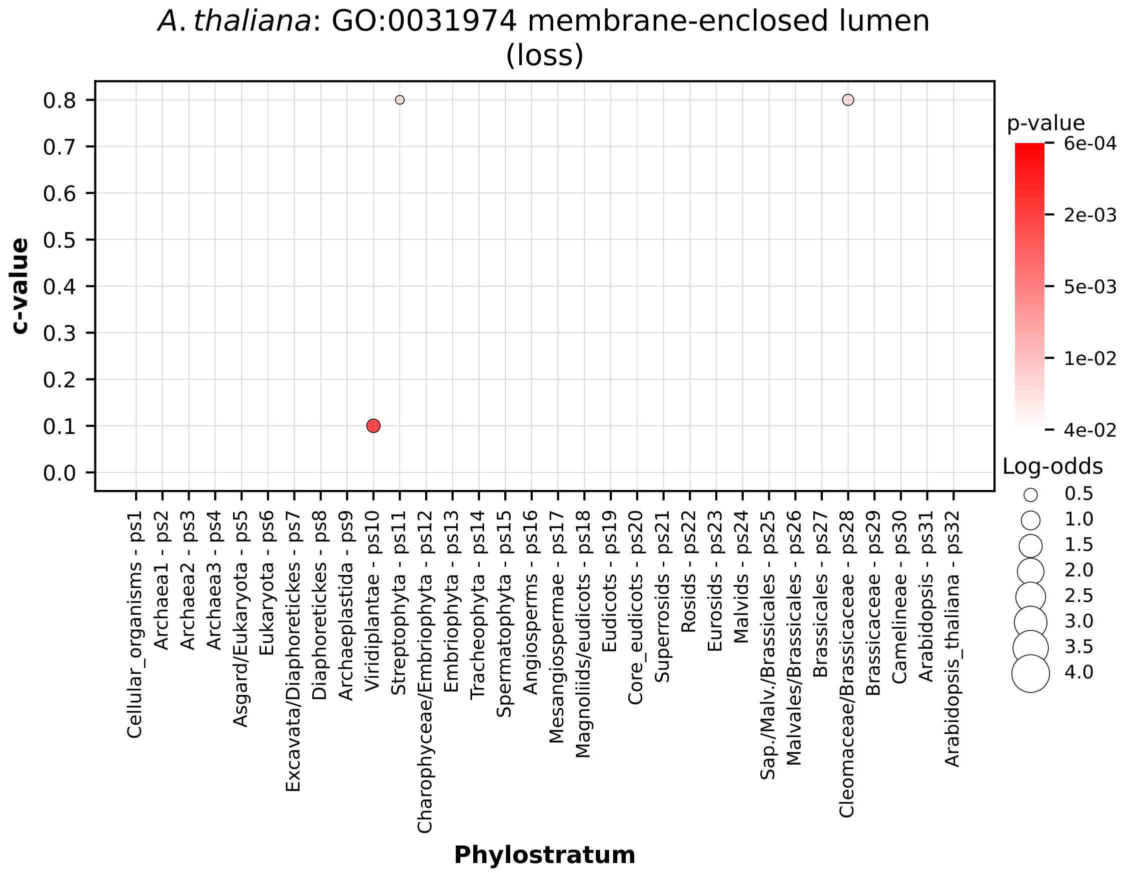

A. thaliana: GO:0031974 membrane-enclosed lumen
(loss)
c-value
0.8
0.7
0.6
0.5
0.4
0.3
0.2
0.1
0.0
p-value
6e-04
2e-03
5e-03
1e-02
4e-02
Log-odds
0.5
1.0
1.5
2.0
2.5
3.0
3.5
4.0
Cellular_organisms - ps1
Archaea1 - ps2
Archaea2 - ps3
Archaea3 - ps4
Asgard/Eukaryota - ps5
Eukaryota - ps6
Excavata/Diaphoretickes - ps7
Diaphoretickes - ps8
Archaeplastida - ps9
Viridiplantae - ps10
Streptophyta - ps11
Charophyceae/Embriophyta - ps12
Embriophyta - ps13
Tracheophyta - ps14
Spermatophyta - ps15
Angiosperms - ps16
Mesangiospermae - ps17
Magnoliids/eudicots - ps18
Eudicots - ps19
Core_eudicots - ps20
Superrosids - ps21
Rosids - ps22
Eurosids - ps23
Malvids - ps24
Sap./Malv./Brassicales - ps25
Malvales/Brassicales - ps26
Brassicales - ps27
Cleomaceae/Brassicaceae - ps28
Brassicaceae - ps29
Camelineae - ps30
Arabidopsis - ps31
Arabidopsis_thaliana - ps32
Phylostratum

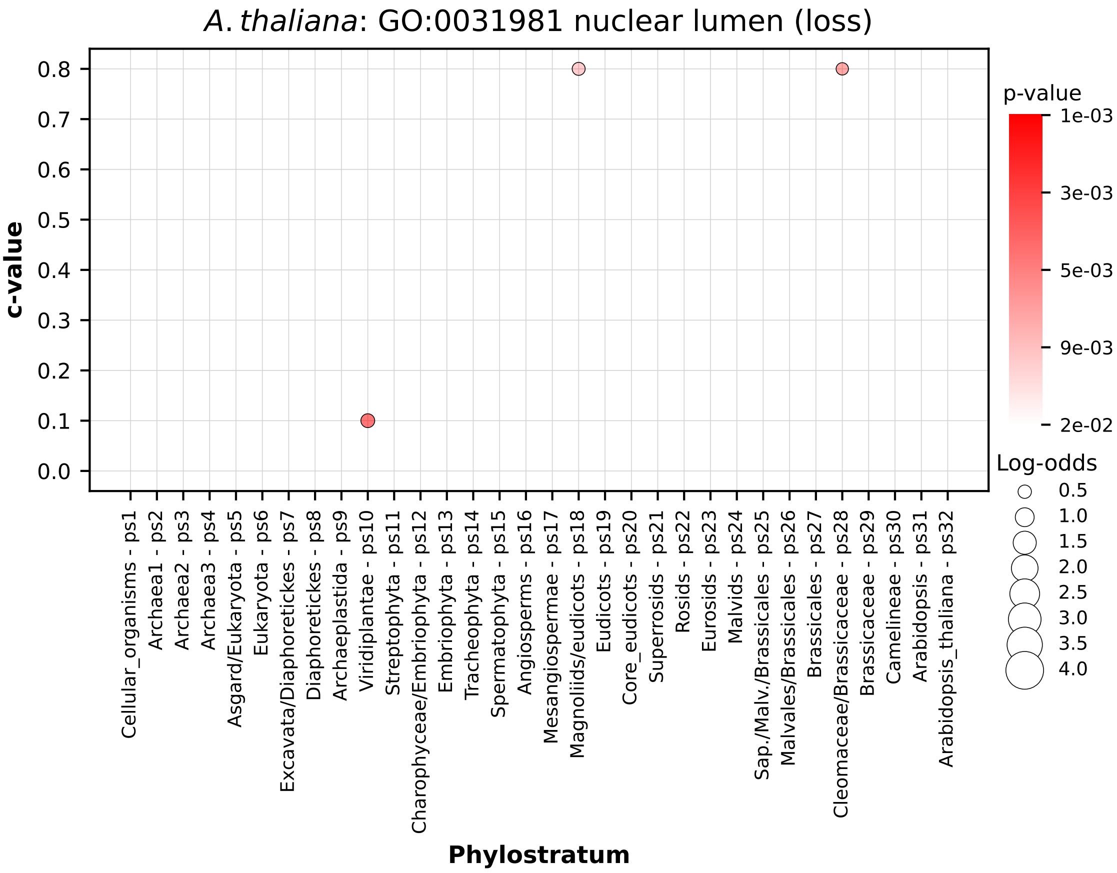

A. thaliana: GO:0031981 nuclear lumen (loss)
c-value
0.0
0.1
0.2
0.3
0.4
0.5
0.6
0.7
0.8
Phylostratum
Cellular_organisms - ps1
Archaea1 - ps2
Archaea2 - ps3
Archaea3 - ps4
Asgard/Eukaryota - ps5
Eukaryota - ps6
Excavata/Diaphoretickes - ps7
Diaphoretickes - ps8
Archaeplastida - ps9
Viridiplantae - ps10
Streptophyta - ps11
Charophyceae/Embriophyta - ps12
Embriophyta - ps13
Tracheophyta - ps14
Spermatophyta - ps15
Angiosperms - ps16
Mesangiospermae - ps17
Magnoliids/eudicots - ps18
Eudicots - ps19
Core_eudicots - ps20
Superrosids - ps21
Rosids - ps22
Eurosids - ps23
Malvids - ps24
Sap./Malv./Brassicales - ps25
Malvales/Brassicales - ps26
Brassicales - ps27
Cleomaceae/Brassicaceae - ps28
Brassicaceae - ps29
Camelineae - ps30
Arabidopsis - ps31
Arabidopsis_thaliana - ps32
p-value
1e-03
3e-03
5e-03
9e-03
2e-02
Log-odds
0.5
1.0
1.5
2.0
2.5
3.0
3.5
4.0

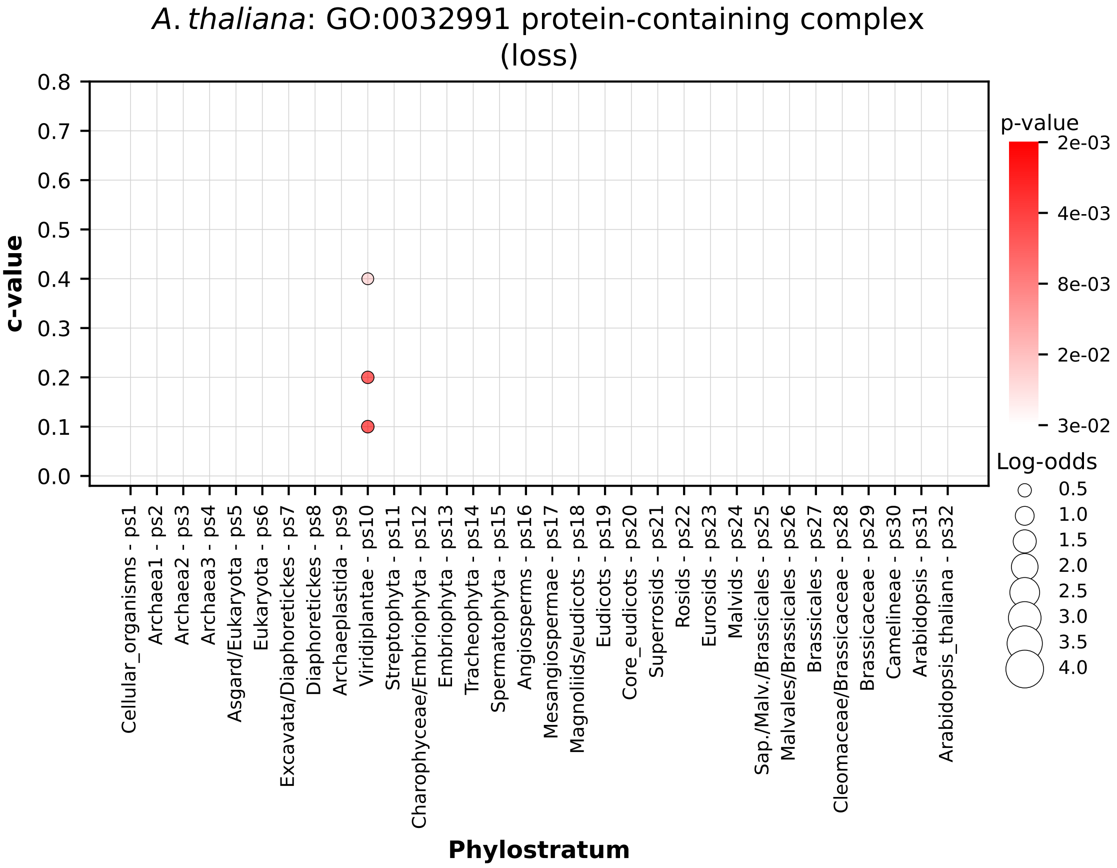

A. thaliana: GO:0032991 protein-containing complex
(loss)
c-value
0.8
0.7
0.6
0.5
0.4
0.3
0.2
0.1
0.0
p-value
2e-03
4e-03
8e-03
2e-02
3e-02
Log-odds
0.5
1.0
1.5
2.0
2.5
3.0
3.5
4.0
Cellular_organisms - ps1
Archaea1 - ps2
Archaea2 - ps3
Archaea3 - ps4
Asgard/Eukaryota - ps5
Eukaryota - ps6
Excavata/Diaphoretickes - ps7
Diaphoretickes - ps8
Archaeplastida - ps9
Viridiplantae - ps10
Streptophyta - ps11
Charophyceae/Embriophyta - ps12
Embriophyta - ps13
Tracheophyta - ps14
Spermatophyta - ps15
Angiosperms - ps16
Mesangiospermae - ps17
Magnoliids/eudicots - ps18
Eudicots - ps19
Core_eudicots - ps20
Superrosids - ps21
Rosids - ps22
Eurosids - ps23
Malvids - ps24
Sap./Malv./Brassicales - ps25
Malvales/Brassicales - ps26
Brassicales - ps27
Cleomaceae/Brassicaceae - ps28
Brassicaceae - ps29
Camelineae - ps30
Arabidopsis - ps31
Arabidopsis_thaliana - ps32
Phylostratum

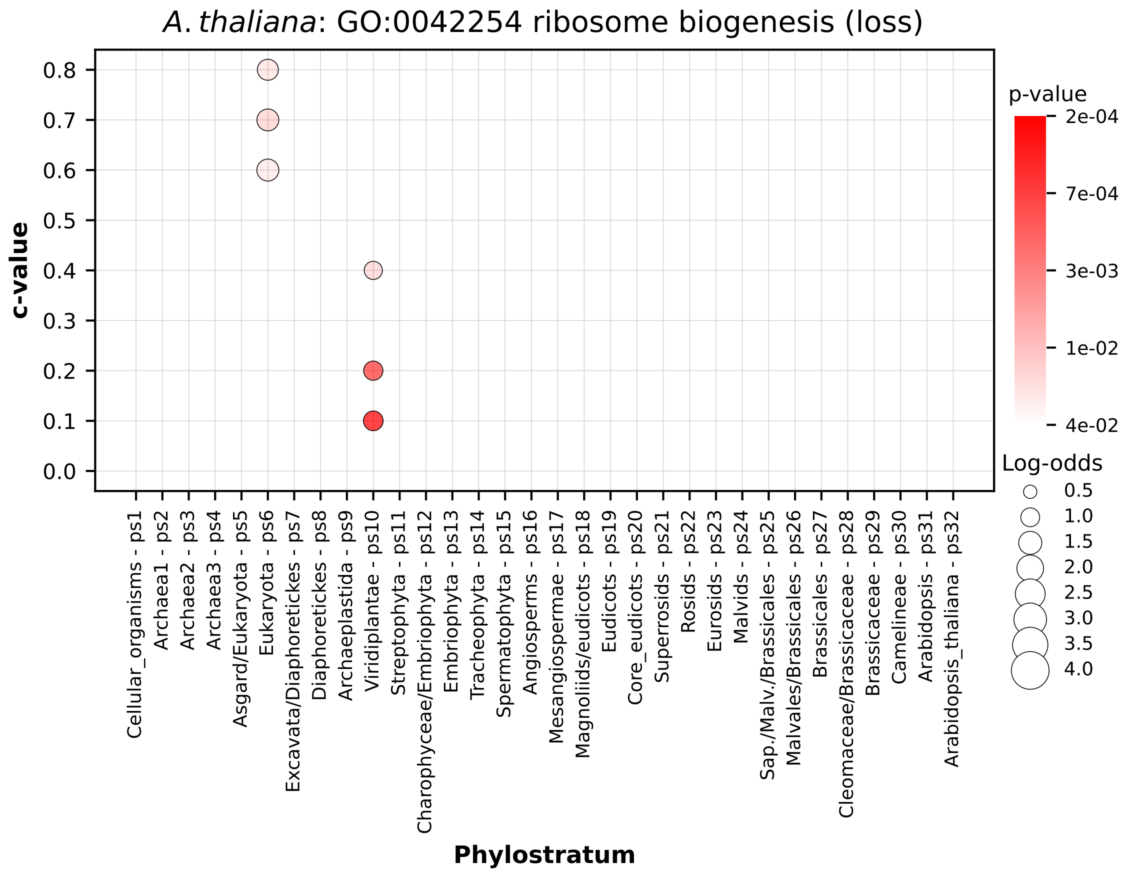
A. thaliana: GO:0042254 ribosome biogenesis (loss)
c-value
0.8
0.7
0.6
0.5
0.4
0.3
0.2
0.1
0.0
Phylostratum
Cellular_organisms - ps1
Archaea1 - ps2
Archaea2 - ps3
Archaea3 - ps4
Asgard/Eukaryota - ps5
Eukaryota - ps6
Excavata/Diaphoretickes - ps7
Diaphoretickes - ps8
Archaeplastida - ps9
Viridiplantae - ps10
Streptophyta - ps11
Charophyceae/Embriophyta - ps12
Embriophyta - ps13
Tracheophyta - ps14
Spermatophyta - ps15
Angiosperms - ps16
Mesangiospermae - ps17
Magnoliids/eudicots - ps18
Eudicots - ps19
Core_eudicots - ps20
Superrosids - ps21
Rosids - ps22
Eurosids - ps23
Malvids - ps24
Sap./Malv./Brassicales - ps25
Malvales/Brassicales - ps26
Brassicales - ps27
Cleomaceae/Brassicaceae - ps28
Brassicaceae - ps29
Camelineae - ps30
Arabidopsis - ps31
Arabidopsis_thaliana - ps32
p-value
2e-04
7e-04
3e-03
1e-02
4e-02
Log-odds
0.5
1.0
1.5
2.0
2.5
3.0
3.5
4.0

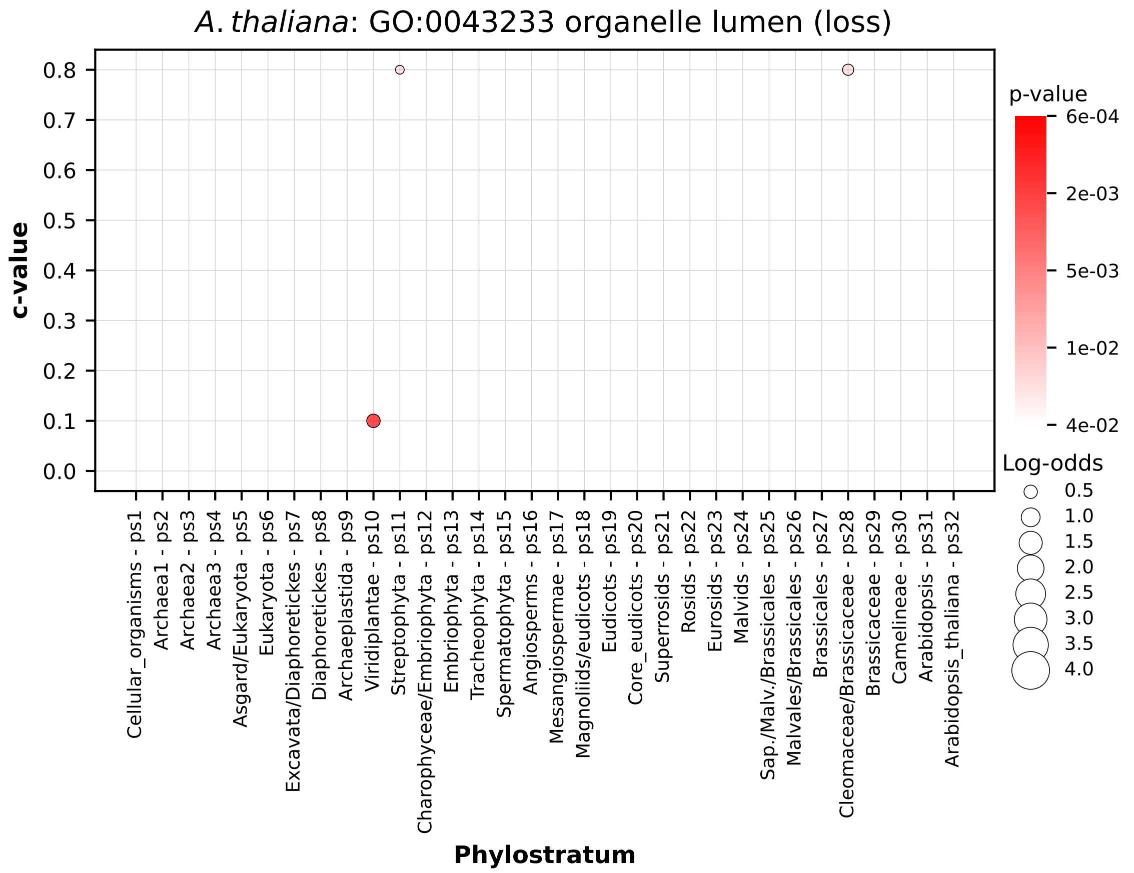

A. thaliana: GO:0043233 organelle lumen (loss)
c-value
0.8
0.7
0.6
0.5
0.4
0.3
0.2
0.1
0.0
p-value
6e-04
2e-03
5e-03
1e-02
4e-02
Log-odds
0.5
1.0
1.5
2.0
2.5
3.0
3.5
4.0
Cellular_organisms - ps1
Archaea1 - ps2
Archaea2 - ps3
Archaea3 - ps4
Asgard/Eukaryota - ps5
Eukaryota - ps6
Excavata/Diaphoretickes - ps7
Diaphoretickes - ps8
Archaeplastida - ps9
Viridiplantae - ps10
Streptophyta - ps11
Charophyceae/Embriophyta - ps12
Embriophyta - ps13
Tracheophyta - ps14
Spermatophyta - ps15
Angiosperms - ps16
Mesangiospermae - ps17
Magnoliids/eudicots - ps18
Eudicots - ps19
Core_eudicots - ps20
Superrosids - ps21
Rosids - ps22
Eurosids - ps23
Malvids - ps24
Sap./Malv./Brassicales - ps25
Malvales/Brassicales - ps26
Brassicales - ps27
Cleomaceae/Brassicaceae - ps28
Brassicaceae - ps29
Camelineae - ps30
Arabidopsis - ps31
Arabidopsis_thaliana - ps32
Phylostratum

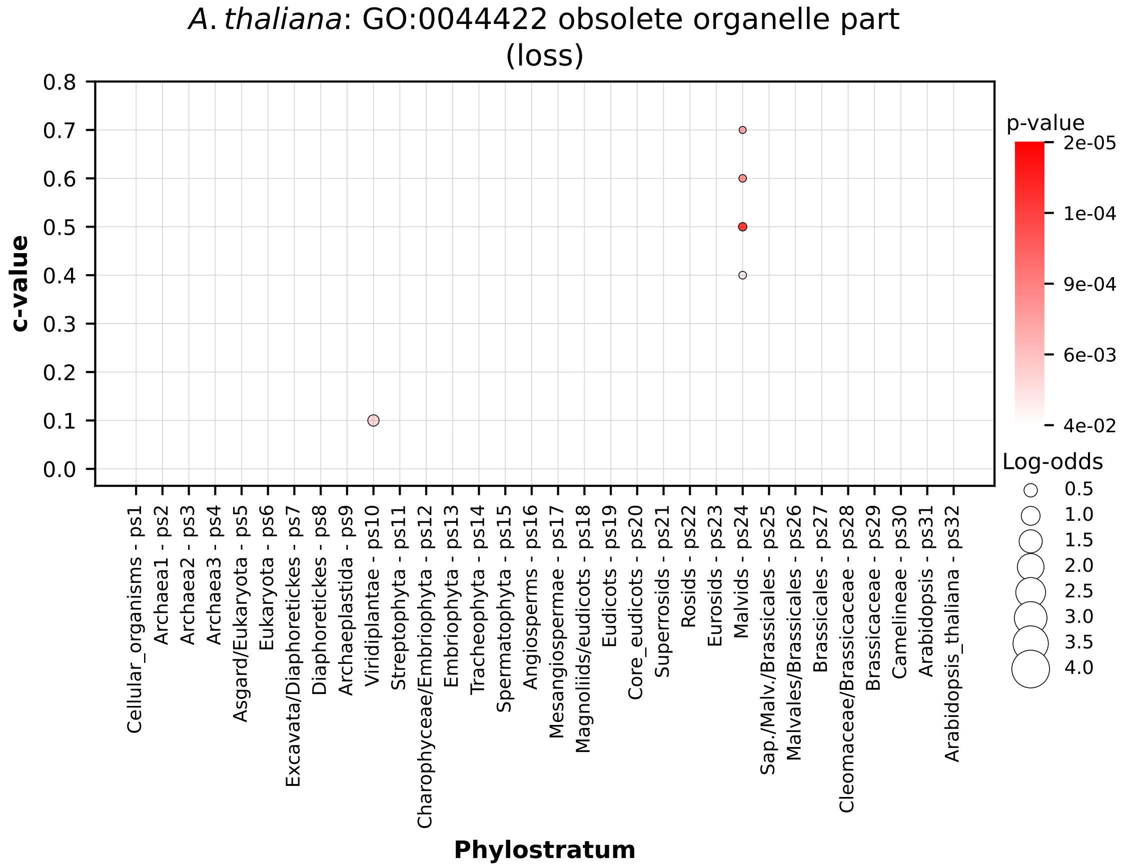

A. thaliana: GO:0044422 obsolete organelle part
(loss)
c-value
0.8
0.7
0.6
0.5
0.4
0.3
0.2
0.1
0.0
Phylostratum
Cellular_organisms - ps1
Archaea1 - ps2
Archaea2 - ps3
Archaea3 - ps4
Asgard/Eukaryota - ps5
Eukaryota - ps6
Excavata/Diaphoretickes - ps7
Diaphoretickes - ps8
Archaeplastida - ps9
Viridiplantae - ps10
Streptophyta - ps11
Charophyceae/Embriophyta - ps12
Embriophyta - ps13
Tracheophyta - ps14
Spermatophyta - ps15
Angiosperms - ps16
Mesangiospermae - ps17
Magnoliids/eudicots - ps18
Eudicots - ps19
Core_eudicots - ps20
Superrosids - ps21
Rosids - ps22
Eurosids - ps23
Malvids - ps24
Sap./Malv./Brassicales - ps25
Malvales/Brassicales - ps26
Brassicales - ps27
Cleomaceae/Brassicaceae - ps28
Brassicaceae - ps29
Camelineae - ps30
Arabidopsis - ps31
Arabidopsis_thaliana - ps32
p-value
2e-05
1e-04
9e-04
6e-03
4e-02
Log-odds
0.5
1.0
1.5
2.0
2.5
3.0
3.5
4.0

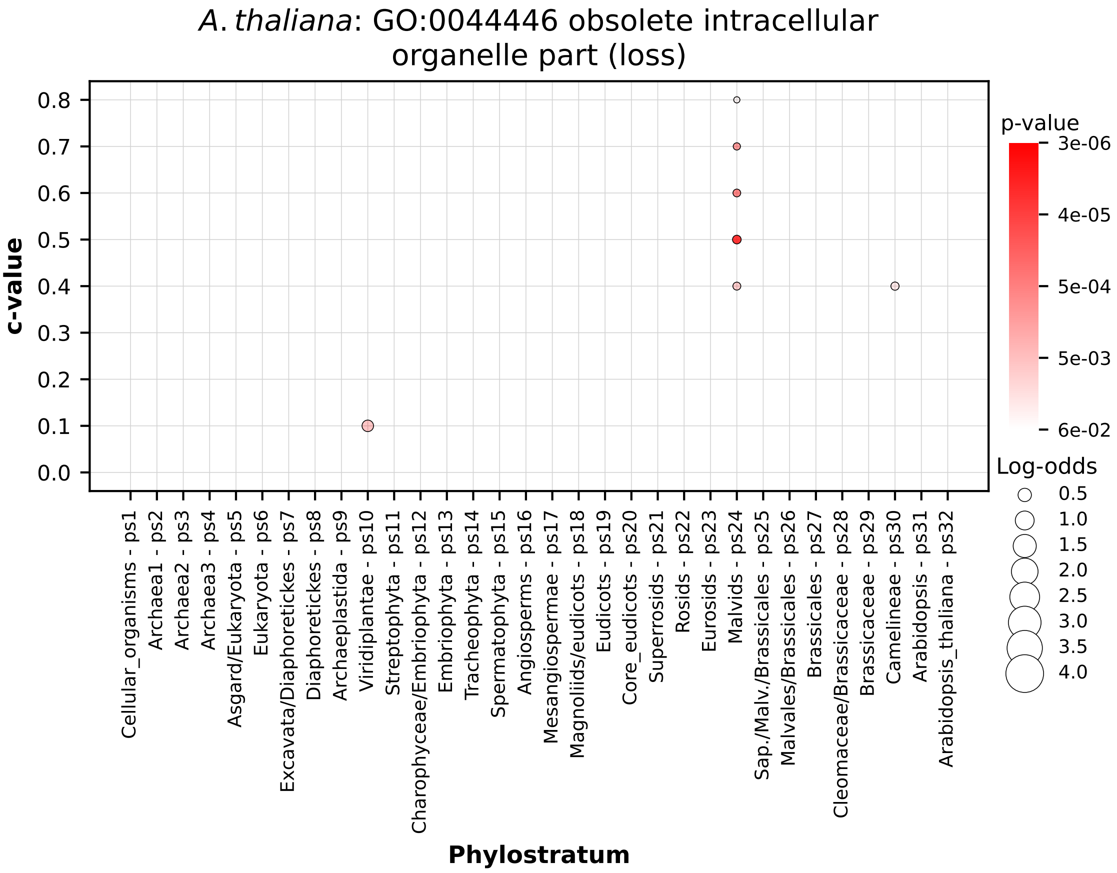
A. thaliana: GO:0044446 obsolete intracellular organelle part (loss)
c-value
0.8
0.7
0.6
0.5
0.4
0.3
0.2
0.1
0.0
p-value
3e-06
4e-05
5e-04
5e-03
6e-02
Log-odds
0.5
1.0
1.5
2.0
2.5
3.0
3.5
4.0
Cellular_organisms - ps1
Archaea1 - ps2
Archaea2 - ps3
Archaea3 - ps4
Asgard/Eukaryota - ps5
Eukaryota - ps6
Excavata/Diaphoretickes - ps7
Diaphoretickes - ps8
Archaeplastida - ps9
Viridiplantae - ps10
Streptophyta - ps11
Charophyceae/Embriophyta - ps12
Embriophyta - ps13
Tracheophyta - ps14
Spermatophyta - ps15
Angiosperms - ps16
Mesangiospermae - ps17
Magnoliids/eudicots - ps18
Eudicots - ps19
Core_eudicots - ps20
Superrosids - ps21
Rosids - ps22
Eurosids - ps23
Malvids - ps24
Sap./Malv./Brassicales - ps25
Malvales/Brassicales - ps26
Brassicales - ps27
Cleomaceae/Brassicaceae - ps28
Brassicaceae - ps29
Camelineae - ps30
Arabidopsis - ps31
Arabidopsis_thaliana - ps32
Phylostratum

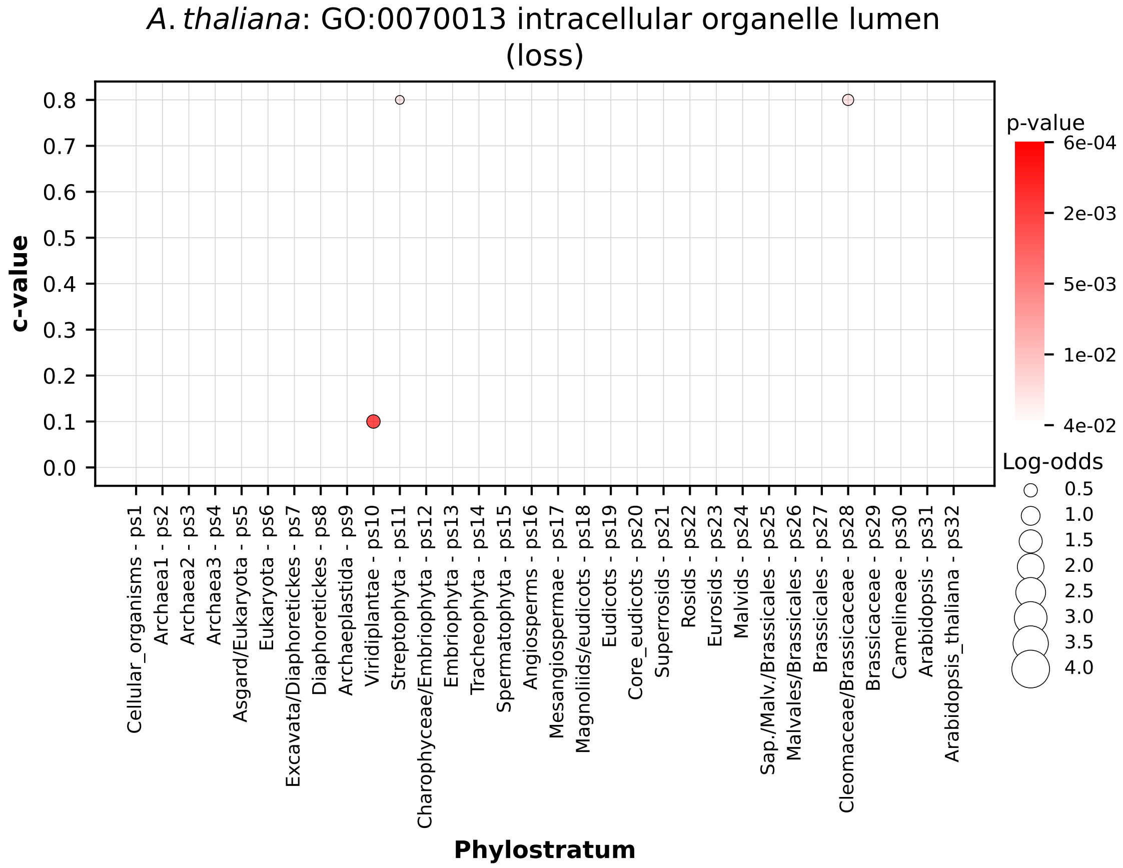
A. thaliana: GO:0070013 intracellular organelle lumen
(loss)
c-value
0.0
0.1
0.2
0.3
0.4
0.5
0.6
0.7
0.8
Phylostratum
Cellular_organisms - ps1
Archaea1 - ps2
Archaea2 - ps3
Archaea3 - ps4
Asgard/Eukaryota - ps5
Eukaryota - ps6
Excavata/Diaphoretickes - ps7
Diaphoretickes - ps8
Archaeplastida - ps9
Viridiplantae - ps10
Streptophyta - ps11
Charophyceae/Embriophyta - ps12
Embriophyta - ps13
Tracheophyta - ps14
Spermatophyta - ps15
Angiosperms - ps16
Mesangiospermae - ps17
Magnoliids/eudicots - ps18
Eudicots - ps19
Core_eudicots - ps20
Superrosids - ps21
Rosids - ps22
Eurosids - ps23
Malvids - ps24
Sap./Malv./Brassicales - ps25
Malvales/Brassicales - ps26
Brassicales - ps27
Cleomaceae/Brassicaceae - ps28
Brassicaceae - ps29
Camelineae - ps30
Arabidopsis - ps31
Arabidopsis_thaliana - ps32
p-value
6e-04
2e-03
5e-03
1e-02
4e-02
Log-odds
0.5
1.0
1.5
2.0
2.5
3.0
3.5
4.0

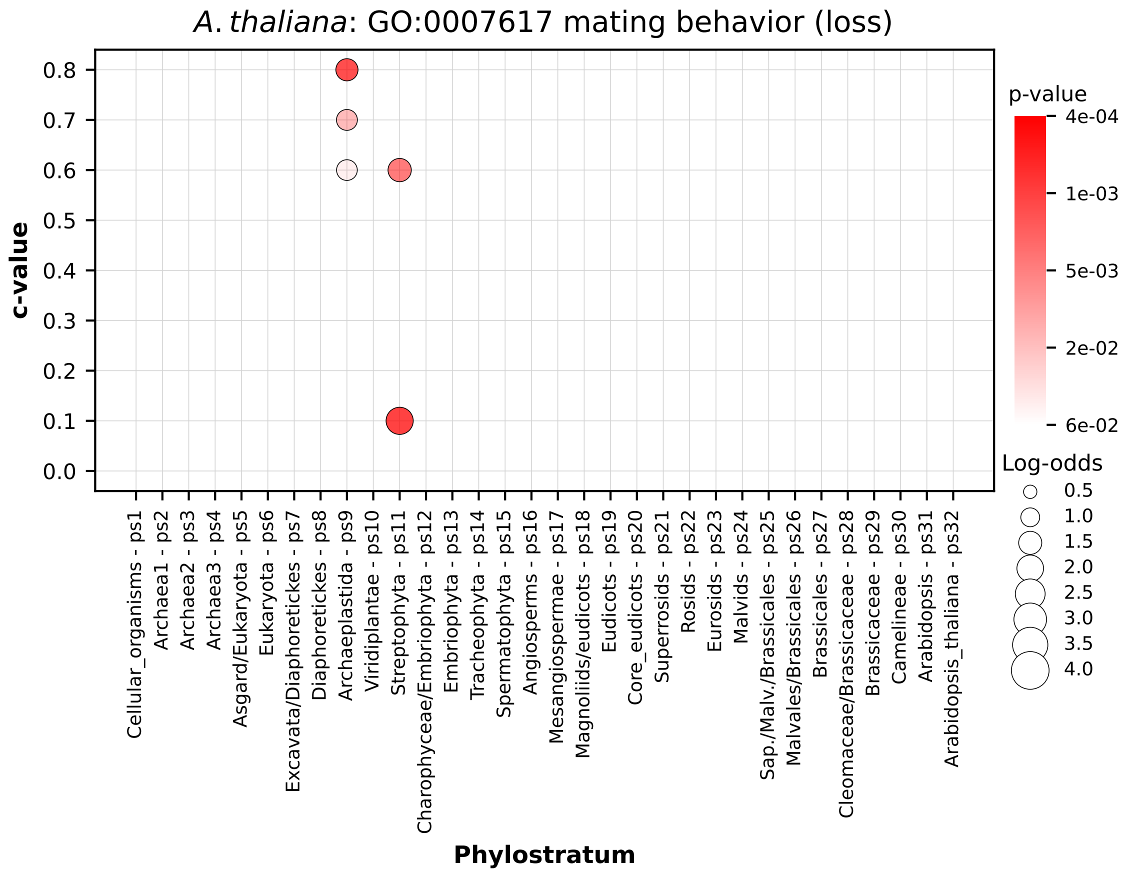

A. thaliana: GO:0007617 mating behavior (loss)
c-value
0.0
0.1
0.2
0.3
0.4
0.5
0.6
0.7
0.8
Phylostratum
Cellular_organisms - ps1
Archaea1 - ps2
Archaea2 - ps3
Archaea3 - ps4
Asgard/Eukaryota - ps5
Eukaryota - ps6
Excavata/Diaphoretickes - ps7
Diaphoretickes - ps8
Archaeplastida - ps9
Viridiplantae - ps10
Streptophyta - ps11
Charophyceae/Embriophyta - ps12
Embriophyta - ps13
Tracheophyta - ps14
Spermatophyta - ps15
Angiosperms - ps16
Mesangiospermae - ps17
Magnoliids/eudicots - ps18
Eudicots - ps19
Core_eudicots - ps20
Superrosids - ps21
Rosids - ps22
Eurosids - ps23
Malvids - ps24
Sap./Malv./Brassicales - ps25
Malvales/Brassicales - ps26
Brassicales - ps27
Cleomaceae/Brassicaceae - ps28
Brassicaceae - ps29
Camelineae - ps30
Arabidopsis - ps31
Arabidopsis_thaliana - ps32
p-value
4e-04
1e-03
5e-03
2e-02
6e-02
Log-odds
0.5
1.0
1.5
2.0
2.5
3.0
3.5
4.0

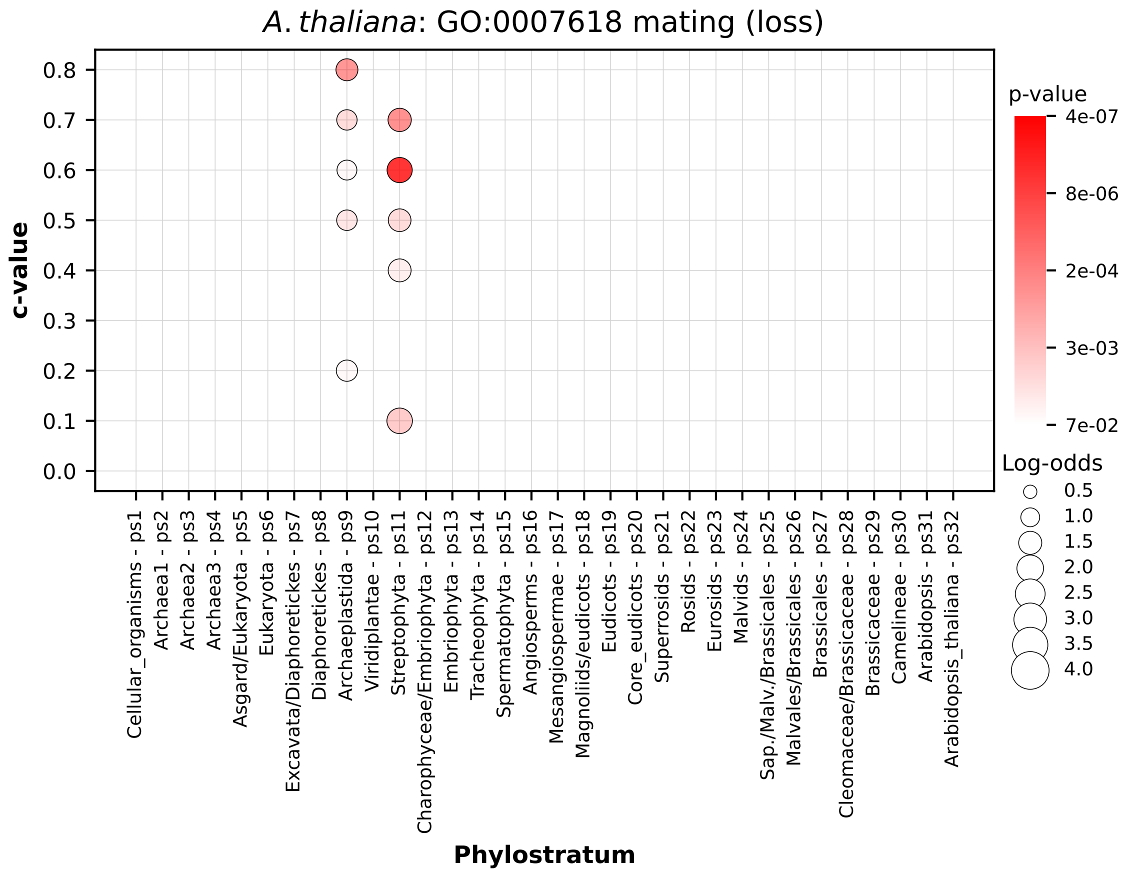

A. thaliana: GO:0007618 mating (loss)
c-value
0.0
0.1
0.2
0.3
0.4
0.5
0.6
0.7
0.8
Phylostratum
Cellular_organisms - ps1
Archaea1 - ps2
Archaea2 - ps3
Archaea3 - ps4
Asgard/Eukaryota - ps5
Eukaryota - ps6
Excavata/Diaphoretickes - ps7
Diaphoretickes - ps8
Archaeplastida - ps9
Viridiplantae - ps10
Streptophyta - ps11
Charophyceae/Embriophyta - ps12
Embriophyta - ps13
Tracheophyta - ps14
Spermatophyta - ps15
Angiosperms - ps16
Mesangiospermae - ps17
Magnoliids/eudicots - ps18
Eudicots - ps19
Core_eudicots - ps20
Superrosids - ps21
Rosids - ps22
Eurosids - ps23
Malvids - ps24
Sap./Malv./Brassicales - ps25
Malvales/Brassicales - ps26
Brassicales - ps27
Cleomaceae/Brassicaceae - ps28
Brassicaceae - ps29
Camelineae - ps30
Arabidopsis - ps31
Arabidopsis_thaliana - ps32
p-value
4e-07
8e-06
2e-04
3e-03
7e-02
Log-odds
0.5
1.0
1.5
2.0
2.5
3.0
3.5
4.0

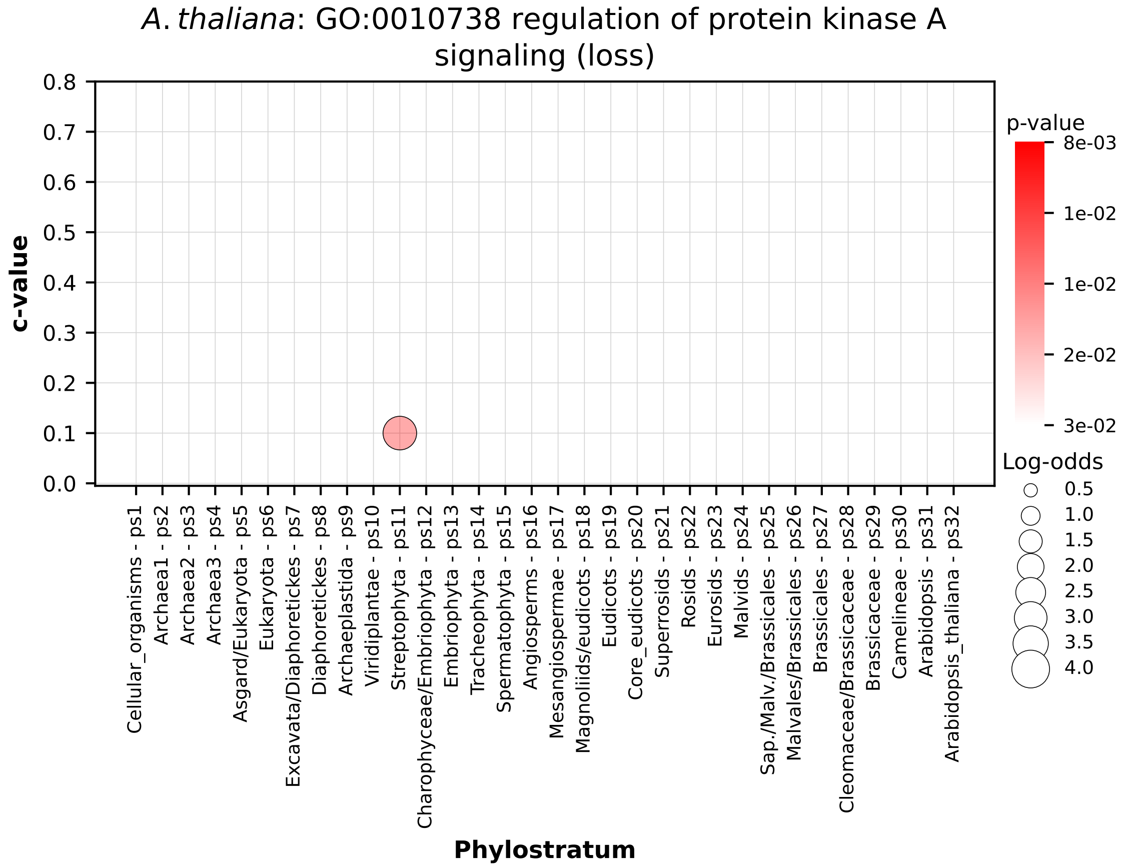

A. thaliana: GO:0010738 regulation of protein kinase A signaling (loss)
c-value
0.8
0.7
0.6
0.5
0.4
0.3
0.2
0.1
0.0
p-value
8e-03
1e-02
1e-02
2e-02
3e-02
Log-odds
0.5
1.0
1.5
2.0
2.5
3.0
3.5
4.0
Cellular_organisms - ps1
Archaea1 - ps2
Archaea2 - ps3
Archaea3 - ps4
Asgard/Eukaryota - ps5
Eukaryota - ps6
Excavata/Diaphoretickes - ps7
Diaphoretickes - ps8
Archaeplastida - ps9
Viridiplantae - ps10
Streptophyta - ps11
Charophyceae/Embriophyta - ps12
Embriophyta - ps13
Tracheophyta - ps14
Spermatophyta - ps15
Angiosperms - ps16
Mesangiospermae - ps17
Magnoliids/eudicots - ps18
Eudicots - ps19
Core_eudicots - ps20
Superrosids - ps21
Rosids - ps22
Eurosids - ps23
Malvids - ps24
Sap./Malv./Brassicales - ps25
Malvales/Brassicales - ps26
Brassicales - ps27
Cleomaceae/Brassicaceae - ps28
Brassicaceae - ps29
Camelineae - ps30
Arabidopsis - ps31
Arabidopsis_thaliana - ps32
Phylostratum

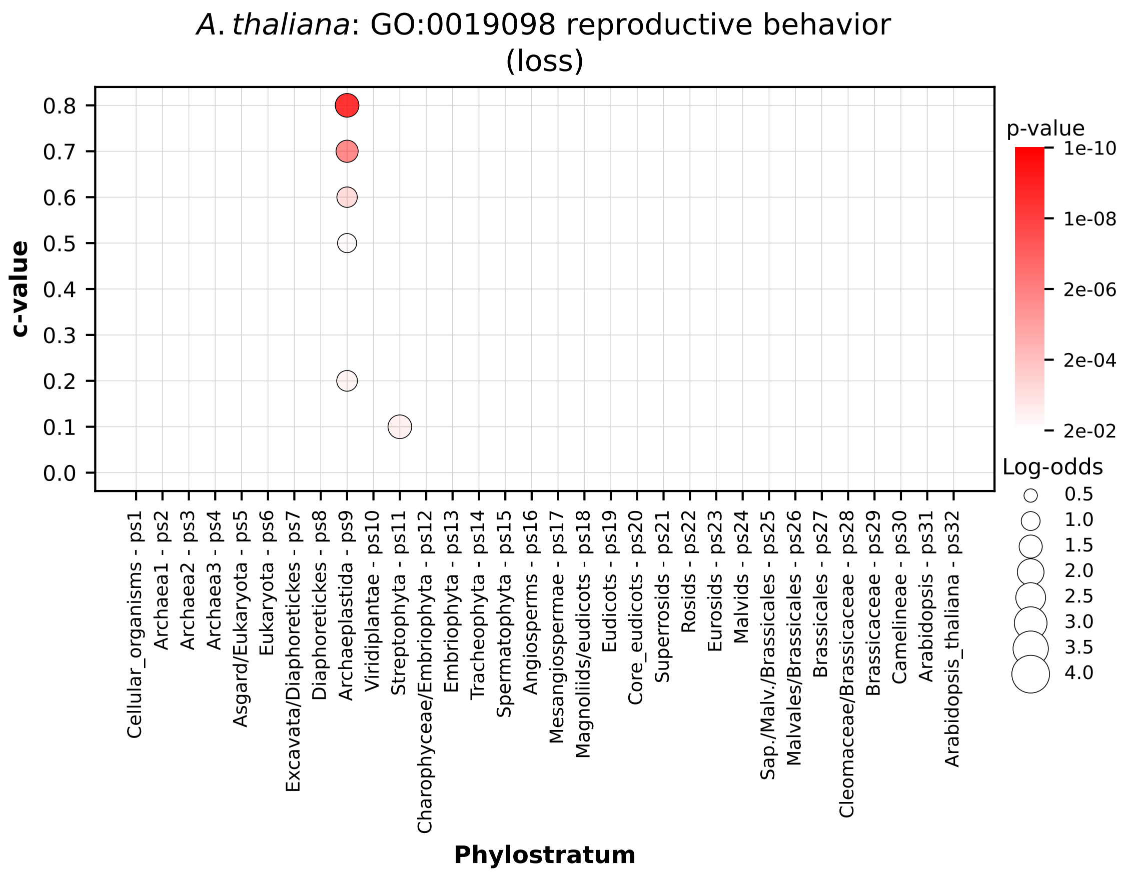
A. thaliana: GO:0019098 reproductive behavior
(loss)
c-value
0.8
0.7
0.6
0.5
0.4
0.3
0.2
0.1
0.0
p-value
1e-10
1e-08
2e-06
2e-04
2e-02
Log-odds
0.5
1.0
1.5
2.0
2.5
3.0
3.5
4.0
Cellular_organisms - ps1
Archaea1 - ps2
Archaea2 - ps3
Archaea3 - ps4
Asgard/Eukaryota - ps5
Eukaryota - ps6
Excavata/Diaphoretickes - ps7
Diaphoretickes - ps8
Archaeplastida - ps9
Viridiplantae - ps10
Streptophyta - ps11
Charophyceae/Embriophyta - ps12
Embriophyta - ps13
Tracheophyta - ps14
Spermatophyta - ps15
Angiosperms - ps16
Mesangiospermae - ps17
Magnoliids/eudicots - ps18
Eudicots - ps19
Core_eudicots - ps20
Superrosids - ps21
Rosids - ps22
Eurosids - ps23
Malvids - ps24
Sap./Malv./Brassicales - ps25
Malvales/Brassicales - ps26
Brassicales - ps27
Cleomaceae/Brassicaceae - ps28
Brassicaceae - ps29
Camelineae - ps30
Arabidopsis - ps31
Arabidopsis_thaliana - ps32
Phylostratum

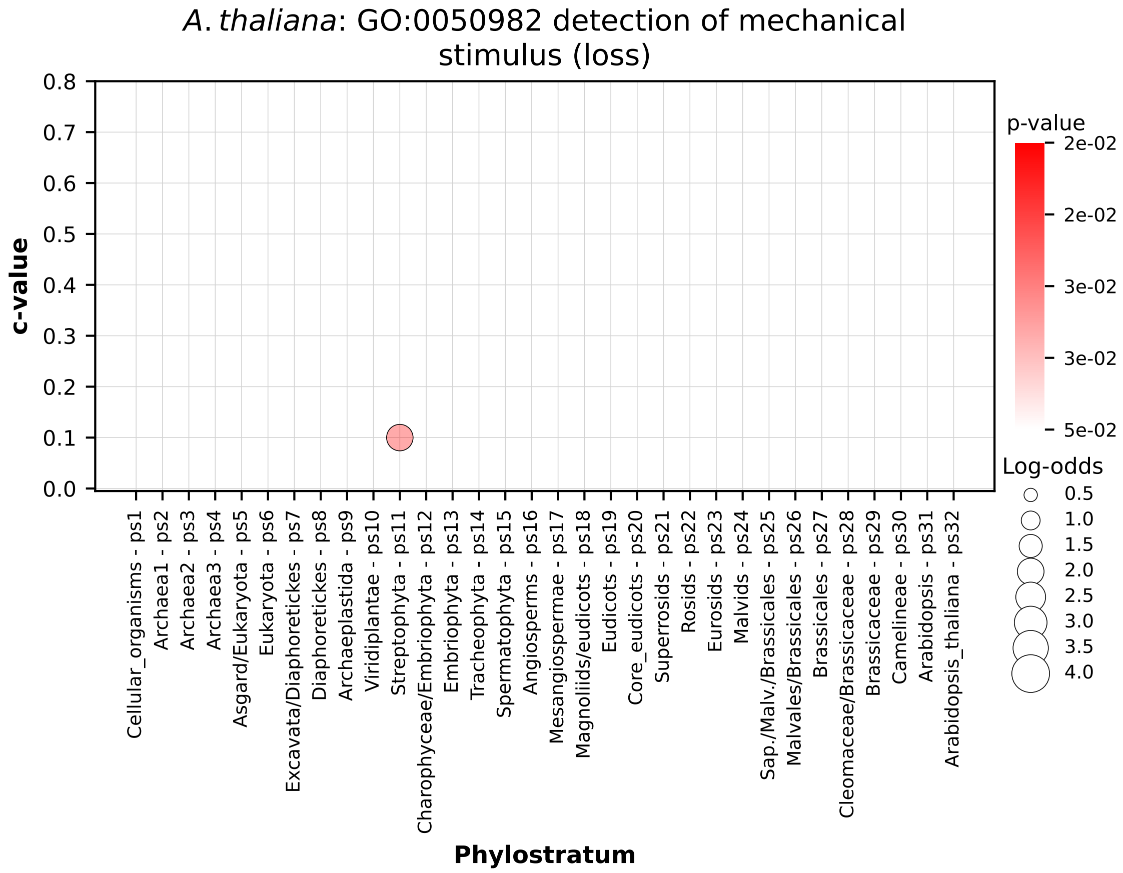

A. thaliana: GO:0050982 detection of mechanical stimulus (loss)
c-value
0.8
0.7
0.6
0.5
0.4
0.3
0.2
0.1
0.0
p-value
2e-02
2e-02
3e-02
3e-02
5e-02
Log-odds
0.5
1.0
1.5
2.0
2.5
3.0
3.5
4.0
Cellular_organisms - ps1
Archaea1 - ps2
Archaea2 - ps3
Archaea3 - ps4
Asgard/Eukaryota - ps5
Eukaryota - ps6
Excavata/Diaphoretickes - ps7
Diaphoretickes - ps8
Archaeplastida - ps9
Viridiplantae - ps10
Streptophyta - ps11
Charophyceae/Embriophyta - ps12
Embriophyta - ps13
Tracheophyta - ps14
Spermatophyta - ps15
Angiosperms - ps16
Mesangiospermae - ps17
Magnoliids/eudicots - ps18
Eudicots - ps19
Core_eudicots - ps20
Superrosids - ps21
Rosids - ps22
Eurosids - ps23
Malvids - ps24
Sap./Malv./Brassicales - ps25
Malvales/Brassicales - ps26
Brassicales - ps27
Cleomaceae/Brassicaceae - ps28
Brassicaceae - ps29
Camelineae - ps30
Arabidopsis - ps31
Arabidopsis_thaliana - ps32
Phylostratum

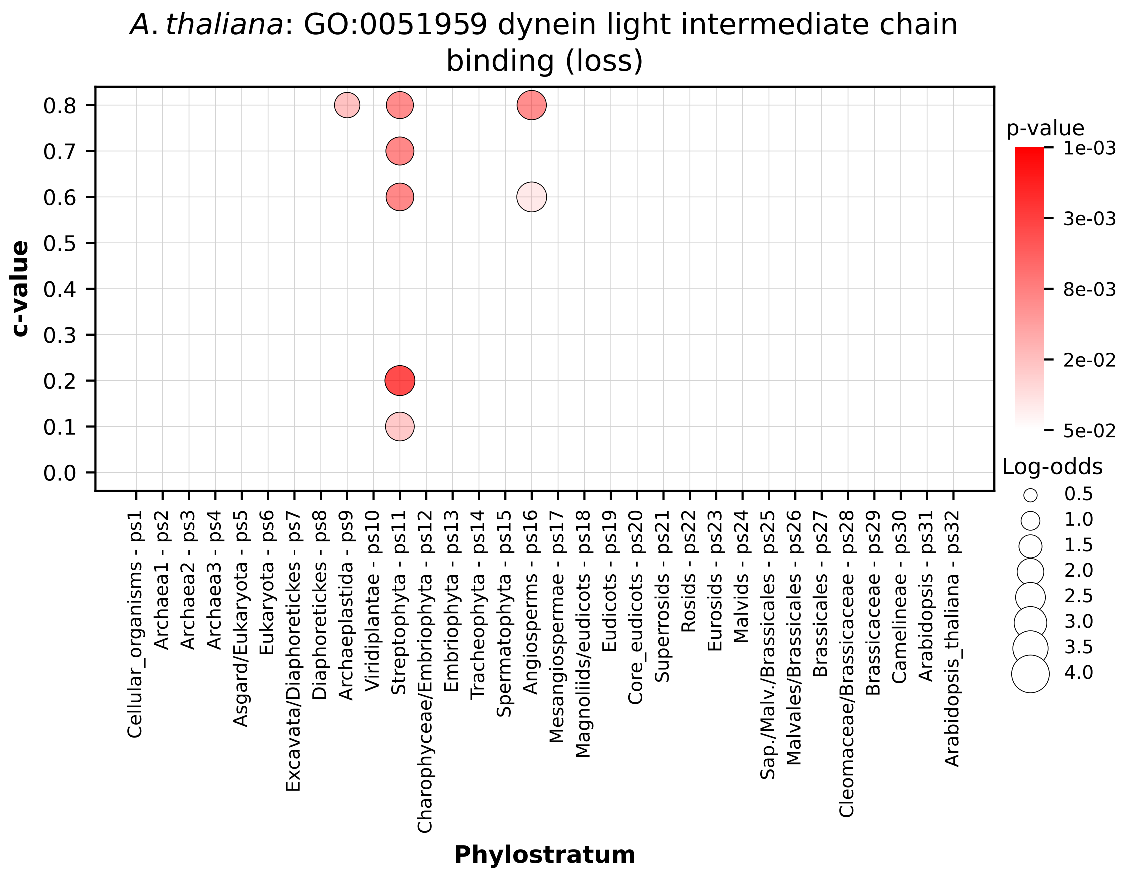

A. thaliana: GO:0051959 dynein light intermediate chain binding (loss)
c-value
0.0
0.1
0.2
0.3
0.4
0.5
0.6
0.7
0.8
Phylostratum
Cellular_organisms - ps1
Archaea1 - ps2
Archaea2 - ps3
Archaea3 - ps4
Asgard/Eukaryota - ps5
Eukaryota - ps6
Excavata/Diaphoretickes - ps7
Diaphoretickes - ps8
Archaeplastida - ps9
Viridiplantae - ps10
Streptophyta - ps11
Charophyceae/Embriophyta - ps12
Embriophyta - ps13
Tracheophyta - ps14
Spermatophyta - ps15
Angiosperms - ps16
Mesangiospermae - ps17
Magnoliids/eudicots - ps18
Eudicots - ps19
Core_eudicots - ps20
Superrosids - ps21
Rosids - ps22
Eurosids - ps23
Malvids - ps24
Sap./Malv./Brassicales - ps25
Malvales/Brassicales - ps26
Brassicales - ps27
Cleomaceae/Brassicaceae - ps28
Brassicaceae - ps29
Camelineae - ps30
Arabidopsis - ps31
Arabidopsis_thaliana - ps32
p-value
1e-03
3e-03
8e-03
2e-02
5e-02
Log-odds
0.5
1.0
1.5
2.0
2.5
3.0
3.5
4.0

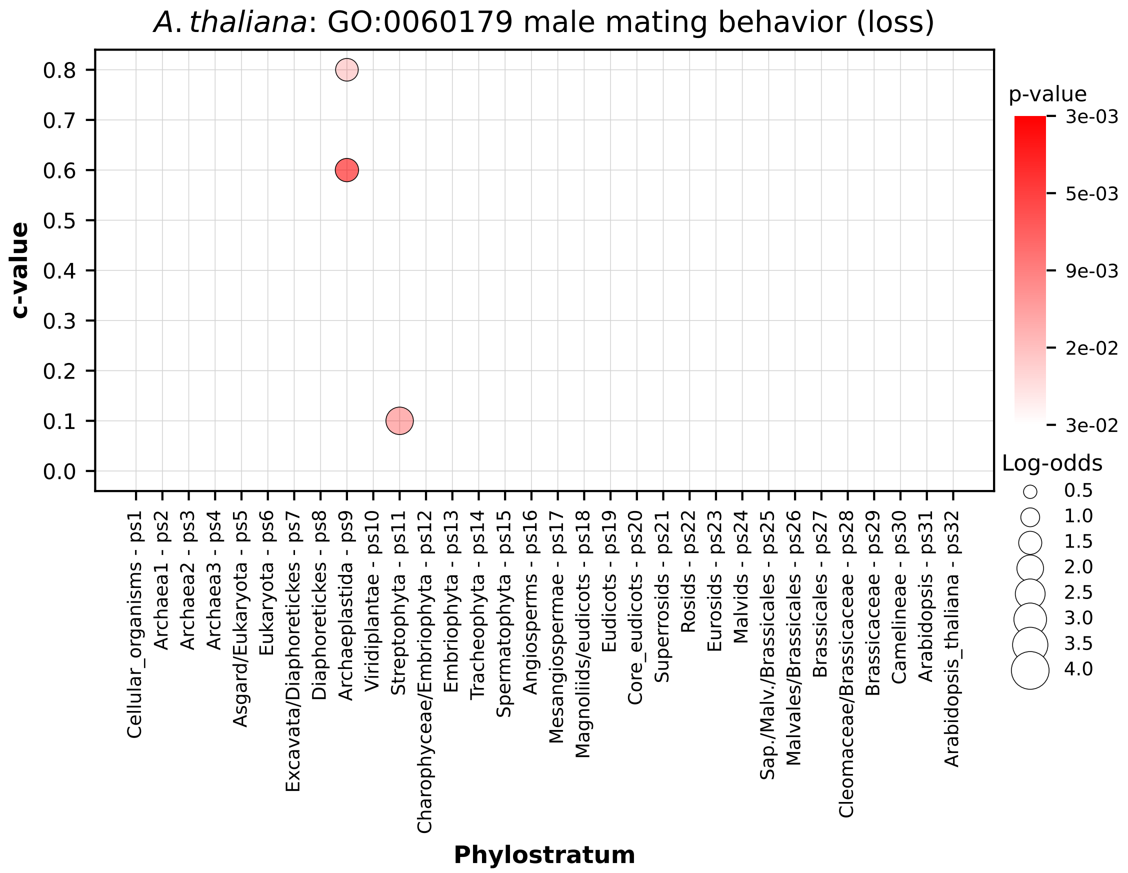

A. thaliana: GO:0060179 male mating behavior (loss)
c-value
0.0
0.1
0.2
0.3
0.4
0.5
0.6
0.7
0.8
Phylostratum
Cellular_organisms - ps1
Archaea1 - ps2
Archaea2 - ps3
Archaea3 - ps4
Asgard/Eukaryota - ps5
Eukaryota - ps6
Excavata/Diaphoretickes - ps7
Diaphoretickes - ps8
Archaeplastida - ps9
Viridiplantae - ps10
Streptophyta - ps11
Charophyceae/Embriophyta - ps12
Embriophyta - ps13
Tracheophyta - ps14
Spermatophyta - ps15
Angiosperms - ps16
Mesangiospermae - ps17
Magnoliids/eudicots - ps18
Eudicots - ps19
Core_eudicots - ps20
Superrosids - ps21
Rosids - ps22
Eurosids - ps23
Malvids - ps24
Sap./Malv./Brassicales - ps25
Malvales/Brassicales - ps26
Brassicales - ps27
Cleomaceae/Brassicaceae - ps28
Brassicaceae - ps29
Camelineae - ps30
Arabidopsis - ps31
Arabidopsis_thaliana - ps32
p-value
3e-03
5e-03
9e-03
2e-02
3e-02
Log-odds
0.5
1.0
1.5
2.0
2.5
3.0
3.5
4.0

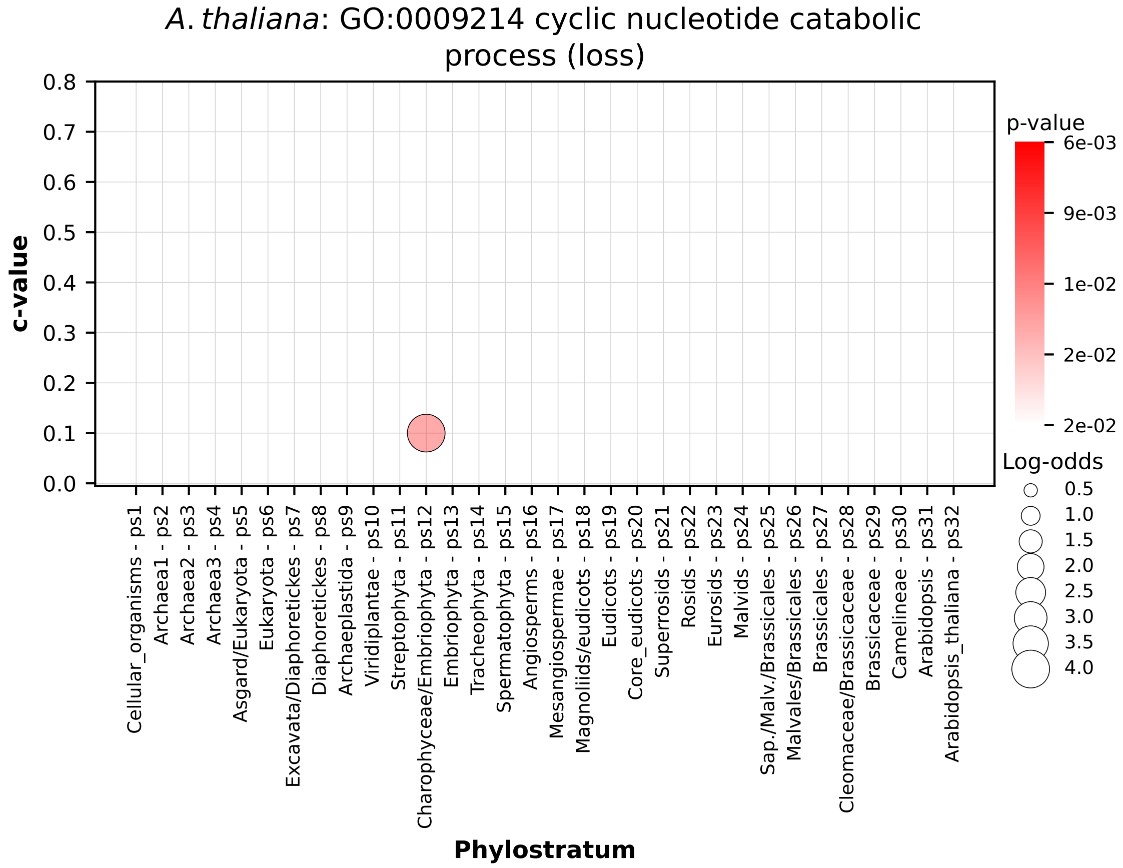

A. thaliana: GO:0009214 cyclic nucleotide catabolic process (loss)
c-value
0.8
0.7
0.6
0.5
0.4
0.3
0.2
0.1
0.0
Phylostratum
Cellular_organisms - ps1
Archaea1 - ps2
Archaea2 - ps3
Archaea3 - ps4
Asgard/Eukaryota - ps5
Eukaryota - ps6
Excavata/Diaphoretickes - ps7
Diaphoretickes - ps8
Archaeplastida - ps9
Viridiplantae - ps10
Streptophyta - ps11
Charophyceae/Embriophyta - ps12
Embriophyta - ps13
Tracheophyta - ps14
Spermatophyta - ps15
Angiosperms - ps16
Mesangiospermae - ps17
Magnoliids/eudicots - ps18
Eudicots - ps19
Core_eudicots - ps20
Superrosids - ps21
Rosids - ps22
Eurosids - ps23
Malvids - ps24
Sap./Malv./Brassicales - ps25
Malvales/Brassicales - ps26
Brassicales - ps27
Cleomaceae/Brassicaceae - ps28
Brassicaceae - ps29
Camelineae - ps30
Arabidopsis - ps31
Arabidopsis_thaliana - ps32
p-value
6e-03
9e-03
1e-02
2e-02
2e-02
Log-odds
0.5
1.0
1.5
2.0
2.5
3.0
3.5
4.0

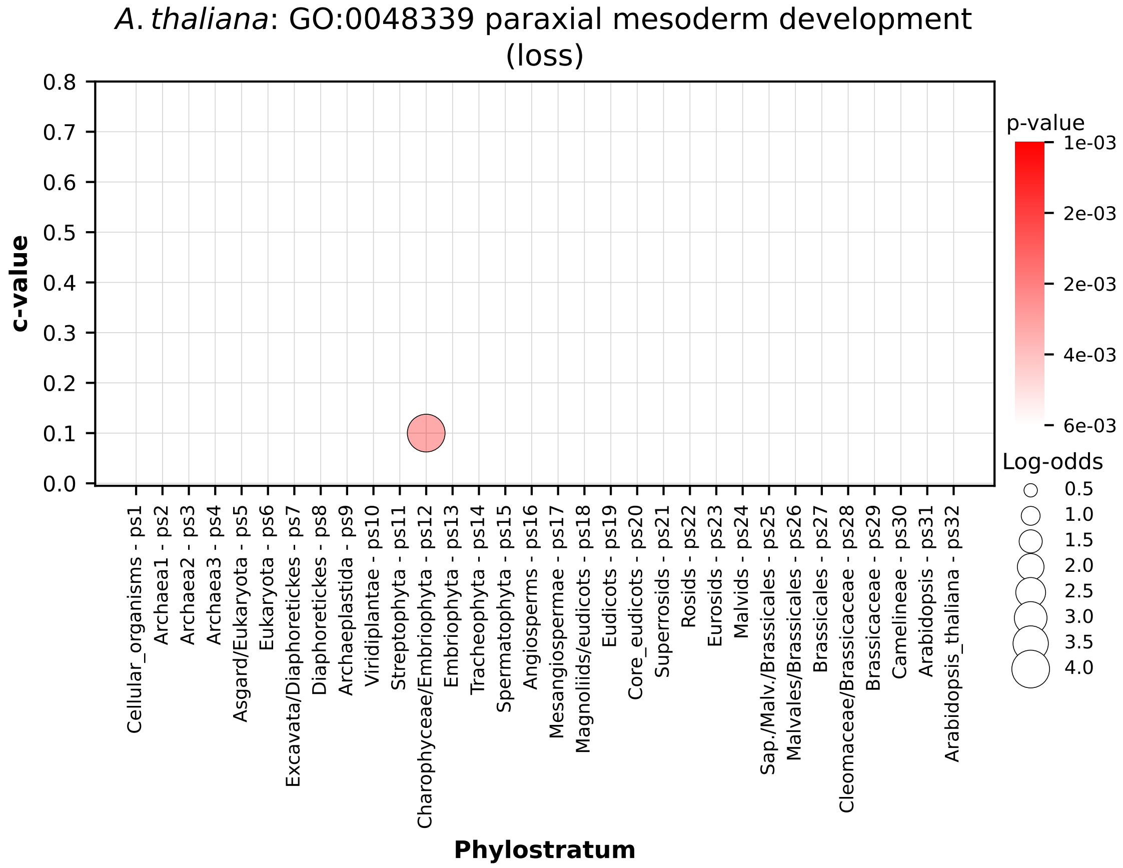

A. thaliana: GO:0048339 paraxial mesoderm development
(loss)
c-value
0.8
0.7
0.6
0.5
0.4
0.3
0.2
0.1
0.0
p-value
1e-03
2e-03
2e-03
4e-03
6e-03
Log-odds
0.5
1.0
1.5
2.0
2.5
3.0
3.5
4.0
Cellular_organisms - ps1
Archaea1 - ps2
Archaea2 - ps3
Archaea3 - ps4
Asgard/Eukaryota - ps5
Eukaryota - ps6
Excavata/Diaphoretickes - ps7
Diaphoretickes - ps8
Archaeplastida - ps9
Viridiplantae - ps10
Streptophyta - ps11
Charophyceae/Embriophyta - ps12
Embriophyta - ps13
Tracheophyta - ps14
Spermatophyta - ps15
Angiosperms - ps16
Mesangiospermae - ps17
Magnoliids/eudicots - ps18
Eudicots - ps19
Core_eudicots - ps20
Superrosids - ps21
Rosids - ps22
Eurosids - ps23
Malvids - ps24
Sap./Malv./Brassicales - ps25
Malvales/Brassicales - ps26
Brassicales - ps27
Cleomaceae/Brassicaceae - ps28
Brassicaceae - ps29
Camelineae - ps30
Arabidopsis - ps31
Arabidopsis_thaliana - ps32
Phylostratum

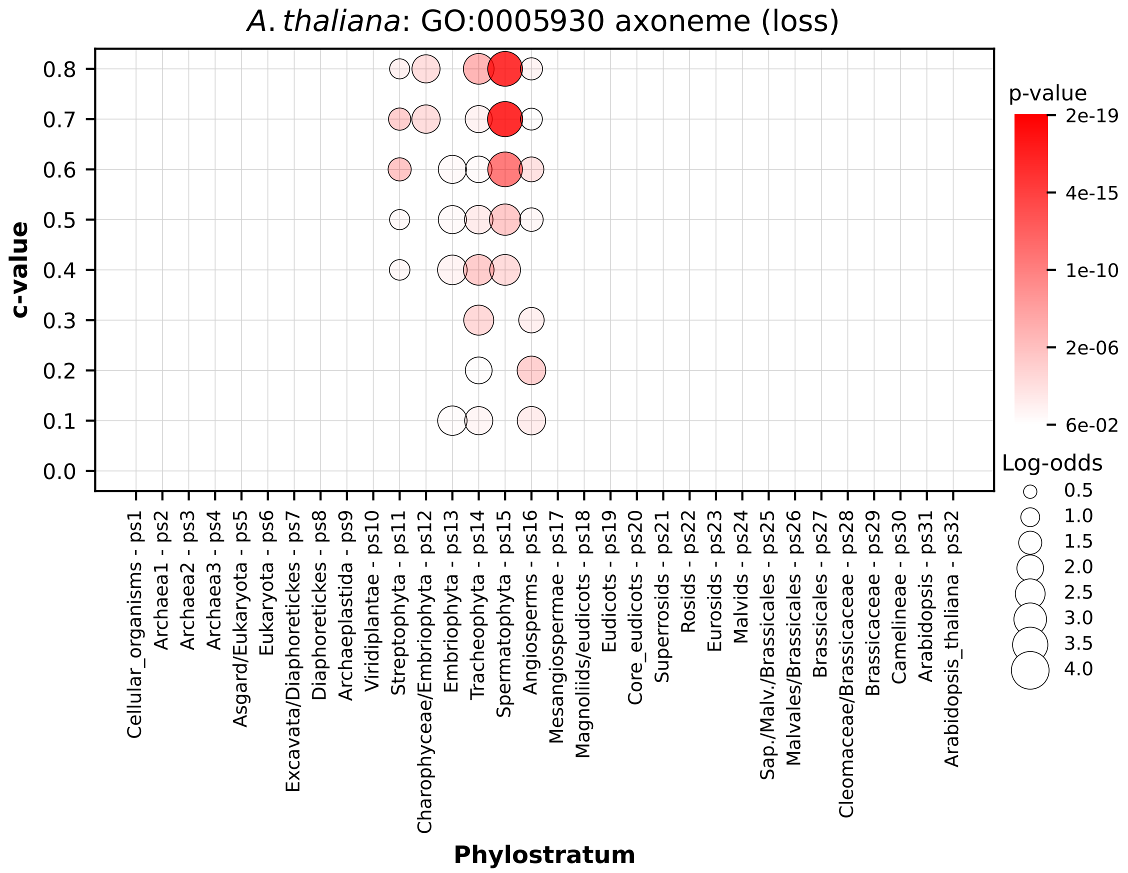

A. thaliana: GO:0005930 axoneme (loss)
c-value
0.0
0.1
0.2
0.3
0.4
0.5
0.6
0.7
0.8
Phylostratum
Cellular_organisms - ps1
Archaea1 - ps2
Archaea2 - ps3
Archaea3 - ps4
Asgard/Eukaryota - ps5
Eukaryota - ps6
Excavata/Diaphoretickes - ps7
Diaphoretickes - ps8
Archaeplastida - ps9
Viridiplantae - ps10
Streptophyta - ps11
Charophyceae/Embriophyta - ps12
Embriophyta - ps13
Tracheophyta - ps14
Spermatophyta - ps15
Angiosperms - ps16
Mesangiospermae - ps17
Magnoliids/eudicots - ps18
Eudicots - ps19
Core_eudicots - ps20
Superrosids - ps21
Rosids - ps22
Eurosids - ps23
Malvids - ps24
Sap./Malv./Brassicales - ps25
Malvales/Brassicales - ps26
Brassicales - ps27
Cleomaceae/Brassicaceae - ps28
Brassicaceae - ps29
Camelineae - ps30
Arabidopsis - ps31
Arabidopsis_thaliana - ps32
p-value
2e-19
4e-15
1e-10
2e-06
6e-02
Log-odds
0.5
1.0
1.5
2.0
2.5
3.0
3.5
4.0

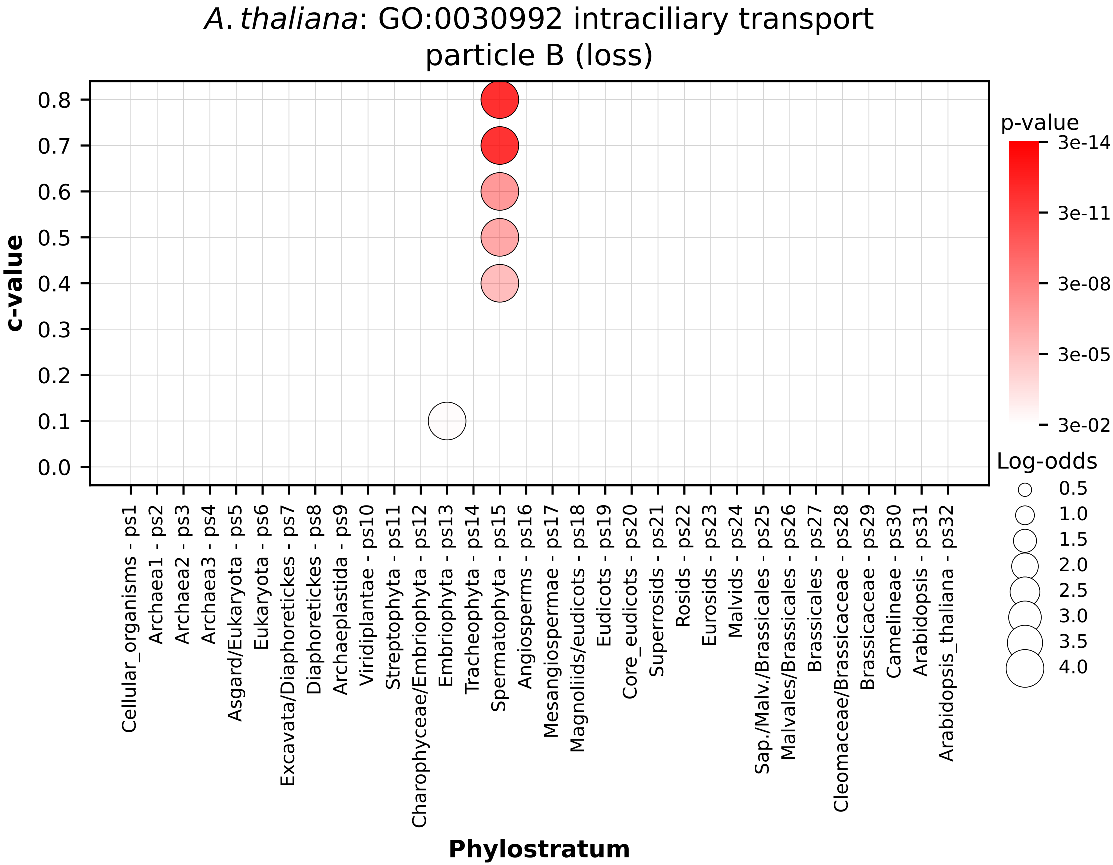

A. thaliana: GO:0030992 intraciliary transport particle B (loss)
c-value
0.0
0.1
0.2
0.3
0.4
0.5
0.6
0.7
0.8
p-value
3e-14
3e-11
3e-08
3e-05
3e-02
Log-odds
0.5
1.0
1.5
2.0
2.5
3.0
3.5
4.0
Cellular_organisms - ps1
Archaea1 - ps2
Archaea2 - ps3
Archaea3 - ps4
Asgard/Eukaryota - ps5
Eukaryota - ps6
Excavata/Diaphoretickes - ps7
Diaphoretickes - ps8
Archaeplastida - ps9
Viridiplantae - ps10
Streptophyta - ps11
Charophyceae/Embriophyta - ps12
Embriophyta - ps13
Tracheophyta - ps14
Spermatophyta - ps15
Angiosperms - ps16
Mesangiospermae - ps17
Magnoliids/eudicots - ps18
Eudicots - ps19
Core_eudicots - ps20
Superrosids - ps21
Rosids - ps22
Eurosids - ps23
Malvids - ps24
Sap./Malv./Brassicales - ps25
Malvales/Brassicales - ps26
Brassicales - ps27
Cleomaceae/Brassicaceae - ps28
Brassicaceae - ps29
Camelineae - ps30
Arabidopsis - ps31
Arabidopsis_thaliana - ps32
Phylostratum

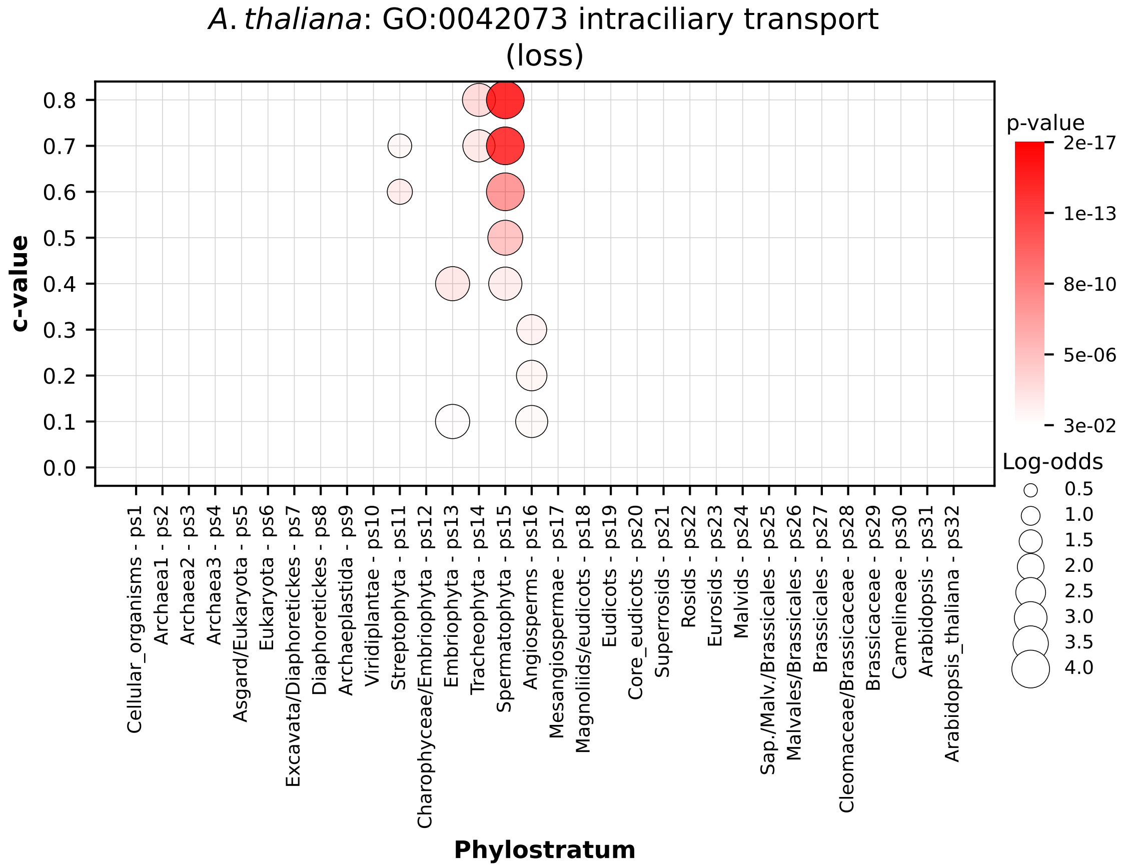

A. thaliana: GO:0042073 intraciliary transport
(loss)
c-value
0.8
0.7
0.6
0.5
0.4
0.3
0.2
0.1
0.0
p-value
2e-17
1e-13
8e-10
5e-06
3e-02
Log-odds
0.5
1.0
1.5
2.0
2.5
3.0
3.5
4.0
Cellular_organisms - ps1
Archaea1 - ps2
Archaea2 - ps3
Archaea3 - ps4
Asgard/Eukaryota - ps5
Eukaryota - ps6
Excavata/Diaphoretickes - ps7
Diaphoretickes - ps8
Archaeplastida - ps9
Viridiplantae - ps10
Streptophyta - ps11
Charophyceae/Embriophyta - ps12
Embriophyta - ps13
Tracheophyta - ps14
Spermatophyta - ps15
Angiosperms - ps16
Mesangiospermae - ps17
Magnoliids/eudicots - ps18
Eudicots - ps19
Core_eudicots - ps20
Superrosids - ps21
Rosids - ps22
Eurosids - ps23
Malvids - ps24
Sap./Malv./Brassicales - ps25
Malvales/Brassicales - ps26
Brassicales - ps27
Cleomaceae/Brassicaceae - ps28
Brassicaceae - ps29
Camelineae - ps30
Arabidopsis - ps31
Arabidopsis_thaliana - ps32
Phylostratum

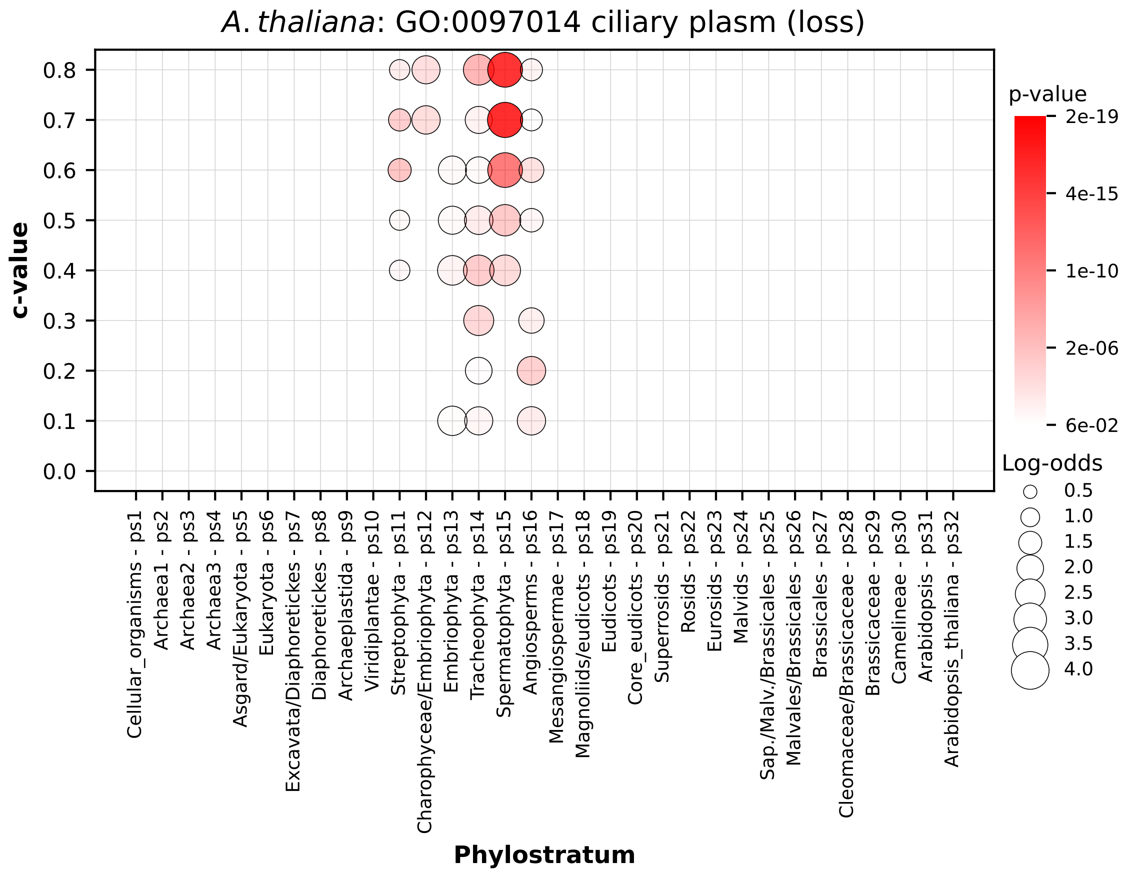

A. thaliana: GO:0097014 ciliary plasm (loss)
c-value
0.0
0.1
0.2
0.3
0.4
0.5
0.6
0.7
0.8
Phylostratum
Cellular_organisms - ps1
Archaea1 - ps2
Archaea2 - ps3
Archaea3 - ps4
Asgard/Eukaryota - ps5
Eukaryota - ps6
Excavata/Diaphoretickes - ps7
Diaphoretickes - ps8
Archaeplastida - ps9
Viridiplantae - ps10
Streptophyta - ps11
Charophyceae/Embriophyta - ps12
Embriophyta - ps13
Tracheophyta - ps14
Spermatophyta - ps15
Angiosperms - ps16
Mesangiospermae - ps17
Magnoliids/eudicots - ps18
Eudicots - ps19
Core_eudicots - ps20
Superrosids - ps21
Rosids - ps22
Eurosids - ps23
Malvids - ps24
Sap./Malv./Brassicales - ps25
Malvales/Brassicales - ps26
Brassicales - ps27
Cleomaceae/Brassicaceae - ps28
Brassicaceae - ps29
Camelineae - ps30
Arabidopsis - ps31
Arabidopsis_thaliana - ps32
p-value
2e-19
4e-15
1e-10
2e-06
6e-02
Log-odds
0.5
1.0
1.5
2.0
2.5
3.0
3.5
4.0

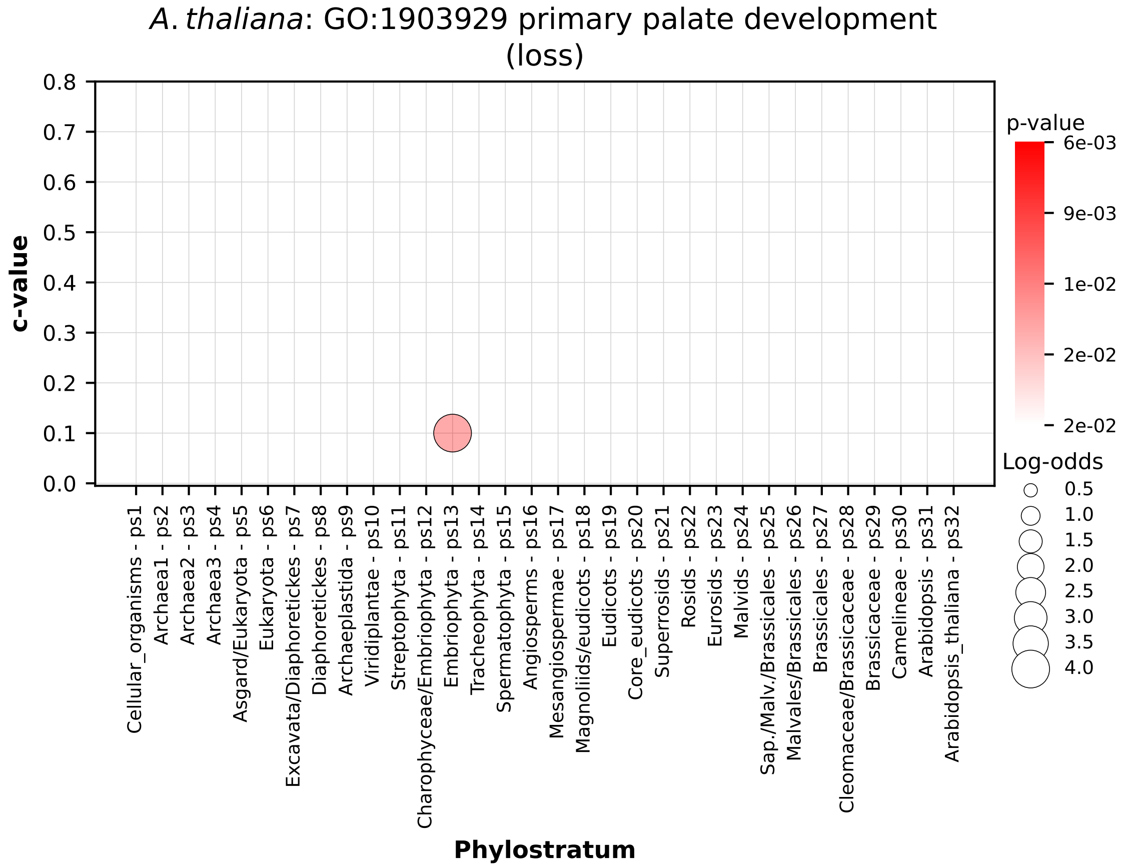

A. thaliana: GO:1903929 primary palate development (loss)
c-value
0.8
0.7
0.6
0.5
0.4
0.3
0.2
0.1
0.0
p-value
6e-03
9e-03
1e-02
2e-02
2e-02
Log-odds
0.5
1.0
1.5
2.0
2.5
3.0
3.5
4.0
Cellular_organisms - ps1
Archaea1 - ps2
Archaea2 - ps3
Archaea3 - ps4
Asgard/Eukaryota - ps5
Eukaryota - ps6
Excavata/Diaphoretickes - ps7
Diaphoretickes - ps8
Archaeplastida - ps9
Viridiplantae - ps10
Streptophyta - ps11
Charophyceae/Embriophyta - ps12
Embriophyta - ps13
Tracheophyta - ps14
Spermatophyta - ps15
Angiosperms - ps16
Mesangiospermae - ps17
Magnoliids/eudicots - ps18
Eudicots - ps19
Core_eudicots - ps20
Superrosids - ps21
Rosids - ps22
Eurosids - ps23
Malvids - ps24
Sap./Malv./Brassicales - ps25
Malvales/Brassicales - ps26
Brassicales - ps27
Cleomaceae/Brassicaceae - ps28
Brassicaceae - ps29
Camelineae - ps30
Arabidopsis - ps31
Arabidopsis_thaliana - ps32
Phylostratum

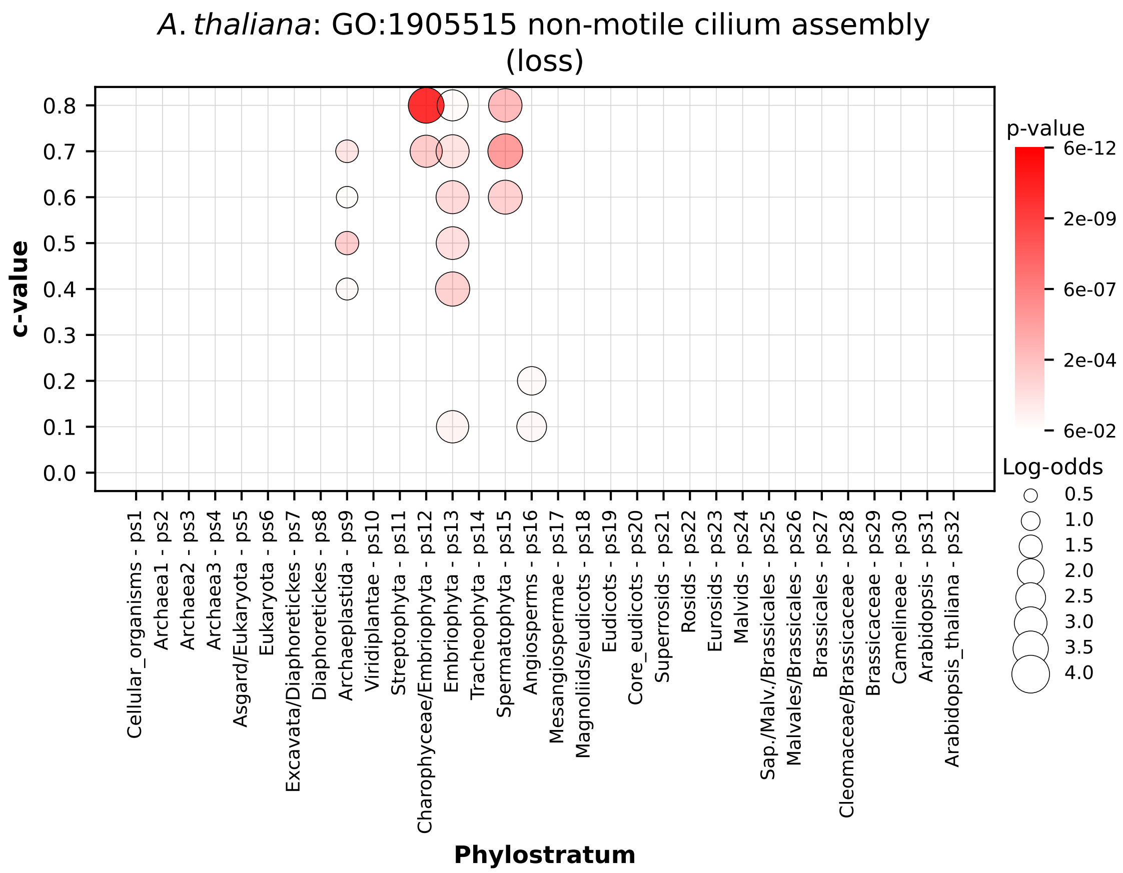
A. thaliana: GO:1905515 non-motile cilium assembly
(loss)
c-value
0.8
0.7
0.6
0.5
0.4
0.3
0.2
0.1
0.0
Phylostratum
Cellular_organisms - ps1
Archaea1 - ps2
Archaea2 - ps3
Archaea3 - ps4
Asgard/Eukaryota - ps5
Eukaryota - ps6
Excavata/Diaphoretickes - ps7
Diaphoretickes - ps8
Archaeplastida - ps9
Viridiplantae - ps10
Streptophyta - ps11
Charophyceae/Embriophyta - ps12
Embriophyta - ps13
Tracheophyta - ps14
Spermatophyta - ps15
Angiosperms - ps16
Mesangiospermae - ps17
Magnoliids/eudicots - ps18
Eudicots - ps19
Core_eudicots - ps20
Superrosids - ps21
Rosids - ps22
Eurosids - ps23
Malvids - ps24
Sap./Malv./Brassicales - ps25
Malvales/Brassicales - ps26
Brassicales - ps27
Cleomaceae/Brassicaceae - ps28
Brassicaceae - ps29
Camelineae - ps30
Arabidopsis - ps31
Arabidopsis_thaliana - ps32
p-value
6e-12
2e-09
6e-07
2e-04
6e-02
Log-odds
0.5
1.0
1.5
2.0
2.5
3.0
3.5
4.0

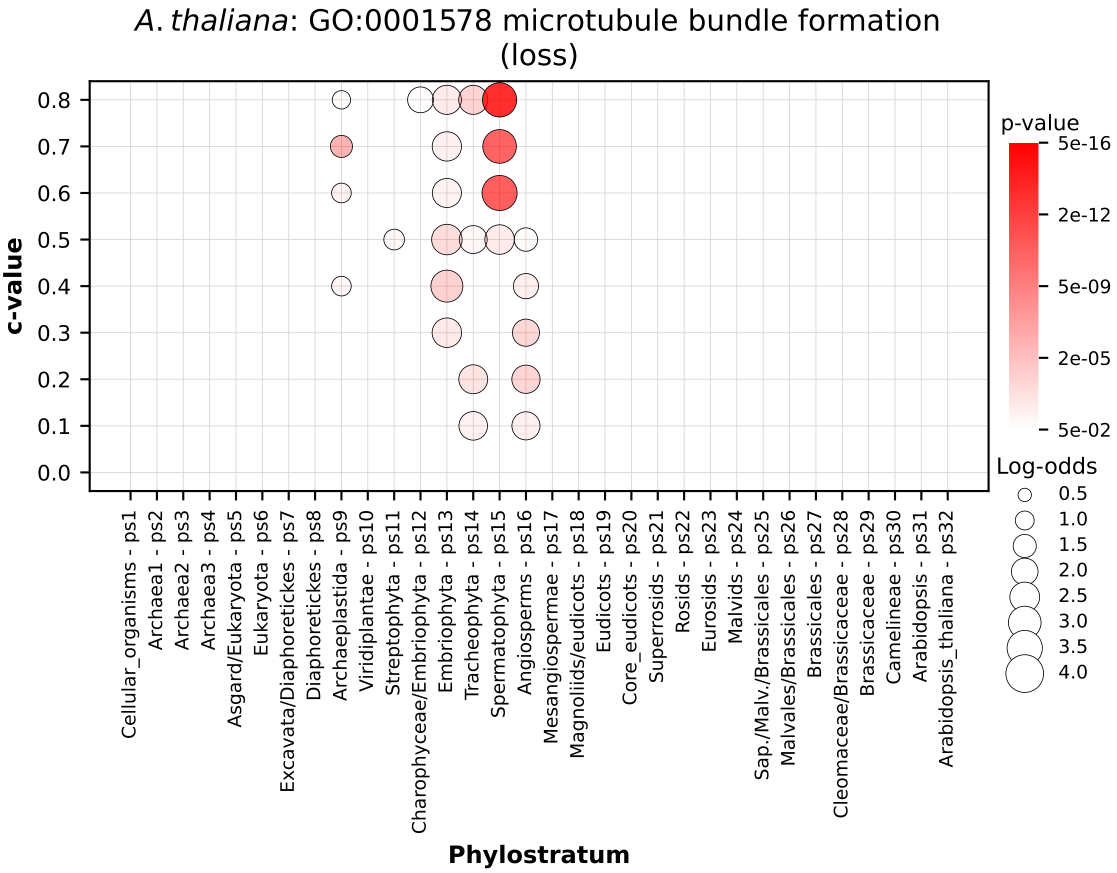
A. thaliana: GO:0001578 microtubule bundle formation
(loss)
c-value
0.8
0.7
0.6
0.5
0.4
0.3
0.2
0.1
0.0
Phylostratum
Cellular_organisms - ps1
Archaea1 - ps2
Archaea2 - ps3
Archaea3 - ps4
Asgard/Eukaryota - ps5
Eukaryota - ps6
Excavata/Diaphoretickes - ps7
Diaphoretickes - ps8
Archaeplastida - ps9
Viridiplantae - ps10
Streptophyta - ps11
Charophyceae/Embriophyta - ps12
Embriophyta - ps13
Tracheophyta - ps14
Spermatophyta - ps15
Angiosperms - ps16
Mesangiospermae - ps17
Magnoliids/eudicots - ps18
Eudicots - ps19
Core_eudicots - ps20
Superrosids - ps21
Rosids - ps22
Eurosids - ps23
Malvids - ps24
Sap./Malv./Brassicales - ps25
Malvales/Brassicales - ps26
Brassicales - ps27
Cleomaceae/Brassicaceae - ps28
Brassicaceae - ps29
Camelineae - ps30
Arabidopsis - ps31
Arabidopsis_thaliana - ps32
p-value
5e-16
2e-12
5e-09
2e-05
5e-02
Log-odds
0.5
1.0
1.5
2.0
2.5
3.0
3.5
4.0

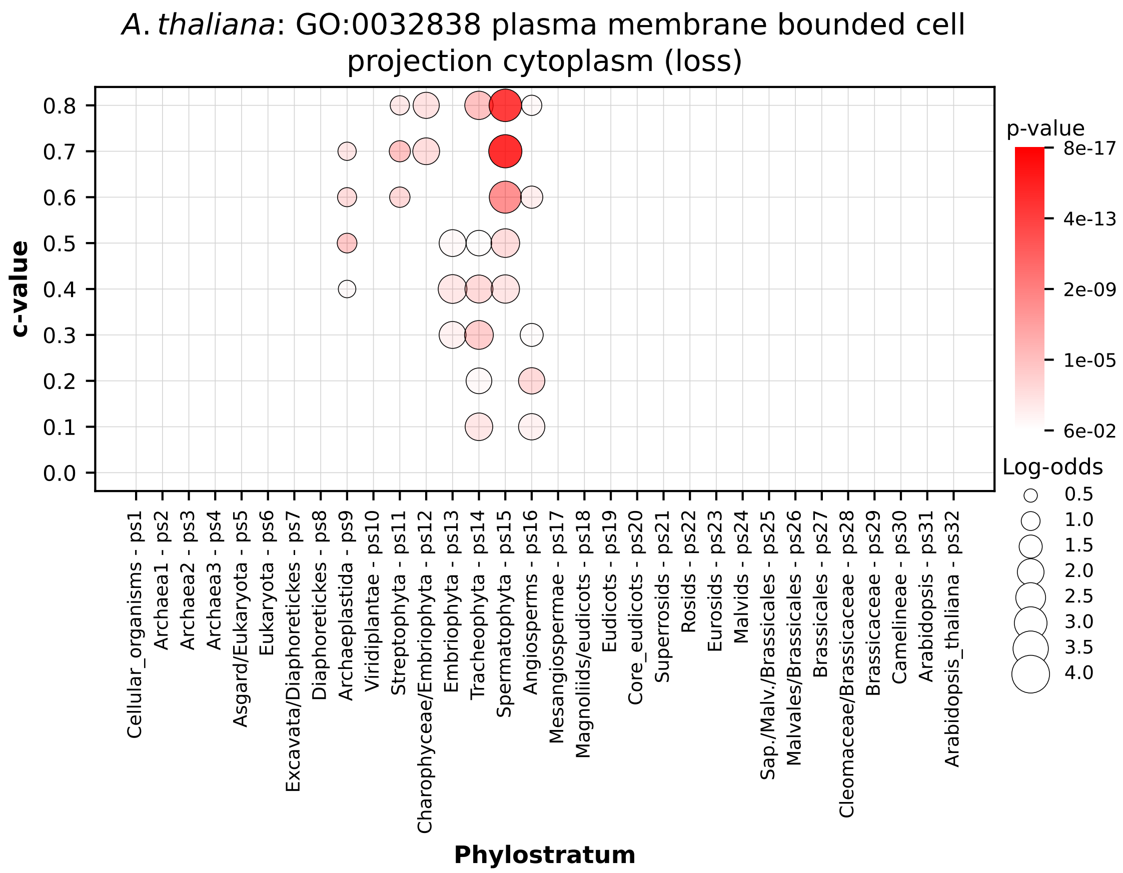

A. thaliana: GO:0032838 plasma membrane bounded cell projection cytoplasm (loss)
c-value
0.8
0.7
0.6
0.5
0.4
0.3
0.2
0.1
0.0
p-value
8e-17
4e-13
2e-09
1e-05
6e-02
Log-odds
0.5
1.0
1.5
2.0
2.5
3.0
3.5
4.0
Cellular_organisms - ps1
Archaea1 - ps2
Archaea2 - ps3
Archaea3 - ps4
Asgard/Eukaryota - ps5
Eukaryota - ps6
Excavata/Diaphoretickes - ps7
Diaphoretickes - ps8
Archaeplastida - ps9
Viridiplantae - ps10
Streptophyta - ps11
Charophyceae/Embriophyta - ps12
Embriophyta - ps13
Tracheophyta - ps14
Spermatophyta - ps15
Angiosperms - ps16
Mesangiospermae - ps17
Magnoliids/eudicots - ps18
Eudicots - ps19
Core_eudicots - ps20
Superrosids - ps21
Rosids - ps22
Eurosids - ps23
Malvids - ps24
Sap./Malv./Brassicales - ps25
Malvales/Brassicales - ps26
Brassicales - ps27
Cleomaceae/Brassicaceae - ps28
Brassicaceae - ps29
Camelineae - ps30
Arabidopsis - ps31
Arabidopsis_thaliana - ps32
Phylostratum

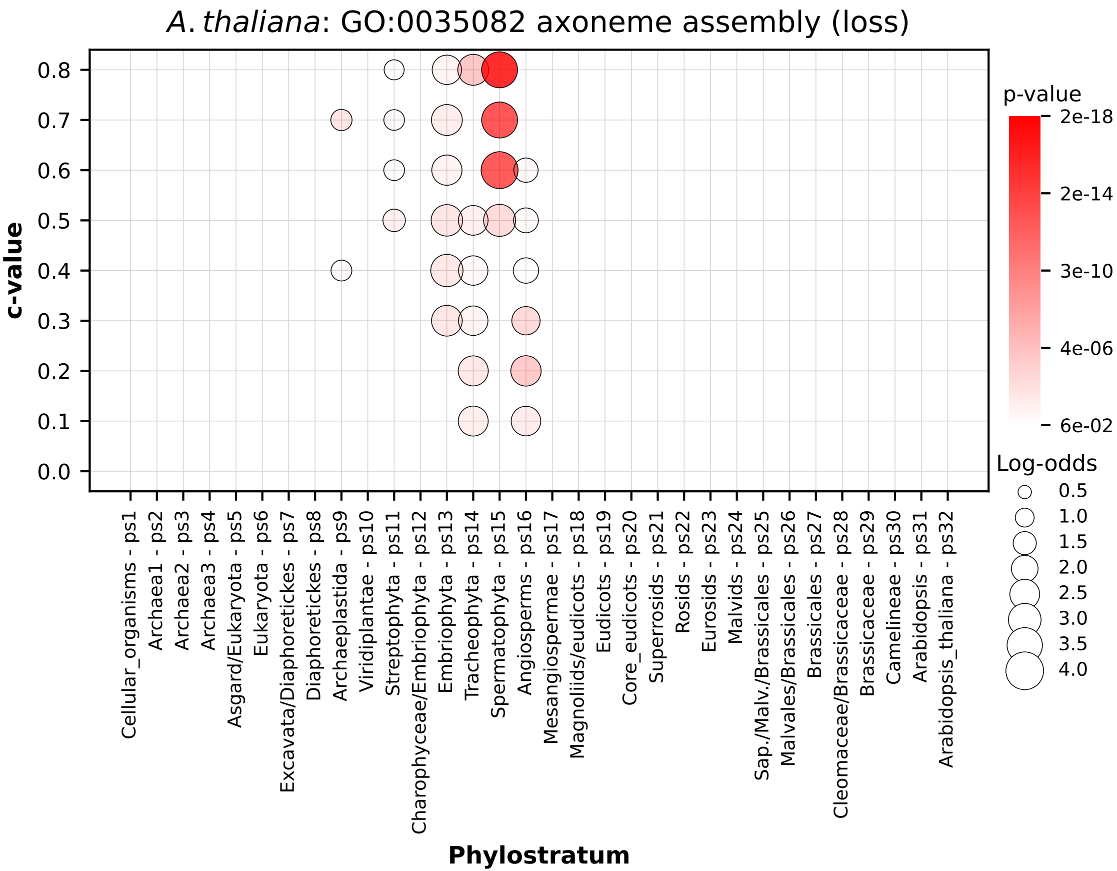
A. thaliana: GO:0035082 axoneme assembly (loss)
c-value
0.8
0.7
0.6
0.5
0.4
0.3
0.2
0.1
0.0
Phylostratum
Cellular_organisms - ps1
Archaea1 - ps2
Archaea2 - ps3
Archaea3 - ps4
Asgard/Eukaryota - ps5
Eukaryota - ps6
Excavata/Diaphoretickes - ps7
Diaphoretickes - ps8
Archaeplastida - ps9
Viridiplantae - ps10
Streptophyta - ps11
Charophyceae/Embriophyta - ps12
Embriophyta - ps13
Tracheophyta - ps14
Spermatophyta - ps15
Angiosperms - ps16
Mesangiospermae - ps17
Magnoliids/eudicots - ps18
Eudicots - ps19
Core_eudicots - ps20
Superrosids - ps21
Rosids - ps22
Eurosids - ps23
Malvids - ps24
Sap./Malv./Brassicales - ps25
Malvales/Brassicales - ps26
Brassicales - ps27
Cleomaceae/Brassicaceae - ps28
Brassicaceae - ps29
Camelineae - ps30
Arabidopsis - ps31
Arabidopsis_thaliana - ps32
p-value
2e-18
2e-14
3e-10
4e-06
6e-02
Log-odds
0.5
1.0
1.5
2.0
2.5
3.0
3.5
4.0

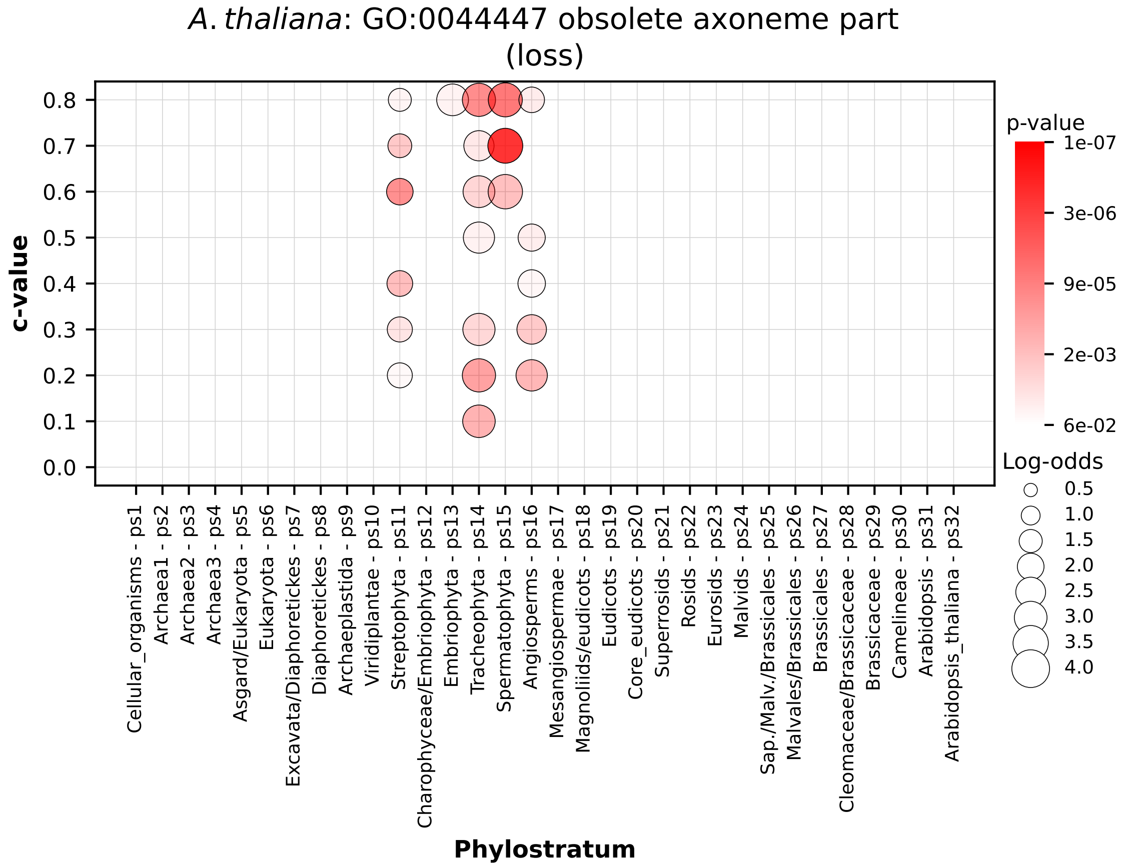

A. thaliana: GO:0044447 obsolete axoneme part
(loss)
c-value
0.0
0.1
0.2
0.3
0.4
0.5
0.6
0.7
0.8
Phylostratum
Cellular_organisms - ps1
Archaea1 - ps2
Archaea2 - ps3
Archaea3 - ps4
Asgard/Eukaryota - ps5
Eukaryota - ps6
Excavata/Diaphoretickes - ps7
Diaphoretickes - ps8
Archaeplastida - ps9
Viridiplantae - ps10
Streptophyta - ps11
Charophyceae/Embriophyta - ps12
Embriophyta - ps13
Tracheophyta - ps14
Spermatophyta - ps15
Angiosperms - ps16
Mesangiospermae - ps17
Magnoliids/eudicots - ps18
Eudicots - ps19
Core_eudicots - ps20
Superrosids - ps21
Rosids - ps22
Eurosids - ps23
Malvids - ps24
Sap./Malv./Brassicales - ps25
Malvales/Brassicales - ps26
Brassicales - ps27
Cleomaceae/Brassicaceae - ps28
Brassicaceae - ps29
Camelineae - ps30
Arabidopsis - ps31
Arabidopsis_thaliana - ps32
p-value
1e-07
3e-06
9e-05
2e-03
6e-02
Log-odds
0.5
1.0
1.5
2.0
2.5
3.0
3.5
4.0

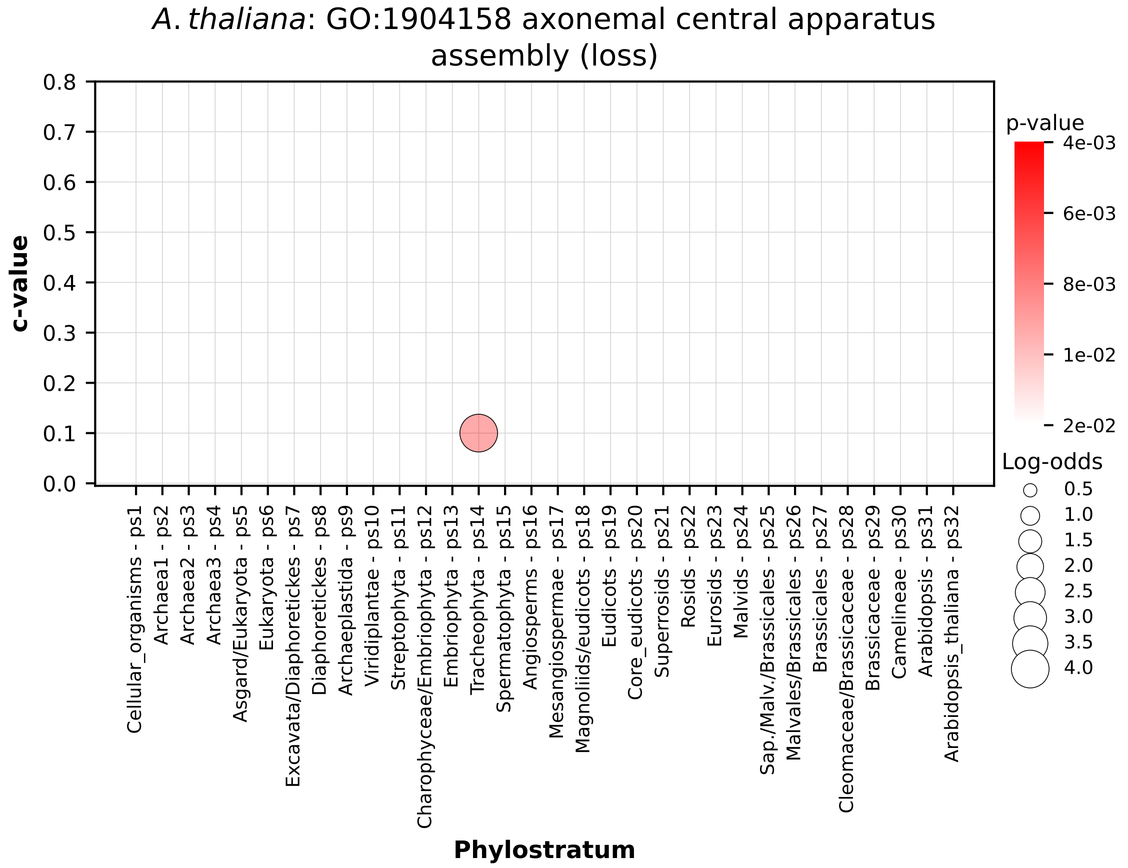

A. thaliana: GO:1904158 axonemal central apparatus assembly (loss)
c-value
0.8
0.7
0.6
0.5
0.4
0.3
0.2
0.1
0.0
Phylostratum
Cellular_organisms - ps1
Archaea1 - ps2
Archaea2 - ps3
Archaea3 - ps4
Asgard/Eukaryota - ps5
Eukaryota - ps6
Excavata/Diaphoretickes - ps7
Diaphoretickes - ps8
Archaeplastida - ps9
Viridiplantae - ps10
Streptophyta - ps11
Charophyceae/Embriophyta - ps12
Embriophyta - ps13
Tracheophyta - ps14
Spermatophyta - ps15
Angiosperms - ps16
Mesangiospermae - ps17
Magnoliids/eudicots - ps18
Eudicots - ps19
Core_eudicots - ps20
Superrosids - ps21
Rosids - ps22
Eurosids - ps23
Malvids - ps24
Sap./Malv./Brassicales - ps25
Malvales/Brassicales - ps26
Brassicales - ps27
Cleomaceae/Brassicaceae - ps28
Brassicaceae - ps29
Camelineae - ps30
Arabidopsis - ps31
Arabidopsis_thaliana - ps32
p-value
4e-03
6e-03
8e-03
1e-02
2e-02
Log-odds
0.5
1.0
1.5
2.0
2.5
3.0
3.5
4.0

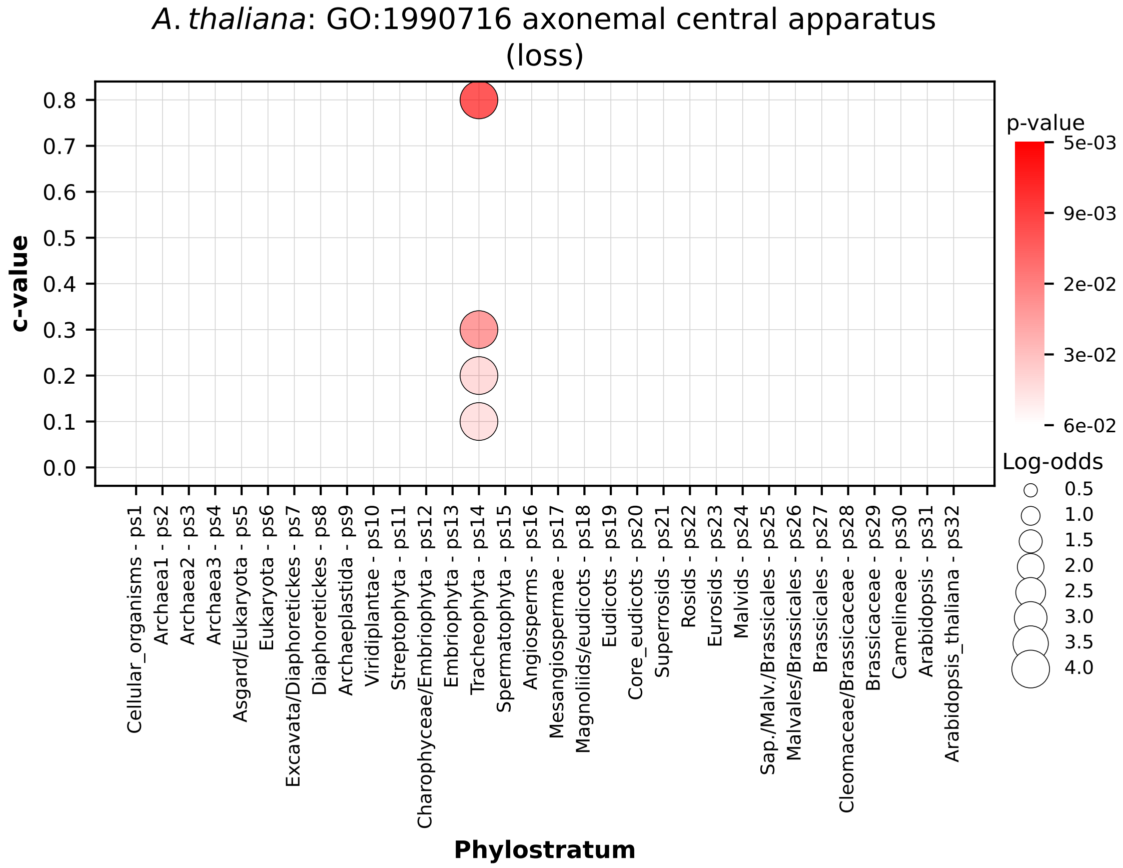

A. thaliana: GO:1990716 axonemal central apparatus (loss)
c-value
0.8
0.7
0.6
0.5
0.4
0.3
0.2
0.1
0.0
p-value
5e-03
9e-03
2e-02
3e-02
6e-02
Log-odds
0.5
1.0
1.5
2.0
2.5
3.0
3.5
4.0
Cellular_organisms - ps1
Archaea1 - ps2
Archaea2 - ps3
Archaea3 - ps4
Asgard/Eukaryota - ps5
Eukaryota - ps6
Excavata/Diaphoretickes - ps7
Diaphoretickes - ps8
Archaeplastida - ps9
Viridiplantae - ps10
Streptophyta - ps11
Charophyceae/Embriophyta - ps12
Embriophyta - ps13
Tracheophyta - ps14
Spermatophyta - ps15
Angiosperms - ps16
Mesangiospermae - ps17
Magnoliids/eudicots - ps18
Eudicots - ps19
Core_eudicots - ps20
Superrosids - ps21
Rosids - ps22
Eurosids - ps23
Malvids - ps24
Sap./Malv./Brassicales - ps25
Malvales/Brassicales - ps26
Brassicales - ps27
Cleomaceae/Brassicaceae - ps28
Brassicaceae - ps29
Camelineae - ps30
Arabidopsis - ps31
Arabidopsis_thaliana - ps32
Phylostratum

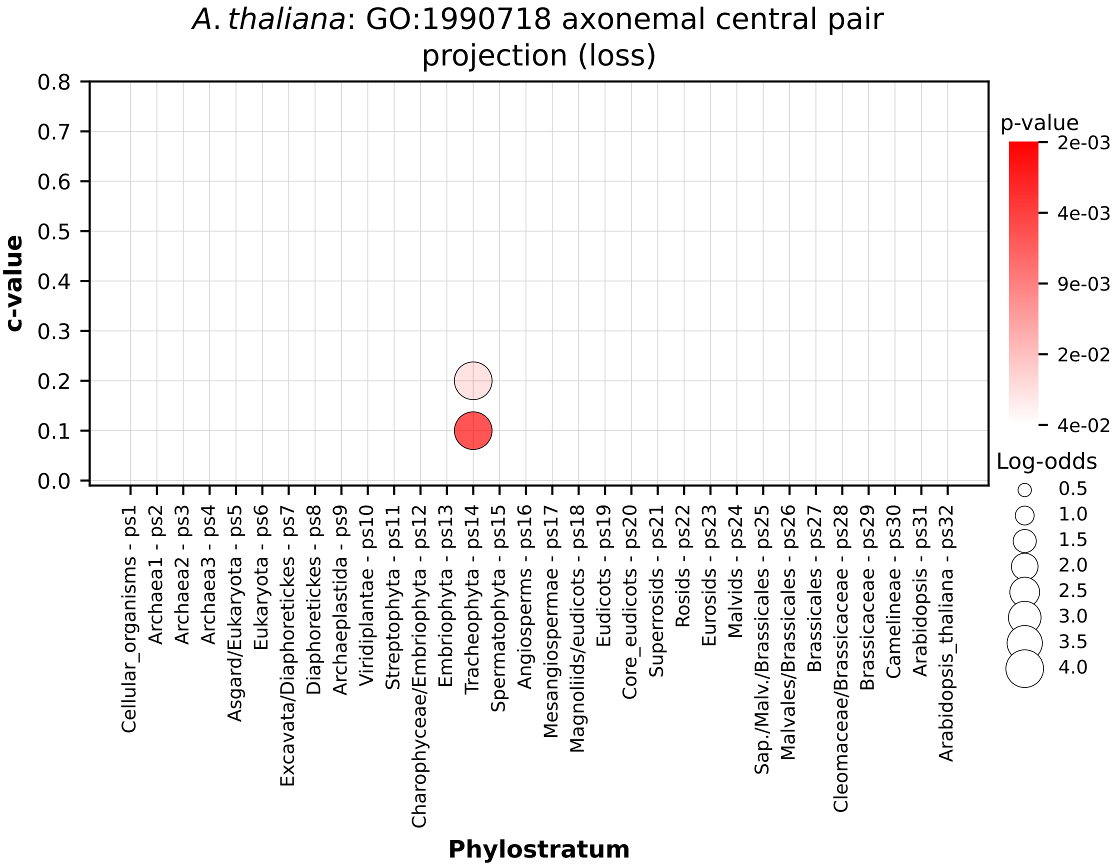

A. thaliana: GO:1990718 axonemal central pair projection (loss)
c-value
0.0
0.1
0.2
0.3
0.4
0.5
0.6
0.7
0.8
Phylostratum
Cellular_organisms - ps1
Archaea1 - ps2
Archaea2 - ps3
Archaea3 - ps4
Asgard/Eukaryota - ps5
Eukaryota - ps6
Excavata/Diaphoretickes - ps7
Diaphoretickes - ps8
Archaeplastida - ps9
Viridiplantae - ps10
Streptophyta - ps11
Charophyceae/Embriophyta - ps12
Embriophyta - ps13
Tracheophyta - ps14
Spermatophyta - ps15
Angiosperms - ps16
Mesangiospermae - ps17
Magnoliids/eudicots - ps18
Eudicots - ps19
Core_eudicots - ps20
Superrosids - ps21
Rosids - ps22
Eurosids - ps23
Malvids - ps24
Sap./Malv./Brassicales - ps25
Malvales/Brassicales - ps26
Brassicales - ps27
Cleomaceae/Brassicaceae - ps28
Brassicaceae - ps29
Camelineae - ps30
Arabidopsis - ps31
Arabidopsis_thaliana - ps32
p-value
2e-03
4e-03
9e-03
2e-02
4e-02
Log-odds
0.5
1.0
1.5
2.0
2.5
3.0
3.5
4.0

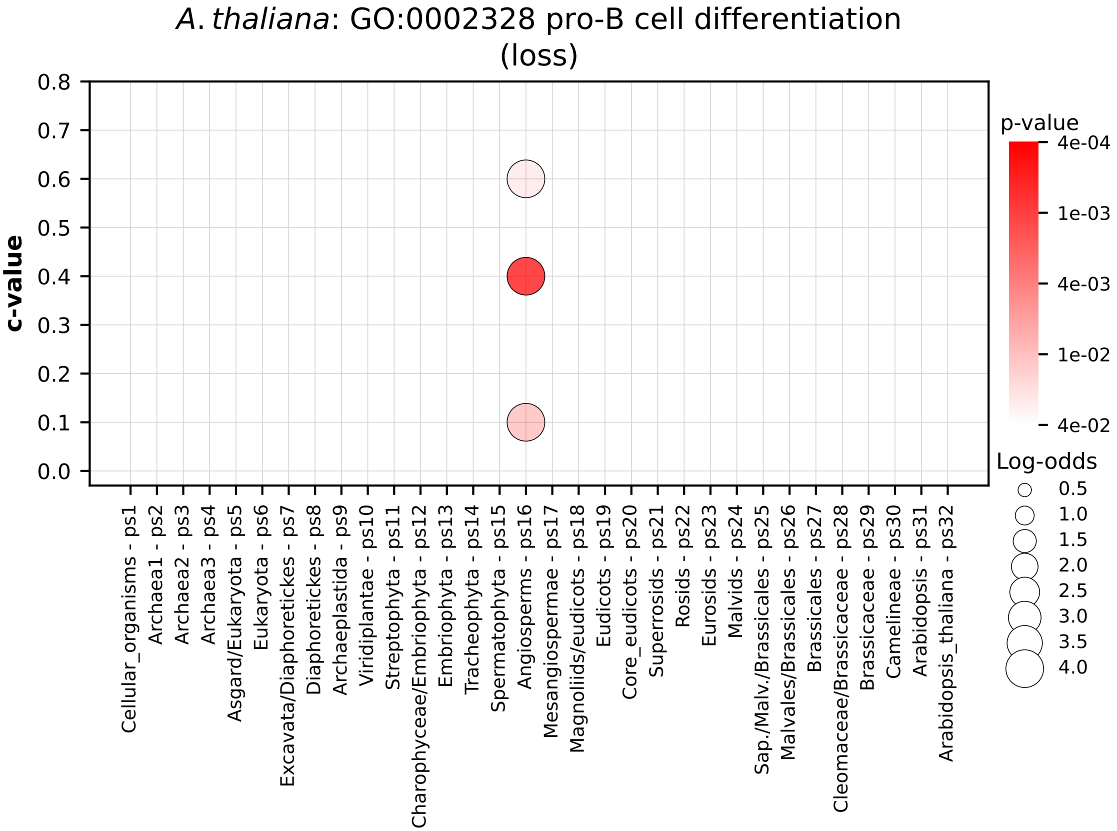
A. thaliana: GO:0002328 pro-B cell differentiation
(loss)
c-value
0.8
0.7
0.6
0.5
0.4
0.3
0.2
0.1
0.0
p-value
4e-04
1e-03
4e-03
1e-02
4e-02
Log-odds
0.5
1.0
1.5
2.0
2.5
3.0
3.5
4.0
Cellular_organisms - ps1
Archaea1 - ps2
Archaea2 - ps3
Archaea3 - ps4
Asgard/Eukaryota - ps5
Eukaryota - ps6
Excavata/Diaphoretickes - ps7
Diaphoretickes - ps8
Archaeplastida - ps9
Viridiplantae - ps10
Streptophyta - ps11
Charophyceae/Embriophyta - ps12
Embriophyta - ps13
Tracheophyta - ps14
Spermatophyta - ps15
Angiosperms - ps16
Mesangiospermae - ps17
Magnoliids/eudicots - ps18
Eudicots - ps19
Core_eudicots - ps20
Superrosids - ps21
Rosids - ps22
Eurosids - ps23
Malvids - ps24
Sap./Malv./Brassicales - ps25
Malvales/Brassicales - ps26
Brassicales - ps27
Cleomaceae/Brassicaceae - ps28
Brassicaceae - ps29
Camelineae - ps30
Arabidopsis - ps31
Arabidopsis_thaliana - ps32
Phylostratum

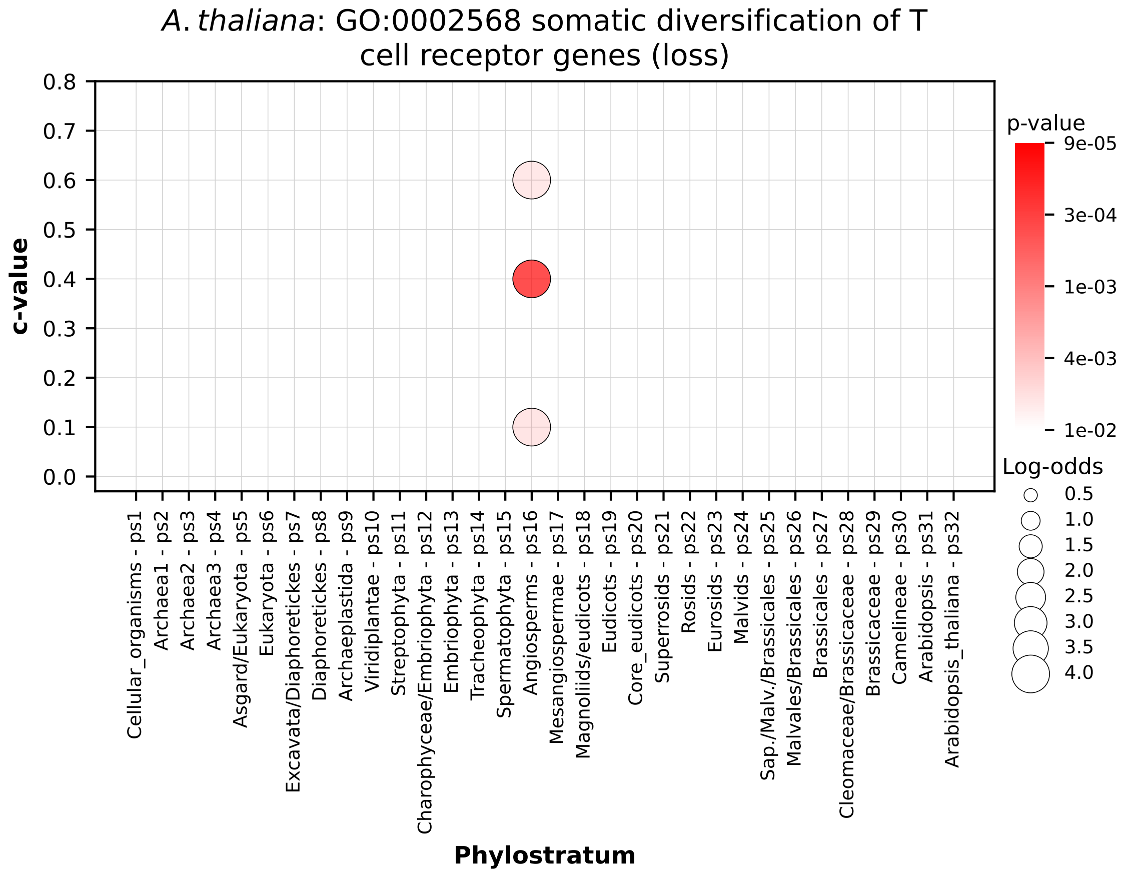
A. thaliana: GO:0002568 somatic diversification of T cell receptor genes (loss)
c-value
0.0
0.1
0.2
0.3
0.4
0.5
0.6
0.7
0.8
p-value
9e-05
3e-04
1e-03
4e-03
1e-02
Log-odds
0.5
1.0
1.5
2.0
2.5
3.0
3.5
4.0
Cellular_organisms - ps1
Archaea1 - ps2
Archaea2 - ps3
Archaea3 - ps4
Asgard/Eukaryota - ps5
Eukaryota - ps6
Excavata/Diaphoretickes - ps7
Diaphoretickes - ps8
Archaeplastida - ps9
Viridiplantae - ps10
Streptophyta - ps11
Charophyceae/Embriophyta - ps12
Embriophyta - ps13
Tracheophyta - ps14
Spermatophyta - ps15
Angiosperms - ps16
Mesangiospermae - ps17
Magnoliids/eudicots - ps18
Eudicots - ps19
Core_eudicots - ps20
Superrosids - ps21
Rosids - ps22
Eurosids - ps23
Malvids - ps24
Sap./Malv./Brassicales - ps25
Malvales/Brassicales - ps26
Brassicales - ps27
Cleomaceae/Brassicaceae - ps28
Brassicaceae - ps29
Camelineae - ps30
Arabidopsis - ps31
Arabidopsis_thaliana - ps32
Phylostratum

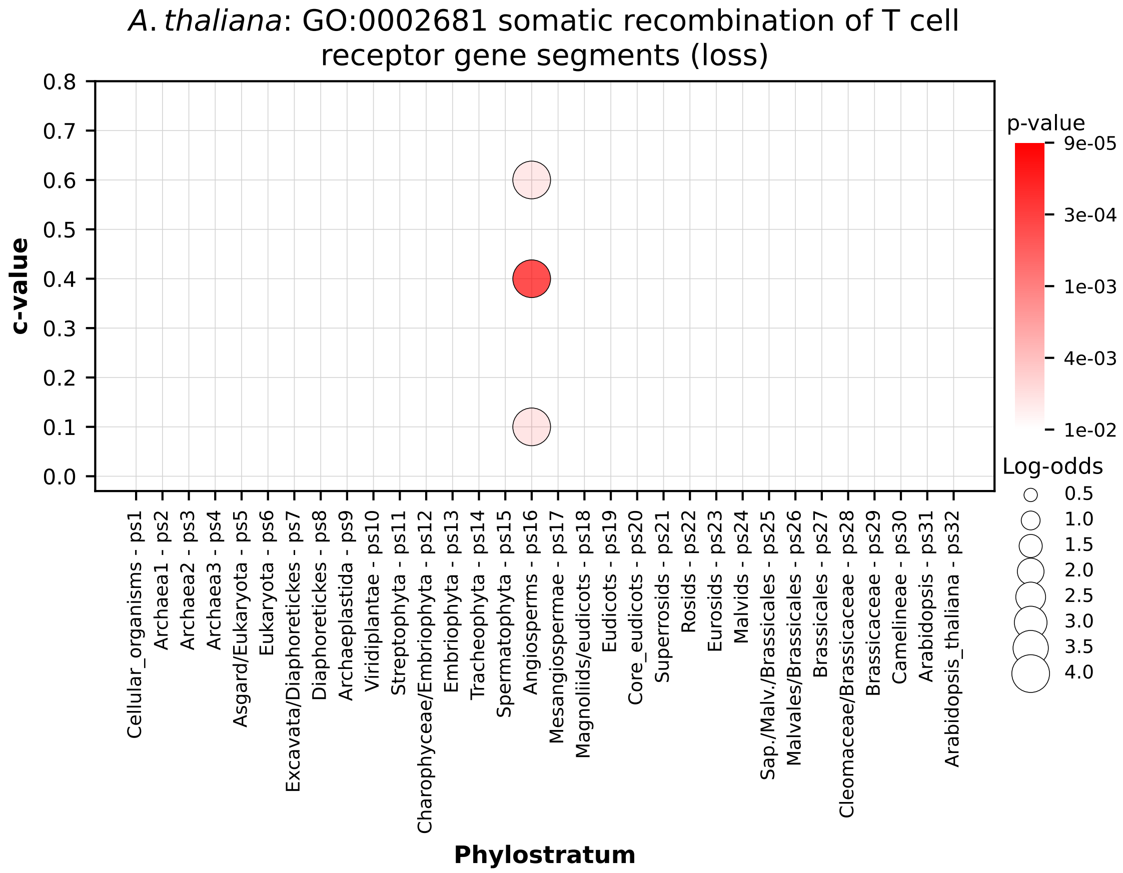
A. thaliana: GO:0002681 somatic recombination of T cell receptor gene segments (loss)
c-value
0.8
0.7
0.6
0.5
0.4
0.3
0.2
0.1
0.0
p-value
9e-05
3e-04
1e-03
4e-03
1e-02
Log-odds
0.5
1.0
1.5
2.0
2.5
3.0
3.5
4.0
Cellular_organisms - ps1
Archaea1 - ps2
Archaea2 - ps3
Archaea3 - ps4
Asgard/Eukaryota - ps5
Eukaryota - ps6
Excavata/Diaphoretickes - ps7
Diaphoretickes - ps8
Archaeplastida - ps9
Viridiplantae - ps10
Streptophyta - ps11
Charophyceae/Embriophyta - ps12
Embriophyta - ps13
Tracheophyta - ps14
Spermatophyta - ps15
Angiosperms - ps16
Mesangiospermae - ps17
Magnoliids/eudicots - ps18
Eudicots - ps19
Core_eudicots - ps20
Superrosids - ps21
Rosids - ps22
Eurosids - ps23
Malvids - ps24
Sap./Malv./Brassicales - ps25
Malvales/Brassicales - ps26
Brassicales - ps27
Cleomaceae/Brassicaceae - ps28
Brassicaceae - ps29
Camelineae - ps30
Arabidopsis - ps31
Arabidopsis_thaliana - ps32
Phylostratum

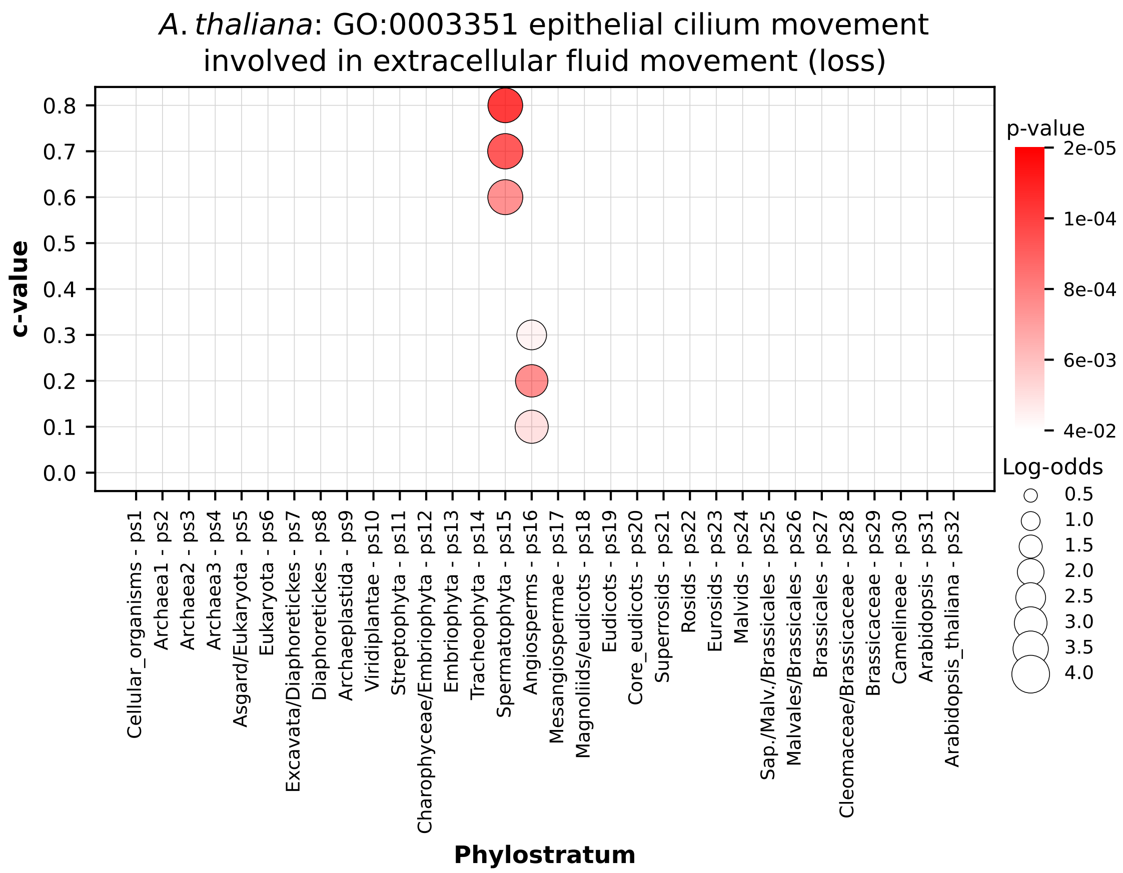
A. thaliana: GO:0003351 epithelial cilium movement involved in extracellular fluid movement (loss)
c-value
0.0
0.1
0.2
0.3
0.4
0.5
0.6
0.7
0.8
Phylostratum
Cellular_organisms - ps1
Archaea1 - ps2
Archaea2 - ps3
Archaea3 - ps4
Asgard/Eukaryota - ps5
Eukaryota - ps6
Excavata/Diaphoretickes - ps7
Diaphoretickes - ps8
Archaeplastida - ps9
Viridiplantae - ps10
Streptophyta - ps11
Charophyceae/Embriophyta - ps12
Embriophyta - ps13
Tracheophyta - ps14
Spermatophyta - ps15
Angiosperms - ps16
Mesangiospermae - ps17
Magnoliids/eudicots - ps18
Eudicots - ps19
Core_eudicots - ps20
Superrosids - ps21
Rosids - ps22
Eurosids - ps23
Malvids - ps24
Sap./Malv./Brassicales - ps25
Malvales/Brassicales - ps26
Brassicales - ps27
Cleomaceae/Brassicaceae - ps28
Brassicaceae - ps29
Camelineae - ps30
Arabidopsis - ps31
Arabidopsis_thaliana - ps32
p-value
2e-05
1e-04
8e-04
6e-03
4e-02
Log-odds
0.5
1.0
1.5
2.0
2.5
3.0
3.5
4.0

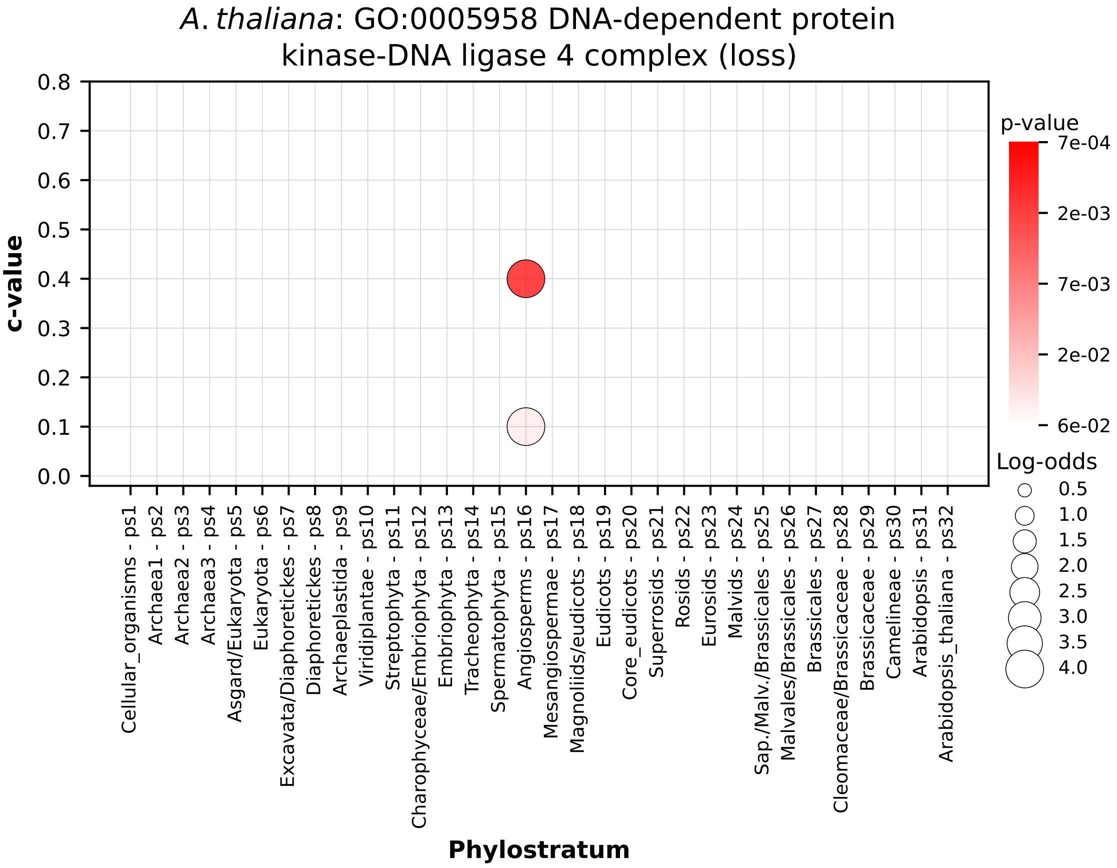

A. thaliana: GO:0005958 DNA-dependent protein kinase-DNA ligase 4 complex (loss)
c-value
0.8
0.7
0.6
0.5
0.4
0.3
0.2
0.1
0.0
Phylostratum
Cellular_organisms - ps1
Archaea1 - ps2
Archaea2 - ps3
Archaea3 - ps4
Asgard/Eukaryota - ps5
Eukaryota - ps6
Excavata/Diaphoretickes - ps7
Diaphoretickes - ps8
Archaeplastida - ps9
Viridiplantae - ps10
Streptophyta - ps11
Charophyceae/Embriophyta - ps12
Embriophyta - ps13
Tracheophyta - ps14
Spermatophyta - ps15
Angiosperms - ps16
Mesangiospermae - ps17
Magnoliids/eudicots - ps18
Eudicots - ps19
Core_eudicots - ps20
Superrosids - ps21
Rosids - ps22
Eurosids - ps23
Malvids - ps24
Sap./Malv./Brassicales - ps25
Malvales/Brassicales - ps26
Brassicales - ps27
Cleomaceae/Brassicaceae - ps28
Brassicaceae - ps29
Camelineae - ps30
Arabidopsis - ps31
Arabidopsis_thaliana - ps32
p-value
7e-04
2e-03
7e-03
2e-02
6e-02
Log-odds
0.5
1.0
1.5
2.0
2.5
3.0
3.5
4.0

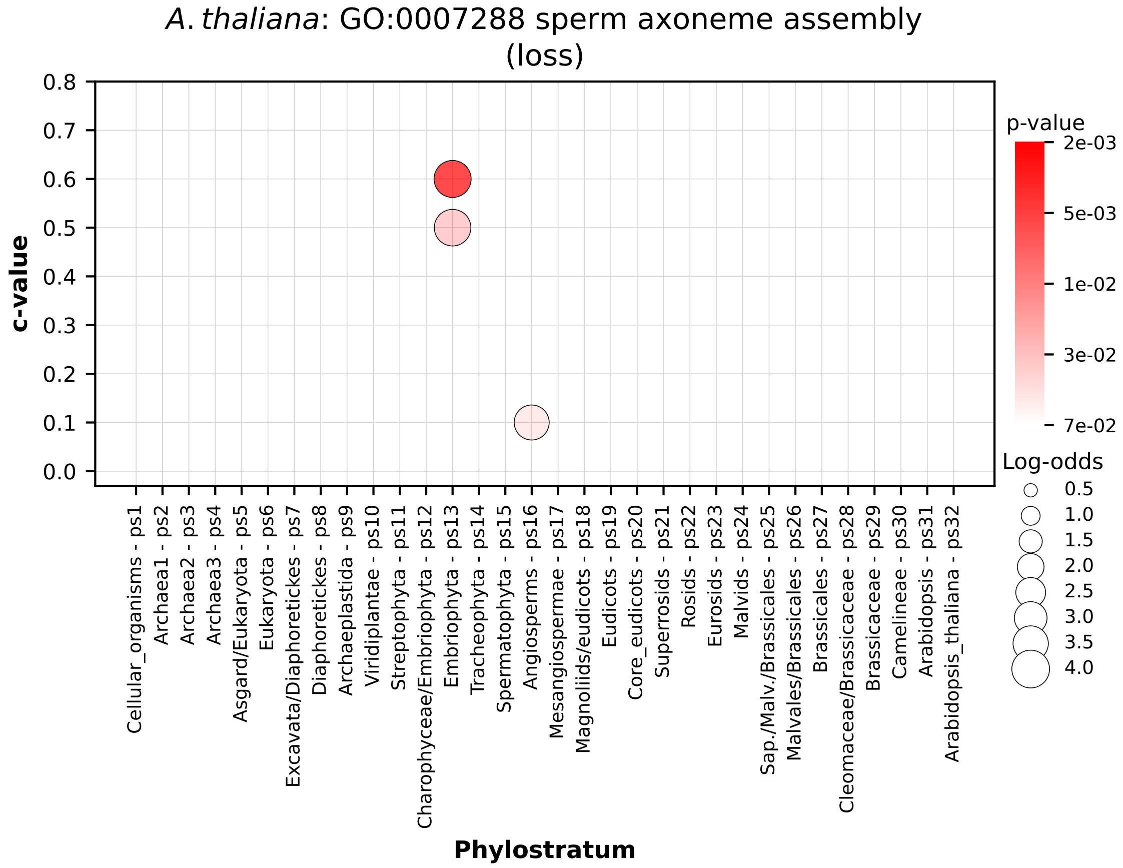
A. thaliana: GO:0007288 sperm axoneme assembly
(loss)
c-value
0.8
0.7
0.6
0.5
0.4
0.3
0.2
0.1
0.0
p-value
2e-03
5e-03
1e-02
3e-02
7e-02
Log-odds
0.5
1.0
1.5
2.0
2.5
3.0
3.5
4.0
Cellular_organisms - ps1
Archaea1 - ps2
Archaea2 - ps3
Archaea3 - ps4
Asgard/Eukaryota - ps5
Eukaryota - ps6
Excavata/Diaphoretickes - ps7
Diaphoretickes - ps8
Archaeplastida - ps9
Viridiplantae - ps10
Streptophyta - ps11
Charophyceae/Embriophyta - ps12
Embriophyta - ps13
Tracheophyta - ps14
Spermatophyta - ps15
Angiosperms - ps16
Mesangiospermae - ps17
Magnoliids/eudicots - ps18
Eudicots - ps19
Core_eudicots - ps20
Superrosids - ps21
Rosids - ps22
Eurosids - ps23
Malvids - ps24
Sap./Malv./Brassicales - ps25
Malvales/Brassicales - ps26
Brassicales - ps27
Cleomaceae/Brassicaceae - ps28
Brassicaceae - ps29
Camelineae - ps30
Arabidopsis - ps31
Arabidopsis_thaliana - ps32
Phylostratum

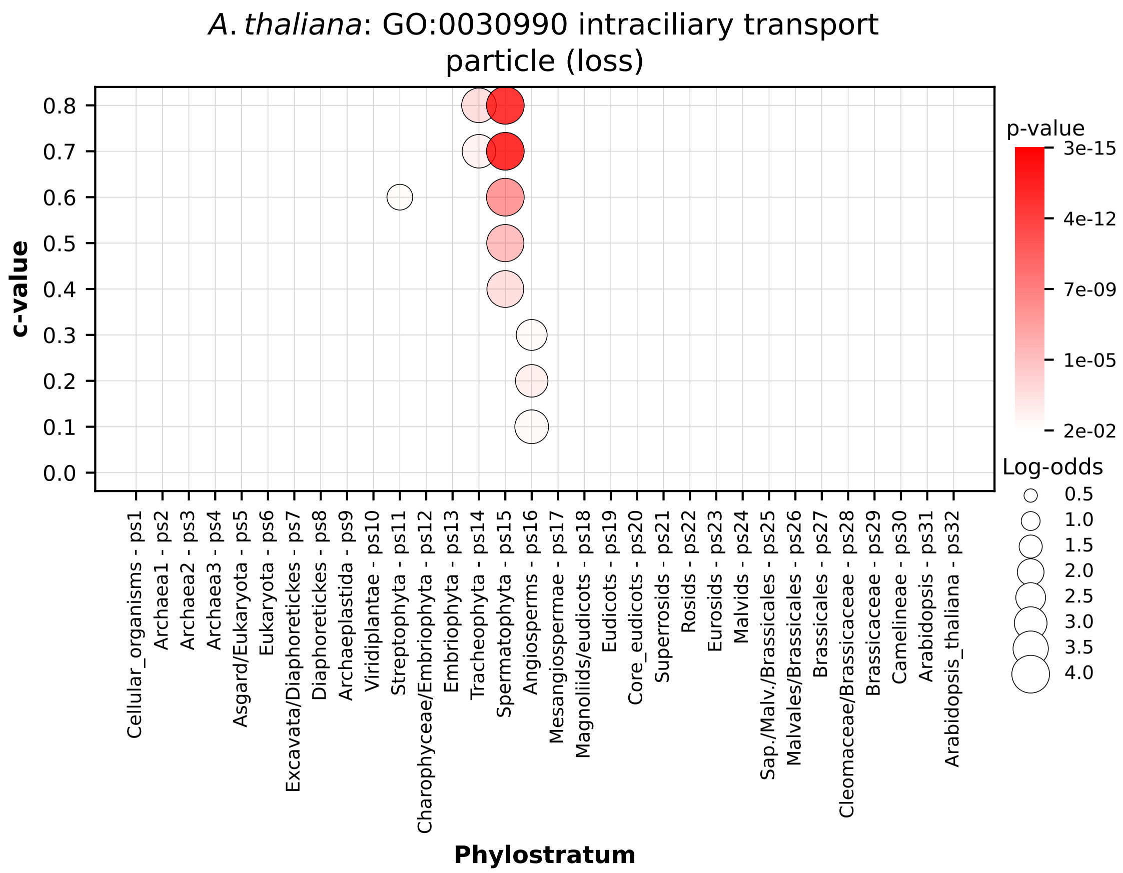

A. thaliana: GO:0030990 intraciliary transport particle (loss)
c-value
0.8
0.7
0.6
0.5
0.4
0.3
0.2
0.1
0.0
p-value
3e-15
4e-12
7e-09
1e-05
2e-02
Log-odds
0.5
1.0
1.5
2.0
2.5
3.0
3.5
4.0
Cellular_organisms - ps1
Archaea1 - ps2
Archaea2 - ps3
Archaea3 - ps4
Asgard/Eukaryota - ps5
Eukaryota - ps6
Excavata/Diaphoretickes - ps7
Diaphoretickes - ps8
Archaeplastida - ps9
Viridiplantae - ps10
Streptophyta - ps11
Charophyceae/Embriophyta - ps12
Embriophyta - ps13
Tracheophyta - ps14
Spermatophyta - ps15
Angiosperms - ps16
Mesangiospermae - ps17
Magnoliids/eudicots - ps18
Eudicots - ps19
Core_eudicots - ps20
Superrosids - ps21
Rosids - ps22
Eurosids - ps23
Malvids - ps24
Sap./Malv./Brassicales - ps25
Malvales/Brassicales - ps26
Brassicales - ps27
Cleomaceae/Brassicaceae - ps28
Brassicaceae - ps29
Camelineae - ps30
Arabidopsis - ps31
Arabidopsis_thaliana - ps32
Phylostratum

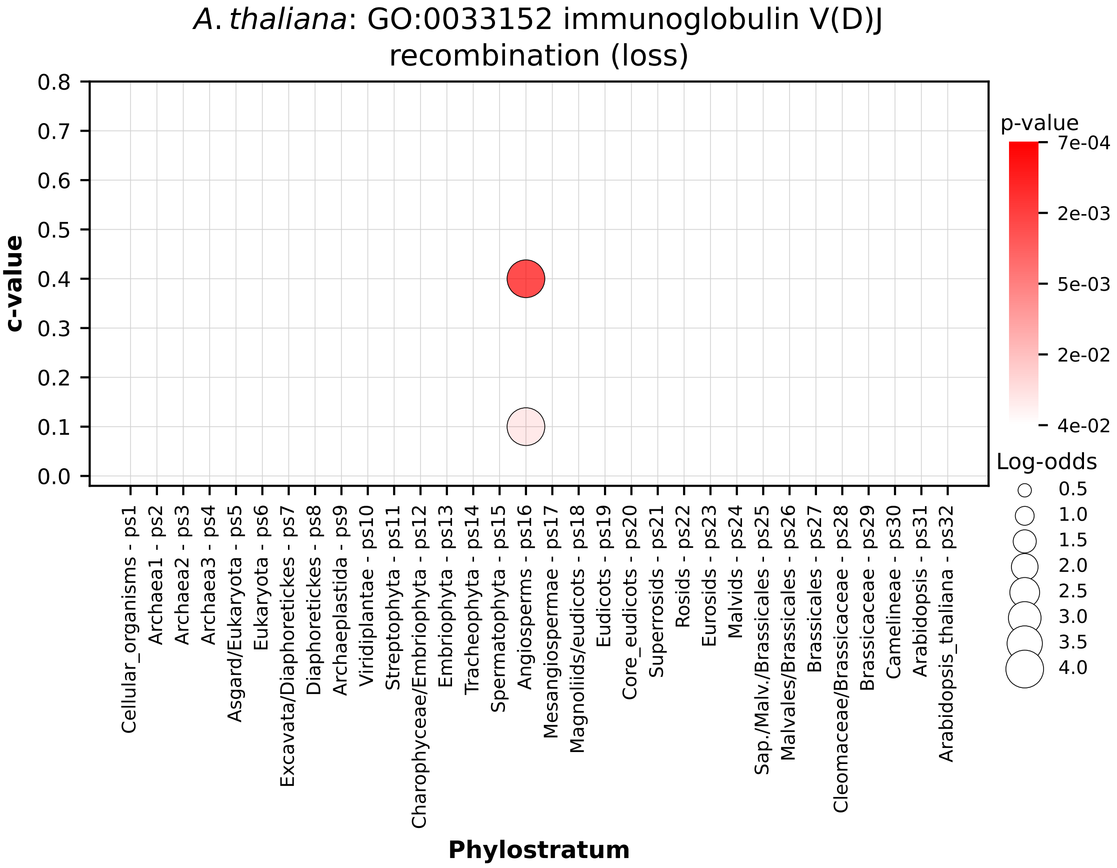

A. thaliana: GO:0033152 immunoglobulin V(D)J recombination (loss)
c-value
0.8
0.7
0.6
0.5
0.4
0.3
0.2
0.1
0.0
Phylostratum
Cellular_organisms - ps1
Archaea1 - ps2
Archaea2 - ps3
Archaea3 - ps4
Asgard/Eukaryota - ps5
Eukaryota - ps6
Excavata/Diaphoretickes - ps7
Diaphoretickes - ps8
Archaeplastida - ps9
Viridiplantae - ps10
Streptophyta - ps11
Charophyceae/Embriophyta - ps12
Embriophyta - ps13
Tracheophyta - ps14
Spermatophyta - ps15
Angiosperms - ps16
Mesangiospermae - ps17
Magnoliids/eudicots - ps18
Eudicots - ps19
Core_eudicots - ps20
Superrosids - ps21
Rosids - ps22
Eurosids - ps23
Malvids - ps24
Sap./Malv./Brassicales - ps25
Malvales/Brassicales - ps26
Brassicales - ps27
Cleomaceae/Brassicaceae - ps28
Brassicaceae - ps29
Camelineae - ps30
Arabidopsis - ps31
Arabidopsis_thaliana - ps32
p-value
7e-04
2e-03
5e-03
2e-02
4e-02
Log-odds
0.5
1.0
1.5
2.0
2.5
3.0
3.5
4.0

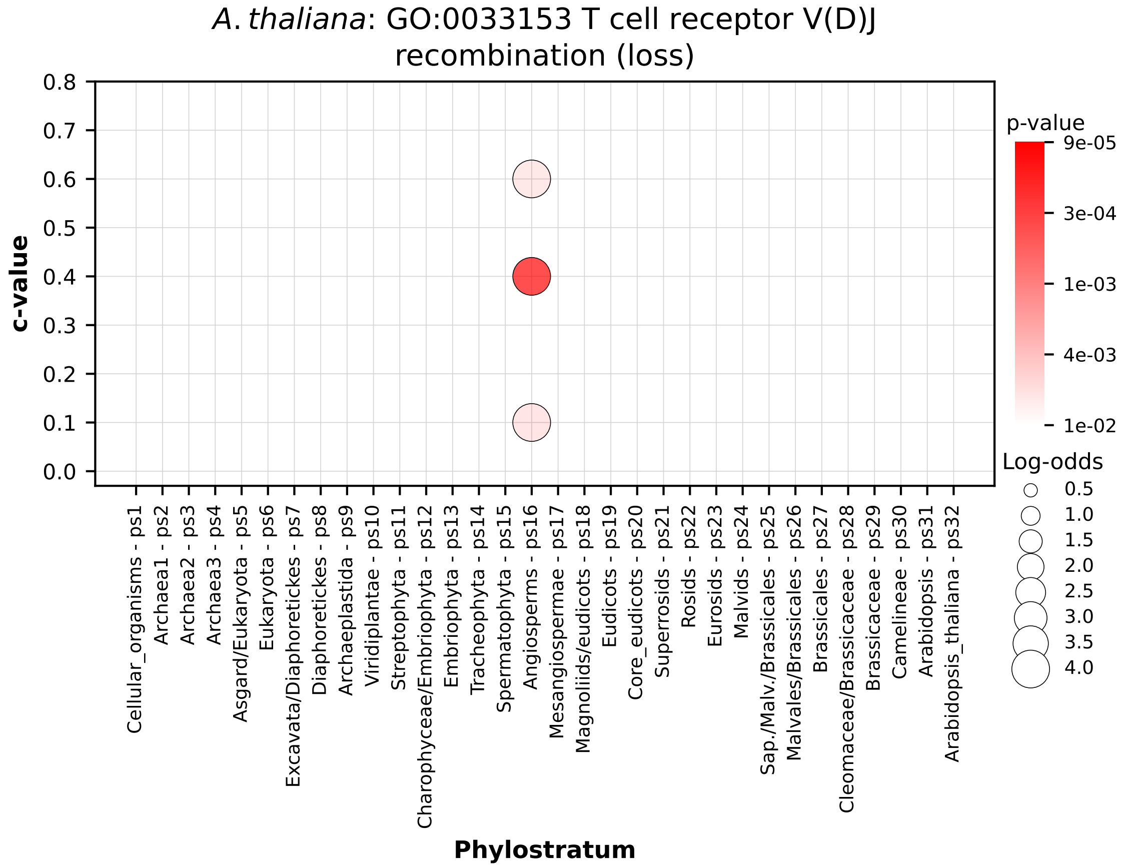

A. thaliana: GO:0033153 T cell receptor V(D)J recombination (loss)
c-value
0.0
0.1
0.2
0.3
0.4
0.5
0.6
0.7
0.8
Phylostratum
Cellular_organisms - ps1
Archaea1 - ps2
Archaea2 - ps3
Archaea3 - ps4
Asgard/Eukaryota - ps5
Eukaryota - ps6
Excavata/Diaphoretickes - ps7
Diaphoretickes - ps8
Archaeplastida - ps9
Viridiplantae - ps10
Streptophyta - ps11
Charophyceae/Embriophyta - ps12
Embriophyta - ps13
Tracheophyta - ps14
Spermatophyta - ps15
Angiosperms - ps16
Mesangiospermae - ps17
Magnoliids/eudicots - ps18
Eudicots - ps19
Core_eudicots - ps20
Superrosids - ps21
Rosids - ps22
Eurosids - ps23
Malvids - ps24
Sap./Malv./Brassicales - ps25
Malvales/Brassicales - ps26
Brassicales - ps27
Cleomaceae/Brassicaceae - ps28
Brassicaceae - ps29
Camelineae - ps30
Arabidopsis - ps31
Arabidopsis_thaliana - ps32
p-value
9e-05
3e-04
1e-03
4e-03
1e-02
Log-odds
0.5
1.0
1.5
2.0
2.5
3.0
3.5
4.0

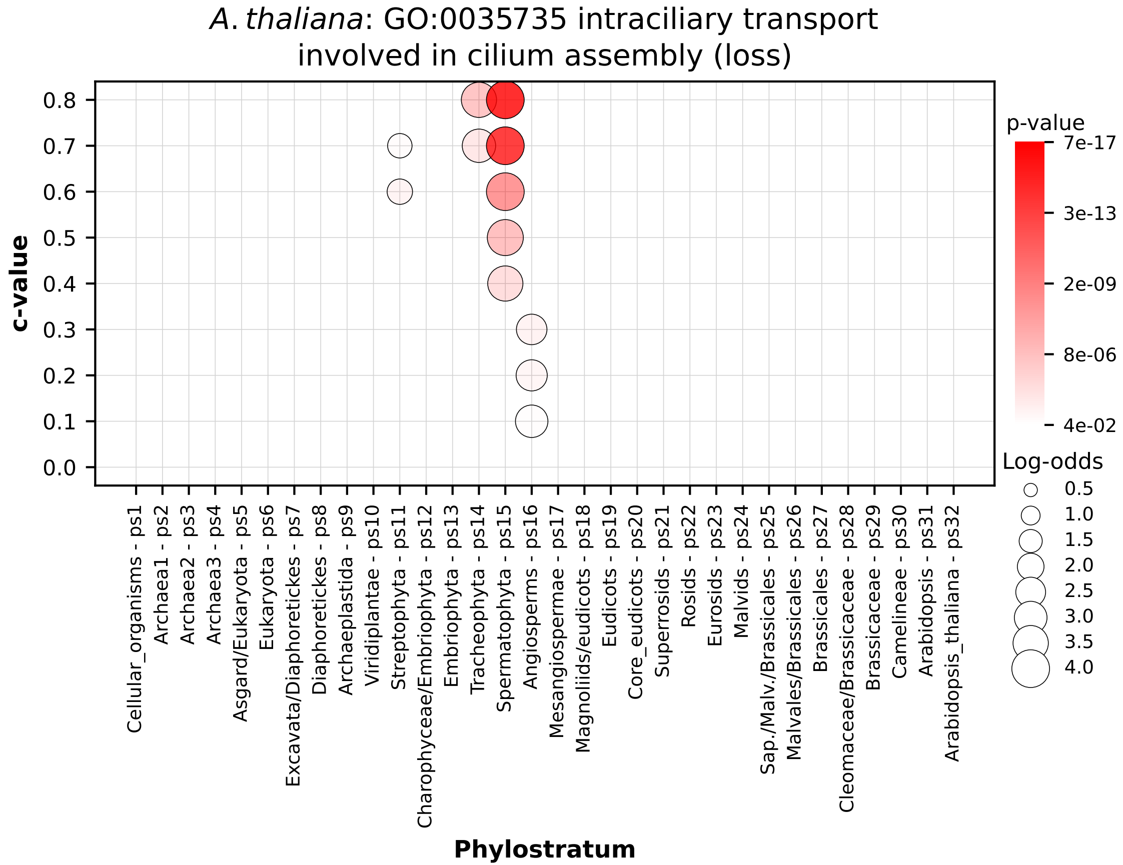

A. thaliana: GO:0035735 intraciliary transport involved in cilium assembly (loss)
c-value
0.0
0.1
0.2
0.3
0.4
0.5
0.6
0.7
0.8
Phylostratum
Cellular_organisms - ps1
Archaea1 - ps2
Archaea2 - ps3
Archaea3 - ps4
Asgard/Eukaryota - ps5
Eukaryota - ps6
Excavata/Diaphoretickes - ps7
Diaphoretickes - ps8
Archaeplastida - ps9
Viridiplantae - ps10
Streptophyta - ps11
Charophyceae/Embriophyta - ps12
Embriophyta - ps13
Tracheophyta - ps14
Spermatophyta - ps15
Angiosperms - ps16
Mesangiospermae - ps17
Magnoliids/eudicots - ps18
Eudicots - ps19
Core_eudicots - ps20
Superrosids - ps21
Rosids - ps22
Eurosids - ps23
Malvids - ps24
Sap./Malv./Brassicales - ps25
Malvales/Brassicales - ps26
Brassicales - ps27
Cleomaceae/Brassicaceae - ps28
Brassicaceae - ps29
Camelineae - ps30
Arabidopsis - ps31
Arabidopsis_thaliana - ps32
p-value
7e-17
3e-13
2e-09
8e-06
4e-02
Log-odds
0.5
1.0
1.5
2.0
2.5
3.0
3.5
4.0

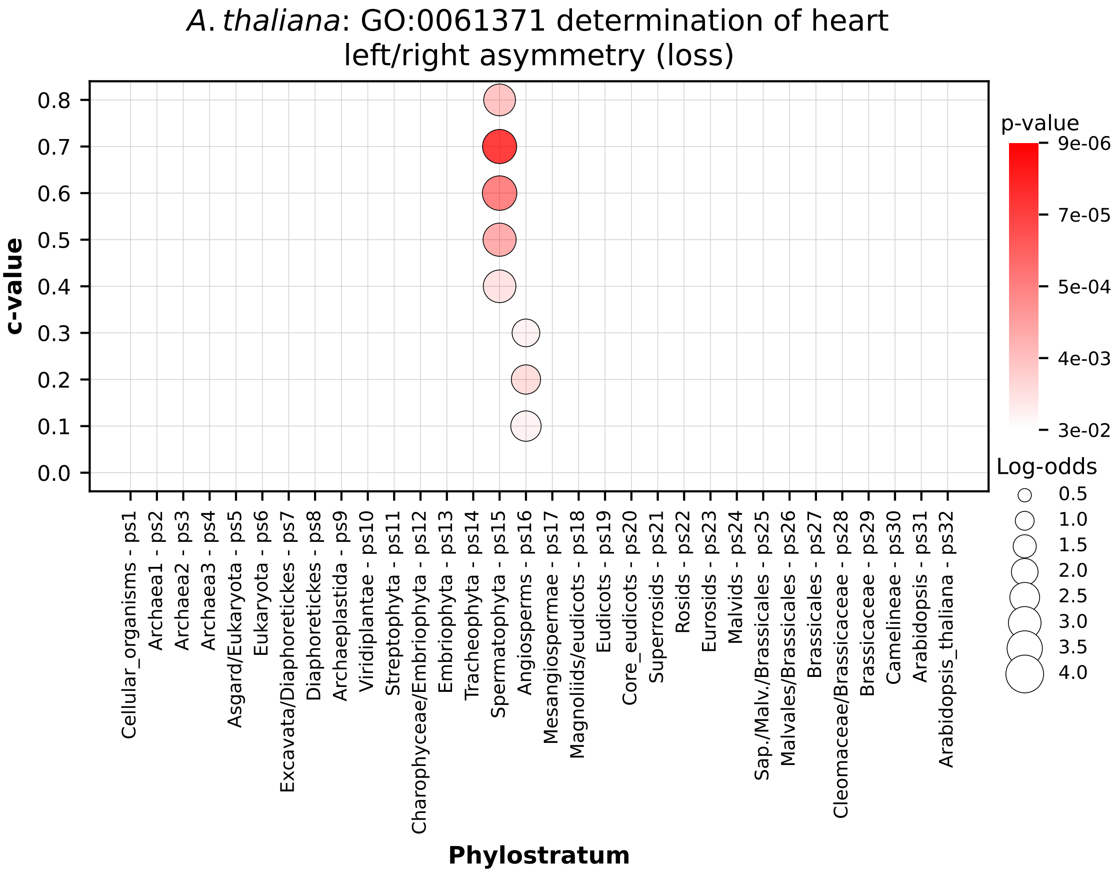

A. thaliana: GO:0061371 determination of heart left/right asymmetry (loss)
c-value
0.0
0.1
0.2
0.3
0.4
0.5
0.6
0.7
0.8
p-value
9e-06
7e-05
5e-04
4e-03
3e-02
Log-odds
0.5
1.0
1.5
2.0
2.5
3.0
3.5
4.0
Cellular_organisms - ps1
Archaea1 - ps2
Archaea2 - ps3
Archaea3 - ps4
Asgard/Eukaryota - ps5
Eukaryota - ps6
Excavata/Diaphoretickes - ps7
Diaphoretickes - ps8
Archaeplastida - ps9
Viridiplantae - ps10
Streptophyta - ps11
Charophyceae/Embriophyta - ps12
Embriophyta - ps13
Tracheophyta - ps14
Spermatophyta - ps15
Angiosperms - ps16
Mesangiospermae - ps17
Magnoliids/eudicots - ps18
Eudicots - ps19
Core_eudicots - ps20
Superrosids - ps21
Rosids - ps22
Eurosids - ps23
Malvids - ps24
Sap./Malv./Brassicales - ps25
Malvales/Brassicales - ps26
Brassicales - ps27
Cleomaceae/Brassicaceae - ps28
Brassicaceae - ps29
Camelineae - ps30
Arabidopsis - ps31
Arabidopsis_thaliana - ps32
Phylostratum

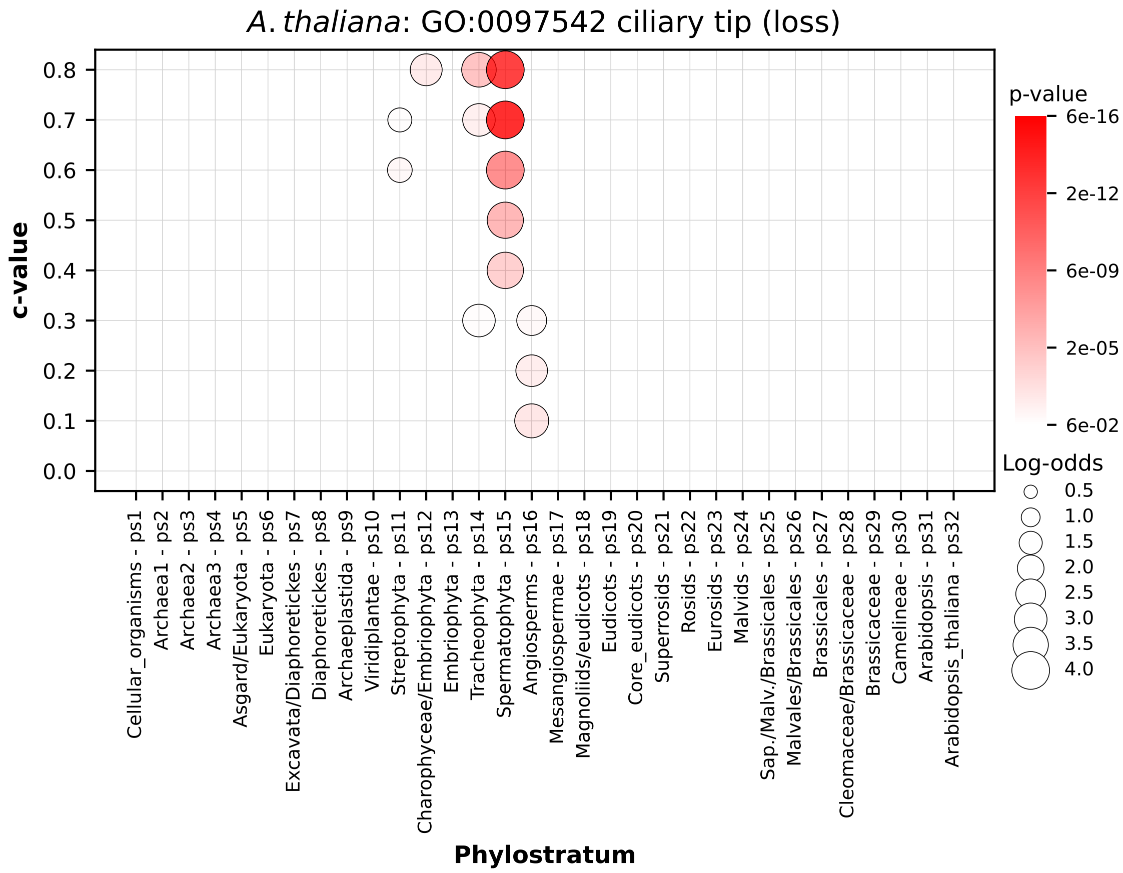

A. thaliana: GO:0097542 ciliary tip (loss)
c-value
0.8
0.7
0.6
0.5
0.4
0.3
0.2
0.1
0.0
p-value
6e-16
2e-12
6e-09
2e-05
6e-02
Log-odds
0.5
1.0
1.5
2.0
2.5
3.0
3.5
4.0
Cellular_organisms - ps1
Archaea1 - ps2
Archaea2 - ps3
Archaea3 - ps4
Asgard/Eukaryota - ps5
Eukaryota - ps6
Excavata/Diaphoretickes - ps7
Diaphoretickes - ps8
Archaeplastida - ps9
Viridiplantae - ps10
Streptophyta - ps11
Charophyceae/Embriophyta - ps12
Embriophyta - ps13
Tracheophyta - ps14
Spermatophyta - ps15
Angiosperms - ps16
Mesangiospermae - ps17
Magnoliids/eudicots - ps18
Eudicots - ps19
Core_eudicots - ps20
Superrosids - ps21
Rosids - ps22
Eurosids - ps23
Malvids - ps24
Sap./Malv./Brassicales - ps25
Malvales/Brassicales - ps26
Brassicales - ps27
Cleomaceae/Brassicaceae - ps28
Brassicaceae - ps29
Camelineae - ps30
Arabidopsis - ps31
Arabidopsis_thaliana - ps32
Phylostratum

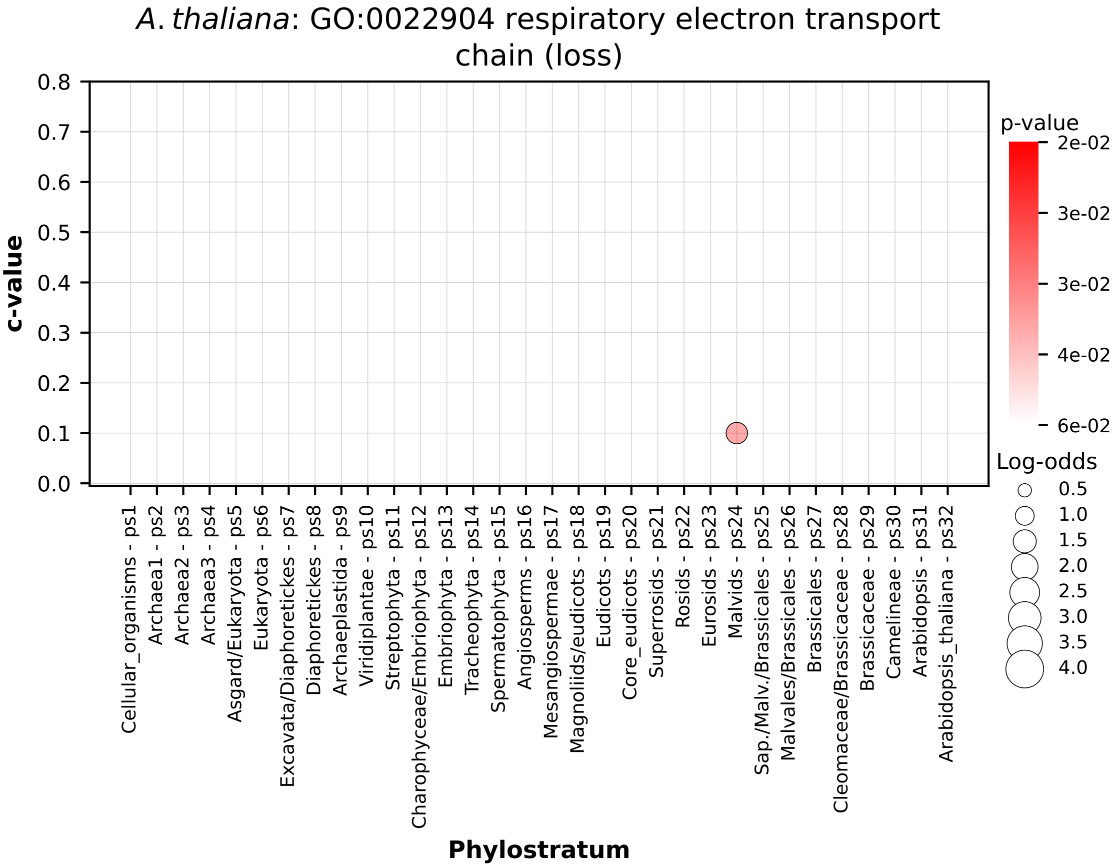
A. thaliana: GO:0022904 respiratory electron transport chain (loss)
c-value
0.0
0.1
0.2
0.3
0.4
0.5
0.6
0.7
0.8
Phylostratum
Cellular_organisms - ps1
Archaea1 - ps2
Archaea2 - ps3
Archaea3 - ps4
Asgard/Eukaryota - ps5
Eukaryota - ps6
Excavata/Diaphoretickes - ps7
Diaphoretickes - ps8
Archaeplastida - ps9
Viridiplantae - ps10
Streptophyta - ps11
Charophyceae/Embriophyta - ps12
Embriophyta - ps13
Tracheophyta - ps14
Spermatophyta - ps15
Angiosperms - ps16
Mesangiospermae - ps17
Magnoliids/eudicots - ps18
Eudicots - ps19
Core_eudicots - ps20
Superrosids - ps21
Rosids - ps22
Eurosids - ps23
Malvids - ps24
Sap./Malv./Brassicales - ps25
Malvales/Brassicales - ps26
Brassicales - ps27
Cleomaceae/Brassicaceae - ps28
Brassicaceae - ps29
Camelineae - ps30
Arabidopsis - ps31
Arabidopsis_thaliana - ps32
p-value
2e-02
3e-02
3e-02
4e-02
6e-02
Log-odds
0.5
1.0
1.5
2.0
2.5
3.0
3.5
4.0

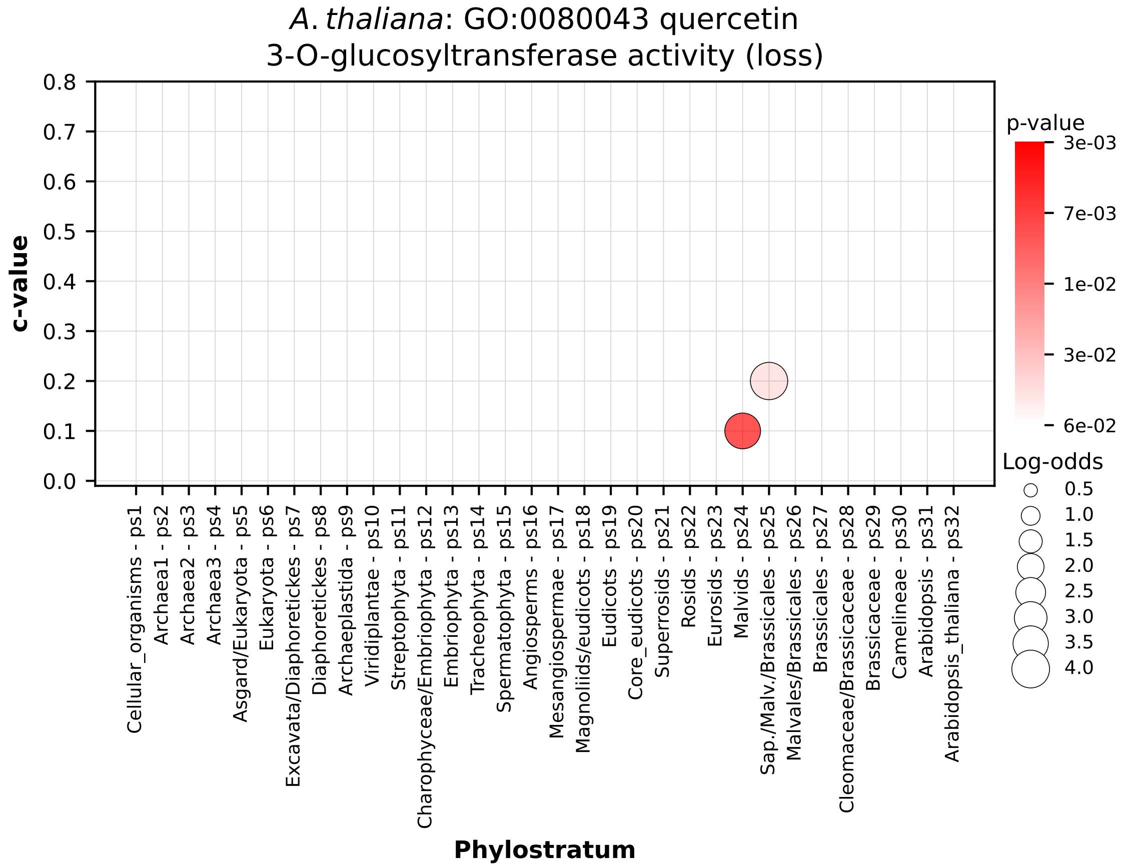

A. thaliana: GO:0080043 quercetin 3-O-glucosyltransferase activity (loss)
c-value
0.8
0.7
0.6
0.5
0.4
0.3
0.2
0.1
0.0
Phylostratum
Cellular_organisms - ps1
Archaea1 - ps2
Archaea2 - ps3
Archaea3 - ps4
Asgard/Eukaryota - ps5
Eukaryota - ps6
Excavata/Diaphoretickes - ps7
Diaphoretickes - ps8
Archaeplastida - ps9
Viridiplantae - ps10
Streptophyta - ps11
Charophyceae/Embriophyta - ps12
Embriophyta - ps13
Tracheophyta - ps14
Spermatophyta - ps15
Angiosperms - ps16
Mesangiospermae - ps17
Magnoliids/eudicots - ps18
Eudicots - ps19
Core_eudicots - ps20
Superrosids - ps21
Rosids - ps22
Eurosids - ps23
Malvids - ps24
Sap./Malv./Brassicales - ps25
Malvales/Brassicales - ps26
Brassicales - ps27
Cleomaceae/Brassicaceae - ps28
Brassicaceae - ps29
Camelineae - ps30
Arabidopsis - ps31
Arabidopsis_thaliana - ps32
p-value
3e-03
7e-03
1e-02
3e-02
6e-02
Log-odds
0.5
1.0
1.5
2.0
2.5
3.0
3.5
4.0

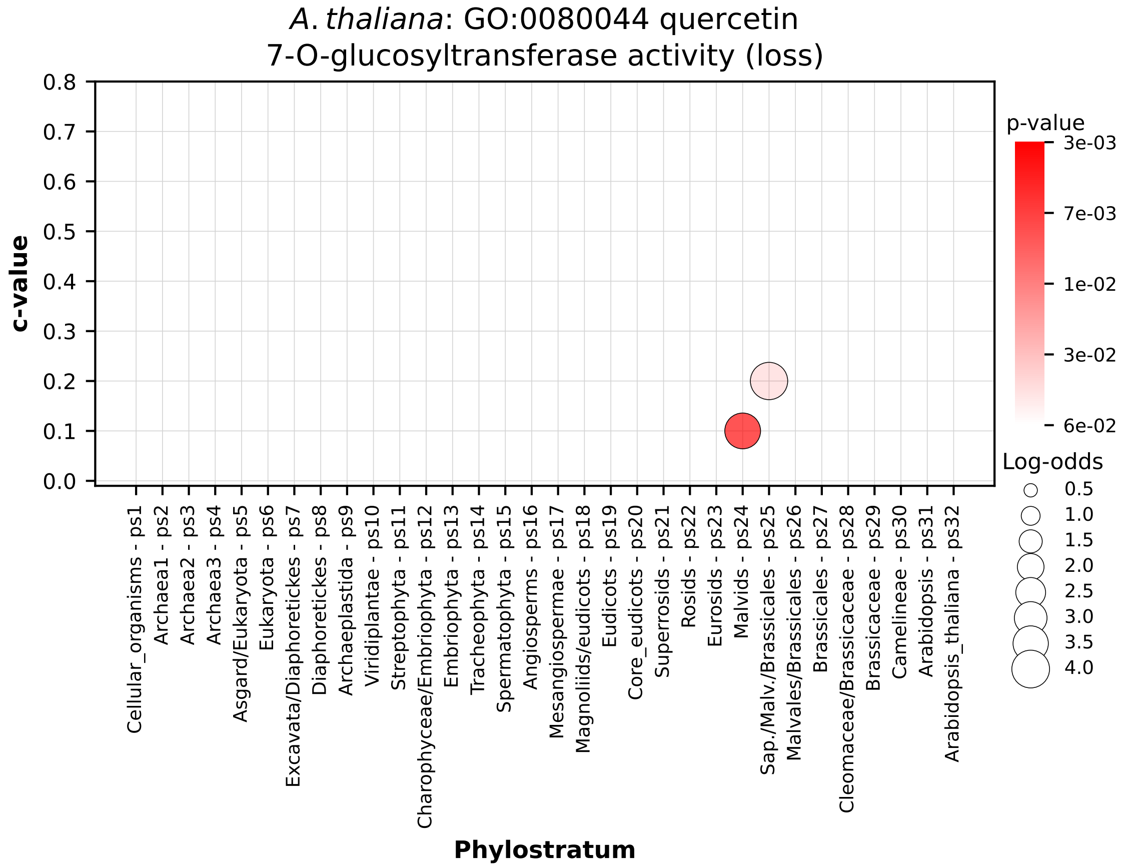

A. thaliana: GO:0080044 quercetin 7-O-glucosyltransferase activity (loss)
c-value
0.8
0.7
0.6
0.5
0.4
0.3
0.2
0.1
0.0
p-value
3e-03
7e-03
1e-02
3e-02
6e-02
Log-odds
0.5
1.0
1.5
2.0
2.5
3.0
3.5
4.0
Cellular_organisms - ps1
Archaea1 - ps2
Archaea2 - ps3
Archaea3 - ps4
Asgard/Eukaryota - ps5
Eukaryota - ps6
Excavata/Diaphoretickes - ps7
Diaphoretickes - ps8
Archaeplastida - ps9
Viridiplantae - ps10
Streptophyta - ps11
Charophyceae/Embriophyta - ps12
Embriophyta - ps13
Tracheophyta - ps14
Spermatophyta - ps15
Angiosperms - ps16
Mesangiospermae - ps17
Magnoliids/eudicots - ps18
Eudicots - ps19
Core_eudicots - ps20
Superrosids - ps21
Rosids - ps22
Eurosids - ps23
Malvids - ps24
Sap./Malv./Brassicales - ps25
Malvales/Brassicales - ps26
Brassicales - ps27
Cleomaceae/Brassicaceae - ps28
Brassicaceae - ps29
Camelineae - ps30
Arabidopsis - ps31
Arabidopsis_thaliana - ps32
Phylostratum

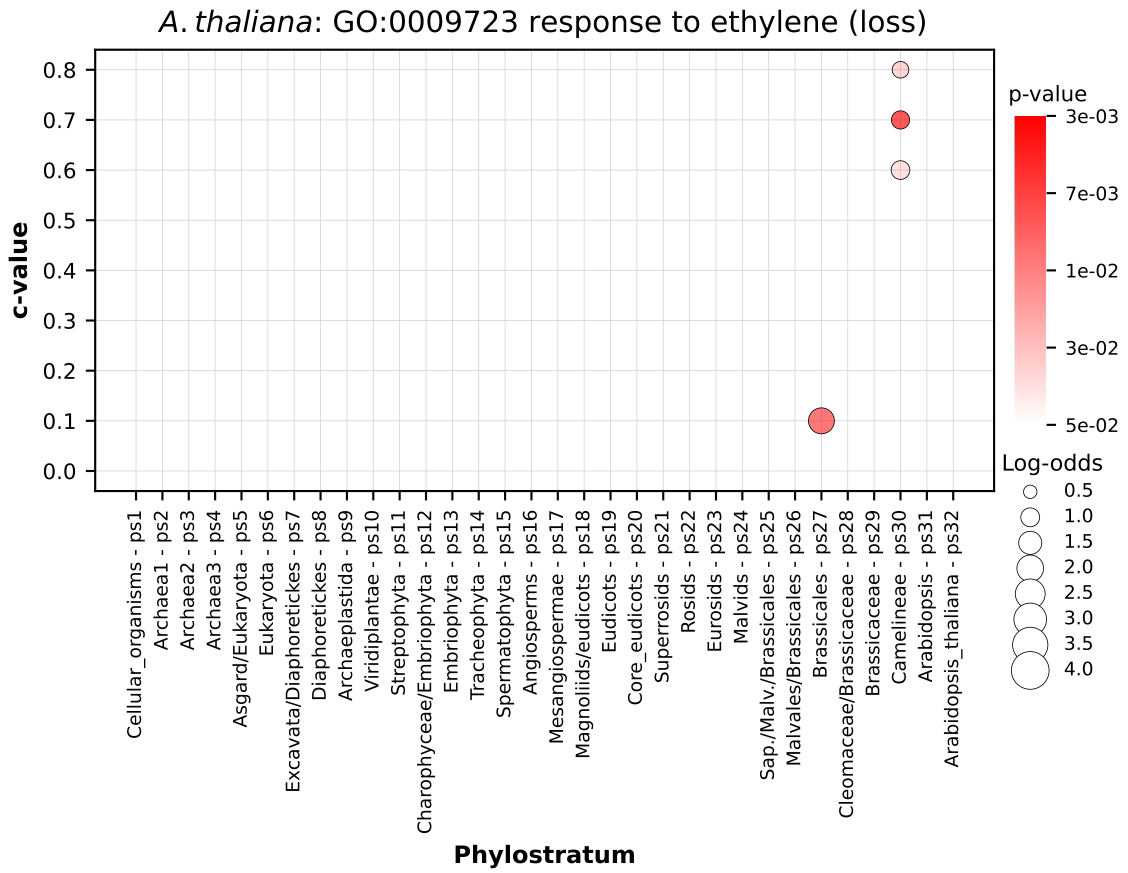

A. thaliana: GO:0009723 response to ethylene (loss)
c-value
0.8
0.7
0.6
0.5
0.4
0.3
0.2
0.1
0.0
p-value
3e-03
7e-03
1e-02
3e-02
5e-02
Log-odds
0.5
1.0
1.5
2.0
2.5
3.0
3.5
4.0
Cellular_organisms - ps1
Archaea1 - ps2
Archaea2 - ps3
Archaea3 - ps4
Asgard/Eukaryota - ps5
Eukaryota - ps6
Excavata/Diaphoretickes - ps7
Diaphoretickes - ps8
Archaeplastida - ps9
Viridiplantae - ps10
Streptophyta - ps11
Charophyceae/Embriophyta - ps12
Embriophyta - ps13
Tracheophyta - ps14
Spermatophyta - ps15
Angiosperms - ps16
Mesangiospermae - ps17
Magnoliids/eudicots - ps18
Eudicots - ps19
Core_eudicots - ps20
Superrosids - ps21
Rosids - ps22
Eurosids - ps23
Malvids - ps24
Sap./Malv./Brassicales - ps25
Malvales/Brassicales - ps26
Brassicales - ps27
Cleomaceae/Brassicaceae - ps28
Brassicaceae - ps29
Camelineae - ps30
Arabidopsis - ps31
Arabidopsis_thaliana - ps32
Phylostratum

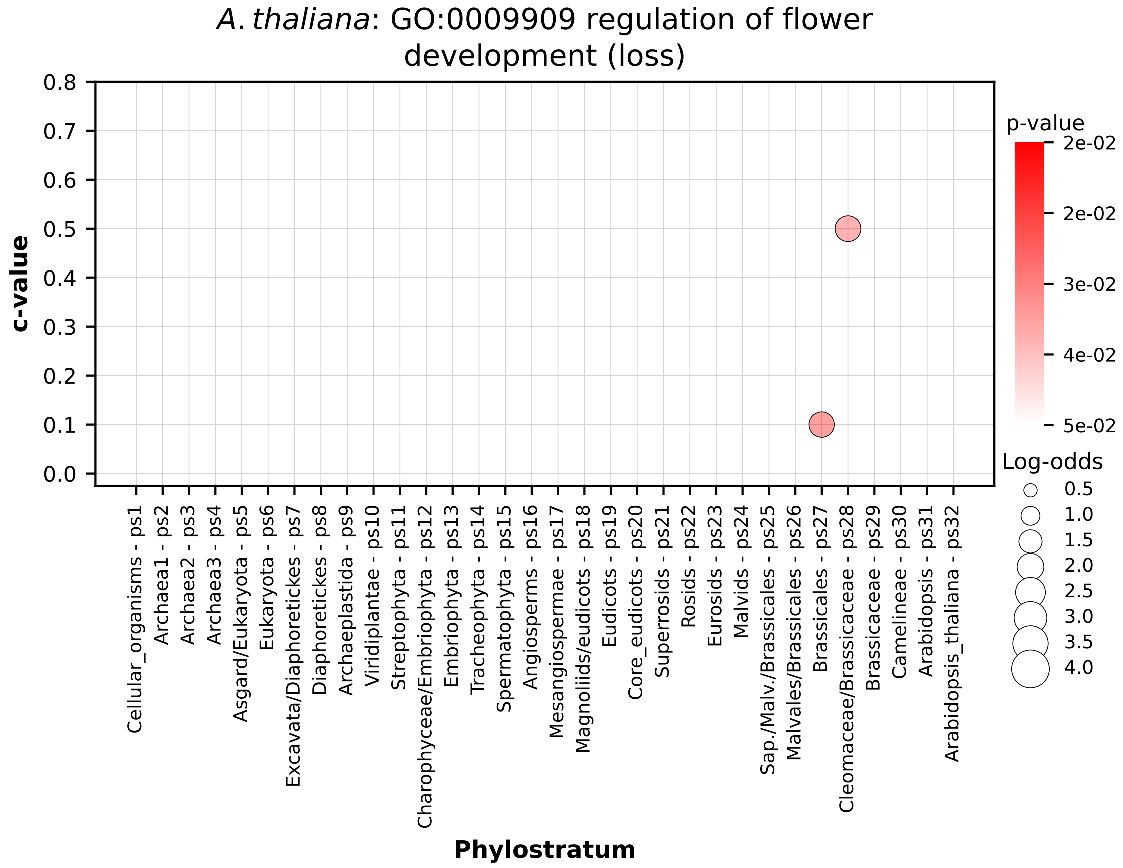

A. thaliana: GO:0009909 regulation of flower development (loss)
c-value
0.8
0.7
0.6
0.5
0.4
0.3
0.2
0.1
0.0
Phylostratum
Cellular_organisms - ps1
Archaea1 - ps2
Archaea2 - ps3
Archaea3 - ps4
Asgard/Eukaryota - ps5
Eukaryota - ps6
Excavata/Diaphoretickes - ps7
Diaphoretickes - ps8
Archaeplastida - ps9
Viridiplantae - ps10
Streptophyta - ps11
Charophyceae/Embriophyta - ps12
Embriophyta - ps13
Tracheophyta - ps14
Spermatophyta - ps15
Angiosperms - ps16
Mesangiospermae - ps17
Magnoliids/eudicots - ps18
Eudicots - ps19
Core_eudicots - ps20
Superrosids - ps21
Rosids - ps22
Eurosids - ps23
Malvids - ps24
Sap./Malv./Brassicales - ps25
Malvales/Brassicales - ps26
Brassicales - ps27
Cleomaceae/Brassicaceae - ps28
Brassicaceae - ps29
Camelineae - ps30
Arabidopsis - ps31
Arabidopsis_thaliana - ps32
p-value
2e-02
2e-02
3e-02
4e-02
5e-02
Log-odds
0.5
1.0
1.5
2.0
2.5
3.0
3.5
4.0

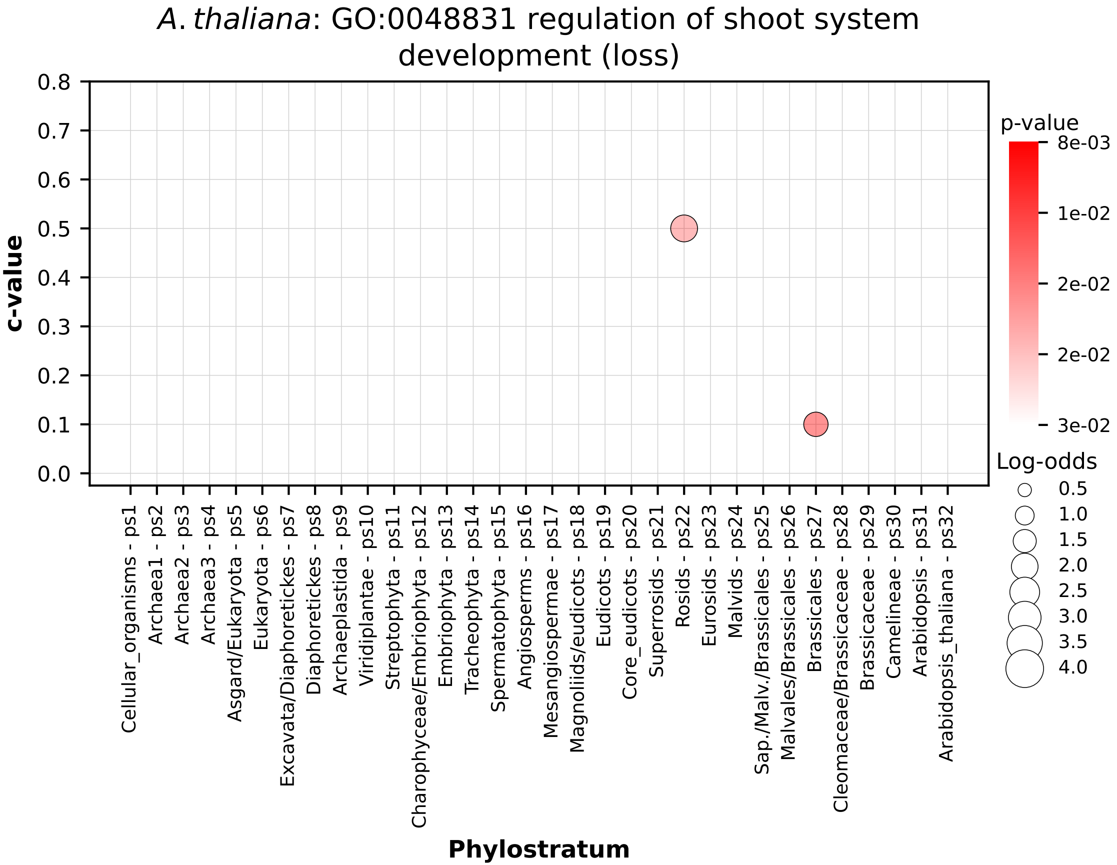

A. thaliana: GO:0048831 regulation of shoot system development (loss)
c-value
0.8
0.7
0.6
0.5
0.4
0.3
0.2
0.1
0.0
Phylostratum
Cellular_organisms - ps1
Archaea1 - ps2
Archaea2 - ps3
Archaea3 - ps4
Asgard/Eukaryota - ps5
Eukaryota - ps6
Excavata/Diaphoretickes - ps7
Diaphoretickes - ps8
Archaeplastida - ps9
Viridiplantae - ps10
Streptophyta - ps11
Charophyceae/Embriophyta - ps12
Embriophyta - ps13
Tracheophyta - ps14
Spermatophyta - ps15
Angiosperms - ps16
Mesangiospermae - ps17
Magnoliids/eudicots - ps18
Eudicots - ps19
Core_eudicots - ps20
Superrosids - ps21
Rosids - ps22
Eurosids - ps23
Malvids - ps24
Sap./Malv./Brassicales - ps25
Malvales/Brassicales - ps26
Brassicales - ps27
Cleomaceae/Brassicaceae - ps28
Brassicaceae - ps29
Camelineae - ps30
Arabidopsis - ps31
Arabidopsis_thaliana - ps32
p-value
8e-03
1e-02
2e-02
2e-02
3e-02
Log-odds
0.5
1.0
1.5
2.0
2.5
3.0
3.5
4.0

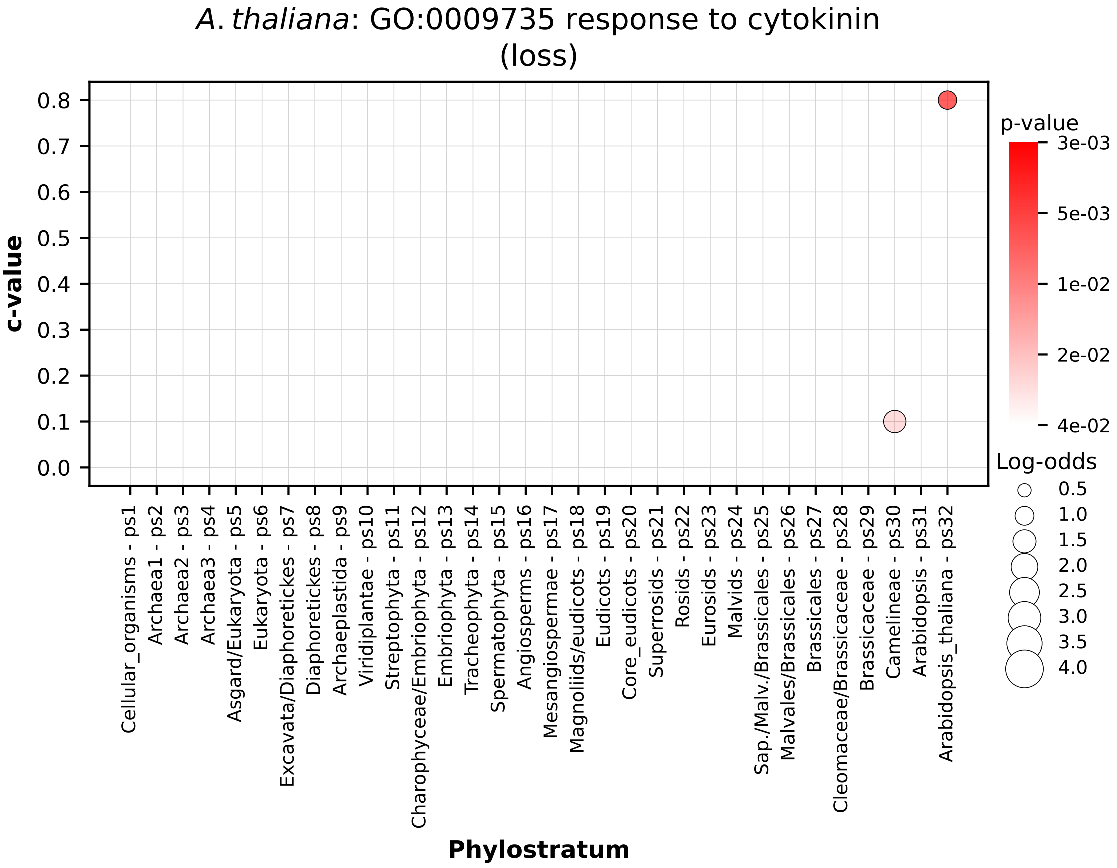

A. thaliana: GO:0009735 response to cytokinin
(loss)
c-value
0.0
0.1
0.2
0.3
0.4
0.5
0.6
0.7
0.8
p-value
3e-03
5e-03
1e-02
2e-02
4e-02
Log-odds
0.5
1.0
1.5
2.0
2.5
3.0
3.5
4.0
Cellular_organisms - ps1
Archaea1 - ps2
Archaea2 - ps3
Archaea3 - ps4
Asgard/Eukaryota - ps5
Eukaryota - ps6
Excavata/Diaphoretickes - ps7
Diaphoretickes - ps8
Archaeplastida - ps9
Viridiplantae - ps10
Streptophyta - ps11
Charophyceae/Embriophyta - ps12
Embriophyta - ps13
Tracheophyta - ps14
Spermatophyta - ps15
Angiosperms - ps16
Mesangiospermae - ps17
Magnoliids/eudicots - ps18
Eudicots - ps19
Core_eudicots - ps20
Superrosids - ps21
Rosids - ps22
Eurosids - ps23
Malvids - ps24
Sap./Malv./Brassicales - ps25
Malvales/Brassicales - ps26
Brassicales - ps27
Cleomaceae/Brassicaceae - ps28
Brassicaceae - ps29
Camelineae - ps30
Arabidopsis - ps31
Arabidopsis_thaliana - ps32
Phylostratum

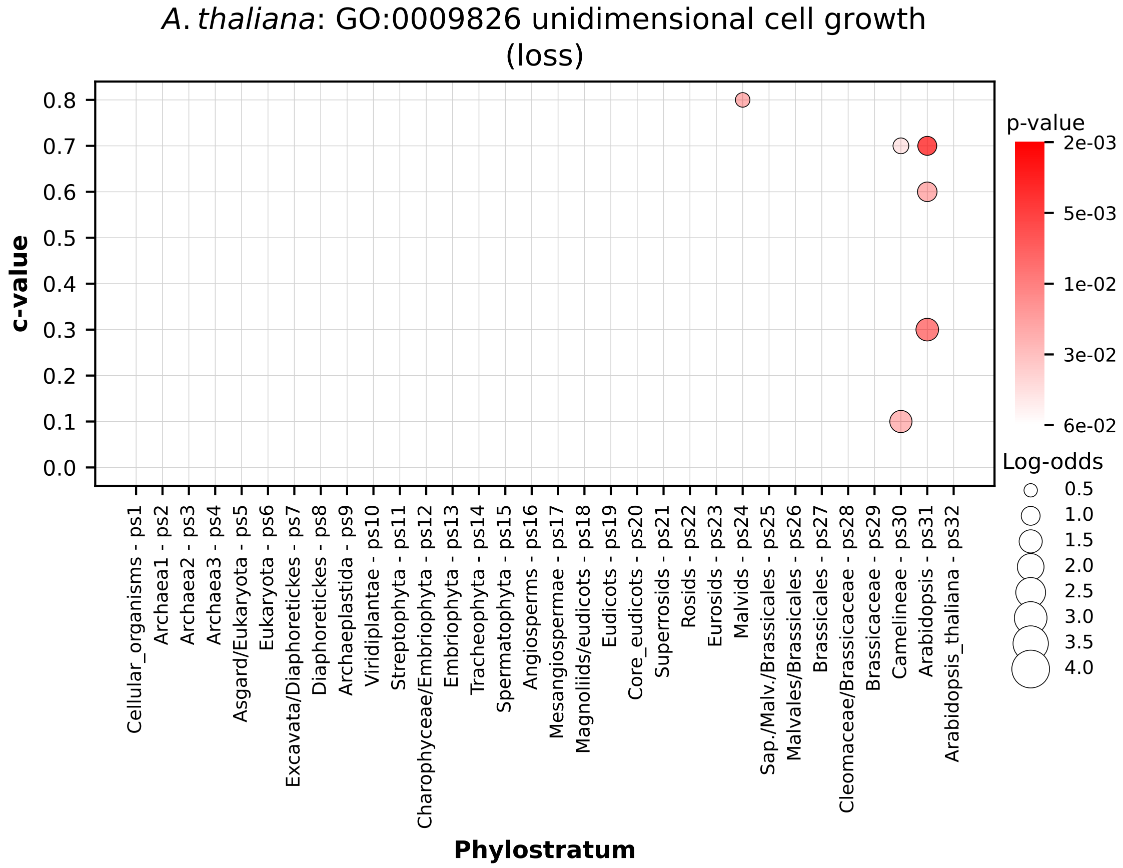
A. thaliana: GO:0009826 unidimensional cell growth
(loss)
c-value
0.8
0.7
0.6
0.5
0.4
0.3
0.2
0.1
0.0
p-value
2e-03
5e-03
1e-02
3e-02
6e-02
Log-odds
0.5
1.0
1.5
2.0
2.5
3.0
3.5
4.0
Cellular_organisms - ps1
Archaea1 - ps2
Archaea2 - ps3
Archaea3 - ps4
Asgard/Eukaryota - ps5
Eukaryota - ps6
Excavata/Diaphoretickes - ps7
Diaphoretickes - ps8
Archaeplastida - ps9
Viridiplantae - ps10
Streptophyta - ps11
Charophyceae/Embriophyta - ps12
Embriophyta - ps13
Tracheophyta - ps14
Spermatophyta - ps15
Angiosperms - ps16
Mesangiospermae - ps17
Magnoliids/eudicots - ps18
Eudicots - ps19
Core_eudicots - ps20
Superrosids - ps21
Rosids - ps22
Eurosids - ps23
Malvids - ps24
Sap./Malv./Brassicales - ps25
Malvales/Brassicales - ps26
Brassicales - ps27
Cleomaceae/Brassicaceae - ps28
Brassicaceae - ps29
Camelineae - ps30
Arabidopsis - ps31
Arabidopsis_thaliana - ps32
Phylostratum

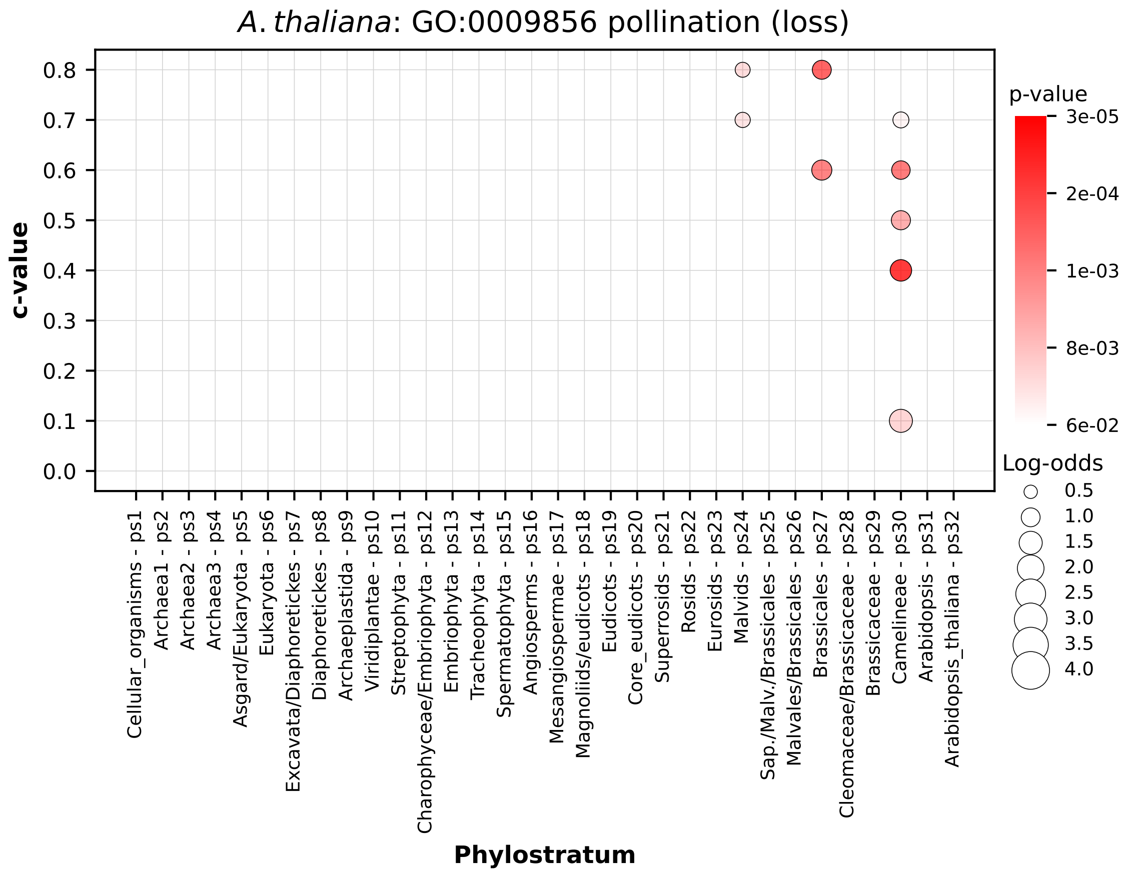
A. thaliana: GO:0009856 pollination (loss)
c-value
0.8
0.7
0.6
0.5
0.4
0.3
0.2
0.1
0.0
Phylostratum
Cellular_organisms - ps1
Archaea1 - ps2
Archaea2 - ps3
Archaea3 - ps4
Asgard/Eukaryota - ps5
Eukaryota - ps6
Excavata/Diaphoretickes - ps7
Diaphoretickes - ps8
Archaeplastida - ps9
Viridiplantae - ps10
Streptophyta - ps11
Charophyceae/Embriophyta - ps12
Embriophyta - ps13
Tracheophyta - ps14
Spermatophyta - ps15
Angiosperms - ps16
Mesangiospermae - ps17
Magnoliids/eudicots - ps18
Eudicots - ps19
Core_eudicots - ps20
Superrosids - ps21
Rosids - ps22
Eurosids - ps23
Malvids - ps24
Sap./Malv./Brassicales - ps25
Malvales/Brassicales - ps26
Brassicales - ps27
Cleomaceae/Brassicaceae - ps28
Brassicaceae - ps29
Camelineae - ps30
Arabidopsis - ps31
Arabidopsis_thaliana - ps32
p-value
3e-05
2e-04
1e-03
8e-03
6e-02
Log-odds
0.5
1.0
1.5
2.0
2.5
3.0
3.5
4.0

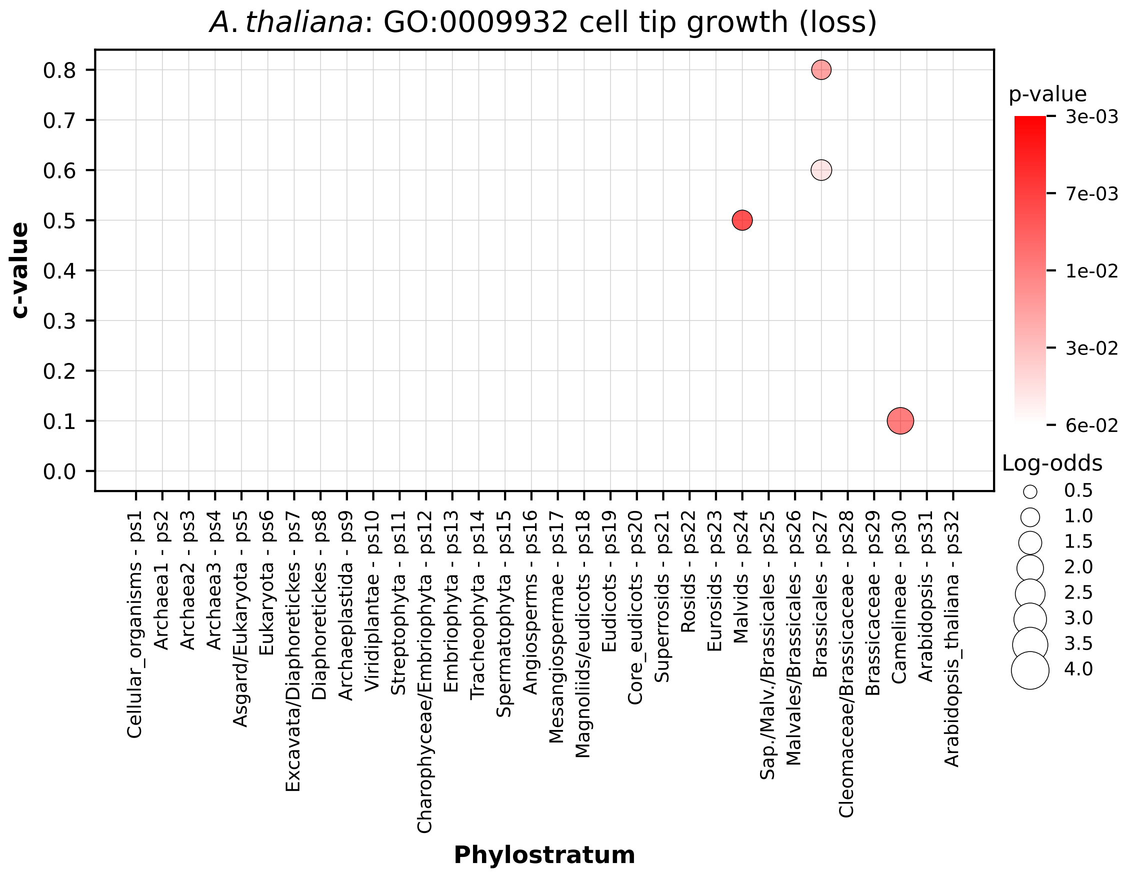
A. thaliana: GO:0009932 cell tip growth (loss)
c-value
0.0
0.1
0.2
0.3
0.4
0.5
0.6
0.7
0.8
p-value
3e-03
7e-03
1e-02
3e-02
6e-02
Log-odds
0.5
1.0
1.5
2.0
2.5
3.0
3.5
4.0
Cellular_organisms - ps1
Archaea1 - ps2
Archaea2 - ps3
Archaea3 - ps4
Asgard/Eukaryota - ps5
Eukaryota - ps6
Excavata/Diaphoretickes - ps7
Diaphoretickes - ps8
Archaeplastida - ps9
Viridiplantae - ps10
Streptophyta - ps11
Charophyceae/Embriophyta - ps12
Embriophyta - ps13
Tracheophyta - ps14
Spermatophyta - ps15
Angiosperms - ps16
Mesangiospermae - ps17
Magnoliids/eudicots - ps18
Eudicots - ps19
Core_eudicots - ps20
Superrosids - ps21
Rosids - ps22
Eurosids - ps23
Malvids - ps24
Sap./Malv./Brassicales - ps25
Malvales/Brassicales - ps26
Brassicales - ps27
Cleomaceae/Brassicaceae - ps28
Brassicaceae - ps29
Camelineae - ps30
Arabidopsis - ps31
Arabidopsis_thaliana - ps32
Phylostratum

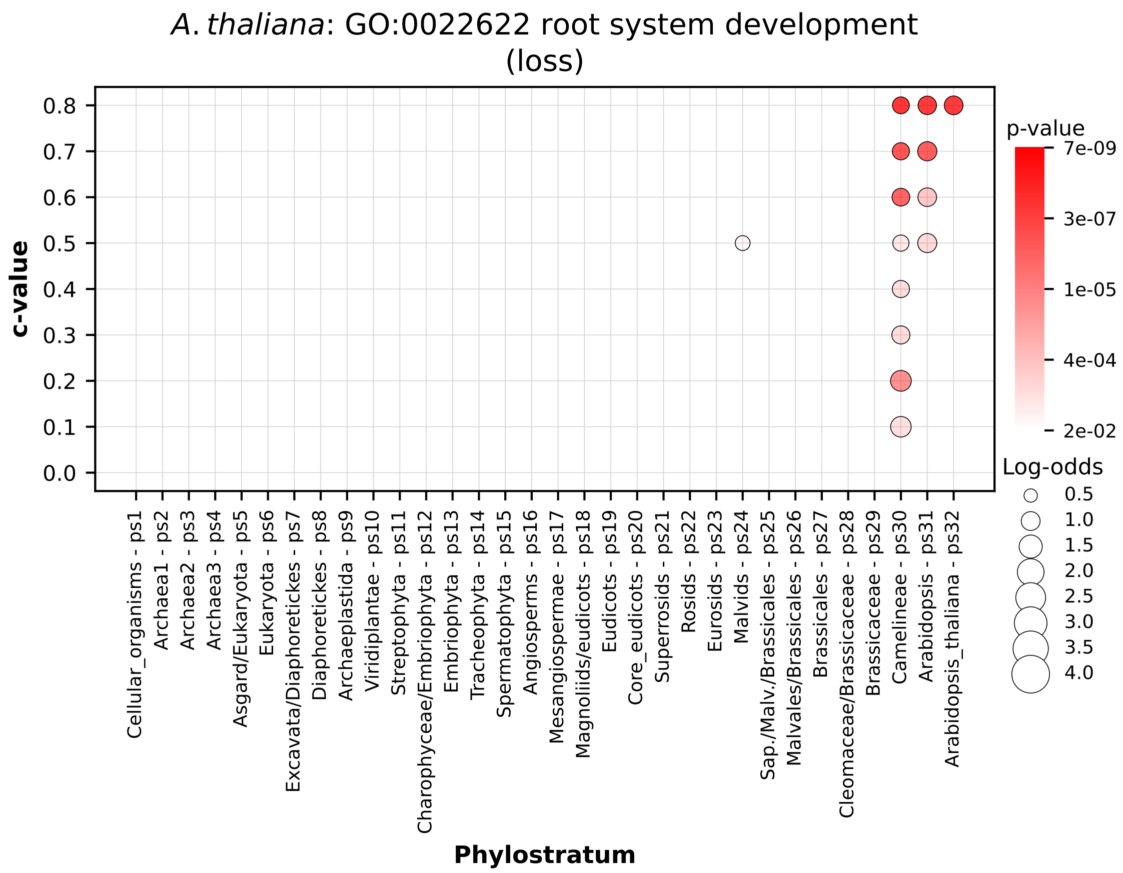

A. thaliana: GO:0022622 root system development
(loss)
c-value
0.8
0.7
0.6
0.5
0.4
0.3
0.2
0.1
0.0
p-value
7e-09
3e-07
1e-05
4e-04
2e-02
Log-odds
0.5
1.0
1.5
2.0
2.5
3.0
3.5
4.0
Cellular_organisms - ps1
Archaea1 - ps2
Archaea2 - ps3
Archaea3 - ps4
Asgard/Eukaryota - ps5
Eukaryota - ps6
Excavata/Diaphoretickes - ps7
Diaphoretickes - ps8
Archaeplastida - ps9
Viridiplantae - ps10
Streptophyta - ps11
Charophyceae/Embriophyta - ps12
Embriophyta - ps13
Tracheophyta - ps14
Spermatophyta - ps15
Angiosperms - ps16
Mesangiospermae - ps17
Magnoliids/eudicots - ps18
Eudicots - ps19
Core_eudicots - ps20
Superrosids - ps21
Rosids - ps22
Eurosids - ps23
Malvids - ps24
Sap./Malv./Brassicales - ps25
Malvales/Brassicales - ps26
Brassicales - ps27
Cleomaceae/Brassicaceae - ps28
Brassicaceae - ps29
Camelineae - ps30
Arabidopsis - ps31
Arabidopsis_thaliana - ps32
Phylostratum

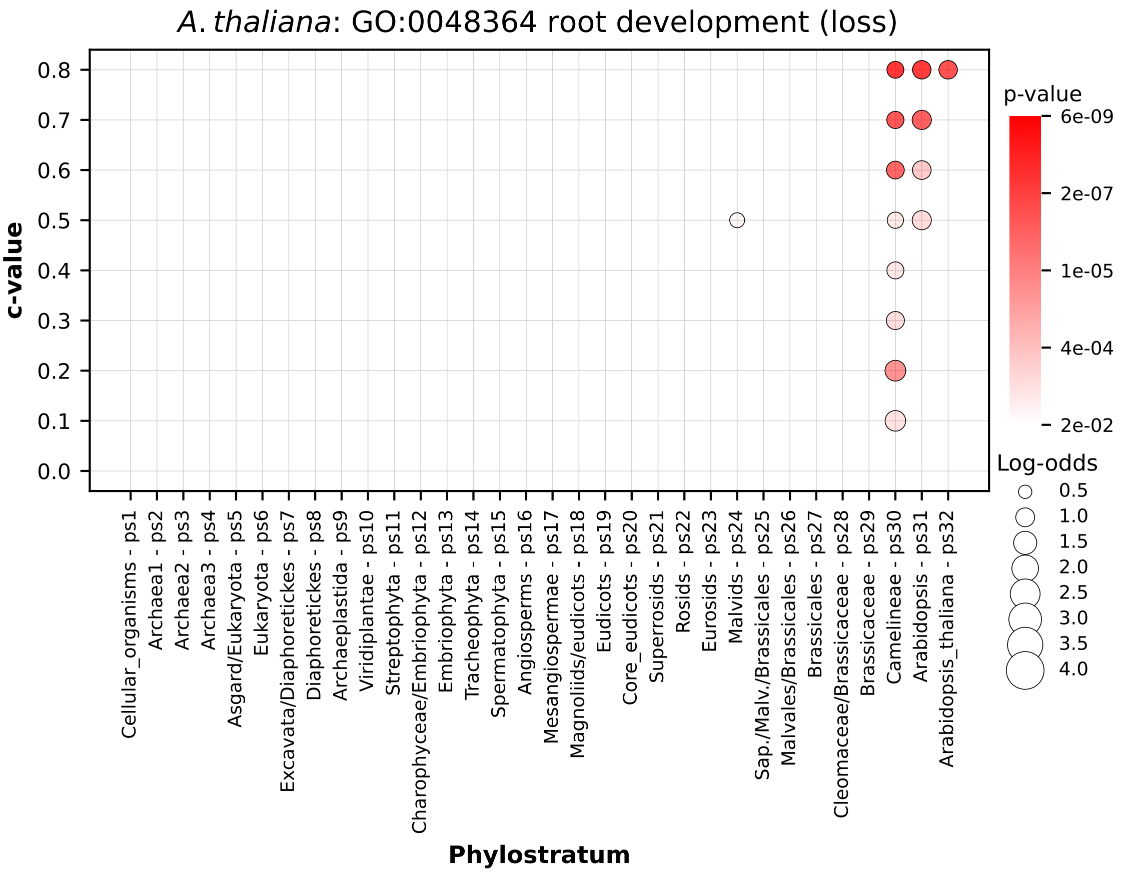
A. thaliana: GO:0048364 root development (loss)
c-value
0.0
0.1
0.2
0.3
0.4
0.5
0.6
0.7
0.8
p-value
6e-09
2e-07
1e-05
4e-04
2e-02
Log-odds
0.5
1.0
1.5
2.0
2.5
3.0
3.5
4.0
Cellular_organisms - ps1
Archaea1 - ps2
Archaea2 - ps3
Archaea3 - ps4
Asgard/Eukaryota - ps5
Eukaryota - ps6
Excavata/Diaphoretickes - ps7
Diaphoretickes - ps8
Archaeplastida - ps9
Viridiplantae - ps10
Streptophyta - ps11
Charophyceae/Embriophyta - ps12
Embriophyta - ps13
Tracheophyta - ps14
Spermatophyta - ps15
Angiosperms - ps16
Mesangiospermae - ps17
Magnoliids/eudicots - ps18
Eudicots - ps19
Core_eudicots - ps20
Superrosids - ps21
Rosids - ps22
Eurosids - ps23
Malvids - ps24
Sap./Malv./Brassicales - ps25
Malvales/Brassicales - ps26
Brassicales - ps27
Cleomaceae/Brassicaceae - ps28
Brassicaceae - ps29
Camelineae - ps30
Arabidopsis - ps31
Arabidopsis_thaliana - ps32
Phylostratum

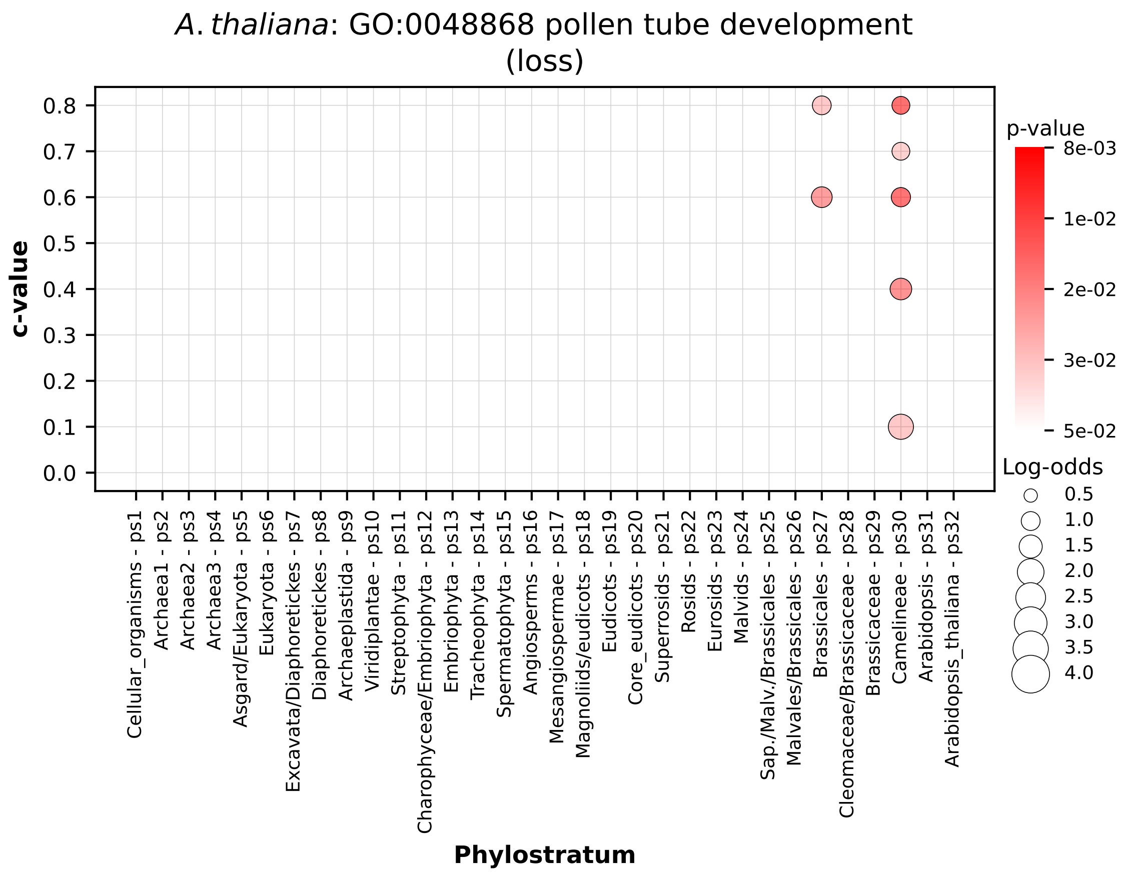

A. thaliana: GO:0048868 pollen tube development
(loss)
c-value
0.8
0.7
0.6
0.5
0.4
0.3
0.2
0.1
0.0
p-value
8e-03
1e-02
2e-02
3e-02
5e-02
Log-odds
0.5
1.0
1.5
2.0
2.5
3.0
3.5
4.0
Cellular_organisms - ps1
Archaea1 - ps2
Archaea2 - ps3
Archaea3 - ps4
Asgard/Eukaryota - ps5
Eukaryota - ps6
Excavata/Diaphoretickes - ps7
Diaphoretickes - ps8
Archaeplastida - ps9
Viridiplantae - ps10
Streptophyta - ps11
Charophyceae/Embriophyta - ps12
Embriophyta - ps13
Tracheophyta - ps14
Spermatophyta - ps15
Angiosperms - ps16
Mesangiospermae - ps17
Magnoliids/eudicots - ps18
Eudicots - ps19
Core_eudicots - ps20
Superrosids - ps21
Rosids - ps22
Eurosids - ps23
Malvids - ps24
Sap./Malv./Brassicales - ps25
Malvales/Brassicales - ps26
Brassicales - ps27
Cleomaceae/Brassicaceae - ps28
Brassicaceae - ps29
Camelineae - ps30
Arabidopsis - ps31
Arabidopsis_thaliana - ps32
Phylostratum

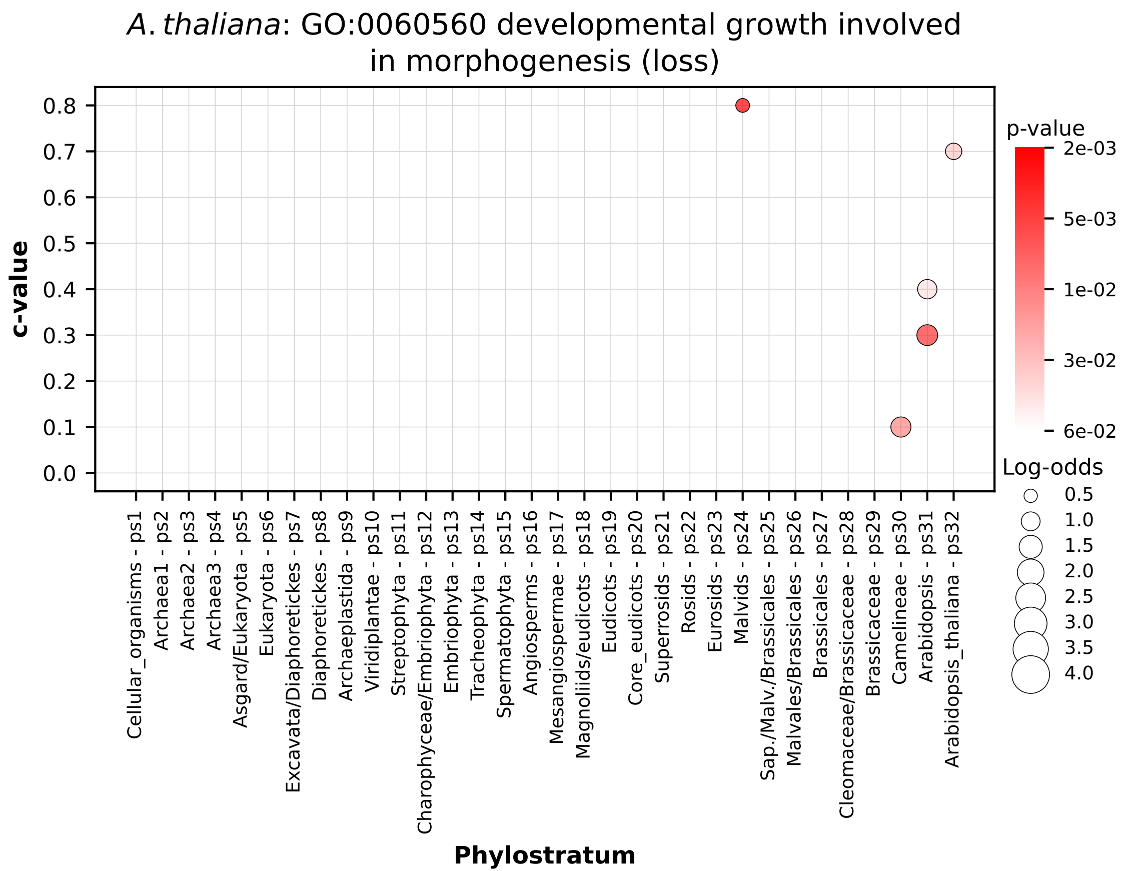

$A. thaliana$: GO:0060560 developmental growth involved in morphogenesis (loss)
c-value
0.8
0.7
0.6
0.5
0.4
0.3
0.2
0.1
0.0
Phylostratum
Cellular_organisms - ps1
Archaea1 - ps2
Archaea2 - ps3
Archaea3 - ps4
Asgard/Eukaryota - ps5
Eukaryota - ps6
Excavata/Diaphoretickes - ps7
Diaphoretickes - ps8
Archaeplastida - ps9
Viridiplantae - ps10
Streptophyta - ps11
Charophyceae/Embriophyta - ps12
Embriophyta - ps13
Tracheophyta - ps14
Spermatophyta - ps15
Angiosperms - ps16
Mesangiospermae - ps17
Magnoliids/eudicots - ps18
Eudicots - ps19
Core_eudicots - ps20
Superrosids - ps21
Rosids - ps22
Eurosids - ps23
Malvids - ps24
Sap./Malv./Brassicales - ps25
Malvales/Brassicales - ps26
Brassicales - ps27
Cleomaceae/Brassicaceae - ps28
Brassicaceae - ps29
Camelineae - ps30
Arabidopsis - ps31
Arabidopsis_thaliana - ps32
p-value
2e-03
5e-03
1e-02
3e-02
6e-02
Log-odds
0.5
1.0
1.5
2.0
2.5
3.0
3.5
4.0

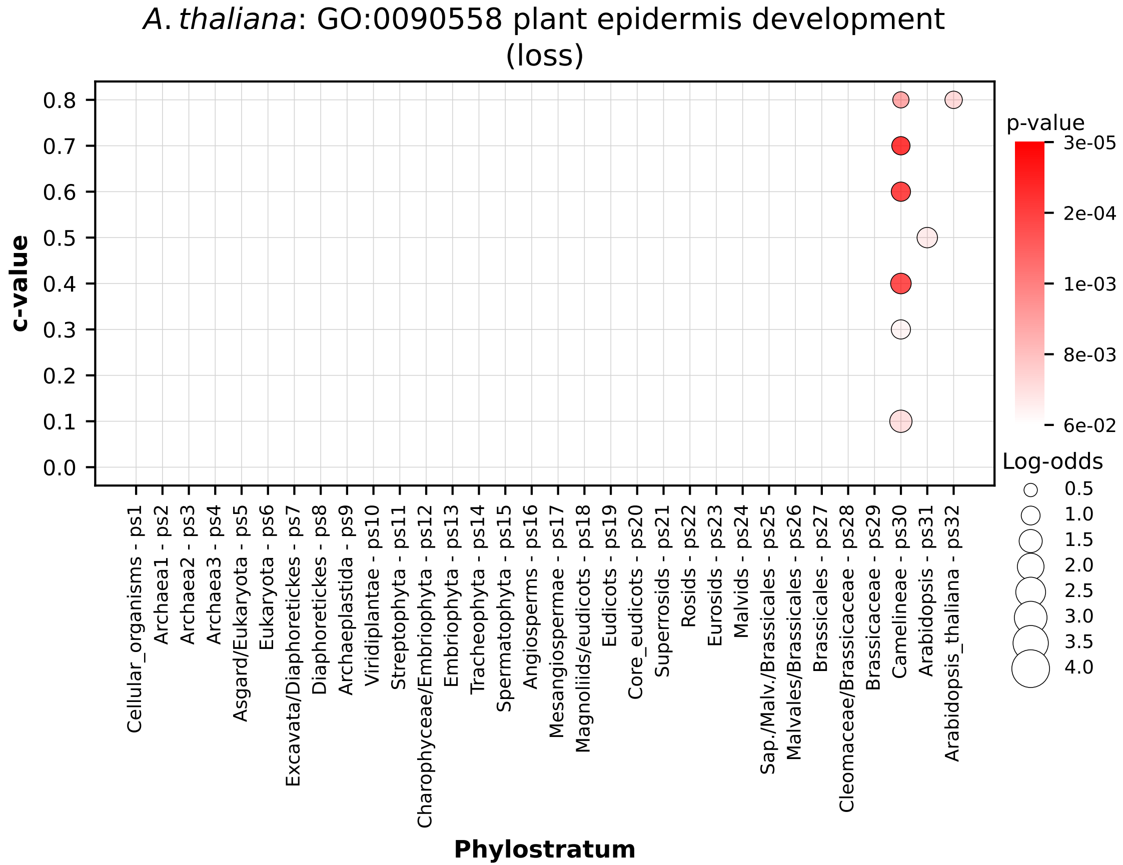

A. thaliana: GO:0090558 plant epidermis development
(loss)
c-value
0.8
0.7
0.6
0.5
0.4
0.3
0.2
0.1
0.0
p-value
3e-05
2e-04
1e-03
8e-03
6e-02
Log-odds
0.5
1.0
1.5
2.0
2.5
3.0
3.5
4.0
Cellular_organisms - ps1
Archaea1 - ps2
Archaea2 - ps3
Archaea3 - ps4
Asgard/Eukaryota - ps5
Eukaryota - ps6
Excavata/Diaphoretickes - ps7
Diaphoretickes - ps8
Archaeplastida - ps9
Viridiplantae - ps10
Streptophyta - ps11
Charophyceae/Embriophyta - ps12
Embriophyta - ps13
Tracheophyta - ps14
Spermatophyta - ps15
Angiosperms - ps16
Mesangiospermae - ps17
Magnoliids/eudicots - ps18
Eudicots - ps19
Core_eudicots - ps20
Superrosids - ps21
Rosids - ps22
Eurosids - ps23
Malvids - ps24
Sap./Malv./Brassicales - ps25
Malvales/Brassicales - ps26
Brassicales - ps27
Cleomaceae/Brassicaceae - ps28
Brassicaceae - ps29
Camelineae - ps30
Arabidopsis - ps31
Arabidopsis_thaliana - ps32
Phylostratum

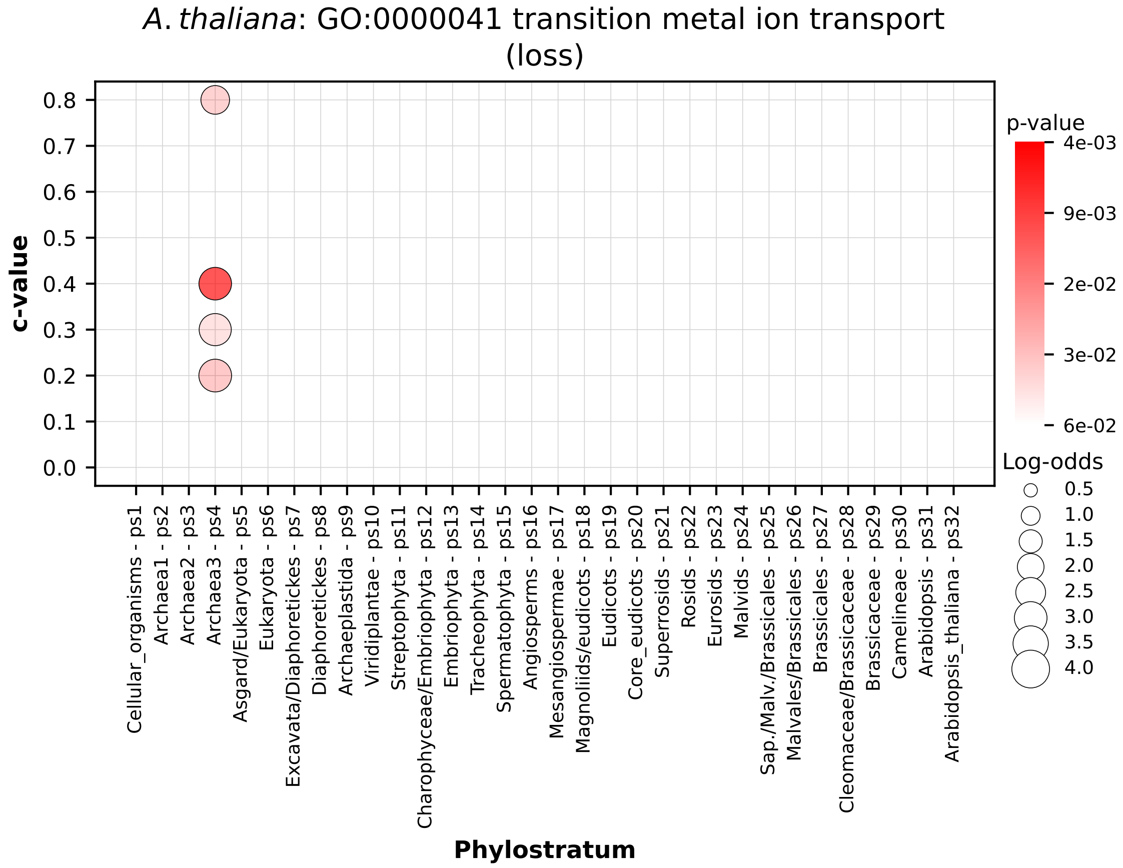
A. thaliana: GO:0000041 transition metal ion transport
(loss)
c-value
0.8
0.7
0.6
0.5
0.4
0.3
0.2
0.1
0.0
p-value
4e-03
9e-03
2e-02
3e-02
6e-02
Log-odds
0.5
1.0
1.5
2.0
2.5
3.0
3.5
4.0
Cellular_organisms - ps1
Archaea1 - ps2
Archaea2 - ps3
Archaea3 - ps4
Asgard/Eukaryota - ps5
Eukaryota - ps6
Excavata/Diaphoretickes - ps7
Diaphoretickes - ps8
Archaeplastida - ps9
Viridiplantae - ps10
Streptophyta - ps11
Charophyceae/Embriophyta - ps12
Embriophyta - ps13
Tracheophyta - ps14
Spermatophyta - ps15
Angiosperms - ps16
Mesangiospermae - ps17
Magnoliids/eudicots - ps18
Eudicots - ps19
Core_eudicots - ps20
Superrosids - ps21
Rosids - ps22
Eurosids - ps23
Malvids - ps24
Sap./Malv./Brassicales - ps25
Malvales/Brassicales - ps26
Brassicales - ps27
Cleomaceae/Brassicaceae - ps28
Brassicaceae - ps29
Camelineae - ps30
Arabidopsis - ps31
Arabidopsis_thaliana - ps32
Phylostratum

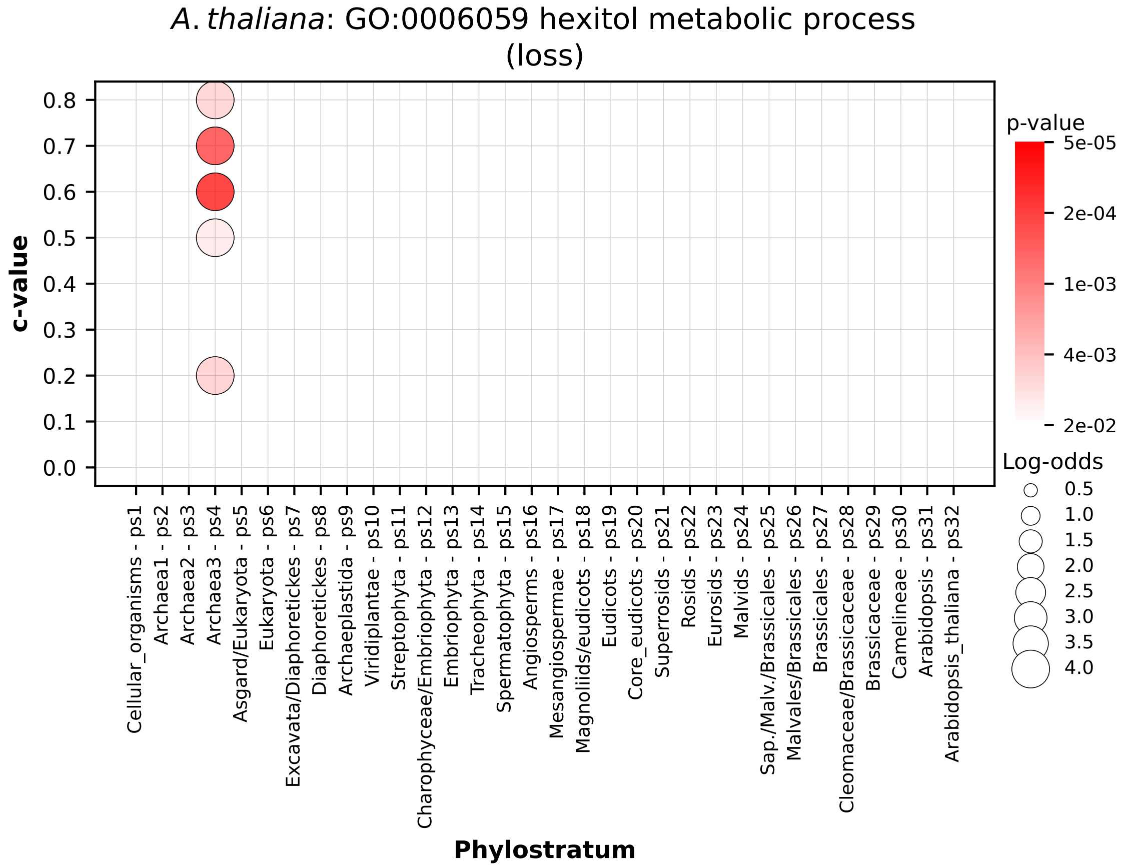
A. thaliana: GO:0006059 hexitol metabolic process
(loss)
c-value
0.0
0.1
0.2
0.3
0.4
0.5
0.6
0.7
0.8
Phylostratum
Cellular_organisms - ps1
Archaea1 - ps2
Archaea2 - ps3
Archaea3 - ps4
Asgard/Eukaryota - ps5
Eukaryota - ps6
Excavata/Diaphoretickes - ps7
Diaphoretickes - ps8
Archaeplastida - ps9
Viridiplantae - ps10
Streptophyta - ps11
Charophyceae/Embriophyta - ps12
Embriophyta - ps13
Tracheophyta - ps14
Spermatophyta - ps15
Angiosperms - ps16
Mesangiospermae - ps17
Magnoliids/eudicots - ps18
Eudicots - ps19
Core_eudicots - ps20
Superrosids - ps21
Rosids - ps22
Eurosids - ps23
Malvids - ps24
Sap./Malv./Brassicales - ps25
Malvales/Brassicales - ps26
Brassicales - ps27
Cleomaceae/Brassicaceae - ps28
Brassicaceae - ps29
Camelineae - ps30
Arabidopsis - ps31
Arabidopsis_thaliana - ps32
p-value
5e-05
2e-04
1e-03
4e-03
2e-02
Log-odds
0.5
1.0
1.5
2.0
2.5
3.0
3.5
4.0

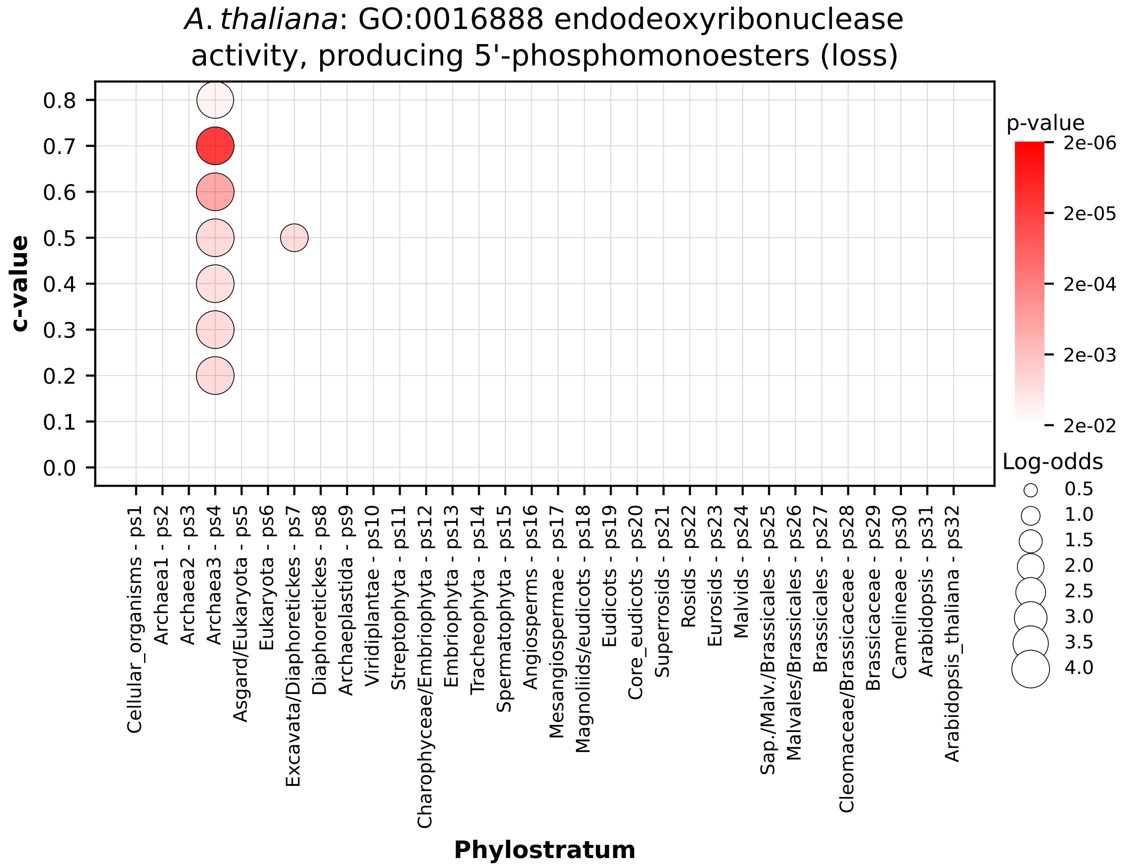
A. thaliana: GO:0016888 endodeoxyribonuclease activity, producing 5'-phosphomonoesters (loss)
c-value
0.0
0.1
0.2
0.3
0.4
0.5
0.6
0.7
0.8
Phylostratum
Cellular_organisms - ps1
Archaea1 - ps2
Archaea2 - ps3
Archaea3 - ps4
Asgard/Eukaryota - ps5
Eukaryota - ps6
Excavata/Diaphoretickes - ps7
Diaphoretickes - ps8
Archaeplastida - ps9
Viridiplantae - ps10
Streptophyta - ps11
Charophyceae/Embriophyta - ps12
Embriophyta - ps13
Tracheophyta - ps14
Spermatophyta - ps15
Angiosperms - ps16
Mesangiospermae - ps17
Magnoliids/eudicots - ps18
Eudicots - ps19
Core_eudicots - ps20
Superrosids - ps21
Rosids - ps22
Eurosids - ps23
Malvids - ps24
Sap./Malv./Brassicales - ps25
Malvales/Brassicales - ps26
Brassicales - ps27
Cleomaceae/Brassicaceae - ps28
Brassicaceae - ps29
Camelineae - ps30
Arabidopsis - ps31
Arabidopsis_thaliana - ps32
p-value
2e-06
2e-05
2e-04
2e-03
2e-02
Log-odds
0.5
1.0
1.5
2.0
2.5
3.0
3.5
4.0

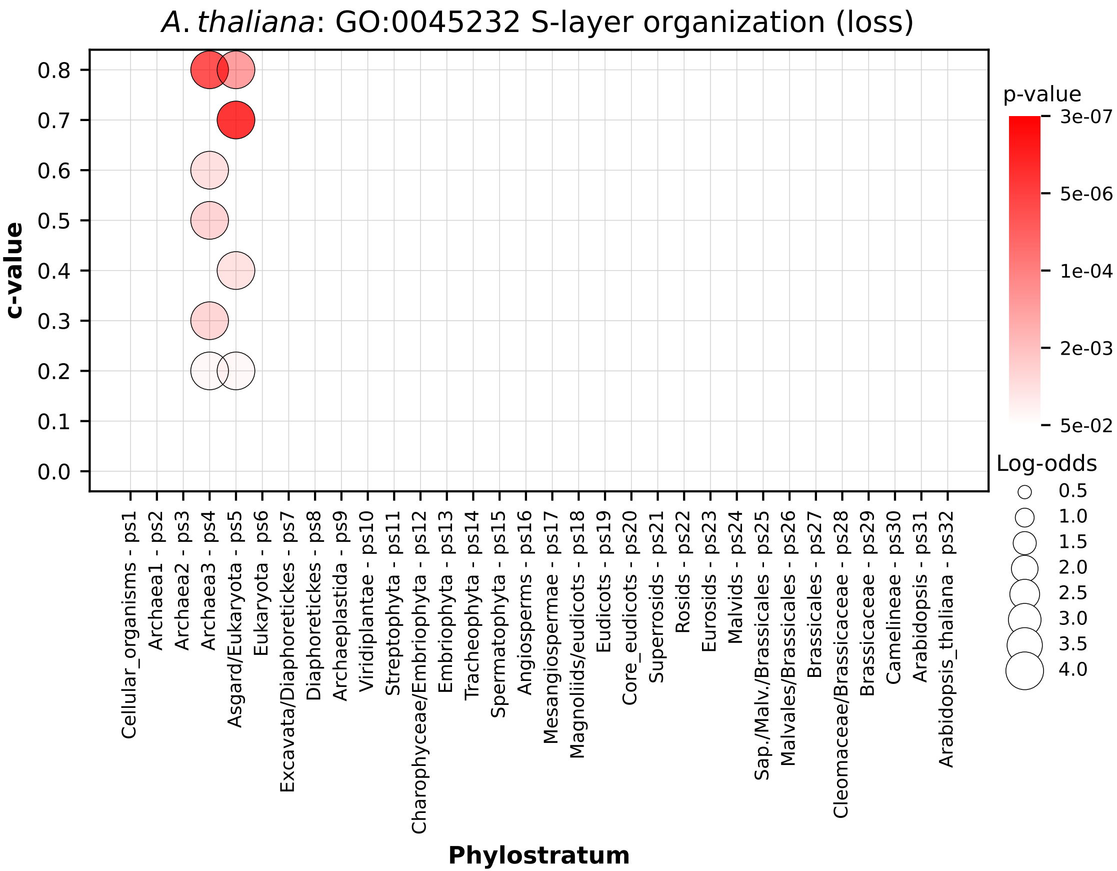

A. thaliana: GO:0045232 S-layer organization (loss)
c-value
0.0
0.1
0.2
0.3
0.4
0.5
0.6
0.7
0.8
Phylostratum
Cellular_organisms - ps1
Archaea1 - ps2
Archaea2 - ps3
Archaea3 - ps4
Asgard/Eukaryota - ps5
Eukaryota - ps6
Excavata/Diaphoretickes - ps7
Diaphoretickes - ps8
Archaeplastida - ps9
Viridiplantae - ps10
Streptophyta - ps11
Charophyceae/Embriophyta - ps12
Embriophyta - ps13
Tracheophyta - ps14
Spermatophyta - ps15
Angiosperms - ps16
Mesangiospermae - ps17
Magnoliids/eudicots - ps18
Eudicots - ps19
Core_eudicots - ps20
Superrosids - ps21
Rosids - ps22
Eurosids - ps23
Malvids - ps24
Sap./Malv./Brassicales - ps25
Malvales/Brassicales - ps26
Brassicales - ps27
Cleomaceae/Brassicaceae - ps28
Brassicaceae - ps29
Camelineae - ps30
Arabidopsis - ps31
Arabidopsis_thaliana - ps32
p-value
3e-07
5e-06
1e-04
2e-03
5e-02
Log-odds
0.5
1.0
1.5
2.0
2.5
3.0
3.5
4.0

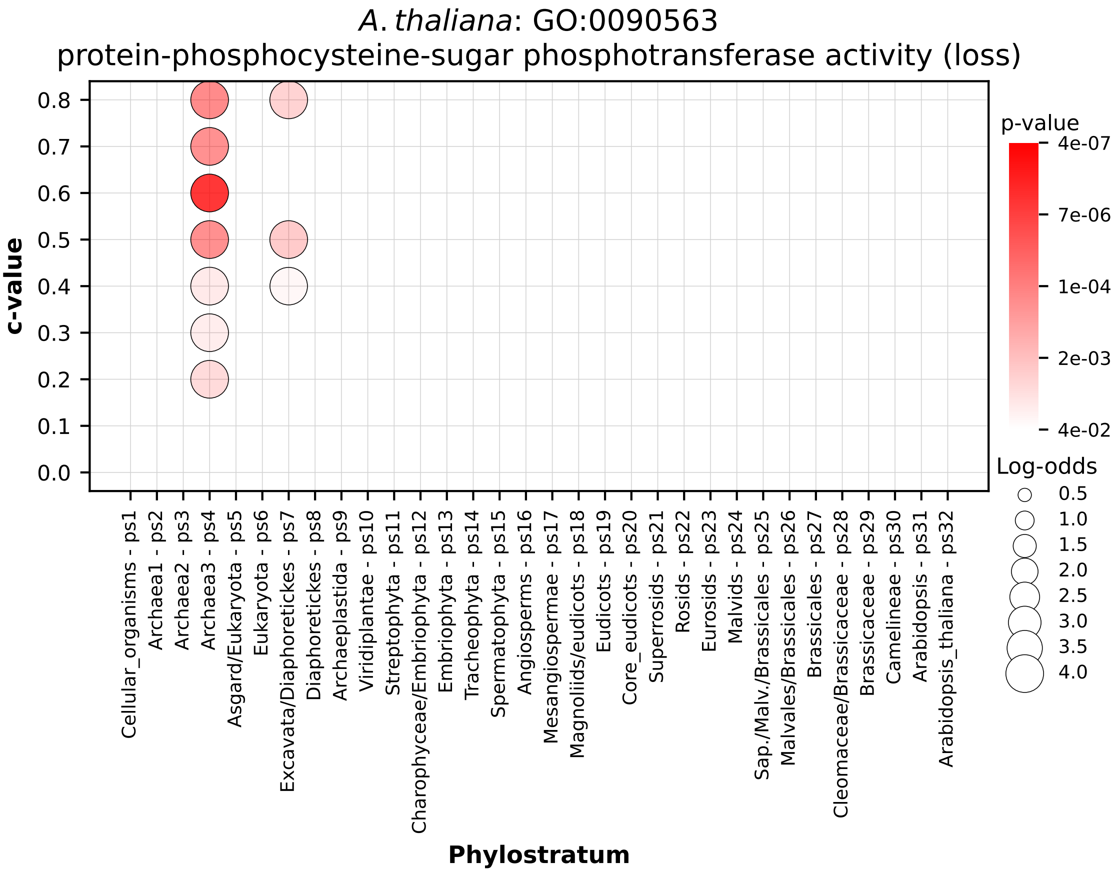

A. thaliana: GO:0090563
protein-phosphocysteine-sugar phosphotransferase activity (loss)
c-value
0.8
0.7
0.6
0.5
0.4
0.3
0.2
0.1
0.0
p-value
4e-07
7e-06
1e-04
2e-03
4e-02
Log-odds
0.5
1.0
1.5
2.0
2.5
3.0
3.5
4.0
Cellular_organisms - ps1
Archaea1 - ps2
Archaea2 - ps3
Archaea3 - ps4
Asgard/Eukaryota - ps5
Eukaryota - ps6
Excavata/Diaphoretickes - ps7
Diaphoretickes - ps8
Archaeplastida - ps9
Viridiplantae - ps10
Streptophyta - ps11
Charophyceae/Embriophyta - ps12
Embriophyta - ps13
Tracheophyta - ps14
Spermatophyta - ps15
Angiosperms - ps16
Mesangiospermae - ps17
Magnoliids/eudicots - ps18
Eudicots - ps19
Core_eudicots - ps20
Superrosids - ps21
Rosids - ps22
Eurosids - ps23
Malvids - ps24
Sap./Malv./Brassicales - ps25
Malvales/Brassicales - ps26
Brassicales - ps27
Cleomaceae/Brassicaceae - ps28
Brassicaceae - ps29
Camelineae - ps30
Arabidopsis - ps31
Arabidopsis_thaliana - ps32
Phylostratum

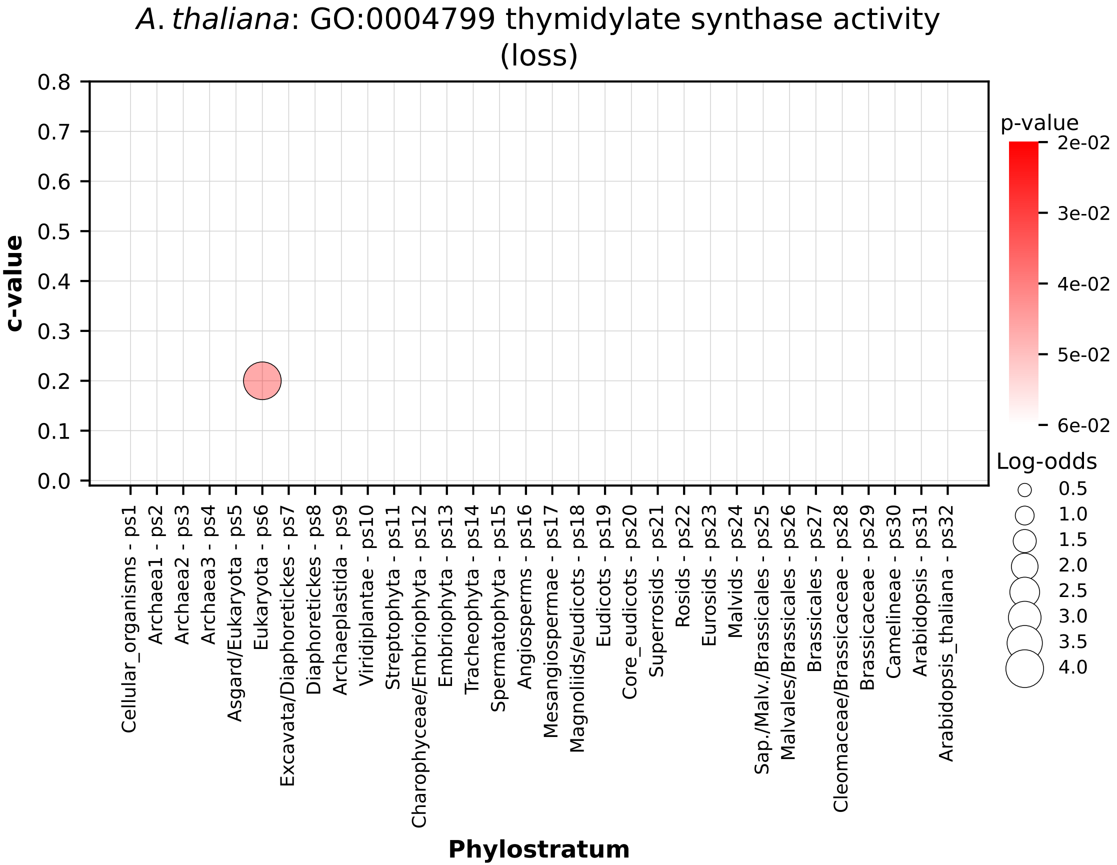

A. thaliana: GO:0004799 thymidylate synthase activity
(loss)
c-value
0.0
0.1
0.2
0.3
0.4
0.5
0.6
0.7
0.8
Phylostratum
Cellular_organisms - ps1
Archaea1 - ps2
Archaea2 - ps3
Archaea3 - ps4
Asgard/Eukaryota - ps5
Eukaryota - ps6
Excavata/Diaphoretickes - ps7
Diaphoretickes - ps8
Archaeplastida - ps9
Viridiplantae - ps10
Streptophyta - ps11
Charophyceae/Embriophyta - ps12
Embriophyta - ps13
Tracheophyta - ps14
Spermatophyta - ps15
Angiosperms - ps16
Mesangiospermae - ps17
Magnoliids/eudicots - ps18
Eudicots - ps19
Core_eudicots - ps20
Superrosids - ps21
Rosids - ps22
Eurosids - ps23
Malvids - ps24
Sap./Malv./Brassicales - ps25
Malvales/Brassicales - ps26
Brassicales - ps27
Cleomaceae/Brassicaceae - ps28
Brassicaceae - ps29
Camelineae - ps30
Arabidopsis - ps31
Arabidopsis_thaliana - ps32
p-value
2e-02
3e-02
4e-02
5e-02
6e-02
Log-odds
0.5
1.0
1.5
2.0
2.5
3.0
3.5
4.0

A. thaliana: GO:0006231 dTMP biosynthetic process (loss)

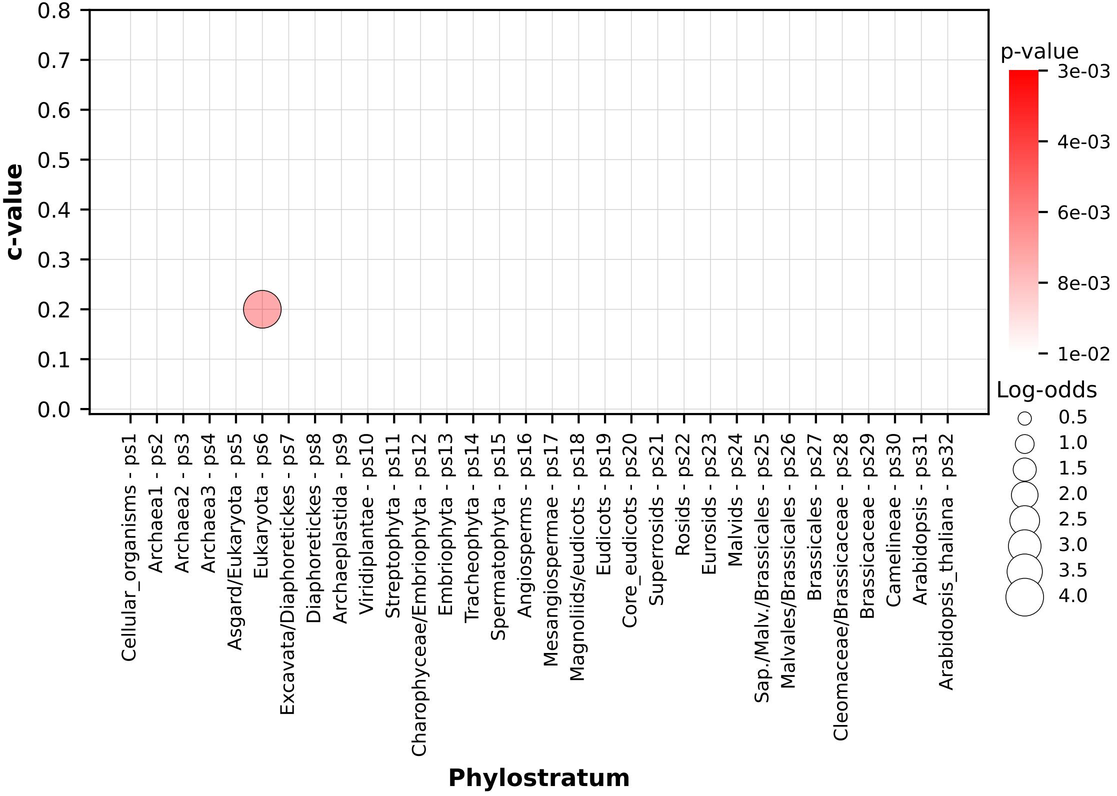

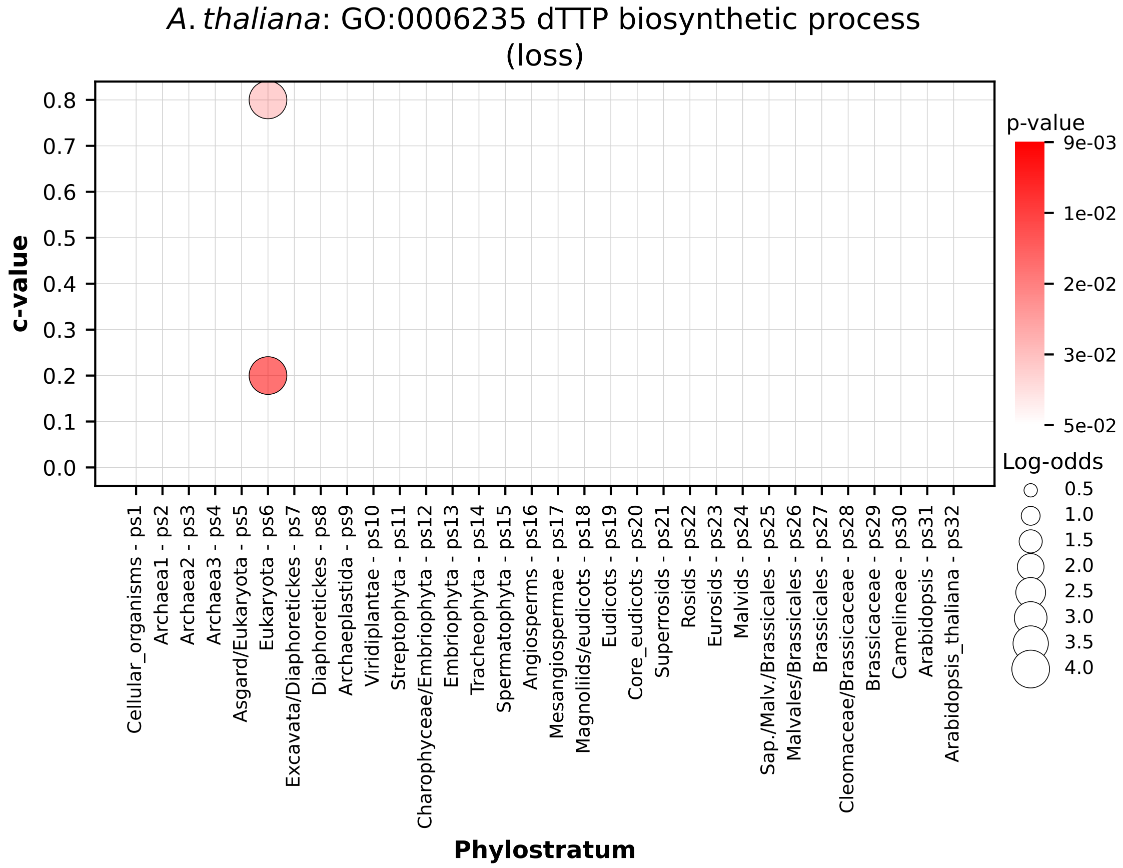

A. thaliana: GO:0006235 dTTP biosynthetic process
(loss)
c-value
0.8
0.7
0.6
0.5
0.4
0.3
0.2
0.1
0.0
p-value
9e-03
1e-02
2e-02
3e-02
5e-02
Log-odds
0.5
1.0
1.5
2.0
2.5
3.0
3.5
4.0
Cellular_organisms - ps1
Archaea1 - ps2
Archaea2 - ps3
Archaea3 - ps4
Asgard/Eukaryota - ps5
Eukaryota - ps6
Excavata/Diaphoretickes - ps7
Diaphoretickes - ps8
Archaeplastida - ps9
Viridiplantae - ps10
Streptophyta - ps11
Charophyceae/Embriophyta - ps12
Embriophyta - ps13
Tracheophyta - ps14
Spermatophyta - ps15
Angiosperms - ps16
Mesangiospermae - ps17
Magnoliids/eudicots - ps18
Eudicots - ps19
Core_eudicots - ps20
Superrosids - ps21
Rosids - ps22
Eurosids - ps23
Malvids - ps24
Sap./Malv./Brassicales - ps25
Malvales/Brassicales - ps26
Brassicales - ps27
Cleomaceae/Brassicaceae - ps28
Brassicaceae - ps29
Camelineae - ps30
Arabidopsis - ps31
Arabidopsis_thaliana - ps32
Phylostratum

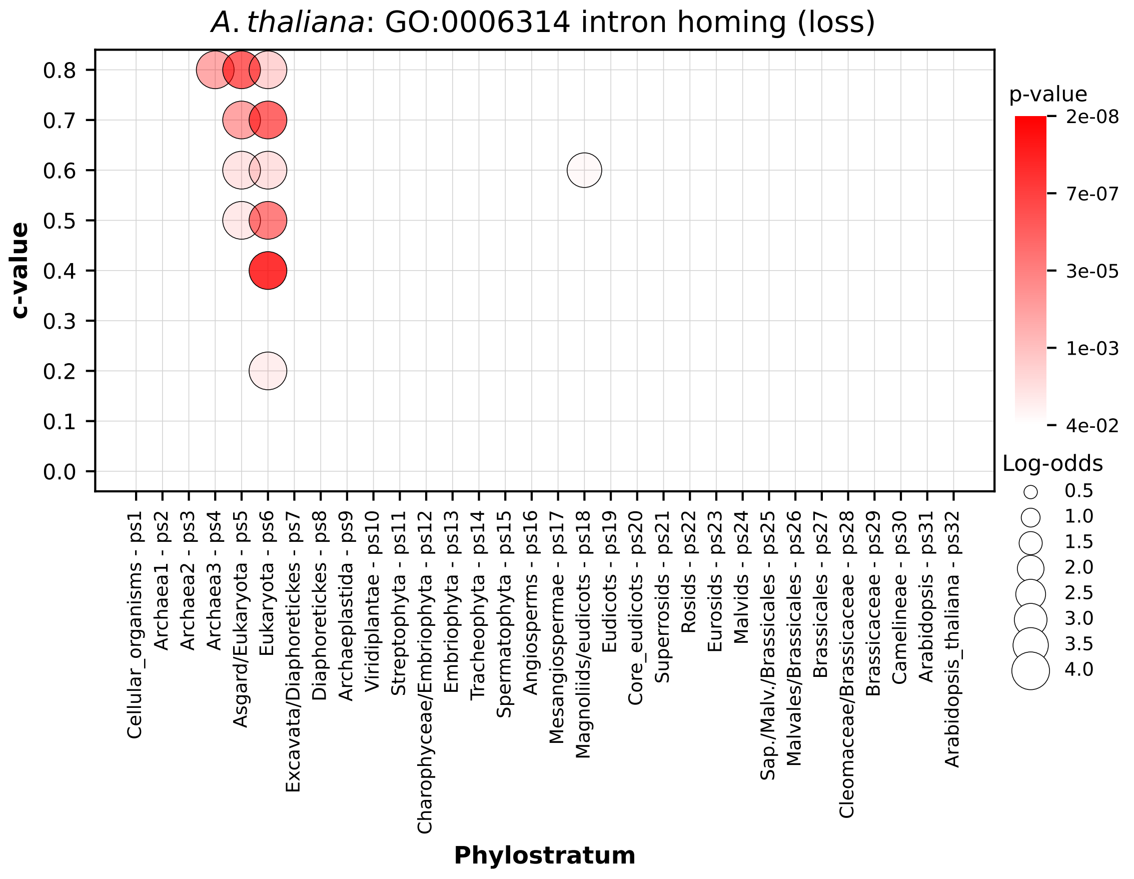

A. thaliana: GO:0006314 intron homing (loss)
c-value
0.0
0.1
0.2
0.3
0.4
0.5
0.6
0.7
0.8
p-value
2e-08
7e-07
3e-05
1e-03
4e-02
Log-odds
0.5
1.0
1.5
2.0
2.5
3.0
3.5
4.0
Cellular_organisms - ps1
Archaea1 - ps2
Archaea2 - ps3
Archaea3 - ps4
Asgard/Eukaryota - ps5
Eukaryota - ps6
Excavata/Diaphoretickes - ps7
Diaphoretickes - ps8
Archaeplastida - ps9
Viridiplantae - ps10
Streptophyta - ps11
Charophyceae/Embriophyta - ps12
Embriophyta - ps13
Tracheophyta - ps14
Spermatophyta - ps15
Angiosperms - ps16
Mesangiospermae - ps17
Magnoliids/eudicots - ps18
Eudicots - ps19
Core_eudicots - ps20
Superrosids - ps21
Rosids - ps22
Eurosids - ps23
Malvids - ps24
Sap./Malv./Brassicales - ps25
Malvales/Brassicales - ps26
Brassicales - ps27
Cleomaceae/Brassicaceae - ps28
Brassicaceae - ps29
Camelineae - ps30
Arabidopsis - ps31
Arabidopsis_thaliana - ps32
Phylostratum

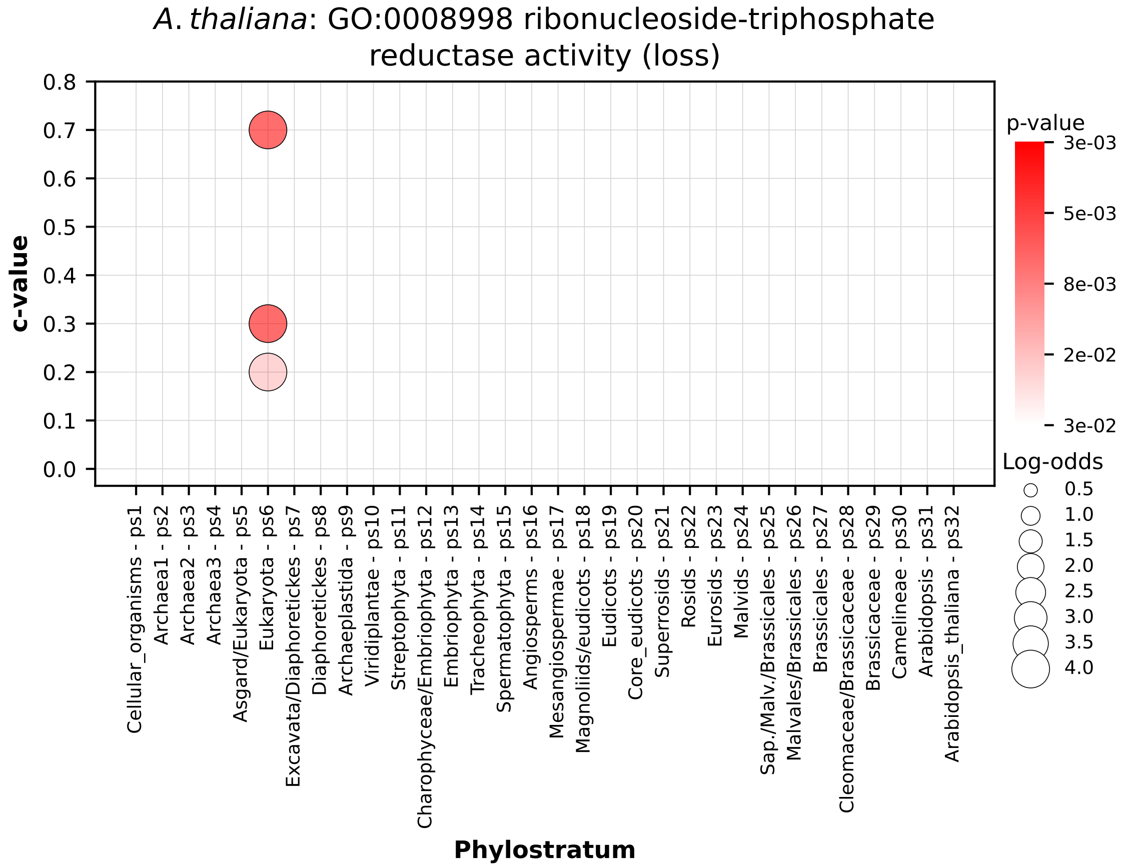

A. thaliana: GO:0008998 ribonucleoside-triphosphate reductase activity (loss)
c-value
0.8
0.7
0.6
0.5
0.4
0.3
0.2
0.1
0.0
Phylostratum
Cellular_organisms - ps1
Archaea1 - ps2
Archaea2 - ps3
Archaea3 - ps4
Asgard/Eukaryota - ps5
Eukaryota - ps6
Excavata/Diaphoretickes - ps7
Diaphoretickes - ps8
Archaeplastida - ps9
Viridiplantae - ps10
Streptophyta - ps11
Charophyceae/Embriophyta - ps12
Embriophyta - ps13
Tracheophyta - ps14
Spermatophyta - ps15
Angiosperms - ps16
Mesangiospermae - ps17
Magnoliids/eudicots - ps18
Eudicots - ps19
Core_eudicots - ps20
Superrosids - ps21
Rosids - ps22
Eurosids - ps23
Malvids - ps24
Sap./Malv./Brassicales - ps25
Malvales/Brassicales - ps26
Brassicales - ps27
Cleomaceae/Brassicaceae - ps28
Brassicaceae - ps29
Camelineae - ps30
Arabidopsis - ps31
Arabidopsis_thaliana - ps32
p-value
3e-03
5e-03
8e-03
2e-02
3e-02
Log-odds
0.5
1.0
1.5
2.0
2.5
3.0
3.5
4.0

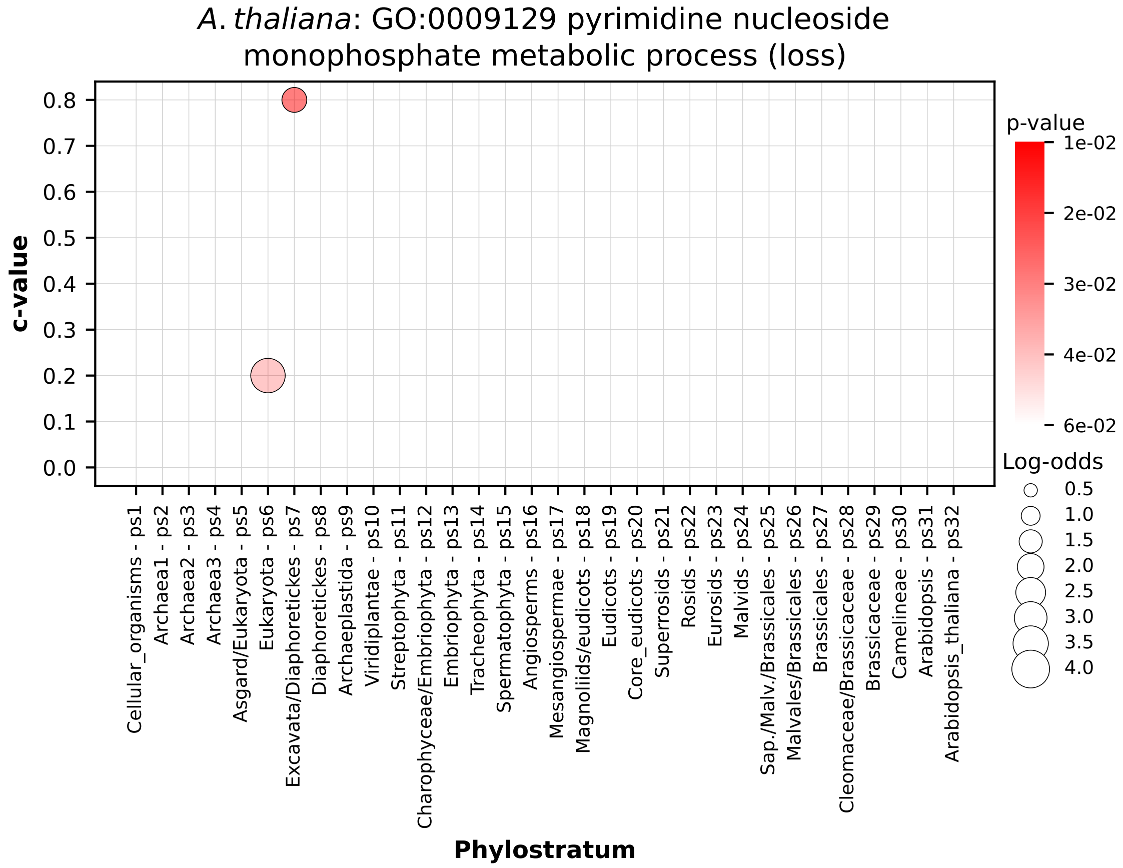

A. thaliana: GO:0009129 pyrimidine nucleoside monophosphate metabolic process (loss)
c-value
0.8
0.7
0.6
0.5
0.4
0.3
0.2
0.1
0.0
p-value
1e-02
2e-02
3e-02
4e-02
6e-02
Log-odds
0.5
1.0
1.5
2.0
2.5
3.0
3.5
4.0
Cellular_organisms - ps1
Archaea1 - ps2
Archaea2 - ps3
Archaea3 - ps4
Asgard/Eukaryota - ps5
Eukaryota - ps6
Excavata/Diaphoretickes - ps7
Diaphoretickes - ps8
Archaeplastida - ps9
Viridiplantae - ps10
Streptophyta - ps11
Charophyceae/Embriophyta - ps12
Embriophyta - ps13
Tracheophyta - ps14
Spermatophyta - ps15
Angiosperms - ps16
Mesangiospermae - ps17
Magnoliids/eudicots - ps18
Eudicots - ps19
Core_eudicots - ps20
Superrosids - ps21
Rosids - ps22
Eurosids - ps23
Malvids - ps24
Sap./Malv./Brassicales - ps25
Malvales/Brassicales - ps26
Brassicales - ps27
Cleomaceae/Brassicaceae - ps28
Brassicaceae - ps29
Camelineae - ps30
Arabidopsis - ps31
Arabidopsis_thaliana - ps32
Phylostratum

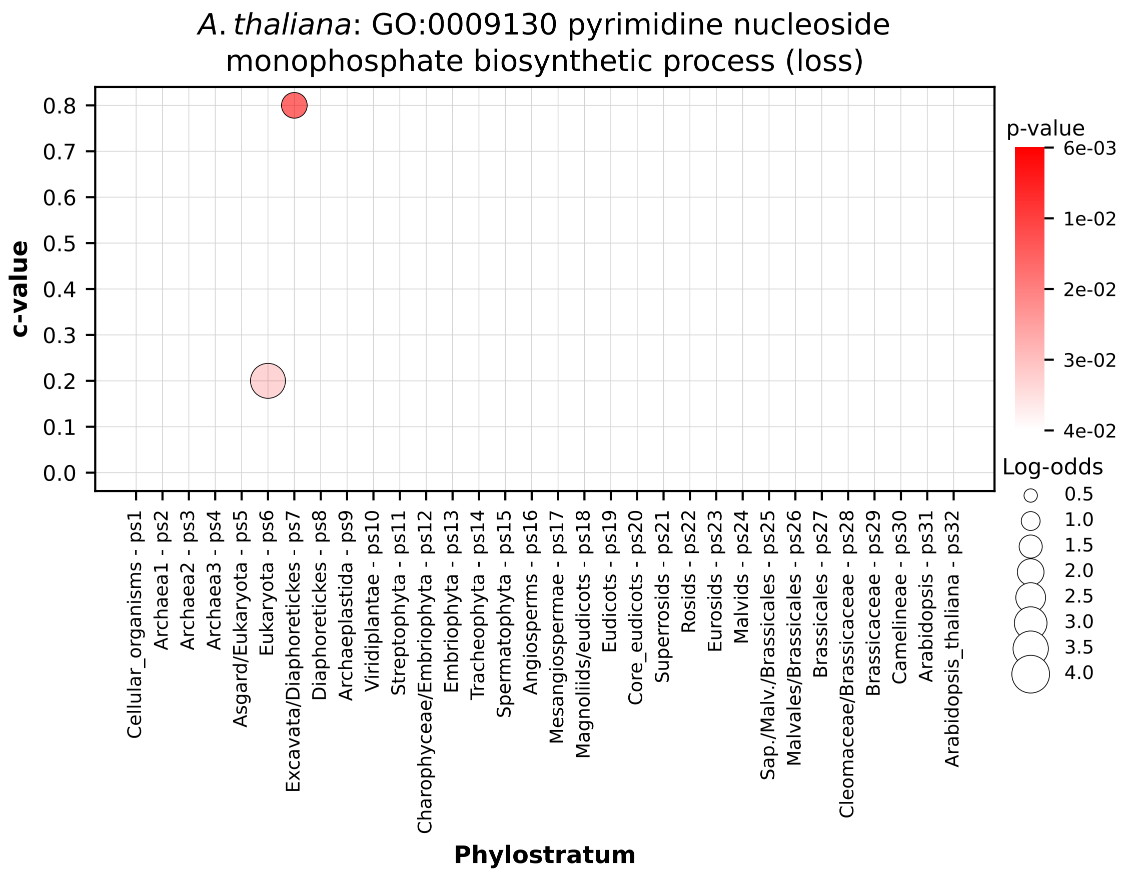
A. thaliana: GO:0009130 pyrimidine nucleoside monophosphate biosynthetic process (loss)
c-value
0.0
0.1
0.2
0.3
0.4
0.5
0.6
0.7
0.8
Phylostratum
Cellular_organisms - ps1
Archaea1 - ps2
Archaea2 - ps3
Archaea3 - ps4
Asgard/Eukaryota - ps5
Eukaryota - ps6
Excavata/Diaphoretickes - ps7
Diaphoretickes - ps8
Archaeplastida - ps9
Viridiplantae - ps10
Streptophyta - ps11
Charophyceae/Embriophyta - ps12
Embriophyta - ps13
Tracheophyta - ps14
Spermatophyta - ps15
Angiosperms - ps16
Mesangiospermae - ps17
Magnoliids/eudicots - ps18
Eudicots - ps19
Core_eudicots - ps20
Superrosids - ps21
Rosids - ps22
Eurosids - ps23
Malvids - ps24
Sap./Malv./Brassicales - ps25
Malvales/Brassicales - ps26
Brassicales - ps27
Cleomaceae/Brassicaceae - ps28
Brassicaceae - ps29
Camelineae - ps30
Arabidopsis - ps31
Arabidopsis_thaliana - ps32
p-value
6e-03
1e-02
2e-02
3e-02
4e-02
Log-odds
0.5
1.0
1.5
2.0
2.5
3.0
3.5
4.0

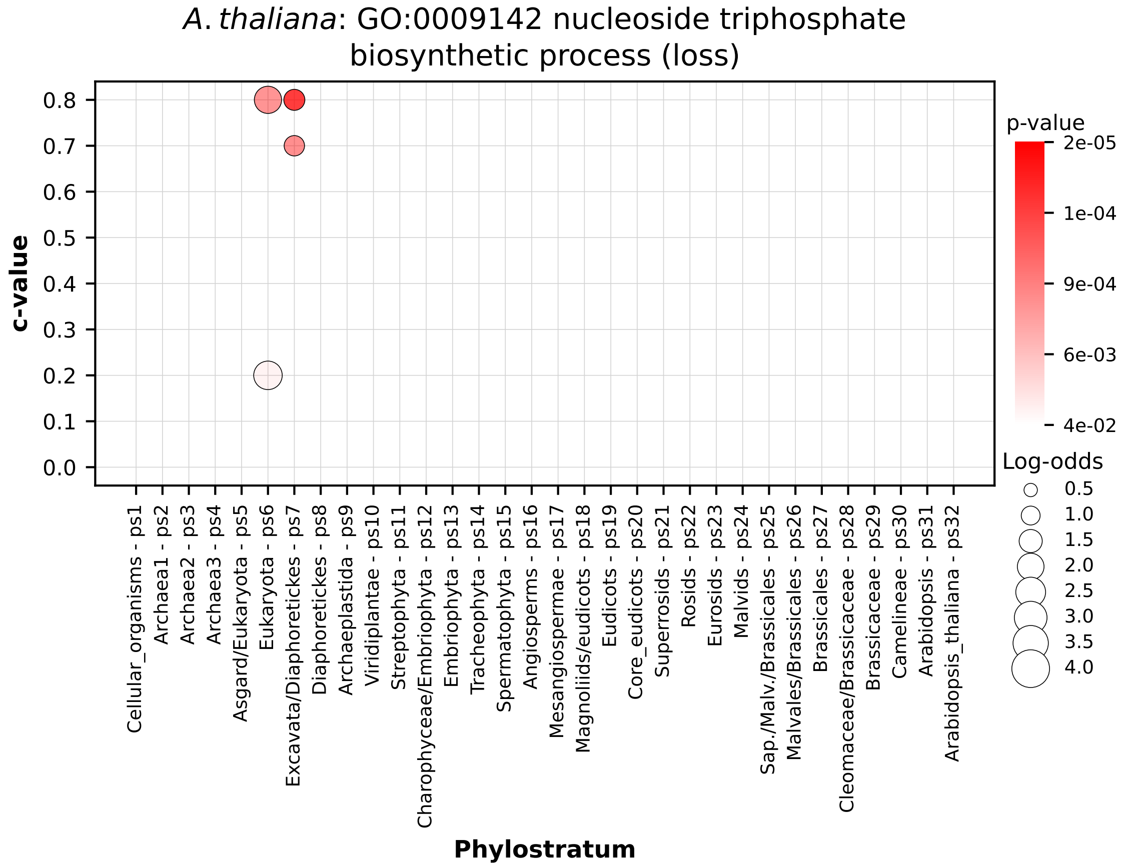

A. thaliana: GO:0009142 nucleoside triphosphate biosynthetic process (loss)
c-value
0.0
0.1
0.2
0.3
0.4
0.5
0.6
0.7
0.8
p-value
2e-05
1e-04
9e-04
6e-03
4e-02
Log-odds
0.5
1.0
1.5
2.0
2.5
3.0
3.5
4.0
Cellular_organisms - ps1
Archaea1 - ps2
Archaea2 - ps3
Archaea3 - ps4
Asgard/Eukaryota - ps5
Eukaryota - ps6
Excavata/Diaphoretickes - ps7
Diaphoretickes - ps8
Archaeplastida - ps9
Viridiplantae - ps10
Streptophyta - ps11
Charophyceae/Embriophyta - ps12
Embriophyta - ps13
Tracheophyta - ps14
Spermatophyta - ps15
Angiosperms - ps16
Mesangiospermae - ps17
Magnoliids/eudicots - ps18
Eudicots - ps19
Core_eudicots - ps20
Superrosids - ps21
Rosids - ps22
Eurosids - ps23
Malvids - ps24
Sap./Malv./Brassicales - ps25
Malvales/Brassicales - ps26
Brassicales - ps27
Cleomaceae/Brassicaceae - ps28
Brassicaceae - ps29
Camelineae - ps30
Arabidopsis - ps31
Arabidopsis_thaliana - ps32
Phylostratum

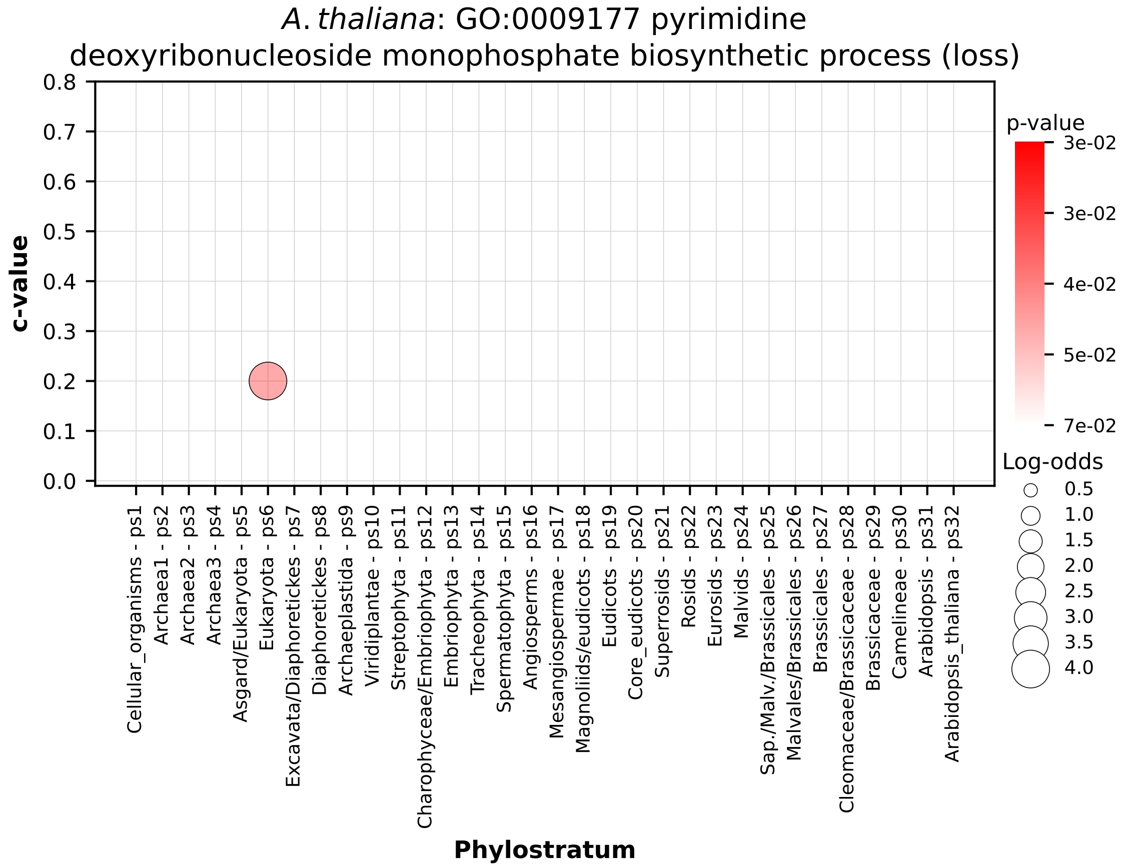
A. thaliana: GO:0009177 pyrimidine deoxyribonucleoside monophosphate biosynthetic process (loss)
c-value
0.0
0.1
0.2
0.3
0.4
0.5
0.6
0.7
0.8
Phylostratum
Cellular_organisms - ps1
Archaea1 - ps2
Archaea2 - ps3
Archaea3 - ps4
Asgard/Eukaryota - ps5
Eukaryota - ps6
Excavata/Diaphoretickes - ps7
Diaphoretickes - ps8
Archaeplastida - ps9
Viridiplantae - ps10
Streptophyta - ps11
Charophyceae/Embriophyta - ps12
Embriophyta - ps13
Tracheophyta - ps14
Spermatophyta - ps15
Angiosperms - ps16
Mesangiospermae - ps17
Magnoliids/eudicots - ps18
Eudicots - ps19
Core_eudicots - ps20
Superrosids - ps21
Rosids - ps22
Eurosids - ps23
Malvids - ps24
Sap./Malv./Brassicales - ps25
Malvales/Brassicales - ps26
Brassicales - ps27
Cleomaceae/Brassicaceae - ps28
Brassicaceae - ps29
Camelineae - ps30
Arabidopsis - ps31
Arabidopsis_thaliana - ps32
p-value
3e-02
3e-02
4e-02
5e-02
7e-02
Log-odds
0.5
1.0
1.5
2.0
2.5
3.0
3.5
4.0

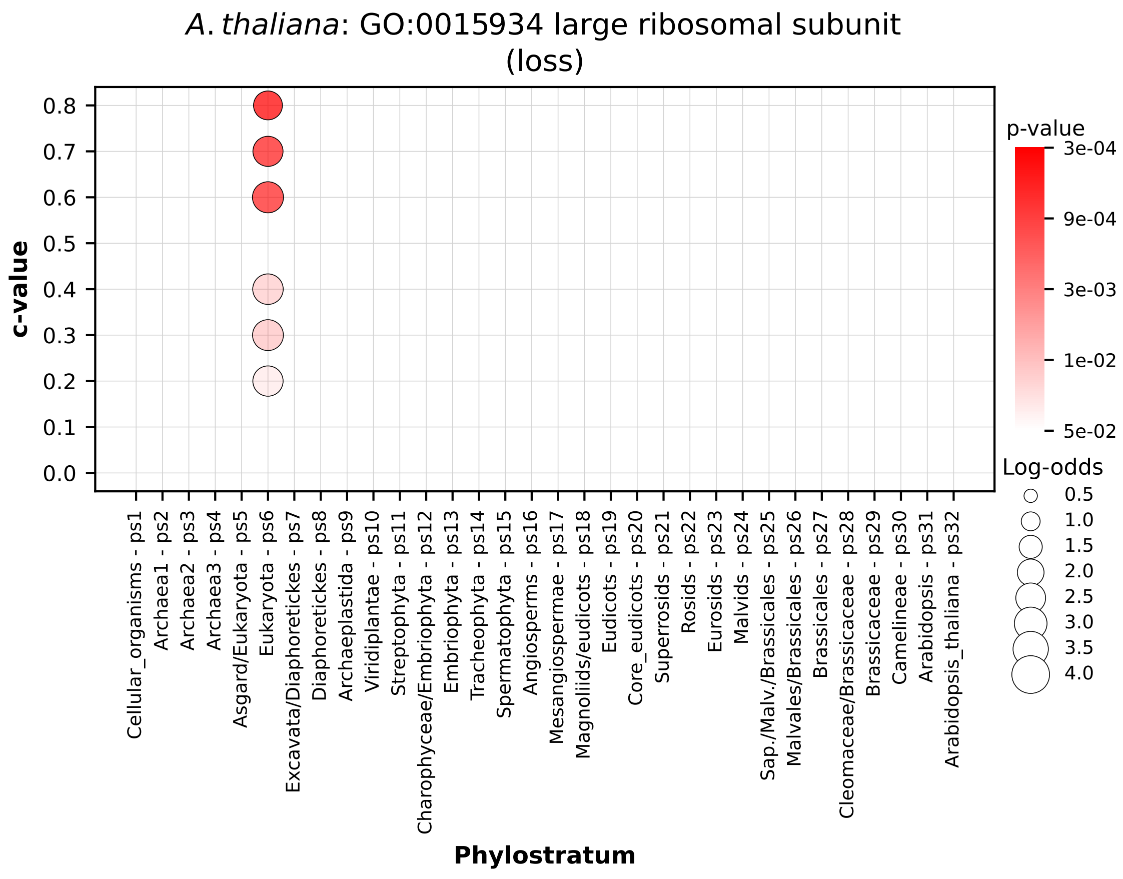
A. thaliana: GO:0015934 large ribosomal subunit
(loss)
c-value
0.8
0.7
0.6
0.5
0.4
0.3
0.2
0.1
0.0
p-value
3e-04
9e-04
3e-03
1e-02
5e-02
Log-odds
0.5
1.0
1.5
2.0
2.5
3.0
3.5
4.0
Cellular_organisms - ps1
Archaea1 - ps2
Archaea2 - ps3
Archaea3 - ps4
Asgard/Eukaryota - ps5
Eukaryota - ps6
Excavata/Diaphoretickes - ps7
Diaphoretickes - ps8
Archaeplastida - ps9
Viridiplantae - ps10
Streptophyta - ps11
Charophyceae/Embriophyta - ps12
Embriophyta - ps13
Tracheophyta - ps14
Spermatophyta - ps15
Angiosperms - ps16
Mesangiospermae - ps17
Magnoliids/eudicots - ps18
Eudicots - ps19
Core_eudicots - ps20
Superrosids - ps21
Rosids - ps22
Eurosids - ps23
Malvids - ps24
Sap./Malv./Brassicales - ps25
Malvales/Brassicales - ps26
Brassicales - ps27
Cleomaceae/Brassicaceae - ps28
Brassicaceae - ps29
Camelineae - ps30
Arabidopsis - ps31
Arabidopsis_thaliana - ps32
Phylostratum

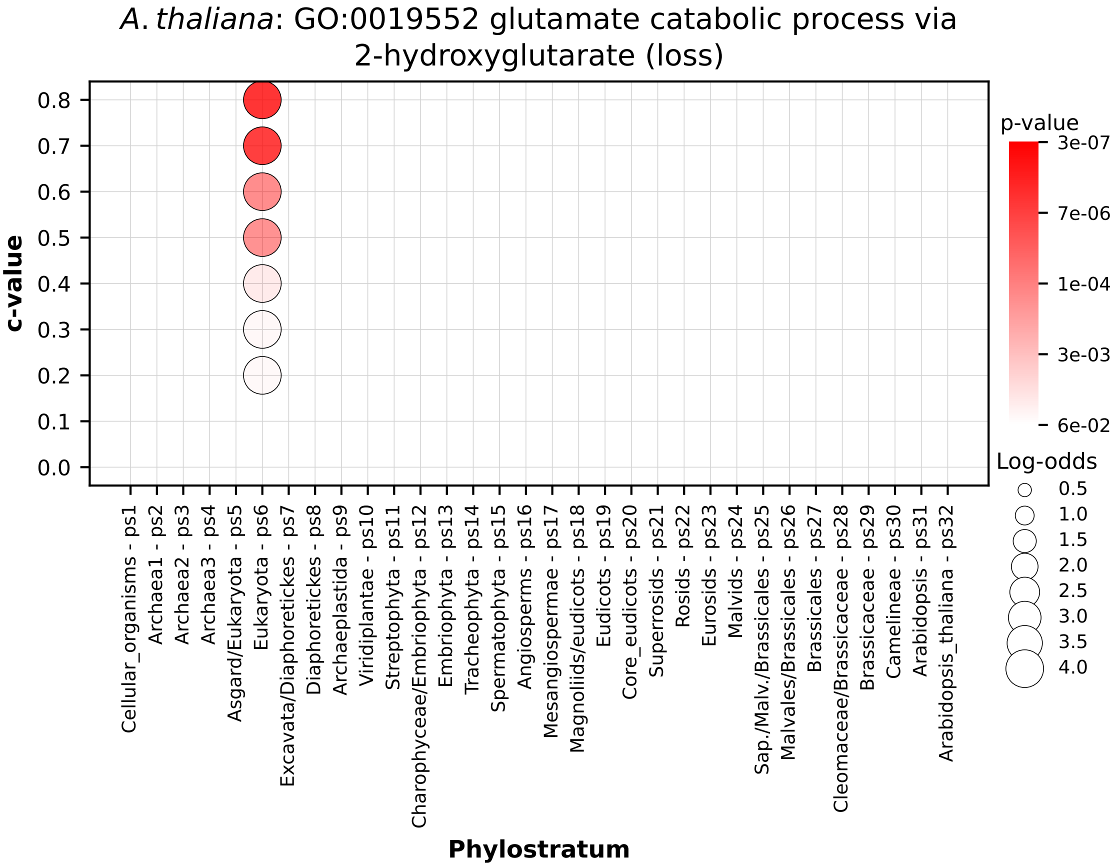

A. thaliana: GO:0019552 glutamate catabolic process via 2-hydroxyglutarate (loss)
c-value
0.8
0.7
0.6
0.5
0.4
0.3
0.2
0.1
0.0
Phylostratum
Cellular_organisms - ps1
Archaea1 - ps2
Archaea2 - ps3
Archaea3 - ps4
Asgard/Eukaryota - ps5
Eukaryota - ps6
Excavata/Diaphoretickes - ps7
Diaphoretickes - ps8
Archaeplastida - ps9
Viridiplantae - ps10
Streptophyta - ps11
Charophyceae/Embriophyta - ps12
Embriophyta - ps13
Tracheophyta - ps14
Spermatophyta - ps15
Angiosperms - ps16
Mesangiospermae - ps17
Magnoliids/eudicots - ps18
Eudicots - ps19
Core_eudicots - ps20
Superrosids - ps21
Rosids - ps22
Eurosids - ps23
Malvids - ps24
Sap./Malv./Brassicales - ps25
Malvales/Brassicales - ps26
Brassicales - ps27
Cleomaceae/Brassicaceae - ps28
Brassicaceae - ps29
Camelineae - ps30
Arabidopsis - ps31
Arabidopsis_thaliana - ps32
p-value
3e-07
7e-06
1e-04
3e-03
6e-02
Log-odds
0.5
1.0
1.5
2.0
2.5
3.0
3.5
4.0

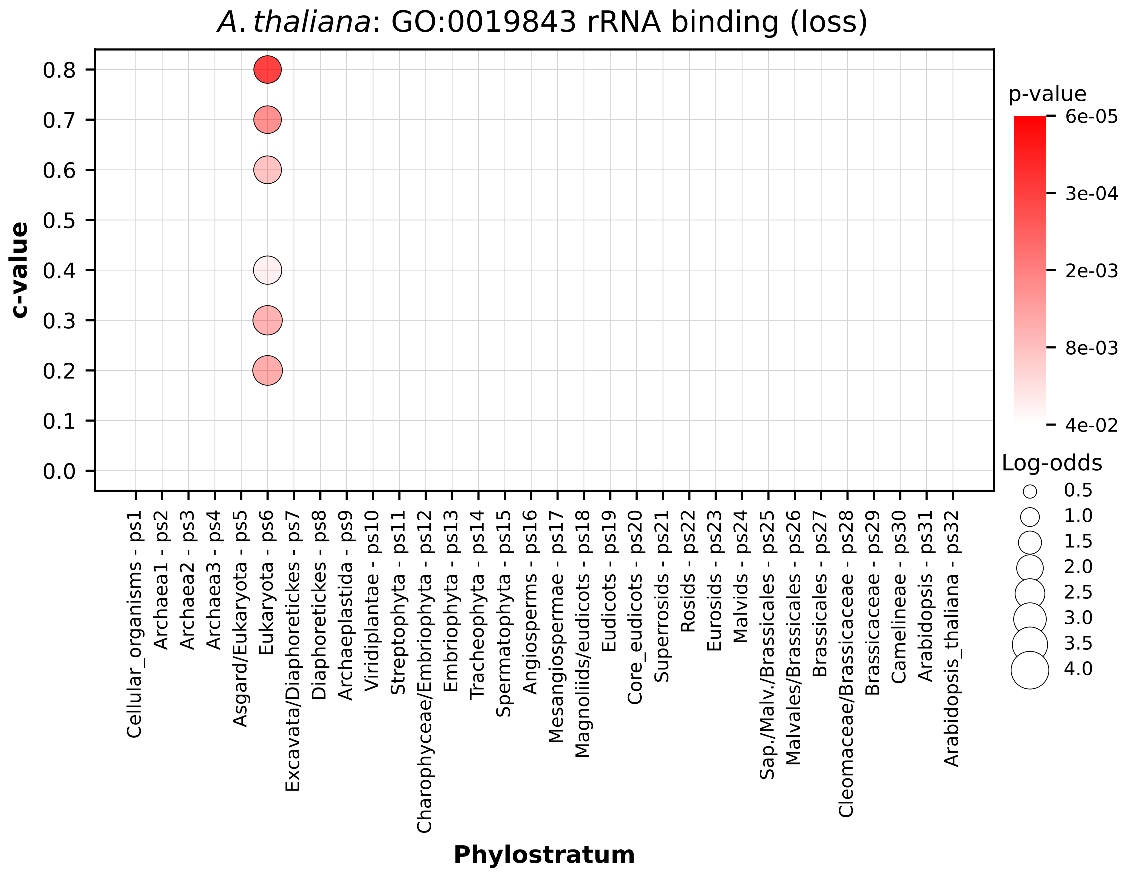

A. thaliana: GO:0019843 rRNA binding (loss)
c-value
0.8
0.7
0.6
0.5
0.4
0.3
0.2
0.1
0.0
p-value
6e-05
3e-04
2e-03
8e-03
4e-02
Log-odds
0.5
1.0
1.5
2.0
2.5
3.0
3.5
4.0
Cellular_organisms - ps1
Archaea1 - ps2
Archaea2 - ps3
Archaea3 - ps4
Asgard/Eukaryota - ps5
Eukaryota - ps6
Excavata/Diaphoretickes - ps7
Diaphoretickes - ps8
Archaeplastida - ps9
Viridiplantae - ps10
Streptophyta - ps11
Charophyceae/Embriophyta - ps12
Embriophyta - ps13
Tracheophyta - ps14
Spermatophyta - ps15
Angiosperms - ps16
Mesangiospermae - ps17
Magnoliids/eudicots - ps18
Eudicots - ps19
Core_eudicots - ps20
Superrosids - ps21
Rosids - ps22
Eurosids - ps23
Malvids - ps24
Sap./Malv./Brassicales - ps25
Malvales/Brassicales - ps26
Brassicales - ps27
Cleomaceae/Brassicaceae - ps28
Brassicaceae - ps29
Camelineae - ps30
Arabidopsis - ps31
Arabidopsis_thaliana - ps32
Phylostratum

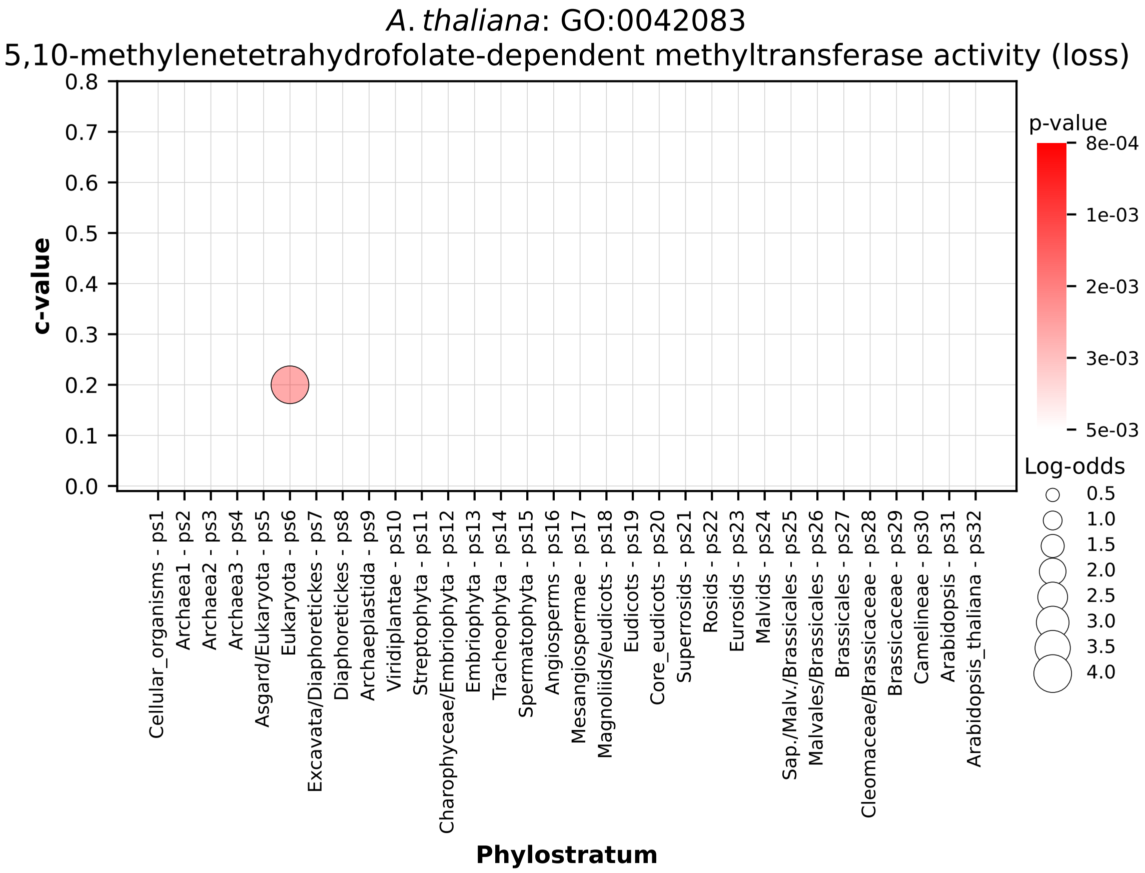

A. thaliana: GO:0042083
5,10-methylenetetrahydrofolate-dependent methyltransferase activity (loss)
c-value
0.8
0.7
0.6
0.5
0.4
0.3
0.2
0.1
0.0
p-value
8e-04
1e-03
2e-03
3e-03
5e-03
Log-odds
0.5
1.0
1.5
2.0
2.5
3.0
3.5
4.0
Cellular_organisms - ps1
Archaea1 - ps2
Archaea2 - ps3
Archaea3 - ps4
Asgard/Eukaryota - ps5
Eukaryota - ps6
Excavata/Diaphoretickes - ps7
Diaphoretickes - ps8
Archaeplastida - ps9
Viridiplantae - ps10
Streptophyta - ps11
Charophyceae/Embriophyta - ps12
Embriophyta - ps13
Tracheophyta - ps14
Spermatophyta - ps15
Angiosperms - ps16
Mesangiospermae - ps17
Magnoliids/eudicots - ps18
Eudicots - ps19
Core_eudicots - ps20
Superrosids - ps21
Rosids - ps22
Eurosids - ps23
Malvids - ps24
Sap./Malv./Brassicales - ps25
Malvales/Brassicales - ps26
Brassicales - ps27
Cleomaceae/Brassicaceae - ps28
Brassicaceae - ps29
Camelineae - ps30
Arabidopsis - ps31
Arabidopsis_thaliana - ps32
Phylostratum

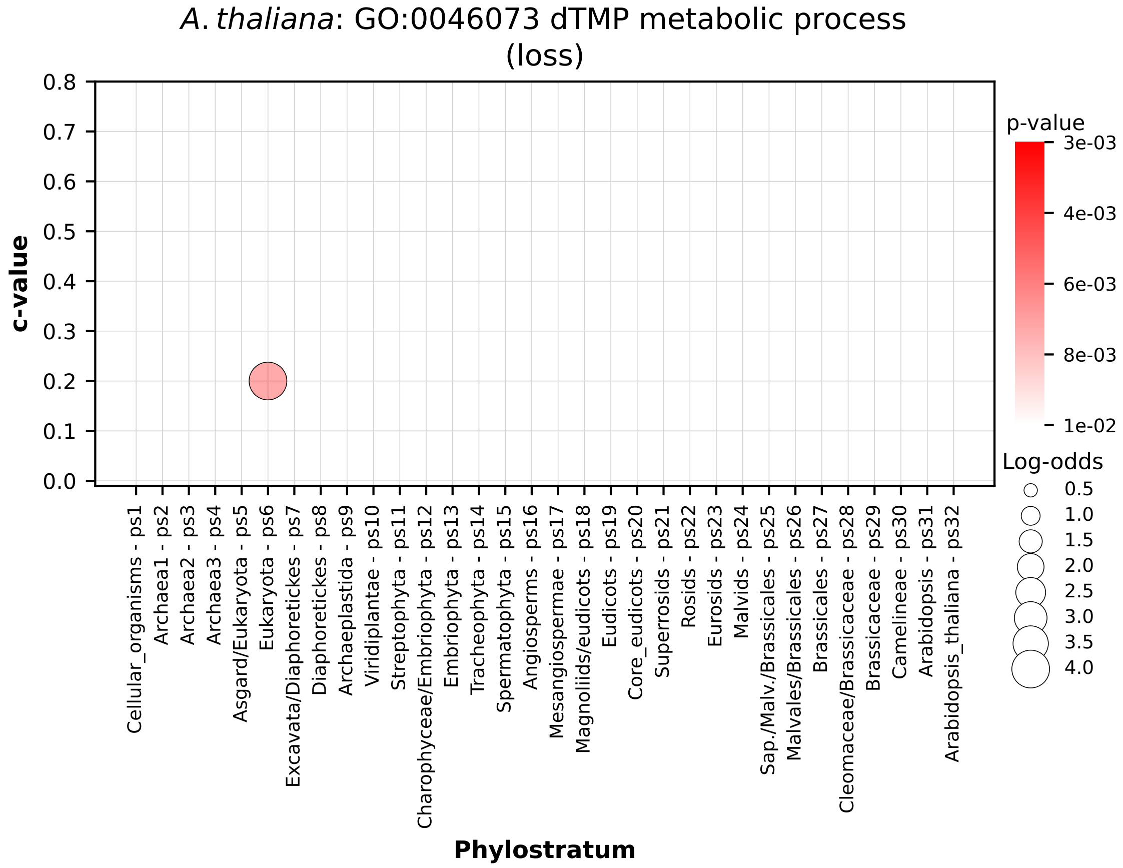

A. thaliana: GO:0046073 dTMP metabolic process
(loss)
c-value
0.0
0.1
0.2
0.3
0.4
0.5
0.6
0.7
0.8
Phylostratum
Cellular_organisms - ps1
Archaea1 - ps2
Archaea2 - ps3
Archaea3 - ps4
Asgard/Eukaryota - ps5
Eukaryota - ps6
Excavata/Diaphoretickes - ps7
Diaphoretickes - ps8
Archaeplastida - ps9
Viridiplantae - ps10
Streptophyta - ps11
Charophyceae/Embriophyta - ps12
Embriophyta - ps13
Tracheophyta - ps14
Spermatophyta - ps15
Angiosperms - ps16
Mesangiospermae - ps17
Magnoliids/eudicots - ps18
Eudicots - ps19
Core_eudicots - ps20
Superrosids - ps21
Rosids - ps22
Eurosids - ps23
Malvids - ps24
Sap./Malv./Brassicales - ps25
Malvales/Brassicales - ps26
Brassicales - ps27
Cleomaceae/Brassicaceae - ps28
Brassicaceae - ps29
Camelineae - ps30
Arabidopsis - ps31
Arabidopsis_thaliana - ps32
p-value
3e-03
4e-03
6e-03
8e-03
1e-02
Log-odds
0.5
1.0
1.5
2.0
2.5
3.0
3.5
4.0

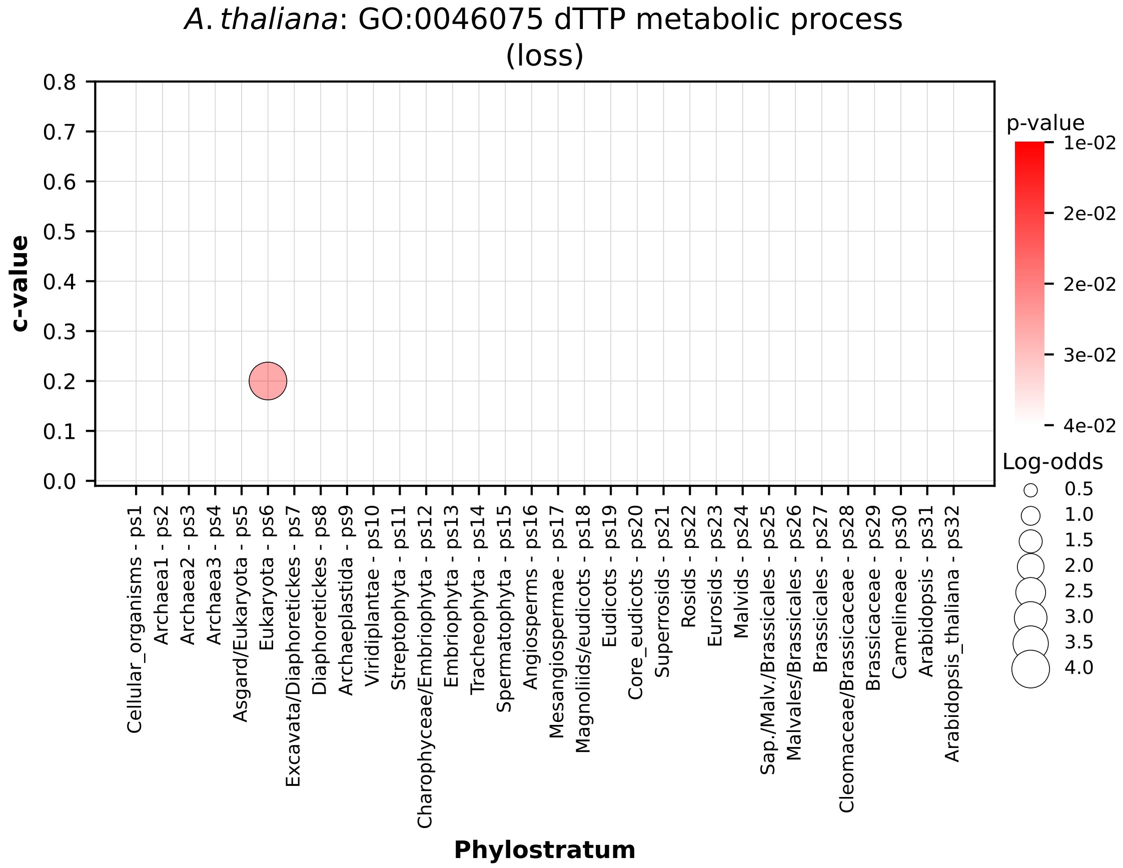

A. thaliana: GO:0046075 dTTP metabolic process
(loss)
c-value
0.8
0.7
0.6
0.5
0.4
0.3
0.2
0.1
0.0
p-value
1e-02
2e-02
2e-02
3e-02
4e-02
Log-odds
0.5
1.0
1.5
2.0
2.5
3.0
3.5
4.0
Cellular_organisms - ps1
Archaea1 - ps2
Archaea2 - ps3
Archaea3 - ps4
Asgard/Eukaryota - ps5
Eukaryota - ps6
Excavata/Diaphoretickes - ps7
Diaphoretickes - ps8
Archaeplastida - ps9
Viridiplantae - ps10
Streptophyta - ps11
Charophyceae/Embriophyta - ps12
Embriophyta - ps13
Tracheophyta - ps14
Spermatophyta - ps15
Angiosperms - ps16
Mesangiospermae - ps17
Magnoliids/eudicots - ps18
Eudicots - ps19
Core_eudicots - ps20
Superrosids - ps21
Rosids - ps22
Eurosids - ps23
Malvids - ps24
Sap./Malv./Brassicales - ps25
Malvales/Brassicales - ps26
Brassicales - ps27
Cleomaceae/Brassicaceae - ps28
Brassicaceae - ps29
Camelineae - ps30
Arabidopsis - ps31
Arabidopsis_thaliana - ps32
Phylostratum

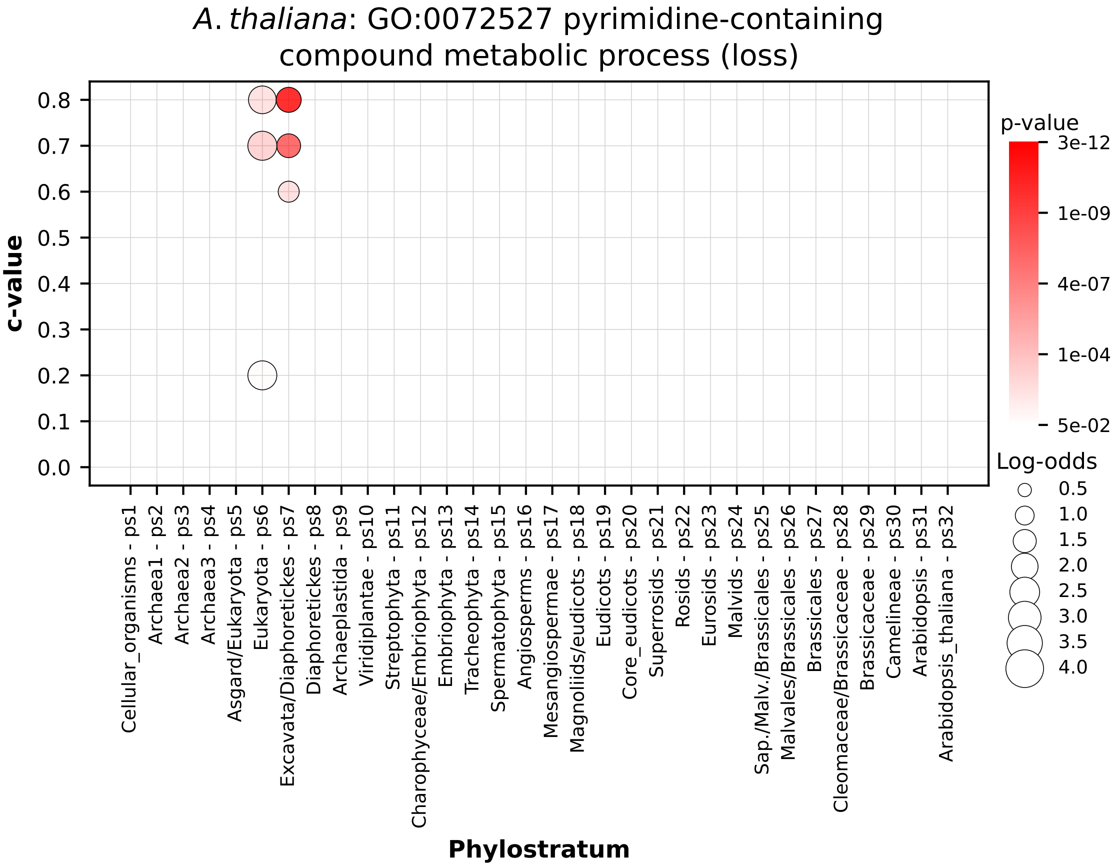

A. thaliana: GO:0072527 pyrimidine-containing compound metabolic process (loss)
c-value
0.8
0.7
0.6
0.5
0.4
0.3
0.2
0.1
0.0
Phylostratum
Cellular_organisms - ps1
Archaea1 - ps2
Archaea2 - ps3
Archaea3 - ps4
Asgard/Eukaryota - ps5
Eukaryota - ps6
Excavata/Diaphoretickes - ps7
Diaphoretickes - ps8
Archaeplastida - ps9
Viridiplantae - ps10
Streptophyta - ps11
Charophyceae/Embriophyta - ps12
Embriophyta - ps13
Tracheophyta - ps14
Spermatophyta - ps15
Angiosperms - ps16
Mesangiospermae - ps17
Magnoliids/eudicots - ps18
Eudicots - ps19
Core_eudicots - ps20
Superrosids - ps21
Rosids - ps22
Eurosids - ps23
Malvids - ps24
Sap./Malv./Brassicales - ps25
Malvales/Brassicales - ps26
Brassicales - ps27
Cleomaceae/Brassicaceae - ps28
Brassicaceae - ps29
Camelineae - ps30
Arabidopsis - ps31
Arabidopsis_thaliana - ps32
p-value
3e-12
1e-09
4e-07
1e-04
5e-02
Log-odds
0.5
1.0
1.5
2.0
2.5
3.0
3.5
4.0

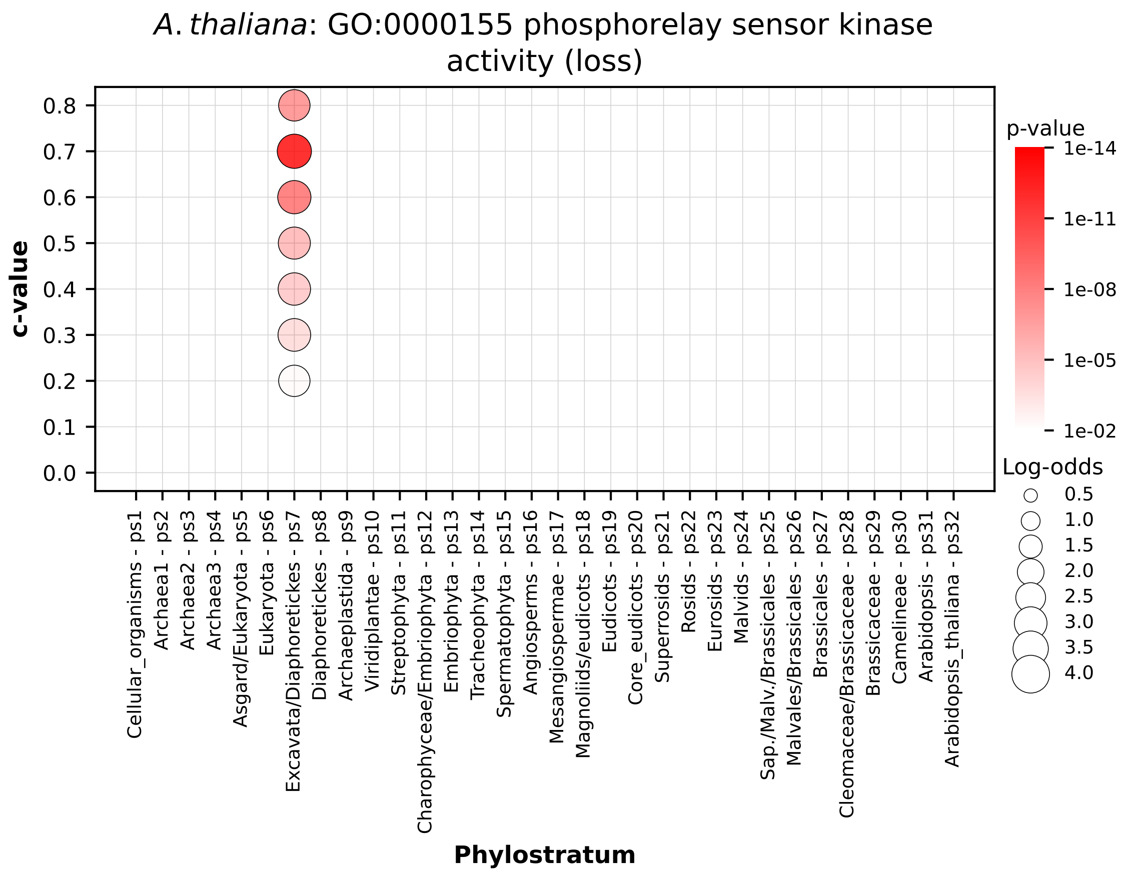

A. thaliana: GO:0000155 phosphorelay sensor kinase activity (loss)
c-value
0.8
0.7
0.6
0.5
0.4
0.3
0.2
0.1
0.0
p-value
1e-14
1e-11
1e-08
1e-05
1e-02
Log-odds
0.5
1.0
1.5
2.0
2.5
3.0
3.5
4.0
Cellular_organisms - ps1
Archaea1 - ps2
Archaea2 - ps3
Archaea3 - ps4
Asgard/Eukaryota - ps5
Eukaryota - ps6
Excavata/Diaphoretickes - ps7
Diaphoretickes - ps8
Archaeplastida - ps9
Viridiplantae - ps10
Streptophyta - ps11
Charophyceae/Embriophyta - ps12
Embriophyta - ps13
Tracheophyta - ps14
Spermatophyta - ps15
Angiosperms - ps16
Mesangiospermae - ps17
Magnoliids/eudicots - ps18
Eudicots - ps19
Core_eudicots - ps20
Superrosids - ps21
Rosids - ps22
Eurosids - ps23
Malvids - ps24
Sap./Malv./Brassicales - ps25
Malvales/Brassicales - ps26
Brassicales - ps27
Cleomaceae/Brassicaceae - ps28
Brassicaceae - ps29
Camelineae - ps30
Arabidopsis - ps31
Arabidopsis_thaliana - ps32
Phylostratum

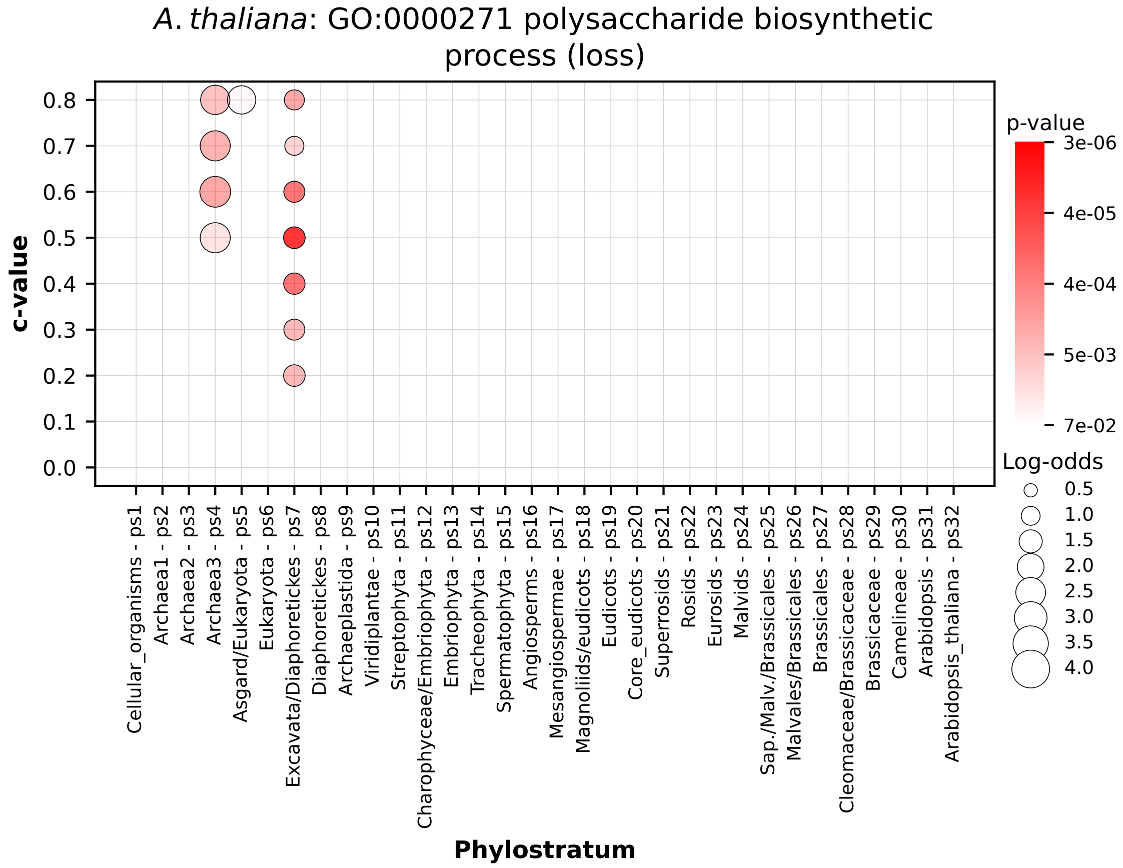

A. thaliana: GO:0000271 polysaccharide biosynthetic process (loss)
c-value
0.0
0.1
0.2
0.3
0.4
0.5
0.6
0.7
0.8
p-value
3e-06
4e-05
4e-04
5e-03
7e-02
Log-odds
0.5
1.0
1.5
2.0
2.5
3.0
3.5
4.0
Cellular_organisms - ps1
Archaea1 - ps2
Archaea2 - ps3
Archaea3 - ps4
Asgard/Eukaryota - ps5
Eukaryota - ps6
Excavata/Diaphoretickes - ps7
Diaphoretickes - ps8
Archaeplastida - ps9
Viridiplantae - ps10
Streptophyta - ps11
Charophyceae/Embriophyta - ps12
Embriophyta - ps13
Tracheophyta - ps14
Spermatophyta - ps15
Angiosperms - ps16
Mesangiospermae - ps17
Magnoliids/eudicots - ps18
Eudicots - ps19
Core_eudicots - ps20
Superrosids - ps21
Rosids - ps22
Eurosids - ps23
Malvids - ps24
Sap./Malv./Brassicales - ps25
Malvales/Brassicales - ps26
Brassicales - ps27
Cleomaceae/Brassicaceae - ps28
Brassicaceae - ps29
Camelineae - ps30
Arabidopsis - ps31
Arabidopsis_thaliana - ps32
Phylostratum

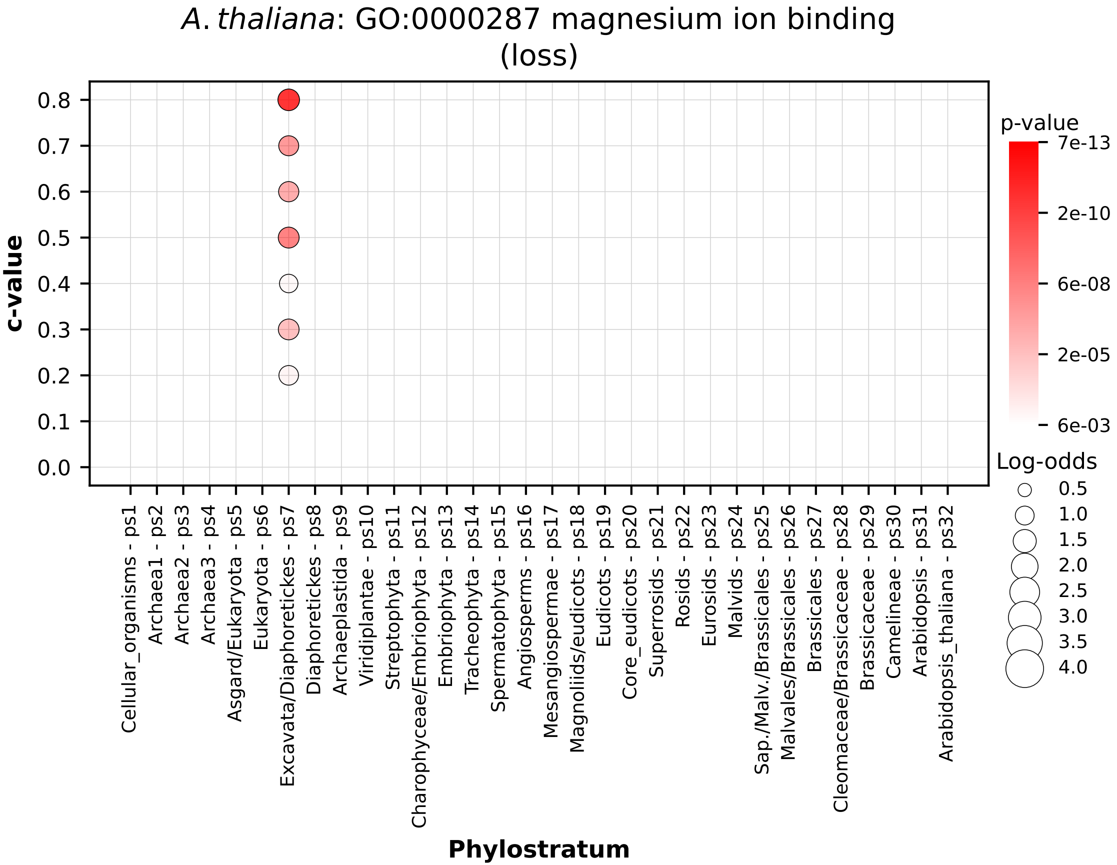

A. thaliana: GO:0000287 magnesium ion binding
(loss)
c-value
0.8
0.7
0.6
0.5
0.4
0.3
0.2
0.1
0.0
Phylostratum
Cellular_organisms - ps1
Archaea1 - ps2
Archaea2 - ps3
Archaea3 - ps4
Asgard/Eukaryota - ps5
Eukaryota - ps6
Excavata/Diaphoretickes - ps7
Diaphoretickes - ps8
Archaeplastida - ps9
Viridiplantae - ps10
Streptophyta - ps11
Charophyceae/Embriophyta - ps12
Embriophyta - ps13
Tracheophyta - ps14
Spermatophyta - ps15
Angiosperms - ps16
Mesangiospermae - ps17
Magnoliids/eudicots - ps18
Eudicots - ps19
Core_eudicots - ps20
Superrosids - ps21
Rosids - ps22
Eurosids - ps23
Malvids - ps24
Sap./Malv./Brassicales - ps25
Malvales/Brassicales - ps26
Brassicales - ps27
Cleomaceae/Brassicaceae - ps28
Brassicaceae - ps29
Camelineae - ps30
Arabidopsis - ps31
Arabidopsis_thaliana - ps32
p-value
7e-13
2e-10
6e-08
2e-05
6e-03
Log-odds
0.5
1.0
1.5
2.0
2.5
3.0
3.5
4.0

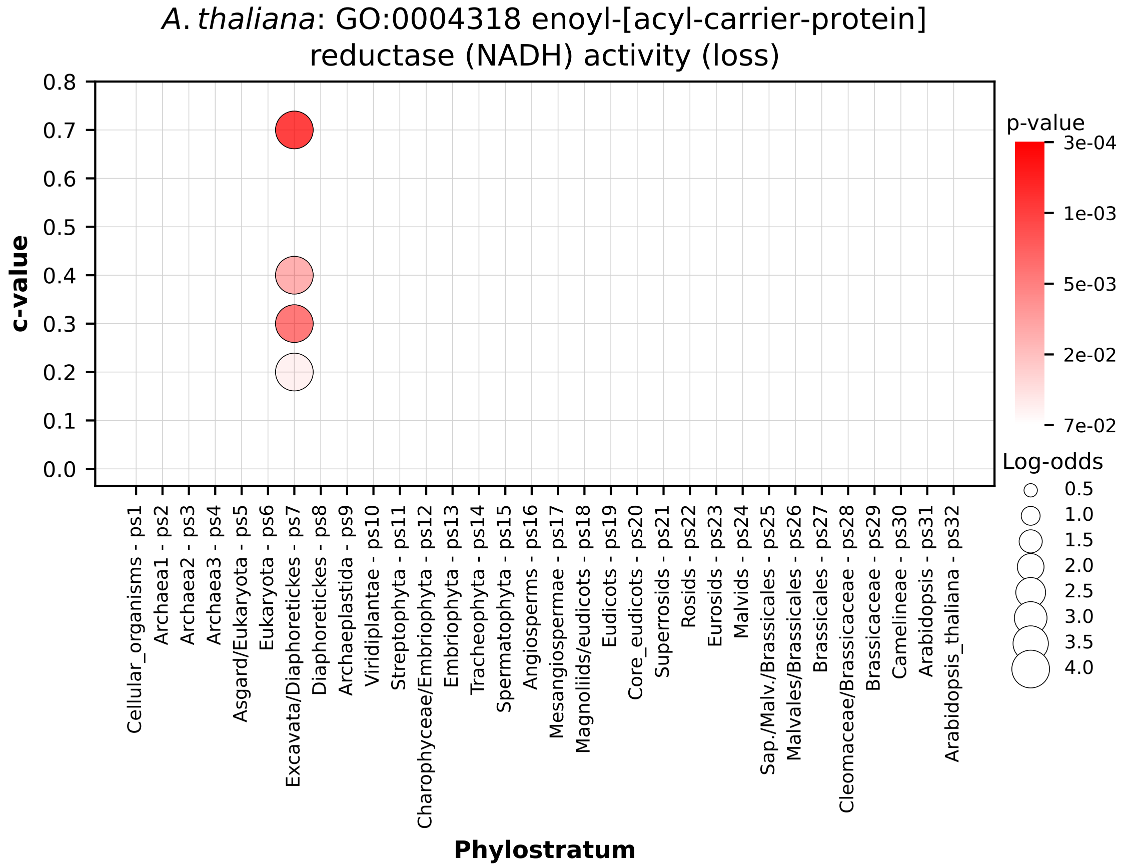

A. thaliana: GO:0004318 enoyl-[acyl-carrier-protein] reductase (NADH) activity (loss)
c-value
0.8
0.7
0.6
0.5
0.4
0.3
0.2
0.1
0.0
p-value
3e-04
1e-03
5e-03
2e-02
7e-02
Log-odds
0.5
1.0
1.5
2.0
2.5
3.0
3.5
4.0
Cellular_organisms - ps1
Archaea1 - ps2
Archaea2 - ps3
Archaea3 - ps4
Asgard/Eukaryota - ps5
Eukaryota - ps6
Excavata/Diaphoretickes - ps7
Diaphoretickes - ps8
Archaeplastida - ps9
Viridiplantae - ps10
Streptophyta - ps11
Charophyceae/Embriophyta - ps12
Embriophyta - ps13
Tracheophyta - ps14
Spermatophyta - ps15
Angiosperms - ps16
Mesangiospermae - ps17
Magnoliids/eudicots - ps18
Eudicots - ps19
Core_eudicots - ps20
Superrosids - ps21
Rosids - ps22
Eurosids - ps23
Malvids - ps24
Sap./Malv./Brassicales - ps25
Malvales/Brassicales - ps26
Brassicales - ps27
Cleomaceae/Brassicaceae - ps28
Brassicaceae - ps29
Camelineae - ps30
Arabidopsis - ps31
Arabidopsis_thaliana - ps32
Phylostratum

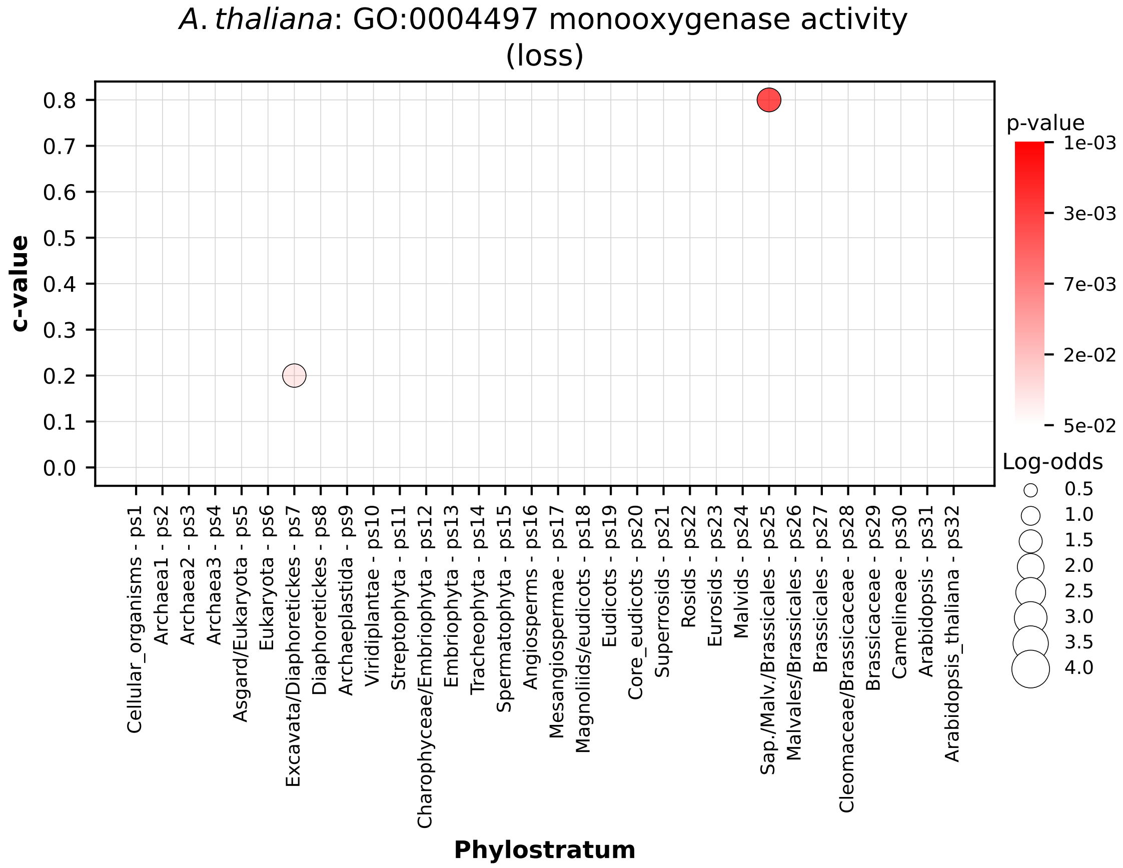

A. thaliana: GO:0004497 monooxygenase activity
(loss)
c-value
0.0
0.1
0.2
0.3
0.4
0.5
0.6
0.7
0.8
Phylostratum
Cellular_organisms - ps1
Archaea1 - ps2
Archaea2 - ps3
Archaea3 - ps4
Asgard/Eukaryota - ps5
Eukaryota - ps6
Excavata/Diaphoretickes - ps7
Diaphoretickes - ps8
Archaeplastida - ps9
Viridiplantae - ps10
Streptophyta - ps11
Charophyceae/Embriophyta - ps12
Embriophyta - ps13
Tracheophyta - ps14
Spermatophyta - ps15
Angiosperms - ps16
Mesangiospermae - ps17
Magnoliids/eudicots - ps18
Eudicots - ps19
Core_eudicots - ps20
Superrosids - ps21
Rosids - ps22
Eurosids - ps23
Malvids - ps24
Sap./Malv./Brassicales - ps25
Malvales/Brassicales - ps26
Brassicales - ps27
Cleomaceae/Brassicaceae - ps28
Brassicaceae - ps29
Camelineae - ps30
Arabidopsis - ps31
Arabidopsis_thaliana - ps32
p-value
1e-03
3e-03
7e-03
2e-02
5e-02
Log-odds
0.5
1.0
1.5
2.0
2.5
3.0
3.5
4.0

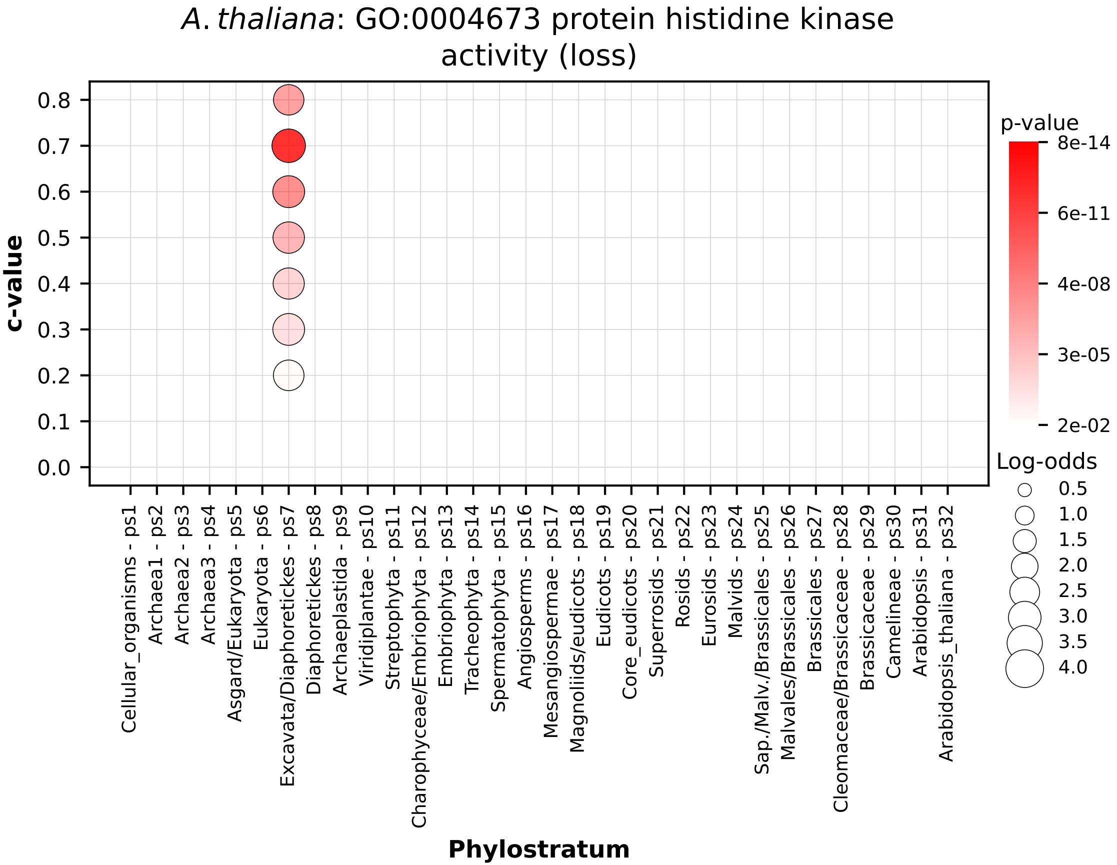
A. thaliana: GO:0004673 protein histidine kinase activity (loss)
c-value
0.8
0.7
0.6
0.5
0.4
0.3
0.2
0.1
0.0
p-value
8e-14
6e-11
4e-08
3e-05
2e-02
Log-odds
0.5
1.0
1.5
2.0
2.5
3.0
3.5
4.0
Cellular_organisms - ps1
Archaea1 - ps2
Archaea2 - ps3
Archaea3 - ps4
Asgard/Eukaryota - ps5
Eukaryota - ps6
Excavata/Diaphoretickes - ps7
Diaphoretickes - ps8
Archaeplastida - ps9
Viridiplantae - ps10
Streptophyta - ps11
Charophyceae/Embriophyta - ps12
Embriophyta - ps13
Tracheophyta - ps14
Spermatophyta - ps15
Angiosperms - ps16
Mesangiospermae - ps17
Magnoliids/eudicots - ps18
Eudicots - ps19
Core_eudicots - ps20
Superrosids - ps21
Rosids - ps22
Eurosids - ps23
Malvids - ps24
Sap./Malv./Brassicales - ps25
Malvales/Brassicales - ps26
Brassicales - ps27
Cleomaceae/Brassicaceae - ps28
Brassicaceae - ps29
Camelineae - ps30
Arabidopsis - ps31
Arabidopsis_thaliana - ps32
Phylostratum

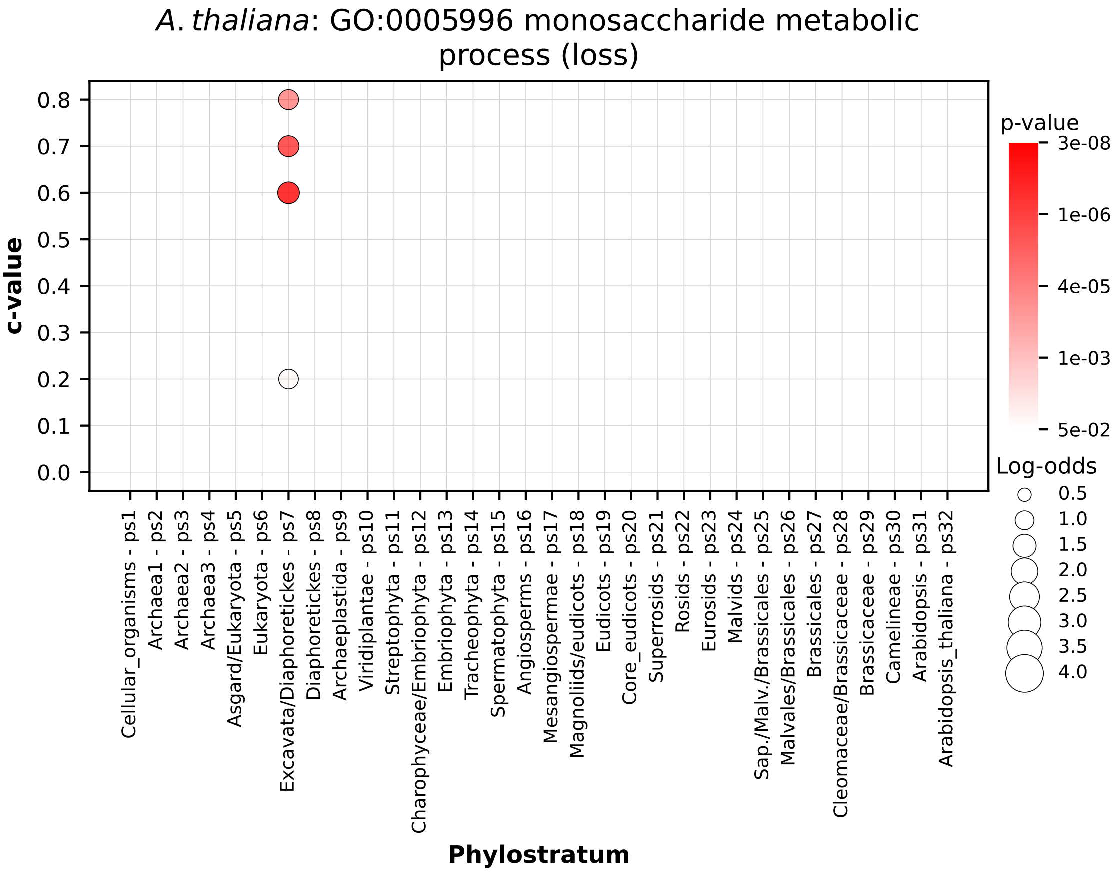

A. thaliana: GO:0005996 monosaccharide metabolic process (loss)
c-value
0.0
0.1
0.2
0.3
0.4
0.5
0.6
0.7
0.8
Phylostratum
Cellular_organisms - ps1
Archaea1 - ps2
Archaea2 - ps3
Archaea3 - ps4
Asgard/Eukaryota - ps5
Eukaryota - ps6
Excavata/Diaphoretickes - ps7
Diaphoretickes - ps8
Archaeplastida - ps9
Viridiplantae - ps10
Streptophyta - ps11
Charophyceae/Embriophyta - ps12
Embriophyta - ps13
Tracheophyta - ps14
Spermatophyta - ps15
Angiosperms - ps16
Mesangiospermae - ps17
Magnoliids/eudicots - ps18
Eudicots - ps19
Core_eudicots - ps20
Superrosids - ps21
Rosids - ps22
Eurosids - ps23
Malvids - ps24
Sap./Malv./Brassicales - ps25
Malvales/Brassicales - ps26
Brassicales - ps27
Cleomaceae/Brassicaceae - ps28
Brassicaceae - ps29
Camelineae - ps30
Arabidopsis - ps31
Arabidopsis_thaliana - ps32
p-value
3e-08
1e-06
4e-05
1e-03
5e-02
Log-odds
0.5
1.0
1.5
2.0
2.5
3.0
3.5
4.0

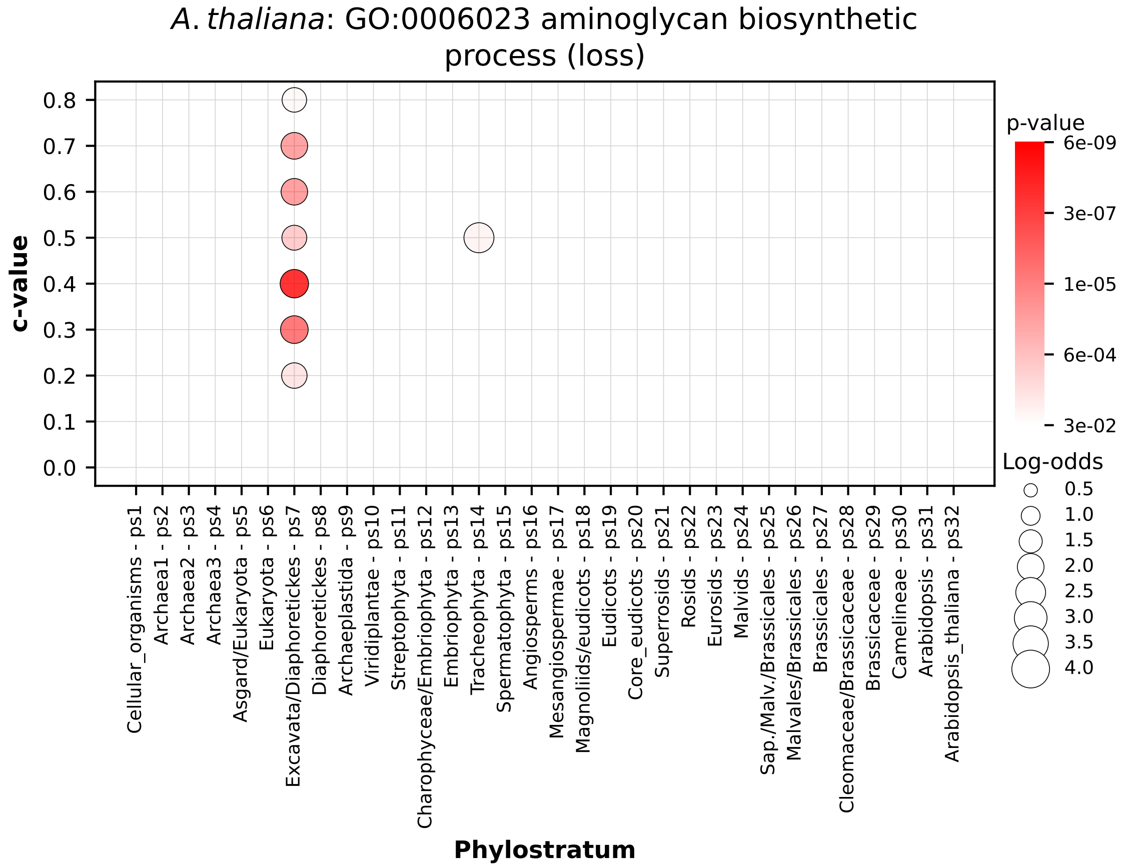

A. thaliana: GO:0006023 aminoglycan biosynthetic process (loss)
c-value
0.0
0.1
0.2
0.3
0.4
0.5
0.6
0.7
0.8
Phylostratum
Cellular_organisms - ps1
Archaea1 - ps2
Archaea2 - ps3
Archaea3 - ps4
Asgard/Eukaryota - ps5
Eukaryota - ps6
Excavata/Diaphoretickes - ps7
Diaphoretickes - ps8
Archaeplastida - ps9
Viridiplantae - ps10
Streptophyta - ps11
Charophyceae/Embriophyta - ps12
Embriophyta - ps13
Tracheophyta - ps14
Spermatophyta - ps15
Angiosperms - ps16
Mesangiospermae - ps17
Magnoliids/eudicots - ps18
Eudicots - ps19
Core_eudicots - ps20
Superrosids - ps21
Rosids - ps22
Eurosids - ps23
Malvids - ps24
Sap./Malv./Brassicales - ps25
Malvales/Brassicales - ps26
Brassicales - ps27
Cleomaceae/Brassicaceae - ps28
Brassicaceae - ps29
Camelineae - ps30
Arabidopsis - ps31
Arabidopsis_thaliana - ps32
p-value
6e-09
3e-07
1e-05
6e-04
3e-02
Log-odds
0.5
1.0
1.5
2.0
2.5
3.0
3.5
4.0

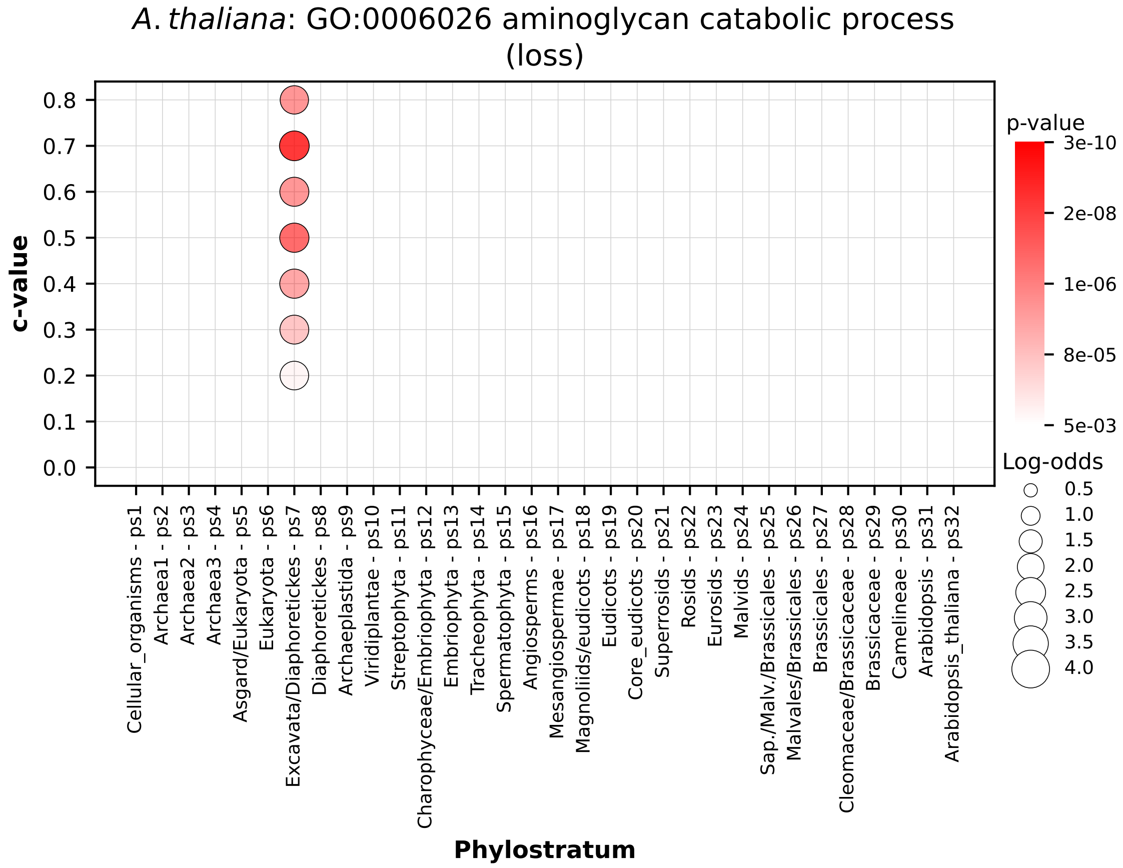

A. thaliana: GO:0006026 aminoglycan catabolic process
(loss)
c-value
0.8
0.7
0.6
0.5
0.4
0.3
0.2
0.1
0.0
p-value
3e-10
2e-08
1e-06
8e-05
5e-03
Log-odds
0.5
1.0
1.5
2.0
2.5
3.0
3.5
4.0
Cellular_organisms - ps1
Archaea1 - ps2
Archaea2 - ps3
Archaea3 - ps4
Asgard/Eukaryota - ps5
Eukaryota - ps6
Excavata/Diaphoretickes - ps7
Diaphoretickes - ps8
Archaeplastida - ps9
Viridiplantae - ps10
Streptophyta - ps11
Charophyceae/Embriophyta - ps12
Embriophyta - ps13
Tracheophyta - ps14
Spermatophyta - ps15
Angiosperms - ps16
Mesangiospermae - ps17
Magnoliids/eudicots - ps18
Eudicots - ps19
Core_eudicots - ps20
Superrosids - ps21
Rosids - ps22
Eurosids - ps23
Malvids - ps24
Sap./Malv./Brassicales - ps25
Malvales/Brassicales - ps26
Brassicales - ps27
Cleomaceae/Brassicaceae - ps28
Brassicaceae - ps29
Camelineae - ps30
Arabidopsis - ps31
Arabidopsis_thaliana - ps32
Phylostratum

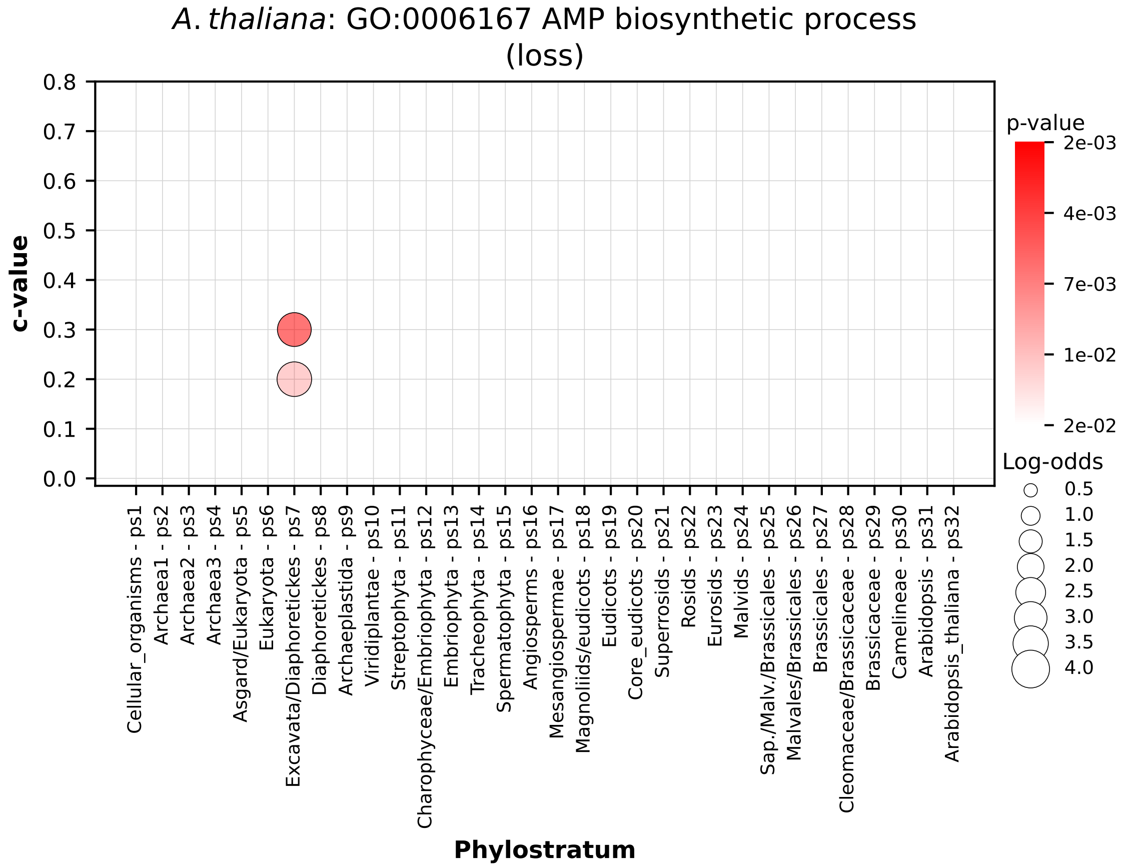

A. thaliana: GO:0006167 AMP biosynthetic process
(loss)
c-value
0.8
0.7
0.6
0.5
0.4
0.3
0.2
0.1
0.0
p-value
2e-03
4e-03
7e-03
1e-02
2e-02
Log-odds
0.5
1.0
1.5
2.0
2.5
3.0
3.5
4.0
Cellular_organisms - ps1
Archaea1 - ps2
Archaea2 - ps3
Archaea3 - ps4
Asgard/Eukaryota - ps5
Eukaryota - ps6
Excavata/Diaphoretickes - ps7
Diaphoretickes - ps8
Archaeplastida - ps9
Viridiplantae - ps10
Streptophyta - ps11
Charophyceae/Embriophyta - ps12
Embriophyta - ps13
Tracheophyta - ps14
Spermatophyta - ps15
Angiosperms - ps16
Mesangiospermae - ps17
Magnoliids/eudicots - ps18
Eudicots - ps19
Core_eudicots - ps20
Superrosids - ps21
Rosids - ps22
Eurosids - ps23
Malvids - ps24
Sap./Malv./Brassicales - ps25
Malvales/Brassicales - ps26
Brassicales - ps27
Cleomaceae/Brassicaceae - ps28
Brassicaceae - ps29
Camelineae - ps30
Arabidopsis - ps31
Arabidopsis_thaliana - ps32
Phylostratum

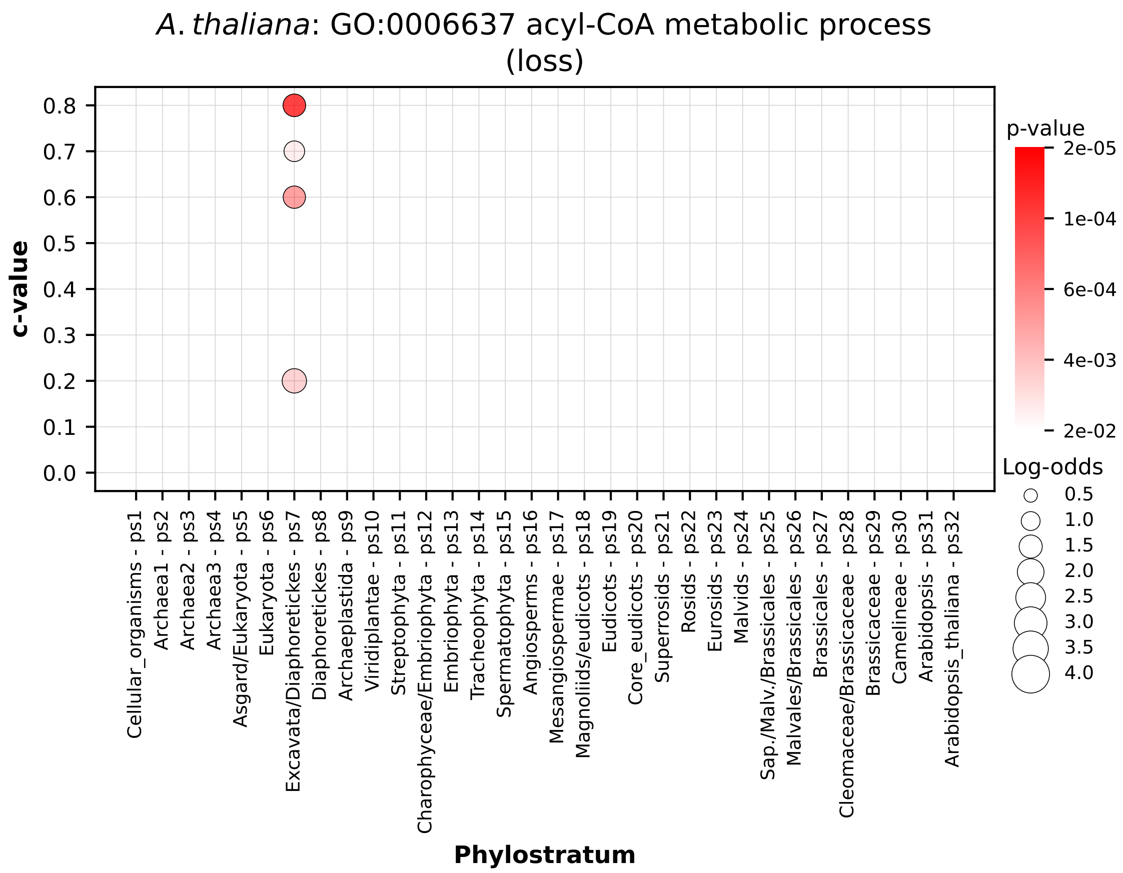

A. thaliana: GO:0006637 acyl-CoA metabolic process (loss)
c-value
0.0
0.1
0.2
0.3
0.4
0.5
0.6
0.7
0.8
p-value
2e-05
1e-04
6e-04
4e-03
2e-02
Log-odds
0.5
1.0
1.5
2.0
2.5
3.0
3.5
4.0
Cellular_organisms - ps1
Archaea1 - ps2
Archaea2 - ps3
Archaea3 - ps4
Asgard/Eukaryota - ps5
Eukaryota - ps6
Excavata/Diaphoretickes - ps7
Diaphoretickes - ps8
Archaeplastida - ps9
Viridiplantae - ps10
Streptophyta - ps11
Charophyceae/Embriophyta - ps12
Embriophyta - ps13
Tracheophyta - ps14
Spermatophyta - ps15
Angiosperms - ps16
Mesangiospermae - ps17
Magnoliids/eudicots - ps18
Eudicots - ps19
Core_eudicots - ps20
Superrosids - ps21
Rosids - ps22
Eurosids - ps23
Malvids - ps24
Sap./Malv./Brassicales - ps25
Malvales/Brassicales - ps26
Brassicales - ps27
Cleomaceae/Brassicaceae - ps28
Brassicaceae - ps29
Camelineae - ps30
Arabidopsis - ps31
Arabidopsis_thaliana - ps32
Phylostratum

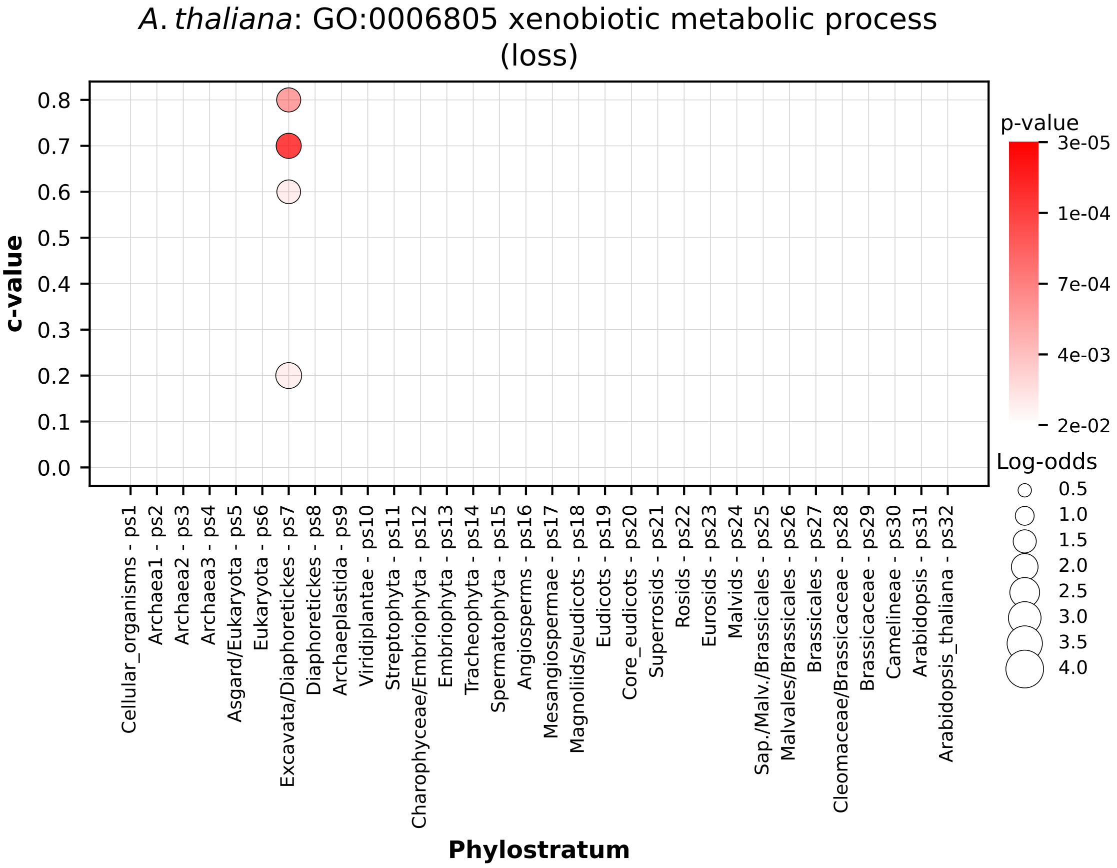
A. thaliana: GO:0006805 xenobiotic metabolic process (loss)
c-value
0.8
0.7
0.6
0.5
0.4
0.3
0.2
0.1
0.0
Phylostratum
Cellular_organisms - ps1
Archaea1 - ps2
Archaea2 - ps3
Archaea3 - ps4
Asgard/Eukaryota - ps5
Eukaryota - ps6
Excavata/Diaphoretickes - ps7
Diaphoretickes - ps8
Archaeplastida - ps9
Viridiplantae - ps10
Streptophyta - ps11
Charophyceae/Embriophyta - ps12
Embriophyta - ps13
Tracheophyta - ps14
Spermatophyta - ps15
Angiosperms - ps16
Mesangiospermae - ps17
Magnoliids/eudicots - ps18
Eudicots - ps19
Core_eudicots - ps20
Superrosids - ps21
Rosids - ps22
Eurosids - ps23
Malvids - ps24
Sap./Malv./Brassicales - ps25
Malvales/Brassicales - ps26
Brassicales - ps27
Cleomaceae/Brassicaceae - ps28
Brassicaceae - ps29
Camelineae - ps30
Arabidopsis - ps31
Arabidopsis_thaliana - ps32
p-value
3e-05
1e-04
7e-04
4e-03
2e-02
Log-odds
0.5
1.0
1.5
2.0
2.5
3.0
3.5
4.0

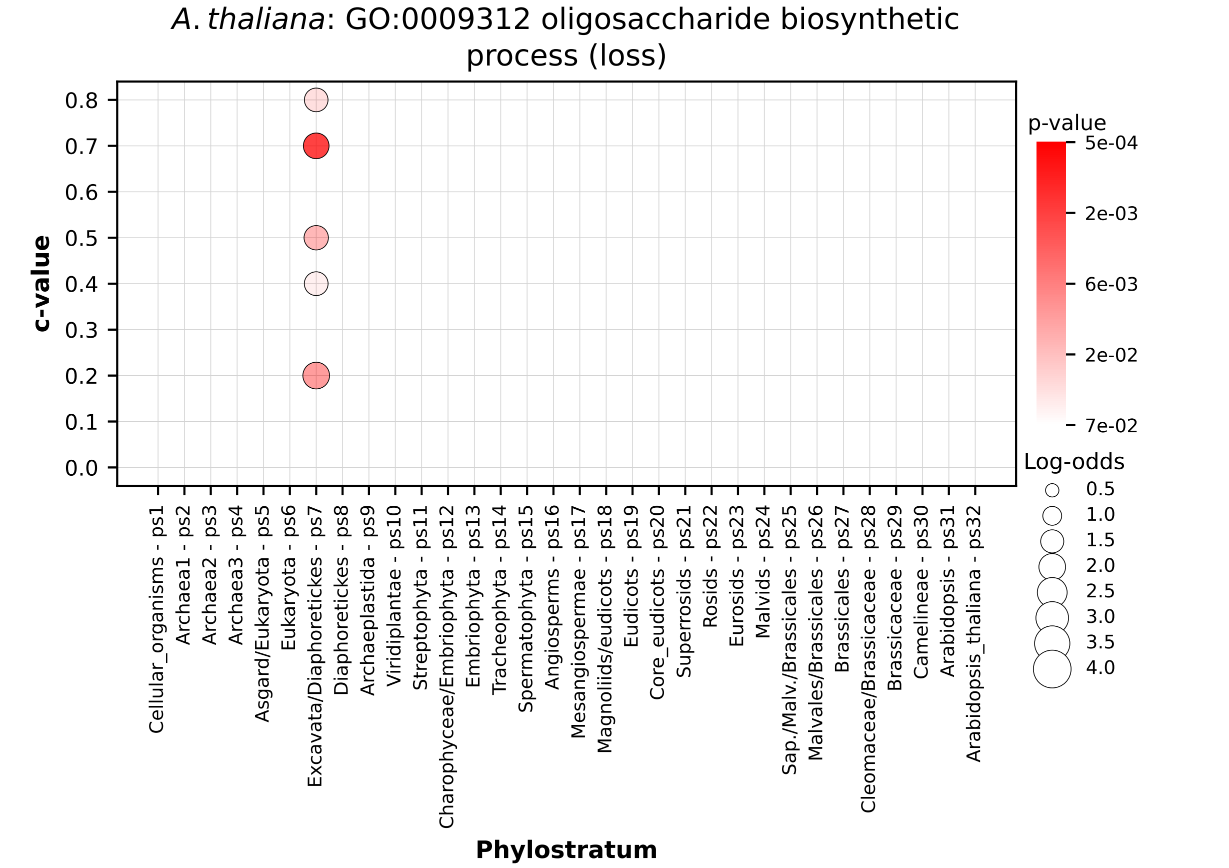

A. thaliana: GO:0009312 oligosaccharide biosynthetic process (loss)
c-value
0.8
0.7
0.6
0.5
0.4
0.3
0.2
0.1
0.0
p-value
5e-04
2e-03
6e-03
2e-02
7e-02
Log-odds
0.5
1.0
1.5
2.0
2.5
3.0
3.5
4.0
Cellular_organisms - ps1
Archaea1 - ps2
Archaea2 - ps3
Archaea3 - ps4
Asgard/Eukaryota - ps5
Eukaryota - ps6
Excavata/Diaphoretickes - ps7
Diaphoretickes - ps8
Archaeplastida - ps9
Viridiplantae - ps10
Streptophyta - ps11
Charophyceae/Embriophyta - ps12
Embriophyta - ps13
Tracheophyta - ps14
Spermatophyta - ps15
Angiosperms - ps16
Mesangiospermae - ps17
Magnoliids/eudicots - ps18
Eudicots - ps19
Core_eudicots - ps20
Superrosids - ps21
Rosids - ps22
Eurosids - ps23
Malvids - ps24
Sap./Malv./Brassicales - ps25
Malvales/Brassicales - ps26
Brassicales - ps27
Cleomaceae/Brassicaceae - ps28
Brassicaceae - ps29
Camelineae - ps30
Arabidopsis - ps31
Arabidopsis_thaliana - ps32
Phylostratum

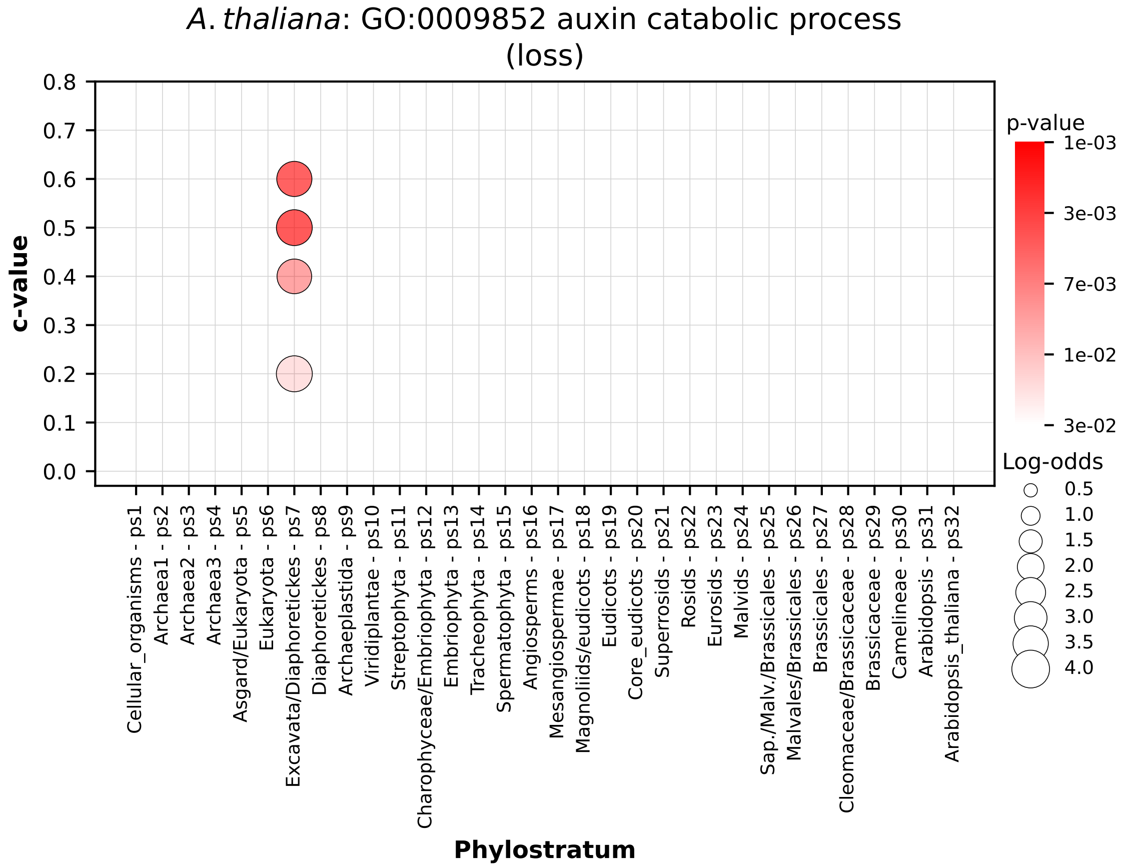

A. thaliana: GO:0009852 auxin catabolic process
(loss)
c-value
0.8
0.7
0.6
0.5
0.4
0.3
0.2
0.1
0.0
p-value
1e-03
3e-03
7e-03
1e-02
3e-02
Log-odds
0.5
1.0
1.5
2.0
2.5
3.0
3.5
4.0
Cellular_organisms - ps1
Archaea1 - ps2
Archaea2 - ps3
Archaea3 - ps4
Asgard/Eukaryota - ps5
Eukaryota - ps6
Excavata/Diaphoretickes - ps7
Diaphoretickes - ps8
Archaeplastida - ps9
Viridiplantae - ps10
Streptophyta - ps11
Charophyceae/Embriophyta - ps12
Embriophyta - ps13
Tracheophyta - ps14
Spermatophyta - ps15
Angiosperms - ps16
Mesangiospermae - ps17
Magnoliids/eudicots - ps18
Eudicots - ps19
Core_eudicots - ps20
Superrosids - ps21
Rosids - ps22
Eurosids - ps23
Malvids - ps24
Sap./Malv./Brassicales - ps25
Malvales/Brassicales - ps26
Brassicales - ps27
Cleomaceae/Brassicaceae - ps28
Brassicaceae - ps29
Camelineae - ps30
Arabidopsis - ps31
Arabidopsis_thaliana - ps32
Phylostratum

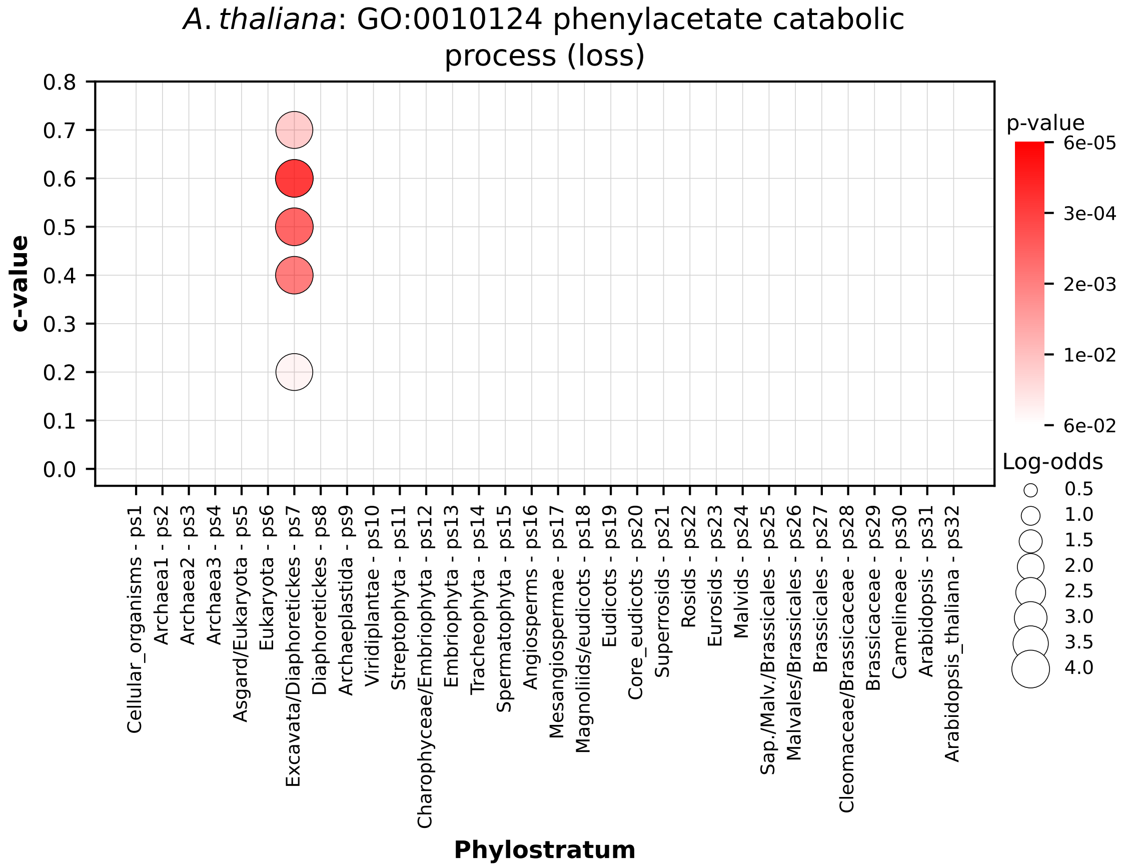
A. thaliana: GO:0010124 phenylacetate catabolic process (loss)
c-value
0.0
0.1
0.2
0.3
0.4
0.5
0.6
0.7
0.8
p-value
6e-05
3e-04
2e-03
1e-02
6e-02
Log-odds
0.5
1.0
1.5
2.0
2.5
3.0
3.5
4.0
Cellular_organisms - ps1
Archaea1 - ps2
Archaea2 - ps3
Archaea3 - ps4
Asgard/Eukaryota - ps5
Eukaryota - ps6
Excavata/Diaphoretickes - ps7
Diaphoretickes - ps8
Archaeplastida - ps9
Viridiplantae - ps10
Streptophyta - ps11
Charophyceae/Embriophyta - ps12
Embriophyta - ps13
Tracheophyta - ps14
Spermatophyta - ps15
Angiosperms - ps16
Mesangiospermae - ps17
Magnoliids/eudicots - ps18
Eudicots - ps19
Core_eudicots - ps20
Superrosids - ps21
Rosids - ps22
Eurosids - ps23
Malvids - ps24
Sap./Malv./Brassicales - ps25
Malvales/Brassicales - ps26
Brassicales - ps27
Cleomaceae/Brassicaceae - ps28
Brassicaceae - ps29
Camelineae - ps30
Arabidopsis - ps31
Arabidopsis_thaliana - ps32
Phylostratum

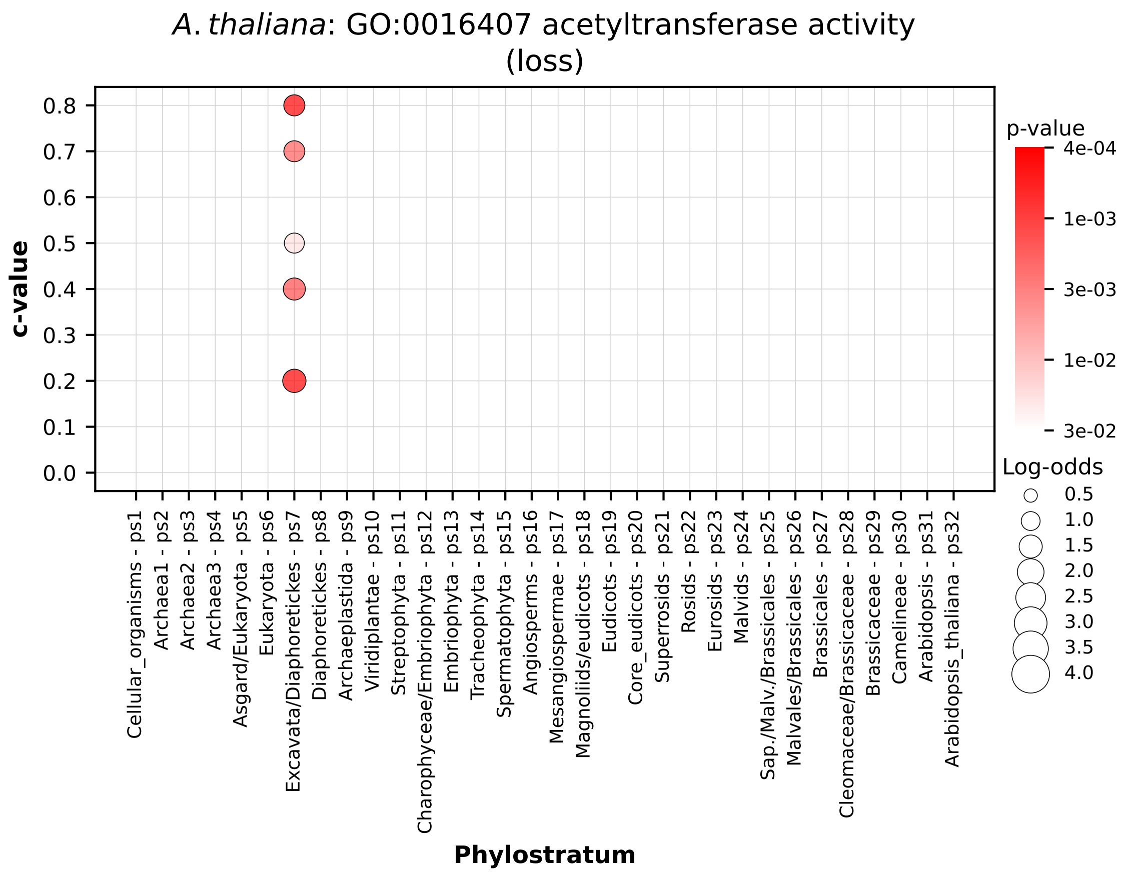

A. thaliana: GO:0016407 acetyltransferase activity
(loss)
c-value
0.0
0.1
0.2
0.3
0.4
0.5
0.6
0.7
0.8
Phylostratum
Cellular_organisms - ps1
Archaea1 - ps2
Archaea2 - ps3
Archaea3 - ps4
Asgard/Eukaryota - ps5
Eukaryota - ps6
Excavata/Diaphoretickes - ps7
Diaphoretickes - ps8
Archaeplastida - ps9
Viridiplantae - ps10
Streptophyta - ps11
Charophyceae/Embriophyta - ps12
Embriophyta - ps13
Tracheophyta - ps14
Spermatophyta - ps15
Angiosperms - ps16
Mesangiospermae - ps17
Magnoliids/eudicots - ps18
Eudicots - ps19
Core_eudicots - ps20
Superrosids - ps21
Rosids - ps22
Eurosids - ps23
Malvids - ps24
Sap./Malv./Brassicales - ps25
Malvales/Brassicales - ps26
Brassicales - ps27
Cleomaceae/Brassicaceae - ps28
Brassicaceae - ps29
Camelineae - ps30
Arabidopsis - ps31
Arabidopsis_thaliana - ps32
p-value
4e-04
1e-03
3e-03
1e-02
3e-02
Log-odds
0.5
1.0
1.5
2.0
2.5
3.0
3.5
4.0

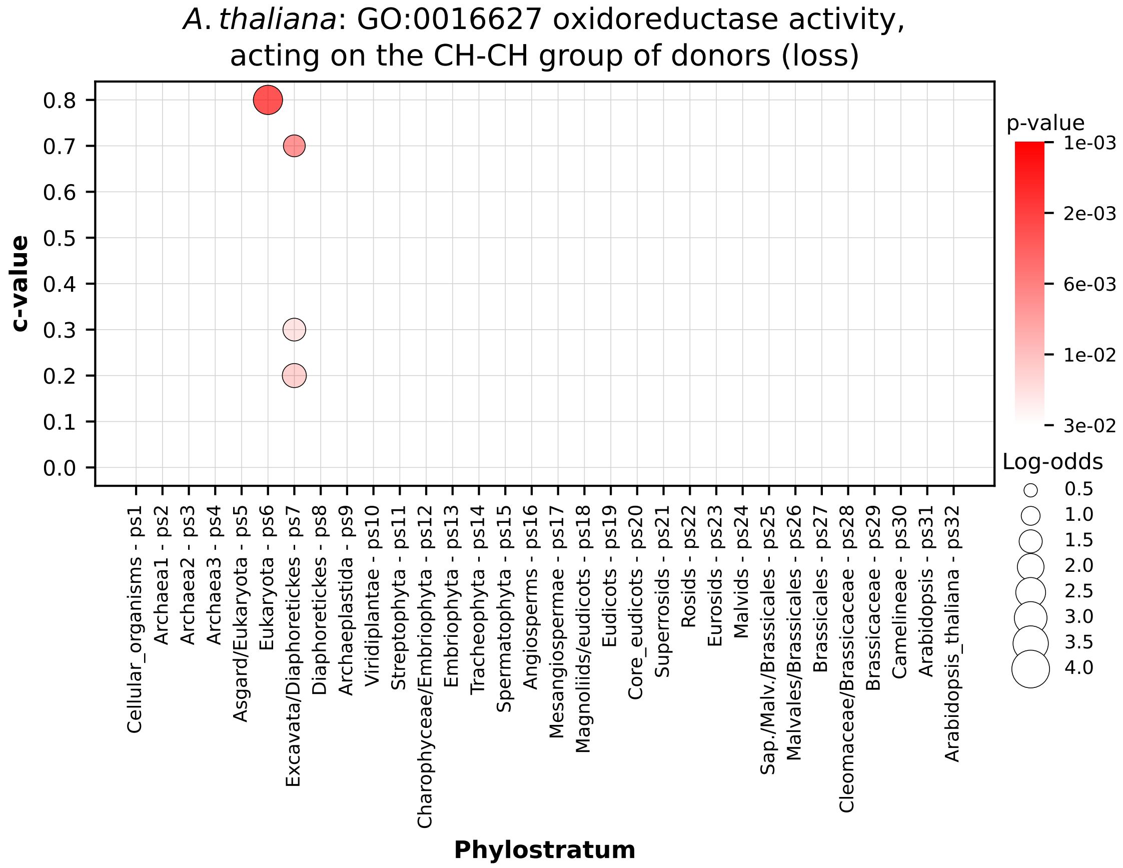

A. thaliana: GO:0016627 oxidoreductase activity, acting on the CH-CH group of donors (loss)
c-value
0.0
0.1
0.2
0.3
0.4
0.5
0.6
0.7
0.8
Phylostratum
Cellular_organisms - ps1
Archaea1 - ps2
Archaea2 - ps3
Archaea3 - ps4
Asgard/Eukaryota - ps5
Eukaryota - ps6
Excavata/Diaphoretickes - ps7
Diaphoretickes - ps8
Archaeplastida - ps9
Viridiplantae - ps10
Streptophyta - ps11
Charophyceae/Embriophyta - ps12
Embriophyta - ps13
Tracheophyta - ps14
Spermatophyta - ps15
Angiosperms - ps16
Mesangiospermae - ps17
Magnoliids/eudicots - ps18
Eudicots - ps19
Core_eudicots - ps20
Superrosids - ps21
Rosids - ps22
Eurosids - ps23
Malvids - ps24
Sap./Malv./Brassicales - ps25
Malvales/Brassicales - ps26
Brassicales - ps27
Cleomaceae/Brassicaceae - ps28
Brassicaceae - ps29
Camelineae - ps30
Arabidopsis - ps31
Arabidopsis_thaliana - ps32
p-value
1e-03
2e-03
6e-03
1e-02
3e-02
Log-odds
0.5
1.0
1.5
2.0
2.5
3.0
3.5
4.0

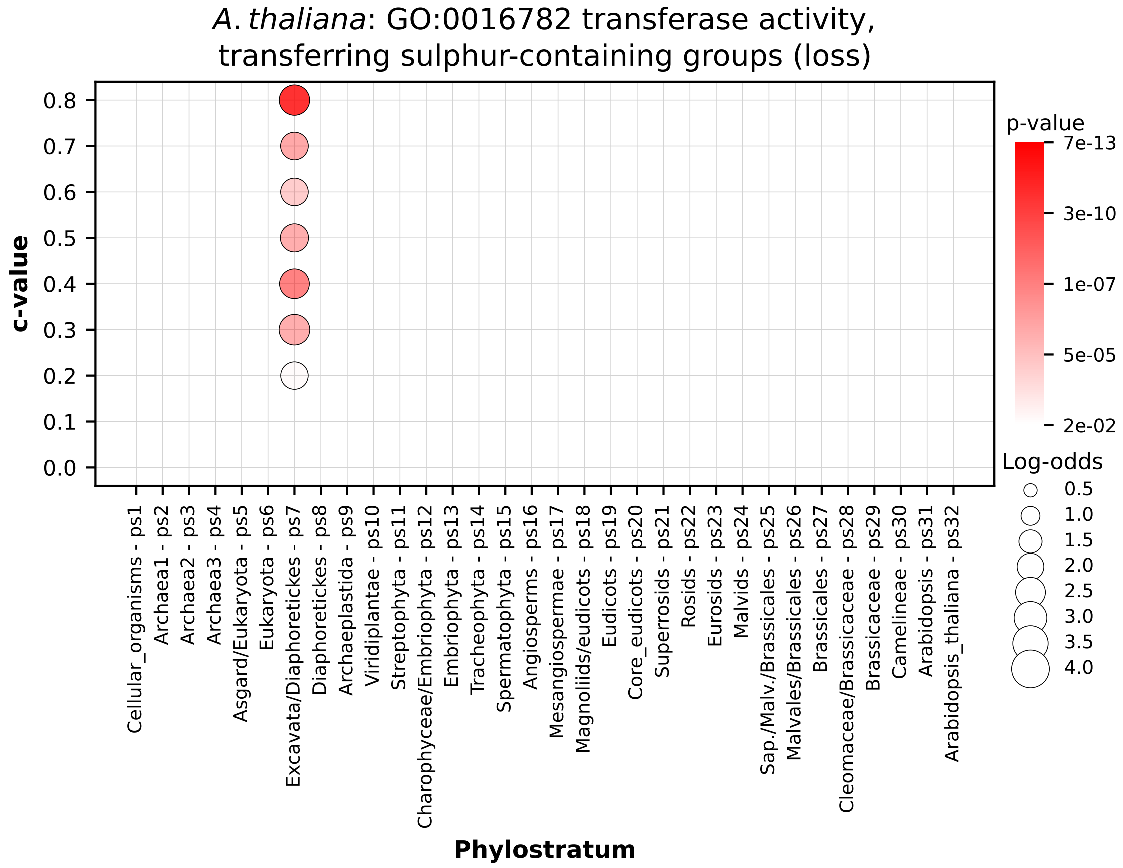
A. thaliana: GO:0016782 transferase activity, transferring sulphur-containing groups (loss)
c-value
0.8
0.7
0.6
0.5
0.4
0.3
0.2
0.1
0.0
p-value
7e-13
3e-10
1e-07
5e-05
2e-02
Log-odds
0.5
1.0
1.5
2.0
2.5
3.0
3.5
4.0
Cellular_organisms - ps1
Archaea1 - ps2
Archaea2 - ps3
Archaea3 - ps4
Asgard/Eukaryota - ps5
Eukaryota - ps6
Excavata/Diaphoretickes - ps7
Diaphoretickes - ps8
Archaeplastida - ps9
Viridiplantae - ps10
Streptophyta - ps11
Charophyceae/Embriophyta - ps12
Embriophyta - ps13
Tracheophyta - ps14
Spermatophyta - ps15
Angiosperms - ps16
Mesangiospermae - ps17
Magnoliids/eudicots - ps18
Eudicots - ps19
Core_eudicots - ps20
Superrosids - ps21
Rosids - ps22
Eurosids - ps23
Malvids - ps24
Sap./Malv./Brassicales - ps25
Malvales/Brassicales - ps26
Brassicales - ps27
Cleomaceae/Brassicaceae - ps28
Brassicaceae - ps29
Camelineae - ps30
Arabidopsis - ps31
Arabidopsis_thaliana - ps32
Phylostratum

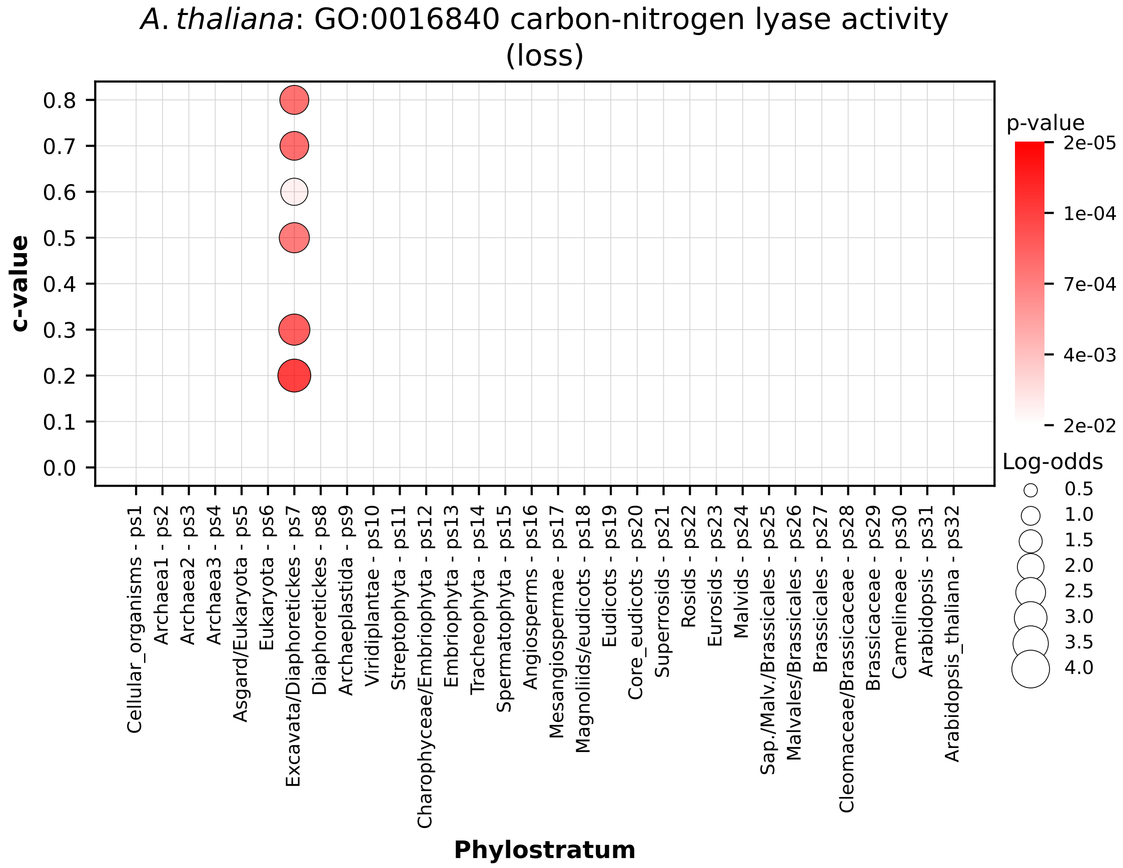
A. thaliana: GO:0016840 carbon-nitrogen lyase activity
(loss)
c-value
0.8
0.7
0.6
0.5
0.4
0.3
0.2
0.1
0.0
p-value
2e-05
1e-04
7e-04
4e-03
2e-02
Log-odds
0.5
1.0
1.5
2.0
2.5
3.0
3.5
4.0
Cellular_organisms - ps1
Archaea1 - ps2
Archaea2 - ps3
Archaea3 - ps4
Asgard/Eukaryota - ps5
Eukaryota - ps6
Excavata/Diaphoretickes - ps7
Diaphoretickes - ps8
Archaeplastida - ps9
Viridiplantae - ps10
Streptophyta - ps11
Charophyceae/Embriophyta - ps12
Embriophyta - ps13
Tracheophyta - ps14
Spermatophyta - ps15
Angiosperms - ps16
Mesangiospermae - ps17
Magnoliids/eudicots - ps18
Eudicots - ps19
Core_eudicots - ps20
Superrosids - ps21
Rosids - ps22
Eurosids - ps23
Malvids - ps24
Sap./Malv./Brassicales - ps25
Malvales/Brassicales - ps26
Brassicales - ps27
Cleomaceae/Brassicaceae - ps28
Brassicaceae - ps29
Camelineae - ps30
Arabidopsis - ps31
Arabidopsis_thaliana - ps32
Phylostratum

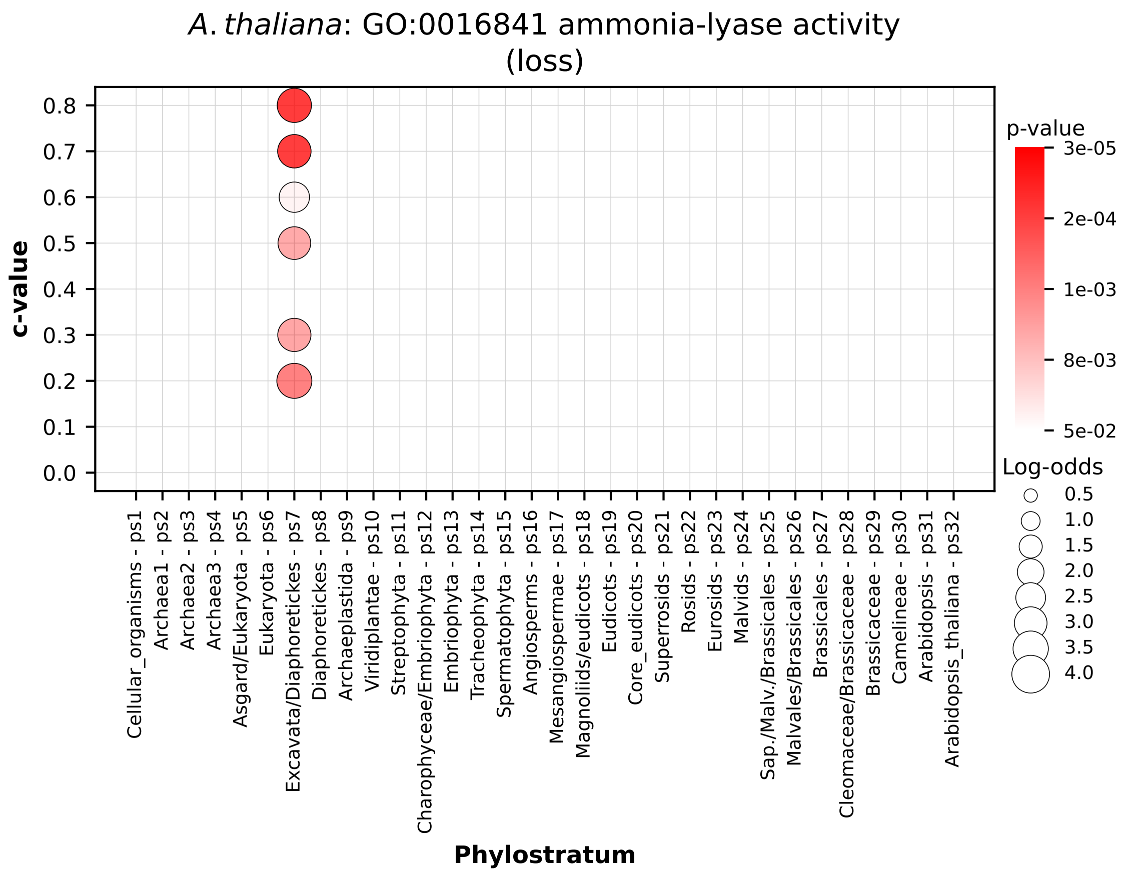
A. thaliana: GO:0016841 ammonia-lyase activity
(loss)
c-value
0.8
0.7
0.6
0.5
0.4
0.3
0.2
0.1
0.0
p-value
3e-05
2e-04
1e-03
8e-03
5e-02
Log-odds
0.5
1.0
1.5
2.0
2.5
3.0
3.5
4.0
Cellular_organisms - ps1
Archaea1 - ps2
Archaea2 - ps3
Archaea3 - ps4
Asgard/Eukaryota - ps5
Eukaryota - ps6
Excavata/Diaphoretickes - ps7
Diaphoretickes - ps8
Archaeplastida - ps9
Viridiplantae - ps10
Streptophyta - ps11
Charophyceae/Embriophyta - ps12
Embriophyta - ps13
Tracheophyta - ps14
Spermatophyta - ps15
Angiosperms - ps16
Mesangiospermae - ps17
Magnoliids/eudicots - ps18
Eudicots - ps19
Core_eudicots - ps20
Superrosids - ps21
Rosids - ps22
Eurosids - ps23
Malvids - ps24
Sap./Malv./Brassicales - ps25
Malvales/Brassicales - ps26
Brassicales - ps27
Cleomaceae/Brassicaceae - ps28
Brassicaceae - ps29
Camelineae - ps30
Arabidopsis - ps31
Arabidopsis_thaliana - ps32
Phylostratum

A. thaliana: GO:0018106 peptidyl-histidine phosphorylation (loss)

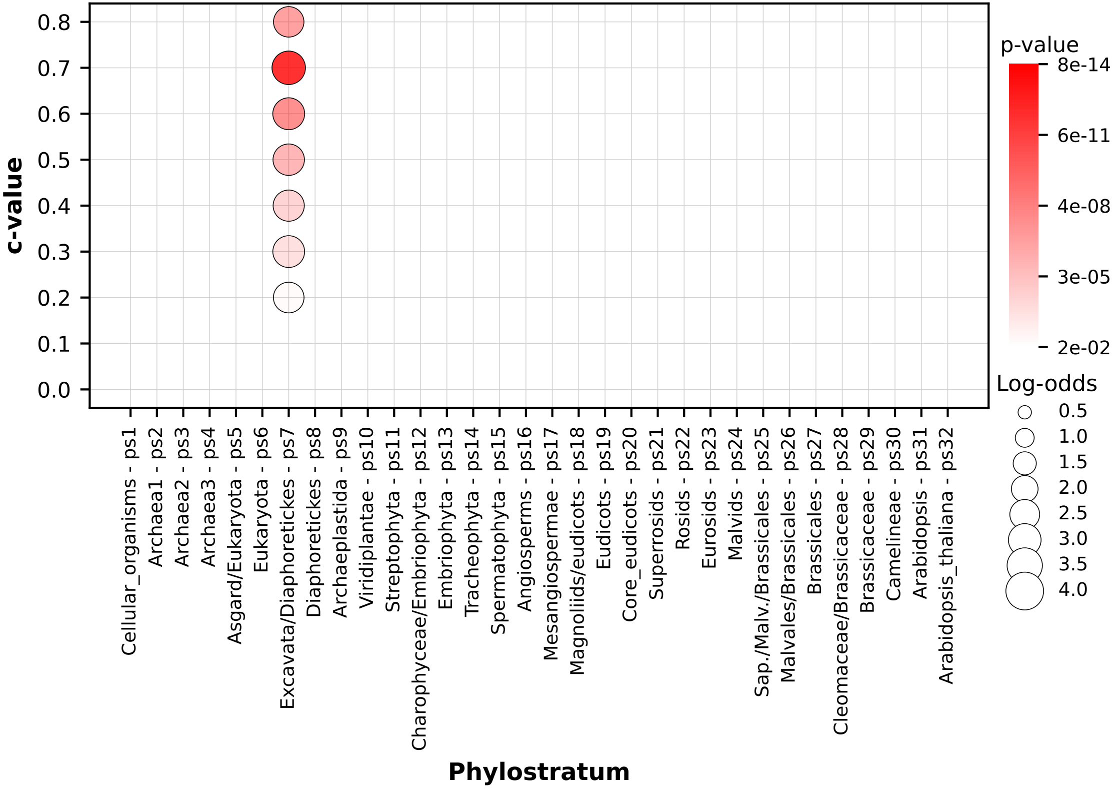

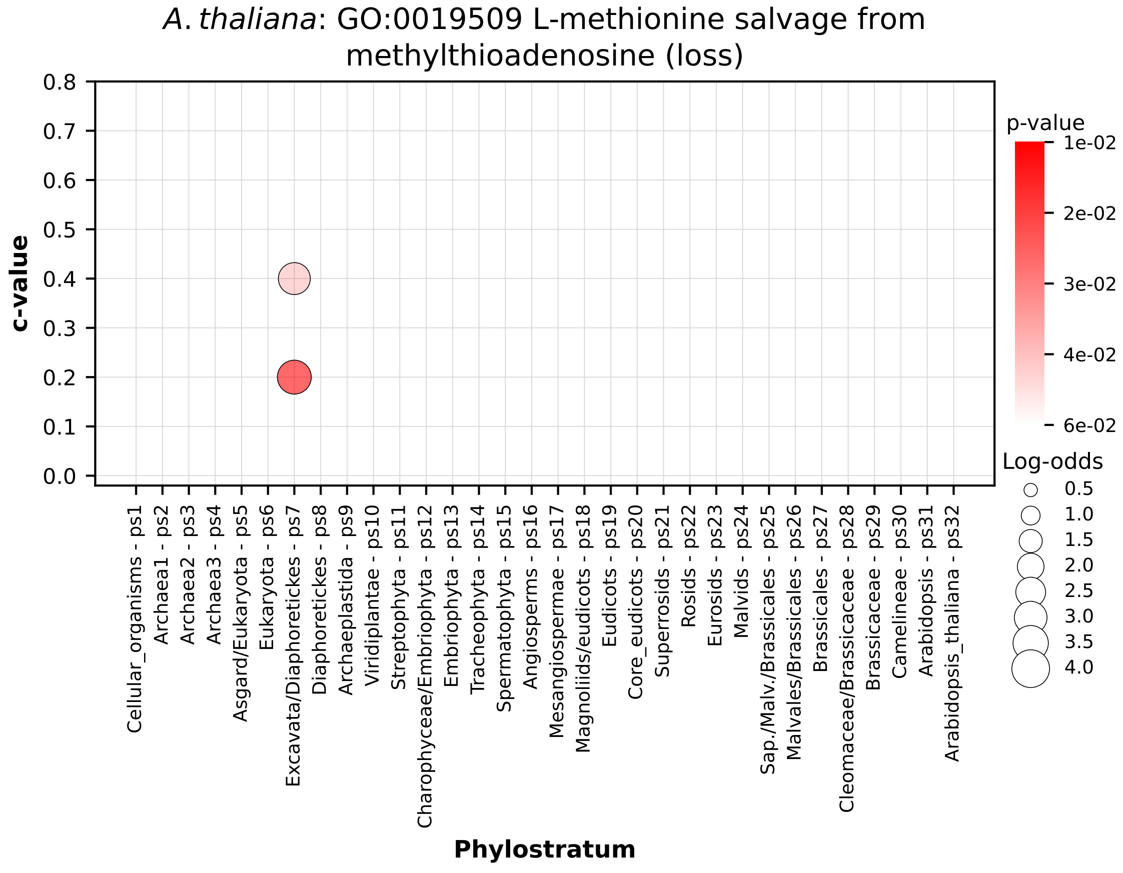

A. thaliana: GO:0019509 L-methionine salvage from methylthioadenosine (loss)
c-value
0.0
0.1
0.2
0.3
0.4
0.5
0.6
0.7
0.8
Phylostratum
Cellular_organisms - ps1
Archaea1 - ps2
Archaea2 - ps3
Archaea3 - ps4
Asgard/Eukaryota - ps5
Eukaryota - ps6
Excavata/Diaphoretickes - ps7
Diaphoretickes - ps8
Archaeplastida - ps9
Viridiplantae - ps10
Streptophyta - ps11
Charophyceae/Embriophyta - ps12
Embriophyta - ps13
Tracheophyta - ps14
Spermatophyta - ps15
Angiosperms - ps16
Mesangiospermae - ps17
Magnoliids/eudicots - ps18
Eudicots - ps19
Core_eudicots - ps20
Superrosids - ps21
Rosids - ps22
Eurosids - ps23
Malvids - ps24
Sap./Malv./Brassicales - ps25
Malvales/Brassicales - ps26
Brassicales - ps27
Cleomaceae/Brassicaceae - ps28
Brassicaceae - ps29
Camelineae - ps30
Arabidopsis - ps31
Arabidopsis_thaliana - ps32
p-value
1e-02
2e-02
3e-02
4e-02
6e-02
Log-odds
0.5
1.0
1.5
2.0
2.5
3.0
3.5
4.0

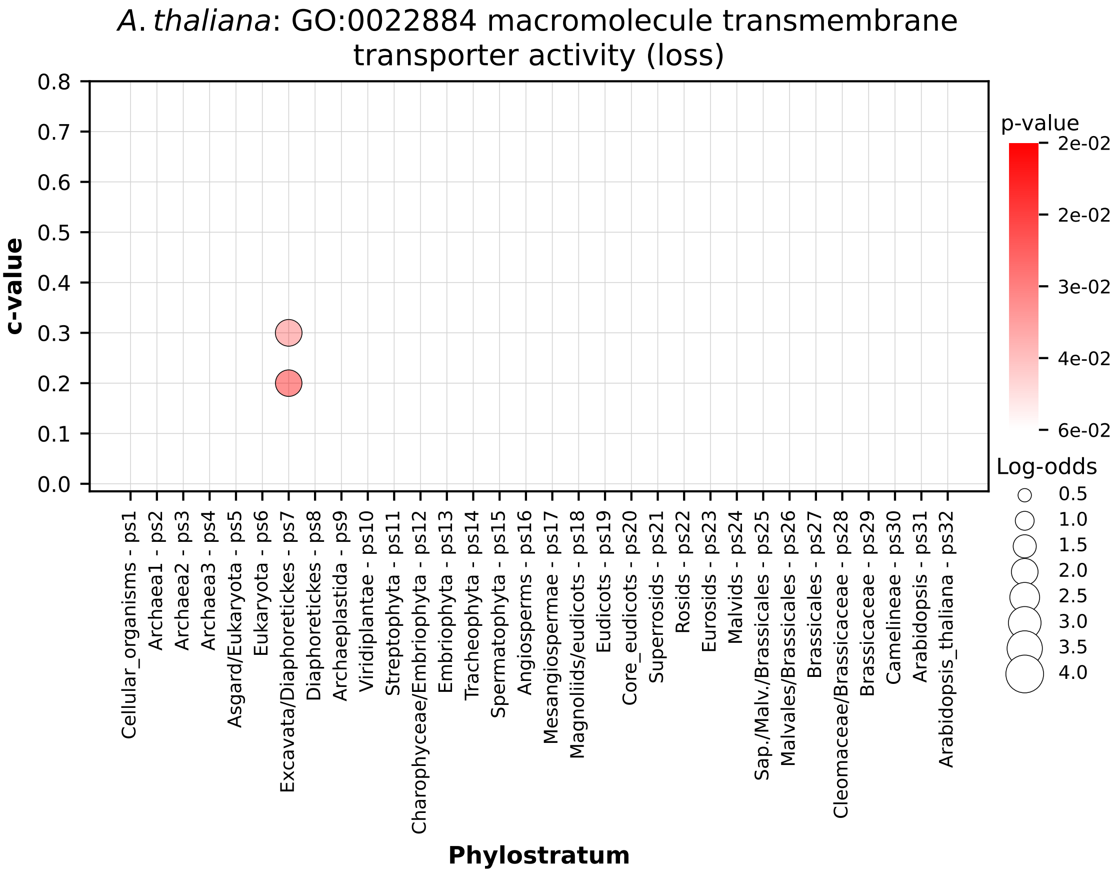

A. thaliana: GO:0022884 macromolecule transmembrane transporter activity (loss)
c-value
0.0
0.1
0.2
0.3
0.4
0.5
0.6
0.7
0.8
Phylostratum
Cellular_organisms - ps1
Archaea1 - ps2
Archaea2 - ps3
Archaea3 - ps4
Asgard/Eukaryota - ps5
Eukaryota - ps6
Excavata/Diaphoretickes - ps7
Diaphoretickes - ps8
Archaeplastida - ps9
Viridiplantae - ps10
Streptophyta - ps11
Charophyceae/Embriophyta - ps12
Embriophyta - ps13
Tracheophyta - ps14
Spermatophyta - ps15
Angiosperms - ps16
Mesangiospermae - ps17
Magnoliids/eudicots - ps18
Eudicots - ps19
Core_eudicots - ps20
Superrosids - ps21
Rosids - ps22
Eurosids - ps23
Malvids - ps24
Sap./Malv./Brassicales - ps25
Malvales/Brassicales - ps26
Brassicales - ps27
Cleomaceae/Brassicaceae - ps28
Brassicaceae - ps29
Camelineae - ps30
Arabidopsis - ps31
Arabidopsis_thaliana - ps32
p-value
2e-02
2e-02
3e-02
4e-02
6e-02
Log-odds
0.5
1.0
1.5
2.0
2.5
3.0
3.5
4.0

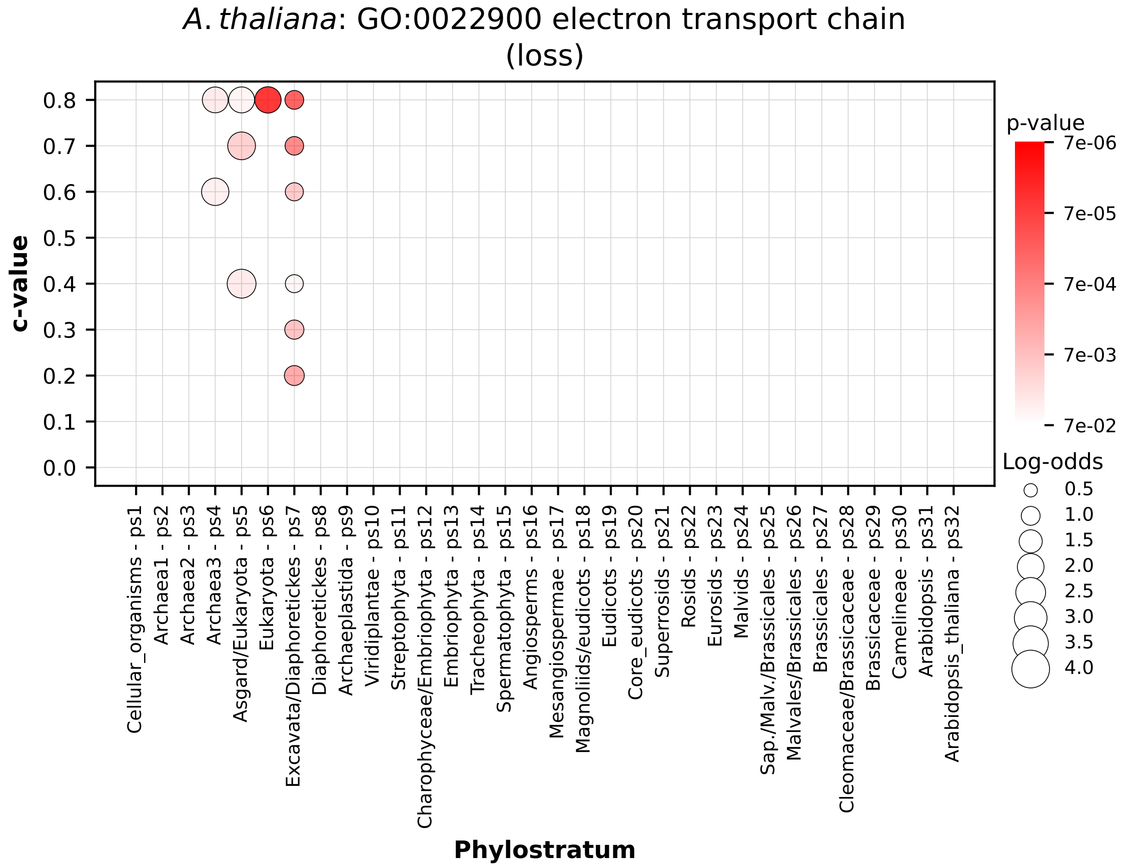

A. thaliana: GO:0022900 electron transport chain
(loss)
c-value
0.8
0.7
0.6
0.5
0.4
0.3
0.2
0.1
0.0
p-value
7e-06
7e-05
7e-04
7e-03
7e-02
Log-odds
0.5
1.0
1.5
2.0
2.5
3.0
3.5
4.0
Cellular_organisms - ps1
Archaea1 - ps2
Archaea2 - ps3
Archaea3 - ps4
Asgard/Eukaryota - ps5
Eukaryota - ps6
Excavata/Diaphoretickes - ps7
Diaphoretickes - ps8
Archaeplastida - ps9
Viridiplantae - ps10
Streptophyta - ps11
Charophyceae/Embriophyta - ps12
Embriophyta - ps13
Tracheophyta - ps14
Spermatophyta - ps15
Angiosperms - ps16
Mesangiospermae - ps17
Magnoliids/eudicots - ps18
Eudicots - ps19
Core_eudicots - ps20
Superrosids - ps21
Rosids - ps22
Eurosids - ps23
Malvids - ps24
Sap./Malv./Brassicales - ps25
Malvales/Brassicales - ps26
Brassicales - ps27
Cleomaceae/Brassicaceae - ps28
Brassicaceae - ps29
Camelineae - ps30
Arabidopsis - ps31
Arabidopsis_thaliana - ps32
Phylostratum

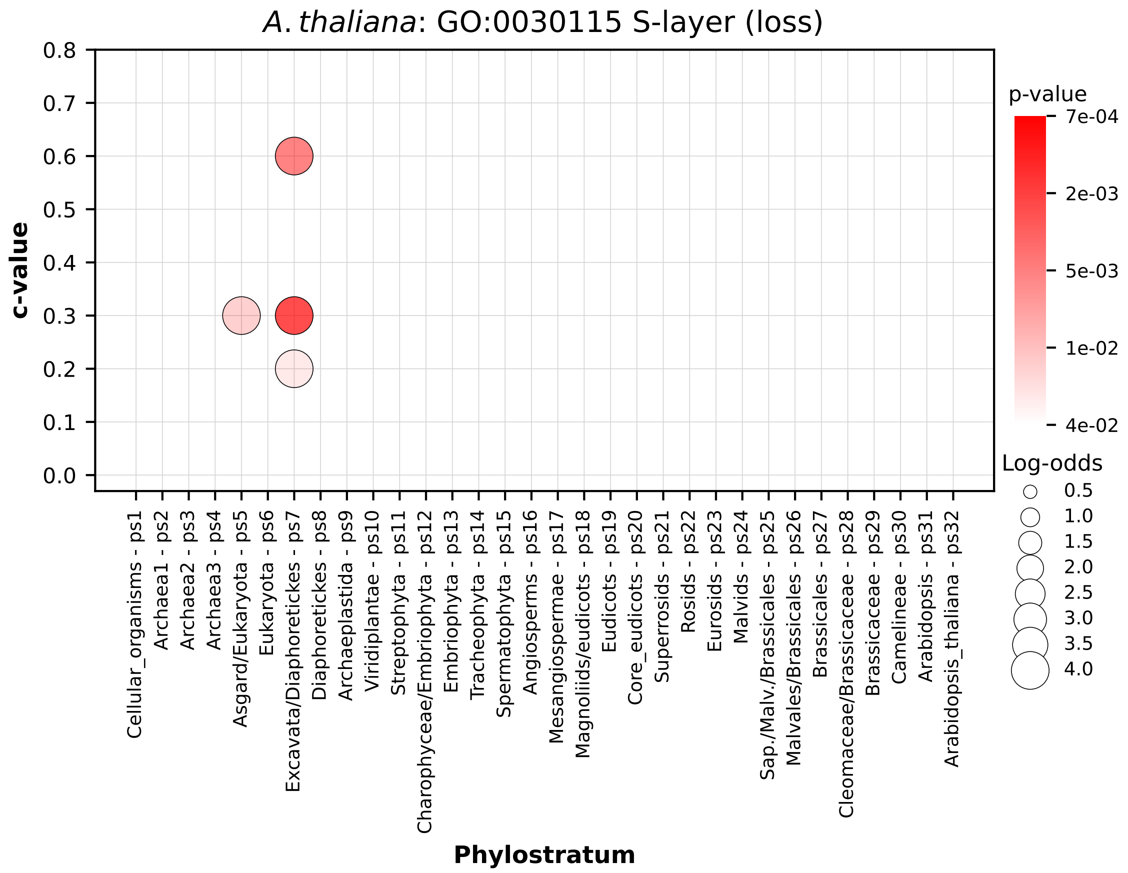
A. thaliana: GO:0030115 S-layer (loss)
c-value
0.0
0.1
0.2
0.3
0.4
0.5
0.6
0.7
0.8
Phylostratum
Cellular_organisms - ps1
Archaea1 - ps2
Archaea2 - ps3
Archaea3 - ps4
Asgard/Eukaryota - ps5
Eukaryota - ps6
Excavata/Diaphoretickes - ps7
Diaphoretickes - ps8
Archaeplastida - ps9
Viridiplantae - ps10
Streptophyta - ps11
Charophyceae/Embriophyta - ps12
Embriophyta - ps13
Tracheophyta - ps14
Spermatophyta - ps15
Angiosperms - ps16
Mesangiospermae - ps17
Magnoliids/eudicots - ps18
Eudicots - ps19
Core_eudicots - ps20
Superrosids - ps21
Rosids - ps22
Eurosids - ps23
Malvids - ps24
Sap./Malv./Brassicales - ps25
Malvales/Brassicales - ps26
Brassicales - ps27
Cleomaceae/Brassicaceae - ps28
Brassicaceae - ps29
Camelineae - ps30
Arabidopsis - ps31
Arabidopsis_thaliana - ps32
p-value
7e-04
2e-03
5e-03
1e-02
4e-02
Log-odds
0.5
1.0
1.5
2.0
2.5
3.0
3.5
4.0

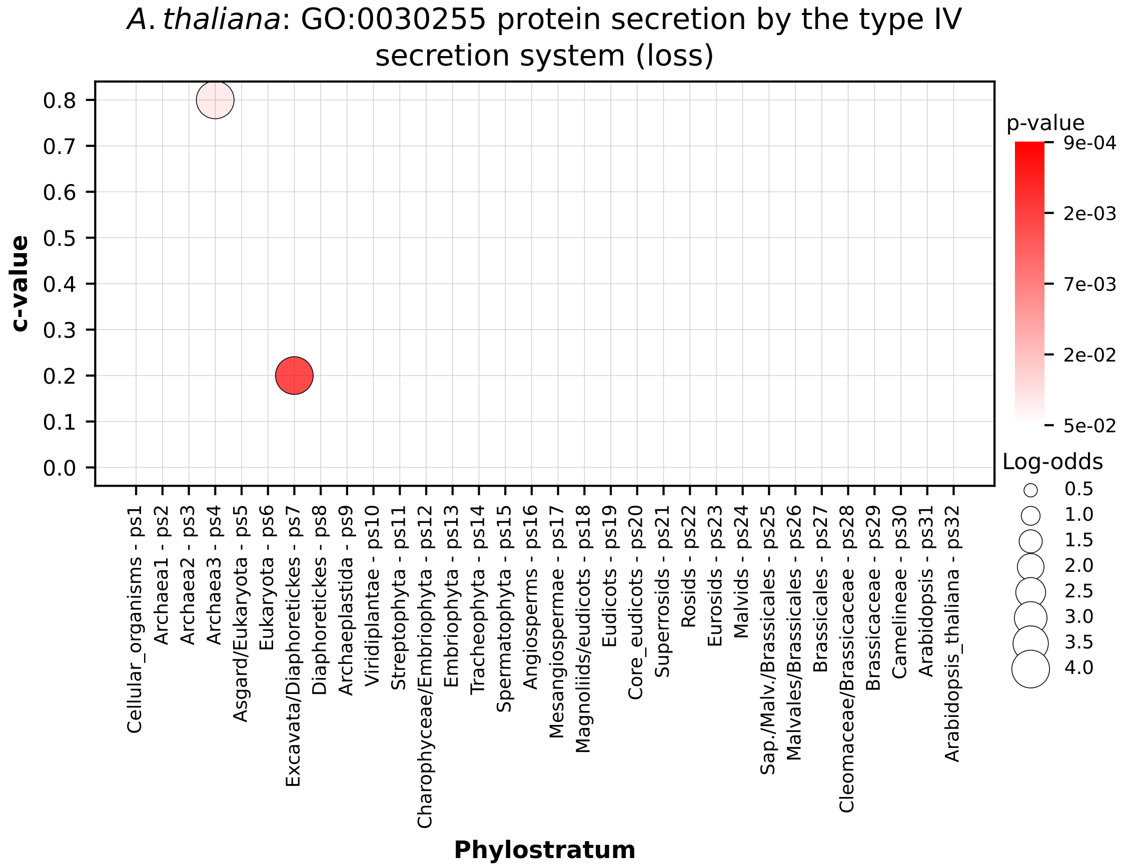

A. thaliana: GO:0030255 protein secretion by the type IV secretion system (loss)
c-value
0.0
0.1
0.2
0.3
0.4
0.5
0.6
0.7
0.8
Phylostratum
Cellular_organisms - ps1
Archaea1 - ps2
Archaea2 - ps3
Archaea3 - ps4
Asgard/Eukaryota - ps5
Eukaryota - ps6
Excavata/Diaphoretickes - ps7
Diaphoretickes - ps8
Archaeplastida - ps9
Viridiplantae - ps10
Streptophyta - ps11
Charophyceae/Embriophyta - ps12
Embriophyta - ps13
Tracheophyta - ps14
Spermatophyta - ps15
Angiosperms - ps16
Mesangiospermae - ps17
Magnoliids/eudicots - ps18
Eudicots - ps19
Core_eudicots - ps20
Superrosids - ps21
Rosids - ps22
Eurosids - ps23
Malvids - ps24
Sap./Malv./Brassicales - ps25
Malvales/Brassicales - ps26
Brassicales - ps27
Cleomaceae/Brassicaceae - ps28
Brassicaceae - ps29
Camelineae - ps30
Arabidopsis - ps31
Arabidopsis_thaliana - ps32
p-value
9e-04
2e-03
7e-03
2e-02
5e-02
Log-odds
0.5
1.0
1.5
2.0
2.5
3.0
3.5
4.0

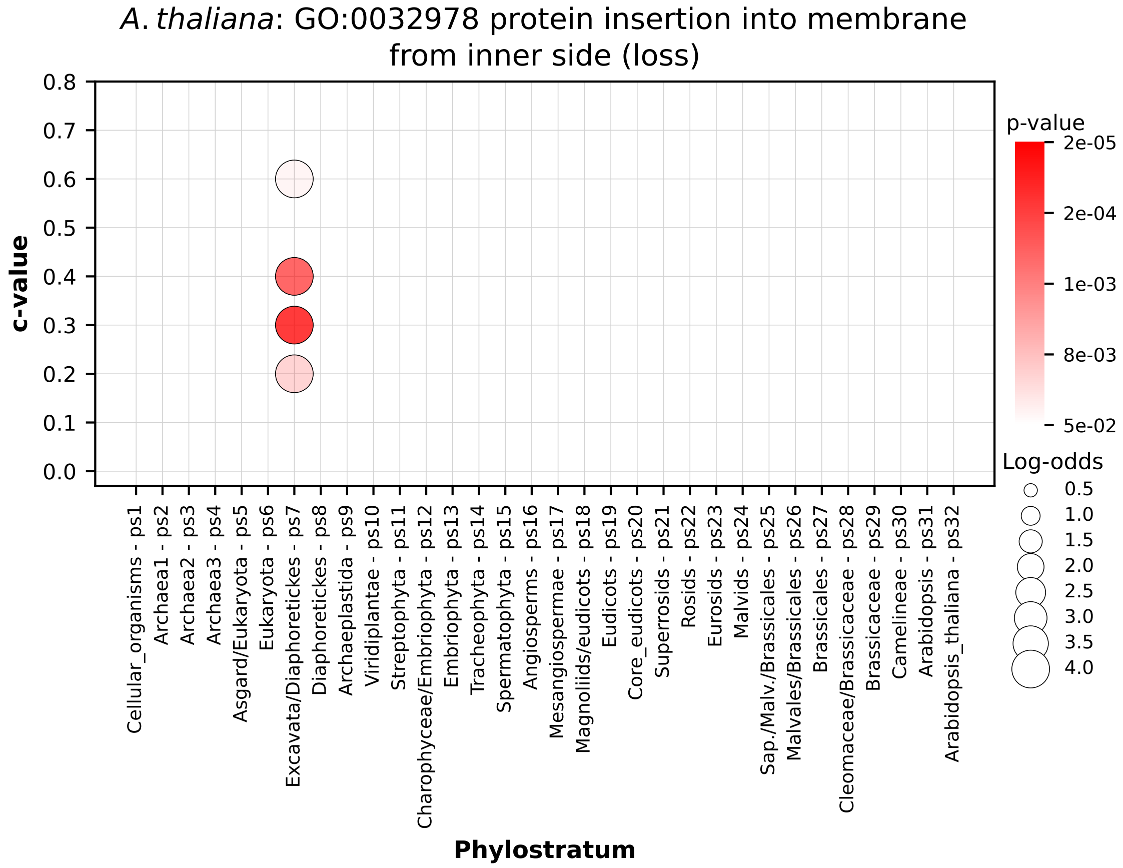

A. thaliana: GO:0032978 protein insertion into membrane from inner side (loss)
c-value
0.0
0.1
0.2
0.3
0.4
0.5
0.6
0.7
0.8
Phylostratum
Cellular_organisms - ps1
Archaea1 - ps2
Archaea2 - ps3
Archaea3 - ps4
Asgard/Eukaryota - ps5
Eukaryota - ps6
Excavata/Diaphoretickes - ps7
Diaphoretickes - ps8
Archaeplastida - ps9
Viridiplantae - ps10
Streptophyta - ps11
Charophyceae/Embriophyta - ps12
Embriophyta - ps13
Tracheophyta - ps14
Spermatophyta - ps15
Angiosperms - ps16
Mesangiospermae - ps17
Magnoliids/eudicots - ps18
Eudicots - ps19
Core_eudicots - ps20
Superrosids - ps21
Rosids - ps22
Eurosids - ps23
Malvids - ps24
Sap./Malv./Brassicales - ps25
Malvales/Brassicales - ps26
Brassicales - ps27
Cleomaceae/Brassicaceae - ps28
Brassicaceae - ps29
Camelineae - ps30
Arabidopsis - ps31
Arabidopsis_thaliana - ps32
p-value
2e-05
2e-04
1e-03
8e-03
5e-02
Log-odds
0.5
1.0
1.5
2.0
2.5
3.0
3.5
4.0

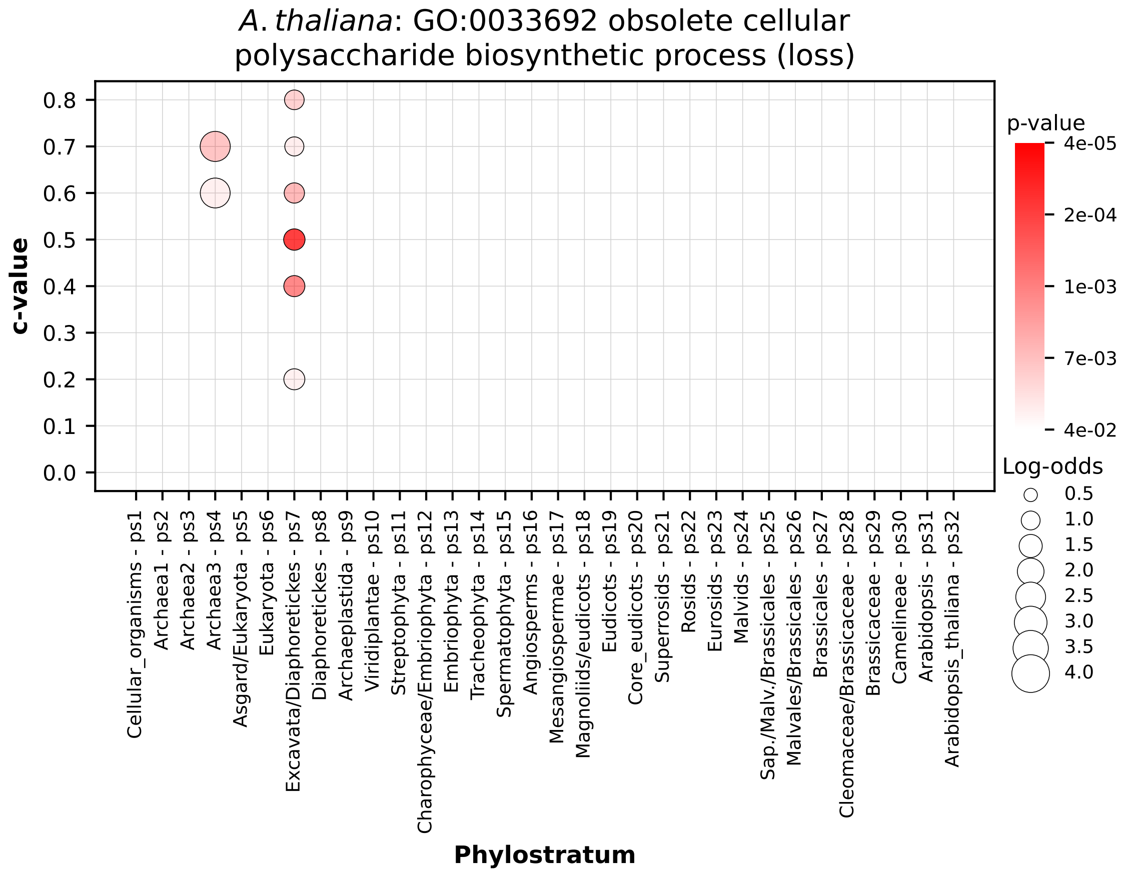

A. thaliana: GO:0033692 obsolete cellular polysaccharide biosynthetic process (loss)
c-value
0.8
0.7
0.6
0.5
0.4
0.3
0.2
0.1
0.0
Phylostratum
Cellular_organisms - ps1
Archaea1 - ps2
Archaea2 - ps3
Archaea3 - ps4
Asgard/Eukaryota - ps5
Eukaryota - ps6
Excavata/Diaphoretickes - ps7
Diaphoretickes - ps8
Archaeplastida - ps9
Viridiplantae - ps10
Streptophyta - ps11
Charophyceae/Embriophyta - ps12
Embriophyta - ps13
Tracheophyta - ps14
Spermatophyta - ps15
Angiosperms - ps16
Mesangiospermae - ps17
Magnoliids/eudicots - ps18
Eudicots - ps19
Core_eudicots - ps20
Superrosids - ps21
Rosids - ps22
Eurosids - ps23
Malvids - ps24
Sap./Malv./Brassicales - ps25
Malvales/Brassicales - ps26
Brassicales - ps27
Cleomaceae/Brassicaceae - ps28
Brassicaceae - ps29
Camelineae - ps30
Arabidopsis - ps31
Arabidopsis_thaliana - ps32
p-value
4e-05
2e-04
1e-03
7e-03
4e-02
Log-odds
0.5
1.0
1.5
2.0
2.5
3.0
3.5
4.0

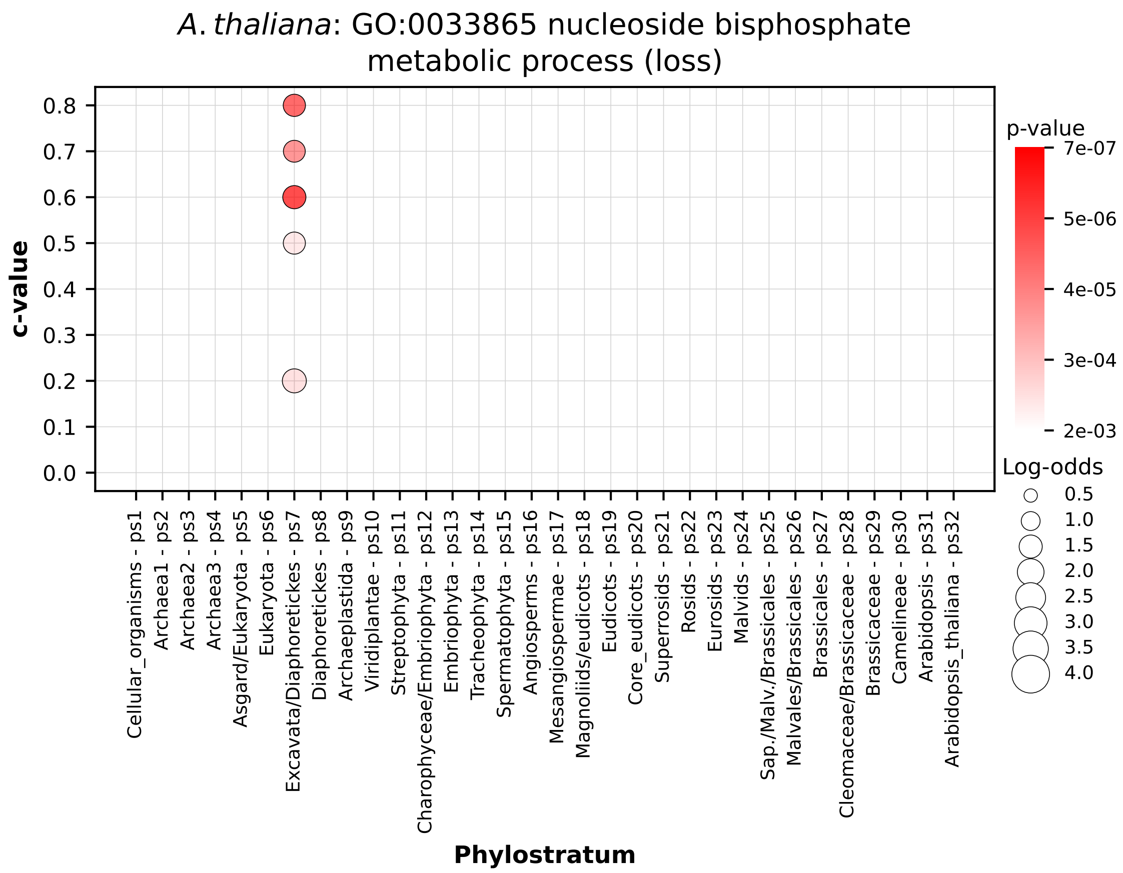
A. thaliana: GO:0033865 nucleoside bisphosphate metabolic process (loss)
c-value
Phylostratum
p-value
7e-07
5e-06
4e-05
3e-04
2e-03
Log-odds
0.5
1.0
1.5
2.0
2.5
3.0
3.5
4.0
Cellular_organisms - ps1
Archaea1 - ps2
Archaea2 - ps3
Archaea3 - ps4
Asgard/Eukaryota - ps5
Eukaryota - ps6
Excavata/Diaphoretickes - ps7
Diaphoretickes - ps8
Archaeplastida - ps9
Viridiplantae - ps10
Streptophyta - ps11
Charophyceae/Embriophyta - ps12
Embriophyta - ps13
Tracheophyta - ps14
Spermatophyta - ps15
Angiosperms - ps16
Mesangiospermae - ps17
Magnoliids/eudicots - ps18
Eudicots - ps19
Core_eudicots - ps20
Superrosids - ps21
Rosids - ps22
Eurosids - ps23
Malvids - ps24
Sap./Malv./Brassicales - ps25
Malvales/Brassicales - ps26
Brassicales - ps27
Cleomaceae/Brassicaceae - ps28
Brassicaceae - ps29
Camelineae - ps30
Arabidopsis - ps31
Arabidopsis_thaliana - ps32

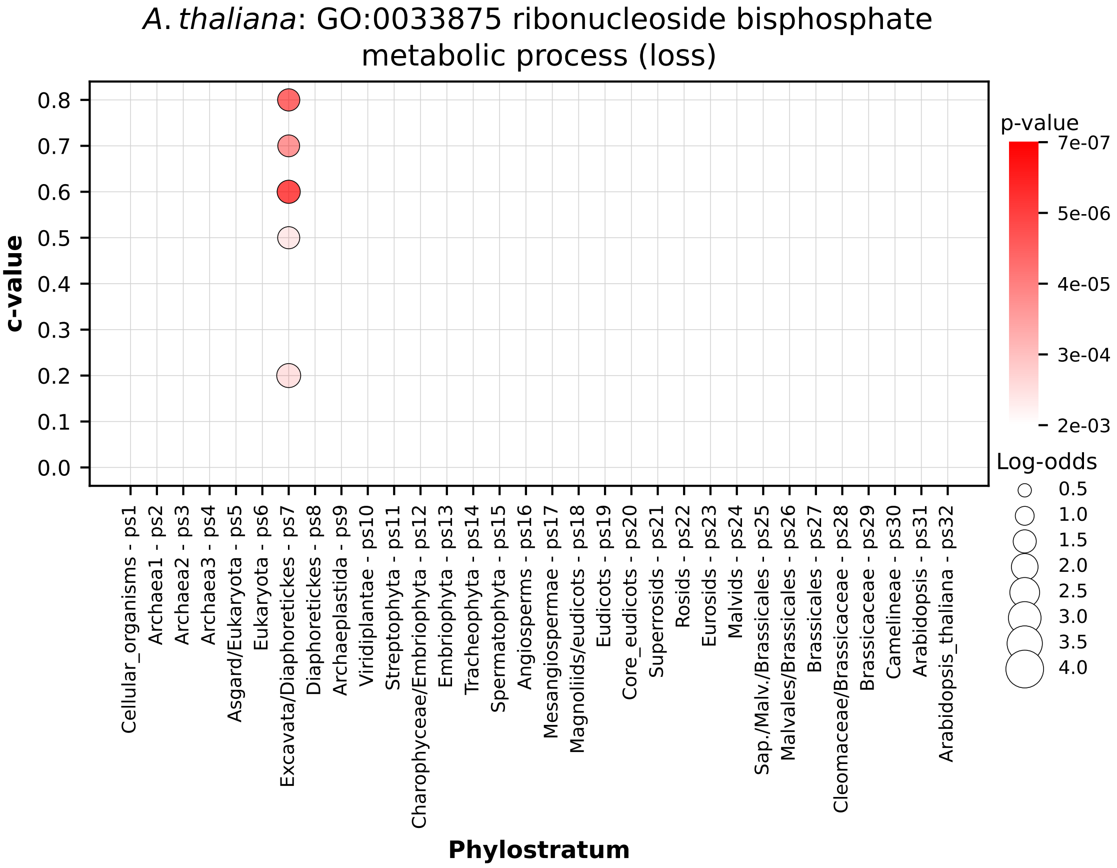

A. thaliana: GO:0033875 ribonucleoside bisphosphate metabolic process (loss)
c-value
0.8
0.7
0.6
0.5
0.4
0.3
0.2
0.1
0.0
p-value
7e-07
5e-06
4e-05
3e-04
2e-03
Log-odds
0.5
1.0
1.5
2.0
2.5
3.0
3.5
4.0
Cellular_organisms - ps1
Archaea1 - ps2
Archaea2 - ps3
Archaea3 - ps4
Asgard/Eukaryota - ps5
Eukaryota - ps6
Excavata/Diaphoretickes - ps7
Diaphoretickes - ps8
Archaeplastida - ps9
Viridiplantae - ps10
Streptophyta - ps11
Charophyceae/Embriophyta - ps12
Embriophyta - ps13
Tracheophyta - ps14
Spermatophyta - ps15
Angiosperms - ps16
Mesangiospermae - ps17
Magnoliids/eudicots - ps18
Eudicots - ps19
Core_eudicots - ps20
Superrosids - ps21
Rosids - ps22
Eurosids - ps23
Malvids - ps24
Sap./Malv./Brassicales - ps25
Malvales/Brassicales - ps26
Brassicales - ps27
Cleomaceae/Brassicaceae - ps28
Brassicaceae - ps29
Camelineae - ps30
Arabidopsis - ps31
Arabidopsis_thaliana - ps32
Phylostratum

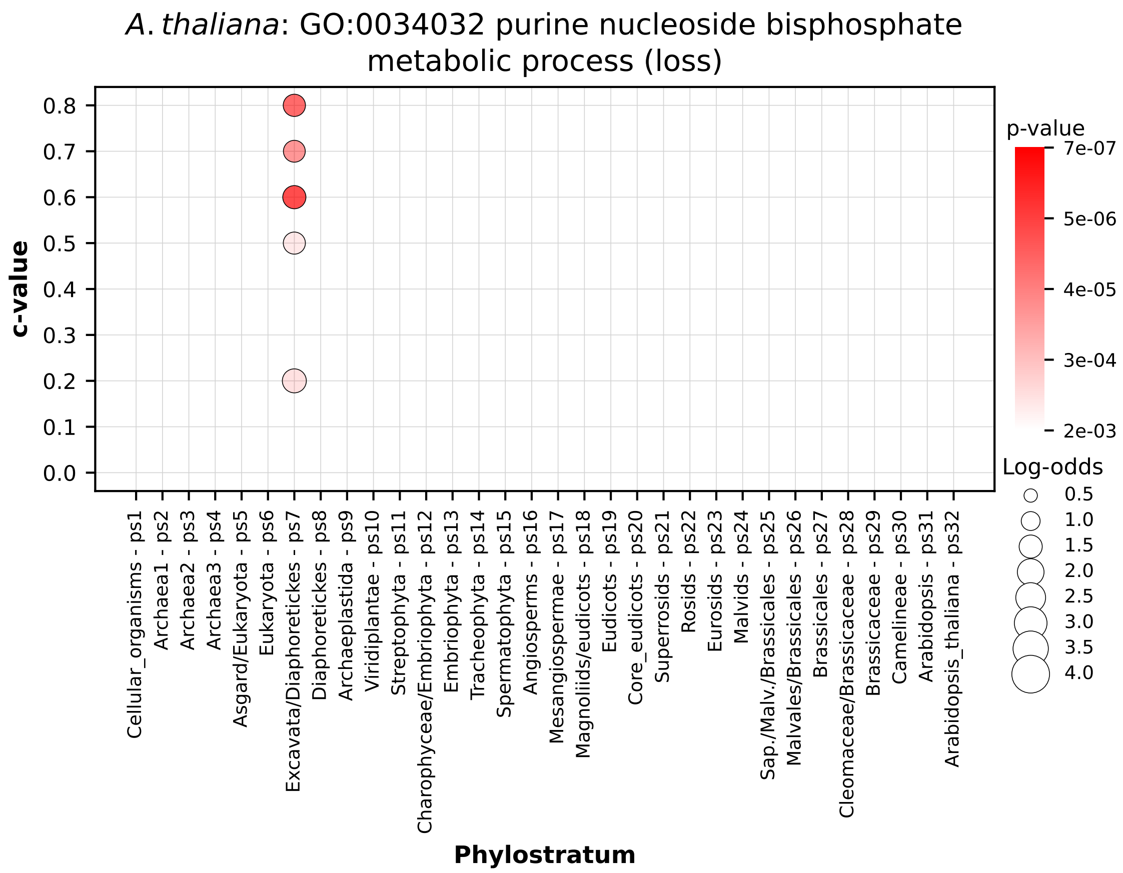

A. thaliana: GO:0034032 purine nucleoside bisphosphate metabolic process (loss)
c-value
0.0
0.1
0.2
0.3
0.4
0.5
0.6
0.7
0.8
Phylostratum
Cellular_organisms - ps1
Archaea1 - ps2
Archaea2 - ps3
Archaea3 - ps4
Asgard/Eukaryota - ps5
Eukaryota - ps6
Excavata/Diaphoretickes - ps7
Diaphoretickes - ps8
Archaeplastida - ps9
Viridiplantae - ps10
Streptophyta - ps11
Charophyceae/Embriophyta - ps12
Embriophyta - ps13
Tracheophyta - ps14
Spermatophyta - ps15
Angiosperms - ps16
Mesangiospermae - ps17
Magnoliids/eudicots - ps18
Eudicots - ps19
Core_eudicots - ps20
Superrosids - ps21
Rosids - ps22
Eurosids - ps23
Malvids - ps24
Sap./Malv./Brassicales - ps25
Malvales/Brassicales - ps26
Brassicales - ps27
Cleomaceae/Brassicaceae - ps28
Brassicaceae - ps29
Camelineae - ps30
Arabidopsis - ps31
Arabidopsis_thaliana - ps32
p-value
7e-07
5e-06
4e-05
3e-04
2e-03
Log-odds
0.5
1.0
1.5
2.0
2.5
3.0
3.5
4.0

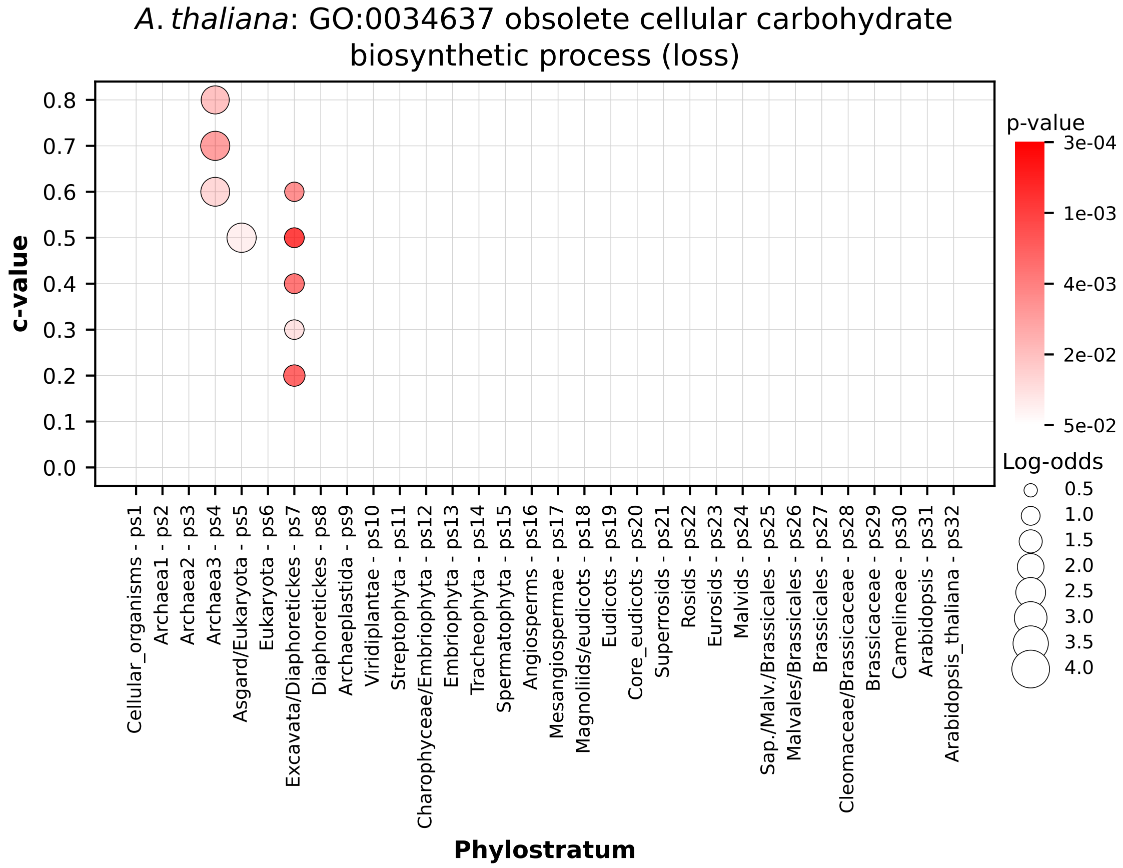
A. thaliana: GO:0034637 obsolete cellular carbohydrate biosynthetic process (loss)
c-value
0.0
0.1
0.2
0.3
0.4
0.5
0.6
0.7
0.8
Phylostratum
Cellular_organisms - ps1
Archaea1 - ps2
Archaea2 - ps3
Archaea3 - ps4
Asgard/Eukaryota - ps5
Eukaryota - ps6
Excavata/Diaphoretickes - ps7
Diaphoretickes - ps8
Archaeplastida - ps9
Viridiplantae - ps10
Streptophyta - ps11
Charophyceae/Embriophyta - ps12
Embriophyta - ps13
Tracheophyta - ps14
Spermatophyta - ps15
Angiosperms - ps16
Mesangiospermae - ps17
Magnoliids/eudicots - ps18
Eudicots - ps19
Core_eudicots - ps20
Superrosids - ps21
Rosids - ps22
Eurosids - ps23
Malvids - ps24
Sap./Malv./Brassicales - ps25
Malvales/Brassicales - ps26
Brassicales - ps27
Cleomaceae/Brassicaceae - ps28
Brassicaceae - ps29
Camelineae - ps30
Arabidopsis - ps31
Arabidopsis_thaliana - ps32
p-value
3e-04
1e-03
4e-03
2e-02
5e-02
Log-odds
0.5
1.0
1.5
2.0
2.5
3.0
3.5
4.0

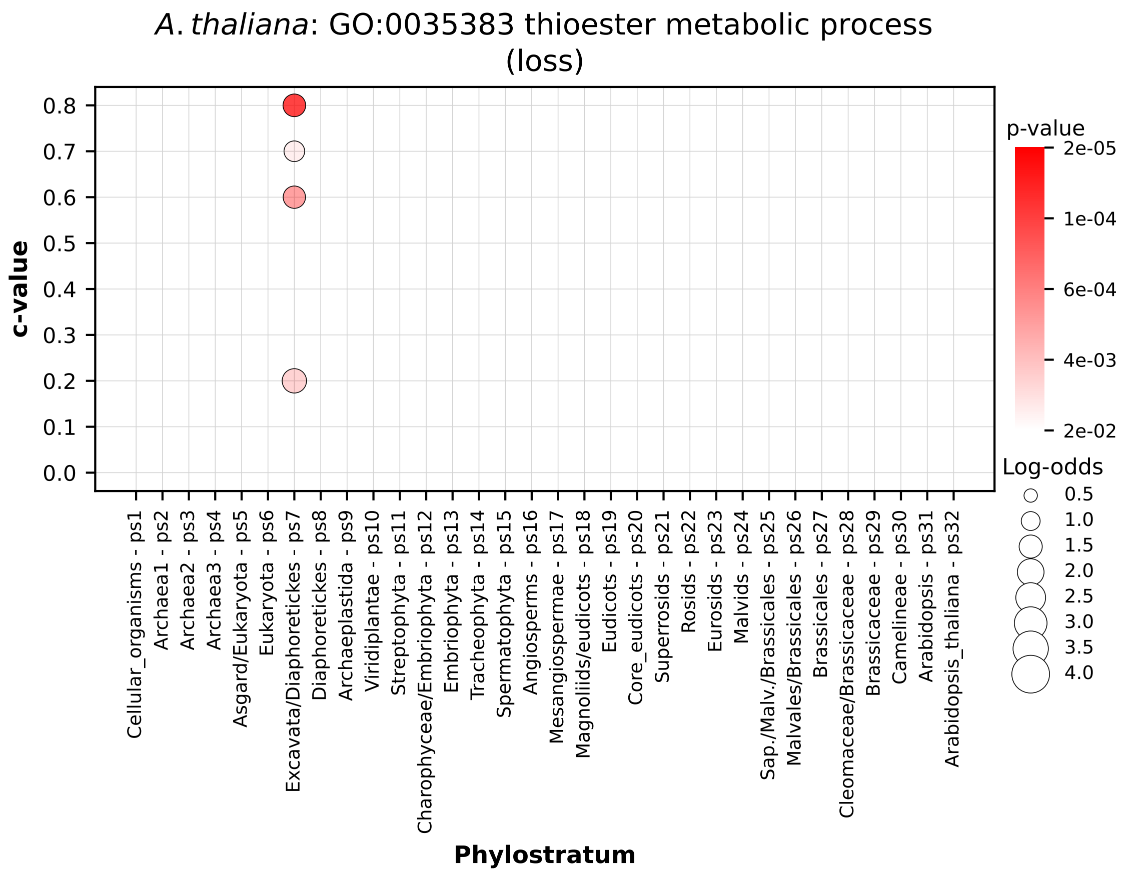

A. thaliana: GO:0035383 thioester metabolic process
(loss)
c-value
Phylostratum
p-value
2e-05
1e-04
6e-04
4e-03
2e-02
Log-odds
0.5
1.0
1.5
2.0
2.5
3.0
3.5
4.0
Cellular_organisms - ps1
Archaea1 - ps2
Archaea2 - ps3
Archaea3 - ps4
Asgard/Eukaryota - ps5
Eukaryota - ps6
Excavata/Diaphoretickes - ps7
Diaphoretickes - ps8
Archaeplastida - ps9
Viridiplantae - ps10
Streptophyta - ps11
Charophyceae/Embriophyta - ps12
Embriophyta - ps13
Tracheophyta - ps14
Spermatophyta - ps15
Angiosperms - ps16
Mesangiospermae - ps17
Magnoliids/eudicots - ps18
Eudicots - ps19
Core_eudicots - ps20
Superrosids - ps21
Rosids - ps22
Eurosids - ps23
Malvids - ps24
Sap./Malv./Brassicales - ps25
Malvales/Brassicales - ps26
Brassicales - ps27
Cleomaceae/Brassicaceae - ps28
Brassicaceae - ps29
Camelineae - ps30
Arabidopsis - ps31
Arabidopsis_thaliana - ps32

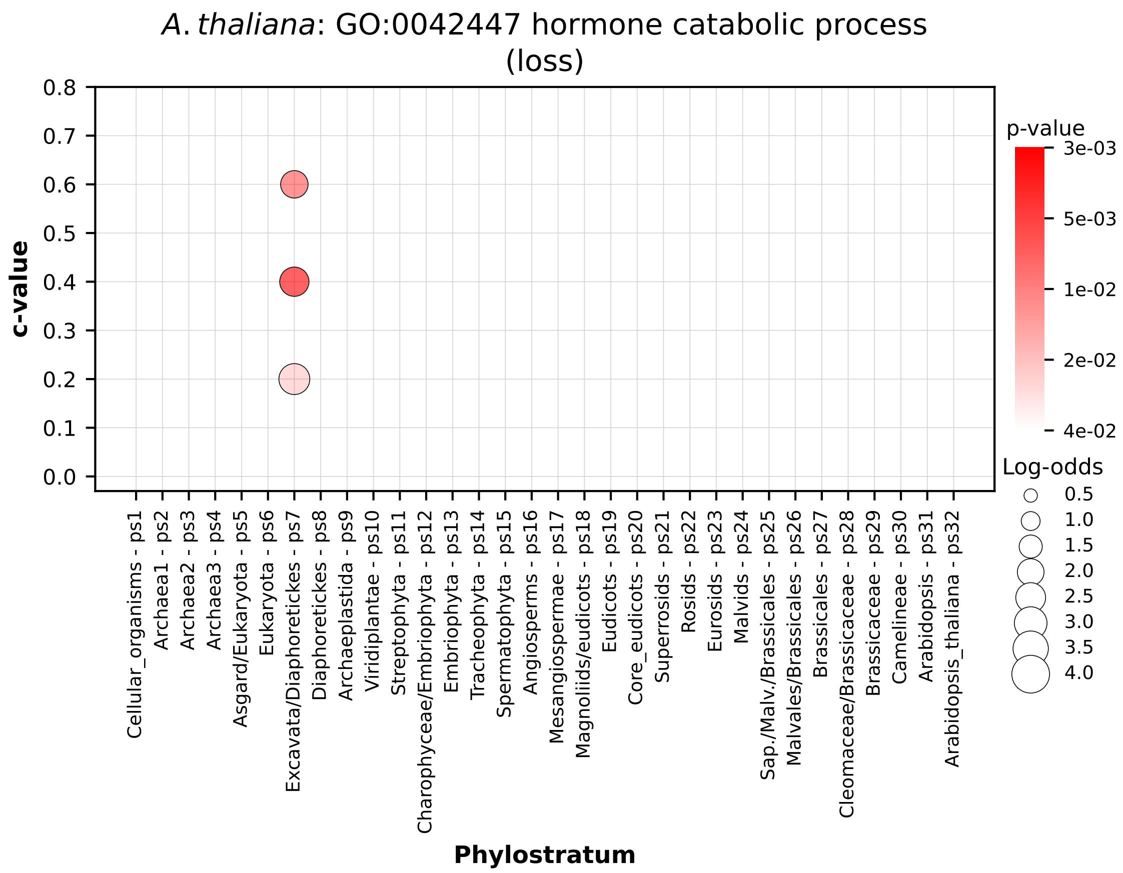

$A. thaliana$: GO:0042447 hormone catabolic process
(loss)
c-value
0.8
0.7
0.6
0.5
0.4
0.3
0.2
0.1
0.0
p-value
3e-03
5e-03
1e-02
2e-02
4e-02
Log-odds
0.5
1.0
1.5
2.0
2.5
3.0
3.5
4.0
Cellular_organisms - ps1
Archaea1 - ps2
Archaea2 - ps3
Archaea3 - ps4
Asgard/Eukaryota - ps5
Eukaryota - ps6
Excavata/Diaphoretickes - ps7
Diaphoretickes - ps8
Archaeplastida - ps9
Viridiplantae - ps10
Streptophyta - ps11
Charophyceae/Embriophyta - ps12
Embriophyta - ps13
Tracheophyta - ps14
Spermatophyta - ps15
Angiosperms - ps16
Mesangiospermae - ps17
Magnoliids/eudicots - ps18
Eudicots - ps19
Core_eudicots - ps20
Superrosids - ps21
Rosids - ps22
Eurosids - ps23
Malvids - ps24
Sap./Malv./Brassicales - ps25
Malvales/Brassicales - ps26
Brassicales - ps27
Cleomaceae/Brassicaceae - ps28
Brassicaceae - ps29
Camelineae - ps30
Arabidopsis - ps31
Arabidopsis_thaliana - ps32
Phylostratum

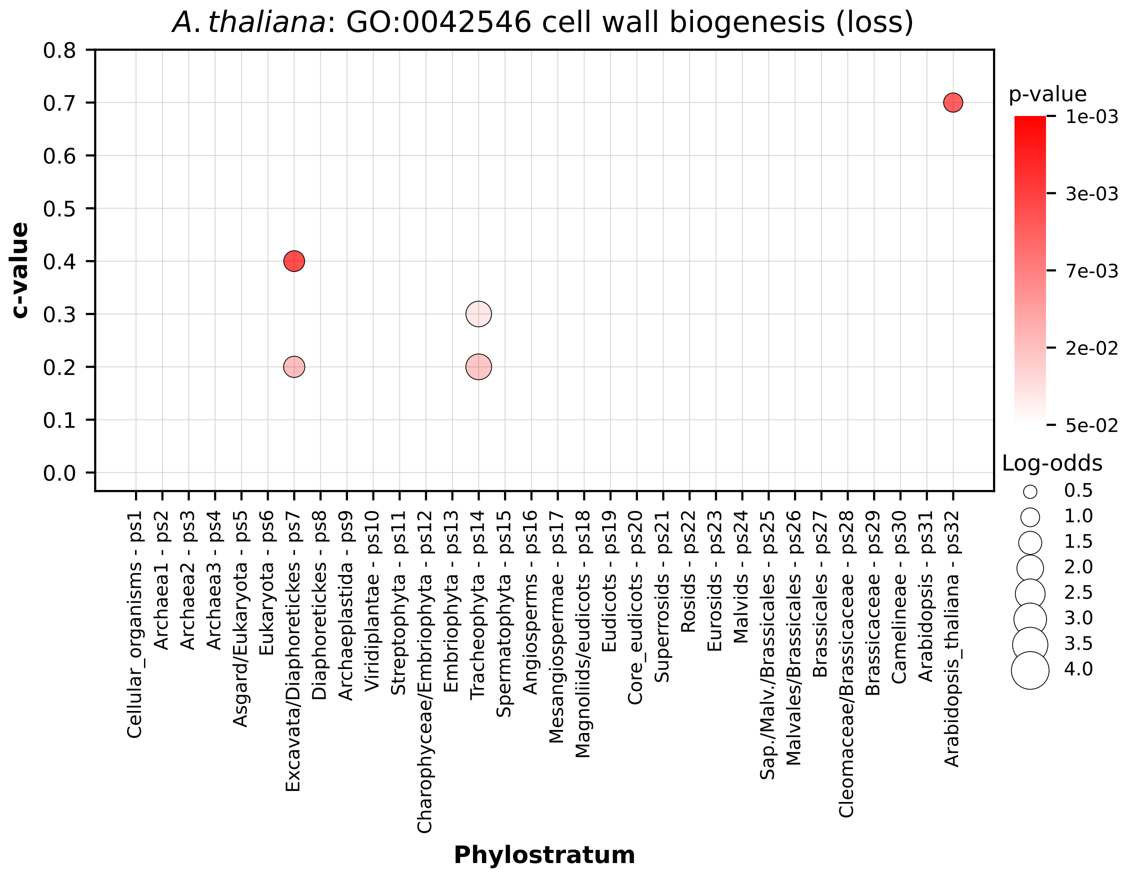

A. thaliana: GO:0042546 cell wall biogenesis (loss)
c-value
0.8
0.7
0.6
0.5
0.4
0.3
0.2
0.1
0.0
p-value
1e-03
3e-03
7e-03
2e-02
5e-02
Log-odds
0.5
1.0
1.5
2.0
2.5
3.0
3.5
4.0
Cellular_organisms - ps1
Archaea1 - ps2
Archaea2 - ps3
Archaea3 - ps4
Asgard/Eukaryota - ps5
Eukaryota - ps6
Excavata/Diaphoretickes - ps7
Diaphoretickes - ps8
Archaeplastida - ps9
Viridiplantae - ps10
Streptophyta - ps11
Charophyceae/Embriophyta - ps12
Embriophyta - ps13
Tracheophyta - ps14
Spermatophyta - ps15
Angiosperms - ps16
Mesangiospermae - ps17
Magnoliids/eudicots - ps18
Eudicots - ps19
Core_eudicots - ps20
Superrosids - ps21
Rosids - ps22
Eurosids - ps23
Malvids - ps24
Sap./Malv./Brassicales - ps25
Malvales/Brassicales - ps26
Brassicales - ps27
Cleomaceae/Brassicaceae - ps28
Brassicaceae - ps29
Camelineae - ps30
Arabidopsis - ps31
Arabidopsis_thaliana - ps32
Phylostratum

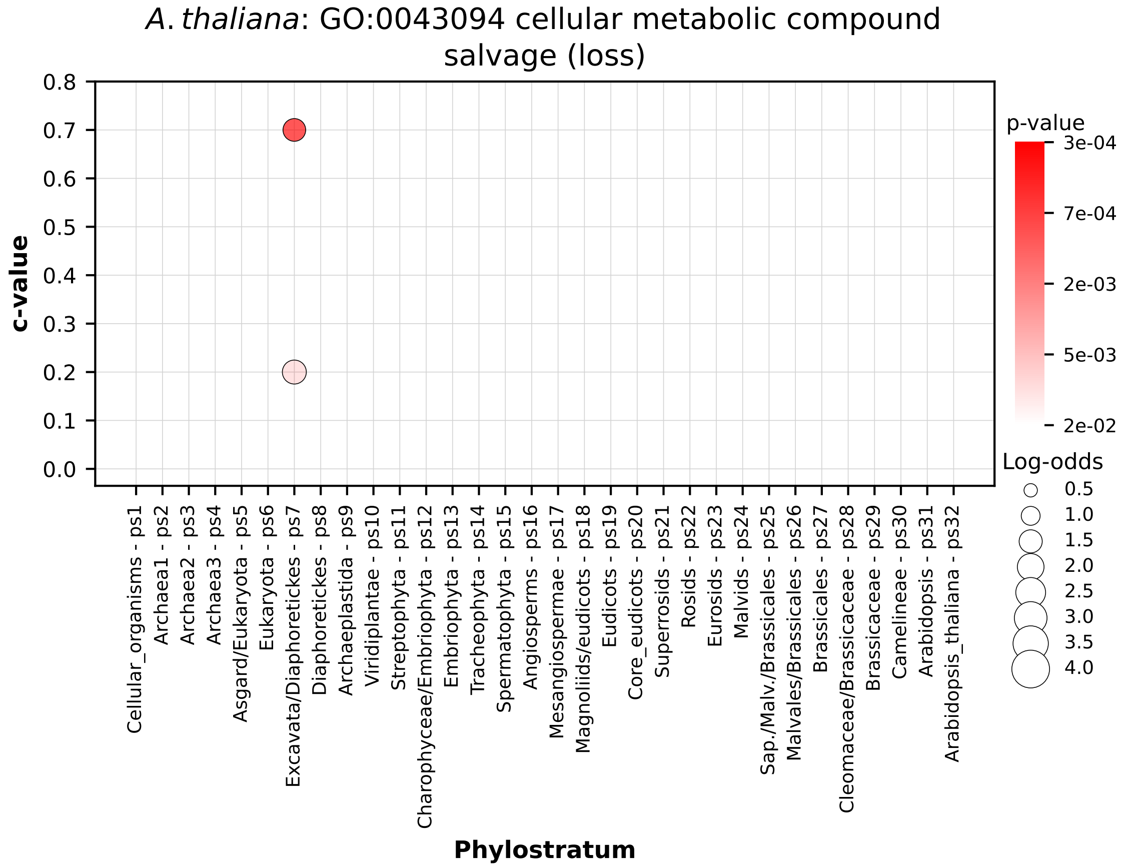

A. thaliana: GO:0043094 cellular metabolic compound salvage (loss)
c-value
0.8
0.7
0.6
0.5
0.4
0.3
0.2
0.1
0.0
Phylostratum
Cellular_organisms - ps1
Archaea1 - ps2
Archaea2 - ps3
Archaea3 - ps4
Asgard/Eukaryota - ps5
Eukaryota - ps6
Excavata/Diaphoretickes - ps7
Diaphoretickes - ps8
Archaeplastida - ps9
Viridiplantae - ps10
Streptophyta - ps11
Charophyceae/Embriophyta - ps12
Embriophyta - ps13
Tracheophyta - ps14
Spermatophyta - ps15
Angiosperms - ps16
Mesangiospermae - ps17
Magnoliids/eudicots - ps18
Eudicots - ps19
Core_eudicots - ps20
Superrosids - ps21
Rosids - ps22
Eurosids - ps23
Malvids - ps24
Sap./Malv./Brassicales - ps25
Malvales/Brassicales - ps26
Brassicales - ps27
Cleomaceae/Brassicaceae - ps28
Brassicaceae - ps29
Camelineae - ps30
Arabidopsis - ps31
Arabidopsis_thaliana - ps32
p-value
3e-04
7e-04
2e-03
5e-03
2e-02
Log-odds
0.5
1.0
1.5
2.0
2.5
3.0
3.5
4.0

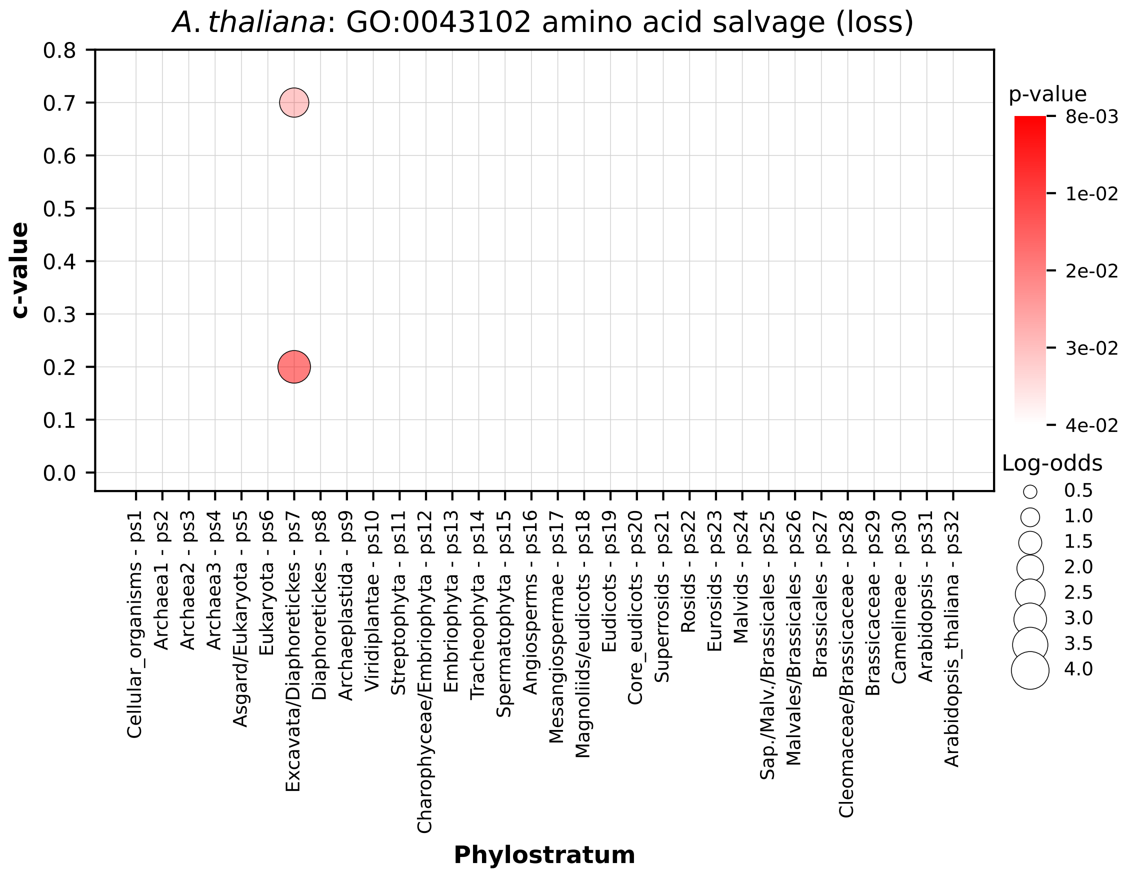

A. thaliana: GO:0043102 amino acid salvage (loss)
c-value
0.8
0.7
0.6
0.5
0.4
0.3
0.2
0.1
0.0
Phylostratum
Cellular_organisms - ps1
Archaea1 - ps2
Archaea2 - ps3
Archaea3 - ps4
Asgard/Eukaryota - ps5
Eukaryota - ps6
Excavata/Diaphoretickes - ps7
Diaphoretickes - ps8
Archaeplastida - ps9
Viridiplantae - ps10
Streptophyta - ps11
Charophyceae/Embriophyta - ps12
Embriophyta - ps13
Tracheophyta - ps14
Spermatophyta - ps15
Angiosperms - ps16
Mesangiospermae - ps17
Magnoliids/eudicots - ps18
Eudicots - ps19
Core_eudicots - ps20
Superrosids - ps21
Rosids - ps22
Eurosids - ps23
Malvids - ps24
Sap./Malv./Brassicales - ps25
Malvales/Brassicales - ps26
Brassicales - ps27
Cleomaceae/Brassicaceae - ps28
Brassicaceae - ps29
Camelineae - ps30
Arabidopsis - ps31
Arabidopsis_thaliana - ps32
p-value
8e-03
1e-02
2e-02
3e-02
4e-02
Log-odds
0.5
1.0
1.5
2.0
2.5
3.0
3.5
4.0

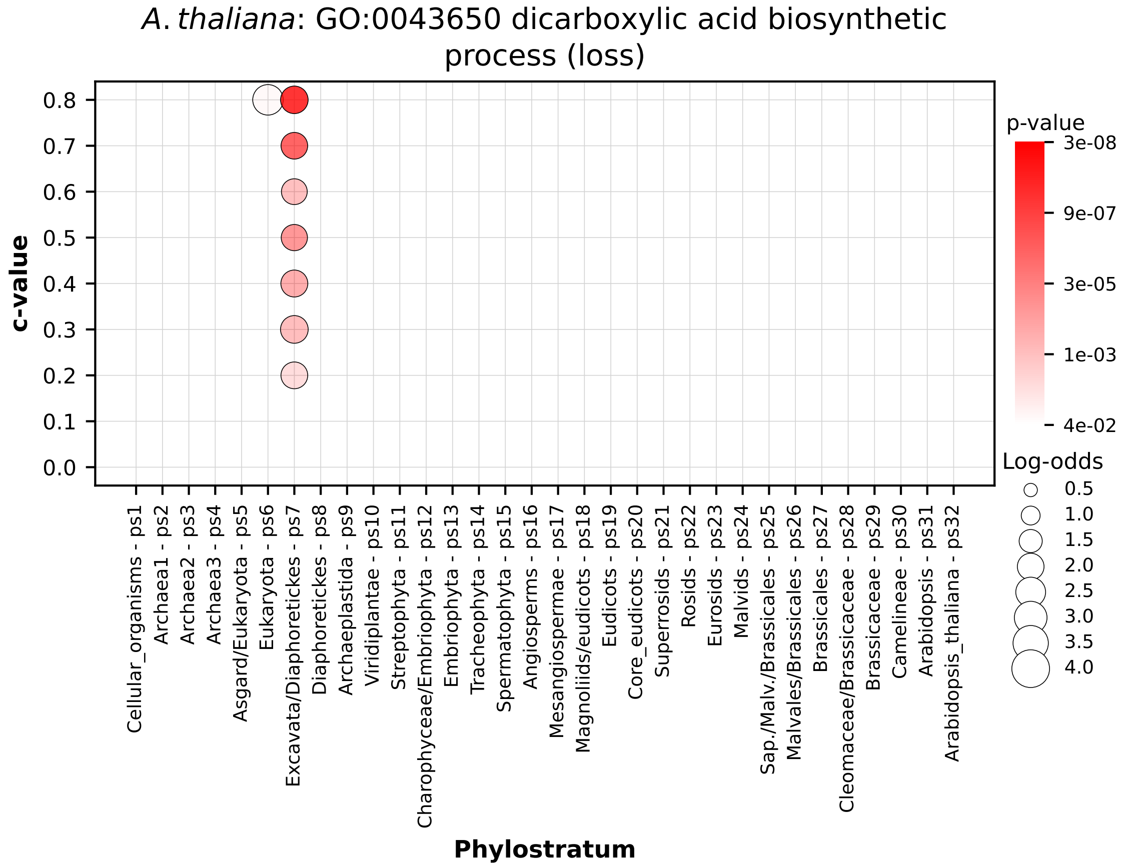

A. thaliana: GO:0043650 dicarboxylic acid biosynthetic process (loss)
c-value
0.8
0.7
0.6
0.5
0.4
0.3
0.2
0.1
0.0
Phylostratum
Cellular_organisms - ps1
Archaea1 - ps2
Archaea2 - ps3
Archaea3 - ps4
Asgard/Eukaryota - ps5
Eukaryota - ps6
Excavata/Diaphoretickes - ps7
Diaphoretickes - ps8
Archaeplastida - ps9
Viridiplantae - ps10
Streptophyta - ps11
Charophyceae/Embriophyta - ps12
Embriophyta - ps13
Tracheophyta - ps14
Spermatophyta - ps15
Angiosperms - ps16
Mesangiospermae - ps17
Magnoliids/eudicots - ps18
Eudicots - ps19
Core_eudicots - ps20
Superrosids - ps21
Rosids - ps22
Eurosids - ps23
Malvids - ps24
Sap./Malv./Brassicales - ps25
Malvales/Brassicales - ps26
Brassicales - ps27
Cleomaceae/Brassicaceae - ps28
Brassicaceae - ps29
Camelineae - ps30
Arabidopsis - ps31
Arabidopsis_thaliana - ps32
p-value
3e-08
9e-07
3e-05
1e-03
4e-02
Log-odds
0.5
1.0
1.5
2.0
2.5
3.0
3.5
4.0

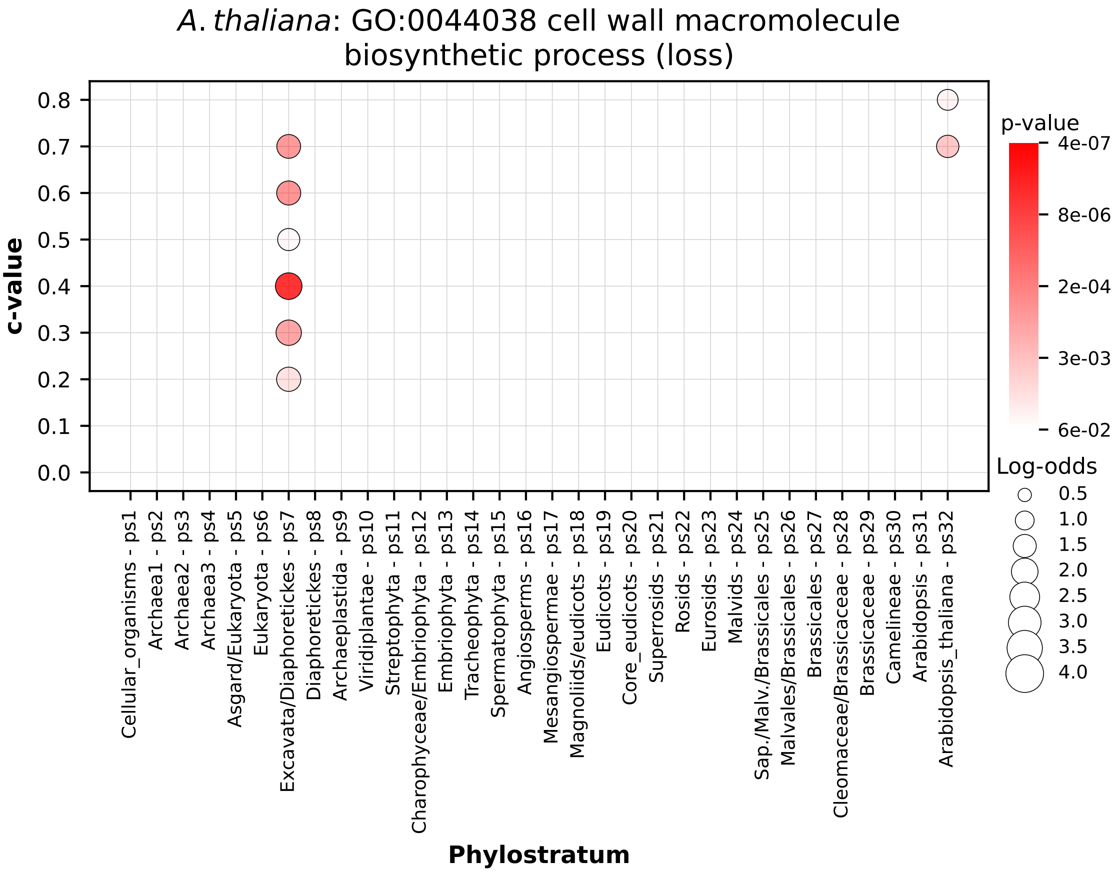

A. thaliana: GO:0044038 cell wall macromolecule biosynthetic process (loss)
c-value
0.0
0.1
0.2
0.3
0.4
0.5
0.6
0.7
0.8
p-value
4e-07
8e-06
2e-04
3e-03
6e-02
Log-odds
0.5
1.0
1.5
2.0
2.5
3.0
3.5
4.0
Cellular_organisms - ps1
Archaea1 - ps2
Archaea2 - ps3
Archaea3 - ps4
Asgard/Eukaryota - ps5
Eukaryota - ps6
Excavata/Diaphoretickes - ps7
Diaphoretickes - ps8
Archaeplastida - ps9
Viridiplantae - ps10
Streptophyta - ps11
Charophyceae/Embriophyta - ps12
Embriophyta - ps13
Tracheophyta - ps14
Spermatophyta - ps15
Angiosperms - ps16
Mesangiospermae - ps17
Magnoliids/eudicots - ps18
Eudicots - ps19
Core_eudicots - ps20
Superrosids - ps21
Rosids - ps22
Eurosids - ps23
Malvids - ps24
Sap./Malv./Brassicales - ps25
Malvales/Brassicales - ps26
Brassicales - ps27
Cleomaceae/Brassicaceae - ps28
Brassicaceae - ps29
Camelineae - ps30
Arabidopsis - ps31
Arabidopsis_thaliana - ps32
Phylostratum

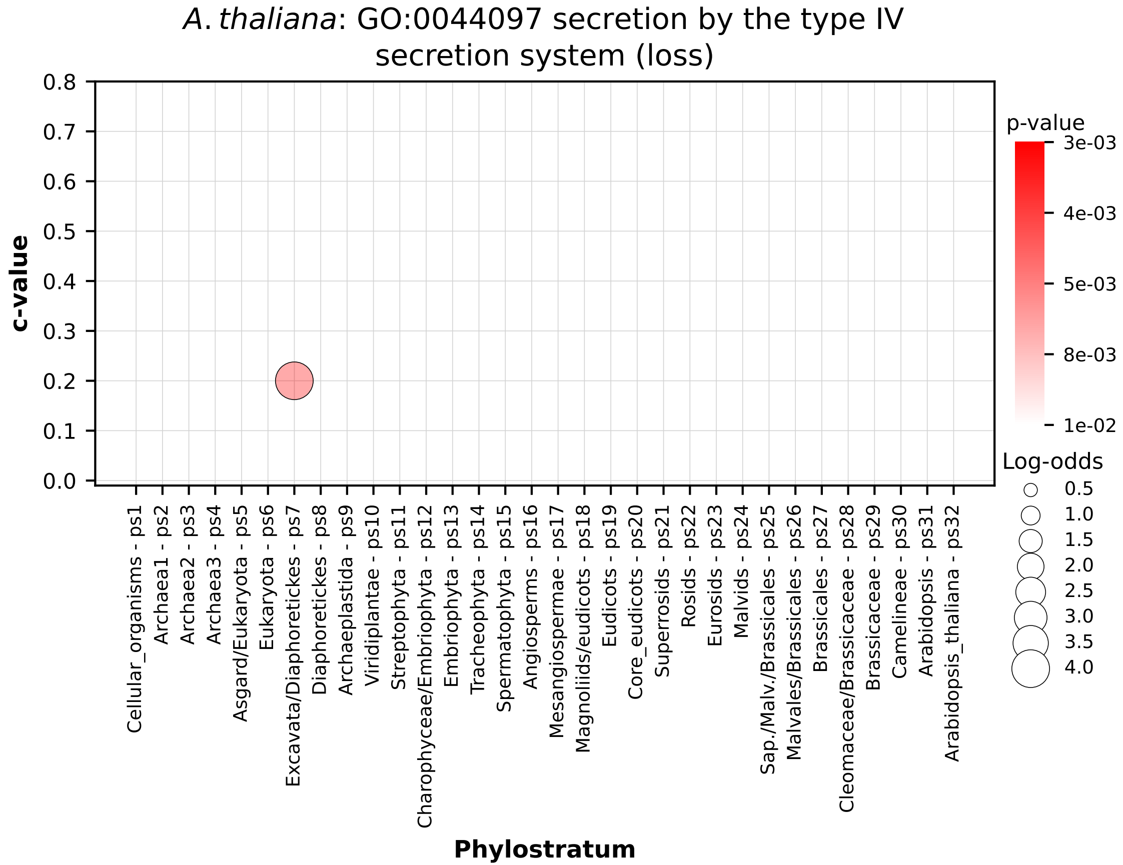

A. thaliana: GO:0044097 secretion by the type IV secretion system (loss)
c-value
0.0
0.1
0.2
0.3
0.4
0.5
0.6
0.7
0.8
Phylostratum
Cellular_organisms - ps1
Archaea1 - ps2
Archaea2 - ps3
Archaea3 - ps4
Asgard/Eukaryota - ps5
Eukaryota - ps6
Excavata/Diaphoretickes - ps7
Diaphoretickes - ps8
Archaeplastida - ps9
Viridiplantae - ps10
Streptophyta - ps11
Charophyceae/Embriophyta - ps12
Embriophyta - ps13
Tracheophyta - ps14
Spermatophyta - ps15
Angiosperms - ps16
Mesangiospermae - ps17
Magnoliids/eudicots - ps18
Eudicots - ps19
Core_eudicots - ps20
Superrosids - ps21
Rosids - ps22
Eurosids - ps23
Malvids - ps24
Sap./Malv./Brassicales - ps25
Malvales/Brassicales - ps26
Brassicales - ps27
Cleomaceae/Brassicaceae - ps28
Brassicaceae - ps29
Camelineae - ps30
Arabidopsis - ps31
Arabidopsis_thaliana - ps32
p-value
3e-03
4e-03
5e-03
8e-03
1e-02
Log-odds
0.5
1.0
1.5
2.0
2.5
3.0
3.5
4.0

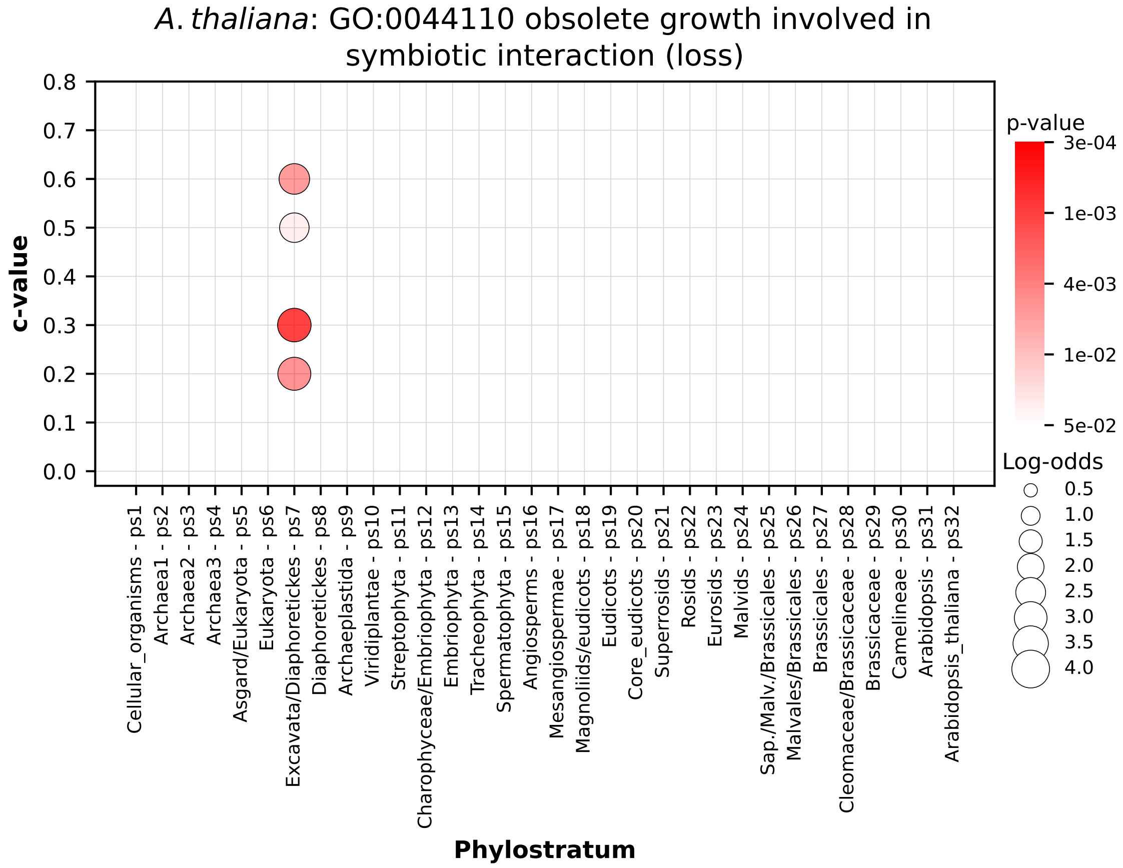

A. thaliana: GO:0044110 obsolete growth involved in symbiotic interaction (loss)
c-value
0.0
0.1
0.2
0.3
0.4
0.5
0.6
0.7
0.8
Phylostratum
Cellular_organisms - ps1
Archaea1 - ps2
Archaea2 - ps3
Archaea3 - ps4
Asgard/Eukaryota - ps5
Eukaryota - ps6
Excavata/Diaphoretickes - ps7
Diaphoretickes - ps8
Archaeplastida - ps9
Viridiplantae - ps10
Streptophyta - ps11
Charophyceae/Embriophyta - ps12
Embriophyta - ps13
Tracheophyta - ps14
Spermatophyta - ps15
Angiosperms - ps16
Mesangiospermae - ps17
Magnoliids/eudicots - ps18
Eudicots - ps19
Core_eudicots - ps20
Superrosids - ps21
Rosids - ps22
Eurosids - ps23
Malvids - ps24
Sap./Malv./Brassicales - ps25
Malvales/Brassicales - ps26
Brassicales - ps27
Cleomaceae/Brassicaceae - ps28
Brassicaceae - ps29
Camelineae - ps30
Arabidopsis - ps31
Arabidopsis_thaliana - ps32
p-value
3e-04
1e-03
4e-03
1e-02
5e-02
Log-odds
0.5
1.0
1.5
2.0
2.5
3.0
3.5
4.0

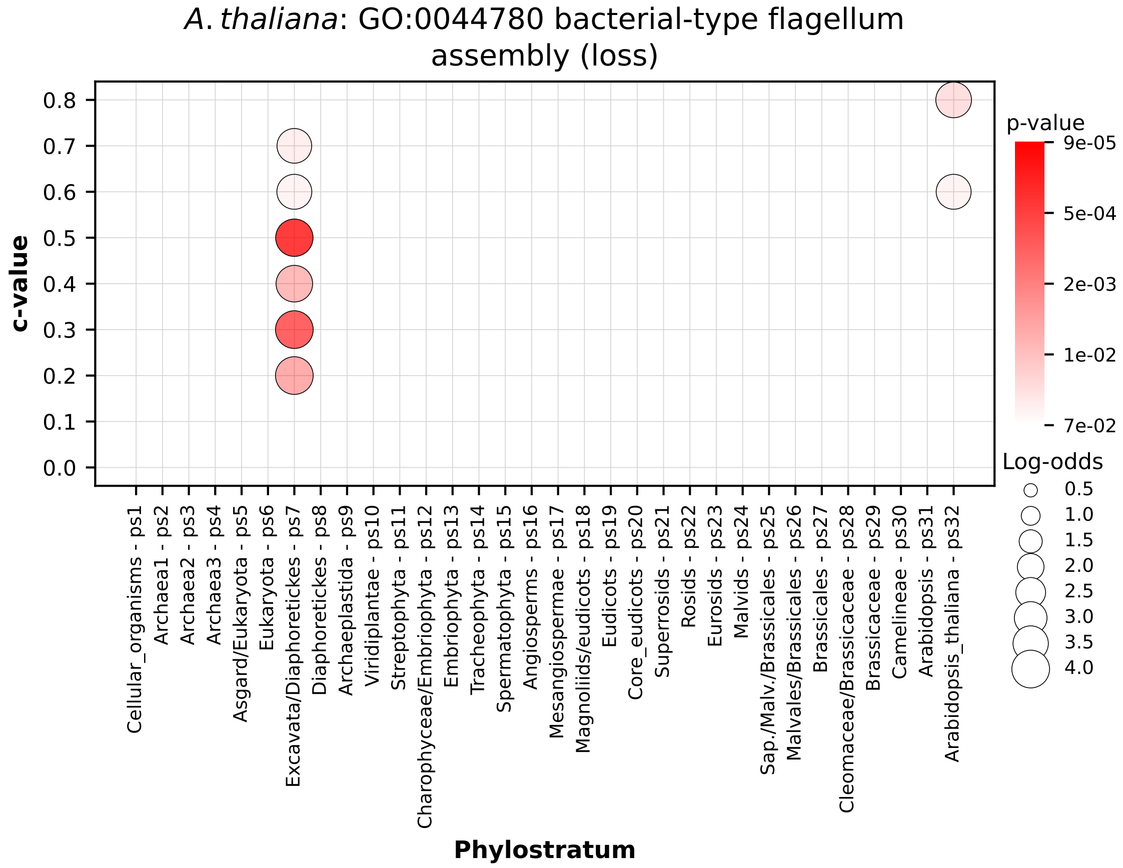

A. thaliana: GO:0044780 bacterial-type flagellum assembly (loss)
c-value
0.0
0.1
0.2
0.3
0.4
0.5
0.6
0.7
0.8
p-value
9e-05
5e-04
2e-03
1e-02
7e-02
Log-odds
0.5
1.0
1.5
2.0
2.5
3.0
3.5
4.0
Cellular_organisms - ps1
Archaea1 - ps2
Archaea2 - ps3
Archaea3 - ps4
Asgard/Eukaryota - ps5
Eukaryota - ps6
Excavata/Diaphoretickes - ps7
Diaphoretickes - ps8
Archaeplastida - ps9
Viridiplantae - ps10
Streptophyta - ps11
Charophyceae/Embriophyta - ps12
Embriophyta - ps13
Tracheophyta - ps14
Spermatophyta - ps15
Angiosperms - ps16
Mesangiospermae - ps17
Magnoliids/eudicots - ps18
Eudicots - ps19
Core_eudicots - ps20
Superrosids - ps21
Rosids - ps22
Eurosids - ps23
Malvids - ps24
Sap./Malv./Brassicales - ps25
Malvales/Brassicales - ps26
Brassicales - ps27
Cleomaceae/Brassicaceae - ps28
Brassicaceae - ps29
Camelineae - ps30
Arabidopsis - ps31
Arabidopsis_thaliana - ps32
Phylostratum

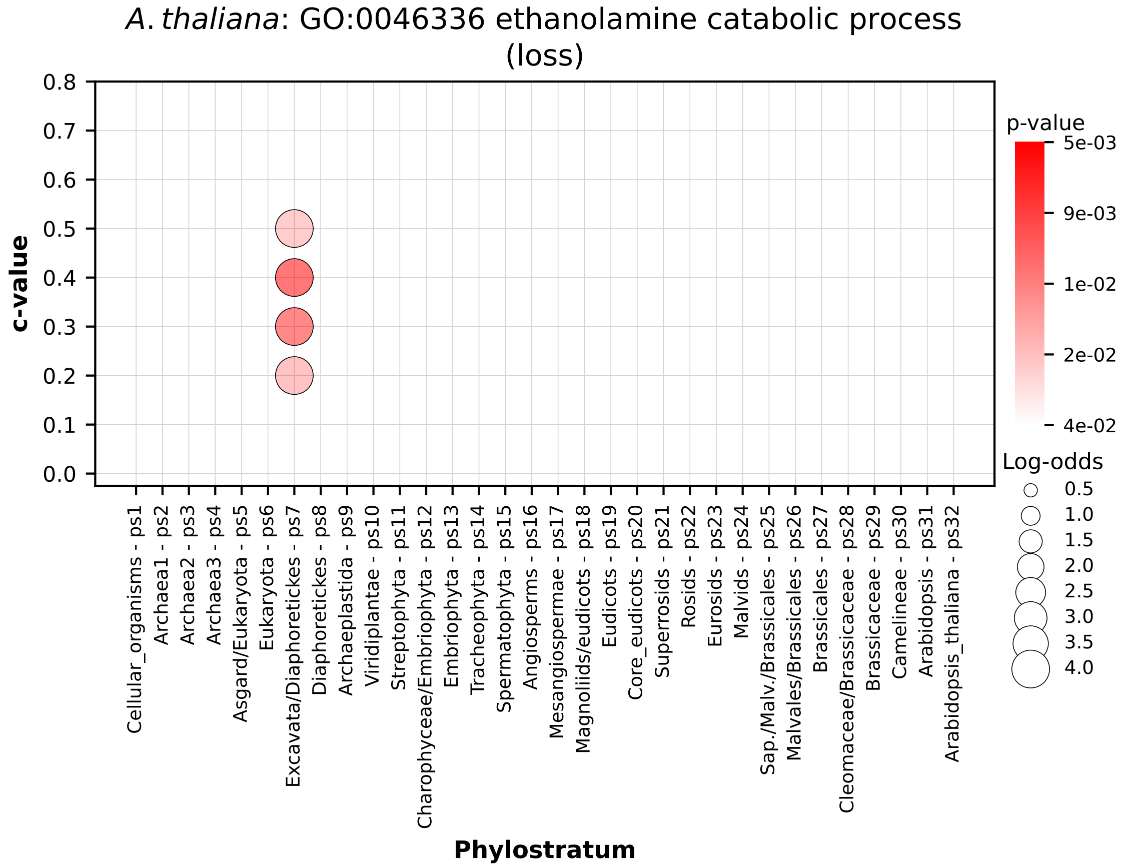

A. thaliana: GO:0046336 ethanolamine catabolic process
(loss)
c-value
0.8
0.7
0.6
0.5
0.4
0.3
0.2
0.1
0.0
p-value
5e-03
9e-03
1e-02
2e-02
4e-02
Log-odds
0.5
1.0
1.5
2.0
2.5
3.0
3.5
4.0
Cellular_organisms - ps1
Archaea1 - ps2
Archaea2 - ps3
Archaea3 - ps4
Asgard/Eukaryota - ps5
Eukaryota - ps6
Excavata/Diaphoretickes - ps7
Diaphoretickes - ps8
Archaeplastida - ps9
Viridiplantae - ps10
Streptophyta - ps11
Charophyceae/Embriophyta - ps12
Embriophyta - ps13
Tracheophyta - ps14
Spermatophyta - ps15
Angiosperms - ps16
Mesangiospermae - ps17
Magnoliids/eudicots - ps18
Eudicots - ps19
Core_eudicots - ps20
Superrosids - ps21
Rosids - ps22
Eurosids - ps23
Malvids - ps24
Sap./Malv./Brassicales - ps25
Malvales/Brassicales - ps26
Brassicales - ps27
Cleomaceae/Brassicaceae - ps28
Brassicaceae - ps29
Camelineae - ps30
Arabidopsis - ps31
Arabidopsis_thaliana - ps32
Phylostratum

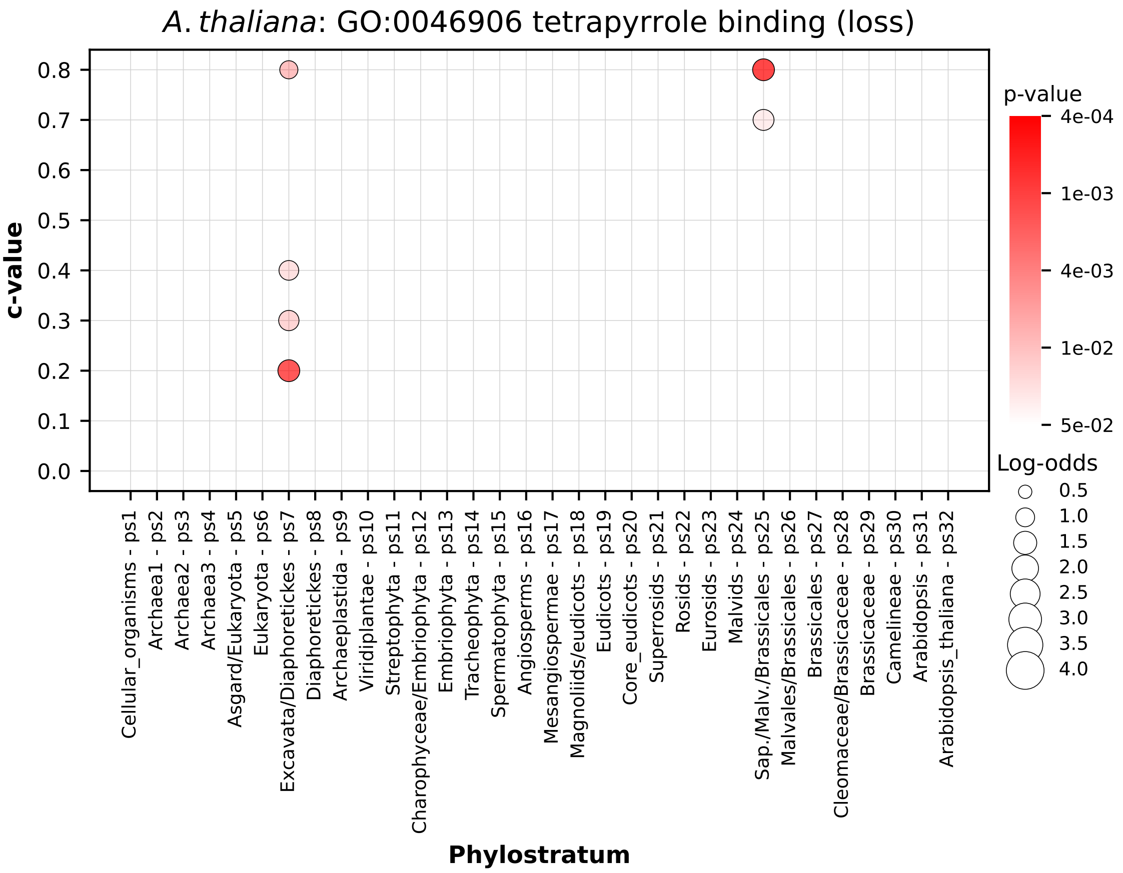

A. thaliana: GO:0046906 tetrapyrrole binding (loss)
c-value
0.0
0.1
0.2
0.3
0.4
0.5
0.6
0.7
0.8
p-value
4e-04
1e-03
4e-03
1e-02
5e-02
Log-odds
0.5
1.0
1.5
2.0
2.5
3.0
3.5
4.0
Cellular_organisms - ps1
Archaea1 - ps2
Archaea2 - ps3
Archaea3 - ps4
Asgard/Eukaryota - ps5
Eukaryota - ps6
Excavata/Diaphoretickes - ps7
Diaphoretickes - ps8
Archaeplastida - ps9
Viridiplantae - ps10
Streptophyta - ps11
Charophyceae/Embriophyta - ps12
Embriophyta - ps13
Tracheophyta - ps14
Spermatophyta - ps15
Angiosperms - ps16
Mesangiospermae - ps17
Magnoliids/eudicots - ps18
Eudicots - ps19
Core_eudicots - ps20
Superrosids - ps21
Rosids - ps22
Eurosids - ps23
Malvids - ps24
Sap./Malv./Brassicales - ps25
Malvales/Brassicales - ps26
Brassicales - ps27
Cleomaceae/Brassicaceae - ps28
Brassicaceae - ps29
Camelineae - ps30
Arabidopsis - ps31
Arabidopsis_thaliana - ps32
Phylostratum

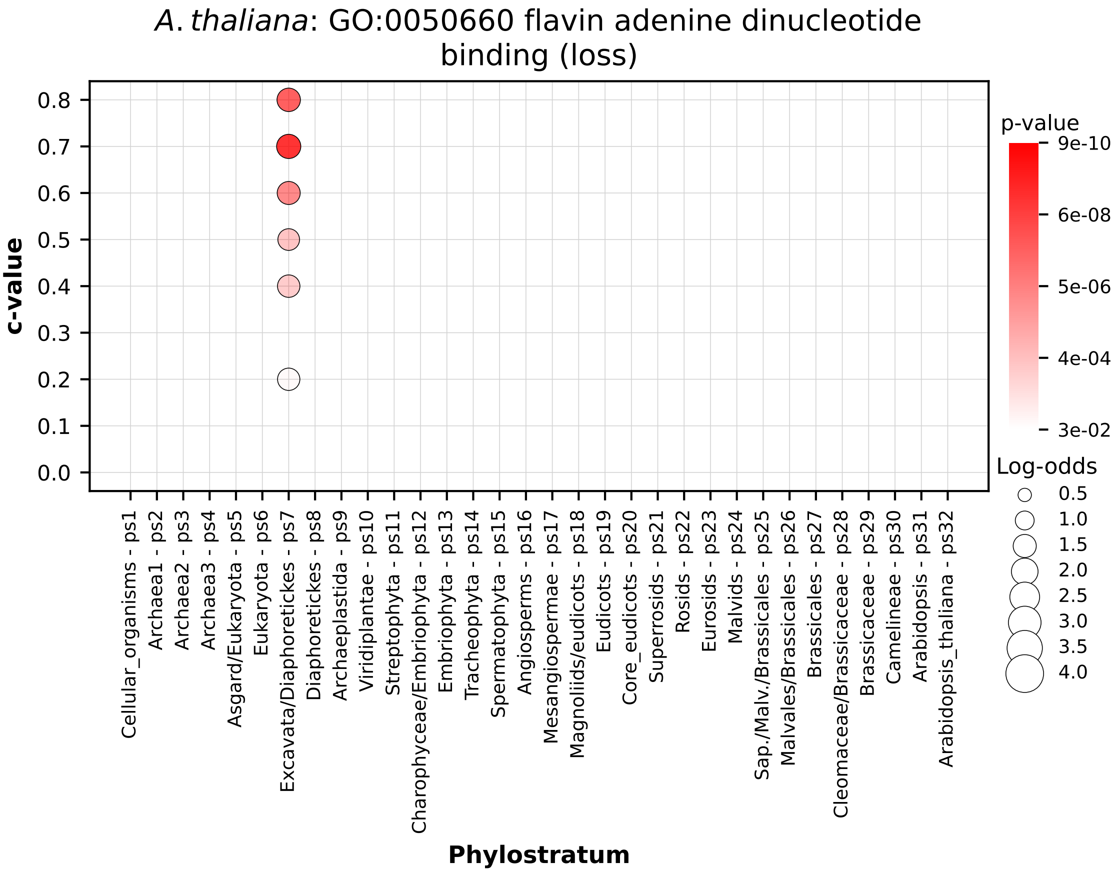
A. thaliana: GO:0050660 flavin adenine dinucleotide binding (loss)
c-value
0.8
0.7
0.6
0.5
0.4
0.3
0.2
0.1
0.0
p-value
9e-10
6e-08
5e-06
4e-04
3e-02
Log-odds
0.5
1.0
1.5
2.0
2.5
3.0
3.5
4.0
Cellular_organisms - ps1
Archaea1 - ps2
Archaea2 - ps3
Archaea3 - ps4
Asgard/Eukaryota - ps5
Eukaryota - ps6
Excavata/Diaphoretickes - ps7
Diaphoretickes - ps8
Archaeplastida - ps9
Viridiplantae - ps10
Streptophyta - ps11
Charophyceae/Embriophyta - ps12
Embriophyta - ps13
Tracheophyta - ps14
Spermatophyta - ps15
Angiosperms - ps16
Mesangiospermae - ps17
Magnoliids/eudicots - ps18
Eudicots - ps19
Core_eudicots - ps20
Superrosids - ps21
Rosids - ps22
Eurosids - ps23
Malvids - ps24
Sap./Malv./Brassicales - ps25
Malvales/Brassicales - ps26
Brassicales - ps27
Cleomaceae/Brassicaceae - ps28
Brassicaceae - ps29
Camelineae - ps30
Arabidopsis - ps31
Arabidopsis_thaliana - ps32
Phylostratum

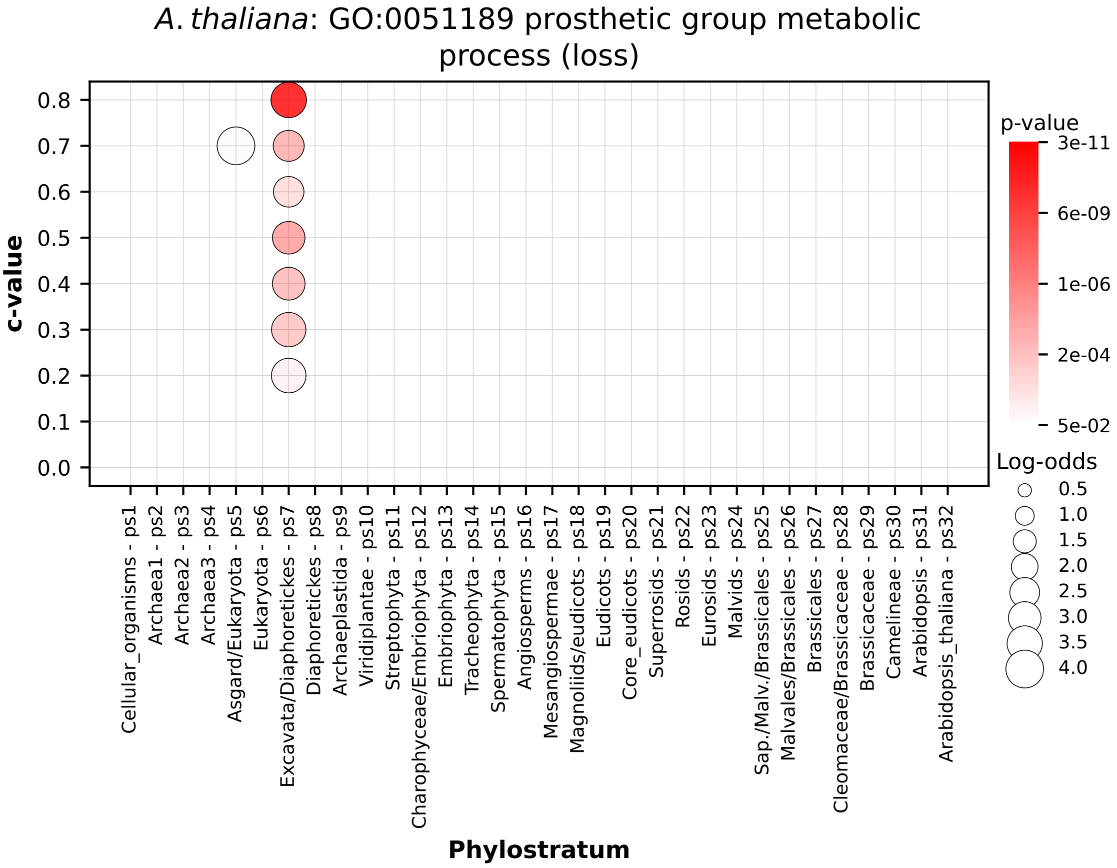

A. thaliana: GO:0051189 prosthetic group metabolic process (loss)
c-value
0.0
0.1
0.2
0.3
0.4
0.5
0.6
0.7
0.8
p-value
3e-11
6e-09
1e-06
2e-04
5e-02
Log-odds
0.5
1.0
1.5
2.0
2.5
3.0
3.5
4.0
Cellular_organisms - ps1
Archaea1 - ps2
Archaea2 - ps3
Archaea3 - ps4
Asgard/Eukaryota - ps5
Eukaryota - ps6
Excavata/Diaphoretickes - ps7
Diaphoretickes - ps8
Archaeplastida - ps9
Viridiplantae - ps10
Streptophyta - ps11
Charophyceae/Embriophyta - ps12
Embriophyta - ps13
Tracheophyta - ps14
Spermatophyta - ps15
Angiosperms - ps16
Mesangiospermae - ps17
Magnoliids/eudicots - ps18
Eudicots - ps19
Core_eudicots - ps20
Superrosids - ps21
Rosids - ps22
Eurosids - ps23
Malvids - ps24
Sap./Malv./Brassicales - ps25
Malvales/Brassicales - ps26
Brassicales - ps27
Cleomaceae/Brassicaceae - ps28
Brassicaceae - ps29
Camelineae - ps30
Arabidopsis - ps31
Arabidopsis_thaliana - ps32
Phylostratum

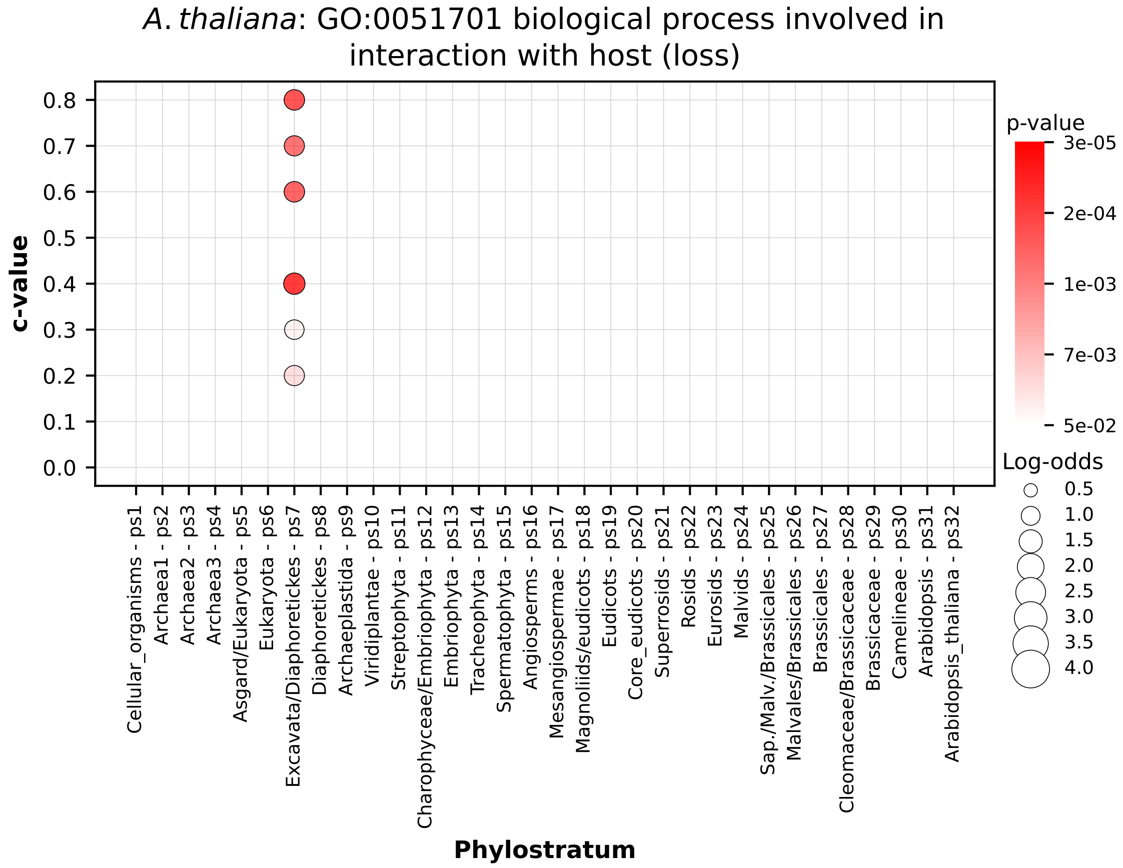

A. thaliana: GO:0051701 biological process involved in interaction with host (loss)
c-value
0.0
0.1
0.2
0.3
0.4
0.5
0.6
0.7
0.8
p-value
3e-05
2e-04
1e-03
7e-03
5e-02
Log-odds
0.5
1.0
1.5
2.0
2.5
3.0
3.5
4.0
Cellular_organisms - ps1
Archaea1 - ps2
Archaea2 - ps3
Archaea3 - ps4
Asgard/Eukaryota - ps5
Eukaryota - ps6
Excavata/Diaphoretickes - ps7
Diaphoretickes - ps8
Archaeplastida - ps9
Viridiplantae - ps10
Streptophyta - ps11
Charophyceae/Embriophyta - ps12
Embriophyta - ps13
Tracheophyta - ps14
Spermatophyta - ps15
Angiosperms - ps16
Mesangiospermae - ps17
Magnoliids/eudicots - ps18
Eudicots - ps19
Core_eudicots - ps20
Superrosids - ps21
Rosids - ps22
Eurosids - ps23
Malvids - ps24
Sap./Malv./Brassicales - ps25
Malvales/Brassicales - ps26
Brassicales - ps27
Cleomaceae/Brassicaceae - ps28
Brassicaceae - ps29
Camelineae - ps30
Arabidopsis - ps31
Arabidopsis_thaliana - ps32
Phylostratum

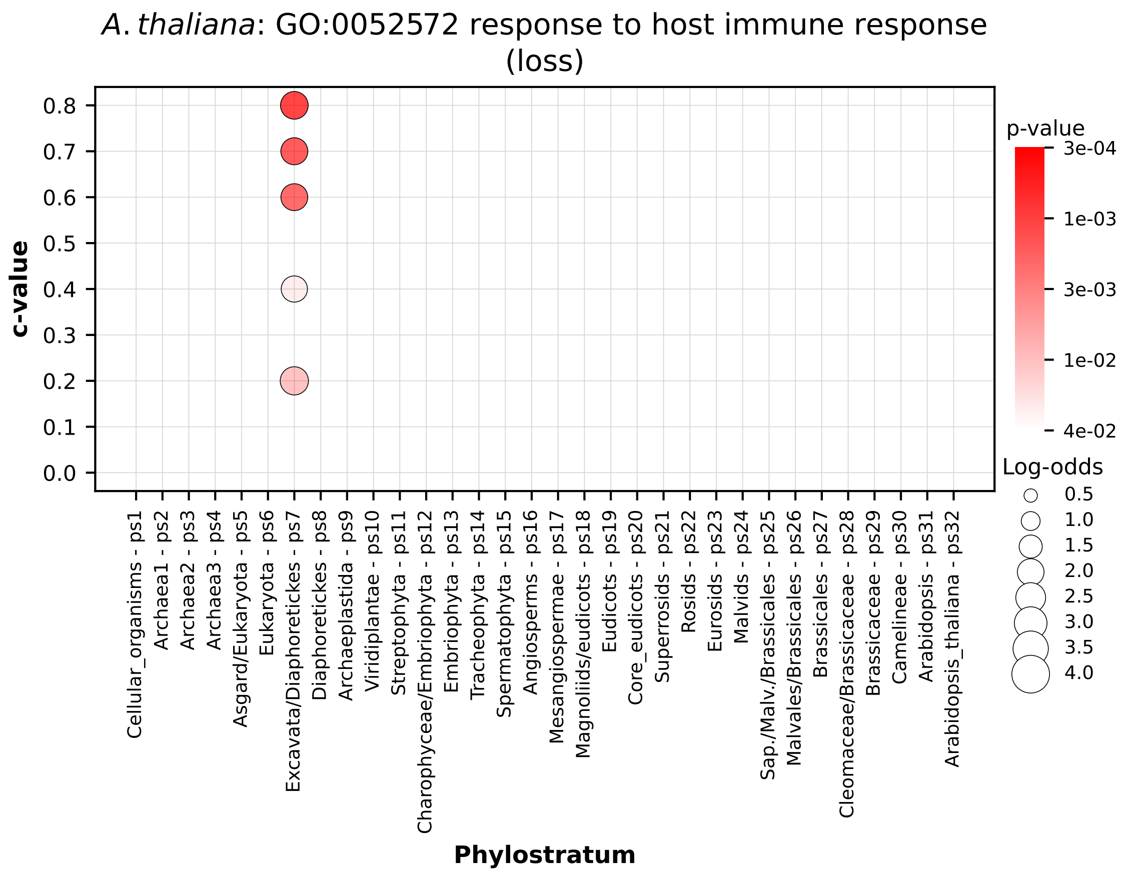

A. thaliana: GO:0052572 response to host immune response
(loss)
c-value
0.8
0.7
0.6
0.5
0.4
0.3
0.2
0.1
0.0
Phylostratum
Cellular_organisms - ps1
Archaea1 - ps2
Archaea2 - ps3
Archaea3 - ps4
Asgard/Eukaryota - ps5
Eukaryota - ps6
Excavata/Diaphoretickes - ps7
Diaphoretickes - ps8
Archaeplastida - ps9
Viridiplantae - ps10
Streptophyta - ps11
Charophyceae/Embriophyta - ps12
Embriophyta - ps13
Tracheophyta - ps14
Spermatophyta - ps15
Angiosperms - ps16
Mesangiospermae - ps17
Magnoliids/eudicots - ps18
Eudicots - ps19
Core_eudicots - ps20
Superrosids - ps21
Rosids - ps22
Eurosids - ps23
Malvids - ps24
Sap./Malv./Brassicales - ps25
Malvales/Brassicales - ps26
Brassicales - ps27
Cleomaceae/Brassicaceae - ps28
Brassicaceae - ps29
Camelineae - ps30
Arabidopsis - ps31
Arabidopsis_thaliana - ps32
p-value
3e-04
1e-03
3e-03
1e-02
4e-02
Log-odds
0.5
1.0
1.5
2.0
2.5
3.0
3.5
4.0

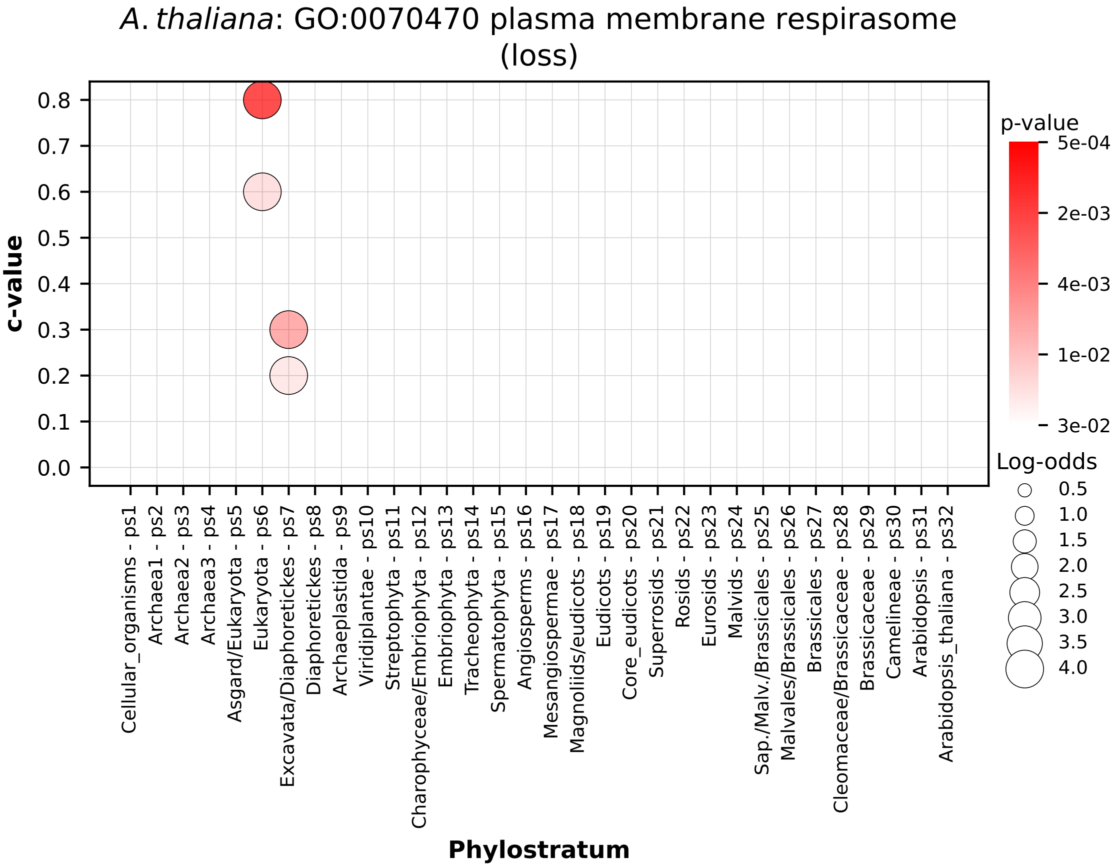

A. thaliana: GO:0070470 plasma membrane respirasome
(loss)
c-value
0.8
0.7
0.6
0.5
0.4
0.3
0.2
0.1
0.0
Phylostratum
Cellular_organisms - ps1
Archaea1 - ps2
Archaea2 - ps3
Archaea3 - ps4
Asgard/Eukaryota - ps5
Eukaryota - ps6
Excavata/Diaphoretickes - ps7
Diaphoretickes - ps8
Archaeplastida - ps9
Viridiplantae - ps10
Streptophyta - ps11
Charophyceae/Embriophyta - ps12
Embriophyta - ps13
Tracheophyta - ps14
Spermatophyta - ps15
Angiosperms - ps16
Mesangiospermae - ps17
Magnoliids/eudicots - ps18
Eudicots - ps19
Core_eudicots - ps20
Superrosids - ps21
Rosids - ps22
Eurosids - ps23
Malvids - ps24
Sap./Malv./Brassicales - ps25
Malvales/Brassicales - ps26
Brassicales - ps27
Cleomaceae/Brassicaceae - ps28
Brassicaceae - ps29
Camelineae - ps30
Arabidopsis - ps31
Arabidopsis_thaliana - ps32
p-value
5e-04
2e-03
4e-03
1e-02
3e-02
Log-odds
0.5
1.0
1.5
2.0
2.5
3.0
3.5
4.0

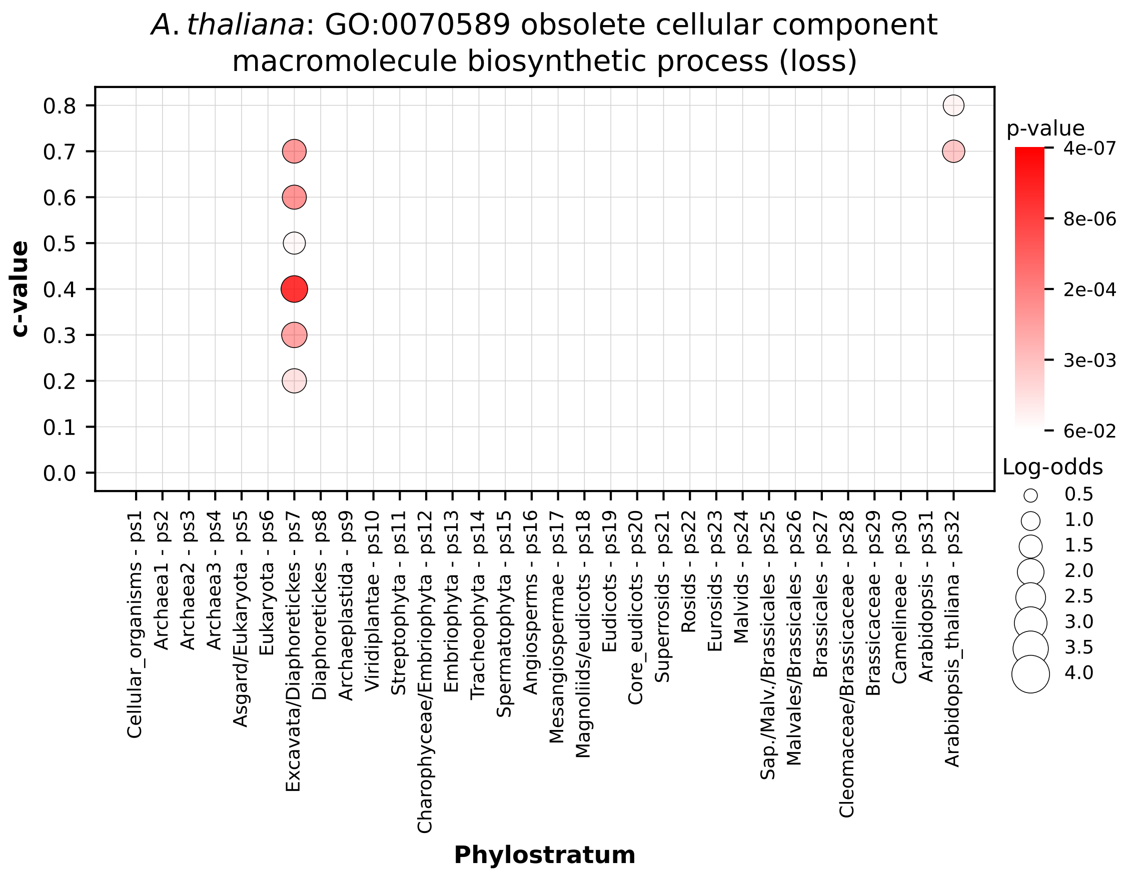

A. thaliana: GO:0070589 obsolete cellular component macromolecule biosynthetic process (loss)
c-value
0.0
0.1
0.2
0.3
0.4
0.5
0.6
0.7
0.8
p-value
4e-07
8e-06
2e-04
3e-03
6e-02
Log-odds
0.5
1.0
1.5
2.0
2.5
3.0
3.5
4.0
Cellular_organisms - ps1
Archaea1 - ps2
Archaea2 - ps3
Archaea3 - ps4
Asgard/Eukaryota - ps5
Eukaryota - ps6
Excavata/Diaphoretickes - ps7
Diaphoretickes - ps8
Archaeplastida - ps9
Viridiplantae - ps10
Streptophyta - ps11
Charophyceae/Embriophyta - ps12
Embriophyta - ps13
Tracheophyta - ps14
Spermatophyta - ps15
Angiosperms - ps16
Mesangiospermae - ps17
Magnoliids/eudicots - ps18
Eudicots - ps19
Core_eudicots - ps20
Superrosids - ps21
Rosids - ps22
Eurosids - ps23
Malvids - ps24
Sap./Malv./Brassicales - ps25
Malvales/Brassicales - ps26
Brassicales - ps27
Cleomaceae/Brassicaceae - ps28
Brassicaceae - ps29
Camelineae - ps30
Arabidopsis - ps31
Arabidopsis_thaliana - ps32
Phylostratum

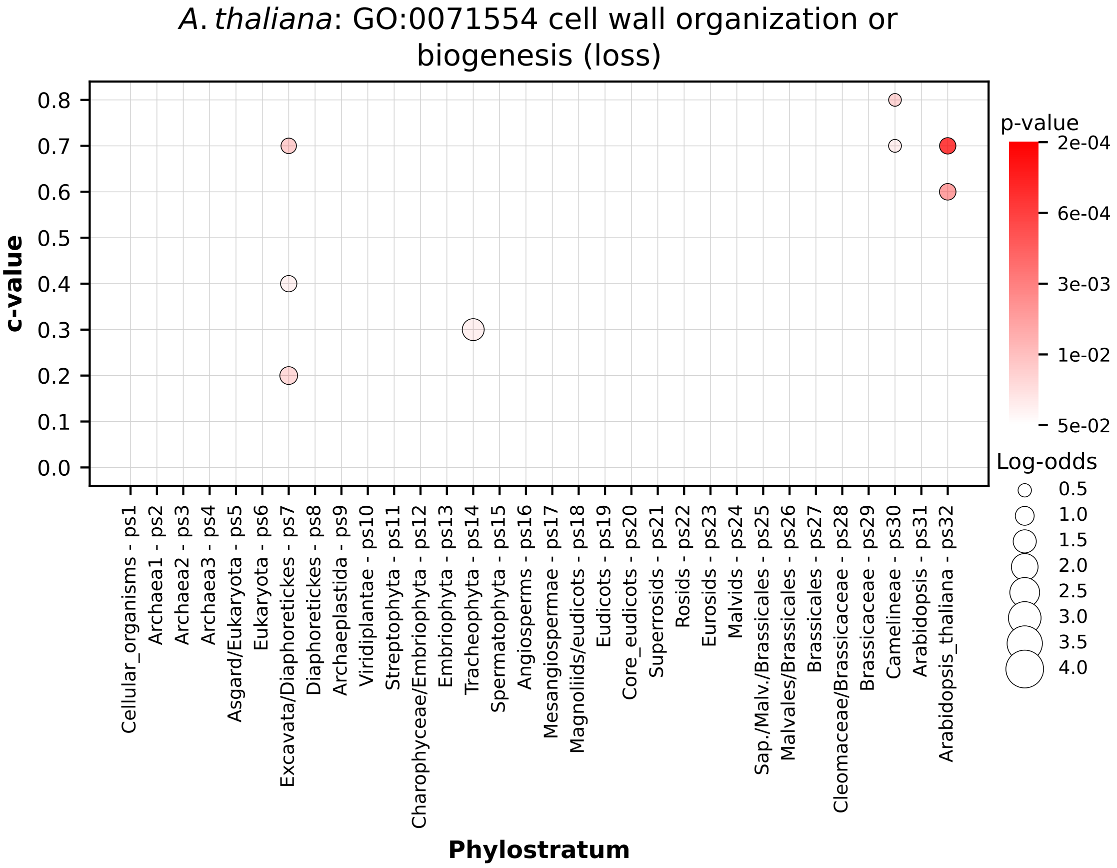
A. thaliana: GO:0071554 cell wall organization or biogenesis (loss)
c-value
0.8
0.7
0.6
0.5
0.4
0.3
0.2
0.1
0.0
Phylostratum
Cellular_organisms - ps1
Archaea1 - ps2
Archaea2 - ps3
Archaea3 - ps4
Asgard/Eukaryota - ps5
Eukaryota - ps6
Excavata/Diaphoretickes - ps7
Diaphoretickes - ps8
Archaeplastida - ps9
Viridiplantae - ps10
Streptophyta - ps11
Charophyceae/Embriophyta - ps12
Embriophyta - ps13
Tracheophyta - ps14
Spermatophyta - ps15
Angiosperms - ps16
Mesangiospermae - ps17
Magnoliids/eudicots - ps18
Eudicots - ps19
Core_eudicots - ps20
Superrosids - ps21
Rosids - ps22
Eurosids - ps23
Malvids - ps24
Sap./Malv./Brassicales - ps25
Malvales/Brassicales - ps26
Brassicales - ps27
Cleomaceae/Brassicaceae - ps28
Brassicaceae - ps29
Camelineae - ps30
Arabidopsis - ps31
Arabidopsis_thaliana - ps32
p-value
2e-04
6e-04
3e-03
1e-02
5e-02
Log-odds
0.5
1.0
1.5
2.0
2.5
3.0
3.5
4.0

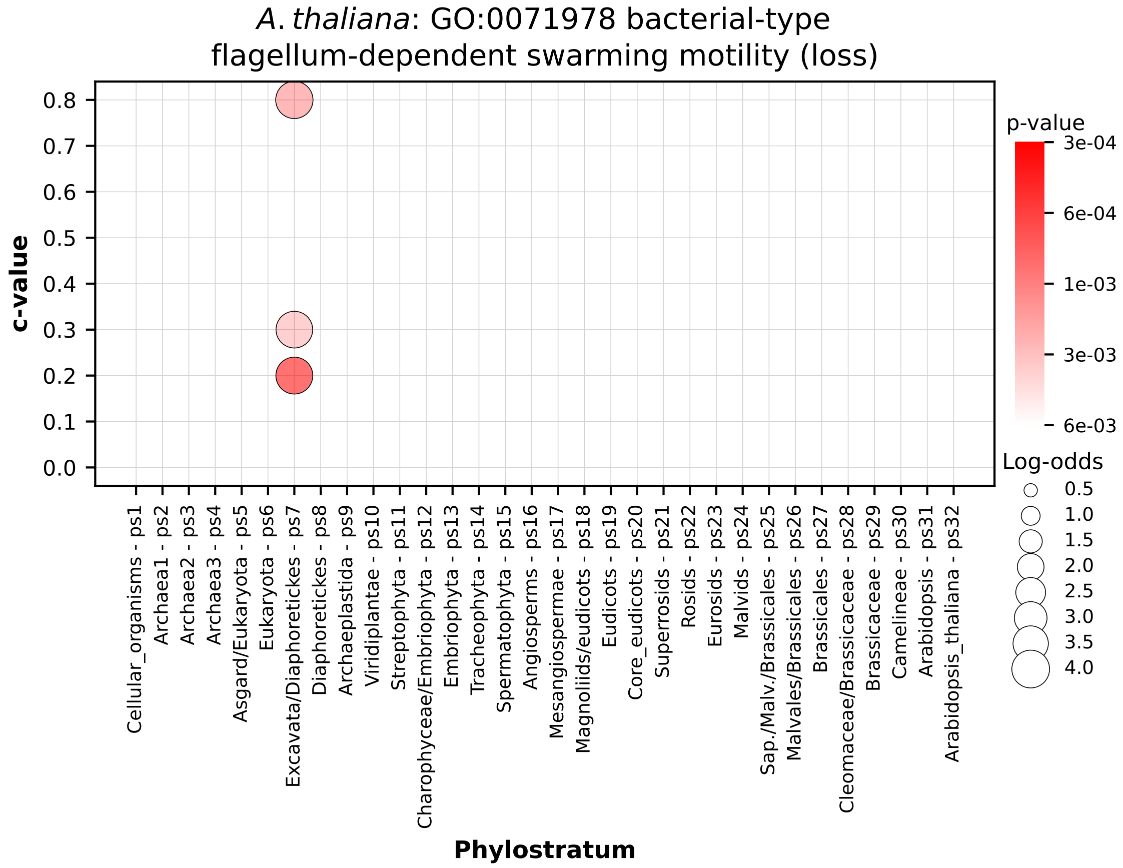

A. thaliana: GO:0071978 bacterial-type flagellum-dependent swarming motility (loss)
c-value
0.0
0.1
0.2
0.3
0.4
0.5
0.6
0.7
0.8
p-value
3e-04
6e-04
1e-03
3e-03
6e-03
Log-odds
0.5
1.0
1.5
2.0
2.5
3.0
3.5
4.0
Cellular_organisms - ps1
Archaea1 - ps2
Archaea2 - ps3
Archaea3 - ps4
Asgard/Eukaryota - ps5
Eukaryota - ps6
Excavata/Diaphoretickes - ps7
Diaphoretickes - ps8
Archaeplastida - ps9
Viridiplantae - ps10
Streptophyta - ps11
Charophyceae/Embriophyta - ps12
Embriophyta - ps13
Tracheophyta - ps14
Spermatophyta - ps15
Angiosperms - ps16
Mesangiospermae - ps17
Magnoliids/eudicots - ps18
Eudicots - ps19
Core_eudicots - ps20
Superrosids - ps21
Rosids - ps22
Eurosids - ps23
Malvids - ps24
Sap./Malv./Brassicales - ps25
Malvales/Brassicales - ps26
Brassicales - ps27
Cleomaceae/Brassicaceae - ps28
Brassicaceae - ps29
Camelineae - ps30
Arabidopsis - ps31
Arabidopsis_thaliana - ps32
Phylostratum

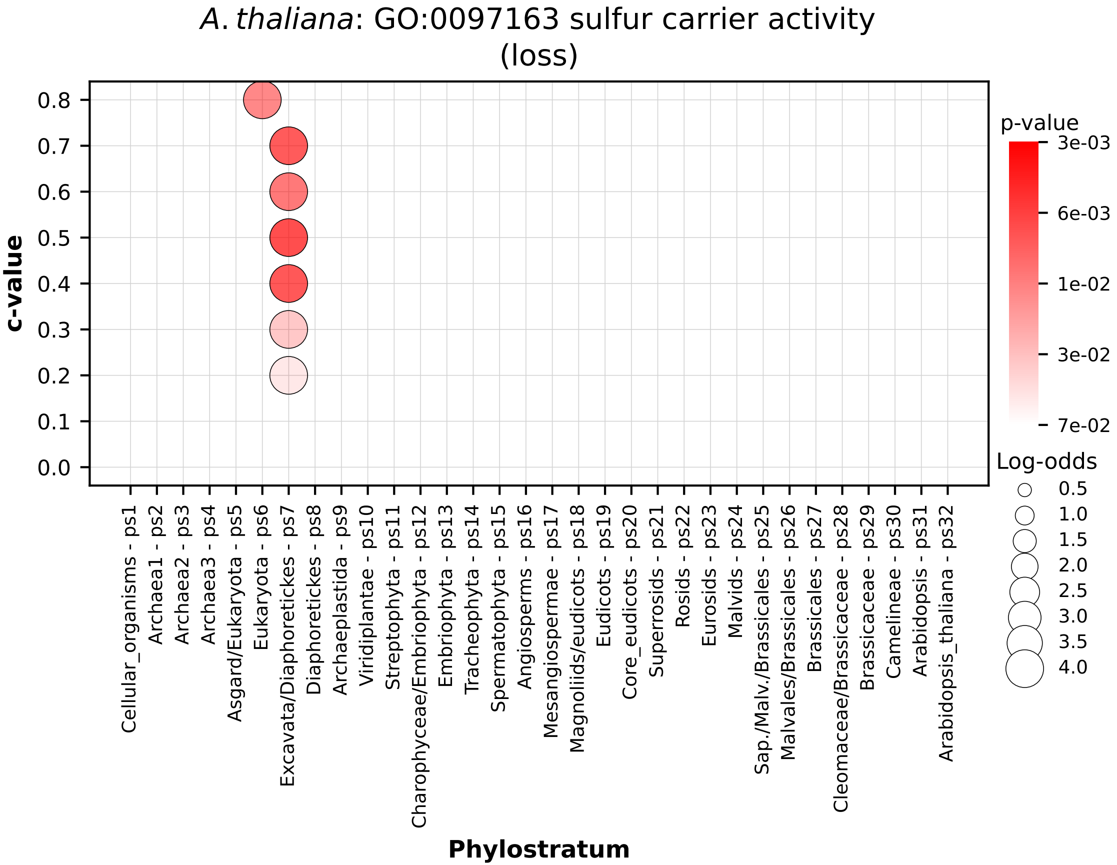

A. thaliana: GO:0097163 sulfur carrier activity
(loss)
c-value
0.0
0.1
0.2
0.3
0.4
0.5
0.6
0.7
0.8
Phylostratum
Cellular_organisms - ps1
Archaea1 - ps2
Archaea2 - ps3
Archaea3 - ps4
Asgard/Eukaryota - ps5
Eukaryota - ps6
Excavata/Diaphoretickes - ps7
Diaphoretickes - ps8
Archaeplastida - ps9
Viridiplantae - ps10
Streptophyta - ps11
Charophyceae/Embriophyta - ps12
Embriophyta - ps13
Tracheophyta - ps14
Spermatophyta - ps15
Angiosperms - ps16
Mesangiospermae - ps17
Magnoliids/eudicots - ps18
Eudicots - ps19
Core_eudicots - ps20
Superrosids - ps21
Rosids - ps22
Eurosids - ps23
Malvids - ps24
Sap./Malv./Brassicales - ps25
Malvales/Brassicales - ps26
Brassicales - ps27
Cleomaceae/Brassicaceae - ps28
Brassicaceae - ps29
Camelineae - ps30
Arabidopsis - ps31
Arabidopsis_thaliana - ps32
p-value
3e-03
6e-03
1e-02
3e-02
7e-02
Log-odds
0.5
1.0
1.5
2.0
2.5
3.0
3.5
4.0

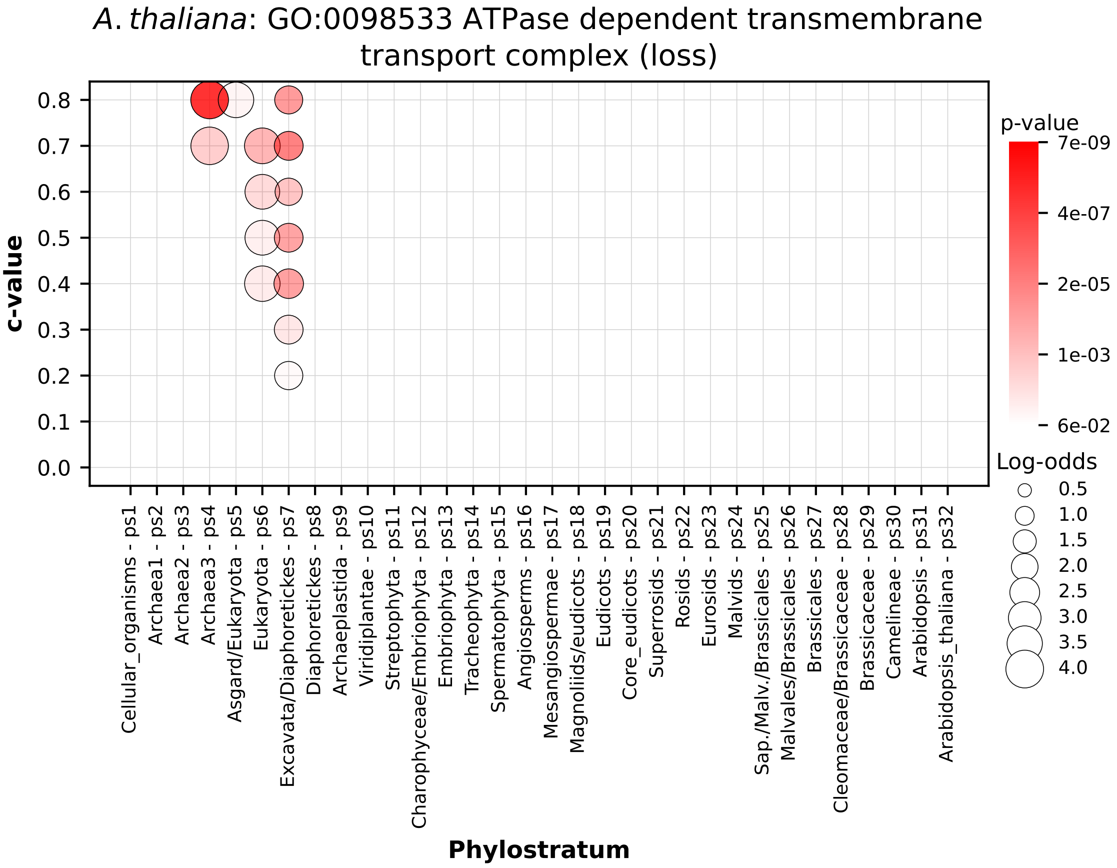

A. thaliana: GO:0098533 ATPase dependent transmembrane transport complex (loss)
c-value
0.0
0.1
0.2
0.3
0.4
0.5
0.6
0.7
0.8
Phylostratum
Cellular_organisms - ps1
Archaea1 - ps2
Archaea2 - ps3
Archaea3 - ps4
Asgard/Eukaryota - ps5
Eukaryota - ps6
Excavata/Diaphoretickes - ps7
Diaphoretickes - ps8
Archaeplastida - ps9
Viridiplantae - ps10
Streptophyta - ps11
Charophyceae/Embriophyta - ps12
Embriophyta - ps13
Tracheophyta - ps14
Spermatophyta - ps15
Angiosperms - ps16
Mesangiospermae - ps17
Magnoliids/eudicots - ps18
Eudicots - ps19
Core_eudicots - ps20
Superrosids - ps21
Rosids - ps22
Eurosids - ps23
Malvids - ps24
Sap./Malv./Brassicales - ps25
Malvales/Brassicales - ps26
Brassicales - ps27
Cleomaceae/Brassicaceae - ps28
Brassicaceae - ps29
Camelineae - ps30
Arabidopsis - ps31
Arabidopsis_thaliana - ps32
p-value
7e-09
4e-07
2e-05
1e-03
6e-02
Log-odds
0.5
1.0
1.5
2.0
2.5
3.0
3.5
4.0

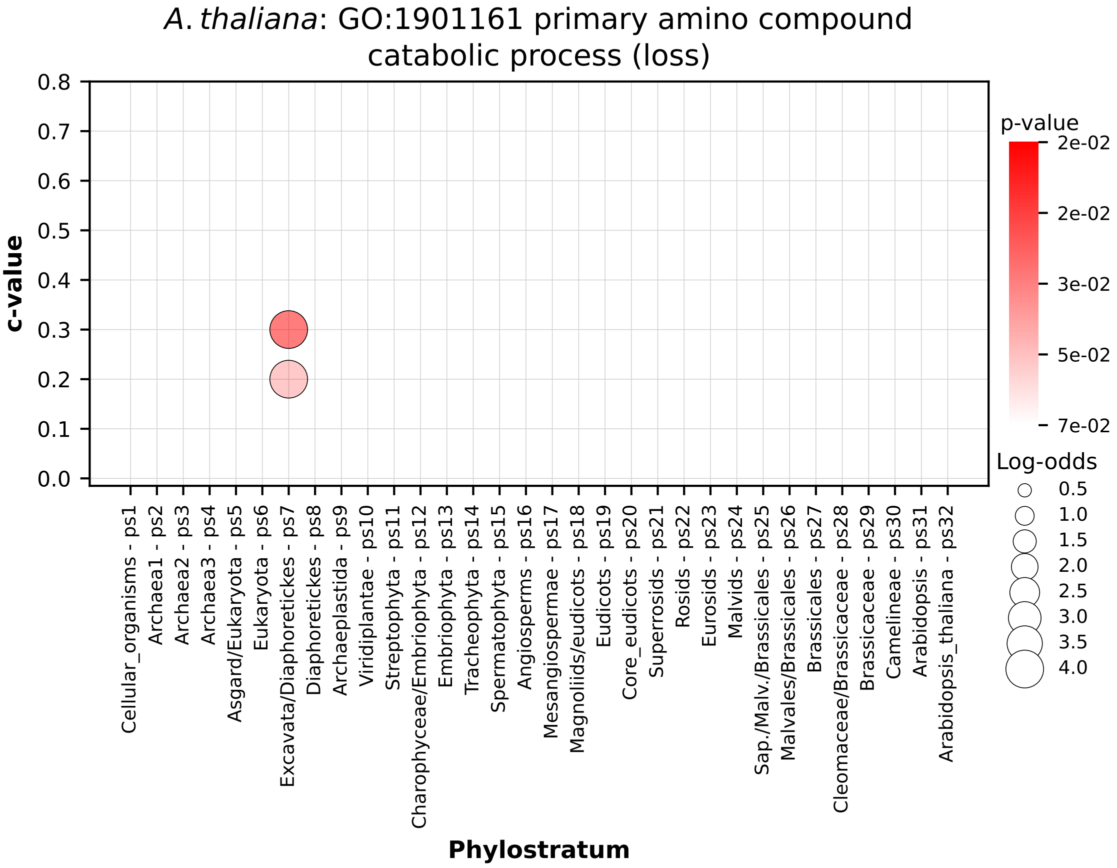
A. thaliana: GO:1901161 primary amino compound catabolic process (loss)
c-value
0.0
0.1
0.2
0.3
0.4
0.5
0.6
0.7
0.8
Cellular_organisms - ps1
Archaea1 - ps2
Archaea2 - ps3
Archaea3 - ps4
Asgard/Eukaryota - ps5
Eukaryota - ps6
Excavata/Diaphoretickes - ps7
Diaphoretickes - ps8
Archaeplastida - ps9
Viridiplantae - ps10
Streptophyta - ps11
Charophyceae/Embriophyta - ps12
Embriophyta - ps13
Tracheophyta - ps14
Spermatophyta - ps15
Angiosperms - ps16
Mesangiospermae - ps17
Magnoliids/eudicots - ps18
Eudicots - ps19
Core_eudicots - ps20
Superrosids - ps21
Rosids - ps22
Eurosids - ps23
Malvids - ps24
Sap./Malv./Brassicales - ps25
Malvales/Brassicales - ps26
Brassicales - ps27
Cleomaceae/Brassicaceae - ps28
Brassicaceae - ps29
Camelineae - ps30
Arabidopsis - ps31
Arabidopsis_thaliana - ps32
Phylostratum
p-value
2e-02
2e-02
3e-02
5e-02
7e-02
Log-odds
0.5
1.0
1.5
2.0
2.5
3.0
3.5
4.0

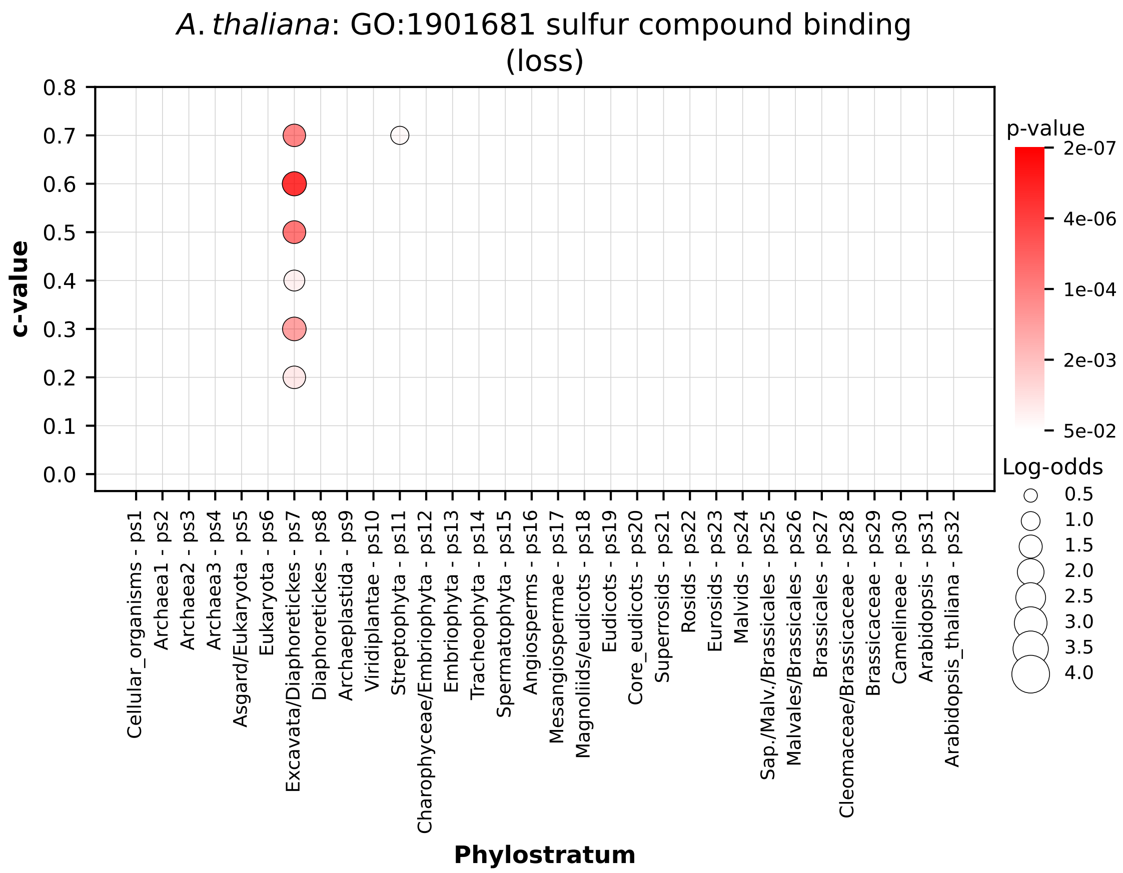
A. thaliana: GO:1901681 sulfur compound binding
(loss)
c-value
0.8
0.7
0.6
0.5
0.4
0.3
0.2
0.1
0.0
Phylostratum
Cellular_organisms - ps1
Archaea1 - ps2
Archaea2 - ps3
Archaea3 - ps4
Asgard/Eukaryota - ps5
Eukaryota - ps6
Excavata/Diaphoretickes - ps7
Diaphoretickes - ps8
Archaeplastida - ps9
Viridiplantae - ps10
Streptophyta - ps11
Charophyceae/Embriophyta - ps12
Embriophyta - ps13
Tracheophyta - ps14
Spermatophyta - ps15
Angiosperms - ps16
Mesangiospermae - ps17
Magnoliids/eudicots - ps18
Eudicots - ps19
Core_eudicots - ps20
Superrosids - ps21
Rosids - ps22
Eurosids - ps23
Malvids - ps24
Sap./Malv./Brassicales - ps25
Malvales/Brassicales - ps26
Brassicales - ps27
Cleomaceae/Brassicaceae - ps28
Brassicaceae - ps29
Camelineae - ps30
Arabidopsis - ps31
Arabidopsis_thaliana - ps32
p-value
2e-07
4e-06
1e-04
2e-03
5e-02
Log-odds
0.5
1.0
1.5
2.0
2.5
3.0
3.5
4.0

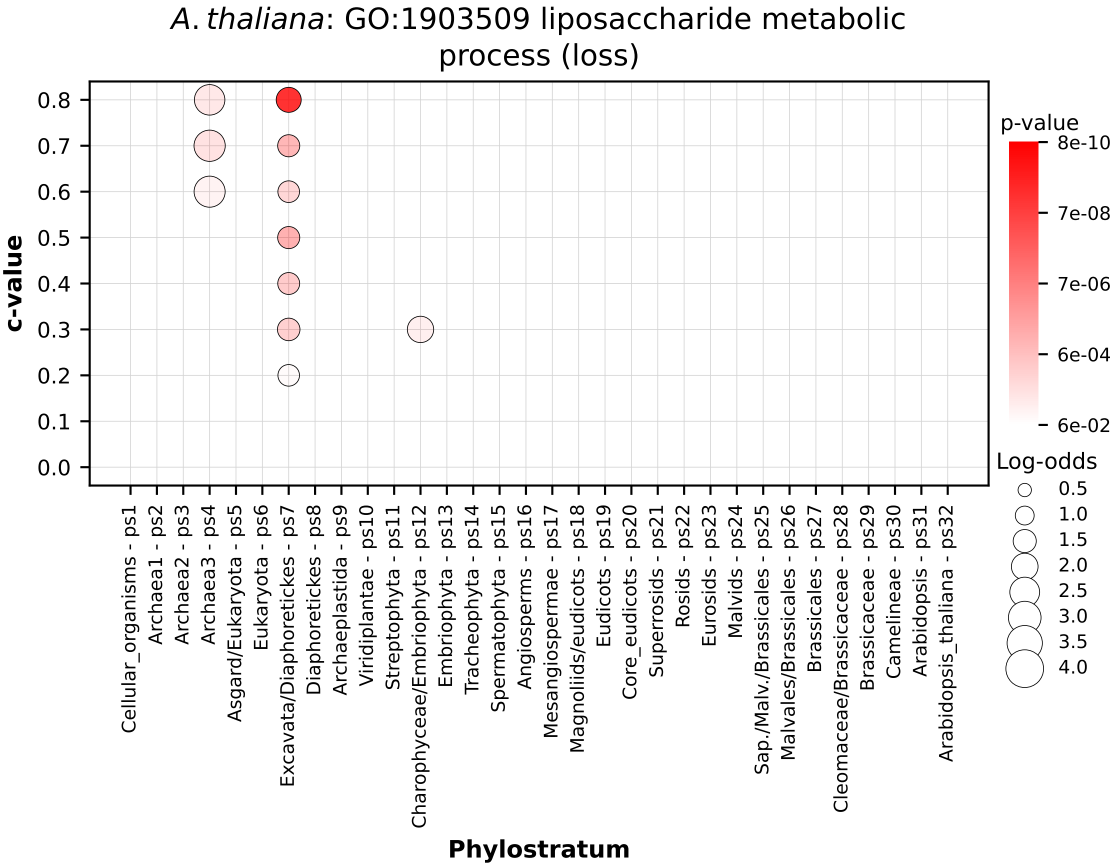

A. thaliana: GO:1903509 liposaccharide metabolic process (loss)
c-value
0.0
0.1
0.2
0.3
0.4
0.5
0.6
0.7
0.8
p-value
8e-10
7e-08
7e-06
6e-04
6e-02
Log-odds
0.5
1.0
1.5
2.0
2.5
3.0
3.5
4.0
Cellular_organisms - ps1
Archaea1 - ps2
Archaea2 - ps3
Archaea3 - ps4
Asgard/Eukaryota - ps5
Eukaryota - ps6
Excavata/Diaphoretickes - ps7
Diaphoretickes - ps8
Archaeplastida - ps9
Viridiplantae - ps10
Streptophyta - ps11
Charophyceae/Embriophyta - ps12
Embriophyta - ps13
Tracheophyta - ps14
Spermatophyta - ps15
Angiosperms - ps16
Mesangiospermae - ps17
Magnoliids/eudicots - ps18
Eudicots - ps19
Core_eudicots - ps20
Superrosids - ps21
Rosids - ps22
Eurosids - ps23
Malvids - ps24
Sap./Malv./Brassicales - ps25
Malvales/Brassicales - ps26
Brassicales - ps27
Cleomaceae/Brassicaceae - ps28
Brassicaceae - ps29
Camelineae - ps30
Arabidopsis - ps31
Arabidopsis_thaliana - ps32
Phylostratum

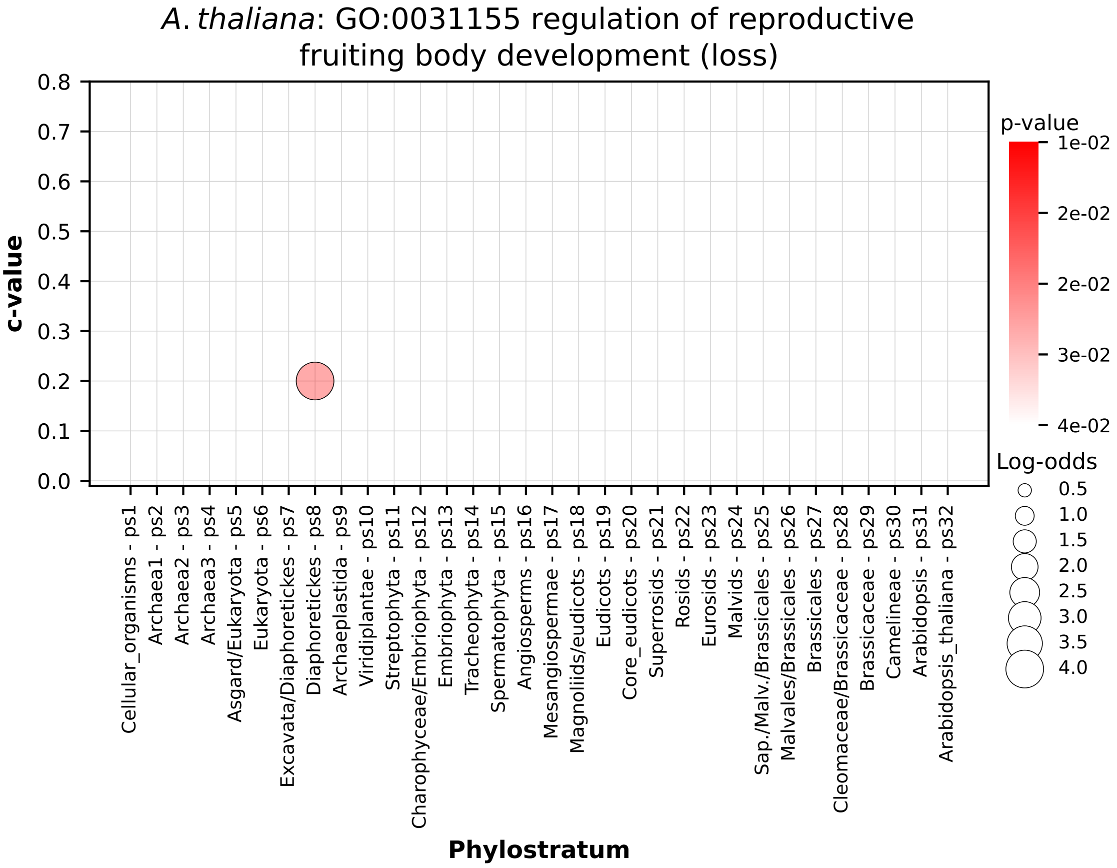

A. thaliana: GO:0031155 regulation of reproductive fruiting body development (loss)
c-value
0.8
0.7
0.6
0.5
0.4
0.3
0.2
0.1
0.0
Phylostratum
Cellular_organisms - ps1
Archaea1 - ps2
Archaea2 - ps3
Archaea3 - ps4
Asgard/Eukaryota - ps5
Eukaryota - ps6
Excavata/Diaphoretickes - ps7
Diaphoretickes - ps8
Archaeplastida - ps9
Viridiplantae - ps10
Streptophyta - ps11
Charophyceae/Embriophyta - ps12
Embriophyta - ps13
Tracheophyta - ps14
Spermatophyta - ps15
Angiosperms - ps16
Mesangiospermae - ps17
Magnoliids/eudicots - ps18
Eudicots - ps19
Core_eudicots - ps20
Superrosids - ps21
Rosids - ps22
Eurosids - ps23
Malvids - ps24
Sap./Malv./Brassicales - ps25
Malvales/Brassicales - ps26
Brassicales - ps27
Cleomaceae/Brassicaceae - ps28
Brassicaceae - ps29
Camelineae - ps30
Arabidopsis - ps31
Arabidopsis_thaliana - ps32
p-value
1e-02
2e-02
2e-02
3e-02
4e-02
Log-odds
0.5
1.0
1.5
2.0
2.5
3.0
3.5
4.0

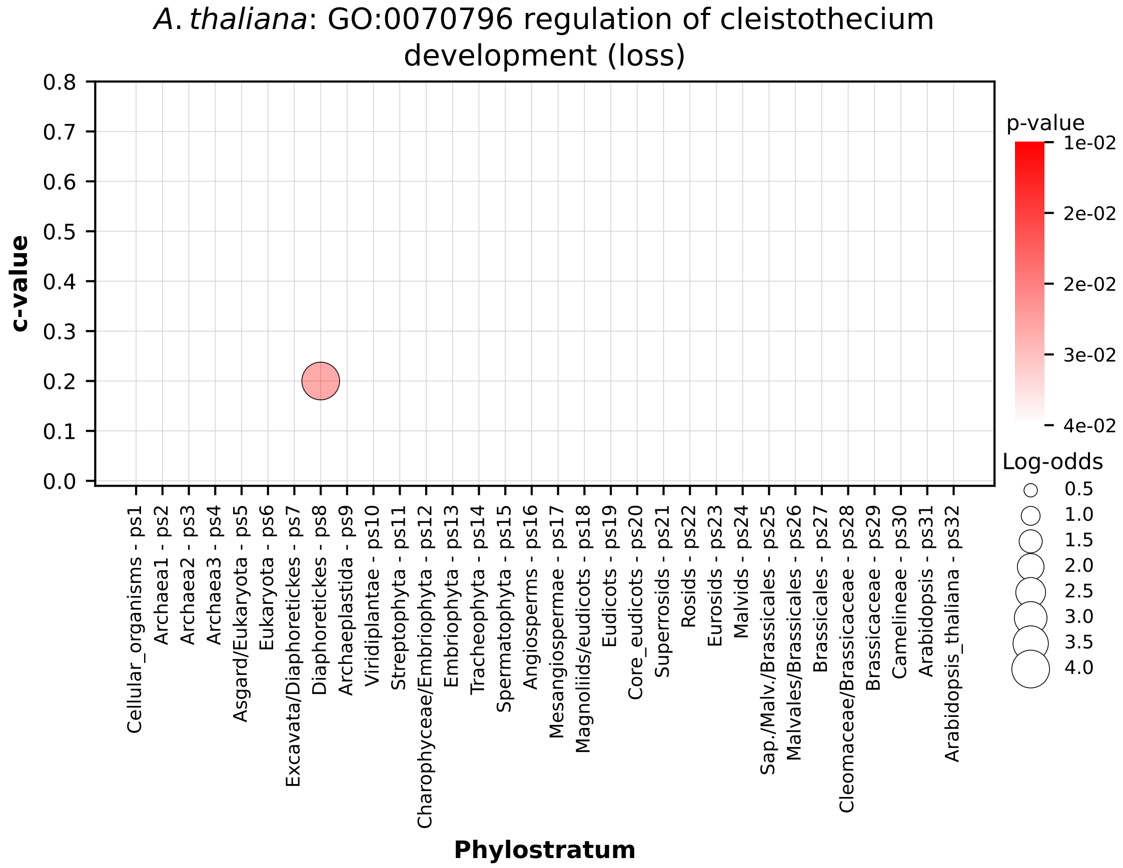
A. thaliana: GO:0070796 regulation of cleistothecium development (loss)
c-value
0.0
0.1
0.2
0.3
0.4
0.5
0.6
0.7
0.8
Phylostratum
Cellular_organisms - ps1
Archaea1 - ps2
Archaea2 - ps3
Archaea3 - ps4
Asgard/Eukaryota - ps5
Eukaryota - ps6
Excavata/Diaphoretickes - ps7
Diaphoretickes - ps8
Archaeplastida - ps9
Viridiplantae - ps10
Streptophyta - ps11
Charophyceae/Embriophyta - ps12
Embriophyta - ps13
Tracheophyta - ps14
Spermatophyta - ps15
Angiosperms - ps16
Mesangiospermae - ps17
Magnoliids/eudicots - ps18
Eudicots - ps19
Core_eudicots - ps20
Superrosids - ps21
Rosids - ps22
Eurosids - ps23
Malvids - ps24
Sap./Malv./Brassicales - ps25
Malvales/Brassicales - ps26
Brassicales - ps27
Cleomaceae/Brassicaceae - ps28
Brassicaceae - ps29
Camelineae - ps30
Arabidopsis - ps31
Arabidopsis_thaliana - ps32
p-value
1e-02
2e-02
2e-02
3e-02
4e-02
Log-odds
0.5
1.0
1.5
2.0
2.5
3.0
3.5
4.0

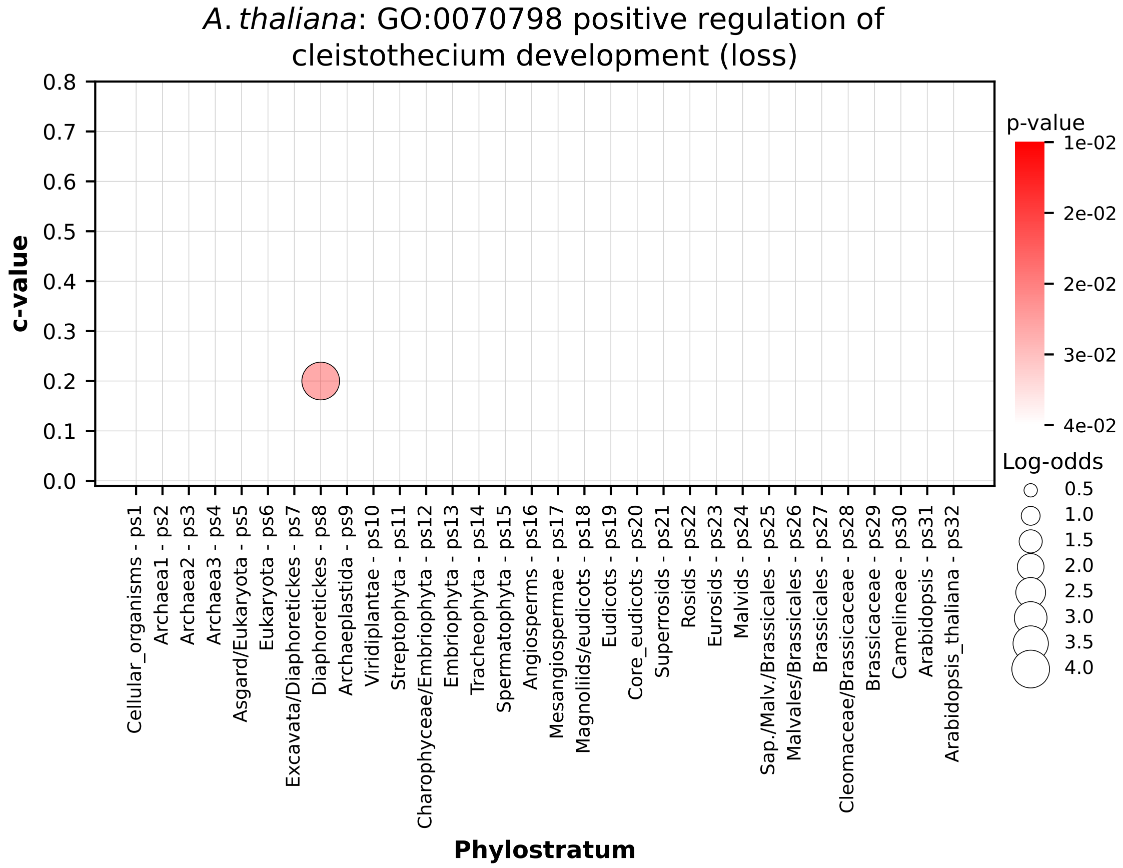

A. thaliana: GO:0070798 positive regulation of cleistothecium development (loss)
c-value
0.8
0.7
0.6
0.5
0.4
0.3
0.2
0.1
0.0
p-value
1e-02
2e-02
2e-02
3e-02
4e-02
Log-odds
0.5
1.0
1.5
2.0
2.5
3.0
3.5
4.0
Cellular_organisms - ps1
Archaea1 - ps2
Archaea2 - ps3
Archaea3 - ps4
Asgard/Eukaryota - ps5
Eukaryota - ps6
Excavata/Diaphoretickes - ps7
Diaphoretickes - ps8
Archaeplastida - ps9
Viridiplantae - ps10
Streptophyta - ps11
Charophyceae/Embriophyta - ps12
Embriophyta - ps13
Tracheophyta - ps14
Spermatophyta - ps15
Angiosperms - ps16
Mesangiospermae - ps17
Magnoliids/eudicots - ps18
Eudicots - ps19
Core_eudicots - ps20
Superrosids - ps21
Rosids - ps22
Eurosids - ps23
Malvids - ps24
Sap./Malv./Brassicales - ps25
Malvales/Brassicales - ps26
Brassicales - ps27
Cleomaceae/Brassicaceae - ps28
Brassicaceae - ps29
Camelineae - ps30
Arabidopsis - ps31
Arabidopsis_thaliana - ps32
Phylostratum

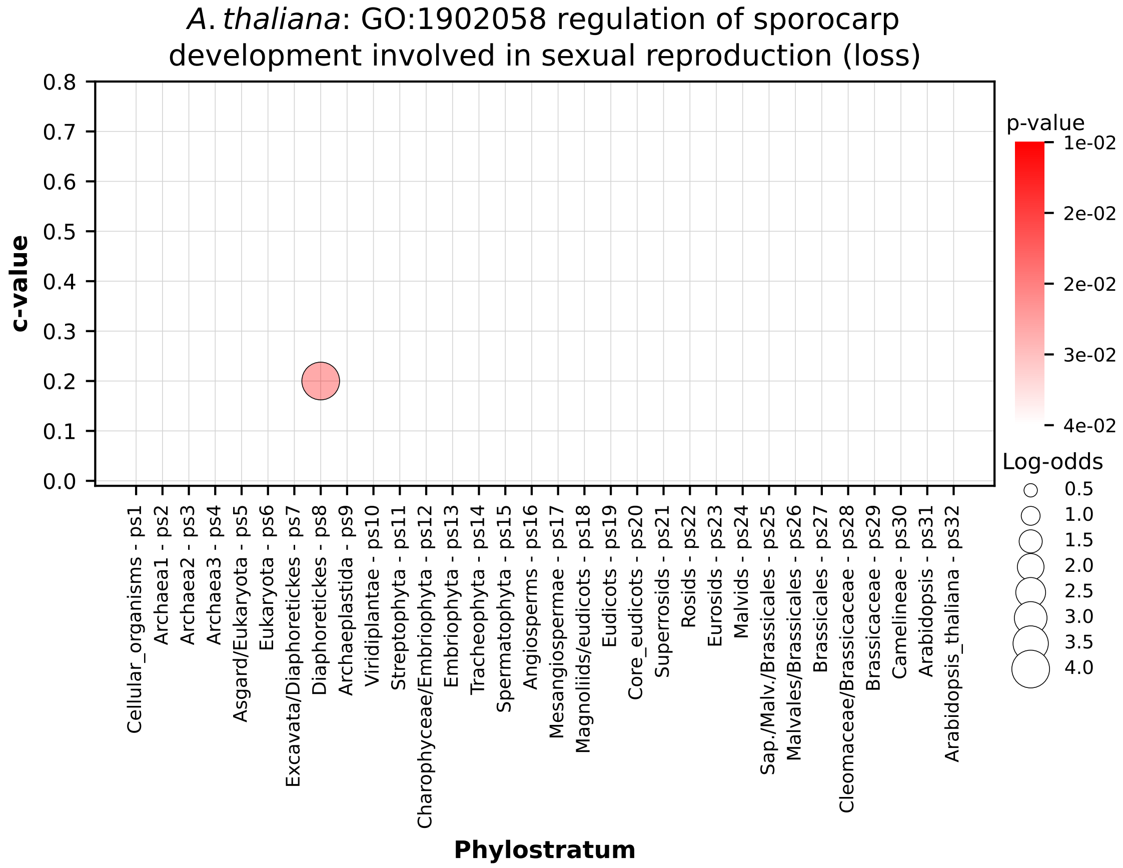

A. thaliana: GO:1902058 regulation of sporocarp development involved in sexual reproduction (loss)
c-value
0.8
0.7
0.6
0.5
0.4
0.3
0.2
0.1
0.0
p-value
1e-02
2e-02
2e-02
3e-02
4e-02
Log-odds
0.5
1.0
1.5
2.0
2.5
3.0
3.5
4.0
Cellular_organisms - ps1
Archaea1 - ps2
Archaea2 - ps3
Archaea3 - ps4
Asgard/Eukaryota - ps5
Eukaryota - ps6
Excavata/Diaphoretickes - ps7
Diaphoretickes - ps8
Archaeplastida - ps9
Viridiplantae - ps10
Streptophyta - ps11
Charophyceae/Embriophyta - ps12
Embriophyta - ps13
Tracheophyta - ps14
Spermatophyta - ps15
Angiosperms - ps16
Mesangiospermae - ps17
Magnoliids/eudicots - ps18
Eudicots - ps19
Core_eudicots - ps20
Superrosids - ps21
Rosids - ps22
Eurosids - ps23
Malvids - ps24
Sap./Malv./Brassicales - ps25
Malvales/Brassicales - ps26
Brassicales - ps27
Cleomaceae/Brassicaceae - ps28
Brassicaceae - ps29
Camelineae - ps30
Arabidopsis - ps31
Arabidopsis_thaliana - ps32
Phylostratum

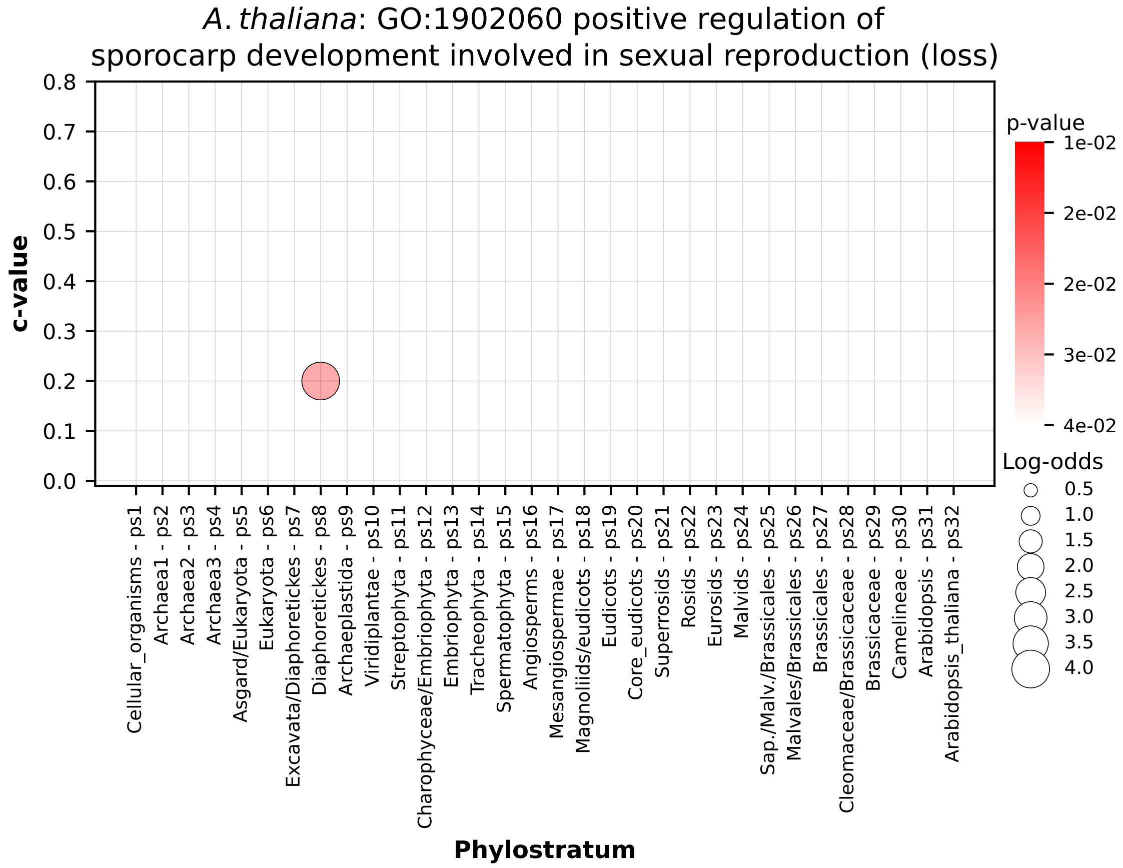
A. thaliana: GO:1902060 positive regulation of sporocarp development involved in sexual reproduction (loss)
c-value
0.0
0.1
0.2
0.3
0.4
0.5
0.6
0.7
0.8
Phylostratum
Cellular_organisms - ps1
Archaea1 - ps2
Archaea2 - ps3
Archaea3 - ps4
Asgard/Eukaryota - ps5
Eukaryota - ps6
Excavata/Diaphoretickes - ps7
Diaphoretickes - ps8
Archaeplastida - ps9
Viridiplantae - ps10
Streptophyta - ps11
Charophyceae/Embriophyta - ps12
Embriophyta - ps13
Tracheophyta - ps14
Spermatophyta - ps15
Angiosperms - ps16
Mesangiospermae - ps17
Magnoliids/eudicots - ps18
Eudicots - ps19
Core_eudicots - ps20
Superrosids - ps21
Rosids - ps22
Eurosids - ps23
Malvids - ps24
Sap./Malv./Brassicales - ps25
Malvales/Brassicales - ps26
Brassicales - ps27
Cleomaceae/Brassicaceae - ps28
Brassicaceae - ps29
Camelineae - ps30
Arabidopsis - ps31
Arabidopsis_thaliana - ps32
p-value
1e-02
2e-02
2e-02
3e-02
4e-02
Log-odds
0.5
1.0
1.5
2.0
2.5
3.0
3.5
4.0

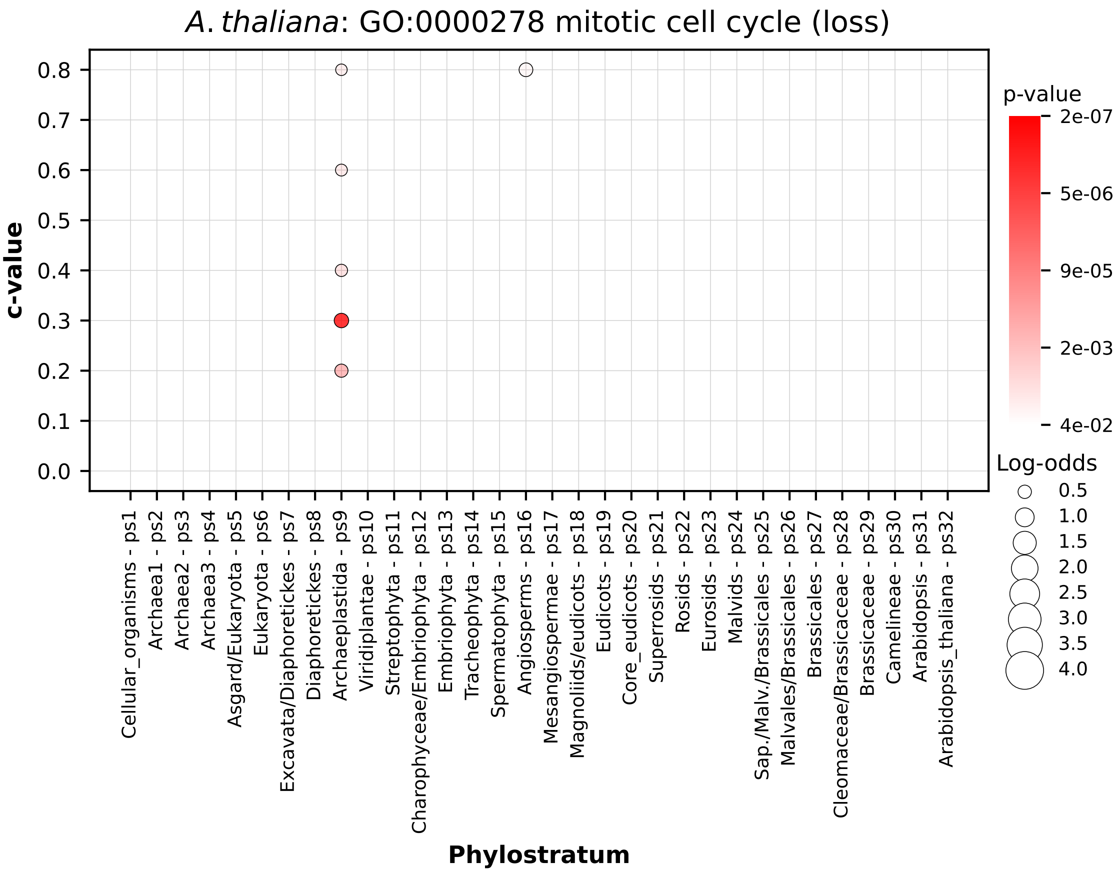
A. thaliana: GO:0000278 mitotic cell cycle (loss)
c-value
0.0
0.1
0.2
0.3
0.4
0.5
0.6
0.7
0.8
Phylostratum
Cellular_organisms - ps1
Archaea1 - ps2
Archaea2 - ps3
Archaea3 - ps4
Asgard/Eukaryota - ps5
Eukaryota - ps6
Excavata/Diaphoretickes - ps7
Diaphoretickes - ps8
Archaeplastida - ps9
Viridiplantae - ps10
Streptophyta - ps11
Charophyceae/Embriophyta - ps12
Embriophyta - ps13
Tracheophyta - ps14
Spermatophyta - ps15
Angiosperms - ps16
Mesangiospermae - ps17
Magnoliids/eudicots - ps18
Eudicots - ps19
Core_eudicots - ps20
Superrosids - ps21
Rosids - ps22
Eurosids - ps23
Malvids - ps24
Sap./Malv./Brassicales - ps25
Malvales/Brassicales - ps26
Brassicales - ps27
Cleomaceae/Brassicaceae - ps28
Brassicaceae - ps29
Camelineae - ps30
Arabidopsis - ps31
Arabidopsis_thaliana - ps32
p-value
2e-07
5e-06
9e-05
2e-03
4e-02
Log-odds
0.5
1.0
1.5
2.0
2.5
3.0
3.5
4.0

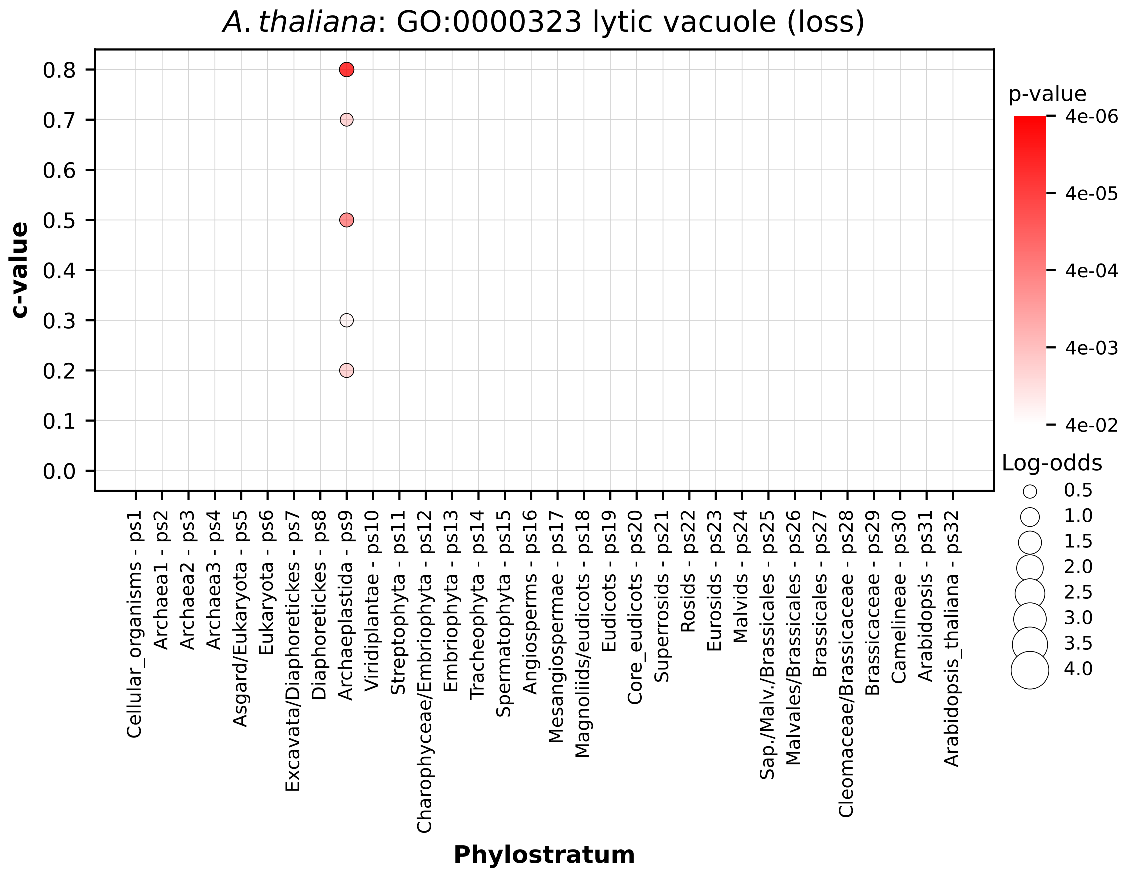

A. thaliana: GO:0000323 lytic vacuole (loss)
c-value
0.0
0.1
0.2
0.3
0.4
0.5
0.6
0.7
0.8
p-value
4e-06
4e-05
4e-04
4e-03
4e-02
Log-odds
0.5
1.0
1.5
2.0
2.5
3.0
3.5
4.0
Cellular_organisms - ps1
Archaea1 - ps2
Archaea2 - ps3
Archaea3 - ps4
Asgard/Eukaryota - ps5
Eukaryota - ps6
Excavata/Diaphoretickes - ps7
Diaphoretickes - ps8
Archaeplastida - ps9
Viridiplantae - ps10
Streptophyta - ps11
Charophyceae/Embriophyta - ps12
Embriophyta - ps13
Tracheophyta - ps14
Spermatophyta - ps15
Angiosperms - ps16
Mesangiospermae - ps17
Magnoliids/eudicots - ps18
Eudicots - ps19
Core_eudicots - ps20
Superrosids - ps21
Rosids - ps22
Eurosids - ps23
Malvids - ps24
Sap./Malv./Brassicales - ps25
Malvales/Brassicales - ps26
Brassicales - ps27
Cleomaceae/Brassicaceae - ps28
Brassicaceae - ps29
Camelineae - ps30
Arabidopsis - ps31
Arabidopsis_thaliana - ps32
Phylostratum

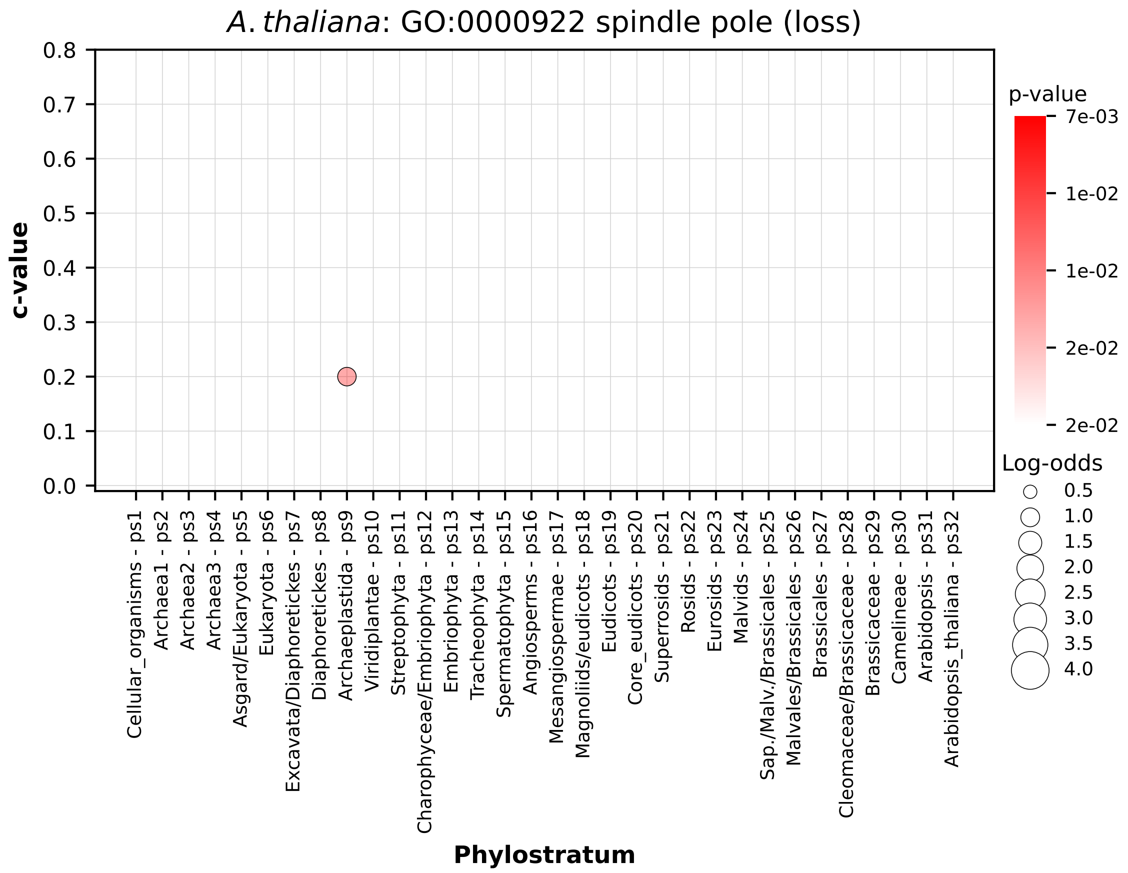
A. thaliana: GO:0000922 spindle pole (loss)
c-value
0.0
0.1
0.2
0.3
0.4
0.5
0.6
0.7
0.8
Phylostratum
Cellular_organisms - ps1
Archaea1 - ps2
Archaea2 - ps3
Archaea3 - ps4
Asgard/Eukaryota - ps5
Eukaryota - ps6
Excavata/Diaphoretickes - ps7
Diaphoretickes - ps8
Archaeplastida - ps9
Viridiplantae - ps10
Streptophyta - ps11
Charophyceae/Embriophyta - ps12
Embriophyta - ps13
Tracheophyta - ps14
Spermatophyta - ps15
Angiosperms - ps16
Mesangiospermae - ps17
Magnoliids/eudicots - ps18
Eudicots - ps19
Core_eudicots - ps20
Superrosids - ps21
Rosids - ps22
Eurosids - ps23
Malvids - ps24
Sap./Malv./Brassicales - ps25
Malvales/Brassicales - ps26
Brassicales - ps27
Cleomaceae/Brassicaceae - ps28
Brassicaceae - ps29
Camelineae - ps30
Arabidopsis - ps31
Arabidopsis_thaliana - ps32
p-value
7e-03
1e-02
1e-02
2e-02
2e-02
Log-odds
0.5
1.0
1.5
2.0
2.5
3.0
3.5
4.0

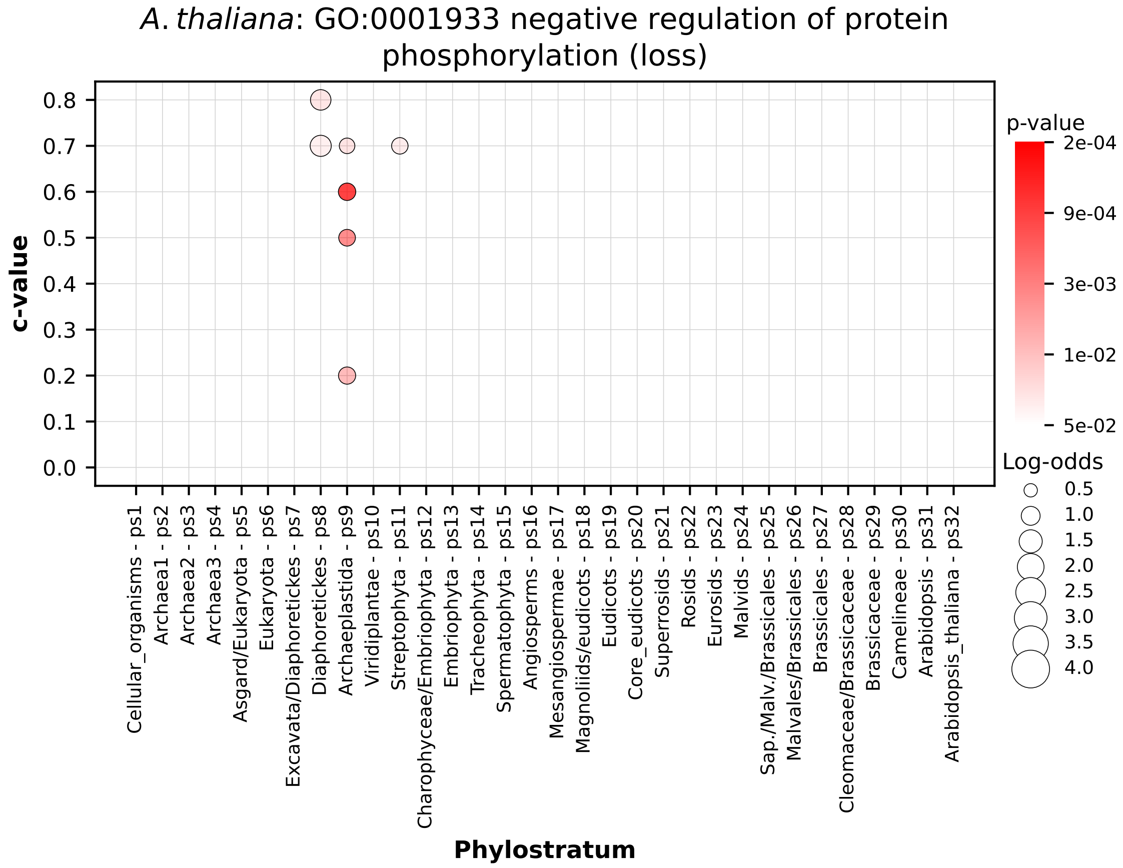

A. thaliana: GO:0001933 negative regulation of protein phosphorylation (loss)
c-value
0.0
0.1
0.2
0.3
0.4
0.5
0.6
0.7
0.8
Phylostratum
Cellular_organisms - ps1
Archaea1 - ps2
Archaea2 - ps3
Archaea3 - ps4
Asgard/Eukaryota - ps5
Eukaryota - ps6
Excavata/Diaphoretickes - ps7
Diaphoretickes - ps8
Archaeplastida - ps9
Viridiplantae - ps10
Streptophyta - ps11
Charophyceae/Embriophyta - ps12
Embriophyta - ps13
Tracheophyta - ps14
Spermatophyta - ps15
Angiosperms - ps16
Mesangiospermae - ps17
Magnoliids/eudicots - ps18
Eudicots - ps19
Core_eudicots - ps20
Superrosids - ps21
Rosids - ps22
Eurosids - ps23
Malvids - ps24
Sap./Malv./Brassicales - ps25
Malvales/Brassicales - ps26
Brassicales - ps27
Cleomaceae/Brassicaceae - ps28
Brassicaceae - ps29
Camelineae - ps30
Arabidopsis - ps31
Arabidopsis_thaliana - ps32
p-value
2e-04
9e-04
3e-03
1e-02
5e-02
Log-odds
0.5
1.0
1.5
2.0
2.5
3.0
3.5
4.0

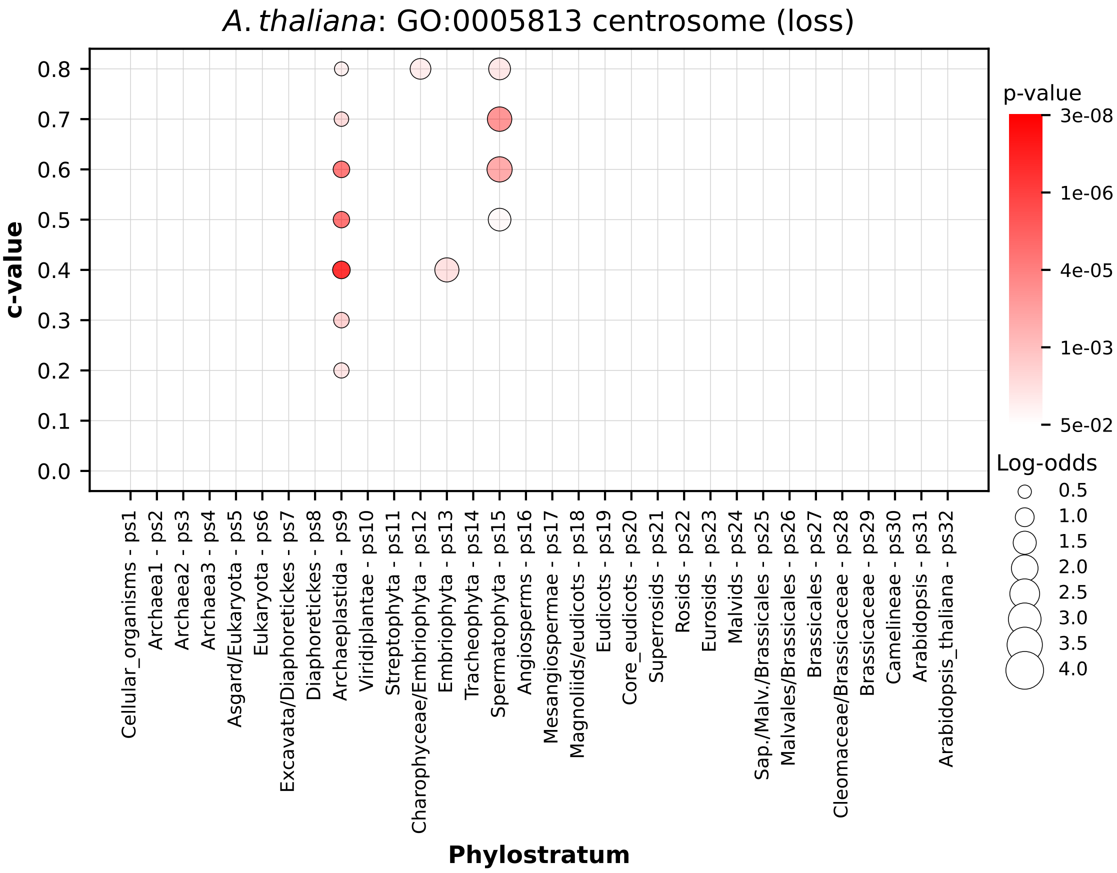
A. thaliana: GO:0005813 centrosome (loss)
c-value
0.8
0.7
0.6
0.5
0.4
0.3
0.2
0.1
0.0
Phylostratum
Cellular_organisms - ps1
Archaea1 - ps2
Archaea2 - ps3
Archaea3 - ps4
Asgard/Eukaryota - ps5
Eukaryota - ps6
Excavata/Diaphoretickes - ps7
Diaphoretickes - ps8
Archaeplastida - ps9
Viridiplantae - ps10
Streptophyta - ps11
Charophyceae/Embriophyta - ps12
Embriophyta - ps13
Tracheophyta - ps14
Spermatophyta - ps15
Angiosperms - ps16
Mesangiospermae - ps17
Magnoliids/eudicots - ps18
Eudicots - ps19
Core_eudicots - ps20
Superrosids - ps21
Rosids - ps22
Eurosids - ps23
Malvids - ps24
Sap./Malv./Brassicales - ps25
Malvales/Brassicales - ps26
Brassicales - ps27
Cleomaceae/Brassicaceae - ps28
Brassicaceae - ps29
Camelineae - ps30
Arabidopsis - ps31
Arabidopsis_thaliana - ps32
p-value
3e-08
1e-06
4e-05
1e-03
5e-02
Log-odds
0.5
1.0
1.5
2.0
2.5
3.0
3.5
4.0

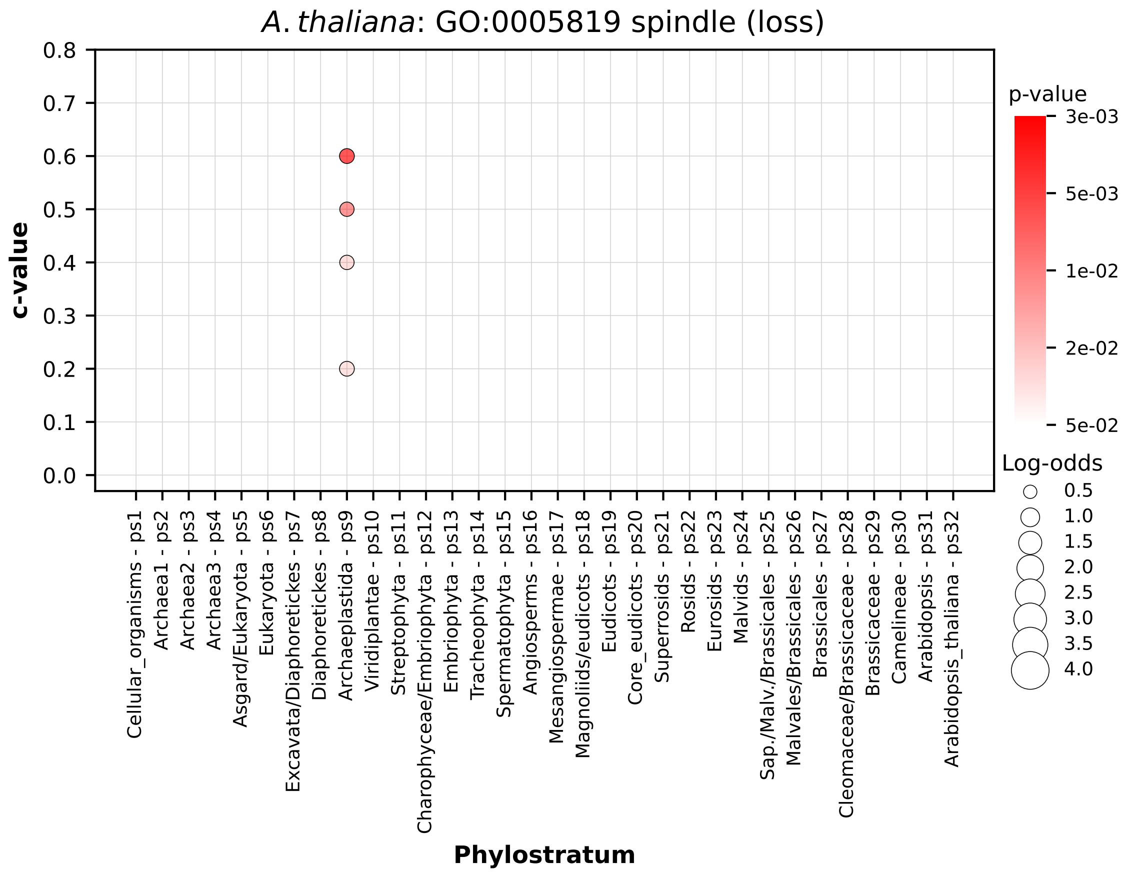

A. thaliana: GO:0005819 spindle (loss)
c-value
0.0
0.1
0.2
0.3
0.4
0.5
0.6
0.7
0.8
Phylostratum
Cellular_organisms - ps1
Archaea1 - ps2
Archaea2 - ps3
Archaea3 - ps4
Asgard/Eukaryota - ps5
Eukaryota - ps6
Excavata/Diaphoretickes - ps7
Diaphoretickes - ps8
Archaeplastida - ps9
Viridiplantae - ps10
Streptophyta - ps11
Charophyceae/Embriophyta - ps12
Embriophyta - ps13
Tracheophyta - ps14
Spermatophyta - ps15
Angiosperms - ps16
Mesangiospermae - ps17
Magnoliids/eudicots - ps18
Eudicots - ps19
Core_eudicots - ps20
Superrosids - ps21
Rosids - ps22
Eurosids - ps23
Malvids - ps24
Sap./Malv./Brassicales - ps25
Malvales/Brassicales - ps26
Brassicales - ps27
Cleomaceae/Brassicaceae - ps28
Brassicaceae - ps29
Camelineae - ps30
Arabidopsis - ps31
Arabidopsis_thaliana - ps32
p-value
3e-03
5e-03
1e-02
2e-02
5e-02
Log-odds
0.5
1.0
1.5
2.0
2.5
3.0
3.5
4.0

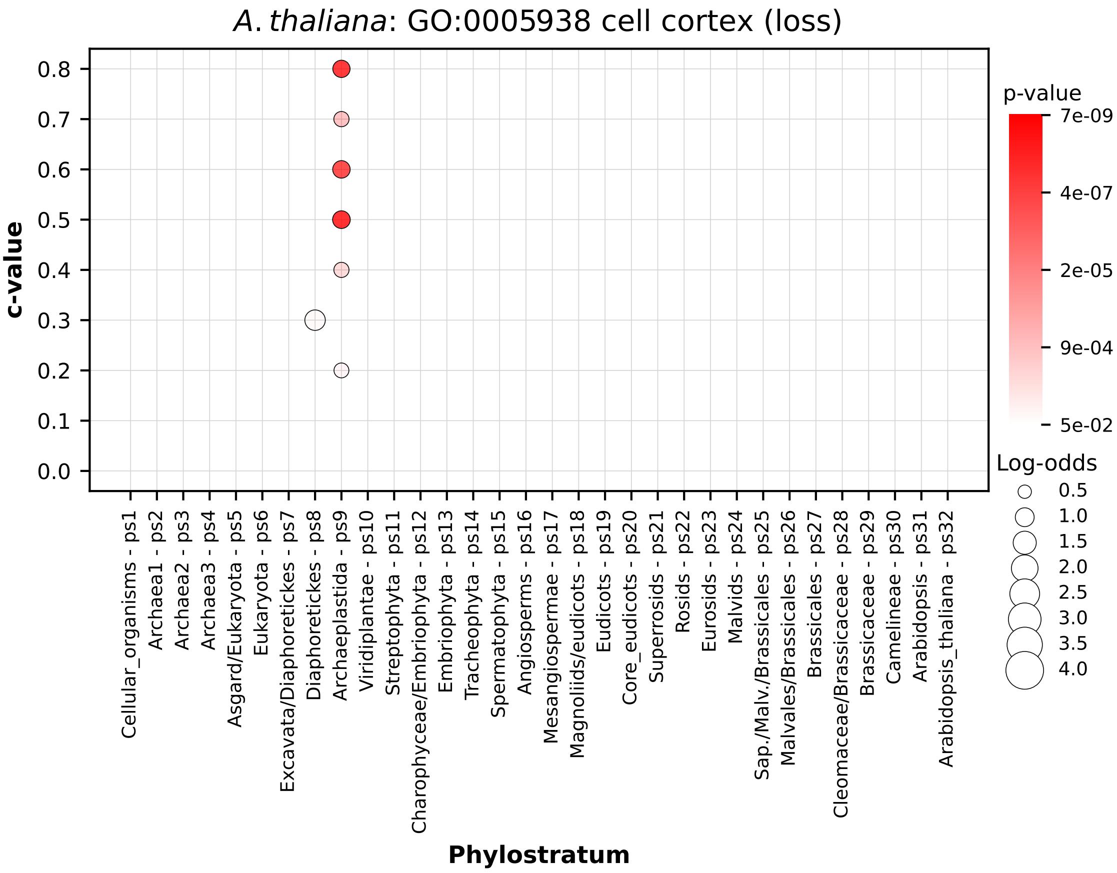
A. thaliana: GO:0005938 cell cortex (loss)
c-value
0.0
0.1
0.2
0.3
0.4
0.5
0.6
0.7
0.8
Phylostratum
Cellular_organisms - ps1
Archaea1 - ps2
Archaea2 - ps3
Archaea3 - ps4
Asgard/Eukaryota - ps5
Eukaryota - ps6
Excavata/Diaphoretickes - ps7
Diaphoretickes - ps8
Archaeplastida - ps9
Viridiplantae - ps10
Streptophyta - ps11
Charophyceae/Embriophyta - ps12
Embriophyta - ps13
Tracheophyta - ps14
Spermatophyta - ps15
Angiosperms - ps16
Mesangiospermae - ps17
Magnoliids/eudicots - ps18
Eudicots - ps19
Core_eudicots - ps20
Superrosids - ps21
Rosids - ps22
Eurosids - ps23
Malvids - ps24
Sap./Malv./Brassicales - ps25
Malvales/Brassicales - ps26
Brassicales - ps27
Cleomaceae/Brassicaceae - ps28
Brassicaceae - ps29
Camelineae - ps30
Arabidopsis - ps31
Arabidopsis_thaliana - ps32
p-value
7e-09
4e-07
2e-05
9e-04
5e-02
Log-odds
0.5
1.0
1.5
2.0
2.5
3.0
3.5
4.0

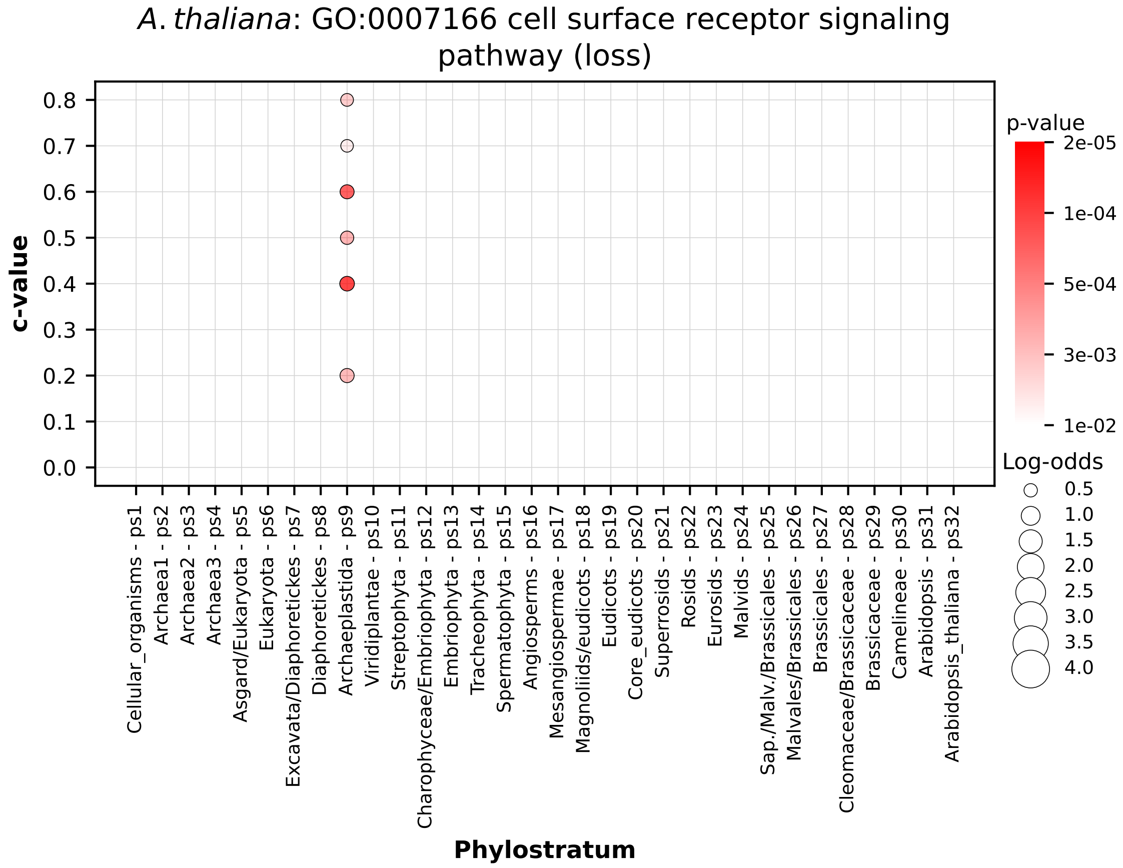

A. thaliana: GO:0007166 cell surface receptor signaling pathway (loss)
c-value
0.0
0.1
0.2
0.3
0.4
0.5
0.6
0.7
0.8
p-value
2e-05
1e-04
5e-04
3e-03
1e-02
Log-odds
0.5
1.0
1.5
2.0
2.5
3.0
3.5
4.0
Cellular_organisms - ps1
Archaea1 - ps2
Archaea2 - ps3
Archaea3 - ps4
Asgard/Eukaryota - ps5
Eukaryota - ps6
Excavata/Diaphoretickes - ps7
Diaphoretickes - ps8
Archaeplastida - ps9
Viridiplantae - ps10
Streptophyta - ps11
Charophyceae/Embriophyta - ps12
Embriophyta - ps13
Tracheophyta - ps14
Spermatophyta - ps15
Angiosperms - ps16
Mesangiospermae - ps17
Magnoliids/eudicots - ps18
Eudicots - ps19
Core_eudicots - ps20
Superrosids - ps21
Rosids - ps22
Eurosids - ps23
Malvids - ps24
Sap./Malv./Brassicales - ps25
Malvales/Brassicales - ps26
Brassicales - ps27
Cleomaceae/Brassicaceae - ps28
Brassicaceae - ps29
Camelineae - ps30
Arabidopsis - ps31
Arabidopsis_thaliana - ps32
Phylostratum

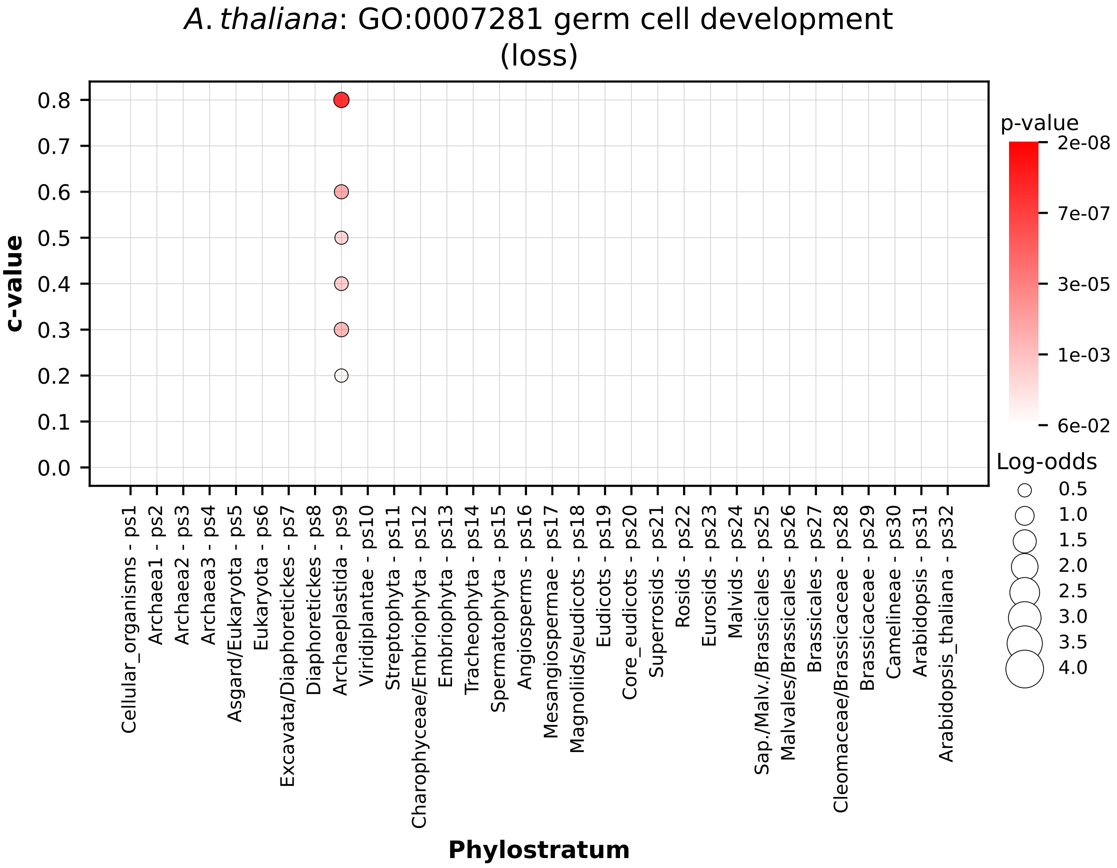

A. thaliana: GO:0007281 germ cell development
(loss)
c-value
0.0
0.1
0.2
0.3
0.4
0.5
0.6
0.7
0.8
p-value
2e-08
7e-07
3e-05
1e-03
6e-02
Log-odds
0.5
1.0
1.5
2.0
2.5
3.0
3.5
4.0
Cellular_organisms - ps1
Archaea1 - ps2
Archaea2 - ps3
Archaea3 - ps4
Asgard/Eukaryota - ps5
Eukaryota - ps6
Excavata/Diaphoretickes - ps7
Diaphoretickes - ps8
Archaeplastida - ps9
Viridiplantae - ps10
Streptophyta - ps11
Charophyceae/Embriophyta - ps12
Embriophyta - ps13
Tracheophyta - ps14
Spermatophyta - ps15
Angiosperms - ps16
Mesangiospermae - ps17
Magnoliids/eudicots - ps18
Eudicots - ps19
Core_eudicots - ps20
Superrosids - ps21
Rosids - ps22
Eurosids - ps23
Malvids - ps24
Sap./Malv./Brassicales - ps25
Malvales/Brassicales - ps26
Brassicales - ps27
Cleomaceae/Brassicaceae - ps28
Brassicaceae - ps29
Camelineae - ps30
Arabidopsis - ps31
Arabidopsis_thaliana - ps32
Phylostratum

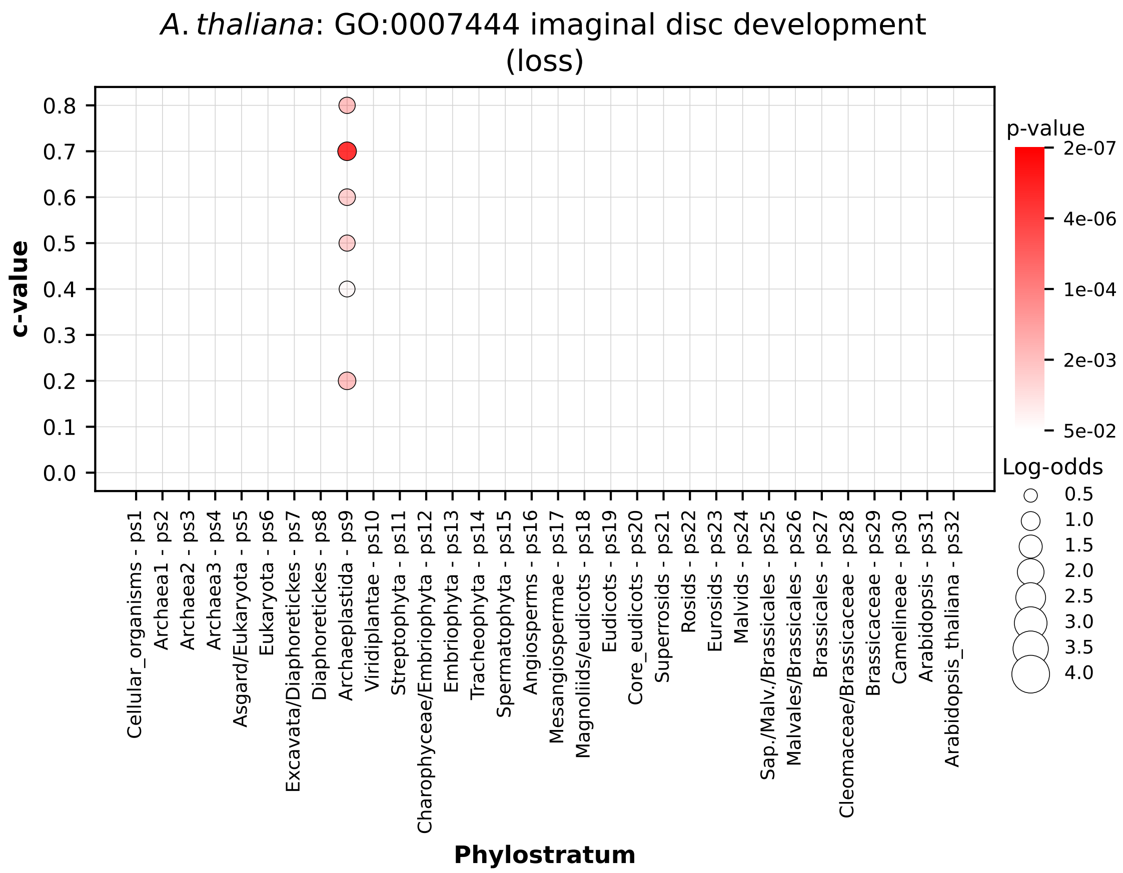
A. thaliana: GO:0007444 imaginal disc development
(loss)
c-value
0.8
0.7
0.6
0.5
0.4
0.3
0.2
0.1
0.0
p-value
2e-07
4e-06
1e-04
2e-03
5e-02
Log-odds
0.5
1.0
1.5
2.0
2.5
3.0
3.5
4.0
Cellular_organisms - ps1
Archaea1 - ps2
Archaea2 - ps3
Archaea3 - ps4
Asgard/Eukaryota - ps5
Eukaryota - ps6
Excavata/Diaphoretickes - ps7
Diaphoretickes - ps8
Archaeplastida - ps9
Viridiplantae - ps10
Streptophyta - ps11
Charophyceae/Embriophyta - ps12
Embriophyta - ps13
Tracheophyta - ps14
Spermatophyta - ps15
Angiosperms - ps16
Mesangiospermae - ps17
Magnoliids/eudicots - ps18
Eudicots - ps19
Core_eudicots - ps20
Superrosids - ps21
Rosids - ps22
Eurosids - ps23
Malvids - ps24
Sap./Malv./Brassicales - ps25
Malvales/Brassicales - ps26
Brassicales - ps27
Cleomaceae/Brassicaceae - ps28
Brassicaceae - ps29
Camelineae - ps30
Arabidopsis - ps31
Arabidopsis_thaliana - ps32
Phylostratum

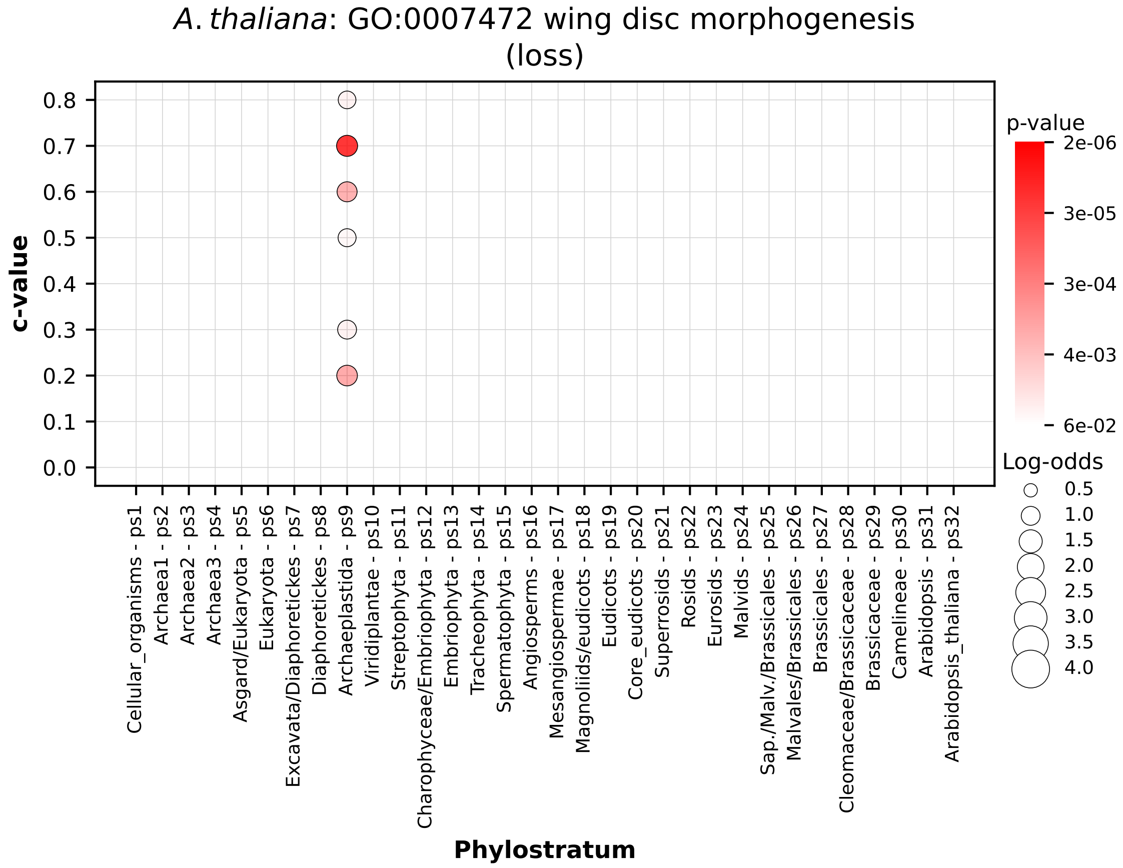
A. thaliana: GO:0007472 wing disc morphogenesis
(loss)
c-value
0.8
0.7
0.6
0.5
0.4
0.3
0.2
0.1
0.0
p-value
2e-06
3e-05
3e-04
4e-03
6e-02
Log-odds
0.5
1.0
1.5
2.0
2.5
3.0
3.5
4.0
Cellular_organisms - ps1
Archaea1 - ps2
Archaea2 - ps3
Archaea3 - ps4
Asgard/Eukaryota - ps5
Eukaryota - ps6
Excavata/Diaphoretickes - ps7
Diaphoretickes - ps8
Archaeplastida - ps9
Viridiplantae - ps10
Streptophyta - ps11
Charophyceae/Embriophyta - ps12
Embriophyta - ps13
Tracheophyta - ps14
Spermatophyta - ps15
Angiosperms - ps16
Mesangiospermae - ps17
Magnoliids/eudicots - ps18
Eudicots - ps19
Core_eudicots - ps20
Superrosids - ps21
Rosids - ps22
Eurosids - ps23
Malvids - ps24
Sap./Malv./Brassicales - ps25
Malvales/Brassicales - ps26
Brassicales - ps27
Cleomaceae/Brassicaceae - ps28
Brassicaceae - ps29
Camelineae - ps30
Arabidopsis - ps31
Arabidopsis_thaliana - ps32
Phylostratum

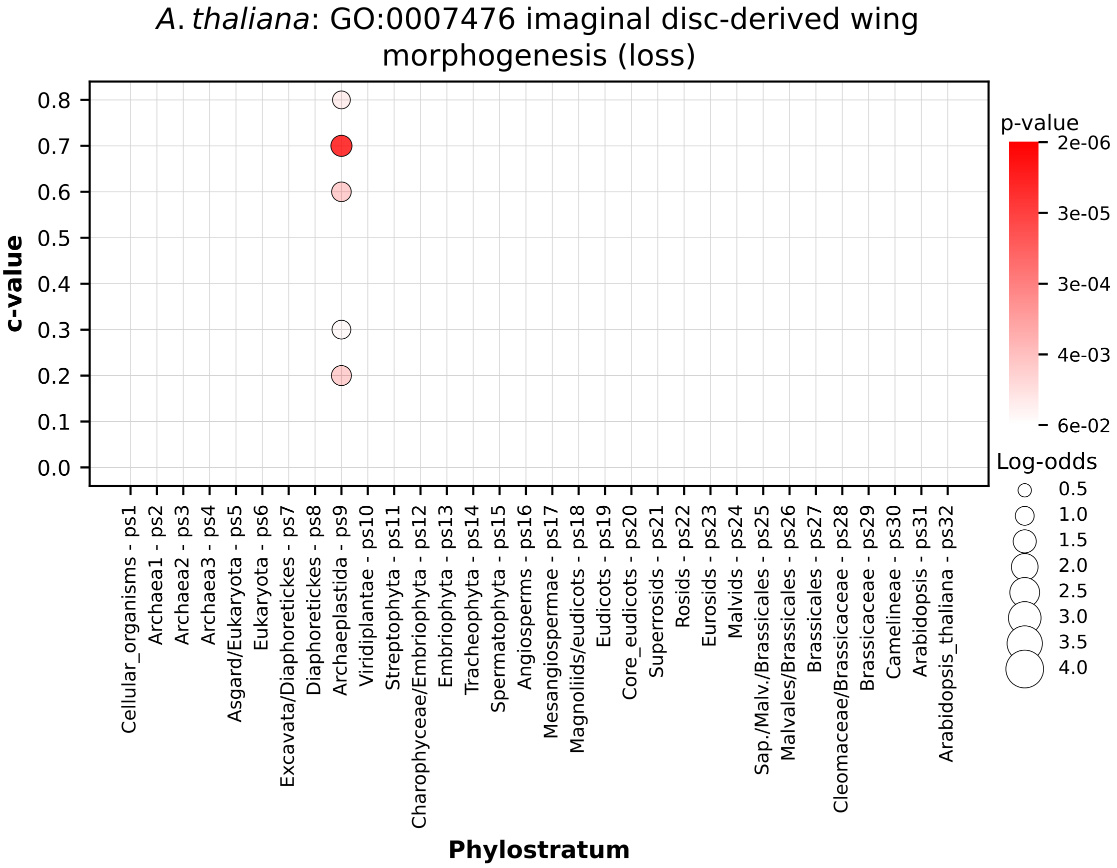

A. thaliana: GO:0007476 imaginal disc-derived wing morphogenesis (loss)
c-value
0.8
0.7
0.6
0.5
0.4
0.3
0.2
0.1
0.0
p-value
2e-06
3e-05
3e-04
4e-03
6e-02
Log-odds
0.5
1.0
1.5
2.0
2.5
3.0
3.5
4.0
Cellular_organisms - ps1
Archaea1 - ps2
Archaea2 - ps3
Archaea3 - ps4
Asgard/Eukaryota - ps5
Eukaryota - ps6
Excavata/Diaphoretickes - ps7
Diaphoretickes - ps8
Archaeplastida - ps9
Viridiplantae - ps10
Streptophyta - ps11
Charophyceae/Embriophyta - ps12
Embriophyta - ps13
Tracheophyta - ps14
Spermatophyta - ps15
Angiosperms - ps16
Mesangiospermae - ps17
Magnoliids/eudicots - ps18
Eudicots - ps19
Core_eudicots - ps20
Superrosids - ps21
Rosids - ps22
Eurosids - ps23
Malvids - ps24
Sap./Malv./Brassicales - ps25
Malvales/Brassicales - ps26
Brassicales - ps27
Cleomaceae/Brassicaceae - ps28
Brassicaceae - ps29
Camelineae - ps30
Arabidopsis - ps31
Arabidopsis_thaliana - ps32
Phylostratum

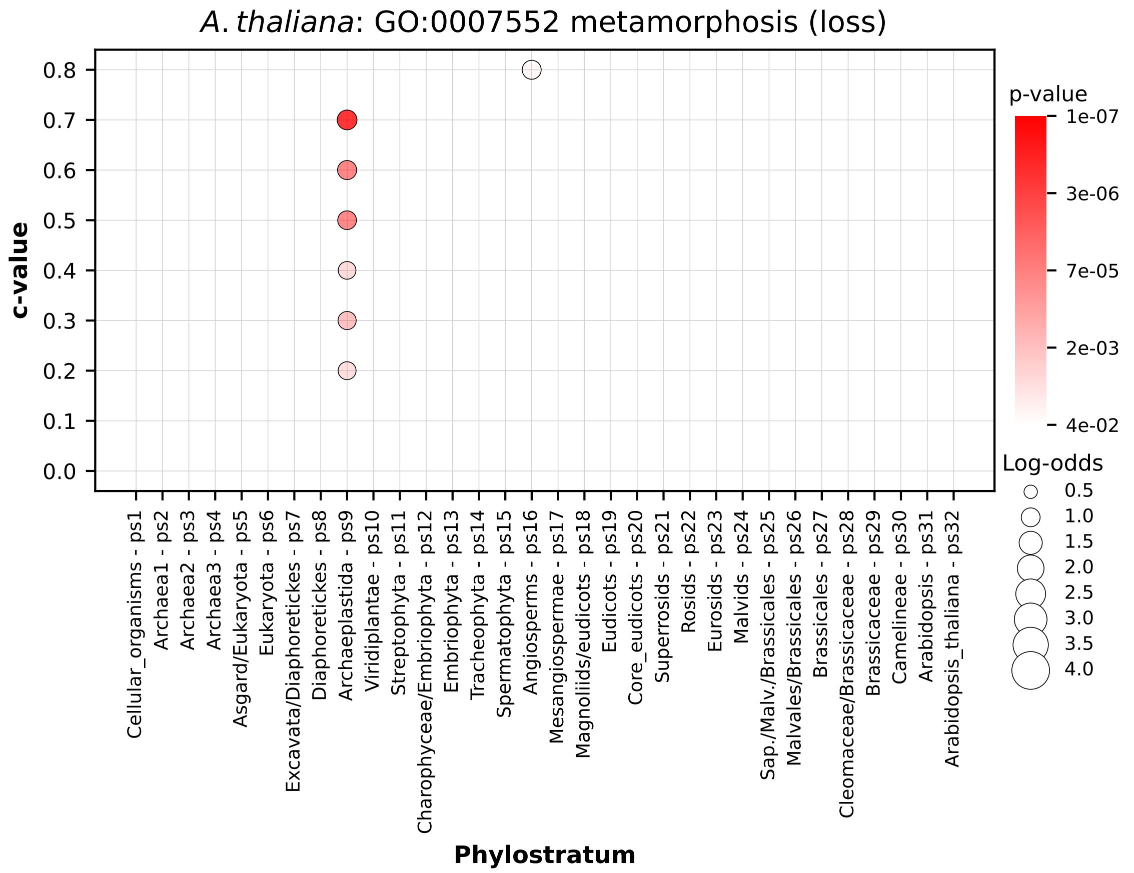
A. thaliana: GO:0007552 metamorphosis (loss)
c-value
0.8
0.7
0.6
0.5
0.4
0.3
0.2
0.1
0.0
p-value
1e-07
3e-06
7e-05
2e-03
4e-02
Log-odds
0.5
1.0
1.5
2.0
2.5
3.0
3.5
4.0
Cellular_organisms - ps1
Archaea1 - ps2
Archaea2 - ps3
Archaea3 - ps4
Asgard/Eukaryota - ps5
Eukaryota - ps6
Excavata/Diaphoretickes - ps7
Diaphoretickes - ps8
Archaeplastida - ps9
Viridiplantae - ps10
Streptophyta - ps11
Charophyceae/Embriophyta - ps12
Embriophyta - ps13
Tracheophyta - ps14
Spermatophyta - ps15
Angiosperms - ps16
Mesangiospermae - ps17
Magnoliids/eudicots - ps18
Eudicots - ps19
Core_eudicots - ps20
Superrosids - ps21
Rosids - ps22
Eurosids - ps23
Malvids - ps24
Sap./Malv./Brassicales - ps25
Malvales/Brassicales - ps26
Brassicales - ps27
Cleomaceae/Brassicaceae - ps28
Brassicaceae - ps29
Camelineae - ps30
Arabidopsis - ps31
Arabidopsis_thaliana - ps32
Phylostratum

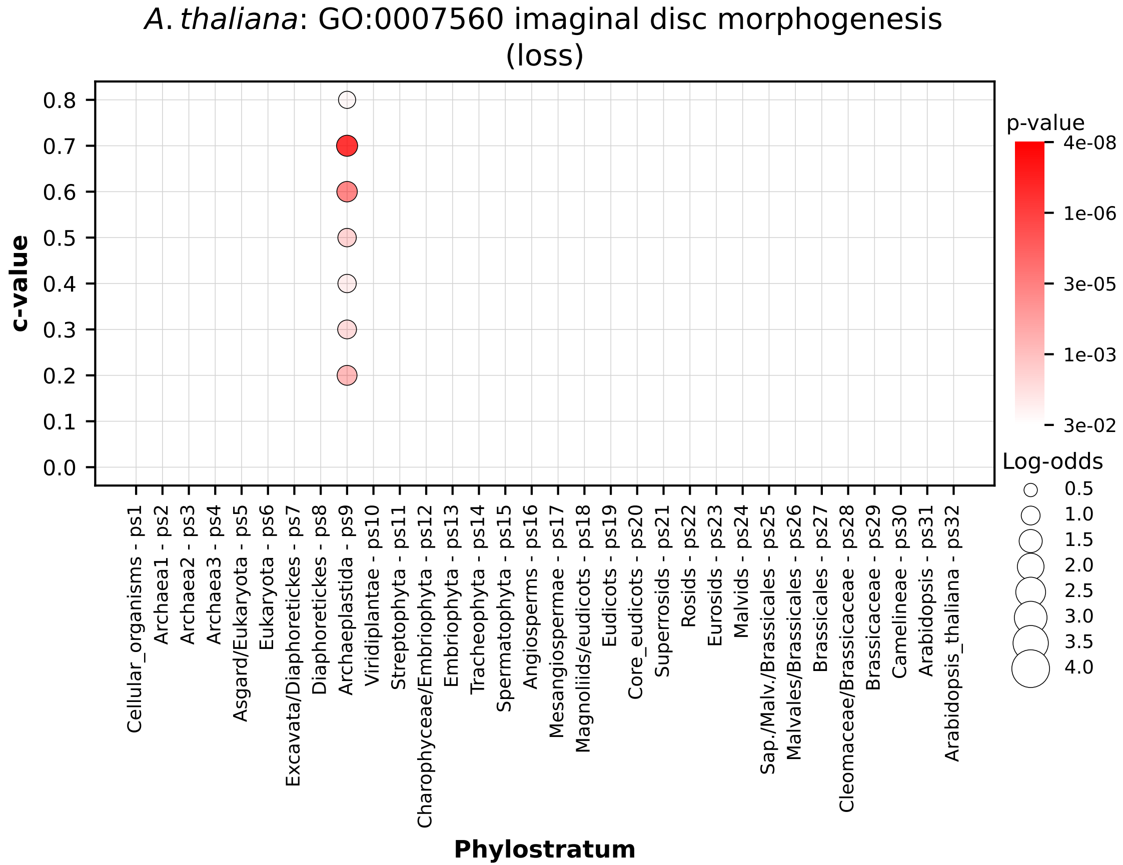

A. thaliana: GO:0007560 imaginal disc morphogenesis
(loss)
c-value
0.8
0.7
0.6
0.5
0.4
0.3
0.2
0.1
0.0
Phylostratum
Cellular_organisms - ps1
Archaea1 - ps2
Archaea2 - ps3
Archaea3 - ps4
Asgard/Eukaryota - ps5
Eukaryota - ps6
Excavata/Diaphoretickes - ps7
Diaphoretickes - ps8
Archaeplastida - ps9
Viridiplantae - ps10
Streptophyta - ps11
Charophyceae/Embriophyta - ps12
Embriophyta - ps13
Tracheophyta - ps14
Spermatophyta - ps15
Angiosperms - ps16
Mesangiospermae - ps17
Magnoliids/eudicots - ps18
Eudicots - ps19
Core_eudicots - ps20
Superrosids - ps21
Rosids - ps22
Eurosids - ps23
Malvids - ps24
Sap./Malv./Brassicales - ps25
Malvales/Brassicales - ps26
Brassicales - ps27
Cleomaceae/Brassicaceae - ps28
Brassicaceae - ps29
Camelineae - ps30
Arabidopsis - ps31
Arabidopsis_thaliana - ps32
p-value
4e-08
1e-06
3e-05
1e-03
3e-02
Log-odds
0.5
1.0
1.5
2.0
2.5
3.0
3.5
4.0

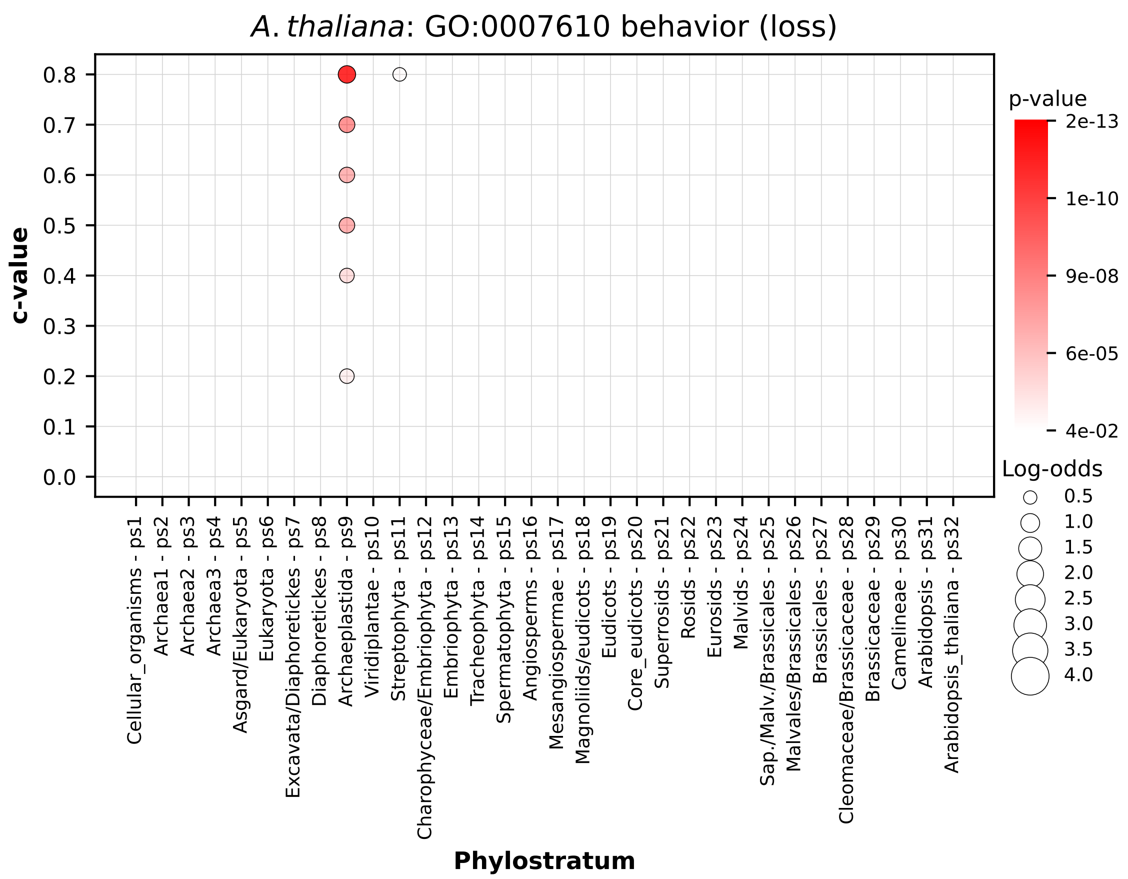

A. thaliana: GO:0007610 behavior (loss)
c-value
0.0
0.1
0.2
0.3
0.4
0.5
0.6
0.7
0.8
Phylostratum
Cellular_organisms - ps1
Archaea1 - ps2
Archaea2 - ps3
Archaea3 - ps4
Asgard/Eukaryota - ps5
Eukaryota - ps6
Excavata/Diaphoretickes - ps7
Diaphoretickes - ps8
Archaeplastida - ps9
Viridiplantae - ps10
Streptophyta - ps11
Charophyceae/Embriophyta - ps12
Embriophyta - ps13
Tracheophyta - ps14
Spermatophyta - ps15
Angiosperms - ps16
Mesangiospermae - ps17
Magnoliids/eudicots - ps18
Eudicots - ps19
Core_eudicots - ps20
Superrosids - ps21
Rosids - ps22
Eurosids - ps23
Malvids - ps24
Sap./Malv./Brassicales - ps25
Malvales/Brassicales - ps26
Brassicales - ps27
Cleomaceae/Brassicaceae - ps28
Brassicaceae - ps29
Camelineae - ps30
Arabidopsis - ps31
Arabidopsis_thaliana - ps32
p-value
2e-13
1e-10
9e-08
6e-05
4e-02
Log-odds
0.5
1.0
1.5
2.0
2.5
3.0
3.5
4.0

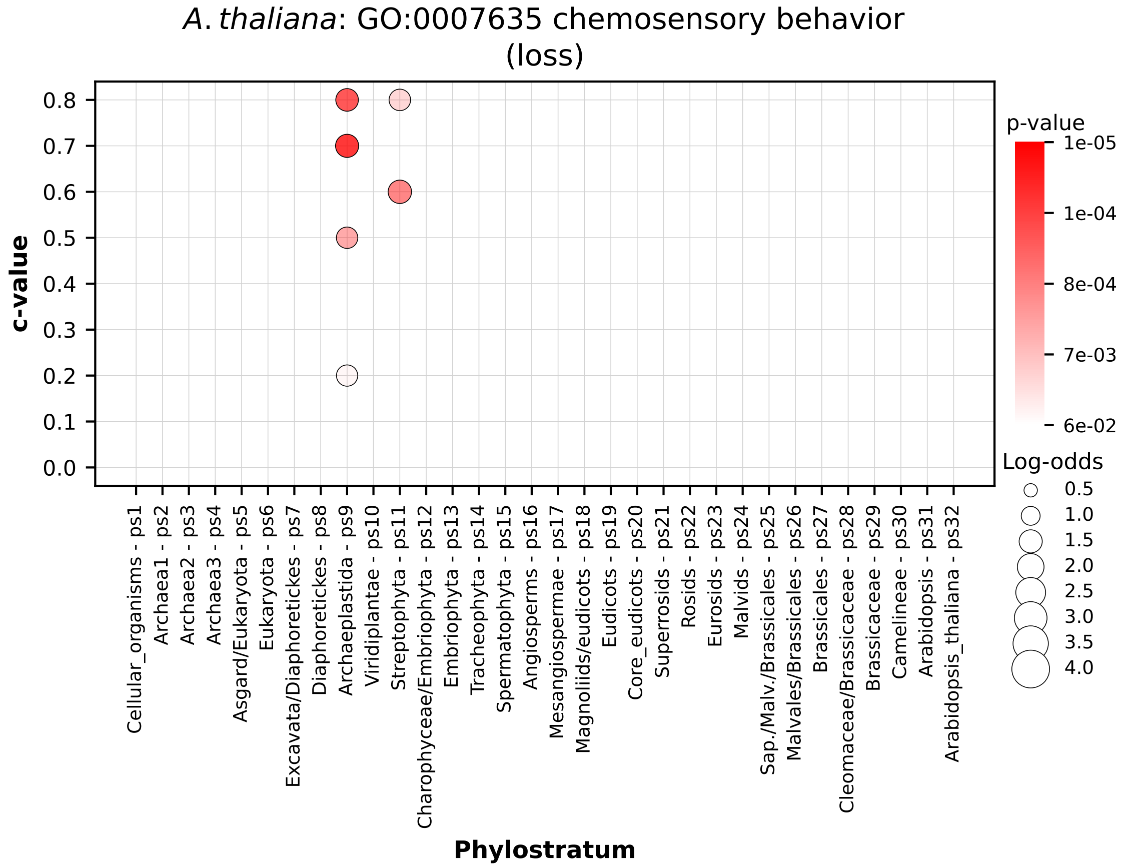
A. thaliana: GO:0007635 chemosensory behavior
(loss)
c-value
0.8
0.7
0.6
0.5
0.4
0.3
0.2
0.1
0.0
Phylostratum
Cellular_organisms - ps1
Archaea1 - ps2
Archaea2 - ps3
Archaea3 - ps4
Asgard/Eukaryota - ps5
Eukaryota - ps6
Excavata/Diaphoretickes - ps7
Diaphoretickes - ps8
Archaeplastida - ps9
Viridiplantae - ps10
Streptophyta - ps11
Charophyceae/Embriophyta - ps12
Embriophyta - ps13
Tracheophyta - ps14
Spermatophyta - ps15
Angiosperms - ps16
Mesangiospermae - ps17
Magnoliids/eudicots - ps18
Eudicots - ps19
Core_eudicots - ps20
Superrosids - ps21
Rosids - ps22
Eurosids - ps23
Malvids - ps24
Sap./Malv./Brassicales - ps25
Malvales/Brassicales - ps26
Brassicales - ps27
Cleomaceae/Brassicaceae - ps28
Brassicaceae - ps29
Camelineae - ps30
Arabidopsis - ps31
Arabidopsis_thaliana - ps32
p-value
1e-05
1e-04
8e-04
7e-03
6e-02
Log-odds
0.5
1.0
1.5
2.0
2.5
3.0
3.5
4.0

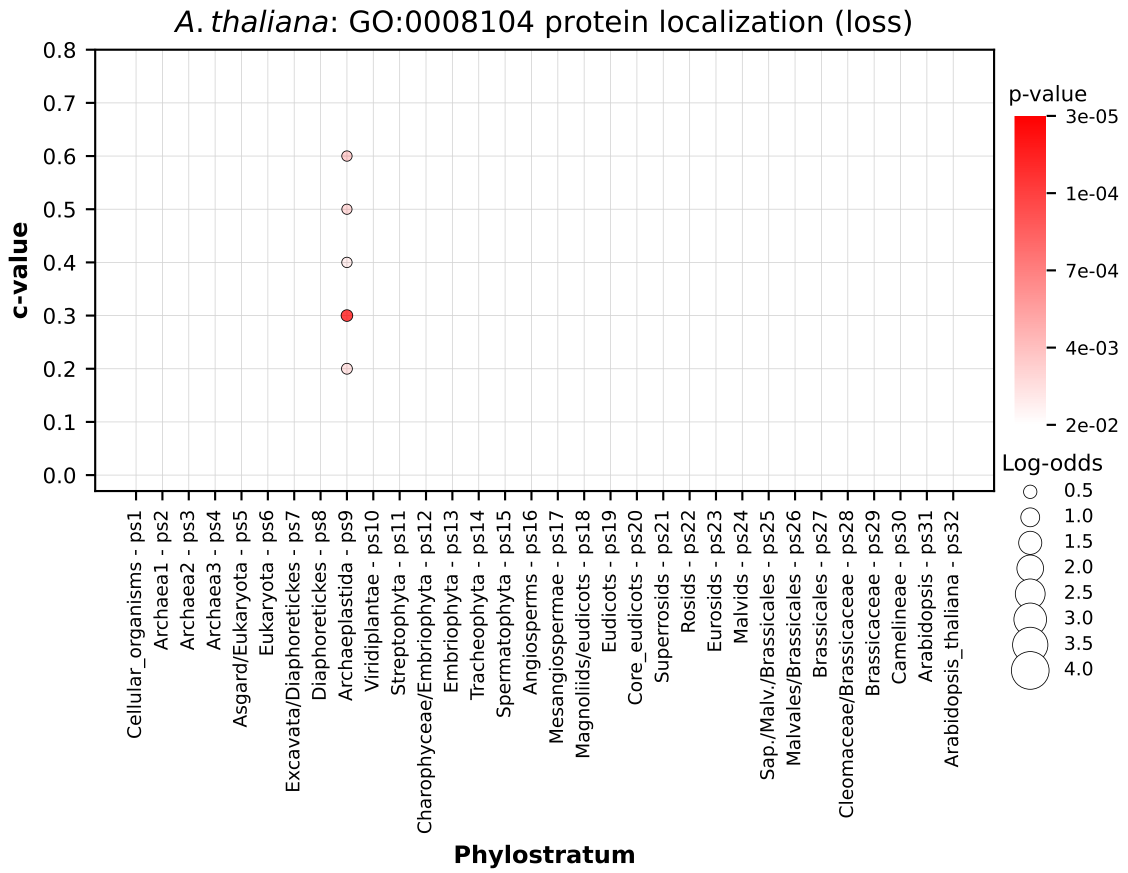

A. thaliana: GO:0008104 protein localization (loss)
c-value
0.0
0.1
0.2
0.3
0.4
0.5
0.6
0.7
0.8
p-value
3e-05
1e-04
7e-04
4e-03
2e-02
Log-odds
0.5
1.0
1.5
2.0
2.5
3.0
3.5
4.0
Cellular_organisms - ps1
Archaea1 - ps2
Archaea2 - ps3
Archaea3 - ps4
Asgard/Eukaryota - ps5
Eukaryota - ps6
Excavata/Diaphoretickes - ps7
Diaphoretickes - ps8
Archaeplastida - ps9
Viridiplantae - ps10
Streptophyta - ps11
Charophyceae/Embriophyta - ps12
Embriophyta - ps13
Tracheophyta - ps14
Spermatophyta - ps15
Angiosperms - ps16
Mesangiospermae - ps17
Magnoliids/eudicots - ps18
Eudicots - ps19
Core_eudicots - ps20
Superrosids - ps21
Rosids - ps22
Eurosids - ps23
Malvids - ps24
Sap./Malv./Brassicales - ps25
Malvales/Brassicales - ps26
Brassicales - ps27
Cleomaceae/Brassicaceae - ps28
Brassicaceae - ps29
Camelineae - ps30
Arabidopsis - ps31
Arabidopsis_thaliana - ps32
Phylostratum

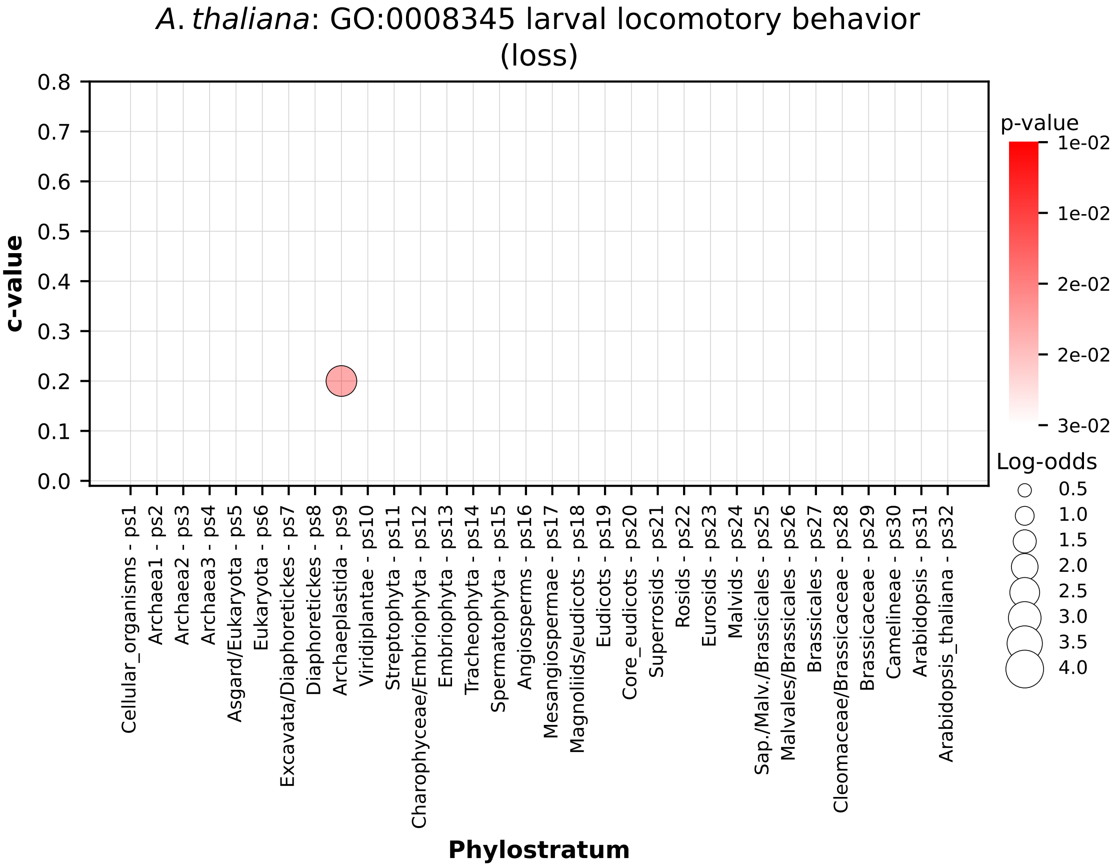

A. thaliana: GO:0008345 larval locomotory behavior
(loss)
c-value
0.8
0.7
0.6
0.5
0.4
0.3
0.2
0.1
0.0
Phylostratum
Cellular_organisms - ps1
Archaea1 - ps2
Archaea2 - ps3
Archaea3 - ps4
Asgard/Eukaryota - ps5
Eukaryota - ps6
Excavata/Diaphoretickes - ps7
Diaphoretickes - ps8
Archaeplastida - ps9
Viridiplantae - ps10
Streptophyta - ps11
Charophyceae/Embriophyta - ps12
Embriophyta - ps13
Tracheophyta - ps14
Spermatophyta - ps15
Angiosperms - ps16
Mesangiospermae - ps17
Magnoliids/eudicots - ps18
Eudicots - ps19
Core_eudicots - ps20
Superrosids - ps21
Rosids - ps22
Eurosids - ps23
Malvids - ps24
Sap./Malv./Brassicales - ps25
Malvales/Brassicales - ps26
Brassicales - ps27
Cleomaceae/Brassicaceae - ps28
Brassicaceae - ps29
Camelineae - ps30
Arabidopsis - ps31
Arabidopsis_thaliana - ps32
p-value
1e-02
1e-02
2e-02
2e-02
3e-02
Log-odds
0.5
1.0
1.5
2.0
2.5
3.0
3.5
4.0

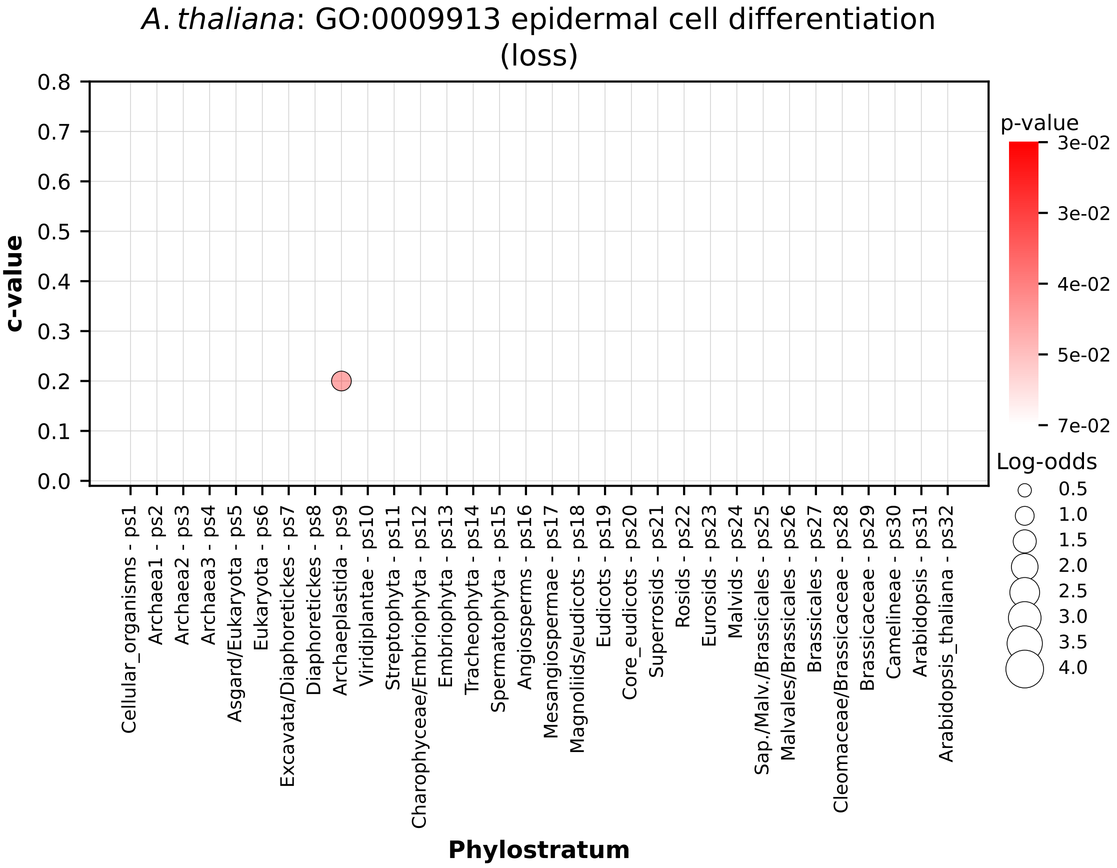

A. thaliana: GO:0009913 epidermal cell differentiation (loss)
c-value
0.8
0.7
0.6
0.5
0.4
0.3
0.2
0.1
0.0
Phylostratum
Cellular_organisms - ps1
Archaea1 - ps2
Archaea2 - ps3
Archaea3 - ps4
Asgard/Eukaryota - ps5
Eukaryota - ps6
Excavata/Diaphoretickes - ps7
Diaphoretickes - ps8
Archaeplastida - ps9
Viridiplantae - ps10
Streptophyta - ps11
Charophyceae/Embriophyta - ps12
Embriophyta - ps13
Tracheophyta - ps14
Spermatophyta - ps15
Angiosperms - ps16
Mesangiospermae - ps17
Magnoliids/eudicots - ps18
Eudicots - ps19
Core_eudicots - ps20
Superrosids - ps21
Rosids - ps22
Eurosids - ps23
Malvids - ps24
Sap./Malv./Brassicales - ps25
Malvales/Brassicales - ps26
Brassicales - ps27
Cleomaceae/Brassicaceae - ps28
Brassicaceae - ps29
Camelineae - ps30
Arabidopsis - ps31
Arabidopsis_thaliana - ps32
p-value
3e-02
3e-02
4e-02
5e-02
7e-02
Log-odds
0.5
1.0
1.5
2.0
2.5
3.0
3.5
4.0

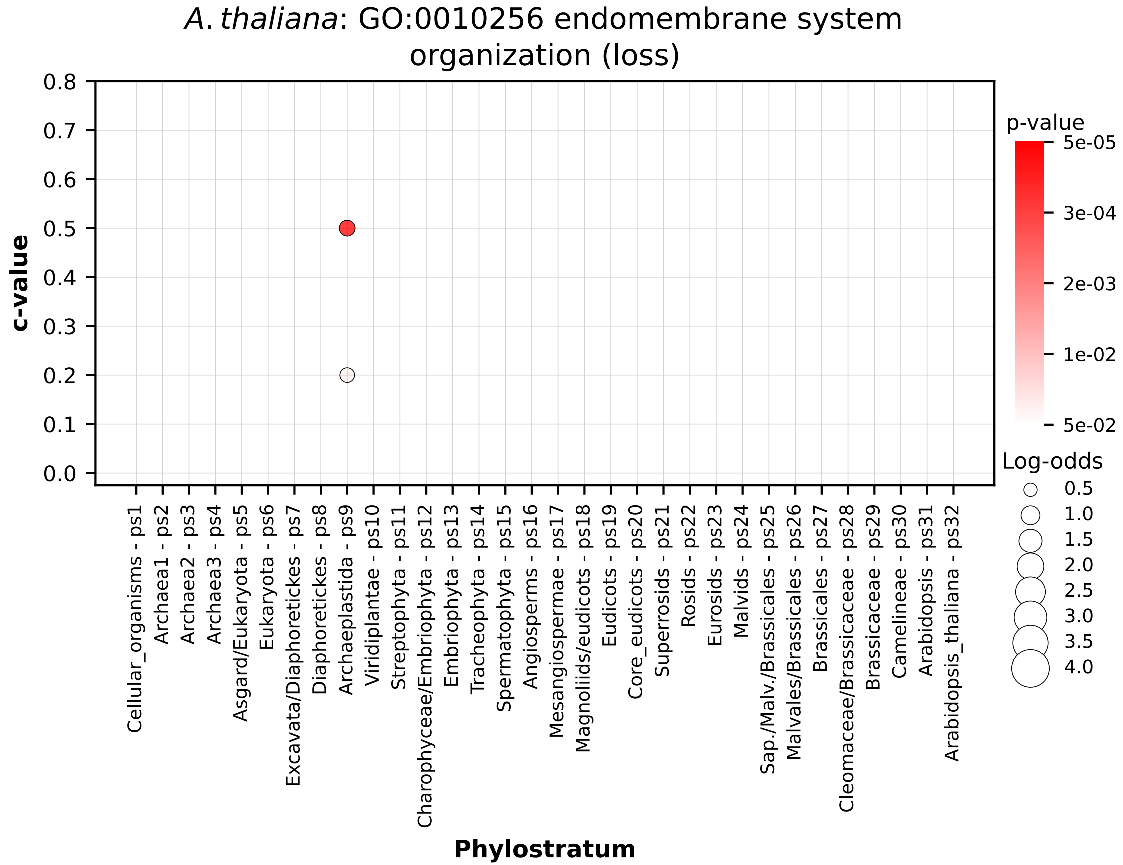

A. thaliana: GO:0010256 endomembrane system organization (loss)
c-value
0.8
0.7
0.6
0.5
0.4
0.3
0.2
0.1
0.0
Phylostratum
Cellular_organisms - ps1
Archaea1 - ps2
Archaea2 - ps3
Archaea3 - ps4
Asgard/Eukaryota - ps5
Eukaryota - ps6
Excavata/Diaphoretickes - ps7
Diaphoretickes - ps8
Archaeplastida - ps9
Viridiplantae - ps10
Streptophyta - ps11
Charophyceae/Embriophyta - ps12
Embriophyta - ps13
Tracheophyta - ps14
Spermatophyta - ps15
Angiosperms - ps16
Mesangiospermae - ps17
Magnoliids/eudicots - ps18
Eudicots - ps19
Core_eudicots - ps20
Superrosids - ps21
Rosids - ps22
Eurosids - ps23
Malvids - ps24
Sap./Malv./Brassicales - ps25
Malvales/Brassicales - ps26
Brassicales - ps27
Cleomaceae/Brassicaceae - ps28
Brassicaceae - ps29
Camelineae - ps30
Arabidopsis - ps31
Arabidopsis_thaliana - ps32
p-value
5e-05
3e-04
2e-03
1e-02
5e-02
Log-odds
0.5
1.0
1.5
2.0
2.5
3.0
3.5
4.0

A. thaliana: GO:0010563 negative regulation of phosphorus metabolic process (loss)

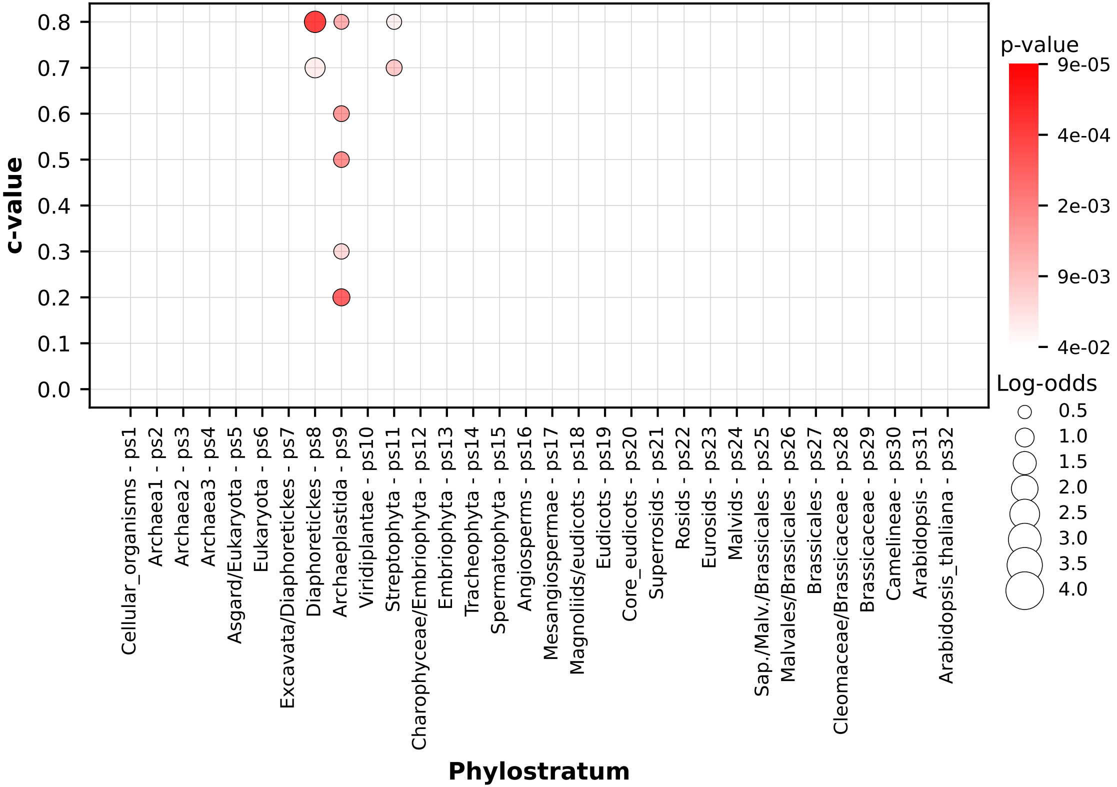

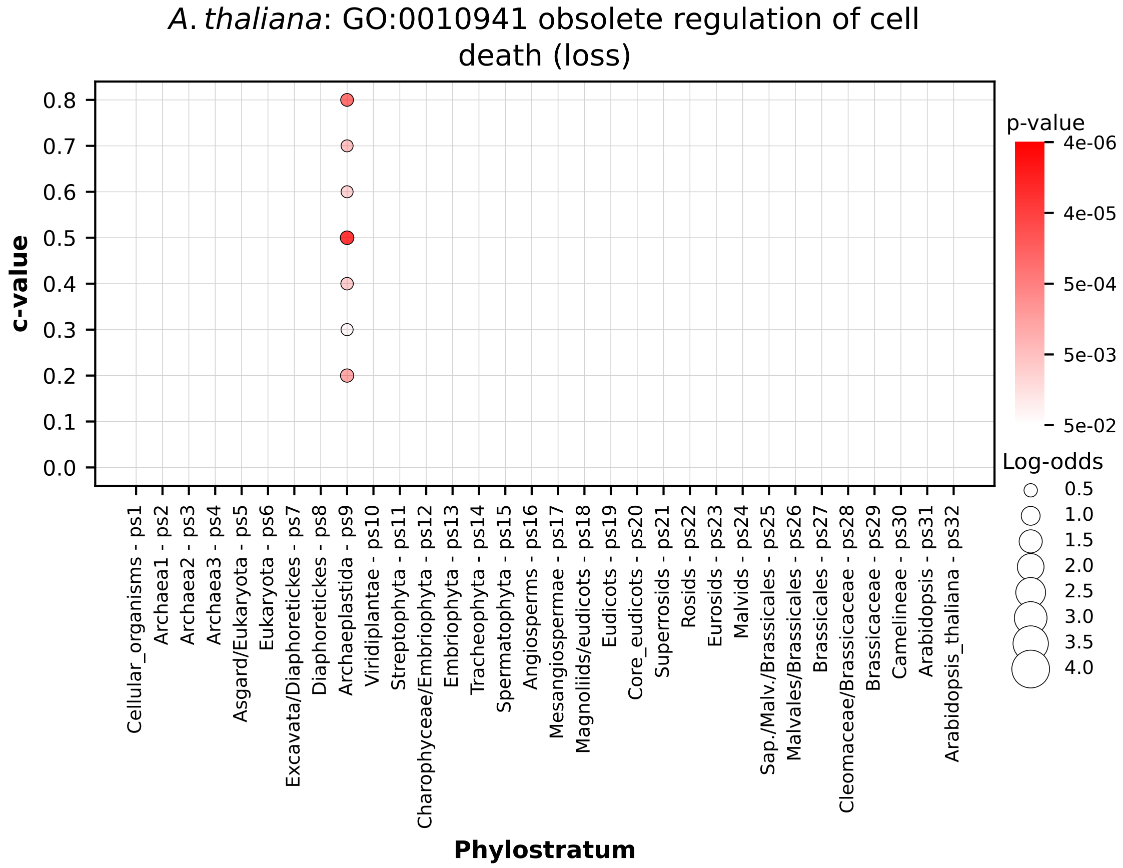

A. thaliana: GO:0010941 obsolete regulation of cell death (loss)
c-value
0.0
0.1
0.2
0.3
0.4
0.5
0.6
0.7
0.8
Phylostratum
Cellular_organisms - ps1
Archaea1 - ps2
Archaea2 - ps3
Archaea3 - ps4
Asgard/Eukaryota - ps5
Eukaryota - ps6
Excavata/Diaphoretickes - ps7
Diaphoretickes - ps8
Archaeplastida - ps9
Viridiplantae - ps10
Streptophyta - ps11
Charophyceae/Embriophyta - ps12
Embriophyta - ps13
Tracheophyta - ps14
Spermatophyta - ps15
Angiosperms - ps16
Mesangiospermae - ps17
Magnoliids/eudicots - ps18
Eudicots - ps19
Core_eudicots - ps20
Superrosids - ps21
Rosids - ps22
Eurosids - ps23
Malvids - ps24
Sap./Malv./Brassicales - ps25
Malvales/Brassicales - ps26
Brassicales - ps27
Cleomaceae/Brassicaceae - ps28
Brassicaceae - ps29
Camelineae - ps30
Arabidopsis - ps31
Arabidopsis_thaliana - ps32
p-value
4e-06
4e-05
5e-04
5e-03
5e-02
Log-odds
0.5
1.0
1.5
2.0
2.5
3.0
3.5
4.0

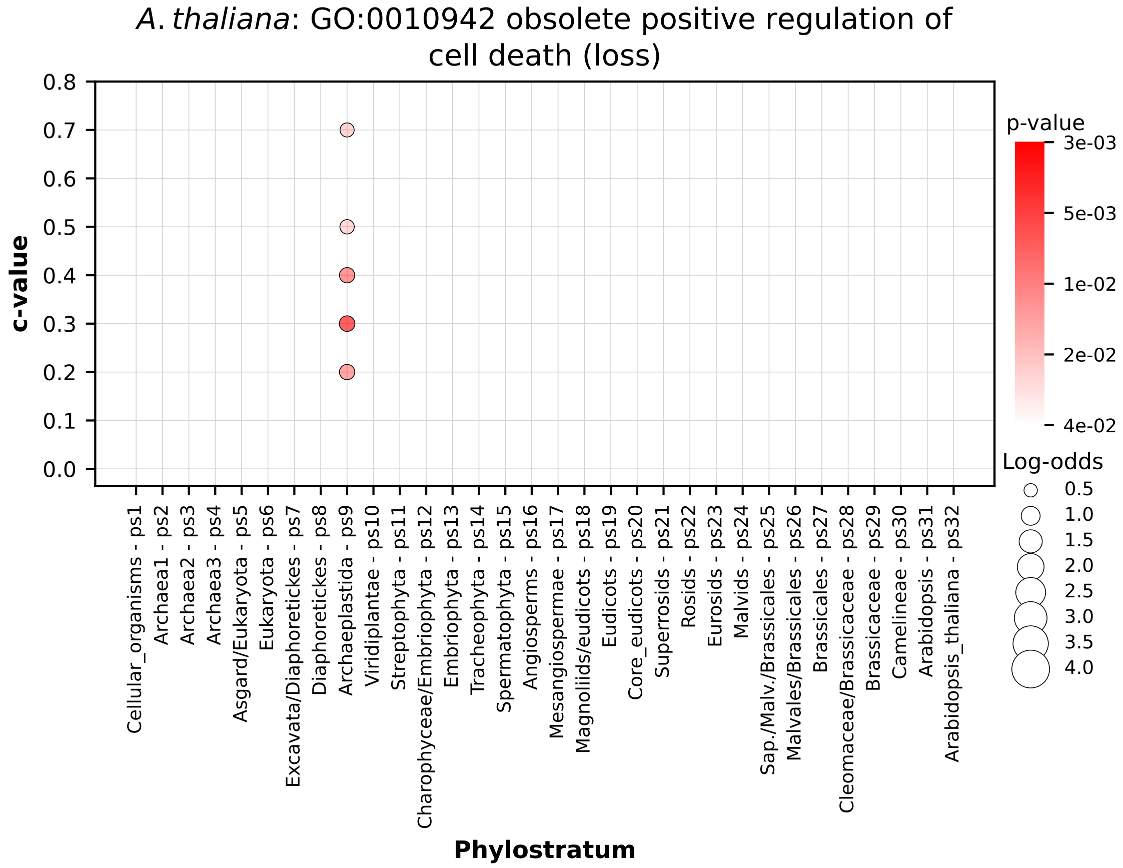
A. thaliana: GO:0010942 obsolete positive regulation of cell death (loss)
c-value
0.8
0.7
0.6
0.5
0.4
0.3
0.2
0.1
0.0
Phylostratum
Cellular_organisms - ps1
Archaea1 - ps2
Archaea2 - ps3
Archaea3 - ps4
Asgard/Eukaryota - ps5
Eukaryota - ps6
Excavata/Diaphoretickes - ps7
Diaphoretickes - ps8
Archaeplastida - ps9
Viridiplantae - ps10
Streptophyta - ps11
Charophyceae/Embriophyta - ps12
Embriophyta - ps13
Tracheophyta - ps14
Spermatophyta - ps15
Angiosperms - ps16
Mesangiospermae - ps17
Magnoliids/eudicots - ps18
Eudicots - ps19
Core_eudicots - ps20
Superrosids - ps21
Rosids - ps22
Eurosids - ps23
Malvids - ps24
Sap./Malv./Brassicales - ps25
Malvales/Brassicales - ps26
Brassicales - ps27
Cleomaceae/Brassicaceae - ps28
Brassicaceae - ps29
Camelineae - ps30
Arabidopsis - ps31
Arabidopsis_thaliana - ps32
p-value
3e-03
5e-03
1e-02
2e-02
4e-02
Log-odds
0.5
1.0
1.5
2.0
2.5
3.0
3.5
4.0

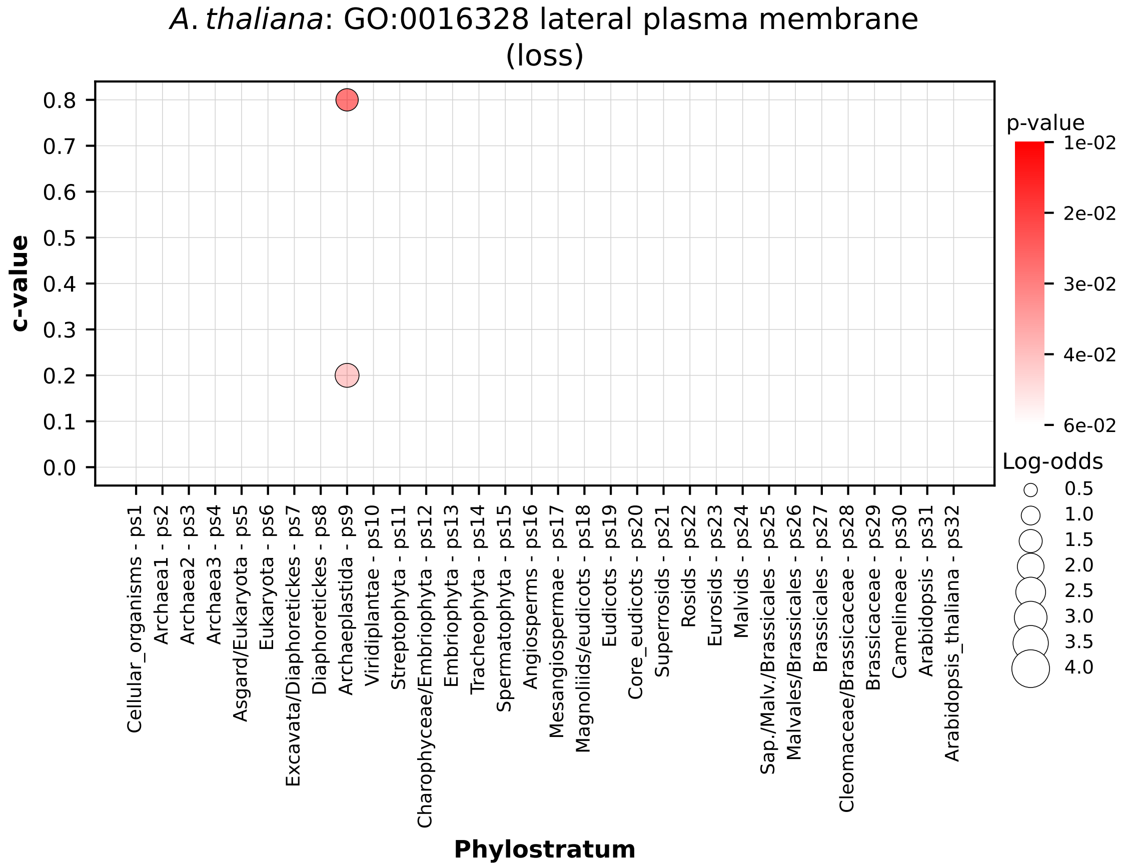
A. thaliana: GO:0016328 lateral plasma membrane (loss)
c-value
0.0
0.1
0.2
0.3
0.4
0.5
0.6
0.7
0.8
Phylostratum
Cellular_organisms - ps1
Archaea1 - ps2
Archaea2 - ps3
Archaea3 - ps4
Asgard/Eukaryota - ps5
Eukaryota - ps6
Excavata/Diaphoretickes - ps7
Diaphoretickes - ps8
Archaeplastida - ps9
Viridiplantae - ps10
Streptophyta - ps11
Charophyceae/Embriophyta - ps12
Embriophyta - ps13
Tracheophyta - ps14
Spermatophyta - ps15
Angiosperms - ps16
Mesangiospermae - ps17
Magnoliids/eudicots - ps18
Eudicots - ps19
Core_eudicots - ps20
Superrosids - ps21
Rosids - ps22
Eurosids - ps23
Malvids - ps24
Sap./Malv./Brassicales - ps25
Malvales/Brassicales - ps26
Brassicales - ps27
Cleomaceae/Brassicaceae - ps28
Brassicaceae - ps29
Camelineae - ps30
Arabidopsis - ps31
Arabidopsis_thaliana - ps32
p-value
1e-02
2e-02
3e-02
4e-02
6e-02
Log-odds
0.5
1.0
1.5
2.0
2.5
3.0
3.5
4.0

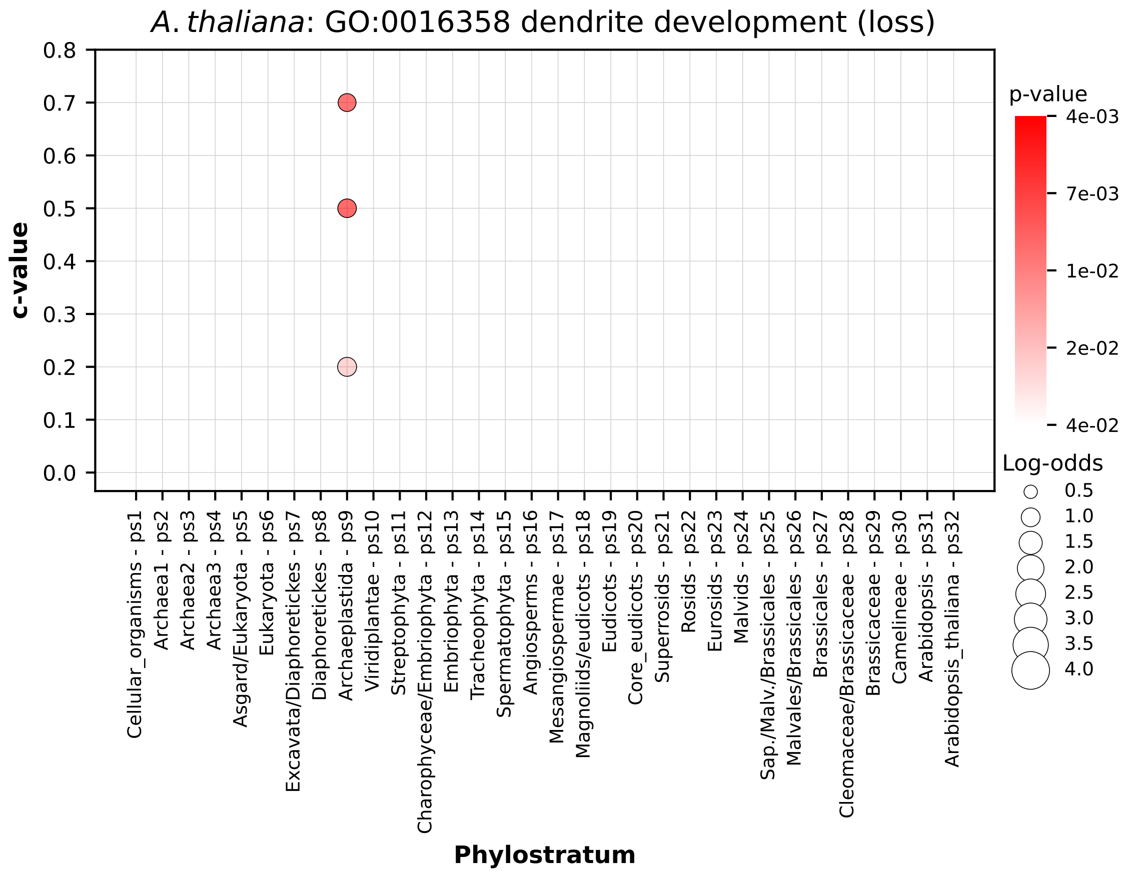
A. thaliana: GO:0016358 dendrite development (loss)
c-value
0.8
0.7
0.6
0.5
0.4
0.3
0.2
0.1
0.0
Phylostratum
Cellular_organisms - ps1
Archaea1 - ps2
Archaea2 - ps3
Archaea3 - ps4
Asgard/Eukaryota - ps5
Eukaryota - ps6
Excavata/Diaphoretickes - ps7
Diaphoretickes - ps8
Archaeplastida - ps9
Viridiplantae - ps10
Streptophyta - ps11
Charophyceae/Embriophyta - ps12
Embriophyta - ps13
Tracheophyta - ps14
Spermatophyta - ps15
Angiosperms - ps16
Mesangiospermae - ps17
Magnoliids/eudicots - ps18
Eudicots - ps19
Core_eudicots - ps20
Superrosids - ps21
Rosids - ps22
Eurosids - ps23
Malvids - ps24
Sap./Malv./Brassicales - ps25
Malvales/Brassicales - ps26
Brassicales - ps27
Cleomaceae/Brassicaceae - ps28
Brassicaceae - ps29
Camelineae - ps30
Arabidopsis - ps31
Arabidopsis_thaliana - ps32
p-value
4e-03
7e-03
1e-02
2e-02
4e-02
Log-odds
0.5
1.0
1.5
2.0
2.5
3.0
3.5
4.0

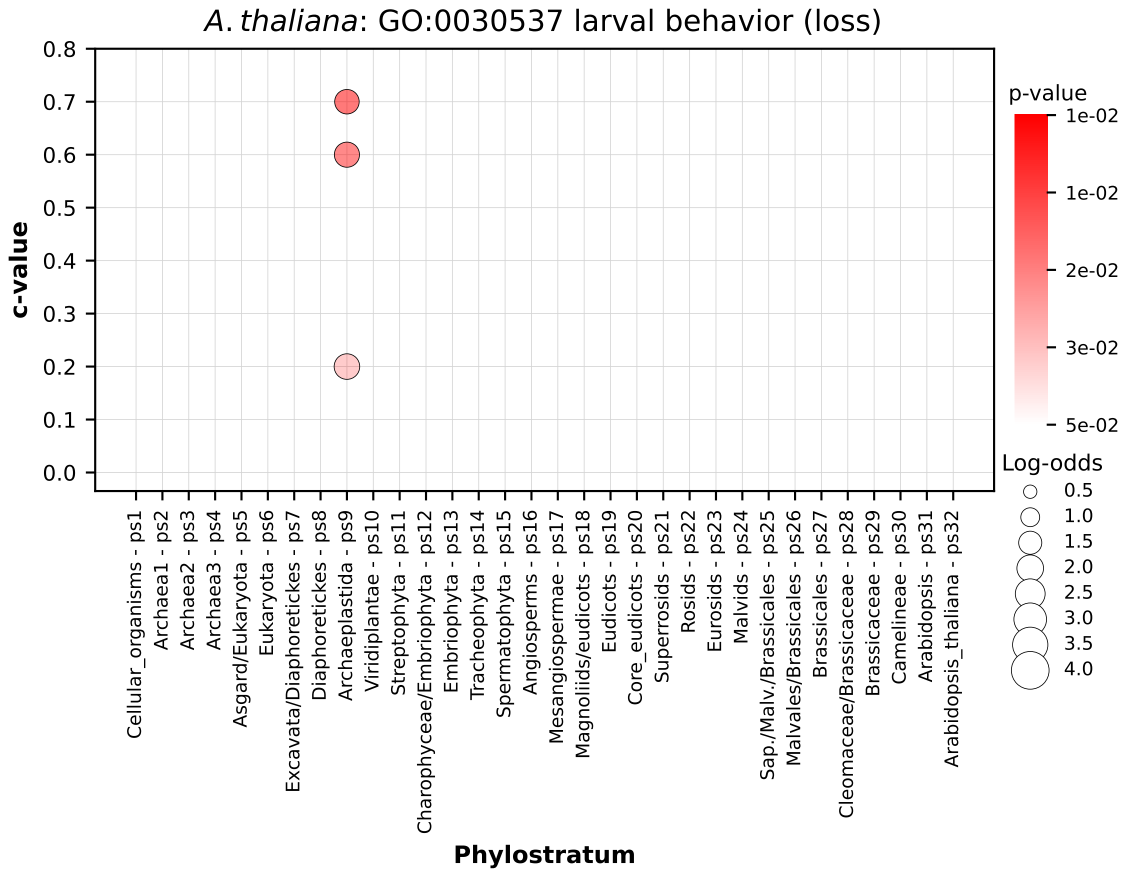

A. thaliana: GO:0030537 larval behavior (loss)
c-value
0.8
0.7
0.6
0.5
0.4
0.3
0.2
0.1
0.0
Phylostratum
Cellular_organisms - ps1
Archaea1 - ps2
Archaea2 - ps3
Archaea3 - ps4
Asgard/Eukaryota - ps5
Eukaryota - ps6
Excavata/Diaphoretickes - ps7
Diaphoretickes - ps8
Archaeplastida - ps9
Viridiplantae - ps10
Streptophyta - ps11
Charophyceae/Embriophyta - ps12
Embriophyta - ps13
Tracheophyta - ps14
Spermatophyta - ps15
Angiosperms - ps16
Mesangiospermae - ps17
Magnoliids/eudicots - ps18
Eudicots - ps19
Core_eudicots - ps20
Superrosids - ps21
Rosids - ps22
Eurosids - ps23
Malvids - ps24
Sap./Malv./Brassicales - ps25
Malvales/Brassicales - ps26
Brassicales - ps27
Cleomaceae/Brassicaceae - ps28
Brassicaceae - ps29
Camelineae - ps30
Arabidopsis - ps31
Arabidopsis_thaliana - ps32
p-value
1e-02
1e-02
2e-02
3e-02
5e-02
Log-odds
0.5
1.0
1.5
2.0
2.5
3.0
3.5
4.0

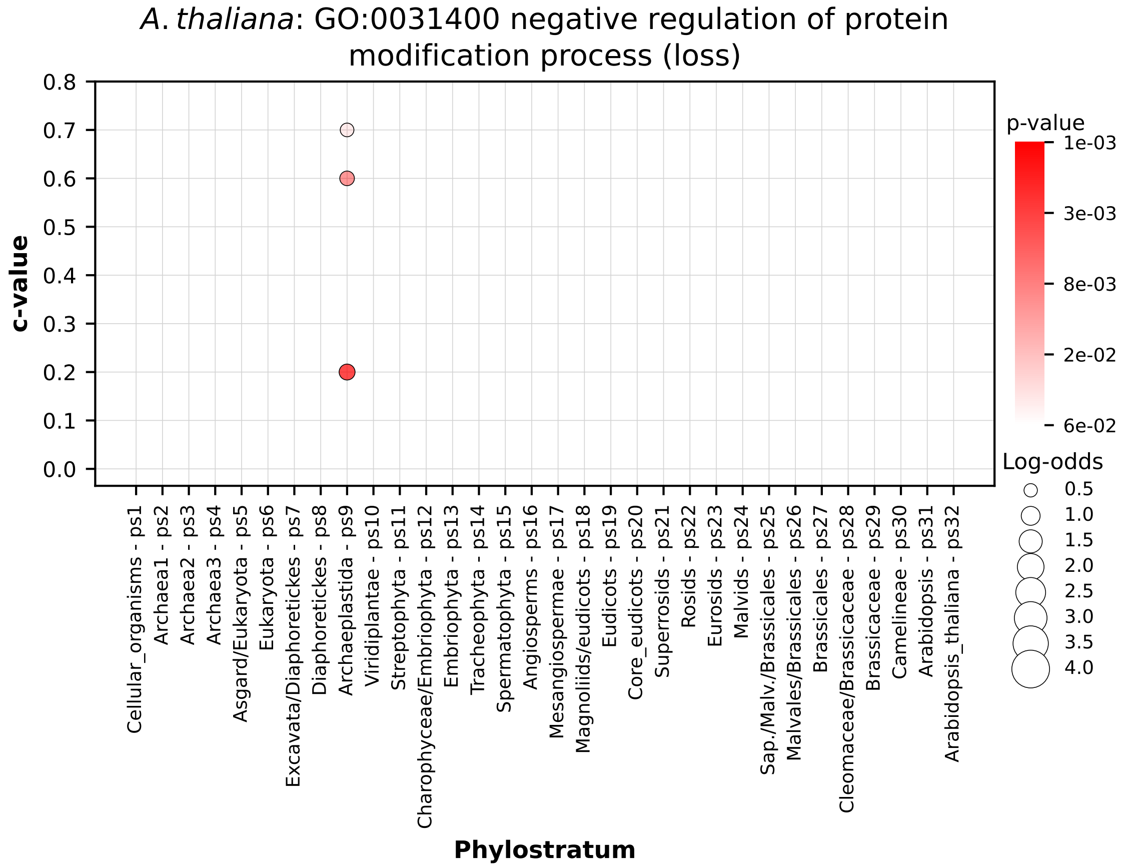
A. thaliana: GO:0031400 negative regulation of protein modification process (loss)
c-value
0.8
0.7
0.6
0.5
0.4
0.3
0.2
0.1
0.0
Phylostratum
Cellular_organisms - ps1
Archaea1 - ps2
Archaea2 - ps3
Archaea3 - ps4
Asgard/Eukaryota - ps5
Eukaryota - ps6
Excavata/Diaphoretickes - ps7
Diaphoretickes - ps8
Archaeplastida - ps9
Viridiplantae - ps10
Streptophyta - ps11
Charophyceae/Embriophyta - ps12
Embriophyta - ps13
Tracheophyta - ps14
Spermatophyta - ps15
Angiosperms - ps16
Mesangiospermae - ps17
Magnoliids/eudicots - ps18
Eudicots - ps19
Core_eudicots - ps20
Superrosids - ps21
Rosids - ps22
Eurosids - ps23
Malvids - ps24
Sap./Malv./Brassicales - ps25
Malvales/Brassicales - ps26
Brassicales - ps27
Cleomaceae/Brassicaceae - ps28
Brassicaceae - ps29
Camelineae - ps30
Arabidopsis - ps31
Arabidopsis_thaliana - ps32
p-value
1e-03
3e-03
8e-03
2e-02
6e-02
Log-odds
0.5
1.0
1.5
2.0
2.5
3.0
3.5
4.0

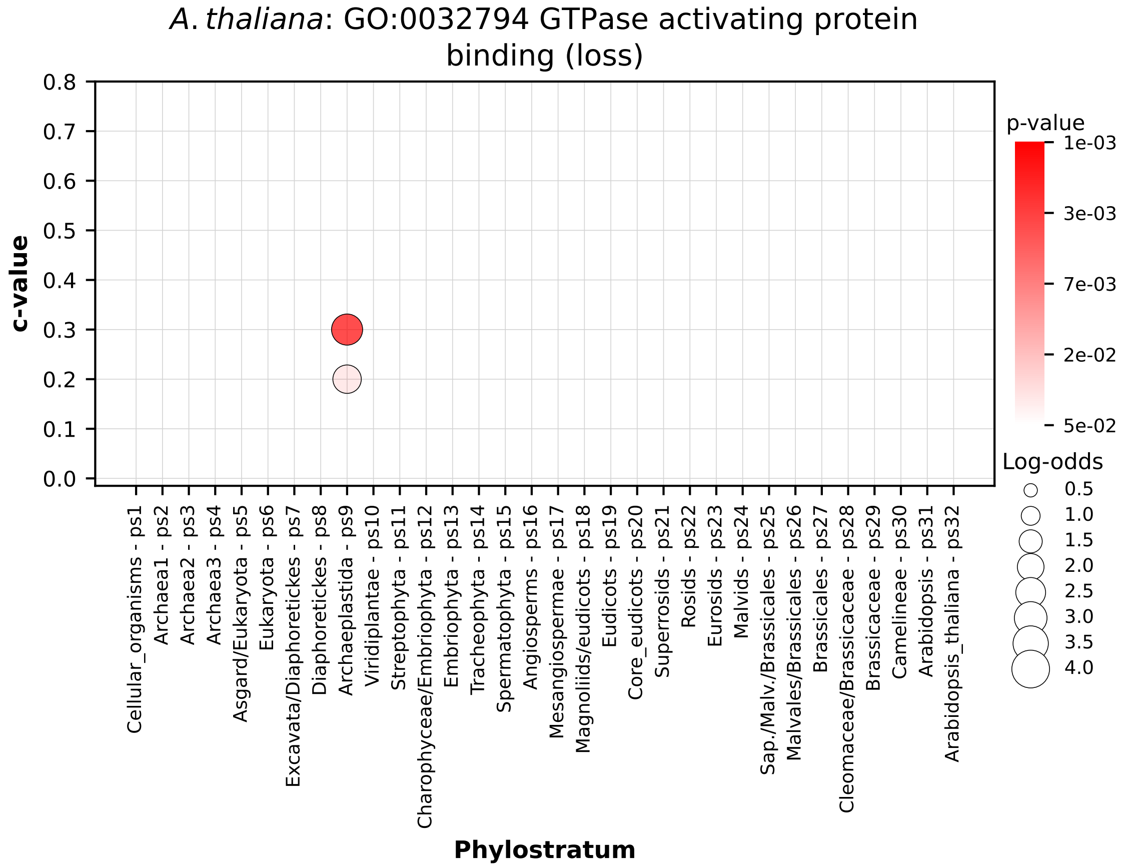

A. thaliana: GO:0032794 GTPase activating protein binding (loss)
c-value
0.8
0.7
0.6
0.5
0.4
0.3
0.2
0.1
0.0
p-value
1e-03
3e-03
7e-03
2e-02
5e-02
Log-odds
0.5
1.0
1.5
2.0
2.5
3.0
3.5
4.0
Cellular_organisms - ps1
Archaea1 - ps2
Archaea2 - ps3
Archaea3 - ps4
Asgard/Eukaryota - ps5
Eukaryota - ps6
Excavata/Diaphoretickes - ps7
Diaphoretickes - ps8
Archaeplastida - ps9
Viridiplantae - ps10
Streptophyta - ps11
Charophyceae/Embriophyta - ps12
Embriophyta - ps13
Tracheophyta - ps14
Spermatophyta - ps15
Angiosperms - ps16
Mesangiospermae - ps17
Magnoliids/eudicots - ps18
Eudicots - ps19
Core_eudicots - ps20
Superrosids - ps21
Rosids - ps22
Eurosids - ps23
Malvids - ps24
Sap./Malv./Brassicales - ps25
Malvales/Brassicales - ps26
Brassicales - ps27
Cleomaceae/Brassicaceae - ps28
Brassicaceae - ps29
Camelineae - ps30
Arabidopsis - ps31
Arabidopsis_thaliana - ps32
Phylostratum

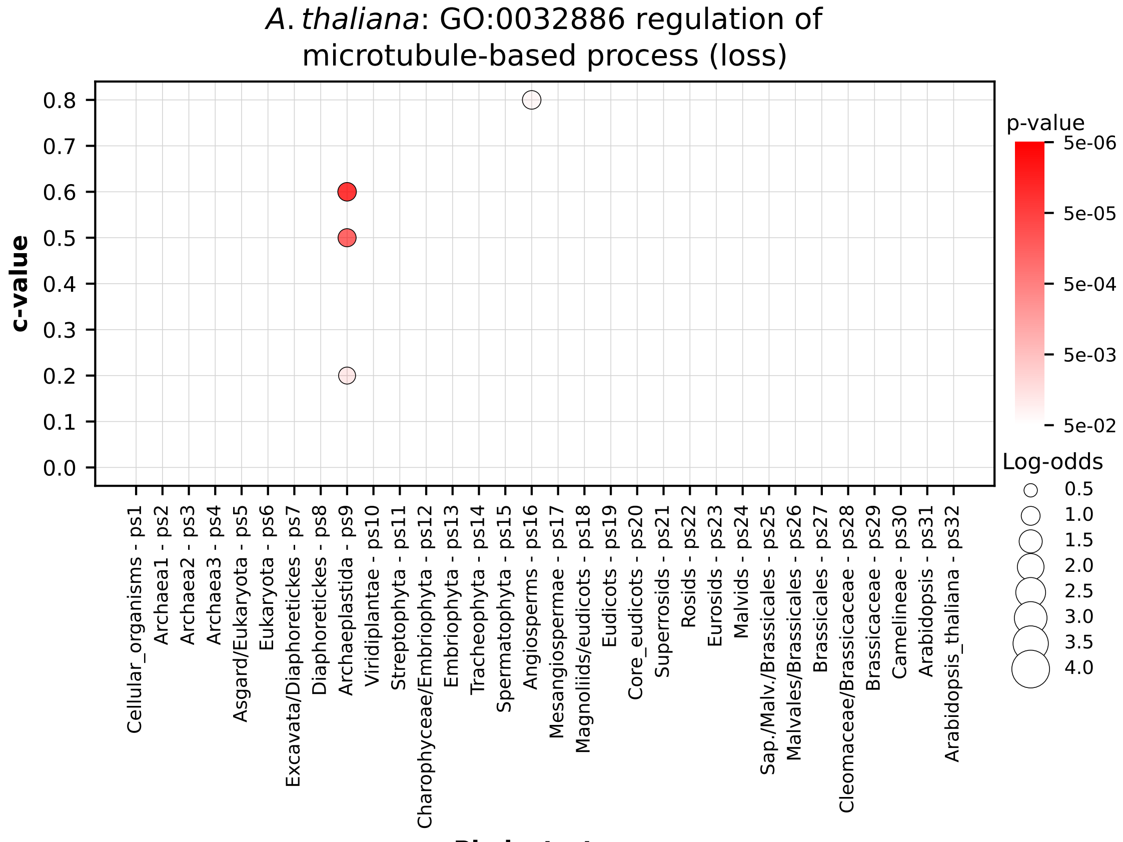

A. thaliana: GO:0032886 regulation of microtubule-based process (loss)
c-value
0.8
0.7
0.6
0.5
0.4
0.3
0.2
0.1
0.0
p-value
5e-06
5e-05
5e-04
5e-03
5e-02
Log-odds
0.5
1.0
1.5
2.0
2.5
3.0
3.5
4.0
Cellular_organisms - ps1
Archaea1 - ps2
Archaea2 - ps3
Archaea3 - ps4
Asgard/Eukaryota - ps5
Eukaryota - ps6
Excavata/Diaphoretickes - ps7
Diaphoretickes - ps8
Archaeplastida - ps9
Viridiplantae - ps10
Streptophyta - ps11
Charophyceae/Embriophyta - ps12
Embriophyta - ps13
Tracheophyta - ps14
Spermatophyta - ps15
Angiosperms - ps16
Mesangiospermae - ps17
Magnoliids/eudicots - ps18
Eudicots - ps19
Core_eudicots - ps20
Superrosids - ps21
Rosids - ps22
Eurosids - ps23
Malvids - ps24
Sap./Malv./Brassicales - ps25
Malvales/Brassicales - ps26
Brassicales - ps27
Cleomaceae/Brassicaceae - ps28
Brassicaceae - ps29
Camelineae - ps30
Arabidopsis - ps31
Arabidopsis_thaliana - ps32
Phylostratum

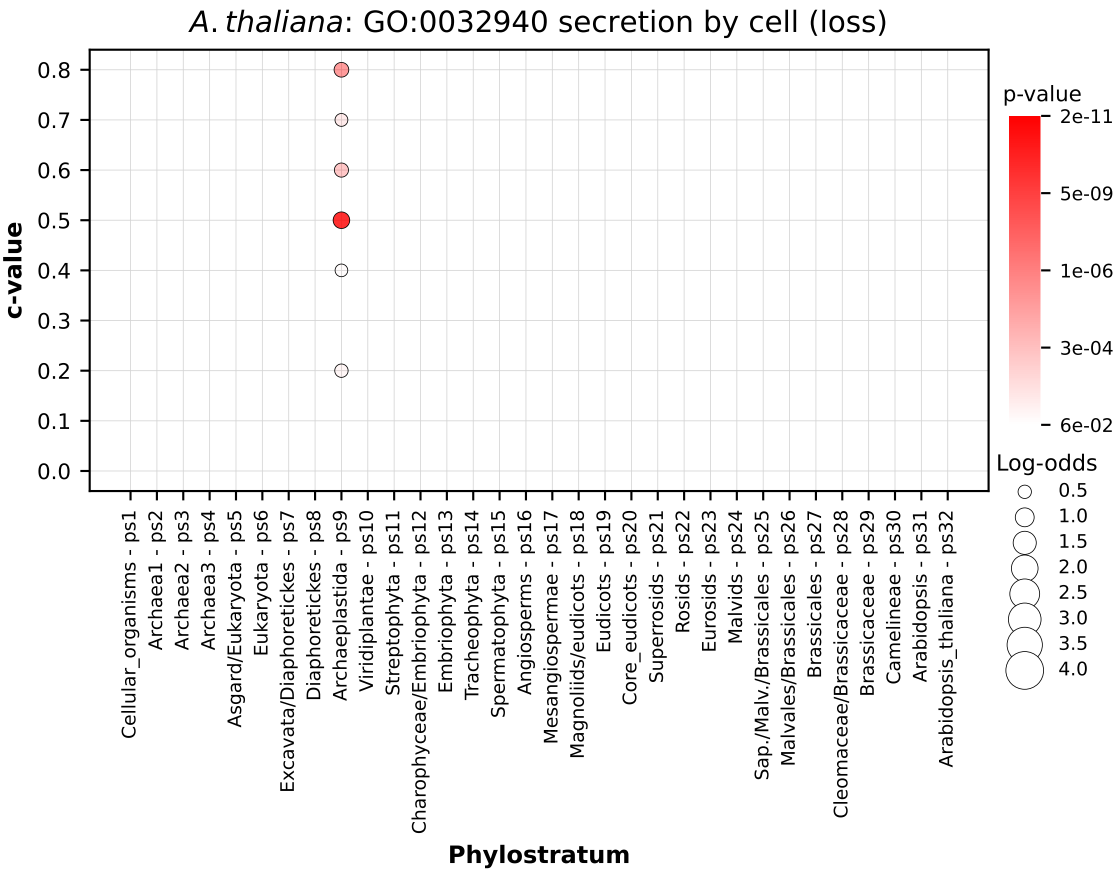

A. thaliana: GO:0032940 secretion by cell (loss)
c-value
0.0
0.1
0.2
0.3
0.4
0.5
0.6
0.7
0.8
p-value
2e-11
5e-09
1e-06
3e-04
6e-02
Log-odds
0.5
1.0
1.5
2.0
2.5
3.0
3.5
4.0
Cellular_organisms - ps1
Archaea1 - ps2
Archaea2 - ps3
Archaea3 - ps4
Asgard/Eukaryota - ps5
Eukaryota - ps6
Excavata/Diaphoretickes - ps7
Diaphoretickes - ps8
Archaeplastida - ps9
Viridiplantae - ps10
Streptophyta - ps11
Charophyceae/Embriophyta - ps12
Embriophyta - ps13
Tracheophyta - ps14
Spermatophyta - ps15
Angiosperms - ps16
Mesangiospermae - ps17
Magnoliids/eudicots - ps18
Eudicots - ps19
Core_eudicots - ps20
Superrosids - ps21
Rosids - ps22
Eurosids - ps23
Malvids - ps24
Sap./Malv./Brassicales - ps25
Malvales/Brassicales - ps26
Brassicales - ps27
Cleomaceae/Brassicaceae - ps28
Brassicaceae - ps29
Camelineae - ps30
Arabidopsis - ps31
Arabidopsis_thaliana - ps32
Phylostratum

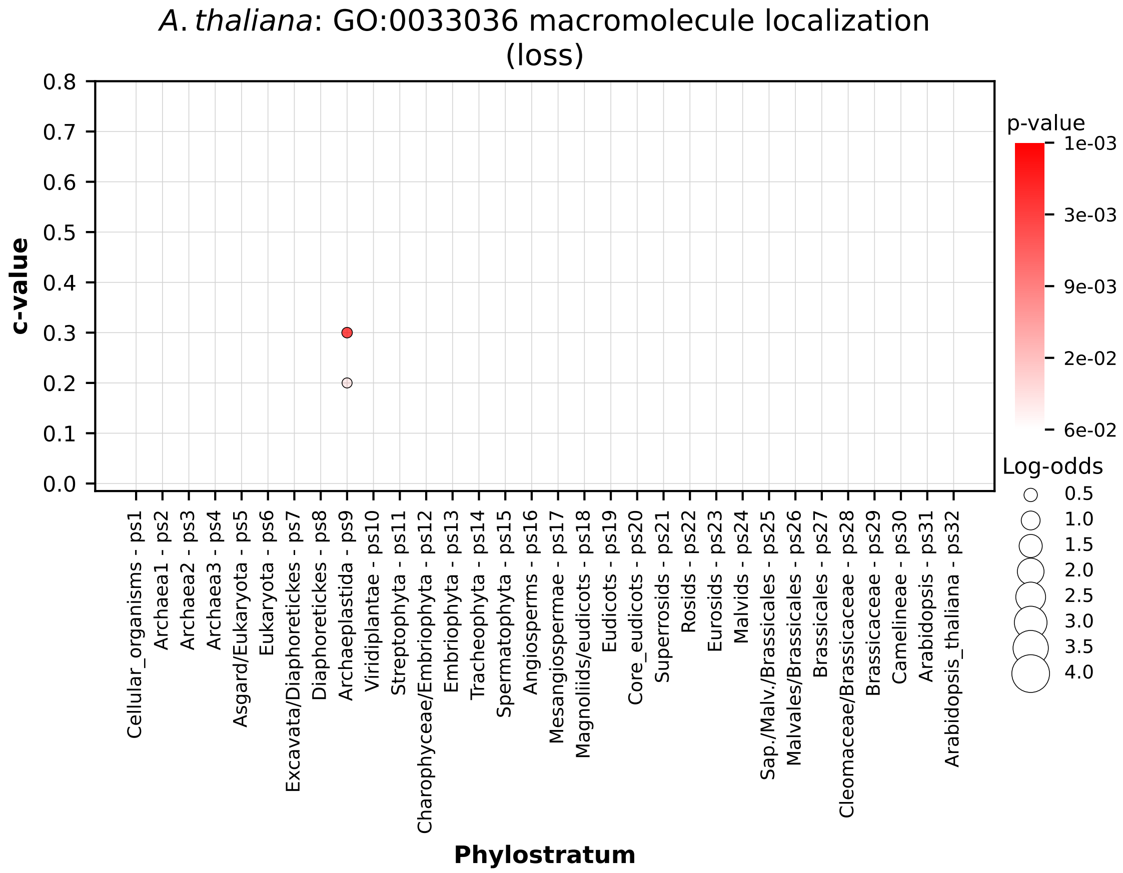

A. thaliana: GO:0033036 macromolecule localization (loss)
c-value
0.8
0.7
0.6
0.5
0.4
0.3
0.2
0.1
0.0
p-value
1e-03
3e-03
9e-03
2e-02
6e-02
Log-odds
0.5
1.0
1.5
2.0
2.5
3.0
3.5
4.0
Cellular_organisms - ps1
Archaea1 - ps2
Archaea2 - ps3
Archaea3 - ps4
Asgard/Eukaryota - ps5
Eukaryota - ps6
Excavata/Diaphoretickes - ps7
Diaphoretickes - ps8
Archaeplastida - ps9
Viridiplantae - ps10
Streptophyta - ps11
Charophyceae/Embriophyta - ps12
Embriophyta - ps13
Tracheophyta - ps14
Spermatophyta - ps15
Angiosperms - ps16
Mesangiospermae - ps17
Magnoliids/eudicots - ps18
Eudicots - ps19
Core_eudicots - ps20
Superrosids - ps21
Rosids - ps22
Eurosids - ps23
Malvids - ps24
Sap./Malv./Brassicales - ps25
Malvales/Brassicales - ps26
Brassicales - ps27
Cleomaceae/Brassicaceae - ps28
Brassicaceae - ps29
Camelineae - ps30
Arabidopsis - ps31
Arabidopsis_thaliana - ps32
Phylostratum

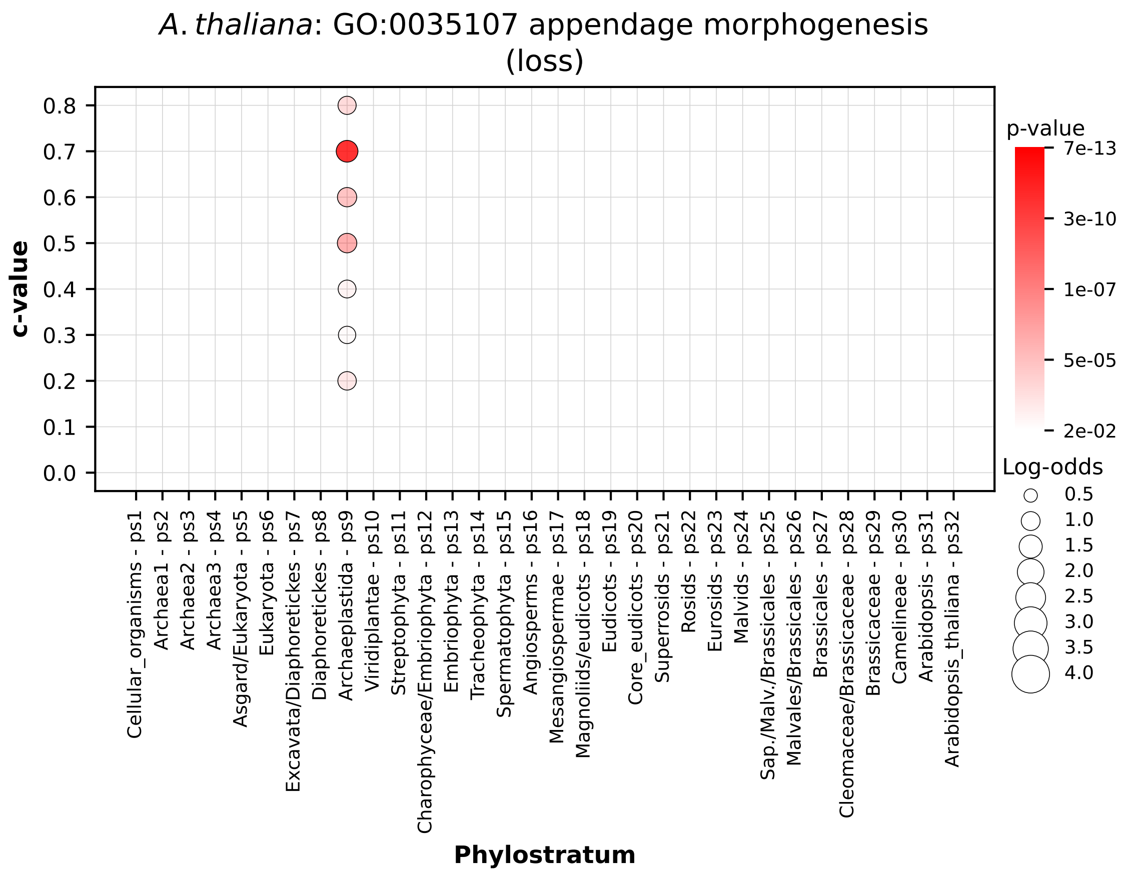
A. thaliana: GO:0035107 appendage morphogenesis
(loss)
c-value
0.8
0.7
0.6
0.5
0.4
0.3
0.2
0.1
0.0
p-value
7e-13
3e-10
1e-07
5e-05
2e-02
Log-odds
0.5
1.0
1.5
2.0
2.5
3.0
3.5
4.0
Cellular_organisms - ps1
Archaea1 - ps2
Archaea2 - ps3
Archaea3 - ps4
Asgard/Eukaryota - ps5
Eukaryota - ps6
Excavata/Diaphoretickes - ps7
Diaphoretickes - ps8
Archaeplastida - ps9
Viridiplantae - ps10
Streptophyta - ps11
Charophyceae/Embriophyta - ps12
Embriophyta - ps13
Tracheophyta - ps14
Spermatophyta - ps15
Angiosperms - ps16
Mesangiospermae - ps17
Magnoliids/eudicots - ps18
Eudicots - ps19
Core_eudicots - ps20
Superrosids - ps21
Rosids - ps22
Eurosids - ps23
Malvids - ps24
Sap./Malv./Brassicales - ps25
Malvales/Brassicales - ps26
Brassicales - ps27
Cleomaceae/Brassicaceae - ps28
Brassicaceae - ps29
Camelineae - ps30
Arabidopsis - ps31
Arabidopsis_thaliana - ps32
Phylostratum

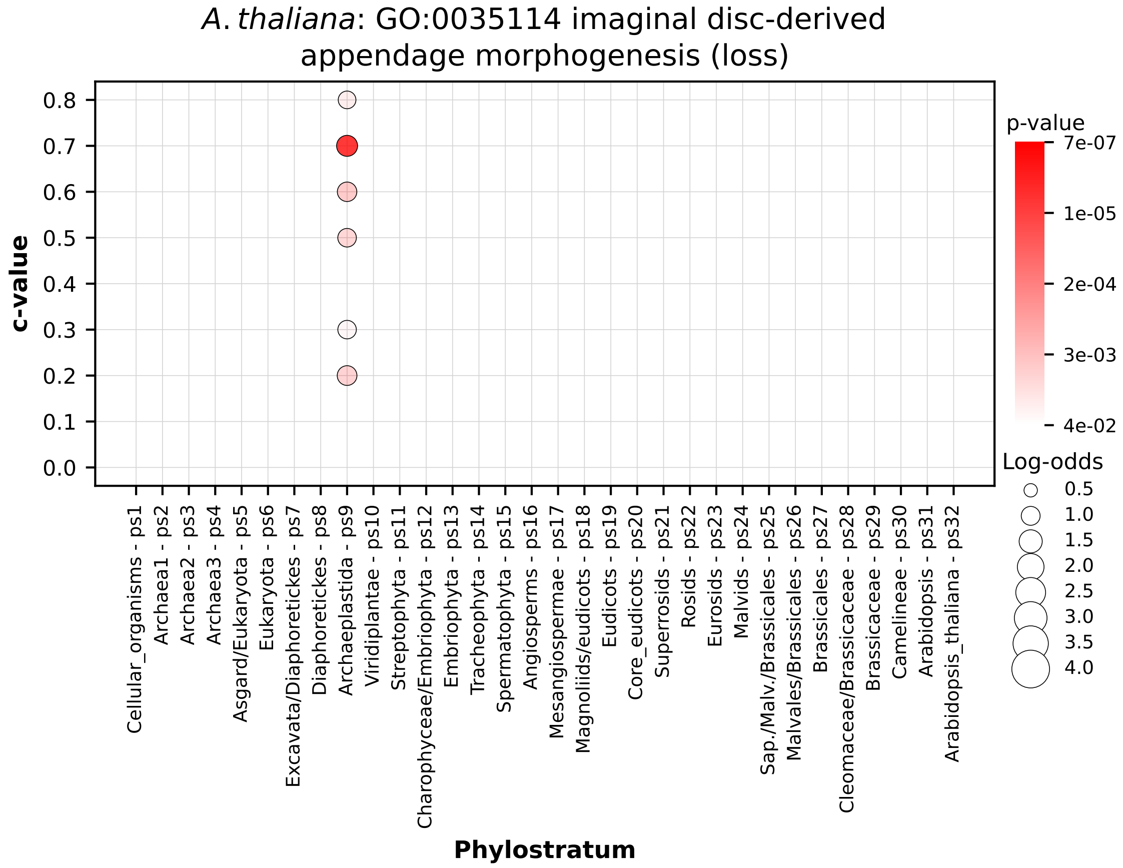
A. thaliana: GO:0035114 imaginal disc-derived appendage morphogenesis (loss)
c-value
0.8
0.7
0.6
0.5
0.4
0.3
0.2
0.1
0.0
Phylostratum
Cellular_organisms - ps1
Archaea1 - ps2
Archaea2 - ps3
Archaea3 - ps4
Asgard/Eukaryota - ps5
Eukaryota - ps6
Excavata/Diaphoretickes - ps7
Diaphoretickes - ps8
Archaeplastida - ps9
Viridiplantae - ps10
Streptophyta - ps11
Charophyceae/Embriophyta - ps12
Embriophyta - ps13
Tracheophyta - ps14
Spermatophyta - ps15
Angiosperms - ps16
Mesangiospermae - ps17
Magnoliids/eudicots - ps18
Eudicots - ps19
Core_eudicots - ps20
Superrosids - ps21
Rosids - ps22
Eurosids - ps23
Malvids - ps24
Sap./Malv./Brassicales - ps25
Malvales/Brassicales - ps26
Brassicales - ps27
Cleomaceae/Brassicaceae - ps28
Brassicaceae - ps29
Camelineae - ps30
Arabidopsis - ps31
Arabidopsis_thaliana - ps32
p-value
7e-07
1e-05
2e-04
3e-03
4e-02
Log-odds
0.5
1.0
1.5
2.0
2.5
3.0
3.5
4.0

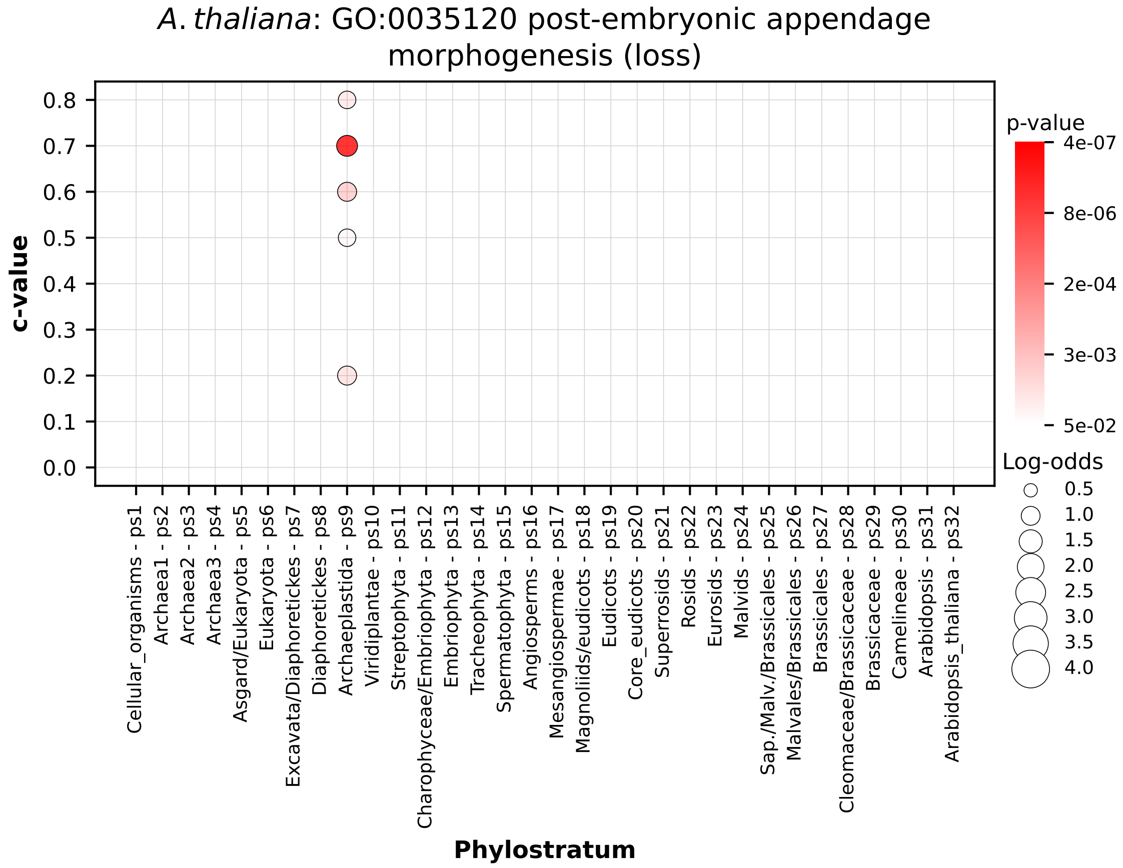

A. thaliana: GO:0035120 post-embryonic appendage morphogenesis (loss)
c-value
0.8
0.7
0.6
0.5
0.4
0.3
0.2
0.1
0.0
p-value
4e-07
8e-06
2e-04
3e-03
5e-02
Log-odds
0.5
1.0
1.5
2.0
2.5
3.0
3.5
4.0
Cellular_organisms - ps1
Archaea1 - ps2
Archaea2 - ps3
Archaea3 - ps4
Asgard/Eukaryota - ps5
Eukaryota - ps6
Excavata/Diaphoretickes - ps7
Diaphoretickes - ps8
Archaeplastida - ps9
Viridiplantae - ps10
Streptophyta - ps11
Charophyceae/Embriophyta - ps12
Embriophyta - ps13
Tracheophyta - ps14
Spermatophyta - ps15
Angiosperms - ps16
Mesangiospermae - ps17
Magnoliids/eudicots - ps18
Eudicots - ps19
Core_eudicots - ps20
Superrosids - ps21
Rosids - ps22
Eurosids - ps23
Malvids - ps24
Sap./Malv./Brassicales - ps25
Malvales/Brassicales - ps26
Brassicales - ps27
Cleomaceae/Brassicaceae - ps28
Brassicaceae - ps29
Camelineae - ps30
Arabidopsis - ps31
Arabidopsis_thaliana - ps32
Phylostratum

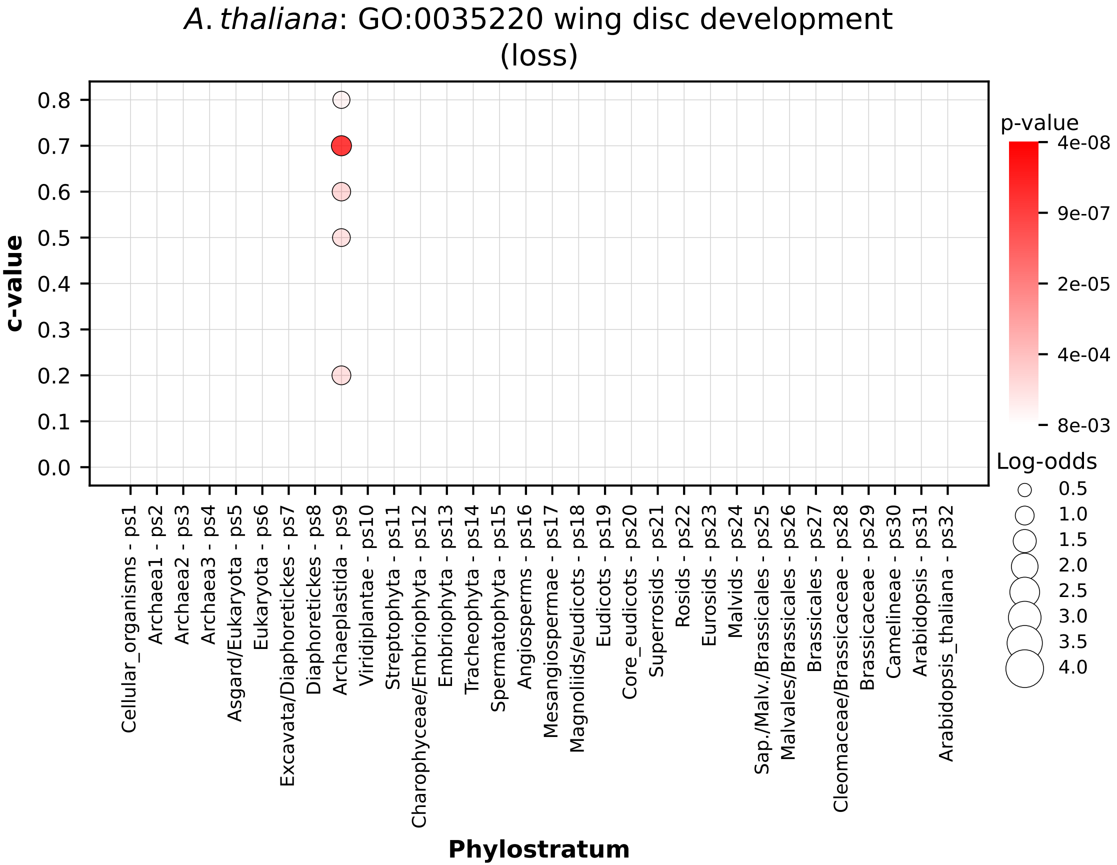

A. thaliana: GO:0035220 wing disc development
(loss)
c-value
0.8
0.7
0.6
0.5
0.4
0.3
0.2
0.1
0.0
Phylostratum
Cellular_organisms - ps1
Archaea1 - ps2
Archaea2 - ps3
Archaea3 - ps4
Asgard/Eukaryota - ps5
Eukaryota - ps6
Excavata/Diaphoretickes - ps7
Diaphoretickes - ps8
Archaeplastida - ps9
Viridiplantae - ps10
Streptophyta - ps11
Charophyceae/Embriophyta - ps12
Embriophyta - ps13
Tracheophyta - ps14
Spermatophyta - ps15
Angiosperms - ps16
Mesangiospermae - ps17
Magnoliids/eudicots - ps18
Eudicots - ps19
Core_eudicots - ps20
Superrosids - ps21
Rosids - ps22
Eurosids - ps23
Malvids - ps24
Sap./Malv./Brassicales - ps25
Malvales/Brassicales - ps26
Brassicales - ps27
Cleomaceae/Brassicaceae - ps28
Brassicaceae - ps29
Camelineae - ps30
Arabidopsis - ps31
Arabidopsis_thaliana - ps32
p-value
4e-08
9e-07
2e-05
4e-04
8e-03
Log-odds
0.5
1.0
1.5
2.0
2.5
3.0
3.5
4.0

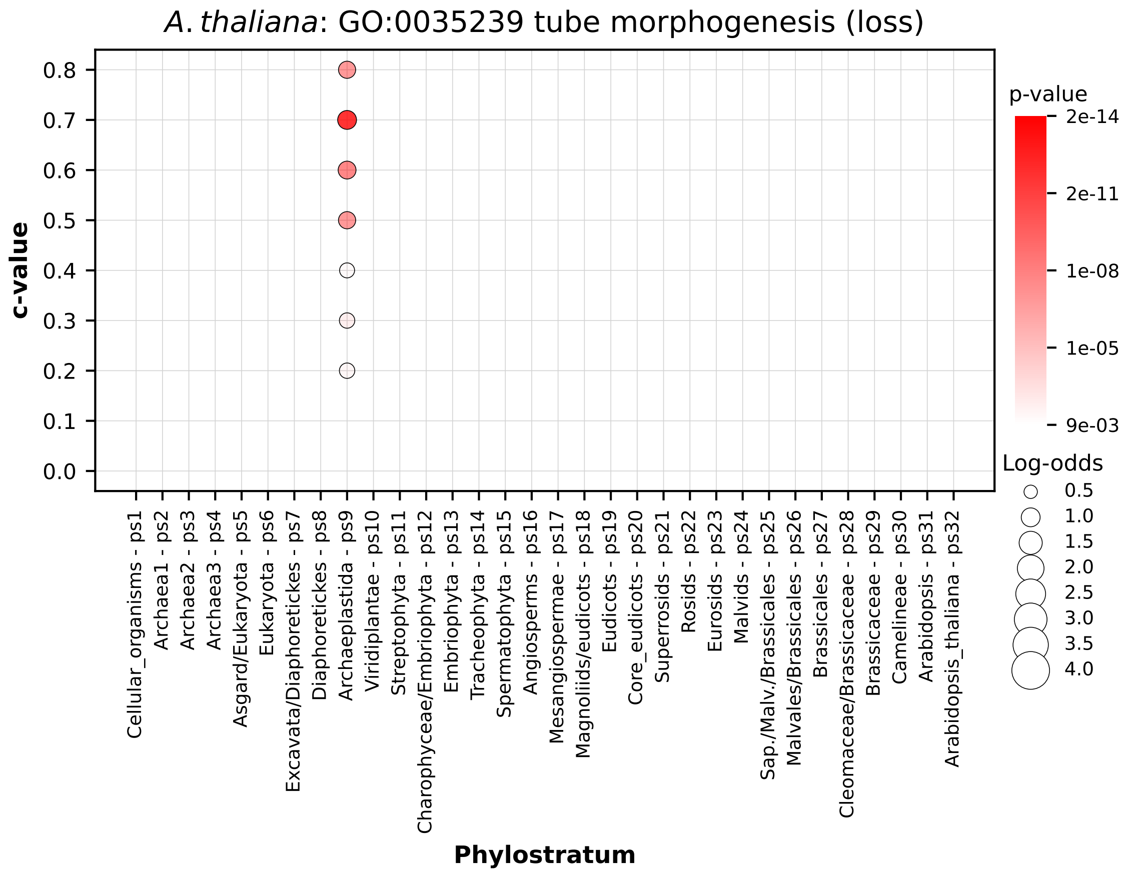
A. thaliana: GO:0035239 tube morphogenesis (loss)
c-value
0.8
0.7
0.6
0.5
0.4
0.3
0.2
0.1
0.0
Phylostratum
Cellular_organisms - ps1
Archaea1 - ps2
Archaea2 - ps3
Archaea3 - ps4
Asgard/Eukaryota - ps5
Eukaryota - ps6
Excavata/Diaphoretickes - ps7
Diaphoretickes - ps8
Archaeplastida - ps9
Viridiplantae - ps10
Streptophyta - ps11
Charophyceae/Embriophyta - ps12
Embriophyta - ps13
Tracheophyta - ps14
Spermatophyta - ps15
Angiosperms - ps16
Mesangiospermae - ps17
Magnoliids/eudicots - ps18
Eudicots - ps19
Core_eudicots - ps20
Superrosids - ps21
Rosids - ps22
Eurosids - ps23
Malvids - ps24
Sap./Malv./Brassicales - ps25
Malvales/Brassicales - ps26
Brassicales - ps27
Cleomaceae/Brassicaceae - ps28
Brassicaceae - ps29
Camelineae - ps30
Arabidopsis - ps31
Arabidopsis_thaliana - ps32
p-value
2e-14
2e-11
1e-08
1e-05
9e-03
Log-odds
0.5
1.0
1.5
2.0
2.5
3.0
3.5
4.0

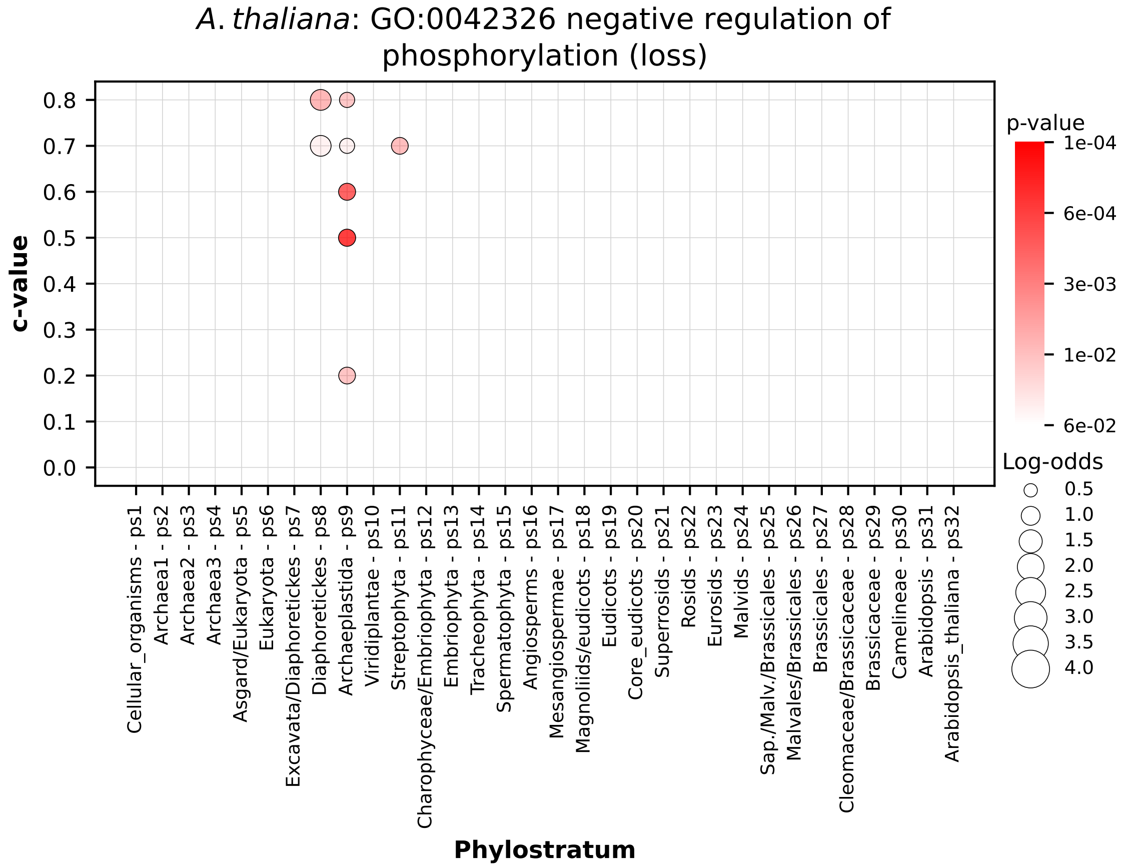

A. thaliana: GO:0042326 negative regulation of phosphorylation (loss)
c-value
0.0
0.1
0.2
0.3
0.4
0.5
0.6
0.7
0.8
p-value
1e-04
6e-04
3e-03
1e-02
6e-02
Log-odds
0.5
1.0
1.5
2.0
2.5
3.0
3.5
4.0
Cellular_organisms - ps1
Archaea1 - ps2
Archaea2 - ps3
Archaea3 - ps4
Asgard/Eukaryota - ps5
Eukaryota - ps6
Excavata/Diaphoretickes - ps7
Diaphoretickes - ps8
Archaeplastida - ps9
Viridiplantae - ps10
Streptophyta - ps11
Charophyceae/Embriophyta - ps12
Embriophyta - ps13
Tracheophyta - ps14
Spermatophyta - ps15
Angiosperms - ps16
Mesangiospermae - ps17
Magnoliids/eudicots - ps18
Eudicots - ps19
Core_eudicots - ps20
Superrosids - ps21
Rosids - ps22
Eurosids - ps23
Malvids - ps24
Sap./Malv./Brassicales - ps25
Malvales/Brassicales - ps26
Brassicales - ps27
Cleomaceae/Brassicaceae - ps28
Brassicaceae - ps29
Camelineae - ps30
Arabidopsis - ps31
Arabidopsis_thaliana - ps32
Phylostratum

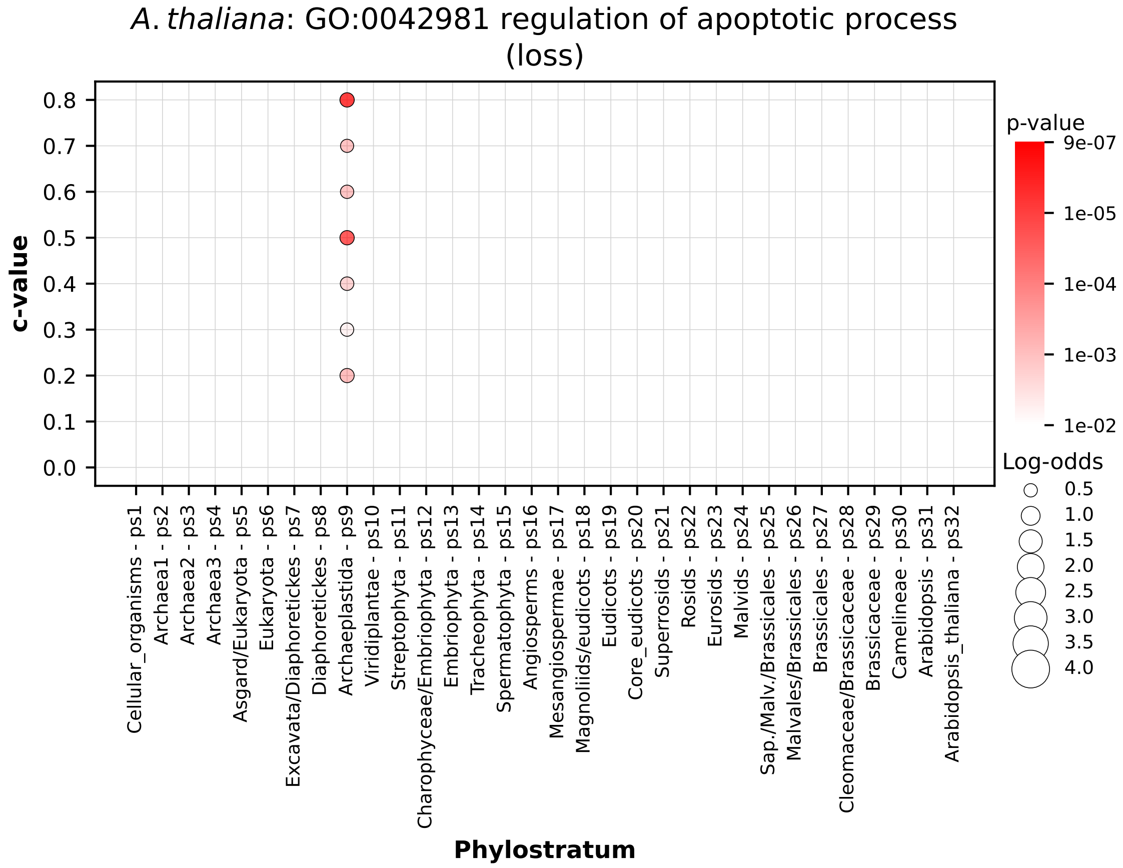

A. thaliana: GO:0042981 regulation of apoptotic process
(loss)
c-value
0.8
0.7
0.6
0.5
0.4
0.3
0.2
0.1
0.0
Phylostratum
Cellular_organisms - ps1
Archaea1 - ps2
Archaea2 - ps3
Archaea3 - ps4
Asgard/Eukaryota - ps5
Eukaryota - ps6
Excavata/Diaphoretickes - ps7
Diaphoretickes - ps8
Archaeplastida - ps9
Viridiplantae - ps10
Streptophyta - ps11
Charophyceae/Embriophyta - ps12
Embriophyta - ps13
Tracheophyta - ps14
Spermatophyta - ps15
Angiosperms - ps16
Mesangiospermae - ps17
Magnoliids/eudicots - ps18
Eudicots - ps19
Core_eudicots - ps20
Superrosids - ps21
Rosids - ps22
Eurosids - ps23
Malvids - ps24
Sap./Malv./Brassicales - ps25
Malvales/Brassicales - ps26
Brassicales - ps27
Cleomaceae/Brassicaceae - ps28
Brassicaceae - ps29
Camelineae - ps30
Arabidopsis - ps31
Arabidopsis_thaliana - ps32
p-value
9e-07
1e-05
1e-04
1e-03
1e-02
Log-odds
0.5
1.0
1.5
2.0
2.5
3.0
3.5
4.0

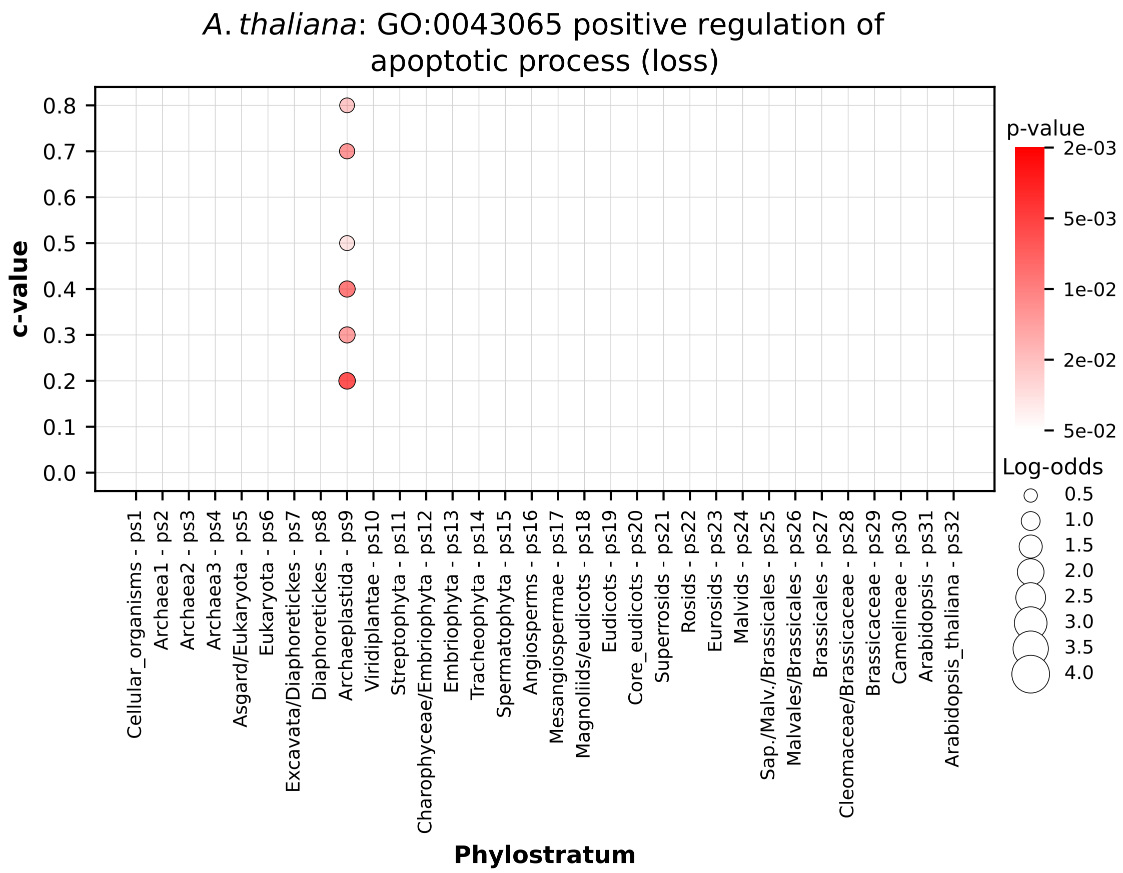

A. thaliana: GO:0043065 positive regulation of apoptotic process (loss)
c-value
0.8
0.7
0.6
0.5
0.4
0.3
0.2
0.1
0.0
Phylostratum
Cellular_organisms - ps1
Archaea1 - ps2
Archaea2 - ps3
Archaea3 - ps4
Asgard/Eukaryota - ps5
Eukaryota - ps6
Excavata/Diaphoretickes - ps7
Diaphoretickes - ps8
Archaeplastida - ps9
Viridiplantae - ps10
Streptophyta - ps11
Charophyceae/Embriophyta - ps12
Embriophyta - ps13
Tracheophyta - ps14
Spermatophyta - ps15
Angiosperms - ps16
Mesangiospermae - ps17
Magnoliids/eudicots - ps18
Eudicots - ps19
Core_eudicots - ps20
Superrosids - ps21
Rosids - ps22
Eurosids - ps23
Malvids - ps24
Sap./Malv./Brassicales - ps25
Malvales/Brassicales - ps26
Brassicales - ps27
Cleomaceae/Brassicaceae - ps28
Brassicaceae - ps29
Camelineae - ps30
Arabidopsis - ps31
Arabidopsis_thaliana - ps32
p-value
2e-03
5e-03
1e-02
2e-02
5e-02
Log-odds
0.5
1.0
1.5
2.0
2.5
3.0
3.5
4.0

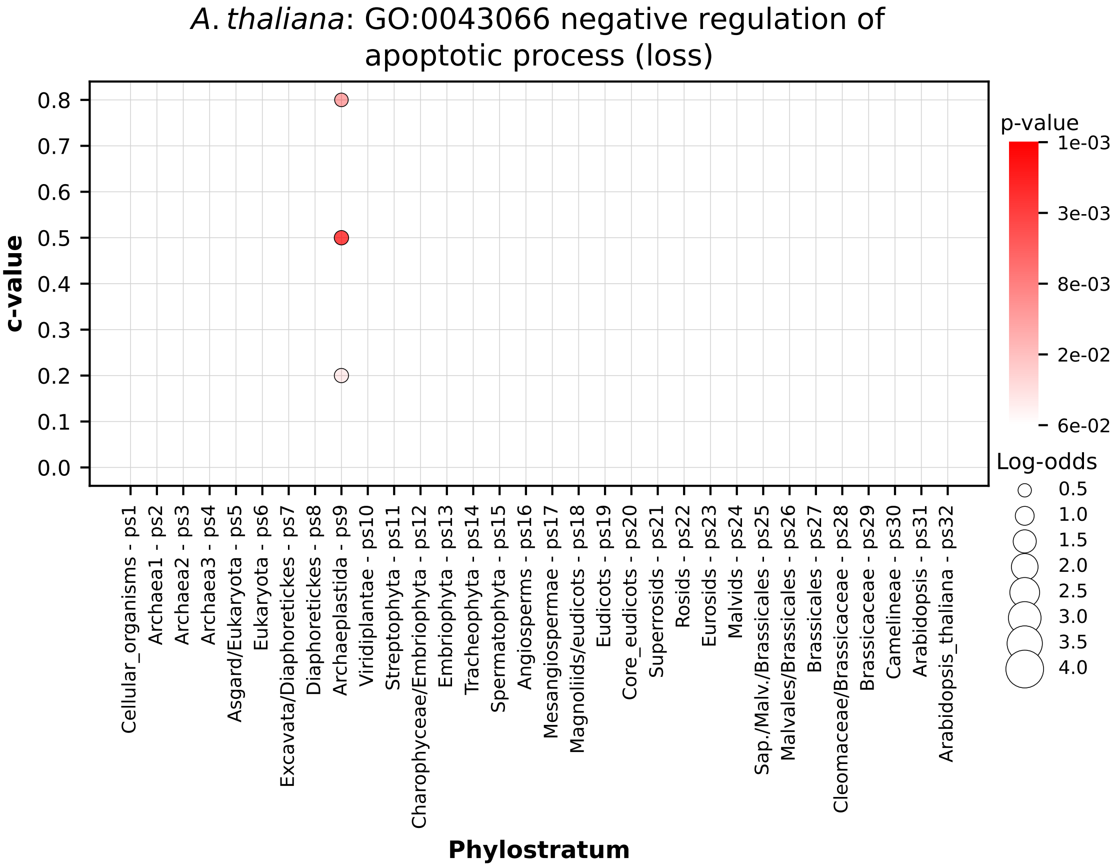
A. thaliana: GO:0043066 negative regulation of apoptotic process (loss)
c-value
0.0
0.1
0.2
0.3
0.4
0.5
0.6
0.7
0.8
p-value
1e-03
3e-03
8e-03
2e-02
6e-02
Log-odds
0.5
1.0
1.5
2.0
2.5
3.0
3.5
4.0
Cellular_organisms - ps1
Archaea1 - ps2
Archaea2 - ps3
Archaea3 - ps4
Asgard/Eukaryota - ps5
Eukaryota - ps6
Excavata/Diaphoretickes - ps7
Diaphoretickes - ps8
Archaeplastida - ps9
Viridiplantae - ps10
Streptophyta - ps11
Charophyceae/Embriophyta - ps12
Embriophyta - ps13
Tracheophyta - ps14
Spermatophyta - ps15
Angiosperms - ps16
Mesangiospermae - ps17
Magnoliids/eudicots - ps18
Eudicots - ps19
Core_eudicots - ps20
Superrosids - ps21
Rosids - ps22
Eurosids - ps23
Malvids - ps24
Sap./Malv./Brassicales - ps25
Malvales/Brassicales - ps26
Brassicales - ps27
Cleomaceae/Brassicaceae - ps28
Brassicaceae - ps29
Camelineae - ps30
Arabidopsis - ps31
Arabidopsis_thaliana - ps32
Phylostratum

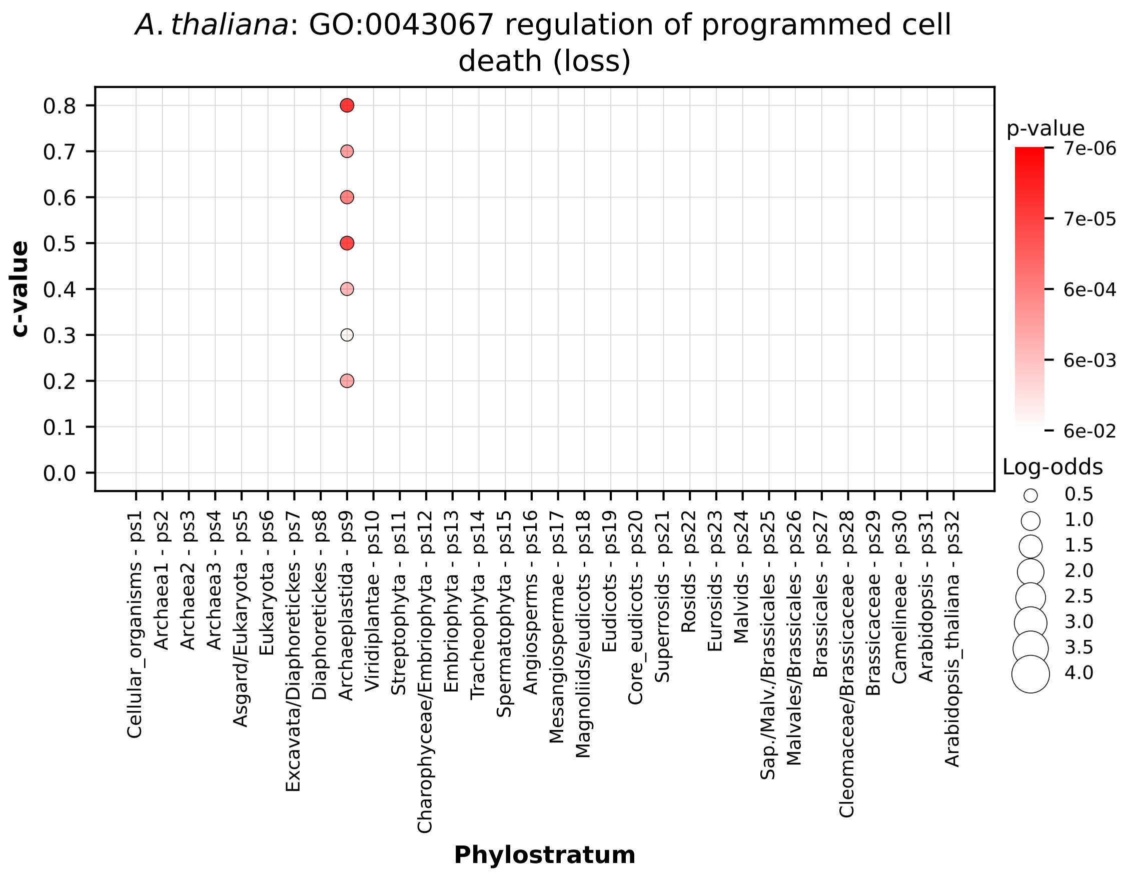

A. thaliana: GO:0043067 regulation of programmed cell death (loss)
c-value
0.0
0.1
0.2
0.3
0.4
0.5
0.6
0.7
0.8
p-value
7e-06
7e-05
6e-04
6e-03
6e-02
Log-odds
0.5
1.0
1.5
2.0
2.5
3.0
3.5
4.0
Cellular_organisms - ps1
Archaea1 - ps2
Archaea2 - ps3
Archaea3 - ps4
Asgard/Eukaryota - ps5
Eukaryota - ps6
Excavata/Diaphoretickes - ps7
Diaphoretickes - ps8
Archaeplastida - ps9
Viridiplantae - ps10
Streptophyta - ps11
Charophyceae/Embriophyta - ps12
Embriophyta - ps13
Tracheophyta - ps14
Spermatophyta - ps15
Angiosperms - ps16
Mesangiospermae - ps17
Magnoliids/eudicots - ps18
Eudicots - ps19
Core_eudicots - ps20
Superrosids - ps21
Rosids - ps22
Eurosids - ps23
Malvids - ps24
Sap./Malv./Brassicales - ps25
Malvales/Brassicales - ps26
Brassicales - ps27
Cleomaceae/Brassicaceae - ps28
Brassicaceae - ps29
Camelineae - ps30
Arabidopsis - ps31
Arabidopsis_thaliana - ps32
Phylostratum

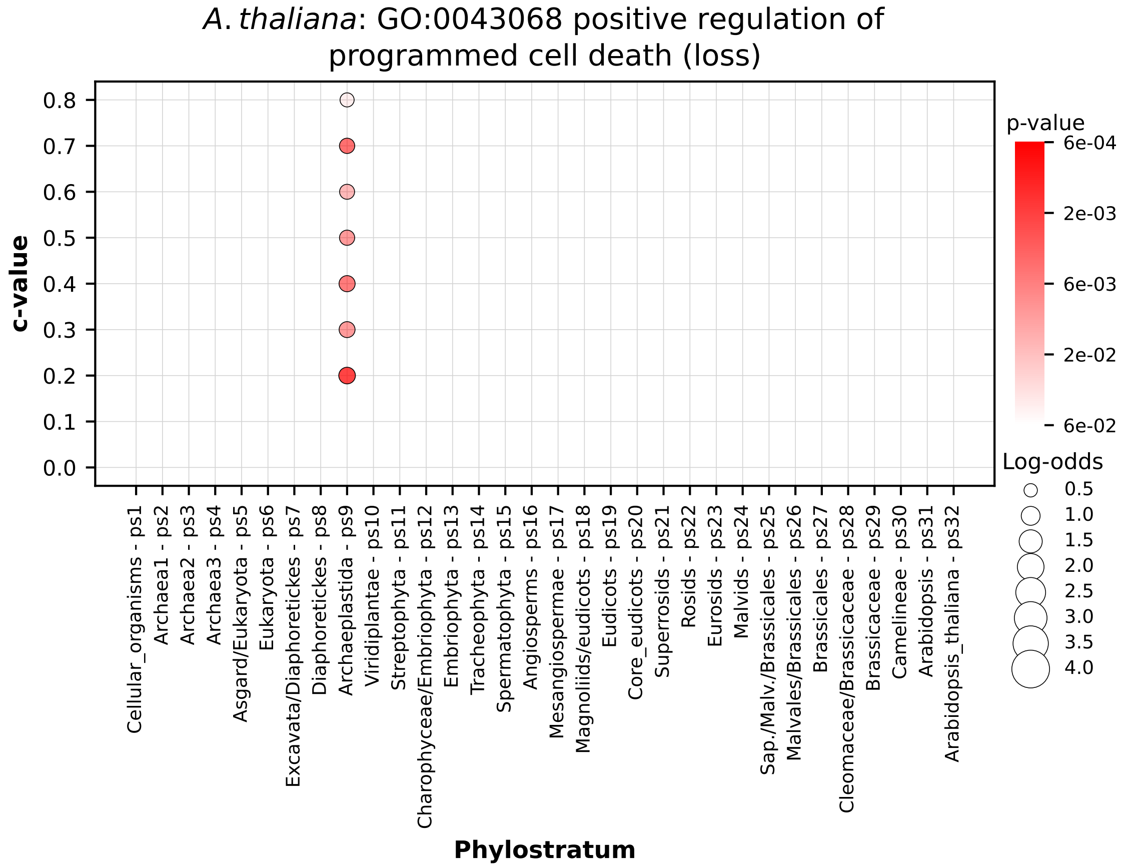

A. thaliana: GO:0043068 positive regulation of programmed cell death (loss)
c-value
0.8
0.7
0.6
0.5
0.4
0.3
0.2
0.1
0.0
p-value
6e-04
2e-03
6e-03
2e-02
6e-02
Log-odds
0.5
1.0
1.5
2.0
2.5
3.0
3.5
4.0
Cellular_organisms - ps1
Archaea1 - ps2
Archaea2 - ps3
Archaea3 - ps4
Asgard/Eukaryota - ps5
Eukaryota - ps6
Excavata/Diaphoretickes - ps7
Diaphoretickes - ps8
Archaeplastida - ps9
Viridiplantae - ps10
Streptophyta - ps11
Charophyceae/Embriophyta - ps12
Embriophyta - ps13
Tracheophyta - ps14
Spermatophyta - ps15
Angiosperms - ps16
Mesangiospermae - ps17
Magnoliids/eudicots - ps18
Eudicots - ps19
Core_eudicots - ps20
Superrosids - ps21
Rosids - ps22
Eurosids - ps23
Malvids - ps24
Sap./Malv./Brassicales - ps25
Malvales/Brassicales - ps26
Brassicales - ps27
Cleomaceae/Brassicaceae - ps28
Brassicaceae - ps29
Camelineae - ps30
Arabidopsis - ps31
Arabidopsis_thaliana - ps32
Phylostratum

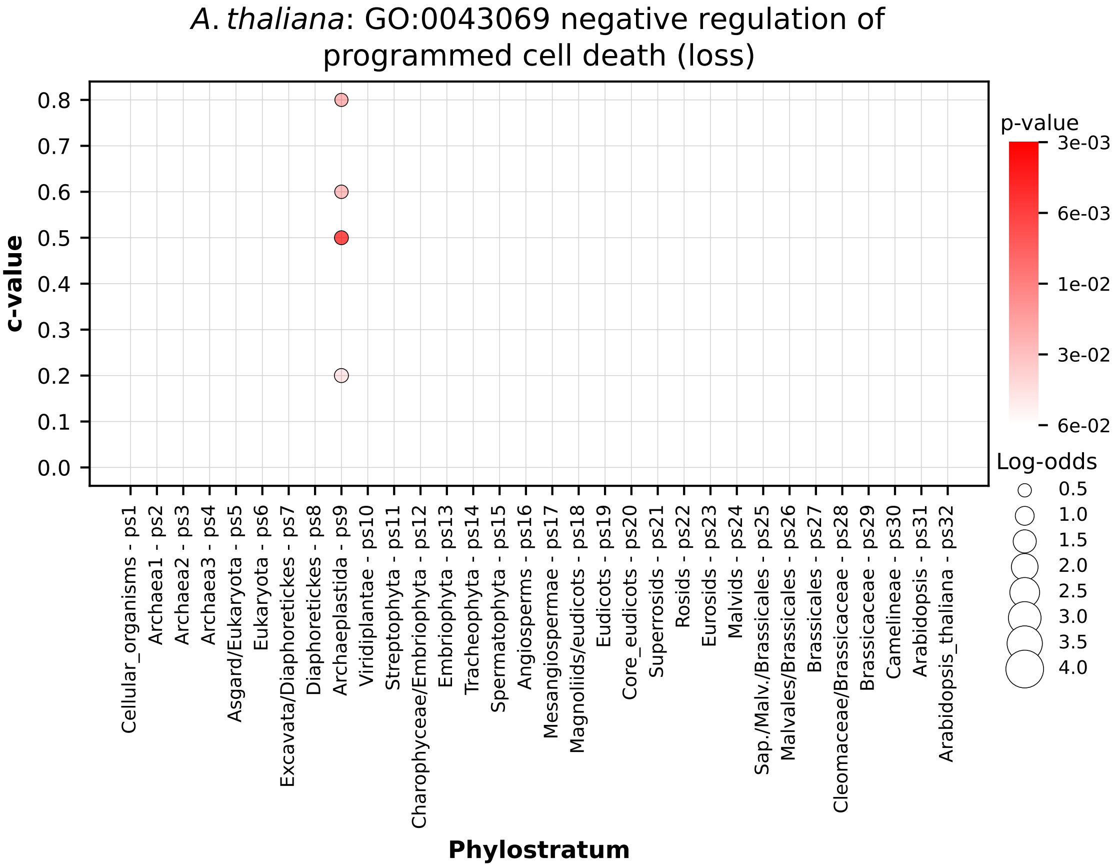

A. thaliana: GO:0043069 negative regulation of programmed cell death (loss)
c-value
0.8
0.7
0.6
0.5
0.4
0.3
0.2
0.1
0.0
p-value
3e-03
6e-03
1e-02
3e-02
6e-02
Log-odds
0.5
1.0
1.5
2.0
2.5
3.0
3.5
4.0
Cellular_organisms - ps1
Archaea1 - ps2
Archaea2 - ps3
Archaea3 - ps4
Asgard/Eukaryota - ps5
Eukaryota - ps6
Excavata/Diaphoretickes - ps7
Diaphoretickes - ps8
Archaeplastida - ps9
Viridiplantae - ps10
Streptophyta - ps11
Charophyceae/Embriophyta - ps12
Embriophyta - ps13
Tracheophyta - ps14
Spermatophyta - ps15
Angiosperms - ps16
Mesangiospermae - ps17
Magnoliids/eudicots - ps18
Eudicots - ps19
Core_eudicots - ps20
Superrosids - ps21
Rosids - ps22
Eurosids - ps23
Malvids - ps24
Sap./Malv./Brassicales - ps25
Malvales/Brassicales - ps26
Brassicales - ps27
Cleomaceae/Brassicaceae - ps28
Brassicaceae - ps29
Camelineae - ps30
Arabidopsis - ps31
Arabidopsis_thaliana - ps32
Phylostratum

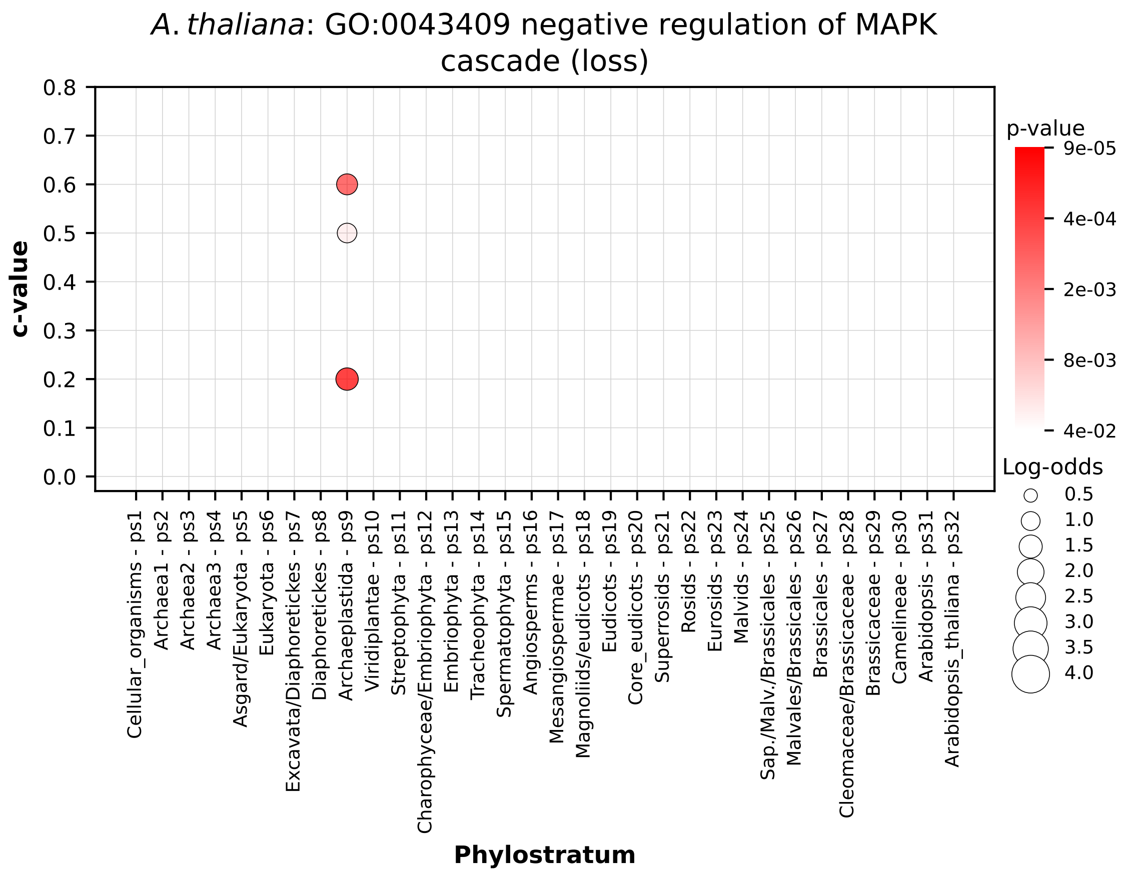
A. thaliana: GO:0043409 negative regulation of MAPK cascade (loss)
c-value
0.8
0.7
0.6
0.5
0.4
0.3
0.2
0.1
0.0
Phylostratum
Cellular_organisms - ps1
Archaea1 - ps2
Archaea2 - ps3
Archaea3 - ps4
Asgard/Eukaryota - ps5
Eukaryota - ps6
Excavata/Diaphoretickes - ps7
Diaphoretickes - ps8
Archaeplastida - ps9
Viridiplantae - ps10
Streptophyta - ps11
Charophyceae/Embriophyta - ps12
Embriophyta - ps13
Tracheophyta - ps14
Spermatophyta - ps15
Angiosperms - ps16
Mesangiospermae - ps17
Magnoliids/eudicots - ps18
Eudicots - ps19
Core_eudicots - ps20
Superrosids - ps21
Rosids - ps22
Eurosids - ps23
Malvids - ps24
Sap./Malv./Brassicales - ps25
Malvales/Brassicales - ps26
Brassicales - ps27
Cleomaceae/Brassicaceae - ps28
Brassicaceae - ps29
Camelineae - ps30
Arabidopsis - ps31
Arabidopsis_thaliana - ps32
p-value
9e-05
4e-04
2e-03
8e-03
4e-02
Log-odds
0.5
1.0
1.5
2.0
2.5
3.0
3.5
4.0

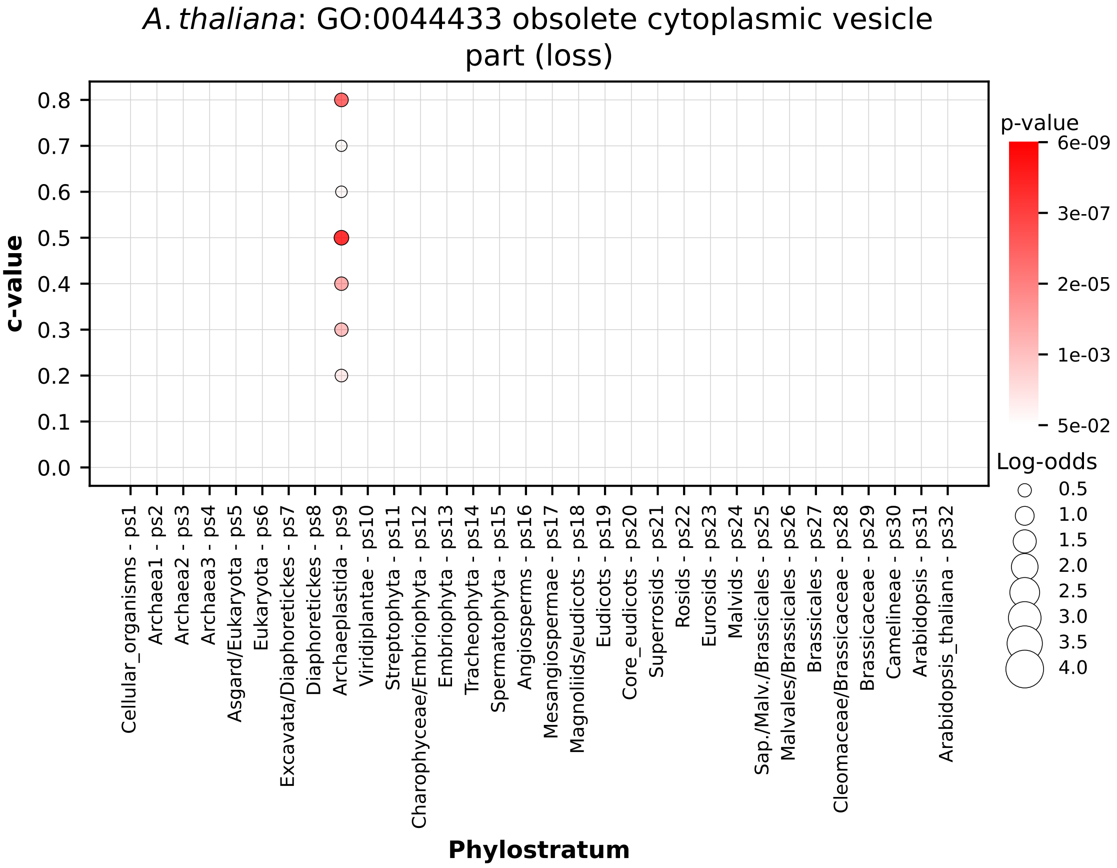
A. thaliana: GO:0044433 obsolete cytoplasmic vesicle part (loss)
c-value
0.0
0.1
0.2
0.3
0.4
0.5
0.6
0.7
0.8
Phylostratum
Cellular_organisms - ps1
Archaea1 - ps2
Archaea2 - ps3
Archaea3 - ps4
Asgard/Eukaryota - ps5
Eukaryota - ps6
Excavata/Diaphoretickes - ps7
Diaphoretickes - ps8
Archaeplastida - ps9
Viridiplantae - ps10
Streptophyta - ps11
Charophyceae/Embriophyta - ps12
Embriophyta - ps13
Tracheophyta - ps14
Spermatophyta - ps15
Angiosperms - ps16
Mesangiospermae - ps17
Magnoliids/eudicots - ps18
Eudicots - ps19
Core_eudicots - ps20
Superrosids - ps21
Rosids - ps22
Eurosids - ps23
Malvids - ps24
Sap./Malv./Brassicales - ps25
Malvales/Brassicales - ps26
Brassicales - ps27
Cleomaceae/Brassicaceae - ps28
Brassicaceae - ps29
Camelineae - ps30
Arabidopsis - ps31
Arabidopsis_thaliana - ps32
p-value
6e-09
3e-07
2e-05
1e-03
5e-02
Log-odds
0.5
1.0
1.5
2.0
2.5
3.0
3.5
4.0

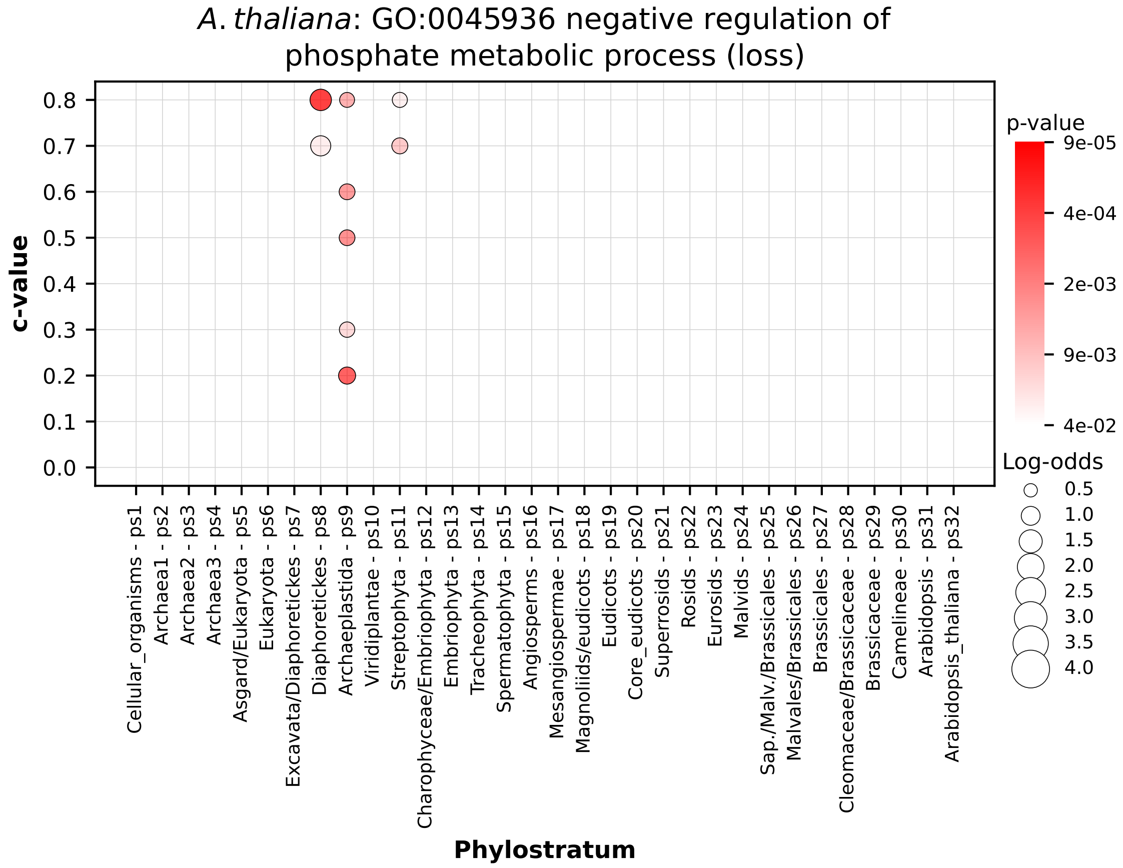

A. thaliana: GO:0045936 negative regulation of phosphate metabolic process (loss)
c-value
0.8
0.7
0.6
0.5
0.4
0.3
0.2
0.1
0.0
p-value
9e-05
4e-04
2e-03
9e-03
4e-02
Log-odds
0.5
1.0
1.5
2.0
2.5
3.0
3.5
4.0
Cellular_organisms - ps1
Archaea1 - ps2
Archaea2 - ps3
Archaea3 - ps4
Asgard/Eukaryota - ps5
Eukaryota - ps6
Excavata/Diaphoretickes - ps7
Diaphoretickes - ps8
Archaeplastida - ps9
Viridiplantae - ps10
Streptophyta - ps11
Charophyceae/Embriophyta - ps12
Embriophyta - ps13
Tracheophyta - ps14
Spermatophyta - ps15
Angiosperms - ps16
Mesangiospermae - ps17
Magnoliids/eudicots - ps18
Eudicots - ps19
Core_eudicots - ps20
Superrosids - ps21
Rosids - ps22
Eurosids - ps23
Malvids - ps24
Sap./Malv./Brassicales - ps25
Malvales/Brassicales - ps26
Brassicales - ps27
Cleomaceae/Brassicaceae - ps28
Brassicaceae - ps29
Camelineae - ps30
Arabidopsis - ps31
Arabidopsis_thaliana - ps32
Phylostratum

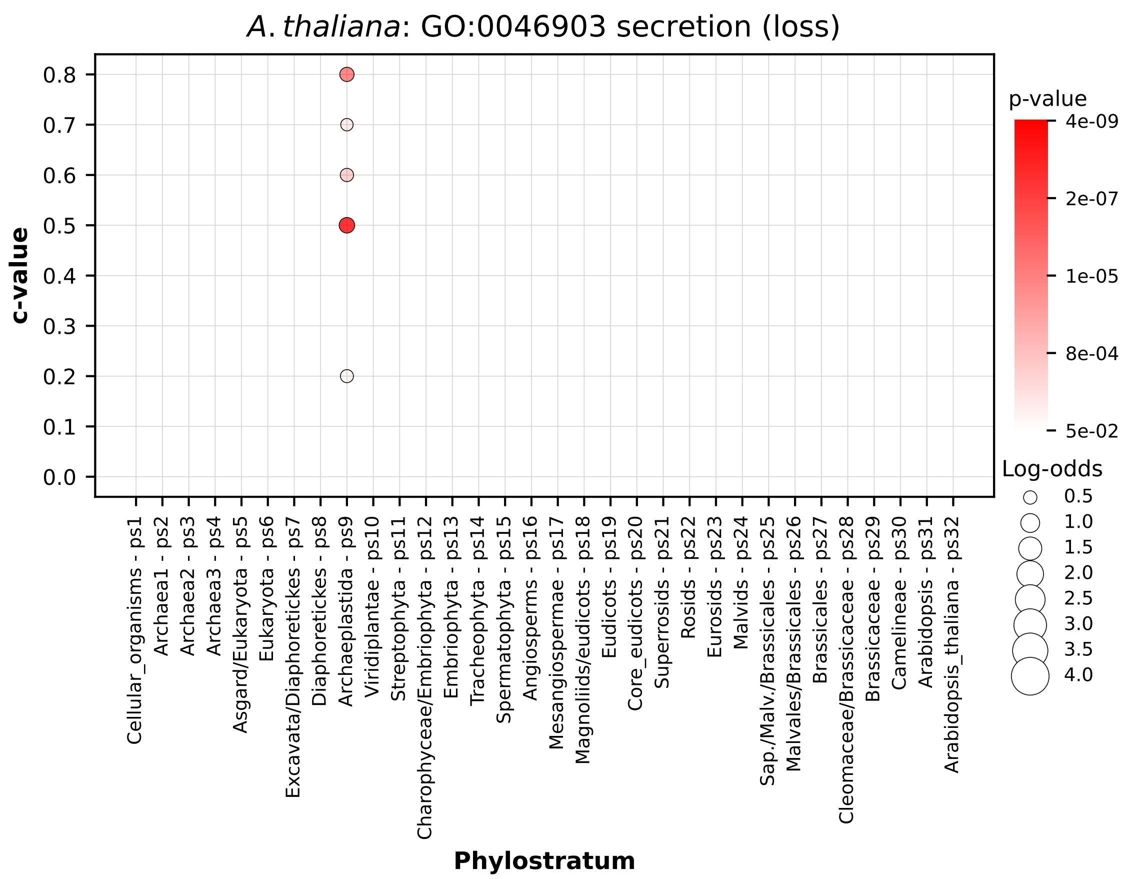

A. thaliana: GO:0046903 secretion (loss)
c-value
0.0
0.1
0.2
0.3
0.4
0.5
0.6
0.7
0.8
p-value
4e-09
2e-07
1e-05
8e-04
5e-02
Log-odds
0.5
1.0
1.5
2.0
2.5
3.0
3.5
4.0
Cellular_organisms - ps1
Archaea1 - ps2
Archaea2 - ps3
Archaea3 - ps4
Asgard/Eukaryota - ps5
Eukaryota - ps6
Excavata/Diaphoretickes - ps7
Diaphoretickes - ps8
Archaeplastida - ps9
Viridiplantae - ps10
Streptophyta - ps11
Charophyceae/Embriophyta - ps12
Embriophyta - ps13
Tracheophyta - ps14
Spermatophyta - ps15
Angiosperms - ps16
Mesangiospermae - ps17
Magnoliids/eudicots - ps18
Eudicots - ps19
Core_eudicots - ps20
Superrosids - ps21
Rosids - ps22
Eurosids - ps23
Malvids - ps24
Sap./Malv./Brassicales - ps25
Malvales/Brassicales - ps26
Brassicales - ps27
Cleomaceae/Brassicaceae - ps28
Brassicaceae - ps29
Camelineae - ps30
Arabidopsis - ps31
Arabidopsis_thaliana - ps32
Phylostratum

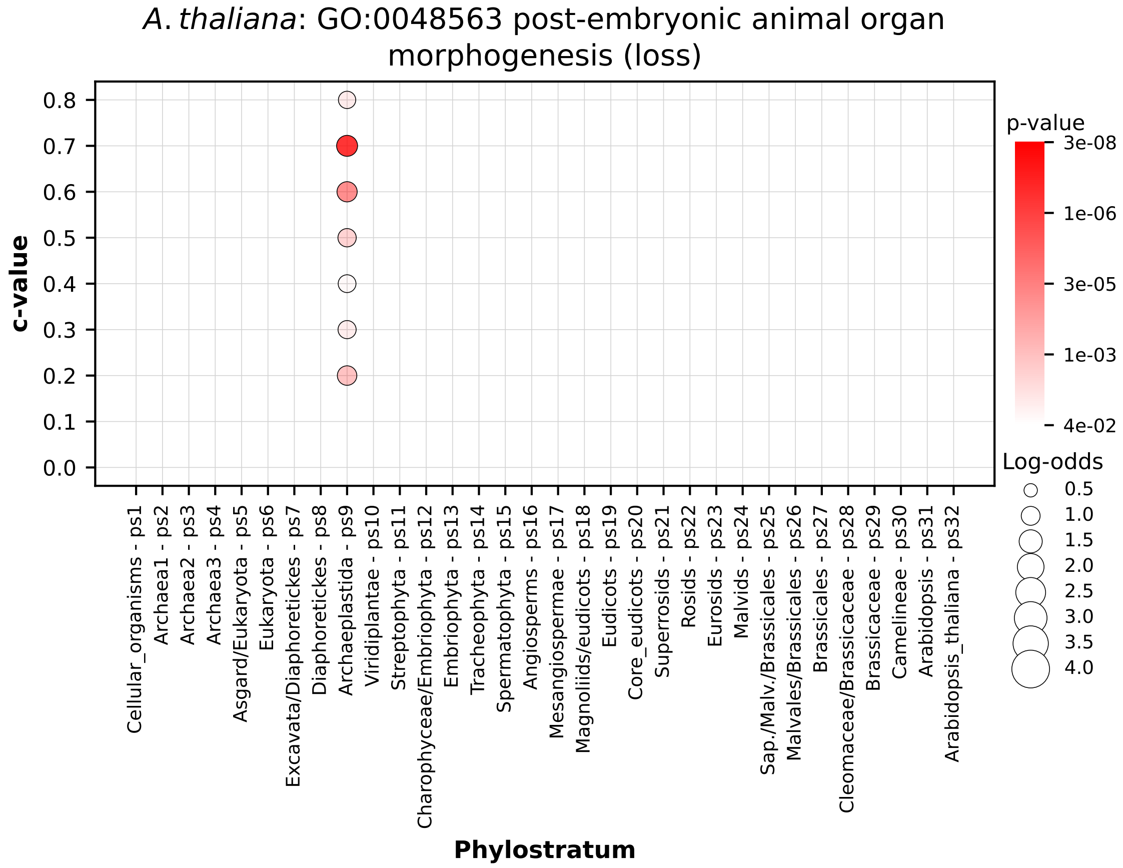

A. thaliana: GO:0048563 post-embryonic animal organ morphogenesis (loss)
c-value
0.8
0.7
0.6
0.5
0.4
0.3
0.2
0.1
0.0
Phylostratum
Cellular_organisms - ps1
Archaea1 - ps2
Archaea2 - ps3
Archaea3 - ps4
Asgard/Eukaryota - ps5
Eukaryota - ps6
Excavata/Diaphoretickes - ps7
Diaphoretickes - ps8
Archaeplastida - ps9
Viridiplantae - ps10
Streptophyta - ps11
Charophyceae/Embriophyta - ps12
Embriophyta - ps13
Tracheophyta - ps14
Spermatophyta - ps15
Angiosperms - ps16
Mesangiospermae - ps17
Magnoliids/eudicots - ps18
Eudicots - ps19
Core_eudicots - ps20
Superrosids - ps21
Rosids - ps22
Eurosids - ps23
Malvids - ps24
Sap./Malv./Brassicales - ps25
Malvales/Brassicales - ps26
Brassicales - ps27
Cleomaceae/Brassicaceae - ps28
Brassicaceae - ps29
Camelineae - ps30
Arabidopsis - ps31
Arabidopsis_thaliana - ps32
p-value
3e-08
1e-06
3e-05
1e-03
4e-02
Log-odds
0.5
1.0
1.5
2.0
2.5
3.0
3.5
4.0

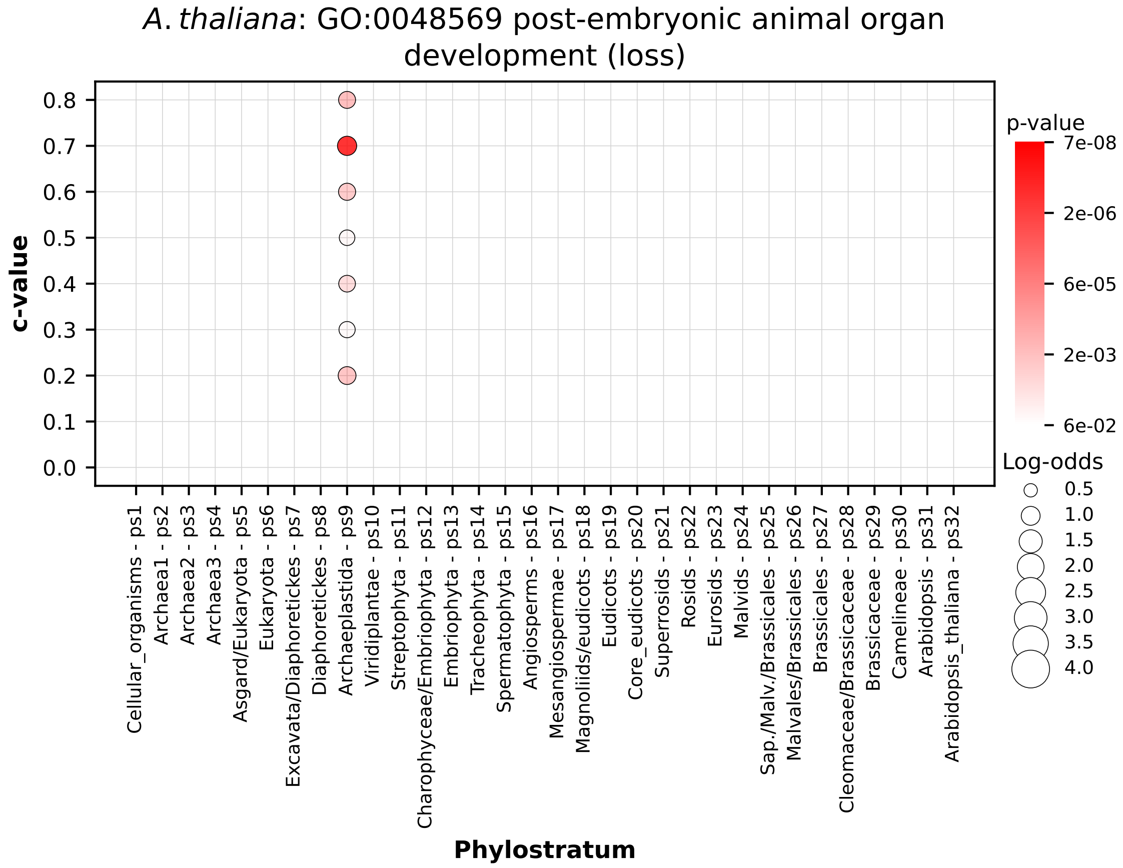

A. thaliana: GO:0048569 post-embryonic animal organ development (loss)
c-value
0.0
0.1
0.2
0.3
0.4
0.5
0.6
0.7
0.8
p-value
7e-08
2e-06
6e-05
2e-03
6e-02
Log-odds
0.5
1.0
1.5
2.0
2.5
3.0
3.5
4.0
Cellular_organisms - ps1
Archaea1 - ps2
Archaea2 - ps3
Archaea3 - ps4
Asgard/Eukaryota - ps5
Eukaryota - ps6
Excavata/Diaphoretickes - ps7
Diaphoretickes - ps8
Archaeplastida - ps9
Viridiplantae - ps10
Streptophyta - ps11
Charophyceae/Embriophyta - ps12
Embriophyta - ps13
Tracheophyta - ps14
Spermatophyta - ps15
Angiosperms - ps16
Mesangiospermae - ps17
Magnoliids/eudicots - ps18
Eudicots - ps19
Core_eudicots - ps20
Superrosids - ps21
Rosids - ps22
Eurosids - ps23
Malvids - ps24
Sap./Malv./Brassicales - ps25
Malvales/Brassicales - ps26
Brassicales - ps27
Cleomaceae/Brassicaceae - ps28
Brassicaceae - ps29
Camelineae - ps30
Arabidopsis - ps31
Arabidopsis_thaliana - ps32
Phylostratum

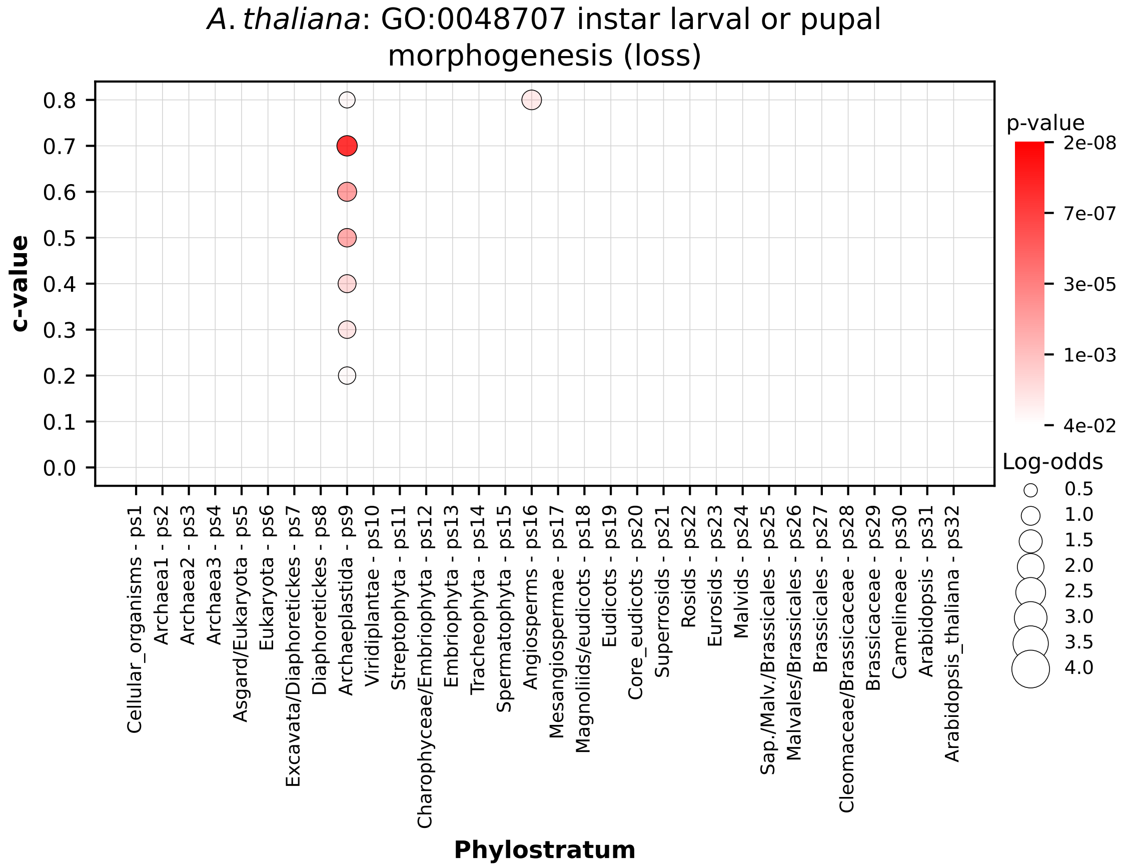

A. thaliana: GO:0048707 instar larval or pupal morphogenesis (loss)
c-value
0.0
0.1
0.2
0.3
0.4
0.5
0.6
0.7
0.8
p-value
2e-08
7e-07
3e-05
1e-03
4e-02
Log-odds
0.5
1.0
1.5
2.0
2.5
3.0
3.5
4.0
Cellular_organisms - ps1
Archaea1 - ps2
Archaea2 - ps3
Archaea3 - ps4
Asgard/Eukaryota - ps5
Eukaryota - ps6
Excavata/Diaphoretickes - ps7
Diaphoretickes - ps8
Archaeplastida - ps9
Viridiplantae - ps10
Streptophyta - ps11
Charophyceae/Embriophyta - ps12
Embriophyta - ps13
Tracheophyta - ps14
Spermatophyta - ps15
Angiosperms - ps16
Mesangiospermae - ps17
Magnoliids/eudicots - ps18
Eudicots - ps19
Core_eudicots - ps20
Superrosids - ps21
Rosids - ps22
Eurosids - ps23
Malvids - ps24
Sap./Malv./Brassicales - ps25
Malvales/Brassicales - ps26
Brassicales - ps27
Cleomaceae/Brassicaceae - ps28
Brassicaceae - ps29
Camelineae - ps30
Arabidopsis - ps31
Arabidopsis_thaliana - ps32
Phylostratum

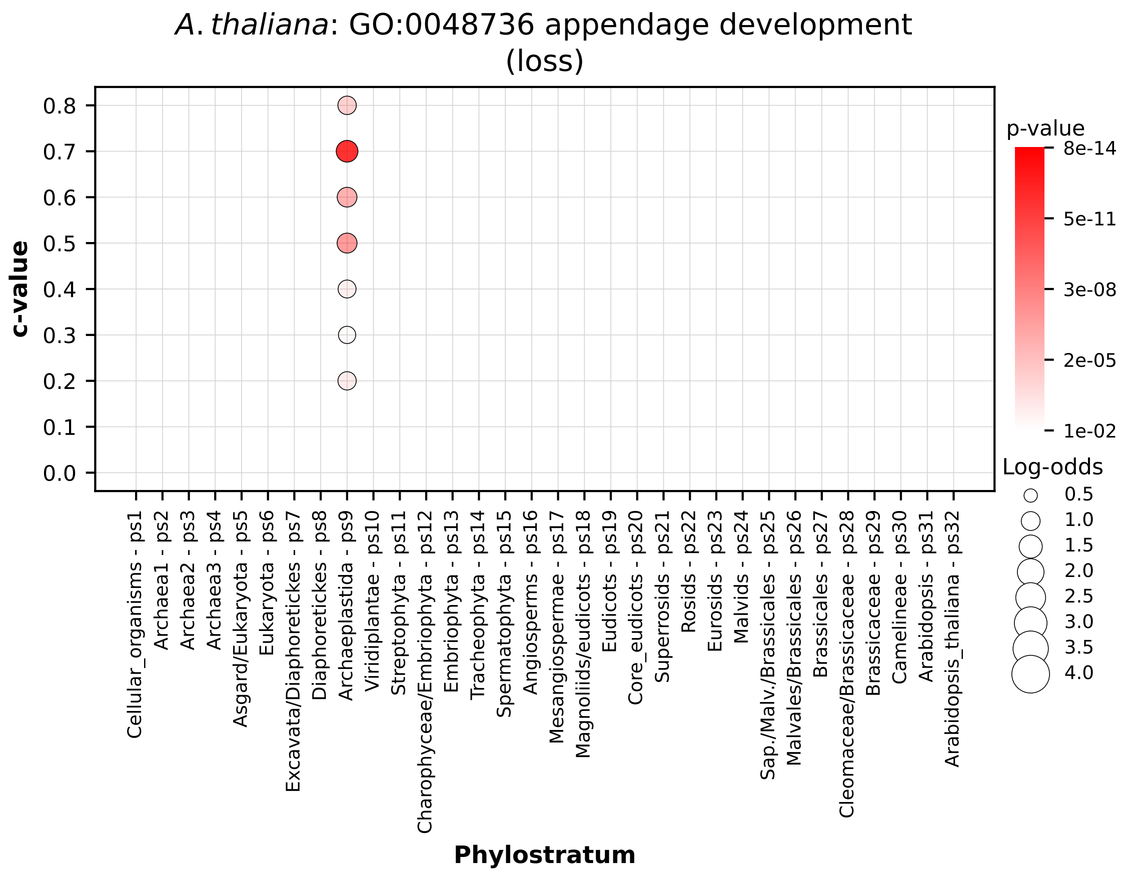

A. thaliana: GO:0048736 appendage development
(loss)
c-value
0.0
0.1
0.2
0.3
0.4
0.5
0.6
0.7
0.8
p-value
8e-14
5e-11
3e-08
2e-05
1e-02
Log-odds
0.5
1.0
1.5
2.0
2.5
3.0
3.5
4.0
Cellular_organisms - ps1
Archaea1 - ps2
Archaea2 - ps3
Archaea3 - ps4
Asgard/Eukaryota - ps5
Eukaryota - ps6
Excavata/Diaphoretickes - ps7
Diaphoretickes - ps8
Archaeplastida - ps9
Viridiplantae - ps10
Streptophyta - ps11
Charophyceae/Embriophyta - ps12
Embriophyta - ps13
Tracheophyta - ps14
Spermatophyta - ps15
Angiosperms - ps16
Mesangiospermae - ps17
Magnoliids/eudicots - ps18
Eudicots - ps19
Core_eudicots - ps20
Superrosids - ps21
Rosids - ps22
Eurosids - ps23
Malvids - ps24
Sap./Malv./Brassicales - ps25
Malvales/Brassicales - ps26
Brassicales - ps27
Cleomaceae/Brassicaceae - ps28
Brassicaceae - ps29
Camelineae - ps30
Arabidopsis - ps31
Arabidopsis_thaliana - ps32
Phylostratum

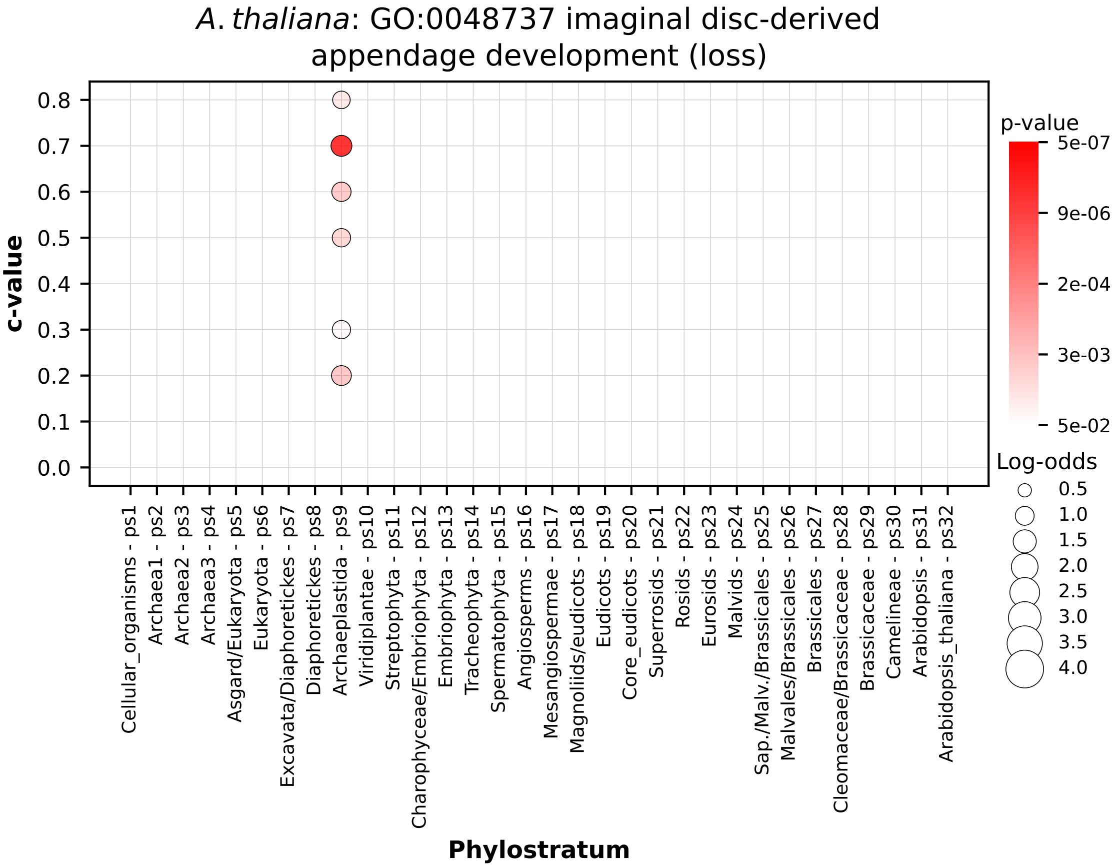

A. thaliana: GO:0048737 imaginal disc-derived appendage development (loss)
c-value
0.8
0.7
0.6
0.5
0.4
0.3
0.2
0.1
0.0
Phylostratum
Cellular_organisms - ps1
Archaea1 - ps2
Archaea2 - ps3
Archaea3 - ps4
Asgard/Eukaryota - ps5
Eukaryota - ps6
Excavata/Diaphoretickes - ps7
Diaphoretickes - ps8
Archaeplastida - ps9
Viridiplantae - ps10
Streptophyta - ps11
Charophyceae/Embriophyta - ps12
Embriophyta - ps13
Tracheophyta - ps14
Spermatophyta - ps15
Angiosperms - ps16
Mesangiospermae - ps17
Magnoliids/eudicots - ps18
Eudicots - ps19
Core_eudicots - ps20
Superrosids - ps21
Rosids - ps22
Eurosids - ps23
Malvids - ps24
Sap./Malv./Brassicales - ps25
Malvales/Brassicales - ps26
Brassicales - ps27
Cleomaceae/Brassicaceae - ps28
Brassicaceae - ps29
Camelineae - ps30
Arabidopsis - ps31
Arabidopsis_thaliana - ps32
p-value
5e-07
9e-06
2e-04
3e-03
5e-02
Log-odds
0.5
1.0
1.5
2.0
2.5
3.0
3.5
4.0

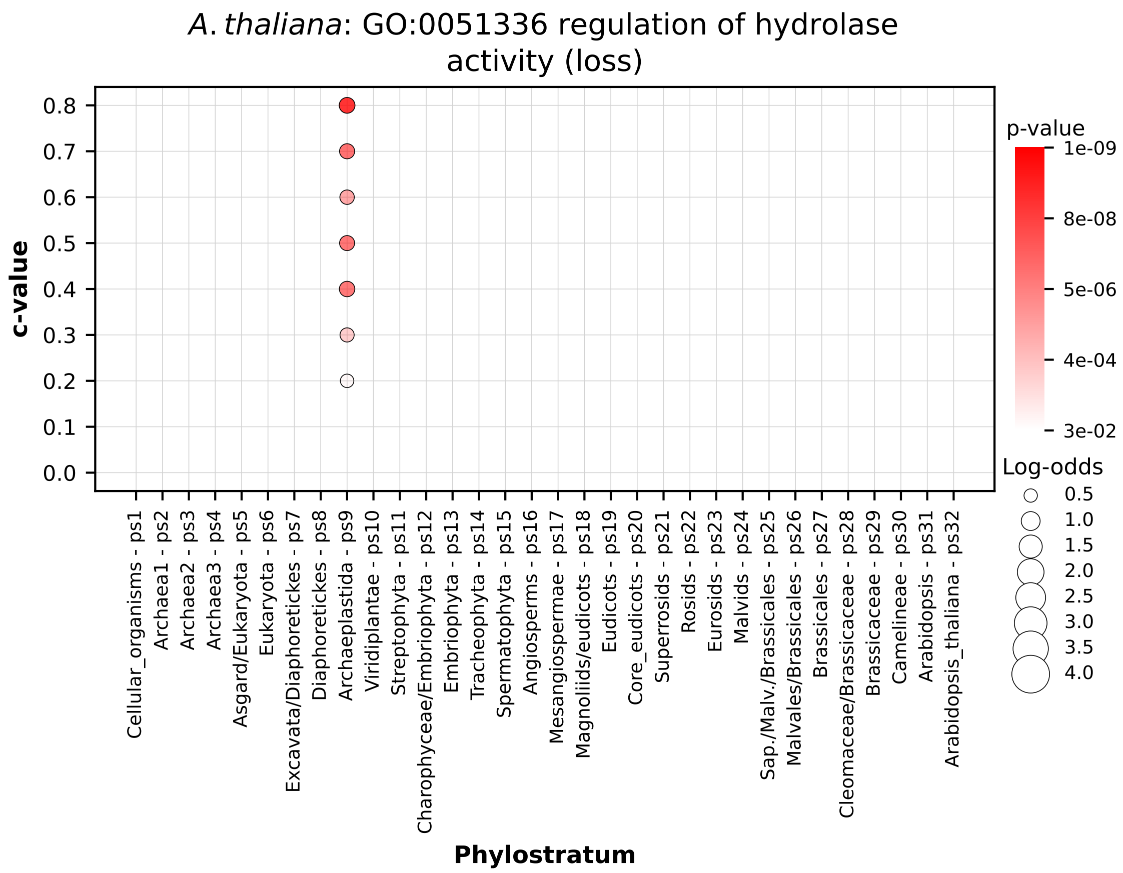

A. thaliana: GO:0051336 regulation of hydrolase activity (loss)
c-value
0.8
0.7
0.6
0.5
0.4
0.3
0.2
0.1
0.0
Phylostratum
Cellular_organisms - ps1
Archaea1 - ps2
Archaea2 - ps3
Archaea3 - ps4
Asgard/Eukaryota - ps5
Eukaryota - ps6
Excavata/Diaphoretickes - ps7
Diaphoretickes - ps8
Archaeplastida - ps9
Viridiplantae - ps10
Streptophyta - ps11
Charophyceae/Embriophyta - ps12
Embriophyta - ps13
Tracheophyta - ps14
Spermatophyta - ps15
Angiosperms - ps16
Mesangiospermae - ps17
Magnoliids/eudicots - ps18
Eudicots - ps19
Core_eudicots - ps20
Superrosids - ps21
Rosids - ps22
Eurosids - ps23
Malvids - ps24
Sap./Malv./Brassicales - ps25
Malvales/Brassicales - ps26
Brassicales - ps27
Cleomaceae/Brassicaceae - ps28
Brassicaceae - ps29
Camelineae - ps30
Arabidopsis - ps31
Arabidopsis_thaliana - ps32
p-value
1e-09
8e-08
5e-06
4e-04
3e-02
Log-odds
0.5
1.0
1.5
2.0
2.5
3.0
3.5
4.0

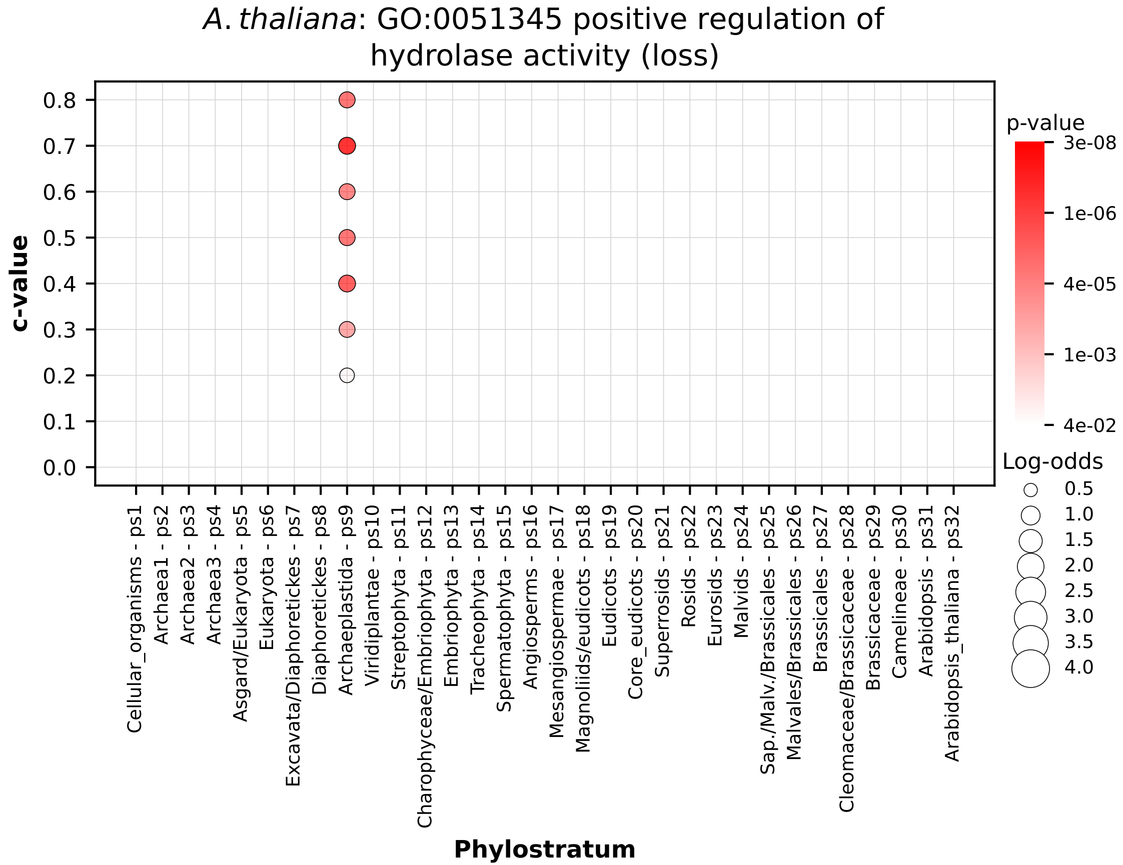

A. thaliana: GO:0051345 positive regulation of hydrolase activity (loss)
c-value
0.0
0.1
0.2
0.3
0.4
0.5
0.6
0.7
0.8
Phylostratum
Cellular_organisms - ps1
Archaea1 - ps2
Archaea2 - ps3
Archaea3 - ps4
Asgard/Eukaryota - ps5
Eukaryota - ps6
Excavata/Diaphoretickes - ps7
Diaphoretickes - ps8
Archaeplastida - ps9
Viridiplantae - ps10
Streptophyta - ps11
Charophyceae/Embriophyta - ps12
Embriophyta - ps13
Tracheophyta - ps14
Spermatophyta - ps15
Angiosperms - ps16
Mesangiospermae - ps17
Magnoliids/eudicots - ps18
Eudicots - ps19
Core_eudicots - ps20
Superrosids - ps21
Rosids - ps22
Eurosids - ps23
Malvids - ps24
Sap./Malv./Brassicales - ps25
Malvales/Brassicales - ps26
Brassicales - ps27
Cleomaceae/Brassicaceae - ps28
Brassicaceae - ps29
Camelineae - ps30
Arabidopsis - ps31
Arabidopsis_thaliana - ps32
p-value
3e-08
1e-06
4e-05
1e-03
4e-02
Log-odds
0.5
1.0
1.5
2.0
2.5
3.0
3.5
4.0

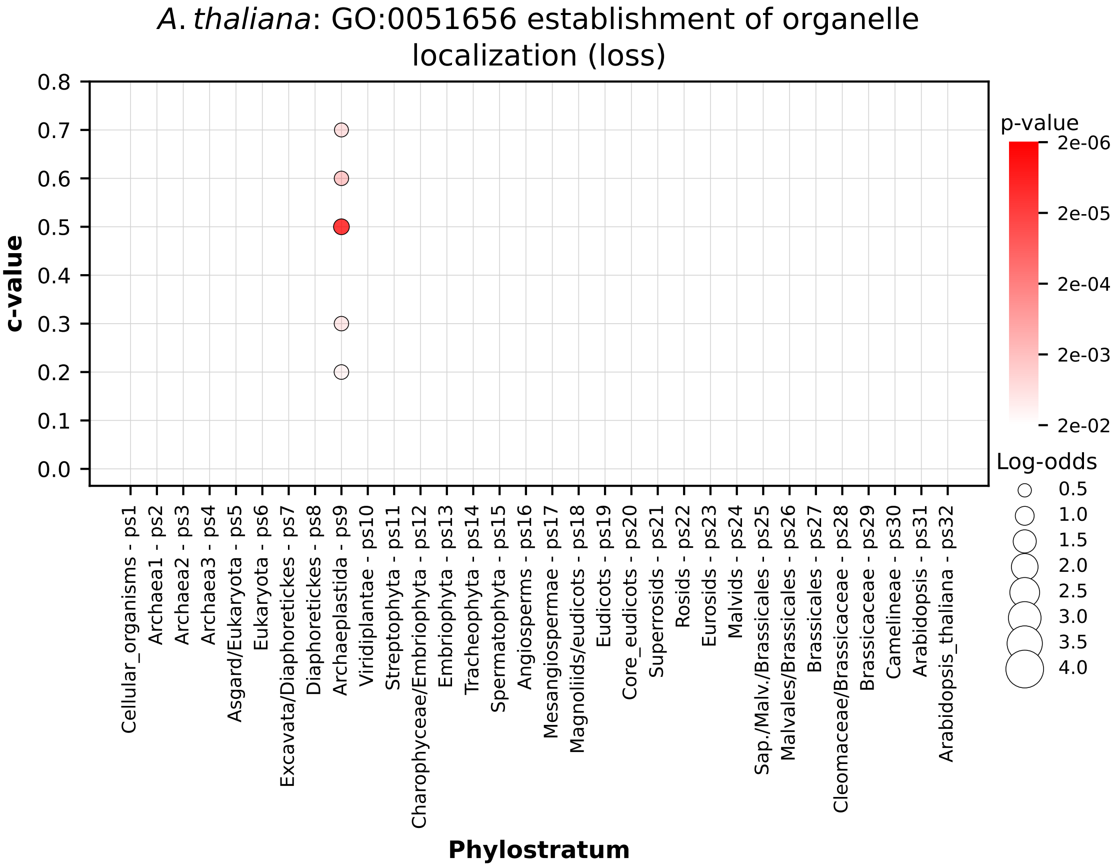
A. thaliana: GO:0051656 establishment of organelle localization (loss)
c-value
0.8
0.7
0.6
0.5
0.4
0.3
0.2
0.1
0.0
p-value
2e-06
2e-05
2e-04
2e-03
2e-02
Log-odds
0.5
1.0
1.5
2.0
2.5
3.0
3.5
4.0
Cellular_organisms - ps1
Archaea1 - ps2
Archaea2 - ps3
Archaea3 - ps4
Asgard/Eukaryota - ps5
Eukaryota - ps6
Excavata/Diaphoretickes - ps7
Diaphoretickes - ps8
Archaeplastida - ps9
Viridiplantae - ps10
Streptophyta - ps11
Charophyceae/Embriophyta - ps12
Embriophyta - ps13
Tracheophyta - ps14
Spermatophyta - ps15
Angiosperms - ps16
Mesangiospermae - ps17
Magnoliids/eudicots - ps18
Eudicots - ps19
Core_eudicots - ps20
Superrosids - ps21
Rosids - ps22
Eurosids - ps23
Malvids - ps24
Sap./Malv./Brassicales - ps25
Malvales/Brassicales - ps26
Brassicales - ps27
Cleomaceae/Brassicaceae - ps28
Brassicaceae - ps29
Camelineae - ps30
Arabidopsis - ps31
Arabidopsis_thaliana - ps32
Phylostratum

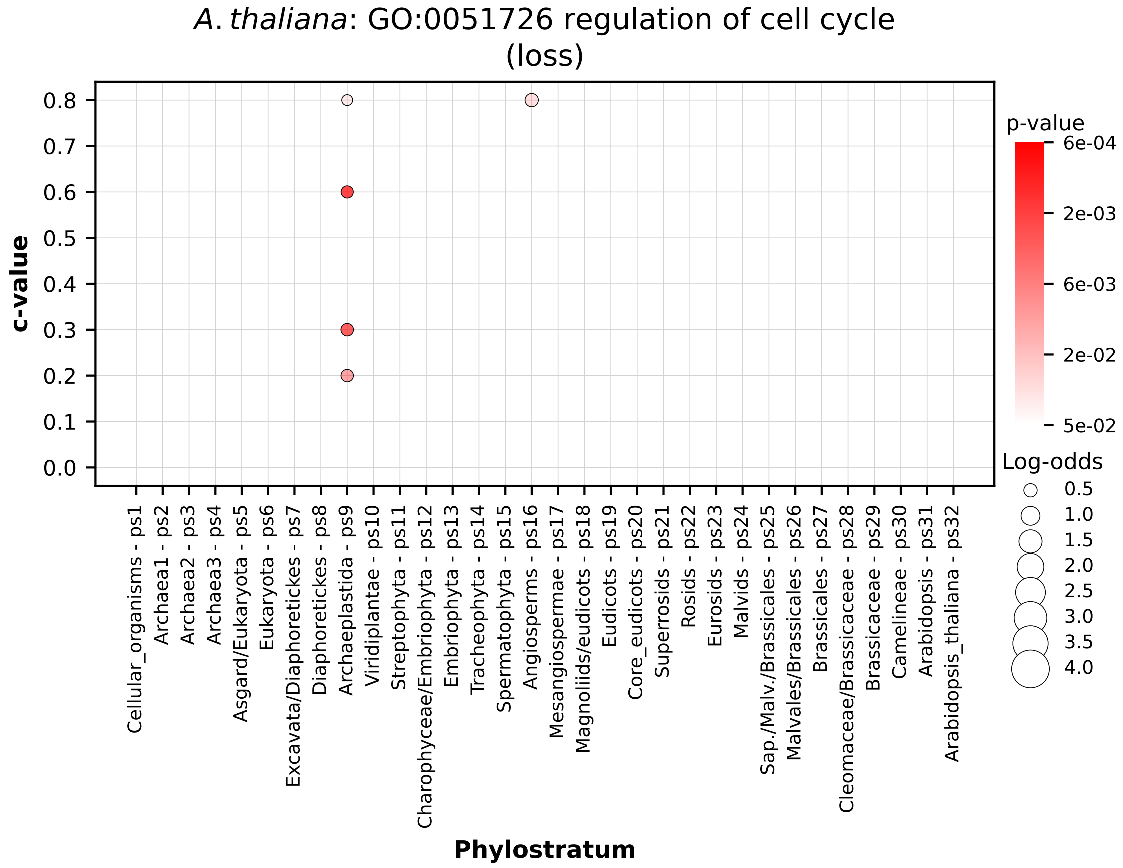
A. thaliana: GO:0051726 regulation of cell cycle
(loss)
c-value
0.8
0.7
0.6
0.5
0.4
0.3
0.2
0.1
0.0
p-value
6e-04
2e-03
6e-03
2e-02
5e-02
Log-odds
0.5
1.0
1.5
2.0
2.5
3.0
3.5
4.0
Cellular_organisms - ps1
Archaea1 - ps2
Archaea2 - ps3
Archaea3 - ps4
Asgard/Eukaryota - ps5
Eukaryota - ps6
Excavata/Diaphoretickes - ps7
Diaphoretickes - ps8
Archaeplastida - ps9
Viridiplantae - ps10
Streptophyta - ps11
Charophyceae/Embriophyta - ps12
Embriophyta - ps13
Tracheophyta - ps14
Spermatophyta - ps15
Angiosperms - ps16
Mesangiospermae - ps17
Magnoliids/eudicots - ps18
Eudicots - ps19
Core_eudicots - ps20
Superrosids - ps21
Rosids - ps22
Eurosids - ps23
Malvids - ps24
Sap./Malv./Brassicales - ps25
Malvales/Brassicales - ps26
Brassicales - ps27
Cleomaceae/Brassicaceae - ps28
Brassicaceae - ps29
Camelineae - ps30
Arabidopsis - ps31
Arabidopsis_thaliana - ps32
Phylostratum

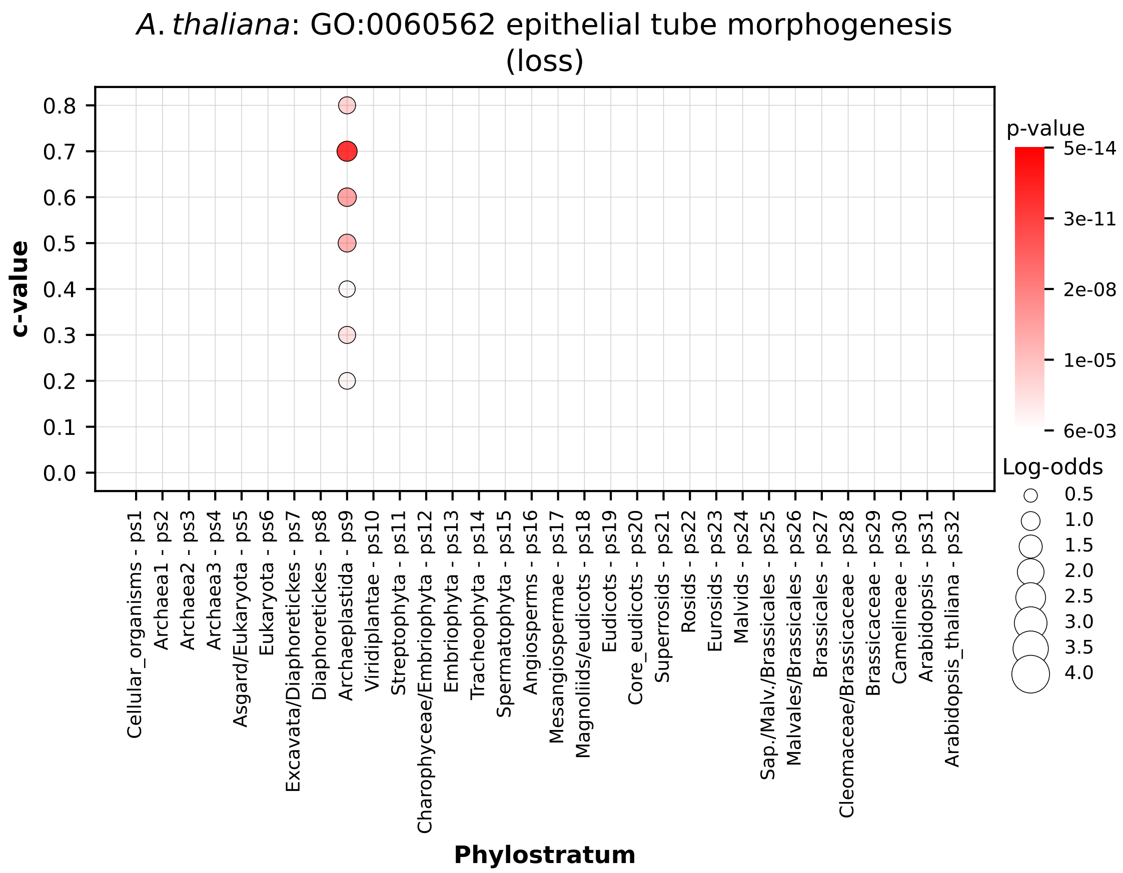
A. thaliana: GO:0060562 epithelial tube morphogenesis
(loss)
c-value
0.8
0.7
0.6
0.5
0.4
0.3
0.2
0.1
0.0
p-value
5e-14
3e-11
2e-08
1e-05
6e-03
Log-odds
0.5
1.0
1.5
2.0
2.5
3.0
3.5
4.0
Cellular_organisms - ps1
Archaea1 - ps2
Archaea2 - ps3
Archaea3 - ps4
Asgard/Eukaryota - ps5
Eukaryota - ps6
Excavata/Diaphoretickes - ps7
Diaphoretickes - ps8
Archaeplastida - ps9
Viridiplantae - ps10
Streptophyta - ps11
Charophyceae/Embriophyta - ps12
Embriophyta - ps13
Tracheophyta - ps14
Spermatophyta - ps15
Angiosperms - ps16
Mesangiospermae - ps17
Magnoliids/eudicots - ps18
Eudicots - ps19
Core_eudicots - ps20
Superrosids - ps21
Rosids - ps22
Eurosids - ps23
Malvids - ps24
Sap./Malv./Brassicales - ps25
Malvales/Brassicales - ps26
Brassicales - ps27
Cleomaceae/Brassicaceae - ps28
Brassicaceae - ps29
Camelineae - ps30
Arabidopsis - ps31
Arabidopsis_thaliana - ps32
Phylostratum

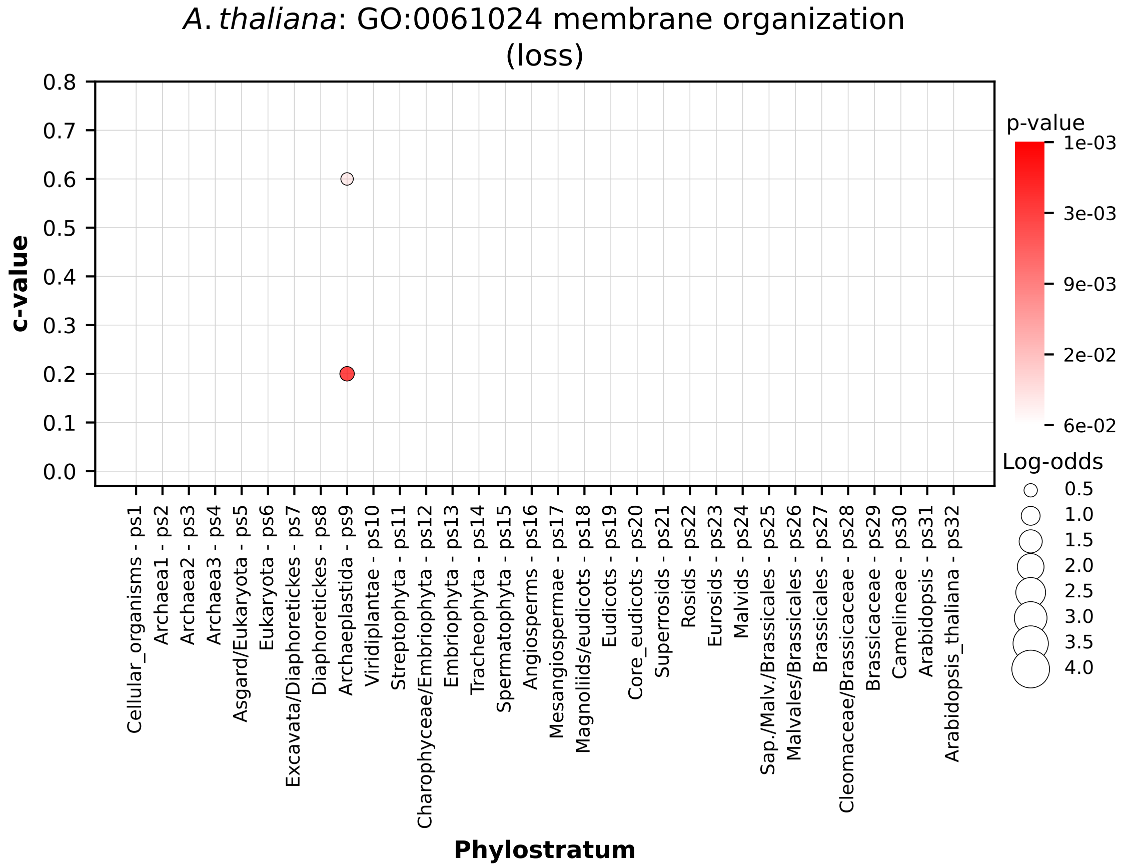

A. thaliana: GO:0061024 membrane organization
(loss)
c-value
0.0
0.1
0.2
0.3
0.4
0.5
0.6
0.7
0.8
p-value
1e-03
3e-03
9e-03
2e-02
6e-02
Log-odds
0.5
1.0
1.5
2.0
2.5
3.0
3.5
4.0
Cellular_organisms - ps1
Archaea1 - ps2
Archaea2 - ps3
Archaea3 - ps4
Asgard/Eukaryota - ps5
Eukaryota - ps6
Excavata/Diaphoretickes - ps7
Diaphoretickes - ps8
Archaeplastida - ps9
Viridiplantae - ps10
Streptophyta - ps11
Charophyceae/Embriophyta - ps12
Embriophyta - ps13
Tracheophyta - ps14
Spermatophyta - ps15
Angiosperms - ps16
Mesangiospermae - ps17
Magnoliids/eudicots - ps18
Eudicots - ps19
Core_eudicots - ps20
Superrosids - ps21
Rosids - ps22
Eurosids - ps23
Malvids - ps24
Sap./Malv./Brassicales - ps25
Malvales/Brassicales - ps26
Brassicales - ps27
Cleomaceae/Brassicaceae - ps28
Brassicaceae - ps29
Camelineae - ps30
Arabidopsis - ps31
Arabidopsis_thaliana - ps32
Phylostratum

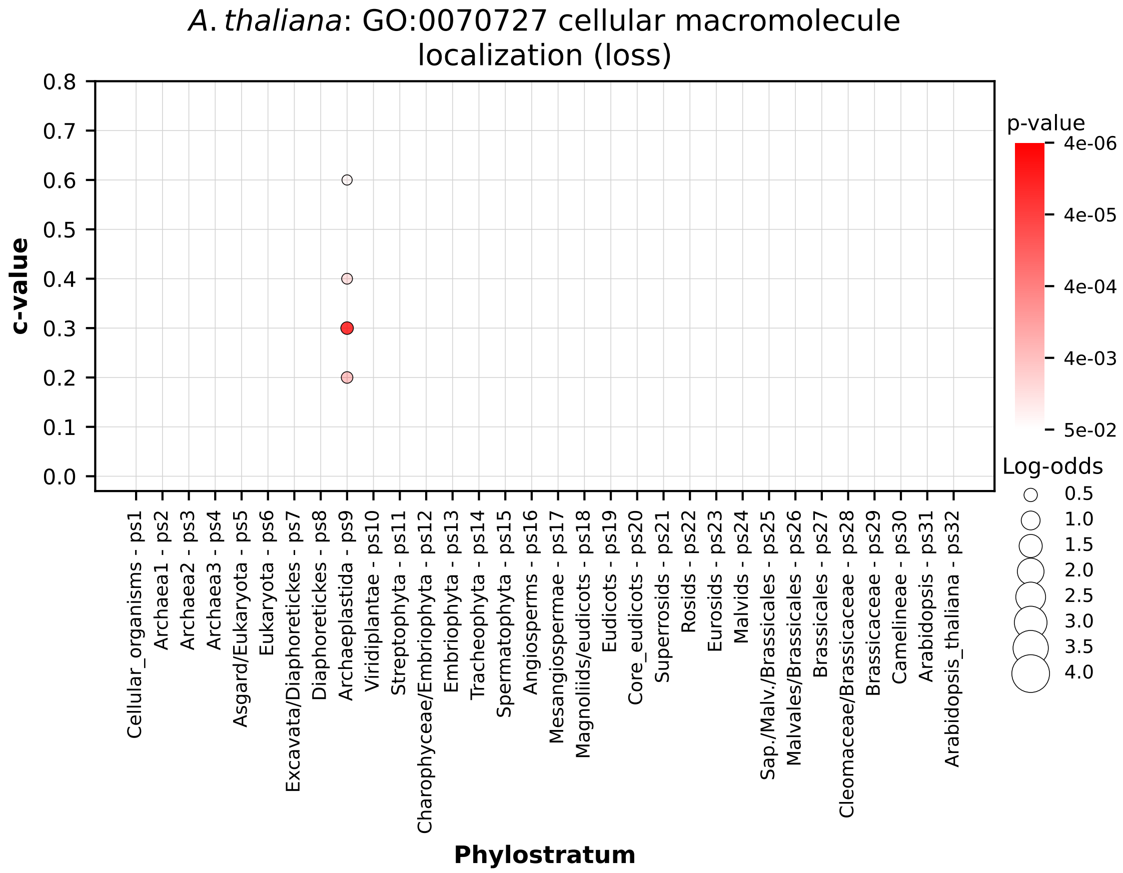

A. thaliana: GO:0070727 cellular macromolecule localization (loss)
c-value
0.8
0.7
0.6
0.5
0.4
0.3
0.2
0.1
0.0
Phylostratum
Cellular_organisms - ps1
Archaea1 - ps2
Archaea2 - ps3
Archaea3 - ps4
Asgard/Eukaryota - ps5
Eukaryota - ps6
Excavata/Diaphoretickes - ps7
Diaphoretickes - ps8
Archaeplastida - ps9
Viridiplantae - ps10
Streptophyta - ps11
Charophyceae/Embriophyta - ps12
Embriophyta - ps13
Tracheophyta - ps14
Spermatophyta - ps15
Angiosperms - ps16
Mesangiospermae - ps17
Magnoliids/eudicots - ps18
Eudicots - ps19
Core_eudicots - ps20
Superrosids - ps21
Rosids - ps22
Eurosids - ps23
Malvids - ps24
Sap./Malv./Brassicales - ps25
Malvales/Brassicales - ps26
Brassicales - ps27
Cleomaceae/Brassicaceae - ps28
Brassicaceae - ps29
Camelineae - ps30
Arabidopsis - ps31
Arabidopsis_thaliana - ps32
p-value
4e-06
4e-05
4e-04
4e-03
5e-02
Log-odds
0.5
1.0
1.5
2.0
2.5
3.0
3.5
4.0

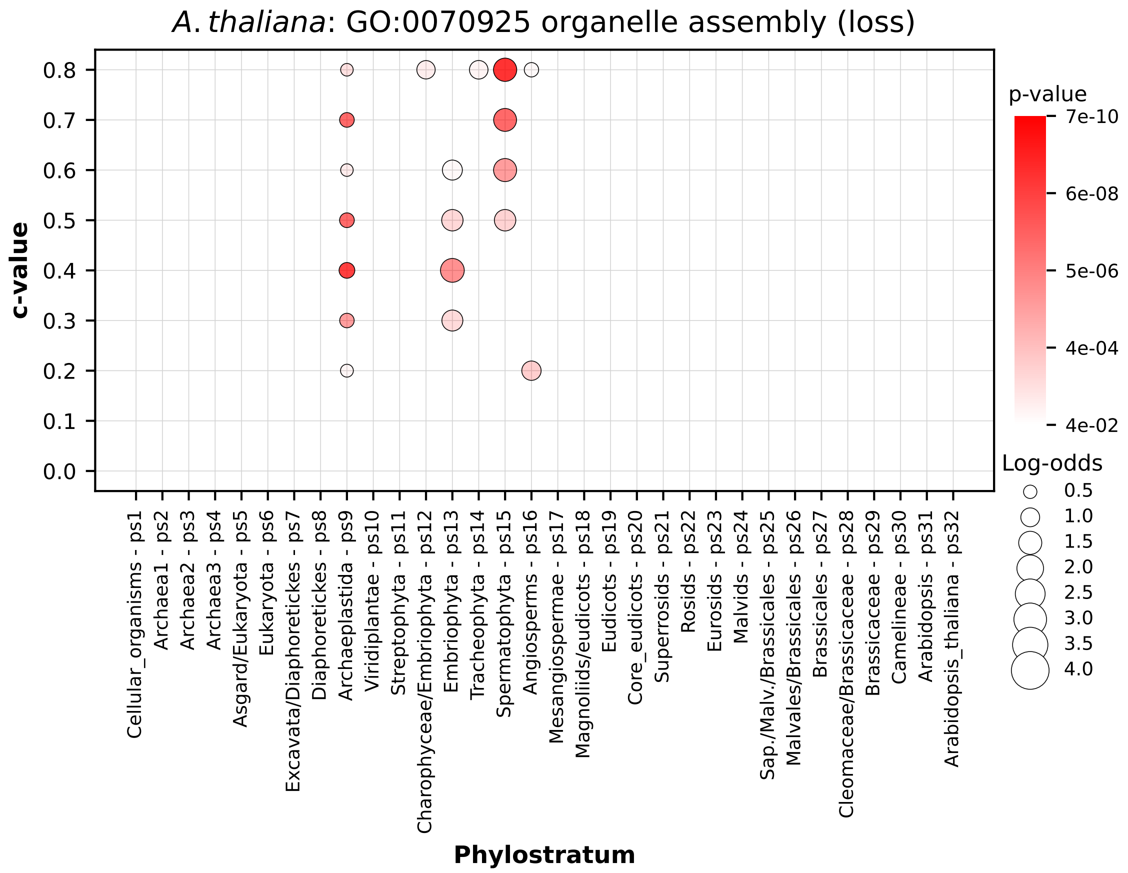

A. thaliana: GO:0070925 organelle assembly (loss)
c-value
0.8
0.7
0.6
0.5
0.4
0.3
0.2
0.1
0.0
p-value
7e-10
6e-08
5e-06
4e-04
4e-02
Log-odds
0.5
1.0
1.5
2.0
2.5
3.0
3.5
4.0
Cellular_organisms - ps1
Archaea1 - ps2
Archaea2 - ps3
Archaea3 - ps4
Asgard/Eukaryota - ps5
Eukaryota - ps6
Excavata/Diaphoretickes - ps7
Diaphoretickes - ps8
Archaeplastida - ps9
Viridiplantae - ps10
Streptophyta - ps11
Charophyceae/Embriophyta - ps12
Embriophyta - ps13
Tracheophyta - ps14
Spermatophyta - ps15
Angiosperms - ps16
Mesangiospermae - ps17
Magnoliids/eudicots - ps18
Eudicots - ps19
Core_eudicots - ps20
Superrosids - ps21
Rosids - ps22
Eurosids - ps23
Malvids - ps24
Sap./Malv./Brassicales - ps25
Malvales/Brassicales - ps26
Brassicales - ps27
Cleomaceae/Brassicaceae - ps28
Brassicaceae - ps29
Camelineae - ps30
Arabidopsis - ps31
Arabidopsis_thaliana - ps32
Phylostratum

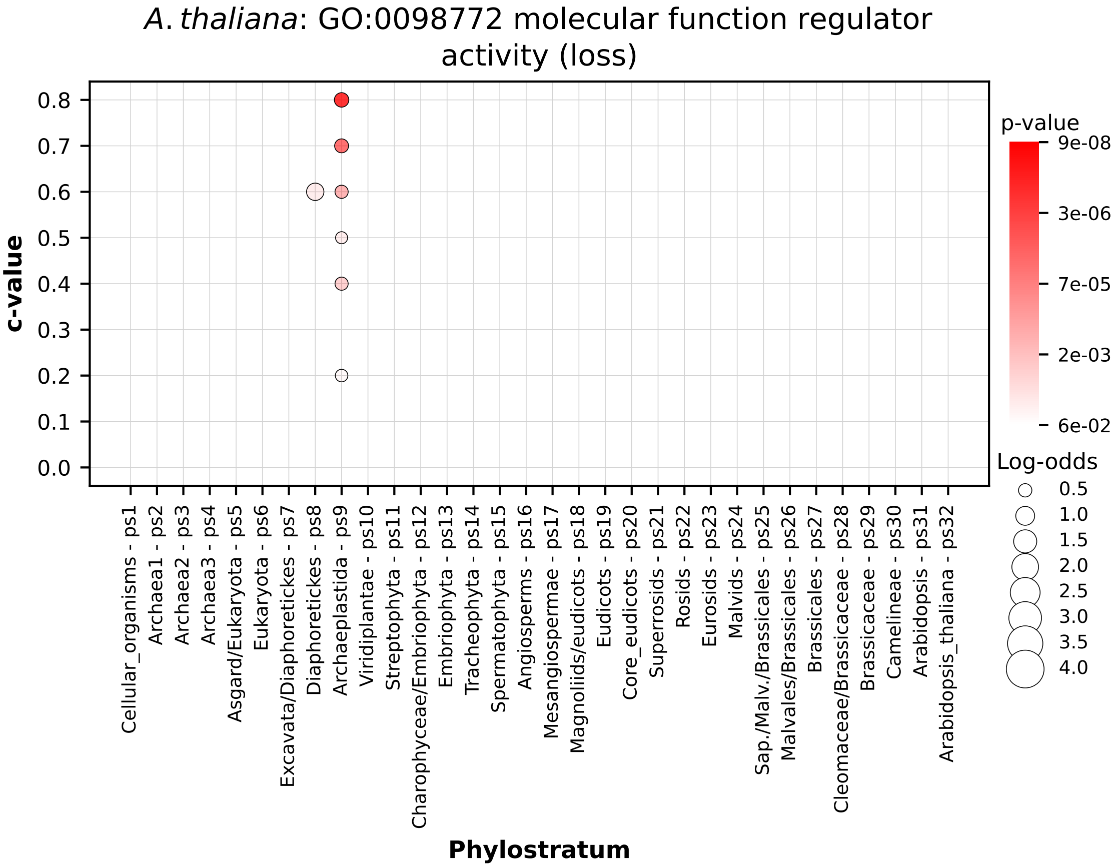
A. thaliana: GO:0098772 molecular function regulator activity (loss)
c-value
0.8
0.7
0.6
0.5
0.4
0.3
0.2
0.1
0.0
p-value
9e-08
3e-06
7e-05
2e-03
6e-02
Log-odds
0.5
1.0
1.5
2.0
2.5
3.0
3.5
4.0
Cellular_organisms - ps1
Archaea1 - ps2
Archaea2 - ps3
Archaea3 - ps4
Asgard/Eukaryota - ps5
Eukaryota - ps6
Excavata/Diaphoretickes - ps7
Diaphoretickes - ps8
Archaeplastida - ps9
Viridiplantae - ps10
Streptophyta - ps11
Charophyceae/Embriophyta - ps12
Embriophyta - ps13
Tracheophyta - ps14
Spermatophyta - ps15
Angiosperms - ps16
Mesangiospermae - ps17
Magnoliids/eudicots - ps18
Eudicots - ps19
Core_eudicots - ps20
Superrosids - ps21
Rosids - ps22
Eurosids - ps23
Malvids - ps24
Sap./Malv./Brassicales - ps25
Malvales/Brassicales - ps26
Brassicales - ps27
Cleomaceae/Brassicaceae - ps28
Brassicaceae - ps29
Camelineae - ps30
Arabidopsis - ps31
Arabidopsis_thaliana - ps32
Phylostratum

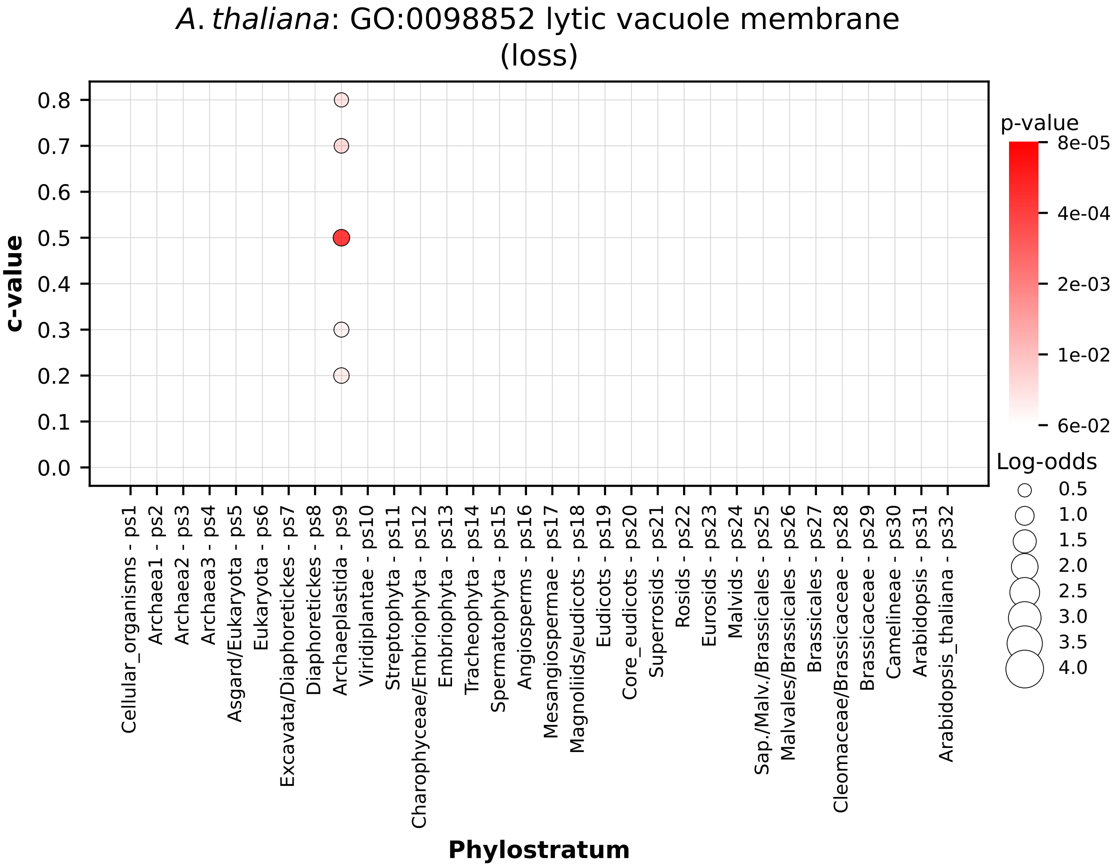

A. thaliana: GO:0098852 lytic vacuole membrane
(loss)
c-value
0.8
0.7
0.6
0.5
0.4
0.3
0.2
0.1
0.0
p-value
8e-05
4e-04
2e-03
1e-02
6e-02
Log-odds
0.5
1.0
1.5
2.0
2.5
3.0
3.5
4.0
Cellular_organisms - ps1
Archaea1 - ps2
Archaea2 - ps3
Archaea3 - ps4
Asgard/Eukaryota - ps5
Eukaryota - ps6
Excavata/Diaphoretickes - ps7
Diaphoretickes - ps8
Archaeplastida - ps9
Viridiplantae - ps10
Streptophyta - ps11
Charophyceae/Embriophyta - ps12
Embriophyta - ps13
Tracheophyta - ps14
Spermatophyta - ps15
Angiosperms - ps16
Mesangiospermae - ps17
Magnoliids/eudicots - ps18
Eudicots - ps19
Core_eudicots - ps20
Superrosids - ps21
Rosids - ps22
Eurosids - ps23
Malvids - ps24
Sap./Malv./Brassicales - ps25
Malvales/Brassicales - ps26
Brassicales - ps27
Cleomaceae/Brassicaceae - ps28
Brassicaceae - ps29
Camelineae - ps30
Arabidopsis - ps31
Arabidopsis_thaliana - ps32
Phylostratum

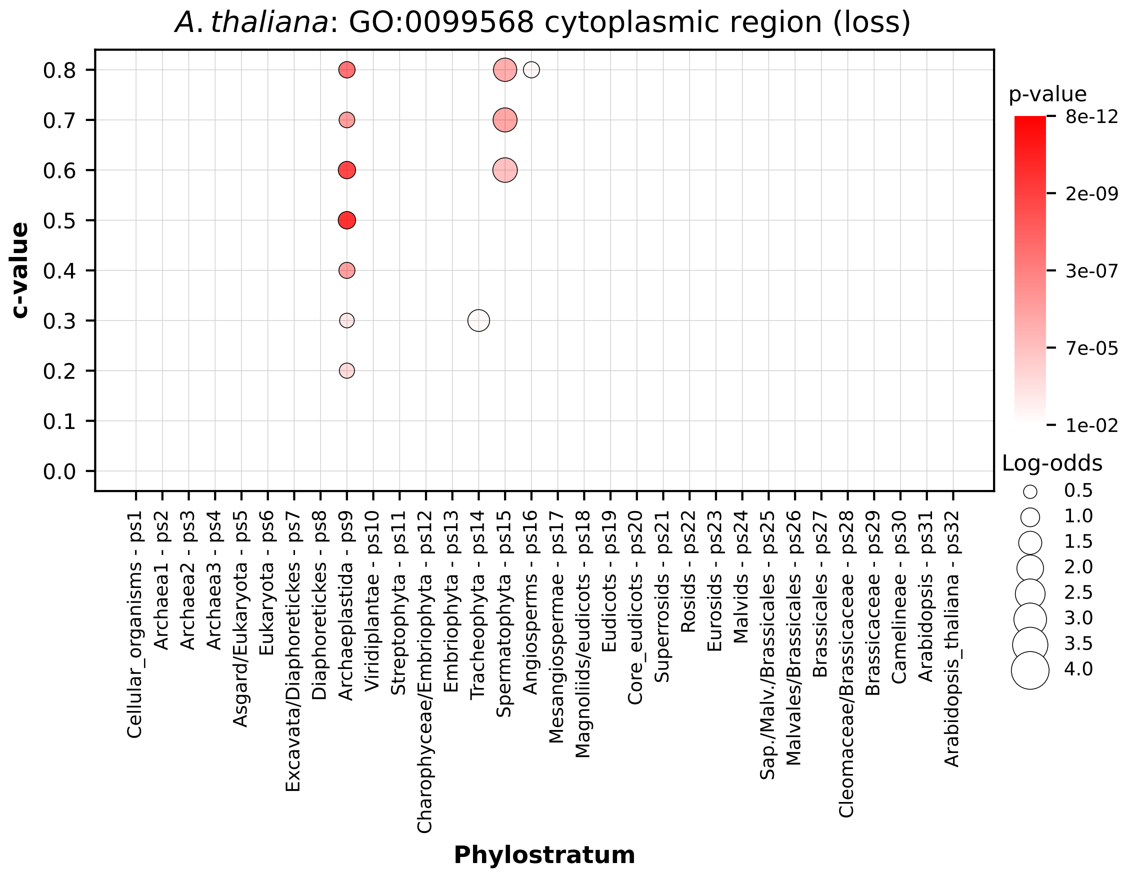

A. thaliana: GO:0099568 cytoplasmic region (loss)
c-value
0.8
0.7
0.6
0.5
0.4
0.3
0.2
0.1
0.0
Phylostratum
Cellular_organisms - ps1
Archaea1 - ps2
Archaea2 - ps3
Archaea3 - ps4
Asgard/Eukaryota - ps5
Eukaryota - ps6
Excavata/Diaphoretickes - ps7
Diaphoretickes - ps8
Archaeplastida - ps9
Viridiplantae - ps10
Streptophyta - ps11
Charophyceae/Embriophyta - ps12
Embriophyta - ps13
Tracheophyta - ps14
Spermatophyta - ps15
Angiosperms - ps16
Mesangiospermae - ps17
Magnoliids/eudicots - ps18
Eudicots - ps19
Core_eudicots - ps20
Superrosids - ps21
Rosids - ps22
Eurosids - ps23
Malvids - ps24
Sap./Malv./Brassicales - ps25
Malvales/Brassicales - ps26
Brassicales - ps27
Cleomaceae/Brassicaceae - ps28
Brassicaceae - ps29
Camelineae - ps30
Arabidopsis - ps31
Arabidopsis_thaliana - ps32
p-value
8e-12
2e-09
3e-07
7e-05
1e-02
Log-odds
0.5
1.0
1.5
2.0
2.5
3.0
3.5
4.0

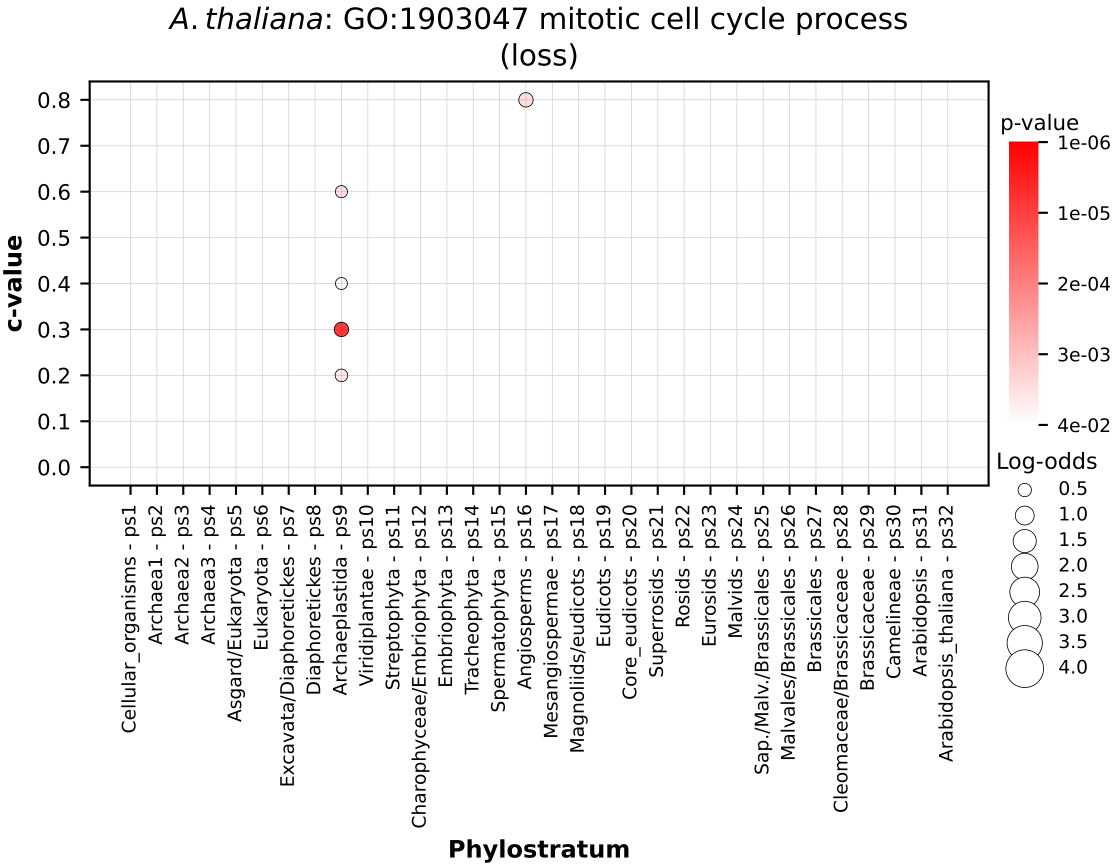

A. thaliana: GO:1903047 mitotic cell cycle process
(loss)
c-value
0.0
0.1
0.2
0.3
0.4
0.5
0.6
0.7
0.8
Phylostratum
Cellular_organisms - ps1
Archaea1 - ps2
Archaea2 - ps3
Archaea3 - ps4
Asgard/Eukaryota - ps5
Eukaryota - ps6
Excavata/Diaphoretickes - ps7
Diaphoretickes - ps8
Archaeplastida - ps9
Viridiplantae - ps10
Streptophyta - ps11
Charophyceae/Embriophyta - ps12
Embriophyta - ps13
Tracheophyta - ps14
Spermatophyta - ps15
Angiosperms - ps16
Mesangiospermae - ps17
Magnoliids/eudicots - ps18
Eudicots - ps19
Core_eudicots - ps20
Superrosids - ps21
Rosids - ps22
Eurosids - ps23
Malvids - ps24
Sap./Malv./Brassicales - ps25
Malvales/Brassicales - ps26
Brassicales - ps27
Cleomaceae/Brassicaceae - ps28
Brassicaceae - ps29
Camelineae - ps30
Arabidopsis - ps31
Arabidopsis_thaliana - ps32
p-value
1e-06
1e-05
2e-04
3e-03
4e-02
Log-odds
0.5
1.0
1.5
2.0
2.5
3.0
3.5
4.0

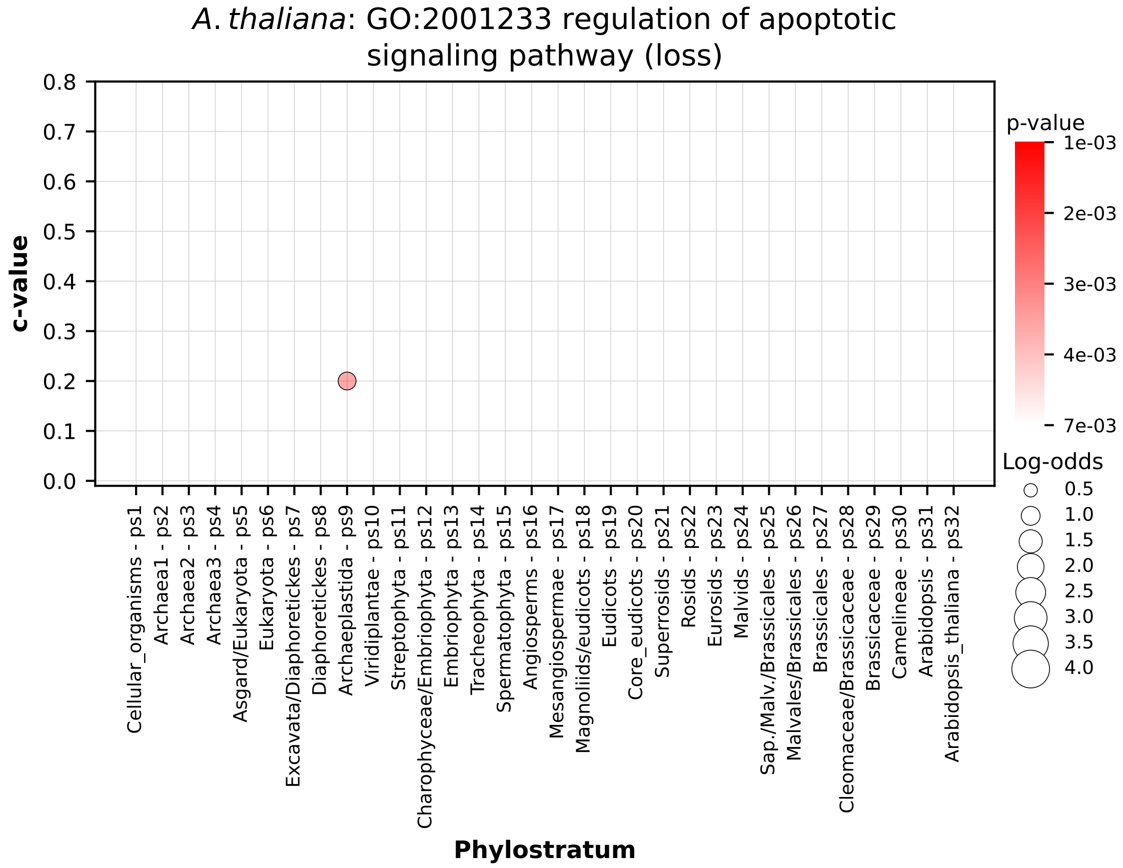

A. thaliana: GO:2001233 regulation of apoptotic signaling pathway (loss)
c-value
0.0
0.1
0.2
0.3
0.4
0.5
0.6
0.7
0.8
Phylostratum
Cellular_organisms - ps1
Archaea1 - ps2
Archaea2 - ps3
Archaea3 - ps4
Asgard/Eukaryota - ps5
Eukaryota - ps6
Excavata/Diaphoretickes - ps7
Diaphoretickes - ps8
Archaeplastida - ps9
Viridiplantae - ps10
Streptophyta - ps11
Charophyceae/Embriophyta - ps12
Embriophyta - ps13
Tracheophyta - ps14
Spermatophyta - ps15
Angiosperms - ps16
Mesangiospermae - ps17
Magnoliids/eudicots - ps18
Eudicots - ps19
Core_eudicots - ps20
Superrosids - ps21
Rosids - ps22
Eurosids - ps23
Malvids - ps24
Sap./Malv./Brassicales - ps25
Malvales/Brassicales - ps26
Brassicales - ps27
Cleomaceae/Brassicaceae - ps28
Brassicaceae - ps29
Camelineae - ps30
Arabidopsis - ps31
Arabidopsis_thaliana - ps32
p-value
1e-03
2e-03
3e-03
4e-03
7e-03
Log-odds
0.5
1.0
1.5
2.0
2.5
3.0
3.5
4.0

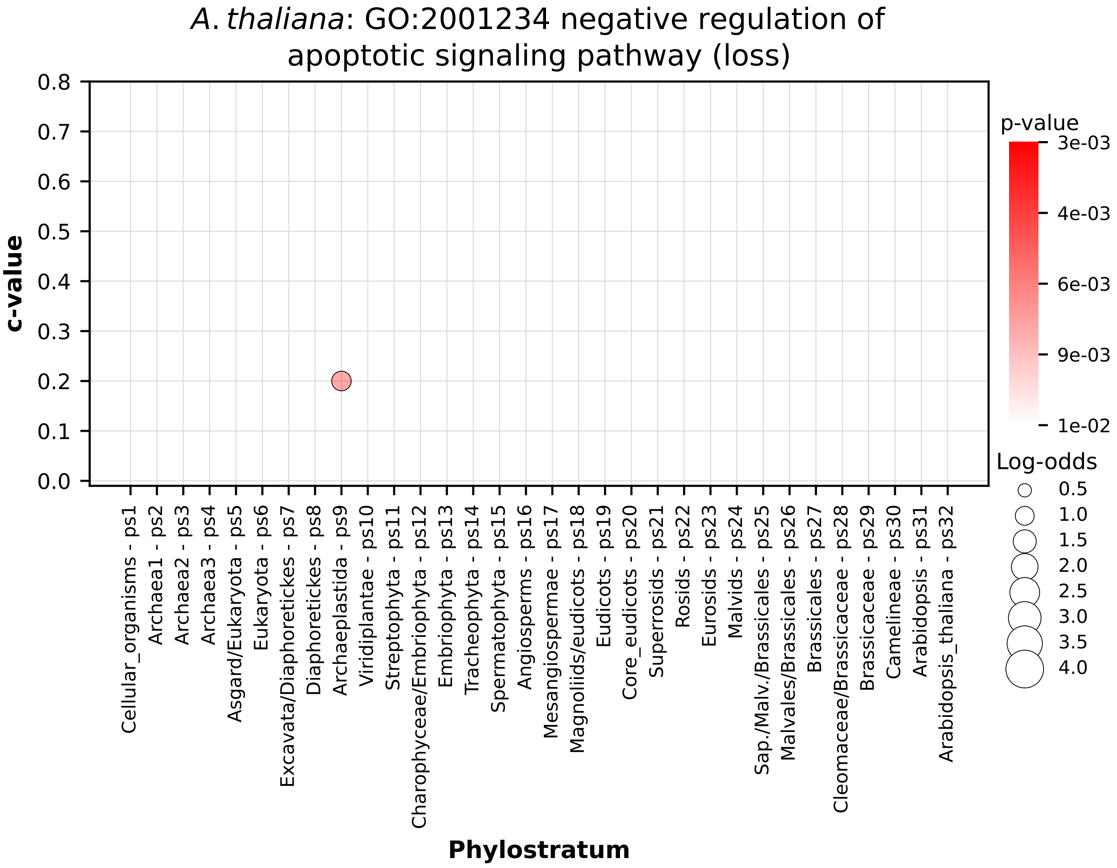
A. thaliana: GO:2001234 negative regulation of apoptotic signaling pathway (loss)
c-value
0.8
0.7
0.6
0.5
0.4
0.3
0.2
0.1
0.0
p-value
3e-03
4e-03
6e-03
9e-03
1e-02
Log-odds
0.5
1.0
1.5
2.0
2.5
3.0
3.5
4.0
Cellular_organisms - ps1
Archaea1 - ps2
Archaea2 - ps3
Archaea3 - ps4
Asgard/Eukaryota - ps5
Eukaryota - ps6
Excavata/Diaphoretickes - ps7
Diaphoretickes - ps8
Archaeplastida - ps9
Viridiplantae - ps10
Streptophyta - ps11
Charophyceae/Embriophyta - ps12
Embriophyta - ps13
Tracheophyta - ps14
Spermatophyta - ps15
Angiosperms - ps16
Mesangiospermae - ps17
Magnoliids/eudicots - ps18
Eudicots - ps19
Core_eudicots - ps20
Superrosids - ps21
Rosids - ps22
Eurosids - ps23
Malvids - ps24
Sap./Malv./Brassicales - ps25
Malvales/Brassicales - ps26
Brassicales - ps27
Cleomaceae/Brassicaceae - ps28
Brassicaceae - ps29
Camelineae - ps30
Arabidopsis - ps31
Arabidopsis_thaliana - ps32
Phylostratum

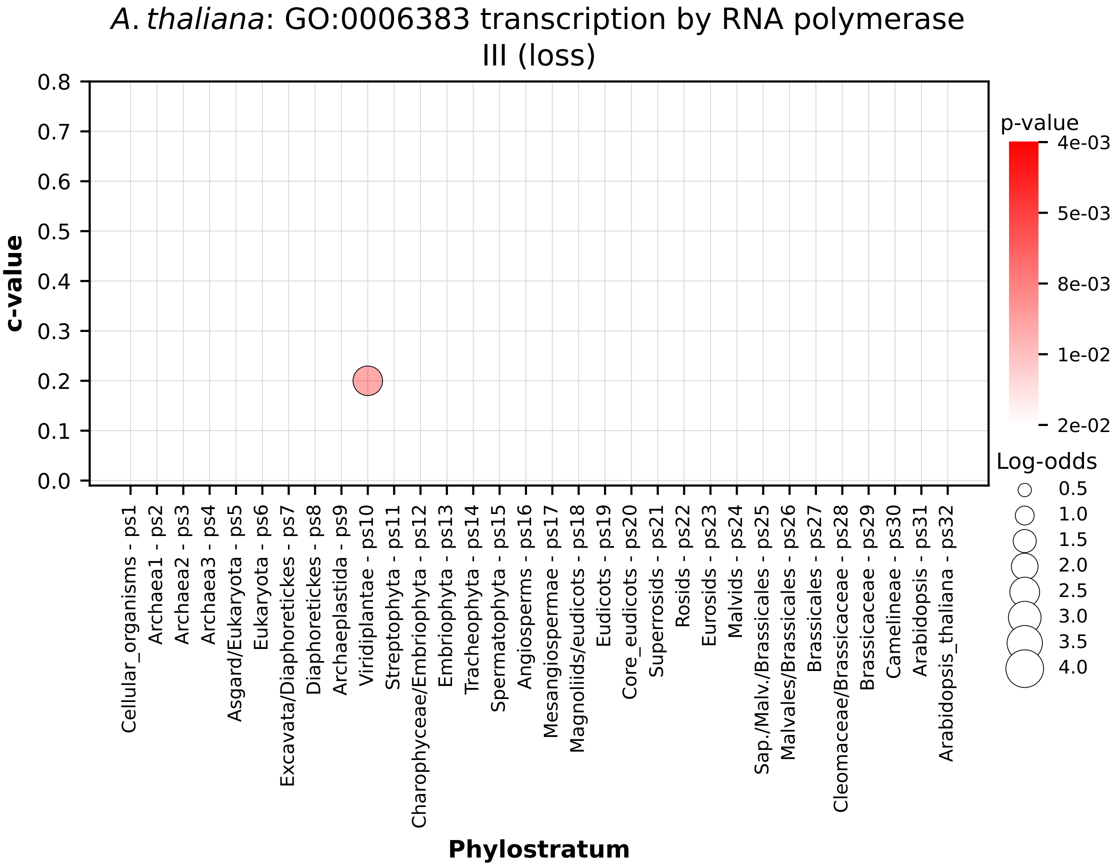

A. thaliana: GO:0006383 transcription by RNA polymerase III (loss)
c-value
0.8
0.7
0.6
0.5
0.4
0.3
0.2
0.1
0.0
p-value
4e-03
5e-03
8e-03
1e-02
2e-02
Log-odds
0.5
1.0
1.5
2.0
2.5
3.0
3.5
4.0
Cellular_organisms - ps1
Archaea1 - ps2
Archaea2 - ps3
Archaea3 - ps4
Asgard/Eukaryota - ps5
Eukaryota - ps6
Excavata/Diaphoretickes - ps7
Diaphoretickes - ps8
Archaeplastida - ps9
Viridiplantae - ps10
Streptophyta - ps11
Charophyceae/Embriophyta - ps12
Embriophyta - ps13
Tracheophyta - ps14
Spermatophyta - ps15
Angiosperms - ps16
Mesangiospermae - ps17
Magnoliids/eudicots - ps18
Eudicots - ps19
Core_eudicots - ps20
Superrosids - ps21
Rosids - ps22
Eurosids - ps23
Malvids - ps24
Sap./Malv./Brassicales - ps25
Malvales/Brassicales - ps26
Brassicales - ps27
Cleomaceae/Brassicaceae - ps28
Brassicaceae - ps29
Camelineae - ps30
Arabidopsis - ps31
Arabidopsis_thaliana - ps32
Phylostratum

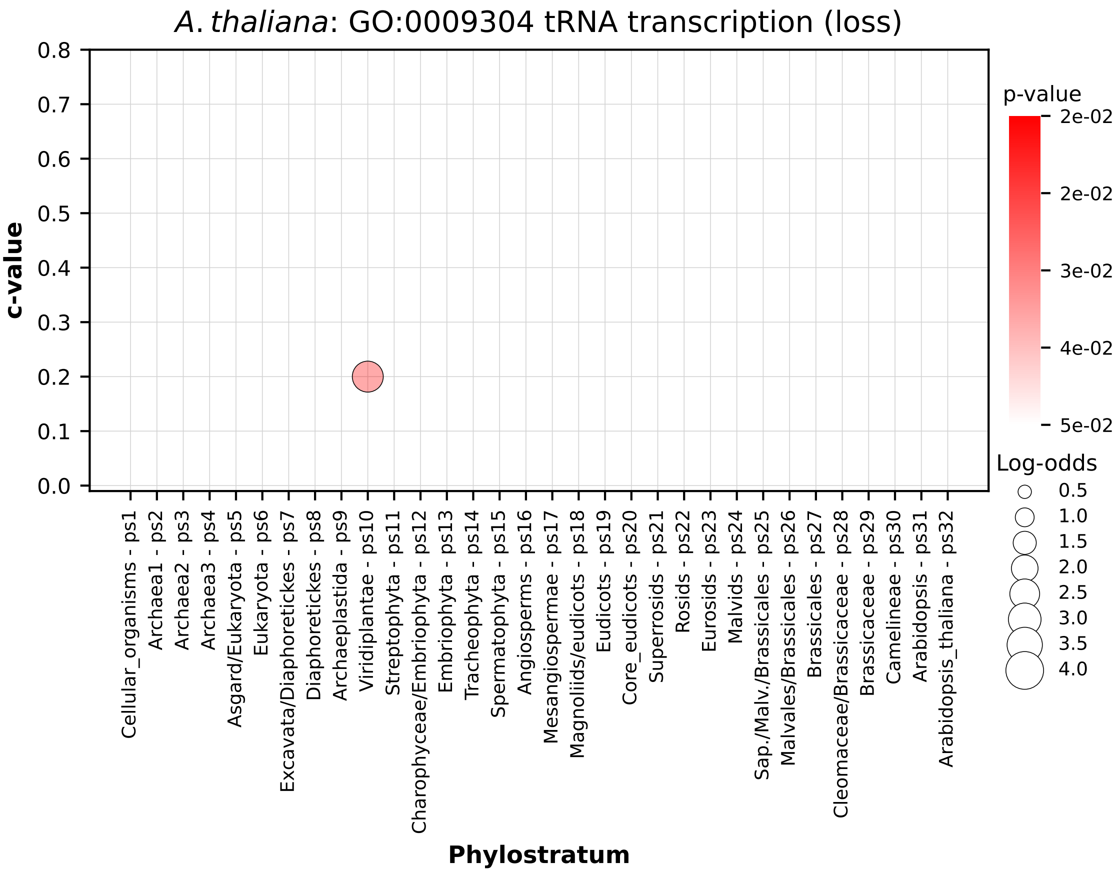

A. thaliana: GO:0009304 tRNA transcription (loss)
c-value
0.0
0.1
0.2
0.3
0.4
0.5
0.6
0.7
0.8
Phylostratum
Cellular_organisms - ps1
Archaea1 - ps2
Archaea2 - ps3
Archaea3 - ps4
Asgard/Eukaryota - ps5
Eukaryota - ps6
Excavata/Diaphoretickes - ps7
Diaphoretickes - ps8
Archaeplastida - ps9
Viridiplantae - ps10
Streptophyta - ps11
Charophyceae/Embriophyta - ps12
Embriophyta - ps13
Tracheophyta - ps14
Spermatophyta - ps15
Angiosperms - ps16
Mesangiospermae - ps17
Magnoliids/eudicots - ps18
Eudicots - ps19
Core_eudicots - ps20
Superrosids - ps21
Rosids - ps22
Eurosids - ps23
Malvids - ps24
Sap./Malv./Brassicales - ps25
Malvales/Brassicales - ps26
Brassicales - ps27
Cleomaceae/Brassicaceae - ps28
Brassicaceae - ps29
Camelineae - ps30
Arabidopsis - ps31
Arabidopsis_thaliana - ps32
p-value
2e-02
2e-02
3e-02
4e-02
5e-02
Log-odds
0.5
1.0
1.5
2.0
2.5
3.0
3.5
4.0

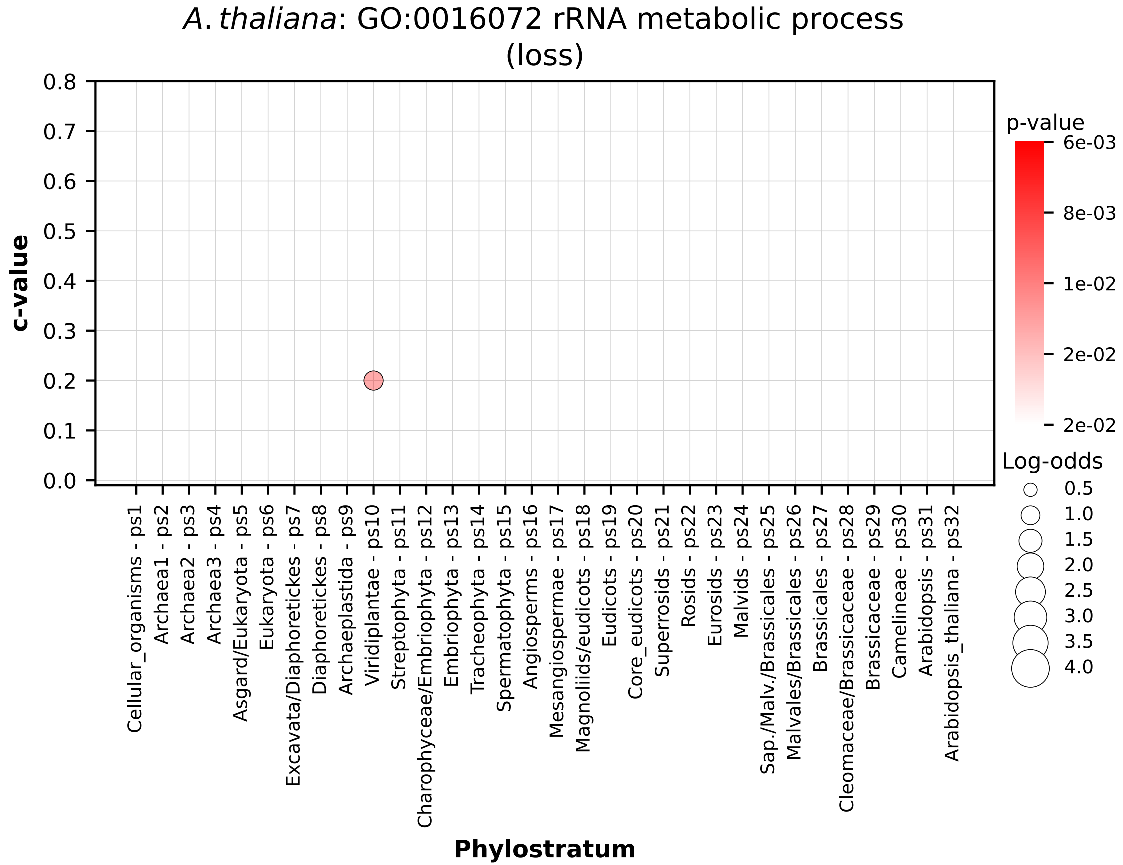

A. thaliana: GO:0016072 rRNA metabolic process
(loss)
c-value
0.0
0.1
0.2
0.3
0.4
0.5
0.6
0.7
0.8
Phylostratum
Cellular_organisms - ps1
Archaea1 - ps2
Archaea2 - ps3
Archaea3 - ps4
Asgard/Eukaryota - ps5
Eukaryota - ps6
Excavata/Diaphoretickes - ps7
Diaphoretickes - ps8
Archaeplastida - ps9
Viridiplantae - ps10
Streptophyta - ps11
Charophyceae/Embriophyta - ps12
Embriophyta - ps13
Tracheophyta - ps14
Spermatophyta - ps15
Angiosperms - ps16
Mesangiospermae - ps17
Magnoliids/eudicots - ps18
Eudicots - ps19
Core_eudicots - ps20
Superrosids - ps21
Rosids - ps22
Eurosids - ps23
Malvids - ps24
Sap./Malv./Brassicales - ps25
Malvales/Brassicales - ps26
Brassicales - ps27
Cleomaceae/Brassicaceae - ps28
Brassicaceae - ps29
Camelineae - ps30
Arabidopsis - ps31
Arabidopsis_thaliana - ps32
p-value
6e-03
8e-03
1e-02
2e-02
2e-02
Log-odds
0.5
1.0
1.5
2.0
2.5
3.0
3.5
4.0

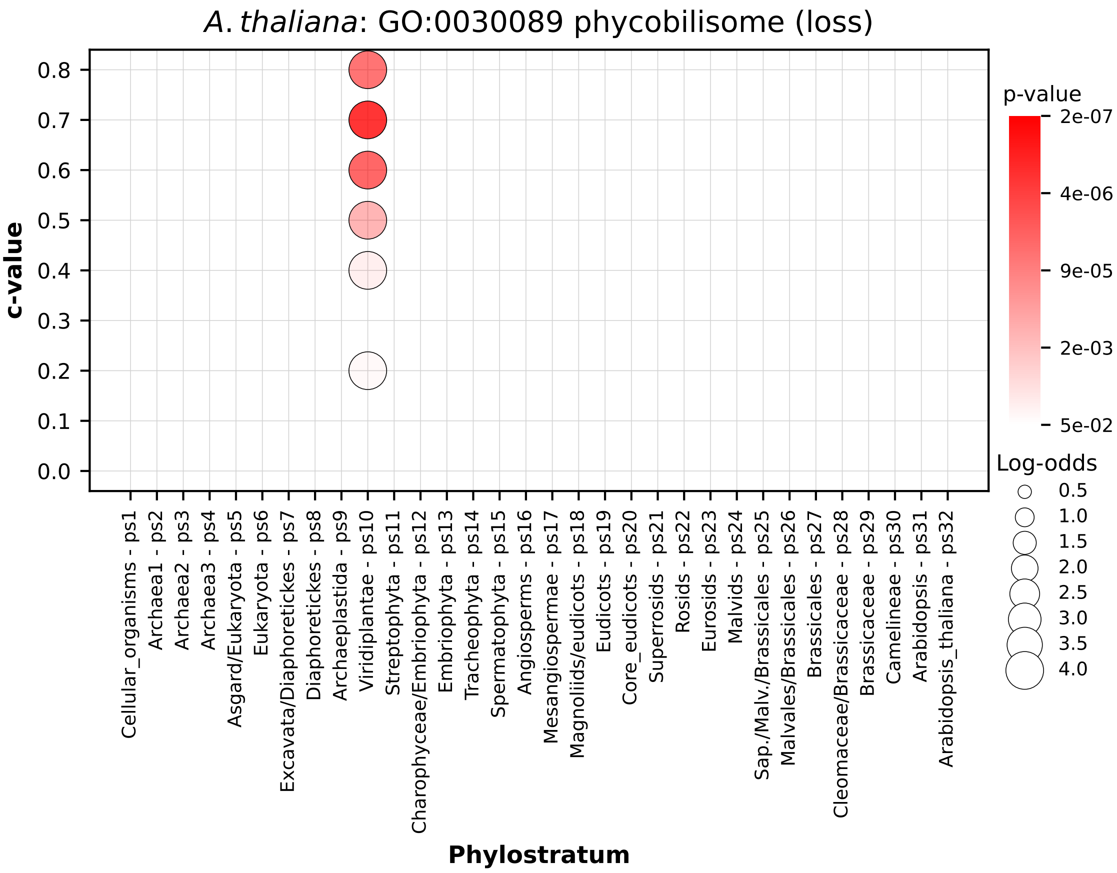

A. thaliana: GO:0030089 phycobilisome (loss)
c-value
0.8
0.7
0.6
0.5
0.4
0.3
0.2
0.1
0.0
p-value
2e-07
4e-06
9e-05
2e-03
5e-02
Log-odds
0.5
1.0
1.5
2.0
2.5
3.0
3.5
4.0
Cellular_organisms - ps1
Archaea1 - ps2
Archaea2 - ps3
Archaea3 - ps4
Asgard/Eukaryota - ps5
Eukaryota - ps6
Excavata/Diaphoretickes - ps7
Diaphoretickes - ps8
Archaeplastida - ps9
Viridiplantae - ps10
Streptophyta - ps11
Charophyceae/Embriophyta - ps12
Embriophyta - ps13
Tracheophyta - ps14
Spermatophyta - ps15
Angiosperms - ps16
Mesangiospermae - ps17
Magnoliids/eudicots - ps18
Eudicots - ps19
Core_eudicots - ps20
Superrosids - ps21
Rosids - ps22
Eurosids - ps23
Malvids - ps24
Sap./Malv./Brassicales - ps25
Malvales/Brassicales - ps26
Brassicales - ps27
Cleomaceae/Brassicaceae - ps28
Brassicaceae - ps29
Camelineae - ps30
Arabidopsis - ps31
Arabidopsis_thaliana - ps32
Phylostratum

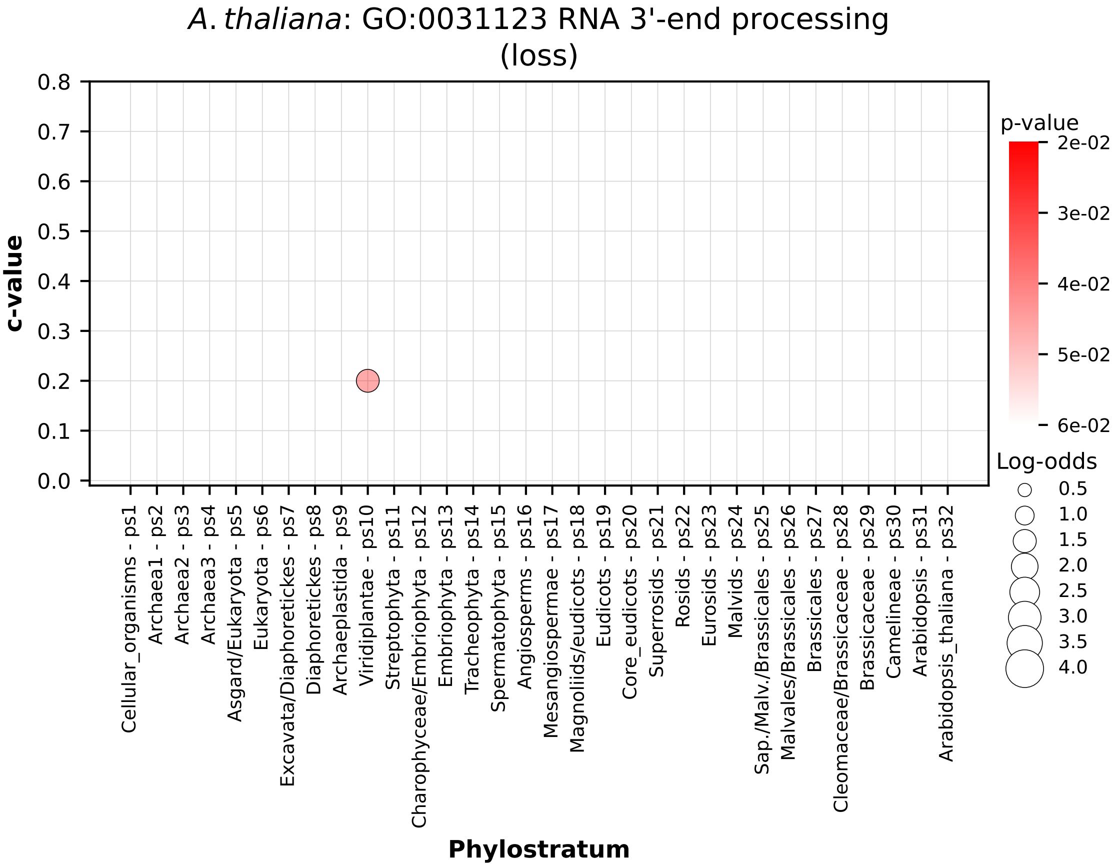

A. thaliana: GO:0031123 RNA 3'-end processing
(loss)
c-value
0.8
0.7
0.6
0.5
0.4
0.3
0.2
0.1
0.0
p-value
2e-02
3e-02
4e-02
5e-02
6e-02
Log-odds
0.5
1.0
1.5
2.0
2.5
3.0
3.5
4.0
Cellular_organisms - ps1
Archaea1 - ps2
Archaea2 - ps3
Archaea3 - ps4
Asgard/Eukaryota - ps5
Eukaryota - ps6
Excavata/Diaphoretickes - ps7
Diaphoretickes - ps8
Archaeplastida - ps9
Viridiplantae - ps10
Streptophyta - ps11
Charophyceae/Embriophyta - ps12
Embriophyta - ps13
Tracheophyta - ps14
Spermatophyta - ps15
Angiosperms - ps16
Mesangiospermae - ps17
Magnoliids/eudicots - ps18
Eudicots - ps19
Core_eudicots - ps20
Superrosids - ps21
Rosids - ps22
Eurosids - ps23
Malvids - ps24
Sap./Malv./Brassicales - ps25
Malvales/Brassicales - ps26
Brassicales - ps27
Cleomaceae/Brassicaceae - ps28
Brassicaceae - ps29
Camelineae - ps30
Arabidopsis - ps31
Arabidopsis_thaliana - ps32
Phylostratum

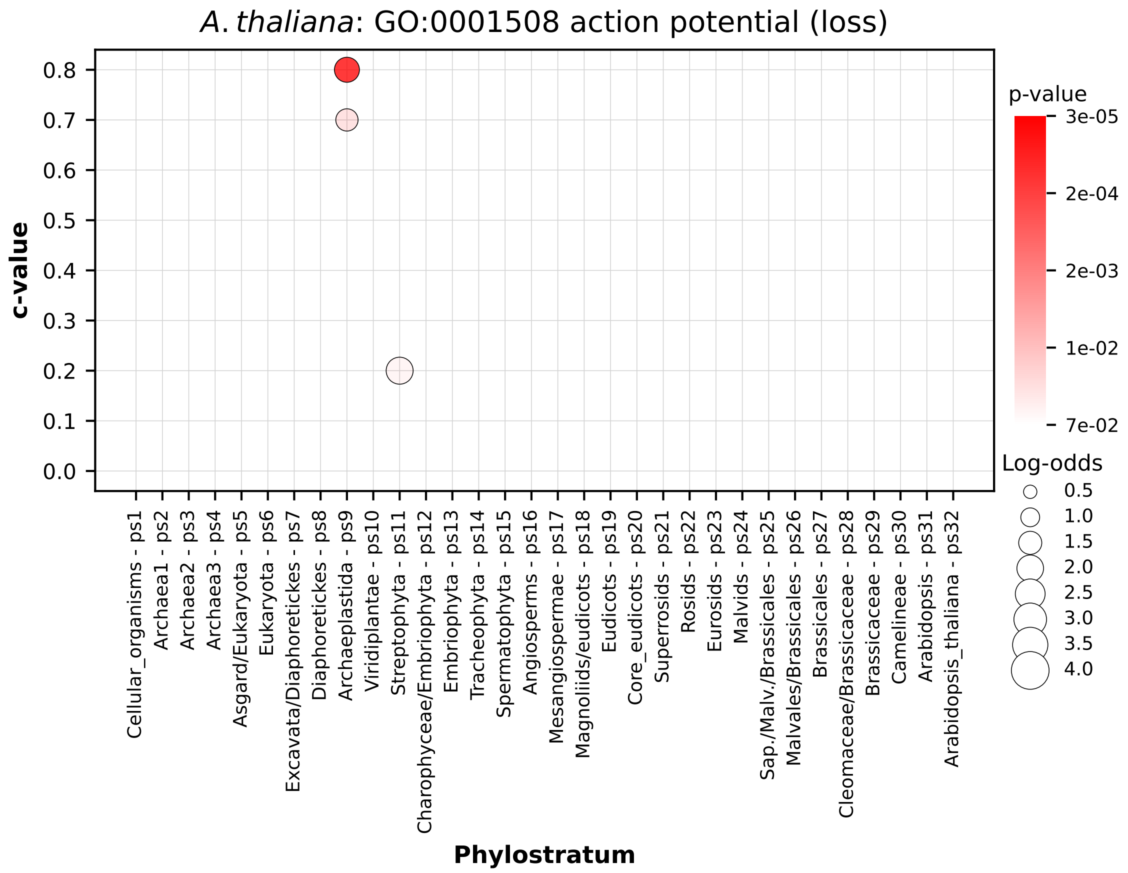

A. thaliana: GO:0001508 action potential (loss)
c-value
0.8
0.7
0.6
0.5
0.4
0.3
0.2
0.1
0.0
p-value
3e-05
2e-04
2e-03
1e-02
7e-02
Log-odds
0.5
1.0
1.5
2.0
2.5
3.0
3.5
4.0
Cellular_organisms - ps1
Archaea1 - ps2
Archaea2 - ps3
Archaea3 - ps4
Asgard/Eukaryota - ps5
Eukaryota - ps6
Excavata/Diaphoretickes - ps7
Diaphoretickes - ps8
Archaeplastida - ps9
Viridiplantae - ps10
Streptophyta - ps11
Charophyceae/Embriophyta - ps12
Embriophyta - ps13
Tracheophyta - ps14
Spermatophyta - ps15
Angiosperms - ps16
Mesangiospermae - ps17
Magnoliids/eudicots - ps18
Eudicots - ps19
Core_eudicots - ps20
Superrosids - ps21
Rosids - ps22
Eurosids - ps23
Malvids - ps24
Sap./Malv./Brassicales - ps25
Malvales/Brassicales - ps26
Brassicales - ps27
Cleomaceae/Brassicaceae - ps28
Brassicaceae - ps29
Camelineae - ps30
Arabidopsis - ps31
Arabidopsis_thaliana - ps32
Phylostratum

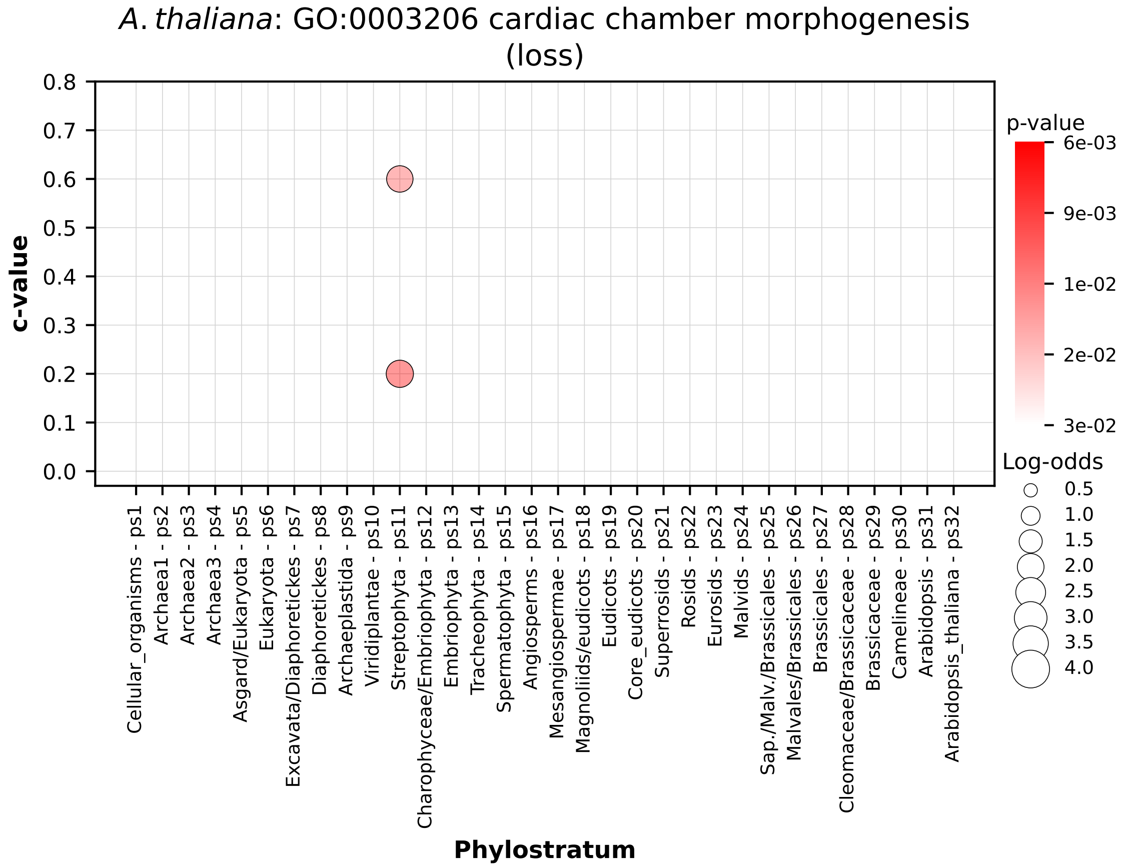
A. thaliana: GO:0003206 cardiac chamber morphogenesis
(loss)
c-value
0.8
0.7
0.6
0.5
0.4
0.3
0.2
0.1
0.0
p-value
6e-03
9e-03
1e-02
2e-02
3e-02
Log-odds
0.5
1.0
1.5
2.0
2.5
3.0
3.5
4.0
Cellular_organisms - ps1
Archaea1 - ps2
Archaea2 - ps3
Archaea3 - ps4
Asgard/Eukaryota - ps5
Eukaryota - ps6
Excavata/Diaphoretickes - ps7
Diaphoretickes - ps8
Archaeplastida - ps9
Viridiplantae - ps10
Streptophyta - ps11
Charophyceae/Embriophyta - ps12
Embriophyta - ps13
Tracheophyta - ps14
Spermatophyta - ps15
Angiosperms - ps16
Mesangiospermae - ps17
Magnoliids/eudicots - ps18
Eudicots - ps19
Core_eudicots - ps20
Superrosids - ps21
Rosids - ps22
Eurosids - ps23
Malvids - ps24
Sap./Malv./Brassicales - ps25
Malvales/Brassicales - ps26
Brassicales - ps27
Cleomaceae/Brassicaceae - ps28
Brassicaceae - ps29
Camelineae - ps30
Arabidopsis - ps31
Arabidopsis_thaliana - ps32
Phylostratum

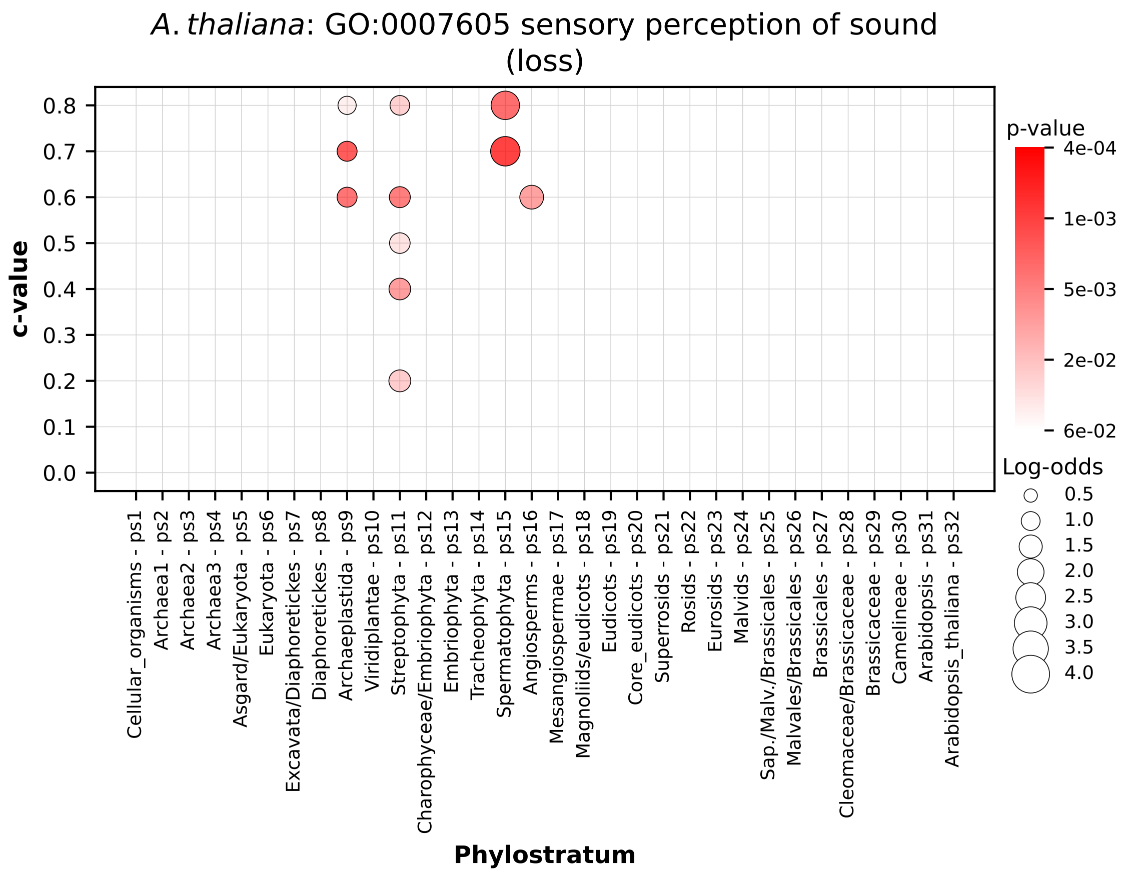
A. thaliana: GO:0007605 sensory perception of sound
(loss)
c-value
0.0
0.1
0.2
0.3
0.4
0.5
0.6
0.7
0.8
Phylostratum
Cellular_organisms - ps1
Archaea1 - ps2
Archaea2 - ps3
Archaea3 - ps4
Asgard/Eukaryota - ps5
Eukaryota - ps6
Excavata/Diaphoretickes - ps7
Diaphoretickes - ps8
Archaeplastida - ps9
Viridiplantae - ps10
Streptophyta - ps11
Charophyceae/Embriophyta - ps12
Embriophyta - ps13
Tracheophyta - ps14
Spermatophyta - ps15
Angiosperms - ps16
Mesangiospermae - ps17
Magnoliids/eudicots - ps18
Eudicots - ps19
Core_eudicots - ps20
Superrosids - ps21
Rosids - ps22
Eurosids - ps23
Malvids - ps24
Sap./Malv./Brassicales - ps25
Malvales/Brassicales - ps26
Brassicales - ps27
Cleomaceae/Brassicaceae - ps28
Brassicaceae - ps29
Camelineae - ps30
Arabidopsis - ps31
Arabidopsis_thaliana - ps32
p-value
4e-04
1e-03
5e-03
2e-02
6e-02
Log-odds
0.5
1.0
1.5
2.0
2.5
3.0
3.5
4.0

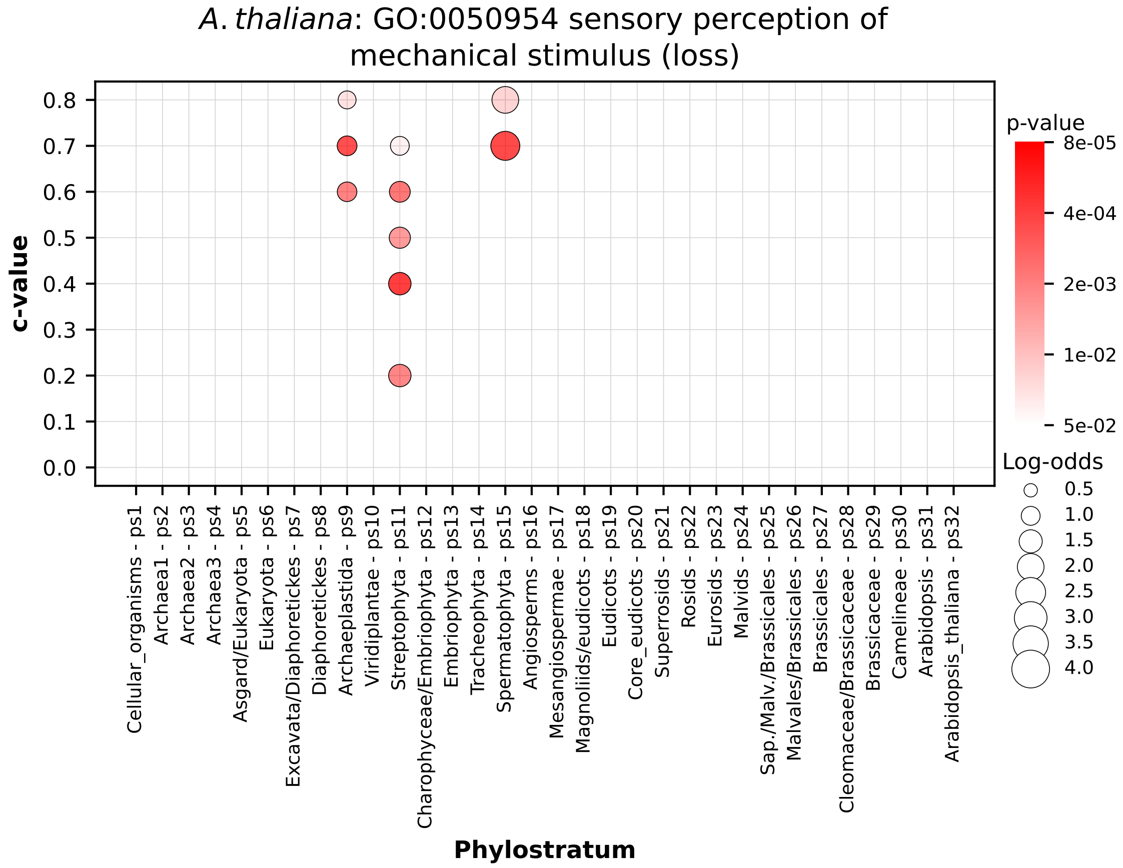

A. thaliana: GO:0050954 sensory perception of mechanical stimulus (loss)
c-value
0.8
0.7
0.6
0.5
0.4
0.3
0.2
0.1
0.0
p-value
8e-05
4e-04
2e-03
1e-02
5e-02
Log-odds
0.5
1.0
1.5
2.0
2.5
3.0
3.5
4.0
Cellular_organisms - ps1
Archaea1 - ps2
Archaea2 - ps3
Archaea3 - ps4
Asgard/Eukaryota - ps5
Eukaryota - ps6
Excavata/Diaphoretickes - ps7
Diaphoretickes - ps8
Archaeplastida - ps9
Viridiplantae - ps10
Streptophyta - ps11
Charophyceae/Embriophyta - ps12
Embriophyta - ps13
Tracheophyta - ps14
Spermatophyta - ps15
Angiosperms - ps16
Mesangiospermae - ps17
Magnoliids/eudicots - ps18
Eudicots - ps19
Core_eudicots - ps20
Superrosids - ps21
Rosids - ps22
Eurosids - ps23
Malvids - ps24
Sap./Malv./Brassicales - ps25
Malvales/Brassicales - ps26
Brassicales - ps27
Cleomaceae/Brassicaceae - ps28
Brassicaceae - ps29
Camelineae - ps30
Arabidopsis - ps31
Arabidopsis_thaliana - ps32
Phylostratum

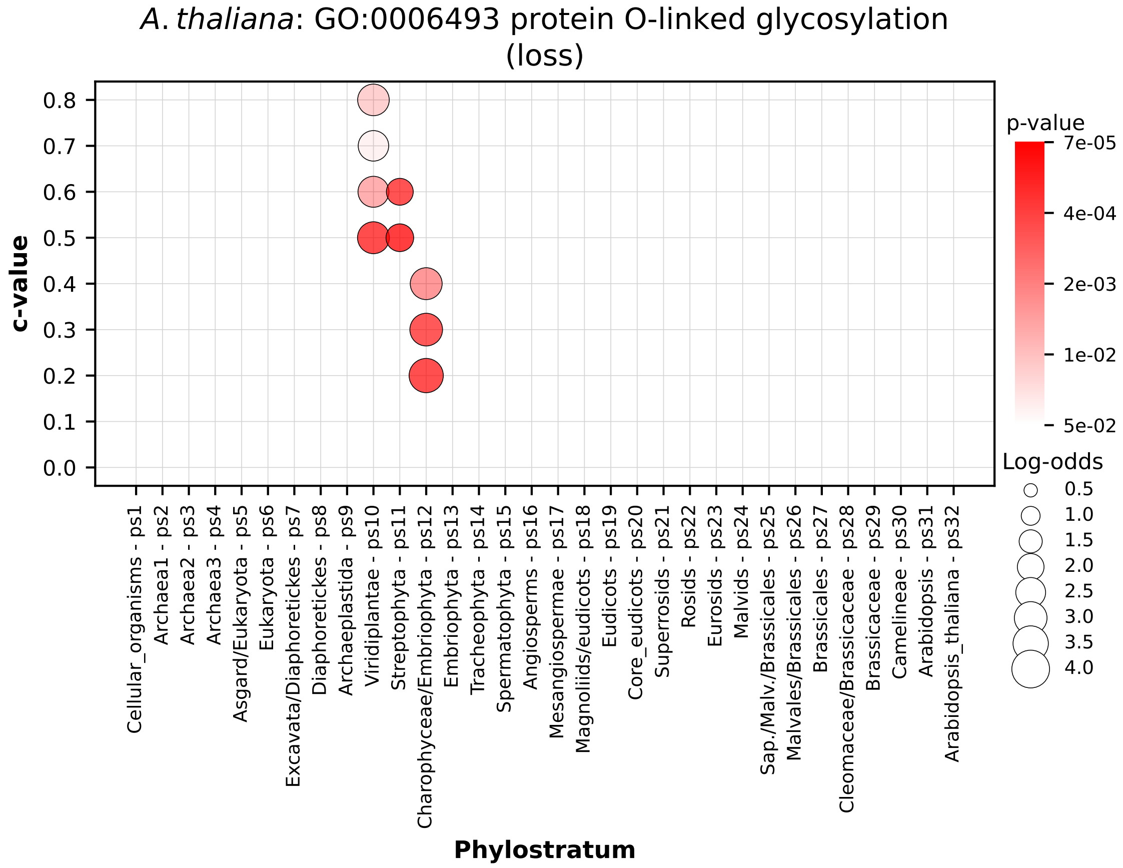

A. thaliana: GO:0006493 protein O-linked glycosylation (loss)
c-value
0.0
0.1
0.2
0.3
0.4
0.5
0.6
0.7
0.8
Phylostratum
Cellular_organisms - ps1
Archaea1 - ps2
Archaea2 - ps3
Archaea3 - ps4
Asgard/Eukaryota - ps5
Eukaryota - ps6
Excavata/Diaphoretickes - ps7
Diaphoretickes - ps8
Archaeplastida - ps9
Viridiplantae - ps10
Streptophyta - ps11
Charophyceae/Embriophyta - ps12
Embriophyta - ps13
Tracheophyta - ps14
Spermatophyta - ps15
Angiosperms - ps16
Mesangiospermae - ps17
Magnoliids/eudicots - ps18
Eudicots - ps19
Core_eudicots - ps20
Superrosids - ps21
Rosids - ps22
Eurosids - ps23
Malvids - ps24
Sap./Malv./Brassicales - ps25
Malvales/Brassicales - ps26
Brassicales - ps27
Cleomaceae/Brassicaceae - ps28
Brassicaceae - ps29
Camelineae - ps30
Arabidopsis - ps31
Arabidopsis_thaliana - ps32
p-value
7e-05
4e-04
2e-03
1e-02
5e-02
Log-odds
0.5
1.0
1.5
2.0
2.5
3.0
3.5
4.0

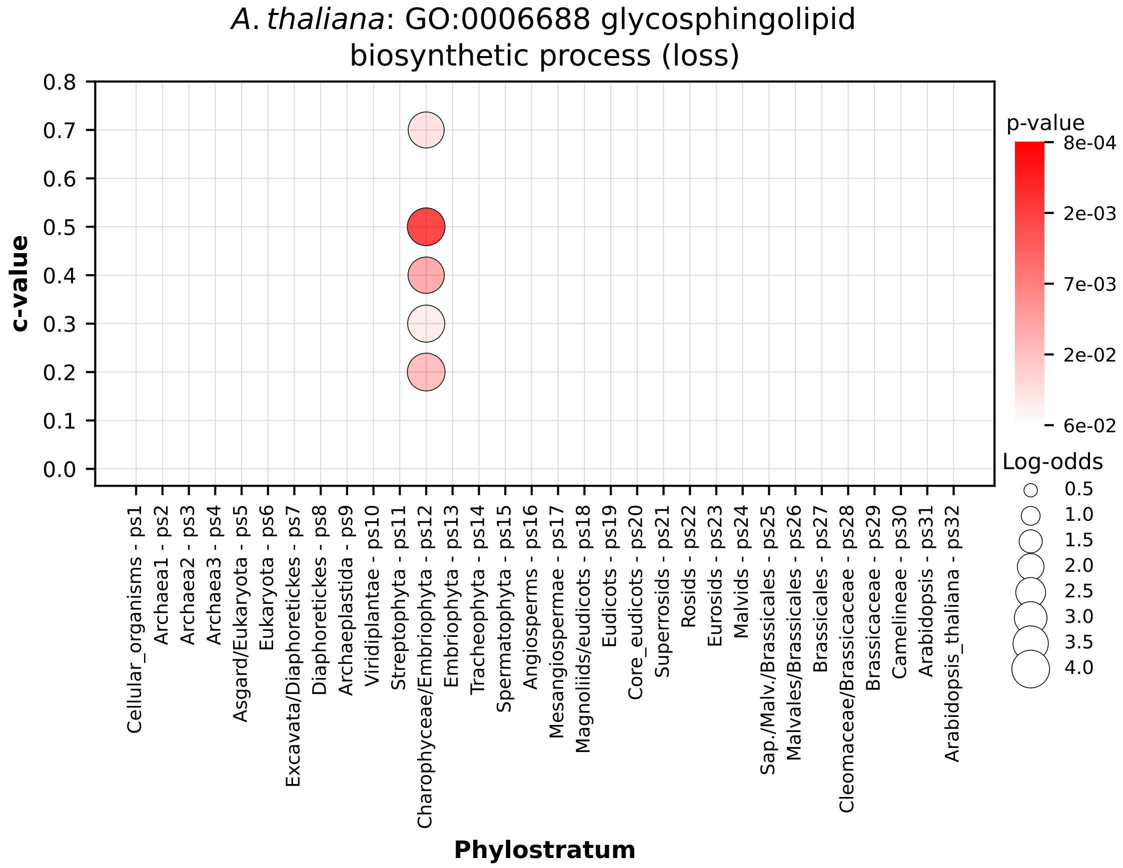
A. thaliana: GO:0006688 glycosphingolipid biosynthetic process (loss)
c-value
0.8
0.7
0.6
0.5
0.4
0.3
0.2
0.1
0.0
p-value
8e-04
2e-03
7e-03
2e-02
6e-02
Log-odds
0.5
1.0
1.5
2.0
2.5
3.0
3.5
4.0
Cellular_organisms - ps1
Archaea1 - ps2
Archaea2 - ps3
Archaea3 - ps4
Asgard/Eukaryota - ps5
Eukaryota - ps6
Excavata/Diaphoretickes - ps7
Diaphoretickes - ps8
Archaeplastida - ps9
Viridiplantae - ps10
Streptophyta - ps11
Charophyceae/Embriophyta - ps12
Embriophyta - ps13
Tracheophyta - ps14
Spermatophyta - ps15
Angiosperms - ps16
Mesangiospermae - ps17
Magnoliids/eudicots - ps18
Eudicots - ps19
Core_eudicots - ps20
Superrosids - ps21
Rosids - ps22
Eurosids - ps23
Malvids - ps24
Sap./Malv./Brassicales - ps25
Malvales/Brassicales - ps26
Brassicales - ps27
Cleomaceae/Brassicaceae - ps28
Brassicaceae - ps29
Camelineae - ps30
Arabidopsis - ps31
Arabidopsis_thaliana - ps32
Phylostratum

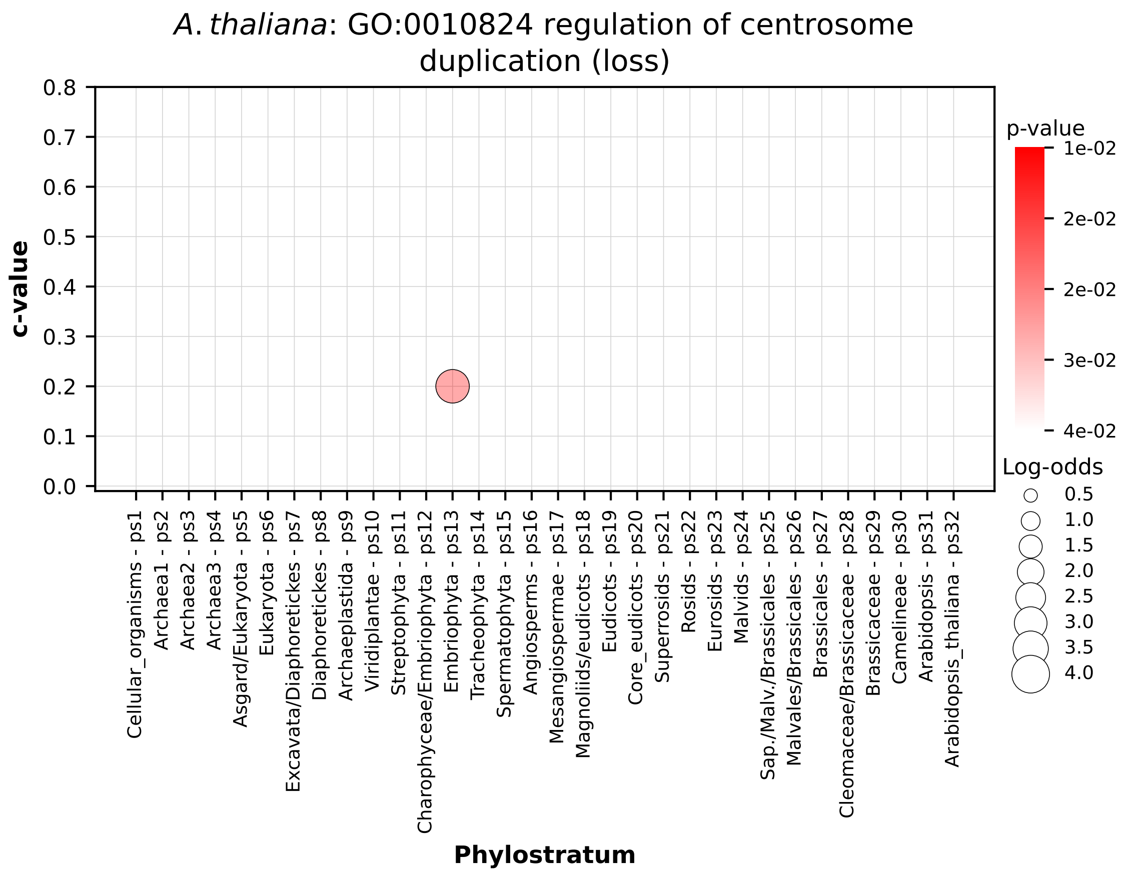

A. thaliana: GO:0010824 regulation of centrosome duplication (loss)
c-value
0.8
0.7
0.6
0.5
0.4
0.3
0.2
0.1
0.0
p-value
1e-02
2e-02
2e-02
3e-02
4e-02
Log-odds
0.5
1.0
1.5
2.0
2.5
3.0
3.5
4.0
Cellular_organisms - ps1
Archaea1 - ps2
Archaea2 - ps3
Archaea3 - ps4
Asgard/Eukaryota - ps5
Eukaryota - ps6
Excavata/Diaphoretickes - ps7
Diaphoretickes - ps8
Archaeplastida - ps9
Viridiplantae - ps10
Streptophyta - ps11
Charophyceae/Embriophyta - ps12
Embriophyta - ps13
Tracheophyta - ps14
Spermatophyta - ps15
Angiosperms - ps16
Mesangiospermae - ps17
Magnoliids/eudicots - ps18
Eudicots - ps19
Core_eudicots - ps20
Superrosids - ps21
Rosids - ps22
Eurosids - ps23
Malvids - ps24
Sap./Malv./Brassicales - ps25
Malvales/Brassicales - ps26
Brassicales - ps27
Cleomaceae/Brassicaceae - ps28
Brassicaceae - ps29
Camelineae - ps30
Arabidopsis - ps31
Arabidopsis_thaliana - ps32
Phylostratum

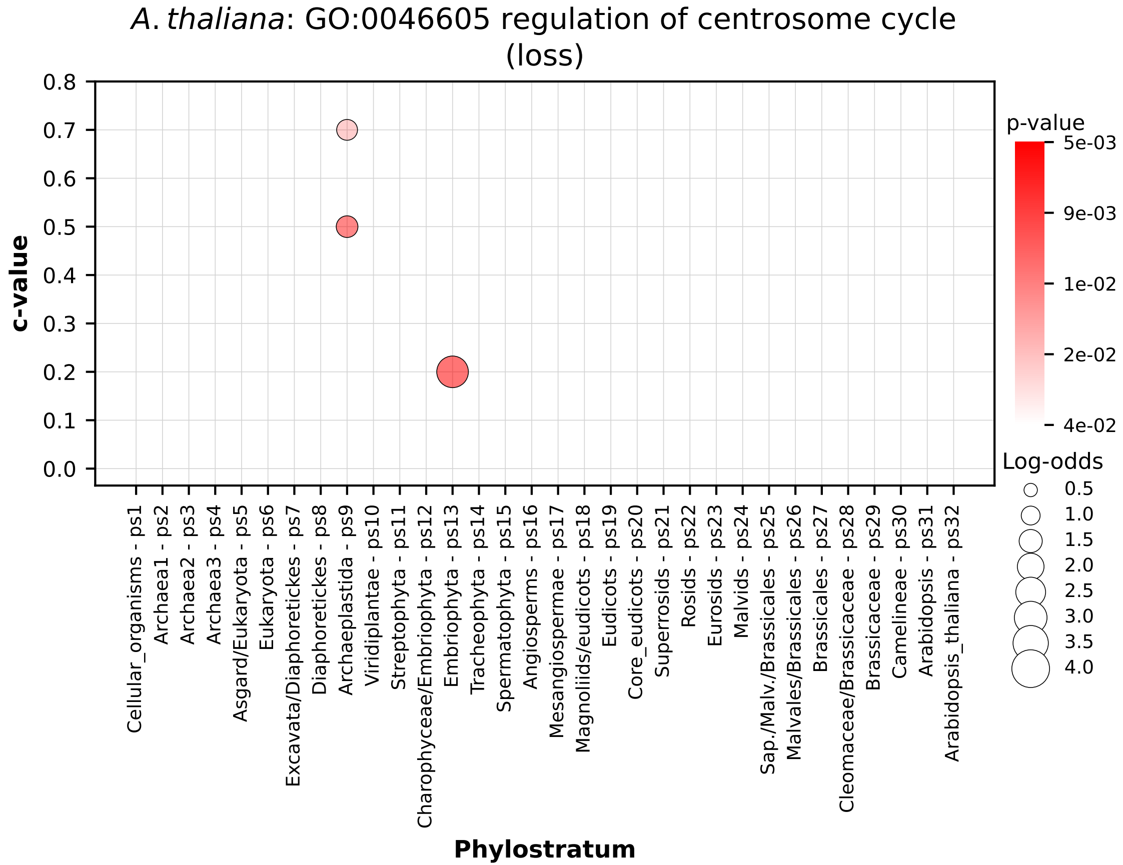
A. thaliana: GO:0046605 regulation of centrosome cycle (loss)
c-value
0.8
0.7
0.6
0.5
0.4
0.3
0.2
0.1
0.0
Phylostratum
Cellular_organisms - ps1
Archaea1 - ps2
Archaea2 - ps3
Archaea3 - ps4
Asgard/Eukaryota - ps5
Eukaryota - ps6
Excavata/Diaphoretickes - ps7
Diaphoretickes - ps8
Archaeplastida - ps9
Viridiplantae - ps10
Streptophyta - ps11
Charophyceae/Embriophyta - ps12
Embriophyta - ps13
Tracheophyta - ps14
Spermatophyta - ps15
Angiosperms - ps16
Mesangiospermae - ps17
Magnoliids/eudicots - ps18
Eudicots - ps19
Core_eudicots - ps20
Superrosids - ps21
Rosids - ps22
Eurosids - ps23
Malvids - ps24
Sap./Malv./Brassicales - ps25
Malvales/Brassicales - ps26
Brassicales - ps27
Cleomaceae/Brassicaceae - ps28
Brassicaceae - ps29
Camelineae - ps30
Arabidopsis - ps31
Arabidopsis_thaliana - ps32
p-value
5e-03
9e-03
1e-02
2e-02
4e-02
Log-odds
0.5
1.0
1.5
2.0
2.5
3.0
3.5
4.0

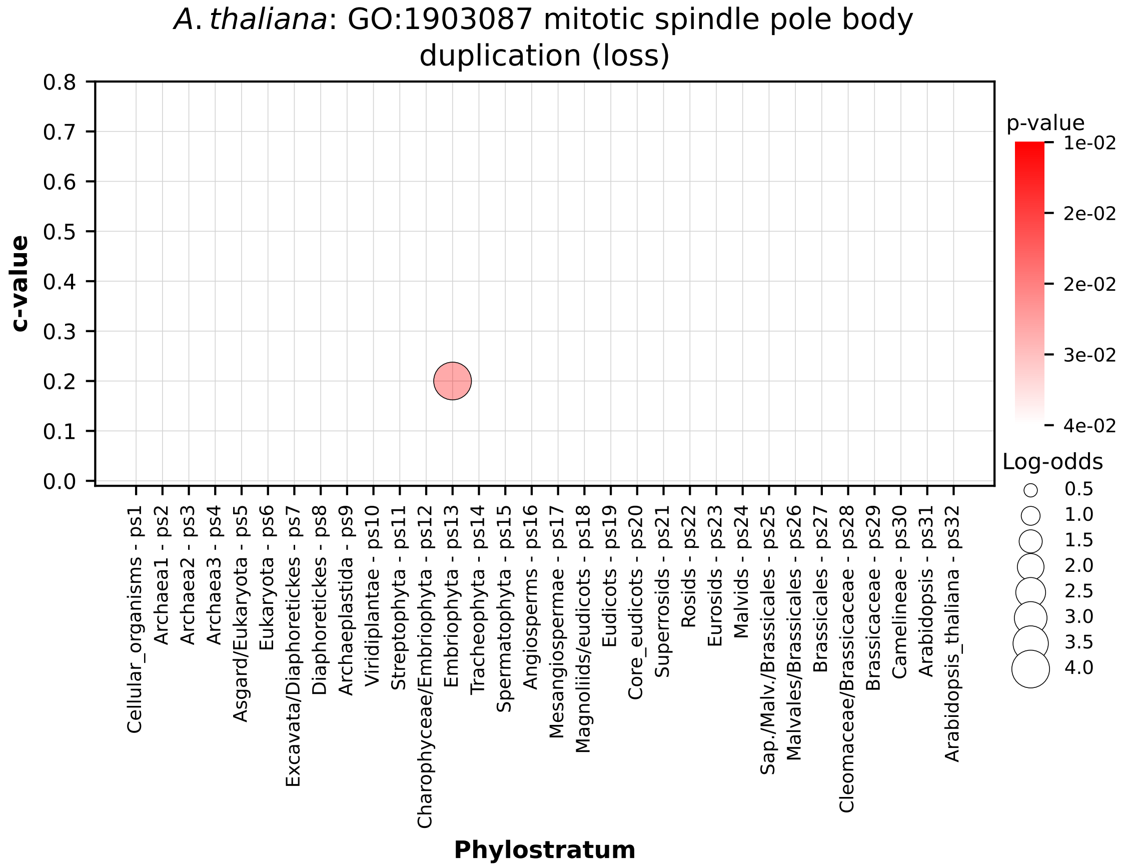

A. thaliana: GO:1903087 mitotic spindle pole body duplication (loss)
c-value
0.8
0.7
0.6
0.5
0.4
0.3
0.2
0.1
0.0
p-value
1e-02
2e-02
2e-02
3e-02
4e-02
Log-odds
0.5
1.0
1.5
2.0
2.5
3.0
3.5
4.0
Cellular_organisms - ps1
Archaea1 - ps2
Archaea2 - ps3
Archaea3 - ps4
Asgard/Eukaryota - ps5
Eukaryota - ps6
Excavata/Diaphoretickes - ps7
Diaphoretickes - ps8
Archaeplastida - ps9
Viridiplantae - ps10
Streptophyta - ps11
Charophyceae/Embriophyta - ps12
Embriophyta - ps13
Tracheophyta - ps14
Spermatophyta - ps15
Angiosperms - ps16
Mesangiospermae - ps17
Magnoliids/eudicots - ps18
Eudicots - ps19
Core_eudicots - ps20
Superrosids - ps21
Rosids - ps22
Eurosids - ps23
Malvids - ps24
Sap./Malv./Brassicales - ps25
Malvales/Brassicales - ps26
Brassicales - ps27
Cleomaceae/Brassicaceae - ps28
Brassicaceae - ps29
Camelineae - ps30
Arabidopsis - ps31
Arabidopsis_thaliana - ps32
Phylostratum

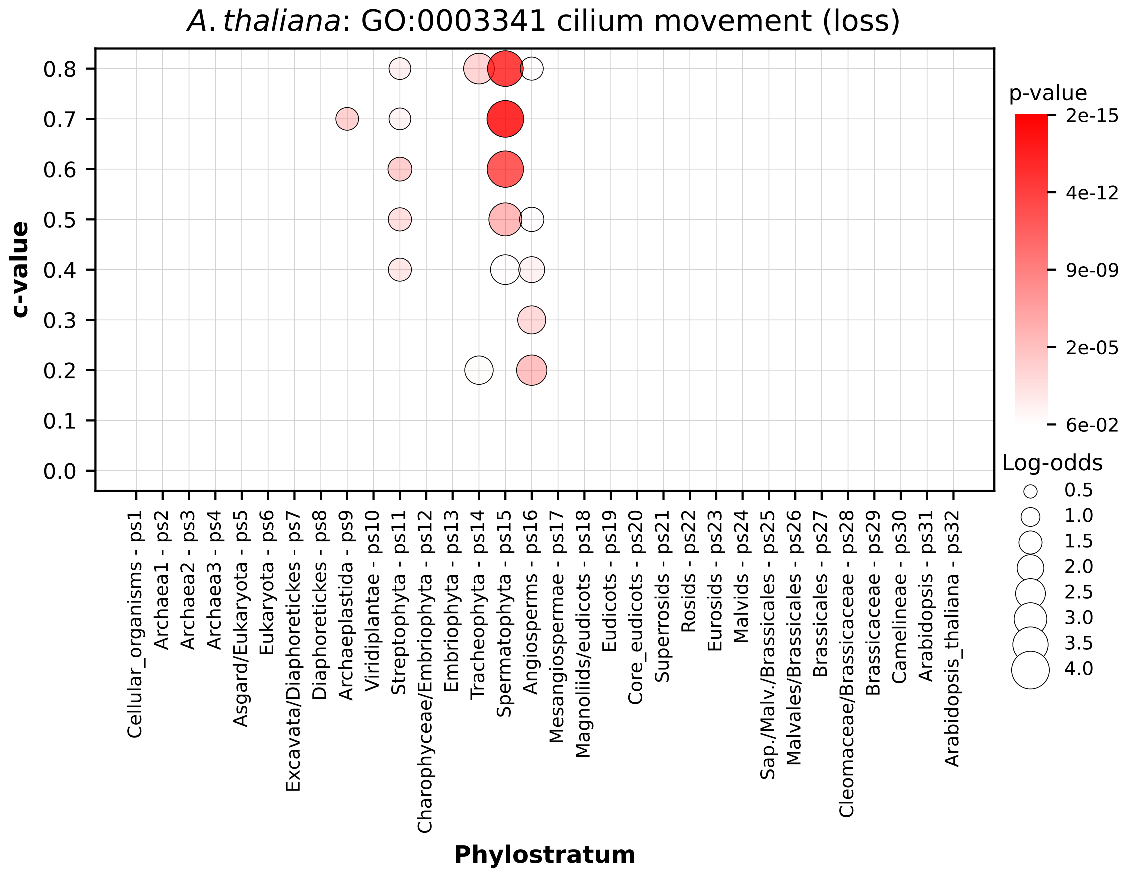
A. thaliana: GO:0003341 cilium movement (loss)
c-value
0.8
0.7
0.6
0.5
0.4
0.3
0.2
0.1
0.0
Phylostratum
Cellular_organisms - ps1
Archaea1 - ps2
Archaea2 - ps3
Archaea3 - ps4
Asgard/Eukaryota - ps5
Eukaryota - ps6
Excavata/Diaphoretickes - ps7
Diaphoretickes - ps8
Archaeplastida - ps9
Viridiplantae - ps10
Streptophyta - ps11
Charophyceae/Embriophyta - ps12
Embriophyta - ps13
Tracheophyta - ps14
Spermatophyta - ps15
Angiosperms - ps16
Mesangiospermae - ps17
Magnoliids/eudicots - ps18
Eudicots - ps19
Core_eudicots - ps20
Superrosids - ps21
Rosids - ps22
Eurosids - ps23
Malvids - ps24
Sap./Malv./Brassicales - ps25
Malvales/Brassicales - ps26
Brassicales - ps27
Cleomaceae/Brassicaceae - ps28
Brassicaceae - ps29
Camelineae - ps30
Arabidopsis - ps31
Arabidopsis_thaliana - ps32
p-value
2e-15
4e-12
9e-09
2e-05
6e-02
Log-odds
0.5
1.0
1.5
2.0
2.5
3.0
3.5
4.0

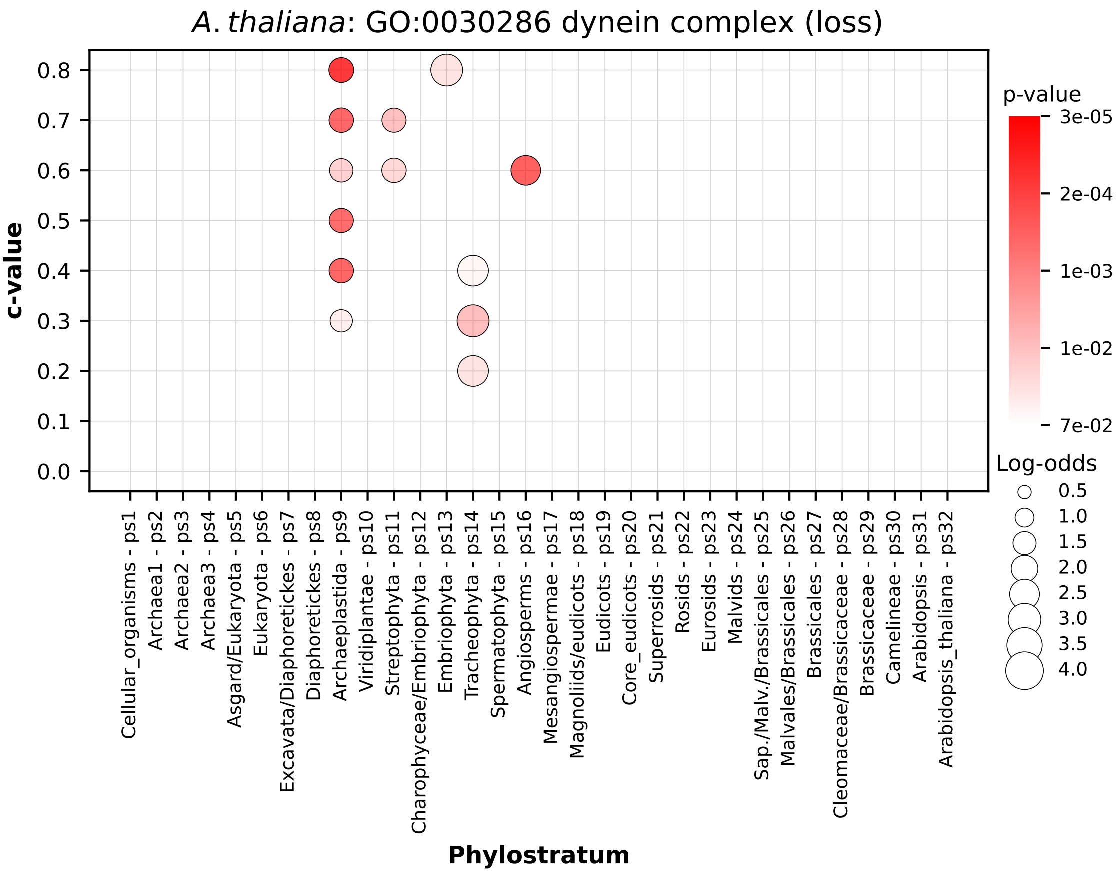
A. thaliana: GO:0030286 dynein complex (loss)
c-value
0.8
0.7
0.6
0.5
0.4
0.3
0.2
0.1
0.0
p-value
3e-05
2e-04
1e-03
1e-02
7e-02
Log-odds
0.5
1.0
1.5
2.0
2.5
3.0
3.5
4.0
Cellular_organisms - ps1
Archaea1 - ps2
Archaea2 - ps3
Archaea3 - ps4
Asgard/Eukaryota - ps5
Eukaryota - ps6
Excavata/Diaphoretickes - ps7
Diaphoretickes - ps8
Archaeplastida - ps9
Viridiplantae - ps10
Streptophyta - ps11
Charophyceae/Embriophyta - ps12
Embriophyta - ps13
Tracheophyta - ps14
Spermatophyta - ps15
Angiosperms - ps16
Mesangiospermae - ps17
Magnoliids/eudicots - ps18
Eudicots - ps19
Core_eudicots - ps20
Superrosids - ps21
Rosids - ps22
Eurosids - ps23
Malvids - ps24
Sap./Malv./Brassicales - ps25
Malvales/Brassicales - ps26
Brassicales - ps27
Cleomaceae/Brassicaceae - ps28
Brassicaceae - ps29
Camelineae - ps30
Arabidopsis - ps31
Arabidopsis_thaliana - ps32
Phylostratum

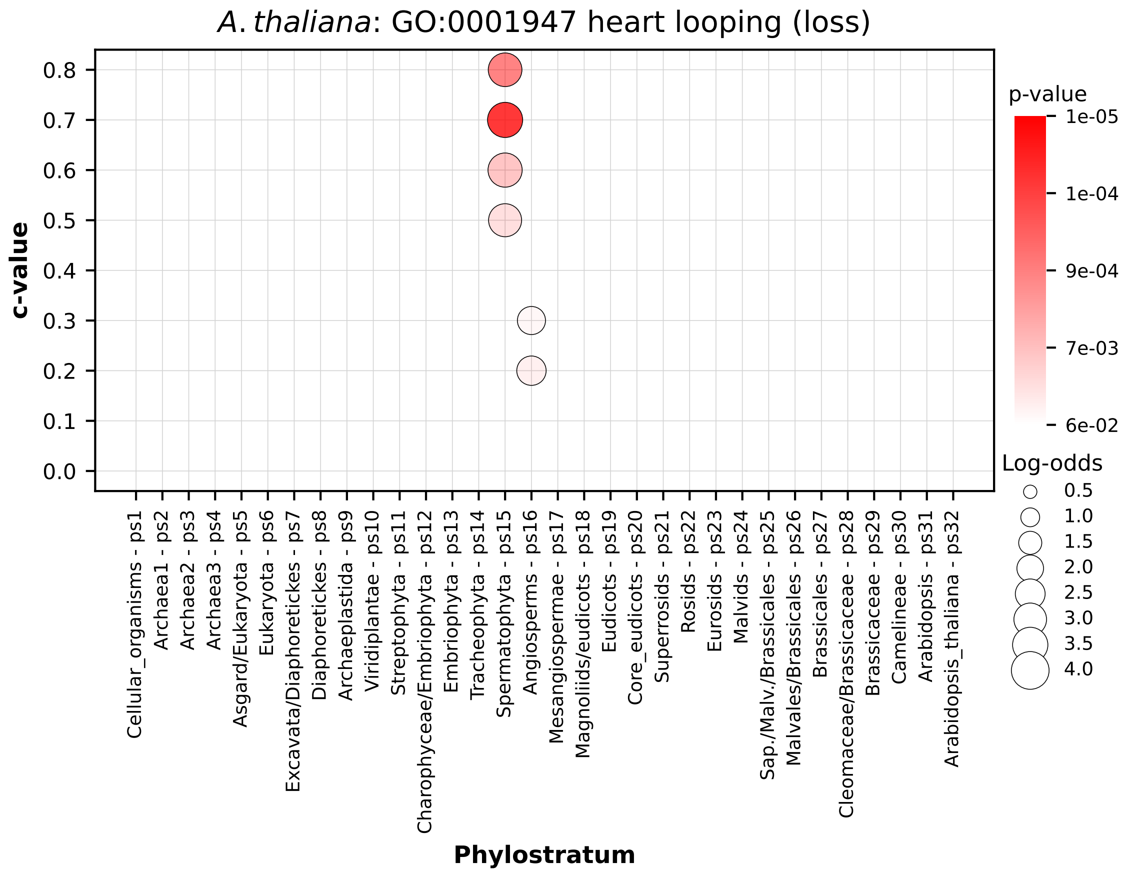

A. thaliana: GO:0001947 heart looping (loss)
c-value
0.8
0.7
0.6
0.5
0.4
0.3
0.2
0.1
0.0
p-value
1e-05
1e-04
9e-04
7e-03
6e-02
Log-odds
0.5
1.0
1.5
2.0
2.5
3.0
3.5
4.0
Cellular_organisms - ps1
Archaea1 - ps2
Archaea2 - ps3
Archaea3 - ps4
Asgard/Eukaryota - ps5
Eukaryota - ps6
Excavata/Diaphoretickes - ps7
Diaphoretickes - ps8
Archaeplastida - ps9
Viridiplantae - ps10
Streptophyta - ps11
Charophyceae/Embriophyta - ps12
Embriophyta - ps13
Tracheophyta - ps14
Spermatophyta - ps15
Angiosperms - ps16
Mesangiospermae - ps17
Magnoliids/eudicots - ps18
Eudicots - ps19
Core_eudicots - ps20
Superrosids - ps21
Rosids - ps22
Eurosids - ps23
Malvids - ps24
Sap./Malv./Brassicales - ps25
Malvales/Brassicales - ps26
Brassicales - ps27
Cleomaceae/Brassicaceae - ps28
Brassicaceae - ps29
Camelineae - ps30
Arabidopsis - ps31
Arabidopsis_thaliana - ps32
Phylostratum

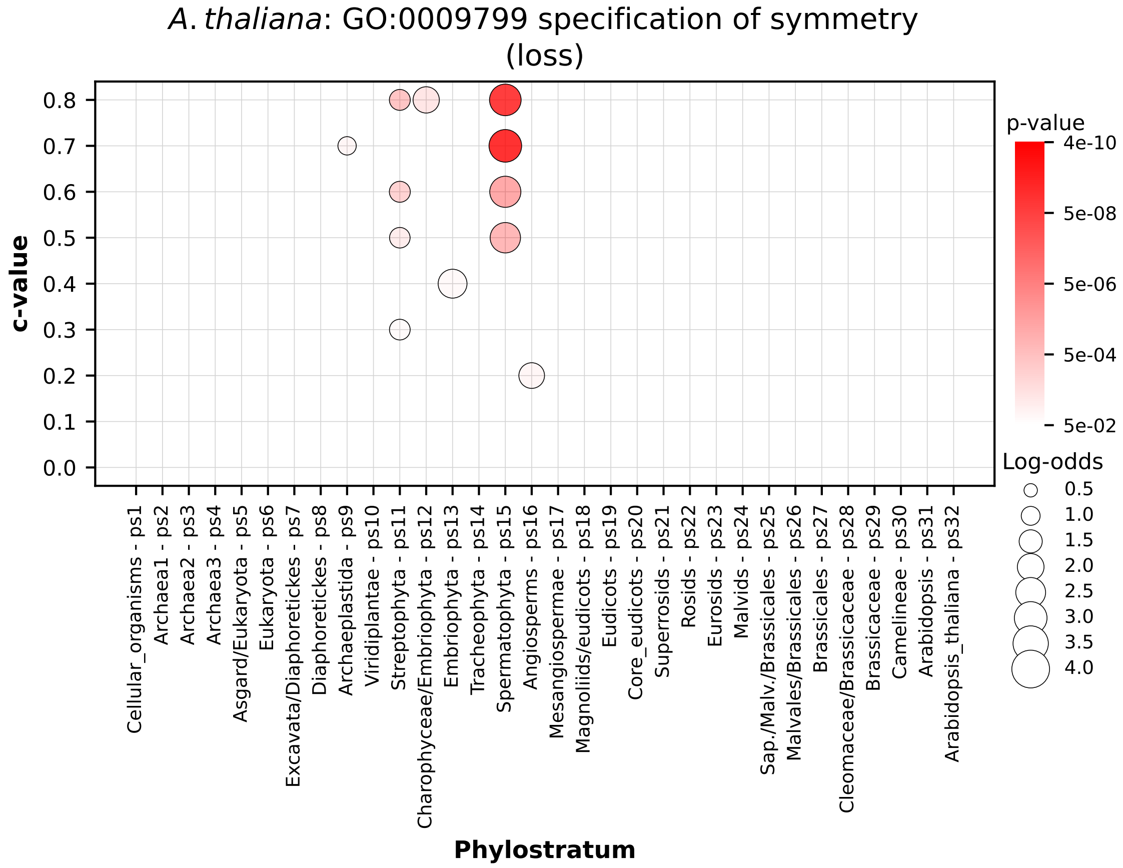

A. thaliana: GO:0009799 specification of symmetry
(loss)
c-value
0.8
0.7
0.6
0.5
0.4
0.3
0.2
0.1
0.0
p-value
4e-10
5e-08
5e-06
5e-04
5e-02
Log-odds
0.5
1.0
1.5
2.0
2.5
3.0
3.5
4.0
Cellular_organisms - ps1
Archaea1 - ps2
Archaea2 - ps3
Archaea3 - ps4
Asgard/Eukaryota - ps5
Eukaryota - ps6
Excavata/Diaphoretickes - ps7
Diaphoretickes - ps8
Archaeplastida - ps9
Viridiplantae - ps10
Streptophyta - ps11
Charophyceae/Embriophyta - ps12
Embriophyta - ps13
Tracheophyta - ps14
Spermatophyta - ps15
Angiosperms - ps16
Mesangiospermae - ps17
Magnoliids/eudicots - ps18
Eudicots - ps19
Core_eudicots - ps20
Superrosids - ps21
Rosids - ps22
Eurosids - ps23
Malvids - ps24
Sap./Malv./Brassicales - ps25
Malvales/Brassicales - ps26
Brassicales - ps27
Cleomaceae/Brassicaceae - ps28
Brassicaceae - ps29
Camelineae - ps30
Arabidopsis - ps31
Arabidopsis_thaliana - ps32
Phylostratum

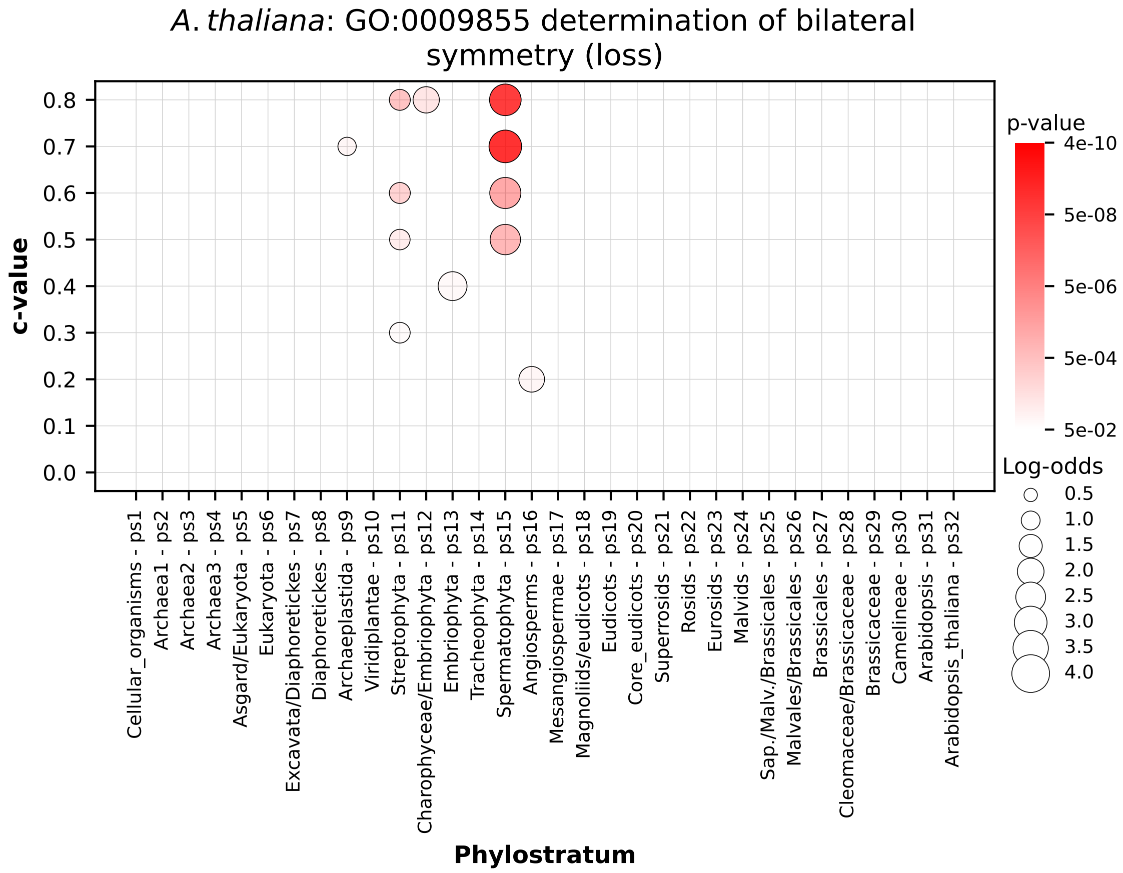

A. thaliana: GO:0009855 determination of bilateral symmetry (loss)
c-value
0.0
0.1
0.2
0.3
0.4
0.5
0.6
0.7
0.8
p-value
4e-10
5e-08
5e-06
5e-04
5e-02
Log-odds
0.5
1.0
1.5
2.0
2.5
3.0
3.5
4.0
Cellular_organisms - ps1
Archaea1 - ps2
Archaea2 - ps3
Archaea3 - ps4
Asgard/Eukaryota - ps5
Eukaryota - ps6
Excavata/Diaphoretickes - ps7
Diaphoretickes - ps8
Archaeplastida - ps9
Viridiplantae - ps10
Streptophyta - ps11
Charophyceae/Embriophyta - ps12
Embriophyta - ps13
Tracheophyta - ps14
Spermatophyta - ps15
Angiosperms - ps16
Mesangiospermae - ps17
Magnoliids/eudicots - ps18
Eudicots - ps19
Core_eudicots - ps20
Superrosids - ps21
Rosids - ps22
Eurosids - ps23
Malvids - ps24
Sap./Malv./Brassicales - ps25
Malvales/Brassicales - ps26
Brassicales - ps27
Cleomaceae/Brassicaceae - ps28
Brassicaceae - ps29
Camelineae - ps30
Arabidopsis - ps31
Arabidopsis_thaliana - ps32
Phylostratum

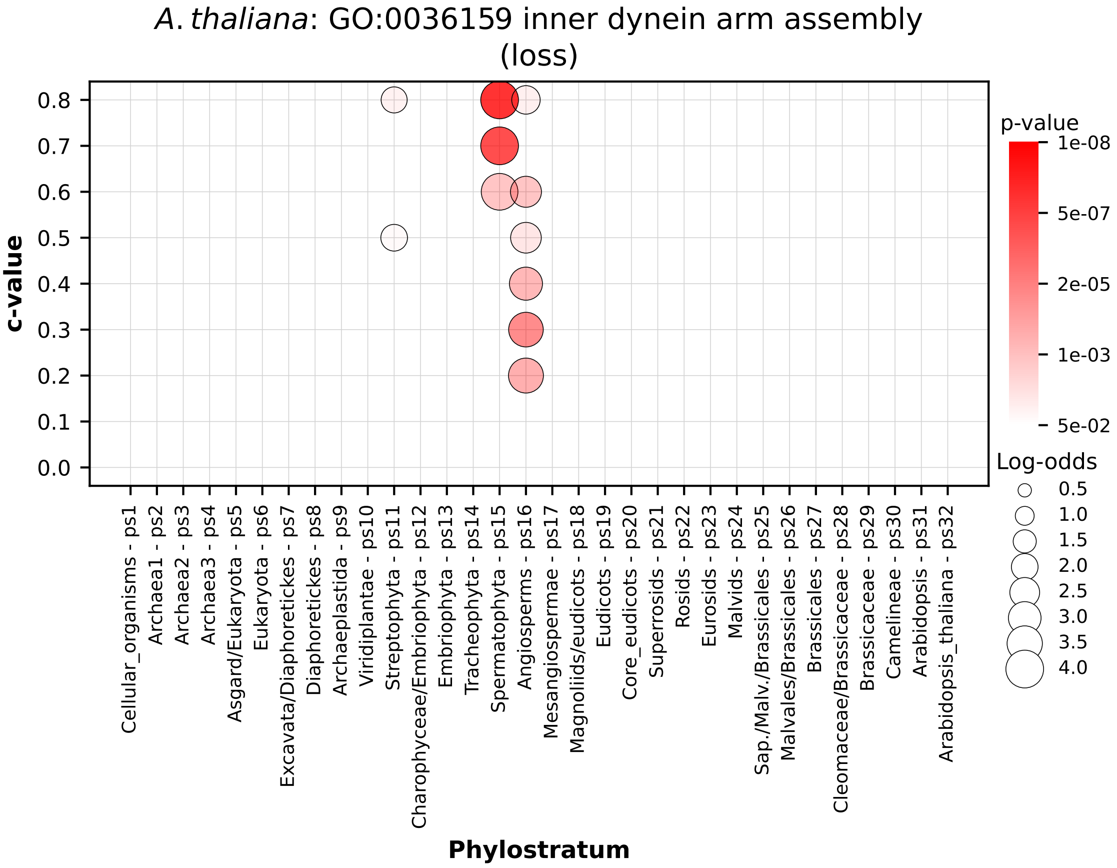
A. thaliana: GO:0036159 inner dynein arm assembly
(loss)
c-value
0.8
0.7
0.6
0.5
0.4
0.3
0.2
0.1
0.0
p-value
1e-08
5e-07
2e-05
1e-03
5e-02
Log-odds
0.5
1.0
1.5
2.0
2.5
3.0
3.5
4.0
Cellular_organisms - ps1
Archaea1 - ps2
Archaea2 - ps3
Archaea3 - ps4
Asgard/Eukaryota - ps5
Eukaryota - ps6
Excavata/Diaphoretickes - ps7
Diaphoretickes - ps8
Archaeplastida - ps9
Viridiplantae - ps10
Streptophyta - ps11
Charophyceae/Embriophyta - ps12
Embriophyta - ps13
Tracheophyta - ps14
Spermatophyta - ps15
Angiosperms - ps16
Mesangiospermae - ps17
Magnoliids/eudicots - ps18
Eudicots - ps19
Core_eudicots - ps20
Superrosids - ps21
Rosids - ps22
Eurosids - ps23
Malvids - ps24
Sap./Malv./Brassicales - ps25
Malvales/Brassicales - ps26
Brassicales - ps27
Cleomaceae/Brassicaceae - ps28
Brassicaceae - ps29
Camelineae - ps30
Arabidopsis - ps31
Arabidopsis_thaliana - ps32
Phylostratum

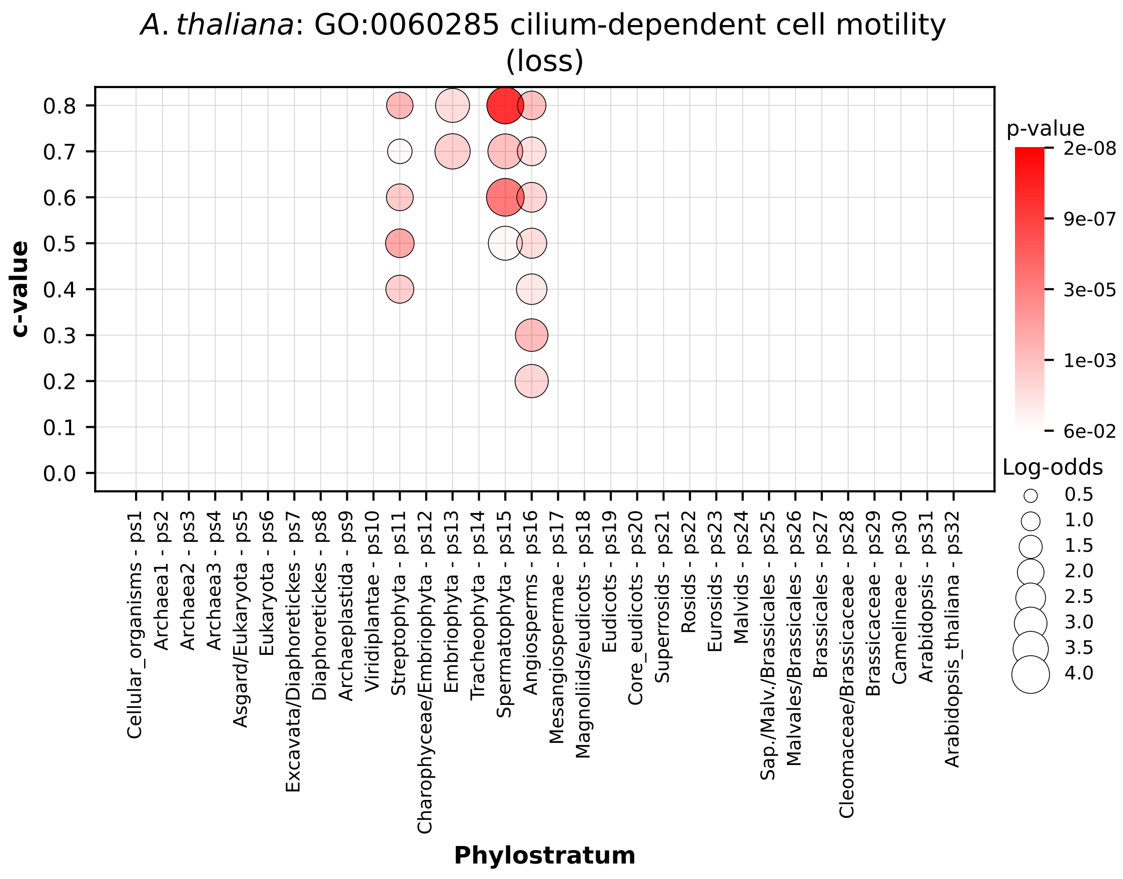

A. thaliana: GO:0060285 cilium-dependent cell motility (loss)
c-value
0.8
0.7
0.6
0.5
0.4
0.3
0.2
0.1
0.0
p-value
2e-08
9e-07
3e-05
1e-03
6e-02
Log-odds
0.5
1.0
1.5
2.0
2.5
3.0
3.5
4.0
Cellular_organisms - ps1
Archaea1 - ps2
Archaea2 - ps3
Archaea3 - ps4
Asgard/Eukaryota - ps5
Eukaryota - ps6
Excavata/Diaphoretickes - ps7
Diaphoretickes - ps8
Archaeplastida - ps9
Viridiplantae - ps10
Streptophyta - ps11
Charophyceae/Embriophyta - ps12
Embriophyta - ps13
Tracheophyta - ps14
Spermatophyta - ps15
Angiosperms - ps16
Mesangiospermae - ps17
Magnoliids/eudicots - ps18
Eudicots - ps19
Core_eudicots - ps20
Superrosids - ps21
Rosids - ps22
Eurosids - ps23
Malvids - ps24
Sap./Malv./Brassicales - ps25
Malvales/Brassicales - ps26
Brassicales - ps27
Cleomaceae/Brassicaceae - ps28
Brassicaceae - ps29
Camelineae - ps30
Arabidopsis - ps31
Arabidopsis_thaliana - ps32
Phylostratum

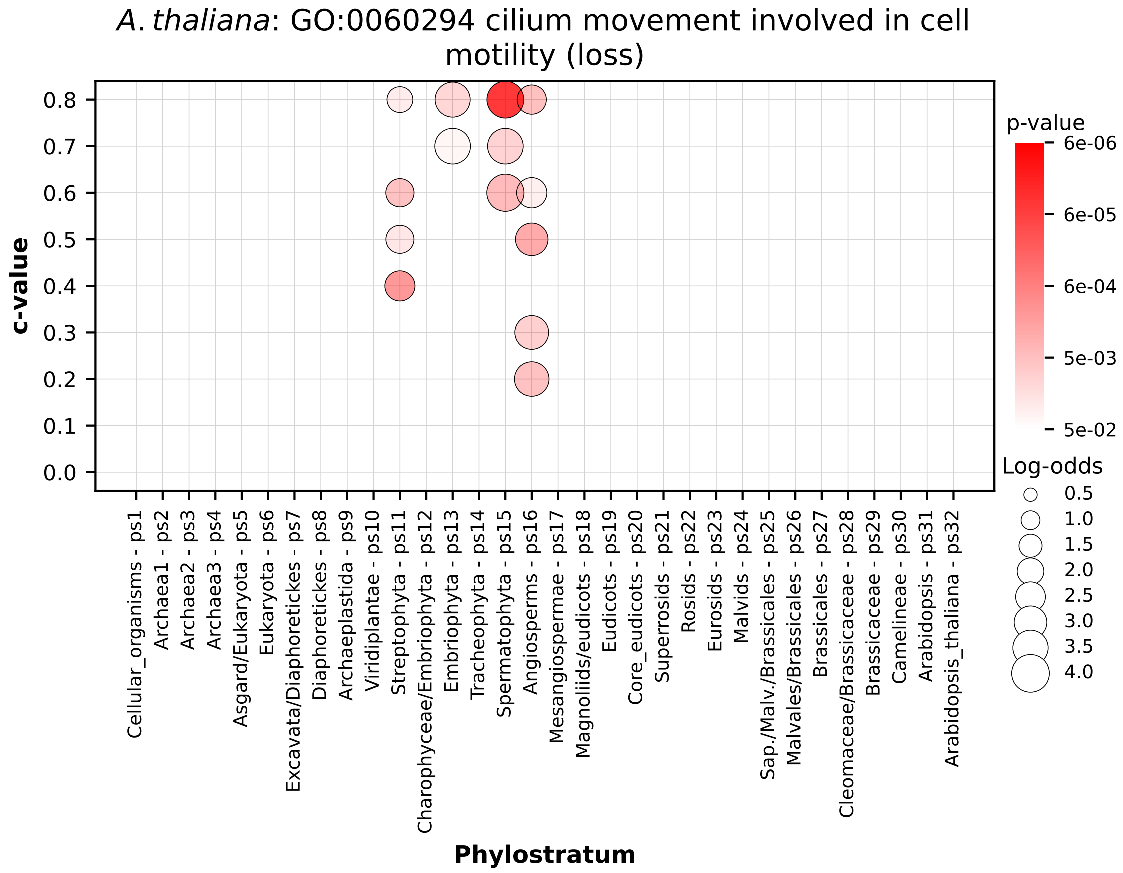
A. thaliana: GO:0060294 cilium movement involved in cell motility (loss)
c-value
0.8
0.7
0.6
0.5
0.4
0.3
0.2
0.1
0.0
p-value
6e-06
6e-05
6e-04
5e-03
5e-02
Log-odds
0.5
1.0
1.5
2.0
2.5
3.0
3.5
4.0
Cellular_organisms - ps1
Archaea1 - ps2
Archaea2 - ps3
Archaea3 - ps4
Asgard/Eukaryota - ps5
Eukaryota - ps6
Excavata/Diaphoretickes - ps7
Diaphoretickes - ps8
Archaeplastida - ps9
Viridiplantae - ps10
Streptophyta - ps11
Charophyceae/Embriophyta - ps12
Embriophyta - ps13
Tracheophyta - ps14
Spermatophyta - ps15
Angiosperms - ps16
Mesangiospermae - ps17
Magnoliids/eudicots - ps18
Eudicots - ps19
Core_eudicots - ps20
Superrosids - ps21
Rosids - ps22
Eurosids - ps23
Malvids - ps24
Sap./Malv./Brassicales - ps25
Malvales/Brassicales - ps26
Brassicales - ps27
Cleomaceae/Brassicaceae - ps28
Brassicaceae - ps29
Camelineae - ps30
Arabidopsis - ps31
Arabidopsis_thaliana - ps32
Phylostratum

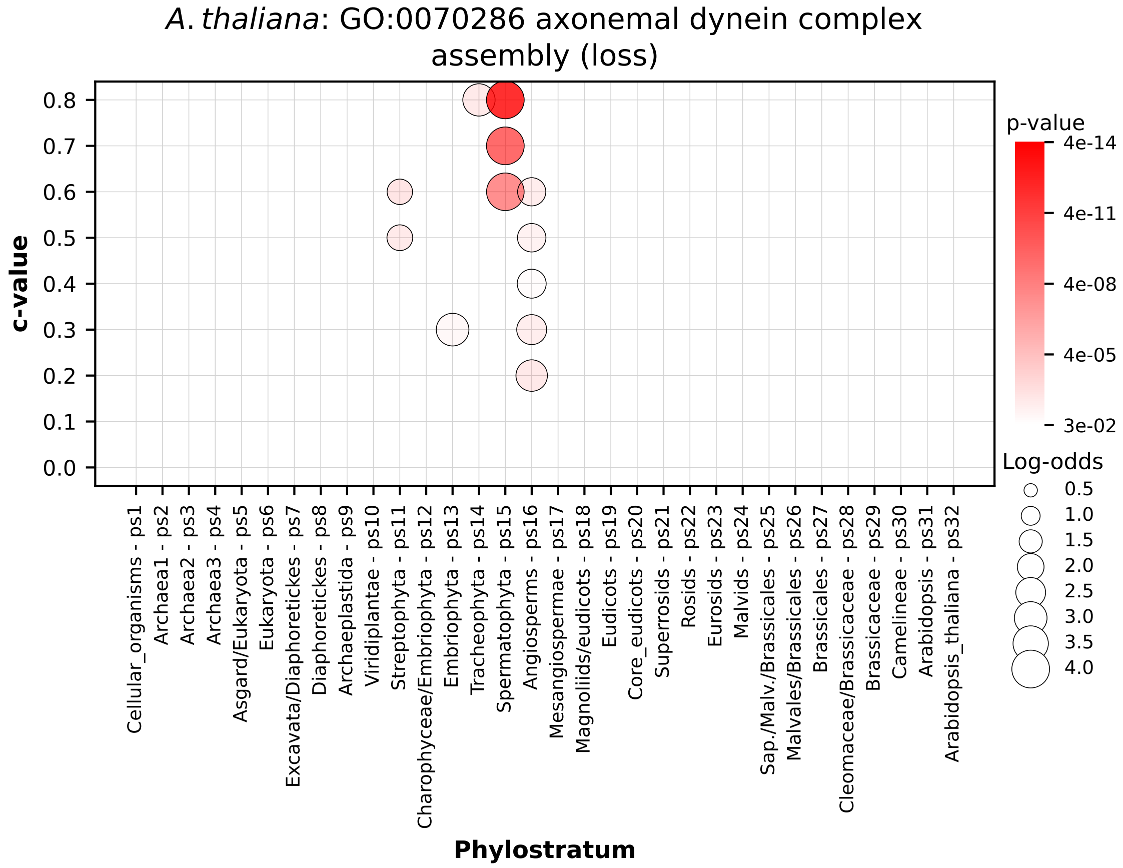

A. thaliana: GO:0070286 axonemal dynein complex assembly (loss)
c-value
0.8
0.7
0.6
0.5
0.4
0.3
0.2
0.1
0.0
p-value
4e-14
4e-11
4e-08
4e-05
3e-02
Log-odds
0.5
1.0
1.5
2.0
2.5
3.0
3.5
4.0
Cellular_organisms - ps1
Archaea1 - ps2
Archaea2 - ps3
Archaea3 - ps4
Asgard/Eukaryota - ps5
Eukaryota - ps6
Excavata/Diaphoretickes - ps7
Diaphoretickes - ps8
Archaeplastida - ps9
Viridiplantae - ps10
Streptophyta - ps11
Charophyceae/Embriophyta - ps12
Embriophyta - ps13
Tracheophyta - ps14
Spermatophyta - ps15
Angiosperms - ps16
Mesangiospermae - ps17
Magnoliids/eudicots - ps18
Eudicots - ps19
Core_eudicots - ps20
Superrosids - ps21
Rosids - ps22
Eurosids - ps23
Malvids - ps24
Sap./Malv./Brassicales - ps25
Malvales/Brassicales - ps26
Brassicales - ps27
Cleomaceae/Brassicaceae - ps28
Brassicaceae - ps29
Camelineae - ps30
Arabidopsis - ps31
Arabidopsis_thaliana - ps32
Phylostratum

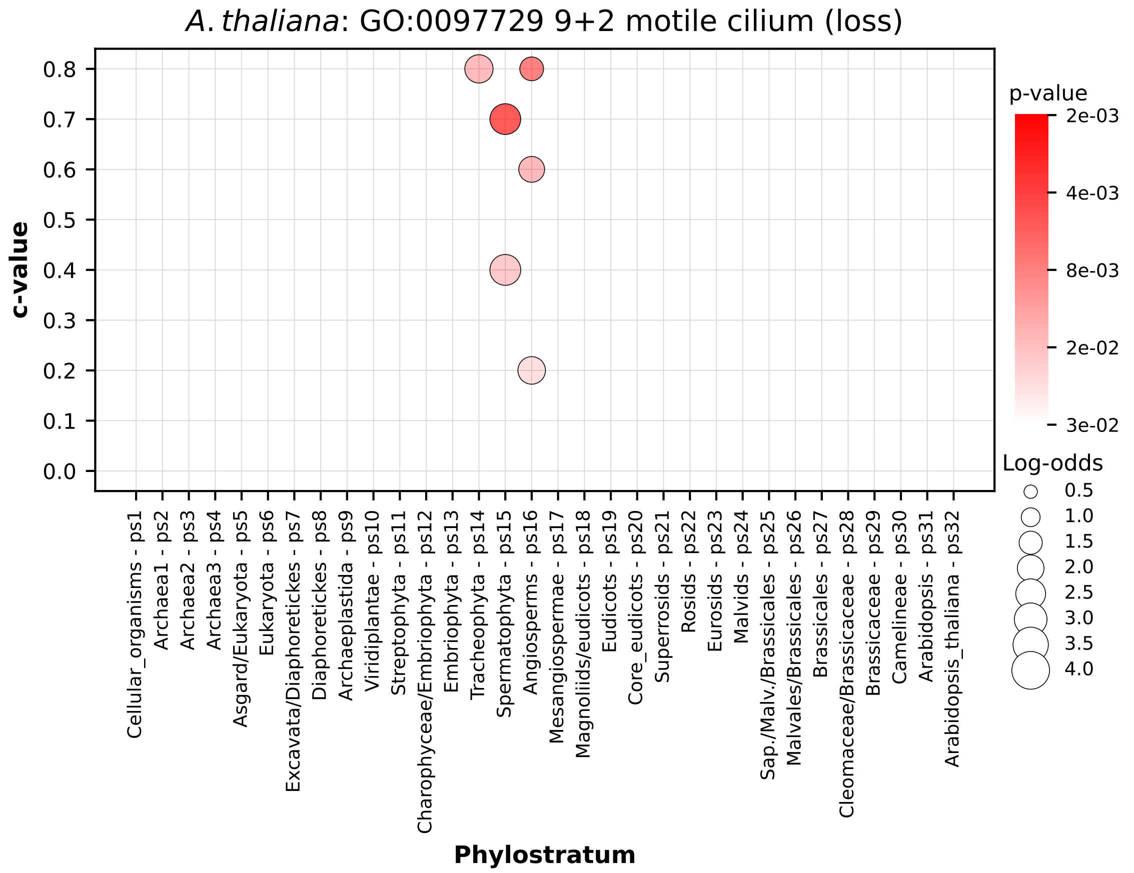

A. thaliana: GO:0097729 9+2 motile cilium (loss)
c-value
0.8
0.7
0.6
0.5
0.4
0.3
0.2
0.1
0.0
p-value
2e-03
4e-03
8e-03
2e-02
3e-02
Log-odds
0.5
1.0
1.5
2.0
2.5
3.0
3.5
4.0
Cellular_organisms - ps1
Archaea1 - ps2
Archaea2 - ps3
Archaea3 - ps4
Asgard/Eukaryota - ps5
Eukaryota - ps6
Excavata/Diaphoretickes - ps7
Diaphoretickes - ps8
Archaeplastida - ps9
Viridiplantae - ps10
Streptophyta - ps11
Charophyceae/Embriophyta - ps12
Embriophyta - ps13
Tracheophyta - ps14
Spermatophyta - ps15
Angiosperms - ps16
Mesangiospermae - ps17
Magnoliids/eudicots - ps18
Eudicots - ps19
Core_eudicots - ps20
Superrosids - ps21
Rosids - ps22
Eurosids - ps23
Malvids - ps24
Sap./Malv./Brassicales - ps25
Malvales/Brassicales - ps26
Brassicales - ps27
Cleomaceae/Brassicaceae - ps28
Brassicaceae - ps29
Camelineae - ps30
Arabidopsis - ps31
Arabidopsis_thaliana - ps32
Phylostratum

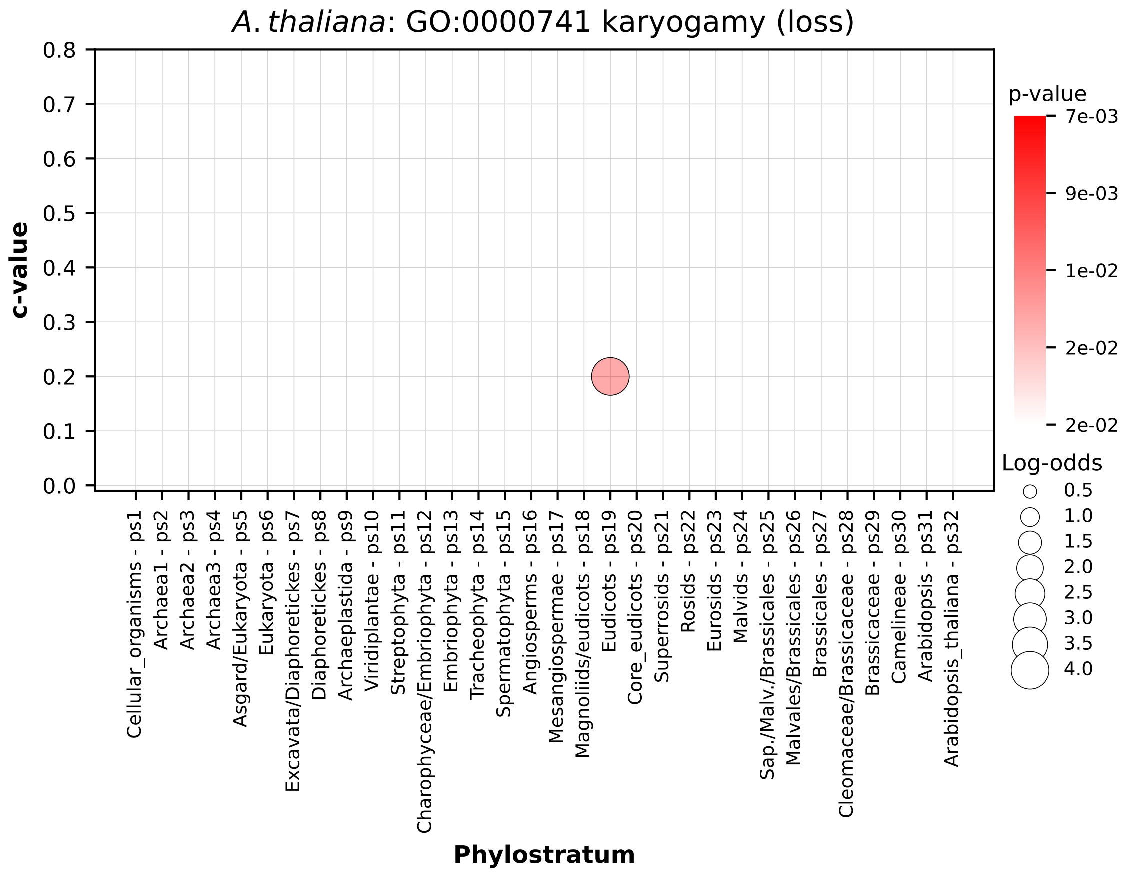

A. thaliana: GO:0000741 karyogamy (loss)
c-value
0.8
0.7
0.6
0.5
0.4
0.3
0.2
0.1
0.0
p-value
7e-03
9e-03
1e-02
2e-02
2e-02
Log-odds
0.5
1.0
1.5
2.0
2.5
3.0
3.5
4.0
Cellular_organisms - ps1
Archaea1 - ps2
Archaea2 - ps3
Archaea3 - ps4
Asgard/Eukaryota - ps5
Eukaryota - ps6
Excavata/Diaphoretickes - ps7
Diaphoretickes - ps8
Archaeplastida - ps9
Viridiplantae - ps10
Streptophyta - ps11
Charophyceae/Embriophyta - ps12
Embriophyta - ps13
Tracheophyta - ps14
Spermatophyta - ps15
Angiosperms - ps16
Mesangiospermae - ps17
Magnoliids/eudicots - ps18
Eudicots - ps19
Core_eudicots - ps20
Superrosids - ps21
Rosids - ps22
Eurosids - ps23
Malvids - ps24
Sap./Malv./Brassicales - ps25
Malvales/Brassicales - ps26
Brassicales - ps27
Cleomaceae/Brassicaceae - ps28
Brassicaceae - ps29
Camelineae - ps30
Arabidopsis - ps31
Arabidopsis_thaliana - ps32
Phylostratum

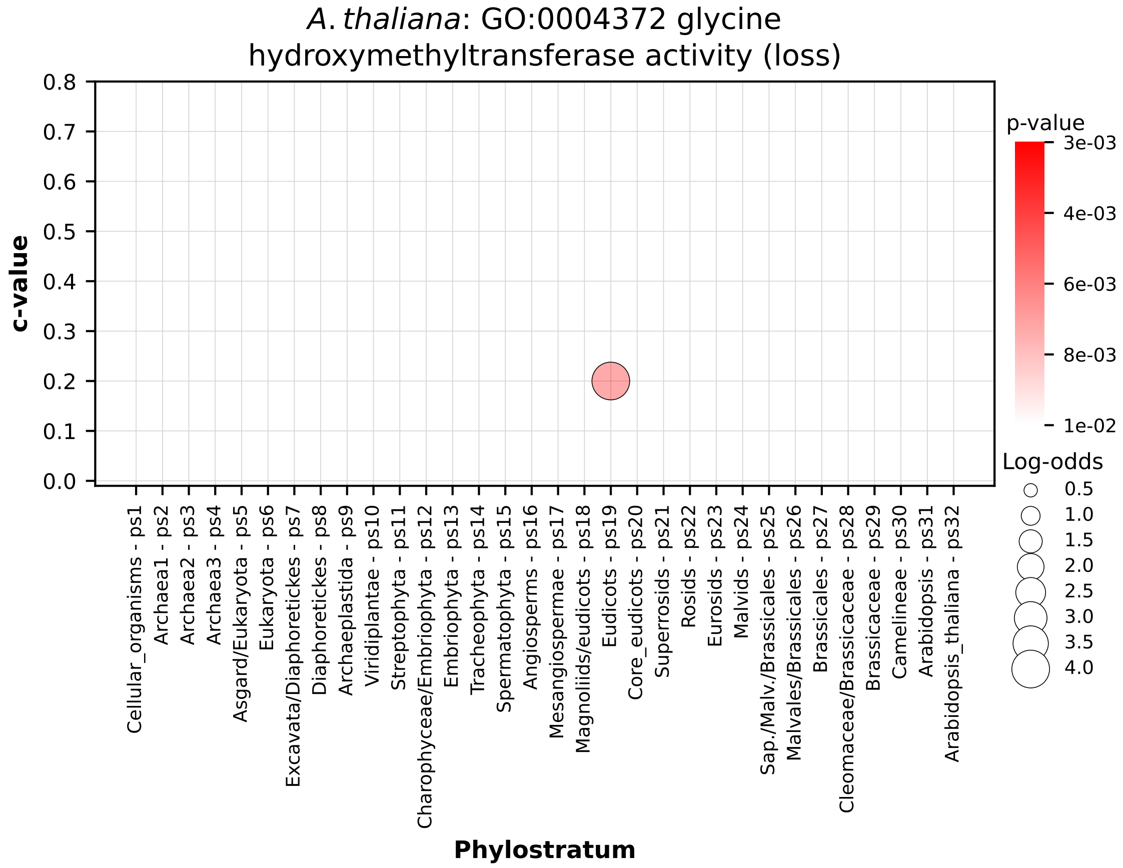

A. thaliana: GO:0004372 glycine hydroxymethyltransferase activity (loss)
c-value
0.8
0.7
0.6
0.5
0.4
0.3
0.2
0.1
0.0
p-value
3e-03
4e-03
6e-03
8e-03
1e-02
Log-odds
0.5
1.0
1.5
2.0
2.5
3.0
3.5
4.0
Cellular_organisms - ps1
Archaea1 - ps2
Archaea2 - ps3
Archaea3 - ps4
Asgard/Eukaryota - ps5
Eukaryota - ps6
Excavata/Diaphoretickes - ps7
Diaphoretickes - ps8
Archaeplastida - ps9
Viridiplantae - ps10
Streptophyta - ps11
Charophyceae/Embriophyta - ps12
Embriophyta - ps13
Tracheophyta - ps14
Spermatophyta - ps15
Angiosperms - ps16
Mesangiospermae - ps17
Magnoliids/eudicots - ps18
Eudicots - ps19
Core_eudicots - ps20
Superrosids - ps21
Rosids - ps22
Eurosids - ps23
Malvids - ps24
Sap./Malv./Brassicales - ps25
Malvales/Brassicales - ps26
Brassicales - ps27
Cleomaceae/Brassicaceae - ps28
Brassicaceae - ps29
Camelineae - ps30
Arabidopsis - ps31
Arabidopsis_thaliana - ps32
Phylostratum

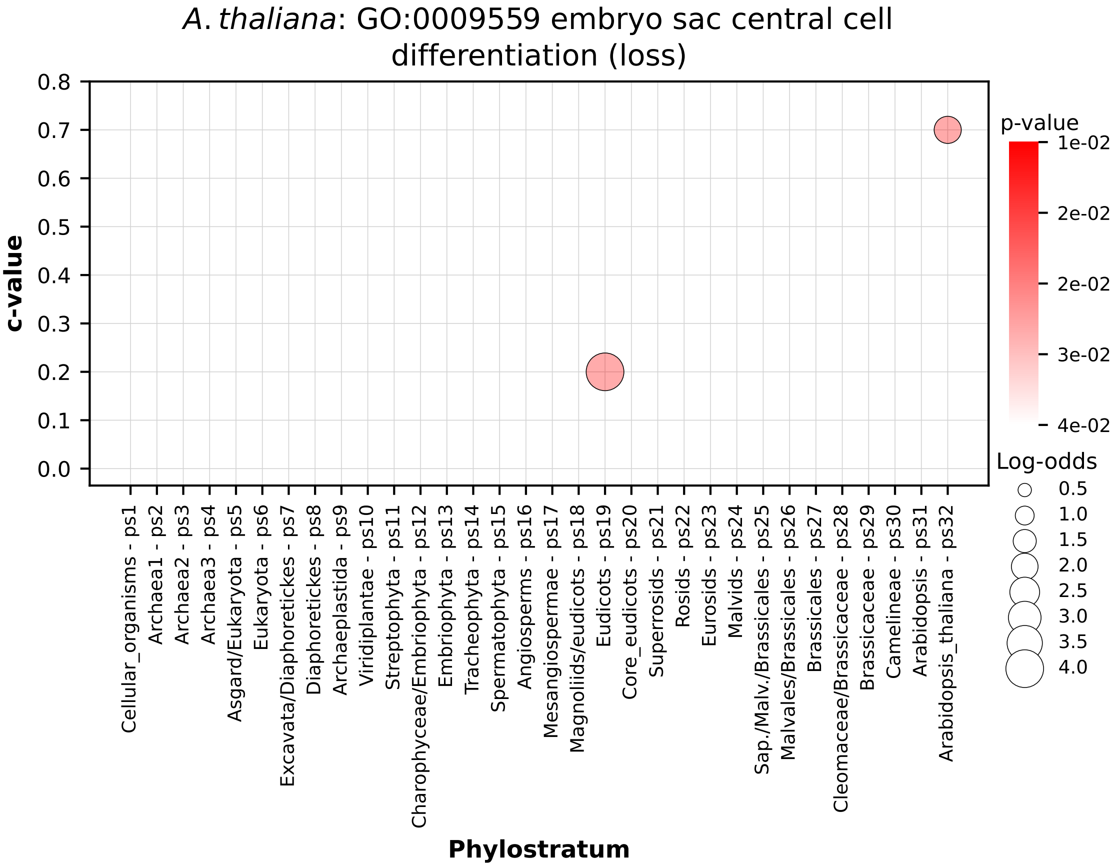

A. thaliana: GO:0009559 embryo sac central cell differentiation (loss)
c-value
0.0
0.1
0.2
0.3
0.4
0.5
0.6
0.7
0.8
Phylostratum
Cellular_organisms - ps1
Archaea1 - ps2
Archaea2 - ps3
Archaea3 - ps4
Asgard/Eukaryota - ps5
Eukaryota - ps6
Excavata/Diaphoretickes - ps7
Diaphoretickes - ps8
Archaeplastida - ps9
Viridiplantae - ps10
Streptophyta - ps11
Charophyceae/Embriophyta - ps12
Embriophyta - ps13
Tracheophyta - ps14
Spermatophyta - ps15
Angiosperms - ps16
Mesangiospermae - ps17
Magnoliids/eudicots - ps18
Eudicots - ps19
Core_eudicots - ps20
Superrosids - ps21
Rosids - ps22
Eurosids - ps23
Malvids - ps24
Sap./Malv./Brassicales - ps25
Malvales/Brassicales - ps26
Brassicales - ps27
Cleomaceae/Brassicaceae - ps28
Brassicaceae - ps29
Camelineae - ps30
Arabidopsis - ps31
Arabidopsis_thaliana - ps32
p-value
1e-02
2e-02
2e-02
3e-02
4e-02
Log-odds
0.5
1.0
1.5
2.0
2.5
3.0
3.5
4.0

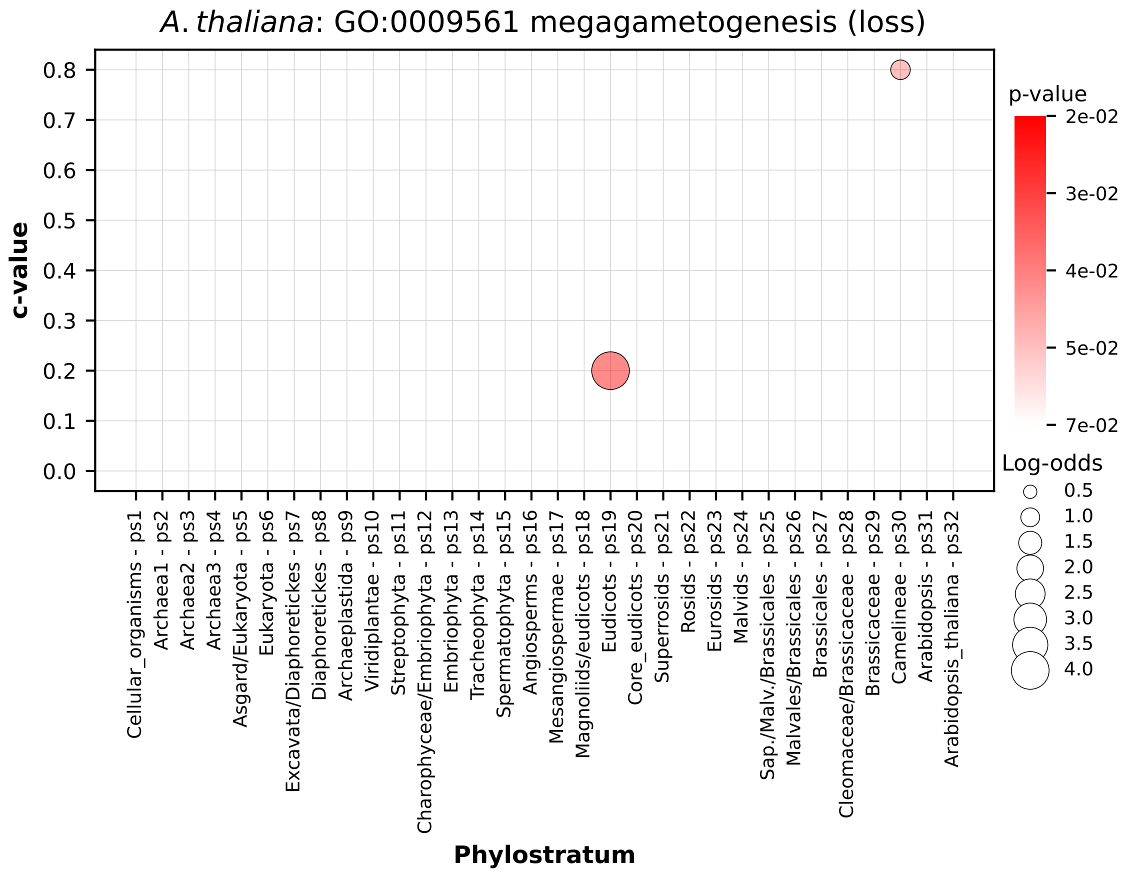

A. thaliana: GO:0009561 megagametogenesis (loss)
c-value
0.0
0.1
0.2
0.3
0.4
0.5
0.6
0.7
0.8
Phylostratum
Cellular_organisms - ps1
Archaea1 - ps2
Archaea2 - ps3
Archaea3 - ps4
Asgard/Eukaryota - ps5
Eukaryota - ps6
Excavata/Diaphoretickes - ps7
Diaphoretickes - ps8
Archaeplastida - ps9
Viridiplantae - ps10
Streptophyta - ps11
Charophyceae/Embriophyta - ps12
Embriophyta - ps13
Tracheophyta - ps14
Spermatophyta - ps15
Angiosperms - ps16
Mesangiospermae - ps17
Magnoliids/eudicots - ps18
Eudicots - ps19
Core_eudicots - ps20
Superrosids - ps21
Rosids - ps22
Eurosids - ps23
Malvids - ps24
Sap./Malv./Brassicales - ps25
Malvales/Brassicales - ps26
Brassicales - ps27
Cleomaceae/Brassicaceae - ps28
Brassicaceae - ps29
Camelineae - ps30
Arabidopsis - ps31
Arabidopsis_thaliana - ps32
p-value
2e-02
3e-02
4e-02
5e-02
7e-02
Log-odds
0.5
1.0
1.5
2.0
2.5
3.0
3.5
4.0

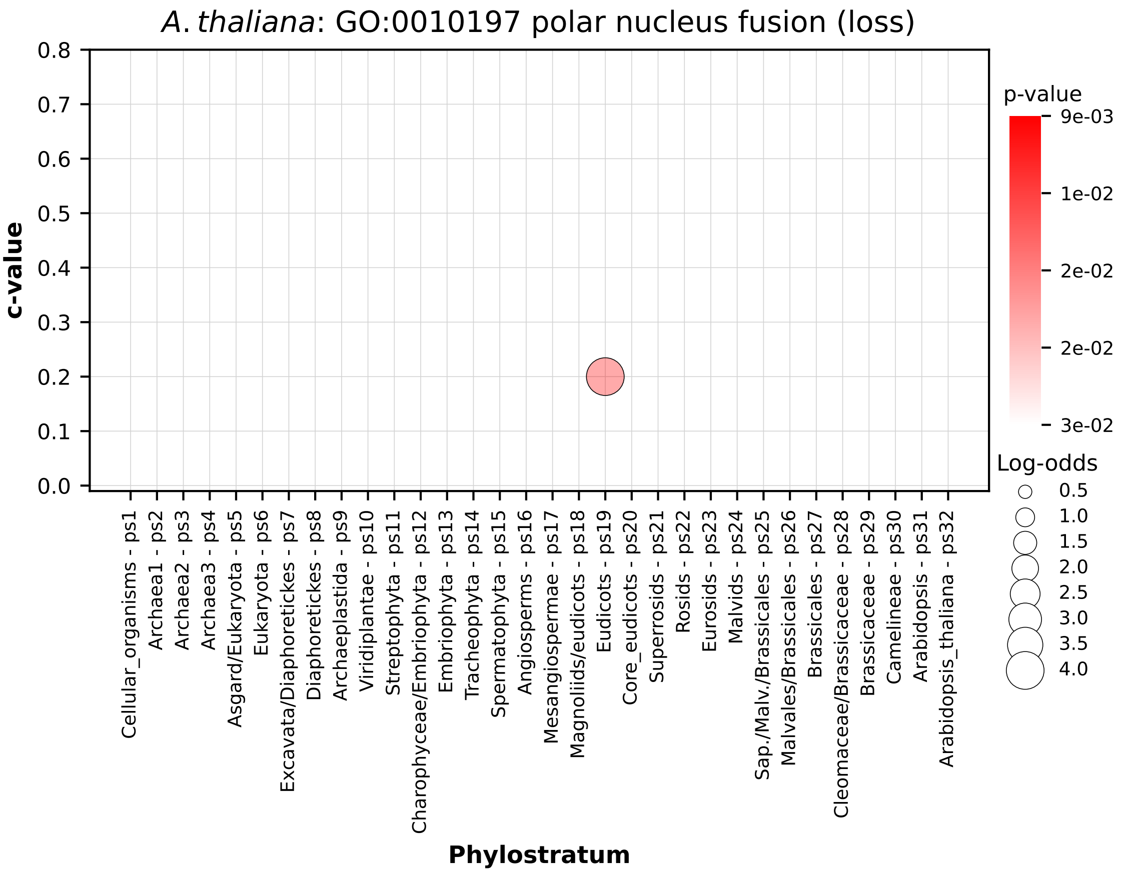

A. thaliana: GO:0010197 polar nucleus fusion (loss)
c-value
0.8
0.7
0.6
0.5
0.4
0.3
0.2
0.1
0.0
Phylostratum
Cellular_organisms - ps1
Archaea1 - ps2
Archaea2 - ps3
Archaea3 - ps4
Asgard/Eukaryota - ps5
Eukaryota - ps6
Excavata/Diaphoretickes - ps7
Diaphoretickes - ps8
Archaeplastida - ps9
Viridiplantae - ps10
Streptophyta - ps11
Charophyceae/Embriophyta - ps12
Embriophyta - ps13
Tracheophyta - ps14
Spermatophyta - ps15
Angiosperms - ps16
Mesangiospermae - ps17
Magnoliids/eudicots - ps18
Eudicots - ps19
Core_eudicots - ps20
Superrosids - ps21
Rosids - ps22
Eurosids - ps23
Malvids - ps24
Sap./Malv./Brassicales - ps25
Malvales/Brassicales - ps26
Brassicales - ps27
Cleomaceae/Brassicaceae - ps28
Brassicaceae - ps29
Camelineae - ps30
Arabidopsis - ps31
Arabidopsis_thaliana - ps32
p-value
9e-03
1e-02
2e-02
2e-02
3e-02
Log-odds
0.5
1.0
1.5
2.0
2.5
3.0
3.5
4.0

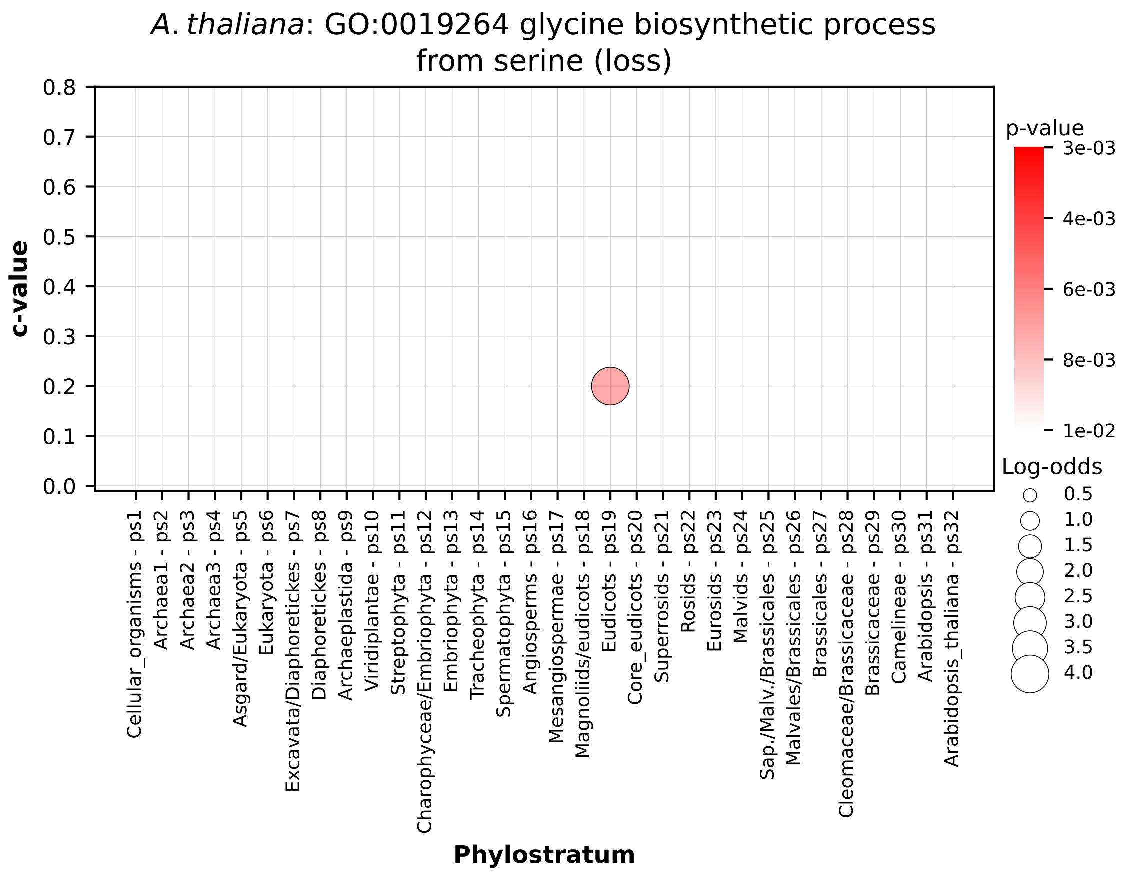

A. thaliana: GO:0019264 glycine biosynthetic process from serine (loss)
c-value
0.0
0.1
0.2
0.3
0.4
0.5
0.6
0.7
0.8
Phylostratum
Cellular_organisms - ps1
Archaea1 - ps2
Archaea2 - ps3
Archaea3 - ps4
Asgard/Eukaryota - ps5
Eukaryota - ps6
Excavata/Diaphoretickes - ps7
Diaphoretickes - ps8
Archaeplastida - ps9
Viridiplantae - ps10
Streptophyta - ps11
Charophyceae/Embriophyta - ps12
Embriophyta - ps13
Tracheophyta - ps14
Spermatophyta - ps15
Angiosperms - ps16
Mesangiospermae - ps17
Magnoliids/eudicots - ps18
Eudicots - ps19
Core_eudicots - ps20
Superrosids - ps21
Rosids - ps22
Eurosids - ps23
Malvids - ps24
Sap./Malv./Brassicales - ps25
Malvales/Brassicales - ps26
Brassicales - ps27
Cleomaceae/Brassicaceae - ps28
Brassicaceae - ps29
Camelineae - ps30
Arabidopsis - ps31
Arabidopsis_thaliana - ps32
p-value
3e-03
4e-03
6e-03
8e-03
1e-02
Log-odds
0.5
1.0
1.5
2.0
2.5
3.0
3.5
4.0

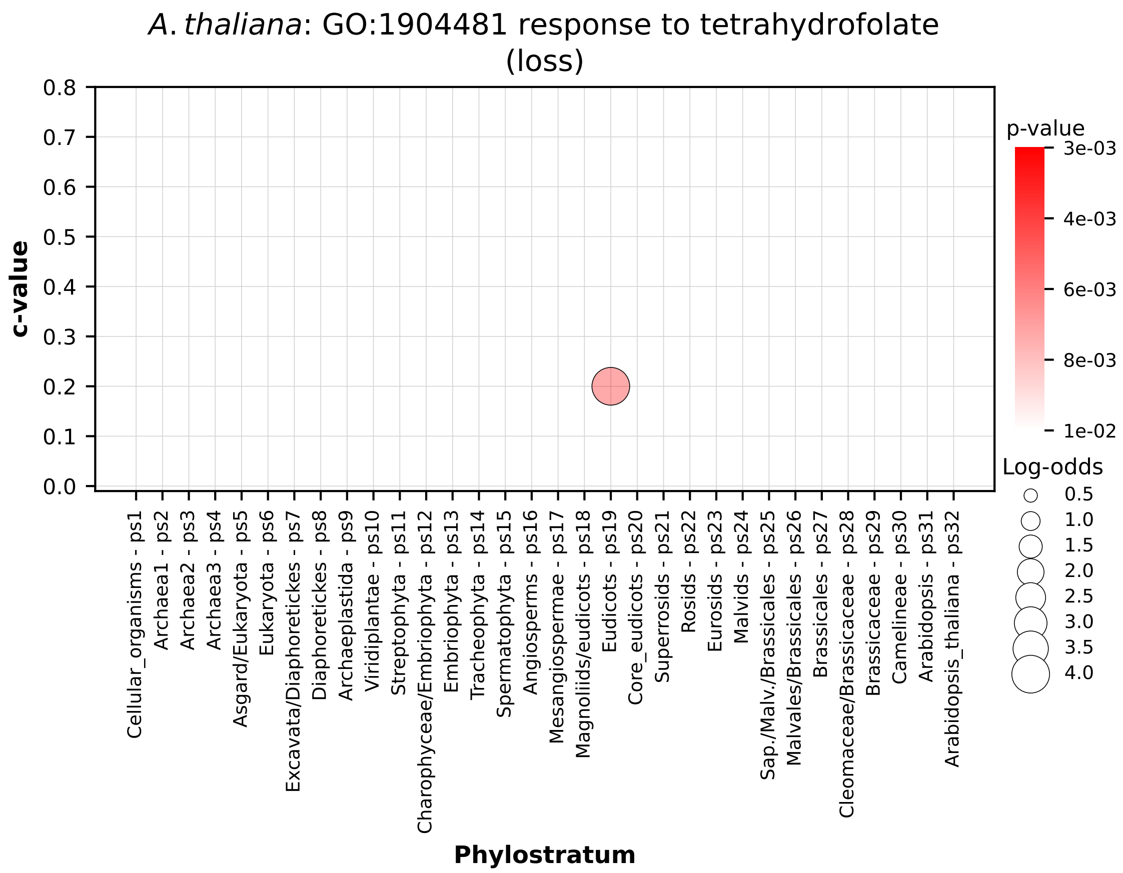

A. thaliana: GO:1904481 response to tetrahydrofolate (loss)
c-value
0.8
0.7
0.6
0.5
0.4
0.3
0.2
0.1
0.0
Phylostratum
Cellular_organisms - ps1
Archaea1 - ps2
Archaea2 - ps3
Archaea3 - ps4
Asgard/Eukaryota - ps5
Eukaryota - ps6
Excavata/Diaphoretickes - ps7
Diaphoretickes - ps8
Archaeplastida - ps9
Viridiplantae - ps10
Streptophyta - ps11
Charophyceae/Embriophyta - ps12
Embriophyta - ps13
Tracheophyta - ps14
Spermatophyta - ps15
Angiosperms - ps16
Mesangiospermae - ps17
Magnoliids/eudicots - ps18
Eudicots - ps19
Core_eudicots - ps20
Superrosids - ps21
Rosids - ps22
Eurosids - ps23
Malvids - ps24
Sap./Malv./Brassicales - ps25
Malvales/Brassicales - ps26
Brassicales - ps27
Cleomaceae/Brassicaceae - ps28
Brassicaceae - ps29
Camelineae - ps30
Arabidopsis - ps31
Arabidopsis_thaliana - ps32
p-value
3e-03
4e-03
6e-03
8e-03
1e-02
Log-odds
0.5
1.0
1.5
2.0
2.5
3.0
3.5
4.0

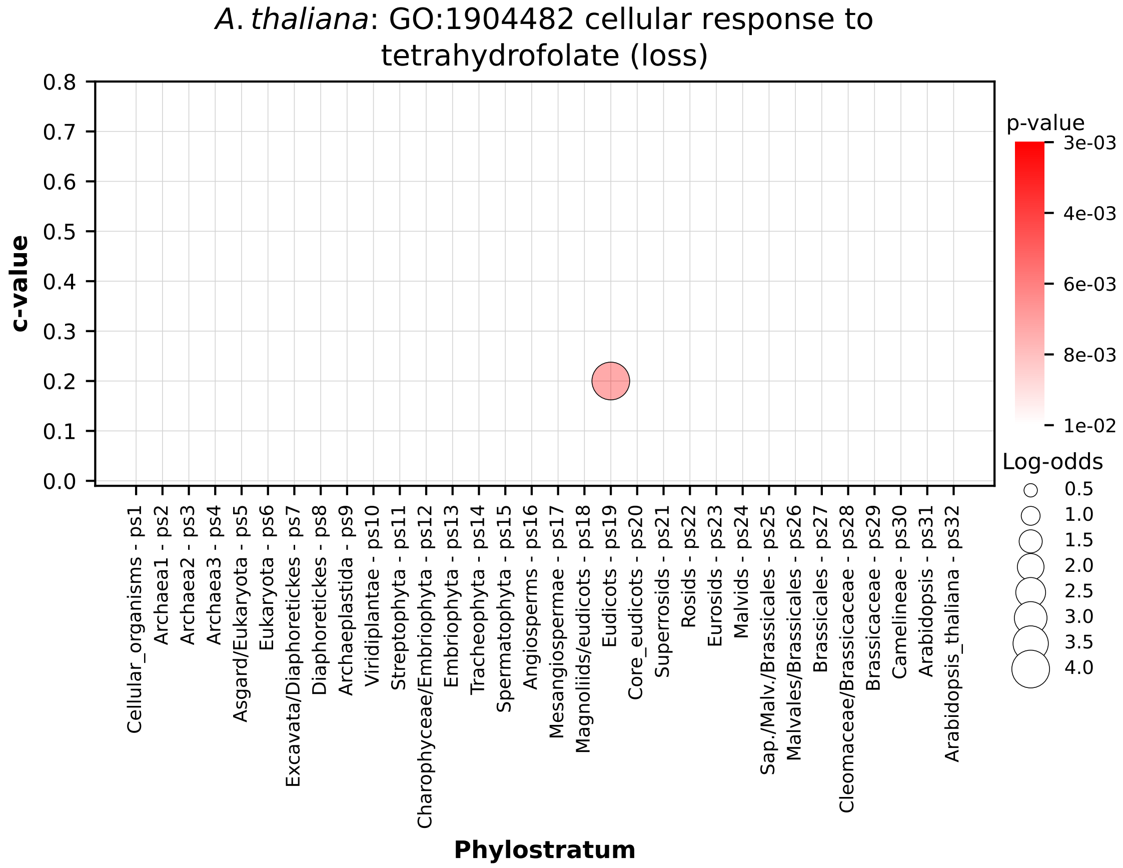
A. thaliana: GO:1904482 cellular response to tetrahydrofolate (loss)
c-value
0.0
0.1
0.2
0.3
0.4
0.5
0.6
0.7
0.8
Phylostratum
Cellular_organisms - ps1
Archaea1 - ps2
Archaea2 - ps3
Archaea3 - ps4
Asgard/Eukaryota - ps5
Eukaryota - ps6
Excavata/Diaphoretickes - ps7
Diaphoretickes - ps8
Archaeplastida - ps9
Viridiplantae - ps10
Streptophyta - ps11
Charophyceae/Embriophyta - ps12
Embriophyta - ps13
Tracheophyta - ps14
Spermatophyta - ps15
Angiosperms - ps16
Mesangiospermae - ps17
Magnoliids/eudicots - ps18
Eudicots - ps19
Core_eudicots - ps20
Superrosids - ps21
Rosids - ps22
Eurosids - ps23
Malvids - ps24
Sap./Malv./Brassicales - ps25
Malvales/Brassicales - ps26
Brassicales - ps27
Cleomaceae/Brassicaceae - ps28
Brassicaceae - ps29
Camelineae - ps30
Arabidopsis - ps31
Arabidopsis_thaliana - ps32
p-value
3e-03
4e-03
6e-03
8e-03
1e-02
Log-odds
0.5
1.0
1.5
2.0
2.5
3.0
3.5
4.0

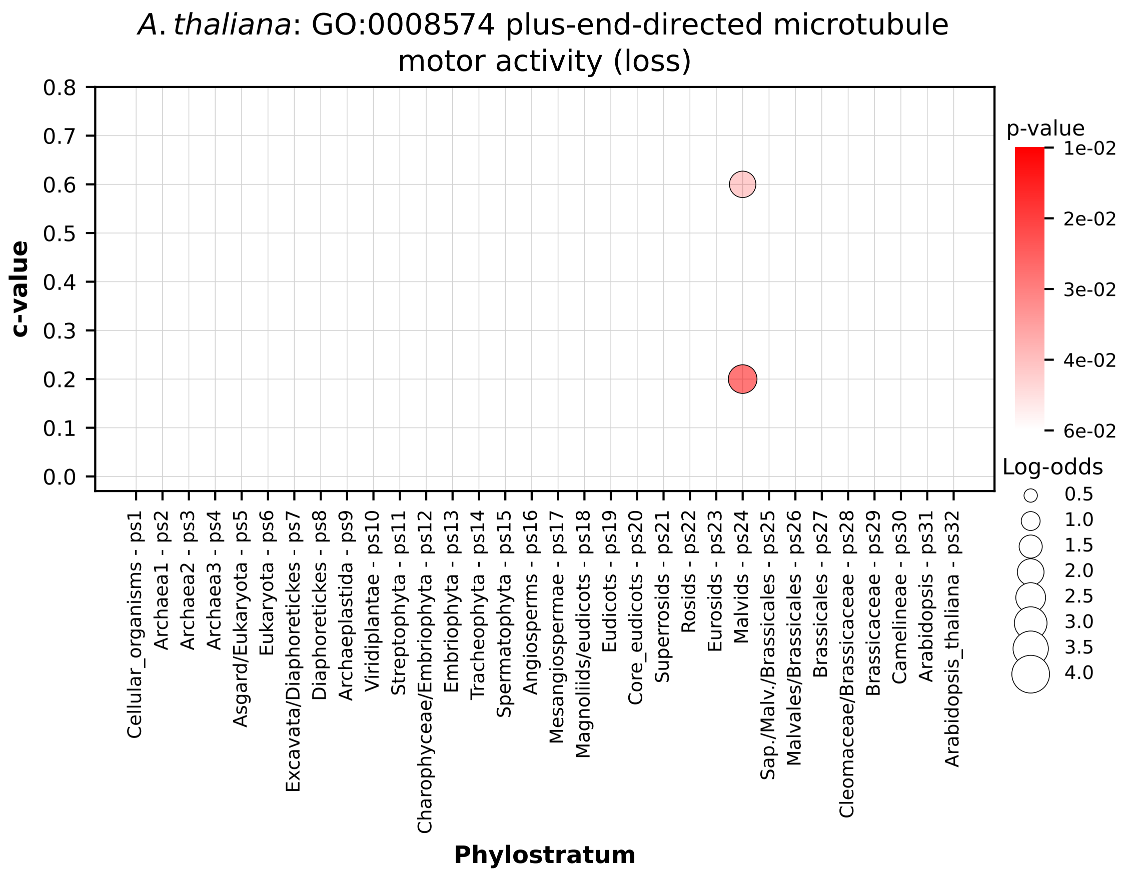
A. thaliana: GO:0008574 plus-end-directed microtubule motor activity (loss)
c-value
0.8
0.7
0.6
0.5
0.4
0.3
0.2
0.1
0.0
p-value
1e-02
2e-02
3e-02
4e-02
6e-02
Log-odds
0.5
1.0
1.5
2.0
2.5
3.0
3.5
4.0
Cellular_organisms - ps1
Archaea1 - ps2
Archaea2 - ps3
Archaea3 - ps4
Asgard/Eukaryota - ps5
Eukaryota - ps6
Excavata/Diaphoretickes - ps7
Diaphoretickes - ps8
Archaeplastida - ps9
Viridiplantae - ps10
Streptophyta - ps11
Charophyceae/Embriophyta - ps12
Embriophyta - ps13
Tracheophyta - ps14
Spermatophyta - ps15
Angiosperms - ps16
Mesangiospermae - ps17
Magnoliids/eudicots - ps18
Eudicots - ps19
Core_eudicots - ps20
Superrosids - ps21
Rosids - ps22
Eurosids - ps23
Malvids - ps24
Sap./Malv./Brassicales - ps25
Malvales/Brassicales - ps26
Brassicales - ps27
Cleomaceae/Brassicaceae - ps28
Brassicaceae - ps29
Camelineae - ps30
Arabidopsis - ps31
Arabidopsis_thaliana - ps32
Phylostratum

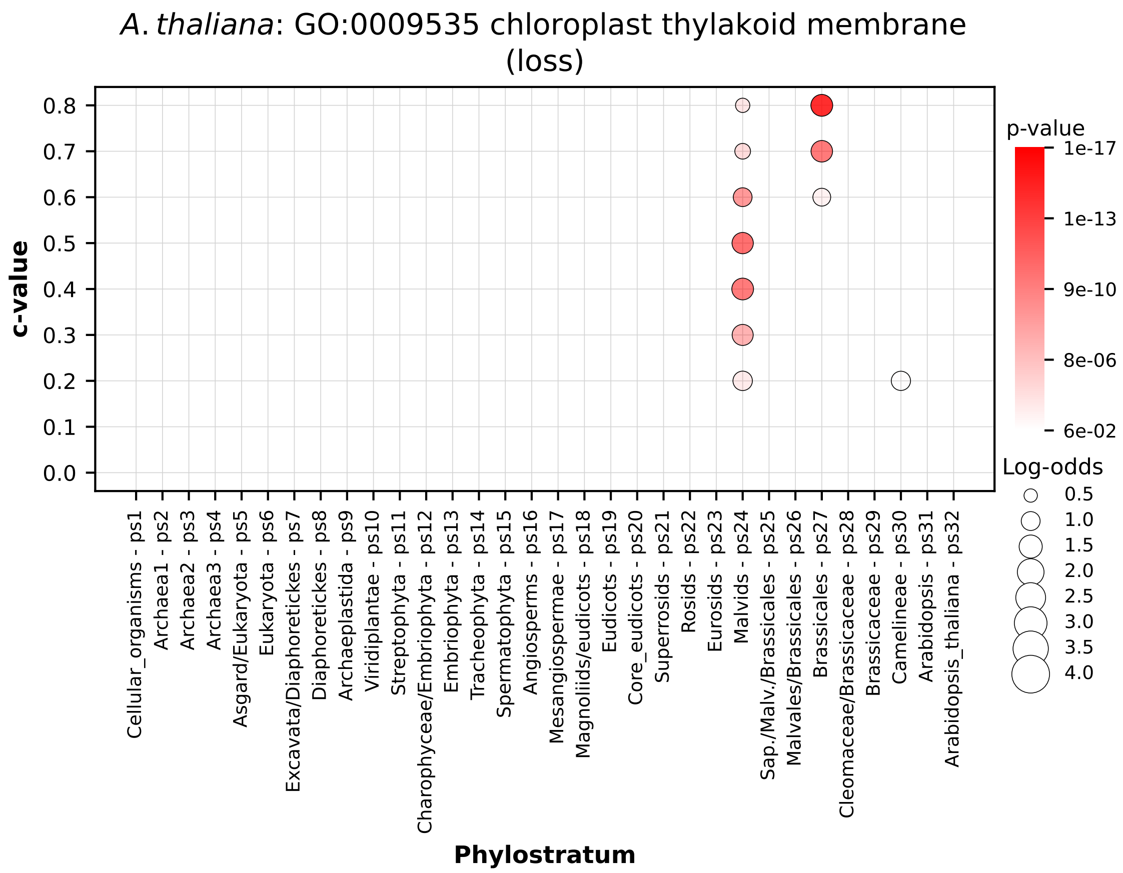
A. thaliana: GO:0009535 chloroplast thylakoid membrane
(loss)
c-value
0.8
0.7
0.6
0.5
0.4
0.3
0.2
0.1
0.0
p-value
1e-17
1e-13
9e-10
8e-06
6e-02
Log-odds
0.5
1.0
1.5
2.0
2.5
3.0
3.5
4.0
Cellular_organisms - ps1
Archaea1 - ps2
Archaea2 - ps3
Archaea3 - ps4
Asgard/Eukaryota - ps5
Eukaryota - ps6
Excavata/Diaphoretickes - ps7
Diaphoretickes - ps8
Archaeplastida - ps9
Viridiplantae - ps10
Streptophyta - ps11
Charophyceae/Embriophyta - ps12
Embriophyta - ps13
Tracheophyta - ps14
Spermatophyta - ps15
Angiosperms - ps16
Mesangiospermae - ps17
Magnoliids/eudicots - ps18
Eudicots - ps19
Core_eudicots - ps20
Superrosids - ps21
Rosids - ps22
Eurosids - ps23
Malvids - ps24
Sap./Malv./Brassicales - ps25
Malvales/Brassicales - ps26
Brassicales - ps27
Cleomaceae/Brassicaceae - ps28
Brassicaceae - ps29
Camelineae - ps30
Arabidopsis - ps31
Arabidopsis_thaliana - ps32
Phylostratum

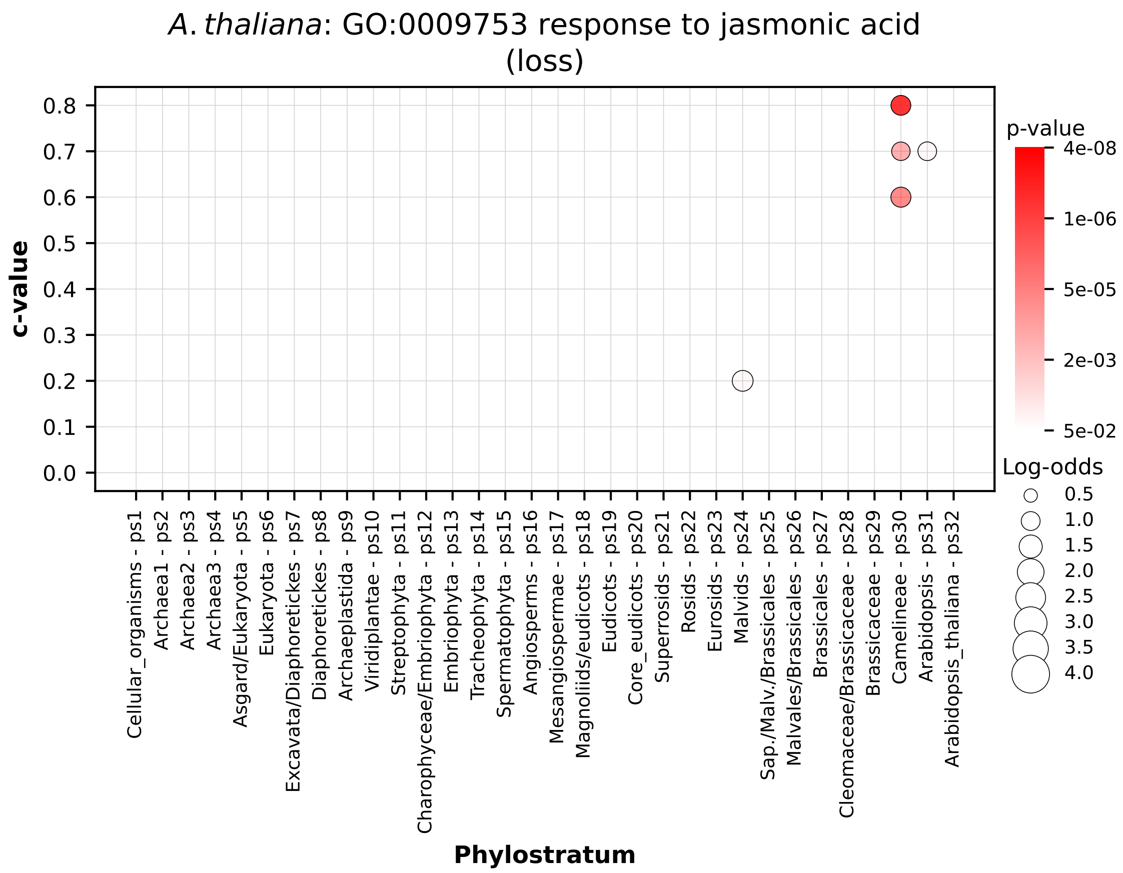

A. thaliana: GO:0009753 response to jasmonic acid
(loss)
c-value
0.0
0.1
0.2
0.3
0.4
0.5
0.6
0.7
0.8
Phylostratum
Cellular_organisms - ps1
Archaea1 - ps2
Archaea2 - ps3
Archaea3 - ps4
Asgard/Eukaryota - ps5
Eukaryota - ps6
Excavata/Diaphoretickes - ps7
Diaphoretickes - ps8
Archaeplastida - ps9
Viridiplantae - ps10
Streptophyta - ps11
Charophyceae/Embriophyta - ps12
Embriophyta - ps13
Tracheophyta - ps14
Spermatophyta - ps15
Angiosperms - ps16
Mesangiospermae - ps17
Magnoliids/eudicots - ps18
Eudicots - ps19
Core_eudicots - ps20
Superrosids - ps21
Rosids - ps22
Eurosids - ps23
Malvids - ps24
Sap./Malv./Brassicales - ps25
Malvales/Brassicales - ps26
Brassicales - ps27
Cleomaceae/Brassicaceae - ps28
Brassicaceae - ps29
Camelineae - ps30
Arabidopsis - ps31
Arabidopsis_thaliana - ps32
p-value
4e-08
1e-06
5e-05
2e-03
5e-02
Log-odds
0.5
1.0
1.5
2.0
2.5
3.0
3.5
4.0

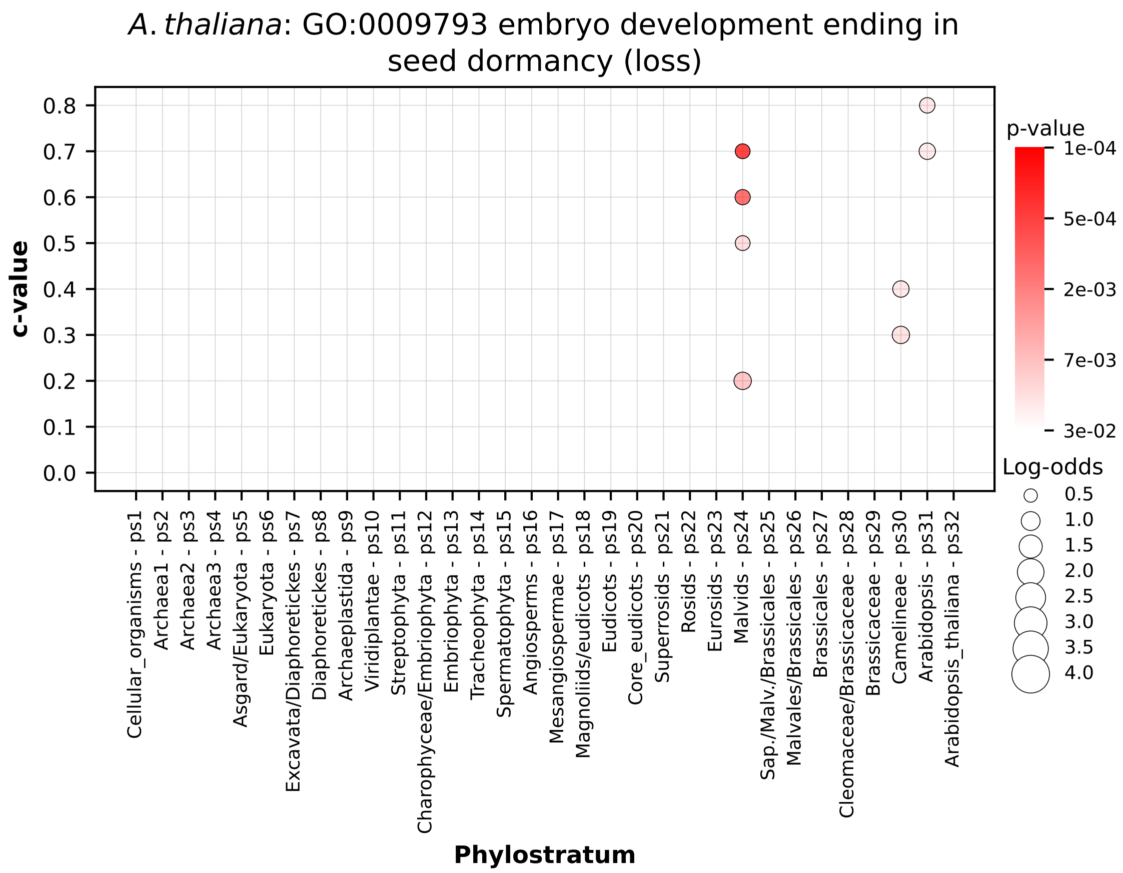

A. thaliana: GO:0009793 embryo development ending in seed dormancy (loss)
c-value
0.0
0.1
0.2
0.3
0.4
0.5
0.6
0.7
0.8
Phylostratum
Cellular_organisms - ps1
Archaea1 - ps2
Archaea2 - ps3
Archaea3 - ps4
Asgard/Eukaryota - ps5
Eukaryota - ps6
Excavata/Diaphoretickes - ps7
Diaphoretickes - ps8
Archaeplastida - ps9
Viridiplantae - ps10
Streptophyta - ps11
Charophyceae/Embriophyta - ps12
Embriophyta - ps13
Tracheophyta - ps14
Spermatophyta - ps15
Angiosperms - ps16
Mesangiospermae - ps17
Magnoliids/eudicots - ps18
Eudicots - ps19
Core_eudicots - ps20
Superrosids - ps21
Rosids - ps22
Eurosids - ps23
Malvids - ps24
Sap./Malv./Brassicales - ps25
Malvales/Brassicales - ps26
Brassicales - ps27
Cleomaceae/Brassicaceae - ps28
Brassicaceae - ps29
Camelineae - ps30
Arabidopsis - ps31
Arabidopsis_thaliana - ps32
p-value
1e-04
5e-04
2e-03
7e-03
3e-02
Log-odds
0.5
1.0
1.5
2.0
2.5
3.0
3.5
4.0

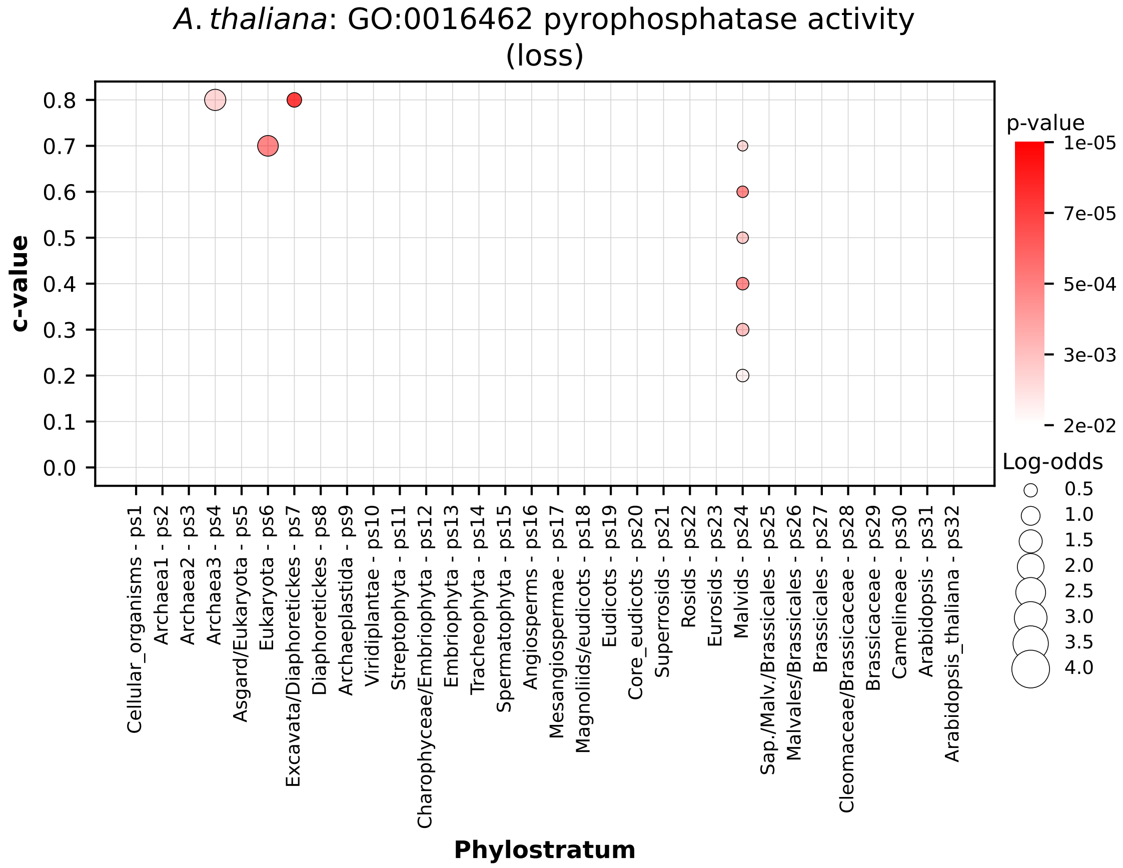

A. thaliana: GO:0016462 pyrophosphatase activity
(loss)
c-value
0.8
0.7
0.6
0.5
0.4
0.3
0.2
0.1
0.0
p-value
1e-05
7e-05
5e-04
3e-03
2e-02
Log-odds
0.5
1.0
1.5
2.0
2.5
3.0
3.5
4.0
Cellular_organisms - ps1
Archaea1 - ps2
Archaea2 - ps3
Archaea3 - ps4
Asgard/Eukaryota - ps5
Eukaryota - ps6
Excavata/Diaphoretickes - ps7
Diaphoretickes - ps8
Archaeplastida - ps9
Viridiplantae - ps10
Streptophyta - ps11
Charophyceae/Embriophyta - ps12
Embriophyta - ps13
Tracheophyta - ps14
Spermatophyta - ps15
Angiosperms - ps16
Mesangiospermae - ps17
Magnoliids/eudicots - ps18
Eudicots - ps19
Core_eudicots - ps20
Superrosids - ps21
Rosids - ps22
Eurosids - ps23
Malvids - ps24
Sap./Malv./Brassicales - ps25
Malvales/Brassicales - ps26
Brassicales - ps27
Cleomaceae/Brassicaceae - ps28
Brassicaceae - ps29
Camelineae - ps30
Arabidopsis - ps31
Arabidopsis_thaliana - ps32
Phylostratum

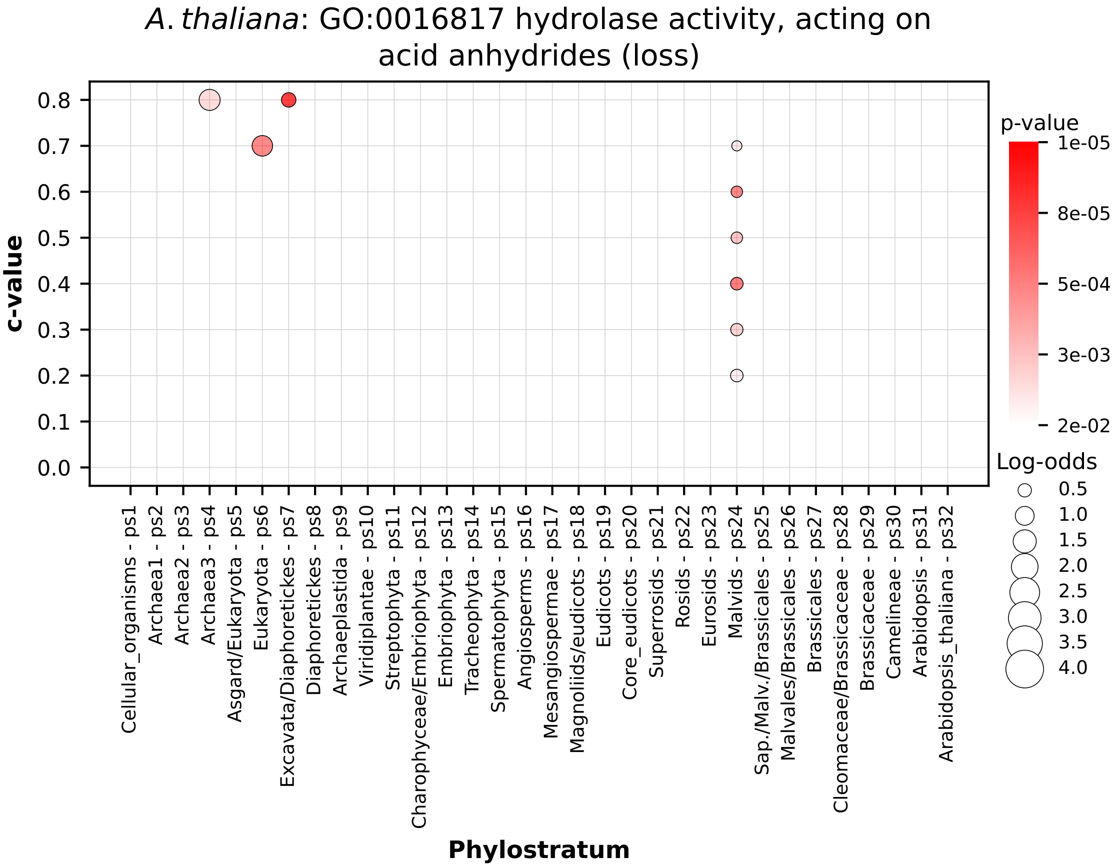

A. thaliana: GO:0016817 hydrolase activity, acting on acid anhydrides (loss)
c-value
0.0
0.1
0.2
0.3
0.4
0.5
0.6
0.7
0.8
p-value
1e-05
8e-05
5e-04
3e-03
2e-02
Log-odds
0.5
1.0
1.5
2.0
2.5
3.0
3.5
4.0
Cellular_organisms - ps1
Archaea1 - ps2
Archaea2 - ps3
Archaea3 - ps4
Asgard/Eukaryota - ps5
Eukaryota - ps6
Excavata/Diaphoretickes - ps7
Diaphoretickes - ps8
Archaeplastida - ps9
Viridiplantae - ps10
Streptophyta - ps11
Charophyceae/Embriophyta - ps12
Embriophyta - ps13
Tracheophyta - ps14
Spermatophyta - ps15
Angiosperms - ps16
Mesangiospermae - ps17
Magnoliids/eudicots - ps18
Eudicots - ps19
Core_eudicots - ps20
Superrosids - ps21
Rosids - ps22
Eurosids - ps23
Malvids - ps24
Sap./Malv./Brassicales - ps25
Malvales/Brassicales - ps26
Brassicales - ps27
Cleomaceae/Brassicaceae - ps28
Brassicaceae - ps29
Camelineae - ps30
Arabidopsis - ps31
Arabidopsis_thaliana - ps32
Phylostratum

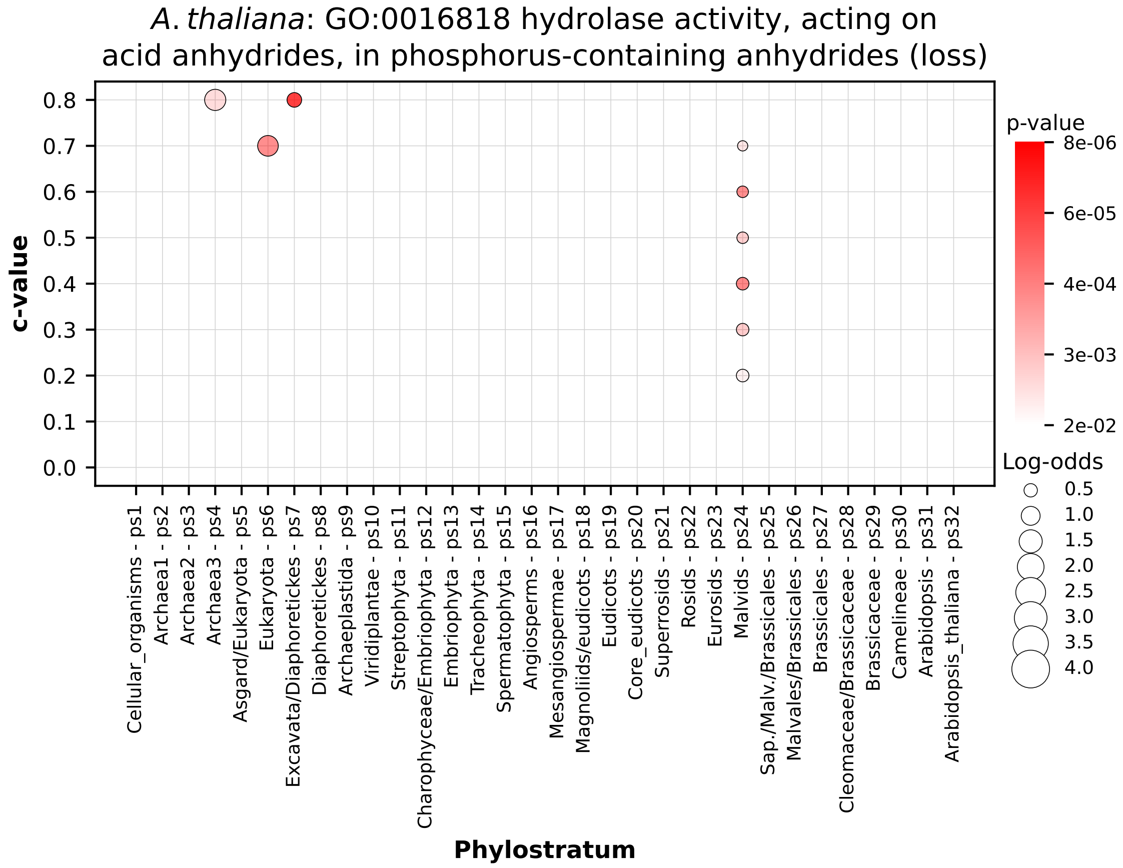
A. thaliana: GO:0016818 hydrolase activity, acting on acid anhydrides, in phosphorus-containing anhydrides (loss)
c-value
0.0
0.1
0.2
0.3
0.4
0.5
0.6
0.7
0.8
p-value
8e-06
6e-05
4e-04
3e-03
2e-02
Log-odds
0.5
1.0
1.5
2.0
2.5
3.0
3.5
4.0
Cellular_organisms - ps1
Archaea1 - ps2
Archaea2 - ps3
Archaea3 - ps4
Asgard/Eukaryota - ps5
Eukaryota - ps6
Excavata/Diaphoretickes - ps7
Diaphoretickes - ps8
Archaeplastida - ps9
Viridiplantae - ps10
Streptophyta - ps11
Charophyceae/Embriophyta - ps12
Embriophyta - ps13
Tracheophyta - ps14
Spermatophyta - ps15
Angiosperms - ps16
Mesangiospermae - ps17
Magnoliids/eudicots - ps18
Eudicots - ps19
Core_eudicots - ps20
Superrosids - ps21
Rosids - ps22
Eurosids - ps23
Malvids - ps24
Sap./Malv./Brassicales - ps25
Malvales/Brassicales - ps26
Brassicales - ps27
Cleomaceae/Brassicaceae - ps28
Brassicaceae - ps29
Camelineae - ps30
Arabidopsis - ps31
Arabidopsis_thaliana - ps32
Phylostratum

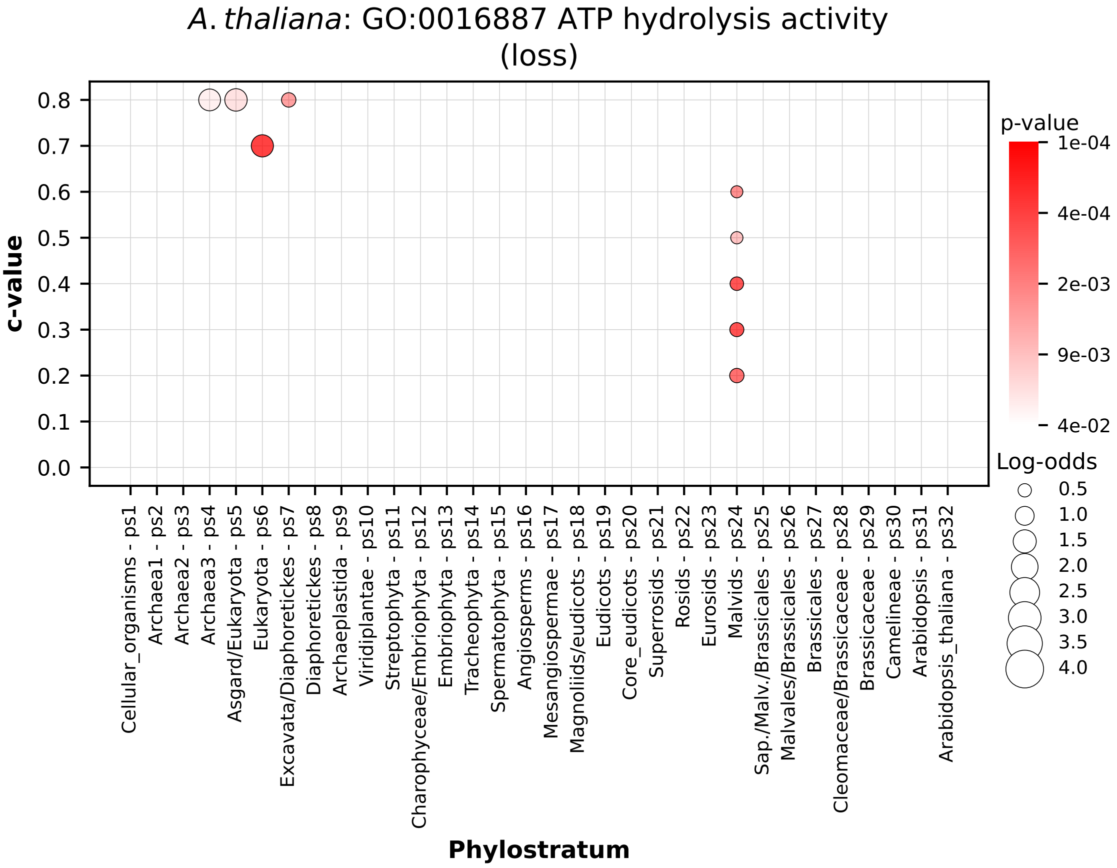

A. thaliana: GO:0016887 ATP hydrolysis activity (loss)
c-value
0.0
0.1
0.2
0.3
0.4
0.5
0.6
0.7
0.8
Phylostratum
Cellular_organisms - ps1
Archaea1 - ps2
Archaea2 - ps3
Archaea3 - ps4
Asgard/Eukaryota - ps5
Eukaryota - ps6
Excavata/Diaphoretickes - ps7
Diaphoretickes - ps8
Archaeplastida - ps9
Viridiplantae - ps10
Streptophyta - ps11
Charophyceae/Embriophyta - ps12
Embriophyta - ps13
Tracheophyta - ps14
Spermatophyta - ps15
Angiosperms - ps16
Mesangiospermae - ps17
Magnoliids/eudicots - ps18
Eudicots - ps19
Core_eudicots - ps20
Superrosids - ps21
Rosids - ps22
Eurosids - ps23
Malvids - ps24
Sap./Malv./Brassicales - ps25
Malvales/Brassicales - ps26
Brassicales - ps27
Cleomaceae/Brassicaceae - ps28
Brassicaceae - ps29
Camelineae - ps30
Arabidopsis - ps31
Arabidopsis_thaliana - ps32
p-value
1e-04
4e-04
2e-03
9e-03
4e-02
Log-odds
0.5
1.0
1.5
2.0
2.5
3.0
3.5
4.0

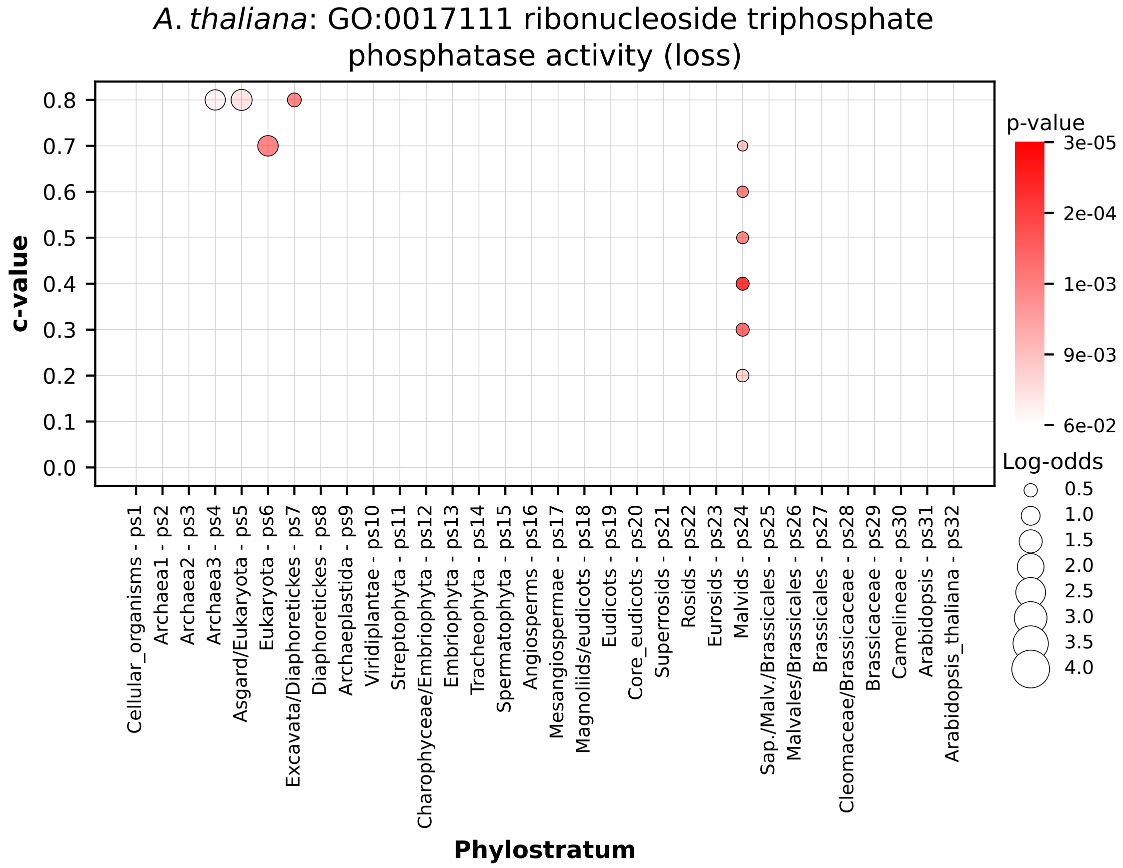
A. thaliana: GO:0017111 ribonucleoside triphosphate phosphatase activity (loss)
c-value
0.8
0.7
0.6
0.5
0.4
0.3
0.2
0.1
0.0
Phylostratum
Cellular_organisms - ps1
Archaea1 - ps2
Archaea2 - ps3
Archaea3 - ps4
Asgard/Eukaryota - ps5
Eukaryota - ps6
Excavata/Diaphoretickes - ps7
Diaphoretickes - ps8
Archaeplastida - ps9
Viridiplantae - ps10
Streptophyta - ps11
Charophyceae/Embriophyta - ps12
Embriophyta - ps13
Tracheophyta - ps14
Spermatophyta - ps15
Angiosperms - ps16
Mesangiospermae - ps17
Magnoliids/eudicots - ps18
Eudicots - ps19
Core_eudicots - ps20
Superrosids - ps21
Rosids - ps22
Eurosids - ps23
Malvids - ps24
Sap./Malv./Brassicales - ps25
Malvales/Brassicales - ps26
Brassicales - ps27
Cleomaceae/Brassicaceae - ps28
Brassicaceae - ps29
Camelineae - ps30
Arabidopsis - ps31
Arabidopsis_thaliana - ps32
p-value
3e-05
2e-04
1e-03
9e-03
6e-02
Log-odds
0.5
1.0
1.5
2.0
2.5
3.0
3.5
4.0

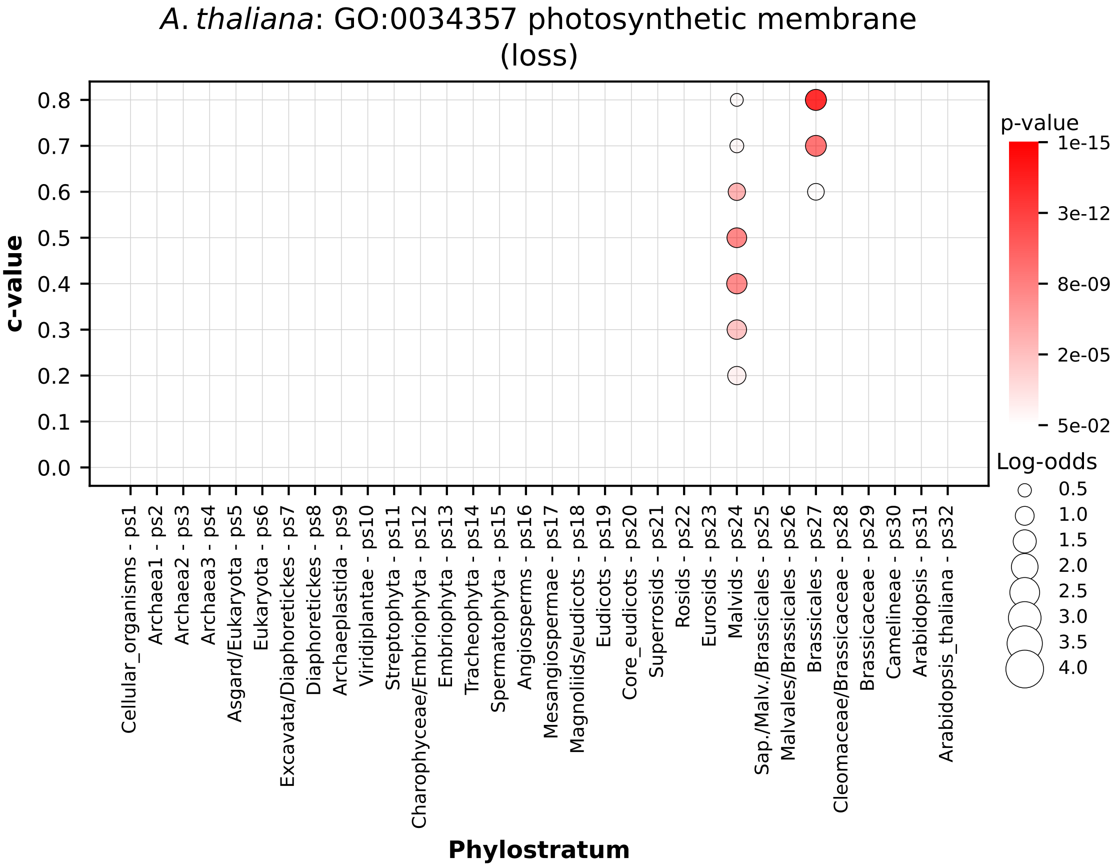
A. thaliana: GO:0034357 photosynthetic membrane
(loss)
c-value
0.8
0.7
0.6
0.5
0.4
0.3
0.2
0.1
0.0
Phylostratum
Cellular_organisms - ps1
Archaea1 - ps2
Archaea2 - ps3
Archaea3 - ps4
Asgard/Eukaryota - ps5
Eukaryota - ps6
Excavata/Diaphoretickes - ps7
Diaphoretickes - ps8
Archaeplastida - ps9
Viridiplantae - ps10
Streptophyta - ps11
Charophyceae/Embriophyta - ps12
Embriophyta - ps13
Tracheophyta - ps14
Spermatophyta - ps15
Angiosperms - ps16
Mesangiospermae - ps17
Magnoliids/eudicots - ps18
Eudicots - ps19
Core_eudicots - ps20
Superrosids - ps21
Rosids - ps22
Eurosids - ps23
Malvids - ps24
Sap./Malv./Brassicales - ps25
Malvales/Brassicales - ps26
Brassicales - ps27
Cleomaceae/Brassicaceae - ps28
Brassicaceae - ps29
Camelineae - ps30
Arabidopsis - ps31
Arabidopsis_thaliana - ps32
p-value
1e-15
3e-12
8e-09
2e-05
5e-02
Log-odds
0.5
1.0
1.5
2.0
2.5
3.0
3.5
4.0

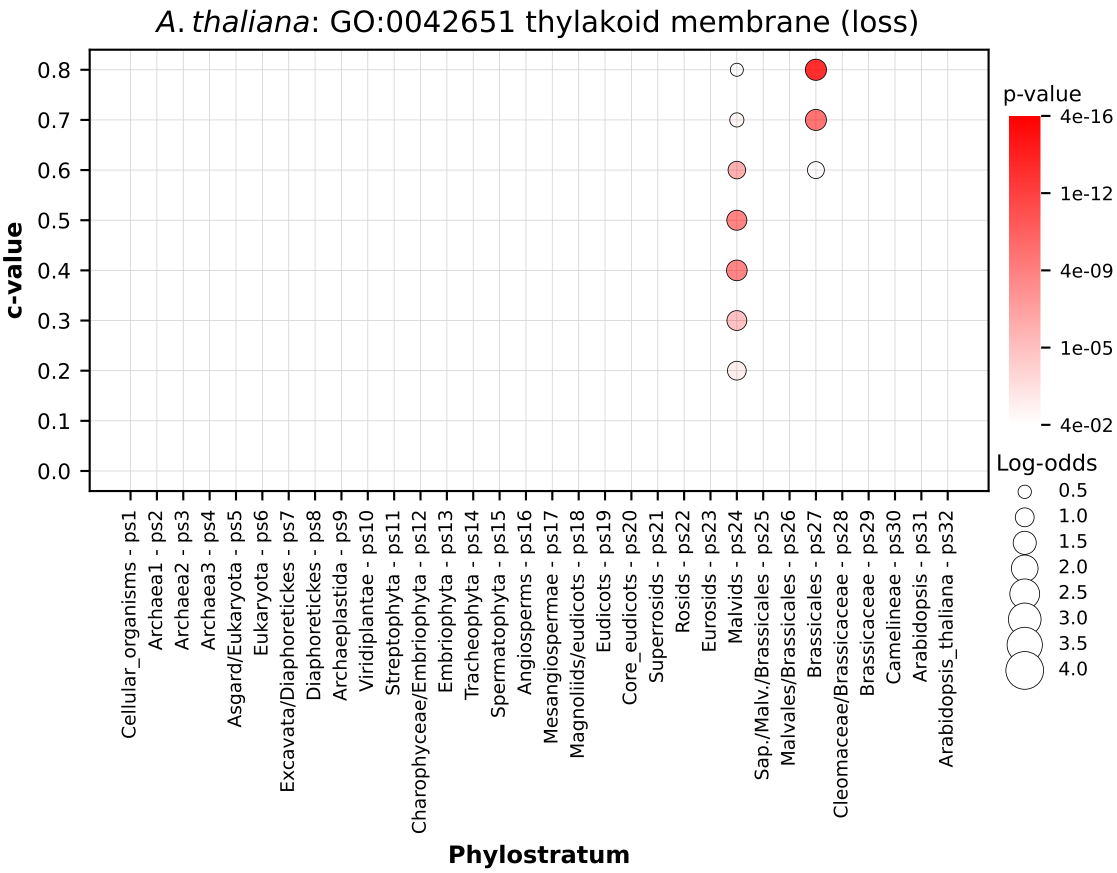
A. thaliana: GO:0042651 thylakoid membrane (loss)
c-value
0.0
0.1
0.2
0.3
0.4
0.5
0.6
0.7
0.8
p-value
4e-16
1e-12
4e-09
1e-05
4e-02
Log-odds
0.5
1.0
1.5
2.0
2.5
3.0
3.5
4.0
Cellular_organisms - ps1
Archaea1 - ps2
Archaea2 - ps3
Archaea3 - ps4
Asgard/Eukaryota - ps5
Eukaryota - ps6
Excavata/Diaphoretickes - ps7
Diaphoretickes - ps8
Archaeplastida - ps9
Viridiplantae - ps10
Streptophyta - ps11
Charophyceae/Embriophyta - ps12
Embriophyta - ps13
Tracheophyta - ps14
Spermatophyta - ps15
Angiosperms - ps16
Mesangiospermae - ps17
Magnoliids/eudicots - ps18
Eudicots - ps19
Core_eudicots - ps20
Superrosids - ps21
Rosids - ps22
Eurosids - ps23
Malvids - ps24
Sap./Malv./Brassicales - ps25
Malvales/Brassicales - ps26
Brassicales - ps27
Cleomaceae/Brassicaceae - ps28
Brassicaceae - ps29
Camelineae - ps30
Arabidopsis - ps31
Arabidopsis_thaliana - ps32
Phylostratum

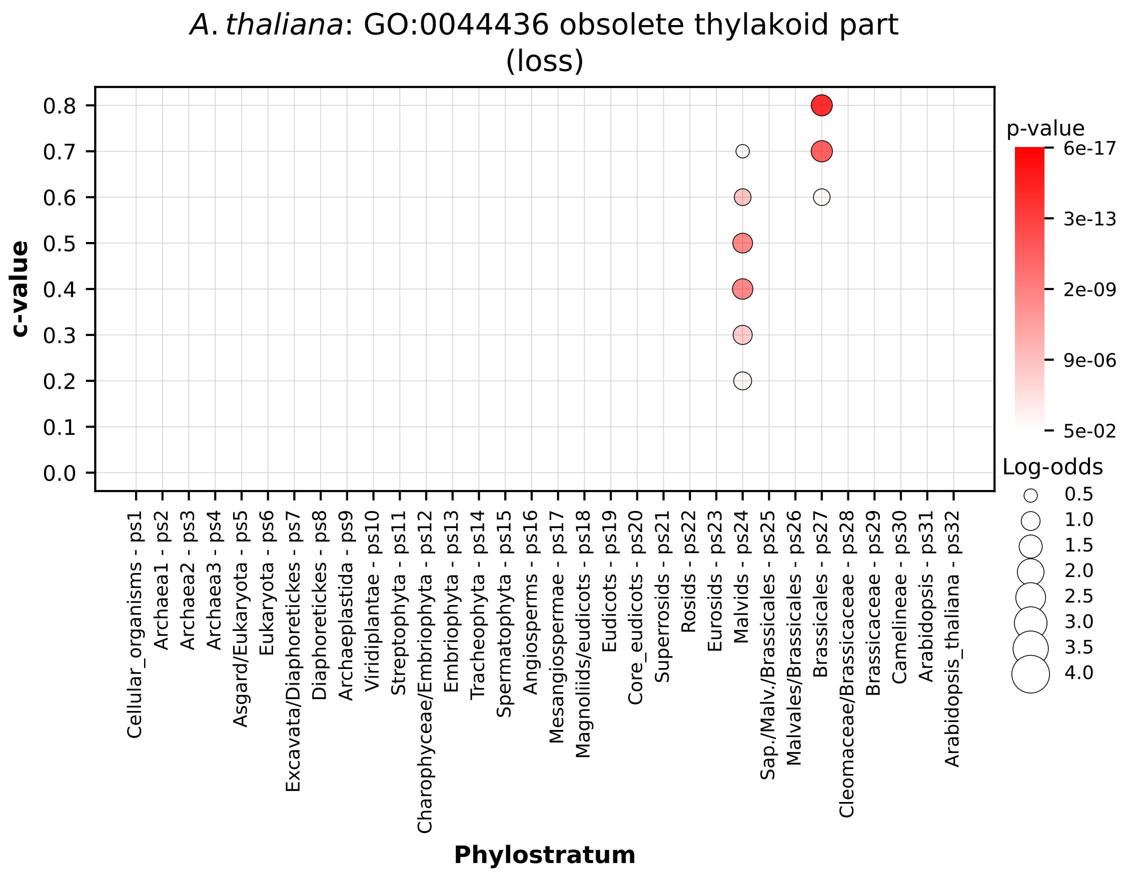

A. thaliana: GO:0044436 obsolete thylakoid part
(loss)
c-value
0.8
0.7
0.6
0.5
0.4
0.3
0.2
0.1
0.0
p-value
6e-17
3e-13
2e-09
9e-06
5e-02
Log-odds
0.5
1.0
1.5
2.0
2.5
3.0
3.5
4.0
Cellular_organisms - ps1
Archaea1 - ps2
Archaea2 - ps3
Archaea3 - ps4
Asgard/Eukaryota - ps5
Eukaryota - ps6
Excavata/Diaphoretickes - ps7
Diaphoretickes - ps8
Archaeplastida - ps9
Viridiplantae - ps10
Streptophyta - ps11
Charophyceae/Embriophyta - ps12
Embriophyta - ps13
Tracheophyta - ps14
Spermatophyta - ps15
Angiosperms - ps16
Mesangiospermae - ps17
Magnoliids/eudicots - ps18
Eudicots - ps19
Core_eudicots - ps20
Superrosids - ps21
Rosids - ps22
Eurosids - ps23
Malvids - ps24
Sap./Malv./Brassicales - ps25
Malvales/Brassicales - ps26
Brassicales - ps27
Cleomaceae/Brassicaceae - ps28
Brassicaceae - ps29
Camelineae - ps30
Arabidopsis - ps31
Arabidopsis_thaliana - ps32
Phylostratum

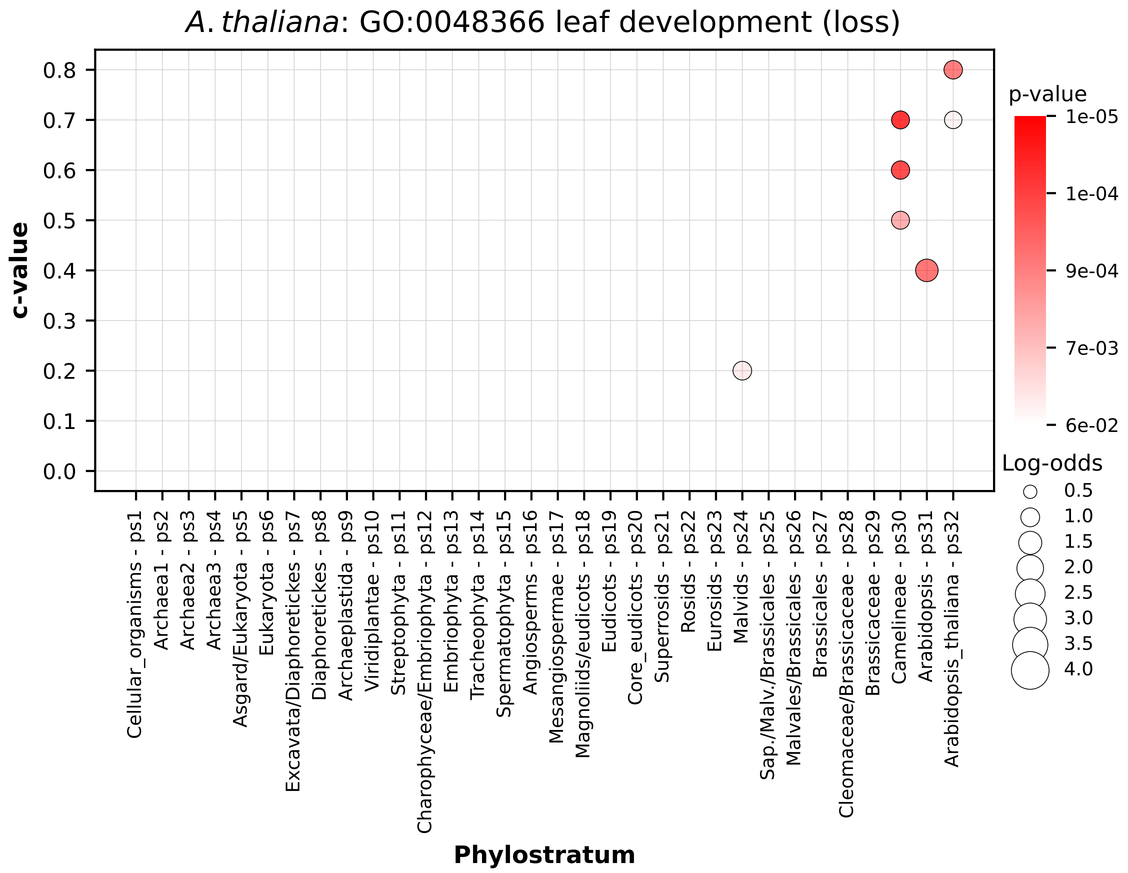

A. thaliana: GO:0048366 leaf development (loss)
c-value
0.8
0.7
0.6
0.5
0.4
0.3
0.2
0.1
0.0
p-value
1e-05
1e-04
9e-04
7e-03
6e-02
Log-odds
0.5
1.0
1.5
2.0
2.5
3.0
3.5
4.0
Cellular_organisms - ps1
Archaea1 - ps2
Archaea2 - ps3
Archaea3 - ps4
Asgard/Eukaryota - ps5
Eukaryota - ps6
Excavata/Diaphoretickes - ps7
Diaphoretickes - ps8
Archaeplastida - ps9
Viridiplantae - ps10
Streptophyta - ps11
Charophyceae/Embriophyta - ps12
Embriophyta - ps13
Tracheophyta - ps14
Spermatophyta - ps15
Angiosperms - ps16
Mesangiospermae - ps17
Magnoliids/eudicots - ps18
Eudicots - ps19
Core_eudicots - ps20
Superrosids - ps21
Rosids - ps22
Eurosids - ps23
Malvids - ps24
Sap./Malv./Brassicales - ps25
Malvales/Brassicales - ps26
Brassicales - ps27
Cleomaceae/Brassicaceae - ps28
Brassicaceae - ps29
Camelineae - ps30
Arabidopsis - ps31
Arabidopsis_thaliana - ps32
Phylostratum

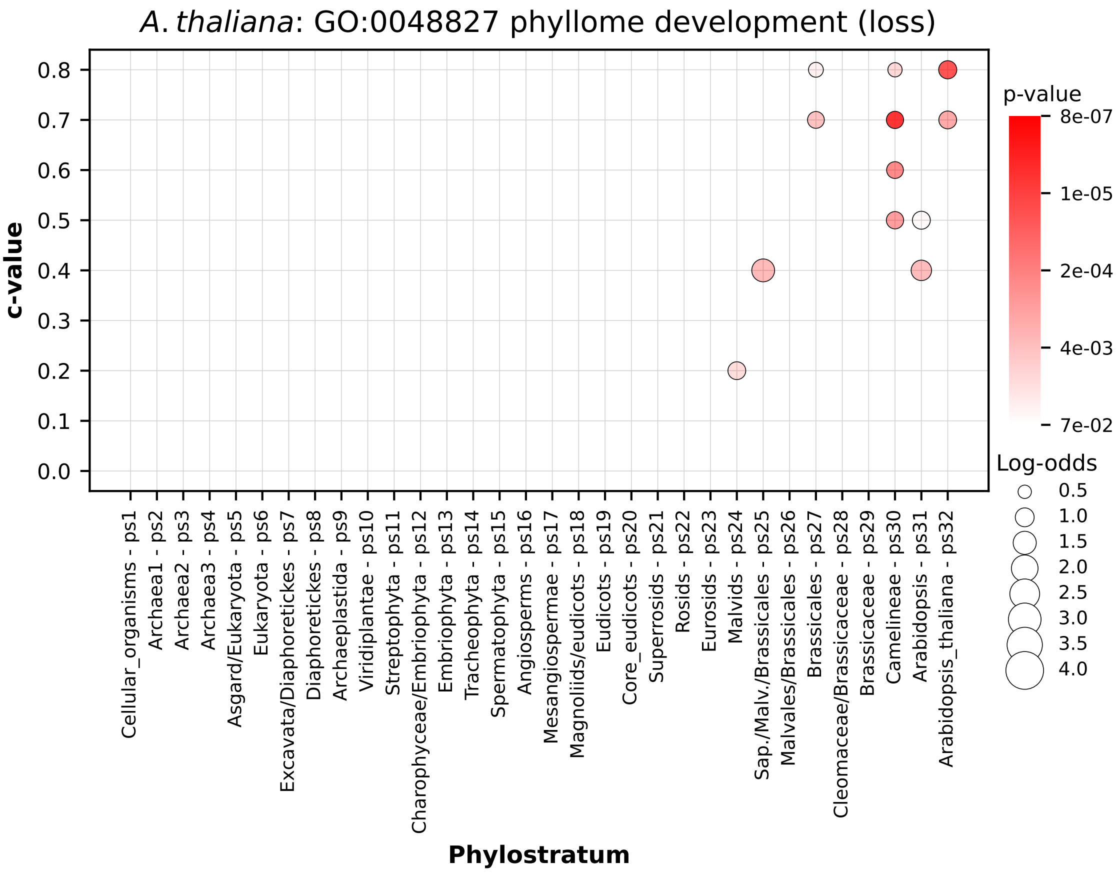

A. thaliana: GO:0048827 phyllome development (loss)
c-value
0.0
0.1
0.2
0.3
0.4
0.5
0.6
0.7
0.8
Phylostratum
Cellular_organisms - ps1
Archaea1 - ps2
Archaea2 - ps3
Archaea3 - ps4
Asgard/Eukaryota - ps5
Eukaryota - ps6
Excavata/Diaphoretickes - ps7
Diaphoretickes - ps8
Archaeplastida - ps9
Viridiplantae - ps10
Streptophyta - ps11
Charophyceae/Embriophyta - ps12
Embriophyta - ps13
Tracheophyta - ps14
Spermatophyta - ps15
Angiosperms - ps16
Mesangiospermae - ps17
Magnoliids/eudicots - ps18
Eudicots - ps19
Core_eudicots - ps20
Superrosids - ps21
Rosids - ps22
Eurosids - ps23
Malvids - ps24
Sap./Malv./Brassicales - ps25
Malvales/Brassicales - ps26
Brassicales - ps27
Cleomaceae/Brassicaceae - ps28
Brassicaceae - ps29
Camelineae - ps30
Arabidopsis - ps31
Arabidopsis_thaliana - ps32
p-value
8e-07
1e-05
2e-04
4e-03
7e-02
Log-odds
0.5
1.0
1.5
2.0
2.5
3.0
3.5
4.0

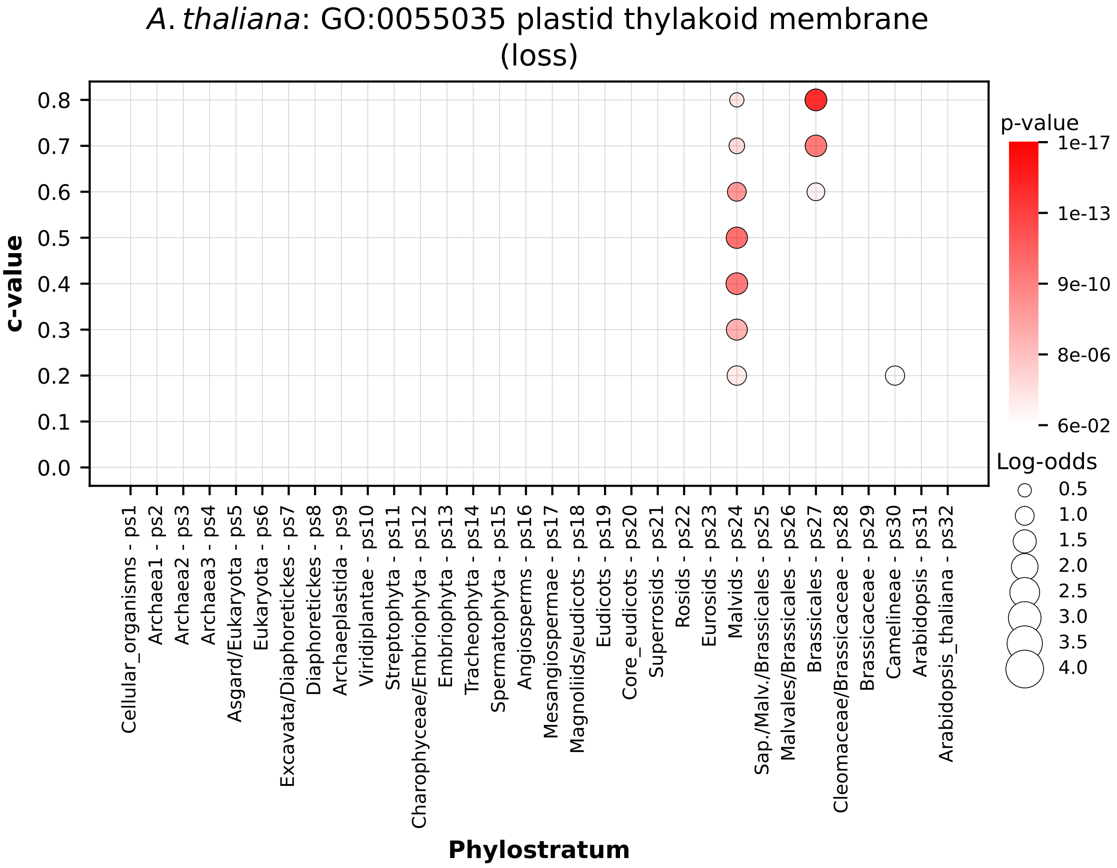

A. thaliana: GO:0055035 plastid thylakoid membrane
(loss)
c-value
0.0
0.1
0.2
0.3
0.4
0.5
0.6
0.7
0.8
Phylostratum
Cellular_organisms - ps1
Archaea1 - ps2
Archaea2 - ps3
Archaea3 - ps4
Asgard/Eukaryota - ps5
Eukaryota - ps6
Excavata/Diaphoretickes - ps7
Diaphoretickes - ps8
Archaeplastida - ps9
Viridiplantae - ps10
Streptophyta - ps11
Charophyceae/Embriophyta - ps12
Embriophyta - ps13
Tracheophyta - ps14
Spermatophyta - ps15
Angiosperms - ps16
Mesangiospermae - ps17
Magnoliids/eudicots - ps18
Eudicots - ps19
Core_eudicots - ps20
Superrosids - ps21
Rosids - ps22
Eurosids - ps23
Malvids - ps24
Sap./Malv./Brassicales - ps25
Malvales/Brassicales - ps26
Brassicales - ps27
Cleomaceae/Brassicaceae - ps28
Brassicaceae - ps29
Camelineae - ps30
Arabidopsis - ps31
Arabidopsis_thaliana - ps32
p-value
1e-17
1e-13
9e-10
8e-06
6e-02
Log-odds
0.5
1.0
1.5
2.0
2.5
3.0
3.5
4.0

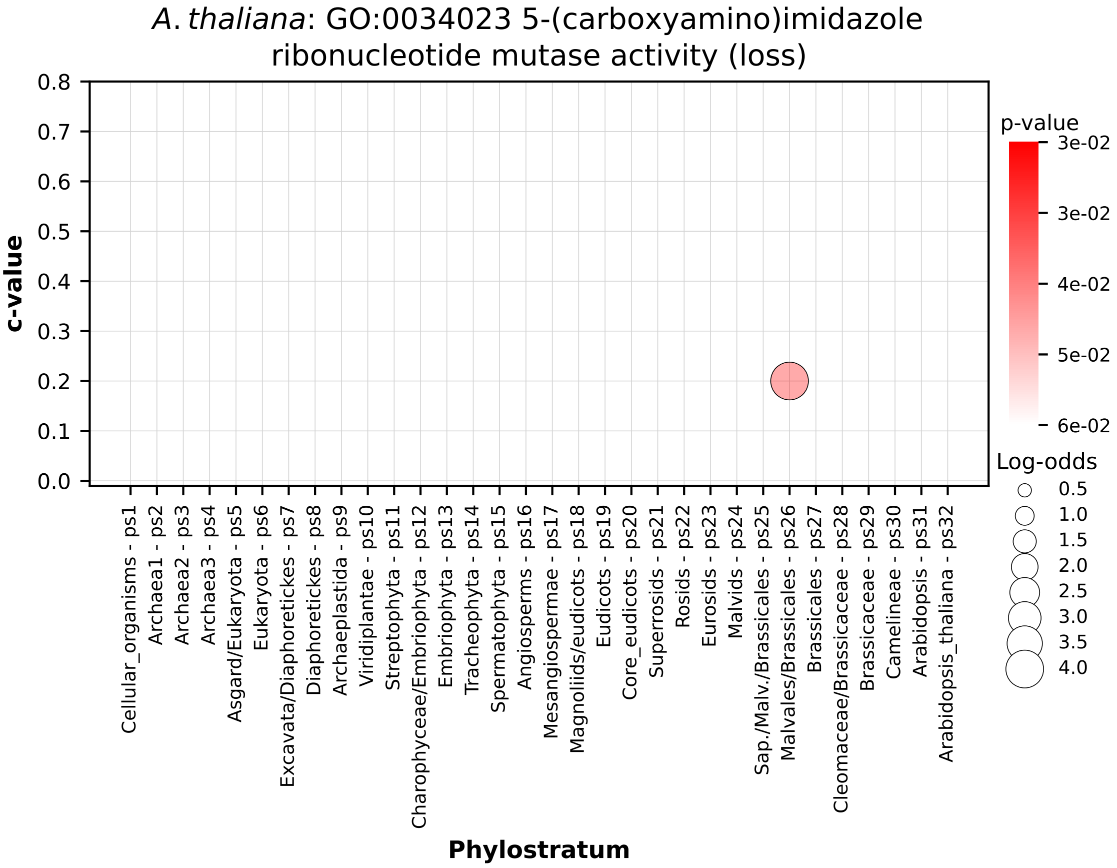

A. thaliana: GO:0034023 5-(carboxyamino)imidazole ribonucleotide mutase activity (loss)
c-value
0.8
0.7
0.6
0.5
0.4
0.3
0.2
0.1
0.0
p-value
3e-02
3e-02
4e-02
5e-02
6e-02
Log-odds
0.5
1.0
1.5
2.0
2.5
3.0
3.5
4.0
Cellular_organisms - ps1
Archaea1 - ps2
Archaea2 - ps3
Archaea3 - ps4
Asgard/Eukaryota - ps5
Eukaryota - ps6
Excavata/Diaphoretickes - ps7
Diaphoretickes - ps8
Archaeplastida - ps9
Viridiplantae - ps10
Streptophyta - ps11
Charophyceae/Embriophyta - ps12
Embriophyta - ps13
Tracheophyta - ps14
Spermatophyta - ps15
Angiosperms - ps16
Mesangiospermae - ps17
Magnoliids/eudicots - ps18
Eudicots - ps19
Core_eudicots - ps20
Superrosids - ps21
Rosids - ps22
Eurosids - ps23
Malvids - ps24
Sap./Malv./Brassicales - ps25
Malvales/Brassicales - ps26
Brassicales - ps27
Cleomaceae/Brassicaceae - ps28
Brassicaceae - ps29
Camelineae - ps30
Arabidopsis - ps31
Arabidopsis_thaliana - ps32
Phylostratum

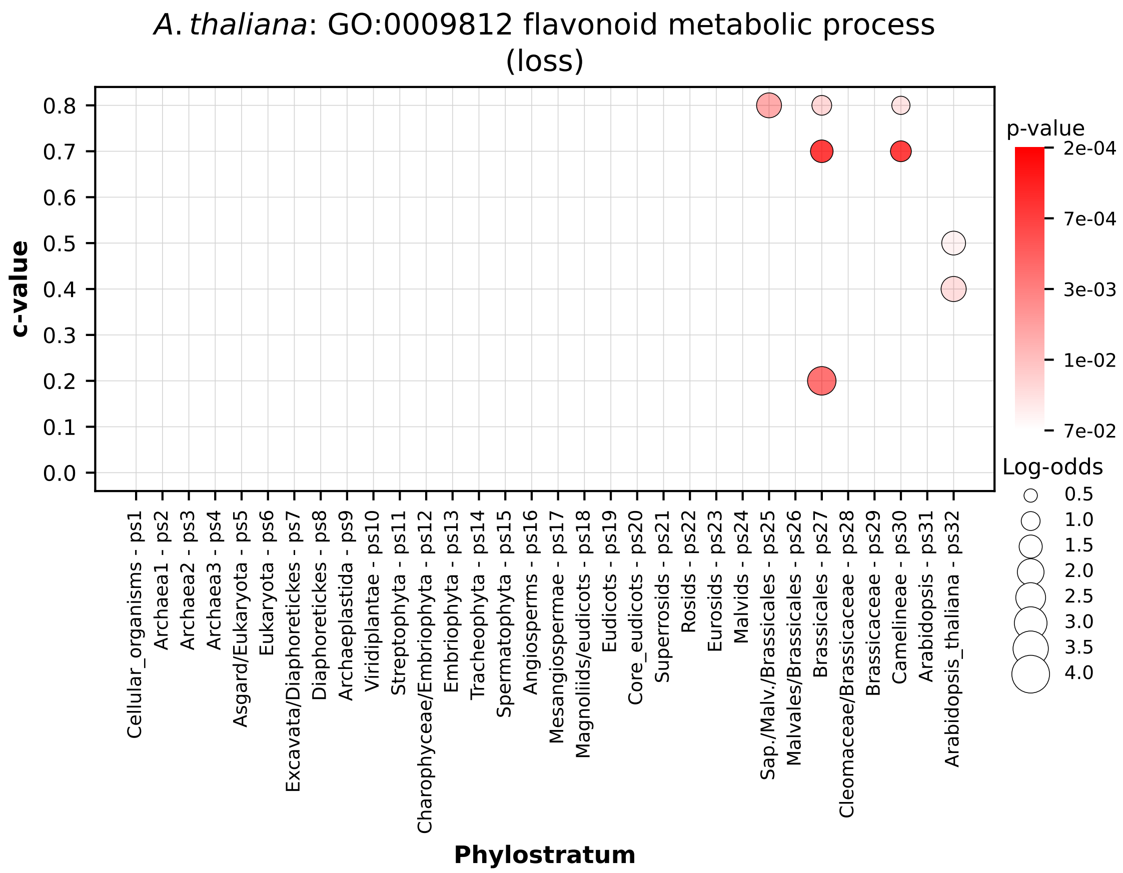

A. thaliana: GO:0009812 flavonoid metabolic process
(loss)
c-value
0.0
0.1
0.2
0.3
0.4
0.5
0.6
0.7
0.8
Phylostratum
Cellular_organisms - ps1
Archaea1 - ps2
Archaea2 - ps3
Archaea3 - ps4
Asgard/Eukaryota - ps5
Eukaryota - ps6
Excavata/Diaphoretickes - ps7
Diaphoretickes - ps8
Archaeplastida - ps9
Viridiplantae - ps10
Streptophyta - ps11
Charophyceae/Embriophyta - ps12
Embriophyta - ps13
Tracheophyta - ps14
Spermatophyta - ps15
Angiosperms - ps16
Mesangiospermae - ps17
Magnoliids/eudicots - ps18
Eudicots - ps19
Core_eudicots - ps20
Superrosids - ps21
Rosids - ps22
Eurosids - ps23
Malvids - ps24
Sap./Malv./Brassicales - ps25
Malvales/Brassicales - ps26
Brassicales - ps27
Cleomaceae/Brassicaceae - ps28
Brassicaceae - ps29
Camelineae - ps30
Arabidopsis - ps31
Arabidopsis_thaliana - ps32
p-value
2e-04
7e-04
3e-03
1e-02
7e-02
Log-odds
0.5
1.0
1.5
2.0
2.5
3.0
3.5
4.0

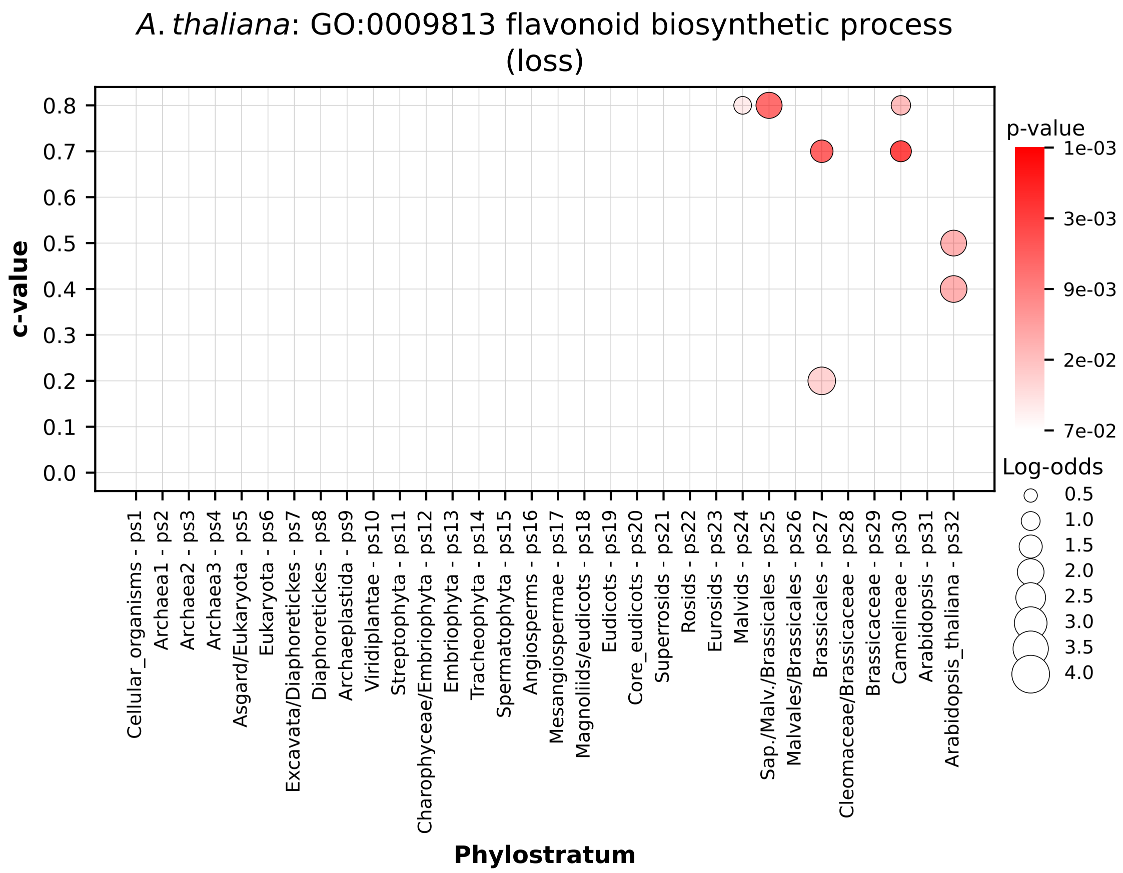

A. thaliana: GO:0009813 flavonoid biosynthetic process
(loss)
c-value
0.8
0.7
0.6
0.5
0.4
0.3
0.2
0.1
0.0
p-value
1e-03
3e-03
9e-03
2e-02
7e-02
Log-odds
0.5
1.0
1.5
2.0
2.5
3.0
3.5
4.0
Cellular_organisms - ps1
Archaea1 - ps2
Archaea2 - ps3
Archaea3 - ps4
Asgard/Eukaryota - ps5
Eukaryota - ps6
Excavata/Diaphoretickes - ps7
Diaphoretickes - ps8
Archaeplastida - ps9
Viridiplantae - ps10
Streptophyta - ps11
Charophyceae/Embriophyta - ps12
Embriophyta - ps13
Tracheophyta - ps14
Spermatophyta - ps15
Angiosperms - ps16
Mesangiospermae - ps17
Magnoliids/eudicots - ps18
Eudicots - ps19
Core_eudicots - ps20
Superrosids - ps21
Rosids - ps22
Eurosids - ps23
Malvids - ps24
Sap./Malv./Brassicales - ps25
Malvales/Brassicales - ps26
Brassicales - ps27
Cleomaceae/Brassicaceae - ps28
Brassicaceae - ps29
Camelineae - ps30
Arabidopsis - ps31
Arabidopsis_thaliana - ps32
Phylostratum

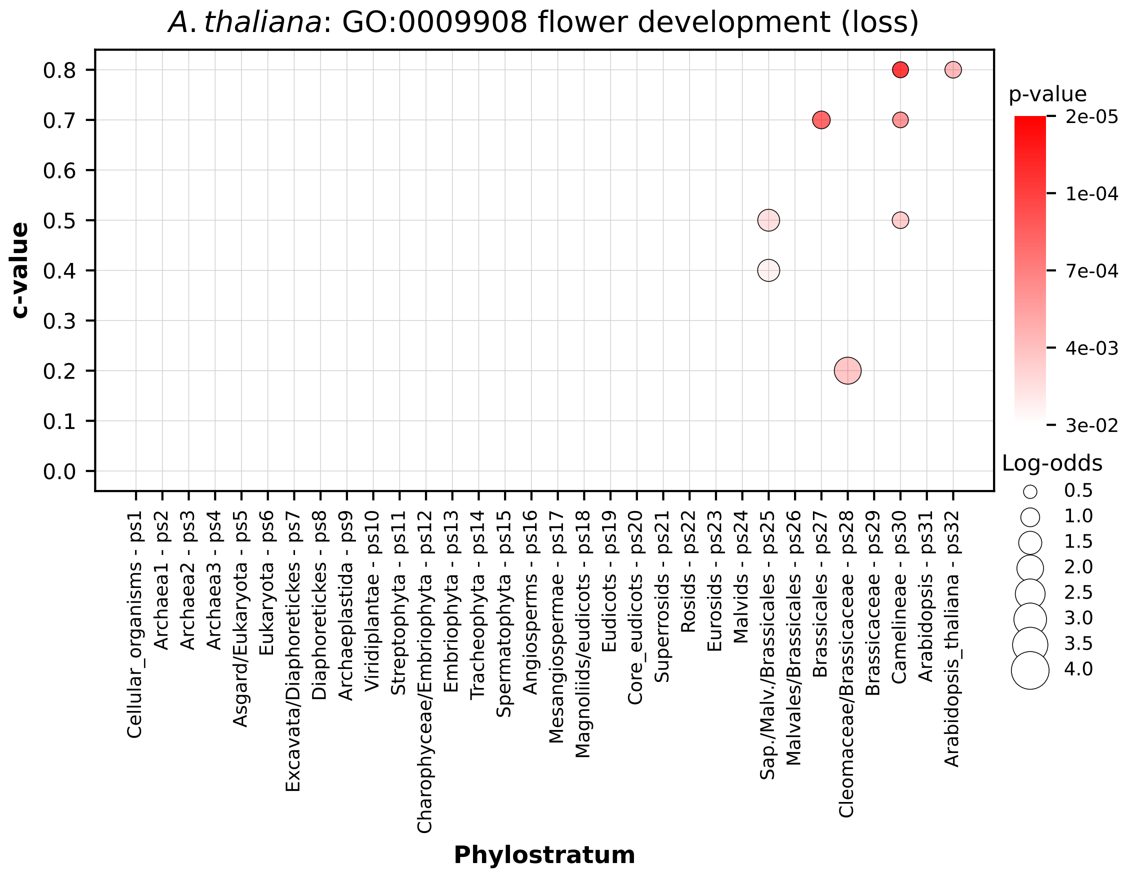

A. thaliana: GO:0009908 flower development (loss)
c-value
0.0
0.1
0.2
0.3
0.4
0.5
0.6
0.7
0.8
Phylostratum
Cellular_organisms - ps1
Archaea1 - ps2
Archaea2 - ps3
Archaea3 - ps4
Asgard/Eukaryota - ps5
Eukaryota - ps6
Excavata/Diaphoretickes - ps7
Diaphoretickes - ps8
Archaeplastida - ps9
Viridiplantae - ps10
Streptophyta - ps11
Charophyceae/Embriophyta - ps12
Embriophyta - ps13
Tracheophyta - ps14
Spermatophyta - ps15
Angiosperms - ps16
Mesangiospermae - ps17
Magnoliids/eudicots - ps18
Eudicots - ps19
Core_eudicots - ps20
Superrosids - ps21
Rosids - ps22
Eurosids - ps23
Malvids - ps24
Sap./Malv./Brassicales - ps25
Malvales/Brassicales - ps26
Brassicales - ps27
Cleomaceae/Brassicaceae - ps28
Brassicaceae - ps29
Camelineae - ps30
Arabidopsis - ps31
Arabidopsis_thaliana - ps32
p-value
2e-05
1e-04
7e-04
4e-03
3e-02
Log-odds
0.5
1.0
1.5
2.0
2.5
3.0
3.5
4.0

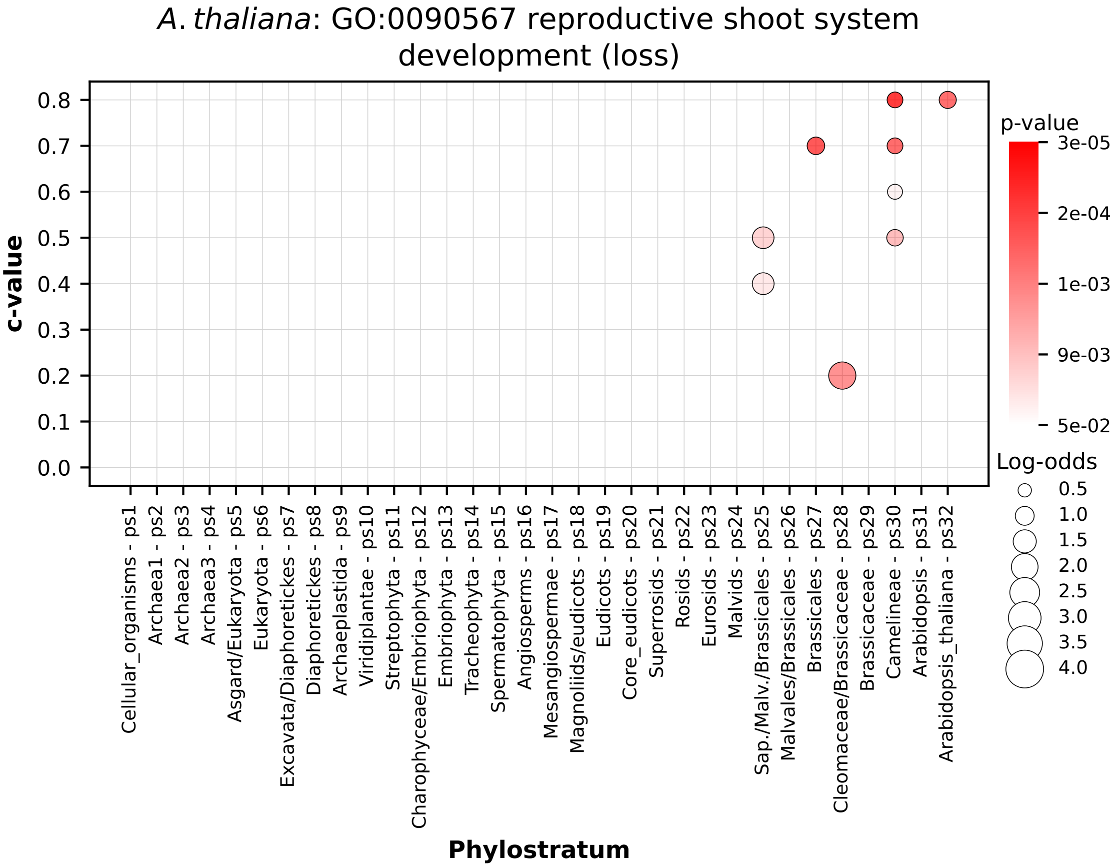

A. thaliana: GO:0090567 reproductive shoot system development (loss)
c-value
0.0
0.1
0.2
0.3
0.4
0.5
0.6
0.7
0.8
Phylostratum
Cellular_organisms - ps1
Archaea1 - ps2
Archaea2 - ps3
Archaea3 - ps4
Asgard/Eukaryota - ps5
Eukaryota - ps6
Excavata/Diaphoretickes - ps7
Diaphoretickes - ps8
Archaeplastida - ps9
Viridiplantae - ps10
Streptophyta - ps11
Charophyceae/Embriophyta - ps12
Embriophyta - ps13
Tracheophyta - ps14
Spermatophyta - ps15
Angiosperms - ps16
Mesangiospermae - ps17
Magnoliids/eudicots - ps18
Eudicots - ps19
Core_eudicots - ps20
Superrosids - ps21
Rosids - ps22
Eurosids - ps23
Malvids - ps24
Sap./Malv./Brassicales - ps25
Malvales/Brassicales - ps26
Brassicales - ps27
Cleomaceae/Brassicaceae - ps28
Brassicaceae - ps29
Camelineae - ps30
Arabidopsis - ps31
Arabidopsis_thaliana - ps32
p-value
3e-05
2e-04
1e-03
9e-03
5e-02
Log-odds
0.5
1.0
1.5
2.0
2.5
3.0
3.5
4.0

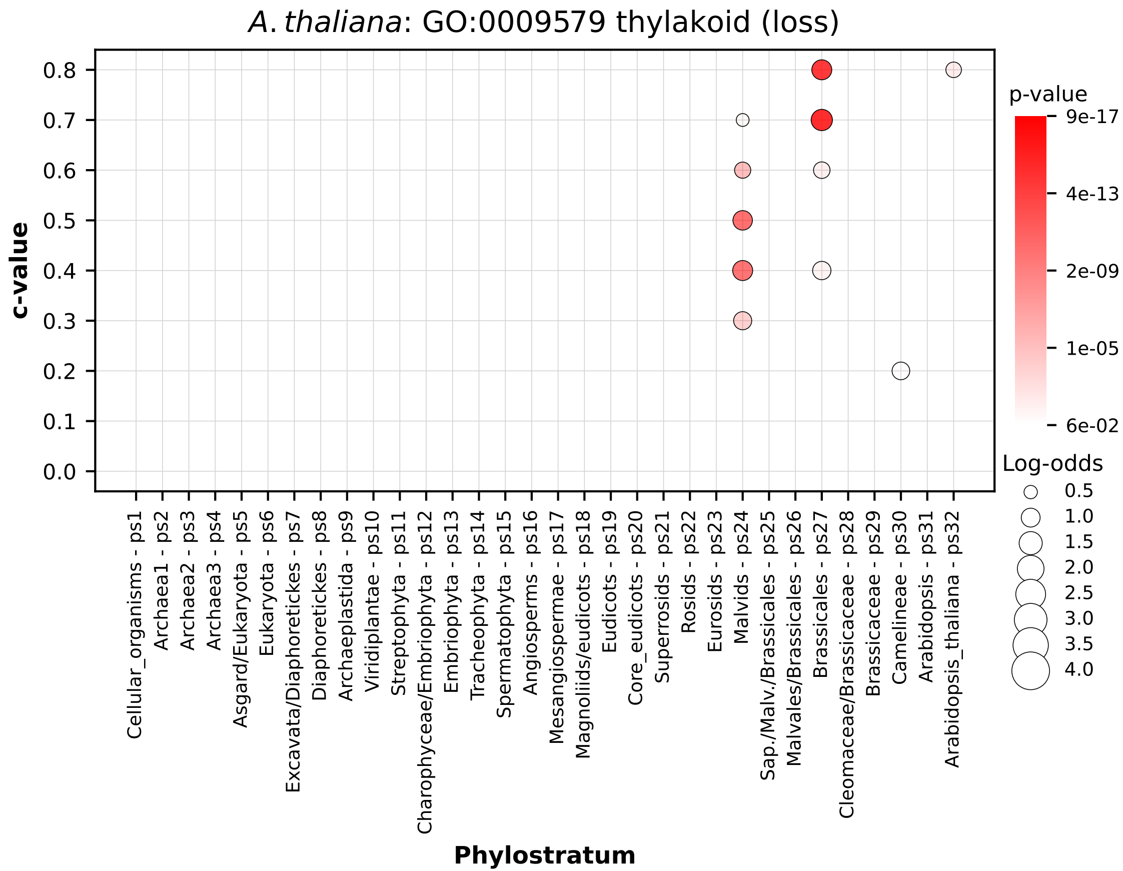

A. thaliana: GO:0009579 thylakoid (loss)
c-value
0.0
0.1
0.2
0.3
0.4
0.5
0.6
0.7
0.8
p-value
9e-17
4e-13
2e-09
1e-05
6e-02
Log-odds
0.5
1.0
1.5
2.0
2.5
3.0
3.5
4.0
Cellular_organisms - ps1
Archaea1 - ps2
Archaea2 - ps3
Archaea3 - ps4
Asgard/Eukaryota - ps5
Eukaryota - ps6
Excavata/Diaphoretickes - ps7
Diaphoretickes - ps8
Archaeplastida - ps9
Viridiplantae - ps10
Streptophyta - ps11
Charophyceae/Embriophyta - ps12
Embriophyta - ps13
Tracheophyta - ps14
Spermatophyta - ps15
Angiosperms - ps16
Mesangiospermae - ps17
Magnoliids/eudicots - ps18
Eudicots - ps19
Core_eudicots - ps20
Superrosids - ps21
Rosids - ps22
Eurosids - ps23
Malvids - ps24
Sap./Malv./Brassicales - ps25
Malvales/Brassicales - ps26
Brassicales - ps27
Cleomaceae/Brassicaceae - ps28
Brassicaceae - ps29
Camelineae - ps30
Arabidopsis - ps31
Arabidopsis_thaliana - ps32
Phylostratum

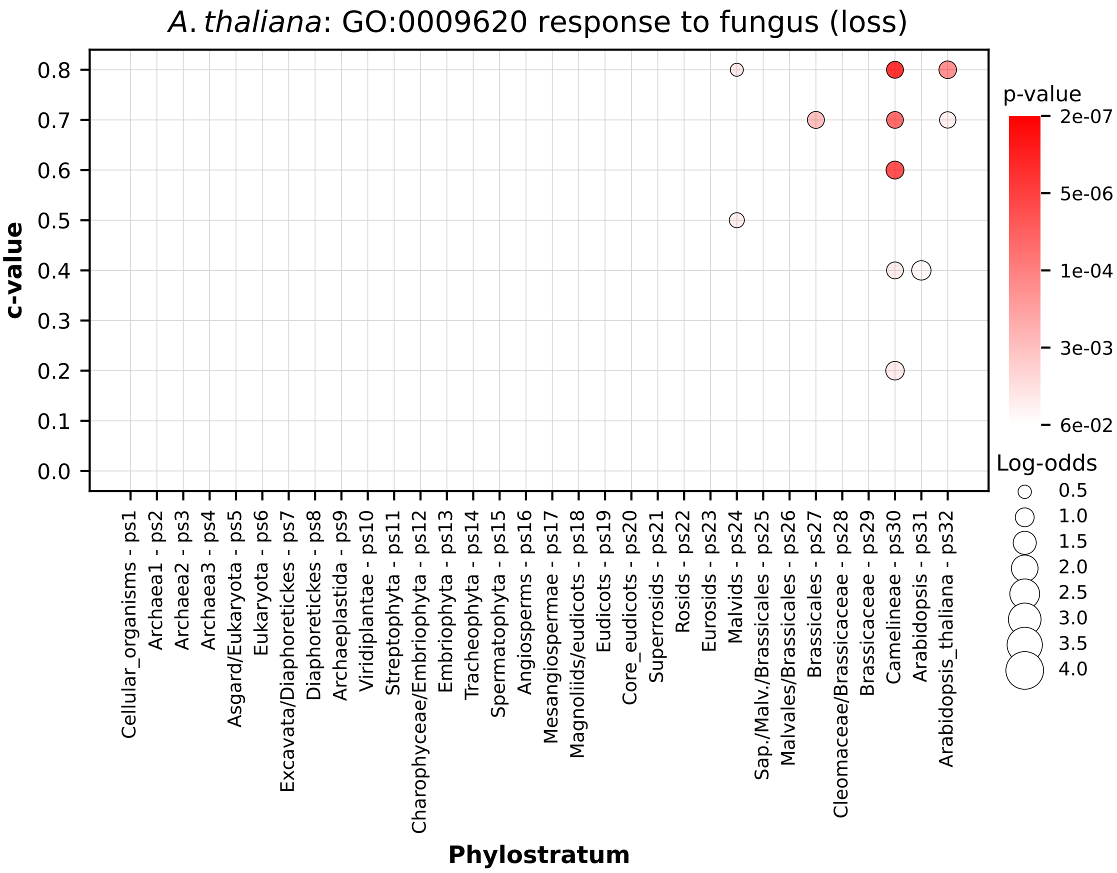

A. thaliana: GO:0009620 response to fungus (loss)
c-value
0.0
0.1
0.2
0.3
0.4
0.5
0.6
0.7
0.8
Phylostratum
Cellular_organisms - ps1
Archaea1 - ps2
Archaea2 - ps3
Archaea3 - ps4
Asgard/Eukaryota - ps5
Eukaryota - ps6
Excavata/Diaphoretickes - ps7
Diaphoretickes - ps8
Archaeplastida - ps9
Viridiplantae - ps10
Streptophyta - ps11
Charophyceae/Embriophyta - ps12
Embriophyta - ps13
Tracheophyta - ps14
Spermatophyta - ps15
Angiosperms - ps16
Mesangiospermae - ps17
Magnoliids/eudicots - ps18
Eudicots - ps19
Core_eudicots - ps20
Superrosids - ps21
Rosids - ps22
Eurosids - ps23
Malvids - ps24
Sap./Malv./Brassicales - ps25
Malvales/Brassicales - ps26
Brassicales - ps27
Cleomaceae/Brassicaceae - ps28
Brassicaceae - ps29
Camelineae - ps30
Arabidopsis - ps31
Arabidopsis_thaliana - ps32
p-value
2e-07
5e-06
1e-04
3e-03
6e-02
Log-odds
0.5
1.0
1.5
2.0
2.5
3.0
3.5
4.0

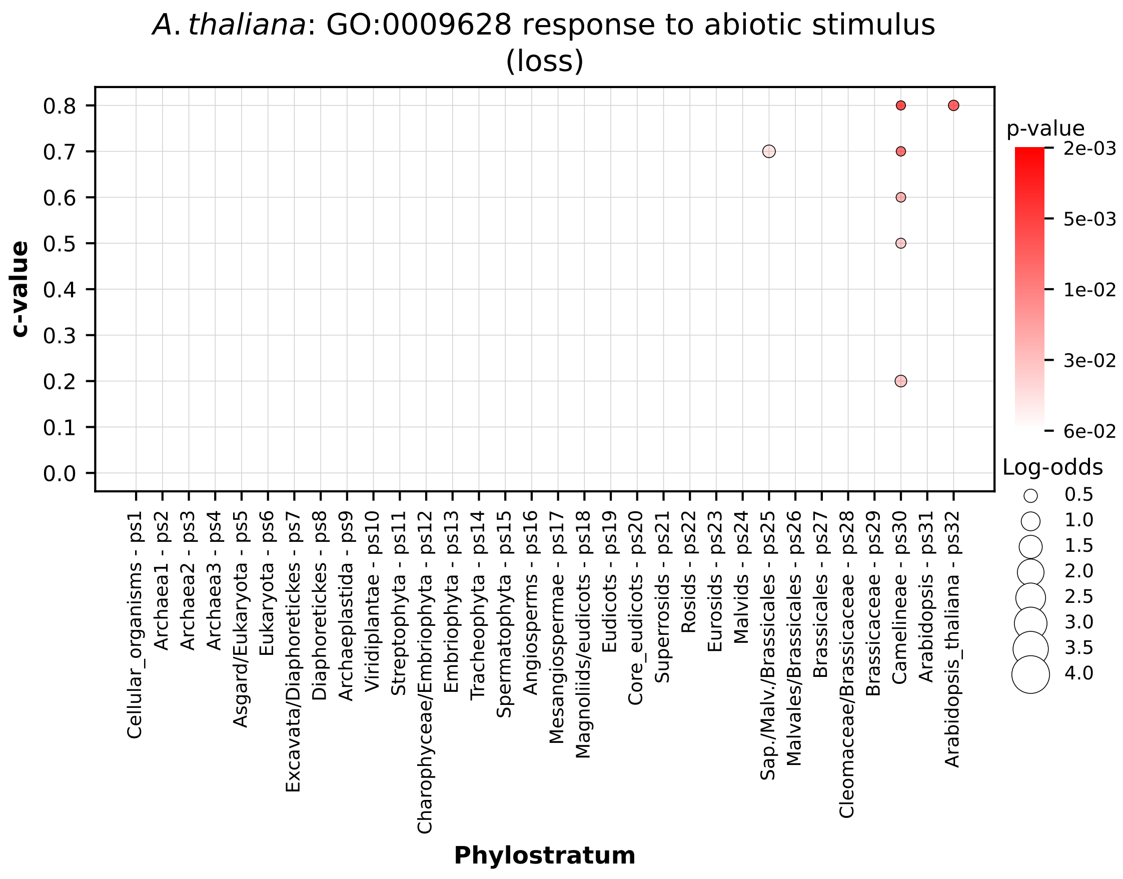
A. thaliana: GO:0009628 response to abiotic stimulus
(loss)
c-value
0.0
0.1
0.2
0.3
0.4
0.5
0.6
0.7
0.8
Phylostratum
Cellular_organisms - ps1
Archaea1 - ps2
Archaea2 - ps3
Archaea3 - ps4
Asgard/Eukaryota - ps5
Eukaryota - ps6
Excavata/Diaphoretickes - ps7
Diaphoretickes - ps8
Archaeplastida - ps9
Viridiplantae - ps10
Streptophyta - ps11
Charophyceae/Embriophyta - ps12
Embriophyta - ps13
Tracheophyta - ps14
Spermatophyta - ps15
Angiosperms - ps16
Mesangiospermae - ps17
Magnoliids/eudicots - ps18
Eudicots - ps19
Core_eudicots - ps20
Superrosids - ps21
Rosids - ps22
Eurosids - ps23
Malvids - ps24
Sap./Malv./Brassicales - ps25
Malvales/Brassicales - ps26
Brassicales - ps27
Cleomaceae/Brassicaceae - ps28
Brassicaceae - ps29
Camelineae - ps30
Arabidopsis - ps31
Arabidopsis_thaliana - ps32
p-value
2e-03
5e-03
1e-02
3e-02
6e-02
Log-odds
0.5
1.0
1.5
2.0
2.5
3.0
3.5
4.0

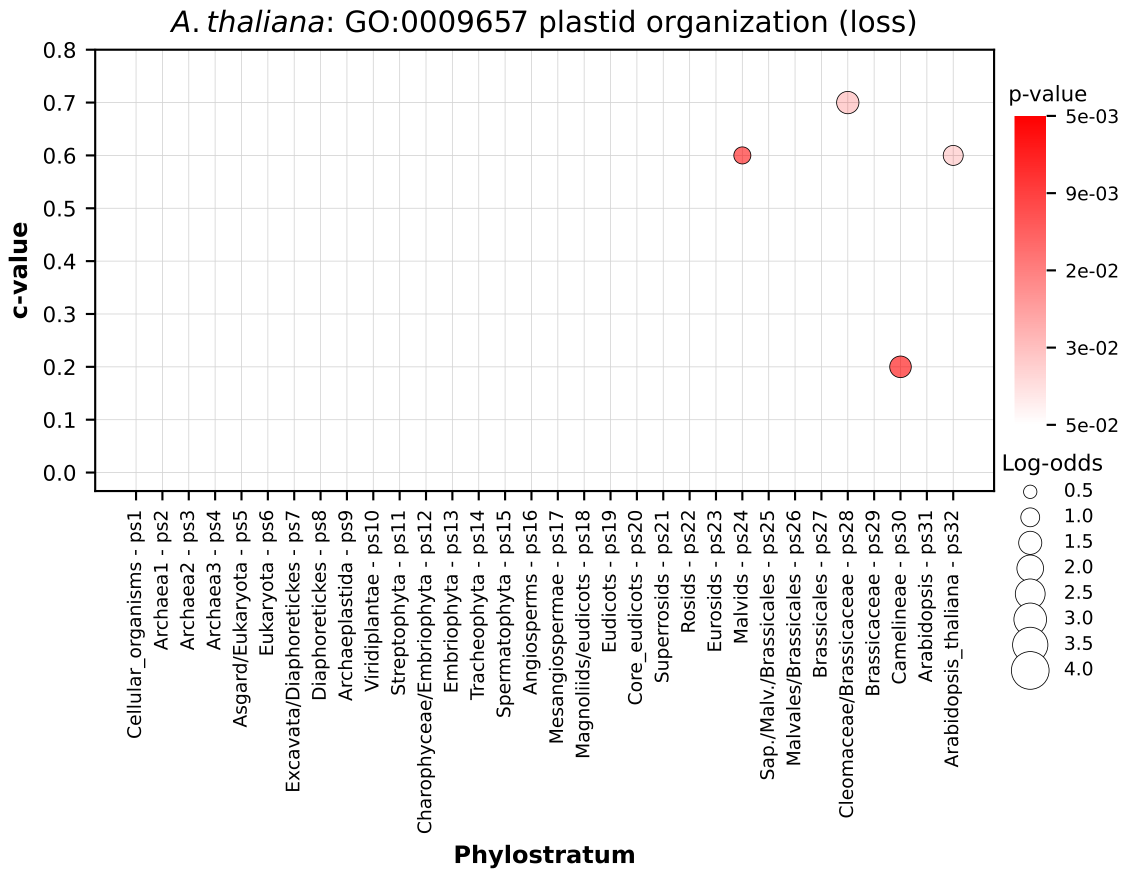

A. thaliana: GO:0009657 plastid organization (loss)
c-value
0.8
0.7
0.6
0.5
0.4
0.3
0.2
0.1
0.0
Phylostratum
Cellular_organisms - ps1
Archaea1 - ps2
Archaea2 - ps3
Archaea3 - ps4
Asgard/Eukaryota - ps5
Eukaryota - ps6
Excavata/Diaphoretickes - ps7
Diaphoretickes - ps8
Archaeplastida - ps9
Viridiplantae - ps10
Streptophyta - ps11
Charophyceae/Embriophyta - ps12
Embriophyta - ps13
Tracheophyta - ps14
Spermatophyta - ps15
Angiosperms - ps16
Mesangiospermae - ps17
Magnoliids/eudicots - ps18
Eudicots - ps19
Core_eudicots - ps20
Superrosids - ps21
Rosids - ps22
Eurosids - ps23
Malvids - ps24
Sap./Malv./Brassicales - ps25
Malvales/Brassicales - ps26
Brassicales - ps27
Cleomaceae/Brassicaceae - ps28
Brassicaceae - ps29
Camelineae - ps30
Arabidopsis - ps31
Arabidopsis_thaliana - ps32
p-value
5e-03
9e-03
2e-02
3e-02
5e-02
Log-odds
0.5
1.0
1.5
2.0
2.5
3.0
3.5
4.0

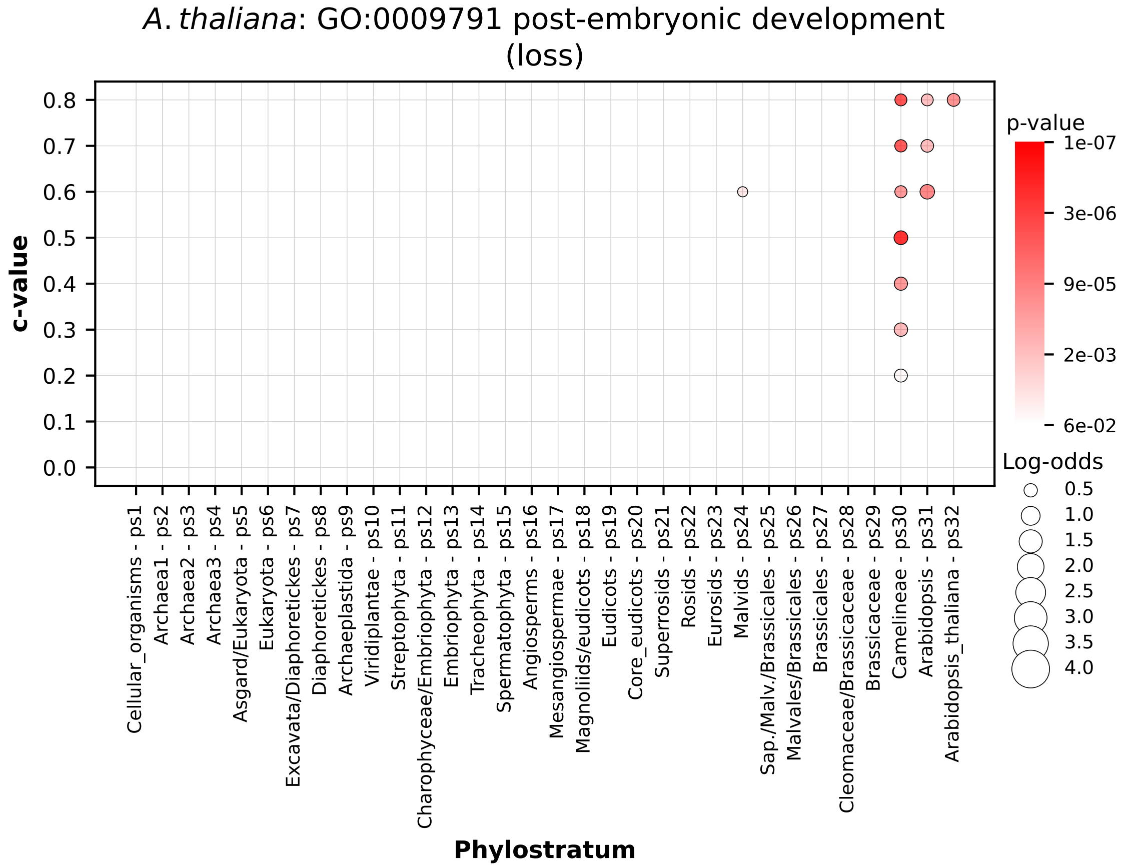

$A. thaliana$: GO:0009791 post-embryonic development (loss)
c-value
0.0
0.1
0.2
0.3
0.4
0.5
0.6
0.7
0.8
Phylostratum
Cellular_organisms - ps1
Archaea1 - ps2
Archaea2 - ps3
Archaea3 - ps4
Asgard/Eukaryota - ps5
Eukaryota - ps6
Excavata/Diaphoretickes - ps7
Diaphoretickes - ps8
Archaeplastida - ps9
Viridiplantae - ps10
Streptophyta - ps11
Charophyceae/Embriophyta - ps12
Embriophyta - ps13
Tracheophyta - ps14
Spermatophyta - ps15
Angiosperms - ps16
Mesangiospermae - ps17
Magnoliids/eudicots - ps18
Eudicots - ps19
Core_eudicots - ps20
Superrosids - ps21
Rosids - ps22
Eurosids - ps23
Malvids - ps24
Sap./Malv./Brassicales - ps25
Malvales/Brassicales - ps26
Brassicales - ps27
Cleomaceae/Brassicaceae - ps28
Brassicaceae - ps29
Camelineae - ps30
Arabidopsis - ps31
Arabidopsis_thaliana - ps32
p-value
1e-07
3e-06
9e-05
2e-03
6e-02
Log-odds
0.5
1.0
1.5
2.0
2.5
3.0
3.5
4.0

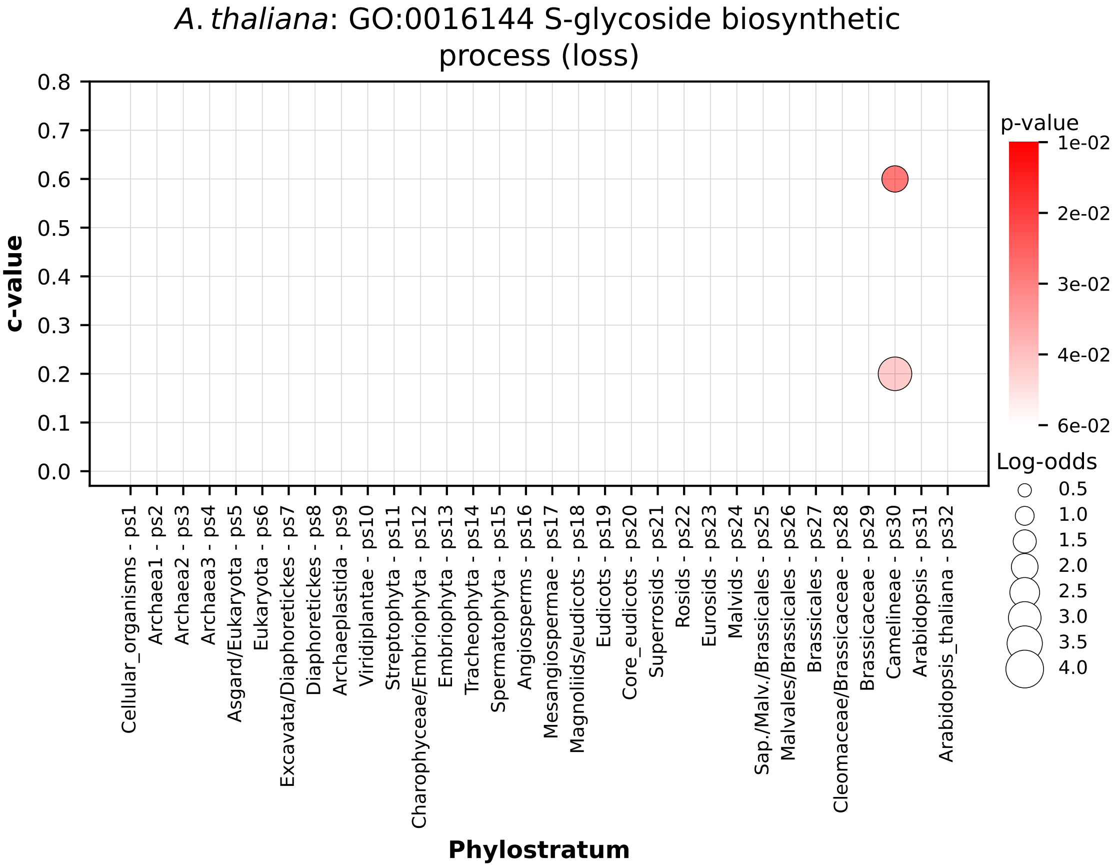

A. thaliana: GO:0016144 S-glycoside biosynthetic process (loss)
c-value
0.8
0.7
0.6
0.5
0.4
0.3
0.2
0.1
0.0
p-value
1e-02
2e-02
3e-02
4e-02
6e-02
Log-odds
0.5
1.0
1.5
2.0
2.5
3.0
3.5
4.0
Cellular_organisms - ps1
Archaea1 - ps2
Archaea2 - ps3
Archaea3 - ps4
Asgard/Eukaryota - ps5
Eukaryota - ps6
Excavata/Diaphoretickes - ps7
Diaphoretickes - ps8
Archaeplastida - ps9
Viridiplantae - ps10
Streptophyta - ps11
Charophyceae/Embriophyta - ps12
Embriophyta - ps13
Tracheophyta - ps14
Spermatophyta - ps15
Angiosperms - ps16
Mesangiospermae - ps17
Magnoliids/eudicots - ps18
Eudicots - ps19
Core_eudicots - ps20
Superrosids - ps21
Rosids - ps22
Eurosids - ps23
Malvids - ps24
Sap./Malv./Brassicales - ps25
Malvales/Brassicales - ps26
Brassicales - ps27
Cleomaceae/Brassicaceae - ps28
Brassicaceae - ps29
Camelineae - ps30
Arabidopsis - ps31
Arabidopsis_thaliana - ps32
Phylostratum

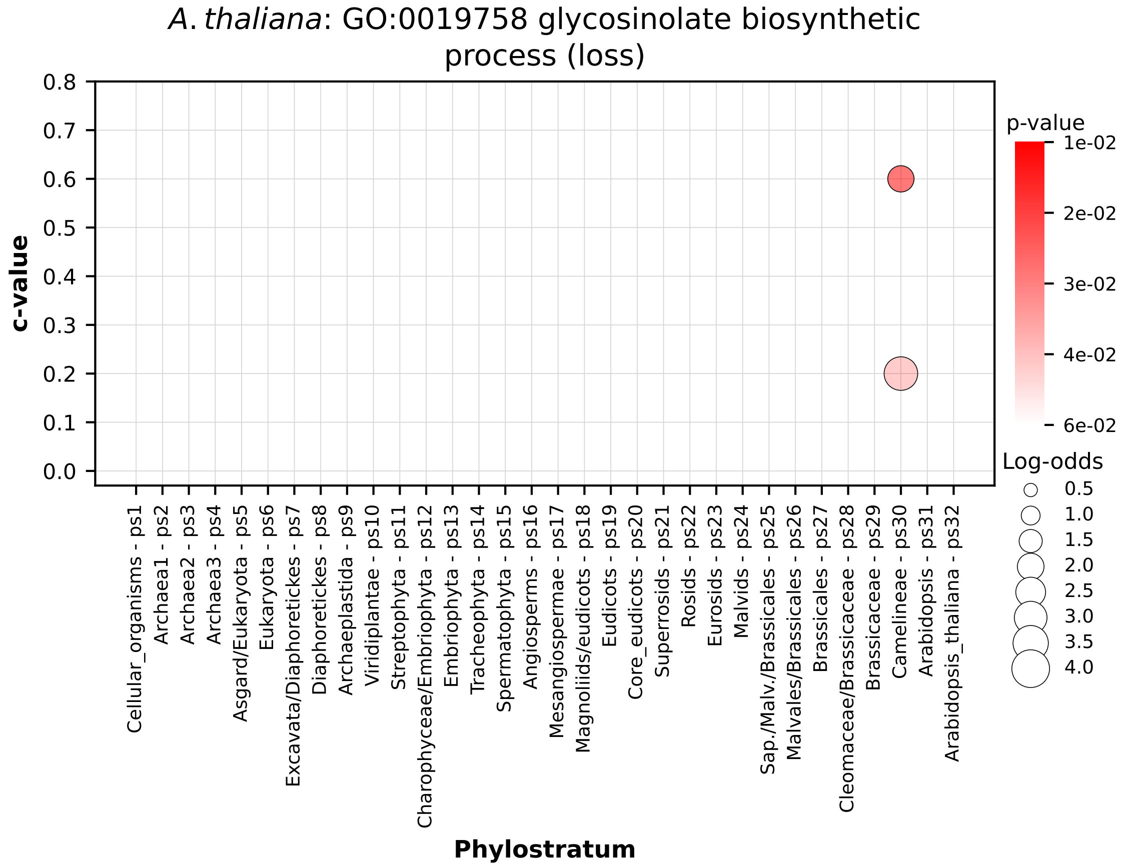

A. thaliana: GO:0019758 glycosinolate biosynthetic process (loss)
c-value
0.8
0.7
0.6
0.5
0.4
0.3
0.2
0.1
0.0
p-value
1e-02
2e-02
3e-02
4e-02
6e-02
Log-odds
0.5
1.0
1.5
2.0
2.5
3.0
3.5
4.0
Cellular_organisms - ps1
Archaea1 - ps2
Archaea2 - ps3
Archaea3 - ps4
Asgard/Eukaryota - ps5
Eukaryota - ps6
Excavata/Diaphoretickes - ps7
Diaphoretickes - ps8
Archaeplastida - ps9
Viridiplantae - ps10
Streptophyta - ps11
Charophyceae/Embriophyta - ps12
Embriophyta - ps13
Tracheophyta - ps14
Spermatophyta - ps15
Angiosperms - ps16
Mesangiospermae - ps17
Magnoliids/eudicots - ps18
Eudicots - ps19
Core_eudicots - ps20
Superrosids - ps21
Rosids - ps22
Eurosids - ps23
Malvids - ps24
Sap./Malv./Brassicales - ps25
Malvales/Brassicales - ps26
Brassicales - ps27
Cleomaceae/Brassicaceae - ps28
Brassicaceae - ps29
Camelineae - ps30
Arabidopsis - ps31
Arabidopsis_thaliana - ps32
Phylostratum

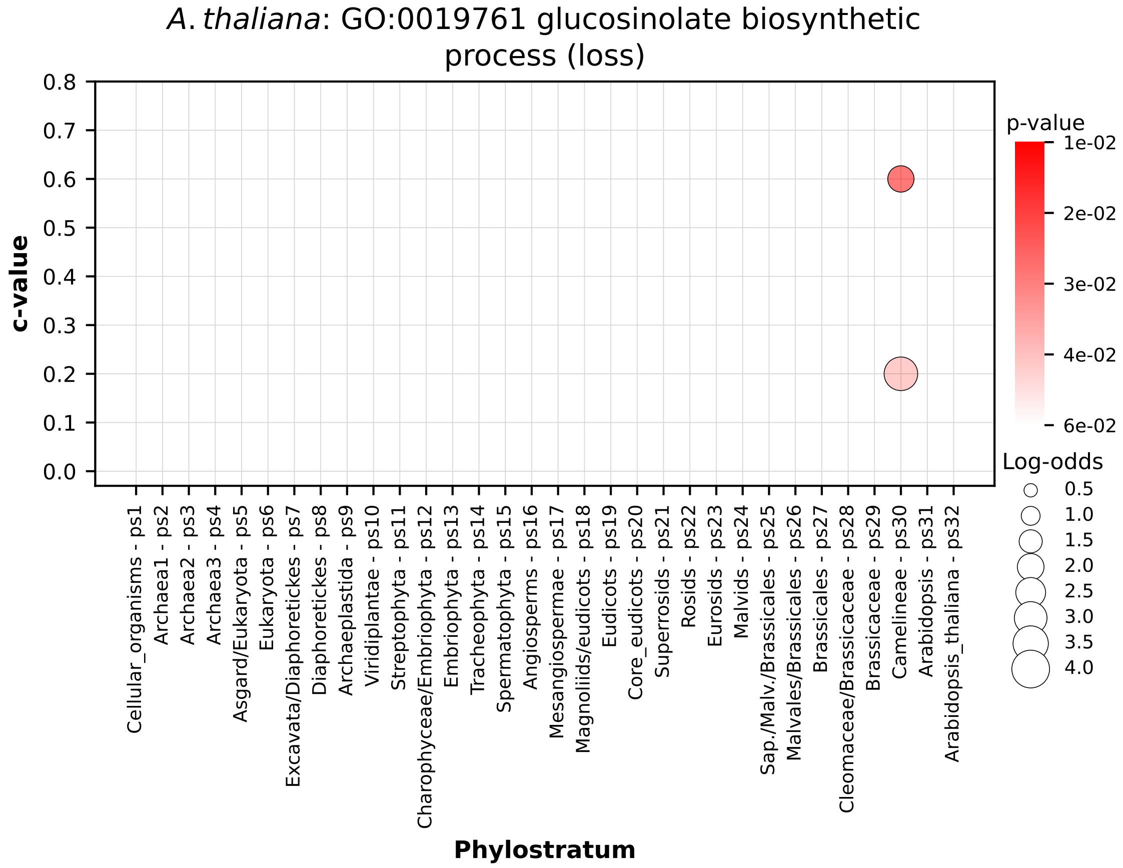

A. thaliana: GO:0019761 glucosinolate biosynthetic process (loss)
c-value
0.8
0.7
0.6
0.5
0.4
0.3
0.2
0.1
0.0
Phylostratum
Cellular_organisms - ps1
Archaea1 - ps2
Archaea2 - ps3
Archaea3 - ps4
Asgard/Eukaryota - ps5
Eukaryota - ps6
Excavata/Diaphoretickes - ps7
Diaphoretickes - ps8
Archaeplastida - ps9
Viridiplantae - ps10
Streptophyta - ps11
Charophyceae/Embriophyta - ps12
Embriophyta - ps13
Tracheophyta - ps14
Spermatophyta - ps15
Angiosperms - ps16
Mesangiospermae - ps17
Magnoliids/eudicots - ps18
Eudicots - ps19
Core_eudicots - ps20
Superrosids - ps21
Rosids - ps22
Eurosids - ps23
Malvids - ps24
Sap./Malv./Brassicales - ps25
Malvales/Brassicales - ps26
Brassicales - ps27
Cleomaceae/Brassicaceae - ps28
Brassicaceae - ps29
Camelineae - ps30
Arabidopsis - ps31
Arabidopsis_thaliana - ps32
p-value
1e-02
2e-02
3e-02
4e-02
6e-02
Log-odds
0.5
1.0
1.5
2.0
2.5
3.0
3.5
4.0

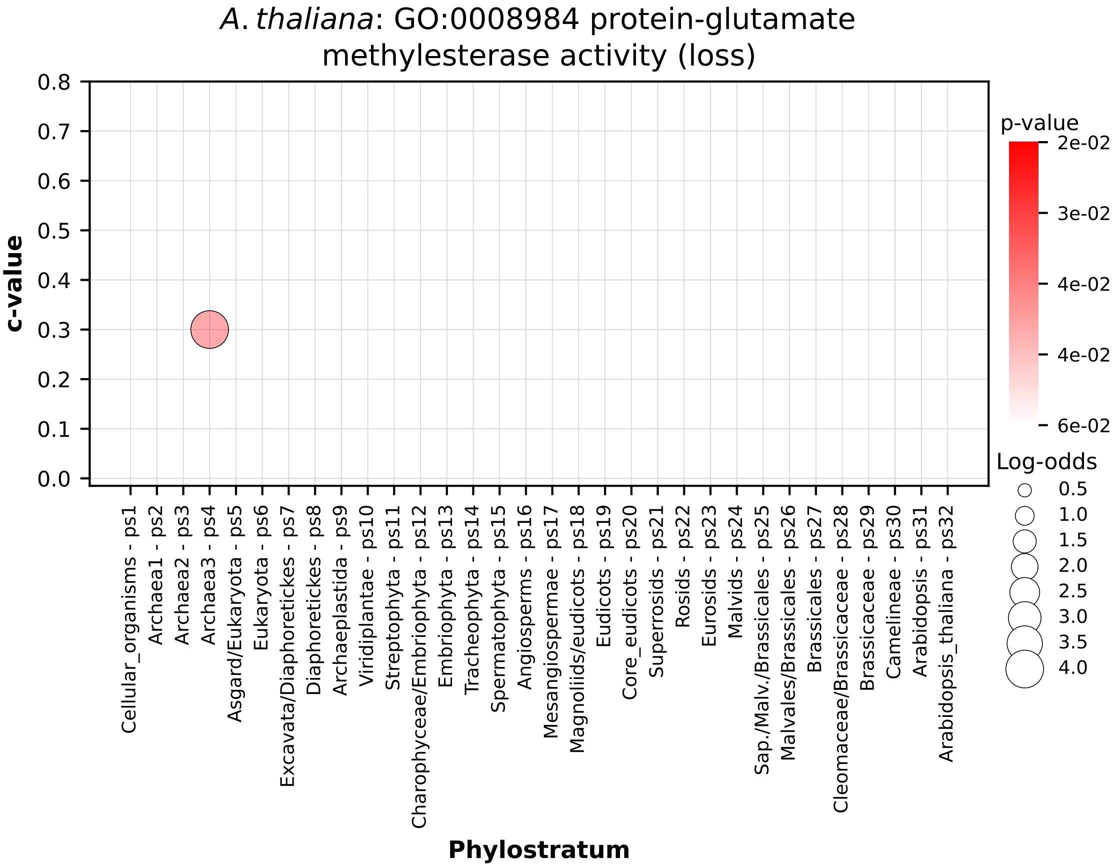

A. thaliana: GO:0008984 protein-glutamate methylesterase activity (loss)
c-value
0.0
0.1
0.2
0.3
0.4
0.5
0.6
0.7
0.8
Phylostratum
Cellular_organisms - ps1
Archaea1 - ps2
Archaea2 - ps3
Archaea3 - ps4
Asgard/Eukaryota - ps5
Eukaryota - ps6
Excavata/Diaphoretickes - ps7
Diaphoretickes - ps8
Archaeplastida - ps9
Viridiplantae - ps10
Streptophyta - ps11
Charophyceae/Embriophyta - ps12
Embriophyta - ps13
Tracheophyta - ps14
Spermatophyta - ps15
Angiosperms - ps16
Mesangiospermae - ps17
Magnoliids/eudicots - ps18
Eudicots - ps19
Core_eudicots - ps20
Superrosids - ps21
Rosids - ps22
Eurosids - ps23
Malvids - ps24
Sap./Malv./Brassicales - ps25
Malvales/Brassicales - ps26
Brassicales - ps27
Cleomaceae/Brassicaceae - ps28
Brassicaceae - ps29
Camelineae - ps30
Arabidopsis - ps31
Arabidopsis_thaliana - ps32
p-value
2e-02
3e-02
4e-02
4e-02
6e-02
Log-odds
0.5
1.0
1.5
2.0
2.5
3.0
3.5
4.0

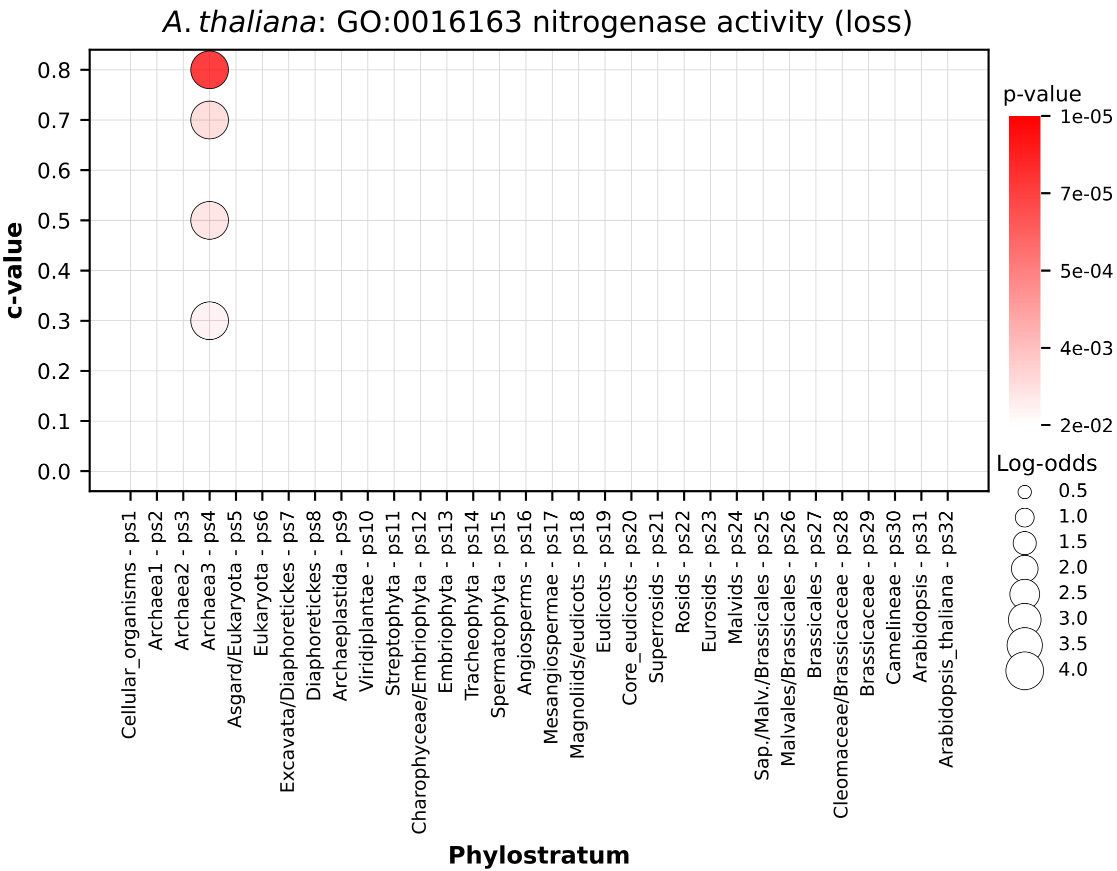

A. thaliana: GO:0016163 nitrogenase activity (loss)
c-value
0.8
0.7
0.6
0.5
0.4
0.3
0.2
0.1
0.0
p-value
1e-05
7e-05
5e-04
4e-03
2e-02
Log-odds
0.5
1.0
1.5
2.0
2.5
3.0
3.5
4.0
Cellular_organisms - ps1
Archaea1 - ps2
Archaea2 - ps3
Archaea3 - ps4
Asgard/Eukaryota - ps5
Eukaryota - ps6
Excavata/Diaphoretickes - ps7
Diaphoretickes - ps8
Archaeplastida - ps9
Viridiplantae - ps10
Streptophyta - ps11
Charophyceae/Embriophyta - ps12
Embriophyta - ps13
Tracheophyta - ps14
Spermatophyta - ps15
Angiosperms - ps16
Mesangiospermae - ps17
Magnoliids/eudicots - ps18
Eudicots - ps19
Core_eudicots - ps20
Superrosids - ps21
Rosids - ps22
Eurosids - ps23
Malvids - ps24
Sap./Malv./Brassicales - ps25
Malvales/Brassicales - ps26
Brassicales - ps27
Cleomaceae/Brassicaceae - ps28
Brassicaceae - ps29
Camelineae - ps30
Arabidopsis - ps31
Arabidopsis_thaliana - ps32
Phylostratum

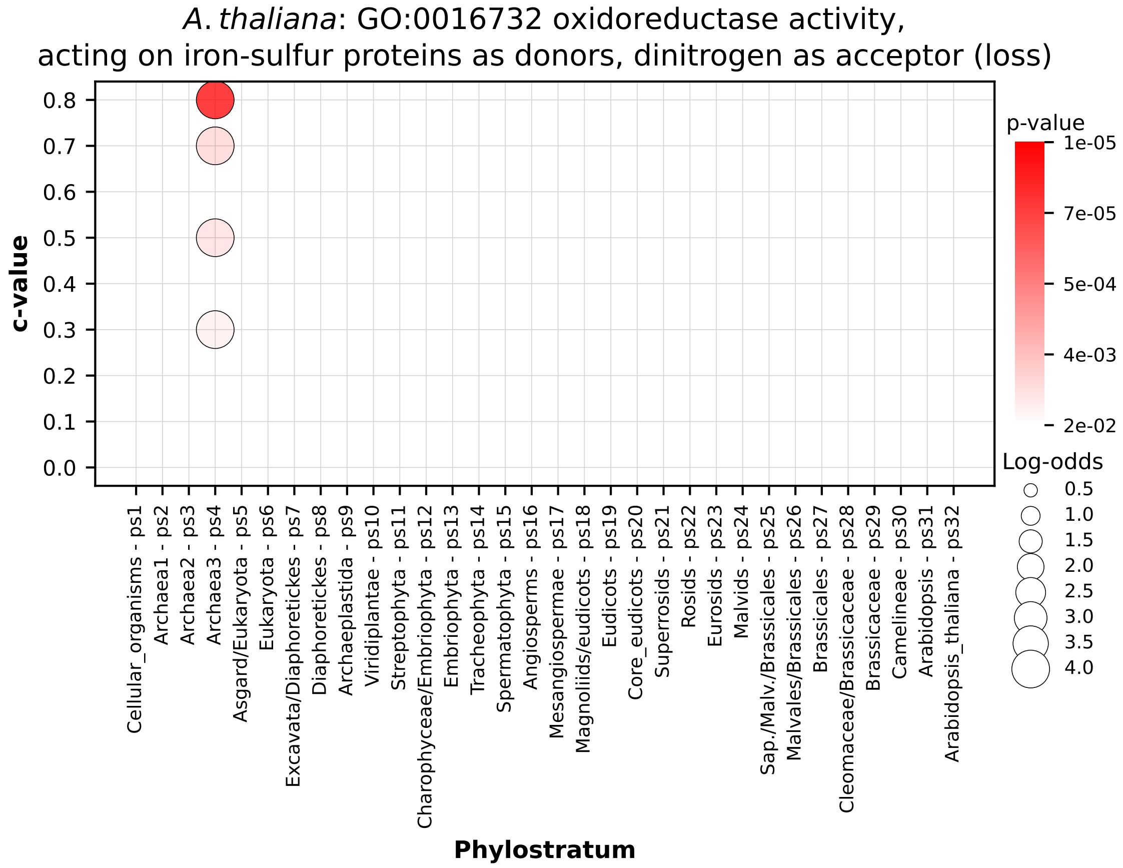

A. thaliana: GO:0016732 oxidoreductase activity,
acting on iron-sulfur proteins as donors, dinitrogen as acceptor (loss)
c-value
0.8
0.7
0.6
0.5
0.4
0.3
0.2
0.1
0.0
p-value
1e-05
7e-05
5e-04
4e-03
2e-02
Log-odds
0.5
1.0
1.5
2.0
2.5
3.0
3.5
4.0
Cellular_organisms - ps1
Archaea1 - ps2
Archaea2 - ps3
Archaea3 - ps4
Asgard/Eukaryota - ps5
Eukaryota - ps6
Excavata/Diaphoretickes - ps7
Diaphoretickes - ps8
Archaeplastida - ps9
Viridiplantae - ps10
Streptophyta - ps11
Charophyceae/Embriophyta - ps12
Embriophyta - ps13
Tracheophyta - ps14
Spermatophyta - ps15
Angiosperms - ps16
Mesangiospermae - ps17
Magnoliids/eudicots - ps18
Eudicots - ps19
Core_eudicots - ps20
Superrosids - ps21
Rosids - ps22
Eurosids - ps23
Malvids - ps24
Sap./Malv./Brassicales - ps25
Malvales/Brassicales - ps26
Brassicales - ps27
Cleomaceae/Brassicaceae - ps28
Brassicaceae - ps29
Camelineae - ps30
Arabidopsis - ps31
Arabidopsis_thaliana - ps32
Phylostratum

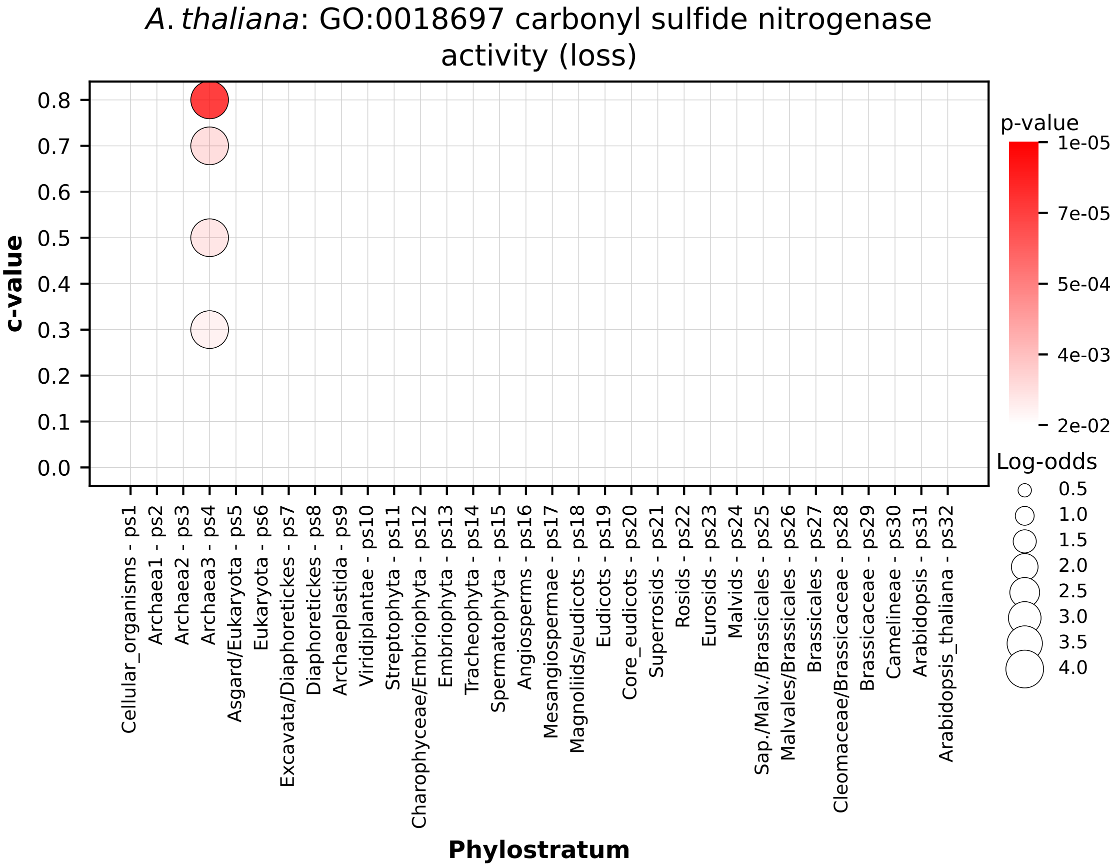

A. thaliana: GO:0018697 carbonyl sulfide nitrogenase activity (loss)
c-value
0.8
0.7
0.6
0.5
0.4
0.3
0.2
0.1
0.0
p-value
1e-05
7e-05
5e-04
4e-03
2e-02
Log-odds
0.5
1.0
1.5
2.0
2.5
3.0
3.5
4.0
Cellular_organisms - ps1
Archaea1 - ps2
Archaea2 - ps3
Archaea3 - ps4
Asgard/Eukaryota - ps5
Eukaryota - ps6
Excavata/Diaphoretickes - ps7
Diaphoretickes - ps8
Archaeplastida - ps9
Viridiplantae - ps10
Streptophyta - ps11
Charophyceae/Embriophyta - ps12
Embriophyta - ps13
Tracheophyta - ps14
Spermatophyta - ps15
Angiosperms - ps16
Mesangiospermae - ps17
Magnoliids/eudicots - ps18
Eudicots - ps19
Core_eudicots - ps20
Superrosids - ps21
Rosids - ps22
Eurosids - ps23
Malvids - ps24
Sap./Malv./Brassicales - ps25
Malvales/Brassicales - ps26
Brassicales - ps27
Cleomaceae/Brassicaceae - ps28
Brassicaceae - ps29
Camelineae - ps30
Arabidopsis - ps31
Arabidopsis_thaliana - ps32
Phylostratum

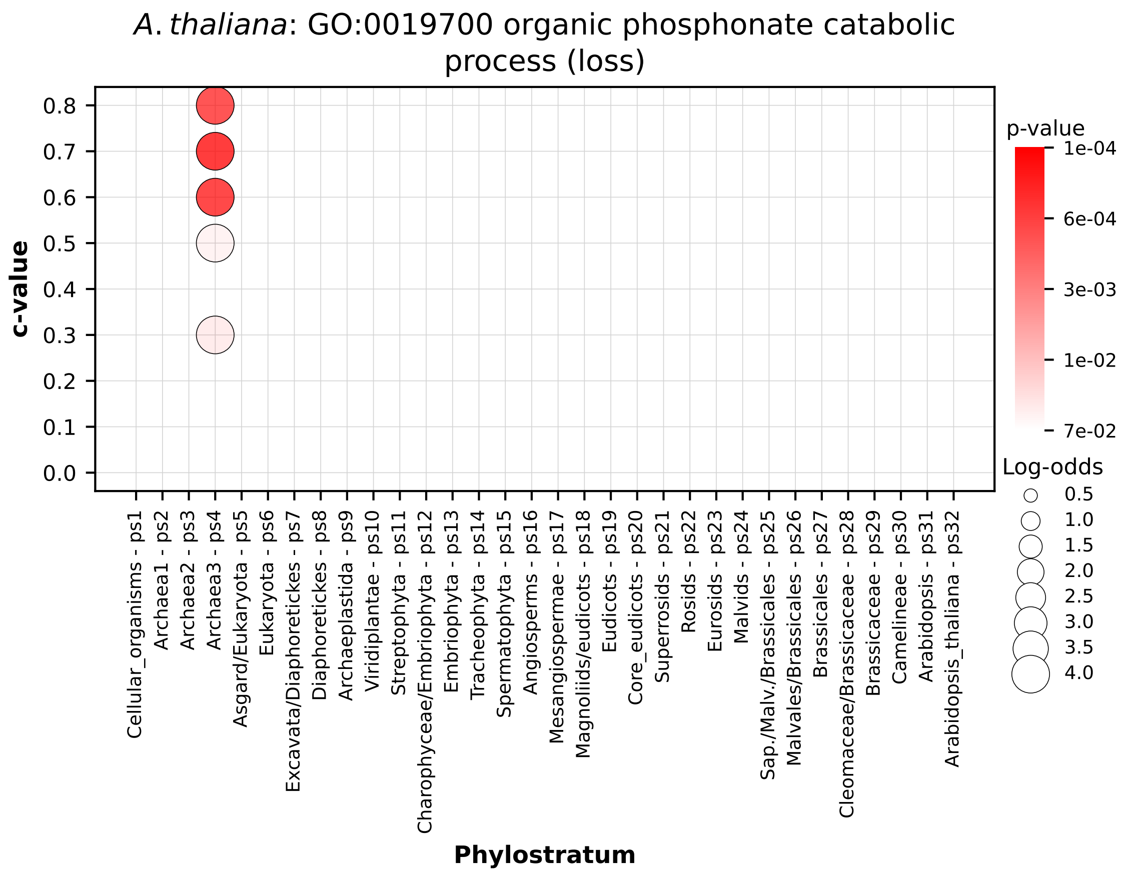

A. thaliana: GO:0019700 organic phosphonate catabolic process (loss)
c-value
0.0
0.1
0.2
0.3
0.4
0.5
0.6
0.7
0.8
p-value
1e-04
6e-04
3e-03
1e-02
7e-02
Log-odds
0.5
1.0
1.5
2.0
2.5
3.0
3.5
4.0
Cellular_organisms - ps1
Archaea1 - ps2
Archaea2 - ps3
Archaea3 - ps4
Asgard/Eukaryota - ps5
Eukaryota - ps6
Excavata/Diaphoretickes - ps7
Diaphoretickes - ps8
Archaeplastida - ps9
Viridiplantae - ps10
Streptophyta - ps11
Charophyceae/Embriophyta - ps12
Embriophyta - ps13
Tracheophyta - ps14
Spermatophyta - ps15
Angiosperms - ps16
Mesangiospermae - ps17
Magnoliids/eudicots - ps18
Eudicots - ps19
Core_eudicots - ps20
Superrosids - ps21
Rosids - ps22
Eurosids - ps23
Malvids - ps24
Sap./Malv./Brassicales - ps25
Malvales/Brassicales - ps26
Brassicales - ps27
Cleomaceae/Brassicaceae - ps28
Brassicaceae - ps29
Camelineae - ps30
Arabidopsis - ps31
Arabidopsis_thaliana - ps32
Phylostratum

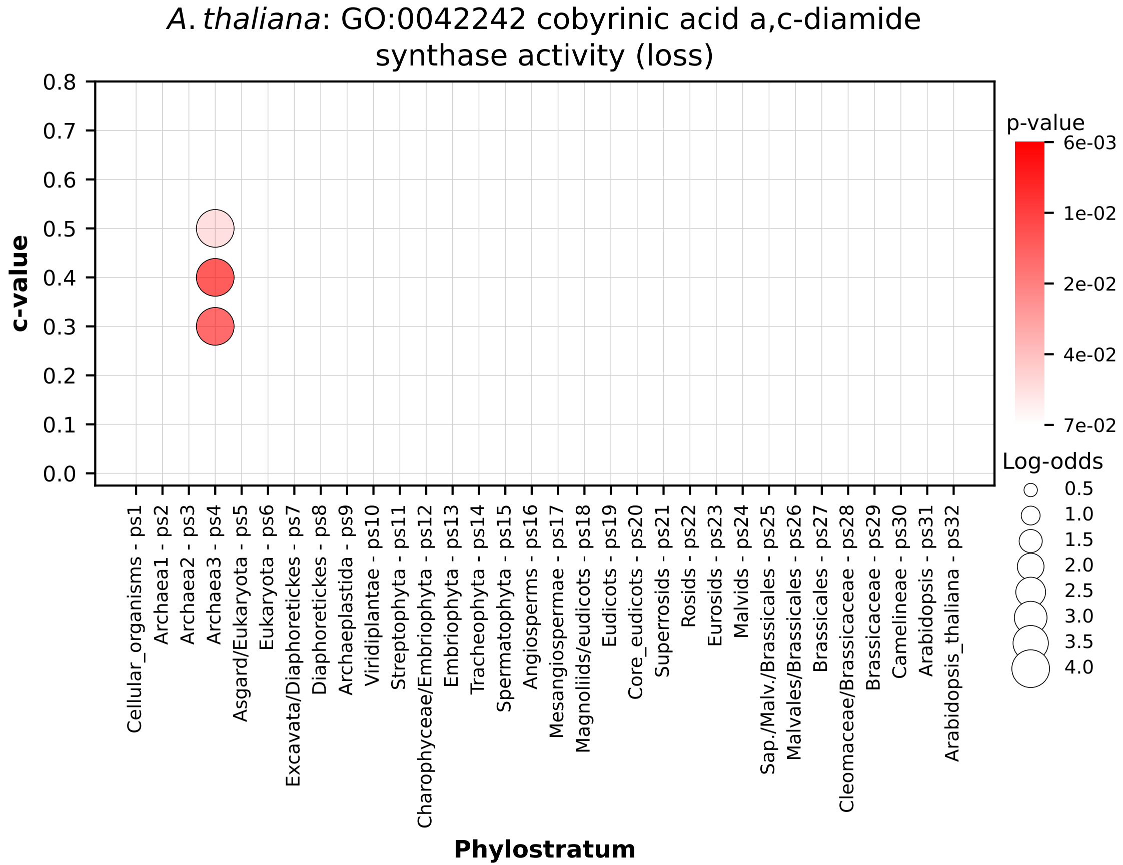

A. thaliana: GO:0042242 cobyrinic acid a,c-diamide synthase activity (loss)
c-value
0.8
0.7
0.6
0.5
0.4
0.3
0.2
0.1
0.0
p-value
6e-03
1e-02
2e-02
4e-02
7e-02
Log-odds
0.5
1.0
1.5
2.0
2.5
3.0
3.5
4.0
Cellular_organisms - ps1
Archaea1 - ps2
Archaea2 - ps3
Archaea3 - ps4
Asgard/Eukaryota - ps5
Eukaryota - ps6
Excavata/Diaphoretickes - ps7
Diaphoretickes - ps8
Archaeplastida - ps9
Viridiplantae - ps10
Streptophyta - ps11
Charophyceae/Embriophyta - ps12
Embriophyta - ps13
Tracheophyta - ps14
Spermatophyta - ps15
Angiosperms - ps16
Mesangiospermae - ps17
Magnoliids/eudicots - ps18
Eudicots - ps19
Core_eudicots - ps20
Superrosids - ps21
Rosids - ps22
Eurosids - ps23
Malvids - ps24
Sap./Malv./Brassicales - ps25
Malvales/Brassicales - ps26
Brassicales - ps27
Cleomaceae/Brassicaceae - ps28
Brassicaceae - ps29
Camelineae - ps30
Arabidopsis - ps31
Arabidopsis_thaliana - ps32
Phylostratum

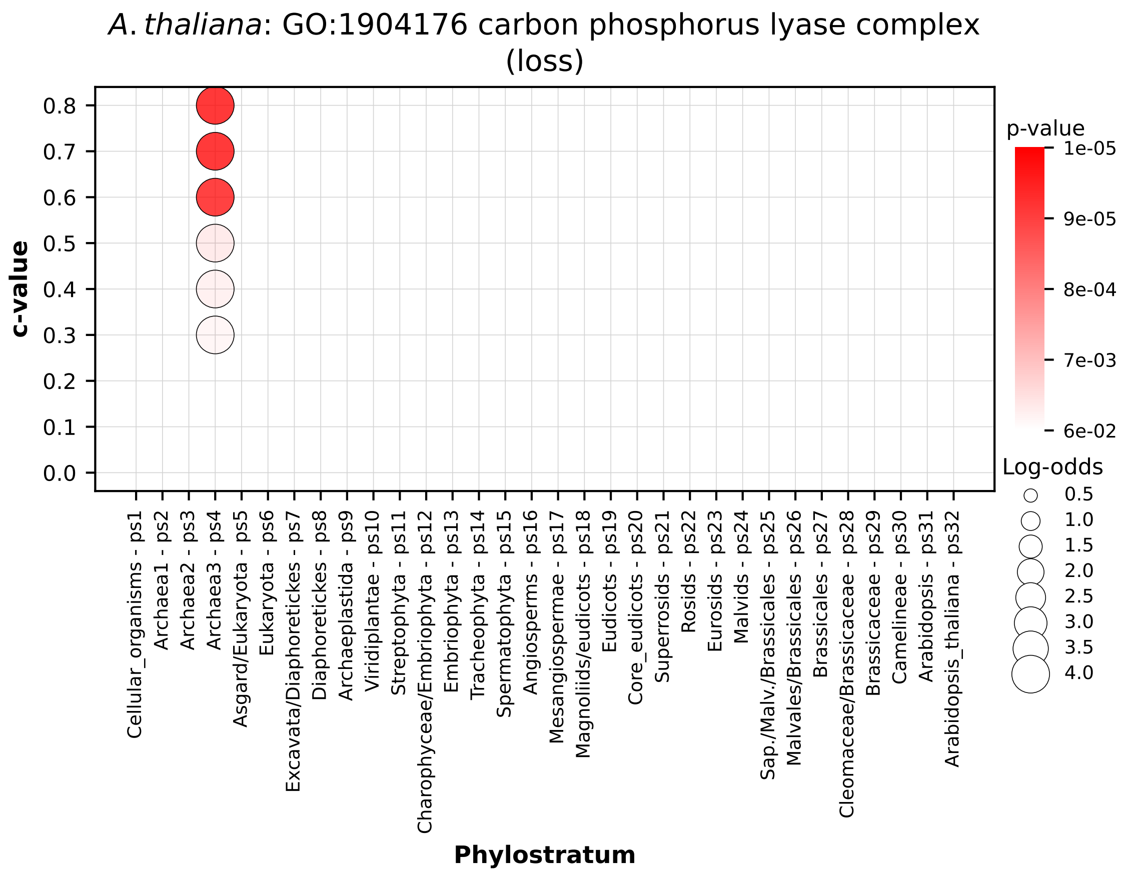

A. thaliana: GO:1904176 carbon phosphorus lyase complex
(loss)
c-value
0.8
0.7
0.6
0.5
0.4
0.3
0.2
0.1
0.0
p-value
1e-05
9e-05
8e-04
7e-03
6e-02
Log-odds
0.5
1.0
1.5
2.0
2.5
3.0
3.5
4.0
Cellular_organisms - ps1
Archaea1 - ps2
Archaea2 - ps3
Archaea3 - ps4
Asgard/Eukaryota - ps5
Eukaryota - ps6
Excavata/Diaphoretickes - ps7
Diaphoretickes - ps8
Archaeplastida - ps9
Viridiplantae - ps10
Streptophyta - ps11
Charophyceae/Embriophyta - ps12
Embriophyta - ps13
Tracheophyta - ps14
Spermatophyta - ps15
Angiosperms - ps16
Mesangiospermae - ps17
Magnoliids/eudicots - ps18
Eudicots - ps19
Core_eudicots - ps20
Superrosids - ps21
Rosids - ps22
Eurosids - ps23
Malvids - ps24
Sap./Malv./Brassicales - ps25
Malvales/Brassicales - ps26
Brassicales - ps27
Cleomaceae/Brassicaceae - ps28
Brassicaceae - ps29
Camelineae - ps30
Arabidopsis - ps31
Arabidopsis_thaliana - ps32
Phylostratum

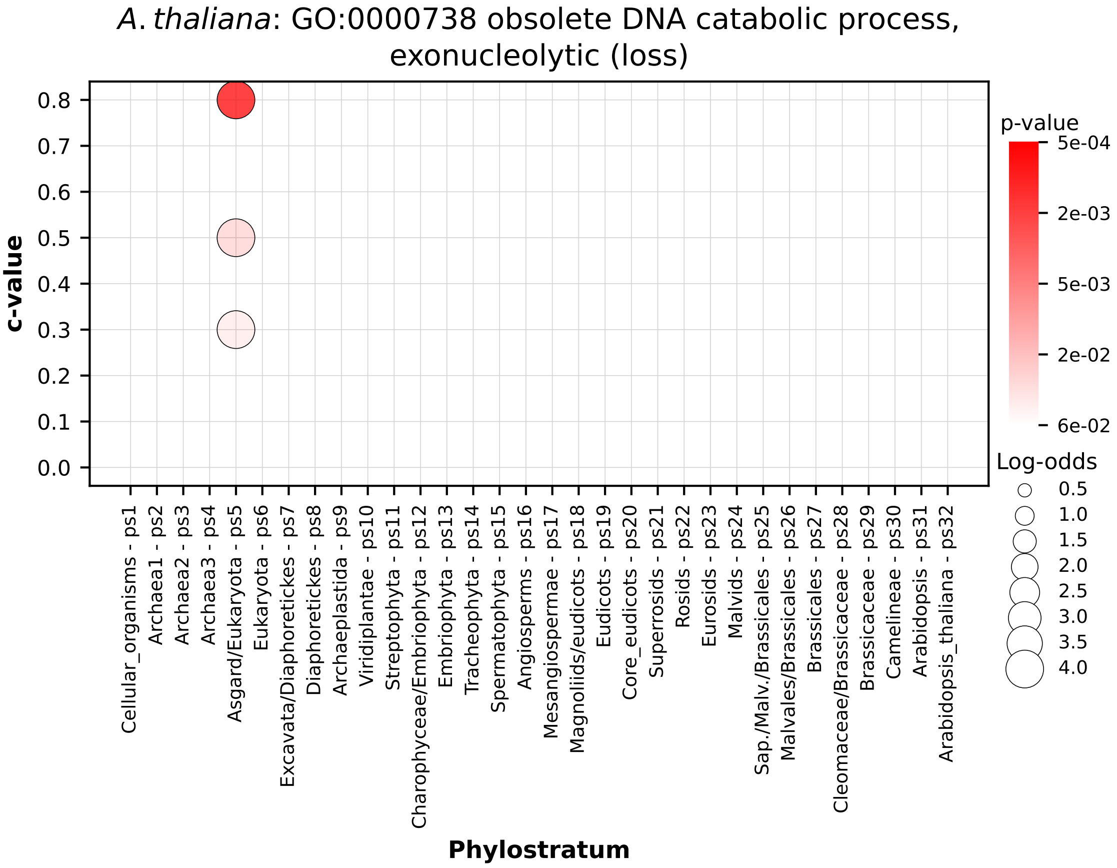
A. thaliana: GO:0000738 obsolete DNA catabolic process, exonucleolytic (loss)
c-value
0.0
0.1
0.2
0.3
0.4
0.5
0.6
0.7
0.8
Phylostratum
Cellular_organisms - ps1
Archaea1 - ps2
Archaea2 - ps3
Archaea3 - ps4
Asgard/Eukaryota - ps5
Eukaryota - ps6
Excavata/Diaphoretickes - ps7
Diaphoretickes - ps8
Archaeplastida - ps9
Viridiplantae - ps10
Streptophyta - ps11
Charophyceae/Embriophyta - ps12
Embriophyta - ps13
Tracheophyta - ps14
Spermatophyta - ps15
Angiosperms - ps16
Mesangiospermae - ps17
Magnoliids/eudicots - ps18
Eudicots - ps19
Core_eudicots - ps20
Superrosids - ps21
Rosids - ps22
Eurosids - ps23
Malvids - ps24
Sap./Malv./Brassicales - ps25
Malvales/Brassicales - ps26
Brassicales - ps27
Cleomaceae/Brassicaceae - ps28
Brassicaceae - ps29
Camelineae - ps30
Arabidopsis - ps31
Arabidopsis_thaliana - ps32
p-value
5e-04
2e-03
5e-03
2e-02
6e-02
Log-odds
0.5
1.0
1.5
2.0
2.5
3.0
3.5
4.0

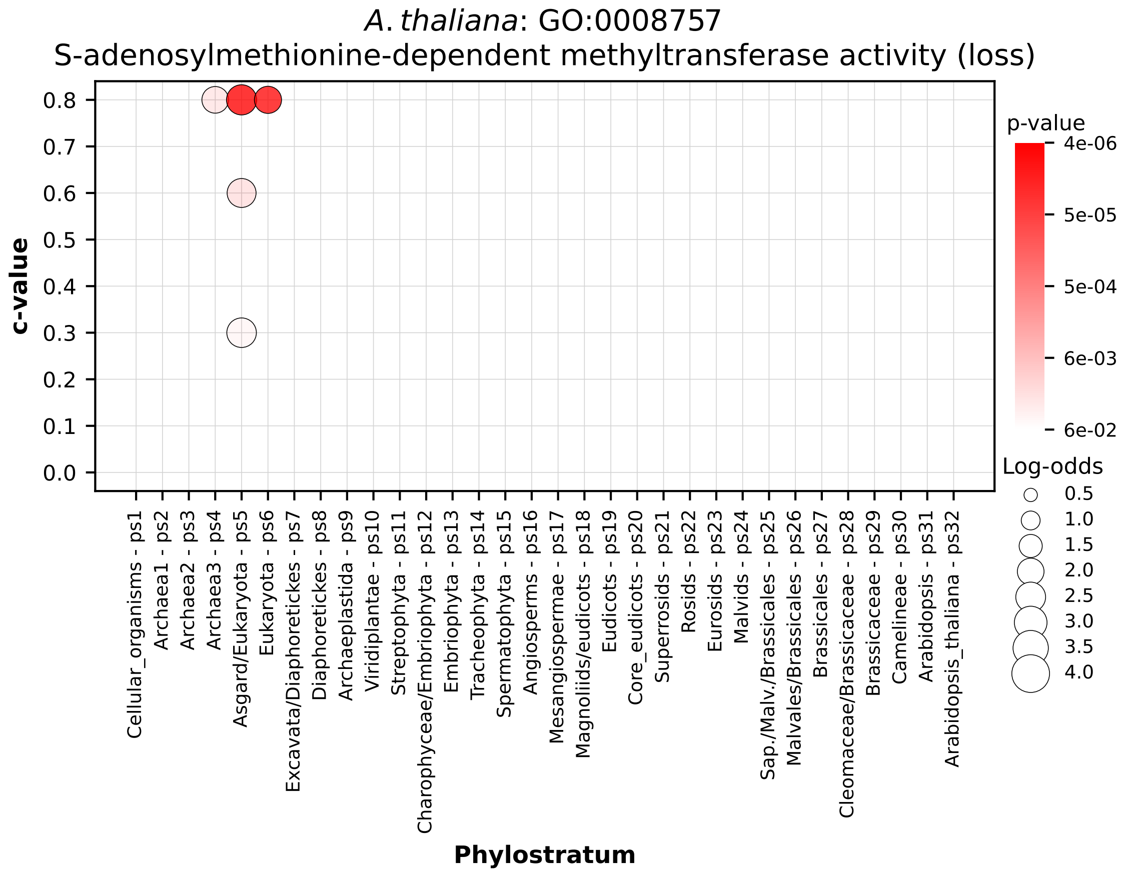

A. thaliana: GO:0008757
S-adenosylmethionine-dependent methyltransferase activity (loss)
c-value
0.8
0.7
0.6
0.5
0.4
0.3
0.2
0.1
0.0
p-value
4e-06
5e-05
5e-04
6e-03
6e-02
Log-odds
0.5
1.0
1.5
2.0
2.5
3.0
3.5
4.0
Cellular_organisms - ps1
Archaea1 - ps2
Archaea2 - ps3
Archaea3 - ps4
Asgard/Eukaryota - ps5
Eukaryota - ps6
Excavata/Diaphoretickes - ps7
Diaphoretickes - ps8
Archaeplastida - ps9
Viridiplantae - ps10
Streptophyta - ps11
Charophyceae/Embriophyta - ps12
Embriophyta - ps13
Tracheophyta - ps14
Spermatophyta - ps15
Angiosperms - ps16
Mesangiospermae - ps17
Magnoliids/eudicots - ps18
Eudicots - ps19
Core_eudicots - ps20
Superrosids - ps21
Rosids - ps22
Eurosids - ps23
Malvids - ps24
Sap./Malv./Brassicales - ps25
Malvales/Brassicales - ps26
Brassicales - ps27
Cleomaceae/Brassicaceae - ps28
Brassicaceae - ps29
Camelineae - ps30
Arabidopsis - ps31
Arabidopsis_thaliana - ps32
Phylostratum

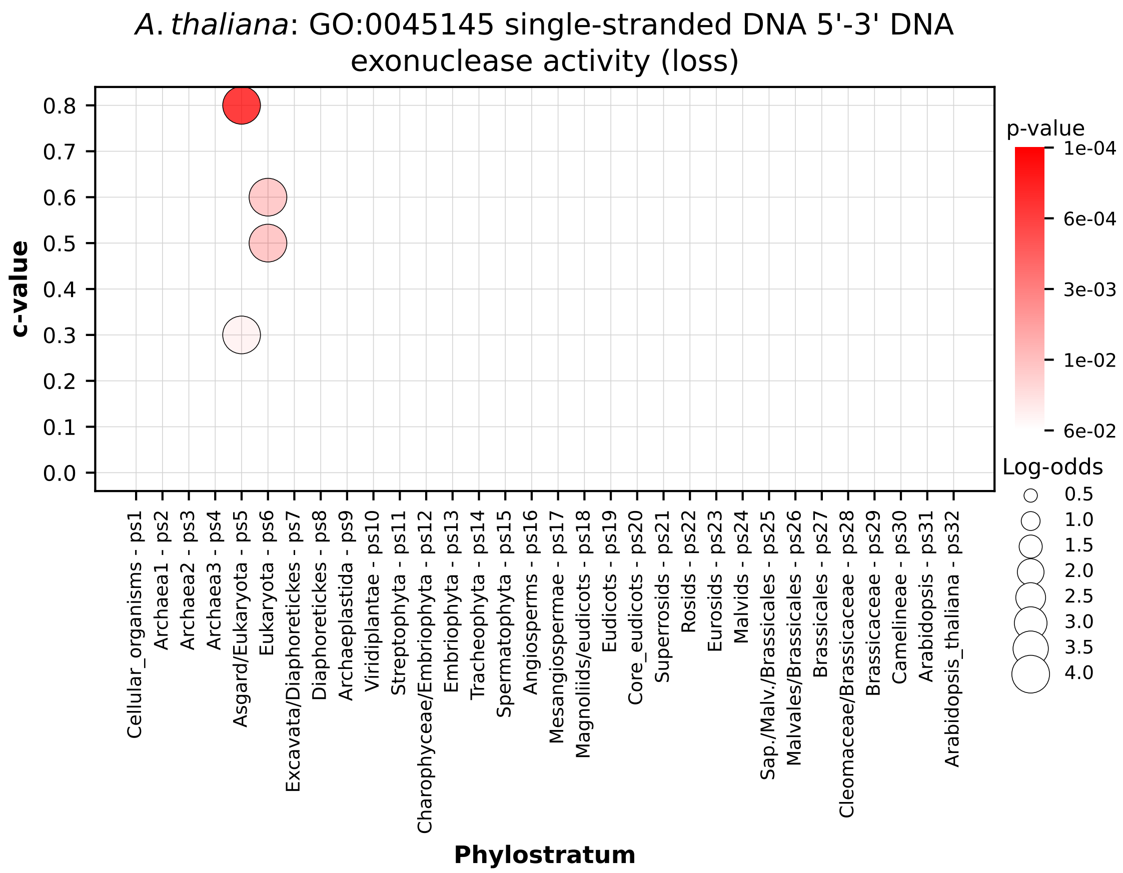

A. thaliana: GO:0045145 single-stranded DNA 5'-3' DNA exonuclease activity (loss)
c-value
0.8
0.7
0.6
0.5
0.4
0.3
0.2
0.1
0.0
p-value
1e-04
6e-04
3e-03
1e-02
6e-02
Log-odds
0.5
1.0
1.5
2.0
2.5
3.0
3.5
4.0
Cellular_organisms - ps1
Archaea1 - ps2
Archaea2 - ps3
Archaea3 - ps4
Asgard/Eukaryota - ps5
Eukaryota - ps6
Excavata/Diaphoretickes - ps7
Diaphoretickes - ps8
Archaeplastida - ps9
Viridiplantae - ps10
Streptophyta - ps11
Charophyceae/Embriophyta - ps12
Embriophyta - ps13
Tracheophyta - ps14
Spermatophyta - ps15
Angiosperms - ps16
Mesangiospermae - ps17
Magnoliids/eudicots - ps18
Eudicots - ps19
Core_eudicots - ps20
Superrosids - ps21
Rosids - ps22
Eurosids - ps23
Malvids - ps24
Sap./Malv./Brassicales - ps25
Malvales/Brassicales - ps26
Brassicales - ps27
Cleomaceae/Brassicaceae - ps28
Brassicaceae - ps29
Camelineae - ps30
Arabidopsis - ps31
Arabidopsis_thaliana - ps32
Phylostratum

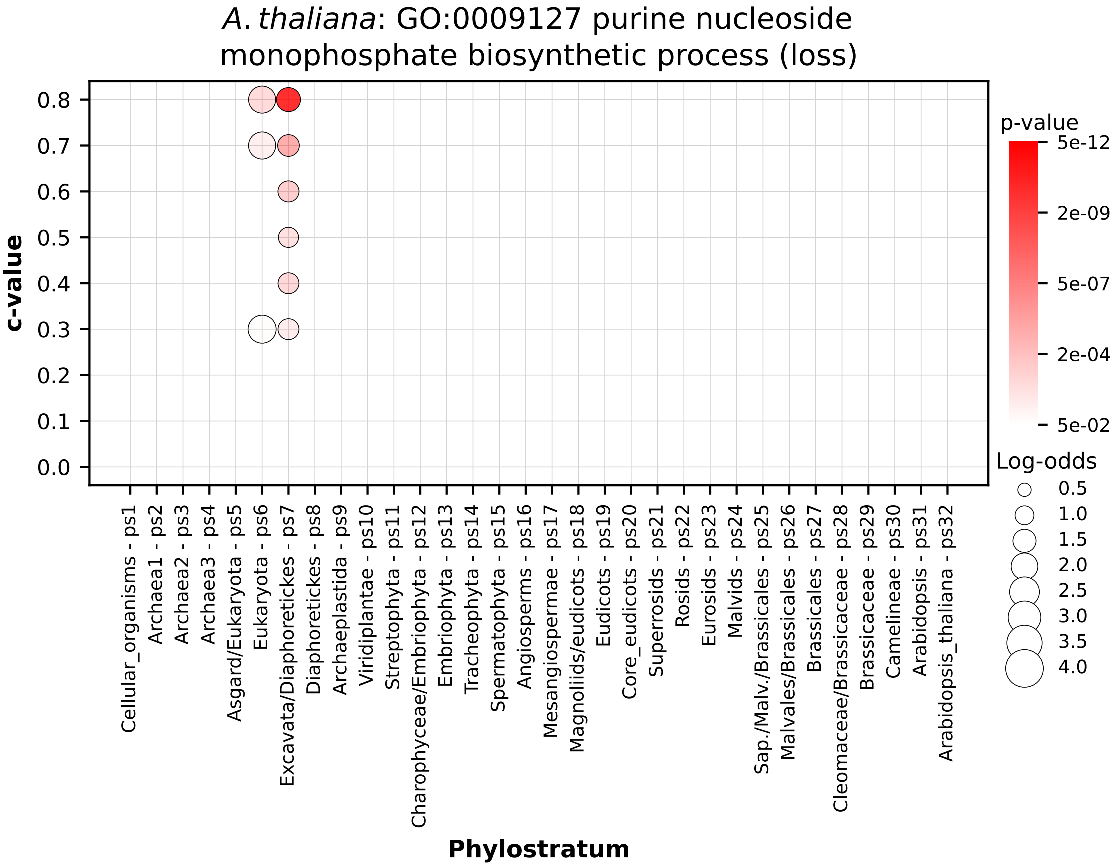

A. thaliana: GO:0009127 purine nucleoside monophosphate biosynthetic process (loss)
c-value
0.8
0.7
0.6
0.5
0.4
0.3
0.2
0.1
0.0
Phylostratum
Cellular_organisms - ps1
Archaea1 - ps2
Archaea2 - ps3
Archaea3 - ps4
Asgard/Eukaryota - ps5
Eukaryota - ps6
Excavata/Diaphoretickes - ps7
Diaphoretickes - ps8
Archaeplastida - ps9
Viridiplantae - ps10
Streptophyta - ps11
Charophyceae/Embriophyta - ps12
Embriophyta - ps13
Tracheophyta - ps14
Spermatophyta - ps15
Angiosperms - ps16
Mesangiospermae - ps17
Magnoliids/eudicots - ps18
Eudicots - ps19
Core_eudicots - ps20
Superrosids - ps21
Rosids - ps22
Eurosids - ps23
Malvids - ps24
Sap./Malv./Brassicales - ps25
Malvales/Brassicales - ps26
Brassicales - ps27
Cleomaceae/Brassicaceae - ps28
Brassicaceae - ps29
Camelineae - ps30
Arabidopsis - ps31
Arabidopsis_thaliana - ps32
p-value
5e-12
2e-09
5e-07
2e-04
5e-02
Log-odds
0.5
1.0
1.5
2.0
2.5
3.0
3.5
4.0

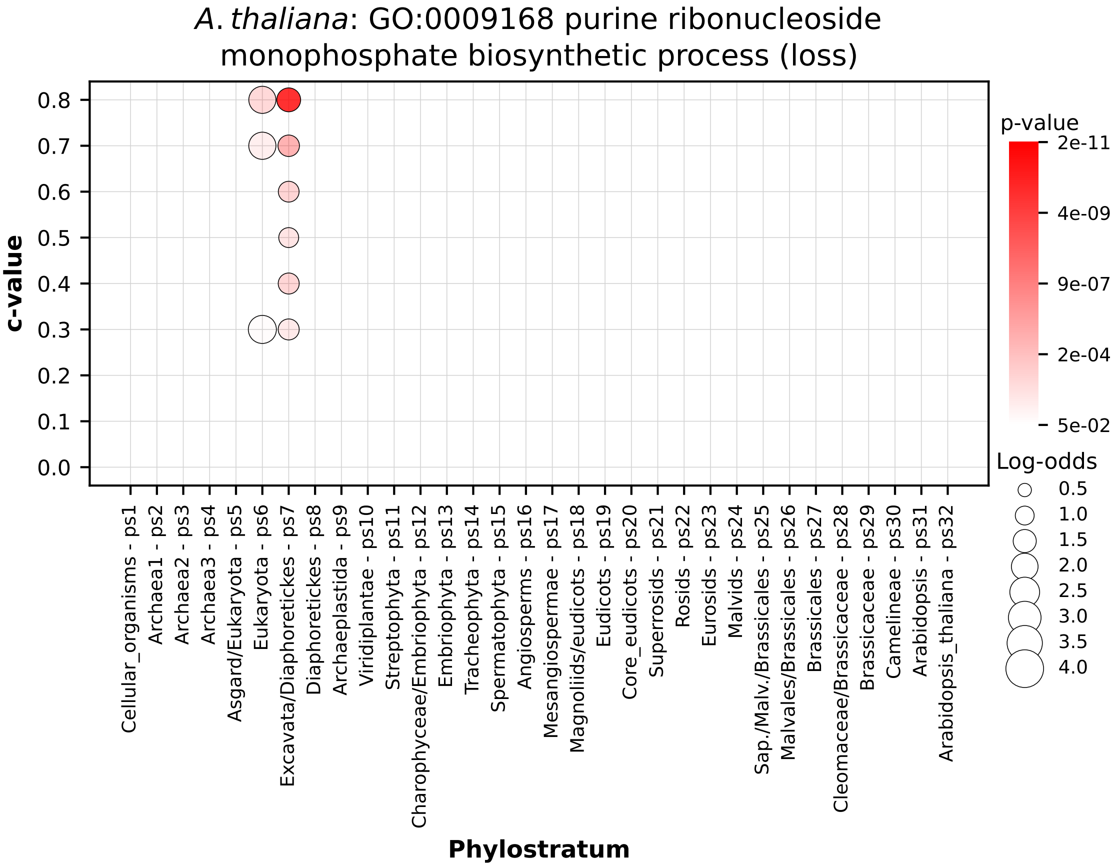

A. thaliana: GO:0009168 purine ribonucleoside monophosphate biosynthetic process (loss)
c-value
0.8
0.7
0.6
0.5
0.4
0.3
0.2
0.1
0.0
p-value
2e-11
4e-09
9e-07
2e-04
5e-02
Log-odds
0.5
1.0
1.5
2.0
2.5
3.0
3.5
4.0
Cellular_organisms - ps1
Archaea1 - ps2
Archaea2 - ps3
Archaea3 - ps4
Asgard/Eukaryota - ps5
Eukaryota - ps6
Excavata/Diaphoretickes - ps7
Diaphoretickes - ps8
Archaeplastida - ps9
Viridiplantae - ps10
Streptophyta - ps11
Charophyceae/Embriophyta - ps12
Embriophyta - ps13
Tracheophyta - ps14
Spermatophyta - ps15
Angiosperms - ps16
Mesangiospermae - ps17
Magnoliids/eudicots - ps18
Eudicots - ps19
Core_eudicots - ps20
Superrosids - ps21
Rosids - ps22
Eurosids - ps23
Malvids - ps24
Sap./Malv./Brassicales - ps25
Malvales/Brassicales - ps26
Brassicales - ps27
Cleomaceae/Brassicaceae - ps28
Brassicaceae - ps29
Camelineae - ps30
Arabidopsis - ps31
Arabidopsis_thaliana - ps32
Phylostratum

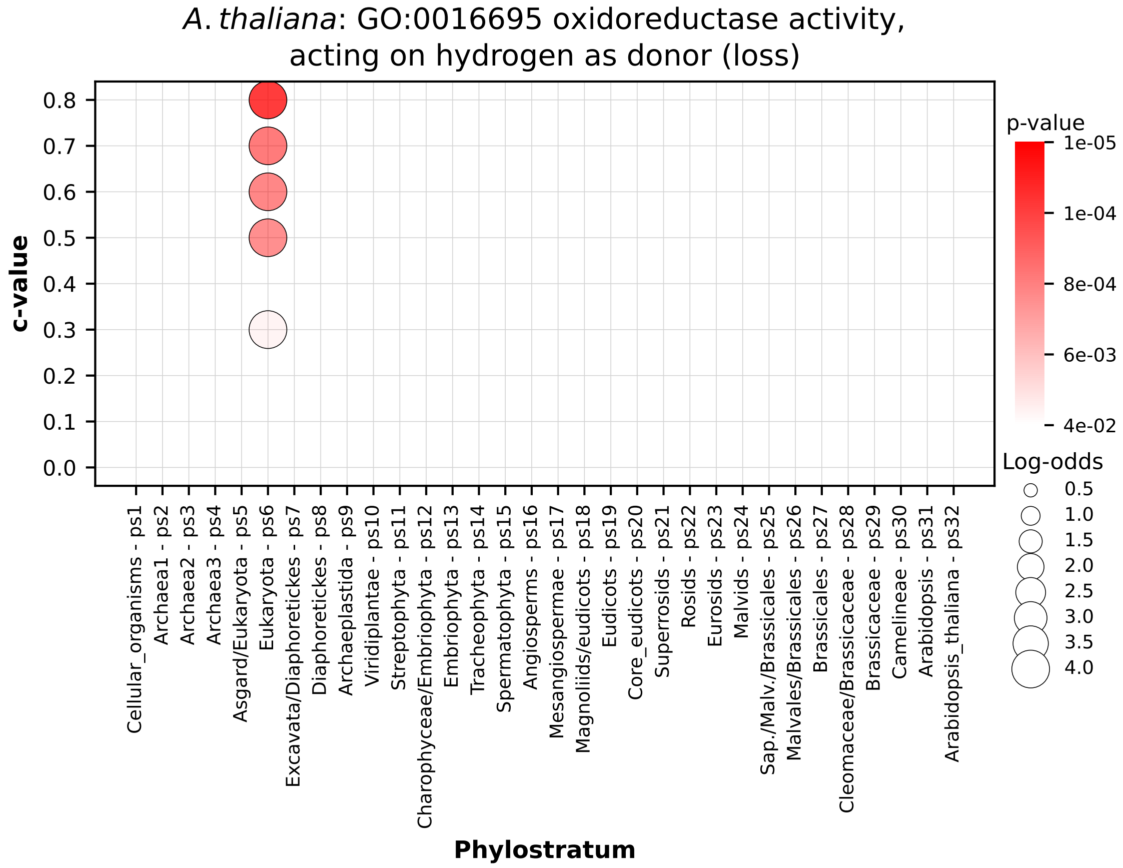

A. thaliana: GO:0016695 oxidoreductase activity, acting on hydrogen as donor (loss)
c-value
0.8
0.7
0.6
0.5
0.4
0.3
0.2
0.1
0.0
p-value
1e-05
1e-04
8e-04
6e-03
4e-02
Log-odds
0.5
1.0
1.5
2.0
2.5
3.0
3.5
4.0
Cellular_organisms - ps1
Archaea1 - ps2
Archaea2 - ps3
Archaea3 - ps4
Asgard/Eukaryota - ps5
Eukaryota - ps6
Excavata/Diaphoretickes - ps7
Diaphoretickes - ps8
Archaeplastida - ps9
Viridiplantae - ps10
Streptophyta - ps11
Charophyceae/Embriophyta - ps12
Embriophyta - ps13
Tracheophyta - ps14
Spermatophyta - ps15
Angiosperms - ps16
Mesangiospermae - ps17
Magnoliids/eudicots - ps18
Eudicots - ps19
Core_eudicots - ps20
Superrosids - ps21
Rosids - ps22
Eurosids - ps23
Malvids - ps24
Sap./Malv./Brassicales - ps25
Malvales/Brassicales - ps26
Brassicales - ps27
Cleomaceae/Brassicaceae - ps28
Brassicaceae - ps29
Camelineae - ps30
Arabidopsis - ps31
Arabidopsis_thaliana - ps32
Phylostratum

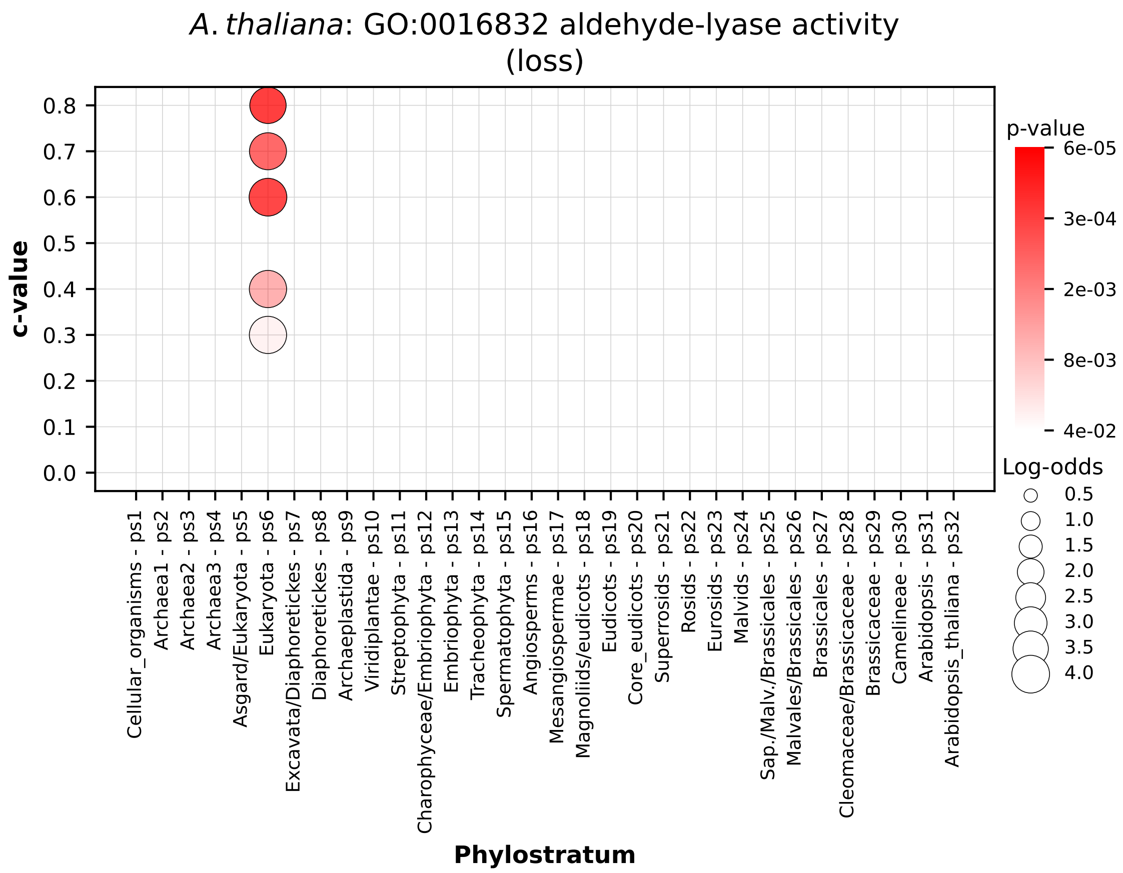

A. thaliana: GO:0016832 aldehyde-lyase activity
(loss)
c-value
0.0
0.1
0.2
0.3
0.4
0.5
0.6
0.7
0.8
p-value
6e-05
3e-04
2e-03
8e-03
4e-02
Log-odds
0.5
1.0
1.5
2.0
2.5
3.0
3.5
4.0
Cellular_organisms - ps1
Archaea1 - ps2
Archaea2 - ps3
Archaea3 - ps4
Asgard/Eukaryota - ps5
Eukaryota - ps6
Excavata/Diaphoretickes - ps7
Diaphoretickes - ps8
Archaeplastida - ps9
Viridiplantae - ps10
Streptophyta - ps11
Charophyceae/Embriophyta - ps12
Embriophyta - ps13
Tracheophyta - ps14
Spermatophyta - ps15
Angiosperms - ps16
Mesangiospermae - ps17
Magnoliids/eudicots - ps18
Eudicots - ps19
Core_eudicots - ps20
Superrosids - ps21
Rosids - ps22
Eurosids - ps23
Malvids - ps24
Sap./Malv./Brassicales - ps25
Malvales/Brassicales - ps26
Brassicales - ps27
Cleomaceae/Brassicaceae - ps28
Brassicaceae - ps29
Camelineae - ps30
Arabidopsis - ps31
Arabidopsis_thaliana - ps32
Phylostratum

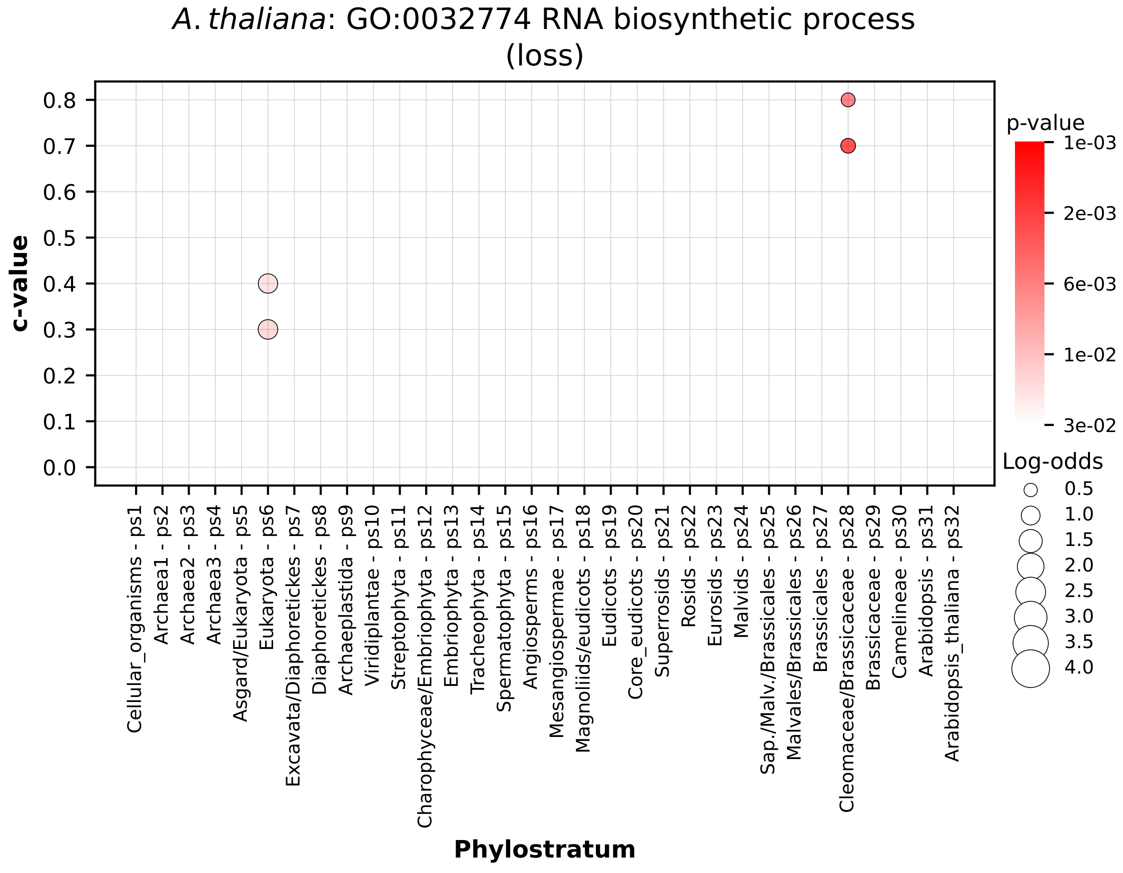
A. thaliana: GO:0032774 RNA biosynthetic process
(loss)
c-value
0.8
0.7
0.6
0.5
0.4
0.3
0.2
0.1
0.0
p-value
1e-03
2e-03
6e-03
1e-02
3e-02
Log-odds
0.5
1.0
1.5
2.0
2.5
3.0
3.5
4.0
Cellular_organisms - ps1
Archaea1 - ps2
Archaea2 - ps3
Archaea3 - ps4
Asgard/Eukaryota - ps5
Eukaryota - ps6
Excavata/Diaphoretickes - ps7
Diaphoretickes - ps8
Archaeplastida - ps9
Viridiplantae - ps10
Streptophyta - ps11
Charophyceae/Embriophyta - ps12
Embriophyta - ps13
Tracheophyta - ps14
Spermatophyta - ps15
Angiosperms - ps16
Mesangiospermae - ps17
Magnoliids/eudicots - ps18
Eudicots - ps19
Core_eudicots - ps20
Superrosids - ps21
Rosids - ps22
Eurosids - ps23
Malvids - ps24
Sap./Malv./Brassicales - ps25
Malvales/Brassicales - ps26
Brassicales - ps27
Cleomaceae/Brassicaceae - ps28
Brassicaceae - ps29
Camelineae - ps30
Arabidopsis - ps31
Arabidopsis_thaliana - ps32
Phylostratum

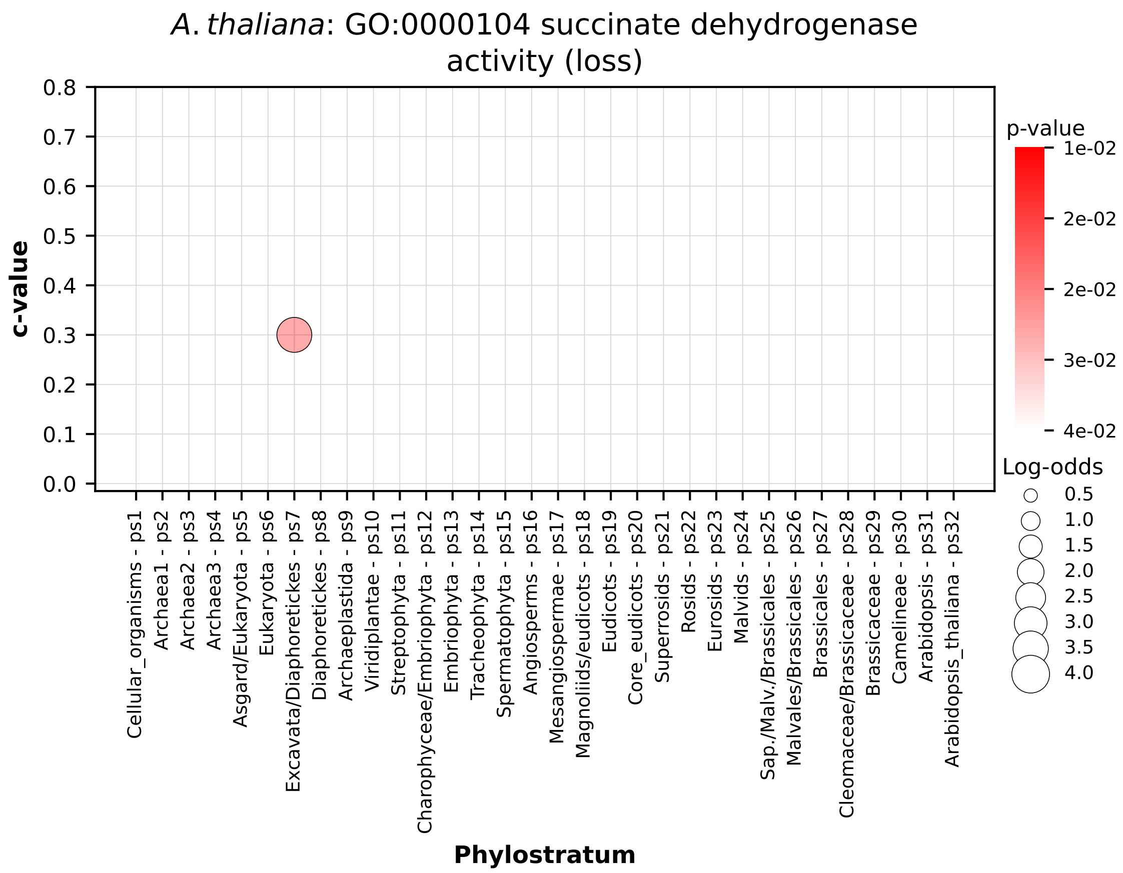
A. thaliana: GO:0000104 succinate dehydrogenase activity (loss)
c-value
0.8
0.7
0.6
0.5
0.4
0.3
0.2
0.1
0.0
p-value
1e-02
2e-02
2e-02
3e-02
4e-02
Log-odds
0.5
1.0
1.5
2.0
2.5
3.0
3.5
4.0
Cellular_organisms - ps1
Archaea1 - ps2
Archaea2 - ps3
Archaea3 - ps4
Asgard/Eukaryota - ps5
Eukaryota - ps6
Excavata/Diaphoretickes - ps7
Diaphoretickes - ps8
Archaeplastida - ps9
Viridiplantae - ps10
Streptophyta - ps11
Charophyceae/Embriophyta - ps12
Embriophyta - ps13
Tracheophyta - ps14
Spermatophyta - ps15
Angiosperms - ps16
Mesangiospermae - ps17
Magnoliids/eudicots - ps18
Eudicots - ps19
Core_eudicots - ps20
Superrosids - ps21
Rosids - ps22
Eurosids - ps23
Malvids - ps24
Sap./Malv./Brassicales - ps25
Malvales/Brassicales - ps26
Brassicales - ps27
Cleomaceae/Brassicaceae - ps28
Brassicaceae - ps29
Camelineae - ps30
Arabidopsis - ps31
Arabidopsis_thaliana - ps32
Phylostratum

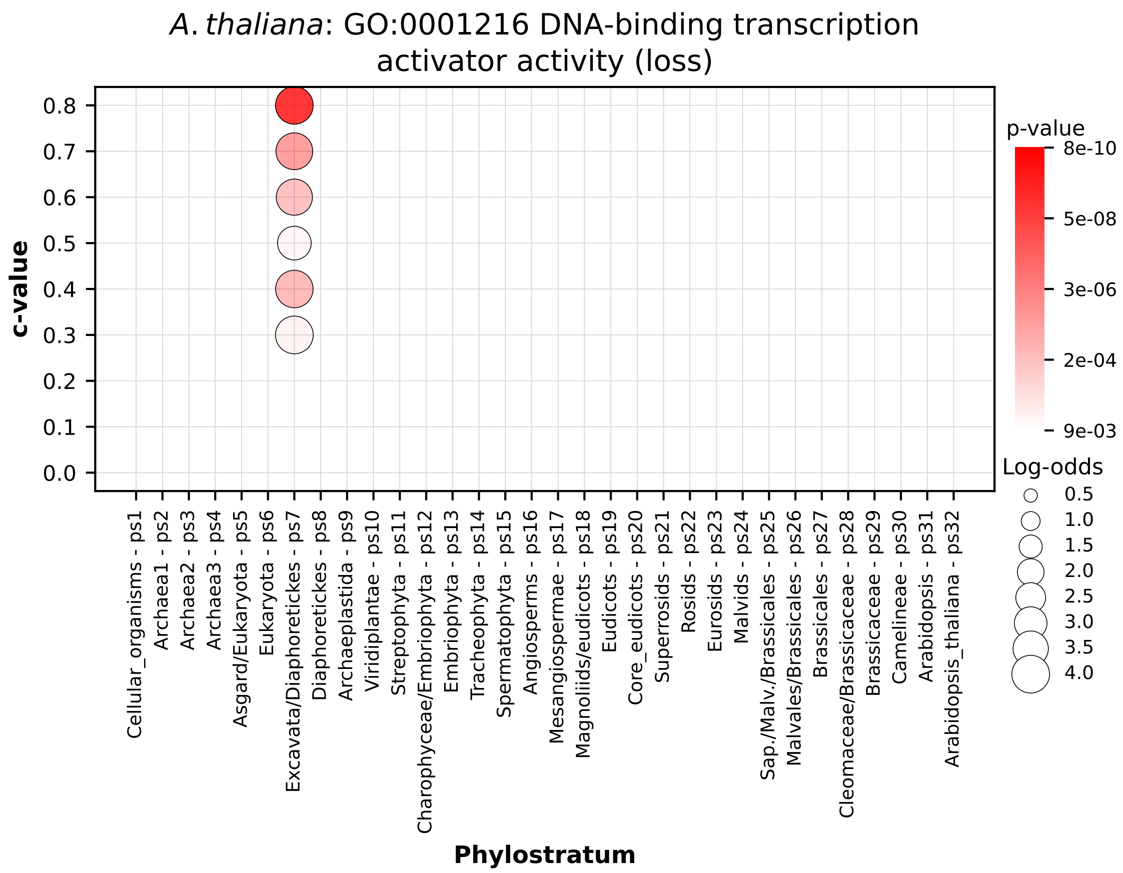

A. thaliana: GO:0001216 DNA-binding transcription activator activity (loss)
c-value
0.0
0.1
0.2
0.3
0.4
0.5
0.6
0.7
0.8
p-value
8e-10
5e-08
3e-06
2e-04
9e-03
Log-odds
0.5
1.0
1.5
2.0
2.5
3.0
3.5
4.0
Cellular_organisms - ps1
Archaea1 - ps2
Archaea2 - ps3
Archaea3 - ps4
Asgard/Eukaryota - ps5
Eukaryota - ps6
Excavata/Diaphoretickes - ps7
Diaphoretickes - ps8
Archaeplastida - ps9
Viridiplantae - ps10
Streptophyta - ps11
Charophyceae/Embriophyta - ps12
Embriophyta - ps13
Tracheophyta - ps14
Spermatophyta - ps15
Angiosperms - ps16
Mesangiospermae - ps17
Magnoliids/eudicots - ps18
Eudicots - ps19
Core_eudicots - ps20
Superrosids - ps21
Rosids - ps22
Eurosids - ps23
Malvids - ps24
Sap./Malv./Brassicales - ps25
Malvales/Brassicales - ps26
Brassicales - ps27
Cleomaceae/Brassicaceae - ps28
Brassicaceae - ps29
Camelineae - ps30
Arabidopsis - ps31
Arabidopsis_thaliana - ps32
Phylostratum

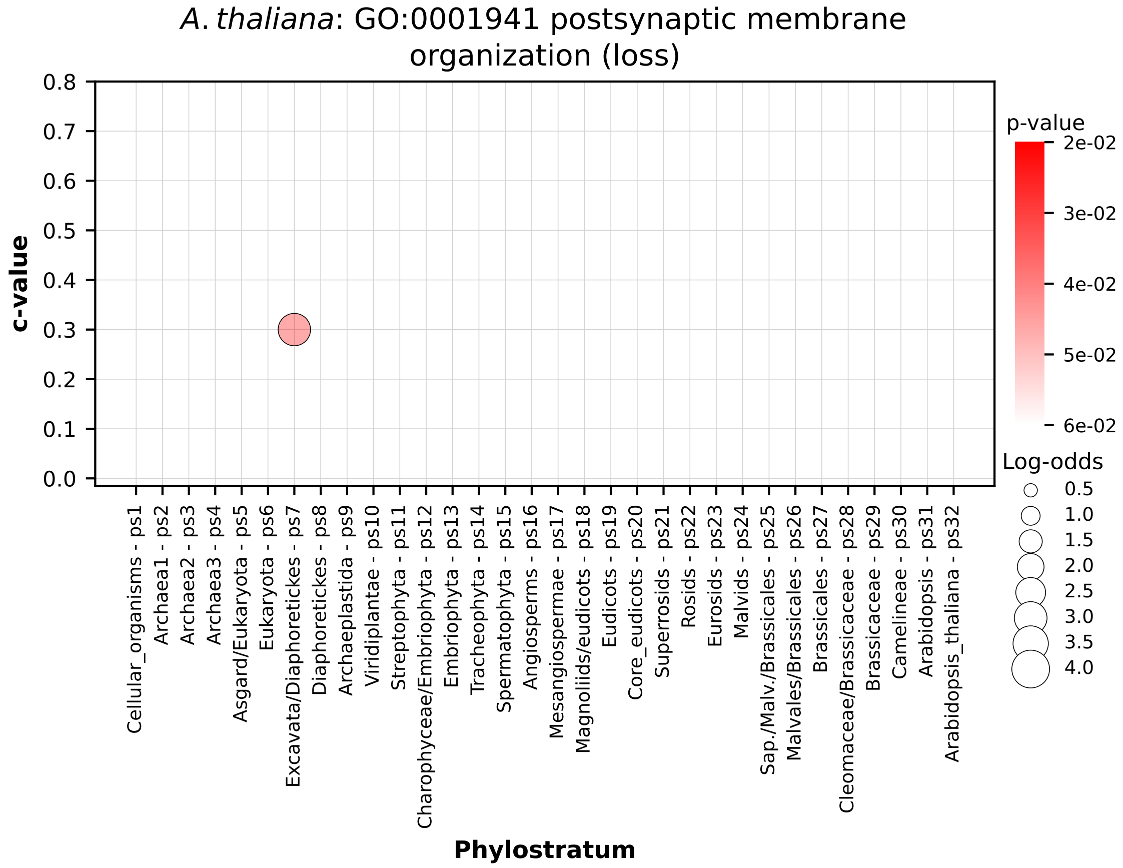
A. thaliana: GO:0001941 postsynaptic membrane organization (loss)
c-value
0.8
0.7
0.6
0.5
0.4
0.3
0.2
0.1
0.0
p-value
2e-02
3e-02
4e-02
5e-02
6e-02
Log-odds
0.5
1.0
1.5
2.0
2.5
3.0
3.5
4.0
Cellular_organisms - ps1
Archaea1 - ps2
Archaea2 - ps3
Archaea3 - ps4
Asgard/Eukaryota - ps5
Eukaryota - ps6
Excavata/Diaphoretickes - ps7
Diaphoretickes - ps8
Archaeplastida - ps9
Viridiplantae - ps10
Streptophyta - ps11
Charophyceae/Embriophyta - ps12
Embriophyta - ps13
Tracheophyta - ps14
Spermatophyta - ps15
Angiosperms - ps16
Mesangiospermae - ps17
Magnoliids/eudicots - ps18
Eudicots - ps19
Core_eudicots - ps20
Superrosids - ps21
Rosids - ps22
Eurosids - ps23
Malvids - ps24
Sap./Malv./Brassicales - ps25
Malvales/Brassicales - ps26
Brassicales - ps27
Cleomaceae/Brassicaceae - ps28
Brassicaceae - ps29
Camelineae - ps30
Arabidopsis - ps31
Arabidopsis_thaliana - ps32
Phylostratum

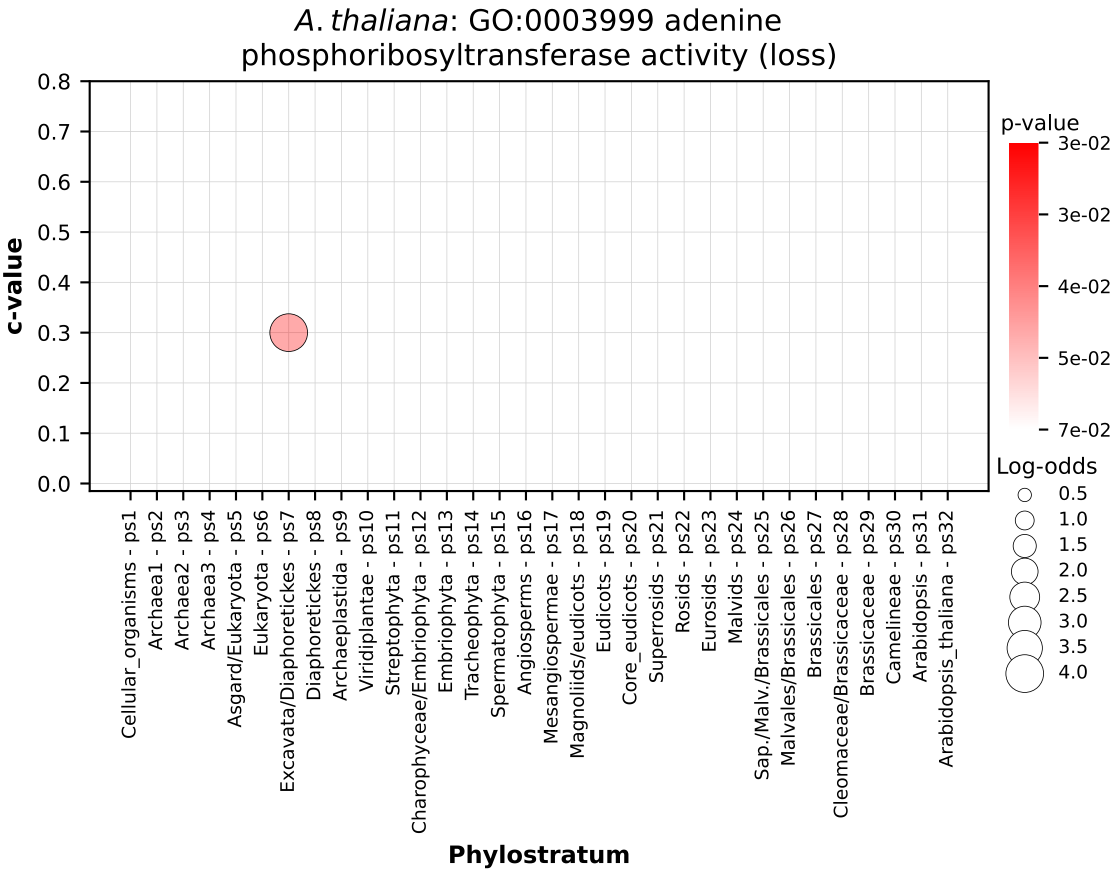

A. thaliana: GO:0003999 adenine phosphoribosyltransferase activity (loss)
c-value
0.0
0.1
0.2
0.3
0.4
0.5
0.6
0.7
0.8
Phylostratum
Cellular_organisms - ps1
Archaea1 - ps2
Archaea2 - ps3
Archaea3 - ps4
Asgard/Eukaryota - ps5
Eukaryota - ps6
Excavata/Diaphoretickes - ps7
Diaphoretickes - ps8
Archaeplastida - ps9
Viridiplantae - ps10
Streptophyta - ps11
Charophyceae/Embriophyta - ps12
Embriophyta - ps13
Tracheophyta - ps14
Spermatophyta - ps15
Angiosperms - ps16
Mesangiospermae - ps17
Magnoliids/eudicots - ps18
Eudicots - ps19
Core_eudicots - ps20
Superrosids - ps21
Rosids - ps22
Eurosids - ps23
Malvids - ps24
Sap./Malv./Brassicales - ps25
Malvales/Brassicales - ps26
Brassicales - ps27
Cleomaceae/Brassicaceae - ps28
Brassicaceae - ps29
Camelineae - ps30
Arabidopsis - ps31
Arabidopsis_thaliana - ps32
p-value
3e-02
3e-02
4e-02
5e-02
7e-02
Log-odds
0.5
1.0
1.5
2.0
2.5
3.0
3.5
4.0

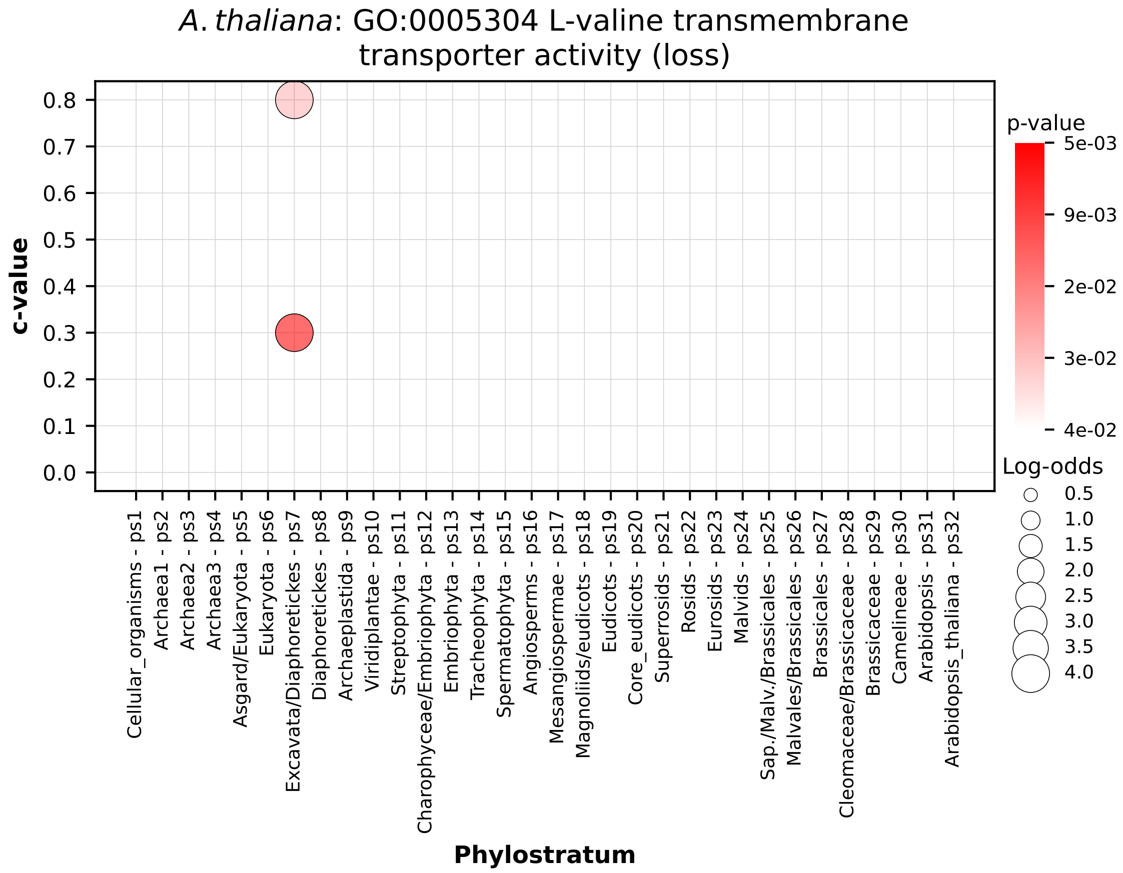
A. thaliana: GO:0005304 L-valine transmembrane transporter activity (loss)
c-value
0.0
0.1
0.2
0.3
0.4
0.5
0.6
0.7
0.8
Phylostratum
Cellular_organisms - ps1
Archaea1 - ps2
Archaea2 - ps3
Archaea3 - ps4
Asgard/Eukaryota - ps5
Eukaryota - ps6
Excavata/Diaphoretickes - ps7
Diaphoretickes - ps8
Archaeplastida - ps9
Viridiplantae - ps10
Streptophyta - ps11
Charophyceae/Embriophyta - ps12
Embriophyta - ps13
Tracheophyta - ps14
Spermatophyta - ps15
Angiosperms - ps16
Mesangiospermae - ps17
Magnoliids/eudicots - ps18
Eudicots - ps19
Core_eudicots - ps20
Superrosids - ps21
Rosids - ps22
Eurosids - ps23
Malvids - ps24
Sap./Malv./Brassicales - ps25
Malvales/Brassicales - ps26
Brassicales - ps27
Cleomaceae/Brassicaceae - ps28
Brassicaceae - ps29
Camelineae - ps30
Arabidopsis - ps31
Arabidopsis_thaliana - ps32
p-value
5e-03
9e-03
2e-02
3e-02
4e-02
Log-odds
0.5
1.0
1.5
2.0
2.5
3.0
3.5
4.0

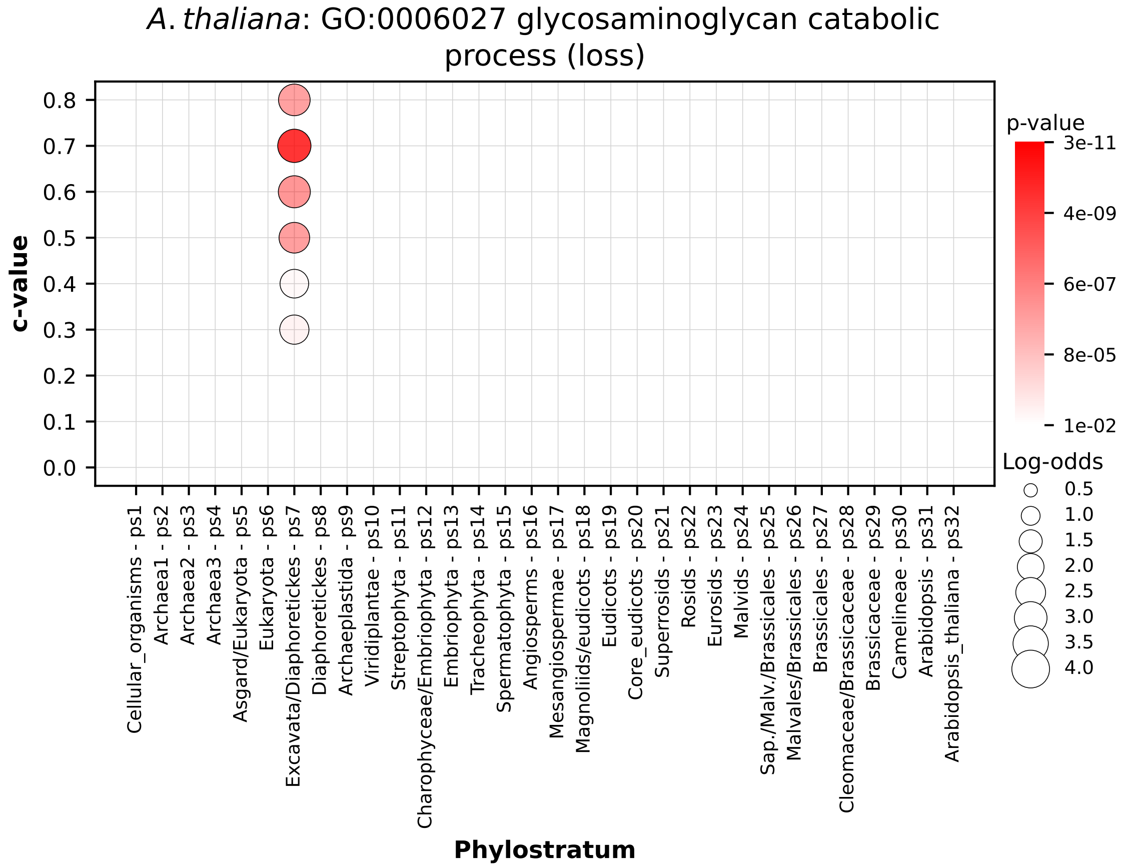

A. thaliana: GO:0006027 glycosaminoglycan catabolic process (loss)
c-value
0.8
0.7
0.6
0.5
0.4
0.3
0.2
0.1
0.0
p-value
3e-11
4e-09
6e-07
8e-05
1e-02
Log-odds
0.5
1.0
1.5
2.0
2.5
3.0
3.5
4.0
Cellular_organisms - ps1
Archaea1 - ps2
Archaea2 - ps3
Archaea3 - ps4
Asgard/Eukaryota - ps5
Eukaryota - ps6
Excavata/Diaphoretickes - ps7
Diaphoretickes - ps8
Archaeplastida - ps9
Viridiplantae - ps10
Streptophyta - ps11
Charophyceae/Embriophyta - ps12
Embriophyta - ps13
Tracheophyta - ps14
Spermatophyta - ps15
Angiosperms - ps16
Mesangiospermae - ps17
Magnoliids/eudicots - ps18
Eudicots - ps19
Core_eudicots - ps20
Superrosids - ps21
Rosids - ps22
Eurosids - ps23
Malvids - ps24
Sap./Malv./Brassicales - ps25
Malvales/Brassicales - ps26
Brassicales - ps27
Cleomaceae/Brassicaceae - ps28
Brassicaceae - ps29
Camelineae - ps30
Arabidopsis - ps31
Arabidopsis_thaliana - ps32
Phylostratum

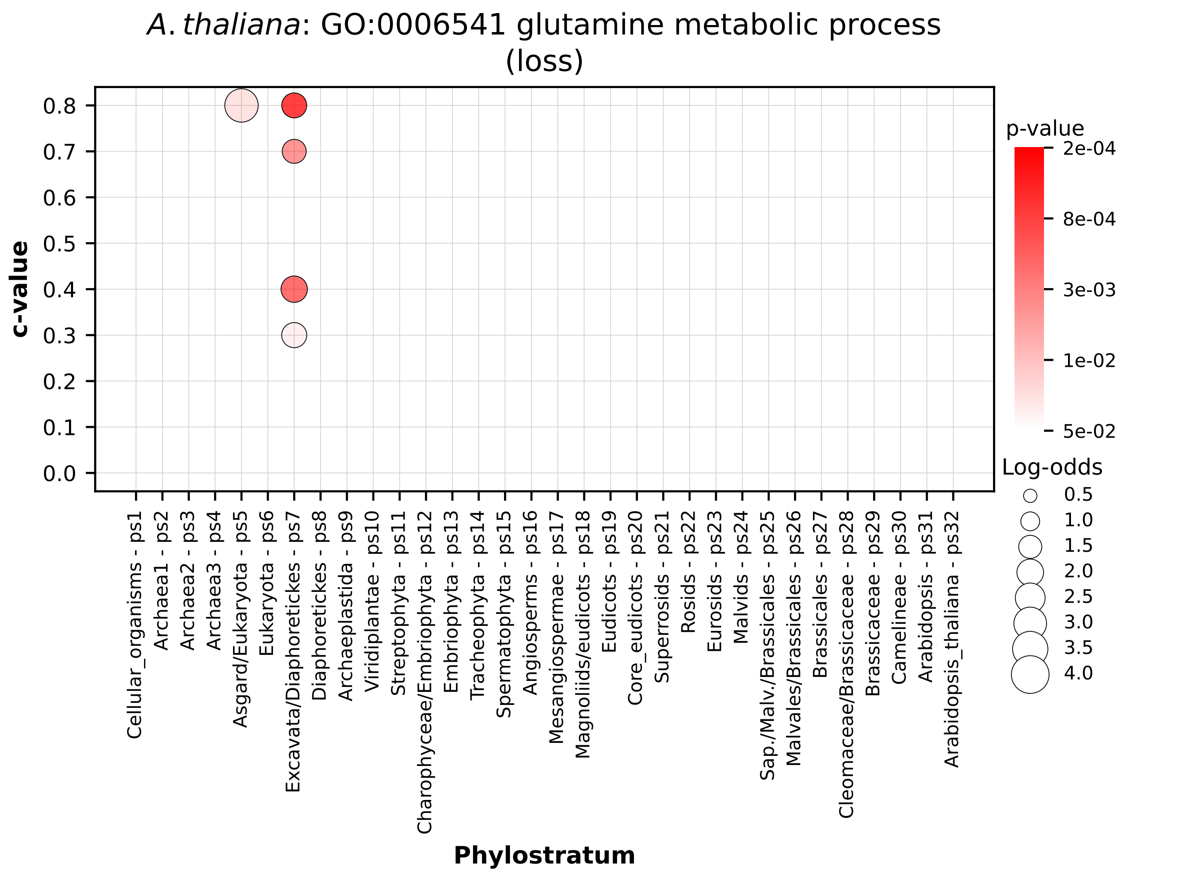

A. thaliana: GO:0006541 glutamine metabolic process
(loss)
c-value
0.8
0.7
0.6
0.5
0.4
0.3
0.2
0.1
0.0
p-value
2e-04
8e-04
3e-03
1e-02
5e-02
Log-odds
0.5
1.0
1.5
2.0
2.5
3.0
3.5
4.0
Cellular_organisms - ps1
Archaea1 - ps2
Archaea2 - ps3
Archaea3 - ps4
Asgard/Eukaryota - ps5
Eukaryota - ps6
Excavata/Diaphoretickes - ps7
Diaphoretickes - ps8
Archaeplastida - ps9
Viridiplantae - ps10
Streptophyta - ps11
Charophyceae/Embriophyta - ps12
Embriophyta - ps13
Tracheophyta - ps14
Spermatophyta - ps15
Angiosperms - ps16
Mesangiospermae - ps17
Magnoliids/eudicots - ps18
Eudicots - ps19
Core_eudicots - ps20
Superrosids - ps21
Rosids - ps22
Eurosids - ps23
Malvids - ps24
Sap./Malv./Brassicales - ps25
Malvales/Brassicales - ps26
Brassicales - ps27
Cleomaceae/Brassicaceae - ps28
Brassicaceae - ps29
Camelineae - ps30
Arabidopsis - ps31
Arabidopsis_thaliana - ps32
Phylostratum

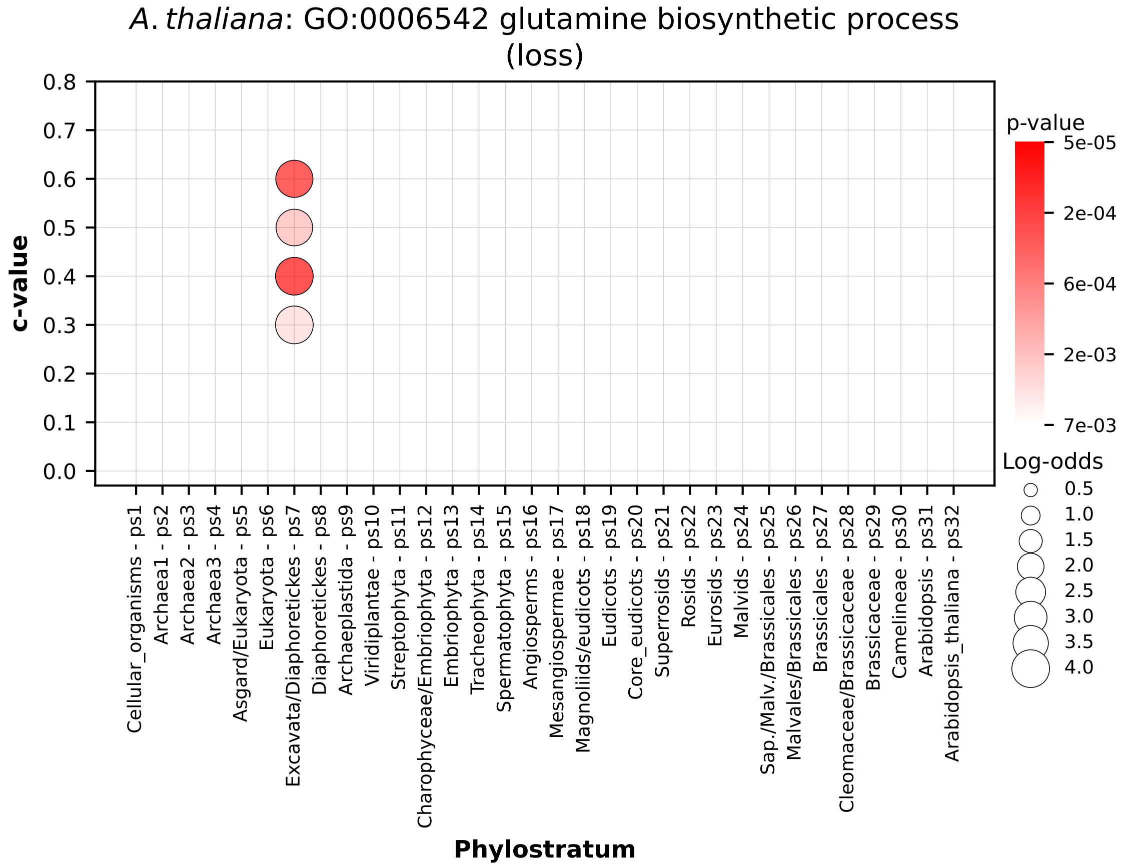

A. thaliana: GO:0006542 glutamine biosynthetic process (loss)
c-value
0.0
0.1
0.2
0.3
0.4
0.5
0.6
0.7
0.8
Phylostratum
Cellular_organisms - ps1
Archaea1 - ps2
Archaea2 - ps3
Archaea3 - ps4
Asgard/Eukaryota - ps5
Eukaryota - ps6
Excavata/Diaphoretickes - ps7
Diaphoretickes - ps8
Archaeplastida - ps9
Viridiplantae - ps10
Streptophyta - ps11
Charophyceae/Embriophyta - ps12
Embriophyta - ps13
Tracheophyta - ps14
Spermatophyta - ps15
Angiosperms - ps16
Mesangiospermae - ps17
Magnoliids/eudicots - ps18
Eudicots - ps19
Core_eudicots - ps20
Superrosids - ps21
Rosids - ps22
Eurosids - ps23
Malvids - ps24
Sap./Malv./Brassicales - ps25
Malvales/Brassicales - ps26
Brassicales - ps27
Cleomaceae/Brassicaceae - ps28
Brassicaceae - ps29
Camelineae - ps30
Arabidopsis - ps31
Arabidopsis_thaliana - ps32
p-value
5e-05
2e-04
6e-04
2e-03
7e-03
Log-odds
0.5
1.0
1.5
2.0
2.5
3.0
3.5
4.0

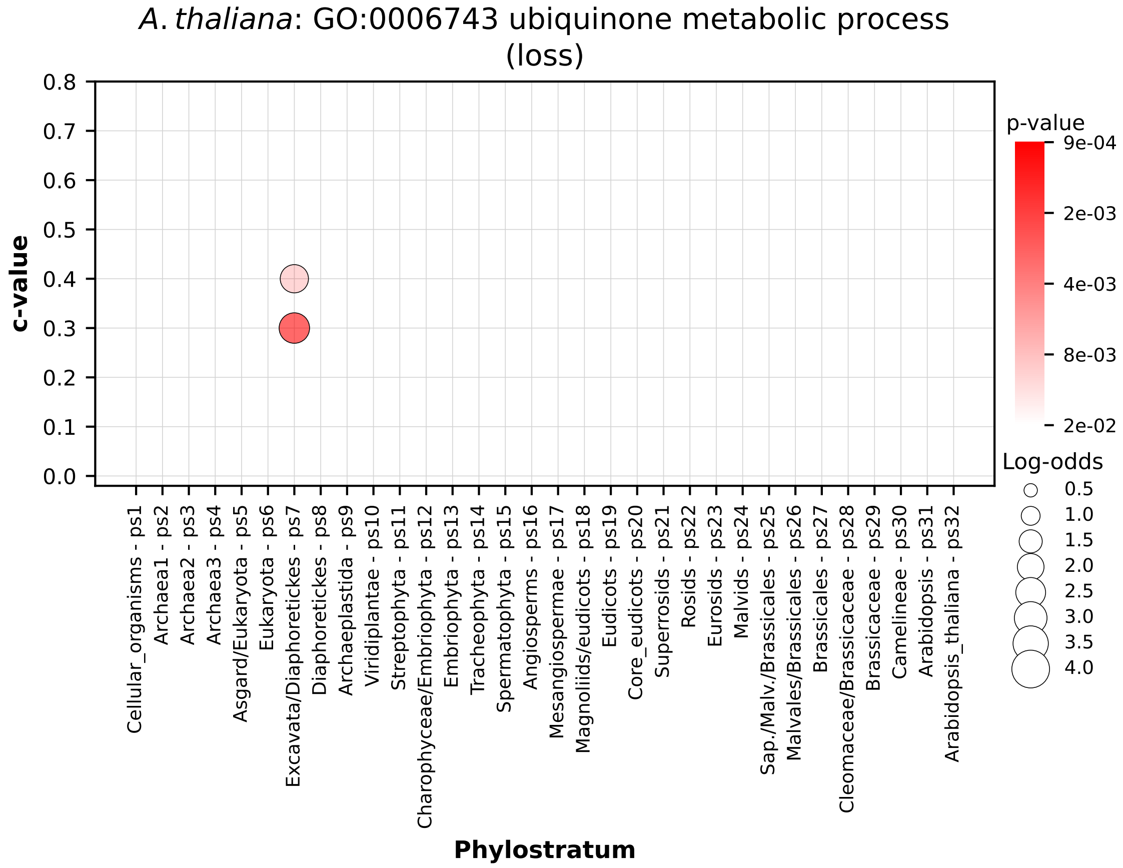
A. thaliana: GO:0006743 ubiquinone metabolic process
(loss)
c-value
0.8
0.7
0.6
0.5
0.4
0.3
0.2
0.1
0.0
p-value
9e-04
2e-03
4e-03
8e-03
2e-02
Log-odds
0.5
1.0
1.5
2.0
2.5
3.0
3.5
4.0
Cellular_organisms - ps1
Archaea1 - ps2
Archaea2 - ps3
Archaea3 - ps4
Asgard/Eukaryota - ps5
Eukaryota - ps6
Excavata/Diaphoretickes - ps7
Diaphoretickes - ps8
Archaeplastida - ps9
Viridiplantae - ps10
Streptophyta - ps11
Charophyceae/Embriophyta - ps12
Embriophyta - ps13
Tracheophyta - ps14
Spermatophyta - ps15
Angiosperms - ps16
Mesangiospermae - ps17
Magnoliids/eudicots - ps18
Eudicots - ps19
Core_eudicots - ps20
Superrosids - ps21
Rosids - ps22
Eurosids - ps23
Malvids - ps24
Sap./Malv./Brassicales - ps25
Malvales/Brassicales - ps26
Brassicales - ps27
Cleomaceae/Brassicaceae - ps28
Brassicaceae - ps29
Camelineae - ps30
Arabidopsis - ps31
Arabidopsis_thaliana - ps32
Phylostratum

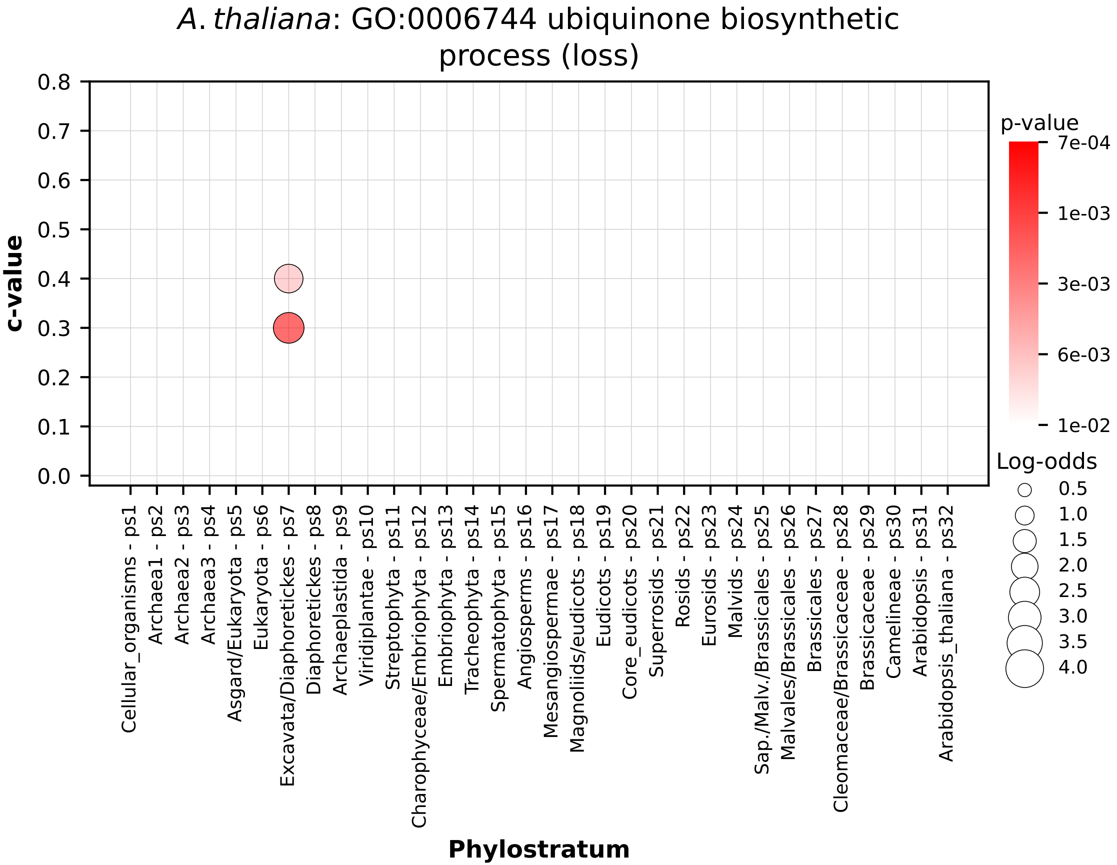

A. thaliana: GO:0006744 ubiquinone biosynthetic process (loss)
c-value
0.8
0.7
0.6
0.5
0.4
0.3
0.2
0.1
0.0
Phylostratum
Cellular_organisms - ps1
Archaea1 - ps2
Archaea2 - ps3
Archaea3 - ps4
Asgard/Eukaryota - ps5
Eukaryota - ps6
Excavata/Diaphoretickes - ps7
Diaphoretickes - ps8
Archaeplastida - ps9
Viridiplantae - ps10
Streptophyta - ps11
Charophyceae/Embriophyta - ps12
Embriophyta - ps13
Tracheophyta - ps14
Spermatophyta - ps15
Angiosperms - ps16
Mesangiospermae - ps17
Magnoliids/eudicots - ps18
Eudicots - ps19
Core_eudicots - ps20
Superrosids - ps21
Rosids - ps22
Eurosids - ps23
Malvids - ps24
Sap./Malv./Brassicales - ps25
Malvales/Brassicales - ps26
Brassicales - ps27
Cleomaceae/Brassicaceae - ps28
Brassicaceae - ps29
Camelineae - ps30
Arabidopsis - ps31
Arabidopsis_thaliana - ps32
p-value
7e-04
1e-03
3e-03
6e-03
1e-02
Log-odds
0.5
1.0
1.5
2.0
2.5
3.0
3.5
4.0

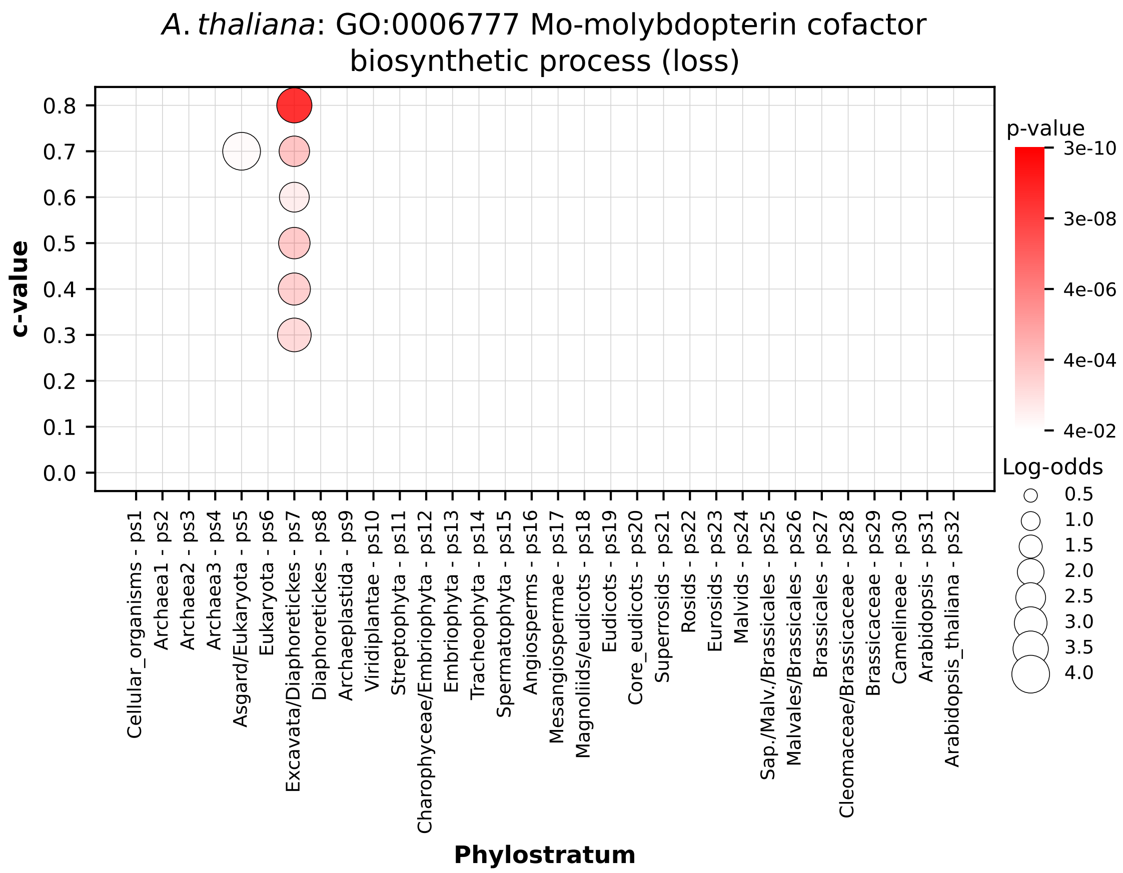

A. thaliana: GO:0006777 Mo-molybdopterin cofactor biosynthetic process (loss)
c-value
0.0
0.1
0.2
0.3
0.4
0.5
0.6
0.7
0.8
p-value
3e-10
3e-08
4e-06
4e-04
4e-02
Log-odds
0.5
1.0
1.5
2.0
2.5
3.0
3.5
4.0
Cellular_organisms - ps1
Archaea1 - ps2
Archaea2 - ps3
Archaea3 - ps4
Asgard/Eukaryota - ps5
Eukaryota - ps6
Excavata/Diaphoretickes - ps7
Diaphoretickes - ps8
Archaeplastida - ps9
Viridiplantae - ps10
Streptophyta - ps11
Charophyceae/Embriophyta - ps12
Embriophyta - ps13
Tracheophyta - ps14
Spermatophyta - ps15
Angiosperms - ps16
Mesangiospermae - ps17
Magnoliids/eudicots - ps18
Eudicots - ps19
Core_eudicots - ps20
Superrosids - ps21
Rosids - ps22
Eurosids - ps23
Malvids - ps24
Sap./Malv./Brassicales - ps25
Malvales/Brassicales - ps26
Brassicales - ps27
Cleomaceae/Brassicaceae - ps28
Brassicaceae - ps29
Camelineae - ps30
Arabidopsis - ps31
Arabidopsis_thaliana - ps32
Phylostratum

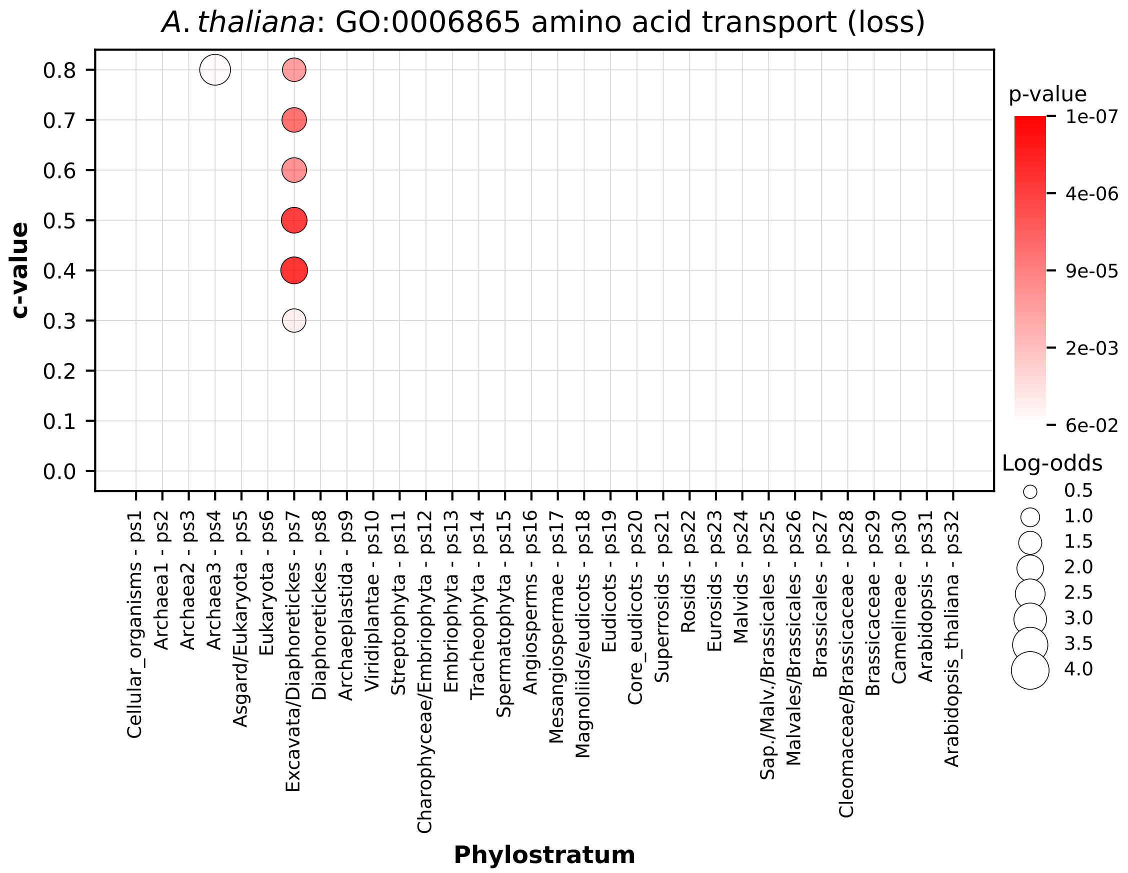

A. thaliana: GO:0006865 amino acid transport (loss)
c-value
0.8
0.7
0.6
0.5
0.4
0.3
0.2
0.1
0.0
p-value
1e-07
4e-06
9e-05
2e-03
6e-02
Log-odds
0.5
1.0
1.5
2.0
2.5
3.0
3.5
4.0
Cellular_organisms - ps1
Archaea1 - ps2
Archaea2 - ps3
Archaea3 - ps4
Asgard/Eukaryota - ps5
Eukaryota - ps6
Excavata/Diaphoretickes - ps7
Diaphoretickes - ps8
Archaeplastida - ps9
Viridiplantae - ps10
Streptophyta - ps11
Charophyceae/Embriophyta - ps12
Embriophyta - ps13
Tracheophyta - ps14
Spermatophyta - ps15
Angiosperms - ps16
Mesangiospermae - ps17
Magnoliids/eudicots - ps18
Eudicots - ps19
Core_eudicots - ps20
Superrosids - ps21
Rosids - ps22
Eurosids - ps23
Malvids - ps24
Sap./Malv./Brassicales - ps25
Malvales/Brassicales - ps26
Brassicales - ps27
Cleomaceae/Brassicaceae - ps28
Brassicaceae - ps29
Camelineae - ps30
Arabidopsis - ps31
Arabidopsis_thaliana - ps32
Phylostratum

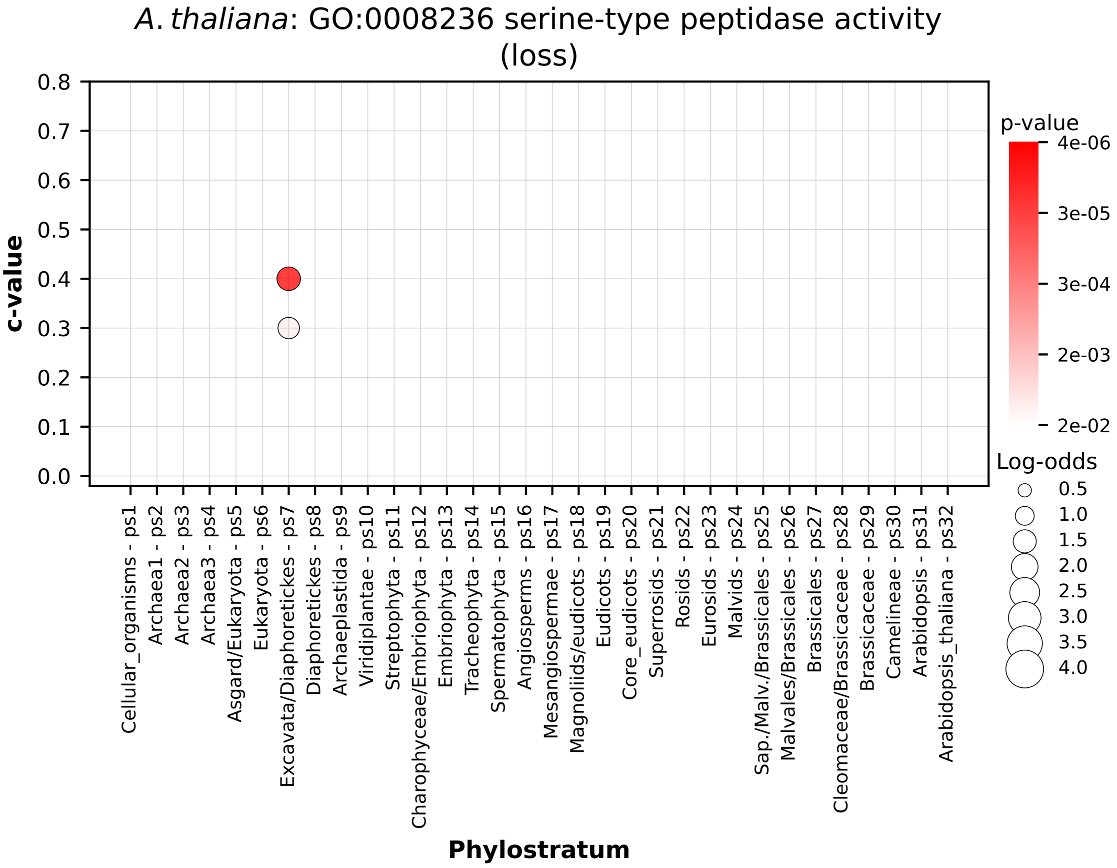
A. thaliana: GO:0008236 serine-type peptidase activity (loss)
c-value
0.8
0.7
0.6
0.5
0.4
0.3
0.2
0.1
0.0
Phylostratum
Cellular_organisms - ps1
Archaea1 - ps2
Archaea2 - ps3
Archaea3 - ps4
Asgard/Eukaryota - ps5
Eukaryota - ps6
Excavata/Diaphoretickes - ps7
Diaphoretickes - ps8
Archaeplastida - ps9
Viridiplantae - ps10
Streptophyta - ps11
Charophyceae/Embriophyta - ps12
Embriophyta - ps13
Tracheophyta - ps14
Spermatophyta - ps15
Angiosperms - ps16
Mesangiospermae - ps17
Magnoliids/eudicots - ps18
Eudicots - ps19
Core_eudicots - ps20
Superrosids - ps21
Rosids - ps22
Eurosids - ps23
Malvids - ps24
Sap./Malv./Brassicales - ps25
Malvales/Brassicales - ps26
Brassicales - ps27
Cleomaceae/Brassicaceae - ps28
Brassicaceae - ps29
Camelineae - ps30
Arabidopsis - ps31
Arabidopsis_thaliana - ps32
p-value
4e-06
3e-05
3e-04
2e-03
2e-02
Log-odds
0.5
1.0
1.5
2.0
2.5
3.0
3.5
4.0

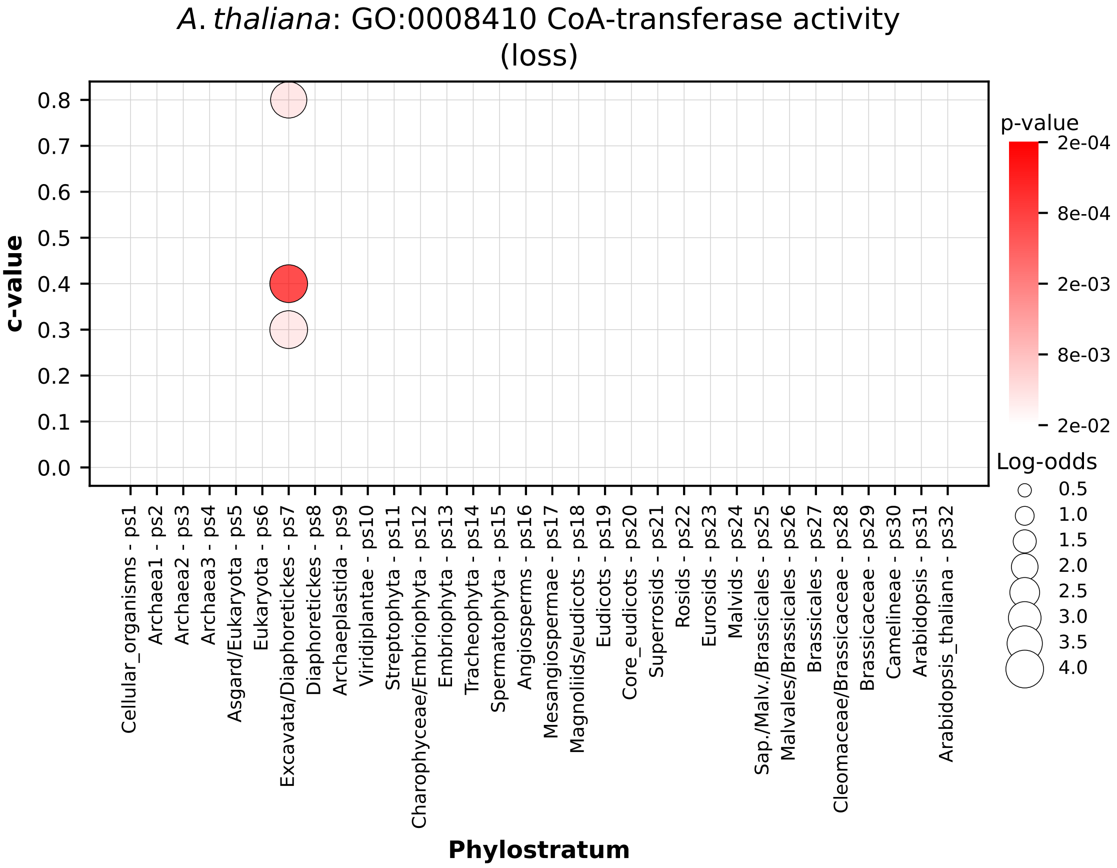

A. thaliana: GO:0008410 CoA-transferase activity
(loss)
c-value
0.8
0.7
0.6
0.5
0.4
0.3
0.2
0.1
0.0
Phylostratum
Cellular_organisms - ps1
Archaea1 - ps2
Archaea2 - ps3
Archaea3 - ps4
Asgard/Eukaryota - ps5
Eukaryota - ps6
Excavata/Diaphoretickes - ps7
Diaphoretickes - ps8
Archaeplastida - ps9
Viridiplantae - ps10
Streptophyta - ps11
Charophyceae/Embriophyta - ps12
Embriophyta - ps13
Tracheophyta - ps14
Spermatophyta - ps15
Angiosperms - ps16
Mesangiospermae - ps17
Magnoliids/eudicots - ps18
Eudicots - ps19
Core_eudicots - ps20
Superrosids - ps21
Rosids - ps22
Eurosids - ps23
Malvids - ps24
Sap./Malv./Brassicales - ps25
Malvales/Brassicales - ps26
Brassicales - ps27
Cleomaceae/Brassicaceae - ps28
Brassicaceae - ps29
Camelineae - ps30
Arabidopsis - ps31
Arabidopsis_thaliana - ps32
p-value
2e-04
8e-04
2e-03
8e-03
2e-02
Log-odds
0.5
1.0
1.5
2.0
2.5
3.0
3.5
4.0

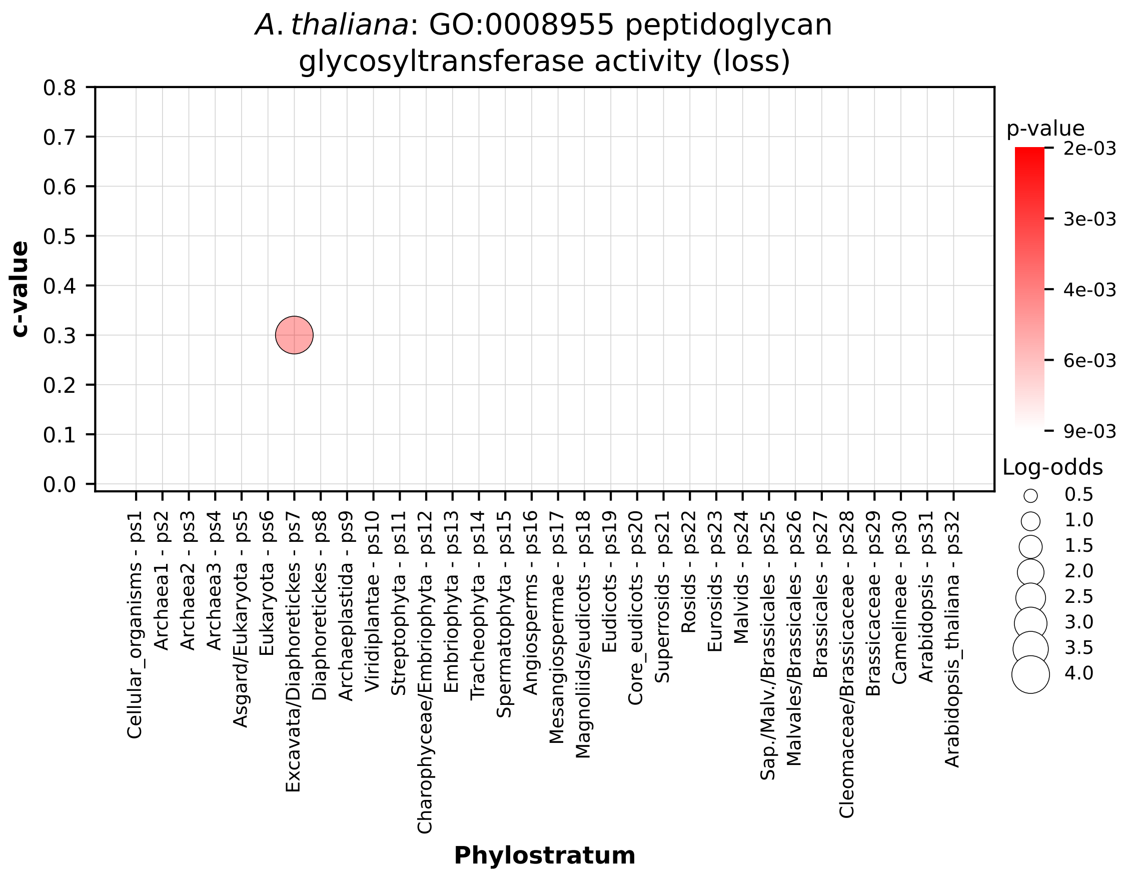

A. thaliana: GO:0008955 peptidoglycan glycosyltransferase activity (loss)
c-value
0.8
0.7
0.6
0.5
0.4
0.3
0.2
0.1
0.0
Phylostratum
Cellular_organisms - ps1
Archaea1 - ps2
Archaea2 - ps3
Archaea3 - ps4
Asgard/Eukaryota - ps5
Eukaryota - ps6
Excavata/Diaphoretickes - ps7
Diaphoretickes - ps8
Archaeplastida - ps9
Viridiplantae - ps10
Streptophyta - ps11
Charophyceae/Embriophyta - ps12
Embriophyta - ps13
Tracheophyta - ps14
Spermatophyta - ps15
Angiosperms - ps16
Mesangiospermae - ps17
Magnoliids/eudicots - ps18
Eudicots - ps19
Core_eudicots - ps20
Superrosids - ps21
Rosids - ps22
Eurosids - ps23
Malvids - ps24
Sap./Malv./Brassicales - ps25
Malvales/Brassicales - ps26
Brassicales - ps27
Cleomaceae/Brassicaceae - ps28
Brassicaceae - ps29
Camelineae - ps30
Arabidopsis - ps31
Arabidopsis_thaliana - ps32
p-value
2e-03
3e-03
4e-03
6e-03
9e-03
Log-odds
0.5
1.0
1.5
2.0
2.5
3.0
3.5
4.0

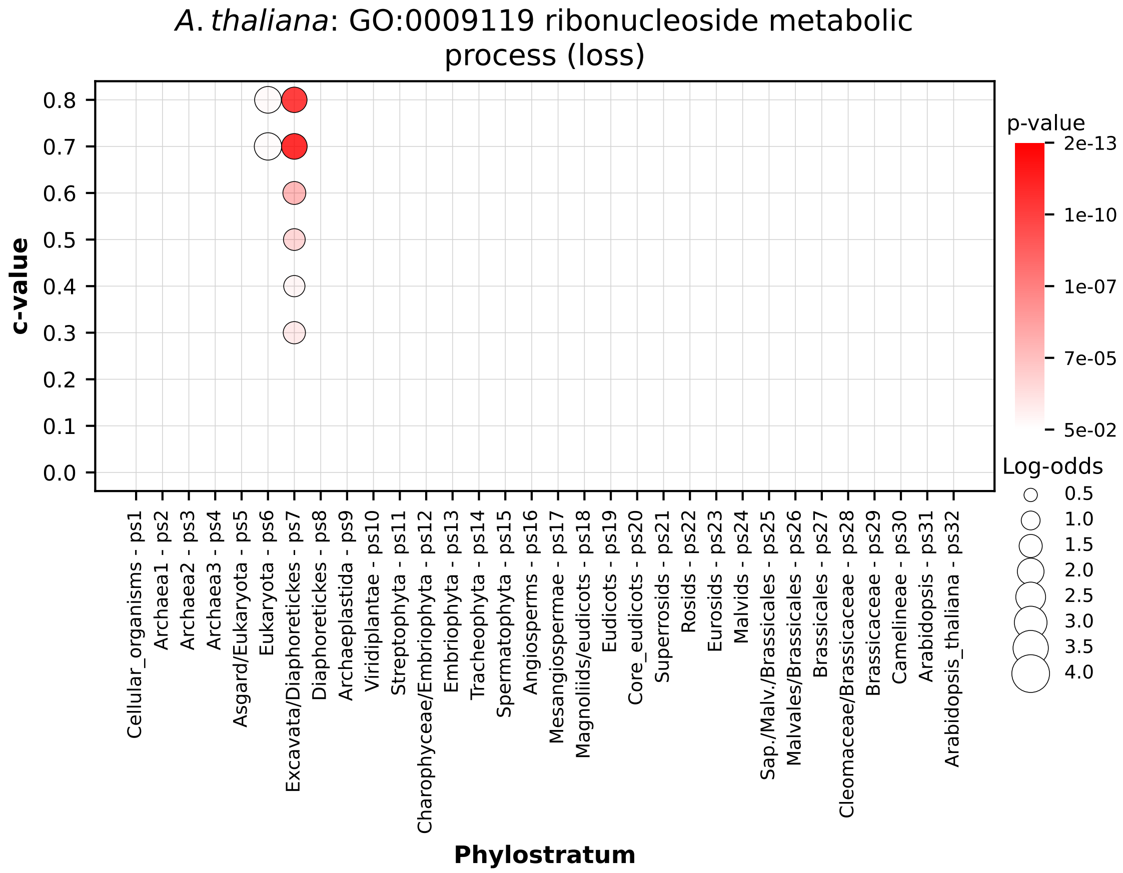
A. thaliana: GO:0009119 ribonucleoside metabolic process (loss)
c-value
0.0
0.1
0.2
0.3
0.4
0.5
0.6
0.7
0.8
Phylostratum
Cellular_organisms - ps1
Archaea1 - ps2
Archaea2 - ps3
Archaea3 - ps4
Asgard/Eukaryota - ps5
Eukaryota - ps6
Excavata/Diaphoretickes - ps7
Diaphoretickes - ps8
Archaeplastida - ps9
Viridiplantae - ps10
Streptophyta - ps11
Charophyceae/Embriophyta - ps12
Embriophyta - ps13
Tracheophyta - ps14
Spermatophyta - ps15
Angiosperms - ps16
Mesangiospermae - ps17
Magnoliids/eudicots - ps18
Eudicots - ps19
Core_eudicots - ps20
Superrosids - ps21
Rosids - ps22
Eurosids - ps23
Malvids - ps24
Sap./Malv./Brassicales - ps25
Malvales/Brassicales - ps26
Brassicales - ps27
Cleomaceae/Brassicaceae - ps28
Brassicaceae - ps29
Camelineae - ps30
Arabidopsis - ps31
Arabidopsis_thaliana - ps32
p-value
2e-13
1e-10
1e-07
7e-05
5e-02
Log-odds
0.5
1.0
1.5
2.0
2.5
3.0
3.5
4.0

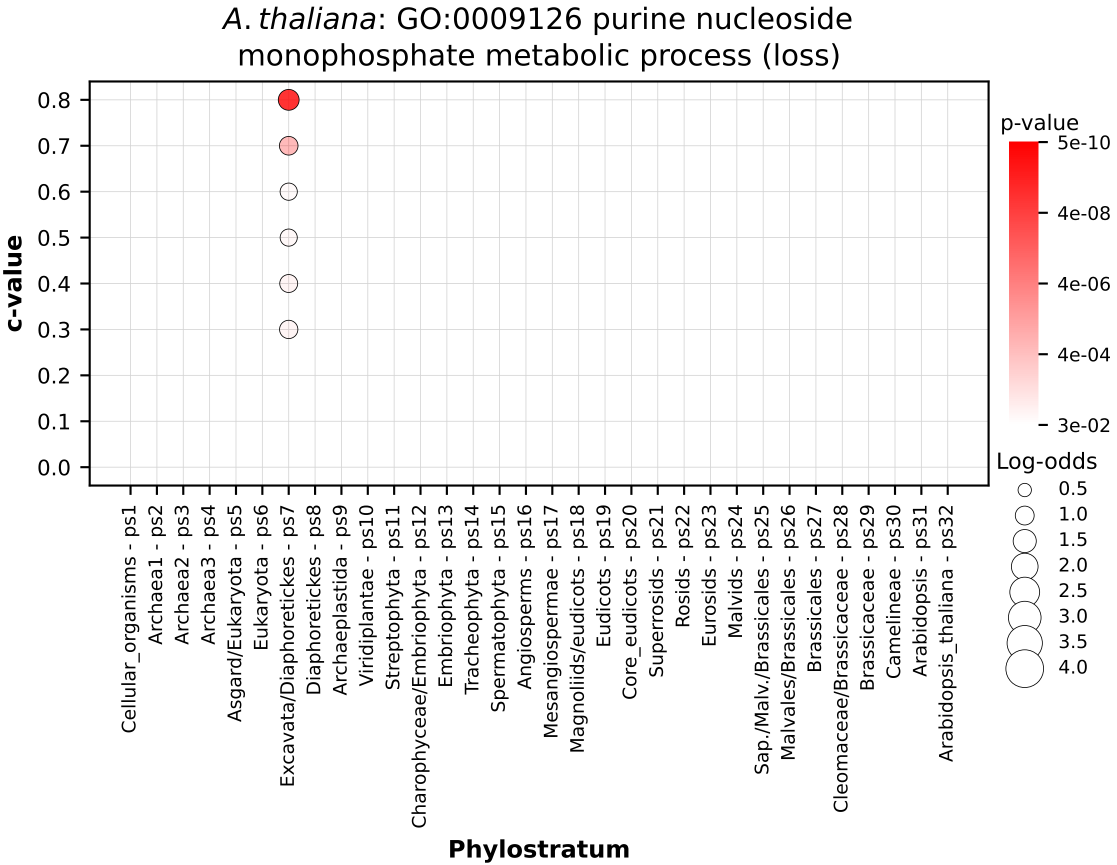

A. thaliana: GO:0009126 purine nucleoside monophosphate metabolic process (loss)
c-value
0.0
0.1
0.2
0.3
0.4
0.5
0.6
0.7
0.8
Phylostratum
Cellular_organisms - ps1
Archaea1 - ps2
Archaea2 - ps3
Archaea3 - ps4
Asgard/Eukaryota - ps5
Eukaryota - ps6
Excavata/Diaphoretickes - ps7
Diaphoretickes - ps8
Archaeplastida - ps9
Viridiplantae - ps10
Streptophyta - ps11
Charophyceae/Embriophyta - ps12
Embriophyta - ps13
Tracheophyta - ps14
Spermatophyta - ps15
Angiosperms - ps16
Mesangiospermae - ps17
Magnoliids/eudicots - ps18
Eudicots - ps19
Core_eudicots - ps20
Superrosids - ps21
Rosids - ps22
Eurosids - ps23
Malvids - ps24
Sap./Malv./Brassicales - ps25
Malvales/Brassicales - ps26
Brassicales - ps27
Cleomaceae/Brassicaceae - ps28
Brassicaceae - ps29
Camelineae - ps30
Arabidopsis - ps31
Arabidopsis_thaliana - ps32
p-value
5e-10
4e-08
4e-06
4e-04
3e-02
Log-odds
0.5
1.0
1.5
2.0
2.5
3.0
3.5
4.0

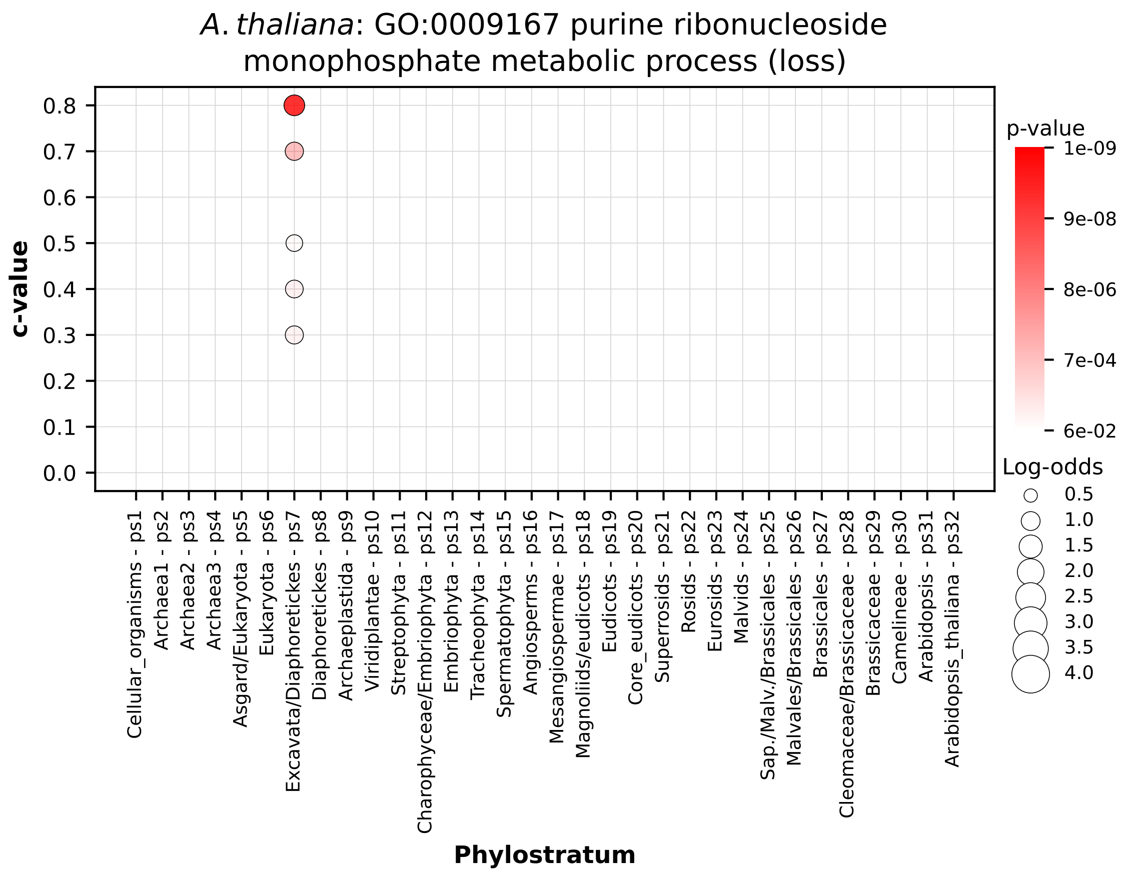
A. thaliana: GO:0009167 purine ribonucleoside monophosphate metabolic process (loss)
c-value
0.8
0.7
0.6
0.5
0.4
0.3
0.2
0.1
0.0
p-value
1e-09
9e-08
8e-06
7e-04
6e-02
Log-odds
0.5
1.0
1.5
2.0
2.5
3.0
3.5
4.0
Cellular_organisms - ps1
Archaea1 - ps2
Archaea2 - ps3
Archaea3 - ps4
Asgard/Eukaryota - ps5
Eukaryota - ps6
Excavata/Diaphoretickes - ps7
Diaphoretickes - ps8
Archaeplastida - ps9
Viridiplantae - ps10
Streptophyta - ps11
Charophyceae/Embriophyta - ps12
Embriophyta - ps13
Tracheophyta - ps14
Spermatophyta - ps15
Angiosperms - ps16
Mesangiospermae - ps17
Magnoliids/eudicots - ps18
Eudicots - ps19
Core_eudicots - ps20
Superrosids - ps21
Rosids - ps22
Eurosids - ps23
Malvids - ps24
Sap./Malv./Brassicales - ps25
Malvales/Brassicales - ps26
Brassicales - ps27
Cleomaceae/Brassicaceae - ps28
Brassicaceae - ps29
Camelineae - ps30
Arabidopsis - ps31
Arabidopsis_thaliana - ps32
Phylostratum

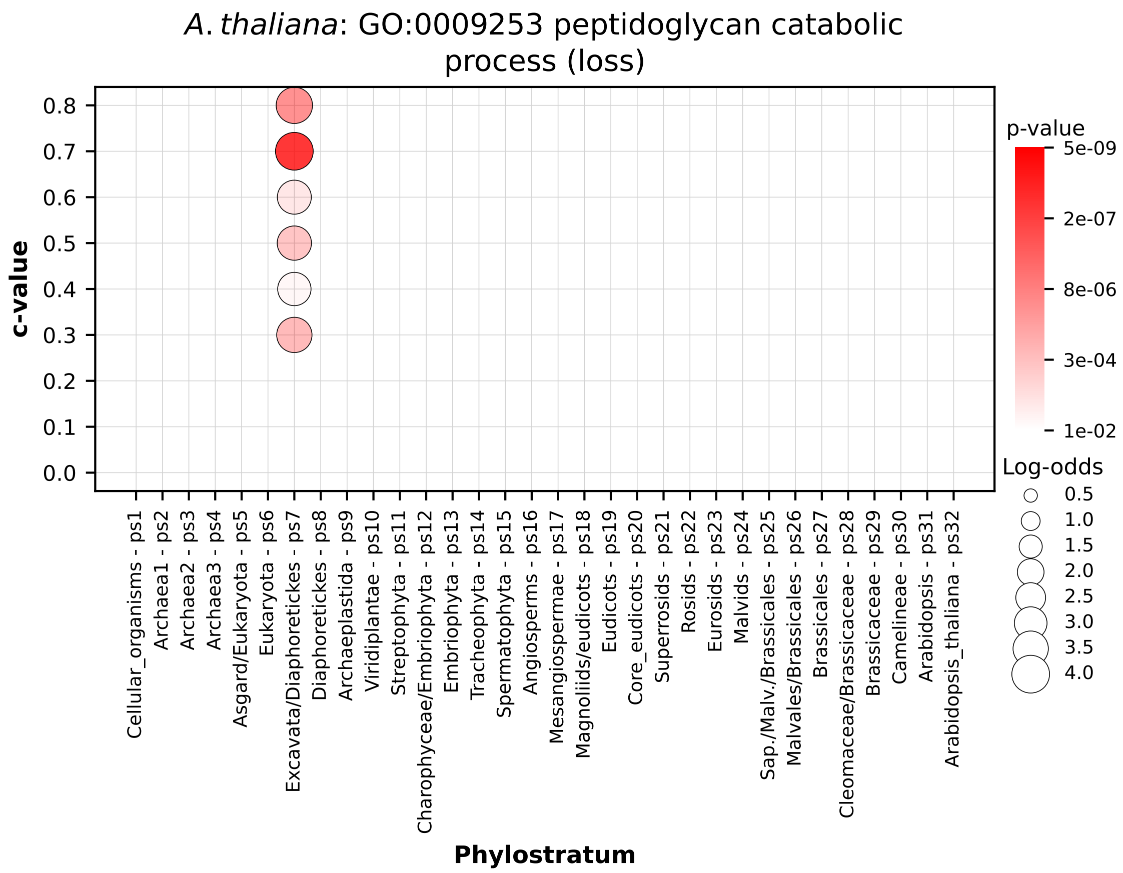

A. thaliana: GO:0009253 peptidoglycan catabolic process (loss)
c-value
0.8
0.7
0.6
0.5
0.4
0.3
0.2
0.1
0.0
p-value
5e-09
2e-07
8e-06
3e-04
1e-02
Log-odds
0.5
1.0
1.5
2.0
2.5
3.0
3.5
4.0
Cellular_organisms - ps1
Archaea1 - ps2
Archaea2 - ps3
Archaea3 - ps4
Asgard/Eukaryota - ps5
Eukaryota - ps6
Excavata/Diaphoretickes - ps7
Diaphoretickes - ps8
Archaeplastida - ps9
Viridiplantae - ps10
Streptophyta - ps11
Charophyceae/Embriophyta - ps12
Embriophyta - ps13
Tracheophyta - ps14
Spermatophyta - ps15
Angiosperms - ps16
Mesangiospermae - ps17
Magnoliids/eudicots - ps18
Eudicots - ps19
Core_eudicots - ps20
Superrosids - ps21
Rosids - ps22
Eurosids - ps23
Malvids - ps24
Sap./Malv./Brassicales - ps25
Malvales/Brassicales - ps26
Brassicales - ps27
Cleomaceae/Brassicaceae - ps28
Brassicaceae - ps29
Camelineae - ps30
Arabidopsis - ps31
Arabidopsis_thaliana - ps32
Phylostratum

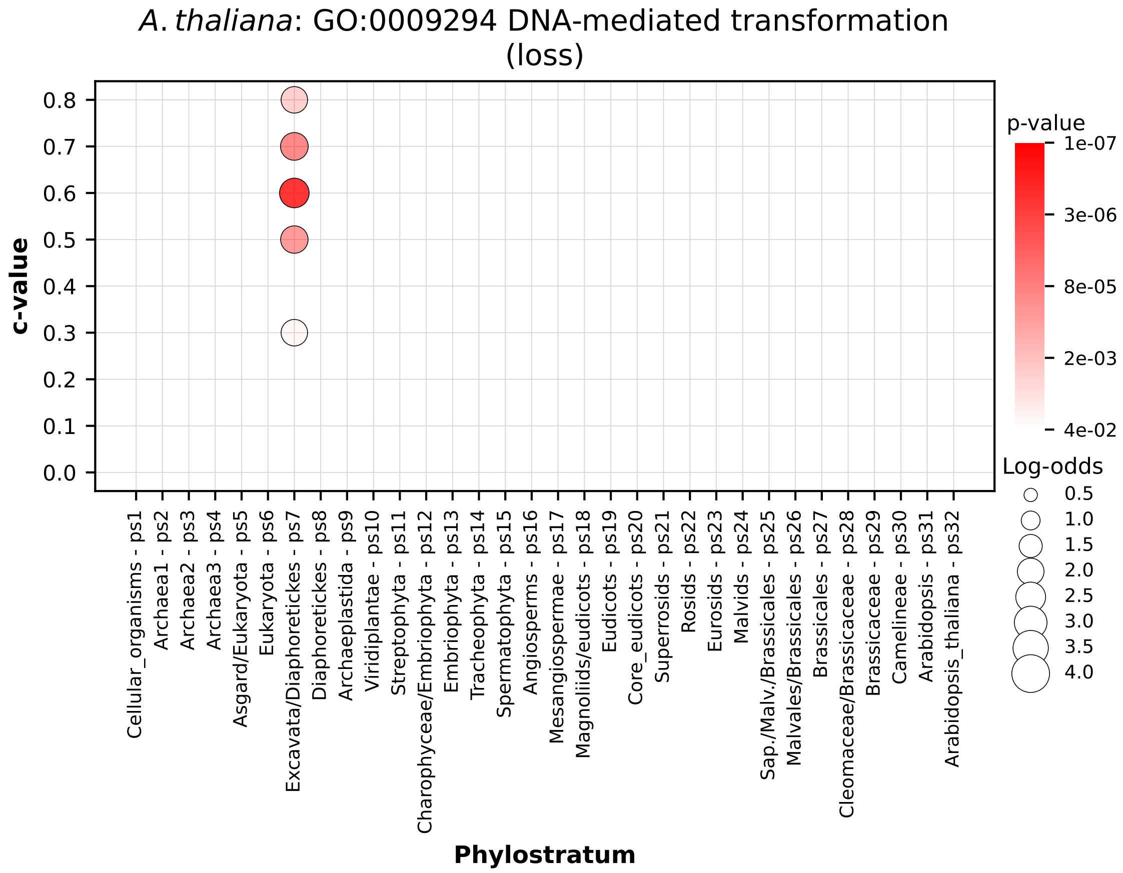
A. thaliana: GO:0009294 DNA-mediated transformation
(loss)
c-value
0.0
0.1
0.2
0.3
0.4
0.5
0.6
0.7
0.8
Phylostratum
Cellular_organisms - ps1
Archaea1 - ps2
Archaea2 - ps3
Archaea3 - ps4
Asgard/Eukaryota - ps5
Eukaryota - ps6
Excavata/Diaphoretickes - ps7
Diaphoretickes - ps8
Archaeplastida - ps9
Viridiplantae - ps10
Streptophyta - ps11
Charophyceae/Embriophyta - ps12
Embriophyta - ps13
Tracheophyta - ps14
Spermatophyta - ps15
Angiosperms - ps16
Mesangiospermae - ps17
Magnoliids/eudicots - ps18
Eudicots - ps19
Core_eudicots - ps20
Superrosids - ps21
Rosids - ps22
Eurosids - ps23
Malvids - ps24
Sap./Malv./Brassicales - ps25
Malvales/Brassicales - ps26
Brassicales - ps27
Cleomaceae/Brassicaceae - ps28
Brassicaceae - ps29
Camelineae - ps30
Arabidopsis - ps31
Arabidopsis_thaliana - ps32
p-value
1e-07
3e-06
8e-05
2e-03
4e-02
Log-odds
0.5
1.0
1.5
2.0
2.5
3.0
3.5
4.0

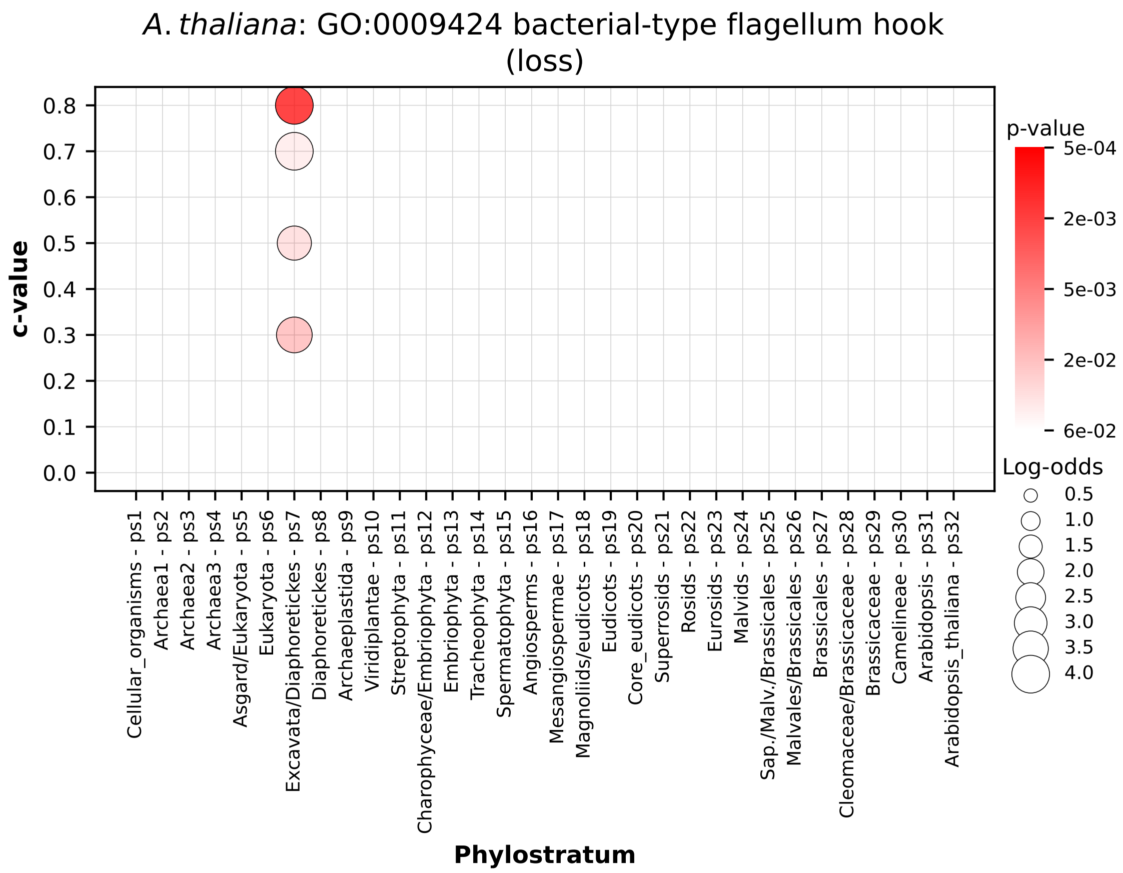

A. thaliana: GO:0009424 bacterial-type flagellum hook
(loss)
c-value
0.8
0.7
0.6
0.5
0.4
0.3
0.2
0.1
0.0
p-value
5e-04
2e-03
5e-03
2e-02
6e-02
Log-odds
0.5
1.0
1.5
2.0
2.5
3.0
3.5
4.0
Cellular_organisms - ps1
Archaea1 - ps2
Archaea2 - ps3
Archaea3 - ps4
Asgard/Eukaryota - ps5
Eukaryota - ps6
Excavata/Diaphoretickes - ps7
Diaphoretickes - ps8
Archaeplastida - ps9
Viridiplantae - ps10
Streptophyta - ps11
Charophyceae/Embriophyta - ps12
Embriophyta - ps13
Tracheophyta - ps14
Spermatophyta - ps15
Angiosperms - ps16
Mesangiospermae - ps17
Magnoliids/eudicots - ps18
Eudicots - ps19
Core_eudicots - ps20
Superrosids - ps21
Rosids - ps22
Eurosids - ps23
Malvids - ps24
Sap./Malv./Brassicales - ps25
Malvales/Brassicales - ps26
Brassicales - ps27
Cleomaceae/Brassicaceae - ps28
Brassicaceae - ps29
Camelineae - ps30
Arabidopsis - ps31
Arabidopsis_thaliana - ps32
Phylostratum

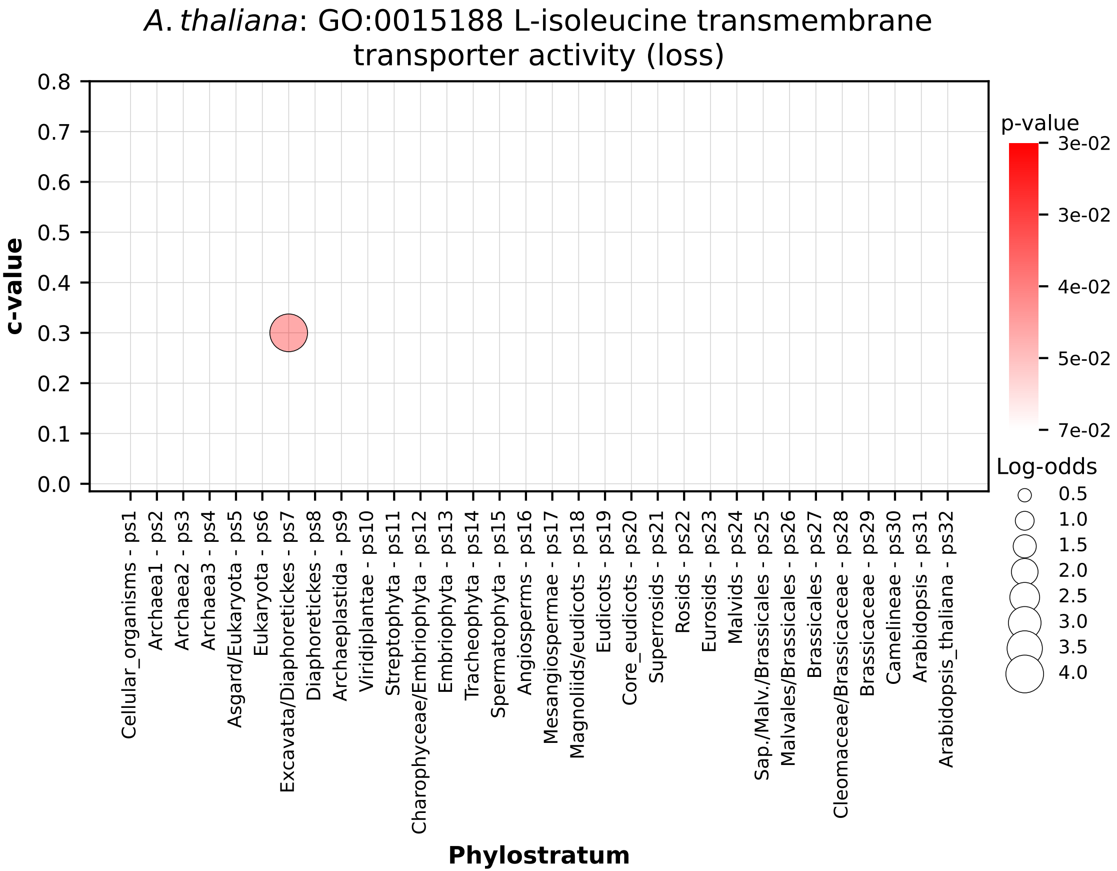

A. thaliana: GO:0015188 L-isoleucine transmembrane transporter activity (loss)
c-value
0.8
0.7
0.6
0.5
0.4
0.3
0.2
0.1
0.0
p-value
3e-02
3e-02
4e-02
5e-02
7e-02
Log-odds
0.5
1.0
1.5
2.0
2.5
3.0
3.5
4.0
Cellular_organisms - ps1
Archaea1 - ps2
Archaea2 - ps3
Archaea3 - ps4
Asgard/Eukaryota - ps5
Eukaryota - ps6
Excavata/Diaphoretickes - ps7
Diaphoretickes - ps8
Archaeplastida - ps9
Viridiplantae - ps10
Streptophyta - ps11
Charophyceae/Embriophyta - ps12
Embriophyta - ps13
Tracheophyta - ps14
Spermatophyta - ps15
Angiosperms - ps16
Mesangiospermae - ps17
Magnoliids/eudicots - ps18
Eudicots - ps19
Core_eudicots - ps20
Superrosids - ps21
Rosids - ps22
Eurosids - ps23
Malvids - ps24
Sap./Malv./Brassicales - ps25
Malvales/Brassicales - ps26
Brassicales - ps27
Cleomaceae/Brassicaceae - ps28
Brassicaceae - ps29
Camelineae - ps30
Arabidopsis - ps31
Arabidopsis_thaliana - ps32
Phylostratum

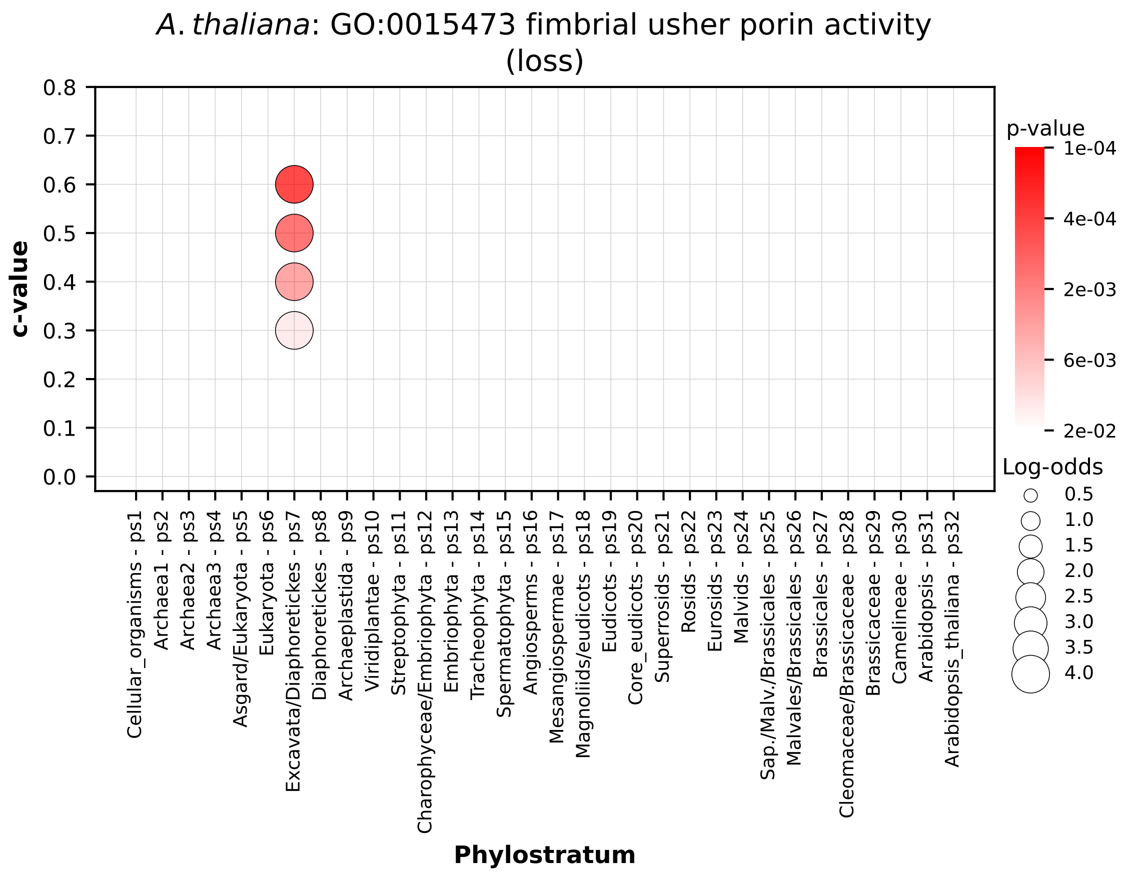

A. thaliana: GO:0015473 fimbrial usher porin activity (loss)
c-value
0.8
0.7
0.6
0.5
0.4
0.3
0.2
0.1
0.0
p-value
1e-04
4e-04
2e-03
6e-03
2e-02
Log-odds
0.5
1.0
1.5
2.0
2.5
3.0
3.5
4.0
Cellular_organisms - ps1
Archaea1 - ps2
Archaea2 - ps3
Archaea3 - ps4
Asgard/Eukaryota - ps5
Eukaryota - ps6
Excavata/Diaphoretickes - ps7
Diaphoretickes - ps8
Archaeplastida - ps9
Viridiplantae - ps10
Streptophyta - ps11
Charophyceae/Embriophyta - ps12
Embriophyta - ps13
Tracheophyta - ps14
Spermatophyta - ps15
Angiosperms - ps16
Mesangiospermae - ps17
Magnoliids/eudicots - ps18
Eudicots - ps19
Core_eudicots - ps20
Superrosids - ps21
Rosids - ps22
Eurosids - ps23
Malvids - ps24
Sap./Malv./Brassicales - ps25
Malvales/Brassicales - ps26
Brassicales - ps27
Cleomaceae/Brassicaceae - ps28
Brassicaceae - ps29
Camelineae - ps30
Arabidopsis - ps31
Arabidopsis_thaliana - ps32
Phylostratum

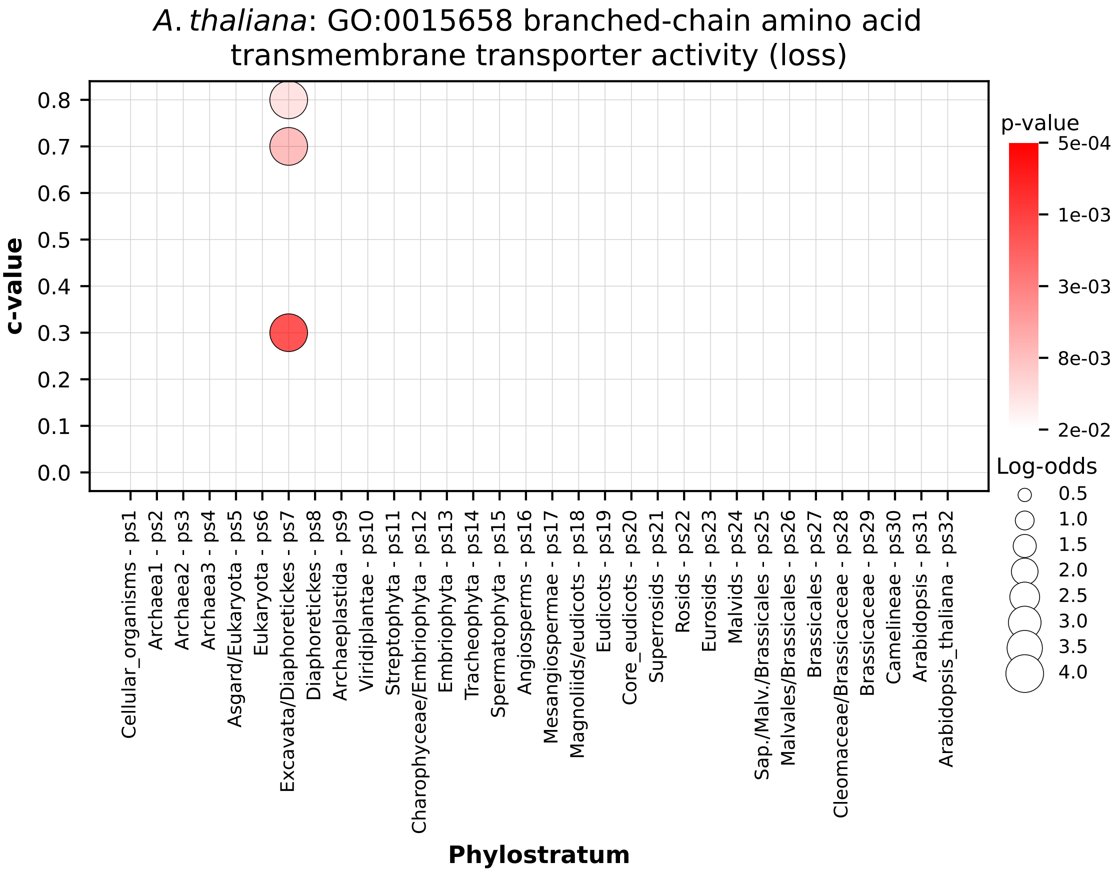

A. thaliana: GO:0015658 branched-chain amino acid transmembrane transporter activity (loss)
c-value
0.8
0.7
0.6
0.5
0.4
0.3
0.2
0.1
0.0
Phylostratum
Cellular_organisms - ps1
Archaea1 - ps2
Archaea2 - ps3
Archaea3 - ps4
Asgard/Eukaryota - ps5
Eukaryota - ps6
Excavata/Diaphoretickes - ps7
Diaphoretickes - ps8
Archaeplastida - ps9
Viridiplantae - ps10
Streptophyta - ps11
Charophyceae/Embriophyta - ps12
Embriophyta - ps13
Tracheophyta - ps14
Spermatophyta - ps15
Angiosperms - ps16
Mesangiospermae - ps17
Magnoliids/eudicots - ps18
Eudicots - ps19
Core_eudicots - ps20
Superrosids - ps21
Rosids - ps22
Eurosids - ps23
Malvids - ps24
Sap./Malv./Brassicales - ps25
Malvales/Brassicales - ps26
Brassicales - ps27
Cleomaceae/Brassicaceae - ps28
Brassicaceae - ps29
Camelineae - ps30
Arabidopsis - ps31
Arabidopsis_thaliana - ps32
p-value
5e-04
1e-03
3e-03
8e-03
2e-02
Log-odds
0.5
1.0
1.5
2.0
2.5
3.0
3.5
4.0

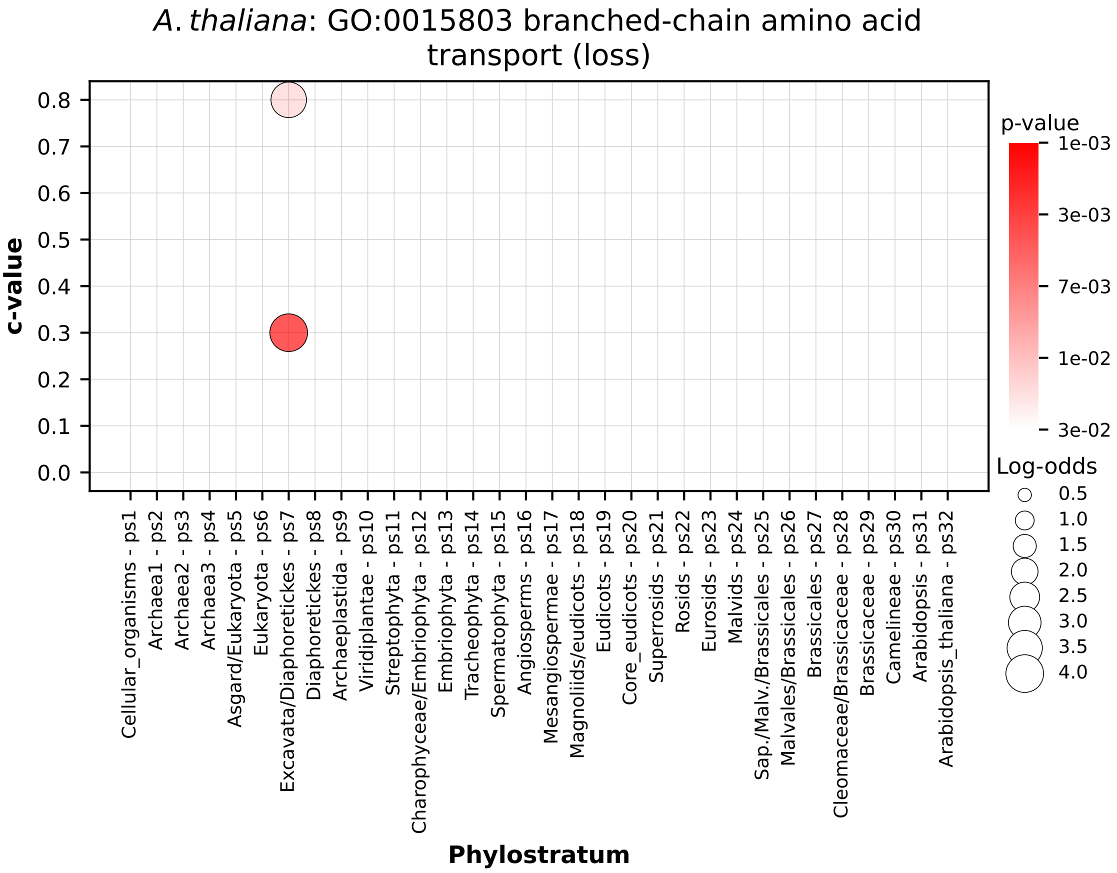

A. thaliana: GO:0015803 branched-chain amino acid transport (loss)
c-value
0.0
0.1
0.2
0.3
0.4
0.5
0.6
0.7
0.8
p-value
1e-03
3e-03
7e-03
1e-02
3e-02
Log-odds
0.5
1.0
1.5
2.0
2.5
3.0
3.5
4.0
Cellular_organisms - ps1
Archaea1 - ps2
Archaea2 - ps3
Archaea3 - ps4
Asgard/Eukaryota - ps5
Eukaryota - ps6
Excavata/Diaphoretickes - ps7
Diaphoretickes - ps8
Archaeplastida - ps9
Viridiplantae - ps10
Streptophyta - ps11
Charophyceae/Embriophyta - ps12
Embriophyta - ps13
Tracheophyta - ps14
Spermatophyta - ps15
Angiosperms - ps16
Mesangiospermae - ps17
Magnoliids/eudicots - ps18
Eudicots - ps19
Core_eudicots - ps20
Superrosids - ps21
Rosids - ps22
Eurosids - ps23
Malvids - ps24
Sap./Malv./Brassicales - ps25
Malvales/Brassicales - ps26
Brassicales - ps27
Cleomaceae/Brassicaceae - ps28
Brassicaceae - ps29
Camelineae - ps30
Arabidopsis - ps31
Arabidopsis_thaliana - ps32
Phylostratum

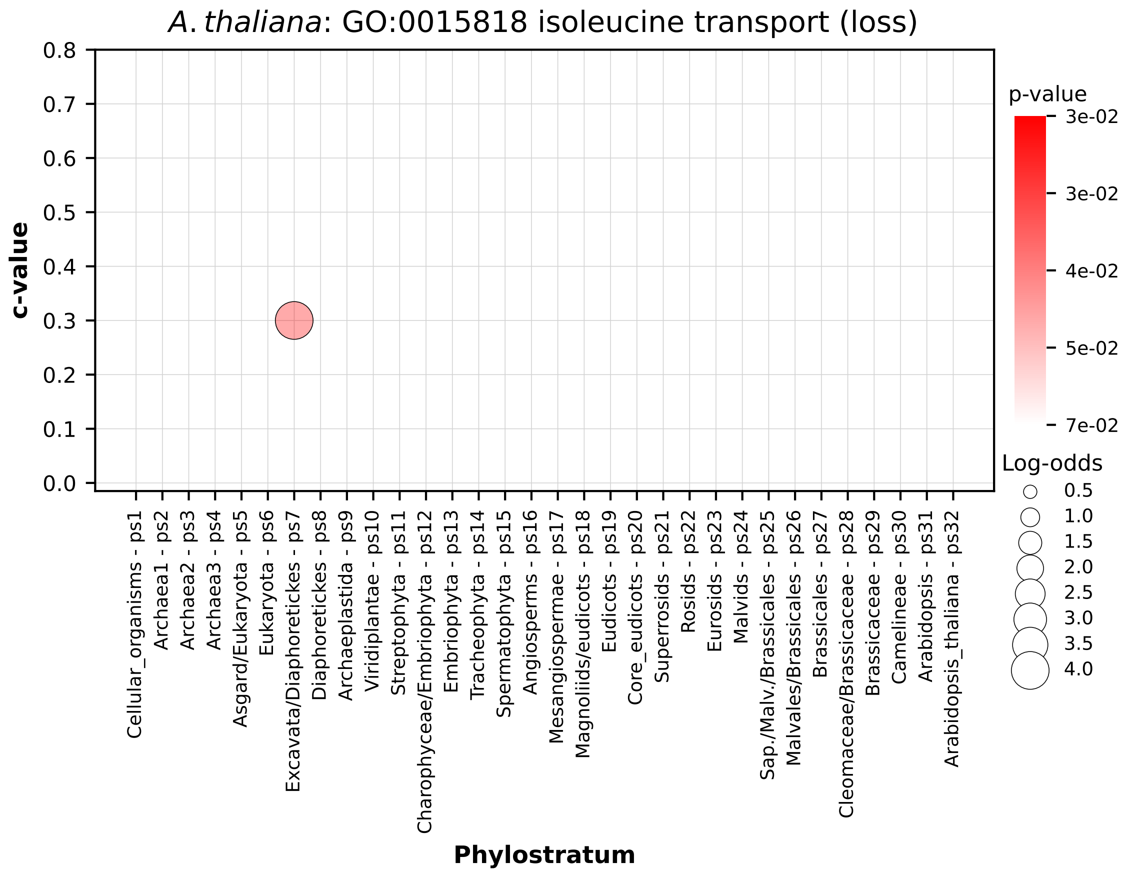
A. thaliana: GO:0015818 isoleucine transport (loss)
c-value
0.8
0.7
0.6
0.5
0.4
0.3
0.2
0.1
0.0
Phylostratum
Cellular_organisms - ps1
Archaea1 - ps2
Archaea2 - ps3
Archaea3 - ps4
Asgard/Eukaryota - ps5
Eukaryota - ps6
Excavata/Diaphoretickes - ps7
Diaphoretickes - ps8
Archaeplastida - ps9
Viridiplantae - ps10
Streptophyta - ps11
Charophyceae/Embriophyta - ps12
Embriophyta - ps13
Tracheophyta - ps14
Spermatophyta - ps15
Angiosperms - ps16
Mesangiospermae - ps17
Magnoliids/eudicots - ps18
Eudicots - ps19
Core_eudicots - ps20
Superrosids - ps21
Rosids - ps22
Eurosids - ps23
Malvids - ps24
Sap./Malv./Brassicales - ps25
Malvales/Brassicales - ps26
Brassicales - ps27
Cleomaceae/Brassicaceae - ps28
Brassicaceae - ps29
Camelineae - ps30
Arabidopsis - ps31
Arabidopsis_thaliana - ps32
p-value
3e-02
3e-02
4e-02
5e-02
7e-02
Log-odds
0.5
1.0
1.5
2.0
2.5
3.0
3.5
4.0

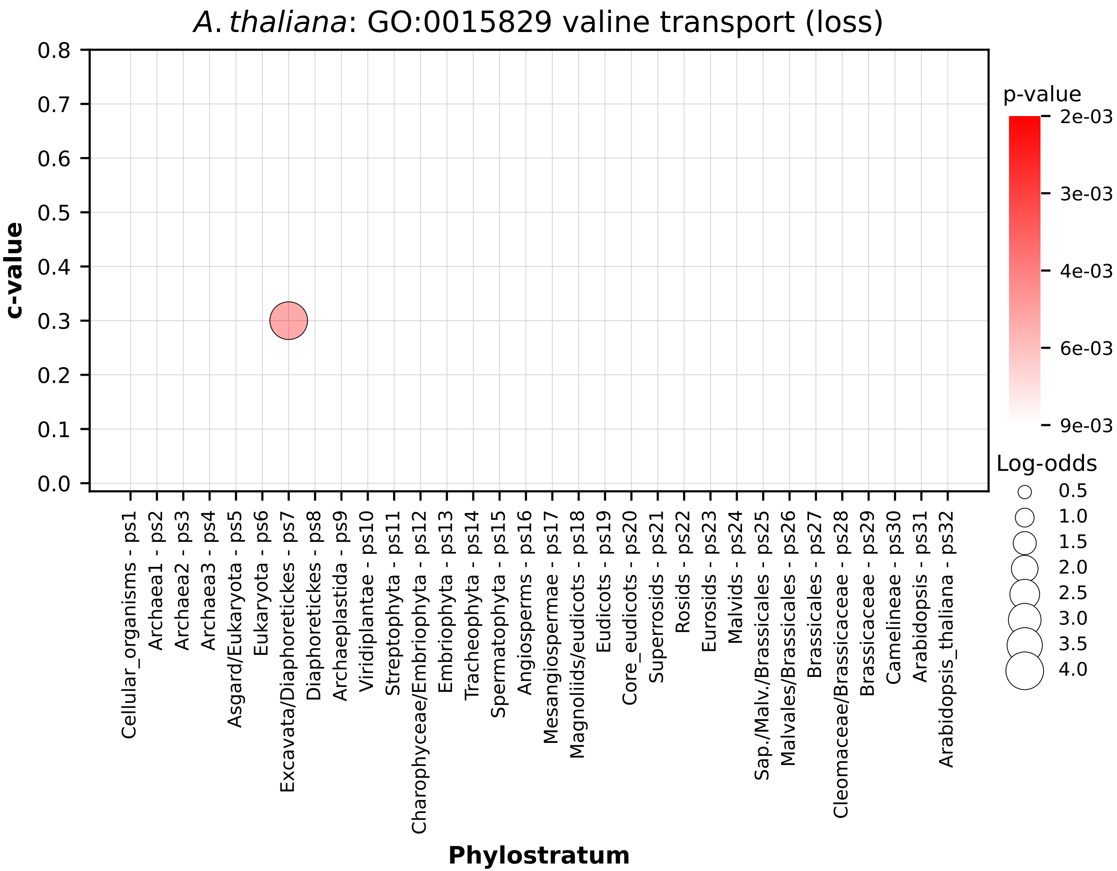

A. thaliana: GO:0015829 valine transport (loss)
c-value
0.0
0.1
0.2
0.3
0.4
0.5
0.6
0.7
0.8
Phylostratum
Cellular_organisms - ps1
Archaea1 - ps2
Archaea2 - ps3
Archaea3 - ps4
Asgard/Eukaryota - ps5
Eukaryota - ps6
Excavata/Diaphoretickes - ps7
Diaphoretickes - ps8
Archaeplastida - ps9
Viridiplantae - ps10
Streptophyta - ps11
Charophyceae/Embriophyta - ps12
Embriophyta - ps13
Tracheophyta - ps14
Spermatophyta - ps15
Angiosperms - ps16
Mesangiospermae - ps17
Magnoliids/eudicots - ps18
Eudicots - ps19
Core_eudicots - ps20
Superrosids - ps21
Rosids - ps22
Eurosids - ps23
Malvids - ps24
Sap./Malv./Brassicales - ps25
Malvales/Brassicales - ps26
Brassicales - ps27
Cleomaceae/Brassicaceae - ps28
Brassicaceae - ps29
Camelineae - ps30
Arabidopsis - ps31
Arabidopsis_thaliana - ps32
p-value
2e-03
3e-03
4e-03
6e-03
9e-03
Log-odds
0.5
1.0
1.5
2.0
2.5
3.0
3.5
4.0

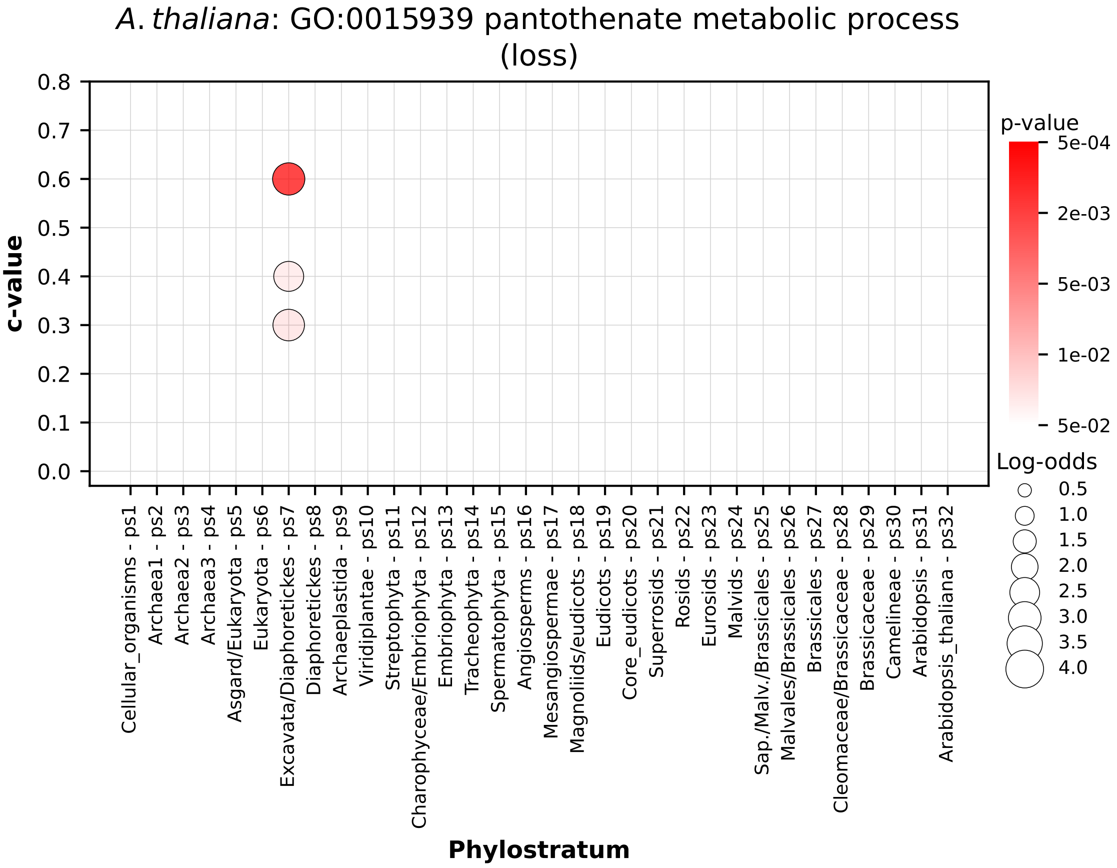
A. thaliana: GO:0015939 pantothenate metabolic process
(loss)
c-value
0.8
0.7
0.6
0.5
0.4
0.3
0.2
0.1
0.0
Cellular_organisms - ps1
Archaea1 - ps2
Archaea2 - ps3
Archaea3 - ps4
Asgard/Eukaryota - ps5
Eukaryota - ps6
Excavata/Diaphoretickes - ps7
Diaphoretickes - ps8
Archaeplastida - ps9
Viridiplantae - ps10
Streptophyta - ps11
Charophyceae/Embriophyta - ps12
Embriophyta - ps13
Tracheophyta - ps14
Spermatophyta - ps15
Angiosperms - ps16
Mesangiospermae - ps17
Magnoliids/eudicots - ps18
Eudicots - ps19
Core_eudicots - ps20
Superrosids - ps21
Rosids - ps22
Eurosids - ps23
Malvids - ps24
Sap./Malv./Brassicales - ps25
Malvales/Brassicales - ps26
Brassicales - ps27
Cleomaceae/Brassicaceae - ps28
Brassicaceae - ps29
Camelineae - ps30
Arabidopsis - ps31
Arabidopsis_thaliana - ps32
Phylostratum
p-value
5e-04
2e-03
5e-03
1e-02
5e-02
Log-odds
0.5
1.0
1.5
2.0
2.5
3.0
3.5
4.0

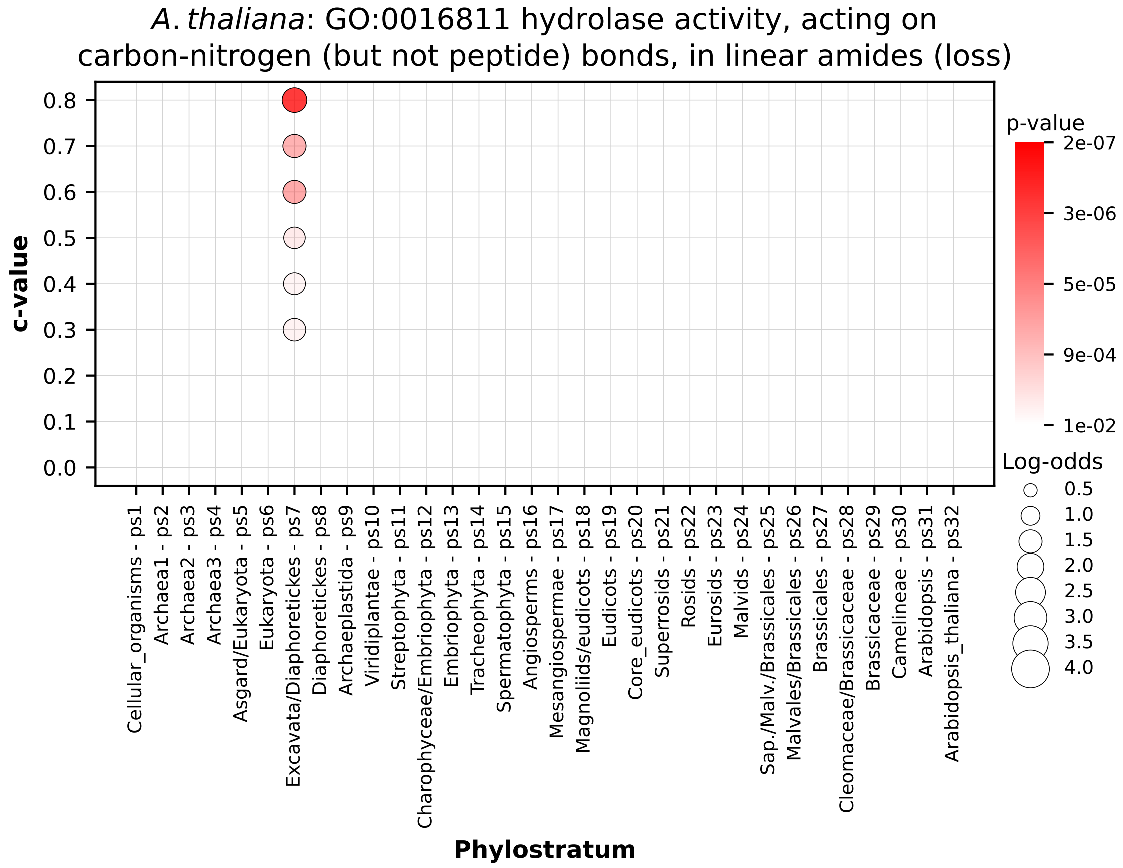

A. thaliana: GO:0016811 hydrolase activity, acting on carbon-nitrogen (but not peptide) bonds, in linear amides (loss)
c-value
0.8
0.7
0.6
0.5
0.4
0.3
0.2
0.1
0.0
Phylostratum
Cellular_organisms - ps1
Archaea1 - ps2
Archaea2 - ps3
Archaea3 - ps4
Asgard/Eukaryota - ps5
Eukaryota - ps6
Excavata/Diaphoretickes - ps7
Diaphoretickes - ps8
Archaeplastida - ps9
Viridiplantae - ps10
Streptophyta - ps11
Charophyceae/Embriophyta - ps12
Embriophyta - ps13
Tracheophyta - ps14
Spermatophyta - ps15
Angiosperms - ps16
Mesangiospermae - ps17
Magnoliids/eudicots - ps18
Eudicots - ps19
Core_eudicots - ps20
Superrosids - ps21
Rosids - ps22
Eurosids - ps23
Malvids - ps24
Sap./Malv./Brassicales - ps25
Malvales/Brassicales - ps26
Brassicales - ps27
Cleomaceae/Brassicaceae - ps28
Brassicaceae - ps29
Camelineae - ps30
Arabidopsis - ps31
Arabidopsis_thaliana - ps32
p-value
2e-07
3e-06
5e-05
9e-04
1e-02
Log-odds
0.5
1.0
1.5
2.0
2.5
3.0
3.5
4.0

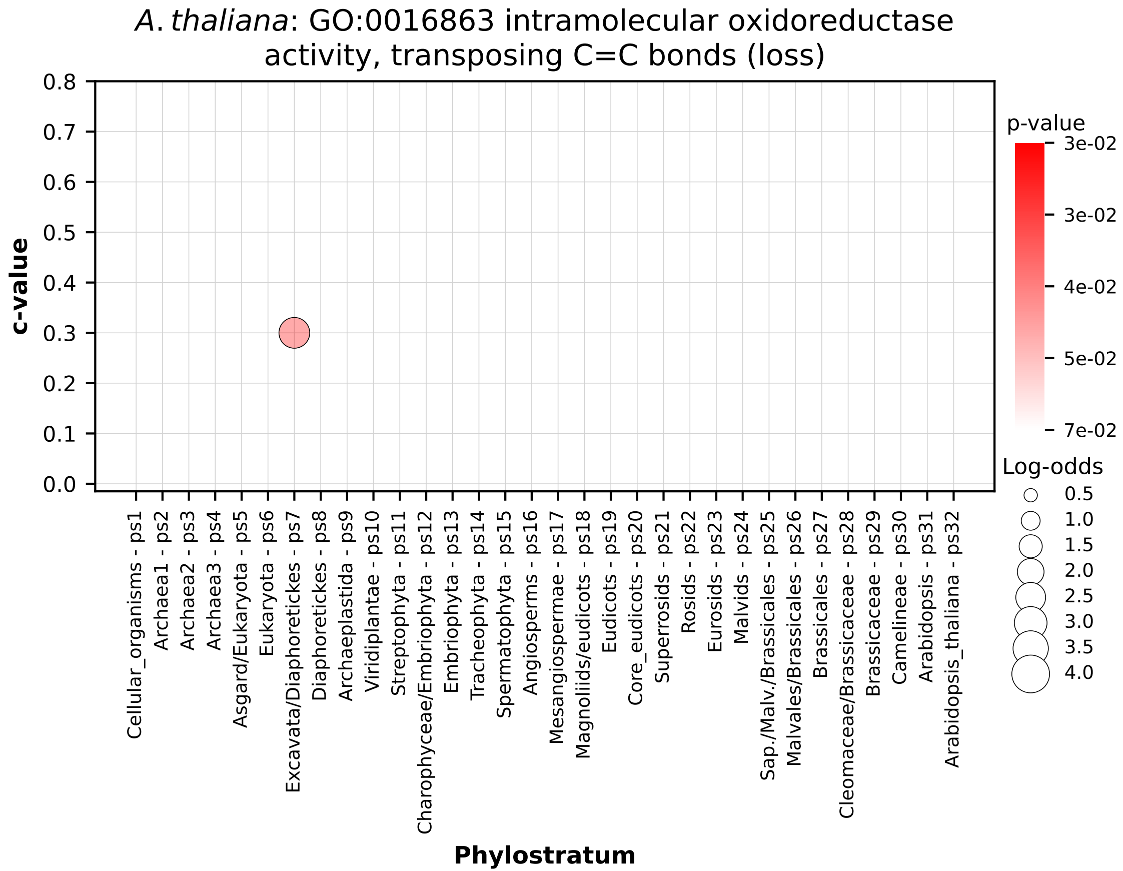

A. thaliana: GO:0016863 intramolecular oxidoreductase activity, transposing C=C bonds (loss)
c-value
0.8
0.7
0.6
0.5
0.4
0.3
0.2
0.1
0.0
Phylostratum
Cellular_organisms - ps1
Archaea1 - ps2
Archaea2 - ps3
Archaea3 - ps4
Asgard/Eukaryota - ps5
Eukaryota - ps6
Excavata/Diaphoretickes - ps7
Diaphoretickes - ps8
Archaeplastida - ps9
Viridiplantae - ps10
Streptophyta - ps11
Charophyceae/Embriophyta - ps12
Embriophyta - ps13
Tracheophyta - ps14
Spermatophyta - ps15
Angiosperms - ps16
Mesangiospermae - ps17
Magnoliids/eudicots - ps18
Eudicots - ps19
Core_eudicots - ps20
Superrosids - ps21
Rosids - ps22
Eurosids - ps23
Malvids - ps24
Sap./Malv./Brassicales - ps25
Malvales/Brassicales - ps26
Brassicales - ps27
Cleomaceae/Brassicaceae - ps28
Brassicaceae - ps29
Camelineae - ps30
Arabidopsis - ps31
Arabidopsis_thaliana - ps32
p-value
3e-02
3e-02
4e-02
5e-02
7e-02
Log-odds
0.5
1.0
1.5
2.0
2.5
3.0
3.5
4.0

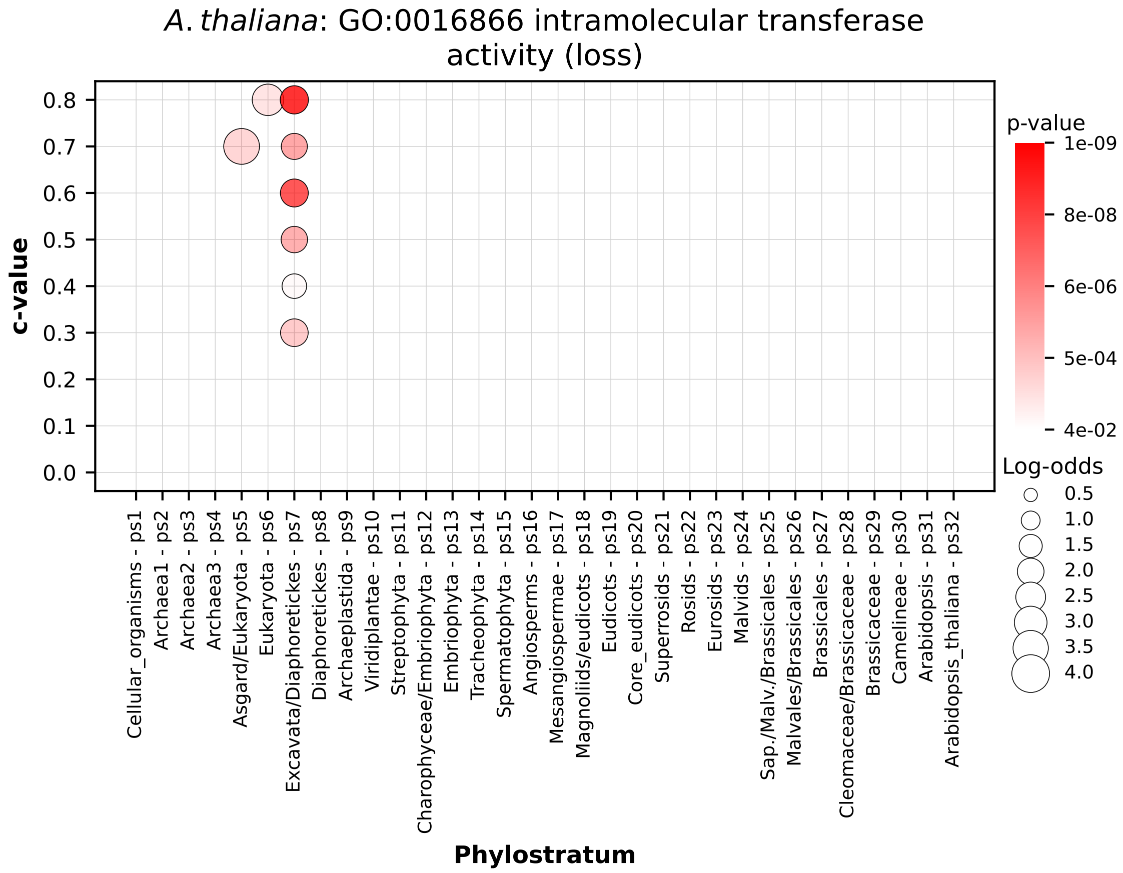

A. thaliana: GO:0016866 intramolecular transferase activity (loss)
c-value
0.0
0.1
0.2
0.3
0.4
0.5
0.6
0.7
0.8
Phylostratum
Cellular_organisms - ps1
Archaea1 - ps2
Archaea2 - ps3
Archaea3 - ps4
Asgard/Eukaryota - ps5
Eukaryota - ps6
Excavata/Diaphoretickes - ps7
Diaphoretickes - ps8
Archaeplastida - ps9
Viridiplantae - ps10
Streptophyta - ps11
Charophyceae/Embriophyta - ps12
Embriophyta - ps13
Tracheophyta - ps14
Spermatophyta - ps15
Angiosperms - ps16
Mesangiospermae - ps17
Magnoliids/eudicots - ps18
Eudicots - ps19
Core_eudicots - ps20
Superrosids - ps21
Rosids - ps22
Eurosids - ps23
Malvids - ps24
Sap./Malv./Brassicales - ps25
Malvales/Brassicales - ps26
Brassicales - ps27
Cleomaceae/Brassicaceae - ps28
Brassicaceae - ps29
Camelineae - ps30
Arabidopsis - ps31
Arabidopsis_thaliana - ps32
p-value
1e-09
8e-08
6e-06
5e-04
4e-02
Log-odds
0.5
1.0
1.5
2.0
2.5
3.0
3.5
4.0

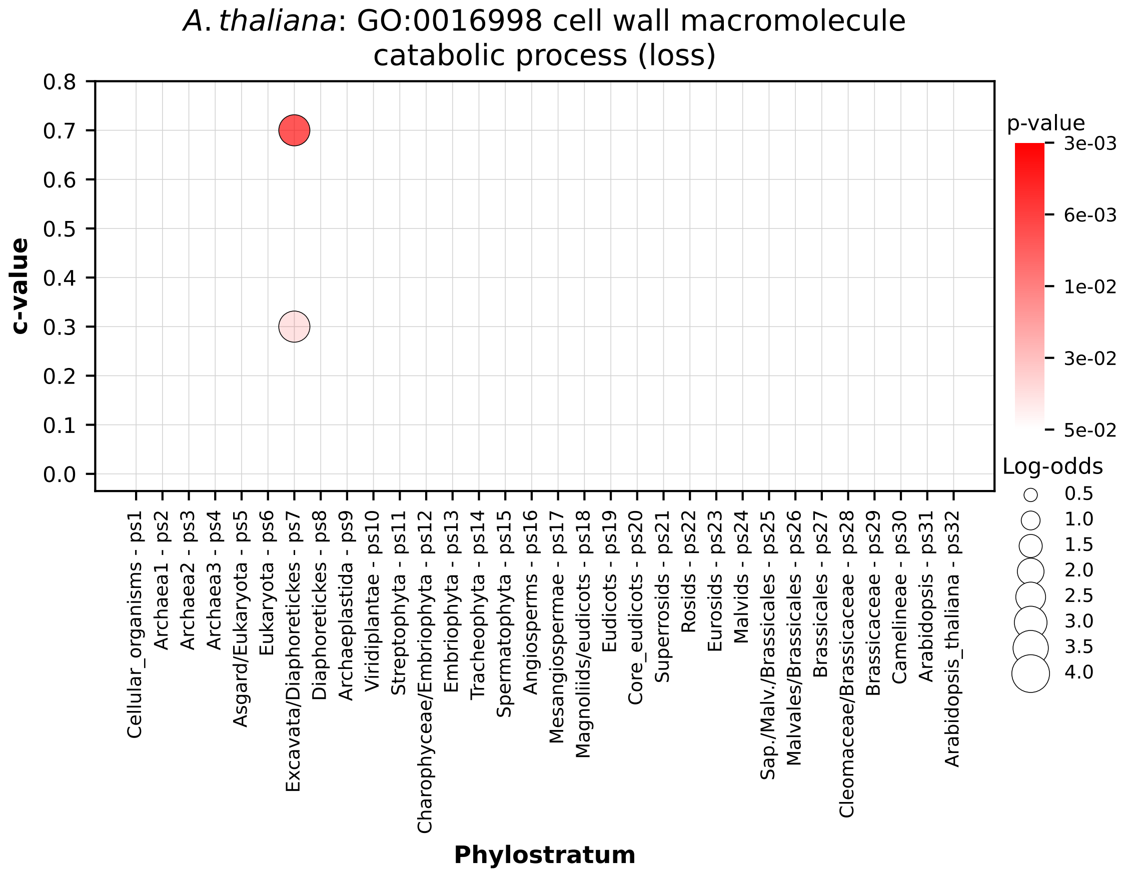

A. thaliana: GO:0016998 cell wall macromolecule catabolic process (loss)
c-value
0.8
0.7
0.6
0.5
0.4
0.3
0.2
0.1
0.0
p-value
3e-03
6e-03
1e-02
3e-02
5e-02
Log-odds
0.5
1.0
1.5
2.0
2.5
3.0
3.5
4.0
Cellular_organisms - ps1
Archaea1 - ps2
Archaea2 - ps3
Archaea3 - ps4
Asgard/Eukaryota - ps5
Eukaryota - ps6
Excavata/Diaphoretickes - ps7
Diaphoretickes - ps8
Archaeplastida - ps9
Viridiplantae - ps10
Streptophyta - ps11
Charophyceae/Embriophyta - ps12
Embriophyta - ps13
Tracheophyta - ps14
Spermatophyta - ps15
Angiosperms - ps16
Mesangiospermae - ps17
Magnoliids/eudicots - ps18
Eudicots - ps19
Core_eudicots - ps20
Superrosids - ps21
Rosids - ps22
Eurosids - ps23
Malvids - ps24
Sap./Malv./Brassicales - ps25
Malvales/Brassicales - ps26
Brassicales - ps27
Cleomaceae/Brassicaceae - ps28
Brassicaceae - ps29
Camelineae - ps30
Arabidopsis - ps31
Arabidopsis_thaliana - ps32
Phylostratum

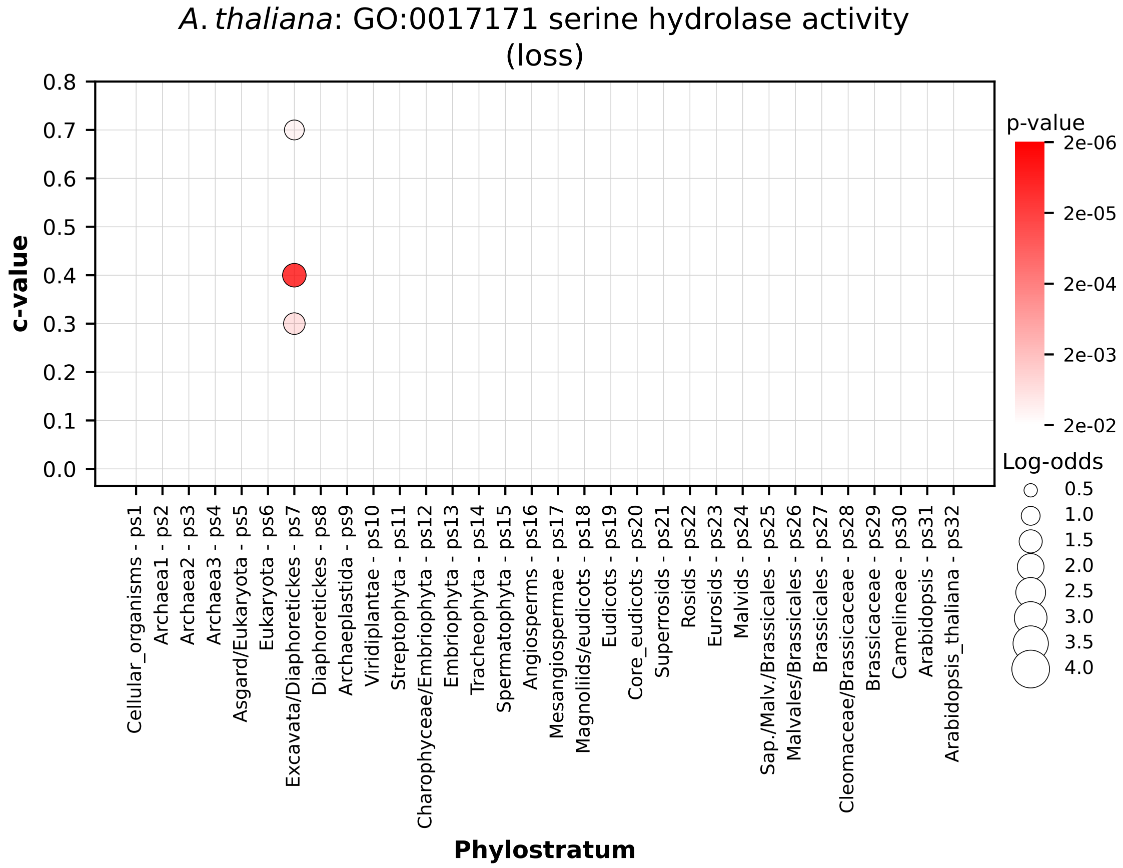

A. thaliana: GO:0017171 serine hydrolase activity
(loss)
c-value
0.8
0.7
0.6
0.5
0.4
0.3
0.2
0.1
0.0
p-value
2e-06
2e-05
2e-04
2e-03
2e-02
Log-odds
0.5
1.0
1.5
2.0
2.5
3.0
3.5
4.0
Cellular_organisms - ps1
Archaea1 - ps2
Archaea2 - ps3
Archaea3 - ps4
Asgard/Eukaryota - ps5
Eukaryota - ps6
Excavata/Diaphoretickes - ps7
Diaphoretickes - ps8
Archaeplastida - ps9
Viridiplantae - ps10
Streptophyta - ps11
Charophyceae/Embriophyta - ps12
Embriophyta - ps13
Tracheophyta - ps14
Spermatophyta - ps15
Angiosperms - ps16
Mesangiospermae - ps17
Magnoliids/eudicots - ps18
Eudicots - ps19
Core_eudicots - ps20
Superrosids - ps21
Rosids - ps22
Eurosids - ps23
Malvids - ps24
Sap./Malv./Brassicales - ps25
Malvales/Brassicales - ps26
Brassicales - ps27
Cleomaceae/Brassicaceae - ps28
Brassicaceae - ps29
Camelineae - ps30
Arabidopsis - ps31
Arabidopsis_thaliana - ps32
Phylostratum

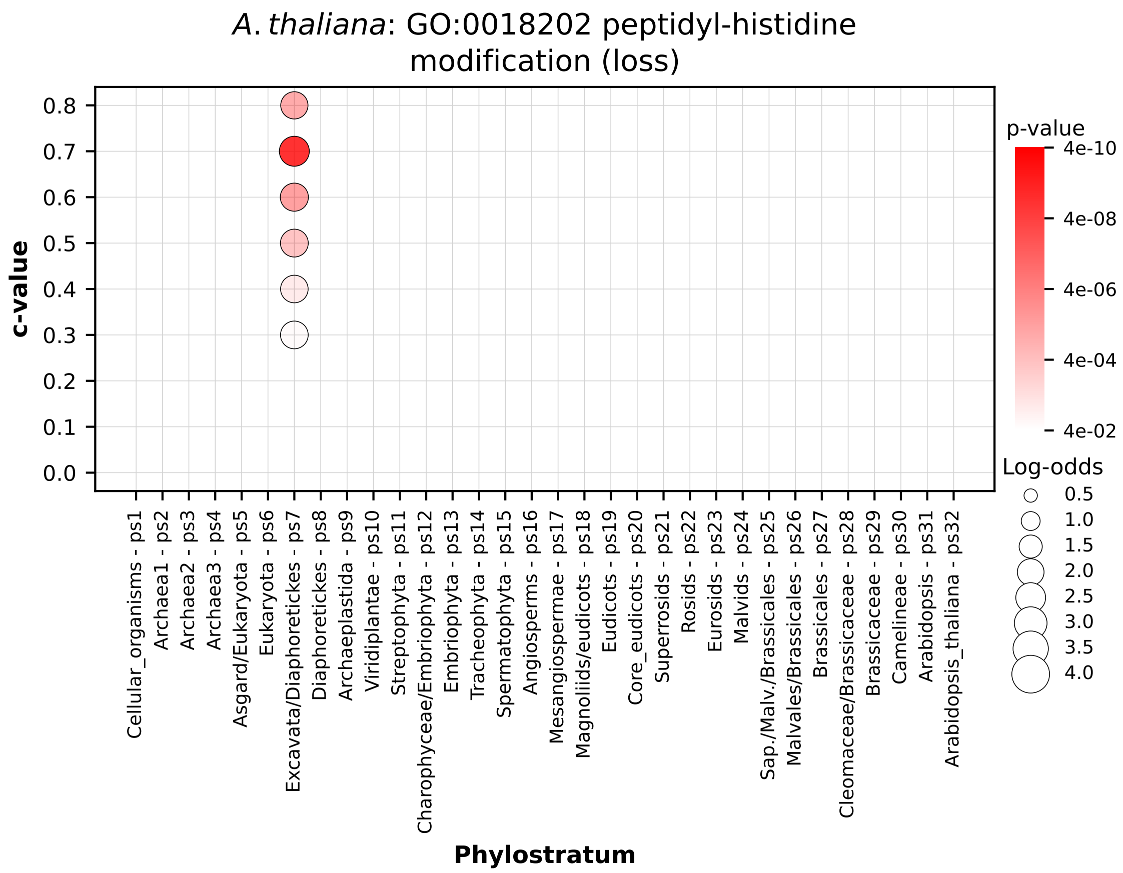

A. thaliana: GO:0018202 peptidyl-histidine modification (loss)
c-value
0.0
0.1
0.2
0.3
0.4
0.5
0.6
0.7
0.8
p-value
4e-10
4e-08
4e-06
4e-04
4e-02
Log-odds
0.5
1.0
1.5
2.0
2.5
3.0
3.5
4.0
Cellular_organisms - ps1
Archaea1 - ps2
Archaea2 - ps3
Archaea3 - ps4
Asgard/Eukaryota - ps5
Eukaryota - ps6
Excavata/Diaphoretickes - ps7
Diaphoretickes - ps8
Archaeplastida - ps9
Viridiplantae - ps10
Streptophyta - ps11
Charophyceae/Embriophyta - ps12
Embriophyta - ps13
Tracheophyta - ps14
Spermatophyta - ps15
Angiosperms - ps16
Mesangiospermae - ps17
Magnoliids/eudicots - ps18
Eudicots - ps19
Core_eudicots - ps20
Superrosids - ps21
Rosids - ps22
Eurosids - ps23
Malvids - ps24
Sap./Malv./Brassicales - ps25
Malvales/Brassicales - ps26
Brassicales - ps27
Cleomaceae/Brassicaceae - ps28
Brassicaceae - ps29
Camelineae - ps30
Arabidopsis - ps31
Arabidopsis_thaliana - ps32
Phylostratum

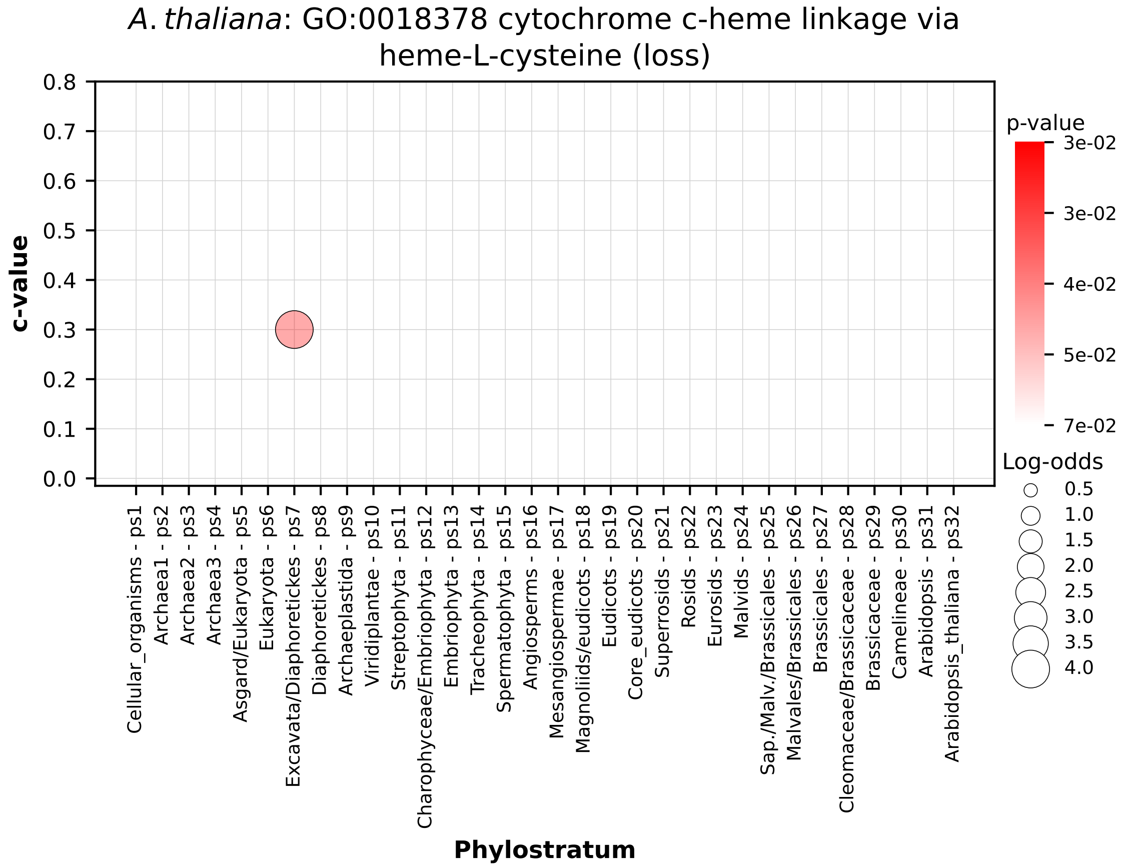

A. thaliana: GO:0018378 cytochrome c-heme linkage via heme-L-cysteine (loss)
c-value
0.8
0.7
0.6
0.5
0.4
0.3
0.2
0.1
0.0
Phylostratum
Cellular_organisms - ps1
Archaea1 - ps2
Archaea2 - ps3
Archaea3 - ps4
Asgard/Eukaryota - ps5
Eukaryota - ps6
Excavata/Diaphoretickes - ps7
Diaphoretickes - ps8
Archaeplastida - ps9
Viridiplantae - ps10
Streptophyta - ps11
Charophyceae/Embriophyta - ps12
Embriophyta - ps13
Tracheophyta - ps14
Spermatophyta - ps15
Angiosperms - ps16
Mesangiospermae - ps17
Magnoliids/eudicots - ps18
Eudicots - ps19
Core_eudicots - ps20
Superrosids - ps21
Rosids - ps22
Eurosids - ps23
Malvids - ps24
Sap./Malv./Brassicales - ps25
Malvales/Brassicales - ps26
Brassicales - ps27
Cleomaceae/Brassicaceae - ps28
Brassicaceae - ps29
Camelineae - ps30
Arabidopsis - ps31
Arabidopsis_thaliana - ps32
p-value
3e-02
3e-02
4e-02
5e-02
7e-02
Log-odds
0.5
1.0
1.5
2.0
2.5
3.0
3.5
4.0

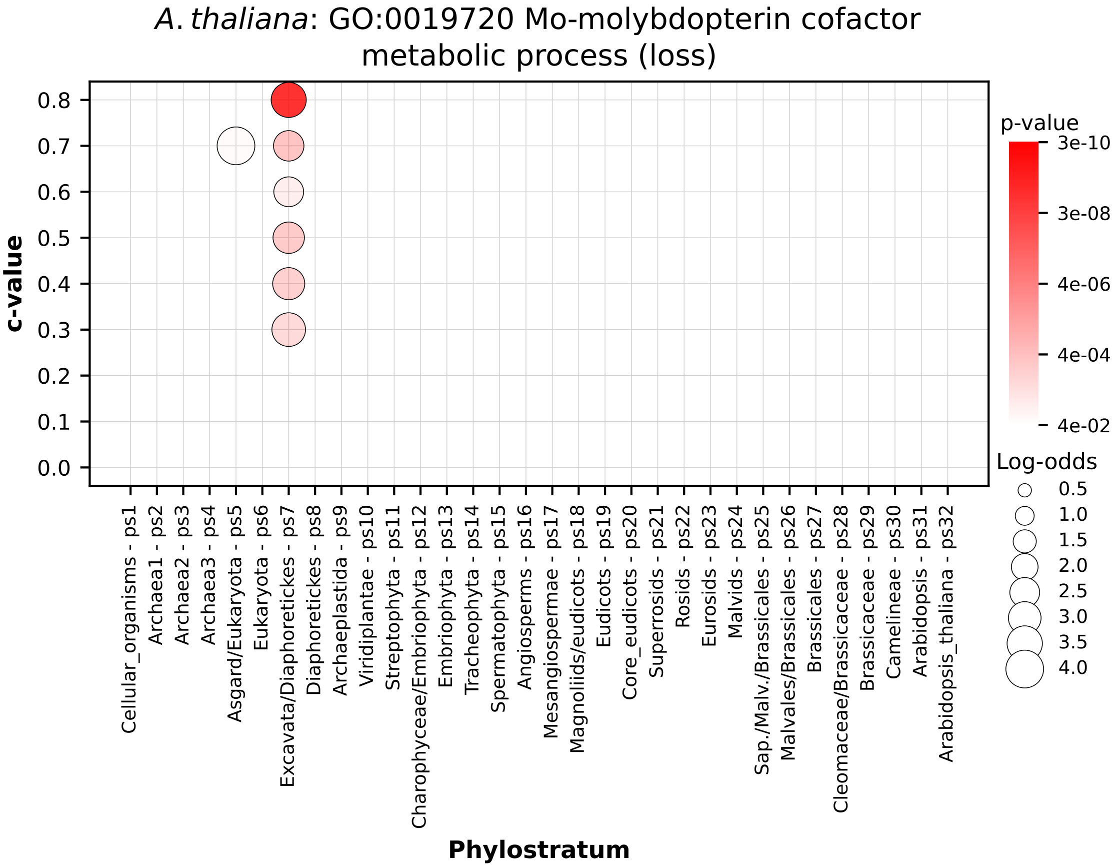

A. thaliana: GO:0019720 Mo-molybdopterin cofactor metabolic process (loss)
c-value
0.8
0.7
0.6
0.5
0.4
0.3
0.2
0.1
0.0
Phylostratum
Cellular_organisms - ps1
Archaea1 - ps2
Archaea2 - ps3
Archaea3 - ps4
Asgard/Eukaryota - ps5
Eukaryota - ps6
Excavata/Diaphoretickes - ps7
Diaphoretickes - ps8
Archaeplastida - ps9
Viridiplantae - ps10
Streptophyta - ps11
Charophyceae/Embriophyta - ps12
Embriophyta - ps13
Tracheophyta - ps14
Spermatophyta - ps15
Angiosperms - ps16
Mesangiospermae - ps17
Magnoliids/eudicots - ps18
Eudicots - ps19
Core_eudicots - ps20
Superrosids - ps21
Rosids - ps22
Eurosids - ps23
Malvids - ps24
Sap./Malv./Brassicales - ps25
Malvales/Brassicales - ps26
Brassicales - ps27
Cleomaceae/Brassicaceae - ps28
Brassicaceae - ps29
Camelineae - ps30
Arabidopsis - ps31
Arabidopsis_thaliana - ps32
p-value
3e-10
3e-08
4e-06
4e-04
4e-02
Log-odds
0.5
1.0
1.5
2.0
2.5
3.0
3.5
4.0

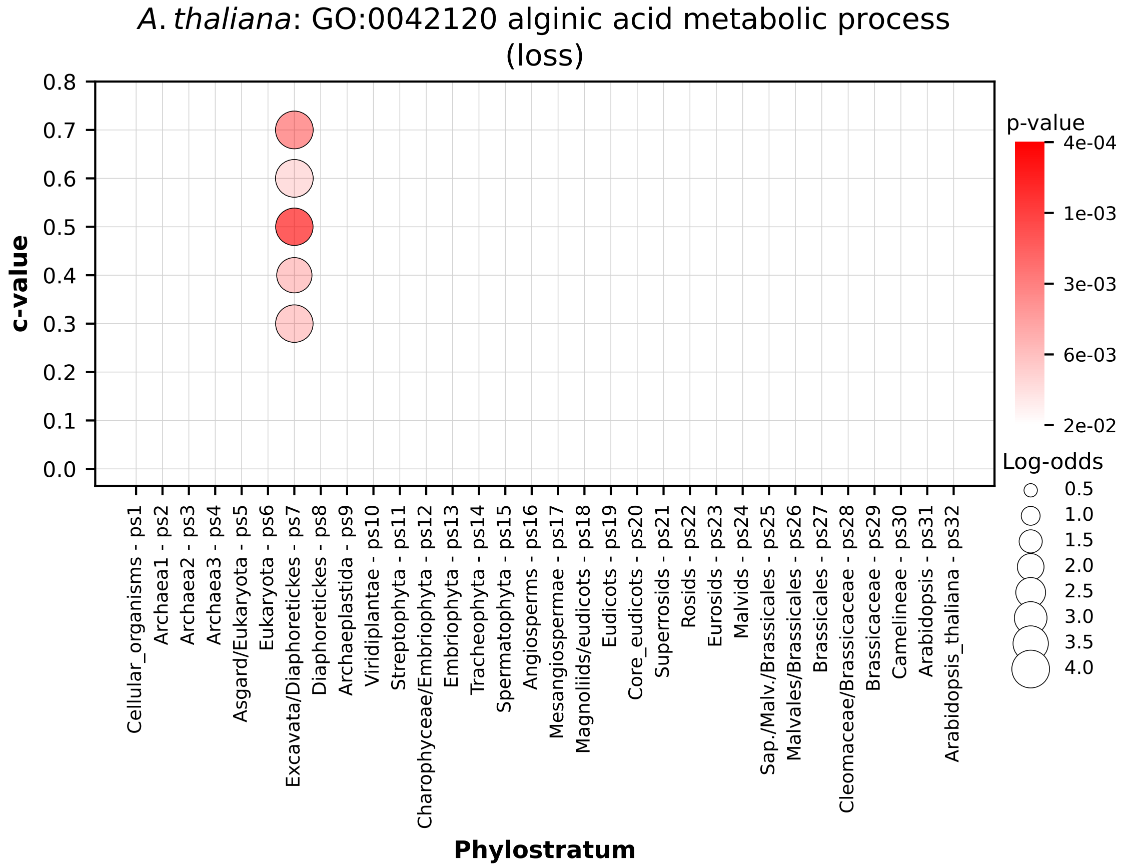

A. thaliana: GO:0042120 alginic acid metabolic process (loss)
c-value
0.8
0.7
0.6
0.5
0.4
0.3
0.2
0.1
0.0
p-value
4e-04
1e-03
3e-03
6e-03
2e-02
Log-odds
0.5
1.0
1.5
2.0
2.5
3.0
3.5
4.0
Cellular_organisms - ps1
Archaea1 - ps2
Archaea2 - ps3
Archaea3 - ps4
Asgard/Eukaryota - ps5
Eukaryota - ps6
Excavata/Diaphoretickes - ps7
Diaphoretickes - ps8
Archaeplastida - ps9
Viridiplantae - ps10
Streptophyta - ps11
Charophyceae/Embriophyta - ps12
Embriophyta - ps13
Tracheophyta - ps14
Spermatophyta - ps15
Angiosperms - ps16
Mesangiospermae - ps17
Magnoliids/eudicots - ps18
Eudicots - ps19
Core_eudicots - ps20
Superrosids - ps21
Rosids - ps22
Eurosids - ps23
Malvids - ps24
Sap./Malv./Brassicales - ps25
Malvales/Brassicales - ps26
Brassicales - ps27
Cleomaceae/Brassicaceae - ps28
Brassicaceae - ps29
Camelineae - ps30
Arabidopsis - ps31
Arabidopsis_thaliana - ps32
Phylostratum

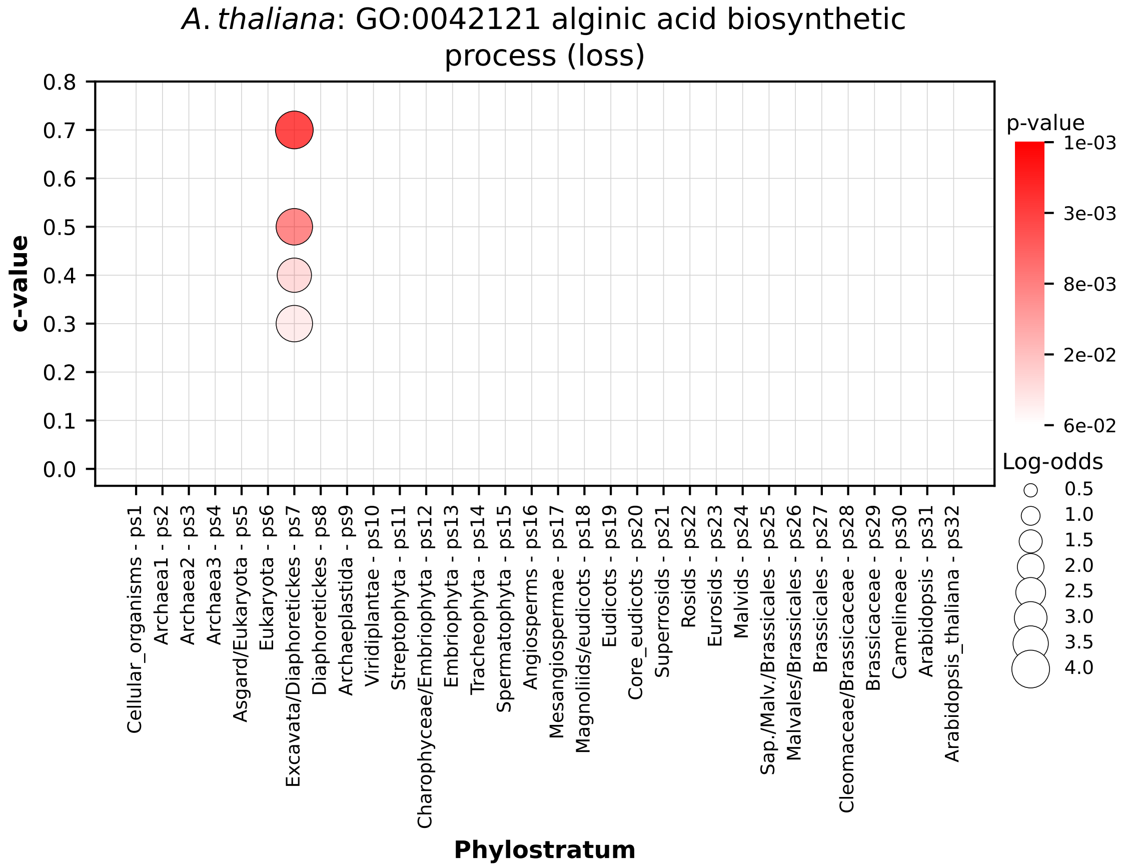

A. thaliana: GO:0042121 alginic acid biosynthetic process (loss)
c-value
0.8
0.7
0.6
0.5
0.4
0.3
0.2
0.1
0.0
p-value
1e-03
3e-03
8e-03
2e-02
6e-02
Log-odds
0.5
1.0
1.5
2.0
2.5
3.0
3.5
4.0
Cellular_organisms - ps1
Archaea1 - ps2
Archaea2 - ps3
Archaea3 - ps4
Asgard/Eukaryota - ps5
Eukaryota - ps6
Excavata/Diaphoretickes - ps7
Diaphoretickes - ps8
Archaeplastida - ps9
Viridiplantae - ps10
Streptophyta - ps11
Charophyceae/Embriophyta - ps12
Embriophyta - ps13
Tracheophyta - ps14
Spermatophyta - ps15
Angiosperms - ps16
Mesangiospermae - ps17
Magnoliids/eudicots - ps18
Eudicots - ps19
Core_eudicots - ps20
Superrosids - ps21
Rosids - ps22
Eurosids - ps23
Malvids - ps24
Sap./Malv./Brassicales - ps25
Malvales/Brassicales - ps26
Brassicales - ps27
Cleomaceae/Brassicaceae - ps28
Brassicaceae - ps29
Camelineae - ps30
Arabidopsis - ps31
Arabidopsis_thaliana - ps32
Phylostratum

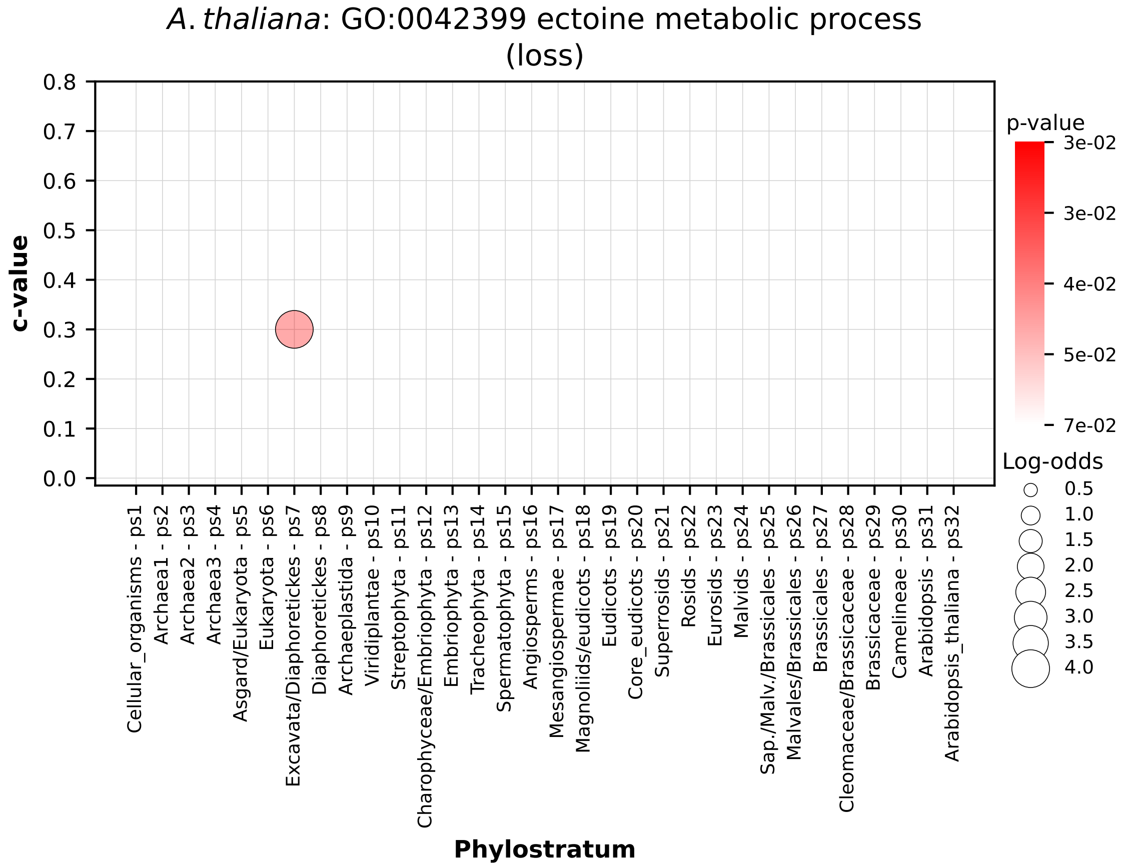

A. thaliana: GO:0042399 ectoine metabolic process (loss)
c-value
0.8
0.7
0.6
0.5
0.4
0.3
0.2
0.1
0.0
Phylostratum
Cellular_organisms - ps1
Archaea1 - ps2
Archaea2 - ps3
Archaea3 - ps4
Asgard/Eukaryota - ps5
Eukaryota - ps6
Excavata/Diaphoretickes - ps7
Diaphoretickes - ps8
Archaeplastida - ps9
Viridiplantae - ps10
Streptophyta - ps11
Charophyceae/Embriophyta - ps12
Embriophyta - ps13
Tracheophyta - ps14
Spermatophyta - ps15
Angiosperms - ps16
Mesangiospermae - ps17
Magnoliids/eudicots - ps18
Eudicots - ps19
Core_eudicots - ps20
Superrosids - ps21
Rosids - ps22
Eurosids - ps23
Malvids - ps24
Sap./Malv./Brassicales - ps25
Malvales/Brassicales - ps26
Brassicales - ps27
Cleomaceae/Brassicaceae - ps28
Brassicaceae - ps29
Camelineae - ps30
Arabidopsis - ps31
Arabidopsis_thaliana - ps32
p-value
3e-02
3e-02
4e-02
5e-02
7e-02
Log-odds
0.5
1.0
1.5
2.0
2.5
3.0
3.5
4.0

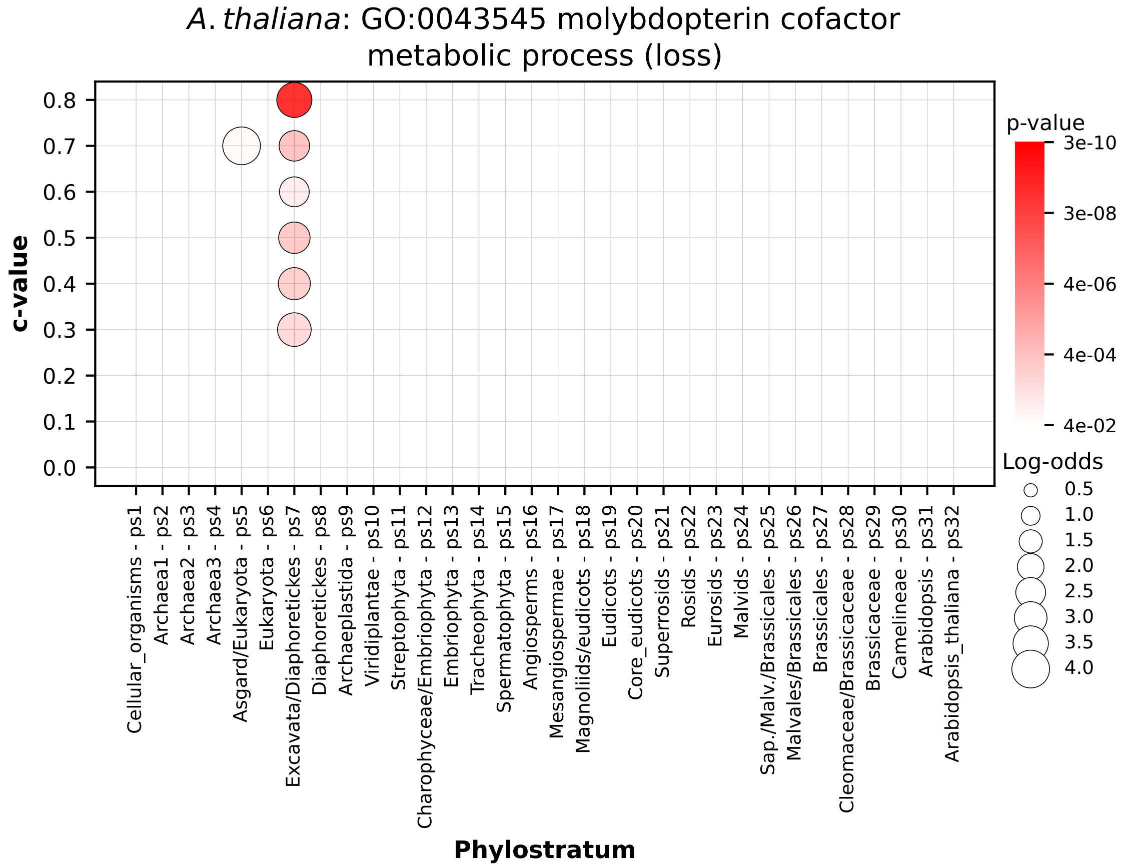

A. thaliana: GO:0043545 molybdopterin cofactor metabolic process (loss)
c-value
0.8
0.7
0.6
0.5
0.4
0.3
0.2
0.1
0.0
p-value
3e-10
3e-08
4e-06
4e-04
4e-02
Log-odds
0.5
1.0
1.5
2.0
2.5
3.0
3.5
4.0
Cellular_organisms - ps1
Archaea1 - ps2
Archaea2 - ps3
Archaea3 - ps4
Asgard/Eukaryota - ps5
Eukaryota - ps6
Excavata/Diaphoretickes - ps7
Diaphoretickes - ps8
Archaeplastida - ps9
Viridiplantae - ps10
Streptophyta - ps11
Charophyceae/Embriophyta - ps12
Embriophyta - ps13
Tracheophyta - ps14
Spermatophyta - ps15
Angiosperms - ps16
Mesangiospermae - ps17
Magnoliids/eudicots - ps18
Eudicots - ps19
Core_eudicots - ps20
Superrosids - ps21
Rosids - ps22
Eurosids - ps23
Malvids - ps24
Sap./Malv./Brassicales - ps25
Malvales/Brassicales - ps26
Brassicales - ps27
Cleomaceae/Brassicaceae - ps28
Brassicaceae - ps29
Camelineae - ps30
Arabidopsis - ps31
Arabidopsis_thaliana - ps32
Phylostratum

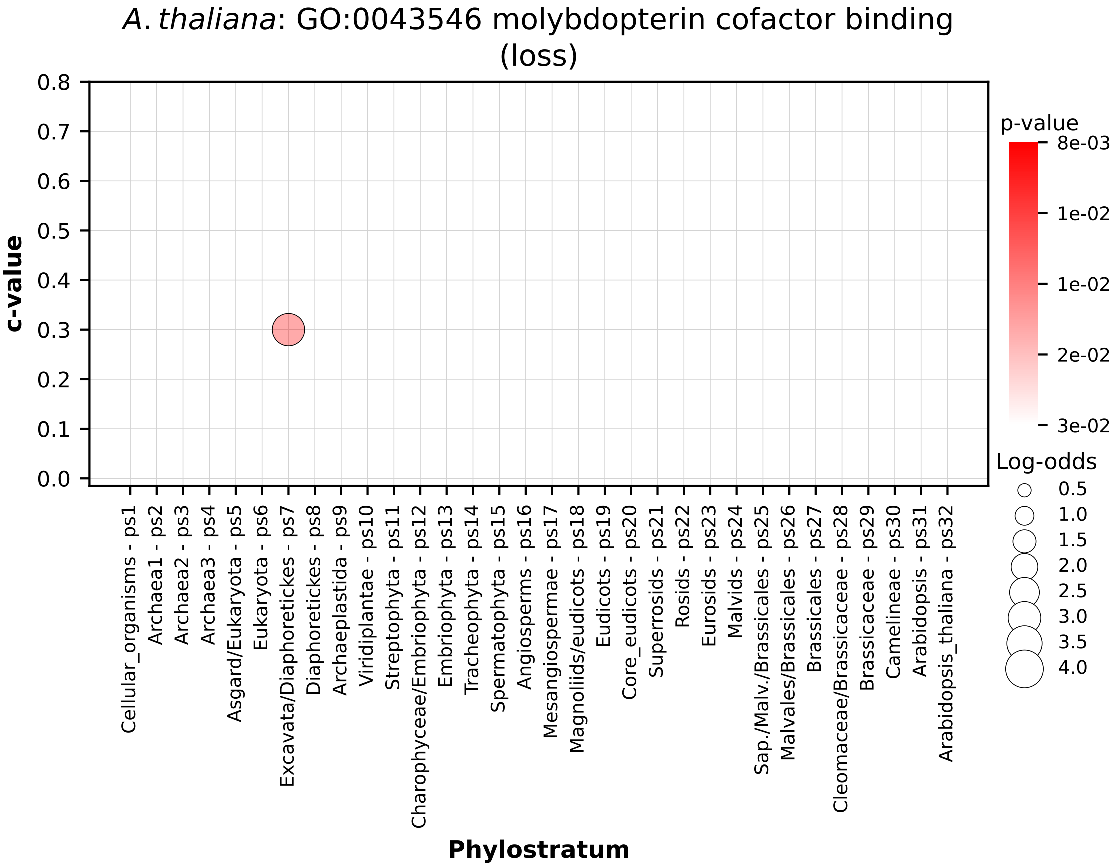

A. thaliana: GO:0043546 molybdopterin cofactor binding (loss)
c-value
0.8
0.7
0.6
0.5
0.4
0.3
0.2
0.1
0.0
Phylostratum
Cellular_organisms - ps1
Archaea1 - ps2
Archaea2 - ps3
Archaea3 - ps4
Asgard/Eukaryota - ps5
Eukaryota - ps6
Excavata/Diaphoretickes - ps7
Diaphoretickes - ps8
Archaeplastida - ps9
Viridiplantae - ps10
Streptophyta - ps11
Charophyceae/Embriophyta - ps12
Embriophyta - ps13
Tracheophyta - ps14
Spermatophyta - ps15
Angiosperms - ps16
Mesangiospermae - ps17
Magnoliids/eudicots - ps18
Eudicots - ps19
Core_eudicots - ps20
Superrosids - ps21
Rosids - ps22
Eurosids - ps23
Malvids - ps24
Sap./Malv./Brassicales - ps25
Malvales/Brassicales - ps26
Brassicales - ps27
Cleomaceae/Brassicaceae - ps28
Brassicaceae - ps29
Camelineae - ps30
Arabidopsis - ps31
Arabidopsis_thaliana - ps32
p-value
8e-03
1e-02
1e-02
2e-02
3e-02
Log-odds
0.5
1.0
1.5
2.0
2.5
3.0
3.5
4.0

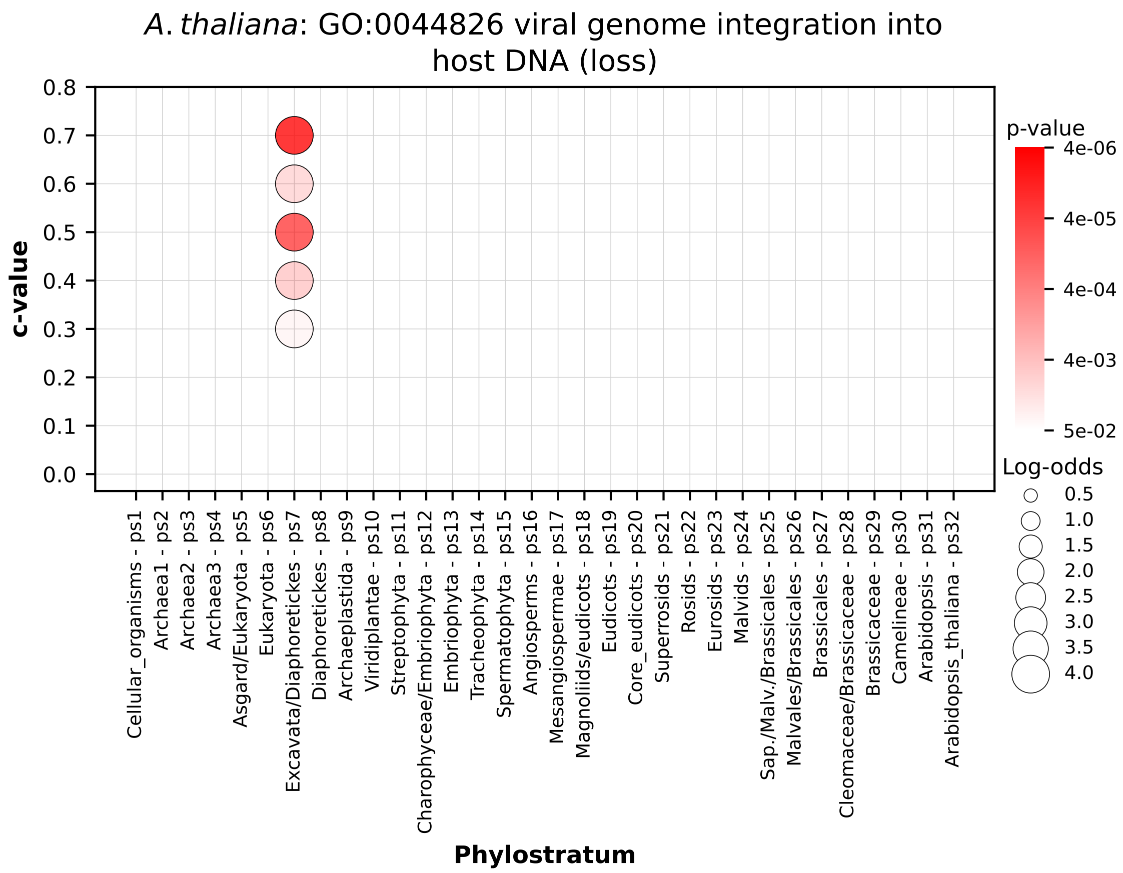
A. thaliana: GO:0044826 viral genome integration into host DNA (loss)
c-value
0.8
0.7
0.6
0.5
0.4
0.3
0.2
0.1
0.0
p-value
4e-06
4e-05
4e-04
4e-03
5e-02
Log-odds
0.5
1.0
1.5
2.0
2.5
3.0
3.5
4.0
Cellular_organisms - ps1
Archaea1 - ps2
Archaea2 - ps3
Archaea3 - ps4
Asgard/Eukaryota - ps5
Eukaryota - ps6
Excavata/Diaphoretickes - ps7
Diaphoretickes - ps8
Archaeplastida - ps9
Viridiplantae - ps10
Streptophyta - ps11
Charophyceae/Embriophyta - ps12
Embriophyta - ps13
Tracheophyta - ps14
Spermatophyta - ps15
Angiosperms - ps16
Mesangiospermae - ps17
Magnoliids/eudicots - ps18
Eudicots - ps19
Core_eudicots - ps20
Superrosids - ps21
Rosids - ps22
Eurosids - ps23
Malvids - ps24
Sap./Malv./Brassicales - ps25
Malvales/Brassicales - ps26
Brassicales - ps27
Cleomaceae/Brassicaceae - ps28
Brassicaceae - ps29
Camelineae - ps30
Arabidopsis - ps31
Arabidopsis_thaliana - ps32
Phylostratum

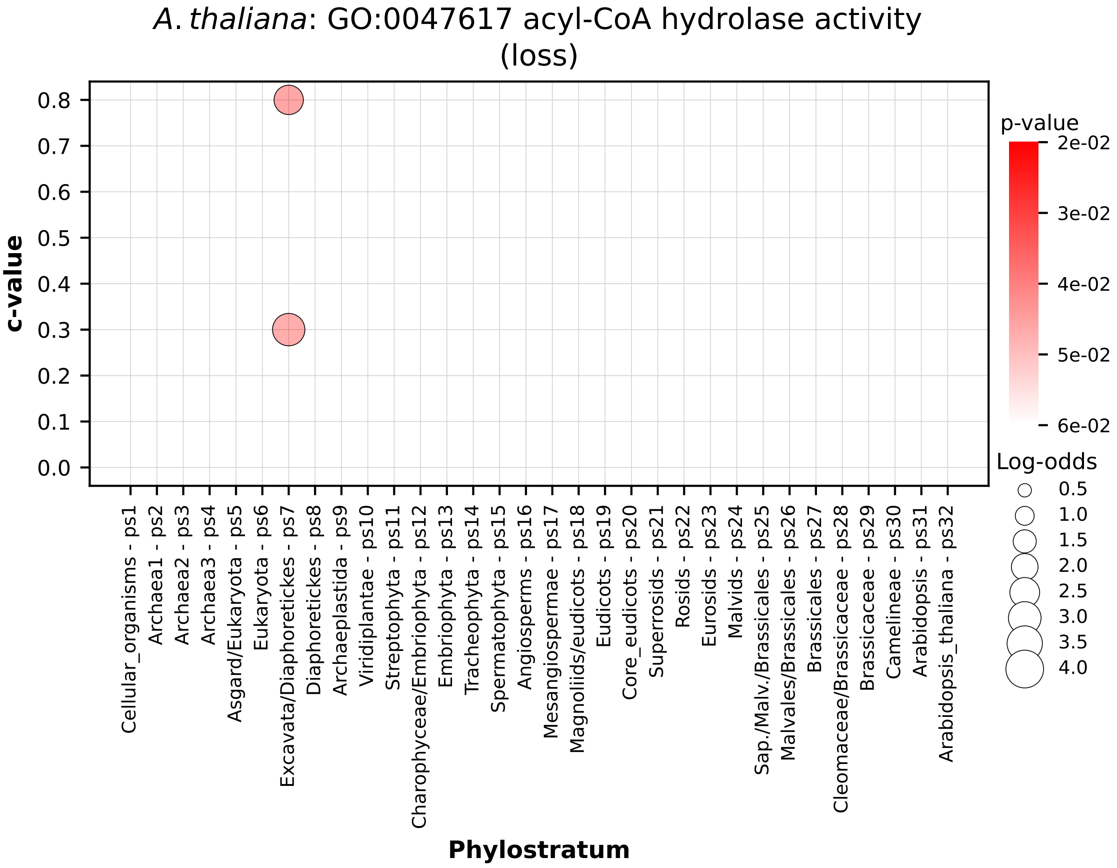

A. thaliana: GO:0047617 acyl-CoA hydrolase activity
(loss)
c-value
0.0
0.1
0.2
0.3
0.4
0.5
0.6
0.7
0.8
p-value
2e-02
3e-02
4e-02
5e-02
6e-02
Log-odds
0.5
1.0
1.5
2.0
2.5
3.0
3.5
4.0
Cellular_organisms - ps1
Archaea1 - ps2
Archaea2 - ps3
Archaea3 - ps4
Asgard/Eukaryota - ps5
Eukaryota - ps6
Excavata/Diaphoretickes - ps7
Diaphoretickes - ps8
Archaeplastida - ps9
Viridiplantae - ps10
Streptophyta - ps11
Charophyceae/Embriophyta - ps12
Embriophyta - ps13
Tracheophyta - ps14
Spermatophyta - ps15
Angiosperms - ps16
Mesangiospermae - ps17
Magnoliids/eudicots - ps18
Eudicots - ps19
Core_eudicots - ps20
Superrosids - ps21
Rosids - ps22
Eurosids - ps23
Malvids - ps24
Sap./Malv./Brassicales - ps25
Malvales/Brassicales - ps26
Brassicales - ps27
Cleomaceae/Brassicaceae - ps28
Brassicaceae - ps29
Camelineae - ps30
Arabidopsis - ps31
Arabidopsis_thaliana - ps32
Phylostratum

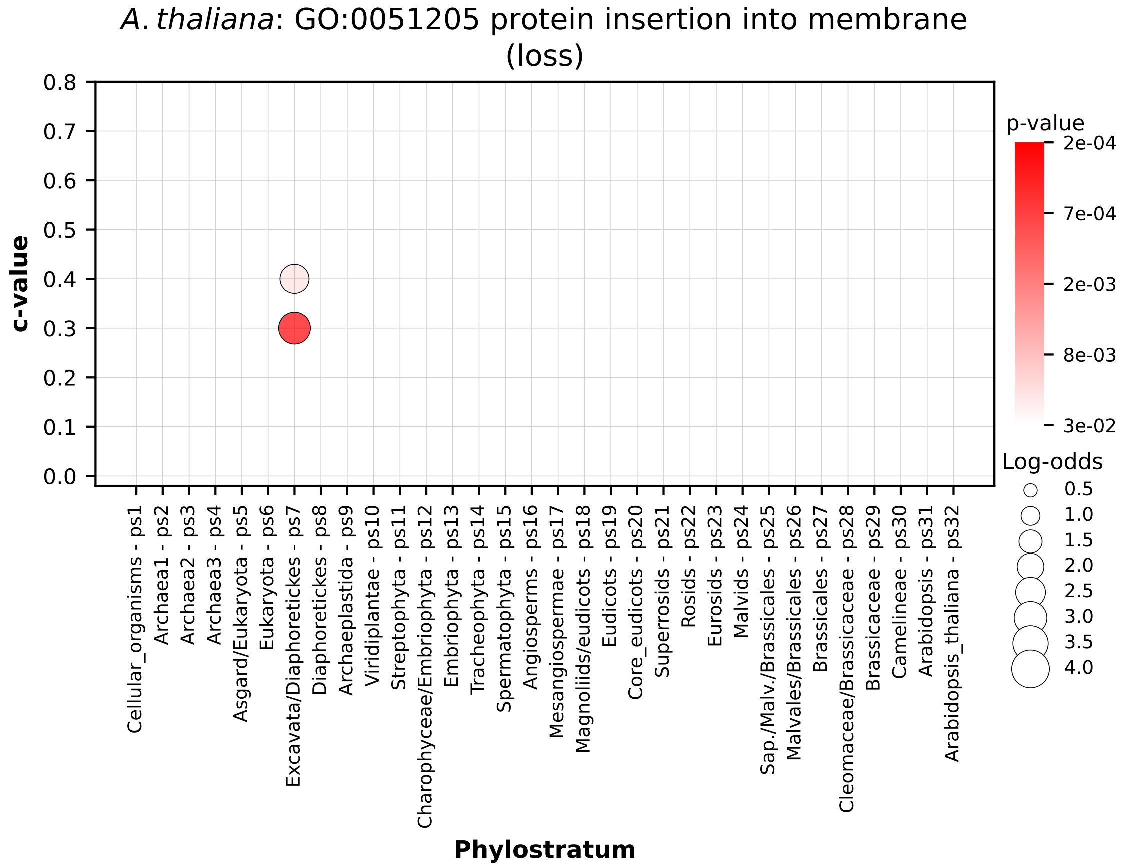
A. thaliana: GO:0051205 protein insertion into membrane
(loss)
c-value
0.0
0.1
0.2
0.3
0.4
0.5
0.6
0.7
0.8
Phylostratum
Cellular_organisms - ps1
Archaea1 - ps2
Archaea2 - ps3
Archaea3 - ps4
Asgard/Eukaryota - ps5
Eukaryota - ps6
Excavata/Diaphoretickes - ps7
Diaphoretickes - ps8
Archaeplastida - ps9
Viridiplantae - ps10
Streptophyta - ps11
Charophyceae/Embriophyta - ps12
Embriophyta - ps13
Tracheophyta - ps14
Spermatophyta - ps15
Angiosperms - ps16
Mesangiospermae - ps17
Magnoliids/eudicots - ps18
Eudicots - ps19
Core_eudicots - ps20
Superrosids - ps21
Rosids - ps22
Eurosids - ps23
Malvids - ps24
Sap./Malv./Brassicales - ps25
Malvales/Brassicales - ps26
Brassicales - ps27
Cleomaceae/Brassicaceae - ps28
Brassicaceae - ps29
Camelineae - ps30
Arabidopsis - ps31
Arabidopsis_thaliana - ps32
p-value
2e-04
7e-04
2e-03
8e-03
3e-02
Log-odds
0.5
1.0
1.5
2.0
2.5
3.0
3.5
4.0

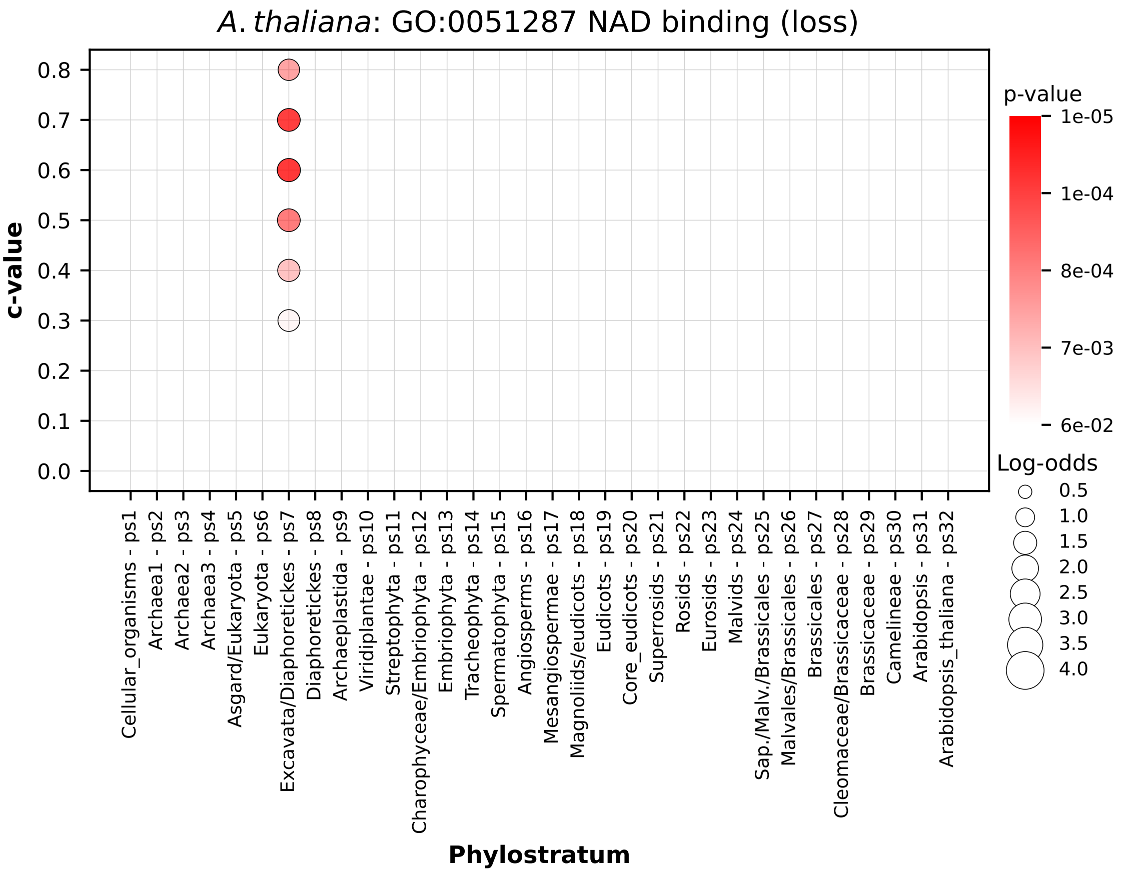
A. thaliana: GO:0051287 NAD binding (loss)
c-value
0.0
0.1
0.2
0.3
0.4
0.5
0.6
0.7
0.8
p-value
1e-05
1e-04
8e-04
7e-03
6e-02
Log-odds
0.5
1.0
1.5
2.0
2.5
3.0
3.5
4.0
Cellular_organisms - ps1
Archaea1 - ps2
Archaea2 - ps3
Archaea3 - ps4
Asgard/Eukaryota - ps5
Eukaryota - ps6
Excavata/Diaphoretickes - ps7
Diaphoretickes - ps8
Archaeplastida - ps9
Viridiplantae - ps10
Streptophyta - ps11
Charophyceae/Embriophyta - ps12
Embriophyta - ps13
Tracheophyta - ps14
Spermatophyta - ps15
Angiosperms - ps16
Mesangiospermae - ps17
Magnoliids/eudicots - ps18
Eudicots - ps19
Core_eudicots - ps20
Superrosids - ps21
Rosids - ps22
Eurosids - ps23
Malvids - ps24
Sap./Malv./Brassicales - ps25
Malvales/Brassicales - ps26
Brassicales - ps27
Cleomaceae/Brassicaceae - ps28
Brassicaceae - ps29
Camelineae - ps30
Arabidopsis - ps31
Arabidopsis_thaliana - ps32
Phylostratum

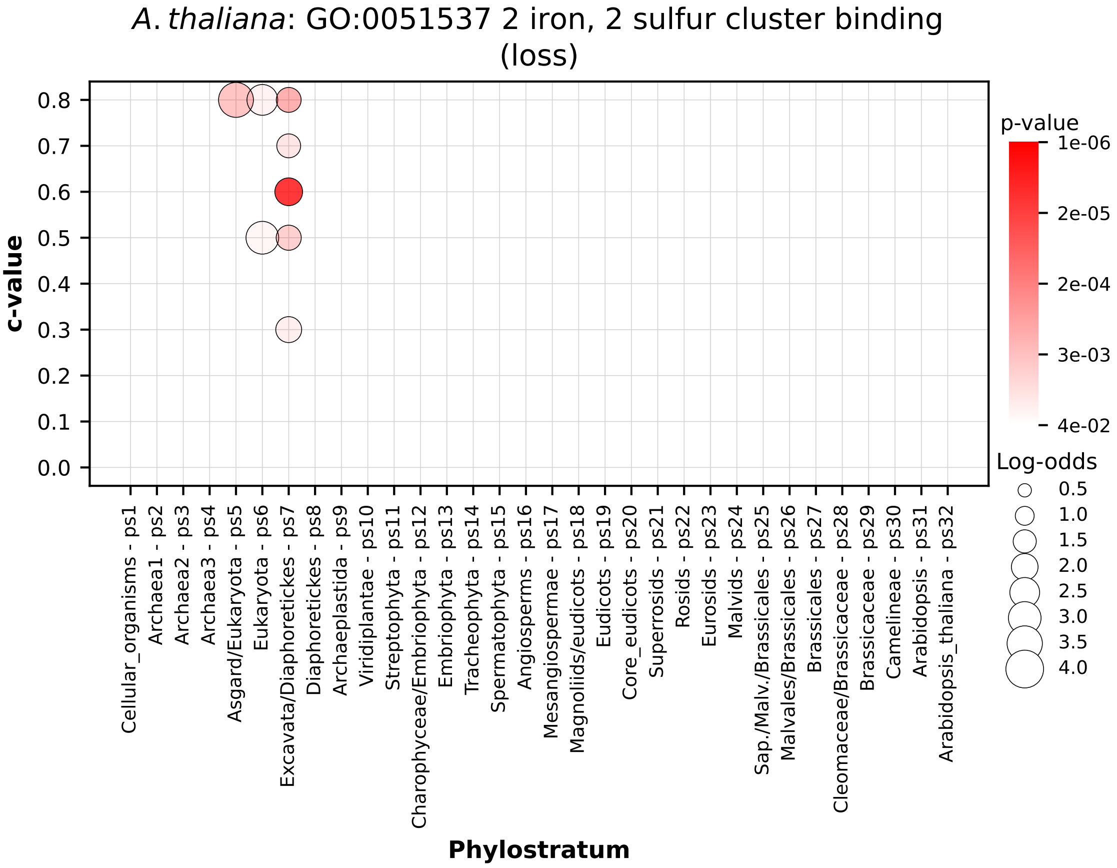
A. thaliana: GO:0051537 2 iron, 2 sulfur cluster binding
(loss)
c-value
0.8
0.7
0.6
0.5
0.4
0.3
0.2
0.1
0.0
p-value
1e-06
2e-05
2e-04
3e-03
4e-02
Log-odds
0.5
1.0
1.5
2.0
2.5
3.0
3.5
4.0
Cellular_organisms - ps1
Archaea1 - ps2
Archaea2 - ps3
Archaea3 - ps4
Asgard/Eukaryota - ps5
Eukaryota - ps6
Excavata/Diaphoretickes - ps7
Diaphoretickes - ps8
Archaeplastida - ps9
Viridiplantae - ps10
Streptophyta - ps11
Charophyceae/Embriophyta - ps12
Embriophyta - ps13
Tracheophyta - ps14
Spermatophyta - ps15
Angiosperms - ps16
Mesangiospermae - ps17
Magnoliids/eudicots - ps18
Eudicots - ps19
Core_eudicots - ps20
Superrosids - ps21
Rosids - ps22
Eurosids - ps23
Malvids - ps24
Sap./Malv./Brassicales - ps25
Malvales/Brassicales - ps26
Brassicales - ps27
Cleomaceae/Brassicaceae - ps28
Brassicaceae - ps29
Camelineae - ps30
Arabidopsis - ps31
Arabidopsis_thaliana - ps32
Phylostratum

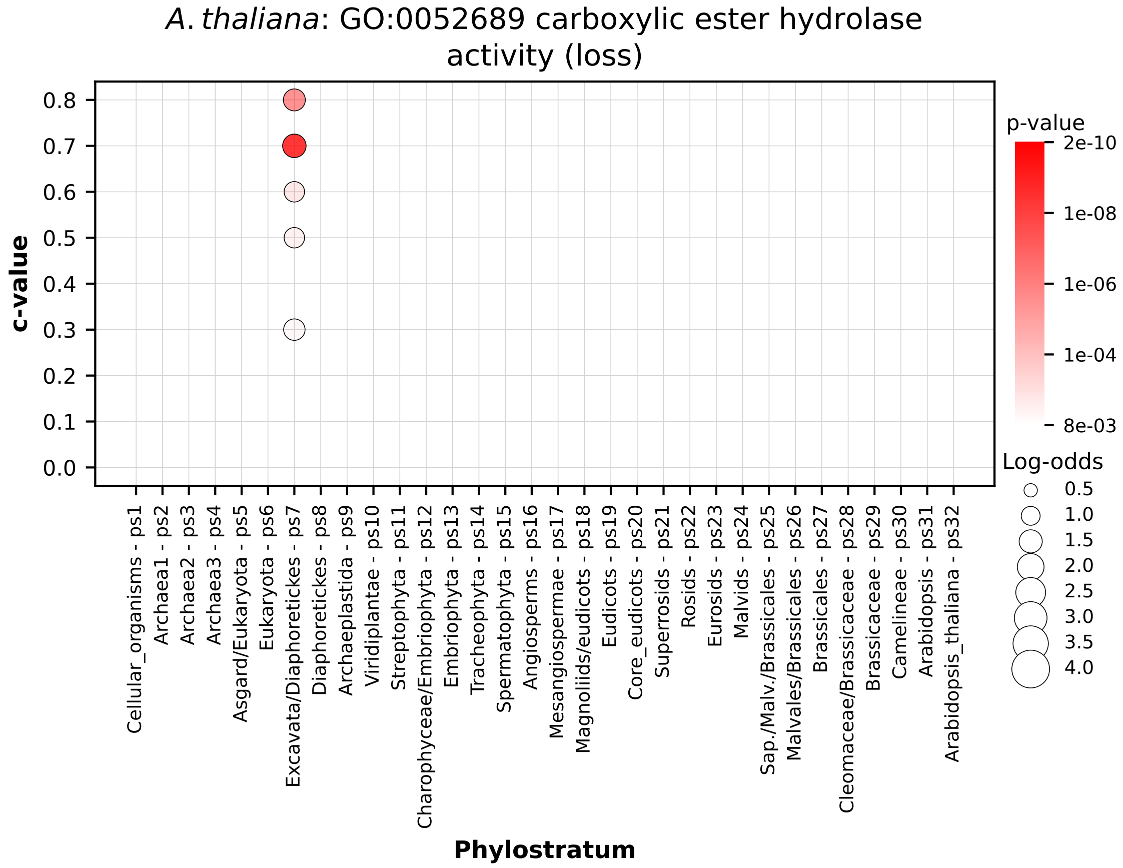

A. thaliana: GO:0052689 carboxylic ester hydrolase activity (loss)
c-value
0.8
0.7
0.6
0.5
0.4
0.3
0.2
0.1
0.0
p-value
2e-10
1e-08
1e-06
1e-04
8e-03
Log-odds
0.5
1.0
1.5
2.0
2.5
3.0
3.5
4.0
Cellular_organisms - ps1
Archaea1 - ps2
Archaea2 - ps3
Archaea3 - ps4
Asgard/Eukaryota - ps5
Eukaryota - ps6
Excavata/Diaphoretickes - ps7
Diaphoretickes - ps8
Archaeplastida - ps9
Viridiplantae - ps10
Streptophyta - ps11
Charophyceae/Embriophyta - ps12
Embriophyta - ps13
Tracheophyta - ps14
Spermatophyta - ps15
Angiosperms - ps16
Mesangiospermae - ps17
Magnoliids/eudicots - ps18
Eudicots - ps19
Core_eudicots - ps20
Superrosids - ps21
Rosids - ps22
Eurosids - ps23
Malvids - ps24
Sap./Malv./Brassicales - ps25
Malvales/Brassicales - ps26
Brassicales - ps27
Cleomaceae/Brassicaceae - ps28
Brassicaceae - ps29
Camelineae - ps30
Arabidopsis - ps31
Arabidopsis_thaliana - ps32
Phylostratum

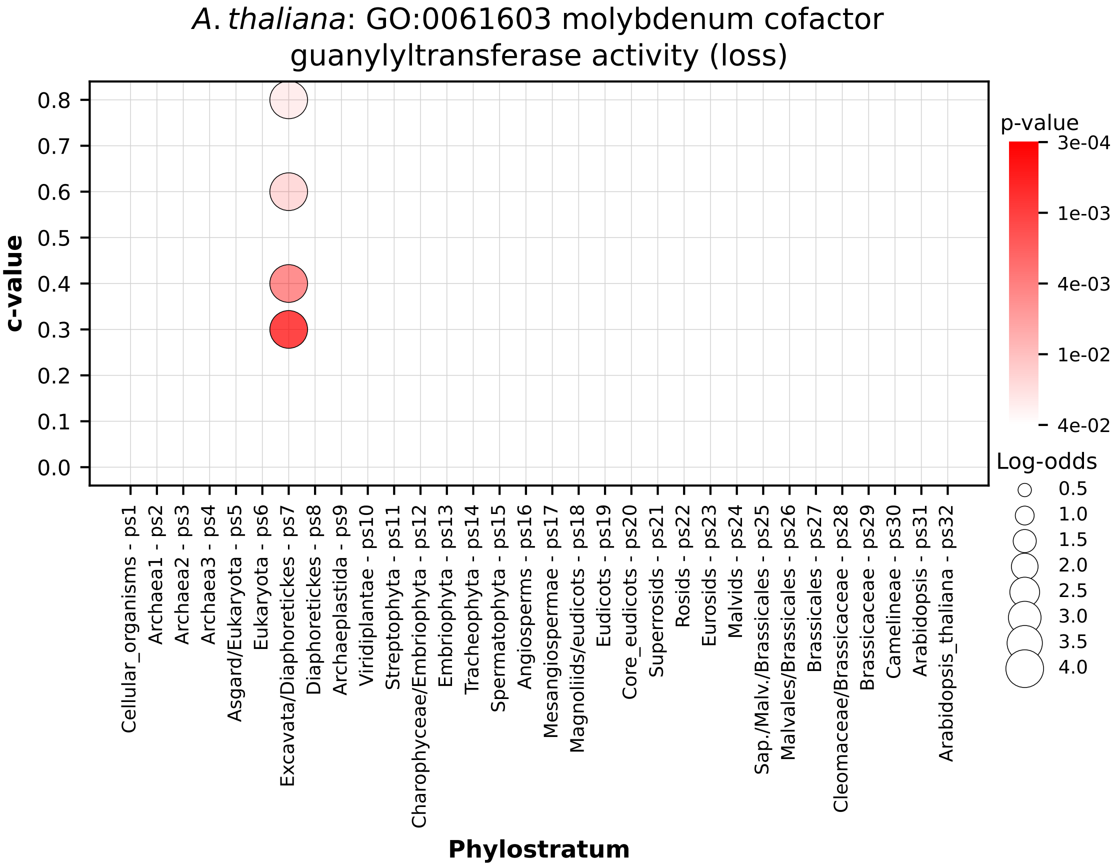
A. thaliana: GO:0061603 molybdenum cofactor guanylyltransferase activity (loss)
c-value
0.0
0.1
0.2
0.3
0.4
0.5
0.6
0.7
0.8
Phylostratum
Cellular_organisms - ps1
Archaea1 - ps2
Archaea2 - ps3
Archaea3 - ps4
Asgard/Eukaryota - ps5
Eukaryota - ps6
Excavata/Diaphoretickes - ps7
Diaphoretickes - ps8
Archaeplastida - ps9
Viridiplantae - ps10
Streptophyta - ps11
Charophyceae/Embriophyta - ps12
Embriophyta - ps13
Tracheophyta - ps14
Spermatophyta - ps15
Angiosperms - ps16
Mesangiospermae - ps17
Magnoliids/eudicots - ps18
Eudicots - ps19
Core_eudicots - ps20
Superrosids - ps21
Rosids - ps22
Eurosids - ps23
Malvids - ps24
Sap./Malv./Brassicales - ps25
Malvales/Brassicales - ps26
Brassicales - ps27
Cleomaceae/Brassicaceae - ps28
Brassicaceae - ps29
Camelineae - ps30
Arabidopsis - ps31
Arabidopsis_thaliana - ps32
p-value
3e-04
1e-03
4e-03
1e-02
4e-02
Log-odds
0.5
1.0
1.5
2.0
2.5
3.0
3.5
4.0

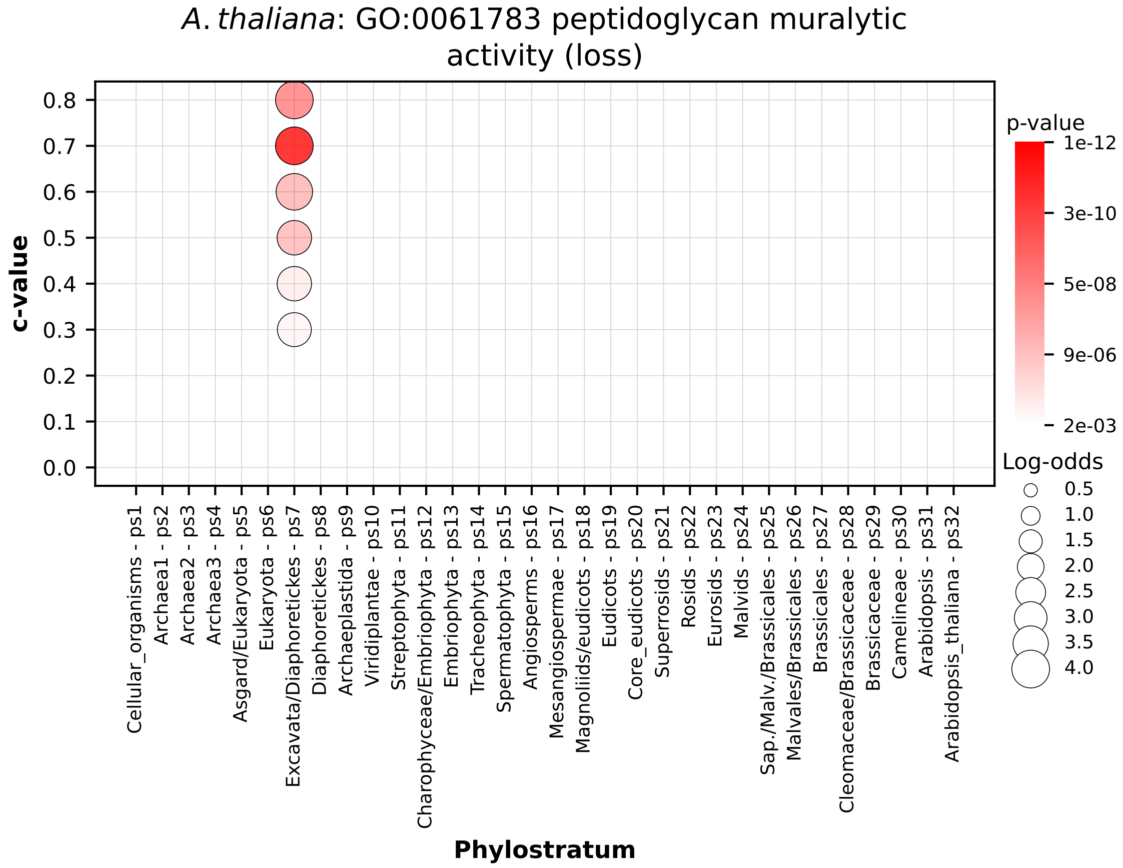

A. thaliana: GO:0061783 peptidoglycan muralytic activity (loss)
c-value
0.8
0.7
0.6
0.5
0.4
0.3
0.2
0.1
0.0
Phylostratum
Cellular_organisms - ps1
Archaea1 - ps2
Archaea2 - ps3
Archaea3 - ps4
Asgard/Eukaryota - ps5
Eukaryota - ps6
Excavata/Diaphoretickes - ps7
Diaphoretickes - ps8
Archaeplastida - ps9
Viridiplantae - ps10
Streptophyta - ps11
Charophyceae/Embriophyta - ps12
Embriophyta - ps13
Tracheophyta - ps14
Spermatophyta - ps15
Angiosperms - ps16
Mesangiospermae - ps17
Magnoliids/eudicots - ps18
Eudicots - ps19
Core_eudicots - ps20
Superrosids - ps21
Rosids - ps22
Eurosids - ps23
Malvids - ps24
Sap./Malv./Brassicales - ps25
Malvales/Brassicales - ps26
Brassicales - ps27
Cleomaceae/Brassicaceae - ps28
Brassicaceae - ps29
Camelineae - ps30
Arabidopsis - ps31
Arabidopsis_thaliana - ps32
p-value
1e-12
3e-10
5e-08
9e-06
2e-03
Log-odds
0.5
1.0
1.5
2.0
2.5
3.0
3.5
4.0

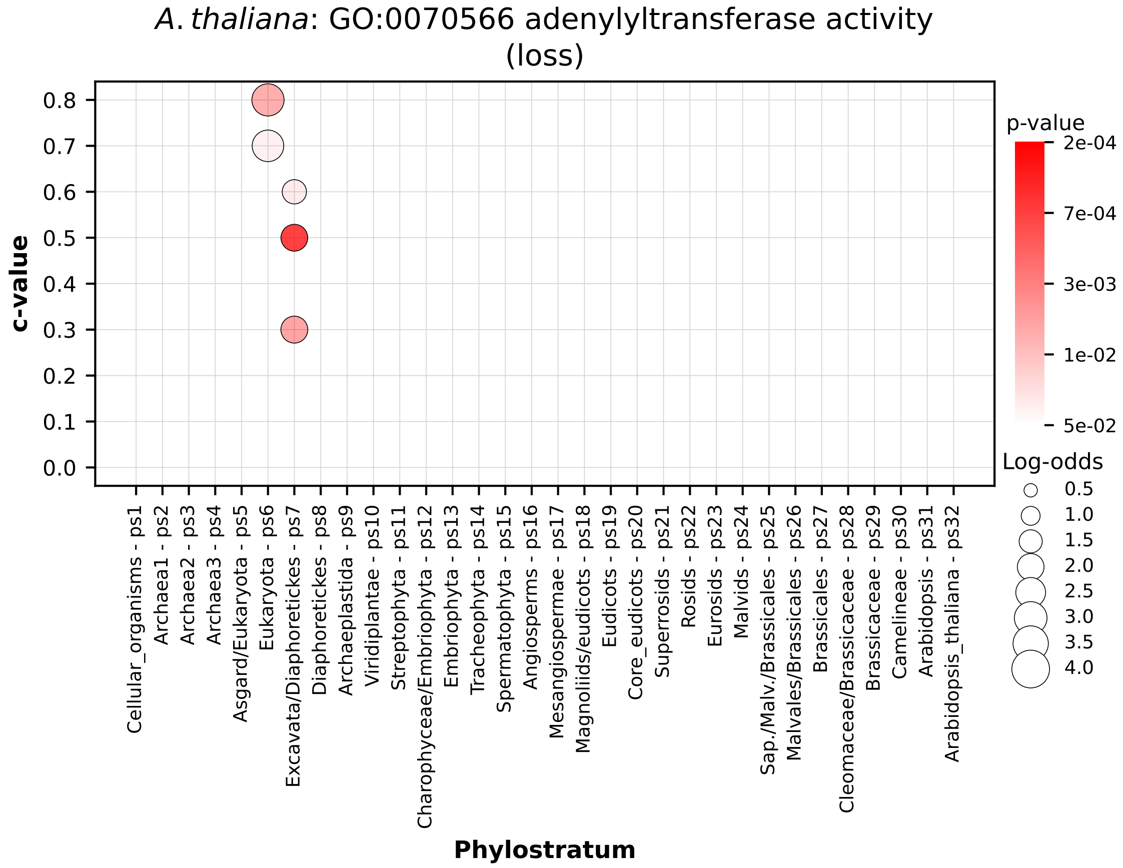
A. thaliana: GO:0070566 adenylyltransferase activity
(loss)
c-value
0.8
0.7
0.6
0.5
0.4
0.3
0.2
0.1
0.0
Phylostratum
Cellular_organisms - ps1
Archaea1 - ps2
Archaea2 - ps3
Archaea3 - ps4
Asgard/Eukaryota - ps5
Eukaryota - ps6
Excavata/Diaphoretickes - ps7
Diaphoretickes - ps8
Archaeplastida - ps9
Viridiplantae - ps10
Streptophyta - ps11
Charophyceae/Embriophyta - ps12
Embriophyta - ps13
Tracheophyta - ps14
Spermatophyta - ps15
Angiosperms - ps16
Mesangiospermae - ps17
Magnoliids/eudicots - ps18
Eudicots - ps19
Core_eudicots - ps20
Superrosids - ps21
Rosids - ps22
Eurosids - ps23
Malvids - ps24
Sap./Malv./Brassicales - ps25
Malvales/Brassicales - ps26
Brassicales - ps27
Cleomaceae/Brassicaceae - ps28
Brassicaceae - ps29
Camelineae - ps30
Arabidopsis - ps31
Arabidopsis_thaliana - ps32
p-value
2e-04
7e-04
3e-03
1e-02
5e-02
Log-odds
0.5
1.0
1.5
2.0
2.5
3.0
3.5
4.0

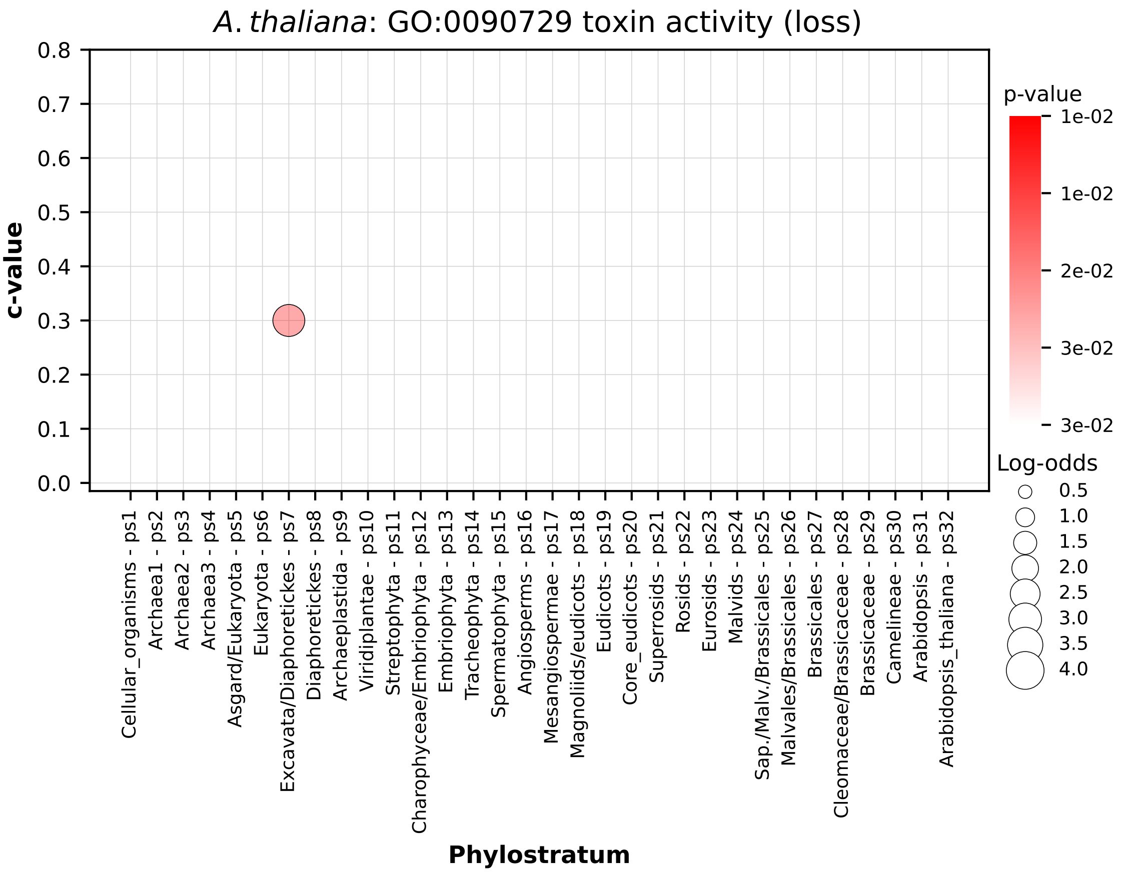

A. thaliana: GO:0090729 toxin activity (loss)
c-value
0.8
0.7
0.6
0.5
0.4
0.3
0.2
0.1
0.0
Phylostratum
Cellular_organisms - ps1
Archaea1 - ps2
Archaea2 - ps3
Archaea3 - ps4
Asgard/Eukaryota - ps5
Eukaryota - ps6
Excavata/Diaphoretickes - ps7
Diaphoretickes - ps8
Archaeplastida - ps9
Viridiplantae - ps10
Streptophyta - ps11
Charophyceae/Embriophyta - ps12
Embriophyta - ps13
Tracheophyta - ps14
Spermatophyta - ps15
Angiosperms - ps16
Mesangiospermae - ps17
Magnoliids/eudicots - ps18
Eudicots - ps19
Core_eudicots - ps20
Superrosids - ps21
Rosids - ps22
Eurosids - ps23
Malvids - ps24
Sap./Malv./Brassicales - ps25
Malvales/Brassicales - ps26
Brassicales - ps27
Cleomaceae/Brassicaceae - ps28
Brassicaceae - ps29
Camelineae - ps30
Arabidopsis - ps31
Arabidopsis_thaliana - ps32
p-value
1e-02
1e-02
2e-02
3e-02
3e-02
Log-odds
0.5
1.0
1.5
2.0
2.5
3.0
3.5
4.0

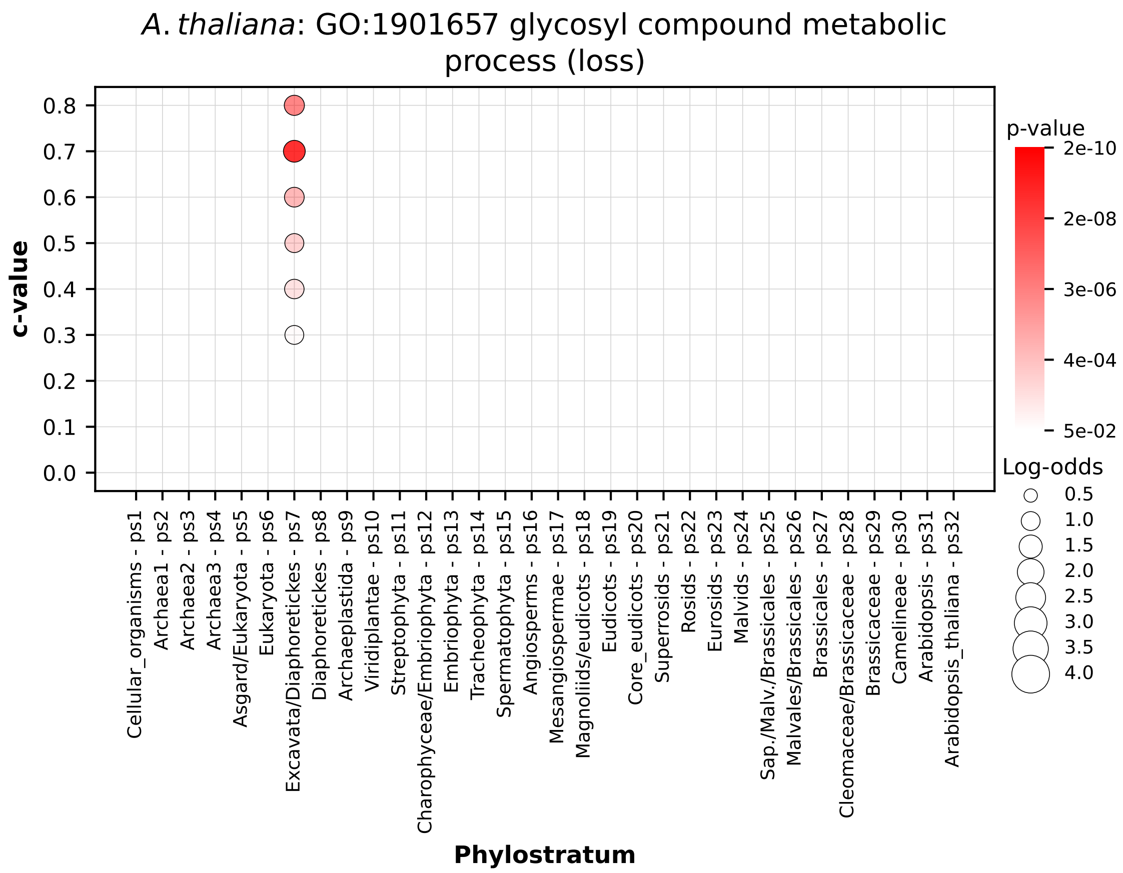

A. thaliana: GO:1901657 glycosyl compound metabolic process (loss)
c-value
0.0
0.1
0.2
0.3
0.4
0.5
0.6
0.7
0.8
Phylostratum
Cellular_organisms - ps1
Archaea1 - ps2
Archaea2 - ps3
Archaea3 - ps4
Asgard/Eukaryota - ps5
Eukaryota - ps6
Excavata/Diaphoretickes - ps7
Diaphoretickes - ps8
Archaeplastida - ps9
Viridiplantae - ps10
Streptophyta - ps11
Charophyceae/Embriophyta - ps12
Embriophyta - ps13
Tracheophyta - ps14
Spermatophyta - ps15
Angiosperms - ps16
Mesangiospermae - ps17
Magnoliids/eudicots - ps18
Eudicots - ps19
Core_eudicots - ps20
Superrosids - ps21
Rosids - ps22
Eurosids - ps23
Malvids - ps24
Sap./Malv./Brassicales - ps25
Malvales/Brassicales - ps26
Brassicales - ps27
Cleomaceae/Brassicaceae - ps28
Brassicaceae - ps29
Camelineae - ps30
Arabidopsis - ps31
Arabidopsis_thaliana - ps32
p-value
2e-10
2e-08
3e-06
4e-04
5e-02
Log-odds
0.5
1.0
1.5
2.0
2.5
3.0
3.5
4.0

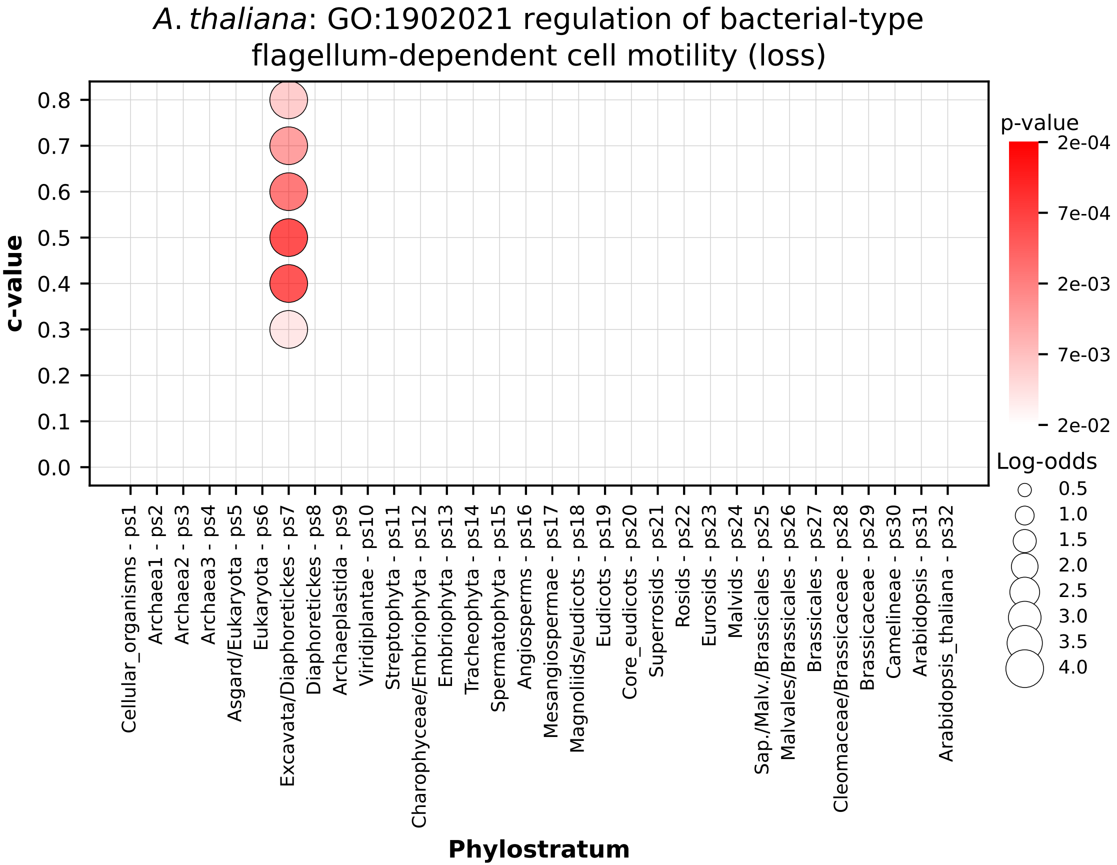
A. thaliana: GO:1902021 regulation of bacterial-type flagellum-dependent cell motility (loss)
c-value
0.0
0.1
0.2
0.3
0.4
0.5
0.6
0.7
0.8
Phylostratum
Cellular_organisms - ps1
Archaea1 - ps2
Archaea2 - ps3
Archaea3 - ps4
Asgard/Eukaryota - ps5
Eukaryota - ps6
Excavata/Diaphoretickes - ps7
Diaphoretickes - ps8
Archaeplastida - ps9
Viridiplantae - ps10
Streptophyta - ps11
Charophyceae/Embriophyta - ps12
Embriophyta - ps13
Tracheophyta - ps14
Spermatophyta - ps15
Angiosperms - ps16
Mesangiospermae - ps17
Magnoliids/eudicots - ps18
Eudicots - ps19
Core_eudicots - ps20
Superrosids - ps21
Rosids - ps22
Eurosids - ps23
Malvids - ps24
Sap./Malv./Brassicales - ps25
Malvales/Brassicales - ps26
Brassicales - ps27
Cleomaceae/Brassicaceae - ps28
Brassicaceae - ps29
Camelineae - ps30
Arabidopsis - ps31
Arabidopsis_thaliana - ps32
p-value
2e-04
7e-04
2e-03
7e-03
2e-02
Log-odds
0.5
1.0
1.5
2.0
2.5
3.0
3.5
4.0

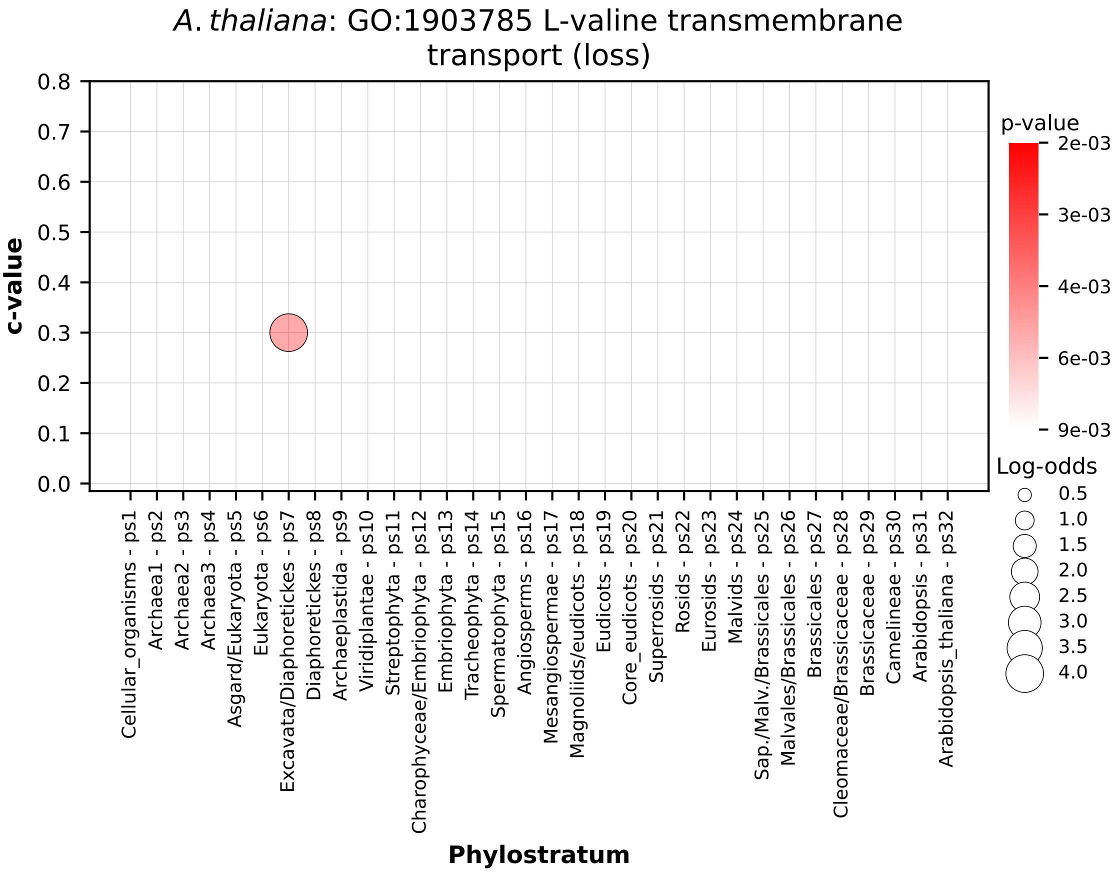

A. thaliana: GO:1903785 L-valine transmembrane transport (loss)
c-value
0.0
0.1
0.2
0.3
0.4
0.5
0.6
0.7
0.8
Phylostratum
Cellular_organisms - ps1
Archaea1 - ps2
Archaea2 - ps3
Archaea3 - ps4
Asgard/Eukaryota - ps5
Eukaryota - ps6
Excavata/Diaphoretickes - ps7
Diaphoretickes - ps8
Archaeplastida - ps9
Viridiplantae - ps10
Streptophyta - ps11
Charophyceae/Embriophyta - ps12
Embriophyta - ps13
Tracheophyta - ps14
Spermatophyta - ps15
Angiosperms - ps16
Mesangiospermae - ps17
Magnoliids/eudicots - ps18
Eudicots - ps19
Core_eudicots - ps20
Superrosids - ps21
Rosids - ps22
Eurosids - ps23
Malvids - ps24
Sap./Malv./Brassicales - ps25
Malvales/Brassicales - ps26
Brassicales - ps27
Cleomaceae/Brassicaceae - ps28
Brassicaceae - ps29
Camelineae - ps30
Arabidopsis - ps31
Arabidopsis_thaliana - ps32
p-value
2e-03
3e-03
4e-03
6e-03
9e-03
Log-odds
0.5
1.0
1.5
2.0
2.5
3.0
3.5
4.0

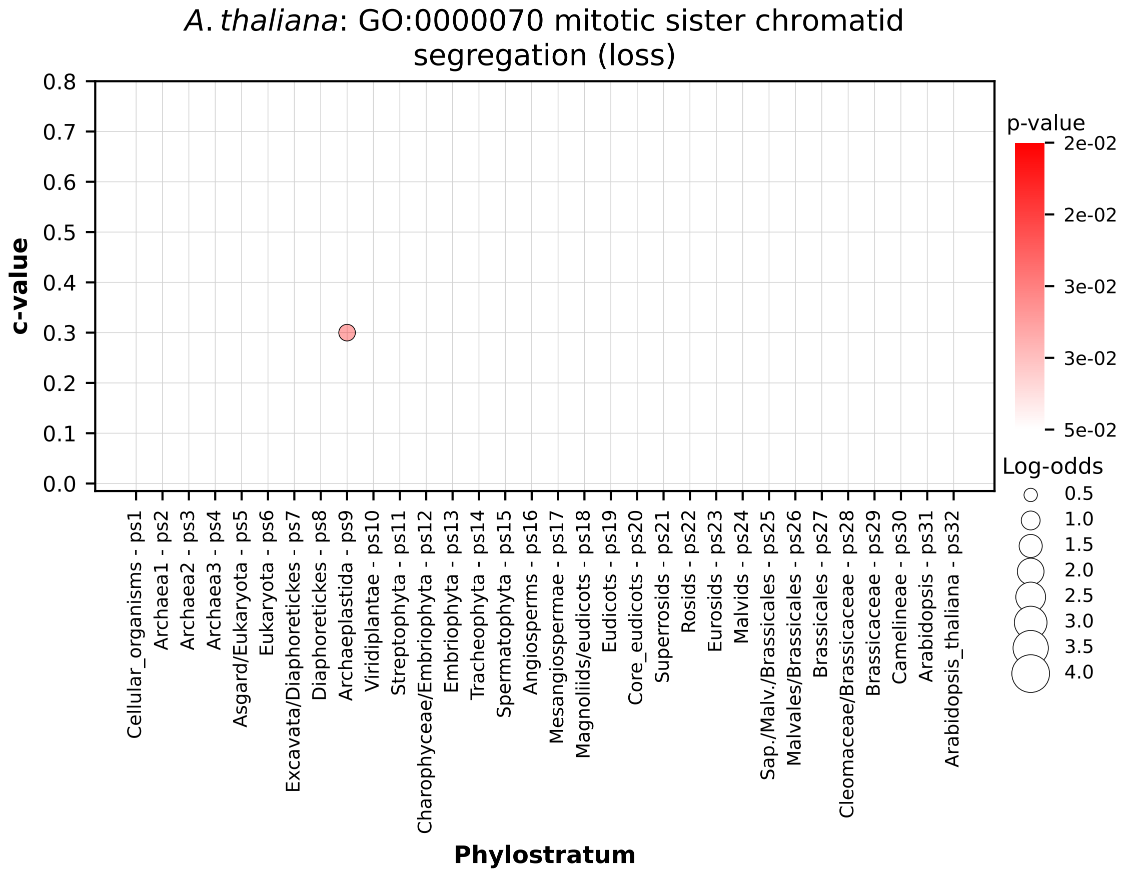

A. thaliana: GO:0000070 mitotic sister chromatid segregation (loss)
c-value
0.0
0.1
0.2
0.3
0.4
0.5
0.6
0.7
0.8
Phylostratum
Cellular_organisms - ps1
Archaea1 - ps2
Archaea2 - ps3
Archaea3 - ps4
Asgard/Eukaryota - ps5
Eukaryota - ps6
Excavata/Diaphoretickes - ps7
Diaphoretickes - ps8
Archaeplastida - ps9
Viridiplantae - ps10
Streptophyta - ps11
Charophyceae/Embriophyta - ps12
Embriophyta - ps13
Tracheophyta - ps14
Spermatophyta - ps15
Angiosperms - ps16
Mesangiospermae - ps17
Magnoliids/eudicots - ps18
Eudicots - ps19
Core_eudicots - ps20
Superrosids - ps21
Rosids - ps22
Eurosids - ps23
Malvids - ps24
Sap./Malv./Brassicales - ps25
Malvales/Brassicales - ps26
Brassicales - ps27
Cleomaceae/Brassicaceae - ps28
Brassicaceae - ps29
Camelineae - ps30
Arabidopsis - ps31
Arabidopsis_thaliana - ps32
p-value
2e-02
2e-02
3e-02
3e-02
5e-02
Log-odds
0.5
1.0
1.5
2.0
2.5
3.0
3.5
4.0

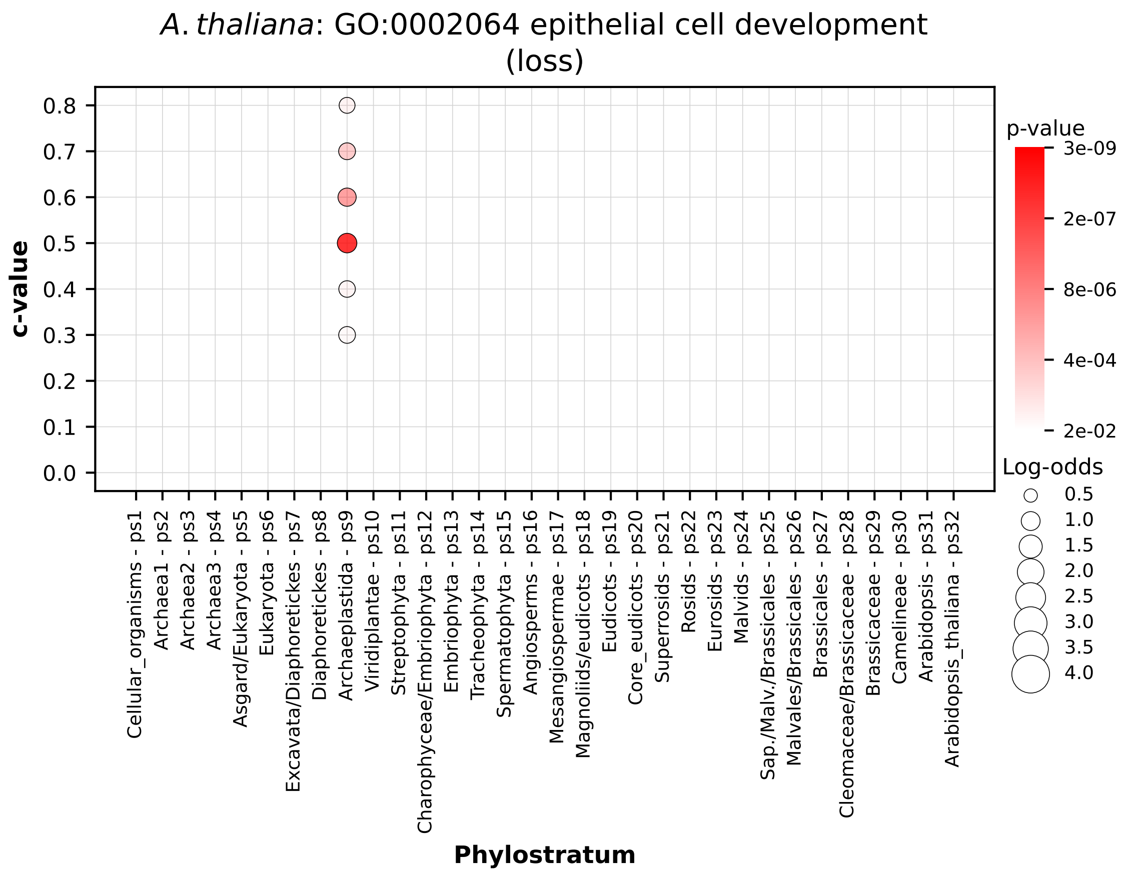
A. thaliana: GO:0002064 epithelial cell development
(loss)
c-value
0.8
0.7
0.6
0.5
0.4
0.3
0.2
0.1
0.0
p-value
3e-09
2e-07
8e-06
4e-04
2e-02
Log-odds
0.5
1.0
1.5
2.0
2.5
3.0
3.5
4.0
Cellular_organisms - ps1
Archaea1 - ps2
Archaea2 - ps3
Archaea3 - ps4
Asgard/Eukaryota - ps5
Eukaryota - ps6
Excavata/Diaphoretickes - ps7
Diaphoretickes - ps8
Archaeplastida - ps9
Viridiplantae - ps10
Streptophyta - ps11
Charophyceae/Embriophyta - ps12
Embriophyta - ps13
Tracheophyta - ps14
Spermatophyta - ps15
Angiosperms - ps16
Mesangiospermae - ps17
Magnoliids/eudicots - ps18
Eudicots - ps19
Core_eudicots - ps20
Superrosids - ps21
Rosids - ps22
Eurosids - ps23
Malvids - ps24
Sap./Malv./Brassicales - ps25
Malvales/Brassicales - ps26
Brassicales - ps27
Cleomaceae/Brassicaceae - ps28
Brassicaceae - ps29
Camelineae - ps30
Arabidopsis - ps31
Arabidopsis_thaliana - ps32
Phylostratum

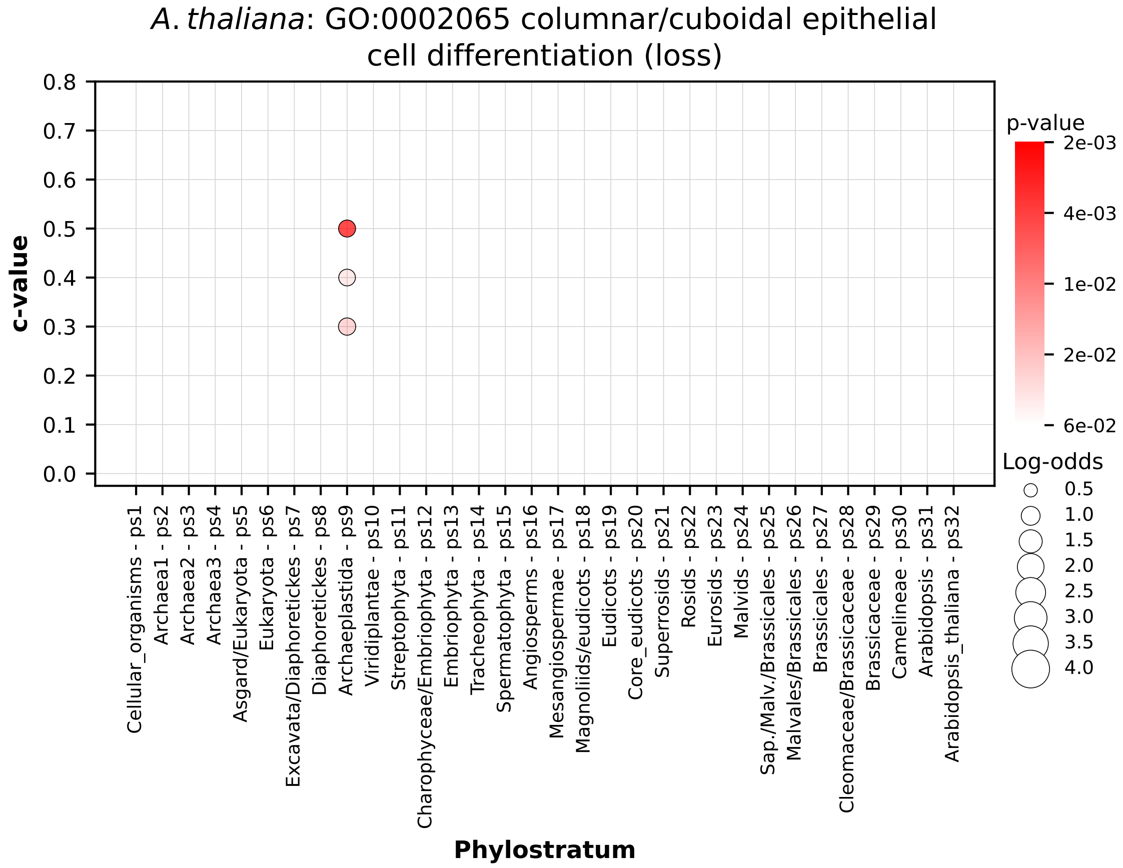

A. thaliana: GO:0002065 columnar/cuboidal epithelial cell differentiation (loss)
c-value
0.8
0.7
0.6
0.5
0.4
0.3
0.2
0.1
0.0
p-value
2e-03
4e-03
1e-02
2e-02
6e-02
Log-odds
0.5
1.0
1.5
2.0
2.5
3.0
3.5
4.0
Cellular_organisms - ps1
Archaea1 - ps2
Archaea2 - ps3
Archaea3 - ps4
Asgard/Eukaryota - ps5
Eukaryota - ps6
Excavata/Diaphoretickes - ps7
Diaphoretickes - ps8
Archaeplastida - ps9
Viridiplantae - ps10
Streptophyta - ps11
Charophyceae/Embriophyta - ps12
Embriophyta - ps13
Tracheophyta - ps14
Spermatophyta - ps15
Angiosperms - ps16
Mesangiospermae - ps17
Magnoliids/eudicots - ps18
Eudicots - ps19
Core_eudicots - ps20
Superrosids - ps21
Rosids - ps22
Eurosids - ps23
Malvids - ps24
Sap./Malv./Brassicales - ps25
Malvales/Brassicales - ps26
Brassicales - ps27
Cleomaceae/Brassicaceae - ps28
Brassicaceae - ps29
Camelineae - ps30
Arabidopsis - ps31
Arabidopsis_thaliana - ps32
Phylostratum

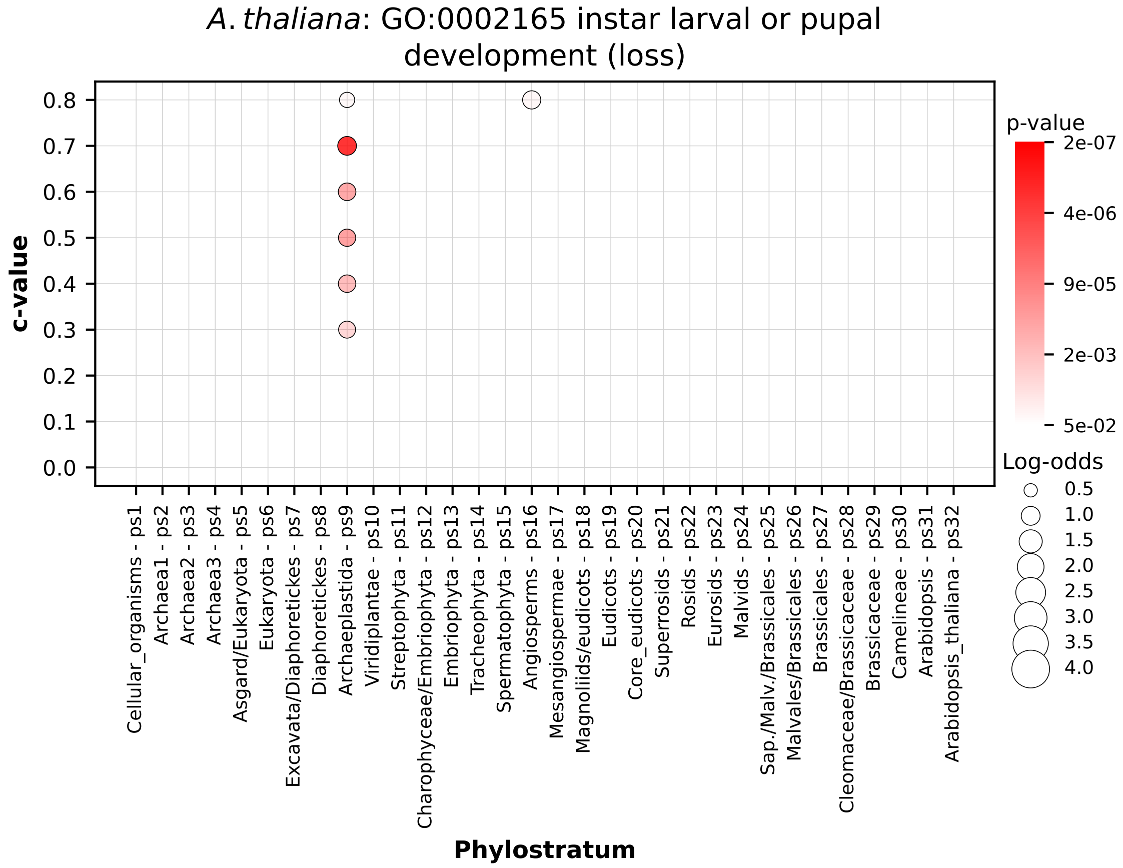

A. thaliana: GO:0002165 instar larval or pupal development (loss)
c-value
0.8
0.7
0.6
0.5
0.4
0.3
0.2
0.1
0.0
Phylostratum
Cellular_organisms - ps1
Archaea1 - ps2
Archaea2 - ps3
Archaea3 - ps4
Asgard/Eukaryota - ps5
Eukaryota - ps6
Excavata/Diaphoretickes - ps7
Diaphoretickes - ps8
Archaeplastida - ps9
Viridiplantae - ps10
Streptophyta - ps11
Charophyceae/Embriophyta - ps12
Embriophyta - ps13
Tracheophyta - ps14
Spermatophyta - ps15
Angiosperms - ps16
Mesangiospermae - ps17
Magnoliids/eudicots - ps18
Eudicots - ps19
Core_eudicots - ps20
Superrosids - ps21
Rosids - ps22
Eurosids - ps23
Malvids - ps24
Sap./Malv./Brassicales - ps25
Malvales/Brassicales - ps26
Brassicales - ps27
Cleomaceae/Brassicaceae - ps28
Brassicaceae - ps29
Camelineae - ps30
Arabidopsis - ps31
Arabidopsis_thaliana - ps32
p-value
2e-07
4e-06
9e-05
2e-03
5e-02
Log-odds
0.5
1.0
1.5
2.0
2.5
3.0
3.5
4.0

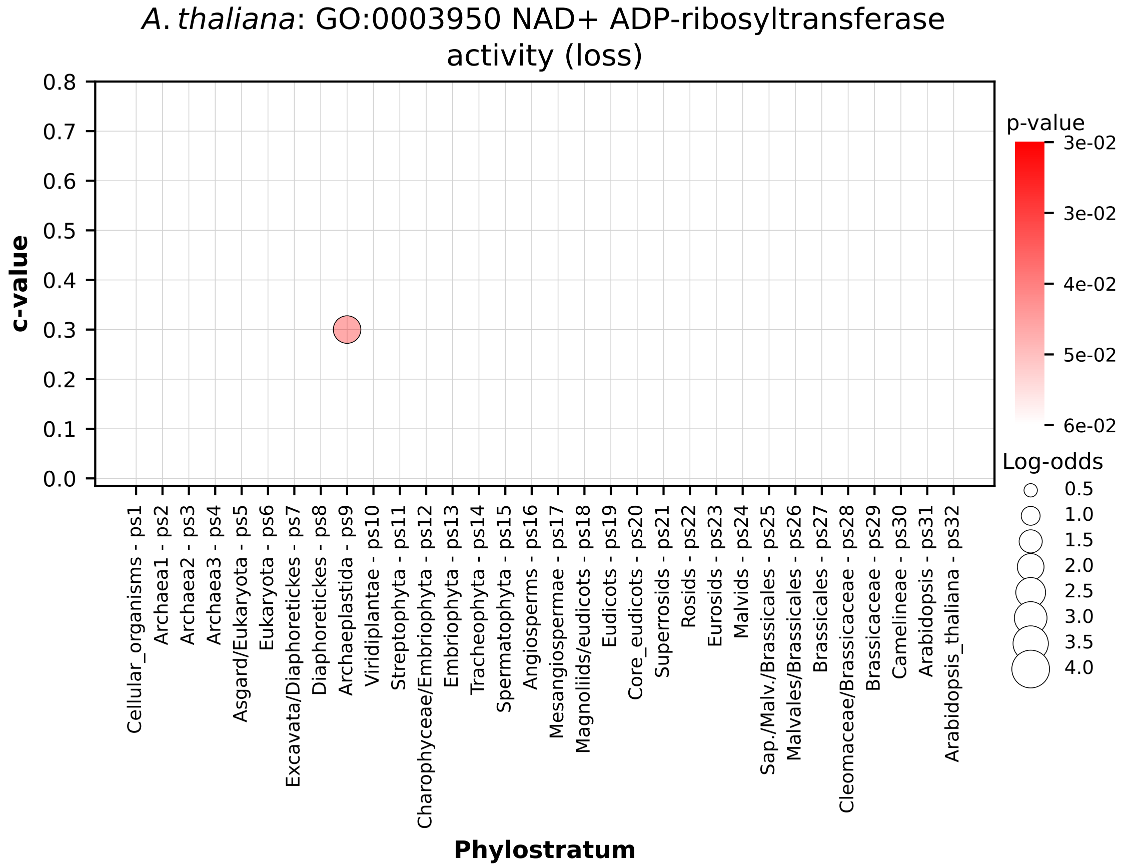

A. thaliana: GO:0003950 NAD+ ADP-ribosyltransferase activity (loss)
c-value
0.8
0.7
0.6
0.5
0.4
0.3
0.2
0.1
0.0
Phylostratum
Cellular_organisms - ps1
Archaea1 - ps2
Archaea2 - ps3
Archaea3 - ps4
Asgard/Eukaryota - ps5
Eukaryota - ps6
Excavata/Diaphoretickes - ps7
Diaphoretickes - ps8
Archaeplastida - ps9
Viridiplantae - ps10
Streptophyta - ps11
Charophyceae/Embriophyta - ps12
Embriophyta - ps13
Tracheophyta - ps14
Spermatophyta - ps15
Angiosperms - ps16
Mesangiospermae - ps17
Magnoliids/eudicots - ps18
Eudicots - ps19
Core_eudicots - ps20
Superrosids - ps21
Rosids - ps22
Eurosids - ps23
Malvids - ps24
Sap./Malv./Brassicales - ps25
Malvales/Brassicales - ps26
Brassicales - ps27
Cleomaceae/Brassicaceae - ps28
Brassicaceae - ps29
Camelineae - ps30
Arabidopsis - ps31
Arabidopsis_thaliana - ps32
p-value
3e-02
3e-02
4e-02
5e-02
6e-02
Log-odds
0.5
1.0
1.5
2.0
2.5
3.0
3.5
4.0

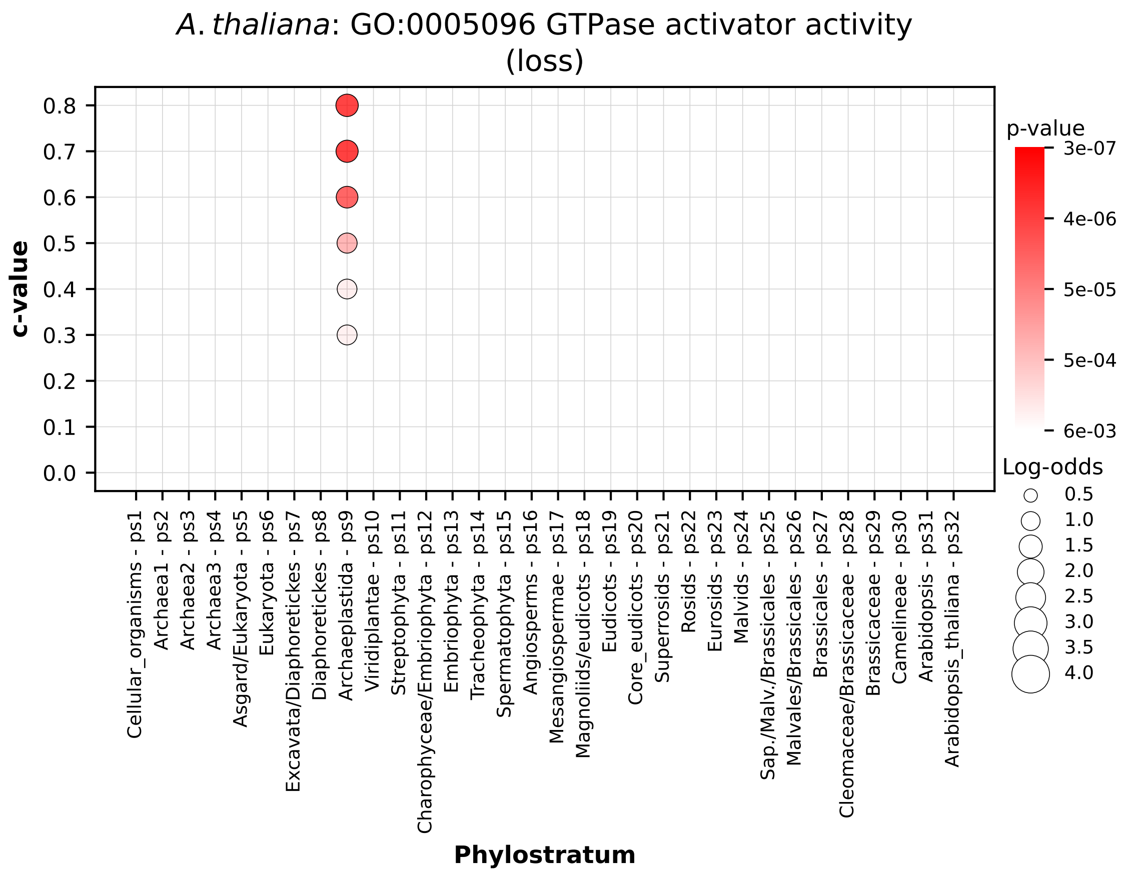

A. thaliana: GO:0005096 GTPase activator activity
(loss)
c-value
0.8
0.7
0.6
0.5
0.4
0.3
0.2
0.1
0.0
p-value
3e-07
4e-06
5e-05
5e-04
6e-03
Log-odds
0.5
1.0
1.5
2.0
2.5
3.0
3.5
4.0
Cellular_organisms - ps1
Archaea1 - ps2
Archaea2 - ps3
Archaea3 - ps4
Asgard/Eukaryota - ps5
Eukaryota - ps6
Excavata/Diaphoretickes - ps7
Diaphoretickes - ps8
Archaeplastida - ps9
Viridiplantae - ps10
Streptophyta - ps11
Charophyceae/Embriophyta - ps12
Embriophyta - ps13
Tracheophyta - ps14
Spermatophyta - ps15
Angiosperms - ps16
Mesangiospermae - ps17
Magnoliids/eudicots - ps18
Eudicots - ps19
Core_eudicots - ps20
Superrosids - ps21
Rosids - ps22
Eurosids - ps23
Malvids - ps24
Sap./Malv./Brassicales - ps25
Malvales/Brassicales - ps26
Brassicales - ps27
Cleomaceae/Brassicaceae - ps28
Brassicaceae - ps29
Camelineae - ps30
Arabidopsis - ps31
Arabidopsis_thaliana - ps32
Phylostratum

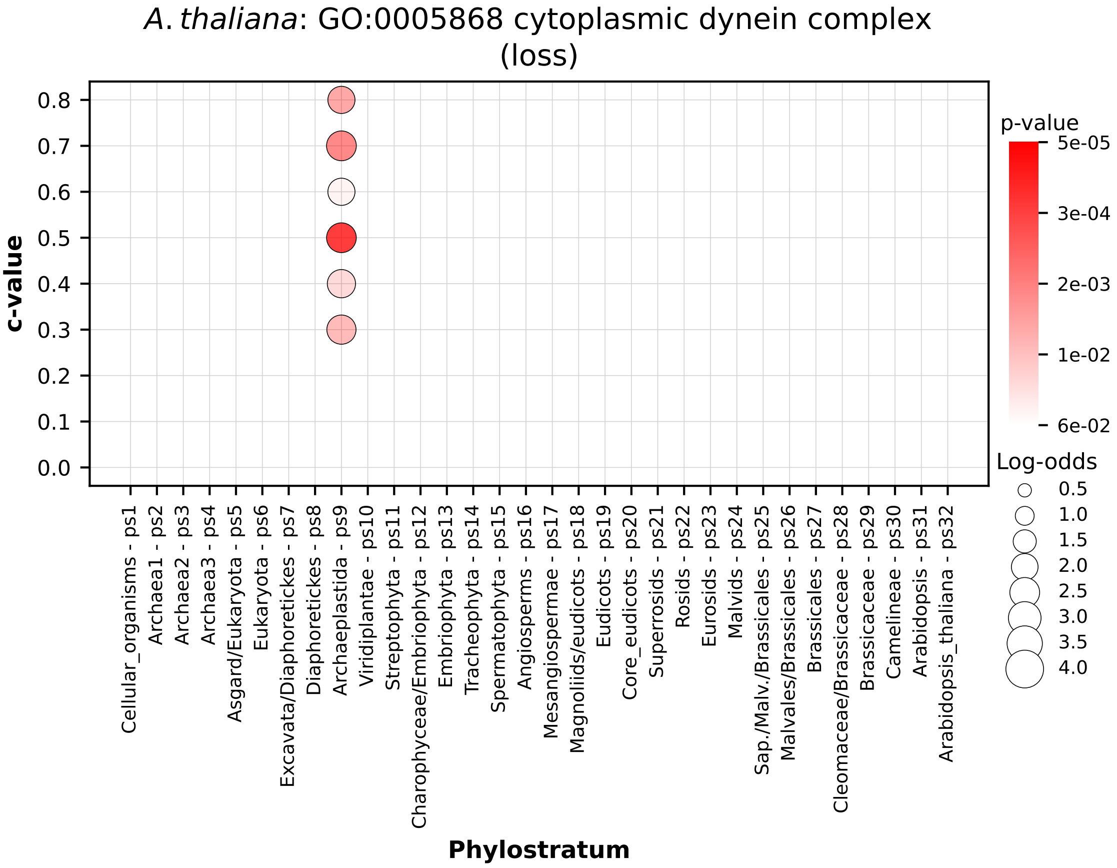

A. thaliana: GO:0005868 cytoplasmic dynein complex
(loss)
c-value
0.8
0.7
0.6
0.5
0.4
0.3
0.2
0.1
0.0
p-value
5e-05
3e-04
2e-03
1e-02
6e-02
Log-odds
0.5
1.0
1.5
2.0
2.5
3.0
3.5
4.0
Cellular_organisms - ps1
Archaea1 - ps2
Archaea2 - ps3
Archaea3 - ps4
Asgard/Eukaryota - ps5
Eukaryota - ps6
Excavata/Diaphoretickes - ps7
Diaphoretickes - ps8
Archaeplastida - ps9
Viridiplantae - ps10
Streptophyta - ps11
Charophyceae/Embriophyta - ps12
Embriophyta - ps13
Tracheophyta - ps14
Spermatophyta - ps15
Angiosperms - ps16
Mesangiospermae - ps17
Magnoliids/eudicots - ps18
Eudicots - ps19
Core_eudicots - ps20
Superrosids - ps21
Rosids - ps22
Eurosids - ps23
Malvids - ps24
Sap./Malv./Brassicales - ps25
Malvales/Brassicales - ps26
Brassicales - ps27
Cleomaceae/Brassicaceae - ps28
Brassicaceae - ps29
Camelineae - ps30
Arabidopsis - ps31
Arabidopsis_thaliana - ps32
Phylostratum

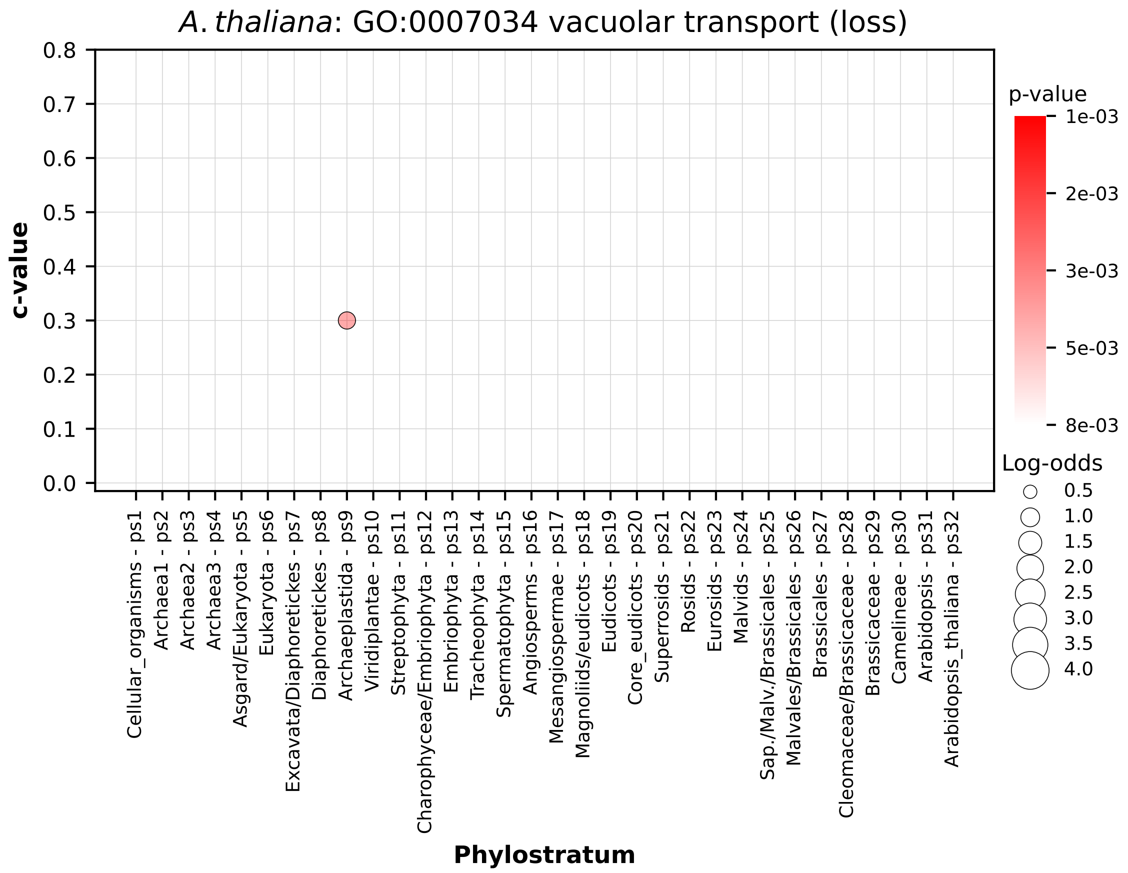

A. thaliana: GO:0007034 vacuolar transport (loss)
c-value
0.8
0.7
0.6
0.5
0.4
0.3
0.2
0.1
0.0
p-value
1e-03
2e-03
3e-03
5e-03
8e-03
Log-odds
0.5
1.0
1.5
2.0
2.5
3.0
3.5
4.0
Cellular_organisms - ps1
Archaea1 - ps2
Archaea2 - ps3
Archaea3 - ps4
Asgard/Eukaryota - ps5
Eukaryota - ps6
Excavata/Diaphoretickes - ps7
Diaphoretickes - ps8
Archaeplastida - ps9
Viridiplantae - ps10
Streptophyta - ps11
Charophyceae/Embriophyta - ps12
Embriophyta - ps13
Tracheophyta - ps14
Spermatophyta - ps15
Angiosperms - ps16
Mesangiospermae - ps17
Magnoliids/eudicots - ps18
Eudicots - ps19
Core_eudicots - ps20
Superrosids - ps21
Rosids - ps22
Eurosids - ps23
Malvids - ps24
Sap./Malv./Brassicales - ps25
Malvales/Brassicales - ps26
Brassicales - ps27
Cleomaceae/Brassicaceae - ps28
Brassicaceae - ps29
Camelineae - ps30
Arabidopsis - ps31
Arabidopsis_thaliana - ps32
Phylostratum

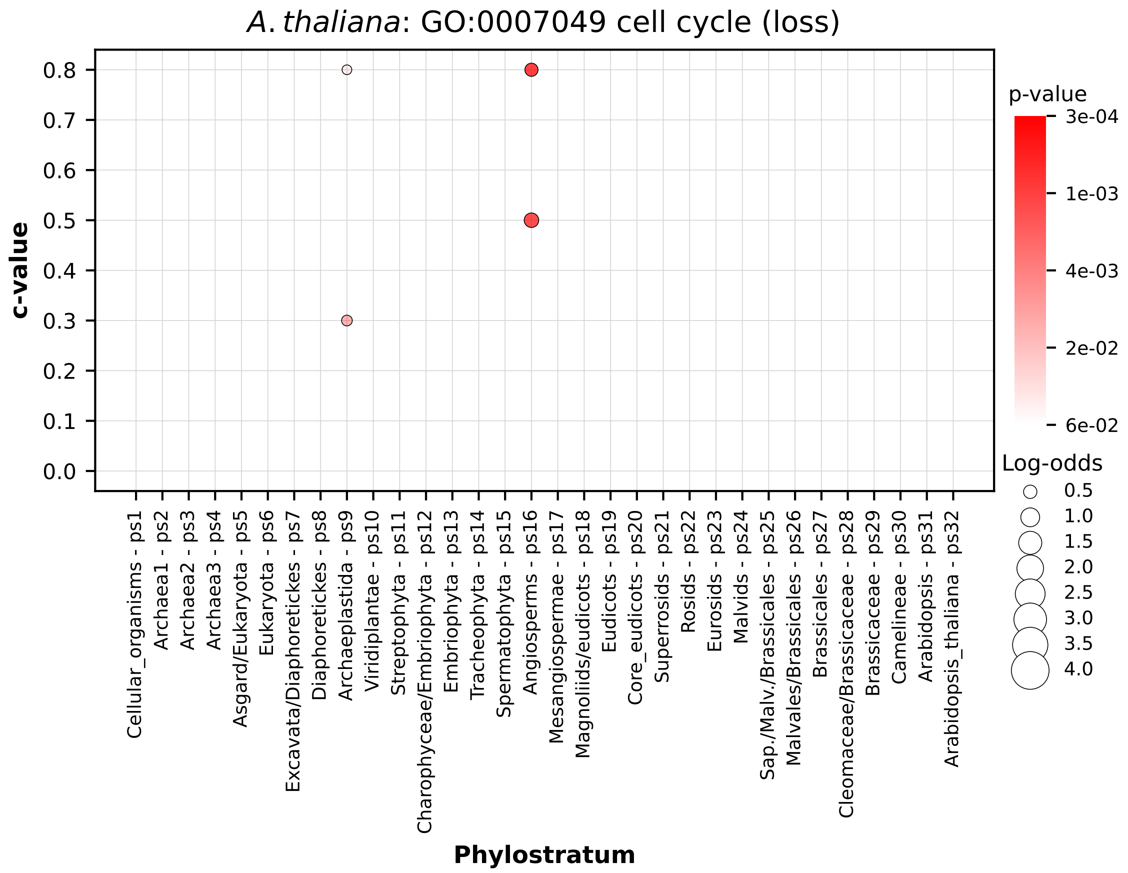

A. thaliana: GO:0007049 cell cycle (loss)
c-value
0.0
0.1
0.2
0.3
0.4
0.5
0.6
0.7
0.8
p-value
3e-04
1e-03
4e-03
2e-02
6e-02
Log-odds
0.5
1.0
1.5
2.0
2.5
3.0
3.5
4.0
Cellular_organisms - ps1
Archaea1 - ps2
Archaea2 - ps3
Archaea3 - ps4
Asgard/Eukaryota - ps5
Eukaryota - ps6
Excavata/Diaphoretickes - ps7
Diaphoretickes - ps8
Archaeplastida - ps9
Viridiplantae - ps10
Streptophyta - ps11
Charophyceae/Embriophyta - ps12
Embriophyta - ps13
Tracheophyta - ps14
Spermatophyta - ps15
Angiosperms - ps16
Mesangiospermae - ps17
Magnoliids/eudicots - ps18
Eudicots - ps19
Core_eudicots - ps20
Superrosids - ps21
Rosids - ps22
Eurosids - ps23
Malvids - ps24
Sap./Malv./Brassicales - ps25
Malvales/Brassicales - ps26
Brassicales - ps27
Cleomaceae/Brassicaceae - ps28
Brassicaceae - ps29
Camelineae - ps30
Arabidopsis - ps31
Arabidopsis_thaliana - ps32
Phylostratum

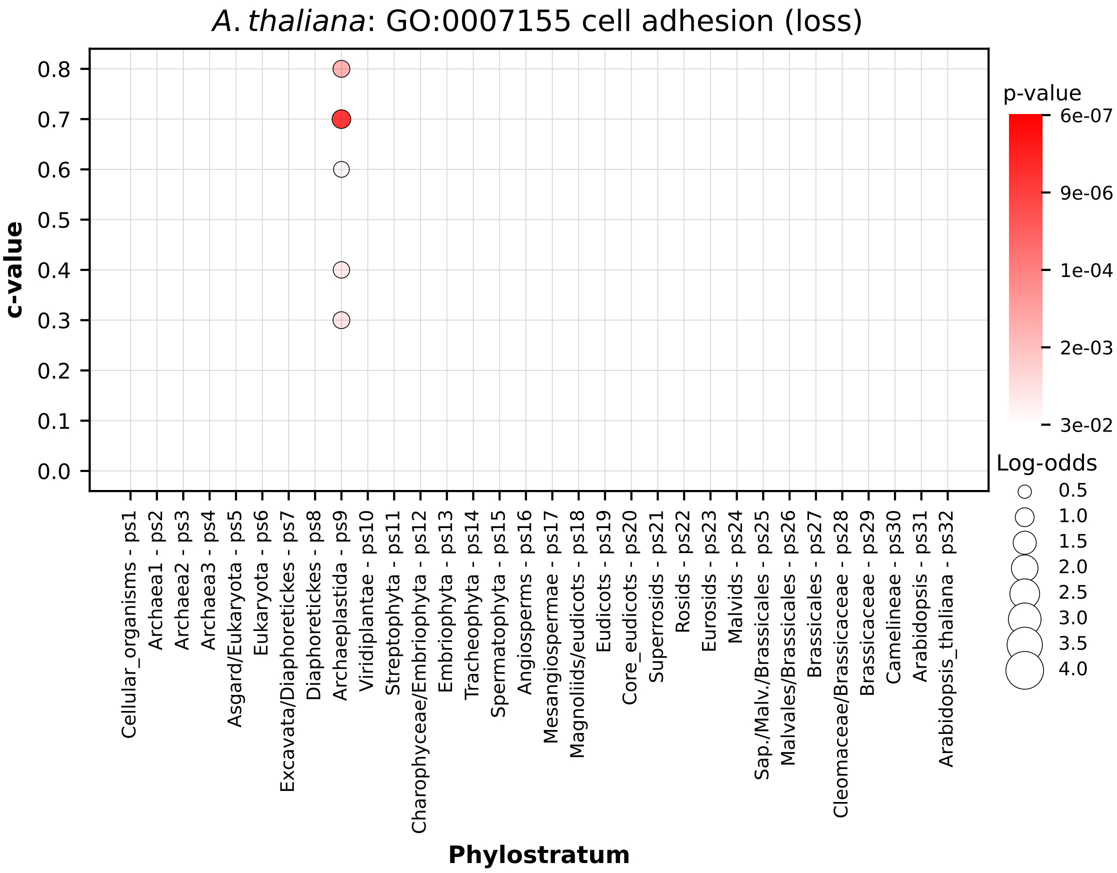

A. thaliana: GO:0007155 cell adhesion (loss)
c-value
0.8
0.7
0.6
0.5
0.4
0.3
0.2
0.1
0.0
p-value
6e-07
9e-06
1e-04
2e-03
3e-02
Log-odds
0.5
1.0
1.5
2.0
2.5
3.0
3.5
4.0
Cellular_organisms - ps1
Archaea1 - ps2
Archaea2 - ps3
Archaea3 - ps4
Asgard/Eukaryota - ps5
Eukaryota - ps6
Excavata/Diaphoretickes - ps7
Diaphoretickes - ps8
Archaeplastida - ps9
Viridiplantae - ps10
Streptophyta - ps11
Charophyceae/Embriophyta - ps12
Embriophyta - ps13
Tracheophyta - ps14
Spermatophyta - ps15
Angiosperms - ps16
Mesangiospermae - ps17
Magnoliids/eudicots - ps18
Eudicots - ps19
Core_eudicots - ps20
Superrosids - ps21
Rosids - ps22
Eurosids - ps23
Malvids - ps24
Sap./Malv./Brassicales - ps25
Malvales/Brassicales - ps26
Brassicales - ps27
Cleomaceae/Brassicaceae - ps28
Brassicaceae - ps29
Camelineae - ps30
Arabidopsis - ps31
Arabidopsis_thaliana - ps32
Phylostratum

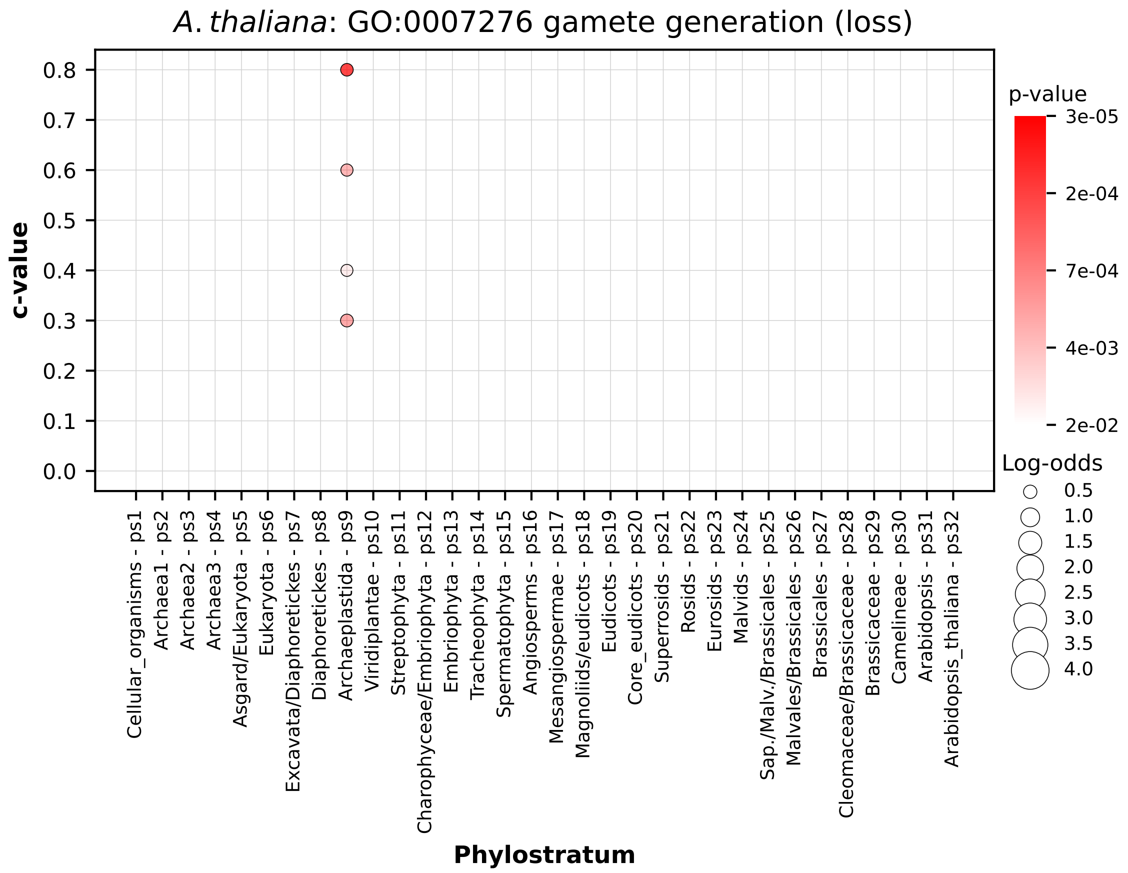

A. thaliana: GO:0007276 gamete generation (loss)
c-value
0.0
0.1
0.2
0.3
0.4
0.5
0.6
0.7
0.8
p-value
3e-05
2e-04
7e-04
4e-03
2e-02
Log-odds
0.5
1.0
1.5
2.0
2.5
3.0
3.5
4.0
Cellular_organisms - ps1
Archaea1 - ps2
Archaea2 - ps3
Archaea3 - ps4
Asgard/Eukaryota - ps5
Eukaryota - ps6
Excavata/Diaphoretickes - ps7
Diaphoretickes - ps8
Archaeplastida - ps9
Viridiplantae - ps10
Streptophyta - ps11
Charophyceae/Embriophyta - ps12
Embriophyta - ps13
Tracheophyta - ps14
Spermatophyta - ps15
Angiosperms - ps16
Mesangiospermae - ps17
Magnoliids/eudicots - ps18
Eudicots - ps19
Core_eudicots - ps20
Superrosids - ps21
Rosids - ps22
Eurosids - ps23
Malvids - ps24
Sap./Malv./Brassicales - ps25
Malvales/Brassicales - ps26
Brassicales - ps27
Cleomaceae/Brassicaceae - ps28
Brassicaceae - ps29
Camelineae - ps30
Arabidopsis - ps31
Arabidopsis_thaliana - ps32
Phylostratum

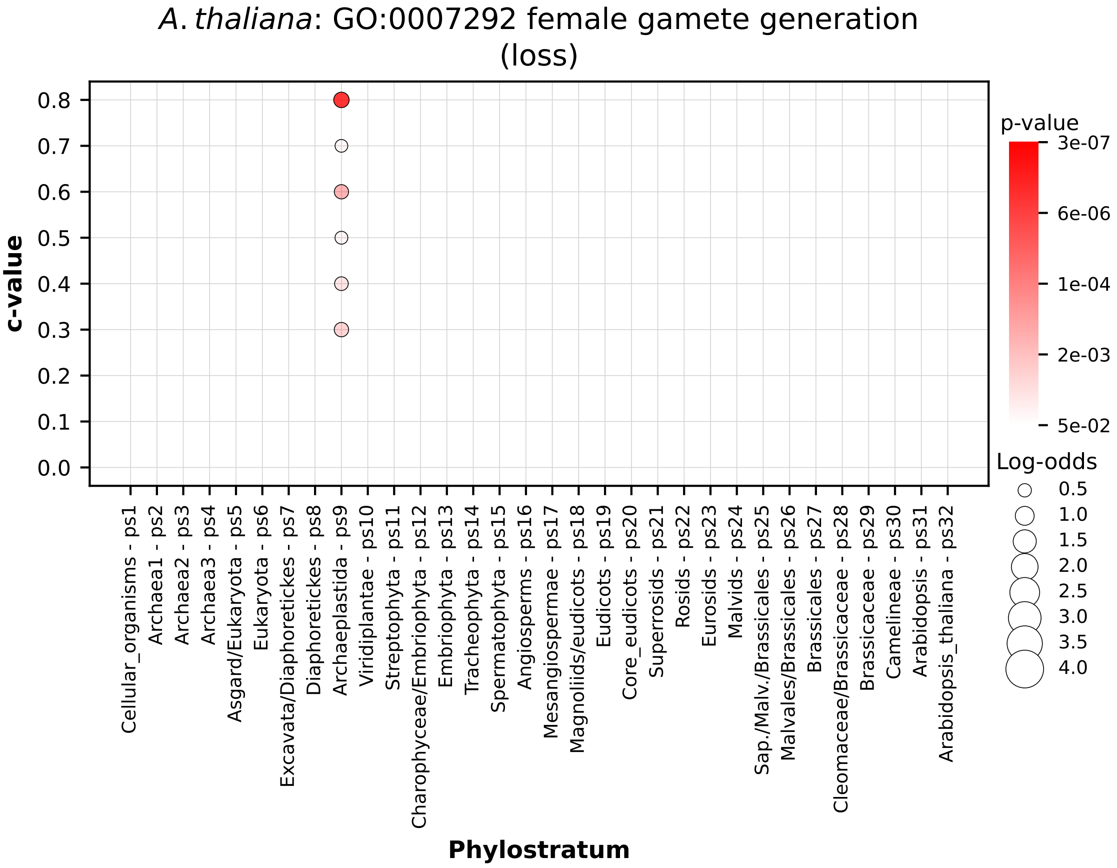

A. thaliana: GO:0007292 female gamete generation
(loss)
c-value
0.8
0.7
0.6
0.5
0.4
0.3
0.2
0.1
0.0
Phylostratum
Cellular_organisms - ps1
Archaea1 - ps2
Archaea2 - ps3
Archaea3 - ps4
Asgard/Eukaryota - ps5
Eukaryota - ps6
Excavata/Diaphoretickes - ps7
Diaphoretickes - ps8
Archaeplastida - ps9
Viridiplantae - ps10
Streptophyta - ps11
Charophyceae/Embriophyta - ps12
Embriophyta - ps13
Tracheophyta - ps14
Spermatophyta - ps15
Angiosperms - ps16
Mesangiospermae - ps17
Magnoliids/eudicots - ps18
Eudicots - ps19
Core_eudicots - ps20
Superrosids - ps21
Rosids - ps22
Eurosids - ps23
Malvids - ps24
Sap./Malv./Brassicales - ps25
Malvales/Brassicales - ps26
Brassicales - ps27
Cleomaceae/Brassicaceae - ps28
Brassicaceae - ps29
Camelineae - ps30
Arabidopsis - ps31
Arabidopsis_thaliana - ps32
p-value
3e-07
6e-06
1e-04
2e-03
5e-02
Log-odds
0.5
1.0
1.5
2.0
2.5
3.0
3.5
4.0

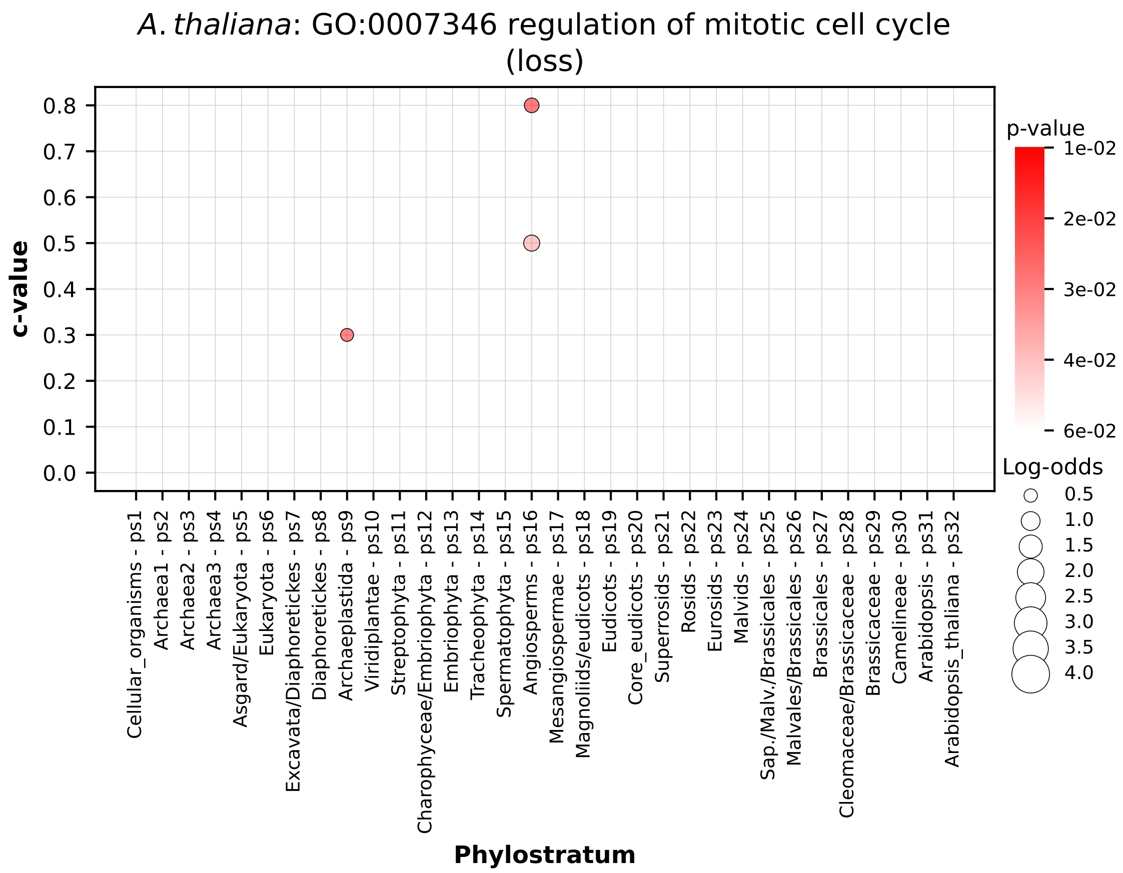

A. thaliana: GO:0007346 regulation of mitotic cell cycle (loss)
c-value
0.0
0.1
0.2
0.3
0.4
0.5
0.6
0.7
0.8
Phylostratum
Cellular_organisms - ps1
Archaea1 - ps2
Archaea2 - ps3
Archaea3 - ps4
Asgard/Eukaryota - ps5
Eukaryota - ps6
Excavata/Diaphoretickes - ps7
Diaphoretickes - ps8
Archaeplastida - ps9
Viridiplantae - ps10
Streptophyta - ps11
Charophyceae/Embriophyta - ps12
Embriophyta - ps13
Tracheophyta - ps14
Spermatophyta - ps15
Angiosperms - ps16
Mesangiospermae - ps17
Magnoliids/eudicots - ps18
Eudicots - ps19
Core_eudicots - ps20
Superrosids - ps21
Rosids - ps22
Eurosids - ps23
Malvids - ps24
Sap./Malv./Brassicales - ps25
Malvales/Brassicales - ps26
Brassicales - ps27
Cleomaceae/Brassicaceae - ps28
Brassicaceae - ps29
Camelineae - ps30
Arabidopsis - ps31
Arabidopsis_thaliana - ps32
p-value
1e-02
2e-02
3e-02
4e-02
6e-02
Log-odds
0.5
1.0
1.5
2.0
2.5
3.0
3.5
4.0

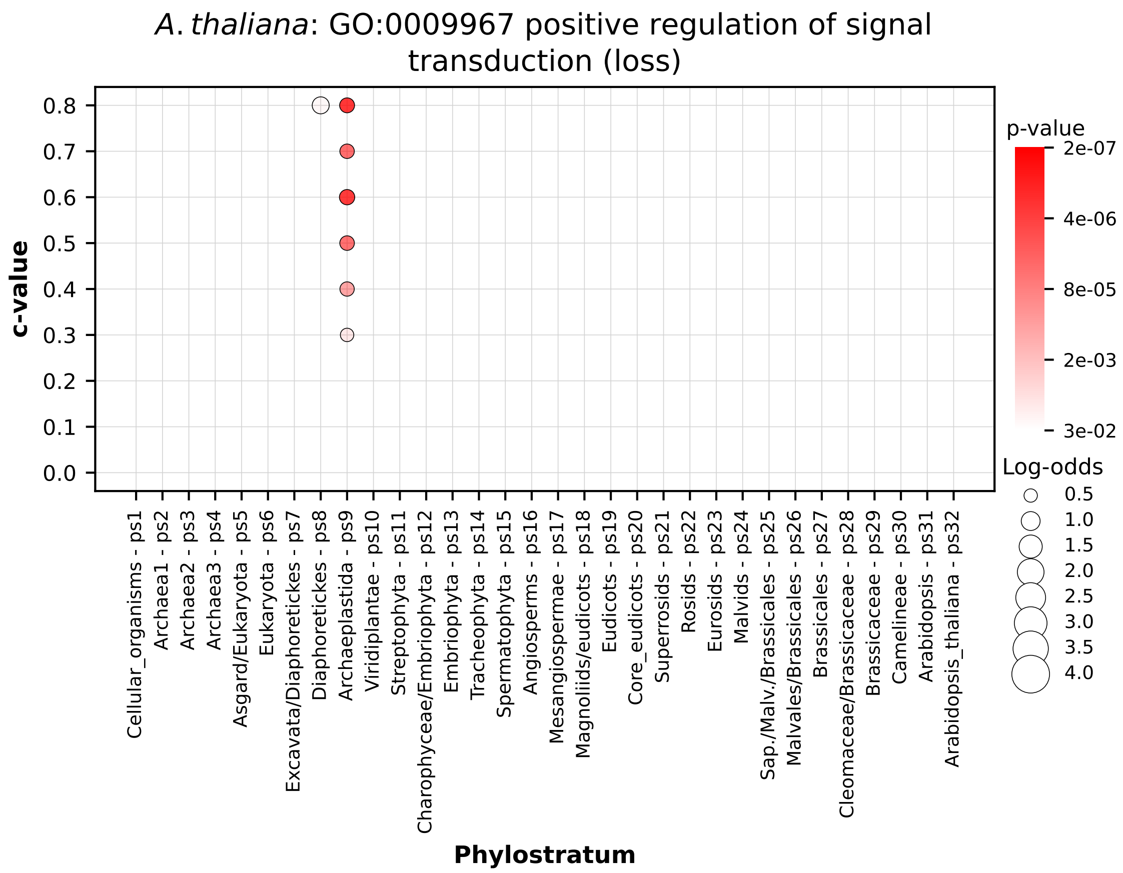
A. thaliana: GO:0009967 positive regulation of signal transduction (loss)
c-value
0.8
0.7
0.6
0.5
0.4
0.3
0.2
0.1
0.0
Phylostratum
Cellular_organisms - ps1
Archaea1 - ps2
Archaea2 - ps3
Archaea3 - ps4
Asgard/Eukaryota - ps5
Eukaryota - ps6
Excavata/Diaphoretickes - ps7
Diaphoretickes - ps8
Archaeplastida - ps9
Viridiplantae - ps10
Streptophyta - ps11
Charophyceae/Embriophyta - ps12
Embriophyta - ps13
Tracheophyta - ps14
Spermatophyta - ps15
Angiosperms - ps16
Mesangiospermae - ps17
Magnoliids/eudicots - ps18
Eudicots - ps19
Core_eudicots - ps20
Superrosids - ps21
Rosids - ps22
Eurosids - ps23
Malvids - ps24
Sap./Malv./Brassicales - ps25
Malvales/Brassicales - ps26
Brassicales - ps27
Cleomaceae/Brassicaceae - ps28
Brassicaceae - ps29
Camelineae - ps30
Arabidopsis - ps31
Arabidopsis_thaliana - ps32
p-value
2e-07
4e-06
8e-05
2e-03
3e-02
Log-odds
0.5
1.0
1.5
2.0
2.5
3.0
3.5
4.0

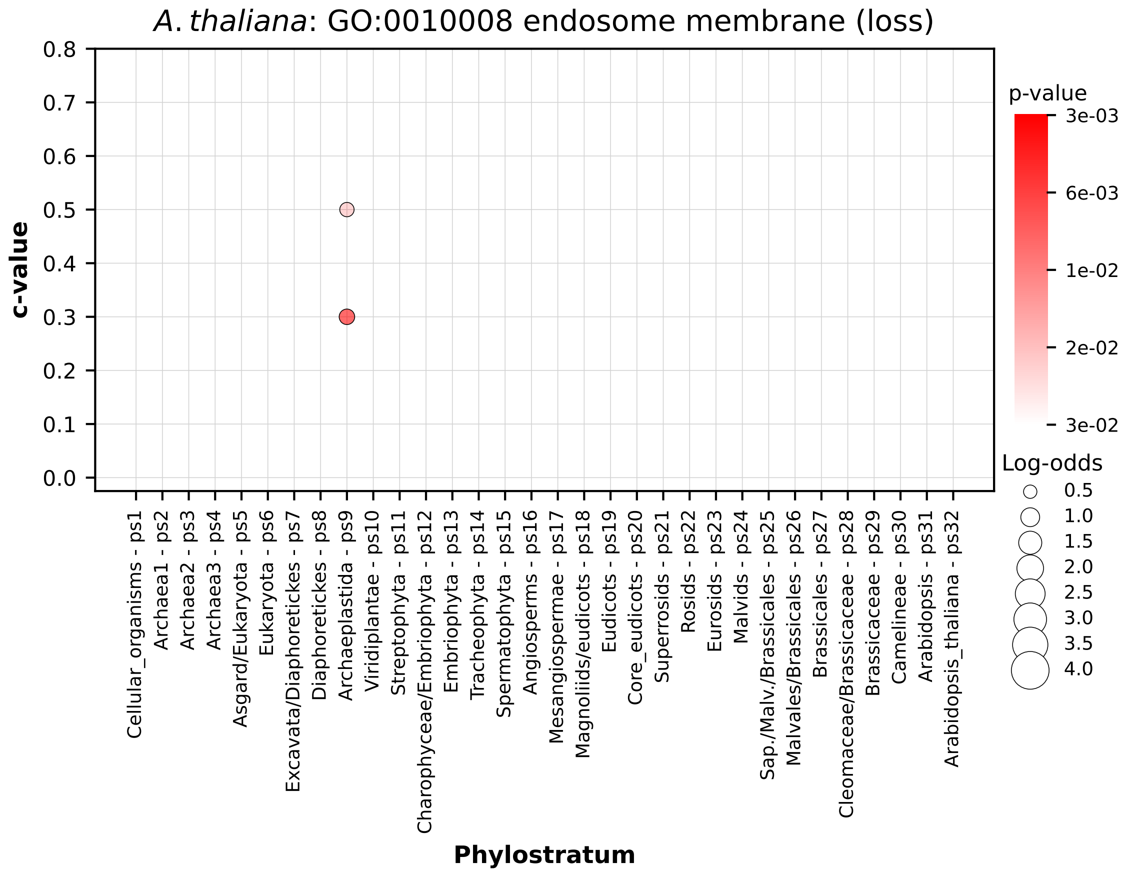
A. thaliana: GO:0010008 endosome membrane (loss)
c-value
0.8
0.7
0.6
0.5
0.4
0.3
0.2
0.1
0.0
Phylostratum
Cellular_organisms - ps1
Archaea1 - ps2
Archaea2 - ps3
Archaea3 - ps4
Asgard/Eukaryota - ps5
Eukaryota - ps6
Excavata/Diaphoretickes - ps7
Diaphoretickes - ps8
Archaeplastida - ps9
Viridiplantae - ps10
Streptophyta - ps11
Charophyceae/Embriophyta - ps12
Embriophyta - ps13
Tracheophyta - ps14
Spermatophyta - ps15
Angiosperms - ps16
Mesangiospermae - ps17
Magnoliids/eudicots - ps18
Eudicots - ps19
Core_eudicots - ps20
Superrosids - ps21
Rosids - ps22
Eurosids - ps23
Malvids - ps24
Sap./Malv./Brassicales - ps25
Malvales/Brassicales - ps26
Brassicales - ps27
Cleomaceae/Brassicaceae - ps28
Brassicaceae - ps29
Camelineae - ps30
Arabidopsis - ps31
Arabidopsis_thaliana - ps32
p-value
3e-03
6e-03
1e-02
2e-02
3e-02
Log-odds
0.5
1.0
1.5
2.0
2.5
3.0
3.5
4.0

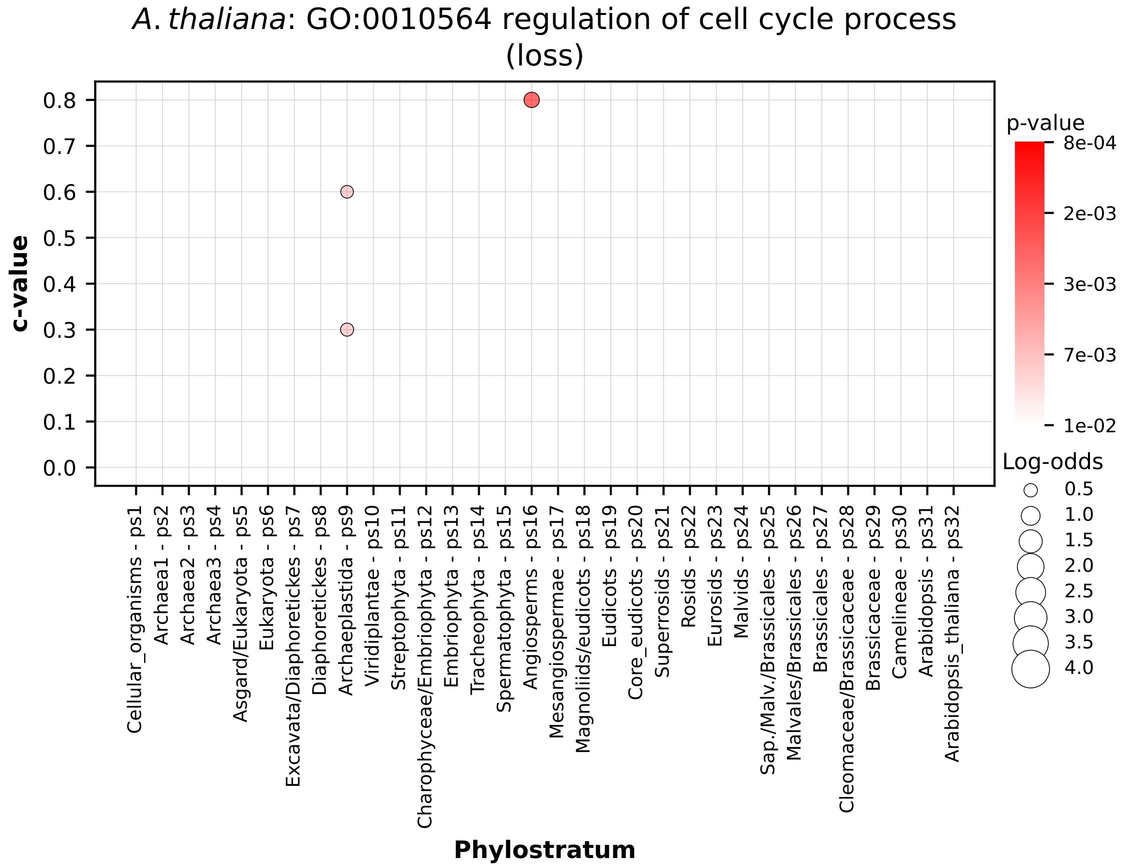

A. thaliana: GO:0010564 regulation of cell cycle process
(loss)
c-value
0.8
0.7
0.6
0.5
0.4
0.3
0.2
0.1
0.0
Phylostratum
Cellular_organisms - ps1
Archaea1 - ps2
Archaea2 - ps3
Archaea3 - ps4
Asgard/Eukaryota - ps5
Eukaryota - ps6
Excavata/Diaphoretickes - ps7
Diaphoretickes - ps8
Archaeplastida - ps9
Viridiplantae - ps10
Streptophyta - ps11
Charophyceae/Embriophyta - ps12
Embriophyta - ps13
Tracheophyta - ps14
Spermatophyta - ps15
Angiosperms - ps16
Mesangiospermae - ps17
Magnoliids/eudicots - ps18
Eudicots - ps19
Core_eudicots - ps20
Superrosids - ps21
Rosids - ps22
Eurosids - ps23
Malvids - ps24
Sap./Malv./Brassicales - ps25
Malvales/Brassicales - ps26
Brassicales - ps27
Cleomaceae/Brassicaceae - ps28
Brassicaceae - ps29
Camelineae - ps30
Arabidopsis - ps31
Arabidopsis_thaliana - ps32
p-value
8e-04
2e-03
3e-03
7e-03
1e-02
Log-odds
0.5
1.0
1.5
2.0
2.5
3.0
3.5
4.0

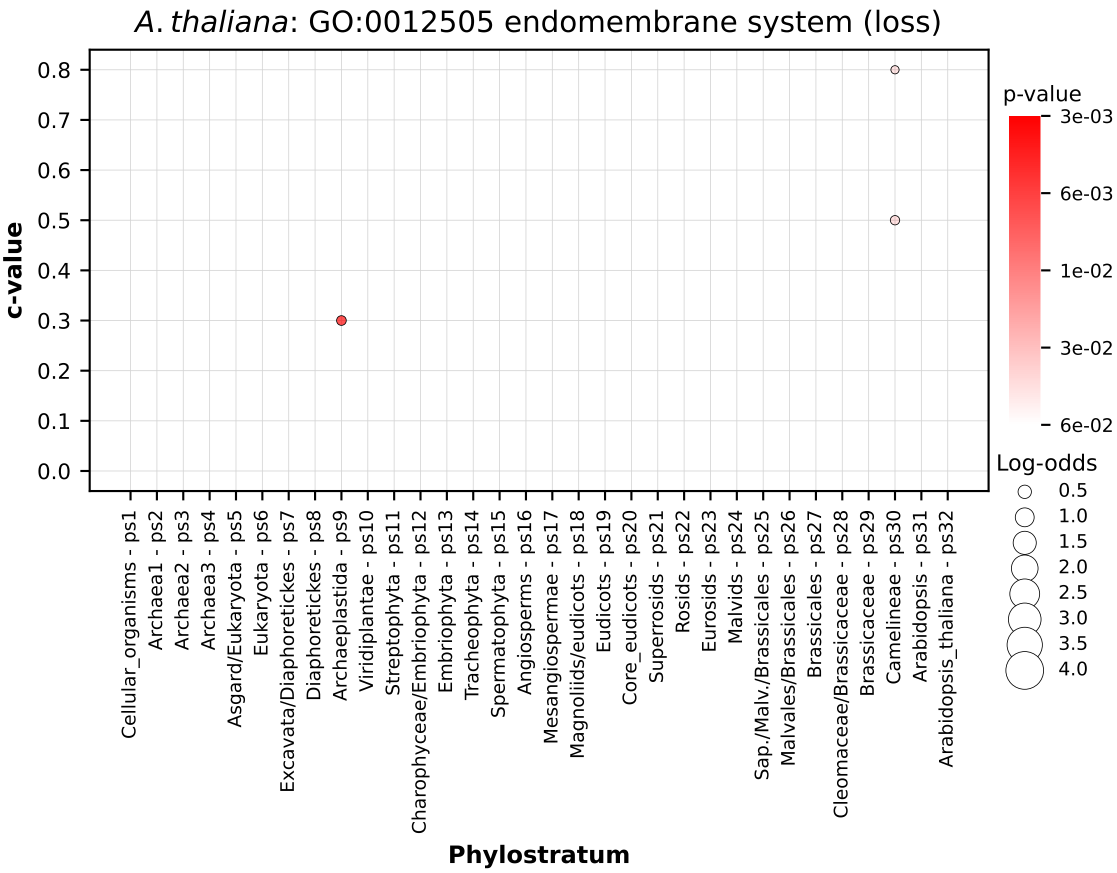

A. thaliana: GO:0012505 endomembrane system (loss)
c-value
0.8
0.7
0.6
0.5
0.4
0.3
0.2
0.1
0.0
Phylostratum
Cellular_organisms - ps1
Archaea1 - ps2
Archaea2 - ps3
Archaea3 - ps4
Asgard/Eukaryota - ps5
Eukaryota - ps6
Excavata/Diaphoretickes - ps7
Diaphoretickes - ps8
Archaeplastida - ps9
Viridiplantae - ps10
Streptophyta - ps11
Charophyceae/Embriophyta - ps12
Embriophyta - ps13
Tracheophyta - ps14
Spermatophyta - ps15
Angiosperms - ps16
Mesangiospermae - ps17
Magnoliids/eudicots - ps18
Eudicots - ps19
Core_eudicots - ps20
Superrosids - ps21
Rosids - ps22
Eurosids - ps23
Malvids - ps24
Sap./Malv./Brassicales - ps25
Malvales/Brassicales - ps26
Brassicales - ps27
Cleomaceae/Brassicaceae - ps28
Brassicaceae - ps29
Camelineae - ps30
Arabidopsis - ps31
Arabidopsis_thaliana - ps32
p-value
3e-03
6e-03
1e-02
3e-02
6e-02
Log-odds
0.5
1.0
1.5
2.0
2.5
3.0
3.5
4.0

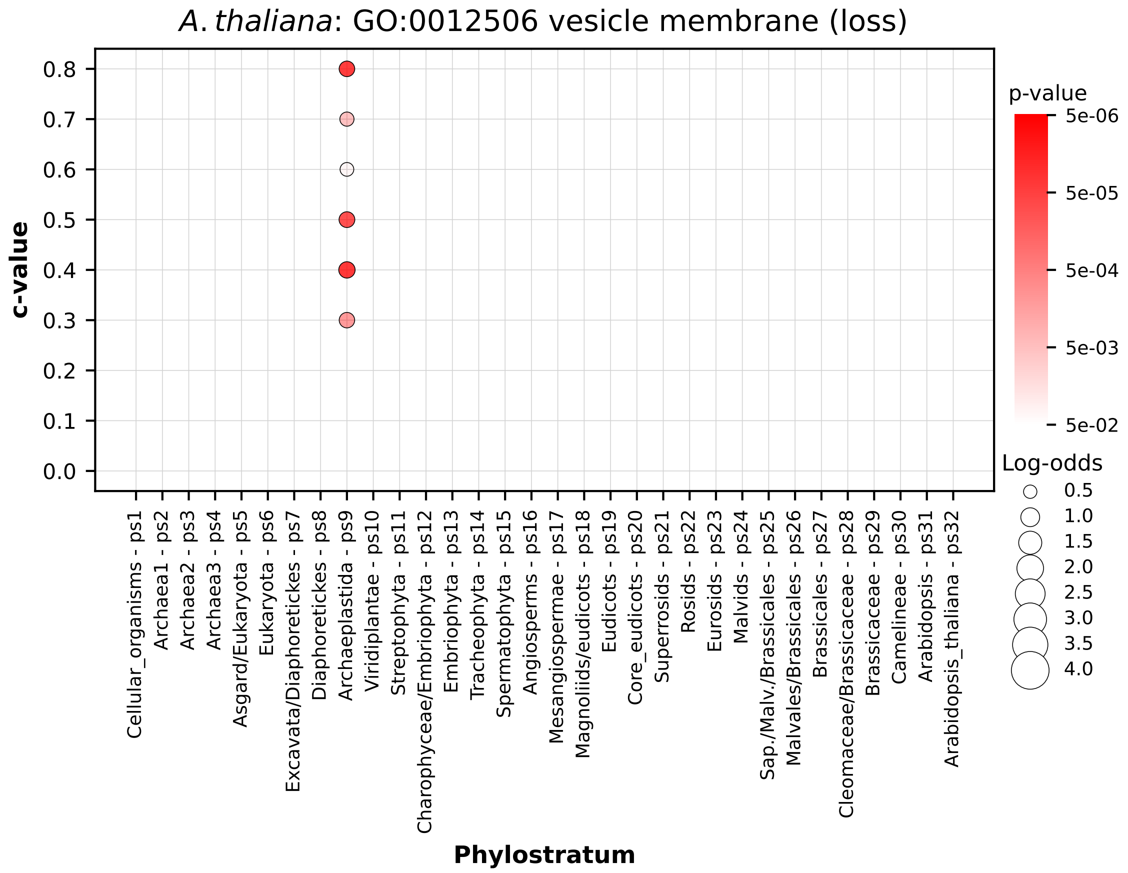

A. thaliana: GO:0012506 vesicle membrane (loss)
c-value
0.0
0.1
0.2
0.3
0.4
0.5
0.6
0.7
0.8
Phylostratum
Cellular_organisms - ps1
Archaea1 - ps2
Archaea2 - ps3
Archaea3 - ps4
Asgard/Eukaryota - ps5
Eukaryota - ps6
Excavata/Diaphoretickes - ps7
Diaphoretickes - ps8
Archaeplastida - ps9
Viridiplantae - ps10
Streptophyta - ps11
Charophyceae/Embriophyta - ps12
Embriophyta - ps13
Tracheophyta - ps14
Spermatophyta - ps15
Angiosperms - ps16
Mesangiospermae - ps17
Magnoliids/eudicots - ps18
Eudicots - ps19
Core_eudicots - ps20
Superrosids - ps21
Rosids - ps22
Eurosids - ps23
Malvids - ps24
Sap./Malv./Brassicales - ps25
Malvales/Brassicales - ps26
Brassicales - ps27
Cleomaceae/Brassicaceae - ps28
Brassicaceae - ps29
Camelineae - ps30
Arabidopsis - ps31
Arabidopsis_thaliana - ps32
p-value
5e-06
5e-05
5e-04
5e-03
5e-02
Log-odds
0.5
1.0
1.5
2.0
2.5
3.0
3.5
4.0

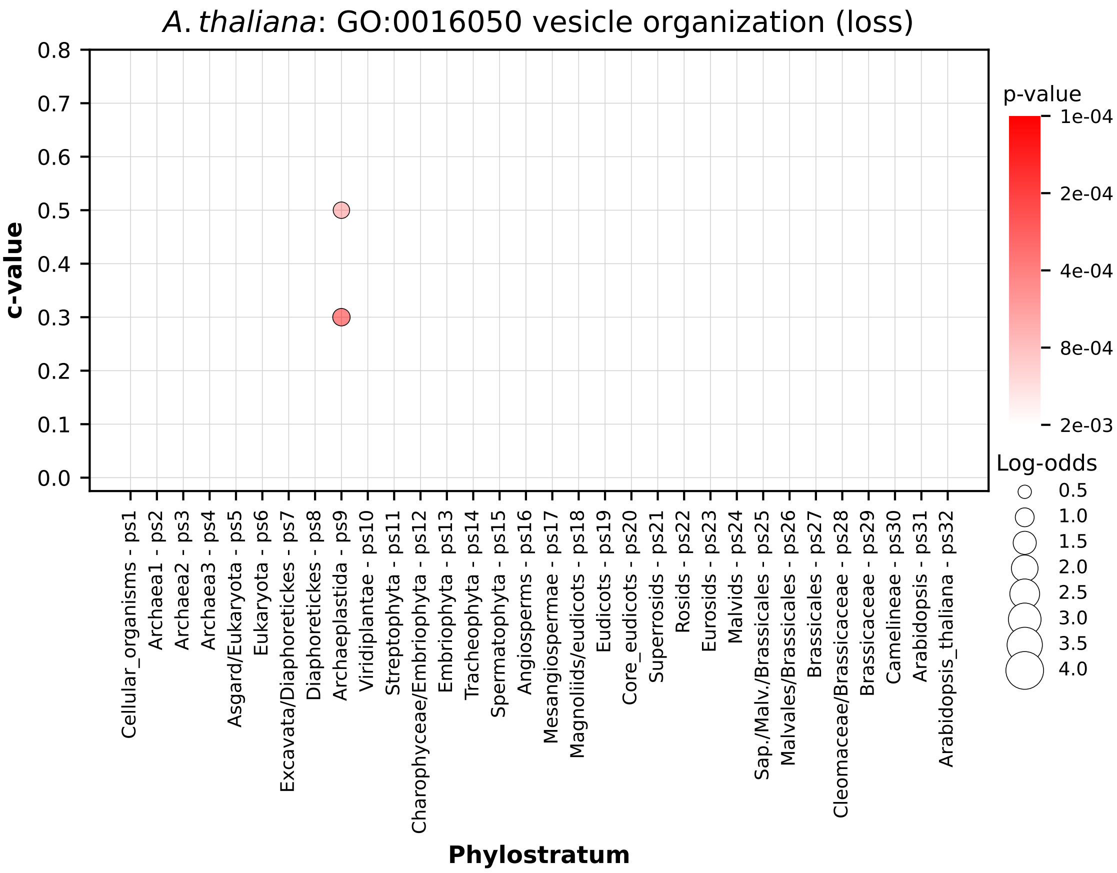

A. thaliana: GO:0016050 vesicle organization (loss)
c-value
0.8
0.7
0.6
0.5
0.4
0.3
0.2
0.1
0.0
p-value
1e-04
2e-04
4e-04
8e-04
2e-03
Log-odds
0.5
1.0
1.5
2.0
2.5
3.0
3.5
4.0
Cellular_organisms - ps1
Archaea1 - ps2
Archaea2 - ps3
Archaea3 - ps4
Asgard/Eukaryota - ps5
Eukaryota - ps6
Excavata/Diaphoretickes - ps7
Diaphoretickes - ps8
Archaeplastida - ps9
Viridiplantae - ps10
Streptophyta - ps11
Charophyceae/Embriophyta - ps12
Embriophyta - ps13
Tracheophyta - ps14
Spermatophyta - ps15
Angiosperms - ps16
Mesangiospermae - ps17
Magnoliids/eudicots - ps18
Eudicots - ps19
Core_eudicots - ps20
Superrosids - ps21
Rosids - ps22
Eurosids - ps23
Malvids - ps24
Sap./Malv./Brassicales - ps25
Malvales/Brassicales - ps26
Brassicales - ps27
Cleomaceae/Brassicaceae - ps28
Brassicaceae - ps29
Camelineae - ps30
Arabidopsis - ps31
Arabidopsis_thaliana - ps32
Phylostratum

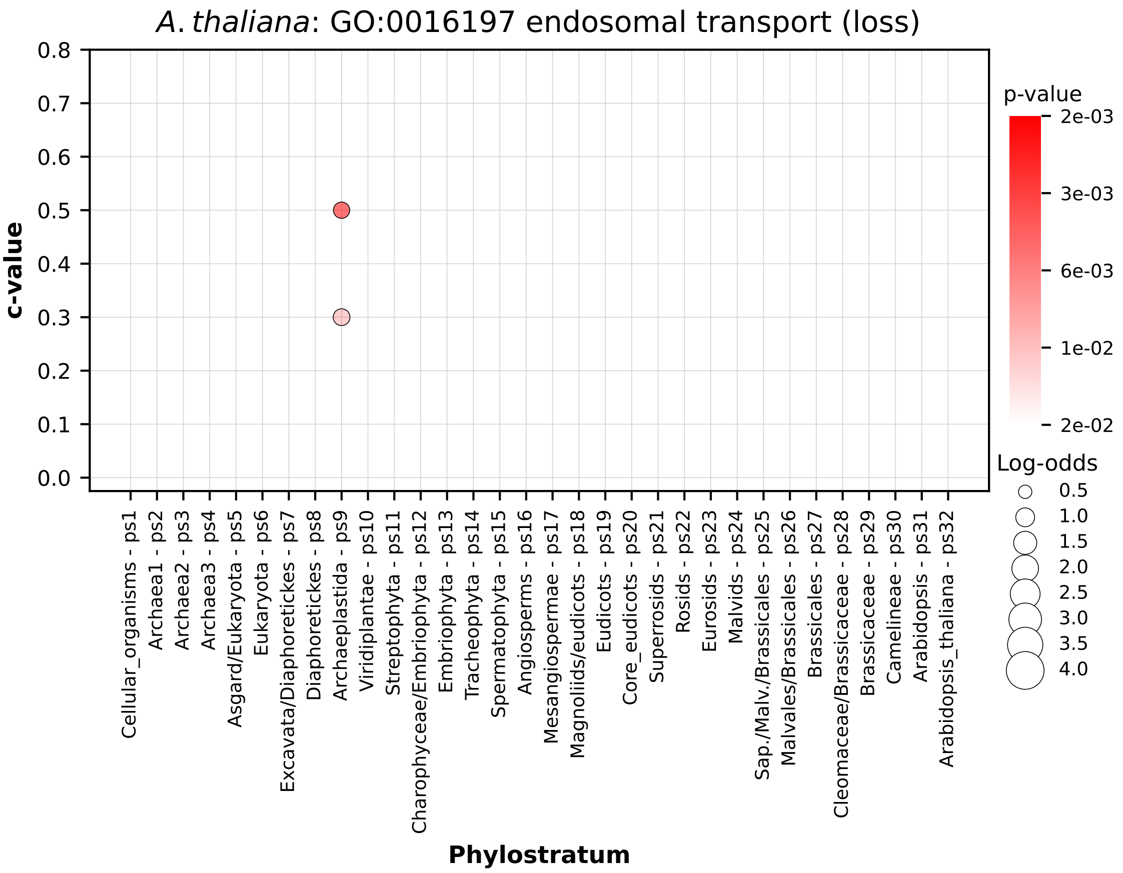

A. thaliana: GO:0016197 endosomal transport (loss)
c-value
0.8
0.7
0.6
0.5
0.4
0.3
0.2
0.1
0.0
Phylostratum
Cellular_organisms - ps1
Archaea1 - ps2
Archaea2 - ps3
Archaea3 - ps4
Asgard/Eukaryota - ps5
Eukaryota - ps6
Excavata/Diaphoretickes - ps7
Diaphoretickes - ps8
Archaeplastida - ps9
Viridiplantae - ps10
Streptophyta - ps11
Charophyceae/Embriophyta - ps12
Embriophyta - ps13
Tracheophyta - ps14
Spermatophyta - ps15
Angiosperms - ps16
Mesangiospermae - ps17
Magnoliids/eudicots - ps18
Eudicots - ps19
Core_eudicots - ps20
Superrosids - ps21
Rosids - ps22
Eurosids - ps23
Malvids - ps24
Sap./Malv./Brassicales - ps25
Malvales/Brassicales - ps26
Brassicales - ps27
Cleomaceae/Brassicaceae - ps28
Brassicaceae - ps29
Camelineae - ps30
Arabidopsis - ps31
Arabidopsis_thaliana - ps32
p-value
2e-03
3e-03
6e-03
1e-02
2e-02
Log-odds
0.5
1.0
1.5
2.0
2.5
3.0
3.5
4.0

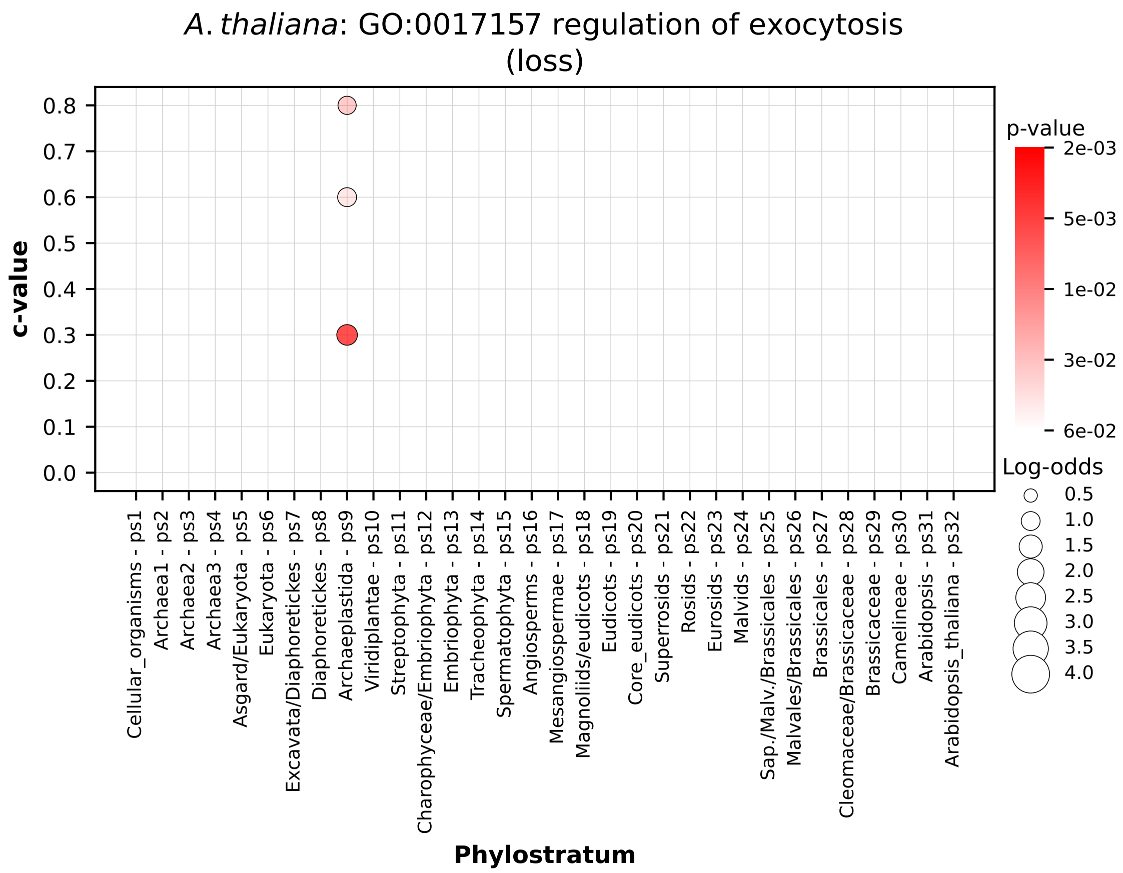
A. thaliana: GO:0017157 regulation of exocytosis
(loss)
c-value
0.0
0.1
0.2
0.3
0.4
0.5
0.6
0.7
0.8
Phylostratum
Cellular_organisms - ps1
Archaea1 - ps2
Archaea2 - ps3
Archaea3 - ps4
Asgard/Eukaryota - ps5
Eukaryota - ps6
Excavata/Diaphoretickes - ps7
Diaphoretickes - ps8
Archaeplastida - ps9
Viridiplantae - ps10
Streptophyta - ps11
Charophyceae/Embriophyta - ps12
Embriophyta - ps13
Tracheophyta - ps14
Spermatophyta - ps15
Angiosperms - ps16
Mesangiospermae - ps17
Magnoliids/eudicots - ps18
Eudicots - ps19
Core_eudicots - ps20
Superrosids - ps21
Rosids - ps22
Eurosids - ps23
Malvids - ps24
Sap./Malv./Brassicales - ps25
Malvales/Brassicales - ps26
Brassicales - ps27
Cleomaceae/Brassicaceae - ps28
Brassicaceae - ps29
Camelineae - ps30
Arabidopsis - ps31
Arabidopsis_thaliana - ps32
p-value
2e-03
5e-03
1e-02
3e-02
6e-02
Log-odds
0.5
1.0
1.5
2.0
2.5
3.0
3.5
4.0

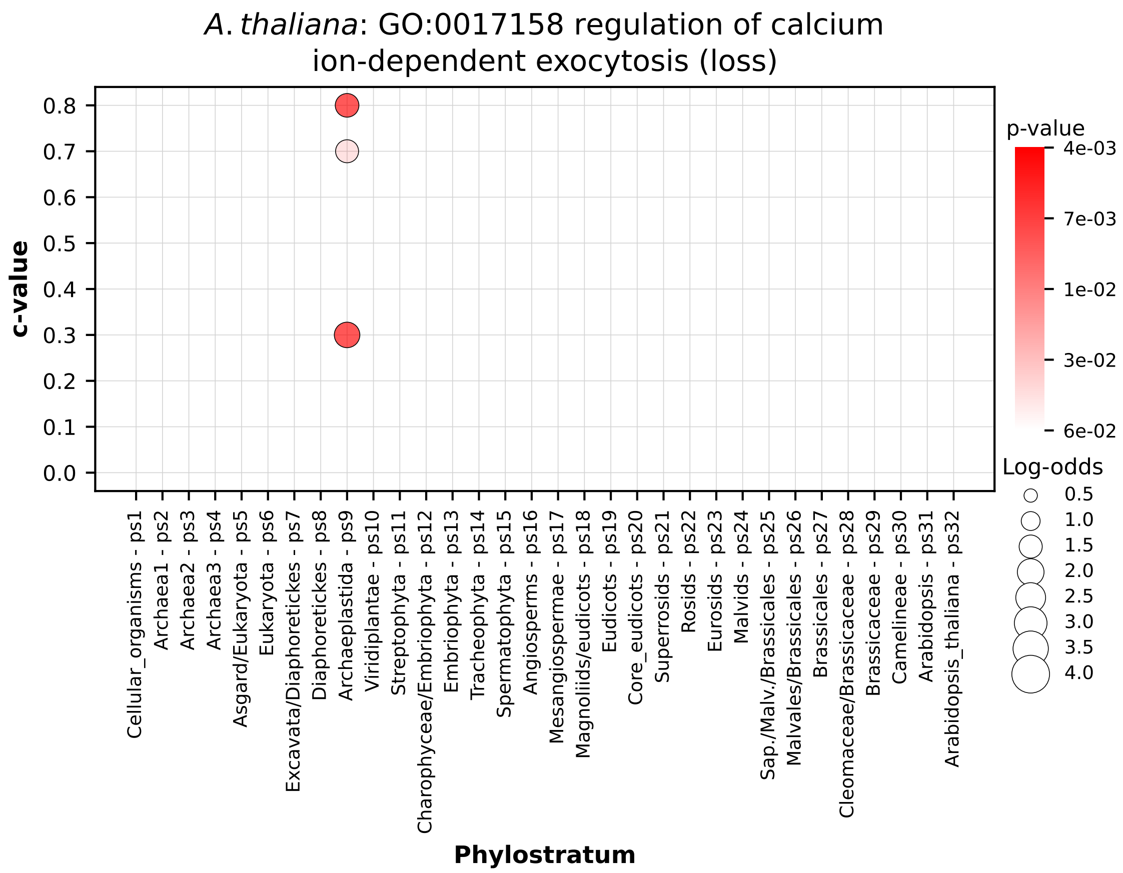

A. thaliana: GO:0017158 regulation of calcium ion-dependent exocytosis (loss)
c-value
0.0
0.1
0.2
0.3
0.4
0.5
0.6
0.7
0.8
Phylostratum
Cellular_organisms - ps1
Archaea1 - ps2
Archaea2 - ps3
Archaea3 - ps4
Asgard/Eukaryota - ps5
Eukaryota - ps6
Excavata/Diaphoretickes - ps7
Diaphoretickes - ps8
Archaeplastida - ps9
Viridiplantae - ps10
Streptophyta - ps11
Charophyceae/Embriophyta - ps12
Embriophyta - ps13
Tracheophyta - ps14
Spermatophyta - ps15
Angiosperms - ps16
Mesangiospermae - ps17
Magnoliids/eudicots - ps18
Eudicots - ps19
Core_eudicots - ps20
Superrosids - ps21
Rosids - ps22
Eurosids - ps23
Malvids - ps24
Sap./Malv./Brassicales - ps25
Malvales/Brassicales - ps26
Brassicales - ps27
Cleomaceae/Brassicaceae - ps28
Brassicaceae - ps29
Camelineae - ps30
Arabidopsis - ps31
Arabidopsis_thaliana - ps32
p-value
4e-03
7e-03
1e-02
3e-02
6e-02
Log-odds
0.5
1.0
1.5
2.0
2.5
3.0
3.5
4.0

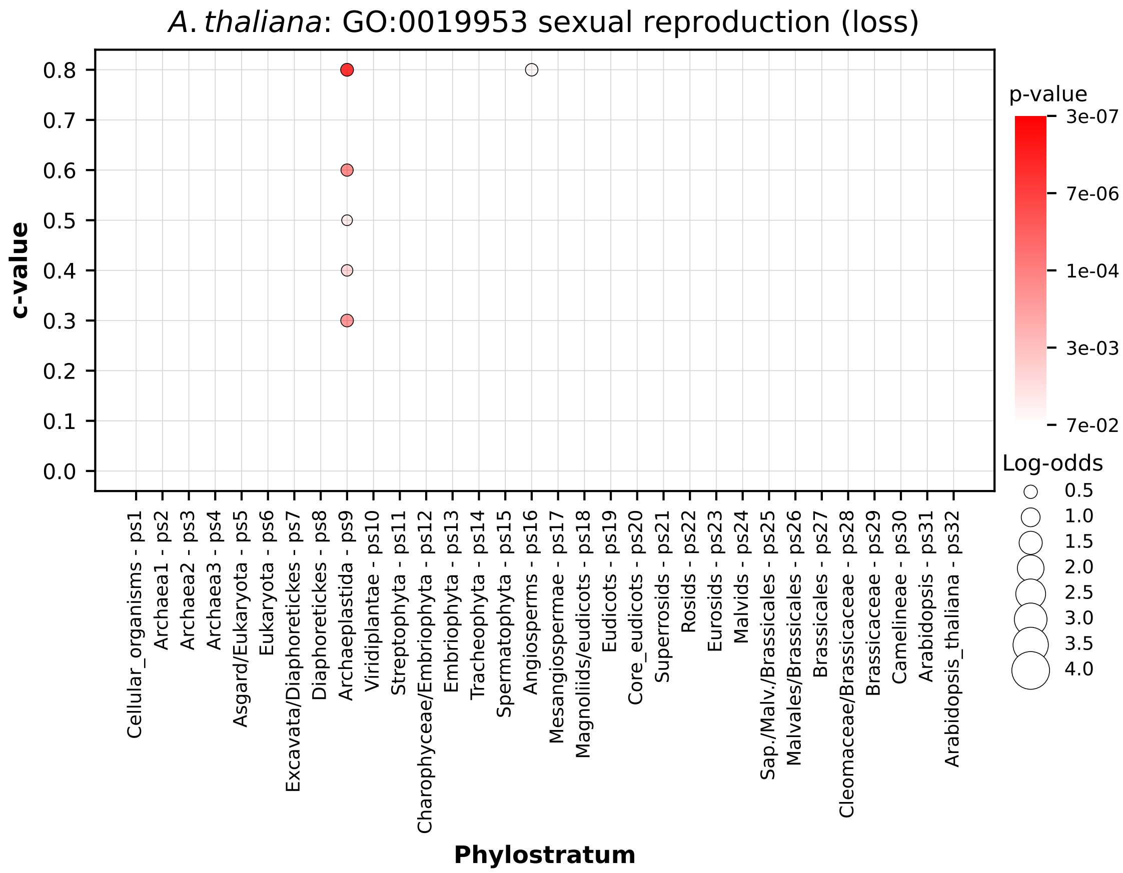
A. thaliana: GO:0019953 sexual reproduction (loss)
c-value
0.0
0.1
0.2
0.3
0.4
0.5
0.6
0.7
0.8
Phylostratum
Cellular_organisms - ps1
Archaea1 - ps2
Archaea2 - ps3
Archaea3 - ps4
Asgard/Eukaryota - ps5
Eukaryota - ps6
Excavata/Diaphoretickes - ps7
Diaphoretickes - ps8
Archaeplastida - ps9
Viridiplantae - ps10
Streptophyta - ps11
Charophyceae/Embriophyta - ps12
Embriophyta - ps13
Tracheophyta - ps14
Spermatophyta - ps15
Angiosperms - ps16
Mesangiospermae - ps17
Magnoliids/eudicots - ps18
Eudicots - ps19
Core_eudicots - ps20
Superrosids - ps21
Rosids - ps22
Eurosids - ps23
Malvids - ps24
Sap./Malv./Brassicales - ps25
Malvales/Brassicales - ps26
Brassicales - ps27
Cleomaceae/Brassicaceae - ps28
Brassicaceae - ps29
Camelineae - ps30
Arabidopsis - ps31
Arabidopsis_thaliana - ps32
p-value
3e-07
7e-06
1e-04
3e-03
7e-02
Log-odds
0.5
1.0
1.5
2.0
2.5
3.0
3.5
4.0

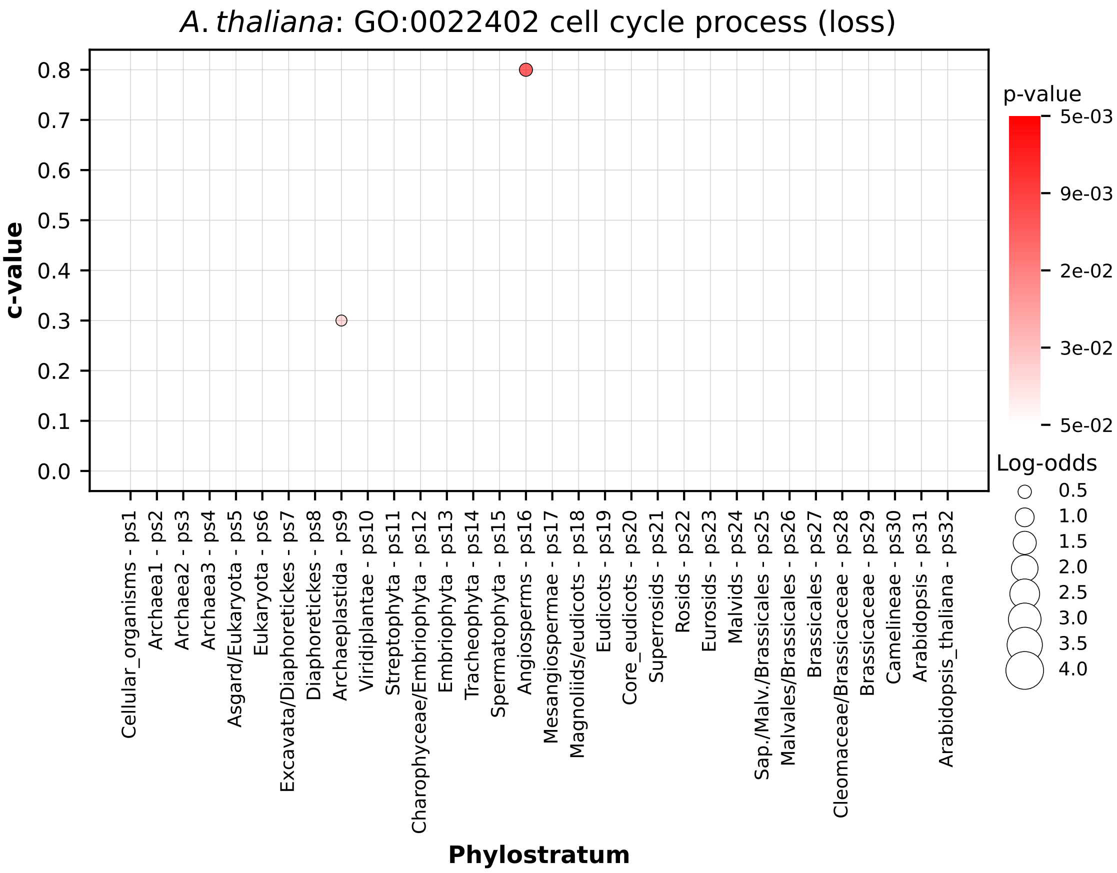

A. thaliana: GO:0022402 cell cycle process (loss)
c-value
0.8
0.7
0.6
0.5
0.4
0.3
0.2
0.1
0.0
p-value
5e-03
9e-03
2e-02
3e-02
5e-02
Log-odds
0.5
1.0
1.5
2.0
2.5
3.0
3.5
4.0
Cellular_organisms - ps1
Archaea1 - ps2
Archaea2 - ps3
Archaea3 - ps4
Asgard/Eukaryota - ps5
Eukaryota - ps6
Excavata/Diaphoretickes - ps7
Diaphoretickes - ps8
Archaeplastida - ps9
Viridiplantae - ps10
Streptophyta - ps11
Charophyceae/Embriophyta - ps12
Embriophyta - ps13
Tracheophyta - ps14
Spermatophyta - ps15
Angiosperms - ps16
Mesangiospermae - ps17
Magnoliids/eudicots - ps18
Eudicots - ps19
Core_eudicots - ps20
Superrosids - ps21
Rosids - ps22
Eurosids - ps23
Malvids - ps24
Sap./Malv./Brassicales - ps25
Malvales/Brassicales - ps26
Brassicales - ps27
Cleomaceae/Brassicaceae - ps28
Brassicaceae - ps29
Camelineae - ps30
Arabidopsis - ps31
Arabidopsis_thaliana - ps32
Phylostratum

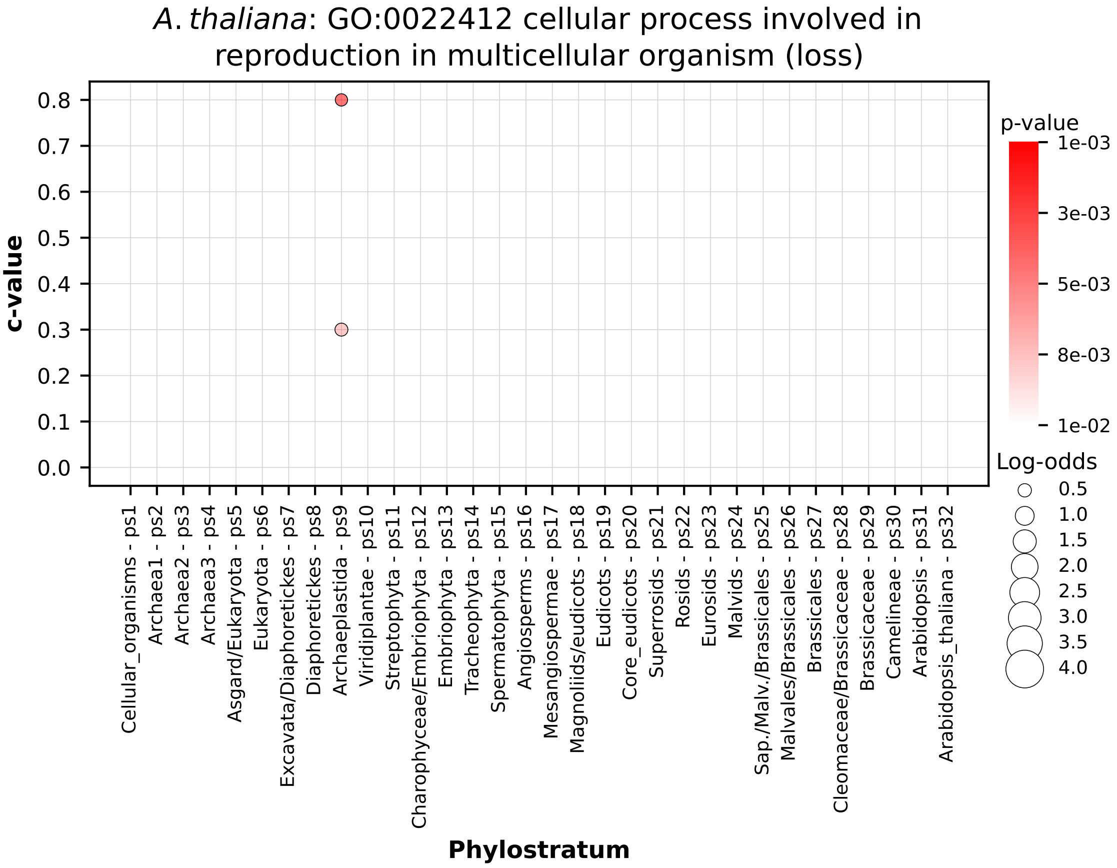

A. thaliana: GO:0022412 cellular process involved in reproduction in multicellular organism (loss)
c-value
0.8
0.7
0.6
0.5
0.4
0.3
0.2
0.1
0.0
Phylostratum
Cellular_organisms - ps1
Archaea1 - ps2
Archaea2 - ps3
Archaea3 - ps4
Asgard/Eukaryota - ps5
Eukaryota - ps6
Excavata/Diaphoretickes - ps7
Diaphoretickes - ps8
Archaeplastida - ps9
Viridiplantae - ps10
Streptophyta - ps11
Charophyceae/Embriophyta - ps12
Embriophyta - ps13
Tracheophyta - ps14
Spermatophyta - ps15
Angiosperms - ps16
Mesangiospermae - ps17
Magnoliids/eudicots - ps18
Eudicots - ps19
Core_eudicots - ps20
Superrosids - ps21
Rosids - ps22
Eurosids - ps23
Malvids - ps24
Sap./Malv./Brassicales - ps25
Malvales/Brassicales - ps26
Brassicales - ps27
Cleomaceae/Brassicaceae - ps28
Brassicaceae - ps29
Camelineae - ps30
Arabidopsis - ps31
Arabidopsis_thaliana - ps32
p-value
1e-03
3e-03
5e-03
8e-03
1e-02
Log-odds
0.5
1.0
1.5
2.0
2.5
3.0
3.5
4.0

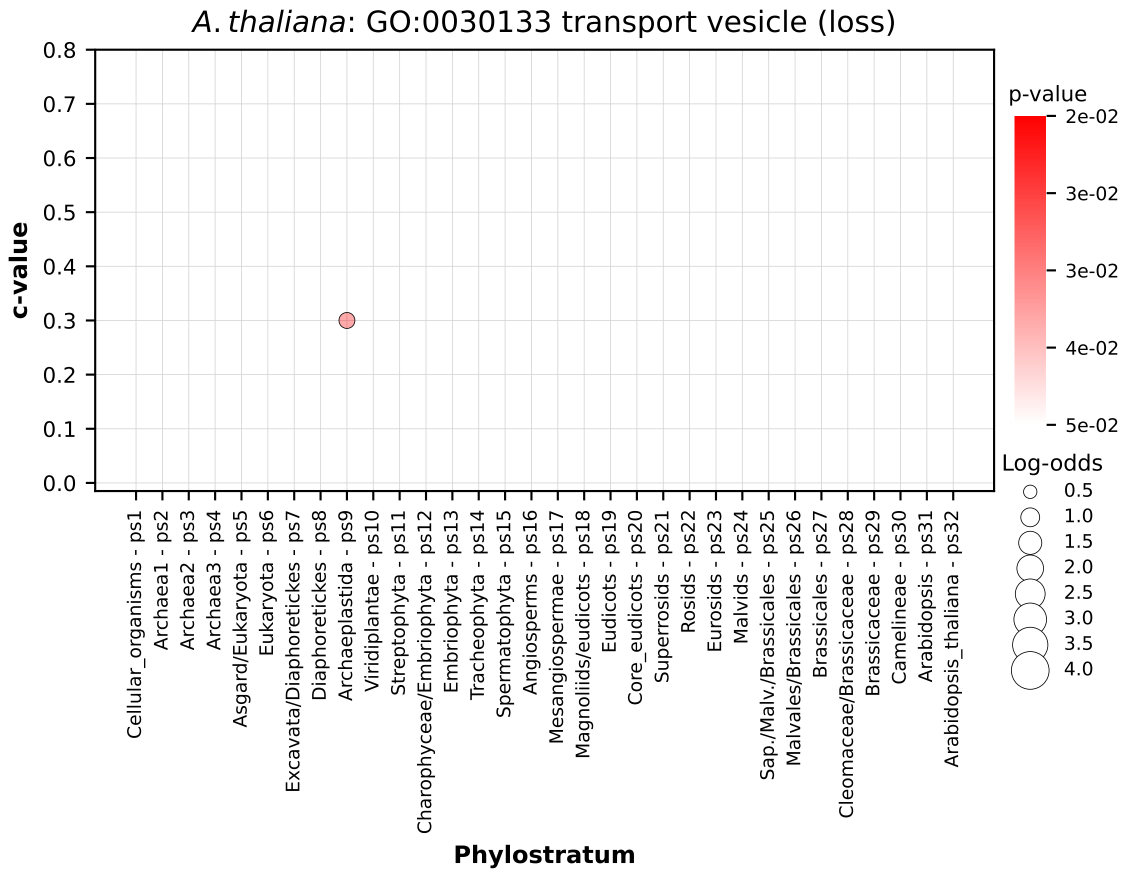

A. thaliana: GO:0030133 transport vesicle (loss)
c-value
0.8
0.7
0.6
0.5
0.4
0.3
0.2
0.1
0.0
p-value
2e-02
3e-02
3e-02
4e-02
5e-02
Log-odds
0.5
1.0
1.5
2.0
2.5
3.0
3.5
4.0
Cellular_organisms - ps1
Archaea1 - ps2
Archaea2 - ps3
Archaea3 - ps4
Asgard/Eukaryota - ps5
Eukaryota - ps6
Excavata/Diaphoretickes - ps7
Diaphoretickes - ps8
Archaeplastida - ps9
Viridiplantae - ps10
Streptophyta - ps11
Charophyceae/Embriophyta - ps12
Embriophyta - ps13
Tracheophyta - ps14
Spermatophyta - ps15
Angiosperms - ps16
Mesangiospermae - ps17
Magnoliids/eudicots - ps18
Eudicots - ps19
Core_eudicots - ps20
Superrosids - ps21
Rosids - ps22
Eurosids - ps23
Malvids - ps24
Sap./Malv./Brassicales - ps25
Malvales/Brassicales - ps26
Brassicales - ps27
Cleomaceae/Brassicaceae - ps28
Brassicaceae - ps29
Camelineae - ps30
Arabidopsis - ps31
Arabidopsis_thaliana - ps32
Phylostratum

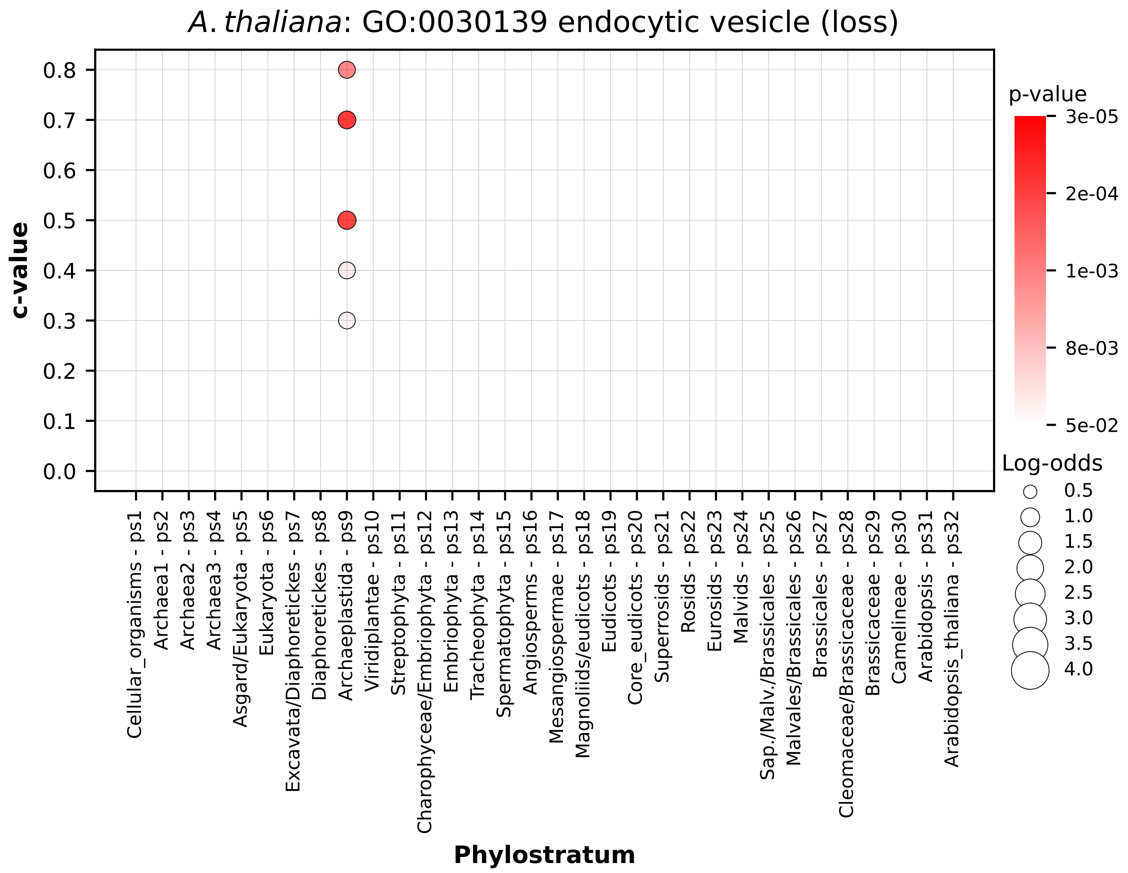

A. thaliana: GO:0030139 endocytic vesicle (loss)
c-value
0.8
0.7
0.6
0.5
0.4
0.3
0.2
0.1
0.0
p-value
3e-05
2e-04
1e-03
8e-03
5e-02
Log-odds
0.5
1.0
1.5
2.0
2.5
3.0
3.5
4.0
Cellular_organisms - ps1
Archaea1 - ps2
Archaea2 - ps3
Archaea3 - ps4
Asgard/Eukaryota - ps5
Eukaryota - ps6
Excavata/Diaphoretickes - ps7
Diaphoretickes - ps8
Archaeplastida - ps9
Viridiplantae - ps10
Streptophyta - ps11
Charophyceae/Embriophyta - ps12
Embriophyta - ps13
Tracheophyta - ps14
Spermatophyta - ps15
Angiosperms - ps16
Mesangiospermae - ps17
Magnoliids/eudicots - ps18
Eudicots - ps19
Core_eudicots - ps20
Superrosids - ps21
Rosids - ps22
Eurosids - ps23
Malvids - ps24
Sap./Malv./Brassicales - ps25
Malvales/Brassicales - ps26
Brassicales - ps27
Cleomaceae/Brassicaceae - ps28
Brassicaceae - ps29
Camelineae - ps30
Arabidopsis - ps31
Arabidopsis_thaliana - ps32
Phylostratum

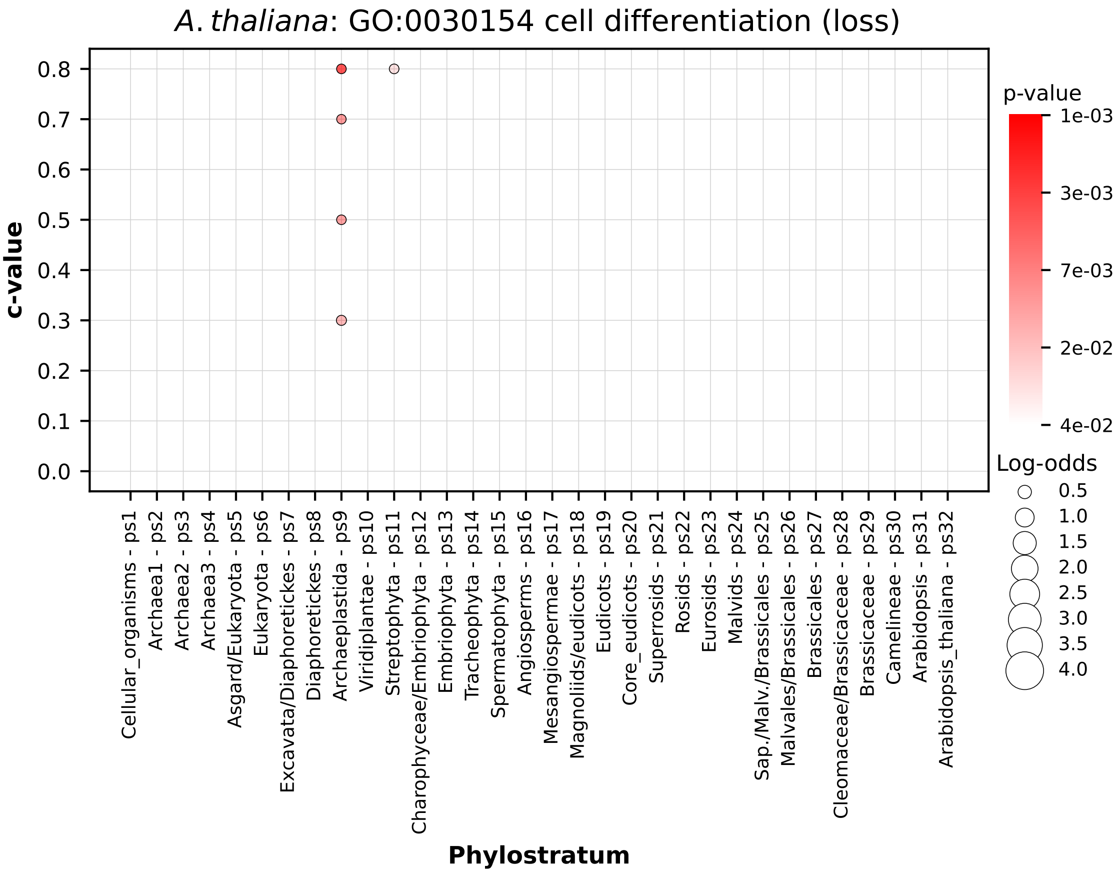

A. thaliana: GO:0030154 cell differentiation (loss)
c-value
0.0
0.1
0.2
0.3
0.4
0.5
0.6
0.7
0.8
Phylostratum
Cellular_organisms - ps1
Archaea1 - ps2
Archaea2 - ps3
Archaea3 - ps4
Asgard/Eukaryota - ps5
Eukaryota - ps6
Excavata/Diaphoretickes - ps7
Diaphoretickes - ps8
Archaeplastida - ps9
Viridiplantae - ps10
Streptophyta - ps11
Charophyceae/Embriophyta - ps12
Embriophyta - ps13
Tracheophyta - ps14
Spermatophyta - ps15
Angiosperms - ps16
Mesangiospermae - ps17
Magnoliids/eudicots - ps18
Eudicots - ps19
Core_eudicots - ps20
Superrosids - ps21
Rosids - ps22
Eurosids - ps23
Malvids - ps24
Sap./Malv./Brassicales - ps25
Malvales/Brassicales - ps26
Brassicales - ps27
Cleomaceae/Brassicaceae - ps28
Brassicaceae - ps29
Camelineae - ps30
Arabidopsis - ps31
Arabidopsis_thaliana - ps32
p-value
1e-03
3e-03
7e-03
2e-02
4e-02
Log-odds
0.5
1.0
1.5
2.0
2.5
3.0
3.5
4.0

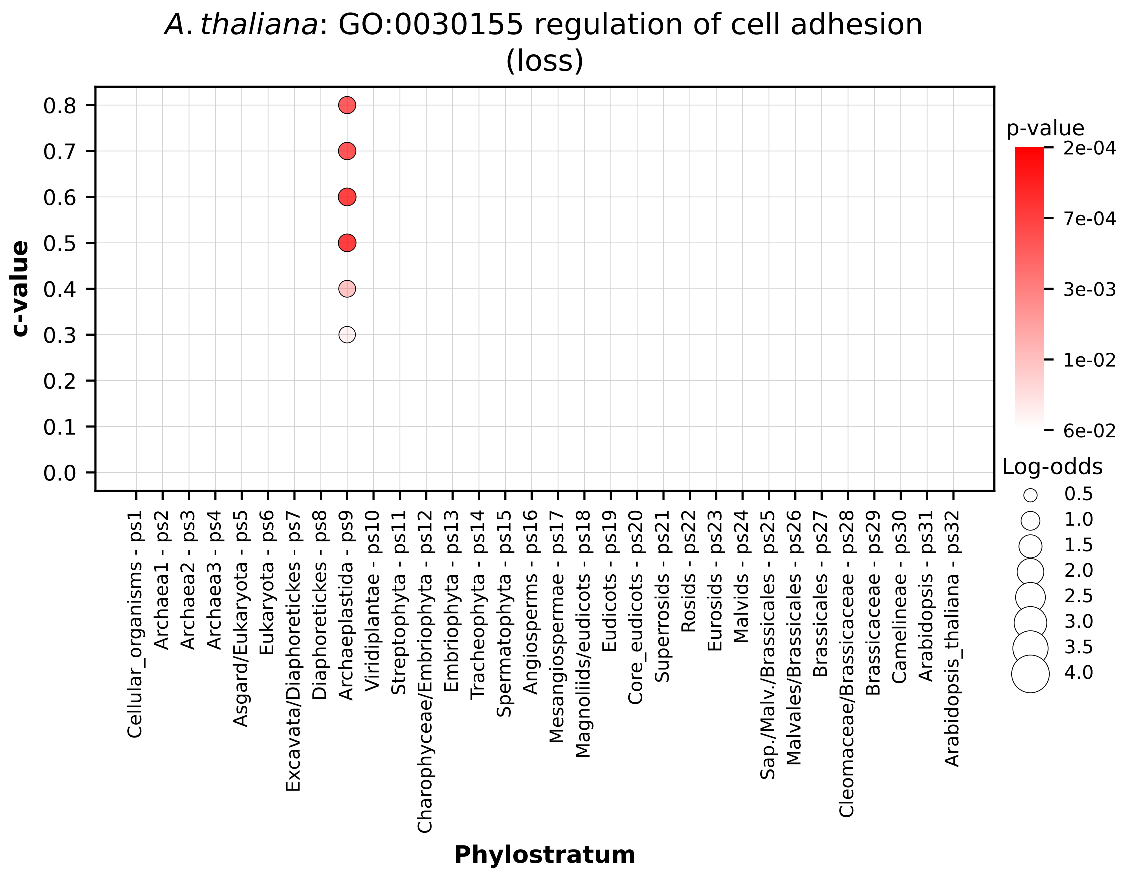

A. thaliana: GO:0030155 regulation of cell adhesion
(loss)
c-value
0.8
0.7
0.6
0.5
0.4
0.3
0.2
0.1
0.0
p-value
2e-04
7e-04
3e-03
1e-02
6e-02
Log-odds
0.5
1.0
1.5
2.0
2.5
3.0
3.5
4.0
Cellular_organisms - ps1
Archaea1 - ps2
Archaea2 - ps3
Archaea3 - ps4
Asgard/Eukaryota - ps5
Eukaryota - ps6
Excavata/Diaphoretickes - ps7
Diaphoretickes - ps8
Archaeplastida - ps9
Viridiplantae - ps10
Streptophyta - ps11
Charophyceae/Embriophyta - ps12
Embriophyta - ps13
Tracheophyta - ps14
Spermatophyta - ps15
Angiosperms - ps16
Mesangiospermae - ps17
Magnoliids/eudicots - ps18
Eudicots - ps19
Core_eudicots - ps20
Superrosids - ps21
Rosids - ps22
Eurosids - ps23
Malvids - ps24
Sap./Malv./Brassicales - ps25
Malvales/Brassicales - ps26
Brassicales - ps27
Cleomaceae/Brassicaceae - ps28
Brassicaceae - ps29
Camelineae - ps30
Arabidopsis - ps31
Arabidopsis_thaliana - ps32
Phylostratum

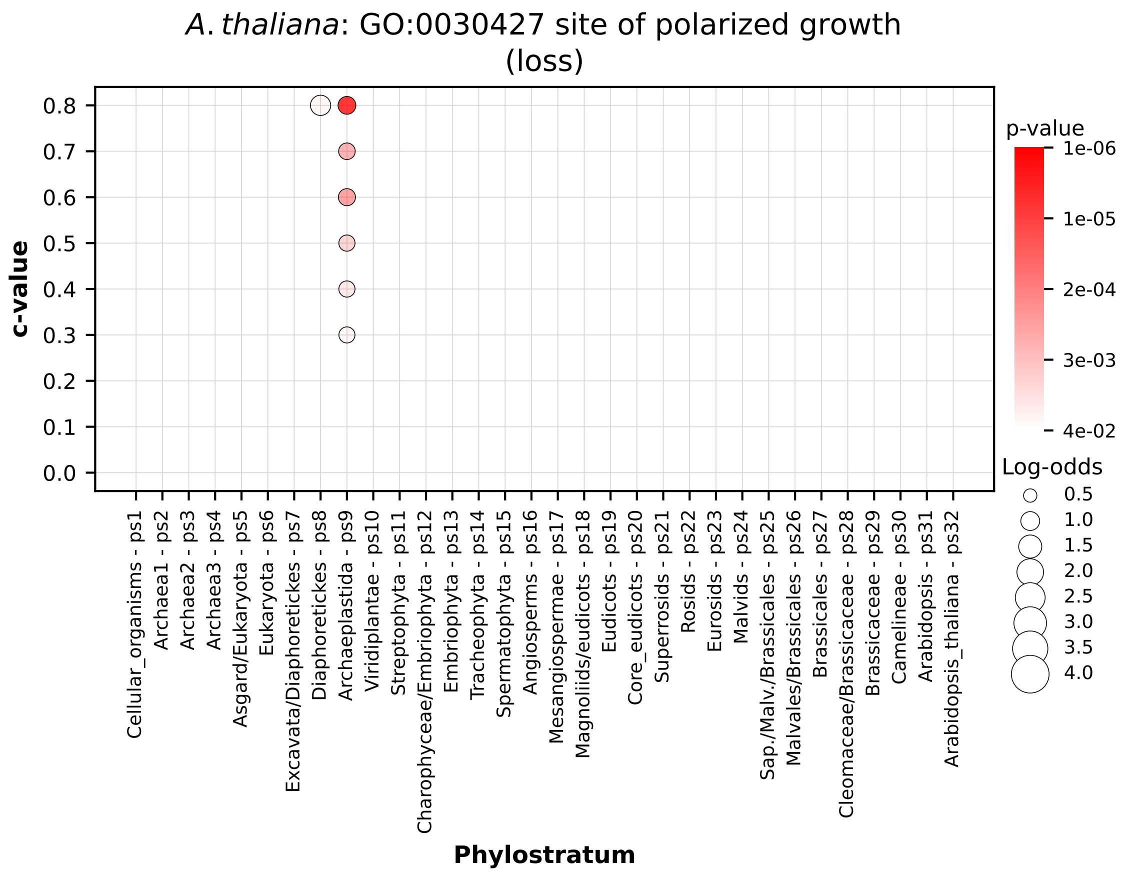
A. thaliana: GO:0030427 site of polarized growth
(loss)
c-value
0.8
0.7
0.6
0.5
0.4
0.3
0.2
0.1
0.0
p-value
1e-06
1e-05
2e-04
3e-03
4e-02
Log-odds
0.5
1.0
1.5
2.0
2.5
3.0
3.5
4.0
Cellular_organisms - ps1
Archaea1 - ps2
Archaea2 - ps3
Archaea3 - ps4
Asgard/Eukaryota - ps5
Eukaryota - ps6
Excavata/Diaphoretickes - ps7
Diaphoretickes - ps8
Archaeplastida - ps9
Viridiplantae - ps10
Streptophyta - ps11
Charophyceae/Embriophyta - ps12
Embriophyta - ps13
Tracheophyta - ps14
Spermatophyta - ps15
Angiosperms - ps16
Mesangiospermae - ps17
Magnoliids/eudicots - ps18
Eudicots - ps19
Core_eudicots - ps20
Superrosids - ps21
Rosids - ps22
Eurosids - ps23
Malvids - ps24
Sap./Malv./Brassicales - ps25
Malvales/Brassicales - ps26
Brassicales - ps27
Cleomaceae/Brassicaceae - ps28
Brassicaceae - ps29
Camelineae - ps30
Arabidopsis - ps31
Arabidopsis_thaliana - ps32
Phylostratum

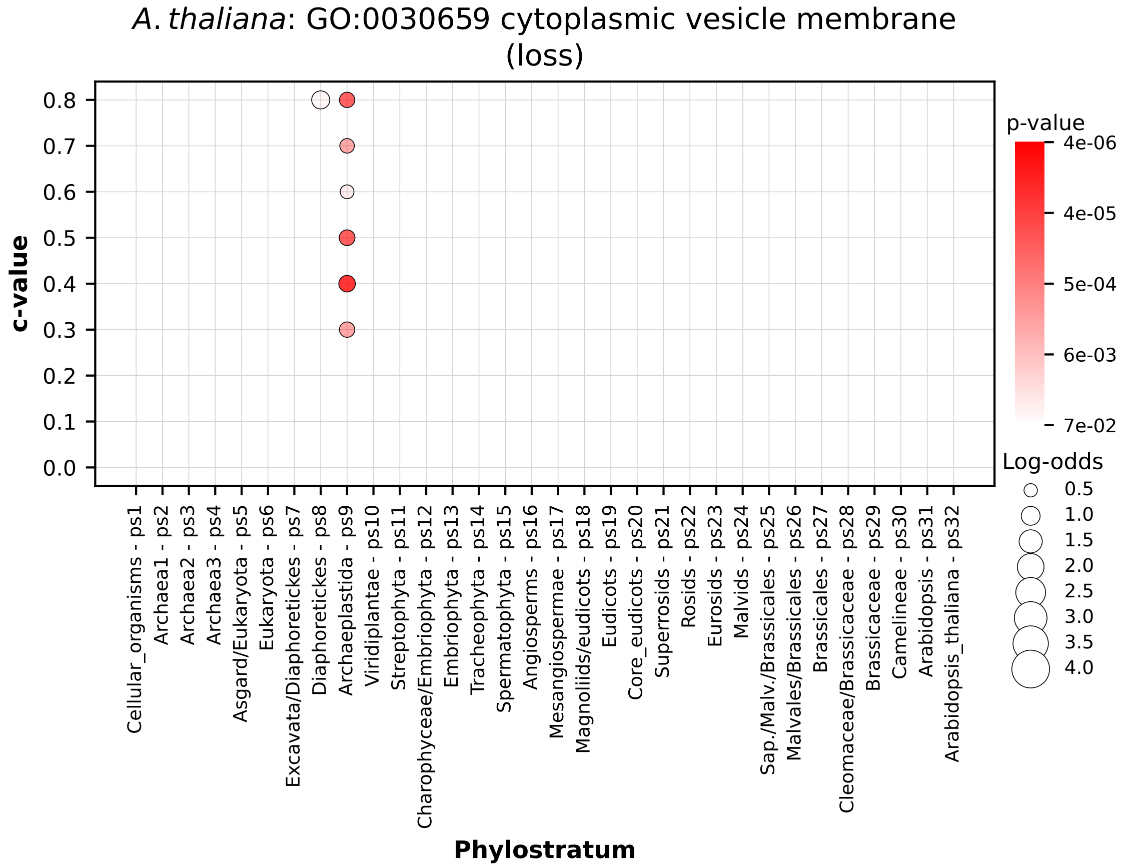

A. thaliana: GO:0030659 cytoplasmic vesicle membrane
(loss)
c-value
0.0
0.1
0.2
0.3
0.4
0.5
0.6
0.7
0.8
p-value
4e-06
4e-05
5e-04
6e-03
7e-02
Log-odds
0.5
1.0
1.5
2.0
2.5
3.0
3.5
4.0
Cellular_organisms - ps1
Archaea1 - ps2
Archaea2 - ps3
Archaea3 - ps4
Asgard/Eukaryota - ps5
Eukaryota - ps6
Excavata/Diaphoretickes - ps7
Diaphoretickes - ps8
Archaeplastida - ps9
Viridiplantae - ps10
Streptophyta - ps11
Charophyceae/Embriophyta - ps12
Embriophyta - ps13
Tracheophyta - ps14
Spermatophyta - ps15
Angiosperms - ps16
Mesangiospermae - ps17
Magnoliids/eudicots - ps18
Eudicots - ps19
Core_eudicots - ps20
Superrosids - ps21
Rosids - ps22
Eurosids - ps23
Malvids - ps24
Sap./Malv./Brassicales - ps25
Malvales/Brassicales - ps26
Brassicales - ps27
Cleomaceae/Brassicaceae - ps28
Brassicaceae - ps29
Camelineae - ps30
Arabidopsis - ps31
Arabidopsis_thaliana - ps32
Phylostratum

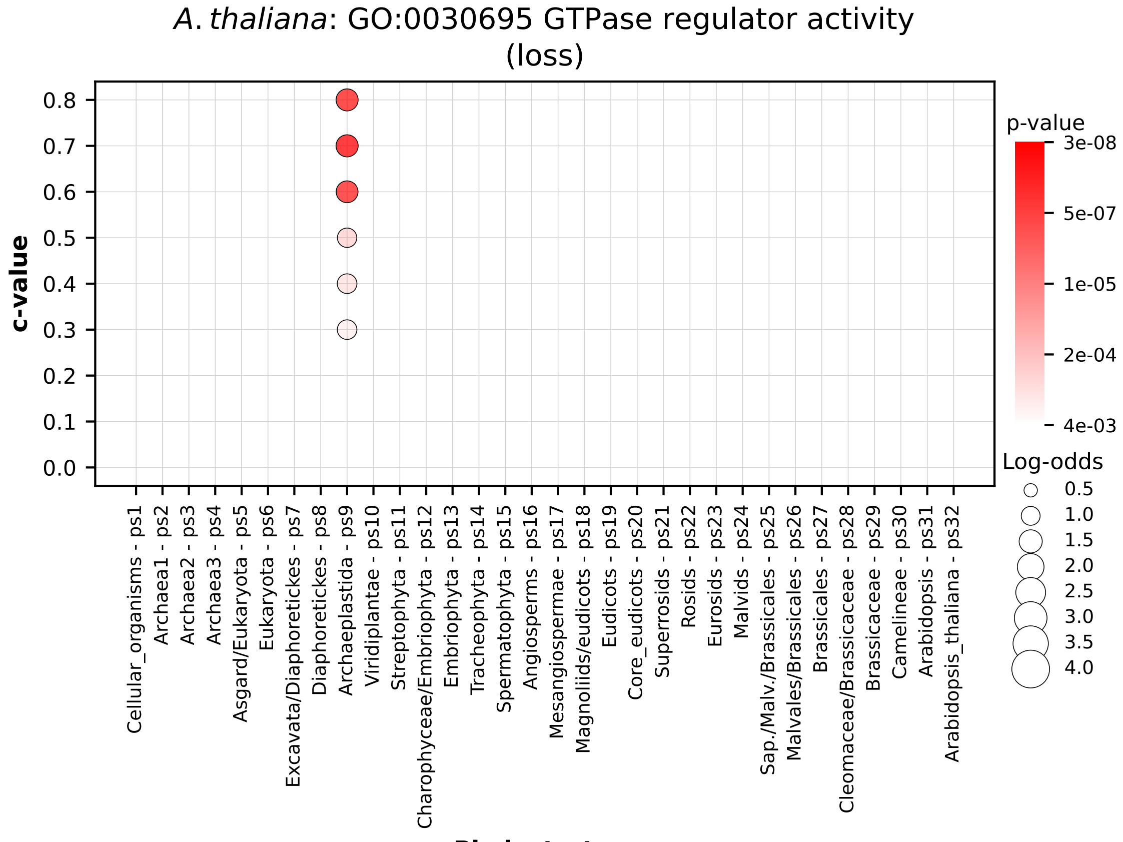

$A. thaliana$: GO:0030695 GTPase regulator activity
(loss)
c-value
0.8
0.7
0.6
0.5
0.4
0.3
0.2
0.1
0.0
p-value
3e-08
5e-07
1e-05
2e-04
4e-03
Log-odds
0.5
1.0
1.5
2.0
2.5
3.0
3.5
4.0
Cellular_organisms - ps1
Archaea1 - ps2
Archaea2 - ps3
Archaea3 - ps4
Asgard/Eukaryota - ps5
Eukaryota - ps6
Excavata/Diaphoretickes - ps7
Diaphoretickes - ps8
Archaeplastida - ps9
Viridiplantae - ps10
Streptophyta - ps11
Charophyceae/Embriophyta - ps12
Embriophyta - ps13
Tracheophyta - ps14
Spermatophyta - ps15
Angiosperms - ps16
Mesangiospermae - ps17
Magnoliids/eudicots - ps18
Eudicots - ps19
Core_eudicots - ps20
Superrosids - ps21
Rosids - ps22
Eurosids - ps23
Malvids - ps24
Sap./Malv./Brassicales - ps25
Malvales/Brassicales - ps26
Brassicales - ps27
Cleomaceae/Brassicaceae - ps28
Brassicaceae - ps29
Camelineae - ps30
Arabidopsis - ps31
Arabidopsis_thaliana - ps32
Phylostratum

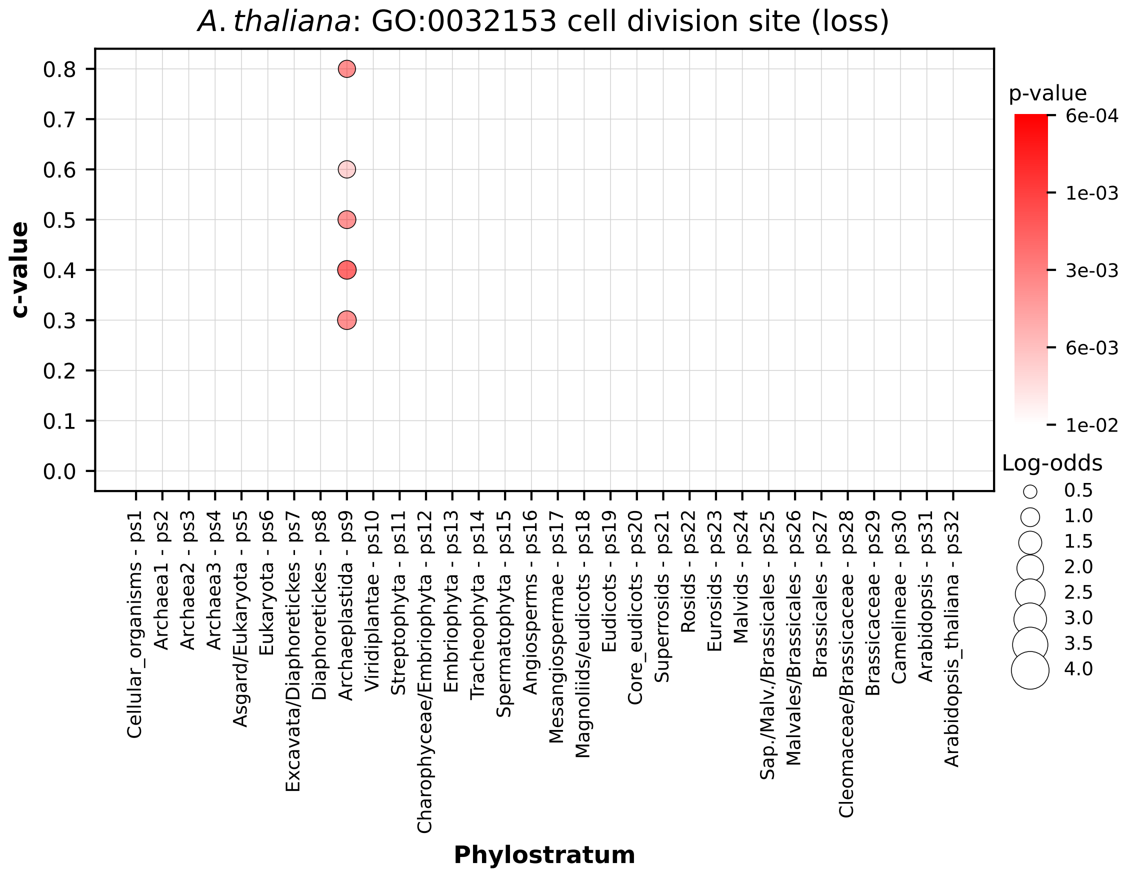

A. thaliana: GO:0032153 cell division site (loss)
c-value
0.8
0.7
0.6
0.5
0.4
0.3
0.2
0.1
0.0
Phylostratum
Cellular_organisms - ps1
Archaea1 - ps2
Archaea2 - ps3
Archaea3 - ps4
Asgard/Eukaryota - ps5
Eukaryota - ps6
Excavata/Diaphoretickes - ps7
Diaphoretickes - ps8
Archaeplastida - ps9
Viridiplantae - ps10
Streptophyta - ps11
Charophyceae/Embriophyta - ps12
Embriophyta - ps13
Tracheophyta - ps14
Spermatophyta - ps15
Angiosperms - ps16
Mesangiospermae - ps17
Magnoliids/eudicots - ps18
Eudicots - ps19
Core_eudicots - ps20
Superrosids - ps21
Rosids - ps22
Eurosids - ps23
Malvids - ps24
Sap./Malv./Brassicales - ps25
Malvales/Brassicales - ps26
Brassicales - ps27
Cleomaceae/Brassicaceae - ps28
Brassicaceae - ps29
Camelineae - ps30
Arabidopsis - ps31
Arabidopsis_thaliana - ps32
p-value
6e-04
1e-03
3e-03
6e-03
1e-02
Log-odds
0.5
1.0
1.5
2.0
2.5
3.0
3.5
4.0

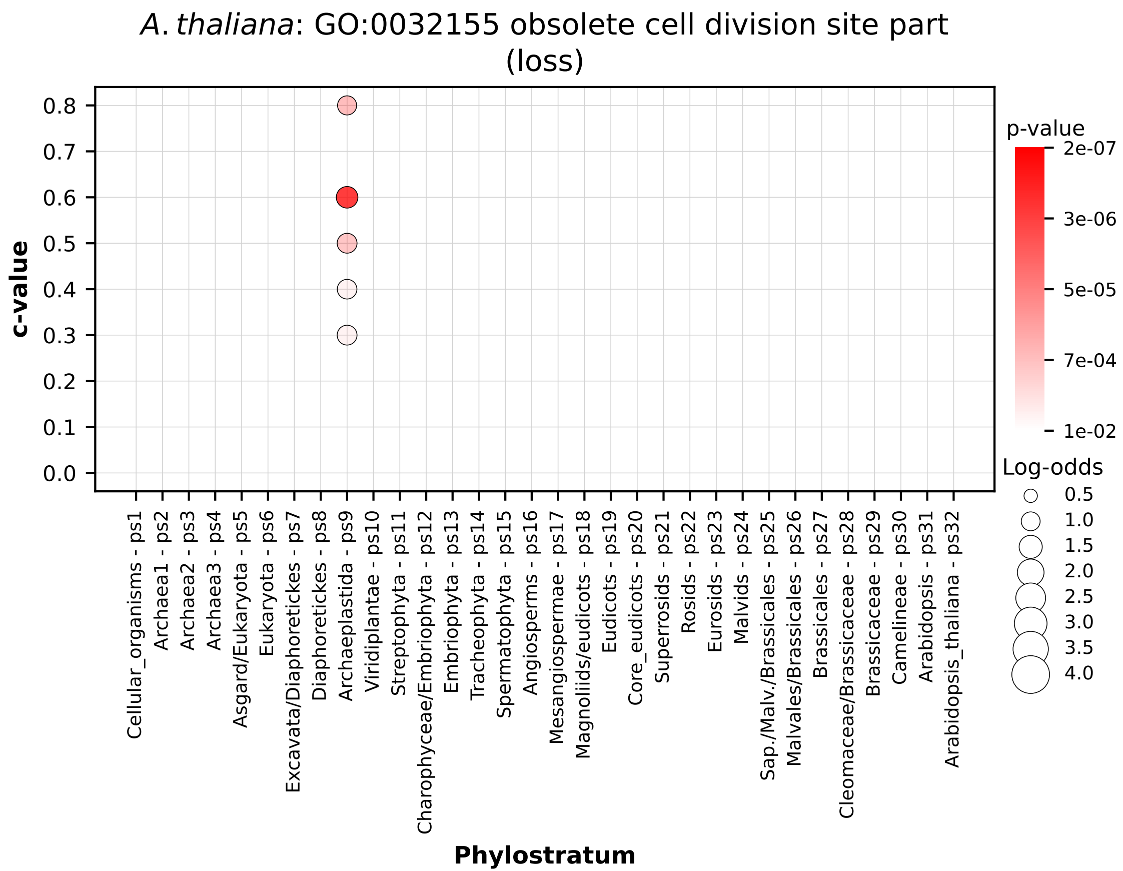

A. thaliana: GO:0032155 obsolete cell division site part (loss)
c-value
0.0
0.1
0.2
0.3
0.4
0.5
0.6
0.7
0.8
Phylostratum
Cellular_organisms - ps1
Archaea1 - ps2
Archaea2 - ps3
Archaea3 - ps4
Asgard/Eukaryota - ps5
Eukaryota - ps6
Excavata/Diaphoretickes - ps7
Diaphoretickes - ps8
Archaeplastida - ps9
Viridiplantae - ps10
Streptophyta - ps11
Charophyceae/Embriophyta - ps12
Embriophyta - ps13
Tracheophyta - ps14
Spermatophyta - ps15
Angiosperms - ps16
Mesangiospermae - ps17
Magnoliids/eudicots - ps18
Eudicots - ps19
Core_eudicots - ps20
Superrosids - ps21
Rosids - ps22
Eurosids - ps23
Malvids - ps24
Sap./Malv./Brassicales - ps25
Malvales/Brassicales - ps26
Brassicales - ps27
Cleomaceae/Brassicaceae - ps28
Brassicaceae - ps29
Camelineae - ps30
Arabidopsis - ps31
Arabidopsis_thaliana - ps32
p-value
2e-07
3e-06
5e-05
7e-04
1e-02
Log-odds
0.5
1.0
1.5
2.0
2.5
3.0
3.5
4.0

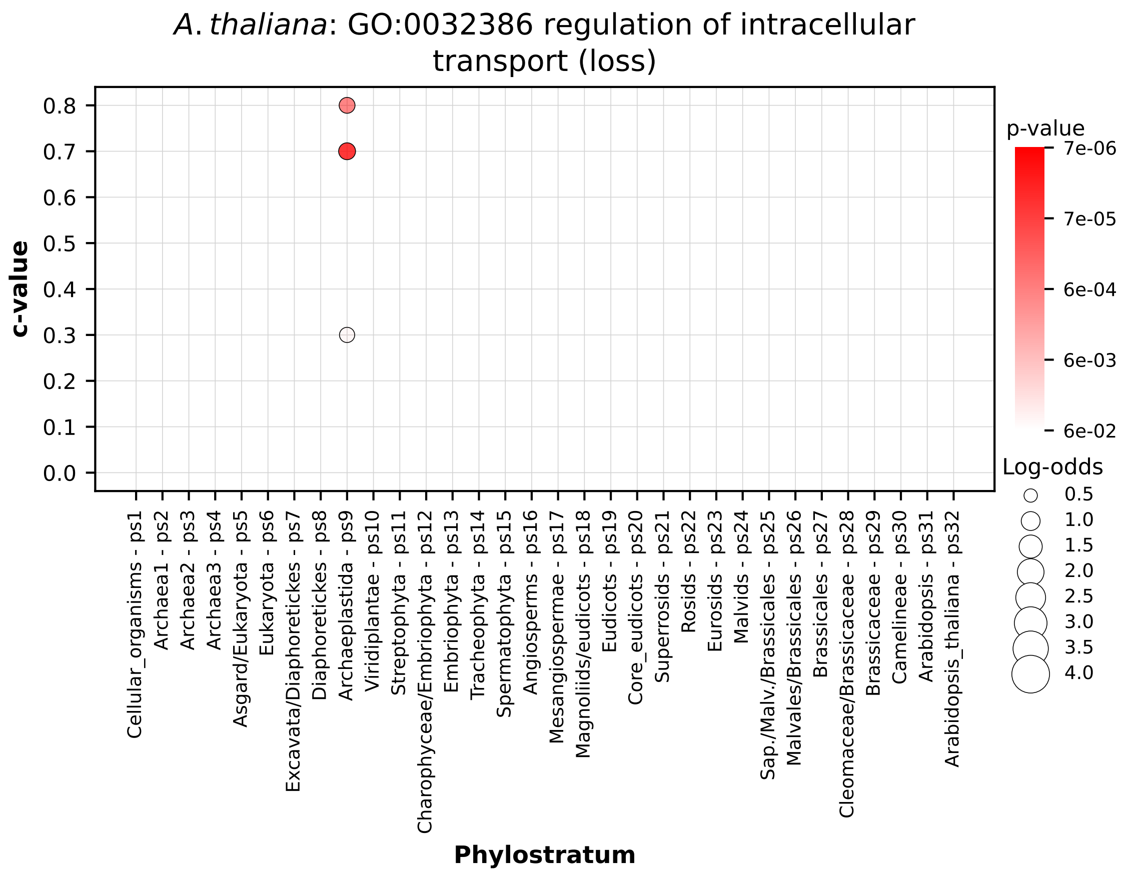
A. thaliana: GO:0032386 regulation of intracellular transport (loss)
c-value
0.8
0.7
0.6
0.5
0.4
0.3
0.2
0.1
0.0
Phylostratum
Cellular_organisms - ps1
Archaea1 - ps2
Archaea2 - ps3
Archaea3 - ps4
Asgard/Eukaryota - ps5
Eukaryota - ps6
Excavata/Diaphoretickes - ps7
Diaphoretickes - ps8
Archaeplastida - ps9
Viridiplantae - ps10
Streptophyta - ps11
Charophyceae/Embriophyta - ps12
Embriophyta - ps13
Tracheophyta - ps14
Spermatophyta - ps15
Angiosperms - ps16
Mesangiospermae - ps17
Magnoliids/eudicots - ps18
Eudicots - ps19
Core_eudicots - ps20
Superrosids - ps21
Rosids - ps22
Eurosids - ps23
Malvids - ps24
Sap./Malv./Brassicales - ps25
Malvales/Brassicales - ps26
Brassicales - ps27
Cleomaceae/Brassicaceae - ps28
Brassicaceae - ps29
Camelineae - ps30
Arabidopsis - ps31
Arabidopsis_thaliana - ps32
p-value
7e-06
7e-05
6e-04
6e-03
6e-02
Log-odds
0.5
1.0
1.5
2.0
2.5
3.0
3.5
4.0

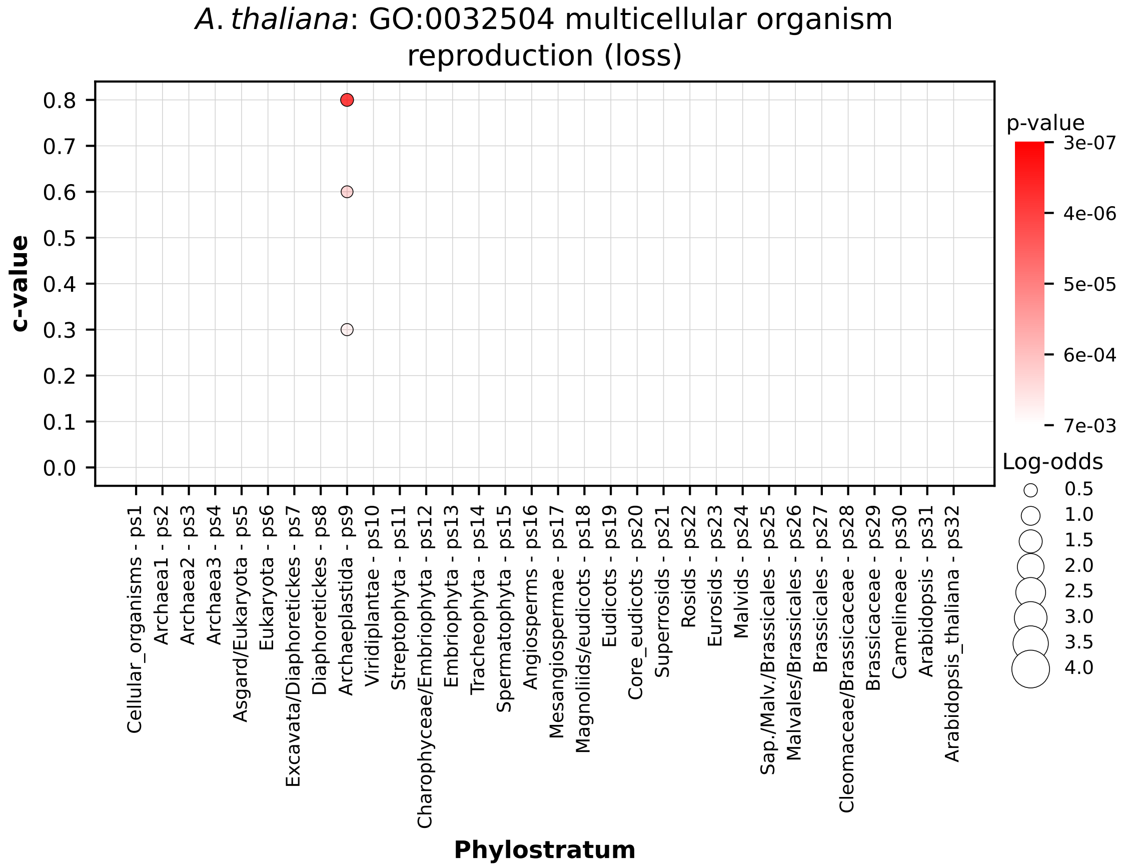

A. thaliana: GO:0032504 multicellular organism reproduction (loss)
c-value
0.8
0.7
0.6
0.5
0.4
0.3
0.2
0.1
0.0
p-value
3e-07
4e-06
5e-05
6e-04
7e-03
Log-odds
0.5
1.0
1.5
2.0
2.5
3.0
3.5
4.0
Cellular_organisms - ps1
Archaea1 - ps2
Archaea2 - ps3
Archaea3 - ps4
Asgard/Eukaryota - ps5
Eukaryota - ps6
Excavata/Diaphoretickes - ps7
Diaphoretickes - ps8
Archaeplastida - ps9
Viridiplantae - ps10
Streptophyta - ps11
Charophyceae/Embriophyta - ps12
Embriophyta - ps13
Tracheophyta - ps14
Spermatophyta - ps15
Angiosperms - ps16
Mesangiospermae - ps17
Magnoliids/eudicots - ps18
Eudicots - ps19
Core_eudicots - ps20
Superrosids - ps21
Rosids - ps22
Eurosids - ps23
Malvids - ps24
Sap./Malv./Brassicales - ps25
Malvales/Brassicales - ps26
Brassicales - ps27
Cleomaceae/Brassicaceae - ps28
Brassicaceae - ps29
Camelineae - ps30
Arabidopsis - ps31
Arabidopsis_thaliana - ps32
Phylostratum

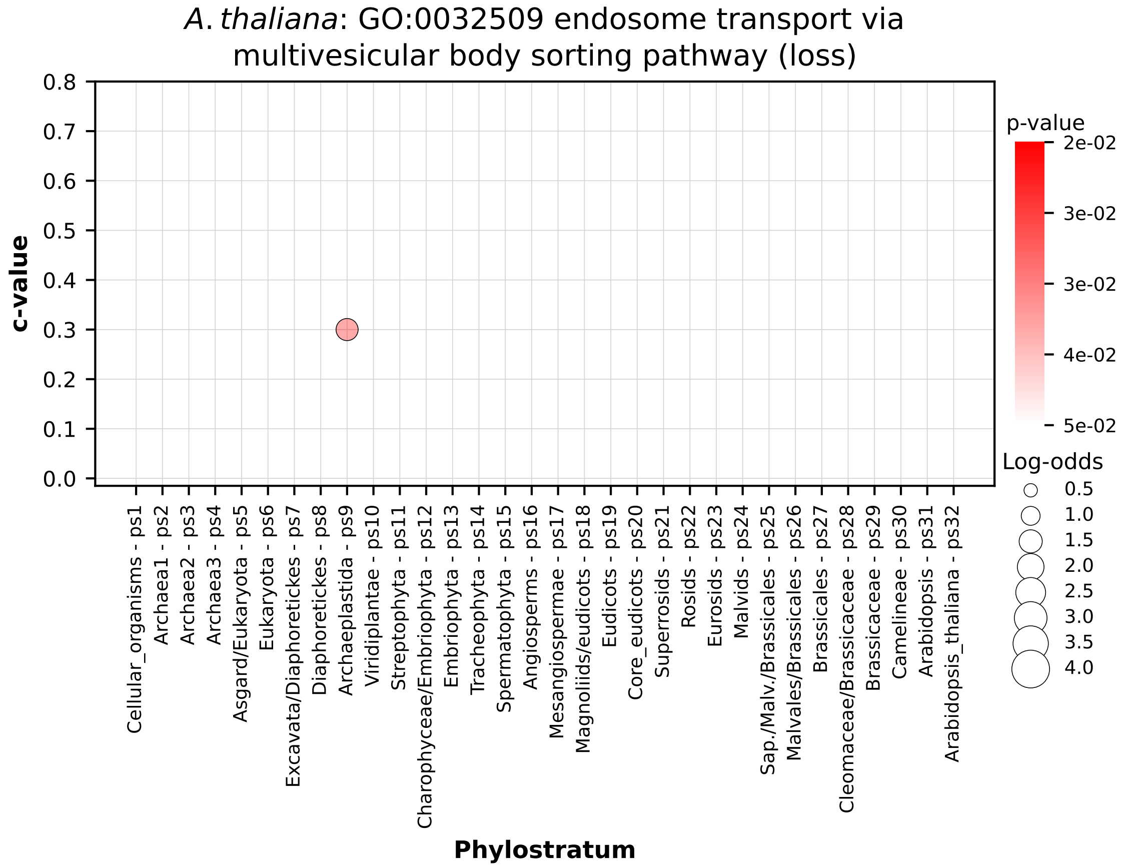
A. thaliana: GO:0032509 endosome transport via multivesicular body sorting pathway (loss)
c-value
0.0
0.1
0.2
0.3
0.4
0.5
0.6
0.7
0.8
Phylostratum
Cellular_organisms - ps1
Archaea1 - ps2
Archaea2 - ps3
Archaea3 - ps4
Asgard/Eukaryota - ps5
Eukaryota - ps6
Excavata/Diaphoretickes - ps7
Diaphoretickes - ps8
Archaeplastida - ps9
Viridiplantae - ps10
Streptophyta - ps11
Charophyceae/Embriophyta - ps12
Embriophyta - ps13
Tracheophyta - ps14
Spermatophyta - ps15
Angiosperms - ps16
Mesangiospermae - ps17
Magnoliids/eudicots - ps18
Eudicots - ps19
Core_eudicots - ps20
Superrosids - ps21
Rosids - ps22
Eurosids - ps23
Malvids - ps24
Sap./Malv./Brassicales - ps25
Malvales/Brassicales - ps26
Brassicales - ps27
Cleomaceae/Brassicaceae - ps28
Brassicaceae - ps29
Camelineae - ps30
Arabidopsis - ps31
Arabidopsis_thaliana - ps32
p-value
2e-02
3e-02
3e-02
4e-02
5e-02
Log-odds
0.5
1.0
1.5
2.0
2.5
3.0
3.5
4.0

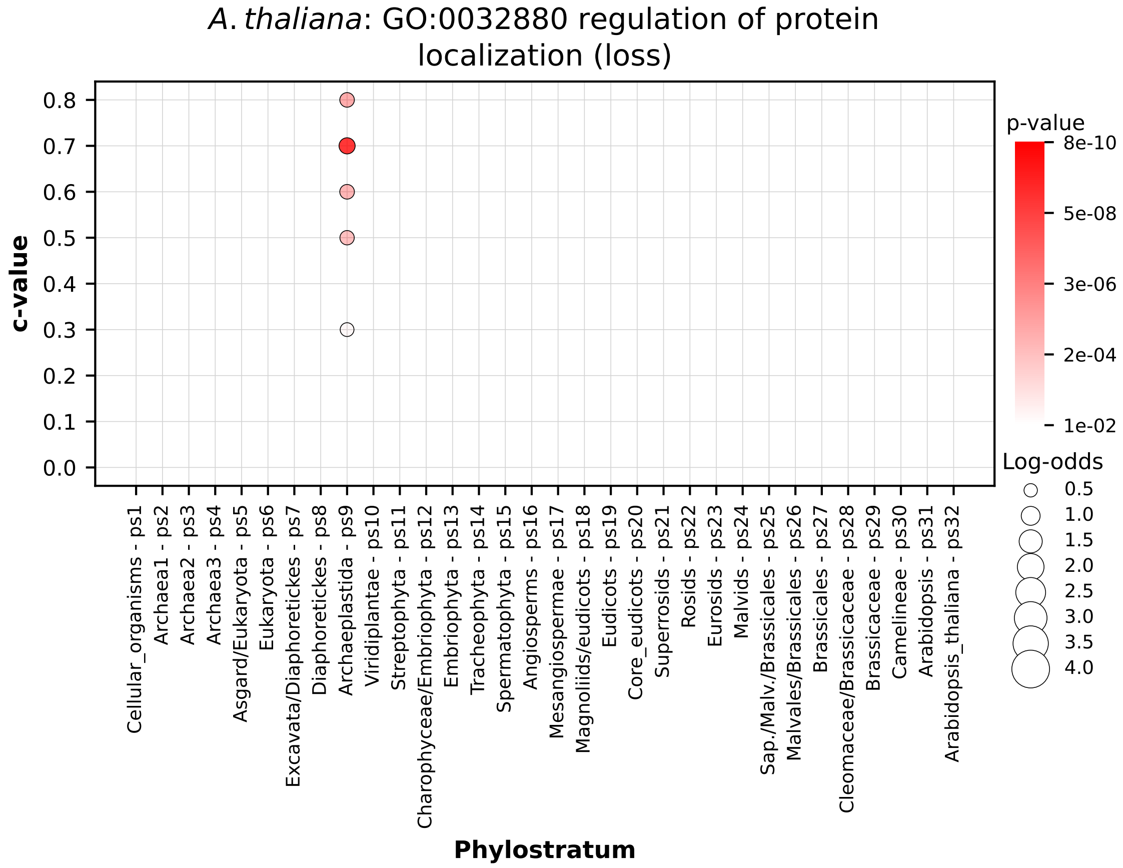

A. thaliana: GO:0032880 regulation of protein localization (loss)
c-value
0.8
0.7
0.6
0.5
0.4
0.3
0.2
0.1
0.0
p-value
8e-10
5e-08
3e-06
2e-04
1e-02
Log-odds
0.5
1.0
1.5
2.0
2.5
3.0
3.5
4.0
Cellular_organisms - ps1
Archaea1 - ps2
Archaea2 - ps3
Archaea3 - ps4
Asgard/Eukaryota - ps5
Eukaryota - ps6
Excavata/Diaphoretickes - ps7
Diaphoretickes - ps8
Archaeplastida - ps9
Viridiplantae - ps10
Streptophyta - ps11
Charophyceae/Embriophyta - ps12
Embriophyta - ps13
Tracheophyta - ps14
Spermatophyta - ps15
Angiosperms - ps16
Mesangiospermae - ps17
Magnoliids/eudicots - ps18
Eudicots - ps19
Core_eudicots - ps20
Superrosids - ps21
Rosids - ps22
Eurosids - ps23
Malvids - ps24
Sap./Malv./Brassicales - ps25
Malvales/Brassicales - ps26
Brassicales - ps27
Cleomaceae/Brassicaceae - ps28
Brassicaceae - ps29
Camelineae - ps30
Arabidopsis - ps31
Arabidopsis_thaliana - ps32
Phylostratum

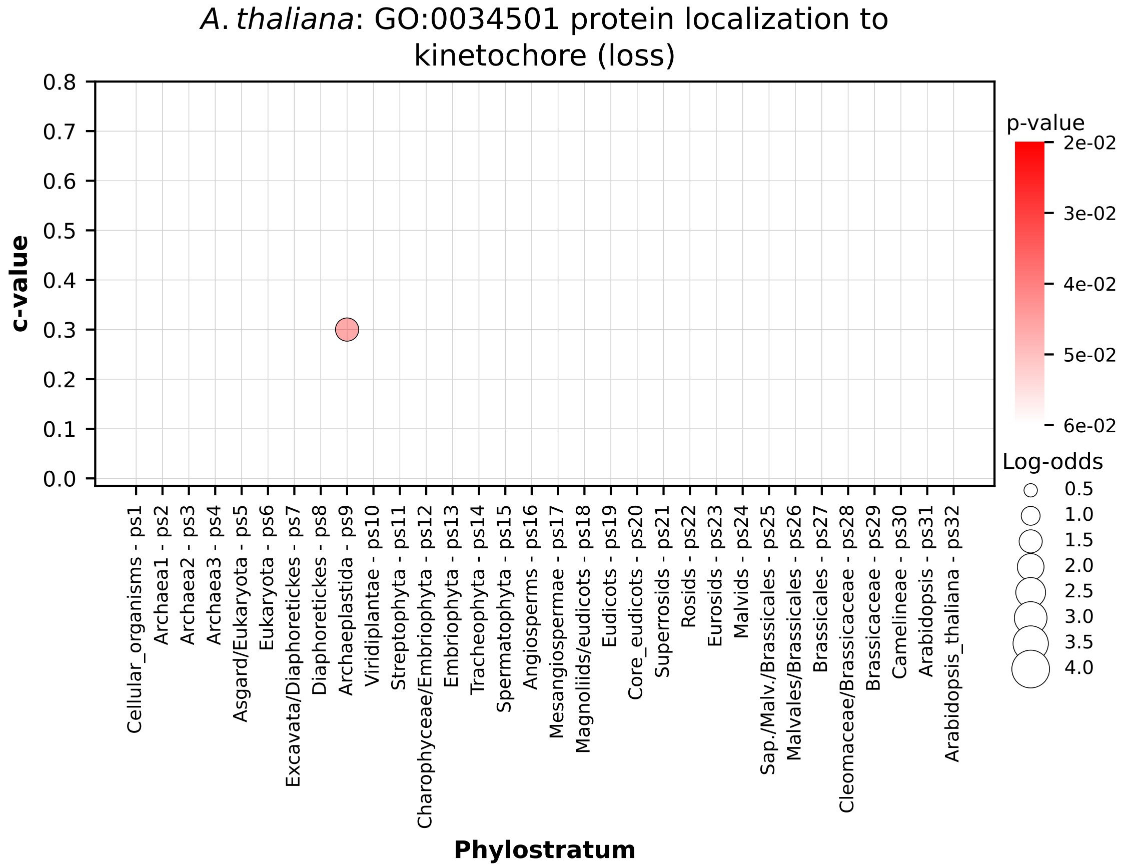

A. thaliana: GO:0034501 protein localization to kinetochore (loss)
c-value
0.8
0.7
0.6
0.5
0.4
0.3
0.2
0.1
0.0
p-value
2e-02
3e-02
4e-02
5e-02
6e-02
Log-odds
0.5
1.0
1.5
2.0
2.5
3.0
3.5
4.0
Cellular_organisms - ps1
Archaea1 - ps2
Archaea2 - ps3
Archaea3 - ps4
Asgard/Eukaryota - ps5
Eukaryota - ps6
Excavata/Diaphoretickes - ps7
Diaphoretickes - ps8
Archaeplastida - ps9
Viridiplantae - ps10
Streptophyta - ps11
Charophyceae/Embriophyta - ps12
Embriophyta - ps13
Tracheophyta - ps14
Spermatophyta - ps15
Angiosperms - ps16
Mesangiospermae - ps17
Magnoliids/eudicots - ps18
Eudicots - ps19
Core_eudicots - ps20
Superrosids - ps21
Rosids - ps22
Eurosids - ps23
Malvids - ps24
Sap./Malv./Brassicales - ps25
Malvales/Brassicales - ps26
Brassicales - ps27
Cleomaceae/Brassicaceae - ps28
Brassicaceae - ps29
Camelineae - ps30
Arabidopsis - ps31
Arabidopsis_thaliana - ps32
Phylostratum

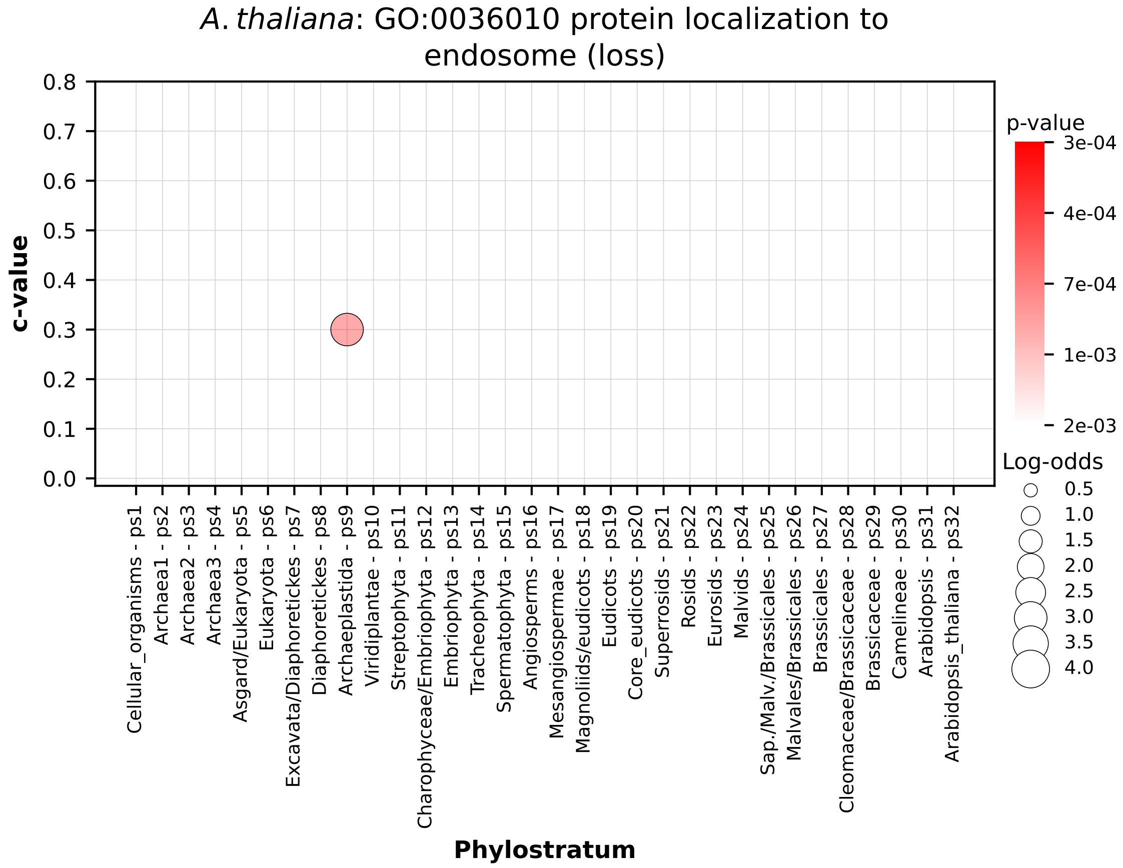

A. thaliana: GO:0036010 protein localization to endosome (loss)
c-value
0.0
0.1
0.2
0.3
0.4
0.5
0.6
0.7
0.8
Phylostratum
Cellular_organisms - ps1
Archaea1 - ps2
Archaea2 - ps3
Archaea3 - ps4
Asgard/Eukaryota - ps5
Eukaryota - ps6
Excavata/Diaphoretickes - ps7
Diaphoretickes - ps8
Archaeplastida - ps9
Viridiplantae - ps10
Streptophyta - ps11
Charophyceae/Embriophyta - ps12
Embriophyta - ps13
Tracheophyta - ps14
Spermatophyta - ps15
Angiosperms - ps16
Mesangiospermae - ps17
Magnoliids/eudicots - ps18
Eudicots - ps19
Core_eudicots - ps20
Superrosids - ps21
Rosids - ps22
Eurosids - ps23
Malvids - ps24
Sap./Malv./Brassicales - ps25
Malvales/Brassicales - ps26
Brassicales - ps27
Cleomaceae/Brassicaceae - ps28
Brassicaceae - ps29
Camelineae - ps30
Arabidopsis - ps31
Arabidopsis_thaliana - ps32
p-value
3e-04
4e-04
7e-04
1e-03
2e-03
Log-odds
0.5
1.0
1.5
2.0
2.5
3.0
3.5
4.0

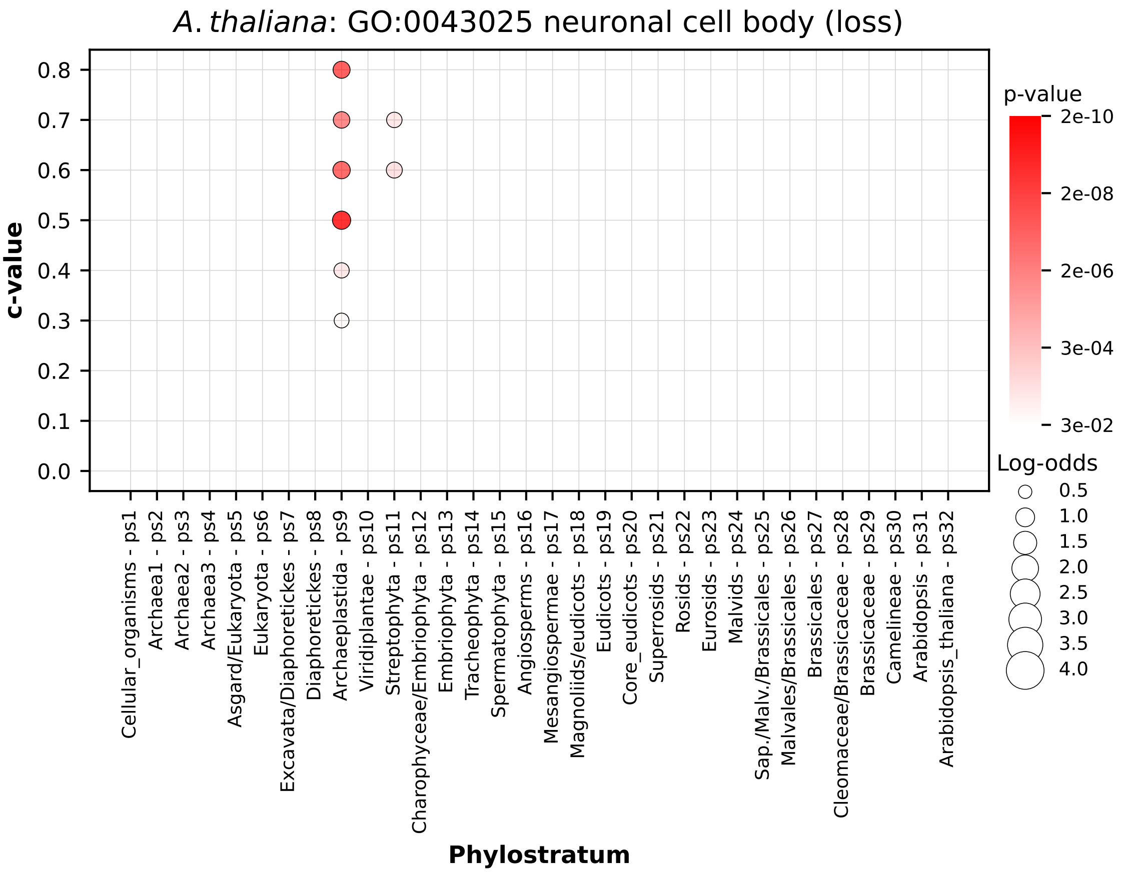

A. thaliana: GO:0043025 neuronal cell body (loss)
c-value
0.8
0.7
0.6
0.5
0.4
0.3
0.2
0.1
0.0
Phylostratum
Cellular_organisms - ps1
Archaea1 - ps2
Archaea2 - ps3
Archaea3 - ps4
Asgard/Eukaryota - ps5
Eukaryota - ps6
Excavata/Diaphoretickes - ps7
Diaphoretickes - ps8
Archaeplastida - ps9
Viridiplantae - ps10
Streptophyta - ps11
Charophyceae/Embriophyta - ps12
Embriophyta - ps13
Tracheophyta - ps14
Spermatophyta - ps15
Angiosperms - ps16
Mesangiospermae - ps17
Magnoliids/eudicots - ps18
Eudicots - ps19
Core_eudicots - ps20
Superrosids - ps21
Rosids - ps22
Eurosids - ps23
Malvids - ps24
Sap./Malv./Brassicales - ps25
Malvales/Brassicales - ps26
Brassicales - ps27
Cleomaceae/Brassicaceae - ps28
Brassicaceae - ps29
Camelineae - ps30
Arabidopsis - ps31
Arabidopsis_thaliana - ps32
p-value
2e-10
2e-08
2e-06
3e-04
3e-02
Log-odds
0.5
1.0
1.5
2.0
2.5
3.0
3.5
4.0

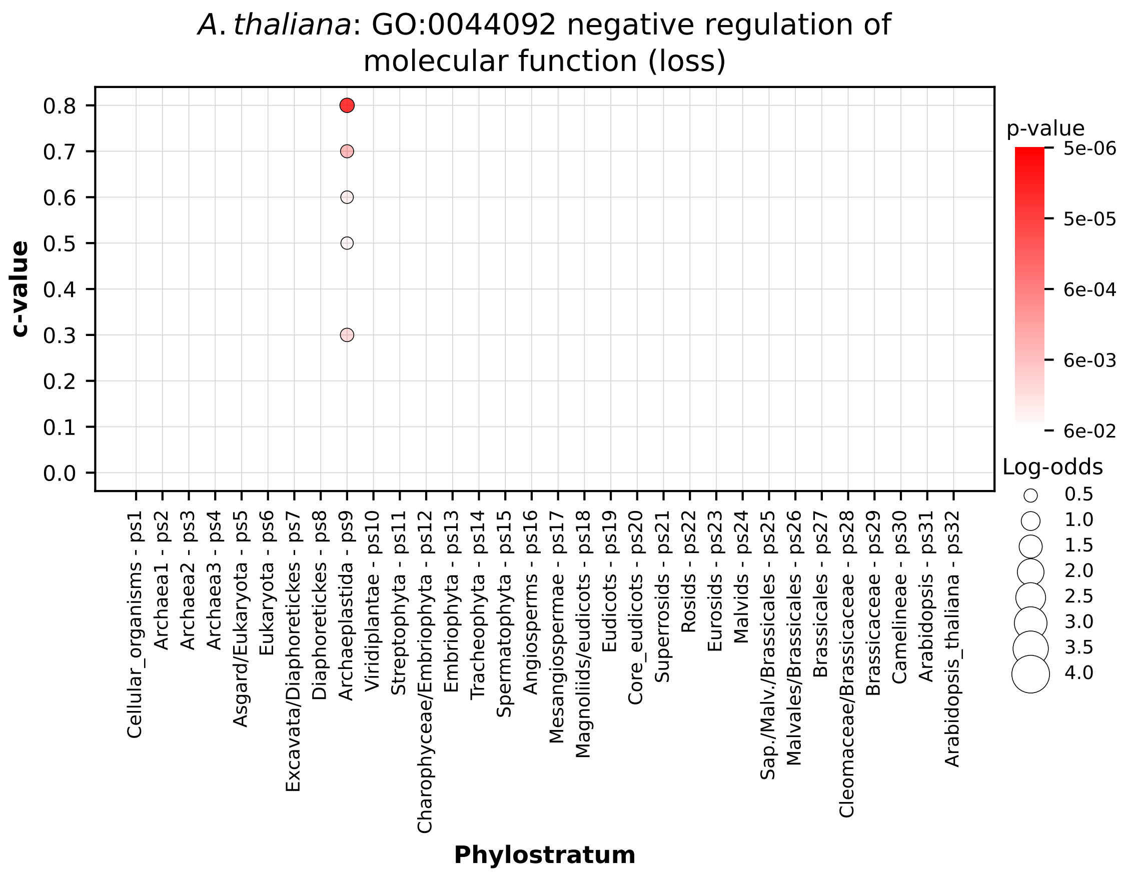
A. thaliana: GO:0044092 negative regulation of molecular function (loss)
c-value
0.8
0.7
0.6
0.5
0.4
0.3
0.2
0.1
0.0
Phylostratum
Cellular_organisms - ps1
Archaea1 - ps2
Archaea2 - ps3
Archaea3 - ps4
Asgard/Eukaryota - ps5
Eukaryota - ps6
Excavata/Diaphoretickes - ps7
Diaphoretickes - ps8
Archaeplastida - ps9
Viridiplantae - ps10
Streptophyta - ps11
Charophyceae/Embriophyta - ps12
Embriophyta - ps13
Tracheophyta - ps14
Spermatophyta - ps15
Angiosperms - ps16
Mesangiospermae - ps17
Magnoliids/eudicots - ps18
Eudicots - ps19
Core_eudicots - ps20
Superrosids - ps21
Rosids - ps22
Eurosids - ps23
Malvids - ps24
Sap./Malv./Brassicales - ps25
Malvales/Brassicales - ps26
Brassicales - ps27
Cleomaceae/Brassicaceae - ps28
Brassicaceae - ps29
Camelineae - ps30
Arabidopsis - ps31
Arabidopsis_thaliana - ps32
p-value
5e-06
5e-05
6e-04
6e-03
6e-02
Log-odds
0.5
1.0
1.5
2.0
2.5
3.0
3.5
4.0

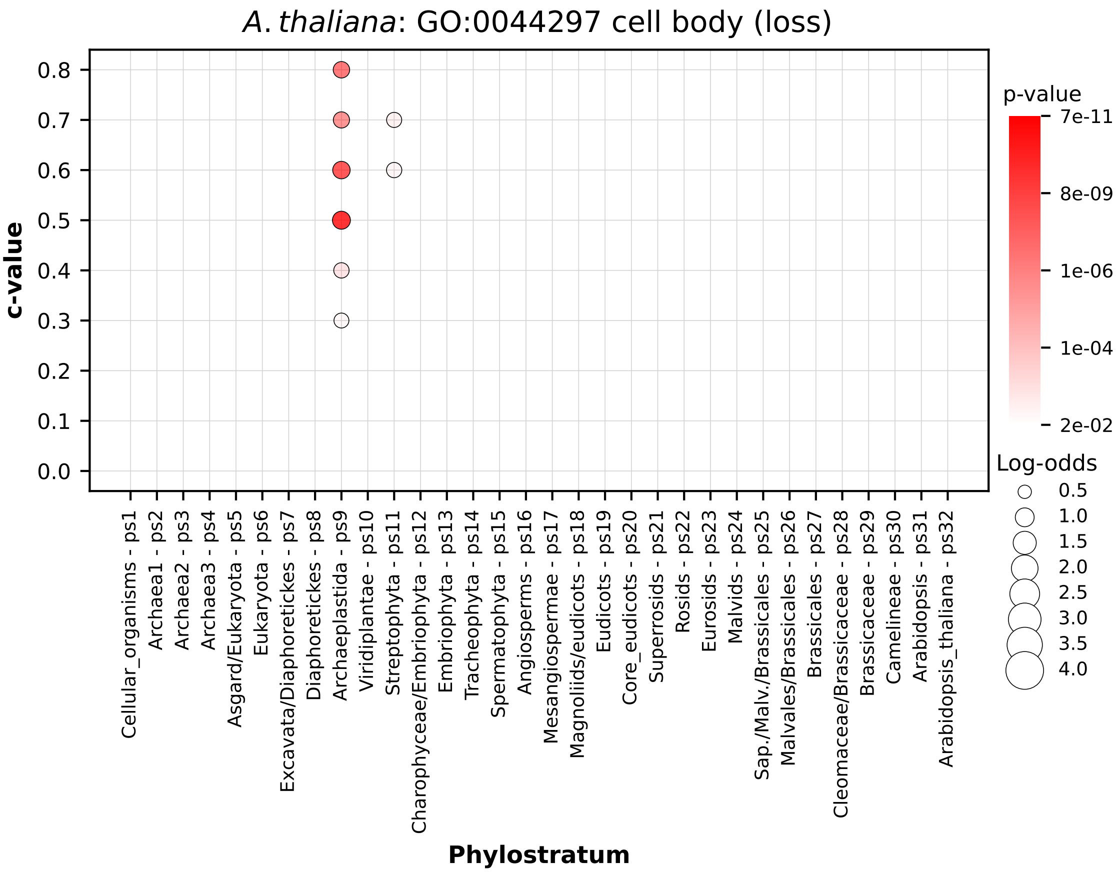
A. thaliana: GO:0044297 cell body (loss)
c-value
0.0
0.1
0.2
0.3
0.4
0.5
0.6
0.7
0.8
Phylostratum
Cellular_organisms - ps1
Archaea1 - ps2
Archaea2 - ps3
Archaea3 - ps4
Asgard/Eukaryota - ps5
Eukaryota - ps6
Excavata/Diaphoretickes - ps7
Diaphoretickes - ps8
Archaeplastida - ps9
Viridiplantae - ps10
Streptophyta - ps11
Charophyceae/Embriophyta - ps12
Embriophyta - ps13
Tracheophyta - ps14
Spermatophyta - ps15
Angiosperms - ps16
Mesangiospermae - ps17
Magnoliids/eudicots - ps18
Eudicots - ps19
Core_eudicots - ps20
Superrosids - ps21
Rosids - ps22
Eurosids - ps23
Malvids - ps24
Sap./Malv./Brassicales - ps25
Malvales/Brassicales - ps26
Brassicales - ps27
Cleomaceae/Brassicaceae - ps28
Brassicaceae - ps29
Camelineae - ps30
Arabidopsis - ps31
Arabidopsis_thaliana - ps32
p-value
7e-11
8e-09
1e-06
1e-04
2e-02
Log-odds
0.5
1.0
1.5
2.0
2.5
3.0
3.5
4.0

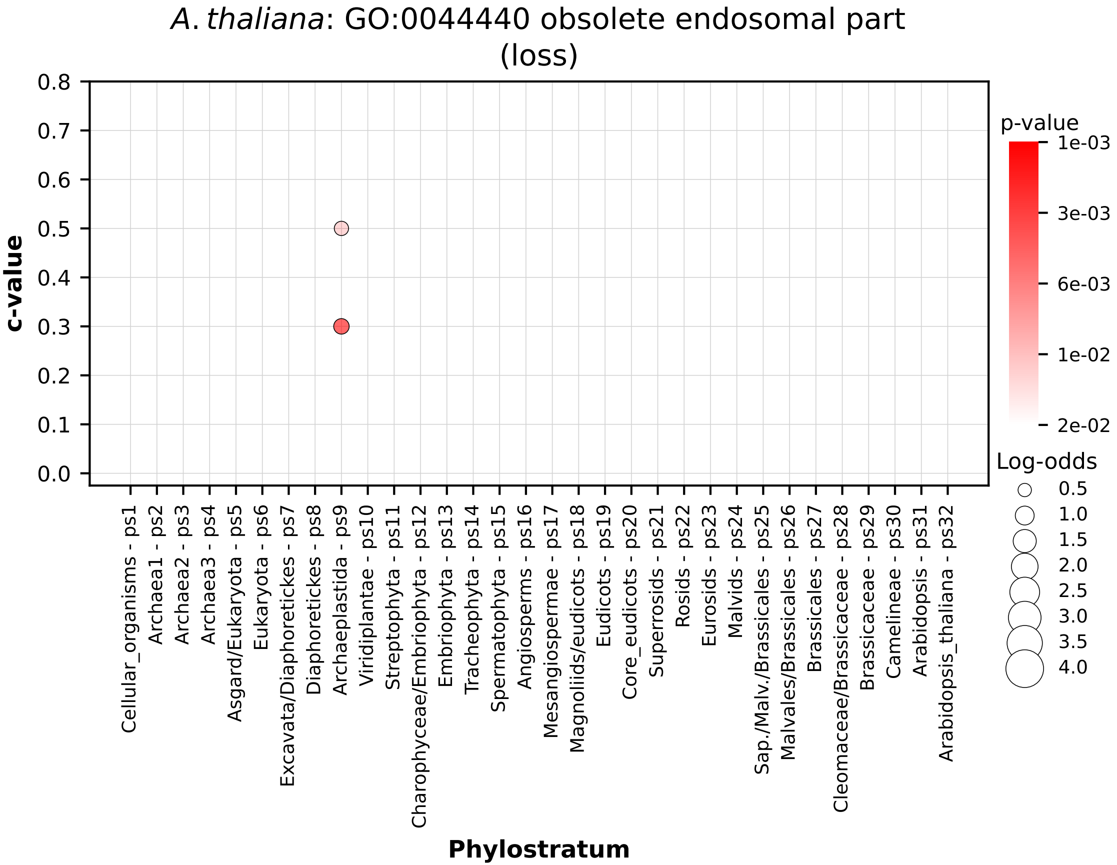

A. thaliana: GO:0044440 obsolete endosomal part
(loss)
c-value
0.8
0.7
0.6
0.5
0.4
0.3
0.2
0.1
0.0
Phylostratum
Cellular_organisms - ps1
Archaea1 - ps2
Archaea2 - ps3
Archaea3 - ps4
Asgard/Eukaryota - ps5
Eukaryota - ps6
Excavata/Diaphoretickes - ps7
Diaphoretickes - ps8
Archaeplastida - ps9
Viridiplantae - ps10
Streptophyta - ps11
Charophyceae/Embriophyta - ps12
Embriophyta - ps13
Tracheophyta - ps14
Spermatophyta - ps15
Angiosperms - ps16
Mesangiospermae - ps17
Magnoliids/eudicots - ps18
Eudicots - ps19
Core_eudicots - ps20
Superrosids - ps21
Rosids - ps22
Eurosids - ps23
Malvids - ps24
Sap./Malv./Brassicales - ps25
Malvales/Brassicales - ps26
Brassicales - ps27
Cleomaceae/Brassicaceae - ps28
Brassicaceae - ps29
Camelineae - ps30
Arabidopsis - ps31
Arabidopsis_thaliana - ps32
p-value
1e-03
3e-03
6e-03
1e-02
2e-02
Log-odds
0.5
1.0
1.5
2.0
2.5
3.0
3.5
4.0

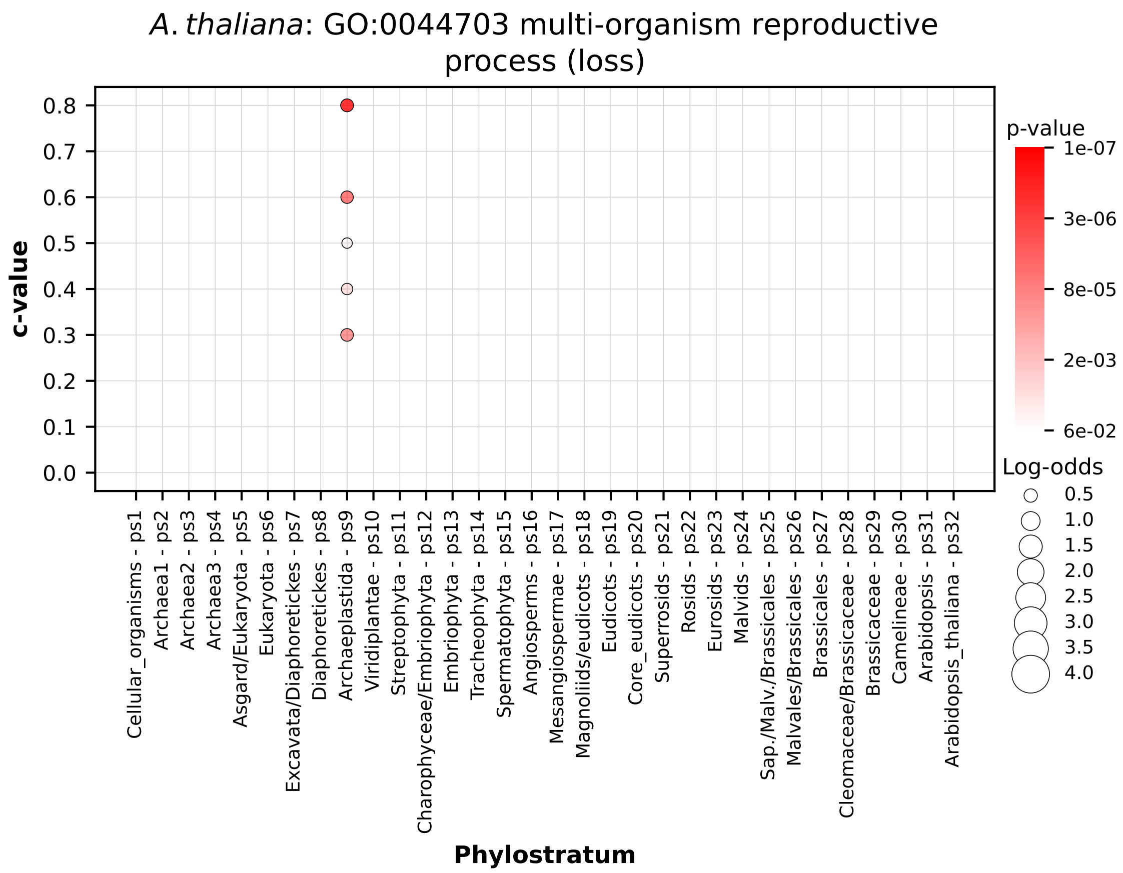

A. thaliana: GO:0044703 multi-organism reproductive process (loss)
c-value
0.8
0.7
0.6
0.5
0.4
0.3
0.2
0.1
0.0
Phylostratum
Cellular_organisms - ps1
Archaea1 - ps2
Archaea2 - ps3
Archaea3 - ps4
Asgard/Eukaryota - ps5
Eukaryota - ps6
Excavata/Diaphoretickes - ps7
Diaphoretickes - ps8
Archaeplastida - ps9
Viridiplantae - ps10
Streptophyta - ps11
Charophyceae/Embriophyta - ps12
Embriophyta - ps13
Tracheophyta - ps14
Spermatophyta - ps15
Angiosperms - ps16
Mesangiospermae - ps17
Magnoliids/eudicots - ps18
Eudicots - ps19
Core_eudicots - ps20
Superrosids - ps21
Rosids - ps22
Eurosids - ps23
Malvids - ps24
Sap./Malv./Brassicales - ps25
Malvales/Brassicales - ps26
Brassicales - ps27
Cleomaceae/Brassicaceae - ps28
Brassicaceae - ps29
Camelineae - ps30
Arabidopsis - ps31
Arabidopsis_thaliana - ps32
p-value
1e-07
3e-06
8e-05
2e-03
6e-02
Log-odds
0.5
1.0
1.5
2.0
2.5
3.0
3.5
4.0

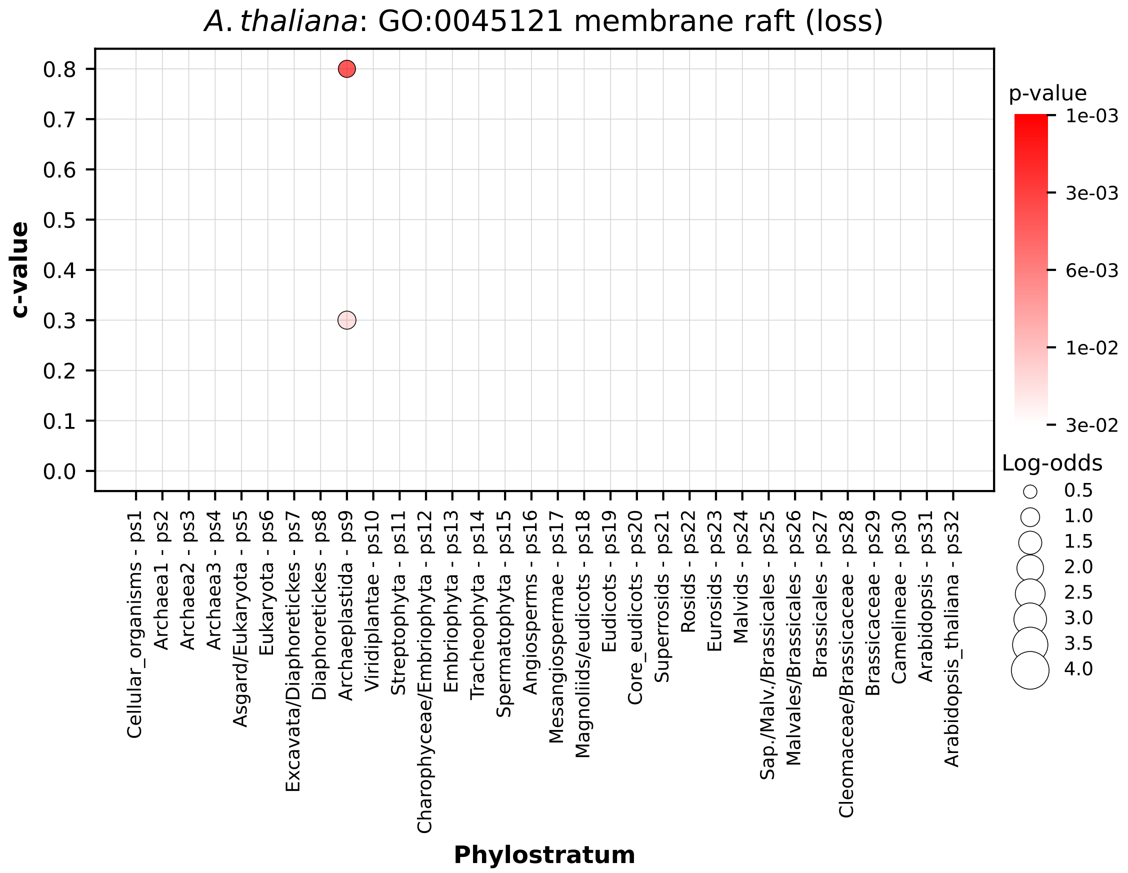

A. thaliana: GO:0045121 membrane raft (loss)
c-value
0.8
0.7
0.6
0.5
0.4
0.3
0.2
0.1
0.0
p-value
1e-03
3e-03
6e-03
1e-02
3e-02
Log-odds
0.5
1.0
1.5
2.0
2.5
3.0
3.5
4.0
Cellular_organisms - ps1
Archaea1 - ps2
Archaea2 - ps3
Archaea3 - ps4
Asgard/Eukaryota - ps5
Eukaryota - ps6
Excavata/Diaphoretickes - ps7
Diaphoretickes - ps8
Archaeplastida - ps9
Viridiplantae - ps10
Streptophyta - ps11
Charophyceae/Embriophyta - ps12
Embriophyta - ps13
Tracheophyta - ps14
Spermatophyta - ps15
Angiosperms - ps16
Mesangiospermae - ps17
Magnoliids/eudicots - ps18
Eudicots - ps19
Core_eudicots - ps20
Superrosids - ps21
Rosids - ps22
Eurosids - ps23
Malvids - ps24
Sap./Malv./Brassicales - ps25
Malvales/Brassicales - ps26
Brassicales - ps27
Cleomaceae/Brassicaceae - ps28
Brassicaceae - ps29
Camelineae - ps30
Arabidopsis - ps31
Arabidopsis_thaliana - ps32
Phylostratum

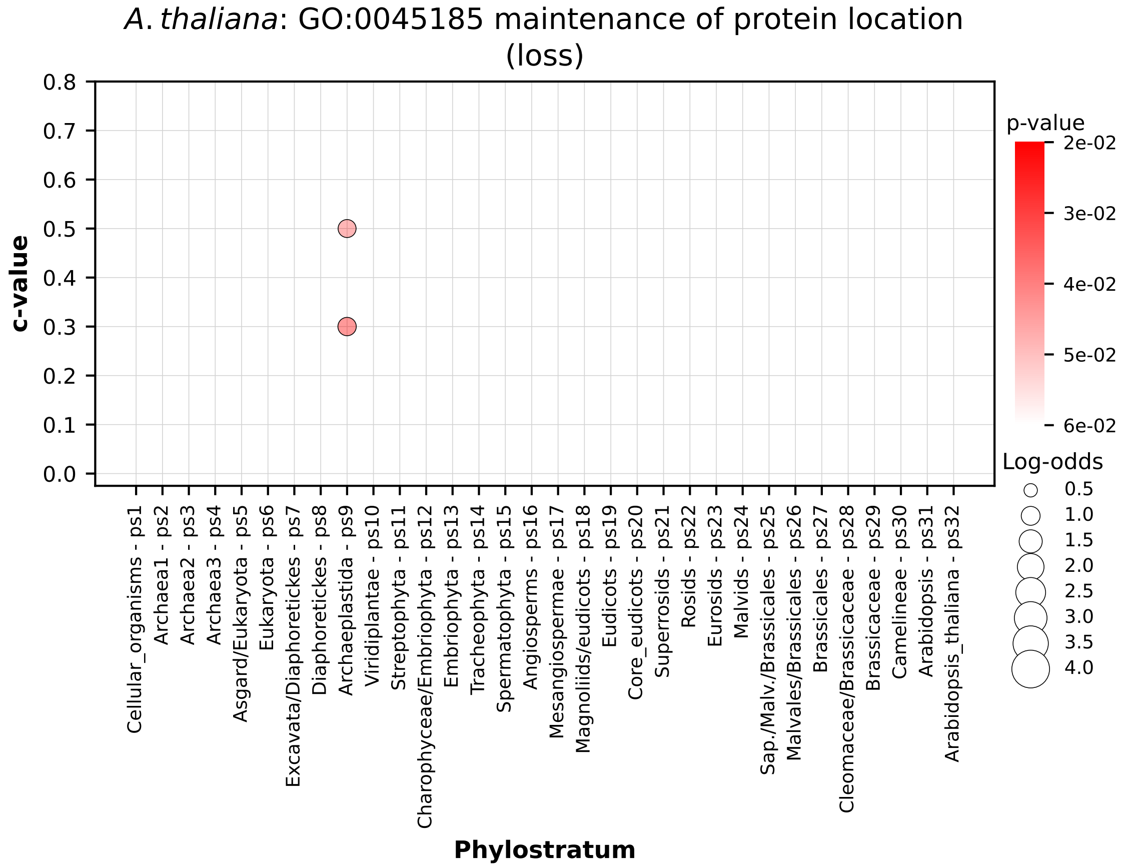
A. thaliana: GO:0045185 maintenance of protein location (loss)
c-value
0.8
0.7
0.6
0.5
0.4
0.3
0.2
0.1
0.0
Phylostratum
Cellular_organisms - ps1
Archaea1 - ps2
Archaea2 - ps3
Archaea3 - ps4
Asgard/Eukaryota - ps5
Eukaryota - ps6
Excavata/Diaphoretickes - ps7
Diaphoretickes - ps8
Archaeplastida - ps9
Viridiplantae - ps10
Streptophyta - ps11
Charophyceae/Embriophyta - ps12
Embriophyta - ps13
Tracheophyta - ps14
Spermatophyta - ps15
Angiosperms - ps16
Mesangiospermae - ps17
Magnoliids/eudicots - ps18
Eudicots - ps19
Core_eudicots - ps20
Superrosids - ps21
Rosids - ps22
Eurosids - ps23
Malvids - ps24
Sap./Malv./Brassicales - ps25
Malvales/Brassicales - ps26
Brassicales - ps27
Cleomaceae/Brassicaceae - ps28
Brassicaceae - ps29
Camelineae - ps30
Arabidopsis - ps31
Arabidopsis_thaliana - ps32
p-value
2e-02
3e-02
4e-02
5e-02
6e-02
Log-odds
0.5
1.0
1.5
2.0
2.5
3.0
3.5
4.0

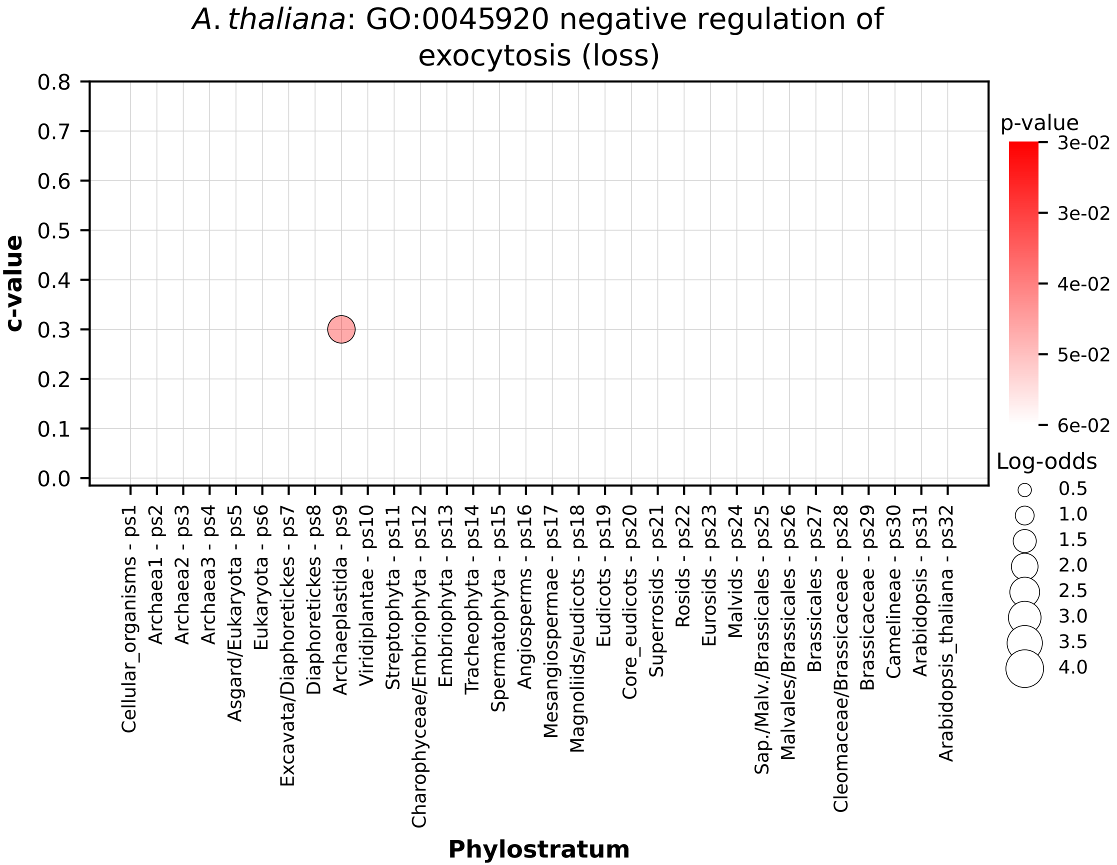
A. thaliana: GO:0045920 negative regulation of exocytosis (loss)
c-value
0.8
0.7
0.6
0.5
0.4
0.3
0.2
0.1
0.0
p-value
3e-02
3e-02
4e-02
5e-02
6e-02
Log-odds
0.5
1.0
1.5
2.0
2.5
3.0
3.5
4.0
Cellular_organisms - ps1
Archaea1 - ps2
Archaea2 - ps3
Archaea3 - ps4
Asgard/Eukaryota - ps5
Eukaryota - ps6
Excavata/Diaphoretickes - ps7
Diaphoretickes - ps8
Archaeplastida - ps9
Viridiplantae - ps10
Streptophyta - ps11
Charophyceae/Embriophyta - ps12
Embriophyta - ps13
Tracheophyta - ps14
Spermatophyta - ps15
Angiosperms - ps16
Mesangiospermae - ps17
Magnoliids/eudicots - ps18
Eudicots - ps19
Core_eudicots - ps20
Superrosids - ps21
Rosids - ps22
Eurosids - ps23
Malvids - ps24
Sap./Malv./Brassicales - ps25
Malvales/Brassicales - ps26
Brassicales - ps27
Cleomaceae/Brassicaceae - ps28
Brassicaceae - ps29
Camelineae - ps30
Arabidopsis - ps31
Arabidopsis_thaliana - ps32
Phylostratum

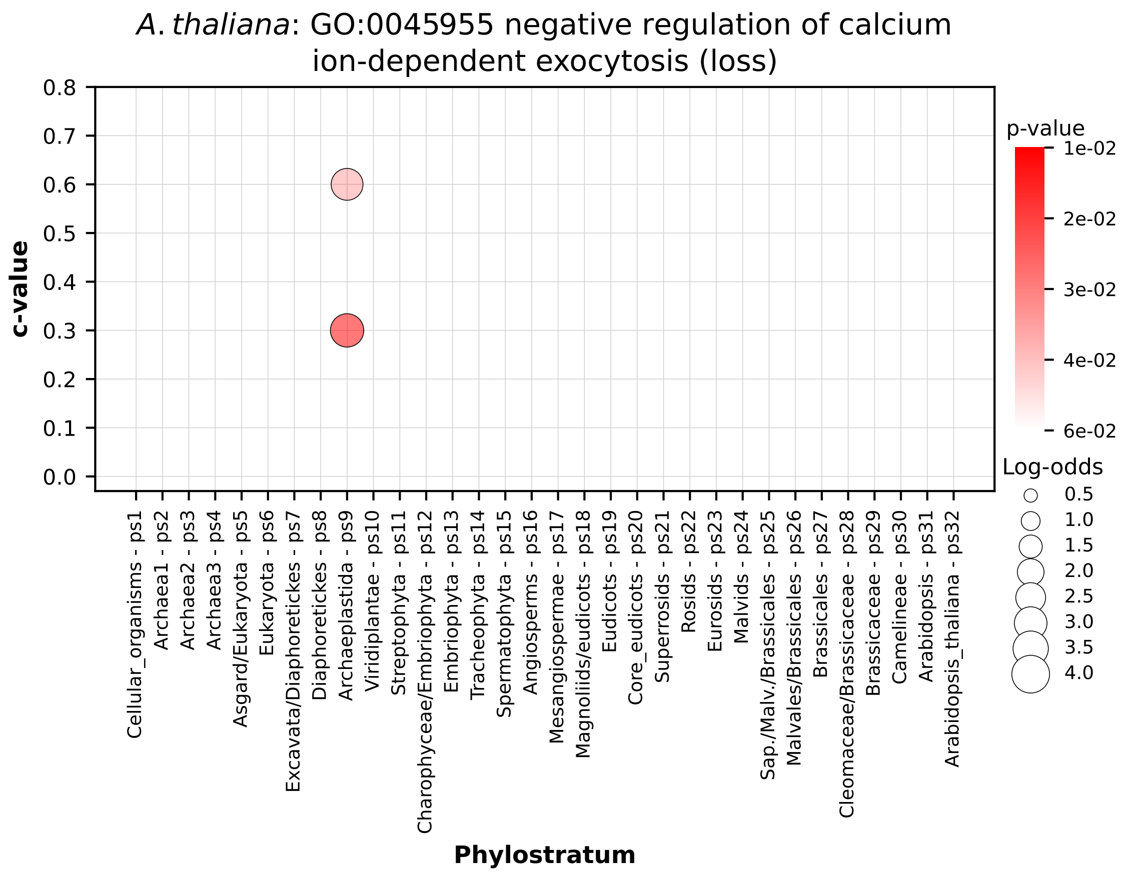
A. thaliana: GO:0045955 negative regulation of calcium ion-dependent exocytosis (loss)
c-value
0.8
0.7
0.6
0.5
0.4
0.3
0.2
0.1
0.0
Phylostratum
Cellular_organisms - ps1
Archaea1 - ps2
Archaea2 - ps3
Archaea3 - ps4
Asgard/Eukaryota - ps5
Eukaryota - ps6
Excavata/Diaphoretickes - ps7
Diaphoretickes - ps8
Archaeplastida - ps9
Viridiplantae - ps10
Streptophyta - ps11
Charophyceae/Embriophyta - ps12
Embriophyta - ps13
Tracheophyta - ps14
Spermatophyta - ps15
Angiosperms - ps16
Mesangiospermae - ps17
Magnoliids/eudicots - ps18
Eudicots - ps19
Core_eudicots - ps20
Superrosids - ps21
Rosids - ps22
Eurosids - ps23
Malvids - ps24
Sap./Malv./Brassicales - ps25
Malvales/Brassicales - ps26
Brassicales - ps27
Cleomaceae/Brassicaceae - ps28
Brassicaceae - ps29
Camelineae - ps30
Arabidopsis - ps31
Arabidopsis_thaliana - ps32
p-value
1e-02
2e-02
3e-02
4e-02
6e-02
Log-odds
0.5
1.0
1.5
2.0
2.5
3.0
3.5
4.0

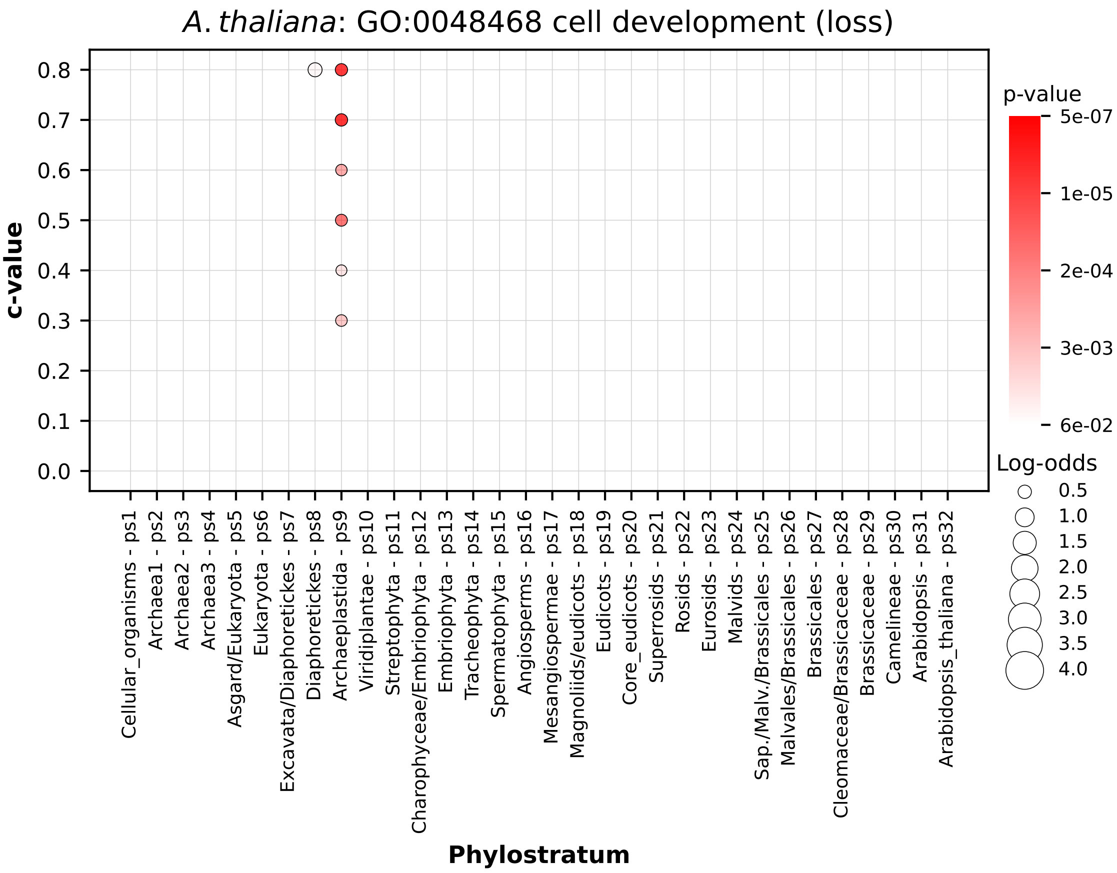

A. thaliana: GO:0048468 cell development (loss)
c-value
0.0
0.1
0.2
0.3
0.4
0.5
0.6
0.7
0.8
p-value
5e-07
1e-05
2e-04
3e-03
6e-02
Log-odds
0.5
1.0
1.5
2.0
2.5
3.0
3.5
4.0
Cellular_organisms - ps1
Archaea1 - ps2
Archaea2 - ps3
Archaea3 - ps4
Asgard/Eukaryota - ps5
Eukaryota - ps6
Excavata/Diaphoretickes - ps7
Diaphoretickes - ps8
Archaeplastida - ps9
Viridiplantae - ps10
Streptophyta - ps11
Charophyceae/Embriophyta - ps12
Embriophyta - ps13
Tracheophyta - ps14
Spermatophyta - ps15
Angiosperms - ps16
Mesangiospermae - ps17
Magnoliids/eudicots - ps18
Eudicots - ps19
Core_eudicots - ps20
Superrosids - ps21
Rosids - ps22
Eurosids - ps23
Malvids - ps24
Sap./Malv./Brassicales - ps25
Malvales/Brassicales - ps26
Brassicales - ps27
Cleomaceae/Brassicaceae - ps28
Brassicaceae - ps29
Camelineae - ps30
Arabidopsis - ps31
Arabidopsis_thaliana - ps32
Phylostratum

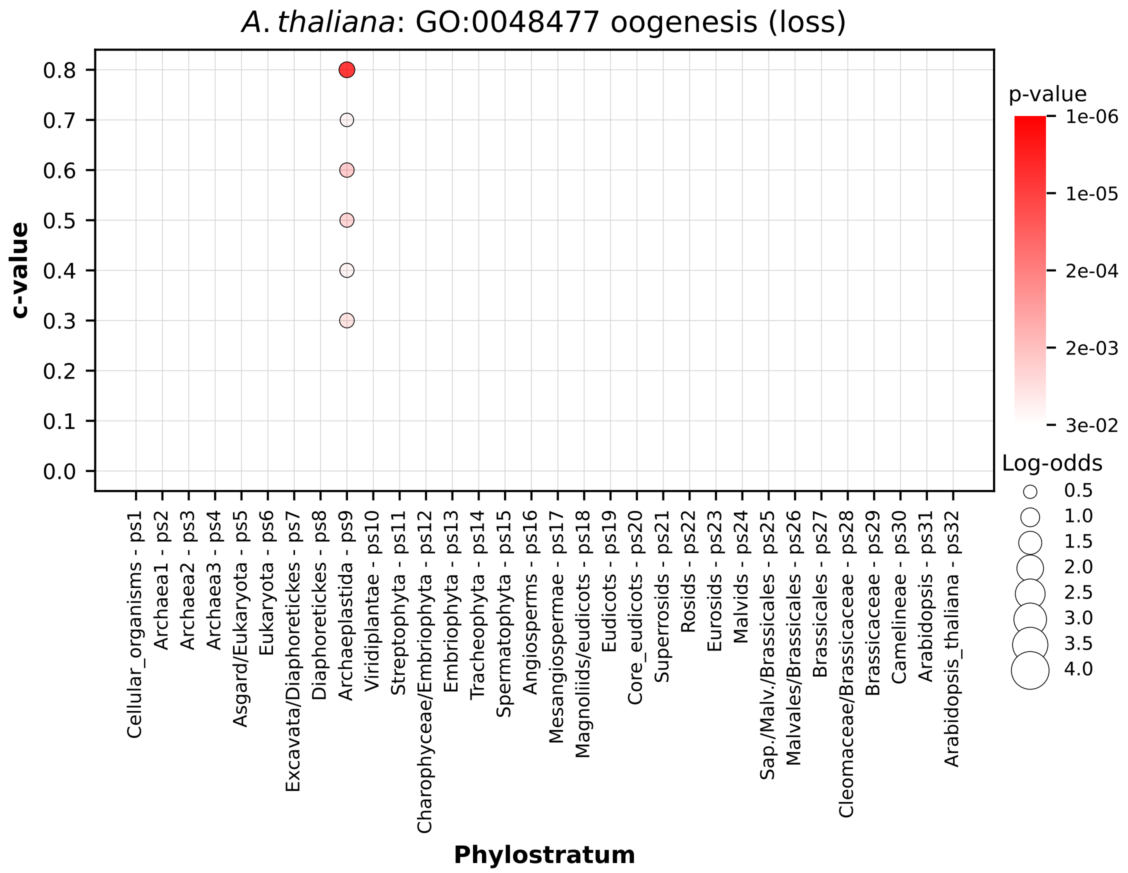

A. thaliana: GO:0048477 oogenesis (loss)
c-value
0.0
0.1
0.2
0.3
0.4
0.5
0.6
0.7
0.8
p-value
1e-06
1e-05
2e-04
2e-03
3e-02
Log-odds
0.5
1.0
1.5
2.0
2.5
3.0
3.5
4.0
Cellular_organisms - ps1
Archaea1 - ps2
Archaea2 - ps3
Archaea3 - ps4
Asgard/Eukaryota - ps5
Eukaryota - ps6
Excavata/Diaphoretickes - ps7
Diaphoretickes - ps8
Archaeplastida - ps9
Viridiplantae - ps10
Streptophyta - ps11
Charophyceae/Embriophyta - ps12
Embriophyta - ps13
Tracheophyta - ps14
Spermatophyta - ps15
Angiosperms - ps16
Mesangiospermae - ps17
Magnoliids/eudicots - ps18
Eudicots - ps19
Core_eudicots - ps20
Superrosids - ps21
Rosids - ps22
Eurosids - ps23
Malvids - ps24
Sap./Malv./Brassicales - ps25
Malvales/Brassicales - ps26
Brassicales - ps27
Cleomaceae/Brassicaceae - ps28
Brassicaceae - ps29
Camelineae - ps30
Arabidopsis - ps31
Arabidopsis_thaliana - ps32
Phylostratum

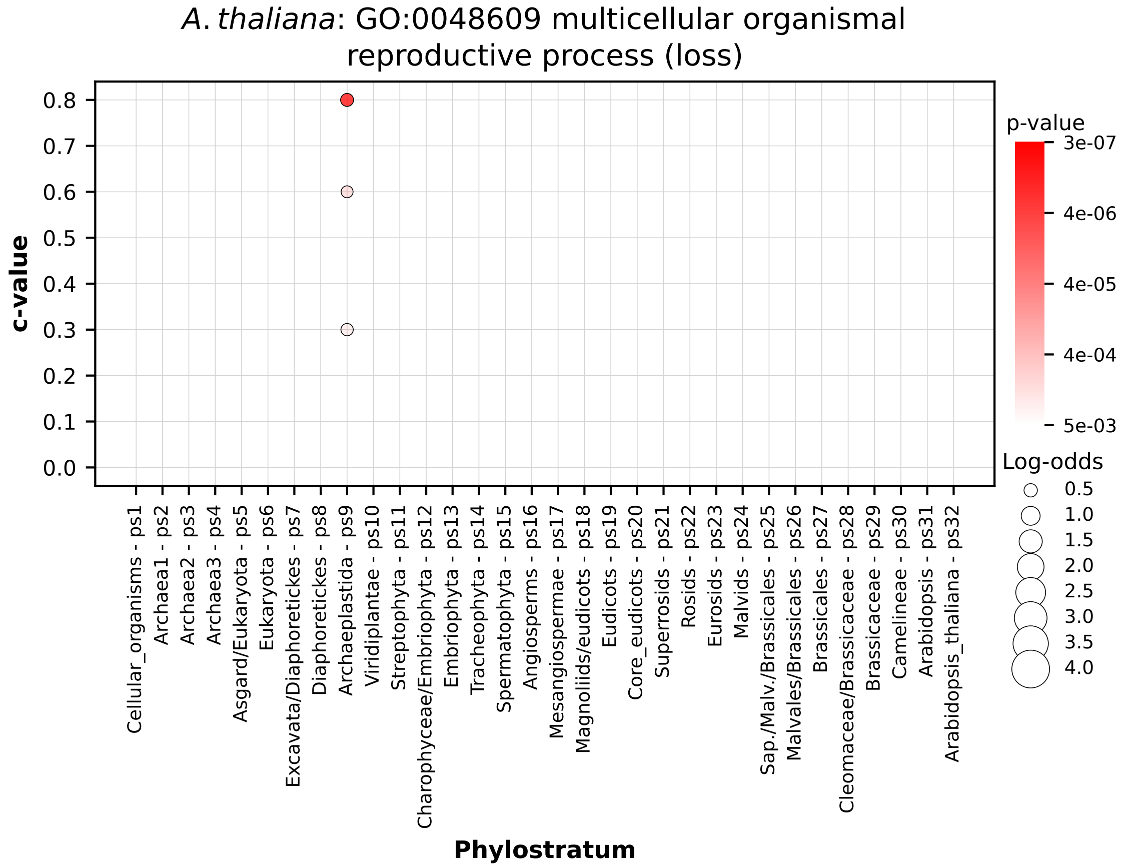

A. thaliana: GO:0048609 multicellular organismal reproductive process (loss)
c-value
0.0
0.1
0.2
0.3
0.4
0.5
0.6
0.7
0.8
p-value
3e-07
4e-06
4e-05
4e-04
5e-03
Log-odds
0.5
1.0
1.5
2.0
2.5
3.0
3.5
4.0
Cellular_organisms - ps1
Archaea1 - ps2
Archaea2 - ps3
Archaea3 - ps4
Asgard/Eukaryota - ps5
Eukaryota - ps6
Excavata/Diaphoretickes - ps7
Diaphoretickes - ps8
Archaeplastida - ps9
Viridiplantae - ps10
Streptophyta - ps11
Charophyceae/Embriophyta - ps12
Embriophyta - ps13
Tracheophyta - ps14
Spermatophyta - ps15
Angiosperms - ps16
Mesangiospermae - ps17
Magnoliids/eudicots - ps18
Eudicots - ps19
Core_eudicots - ps20
Superrosids - ps21
Rosids - ps22
Eurosids - ps23
Malvids - ps24
Sap./Malv./Brassicales - ps25
Malvales/Brassicales - ps26
Brassicales - ps27
Cleomaceae/Brassicaceae - ps28
Brassicaceae - ps29
Camelineae - ps30
Arabidopsis - ps31
Arabidopsis_thaliana - ps32
Phylostratum

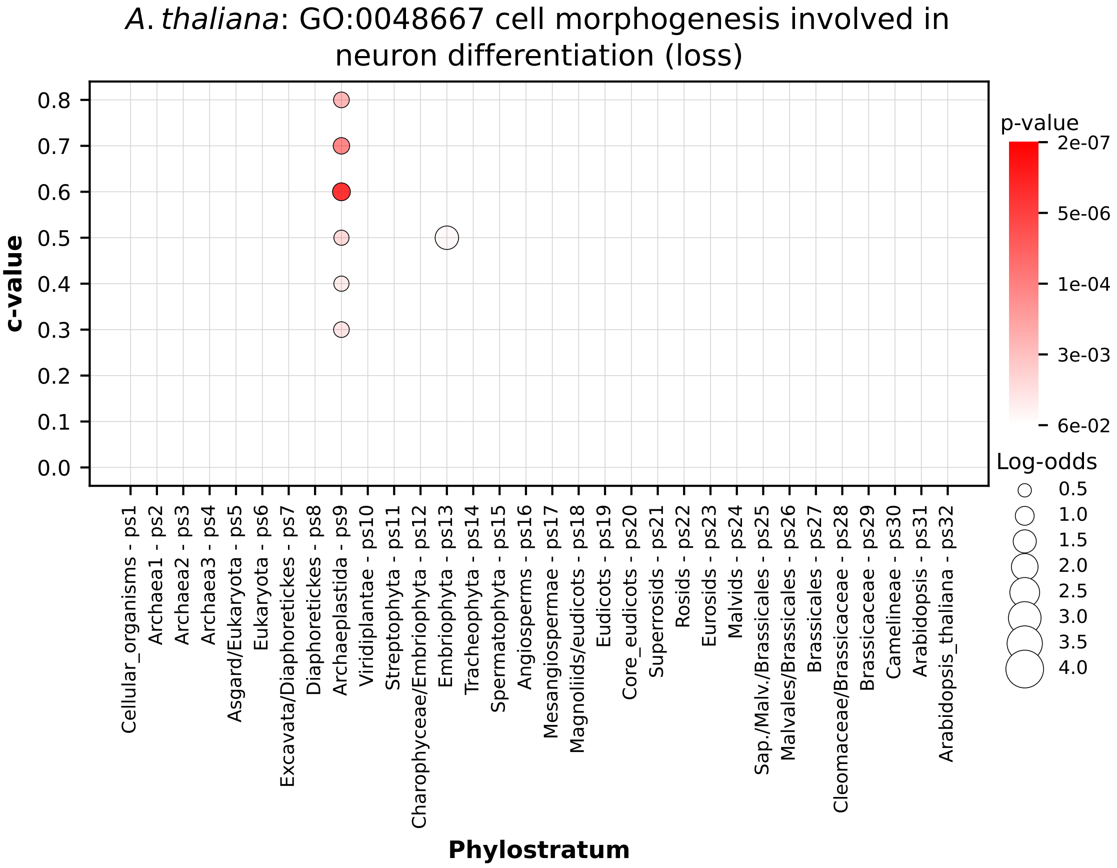

A. thaliana: GO:0048667 cell morphogenesis involved in neuron differentiation (loss)
c-value
0.8
0.7
0.6
0.5
0.4
0.3
0.2
0.1
0.0
p-value
2e-07
5e-06
1e-04
3e-03
6e-02
Log-odds
0.5
1.0
1.5
2.0
2.5
3.0
3.5
4.0
Cellular_organisms - ps1
Archaea1 - ps2
Archaea2 - ps3
Archaea3 - ps4
Asgard/Eukaryota - ps5
Eukaryota - ps6
Excavata/Diaphoretickes - ps7
Diaphoretickes - ps8
Archaeplastida - ps9
Viridiplantae - ps10
Streptophyta - ps11
Charophyceae/Embriophyta - ps12
Embriophyta - ps13
Tracheophyta - ps14
Spermatophyta - ps15
Angiosperms - ps16
Mesangiospermae - ps17
Magnoliids/eudicots - ps18
Eudicots - ps19
Core_eudicots - ps20
Superrosids - ps21
Rosids - ps22
Eurosids - ps23
Malvids - ps24
Sap./Malv./Brassicales - ps25
Malvales/Brassicales - ps26
Brassicales - ps27
Cleomaceae/Brassicaceae - ps28
Brassicaceae - ps29
Camelineae - ps30
Arabidopsis - ps31
Arabidopsis_thaliana - ps32
Phylostratum

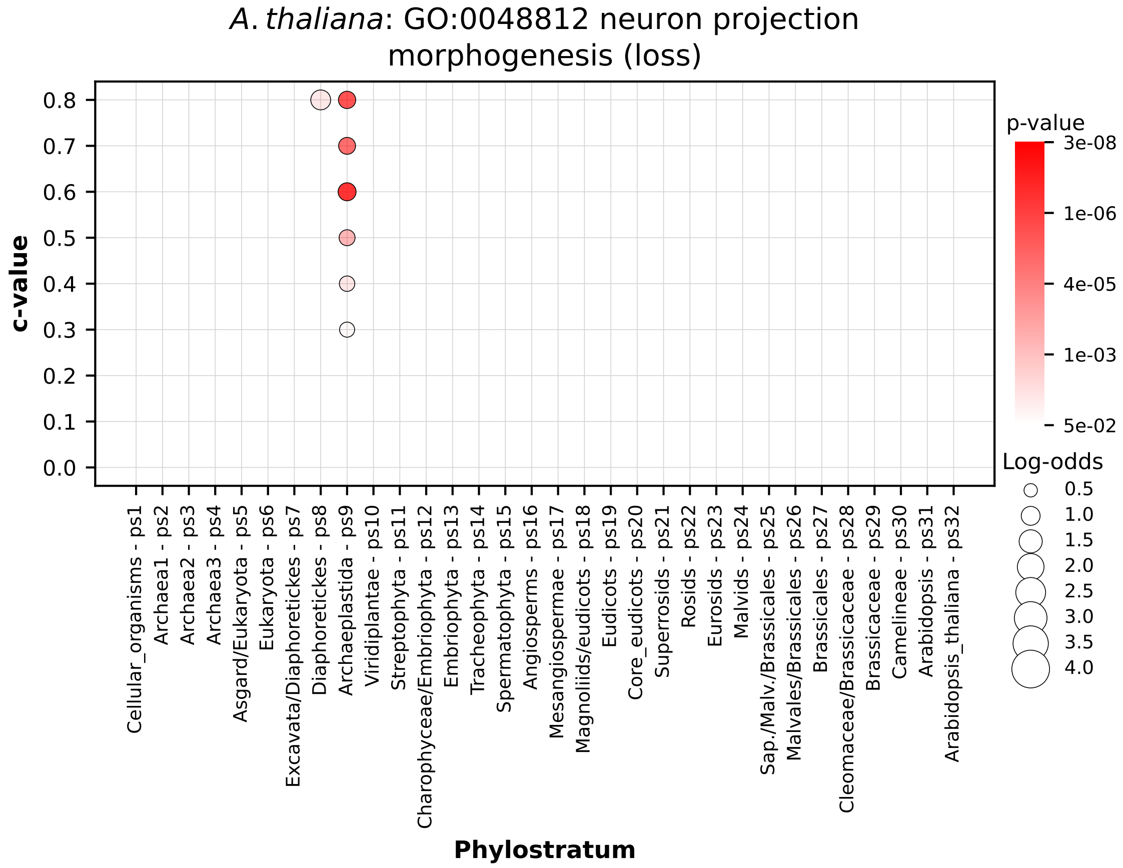
A. thaliana: GO:0048812 neuron projection morphogenesis (loss)
c-value
0.8
0.7
0.6
0.5
0.4
0.3
0.2
0.1
0.0
p-value
3e-08
1e-06
4e-05
1e-03
5e-02
Log-odds
0.5
1.0
1.5
2.0
2.5
3.0
3.5
4.0
Cellular_organisms - ps1
Archaea1 - ps2
Archaea2 - ps3
Archaea3 - ps4
Asgard/Eukaryota - ps5
Eukaryota - ps6
Excavata/Diaphoretickes - ps7
Diaphoretickes - ps8
Archaeplastida - ps9
Viridiplantae - ps10
Streptophyta - ps11
Charophyceae/Embriophyta - ps12
Embriophyta - ps13
Tracheophyta - ps14
Spermatophyta - ps15
Angiosperms - ps16
Mesangiospermae - ps17
Magnoliids/eudicots - ps18
Eudicots - ps19
Core_eudicots - ps20
Superrosids - ps21
Rosids - ps22
Eurosids - ps23
Malvids - ps24
Sap./Malv./Brassicales - ps25
Malvales/Brassicales - ps26
Brassicales - ps27
Cleomaceae/Brassicaceae - ps28
Brassicaceae - ps29
Camelineae - ps30
Arabidopsis - ps31
Arabidopsis_thaliana - ps32
Phylostratum

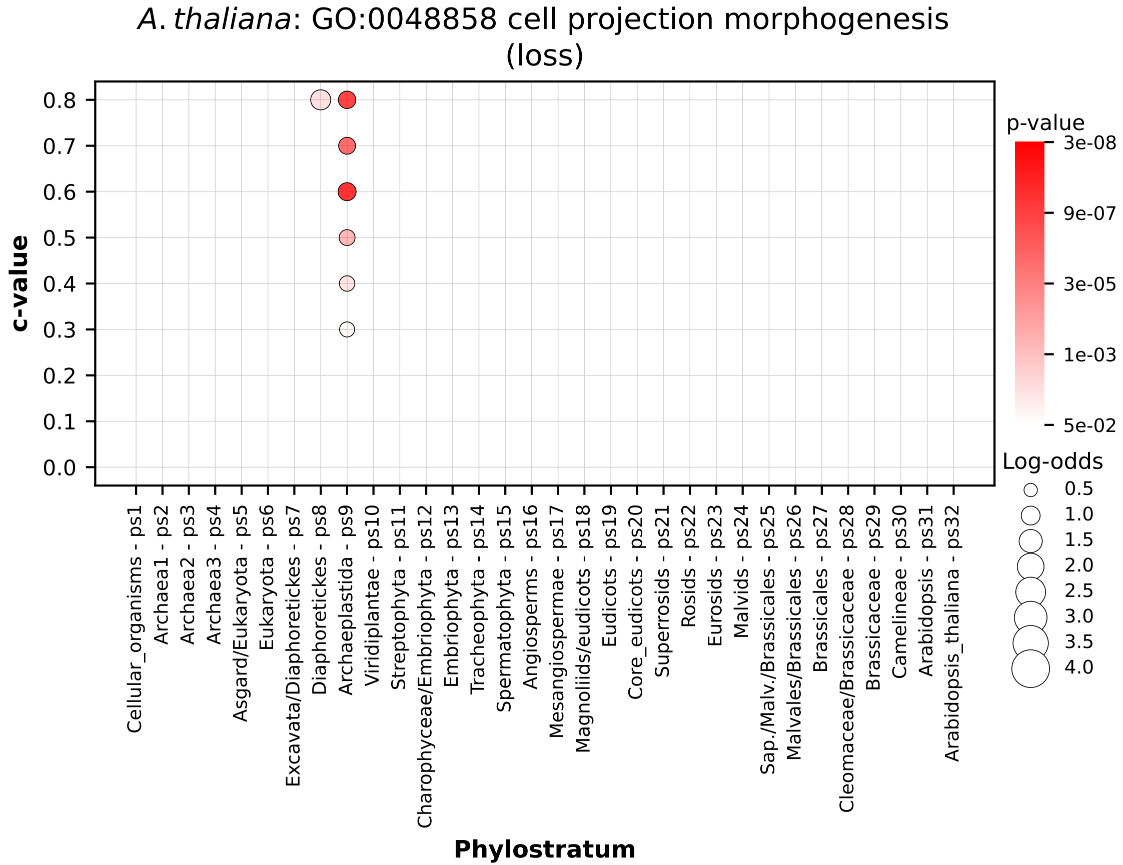
A. thaliana: GO:0048858 cell projection morphogenesis
(loss)
c-value
0.8
0.7
0.6
0.5
0.4
0.3
0.2
0.1
0.0
p-value
3e-08
9e-07
3e-05
1e-03
5e-02
Log-odds
0.5
1.0
1.5
2.0
2.5
3.0
3.5
4.0
Cellular_organisms - ps1
Archaea1 - ps2
Archaea2 - ps3
Archaea3 - ps4
Asgard/Eukaryota - ps5
Eukaryota - ps6
Excavata/Diaphoretickes - ps7
Diaphoretickes - ps8
Archaeplastida - ps9
Viridiplantae - ps10
Streptophyta - ps11
Charophyceae/Embriophyta - ps12
Embriophyta - ps13
Tracheophyta - ps14
Spermatophyta - ps15
Angiosperms - ps16
Mesangiospermae - ps17
Magnoliids/eudicots - ps18
Eudicots - ps19
Core_eudicots - ps20
Superrosids - ps21
Rosids - ps22
Eurosids - ps23
Malvids - ps24
Sap./Malv./Brassicales - ps25
Malvales/Brassicales - ps26
Brassicales - ps27
Cleomaceae/Brassicaceae - ps28
Brassicaceae - ps29
Camelineae - ps30
Arabidopsis - ps31
Arabidopsis_thaliana - ps32
Phylostratum

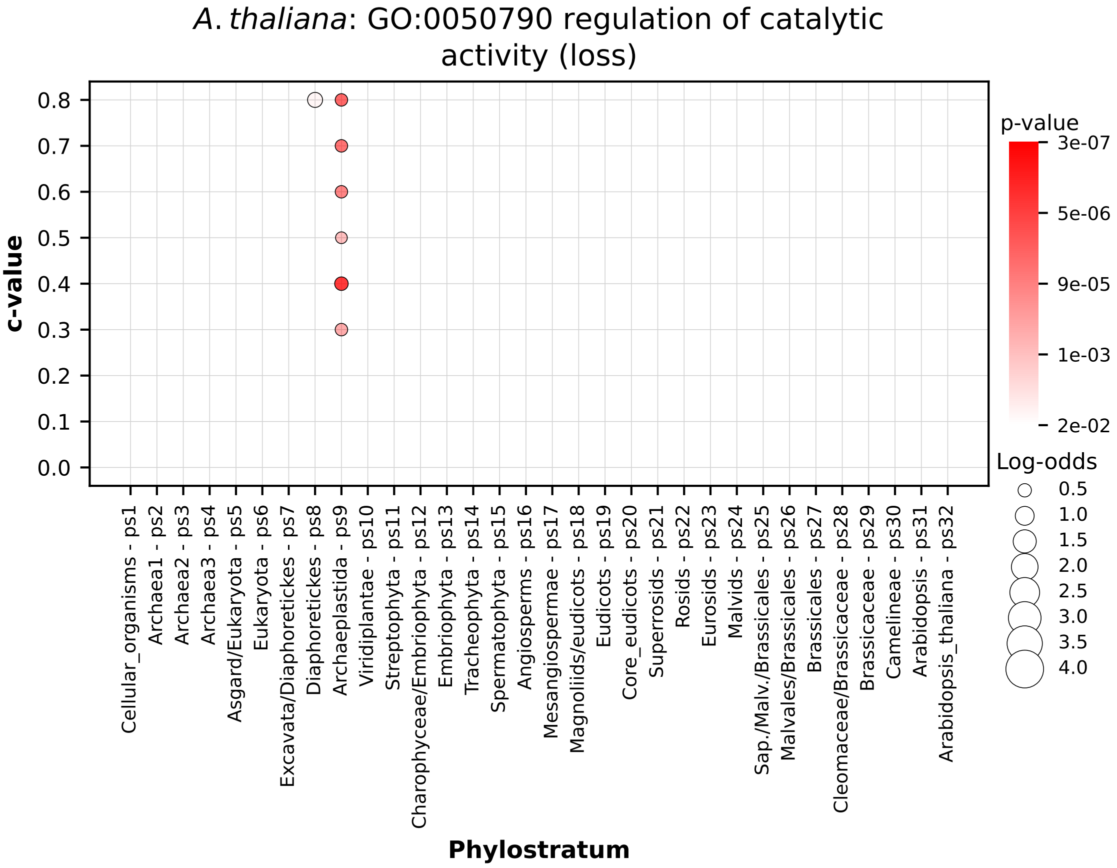

$A. thaliana$: GO:0050790 regulation of catalytic activity (loss)
c-value
0.8
0.7
0.6
0.5
0.4
0.3
0.2
0.1
0.0
p-value
3e-07
5e-06
9e-05
1e-03
2e-02
Log-odds
0.5
1.0
1.5
2.0
2.5
3.0
3.5
4.0
Cellular_organisms - ps1
Archaea1 - ps2
Archaea2 - ps3
Archaea3 - ps4
Asgard/Eukaryota - ps5
Eukaryota - ps6
Excavata/Diaphoretickes - ps7
Diaphoretickes - ps8
Archaeplastida - ps9
Viridiplantae - ps10
Streptophyta - ps11
Charophyceae/Embriophyta - ps12
Embriophyta - ps13
Tracheophyta - ps14
Spermatophyta - ps15
Angiosperms - ps16
Mesangiospermae - ps17
Magnoliids/eudicots - ps18
Eudicots - ps19
Core_eudicots - ps20
Superrosids - ps21
Rosids - ps22
Eurosids - ps23
Malvids - ps24
Sap./Malv./Brassicales - ps25
Malvales/Brassicales - ps26
Brassicales - ps27
Cleomaceae/Brassicaceae - ps28
Brassicaceae - ps29
Camelineae - ps30
Arabidopsis - ps31
Arabidopsis_thaliana - ps32
Phylostratum

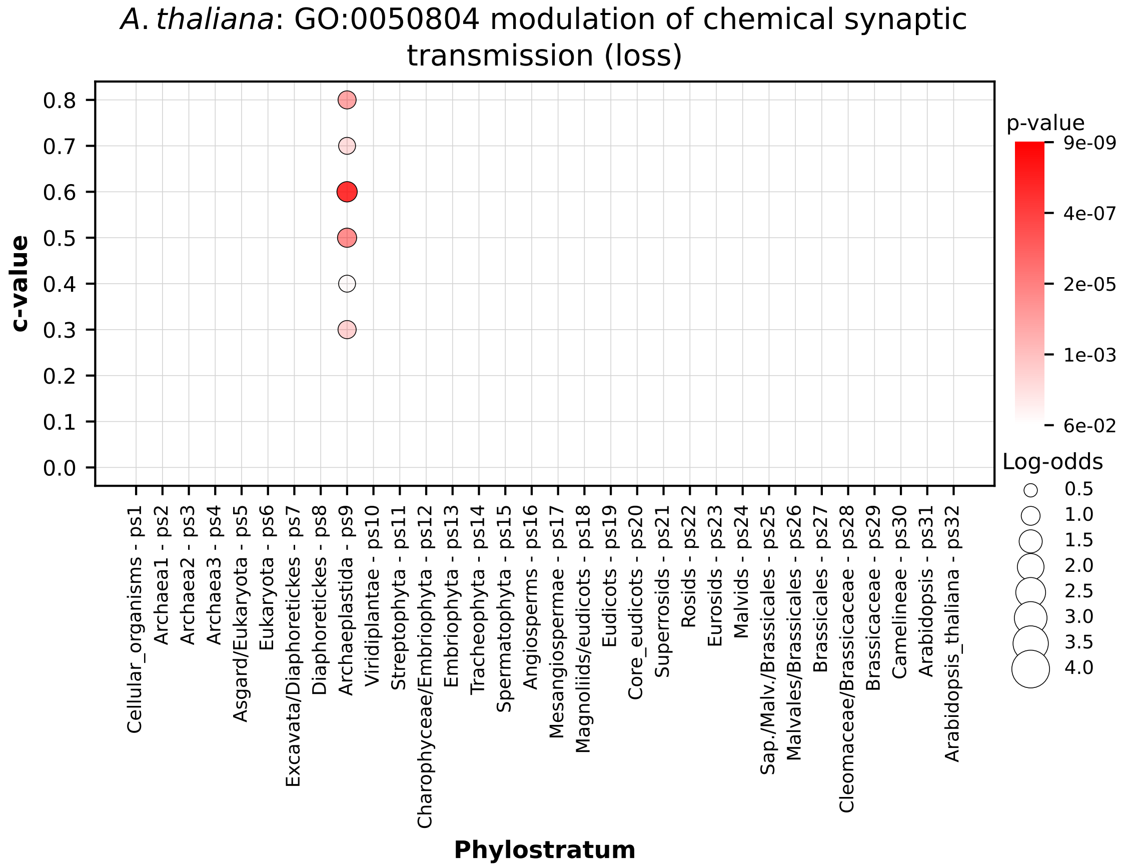
A. thaliana: GO:0050804 modulation of chemical synaptic transmission (loss)
c-value
0.0
0.1
0.2
0.3
0.4
0.5
0.6
0.7
0.8
p-value
9e-09
4e-07
2e-05
1e-03
6e-02
Log-odds
0.5
1.0
1.5
2.0
2.5
3.0
3.5
4.0
Cellular_organisms - ps1
Archaea1 - ps2
Archaea2 - ps3
Archaea3 - ps4
Asgard/Eukaryota - ps5
Eukaryota - ps6
Excavata/Diaphoretickes - ps7
Diaphoretickes - ps8
Archaeplastida - ps9
Viridiplantae - ps10
Streptophyta - ps11
Charophyceae/Embriophyta - ps12
Embriophyta - ps13
Tracheophyta - ps14
Spermatophyta - ps15
Angiosperms - ps16
Mesangiospermae - ps17
Magnoliids/eudicots - ps18
Eudicots - ps19
Core_eudicots - ps20
Superrosids - ps21
Rosids - ps22
Eurosids - ps23
Malvids - ps24
Sap./Malv./Brassicales - ps25
Malvales/Brassicales - ps26
Brassicales - ps27
Cleomaceae/Brassicaceae - ps28
Brassicaceae - ps29
Camelineae - ps30
Arabidopsis - ps31
Arabidopsis_thaliana - ps32
Phylostratum

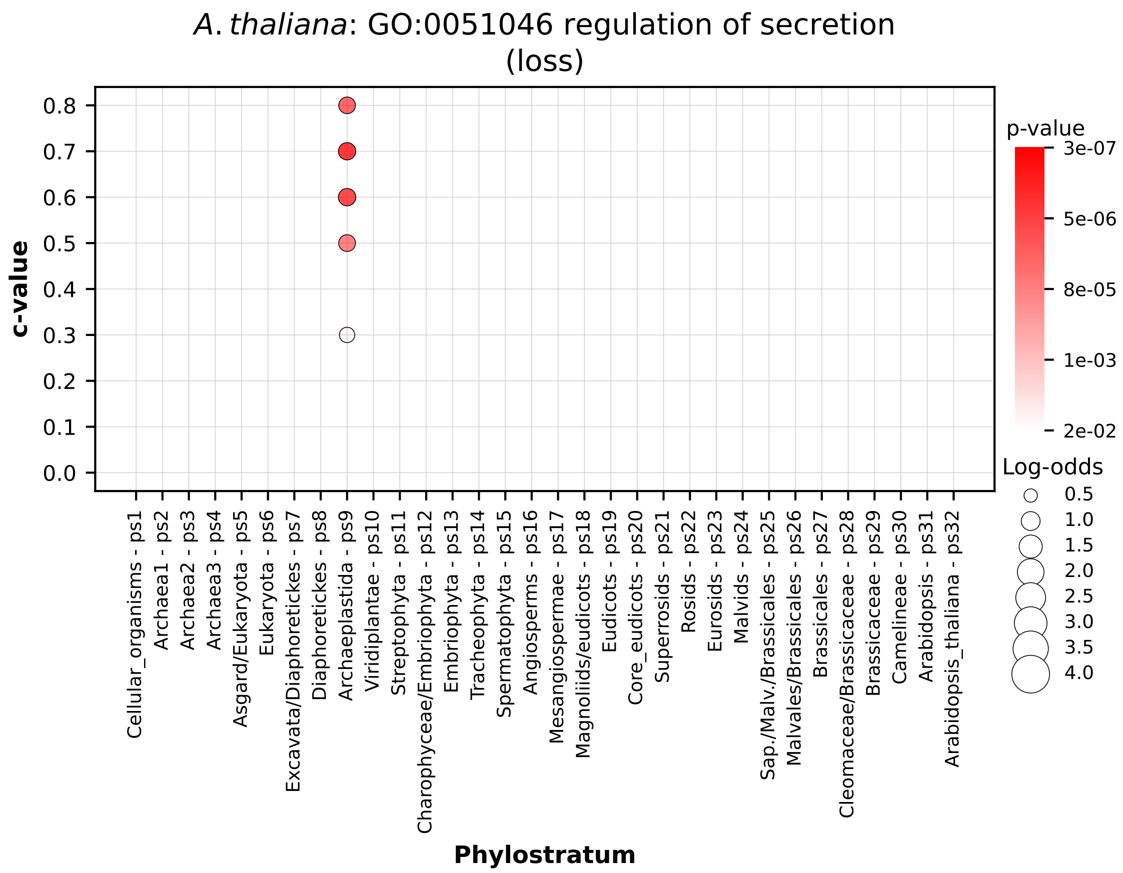

A. thaliana: GO:0051046 regulation of secretion
(loss)
c-value
0.0
0.1
0.2
0.3
0.4
0.5
0.6
0.7
0.8
Phylostratum
Cellular_organisms - ps1
Archaea1 - ps2
Archaea2 - ps3
Archaea3 - ps4
Asgard/Eukaryota - ps5
Eukaryota - ps6
Excavata/Diaphoretickes - ps7
Diaphoretickes - ps8
Archaeplastida - ps9
Viridiplantae - ps10
Streptophyta - ps11
Charophyceae/Embriophyta - ps12
Embriophyta - ps13
Tracheophyta - ps14
Spermatophyta - ps15
Angiosperms - ps16
Mesangiospermae - ps17
Magnoliids/eudicots - ps18
Eudicots - ps19
Core_eudicots - ps20
Superrosids - ps21
Rosids - ps22
Eurosids - ps23
Malvids - ps24
Sap./Malv./Brassicales - ps25
Malvales/Brassicales - ps26
Brassicales - ps27
Cleomaceae/Brassicaceae - ps28
Brassicaceae - ps29
Camelineae - ps30
Arabidopsis - ps31
Arabidopsis_thaliana - ps32
p-value
3e-07
5e-06
8e-05
1e-03
2e-02
Log-odds
0.5
1.0
1.5
2.0
2.5
3.0
3.5
4.0

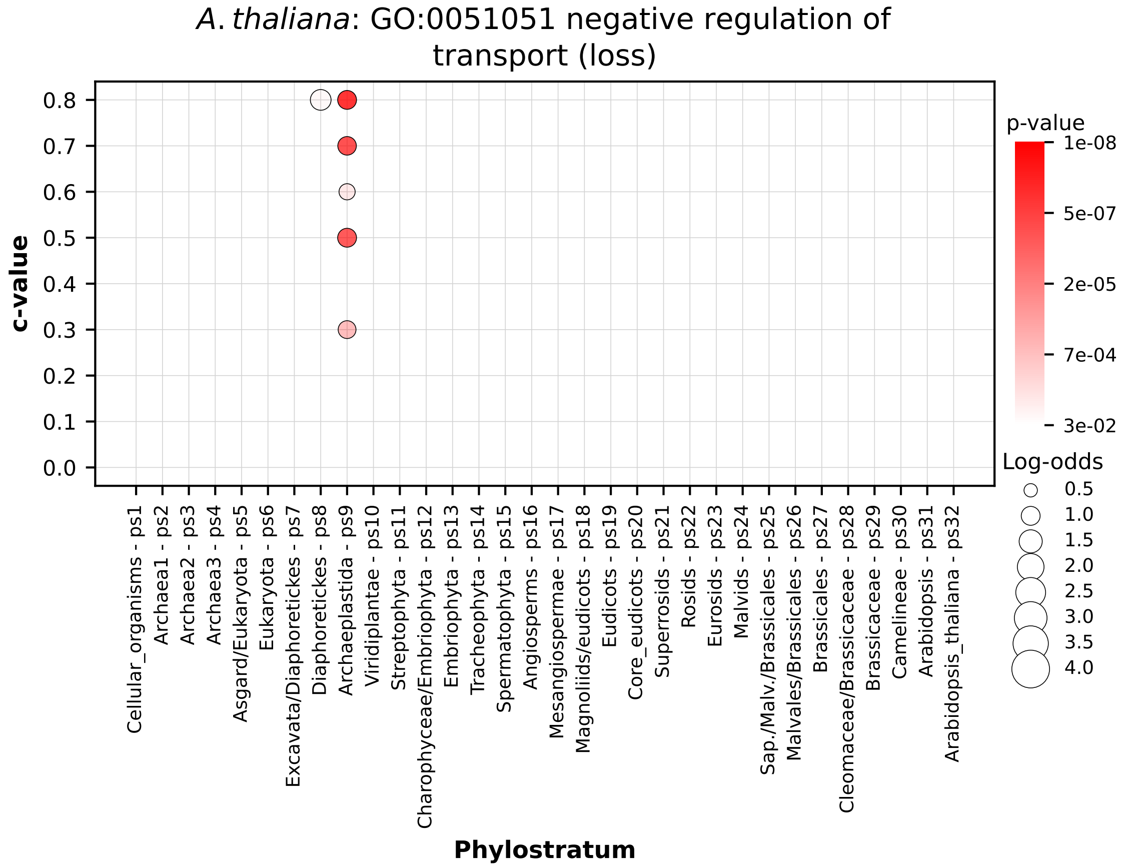

A. thaliana: GO:0051051 negative regulation of transport (loss)
c-value
0.8
0.7
0.6
0.5
0.4
0.3
0.2
0.1
0.0
Phylostratum
Cellular_organisms - ps1
Archaea1 - ps2
Archaea2 - ps3
Archaea3 - ps4
Asgard/Eukaryota - ps5
Eukaryota - ps6
Excavata/Diaphoretickes - ps7
Diaphoretickes - ps8
Archaeplastida - ps9
Viridiplantae - ps10
Streptophyta - ps11
Charophyceae/Embriophyta - ps12
Embriophyta - ps13
Tracheophyta - ps14
Spermatophyta - ps15
Angiosperms - ps16
Mesangiospermae - ps17
Magnoliids/eudicots - ps18
Eudicots - ps19
Core_eudicots - ps20
Superrosids - ps21
Rosids - ps22
Eurosids - ps23
Malvids - ps24
Sap./Malv./Brassicales - ps25
Malvales/Brassicales - ps26
Brassicales - ps27
Cleomaceae/Brassicaceae - ps28
Brassicaceae - ps29
Camelineae - ps30
Arabidopsis - ps31
Arabidopsis_thaliana - ps32
p-value
1e-08
5e-07
2e-05
7e-04
3e-02
Log-odds
0.5
1.0
1.5
2.0
2.5
3.0
3.5
4.0

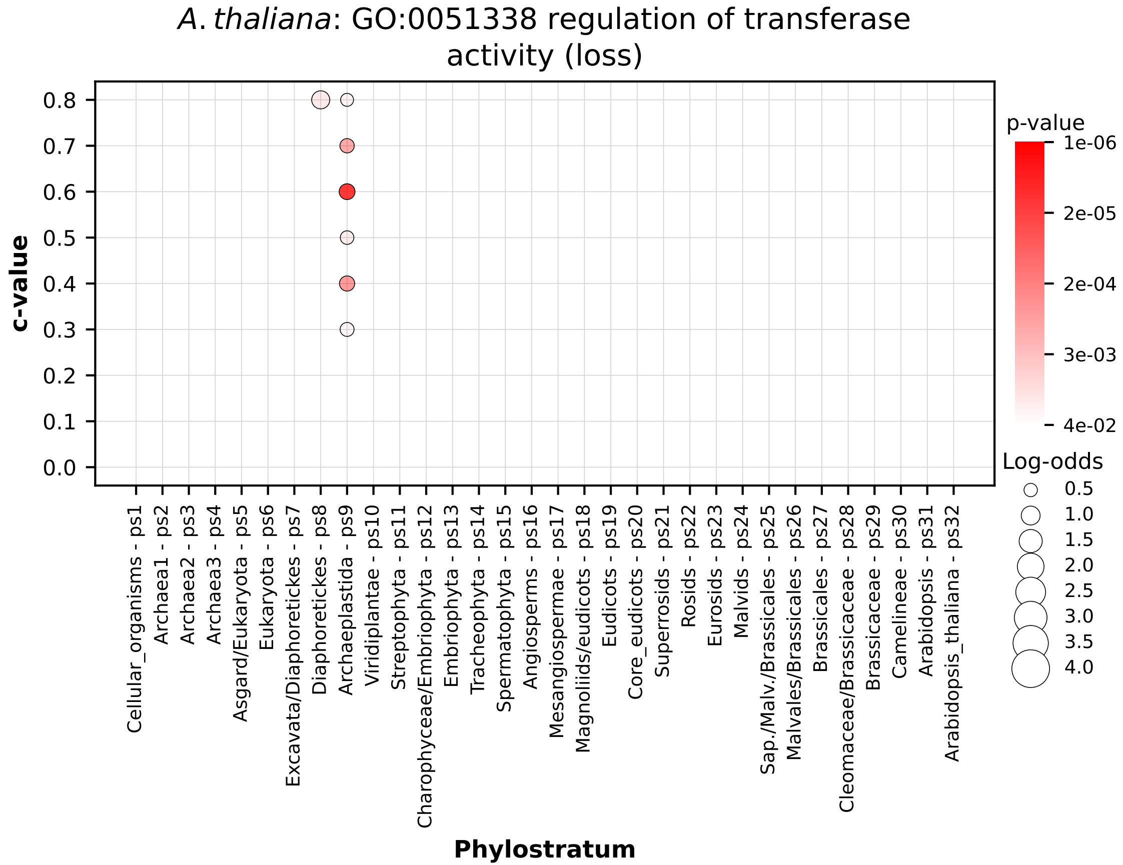

$A. thaliana$: GO:0051338 regulation of transferase activity (loss)
c-value
0.8
0.7
0.6
0.5
0.4
0.3
0.2
0.1
0.0
Phylostratum
Cellular_organisms - ps1
Archaea1 - ps2
Archaea2 - ps3
Archaea3 - ps4
Asgard/Eukaryota - ps5
Eukaryota - ps6
Excavata/Diaphoretickes - ps7
Diaphoretickes - ps8
Archaeplastida - ps9
Viridiplantae - ps10
Streptophyta - ps11
Charophyceae/Embriophyta - ps12
Embriophyta - ps13
Tracheophyta - ps14
Spermatophyta - ps15
Angiosperms - ps16
Mesangiospermae - ps17
Magnoliids/eudicots - ps18
Eudicots - ps19
Core_eudicots - ps20
Superrosids - ps21
Rosids - ps22
Eurosids - ps23
Malvids - ps24
Sap./Malv./Brassicales - ps25
Malvales/Brassicales - ps26
Brassicales - ps27
Cleomaceae/Brassicaceae - ps28
Brassicaceae - ps29
Camelineae - ps30
Arabidopsis - ps31
Arabidopsis_thaliana - ps32
p-value
1e-06
2e-05
2e-04
3e-03
4e-02
Log-odds
0.5
1.0
1.5
2.0
2.5
3.0
3.5
4.0

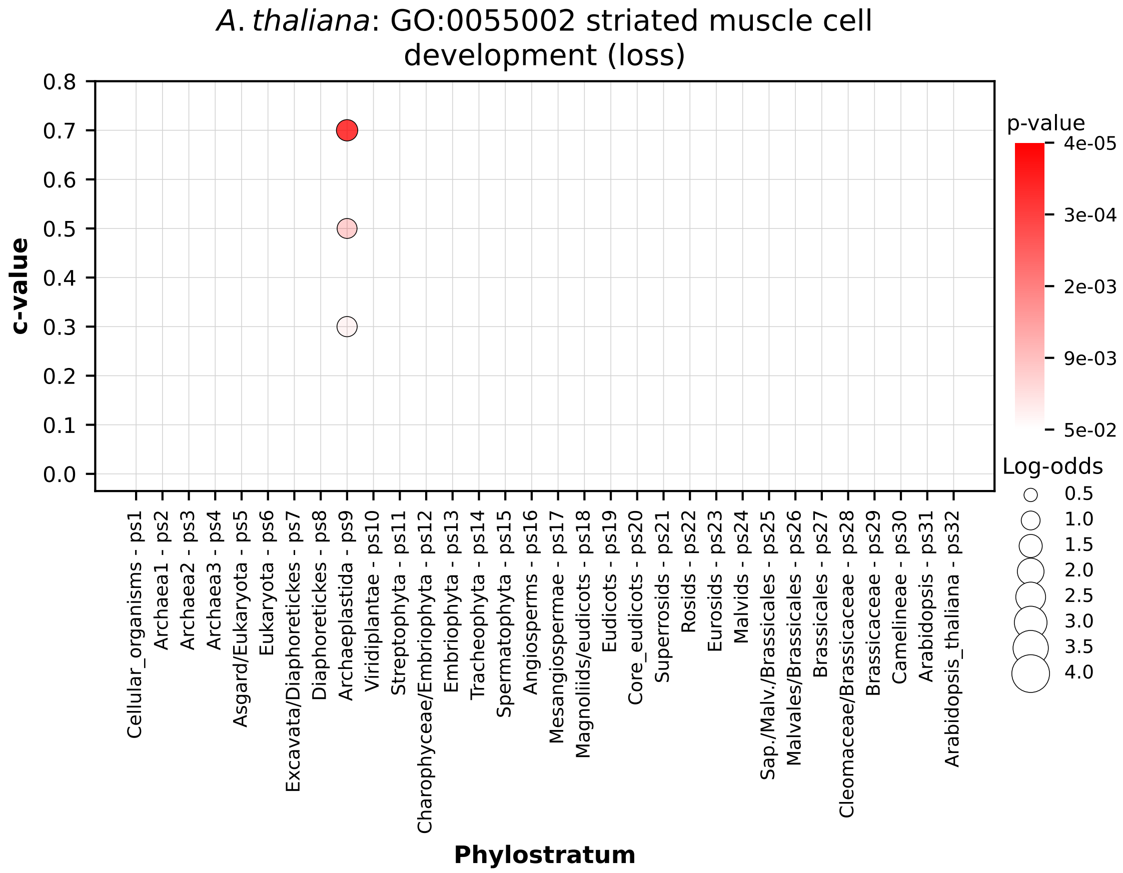

A. thaliana: GO:0055002 striated muscle cell development (loss)
c-value
0.8
0.7
0.6
0.5
0.4
0.3
0.2
0.1
0.0
p-value
4e-05
3e-04
2e-03
9e-03
5e-02
Log-odds
0.5
1.0
1.5
2.0
2.5
3.0
3.5
4.0
Cellular_organisms - ps1
Archaea1 - ps2
Archaea2 - ps3
Archaea3 - ps4
Asgard/Eukaryota - ps5
Eukaryota - ps6
Excavata/Diaphoretickes - ps7
Diaphoretickes - ps8
Archaeplastida - ps9
Viridiplantae - ps10
Streptophyta - ps11
Charophyceae/Embriophyta - ps12
Embriophyta - ps13
Tracheophyta - ps14
Spermatophyta - ps15
Angiosperms - ps16
Mesangiospermae - ps17
Magnoliids/eudicots - ps18
Eudicots - ps19
Core_eudicots - ps20
Superrosids - ps21
Rosids - ps22
Eurosids - ps23
Malvids - ps24
Sap./Malv./Brassicales - ps25
Malvales/Brassicales - ps26
Brassicales - ps27
Cleomaceae/Brassicaceae - ps28
Brassicaceae - ps29
Camelineae - ps30
Arabidopsis - ps31
Arabidopsis_thaliana - ps32
Phylostratum

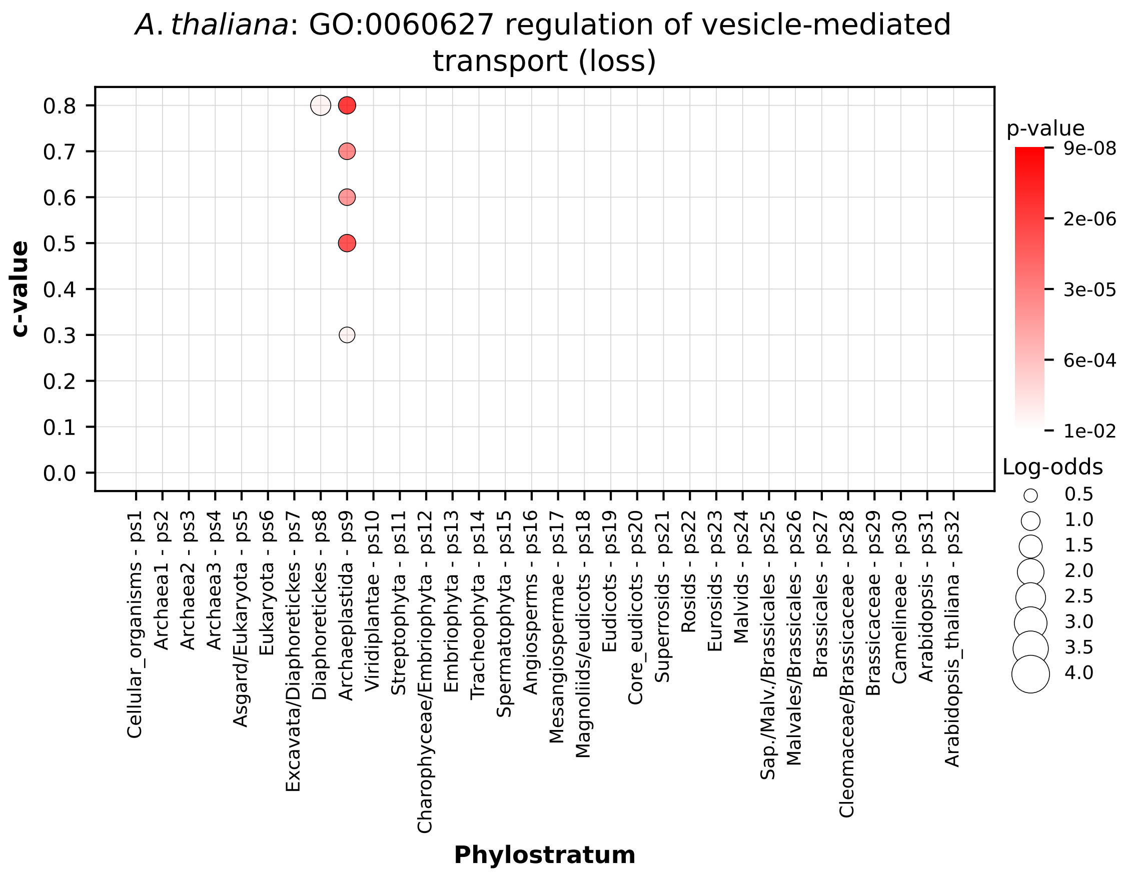

A. thaliana: GO:0060627 regulation of vesicle-mediated transport (loss)
c-value
0.8
0.7
0.6
0.5
0.4
0.3
0.2
0.1
0.0
p-value
9e-08
2e-06
3e-05
6e-04
1e-02
Log-odds
0.5
1.0
1.5
2.0
2.5
3.0
3.5
4.0
Cellular_organisms - ps1
Archaea1 - ps2
Archaea2 - ps3
Archaea3 - ps4
Asgard/Eukaryota - ps5
Eukaryota - ps6
Excavata/Diaphoretickes - ps7
Diaphoretickes - ps8
Archaeplastida - ps9
Viridiplantae - ps10
Streptophyta - ps11
Charophyceae/Embriophyta - ps12
Embriophyta - ps13
Tracheophyta - ps14
Spermatophyta - ps15
Angiosperms - ps16
Mesangiospermae - ps17
Magnoliids/eudicots - ps18
Eudicots - ps19
Core_eudicots - ps20
Superrosids - ps21
Rosids - ps22
Eurosids - ps23
Malvids - ps24
Sap./Malv./Brassicales - ps25
Malvales/Brassicales - ps26
Brassicales - ps27
Cleomaceae/Brassicaceae - ps28
Brassicaceae - ps29
Camelineae - ps30
Arabidopsis - ps31
Arabidopsis_thaliana - ps32
Phylostratum

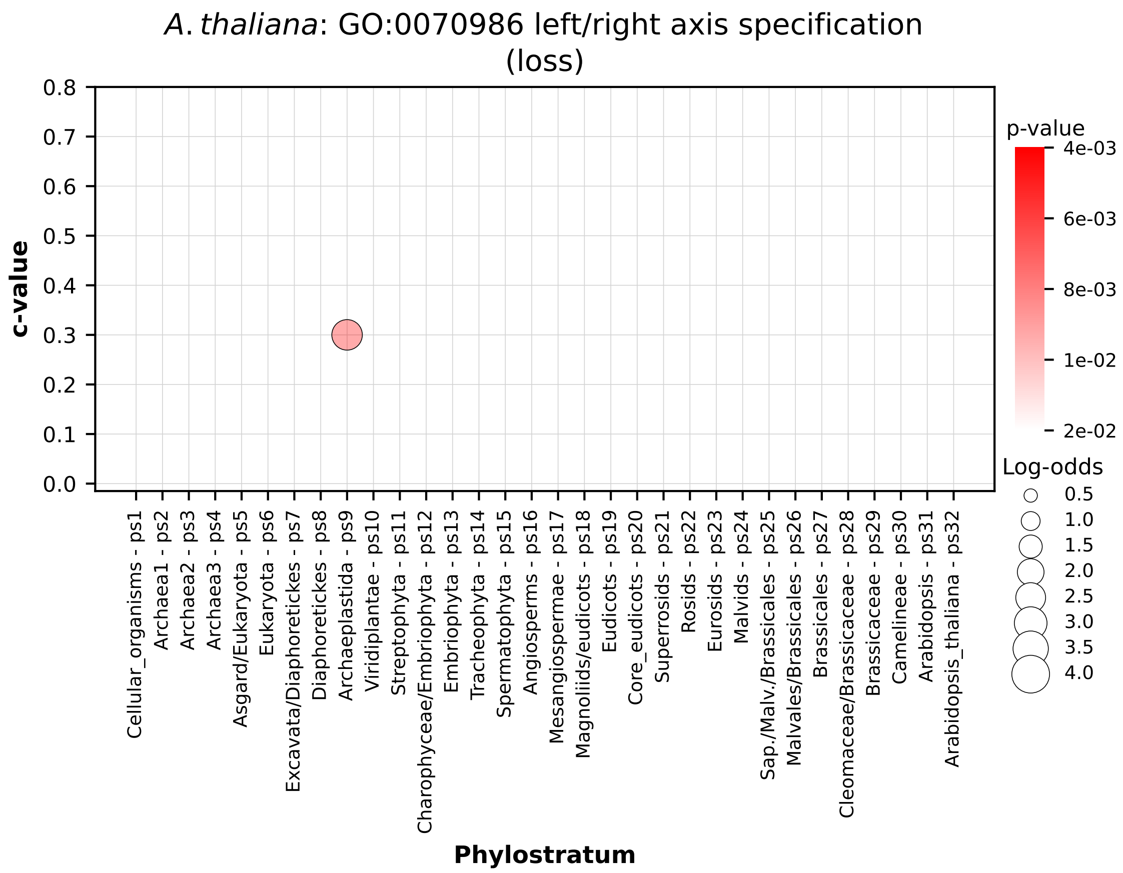

A. thaliana: GO:0070986 left/right axis specification
(loss)
c-value
0.8
0.7
0.6
0.5
0.4
0.3
0.2
0.1
0.0
p-value
4e-03
6e-03
8e-03
1e-02
2e-02
Log-odds
0.5
1.0
1.5
2.0
2.5
3.0
3.5
4.0
Cellular_organisms - ps1
Archaea1 - ps2
Archaea2 - ps3
Archaea3 - ps4
Asgard/Eukaryota - ps5
Eukaryota - ps6
Excavata/Diaphoretickes - ps7
Diaphoretickes - ps8
Archaeplastida - ps9
Viridiplantae - ps10
Streptophyta - ps11
Charophyceae/Embriophyta - ps12
Embriophyta - ps13
Tracheophyta - ps14
Spermatophyta - ps15
Angiosperms - ps16
Mesangiospermae - ps17
Magnoliids/eudicots - ps18
Eudicots - ps19
Core_eudicots - ps20
Superrosids - ps21
Rosids - ps22
Eurosids - ps23
Malvids - ps24
Sap./Malv./Brassicales - ps25
Malvales/Brassicales - ps26
Brassicales - ps27
Cleomaceae/Brassicaceae - ps28
Brassicaceae - ps29
Camelineae - ps30
Arabidopsis - ps31
Arabidopsis_thaliana - ps32
Phylostratum

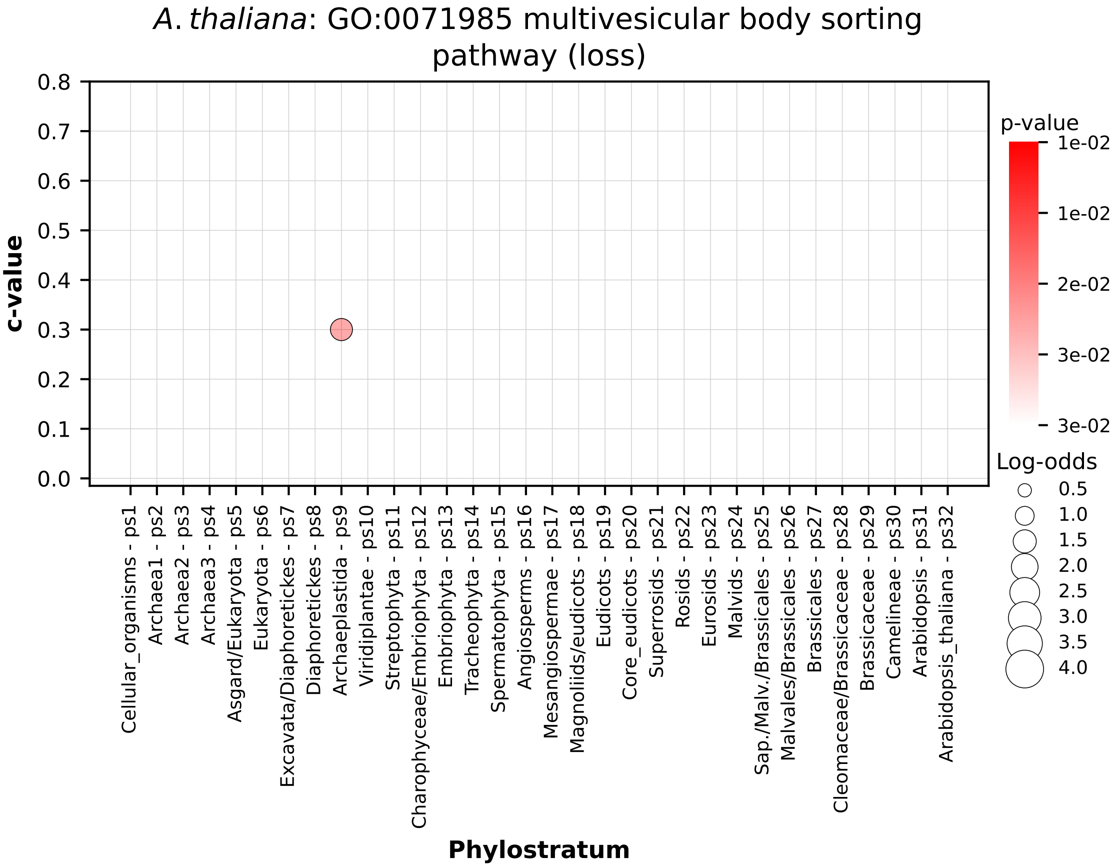

A. thaliana: GO:0071985 multivesicular body sorting pathway (loss)
c-value
0.0
0.1
0.2
0.3
0.4
0.5
0.6
0.7
0.8
p-value
1e-02
1e-02
2e-02
3e-02
3e-02
Log-odds
0.5
1.0
1.5
2.0
2.5
3.0
3.5
4.0
Cellular_organisms - ps1
Archaea1 - ps2
Archaea2 - ps3
Archaea3 - ps4
Asgard/Eukaryota - ps5
Eukaryota - ps6
Excavata/Diaphoretickes - ps7
Diaphoretickes - ps8
Archaeplastida - ps9
Viridiplantae - ps10
Streptophyta - ps11
Charophyceae/Embriophyta - ps12
Embriophyta - ps13
Tracheophyta - ps14
Spermatophyta - ps15
Angiosperms - ps16
Mesangiospermae - ps17
Magnoliids/eudicots - ps18
Eudicots - ps19
Core_eudicots - ps20
Superrosids - ps21
Rosids - ps22
Eurosids - ps23
Malvids - ps24
Sap./Malv./Brassicales - ps25
Malvales/Brassicales - ps26
Brassicales - ps27
Cleomaceae/Brassicaceae - ps28
Brassicaceae - ps29
Camelineae - ps30
Arabidopsis - ps31
Arabidopsis_thaliana - ps32
Phylostratum

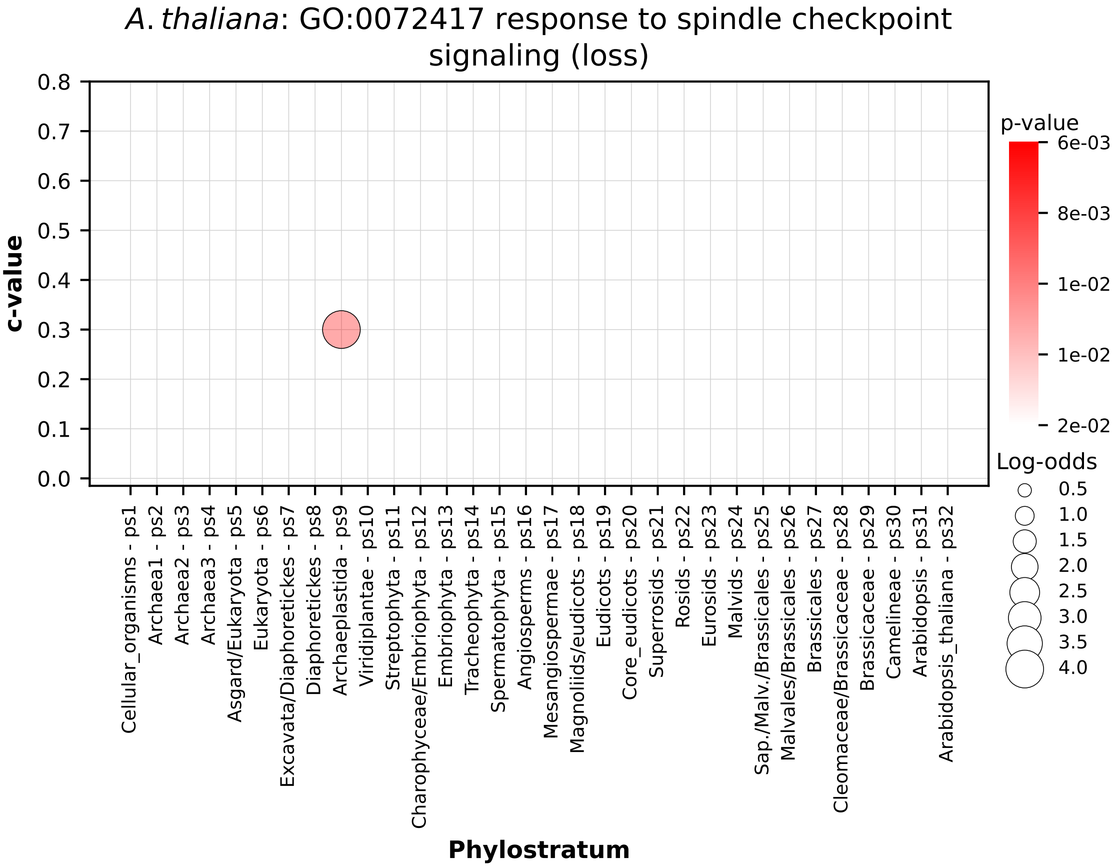

$A. thaliana$: GO:0072417 response to spindle checkpoint signaling (loss)
c-value
0.8
0.7
0.6
0.5
0.4
0.3
0.2
0.1
0.0
p-value
6e-03
8e-03
1e-02
1e-02
2e-02
Log-odds
0.5
1.0
1.5
2.0
2.5
3.0
3.5
4.0
Cellular_organisms - ps1
Archaea1 - ps2
Archaea2 - ps3
Archaea3 - ps4
Asgard/Eukaryota - ps5
Eukaryota - ps6
Excavata/Diaphoretickes - ps7
Diaphoretickes - ps8
Archaeplastida - ps9
Viridiplantae - ps10
Streptophyta - ps11
Charophyceae/Embriophyta - ps12
Embriophyta - ps13
Tracheophyta - ps14
Spermatophyta - ps15
Angiosperms - ps16
Mesangiospermae - ps17
Magnoliids/eudicots - ps18
Eudicots - ps19
Core_eudicots - ps20
Superrosids - ps21
Rosids - ps22
Eurosids - ps23
Malvids - ps24
Sap./Malv./Brassicales - ps25
Malvales/Brassicales - ps26
Brassicales - ps27
Cleomaceae/Brassicaceae - ps28
Brassicaceae - ps29
Camelineae - ps30
Arabidopsis - ps31
Arabidopsis_thaliana - ps32
Phylostratum

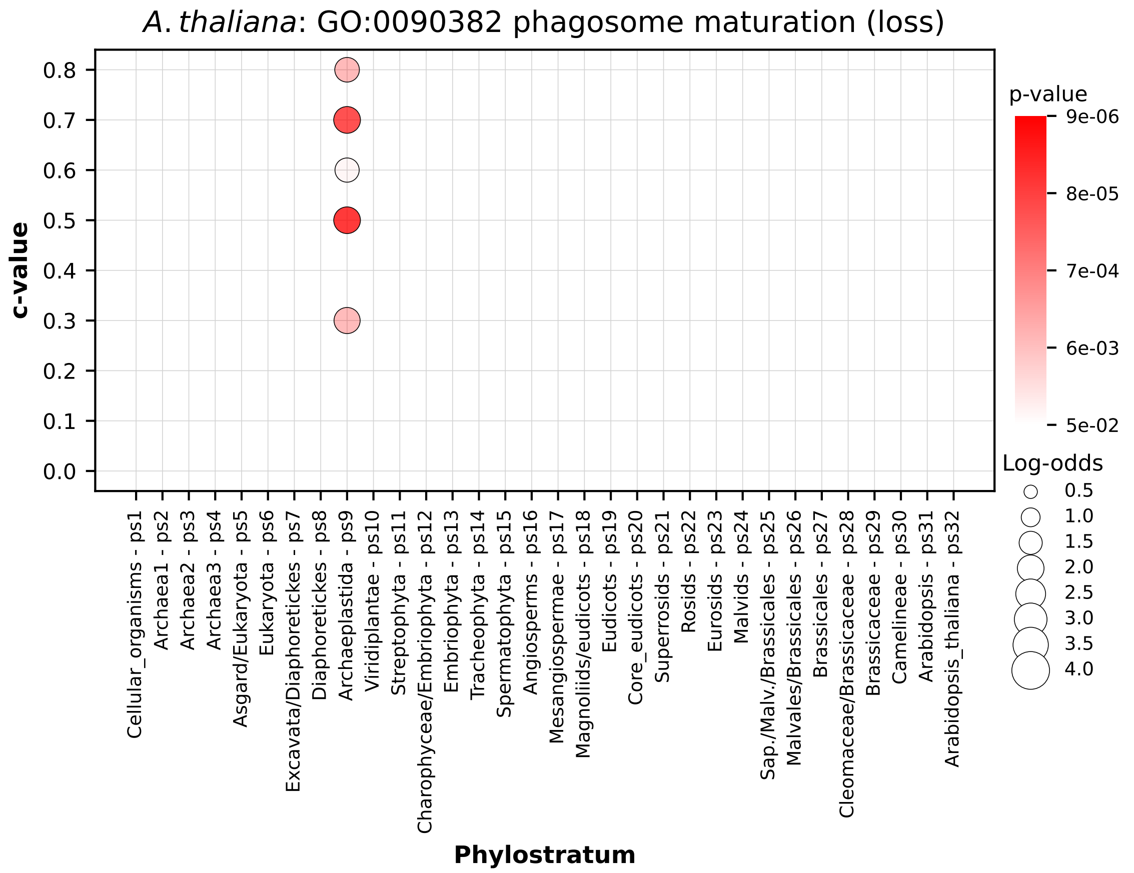

A. thaliana: GO:0090382 phagosome maturation (loss)
c-value
0.8
0.7
0.6
0.5
0.4
0.3
0.2
0.1
0.0
p-value
9e-06
8e-05
7e-04
6e-03
5e-02
Log-odds
0.5
1.0
1.5
2.0
2.5
3.0
3.5
4.0
Cellular_organisms - ps1
Archaea1 - ps2
Archaea2 - ps3
Archaea3 - ps4
Asgard/Eukaryota - ps5
Eukaryota - ps6
Excavata/Diaphoretickes - ps7
Diaphoretickes - ps8
Archaeplastida - ps9
Viridiplantae - ps10
Streptophyta - ps11
Charophyceae/Embriophyta - ps12
Embriophyta - ps13
Tracheophyta - ps14
Spermatophyta - ps15
Angiosperms - ps16
Mesangiospermae - ps17
Magnoliids/eudicots - ps18
Eudicots - ps19
Core_eudicots - ps20
Superrosids - ps21
Rosids - ps22
Eurosids - ps23
Malvids - ps24
Sap./Malv./Brassicales - ps25
Malvales/Brassicales - ps26
Brassicales - ps27
Cleomaceae/Brassicaceae - ps28
Brassicaceae - ps29
Camelineae - ps30
Arabidopsis - ps31
Arabidopsis_thaliana - ps32
Phylostratum

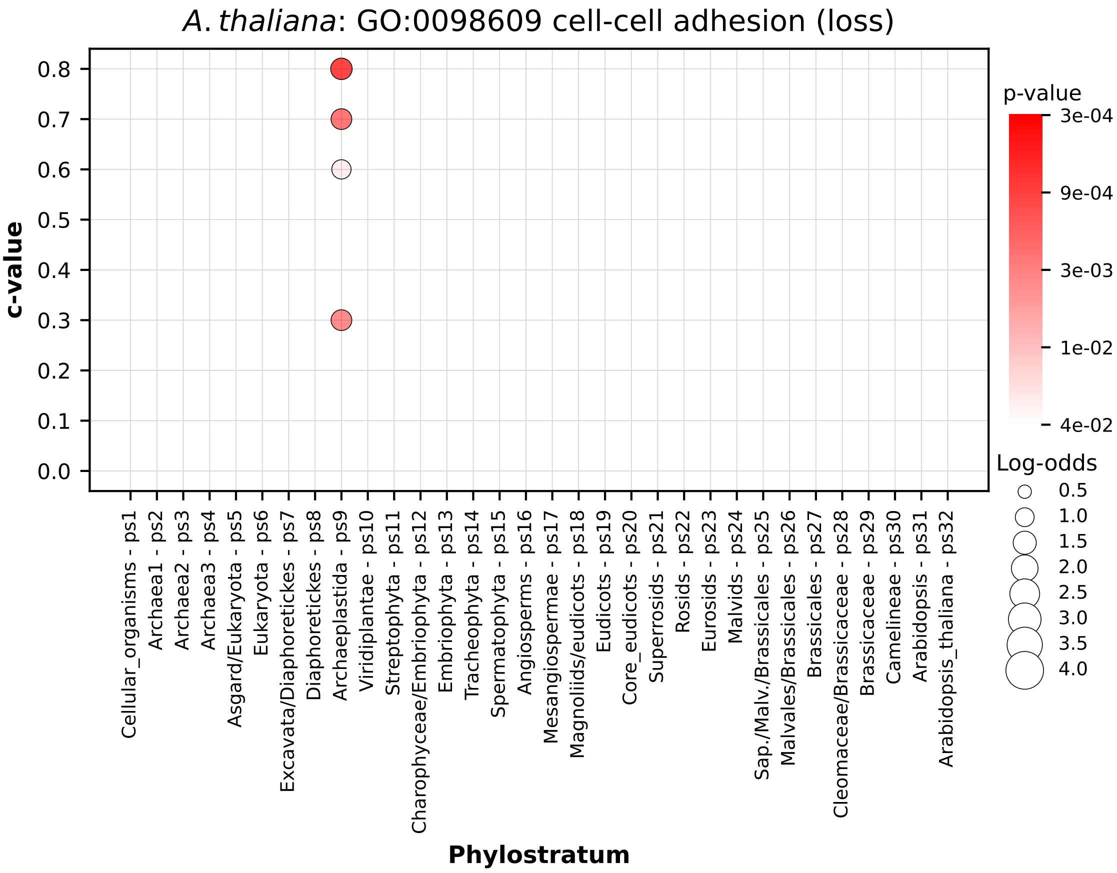

A. thaliana: GO:0098609 cell-cell adhesion (loss)
c-value
0.0
0.1
0.2
0.3
0.4
0.5
0.6
0.7
0.8
Phylostratum
Cellular_organisms - ps1
Archaea1 - ps2
Archaea2 - ps3
Archaea3 - ps4
Asgard/Eukaryota - ps5
Eukaryota - ps6
Excavata/Diaphoretickes - ps7
Diaphoretickes - ps8
Archaeplastida - ps9
Viridiplantae - ps10
Streptophyta - ps11
Charophyceae/Embriophyta - ps12
Embriophyta - ps13
Tracheophyta - ps14
Spermatophyta - ps15
Angiosperms - ps16
Mesangiospermae - ps17
Magnoliids/eudicots - ps18
Eudicots - ps19
Core_eudicots - ps20
Superrosids - ps21
Rosids - ps22
Eurosids - ps23
Malvids - ps24
Sap./Malv./Brassicales - ps25
Malvales/Brassicales - ps26
Brassicales - ps27
Cleomaceae/Brassicaceae - ps28
Brassicaceae - ps29
Camelineae - ps30
Arabidopsis - ps31
Arabidopsis_thaliana - ps32
p-value
3e-04
9e-04
3e-03
1e-02
4e-02
Log-odds
0.5
1.0
1.5
2.0
2.5
3.0
3.5
4.0

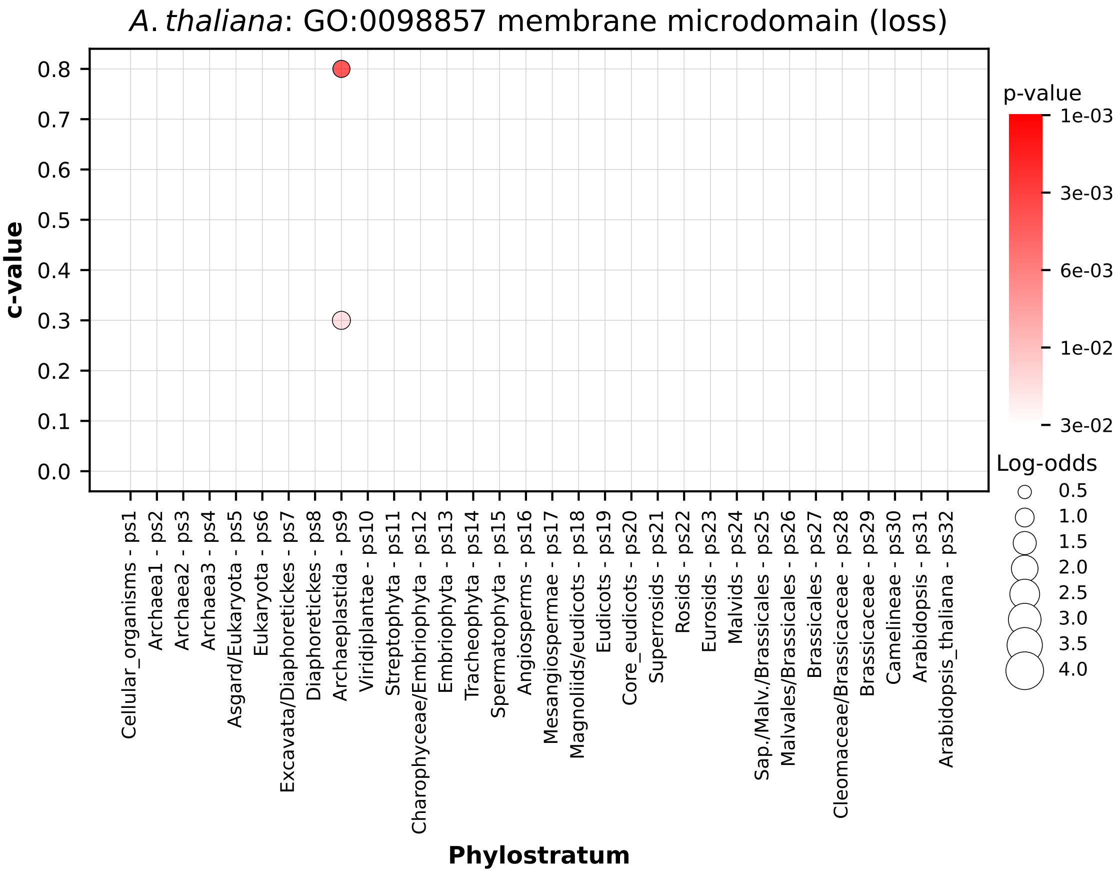

A. thaliana: GO:0098857 membrane microdomain (loss)
c-value
0.0
0.1
0.2
0.3
0.4
0.5
0.6
0.7
0.8
p-value
1e-03
3e-03
6e-03
1e-02
3e-02
Log-odds
0.5
1.0
1.5
2.0
2.5
3.0
3.5
4.0
Cellular_organisms - ps1
Archaea1 - ps2
Archaea2 - ps3
Archaea3 - ps4
Asgard/Eukaryota - ps5
Eukaryota - ps6
Excavata/Diaphoretickes - ps7
Diaphoretickes - ps8
Archaeplastida - ps9
Viridiplantae - ps10
Streptophyta - ps11
Charophyceae/Embriophyta - ps12
Embriophyta - ps13
Tracheophyta - ps14
Spermatophyta - ps15
Angiosperms - ps16
Mesangiospermae - ps17
Magnoliids/eudicots - ps18
Eudicots - ps19
Core_eudicots - ps20
Superrosids - ps21
Rosids - ps22
Eurosids - ps23
Malvids - ps24
Sap./Malv./Brassicales - ps25
Malvales/Brassicales - ps26
Brassicales - ps27
Cleomaceae/Brassicaceae - ps28
Brassicaceae - ps29
Camelineae - ps30
Arabidopsis - ps31
Arabidopsis_thaliana - ps32
Phylostratum

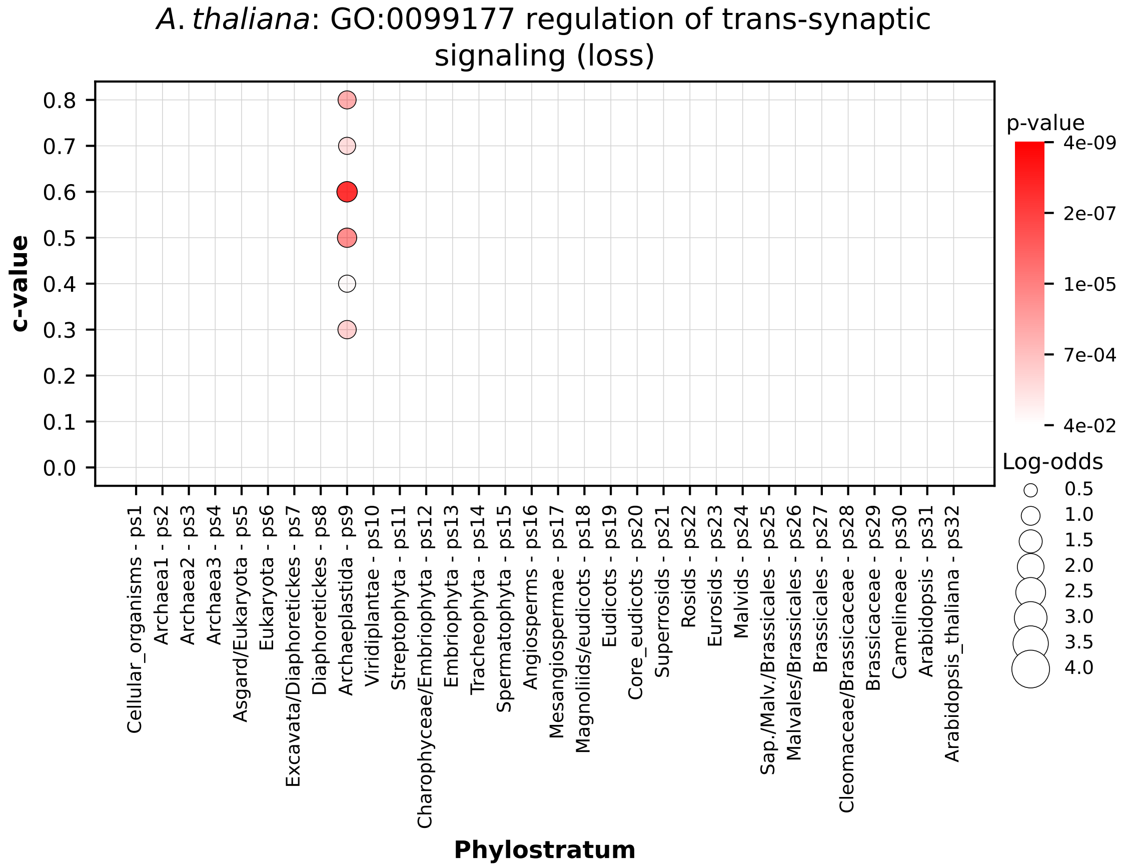

A. thaliana: GO:0099177 regulation of trans-synaptic signaling (loss)
c-value
0.8
0.7
0.6
0.5
0.4
0.3
0.2
0.1
0.0
Phylostratum
Cellular_organisms - ps1
Archaea1 - ps2
Archaea2 - ps3
Archaea3 - ps4
Asgard/Eukaryota - ps5
Eukaryota - ps6
Excavata/Diaphoretickes - ps7
Diaphoretickes - ps8
Archaeplastida - ps9
Viridiplantae - ps10
Streptophyta - ps11
Charophyceae/Embriophyta - ps12
Embriophyta - ps13
Tracheophyta - ps14
Spermatophyta - ps15
Angiosperms - ps16
Mesangiospermae - ps17
Magnoliids/eudicots - ps18
Eudicots - ps19
Core_eudicots - ps20
Superrosids - ps21
Rosids - ps22
Eurosids - ps23
Malvids - ps24
Sap./Malv./Brassicales - ps25
Malvales/Brassicales - ps26
Brassicales - ps27
Cleomaceae/Brassicaceae - ps28
Brassicaceae - ps29
Camelineae - ps30
Arabidopsis - ps31
Arabidopsis_thaliana - ps32
p-value
4e-09
2e-07
1e-05
7e-04
4e-02
Log-odds
0.5
1.0
1.5
2.0
2.5
3.0
3.5
4.0

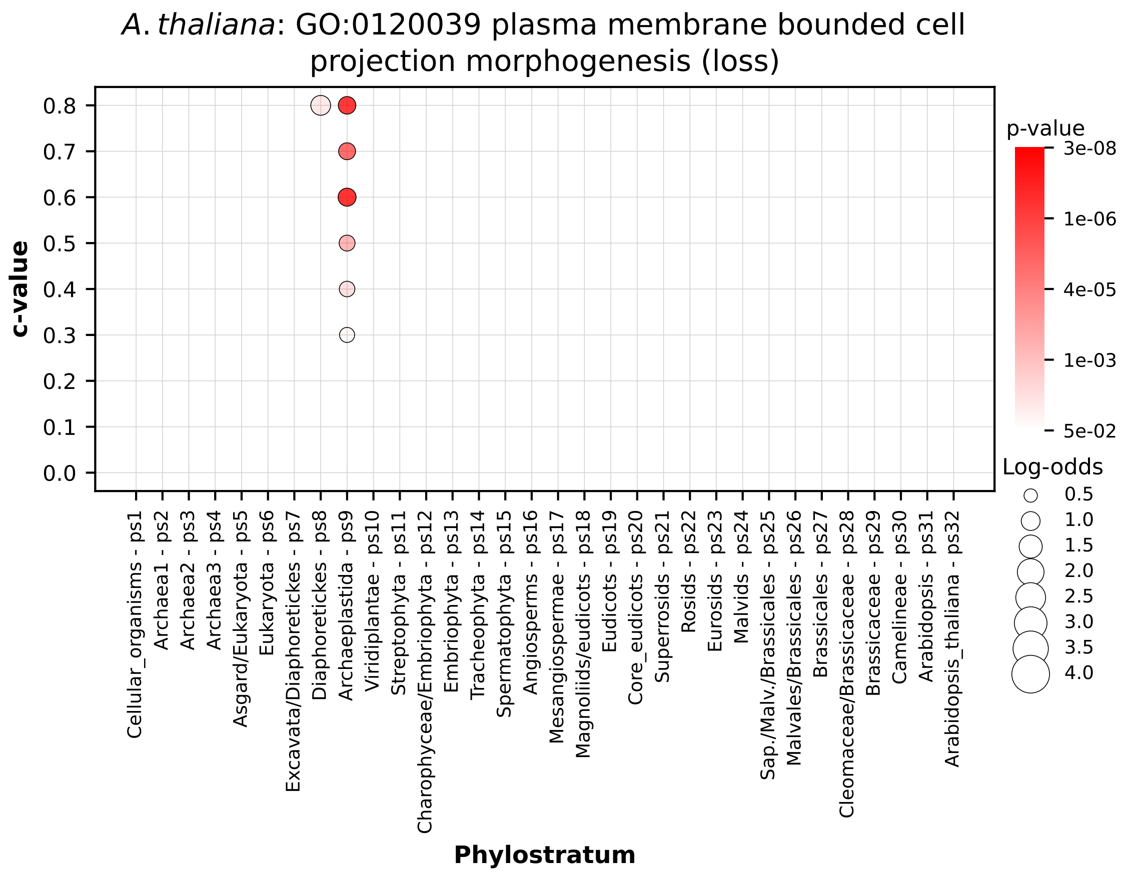

A. thaliana: GO:0120039 plasma membrane bounded cell projection morphogenesis (loss)
c-value
0.0
0.1
0.2
0.3
0.4
0.5
0.6
0.7
0.8
Phylostratum
Cellular_organisms - ps1
Archaea1 - ps2
Archaea2 - ps3
Archaea3 - ps4
Asgard/Eukaryota - ps5
Eukaryota - ps6
Excavata/Diaphoretickes - ps7
Diaphoretickes - ps8
Archaeplastida - ps9
Viridiplantae - ps10
Streptophyta - ps11
Charophyceae/Embriophyta - ps12
Embriophyta - ps13
Tracheophyta - ps14
Spermatophyta - ps15
Angiosperms - ps16
Mesangiospermae - ps17
Magnoliids/eudicots - ps18
Eudicots - ps19
Core_eudicots - ps20
Superrosids - ps21
Rosids - ps22
Eurosids - ps23
Malvids - ps24
Sap./Malv./Brassicales - ps25
Malvales/Brassicales - ps26
Brassicales - ps27
Cleomaceae/Brassicaceae - ps28
Brassicaceae - ps29
Camelineae - ps30
Arabidopsis - ps31
Arabidopsis_thaliana - ps32
p-value
3e-08
1e-06
4e-05
1e-03
5e-02
Log-odds
0.5
1.0
1.5
2.0
2.5
3.0
3.5
4.0

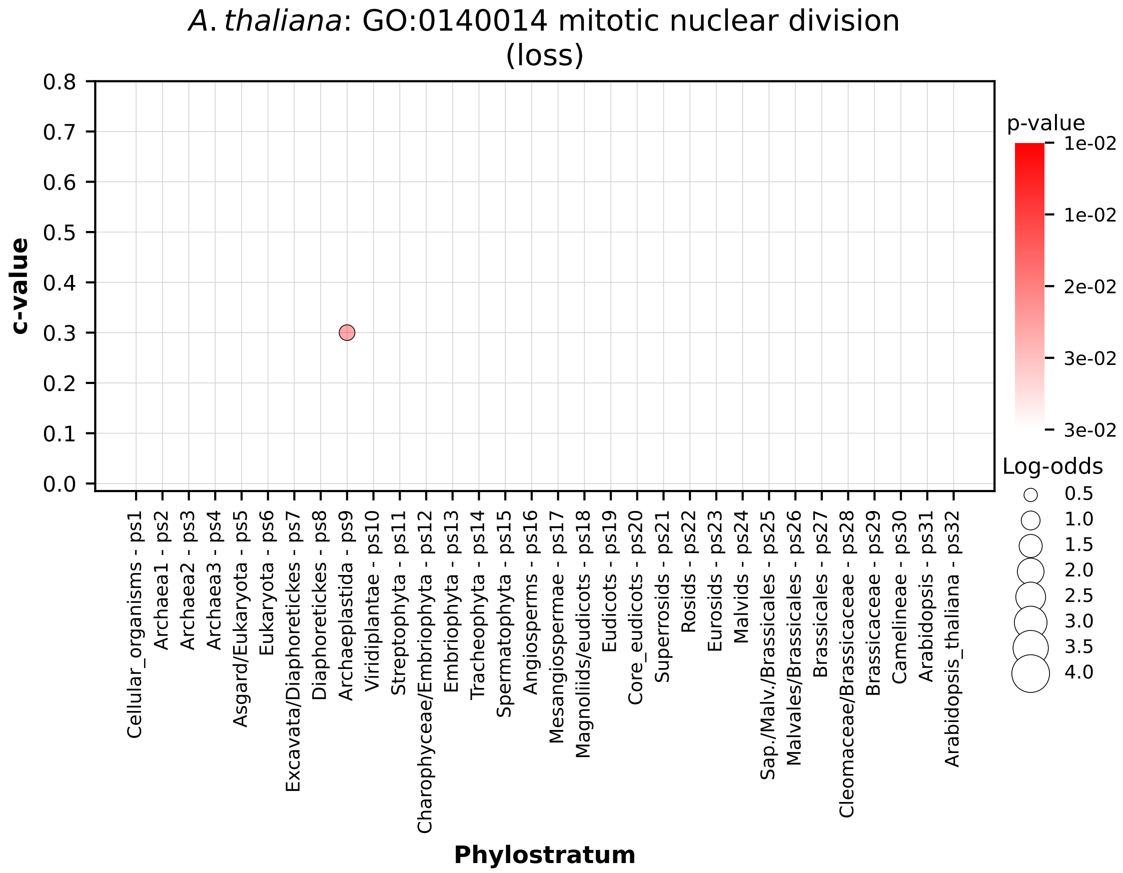

A. thaliana: GO:0140014 mitotic nuclear division
(loss)
c-value
0.8
0.7
0.6
0.5
0.4
0.3
0.2
0.1
0.0
p-value
1e-02
1e-02
2e-02
3e-02
3e-02
Log-odds
0.5
1.0
1.5
2.0
2.5
3.0
3.5
4.0
Cellular_organisms - ps1
Archaea1 - ps2
Archaea2 - ps3
Archaea3 - ps4
Asgard/Eukaryota - ps5
Eukaryota - ps6
Excavata/Diaphoretickes - ps7
Diaphoretickes - ps8
Archaeplastida - ps9
Viridiplantae - ps10
Streptophyta - ps11
Charophyceae/Embriophyta - ps12
Embriophyta - ps13
Tracheophyta - ps14
Spermatophyta - ps15
Angiosperms - ps16
Mesangiospermae - ps17
Magnoliids/eudicots - ps18
Eudicots - ps19
Core_eudicots - ps20
Superrosids - ps21
Rosids - ps22
Eurosids - ps23
Malvids - ps24
Sap./Malv./Brassicales - ps25
Malvales/Brassicales - ps26
Brassicales - ps27
Cleomaceae/Brassicaceae - ps28
Brassicaceae - ps29
Camelineae - ps30
Arabidopsis - ps31
Arabidopsis_thaliana - ps32
Phylostratum

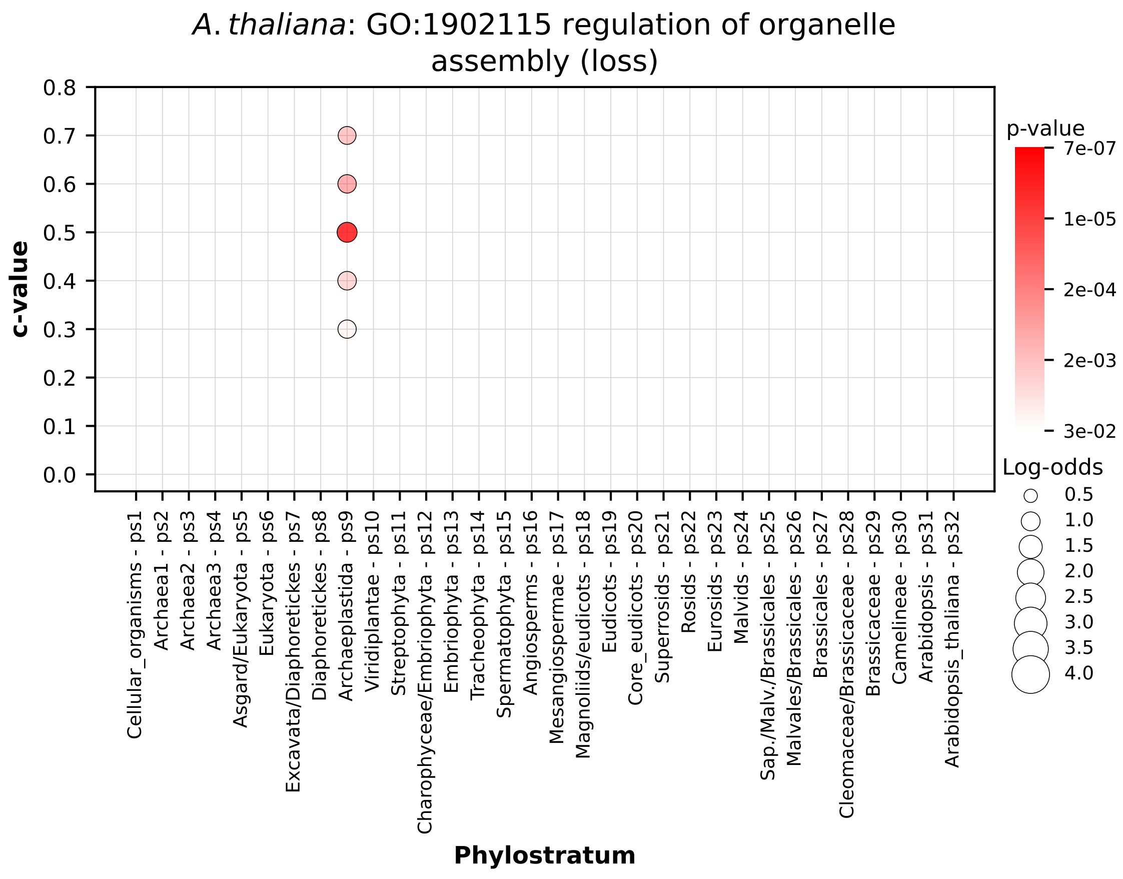

A. thaliana: GO:1902115 regulation of organelle assembly (loss)
c-value
0.8
0.7
0.6
0.5
0.4
0.3
0.2
0.1
0.0
p-value
7e-07
1e-05
2e-04
2e-03
3e-02
Log-odds
0.5
1.0
1.5
2.0
2.5
3.0
3.5
4.0
Cellular_organisms - ps1
Archaea1 - ps2
Archaea2 - ps3
Archaea3 - ps4
Asgard/Eukaryota - ps5
Eukaryota - ps6
Excavata/Diaphoretickes - ps7
Diaphoretickes - ps8
Archaeplastida - ps9
Viridiplantae - ps10
Streptophyta - ps11
Charophyceae/Embriophyta - ps12
Embriophyta - ps13
Tracheophyta - ps14
Spermatophyta - ps15
Angiosperms - ps16
Mesangiospermae - ps17
Magnoliids/eudicots - ps18
Eudicots - ps19
Core_eudicots - ps20
Superrosids - ps21
Rosids - ps22
Eurosids - ps23
Malvids - ps24
Sap./Malv./Brassicales - ps25
Malvales/Brassicales - ps26
Brassicales - ps27
Cleomaceae/Brassicaceae - ps28
Brassicaceae - ps29
Camelineae - ps30
Arabidopsis - ps31
Arabidopsis_thaliana - ps32
Phylostratum

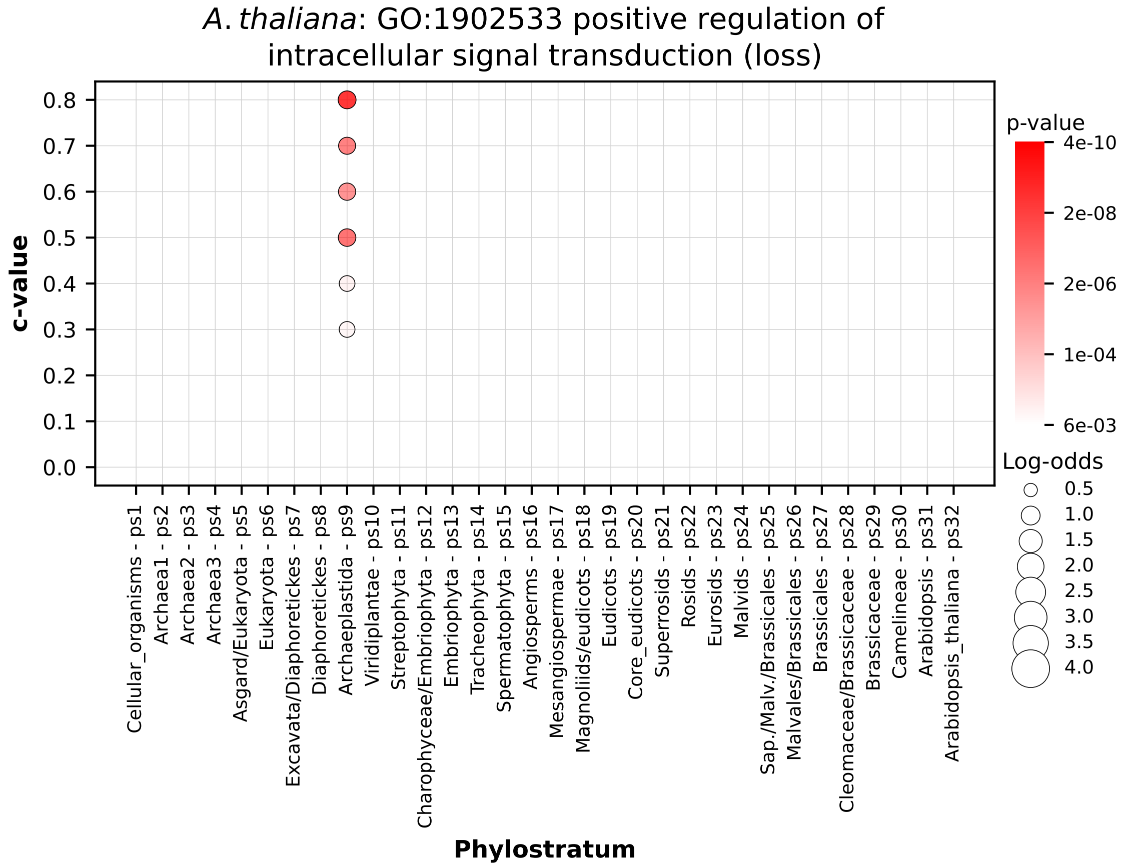

A. thaliana: GO:1902533 positive regulation of intracellular signal transduction (loss)
c-value
0.8
0.7
0.6
0.5
0.4
0.3
0.2
0.1
0.0
Phylostratum
Cellular_organisms - ps1
Archaea1 - ps2
Archaea2 - ps3
Archaea3 - ps4
Asgard/Eukaryota - ps5
Eukaryota - ps6
Excavata/Diaphoretickes - ps7
Diaphoretickes - ps8
Archaeplastida - ps9
Viridiplantae - ps10
Streptophyta - ps11
Charophyceae/Embriophyta - ps12
Embriophyta - ps13
Tracheophyta - ps14
Spermatophyta - ps15
Angiosperms - ps16
Mesangiospermae - ps17
Magnoliids/eudicots - ps18
Eudicots - ps19
Core_eudicots - ps20
Superrosids - ps21
Rosids - ps22
Eurosids - ps23
Malvids - ps24
Sap./Malv./Brassicales - ps25
Malvales/Brassicales - ps26
Brassicales - ps27
Cleomaceae/Brassicaceae - ps28
Brassicaceae - ps29
Camelineae - ps30
Arabidopsis - ps31
Arabidopsis_thaliana - ps32
p-value
4e-10
2e-08
2e-06
1e-04
6e-03
Log-odds
0.5
1.0
1.5
2.0
2.5
3.0
3.5
4.0

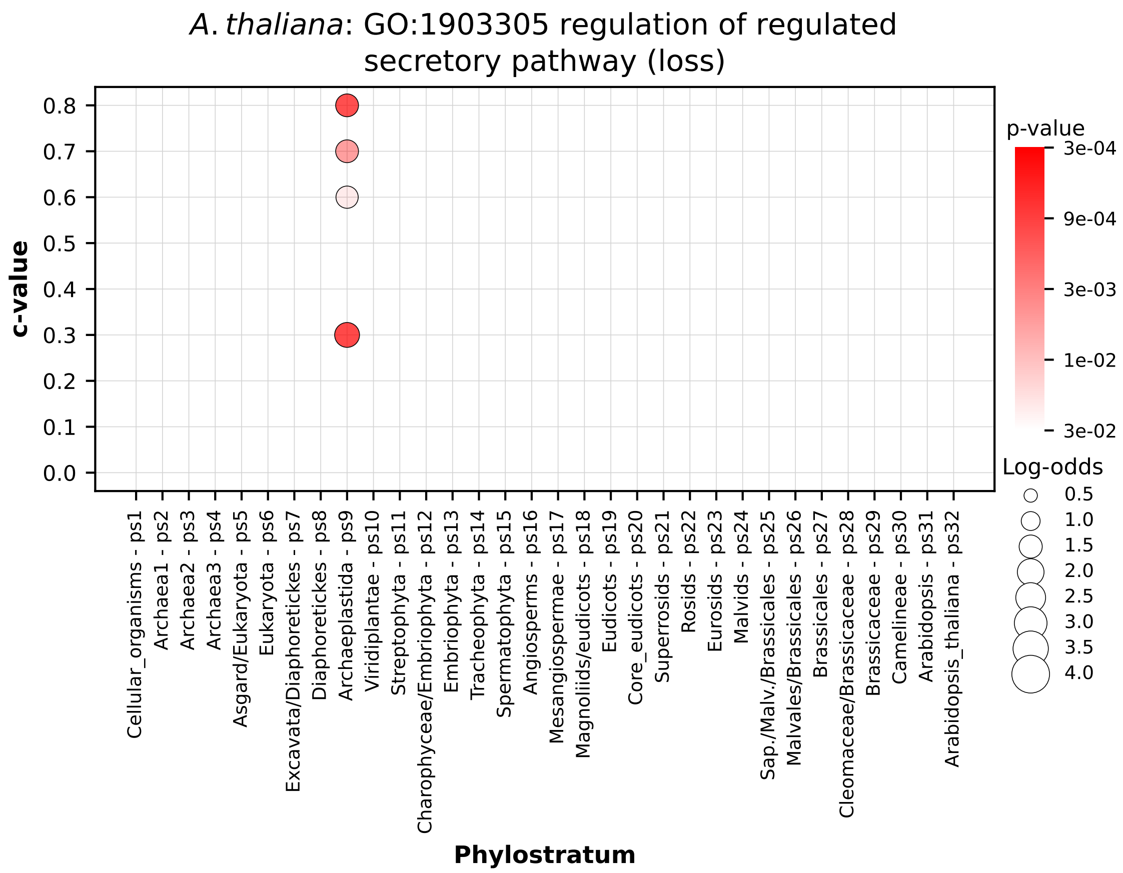

A. thaliana: GO:1903305 regulation of regulated secretory pathway (loss)
c-value
0.8
0.7
0.6
0.5
0.4
0.3
0.2
0.1
0.0
p-value
3e-04
9e-04
3e-03
1e-02
3e-02
Log-odds
0.5
1.0
1.5
2.0
2.5
3.0
3.5
4.0
Cellular_organisms - ps1
Archaea1 - ps2
Archaea2 - ps3
Archaea3 - ps4
Asgard/Eukaryota - ps5
Eukaryota - ps6
Excavata/Diaphoretickes - ps7
Diaphoretickes - ps8
Archaeplastida - ps9
Viridiplantae - ps10
Streptophyta - ps11
Charophyceae/Embriophyta - ps12
Embriophyta - ps13
Tracheophyta - ps14
Spermatophyta - ps15
Angiosperms - ps16
Mesangiospermae - ps17
Magnoliids/eudicots - ps18
Eudicots - ps19
Core_eudicots - ps20
Superrosids - ps21
Rosids - ps22
Eurosids - ps23
Malvids - ps24
Sap./Malv./Brassicales - ps25
Malvales/Brassicales - ps26
Brassicales - ps27
Cleomaceae/Brassicaceae - ps28
Brassicaceae - ps29
Camelineae - ps30
Arabidopsis - ps31
Arabidopsis_thaliana - ps32
Phylostratum

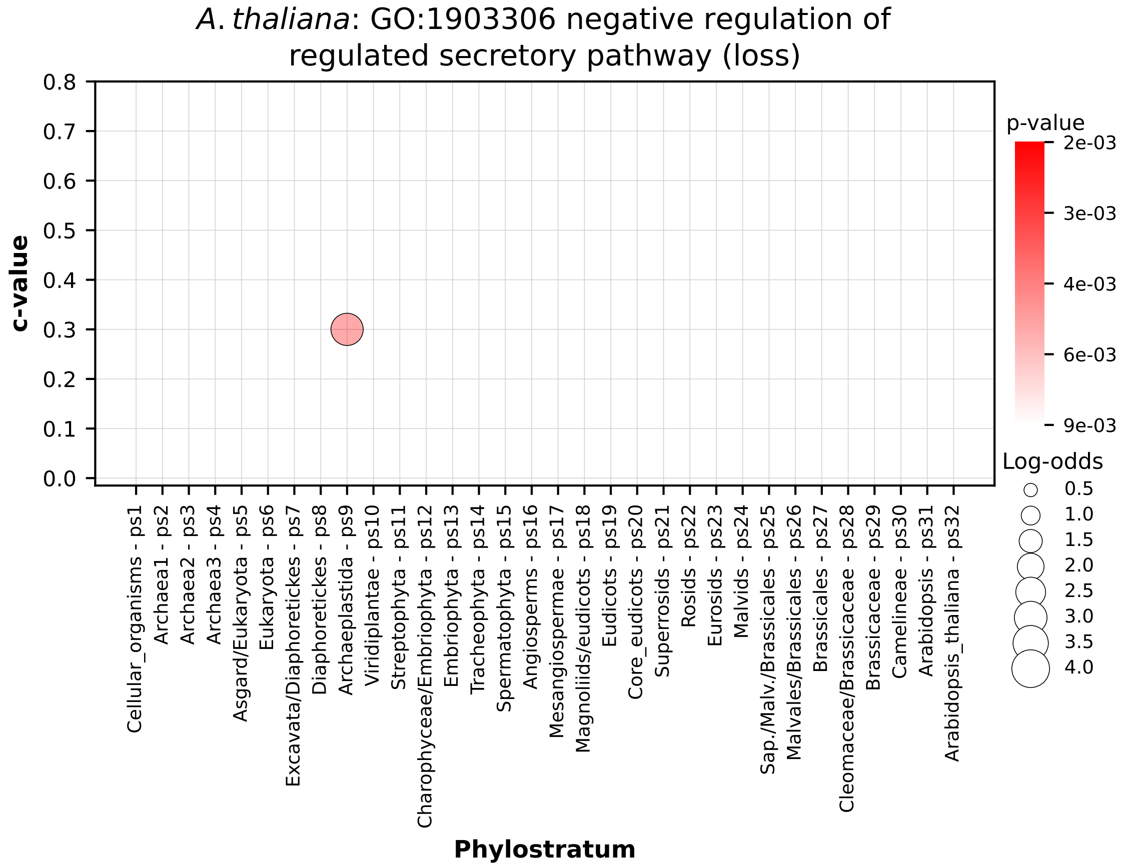

A. thaliana: GO:1903306 negative regulation of regulated secretory pathway (loss)
c-value
0.8
0.7
0.6
0.5
0.4
0.3
0.2
0.1
0.0
Cellular_organisms - ps1
Archaea1 - ps2
Archaea2 - ps3
Archaea3 - ps4
Asgard/Eukaryota - ps5
Eukaryota - ps6
Excavata/Diaphoretickes - ps7
Diaphoretickes - ps8
Archaeplastida - ps9
Viridiplantae - ps10
Streptophyta - ps11
Charophyceae/Embriophyta - ps12
Embriophyta - ps13
Tracheophyta - ps14
Spermatophyta - ps15
Angiosperms - ps16
Mesangiospermae - ps17
Magnoliids/eudicots - ps18
Eudicots - ps19
Core_eudicots - ps20
Superrosids - ps21
Rosids - ps22
Eurosids - ps23
Malvids - ps24
Sap./Malv./Brassicales - ps25
Malvales/Brassicales - ps26
Brassicales - ps27
Cleomaceae/Brassicaceae - ps28
Brassicaceae - ps29
Camelineae - ps30
Arabidopsis - ps31
Arabidopsis_thaliana - ps32
Phylostratum
p-value
2e-03
3e-03
4e-03
6e-03
9e-03
Log-odds
0.5
1.0
1.5
2.0
2.5
3.0
3.5
4.0

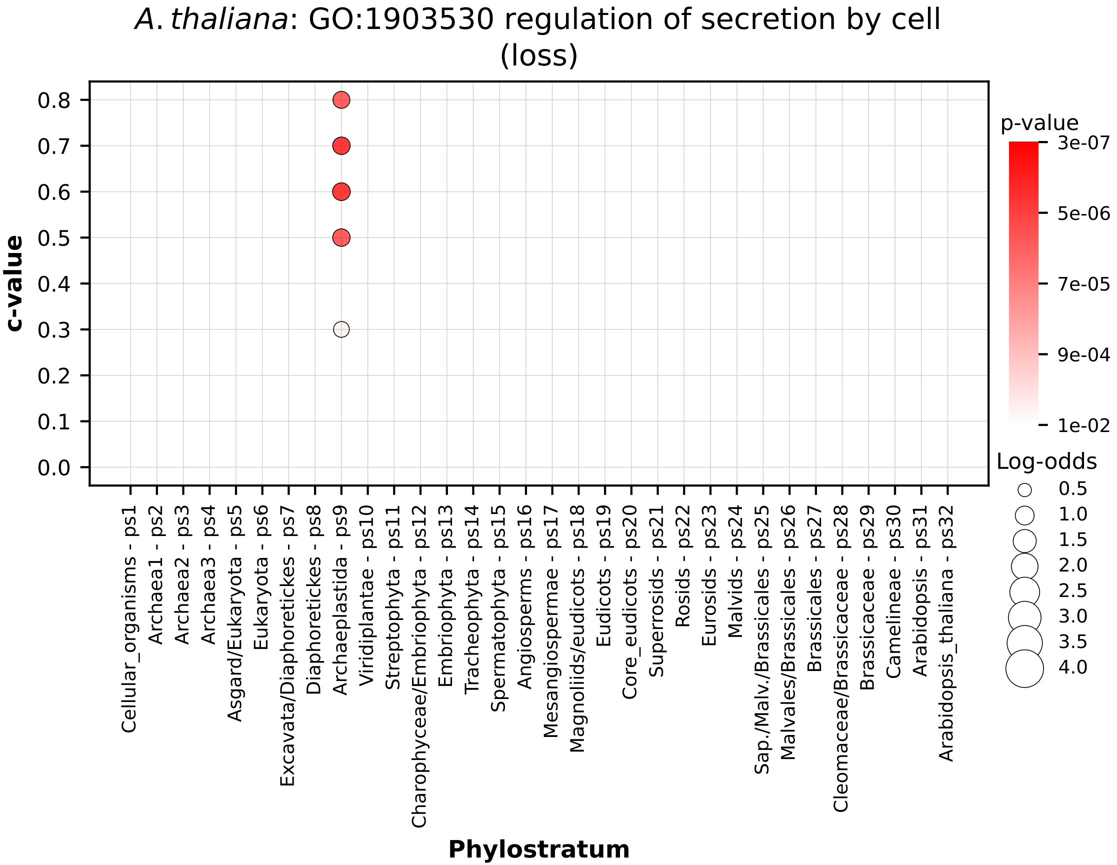

A. thaliana: GO:1903530 regulation of secretion by cell
(loss)
c-value
0.8
0.7
0.6
0.5
0.4
0.3
0.2
0.1
0.0
p-value
3e-07
5e-06
7e-05
9e-04
1e-02
Log-odds
0.5
1.0
1.5
2.0
2.5
3.0
3.5
4.0
Cellular_organisms - ps1
Archaea1 - ps2
Archaea2 - ps3
Archaea3 - ps4
Asgard/Eukaryota - ps5
Eukaryota - ps6
Excavata/Diaphoretickes - ps7
Diaphoretickes - ps8
Archaeplastida - ps9
Viridiplantae - ps10
Streptophyta - ps11
Charophyceae/Embriophyta - ps12
Embriophyta - ps13
Tracheophyta - ps14
Spermatophyta - ps15
Angiosperms - ps16
Mesangiospermae - ps17
Magnoliids/eudicots - ps18
Eudicots - ps19
Core_eudicots - ps20
Superrosids - ps21
Rosids - ps22
Eurosids - ps23
Malvids - ps24
Sap./Malv./Brassicales - ps25
Malvales/Brassicales - ps26
Brassicales - ps27
Cleomaceae/Brassicaceae - ps28
Brassicaceae - ps29
Camelineae - ps30
Arabidopsis - ps31
Arabidopsis_thaliana - ps32
Phylostratum

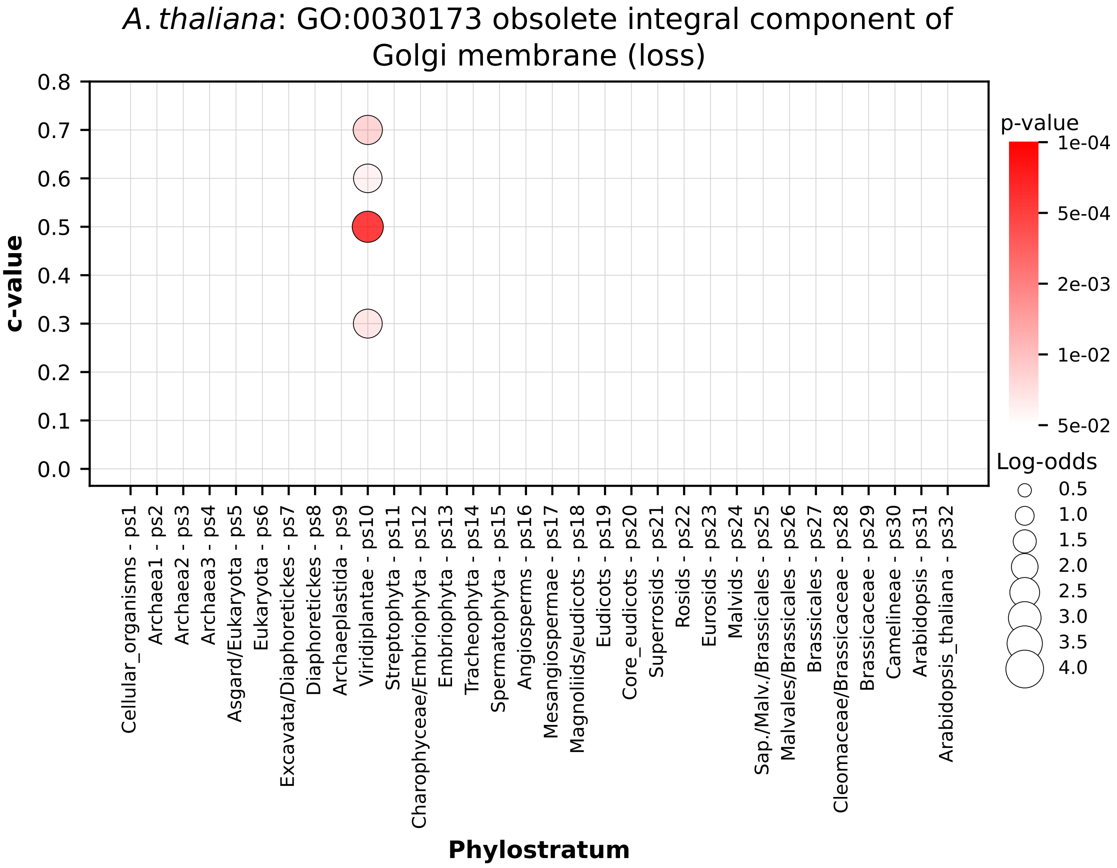

A. thaliana: GO:0030173 obsolete integral component of Golgi membrane (loss)
c-value
0.8
0.7
0.6
0.5
0.4
0.3
0.2
0.1
0.0
Phylostratum
Cellular_organisms - ps1
Archaea1 - ps2
Archaea2 - ps3
Archaea3 - ps4
Asgard/Eukaryota - ps5
Eukaryota - ps6
Excavata/Diaphoretickes - ps7
Diaphoretickes - ps8
Archaeplastida - ps9
Viridiplantae - ps10
Streptophyta - ps11
Charophyceae/Embriophyta - ps12
Embriophyta - ps13
Tracheophyta - ps14
Spermatophyta - ps15
Angiosperms - ps16
Mesangiospermae - ps17
Magnoliids/eudicots - ps18
Eudicots - ps19
Core_eudicots - ps20
Superrosids - ps21
Rosids - ps22
Eurosids - ps23
Malvids - ps24
Sap./Malv./Brassicales - ps25
Malvales/Brassicales - ps26
Brassicales - ps27
Cleomaceae/Brassicaceae - ps28
Brassicaceae - ps29
Camelineae - ps30
Arabidopsis - ps31
Arabidopsis_thaliana - ps32
p-value
1e-04
5e-04
2e-03
1e-02
5e-02
Log-odds
0.5
1.0
1.5
2.0
2.5
3.0
3.5
4.0

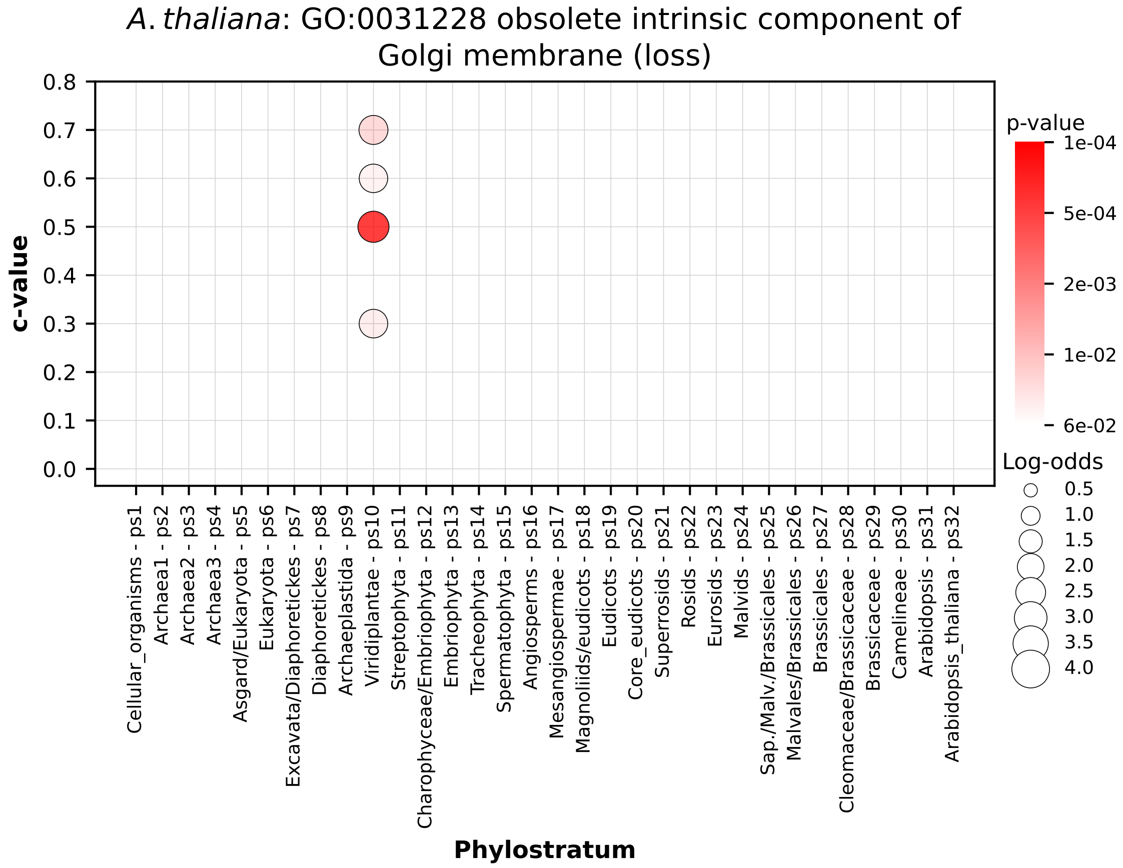

A. thaliana: GO:0031228 obsolete intrinsic component of Golgi membrane (loss)
c-value
0.8
0.7
0.6
0.5
0.4
0.3
0.2
0.1
0.0
p-value
1e-04
5e-04
2e-03
1e-02
6e-02
Log-odds
0.5
1.0
1.5
2.0
2.5
3.0
3.5
4.0
Cellular_organisms - ps1
Archaea1 - ps2
Archaea2 - ps3
Archaea3 - ps4
Asgard/Eukaryota - ps5
Eukaryota - ps6
Excavata/Diaphoretickes - ps7
Diaphoretickes - ps8
Archaeplastida - ps9
Viridiplantae - ps10
Streptophyta - ps11
Charophyceae/Embriophyta - ps12
Embriophyta - ps13
Tracheophyta - ps14
Spermatophyta - ps15
Angiosperms - ps16
Mesangiospermae - ps17
Magnoliids/eudicots - ps18
Eudicots - ps19
Core_eudicots - ps20
Superrosids - ps21
Rosids - ps22
Eurosids - ps23
Malvids - ps24
Sap./Malv./Brassicales - ps25
Malvales/Brassicales - ps26
Brassicales - ps27
Cleomaceae/Brassicaceae - ps28
Brassicaceae - ps29
Camelineae - ps30
Arabidopsis - ps31
Arabidopsis_thaliana - ps32
Phylostratum

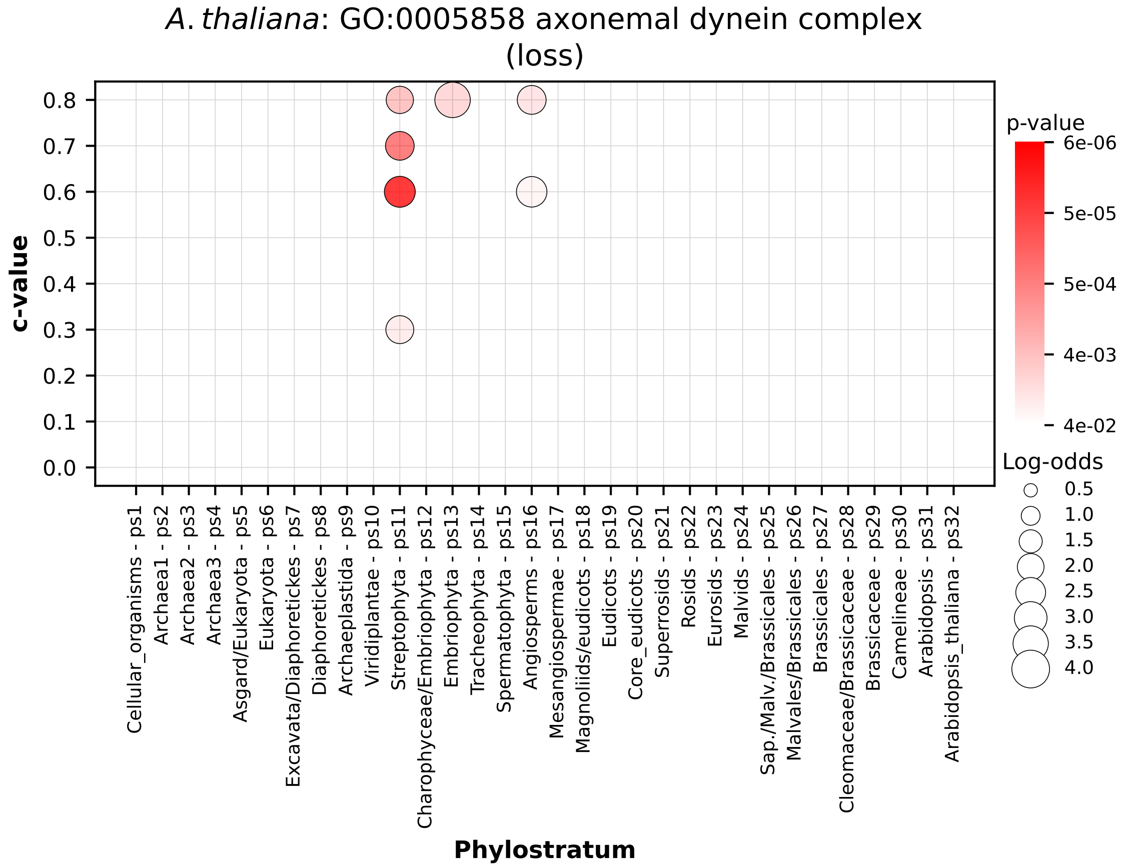
A. thaliana: GO:0005858 axonemal dynein complex
(loss)
c-value
0.8
0.7
0.6
0.5
0.4
0.3
0.2
0.1
0.0
Phylostratum
Cellular_organisms - ps1
Archaea1 - ps2
Archaea2 - ps3
Archaea3 - ps4
Asgard/Eukaryota - ps5
Eukaryota - ps6
Excavata/Diaphoretickes - ps7
Diaphoretickes - ps8
Archaeplastida - ps9
Viridiplantae - ps10
Streptophyta - ps11
Charophyceae/Embriophyta - ps12
Embriophyta - ps13
Tracheophyta - ps14
Spermatophyta - ps15
Angiosperms - ps16
Mesangiospermae - ps17
Magnoliids/eudicots - ps18
Eudicots - ps19
Core_eudicots - ps20
Superrosids - ps21
Rosids - ps22
Eurosids - ps23
Malvids - ps24
Sap./Malv./Brassicales - ps25
Malvales/Brassicales - ps26
Brassicales - ps27
Cleomaceae/Brassicaceae - ps28
Brassicaceae - ps29
Camelineae - ps30
Arabidopsis - ps31
Arabidopsis_thaliana - ps32
p-value
6e-06
5e-05
5e-04
4e-03
4e-02
Log-odds
0.5
1.0
1.5
2.0
2.5
3.0
3.5
4.0

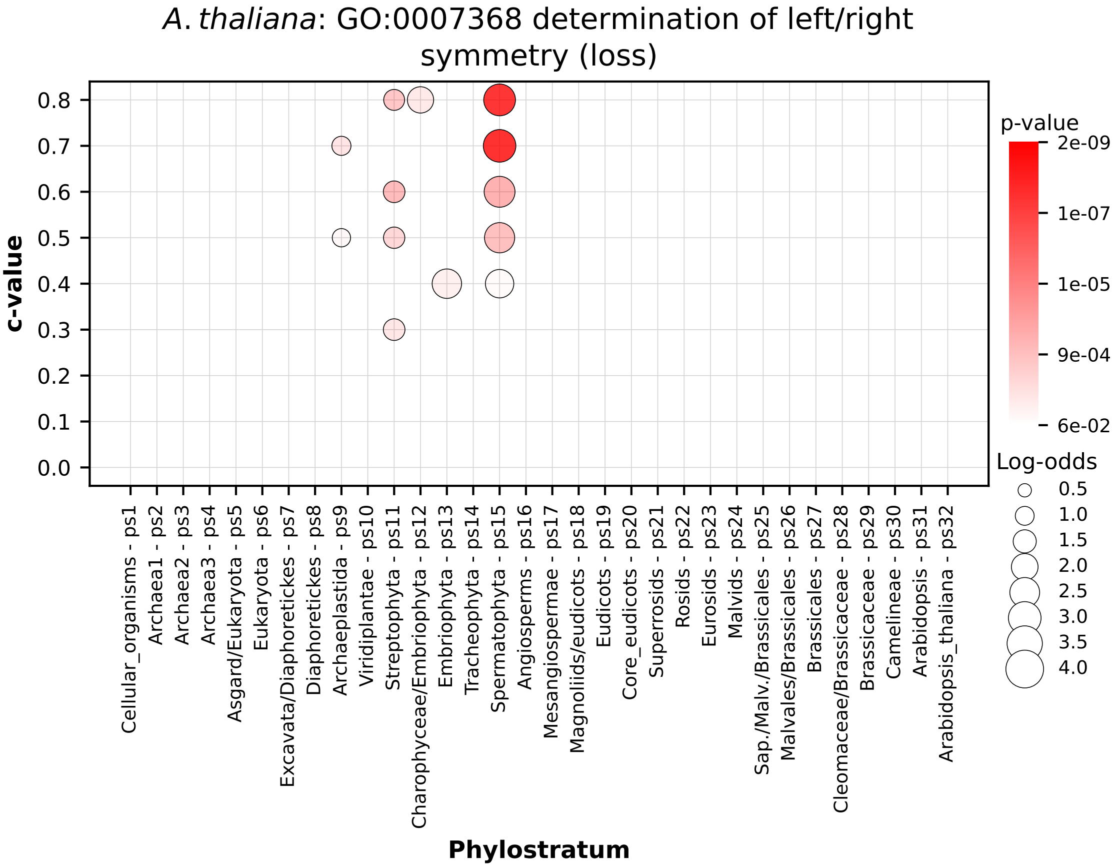

A. thaliana: GO:0007368 determination of left/right symmetry (loss)
c-value
0.8
0.7
0.6
0.5
0.4
0.3
0.2
0.1
0.0
Phylostratum
Cellular_organisms - ps1
Archaea1 - ps2
Archaea2 - ps3
Archaea3 - ps4
Asgard/Eukaryota - ps5
Eukaryota - ps6
Excavata/Diaphoretickes - ps7
Diaphoretickes - ps8
Archaeplastida - ps9
Viridiplantae - ps10
Streptophyta - ps11
Charophyceae/Embriophyta - ps12
Embriophyta - ps13
Tracheophyta - ps14
Spermatophyta - ps15
Angiosperms - ps16
Mesangiospermae - ps17
Magnoliids/eudicots - ps18
Eudicots - ps19
Core_eudicots - ps20
Superrosids - ps21
Rosids - ps22
Eurosids - ps23
Malvids - ps24
Sap./Malv./Brassicales - ps25
Malvales/Brassicales - ps26
Brassicales - ps27
Cleomaceae/Brassicaceae - ps28
Brassicaceae - ps29
Camelineae - ps30
Arabidopsis - ps31
Arabidopsis_thaliana - ps32
p-value
2e-09
1e-07
1e-05
9e-04
6e-02
Log-odds
0.5
1.0
1.5
2.0
2.5
3.0
3.5
4.0

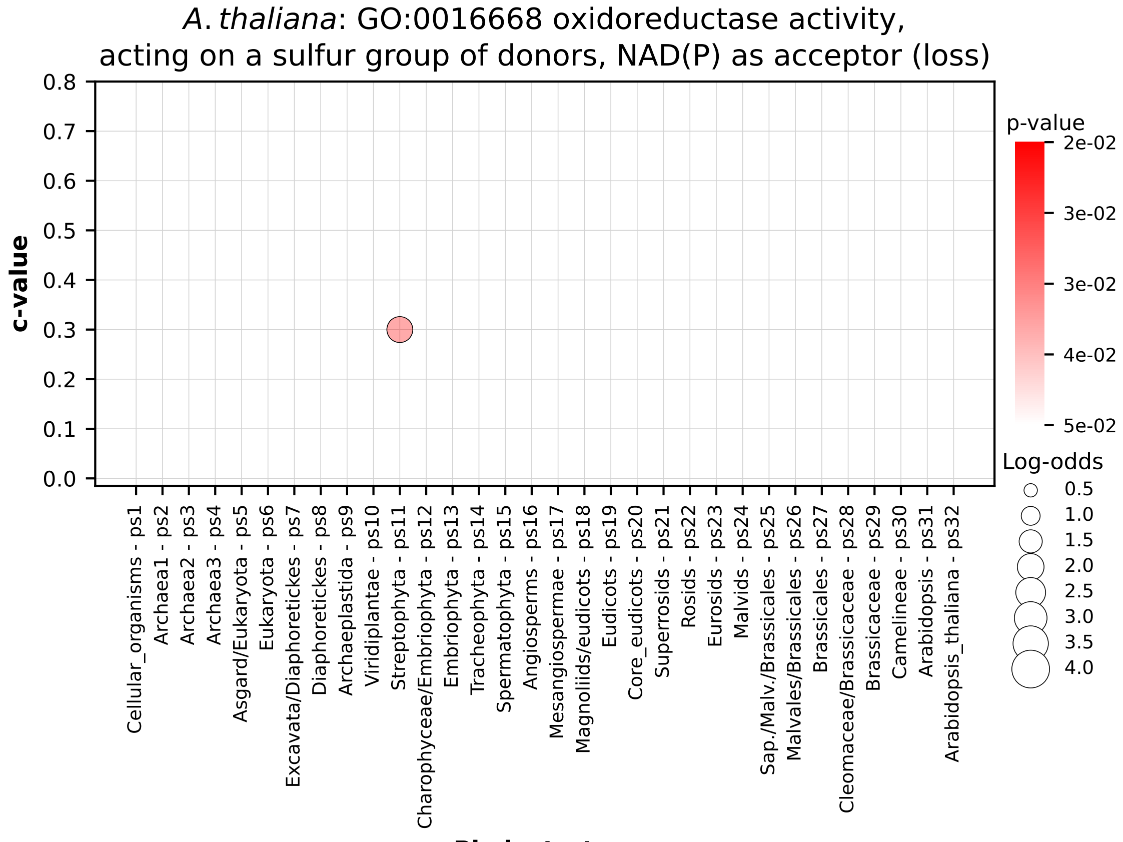

A. thaliana: GO:0016668 oxidoreductase activity, acting on a sulfur group of donors, NAD(P) as acceptor (loss)
c-value
0.8
0.7
0.6
0.5
0.4
0.3
0.2
0.1
0.0
Phylostratum
Cellular_organisms - ps1
Archaea1 - ps2
Archaea2 - ps3
Archaea3 - ps4
Asgard/Eukaryota - ps5
Eukaryota - ps6
Excavata/Diaphoretickes - ps7
Diaphoretickes - ps8
Archaeplastida - ps9
Viridiplantae - ps10
Streptophyta - ps11
Charophyceae/Embriophyta - ps12
Embriophyta - ps13
Tracheophyta - ps14
Spermatophyta - ps15
Angiosperms - ps16
Mesangiospermae - ps17
Magnoliids/eudicots - ps18
Eudicots - ps19
Core_eudicots - ps20
Superrosids - ps21
Rosids - ps22
Eurosids - ps23
Malvids - ps24
Sap./Malv./Brassicales - ps25
Malvales/Brassicales - ps26
Brassicales - ps27
Cleomaceae/Brassicaceae - ps28
Brassicaceae - ps29
Camelineae - ps30
Arabidopsis - ps31
Arabidopsis_thaliana - ps32
p-value
2e-02
3e-02
3e-02
4e-02
5e-02
Log-odds
0.5
1.0
1.5
2.0
2.5
3.0
3.5
4.0

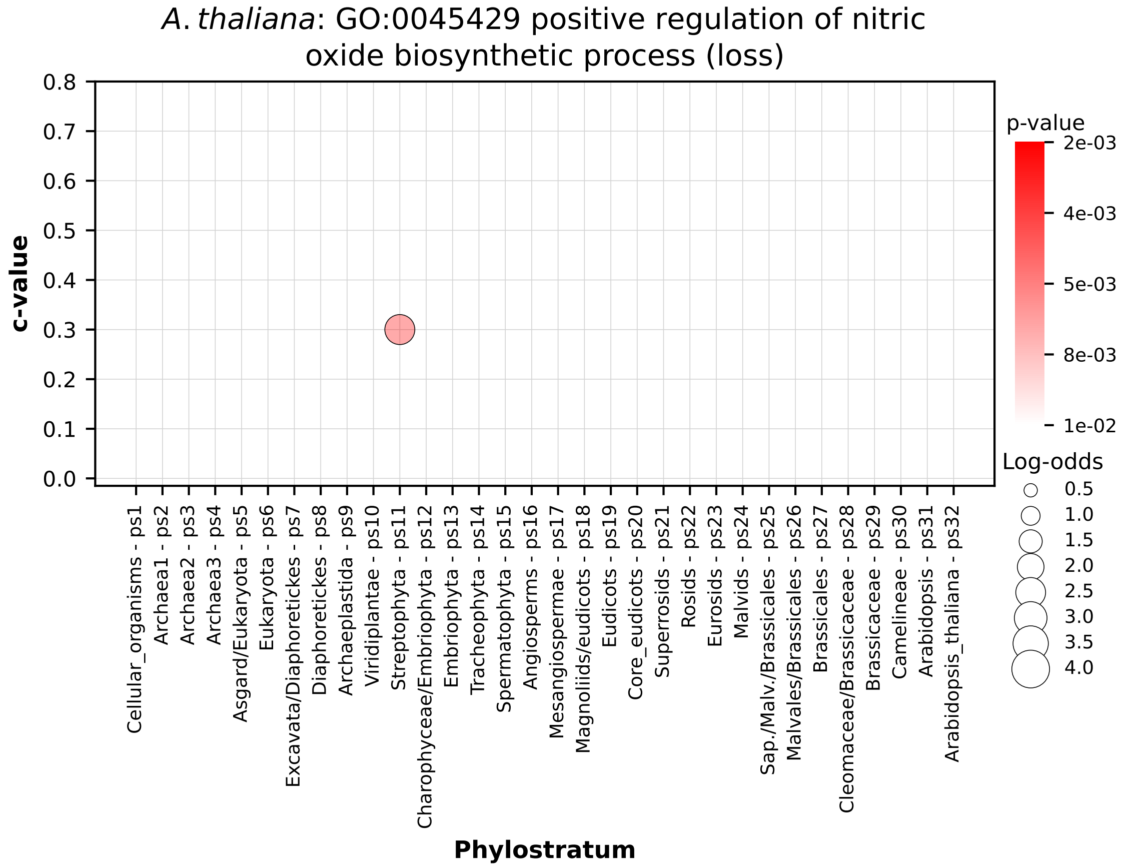

A. thaliana: GO:0045429 positive regulation of nitric oxide biosynthetic process (loss)
c-value
0.8
0.7
0.6
0.5
0.4
0.3
0.2
0.1
0.0
Phylostratum
Cellular_organisms - ps1
Archaea1 - ps2
Archaea2 - ps3
Archaea3 - ps4
Asgard/Eukaryota - ps5
Eukaryota - ps6
Excavata/Diaphoretickes - ps7
Diaphoretickes - ps8
Archaeplastida - ps9
Viridiplantae - ps10
Streptophyta - ps11
Charophyceae/Embriophyta - ps12
Embriophyta - ps13
Tracheophyta - ps14
Spermatophyta - ps15
Angiosperms - ps16
Mesangiospermae - ps17
Magnoliids/eudicots - ps18
Eudicots - ps19
Core_eudicots - ps20
Superrosids - ps21
Rosids - ps22
Eurosids - ps23
Malvids - ps24
Sap./Malv./Brassicales - ps25
Malvales/Brassicales - ps26
Brassicales - ps27
Cleomaceae/Brassicaceae - ps28
Brassicaceae - ps29
Camelineae - ps30
Arabidopsis - ps31
Arabidopsis_thaliana - ps32
p-value
2e-03
4e-03
5e-03
8e-03
1e-02
Log-odds
0.5
1.0
1.5
2.0
2.5
3.0
3.5
4.0

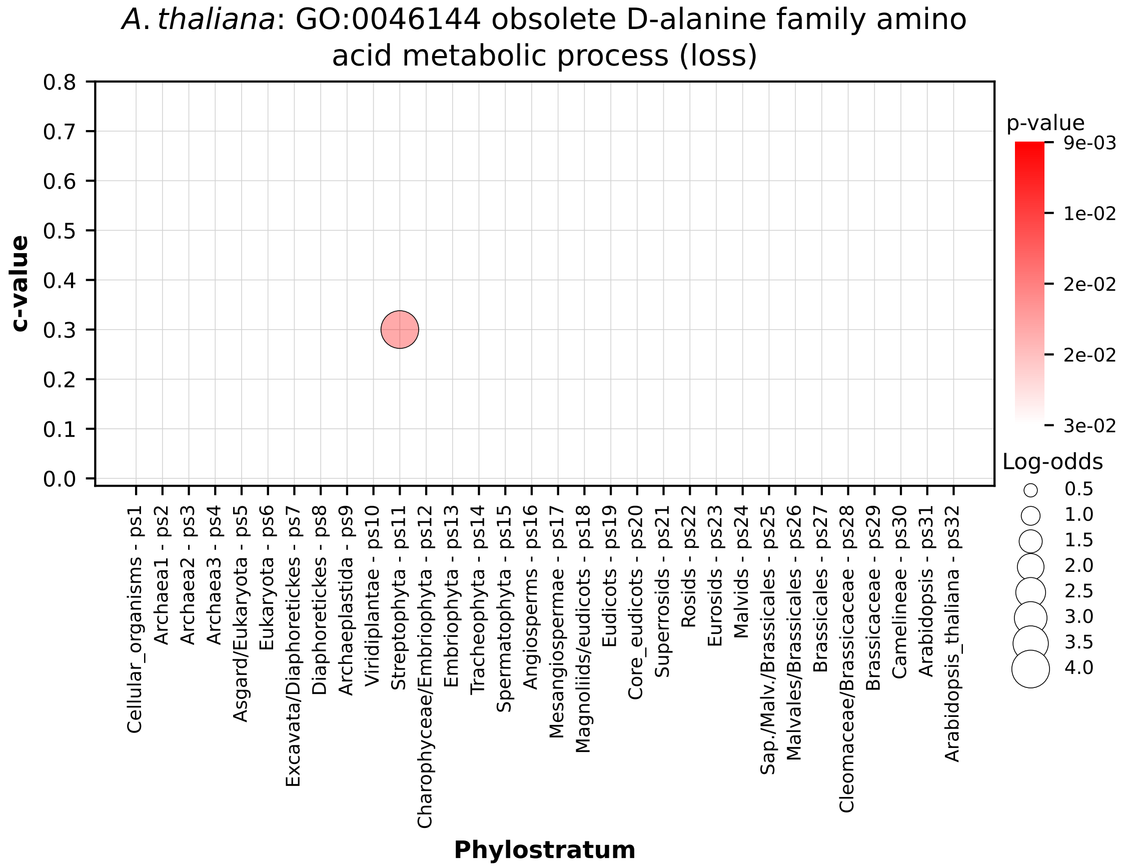

A. thaliana: GO:0046144 obsolete D-alanine family amino acid metabolic process (loss)
c-value
0.0
0.1
0.2
0.3
0.4
0.5
0.6
0.7
0.8
Phylostratum
Cellular_organisms - ps1
Archaea1 - ps2
Archaea2 - ps3
Archaea3 - ps4
Asgard/Eukaryota - ps5
Eukaryota - ps6
Excavata/Diaphoretickes - ps7
Diaphoretickes - ps8
Archaeplastida - ps9
Viridiplantae - ps10
Streptophyta - ps11
Charophyceae/Embriophyta - ps12
Embriophyta - ps13
Tracheophyta - ps14
Spermatophyta - ps15
Angiosperms - ps16
Mesangiospermae - ps17
Magnoliids/eudicots - ps18
Eudicots - ps19
Core_eudicots - ps20
Superrosids - ps21
Rosids - ps22
Eurosids - ps23
Malvids - ps24
Sap./Malv./Brassicales - ps25
Malvales/Brassicales - ps26
Brassicales - ps27
Cleomaceae/Brassicaceae - ps28
Brassicaceae - ps29
Camelineae - ps30
Arabidopsis - ps31
Arabidopsis_thaliana - ps32
p-value
9e-03
1e-02
2e-02
2e-02
3e-02
Log-odds
0.5
1.0
1.5
2.0
2.5
3.0
3.5
4.0

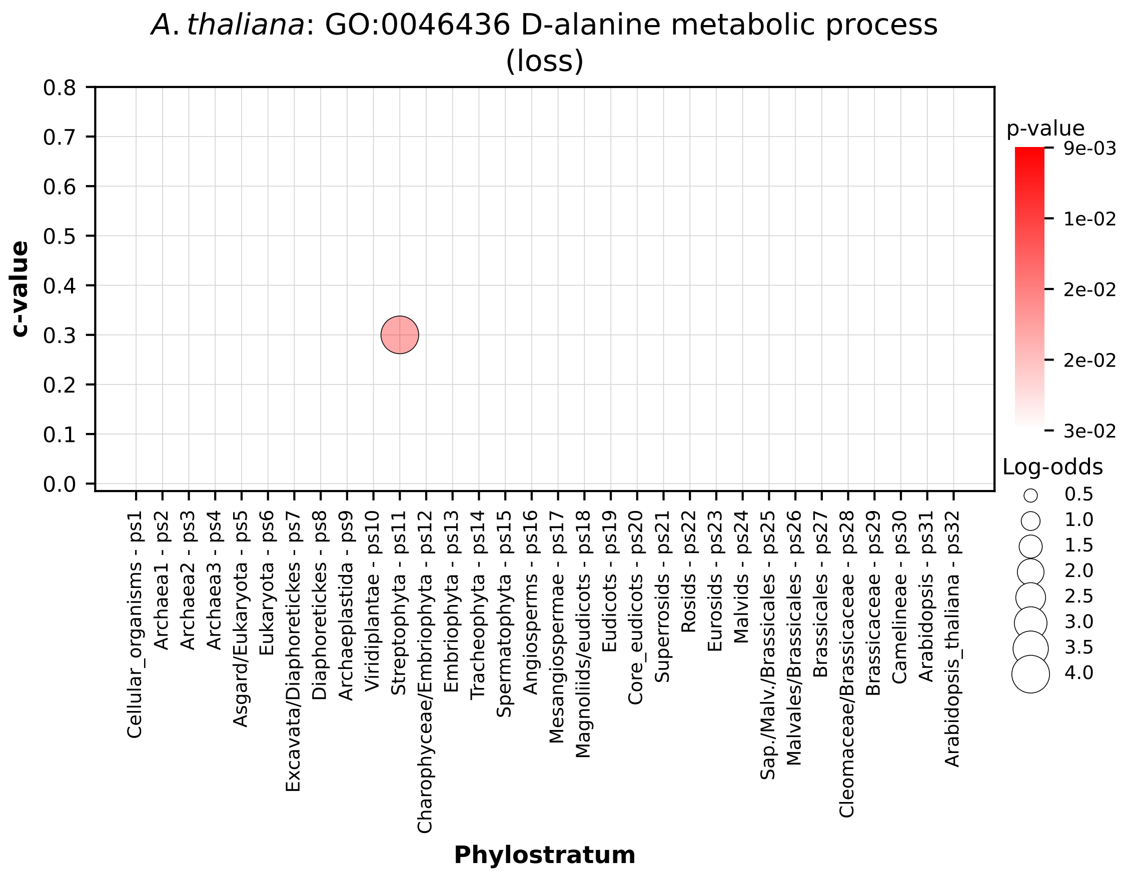
A. thaliana: GO:0046436 D-alanine metabolic process
(loss)
c-value
0.0
0.1
0.2
0.3
0.4
0.5
0.6
0.7
0.8
Phylostratum
Cellular_organisms - ps1
Archaea1 - ps2
Archaea2 - ps3
Archaea3 - ps4
Asgard/Eukaryota - ps5
Eukaryota - ps6
Excavata/Diaphoretickes - ps7
Diaphoretickes - ps8
Archaeplastida - ps9
Viridiplantae - ps10
Streptophyta - ps11
Charophyceae/Embriophyta - ps12
Embriophyta - ps13
Tracheophyta - ps14
Spermatophyta - ps15
Angiosperms - ps16
Mesangiospermae - ps17
Magnoliids/eudicots - ps18
Eudicots - ps19
Core_eudicots - ps20
Superrosids - ps21
Rosids - ps22
Eurosids - ps23
Malvids - ps24
Sap./Malv./Brassicales - ps25
Malvales/Brassicales - ps26
Brassicales - ps27
Cleomaceae/Brassicaceae - ps28
Brassicaceae - ps29
Camelineae - ps30
Arabidopsis - ps31
Arabidopsis_thaliana - ps32
p-value
9e-03
1e-02
2e-02
2e-02
3e-02
Log-odds
0.5
1.0
1.5
2.0
2.5
3.0
3.5
4.0

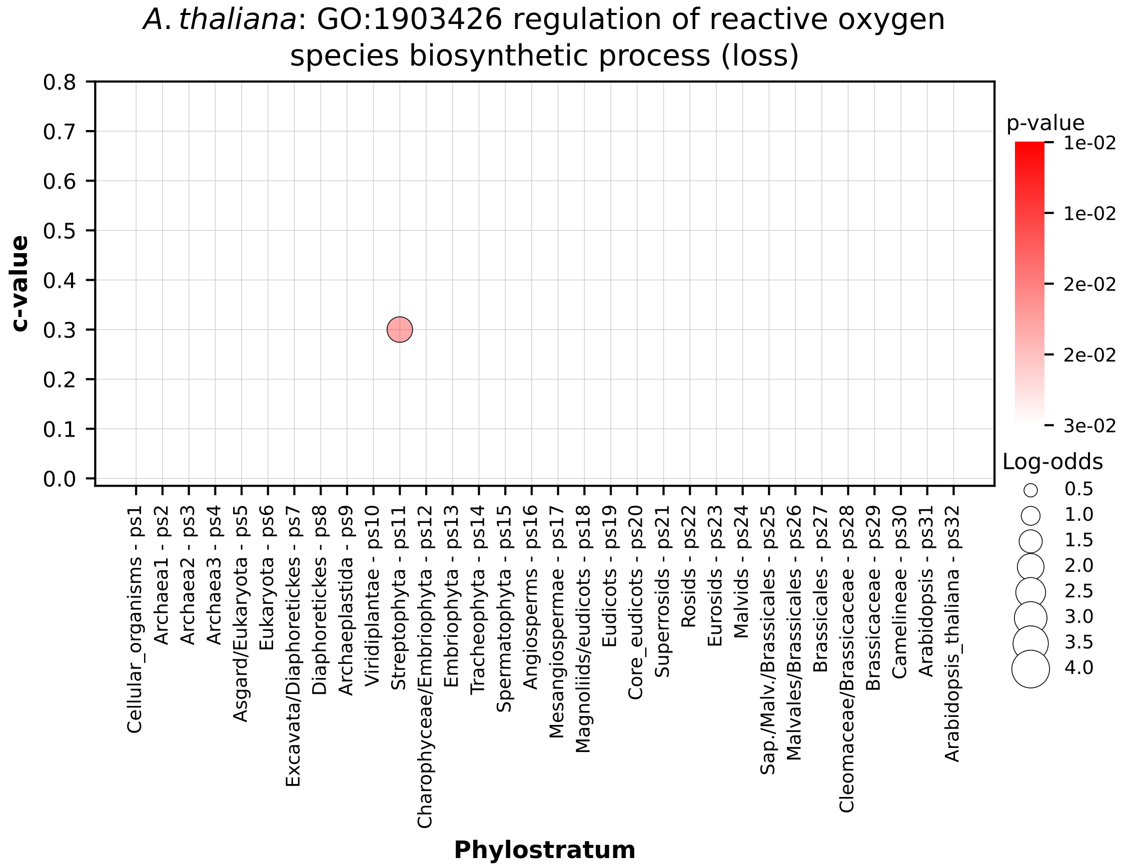

A. thaliana: GO:1903426 regulation of reactive oxygen species biosynthetic process (loss)
c-value
0.8
0.7
0.6
0.5
0.4
0.3
0.2
0.1
0.0
Cellular_organisms - ps1
Archaea1 - ps2
Archaea2 - ps3
Archaea3 - ps4
Asgard/Eukaryota - ps5
Eukaryota - ps6
Excavata/Diaphoretickes - ps7
Diaphoretickes - ps8
Archaeplastida - ps9
Viridiplantae - ps10
Streptophyta - ps11
Charophyceae/Embriophyta - ps12
Embriophyta - ps13
Tracheophyta - ps14
Spermatophyta - ps15
Angiosperms - ps16
Mesangiospermae - ps17
Magnoliids/eudicots - ps18
Eudicots - ps19
Core_eudicots - ps20
Superrosids - ps21
Rosids - ps22
Eurosids - ps23
Malvids - ps24
Sap./Malv./Brassicales - ps25
Malvales/Brassicales - ps26
Brassicales - ps27
Cleomaceae/Brassicaceae - ps28
Brassicaceae - ps29
Camelineae - ps30
Arabidopsis - ps31
Arabidopsis_thaliana - ps32
Phylostratum
p-value
1e-02
1e-02
2e-02
2e-02
3e-02
Log-odds
0.5
1.0
1.5
2.0
2.5
3.0
3.5
4.0

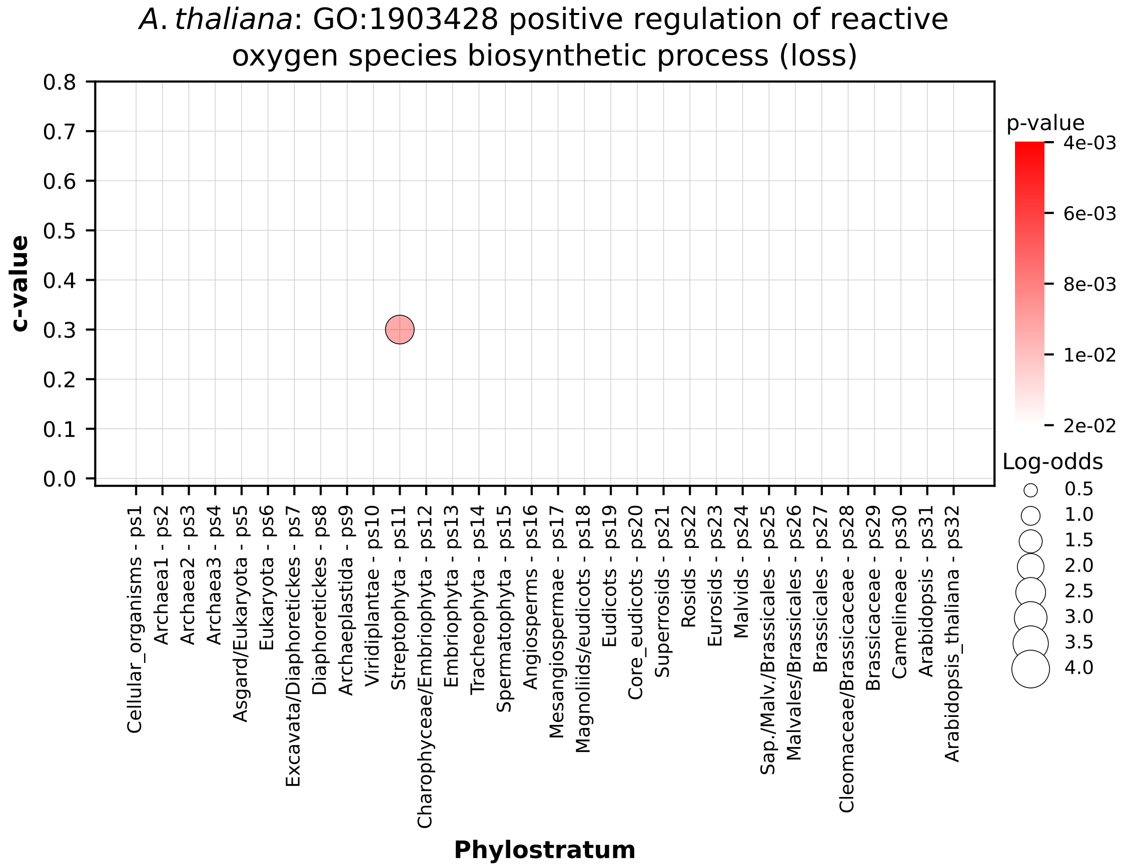
A. thaliana: GO:1903428 positive regulation of reactive oxygen species biosynthetic process (loss)
c-value
0.8
0.7
0.6
0.5
0.4
0.3
0.2
0.1
0.0
p-value
4e-03
6e-03
8e-03
1e-02
2e-02
Log-odds
0.5
1.0
1.5
2.0
2.5
3.0
3.5
4.0
Cellular_organisms - ps1
Archaea1 - ps2
Archaea2 - ps3
Archaea3 - ps4
Asgard/Eukaryota - ps5
Eukaryota - ps6
Excavata/Diaphoretickes - ps7
Diaphoretickes - ps8
Archaeplastida - ps9
Viridiplantae - ps10
Streptophyta - ps11
Charophyceae/Embriophyta - ps12
Embriophyta - ps13
Tracheophyta - ps14
Spermatophyta - ps15
Angiosperms - ps16
Mesangiospermae - ps17
Magnoliids/eudicots - ps18
Eudicots - ps19
Core_eudicots - ps20
Superrosids - ps21
Rosids - ps22
Eurosids - ps23
Malvids - ps24
Sap./Malv./Brassicales - ps25
Malvales/Brassicales - ps26
Brassicales - ps27
Cleomaceae/Brassicaceae - ps28
Brassicaceae - ps29
Camelineae - ps30
Arabidopsis - ps31
Arabidopsis_thaliana - ps32
Phylostratum

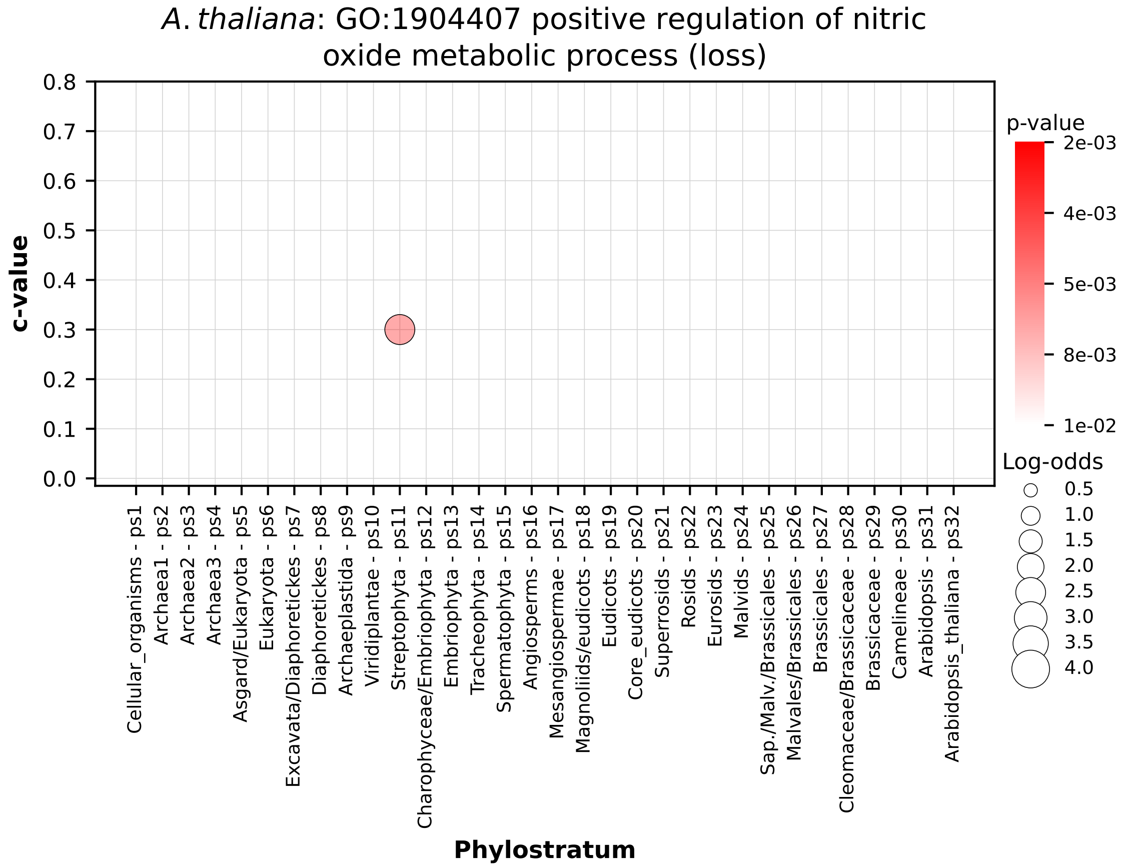

A. thaliana: GO:1904407 positive regulation of nitric oxide metabolic process (loss)
c-value
0.8
0.7
0.6
0.5
0.4
0.3
0.2
0.1
0.0
Phylostratum
Cellular_organisms - ps1
Archaea1 - ps2
Archaea2 - ps3
Archaea3 - ps4
Asgard/Eukaryota - ps5
Eukaryota - ps6
Excavata/Diaphoretickes - ps7
Diaphoretickes - ps8
Archaeplastida - ps9
Viridiplantae - ps10
Streptophyta - ps11
Charophyceae/Embriophyta - ps12
Embriophyta - ps13
Tracheophyta - ps14
Spermatophyta - ps15
Angiosperms - ps16
Mesangiospermae - ps17
Magnoliids/eudicots - ps18
Eudicots - ps19
Core_eudicots - ps20
Superrosids - ps21
Rosids - ps22
Eurosids - ps23
Malvids - ps24
Sap./Malv./Brassicales - ps25
Malvales/Brassicales - ps26
Brassicales - ps27
Cleomaceae/Brassicaceae - ps28
Brassicaceae - ps29
Camelineae - ps30
Arabidopsis - ps31
Arabidopsis_thaliana - ps32
p-value
2e-03
4e-03
5e-03
8e-03
1e-02
Log-odds
0.5
1.0
1.5
2.0
2.5
3.0
3.5
4.0

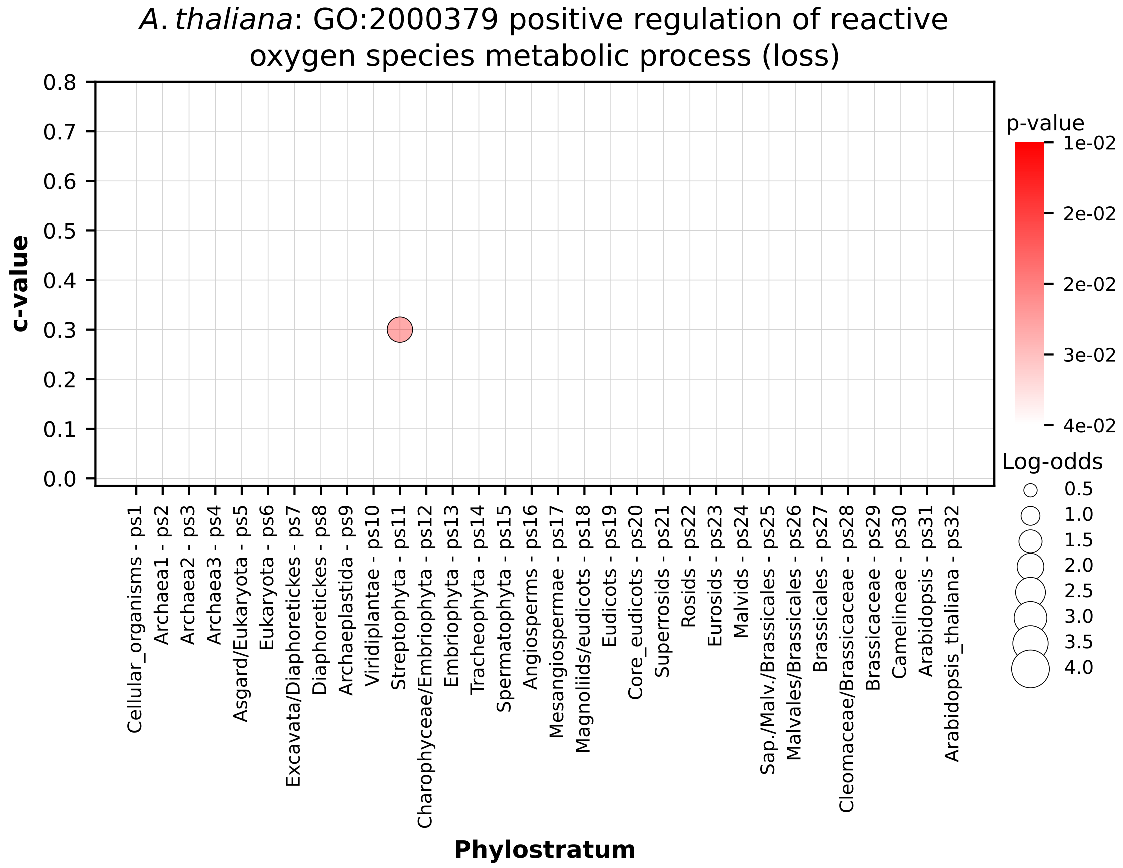

A. thaliana: GO:2000379 positive regulation of reactive oxygen species metabolic process (loss)
c-value
0.8
0.7
0.6
0.5
0.4
0.3
0.2
0.1
0.0
p-value
1e-02
2e-02
2e-02
3e-02
4e-02
Log-odds
0.5
1.0
1.5
2.0
2.5
3.0
3.5
4.0
Cellular_organisms - ps1
Archaea1 - ps2
Archaea2 - ps3
Archaea3 - ps4
Asgard/Eukaryota - ps5
Eukaryota - ps6
Excavata/Diaphoretickes - ps7
Diaphoretickes - ps8
Archaeplastida - ps9
Viridiplantae - ps10
Streptophyta - ps11
Charophyceae/Embriophyta - ps12
Embriophyta - ps13
Tracheophyta - ps14
Spermatophyta - ps15
Angiosperms - ps16
Mesangiospermae - ps17
Magnoliids/eudicots - ps18
Eudicots - ps19
Core_eudicots - ps20
Superrosids - ps21
Rosids - ps22
Eurosids - ps23
Malvids - ps24
Sap./Malv./Brassicales - ps25
Malvales/Brassicales - ps26
Brassicales - ps27
Cleomaceae/Brassicaceae - ps28
Brassicaceae - ps29
Camelineae - ps30
Arabidopsis - ps31
Arabidopsis_thaliana - ps32
Phylostratum

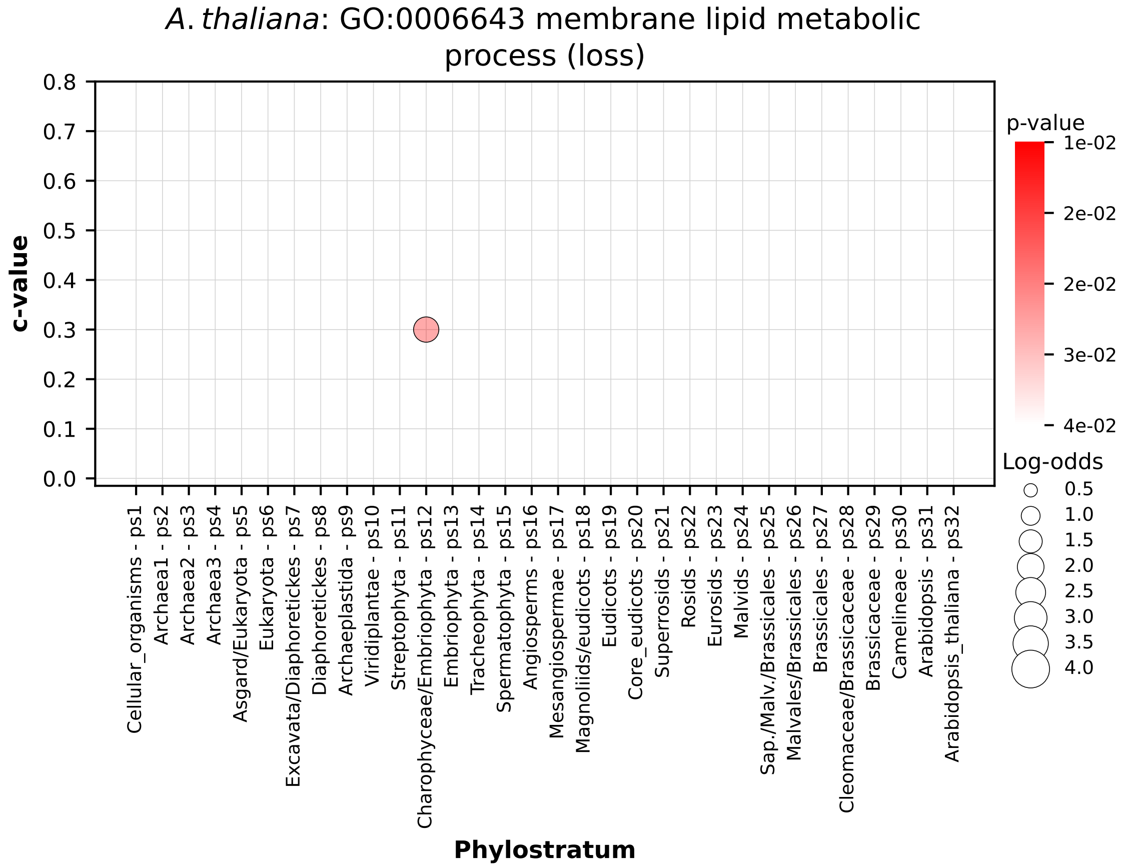
A. thaliana: GO:0006643 membrane lipid metabolic process (loss)
c-value
0.8
0.7
0.6
0.5
0.4
0.3
0.2
0.1
0.0
p-value
1e-02
2e-02
2e-02
3e-02
4e-02
Log-odds
0.5
1.0
1.5
2.0
2.5
3.0
3.5
4.0
Cellular_organisms - ps1
Archaea1 - ps2
Archaea2 - ps3
Archaea3 - ps4
Asgard/Eukaryota - ps5
Eukaryota - ps6
Excavata/Diaphoretickes - ps7
Diaphoretickes - ps8
Archaeplastida - ps9
Viridiplantae - ps10
Streptophyta - ps11
Charophyceae/Embriophyta - ps12
Embriophyta - ps13
Tracheophyta - ps14
Spermatophyta - ps15
Angiosperms - ps16
Mesangiospermae - ps17
Magnoliids/eudicots - ps18
Eudicots - ps19
Core_eudicots - ps20
Superrosids - ps21
Rosids - ps22
Eurosids - ps23
Malvids - ps24
Sap./Malv./Brassicales - ps25
Malvales/Brassicales - ps26
Brassicales - ps27
Cleomaceae/Brassicaceae - ps28
Brassicaceae - ps29
Camelineae - ps30
Arabidopsis - ps31
Arabidopsis_thaliana - ps32
Phylostratum

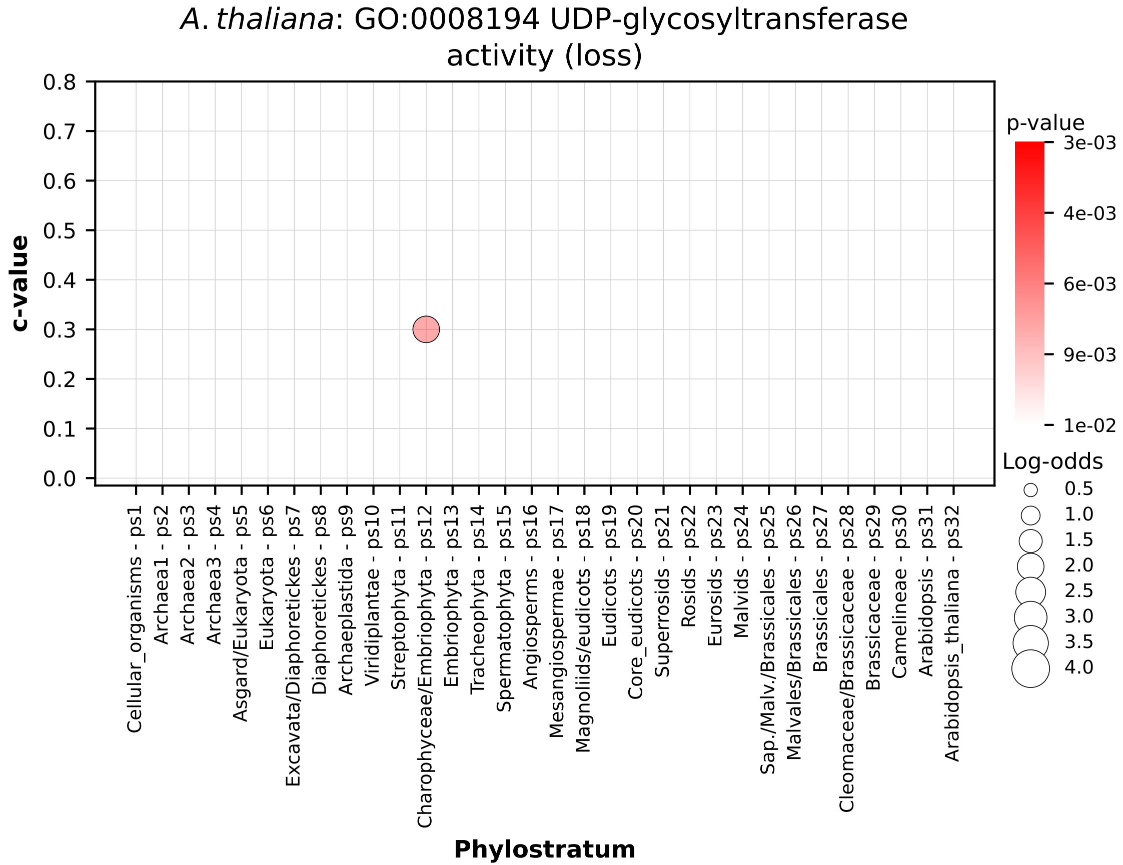
A. thaliana: GO:0008194 UDP-glycosyltransferase activity (loss)
c-value
0.8
0.7
0.6
0.5
0.4
0.3
0.2
0.1
0.0
p-value
3e-03
4e-03
6e-03
9e-03
1e-02
Log-odds
0.5
1.0
1.5
2.0
2.5
3.0
3.5
4.0
Cellular_organisms - ps1
Archaea1 - ps2
Archaea2 - ps3
Archaea3 - ps4
Asgard/Eukaryota - ps5
Eukaryota - ps6
Excavata/Diaphoretickes - ps7
Diaphoretickes - ps8
Archaeplastida - ps9
Viridiplantae - ps10
Streptophyta - ps11
Charophyceae/Embriophyta - ps12
Embriophyta - ps13
Tracheophyta - ps14
Spermatophyta - ps15
Angiosperms - ps16
Mesangiospermae - ps17
Magnoliids/eudicots - ps18
Eudicots - ps19
Core_eudicots - ps20
Superrosids - ps21
Rosids - ps22
Eurosids - ps23
Malvids - ps24
Sap./Malv./Brassicales - ps25
Malvales/Brassicales - ps26
Brassicales - ps27
Cleomaceae/Brassicaceae - ps28
Brassicaceae - ps29
Camelineae - ps30
Arabidopsis - ps31
Arabidopsis_thaliana - ps32
Phylostratum

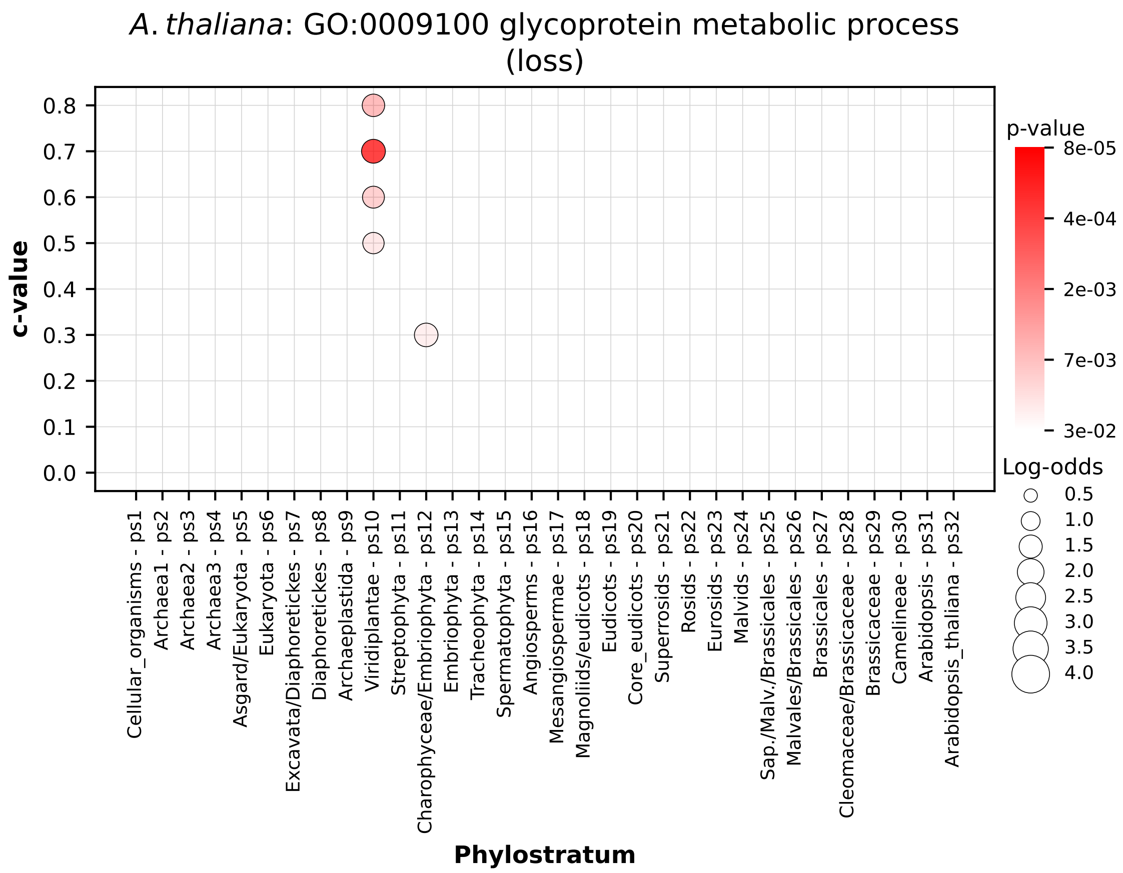

A. thaliana: GO:0009100 glycoprotein metabolic process
(loss)
c-value
0.8
0.7
0.6
0.5
0.4
0.3
0.2
0.1
0.0
p-value
8e-05
4e-04
2e-03
7e-03
3e-02
Log-odds
0.5
1.0
1.5
2.0
2.5
3.0
3.5
4.0
Cellular_organisms - ps1
Archaea1 - ps2
Archaea2 - ps3
Archaea3 - ps4
Asgard/Eukaryota - ps5
Eukaryota - ps6
Excavata/Diaphoretickes - ps7
Diaphoretickes - ps8
Archaeplastida - ps9
Viridiplantae - ps10
Streptophyta - ps11
Charophyceae/Embriophyta - ps12
Embriophyta - ps13
Tracheophyta - ps14
Spermatophyta - ps15
Angiosperms - ps16
Mesangiospermae - ps17
Magnoliids/eudicots - ps18
Eudicots - ps19
Core_eudicots - ps20
Superrosids - ps21
Rosids - ps22
Eurosids - ps23
Malvids - ps24
Sap./Malv./Brassicales - ps25
Malvales/Brassicales - ps26
Brassicales - ps27
Cleomaceae/Brassicaceae - ps28
Brassicaceae - ps29
Camelineae - ps30
Arabidopsis - ps31
Arabidopsis_thaliana - ps32
Phylostratum

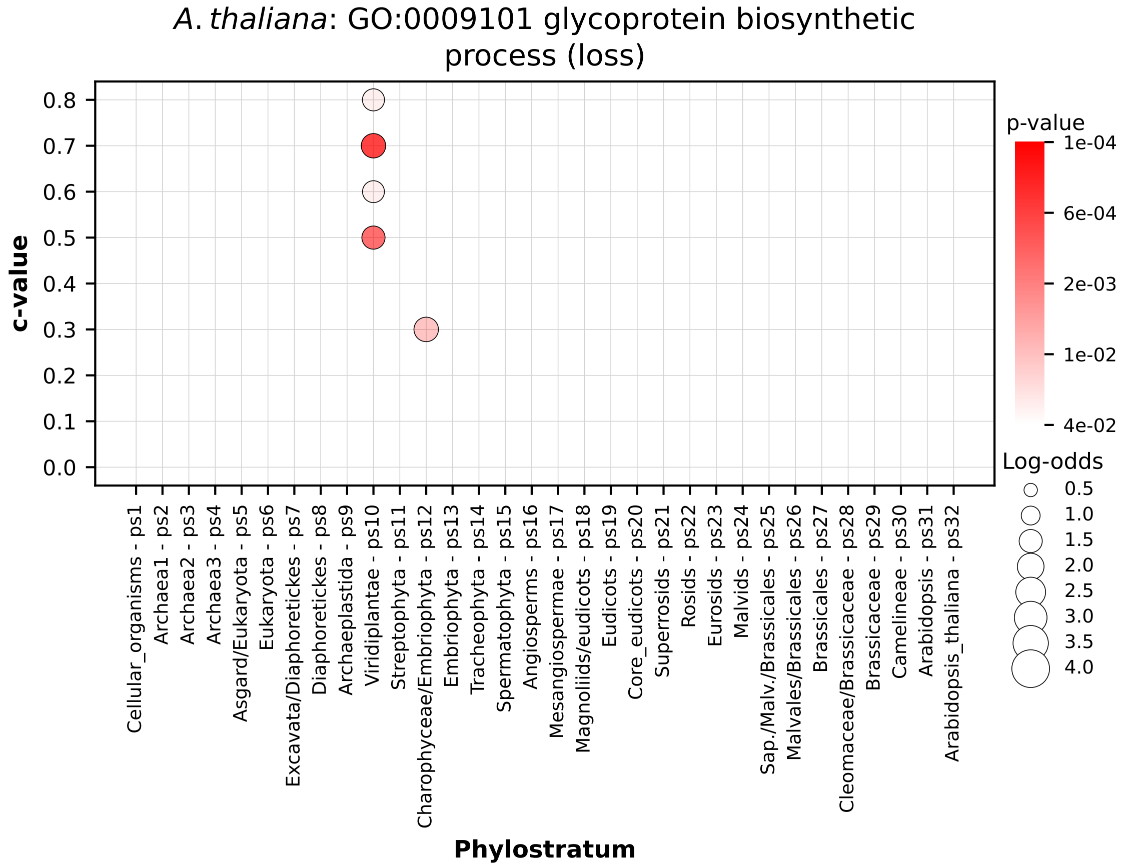

A. thaliana: GO:0009101 glycoprotein biosynthetic process (loss)
c-value
0.8
0.7
0.6
0.5
0.4
0.3
0.2
0.1
0.0
Phylostratum
Cellular_organisms - ps1
Archaea1 - ps2
Archaea2 - ps3
Archaea3 - ps4
Asgard/Eukaryota - ps5
Eukaryota - ps6
Excavata/Diaphoretickes - ps7
Diaphoretickes - ps8
Archaeplastida - ps9
Viridiplantae - ps10
Streptophyta - ps11
Charophyceae/Embriophyta - ps12
Embriophyta - ps13
Tracheophyta - ps14
Spermatophyta - ps15
Angiosperms - ps16
Mesangiospermae - ps17
Magnoliids/eudicots - ps18
Eudicots - ps19
Core_eudicots - ps20
Superrosids - ps21
Rosids - ps22
Eurosids - ps23
Malvids - ps24
Sap./Malv./Brassicales - ps25
Malvales/Brassicales - ps26
Brassicales - ps27
Cleomaceae/Brassicaceae - ps28
Brassicaceae - ps29
Camelineae - ps30
Arabidopsis - ps31
Arabidopsis_thaliana - ps32
p-value
1e-04
6e-04
2e-03
1e-02
4e-02
Log-odds
0.5
1.0
1.5
2.0
2.5
3.0
3.5
4.0

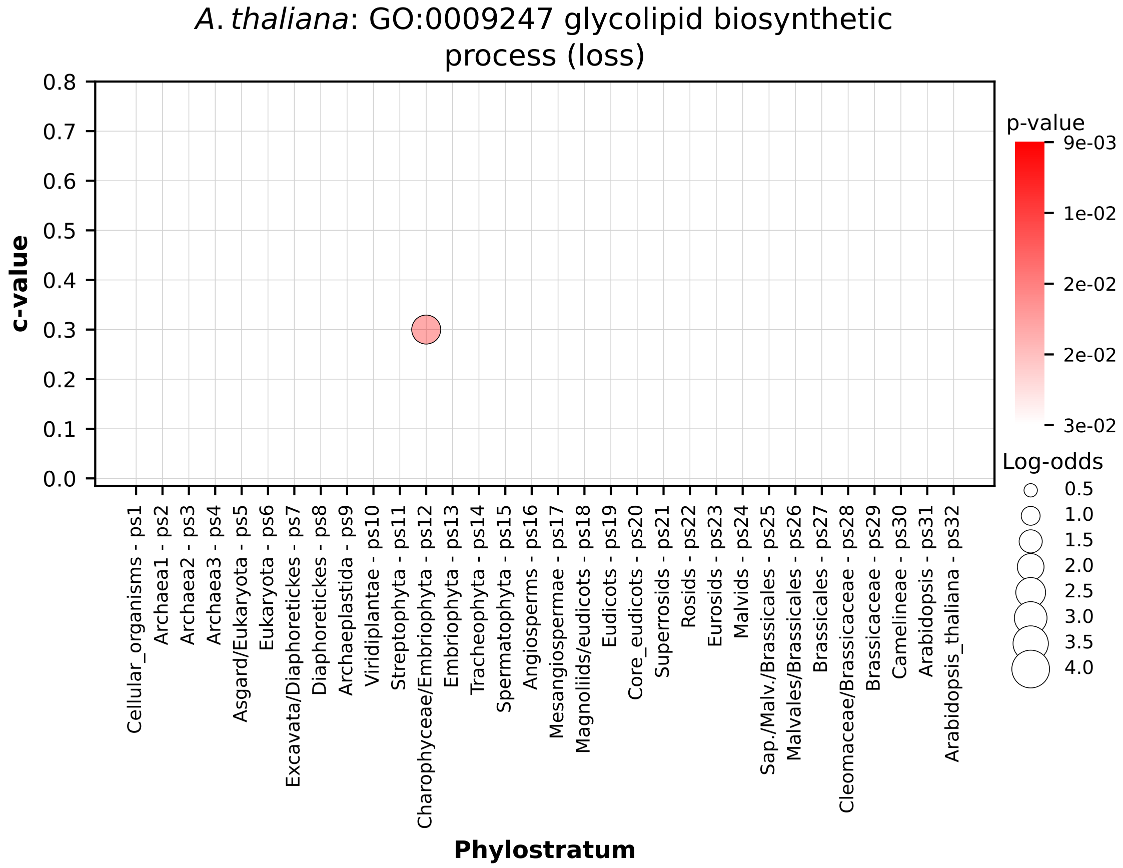

A. thaliana: GO:0009247 glycolipid biosynthetic process (loss)
c-value
0.8
0.7
0.6
0.5
0.4
0.3
0.2
0.1
0.0
Phylostratum
Cellular_organisms - ps1
Archaea1 - ps2
Archaea2 - ps3
Archaea3 - ps4
Asgard/Eukaryota - ps5
Eukaryota - ps6
Excavata/Diaphoretickes - ps7
Diaphoretickes - ps8
Archaeplastida - ps9
Viridiplantae - ps10
Streptophyta - ps11
Charophyceae/Embriophyta - ps12
Embriophyta - ps13
Tracheophyta - ps14
Spermatophyta - ps15
Angiosperms - ps16
Mesangiospermae - ps17
Magnoliids/eudicots - ps18
Eudicots - ps19
Core_eudicots - ps20
Superrosids - ps21
Rosids - ps22
Eurosids - ps23
Malvids - ps24
Sap./Malv./Brassicales - ps25
Malvales/Brassicales - ps26
Brassicales - ps27
Cleomaceae/Brassicaceae - ps28
Brassicaceae - ps29
Camelineae - ps30
Arabidopsis - ps31
Arabidopsis_thaliana - ps32
p-value
9e-03
1e-02
2e-02
2e-02
3e-02
Log-odds
0.5
1.0
1.5
2.0
2.5
3.0
3.5
4.0

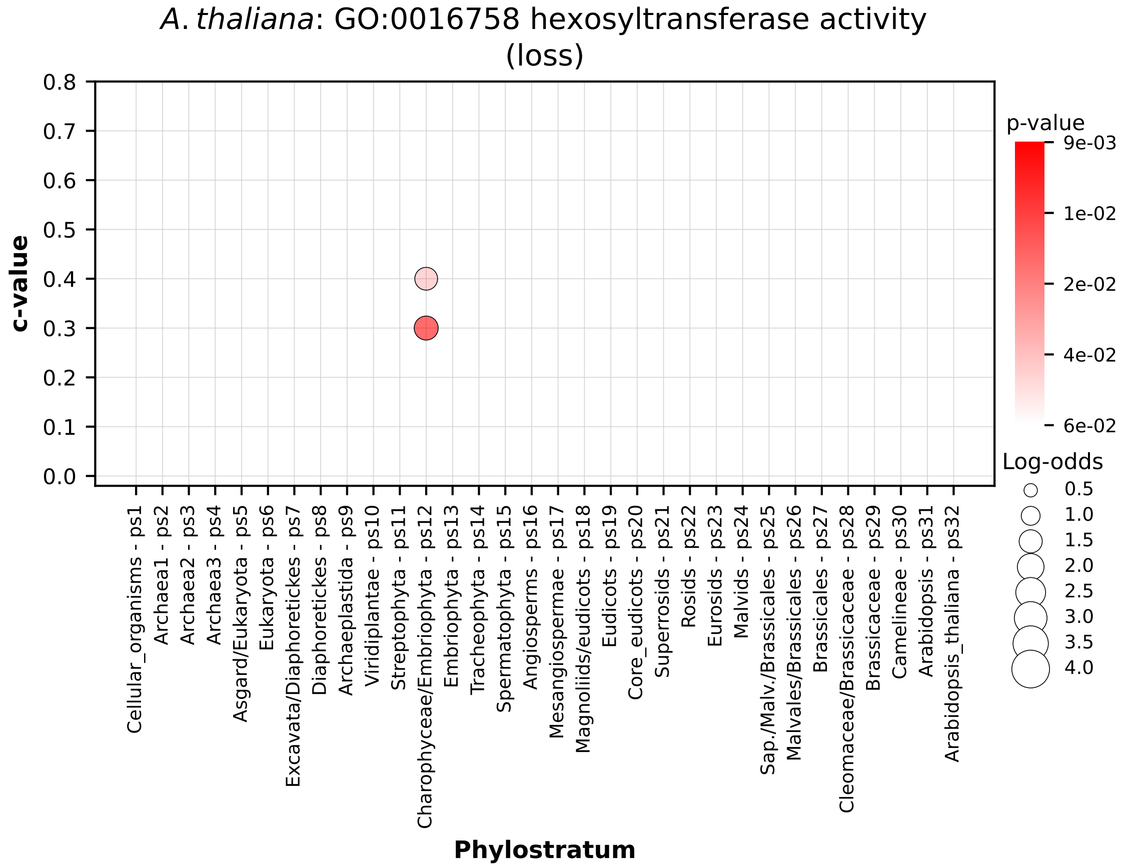

A. thaliana: GO:0016758 hexosyltransferase activity (loss)
c-value
0.8
0.7
0.6
0.5
0.4
0.3
0.2
0.1
0.0
Phylostratum
Cellular_organisms - ps1
Archaea1 - ps2
Archaea2 - ps3
Archaea3 - ps4
Asgard/Eukaryota - ps5
Eukaryota - ps6
Excavata/Diaphoretickes - ps7
Diaphoretickes - ps8
Archaeplastida - ps9
Viridiplantae - ps10
Streptophyta - ps11
Charophyceae/Embriophyta - ps12
Embriophyta - ps13
Tracheophyta - ps14
Spermatophyta - ps15
Angiosperms - ps16
Mesangiospermae - ps17
Magnoliids/eudicots - ps18
Eudicots - ps19
Core_eudicots - ps20
Superrosids - ps21
Rosids - ps22
Eurosids - ps23
Malvids - ps24
Sap./Malv./Brassicales - ps25
Malvales/Brassicales - ps26
Brassicales - ps27
Cleomaceae/Brassicaceae - ps28
Brassicaceae - ps29
Camelineae - ps30
Arabidopsis - ps31
Arabidopsis_thaliana - ps32
p-value
9e-03
1e-02
2e-02
4e-02
6e-02
Log-odds
0.5
1.0
1.5
2.0
2.5
3.0
3.5
4.0

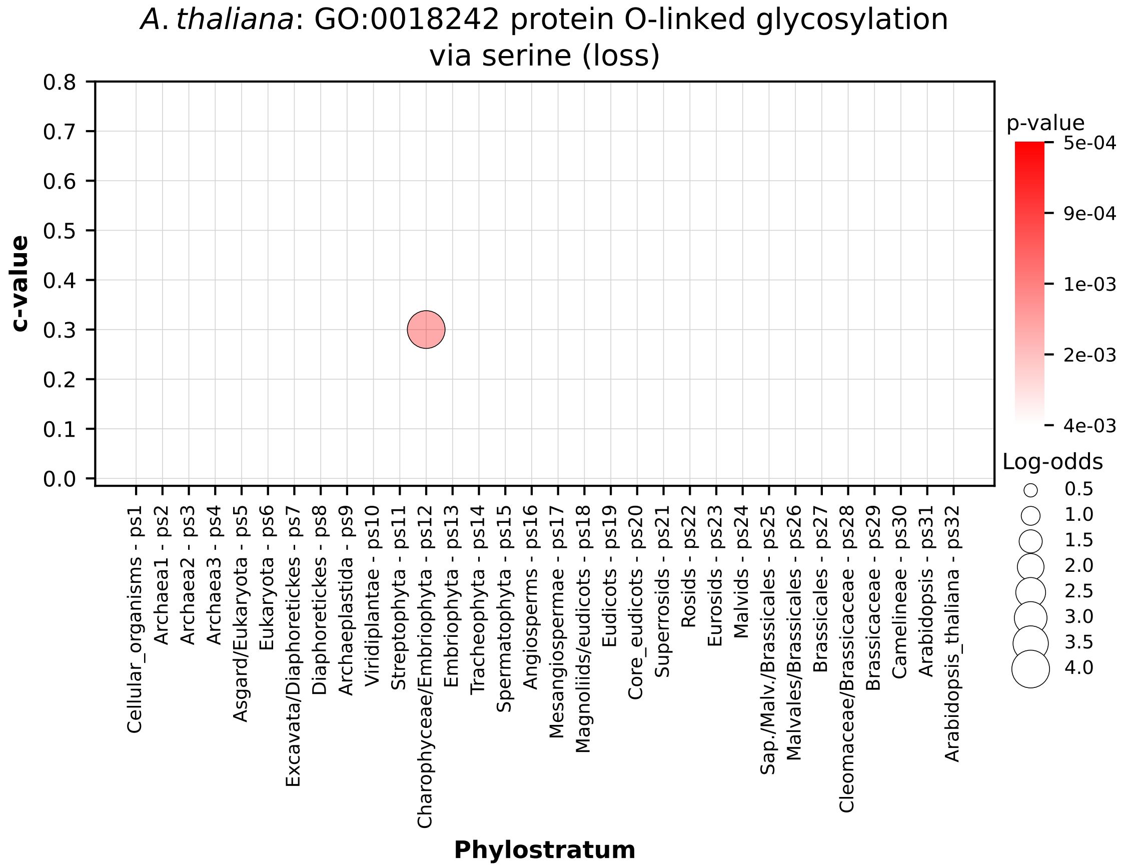
A. thaliana: GO:0018242 protein O-linked glycosylation via serine (loss)
c-value
0.8
0.7
0.6
0.5
0.4
0.3
0.2
0.1
0.0
Phylostratum
Cellular_organisms - ps1
Archaea1 - ps2
Archaea2 - ps3
Archaea3 - ps4
Asgard/Eukaryota - ps5
Eukaryota - ps6
Excavata/Diaphoretickes - ps7
Diaphoretickes - ps8
Archaeplastida - ps9
Viridiplantae - ps10
Streptophyta - ps11
Charophyceae/Embriophyta - ps12
Embriophyta - ps13
Tracheophyta - ps14
Spermatophyta - ps15
Angiosperms - ps16
Mesangiospermae - ps17
Magnoliids/eudicots - ps18
Eudicots - ps19
Core_eudicots - ps20
Superrosids - ps21
Rosids - ps22
Eurosids - ps23
Malvids - ps24
Sap./Malv./Brassicales - ps25
Malvales/Brassicales - ps26
Brassicales - ps27
Cleomaceae/Brassicaceae - ps28
Brassicaceae - ps29
Camelineae - ps30
Arabidopsis - ps31
Arabidopsis_thaliana - ps32
p-value
5e-04
9e-04
1e-03
2e-03
4e-03
Log-odds
0.5
1.0
1.5
2.0
2.5
3.0
3.5
4.0

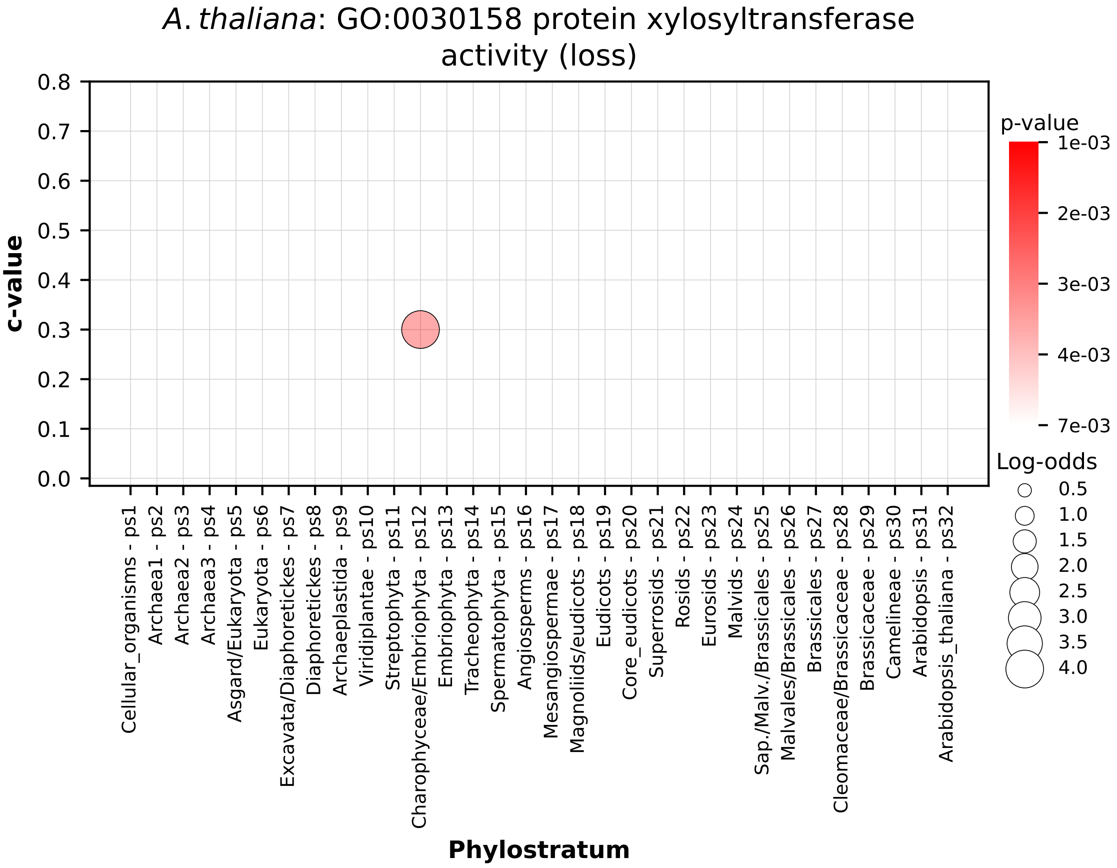
A. thaliana: GO:0030158 protein xylosyltransferase activity (loss)
c-value
0.0
0.1
0.2
0.3
0.4
0.5
0.6
0.7
0.8
Phylostratum
Cellular_organisms - ps1
Archaea1 - ps2
Archaea2 - ps3
Archaea3 - ps4
Asgard/Eukaryota - ps5
Eukaryota - ps6
Excavata/Diaphoretickes - ps7
Diaphoretickes - ps8
Archaeplastida - ps9
Viridiplantae - ps10
Streptophyta - ps11
Charophyceae/Embriophyta - ps12
Embriophyta - ps13
Tracheophyta - ps14
Spermatophyta - ps15
Angiosperms - ps16
Mesangiospermae - ps17
Magnoliids/eudicots - ps18
Eudicots - ps19
Core_eudicots - ps20
Superrosids - ps21
Rosids - ps22
Eurosids - ps23
Malvids - ps24
Sap./Malv./Brassicales - ps25
Malvales/Brassicales - ps26
Brassicales - ps27
Cleomaceae/Brassicaceae - ps28
Brassicaceae - ps29
Camelineae - ps30
Arabidopsis - ps31
Arabidopsis_thaliana - ps32
p-value
1e-03
2e-03
3e-03
4e-03
7e-03
Log-odds
0.5
1.0
1.5
2.0
2.5
3.0
3.5
4.0

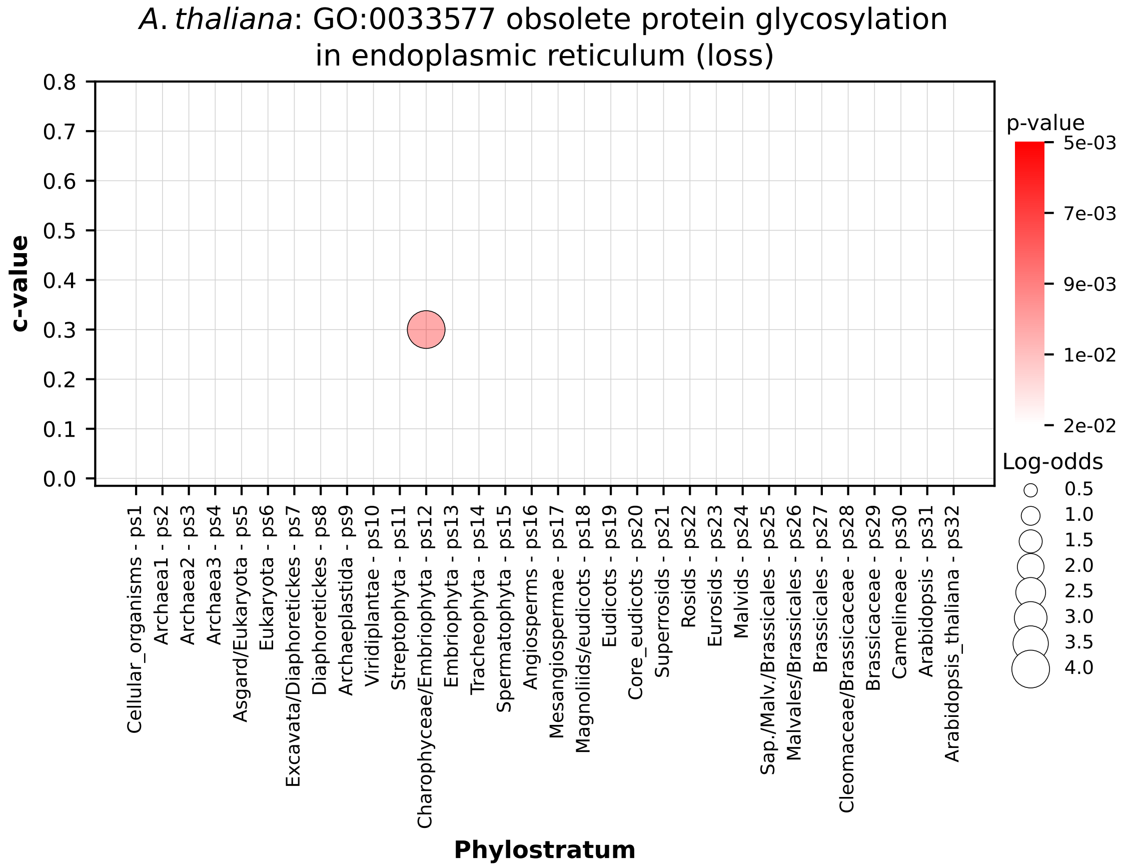

A. thaliana: GO:0033577 obsolete protein glycosylation in endoplasmic reticulum (loss)
c-value
0.8
0.7
0.6
0.5
0.4
0.3
0.2
0.1
0.0
Phylostratum
Cellular_organisms - ps1
Archaea1 - ps2
Archaea2 - ps3
Archaea3 - ps4
Asgard/Eukaryota - ps5
Eukaryota - ps6
Excavata/Diaphoretickes - ps7
Diaphoretickes - ps8
Archaeplastida - ps9
Viridiplantae - ps10
Streptophyta - ps11
Charophyceae/Embriophyta - ps12
Embriophyta - ps13
Tracheophyta - ps14
Spermatophyta - ps15
Angiosperms - ps16
Mesangiospermae - ps17
Magnoliids/eudicots - ps18
Eudicots - ps19
Core_eudicots - ps20
Superrosids - ps21
Rosids - ps22
Eurosids - ps23
Malvids - ps24
Sap./Malv./Brassicales - ps25
Malvales/Brassicales - ps26
Brassicales - ps27
Cleomaceae/Brassicaceae - ps28
Brassicaceae - ps29
Camelineae - ps30
Arabidopsis - ps31
Arabidopsis_thaliana - ps32
p-value
5e-03
7e-03
9e-03
1e-02
2e-02
Log-odds
0.5
1.0
1.5
2.0
2.5
3.0
3.5
4.0

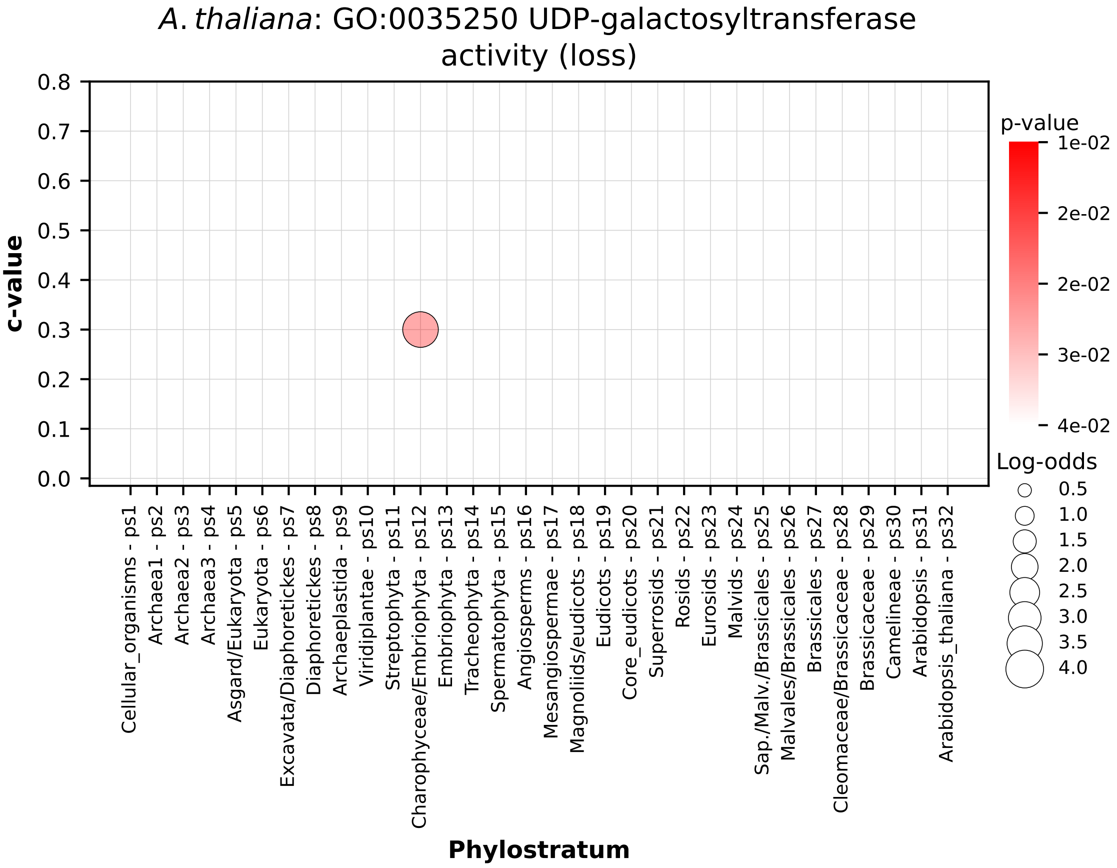
A. thaliana: GO:0035250 UDP-galactosyltransferase activity (loss)
c-value
0.8
0.7
0.6
0.5
0.4
0.3
0.2
0.1
0.0
Phylostratum
Cellular_organisms - ps1
Archaea1 - ps2
Archaea2 - ps3
Archaea3 - ps4
Asgard/Eukaryota - ps5
Eukaryota - ps6
Excavata/Diaphoretickes - ps7
Diaphoretickes - ps8
Archaeplastida - ps9
Viridiplantae - ps10
Streptophyta - ps11
Charophyceae/Embriophyta - ps12
Embriophyta - ps13
Tracheophyta - ps14
Spermatophyta - ps15
Angiosperms - ps16
Mesangiospermae - ps17
Magnoliids/eudicots - ps18
Eudicots - ps19
Core_eudicots - ps20
Superrosids - ps21
Rosids - ps22
Eurosids - ps23
Malvids - ps24
Sap./Malv./Brassicales - ps25
Malvales/Brassicales - ps26
Brassicales - ps27
Cleomaceae/Brassicaceae - ps28
Brassicaceae - ps29
Camelineae - ps30
Arabidopsis - ps31
Arabidopsis_thaliana - ps32
p-value
1e-02
2e-02
2e-02
3e-02
4e-02
Log-odds
0.5
1.0
1.5
2.0
2.5
3.0
3.5
4.0

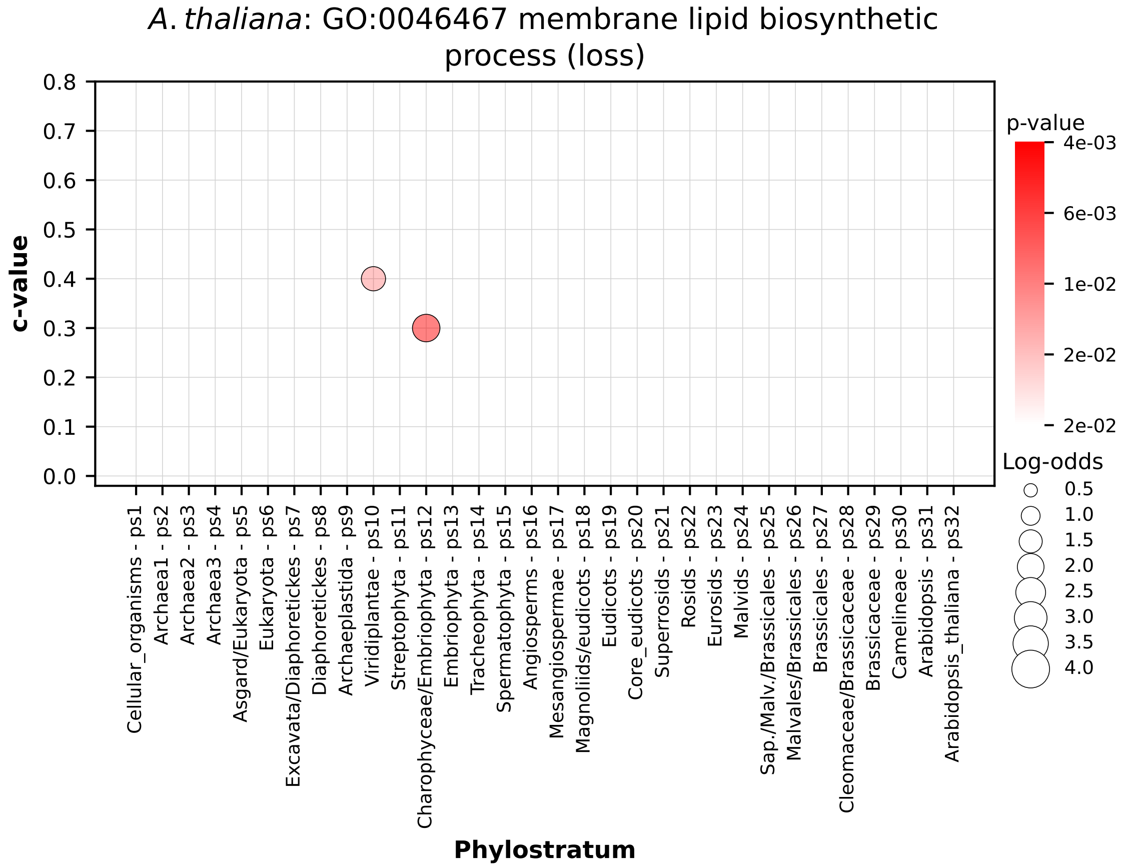

A. thaliana: GO:0046467 membrane lipid biosynthetic process (loss)
c-value
0.8
0.7
0.6
0.5
0.4
0.3
0.2
0.1
0.0
Phylostratum
Cellular_organisms - ps1
Archaea1 - ps2
Archaea2 - ps3
Archaea3 - ps4
Asgard/Eukaryota - ps5
Eukaryota - ps6
Excavata/Diaphoretickes - ps7
Diaphoretickes - ps8
Archaeplastida - ps9
Viridiplantae - ps10
Streptophyta - ps11
Charophyceae/Embriophyta - ps12
Embriophyta - ps13
Tracheophyta - ps14
Spermatophyta - ps15
Angiosperms - ps16
Mesangiospermae - ps17
Magnoliids/eudicots - ps18
Eudicots - ps19
Core_eudicots - ps20
Superrosids - ps21
Rosids - ps22
Eurosids - ps23
Malvids - ps24
Sap./Malv./Brassicales - ps25
Malvales/Brassicales - ps26
Brassicales - ps27
Cleomaceae/Brassicaceae - ps28
Brassicaceae - ps29
Camelineae - ps30
Arabidopsis - ps31
Arabidopsis_thaliana - ps32
p-value
4e-03
6e-03
1e-02
2e-02
2e-02
Log-odds
0.5
1.0
1.5
2.0
2.5
3.0
3.5
4.0

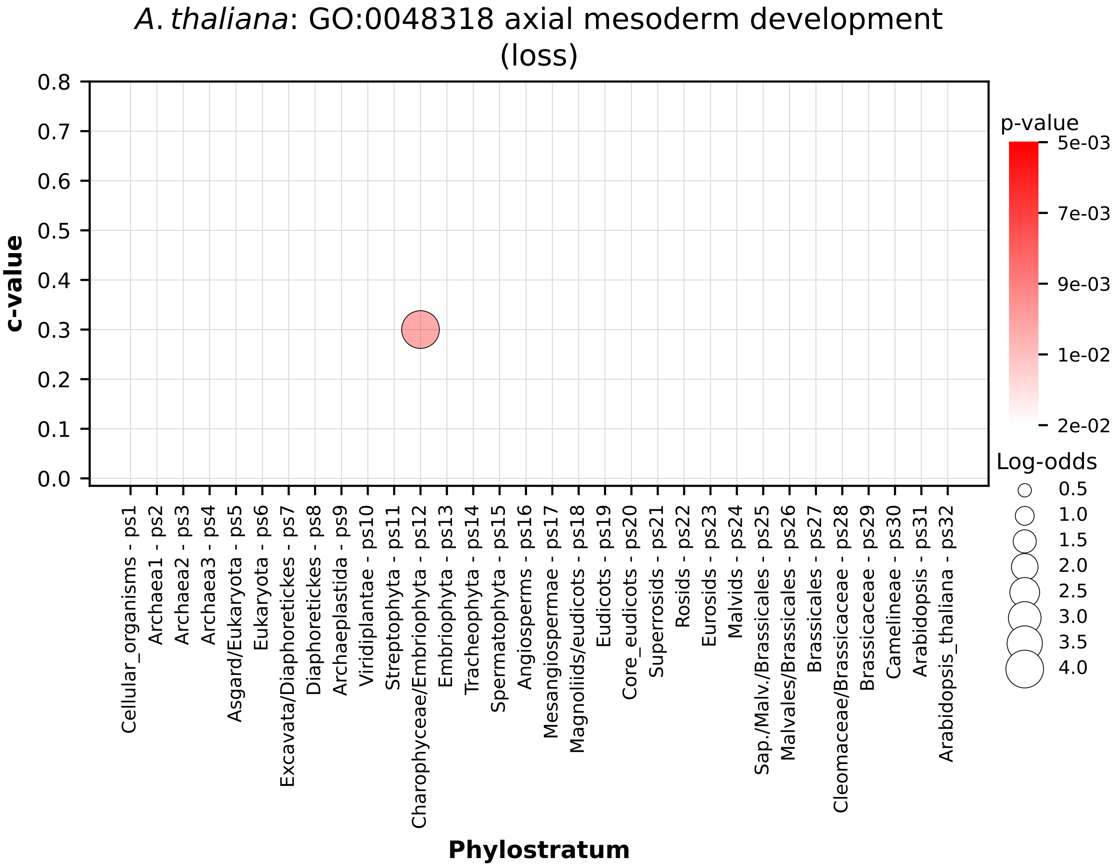

A. thaliana: GO:0048318 axial mesoderm development
(loss)
c-value
0.0
0.1
0.2
0.3
0.4
0.5
0.6
0.7
0.8
Phylostratum
Cellular_organisms - ps1
Archaea1 - ps2
Archaea2 - ps3
Archaea3 - ps4
Asgard/Eukaryota - ps5
Eukaryota - ps6
Excavata/Diaphoretickes - ps7
Diaphoretickes - ps8
Archaeplastida - ps9
Viridiplantae - ps10
Streptophyta - ps11
Charophyceae/Embriophyta - ps12
Embriophyta - ps13
Tracheophyta - ps14
Spermatophyta - ps15
Angiosperms - ps16
Mesangiospermae - ps17
Magnoliids/eudicots - ps18
Eudicots - ps19
Core_eudicots - ps20
Superrosids - ps21
Rosids - ps22
Eurosids - ps23
Malvids - ps24
Sap./Malv./Brassicales - ps25
Malvales/Brassicales - ps26
Brassicales - ps27
Cleomaceae/Brassicaceae - ps28
Brassicaceae - ps29
Camelineae - ps30
Arabidopsis - ps31
Arabidopsis_thaliana - ps32
p-value
5e-03
7e-03
9e-03
1e-02
2e-02
Log-odds
0.5
1.0
1.5
2.0
2.5
3.0
3.5
4.0

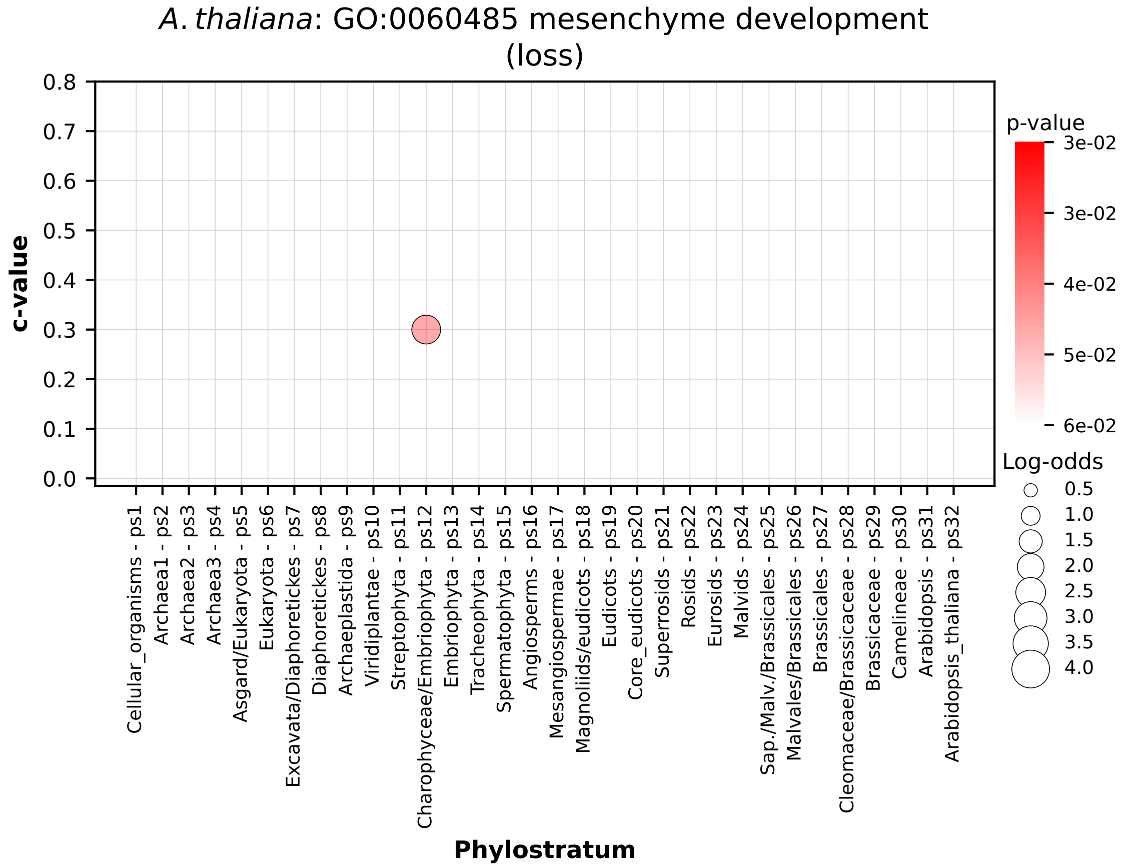

$A. thaliana$: GO:0060485 mesenchyme development
(loss)
c-value
0.0
0.1
0.2
0.3
0.4
0.5
0.6
0.7
0.8
Phylostratum
Cellular_organisms - ps1
Archaea1 - ps2
Archaea2 - ps3
Archaea3 - ps4
Asgard/Eukaryota - ps5
Eukaryota - ps6
Excavata/Diaphoretickes - ps7
Diaphoretickes - ps8
Archaeplastida - ps9
Viridiplantae - ps10
Streptophyta - ps11
Charophyceae/Embriophyta - ps12
Embriophyta - ps13
Tracheophyta - ps14
Spermatophyta - ps15
Angiosperms - ps16
Mesangiospermae - ps17
Magnoliids/eudicots - ps18
Eudicots - ps19
Core_eudicots - ps20
Superrosids - ps21
Rosids - ps22
Eurosids - ps23
Malvids - ps24
Sap./Malv./Brassicales - ps25
Malvales/Brassicales - ps26
Brassicales - ps27
Cleomaceae/Brassicaceae - ps28
Brassicaceae - ps29
Camelineae - ps30
Arabidopsis - ps31
Arabidopsis_thaliana - ps32
p-value
3e-02
3e-02
4e-02
5e-02
6e-02
Log-odds
0.5
1.0
1.5
2.0
2.5
3.0
3.5
4.0

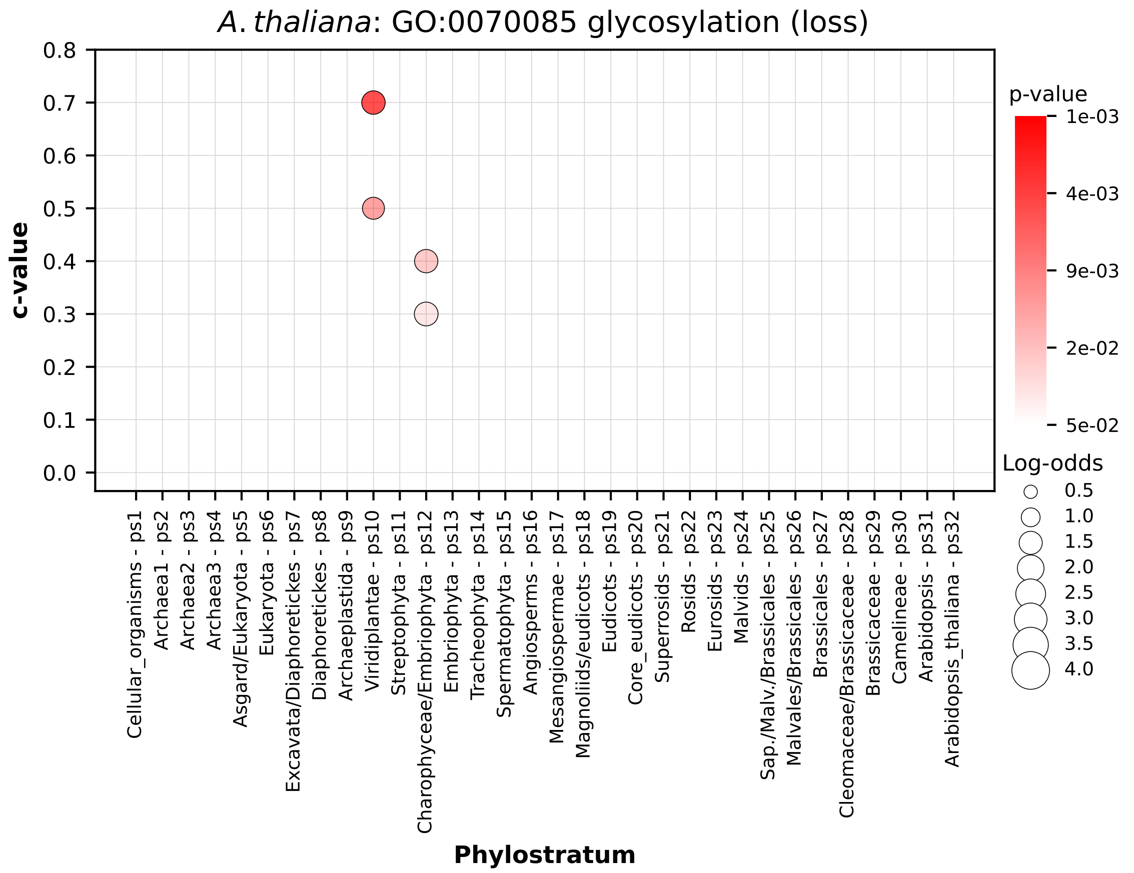

A. thaliana: GO:0070085 glycosylation (loss)
c-value
0.8
0.7
0.6
0.5
0.4
0.3
0.2
0.1
0.0
Phylostratum
Cellular_organisms - ps1
Archaea1 - ps2
Archaea2 - ps3
Archaea3 - ps4
Asgard/Eukaryota - ps5
Eukaryota - ps6
Excavata/Diaphoretickes - ps7
Diaphoretickes - ps8
Archaeplastida - ps9
Viridiplantae - ps10
Streptophyta - ps11
Charophyceae/Embriophyta - ps12
Embriophyta - ps13
Tracheophyta - ps14
Spermatophyta - ps15
Angiosperms - ps16
Mesangiospermae - ps17
Magnoliids/eudicots - ps18
Eudicots - ps19
Core_eudicots - ps20
Superrosids - ps21
Rosids - ps22
Eurosids - ps23
Malvids - ps24
Sap./Malv./Brassicales - ps25
Malvales/Brassicales - ps26
Brassicales - ps27
Cleomaceae/Brassicaceae - ps28
Brassicaceae - ps29
Camelineae - ps30
Arabidopsis - ps31
Arabidopsis_thaliana - ps32
p-value
1e-03
4e-03
9e-03
2e-02
5e-02
Log-odds
0.5
1.0
1.5
2.0
2.5
3.0
3.5
4.0

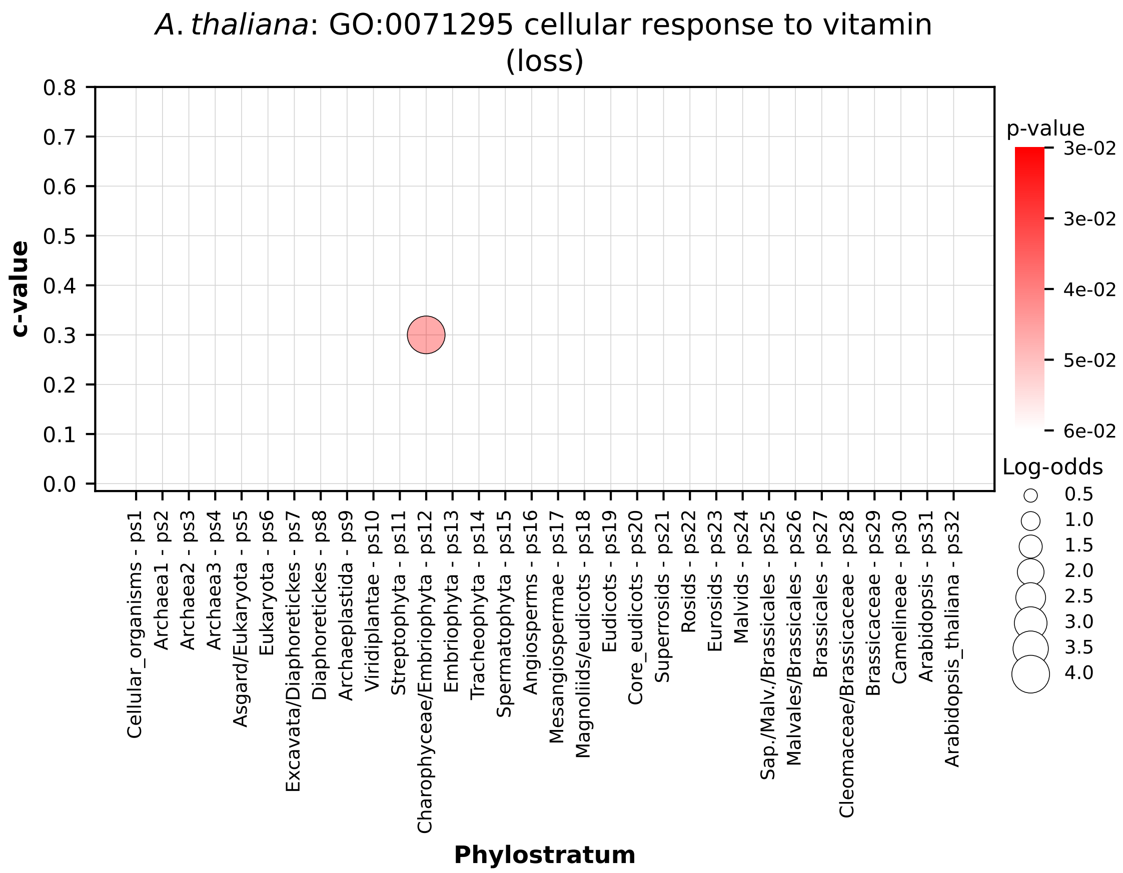

A. thaliana: GO:0071295 cellular response to vitamin (loss)
c-value
0.0
0.1
0.2
0.3
0.4
0.5
0.6
0.7
0.8
Phylostratum
Cellular_organisms - ps1
Archaea1 - ps2
Archaea2 - ps3
Archaea3 - ps4
Asgard/Eukaryota - ps5
Eukaryota - ps6
Excavata/Diaphoretickes - ps7
Diaphoretickes - ps8
Archaeplastida - ps9
Viridiplantae - ps10
Streptophyta - ps11
Charophyceae/Embriophyta - ps12
Embriophyta - ps13
Tracheophyta - ps14
Spermatophyta - ps15
Angiosperms - ps16
Mesangiospermae - ps17
Magnoliids/eudicots - ps18
Eudicots - ps19
Core_eudicots - ps20
Superrosids - ps21
Rosids - ps22
Eurosids - ps23
Malvids - ps24
Sap./Malv./Brassicales - ps25
Malvales/Brassicales - ps26
Brassicales - ps27
Cleomaceae/Brassicaceae - ps28
Brassicaceae - ps29
Camelineae - ps30
Arabidopsis - ps31
Arabidopsis_thaliana - ps32
p-value
3e-02
3e-02
4e-02
5e-02
6e-02
Log-odds
0.5
1.0
1.5
2.0
2.5
3.0
3.5
4.0

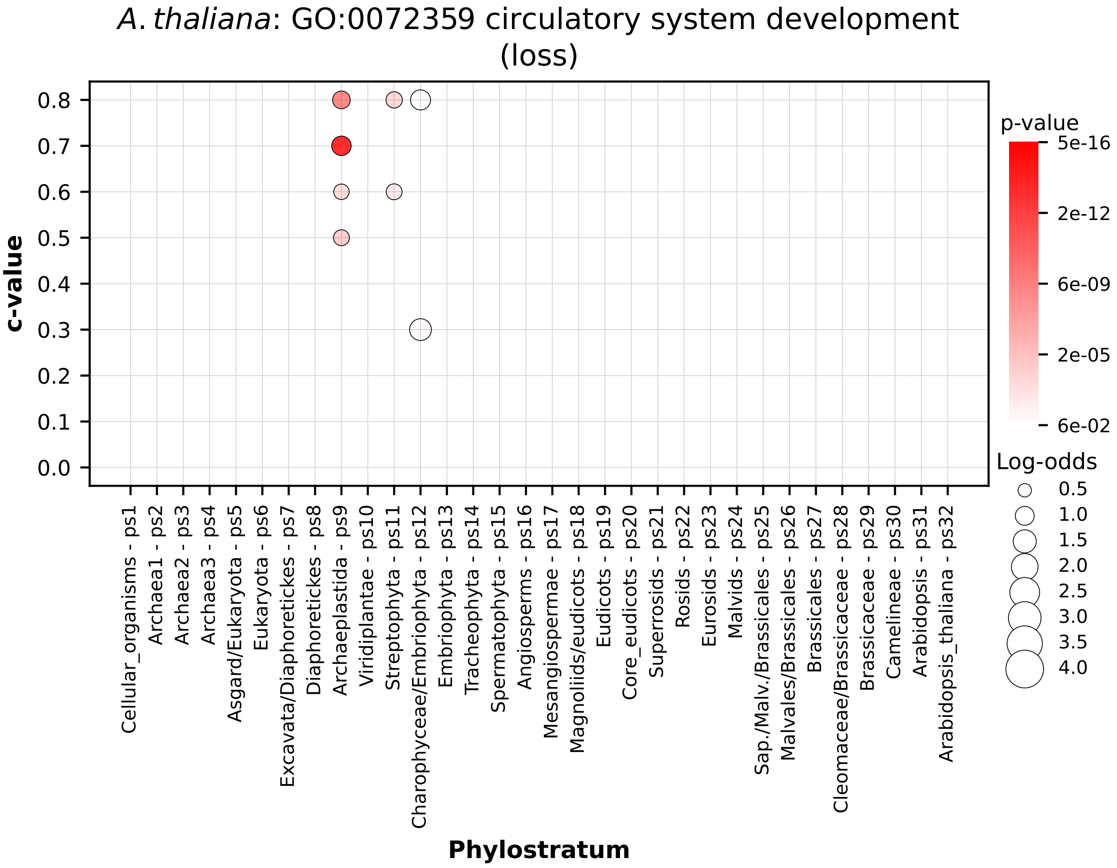
A. thaliana: GO:0072359 circulatory system development (loss)
c-value
0.8
0.7
0.6
0.5
0.4
0.3
0.2
0.1
0.0
Phylostratum
Cellular_organisms - ps1
Archaea1 - ps2
Archaea2 - ps3
Archaea3 - ps4
Asgard/Eukaryota - ps5
Eukaryota - ps6
Excavata/Diaphoretickes - ps7
Diaphoretickes - ps8
Archaeplastida - ps9
Viridiplantae - ps10
Streptophyta - ps11
Charophyceae/Embriophyta - ps12
Embriophyta - ps13
Tracheophyta - ps14
Spermatophyta - ps15
Angiosperms - ps16
Mesangiospermae - ps17
Magnoliids/eudicots - ps18
Eudicots - ps19
Core_eudicots - ps20
Superrosids - ps21
Rosids - ps22
Eurosids - ps23
Malvids - ps24
Sap./Malv./Brassicales - ps25
Malvales/Brassicales - ps26
Brassicales - ps27
Cleomaceae/Brassicaceae - ps28
Brassicaceae - ps29
Camelineae - ps30
Arabidopsis - ps31
Arabidopsis_thaliana - ps32
p-value
5e-16
2e-12
6e-09
2e-05
6e-02
Log-odds
0.5
1.0
1.5
2.0
2.5
3.0
3.5
4.0

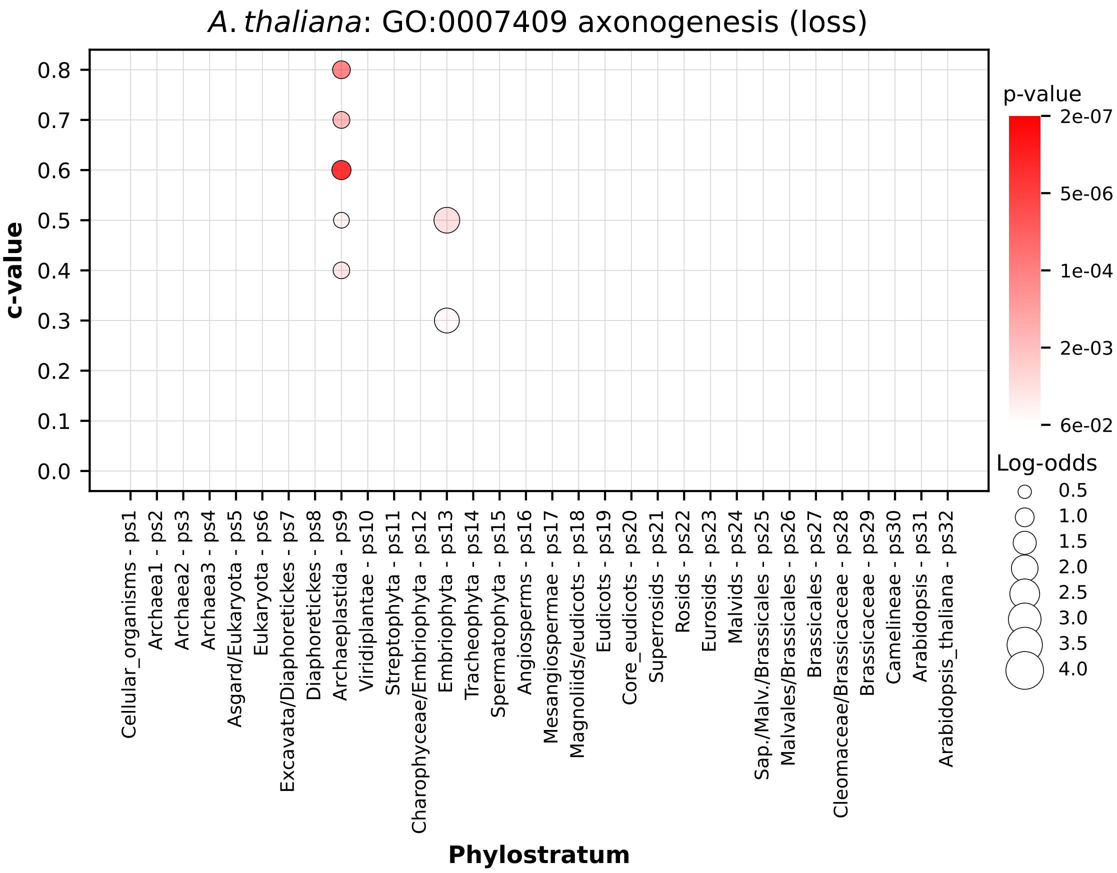

A. thaliana: GO:0007409 axonogenesis (loss)
c-value
0.8
0.7
0.6
0.5
0.4
0.3
0.2
0.1
0.0
Phylostratum
Cellular_organisms - ps1
Archaea1 - ps2
Archaea2 - ps3
Archaea3 - ps4
Asgard/Eukaryota - ps5
Eukaryota - ps6
Excavata/Diaphoretickes - ps7
Diaphoretickes - ps8
Archaeplastida - ps9
Viridiplantae - ps10
Streptophyta - ps11
Charophyceae/Embriophyta - ps12
Embriophyta - ps13
Tracheophyta - ps14
Spermatophyta - ps15
Angiosperms - ps16
Mesangiospermae - ps17
Magnoliids/eudicots - ps18
Eudicots - ps19
Core_eudicots - ps20
Superrosids - ps21
Rosids - ps22
Eurosids - ps23
Malvids - ps24
Sap./Malv./Brassicales - ps25
Malvales/Brassicales - ps26
Brassicales - ps27
Cleomaceae/Brassicaceae - ps28
Brassicaceae - ps29
Camelineae - ps30
Arabidopsis - ps31
Arabidopsis_thaliana - ps32
p-value
2e-07
5e-06
1e-04
2e-03
6e-02
Log-odds
0.5
1.0
1.5
2.0
2.5
3.0
3.5
4.0

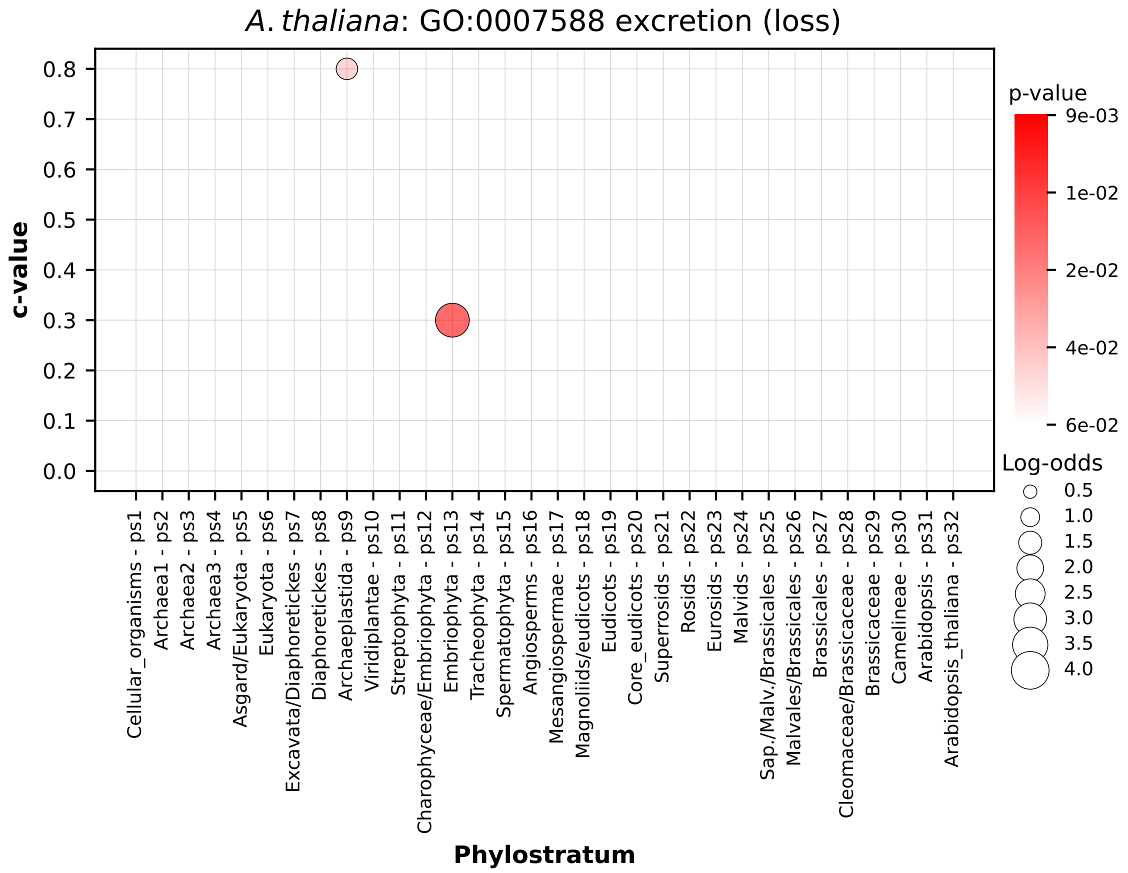
A. thaliana: GO:0007588 excretion (loss)
c-value
0.8
0.7
0.6
0.5
0.4
0.3
0.2
0.1
0.0
Phylostratum
Cellular_organisms - ps1
Archaea1 - ps2
Archaea2 - ps3
Archaea3 - ps4
Asgard/Eukaryota - ps5
Eukaryota - ps6
Excavata/Diaphoretickes - ps7
Diaphoretickes - ps8
Archaeplastida - ps9
Viridiplantae - ps10
Streptophyta - ps11
Charophyceae/Embriophyta - ps12
Embriophyta - ps13
Tracheophyta - ps14
Spermatophyta - ps15
Angiosperms - ps16
Mesangiospermae - ps17
Magnoliids/eudicots - ps18
Eudicots - ps19
Core_eudicots - ps20
Superrosids - ps21
Rosids - ps22
Eurosids - ps23
Malvids - ps24
Sap./Malv./Brassicales - ps25
Malvales/Brassicales - ps26
Brassicales - ps27
Cleomaceae/Brassicaceae - ps28
Brassicaceae - ps29
Camelineae - ps30
Arabidopsis - ps31
Arabidopsis_thaliana - ps32
p-value
9e-03
1e-02
2e-02
4e-02
6e-02
Log-odds
0.5
1.0
1.5
2.0
2.5
3.0
3.5
4.0

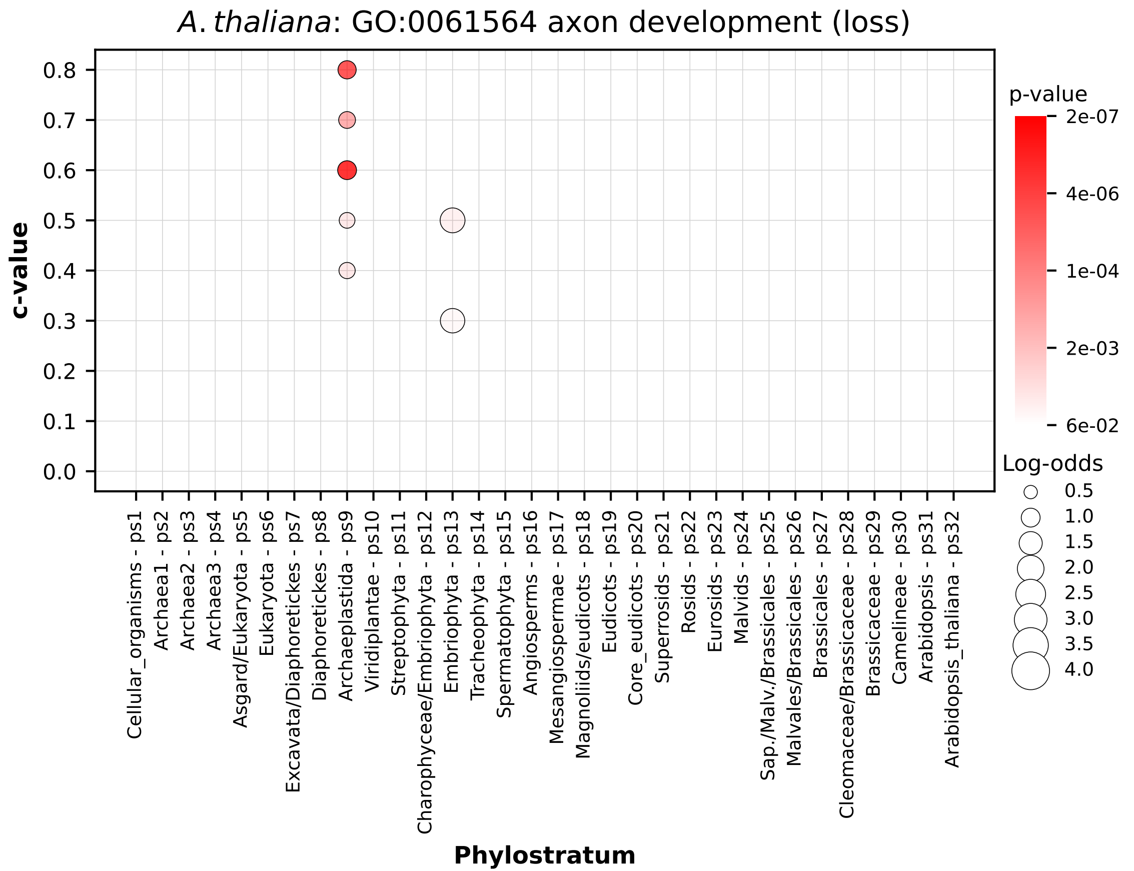
A. thaliana: GO:0061564 axon development (loss)
c-value
0.0
0.1
0.2
0.3
0.4
0.5
0.6
0.7
0.8
Phylostratum
Cellular_organisms - ps1
Archaea1 - ps2
Archaea2 - ps3
Archaea3 - ps4
Asgard/Eukaryota - ps5
Eukaryota - ps6
Excavata/Diaphoretickes - ps7
Diaphoretickes - ps8
Archaeplastida - ps9
Viridiplantae - ps10
Streptophyta - ps11
Charophyceae/Embriophyta - ps12
Embriophyta - ps13
Tracheophyta - ps14
Spermatophyta - ps15
Angiosperms - ps16
Mesangiospermae - ps17
Magnoliids/eudicots - ps18
Eudicots - ps19
Core_eudicots - ps20
Superrosids - ps21
Rosids - ps22
Eurosids - ps23
Malvids - ps24
Sap./Malv./Brassicales - ps25
Malvales/Brassicales - ps26
Brassicales - ps27
Cleomaceae/Brassicaceae - ps28
Brassicaceae - ps29
Camelineae - ps30
Arabidopsis - ps31
Arabidopsis_thaliana - ps32
p-value
2e-07
4e-06
1e-04
2e-03
6e-02
Log-odds
0.5
1.0
1.5
2.0
2.5
3.0
3.5
4.0

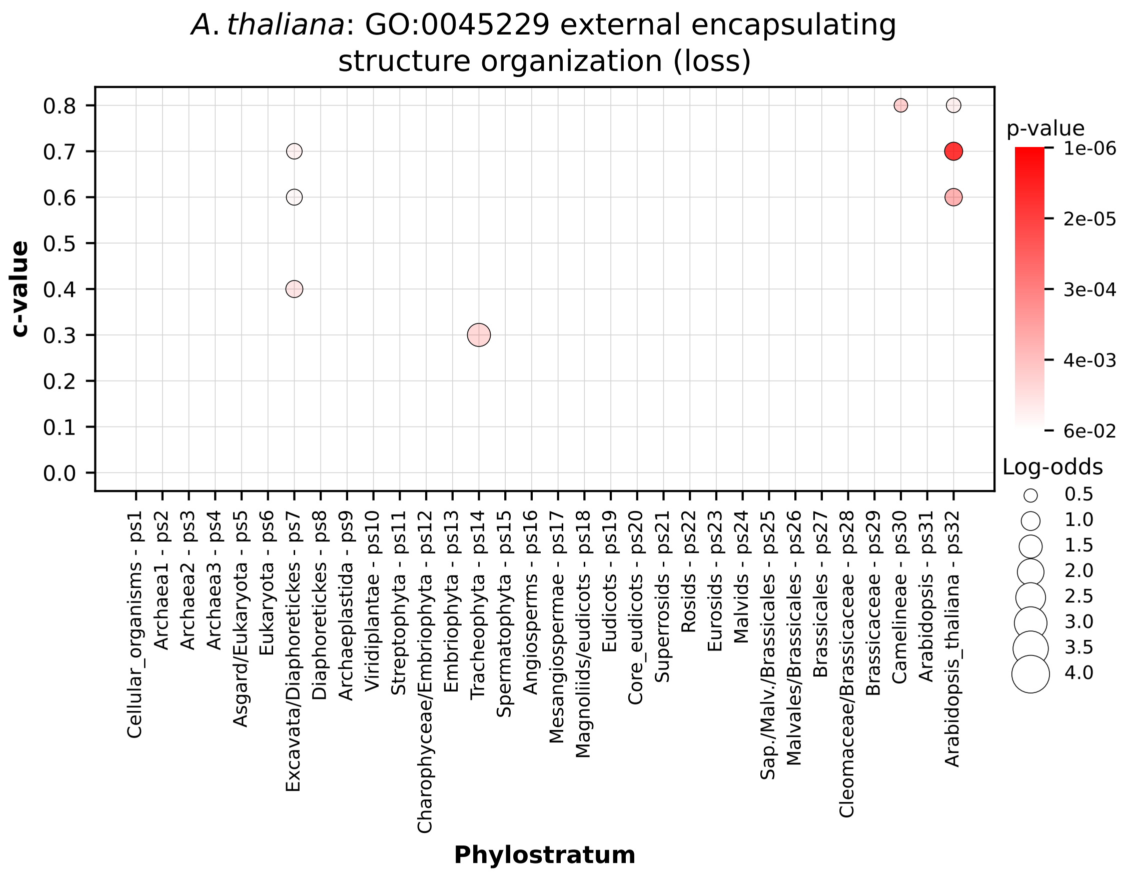

A. thaliana: GO:0045229 external encapsulating structure organization (loss)
c-value
0.0
0.1
0.2
0.3
0.4
0.5
0.6
0.7
0.8
Phylostratum
Cellular_organisms - ps1
Archaea1 - ps2
Archaea2 - ps3
Archaea3 - ps4
Asgard/Eukaryota - ps5
Eukaryota - ps6
Excavata/Diaphoretickes - ps7
Diaphoretickes - ps8
Archaeplastida - ps9
Viridiplantae - ps10
Streptophyta - ps11
Charophyceae/Embriophyta - ps12
Embriophyta - ps13
Tracheophyta - ps14
Spermatophyta - ps15
Angiosperms - ps16
Mesangiospermae - ps17
Magnoliids/eudicots - ps18
Eudicots - ps19
Core_eudicots - ps20
Superrosids - ps21
Rosids - ps22
Eurosids - ps23
Malvids - ps24
Sap./Malv./Brassicales - ps25
Malvales/Brassicales - ps26
Brassicales - ps27
Cleomaceae/Brassicaceae - ps28
Brassicaceae - ps29
Camelineae - ps30
Arabidopsis - ps31
Arabidopsis_thaliana - ps32
p-value
1e-06
2e-05
3e-04
4e-03
6e-02
Log-odds
0.5
1.0
1.5
2.0
2.5
3.0
3.5
4.0

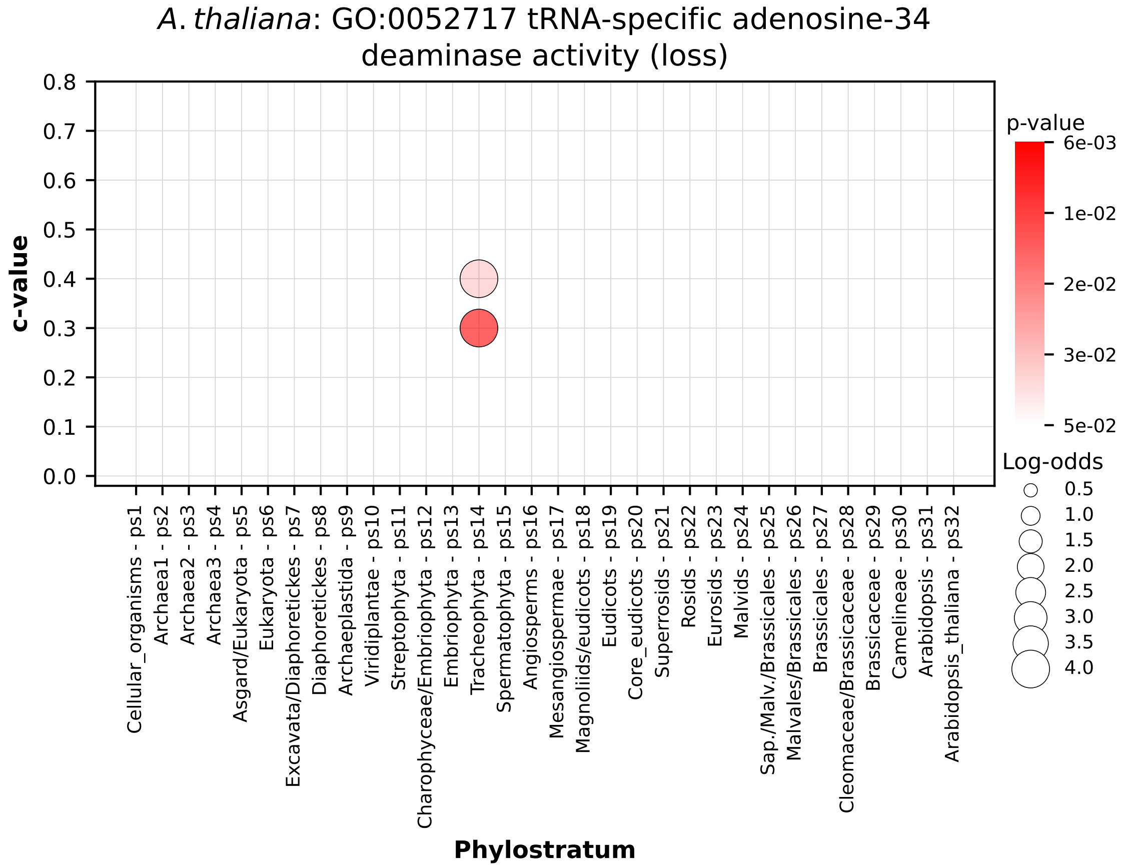

A. thaliana: GO:0052717 tRNA-specific adenosine-34 deaminase activity (loss)
c-value
0.8
0.7
0.6
0.5
0.4
0.3
0.2
0.1
0.0
Phylostratum
Cellular_organisms - ps1
Archaea1 - ps2
Archaea2 - ps3
Archaea3 - ps4
Asgard/Eukaryota - ps5
Eukaryota - ps6
Excavata/Diaphoretickes - ps7
Diaphoretickes - ps8
Archaeplastida - ps9
Viridiplantae - ps10
Streptophyta - ps11
Charophyceae/Embriophyta - ps12
Embriophyta - ps13
Tracheophyta - ps14
Spermatophyta - ps15
Angiosperms - ps16
Mesangiospermae - ps17
Magnoliids/eudicots - ps18
Eudicots - ps19
Core_eudicots - ps20
Superrosids - ps21
Rosids - ps22
Eurosids - ps23
Malvids - ps24
Sap./Malv./Brassicales - ps25
Malvales/Brassicales - ps26
Brassicales - ps27
Cleomaceae/Brassicaceae - ps28
Brassicaceae - ps29
Camelineae - ps30
Arabidopsis - ps31
Arabidopsis_thaliana - ps32
p-value
6e-03
1e-02
2e-02
3e-02
5e-02
Log-odds
0.5
1.0
1.5
2.0
2.5
3.0
3.5
4.0

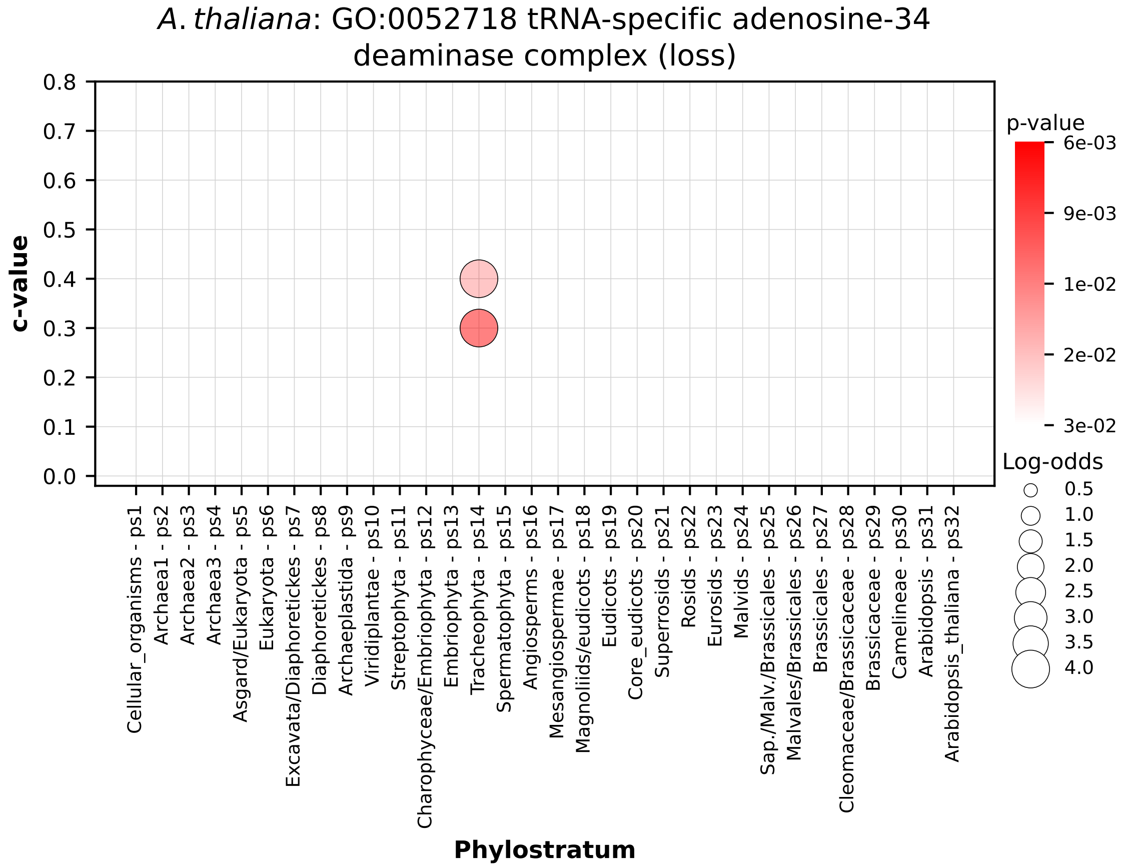

A. thaliana: GO:0052718 tRNA-specific adenosine-34 deaminase complex (loss)
c-value
0.8
0.7
0.6
0.5
0.4
0.3
0.2
0.1
0.0
Phylostratum
Cellular_organisms - ps1
Archaea1 - ps2
Archaea2 - ps3
Archaea3 - ps4
Asgard/Eukaryota - ps5
Eukaryota - ps6
Excavata/Diaphoretickes - ps7
Diaphoretickes - ps8
Archaeplastida - ps9
Viridiplantae - ps10
Streptophyta - ps11
Charophyceae/Embriophyta - ps12
Embriophyta - ps13
Tracheophyta - ps14
Spermatophyta - ps15
Angiosperms - ps16
Mesangiospermae - ps17
Magnoliids/eudicots - ps18
Eudicots - ps19
Core_eudicots - ps20
Superrosids - ps21
Rosids - ps22
Eurosids - ps23
Malvids - ps24
Sap./Malv./Brassicales - ps25
Malvales/Brassicales - ps26
Brassicales - ps27
Cleomaceae/Brassicaceae - ps28
Brassicaceae - ps29
Camelineae - ps30
Arabidopsis - ps31
Arabidopsis_thaliana - ps32
p-value
6e-03
9e-03
1e-02
2e-02
3e-02
Log-odds
0.5
1.0
1.5
2.0
2.5
3.0
3.5
4.0

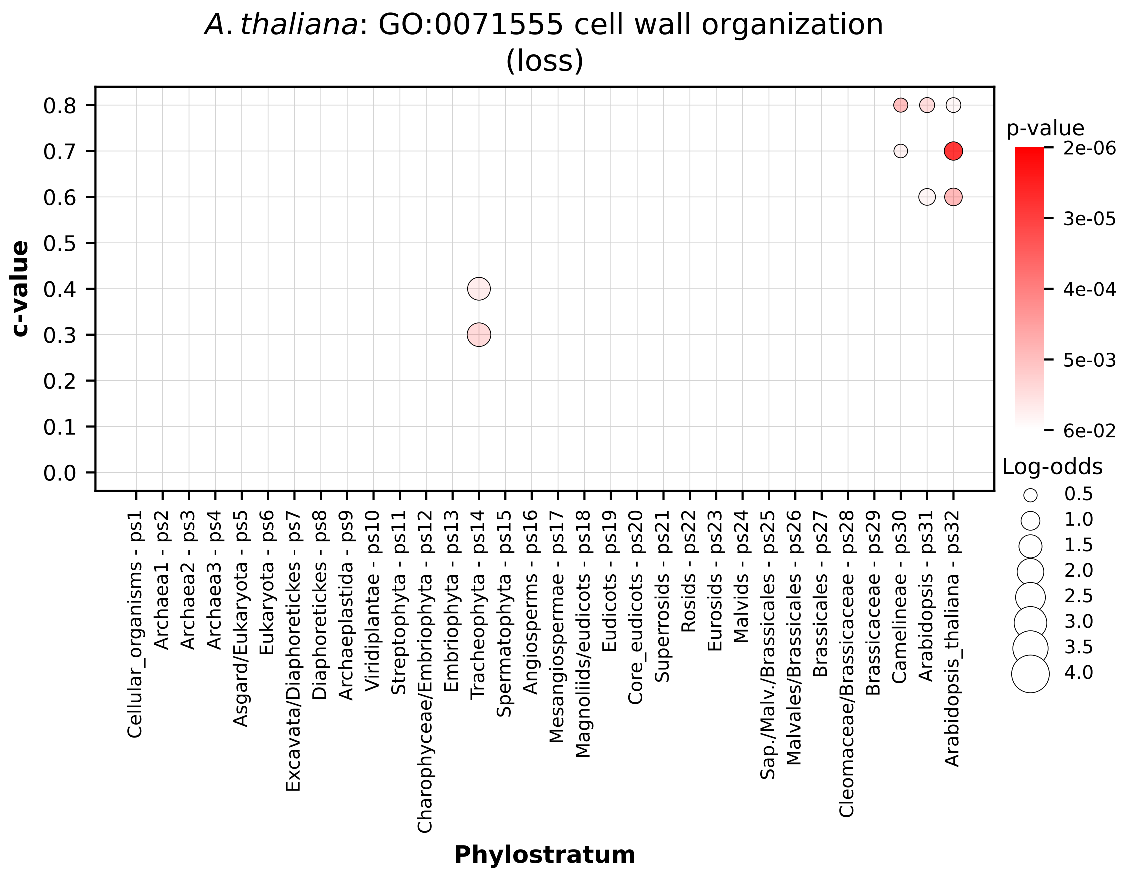

A. thaliana: GO:0071555 cell wall organization
(loss)
c-value
0.8
0.7
0.6
0.5
0.4
0.3
0.2
0.1
0.0
p-value
2e-06
3e-05
4e-04
5e-03
6e-02
Log-odds
0.5
1.0
1.5
2.0
2.5
3.0
3.5
4.0
Cellular_organisms - ps1
Archaea1 - ps2
Archaea2 - ps3
Archaea3 - ps4
Asgard/Eukaryota - ps5
Eukaryota - ps6
Excavata/Diaphoretickes - ps7
Diaphoretickes - ps8
Archaeplastida - ps9
Viridiplantae - ps10
Streptophyta - ps11
Charophyceae/Embriophyta - ps12
Embriophyta - ps13
Tracheophyta - ps14
Spermatophyta - ps15
Angiosperms - ps16
Mesangiospermae - ps17
Magnoliids/eudicots - ps18
Eudicots - ps19
Core_eudicots - ps20
Superrosids - ps21
Rosids - ps22
Eurosids - ps23
Malvids - ps24
Sap./Malv./Brassicales - ps25
Malvales/Brassicales - ps26
Brassicales - ps27
Cleomaceae/Brassicaceae - ps28
Brassicaceae - ps29
Camelineae - ps30
Arabidopsis - ps31
Arabidopsis_thaliana - ps32
Phylostratum

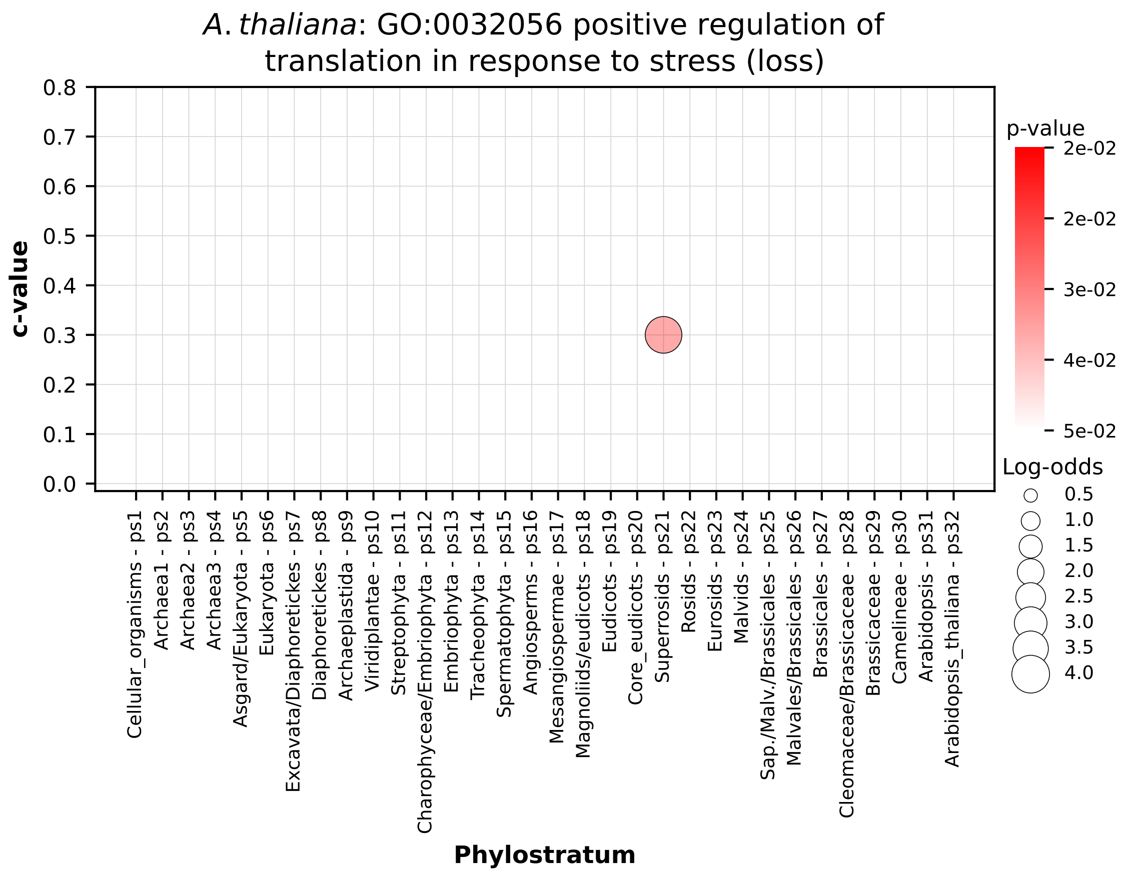

A. thaliana: GO:0032056 positive regulation of translation in response to stress (loss)
c-value
0.8
0.7
0.6
0.5
0.4
0.3
0.2
0.1
0.0
p-value
2e-02
2e-02
3e-02
4e-02
5e-02
Log-odds
0.5
1.0
1.5
2.0
2.5
3.0
3.5
4.0
Cellular_organisms - ps1
Archaea1 - ps2
Archaea2 - ps3
Archaea3 - ps4
Asgard/Eukaryota - ps5
Eukaryota - ps6
Excavata/Diaphoretickes - ps7
Diaphoretickes - ps8
Archaeplastida - ps9
Viridiplantae - ps10
Streptophyta - ps11
Charophyceae/Embriophyta - ps12
Embriophyta - ps13
Tracheophyta - ps14
Spermatophyta - ps15
Angiosperms - ps16
Mesangiospermae - ps17
Magnoliids/eudicots - ps18
Eudicots - ps19
Core_eudicots - ps20
Superrosids - ps21
Rosids - ps22
Eurosids - ps23
Malvids - ps24
Sap./Malv./Brassicales - ps25
Malvales/Brassicales - ps26
Brassicales - ps27
Cleomaceae/Brassicaceae - ps28
Brassicaceae - ps29
Camelineae - ps30
Arabidopsis - ps31
Arabidopsis_thaliana - ps32
Phylostratum

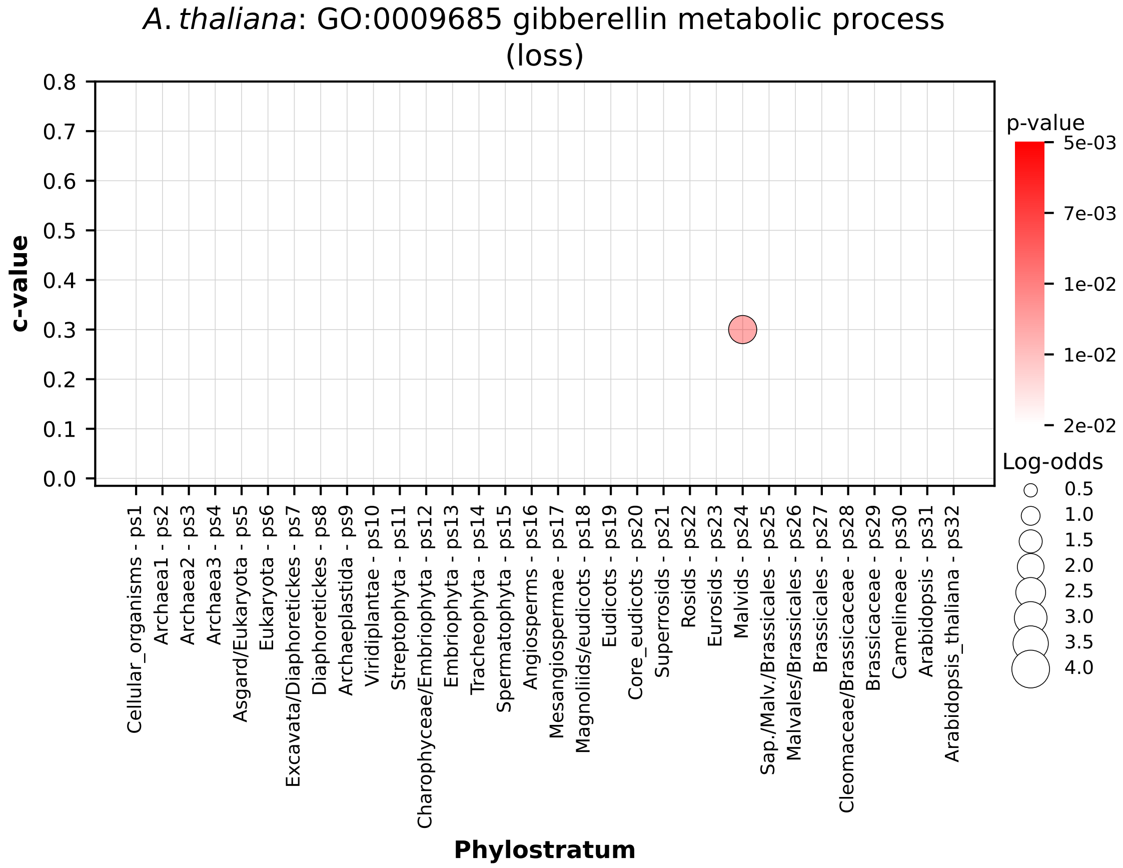

A. thaliana: GO:0009685 gibberellin metabolic process
(loss)
c-value
0.8
0.7
0.6
0.5
0.4
0.3
0.2
0.1
0.0
p-value
5e-03
7e-03
1e-02
1e-02
2e-02
Log-odds
0.5
1.0
1.5
2.0
2.5
3.0
3.5
4.0
Cellular_organisms - ps1
Archaea1 - ps2
Archaea2 - ps3
Archaea3 - ps4
Asgard/Eukaryota - ps5
Eukaryota - ps6
Excavata/Diaphoretickes - ps7
Diaphoretickes - ps8
Archaeplastida - ps9
Viridiplantae - ps10
Streptophyta - ps11
Charophyceae/Embriophyta - ps12
Embriophyta - ps13
Tracheophyta - ps14
Spermatophyta - ps15
Angiosperms - ps16
Mesangiospermae - ps17
Magnoliids/eudicots - ps18
Eudicots - ps19
Core_eudicots - ps20
Superrosids - ps21
Rosids - ps22
Eurosids - ps23
Malvids - ps24
Sap./Malv./Brassicales - ps25
Malvales/Brassicales - ps26
Brassicales - ps27
Cleomaceae/Brassicaceae - ps28
Brassicaceae - ps29
Camelineae - ps30
Arabidopsis - ps31
Arabidopsis_thaliana - ps32
Phylostratum

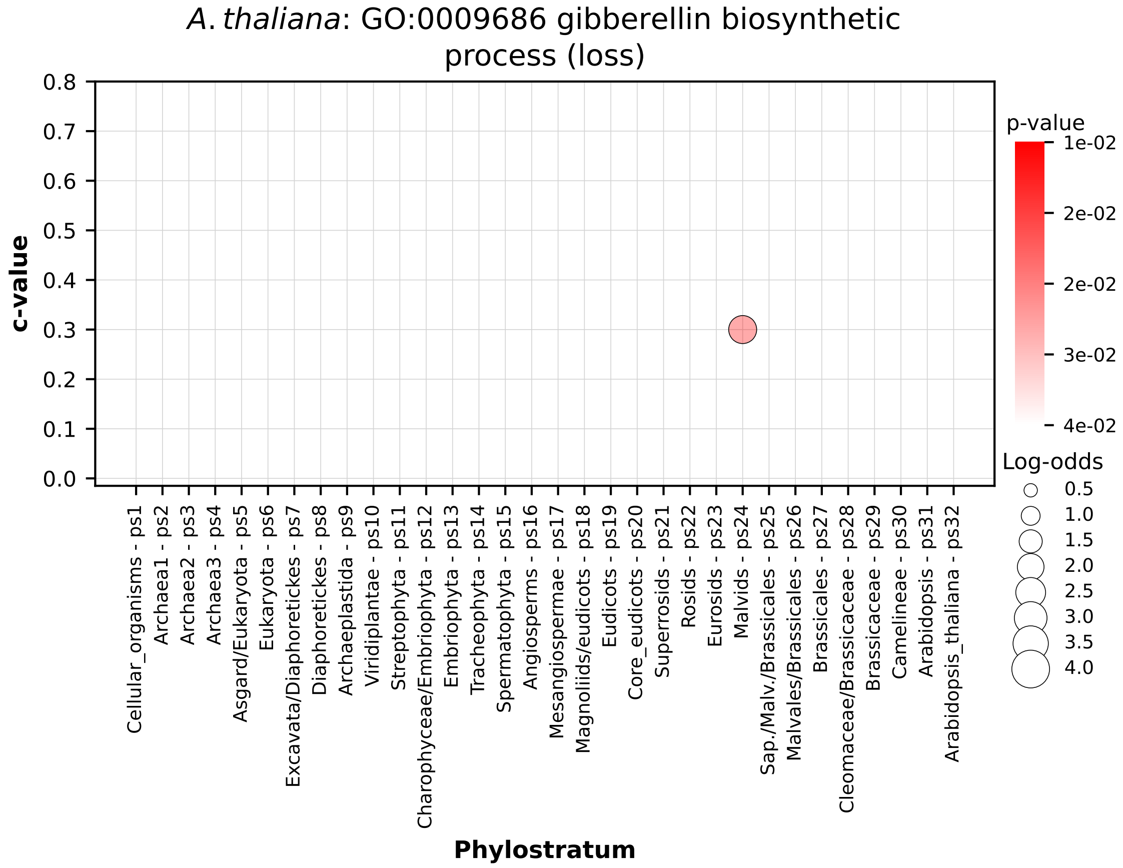

A. thaliana: GO:0009686 gibberellin biosynthetic process (loss)
c-value
0.8
0.7
0.6
0.5
0.4
0.3
0.2
0.1
0.0
p-value
1e-02
2e-02
2e-02
3e-02
4e-02
Log-odds
0.5
1.0
1.5
2.0
2.5
3.0
3.5
4.0
Cellular_organisms - ps1
Archaea1 - ps2
Archaea2 - ps3
Archaea3 - ps4
Asgard/Eukaryota - ps5
Eukaryota - ps6
Excavata/Diaphoretickes - ps7
Diaphoretickes - ps8
Archaeplastida - ps9
Viridiplantae - ps10
Streptophyta - ps11
Charophyceae/Embriophyta - ps12
Embriophyta - ps13
Tracheophyta - ps14
Spermatophyta - ps15
Angiosperms - ps16
Mesangiospermae - ps17
Magnoliids/eudicots - ps18
Eudicots - ps19
Core_eudicots - ps20
Superrosids - ps21
Rosids - ps22
Eurosids - ps23
Malvids - ps24
Sap./Malv./Brassicales - ps25
Malvales/Brassicales - ps26
Brassicales - ps27
Cleomaceae/Brassicaceae - ps28
Brassicaceae - ps29
Camelineae - ps30
Arabidopsis - ps31
Arabidopsis_thaliana - ps32
Phylostratum

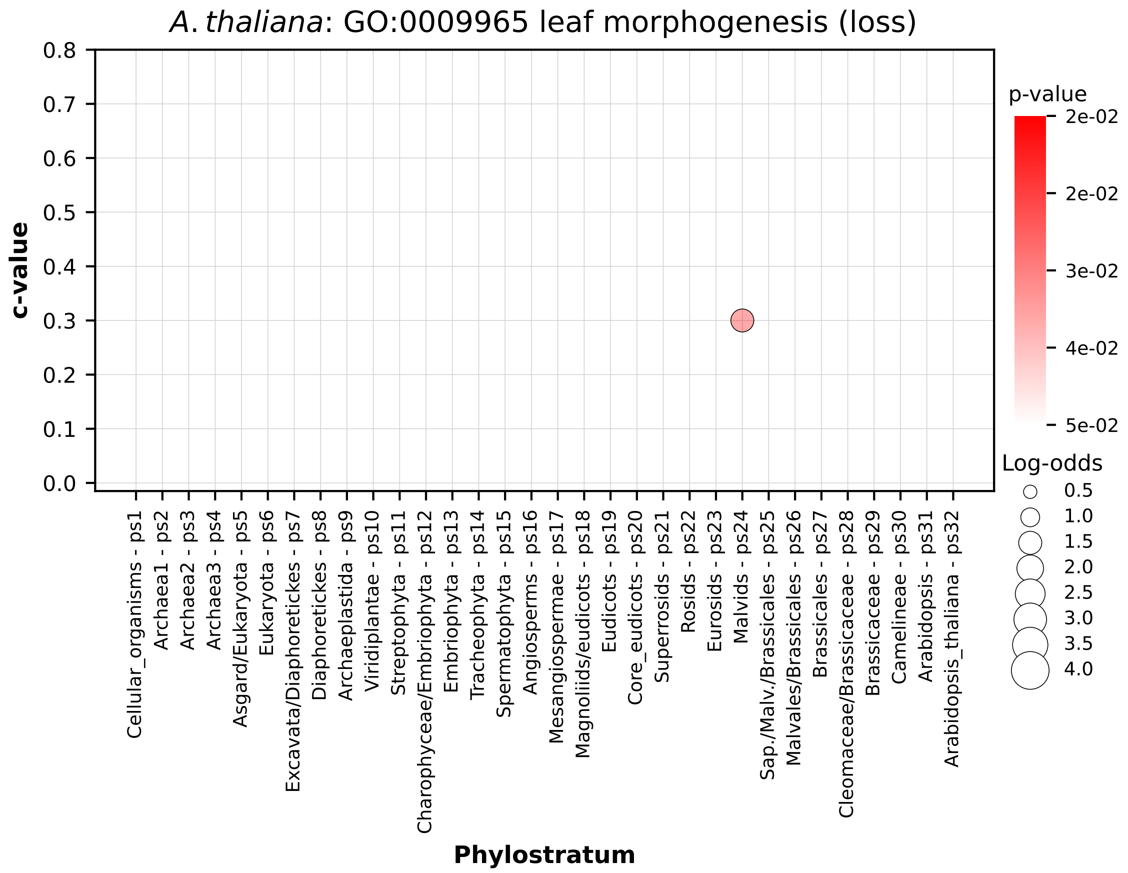

A. thaliana: GO:0009965 leaf morphogenesis (loss)
c-value
0.8
0.7
0.6
0.5
0.4
0.3
0.2
0.1
0.0
p-value
2e-02
2e-02
3e-02
4e-02
5e-02
Log-odds
0.5
1.0
1.5
2.0
2.5
3.0
3.5
4.0
Cellular_organisms - ps1
Archaea1 - ps2
Archaea2 - ps3
Archaea3 - ps4
Asgard/Eukaryota - ps5
Eukaryota - ps6
Excavata/Diaphoretickes - ps7
Diaphoretickes - ps8
Archaeplastida - ps9
Viridiplantae - ps10
Streptophyta - ps11
Charophyceae/Embriophyta - ps12
Embriophyta - ps13
Tracheophyta - ps14
Spermatophyta - ps15
Angiosperms - ps16
Mesangiospermae - ps17
Magnoliids/eudicots - ps18
Eudicots - ps19
Core_eudicots - ps20
Superrosids - ps21
Rosids - ps22
Eurosids - ps23
Malvids - ps24
Sap./Malv./Brassicales - ps25
Malvales/Brassicales - ps26
Brassicales - ps27
Cleomaceae/Brassicaceae - ps28
Brassicaceae - ps29
Camelineae - ps30
Arabidopsis - ps31
Arabidopsis_thaliana - ps32
Phylostratum

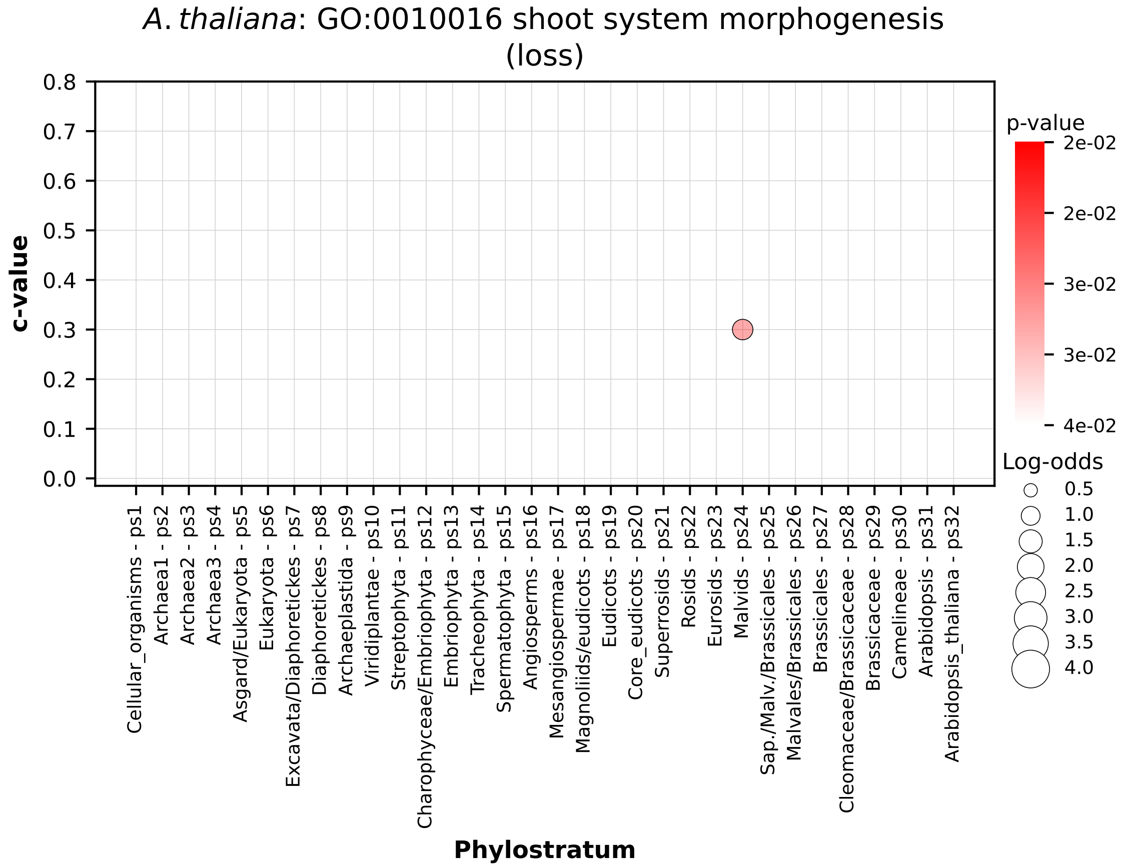
A. thaliana: GO:0010016 shoot system morphogenesis
(loss)
c-value
0.8
0.7
0.6
0.5
0.4
0.3
0.2
0.1
0.0
Phylostratum
Cellular_organisms - ps1
Archaea1 - ps2
Archaea2 - ps3
Archaea3 - ps4
Asgard/Eukaryota - ps5
Eukaryota - ps6
Excavata/Diaphoretickes - ps7
Diaphoretickes - ps8
Archaeplastida - ps9
Viridiplantae - ps10
Streptophyta - ps11
Charophyceae/Embriophyta - ps12
Embriophyta - ps13
Tracheophyta - ps14
Spermatophyta - ps15
Angiosperms - ps16
Mesangiospermae - ps17
Magnoliids/eudicots - ps18
Eudicots - ps19
Core_eudicots - ps20
Superrosids - ps21
Rosids - ps22
Eurosids - ps23
Malvids - ps24
Sap./Malv./Brassicales - ps25
Malvales/Brassicales - ps26
Brassicales - ps27
Cleomaceae/Brassicaceae - ps28
Brassicaceae - ps29
Camelineae - ps30
Arabidopsis - ps31
Arabidopsis_thaliana - ps32
p-value
2e-02
2e-02
3e-02
3e-02
4e-02
Log-odds
0.5
1.0
1.5
2.0
2.5
3.0
3.5
4.0

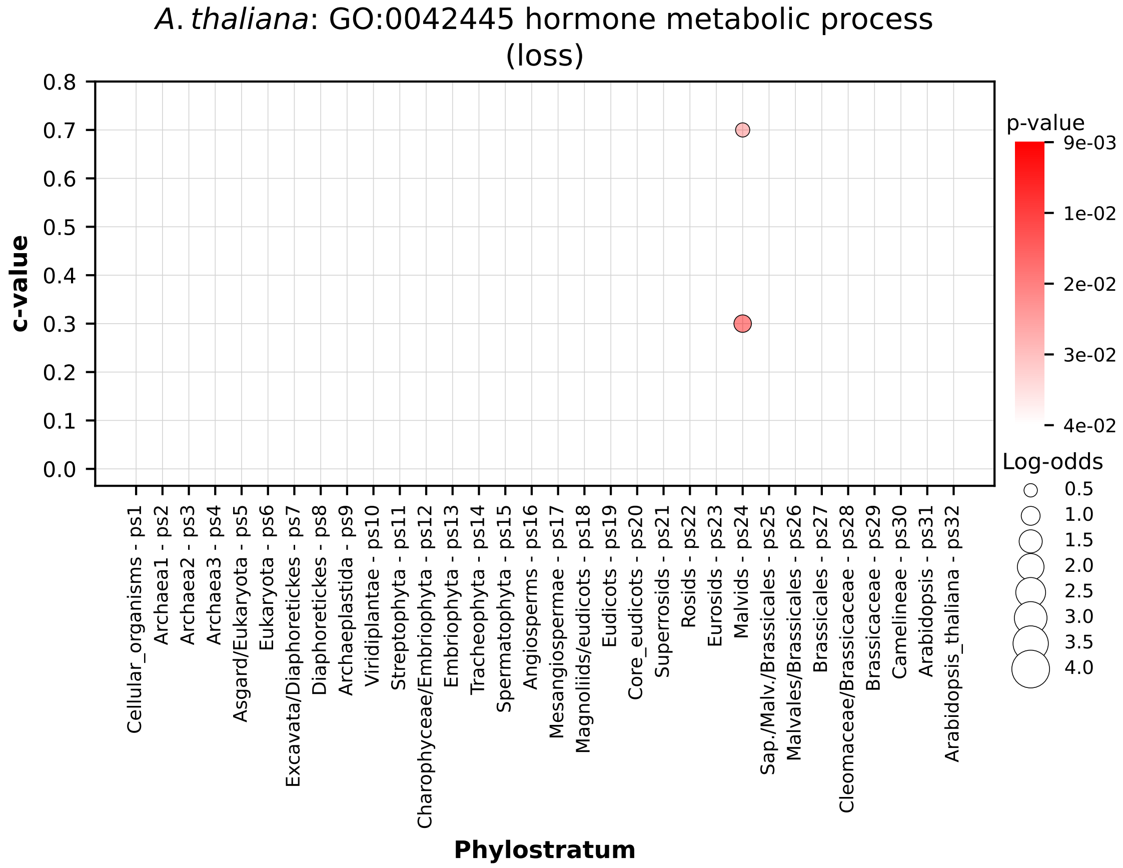
A. thaliana: GO:0042445 hormone metabolic process
(loss)
c-value
0.8
0.7
0.6
0.5
0.4
0.3
0.2
0.1
0.0
p-value
9e-03
1e-02
2e-02
3e-02
4e-02
Log-odds
0.5
1.0
1.5
2.0
2.5
3.0
3.5
4.0
Cellular_organisms - ps1
Archaea1 - ps2
Archaea2 - ps3
Archaea3 - ps4
Asgard/Eukaryota - ps5
Eukaryota - ps6
Excavata/Diaphoretickes - ps7
Diaphoretickes - ps8
Archaeplastida - ps9
Viridiplantae - ps10
Streptophyta - ps11
Charophyceae/Embriophyta - ps12
Embriophyta - ps13
Tracheophyta - ps14
Spermatophyta - ps15
Angiosperms - ps16
Mesangiospermae - ps17
Magnoliids/eudicots - ps18
Eudicots - ps19
Core_eudicots - ps20
Superrosids - ps21
Rosids - ps22
Eurosids - ps23
Malvids - ps24
Sap./Malv./Brassicales - ps25
Malvales/Brassicales - ps26
Brassicales - ps27
Cleomaceae/Brassicaceae - ps28
Brassicaceae - ps29
Camelineae - ps30
Arabidopsis - ps31
Arabidopsis_thaliana - ps32
Phylostratum

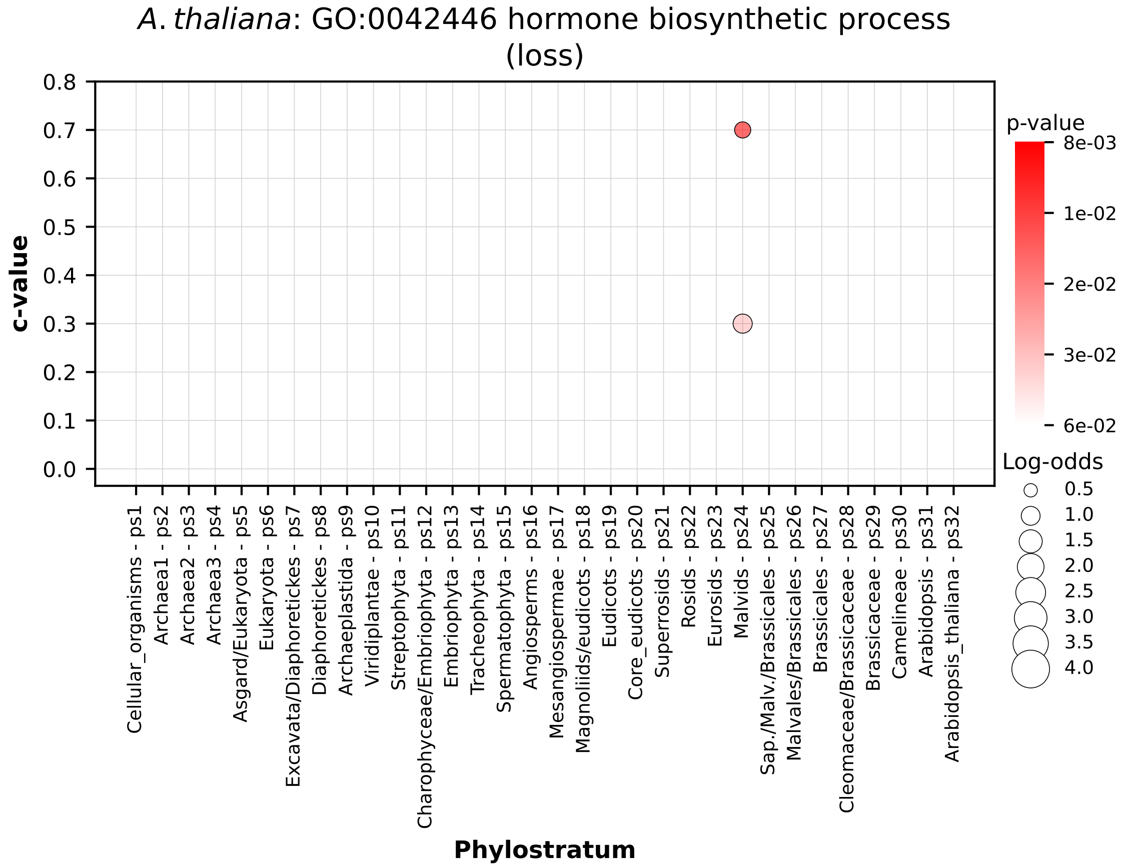

A. thaliana: GO:0042446 hormone biosynthetic process
(loss)
c-value
0.8
0.7
0.6
0.5
0.4
0.3
0.2
0.1
0.0
p-value
8e-03
1e-02
2e-02
3e-02
6e-02
Log-odds
0.5
1.0
1.5
2.0
2.5
3.0
3.5
4.0
Cellular_organisms - ps1
Archaea1 - ps2
Archaea2 - ps3
Archaea3 - ps4
Asgard/Eukaryota - ps5
Eukaryota - ps6
Excavata/Diaphoretickes - ps7
Diaphoretickes - ps8
Archaeplastida - ps9
Viridiplantae - ps10
Streptophyta - ps11
Charophyceae/Embriophyta - ps12
Embriophyta - ps13
Tracheophyta - ps14
Spermatophyta - ps15
Angiosperms - ps16
Mesangiospermae - ps17
Magnoliids/eudicots - ps18
Eudicots - ps19
Core_eudicots - ps20
Superrosids - ps21
Rosids - ps22
Eurosids - ps23
Malvids - ps24
Sap./Malv./Brassicales - ps25
Malvales/Brassicales - ps26
Brassicales - ps27
Cleomaceae/Brassicaceae - ps28
Brassicaceae - ps29
Camelineae - ps30
Arabidopsis - ps31
Arabidopsis_thaliana - ps32
Phylostratum

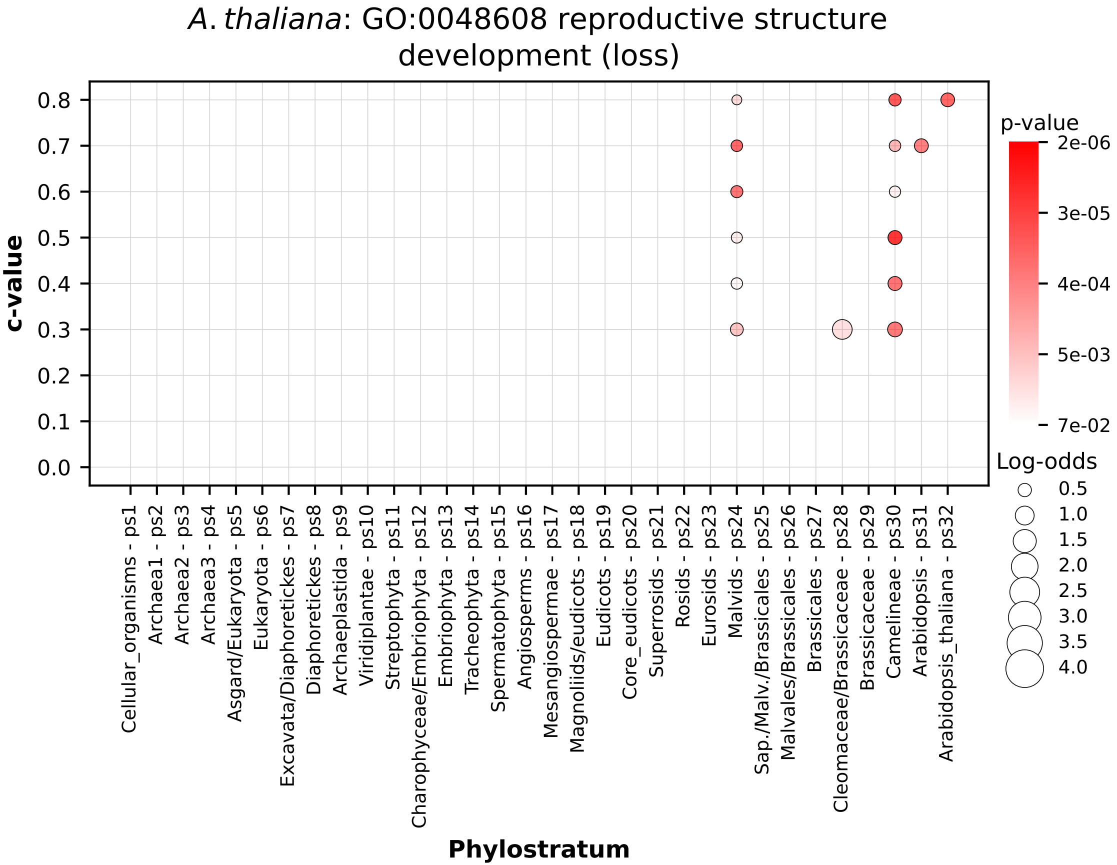

A. thaliana: GO:0048608 reproductive structure development (loss)
c-value
0.8
0.7
0.6
0.5
0.4
0.3
0.2
0.1
0.0
p-value
2e-06
3e-05
4e-04
5e-03
7e-02
Log-odds
0.5
1.0
1.5
2.0
2.5
3.0
3.5
4.0
Cellular_organisms - ps1
Archaea1 - ps2
Archaea2 - ps3
Archaea3 - ps4
Asgard/Eukaryota - ps5
Eukaryota - ps6
Excavata/Diaphoretickes - ps7
Diaphoretickes - ps8
Archaeplastida - ps9
Viridiplantae - ps10
Streptophyta - ps11
Charophyceae/Embriophyta - ps12
Embriophyta - ps13
Tracheophyta - ps14
Spermatophyta - ps15
Angiosperms - ps16
Mesangiospermae - ps17
Magnoliids/eudicots - ps18
Eudicots - ps19
Core_eudicots - ps20
Superrosids - ps21
Rosids - ps22
Eurosids - ps23
Malvids - ps24
Sap./Malv./Brassicales - ps25
Malvales/Brassicales - ps26
Brassicales - ps27
Cleomaceae/Brassicaceae - ps28
Brassicaceae - ps29
Camelineae - ps30
Arabidopsis - ps31
Arabidopsis_thaliana - ps32
Phylostratum

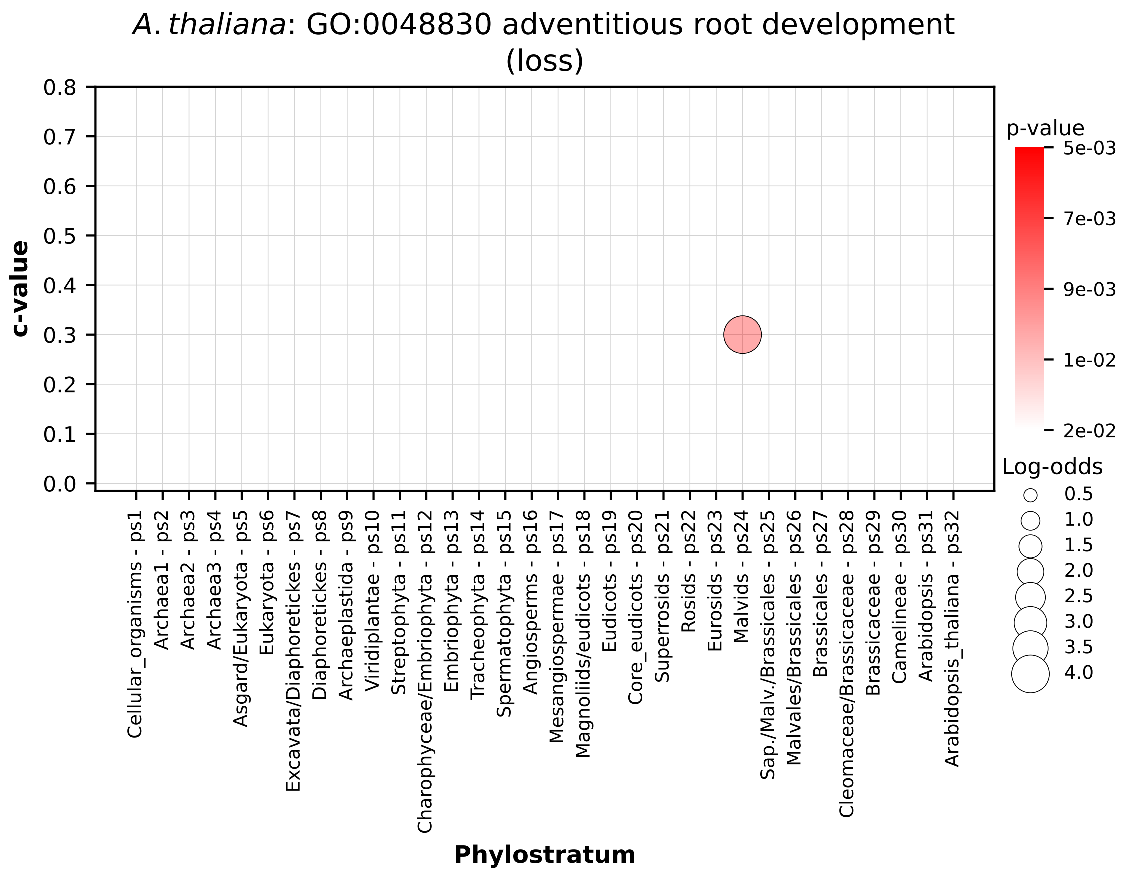

A. thaliana: GO:0048830 adventitious root development
(loss)
c-value
0.8
0.7
0.6
0.5
0.4
0.3
0.2
0.1
0.0
p-value
5e-03
7e-03
9e-03
1e-02
2e-02
Log-odds
0.5
1.0
1.5
2.0
2.5
3.0
3.5
4.0
Cellular_organisms - ps1
Archaea1 - ps2
Archaea2 - ps3
Archaea3 - ps4
Asgard/Eukaryota - ps5
Eukaryota - ps6
Excavata/Diaphoretickes - ps7
Diaphoretickes - ps8
Archaeplastida - ps9
Viridiplantae - ps10
Streptophyta - ps11
Charophyceae/Embriophyta - ps12
Embriophyta - ps13
Tracheophyta - ps14
Spermatophyta - ps15
Angiosperms - ps16
Mesangiospermae - ps17
Magnoliids/eudicots - ps18
Eudicots - ps19
Core_eudicots - ps20
Superrosids - ps21
Rosids - ps22
Eurosids - ps23
Malvids - ps24
Sap./Malv./Brassicales - ps25
Malvales/Brassicales - ps26
Brassicales - ps27
Cleomaceae/Brassicaceae - ps28
Brassicaceae - ps29
Camelineae - ps30
Arabidopsis - ps31
Arabidopsis_thaliana - ps32
Phylostratum

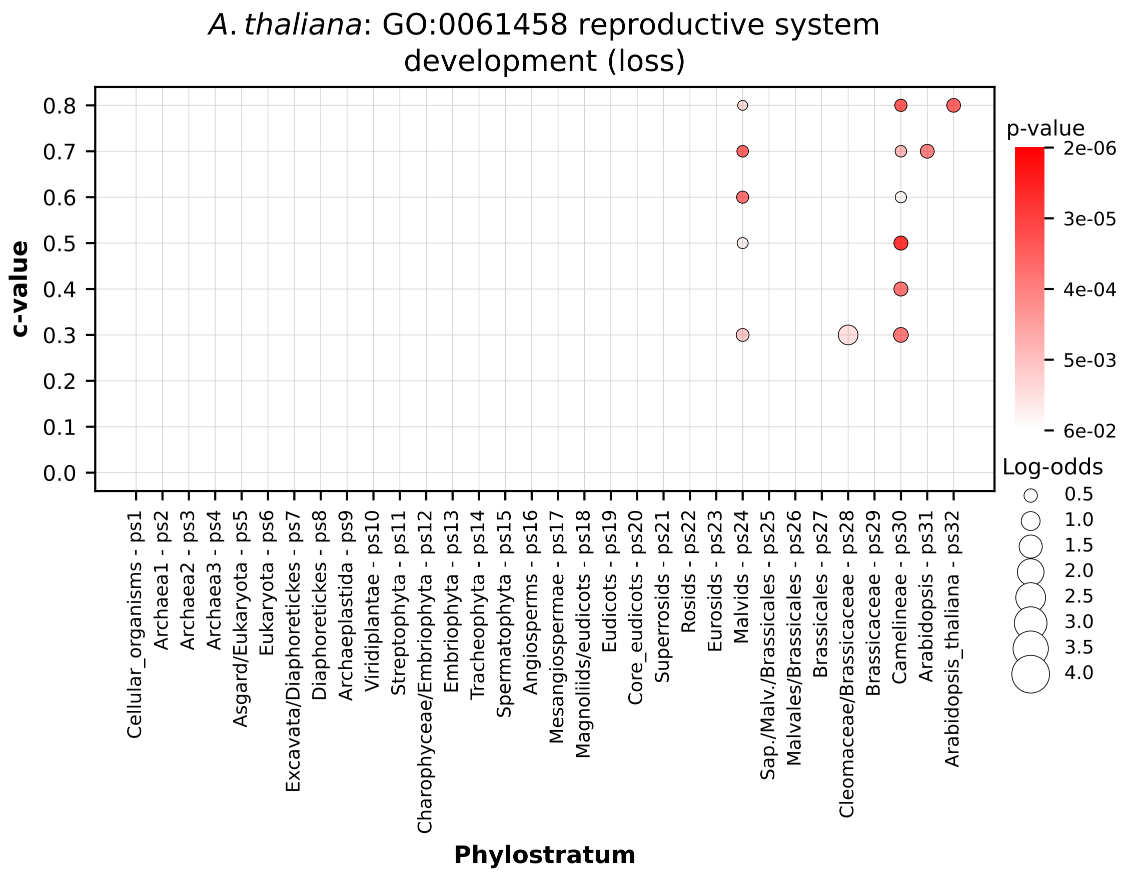

A. thaliana: GO:0061458 reproductive system development (loss)
c-value
0.8
0.7
0.6
0.5
0.4
0.3
0.2
0.1
0.0
p-value
2e-06
3e-05
4e-04
5e-03
6e-02
Log-odds
0.5
1.0
1.5
2.0
2.5
3.0
3.5
4.0
Cellular_organisms - ps1
Archaea1 - ps2
Archaea2 - ps3
Archaea3 - ps4
Asgard/Eukaryota - ps5
Eukaryota - ps6
Excavata/Diaphoretickes - ps7
Diaphoretickes - ps8
Archaeplastida - ps9
Viridiplantae - ps10
Streptophyta - ps11
Charophyceae/Embriophyta - ps12
Embriophyta - ps13
Tracheophyta - ps14
Spermatophyta - ps15
Angiosperms - ps16
Mesangiospermae - ps17
Magnoliids/eudicots - ps18
Eudicots - ps19
Core_eudicots - ps20
Superrosids - ps21
Rosids - ps22
Eurosids - ps23
Malvids - ps24
Sap./Malv./Brassicales - ps25
Malvales/Brassicales - ps26
Brassicales - ps27
Cleomaceae/Brassicaceae - ps28
Brassicaceae - ps29
Camelineae - ps30
Arabidopsis - ps31
Arabidopsis_thaliana - ps32
Phylostratum

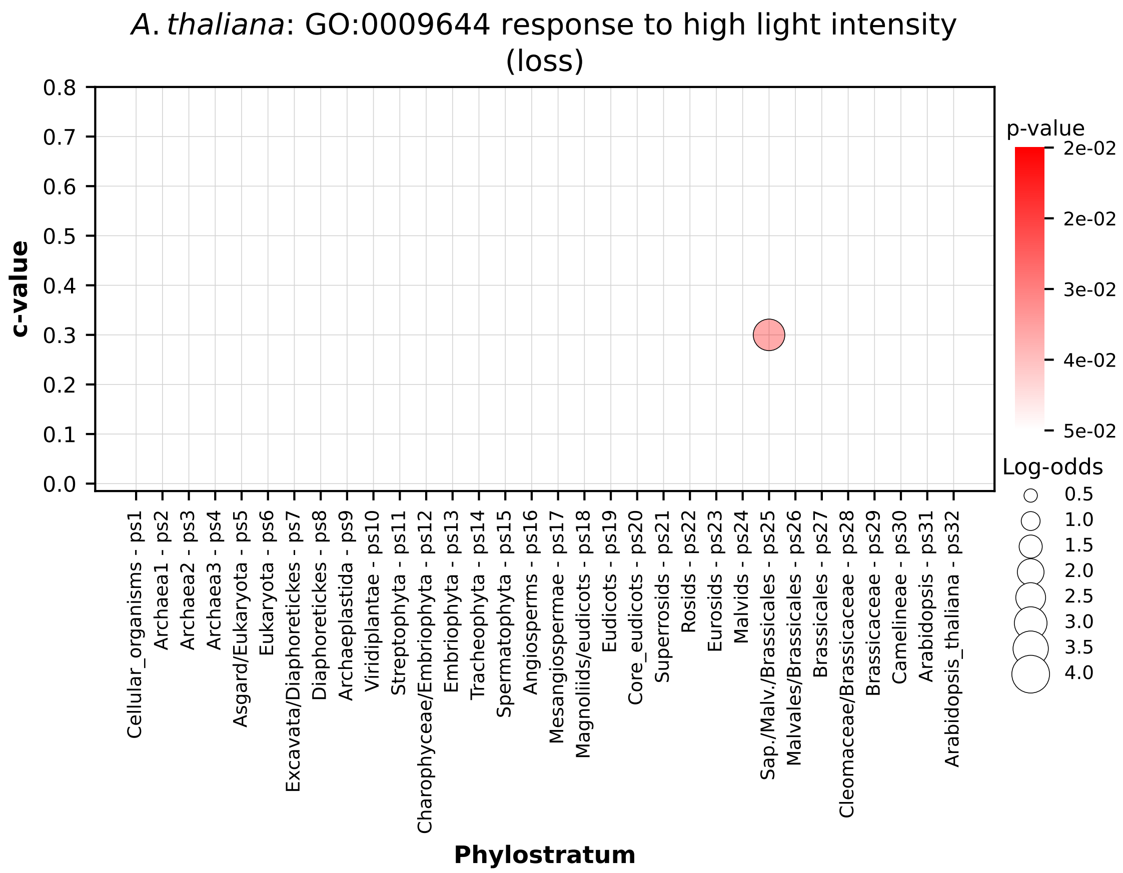

A. thaliana: GO:0009644 response to high light intensity (loss)
c-value
0.8
0.7
0.6
0.5
0.4
0.3
0.2
0.1
0.0
p-value
2e-02
2e-02
3e-02
4e-02
5e-02
Log-odds
0.5
1.0
1.5
2.0
2.5
3.0
3.5
4.0
Cellular_organisms - ps1
Archaea1 - ps2
Archaea2 - ps3
Archaea3 - ps4
Asgard/Eukaryota - ps5
Eukaryota - ps6
Excavata/Diaphoretickes - ps7
Diaphoretickes - ps8
Archaeplastida - ps9
Viridiplantae - ps10
Streptophyta - ps11
Charophyceae/Embriophyta - ps12
Embriophyta - ps13
Tracheophyta - ps14
Spermatophyta - ps15
Angiosperms - ps16
Mesangiospermae - ps17
Magnoliids/eudicots - ps18
Eudicots - ps19
Core_eudicots - ps20
Superrosids - ps21
Rosids - ps22
Eurosids - ps23
Malvids - ps24
Sap./Malv./Brassicales - ps25
Malvales/Brassicales - ps26
Brassicales - ps27
Cleomaceae/Brassicaceae - ps28
Brassicaceae - ps29
Camelineae - ps30
Arabidopsis - ps31
Arabidopsis_thaliana - ps32
Phylostratum

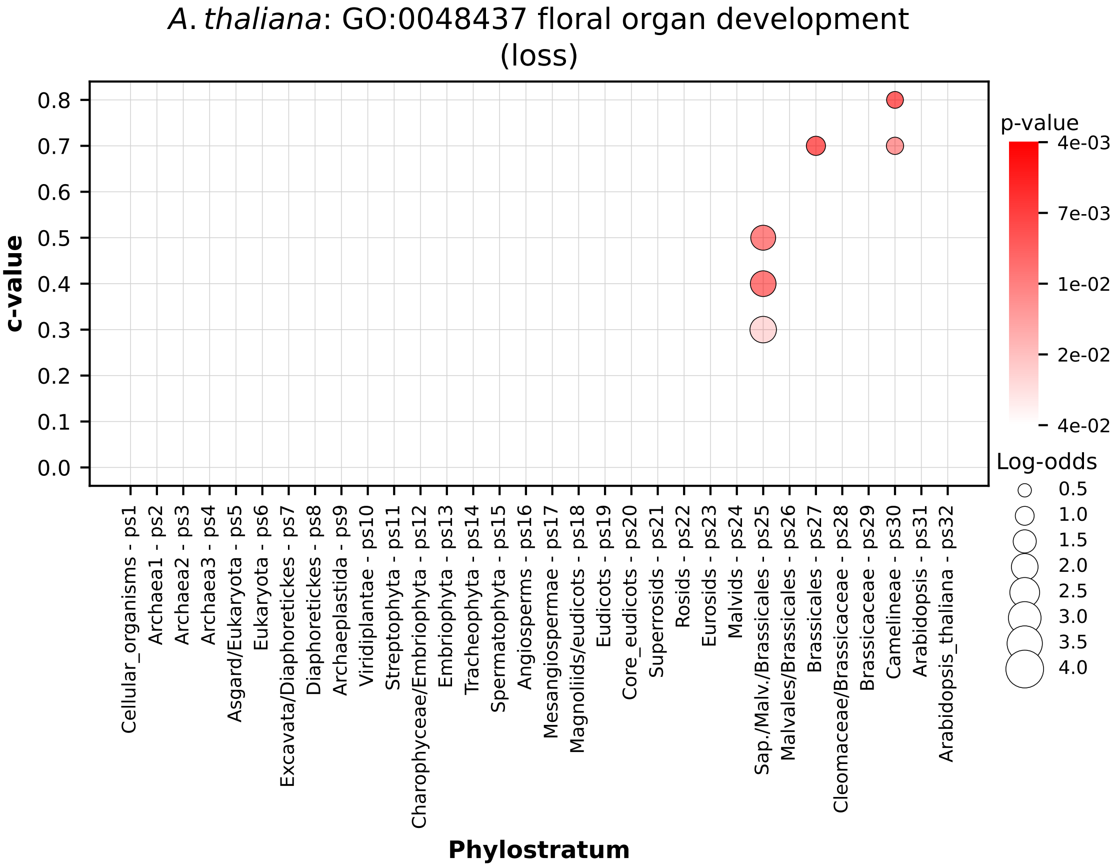
A. thaliana: GO:0048437 floral organ development
(loss)
c-value
0.8
0.7
0.6
0.5
0.4
0.3
0.2
0.1
0.0
p-value
4e-03
7e-03
1e-02
2e-02
4e-02
Log-odds
0.5
1.0
1.5
2.0
2.5
3.0
3.5
4.0
Cellular_organisms - ps1
Archaea1 - ps2
Archaea2 - ps3
Archaea3 - ps4
Asgard/Eukaryota - ps5
Eukaryota - ps6
Excavata/Diaphoretickes - ps7
Diaphoretickes - ps8
Archaeplastida - ps9
Viridiplantae - ps10
Streptophyta - ps11
Charophyceae/Embriophyta - ps12
Embriophyta - ps13
Tracheophyta - ps14
Spermatophyta - ps15
Angiosperms - ps16
Mesangiospermae - ps17
Magnoliids/eudicots - ps18
Eudicots - ps19
Core_eudicots - ps20
Superrosids - ps21
Rosids - ps22
Eurosids - ps23
Malvids - ps24
Sap./Malv./Brassicales - ps25
Malvales/Brassicales - ps26
Brassicales - ps27
Cleomaceae/Brassicaceae - ps28
Brassicaceae - ps29
Camelineae - ps30
Arabidopsis - ps31
Arabidopsis_thaliana - ps32
Phylostratum

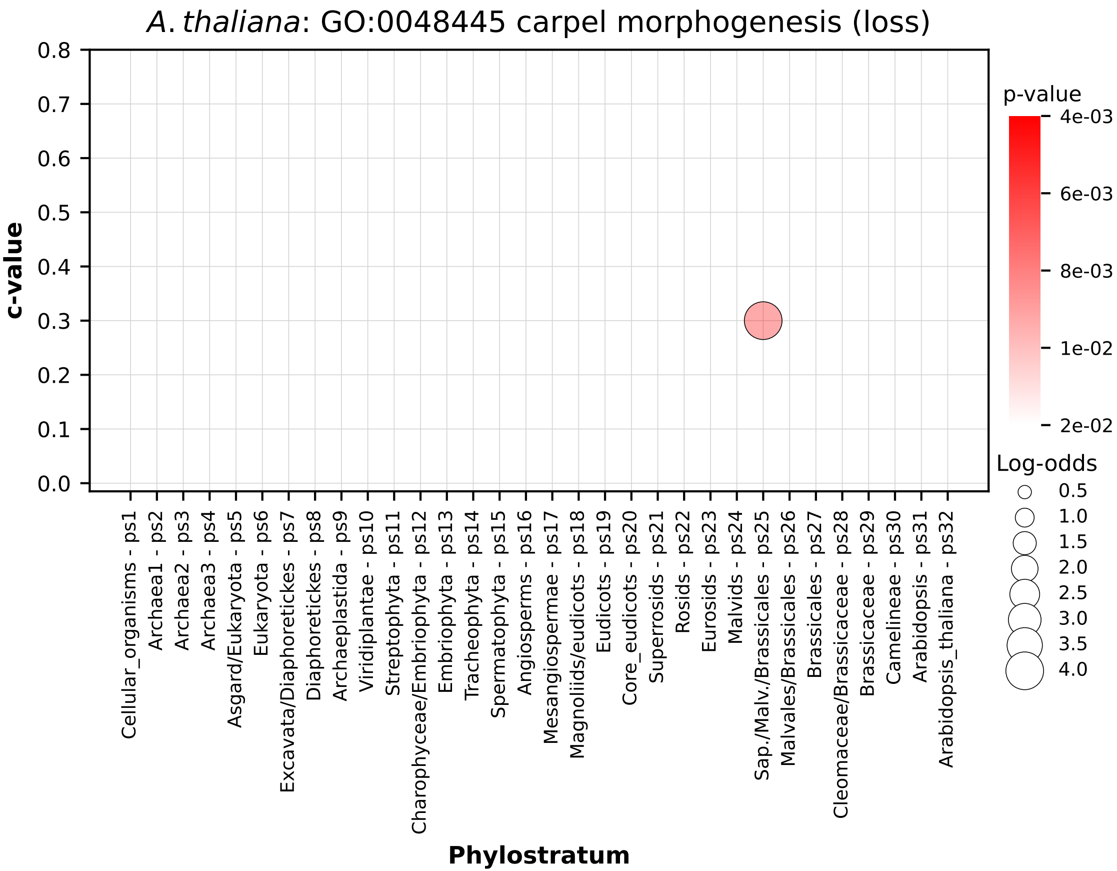

A. thaliana: GO:0048445 carpel morphogenesis (loss)
c-value
0.0
0.1
0.2
0.3
0.4
0.5
0.6
0.7
0.8
p-value
4e-03
6e-03
8e-03
1e-02
2e-02
Log-odds
0.5
1.0
1.5
2.0
2.5
3.0
3.5
4.0
Cellular_organisms - ps1
Archaea1 - ps2
Archaea2 - ps3
Archaea3 - ps4
Asgard/Eukaryota - ps5
Eukaryota - ps6
Excavata/Diaphoretickes - ps7
Diaphoretickes - ps8
Archaeplastida - ps9
Viridiplantae - ps10
Streptophyta - ps11
Charophyceae/Embriophyta - ps12
Embriophyta - ps13
Tracheophyta - ps14
Spermatophyta - ps15
Angiosperms - ps16
Mesangiospermae - ps17
Magnoliids/eudicots - ps18
Eudicots - ps19
Core_eudicots - ps20
Superrosids - ps21
Rosids - ps22
Eurosids - ps23
Malvids - ps24
Sap./Malv./Brassicales - ps25
Malvales/Brassicales - ps26
Brassicales - ps27
Cleomaceae/Brassicaceae - ps28
Brassicaceae - ps29
Camelineae - ps30
Arabidopsis - ps31
Arabidopsis_thaliana - ps32
Phylostratum

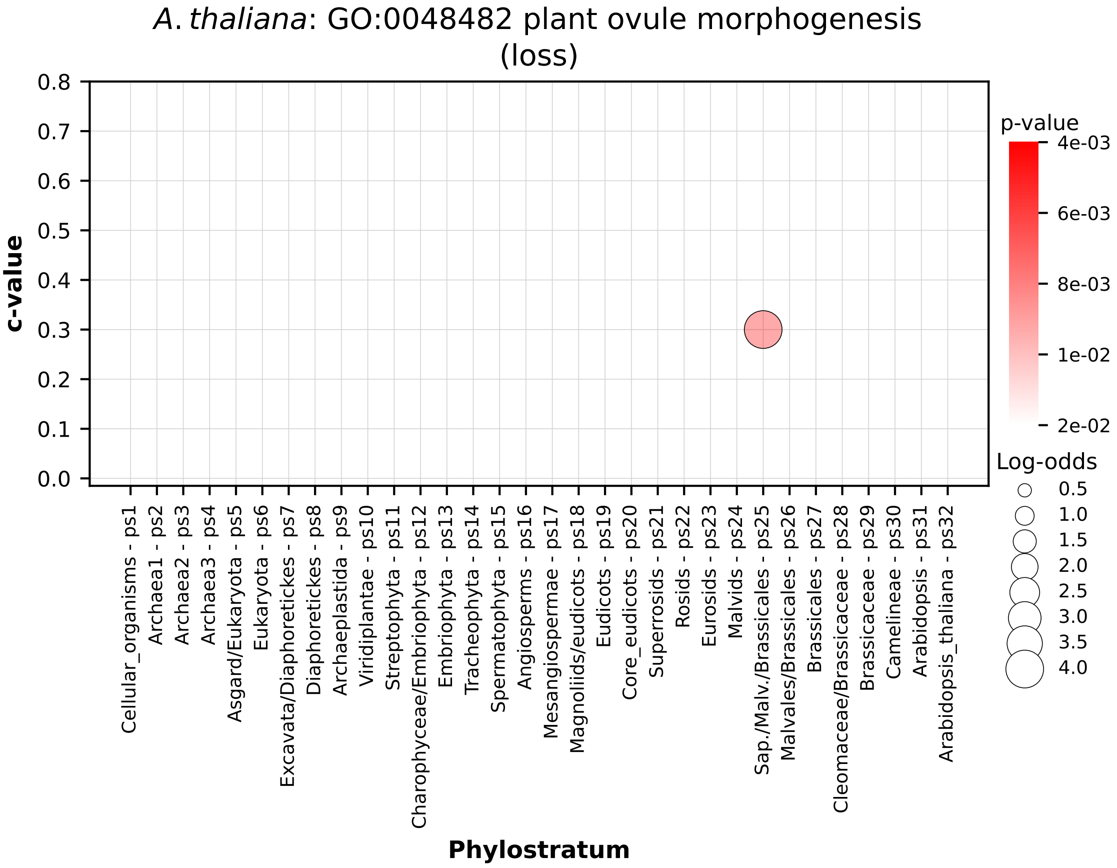
A. thaliana: GO:0048482 plant ovule morphogenesis
(loss)
c-value
0.0
0.1
0.2
0.3
0.4
0.5
0.6
0.7
0.8
Phylostratum
Cellular_organisms - ps1
Archaea1 - ps2
Archaea2 - ps3
Archaea3 - ps4
Asgard/Eukaryota - ps5
Eukaryota - ps6
Excavata/Diaphoretickes - ps7
Diaphoretickes - ps8
Archaeplastida - ps9
Viridiplantae - ps10
Streptophyta - ps11
Charophyceae/Embriophyta - ps12
Embriophyta - ps13
Tracheophyta - ps14
Spermatophyta - ps15
Angiosperms - ps16
Mesangiospermae - ps17
Magnoliids/eudicots - ps18
Eudicots - ps19
Core_eudicots - ps20
Superrosids - ps21
Rosids - ps22
Eurosids - ps23
Malvids - ps24
Sap./Malv./Brassicales - ps25
Malvales/Brassicales - ps26
Brassicales - ps27
Cleomaceae/Brassicaceae - ps28
Brassicaceae - ps29
Camelineae - ps30
Arabidopsis - ps31
Arabidopsis_thaliana - ps32
p-value
4e-03
6e-03
8e-03
1e-02
2e-02
Log-odds
0.5
1.0
1.5
2.0
2.5
3.0
3.5
4.0

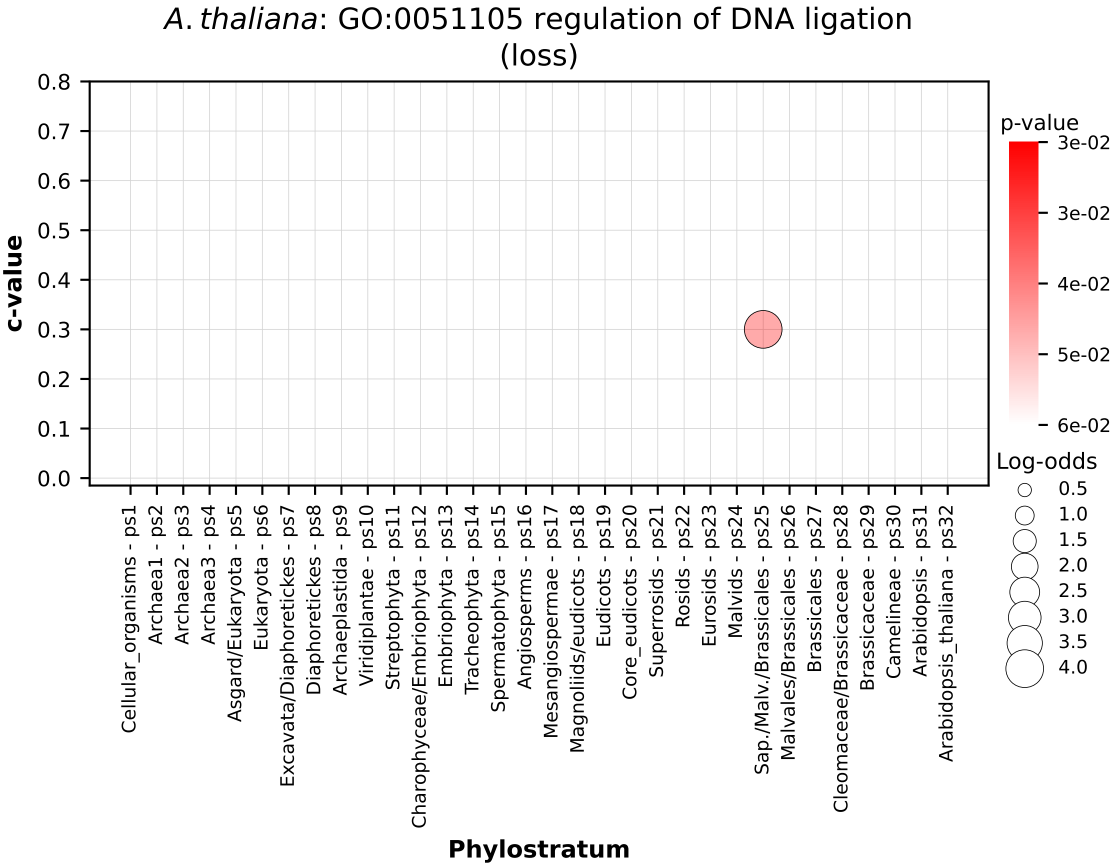
A. thaliana: GO:0051105 regulation of DNA ligation
(loss)
c-value
0.8
0.7
0.6
0.5
0.4
0.3
0.2
0.1
0.0
p-value
3e-02
3e-02
4e-02
5e-02
6e-02
Log-odds
0.5
1.0
1.5
2.0
2.5
3.0
3.5
4.0
Cellular_organisms - ps1
Archaea1 - ps2
Archaea2 - ps3
Archaea3 - ps4
Asgard/Eukaryota - ps5
Eukaryota - ps6
Excavata/Diaphoretickes - ps7
Diaphoretickes - ps8
Archaeplastida - ps9
Viridiplantae - ps10
Streptophyta - ps11
Charophyceae/Embriophyta - ps12
Embriophyta - ps13
Tracheophyta - ps14
Spermatophyta - ps15
Angiosperms - ps16
Mesangiospermae - ps17
Magnoliids/eudicots - ps18
Eudicots - ps19
Core_eudicots - ps20
Superrosids - ps21
Rosids - ps22
Eurosids - ps23
Malvids - ps24
Sap./Malv./Brassicales - ps25
Malvales/Brassicales - ps26
Brassicales - ps27
Cleomaceae/Brassicaceae - ps28
Brassicaceae - ps29
Camelineae - ps30
Arabidopsis - ps31
Arabidopsis_thaliana - ps32
Phylostratum

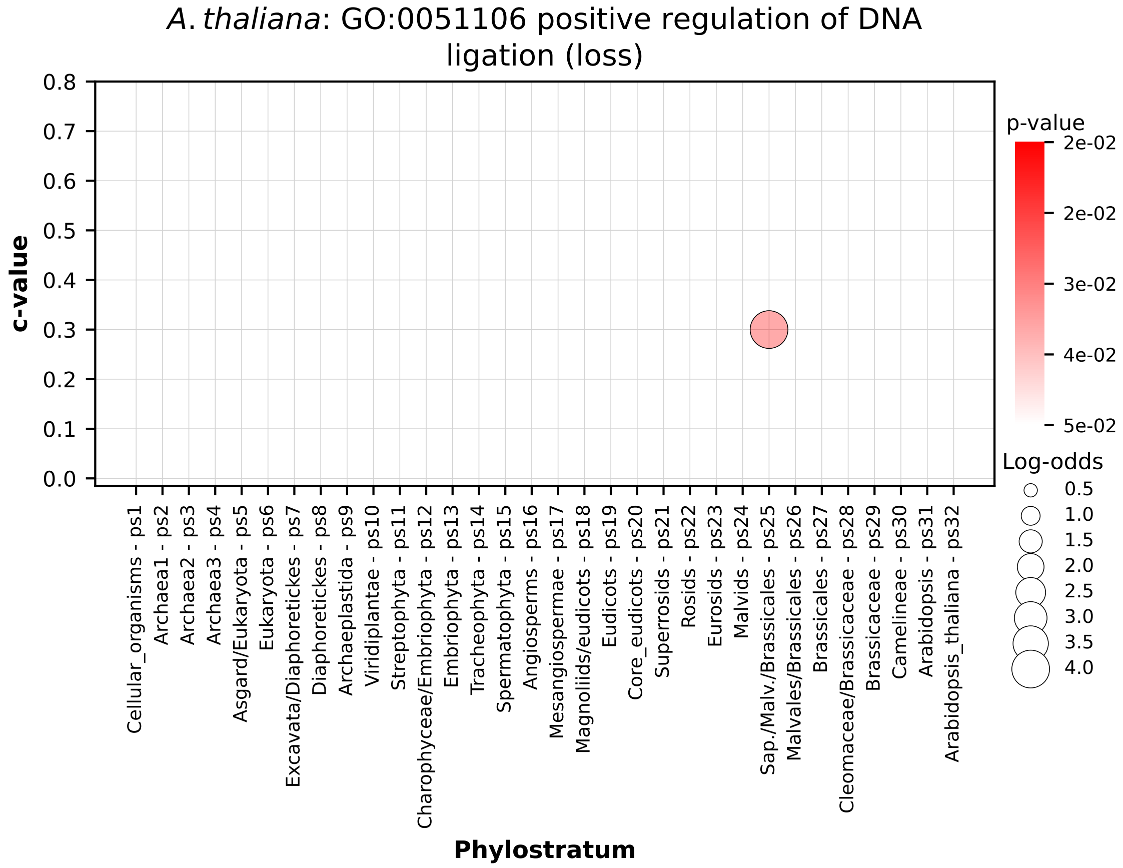

A. thaliana: GO:0051106 positive regulation of DNA ligation (loss)
c-value
0.0
0.1
0.2
0.3
0.4
0.5
0.6
0.7
0.8
Phylostratum
Cellular_organisms - ps1
Archaea1 - ps2
Archaea2 - ps3
Archaea3 - ps4
Asgard/Eukaryota - ps5
Eukaryota - ps6
Excavata/Diaphoretickes - ps7
Diaphoretickes - ps8
Archaeplastida - ps9
Viridiplantae - ps10
Streptophyta - ps11
Charophyceae/Embriophyta - ps12
Embriophyta - ps13
Tracheophyta - ps14
Spermatophyta - ps15
Angiosperms - ps16
Mesangiospermae - ps17
Magnoliids/eudicots - ps18
Eudicots - ps19
Core_eudicots - ps20
Superrosids - ps21
Rosids - ps22
Eurosids - ps23
Malvids - ps24
Sap./Malv./Brassicales - ps25
Malvales/Brassicales - ps26
Brassicales - ps27
Cleomaceae/Brassicaceae - ps28
Brassicaceae - ps29
Camelineae - ps30
Arabidopsis - ps31
Arabidopsis_thaliana - ps32
p-value
2e-02
2e-02
3e-02
4e-02
5e-02
Log-odds
0.5
1.0
1.5
2.0
2.5
3.0
3.5
4.0

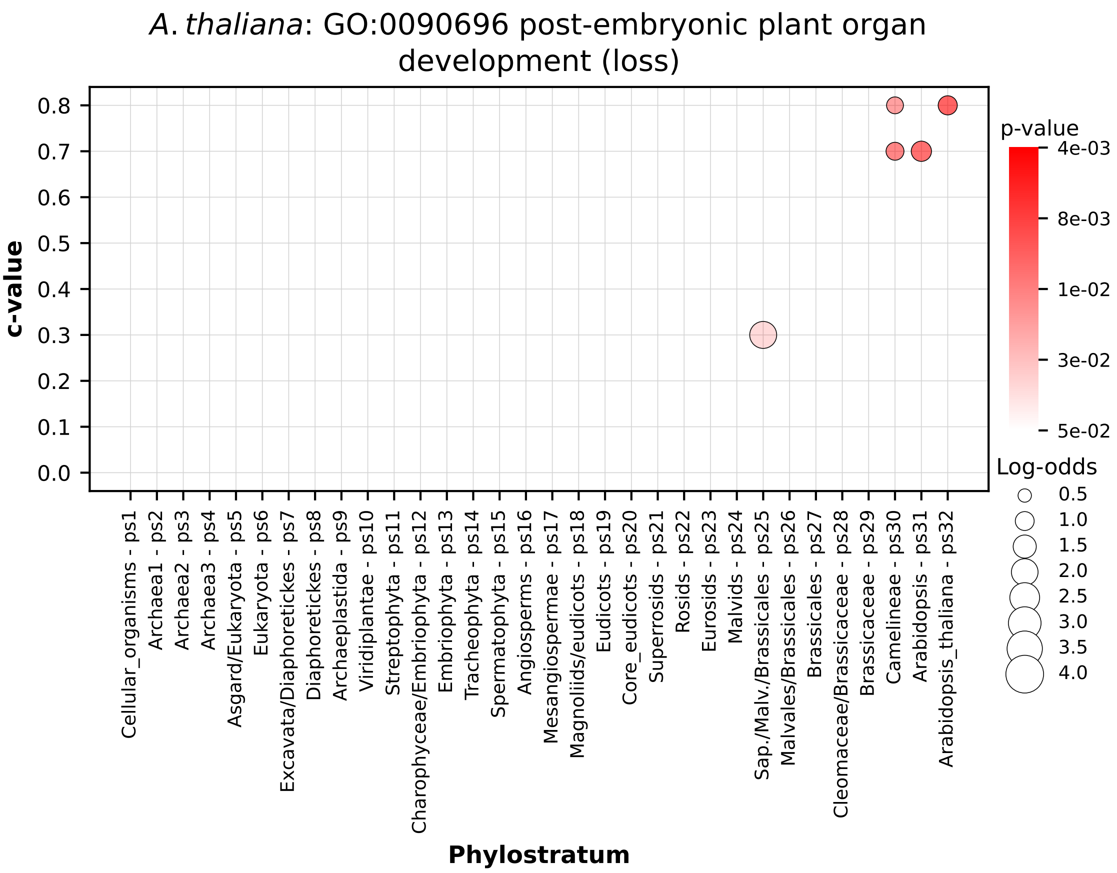

A. thaliana: GO:0090696 post-embryonic plant organ development (loss)
c-value
0.0
0.1
0.2
0.3
0.4
0.5
0.6
0.7
0.8
Phylostratum
Cellular_organisms - ps1
Archaea1 - ps2
Archaea2 - ps3
Archaea3 - ps4
Asgard/Eukaryota - ps5
Eukaryota - ps6
Excavata/Diaphoretickes - ps7
Diaphoretickes - ps8
Archaeplastida - ps9
Viridiplantae - ps10
Streptophyta - ps11
Charophyceae/Embriophyta - ps12
Embriophyta - ps13
Tracheophyta - ps14
Spermatophyta - ps15
Angiosperms - ps16
Mesangiospermae - ps17
Magnoliids/eudicots - ps18
Eudicots - ps19
Core_eudicots - ps20
Superrosids - ps21
Rosids - ps22
Eurosids - ps23
Malvids - ps24
Sap./Malv./Brassicales - ps25
Malvales/Brassicales - ps26
Brassicales - ps27
Cleomaceae/Brassicaceae - ps28
Brassicaceae - ps29
Camelineae - ps30
Arabidopsis - ps31
Arabidopsis_thaliana - ps32
p-value
4e-03
8e-03
1e-02
3e-02
5e-02
Log-odds
0.5
1.0
1.5
2.0
2.5
3.0
3.5
4.0

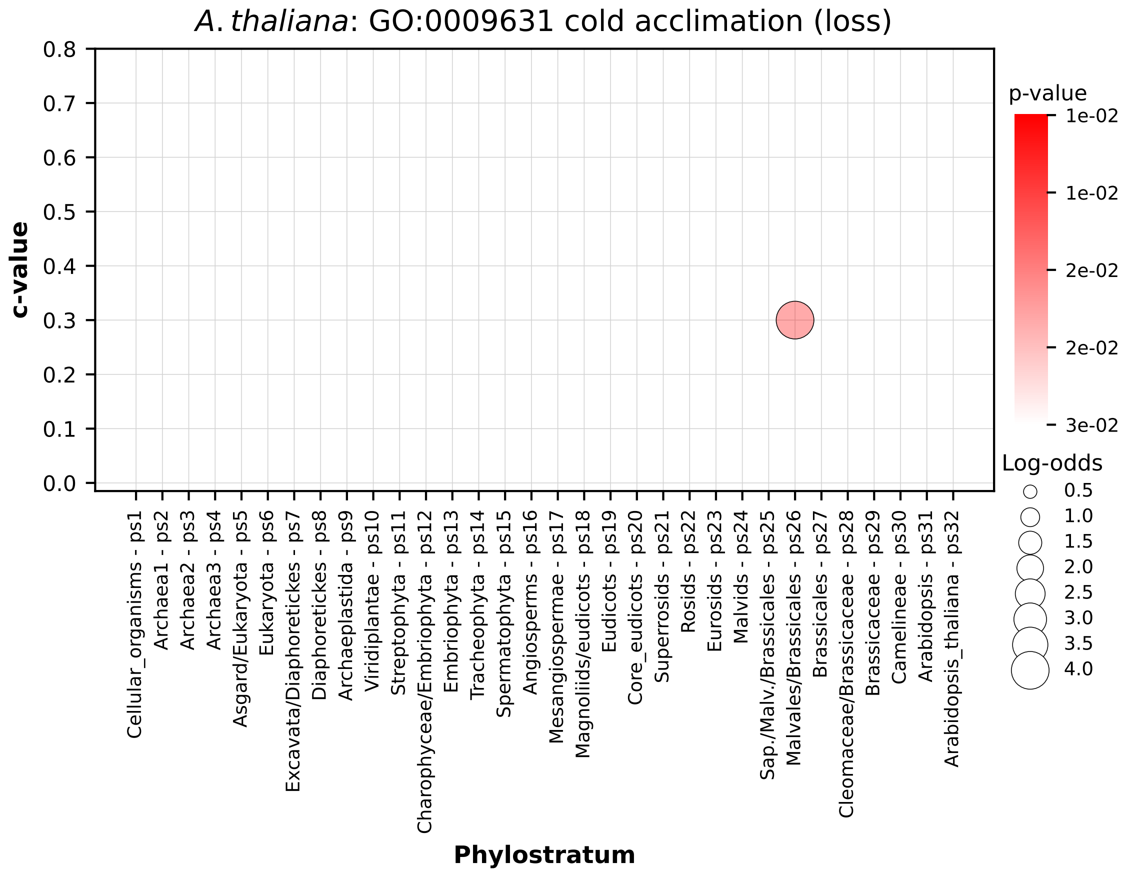
A. thaliana: GO:0009631 cold acclimation (loss)
c-value
0.0
0.1
0.2
0.3
0.4
0.5
0.6
0.7
0.8
Phylostratum
Cellular_organisms - ps1
Archaea1 - ps2
Archaea2 - ps3
Archaea3 - ps4
Asgard/Eukaryota - ps5
Eukaryota - ps6
Excavata/Diaphoretickes - ps7
Diaphoretickes - ps8
Archaeplastida - ps9
Viridiplantae - ps10
Streptophyta - ps11
Charophyceae/Embriophyta - ps12
Embriophyta - ps13
Tracheophyta - ps14
Spermatophyta - ps15
Angiosperms - ps16
Mesangiospermae - ps17
Magnoliids/eudicots - ps18
Eudicots - ps19
Core_eudicots - ps20
Superrosids - ps21
Rosids - ps22
Eurosids - ps23
Malvids - ps24
Sap./Malv./Brassicales - ps25
Malvales/Brassicales - ps26
Brassicales - ps27
Cleomaceae/Brassicaceae - ps28
Brassicaceae - ps29
Camelineae - ps30
Arabidopsis - ps31
Arabidopsis_thaliana - ps32
p-value
1e-02
1e-02
2e-02
2e-02
3e-02
Log-odds
0.5
1.0
1.5
2.0
2.5
3.0
3.5
4.0

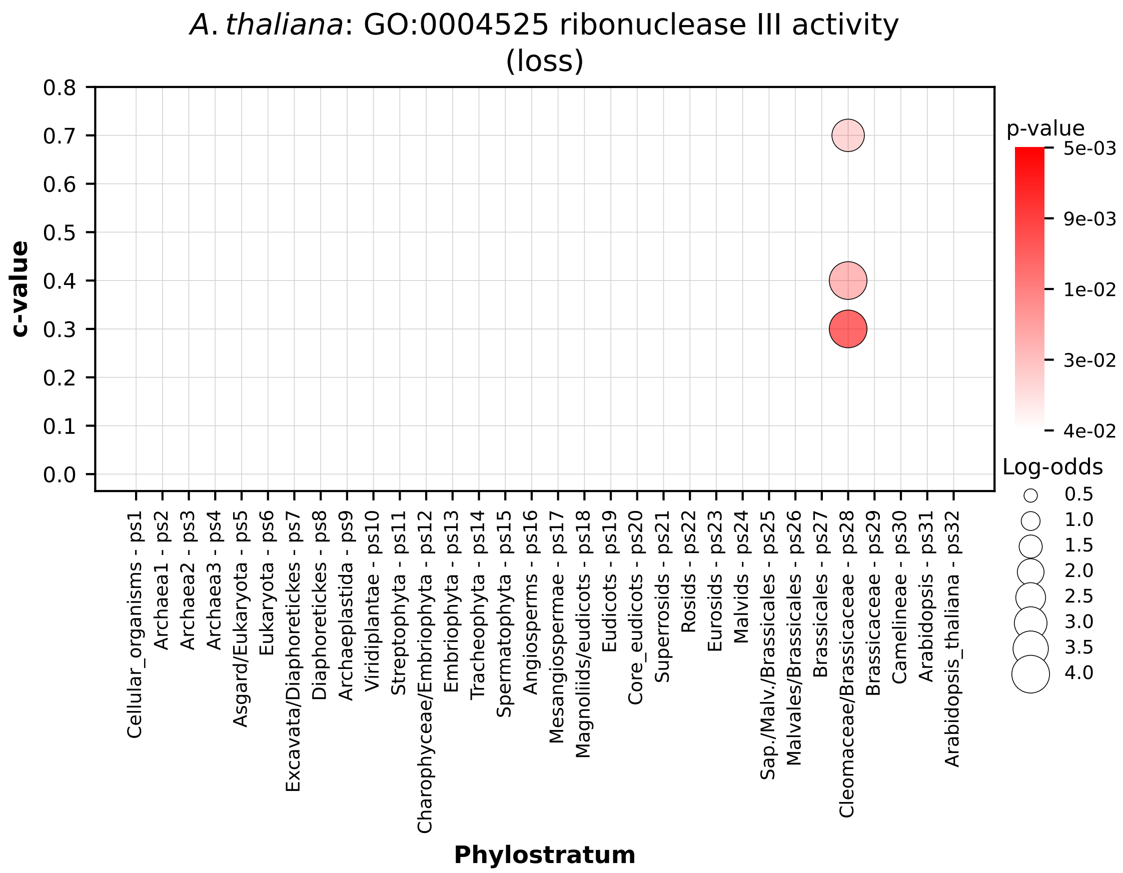

A. thaliana: GO:0004525 ribonuclease III activity
(loss)
c-value
0.8
0.7
0.6
0.5
0.4
0.3
0.2
0.1
0.0
p-value
5e-03
9e-03
1e-02
3e-02
4e-02
Log-odds
0.5
1.0
1.5
2.0
2.5
3.0
3.5
4.0
Cellular_organisms - ps1
Archaea1 - ps2
Archaea2 - ps3
Archaea3 - ps4
Asgard/Eukaryota - ps5
Eukaryota - ps6
Excavata/Diaphoretickes - ps7
Diaphoretickes - ps8
Archaeplastida - ps9
Viridiplantae - ps10
Streptophyta - ps11
Charophyceae/Embriophyta - ps12
Embriophyta - ps13
Tracheophyta - ps14
Spermatophyta - ps15
Angiosperms - ps16
Mesangiospermae - ps17
Magnoliids/eudicots - ps18
Eudicots - ps19
Core_eudicots - ps20
Superrosids - ps21
Rosids - ps22
Eurosids - ps23
Malvids - ps24
Sap./Malv./Brassicales - ps25
Malvales/Brassicales - ps26
Brassicales - ps27
Cleomaceae/Brassicaceae - ps28
Brassicaceae - ps29
Camelineae - ps30
Arabidopsis - ps31
Arabidopsis_thaliana - ps32
Phylostratum

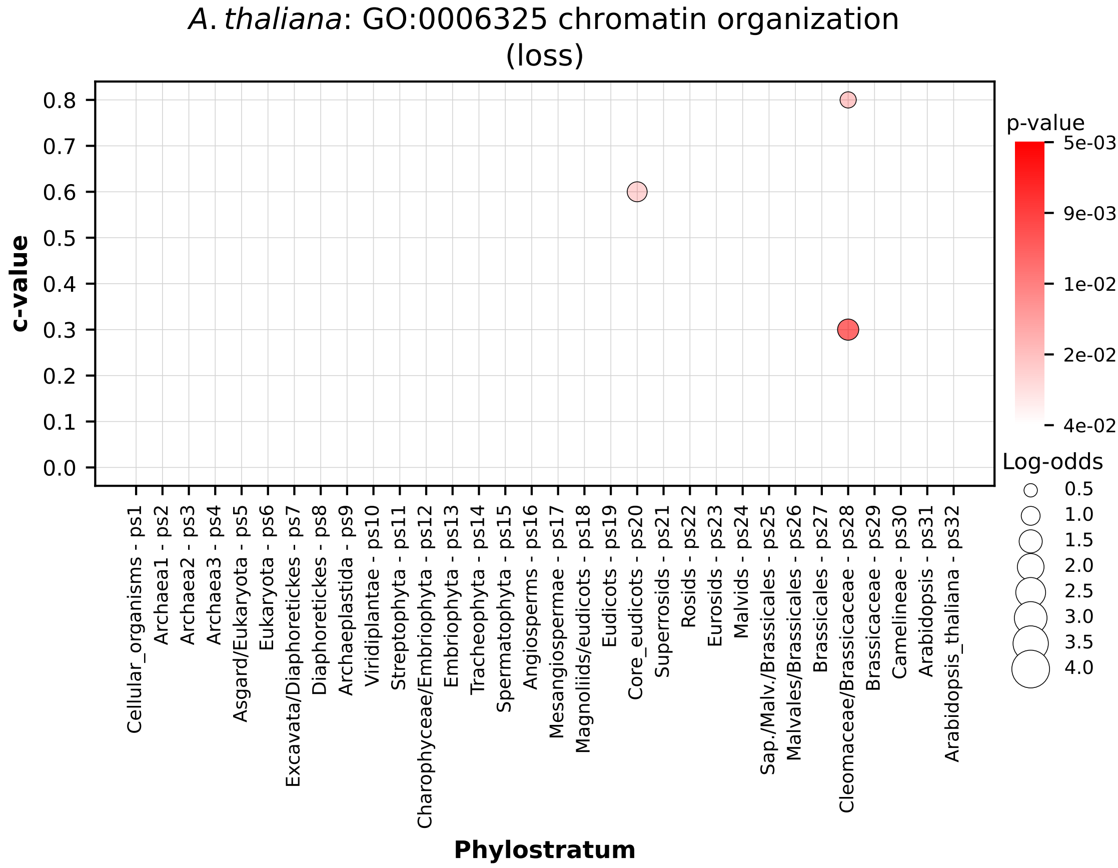
A. thaliana: GO:0006325 chromatin organization
(loss)
c-value
0.8
0.7
0.6
0.5
0.4
0.3
0.2
0.1
0.0
Phylostratum
Cellular_organisms - ps1
Archaea1 - ps2
Archaea2 - ps3
Archaea3 - ps4
Asgard/Eukaryota - ps5
Eukaryota - ps6
Excavata/Diaphoretickes - ps7
Diaphoretickes - ps8
Archaeplastida - ps9
Viridiplantae - ps10
Streptophyta - ps11
Charophyceae/Embriophyta - ps12
Embriophyta - ps13
Tracheophyta - ps14
Spermatophyta - ps15
Angiosperms - ps16
Mesangiospermae - ps17
Magnoliids/eudicots - ps18
Eudicots - ps19
Core_eudicots - ps20
Superrosids - ps21
Rosids - ps22
Eurosids - ps23
Malvids - ps24
Sap./Malv./Brassicales - ps25
Malvales/Brassicales - ps26
Brassicales - ps27
Cleomaceae/Brassicaceae - ps28
Brassicaceae - ps29
Camelineae - ps30
Arabidopsis - ps31
Arabidopsis_thaliana - ps32
p-value
5e-03
9e-03
1e-02
2e-02
4e-02
Log-odds
0.5
1.0
1.5
2.0
2.5
3.0
3.5
4.0

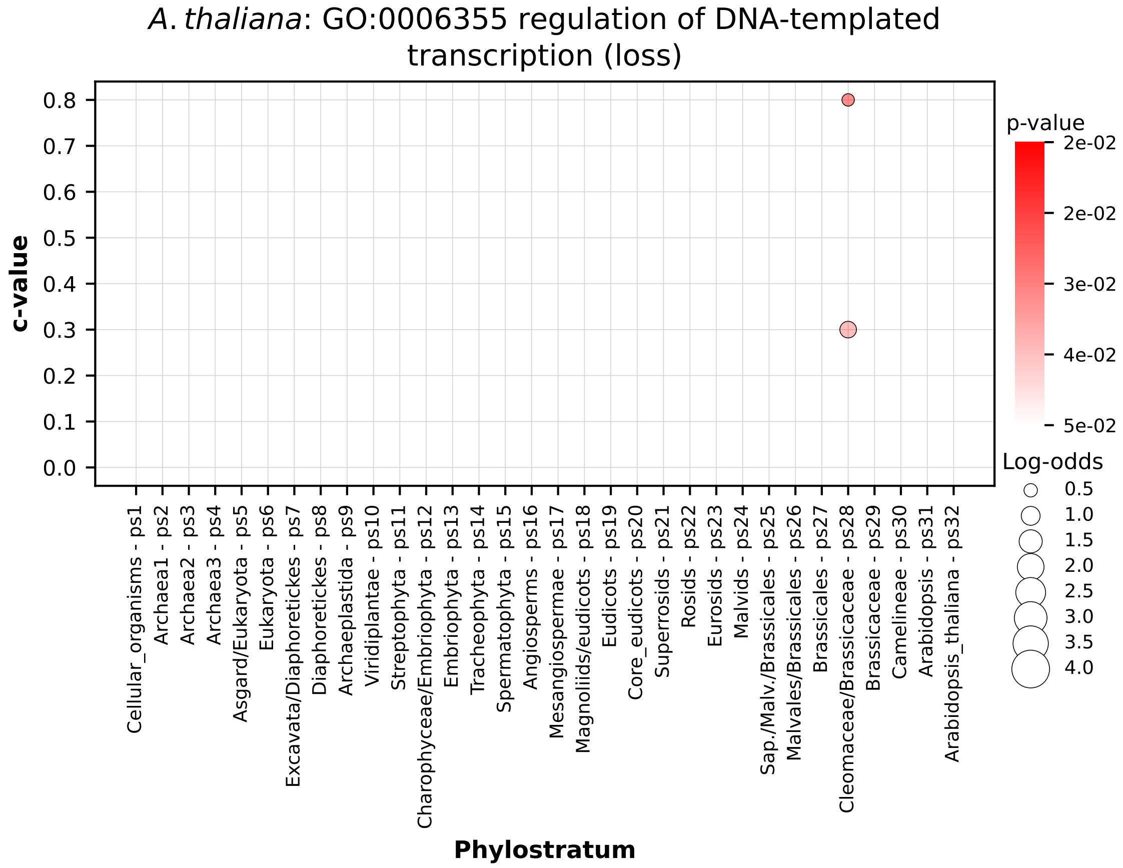
A. thaliana: GO:0006355 regulation of DNA-templated transcription (loss)
c-value
0.0
0.1
0.2
0.3
0.4
0.5
0.6
0.7
0.8
Phylostratum
Cellular_organisms - ps1
Archaea1 - ps2
Archaea2 - ps3
Archaea3 - ps4
Asgard/Eukaryota - ps5
Eukaryota - ps6
Excavata/Diaphoretickes - ps7
Diaphoretickes - ps8
Archaeplastida - ps9
Viridiplantae - ps10
Streptophyta - ps11
Charophyceae/Embriophyta - ps12
Embriophyta - ps13
Tracheophyta - ps14
Spermatophyta - ps15
Angiosperms - ps16
Mesangiospermae - ps17
Magnoliids/eudicots - ps18
Eudicots - ps19
Core_eudicots - ps20
Superrosids - ps21
Rosids - ps22
Eurosids - ps23
Malvids - ps24
Sap./Malv./Brassicales - ps25
Malvales/Brassicales - ps26
Brassicales - ps27
Cleomaceae/Brassicaceae - ps28
Brassicaceae - ps29
Camelineae - ps30
Arabidopsis - ps31
Arabidopsis_thaliana - ps32
p-value
2e-02
2e-02
3e-02
4e-02
5e-02
Log-odds
0.5
1.0
1.5
2.0
2.5
3.0
3.5
4.0

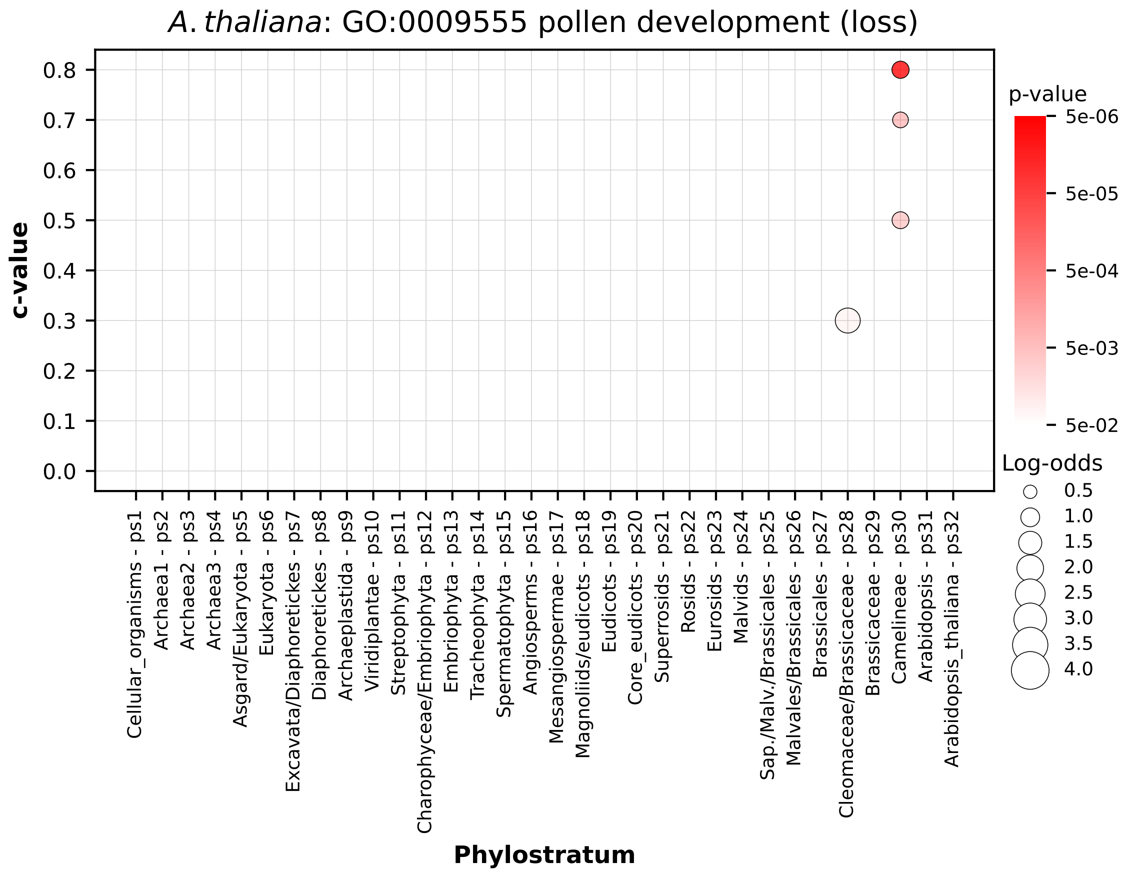

A. thaliana: GO:0009555 pollen development (loss)
c-value
0.0
0.1
0.2
0.3
0.4
0.5
0.6
0.7
0.8
Phylostratum
Cellular_organisms - ps1
Archaea1 - ps2
Archaea2 - ps3
Archaea3 - ps4
Asgard/Eukaryota - ps5
Eukaryota - ps6
Excavata/Diaphoretickes - ps7
Diaphoretickes - ps8
Archaeplastida - ps9
Viridiplantae - ps10
Streptophyta - ps11
Charophyceae/Embriophyta - ps12
Embriophyta - ps13
Tracheophyta - ps14
Spermatophyta - ps15
Angiosperms - ps16
Mesangiospermae - ps17
Magnoliids/eudicots - ps18
Eudicots - ps19
Core_eudicots - ps20
Superrosids - ps21
Rosids - ps22
Eurosids - ps23
Malvids - ps24
Sap./Malv./Brassicales - ps25
Malvales/Brassicales - ps26
Brassicales - ps27
Cleomaceae/Brassicaceae - ps28
Brassicaceae - ps29
Camelineae - ps30
Arabidopsis - ps31
Arabidopsis_thaliana - ps32
p-value
5e-06
5e-05
5e-04
5e-03
5e-02
Log-odds
0.5
1.0
1.5
2.0
2.5
3.0
3.5
4.0

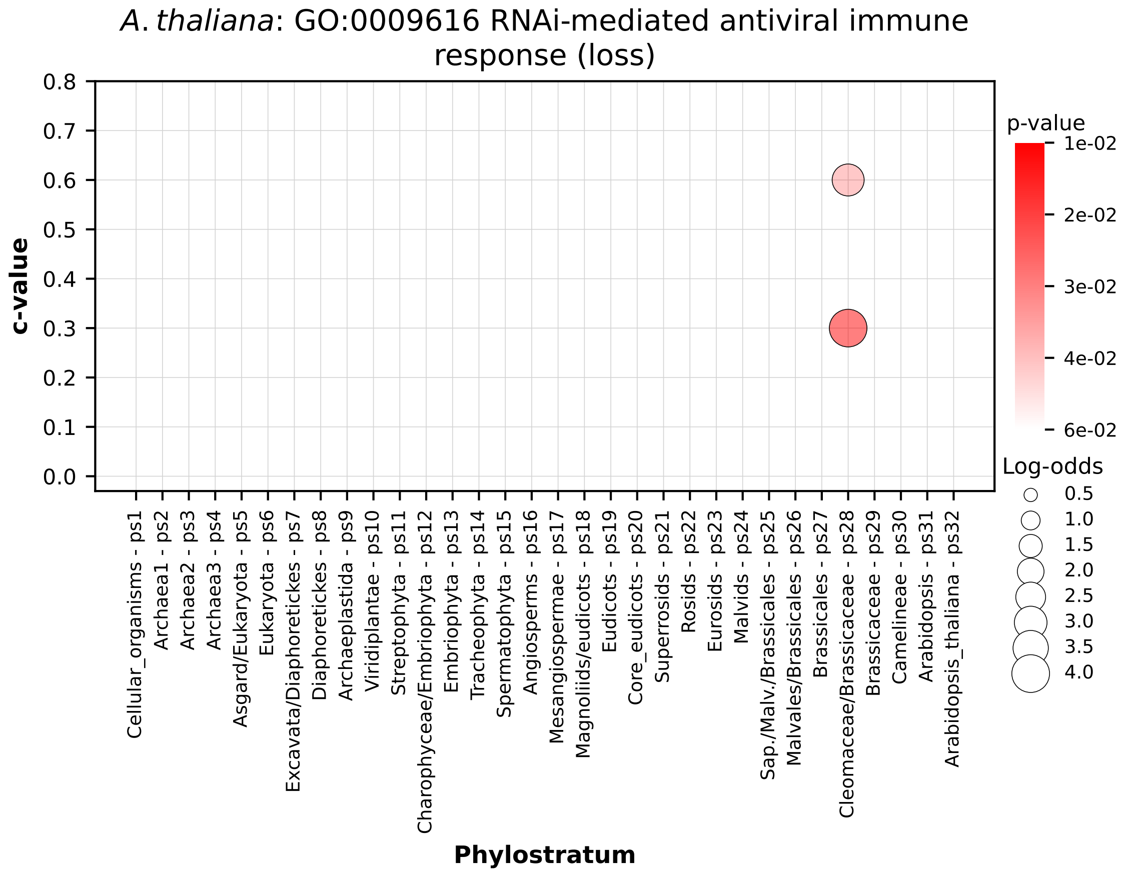

A. thaliana: GO:0009616 RNAi-mediated antiviral immune response (loss)
c-value
0.8
0.7
0.6
0.5
0.4
0.3
0.2
0.1
0.0
Phylostratum
Cellular_organisms - ps1
Archaea1 - ps2
Archaea2 - ps3
Archaea3 - ps4
Asgard/Eukaryota - ps5
Eukaryota - ps6
Excavata/Diaphoretickes - ps7
Diaphoretickes - ps8
Archaeplastida - ps9
Viridiplantae - ps10
Streptophyta - ps11
Charophyceae/Embriophyta - ps12
Embriophyta - ps13
Tracheophyta - ps14
Spermatophyta - ps15
Angiosperms - ps16
Mesangiospermae - ps17
Magnoliids/eudicots - ps18
Eudicots - ps19
Core_eudicots - ps20
Superrosids - ps21
Rosids - ps22
Eurosids - ps23
Malvids - ps24
Sap./Malv./Brassicales - ps25
Malvales/Brassicales - ps26
Brassicales - ps27
Cleomaceae/Brassicaceae - ps28
Brassicaceae - ps29
Camelineae - ps30
Arabidopsis - ps31
Arabidopsis_thaliana - ps32
p-value
1e-02
2e-02
3e-02
4e-02
6e-02
Log-odds
0.5
1.0
1.5
2.0
2.5
3.0
3.5
4.0

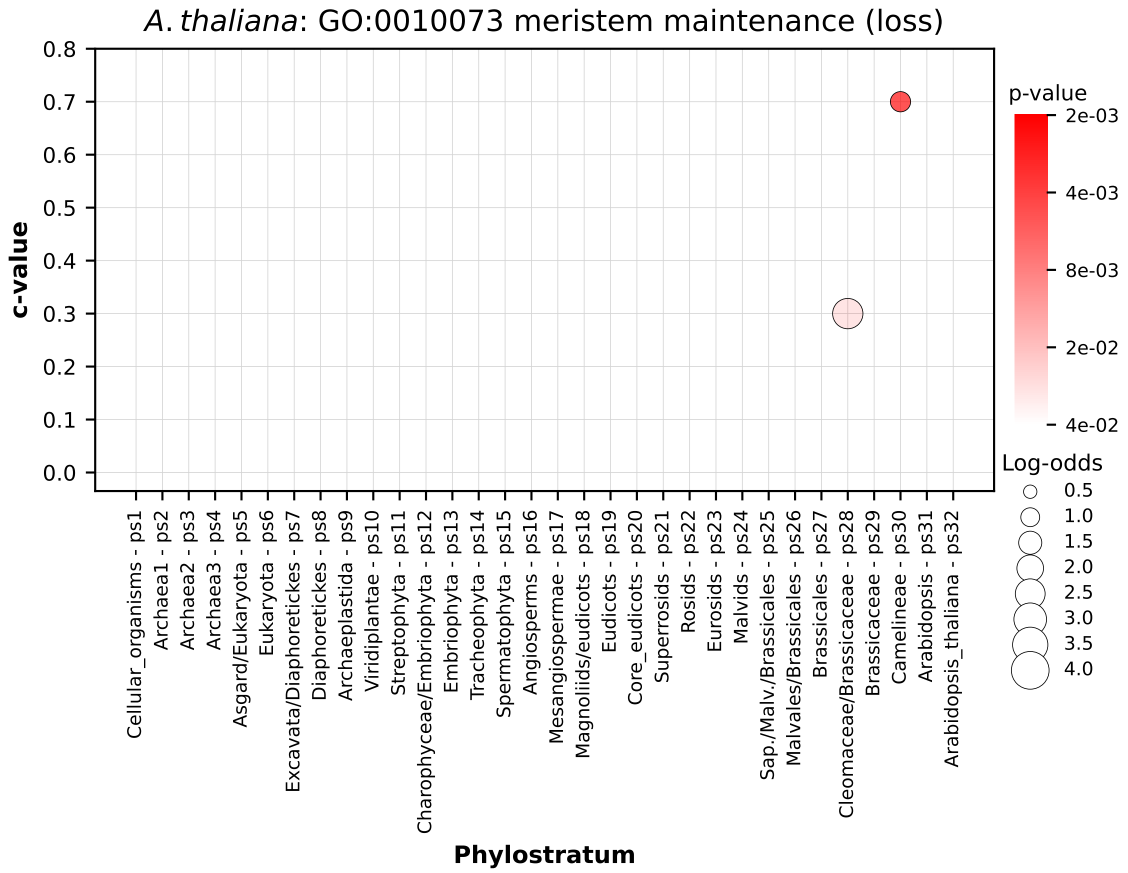

A. thaliana: GO:0010073 meristem maintenance (loss)
c-value
0.8
0.7
0.6
0.5
0.4
0.3
0.2
0.1
0.0
Phylostratum
Cellular_organisms - ps1
Archaea1 - ps2
Archaea2 - ps3
Archaea3 - ps4
Asgard/Eukaryota - ps5
Eukaryota - ps6
Excavata/Diaphoretickes - ps7
Diaphoretickes - ps8
Archaeplastida - ps9
Viridiplantae - ps10
Streptophyta - ps11
Charophyceae/Embriophyta - ps12
Embriophyta - ps13
Tracheophyta - ps14
Spermatophyta - ps15
Angiosperms - ps16
Mesangiospermae - ps17
Magnoliids/eudicots - ps18
Eudicots - ps19
Core_eudicots - ps20
Superrosids - ps21
Rosids - ps22
Eurosids - ps23
Malvids - ps24
Sap./Malv./Brassicales - ps25
Malvales/Brassicales - ps26
Brassicales - ps27
Cleomaceae/Brassicaceae - ps28
Brassicaceae - ps29
Camelineae - ps30
Arabidopsis - ps31
Arabidopsis_thaliana - ps32
p-value
2e-03
4e-03
8e-03
2e-02
4e-02
Log-odds
0.5
1.0
1.5
2.0
2.5
3.0
3.5
4.0

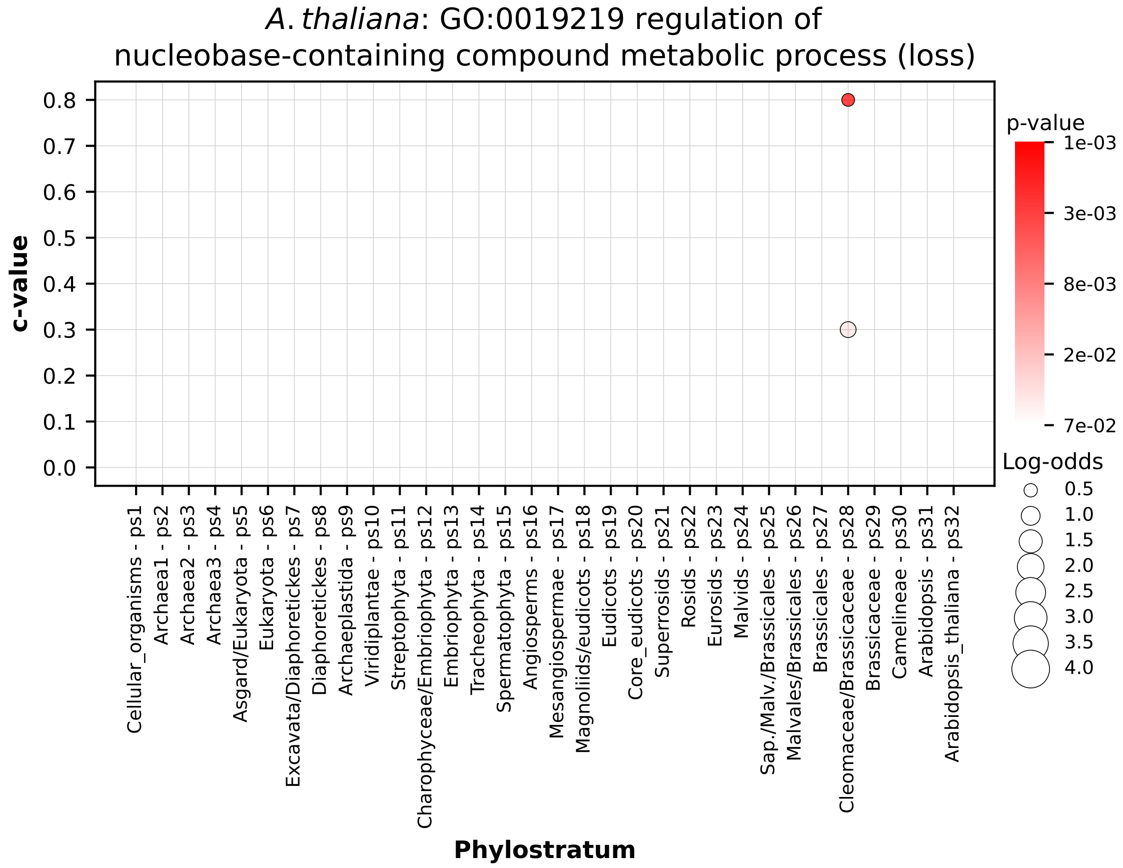

A. thaliana: GO:0019219 regulation of nucleobase-containing compound metabolic process (loss)
c-value
0.0
0.1
0.2
0.3
0.4
0.5
0.6
0.7
0.8
p-value
1e-03
3e-03
8e-03
2e-02
7e-02
Log-odds
0.5
1.0
1.5
2.0
2.5
3.0
3.5
4.0
Cellular_organisms - ps1
Archaea1 - ps2
Archaea2 - ps3
Archaea3 - ps4
Asgard/Eukaryota - ps5
Eukaryota - ps6
Excavata/Diaphoretickes - ps7
Diaphoretickes - ps8
Archaeplastida - ps9
Viridiplantae - ps10
Streptophyta - ps11
Charophyceae/Embriophyta - ps12
Embriophyta - ps13
Tracheophyta - ps14
Spermatophyta - ps15
Angiosperms - ps16
Mesangiospermae - ps17
Magnoliids/eudicots - ps18
Eudicots - ps19
Core_eudicots - ps20
Superrosids - ps21
Rosids - ps22
Eurosids - ps23
Malvids - ps24
Sap./Malv./Brassicales - ps25
Malvales/Brassicales - ps26
Brassicales - ps27
Cleomaceae/Brassicaceae - ps28
Brassicaceae - ps29
Camelineae - ps30
Arabidopsis - ps31
Arabidopsis_thaliana - ps32
Phylostratum

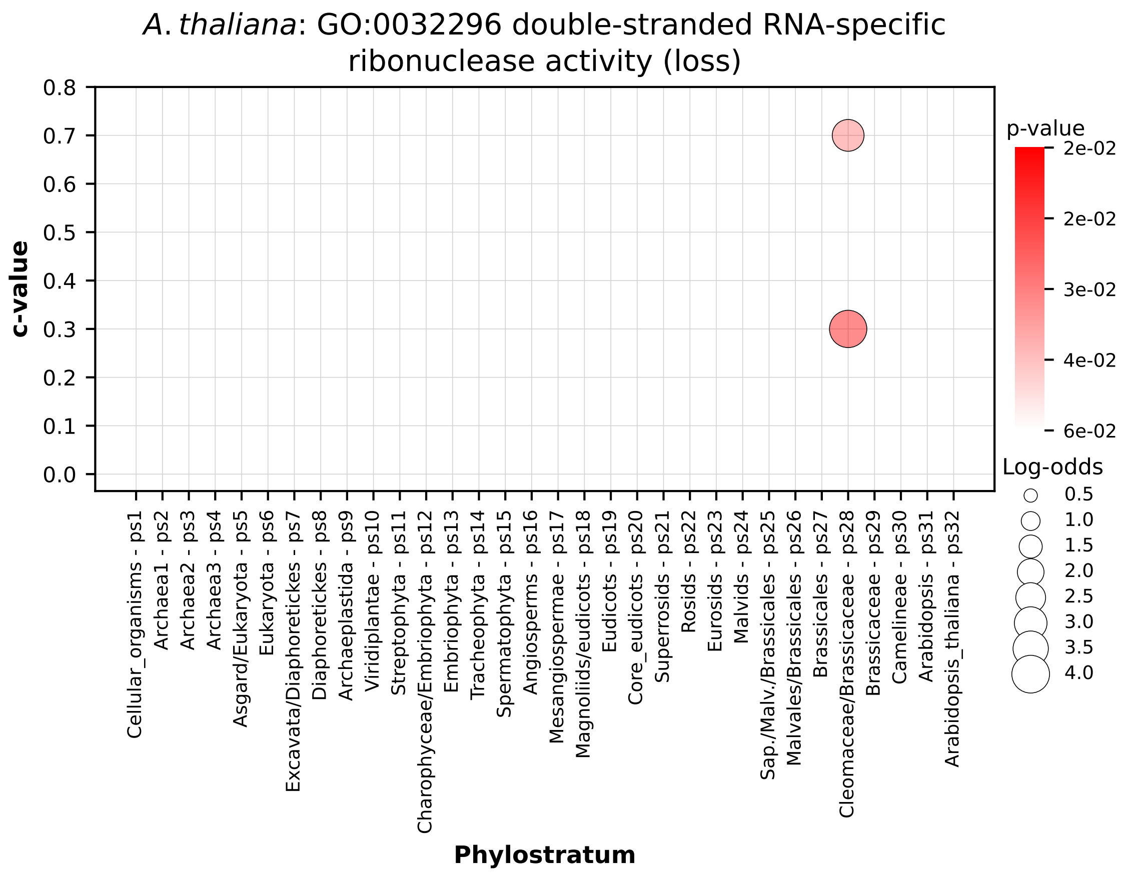
A. thaliana: GO:0032296 double-stranded RNA-specific ribonuclease activity (loss)
c-value
0.8
0.7
0.6
0.5
0.4
0.3
0.2
0.1
0.0
p-value
2e-02
2e-02
3e-02
4e-02
6e-02
Log-odds
0.5
1.0
1.5
2.0
2.5
3.0
3.5
4.0
Cellular_organisms - ps1
Archaea1 - ps2
Archaea2 - ps3
Archaea3 - ps4
Asgard/Eukaryota - ps5
Eukaryota - ps6
Excavata/Diaphoretickes - ps7
Diaphoretickes - ps8
Archaeplastida - ps9
Viridiplantae - ps10
Streptophyta - ps11
Charophyceae/Embriophyta - ps12
Embriophyta - ps13
Tracheophyta - ps14
Spermatophyta - ps15
Angiosperms - ps16
Mesangiospermae - ps17
Magnoliids/eudicots - ps18
Eudicots - ps19
Core_eudicots - ps20
Superrosids - ps21
Rosids - ps22
Eurosids - ps23
Malvids - ps24
Sap./Malv./Brassicales - ps25
Malvales/Brassicales - ps26
Brassicales - ps27
Cleomaceae/Brassicaceae - ps28
Brassicaceae - ps29
Camelineae - ps30
Arabidopsis - ps31
Arabidopsis_thaliana - ps32
Phylostratum

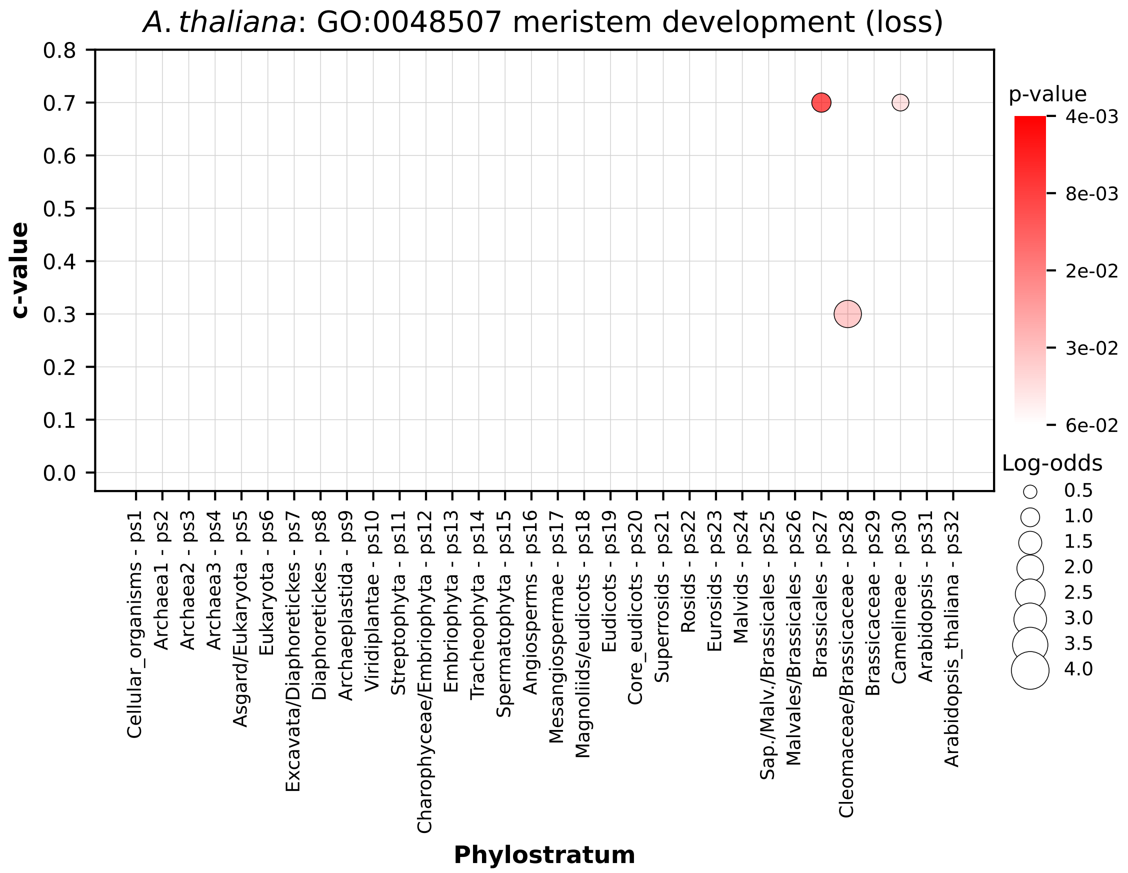

A. thaliana: GO:0048507 meristem development (loss)
c-value
0.8
0.7
0.6
0.5
0.4
0.3
0.2
0.1
0.0
p-value
4e-03
8e-03
2e-02
3e-02
6e-02
Log-odds
0.5
1.0
1.5
2.0
2.5
3.0
3.5
4.0
Cellular_organisms - ps1
Archaea1 - ps2
Archaea2 - ps3
Archaea3 - ps4
Asgard/Eukaryota - ps5
Eukaryota - ps6
Excavata/Diaphoretickes - ps7
Diaphoretickes - ps8
Archaeplastida - ps9
Viridiplantae - ps10
Streptophyta - ps11
Charophyceae/Embriophyta - ps12
Embriophyta - ps13
Tracheophyta - ps14
Spermatophyta - ps15
Angiosperms - ps16
Mesangiospermae - ps17
Magnoliids/eudicots - ps18
Eudicots - ps19
Core_eudicots - ps20
Superrosids - ps21
Rosids - ps22
Eurosids - ps23
Malvids - ps24
Sap./Malv./Brassicales - ps25
Malvales/Brassicales - ps26
Brassicales - ps27
Cleomaceae/Brassicaceae - ps28
Brassicaceae - ps29
Camelineae - ps30
Arabidopsis - ps31
Arabidopsis_thaliana - ps32
Phylostratum

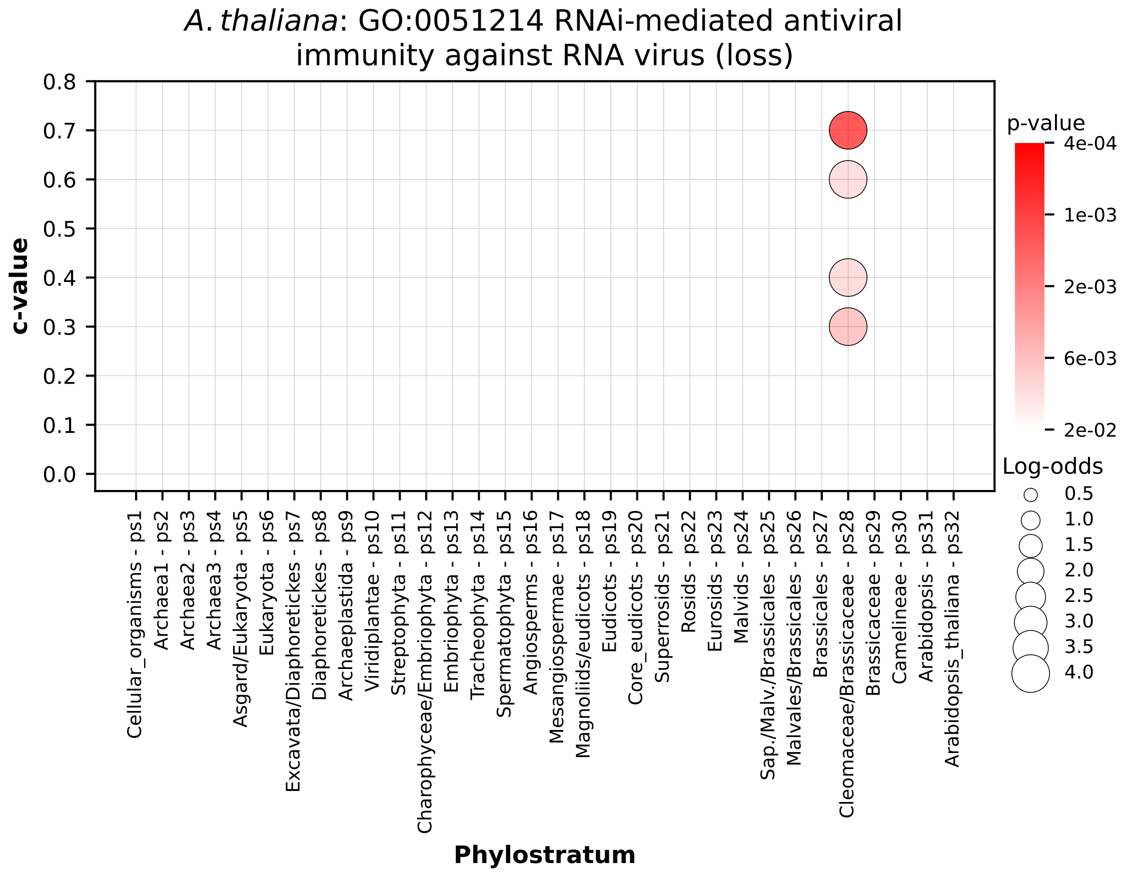

A. thaliana: GO:0051214 RNAi-mediated antiviral immunity against RNA virus (loss)
c-value
0.8
0.7
0.6
0.5
0.4
0.3
0.2
0.1
0.0
p-value
4e-04
1e-03
2e-03
6e-03
2e-02
Log-odds
0.5
1.0
1.5
2.0
2.5
3.0
3.5
4.0
Cellular_organisms - ps1
Archaea1 - ps2
Archaea2 - ps3
Archaea3 - ps4
Asgard/Eukaryota - ps5
Eukaryota - ps6
Excavata/Diaphoretickes - ps7
Diaphoretickes - ps8
Archaeplastida - ps9
Viridiplantae - ps10
Streptophyta - ps11
Charophyceae/Embriophyta - ps12
Embriophyta - ps13
Tracheophyta - ps14
Spermatophyta - ps15
Angiosperms - ps16
Mesangiospermae - ps17
Magnoliids/eudicots - ps18
Eudicots - ps19
Core_eudicots - ps20
Superrosids - ps21
Rosids - ps22
Eurosids - ps23
Malvids - ps24
Sap./Malv./Brassicales - ps25
Malvales/Brassicales - ps26
Brassicales - ps27
Cleomaceae/Brassicaceae - ps28
Brassicaceae - ps29
Camelineae - ps30
Arabidopsis - ps31
Arabidopsis_thaliana - ps32
Phylostratum

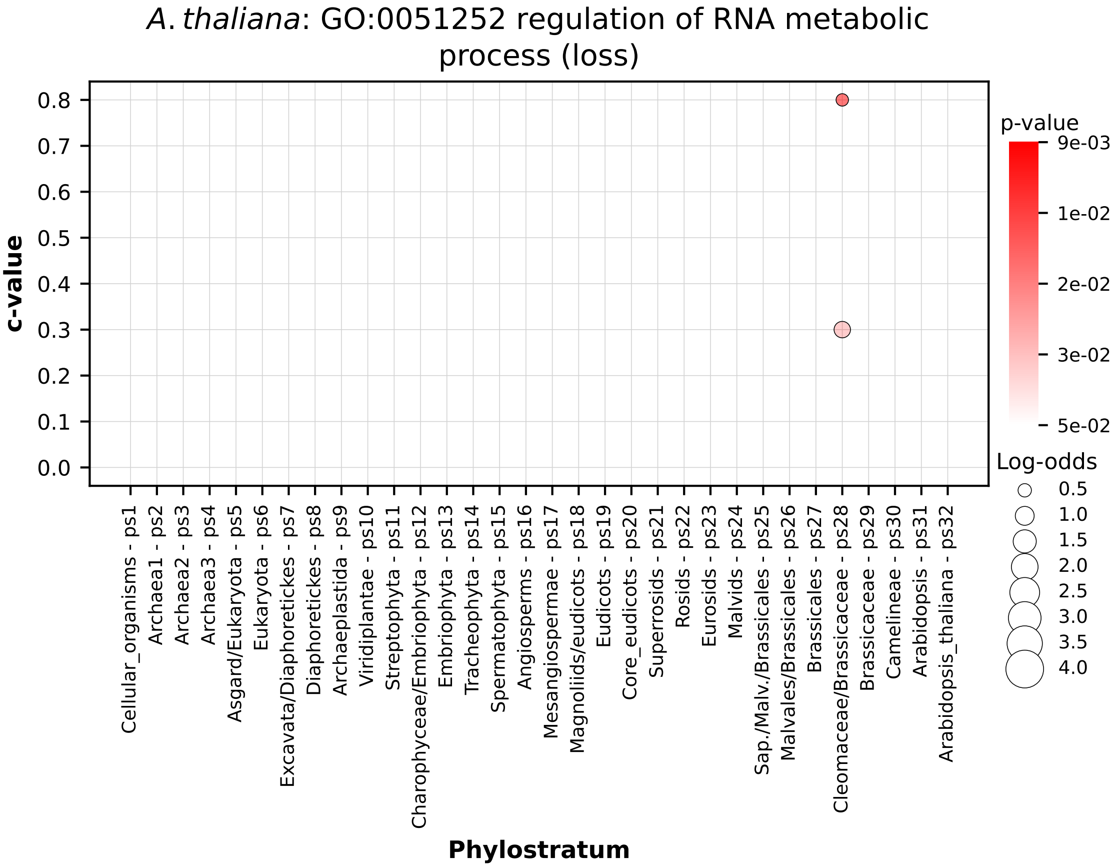

A. thaliana: GO:0051252 regulation of RNA metabolic process (loss)
c-value
0.0
0.1
0.2
0.3
0.4
0.5
0.6
0.7
0.8
Phylostratum
Cellular_organisms - ps1
Archaea1 - ps2
Archaea2 - ps3
Archaea3 - ps4
Asgard/Eukaryota - ps5
Eukaryota - ps6
Excavata/Diaphoretickes - ps7
Diaphoretickes - ps8
Archaeplastida - ps9
Viridiplantae - ps10
Streptophyta - ps11
Charophyceae/Embriophyta - ps12
Embriophyta - ps13
Tracheophyta - ps14
Spermatophyta - ps15
Angiosperms - ps16
Mesangiospermae - ps17
Magnoliids/eudicots - ps18
Eudicots - ps19
Core_eudicots - ps20
Superrosids - ps21
Rosids - ps22
Eurosids - ps23
Malvids - ps24
Sap./Malv./Brassicales - ps25
Malvales/Brassicales - ps26
Brassicales - ps27
Cleomaceae/Brassicaceae - ps28
Brassicaceae - ps29
Camelineae - ps30
Arabidopsis - ps31
Arabidopsis_thaliana - ps32
p-value
9e-03
1e-02
2e-02
3e-02
5e-02
Log-odds
0.5
1.0
1.5
2.0
2.5
3.0
3.5
4.0

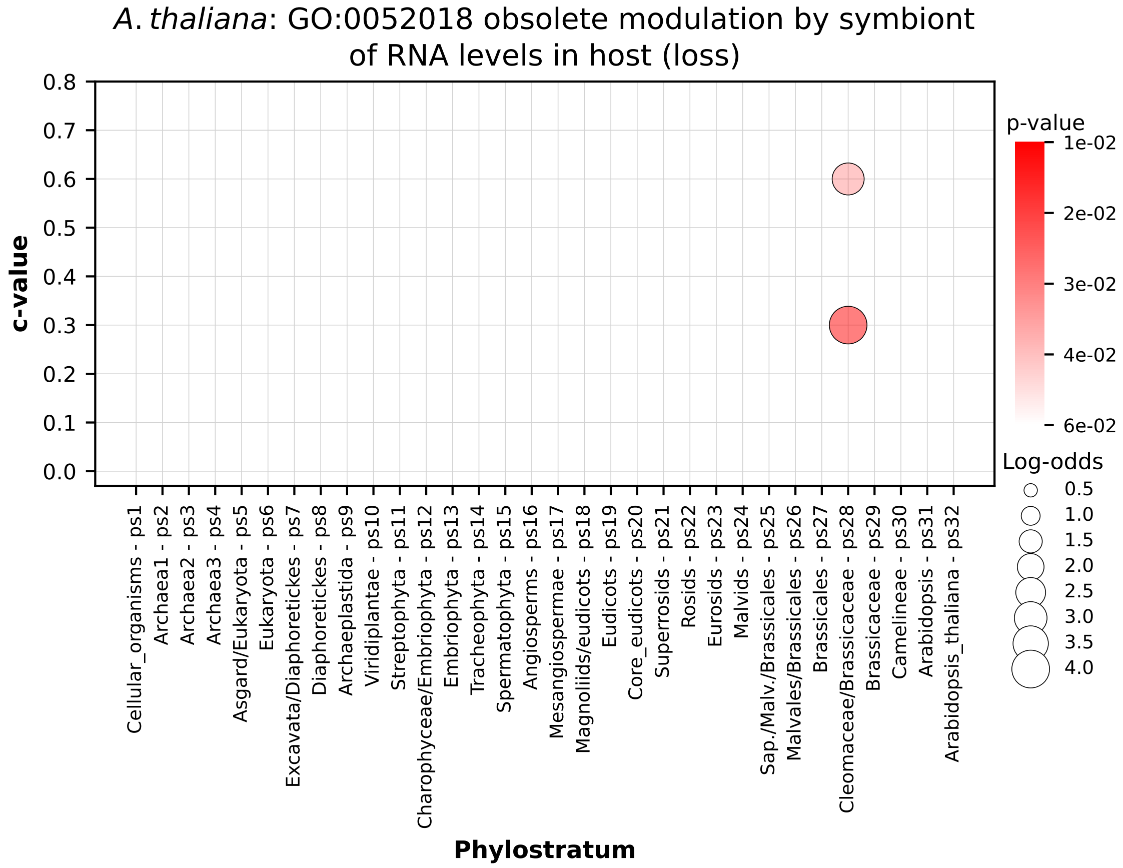

A. thaliana: GO:0052018 obsolete modulation by symbiont of RNA levels in host (loss)
c-value
0.8
0.7
0.6
0.5
0.4
0.3
0.2
0.1
0.0
p-value
1e-02
2e-02
3e-02
4e-02
6e-02
Log-odds
0.5
1.0
1.5
2.0
2.5
3.0
3.5
4.0
Cellular_organisms - ps1
Archaea1 - ps2
Archaea2 - ps3
Archaea3 - ps4
Asgard/Eukaryota - ps5
Eukaryota - ps6
Excavata/Diaphoretickes - ps7
Diaphoretickes - ps8
Archaeplastida - ps9
Viridiplantae - ps10
Streptophyta - ps11
Charophyceae/Embriophyta - ps12
Embriophyta - ps13
Tracheophyta - ps14
Spermatophyta - ps15
Angiosperms - ps16
Mesangiospermae - ps17
Magnoliids/eudicots - ps18
Eudicots - ps19
Core_eudicots - ps20
Superrosids - ps21
Rosids - ps22
Eurosids - ps23
Malvids - ps24
Sap./Malv./Brassicales - ps25
Malvales/Brassicales - ps26
Brassicales - ps27
Cleomaceae/Brassicaceae - ps28
Brassicaceae - ps29
Camelineae - ps30
Arabidopsis - ps31
Arabidopsis_thaliana - ps32
Phylostratum

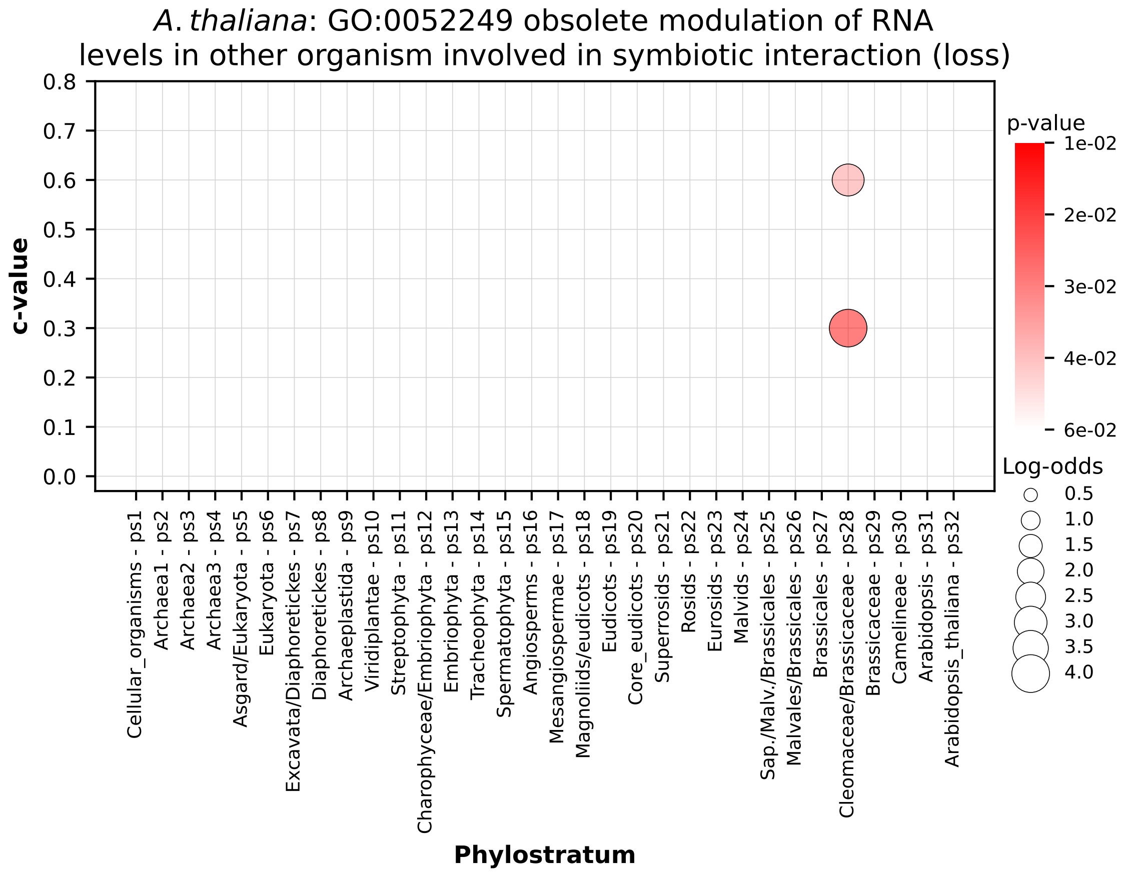

A. thaliana: GO:0052249 obsolete modulation of RNA levels in other organism involved in symbiotic interaction (loss)
c-value
0.8
0.7
0.6
0.5
0.4
0.3
0.2
0.1
0.0
p-value
1e-02
2e-02
3e-02
4e-02
6e-02
Log-odds
0.5
1.0
1.5
2.0
2.5
3.0
3.5
4.0
Cellular_organisms - ps1
Archaea1 - ps2
Archaea2 - ps3
Archaea3 - ps4
Asgard/Eukaryota - ps5
Eukaryota - ps6
Excavata/Diaphoretickes - ps7
Diaphoretickes - ps8
Archaeplastida - ps9
Viridiplantae - ps10
Streptophyta - ps11
Charophyceae/Embriophyta - ps12
Embriophyta - ps13
Tracheophyta - ps14
Spermatophyta - ps15
Angiosperms - ps16
Mesangiospermae - ps17
Magnoliids/eudicots - ps18
Eudicots - ps19
Core_eudicots - ps20
Superrosids - ps21
Rosids - ps22
Eurosids - ps23
Malvids - ps24
Sap./Malv./Brassicales - ps25
Malvales/Brassicales - ps26
Brassicales - ps27
Cleomaceae/Brassicaceae - ps28
Brassicaceae - ps29
Camelineae - ps30
Arabidopsis - ps31
Arabidopsis_thaliana - ps32
Phylostratum

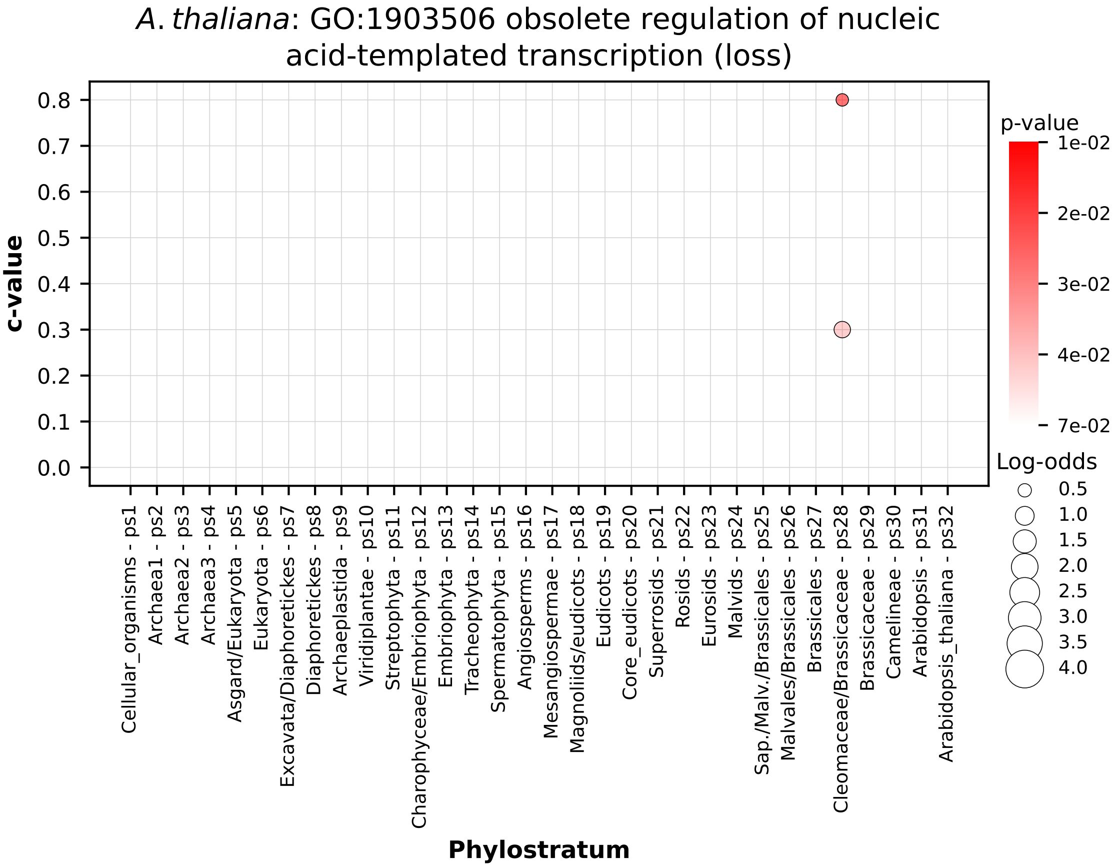

A. thaliana: GO:1903506 obsolete regulation of nucleic acid-templated transcription (loss)
c-value
0.8
0.7
0.6
0.5
0.4
0.3
0.2
0.1
0.0
Phylostratum
Cellular_organisms - ps1
Archaea1 - ps2
Archaea2 - ps3
Archaea3 - ps4
Asgard/Eukaryota - ps5
Eukaryota - ps6
Excavata/Diaphoretickes - ps7
Diaphoretickes - ps8
Archaeplastida - ps9
Viridiplantae - ps10
Streptophyta - ps11
Charophyceae/Embriophyta - ps12
Embriophyta - ps13
Tracheophyta - ps14
Spermatophyta - ps15
Angiosperms - ps16
Mesangiospermae - ps17
Magnoliids/eudicots - ps18
Eudicots - ps19
Core_eudicots - ps20
Superrosids - ps21
Rosids - ps22
Eurosids - ps23
Malvids - ps24
Sap./Malv./Brassicales - ps25
Malvales/Brassicales - ps26
Brassicales - ps27
Cleomaceae/Brassicaceae - ps28
Brassicaceae - ps29
Camelineae - ps30
Arabidopsis - ps31
Arabidopsis_thaliana - ps32
p-value
1e-02
2e-02
3e-02
4e-02
7e-02
Log-odds
0.5
1.0
1.5
2.0
2.5
3.0
3.5
4.0

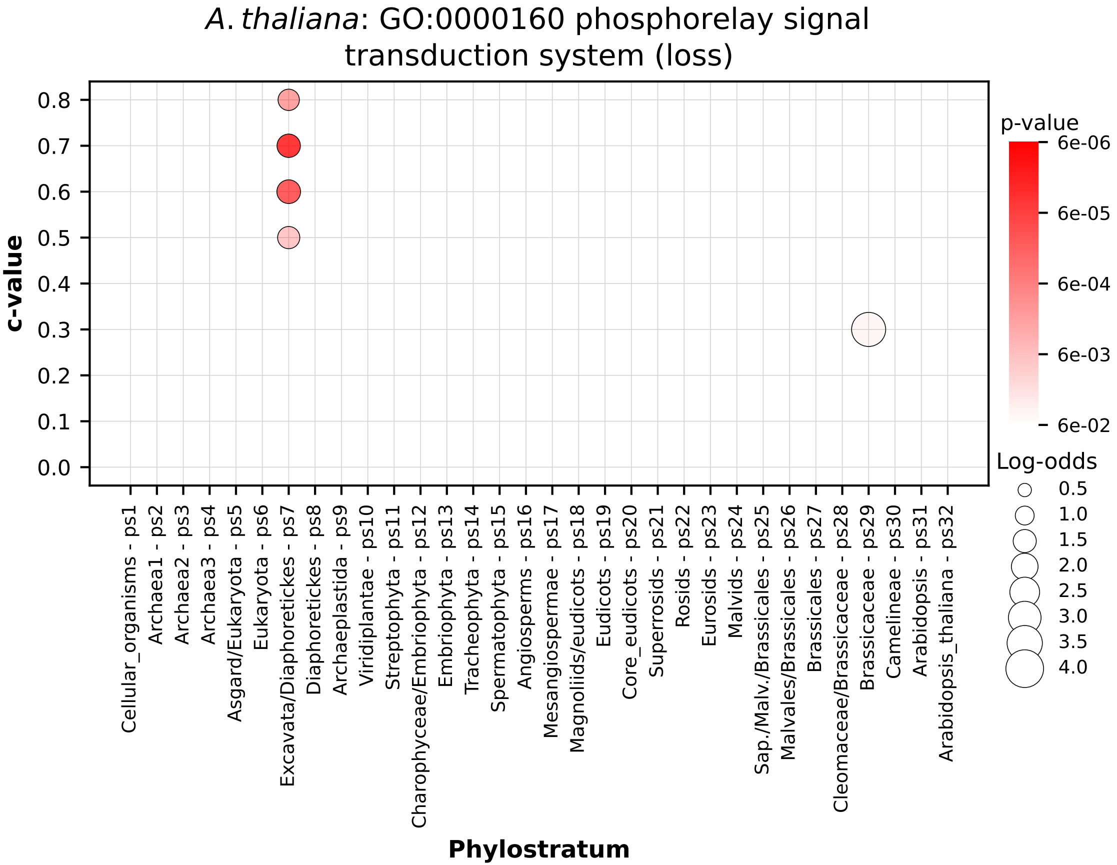

A. thaliana: GO:0000160 phosphorelay signal transduction system (loss)
c-value
0.8
0.7
0.6
0.5
0.4
0.3
0.2
0.1
0.0
Phylostratum
Cellular_organisms - ps1
Archaea1 - ps2
Archaea2 - ps3
Archaea3 - ps4
Asgard/Eukaryota - ps5
Eukaryota - ps6
Excavata/Diaphoretickes - ps7
Diaphoretickes - ps8
Archaeplastida - ps9
Viridiplantae - ps10
Streptophyta - ps11
Charophyceae/Embriophyta - ps12
Embriophyta - ps13
Tracheophyta - ps14
Spermatophyta - ps15
Angiosperms - ps16
Mesangiospermae - ps17
Magnoliids/eudicots - ps18
Eudicots - ps19
Core_eudicots - ps20
Superrosids - ps21
Rosids - ps22
Eurosids - ps23
Malvids - ps24
Sap./Malv./Brassicales - ps25
Malvales/Brassicales - ps26
Brassicales - ps27
Cleomaceae/Brassicaceae - ps28
Brassicaceae - ps29
Camelineae - ps30
Arabidopsis - ps31
Arabidopsis_thaliana - ps32
p-value
6e-06
6e-05
6e-04
6e-03
6e-02
Log-odds
0.5
1.0
1.5
2.0
2.5
3.0
3.5
4.0

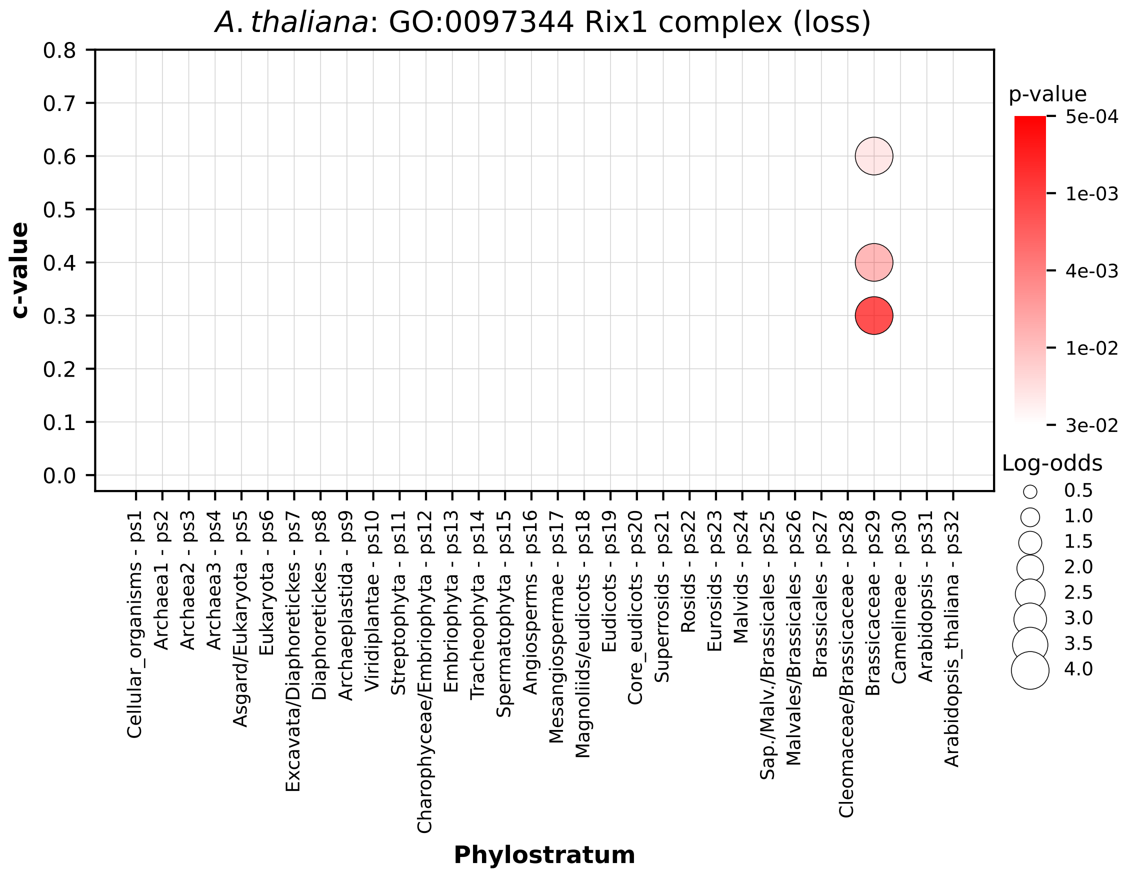

A. thaliana: GO:0097344 Rix1 complex (loss)
c-value
0.8
0.7
0.6
0.5
0.4
0.3
0.2
0.1
0.0
p-value
5e-04
1e-03
4e-03
1e-02
3e-02
Log-odds
0.5
1.0
1.5
2.0
2.5
3.0
3.5
4.0
Cellular_organisms - ps1
Archaea1 - ps2
Archaea2 - ps3
Archaea3 - ps4
Asgard/Eukaryota - ps5
Eukaryota - ps6
Excavata/Diaphoretickes - ps7
Diaphoretickes - ps8
Archaeplastida - ps9
Viridiplantae - ps10
Streptophyta - ps11
Charophyceae/Embriophyta - ps12
Embriophyta - ps13
Tracheophyta - ps14
Spermatophyta - ps15
Angiosperms - ps16
Mesangiospermae - ps17
Magnoliids/eudicots - ps18
Eudicots - ps19
Core_eudicots - ps20
Superrosids - ps21
Rosids - ps22
Eurosids - ps23
Malvids - ps24
Sap./Malv./Brassicales - ps25
Malvales/Brassicales - ps26
Brassicales - ps27
Cleomaceae/Brassicaceae - ps28
Brassicaceae - ps29
Camelineae - ps30
Arabidopsis - ps31
Arabidopsis_thaliana - ps32
Phylostratum

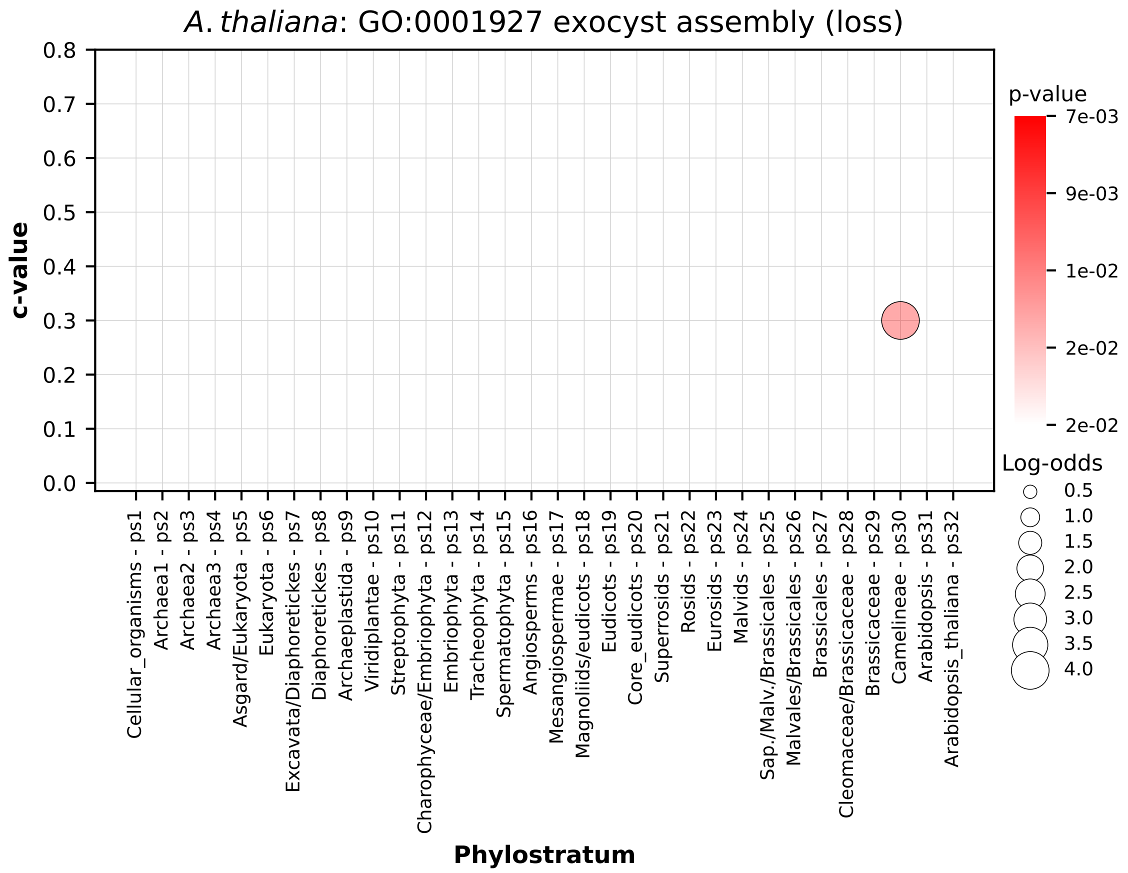

A. thaliana: GO:0001927 exocyst assembly (loss)
c-value
0.0
0.1
0.2
0.3
0.4
0.5
0.6
0.7
0.8
Phylostratum
Cellular_organisms - ps1
Archaea1 - ps2
Archaea2 - ps3
Archaea3 - ps4
Asgard/Eukaryota - ps5
Eukaryota - ps6
Excavata/Diaphoretickes - ps7
Diaphoretickes - ps8
Archaeplastida - ps9
Viridiplantae - ps10
Streptophyta - ps11
Charophyceae/Embriophyta - ps12
Embriophyta - ps13
Tracheophyta - ps14
Spermatophyta - ps15
Angiosperms - ps16
Mesangiospermae - ps17
Magnoliids/eudicots - ps18
Eudicots - ps19
Core_eudicots - ps20
Superrosids - ps21
Rosids - ps22
Eurosids - ps23
Malvids - ps24
Sap./Malv./Brassicales - ps25
Malvales/Brassicales - ps26
Brassicales - ps27
Cleomaceae/Brassicaceae - ps28
Brassicaceae - ps29
Camelineae - ps30
Arabidopsis - ps31
Arabidopsis_thaliana - ps32
p-value
7e-03
9e-03
1e-02
2e-02
2e-02
Log-odds
0.5
1.0
1.5
2.0
2.5
3.0
3.5
4.0

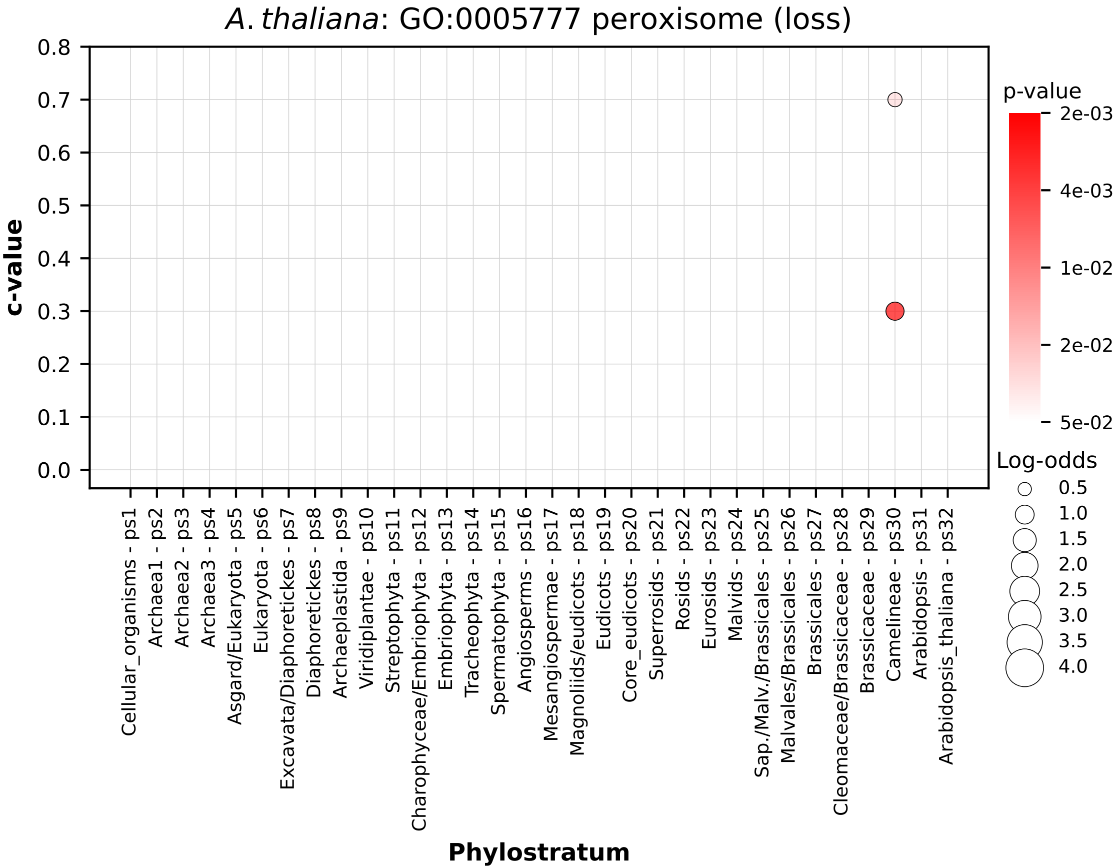
A. thaliana: GO:0005777 peroxisome (loss)
c-value
0.0
0.1
0.2
0.3
0.4
0.5
0.6
0.7
0.8
p-value
2e-03
4e-03
1e-02
2e-02
5e-02
Log-odds
0.5
1.0
1.5
2.0
2.5
3.0
3.5
4.0
Cellular_organisms - ps1
Archaea1 - ps2
Archaea2 - ps3
Archaea3 - ps4
Asgard/Eukaryota - ps5
Eukaryota - ps6
Excavata/Diaphoretickes - ps7
Diaphoretickes - ps8
Archaeplastida - ps9
Viridiplantae - ps10
Streptophyta - ps11
Charophyceae/Embriophyta - ps12
Embriophyta - ps13
Tracheophyta - ps14
Spermatophyta - ps15
Angiosperms - ps16
Mesangiospermae - ps17
Magnoliids/eudicots - ps18
Eudicots - ps19
Core_eudicots - ps20
Superrosids - ps21
Rosids - ps22
Eurosids - ps23
Malvids - ps24
Sap./Malv./Brassicales - ps25
Malvales/Brassicales - ps26
Brassicales - ps27
Cleomaceae/Brassicaceae - ps28
Brassicaceae - ps29
Camelineae - ps30
Arabidopsis - ps31
Arabidopsis_thaliana - ps32
Phylostratum

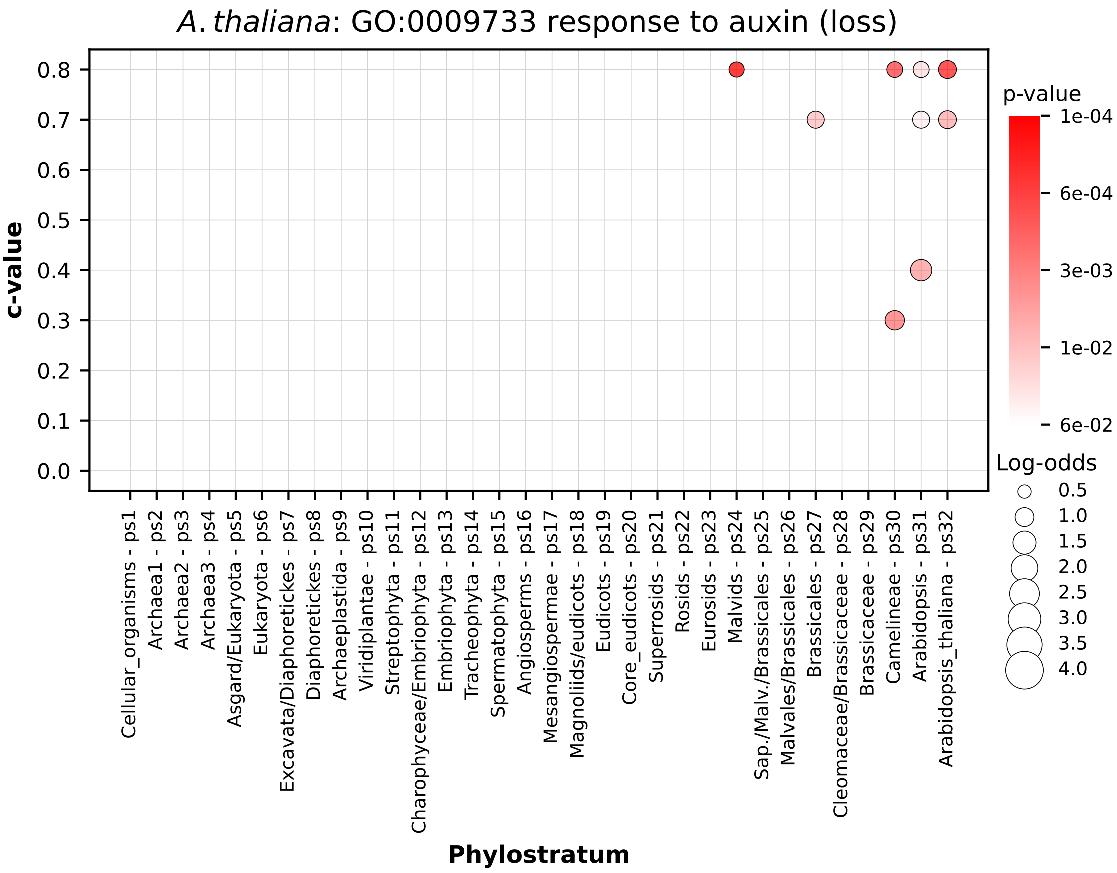
A. thaliana: GO:0009733 response to auxin (loss)
c-value
0.0
0.1
0.2
0.3
0.4
0.5
0.6
0.7
0.8
Phylostratum
Cellular_organisms - ps1
Archaea1 - ps2
Archaea2 - ps3
Archaea3 - ps4
Asgard/Eukaryota - ps5
Eukaryota - ps6
Excavata/Diaphoretickes - ps7
Diaphoretickes - ps8
Archaeplastida - ps9
Viridiplantae - ps10
Streptophyta - ps11
Charophyceae/Embriophyta - ps12
Embriophyta - ps13
Tracheophyta - ps14
Spermatophyta - ps15
Angiosperms - ps16
Mesangiospermae - ps17
Magnoliids/eudicots - ps18
Eudicots - ps19
Core_eudicots - ps20
Superrosids - ps21
Rosids - ps22
Eurosids - ps23
Malvids - ps24
Sap./Malv./Brassicales - ps25
Malvales/Brassicales - ps26
Brassicales - ps27
Cleomaceae/Brassicaceae - ps28
Brassicaceae - ps29
Camelineae - ps30
Arabidopsis - ps31
Arabidopsis_thaliana - ps32
p-value
1e-04
6e-04
3e-03
1e-02
6e-02
Log-odds
0.5
1.0
1.5
2.0
2.5
3.0
3.5
4.0

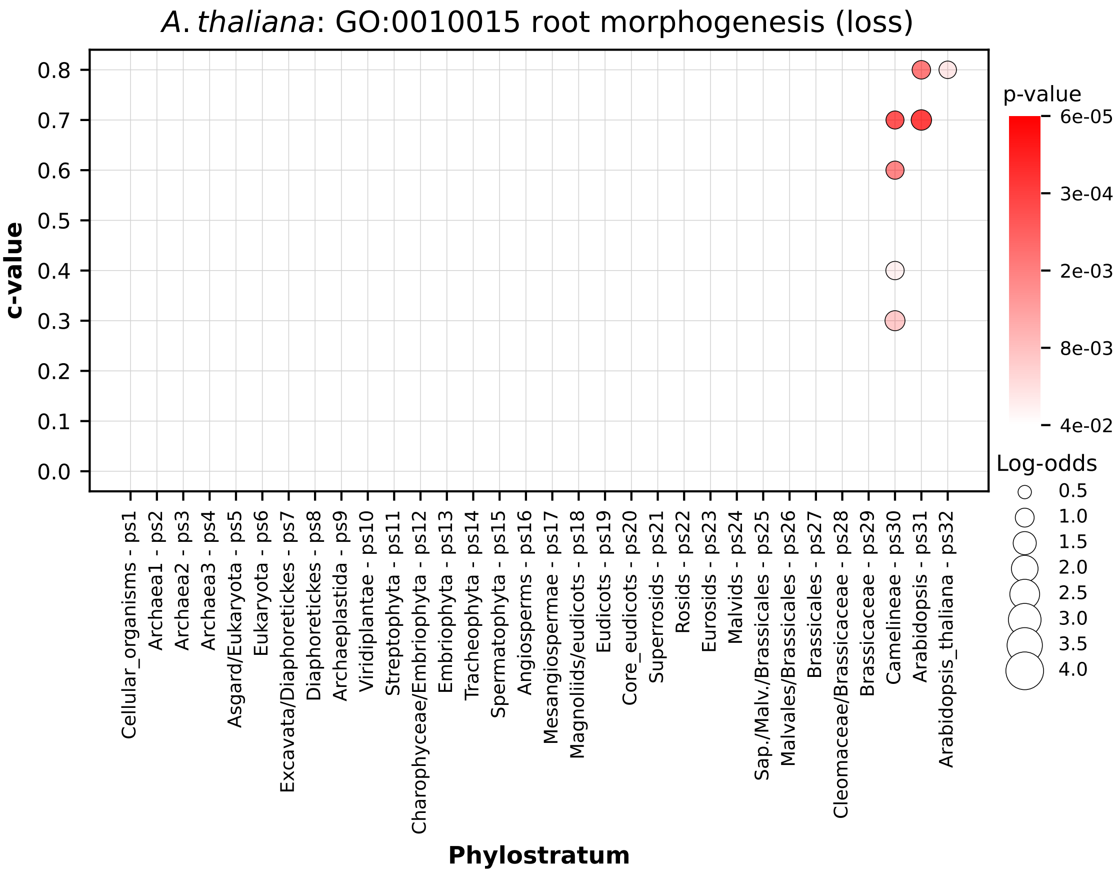

A. thaliana: GO:0010015 root morphogenesis (loss)
c-value
0.0
0.1
0.2
0.3
0.4
0.5
0.6
0.7
0.8
Phylostratum
Cellular_organisms - ps1
Archaea1 - ps2
Archaea2 - ps3
Archaea3 - ps4
Asgard/Eukaryota - ps5
Eukaryota - ps6
Excavata/Diaphoretickes - ps7
Diaphoretickes - ps8
Archaeplastida - ps9
Viridiplantae - ps10
Streptophyta - ps11
Charophyceae/Embriophyta - ps12
Embriophyta - ps13
Tracheophyta - ps14
Spermatophyta - ps15
Angiosperms - ps16
Mesangiospermae - ps17
Magnoliids/eudicots - ps18
Eudicots - ps19
Core_eudicots - ps20
Superrosids - ps21
Rosids - ps22
Eurosids - ps23
Malvids - ps24
Sap./Malv./Brassicales - ps25
Malvales/Brassicales - ps26
Brassicales - ps27
Cleomaceae/Brassicaceae - ps28
Brassicaceae - ps29
Camelineae - ps30
Arabidopsis - ps31
Arabidopsis_thaliana - ps32
p-value
6e-05
3e-04
2e-03
8e-03
4e-02
Log-odds
0.5
1.0
1.5
2.0
2.5
3.0
3.5
4.0

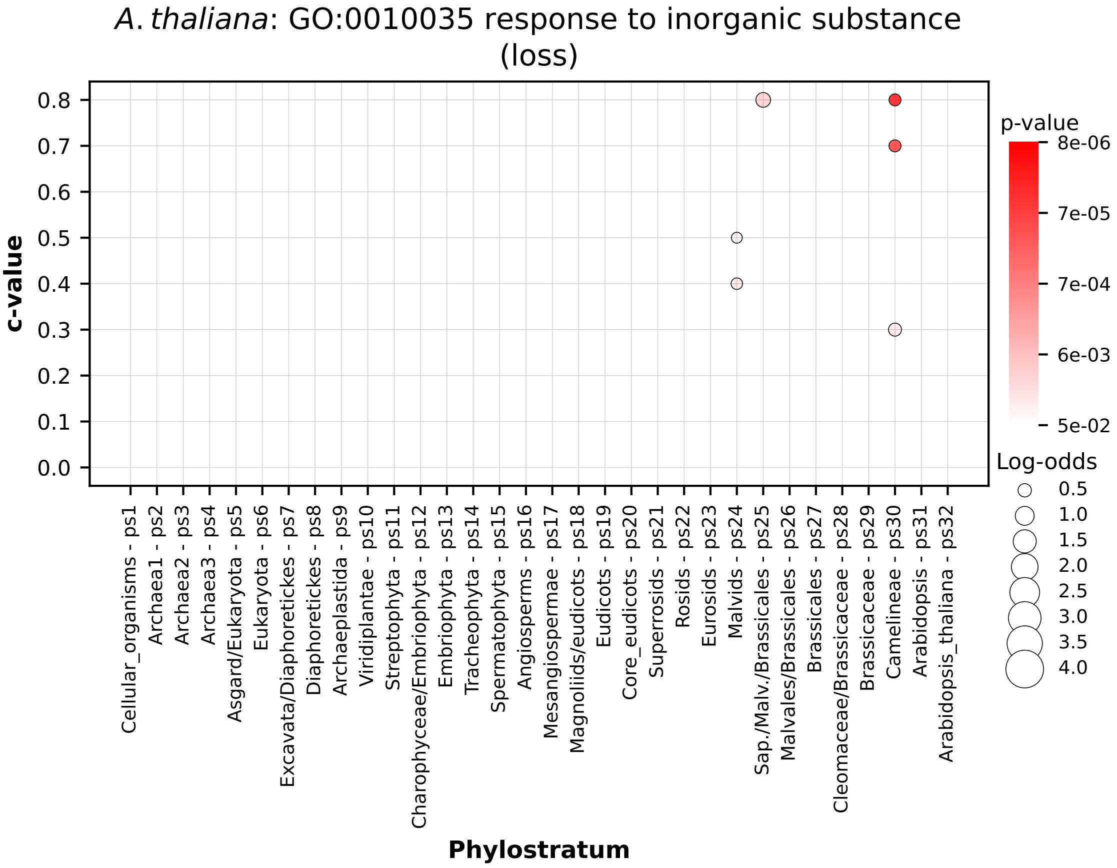

A. thaliana: GO:0010035 response to inorganic substance (loss)
c-value
0.0
0.1
0.2
0.3
0.4
0.5
0.6
0.7
0.8
Phylostratum
Cellular_organisms - ps1
Archaea1 - ps2
Archaea2 - ps3
Archaea3 - ps4
Asgard/Eukaryota - ps5
Eukaryota - ps6
Excavata/Diaphoretickes - ps7
Diaphoretickes - ps8
Archaeplastida - ps9
Viridiplantae - ps10
Streptophyta - ps11
Charophyceae/Embriophyta - ps12
Embriophyta - ps13
Tracheophyta - ps14
Spermatophyta - ps15
Angiosperms - ps16
Mesangiospermae - ps17
Magnoliids/eudicots - ps18
Eudicots - ps19
Core_eudicots - ps20
Superrosids - ps21
Rosids - ps22
Eurosids - ps23
Malvids - ps24
Sap./Malv./Brassicales - ps25
Malvales/Brassicales - ps26
Brassicales - ps27
Cleomaceae/Brassicaceae - ps28
Brassicaceae - ps29
Camelineae - ps30
Arabidopsis - ps31
Arabidopsis_thaliana - ps32
p-value
8e-06
7e-05
7e-04
6e-03
5e-02
Log-odds
0.5
1.0
1.5
2.0
2.5
3.0
3.5
4.0

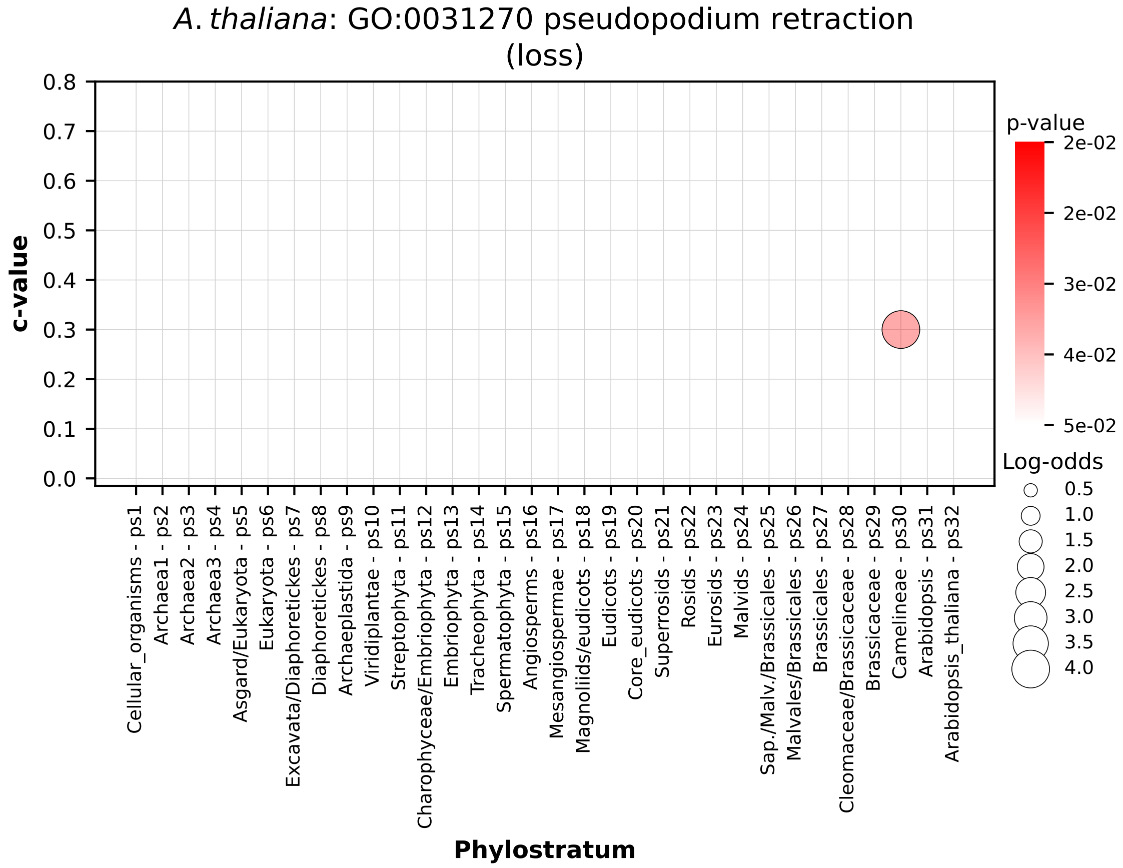
A. thaliana: GO:0031270 pseudopodium retraction
(loss)
c-value
0.8
0.7
0.6
0.5
0.4
0.3
0.2
0.1
0.0
p-value
2e-02
2e-02
3e-02
4e-02
5e-02
Log-odds
0.5
1.0
1.5
2.0
2.5
3.0
3.5
4.0
Cellular_organisms - ps1
Archaea1 - ps2
Archaea2 - ps3
Archaea3 - ps4
Asgard/Eukaryota - ps5
Eukaryota - ps6
Excavata/Diaphoretickes - ps7
Diaphoretickes - ps8
Archaeplastida - ps9
Viridiplantae - ps10
Streptophyta - ps11
Charophyceae/Embriophyta - ps12
Embriophyta - ps13
Tracheophyta - ps14
Spermatophyta - ps15
Angiosperms - ps16
Mesangiospermae - ps17
Magnoliids/eudicots - ps18
Eudicots - ps19
Core_eudicots - ps20
Superrosids - ps21
Rosids - ps22
Eurosids - ps23
Malvids - ps24
Sap./Malv./Brassicales - ps25
Malvales/Brassicales - ps26
Brassicales - ps27
Cleomaceae/Brassicaceae - ps28
Brassicaceae - ps29
Camelineae - ps30
Arabidopsis - ps31
Arabidopsis_thaliana - ps32
Phylostratum

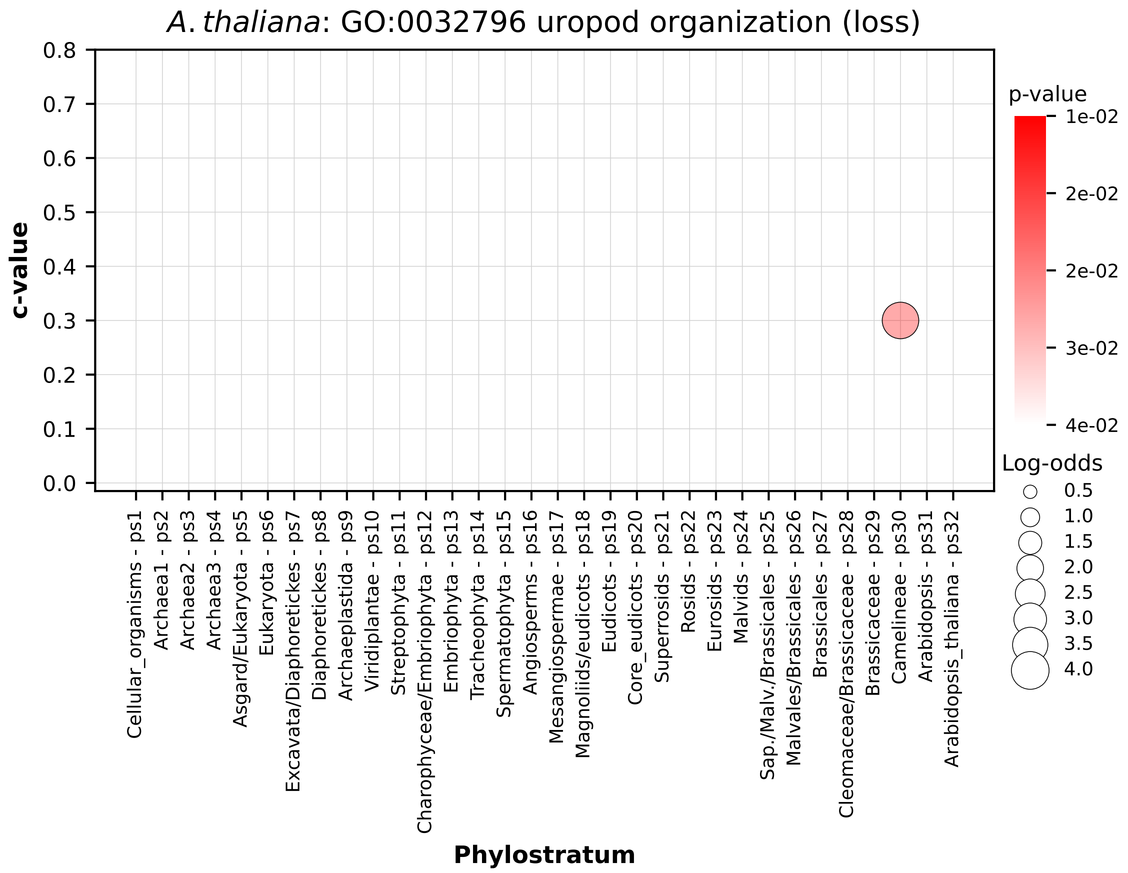

A. thaliana: GO:0032796 uropod organization (loss)
c-value
0.8
0.7
0.6
0.5
0.4
0.3
0.2
0.1
0.0
Phylostratum
Cellular_organisms - ps1
Archaea1 - ps2
Archaea2 - ps3
Archaea3 - ps4
Asgard/Eukaryota - ps5
Eukaryota - ps6
Excavata/Diaphoretickes - ps7
Diaphoretickes - ps8
Archaeplastida - ps9
Viridiplantae - ps10
Streptophyta - ps11
Charophyceae/Embriophyta - ps12
Embriophyta - ps13
Tracheophyta - ps14
Spermatophyta - ps15
Angiosperms - ps16
Mesangiospermae - ps17
Magnoliids/eudicots - ps18
Eudicots - ps19
Core_eudicots - ps20
Superrosids - ps21
Rosids - ps22
Eurosids - ps23
Malvids - ps24
Sap./Malv./Brassicales - ps25
Malvales/Brassicales - ps26
Brassicales - ps27
Cleomaceae/Brassicaceae - ps28
Brassicaceae - ps29
Camelineae - ps30
Arabidopsis - ps31
Arabidopsis_thaliana - ps32
p-value
1e-02
2e-02
2e-02
3e-02
4e-02
Log-odds
0.5
1.0
1.5
2.0
2.5
3.0
3.5
4.0

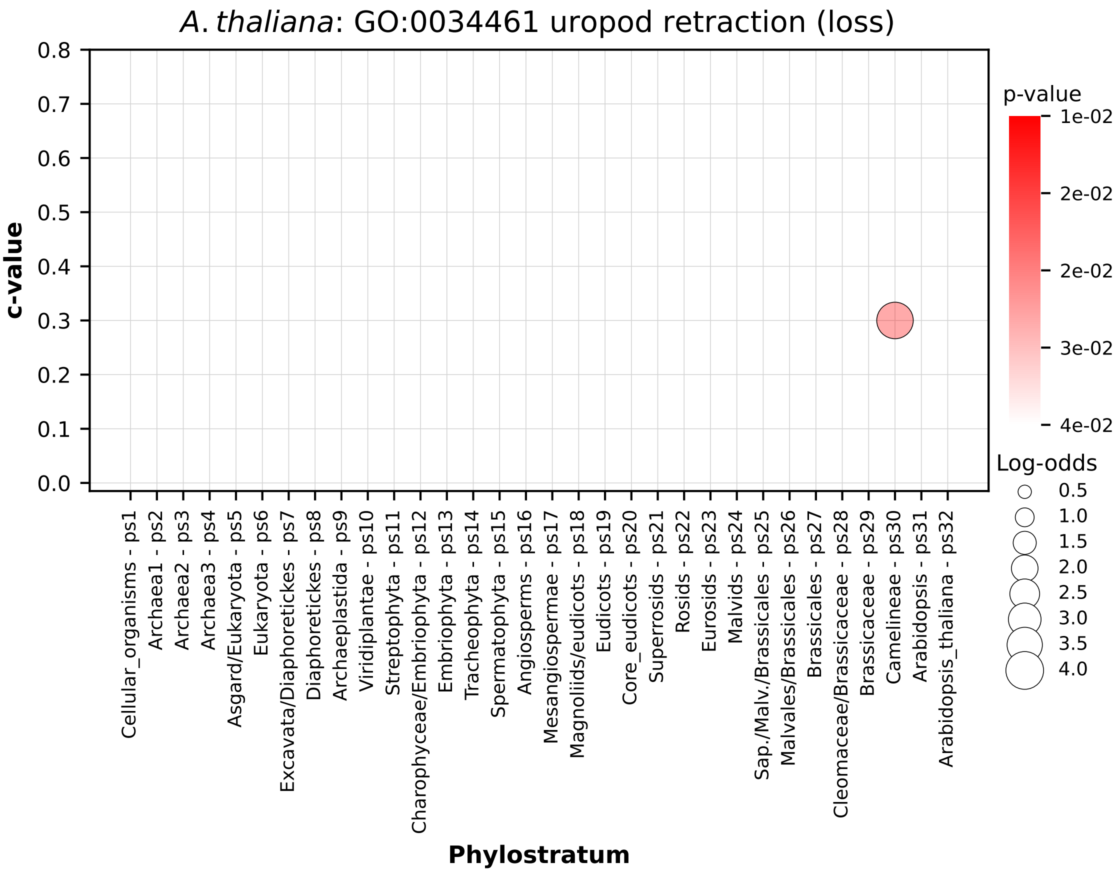

A. thaliana: GO:0034461 uropod retraction (loss)
c-value
0.8
0.7
0.6
0.5
0.4
0.3
0.2
0.1
0.0
p-value
1e-02
2e-02
2e-02
3e-02
4e-02
Log-odds
0.5
1.0
1.5
2.0
2.5
3.0
3.5
4.0
Cellular_organisms - ps1
Archaea1 - ps2
Archaea2 - ps3
Archaea3 - ps4
Asgard/Eukaryota - ps5
Eukaryota - ps6
Excavata/Diaphoretickes - ps7
Diaphoretickes - ps8
Archaeplastida - ps9
Viridiplantae - ps10
Streptophyta - ps11
Charophyceae/Embriophyta - ps12
Embriophyta - ps13
Tracheophyta - ps14
Spermatophyta - ps15
Angiosperms - ps16
Mesangiospermae - ps17
Magnoliids/eudicots - ps18
Eudicots - ps19
Core_eudicots - ps20
Superrosids - ps21
Rosids - ps22
Eurosids - ps23
Malvids - ps24
Sap./Malv./Brassicales - ps25
Malvales/Brassicales - ps26
Brassicales - ps27
Cleomaceae/Brassicaceae - ps28
Brassicaceae - ps29
Camelineae - ps30
Arabidopsis - ps31
Arabidopsis_thaliana - ps32
Phylostratum

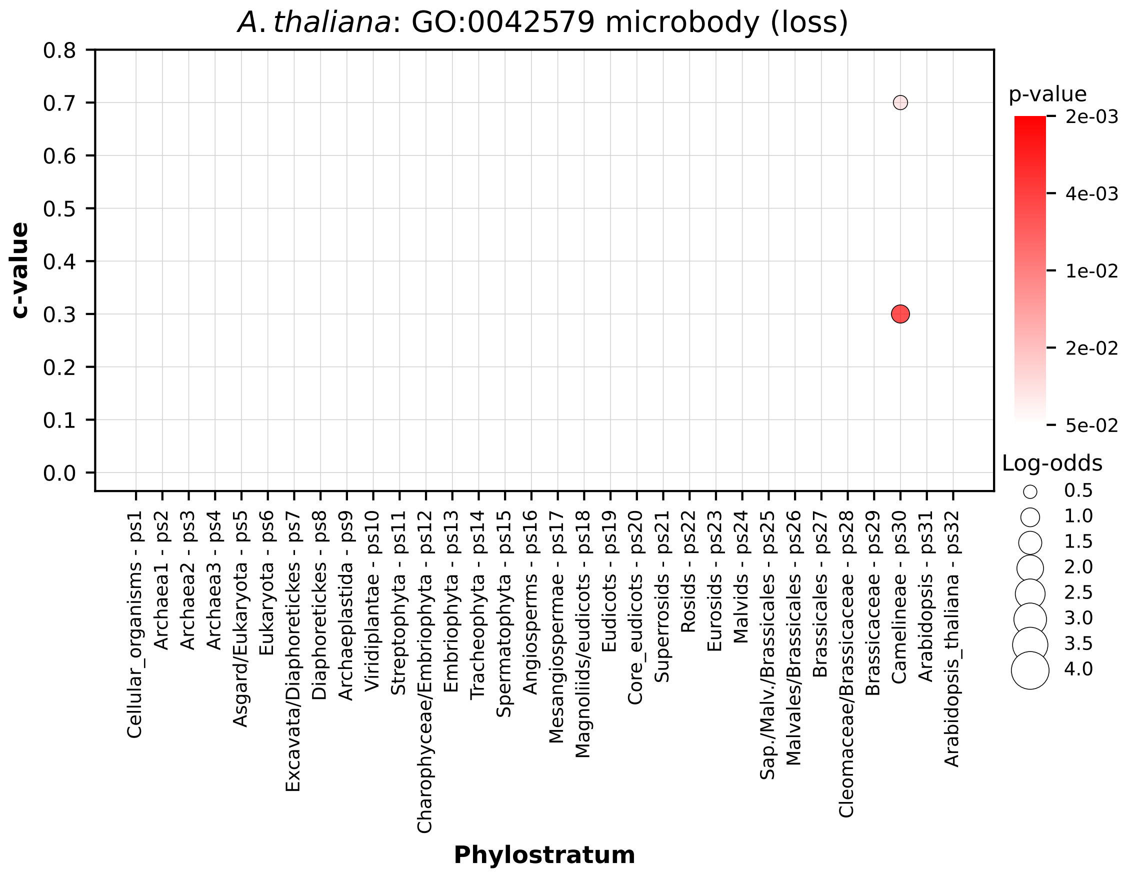

A. thaliana: GO:0042579 microbody (loss)
c-value
0.0
0.1
0.2
0.3
0.4
0.5
0.6
0.7
0.8
p-value
2e-03
4e-03
1e-02
2e-02
5e-02
Log-odds
0.5
1.0
1.5
2.0
2.5
3.0
3.5
4.0
Cellular_organisms - ps1
Archaea1 - ps2
Archaea2 - ps3
Archaea3 - ps4
Asgard/Eukaryota - ps5
Eukaryota - ps6
Excavata/Diaphoretickes - ps7
Diaphoretickes - ps8
Archaeplastida - ps9
Viridiplantae - ps10
Streptophyta - ps11
Charophyceae/Embriophyta - ps12
Embriophyta - ps13
Tracheophyta - ps14
Spermatophyta - ps15
Angiosperms - ps16
Mesangiospermae - ps17
Magnoliids/eudicots - ps18
Eudicots - ps19
Core_eudicots - ps20
Superrosids - ps21
Rosids - ps22
Eurosids - ps23
Malvids - ps24
Sap./Malv./Brassicales - ps25
Malvales/Brassicales - ps26
Brassicales - ps27
Cleomaceae/Brassicaceae - ps28
Brassicaceae - ps29
Camelineae - ps30
Arabidopsis - ps31
Arabidopsis_thaliana - ps32
Phylostratum

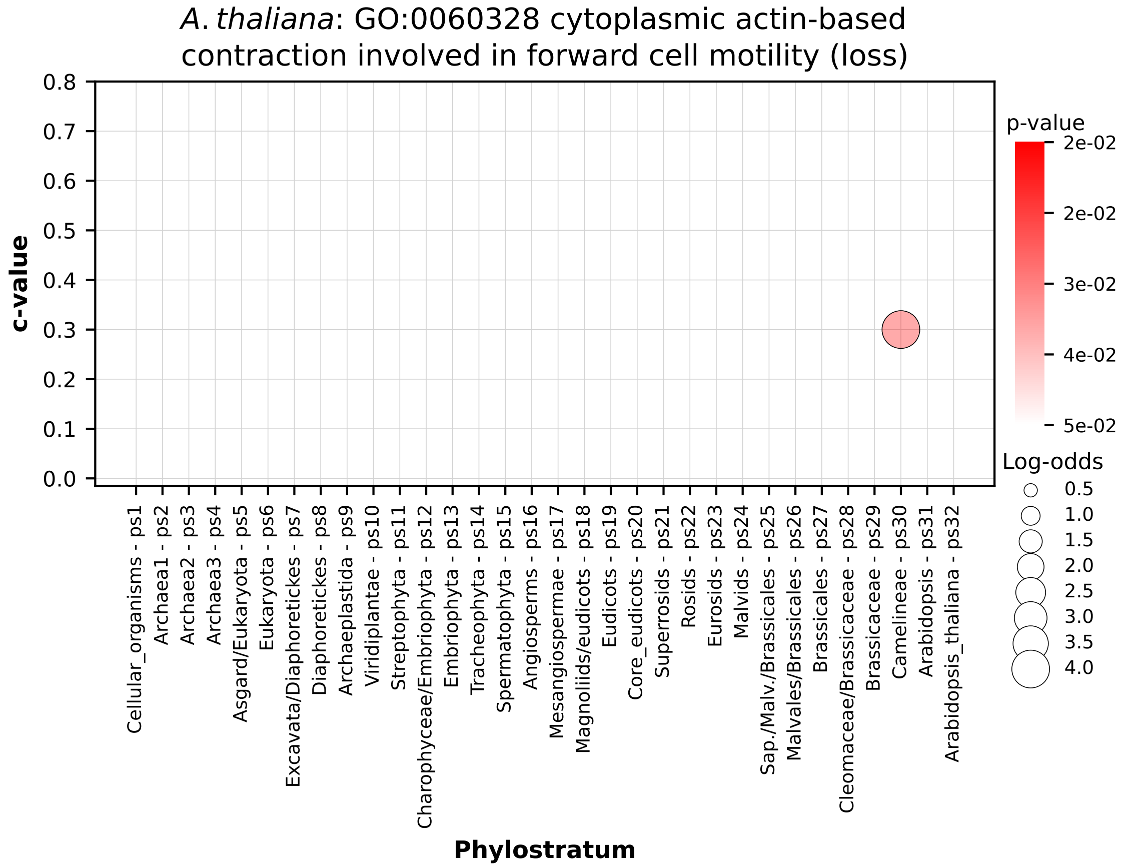

A. thaliana: GO:0060328 cytoplasmic actin-based contraction involved in forward cell motility (loss)
c-value
0.8
0.7
0.6
0.5
0.4
0.3
0.2
0.1
0.0
Phylostratum
Cellular_organisms - ps1
Archaea1 - ps2
Archaea2 - ps3
Archaea3 - ps4
Asgard/Eukaryota - ps5
Eukaryota - ps6
Excavata/Diaphoretickes - ps7
Diaphoretickes - ps8
Archaeplastida - ps9
Viridiplantae - ps10
Streptophyta - ps11
Charophyceae/Embriophyta - ps12
Embriophyta - ps13
Tracheophyta - ps14
Spermatophyta - ps15
Angiosperms - ps16
Mesangiospermae - ps17
Magnoliids/eudicots - ps18
Eudicots - ps19
Core_eudicots - ps20
Superrosids - ps21
Rosids - ps22
Eurosids - ps23
Malvids - ps24
Sap./Malv./Brassicales - ps25
Malvales/Brassicales - ps26
Brassicales - ps27
Cleomaceae/Brassicaceae - ps28
Brassicaceae - ps29
Camelineae - ps30
Arabidopsis - ps31
Arabidopsis_thaliana - ps32
p-value
2e-02
2e-02
3e-02
4e-02
5e-02
Log-odds
0.5
1.0
1.5
2.0
2.5
3.0
3.5
4.0

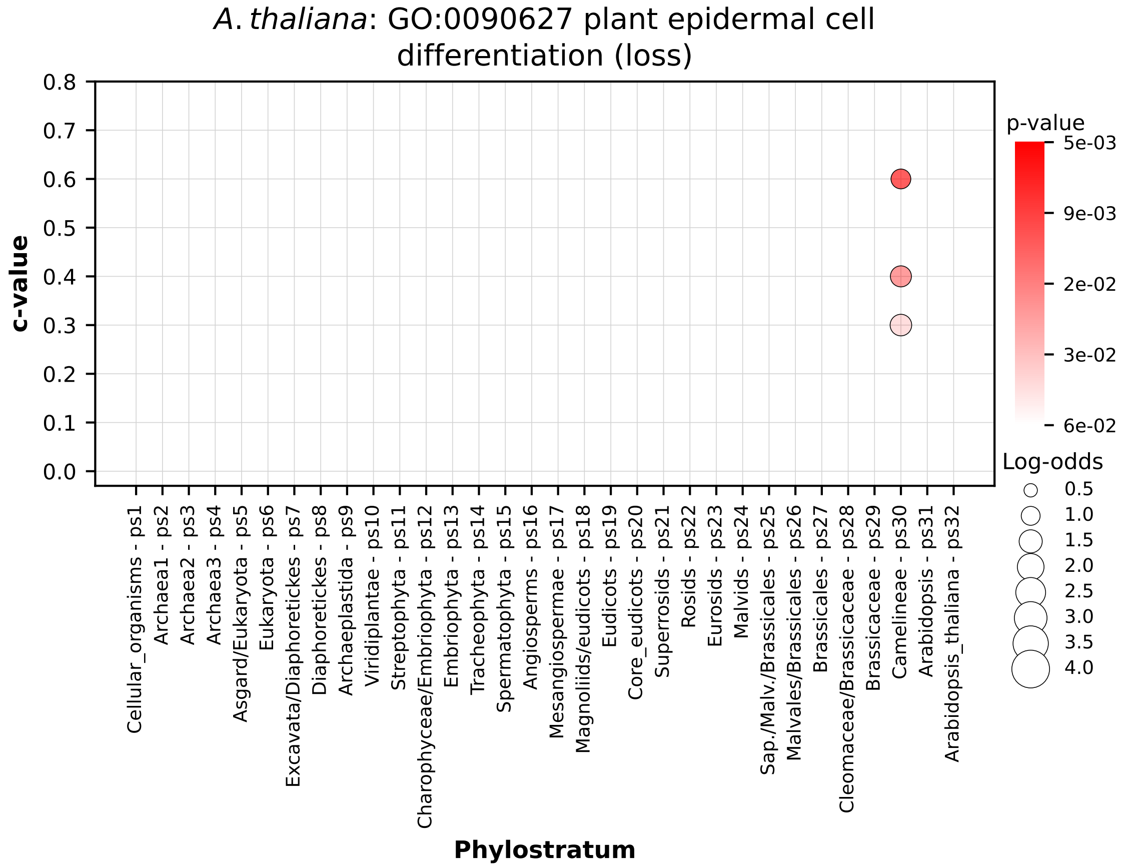

A. thaliana: GO:0090627 plant epidermal cell differentiation (loss)
c-value
0.8
0.7
0.6
0.5
0.4
0.3
0.2
0.1
0.0
Phylostratum
Cellular_organisms - ps1
Archaea1 - ps2
Archaea2 - ps3
Archaea3 - ps4
Asgard/Eukaryota - ps5
Eukaryota - ps6
Excavata/Diaphoretickes - ps7
Diaphoretickes - ps8
Archaeplastida - ps9
Viridiplantae - ps10
Streptophyta - ps11
Charophyceae/Embriophyta - ps12
Embriophyta - ps13
Tracheophyta - ps14
Spermatophyta - ps15
Angiosperms - ps16
Mesangiospermae - ps17
Magnoliids/eudicots - ps18
Eudicots - ps19
Core_eudicots - ps20
Superrosids - ps21
Rosids - ps22
Eurosids - ps23
Malvids - ps24
Sap./Malv./Brassicales - ps25
Malvales/Brassicales - ps26
Brassicales - ps27
Cleomaceae/Brassicaceae - ps28
Brassicaceae - ps29
Camelineae - ps30
Arabidopsis - ps31
Arabidopsis_thaliana - ps32
p-value
5e-03
9e-03
2e-02
3e-02
6e-02
Log-odds
0.5
1.0
1.5
2.0
2.5
3.0
3.5
4.0

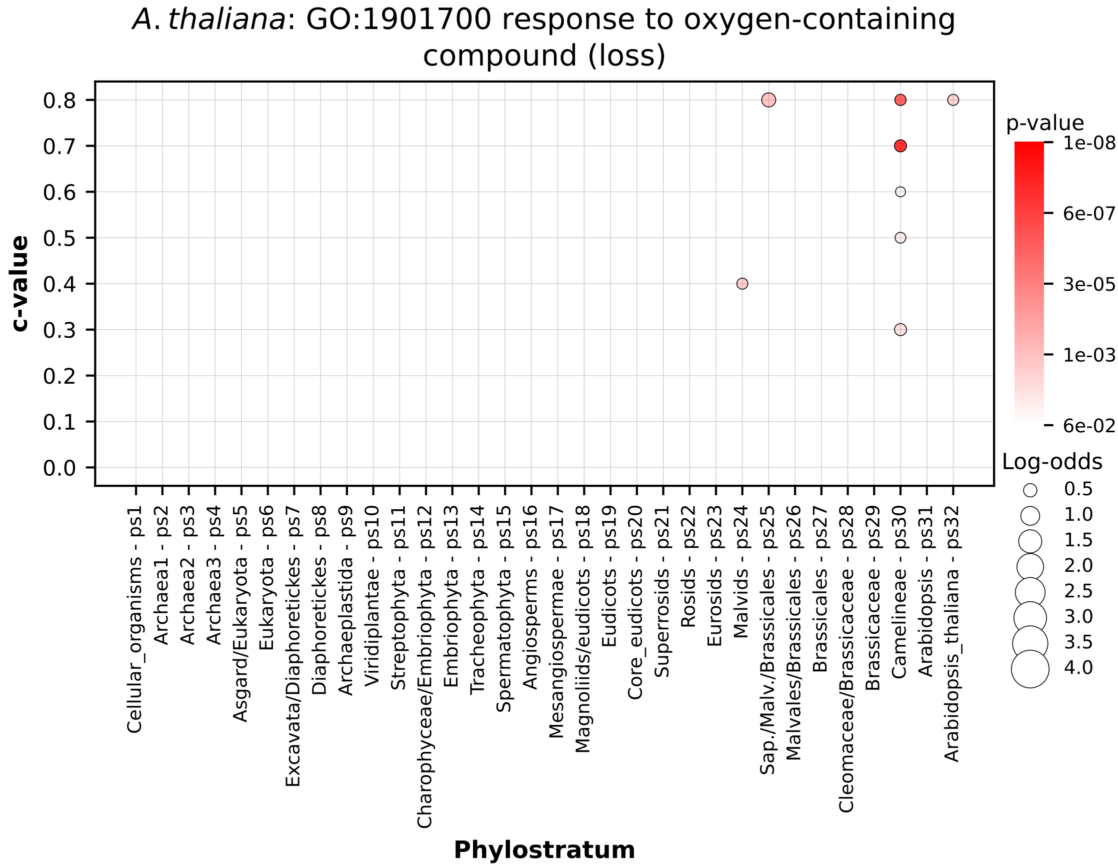

A. thaliana: GO:1901700 response to oxygen-containing compound (loss)
c-value
0.8
0.7
0.6
0.5
0.4
0.3
0.2
0.1
0.0
Phylostratum
Cellular_organisms - ps1
Archaea1 - ps2
Archaea2 - ps3
Archaea3 - ps4
Asgard/Eukaryota - ps5
Eukaryota - ps6
Excavata/Diaphoretickes - ps7
Diaphoretickes - ps8
Archaeplastida - ps9
Viridiplantae - ps10
Streptophyta - ps11
Charophyceae/Embriophyta - ps12
Embriophyta - ps13
Tracheophyta - ps14
Spermatophyta - ps15
Angiosperms - ps16
Mesangiospermae - ps17
Magnoliids/eudicots - ps18
Eudicots - ps19
Core_eudicots - ps20
Superrosids - ps21
Rosids - ps22
Eurosids - ps23
Malvids - ps24
Sap./Malv./Brassicales - ps25
Malvales/Brassicales - ps26
Brassicales - ps27
Cleomaceae/Brassicaceae - ps28
Brassicaceae - ps29
Camelineae - ps30
Arabidopsis - ps31
Arabidopsis_thaliana - ps32
p-value
1e-08
6e-07
3e-05
1e-03
6e-02
Log-odds
0.5
1.0
1.5
2.0
2.5
3.0
3.5
4.0

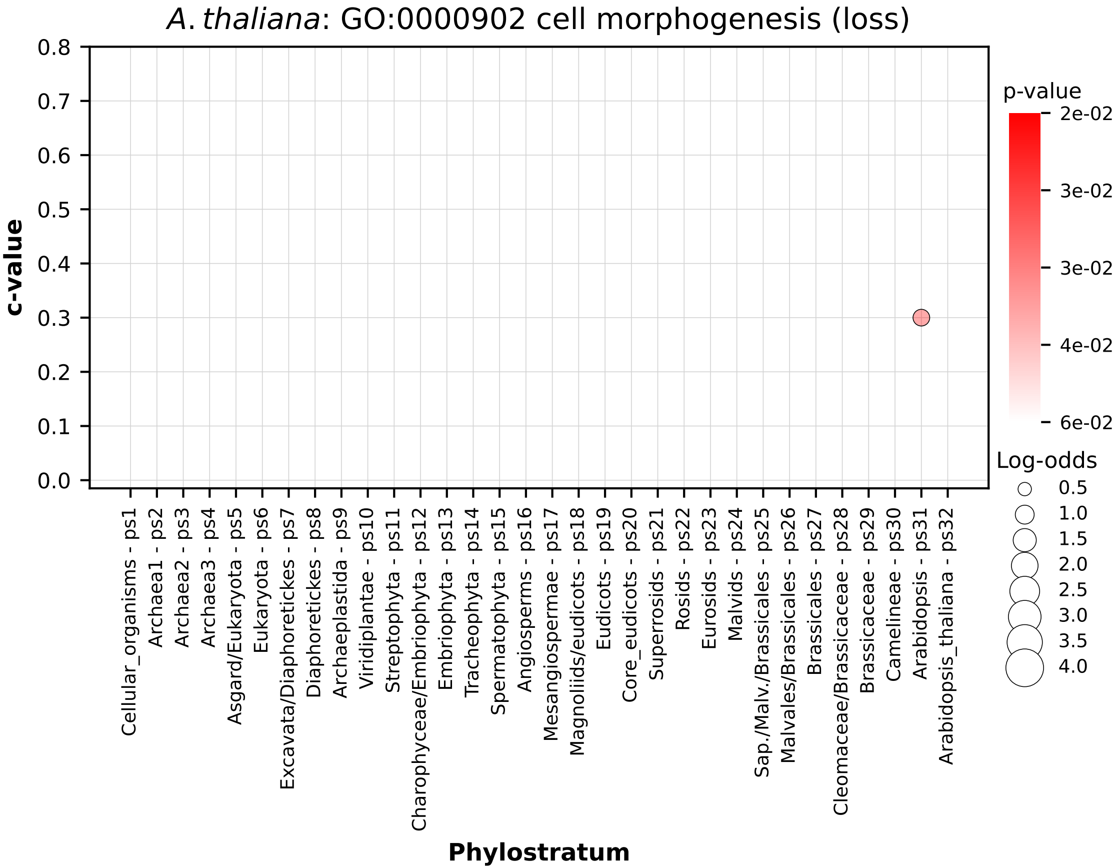

A. thaliana: GO:0000902 cell morphogenesis (loss)
c-value
0.8
0.7
0.6
0.5
0.4
0.3
0.2
0.1
0.0
Phylostratum
Cellular_organisms - ps1
Archaea1 - ps2
Archaea2 - ps3
Archaea3 - ps4
Asgard/Eukaryota - ps5
Eukaryota - ps6
Excavata/Diaphoretickes - ps7
Diaphoretickes - ps8
Archaeplastida - ps9
Viridiplantae - ps10
Streptophyta - ps11
Charophyceae/Embriophyta - ps12
Embriophyta - ps13
Tracheophyta - ps14
Spermatophyta - ps15
Angiosperms - ps16
Mesangiospermae - ps17
Magnoliids/eudicots - ps18
Eudicots - ps19
Core_eudicots - ps20
Superrosids - ps21
Rosids - ps22
Eurosids - ps23
Malvids - ps24
Sap./Malv./Brassicales - ps25
Malvales/Brassicales - ps26
Brassicales - ps27
Cleomaceae/Brassicaceae - ps28
Brassicaceae - ps29
Camelineae - ps30
Arabidopsis - ps31
Arabidopsis_thaliana - ps32
p-value
2e-02
3e-02
3e-02
4e-02
6e-02
Log-odds
0.5
1.0
1.5
2.0
2.5
3.0
3.5
4.0

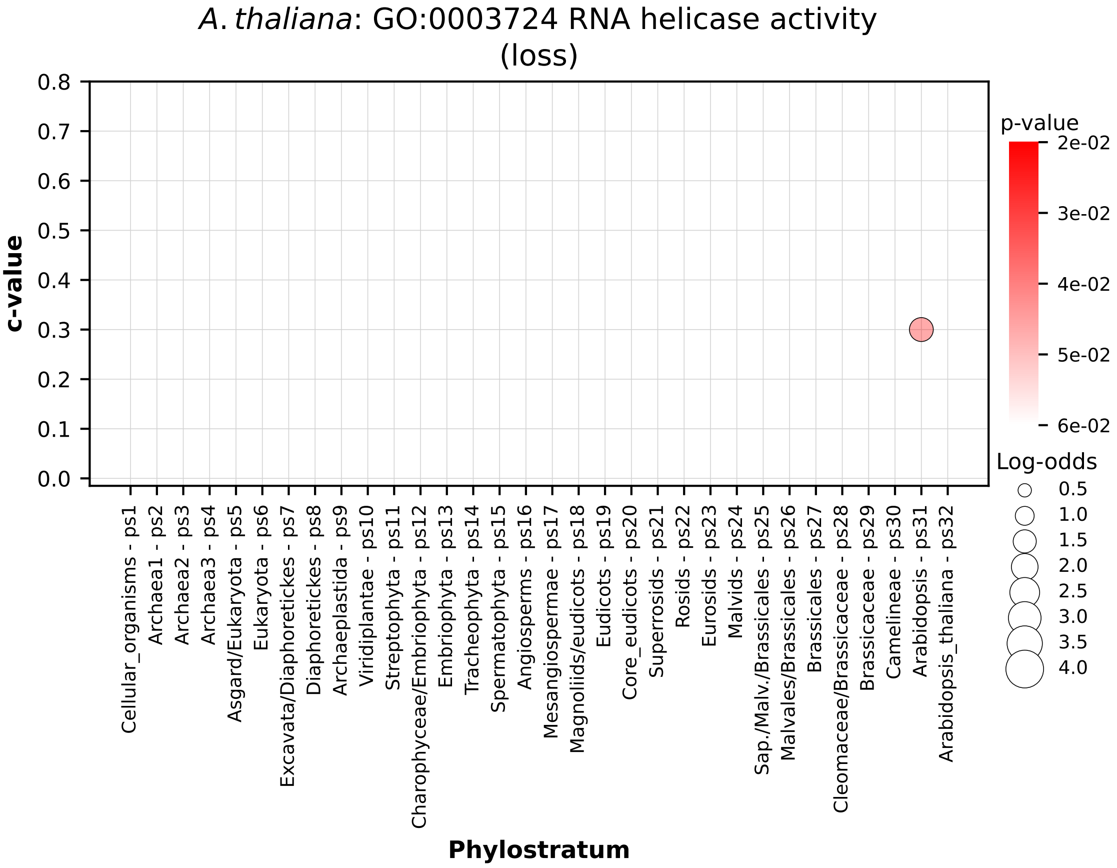

A. thaliana: GO:0003724 RNA helicase activity
(loss)
c-value
0.0
0.1
0.2
0.3
0.4
0.5
0.6
0.7
0.8
Phylostratum
Cellular_organisms - ps1
Archaea1 - ps2
Archaea2 - ps3
Archaea3 - ps4
Asgard/Eukaryota - ps5
Eukaryota - ps6
Excavata/Diaphoretickes - ps7
Diaphoretickes - ps8
Archaeplastida - ps9
Viridiplantae - ps10
Streptophyta - ps11
Charophyceae/Embriophyta - ps12
Embriophyta - ps13
Tracheophyta - ps14
Spermatophyta - ps15
Angiosperms - ps16
Mesangiospermae - ps17
Magnoliids/eudicots - ps18
Eudicots - ps19
Core_eudicots - ps20
Superrosids - ps21
Rosids - ps22
Eurosids - ps23
Malvids - ps24
Sap./Malv./Brassicales - ps25
Malvales/Brassicales - ps26
Brassicales - ps27
Cleomaceae/Brassicaceae - ps28
Brassicaceae - ps29
Camelineae - ps30
Arabidopsis - ps31
Arabidopsis_thaliana - ps32
p-value
2e-02
3e-02
4e-02
5e-02
6e-02
Log-odds
0.5
1.0
1.5
2.0
2.5
3.0
3.5
4.0

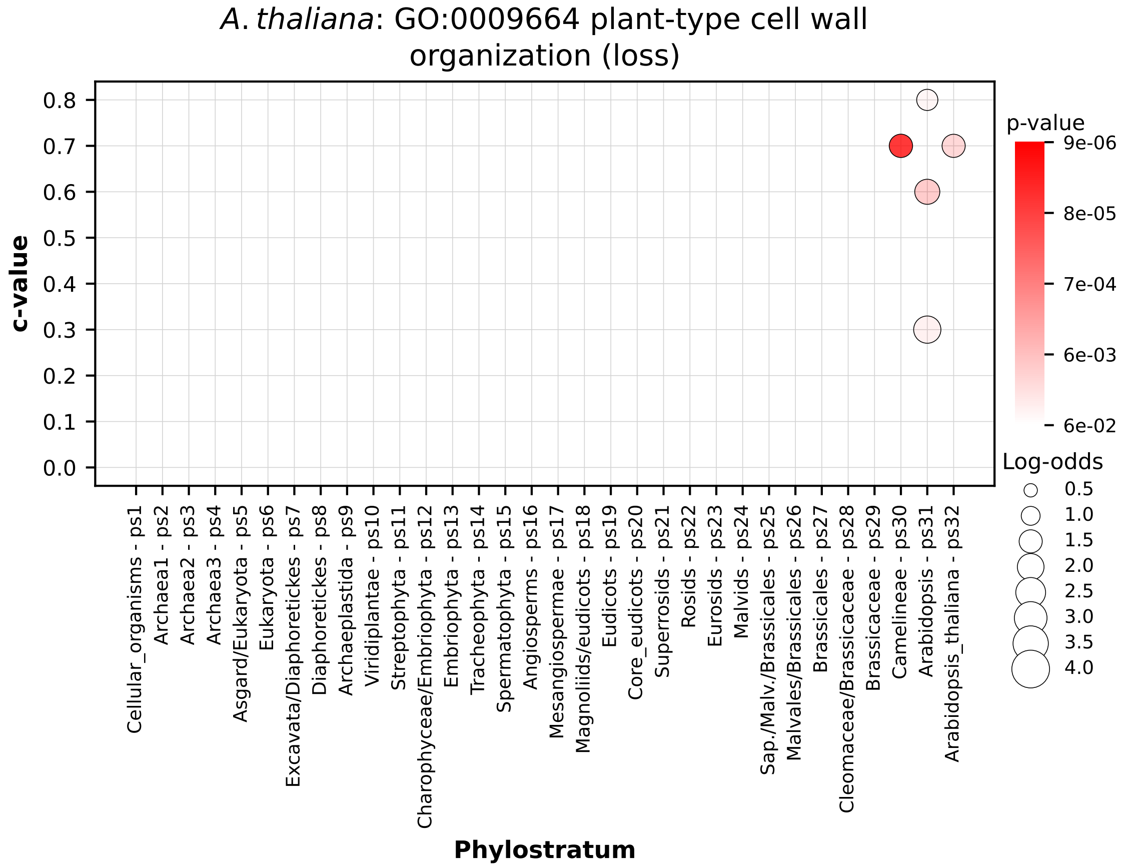

A. thaliana: GO:0009664 plant-type cell wall organization (loss)
c-value
0.8
0.7
0.6
0.5
0.4
0.3
0.2
0.1
0.0
p-value
9e-06
8e-05
7e-04
6e-03
6e-02
Log-odds
0.5
1.0
1.5
2.0
2.5
3.0
3.5
4.0
Cellular_organisms - ps1
Archaea1 - ps2
Archaea2 - ps3
Archaea3 - ps4
Asgard/Eukaryota - ps5
Eukaryota - ps6
Excavata/Diaphoretickes - ps7
Diaphoretickes - ps8
Archaeplastida - ps9
Viridiplantae - ps10
Streptophyta - ps11
Charophyceae/Embriophyta - ps12
Embriophyta - ps13
Tracheophyta - ps14
Spermatophyta - ps15
Angiosperms - ps16
Mesangiospermae - ps17
Magnoliids/eudicots - ps18
Eudicots - ps19
Core_eudicots - ps20
Superrosids - ps21
Rosids - ps22
Eurosids - ps23
Malvids - ps24
Sap./Malv./Brassicales - ps25
Malvales/Brassicales - ps26
Brassicales - ps27
Cleomaceae/Brassicaceae - ps28
Brassicaceae - ps29
Camelineae - ps30
Arabidopsis - ps31
Arabidopsis_thaliana - ps32
Phylostratum

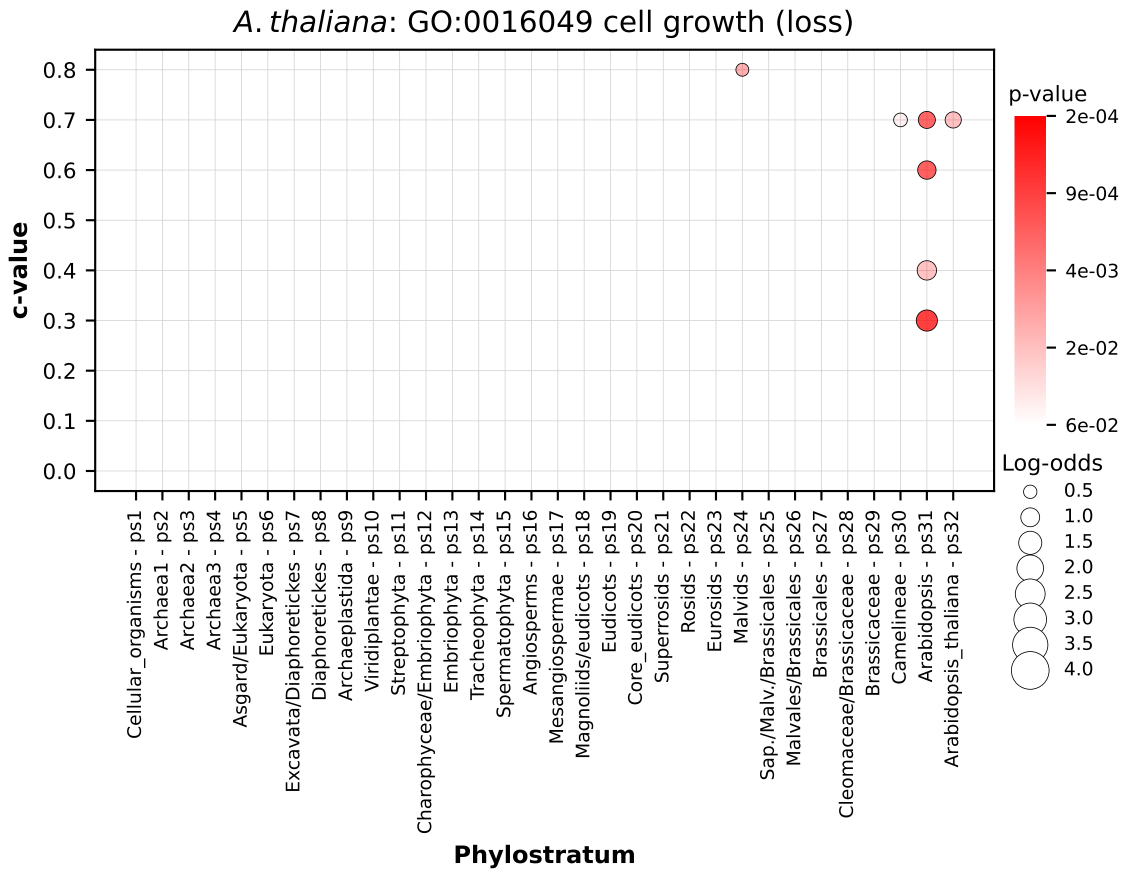
A. thaliana: GO:0016049 cell growth (loss)
c-value
0.8
0.7
0.6
0.5
0.4
0.3
0.2
0.1
0.0
p-value
2e-04
9e-04
4e-03
2e-02
6e-02
Log-odds
0.5
1.0
1.5
2.0
2.5
3.0
3.5
4.0
Cellular_organisms - ps1
Archaea1 - ps2
Archaea2 - ps3
Archaea3 - ps4
Asgard/Eukaryota - ps5
Eukaryota - ps6
Excavata/Diaphoretickes - ps7
Diaphoretickes - ps8
Archaeplastida - ps9
Viridiplantae - ps10
Streptophyta - ps11
Charophyceae/Embriophyta - ps12
Embriophyta - ps13
Tracheophyta - ps14
Spermatophyta - ps15
Angiosperms - ps16
Mesangiospermae - ps17
Magnoliids/eudicots - ps18
Eudicots - ps19
Core_eudicots - ps20
Superrosids - ps21
Rosids - ps22
Eurosids - ps23
Malvids - ps24
Sap./Malv./Brassicales - ps25
Malvales/Brassicales - ps26
Brassicales - ps27
Cleomaceae/Brassicaceae - ps28
Brassicaceae - ps29
Camelineae - ps30
Arabidopsis - ps31
Arabidopsis_thaliana - ps32
Phylostratum

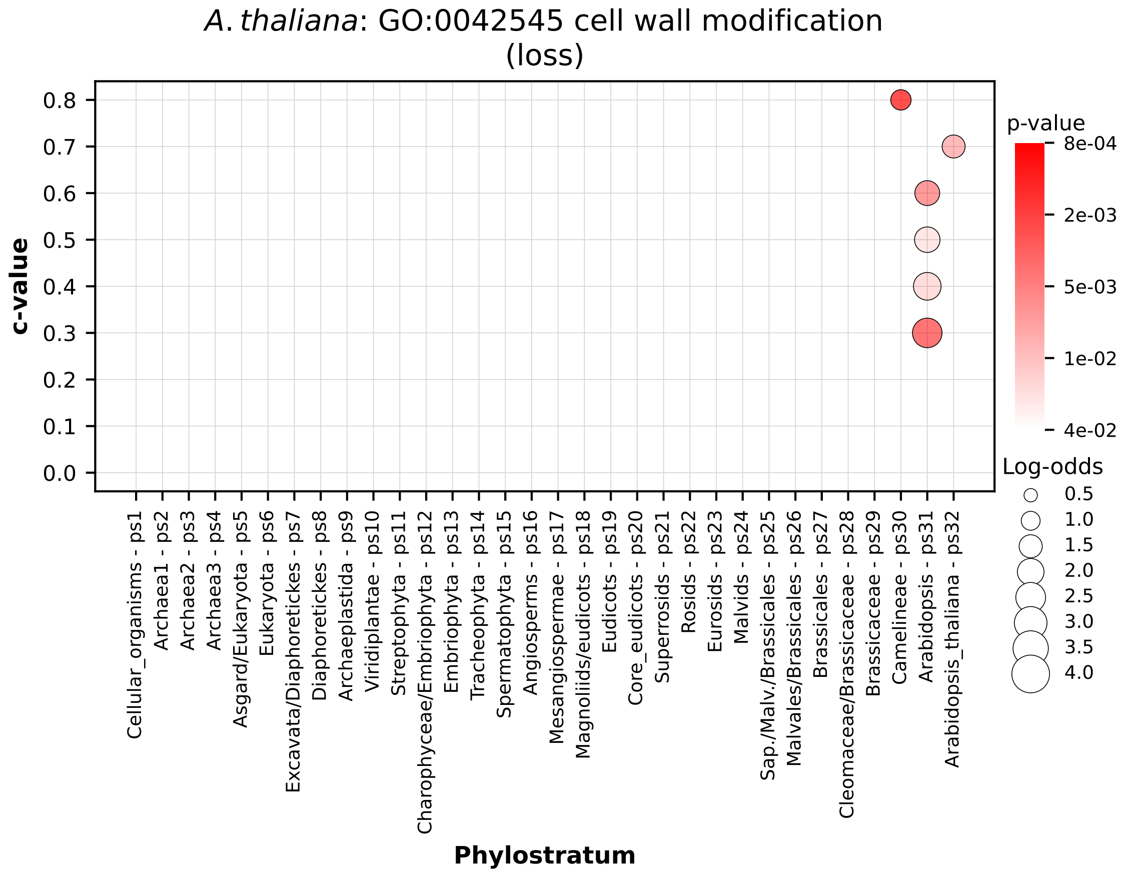

A. thaliana: GO:0042545 cell wall modification
(loss)
c-value
0.8
0.7
0.6
0.5
0.4
0.3
0.2
0.1
0.0
p-value
8e-04
2e-03
5e-03
1e-02
4e-02
Log-odds
0.5
1.0
1.5
2.0
2.5
3.0
3.5
4.0
Cellular_organisms - ps1
Archaea1 - ps2
Archaea2 - ps3
Archaea3 - ps4
Asgard/Eukaryota - ps5
Eukaryota - ps6
Excavata/Diaphoretickes - ps7
Diaphoretickes - ps8
Archaeplastida - ps9
Viridiplantae - ps10
Streptophyta - ps11
Charophyceae/Embriophyta - ps12
Embriophyta - ps13
Tracheophyta - ps14
Spermatophyta - ps15
Angiosperms - ps16
Mesangiospermae - ps17
Magnoliids/eudicots - ps18
Eudicots - ps19
Core_eudicots - ps20
Superrosids - ps21
Rosids - ps22
Eurosids - ps23
Malvids - ps24
Sap./Malv./Brassicales - ps25
Malvales/Brassicales - ps26
Brassicales - ps27
Cleomaceae/Brassicaceae - ps28
Brassicaceae - ps29
Camelineae - ps30
Arabidopsis - ps31
Arabidopsis_thaliana - ps32
Phylostratum

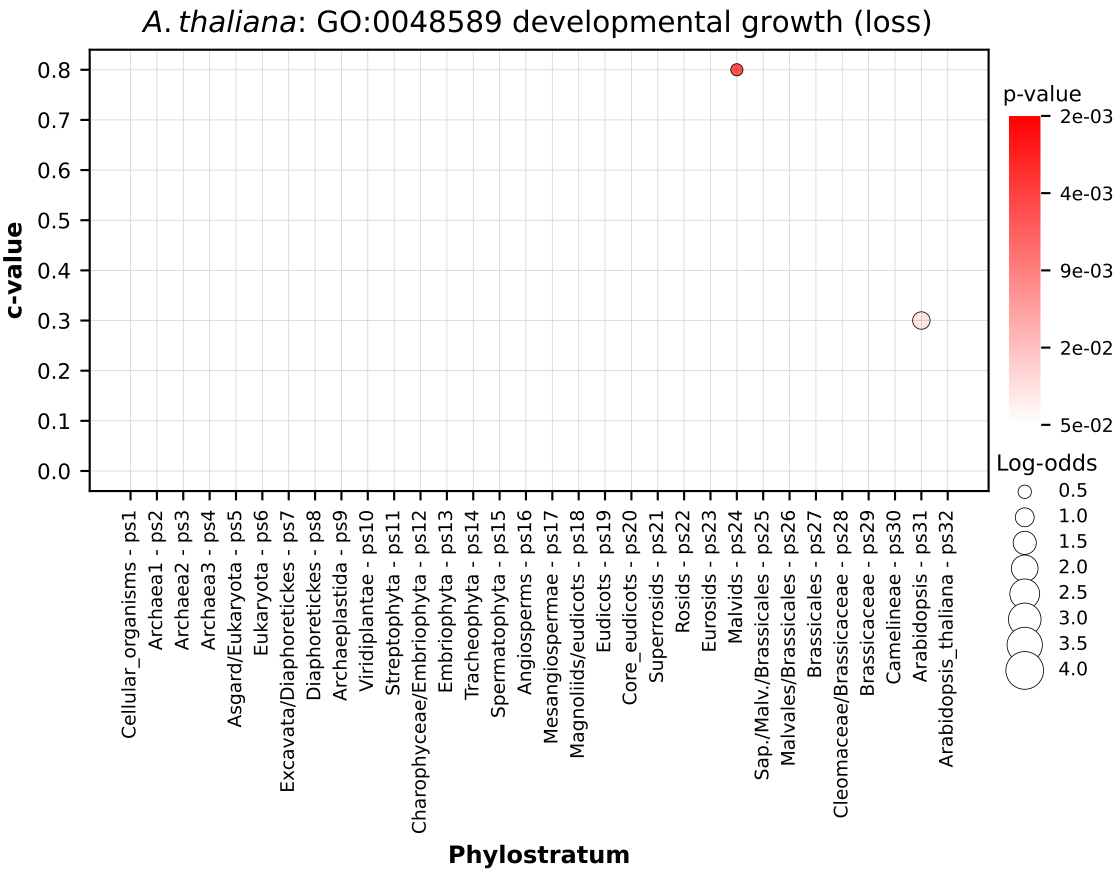

A. thaliana: GO:0048589 developmental growth (loss)
c-value
0.8
0.7
0.6
0.5
0.4
0.3
0.2
0.1
0.0
p-value
2e-03
4e-03
9e-03
2e-02
5e-02
Log-odds
0.5
1.0
1.5
2.0
2.5
3.0
3.5
4.0
Cellular_organisms - ps1
Archaea1 - ps2
Archaea2 - ps3
Archaea3 - ps4
Asgard/Eukaryota - ps5
Eukaryota - ps6
Excavata/Diaphoretickes - ps7
Diaphoretickes - ps8
Archaeplastida - ps9
Viridiplantae - ps10
Streptophyta - ps11
Charophyceae/Embriophyta - ps12
Embriophyta - ps13
Tracheophyta - ps14
Spermatophyta - ps15
Angiosperms - ps16
Mesangiospermae - ps17
Magnoliids/eudicots - ps18
Eudicots - ps19
Core_eudicots - ps20
Superrosids - ps21
Rosids - ps22
Eurosids - ps23
Malvids - ps24
Sap./Malv./Brassicales - ps25
Malvales/Brassicales - ps26
Brassicales - ps27
Cleomaceae/Brassicaceae - ps28
Brassicaceae - ps29
Camelineae - ps30
Arabidopsis - ps31
Arabidopsis_thaliana - ps32
Phylostratum

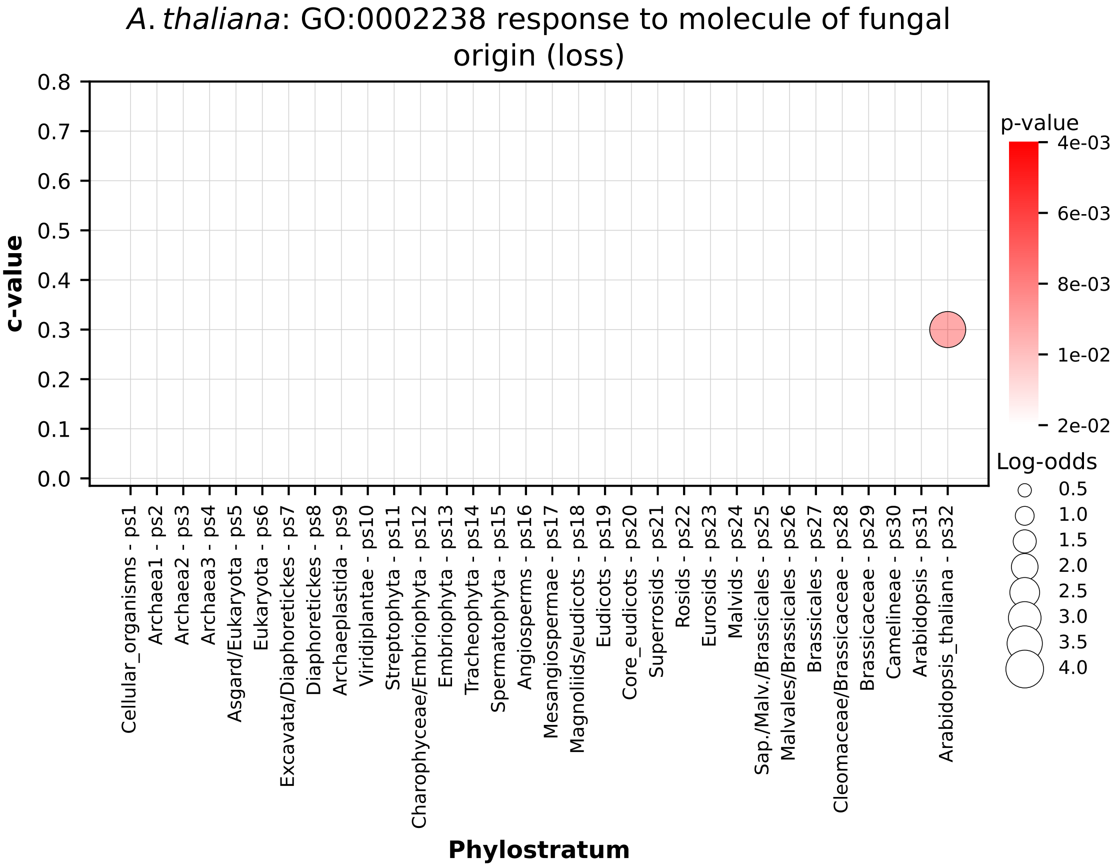
A. thaliana: GO:0002238 response to molecule of fungal origin (loss)
c-value
0.8
0.7
0.6
0.5
0.4
0.3
0.2
0.1
0.0
Phylostratum
Cellular_organisms - ps1
Archaea1 - ps2
Archaea2 - ps3
Archaea3 - ps4
Asgard/Eukaryota - ps5
Eukaryota - ps6
Excavata/Diaphoretickes - ps7
Diaphoretickes - ps8
Archaeplastida - ps9
Viridiplantae - ps10
Streptophyta - ps11
Charophyceae/Embriophyta - ps12
Embriophyta - ps13
Tracheophyta - ps14
Spermatophyta - ps15
Angiosperms - ps16
Mesangiospermae - ps17
Magnoliids/eudicots - ps18
Eudicots - ps19
Core_eudicots - ps20
Superrosids - ps21
Rosids - ps22
Eurosids - ps23
Malvids - ps24
Sap./Malv./Brassicales - ps25
Malvales/Brassicales - ps26
Brassicales - ps27
Cleomaceae/Brassicaceae - ps28
Brassicaceae - ps29
Camelineae - ps30
Arabidopsis - ps31
Arabidopsis_thaliana - ps32
p-value
4e-03
6e-03
8e-03
1e-02
2e-02
Log-odds
0.5
1.0
1.5
2.0
2.5
3.0
3.5
4.0

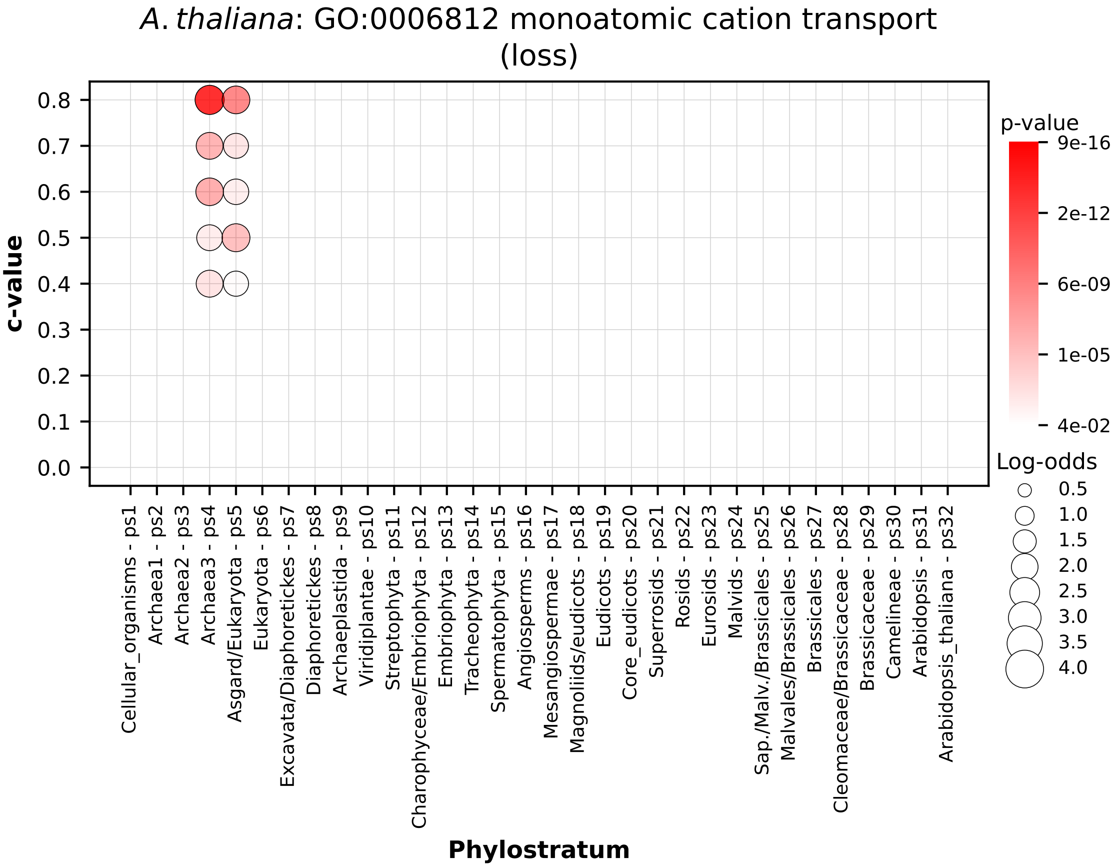

A. thaliana: GO:0006812 monoatomic cation transport
(loss)
c-value
0.8
0.7
0.6
0.5
0.4
0.3
0.2
0.1
0.0
p-value
9e-16
2e-12
6e-09
1e-05
4e-02
Log-odds
0.5
1.0
1.5
2.0
2.5
3.0
3.5
4.0
Cellular_organisms - ps1
Archaea1 - ps2
Archaea2 - ps3
Archaea3 - ps4
Asgard/Eukaryota - ps5
Eukaryota - ps6
Excavata/Diaphoretickes - ps7
Diaphoretickes - ps8
Archaeplastida - ps9
Viridiplantae - ps10
Streptophyta - ps11
Charophyceae/Embriophyta - ps12
Embriophyta - ps13
Tracheophyta - ps14
Spermatophyta - ps15
Angiosperms - ps16
Mesangiospermae - ps17
Magnoliids/eudicots - ps18
Eudicots - ps19
Core_eudicots - ps20
Superrosids - ps21
Rosids - ps22
Eurosids - ps23
Malvids - ps24
Sap./Malv./Brassicales - ps25
Malvales/Brassicales - ps26
Brassicales - ps27
Cleomaceae/Brassicaceae - ps28
Brassicaceae - ps29
Camelineae - ps30
Arabidopsis - ps31
Arabidopsis_thaliana - ps32
Phylostratum

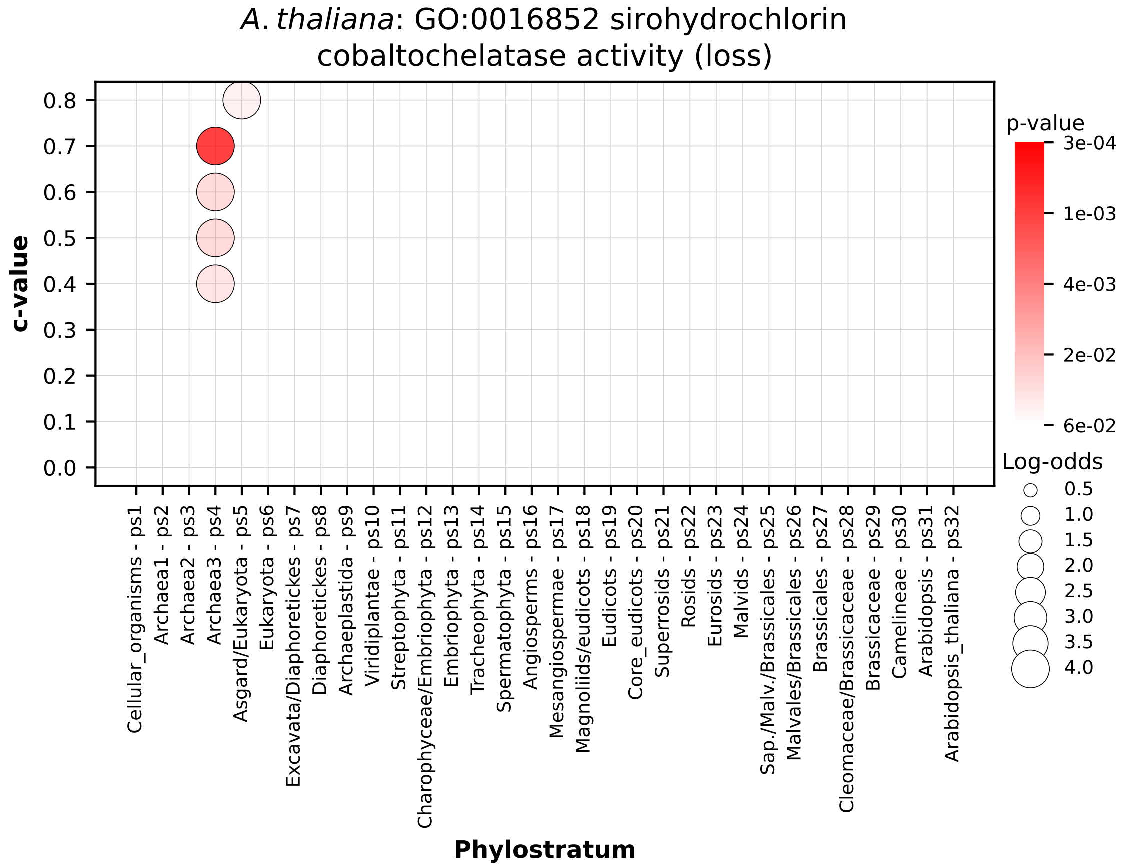

A. thaliana: GO:0016852 sirohydrochlorin cobaltochelatase activity (loss)
c-value
0.0
0.1
0.2
0.3
0.4
0.5
0.6
0.7
0.8
Phylostratum
Cellular_organisms - ps1
Archaea1 - ps2
Archaea2 - ps3
Archaea3 - ps4
Asgard/Eukaryota - ps5
Eukaryota - ps6
Excavata/Diaphoretickes - ps7
Diaphoretickes - ps8
Archaeplastida - ps9
Viridiplantae - ps10
Streptophyta - ps11
Charophyceae/Embriophyta - ps12
Embriophyta - ps13
Tracheophyta - ps14
Spermatophyta - ps15
Angiosperms - ps16
Mesangiospermae - ps17
Magnoliids/eudicots - ps18
Eudicots - ps19
Core_eudicots - ps20
Superrosids - ps21
Rosids - ps22
Eurosids - ps23
Malvids - ps24
Sap./Malv./Brassicales - ps25
Malvales/Brassicales - ps26
Brassicales - ps27
Cleomaceae/Brassicaceae - ps28
Brassicaceae - ps29
Camelineae - ps30
Arabidopsis - ps31
Arabidopsis_thaliana - ps32
p-value
3e-04
1e-03
4e-03
2e-02
6e-02
Log-odds
0.5
1.0
1.5
2.0
2.5
3.0
3.5
4.0

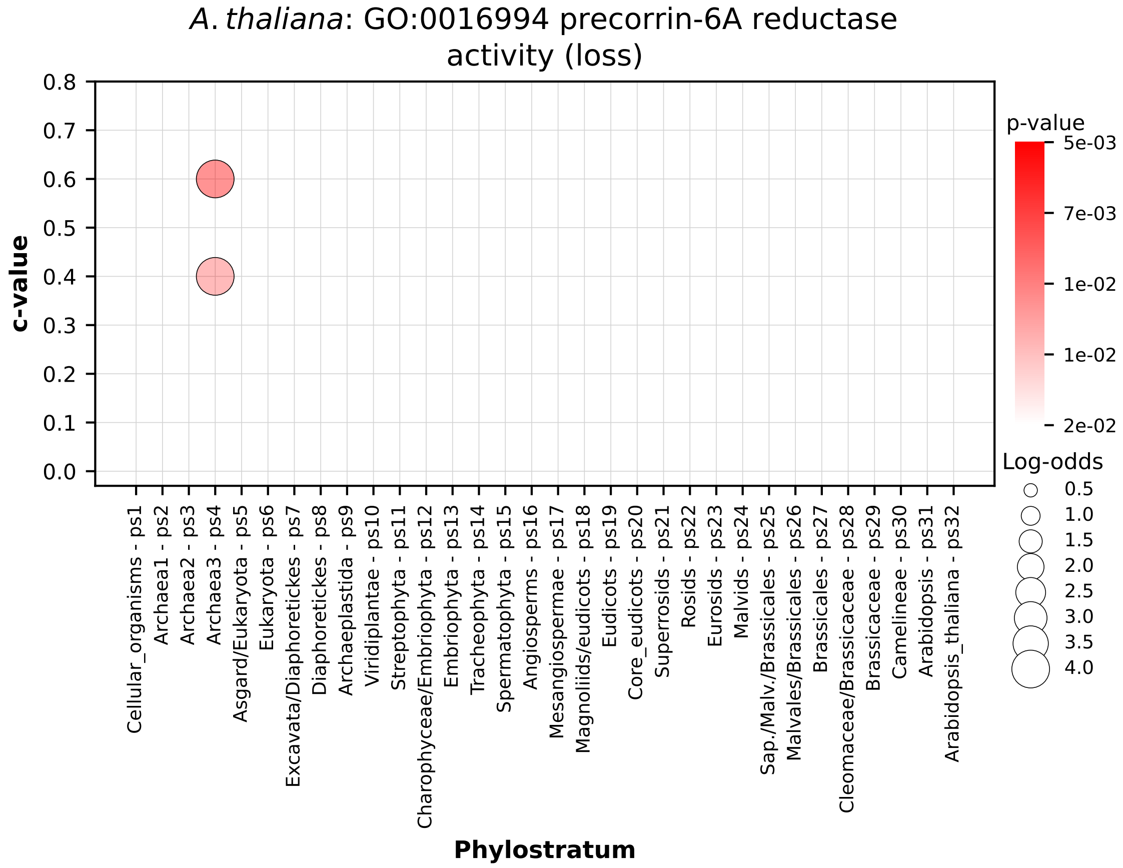

A. thaliana: GO:0016994 precorrin-6A reductase activity (loss)
c-value
0.8
0.7
0.6
0.5
0.4
0.3
0.2
0.1
0.0
Phylostratum
Cellular_organisms - ps1
Archaea1 - ps2
Archaea2 - ps3
Archaea3 - ps4
Asgard/Eukaryota - ps5
Eukaryota - ps6
Excavata/Diaphoretickes - ps7
Diaphoretickes - ps8
Archaeplastida - ps9
Viridiplantae - ps10
Streptophyta - ps11
Charophyceae/Embriophyta - ps12
Embriophyta - ps13
Tracheophyta - ps14
Spermatophyta - ps15
Angiosperms - ps16
Mesangiospermae - ps17
Magnoliids/eudicots - ps18
Eudicots - ps19
Core_eudicots - ps20
Superrosids - ps21
Rosids - ps22
Eurosids - ps23
Malvids - ps24
Sap./Malv./Brassicales - ps25
Malvales/Brassicales - ps26
Brassicales - ps27
Cleomaceae/Brassicaceae - ps28
Brassicaceae - ps29
Camelineae - ps30
Arabidopsis - ps31
Arabidopsis_thaliana - ps32
p-value
5e-03
7e-03
1e-02
1e-02
2e-02
Log-odds
0.5
1.0
1.5
2.0
2.5
3.0
3.5
4.0

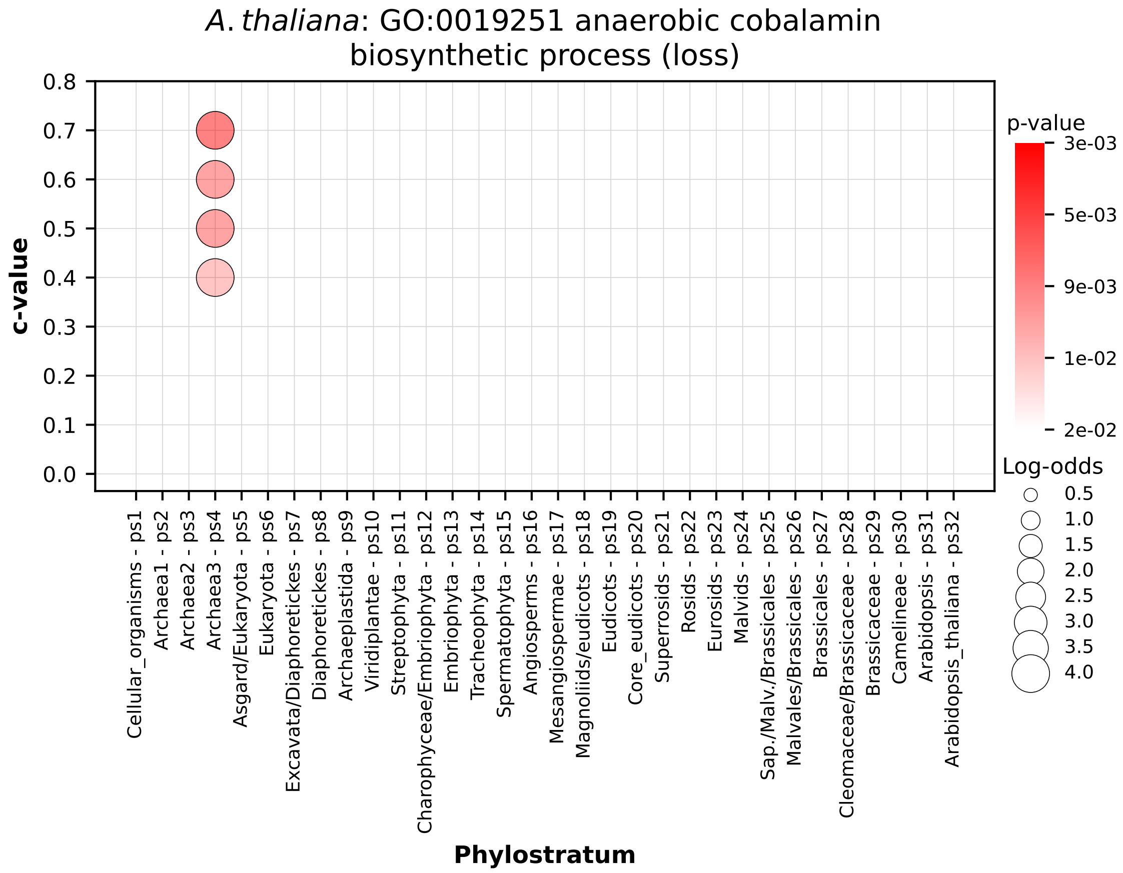

A. thaliana: GO:0019251 anaerobic cobalamin biosynthetic process (loss)
c-value
0.8
0.7
0.6
0.5
0.4
0.3
0.2
0.1
0.0
p-value
3e-03
5e-03
9e-03
1e-02
2e-02
Log-odds
0.5
1.0
1.5
2.0
2.5
3.0
3.5
4.0
Cellular_organisms - ps1
Archaea1 - ps2
Archaea2 - ps3
Archaea3 - ps4
Asgard/Eukaryota - ps5
Eukaryota - ps6
Excavata/Diaphoretickes - ps7
Diaphoretickes - ps8
Archaeplastida - ps9
Viridiplantae - ps10
Streptophyta - ps11
Charophyceae/Embriophyta - ps12
Embriophyta - ps13
Tracheophyta - ps14
Spermatophyta - ps15
Angiosperms - ps16
Mesangiospermae - ps17
Magnoliids/eudicots - ps18
Eudicots - ps19
Core_eudicots - ps20
Superrosids - ps21
Rosids - ps22
Eurosids - ps23
Malvids - ps24
Sap./Malv./Brassicales - ps25
Malvales/Brassicales - ps26
Brassicales - ps27
Cleomaceae/Brassicaceae - ps28
Brassicaceae - ps29
Camelineae - ps30
Arabidopsis - ps31
Arabidopsis_thaliana - ps32
Phylostratum

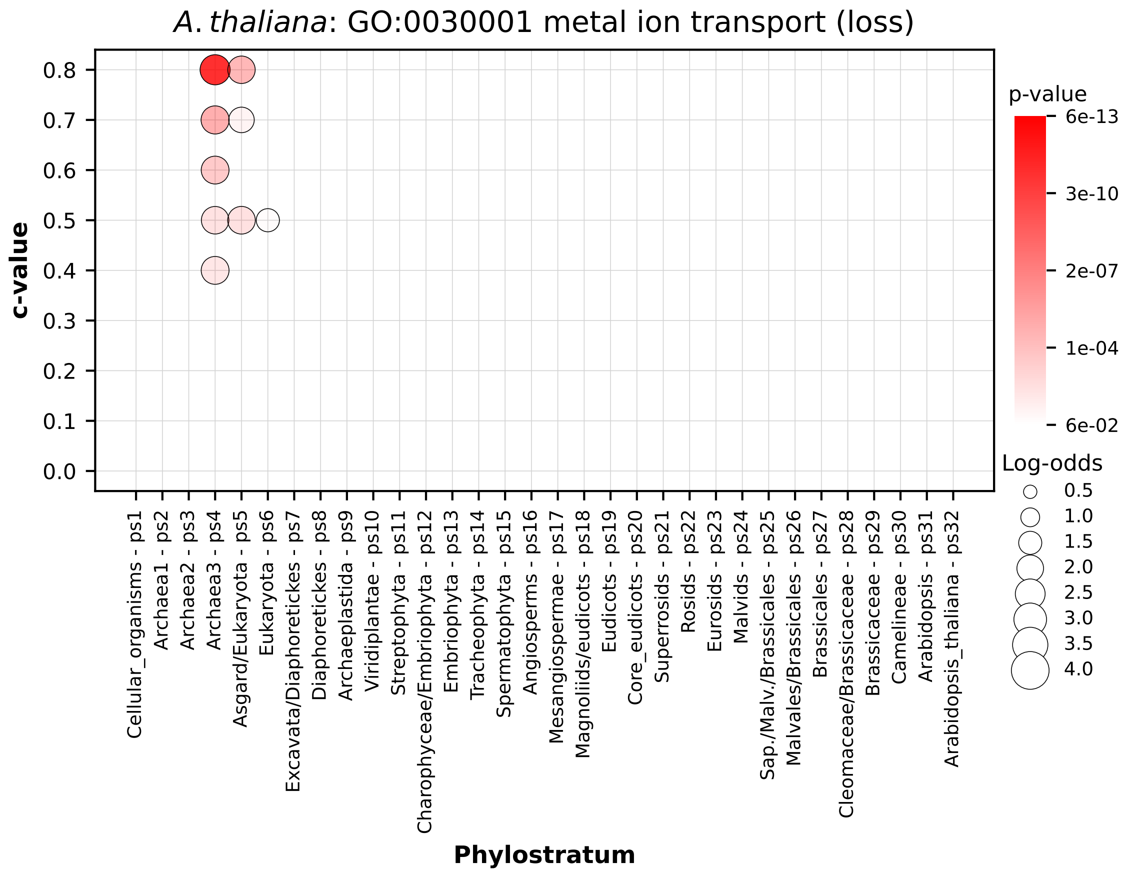

A. thaliana: GO:0030001 metal ion transport (loss)
c-value
0.8
0.7
0.6
0.5
0.4
0.3
0.2
0.1
0.0
Phylostratum
Cellular_organisms - ps1
Archaea1 - ps2
Archaea2 - ps3
Archaea3 - ps4
Asgard/Eukaryota - ps5
Eukaryota - ps6
Excavata/Diaphoretickes - ps7
Diaphoretickes - ps8
Archaeplastida - ps9
Viridiplantae - ps10
Streptophyta - ps11
Charophyceae/Embriophyta - ps12
Embriophyta - ps13
Tracheophyta - ps14
Spermatophyta - ps15
Angiosperms - ps16
Mesangiospermae - ps17
Magnoliids/eudicots - ps18
Eudicots - ps19
Core_eudicots - ps20
Superrosids - ps21
Rosids - ps22
Eurosids - ps23
Malvids - ps24
Sap./Malv./Brassicales - ps25
Malvales/Brassicales - ps26
Brassicales - ps27
Cleomaceae/Brassicaceae - ps28
Brassicaceae - ps29
Camelineae - ps30
Arabidopsis - ps31
Arabidopsis_thaliana - ps32
p-value
6e-13
3e-10
2e-07
1e-04
6e-02
Log-odds
0.5
1.0
1.5
2.0
2.5
3.0
3.5
4.0

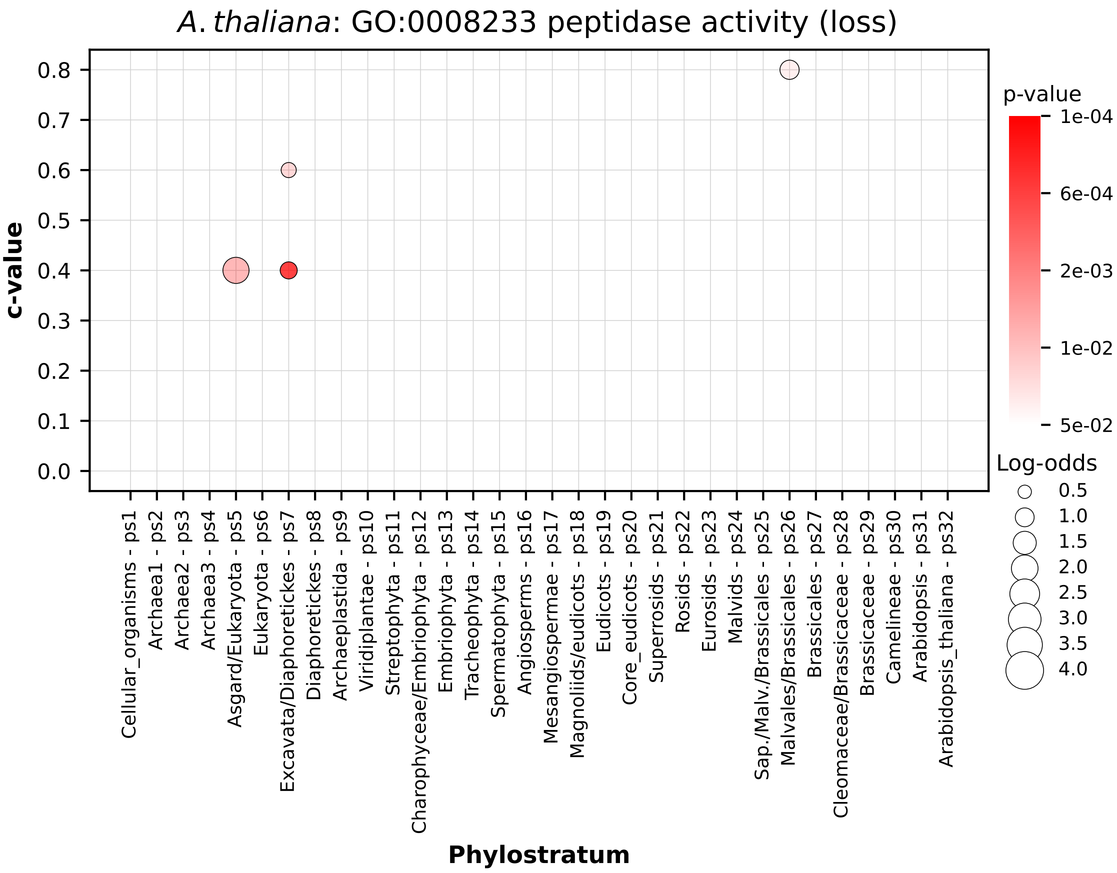

A. thaliana: GO:0008233 peptidase activity (loss)
c-value
0.8
0.7
0.6
0.5
0.4
0.3
0.2
0.1
0.0
p-value
1e-04
6e-04
2e-03
1e-02
5e-02
Log-odds
0.5
1.0
1.5
2.0
2.5
3.0
3.5
4.0
Cellular_organisms - ps1
Archaea1 - ps2
Archaea2 - ps3
Archaea3 - ps4
Asgard/Eukaryota - ps5
Eukaryota - ps6
Excavata/Diaphoretickes - ps7
Diaphoretickes - ps8
Archaeplastida - ps9
Viridiplantae - ps10
Streptophyta - ps11
Charophyceae/Embriophyta - ps12
Embriophyta - ps13
Tracheophyta - ps14
Spermatophyta - ps15
Angiosperms - ps16
Mesangiospermae - ps17
Magnoliids/eudicots - ps18
Eudicots - ps19
Core_eudicots - ps20
Superrosids - ps21
Rosids - ps22
Eurosids - ps23
Malvids - ps24
Sap./Malv./Brassicales - ps25
Malvales/Brassicales - ps26
Brassicales - ps27
Cleomaceae/Brassicaceae - ps28
Brassicaceae - ps29
Camelineae - ps30
Arabidopsis - ps31
Arabidopsis_thaliana - ps32
Phylostratum

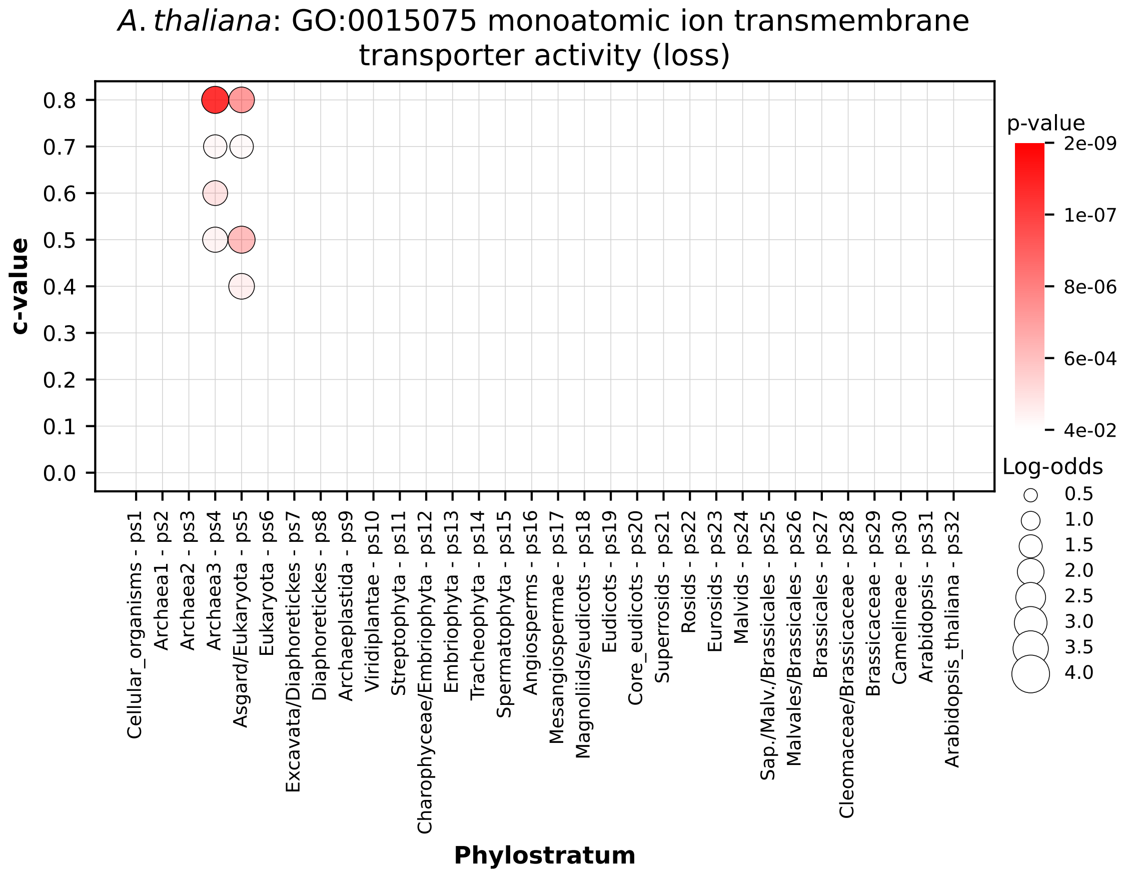

A. thaliana: GO:0015075 monoatomic ion transmembrane transporter activity (loss)
c-value
0.8
0.7
0.6
0.5
0.4
0.3
0.2
0.1
0.0
p-value
2e-09
1e-07
8e-06
6e-04
4e-02
Log-odds
0.5
1.0
1.5
2.0
2.5
3.0
3.5
4.0
Cellular_organisms - ps1
Archaea1 - ps2
Archaea2 - ps3
Archaea3 - ps4
Asgard/Eukaryota - ps5
Eukaryota - ps6
Excavata/Diaphoretickes - ps7
Diaphoretickes - ps8
Archaeplastida - ps9
Viridiplantae - ps10
Streptophyta - ps11
Charophyceae/Embriophyta - ps12
Embriophyta - ps13
Tracheophyta - ps14
Spermatophyta - ps15
Angiosperms - ps16
Mesangiospermae - ps17
Magnoliids/eudicots - ps18
Eudicots - ps19
Core_eudicots - ps20
Superrosids - ps21
Rosids - ps22
Eurosids - ps23
Malvids - ps24
Sap./Malv./Brassicales - ps25
Malvales/Brassicales - ps26
Brassicales - ps27
Cleomaceae/Brassicaceae - ps28
Brassicaceae - ps29
Camelineae - ps30
Arabidopsis - ps31
Arabidopsis_thaliana - ps32
Phylostratum

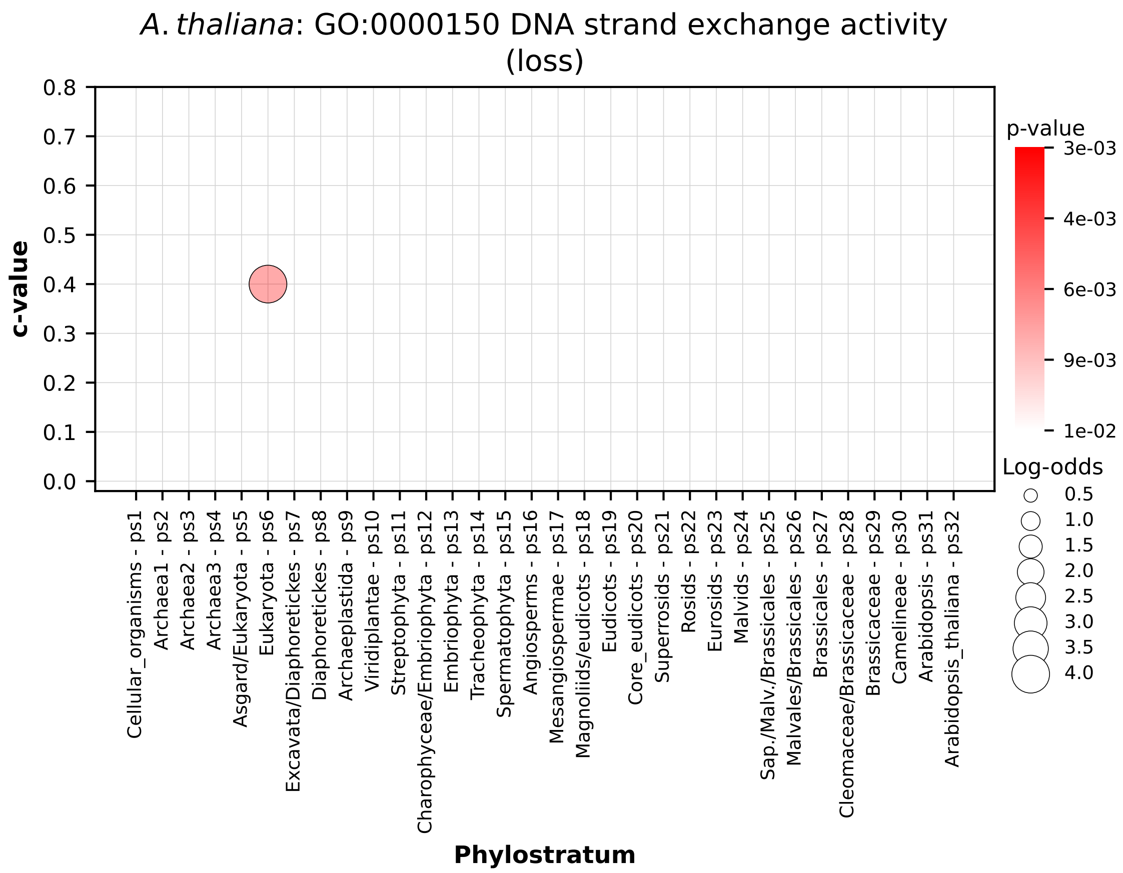

A. thaliana: GO:0000150 DNA strand exchange activity
(loss)
c-value
0.8
0.7
0.6
0.5
0.4
0.3
0.2
0.1
0.0
p-value
3e-03
4e-03
6e-03
9e-03
1e-02
Log-odds
0.5
1.0
1.5
2.0
2.5
3.0
3.5
4.0
Cellular_organisms - ps1
Archaea1 - ps2
Archaea2 - ps3
Archaea3 - ps4
Asgard/Eukaryota - ps5
Eukaryota - ps6
Excavata/Diaphoretickes - ps7
Diaphoretickes - ps8
Archaeplastida - ps9
Viridiplantae - ps10
Streptophyta - ps11
Charophyceae/Embriophyta - ps12
Embriophyta - ps13
Tracheophyta - ps14
Spermatophyta - ps15
Angiosperms - ps16
Mesangiospermae - ps17
Magnoliids/eudicots - ps18
Eudicots - ps19
Core_eudicots - ps20
Superrosids - ps21
Rosids - ps22
Eurosids - ps23
Malvids - ps24
Sap./Malv./Brassicales - ps25
Malvales/Brassicales - ps26
Brassicales - ps27
Cleomaceae/Brassicaceae - ps28
Brassicaceae - ps29
Camelineae - ps30
Arabidopsis - ps31
Arabidopsis_thaliana - ps32
Phylostratum

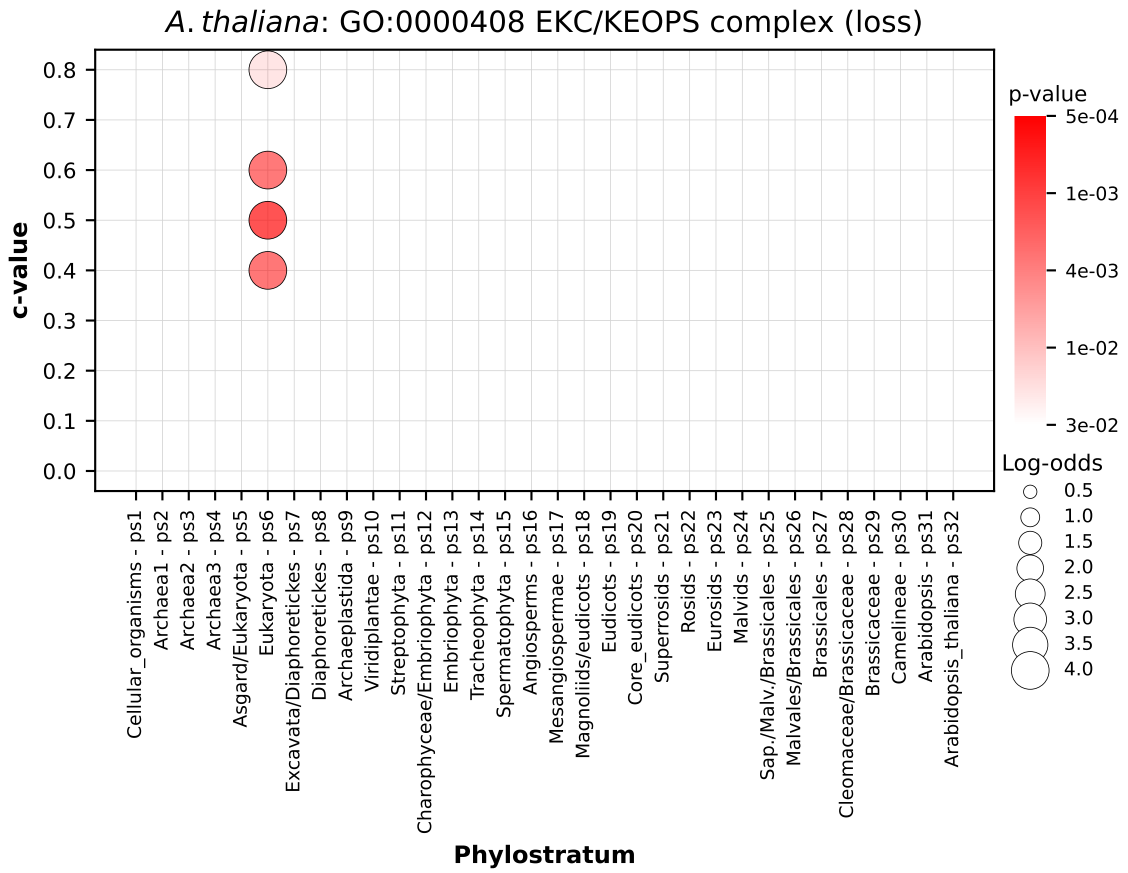

A. thaliana: GO:0000408 EKC/KEOPS complex (loss)
c-value
0.0
0.1
0.2
0.3
0.4
0.5
0.6
0.7
0.8
p-value
5e-04
1e-03
4e-03
1e-02
3e-02
Log-odds
0.5
1.0
1.5
2.0
2.5
3.0
3.5
4.0
Cellular_organisms - ps1
Archaea1 - ps2
Archaea2 - ps3
Archaea3 - ps4
Asgard/Eukaryota - ps5
Eukaryota - ps6
Excavata/Diaphoretickes - ps7
Diaphoretickes - ps8
Archaeplastida - ps9
Viridiplantae - ps10
Streptophyta - ps11
Charophyceae/Embriophyta - ps12
Embriophyta - ps13
Tracheophyta - ps14
Spermatophyta - ps15
Angiosperms - ps16
Mesangiospermae - ps17
Magnoliids/eudicots - ps18
Eudicots - ps19
Core_eudicots - ps20
Superrosids - ps21
Rosids - ps22
Eurosids - ps23
Malvids - ps24
Sap./Malv./Brassicales - ps25
Malvales/Brassicales - ps26
Brassicales - ps27
Cleomaceae/Brassicaceae - ps28
Brassicaceae - ps29
Camelineae - ps30
Arabidopsis - ps31
Arabidopsis_thaliana - ps32
Phylostratum

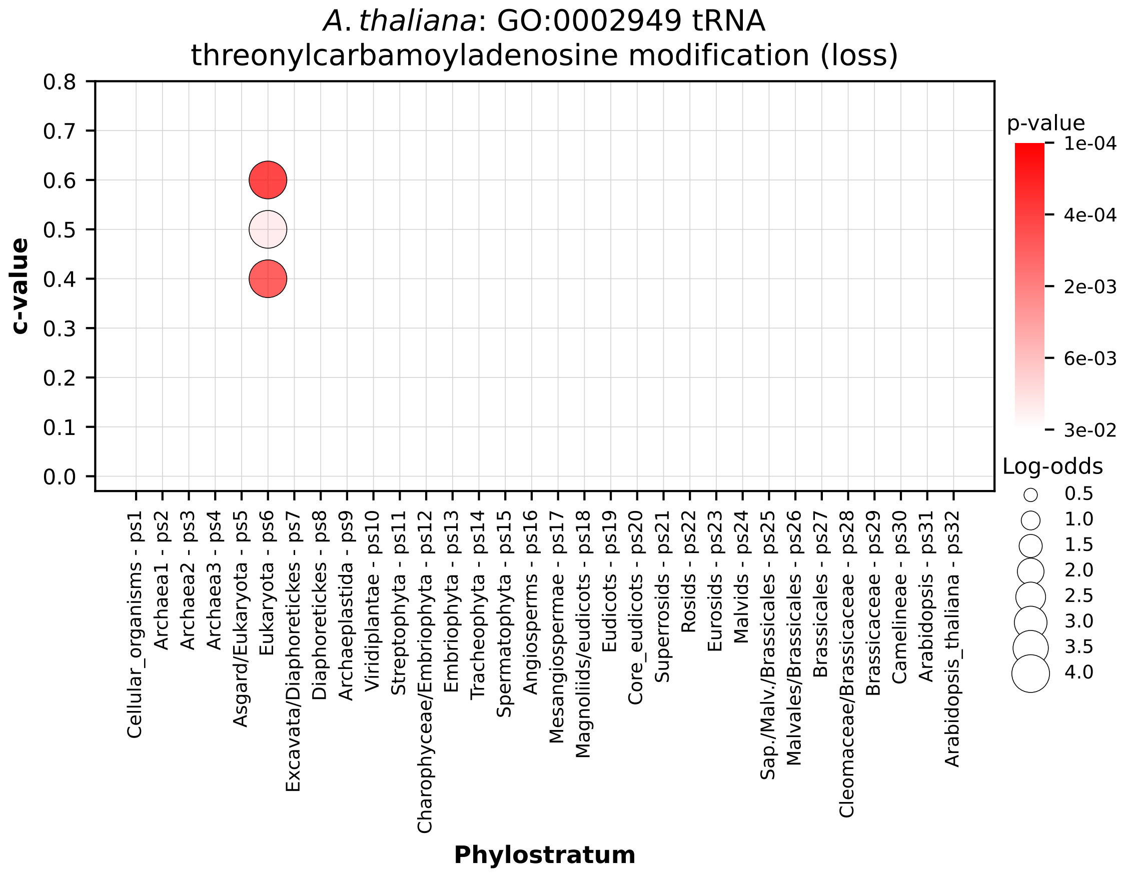

A. thaliana: GO:0002949 tRNA threonylcarbamoyladenosine modification (loss)
c-value
0.8
0.7
0.6
0.5
0.4
0.3
0.2
0.1
0.0
Phylostratum
Cellular_organisms - ps1
Archaea1 - ps2
Archaea2 - ps3
Archaea3 - ps4
Asgard/Eukaryota - ps5
Eukaryota - ps6
Excavata/Diaphoretickes - ps7
Diaphoretickes - ps8
Archaeplastida - ps9
Viridiplantae - ps10
Streptophyta - ps11
Charophyceae/Embriophyta - ps12
Embriophyta - ps13
Tracheophyta - ps14
Spermatophyta - ps15
Angiosperms - ps16
Mesangiospermae - ps17
Magnoliids/eudicots - ps18
Eudicots - ps19
Core_eudicots - ps20
Superrosids - ps21
Rosids - ps22
Eurosids - ps23
Malvids - ps24
Sap./Malv./Brassicales - ps25
Malvales/Brassicales - ps26
Brassicales - ps27
Cleomaceae/Brassicaceae - ps28
Brassicaceae - ps29
Camelineae - ps30
Arabidopsis - ps31
Arabidopsis_thaliana - ps32
p-value
1e-04
4e-04
2e-03
6e-03
3e-02
Log-odds
0.5
1.0
1.5
2.0
2.5
3.0
3.5
4.0

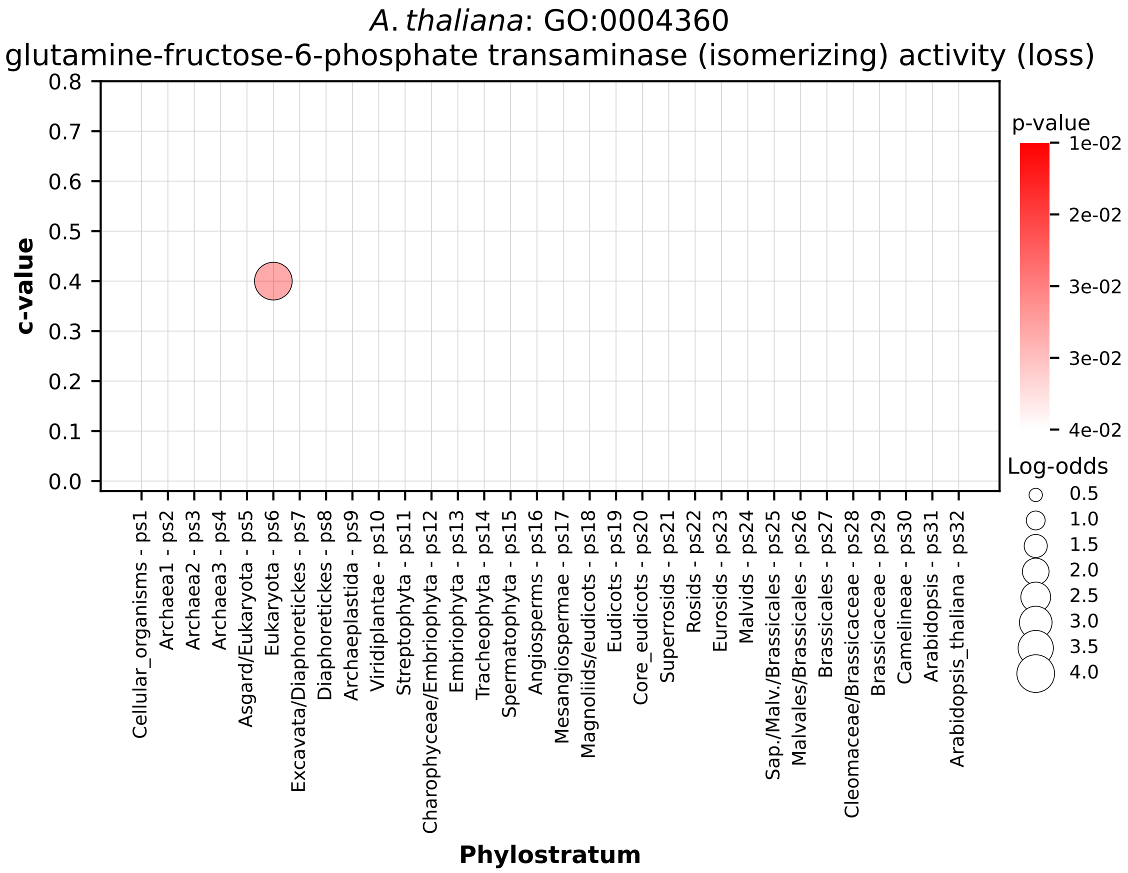

A. thaliana: GO:0004360
glutamine-fructose-6-phosphate transaminase (isomerizing) activity (loss)
c-value
0.0
0.1
0.2
0.3
0.4
0.5
0.6
0.7
0.8
Phylostratum
Cellular_organisms - ps1
Archaea1 - ps2
Archaea2 - ps3
Archaea3 - ps4
Asgard/Eukaryota - ps5
Eukaryota - ps6
Excavata/Diaphoretickes - ps7
Diaphoretickes - ps8
Archaeplastida - ps9
Viridiplantae - ps10
Streptophyta - ps11
Charophyceae/Embriophyta - ps12
Embriophyta - ps13
Tracheophyta - ps14
Spermatophyta - ps15
Angiosperms - ps16
Mesangiospermae - ps17
Magnoliids/eudicots - ps18
Eudicots - ps19
Core_eudicots - ps20
Superrosids - ps21
Rosids - ps22
Eurosids - ps23
Malvids - ps24
Sap./Malv./Brassicales - ps25
Malvales/Brassicales - ps26
Brassicales - ps27
Cleomaceae/Brassicaceae - ps28
Brassicaceae - ps29
Camelineae - ps30
Arabidopsis - ps31
Arabidopsis_thaliana - ps32
p-value
1e-02
2e-02
3e-02
3e-02
4e-02
Log-odds
0.5
1.0
1.5
2.0
2.5
3.0
3.5
4.0

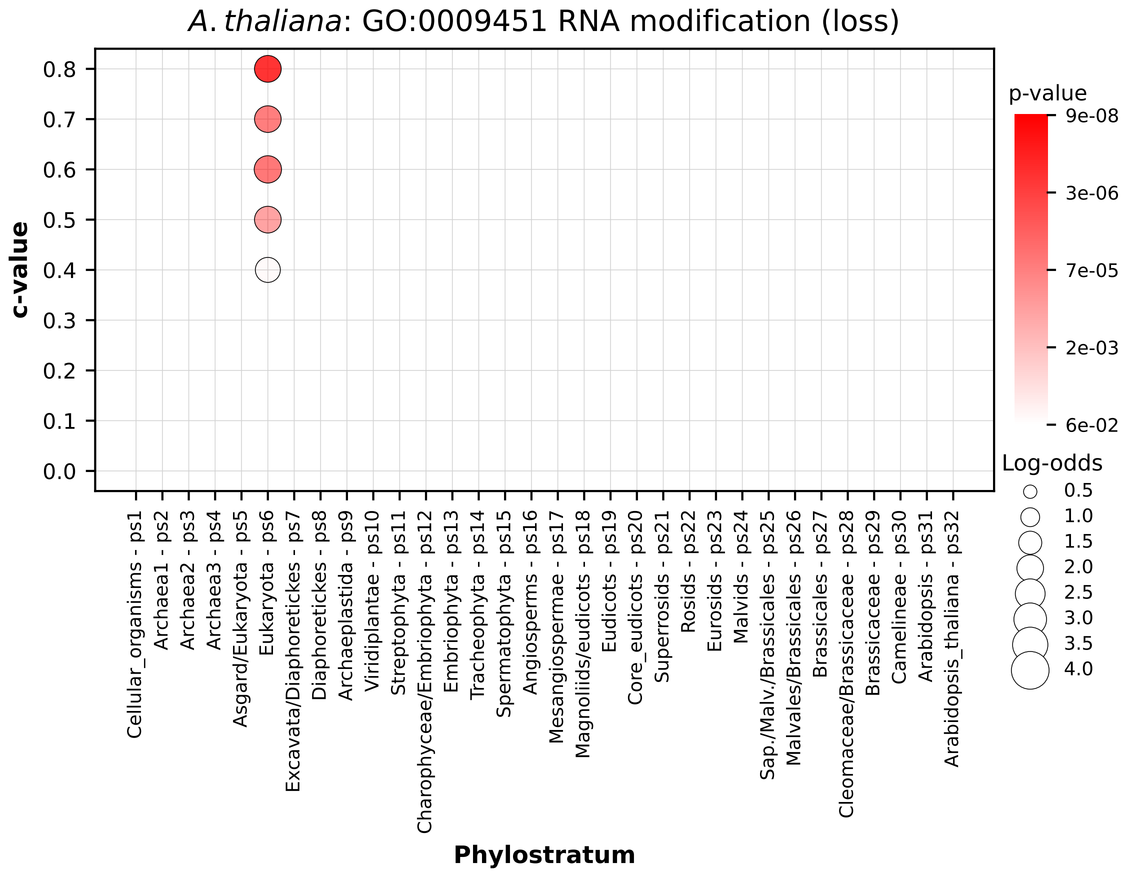

A. thaliana: GO:0009451 RNA modification (loss)
c-value
0.0
0.1
0.2
0.3
0.4
0.5
0.6
0.7
0.8
Phylostratum
Cellular_organisms - ps1
Archaea1 - ps2
Archaea2 - ps3
Archaea3 - ps4
Asgard/Eukaryota - ps5
Eukaryota - ps6
Excavata/Diaphoretickes - ps7
Diaphoretickes - ps8
Archaeplastida - ps9
Viridiplantae - ps10
Streptophyta - ps11
Charophyceae/Embriophyta - ps12
Embriophyta - ps13
Tracheophyta - ps14
Spermatophyta - ps15
Angiosperms - ps16
Mesangiospermae - ps17
Magnoliids/eudicots - ps18
Eudicots - ps19
Core_eudicots - ps20
Superrosids - ps21
Rosids - ps22
Eurosids - ps23
Malvids - ps24
Sap./Malv./Brassicales - ps25
Malvales/Brassicales - ps26
Brassicales - ps27
Cleomaceae/Brassicaceae - ps28
Brassicaceae - ps29
Camelineae - ps30
Arabidopsis - ps31
Arabidopsis_thaliana - ps32
p-value
9e-08
3e-06
7e-05
2e-03
6e-02
Log-odds
0.5
1.0
1.5
2.0
2.5
3.0
3.5
4.0

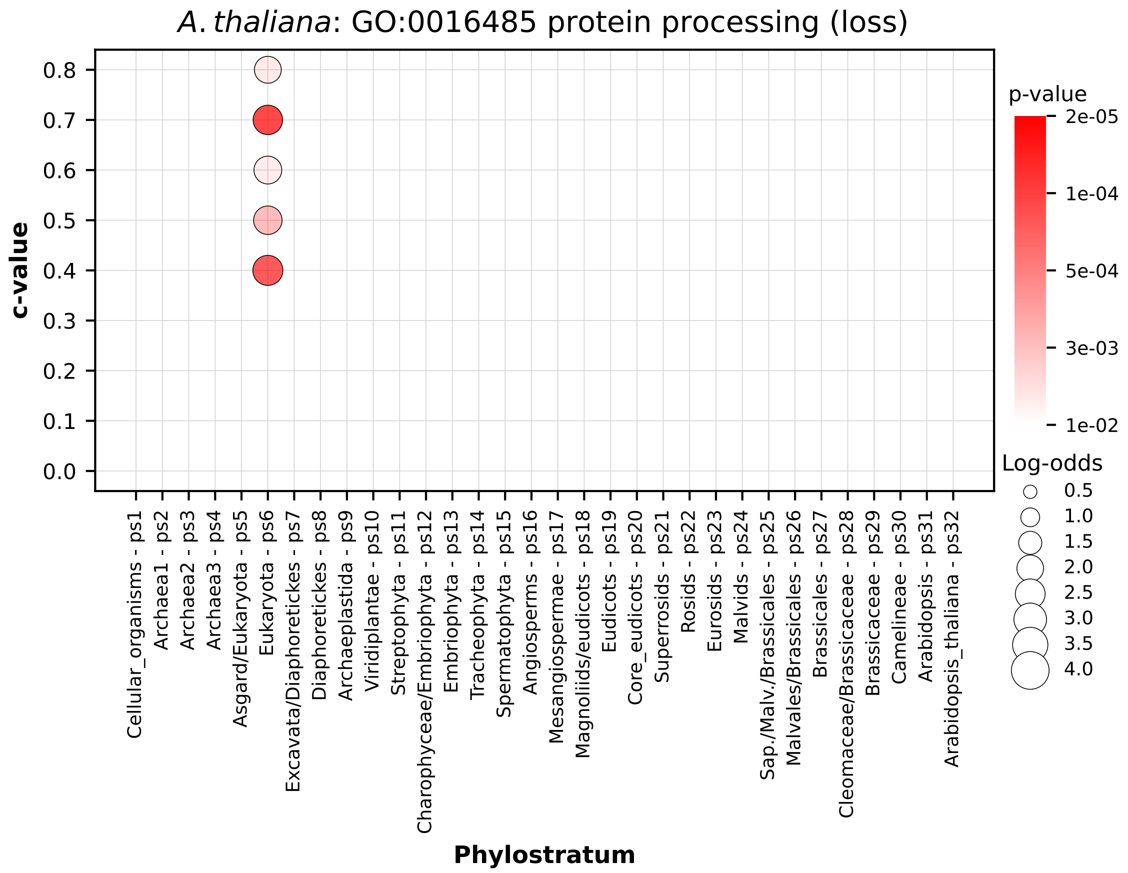

A. thaliana: GO:0016485 protein processing (loss)
c-value
0.8
0.7
0.6
0.5
0.4
0.3
0.2
0.1
0.0
p-value
2e-05
1e-04
5e-04
3e-03
1e-02
Log-odds
0.5
1.0
1.5
2.0
2.5
3.0
3.5
4.0
Cellular_organisms - ps1
Archaea1 - ps2
Archaea2 - ps3
Archaea3 - ps4
Asgard/Eukaryota - ps5
Eukaryota - ps6
Excavata/Diaphoretickes - ps7
Diaphoretickes - ps8
Archaeplastida - ps9
Viridiplantae - ps10
Streptophyta - ps11
Charophyceae/Embriophyta - ps12
Embriophyta - ps13
Tracheophyta - ps14
Spermatophyta - ps15
Angiosperms - ps16
Mesangiospermae - ps17
Magnoliids/eudicots - ps18
Eudicots - ps19
Core_eudicots - ps20
Superrosids - ps21
Rosids - ps22
Eurosids - ps23
Malvids - ps24
Sap./Malv./Brassicales - ps25
Malvales/Brassicales - ps26
Brassicales - ps27
Cleomaceae/Brassicaceae - ps28
Brassicaceae - ps29
Camelineae - ps30
Arabidopsis - ps31
Arabidopsis_thaliana - ps32
Phylostratum

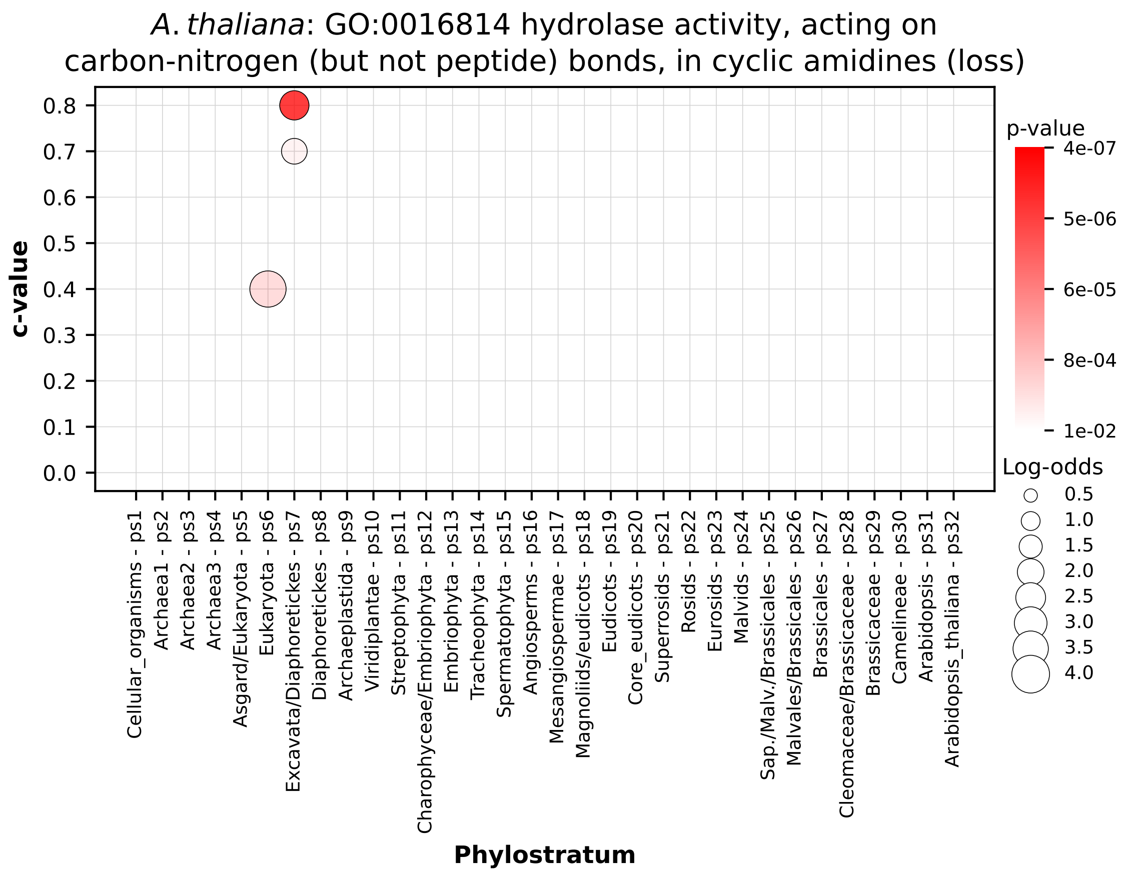

A. thaliana: GO:0016814 hydrolase activity, acting on carbon-nitrogen (but not peptide) bonds, in cyclic amidines (loss)
c-value
0.8
0.7
0.6
0.5
0.4
0.3
0.2
0.1
0.0
p-value
4e-07
5e-06
6e-05
8e-04
1e-02
Log-odds
0.5
1.0
1.5
2.0
2.5
3.0
3.5
4.0
Cellular_organisms - ps1
Archaea1 - ps2
Archaea2 - ps3
Archaea3 - ps4
Asgard/Eukaryota - ps5
Eukaryota - ps6
Excavata/Diaphoretickes - ps7
Diaphoretickes - ps8
Archaeplastida - ps9
Viridiplantae - ps10
Streptophyta - ps11
Charophyceae/Embriophyta - ps12
Embriophyta - ps13
Tracheophyta - ps14
Spermatophyta - ps15
Angiosperms - ps16
Mesangiospermae - ps17
Magnoliids/eudicots - ps18
Eudicots - ps19
Core_eudicots - ps20
Superrosids - ps21
Rosids - ps22
Eurosids - ps23
Malvids - ps24
Sap./Malv./Brassicales - ps25
Malvales/Brassicales - ps26
Brassicales - ps27
Cleomaceae/Brassicaceae - ps28
Brassicaceae - ps29
Camelineae - ps30
Arabidopsis - ps31
Arabidopsis_thaliana - ps32
Phylostratum

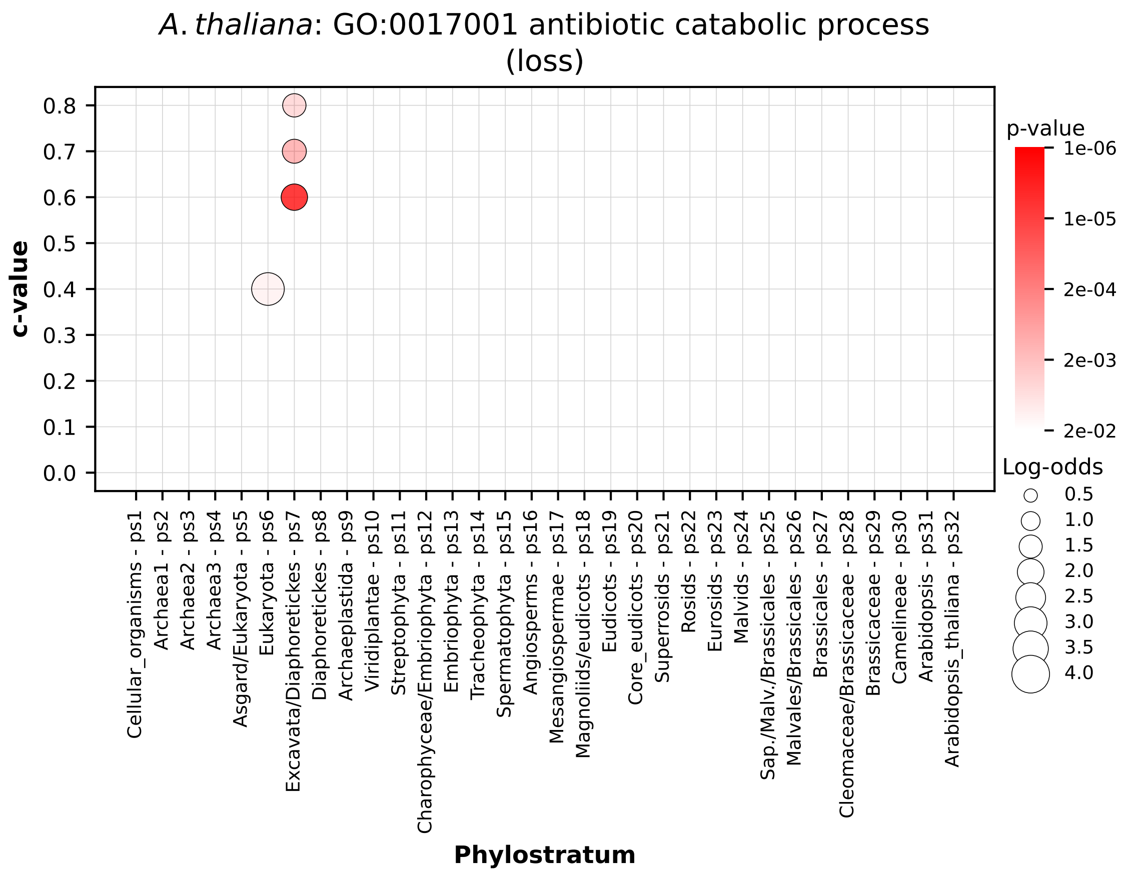

A. thaliana: GO:0017001 antibiotic catabolic process
(loss)
c-value
0.8
0.7
0.6
0.5
0.4
0.3
0.2
0.1
0.0
p-value
1e-06
1e-05
2e-04
2e-03
2e-02
Log-odds
0.5
1.0
1.5
2.0
2.5
3.0
3.5
4.0
Cellular_organisms - ps1
Archaea1 - ps2
Archaea2 - ps3
Archaea3 - ps4
Asgard/Eukaryota - ps5
Eukaryota - ps6
Excavata/Diaphoretickes - ps7
Diaphoretickes - ps8
Archaeplastida - ps9
Viridiplantae - ps10
Streptophyta - ps11
Charophyceae/Embriophyta - ps12
Embriophyta - ps13
Tracheophyta - ps14
Spermatophyta - ps15
Angiosperms - ps16
Mesangiospermae - ps17
Magnoliids/eudicots - ps18
Eudicots - ps19
Core_eudicots - ps20
Superrosids - ps21
Rosids - ps22
Eurosids - ps23
Malvids - ps24
Sap./Malv./Brassicales - ps25
Malvales/Brassicales - ps26
Brassicales - ps27
Cleomaceae/Brassicaceae - ps28
Brassicaceae - ps29
Camelineae - ps30
Arabidopsis - ps31
Arabidopsis_thaliana - ps32
Phylostratum

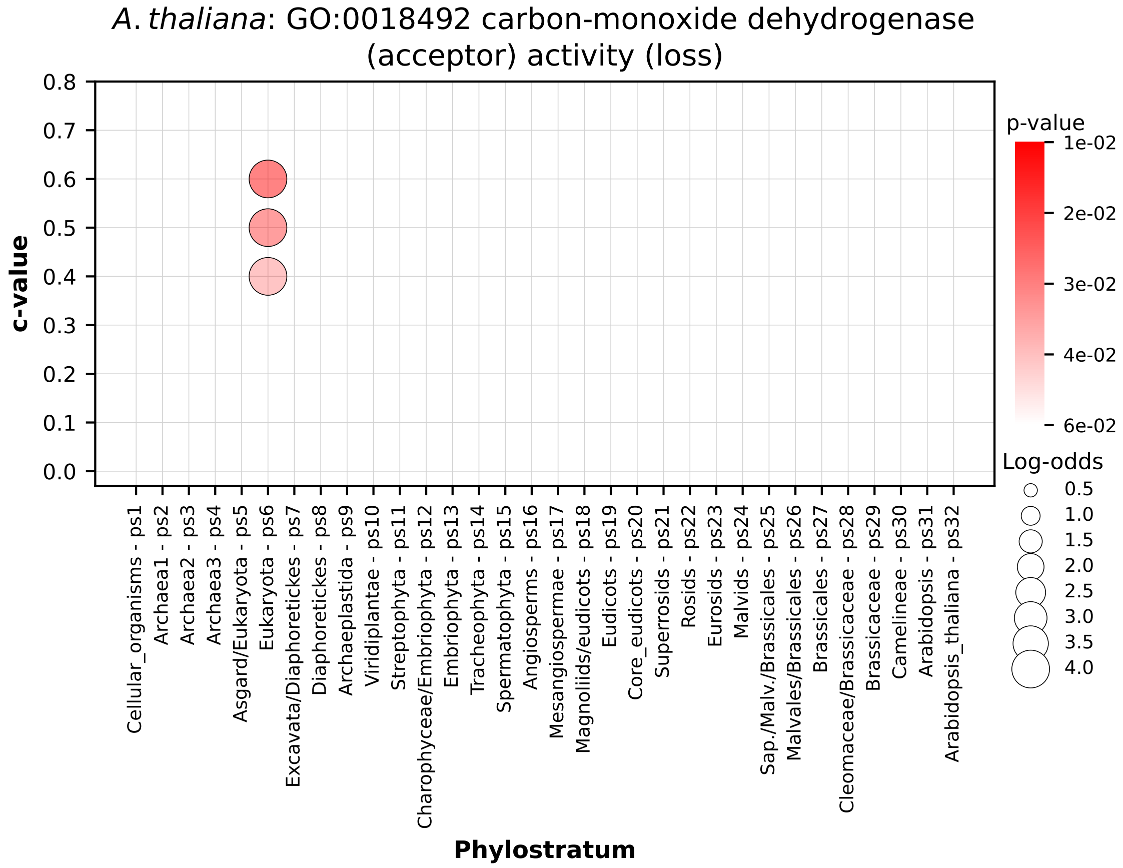

A. thaliana: GO:0018492 carbon-monoxide dehydrogenase (acceptor) activity (loss)
c-value
0.8
0.7
0.6
0.5
0.4
0.3
0.2
0.1
0.0
p-value
1e-02
2e-02
3e-02
4e-02
6e-02
Log-odds
0.5
1.0
1.5
2.0
2.5
3.0
3.5
4.0
Cellular_organisms - ps1
Archaea1 - ps2
Archaea2 - ps3
Archaea3 - ps4
Asgard/Eukaryota - ps5
Eukaryota - ps6
Excavata/Diaphoretickes - ps7
Diaphoretickes - ps8
Archaeplastida - ps9
Viridiplantae - ps10
Streptophyta - ps11
Charophyceae/Embriophyta - ps12
Embriophyta - ps13
Tracheophyta - ps14
Spermatophyta - ps15
Angiosperms - ps16
Mesangiospermae - ps17
Magnoliids/eudicots - ps18
Eudicots - ps19
Core_eudicots - ps20
Superrosids - ps21
Rosids - ps22
Eurosids - ps23
Malvids - ps24
Sap./Malv./Brassicales - ps25
Malvales/Brassicales - ps26
Brassicales - ps27
Cleomaceae/Brassicaceae - ps28
Brassicaceae - ps29
Camelineae - ps30
Arabidopsis - ps31
Arabidopsis_thaliana - ps32
Phylostratum

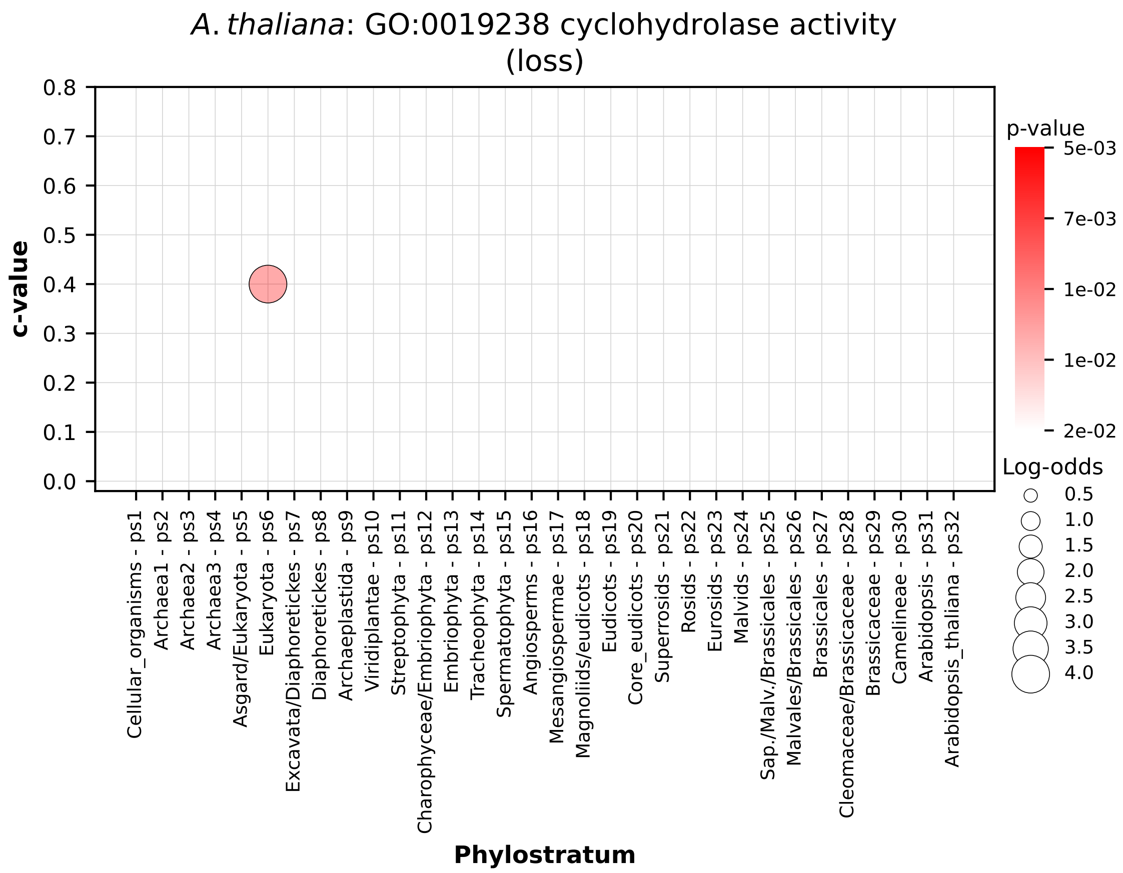

A. thaliana: GO:0019238 cyclohydrolase activity
(loss)
c-value
0.8
0.7
0.6
0.5
0.4
0.3
0.2
0.1
0.0
p-value
5e-03
7e-03
1e-02
1e-02
2e-02
Log-odds
0.5
1.0
1.5
2.0
2.5
3.0
3.5
4.0
Cellular_organisms - ps1
Archaea1 - ps2
Archaea2 - ps3
Archaea3 - ps4
Asgard/Eukaryota - ps5
Eukaryota - ps6
Excavata/Diaphoretickes - ps7
Diaphoretickes - ps8
Archaeplastida - ps9
Viridiplantae - ps10
Streptophyta - ps11
Charophyceae/Embriophyta - ps12
Embriophyta - ps13
Tracheophyta - ps14
Spermatophyta - ps15
Angiosperms - ps16
Mesangiospermae - ps17
Magnoliids/eudicots - ps18
Eudicots - ps19
Core_eudicots - ps20
Superrosids - ps21
Rosids - ps22
Eurosids - ps23
Malvids - ps24
Sap./Malv./Brassicales - ps25
Malvales/Brassicales - ps26
Brassicales - ps27
Cleomaceae/Brassicaceae - ps28
Brassicaceae - ps29
Camelineae - ps30
Arabidopsis - ps31
Arabidopsis_thaliana - ps32
Phylostratum

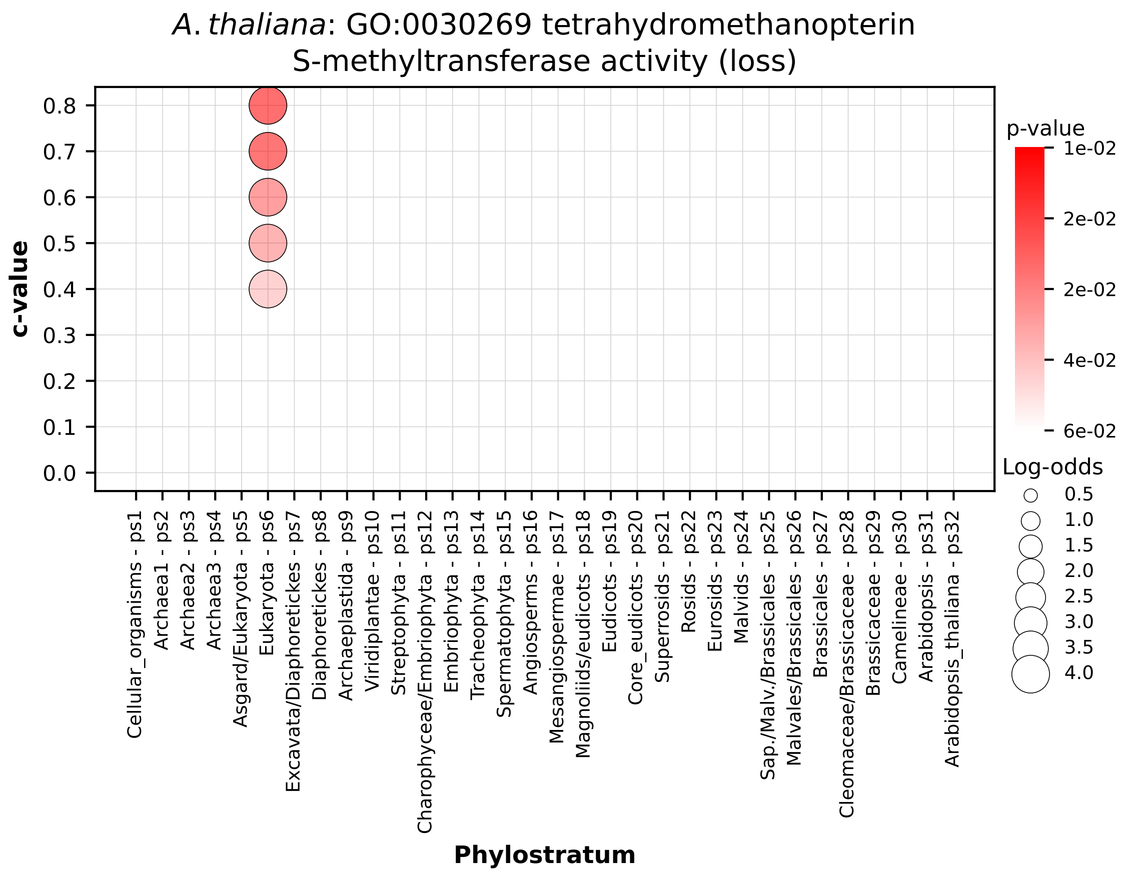

A. thaliana: GO:0030269 tetrahydromethanopterin S-methyltransferase activity (loss)
c-value
0.8
0.7
0.6
0.5
0.4
0.3
0.2
0.1
0.0
p-value
1e-02
2e-02
2e-02
4e-02
6e-02
Log-odds
0.5
1.0
1.5
2.0
2.5
3.0
3.5
4.0
Cellular_organisms - ps1
Archaea1 - ps2
Archaea2 - ps3
Archaea3 - ps4
Asgard/Eukaryota - ps5
Eukaryota - ps6
Excavata/Diaphoretickes - ps7
Diaphoretickes - ps8
Archaeplastida - ps9
Viridiplantae - ps10
Streptophyta - ps11
Charophyceae/Embriophyta - ps12
Embriophyta - ps13
Tracheophyta - ps14
Spermatophyta - ps15
Angiosperms - ps16
Mesangiospermae - ps17
Magnoliids/eudicots - ps18
Eudicots - ps19
Core_eudicots - ps20
Superrosids - ps21
Rosids - ps22
Eurosids - ps23
Malvids - ps24
Sap./Malv./Brassicales - ps25
Malvales/Brassicales - ps26
Brassicales - ps27
Cleomaceae/Brassicaceae - ps28
Brassicaceae - ps29
Camelineae - ps30
Arabidopsis - ps31
Arabidopsis_thaliana - ps32
Phylostratum

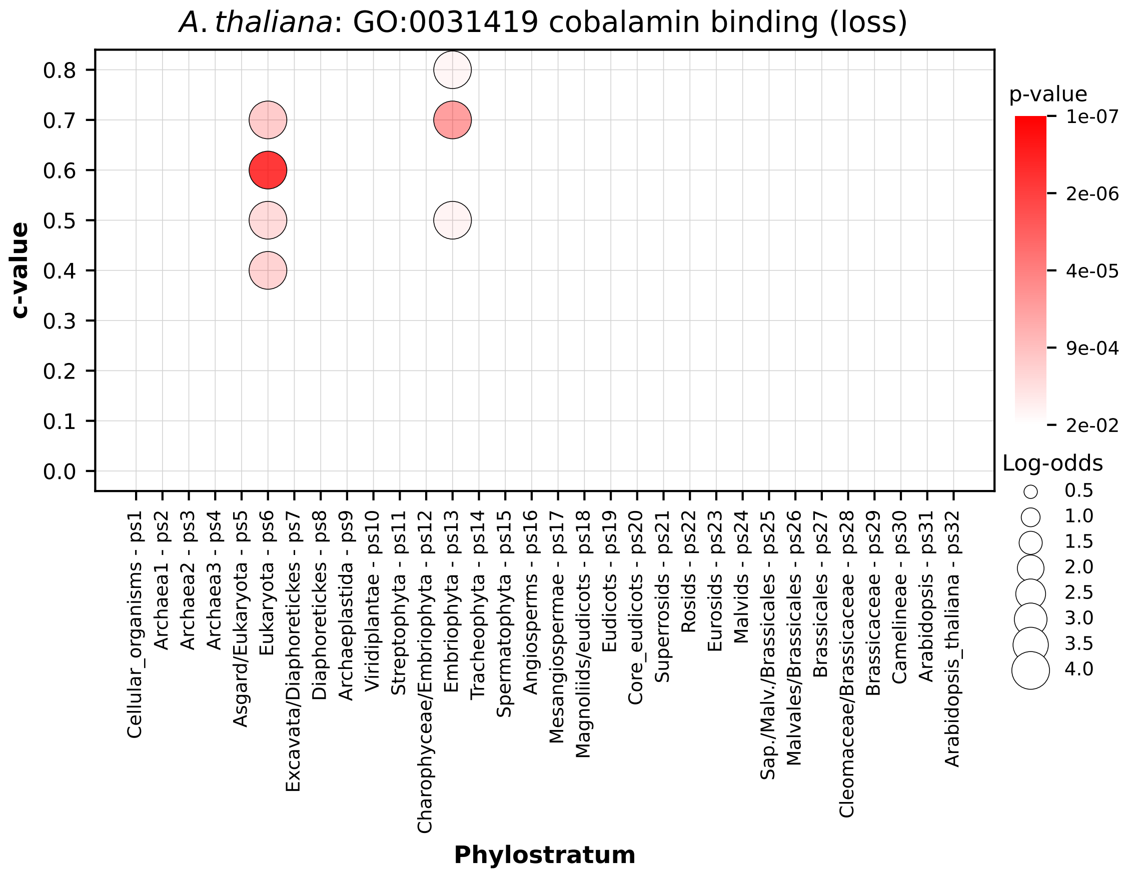
A. thaliana: GO:0031419 cobalamin binding (loss)
c-value
0.0
0.1
0.2
0.3
0.4
0.5
0.6
0.7
0.8
Phylostratum
Cellular_organisms - ps1
Archaea1 - ps2
Archaea2 - ps3
Archaea3 - ps4
Asgard/Eukaryota - ps5
Eukaryota - ps6
Excavata/Diaphoretickes - ps7
Diaphoretickes - ps8
Archaeplastida - ps9
Viridiplantae - ps10
Streptophyta - ps11
Charophyceae/Embriophyta - ps12
Embriophyta - ps13
Tracheophyta - ps14
Spermatophyta - ps15
Angiosperms - ps16
Mesangiospermae - ps17
Magnoliids/eudicots - ps18
Eudicots - ps19
Core_eudicots - ps20
Superrosids - ps21
Rosids - ps22
Eurosids - ps23
Malvids - ps24
Sap./Malv./Brassicales - ps25
Malvales/Brassicales - ps26
Brassicales - ps27
Cleomaceae/Brassicaceae - ps28
Brassicaceae - ps29
Camelineae - ps30
Arabidopsis - ps31
Arabidopsis_thaliana - ps32
p-value
1e-07
2e-06
4e-05
9e-04
2e-02
Log-odds
0.5
1.0
1.5
2.0
2.5
3.0
3.5
4.0

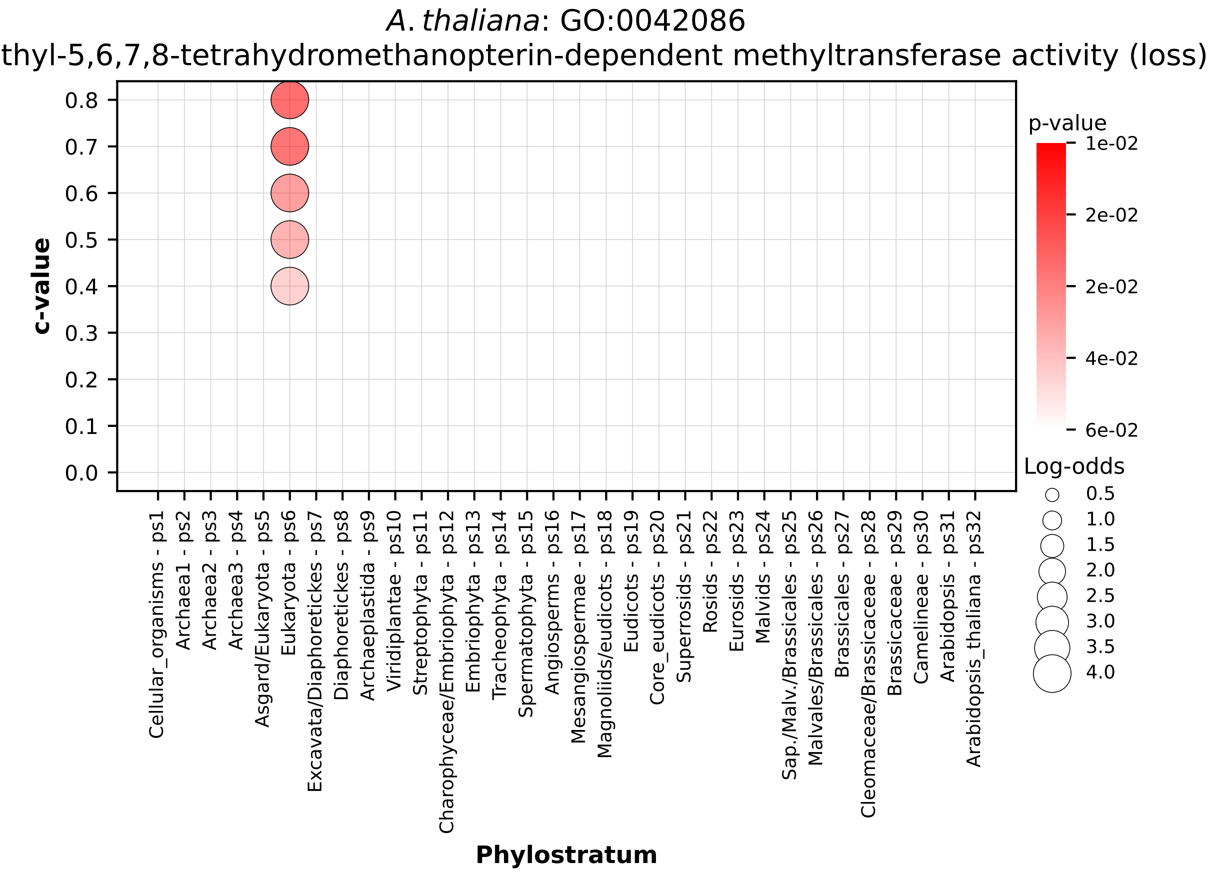

A. thaliana: GO:0042086
thyl-5,6,7,8-tetrahydromethanopterin-dependent methyltransferase activity (loss)
c-value
0.8
0.7
0.6
0.5
0.4
0.3
0.2
0.1
0.0
Phylostratum
Cellular_organisms - ps1
Archaea1 - ps2
Archaea2 - ps3
Archaea3 - ps4
Asgard/Eukaryota - ps5
Eukaryota - ps6
Excavata/Diaphoretickes - ps7
Diaphoretickes - ps8
Archaeplastida - ps9
Viridiplantae - ps10
Streptophyta - ps11
Charophyceae/Embriophyta - ps12
Embriophyta - ps13
Tracheophyta - ps14
Spermatophyta - ps15
Angiosperms - ps16
Mesangiospermae - ps17
Magnoliids/eudicots - ps18
Eudicots - ps19
Core_eudicots - ps20
Superrosids - ps21
Rosids - ps22
Eurosids - ps23
Malvids - ps24
Sap./Malv./Brassicales - ps25
Malvales/Brassicales - ps26
Brassicales - ps27
Cleomaceae/Brassicaceae - ps28
Brassicaceae - ps29
Camelineae - ps30
Arabidopsis - ps31
Arabidopsis_thaliana - ps32
p-value
1e-02
2e-02
2e-02
4e-02
6e-02
Log-odds
0.5
1.0
1.5
2.0
2.5
3.0
3.5
4.0

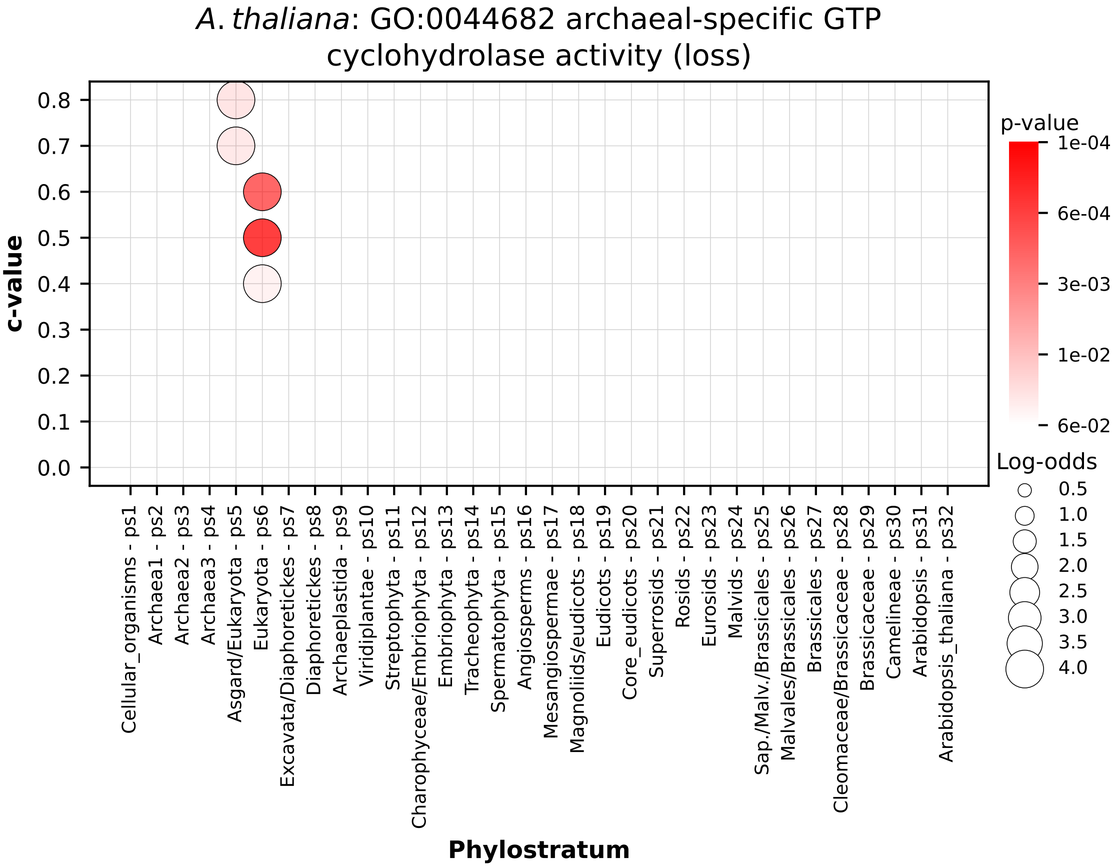

A. thaliana: GO:0044682 archaeal-specific GTP cyclohydrolase activity (loss)
c-value
0.8
0.7
0.6
0.5
0.4
0.3
0.2
0.1
0.0
Phylostratum
Cellular_organisms - ps1
Archaea1 - ps2
Archaea2 - ps3
Archaea3 - ps4
Asgard/Eukaryota - ps5
Eukaryota - ps6
Excavata/Diaphoretickes - ps7
Diaphoretickes - ps8
Archaeplastida - ps9
Viridiplantae - ps10
Streptophyta - ps11
Charophyceae/Embriophyta - ps12
Embriophyta - ps13
Tracheophyta - ps14
Spermatophyta - ps15
Angiosperms - ps16
Mesangiospermae - ps17
Magnoliids/eudicots - ps18
Eudicots - ps19
Core_eudicots - ps20
Superrosids - ps21
Rosids - ps22
Eurosids - ps23
Malvids - ps24
Sap./Malv./Brassicales - ps25
Malvales/Brassicales - ps26
Brassicales - ps27
Cleomaceae/Brassicaceae - ps28
Brassicaceae - ps29
Camelineae - ps30
Arabidopsis - ps31
Arabidopsis_thaliana - ps32
p-value
1e-04
6e-04
3e-03
1e-02
6e-02
Log-odds
0.5
1.0
1.5
2.0
2.5
3.0
3.5
4.0

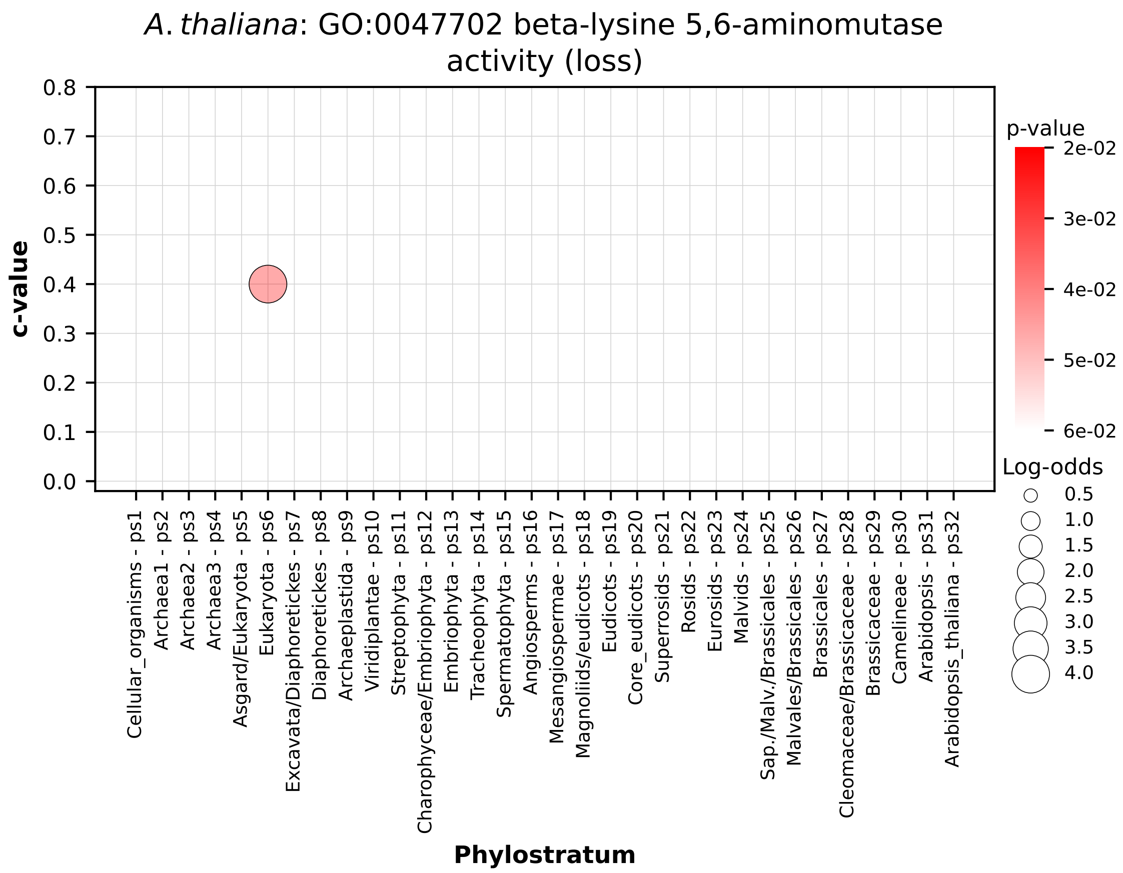
A. thaliana: GO:0047702 beta-lysine 5,6-aminomutase activity (loss)
c-value
0.0
0.1
0.2
0.3
0.4
0.5
0.6
0.7
0.8
p-value
2e-02
3e-02
4e-02
5e-02
6e-02
Log-odds
0.5
1.0
1.5
2.0
2.5
3.0
3.5
4.0
Cellular_organisms - ps1
Archaea1 - ps2
Archaea2 - ps3
Archaea3 - ps4
Asgard/Eukaryota - ps5
Eukaryota - ps6
Excavata/Diaphoretickes - ps7
Diaphoretickes - ps8
Archaeplastida - ps9
Viridiplantae - ps10
Streptophyta - ps11
Charophyceae/Embriophyta - ps12
Embriophyta - ps13
Tracheophyta - ps14
Spermatophyta - ps15
Angiosperms - ps16
Mesangiospermae - ps17
Magnoliids/eudicots - ps18
Eudicots - ps19
Core_eudicots - ps20
Superrosids - ps21
Rosids - ps22
Eurosids - ps23
Malvids - ps24
Sap./Malv./Brassicales - ps25
Malvales/Brassicales - ps26
Brassicales - ps27
Cleomaceae/Brassicaceae - ps28
Brassicaceae - ps29
Camelineae - ps30
Arabidopsis - ps31
Arabidopsis_thaliana - ps32
Phylostratum

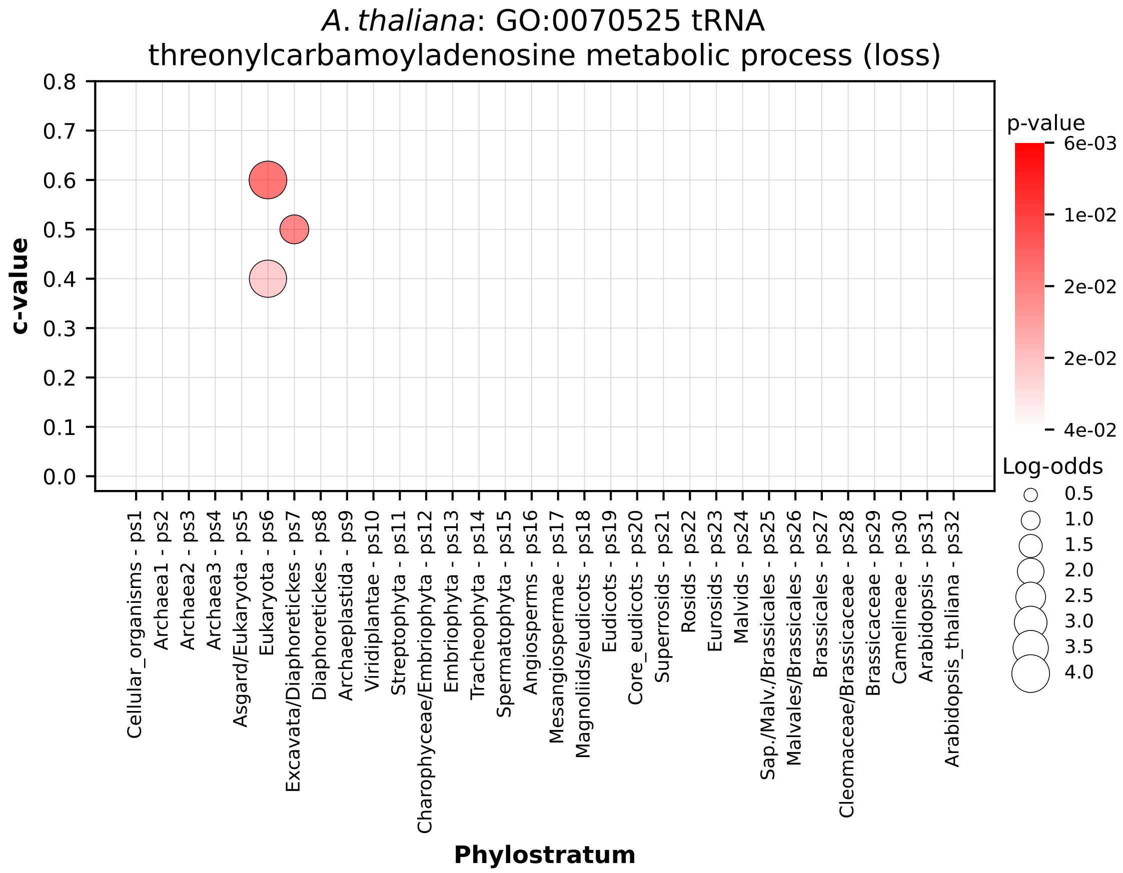

A. thaliana: GO:0070525 tRNA threonylcarbamoyladenosine metabolic process (loss)
c-value
0.0
0.1
0.2
0.3
0.4
0.5
0.6
0.7
0.8
Phylostratum
Cellular_organisms - ps1
Archaea1 - ps2
Archaea2 - ps3
Archaea3 - ps4
Asgard/Eukaryota - ps5
Eukaryota - ps6
Excavata/Diaphoretickes - ps7
Diaphoretickes - ps8
Archaeplastida - ps9
Viridiplantae - ps10
Streptophyta - ps11
Charophyceae/Embriophyta - ps12
Embriophyta - ps13
Tracheophyta - ps14
Spermatophyta - ps15
Angiosperms - ps16
Mesangiospermae - ps17
Magnoliids/eudicots - ps18
Eudicots - ps19
Core_eudicots - ps20
Superrosids - ps21
Rosids - ps22
Eurosids - ps23
Malvids - ps24
Sap./Malv./Brassicales - ps25
Malvales/Brassicales - ps26
Brassicales - ps27
Cleomaceae/Brassicaceae - ps28
Brassicaceae - ps29
Camelineae - ps30
Arabidopsis - ps31
Arabidopsis_thaliana - ps32
p-value
6e-03
1e-02
2e-02
2e-02
4e-02
Log-odds
0.5
1.0
1.5
2.0
2.5
3.0
3.5
4.0

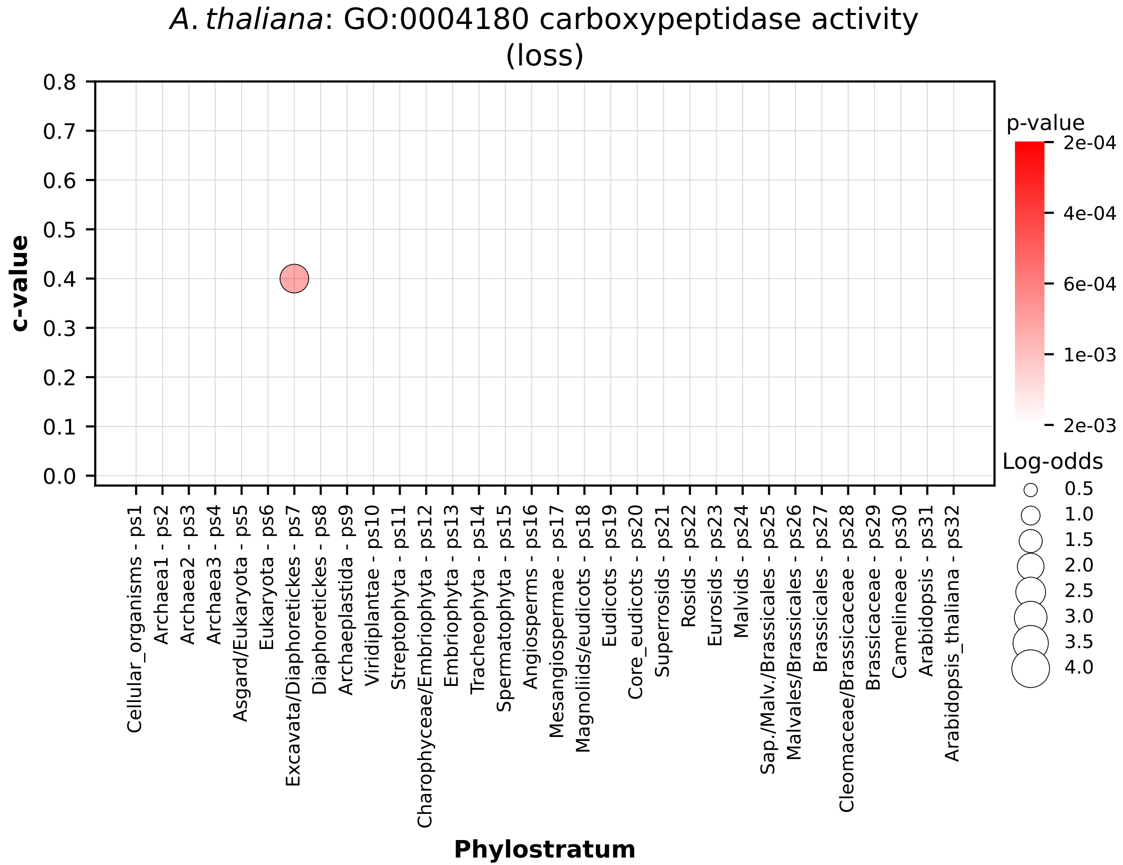

A. thaliana: GO:0004180 carboxypeptidase activity
(loss)
c-value
0.8
0.7
0.6
0.5
0.4
0.3
0.2
0.1
0.0
Phylostratum
Cellular_organisms - ps1
Archaea1 - ps2
Archaea2 - ps3
Archaea3 - ps4
Asgard/Eukaryota - ps5
Eukaryota - ps6
Excavata/Diaphoretickes - ps7
Diaphoretickes - ps8
Archaeplastida - ps9
Viridiplantae - ps10
Streptophyta - ps11
Charophyceae/Embriophyta - ps12
Embriophyta - ps13
Tracheophyta - ps14
Spermatophyta - ps15
Angiosperms - ps16
Mesangiospermae - ps17
Magnoliids/eudicots - ps18
Eudicots - ps19
Core_eudicots - ps20
Superrosids - ps21
Rosids - ps22
Eurosids - ps23
Malvids - ps24
Sap./Malv./Brassicales - ps25
Malvales/Brassicales - ps26
Brassicales - ps27
Cleomaceae/Brassicaceae - ps28
Brassicaceae - ps29
Camelineae - ps30
Arabidopsis - ps31
Arabidopsis_thaliana - ps32
p-value
2e-04
4e-04
6e-04
1e-03
2e-03
Log-odds
0.5
1.0
1.5
2.0
2.5
3.0
3.5
4.0

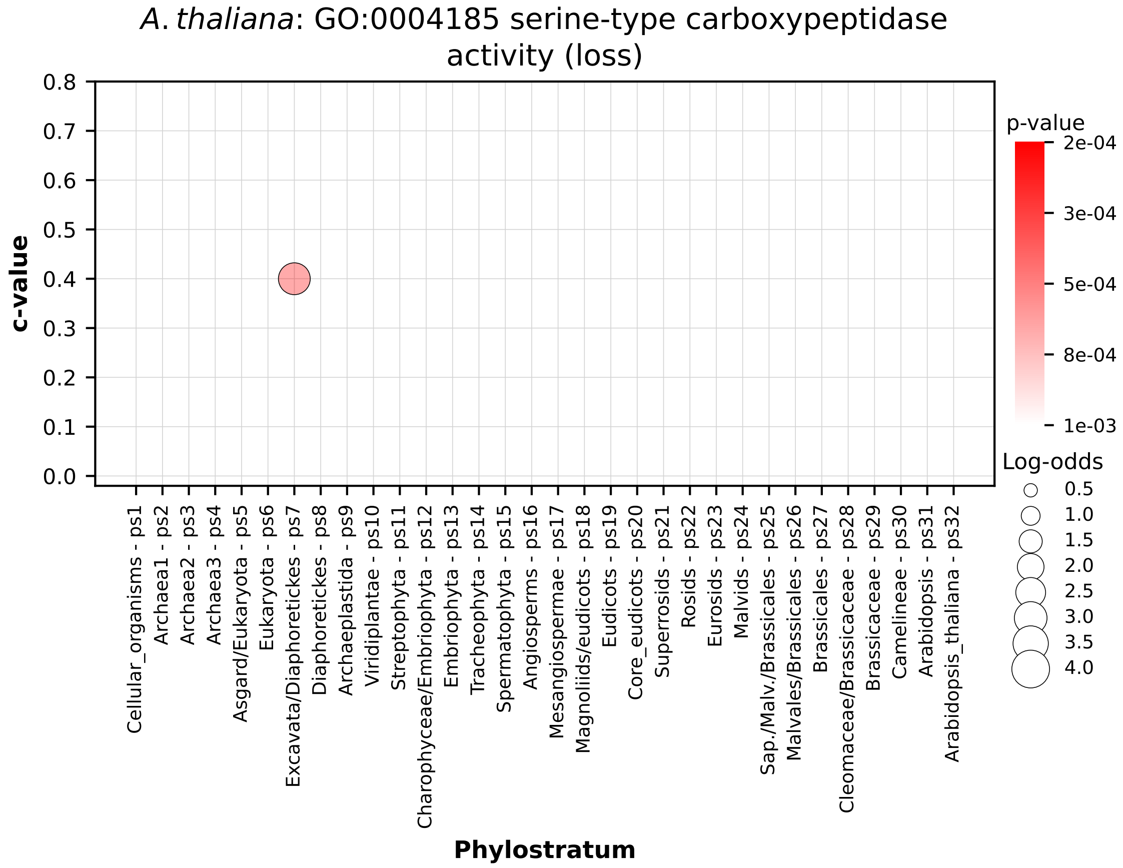

A. thaliana: GO:0004185 serine-type carboxypeptidase activity (loss)
c-value
0.8
0.7
0.6
0.5
0.4
0.3
0.2
0.1
0.0
Phylostratum
Cellular_organisms - ps1
Archaea1 - ps2
Archaea2 - ps3
Archaea3 - ps4
Asgard/Eukaryota - ps5
Eukaryota - ps6
Excavata/Diaphoretickes - ps7
Diaphoretickes - ps8
Archaeplastida - ps9
Viridiplantae - ps10
Streptophyta - ps11
Charophyceae/Embriophyta - ps12
Embriophyta - ps13
Tracheophyta - ps14
Spermatophyta - ps15
Angiosperms - ps16
Mesangiospermae - ps17
Magnoliids/eudicots - ps18
Eudicots - ps19
Core_eudicots - ps20
Superrosids - ps21
Rosids - ps22
Eurosids - ps23
Malvids - ps24
Sap./Malv./Brassicales - ps25
Malvales/Brassicales - ps26
Brassicales - ps27
Cleomaceae/Brassicaceae - ps28
Brassicaceae - ps29
Camelineae - ps30
Arabidopsis - ps31
Arabidopsis_thaliana - ps32
p-value
2e-04
3e-04
5e-04
8e-04
1e-03
Log-odds
0.5
1.0
1.5
2.0
2.5
3.0
3.5
4.0

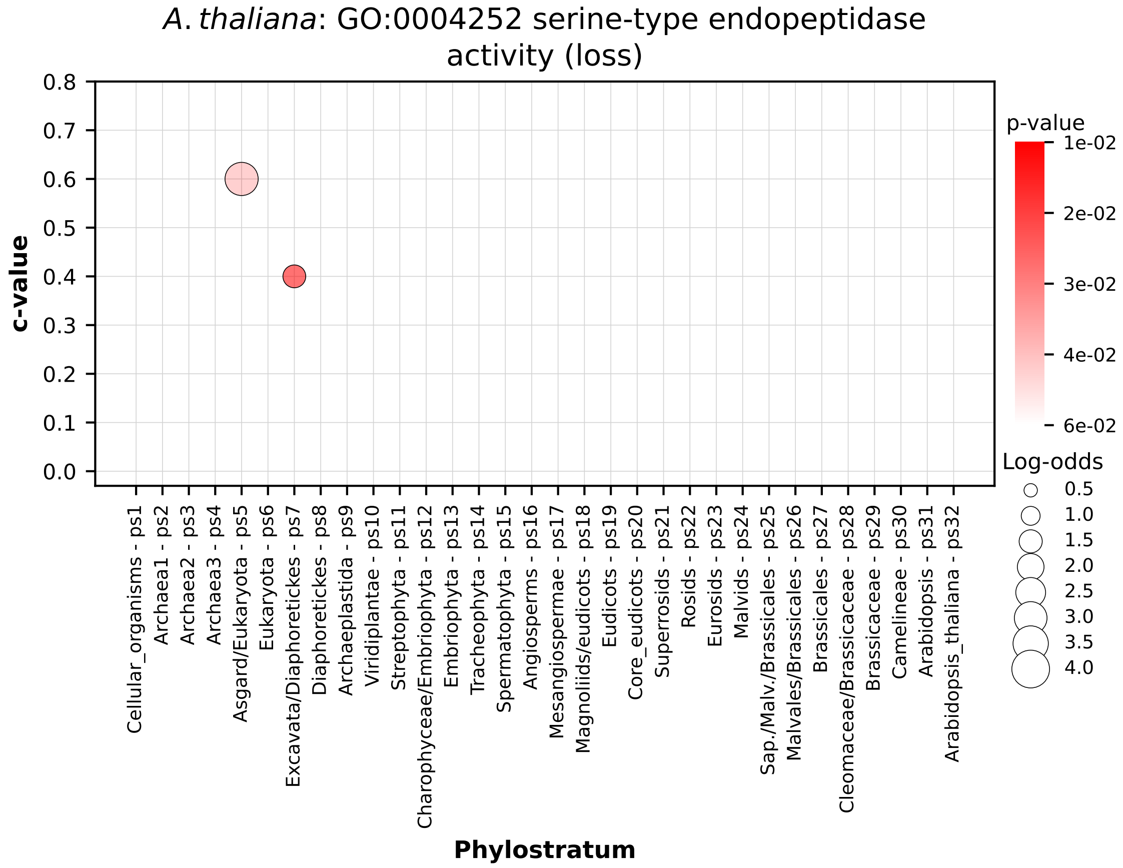

A. thaliana: GO:0004252 serine-type endopeptidase activity (loss)
c-value
0.0
0.1
0.2
0.3
0.4
0.5
0.6
0.7
0.8
Phylostratum
Cellular_organisms - ps1
Archaea1 - ps2
Archaea2 - ps3
Archaea3 - ps4
Asgard/Eukaryota - ps5
Eukaryota - ps6
Excavata/Diaphoretickes - ps7
Diaphoretickes - ps8
Archaeplastida - ps9
Viridiplantae - ps10
Streptophyta - ps11
Charophyceae/Embriophyta - ps12
Embriophyta - ps13
Tracheophyta - ps14
Spermatophyta - ps15
Angiosperms - ps16
Mesangiospermae - ps17
Magnoliids/eudicots - ps18
Eudicots - ps19
Core_eudicots - ps20
Superrosids - ps21
Rosids - ps22
Eurosids - ps23
Malvids - ps24
Sap./Malv./Brassicales - ps25
Malvales/Brassicales - ps26
Brassicales - ps27
Cleomaceae/Brassicaceae - ps28
Brassicaceae - ps29
Camelineae - ps30
Arabidopsis - ps31
Arabidopsis_thaliana - ps32
p-value
1e-02
2e-02
3e-02
4e-02
6e-02
Log-odds
0.5
1.0
1.5
2.0
2.5
3.0
3.5
4.0

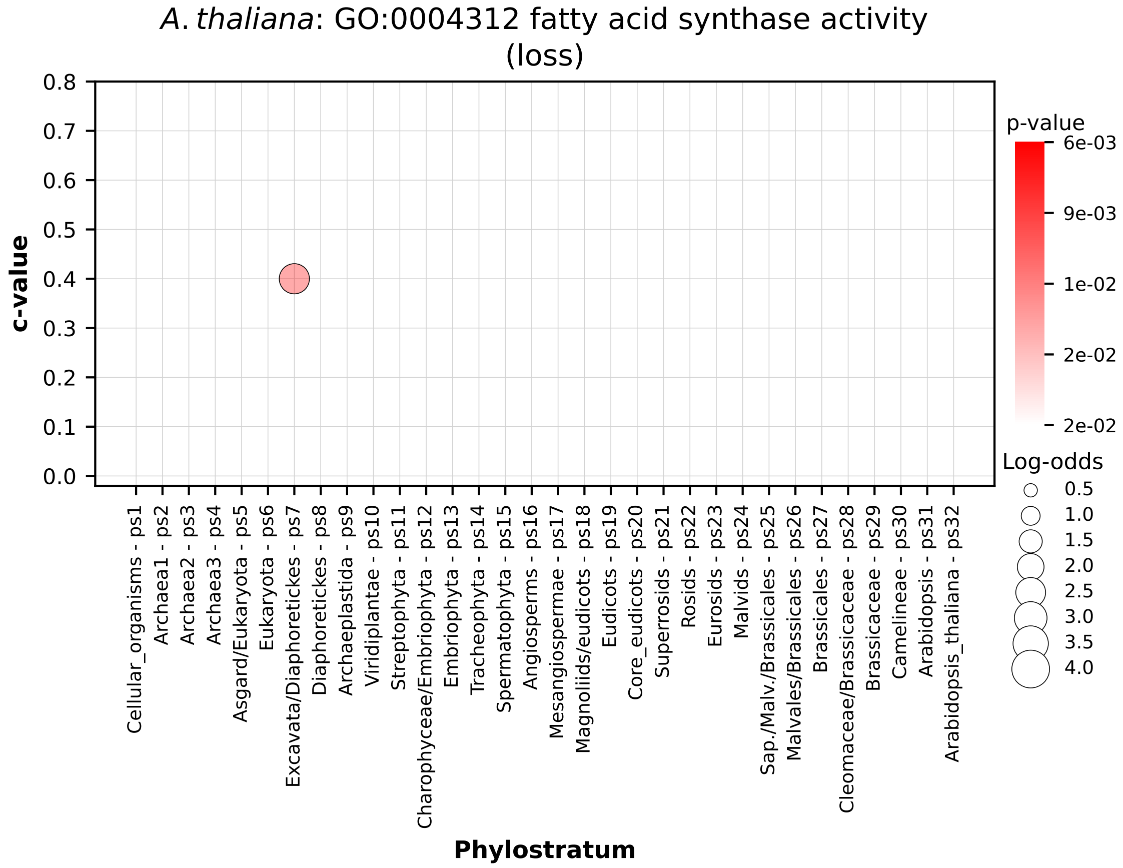

A. thaliana: GO:0004312 fatty acid synthase activity
(loss)
c-value
0.8
0.7
0.6
0.5
0.4
0.3
0.2
0.1
0.0
p-value
6e-03
9e-03
1e-02
2e-02
2e-02
Log-odds
0.5
1.0
1.5
2.0
2.5
3.0
3.5
4.0
Cellular_organisms - ps1
Archaea1 - ps2
Archaea2 - ps3
Archaea3 - ps4
Asgard/Eukaryota - ps5
Eukaryota - ps6
Excavata/Diaphoretickes - ps7
Diaphoretickes - ps8
Archaeplastida - ps9
Viridiplantae - ps10
Streptophyta - ps11
Charophyceae/Embriophyta - ps12
Embriophyta - ps13
Tracheophyta - ps14
Spermatophyta - ps15
Angiosperms - ps16
Mesangiospermae - ps17
Magnoliids/eudicots - ps18
Eudicots - ps19
Core_eudicots - ps20
Superrosids - ps21
Rosids - ps22
Eurosids - ps23
Malvids - ps24
Sap./Malv./Brassicales - ps25
Malvales/Brassicales - ps26
Brassicales - ps27
Cleomaceae/Brassicaceae - ps28
Brassicaceae - ps29
Camelineae - ps30
Arabidopsis - ps31
Arabidopsis_thaliana - ps32
Phylostratum

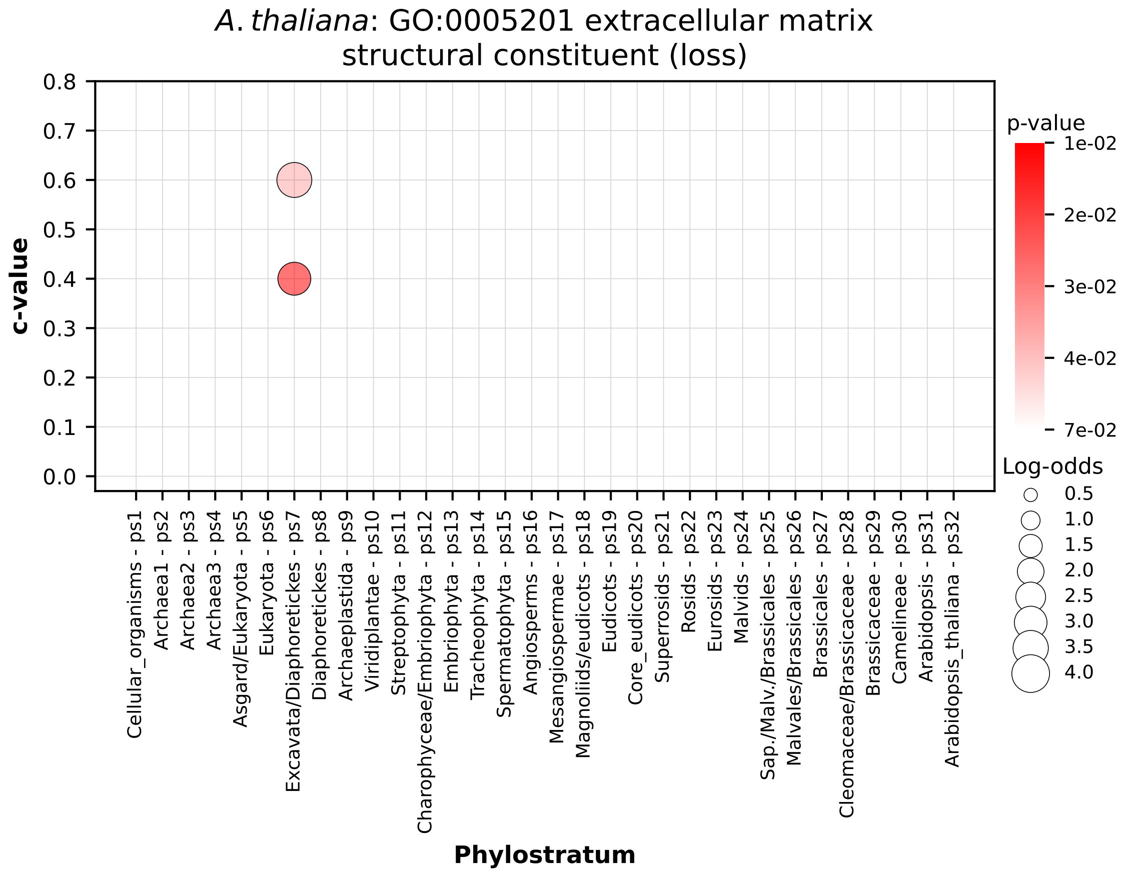

A. thaliana: GO:0005201 extracellular matrix structural constituent (loss)
c-value
0.8
0.7
0.6
0.5
0.4
0.3
0.2
0.1
0.0
p-value
1e-02
2e-02
3e-02
4e-02
7e-02
Log-odds
0.5
1.0
1.5
2.0
2.5
3.0
3.5
4.0
Cellular_organisms - ps1
Archaea1 - ps2
Archaea2 - ps3
Archaea3 - ps4
Asgard/Eukaryota - ps5
Eukaryota - ps6
Excavata/Diaphoretickes - ps7
Diaphoretickes - ps8
Archaeplastida - ps9
Viridiplantae - ps10
Streptophyta - ps11
Charophyceae/Embriophyta - ps12
Embriophyta - ps13
Tracheophyta - ps14
Spermatophyta - ps15
Angiosperms - ps16
Mesangiospermae - ps17
Magnoliids/eudicots - ps18
Eudicots - ps19
Core_eudicots - ps20
Superrosids - ps21
Rosids - ps22
Eurosids - ps23
Malvids - ps24
Sap./Malv./Brassicales - ps25
Malvales/Brassicales - ps26
Brassicales - ps27
Cleomaceae/Brassicaceae - ps28
Brassicaceae - ps29
Camelineae - ps30
Arabidopsis - ps31
Arabidopsis_thaliana - ps32
Phylostratum

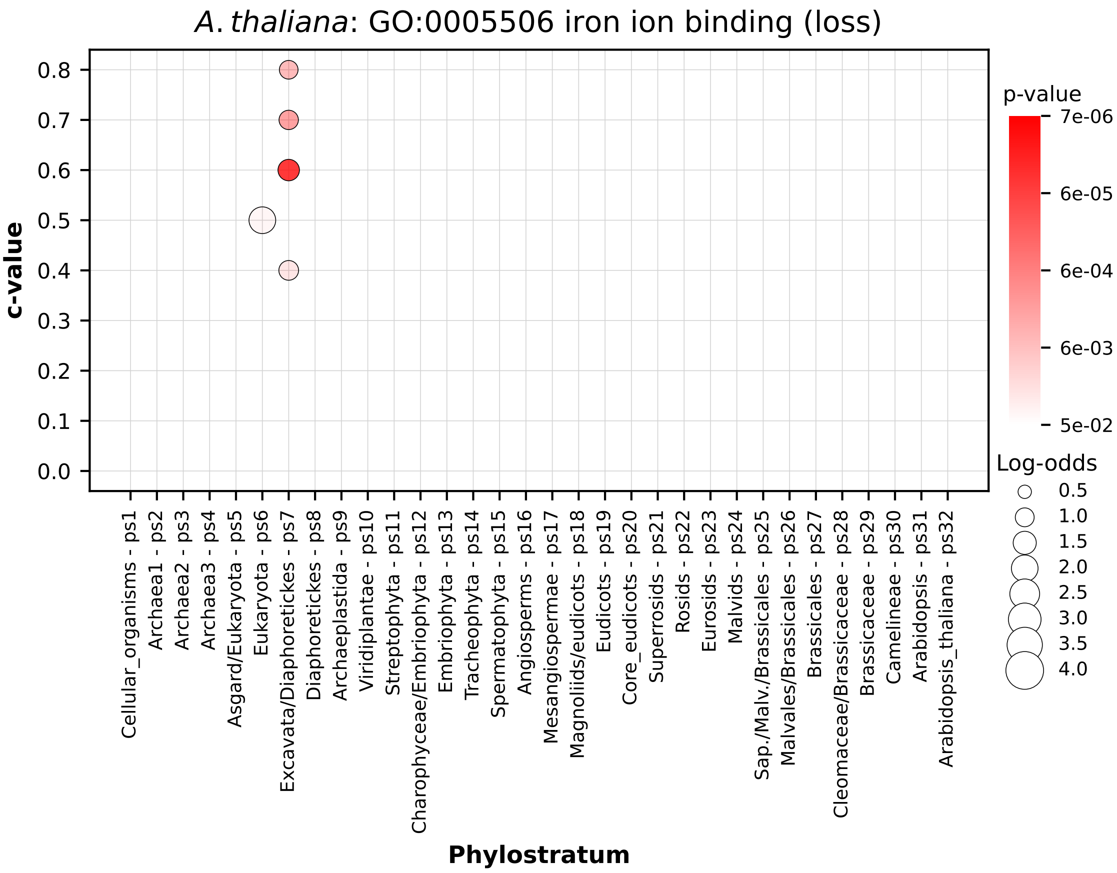

A. thaliana: GO:0005506 iron ion binding (loss)
c-value
0.0
0.1
0.2
0.3
0.4
0.5
0.6
0.7
0.8
Phylostratum
Cellular_organisms - ps1
Archaea1 - ps2
Archaea2 - ps3
Archaea3 - ps4
Asgard/Eukaryota - ps5
Eukaryota - ps6
Excavata/Diaphoretickes - ps7
Diaphoretickes - ps8
Archaeplastida - ps9
Viridiplantae - ps10
Streptophyta - ps11
Charophyceae/Embriophyta - ps12
Embriophyta - ps13
Tracheophyta - ps14
Spermatophyta - ps15
Angiosperms - ps16
Mesangiospermae - ps17
Magnoliids/eudicots - ps18
Eudicots - ps19
Core_eudicots - ps20
Superrosids - ps21
Rosids - ps22
Eurosids - ps23
Malvids - ps24
Sap./Malv./Brassicales - ps25
Malvales/Brassicales - ps26
Brassicales - ps27
Cleomaceae/Brassicaceae - ps28
Brassicaceae - ps29
Camelineae - ps30
Arabidopsis - ps31
Arabidopsis_thaliana - ps32
p-value
7e-06
6e-05
6e-04
6e-03
5e-02
Log-odds
0.5
1.0
1.5
2.0
2.5
3.0
3.5
4.0

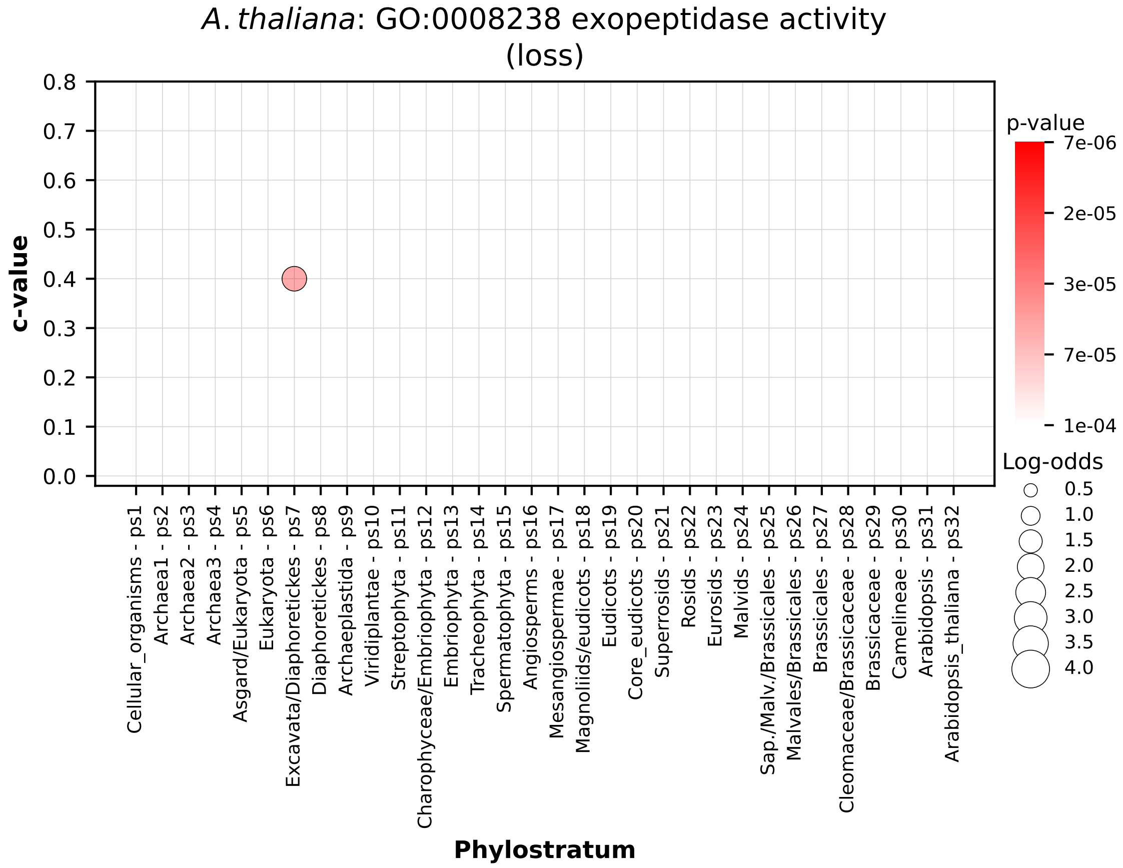

$A. thaliana$: GO:0008238 exopeptidase activity
(loss)
c-value
0.8
0.7
0.6
0.5
0.4
0.3
0.2
0.1
0.0
p-value
7e-06
2e-05
3e-05
7e-05
1e-04
Log-odds
0.5
1.0
1.5
2.0
2.5
3.0
3.5
4.0
Cellular_organisms - ps1
Archaea1 - ps2
Archaea2 - ps3
Archaea3 - ps4
Asgard/Eukaryota - ps5
Eukaryota - ps6
Excavata/Diaphoretickes - ps7
Diaphoretickes - ps8
Archaeplastida - ps9
Viridiplantae - ps10
Streptophyta - ps11
Charophyceae/Embriophyta - ps12
Embriophyta - ps13
Tracheophyta - ps14
Spermatophyta - ps15
Angiosperms - ps16
Mesangiospermae - ps17
Magnoliids/eudicots - ps18
Eudicots - ps19
Core_eudicots - ps20
Superrosids - ps21
Rosids - ps22
Eurosids - ps23
Malvids - ps24
Sap./Malv./Brassicales - ps25
Malvales/Brassicales - ps26
Brassicales - ps27
Cleomaceae/Brassicaceae - ps28
Brassicaceae - ps29
Camelineae - ps30
Arabidopsis - ps31
Arabidopsis_thaliana - ps32
Phylostratum

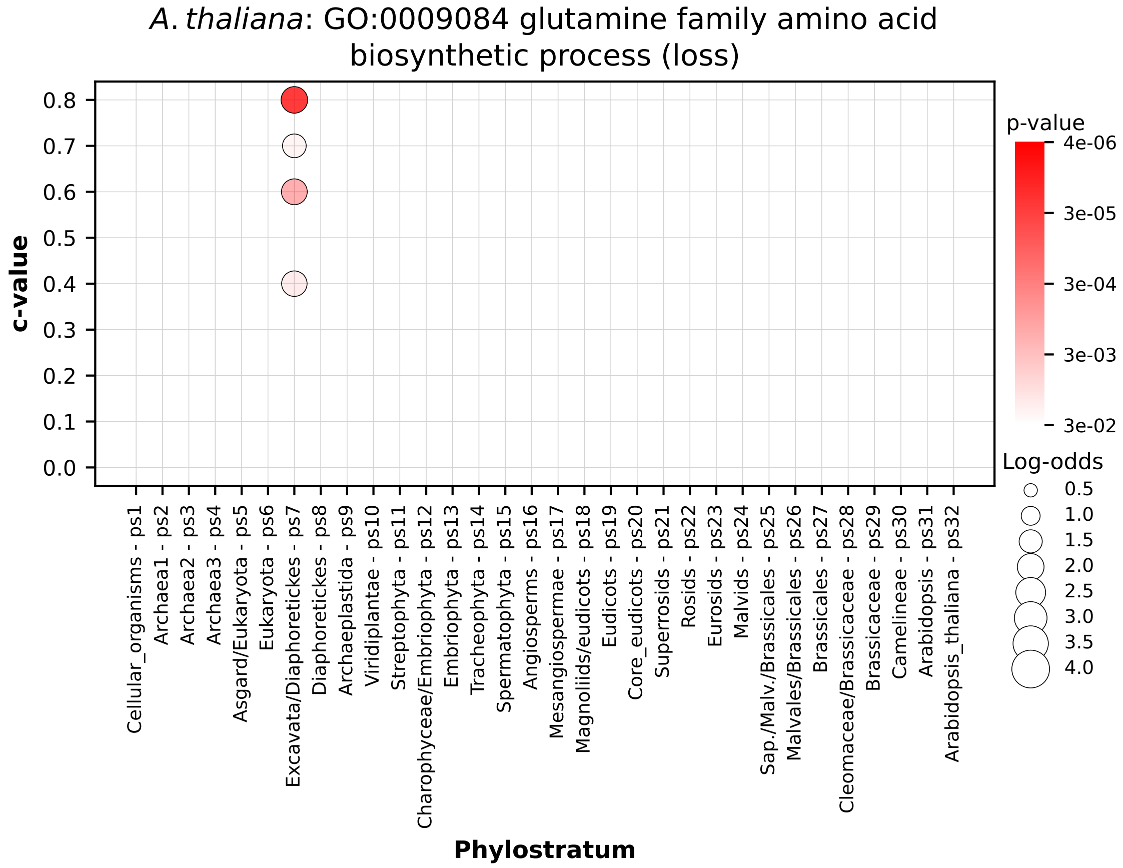

A. thaliana: GO:0009084 glutamine family amino acid biosynthetic process (loss)
c-value
0.8
0.7
0.6
0.5
0.4
0.3
0.2
0.1
0.0
Phylostratum
Cellular_organisms - ps1
Archaea1 - ps2
Archaea2 - ps3
Archaea3 - ps4
Asgard/Eukaryota - ps5
Eukaryota - ps6
Excavata/Diaphoretickes - ps7
Diaphoretickes - ps8
Archaeplastida - ps9
Viridiplantae - ps10
Streptophyta - ps11
Charophyceae/Embriophyta - ps12
Embriophyta - ps13
Tracheophyta - ps14
Spermatophyta - ps15
Angiosperms - ps16
Mesangiospermae - ps17
Magnoliids/eudicots - ps18
Eudicots - ps19
Core_eudicots - ps20
Superrosids - ps21
Rosids - ps22
Eurosids - ps23
Malvids - ps24
Sap./Malv./Brassicales - ps25
Malvales/Brassicales - ps26
Brassicales - ps27
Cleomaceae/Brassicaceae - ps28
Brassicaceae - ps29
Camelineae - ps30
Arabidopsis - ps31
Arabidopsis_thaliana - ps32
p-value
4e-06
3e-05
3e-04
3e-03
3e-02
Log-odds
0.5
1.0
1.5
2.0
2.5
3.0
3.5
4.0

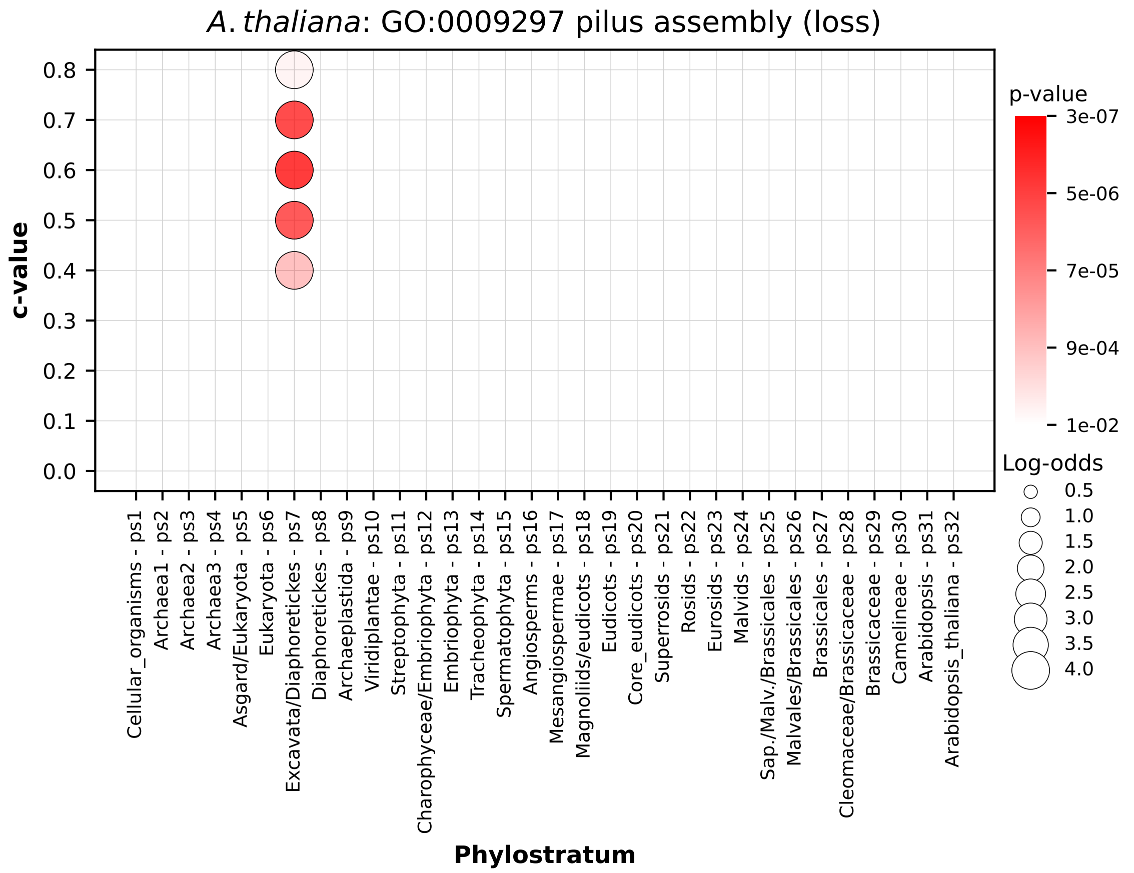
A. thaliana: GO:0009297 pilus assembly (loss)
c-value
0.0
0.1
0.2
0.3
0.4
0.5
0.6
0.7
0.8
p-value
3e-07
5e-06
7e-05
9e-04
1e-02
Log-odds
0.5
1.0
1.5
2.0
2.5
3.0
3.5
4.0
Cellular_organisms - ps1
Archaea1 - ps2
Archaea2 - ps3
Archaea3 - ps4
Asgard/Eukaryota - ps5
Eukaryota - ps6
Excavata/Diaphoretickes - ps7
Diaphoretickes - ps8
Archaeplastida - ps9
Viridiplantae - ps10
Streptophyta - ps11
Charophyceae/Embriophyta - ps12
Embriophyta - ps13
Tracheophyta - ps14
Spermatophyta - ps15
Angiosperms - ps16
Mesangiospermae - ps17
Magnoliids/eudicots - ps18
Eudicots - ps19
Core_eudicots - ps20
Superrosids - ps21
Rosids - ps22
Eurosids - ps23
Malvids - ps24
Sap./Malv./Brassicales - ps25
Malvales/Brassicales - ps26
Brassicales - ps27
Cleomaceae/Brassicaceae - ps28
Brassicaceae - ps29
Camelineae - ps30
Arabidopsis - ps31
Arabidopsis_thaliana - ps32
Phylostratum

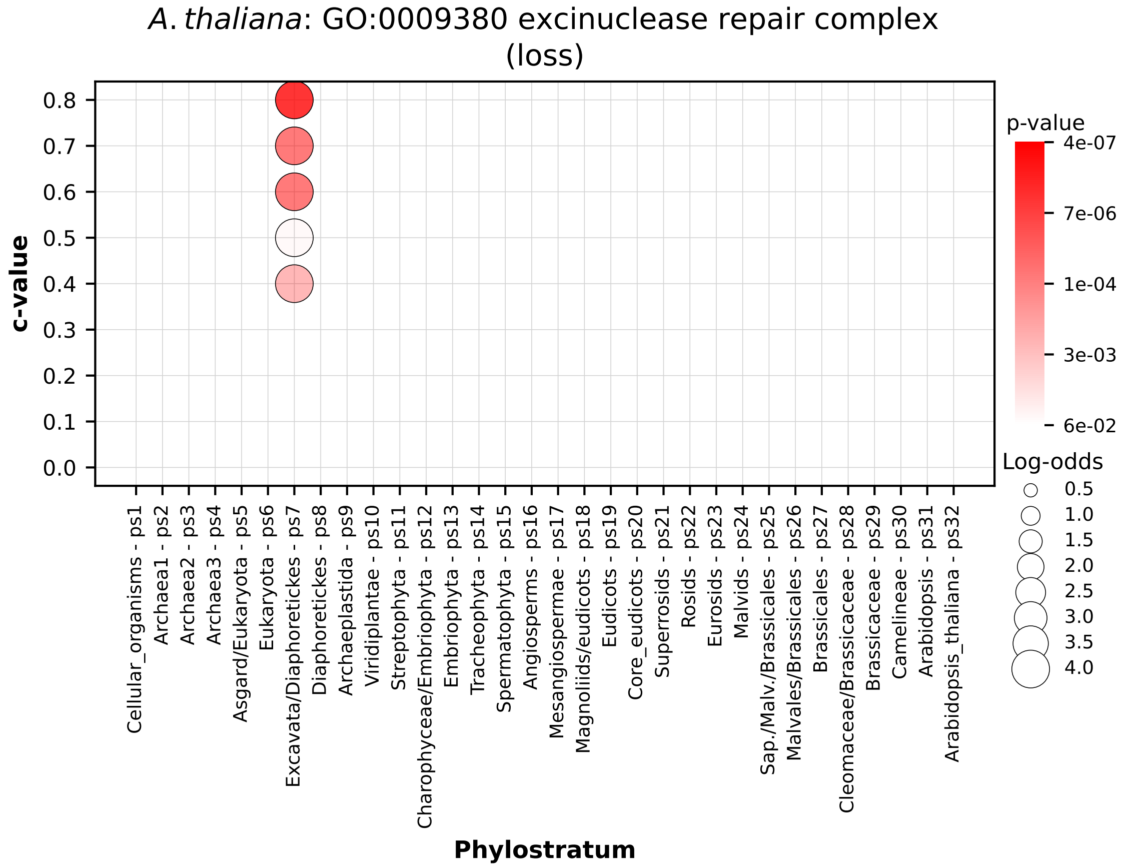

A. thaliana: GO:0009380 excinuclease repair complex
(loss)
c-value
0.8
0.7
0.6
0.5
0.4
0.3
0.2
0.1
0.0
p-value
4e-07
7e-06
1e-04
3e-03
6e-02
Log-odds
0.5
1.0
1.5
2.0
2.5
3.0
3.5
4.0
Cellular_organisms - ps1
Archaea1 - ps2
Archaea2 - ps3
Archaea3 - ps4
Asgard/Eukaryota - ps5
Eukaryota - ps6
Excavata/Diaphoretickes - ps7
Diaphoretickes - ps8
Archaeplastida - ps9
Viridiplantae - ps10
Streptophyta - ps11
Charophyceae/Embriophyta - ps12
Embriophyta - ps13
Tracheophyta - ps14
Spermatophyta - ps15
Angiosperms - ps16
Mesangiospermae - ps17
Magnoliids/eudicots - ps18
Eudicots - ps19
Core_eudicots - ps20
Superrosids - ps21
Rosids - ps22
Eurosids - ps23
Malvids - ps24
Sap./Malv./Brassicales - ps25
Malvales/Brassicales - ps26
Brassicales - ps27
Cleomaceae/Brassicaceae - ps28
Brassicaceae - ps29
Camelineae - ps30
Arabidopsis - ps31
Arabidopsis_thaliana - ps32
Phylostratum

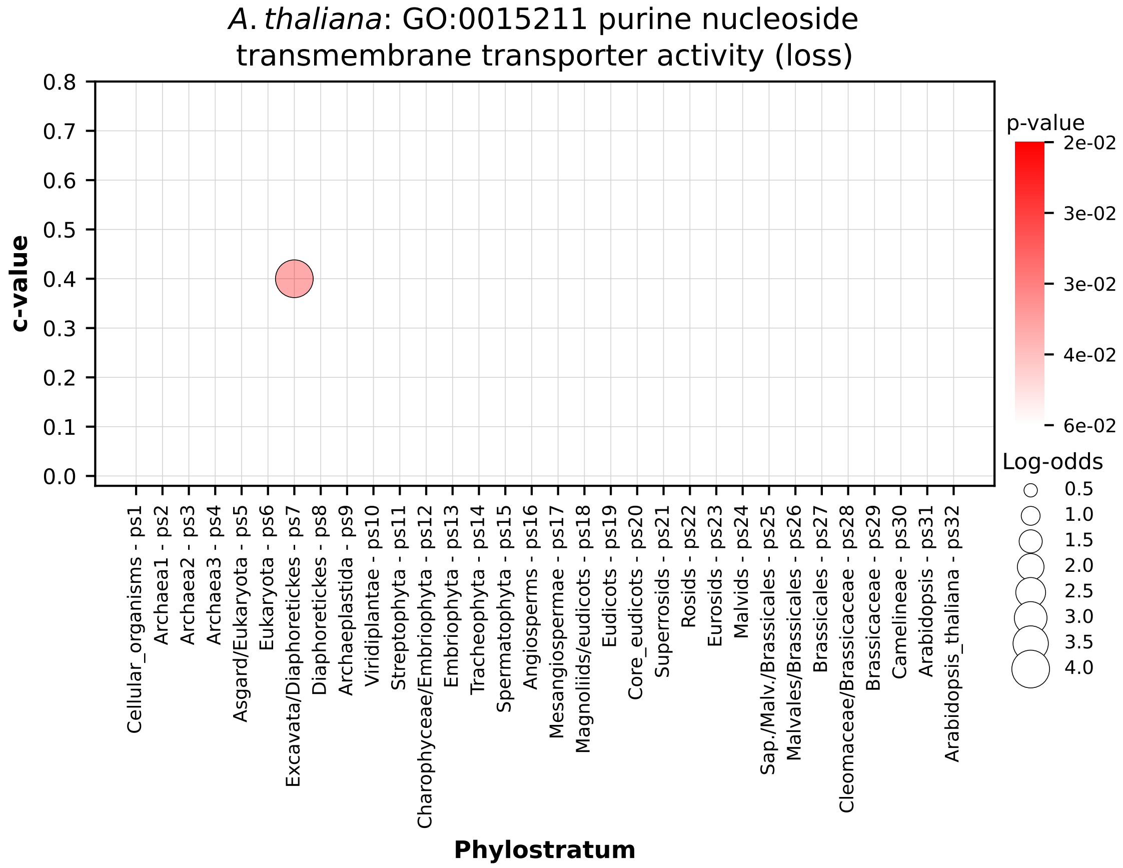

A. thaliana: GO:0015211 purine nucleoside transmembrane transporter activity (loss)
c-value
0.8
0.7
0.6
0.5
0.4
0.3
0.2
0.1
0.0
p-value
2e-02
3e-02
3e-02
4e-02
6e-02
Log-odds
0.5
1.0
1.5
2.0
2.5
3.0
3.5
4.0
Cellular_organisms - ps1
Archaea1 - ps2
Archaea2 - ps3
Archaea3 - ps4
Asgard/Eukaryota - ps5
Eukaryota - ps6
Excavata/Diaphoretickes - ps7
Diaphoretickes - ps8
Archaeplastida - ps9
Viridiplantae - ps10
Streptophyta - ps11
Charophyceae/Embriophyta - ps12
Embriophyta - ps13
Tracheophyta - ps14
Spermatophyta - ps15
Angiosperms - ps16
Mesangiospermae - ps17
Magnoliids/eudicots - ps18
Eudicots - ps19
Core_eudicots - ps20
Superrosids - ps21
Rosids - ps22
Eurosids - ps23
Malvids - ps24
Sap./Malv./Brassicales - ps25
Malvales/Brassicales - ps26
Brassicales - ps27
Cleomaceae/Brassicaceae - ps28
Brassicaceae - ps29
Camelineae - ps30
Arabidopsis - ps31
Arabidopsis_thaliana - ps32
Phylostratum

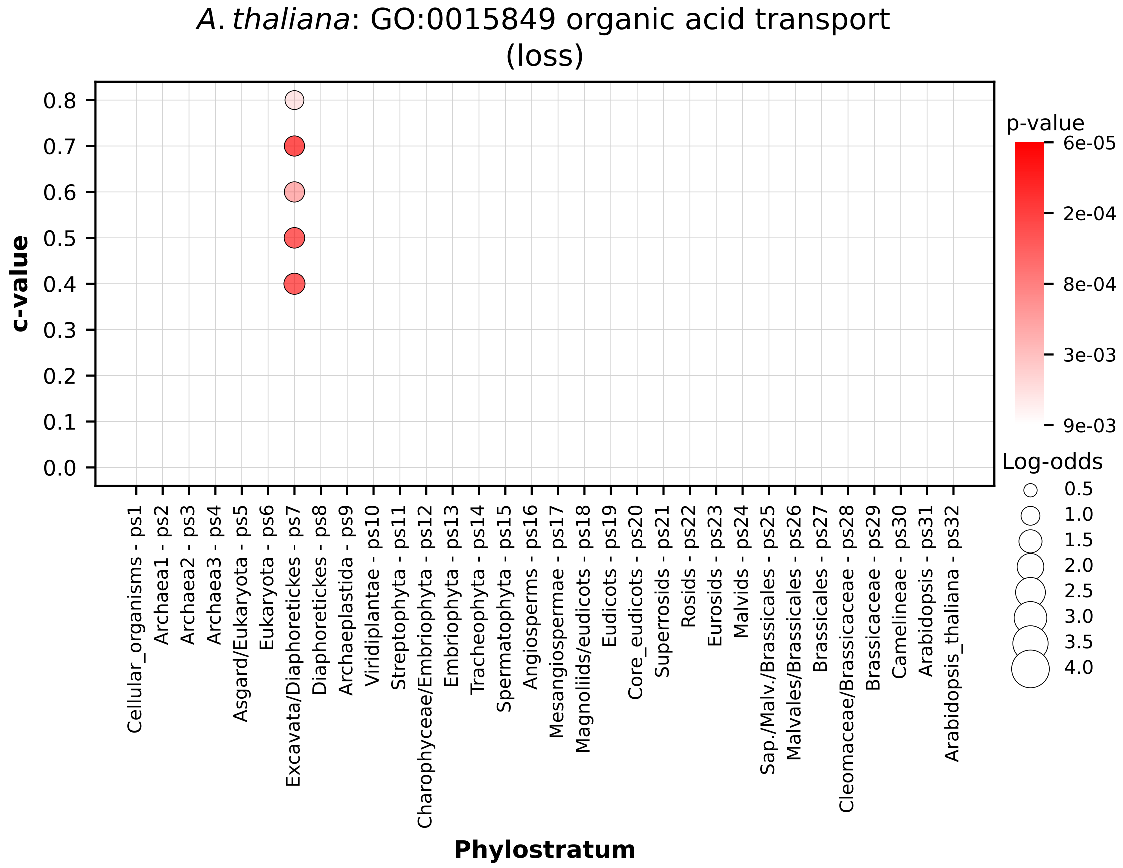

A. thaliana: GO:0015849 organic acid transport
(loss)
c-value
0.8
0.7
0.6
0.5
0.4
0.3
0.2
0.1
0.0
p-value
6e-05
2e-04
8e-04
3e-03
9e-03
Log-odds
0.5
1.0
1.5
2.0
2.5
3.0
3.5
4.0
Cellular_organisms - ps1
Archaea1 - ps2
Archaea2 - ps3
Archaea3 - ps4
Asgard/Eukaryota - ps5
Eukaryota - ps6
Excavata/Diaphoretickes - ps7
Diaphoretickes - ps8
Archaeplastida - ps9
Viridiplantae - ps10
Streptophyta - ps11
Charophyceae/Embriophyta - ps12
Embriophyta - ps13
Tracheophyta - ps14
Spermatophyta - ps15
Angiosperms - ps16
Mesangiospermae - ps17
Magnoliids/eudicots - ps18
Eudicots - ps19
Core_eudicots - ps20
Superrosids - ps21
Rosids - ps22
Eurosids - ps23
Malvids - ps24
Sap./Malv./Brassicales - ps25
Malvales/Brassicales - ps26
Brassicales - ps27
Cleomaceae/Brassicaceae - ps28
Brassicaceae - ps29
Camelineae - ps30
Arabidopsis - ps31
Arabidopsis_thaliana - ps32
Phylostratum

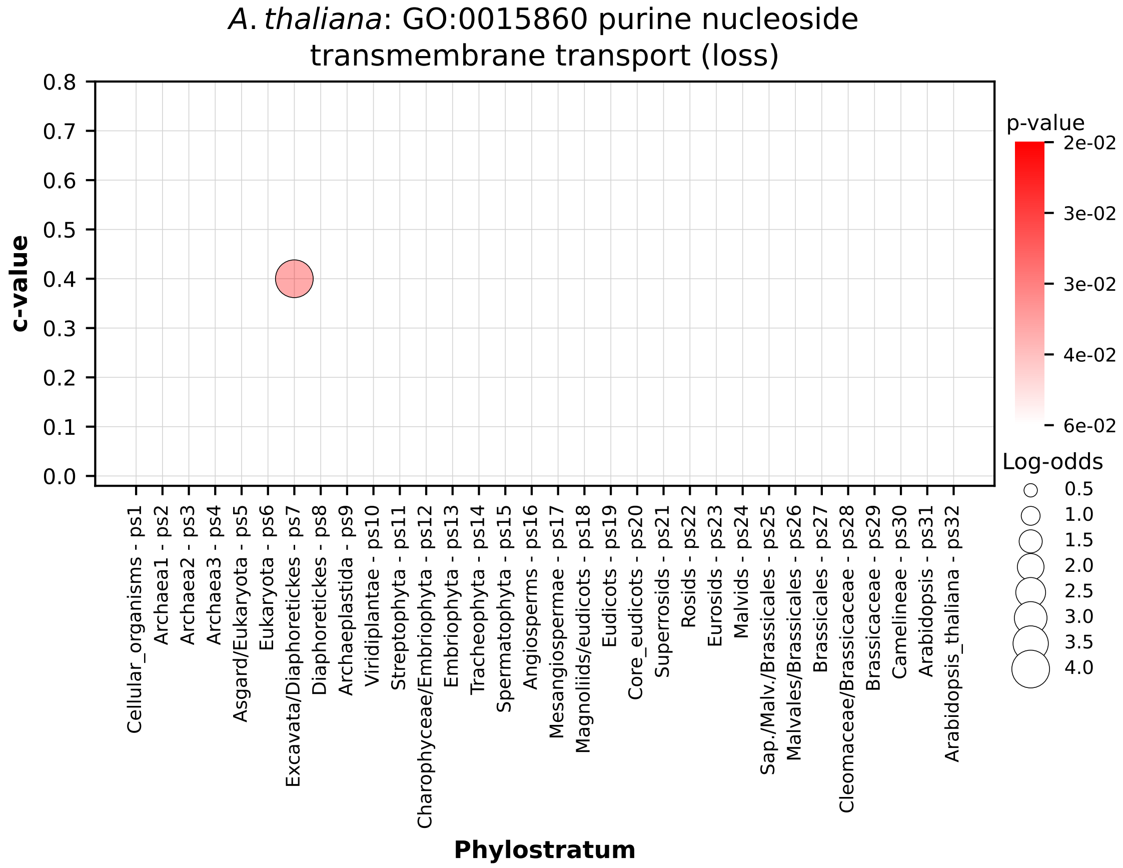
A. thaliana: GO:0015860 purine nucleoside transmembrane transport (loss)
c-value
0.8
0.7
0.6
0.5
0.4
0.3
0.2
0.1
0.0
p-value
2e-02
3e-02
3e-02
4e-02
6e-02
Log-odds
0.5
1.0
1.5
2.0
2.5
3.0
3.5
4.0
Cellular_organisms - ps1
Archaea1 - ps2
Archaea2 - ps3
Archaea3 - ps4
Asgard/Eukaryota - ps5
Eukaryota - ps6
Excavata/Diaphoretickes - ps7
Diaphoretickes - ps8
Archaeplastida - ps9
Viridiplantae - ps10
Streptophyta - ps11
Charophyceae/Embriophyta - ps12
Embriophyta - ps13
Tracheophyta - ps14
Spermatophyta - ps15
Angiosperms - ps16
Mesangiospermae - ps17
Magnoliids/eudicots - ps18
Eudicots - ps19
Core_eudicots - ps20
Superrosids - ps21
Rosids - ps22
Eurosids - ps23
Malvids - ps24
Sap./Malv./Brassicales - ps25
Malvales/Brassicales - ps26
Brassicales - ps27
Cleomaceae/Brassicaceae - ps28
Brassicaceae - ps29
Camelineae - ps30
Arabidopsis - ps31
Arabidopsis_thaliana - ps32
Phylostratum

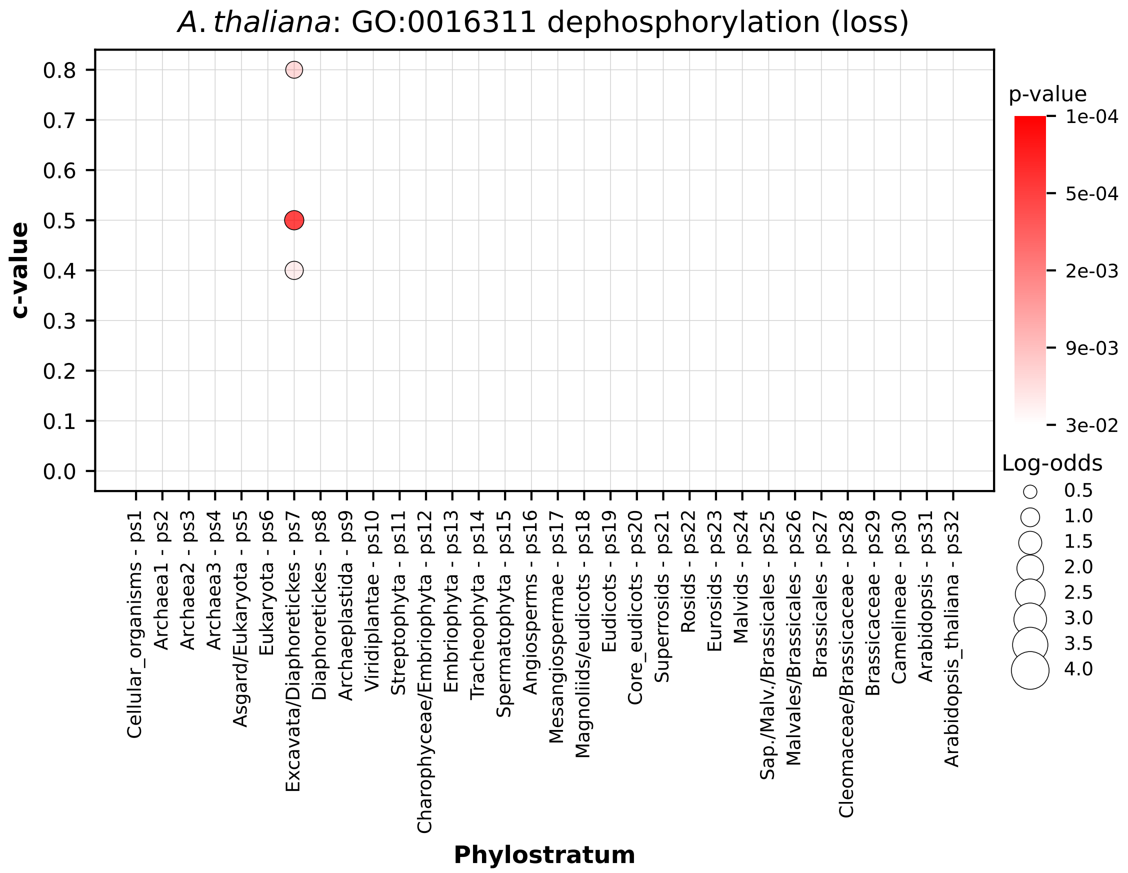

A. thaliana: GO:0016311 dephosphorylation (loss)
c-value
0.0
0.1
0.2
0.3
0.4
0.5
0.6
0.7
0.8
Phylostratum
Cellular_organisms - ps1
Archaea1 - ps2
Archaea2 - ps3
Archaea3 - ps4
Asgard/Eukaryota - ps5
Eukaryota - ps6
Excavata/Diaphoretickes - ps7
Diaphoretickes - ps8
Archaeplastida - ps9
Viridiplantae - ps10
Streptophyta - ps11
Charophyceae/Embriophyta - ps12
Embriophyta - ps13
Tracheophyta - ps14
Spermatophyta - ps15
Angiosperms - ps16
Mesangiospermae - ps17
Magnoliids/eudicots - ps18
Eudicots - ps19
Core_eudicots - ps20
Superrosids - ps21
Rosids - ps22
Eurosids - ps23
Malvids - ps24
Sap./Malv./Brassicales - ps25
Malvales/Brassicales - ps26
Brassicales - ps27
Cleomaceae/Brassicaceae - ps28
Brassicaceae - ps29
Camelineae - ps30
Arabidopsis - ps31
Arabidopsis_thaliana - ps32
p-value
1e-04
5e-04
2e-03
9e-03
3e-02
Log-odds
0.5
1.0
1.5
2.0
2.5
3.0
3.5
4.0

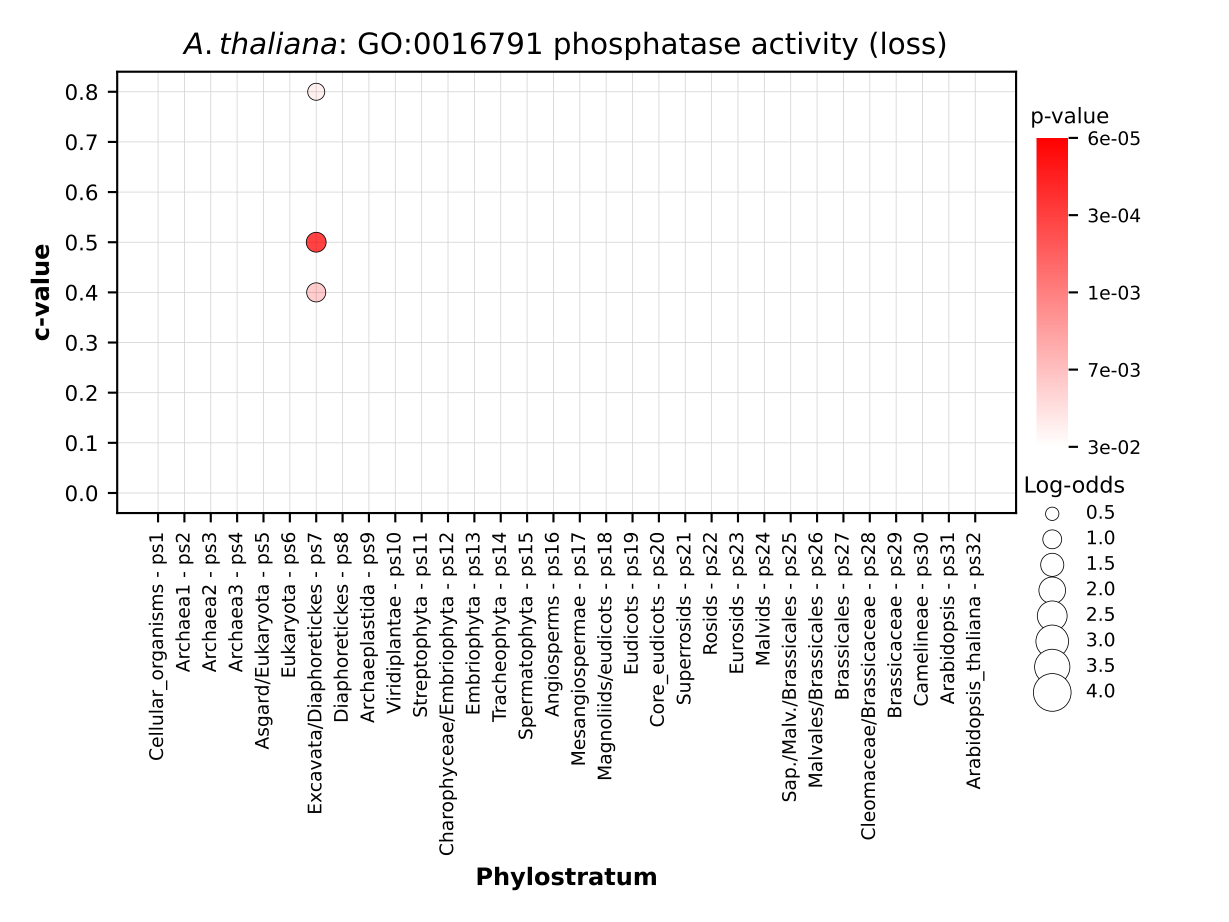
A. thaliana: GO:0016791 phosphatase activity (loss)
c-value
0.0
0.1
0.2
0.3
0.4
0.5
0.6
0.7
0.8
Phylostratum
Cellular_organisms - ps1
Archaea1 - ps2
Archaea2 - ps3
Archaea3 - ps4
Asgard/Eukaryota - ps5
Eukaryota - ps6
Excavata/Diaphoretickes - ps7
Diaphoretickes - ps8
Archaeplastida - ps9
Viridiplantae - ps10
Streptophyta - ps11
Charophyceae/Embriophyta - ps12
Embriophyta - ps13
Tracheophyta - ps14
Spermatophyta - ps15
Angiosperms - ps16
Mesangiospermae - ps17
Magnoliids/eudicots - ps18
Eudicots - ps19
Core_eudicots - ps20
Superrosids - ps21
Rosids - ps22
Eurosids - ps23
Malvids - ps24
Sap./Malv./Brassicales - ps25
Malvales/Brassicales - ps26
Brassicales - ps27
Cleomaceae/Brassicaceae - ps28
Brassicaceae - ps29
Camelineae - ps30
Arabidopsis - ps31
Arabidopsis_thaliana - ps32
p-value
6e-05
3e-04
1e-03
7e-03
3e-02
Log-odds
0.5
1.0
1.5
2.0
2.5
3.0
3.5
4.0

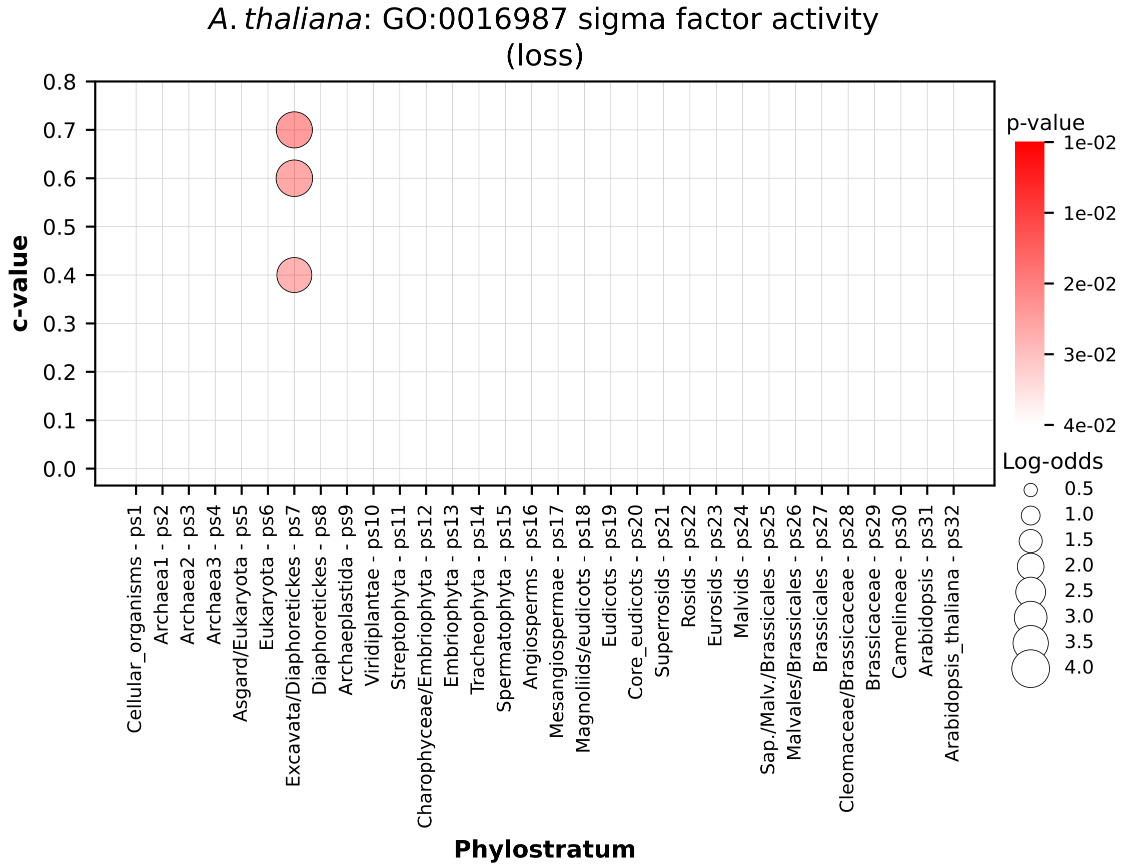

A. thaliana: GO:0016987 sigma factor activity
(loss)
c-value
0.8
0.7
0.6
0.5
0.4
0.3
0.2
0.1
0.0
p-value
1e-02
1e-02
2e-02
3e-02
4e-02
Log-odds
0.5
1.0
1.5
2.0
2.5
3.0
3.5
4.0
Cellular_organisms - ps1
Archaea1 - ps2
Archaea2 - ps3
Archaea3 - ps4
Asgard/Eukaryota - ps5
Eukaryota - ps6
Excavata/Diaphoretickes - ps7
Diaphoretickes - ps8
Archaeplastida - ps9
Viridiplantae - ps10
Streptophyta - ps11
Charophyceae/Embriophyta - ps12
Embriophyta - ps13
Tracheophyta - ps14
Spermatophyta - ps15
Angiosperms - ps16
Mesangiospermae - ps17
Magnoliids/eudicots - ps18
Eudicots - ps19
Core_eudicots - ps20
Superrosids - ps21
Rosids - ps22
Eurosids - ps23
Malvids - ps24
Sap./Malv./Brassicales - ps25
Malvales/Brassicales - ps26
Brassicales - ps27
Cleomaceae/Brassicaceae - ps28
Brassicaceae - ps29
Camelineae - ps30
Arabidopsis - ps31
Arabidopsis_thaliana - ps32
Phylostratum

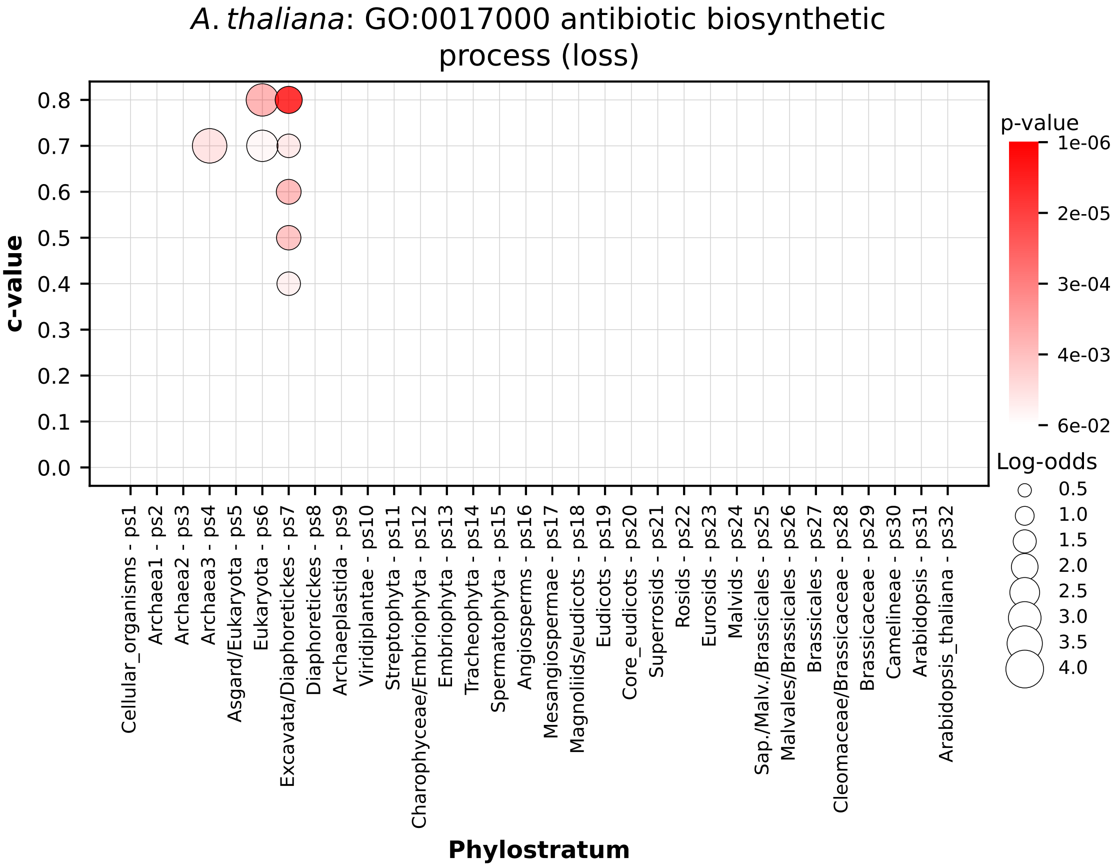

A. thaliana: GO:0017000 antibiotic biosynthetic process (loss)
c-value
0.0
0.1
0.2
0.3
0.4
0.5
0.6
0.7
0.8
p-value
1e-06
2e-05
3e-04
4e-03
6e-02
Log-odds
0.5
1.0
1.5
2.0
2.5
3.0
3.5
4.0
Cellular_organisms - ps1
Archaea1 - ps2
Archaea2 - ps3
Archaea3 - ps4
Asgard/Eukaryota - ps5
Eukaryota - ps6
Excavata/Diaphoretickes - ps7
Diaphoretickes - ps8
Archaeplastida - ps9
Viridiplantae - ps10
Streptophyta - ps11
Charophyceae/Embriophyta - ps12
Embriophyta - ps13
Tracheophyta - ps14
Spermatophyta - ps15
Angiosperms - ps16
Mesangiospermae - ps17
Magnoliids/eudicots - ps18
Eudicots - ps19
Core_eudicots - ps20
Superrosids - ps21
Rosids - ps22
Eurosids - ps23
Malvids - ps24
Sap./Malv./Brassicales - ps25
Malvales/Brassicales - ps26
Brassicales - ps27
Cleomaceae/Brassicaceae - ps28
Brassicaceae - ps29
Camelineae - ps30
Arabidopsis - ps31
Arabidopsis_thaliana - ps32
Phylostratum

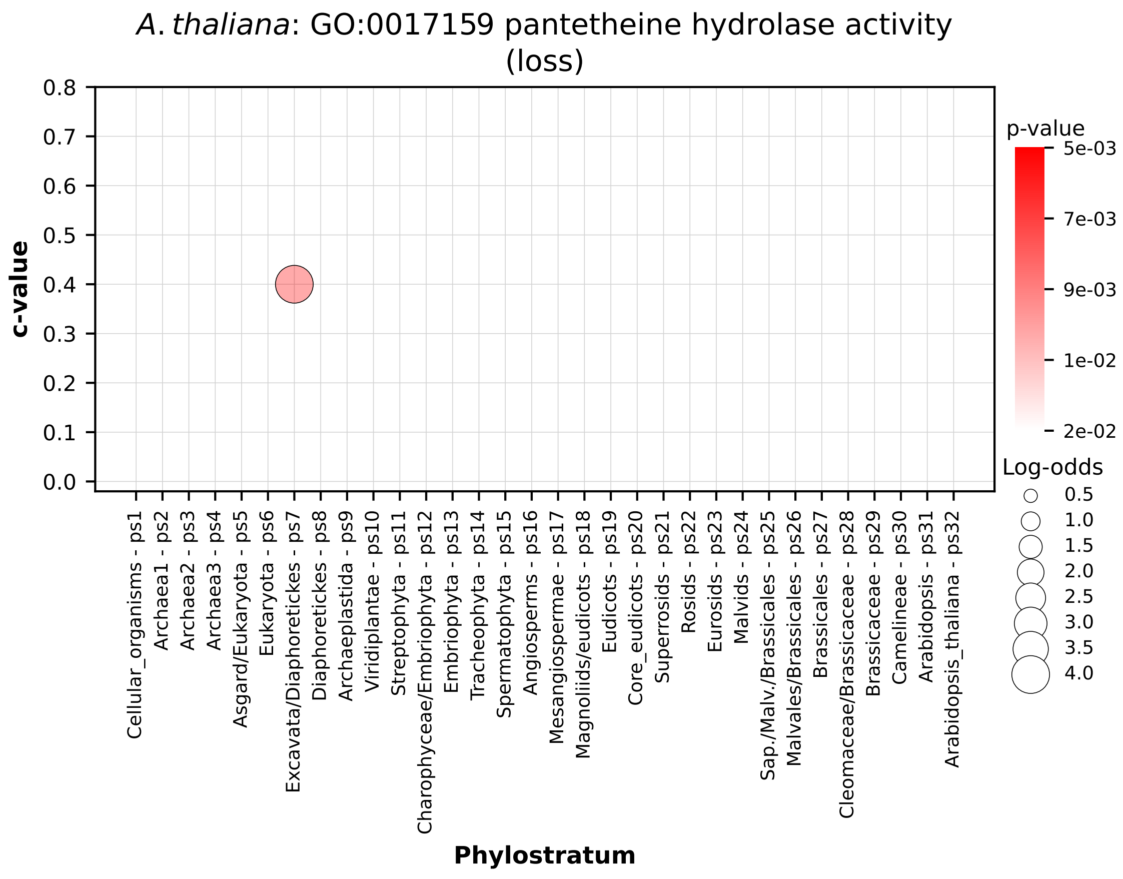
A. thaliana: GO:0017159 pantetheine hydrolase activity
(loss)
c-value
0.8
0.7
0.6
0.5
0.4
0.3
0.2
0.1
0.0
p-value
5e-03
7e-03
9e-03
1e-02
2e-02
Log-odds
0.5
1.0
1.5
2.0
2.5
3.0
3.5
4.0
Cellular_organisms - ps1
Archaea1 - ps2
Archaea2 - ps3
Archaea3 - ps4
Asgard/Eukaryota - ps5
Eukaryota - ps6
Excavata/Diaphoretickes - ps7
Diaphoretickes - ps8
Archaeplastida - ps9
Viridiplantae - ps10
Streptophyta - ps11
Charophyceae/Embriophyta - ps12
Embriophyta - ps13
Tracheophyta - ps14
Spermatophyta - ps15
Angiosperms - ps16
Mesangiospermae - ps17
Magnoliids/eudicots - ps18
Eudicots - ps19
Core_eudicots - ps20
Superrosids - ps21
Rosids - ps22
Eurosids - ps23
Malvids - ps24
Sap./Malv./Brassicales - ps25
Malvales/Brassicales - ps26
Brassicales - ps27
Cleomaceae/Brassicaceae - ps28
Brassicaceae - ps29
Camelineae - ps30
Arabidopsis - ps31
Arabidopsis_thaliana - ps32
Phylostratum

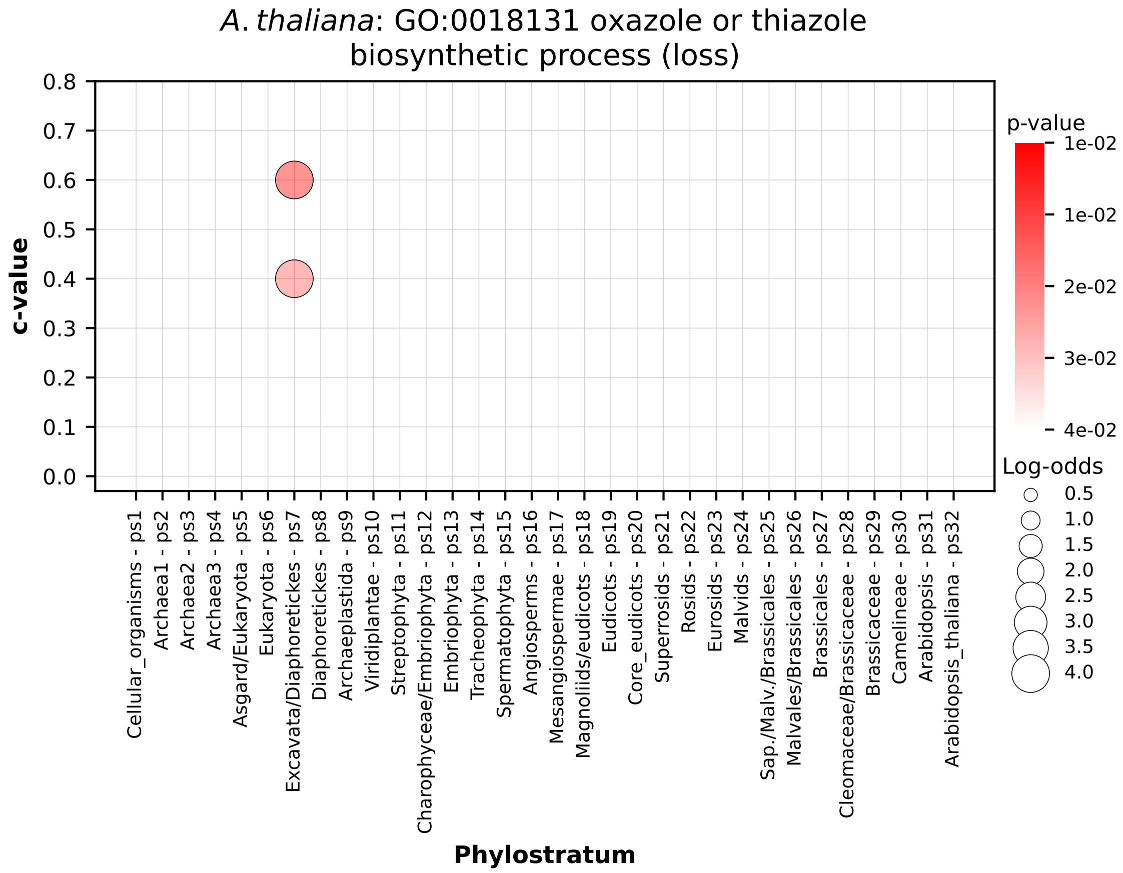

A. thaliana: GO:0018131 oxazole or thiazole biosynthetic process (loss)
c-value
0.8
0.7
0.6
0.5
0.4
0.3
0.2
0.1
0.0
Phylostratum
Cellular_organisms - ps1
Archaea1 - ps2
Archaea2 - ps3
Archaea3 - ps4
Asgard/Eukaryota - ps5
Eukaryota - ps6
Excavata/Diaphoretickes - ps7
Diaphoretickes - ps8
Archaeplastida - ps9
Viridiplantae - ps10
Streptophyta - ps11
Charophyceae/Embriophyta - ps12
Embriophyta - ps13
Tracheophyta - ps14
Spermatophyta - ps15
Angiosperms - ps16
Mesangiospermae - ps17
Magnoliids/eudicots - ps18
Eudicots - ps19
Core_eudicots - ps20
Superrosids - ps21
Rosids - ps22
Eurosids - ps23
Malvids - ps24
Sap./Malv./Brassicales - ps25
Malvales/Brassicales - ps26
Brassicales - ps27
Cleomaceae/Brassicaceae - ps28
Brassicaceae - ps29
Camelineae - ps30
Arabidopsis - ps31
Arabidopsis_thaliana - ps32
p-value
1e-02
1e-02
2e-02
3e-02
4e-02
Log-odds
0.5
1.0
1.5
2.0
2.5
3.0
3.5
4.0

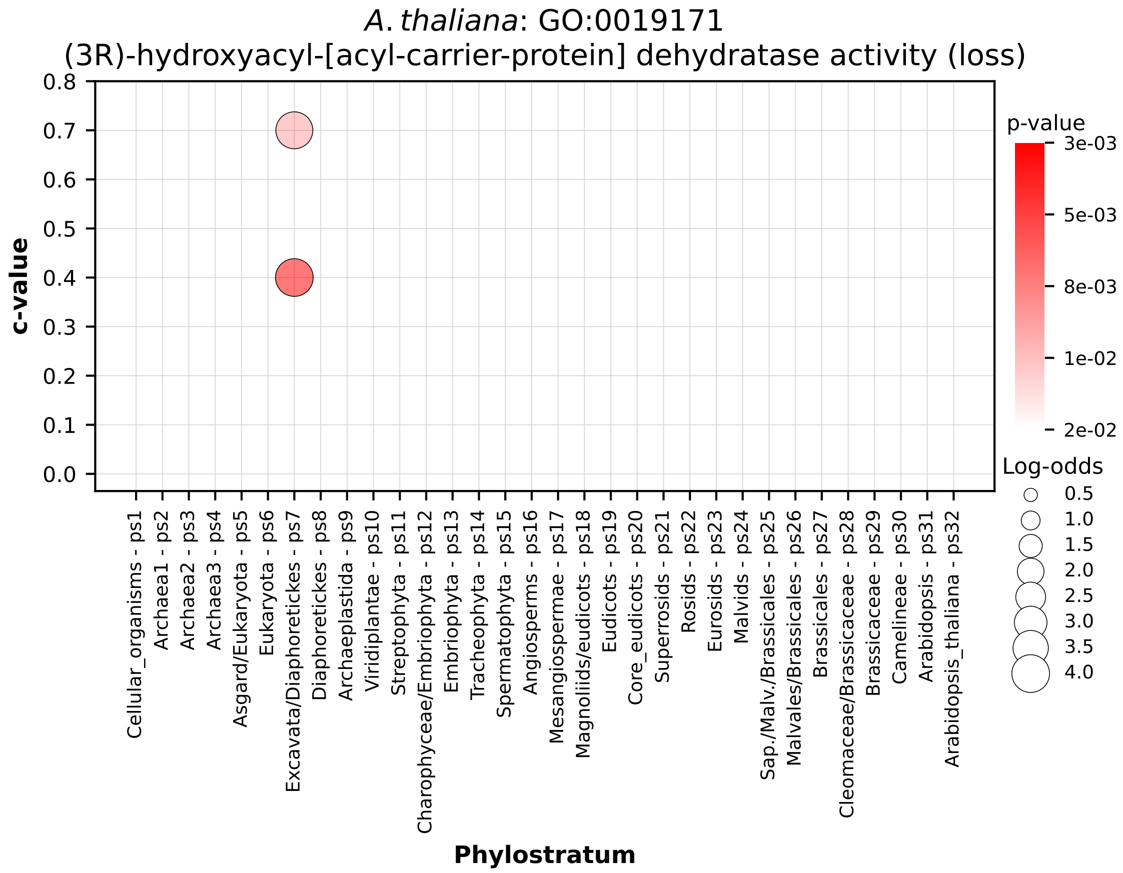

A. thaliana: GO:0019171
(3R)-hydroxyacyl-[acyl-carrier-protein] dehydratase activity (loss)
c-value
0.8
0.7
0.6
0.5
0.4
0.3
0.2
0.1
0.0
p-value
3e-03
5e-03
8e-03
1e-02
2e-02
Log-odds
0.5
1.0
1.5
2.0
2.5
3.0
3.5
4.0
Cellular_organisms - ps1
Archaea1 - ps2
Archaea2 - ps3
Archaea3 - ps4
Asgard/Eukaryota - ps5
Eukaryota - ps6
Excavata/Diaphoretickes - ps7
Diaphoretickes - ps8
Archaeplastida - ps9
Viridiplantae - ps10
Streptophyta - ps11
Charophyceae/Embriophyta - ps12
Embriophyta - ps13
Tracheophyta - ps14
Spermatophyta - ps15
Angiosperms - ps16
Mesangiospermae - ps17
Magnoliids/eudicots - ps18
Eudicots - ps19
Core_eudicots - ps20
Superrosids - ps21
Rosids - ps22
Eurosids - ps23
Malvids - ps24
Sap./Malv./Brassicales - ps25
Malvales/Brassicales - ps26
Brassicales - ps27
Cleomaceae/Brassicaceae - ps28
Brassicaceae - ps29
Camelineae - ps30
Arabidopsis - ps31
Arabidopsis_thaliana - ps32
Phylostratum

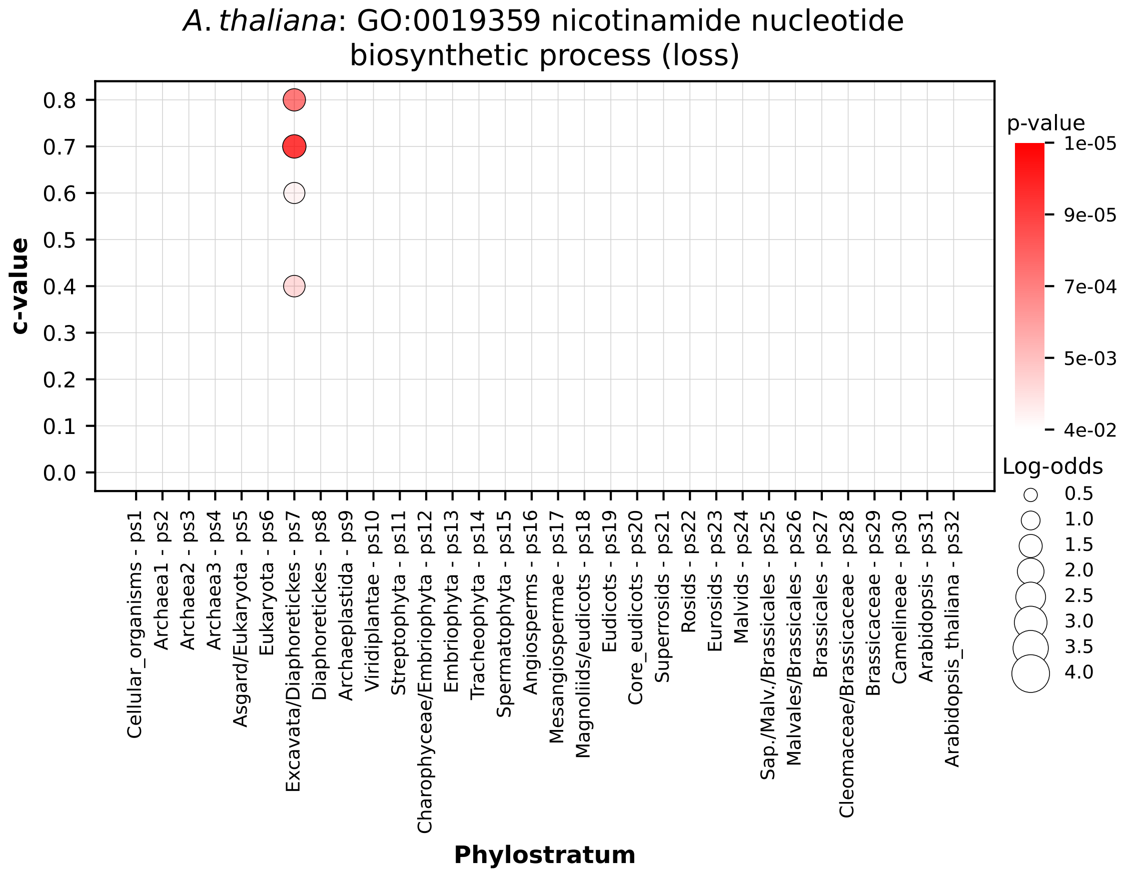
A. thaliana: GO:0019359 nicotinamide nucleotide biosynthetic process (loss)
c-value
0.8
0.7
0.6
0.5
0.4
0.3
0.2
0.1
0.0
p-value
1e-05
9e-05
7e-04
5e-03
4e-02
Log-odds
0.5
1.0
1.5
2.0
2.5
3.0
3.5
4.0
Cellular_organisms - ps1
Archaea1 - ps2
Archaea2 - ps3
Archaea3 - ps4
Asgard/Eukaryota - ps5
Eukaryota - ps6
Excavata/Diaphoretickes - ps7
Diaphoretickes - ps8
Archaeplastida - ps9
Viridiplantae - ps10
Streptophyta - ps11
Charophyceae/Embriophyta - ps12
Embriophyta - ps13
Tracheophyta - ps14
Spermatophyta - ps15
Angiosperms - ps16
Mesangiospermae - ps17
Magnoliids/eudicots - ps18
Eudicots - ps19
Core_eudicots - ps20
Superrosids - ps21
Rosids - ps22
Eurosids - ps23
Malvids - ps24
Sap./Malv./Brassicales - ps25
Malvales/Brassicales - ps26
Brassicales - ps27
Cleomaceae/Brassicaceae - ps28
Brassicaceae - ps29
Camelineae - ps30
Arabidopsis - ps31
Arabidopsis_thaliana - ps32
Phylostratum

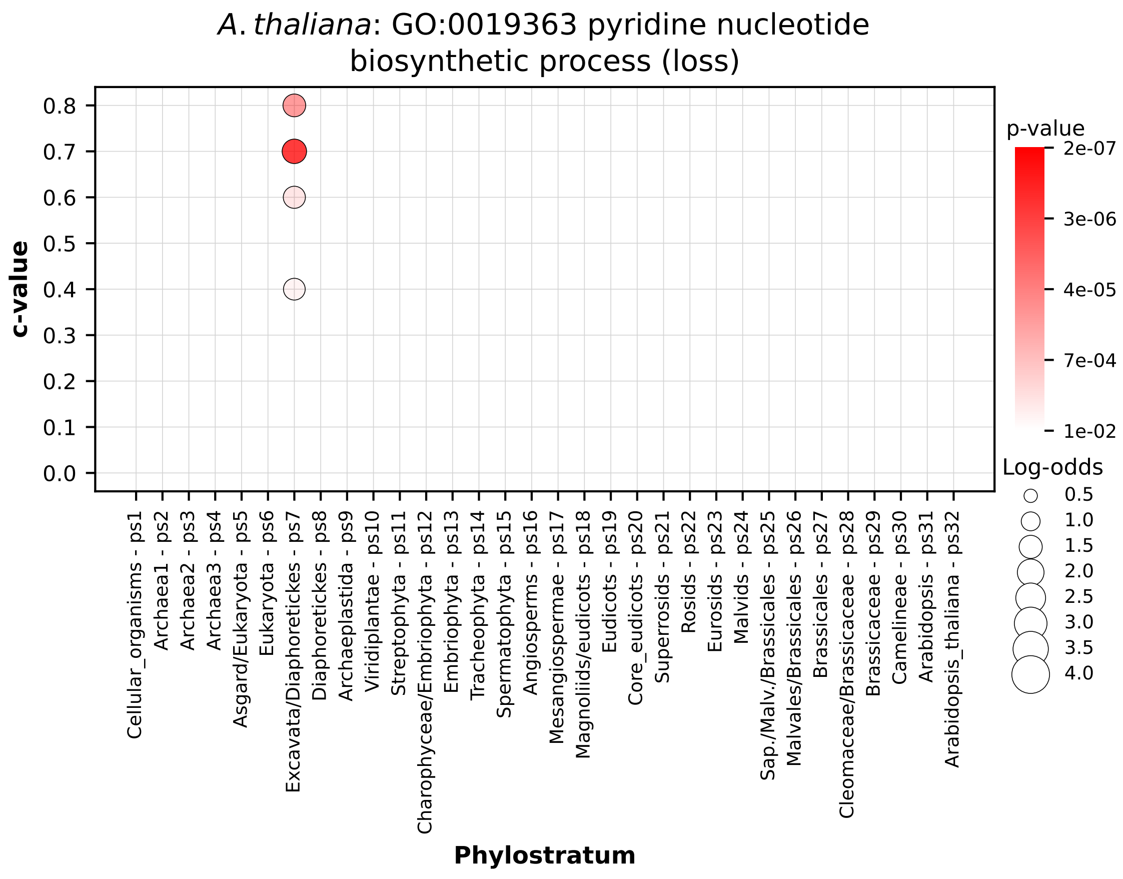

A. thaliana: GO:0019363 pyridine nucleotide biosynthetic process (loss)
c-value
0.8
0.7
0.6
0.5
0.4
0.3
0.2
0.1
0.0
p-value
2e-07
3e-06
4e-05
7e-04
1e-02
Log-odds
0.5
1.0
1.5
2.0
2.5
3.0
3.5
4.0
Cellular_organisms - ps1
Archaea1 - ps2
Archaea2 - ps3
Archaea3 - ps4
Asgard/Eukaryota - ps5
Eukaryota - ps6
Excavata/Diaphoretickes - ps7
Diaphoretickes - ps8
Archaeplastida - ps9
Viridiplantae - ps10
Streptophyta - ps11
Charophyceae/Embriophyta - ps12
Embriophyta - ps13
Tracheophyta - ps14
Spermatophyta - ps15
Angiosperms - ps16
Mesangiospermae - ps17
Magnoliids/eudicots - ps18
Eudicots - ps19
Core_eudicots - ps20
Superrosids - ps21
Rosids - ps22
Eurosids - ps23
Malvids - ps24
Sap./Malv./Brassicales - ps25
Malvales/Brassicales - ps26
Brassicales - ps27
Cleomaceae/Brassicaceae - ps28
Brassicaceae - ps29
Camelineae - ps30
Arabidopsis - ps31
Arabidopsis_thaliana - ps32
Phylostratum

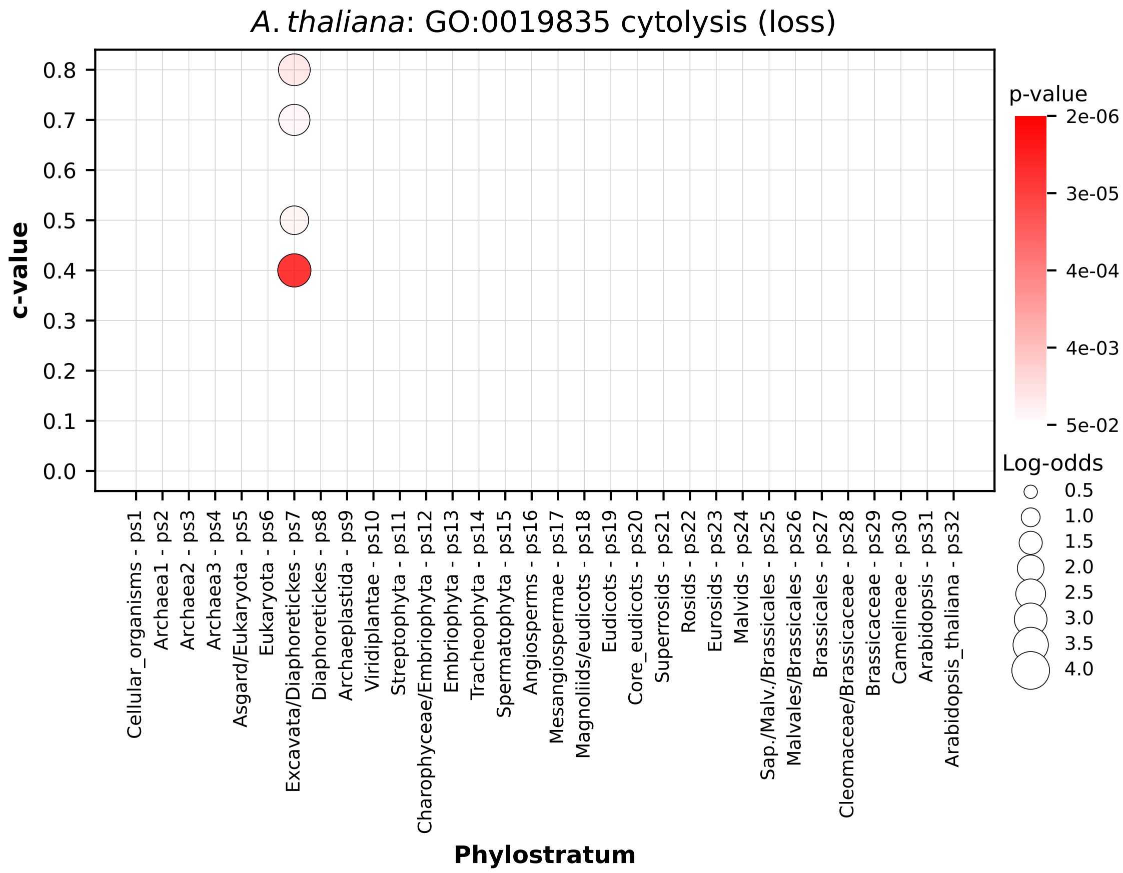

A. thaliana: GO:0019835 cytolysis (loss)
c-value
0.8
0.7
0.6
0.5
0.4
0.3
0.2
0.1
0.0
p-value
2e-06
3e-05
4e-04
4e-03
5e-02
Log-odds
0.5
1.0
1.5
2.0
2.5
3.0
3.5
4.0
Cellular_organisms - ps1
Archaea1 - ps2
Archaea2 - ps3
Archaea3 - ps4
Asgard/Eukaryota - ps5
Eukaryota - ps6
Excavata/Diaphoretickes - ps7
Diaphoretickes - ps8
Archaeplastida - ps9
Viridiplantae - ps10
Streptophyta - ps11
Charophyceae/Embriophyta - ps12
Embriophyta - ps13
Tracheophyta - ps14
Spermatophyta - ps15
Angiosperms - ps16
Mesangiospermae - ps17
Magnoliids/eudicots - ps18
Eudicots - ps19
Core_eudicots - ps20
Superrosids - ps21
Rosids - ps22
Eurosids - ps23
Malvids - ps24
Sap./Malv./Brassicales - ps25
Malvales/Brassicales - ps26
Brassicales - ps27
Cleomaceae/Brassicaceae - ps28
Brassicaceae - ps29
Camelineae - ps30
Arabidopsis - ps31
Arabidopsis_thaliana - ps32
Phylostratum

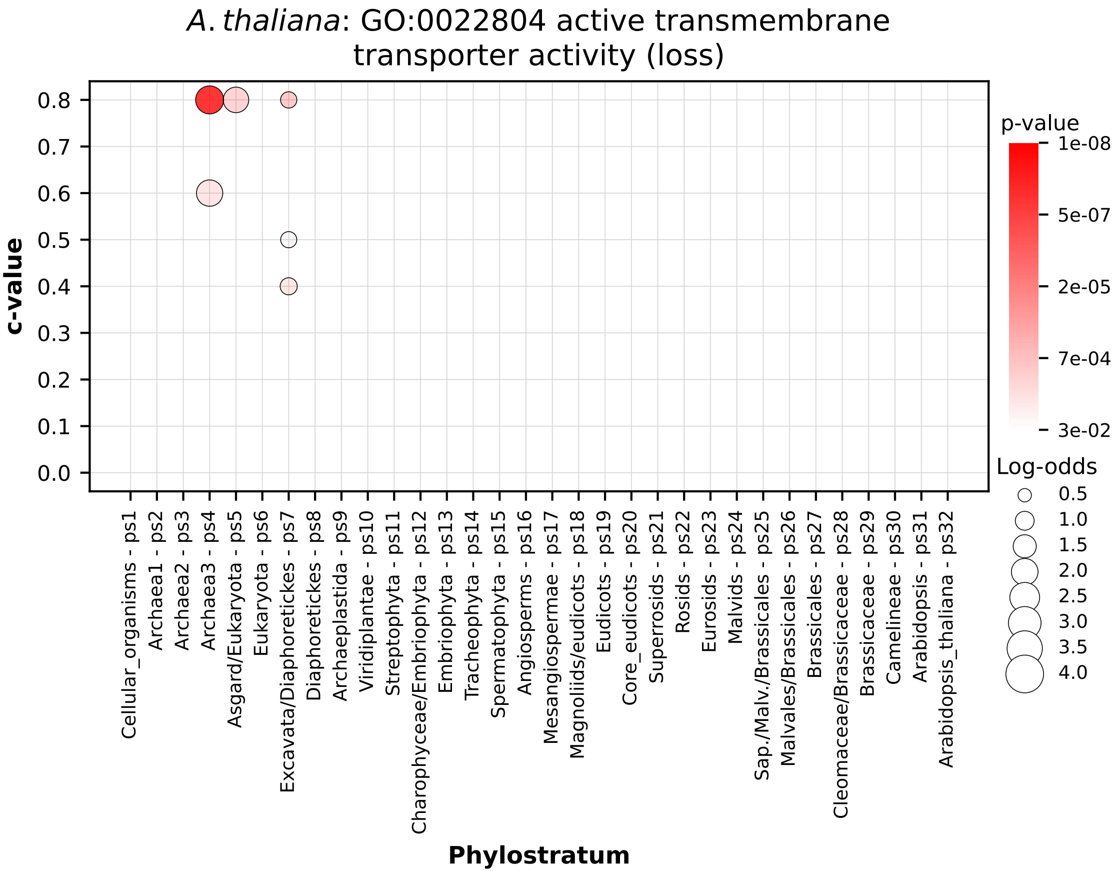
A. thaliana: GO:0022804 active transmembrane transporter activity (loss)
c-value
0.0
0.1
0.2
0.3
0.4
0.5
0.6
0.7
0.8
Phylostratum
Cellular_organisms - ps1
Archaea1 - ps2
Archaea2 - ps3
Archaea3 - ps4
Asgard/Eukaryota - ps5
Eukaryota - ps6
Excavata/Diaphoretickes - ps7
Diaphoretickes - ps8
Archaeplastida - ps9
Viridiplantae - ps10
Streptophyta - ps11
Charophyceae/Embriophyta - ps12
Embriophyta - ps13
Tracheophyta - ps14
Spermatophyta - ps15
Angiosperms - ps16
Mesangiospermae - ps17
Magnoliids/eudicots - ps18
Eudicots - ps19
Core_eudicots - ps20
Superrosids - ps21
Rosids - ps22
Eurosids - ps23
Malvids - ps24
Sap./Malv./Brassicales - ps25
Malvales/Brassicales - ps26
Brassicales - ps27
Cleomaceae/Brassicaceae - ps28
Brassicaceae - ps29
Camelineae - ps30
Arabidopsis - ps31
Arabidopsis_thaliana - ps32
p-value
1e-08
5e-07
2e-05
7e-04
3e-02
Log-odds
0.5
1.0
1.5
2.0
2.5
3.0
3.5
4.0

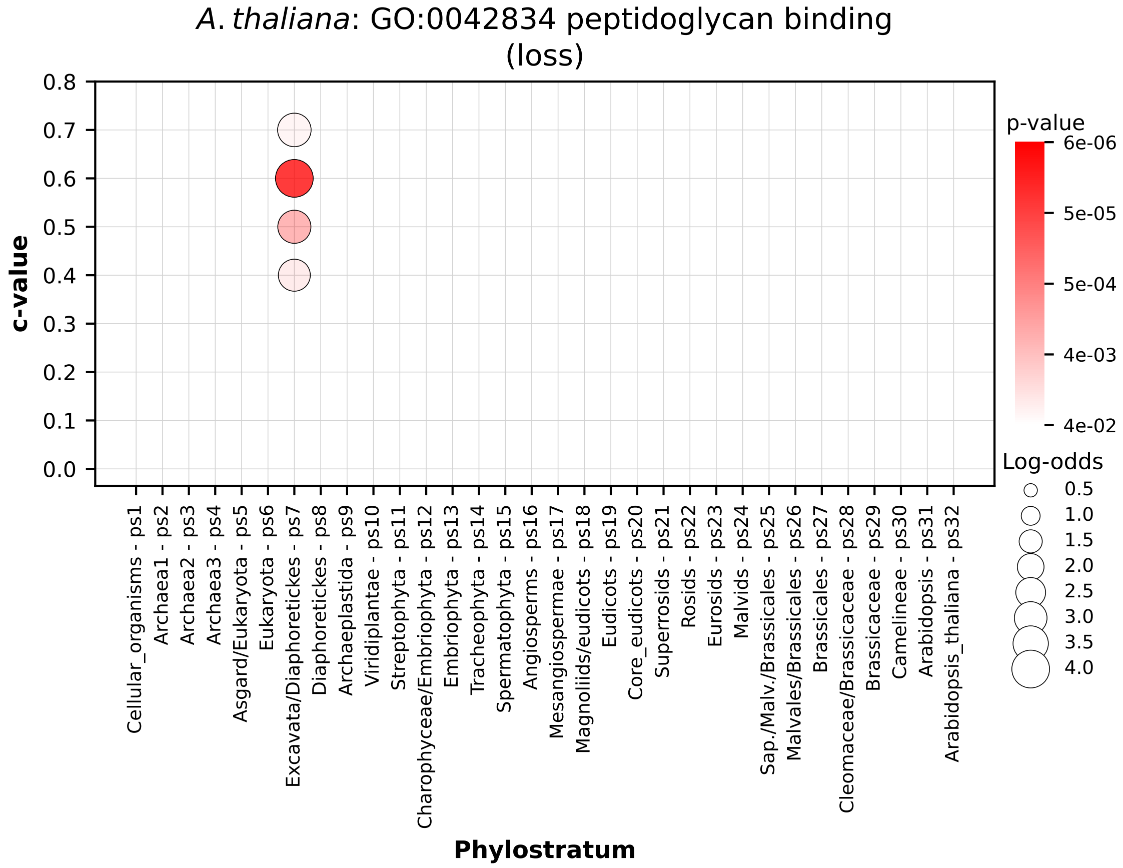

A. thaliana: GO:0042834 peptidoglycan binding
(loss)
c-value
0.0
0.1
0.2
0.3
0.4
0.5
0.6
0.7
0.8
p-value
6e-06
5e-05
5e-04
4e-03
4e-02
Log-odds
0.5
1.0
1.5
2.0
2.5
3.0
3.5
4.0
Cellular_organisms - ps1
Archaea1 - ps2
Archaea2 - ps3
Archaea3 - ps4
Asgard/Eukaryota - ps5
Eukaryota - ps6
Excavata/Diaphoretickes - ps7
Diaphoretickes - ps8
Archaeplastida - ps9
Viridiplantae - ps10
Streptophyta - ps11
Charophyceae/Embriophyta - ps12
Embriophyta - ps13
Tracheophyta - ps14
Spermatophyta - ps15
Angiosperms - ps16
Mesangiospermae - ps17
Magnoliids/eudicots - ps18
Eudicots - ps19
Core_eudicots - ps20
Superrosids - ps21
Rosids - ps22
Eurosids - ps23
Malvids - ps24
Sap./Malv./Brassicales - ps25
Malvales/Brassicales - ps26
Brassicales - ps27
Cleomaceae/Brassicaceae - ps28
Brassicaceae - ps29
Camelineae - ps30
Arabidopsis - ps31
Arabidopsis_thaliana - ps32
Phylostratum

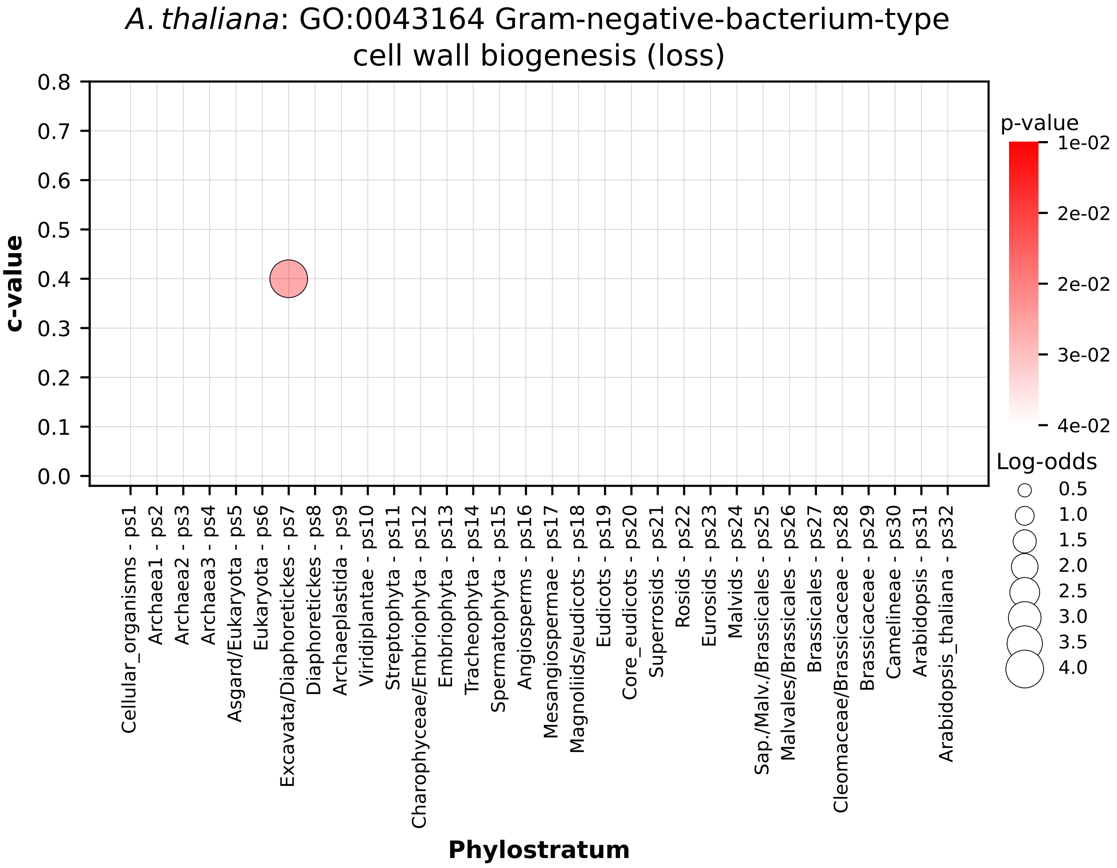

A. thaliana: GO:0043164 Gram-negative-bacterium-type cell wall biogenesis (loss)
c-value
0.8
0.7
0.6
0.5
0.4
0.3
0.2
0.1
0.0
p-value
1e-02
2e-02
2e-02
3e-02
4e-02
Log-odds
0.5
1.0
1.5
2.0
2.5
3.0
3.5
4.0
Cellular_organisms - ps1
Archaea1 - ps2
Archaea2 - ps3
Archaea3 - ps4
Asgard/Eukaryota - ps5
Eukaryota - ps6
Excavata/Diaphoretickes - ps7
Diaphoretickes - ps8
Archaeplastida - ps9
Viridiplantae - ps10
Streptophyta - ps11
Charophyceae/Embriophyta - ps12
Embriophyta - ps13
Tracheophyta - ps14
Spermatophyta - ps15
Angiosperms - ps16
Mesangiospermae - ps17
Magnoliids/eudicots - ps18
Eudicots - ps19
Core_eudicots - ps20
Superrosids - ps21
Rosids - ps22
Eurosids - ps23
Malvids - ps24
Sap./Malv./Brassicales - ps25
Malvales/Brassicales - ps26
Brassicales - ps27
Cleomaceae/Brassicaceae - ps28
Brassicaceae - ps29
Camelineae - ps30
Arabidopsis - ps31
Arabidopsis_thaliana - ps32
Phylostratum

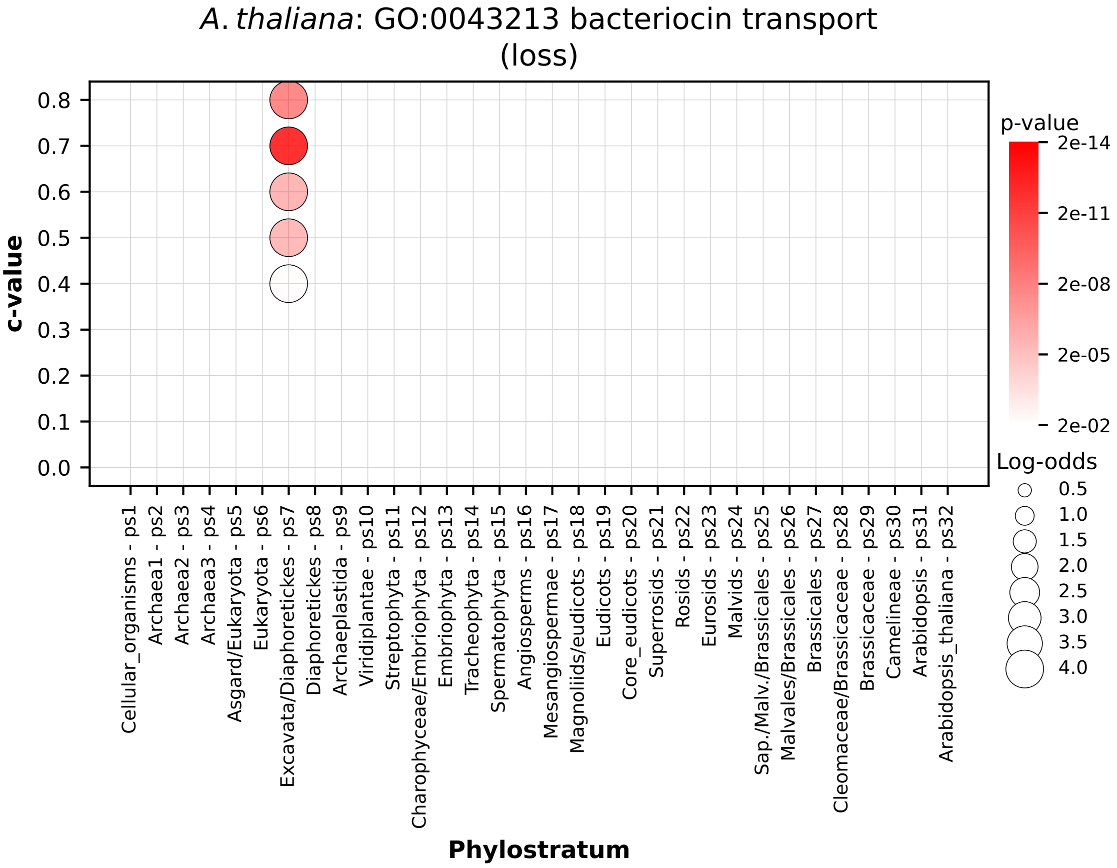

A. thaliana: GO:0043213 bacteriocin transport
(loss)
c-value
0.8
0.7
0.6
0.5
0.4
0.3
0.2
0.1
0.0
p-value
2e-14
2e-11
2e-08
2e-05
2e-02
Log-odds
0.5
1.0
1.5
2.0
2.5
3.0
3.5
4.0
Cellular_organisms - ps1
Archaea1 - ps2
Archaea2 - ps3
Archaea3 - ps4
Asgard/Eukaryota - ps5
Eukaryota - ps6
Excavata/Diaphoretickes - ps7
Diaphoretickes - ps8
Archaeplastida - ps9
Viridiplantae - ps10
Streptophyta - ps11
Charophyceae/Embriophyta - ps12
Embriophyta - ps13
Tracheophyta - ps14
Spermatophyta - ps15
Angiosperms - ps16
Mesangiospermae - ps17
Magnoliids/eudicots - ps18
Eudicots - ps19
Core_eudicots - ps20
Superrosids - ps21
Rosids - ps22
Eurosids - ps23
Malvids - ps24
Sap./Malv./Brassicales - ps25
Malvales/Brassicales - ps26
Brassicales - ps27
Cleomaceae/Brassicaceae - ps28
Brassicaceae - ps29
Camelineae - ps30
Arabidopsis - ps31
Arabidopsis_thaliana - ps32
Phylostratum

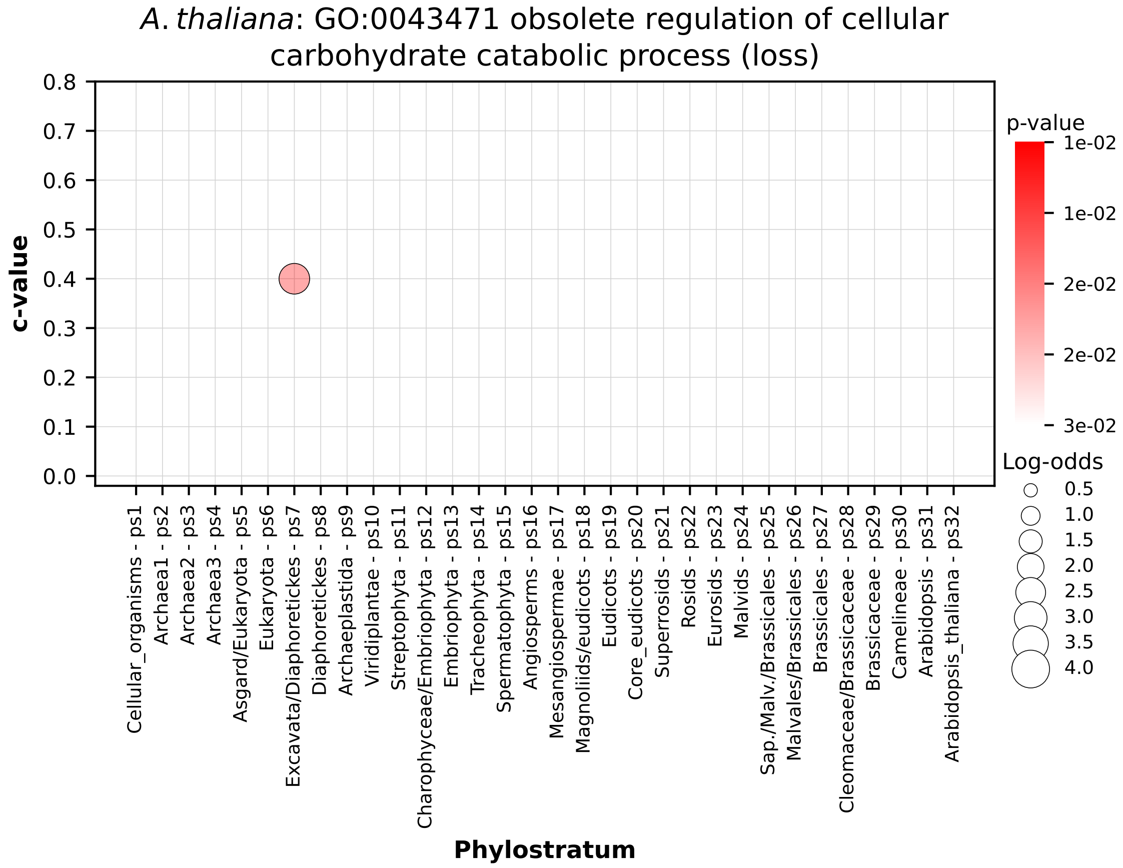

A. thaliana: GO:0043471 obsolete regulation of cellular carbohydrate catabolic process (loss)
c-value
0.8
0.7
0.6
0.5
0.4
0.3
0.2
0.1
0.0
Phylostratum
Cellular_organisms - ps1
Archaea1 - ps2
Archaea2 - ps3
Archaea3 - ps4
Asgard/Eukaryota - ps5
Eukaryota - ps6
Excavata/Diaphoretickes - ps7
Diaphoretickes - ps8
Archaeplastida - ps9
Viridiplantae - ps10
Streptophyta - ps11
Charophyceae/Embriophyta - ps12
Embriophyta - ps13
Tracheophyta - ps14
Spermatophyta - ps15
Angiosperms - ps16
Mesangiospermae - ps17
Magnoliids/eudicots - ps18
Eudicots - ps19
Core_eudicots - ps20
Superrosids - ps21
Rosids - ps22
Eurosids - ps23
Malvids - ps24
Sap./Malv./Brassicales - ps25
Malvales/Brassicales - ps26
Brassicales - ps27
Cleomaceae/Brassicaceae - ps28
Brassicaceae - ps29
Camelineae - ps30
Arabidopsis - ps31
Arabidopsis_thaliana - ps32
p-value
1e-02
1e-02
2e-02
2e-02
3e-02
Log-odds
0.5
1.0
1.5
2.0
2.5
3.0
3.5
4.0

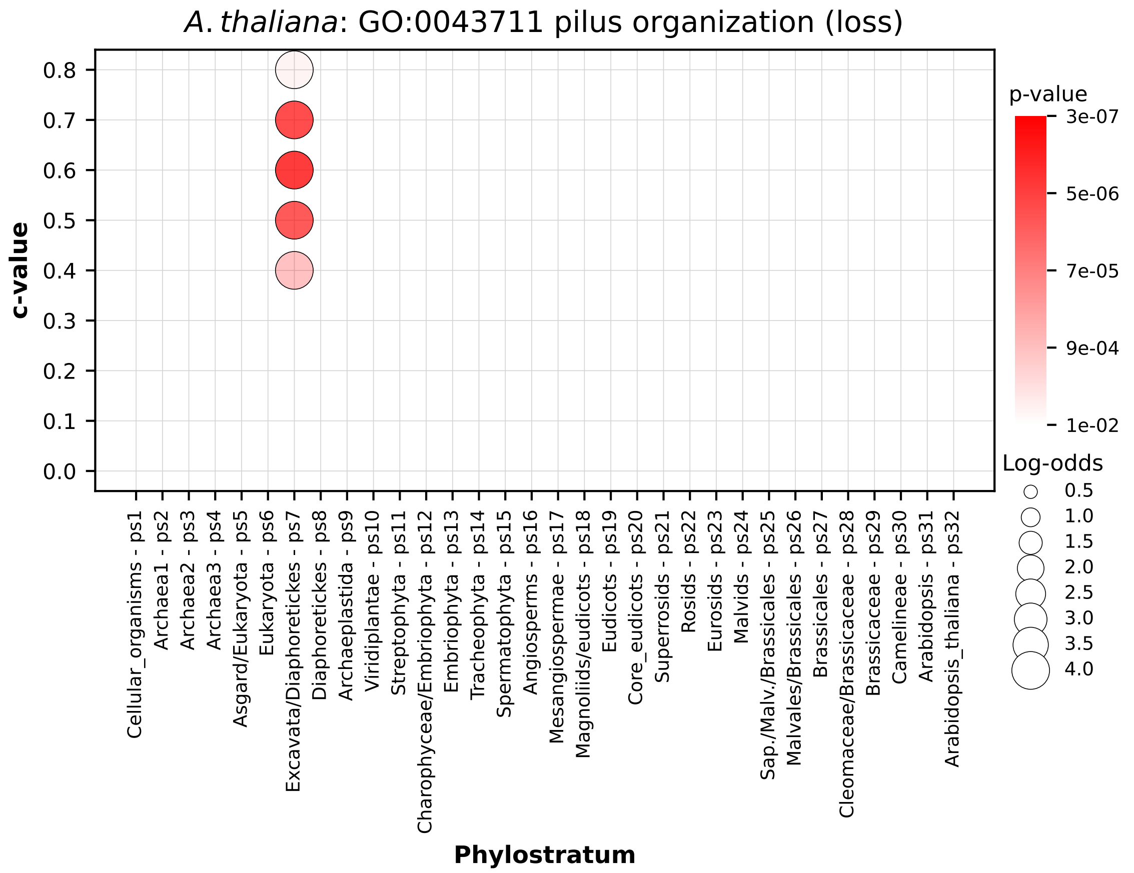

A. thaliana: GO:0043711 pilus organization (loss)
c-value
0.0
0.1
0.2
0.3
0.4
0.5
0.6
0.7
0.8
Phylostratum
Cellular_organisms - ps1
Archaea1 - ps2
Archaea2 - ps3
Archaea3 - ps4
Asgard/Eukaryota - ps5
Eukaryota - ps6
Excavata/Diaphoretickes - ps7
Diaphoretickes - ps8
Archaeplastida - ps9
Viridiplantae - ps10
Streptophyta - ps11
Charophyceae/Embriophyta - ps12
Embriophyta - ps13
Tracheophyta - ps14
Spermatophyta - ps15
Angiosperms - ps16
Mesangiospermae - ps17
Magnoliids/eudicots - ps18
Eudicots - ps19
Core_eudicots - ps20
Superrosids - ps21
Rosids - ps22
Eurosids - ps23
Malvids - ps24
Sap./Malv./Brassicales - ps25
Malvales/Brassicales - ps26
Brassicales - ps27
Cleomaceae/Brassicaceae - ps28
Brassicaceae - ps29
Camelineae - ps30
Arabidopsis - ps31
Arabidopsis_thaliana - ps32
p-value
3e-07
5e-06
7e-05
9e-04
1e-02
Log-odds
0.5
1.0
1.5
2.0
2.5
3.0
3.5
4.0

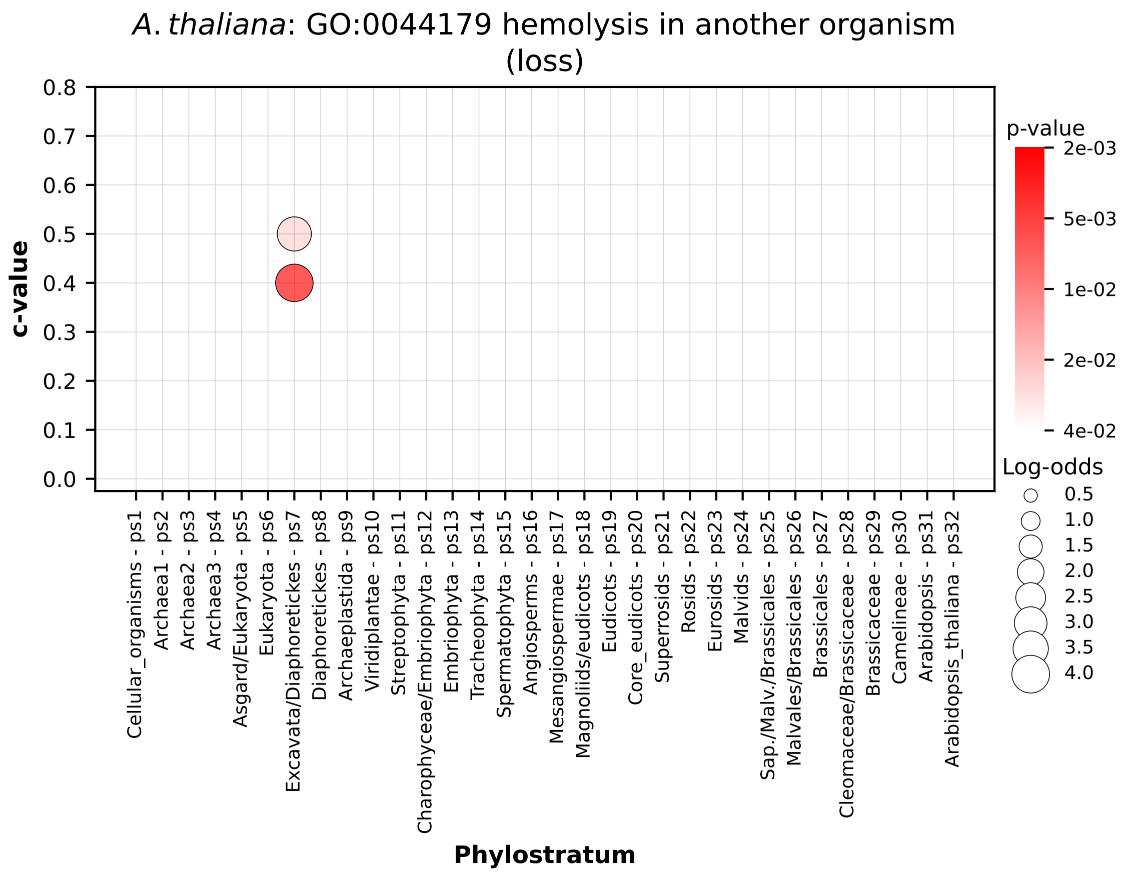
A. thaliana: GO:0044179 hemolysis in another organism (loss)
c-value
0.0
0.1
0.2
0.3
0.4
0.5
0.6
0.7
0.8
Phylostratum
Cellular_organisms - ps1
Archaea1 - ps2
Archaea2 - ps3
Archaea3 - ps4
Asgard/Eukaryota - ps5
Eukaryota - ps6
Excavata/Diaphoretickes - ps7
Diaphoretickes - ps8
Archaeplastida - ps9
Viridiplantae - ps10
Streptophyta - ps11
Charophyceae/Embriophyta - ps12
Embriophyta - ps13
Tracheophyta - ps14
Spermatophyta - ps15
Angiosperms - ps16
Mesangiospermae - ps17
Magnoliids/eudicots - ps18
Eudicots - ps19
Core_eudicots - ps20
Superrosids - ps21
Rosids - ps22
Eurosids - ps23
Malvids - ps24
Sap./Malv./Brassicales - ps25
Malvales/Brassicales - ps26
Brassicales - ps27
Cleomaceae/Brassicaceae - ps28
Brassicaceae - ps29
Camelineae - ps30
Arabidopsis - ps31
Arabidopsis_thaliana - ps32
p-value
2e-03
5e-03
1e-02
2e-02
4e-02
Log-odds
0.5
1.0
1.5
2.0
2.5
3.0
3.5
4.0

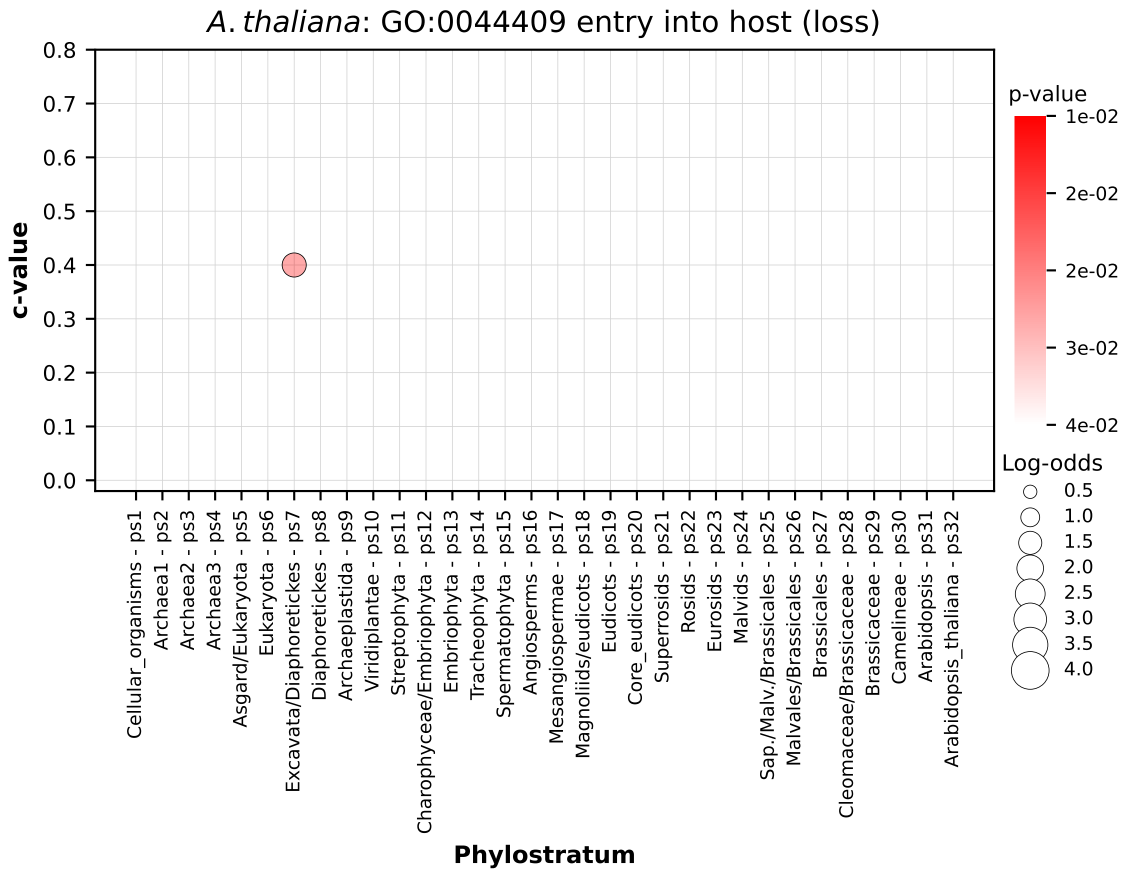
A. thaliana: GO:0044409 entry into host (loss)
c-value
0.8
0.7
0.6
0.5
0.4
0.3
0.2
0.1
0.0
Phylostratum
Cellular_organisms - ps1
Archaea1 - ps2
Archaea2 - ps3
Archaea3 - ps4
Asgard/Eukaryota - ps5
Eukaryota - ps6
Excavata/Diaphoretickes - ps7
Diaphoretickes - ps8
Archaeplastida - ps9
Viridiplantae - ps10
Streptophyta - ps11
Charophyceae/Embriophyta - ps12
Embriophyta - ps13
Tracheophyta - ps14
Spermatophyta - ps15
Angiosperms - ps16
Mesangiospermae - ps17
Magnoliids/eudicots - ps18
Eudicots - ps19
Core_eudicots - ps20
Superrosids - ps21
Rosids - ps22
Eurosids - ps23
Malvids - ps24
Sap./Malv./Brassicales - ps25
Malvales/Brassicales - ps26
Brassicales - ps27
Cleomaceae/Brassicaceae - ps28
Brassicaceae - ps29
Camelineae - ps30
Arabidopsis - ps31
Arabidopsis_thaliana - ps32
p-value
1e-02
2e-02
2e-02
3e-02
4e-02
Log-odds
0.5
1.0
1.5
2.0
2.5
3.0
3.5
4.0

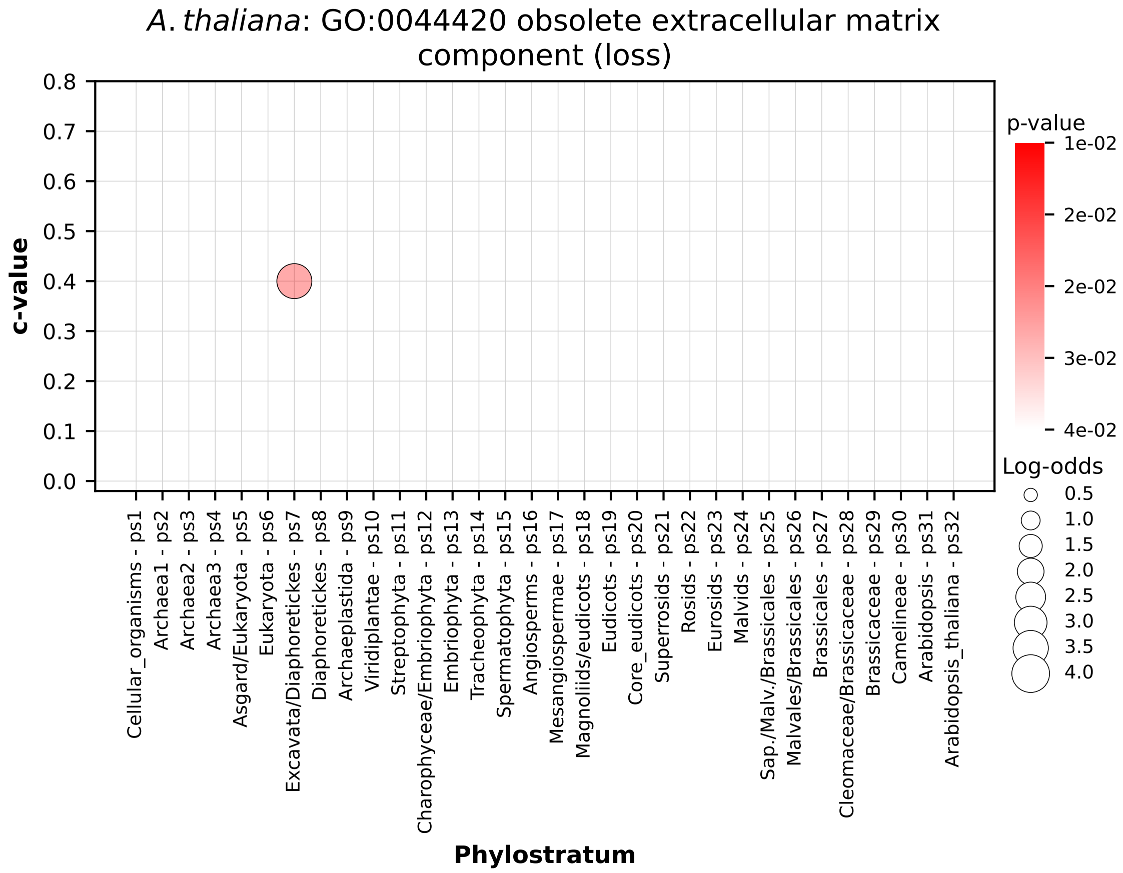

A. thaliana: GO:0044420 obsolete extracellular matrix component (loss)
c-value
0.8
0.7
0.6
0.5
0.4
0.3
0.2
0.1
0.0
Phylostratum
Cellular_organisms - ps1
Archaea1 - ps2
Archaea2 - ps3
Archaea3 - ps4
Asgard/Eukaryota - ps5
Eukaryota - ps6
Excavata/Diaphoretickes - ps7
Diaphoretickes - ps8
Archaeplastida - ps9
Viridiplantae - ps10
Streptophyta - ps11
Charophyceae/Embriophyta - ps12
Embriophyta - ps13
Tracheophyta - ps14
Spermatophyta - ps15
Angiosperms - ps16
Mesangiospermae - ps17
Magnoliids/eudicots - ps18
Eudicots - ps19
Core_eudicots - ps20
Superrosids - ps21
Rosids - ps22
Eurosids - ps23
Malvids - ps24
Sap./Malv./Brassicales - ps25
Malvales/Brassicales - ps26
Brassicales - ps27
Cleomaceae/Brassicaceae - ps28
Brassicaceae - ps29
Camelineae - ps30
Arabidopsis - ps31
Arabidopsis_thaliana - ps32
p-value
1e-02
2e-02
2e-02
3e-02
4e-02
Log-odds
0.5
1.0
1.5
2.0
2.5
3.0
3.5
4.0

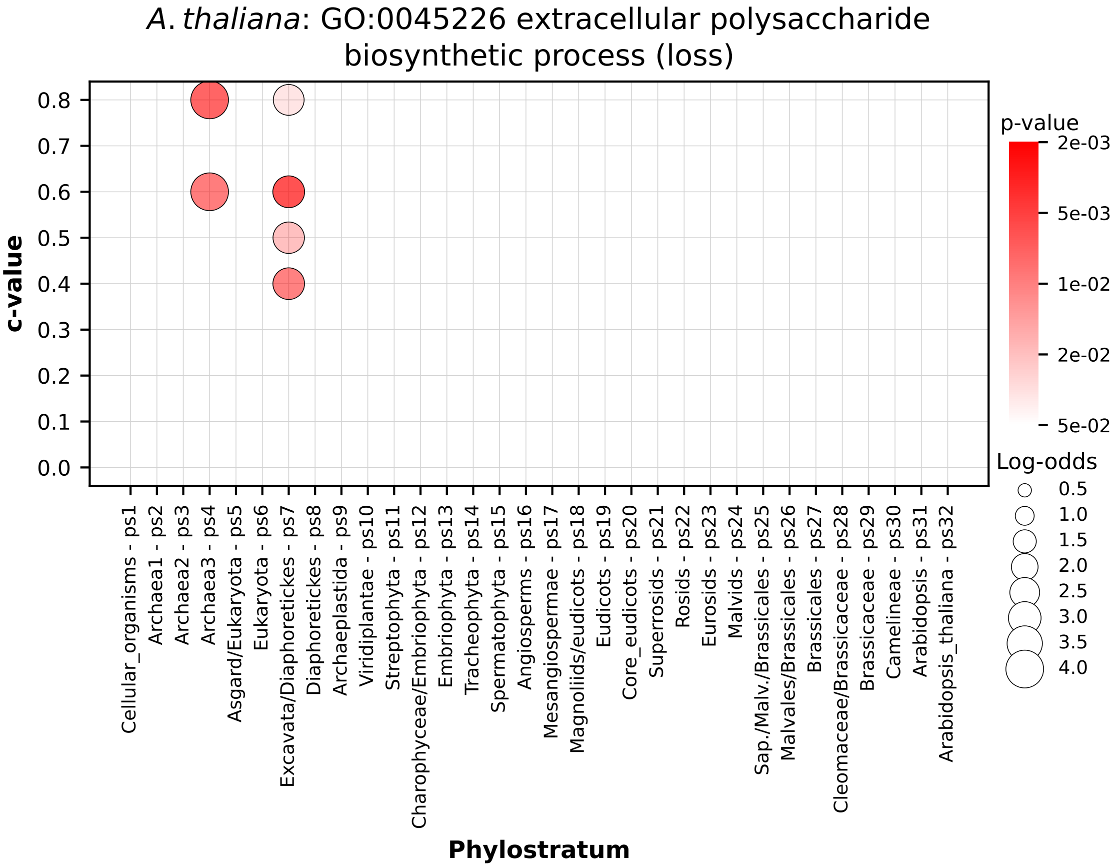

A. thaliana: GO:0045226 extracellular polysaccharide biosynthetic process (loss)
c-value
0.8
0.7
0.6
0.5
0.4
0.3
0.2
0.1
0.0
p-value
2e-03
5e-03
1e-02
2e-02
5e-02
Log-odds
0.5
1.0
1.5
2.0
2.5
3.0
3.5
4.0
Cellular_organisms - ps1
Archaea1 - ps2
Archaea2 - ps3
Archaea3 - ps4
Asgard/Eukaryota - ps5
Eukaryota - ps6
Excavata/Diaphoretickes - ps7
Diaphoretickes - ps8
Archaeplastida - ps9
Viridiplantae - ps10
Streptophyta - ps11
Charophyceae/Embriophyta - ps12
Embriophyta - ps13
Tracheophyta - ps14
Spermatophyta - ps15
Angiosperms - ps16
Mesangiospermae - ps17
Magnoliids/eudicots - ps18
Eudicots - ps19
Core_eudicots - ps20
Superrosids - ps21
Rosids - ps22
Eurosids - ps23
Malvids - ps24
Sap./Malv./Brassicales - ps25
Malvales/Brassicales - ps26
Brassicales - ps27
Cleomaceae/Brassicaceae - ps28
Brassicaceae - ps29
Camelineae - ps30
Arabidopsis - ps31
Arabidopsis_thaliana - ps32
Phylostratum

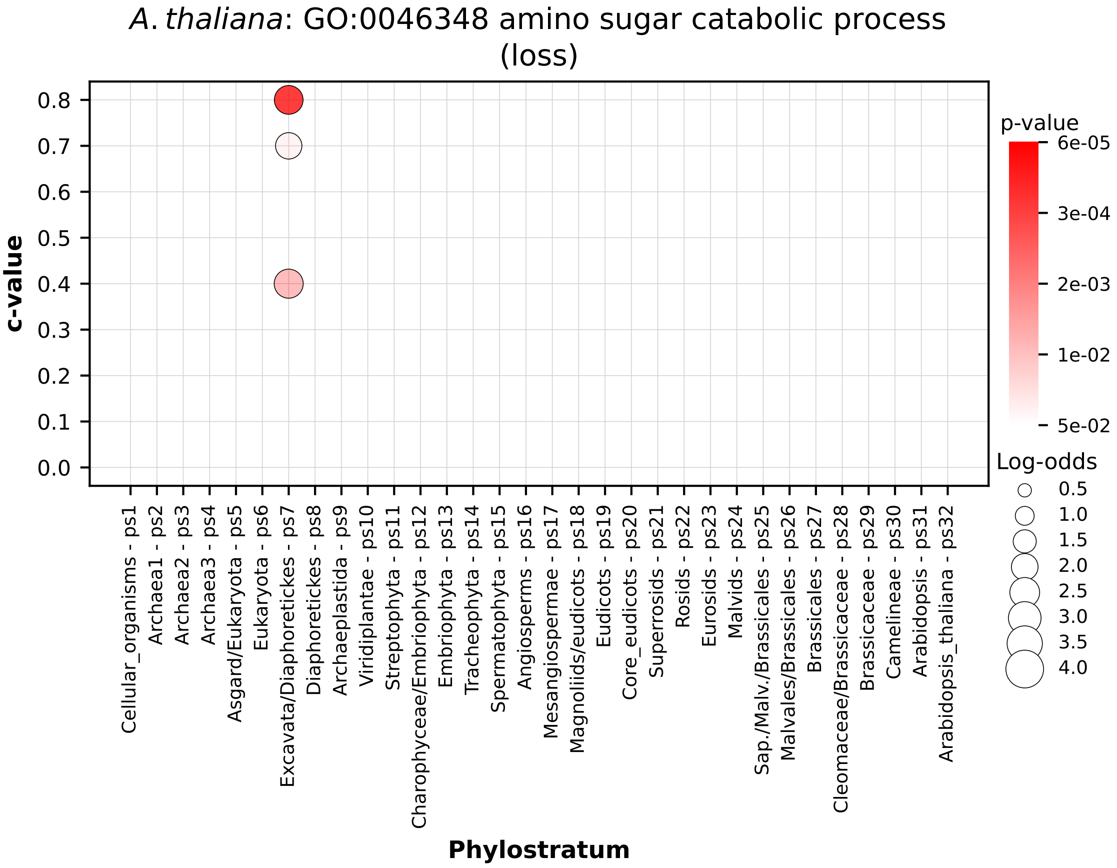
A. thaliana: GO:0046348 amino sugar catabolic process
(loss)
c-value
0.8
0.7
0.6
0.5
0.4
0.3
0.2
0.1
0.0
Phylostratum
Cellular_organisms - ps1
Archaea1 - ps2
Archaea2 - ps3
Archaea3 - ps4
Asgard/Eukaryota - ps5
Eukaryota - ps6
Excavata/Diaphoretickes - ps7
Diaphoretickes - ps8
Archaeplastida - ps9
Viridiplantae - ps10
Streptophyta - ps11
Charophyceae/Embriophyta - ps12
Embriophyta - ps13
Tracheophyta - ps14
Spermatophyta - ps15
Angiosperms - ps16
Mesangiospermae - ps17
Magnoliids/eudicots - ps18
Eudicots - ps19
Core_eudicots - ps20
Superrosids - ps21
Rosids - ps22
Eurosids - ps23
Malvids - ps24
Sap./Malv./Brassicales - ps25
Malvales/Brassicales - ps26
Brassicales - ps27
Cleomaceae/Brassicaceae - ps28
Brassicaceae - ps29
Camelineae - ps30
Arabidopsis - ps31
Arabidopsis_thaliana - ps32
p-value
6e-05
3e-04
2e-03
1e-02
5e-02
Log-odds
0.5
1.0
1.5
2.0
2.5
3.0
3.5
4.0

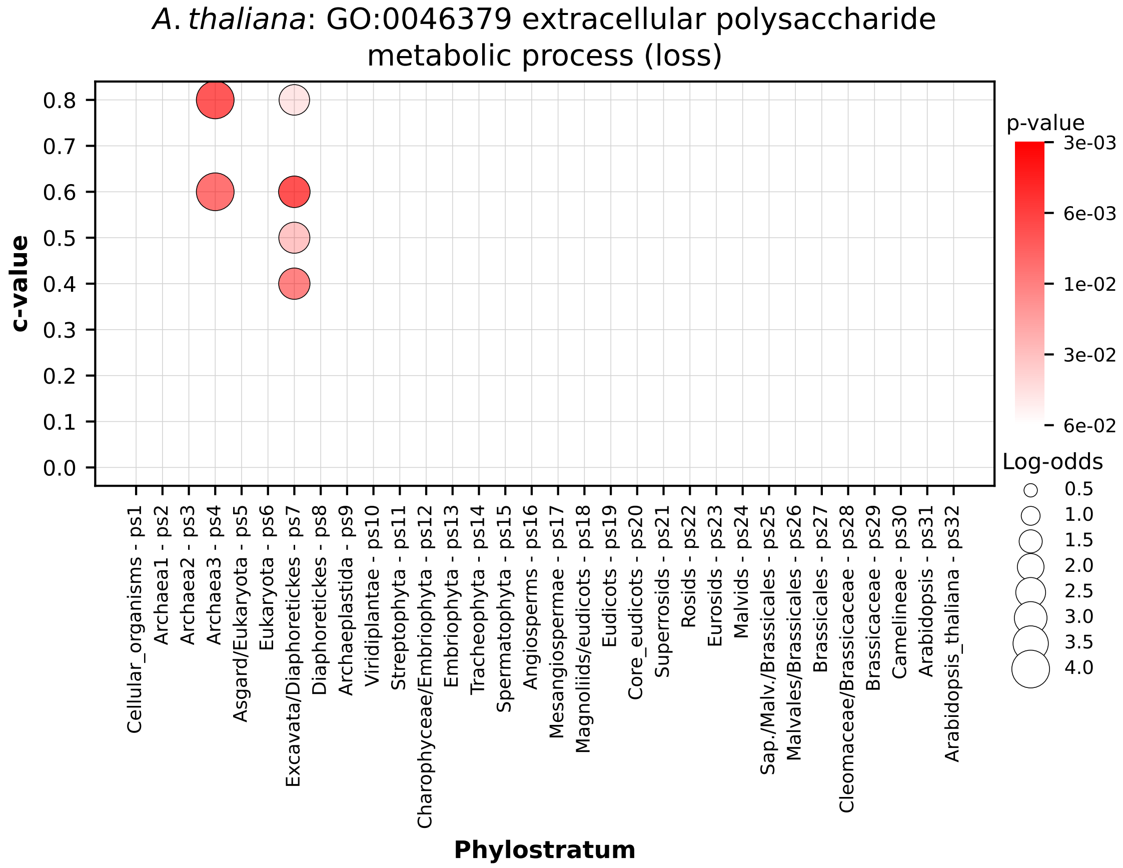

A. thaliana: GO:0046379 extracellular polysaccharide metabolic process (loss)
c-value
0.0
0.1
0.2
0.3
0.4
0.5
0.6
0.7
0.8
p-value
3e-03
6e-03
1e-02
3e-02
6e-02
Log-odds
0.5
1.0
1.5
2.0
2.5
3.0
3.5
4.0
Cellular_organisms - ps1
Archaea1 - ps2
Archaea2 - ps3
Archaea3 - ps4
Asgard/Eukaryota - ps5
Eukaryota - ps6
Excavata/Diaphoretickes - ps7
Diaphoretickes - ps8
Archaeplastida - ps9
Viridiplantae - ps10
Streptophyta - ps11
Charophyceae/Embriophyta - ps12
Embriophyta - ps13
Tracheophyta - ps14
Spermatophyta - ps15
Angiosperms - ps16
Mesangiospermae - ps17
Magnoliids/eudicots - ps18
Eudicots - ps19
Core_eudicots - ps20
Superrosids - ps21
Rosids - ps22
Eurosids - ps23
Malvids - ps24
Sap./Malv./Brassicales - ps25
Malvales/Brassicales - ps26
Brassicales - ps27
Cleomaceae/Brassicaceae - ps28
Brassicaceae - ps29
Camelineae - ps30
Arabidopsis - ps31
Arabidopsis_thaliana - ps32
Phylostratum

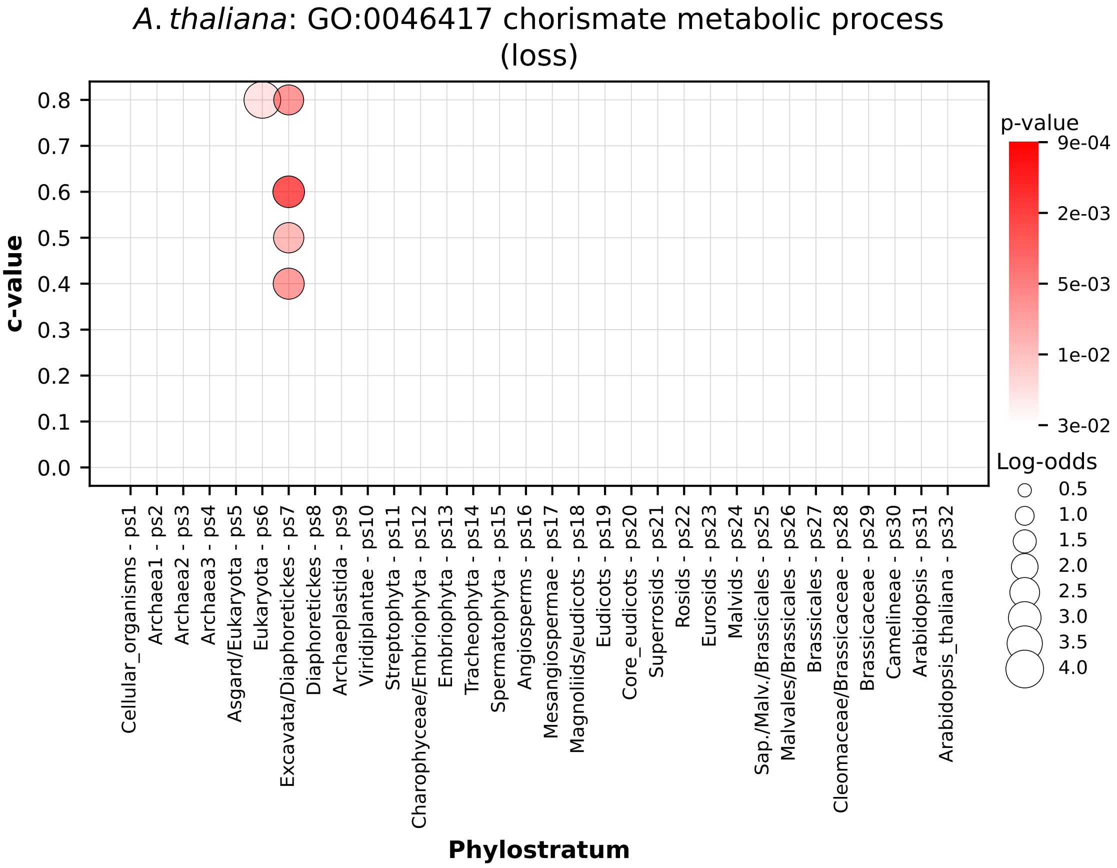
A. thaliana: GO:0046417 chorismate metabolic process
(loss)
c-value
0.8
0.7
0.6
0.5
0.4
0.3
0.2
0.1
0.0
p-value
9e-04
2e-03
5e-03
1e-02
3e-02
Log-odds
0.5
1.0
1.5
2.0
2.5
3.0
3.5
4.0
Cellular_organisms - ps1
Archaea1 - ps2
Archaea2 - ps3
Archaea3 - ps4
Asgard/Eukaryota - ps5
Eukaryota - ps6
Excavata/Diaphoretickes - ps7
Diaphoretickes - ps8
Archaeplastida - ps9
Viridiplantae - ps10
Streptophyta - ps11
Charophyceae/Embriophyta - ps12
Embriophyta - ps13
Tracheophyta - ps14
Spermatophyta - ps15
Angiosperms - ps16
Mesangiospermae - ps17
Magnoliids/eudicots - ps18
Eudicots - ps19
Core_eudicots - ps20
Superrosids - ps21
Rosids - ps22
Eurosids - ps23
Malvids - ps24
Sap./Malv./Brassicales - ps25
Malvales/Brassicales - ps26
Brassicales - ps27
Cleomaceae/Brassicaceae - ps28
Brassicaceae - ps29
Camelineae - ps30
Arabidopsis - ps31
Arabidopsis_thaliana - ps32
Phylostratum

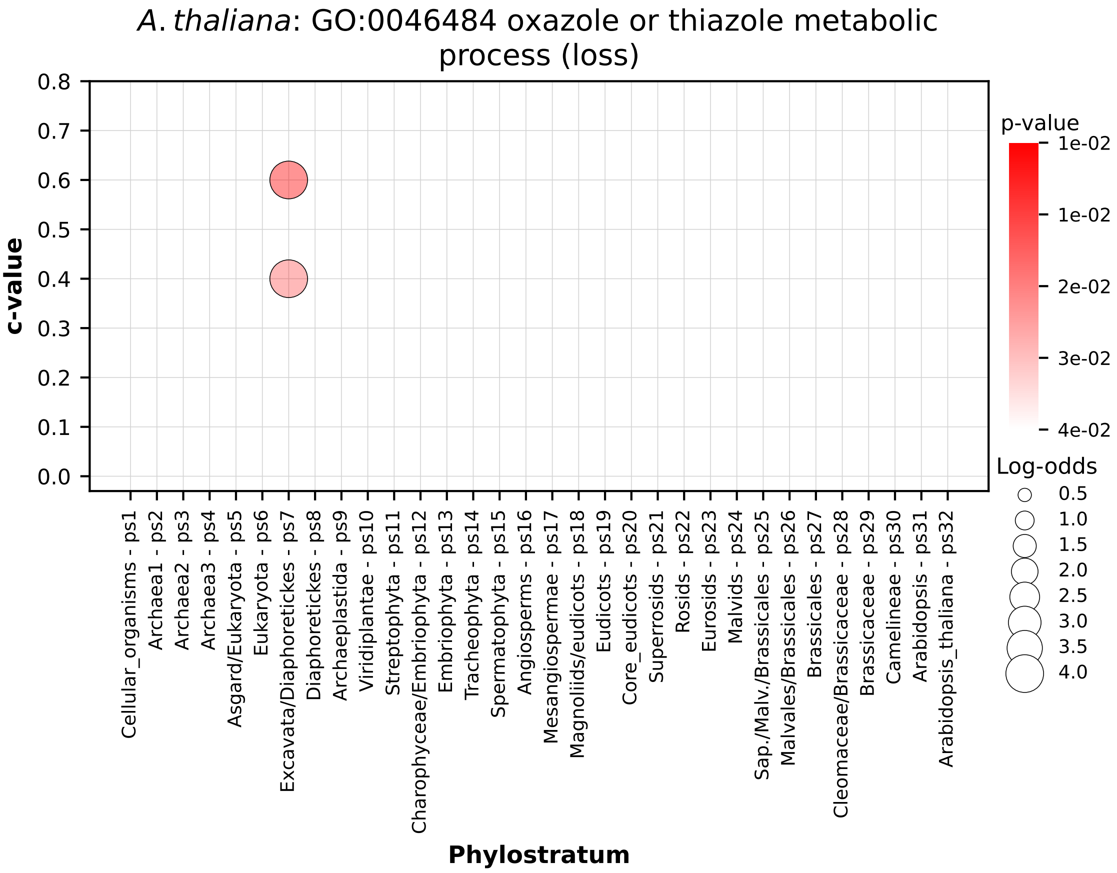

A. thaliana: GO:0046484 oxazole or thiazole metabolic process (loss)
c-value
0.8
0.7
0.6
0.5
0.4
0.3
0.2
0.1
0.0
Phylostratum
Cellular_organisms - ps1
Archaea1 - ps2
Archaea2 - ps3
Archaea3 - ps4
Asgard/Eukaryota - ps5
Eukaryota - ps6
Excavata/Diaphoretickes - ps7
Diaphoretickes - ps8
Archaeplastida - ps9
Viridiplantae - ps10
Streptophyta - ps11
Charophyceae/Embriophyta - ps12
Embriophyta - ps13
Tracheophyta - ps14
Spermatophyta - ps15
Angiosperms - ps16
Mesangiospermae - ps17
Magnoliids/eudicots - ps18
Eudicots - ps19
Core_eudicots - ps20
Superrosids - ps21
Rosids - ps22
Eurosids - ps23
Malvids - ps24
Sap./Malv./Brassicales - ps25
Malvales/Brassicales - ps26
Brassicales - ps27
Cleomaceae/Brassicaceae - ps28
Brassicaceae - ps29
Camelineae - ps30
Arabidopsis - ps31
Arabidopsis_thaliana - ps32
p-value
1e-02
1e-02
2e-02
3e-02
4e-02
Log-odds
0.5
1.0
1.5
2.0
2.5
3.0
3.5
4.0

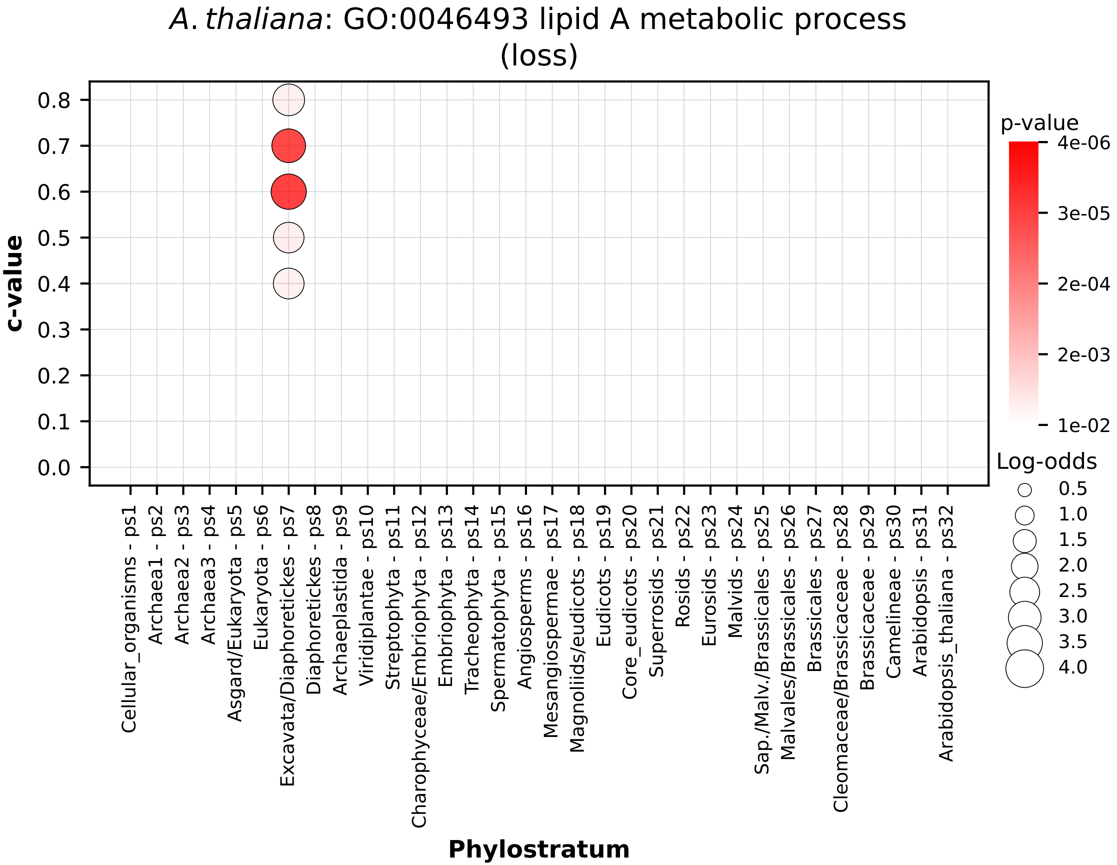

A. thaliana: GO:0046493 lipid A metabolic process
(loss)
c-value
0.8
0.7
0.6
0.5
0.4
0.3
0.2
0.1
0.0
p-value
4e-06
3e-05
2e-04
2e-03
1e-02
Log-odds
0.5
1.0
1.5
2.0
2.5
3.0
3.5
4.0
Cellular_organisms - ps1
Archaea1 - ps2
Archaea2 - ps3
Archaea3 - ps4
Asgard/Eukaryota - ps5
Eukaryota - ps6
Excavata/Diaphoretickes - ps7
Diaphoretickes - ps8
Archaeplastida - ps9
Viridiplantae - ps10
Streptophyta - ps11
Charophyceae/Embriophyta - ps12
Embriophyta - ps13
Tracheophyta - ps14
Spermatophyta - ps15
Angiosperms - ps16
Mesangiospermae - ps17
Magnoliids/eudicots - ps18
Eudicots - ps19
Core_eudicots - ps20
Superrosids - ps21
Rosids - ps22
Eurosids - ps23
Malvids - ps24
Sap./Malv./Brassicales - ps25
Malvales/Brassicales - ps26
Brassicales - ps27
Cleomaceae/Brassicaceae - ps28
Brassicaceae - ps29
Camelineae - ps30
Arabidopsis - ps31
Arabidopsis_thaliana - ps32
Phylostratum

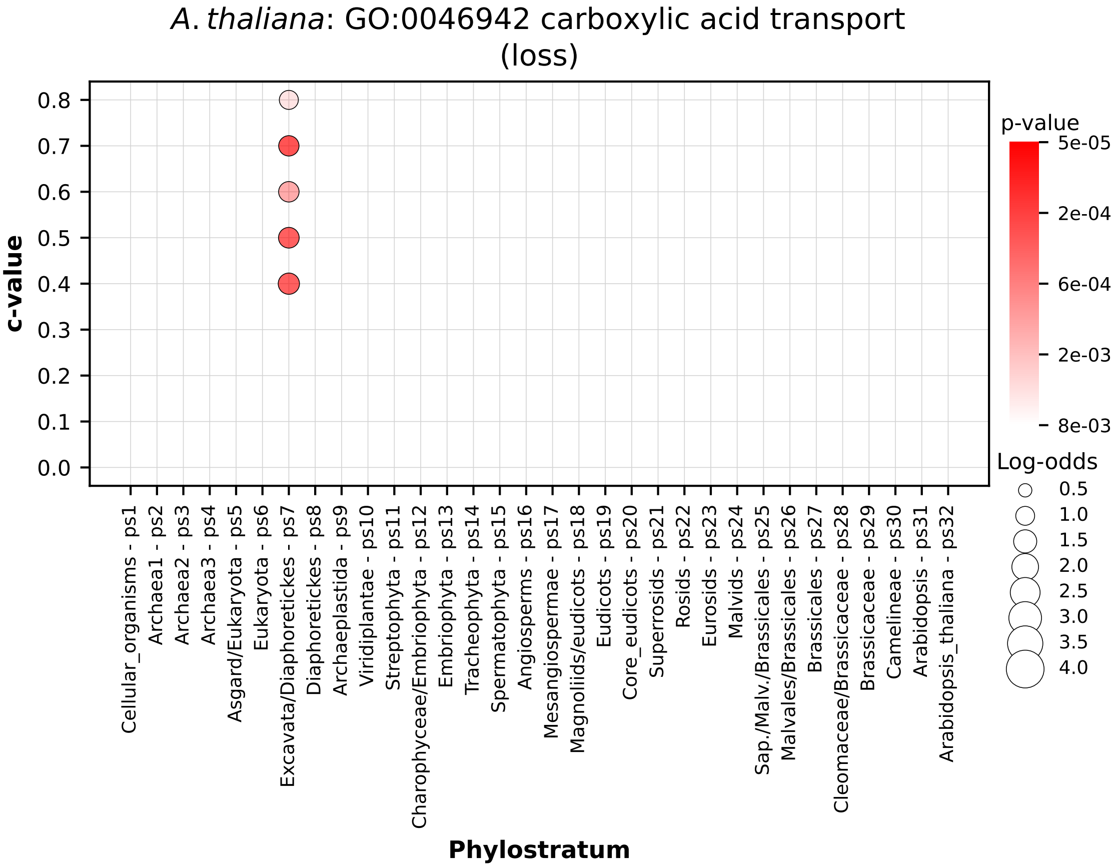

A. thaliana: GO:0046942 carboxylic acid transport
(loss)
c-value
0.8
0.7
0.6
0.5
0.4
0.3
0.2
0.1
0.0
p-value
5e-05
2e-04
6e-04
2e-03
8e-03
Log-odds
0.5
1.0
1.5
2.0
2.5
3.0
3.5
4.0
Cellular_organisms - ps1
Archaea1 - ps2
Archaea2 - ps3
Archaea3 - ps4
Asgard/Eukaryota - ps5
Eukaryota - ps6
Excavata/Diaphoretickes - ps7
Diaphoretickes - ps8
Archaeplastida - ps9
Viridiplantae - ps10
Streptophyta - ps11
Charophyceae/Embriophyta - ps12
Embriophyta - ps13
Tracheophyta - ps14
Spermatophyta - ps15
Angiosperms - ps16
Mesangiospermae - ps17
Magnoliids/eudicots - ps18
Eudicots - ps19
Core_eudicots - ps20
Superrosids - ps21
Rosids - ps22
Eurosids - ps23
Malvids - ps24
Sap./Malv./Brassicales - ps25
Malvales/Brassicales - ps26
Brassicales - ps27
Cleomaceae/Brassicaceae - ps28
Brassicaceae - ps29
Camelineae - ps30
Arabidopsis - ps31
Arabidopsis_thaliana - ps32
Phylostratum

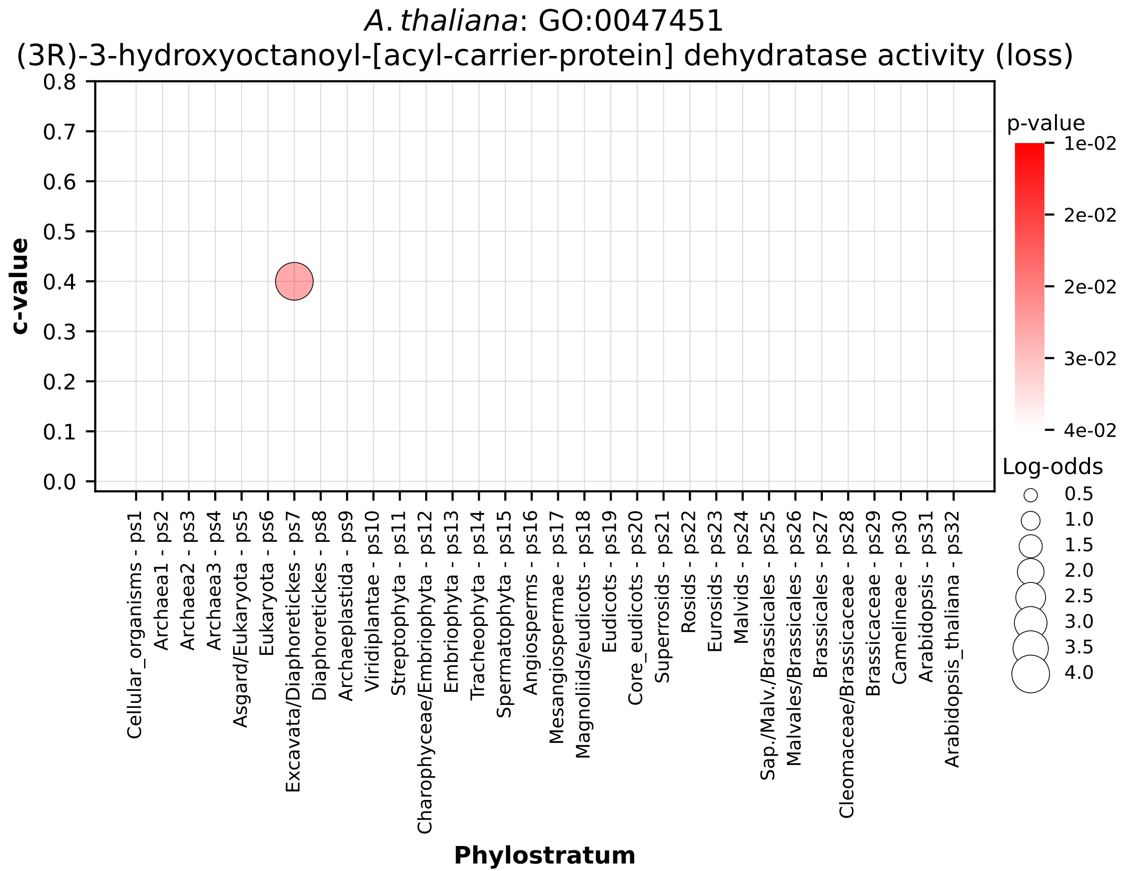

A. thaliana: GO:0047451
(3R)-3-hydroxyoctanoyl-[acyl-carrier-protein] dehydratase activity (loss)
c-value
0.0
0.1
0.2
0.3
0.4
0.5
0.6
0.7
0.8
p-value
1e-02
2e-02
2e-02
3e-02
4e-02
Log-odds
0.5
1.0
1.5
2.0
2.5
3.0
3.5
4.0
Cellular_organisms - ps1
Archaea1 - ps2
Archaea2 - ps3
Archaea3 - ps4
Asgard/Eukaryota - ps5
Eukaryota - ps6
Excavata/Diaphoretickes - ps7
Diaphoretickes - ps8
Archaeplastida - ps9
Viridiplantae - ps10
Streptophyta - ps11
Charophyceae/Embriophyta - ps12
Embriophyta - ps13
Tracheophyta - ps14
Spermatophyta - ps15
Angiosperms - ps16
Mesangiospermae - ps17
Magnoliids/eudicots - ps18
Eudicots - ps19
Core_eudicots - ps20
Superrosids - ps21
Rosids - ps22
Eurosids - ps23
Malvids - ps24
Sap./Malv./Brassicales - ps25
Malvales/Brassicales - ps26
Brassicales - ps27
Cleomaceae/Brassicaceae - ps28
Brassicaceae - ps29
Camelineae - ps30
Arabidopsis - ps31
Arabidopsis_thaliana - ps32
Phylostratum

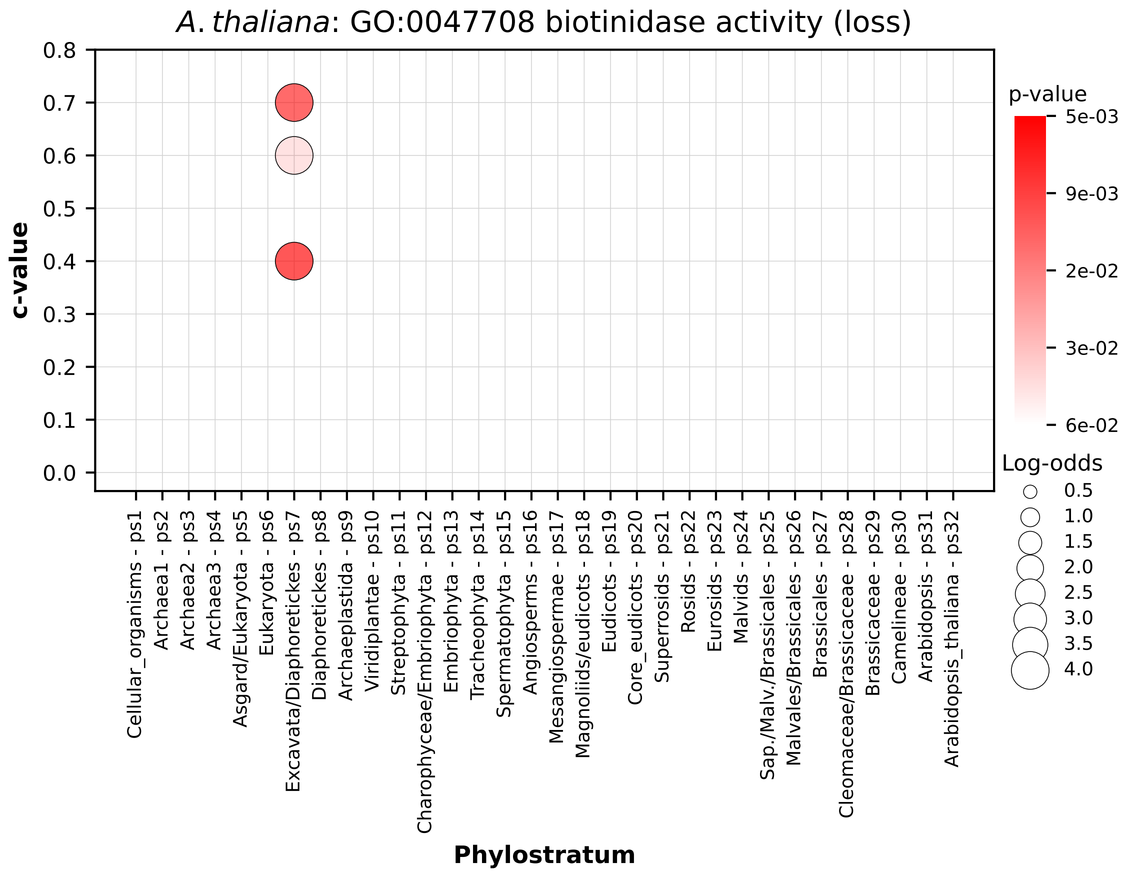
A. thaliana: GO:0047708 biotinidase activity (loss)
c-value
0.8
0.7
0.6
0.5
0.4
0.3
0.2
0.1
0.0
p-value
5e-03
9e-03
2e-02
3e-02
6e-02
Log-odds
0.5
1.0
1.5
2.0
2.5
3.0
3.5
4.0
Cellular_organisms - ps1
Archaea1 - ps2
Archaea2 - ps3
Archaea3 - ps4
Asgard/Eukaryota - ps5
Eukaryota - ps6
Excavata/Diaphoretickes - ps7
Diaphoretickes - ps8
Archaeplastida - ps9
Viridiplantae - ps10
Streptophyta - ps11
Charophyceae/Embriophyta - ps12
Embriophyta - ps13
Tracheophyta - ps14
Spermatophyta - ps15
Angiosperms - ps16
Mesangiospermae - ps17
Magnoliids/eudicots - ps18
Eudicots - ps19
Core_eudicots - ps20
Superrosids - ps21
Rosids - ps22
Eurosids - ps23
Malvids - ps24
Sap./Malv./Brassicales - ps25
Malvales/Brassicales - ps26
Brassicales - ps27
Cleomaceae/Brassicaceae - ps28
Brassicaceae - ps29
Camelineae - ps30
Arabidopsis - ps31
Arabidopsis_thaliana - ps32
Phylostratum

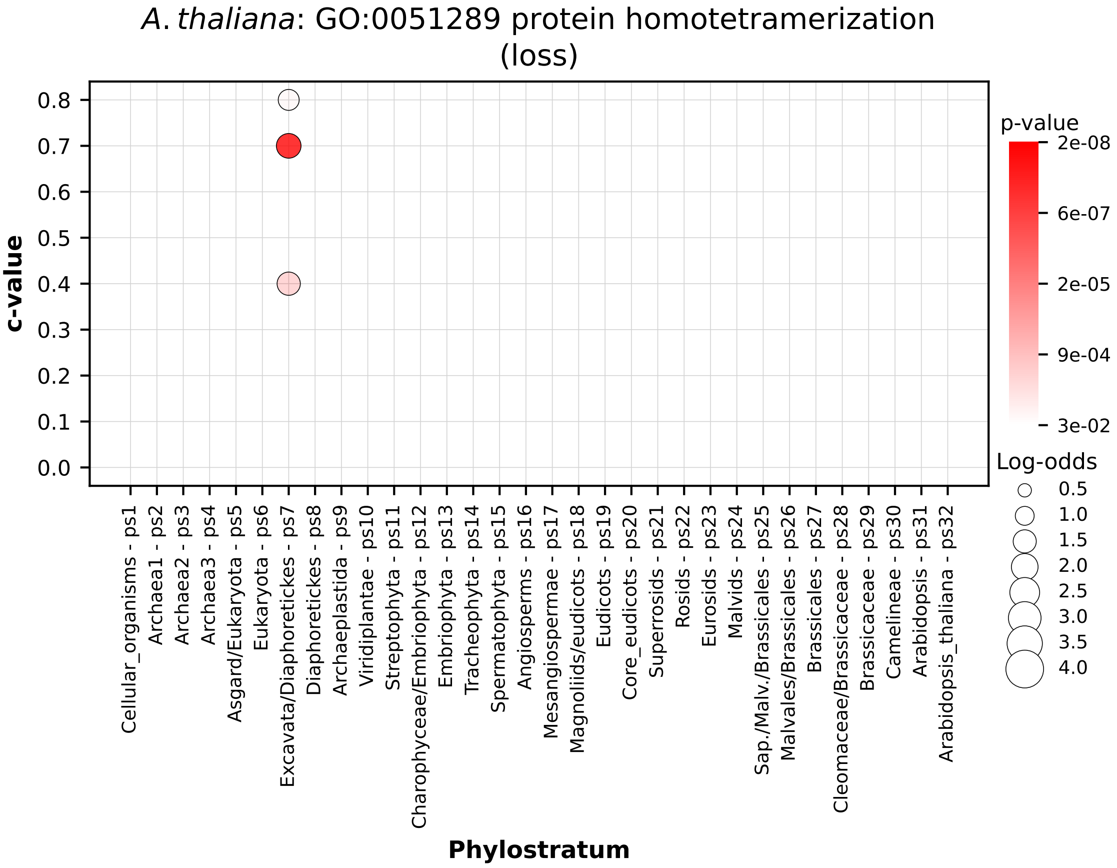

A. thaliana: GO:0051289 protein homotetramerization
(loss)
c-value
0.8
0.7
0.6
0.5
0.4
0.3
0.2
0.1
0.0
p-value
2e-08
6e-07
2e-05
9e-04
3e-02
Log-odds
0.5
1.0
1.5
2.0
2.5
3.0
3.5
4.0
Cellular_organisms - ps1
Archaea1 - ps2
Archaea2 - ps3
Archaea3 - ps4
Asgard/Eukaryota - ps5
Eukaryota - ps6
Excavata/Diaphoretickes - ps7
Diaphoretickes - ps8
Archaeplastida - ps9
Viridiplantae - ps10
Streptophyta - ps11
Charophyceae/Embriophyta - ps12
Embriophyta - ps13
Tracheophyta - ps14
Spermatophyta - ps15
Angiosperms - ps16
Mesangiospermae - ps17
Magnoliids/eudicots - ps18
Eudicots - ps19
Core_eudicots - ps20
Superrosids - ps21
Rosids - ps22
Eurosids - ps23
Malvids - ps24
Sap./Malv./Brassicales - ps25
Malvales/Brassicales - ps26
Brassicales - ps27
Cleomaceae/Brassicaceae - ps28
Brassicaceae - ps29
Camelineae - ps30
Arabidopsis - ps31
Arabidopsis_thaliana - ps32
Phylostratum

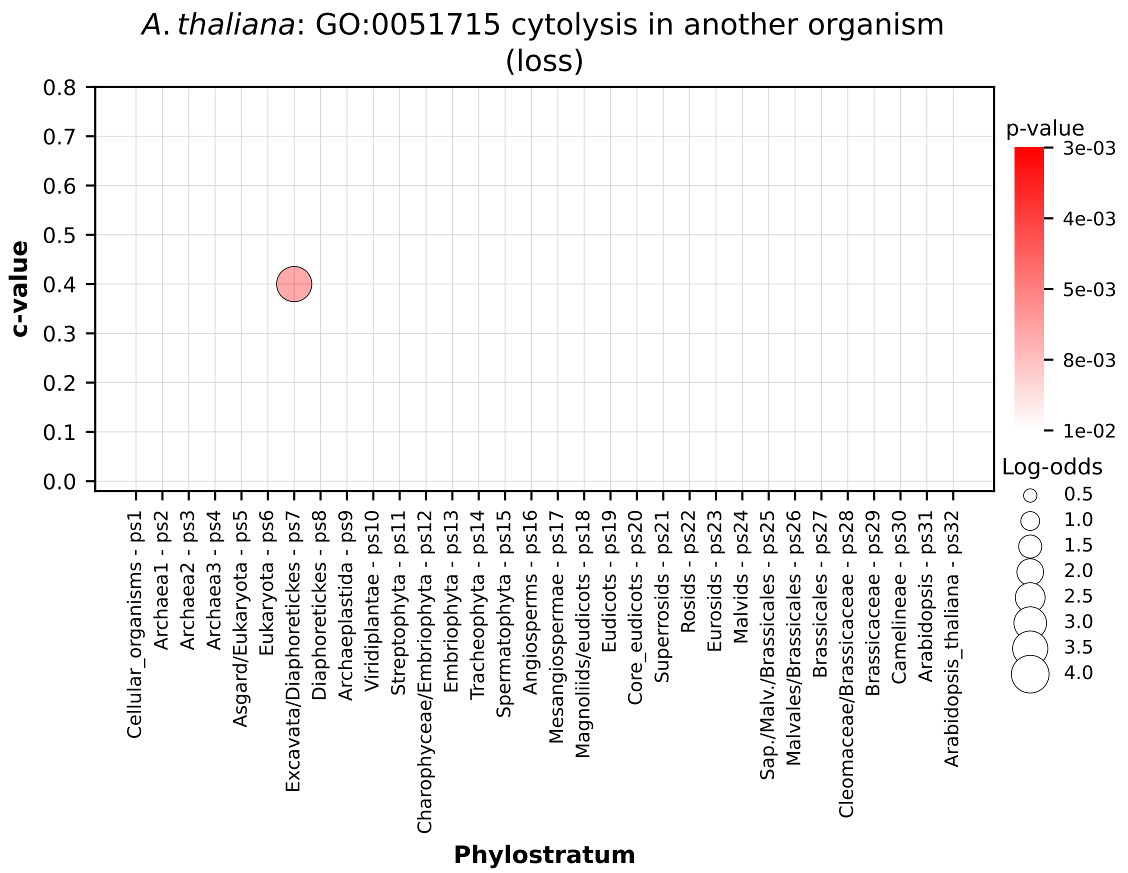

A. thaliana: GO:0051715 cytolysis in another organism (loss)
c-value
0.8
0.7
0.6
0.5
0.4
0.3
0.2
0.1
0.0
p-value
3e-03
4e-03
5e-03
8e-03
1e-02
Log-odds
0.5
1.0
1.5
2.0
2.5
3.0
3.5
4.0
Cellular_organisms - ps1
Archaea1 - ps2
Archaea2 - ps3
Archaea3 - ps4
Asgard/Eukaryota - ps5
Eukaryota - ps6
Excavata/Diaphoretickes - ps7
Diaphoretickes - ps8
Archaeplastida - ps9
Viridiplantae - ps10
Streptophyta - ps11
Charophyceae/Embriophyta - ps12
Embriophyta - ps13
Tracheophyta - ps14
Spermatophyta - ps15
Angiosperms - ps16
Mesangiospermae - ps17
Magnoliids/eudicots - ps18
Eudicots - ps19
Core_eudicots - ps20
Superrosids - ps21
Rosids - ps22
Eurosids - ps23
Malvids - ps24
Sap./Malv./Brassicales - ps25
Malvales/Brassicales - ps26
Brassicales - ps27
Cleomaceae/Brassicaceae - ps28
Brassicaceae - ps29
Camelineae - ps30
Arabidopsis - ps31
Arabidopsis_thaliana - ps32
Phylostratum

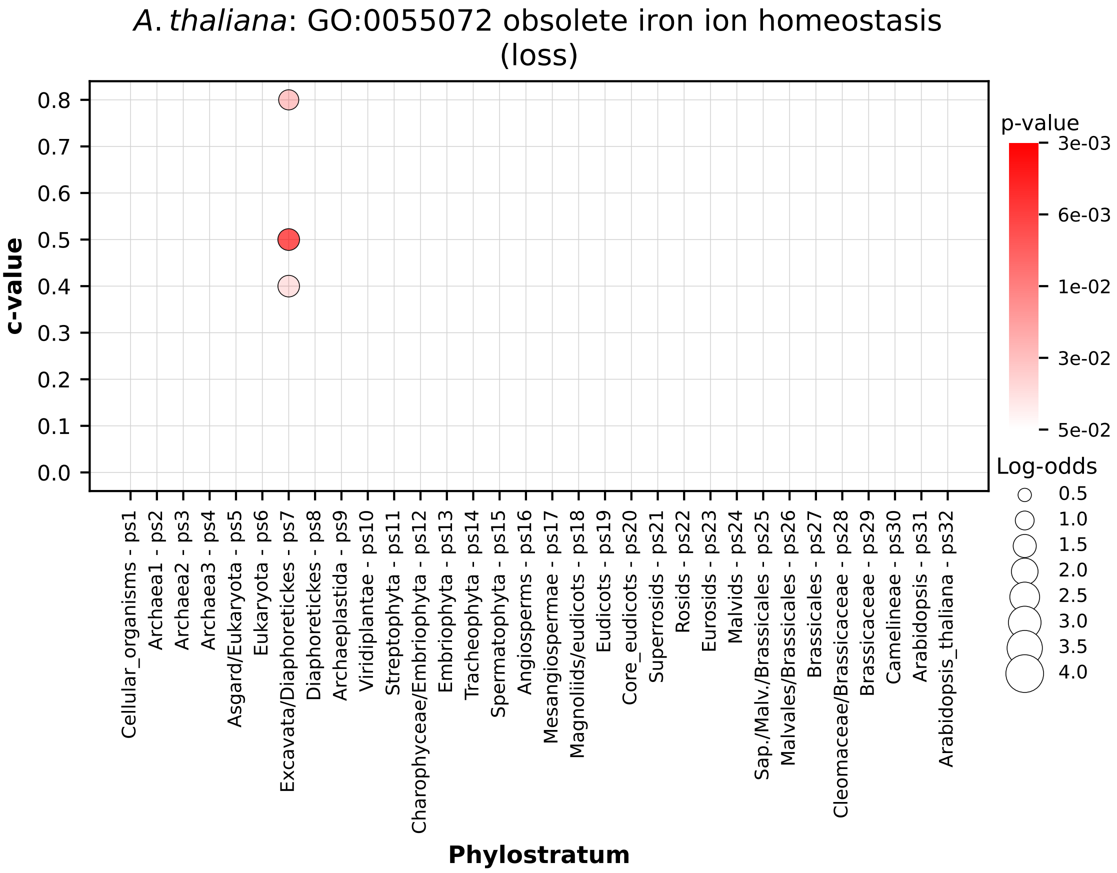

A. thaliana: GO:0055072 obsolete iron ion homeostasis
(loss)
c-value
0.8
0.7
0.6
0.5
0.4
0.3
0.2
0.1
0.0
p-value
3e-03
6e-03
1e-02
3e-02
5e-02
Log-odds
0.5
1.0
1.5
2.0
2.5
3.0
3.5
4.0
Cellular_organisms - ps1
Archaea1 - ps2
Archaea2 - ps3
Archaea3 - ps4
Asgard/Eukaryota - ps5
Eukaryota - ps6
Excavata/Diaphoretickes - ps7
Diaphoretickes - ps8
Archaeplastida - ps9
Viridiplantae - ps10
Streptophyta - ps11
Charophyceae/Embriophyta - ps12
Embriophyta - ps13
Tracheophyta - ps14
Spermatophyta - ps15
Angiosperms - ps16
Mesangiospermae - ps17
Magnoliids/eudicots - ps18
Eudicots - ps19
Core_eudicots - ps20
Superrosids - ps21
Rosids - ps22
Eurosids - ps23
Malvids - ps24
Sap./Malv./Brassicales - ps25
Malvales/Brassicales - ps26
Brassicales - ps27
Cleomaceae/Brassicaceae - ps28
Brassicaceae - ps29
Camelineae - ps30
Arabidopsis - ps31
Arabidopsis_thaliana - ps32
Phylostratum

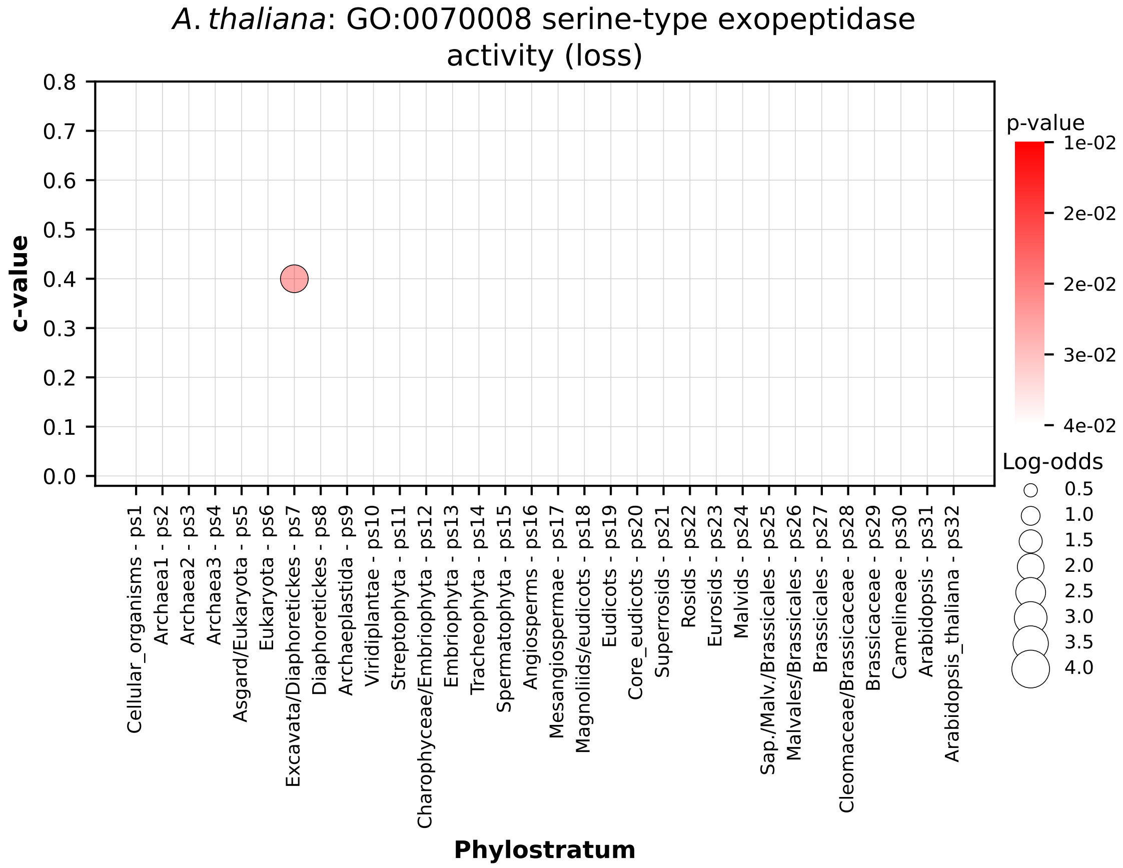
A. thaliana: GO:0070008 serine-type exopeptidase activity (loss)
c-value
0.8
0.7
0.6
0.5
0.4
0.3
0.2
0.1
0.0
Phylostratum
Cellular_organisms - ps1
Archaea1 - ps2
Archaea2 - ps3
Archaea3 - ps4
Asgard/Eukaryota - ps5
Eukaryota - ps6
Excavata/Diaphoretickes - ps7
Diaphoretickes - ps8
Archaeplastida - ps9
Viridiplantae - ps10
Streptophyta - ps11
Charophyceae/Embriophyta - ps12
Embriophyta - ps13
Tracheophyta - ps14
Spermatophyta - ps15
Angiosperms - ps16
Mesangiospermae - ps17
Magnoliids/eudicots - ps18
Eudicots - ps19
Core_eudicots - ps20
Superrosids - ps21
Rosids - ps22
Eurosids - ps23
Malvids - ps24
Sap./Malv./Brassicales - ps25
Malvales/Brassicales - ps26
Brassicales - ps27
Cleomaceae/Brassicaceae - ps28
Brassicaceae - ps29
Camelineae - ps30
Arabidopsis - ps31
Arabidopsis_thaliana - ps32
p-value
1e-02
2e-02
2e-02
3e-02
4e-02
Log-odds
0.5
1.0
1.5
2.0
2.5
3.0
3.5
4.0

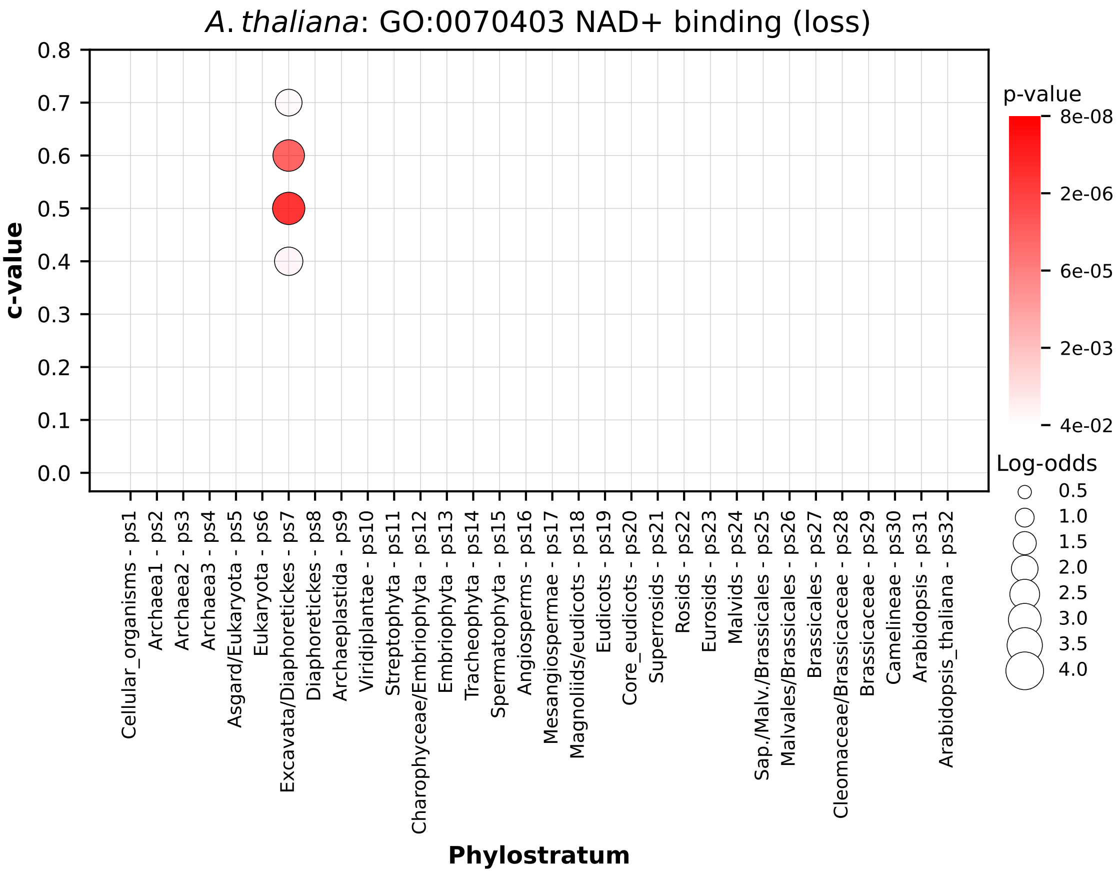
A. thaliana: GO:0070403 NAD+ binding (loss)
c-value
0.8
0.7
0.6
0.5
0.4
0.3
0.2
0.1
0.0
p-value
8e-08
2e-06
6e-05
2e-03
4e-02
Log-odds
0.5
1.0
1.5
2.0
2.5
3.0
3.5
4.0
Cellular_organisms - ps1
Archaea1 - ps2
Archaea2 - ps3
Archaea3 - ps4
Asgard/Eukaryota - ps5
Eukaryota - ps6
Excavata/Diaphoretickes - ps7
Diaphoretickes - ps8
Archaeplastida - ps9
Viridiplantae - ps10
Streptophyta - ps11
Charophyceae/Embriophyta - ps12
Embriophyta - ps13
Tracheophyta - ps14
Spermatophyta - ps15
Angiosperms - ps16
Mesangiospermae - ps17
Magnoliids/eudicots - ps18
Eudicots - ps19
Core_eudicots - ps20
Superrosids - ps21
Rosids - ps22
Eurosids - ps23
Malvids - ps24
Sap./Malv./Brassicales - ps25
Malvales/Brassicales - ps26
Brassicales - ps27
Cleomaceae/Brassicaceae - ps28
Brassicaceae - ps29
Camelineae - ps30
Arabidopsis - ps31
Arabidopsis_thaliana - ps32
Phylostratum

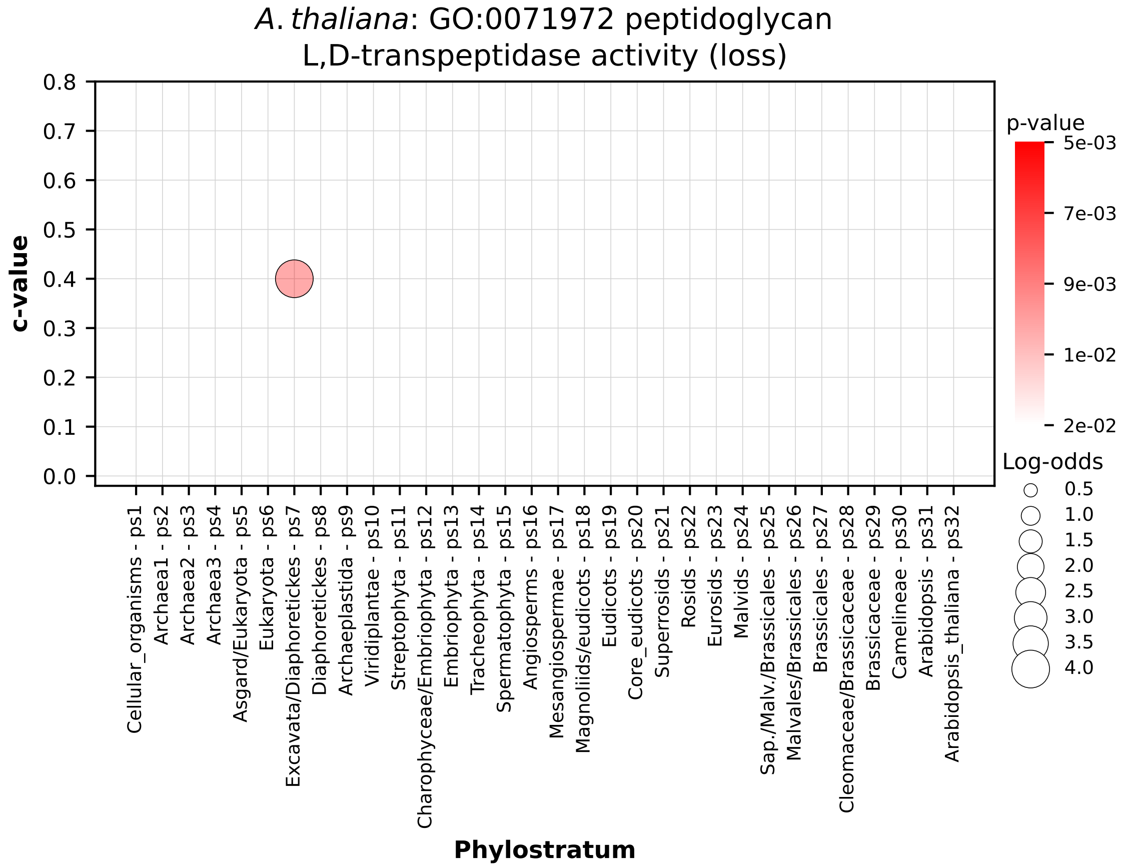

A. thaliana: GO:0071972 peptidoglycan
L,D-transpeptidase activity (loss)
c-value
0.8
0.7
0.6
0.5
0.4
0.3
0.2
0.1
0.0
p-value
5e-03
7e-03
9e-03
1e-02
2e-02
Log-odds
0.5
1.0
1.5
2.0
2.5
3.0
3.5
4.0
Cellular_organisms - ps1
Archaea1 - ps2
Archaea2 - ps3
Archaea3 - ps4
Asgard/Eukaryota - ps5
Eukaryota - ps6
Excavata/Diaphoretickes - ps7
Diaphoretickes - ps8
Archaeplastida - ps9
Viridiplantae - ps10
Streptophyta - ps11
Charophyceae/Embriophyta - ps12
Embriophyta - ps13
Tracheophyta - ps14
Spermatophyta - ps15
Angiosperms - ps16
Mesangiospermae - ps17
Magnoliids/eudicots - ps18
Eudicots - ps19
Core_eudicots - ps20
Superrosids - ps21
Rosids - ps22
Eurosids - ps23
Malvids - ps24
Sap./Malv./Brassicales - ps25
Malvales/Brassicales - ps26
Brassicales - ps27
Cleomaceae/Brassicaceae - ps28
Brassicaceae - ps29
Camelineae - ps30
Arabidopsis - ps31
Arabidopsis_thaliana - ps32
Phylostratum

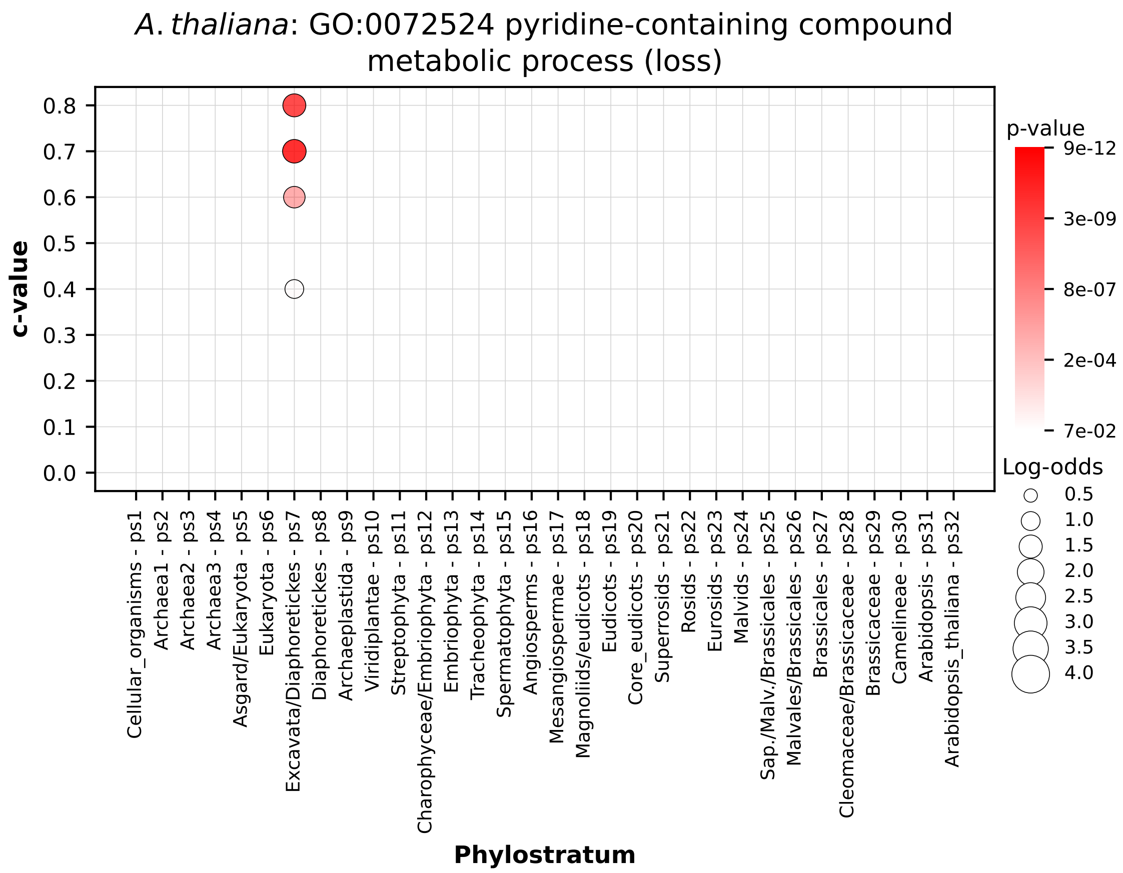

A. thaliana: GO:0072524 pyridine-containing compound metabolic process (loss)
c-value
0.0
0.1
0.2
0.3
0.4
0.5
0.6
0.7
0.8
p-value
9e-12
3e-09
8e-07
2e-04
7e-02
Log-odds
0.5
1.0
1.5
2.0
2.5
3.0
3.5
4.0
Cellular_organisms - ps1
Archaea1 - ps2
Archaea2 - ps3
Archaea3 - ps4
Asgard/Eukaryota - ps5
Eukaryota - ps6
Excavata/Diaphoretickes - ps7
Diaphoretickes - ps8
Archaeplastida - ps9
Viridiplantae - ps10
Streptophyta - ps11
Charophyceae/Embriophyta - ps12
Embriophyta - ps13
Tracheophyta - ps14
Spermatophyta - ps15
Angiosperms - ps16
Mesangiospermae - ps17
Magnoliids/eudicots - ps18
Eudicots - ps19
Core_eudicots - ps20
Superrosids - ps21
Rosids - ps22
Eurosids - ps23
Malvids - ps24
Sap./Malv./Brassicales - ps25
Malvales/Brassicales - ps26
Brassicales - ps27
Cleomaceae/Brassicaceae - ps28
Brassicaceae - ps29
Camelineae - ps30
Arabidopsis - ps31
Arabidopsis_thaliana - ps32
Phylostratum

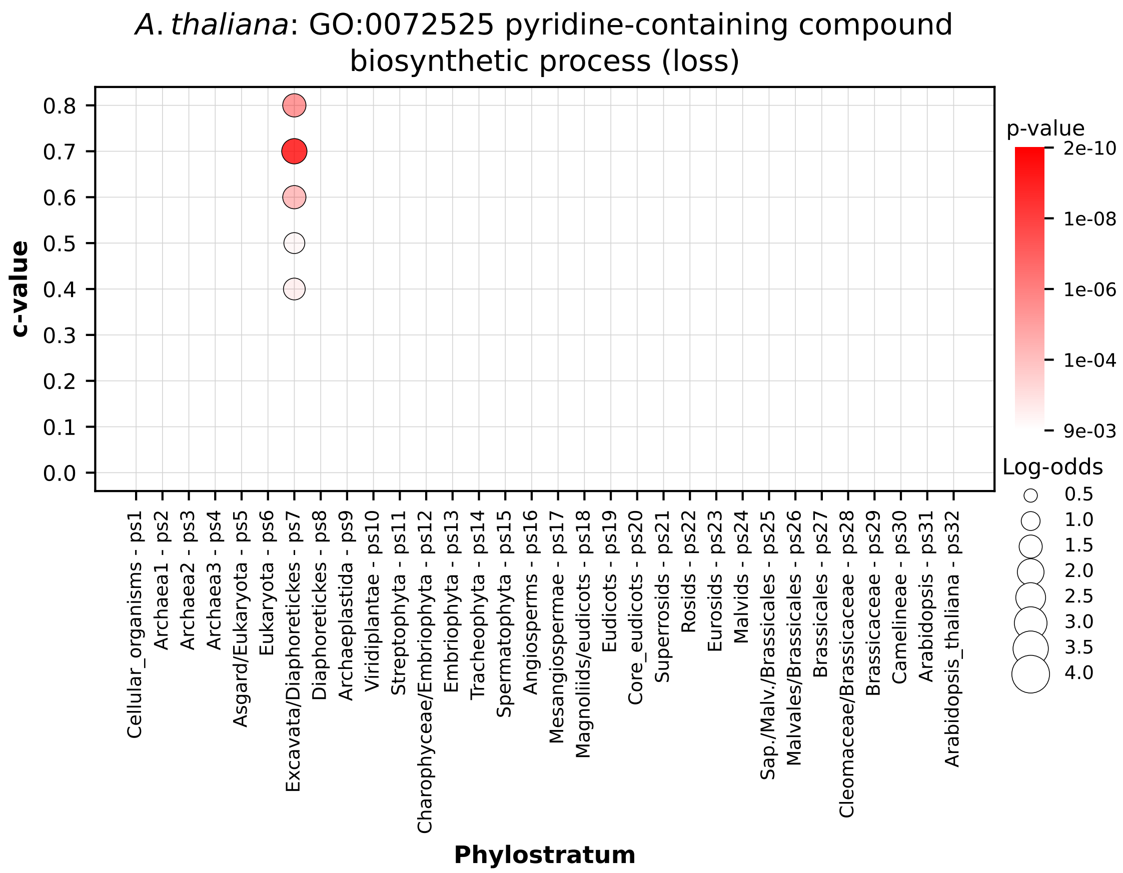

A. thaliana: GO:0072525 pyridine-containing compound biosynthetic process (loss)
c-value
0.8
0.7
0.6
0.5
0.4
0.3
0.2
0.1
0.0
Phylostratum
Cellular_organisms - ps1
Archaea1 - ps2
Archaea2 - ps3
Archaea3 - ps4
Asgard/Eukaryota - ps5
Eukaryota - ps6
Excavata/Diaphoretickes - ps7
Diaphoretickes - ps8
Archaeplastida - ps9
Viridiplantae - ps10
Streptophyta - ps11
Charophyceae/Embriophyta - ps12
Embriophyta - ps13
Tracheophyta - ps14
Spermatophyta - ps15
Angiosperms - ps16
Mesangiospermae - ps17
Magnoliids/eudicots - ps18
Eudicots - ps19
Core_eudicots - ps20
Superrosids - ps21
Rosids - ps22
Eurosids - ps23
Malvids - ps24
Sap./Malv./Brassicales - ps25
Malvales/Brassicales - ps26
Brassicales - ps27
Cleomaceae/Brassicaceae - ps28
Brassicaceae - ps29
Camelineae - ps30
Arabidopsis - ps31
Arabidopsis_thaliana - ps32
p-value
2e-10
1e-08
1e-06
1e-04
9e-03
Log-odds
0.5
1.0
1.5
2.0
2.5
3.0
3.5
4.0

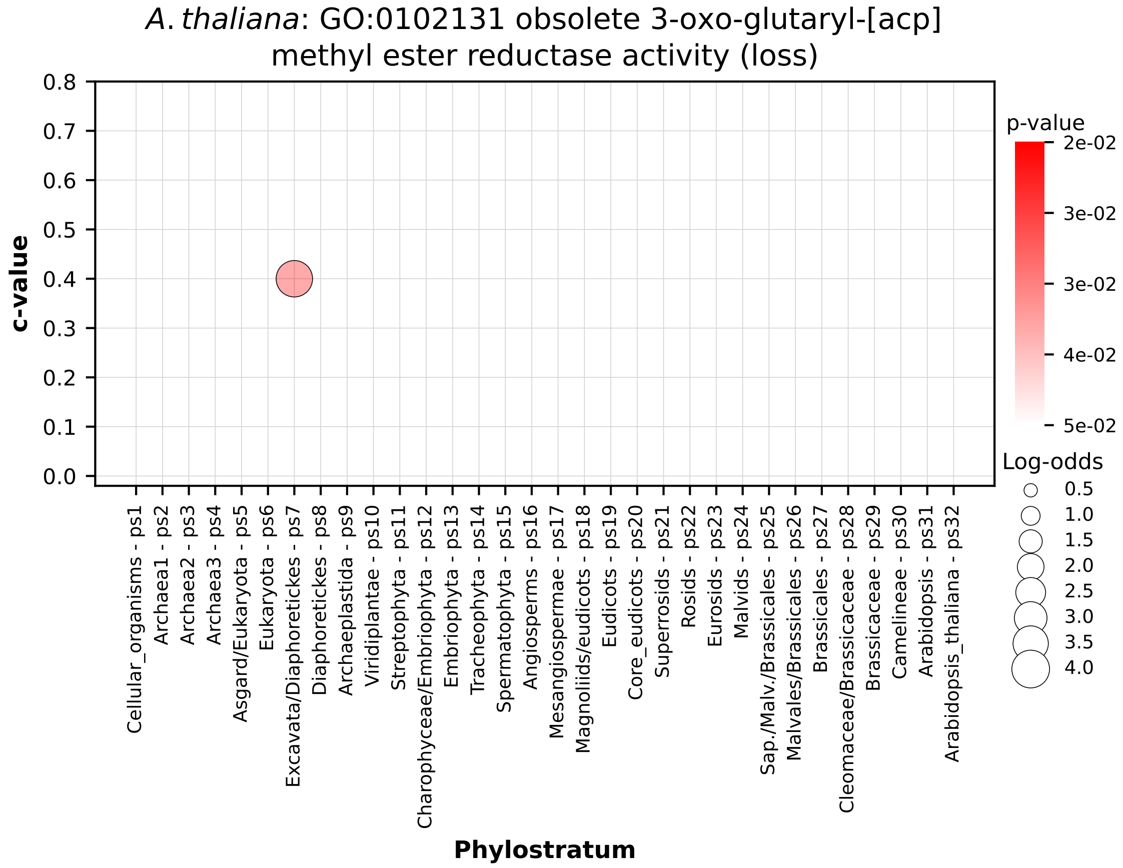
A. thaliana: GO:0102131 obsolete 3-oxo-glutaryl-[acp] methyl ester reductase activity (loss)
c-value
0.8
0.7
0.6
0.5
0.4
0.3
0.2
0.1
0.0
Phylostratum
Cellular_organisms - ps1
Archaea1 - ps2
Archaea2 - ps3
Archaea3 - ps4
Asgard/Eukaryota - ps5
Eukaryota - ps6
Excavata/Diaphoretickes - ps7
Diaphoretickes - ps8
Archaeplastida - ps9
Viridiplantae - ps10
Streptophyta - ps11
Charophyceae/Embriophyta - ps12
Embriophyta - ps13
Tracheophyta - ps14
Spermatophyta - ps15
Angiosperms - ps16
Mesangiospermae - ps17
Magnoliids/eudicots - ps18
Eudicots - ps19
Core_eudicots - ps20
Superrosids - ps21
Rosids - ps22
Eurosids - ps23
Malvids - ps24
Sap./Malv./Brassicales - ps25
Malvales/Brassicales - ps26
Brassicales - ps27
Cleomaceae/Brassicaceae - ps28
Brassicaceae - ps29
Camelineae - ps30
Arabidopsis - ps31
Arabidopsis_thaliana - ps32
p-value
2e-02
3e-02
3e-02
4e-02
5e-02
Log-odds
0.5
1.0
1.5
2.0
2.5
3.0
3.5
4.0

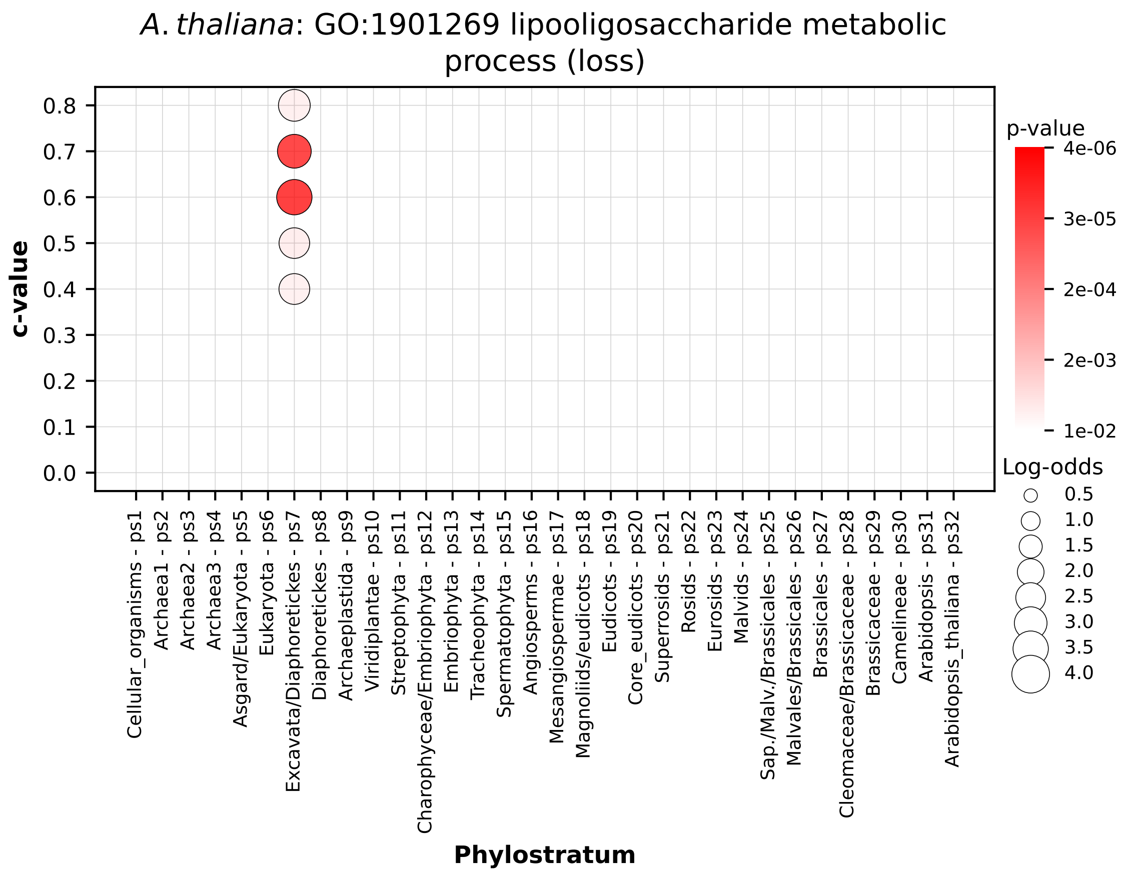
A. thaliana: GO:1901269 lipooligosaccharide metabolic process (loss)
c-value
0.0
0.1
0.2
0.3
0.4
0.5
0.6
0.7
0.8
Phylostratum
Cellular_organisms - ps1
Archaea1 - ps2
Archaea2 - ps3
Archaea3 - ps4
Asgard/Eukaryota - ps5
Eukaryota - ps6
Excavata/Diaphoretickes - ps7
Diaphoretickes - ps8
Archaeplastida - ps9
Viridiplantae - ps10
Streptophyta - ps11
Charophyceae/Embriophyta - ps12
Embriophyta - ps13
Tracheophyta - ps14
Spermatophyta - ps15
Angiosperms - ps16
Mesangiospermae - ps17
Magnoliids/eudicots - ps18
Eudicots - ps19
Core_eudicots - ps20
Superrosids - ps21
Rosids - ps22
Eurosids - ps23
Malvids - ps24
Sap./Malv./Brassicales - ps25
Malvales/Brassicales - ps26
Brassicales - ps27
Cleomaceae/Brassicaceae - ps28
Brassicaceae - ps29
Camelineae - ps30
Arabidopsis - ps31
Arabidopsis_thaliana - ps32
p-value
4e-06
3e-05
2e-04
2e-03
1e-02
Log-odds
0.5
1.0
1.5
2.0
2.5
3.0
3.5
4.0

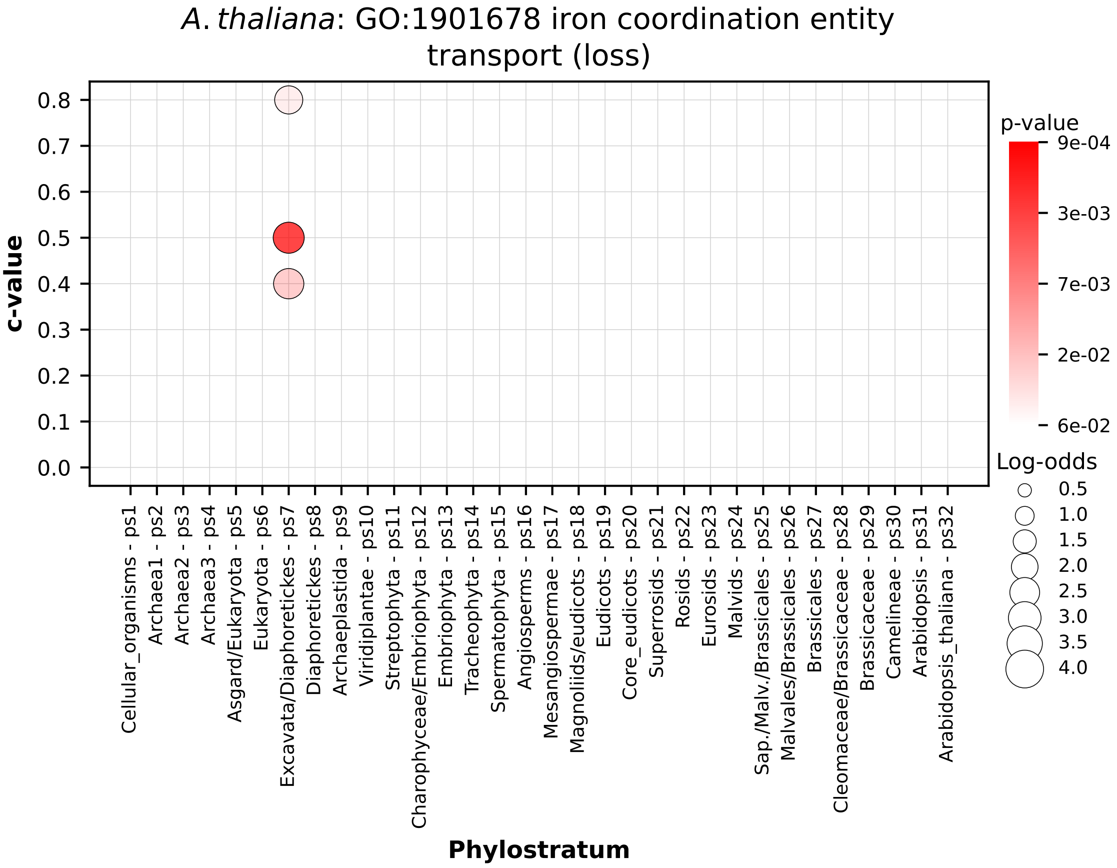

A. thaliana: GO:1901678 iron coordination entity transport (loss)
c-value
0.8
0.7
0.6
0.5
0.4
0.3
0.2
0.1
0.0
p-value
9e-04
3e-03
7e-03
2e-02
6e-02
Log-odds
0.5
1.0
1.5
2.0
2.5
3.0
3.5
4.0
Cellular_organisms - ps1
Archaea1 - ps2
Archaea2 - ps3
Archaea3 - ps4
Asgard/Eukaryota - ps5
Eukaryota - ps6
Excavata/Diaphoretickes - ps7
Diaphoretickes - ps8
Archaeplastida - ps9
Viridiplantae - ps10
Streptophyta - ps11
Charophyceae/Embriophyta - ps12
Embriophyta - ps13
Tracheophyta - ps14
Spermatophyta - ps15
Angiosperms - ps16
Mesangiospermae - ps17
Magnoliids/eudicots - ps18
Eudicots - ps19
Core_eudicots - ps20
Superrosids - ps21
Rosids - ps22
Eurosids - ps23
Malvids - ps24
Sap./Malv./Brassicales - ps25
Malvales/Brassicales - ps26
Brassicales - ps27
Cleomaceae/Brassicaceae - ps28
Brassicaceae - ps29
Camelineae - ps30
Arabidopsis - ps31
Arabidopsis_thaliana - ps32
Phylostratum

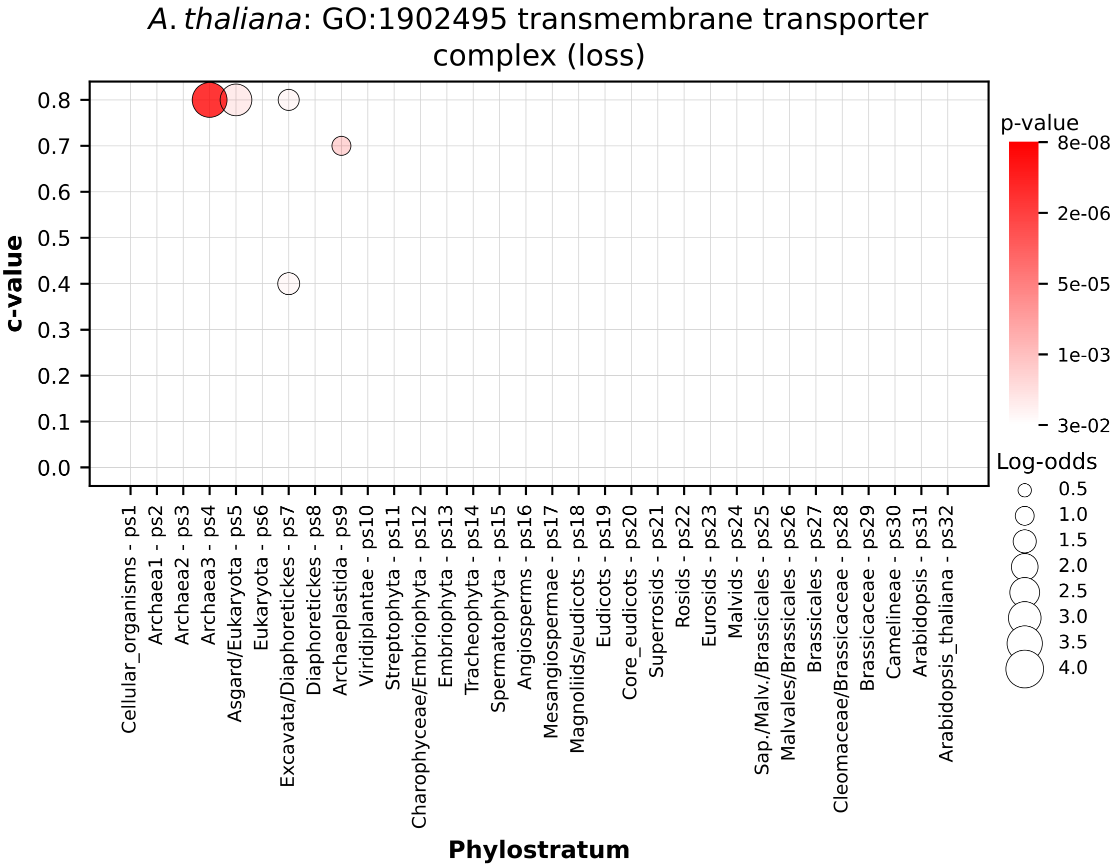

A. thaliana: GO:1902495 transmembrane transporter complex (loss)
c-value
0.8
0.7
0.6
0.5
0.4
0.3
0.2
0.1
0.0
Phylostratum
Cellular_organisms - ps1
Archaea1 - ps2
Archaea2 - ps3
Archaea3 - ps4
Asgard/Eukaryota - ps5
Eukaryota - ps6
Excavata/Diaphoretickes - ps7
Diaphoretickes - ps8
Archaeplastida - ps9
Viridiplantae - ps10
Streptophyta - ps11
Charophyceae/Embriophyta - ps12
Embriophyta - ps13
Tracheophyta - ps14
Spermatophyta - ps15
Angiosperms - ps16
Mesangiospermae - ps17
Magnoliids/eudicots - ps18
Eudicots - ps19
Core_eudicots - ps20
Superrosids - ps21
Rosids - ps22
Eurosids - ps23
Malvids - ps24
Sap./Malv./Brassicales - ps25
Malvales/Brassicales - ps26
Brassicales - ps27
Cleomaceae/Brassicaceae - ps28
Brassicaceae - ps29
Camelineae - ps30
Arabidopsis - ps31
Arabidopsis_thaliana - ps32
p-value
8e-08
2e-06
5e-05
1e-03
3e-02
Log-odds
0.5
1.0
1.5
2.0
2.5
3.0
3.5
4.0

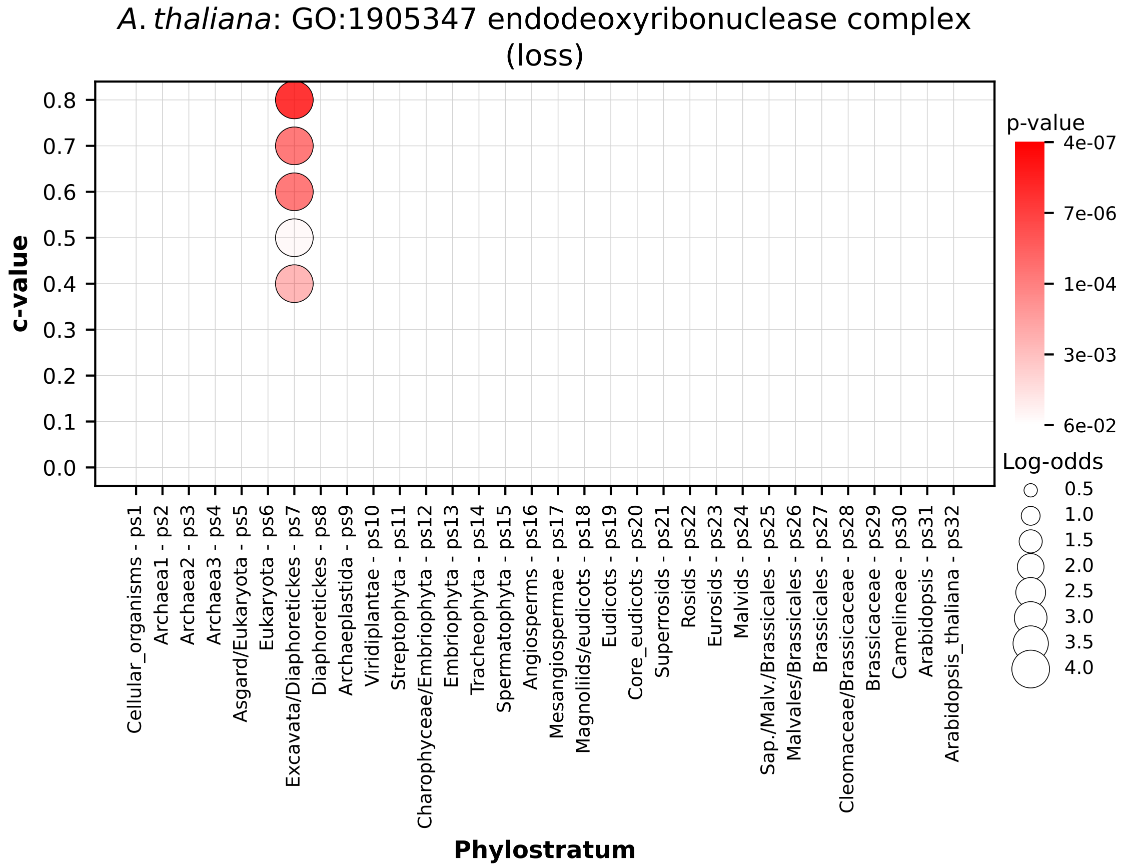

A. thaliana: GO:1905347 endodeoxyribonuclease complex (loss)
c-value
0.0
0.1
0.2
0.3
0.4
0.5
0.6
0.7
0.8
p-value
4e-07
7e-06
1e-04
3e-03
6e-02
Log-odds
0.5
1.0
1.5
2.0
2.5
3.0
3.5
4.0
Cellular_organisms - ps1
Archaea1 - ps2
Archaea2 - ps3
Archaea3 - ps4
Asgard/Eukaryota - ps5
Eukaryota - ps6
Excavata/Diaphoretickes - ps7
Diaphoretickes - ps8
Archaeplastida - ps9
Viridiplantae - ps10
Streptophyta - ps11
Charophyceae/Embriophyta - ps12
Embriophyta - ps13
Tracheophyta - ps14
Spermatophyta - ps15
Angiosperms - ps16
Mesangiospermae - ps17
Magnoliids/eudicots - ps18
Eudicots - ps19
Core_eudicots - ps20
Superrosids - ps21
Rosids - ps22
Eurosids - ps23
Malvids - ps24
Sap./Malv./Brassicales - ps25
Malvales/Brassicales - ps26
Brassicales - ps27
Cleomaceae/Brassicaceae - ps28
Brassicaceae - ps29
Camelineae - ps30
Arabidopsis - ps31
Arabidopsis_thaliana - ps32
Phylostratum

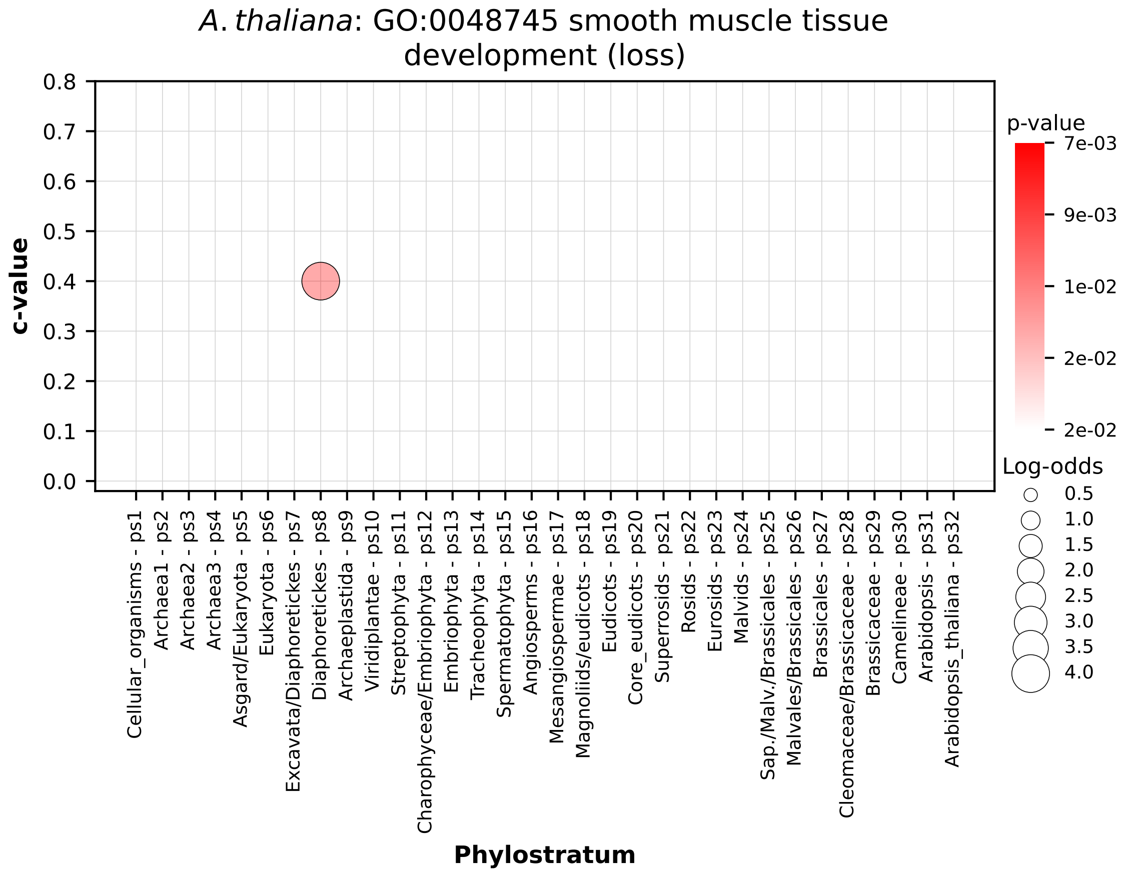
A. thaliana: GO:0048745 smooth muscle tissue development (loss)
c-value
0.0
0.1
0.2
0.3
0.4
0.5
0.6
0.7
0.8
p-value
7e-03
9e-03
1e-02
2e-02
2e-02
Log-odds
0.5
1.0
1.5
2.0
2.5
3.0
3.5
4.0
Cellular_organisms - ps1
Archaea1 - ps2
Archaea2 - ps3
Archaea3 - ps4
Asgard/Eukaryota - ps5
Eukaryota - ps6
Excavata/Diaphoretickes - ps7
Diaphoretickes - ps8
Archaeplastida - ps9
Viridiplantae - ps10
Streptophyta - ps11
Charophyceae/Embriophyta - ps12
Embriophyta - ps13
Tracheophyta - ps14
Spermatophyta - ps15
Angiosperms - ps16
Mesangiospermae - ps17
Magnoliids/eudicots - ps18
Eudicots - ps19
Core_eudicots - ps20
Superrosids - ps21
Rosids - ps22
Eurosids - ps23
Malvids - ps24
Sap./Malv./Brassicales - ps25
Malvales/Brassicales - ps26
Brassicales - ps27
Cleomaceae/Brassicaceae - ps28
Brassicaceae - ps29
Camelineae - ps30
Arabidopsis - ps31
Arabidopsis_thaliana - ps32
Phylostratum

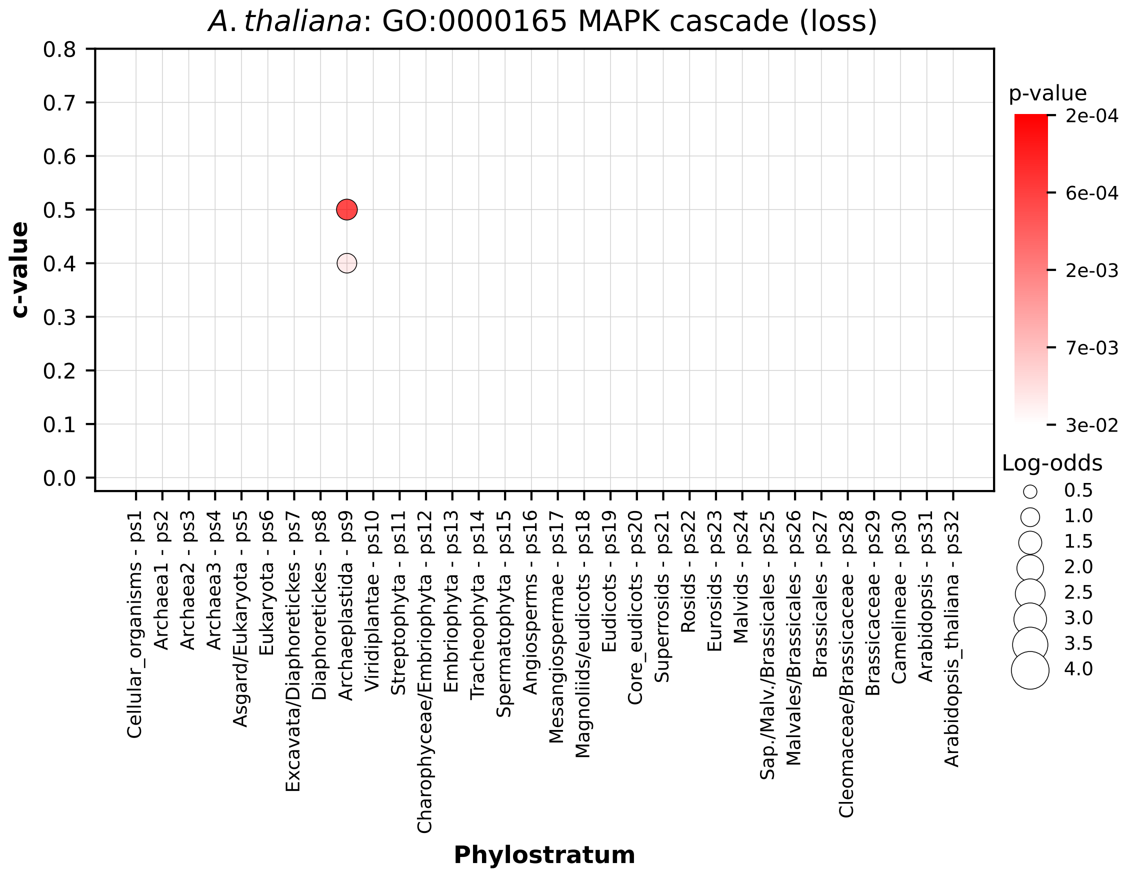

A. thaliana: GO:0000165 MAPK cascade (loss)
c-value
0.8
0.7
0.6
0.5
0.4
0.3
0.2
0.1
0.0
p-value
2e-04
6e-04
2e-03
7e-03
3e-02
Log-odds
0.5
1.0
1.5
2.0
2.5
3.0
3.5
4.0
Cellular_organisms - ps1
Archaea1 - ps2
Archaea2 - ps3
Archaea3 - ps4
Asgard/Eukaryota - ps5
Eukaryota - ps6
Excavata/Diaphoretickes - ps7
Diaphoretickes - ps8
Archaeplastida - ps9
Viridiplantae - ps10
Streptophyta - ps11
Charophyceae/Embriophyta - ps12
Embriophyta - ps13
Tracheophyta - ps14
Spermatophyta - ps15
Angiosperms - ps16
Mesangiospermae - ps17
Magnoliids/eudicots - ps18
Eudicots - ps19
Core_eudicots - ps20
Superrosids - ps21
Rosids - ps22
Eurosids - ps23
Malvids - ps24
Sap./Malv./Brassicales - ps25
Malvales/Brassicales - ps26
Brassicales - ps27
Cleomaceae/Brassicaceae - ps28
Brassicaceae - ps29
Camelineae - ps30
Arabidopsis - ps31
Arabidopsis_thaliana - ps32
Phylostratum

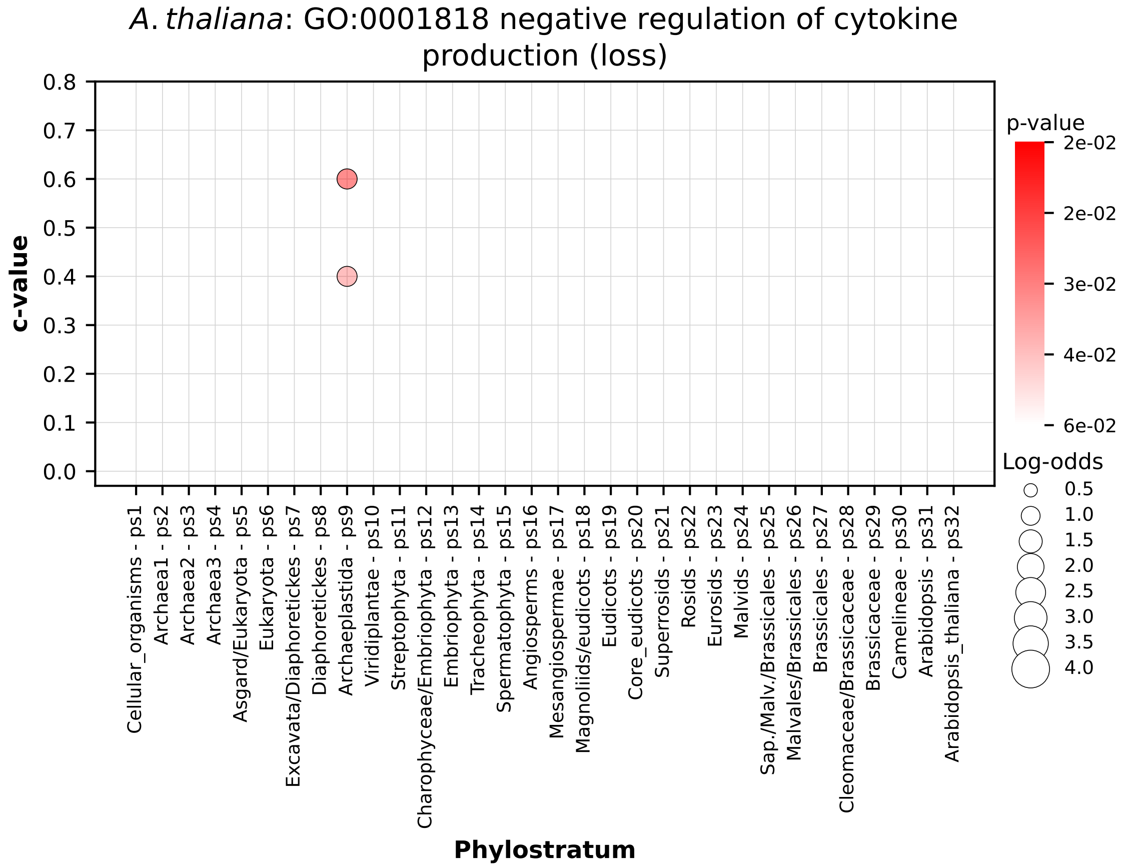

A. thaliana: GO:0001818 negative regulation of cytokine production (loss)
c-value
0.0
0.1
0.2
0.3
0.4
0.5
0.6
0.7
0.8
Phylostratum
Cellular_organisms - ps1
Archaea1 - ps2
Archaea2 - ps3
Archaea3 - ps4
Asgard/Eukaryota - ps5
Eukaryota - ps6
Excavata/Diaphoretickes - ps7
Diaphoretickes - ps8
Archaeplastida - ps9
Viridiplantae - ps10
Streptophyta - ps11
Charophyceae/Embriophyta - ps12
Embriophyta - ps13
Tracheophyta - ps14
Spermatophyta - ps15
Angiosperms - ps16
Mesangiospermae - ps17
Magnoliids/eudicots - ps18
Eudicots - ps19
Core_eudicots - ps20
Superrosids - ps21
Rosids - ps22
Eurosids - ps23
Malvids - ps24
Sap./Malv./Brassicales - ps25
Malvales/Brassicales - ps26
Brassicales - ps27
Cleomaceae/Brassicaceae - ps28
Brassicaceae - ps29
Camelineae - ps30
Arabidopsis - ps31
Arabidopsis_thaliana - ps32
p-value
2e-02
2e-02
3e-02
4e-02
6e-02
Log-odds
0.5
1.0
1.5
2.0
2.5
3.0
3.5
4.0

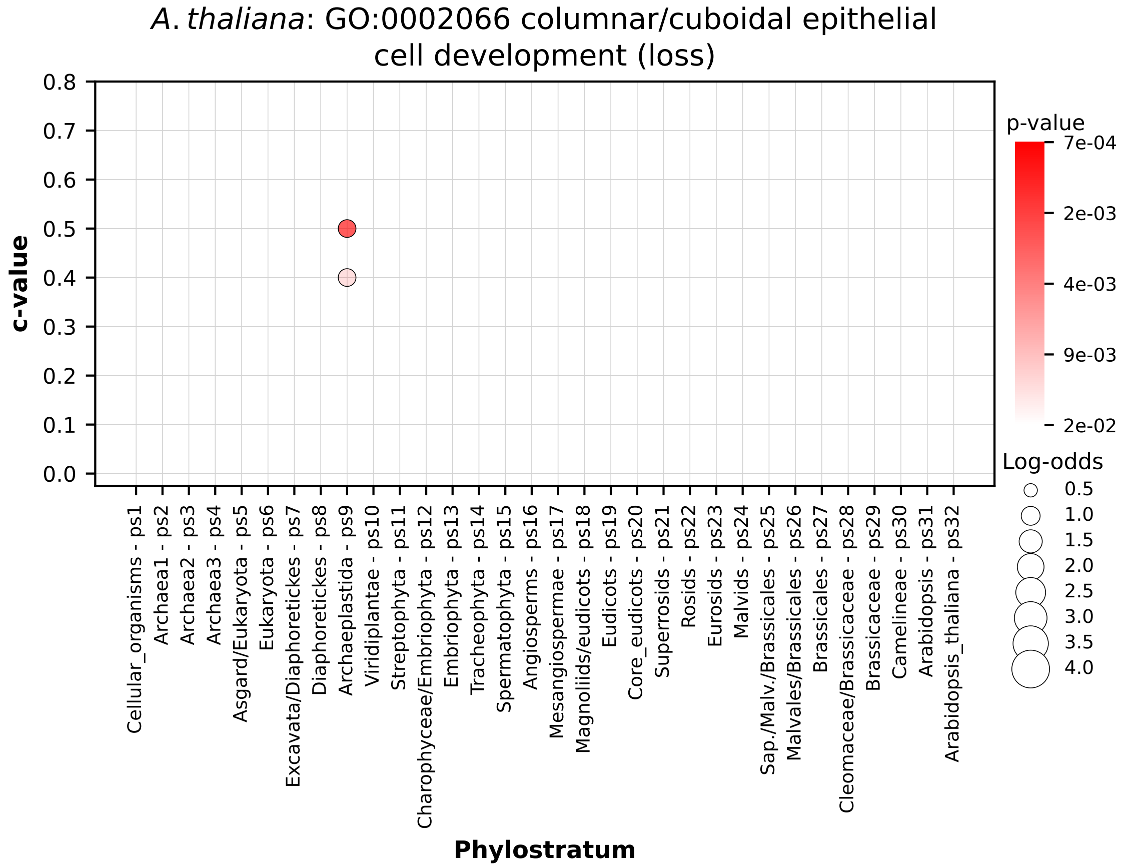
A. thaliana: GO:0002066 columnar/cuboidal epithelial cell development (loss)
c-value
0.0
0.1
0.2
0.3
0.4
0.5
0.6
0.7
0.8
Phylostratum
Cellular_organisms - ps1
Archaea1 - ps2
Archaea2 - ps3
Archaea3 - ps4
Asgard/Eukaryota - ps5
Eukaryota - ps6
Excavata/Diaphoretickes - ps7
Diaphoretickes - ps8
Archaeplastida - ps9
Viridiplantae - ps10
Streptophyta - ps11
Charophyceae/Embriophyta - ps12
Embriophyta - ps13
Tracheophyta - ps14
Spermatophyta - ps15
Angiosperms - ps16
Mesangiospermae - ps17
Magnoliids/eudicots - ps18
Eudicots - ps19
Core_eudicots - ps20
Superrosids - ps21
Rosids - ps22
Eurosids - ps23
Malvids - ps24
Sap./Malv./Brassicales - ps25
Malvales/Brassicales - ps26
Brassicales - ps27
Cleomaceae/Brassicaceae - ps28
Brassicaceae - ps29
Camelineae - ps30
Arabidopsis - ps31
Arabidopsis_thaliana - ps32
p-value
7e-04
2e-03
4e-03
9e-03
2e-02
Log-odds
0.5
1.0
1.5
2.0
2.5
3.0
3.5
4.0

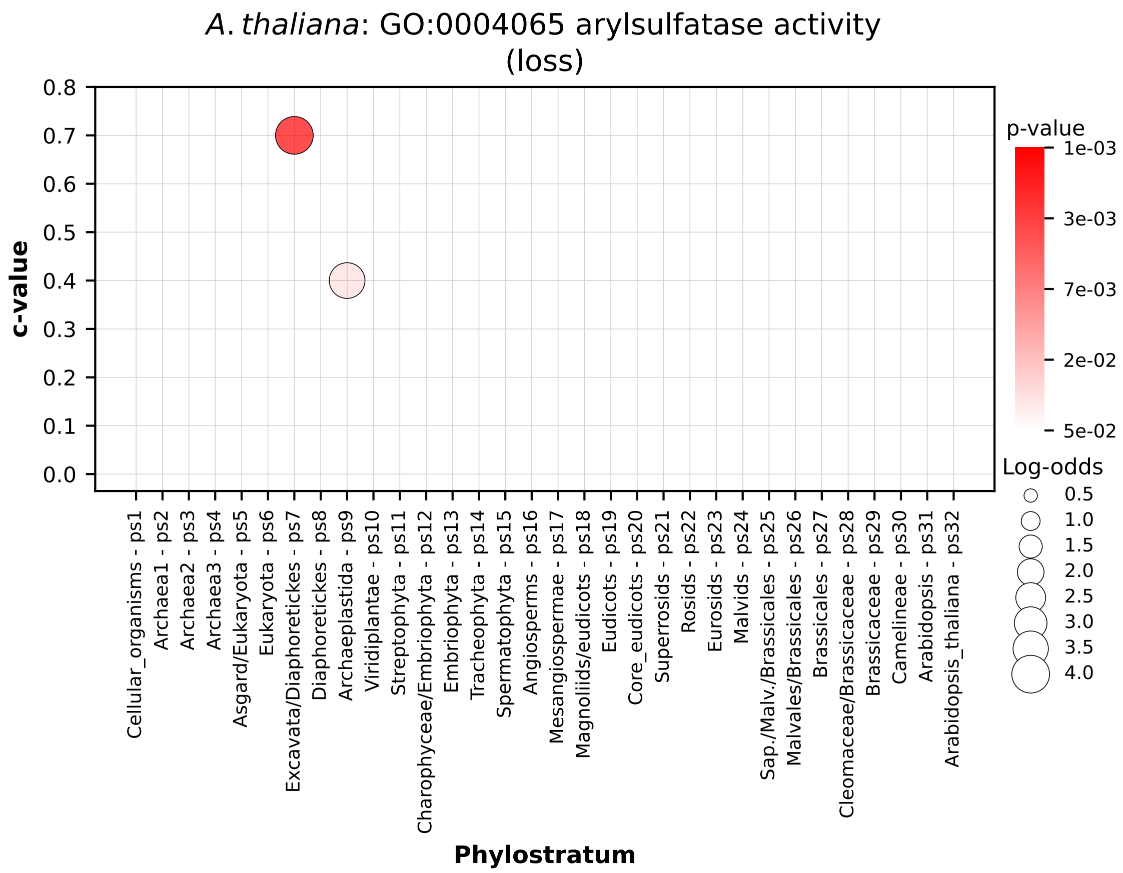

A. thaliana: GO:0004065 arylsulfatase activity (loss)
c-value
0.8
0.7
0.6
0.5
0.4
0.3
0.2
0.1
0.0
p-value
1e-03
3e-03
7e-03
2e-02
5e-02
Log-odds
0.5
1.0
1.5
2.0
2.5
3.0
3.5
4.0
Cellular_organisms - ps1
Archaea1 - ps2
Archaea2 - ps3
Archaea3 - ps4
Asgard/Eukaryota - ps5
Eukaryota - ps6
Excavata/Diaphoretickes - ps7
Diaphoretickes - ps8
Archaeplastida - ps9
Viridiplantae - ps10
Streptophyta - ps11
Charophyceae/Embriophyta - ps12
Embriophyta - ps13
Tracheophyta - ps14
Spermatophyta - ps15
Angiosperms - ps16
Mesangiospermae - ps17
Magnoliids/eudicots - ps18
Eudicots - ps19
Core_eudicots - ps20
Superrosids - ps21
Rosids - ps22
Eurosids - ps23
Malvids - ps24
Sap./Malv./Brassicales - ps25
Malvales/Brassicales - ps26
Brassicales - ps27
Cleomaceae/Brassicaceae - ps28
Brassicaceae - ps29
Camelineae - ps30
Arabidopsis - ps31
Arabidopsis_thaliana - ps32
Phylostratum

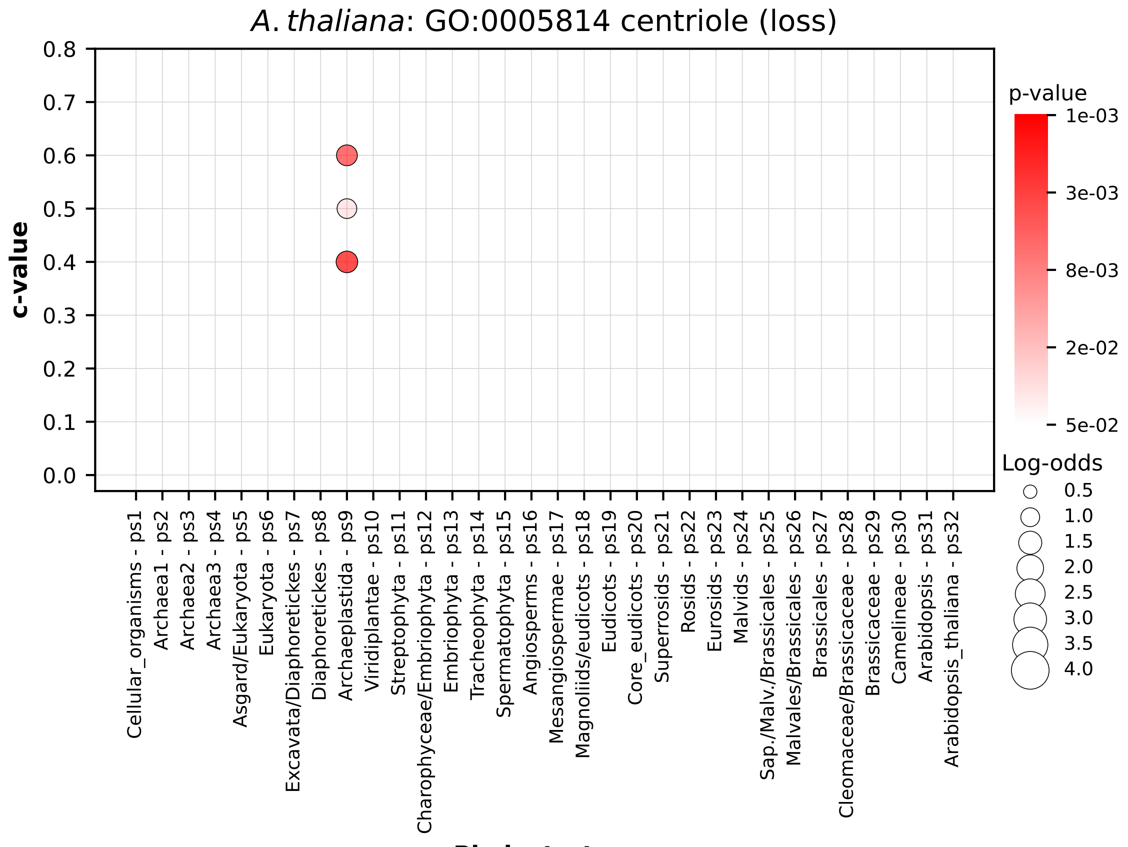

A. thaliana: GO:0005814 centriole (loss)
c-value
0.8
0.7
0.6
0.5
0.4
0.3
0.2
0.1
0.0
p-value
1e-03
3e-03
8e-03
2e-02
5e-02
Log-odds
0.5
1.0
1.5
2.0
2.5
3.0
3.5
4.0
Cellular_organisms - ps1
Archaea1 - ps2
Archaea2 - ps3
Archaea3 - ps4
Asgard/Eukaryota - ps5
Eukaryota - ps6
Excavata/Diaphoretickes - ps7
Diaphoretickes - ps8
Archaeplastida - ps9
Viridiplantae - ps10
Streptophyta - ps11
Charophyceae/Embriophyta - ps12
Embriophyta - ps13
Tracheophyta - ps14
Spermatophyta - ps15
Angiosperms - ps16
Mesangiospermae - ps17
Magnoliids/eudicots - ps18
Eudicots - ps19
Core_eudicots - ps20
Superrosids - ps21
Rosids - ps22
Eurosids - ps23
Malvids - ps24
Sap./Malv./Brassicales - ps25
Malvales/Brassicales - ps26
Brassicales - ps27
Cleomaceae/Brassicaceae - ps28
Brassicaceae - ps29
Camelineae - ps30
Arabidopsis - ps31
Arabidopsis_thaliana - ps32
Phylostratum

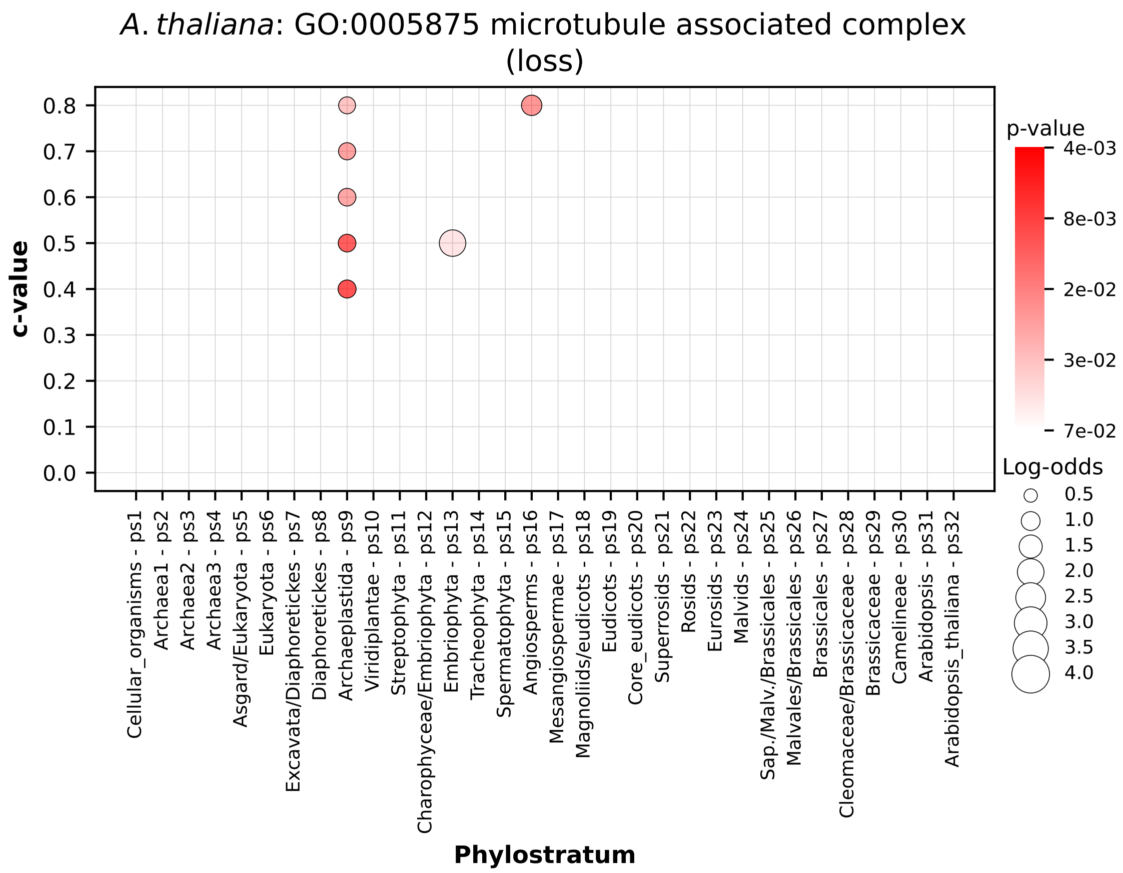

A. thaliana: GO:0005875 microtubule associated complex
(loss)
c-value
0.8
0.7
0.6
0.5
0.4
0.3
0.2
0.1
0.0
Phylostratum
Cellular_organisms - ps1
Archaea1 - ps2
Archaea2 - ps3
Archaea3 - ps4
Asgard/Eukaryota - ps5
Eukaryota - ps6
Excavata/Diaphoretickes - ps7
Diaphoretickes - ps8
Archaeplastida - ps9
Viridiplantae - ps10
Streptophyta - ps11
Charophyceae/Embriophyta - ps12
Embriophyta - ps13
Tracheophyta - ps14
Spermatophyta - ps15
Angiosperms - ps16
Mesangiospermae - ps17
Magnoliids/eudicots - ps18
Eudicots - ps19
Core_eudicots - ps20
Superrosids - ps21
Rosids - ps22
Eurosids - ps23
Malvids - ps24
Sap./Malv./Brassicales - ps25
Malvales/Brassicales - ps26
Brassicales - ps27
Cleomaceae/Brassicaceae - ps28
Brassicaceae - ps29
Camelineae - ps30
Arabidopsis - ps31
Arabidopsis_thaliana - ps32
p-value
4e-03
8e-03
2e-02
3e-02
7e-02
Log-odds
0.5
1.0
1.5
2.0
2.5
3.0
3.5
4.0

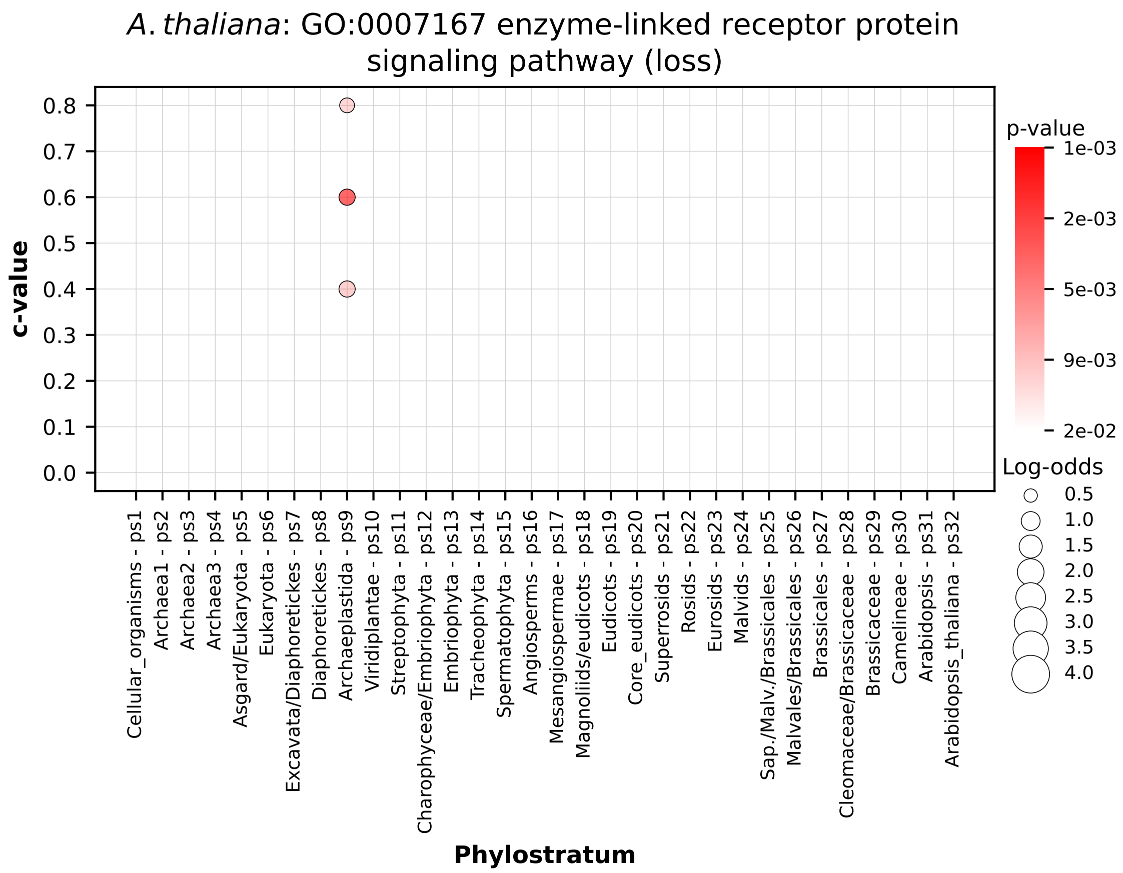

A. thaliana: GO:0007167 enzyme-linked receptor protein signaling pathway (loss)
c-value
0.8
0.7
0.6
0.5
0.4
0.3
0.2
0.1
0.0
p-value
1e-03
2e-03
5e-03
9e-03
2e-02
Log-odds
0.5
1.0
1.5
2.0
2.5
3.0
3.5
4.0
Cellular_organisms - ps1
Archaea1 - ps2
Archaea2 - ps3
Archaea3 - ps4
Asgard/Eukaryota - ps5
Eukaryota - ps6
Excavata/Diaphoretickes - ps7
Diaphoretickes - ps8
Archaeplastida - ps9
Viridiplantae - ps10
Streptophyta - ps11
Charophyceae/Embriophyta - ps12
Embriophyta - ps13
Tracheophyta - ps14
Spermatophyta - ps15
Angiosperms - ps16
Mesangiospermae - ps17
Magnoliids/eudicots - ps18
Eudicots - ps19
Core_eudicots - ps20
Superrosids - ps21
Rosids - ps22
Eurosids - ps23
Malvids - ps24
Sap./Malv./Brassicales - ps25
Malvales/Brassicales - ps26
Brassicales - ps27
Cleomaceae/Brassicaceae - ps28
Brassicaceae - ps29
Camelineae - ps30
Arabidopsis - ps31
Arabidopsis_thaliana - ps32
Phylostratum

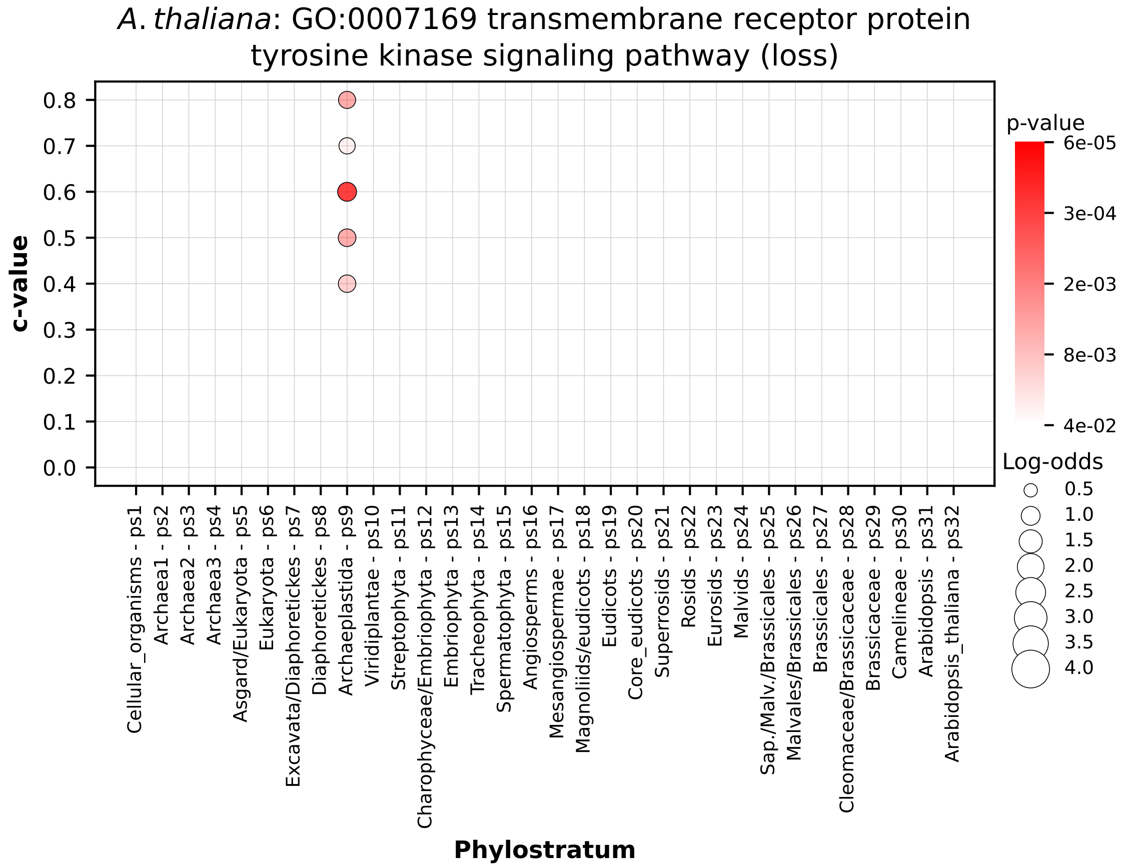

A. thaliana: GO:0007169 transmembrane receptor protein tyrosine kinase signaling pathway (loss)
c-value
0.0
0.1
0.2
0.3
0.4
0.5
0.6
0.7
0.8
Phylostratum
Cellular_organisms - ps1
Archaea1 - ps2
Archaea2 - ps3
Archaea3 - ps4
Asgard/Eukaryota - ps5
Eukaryota - ps6
Excavata/Diaphoretickes - ps7
Diaphoretickes - ps8
Archaeplastida - ps9
Viridiplantae - ps10
Streptophyta - ps11
Charophyceae/Embriophyta - ps12
Embriophyta - ps13
Tracheophyta - ps14
Spermatophyta - ps15
Angiosperms - ps16
Mesangiospermae - ps17
Magnoliids/eudicots - ps18
Eudicots - ps19
Core_eudicots - ps20
Superrosids - ps21
Rosids - ps22
Eurosids - ps23
Malvids - ps24
Sap./Malv./Brassicales - ps25
Malvales/Brassicales - ps26
Brassicales - ps27
Cleomaceae/Brassicaceae - ps28
Brassicaceae - ps29
Camelineae - ps30
Arabidopsis - ps31
Arabidopsis_thaliana - ps32
p-value
6e-05
3e-04
2e-03
8e-03
4e-02
Log-odds
0.5
1.0
1.5
2.0
2.5
3.0
3.5
4.0

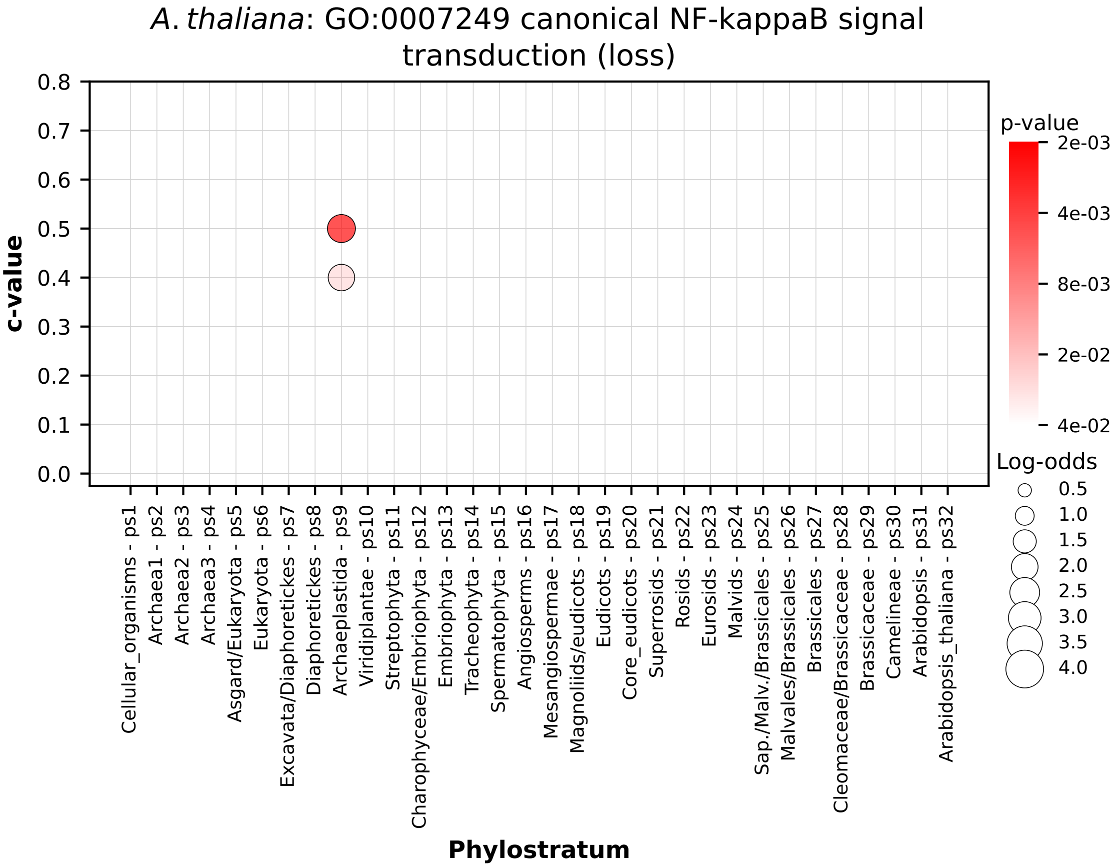

A. thaliana: GO:0007249 canonical NF-kappaB signal transduction (loss)
c-value
0.8
0.7
0.6
0.5
0.4
0.3
0.2
0.1
0.0
p-value
2e-03
4e-03
8e-03
2e-02
4e-02
Log-odds
0.5
1.0
1.5
2.0
2.5
3.0
3.5
4.0
Cellular_organisms - ps1
Archaea1 - ps2
Archaea2 - ps3
Archaea3 - ps4
Asgard/Eukaryota - ps5
Eukaryota - ps6
Excavata/Diaphoretickes - ps7
Diaphoretickes - ps8
Archaeplastida - ps9
Viridiplantae - ps10
Streptophyta - ps11
Charophyceae/Embriophyta - ps12
Embriophyta - ps13
Tracheophyta - ps14
Spermatophyta - ps15
Angiosperms - ps16
Mesangiospermae - ps17
Magnoliids/eudicots - ps18
Eudicots - ps19
Core_eudicots - ps20
Superrosids - ps21
Rosids - ps22
Eurosids - ps23
Malvids - ps24
Sap./Malv./Brassicales - ps25
Malvales/Brassicales - ps26
Brassicales - ps27
Cleomaceae/Brassicaceae - ps28
Brassicaceae - ps29
Camelineae - ps30
Arabidopsis - ps31
Arabidopsis_thaliana - ps32
Phylostratum

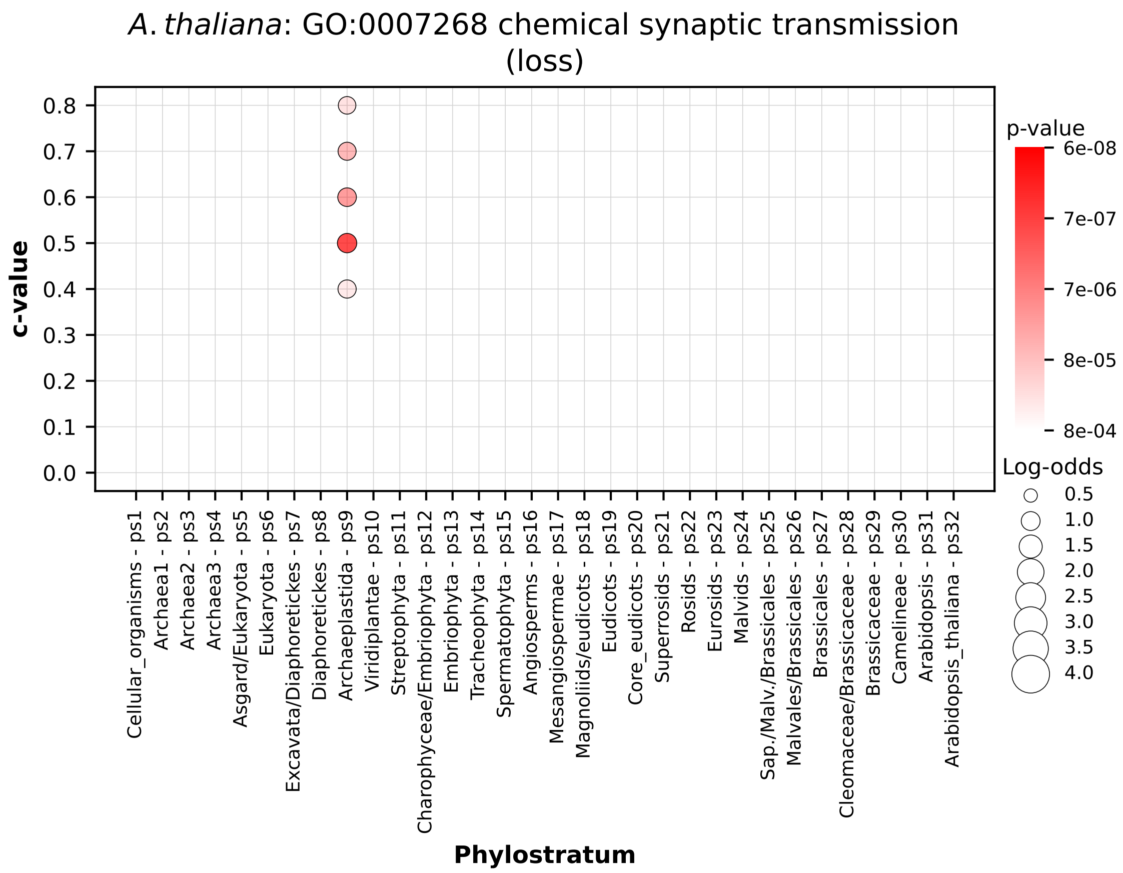

A. thaliana: GO:0007268 chemical synaptic transmission
(loss)
c-value
0.0
0.1
0.2
0.3
0.4
0.5
0.6
0.7
0.8
p-value
6e-08
7e-07
7e-06
8e-05
8e-04
Log-odds
0.5
1.0
1.5
2.0
2.5
3.0
3.5
4.0
Cellular_organisms - ps1
Archaea1 - ps2
Archaea2 - ps3
Archaea3 - ps4
Asgard/Eukaryota - ps5
Eukaryota - ps6
Excavata/Diaphoretickes - ps7
Diaphoretickes - ps8
Archaeplastida - ps9
Viridiplantae - ps10
Streptophyta - ps11
Charophyceae/Embriophyta - ps12
Embriophyta - ps13
Tracheophyta - ps14
Spermatophyta - ps15
Angiosperms - ps16
Mesangiospermae - ps17
Magnoliids/eudicots - ps18
Eudicots - ps19
Core_eudicots - ps20
Superrosids - ps21
Rosids - ps22
Eurosids - ps23
Malvids - ps24
Sap./Malv./Brassicales - ps25
Malvales/Brassicales - ps26
Brassicales - ps27
Cleomaceae/Brassicaceae - ps28
Brassicaceae - ps29
Camelineae - ps30
Arabidopsis - ps31
Arabidopsis_thaliana - ps32
Phylostratum

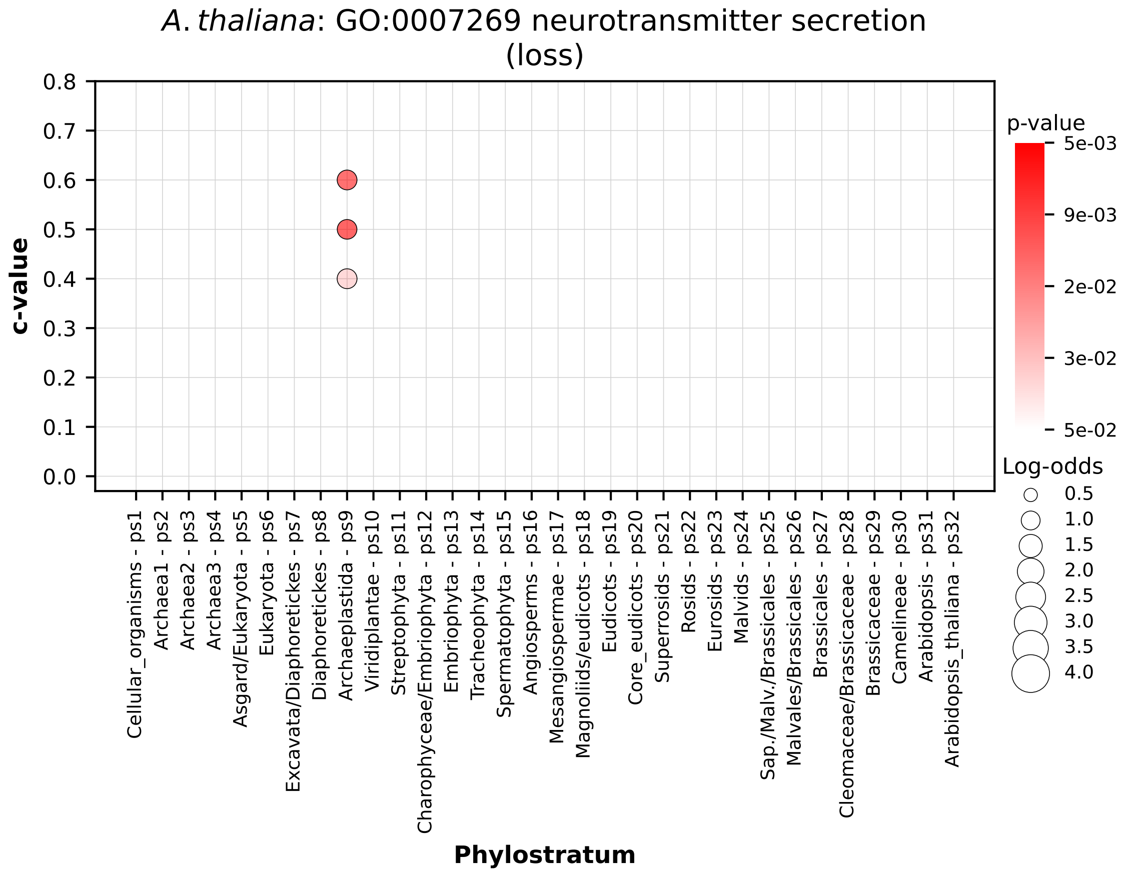

A. thaliana: GO:0007269 neurotransmitter secretion
(loss)
c-value
0.8
0.7
0.6
0.5
0.4
0.3
0.2
0.1
0.0
p-value
5e-03
9e-03
2e-02
3e-02
5e-02
Log-odds
0.5
1.0
1.5
2.0
2.5
3.0
3.5
4.0
Cellular_organisms - ps1
Archaea1 - ps2
Archaea2 - ps3
Archaea3 - ps4
Asgard/Eukaryota - ps5
Eukaryota - ps6
Excavata/Diaphoretickes - ps7
Diaphoretickes - ps8
Archaeplastida - ps9
Viridiplantae - ps10
Streptophyta - ps11
Charophyceae/Embriophyta - ps12
Embriophyta - ps13
Tracheophyta - ps14
Spermatophyta - ps15
Angiosperms - ps16
Mesangiospermae - ps17
Magnoliids/eudicots - ps18
Eudicots - ps19
Core_eudicots - ps20
Superrosids - ps21
Rosids - ps22
Eurosids - ps23
Malvids - ps24
Sap./Malv./Brassicales - ps25
Malvales/Brassicales - ps26
Brassicales - ps27
Cleomaceae/Brassicaceae - ps28
Brassicaceae - ps29
Camelineae - ps30
Arabidopsis - ps31
Arabidopsis_thaliana - ps32
Phylostratum

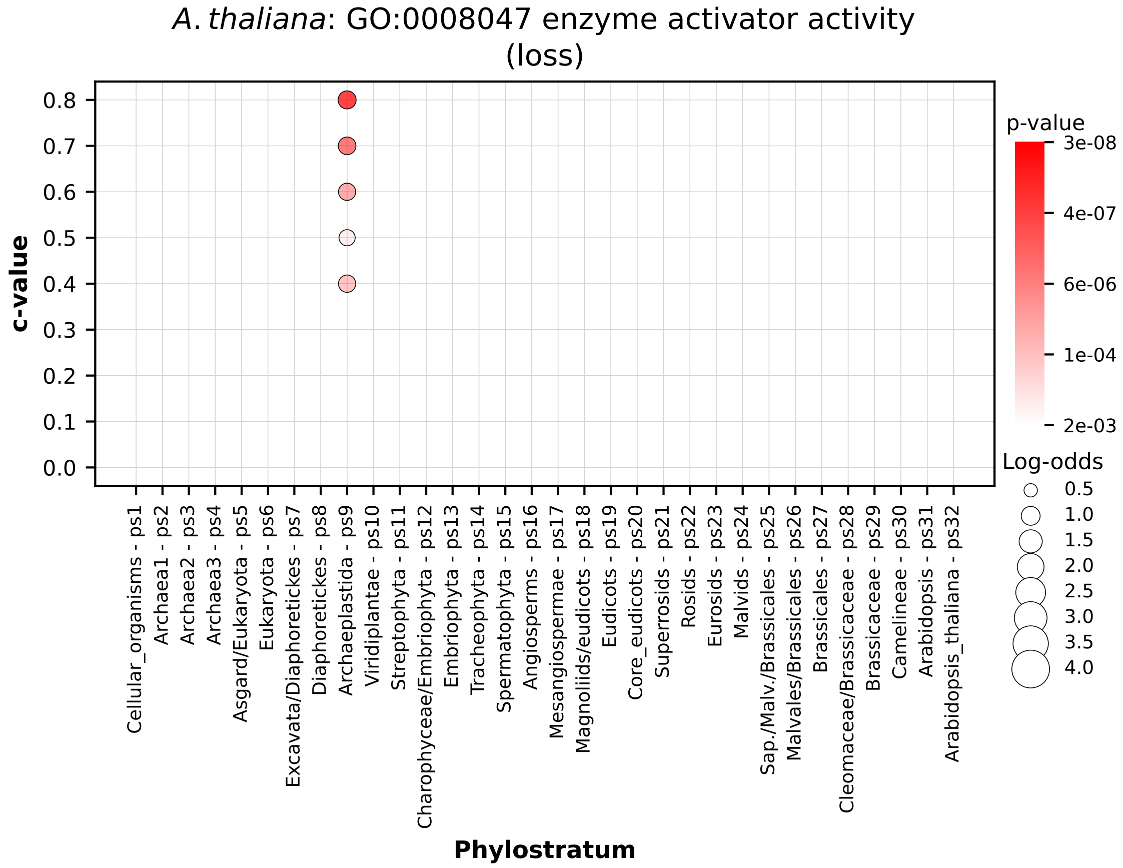

A. thaliana: GO:0008047 enzyme activator activity
(loss)
c-value
0.0
0.1
0.2
0.3
0.4
0.5
0.6
0.7
0.8
Phylostratum
Cellular_organisms - ps1
Archaea1 - ps2
Archaea2 - ps3
Archaea3 - ps4
Asgard/Eukaryota - ps5
Eukaryota - ps6
Excavata/Diaphoretickes - ps7
Diaphoretickes - ps8
Archaeplastida - ps9
Viridiplantae - ps10
Streptophyta - ps11
Charophyceae/Embriophyta - ps12
Embriophyta - ps13
Tracheophyta - ps14
Spermatophyta - ps15
Angiosperms - ps16
Mesangiospermae - ps17
Magnoliids/eudicots - ps18
Eudicots - ps19
Core_eudicots - ps20
Superrosids - ps21
Rosids - ps22
Eurosids - ps23
Malvids - ps24
Sap./Malv./Brassicales - ps25
Malvales/Brassicales - ps26
Brassicales - ps27
Cleomaceae/Brassicaceae - ps28
Brassicaceae - ps29
Camelineae - ps30
Arabidopsis - ps31
Arabidopsis_thaliana - ps32
p-value
3e-08
4e-07
6e-06
1e-04
2e-03
Log-odds
0.5
1.0
1.5
2.0
2.5
3.0
3.5
4.0

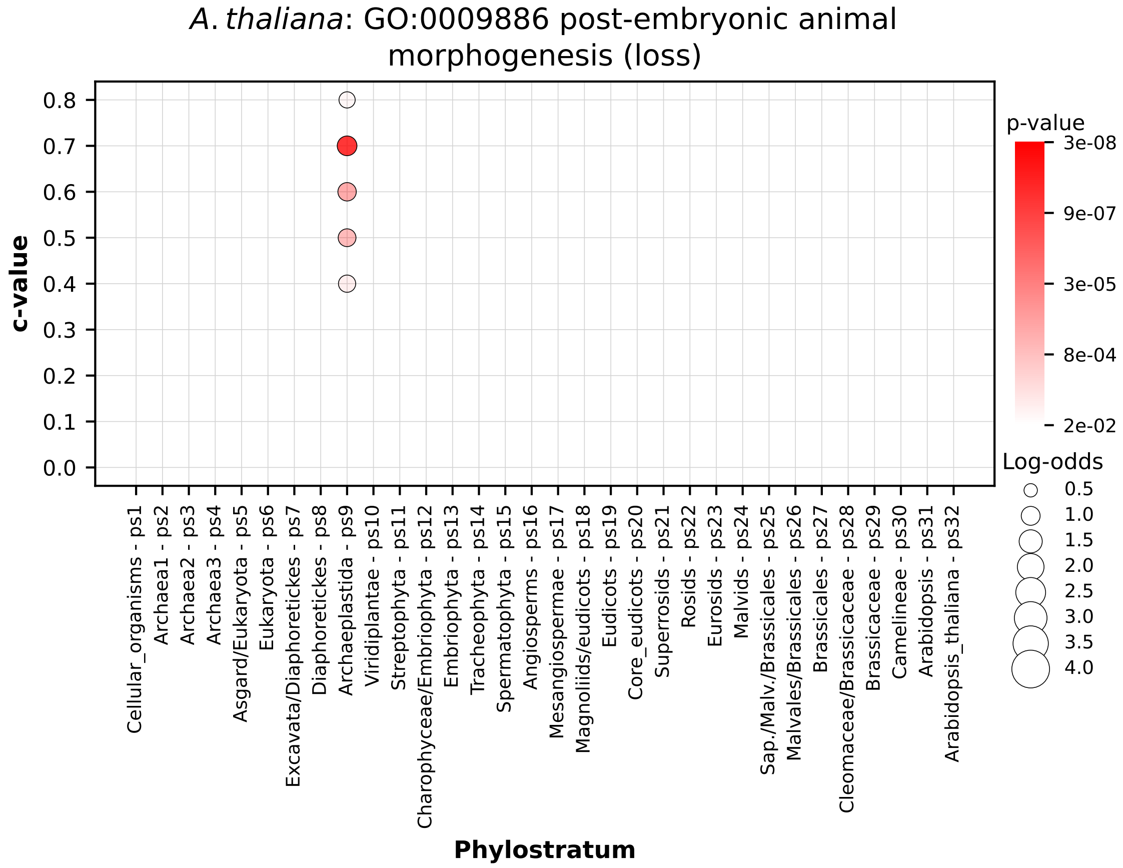

A. thaliana: GO:0009886 post-embryonic animal morphogenesis (loss)
c-value
0.0
0.1
0.2
0.3
0.4
0.5
0.6
0.7
0.8
Phylostratum
Cellular_organisms - ps1
Archaea1 - ps2
Archaea2 - ps3
Archaea3 - ps4
Asgard/Eukaryota - ps5
Eukaryota - ps6
Excavata/Diaphoretickes - ps7
Diaphoretickes - ps8
Archaeplastida - ps9
Viridiplantae - ps10
Streptophyta - ps11
Charophyceae/Embriophyta - ps12
Embriophyta - ps13
Tracheophyta - ps14
Spermatophyta - ps15
Angiosperms - ps16
Mesangiospermae - ps17
Magnoliids/eudicots - ps18
Eudicots - ps19
Core_eudicots - ps20
Superrosids - ps21
Rosids - ps22
Eurosids - ps23
Malvids - ps24
Sap./Malv./Brassicales - ps25
Malvales/Brassicales - ps26
Brassicales - ps27
Cleomaceae/Brassicaceae - ps28
Brassicaceae - ps29
Camelineae - ps30
Arabidopsis - ps31
Arabidopsis_thaliana - ps32
p-value
3e-08
9e-07
3e-05
8e-04
2e-02
Log-odds
0.5
1.0
1.5
2.0
2.5
3.0
3.5
4.0

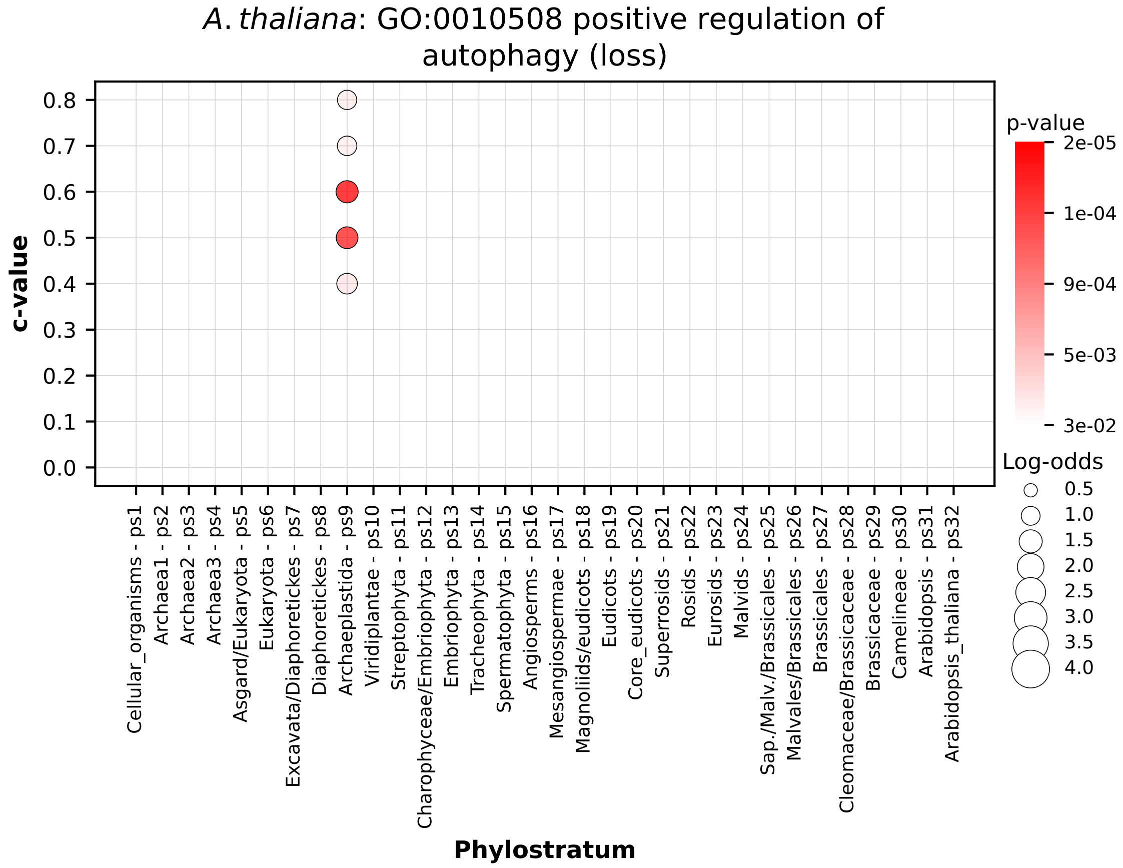

A. thaliana: GO:0010508 positive regulation of autophagy (loss)
c-value
0.8
0.7
0.6
0.5
0.4
0.3
0.2
0.1
0.0
p-value
2e-05
1e-04
9e-04
5e-03
3e-02
Log-odds
0.5
1.0
1.5
2.0
2.5
3.0
3.5
4.0
Cellular_organisms - ps1
Archaea1 - ps2
Archaea2 - ps3
Archaea3 - ps4
Asgard/Eukaryota - ps5
Eukaryota - ps6
Excavata/Diaphoretickes - ps7
Diaphoretickes - ps8
Archaeplastida - ps9
Viridiplantae - ps10
Streptophyta - ps11
Charophyceae/Embriophyta - ps12
Embriophyta - ps13
Tracheophyta - ps14
Spermatophyta - ps15
Angiosperms - ps16
Mesangiospermae - ps17
Magnoliids/eudicots - ps18
Eudicots - ps19
Core_eudicots - ps20
Superrosids - ps21
Rosids - ps22
Eurosids - ps23
Malvids - ps24
Sap./Malv./Brassicales - ps25
Malvales/Brassicales - ps26
Brassicales - ps27
Cleomaceae/Brassicaceae - ps28
Brassicaceae - ps29
Camelineae - ps30
Arabidopsis - ps31
Arabidopsis_thaliana - ps32
Phylostratum

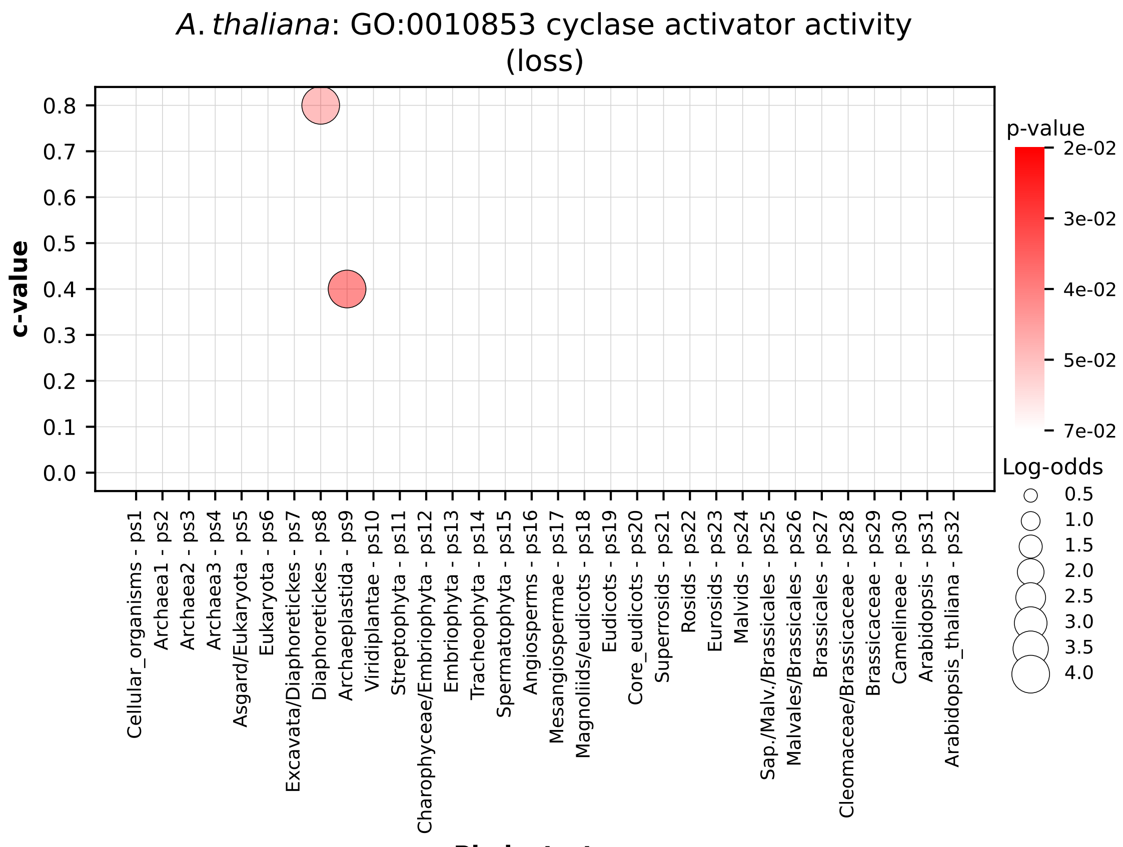

A. thaliana: GO:0010853 cyclase activator activity
(loss)
c-value
0.0
0.1
0.2
0.3
0.4
0.5
0.6
0.7
0.8
p-value
2e-02
3e-02
4e-02
5e-02
7e-02
Log-odds
0.5
1.0
1.5
2.0
2.5
3.0
3.5
4.0
Cellular_organisms - ps1
Archaea1 - ps2
Archaea2 - ps3
Archaea3 - ps4
Asgard/Eukaryota - ps5
Eukaryota - ps6
Excavata/Diaphoretickes - ps7
Diaphoretickes - ps8
Archaeplastida - ps9
Viridiplantae - ps10
Streptophyta - ps11
Charophyceae/Embriophyta - ps12
Embriophyta - ps13
Tracheophyta - ps14
Spermatophyta - ps15
Angiosperms - ps16
Mesangiospermae - ps17
Magnoliids/eudicots - ps18
Eudicots - ps19
Core_eudicots - ps20
Superrosids - ps21
Rosids - ps22
Eurosids - ps23
Malvids - ps24
Sap./Malv./Brassicales - ps25
Malvales/Brassicales - ps26
Brassicales - ps27
Cleomaceae/Brassicaceae - ps28
Brassicaceae - ps29
Camelineae - ps30
Arabidopsis - ps31
Arabidopsis_thaliana - ps32
Phylostratum

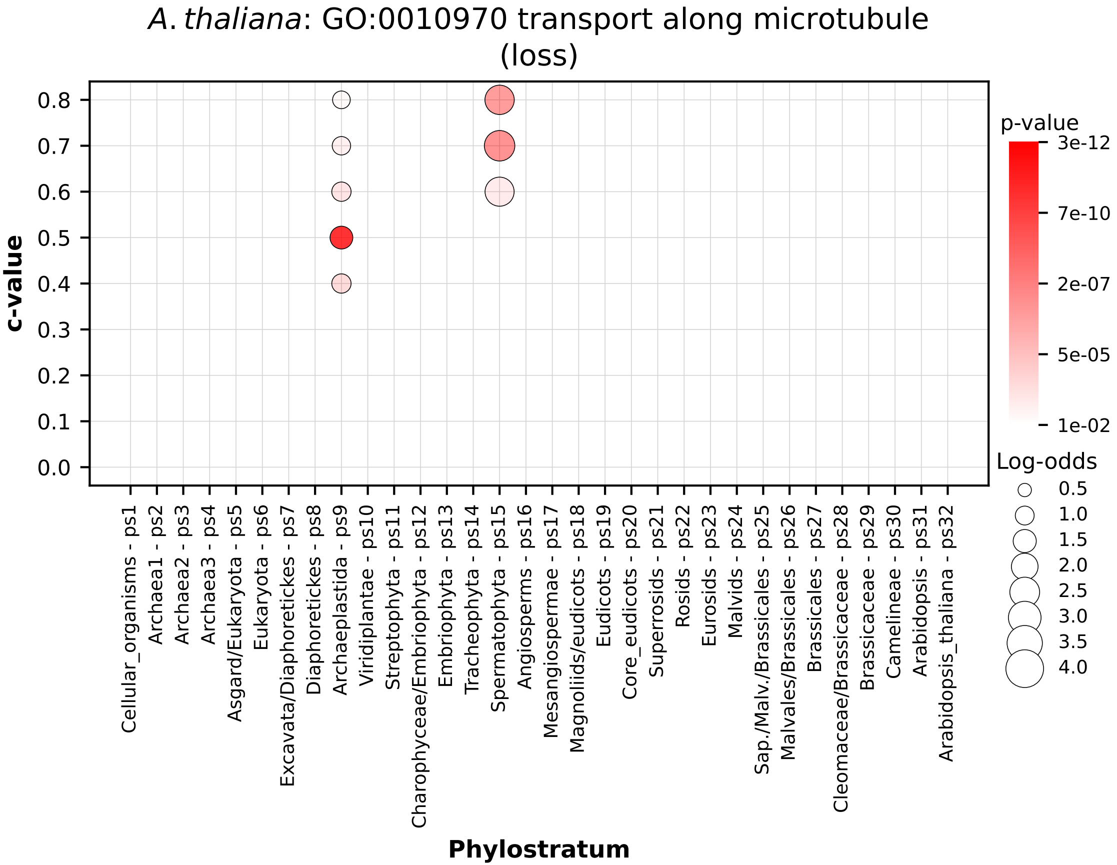

A. thaliana: GO:0010970 transport along microtubule
(loss)
c-value
0.0
0.1
0.2
0.3
0.4
0.5
0.6
0.7
0.8
Phylostratum
Cellular_organisms - ps1
Archaea1 - ps2
Archaea2 - ps3
Archaea3 - ps4
Asgard/Eukaryota - ps5
Eukaryota - ps6
Excavata/Diaphoretickes - ps7
Diaphoretickes - ps8
Archaeplastida - ps9
Viridiplantae - ps10
Streptophyta - ps11
Charophyceae/Embriophyta - ps12
Embriophyta - ps13
Tracheophyta - ps14
Spermatophyta - ps15
Angiosperms - ps16
Mesangiospermae - ps17
Magnoliids/eudicots - ps18
Eudicots - ps19
Core_eudicots - ps20
Superrosids - ps21
Rosids - ps22
Eurosids - ps23
Malvids - ps24
Sap./Malv./Brassicales - ps25
Malvales/Brassicales - ps26
Brassicales - ps27
Cleomaceae/Brassicaceae - ps28
Brassicaceae - ps29
Camelineae - ps30
Arabidopsis - ps31
Arabidopsis_thaliana - ps32
p-value
3e-12
7e-10
2e-07
5e-05
1e-02
Log-odds
0.5
1.0
1.5
2.0
2.5
3.0
3.5
4.0

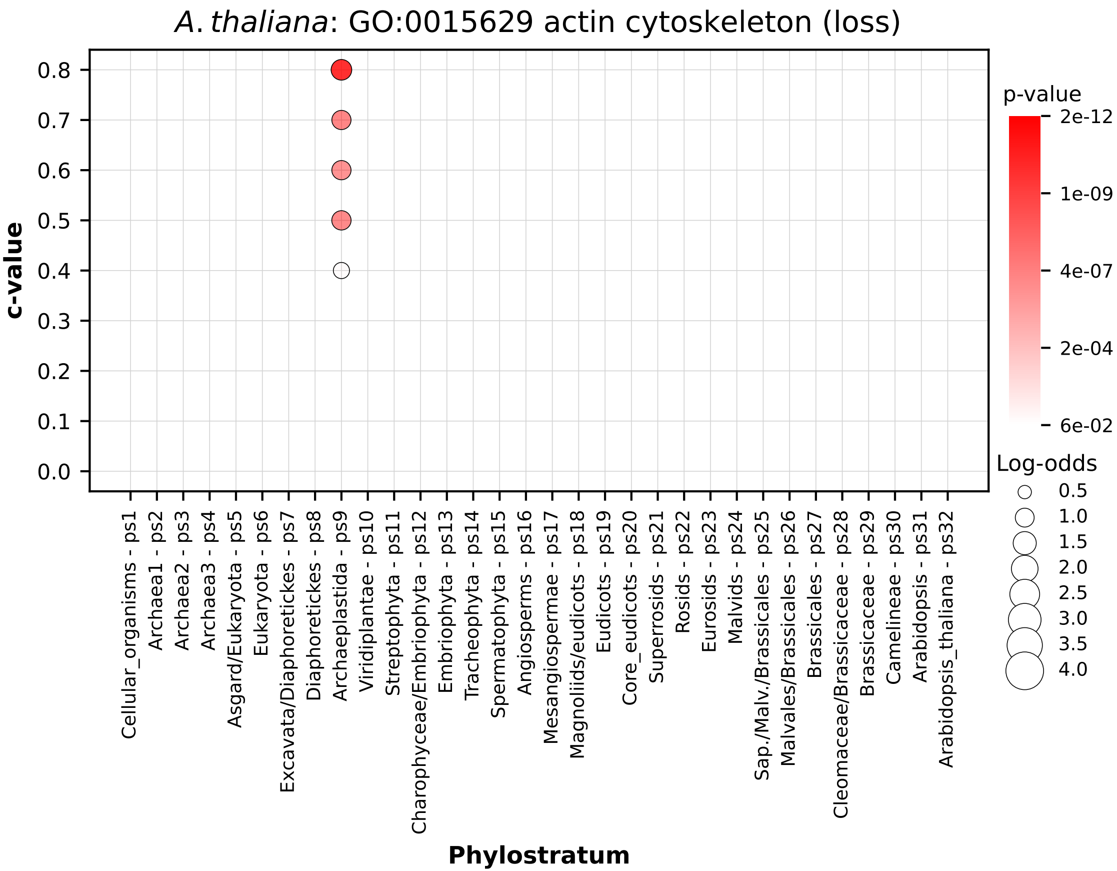

A. thaliana: GO:0015629 actin cytoskeleton (loss)
c-value
0.8
0.7
0.6
0.5
0.4
0.3
0.2
0.1
0.0
Phylostratum
Cellular_organisms - ps1
Archaea1 - ps2
Archaea2 - ps3
Archaea3 - ps4
Asgard/Eukaryota - ps5
Eukaryota - ps6
Excavata/Diaphoretickes - ps7
Diaphoretickes - ps8
Archaeplastida - ps9
Viridiplantae - ps10
Streptophyta - ps11
Charophyceae/Embriophyta - ps12
Embriophyta - ps13
Tracheophyta - ps14
Spermatophyta - ps15
Angiosperms - ps16
Mesangiospermae - ps17
Magnoliids/eudicots - ps18
Eudicots - ps19
Core_eudicots - ps20
Superrosids - ps21
Rosids - ps22
Eurosids - ps23
Malvids - ps24
Sap./Malv./Brassicales - ps25
Malvales/Brassicales - ps26
Brassicales - ps27
Cleomaceae/Brassicaceae - ps28
Brassicaceae - ps29
Camelineae - ps30
Arabidopsis - ps31
Arabidopsis_thaliana - ps32
p-value
2e-12
1e-09
4e-07
2e-04
6e-02
Log-odds
0.5
1.0
1.5
2.0
2.5
3.0
3.5
4.0

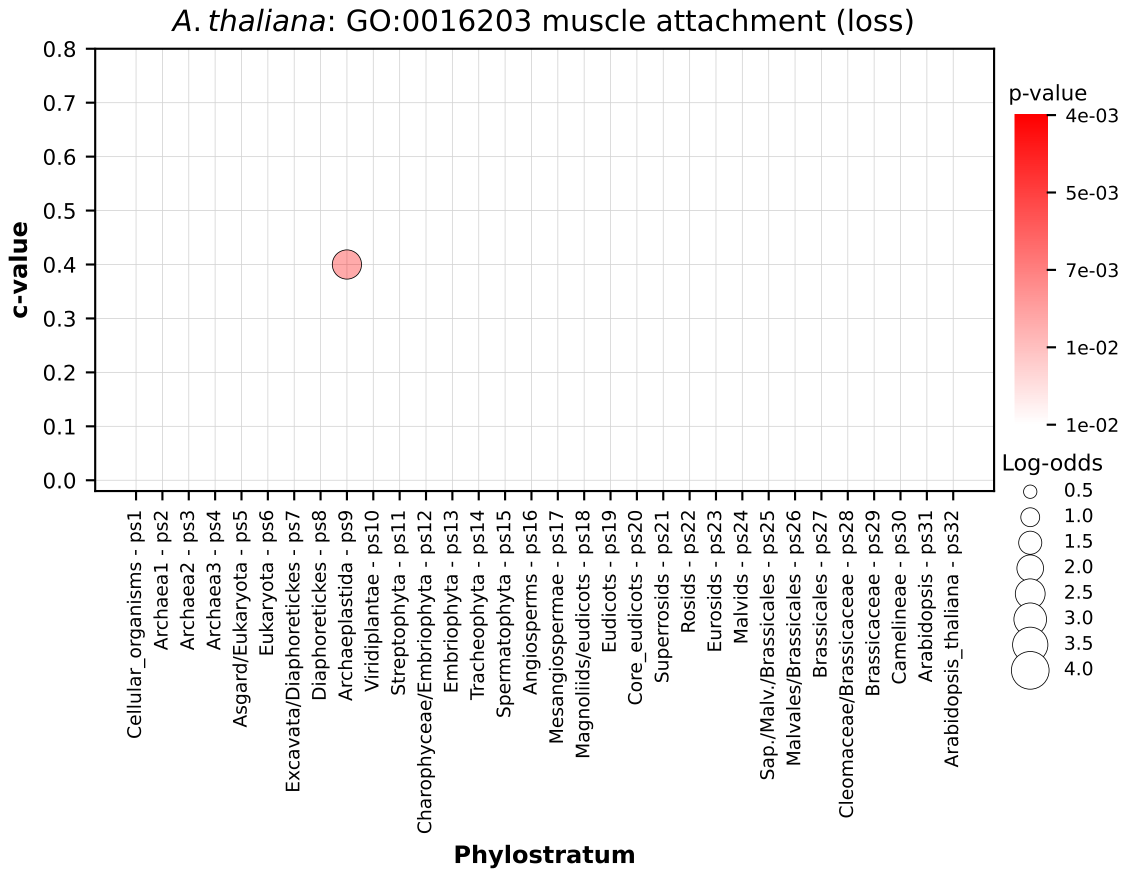

A. thaliana: GO:0016203 muscle attachment (loss)
c-value
0.0
0.1
0.2
0.3
0.4
0.5
0.6
0.7
0.8
Phylostratum
Cellular_organisms - ps1
Archaea1 - ps2
Archaea2 - ps3
Archaea3 - ps4
Asgard/Eukaryota - ps5
Eukaryota - ps6
Excavata/Diaphoretickes - ps7
Diaphoretickes - ps8
Archaeplastida - ps9
Viridiplantae - ps10
Streptophyta - ps11
Charophyceae/Embriophyta - ps12
Embriophyta - ps13
Tracheophyta - ps14
Spermatophyta - ps15
Angiosperms - ps16
Mesangiospermae - ps17
Magnoliids/eudicots - ps18
Eudicots - ps19
Core_eudicots - ps20
Superrosids - ps21
Rosids - ps22
Eurosids - ps23
Malvids - ps24
Sap./Malv./Brassicales - ps25
Malvales/Brassicales - ps26
Brassicales - ps27
Cleomaceae/Brassicaceae - ps28
Brassicaceae - ps29
Camelineae - ps30
Arabidopsis - ps31
Arabidopsis_thaliana - ps32
p-value
4e-03
5e-03
7e-03
1e-02
1e-02
Log-odds
0.5
1.0
1.5
2.0
2.5
3.0
3.5
4.0

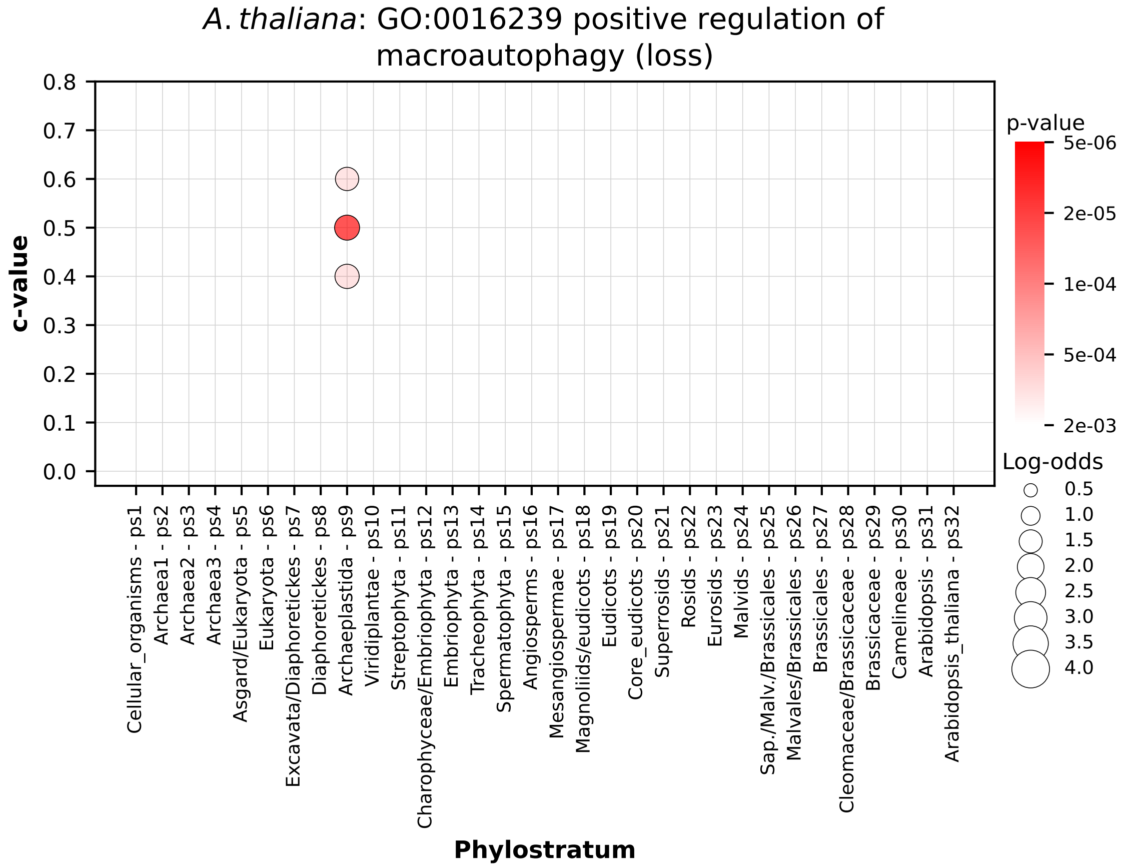

A. thaliana: GO:0016239 positive regulation of macroautophagy (loss)
c-value
0.8
0.7
0.6
0.5
0.4
0.3
0.2
0.1
0.0
p-value
5e-06
2e-05
1e-04
5e-04
2e-03
Log-odds
0.5
1.0
1.5
2.0
2.5
3.0
3.5
4.0
Cellular_organisms - ps1
Archaea1 - ps2
Archaea2 - ps3
Archaea3 - ps4
Asgard/Eukaryota - ps5
Eukaryota - ps6
Excavata/Diaphoretickes - ps7
Diaphoretickes - ps8
Archaeplastida - ps9
Viridiplantae - ps10
Streptophyta - ps11
Charophyceae/Embriophyta - ps12
Embriophyta - ps13
Tracheophyta - ps14
Spermatophyta - ps15
Angiosperms - ps16
Mesangiospermae - ps17
Magnoliids/eudicots - ps18
Eudicots - ps19
Core_eudicots - ps20
Superrosids - ps21
Rosids - ps22
Eurosids - ps23
Malvids - ps24
Sap./Malv./Brassicales - ps25
Malvales/Brassicales - ps26
Brassicales - ps27
Cleomaceae/Brassicaceae - ps28
Brassicaceae - ps29
Camelineae - ps30
Arabidopsis - ps31
Arabidopsis_thaliana - ps32
Phylostratum

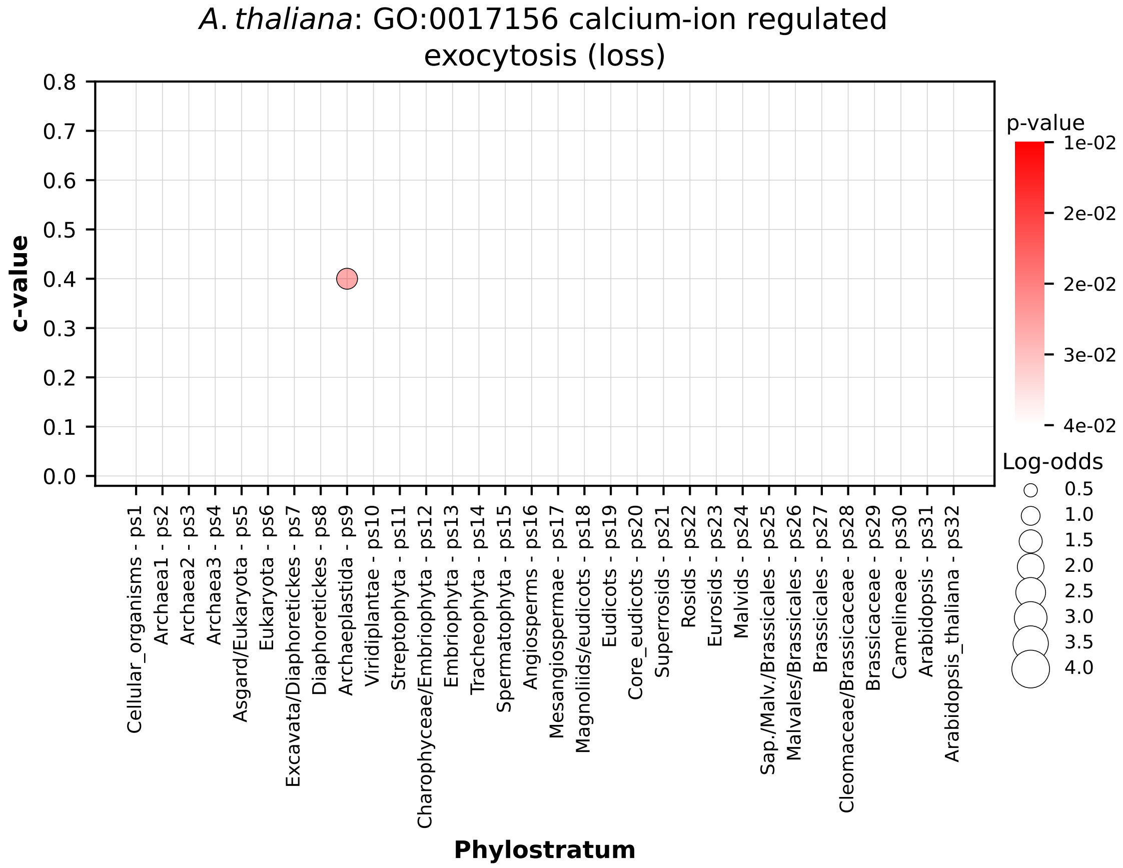

A. thaliana: GO:0017156 calcium-ion regulated exocytosis (loss)
c-value
0.8
0.7
0.6
0.5
0.4
0.3
0.2
0.1
0.0
p-value
1e-02
2e-02
2e-02
3e-02
4e-02
Log-odds
0.5
1.0
1.5
2.0
2.5
3.0
3.5
4.0
Cellular_organisms - ps1
Archaea1 - ps2
Archaea2 - ps3
Archaea3 - ps4
Asgard/Eukaryota - ps5
Eukaryota - ps6
Excavata/Diaphoretickes - ps7
Diaphoretickes - ps8
Archaeplastida - ps9
Viridiplantae - ps10
Streptophyta - ps11
Charophyceae/Embriophyta - ps12
Embriophyta - ps13
Tracheophyta - ps14
Spermatophyta - ps15
Angiosperms - ps16
Mesangiospermae - ps17
Magnoliids/eudicots - ps18
Eudicots - ps19
Core_eudicots - ps20
Superrosids - ps21
Rosids - ps22
Eurosids - ps23
Malvids - ps24
Sap./Malv./Brassicales - ps25
Malvales/Brassicales - ps26
Brassicales - ps27
Cleomaceae/Brassicaceae - ps28
Brassicaceae - ps29
Camelineae - ps30
Arabidopsis - ps31
Arabidopsis_thaliana - ps32
Phylostratum

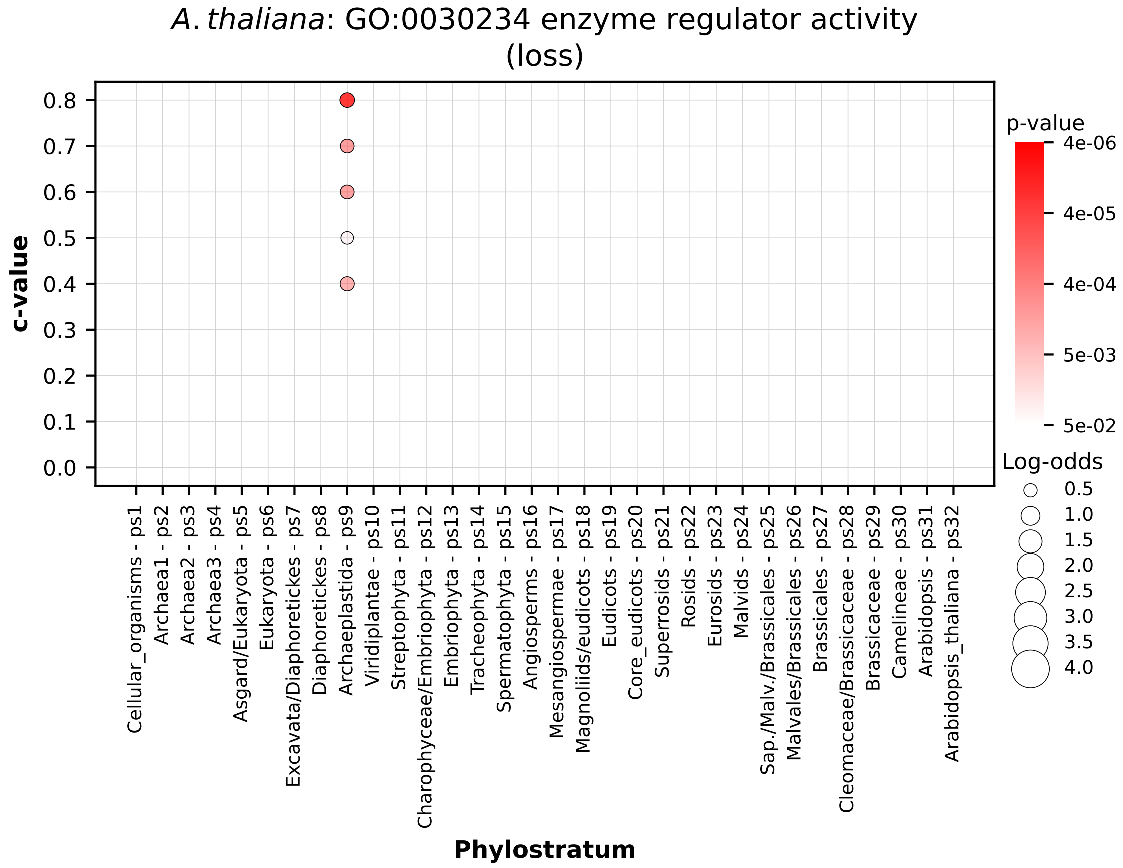

A. thaliana: GO:0030234 enzyme regulator activity
(loss)
c-value
0.0
0.1
0.2
0.3
0.4
0.5
0.6
0.7
0.8
p-value
4e-06
4e-05
4e-04
5e-03
5e-02
Log-odds
0.5
1.0
1.5
2.0
2.5
3.0
3.5
4.0
Cellular_organisms - ps1
Archaea1 - ps2
Archaea2 - ps3
Archaea3 - ps4
Asgard/Eukaryota - ps5
Eukaryota - ps6
Excavata/Diaphoretickes - ps7
Diaphoretickes - ps8
Archaeplastida - ps9
Viridiplantae - ps10
Streptophyta - ps11
Charophyceae/Embriophyta - ps12
Embriophyta - ps13
Tracheophyta - ps14
Spermatophyta - ps15
Angiosperms - ps16
Mesangiospermae - ps17
Magnoliids/eudicots - ps18
Eudicots - ps19
Core_eudicots - ps20
Superrosids - ps21
Rosids - ps22
Eurosids - ps23
Malvids - ps24
Sap./Malv./Brassicales - ps25
Malvales/Brassicales - ps26
Brassicales - ps27
Cleomaceae/Brassicaceae - ps28
Brassicaceae - ps29
Camelineae - ps30
Arabidopsis - ps31
Arabidopsis_thaliana - ps32
Phylostratum

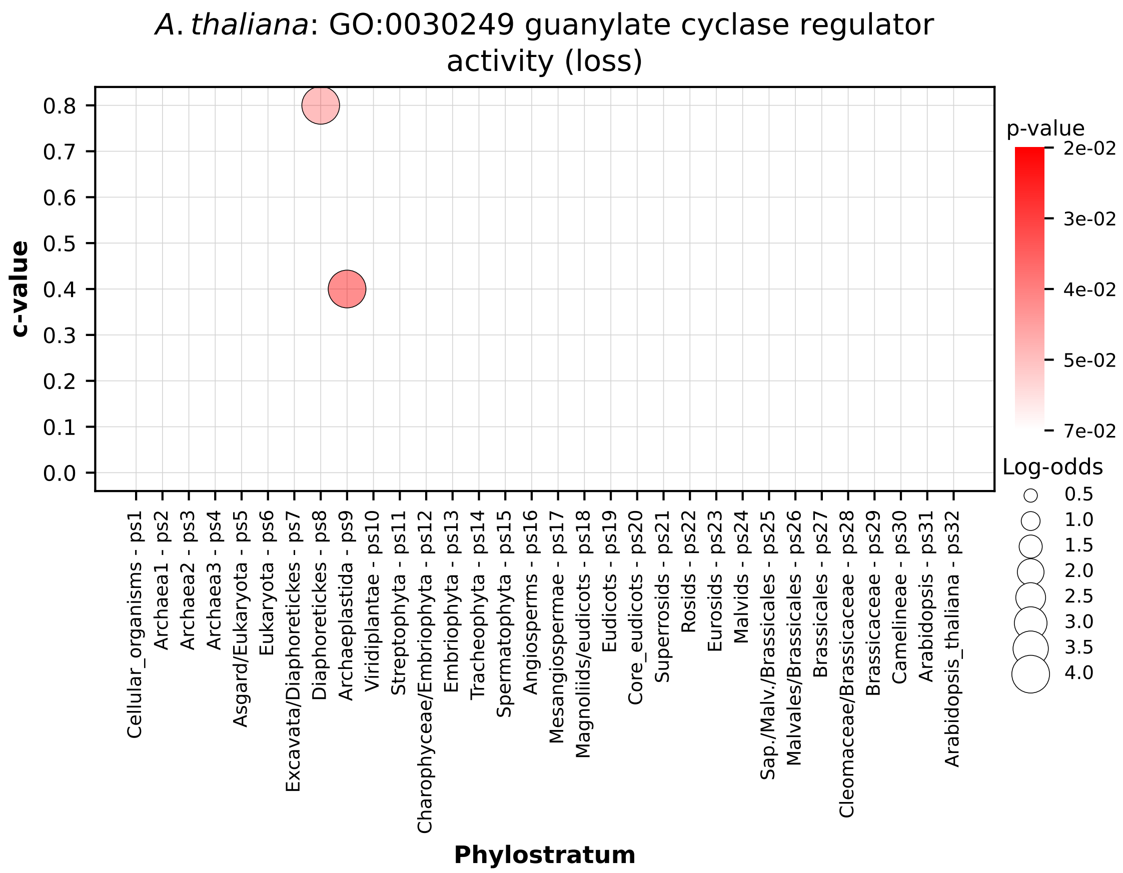

A. thaliana: GO:0030249 guanylate cyclase regulator activity (loss)
c-value
0.0
0.1
0.2
0.3
0.4
0.5
0.6
0.7
0.8
Phylostratum
Cellular_organisms - ps1
Archaea1 - ps2
Archaea2 - ps3
Archaea3 - ps4
Asgard/Eukaryota - ps5
Eukaryota - ps6
Excavata/Diaphoretickes - ps7
Diaphoretickes - ps8
Archaeplastida - ps9
Viridiplantae - ps10
Streptophyta - ps11
Charophyceae/Embriophyta - ps12
Embriophyta - ps13
Tracheophyta - ps14
Spermatophyta - ps15
Angiosperms - ps16
Mesangiospermae - ps17
Magnoliids/eudicots - ps18
Eudicots - ps19
Core_eudicots - ps20
Superrosids - ps21
Rosids - ps22
Eurosids - ps23
Malvids - ps24
Sap./Malv./Brassicales - ps25
Malvales/Brassicales - ps26
Brassicales - ps27
Cleomaceae/Brassicaceae - ps28
Brassicaceae - ps29
Camelineae - ps30
Arabidopsis - ps31
Arabidopsis_thaliana - ps32
p-value
2e-02
3e-02
4e-02
5e-02
7e-02
Log-odds
0.5
1.0
1.5
2.0
2.5
3.0
3.5
4.0

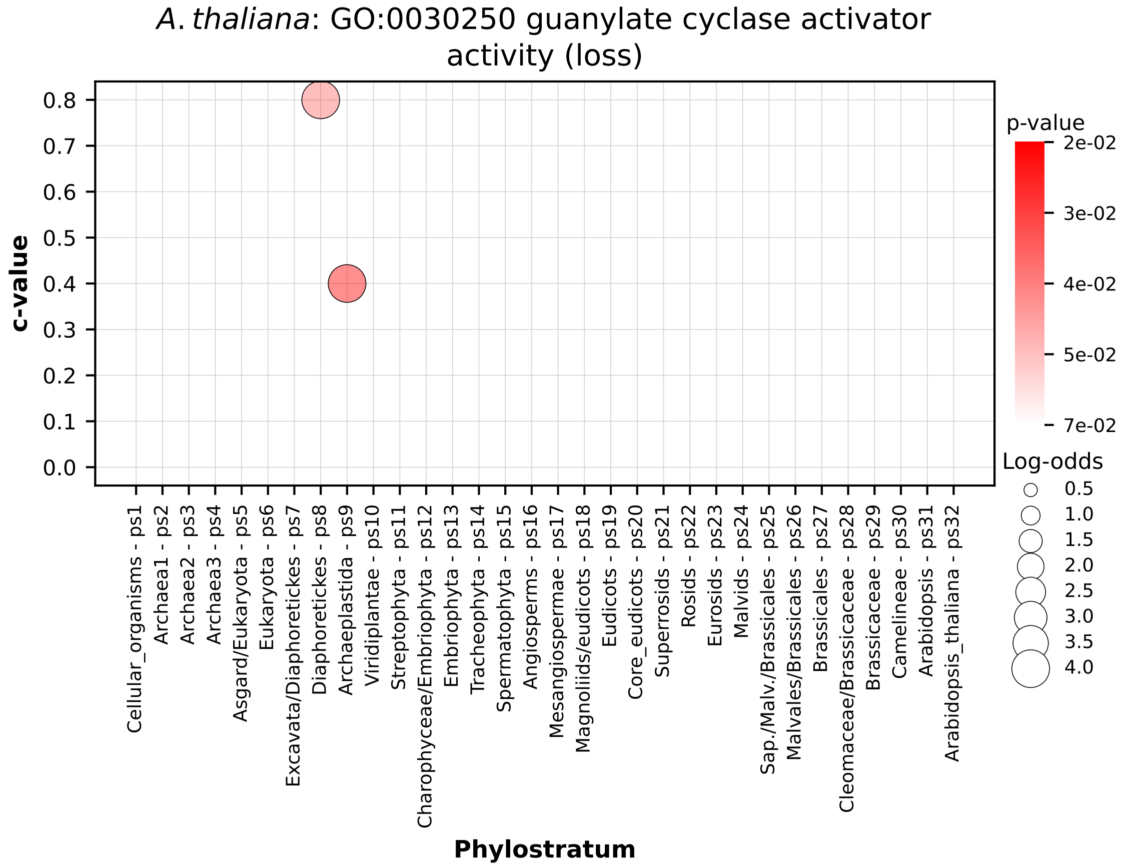

A. thaliana: GO:0030250 guanylate cyclase activator activity (loss)
c-value
0.0
0.1
0.2
0.3
0.4
0.5
0.6
0.7
0.8
Phylostratum
Cellular_organisms - ps1
Archaea1 - ps2
Archaea2 - ps3
Archaea3 - ps4
Asgard/Eukaryota - ps5
Eukaryota - ps6
Excavata/Diaphoretickes - ps7
Diaphoretickes - ps8
Archaeplastida - ps9
Viridiplantae - ps10
Streptophyta - ps11
Charophyceae/Embriophyta - ps12
Embriophyta - ps13
Tracheophyta - ps14
Spermatophyta - ps15
Angiosperms - ps16
Mesangiospermae - ps17
Magnoliids/eudicots - ps18
Eudicots - ps19
Core_eudicots - ps20
Superrosids - ps21
Rosids - ps22
Eurosids - ps23
Malvids - ps24
Sap./Malv./Brassicales - ps25
Malvales/Brassicales - ps26
Brassicales - ps27
Cleomaceae/Brassicaceae - ps28
Brassicaceae - ps29
Camelineae - ps30
Arabidopsis - ps31
Arabidopsis_thaliana - ps32
p-value
2e-02
3e-02
4e-02
5e-02
7e-02
Log-odds
0.5
1.0
1.5
2.0
2.5
3.0
3.5
4.0

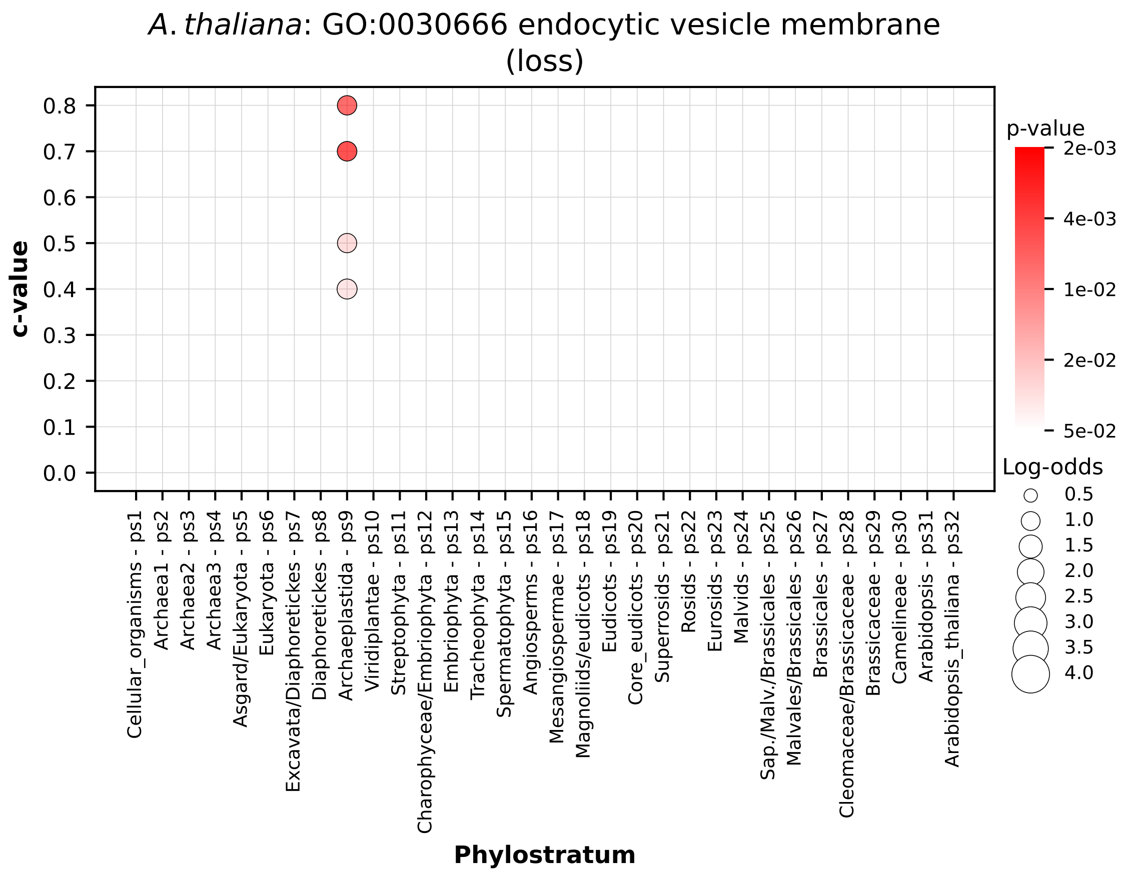

A. thaliana: GO:0030666 endocytic vesicle membrane
(loss)
c-value
0.0
0.1
0.2
0.3
0.4
0.5
0.6
0.7
0.8
p-value
2e-03
4e-03
1e-02
2e-02
5e-02
Log-odds
0.5
1.0
1.5
2.0
2.5
3.0
3.5
4.0
Cellular_organisms - ps1
Archaea1 - ps2
Archaea2 - ps3
Archaea3 - ps4
Asgard/Eukaryota - ps5
Eukaryota - ps6
Excavata/Diaphoretickes - ps7
Diaphoretickes - ps8
Archaeplastida - ps9
Viridiplantae - ps10
Streptophyta - ps11
Charophyceae/Embriophyta - ps12
Embriophyta - ps13
Tracheophyta - ps14
Spermatophyta - ps15
Angiosperms - ps16
Mesangiospermae - ps17
Magnoliids/eudicots - ps18
Eudicots - ps19
Core_eudicots - ps20
Superrosids - ps21
Rosids - ps22
Eurosids - ps23
Malvids - ps24
Sap./Malv./Brassicales - ps25
Malvales/Brassicales - ps26
Brassicales - ps27
Cleomaceae/Brassicaceae - ps28
Brassicaceae - ps29
Camelineae - ps30
Arabidopsis - ps31
Arabidopsis_thaliana - ps32
Phylostratum

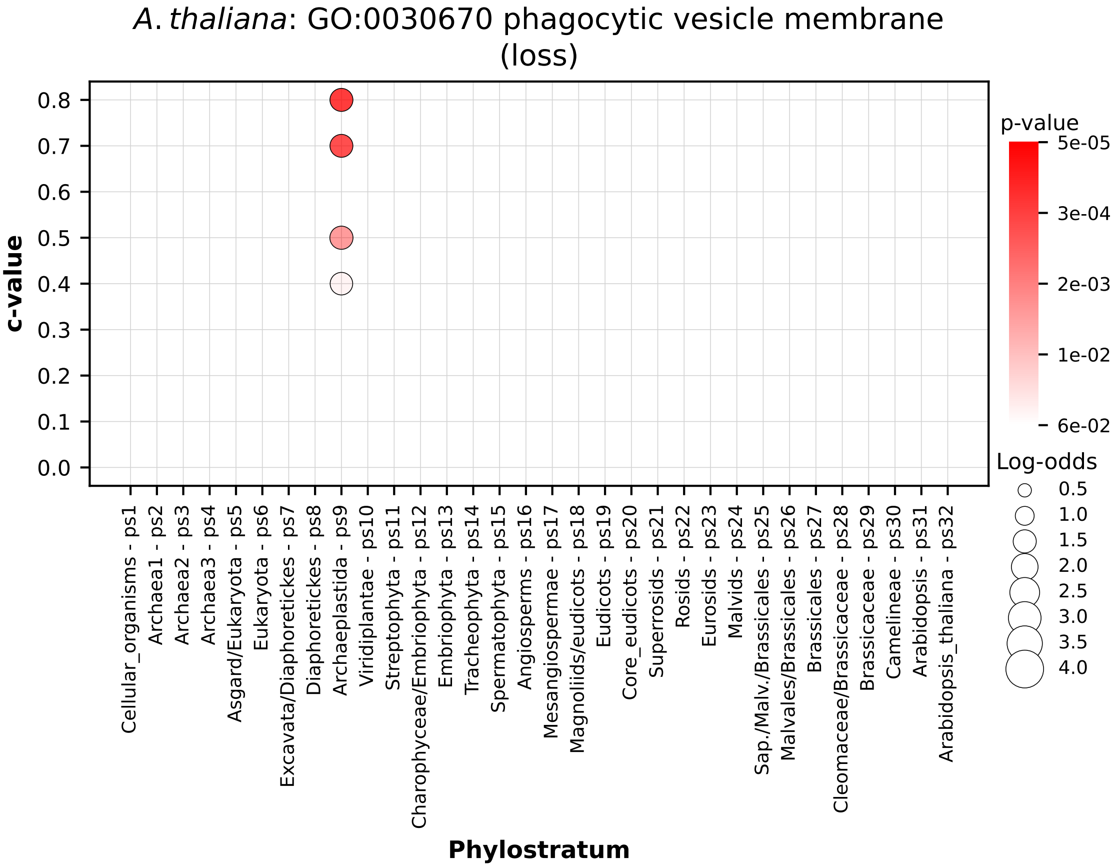
A. thaliana: GO:0030670 phagocytic vesicle membrane
(loss)
c-value
0.8
0.7
0.6
0.5
0.4
0.3
0.2
0.1
0.0
p-value
5e-05
3e-04
2e-03
1e-02
6e-02
Log-odds
0.5
1.0
1.5
2.0
2.5
3.0
3.5
4.0
Cellular_organisms - ps1
Archaea1 - ps2
Archaea2 - ps3
Archaea3 - ps4
Asgard/Eukaryota - ps5
Eukaryota - ps6
Excavata/Diaphoretickes - ps7
Diaphoretickes - ps8
Archaeplastida - ps9
Viridiplantae - ps10
Streptophyta - ps11
Charophyceae/Embriophyta - ps12
Embriophyta - ps13
Tracheophyta - ps14
Spermatophyta - ps15
Angiosperms - ps16
Mesangiospermae - ps17
Magnoliids/eudicots - ps18
Eudicots - ps19
Core_eudicots - ps20
Superrosids - ps21
Rosids - ps22
Eurosids - ps23
Malvids - ps24
Sap./Malv./Brassicales - ps25
Malvales/Brassicales - ps26
Brassicales - ps27
Cleomaceae/Brassicaceae - ps28
Brassicaceae - ps29
Camelineae - ps30
Arabidopsis - ps31
Arabidopsis_thaliana - ps32
Phylostratum

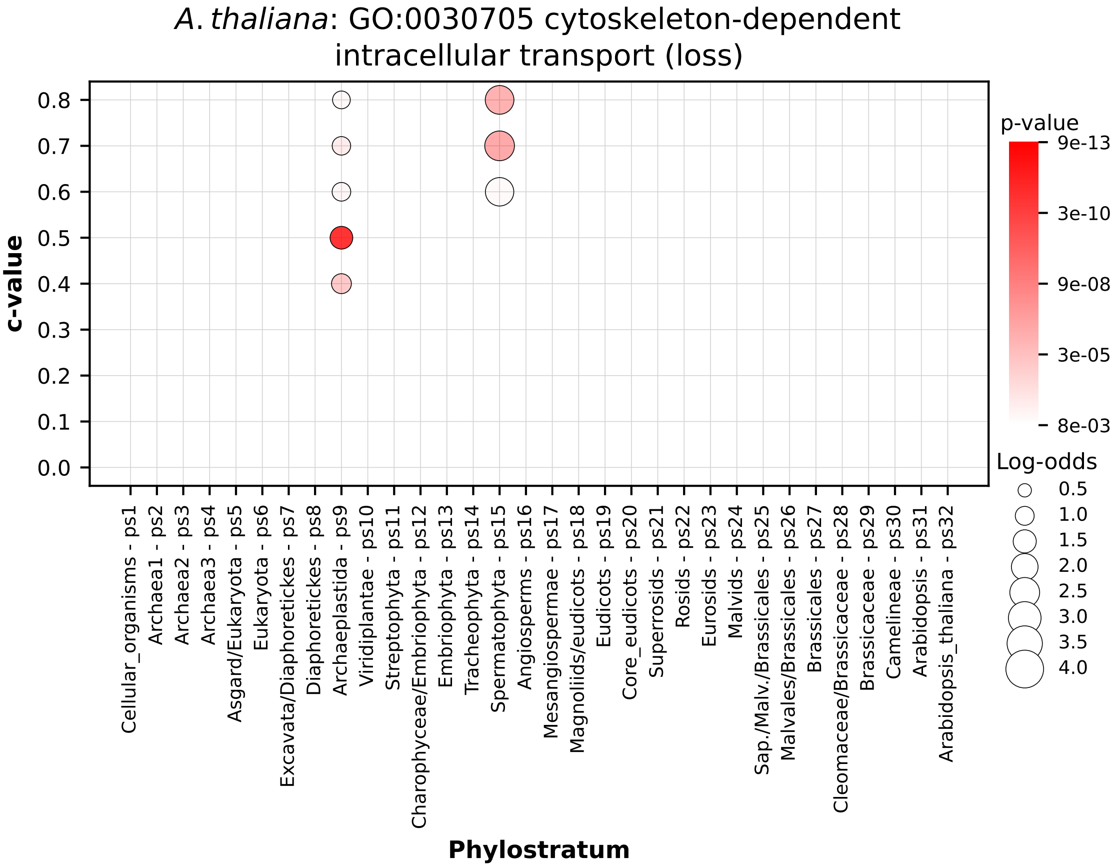

A. thaliana: GO:0030705 cytoskeleton-dependent intracellular transport (loss)
c-value
0.0
0.1
0.2
0.3
0.4
0.5
0.6
0.7
0.8
Phylostratum
Cellular_organisms - ps1
Archaea1 - ps2
Archaea2 - ps3
Archaea3 - ps4
Asgard/Eukaryota - ps5
Eukaryota - ps6
Excavata/Diaphoretickes - ps7
Diaphoretickes - ps8
Archaeplastida - ps9
Viridiplantae - ps10
Streptophyta - ps11
Charophyceae/Embriophyta - ps12
Embriophyta - ps13
Tracheophyta - ps14
Spermatophyta - ps15
Angiosperms - ps16
Mesangiospermae - ps17
Magnoliids/eudicots - ps18
Eudicots - ps19
Core_eudicots - ps20
Superrosids - ps21
Rosids - ps22
Eurosids - ps23
Malvids - ps24
Sap./Malv./Brassicales - ps25
Malvales/Brassicales - ps26
Brassicales - ps27
Cleomaceae/Brassicaceae - ps28
Brassicaceae - ps29
Camelineae - ps30
Arabidopsis - ps31
Arabidopsis_thaliana - ps32
p-value
9e-13
3e-10
9e-08
3e-05
8e-03
Log-odds
0.5
1.0
1.5
2.0
2.5
3.0
3.5
4.0

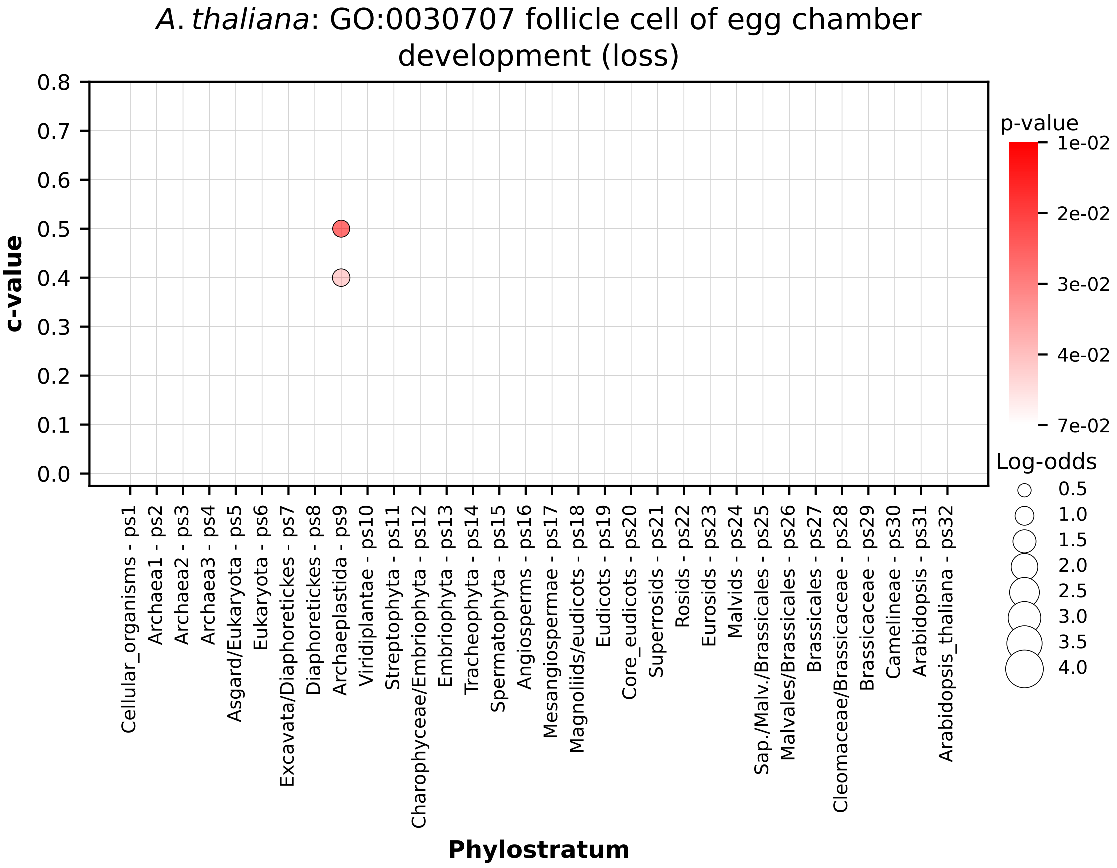

A. thaliana: GO:0030707 follicle cell of egg chamber development (loss)
c-value
0.8
0.7
0.6
0.5
0.4
0.3
0.2
0.1
0.0
p-value
1e-02
2e-02
3e-02
4e-02
7e-02
Log-odds
0.5
1.0
1.5
2.0
2.5
3.0
3.5
4.0
Cellular_organisms - ps1
Archaea1 - ps2
Archaea2 - ps3
Archaea3 - ps4
Asgard/Eukaryota - ps5
Eukaryota - ps6
Excavata/Diaphoretickes - ps7
Diaphoretickes - ps8
Archaeplastida - ps9
Viridiplantae - ps10
Streptophyta - ps11
Charophyceae/Embriophyta - ps12
Embriophyta - ps13
Tracheophyta - ps14
Spermatophyta - ps15
Angiosperms - ps16
Mesangiospermae - ps17
Magnoliids/eudicots - ps18
Eudicots - ps19
Core_eudicots - ps20
Superrosids - ps21
Rosids - ps22
Eurosids - ps23
Malvids - ps24
Sap./Malv./Brassicales - ps25
Malvales/Brassicales - ps26
Brassicales - ps27
Cleomaceae/Brassicaceae - ps28
Brassicaceae - ps29
Camelineae - ps30
Arabidopsis - ps31
Arabidopsis_thaliana - ps32
Phylostratum

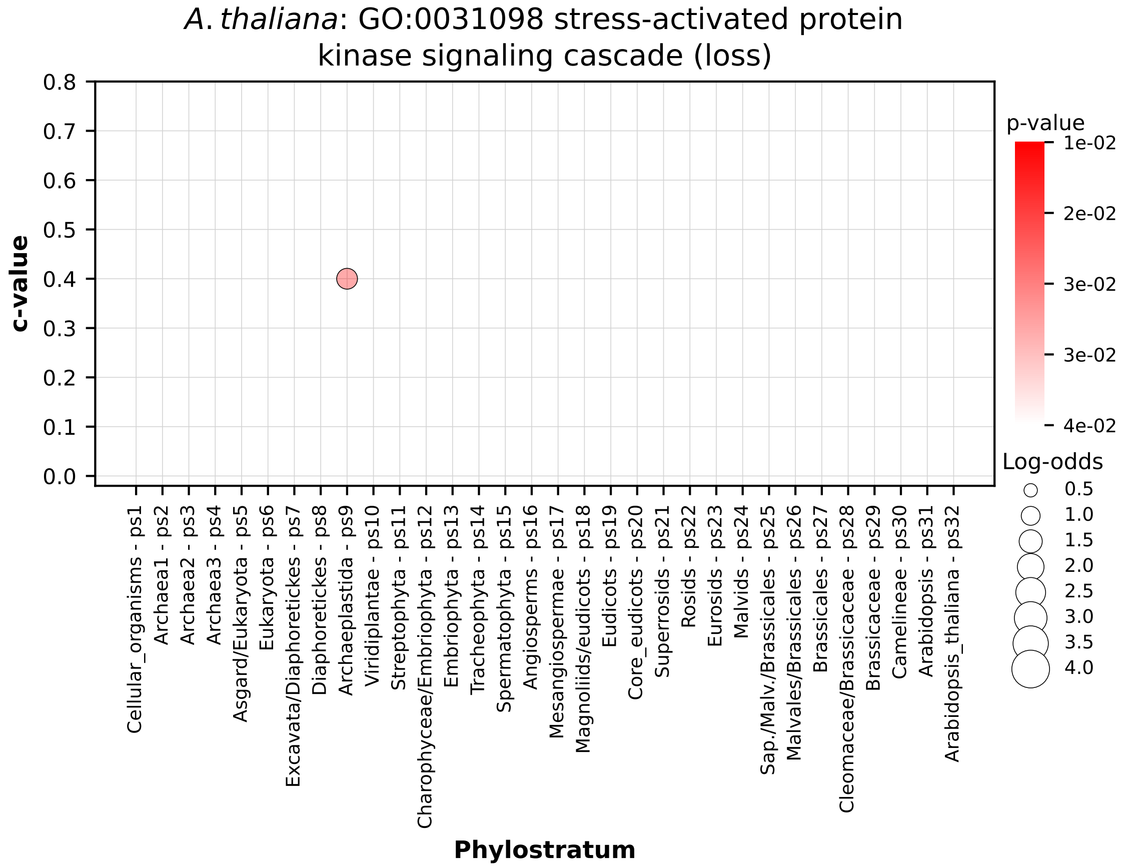

A. thaliana: GO:0031098 stress-activated protein kinase signaling cascade (loss)
c-value
0.8
0.7
0.6
0.5
0.4
0.3
0.2
0.1
0.0
p-value
1e-02
2e-02
3e-02
3e-02
4e-02
Log-odds
0.5
1.0
1.5
2.0
2.5
3.0
3.5
4.0
Cellular_organisms - ps1
Archaea1 - ps2
Archaea2 - ps3
Archaea3 - ps4
Asgard/Eukaryota - ps5
Eukaryota - ps6
Excavata/Diaphoretickes - ps7
Diaphoretickes - ps8
Archaeplastida - ps9
Viridiplantae - ps10
Streptophyta - ps11
Charophyceae/Embriophyta - ps12
Embriophyta - ps13
Tracheophyta - ps14
Spermatophyta - ps15
Angiosperms - ps16
Mesangiospermae - ps17
Magnoliids/eudicots - ps18
Eudicots - ps19
Core_eudicots - ps20
Superrosids - ps21
Rosids - ps22
Eurosids - ps23
Malvids - ps24
Sap./Malv./Brassicales - ps25
Malvales/Brassicales - ps26
Brassicales - ps27
Cleomaceae/Brassicaceae - ps28
Brassicaceae - ps29
Camelineae - ps30
Arabidopsis - ps31
Arabidopsis_thaliana - ps32
Phylostratum

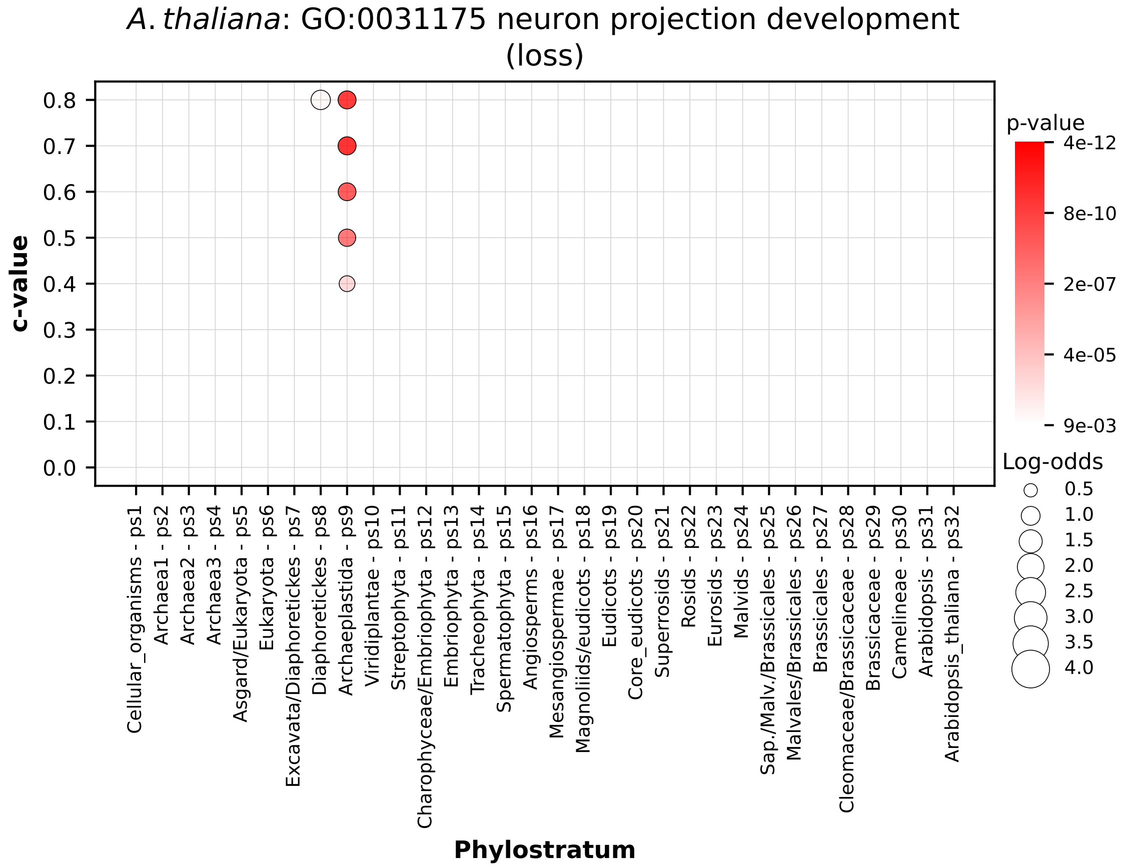
A. thaliana: GO:0031175 neuron projection development
(loss)
c-value
0.8
0.7
0.6
0.5
0.4
0.3
0.2
0.1
0.0
p-value
4e-12
8e-10
2e-07
4e-05
9e-03
Log-odds
0.5
1.0
1.5
2.0
2.5
3.0
3.5
4.0
Cellular_organisms - ps1
Archaea1 - ps2
Archaea2 - ps3
Archaea3 - ps4
Asgard/Eukaryota - ps5
Eukaryota - ps6
Excavata/Diaphoretickes - ps7
Diaphoretickes - ps8
Archaeplastida - ps9
Viridiplantae - ps10
Streptophyta - ps11
Charophyceae/Embriophyta - ps12
Embriophyta - ps13
Tracheophyta - ps14
Spermatophyta - ps15
Angiosperms - ps16
Mesangiospermae - ps17
Magnoliids/eudicots - ps18
Eudicots - ps19
Core_eudicots - ps20
Superrosids - ps21
Rosids - ps22
Eurosids - ps23
Malvids - ps24
Sap./Malv./Brassicales - ps25
Malvales/Brassicales - ps26
Brassicales - ps27
Cleomaceae/Brassicaceae - ps28
Brassicaceae - ps29
Camelineae - ps30
Arabidopsis - ps31
Arabidopsis_thaliana - ps32
Phylostratum

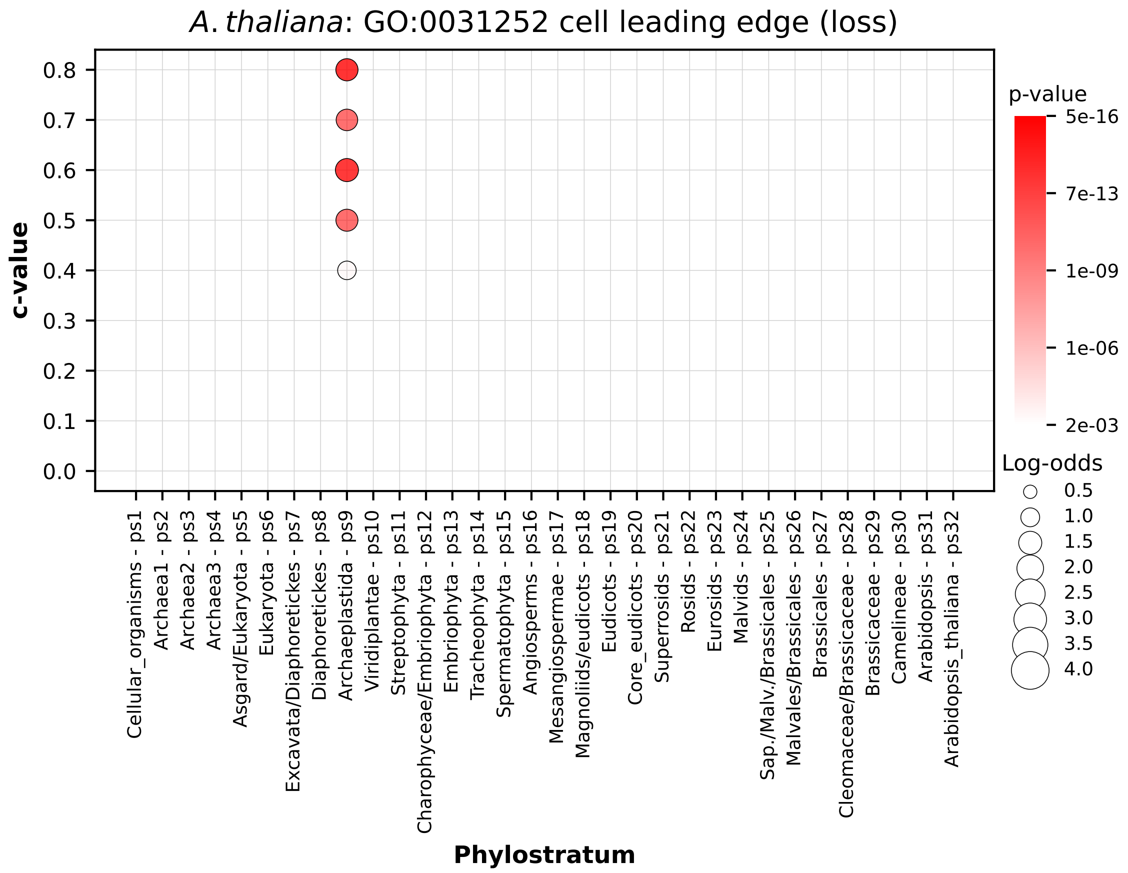

A. thaliana: GO:0031252 cell leading edge (loss)
c-value
0.8
0.7
0.6
0.5
0.4
0.3
0.2
0.1
0.0
p-value
5e-16
7e-13
1e-09
1e-06
2e-03
Log-odds
0.5
1.0
1.5
2.0
2.5
3.0
3.5
4.0
Cellular_organisms - ps1
Archaea1 - ps2
Archaea2 - ps3
Archaea3 - ps4
Asgard/Eukaryota - ps5
Eukaryota - ps6
Excavata/Diaphoretickes - ps7
Diaphoretickes - ps8
Archaeplastida - ps9
Viridiplantae - ps10
Streptophyta - ps11
Charophyceae/Embriophyta - ps12
Embriophyta - ps13
Tracheophyta - ps14
Spermatophyta - ps15
Angiosperms - ps16
Mesangiospermae - ps17
Magnoliids/eudicots - ps18
Eudicots - ps19
Core_eudicots - ps20
Superrosids - ps21
Rosids - ps22
Eurosids - ps23
Malvids - ps24
Sap./Malv./Brassicales - ps25
Malvales/Brassicales - ps26
Brassicales - ps27
Cleomaceae/Brassicaceae - ps28
Brassicaceae - ps29
Camelineae - ps30
Arabidopsis - ps31
Arabidopsis_thaliana - ps32
Phylostratum

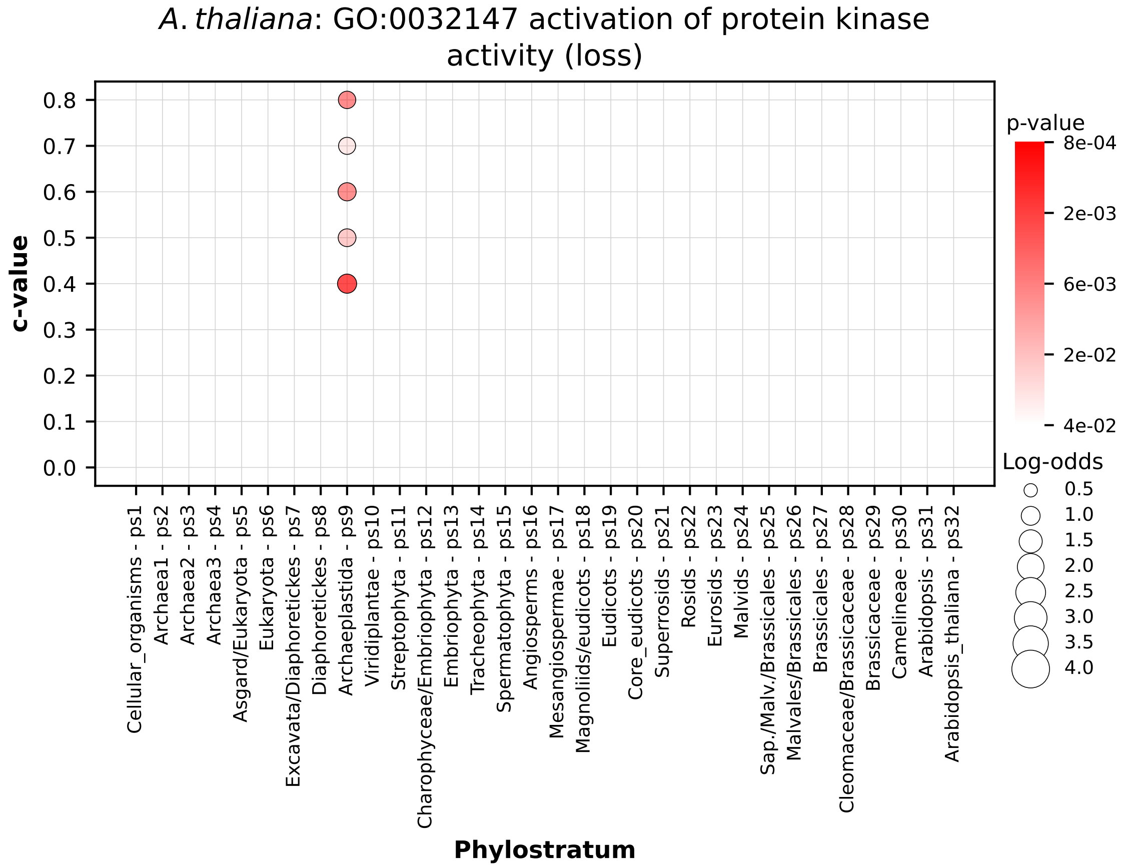

A. thaliana: GO:0032147 activation of protein kinase activity (loss)
c-value
0.0
0.1
0.2
0.3
0.4
0.5
0.6
0.7
0.8
p-value
8e-04
2e-03
6e-03
2e-02
4e-02
Log-odds
0.5
1.0
1.5
2.0
2.5
3.0
3.5
4.0
Cellular_organisms - ps1
Archaea1 - ps2
Archaea2 - ps3
Archaea3 - ps4
Asgard/Eukaryota - ps5
Eukaryota - ps6
Excavata/Diaphoretickes - ps7
Diaphoretickes - ps8
Archaeplastida - ps9
Viridiplantae - ps10
Streptophyta - ps11
Charophyceae/Embriophyta - ps12
Embriophyta - ps13
Tracheophyta - ps14
Spermatophyta - ps15
Angiosperms - ps16
Mesangiospermae - ps17
Magnoliids/eudicots - ps18
Eudicots - ps19
Core_eudicots - ps20
Superrosids - ps21
Rosids - ps22
Eurosids - ps23
Malvids - ps24
Sap./Malv./Brassicales - ps25
Malvales/Brassicales - ps26
Brassicales - ps27
Cleomaceae/Brassicaceae - ps28
Brassicaceae - ps29
Camelineae - ps30
Arabidopsis - ps31
Arabidopsis_thaliana - ps32
Phylostratum

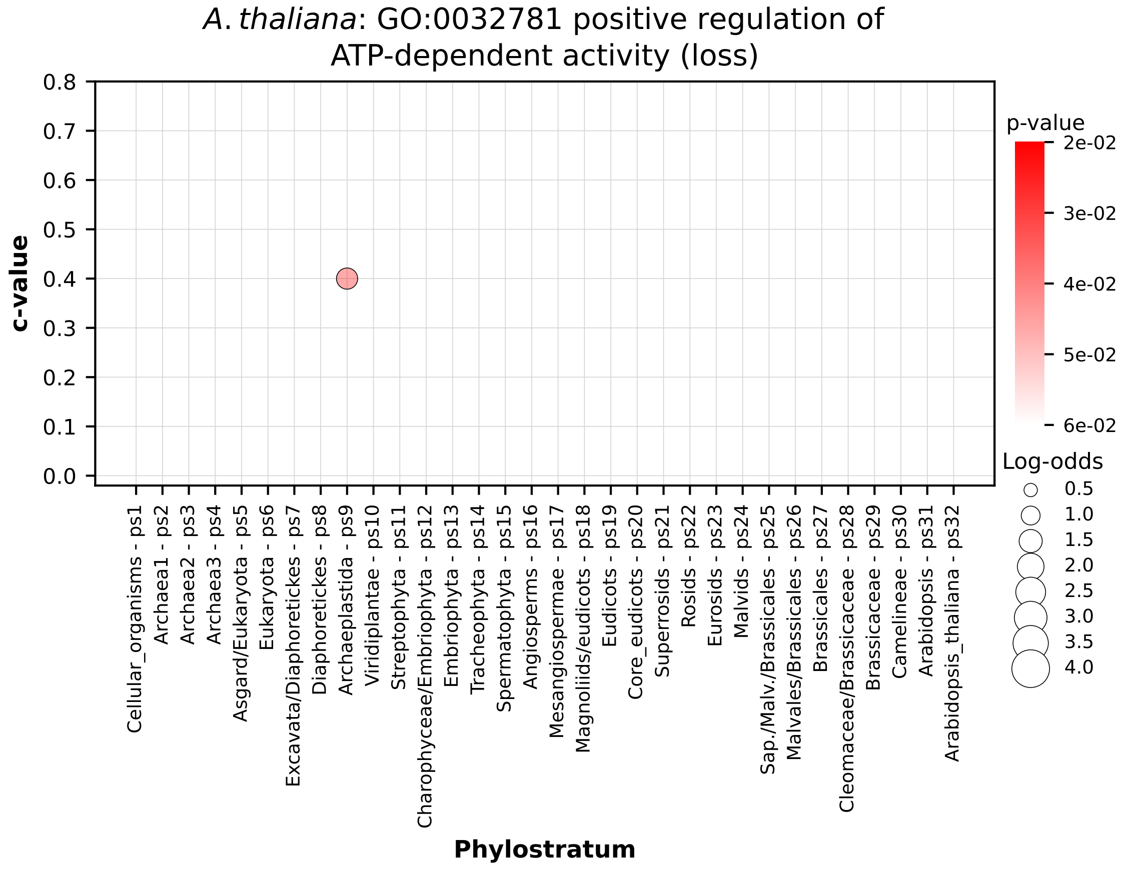

A. thaliana: GO:0032781 positive regulation of ATP-dependent activity (loss)
c-value
0.0
0.1
0.2
0.3
0.4
0.5
0.6
0.7
0.8
Phylostratum
Cellular_organisms - ps1
Archaea1 - ps2
Archaea2 - ps3
Archaea3 - ps4
Asgard/Eukaryota - ps5
Eukaryota - ps6
Excavata/Diaphoretickes - ps7
Diaphoretickes - ps8
Archaeplastida - ps9
Viridiplantae - ps10
Streptophyta - ps11
Charophyceae/Embriophyta - ps12
Embriophyta - ps13
Tracheophyta - ps14
Spermatophyta - ps15
Angiosperms - ps16
Mesangiospermae - ps17
Magnoliids/eudicots - ps18
Eudicots - ps19
Core_eudicots - ps20
Superrosids - ps21
Rosids - ps22
Eurosids - ps23
Malvids - ps24
Sap./Malv./Brassicales - ps25
Malvales/Brassicales - ps26
Brassicales - ps27
Cleomaceae/Brassicaceae - ps28
Brassicaceae - ps29
Camelineae - ps30
Arabidopsis - ps31
Arabidopsis_thaliana - ps32
p-value
2e-02
3e-02
4e-02
5e-02
6e-02
Log-odds
0.5
1.0
1.5
2.0
2.5
3.0
3.5
4.0

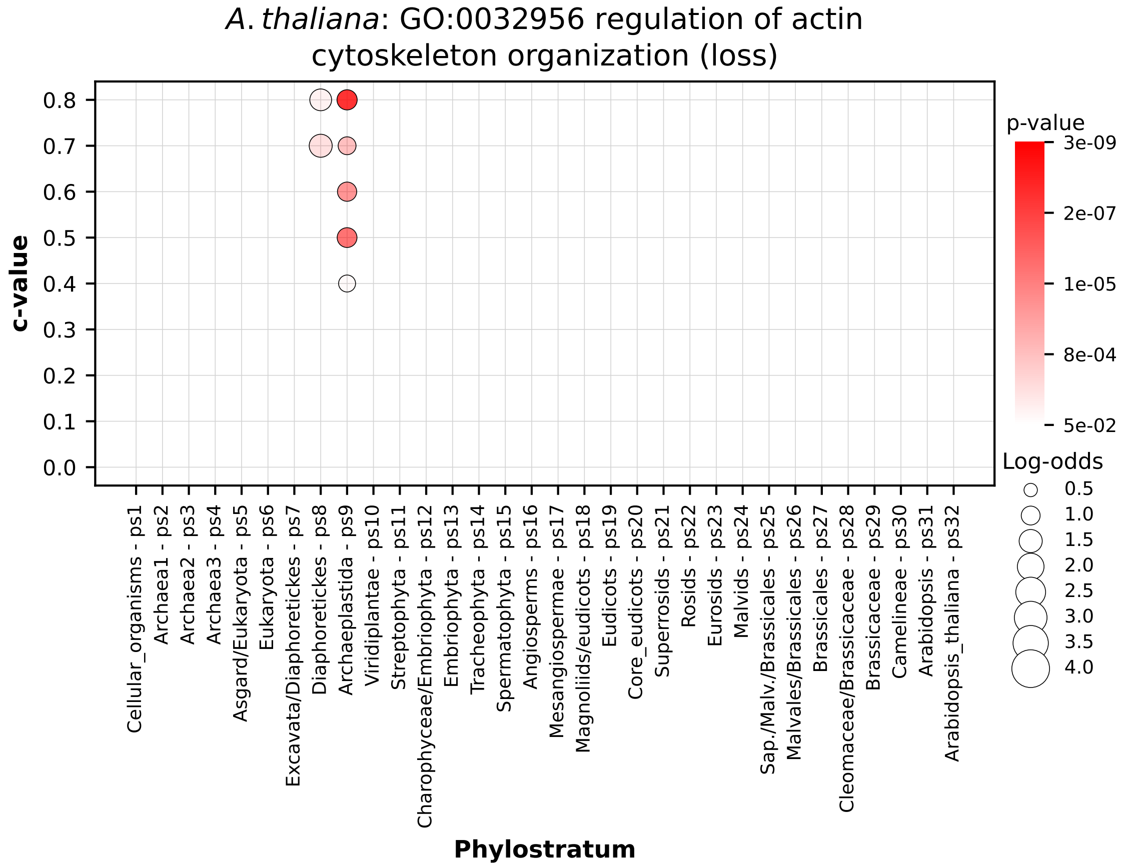

A. thaliana: GO:0032956 regulation of actin cytoskeleton organization (loss)
c-value
0.0
0.1
0.2
0.3
0.4
0.5
0.6
0.7
0.8
Phylostratum
Cellular_organisms - ps1
Archaea1 - ps2
Archaea2 - ps3
Archaea3 - ps4
Asgard/Eukaryota - ps5
Eukaryota - ps6
Excavata/Diaphoretickes - ps7
Diaphoretickes - ps8
Archaeplastida - ps9
Viridiplantae - ps10
Streptophyta - ps11
Charophyceae/Embriophyta - ps12
Embriophyta - ps13
Tracheophyta - ps14
Spermatophyta - ps15
Angiosperms - ps16
Mesangiospermae - ps17
Magnoliids/eudicots - ps18
Eudicots - ps19
Core_eudicots - ps20
Superrosids - ps21
Rosids - ps22
Eurosids - ps23
Malvids - ps24
Sap./Malv./Brassicales - ps25
Malvales/Brassicales - ps26
Brassicales - ps27
Cleomaceae/Brassicaceae - ps28
Brassicaceae - ps29
Camelineae - ps30
Arabidopsis - ps31
Arabidopsis_thaliana - ps32
p-value
3e-09
2e-07
1e-05
8e-04
5e-02
Log-odds
0.5
1.0
1.5
2.0
2.5
3.0
3.5
4.0

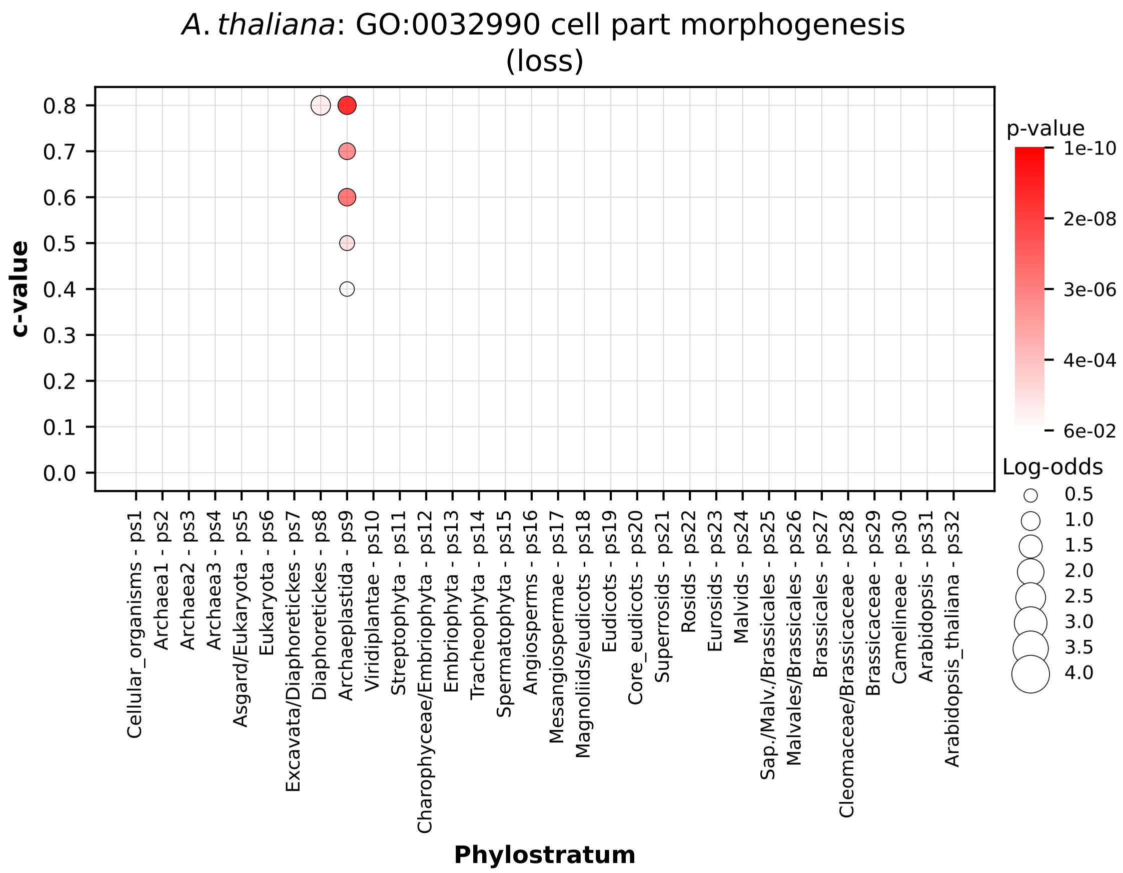
A. thaliana: GO:0032990 cell part morphogenesis
(loss)
c-value
0.8
0.7
0.6
0.5
0.4
0.3
0.2
0.1
0.0
p-value
1e-10
2e-08
3e-06
4e-04
6e-02
Log-odds
0.5
1.0
1.5
2.0
2.5
3.0
3.5
4.0
Cellular_organisms - ps1
Archaea1 - ps2
Archaea2 - ps3
Archaea3 - ps4
Asgard/Eukaryota - ps5
Eukaryota - ps6
Excavata/Diaphoretickes - ps7
Diaphoretickes - ps8
Archaeplastida - ps9
Viridiplantae - ps10
Streptophyta - ps11
Charophyceae/Embriophyta - ps12
Embriophyta - ps13
Tracheophyta - ps14
Spermatophyta - ps15
Angiosperms - ps16
Mesangiospermae - ps17
Magnoliids/eudicots - ps18
Eudicots - ps19
Core_eudicots - ps20
Superrosids - ps21
Rosids - ps22
Eurosids - ps23
Malvids - ps24
Sap./Malv./Brassicales - ps25
Malvales/Brassicales - ps26
Brassicales - ps27
Cleomaceae/Brassicaceae - ps28
Brassicaceae - ps29
Camelineae - ps30
Arabidopsis - ps31
Arabidopsis_thaliana - ps32
Phylostratum

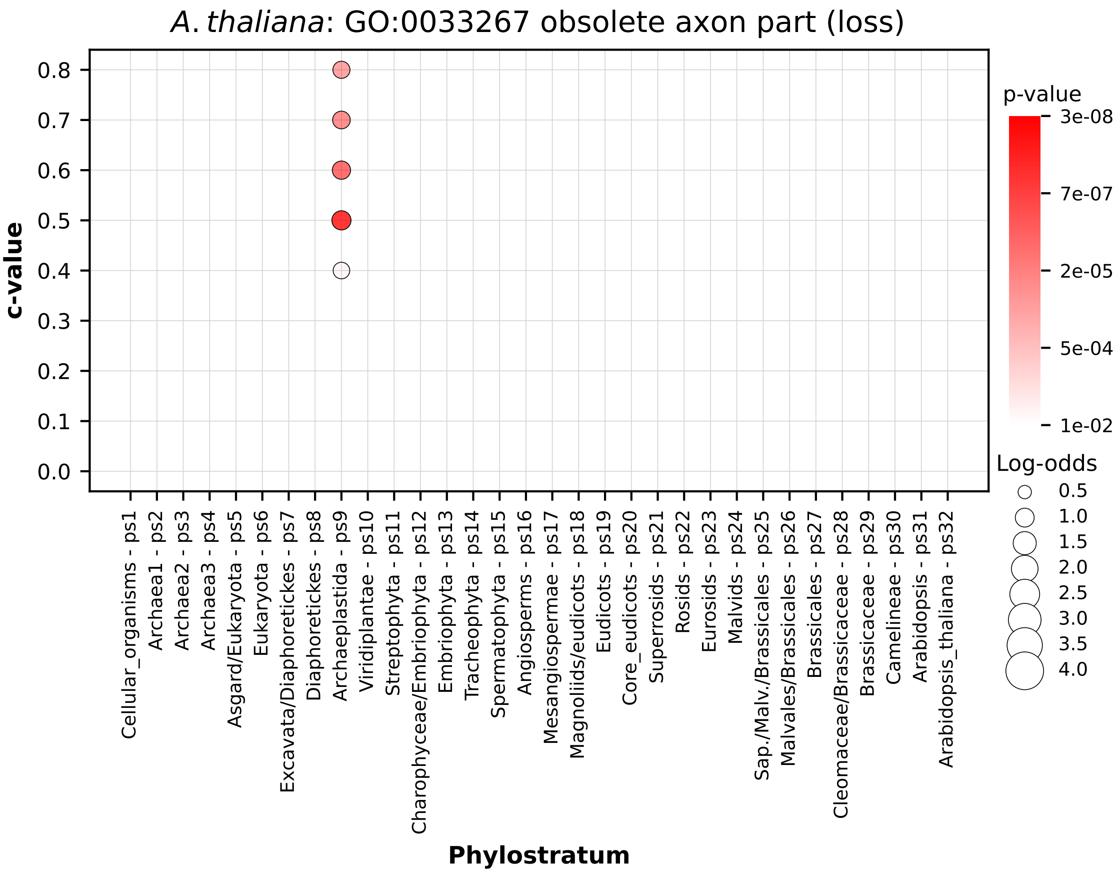

A. thaliana: GO:0033267 obsolete axon part (loss)
c-value
0.8
0.7
0.6
0.5
0.4
0.3
0.2
0.1
0.0
p-value
3e-08
7e-07
2e-05
5e-04
1e-02
Log-odds
0.5
1.0
1.5
2.0
2.5
3.0
3.5
4.0
Cellular_organisms - ps1
Archaea1 - ps2
Archaea2 - ps3
Archaea3 - ps4
Asgard/Eukaryota - ps5
Eukaryota - ps6
Excavata/Diaphoretickes - ps7
Diaphoretickes - ps8
Archaeplastida - ps9
Viridiplantae - ps10
Streptophyta - ps11
Charophyceae/Embriophyta - ps12
Embriophyta - ps13
Tracheophyta - ps14
Spermatophyta - ps15
Angiosperms - ps16
Mesangiospermae - ps17
Magnoliids/eudicots - ps18
Eudicots - ps19
Core_eudicots - ps20
Superrosids - ps21
Rosids - ps22
Eurosids - ps23
Malvids - ps24
Sap./Malv./Brassicales - ps25
Malvales/Brassicales - ps26
Brassicales - ps27
Cleomaceae/Brassicaceae - ps28
Brassicaceae - ps29
Camelineae - ps30
Arabidopsis - ps31
Arabidopsis_thaliana - ps32
Phylostratum

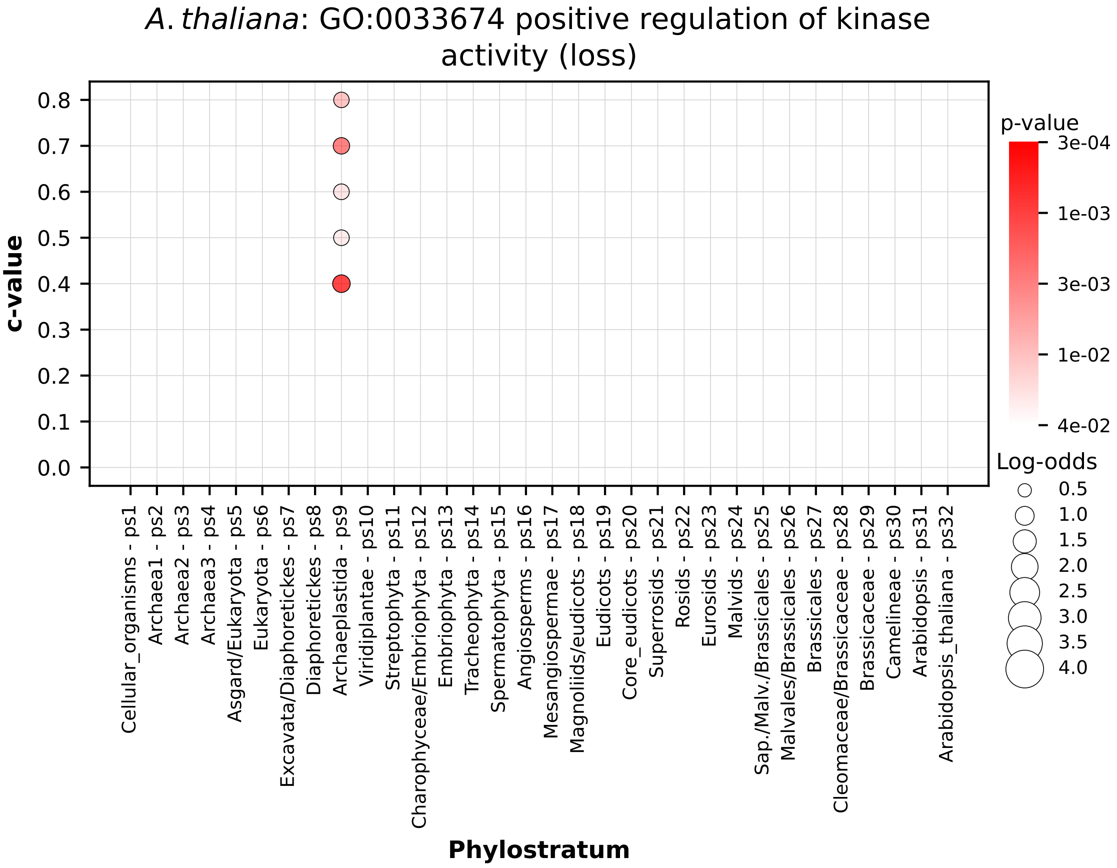

A. thaliana: GO:0033674 positive regulation of kinase activity (loss)
c-value
0.8
0.7
0.6
0.5
0.4
0.3
0.2
0.1
0.0
p-value
3e-04
1e-03
3e-03
1e-02
4e-02
Log-odds
0.5
1.0
1.5
2.0
2.5
3.0
3.5
4.0
Cellular_organisms - ps1
Archaea1 - ps2
Archaea2 - ps3
Archaea3 - ps4
Asgard/Eukaryota - ps5
Eukaryota - ps6
Excavata/Diaphoretickes - ps7
Diaphoretickes - ps8
Archaeplastida - ps9
Viridiplantae - ps10
Streptophyta - ps11
Charophyceae/Embriophyta - ps12
Embriophyta - ps13
Tracheophyta - ps14
Spermatophyta - ps15
Angiosperms - ps16
Mesangiospermae - ps17
Magnoliids/eudicots - ps18
Eudicots - ps19
Core_eudicots - ps20
Superrosids - ps21
Rosids - ps22
Eurosids - ps23
Malvids - ps24
Sap./Malv./Brassicales - ps25
Malvales/Brassicales - ps26
Brassicales - ps27
Cleomaceae/Brassicaceae - ps28
Brassicaceae - ps29
Camelineae - ps30
Arabidopsis - ps31
Arabidopsis_thaliana - ps32
Phylostratum

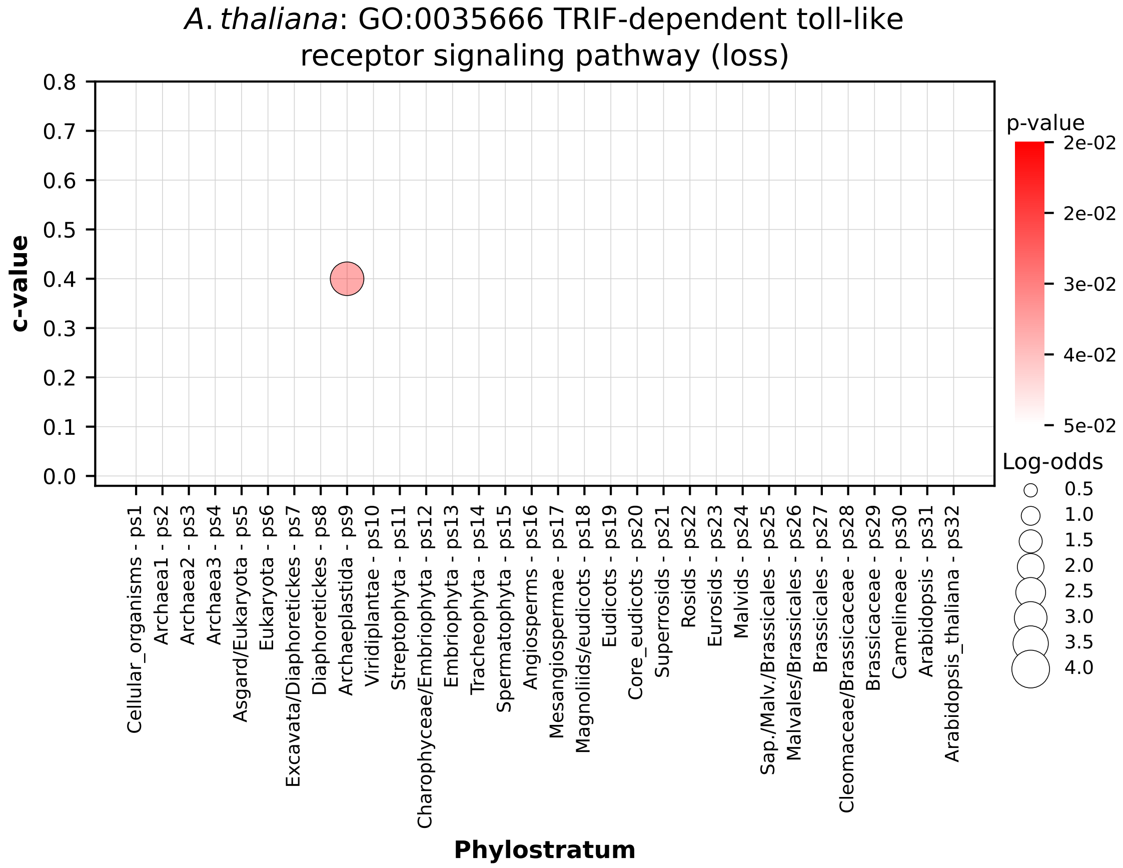

A. thaliana: GO:0035666 TRIF-dependent toll-like receptor signaling pathway (loss)
c-value
0.8
0.7
0.6
0.5
0.4
0.3
0.2
0.1
0.0
p-value
2e-02
2e-02
3e-02
4e-02
5e-02
Log-odds
0.5
1.0
1.5
2.0
2.5
3.0
3.5
4.0
Cellular_organisms - ps1
Archaea1 - ps2
Archaea2 - ps3
Archaea3 - ps4
Asgard/Eukaryota - ps5
Eukaryota - ps6
Excavata/Diaphoretickes - ps7
Diaphoretickes - ps8
Archaeplastida - ps9
Viridiplantae - ps10
Streptophyta - ps11
Charophyceae/Embriophyta - ps12
Embriophyta - ps13
Tracheophyta - ps14
Spermatophyta - ps15
Angiosperms - ps16
Mesangiospermae - ps17
Magnoliids/eudicots - ps18
Eudicots - ps19
Core_eudicots - ps20
Superrosids - ps21
Rosids - ps22
Eurosids - ps23
Malvids - ps24
Sap./Malv./Brassicales - ps25
Malvales/Brassicales - ps26
Brassicales - ps27
Cleomaceae/Brassicaceae - ps28
Brassicaceae - ps29
Camelineae - ps30
Arabidopsis - ps31
Arabidopsis_thaliana - ps32
Phylostratum

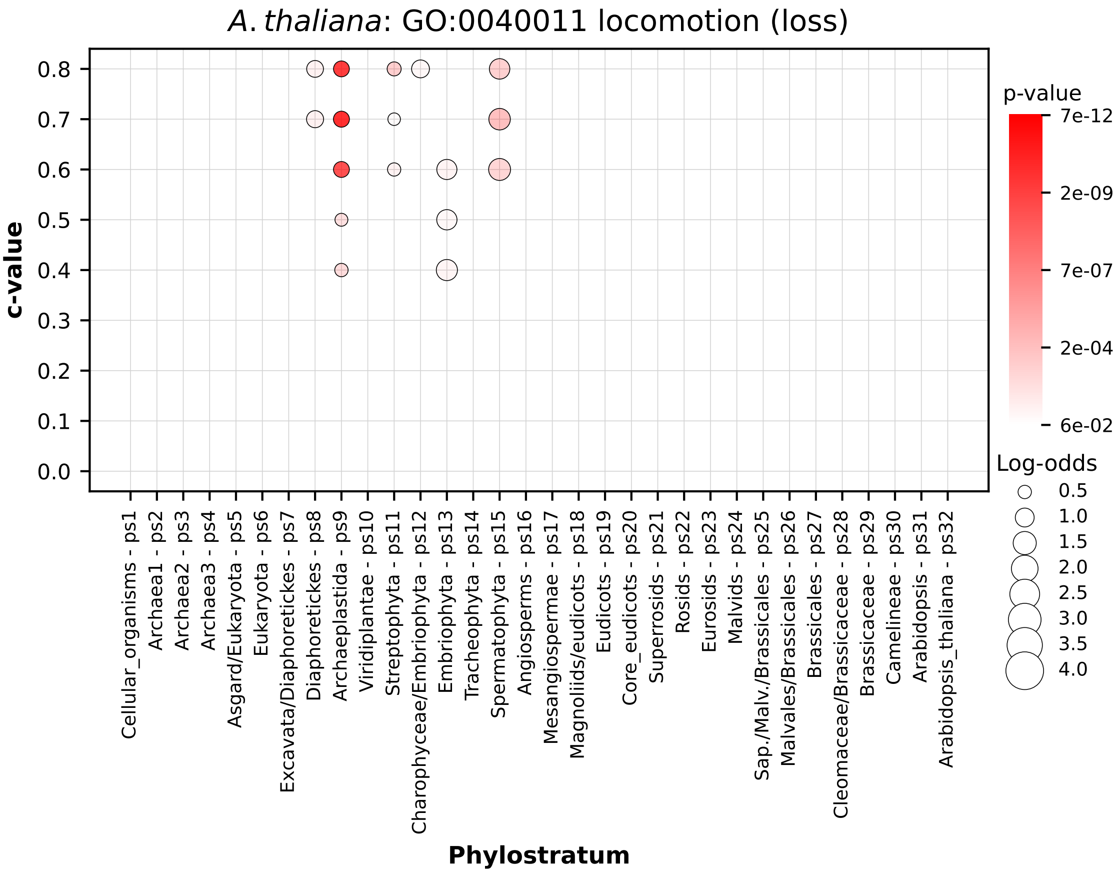

A. thaliana: GO:0040011 locomotion (loss)
c-value
0.8
0.7
0.6
0.5
0.4
0.3
0.2
0.1
0.0
p-value
7e-12
2e-09
7e-07
2e-04
6e-02
Log-odds
0.5
1.0
1.5
2.0
2.5
3.0
3.5
4.0
Cellular_organisms - ps1
Archaea1 - ps2
Archaea2 - ps3
Archaea3 - ps4
Asgard/Eukaryota - ps5
Eukaryota - ps6
Excavata/Diaphoretickes - ps7
Diaphoretickes - ps8
Archaeplastida - ps9
Viridiplantae - ps10
Streptophyta - ps11
Charophyceae/Embriophyta - ps12
Embriophyta - ps13
Tracheophyta - ps14
Spermatophyta - ps15
Angiosperms - ps16
Mesangiospermae - ps17
Magnoliids/eudicots - ps18
Eudicots - ps19
Core_eudicots - ps20
Superrosids - ps21
Rosids - ps22
Eurosids - ps23
Malvids - ps24
Sap./Malv./Brassicales - ps25
Malvales/Brassicales - ps26
Brassicales - ps27
Cleomaceae/Brassicaceae - ps28
Brassicaceae - ps29
Camelineae - ps30
Arabidopsis - ps31
Arabidopsis_thaliana - ps32
Phylostratum

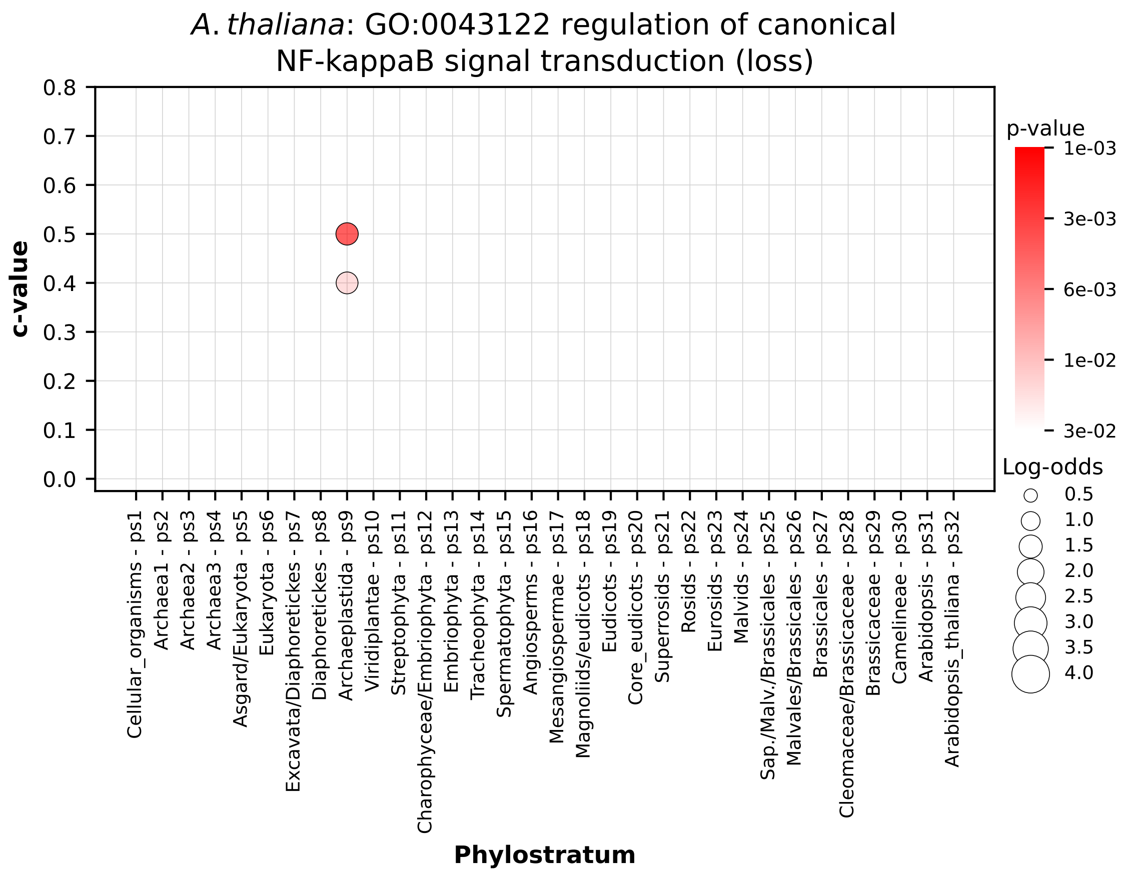

A. thaliana: GO:0043122 regulation of canonical NF-kappaB signal transduction (loss)
c-value
0.0
0.1
0.2
0.3
0.4
0.5
0.6
0.7
0.8
Phylostratum
Cellular_organisms - ps1
Archaea1 - ps2
Archaea2 - ps3
Archaea3 - ps4
Asgard/Eukaryota - ps5
Eukaryota - ps6
Excavata/Diaphoretickes - ps7
Diaphoretickes - ps8
Archaeplastida - ps9
Viridiplantae - ps10
Streptophyta - ps11
Charophyceae/Embriophyta - ps12
Embriophyta - ps13
Tracheophyta - ps14
Spermatophyta - ps15
Angiosperms - ps16
Mesangiospermae - ps17
Magnoliids/eudicots - ps18
Eudicots - ps19
Core_eudicots - ps20
Superrosids - ps21
Rosids - ps22
Eurosids - ps23
Malvids - ps24
Sap./Malv./Brassicales - ps25
Malvales/Brassicales - ps26
Brassicales - ps27
Cleomaceae/Brassicaceae - ps28
Brassicaceae - ps29
Camelineae - ps30
Arabidopsis - ps31
Arabidopsis_thaliana - ps32
p-value
1e-03
3e-03
6e-03
1e-02
3e-02
Log-odds
0.5
1.0
1.5
2.0
2.5
3.0
3.5
4.0

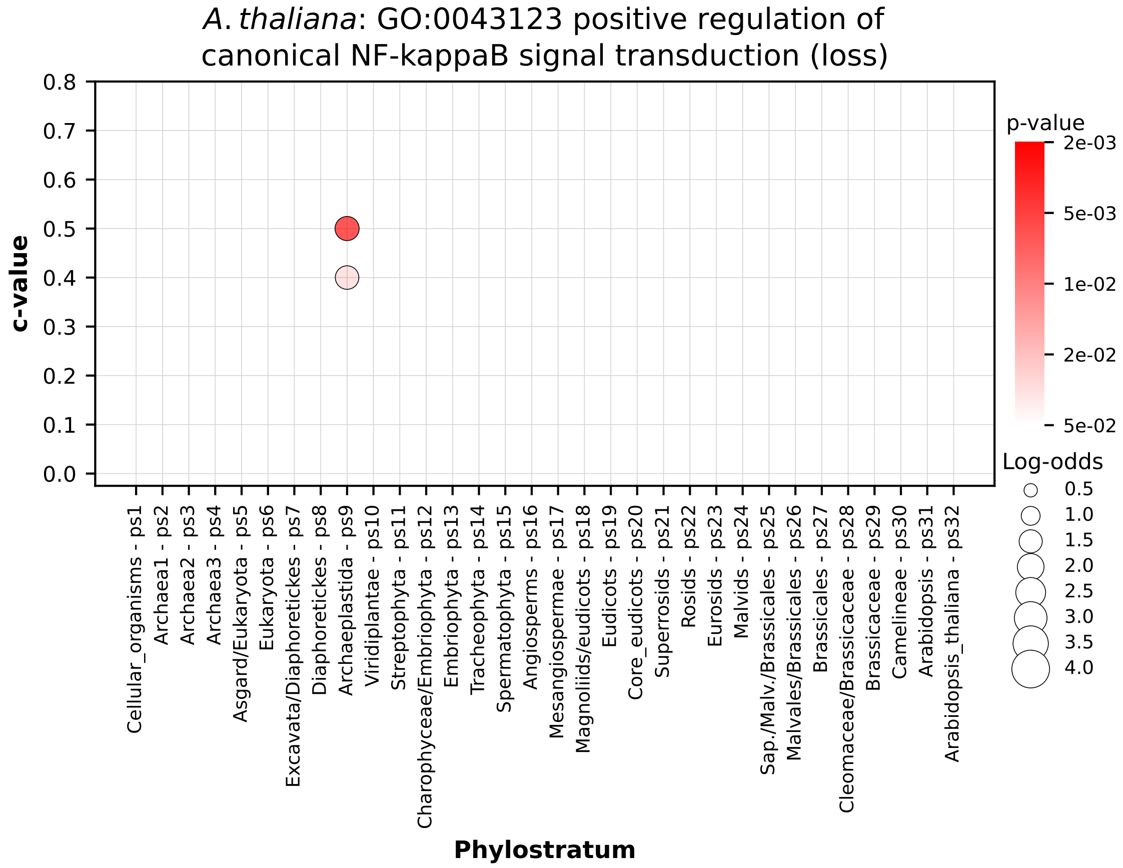

A. thaliana: GO:0043123 positive regulation of canonical NF-kappaB signal transduction (loss)
c-value
0.8
0.7
0.6
0.5
0.4
0.3
0.2
0.1
0.0
p-value
2e-03
5e-03
1e-02
2e-02
5e-02
Log-odds
0.5
1.0
1.5
2.0
2.5
3.0
3.5
4.0
Cellular_organisms - ps1
Archaea1 - ps2
Archaea2 - ps3
Archaea3 - ps4
Asgard/Eukaryota - ps5
Eukaryota - ps6
Excavata/Diaphoretickes - ps7
Diaphoretickes - ps8
Archaeplastida - ps9
Viridiplantae - ps10
Streptophyta - ps11
Charophyceae/Embriophyta - ps12
Embriophyta - ps13
Tracheophyta - ps14
Spermatophyta - ps15
Angiosperms - ps16
Mesangiospermae - ps17
Magnoliids/eudicots - ps18
Eudicots - ps19
Core_eudicots - ps20
Superrosids - ps21
Rosids - ps22
Eurosids - ps23
Malvids - ps24
Sap./Malv./Brassicales - ps25
Malvales/Brassicales - ps26
Brassicales - ps27
Cleomaceae/Brassicaceae - ps28
Brassicaceae - ps29
Camelineae - ps30
Arabidopsis - ps31
Arabidopsis_thaliana - ps32
Phylostratum

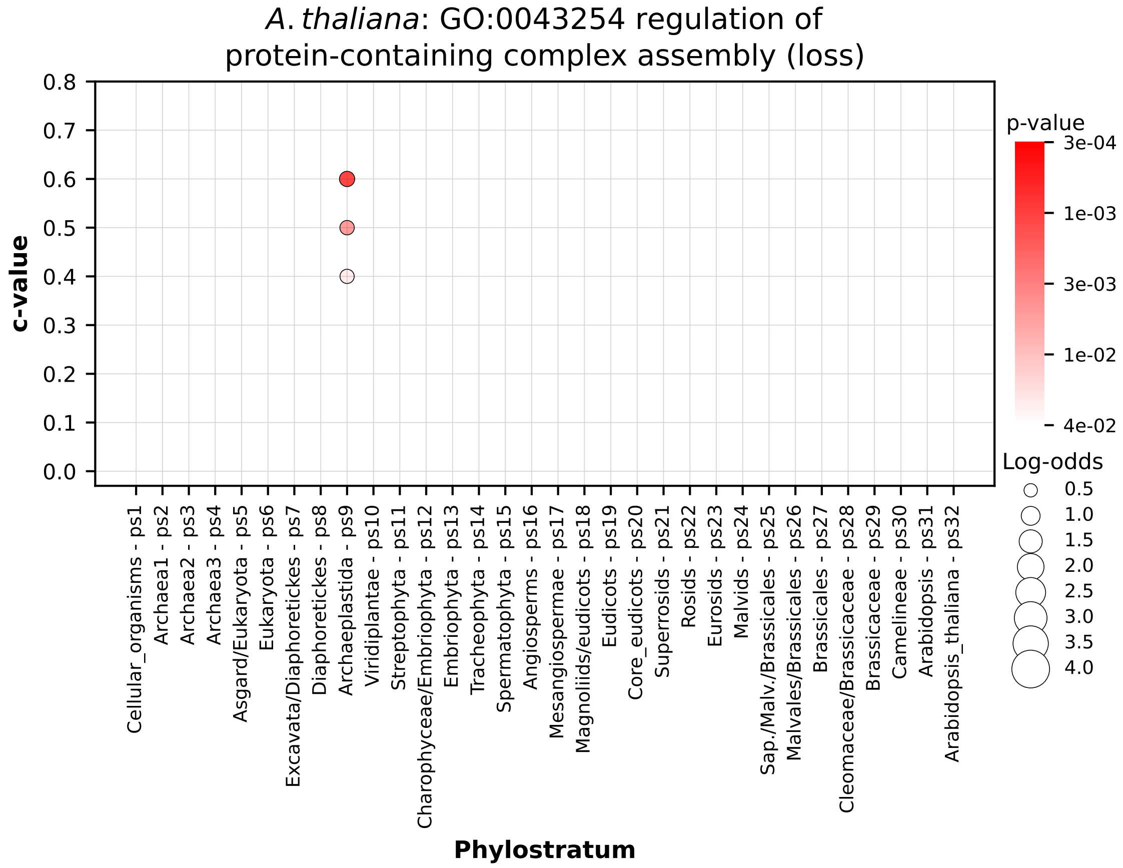

A. thaliana: GO:0043254 regulation of
protein-containing complex assembly (loss)
c-value
0.8
0.7
0.6
0.5
0.4
0.3
0.2
0.1
0.0
p-value
3e-04
1e-03
3e-03
1e-02
4e-02
Log-odds
0.5
1.0
1.5
2.0
2.5
3.0
3.5
4.0
Cellular_organisms - ps1
Archaea1 - ps2
Archaea2 - ps3
Archaea3 - ps4
Asgard/Eukaryota - ps5
Eukaryota - ps6
Excavata/Diaphoretickes - ps7
Diaphoretickes - ps8
Archaeplastida - ps9
Viridiplantae - ps10
Streptophyta - ps11
Charophyceae/Embriophyta - ps12
Embriophyta - ps13
Tracheophyta - ps14
Spermatophyta - ps15
Angiosperms - ps16
Mesangiospermae - ps17
Magnoliids/eudicots - ps18
Eudicots - ps19
Core_eudicots - ps20
Superrosids - ps21
Rosids - ps22
Eurosids - ps23
Malvids - ps24
Sap./Malv./Brassicales - ps25
Malvales/Brassicales - ps26
Brassicales - ps27
Cleomaceae/Brassicaceae - ps28
Brassicaceae - ps29
Camelineae - ps30
Arabidopsis - ps31
Arabidopsis_thaliana - ps32
Phylostratum

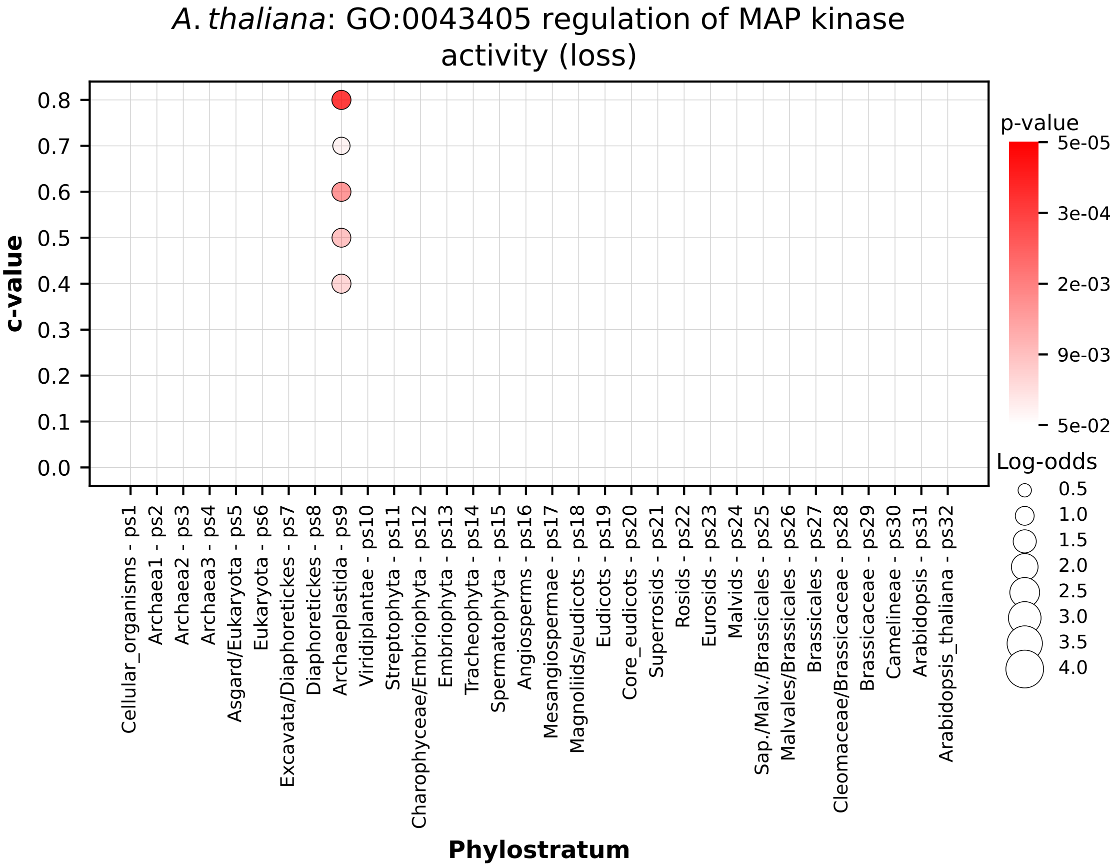
A. thaliana: GO:0043405 regulation of MAP kinase activity (loss)
c-value
0.0
0.1
0.2
0.3
0.4
0.5
0.6
0.7
0.8
p-value
5e-05
3e-04
2e-03
9e-03
5e-02
Log-odds
0.5
1.0
1.5
2.0
2.5
3.0
3.5
4.0
Cellular_organisms - ps1
Archaea1 - ps2
Archaea2 - ps3
Archaea3 - ps4
Asgard/Eukaryota - ps5
Eukaryota - ps6
Excavata/Diaphoretickes - ps7
Diaphoretickes - ps8
Archaeplastida - ps9
Viridiplantae - ps10
Streptophyta - ps11
Charophyceae/Embriophyta - ps12
Embriophyta - ps13
Tracheophyta - ps14
Spermatophyta - ps15
Angiosperms - ps16
Mesangiospermae - ps17
Magnoliids/eudicots - ps18
Eudicots - ps19
Core_eudicots - ps20
Superrosids - ps21
Rosids - ps22
Eurosids - ps23
Malvids - ps24
Sap./Malv./Brassicales - ps25
Malvales/Brassicales - ps26
Brassicales - ps27
Cleomaceae/Brassicaceae - ps28
Brassicaceae - ps29
Camelineae - ps30
Arabidopsis - ps31
Arabidopsis_thaliana - ps32
Phylostratum

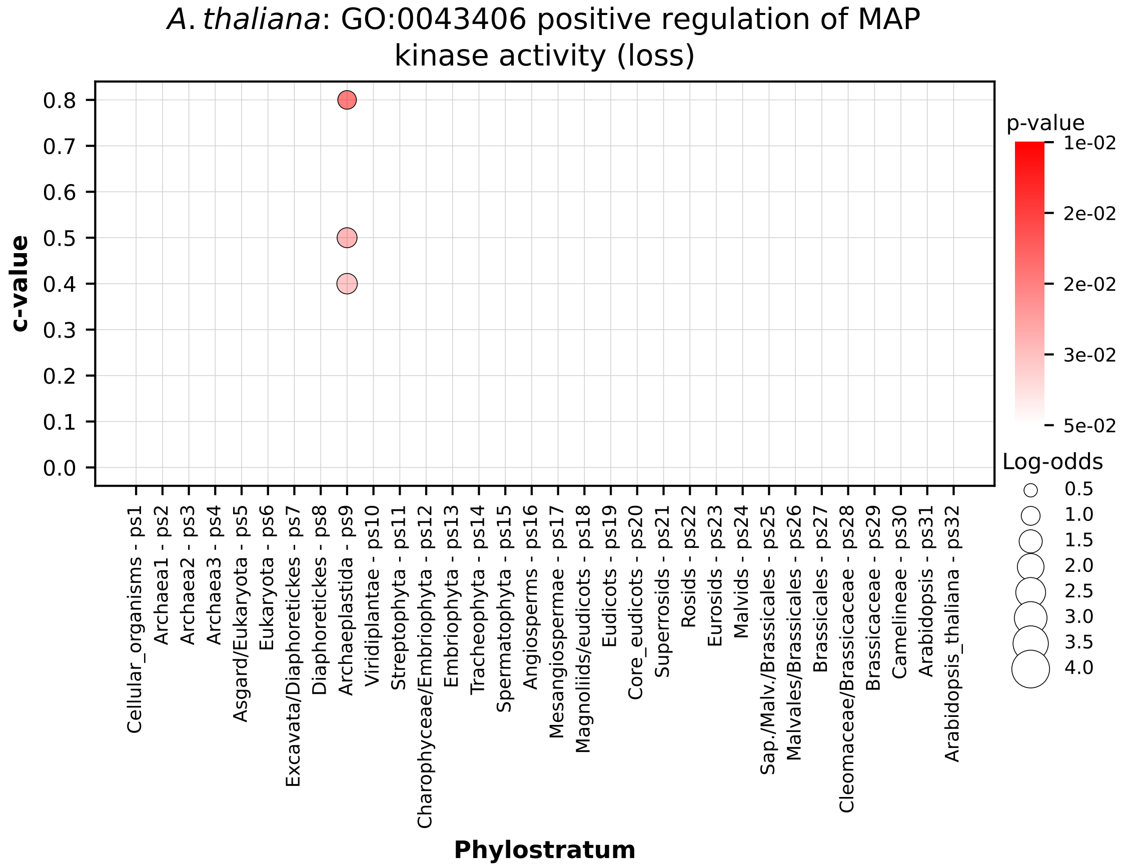

A. thaliana: GO:0043406 positive regulation of MAP kinase activity (loss)
c-value
0.8
0.7
0.6
0.5
0.4
0.3
0.2
0.1
0.0
Phylostratum
Cellular_organisms - ps1
Archaea1 - ps2
Archaea2 - ps3
Archaea3 - ps4
Asgard/Eukaryota - ps5
Eukaryota - ps6
Excavata/Diaphoretickes - ps7
Diaphoretickes - ps8
Archaeplastida - ps9
Viridiplantae - ps10
Streptophyta - ps11
Charophyceae/Embriophyta - ps12
Embriophyta - ps13
Tracheophyta - ps14
Spermatophyta - ps15
Angiosperms - ps16
Mesangiospermae - ps17
Magnoliids/eudicots - ps18
Eudicots - ps19
Core_eudicots - ps20
Superrosids - ps21
Rosids - ps22
Eurosids - ps23
Malvids - ps24
Sap./Malv./Brassicales - ps25
Malvales/Brassicales - ps26
Brassicales - ps27
Cleomaceae/Brassicaceae - ps28
Brassicaceae - ps29
Camelineae - ps30
Arabidopsis - ps31
Arabidopsis_thaliana - ps32
p-value
1e-02
2e-02
2e-02
3e-02
5e-02
Log-odds
0.5
1.0
1.5
2.0
2.5
3.0
3.5
4.0

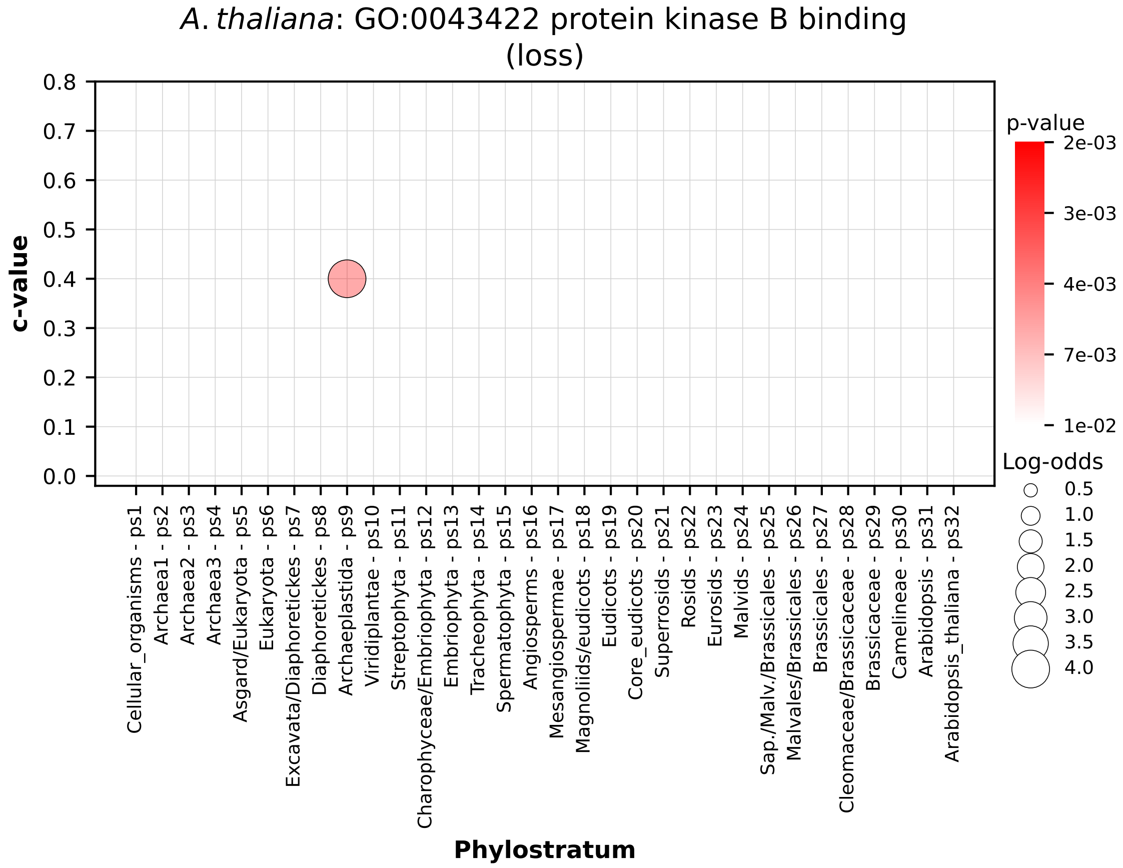

A. thaliana: GO:0043422 protein kinase B binding
(loss)
c-value
0.8
0.7
0.6
0.5
0.4
0.3
0.2
0.1
0.0
Phylostratum
Cellular_organisms - ps1
Archaea1 - ps2
Archaea2 - ps3
Archaea3 - ps4
Asgard/Eukaryota - ps5
Eukaryota - ps6
Excavata/Diaphoretickes - ps7
Diaphoretickes - ps8
Archaeplastida - ps9
Viridiplantae - ps10
Streptophyta - ps11
Charophyceae/Embriophyta - ps12
Embriophyta - ps13
Tracheophyta - ps14
Spermatophyta - ps15
Angiosperms - ps16
Mesangiospermae - ps17
Magnoliids/eudicots - ps18
Eudicots - ps19
Core_eudicots - ps20
Superrosids - ps21
Rosids - ps22
Eurosids - ps23
Malvids - ps24
Sap./Malv./Brassicales - ps25
Malvales/Brassicales - ps26
Brassicales - ps27
Cleomaceae/Brassicaceae - ps28
Brassicaceae - ps29
Camelineae - ps30
Arabidopsis - ps31
Arabidopsis_thaliana - ps32
p-value
2e-03
3e-03
4e-03
7e-03
1e-02
Log-odds
0.5
1.0
1.5
2.0
2.5
3.0
3.5
4.0

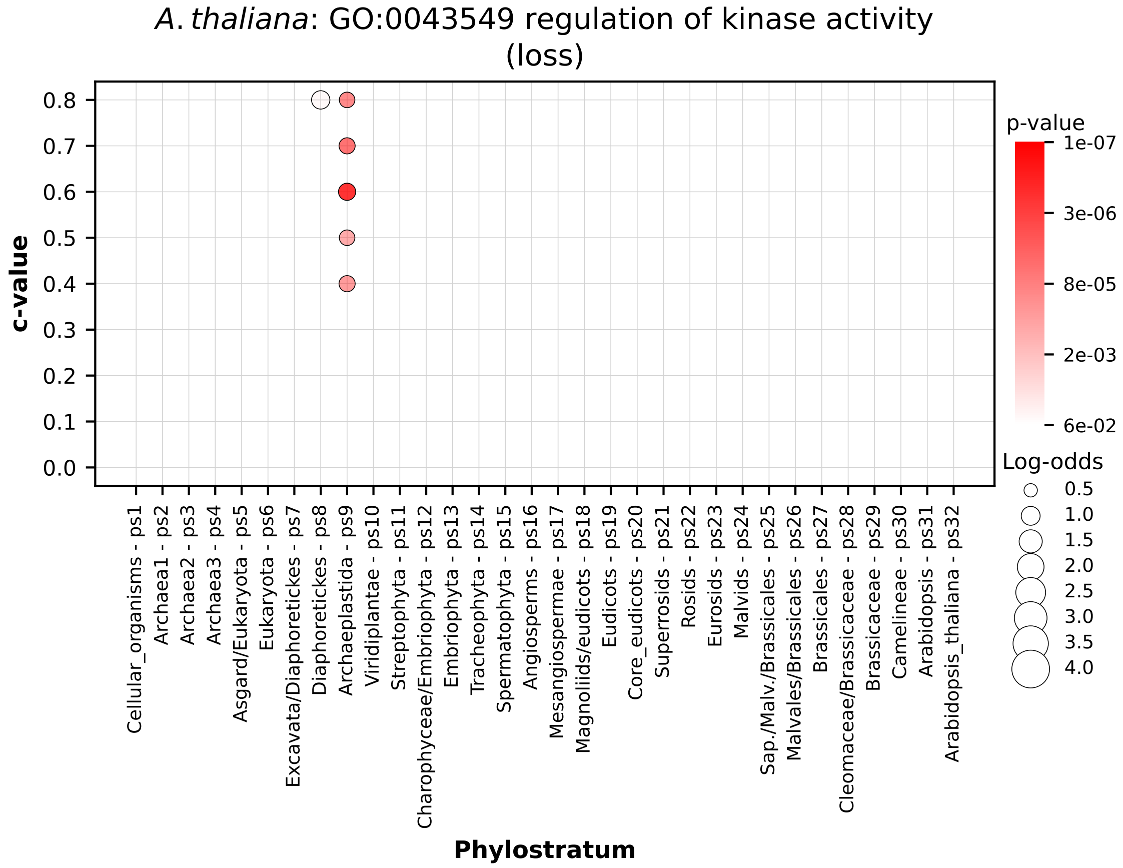

A. thaliana: GO:0043549 regulation of kinase activity
(loss)
c-value
0.8
0.7
0.6
0.5
0.4
0.3
0.2
0.1
0.0
Phylostratum
Cellular_organisms - ps1
Archaea1 - ps2
Archaea2 - ps3
Archaea3 - ps4
Asgard/Eukaryota - ps5
Eukaryota - ps6
Excavata/Diaphoretickes - ps7
Diaphoretickes - ps8
Archaeplastida - ps9
Viridiplantae - ps10
Streptophyta - ps11
Charophyceae/Embriophyta - ps12
Embriophyta - ps13
Tracheophyta - ps14
Spermatophyta - ps15
Angiosperms - ps16
Mesangiospermae - ps17
Magnoliids/eudicots - ps18
Eudicots - ps19
Core_eudicots - ps20
Superrosids - ps21
Rosids - ps22
Eurosids - ps23
Malvids - ps24
Sap./Malv./Brassicales - ps25
Malvales/Brassicales - ps26
Brassicales - ps27
Cleomaceae/Brassicaceae - ps28
Brassicaceae - ps29
Camelineae - ps30
Arabidopsis - ps31
Arabidopsis_thaliana - ps32
p-value
1e-07
3e-06
8e-05
2e-03
6e-02
Log-odds
0.5
1.0
1.5
2.0
2.5
3.0
3.5
4.0

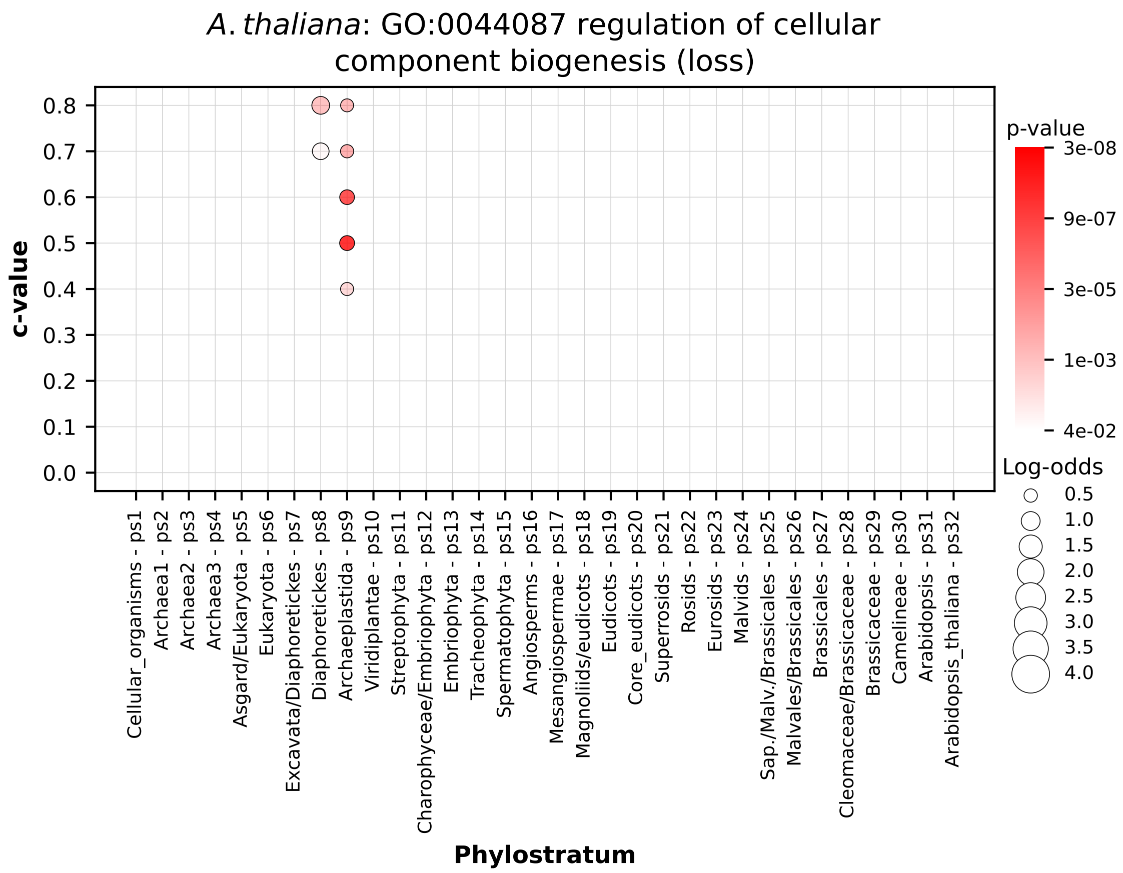

A. thaliana: GO:0044087 regulation of cellular component biogenesis (loss)
c-value
0.8
0.7
0.6
0.5
0.4
0.3
0.2
0.1
0.0
p-value
3e-08
9e-07
3e-05
1e-03
4e-02
Log-odds
0.5
1.0
1.5
2.0
2.5
3.0
3.5
4.0
Cellular_organisms - ps1
Archaea1 - ps2
Archaea2 - ps3
Archaea3 - ps4
Asgard/Eukaryota - ps5
Eukaryota - ps6
Excavata/Diaphoretickes - ps7
Diaphoretickes - ps8
Archaeplastida - ps9
Viridiplantae - ps10
Streptophyta - ps11
Charophyceae/Embriophyta - ps12
Embriophyta - ps13
Tracheophyta - ps14
Spermatophyta - ps15
Angiosperms - ps16
Mesangiospermae - ps17
Magnoliids/eudicots - ps18
Eudicots - ps19
Core_eudicots - ps20
Superrosids - ps21
Rosids - ps22
Eurosids - ps23
Malvids - ps24
Sap./Malv./Brassicales - ps25
Malvales/Brassicales - ps26
Brassicales - ps27
Cleomaceae/Brassicaceae - ps28
Brassicaceae - ps29
Camelineae - ps30
Arabidopsis - ps31
Arabidopsis_thaliana - ps32
Phylostratum

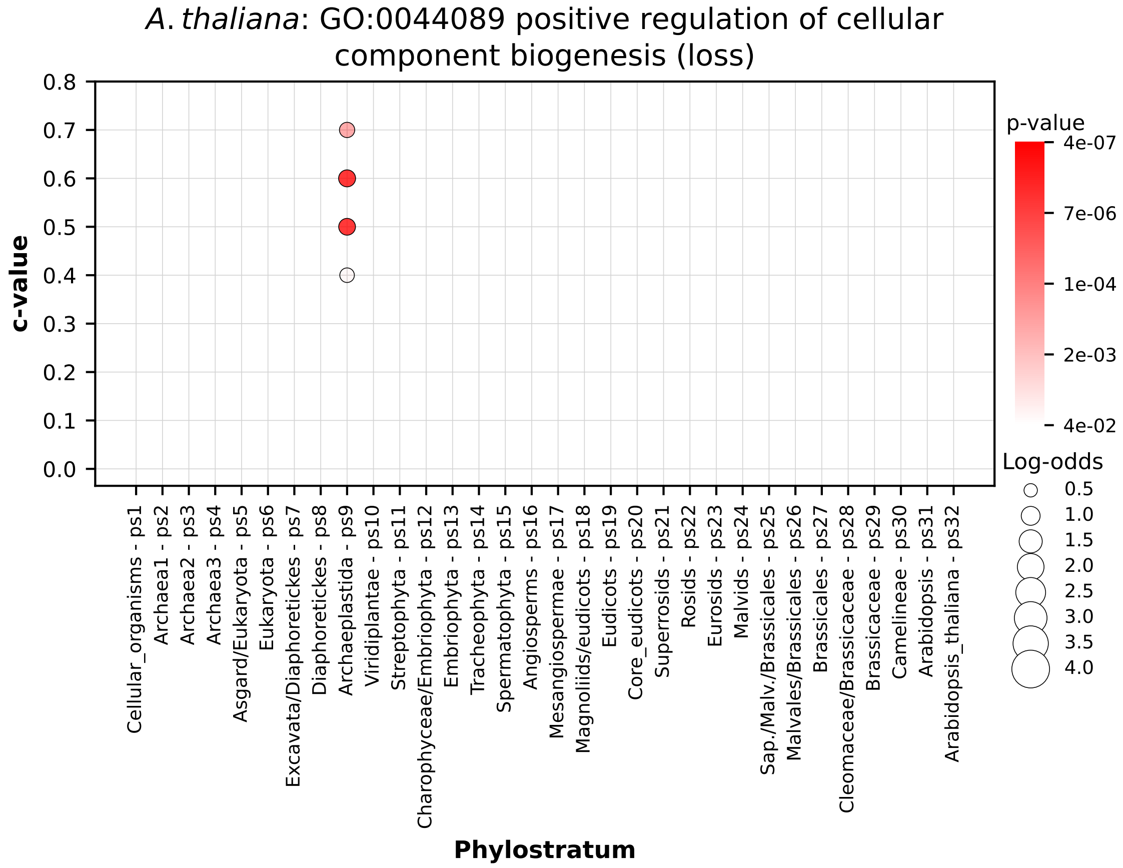

A. thaliana: GO:0044089 positive regulation of cellular component biogenesis (loss)
c-value
0.0
0.1
0.2
0.3
0.4
0.5
0.6
0.7
0.8
Phylostratum
Cellular_organisms - ps1
Archaea1 - ps2
Archaea2 - ps3
Archaea3 - ps4
Asgard/Eukaryota - ps5
Eukaryota - ps6
Excavata/Diaphoretickes - ps7
Diaphoretickes - ps8
Archaeplastida - ps9
Viridiplantae - ps10
Streptophyta - ps11
Charophyceae/Embriophyta - ps12
Embriophyta - ps13
Tracheophyta - ps14
Spermatophyta - ps15
Angiosperms - ps16
Mesangiospermae - ps17
Magnoliids/eudicots - ps18
Eudicots - ps19
Core_eudicots - ps20
Superrosids - ps21
Rosids - ps22
Eurosids - ps23
Malvids - ps24
Sap./Malv./Brassicales - ps25
Malvales/Brassicales - ps26
Brassicales - ps27
Cleomaceae/Brassicaceae - ps28
Brassicaceae - ps29
Camelineae - ps30
Arabidopsis - ps31
Arabidopsis_thaliana - ps32
p-value
4e-07
7e-06
1e-04
2e-03
4e-02
Log-odds
0.5
1.0
1.5
2.0
2.5
3.0
3.5
4.0

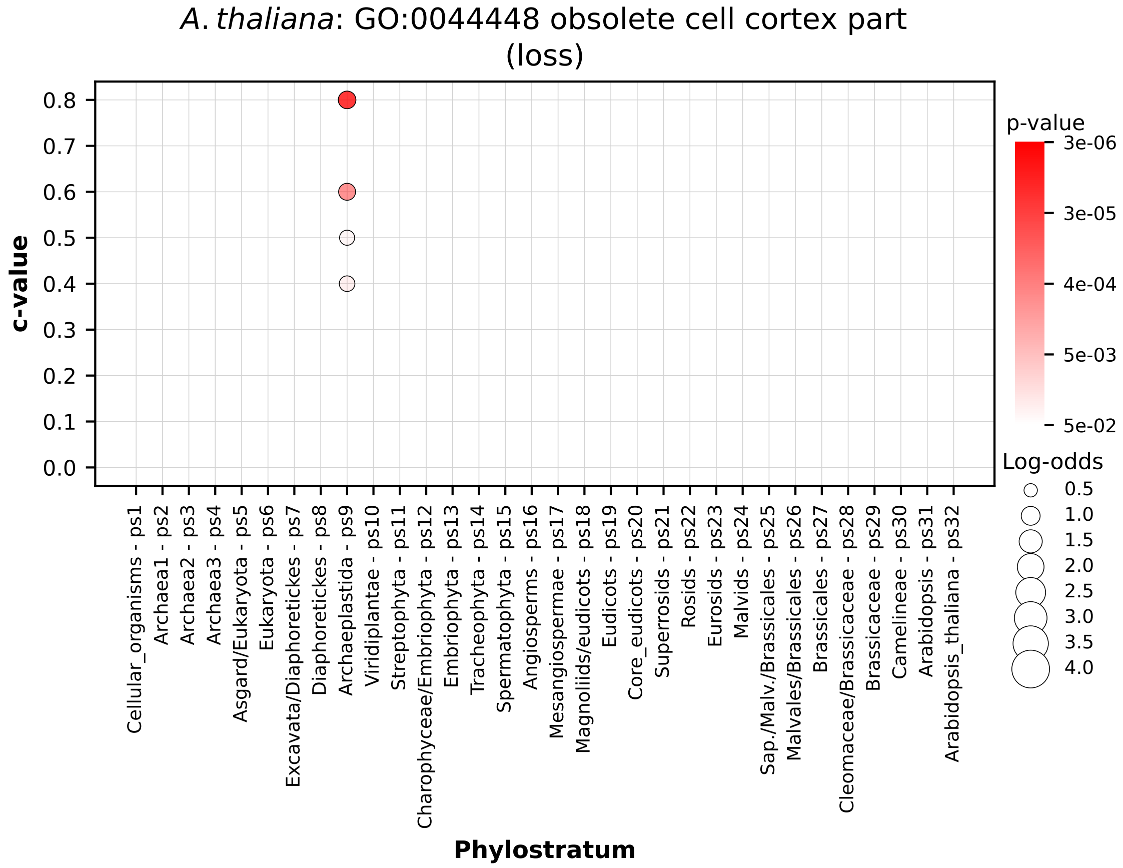

A. thaliana: GO:0044448 obsolete cell cortex part
(loss)
c-value
0.0
0.1
0.2
0.3
0.4
0.5
0.6
0.7
0.8
p-value
3e-06
3e-05
4e-04
5e-03
5e-02
Log-odds
0.5
1.0
1.5
2.0
2.5
3.0
3.5
4.0
Cellular_organisms - ps1
Archaea1 - ps2
Archaea2 - ps3
Archaea3 - ps4
Asgard/Eukaryota - ps5
Eukaryota - ps6
Excavata/Diaphoretickes - ps7
Diaphoretickes - ps8
Archaeplastida - ps9
Viridiplantae - ps10
Streptophyta - ps11
Charophyceae/Embriophyta - ps12
Embriophyta - ps13
Tracheophyta - ps14
Spermatophyta - ps15
Angiosperms - ps16
Mesangiospermae - ps17
Magnoliids/eudicots - ps18
Eudicots - ps19
Core_eudicots - ps20
Superrosids - ps21
Rosids - ps22
Eurosids - ps23
Malvids - ps24
Sap./Malv./Brassicales - ps25
Malvales/Brassicales - ps26
Brassicales - ps27
Cleomaceae/Brassicaceae - ps28
Brassicaceae - ps29
Camelineae - ps30
Arabidopsis - ps31
Arabidopsis_thaliana - ps32
Phylostratum

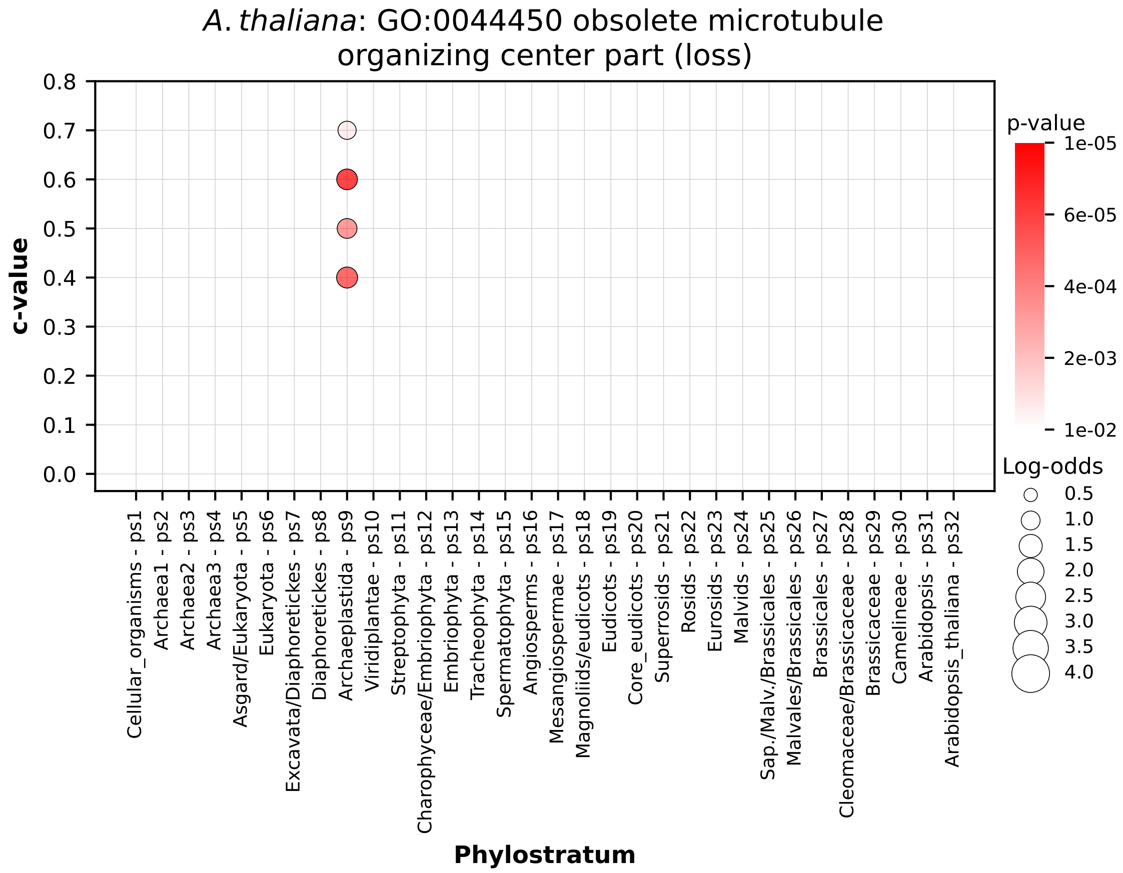
A. thaliana: GO:0044450 obsolete microtubule organizing center part (loss)
c-value
0.8
0.7
0.6
0.5
0.4
0.3
0.2
0.1
0.0
p-value
1e-05
6e-05
4e-04
2e-03
1e-02
Log-odds
0.5
1.0
1.5
2.0
2.5
3.0
3.5
4.0
Cellular_organisms - ps1
Archaea1 - ps2
Archaea2 - ps3
Archaea3 - ps4
Asgard/Eukaryota - ps5
Eukaryota - ps6
Excavata/Diaphoretickes - ps7
Diaphoretickes - ps8
Archaeplastida - ps9
Viridiplantae - ps10
Streptophyta - ps11
Charophyceae/Embriophyta - ps12
Embriophyta - ps13
Tracheophyta - ps14
Spermatophyta - ps15
Angiosperms - ps16
Mesangiospermae - ps17
Magnoliids/eudicots - ps18
Eudicots - ps19
Core_eudicots - ps20
Superrosids - ps21
Rosids - ps22
Eurosids - ps23
Malvids - ps24
Sap./Malv./Brassicales - ps25
Malvales/Brassicales - ps26
Brassicales - ps27
Cleomaceae/Brassicaceae - ps28
Brassicaceae - ps29
Camelineae - ps30
Arabidopsis - ps31
Arabidopsis_thaliana - ps32
Phylostratum

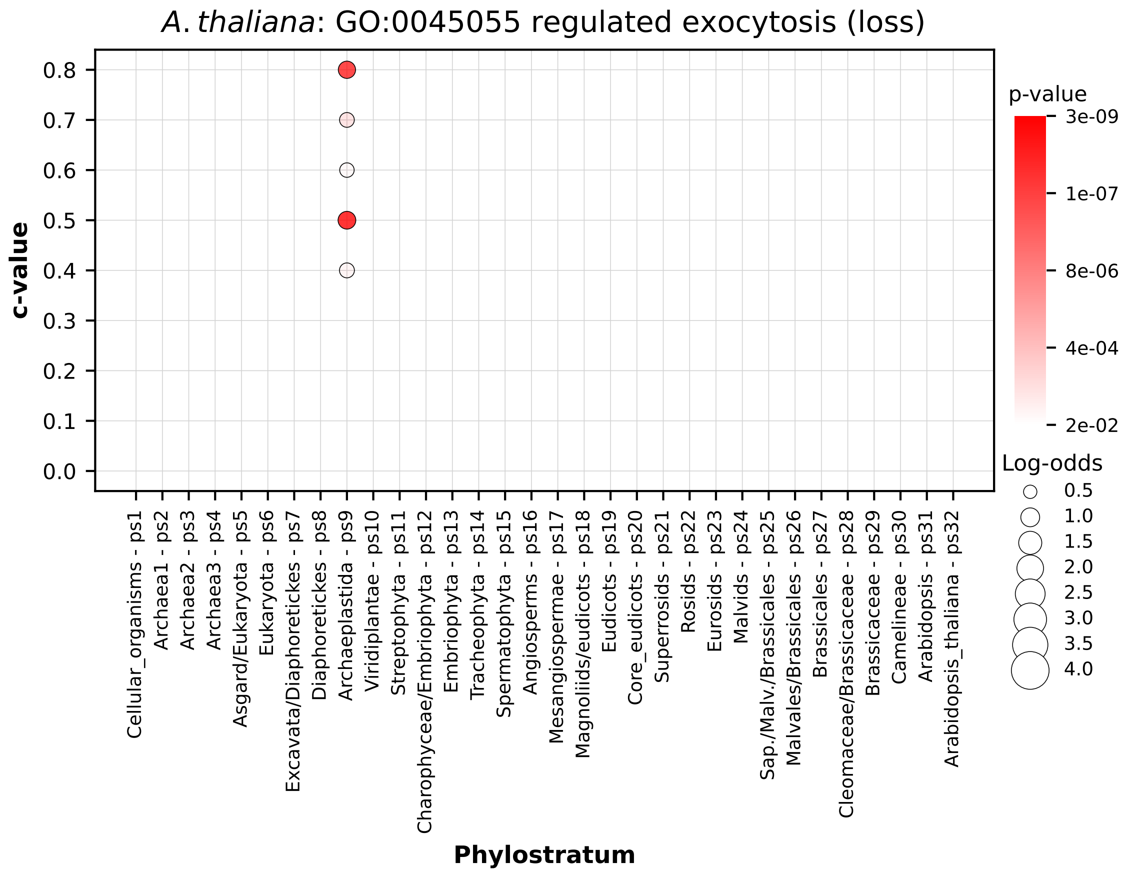
A. thaliana: GO:0045055 regulated exocytosis (loss)
c-value
0.0
0.1
0.2
0.3
0.4
0.5
0.6
0.7
0.8
Phylostratum
Cellular_organisms - ps1
Archaea1 - ps2
Archaea2 - ps3
Archaea3 - ps4
Asgard/Eukaryota - ps5
Eukaryota - ps6
Excavata/Diaphoretickes - ps7
Diaphoretickes - ps8
Archaeplastida - ps9
Viridiplantae - ps10
Streptophyta - ps11
Charophyceae/Embriophyta - ps12
Embriophyta - ps13
Tracheophyta - ps14
Spermatophyta - ps15
Angiosperms - ps16
Mesangiospermae - ps17
Magnoliids/eudicots - ps18
Eudicots - ps19
Core_eudicots - ps20
Superrosids - ps21
Rosids - ps22
Eurosids - ps23
Malvids - ps24
Sap./Malv./Brassicales - ps25
Malvales/Brassicales - ps26
Brassicales - ps27
Cleomaceae/Brassicaceae - ps28
Brassicaceae - ps29
Camelineae - ps30
Arabidopsis - ps31
Arabidopsis_thaliana - ps32
p-value
3e-09
1e-07
8e-06
4e-04
2e-02
Log-odds
0.5
1.0
1.5
2.0
2.5
3.0
3.5
4.0

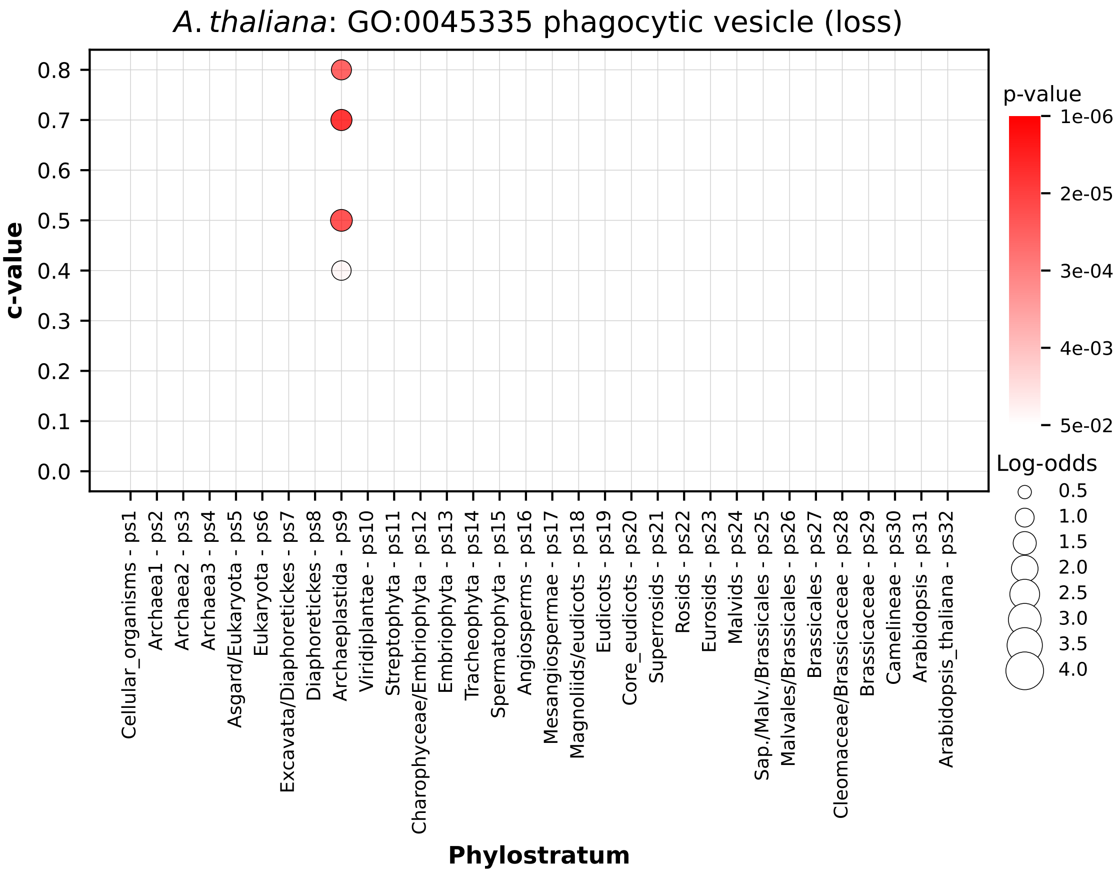
A. thaliana: GO:0045335 phagocytic vesicle (loss)
c-value
0.8
0.7
0.6
0.5
0.4
0.3
0.2
0.1
0.0
p-value
1e-06
2e-05
3e-04
4e-03
5e-02
Log-odds
0.5
1.0
1.5
2.0
2.5
3.0
3.5
4.0
Cellular_organisms - ps1
Archaea1 - ps2
Archaea2 - ps3
Archaea3 - ps4
Asgard/Eukaryota - ps5
Eukaryota - ps6
Excavata/Diaphoretickes - ps7
Diaphoretickes - ps8
Archaeplastida - ps9
Viridiplantae - ps10
Streptophyta - ps11
Charophyceae/Embriophyta - ps12
Embriophyta - ps13
Tracheophyta - ps14
Spermatophyta - ps15
Angiosperms - ps16
Mesangiospermae - ps17
Magnoliids/eudicots - ps18
Eudicots - ps19
Core_eudicots - ps20
Superrosids - ps21
Rosids - ps22
Eurosids - ps23
Malvids - ps24
Sap./Malv./Brassicales - ps25
Malvales/Brassicales - ps26
Brassicales - ps27
Cleomaceae/Brassicaceae - ps28
Brassicaceae - ps29
Camelineae - ps30
Arabidopsis - ps31
Arabidopsis_thaliana - ps32
Phylostratum

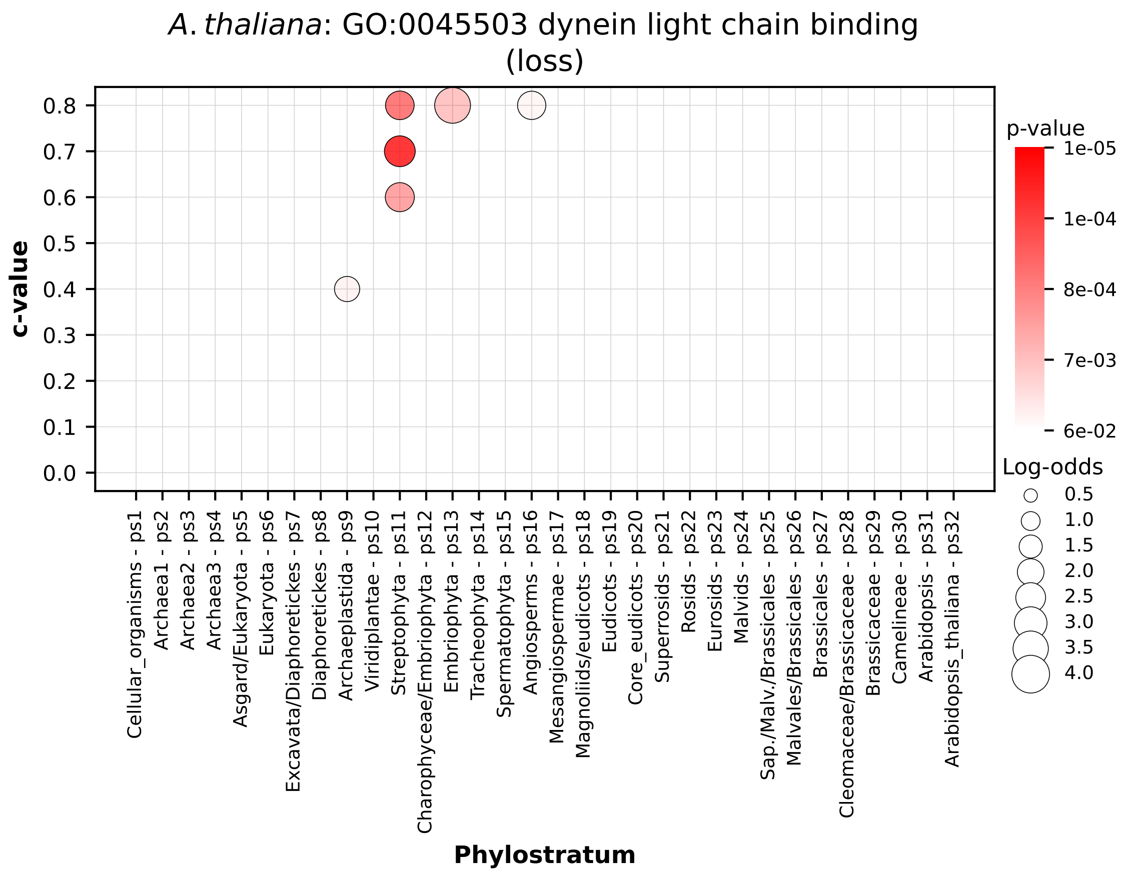

A. thaliana: GO:0045503 dynein light chain binding
(loss)
c-value
0.8
0.7
0.6
0.5
0.4
0.3
0.2
0.1
0.0
p-value
1e-05
1e-04
8e-04
7e-03
6e-02
Log-odds
0.5
1.0
1.5
2.0
2.5
3.0
3.5
4.0
Cellular_organisms - ps1
Archaea1 - ps2
Archaea2 - ps3
Archaea3 - ps4
Asgard/Eukaryota - ps5
Eukaryota - ps6
Excavata/Diaphoretickes - ps7
Diaphoretickes - ps8
Archaeplastida - ps9
Viridiplantae - ps10
Streptophyta - ps11
Charophyceae/Embriophyta - ps12
Embriophyta - ps13
Tracheophyta - ps14
Spermatophyta - ps15
Angiosperms - ps16
Mesangiospermae - ps17
Magnoliids/eudicots - ps18
Eudicots - ps19
Core_eudicots - ps20
Superrosids - ps21
Rosids - ps22
Eurosids - ps23
Malvids - ps24
Sap./Malv./Brassicales - ps25
Malvales/Brassicales - ps26
Brassicales - ps27
Cleomaceae/Brassicaceae - ps28
Brassicaceae - ps29
Camelineae - ps30
Arabidopsis - ps31
Arabidopsis_thaliana - ps32
Phylostratum

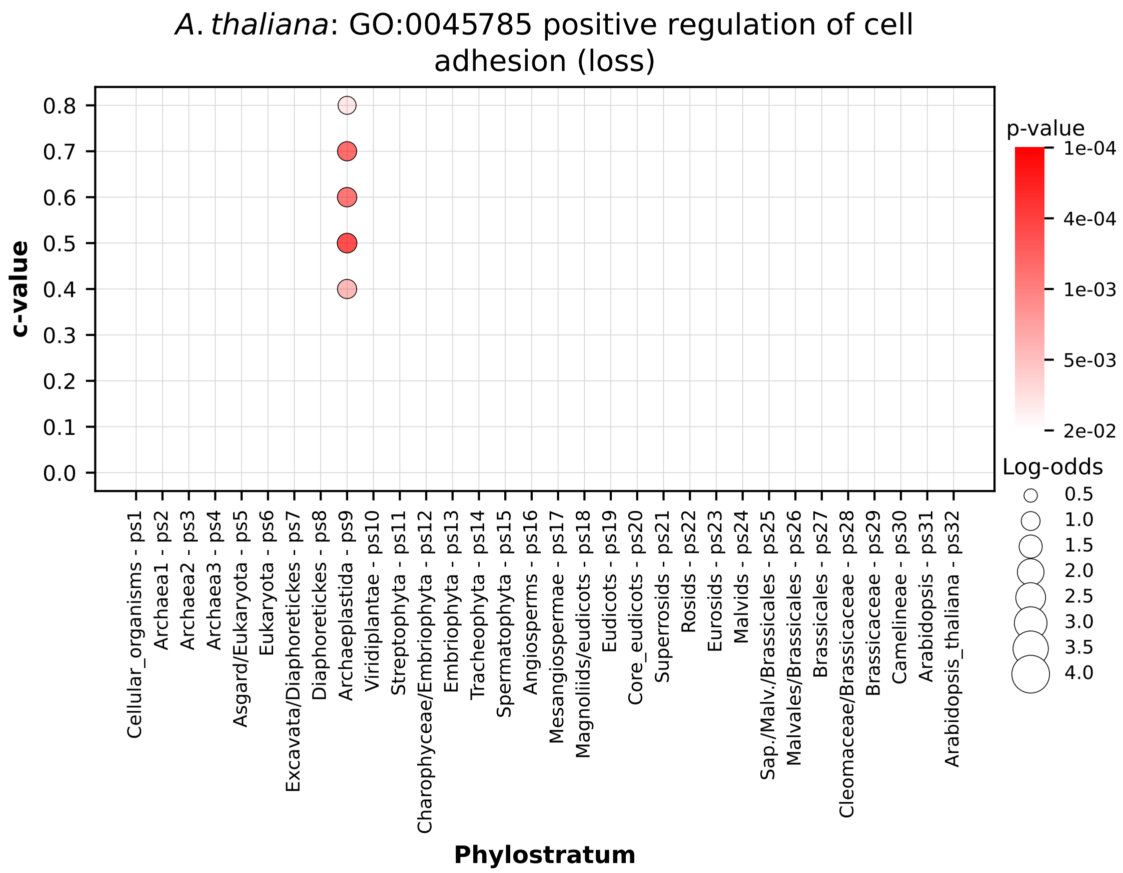

A. thaliana: GO:0045785 positive regulation of cell adhesion (loss)
c-value
0.0
0.1
0.2
0.3
0.4
0.5
0.6
0.7
0.8
Phylostratum
Cellular_organisms - ps1
Archaea1 - ps2
Archaea2 - ps3
Archaea3 - ps4
Asgard/Eukaryota - ps5
Eukaryota - ps6
Excavata/Diaphoretickes - ps7
Diaphoretickes - ps8
Archaeplastida - ps9
Viridiplantae - ps10
Streptophyta - ps11
Charophyceae/Embriophyta - ps12
Embriophyta - ps13
Tracheophyta - ps14
Spermatophyta - ps15
Angiosperms - ps16
Mesangiospermae - ps17
Magnoliids/eudicots - ps18
Eudicots - ps19
Core_eudicots - ps20
Superrosids - ps21
Rosids - ps22
Eurosids - ps23
Malvids - ps24
Sap./Malv./Brassicales - ps25
Malvales/Brassicales - ps26
Brassicales - ps27
Cleomaceae/Brassicaceae - ps28
Brassicaceae - ps29
Camelineae - ps30
Arabidopsis - ps31
Arabidopsis_thaliana - ps32
p-value
1e-04
4e-04
1e-03
5e-03
2e-02
Log-odds
0.5
1.0
1.5
2.0
2.5
3.0
3.5
4.0

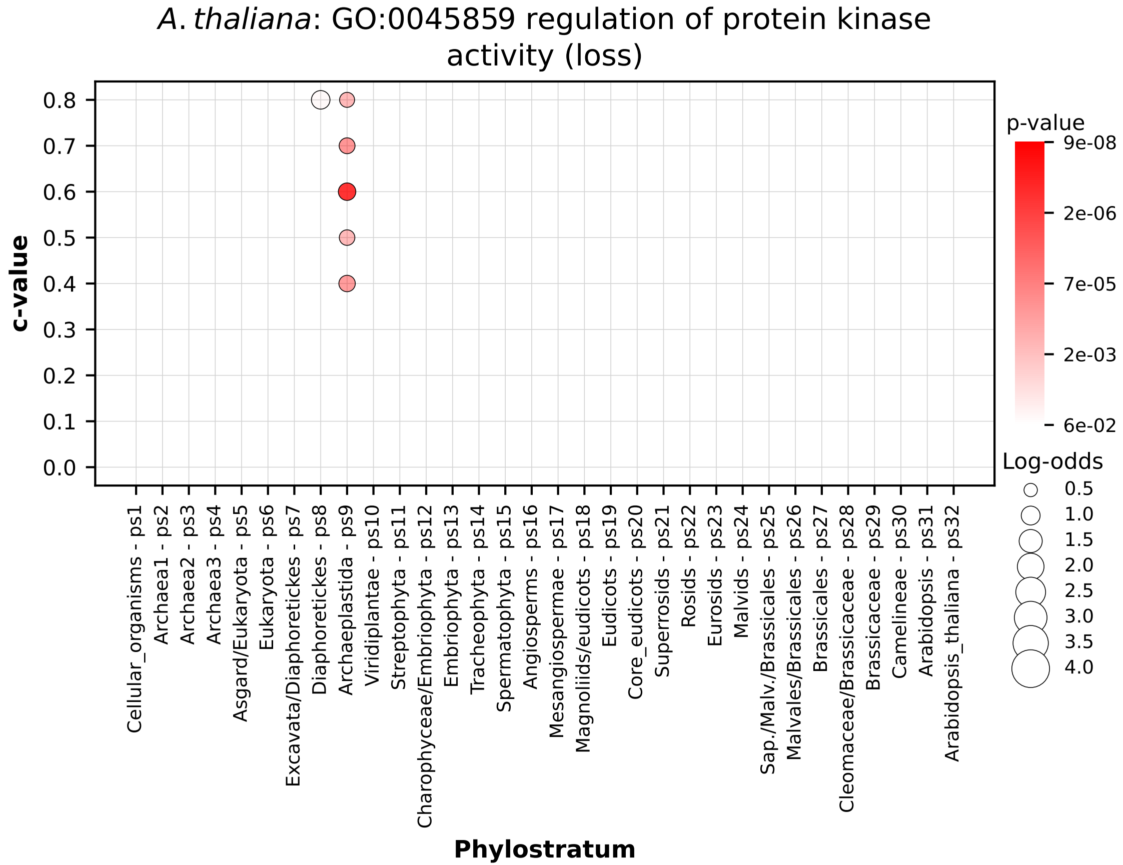

A. thaliana: GO:0045859 regulation of protein kinase activity (loss)
c-value
0.8
0.7
0.6
0.5
0.4
0.3
0.2
0.1
0.0
p-value
9e-08
2e-06
7e-05
2e-03
6e-02
Log-odds
0.5
1.0
1.5
2.0
2.5
3.0
3.5
4.0
Cellular_organisms - ps1
Archaea1 - ps2
Archaea2 - ps3
Archaea3 - ps4
Asgard/Eukaryota - ps5
Eukaryota - ps6
Excavata/Diaphoretickes - ps7
Diaphoretickes - ps8
Archaeplastida - ps9
Viridiplantae - ps10
Streptophyta - ps11
Charophyceae/Embriophyta - ps12
Embriophyta - ps13
Tracheophyta - ps14
Spermatophyta - ps15
Angiosperms - ps16
Mesangiospermae - ps17
Magnoliids/eudicots - ps18
Eudicots - ps19
Core_eudicots - ps20
Superrosids - ps21
Rosids - ps22
Eurosids - ps23
Malvids - ps24
Sap./Malv./Brassicales - ps25
Malvales/Brassicales - ps26
Brassicales - ps27
Cleomaceae/Brassicaceae - ps28
Brassicaceae - ps29
Camelineae - ps30
Arabidopsis - ps31
Arabidopsis_thaliana - ps32
Phylostratum

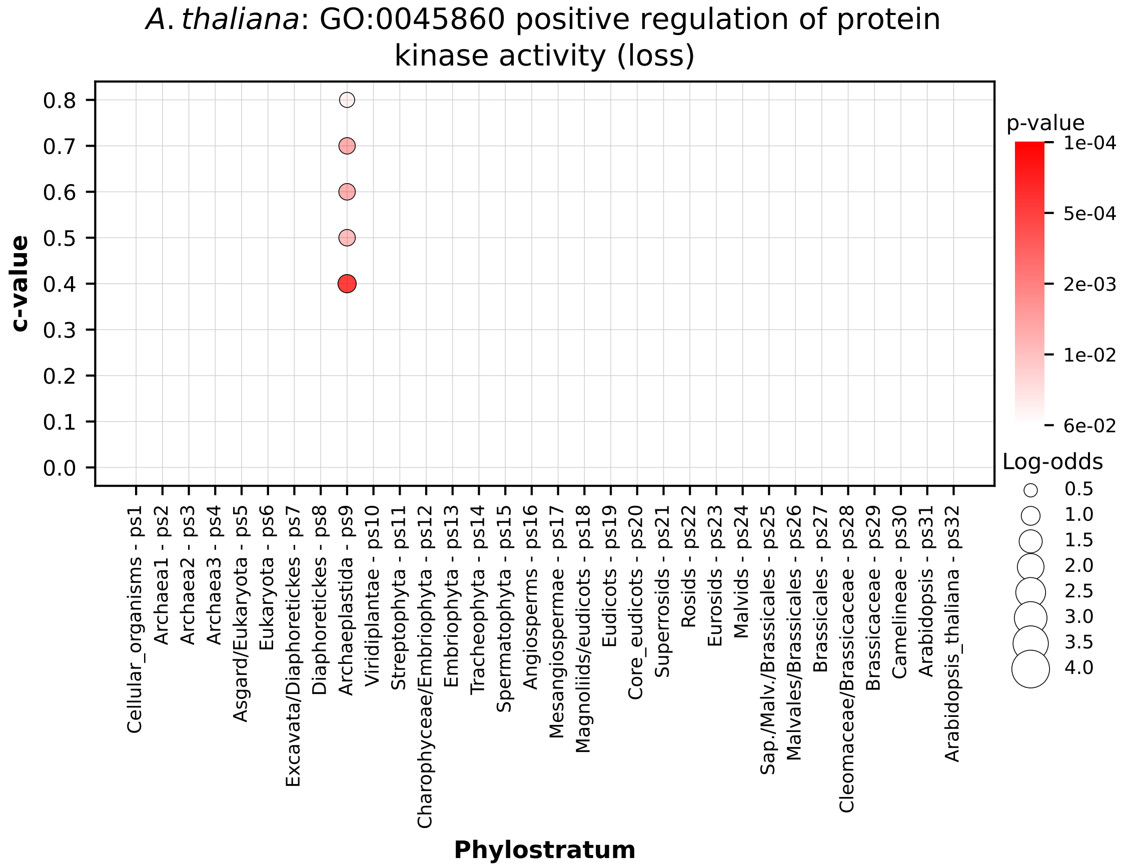

A. thaliana: GO:0045860 positive regulation of protein kinase activity (loss)
c-value
0.8
0.7
0.6
0.5
0.4
0.3
0.2
0.1
0.0
p-value
1e-04
5e-04
2e-03
1e-02
6e-02
Log-odds
0.5
1.0
1.5
2.0
2.5
3.0
3.5
4.0
Cellular_organisms - ps1
Archaea1 - ps2
Archaea2 - ps3
Archaea3 - ps4
Asgard/Eukaryota - ps5
Eukaryota - ps6
Excavata/Diaphoretickes - ps7
Diaphoretickes - ps8
Archaeplastida - ps9
Viridiplantae - ps10
Streptophyta - ps11
Charophyceae/Embriophyta - ps12
Embriophyta - ps13
Tracheophyta - ps14
Spermatophyta - ps15
Angiosperms - ps16
Mesangiospermae - ps17
Magnoliids/eudicots - ps18
Eudicots - ps19
Core_eudicots - ps20
Superrosids - ps21
Rosids - ps22
Eurosids - ps23
Malvids - ps24
Sap./Malv./Brassicales - ps25
Malvales/Brassicales - ps26
Brassicales - ps27
Cleomaceae/Brassicaceae - ps28
Brassicaceae - ps29
Camelineae - ps30
Arabidopsis - ps31
Arabidopsis_thaliana - ps32
Phylostratum

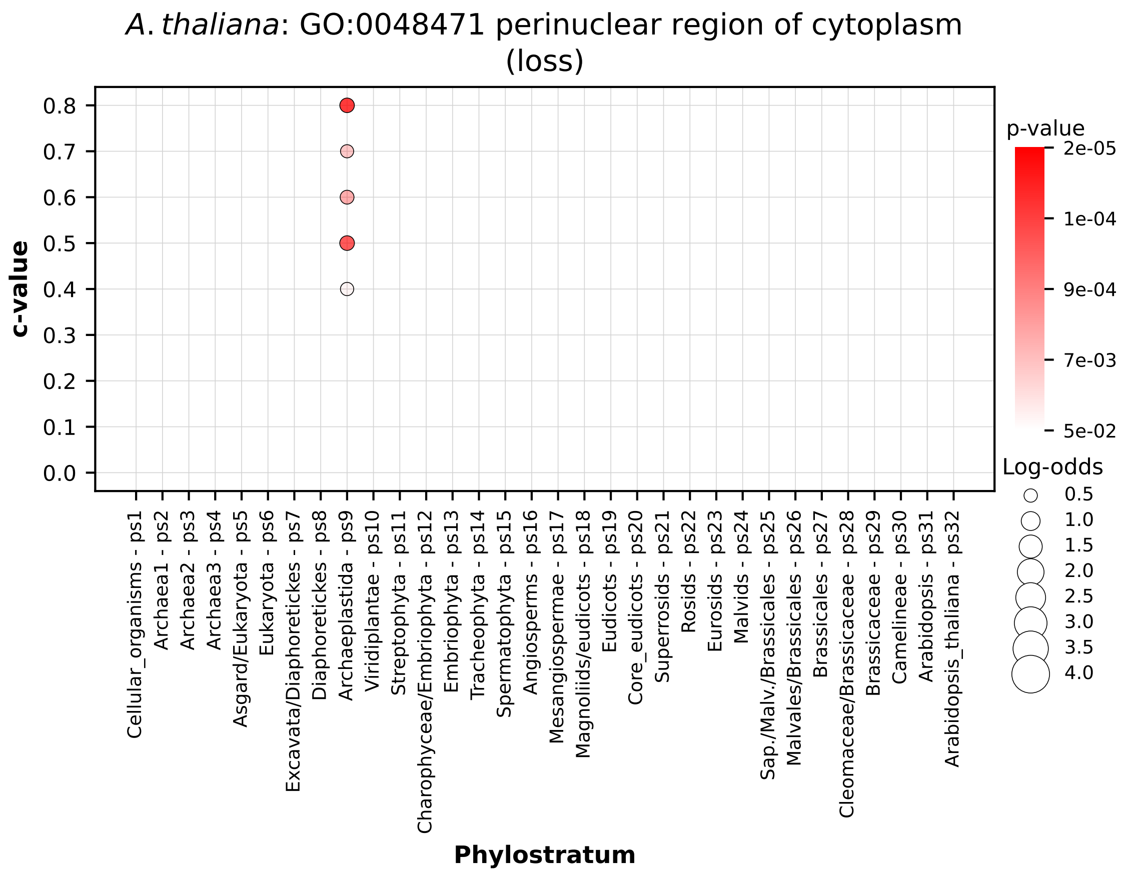
A. thaliana: GO:0048471 perinuclear region of cytoplasm (loss)
c-value
0.0
0.1
0.2
0.3
0.4
0.5
0.6
0.7
0.8
Phylostratum
Cellular_organisms - ps1
Archaea1 - ps2
Archaea2 - ps3
Archaea3 - ps4
Asgard/Eukaryota - ps5
Eukaryota - ps6
Excavata/Diaphoretickes - ps7
Diaphoretickes - ps8
Archaeplastida - ps9
Viridiplantae - ps10
Streptophyta - ps11
Charophyceae/Embriophyta - ps12
Embriophyta - ps13
Tracheophyta - ps14
Spermatophyta - ps15
Angiosperms - ps16
Mesangiospermae - ps17
Magnoliids/eudicots - ps18
Eudicots - ps19
Core_eudicots - ps20
Superrosids - ps21
Rosids - ps22
Eurosids - ps23
Malvids - ps24
Sap./Malv./Brassicales - ps25
Malvales/Brassicales - ps26
Brassicales - ps27
Cleomaceae/Brassicaceae - ps28
Brassicaceae - ps29
Camelineae - ps30
Arabidopsis - ps31
Arabidopsis_thaliana - ps32
p-value
2e-05
1e-04
9e-04
7e-03
5e-02
Log-odds
0.5
1.0
1.5
2.0
2.5
3.0
3.5
4.0

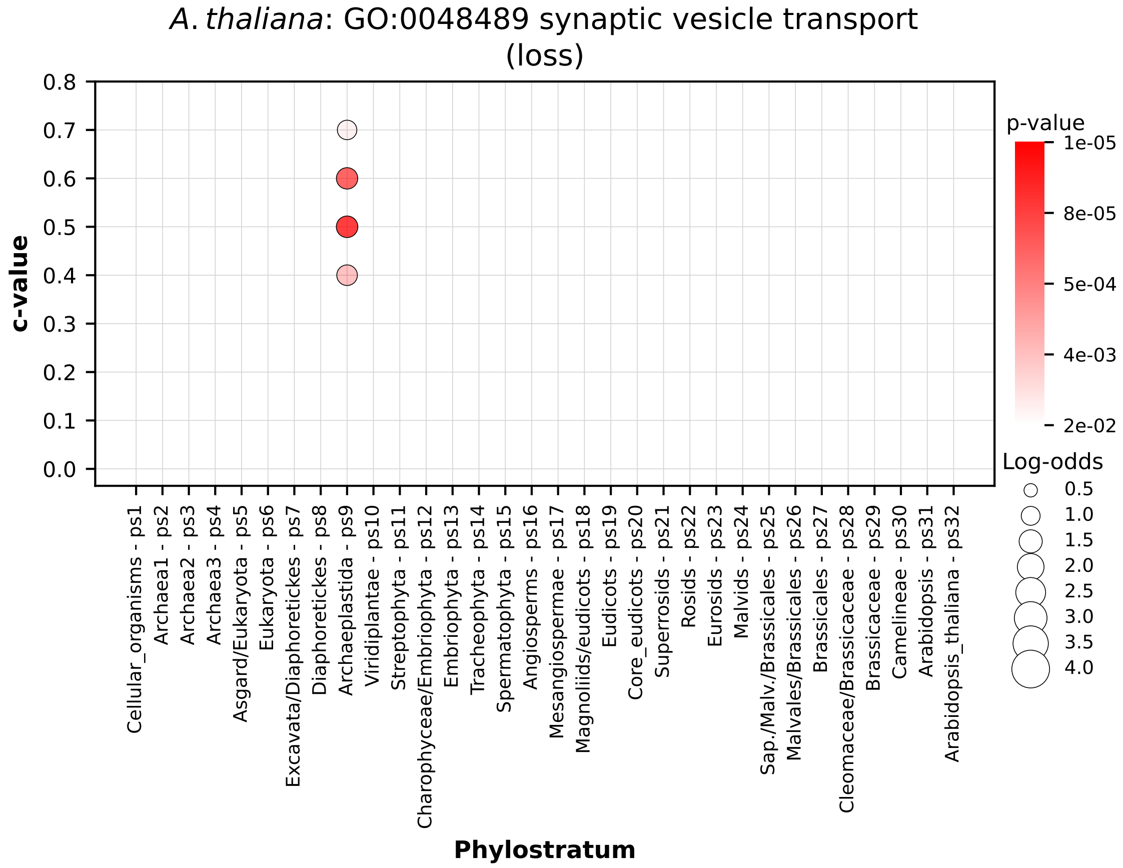

A. thaliana: GO:0048489 synaptic vesicle transport
(loss)
c-value
0.8
0.7
0.6
0.5
0.4
0.3
0.2
0.1
0.0
p-value
1e-05
8e-05
5e-04
4e-03
2e-02
Log-odds
0.5
1.0
1.5
2.0
2.5
3.0
3.5
4.0
Cellular_organisms - ps1
Archaea1 - ps2
Archaea2 - ps3
Archaea3 - ps4
Asgard/Eukaryota - ps5
Eukaryota - ps6
Excavata/Diaphoretickes - ps7
Diaphoretickes - ps8
Archaeplastida - ps9
Viridiplantae - ps10
Streptophyta - ps11
Charophyceae/Embriophyta - ps12
Embriophyta - ps13
Tracheophyta - ps14
Spermatophyta - ps15
Angiosperms - ps16
Mesangiospermae - ps17
Magnoliids/eudicots - ps18
Eudicots - ps19
Core_eudicots - ps20
Superrosids - ps21
Rosids - ps22
Eurosids - ps23
Malvids - ps24
Sap./Malv./Brassicales - ps25
Malvales/Brassicales - ps26
Brassicales - ps27
Cleomaceae/Brassicaceae - ps28
Brassicaceae - ps29
Camelineae - ps30
Arabidopsis - ps31
Arabidopsis_thaliana - ps32
Phylostratum

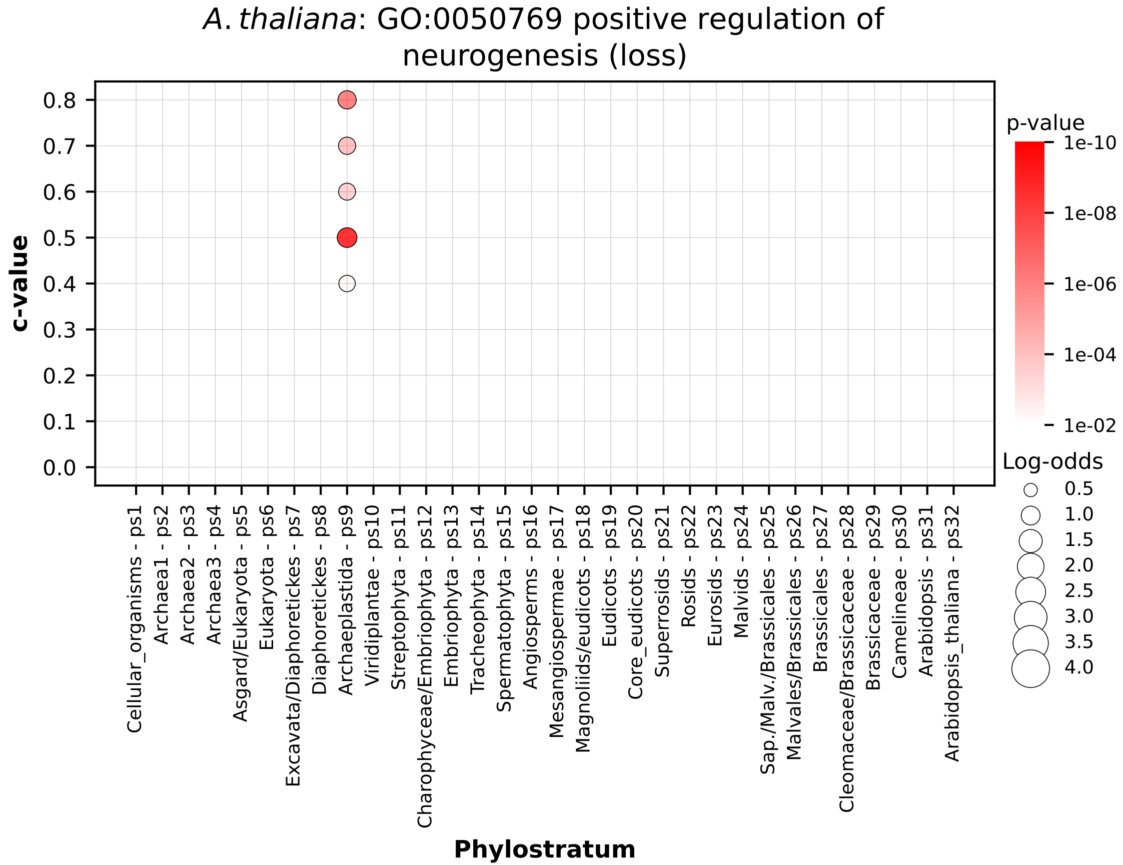

A. thaliana: GO:0050769 positive regulation of neurogenesis (loss)
c-value
0.8
0.7
0.6
0.5
0.4
0.3
0.2
0.1
0.0
p-value
1e-10
1e-08
1e-06
1e-04
1e-02
Log-odds
0.5
1.0
1.5
2.0
2.5
3.0
3.5
4.0
Cellular_organisms - ps1
Archaea1 - ps2
Archaea2 - ps3
Archaea3 - ps4
Asgard/Eukaryota - ps5
Eukaryota - ps6
Excavata/Diaphoretickes - ps7
Diaphoretickes - ps8
Archaeplastida - ps9
Viridiplantae - ps10
Streptophyta - ps11
Charophyceae/Embriophyta - ps12
Embriophyta - ps13
Tracheophyta - ps14
Spermatophyta - ps15
Angiosperms - ps16
Mesangiospermae - ps17
Magnoliids/eudicots - ps18
Eudicots - ps19
Core_eudicots - ps20
Superrosids - ps21
Rosids - ps22
Eurosids - ps23
Malvids - ps24
Sap./Malv./Brassicales - ps25
Malvales/Brassicales - ps26
Brassicales - ps27
Cleomaceae/Brassicaceae - ps28
Brassicaceae - ps29
Camelineae - ps30
Arabidopsis - ps31
Arabidopsis_thaliana - ps32
Phylostratum

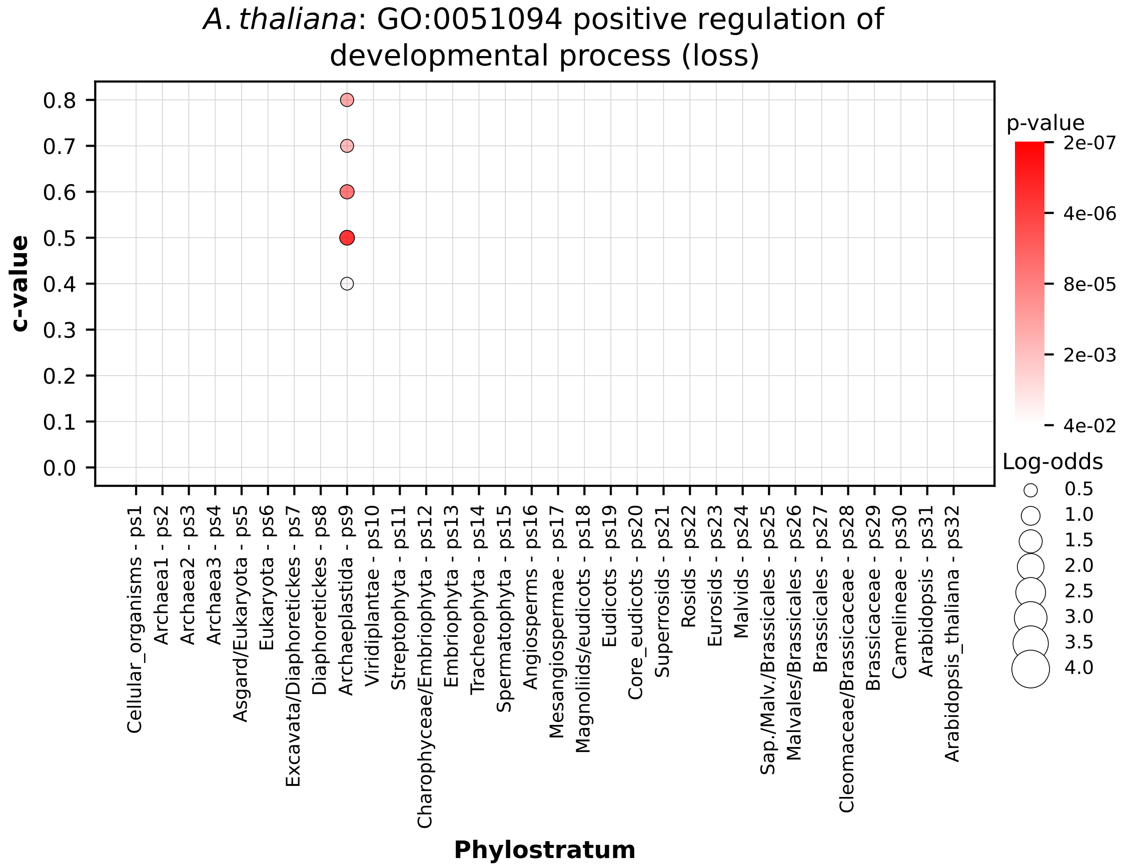

A. thaliana: GO:0051094 positive regulation of developmental process (loss)
c-value
0.0
0.1
0.2
0.3
0.4
0.5
0.6
0.7
0.8
Phylostratum
Cellular_organisms - ps1
Archaea1 - ps2
Archaea2 - ps3
Archaea3 - ps4
Asgard/Eukaryota - ps5
Eukaryota - ps6
Excavata/Diaphoretickes - ps7
Diaphoretickes - ps8
Archaeplastida - ps9
Viridiplantae - ps10
Streptophyta - ps11
Charophyceae/Embriophyta - ps12
Embriophyta - ps13
Tracheophyta - ps14
Spermatophyta - ps15
Angiosperms - ps16
Mesangiospermae - ps17
Magnoliids/eudicots - ps18
Eudicots - ps19
Core_eudicots - ps20
Superrosids - ps21
Rosids - ps22
Eurosids - ps23
Malvids - ps24
Sap./Malv./Brassicales - ps25
Malvales/Brassicales - ps26
Brassicales - ps27
Cleomaceae/Brassicaceae - ps28
Brassicaceae - ps29
Camelineae - ps30
Arabidopsis - ps31
Arabidopsis_thaliana - ps32
p-value
2e-07
4e-06
8e-05
2e-03
4e-02
Log-odds
0.5
1.0
1.5
2.0
2.5
3.0
3.5
4.0

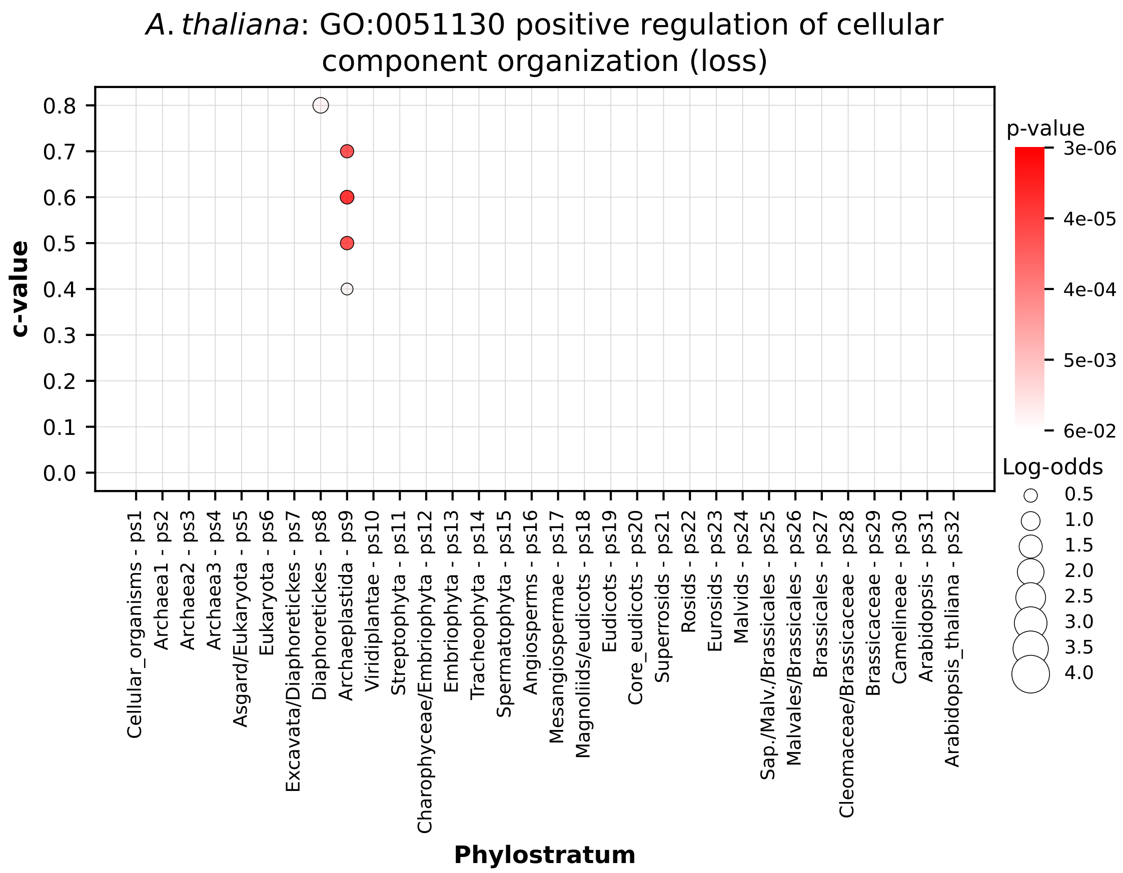

A. thaliana: GO:0051130 positive regulation of cellular component organization (loss)
c-value
0.8
0.7
0.6
0.5
0.4
0.3
0.2
0.1
0.0
Phylostratum
Cellular_organisms - ps1
Archaea1 - ps2
Archaea2 - ps3
Archaea3 - ps4
Asgard/Eukaryota - ps5
Eukaryota - ps6
Excavata/Diaphoretickes - ps7
Diaphoretickes - ps8
Archaeplastida - ps9
Viridiplantae - ps10
Streptophyta - ps11
Charophyceae/Embriophyta - ps12
Embriophyta - ps13
Tracheophyta - ps14
Spermatophyta - ps15
Angiosperms - ps16
Mesangiospermae - ps17
Magnoliids/eudicots - ps18
Eudicots - ps19
Core_eudicots - ps20
Superrosids - ps21
Rosids - ps22
Eurosids - ps23
Malvids - ps24
Sap./Malv./Brassicales - ps25
Malvales/Brassicales - ps26
Brassicales - ps27
Cleomaceae/Brassicaceae - ps28
Brassicaceae - ps29
Camelineae - ps30
Arabidopsis - ps31
Arabidopsis_thaliana - ps32
p-value
3e-06
4e-05
4e-04
5e-03
6e-02
Log-odds
0.5
1.0
1.5
2.0
2.5
3.0
3.5
4.0

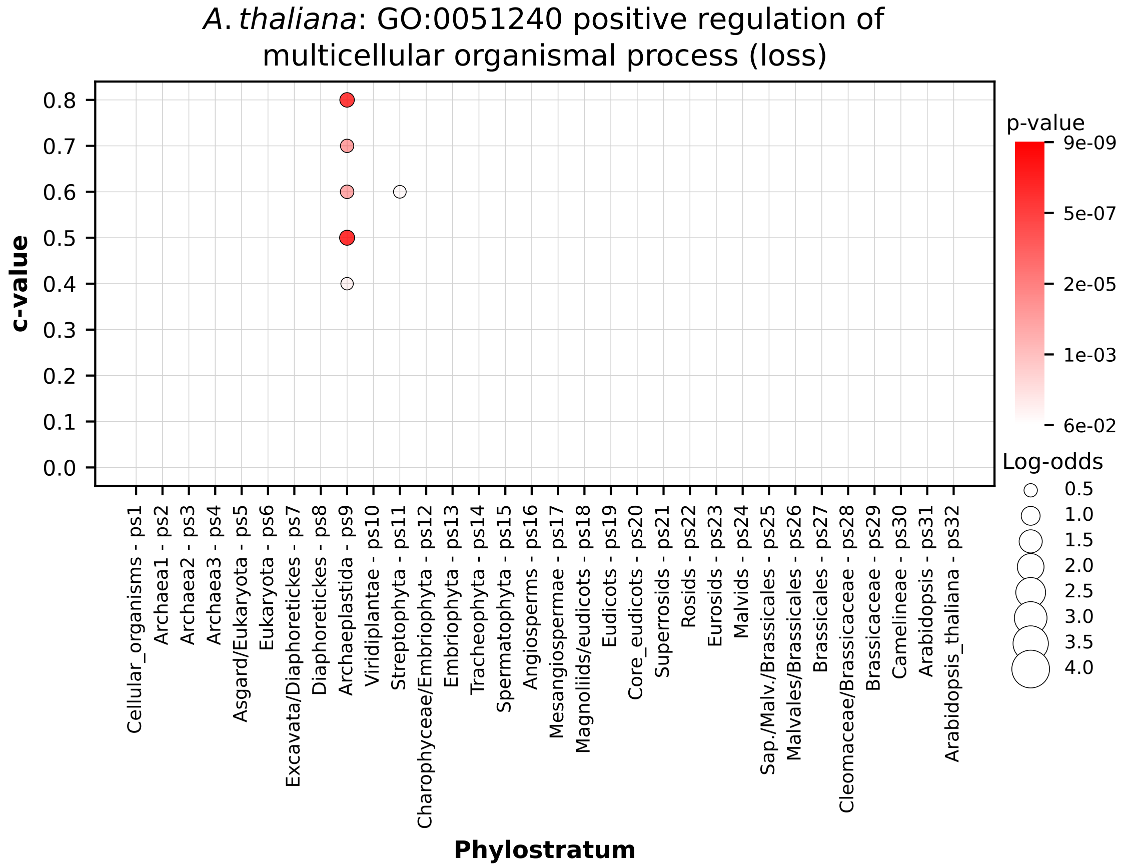
A. thaliana: GO:0051240 positive regulation of multicellular organismal process (loss)
c-value
0.8
0.7
0.6
0.5
0.4
0.3
0.2
0.1
0.0
p-value
9e-09
5e-07
2e-05
1e-03
6e-02
Log-odds
0.5
1.0
1.5
2.0
2.5
3.0
3.5
4.0
Cellular_organisms - ps1
Archaea1 - ps2
Archaea2 - ps3
Archaea3 - ps4
Asgard/Eukaryota - ps5
Eukaryota - ps6
Excavata/Diaphoretickes - ps7
Diaphoretickes - ps8
Archaeplastida - ps9
Viridiplantae - ps10
Streptophyta - ps11
Charophyceae/Embriophyta - ps12
Embriophyta - ps13
Tracheophyta - ps14
Spermatophyta - ps15
Angiosperms - ps16
Mesangiospermae - ps17
Magnoliids/eudicots - ps18
Eudicots - ps19
Core_eudicots - ps20
Superrosids - ps21
Rosids - ps22
Eurosids - ps23
Malvids - ps24
Sap./Malv./Brassicales - ps25
Malvales/Brassicales - ps26
Brassicales - ps27
Cleomaceae/Brassicaceae - ps28
Brassicaceae - ps29
Camelineae - ps30
Arabidopsis - ps31
Arabidopsis_thaliana - ps32
Phylostratum

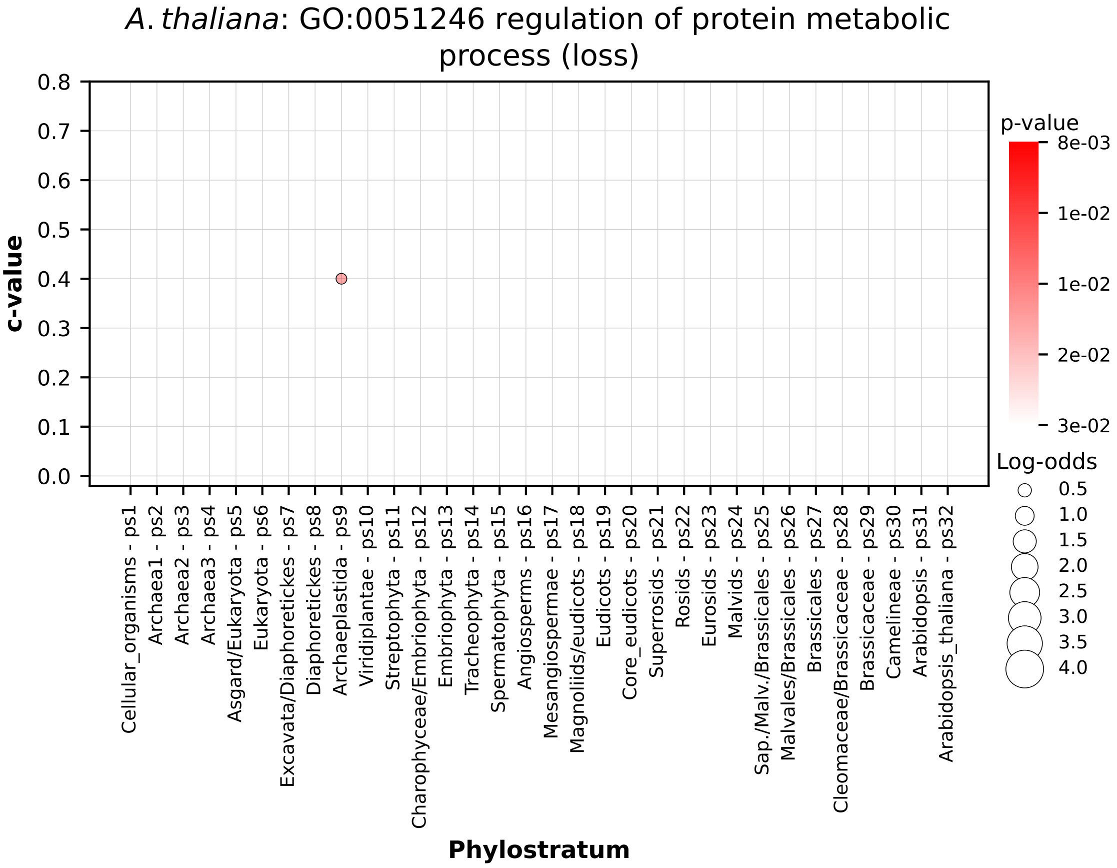

A. thaliana: GO:0051246 regulation of protein metabolic process (loss)
c-value
0.8
0.7
0.6
0.5
0.4
0.3
0.2
0.1
0.0
p-value
8e-03
1e-02
1e-02
2e-02
3e-02
Log-odds
0.5
1.0
1.5
2.0
2.5
3.0
3.5
4.0
Cellular_organisms - ps1
Archaea1 - ps2
Archaea2 - ps3
Archaea3 - ps4
Asgard/Eukaryota - ps5
Eukaryota - ps6
Excavata/Diaphoretickes - ps7
Diaphoretickes - ps8
Archaeplastida - ps9
Viridiplantae - ps10
Streptophyta - ps11
Charophyceae/Embriophyta - ps12
Embriophyta - ps13
Tracheophyta - ps14
Spermatophyta - ps15
Angiosperms - ps16
Mesangiospermae - ps17
Magnoliids/eudicots - ps18
Eudicots - ps19
Core_eudicots - ps20
Superrosids - ps21
Rosids - ps22
Eurosids - ps23
Malvids - ps24
Sap./Malv./Brassicales - ps25
Malvales/Brassicales - ps26
Brassicales - ps27
Cleomaceae/Brassicaceae - ps28
Brassicaceae - ps29
Camelineae - ps30
Arabidopsis - ps31
Arabidopsis_thaliana - ps32
Phylostratum

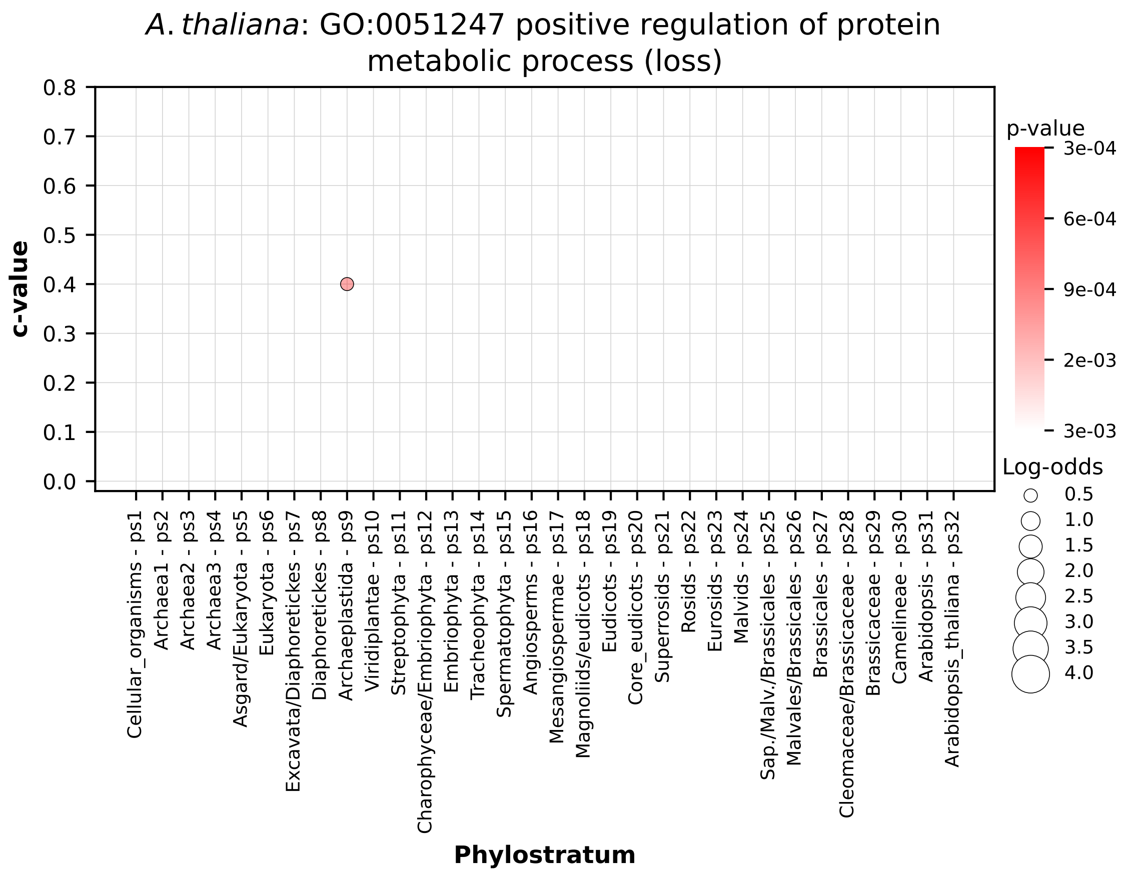

A. thaliana: GO:0051247 positive regulation of protein metabolic process (loss)
c-value
0.8
0.7
0.6
0.5
0.4
0.3
0.2
0.1
0.0
Phylostratum
Cellular_organisms - ps1
Archaea1 - ps2
Archaea2 - ps3
Archaea3 - ps4
Asgard/Eukaryota - ps5
Eukaryota - ps6
Excavata/Diaphoretickes - ps7
Diaphoretickes - ps8
Archaeplastida - ps9
Viridiplantae - ps10
Streptophyta - ps11
Charophyceae/Embriophyta - ps12
Embriophyta - ps13
Tracheophyta - ps14
Spermatophyta - ps15
Angiosperms - ps16
Mesangiospermae - ps17
Magnoliids/eudicots - ps18
Eudicots - ps19
Core_eudicots - ps20
Superrosids - ps21
Rosids - ps22
Eurosids - ps23
Malvids - ps24
Sap./Malv./Brassicales - ps25
Malvales/Brassicales - ps26
Brassicales - ps27
Cleomaceae/Brassicaceae - ps28
Brassicaceae - ps29
Camelineae - ps30
Arabidopsis - ps31
Arabidopsis_thaliana - ps32
p-value
3e-04
6e-04
9e-04
2e-03
3e-03
Log-odds
0.5
1.0
1.5
2.0
2.5
3.0
3.5
4.0

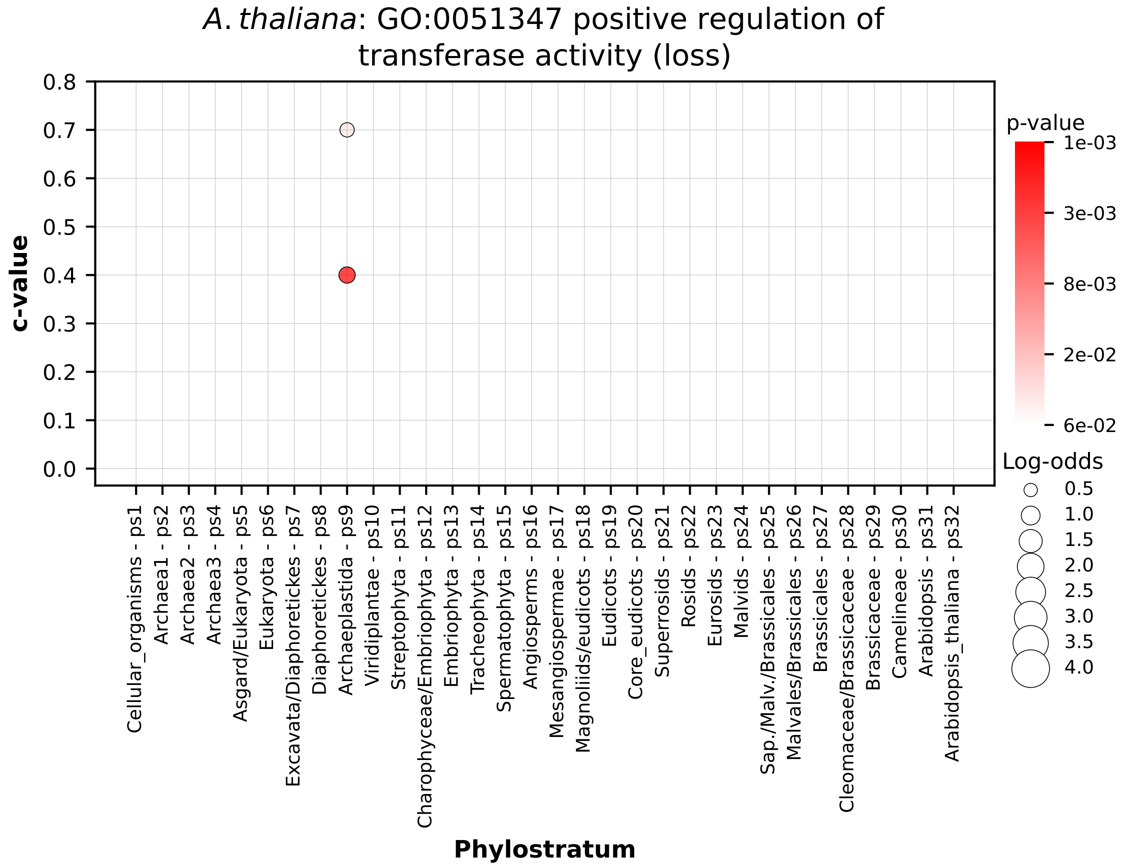
A. thaliana: GO:0051347 positive regulation of transferase activity (loss)
c-value
0.8
0.7
0.6
0.5
0.4
0.3
0.2
0.1
0.0
Phylostratum
Cellular_organisms - ps1
Archaea1 - ps2
Archaea2 - ps3
Archaea3 - ps4
Asgard/Eukaryota - ps5
Eukaryota - ps6
Excavata/Diaphoretickes - ps7
Diaphoretickes - ps8
Archaeplastida - ps9
Viridiplantae - ps10
Streptophyta - ps11
Charophyceae/Embriophyta - ps12
Embriophyta - ps13
Tracheophyta - ps14
Spermatophyta - ps15
Angiosperms - ps16
Mesangiospermae - ps17
Magnoliids/eudicots - ps18
Eudicots - ps19
Core_eudicots - ps20
Superrosids - ps21
Rosids - ps22
Eurosids - ps23
Malvids - ps24
Sap./Malv./Brassicales - ps25
Malvales/Brassicales - ps26
Brassicales - ps27
Cleomaceae/Brassicaceae - ps28
Brassicaceae - ps29
Camelineae - ps30
Arabidopsis - ps31
Arabidopsis_thaliana - ps32
p-value
1e-03
3e-03
8e-03
2e-02
6e-02
Log-odds
0.5
1.0
1.5
2.0
2.5
3.0
3.5
4.0

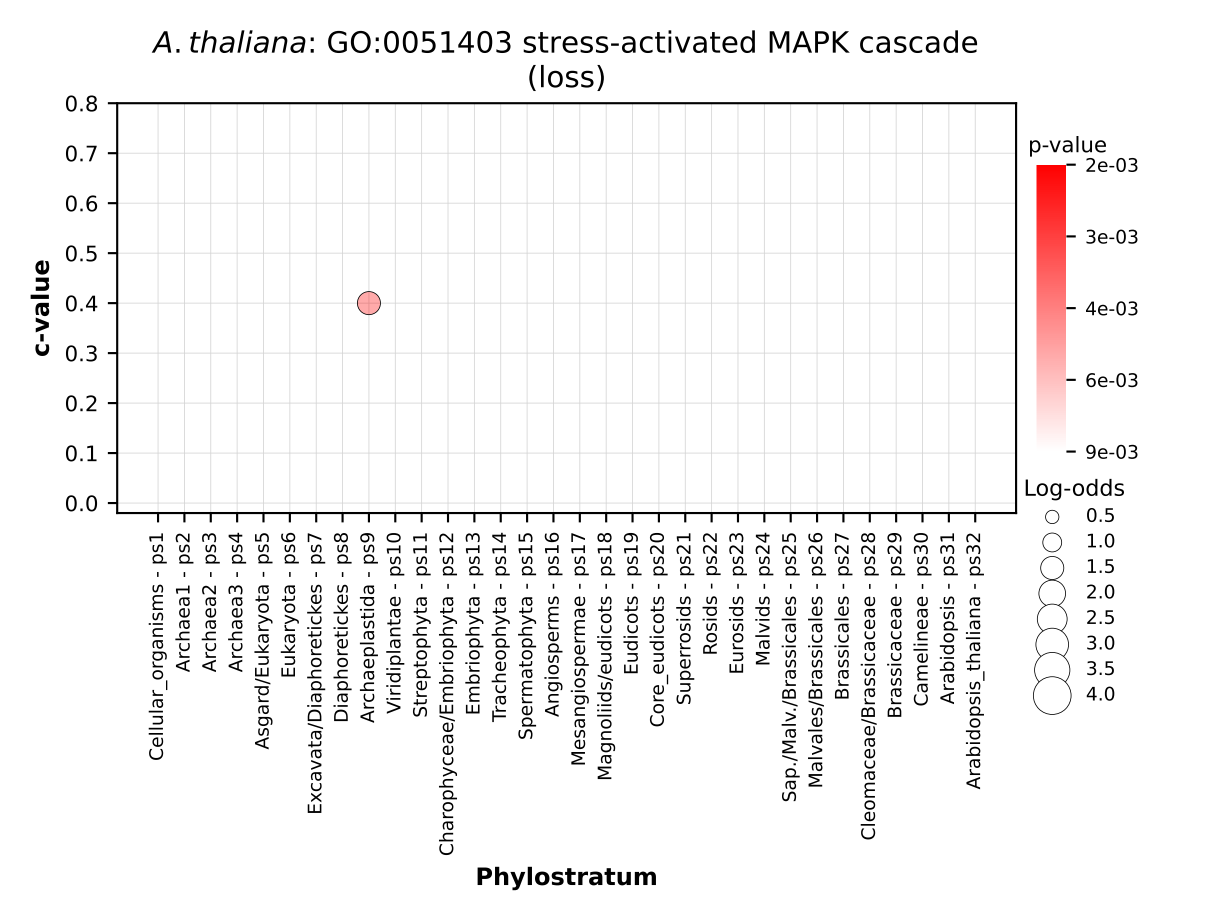
A. thaliana: GO:0051403 stress-activated MAPK cascade
(loss)
c-value
0.8
0.7
0.6
0.5
0.4
0.3
0.2
0.1
0.0
Phylostratum
Cellular_organisms - ps1
Archaea1 - ps2
Archaea2 - ps3
Archaea3 - ps4
Asgard/Eukaryota - ps5
Eukaryota - ps6
Excavata/Diaphoretickes - ps7
Diaphoretickes - ps8
Archaeplastida - ps9
Viridiplantae - ps10
Streptophyta - ps11
Charophyceae/Embriophyta - ps12
Embriophyta - ps13
Tracheophyta - ps14
Spermatophyta - ps15
Angiosperms - ps16
Mesangiospermae - ps17
Magnoliids/eudicots - ps18
Eudicots - ps19
Core_eudicots - ps20
Superrosids - ps21
Rosids - ps22
Eurosids - ps23
Malvids - ps24
Sap./Malv./Brassicales - ps25
Malvales/Brassicales - ps26
Brassicales - ps27
Cleomaceae/Brassicaceae - ps28
Brassicaceae - ps29
Camelineae - ps30
Arabidopsis - ps31
Arabidopsis_thaliana - ps32
p-value
2e-03
3e-03
4e-03
6e-03
9e-03
Log-odds
0.5
1.0
1.5
2.0
2.5
3.0
3.5
4.0

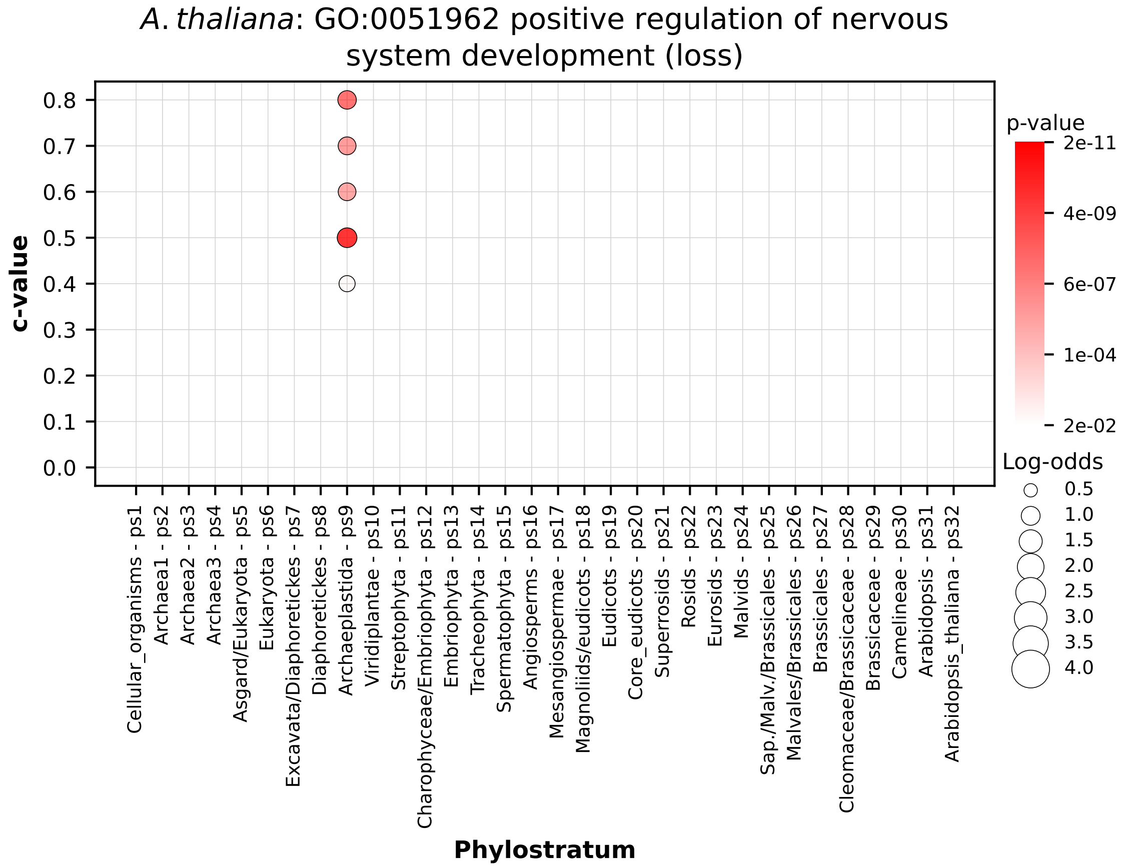

A. thaliana: GO:0051962 positive regulation of nervous system development (loss)
c-value
0.0
0.1
0.2
0.3
0.4
0.5
0.6
0.7
0.8
Phylostratum
Cellular_organisms - ps1
Archaea1 - ps2
Archaea2 - ps3
Archaea3 - ps4
Asgard/Eukaryota - ps5
Eukaryota - ps6
Excavata/Diaphoretickes - ps7
Diaphoretickes - ps8
Archaeplastida - ps9
Viridiplantae - ps10
Streptophyta - ps11
Charophyceae/Embriophyta - ps12
Embriophyta - ps13
Tracheophyta - ps14
Spermatophyta - ps15
Angiosperms - ps16
Mesangiospermae - ps17
Magnoliids/eudicots - ps18
Eudicots - ps19
Core_eudicots - ps20
Superrosids - ps21
Rosids - ps22
Eurosids - ps23
Malvids - ps24
Sap./Malv./Brassicales - ps25
Malvales/Brassicales - ps26
Brassicales - ps27
Cleomaceae/Brassicaceae - ps28
Brassicaceae - ps29
Camelineae - ps30
Arabidopsis - ps31
Arabidopsis_thaliana - ps32
p-value
2e-11
4e-09
6e-07
1e-04
2e-02
Log-odds
0.5
1.0
1.5
2.0
2.5
3.0
3.5
4.0

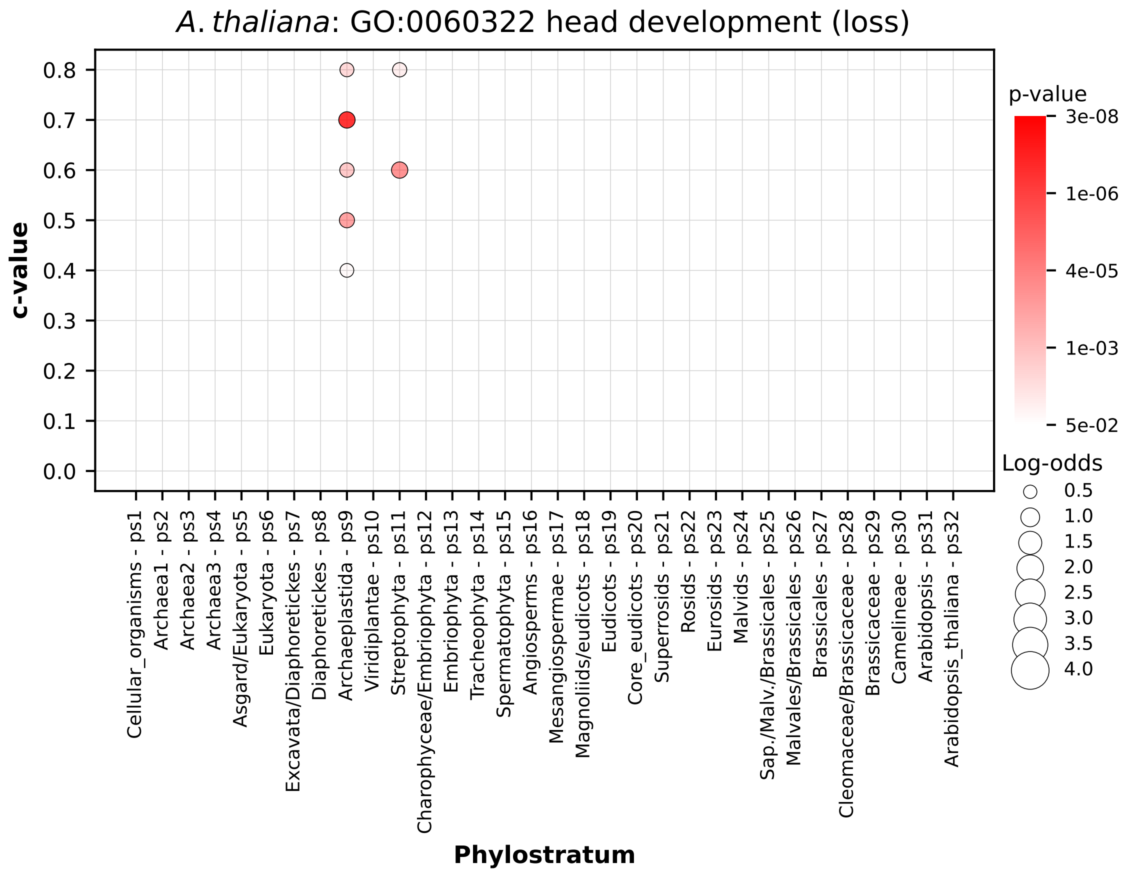

A. thaliana: GO:0060322 head development (loss)
c-value
0.8
0.7
0.6
0.5
0.4
0.3
0.2
0.1
0.0
p-value
3e-08
1e-06
4e-05
1e-03
5e-02
Log-odds
0.5
1.0
1.5
2.0
2.5
3.0
3.5
4.0
Cellular_organisms - ps1
Archaea1 - ps2
Archaea2 - ps3
Archaea3 - ps4
Asgard/Eukaryota - ps5
Eukaryota - ps6
Excavata/Diaphoretickes - ps7
Diaphoretickes - ps8
Archaeplastida - ps9
Viridiplantae - ps10
Streptophyta - ps11
Charophyceae/Embriophyta - ps12
Embriophyta - ps13
Tracheophyta - ps14
Spermatophyta - ps15
Angiosperms - ps16
Mesangiospermae - ps17
Magnoliids/eudicots - ps18
Eudicots - ps19
Core_eudicots - ps20
Superrosids - ps21
Rosids - ps22
Eurosids - ps23
Malvids - ps24
Sap./Malv./Brassicales - ps25
Malvales/Brassicales - ps26
Brassicales - ps27
Cleomaceae/Brassicaceae - ps28
Brassicaceae - ps29
Camelineae - ps30
Arabidopsis - ps31
Arabidopsis_thaliana - ps32
Phylostratum

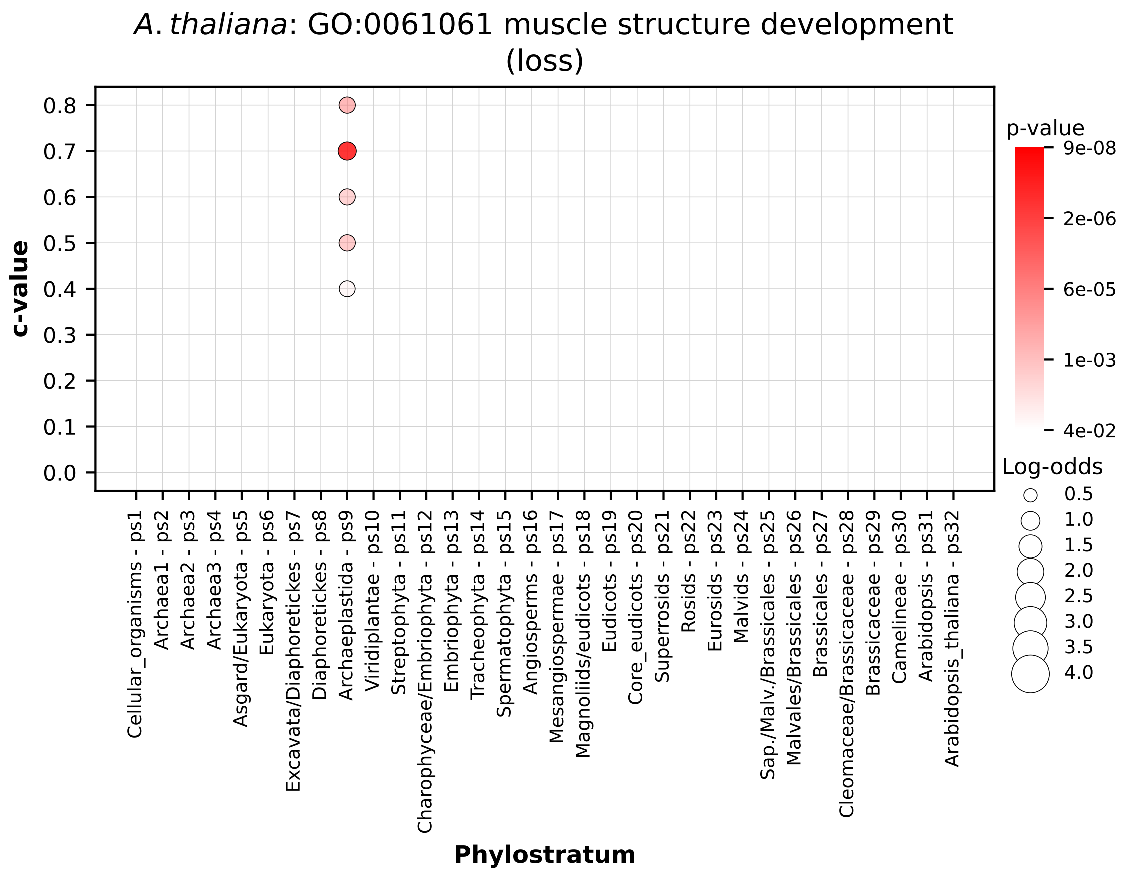

A. thaliana: GO:0061061 muscle structure development (loss)
c-value
0.8
0.7
0.6
0.5
0.4
0.3
0.2
0.1
0.0
p-value
9e-08
2e-06
6e-05
1e-03
4e-02
Log-odds
0.5
1.0
1.5
2.0
2.5
3.0
3.5
4.0
Cellular_organisms - ps1
Archaea1 - ps2
Archaea2 - ps3
Archaea3 - ps4
Asgard/Eukaryota - ps5
Eukaryota - ps6
Excavata/Diaphoretickes - ps7
Diaphoretickes - ps8
Archaeplastida - ps9
Viridiplantae - ps10
Streptophyta - ps11
Charophyceae/Embriophyta - ps12
Embriophyta - ps13
Tracheophyta - ps14
Spermatophyta - ps15
Angiosperms - ps16
Mesangiospermae - ps17
Magnoliids/eudicots - ps18
Eudicots - ps19
Core_eudicots - ps20
Superrosids - ps21
Rosids - ps22
Eurosids - ps23
Malvids - ps24
Sap./Malv./Brassicales - ps25
Malvales/Brassicales - ps26
Brassicales - ps27
Cleomaceae/Brassicaceae - ps28
Brassicaceae - ps29
Camelineae - ps30
Arabidopsis - ps31
Arabidopsis_thaliana - ps32
Phylostratum

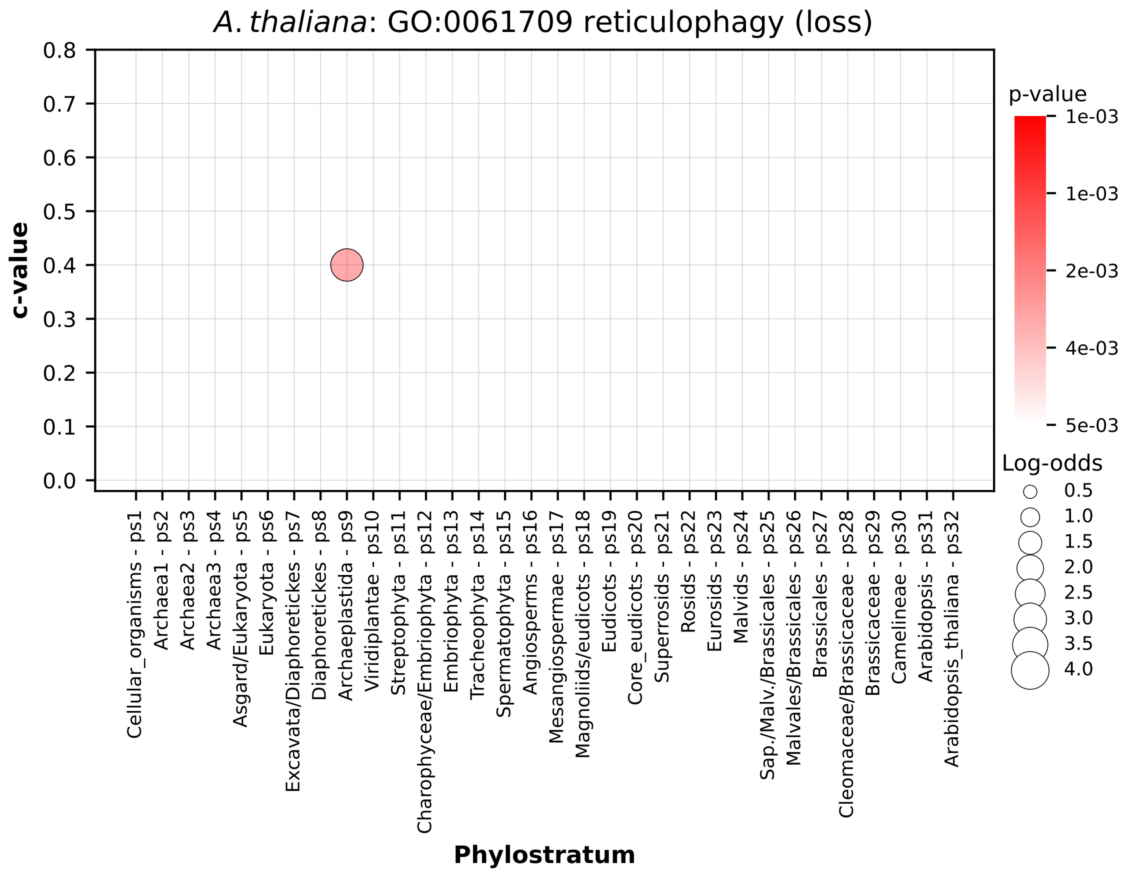

A. thaliana: GO:0061709 reticulophagy (loss)
c-value
0.8
0.7
0.6
0.5
0.4
0.3
0.2
0.1
0.0
Phylostratum
Cellular_organisms - ps1
Archaea1 - ps2
Archaea2 - ps3
Archaea3 - ps4
Asgard/Eukaryota - ps5
Eukaryota - ps6
Excavata/Diaphoretickes - ps7
Diaphoretickes - ps8
Archaeplastida - ps9
Viridiplantae - ps10
Streptophyta - ps11
Charophyceae/Embriophyta - ps12
Embriophyta - ps13
Tracheophyta - ps14
Spermatophyta - ps15
Angiosperms - ps16
Mesangiospermae - ps17
Magnoliids/eudicots - ps18
Eudicots - ps19
Core_eudicots - ps20
Superrosids - ps21
Rosids - ps22
Eurosids - ps23
Malvids - ps24
Sap./Malv./Brassicales - ps25
Malvales/Brassicales - ps26
Brassicales - ps27
Cleomaceae/Brassicaceae - ps28
Brassicaceae - ps29
Camelineae - ps30
Arabidopsis - ps31
Arabidopsis_thaliana - ps32
p-value
1e-03
1e-03
2e-03
4e-03
5e-03
Log-odds
0.5
1.0
1.5
2.0
2.5
3.0
3.5
4.0

A. thaliana: GO:0071902 positive regulation of protein serine/threonine kinase activity (loss)

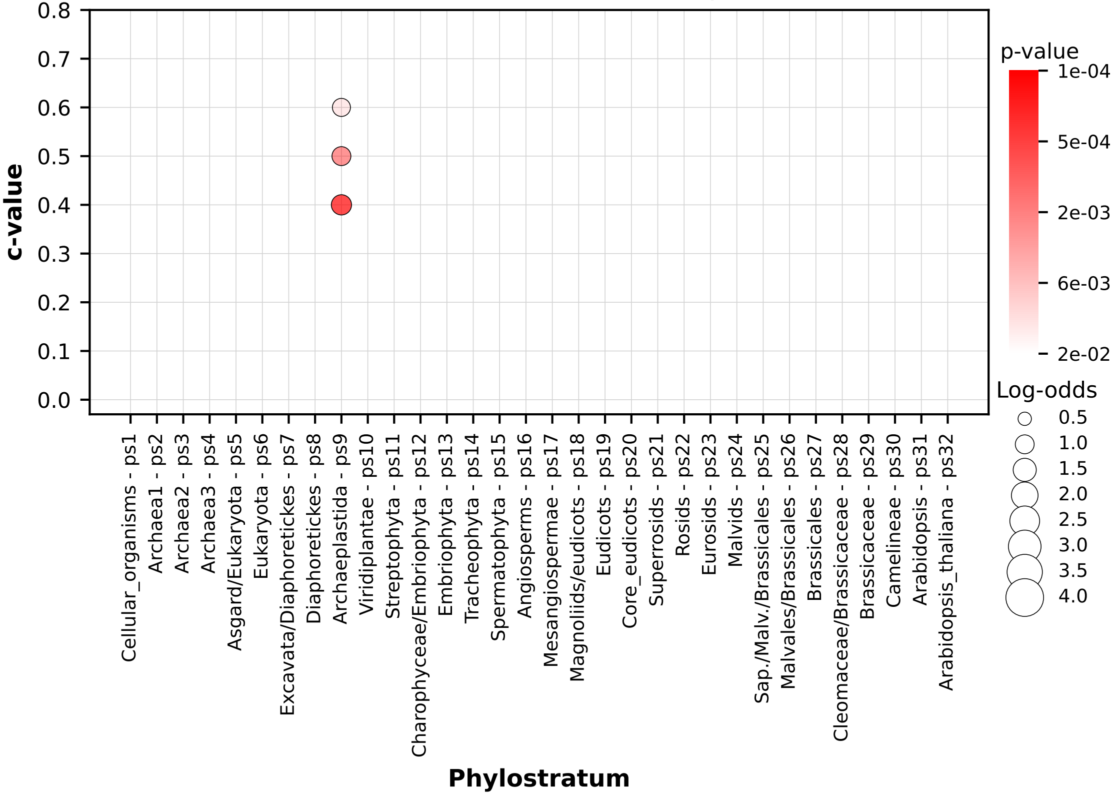

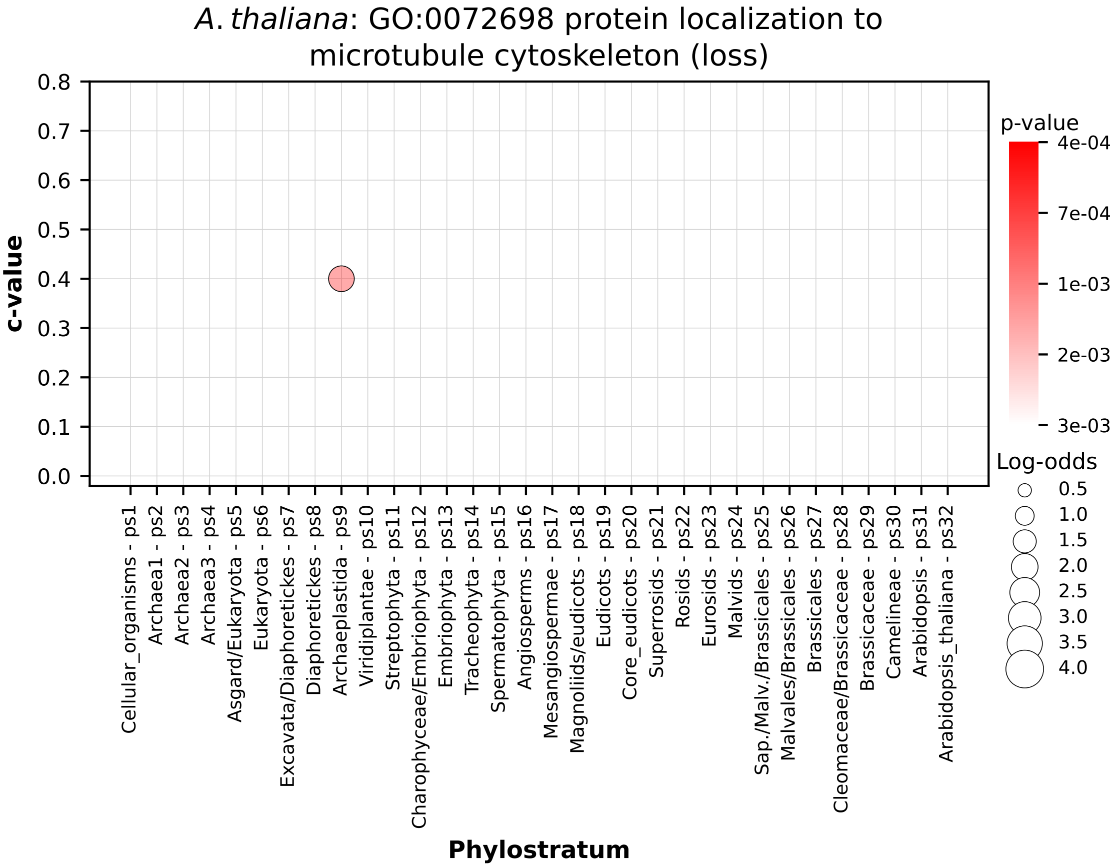

A. thaliana: GO:0072698 protein localization to microtubule cytoskeleton (loss)
c-value
0.8
0.7
0.6
0.5
0.4
0.3
0.2
0.1
0.0
Phylostratum
Cellular_organisms - ps1
Archaea1 - ps2
Archaea2 - ps3
Archaea3 - ps4
Asgard/Eukaryota - ps5
Eukaryota - ps6
Excavata/Diaphoretickes - ps7
Diaphoretickes - ps8
Archaeplastida - ps9
Viridiplantae - ps10
Streptophyta - ps11
Charophyceae/Embriophyta - ps12
Embriophyta - ps13
Tracheophyta - ps14
Spermatophyta - ps15
Angiosperms - ps16
Mesangiospermae - ps17
Magnoliids/eudicots - ps18
Eudicots - ps19
Core_eudicots - ps20
Superrosids - ps21
Rosids - ps22
Eurosids - ps23
Malvids - ps24
Sap./Malv./Brassicales - ps25
Malvales/Brassicales - ps26
Brassicales - ps27
Cleomaceae/Brassicaceae - ps28
Brassicaceae - ps29
Camelineae - ps30
Arabidopsis - ps31
Arabidopsis_thaliana - ps32
p-value
4e-04
7e-04
1e-03
2e-03
3e-03
Log-odds
0.5
1.0
1.5
2.0
2.5
3.0
3.5
4.0

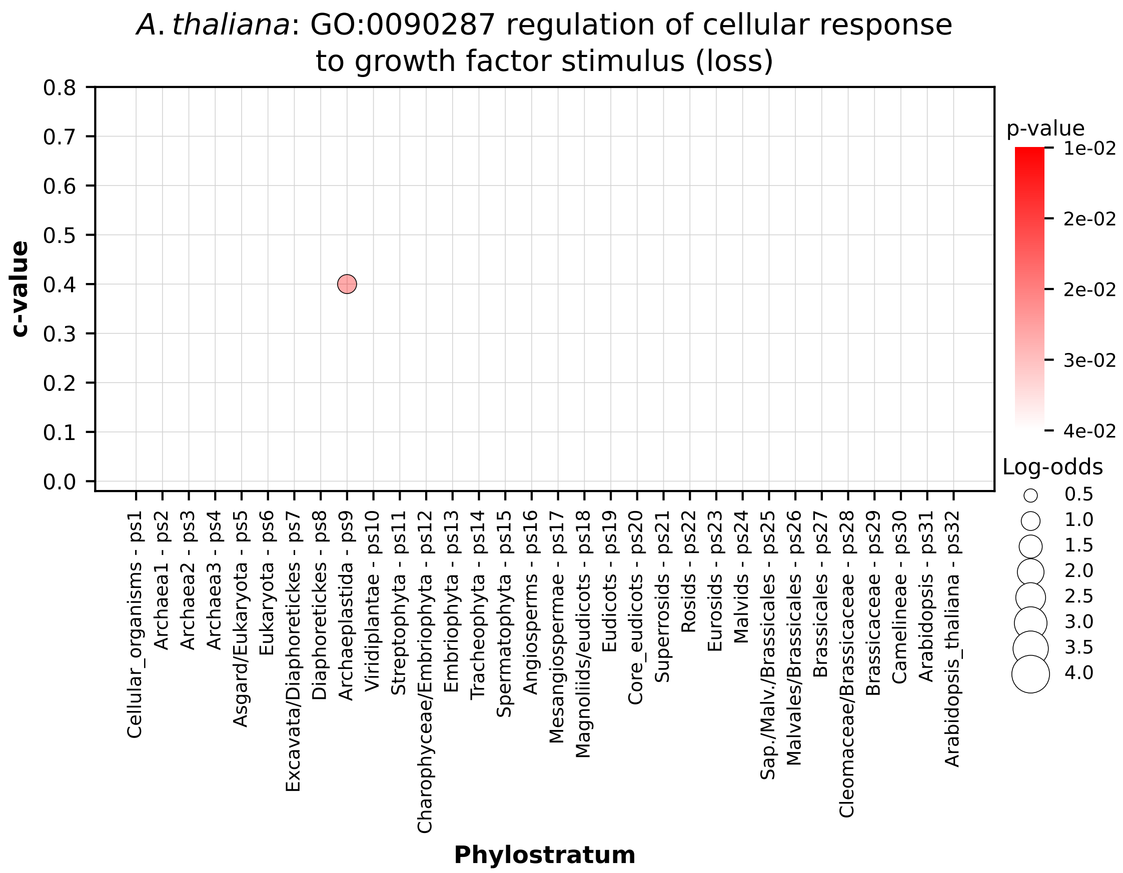

A. thaliana: GO:0090287 regulation of cellular response to growth factor stimulus (loss)
c-value
0.8
0.7
0.6
0.5
0.4
0.3
0.2
0.1
0.0
p-value
1e-02
2e-02
2e-02
3e-02
4e-02
Log-odds
0.5
1.0
1.5
2.0
2.5
3.0
3.5
4.0
Cellular_organisms - ps1
Archaea1 - ps2
Archaea2 - ps3
Archaea3 - ps4
Asgard/Eukaryota - ps5
Eukaryota - ps6
Excavata/Diaphoretickes - ps7
Diaphoretickes - ps8
Archaeplastida - ps9
Viridiplantae - ps10
Streptophyta - ps11
Charophyceae/Embriophyta - ps12
Embriophyta - ps13
Tracheophyta - ps14
Spermatophyta - ps15
Angiosperms - ps16
Mesangiospermae - ps17
Magnoliids/eudicots - ps18
Eudicots - ps19
Core_eudicots - ps20
Superrosids - ps21
Rosids - ps22
Eurosids - ps23
Malvids - ps24
Sap./Malv./Brassicales - ps25
Malvales/Brassicales - ps26
Brassicales - ps27
Cleomaceae/Brassicaceae - ps28
Brassicaceae - ps29
Camelineae - ps30
Arabidopsis - ps31
Arabidopsis_thaliana - ps32
Phylostratum

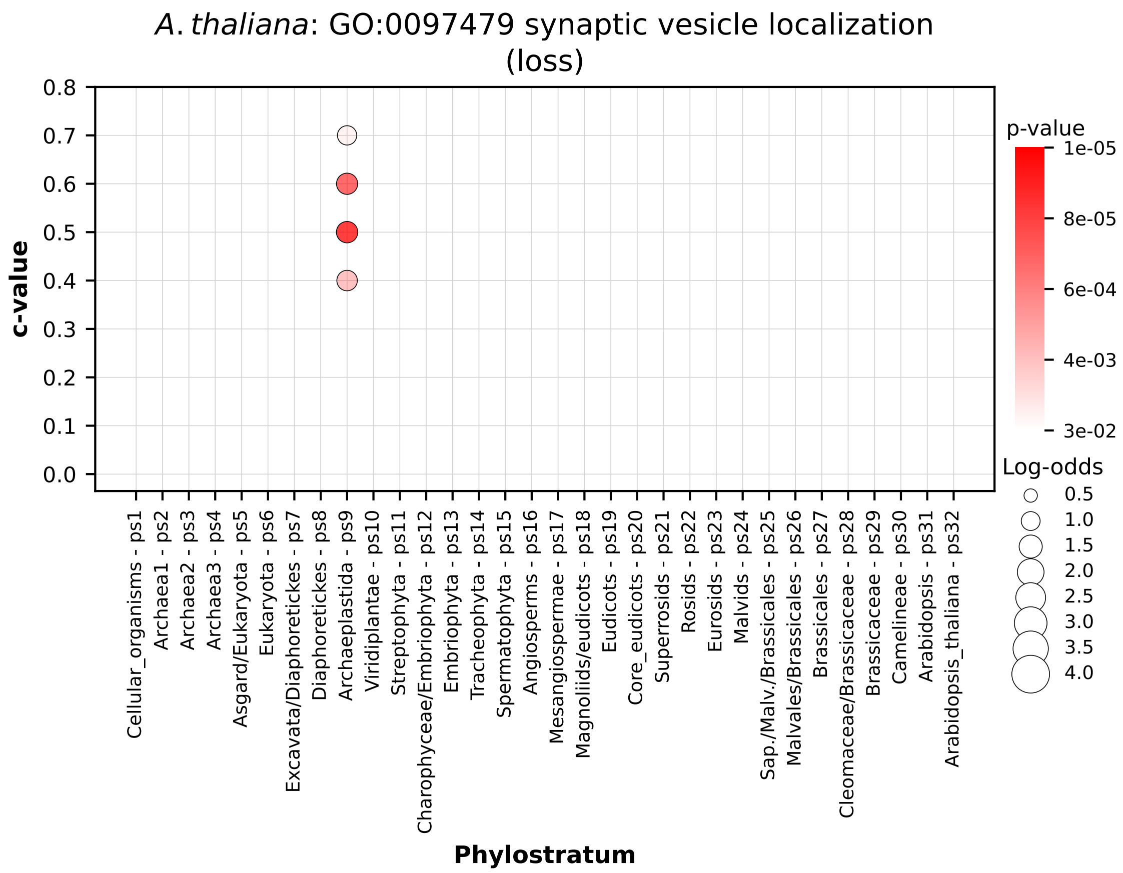

A. thaliana: GO:0097479 synaptic vesicle localization
(loss)
c-value
0.8
0.7
0.6
0.5
0.4
0.3
0.2
0.1
0.0
p-value
1e-05
8e-05
6e-04
4e-03
3e-02
Log-odds
0.5
1.0
1.5
2.0
2.5
3.0
3.5
4.0
Cellular_organisms - ps1
Archaea1 - ps2
Archaea2 - ps3
Archaea3 - ps4
Asgard/Eukaryota - ps5
Eukaryota - ps6
Excavata/Diaphoretickes - ps7
Diaphoretickes - ps8
Archaeplastida - ps9
Viridiplantae - ps10
Streptophyta - ps11
Charophyceae/Embriophyta - ps12
Embriophyta - ps13
Tracheophyta - ps14
Spermatophyta - ps15
Angiosperms - ps16
Mesangiospermae - ps17
Magnoliids/eudicots - ps18
Eudicots - ps19
Core_eudicots - ps20
Superrosids - ps21
Rosids - ps22
Eurosids - ps23
Malvids - ps24
Sap./Malv./Brassicales - ps25
Malvales/Brassicales - ps26
Brassicales - ps27
Cleomaceae/Brassicaceae - ps28
Brassicaceae - ps29
Camelineae - ps30
Arabidopsis - ps31
Arabidopsis_thaliana - ps32
Phylostratum

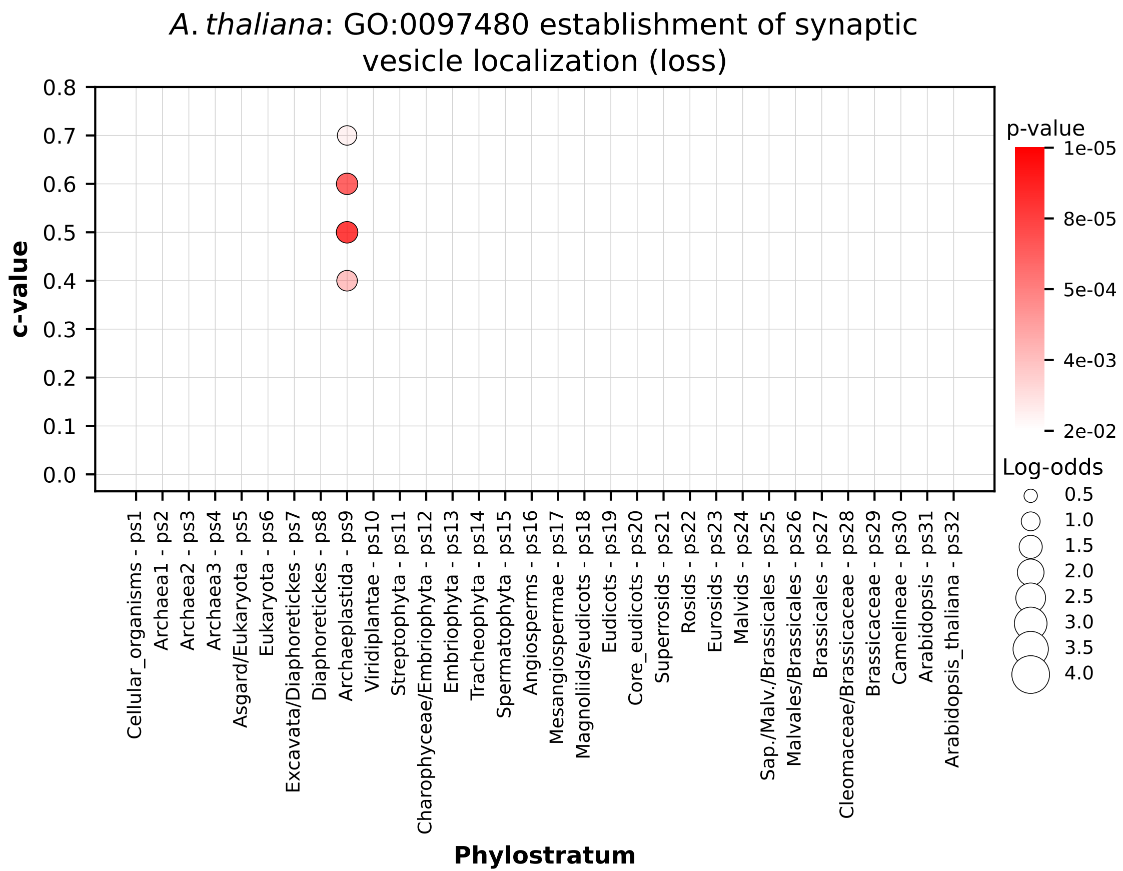

A. thaliana: GO:0097480 establishment of synaptic vesicle localization (loss)
c-value
0.0
0.1
0.2
0.3
0.4
0.5
0.6
0.7
0.8
Phylostratum
Cellular_organisms - ps1
Archaea1 - ps2
Archaea2 - ps3
Archaea3 - ps4
Asgard/Eukaryota - ps5
Eukaryota - ps6
Excavata/Diaphoretickes - ps7
Diaphoretickes - ps8
Archaeplastida - ps9
Viridiplantae - ps10
Streptophyta - ps11
Charophyceae/Embriophyta - ps12
Embriophyta - ps13
Tracheophyta - ps14
Spermatophyta - ps15
Angiosperms - ps16
Mesangiospermae - ps17
Magnoliids/eudicots - ps18
Eudicots - ps19
Core_eudicots - ps20
Superrosids - ps21
Rosids - ps22
Eurosids - ps23
Malvids - ps24
Sap./Malv./Brassicales - ps25
Malvales/Brassicales - ps26
Brassicales - ps27
Cleomaceae/Brassicaceae - ps28
Brassicaceae - ps29
Camelineae - ps30
Arabidopsis - ps31
Arabidopsis_thaliana - ps32
p-value
1e-05
8e-05
5e-04
4e-03
2e-02
Log-odds
0.5
1.0
1.5
2.0
2.5
3.0
3.5
4.0

A. thaliana: GO:0098916 anterograde trans-synaptic signaling (loss)

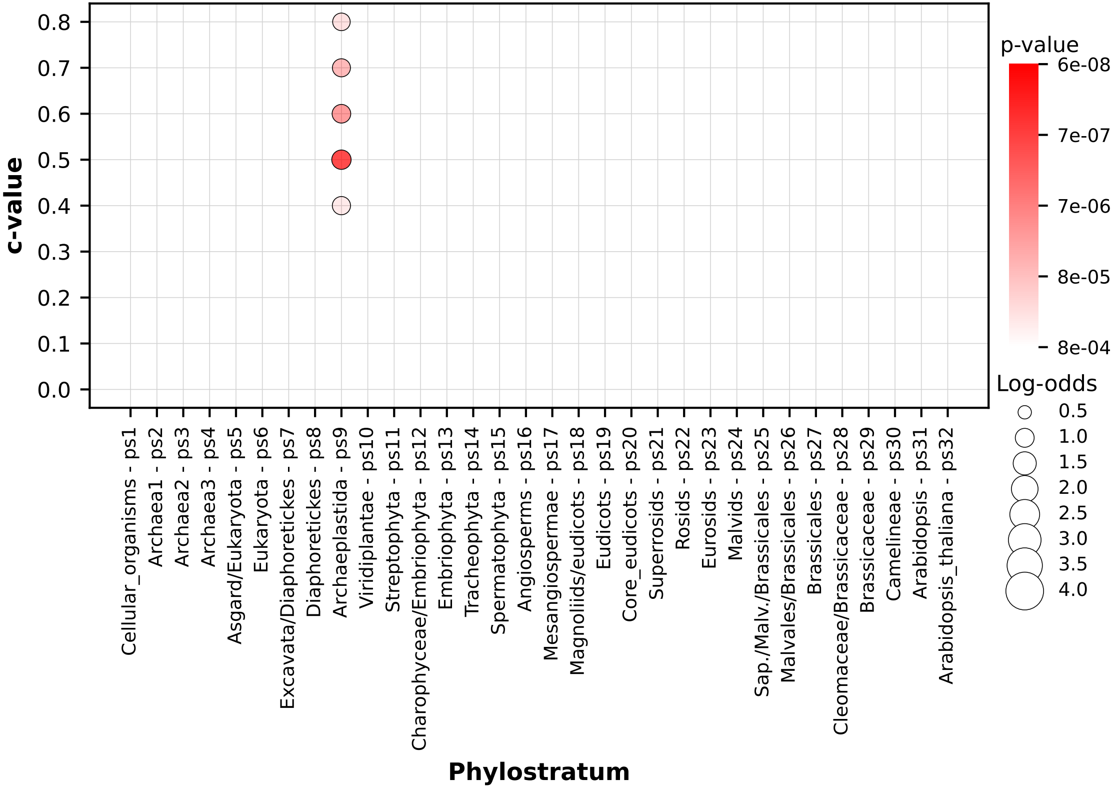

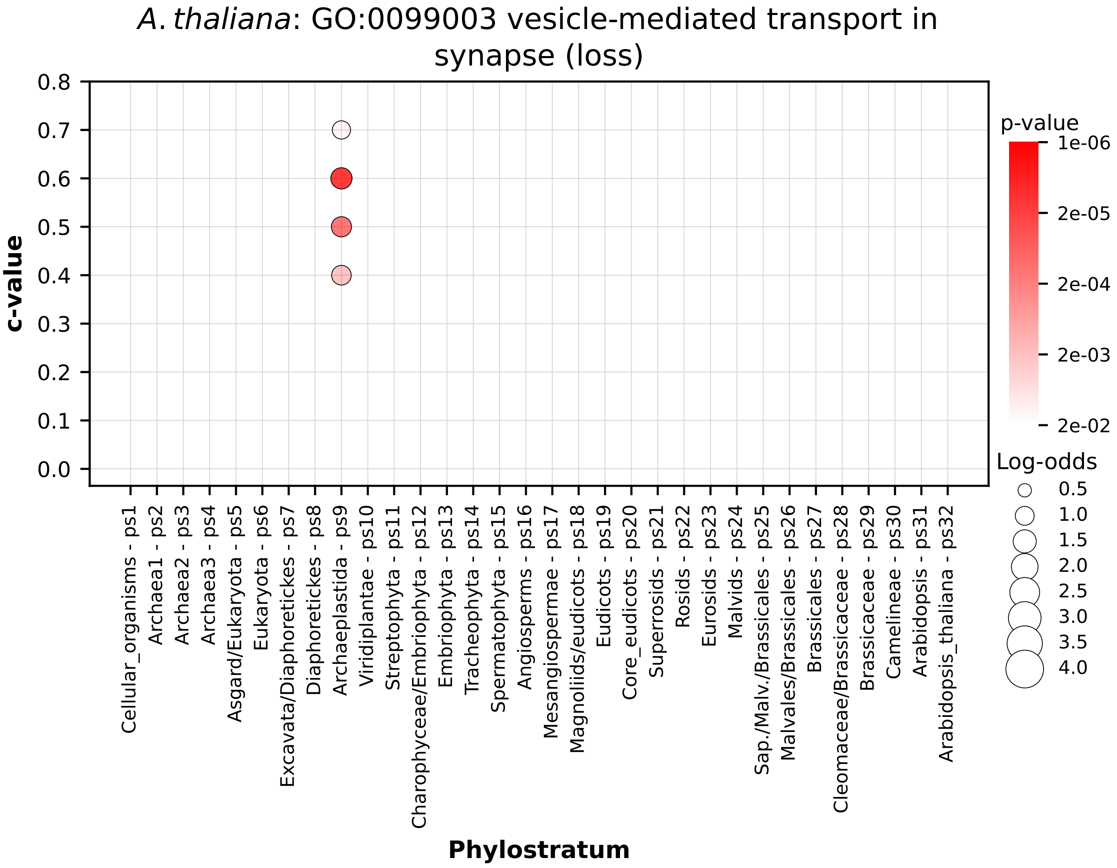

A. thaliana: GO:0099003 vesicle-mediated transport in synapse (loss)
c-value
0.8
0.7
0.6
0.5
0.4
0.3
0.2
0.1
0.0
Phylostratum
Cellular_organisms - ps1
Archaea1 - ps2
Archaea2 - ps3
Archaea3 - ps4
Asgard/Eukaryota - ps5
Eukaryota - ps6
Excavata/Diaphoretickes - ps7
Diaphoretickes - ps8
Archaeplastida - ps9
Viridiplantae - ps10
Streptophyta - ps11
Charophyceae/Embriophyta - ps12
Embriophyta - ps13
Tracheophyta - ps14
Spermatophyta - ps15
Angiosperms - ps16
Mesangiospermae - ps17
Magnoliids/eudicots - ps18
Eudicots - ps19
Core_eudicots - ps20
Superrosids - ps21
Rosids - ps22
Eurosids - ps23
Malvids - ps24
Sap./Malv./Brassicales - ps25
Malvales/Brassicales - ps26
Brassicales - ps27
Cleomaceae/Brassicaceae - ps28
Brassicaceae - ps29
Camelineae - ps30
Arabidopsis - ps31
Arabidopsis_thaliana - ps32
p-value
1e-06
2e-05
2e-04
2e-03
2e-02
Log-odds
0.5
1.0
1.5
2.0
2.5
3.0
3.5
4.0

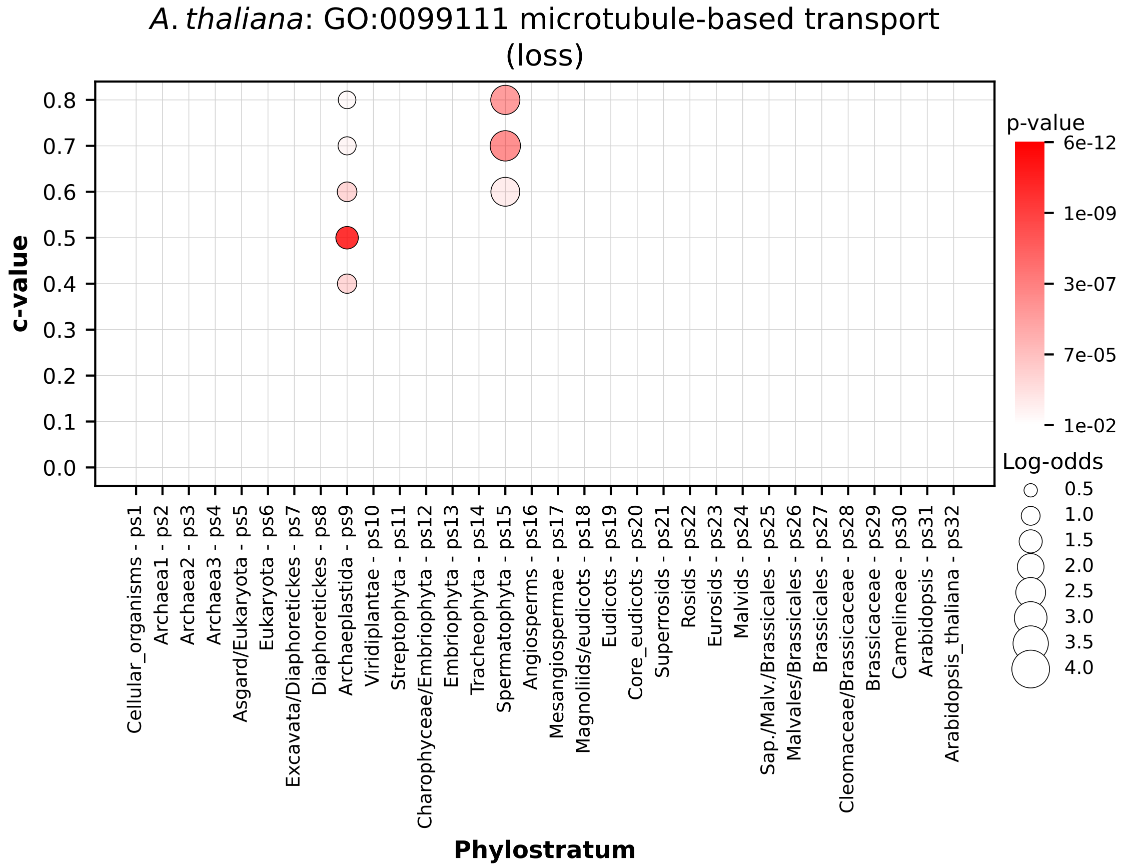

A. thaliana: GO:0099111 microtubule-based transport
(loss)
c-value
0.0
0.1
0.2
0.3
0.4
0.5
0.6
0.7
0.8
Phylostratum
Cellular_organisms - ps1
Archaea1 - ps2
Archaea2 - ps3
Archaea3 - ps4
Asgard/Eukaryota - ps5
Eukaryota - ps6
Excavata/Diaphoretickes - ps7
Diaphoretickes - ps8
Archaeplastida - ps9
Viridiplantae - ps10
Streptophyta - ps11
Charophyceae/Embriophyta - ps12
Embriophyta - ps13
Tracheophyta - ps14
Spermatophyta - ps15
Angiosperms - ps16
Mesangiospermae - ps17
Magnoliids/eudicots - ps18
Eudicots - ps19
Core_eudicots - ps20
Superrosids - ps21
Rosids - ps22
Eurosids - ps23
Malvids - ps24
Sap./Malv./Brassicales - ps25
Malvales/Brassicales - ps26
Brassicales - ps27
Cleomaceae/Brassicaceae - ps28
Brassicaceae - ps29
Camelineae - ps30
Arabidopsis - ps31
Arabidopsis_thaliana - ps32
p-value
6e-12
1e-09
3e-07
7e-05
1e-02
Log-odds
0.5
1.0
1.5
2.0
2.5
3.0
3.5
4.0

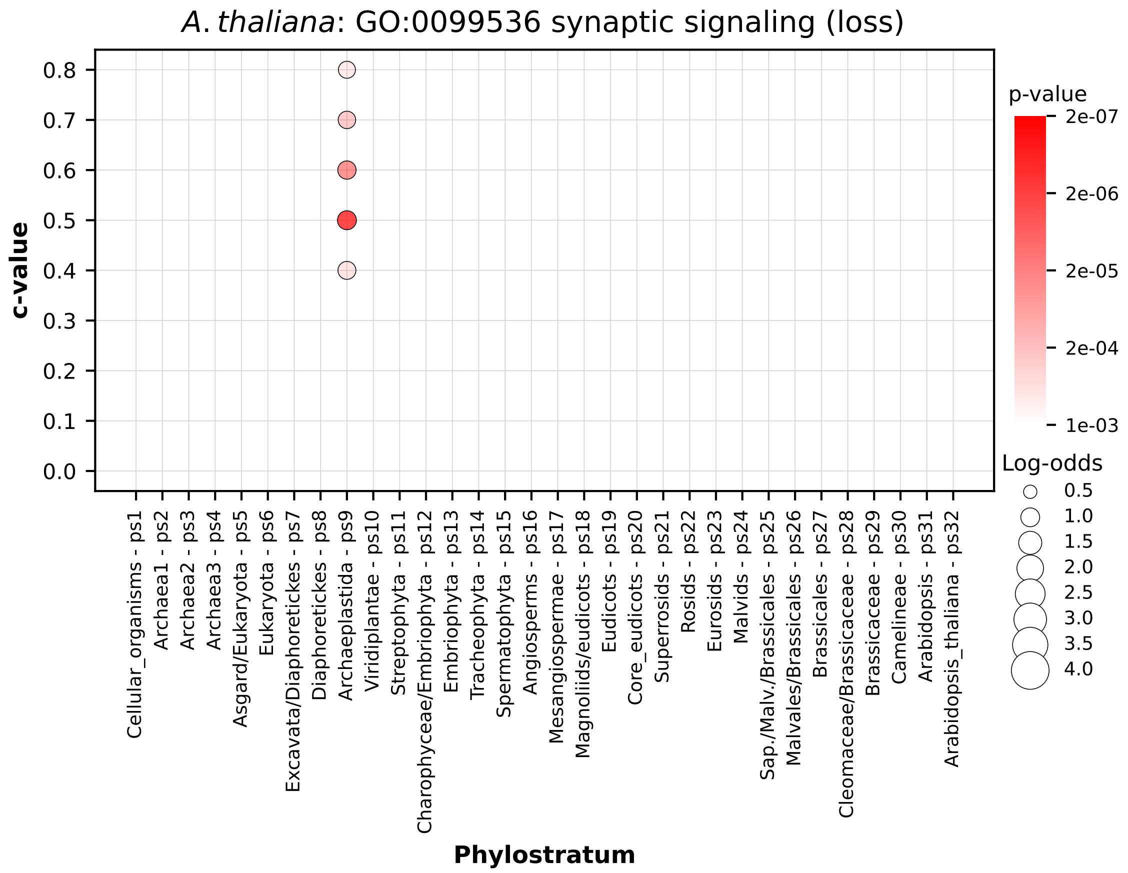
A. thaliana: GO:0099536 synaptic signaling (loss)
c-value
0.8
0.7
0.6
0.5
0.4
0.3
0.2
0.1
0.0
p-value
2e-07
2e-06
2e-05
2e-04
1e-03
Log-odds
0.5
1.0
1.5
2.0
2.5
3.0
3.5
4.0
Cellular_organisms - ps1
Archaea1 - ps2
Archaea2 - ps3
Archaea3 - ps4
Asgard/Eukaryota - ps5
Eukaryota - ps6
Excavata/Diaphoretickes - ps7
Diaphoretickes - ps8
Archaeplastida - ps9
Viridiplantae - ps10
Streptophyta - ps11
Charophyceae/Embriophyta - ps12
Embriophyta - ps13
Tracheophyta - ps14
Spermatophyta - ps15
Angiosperms - ps16
Mesangiospermae - ps17
Magnoliids/eudicots - ps18
Eudicots - ps19
Core_eudicots - ps20
Superrosids - ps21
Rosids - ps22
Eurosids - ps23
Malvids - ps24
Sap./Malv./Brassicales - ps25
Malvales/Brassicales - ps26
Brassicales - ps27
Cleomaceae/Brassicaceae - ps28
Brassicaceae - ps29
Camelineae - ps30
Arabidopsis - ps31
Arabidopsis_thaliana - ps32
Phylostratum

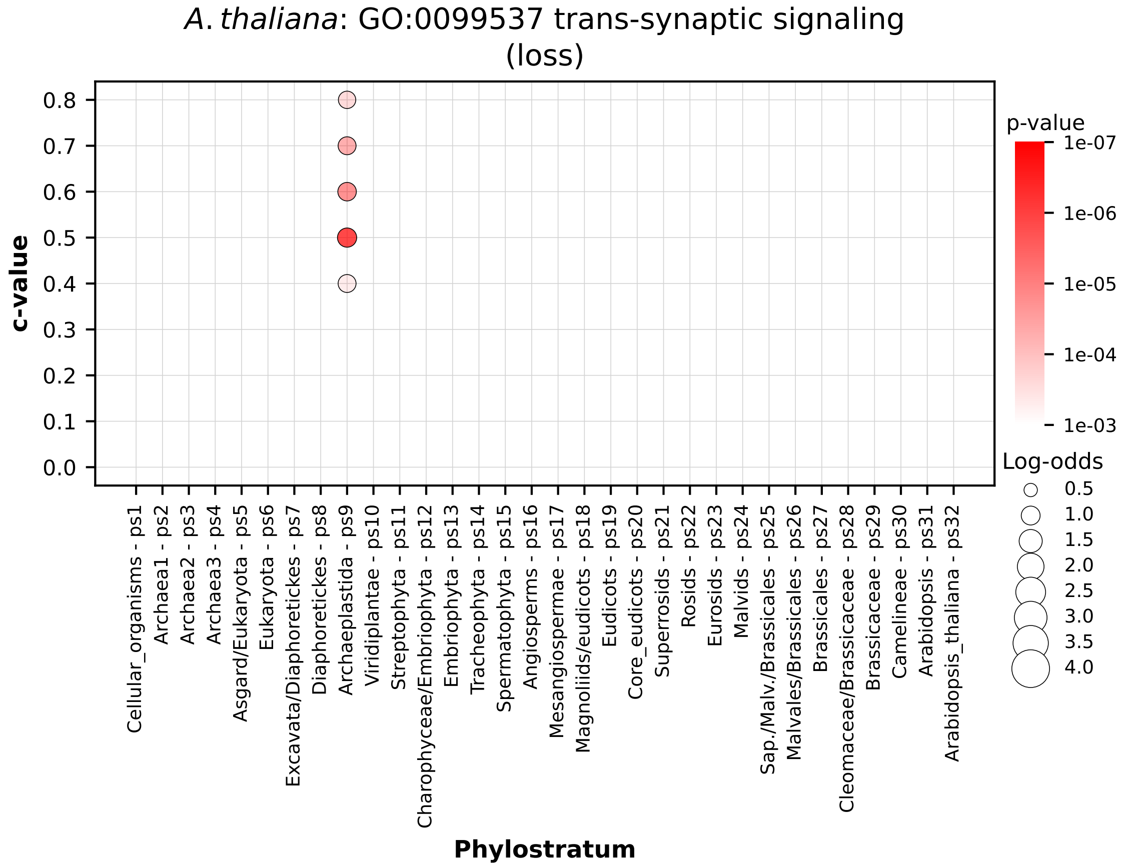
A. thaliana: GO:0099537 trans-synaptic signaling
(loss)
c-value
0.0
0.1
0.2
0.3
0.4
0.5
0.6
0.7
0.8
p-value
1e-07
1e-06
1e-05
1e-04
1e-03
Log-odds
0.5
1.0
1.5
2.0
2.5
3.0
3.5
4.0
Cellular_organisms - ps1
Archaea1 - ps2
Archaea2 - ps3
Archaea3 - ps4
Asgard/Eukaryota - ps5
Eukaryota - ps6
Excavata/Diaphoretickes - ps7
Diaphoretickes - ps8
Archaeplastida - ps9
Viridiplantae - ps10
Streptophyta - ps11
Charophyceae/Embriophyta - ps12
Embriophyta - ps13
Tracheophyta - ps14
Spermatophyta - ps15
Angiosperms - ps16
Mesangiospermae - ps17
Magnoliids/eudicots - ps18
Eudicots - ps19
Core_eudicots - ps20
Superrosids - ps21
Rosids - ps22
Eurosids - ps23
Malvids - ps24
Sap./Malv./Brassicales - ps25
Malvales/Brassicales - ps26
Brassicales - ps27
Cleomaceae/Brassicaceae - ps28
Brassicaceae - ps29
Camelineae - ps30
Arabidopsis - ps31
Arabidopsis_thaliana - ps32
Phylostratum

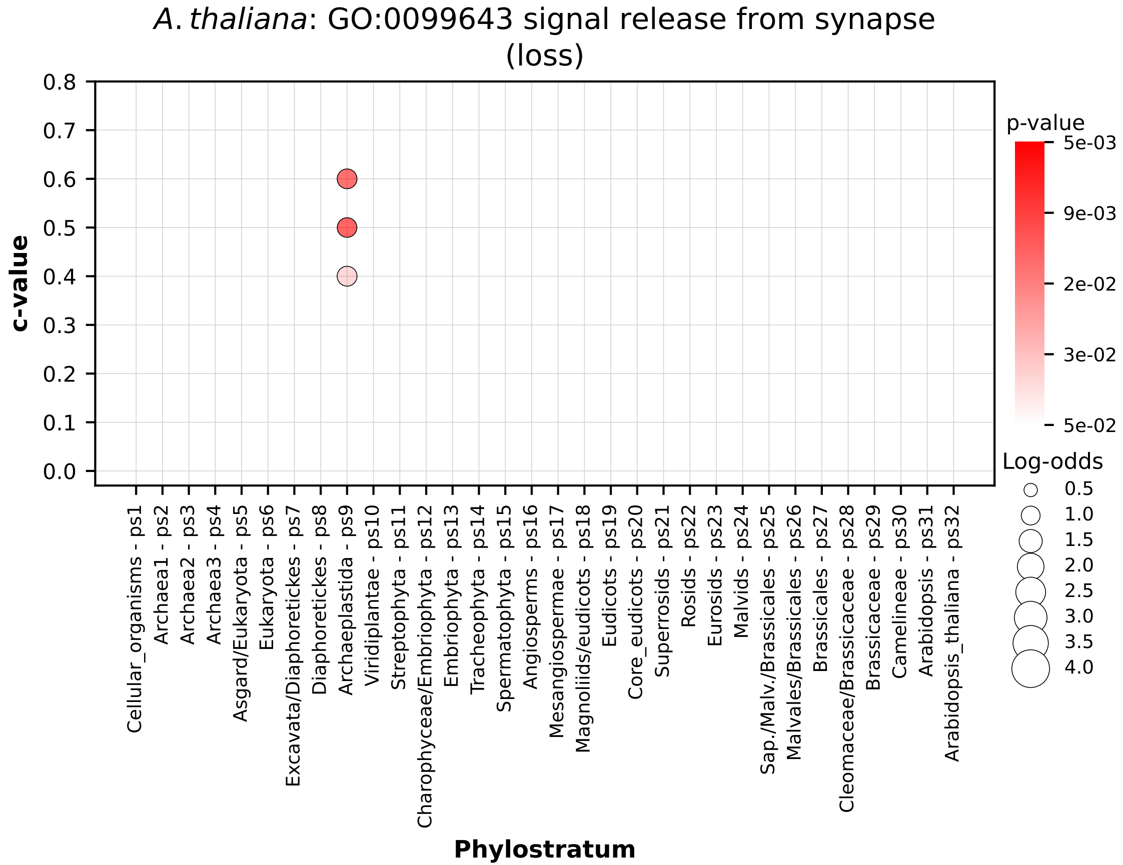

A. thaliana: GO:0099643 signal release from synapse (loss)
c-value
0.8
0.7
0.6
0.5
0.4
0.3
0.2
0.1
0.0
Phylostratum
Cellular_organisms - ps1
Archaea1 - ps2
Archaea2 - ps3
Archaea3 - ps4
Asgard/Eukaryota - ps5
Eukaryota - ps6
Excavata/Diaphoretickes - ps7
Diaphoretickes - ps8
Archaeplastida - ps9
Viridiplantae - ps10
Streptophyta - ps11
Charophyceae/Embriophyta - ps12
Embriophyta - ps13
Tracheophyta - ps14
Spermatophyta - ps15
Angiosperms - ps16
Mesangiospermae - ps17
Magnoliids/eudicots - ps18
Eudicots - ps19
Core_eudicots - ps20
Superrosids - ps21
Rosids - ps22
Eurosids - ps23
Malvids - ps24
Sap./Malv./Brassicales - ps25
Malvales/Brassicales - ps26
Brassicales - ps27
Cleomaceae/Brassicaceae - ps28
Brassicaceae - ps29
Camelineae - ps30
Arabidopsis - ps31
Arabidopsis_thaliana - ps32
p-value
5e-03
9e-03
2e-02
3e-02
5e-02
Log-odds
0.5
1.0
1.5
2.0
2.5
3.0
3.5
4.0

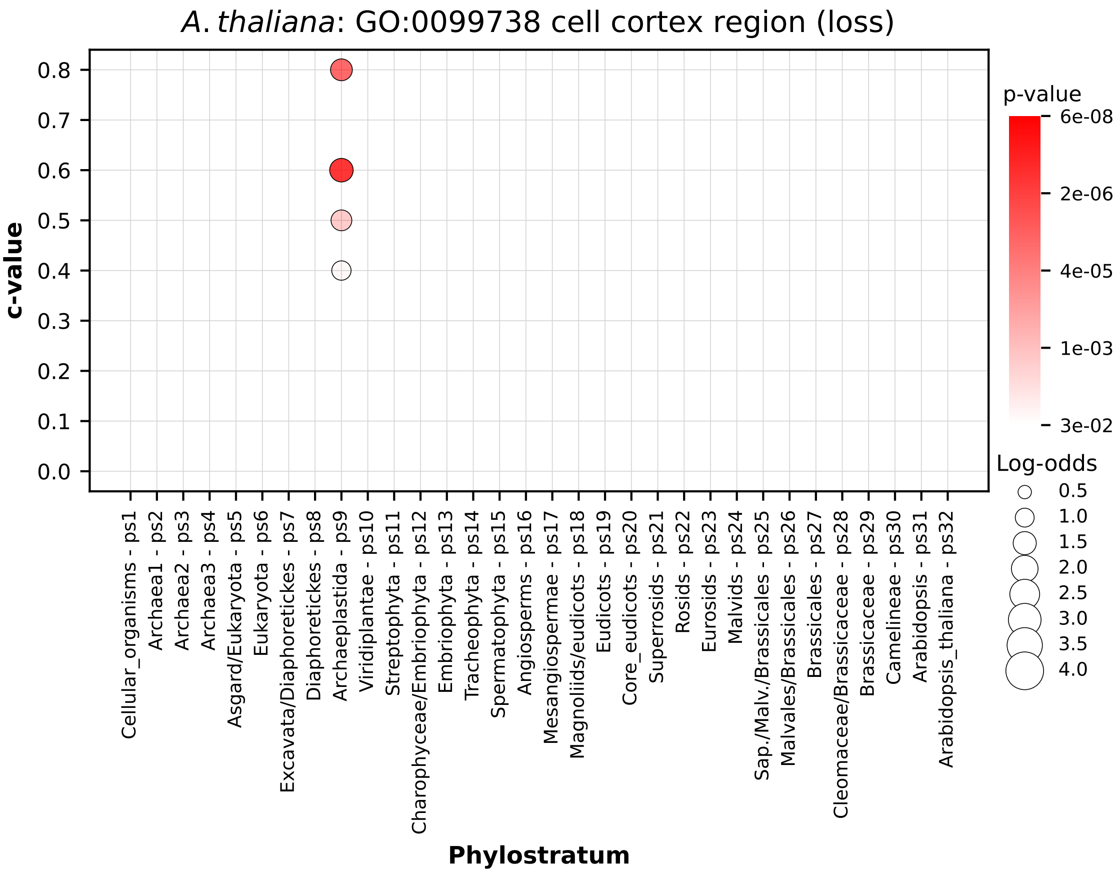

A. thaliana: GO:0099738 cell cortex region (loss)
c-value
0.8
0.7
0.6
0.5
0.4
0.3
0.2
0.1
0.0
p-value
6e-08
2e-06
4e-05
1e-03
3e-02
Log-odds
0.5
1.0
1.5
2.0
2.5
3.0
3.5
4.0
Cellular_organisms - ps1
Archaea1 - ps2
Archaea2 - ps3
Archaea3 - ps4
Asgard/Eukaryota - ps5
Eukaryota - ps6
Excavata/Diaphoretickes - ps7
Diaphoretickes - ps8
Archaeplastida - ps9
Viridiplantae - ps10
Streptophyta - ps11
Charophyceae/Embriophyta - ps12
Embriophyta - ps13
Tracheophyta - ps14
Spermatophyta - ps15
Angiosperms - ps16
Mesangiospermae - ps17
Magnoliids/eudicots - ps18
Eudicots - ps19
Core_eudicots - ps20
Superrosids - ps21
Rosids - ps22
Eurosids - ps23
Malvids - ps24
Sap./Malv./Brassicales - ps25
Malvales/Brassicales - ps26
Brassicales - ps27
Cleomaceae/Brassicaceae - ps28
Brassicaceae - ps29
Camelineae - ps30
Arabidopsis - ps31
Arabidopsis_thaliana - ps32
Phylostratum

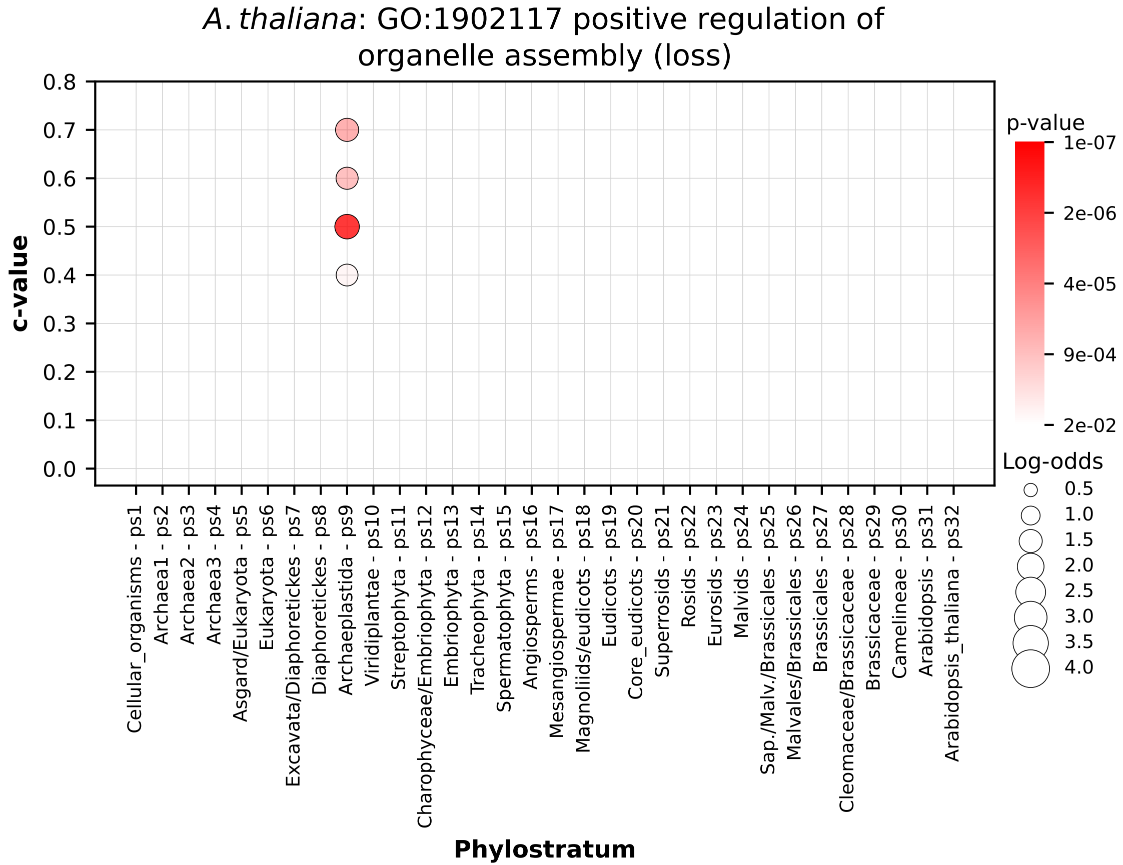

A. thaliana: GO:1902117 positive regulation of organelle assembly (loss)
c-value
0.0
0.1
0.2
0.3
0.4
0.5
0.6
0.7
0.8
p-value
1e-07
2e-06
4e-05
9e-04
2e-02
Log-odds
0.5
1.0
1.5
2.0
2.5
3.0
3.5
4.0
Cellular_organisms - ps1
Archaea1 - ps2
Archaea2 - ps3
Archaea3 - ps4
Asgard/Eukaryota - ps5
Eukaryota - ps6
Excavata/Diaphoretickes - ps7
Diaphoretickes - ps8
Archaeplastida - ps9
Viridiplantae - ps10
Streptophyta - ps11
Charophyceae/Embriophyta - ps12
Embriophyta - ps13
Tracheophyta - ps14
Spermatophyta - ps15
Angiosperms - ps16
Mesangiospermae - ps17
Magnoliids/eudicots - ps18
Eudicots - ps19
Core_eudicots - ps20
Superrosids - ps21
Rosids - ps22
Eurosids - ps23
Malvids - ps24
Sap./Malv./Brassicales - ps25
Malvales/Brassicales - ps26
Brassicales - ps27
Cleomaceae/Brassicaceae - ps28
Brassicaceae - ps29
Camelineae - ps30
Arabidopsis - ps31
Arabidopsis_thaliana - ps32
Phylostratum

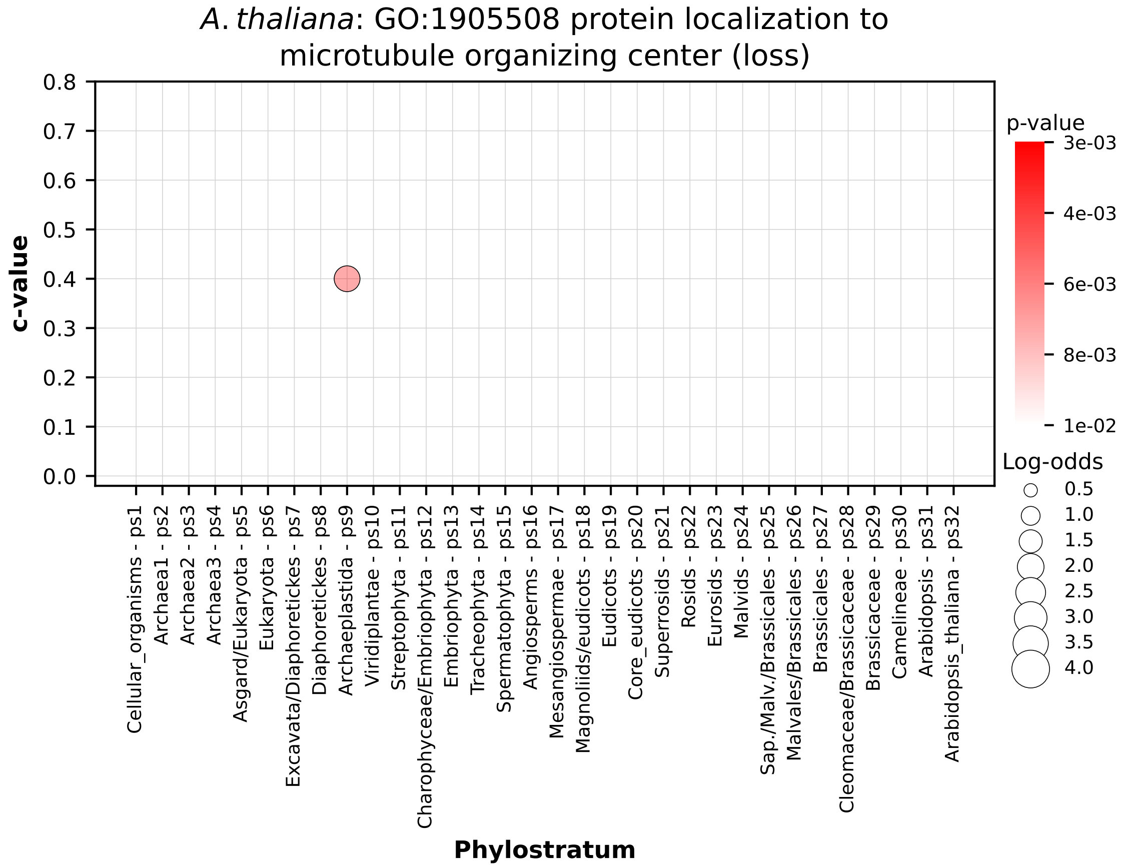

A. thaliana: GO:1905508 protein localization to microtubule organizing center (loss)
c-value
0.0
0.1
0.2
0.3
0.4
0.5
0.6
0.7
0.8
Phylostratum
Cellular_organisms - ps1
Archaea1 - ps2
Archaea2 - ps3
Archaea3 - ps4
Asgard/Eukaryota - ps5
Eukaryota - ps6
Excavata/Diaphoretickes - ps7
Diaphoretickes - ps8
Archaeplastida - ps9
Viridiplantae - ps10
Streptophyta - ps11
Charophyceae/Embriophyta - ps12
Embriophyta - ps13
Tracheophyta - ps14
Spermatophyta - ps15
Angiosperms - ps16
Mesangiospermae - ps17
Magnoliids/eudicots - ps18
Eudicots - ps19
Core_eudicots - ps20
Superrosids - ps21
Rosids - ps22
Eurosids - ps23
Malvids - ps24
Sap./Malv./Brassicales - ps25
Malvales/Brassicales - ps26
Brassicales - ps27
Cleomaceae/Brassicaceae - ps28
Brassicaceae - ps29
Camelineae - ps30
Arabidopsis - ps31
Arabidopsis_thaliana - ps32
p-value
3e-03
4e-03
6e-03
8e-03
1e-02
Log-odds
0.5
1.0
1.5
2.0
2.5
3.0
3.5
4.0

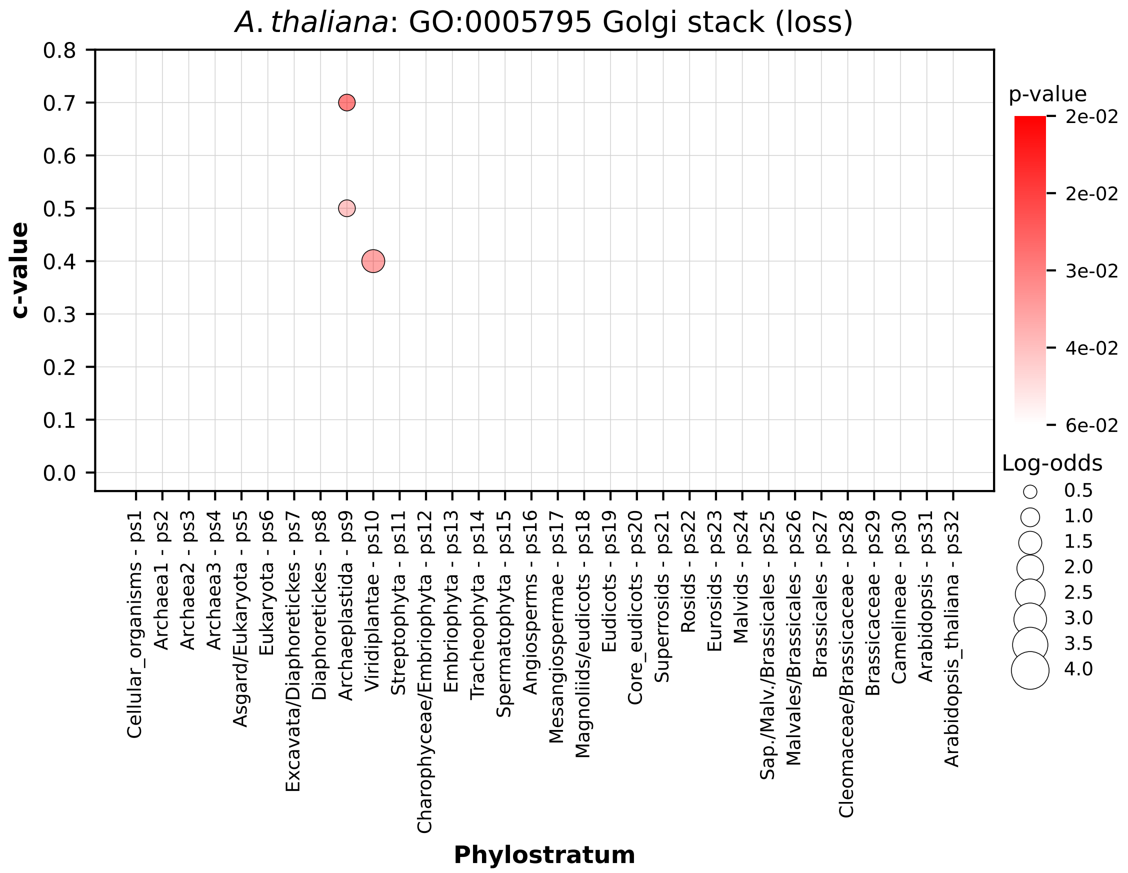

A. thaliana: GO:0005795 Golgi stack (loss)
c-value
0.8
0.7
0.6
0.5
0.4
0.3
0.2
0.1
0.0
p-value
2e-02
2e-02
3e-02
4e-02
6e-02
Log-odds
0.5
1.0
1.5
2.0
2.5
3.0
3.5
4.0
Cellular_organisms - ps1
Archaea1 - ps2
Archaea2 - ps3
Archaea3 - ps4
Asgard/Eukaryota - ps5
Eukaryota - ps6
Excavata/Diaphoretickes - ps7
Diaphoretickes - ps8
Archaeplastida - ps9
Viridiplantae - ps10
Streptophyta - ps11
Charophyceae/Embriophyta - ps12
Embriophyta - ps13
Tracheophyta - ps14
Spermatophyta - ps15
Angiosperms - ps16
Mesangiospermae - ps17
Magnoliids/eudicots - ps18
Eudicots - ps19
Core_eudicots - ps20
Superrosids - ps21
Rosids - ps22
Eurosids - ps23
Malvids - ps24
Sap./Malv./Brassicales - ps25
Malvales/Brassicales - ps26
Brassicales - ps27
Cleomaceae/Brassicaceae - ps28
Brassicaceae - ps29
Camelineae - ps30
Arabidopsis - ps31
Arabidopsis_thaliana - ps32
Phylostratum

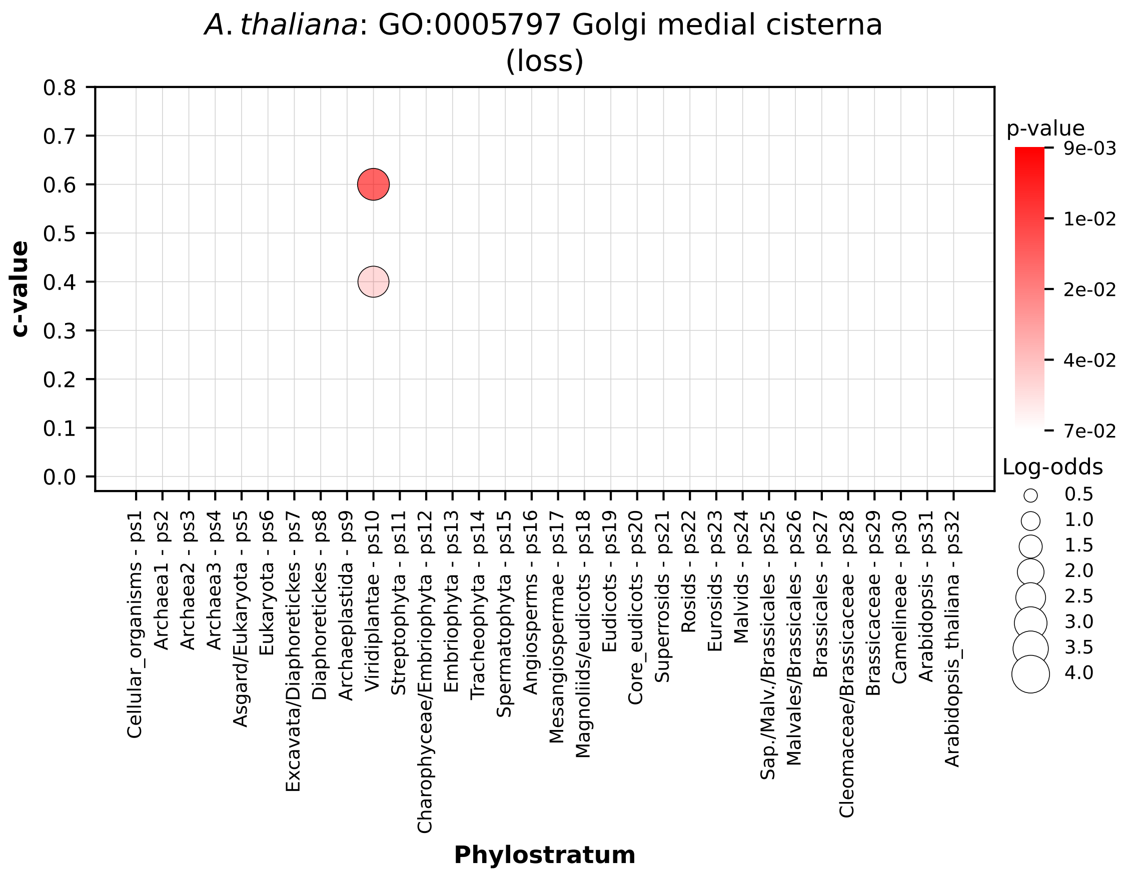

A. thaliana: GO:0005797 Golgi medial cisterna
(loss)
c-value
0.8
0.7
0.6
0.5
0.4
0.3
0.2
0.1
0.0
Phylostratum
Cellular_organisms - ps1
Archaea1 - ps2
Archaea2 - ps3
Archaea3 - ps4
Asgard/Eukaryota - ps5
Eukaryota - ps6
Excavata/Diaphoretickes - ps7
Diaphoretickes - ps8
Archaeplastida - ps9
Viridiplantae - ps10
Streptophyta - ps11
Charophyceae/Embriophyta - ps12
Embriophyta - ps13
Tracheophyta - ps14
Spermatophyta - ps15
Angiosperms - ps16
Mesangiospermae - ps17
Magnoliids/eudicots - ps18
Eudicots - ps19
Core_eudicots - ps20
Superrosids - ps21
Rosids - ps22
Eurosids - ps23
Malvids - ps24
Sap./Malv./Brassicales - ps25
Malvales/Brassicales - ps26
Brassicales - ps27
Cleomaceae/Brassicaceae - ps28
Brassicaceae - ps29
Camelineae - ps30
Arabidopsis - ps31
Arabidopsis_thaliana - ps32
p-value
9e-03
1e-02
2e-02
4e-02
7e-02
Log-odds
0.5
1.0
1.5
2.0
2.5
3.0
3.5
4.0

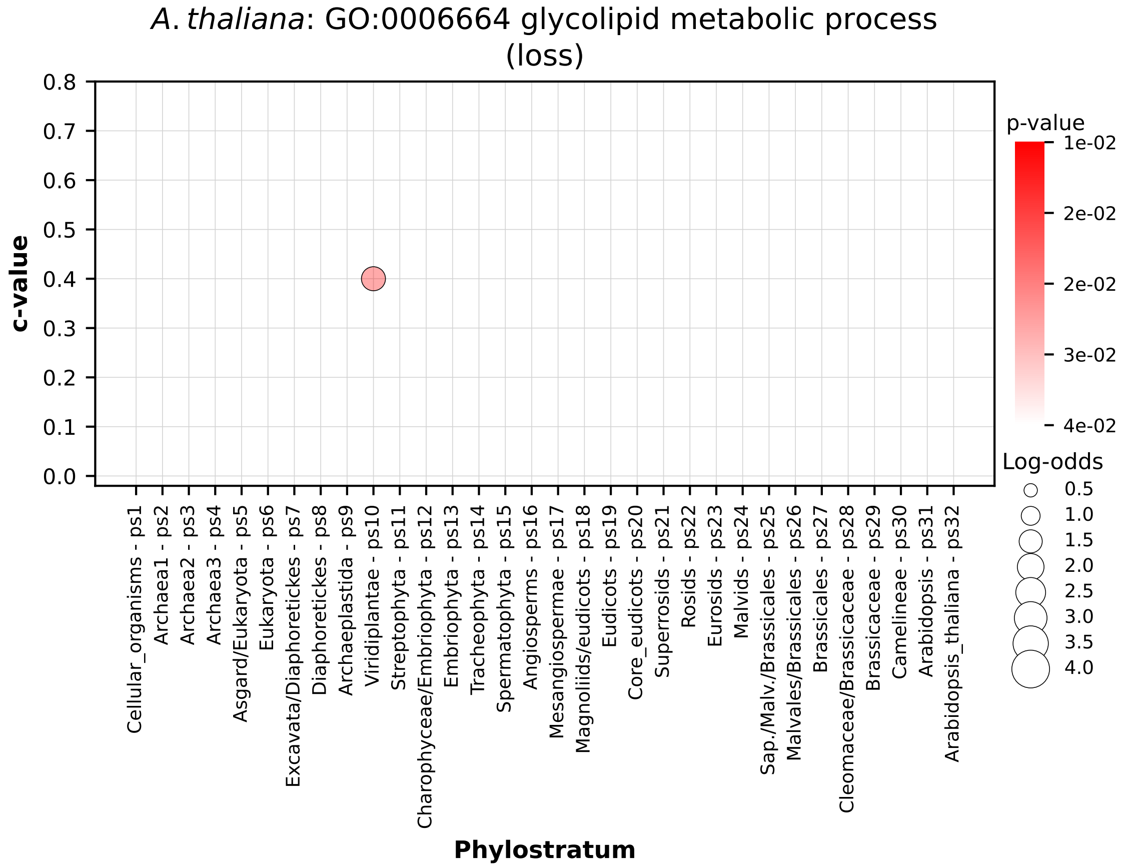

A. thaliana: GO:0006664 glycolipid metabolic process
(loss)
c-value
0.8
0.7
0.6
0.5
0.4
0.3
0.2
0.1
0.0
p-value
1e-02
2e-02
2e-02
3e-02
4e-02
Log-odds
0.5
1.0
1.5
2.0
2.5
3.0
3.5
4.0
Cellular_organisms - ps1
Archaea1 - ps2
Archaea2 - ps3
Archaea3 - ps4
Asgard/Eukaryota - ps5
Eukaryota - ps6
Excavata/Diaphoretickes - ps7
Diaphoretickes - ps8
Archaeplastida - ps9
Viridiplantae - ps10
Streptophyta - ps11
Charophyceae/Embriophyta - ps12
Embriophyta - ps13
Tracheophyta - ps14
Spermatophyta - ps15
Angiosperms - ps16
Mesangiospermae - ps17
Magnoliids/eudicots - ps18
Eudicots - ps19
Core_eudicots - ps20
Superrosids - ps21
Rosids - ps22
Eurosids - ps23
Malvids - ps24
Sap./Malv./Brassicales - ps25
Malvales/Brassicales - ps26
Brassicales - ps27
Cleomaceae/Brassicaceae - ps28
Brassicaceae - ps29
Camelineae - ps30
Arabidopsis - ps31
Arabidopsis_thaliana - ps32
Phylostratum

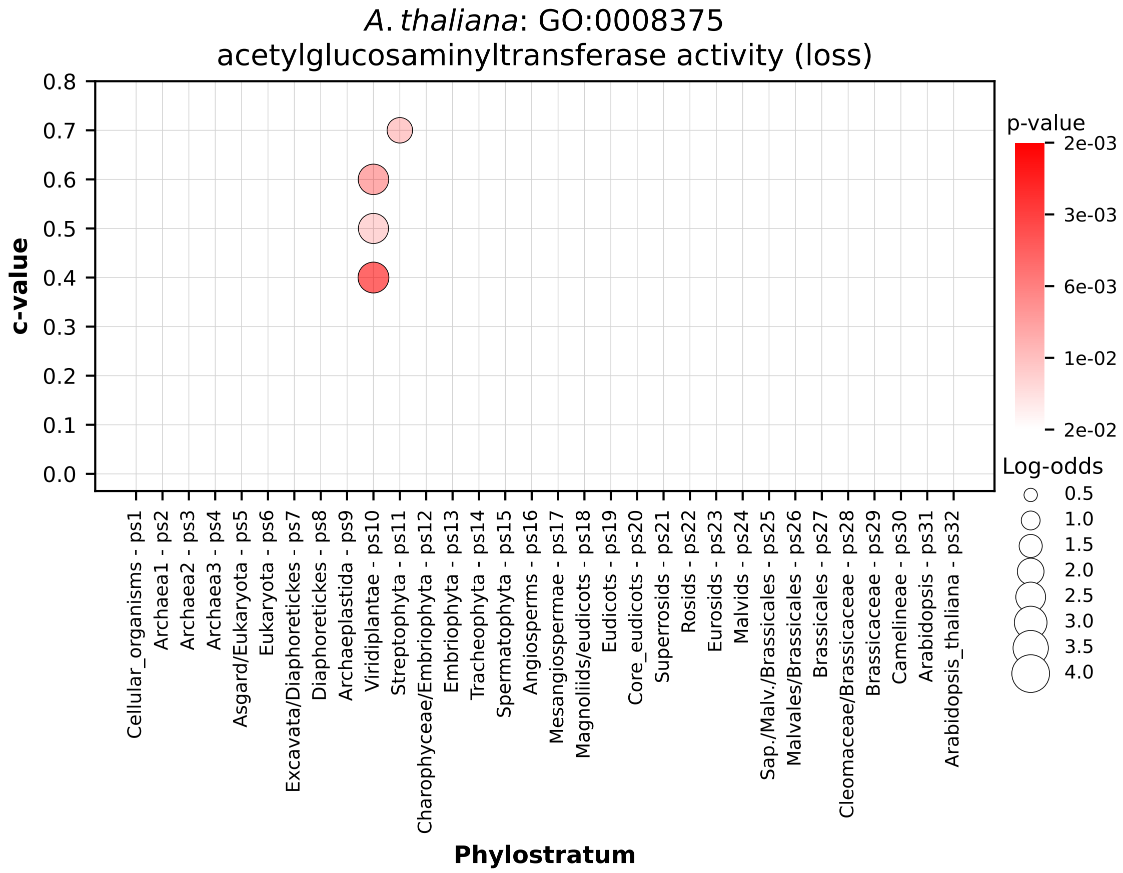

A. thaliana: GO:0008375
acetylglucosaminyltransferase activity (loss)
c-value
0.0
0.1
0.2
0.3
0.4
0.5
0.6
0.7
0.8
p-value
2e-03
3e-03
6e-03
1e-02
2e-02
Log-odds
0.5
1.0
1.5
2.0
2.5
3.0
3.5
4.0
Cellular_organisms - ps1
Archaea1 - ps2
Archaea2 - ps3
Archaea3 - ps4
Asgard/Eukaryota - ps5
Eukaryota - ps6
Excavata/Diaphoretickes - ps7
Diaphoretickes - ps8
Archaeplastida - ps9
Viridiplantae - ps10
Streptophyta - ps11
Charophyceae/Embriophyta - ps12
Embriophyta - ps13
Tracheophyta - ps14
Spermatophyta - ps15
Angiosperms - ps16
Mesangiospermae - ps17
Magnoliids/eudicots - ps18
Eudicots - ps19
Core_eudicots - ps20
Superrosids - ps21
Rosids - ps22
Eurosids - ps23
Malvids - ps24
Sap./Malv./Brassicales - ps25
Malvales/Brassicales - ps26
Brassicales - ps27
Cleomaceae/Brassicaceae - ps28
Brassicaceae - ps29
Camelineae - ps30
Arabidopsis - ps31
Arabidopsis_thaliana - ps32
Phylostratum

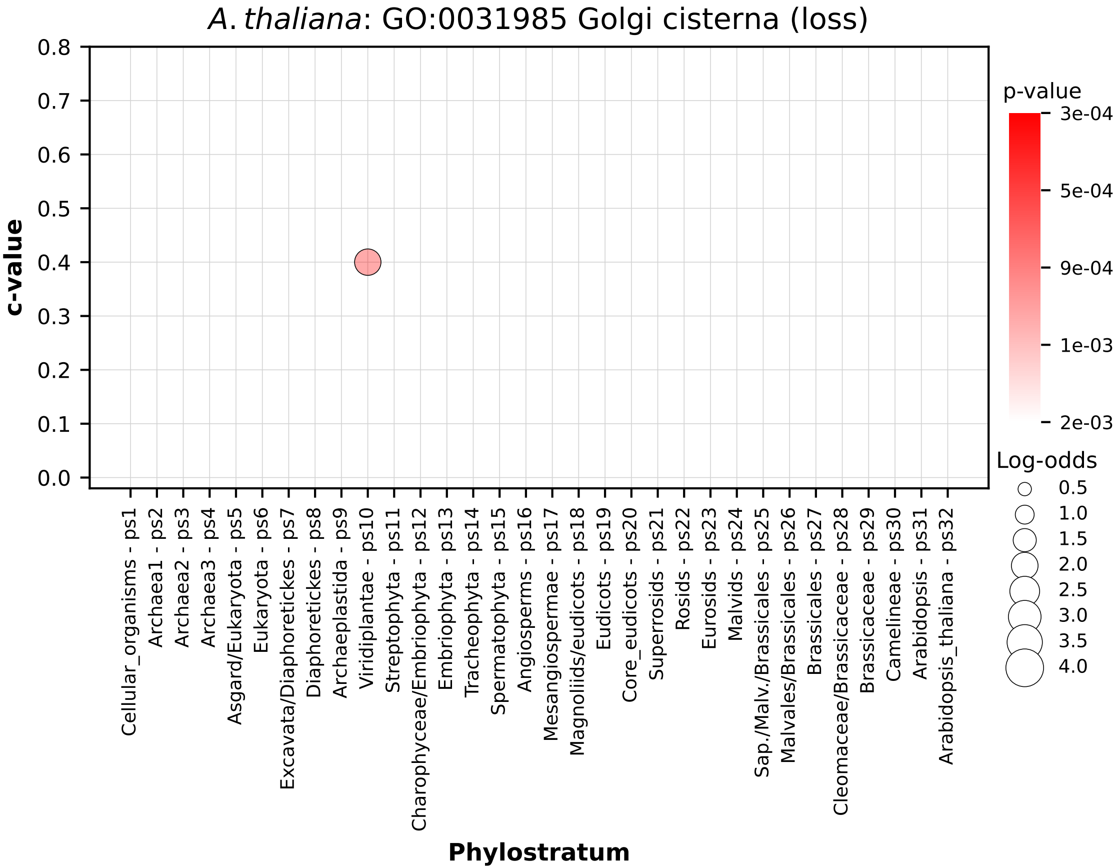

A. thaliana: GO:0031985 Golgi cisterna (loss)
c-value
0.8
0.7
0.6
0.5
0.4
0.3
0.2
0.1
0.0
Phylostratum
Cellular_organisms - ps1
Archaea1 - ps2
Archaea2 - ps3
Archaea3 - ps4
Asgard/Eukaryota - ps5
Eukaryota - ps6
Excavata/Diaphoretickes - ps7
Diaphoretickes - ps8
Archaeplastida - ps9
Viridiplantae - ps10
Streptophyta - ps11
Charophyceae/Embriophyta - ps12
Embriophyta - ps13
Tracheophyta - ps14
Spermatophyta - ps15
Angiosperms - ps16
Mesangiospermae - ps17
Magnoliids/eudicots - ps18
Eudicots - ps19
Core_eudicots - ps20
Superrosids - ps21
Rosids - ps22
Eurosids - ps23
Malvids - ps24
Sap./Malv./Brassicales - ps25
Malvales/Brassicales - ps26
Brassicales - ps27
Cleomaceae/Brassicaceae - ps28
Brassicaceae - ps29
Camelineae - ps30
Arabidopsis - ps31
Arabidopsis_thaliana - ps32
p-value
3e-04
5e-04
9e-04
1e-03
2e-03
Log-odds
0.5
1.0
1.5
2.0
2.5
3.0
3.5
4.0

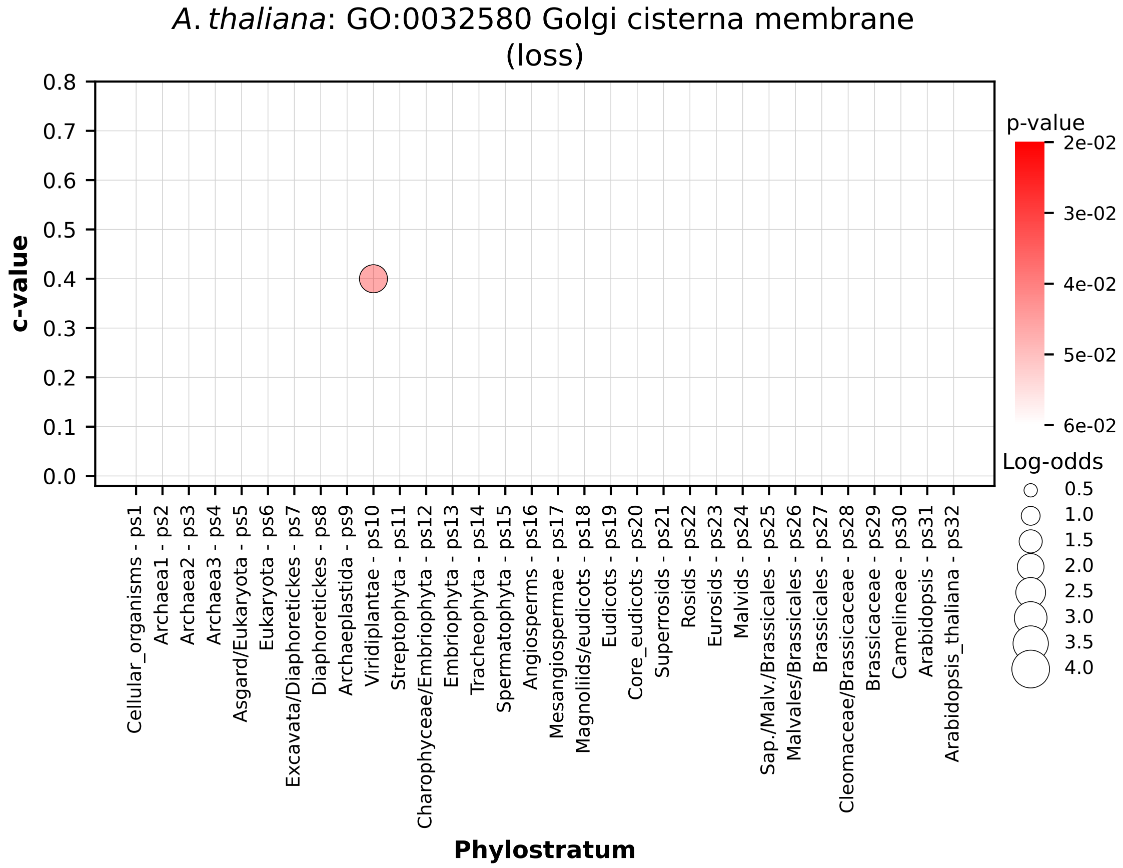

A. thaliana: GO:0032580 Golgi cisterna membrane
(loss)
c-value
0.8
0.7
0.6
0.5
0.4
0.3
0.2
0.1
0.0
Cellular_organisms - ps1
Archaea1 - ps2
Archaea2 - ps3
Archaea3 - ps4
Asgard/Eukaryota - ps5
Eukaryota - ps6
Excavata/Diaphoretickes - ps7
Diaphoretickes - ps8
Archaeplastida - ps9
Viridiplantae - ps10
Streptophyta - ps11
Charophyceae/Embriophyta - ps12
Embriophyta - ps13
Tracheophyta - ps14
Spermatophyta - ps15
Angiosperms - ps16
Mesangiospermae - ps17
Magnoliids/eudicots - ps18
Eudicots - ps19
Core_eudicots - ps20
Superrosids - ps21
Rosids - ps22
Eurosids - ps23
Malvids - ps24
Sap./Malv./Brassicales - ps25
Malvales/Brassicales - ps26
Brassicales - ps27
Cleomaceae/Brassicaceae - ps28
Brassicaceae - ps29
Camelineae - ps30
Arabidopsis - ps31
Arabidopsis_thaliana - ps32
Phylostratum
p-value
2e-02
3e-02
4e-02
5e-02
6e-02
Log-odds
0.5
1.0
1.5
2.0
2.5
3.0
3.5
4.0

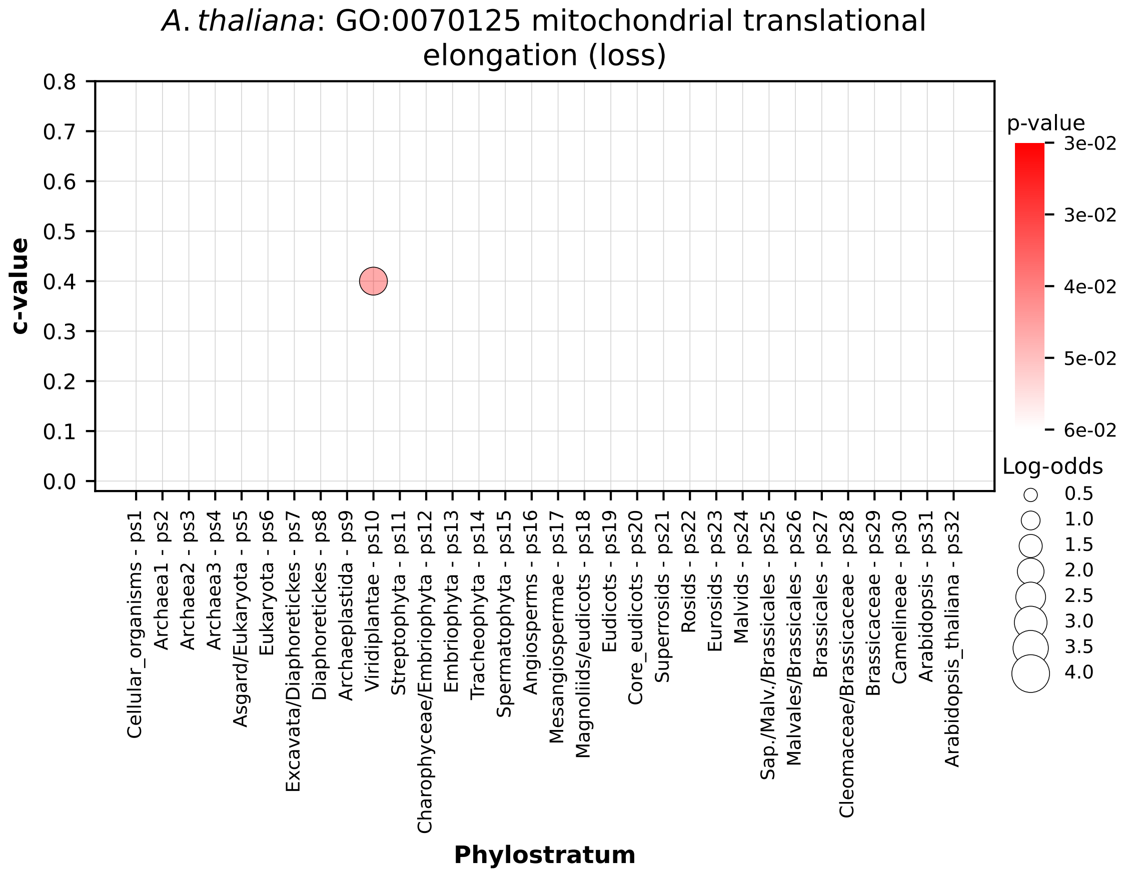
A. thaliana: GO:0070125 mitochondrial translational elongation (loss)
c-value
0.0
0.1
0.2
0.3
0.4
0.5
0.6
0.7
0.8
p-value
3e-02
3e-02
4e-02
5e-02
6e-02
Log-odds
0.5
1.0
1.5
2.0
2.5
3.0
3.5
4.0
Cellular_organisms - ps1
Archaea1 - ps2
Archaea2 - ps3
Archaea3 - ps4
Asgard/Eukaryota - ps5
Eukaryota - ps6
Excavata/Diaphoretickes - ps7
Diaphoretickes - ps8
Archaeplastida - ps9
Viridiplantae - ps10
Streptophyta - ps11
Charophyceae/Embriophyta - ps12
Embriophyta - ps13
Tracheophyta - ps14
Spermatophyta - ps15
Angiosperms - ps16
Mesangiospermae - ps17
Magnoliids/eudicots - ps18
Eudicots - ps19
Core_eudicots - ps20
Superrosids - ps21
Rosids - ps22
Eurosids - ps23
Malvids - ps24
Sap./Malv./Brassicales - ps25
Malvales/Brassicales - ps26
Brassicales - ps27
Cleomaceae/Brassicaceae - ps28
Brassicaceae - ps29
Camelineae - ps30
Arabidopsis - ps31
Arabidopsis_thaliana - ps32
Phylostratum

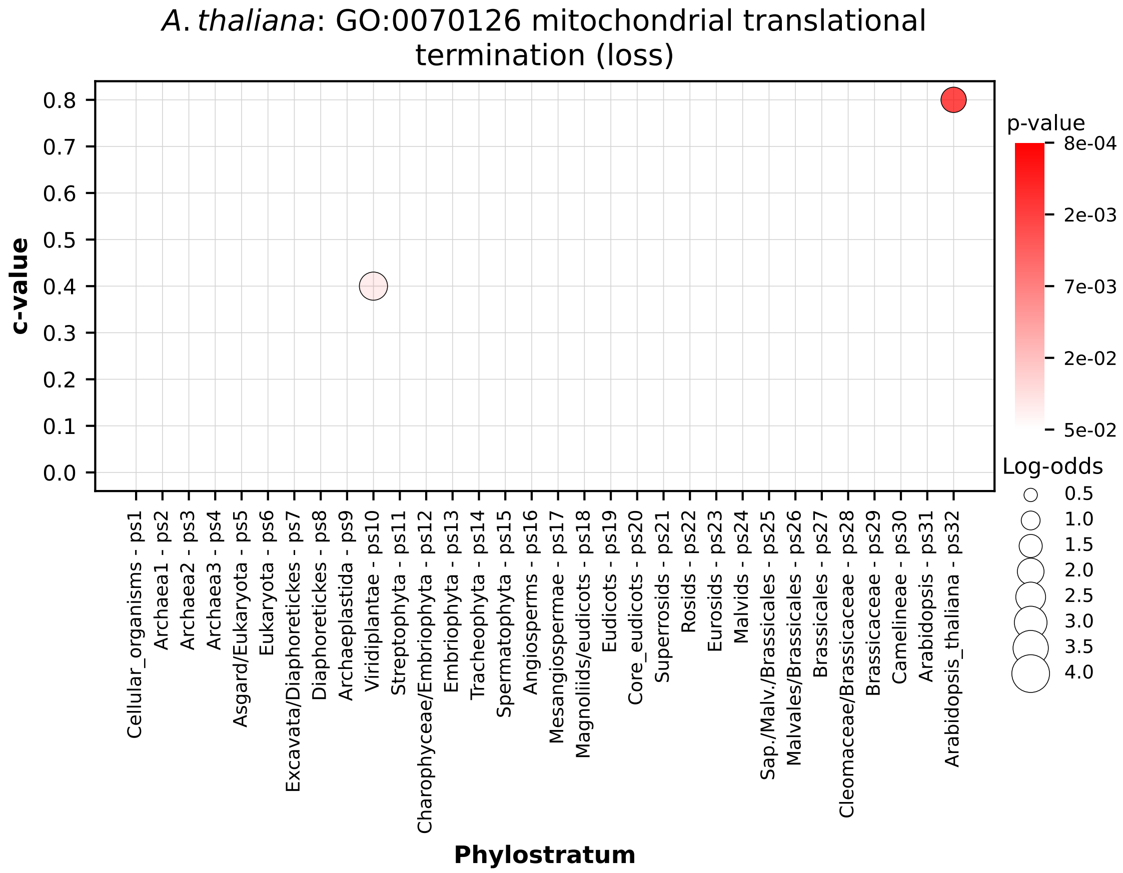

A. thaliana: GO:0070126 mitochondrial translational termination (loss)
c-value
0.8
0.7
0.6
0.5
0.4
0.3
0.2
0.1
0.0
p-value
8e-04
2e-03
7e-03
2e-02
5e-02
Log-odds
0.5
1.0
1.5
2.0
2.5
3.0
3.5
4.0
Cellular_organisms - ps1
Archaea1 - ps2
Archaea2 - ps3
Archaea3 - ps4
Asgard/Eukaryota - ps5
Eukaryota - ps6
Excavata/Diaphoretickes - ps7
Diaphoretickes - ps8
Archaeplastida - ps9
Viridiplantae - ps10
Streptophyta - ps11
Charophyceae/Embriophyta - ps12
Embriophyta - ps13
Tracheophyta - ps14
Spermatophyta - ps15
Angiosperms - ps16
Mesangiospermae - ps17
Magnoliids/eudicots - ps18
Eudicots - ps19
Core_eudicots - ps20
Superrosids - ps21
Rosids - ps22
Eurosids - ps23
Malvids - ps24
Sap./Malv./Brassicales - ps25
Malvales/Brassicales - ps26
Brassicales - ps27
Cleomaceae/Brassicaceae - ps28
Brassicaceae - ps29
Camelineae - ps30
Arabidopsis - ps31
Arabidopsis_thaliana - ps32
Phylostratum

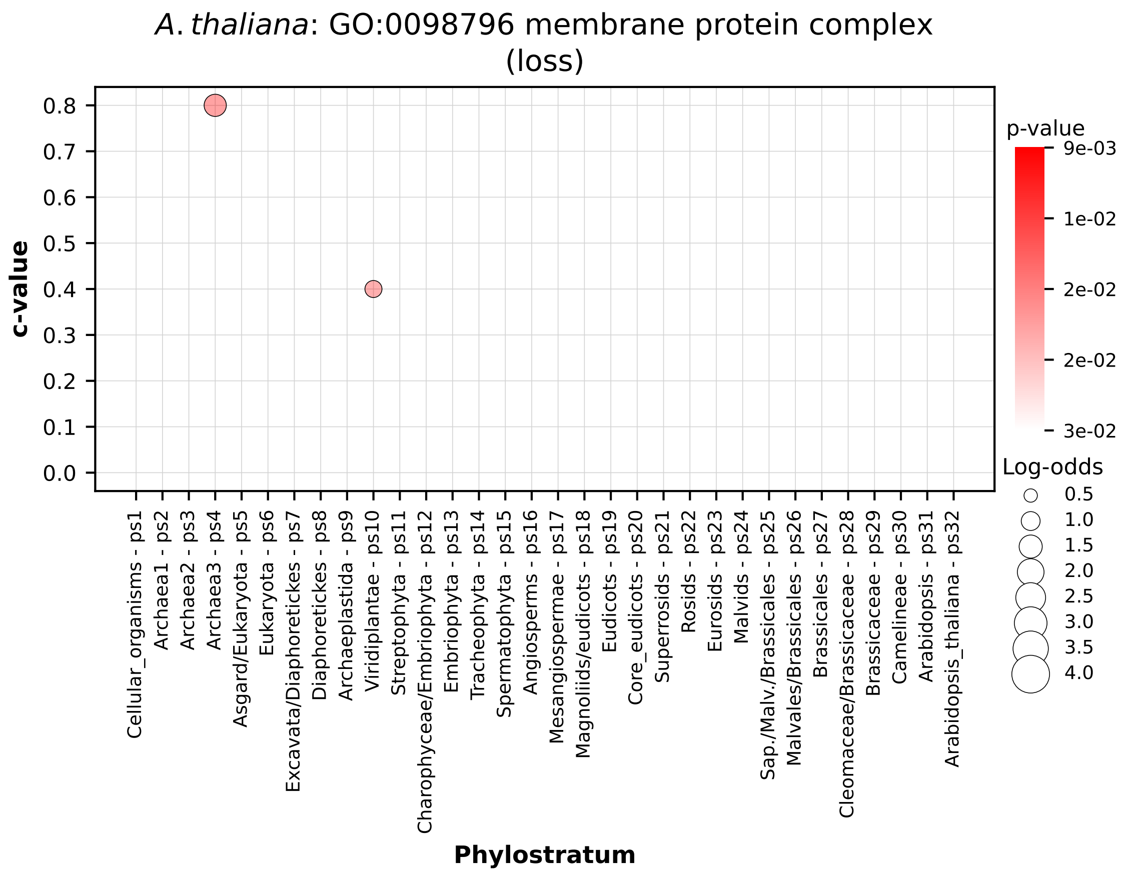

A. thaliana: GO:0098796 membrane protein complex
(loss)
c-value
0.0
0.1
0.2
0.3
0.4
0.5
0.6
0.7
0.8
p-value
9e-03
1e-02
2e-02
2e-02
3e-02
Log-odds
0.5
1.0
1.5
2.0
2.5
3.0
3.5
4.0
Cellular_organisms - ps1
Archaea1 - ps2
Archaea2 - ps3
Archaea3 - ps4
Asgard/Eukaryota - ps5
Eukaryota - ps6
Excavata/Diaphoretickes - ps7
Diaphoretickes - ps8
Archaeplastida - ps9
Viridiplantae - ps10
Streptophyta - ps11
Charophyceae/Embriophyta - ps12
Embriophyta - ps13
Tracheophyta - ps14
Spermatophyta - ps15
Angiosperms - ps16
Mesangiospermae - ps17
Magnoliids/eudicots - ps18
Eudicots - ps19
Core_eudicots - ps20
Superrosids - ps21
Rosids - ps22
Eurosids - ps23
Malvids - ps24
Sap./Malv./Brassicales - ps25
Malvales/Brassicales - ps26
Brassicales - ps27
Cleomaceae/Brassicaceae - ps28
Brassicaceae - ps29
Camelineae - ps30
Arabidopsis - ps31
Arabidopsis_thaliana - ps32
Phylostratum

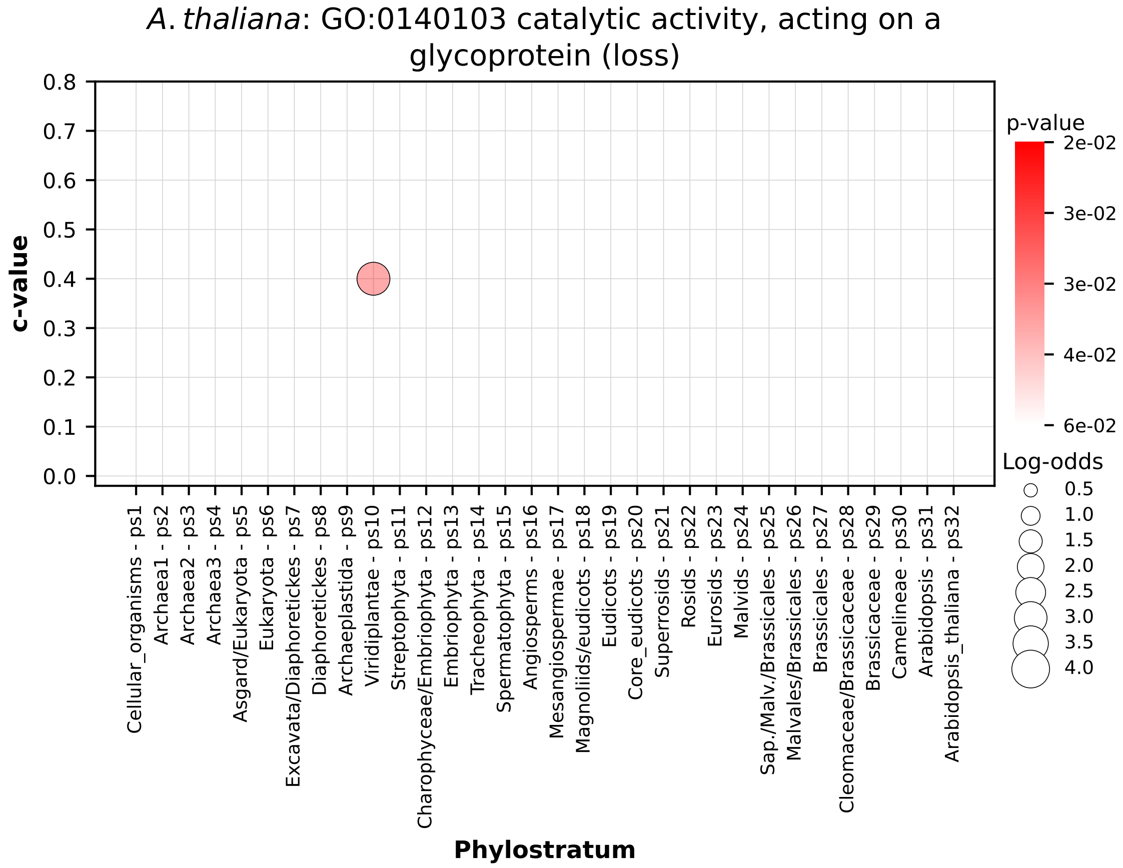

A. thaliana: GO:0140103 catalytic activity, acting on a glycoprotein (loss)
c-value
0.0
0.1
0.2
0.3
0.4
0.5
0.6
0.7
0.8
Phylostratum
Cellular_organisms - ps1
Archaea1 - ps2
Archaea2 - ps3
Archaea3 - ps4
Asgard/Eukaryota - ps5
Eukaryota - ps6
Excavata/Diaphoretickes - ps7
Diaphoretickes - ps8
Archaeplastida - ps9
Viridiplantae - ps10
Streptophyta - ps11
Charophyceae/Embriophyta - ps12
Embriophyta - ps13
Tracheophyta - ps14
Spermatophyta - ps15
Angiosperms - ps16
Mesangiospermae - ps17
Magnoliids/eudicots - ps18
Eudicots - ps19
Core_eudicots - ps20
Superrosids - ps21
Rosids - ps22
Eurosids - ps23
Malvids - ps24
Sap./Malv./Brassicales - ps25
Malvales/Brassicales - ps26
Brassicales - ps27
Cleomaceae/Brassicaceae - ps28
Brassicaceae - ps29
Camelineae - ps30
Arabidopsis - ps31
Arabidopsis_thaliana - ps32
p-value
2e-02
3e-02
3e-02
4e-02
6e-02
Log-odds
0.5
1.0
1.5
2.0
2.5
3.0
3.5
4.0

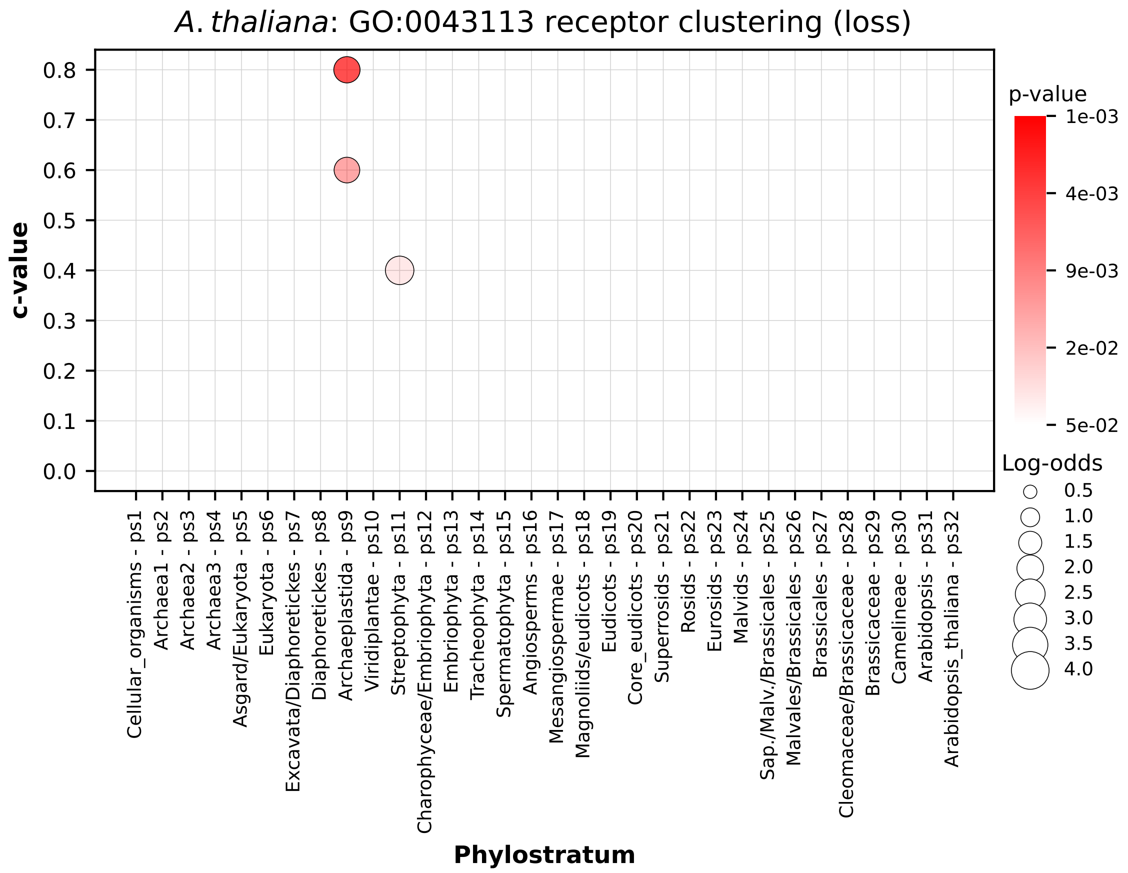
A. thaliana: GO:0043113 receptor clustering (loss)
c-value
0.0
0.1
0.2
0.3
0.4
0.5
0.6
0.7
0.8
p-value
1e-03
4e-03
9e-03
2e-02
5e-02
Log-odds
0.5
1.0
1.5
2.0
2.5
3.0
3.5
4.0
Cellular_organisms - ps1
Archaea1 - ps2
Archaea2 - ps3
Archaea3 - ps4
Asgard/Eukaryota - ps5
Eukaryota - ps6
Excavata/Diaphoretickes - ps7
Diaphoretickes - ps8
Archaeplastida - ps9
Viridiplantae - ps10
Streptophyta - ps11
Charophyceae/Embriophyta - ps12
Embriophyta - ps13
Tracheophyta - ps14
Spermatophyta - ps15
Angiosperms - ps16
Mesangiospermae - ps17
Magnoliids/eudicots - ps18
Eudicots - ps19
Core_eudicots - ps20
Superrosids - ps21
Rosids - ps22
Eurosids - ps23
Malvids - ps24
Sap./Malv./Brassicales - ps25
Malvales/Brassicales - ps26
Brassicales - ps27
Cleomaceae/Brassicaceae - ps28
Brassicaceae - ps29
Camelineae - ps30
Arabidopsis - ps31
Arabidopsis_thaliana - ps32
Phylostratum

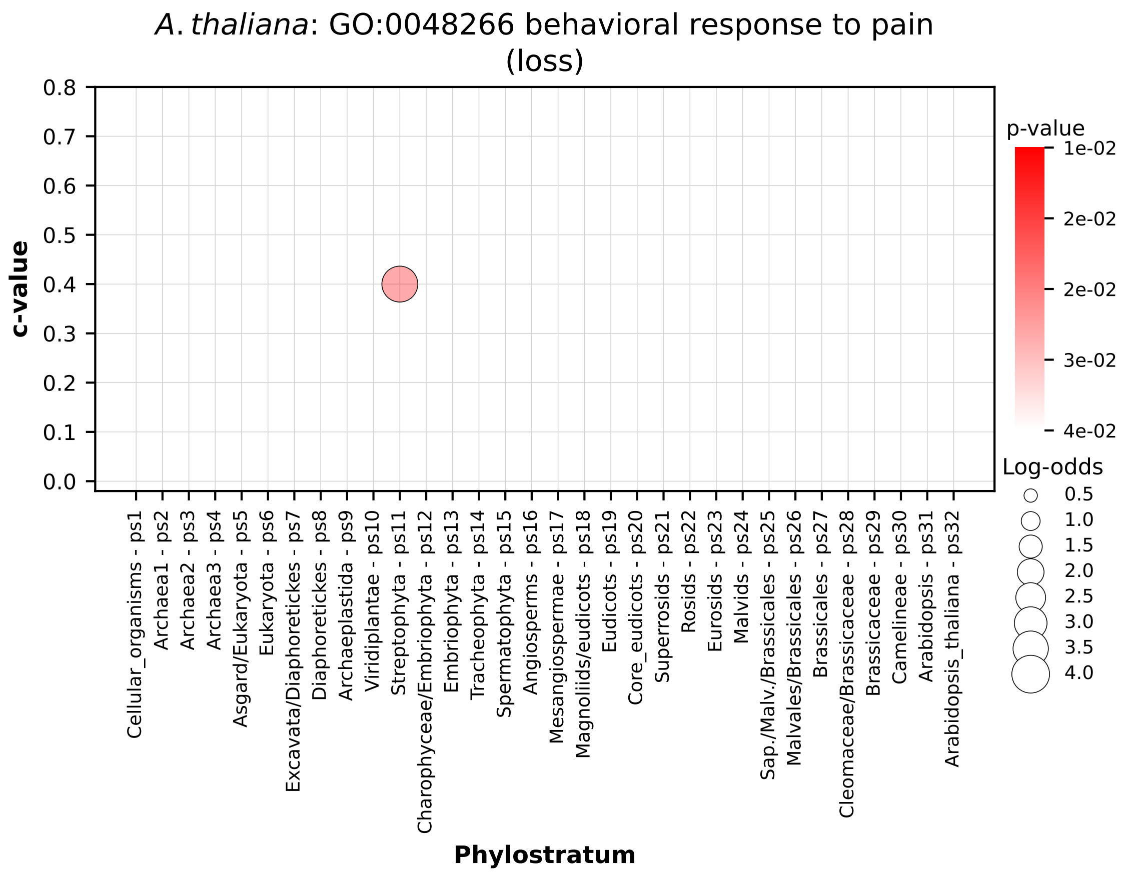

A. thaliana: GO:0048266 behavioral response to pain
(loss)
c-value
0.8
0.7
0.6
0.5
0.4
0.3
0.2
0.1
0.0
p-value
1e-02
2e-02
2e-02
3e-02
4e-02
Log-odds
0.5
1.0
1.5
2.0
2.5
3.0
3.5
4.0
Cellular_organisms - ps1
Archaea1 - ps2
Archaea2 - ps3
Archaea3 - ps4
Asgard/Eukaryota - ps5
Eukaryota - ps6
Excavata/Diaphoretickes - ps7
Diaphoretickes - ps8
Archaeplastida - ps9
Viridiplantae - ps10
Streptophyta - ps11
Charophyceae/Embriophyta - ps12
Embriophyta - ps13
Tracheophyta - ps14
Spermatophyta - ps15
Angiosperms - ps16
Mesangiospermae - ps17
Magnoliids/eudicots - ps18
Eudicots - ps19
Core_eudicots - ps20
Superrosids - ps21
Rosids - ps22
Eurosids - ps23
Malvids - ps24
Sap./Malv./Brassicales - ps25
Malvales/Brassicales - ps26
Brassicales - ps27
Cleomaceae/Brassicaceae - ps28
Brassicaceae - ps29
Camelineae - ps30
Arabidopsis - ps31
Arabidopsis_thaliana - ps32
Phylostratum

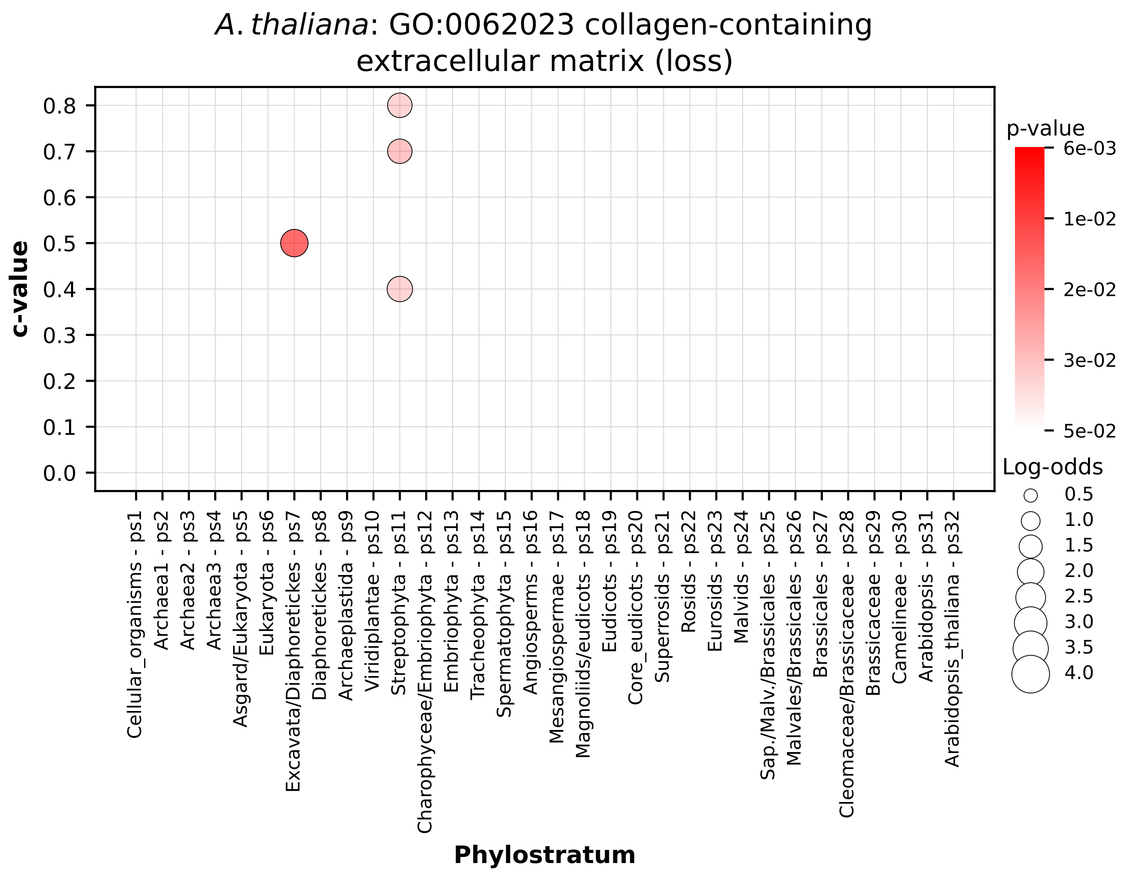

A. thaliana: GO:0062023 collagen-containing extracellular matrix (loss)
c-value
0.8
0.7
0.6
0.5
0.4
0.3
0.2
0.1
0.0
p-value
6e-03
1e-02
2e-02
3e-02
5e-02
Log-odds
0.5
1.0
1.5
2.0
2.5
3.0
3.5
4.0
Cellular_organisms - ps1
Archaea1 - ps2
Archaea2 - ps3
Archaea3 - ps4
Asgard/Eukaryota - ps5
Eukaryota - ps6
Excavata/Diaphoretickes - ps7
Diaphoretickes - ps8
Archaeplastida - ps9
Viridiplantae - ps10
Streptophyta - ps11
Charophyceae/Embriophyta - ps12
Embriophyta - ps13
Tracheophyta - ps14
Spermatophyta - ps15
Angiosperms - ps16
Mesangiospermae - ps17
Magnoliids/eudicots - ps18
Eudicots - ps19
Core_eudicots - ps20
Superrosids - ps21
Rosids - ps22
Eurosids - ps23
Malvids - ps24
Sap./Malv./Brassicales - ps25
Malvales/Brassicales - ps26
Brassicales - ps27
Cleomaceae/Brassicaceae - ps28
Brassicaceae - ps29
Camelineae - ps30
Arabidopsis - ps31
Arabidopsis_thaliana - ps32
Phylostratum

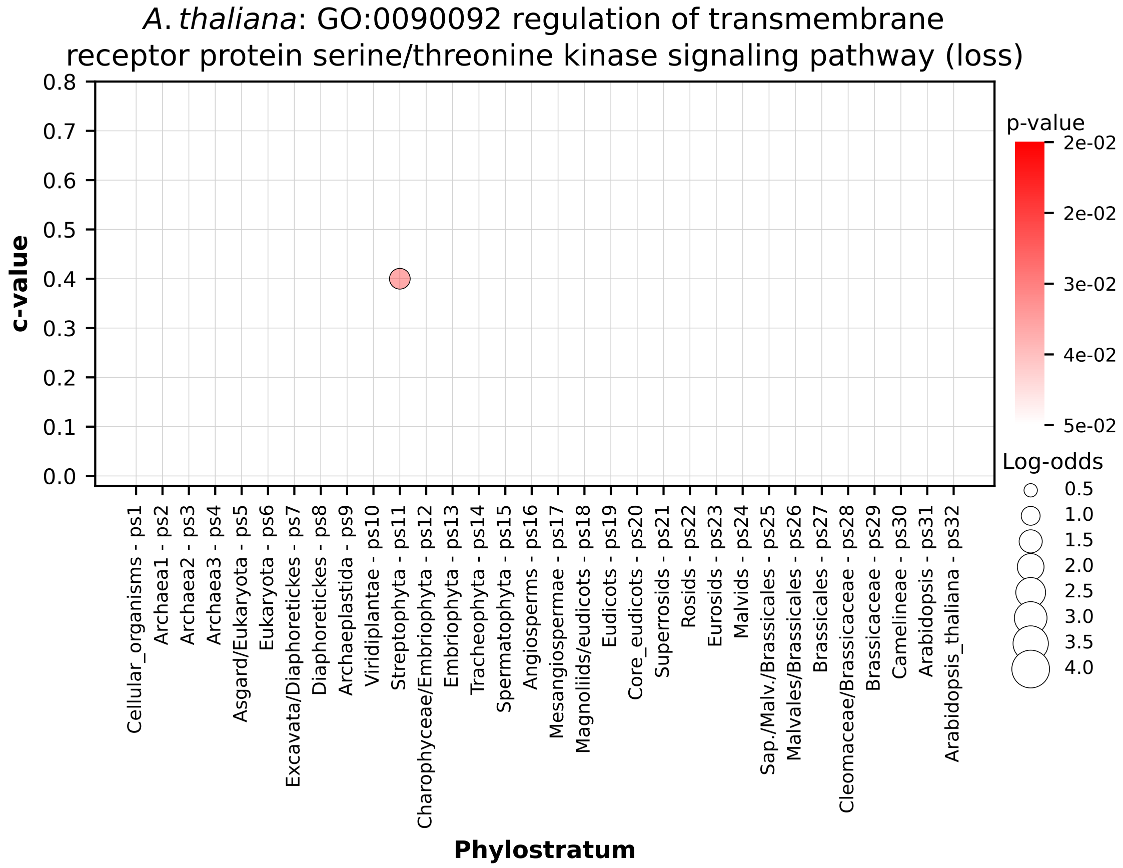

A. thaliana: GO:0090092 regulation of transmembrane receptor protein serine/threonine kinase signaling pathway (loss)
c-value
0.8
0.7
0.6
0.5
0.4
0.3
0.2
0.1
0.0
Phylostratum
Cellular_organisms - ps1
Archaea1 - ps2
Archaea2 - ps3
Archaea3 - ps4
Asgard/Eukaryota - ps5
Eukaryota - ps6
Excavata/Diaphoretickes - ps7
Diaphoretickes - ps8
Archaeplastida - ps9
Viridiplantae - ps10
Streptophyta - ps11
Charophyceae/Embriophyta - ps12
Embriophyta - ps13
Tracheophyta - ps14
Spermatophyta - ps15
Angiosperms - ps16
Mesangiospermae - ps17
Magnoliids/eudicots - ps18
Eudicots - ps19
Core_eudicots - ps20
Superrosids - ps21
Rosids - ps22
Eurosids - ps23
Malvids - ps24
Sap./Malv./Brassicales - ps25
Malvales/Brassicales - ps26
Brassicales - ps27
Cleomaceae/Brassicaceae - ps28
Brassicaceae - ps29
Camelineae - ps30
Arabidopsis - ps31
Arabidopsis_thaliana - ps32
p-value
2e-02
2e-02
3e-02
4e-02
5e-02
Log-odds
0.5
1.0
1.5
2.0
2.5
3.0
3.5
4.0

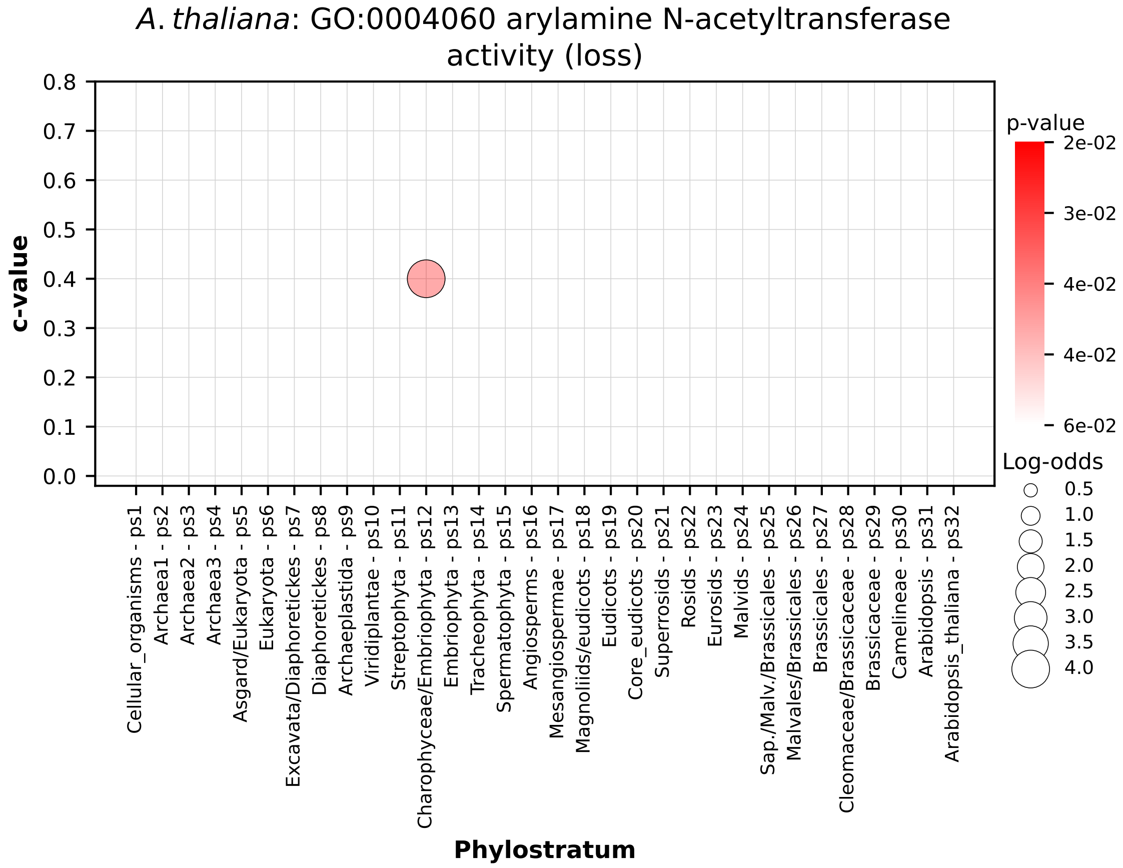

$A. thaliana$: GO:0004060 arylamine N-acetyltransferase activity (loss)
c-value
0.8
0.7
0.6
0.5
0.4
0.3
0.2
0.1
0.0
Phylostratum
Cellular_organisms - ps1
Archaea1 - ps2
Archaea2 - ps3
Archaea3 - ps4
Asgard/Eukaryota - ps5
Eukaryota - ps6
Excavata/Diaphoretickes - ps7
Diaphoretickes - ps8
Archaeplastida - ps9
Viridiplantae - ps10
Streptophyta - ps11
Charophyceae/Embriophyta - ps12
Embriophyta - ps13
Tracheophyta - ps14
Spermatophyta - ps15
Angiosperms - ps16
Mesangiospermae - ps17
Magnoliids/eudicots - ps18
Eudicots - ps19
Core_eudicots - ps20
Superrosids - ps21
Rosids - ps22
Eurosids - ps23
Malvids - ps24
Sap./Malv./Brassicales - ps25
Malvales/Brassicales - ps26
Brassicales - ps27
Cleomaceae/Brassicaceae - ps28
Brassicaceae - ps29
Camelineae - ps30
Arabidopsis - ps31
Arabidopsis_thaliana - ps32
p-value
2e-02
3e-02
4e-02
4e-02
6e-02
Log-odds
0.5
1.0
1.5
2.0
2.5
3.0
3.5
4.0

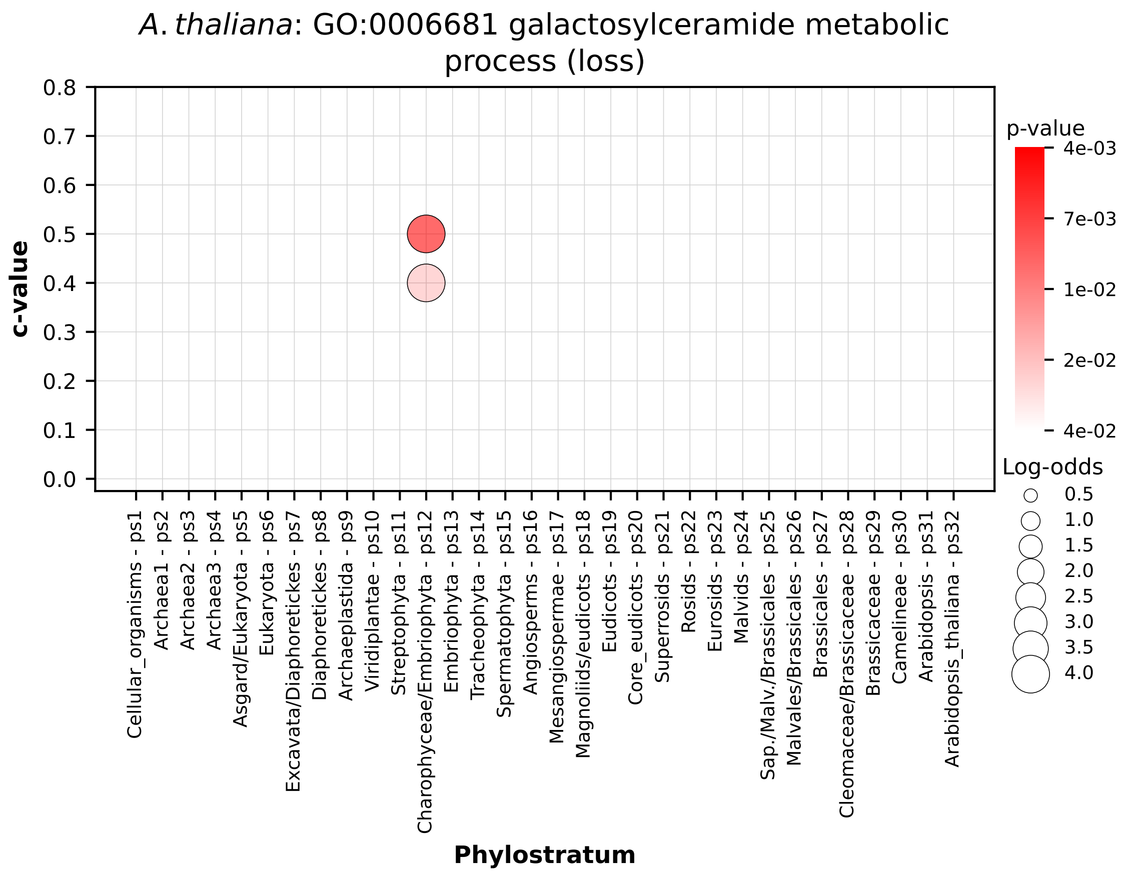

A. thaliana: GO:0006681 galactosylceramide metabolic process (loss)
c-value
0.0
0.1
0.2
0.3
0.4
0.5
0.6
0.7
0.8
Phylostratum
Cellular_organisms - ps1
Archaea1 - ps2
Archaea2 - ps3
Archaea3 - ps4
Asgard/Eukaryota - ps5
Eukaryota - ps6
Excavata/Diaphoretickes - ps7
Diaphoretickes - ps8
Archaeplastida - ps9
Viridiplantae - ps10
Streptophyta - ps11
Charophyceae/Embriophyta - ps12
Embriophyta - ps13
Tracheophyta - ps14
Spermatophyta - ps15
Angiosperms - ps16
Mesangiospermae - ps17
Magnoliids/eudicots - ps18
Eudicots - ps19
Core_eudicots - ps20
Superrosids - ps21
Rosids - ps22
Eurosids - ps23
Malvids - ps24
Sap./Malv./Brassicales - ps25
Malvales/Brassicales - ps26
Brassicales - ps27
Cleomaceae/Brassicaceae - ps28
Brassicaceae - ps29
Camelineae - ps30
Arabidopsis - ps31
Arabidopsis_thaliana - ps32
p-value
4e-03
7e-03
1e-02
2e-02
4e-02
Log-odds
0.5
1.0
1.5
2.0
2.5
3.0
3.5
4.0

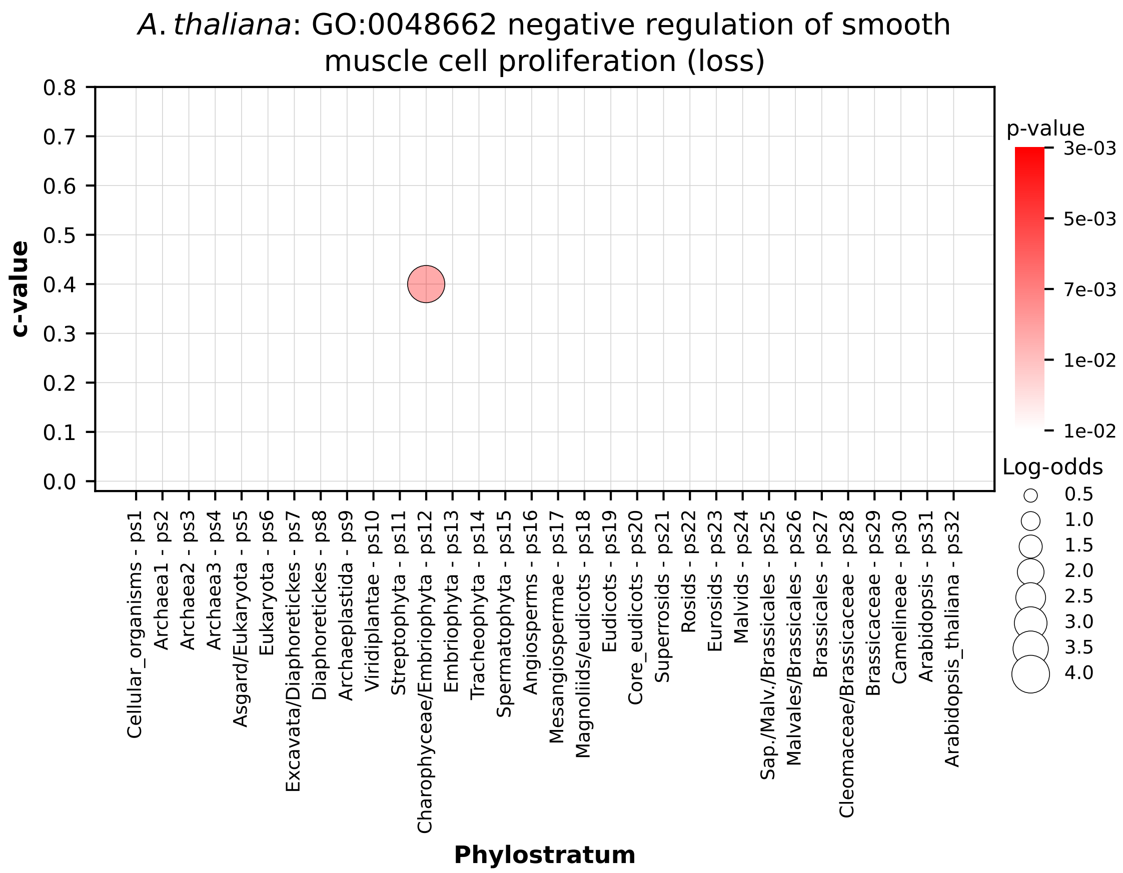

A. thaliana: GO:0048662 negative regulation of smooth muscle cell proliferation (loss)
c-value
0.8
0.7
0.6
0.5
0.4
0.3
0.2
0.1
0.0
p-value
3e-03
5e-03
7e-03
1e-02
1e-02
Log-odds
0.5
1.0
1.5
2.0
2.5
3.0
3.5
4.0
Cellular_organisms - ps1
Archaea1 - ps2
Archaea2 - ps3
Archaea3 - ps4
Asgard/Eukaryota - ps5
Eukaryota - ps6
Excavata/Diaphoretickes - ps7
Diaphoretickes - ps8
Archaeplastida - ps9
Viridiplantae - ps10
Streptophyta - ps11
Charophyceae/Embriophyta - ps12
Embriophyta - ps13
Tracheophyta - ps14
Spermatophyta - ps15
Angiosperms - ps16
Mesangiospermae - ps17
Magnoliids/eudicots - ps18
Eudicots - ps19
Core_eudicots - ps20
Superrosids - ps21
Rosids - ps22
Eurosids - ps23
Malvids - ps24
Sap./Malv./Brassicales - ps25
Malvales/Brassicales - ps26
Brassicales - ps27
Cleomaceae/Brassicaceae - ps28
Brassicaceae - ps29
Camelineae - ps30
Arabidopsis - ps31
Arabidopsis_thaliana - ps32
Phylostratum

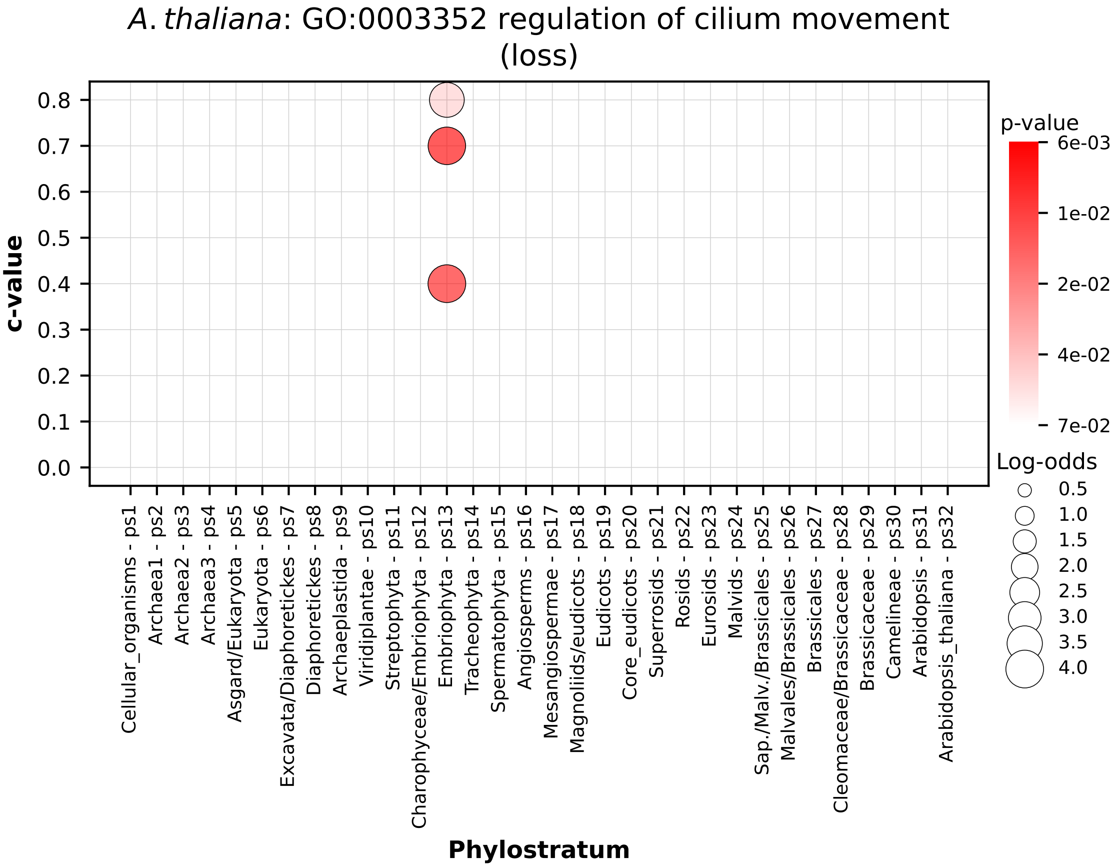
A. thaliana: GO:0003352 regulation of cilium movement
(loss)
c-value
0.8
0.7
0.6
0.5
0.4
0.3
0.2
0.1
0.0
p-value
6e-03
1e-02
2e-02
4e-02
7e-02
Log-odds
0.5
1.0
1.5
2.0
2.5
3.0
3.5
4.0
Cellular_organisms - ps1
Archaea1 - ps2
Archaea2 - ps3
Archaea3 - ps4
Asgard/Eukaryota - ps5
Eukaryota - ps6
Excavata/Diaphoretickes - ps7
Diaphoretickes - ps8
Archaeplastida - ps9
Viridiplantae - ps10
Streptophyta - ps11
Charophyceae/Embriophyta - ps12
Embriophyta - ps13
Tracheophyta - ps14
Spermatophyta - ps15
Angiosperms - ps16
Mesangiospermae - ps17
Magnoliids/eudicots - ps18
Eudicots - ps19
Core_eudicots - ps20
Superrosids - ps21
Rosids - ps22
Eurosids - ps23
Malvids - ps24
Sap./Malv./Brassicales - ps25
Malvales/Brassicales - ps26
Brassicales - ps27
Cleomaceae/Brassicaceae - ps28
Brassicaceae - ps29
Camelineae - ps30
Arabidopsis - ps31
Arabidopsis_thaliana - ps32
Phylostratum

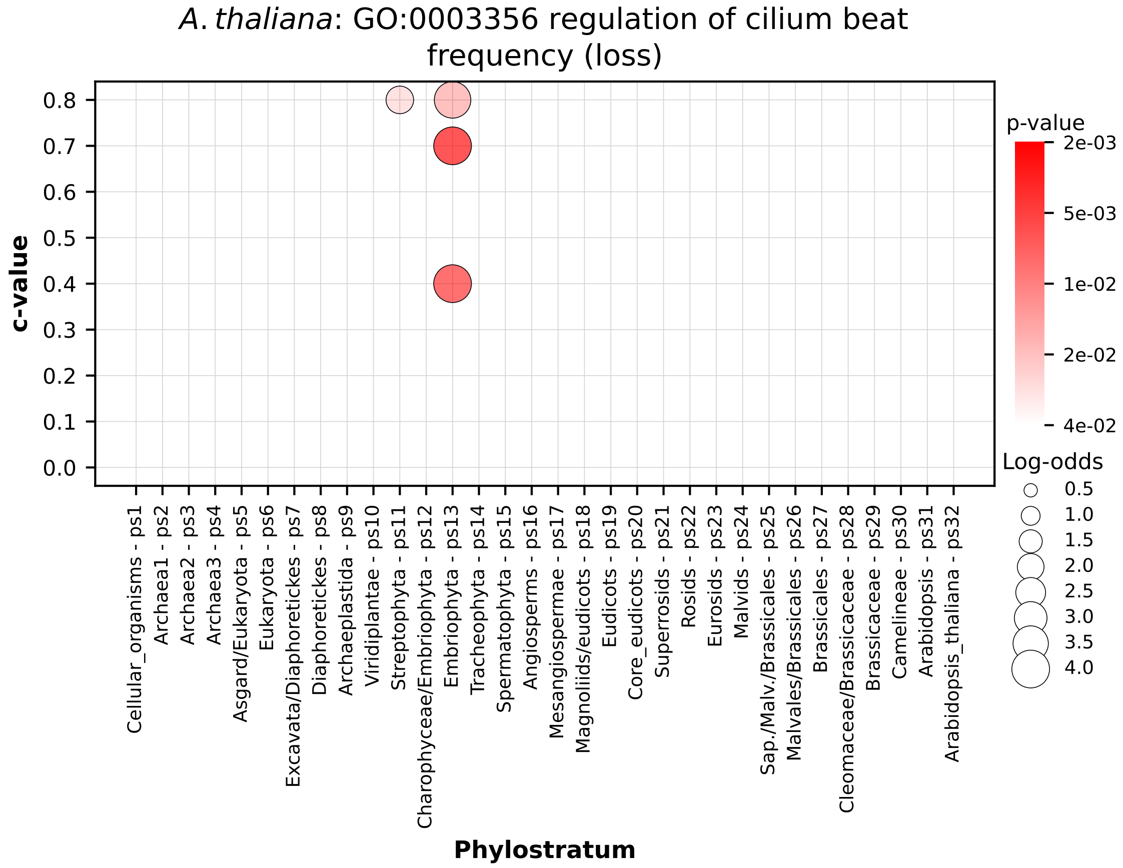

A. thaliana: GO:0003356 regulation of cilium beat frequency (loss)
c-value
0.8
0.7
0.6
0.5
0.4
0.3
0.2
0.1
0.0
p-value
2e-03
5e-03
1e-02
2e-02
4e-02
Log-odds
0.5
1.0
1.5
2.0
2.5
3.0
3.5
4.0
Cellular_organisms - ps1
Archaea1 - ps2
Archaea2 - ps3
Archaea3 - ps4
Asgard/Eukaryota - ps5
Eukaryota - ps6
Excavata/Diaphoretickes - ps7
Diaphoretickes - ps8
Archaeplastida - ps9
Viridiplantae - ps10
Streptophyta - ps11
Charophyceae/Embriophyta - ps12
Embriophyta - ps13
Tracheophyta - ps14
Spermatophyta - ps15
Angiosperms - ps16
Mesangiospermae - ps17
Magnoliids/eudicots - ps18
Eudicots - ps19
Core_eudicots - ps20
Superrosids - ps21
Rosids - ps22
Eurosids - ps23
Malvids - ps24
Sap./Malv./Brassicales - ps25
Malvales/Brassicales - ps26
Brassicales - ps27
Cleomaceae/Brassicaceae - ps28
Brassicaceae - ps29
Camelineae - ps30
Arabidopsis - ps31
Arabidopsis_thaliana - ps32
Phylostratum

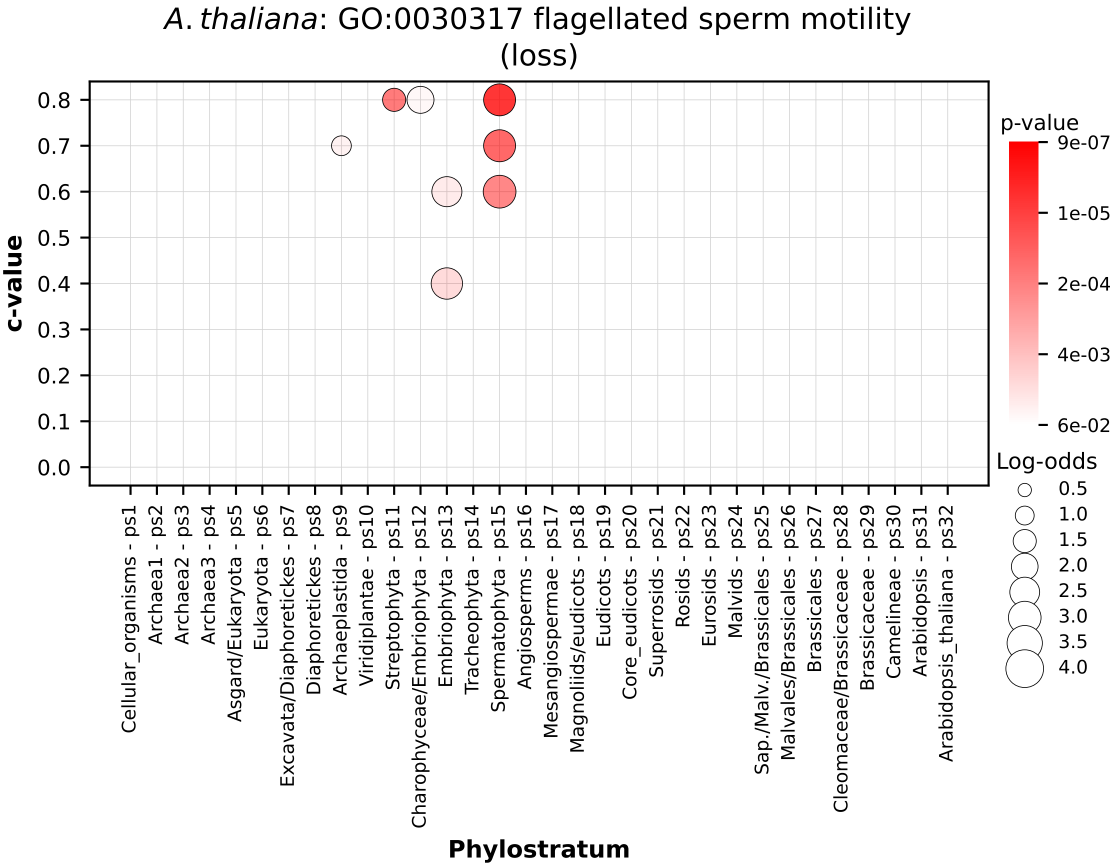

$A. thaliana$: GO:0030317 flagellated sperm motility (loss)
c-value
0.0
0.1
0.2
0.3
0.4
0.5
0.6
0.7
0.8
Phylostratum
Cellular_organisms - ps1
Archaea1 - ps2
Archaea2 - ps3
Archaea3 - ps4
Asgard/Eukaryota - ps5
Eukaryota - ps6
Excavata/Diaphoretickes - ps7
Diaphoretickes - ps8
Archaeplastida - ps9
Viridiplantae - ps10
Streptophyta - ps11
Charophyceae/Embriophyta - ps12
Embriophyta - ps13
Tracheophyta - ps14
Spermatophyta - ps15
Angiosperms - ps16
Mesangiospermae - ps17
Magnoliids/eudicots - ps18
Eudicots - ps19
Core_eudicots - ps20
Superrosids - ps21
Rosids - ps22
Eurosids - ps23
Malvids - ps24
Sap./Malv./Brassicales - ps25
Malvales/Brassicales - ps26
Brassicales - ps27
Cleomaceae/Brassicaceae - ps28
Brassicaceae - ps29
Camelineae - ps30
Arabidopsis - ps31
Arabidopsis_thaliana - ps32
p-value
9e-07
1e-05
2e-04
4e-03
6e-02
Log-odds
0.5
1.0
1.5
2.0
2.5
3.0
3.5
4.0

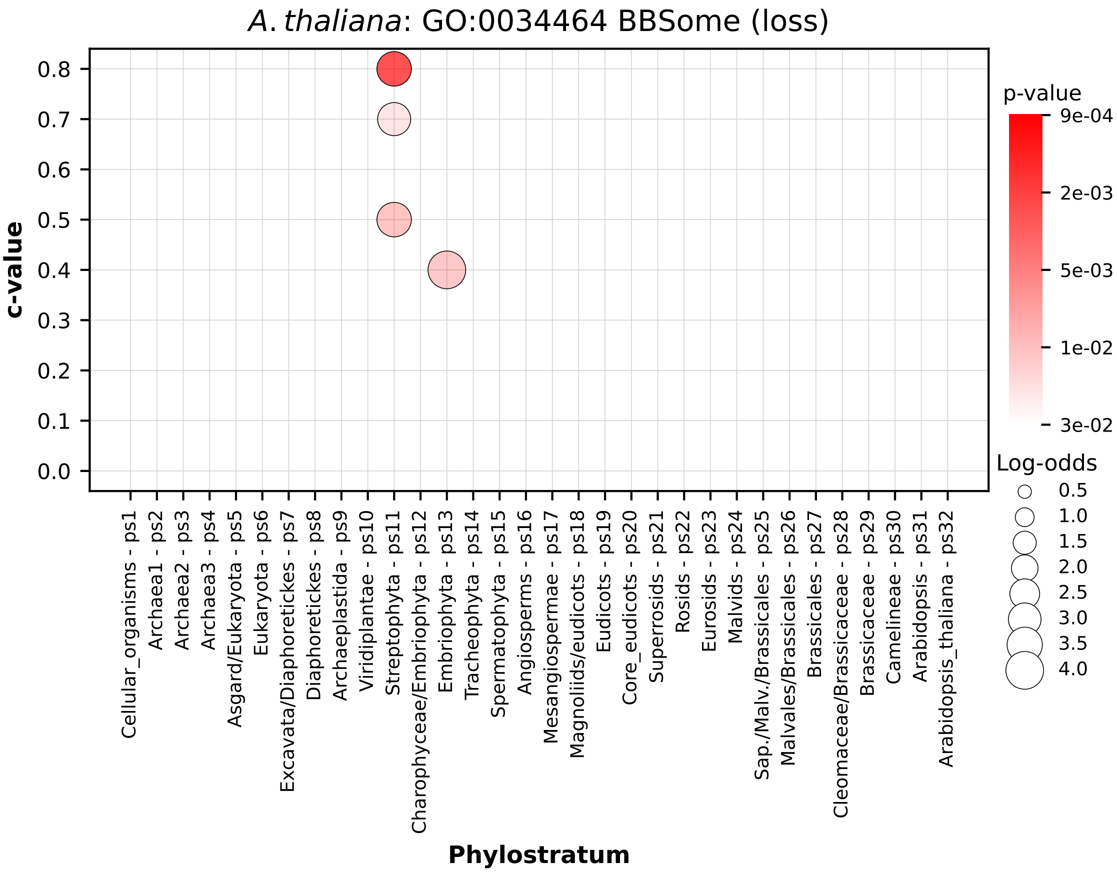

A. thaliana: GO:0034464 BBSome (loss)
c-value
0.8
0.7
0.6
0.5
0.4
0.3
0.2
0.1
0.0
p-value
9e-04
2e-03
5e-03
1e-02
3e-02
Log-odds
0.5
1.0
1.5
2.0
2.5
3.0
3.5
4.0
Cellular_organisms - ps1
Archaea1 - ps2
Archaea2 - ps3
Archaea3 - ps4
Asgard/Eukaryota - ps5
Eukaryota - ps6
Excavata/Diaphoretickes - ps7
Diaphoretickes - ps8
Archaeplastida - ps9
Viridiplantae - ps10
Streptophyta - ps11
Charophyceae/Embriophyta - ps12
Embriophyta - ps13
Tracheophyta - ps14
Spermatophyta - ps15
Angiosperms - ps16
Mesangiospermae - ps17
Magnoliids/eudicots - ps18
Eudicots - ps19
Core_eudicots - ps20
Superrosids - ps21
Rosids - ps22
Eurosids - ps23
Malvids - ps24
Sap./Malv./Brassicales - ps25
Malvales/Brassicales - ps26
Brassicales - ps27
Cleomaceae/Brassicaceae - ps28
Brassicaceae - ps29
Camelineae - ps30
Arabidopsis - ps31
Arabidopsis_thaliana - ps32
Phylostratum

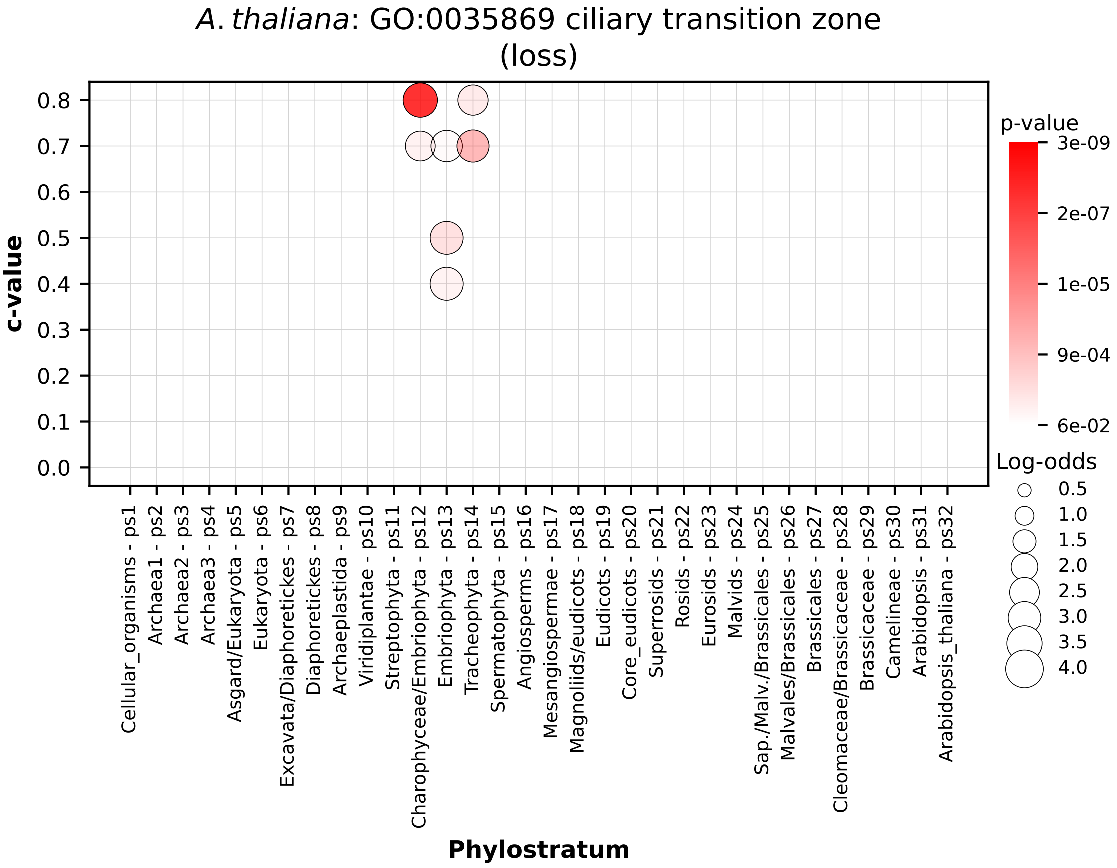

A. thaliana: GO:0035869 ciliary transition zone
(loss)
c-value
0.8
0.7
0.6
0.5
0.4
0.3
0.2
0.1
0.0
Phylostratum
Cellular_organisms - ps1
Archaea1 - ps2
Archaea2 - ps3
Archaea3 - ps4
Asgard/Eukaryota - ps5
Eukaryota - ps6
Excavata/Diaphoretickes - ps7
Diaphoretickes - ps8
Archaeplastida - ps9
Viridiplantae - ps10
Streptophyta - ps11
Charophyceae/Embriophyta - ps12
Embriophyta - ps13
Tracheophyta - ps14
Spermatophyta - ps15
Angiosperms - ps16
Mesangiospermae - ps17
Magnoliids/eudicots - ps18
Eudicots - ps19
Core_eudicots - ps20
Superrosids - ps21
Rosids - ps22
Eurosids - ps23
Malvids - ps24
Sap./Malv./Brassicales - ps25
Malvales/Brassicales - ps26
Brassicales - ps27
Cleomaceae/Brassicaceae - ps28
Brassicaceae - ps29
Camelineae - ps30
Arabidopsis - ps31
Arabidopsis_thaliana - ps32
p-value
3e-09
2e-07
1e-05
9e-04
6e-02
Log-odds
0.5
1.0
1.5
2.0
2.5
3.0
3.5
4.0

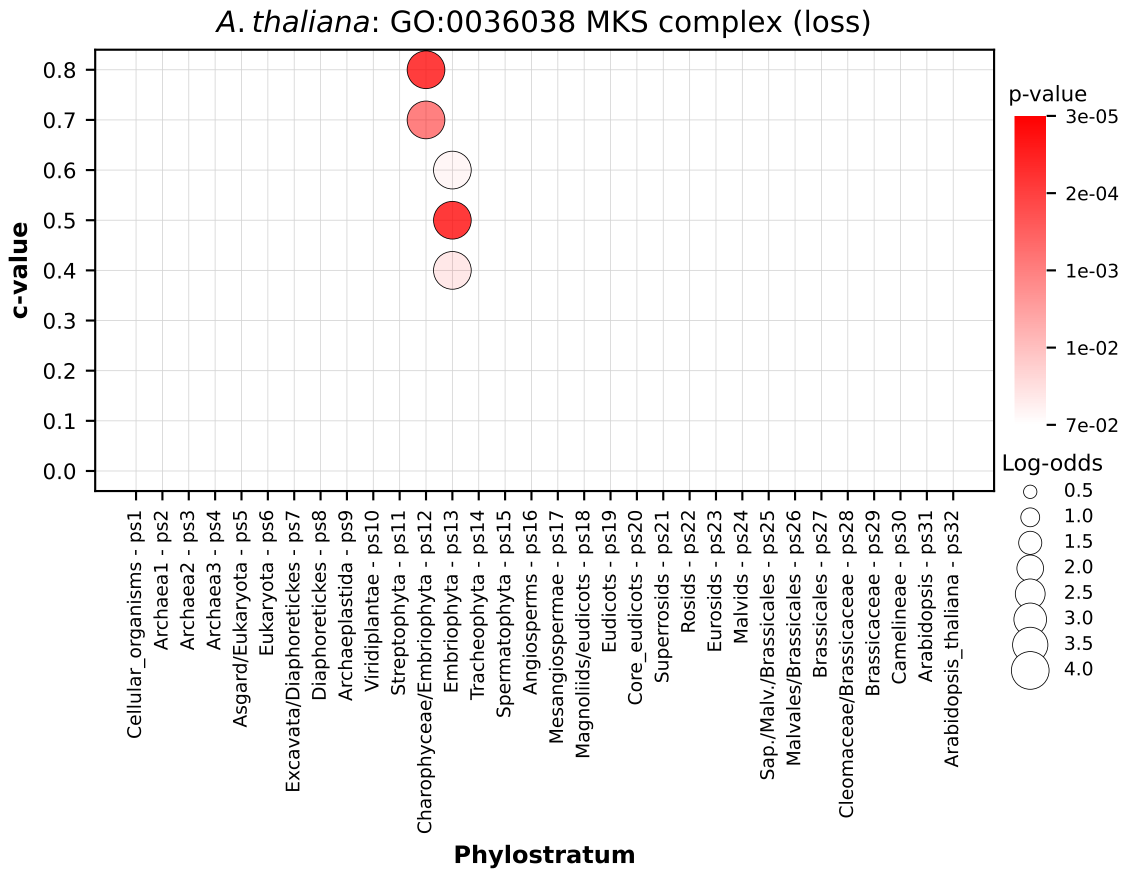

A. thaliana: GO:0036038 MKS complex (loss)
c-value
0.0
0.1
0.2
0.3
0.4
0.5
0.6
0.7
0.8
Phylostratum
Cellular_organisms - ps1
Archaea1 - ps2
Archaea2 - ps3
Archaea3 - ps4
Asgard/Eukaryota - ps5
Eukaryota - ps6
Excavata/Diaphoretickes - ps7
Diaphoretickes - ps8
Archaeplastida - ps9
Viridiplantae - ps10
Streptophyta - ps11
Charophyceae/Embriophyta - ps12
Embriophyta - ps13
Tracheophyta - ps14
Spermatophyta - ps15
Angiosperms - ps16
Mesangiospermae - ps17
Magnoliids/eudicots - ps18
Eudicots - ps19
Core_eudicots - ps20
Superrosids - ps21
Rosids - ps22
Eurosids - ps23
Malvids - ps24
Sap./Malv./Brassicales - ps25
Malvales/Brassicales - ps26
Brassicales - ps27
Cleomaceae/Brassicaceae - ps28
Brassicaceae - ps29
Camelineae - ps30
Arabidopsis - ps31
Arabidopsis_thaliana - ps32
p-value
3e-05
2e-04
1e-03
1e-02
7e-02
Log-odds
0.5
1.0
1.5
2.0
2.5
3.0
3.5
4.0

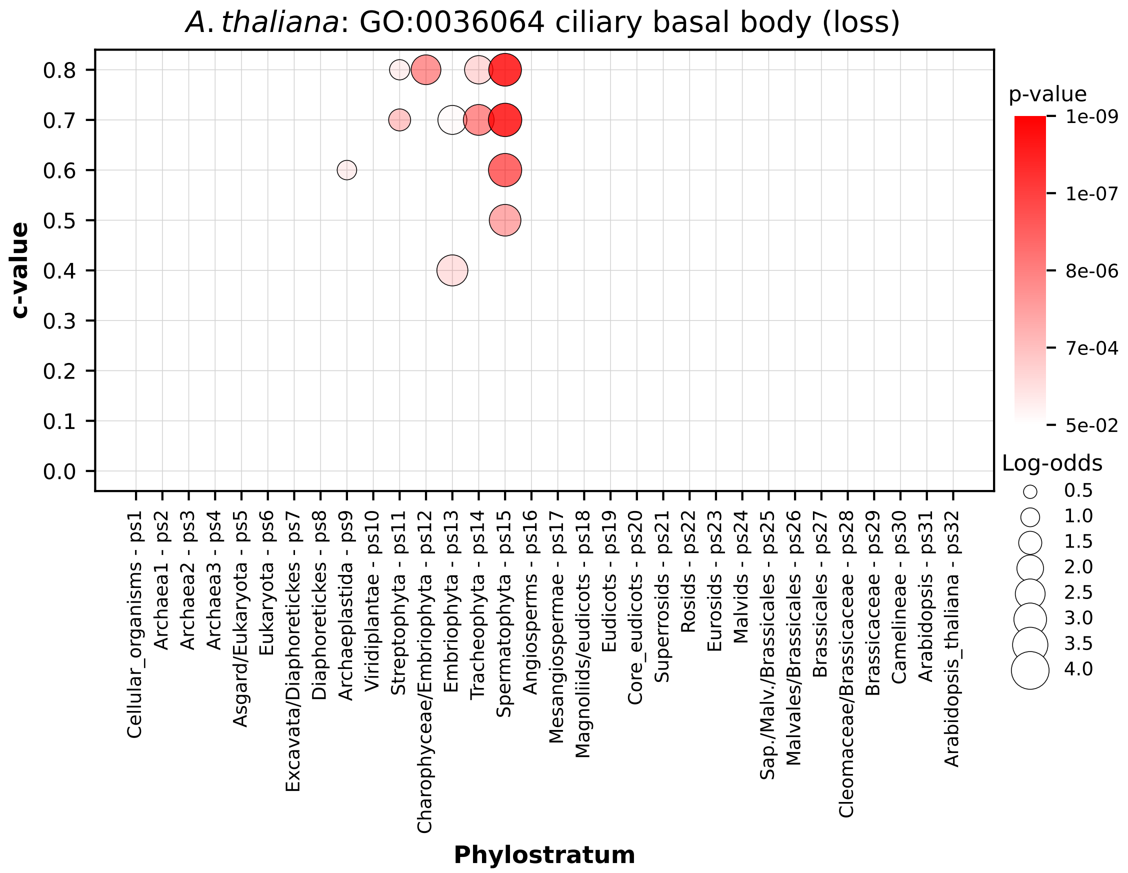
A. thaliana: GO:0036064 ciliary basal body (loss)
c-value
0.0
0.1
0.2
0.3
0.4
0.5
0.6
0.7
0.8
Phylostratum
Cellular_organisms - ps1
Archaea1 - ps2
Archaea2 - ps3
Archaea3 - ps4
Asgard/Eukaryota - ps5
Eukaryota - ps6
Excavata/Diaphoretickes - ps7
Diaphoretickes - ps8
Archaeplastida - ps9
Viridiplantae - ps10
Streptophyta - ps11
Charophyceae/Embriophyta - ps12
Embriophyta - ps13
Tracheophyta - ps14
Spermatophyta - ps15
Angiosperms - ps16
Mesangiospermae - ps17
Magnoliids/eudicots - ps18
Eudicots - ps19
Core_eudicots - ps20
Superrosids - ps21
Rosids - ps22
Eurosids - ps23
Malvids - ps24
Sap./Malv./Brassicales - ps25
Malvales/Brassicales - ps26
Brassicales - ps27
Cleomaceae/Brassicaceae - ps28
Brassicaceae - ps29
Camelineae - ps30
Arabidopsis - ps31
Arabidopsis_thaliana - ps32
p-value
1e-09
1e-07
8e-06
7e-04
5e-02
Log-odds
0.5
1.0
1.5
2.0
2.5
3.0
3.5
4.0

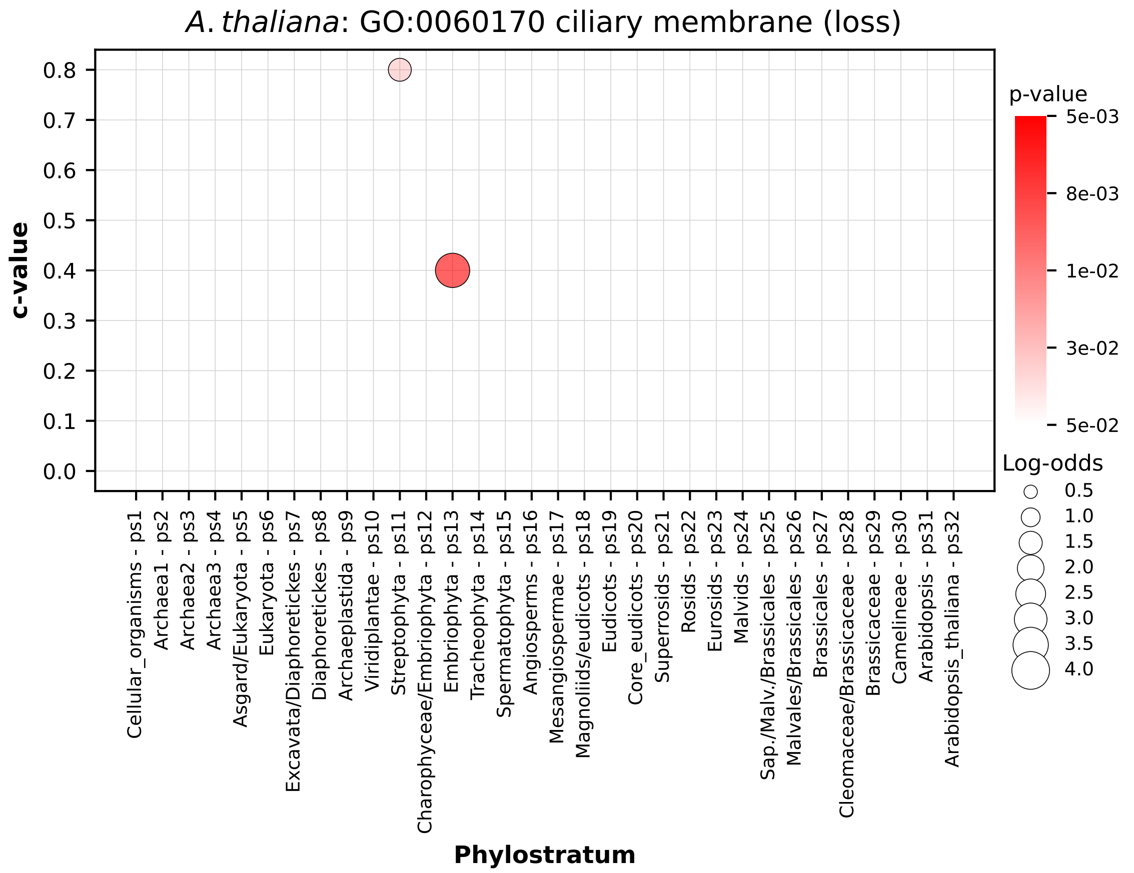
A. thaliana: GO:0060170 ciliary membrane (loss)
c-value
0.0
0.1
0.2
0.3
0.4
0.5
0.6
0.7
0.8
Phylostratum
Cellular_organisms - ps1
Archaea1 - ps2
Archaea2 - ps3
Archaea3 - ps4
Asgard/Eukaryota - ps5
Eukaryota - ps6
Excavata/Diaphoretickes - ps7
Diaphoretickes - ps8
Archaeplastida - ps9
Viridiplantae - ps10
Streptophyta - ps11
Charophyceae/Embriophyta - ps12
Embriophyta - ps13
Tracheophyta - ps14
Spermatophyta - ps15
Angiosperms - ps16
Mesangiospermae - ps17
Magnoliids/eudicots - ps18
Eudicots - ps19
Core_eudicots - ps20
Superrosids - ps21
Rosids - ps22
Eurosids - ps23
Malvids - ps24
Sap./Malv./Brassicales - ps25
Malvales/Brassicales - ps26
Brassicales - ps27
Cleomaceae/Brassicaceae - ps28
Brassicaceae - ps29
Camelineae - ps30
Arabidopsis - ps31
Arabidopsis_thaliana - ps32
p-value
5e-03
8e-03
1e-02
3e-02
5e-02
Log-odds
0.5
1.0
1.5
2.0
2.5
3.0
3.5
4.0

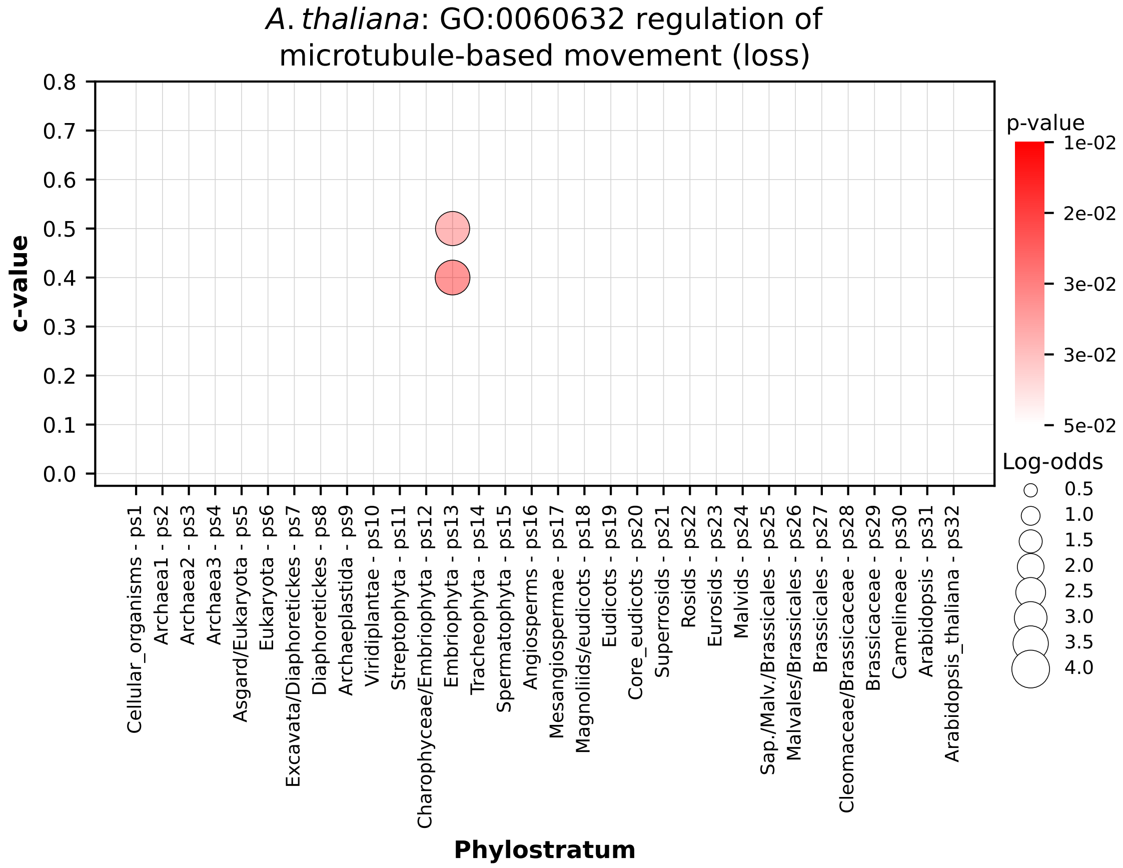

A. thaliana: GO:0060632 regulation of microtubule-based movement (loss)
c-value
0.8
0.7
0.6
0.5
0.4
0.3
0.2
0.1
0.0
p-value
1e-02
2e-02
3e-02
3e-02
5e-02
Log-odds
0.5
1.0
1.5
2.0
2.5
3.0
3.5
4.0
Cellular_organisms - ps1
Archaea1 - ps2
Archaea2 - ps3
Archaea3 - ps4
Asgard/Eukaryota - ps5
Eukaryota - ps6
Excavata/Diaphoretickes - ps7
Diaphoretickes - ps8
Archaeplastida - ps9
Viridiplantae - ps10
Streptophyta - ps11
Charophyceae/Embriophyta - ps12
Embriophyta - ps13
Tracheophyta - ps14
Spermatophyta - ps15
Angiosperms - ps16
Mesangiospermae - ps17
Magnoliids/eudicots - ps18
Eudicots - ps19
Core_eudicots - ps20
Superrosids - ps21
Rosids - ps22
Eurosids - ps23
Malvids - ps24
Sap./Malv./Brassicales - ps25
Malvales/Brassicales - ps26
Brassicales - ps27
Cleomaceae/Brassicaceae - ps28
Brassicaceae - ps29
Camelineae - ps30
Arabidopsis - ps31
Arabidopsis_thaliana - ps32
Phylostratum

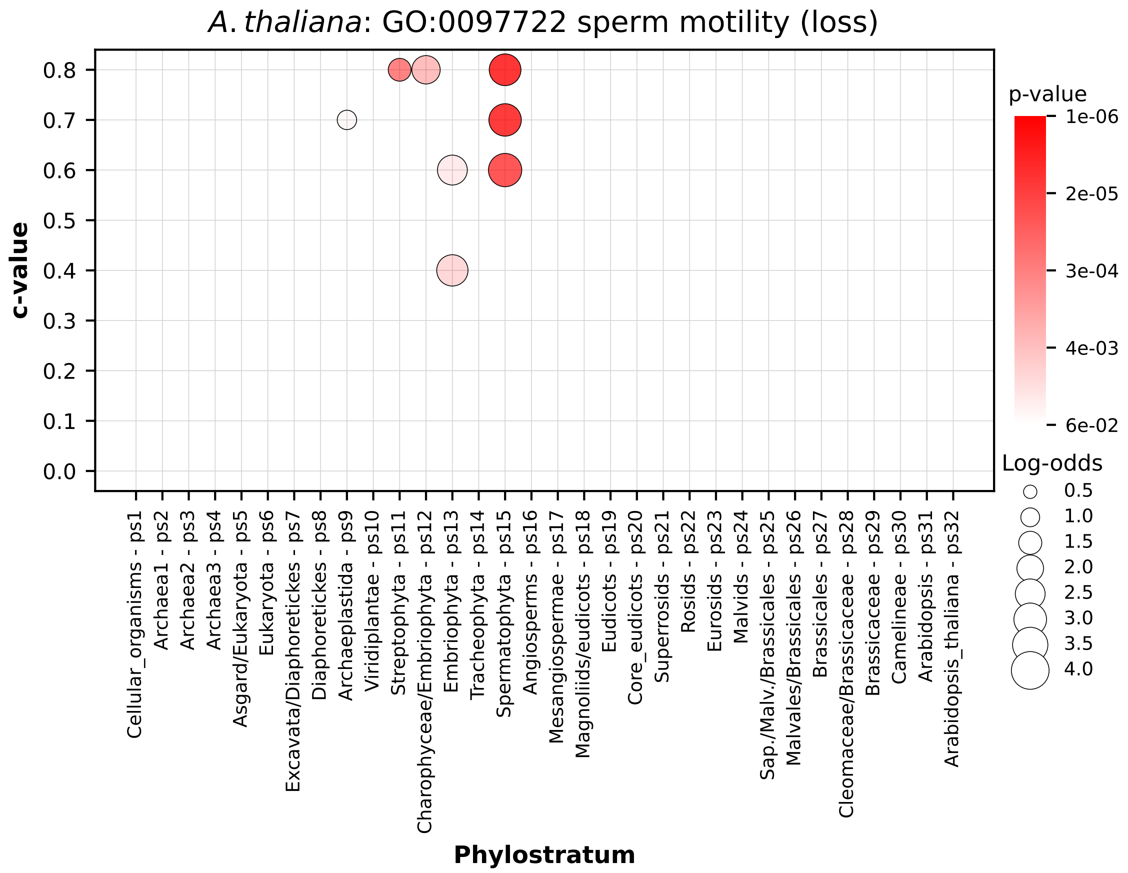

A. thaliana: GO:0097722 sperm motility (loss)
c-value
0.0
0.1
0.2
0.3
0.4
0.5
0.6
0.7
0.8
Phylostratum
Cellular_organisms - ps1
Archaea1 - ps2
Archaea2 - ps3
Archaea3 - ps4
Asgard/Eukaryota - ps5
Eukaryota - ps6
Excavata/Diaphoretickes - ps7
Diaphoretickes - ps8
Archaeplastida - ps9
Viridiplantae - ps10
Streptophyta - ps11
Charophyceae/Embriophyta - ps12
Embriophyta - ps13
Tracheophyta - ps14
Spermatophyta - ps15
Angiosperms - ps16
Mesangiospermae - ps17
Magnoliids/eudicots - ps18
Eudicots - ps19
Core_eudicots - ps20
Superrosids - ps21
Rosids - ps22
Eurosids - ps23
Malvids - ps24
Sap./Malv./Brassicales - ps25
Malvales/Brassicales - ps26
Brassicales - ps27
Cleomaceae/Brassicaceae - ps28
Brassicaceae - ps29
Camelineae - ps30
Arabidopsis - ps31
Arabidopsis_thaliana - ps32
p-value
1e-06
2e-05
3e-04
4e-03
6e-02
Log-odds
0.5
1.0
1.5
2.0
2.5
3.0
3.5
4.0

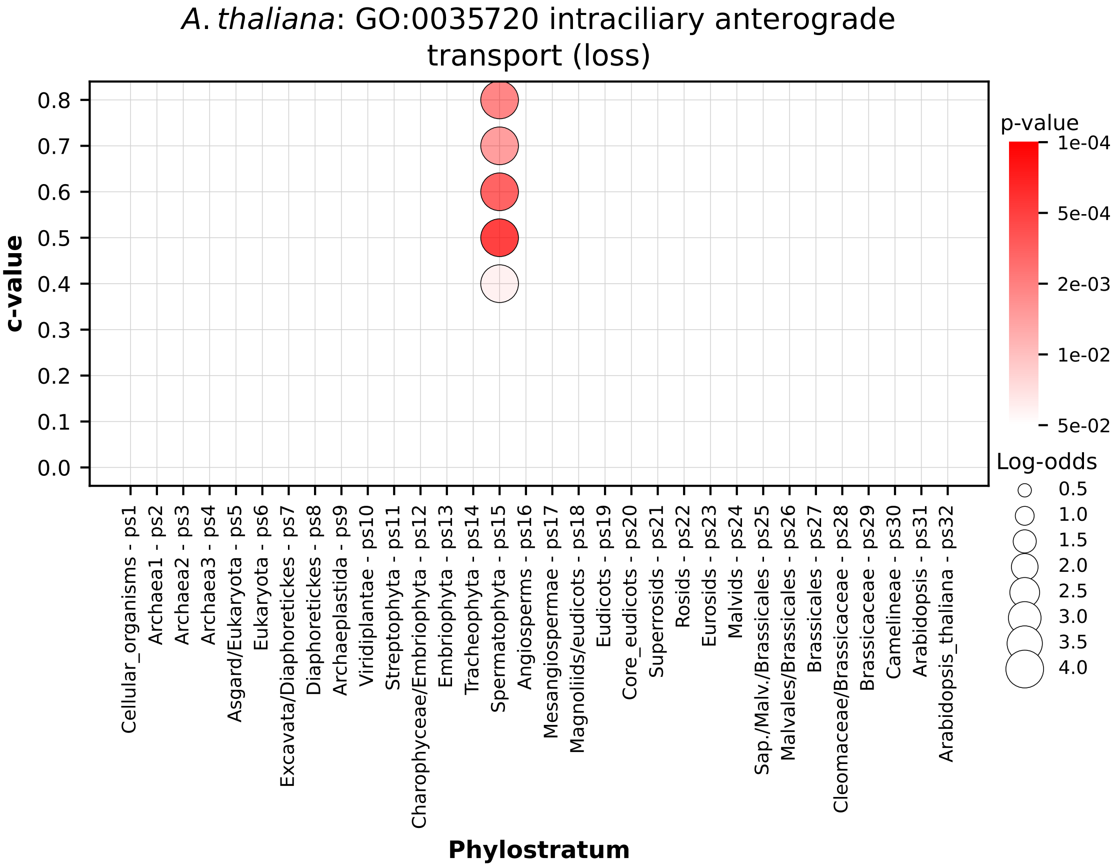
A. thaliana: GO:0035720 intraciliary anterograde transport (loss)
c-value
0.8
0.7
0.6
0.5
0.4
0.3
0.2
0.1
0.0
p-value
1e-04
5e-04
2e-03
1e-02
5e-02
Log-odds
0.5
1.0
1.5
2.0
2.5
3.0
3.5
4.0
Cellular_organisms - ps1
Archaea1 - ps2
Archaea2 - ps3
Archaea3 - ps4
Asgard/Eukaryota - ps5
Eukaryota - ps6
Excavata/Diaphoretickes - ps7
Diaphoretickes - ps8
Archaeplastida - ps9
Viridiplantae - ps10
Streptophyta - ps11
Charophyceae/Embriophyta - ps12
Embriophyta - ps13
Tracheophyta - ps14
Spermatophyta - ps15
Angiosperms - ps16
Mesangiospermae - ps17
Magnoliids/eudicots - ps18
Eudicots - ps19
Core_eudicots - ps20
Superrosids - ps21
Rosids - ps22
Eurosids - ps23
Malvids - ps24
Sap./Malv./Brassicales - ps25
Malvales/Brassicales - ps26
Brassicales - ps27
Cleomaceae/Brassicaceae - ps28
Brassicaceae - ps29
Camelineae - ps30
Arabidopsis - ps31
Arabidopsis_thaliana - ps32
Phylostratum

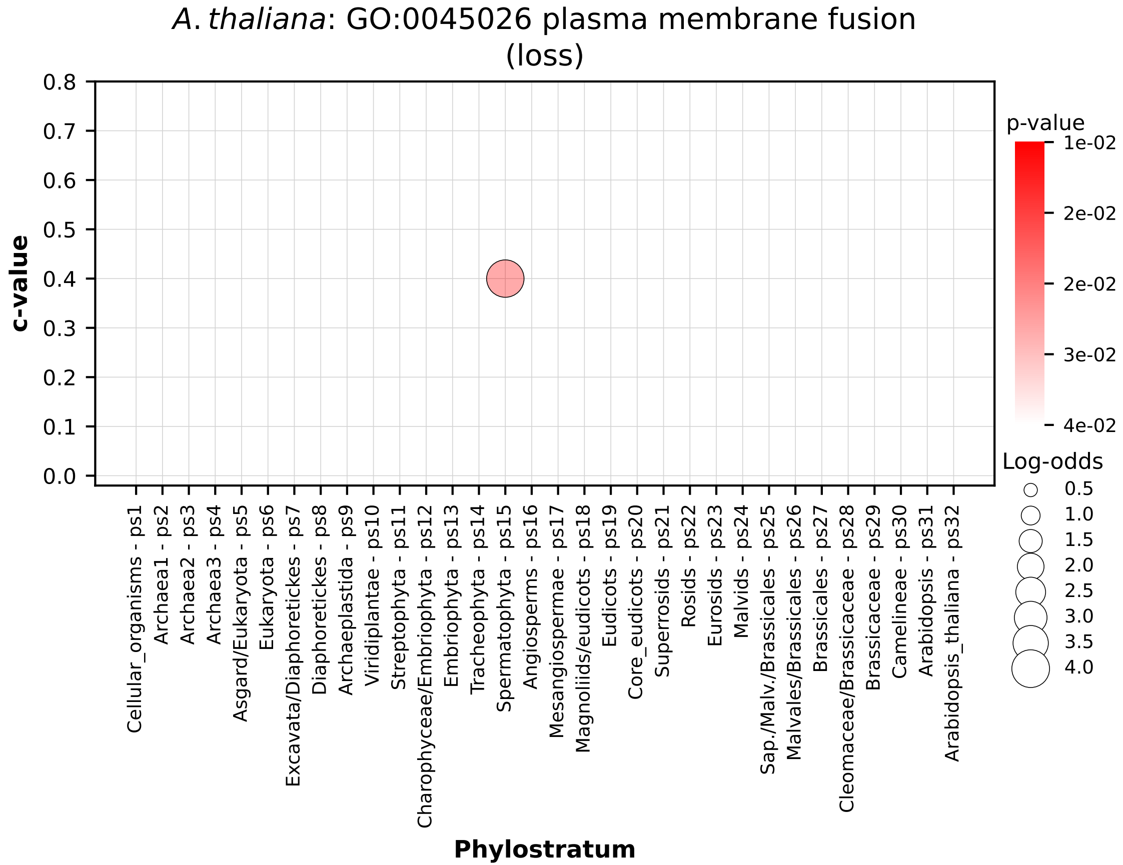

A. thaliana: GO:0045026 plasma membrane fusion
(loss)
c-value
0.8
0.7
0.6
0.5
0.4
0.3
0.2
0.1
0.0
p-value
1e-02
2e-02
2e-02
3e-02
4e-02
Log-odds
0.5
1.0
1.5
2.0
2.5
3.0
3.5
4.0
Cellular_organisms - ps1
Archaea1 - ps2
Archaea2 - ps3
Archaea3 - ps4
Asgard/Eukaryota - ps5
Eukaryota - ps6
Excavata/Diaphoretickes - ps7
Diaphoretickes - ps8
Archaeplastida - ps9
Viridiplantae - ps10
Streptophyta - ps11
Charophyceae/Embriophyta - ps12
Embriophyta - ps13
Tracheophyta - ps14
Spermatophyta - ps15
Angiosperms - ps16
Mesangiospermae - ps17
Magnoliids/eudicots - ps18
Eudicots - ps19
Core_eudicots - ps20
Superrosids - ps21
Rosids - ps22
Eurosids - ps23
Malvids - ps24
Sap./Malv./Brassicales - ps25
Malvales/Brassicales - ps26
Brassicales - ps27
Cleomaceae/Brassicaceae - ps28
Brassicaceae - ps29
Camelineae - ps30
Arabidopsis - ps31
Arabidopsis_thaliana - ps32
Phylostratum

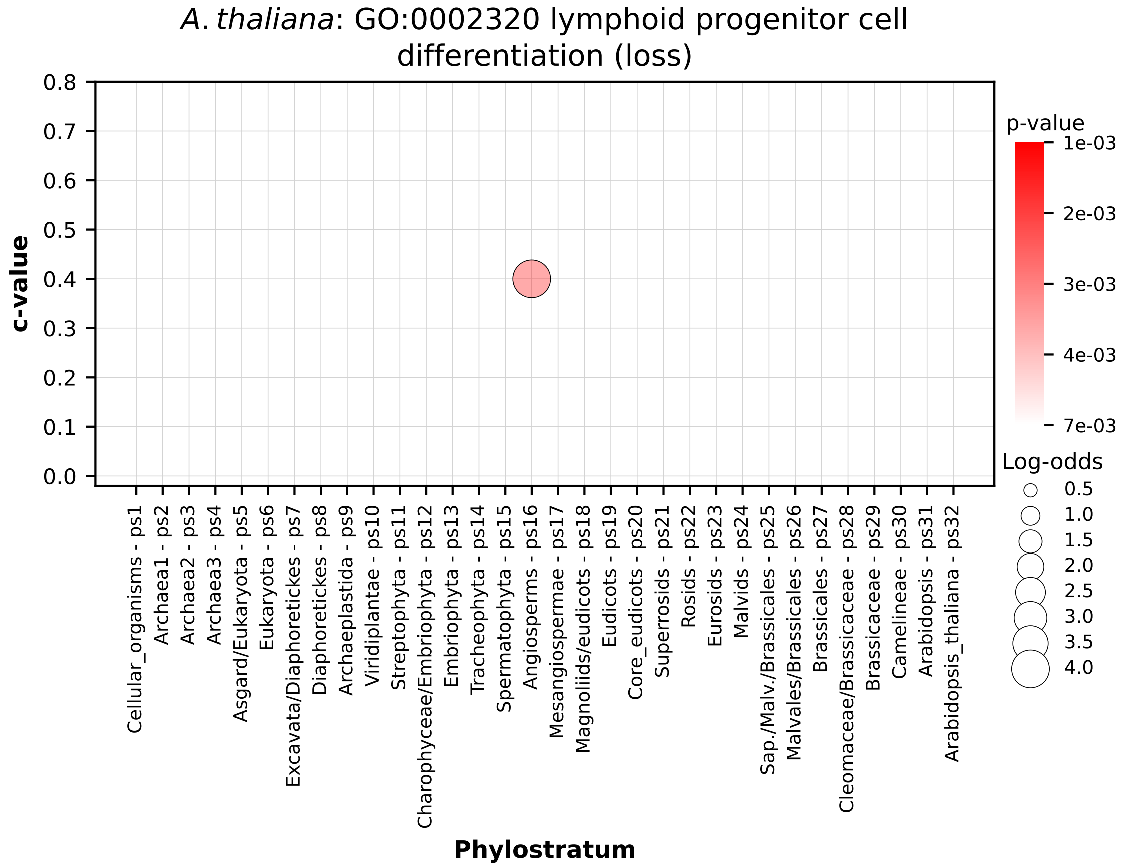

A. thaliana: GO:0002320 lymphoid progenitor cell differentiation (loss)
c-value
0.8
0.7
0.6
0.5
0.4
0.3
0.2
0.1
0.0
Phylostratum
Cellular_organisms - ps1
Archaea1 - ps2
Archaea2 - ps3
Archaea3 - ps4
Asgard/Eukaryota - ps5
Eukaryota - ps6
Excavata/Diaphoretickes - ps7
Diaphoretickes - ps8
Archaeplastida - ps9
Viridiplantae - ps10
Streptophyta - ps11
Charophyceae/Embriophyta - ps12
Embriophyta - ps13
Tracheophyta - ps14
Spermatophyta - ps15
Angiosperms - ps16
Mesangiospermae - ps17
Magnoliids/eudicots - ps18
Eudicots - ps19
Core_eudicots - ps20
Superrosids - ps21
Rosids - ps22
Eurosids - ps23
Malvids - ps24
Sap./Malv./Brassicales - ps25
Malvales/Brassicales - ps26
Brassicales - ps27
Cleomaceae/Brassicaceae - ps28
Brassicaceae - ps29
Camelineae - ps30
Arabidopsis - ps31
Arabidopsis_thaliana - ps32
p-value
1e-03
2e-03
3e-03
4e-03
7e-03
Log-odds
0.5
1.0
1.5
2.0
2.5
3.0
3.5
4.0

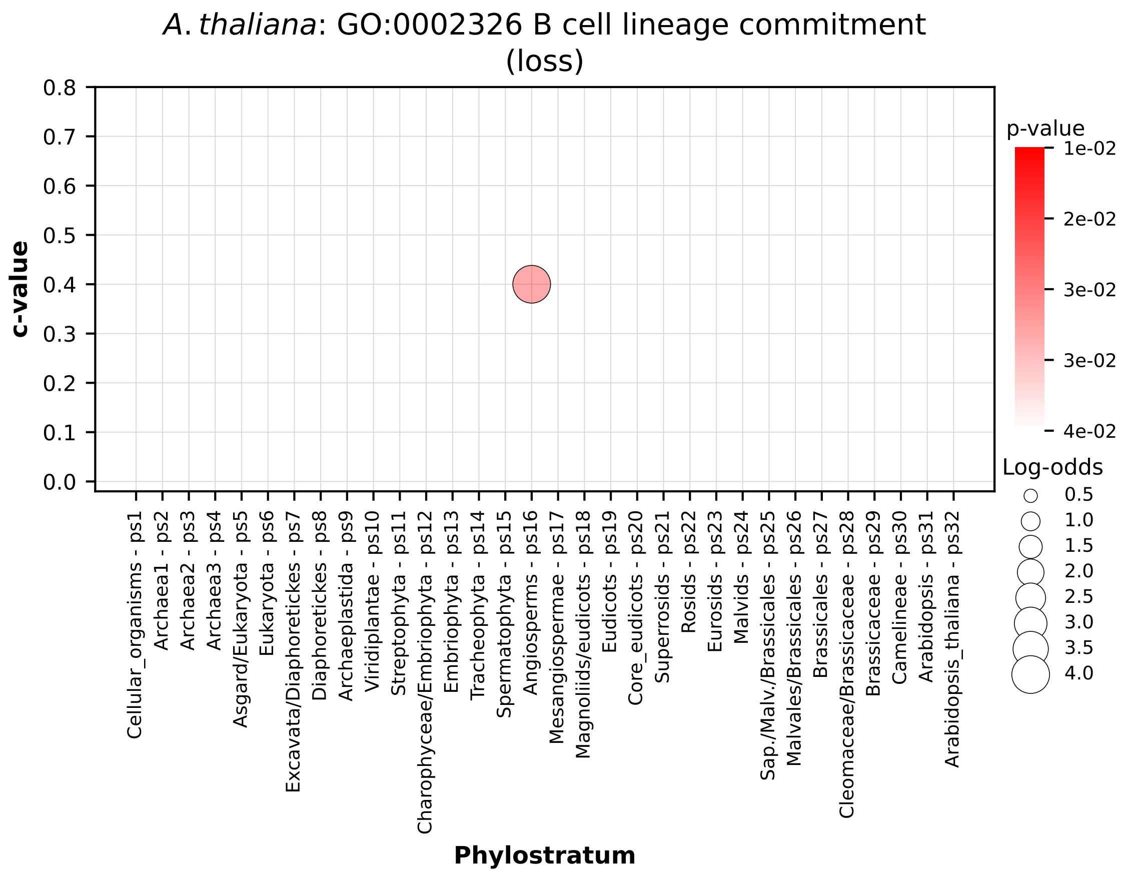

A. thaliana: GO:0002326 B cell lineage commitment
(loss)
c-value
0.8
0.7
0.6
0.5
0.4
0.3
0.2
0.1
0.0
Phylostratum
Cellular_organisms - ps1
Archaea1 - ps2
Archaea2 - ps3
Archaea3 - ps4
Asgard/Eukaryota - ps5
Eukaryota - ps6
Excavata/Diaphoretickes - ps7
Diaphoretickes - ps8
Archaeplastida - ps9
Viridiplantae - ps10
Streptophyta - ps11
Charophyceae/Embriophyta - ps12
Embriophyta - ps13
Tracheophyta - ps14
Spermatophyta - ps15
Angiosperms - ps16
Mesangiospermae - ps17
Magnoliids/eudicots - ps18
Eudicots - ps19
Core_eudicots - ps20
Superrosids - ps21
Rosids - ps22
Eurosids - ps23
Malvids - ps24
Sap./Malv./Brassicales - ps25
Malvales/Brassicales - ps26
Brassicales - ps27
Cleomaceae/Brassicaceae - ps28
Brassicaceae - ps29
Camelineae - ps30
Arabidopsis - ps31
Arabidopsis_thaliana - ps32
p-value
1e-02
2e-02
3e-02
3e-02
4e-02
Log-odds
0.5
1.0
1.5
2.0
2.5
3.0
3.5
4.0

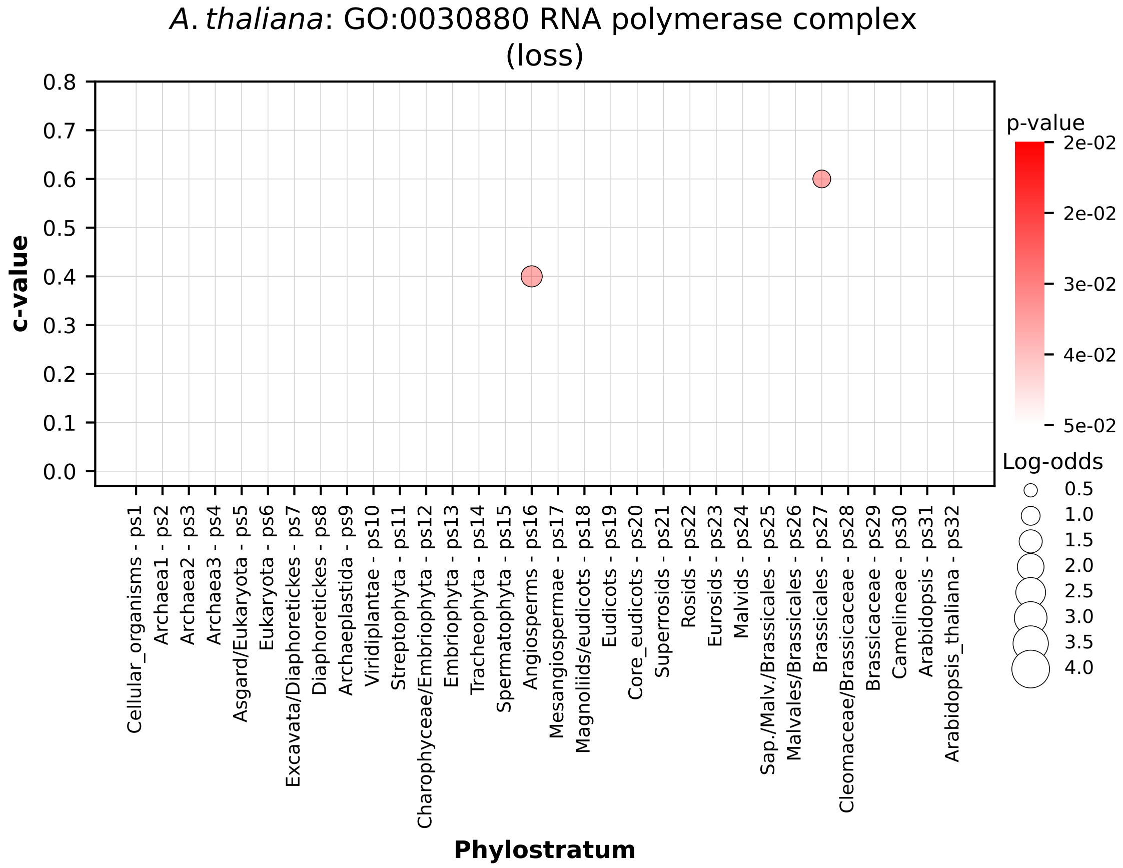

A. thaliana: GO:0030880 RNA polymerase complex
(loss)
c-value
0.8
0.7
0.6
0.5
0.4
0.3
0.2
0.1
0.0
p-value
2e-02
2e-02
3e-02
4e-02
5e-02
Log-odds
0.5
1.0
1.5
2.0
2.5
3.0
3.5
4.0
Cellular_organisms - ps1
Archaea1 - ps2
Archaea2 - ps3
Archaea3 - ps4
Asgard/Eukaryota - ps5
Eukaryota - ps6
Excavata/Diaphoretickes - ps7
Diaphoretickes - ps8
Archaeplastida - ps9
Viridiplantae - ps10
Streptophyta - ps11
Charophyceae/Embriophyta - ps12
Embriophyta - ps13
Tracheophyta - ps14
Spermatophyta - ps15
Angiosperms - ps16
Mesangiospermae - ps17
Magnoliids/eudicots - ps18
Eudicots - ps19
Core_eudicots - ps20
Superrosids - ps21
Rosids - ps22
Eurosids - ps23
Malvids - ps24
Sap./Malv./Brassicales - ps25
Malvales/Brassicales - ps26
Brassicales - ps27
Cleomaceae/Brassicaceae - ps28
Brassicaceae - ps29
Camelineae - ps30
Arabidopsis - ps31
Arabidopsis_thaliana - ps32
Phylostratum

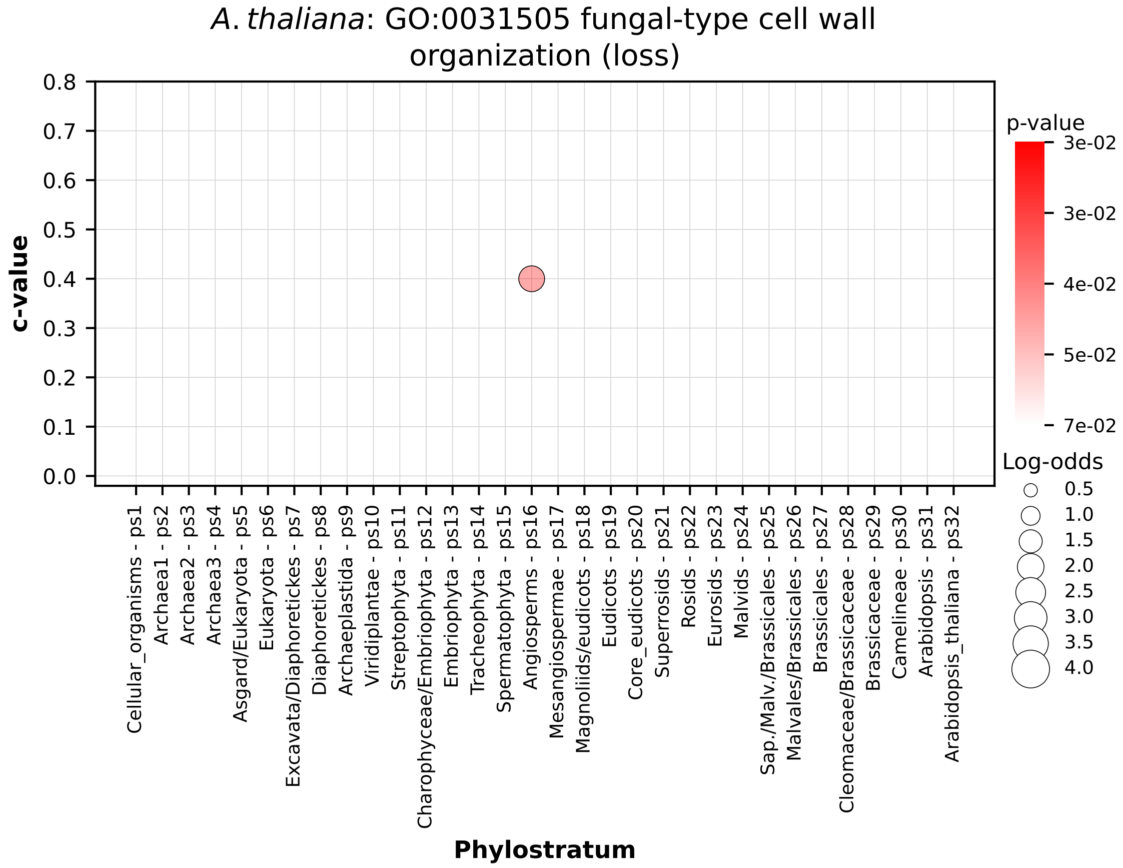

A. thaliana: GO:0031505 fungal-type cell wall organization (loss)
c-value
0.8
0.7
0.6
0.5
0.4
0.3
0.2
0.1
0.0
Phylostratum
Cellular_organisms - ps1
Archaea1 - ps2
Archaea2 - ps3
Archaea3 - ps4
Asgard/Eukaryota - ps5
Eukaryota - ps6
Excavata/Diaphoretickes - ps7
Diaphoretickes - ps8
Archaeplastida - ps9
Viridiplantae - ps10
Streptophyta - ps11
Charophyceae/Embriophyta - ps12
Embriophyta - ps13
Tracheophyta - ps14
Spermatophyta - ps15
Angiosperms - ps16
Mesangiospermae - ps17
Magnoliids/eudicots - ps18
Eudicots - ps19
Core_eudicots - ps20
Superrosids - ps21
Rosids - ps22
Eurosids - ps23
Malvids - ps24
Sap./Malv./Brassicales - ps25
Malvales/Brassicales - ps26
Brassicales - ps27
Cleomaceae/Brassicaceae - ps28
Brassicaceae - ps29
Camelineae - ps30
Arabidopsis - ps31
Arabidopsis_thaliana - ps32
p-value
3e-02
3e-02
4e-02
5e-02
7e-02
Log-odds
0.5
1.0
1.5
2.0
2.5
3.0
3.5
4.0

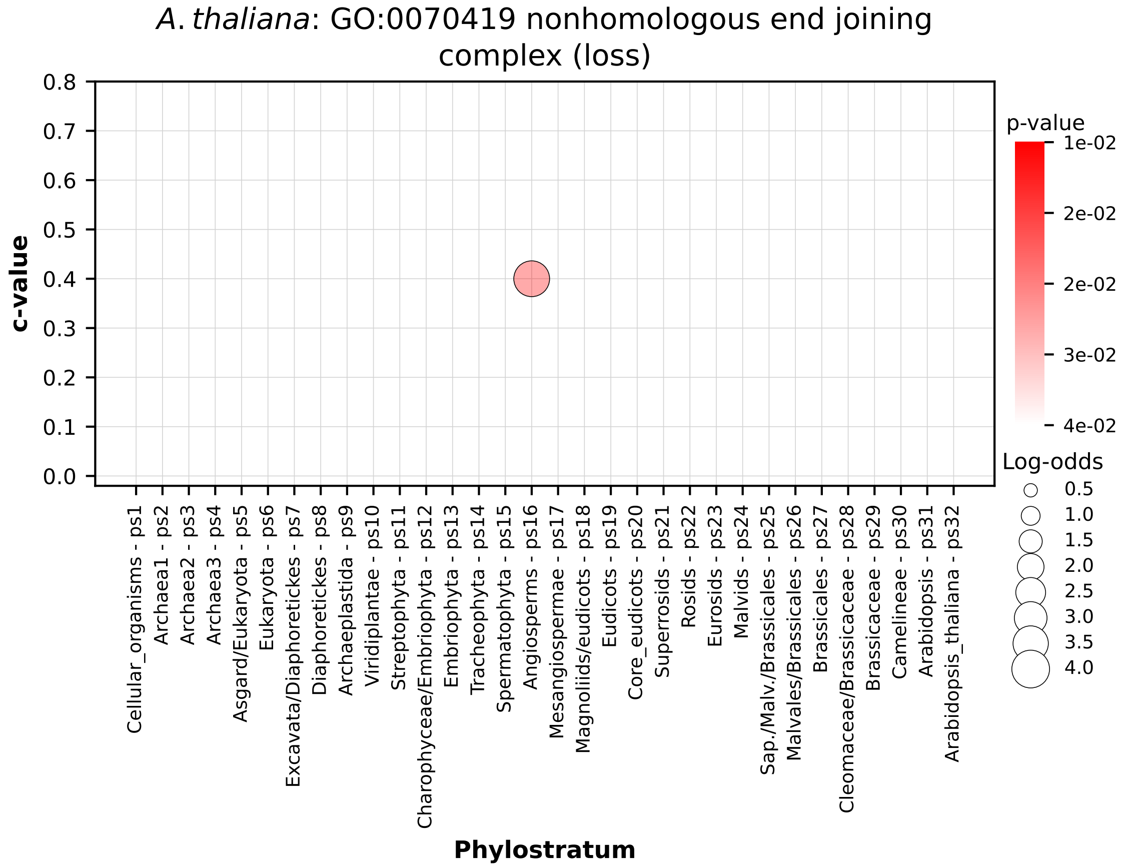
A. thaliana: GO:0070419 nonhomologous end joining complex (loss)
c-value
0.0
0.1
0.2
0.3
0.4
0.5
0.6
0.7
0.8
Phylostratum
Cellular_organisms - ps1
Archaea1 - ps2
Archaea2 - ps3
Archaea3 - ps4
Asgard/Eukaryota - ps5
Eukaryota - ps6
Excavata/Diaphoretickes - ps7
Diaphoretickes - ps8
Archaeplastida - ps9
Viridiplantae - ps10
Streptophyta - ps11
Charophyceae/Embriophyta - ps12
Embriophyta - ps13
Tracheophyta - ps14
Spermatophyta - ps15
Angiosperms - ps16
Mesangiospermae - ps17
Magnoliids/eudicots - ps18
Eudicots - ps19
Core_eudicots - ps20
Superrosids - ps21
Rosids - ps22
Eurosids - ps23
Malvids - ps24
Sap./Malv./Brassicales - ps25
Malvales/Brassicales - ps26
Brassicales - ps27
Cleomaceae/Brassicaceae - ps28
Brassicaceae - ps29
Camelineae - ps30
Arabidopsis - ps31
Arabidopsis_thaliana - ps32
p-value
1e-02
2e-02
2e-02
3e-02
4e-02
Log-odds
0.5
1.0
1.5
2.0
2.5
3.0
3.5
4.0

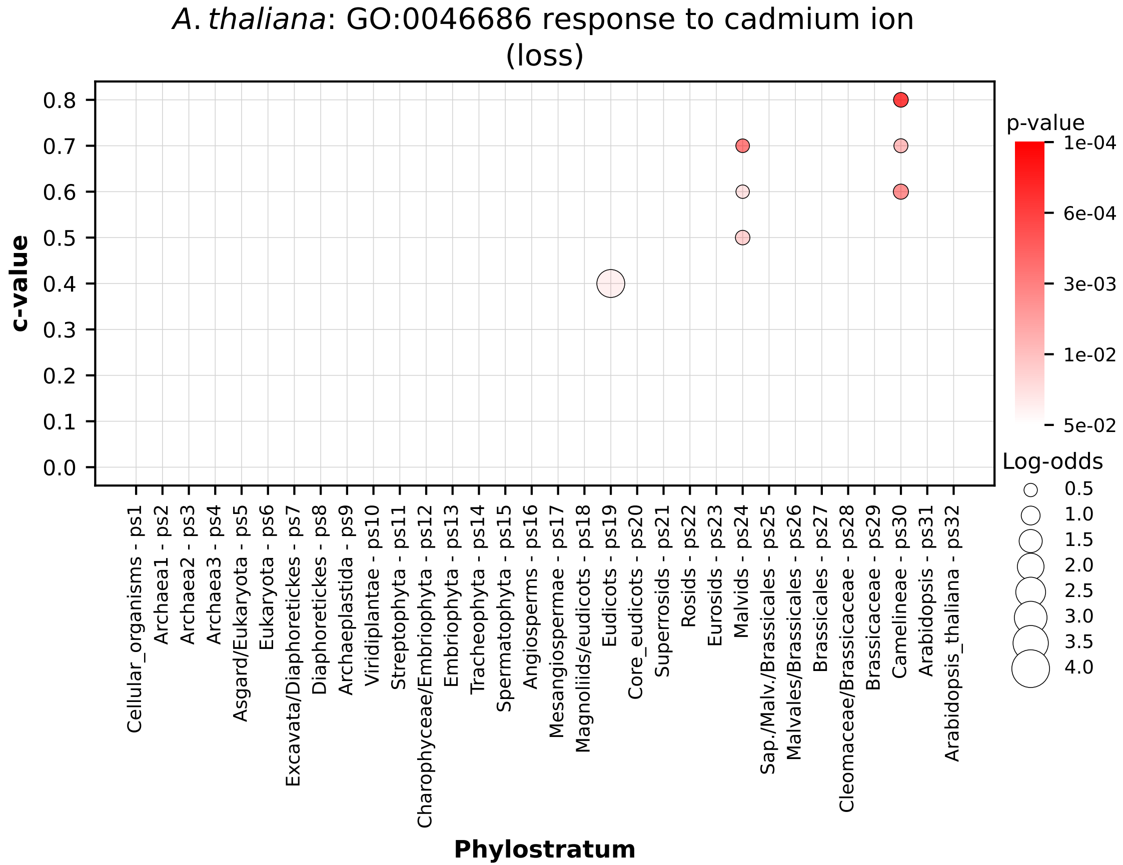

A. thaliana: GO:0046686 response to cadmium ion
(loss)
c-value
0.8
0.7
0.6
0.5
0.4
0.3
0.2
0.1
0.0
p-value
1e-04
6e-04
3e-03
1e-02
5e-02
Log-odds
0.5
1.0
1.5
2.0
2.5
3.0
3.5
4.0
Cellular_organisms - ps1
Archaea1 - ps2
Archaea2 - ps3
Archaea3 - ps4
Asgard/Eukaryota - ps5
Eukaryota - ps6
Excavata/Diaphoretickes - ps7
Diaphoretickes - ps8
Archaeplastida - ps9
Viridiplantae - ps10
Streptophyta - ps11
Charophyceae/Embriophyta - ps12
Embriophyta - ps13
Tracheophyta - ps14
Spermatophyta - ps15
Angiosperms - ps16
Mesangiospermae - ps17
Magnoliids/eudicots - ps18
Eudicots - ps19
Core_eudicots - ps20
Superrosids - ps21
Rosids - ps22
Eurosids - ps23
Malvids - ps24
Sap./Malv./Brassicales - ps25
Malvales/Brassicales - ps26
Brassicales - ps27
Cleomaceae/Brassicaceae - ps28
Brassicaceae - ps29
Camelineae - ps30
Arabidopsis - ps31
Arabidopsis_thaliana - ps32
Phylostratum

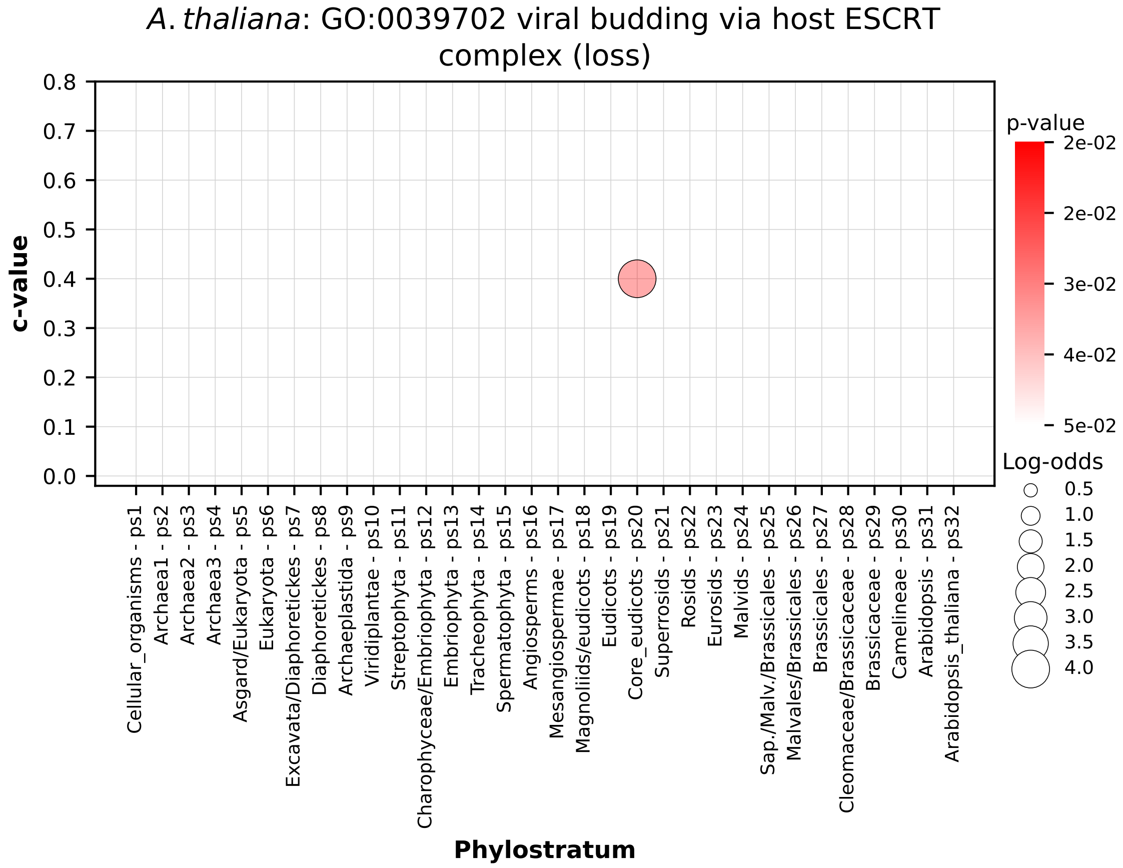

A. thaliana: GO:0039702 viral budding via host ESCRT complex (loss)
c-value
0.8
0.7
0.6
0.5
0.4
0.3
0.2
0.1
0.0
Cellular_organisms - ps1
Archaea1 - ps2
Archaea2 - ps3
Archaea3 - ps4
Asgard/Eukaryota - ps5
Eukaryota - ps6
Excavata/Diaphoretickes - ps7
Diaphoretickes - ps8
Archaeplastida - ps9
Viridiplantae - ps10
Streptophyta - ps11
Charophyceae/Embriophyta - ps12
Embriophyta - ps13
Tracheophyta - ps14
Spermatophyta - ps15
Angiosperms - ps16
Mesangiospermae - ps17
Magnoliids/eudicots - ps18
Eudicots - ps19
Core_eudicots - ps20
Superrosids - ps21
Rosids - ps22
Eurosids - ps23
Malvids - ps24
Sap./Malv./Brassicales - ps25
Malvales/Brassicales - ps26
Brassicales - ps27
Cleomaceae/Brassicaceae - ps28
Brassicaceae - ps29
Camelineae - ps30
Arabidopsis - ps31
Arabidopsis_thaliana - ps32
Phylostratum
p-value
2e-02
2e-02
3e-02
4e-02
5e-02
Log-odds
0.5
1.0
1.5
2.0
2.5
3.0
3.5
4.0

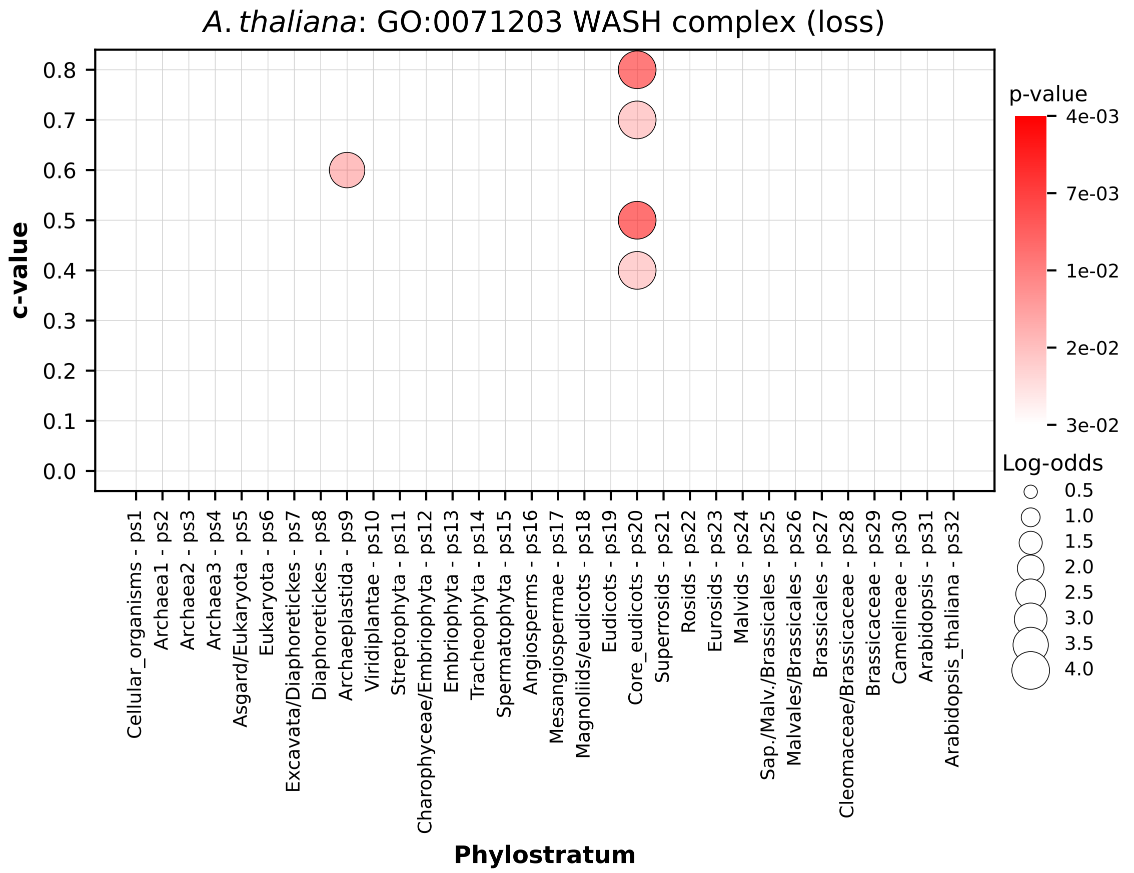

A. thaliana: GO:0071203 WASH complex (loss)
c-value
0.0
0.1
0.2
0.3
0.4
0.5
0.6
0.7
0.8
Phylostratum
Cellular_organisms - ps1
Archaea1 - ps2
Archaea2 - ps3
Archaea3 - ps4
Asgard/Eukaryota - ps5
Eukaryota - ps6
Excavata/Diaphoretickes - ps7
Diaphoretickes - ps8
Archaeplastida - ps9
Viridiplantae - ps10
Streptophyta - ps11
Charophyceae/Embriophyta - ps12
Embriophyta - ps13
Tracheophyta - ps14
Spermatophyta - ps15
Angiosperms - ps16
Mesangiospermae - ps17
Magnoliids/eudicots - ps18
Eudicots - ps19
Core_eudicots - ps20
Superrosids - ps21
Rosids - ps22
Eurosids - ps23
Malvids - ps24
Sap./Malv./Brassicales - ps25
Malvales/Brassicales - ps26
Brassicales - ps27
Cleomaceae/Brassicaceae - ps28
Brassicaceae - ps29
Camelineae - ps30
Arabidopsis - ps31
Arabidopsis_thaliana - ps32
p-value
4e-03
7e-03
1e-02
2e-02
3e-02
Log-odds
0.5
1.0
1.5
2.0
2.5
3.0
3.5
4.0

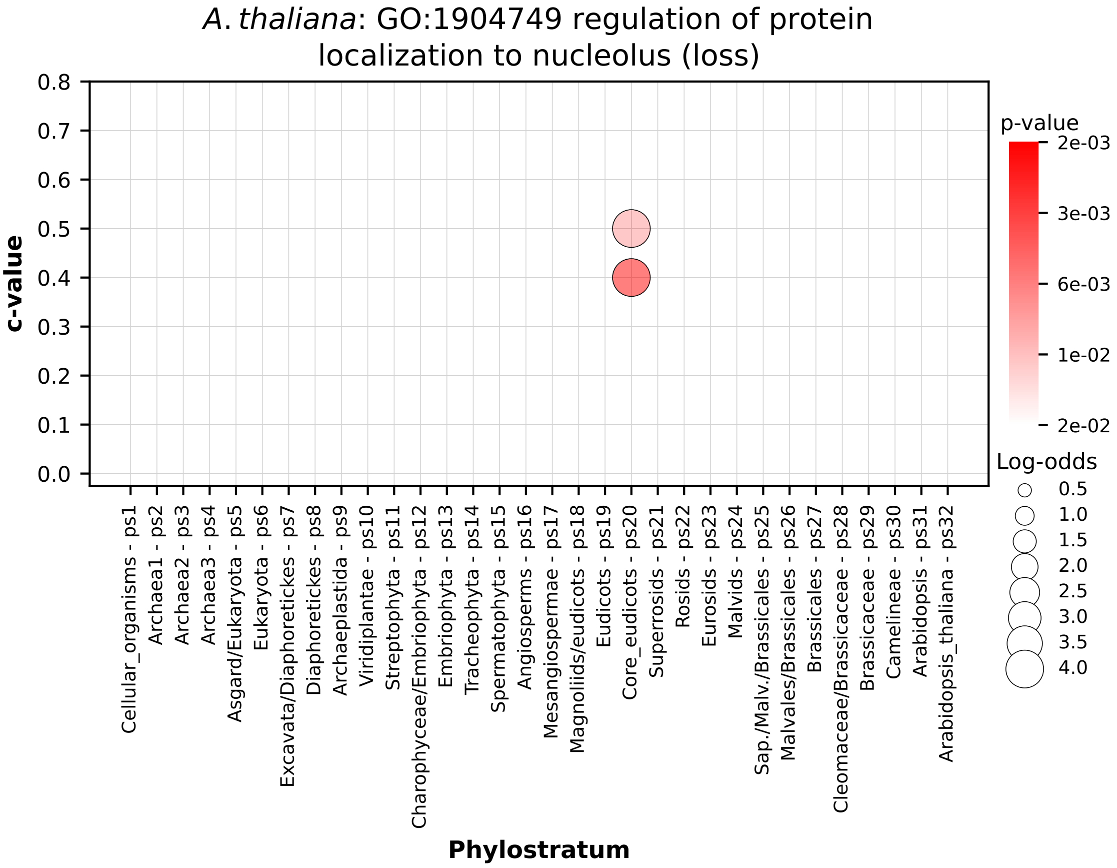

A. thaliana: GO:1904749 regulation of protein localization to nucleolus (loss)
c-value
0.8
0.7
0.6
0.5
0.4
0.3
0.2
0.1
0.0
p-value
2e-03
3e-03
6e-03
1e-02
2e-02
Log-odds
0.5
1.0
1.5
2.0
2.5
3.0
3.5
4.0
Cellular_organisms - ps1
Archaea1 - ps2
Archaea2 - ps3
Archaea3 - ps4
Asgard/Eukaryota - ps5
Eukaryota - ps6
Excavata/Diaphoretickes - ps7
Diaphoretickes - ps8
Archaeplastida - ps9
Viridiplantae - ps10
Streptophyta - ps11
Charophyceae/Embriophyta - ps12
Embriophyta - ps13
Tracheophyta - ps14
Spermatophyta - ps15
Angiosperms - ps16
Mesangiospermae - ps17
Magnoliids/eudicots - ps18
Eudicots - ps19
Core_eudicots - ps20
Superrosids - ps21
Rosids - ps22
Eurosids - ps23
Malvids - ps24
Sap./Malv./Brassicales - ps25
Malvales/Brassicales - ps26
Brassicales - ps27
Cleomaceae/Brassicaceae - ps28
Brassicaceae - ps29
Camelineae - ps30
Arabidopsis - ps31
Arabidopsis_thaliana - ps32
Phylostratum

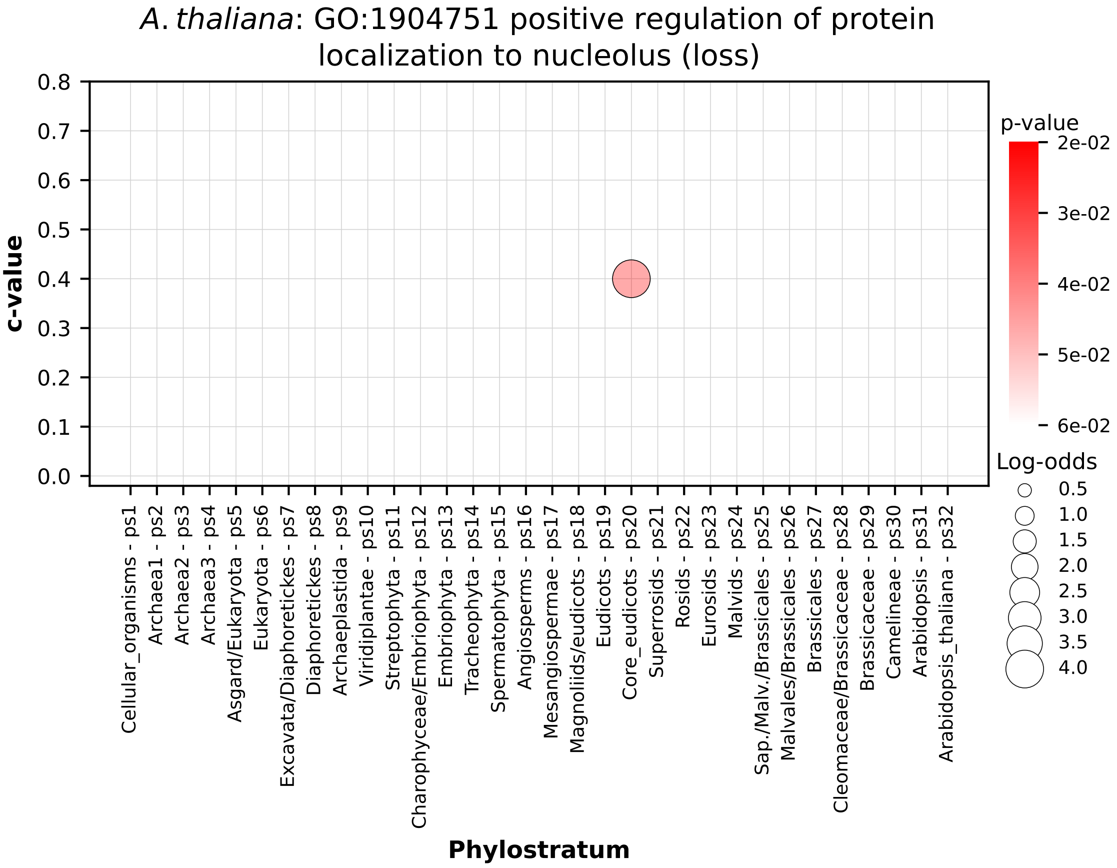
A. thaliana: GO:1904751 positive regulation of protein localization to nucleolus (loss)
c-value
0.8
0.7
0.6
0.5
0.4
0.3
0.2
0.1
0.0
Phylostratum
Cellular_organisms - ps1
Archaea1 - ps2
Archaea2 - ps3
Archaea3 - ps4
Asgard/Eukaryota - ps5
Eukaryota - ps6
Excavata/Diaphoretickes - ps7
Diaphoretickes - ps8
Archaeplastida - ps9
Viridiplantae - ps10
Streptophyta - ps11
Charophyceae/Embriophyta - ps12
Embriophyta - ps13
Tracheophyta - ps14
Spermatophyta - ps15
Angiosperms - ps16
Mesangiospermae - ps17
Magnoliids/eudicots - ps18
Eudicots - ps19
Core_eudicots - ps20
Superrosids - ps21
Rosids - ps22
Eurosids - ps23
Malvids - ps24
Sap./Malv./Brassicales - ps25
Malvales/Brassicales - ps26
Brassicales - ps27
Cleomaceae/Brassicaceae - ps28
Brassicaceae - ps29
Camelineae - ps30
Arabidopsis - ps31
Arabidopsis_thaliana - ps32
p-value
2e-02
3e-02
4e-02
5e-02
6e-02
Log-odds
0.5
1.0
1.5
2.0
2.5
3.0
3.5
4.0

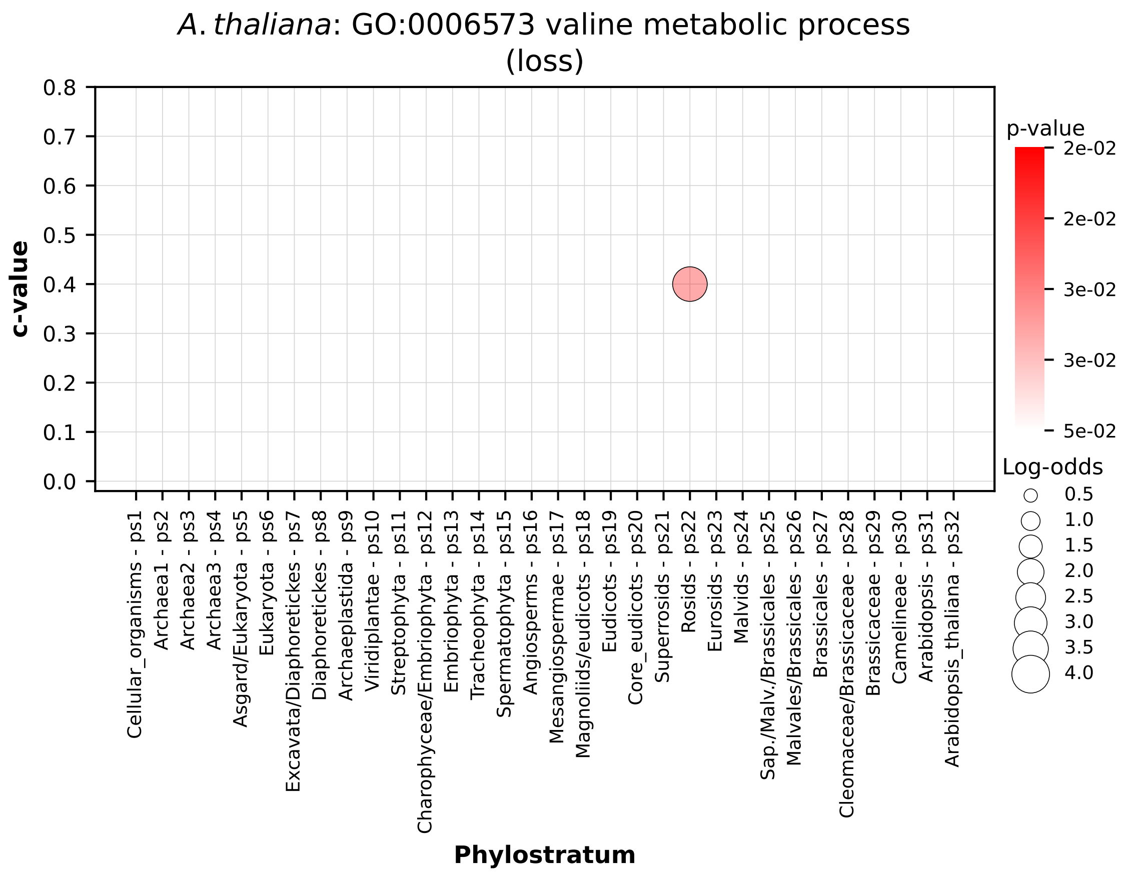

A. thaliana: GO:0006573 valine metabolic process
(loss)
c-value
0.8
0.7
0.6
0.5
0.4
0.3
0.2
0.1
0.0
p-value
2e-02
2e-02
3e-02
3e-02
5e-02
Log-odds
0.5
1.0
1.5
2.0
2.5
3.0
3.5
4.0
Cellular_organisms - ps1
Archaea1 - ps2
Archaea2 - ps3
Archaea3 - ps4
Asgard/Eukaryota - ps5
Eukaryota - ps6
Excavata/Diaphoretickes - ps7
Diaphoretickes - ps8
Archaeplastida - ps9
Viridiplantae - ps10
Streptophyta - ps11
Charophyceae/Embriophyta - ps12
Embriophyta - ps13
Tracheophyta - ps14
Spermatophyta - ps15
Angiosperms - ps16
Mesangiospermae - ps17
Magnoliids/eudicots - ps18
Eudicots - ps19
Core_eudicots - ps20
Superrosids - ps21
Rosids - ps22
Eurosids - ps23
Malvids - ps24
Sap./Malv./Brassicales - ps25
Malvales/Brassicales - ps26
Brassicales - ps27
Cleomaceae/Brassicaceae - ps28
Brassicaceae - ps29
Camelineae - ps30
Arabidopsis - ps31
Arabidopsis_thaliana - ps32
Phylostratum

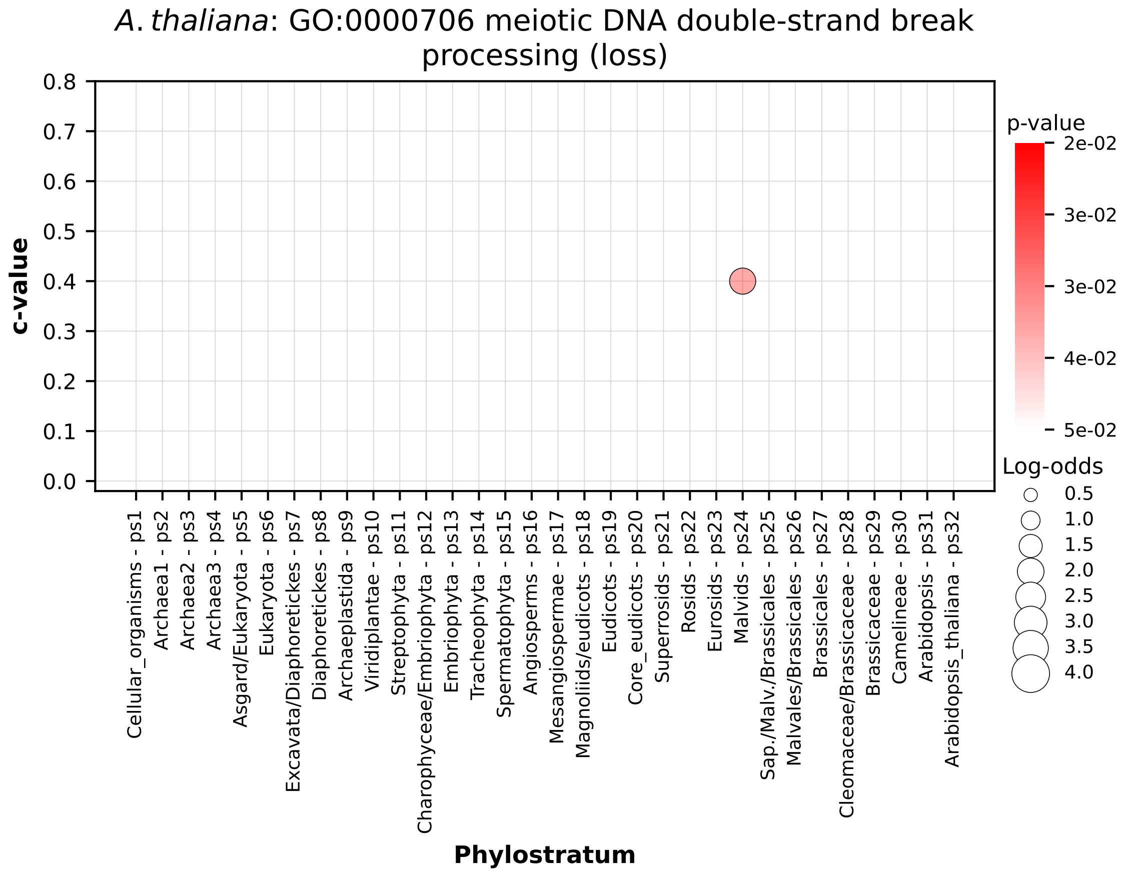

A. thaliana: GO:0000706 meiotic DNA double-strand break processing (loss)
c-value
0.8
0.7
0.6
0.5
0.4
0.3
0.2
0.1
0.0
p-value
2e-02
3e-02
3e-02
4e-02
5e-02
Log-odds
0.5
1.0
1.5
2.0
2.5
3.0
3.5
4.0
Cellular_organisms - ps1
Archaea1 - ps2
Archaea2 - ps3
Archaea3 - ps4
Asgard/Eukaryota - ps5
Eukaryota - ps6
Excavata/Diaphoretickes - ps7
Diaphoretickes - ps8
Archaeplastida - ps9
Viridiplantae - ps10
Streptophyta - ps11
Charophyceae/Embriophyta - ps12
Embriophyta - ps13
Tracheophyta - ps14
Spermatophyta - ps15
Angiosperms - ps16
Mesangiospermae - ps17
Magnoliids/eudicots - ps18
Eudicots - ps19
Core_eudicots - ps20
Superrosids - ps21
Rosids - ps22
Eurosids - ps23
Malvids - ps24
Sap./Malv./Brassicales - ps25
Malvales/Brassicales - ps26
Brassicales - ps27
Cleomaceae/Brassicaceae - ps28
Brassicaceae - ps29
Camelineae - ps30
Arabidopsis - ps31
Arabidopsis_thaliana - ps32
Phylostratum

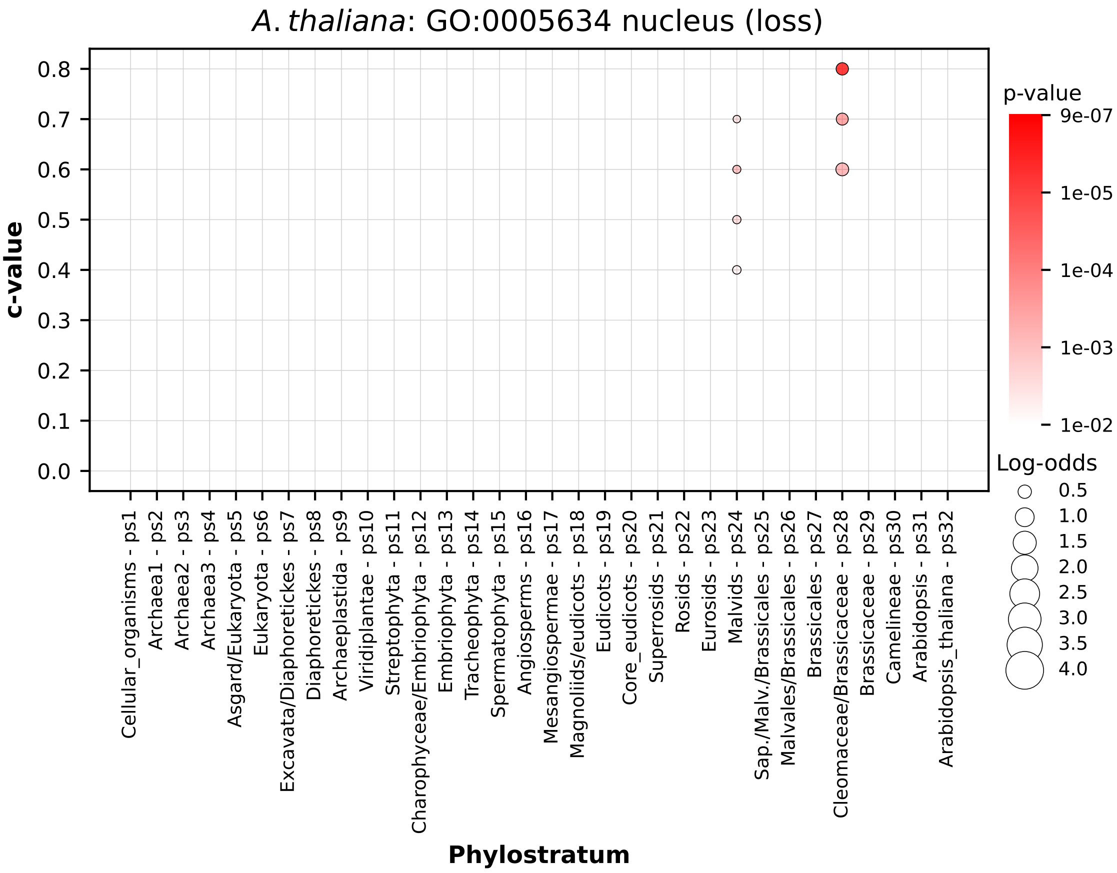

A. thaliana: GO:0005634 nucleus (loss)
c-value
0.0
0.1
0.2
0.3
0.4
0.5
0.6
0.7
0.8
Phylostratum
Cellular_organisms - ps1
Archaea1 - ps2
Archaea2 - ps3
Archaea3 - ps4
Asgard/Eukaryota - ps5
Eukaryota - ps6
Excavata/Diaphoretickes - ps7
Diaphoretickes - ps8
Archaeplastida - ps9
Viridiplantae - ps10
Streptophyta - ps11
Charophyceae/Embriophyta - ps12
Embriophyta - ps13
Tracheophyta - ps14
Spermatophyta - ps15
Angiosperms - ps16
Mesangiospermae - ps17
Magnoliids/eudicots - ps18
Eudicots - ps19
Core_eudicots - ps20
Superrosids - ps21
Rosids - ps22
Eurosids - ps23
Malvids - ps24
Sap./Malv./Brassicales - ps25
Malvales/Brassicales - ps26
Brassicales - ps27
Cleomaceae/Brassicaceae - ps28
Brassicaceae - ps29
Camelineae - ps30
Arabidopsis - ps31
Arabidopsis_thaliana - ps32
p-value
9e-07
1e-05
1e-04
1e-03
1e-02
Log-odds
0.5
1.0
1.5
2.0
2.5
3.0
3.5
4.0

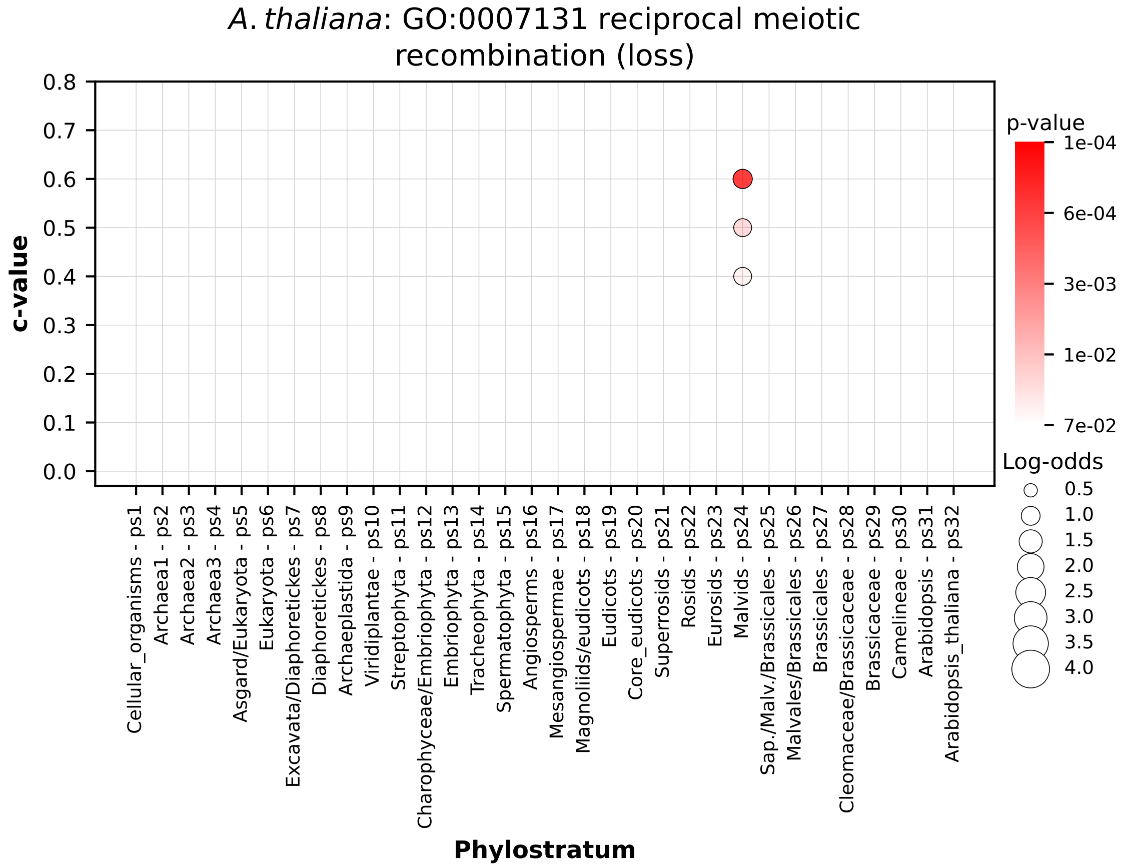

A. thaliana: GO:0007131 reciprocal meiotic recombination (loss)
c-value
0.8
0.7
0.6
0.5
0.4
0.3
0.2
0.1
0.0
Phylostratum
Cellular_organisms - ps1
Archaea1 - ps2
Archaea2 - ps3
Archaea3 - ps4
Asgard/Eukaryota - ps5
Eukaryota - ps6
Excavata/Diaphoretickes - ps7
Diaphoretickes - ps8
Archaeplastida - ps9
Viridiplantae - ps10
Streptophyta - ps11
Charophyceae/Embriophyta - ps12
Embriophyta - ps13
Tracheophyta - ps14
Spermatophyta - ps15
Angiosperms - ps16
Mesangiospermae - ps17
Magnoliids/eudicots - ps18
Eudicots - ps19
Core_eudicots - ps20
Superrosids - ps21
Rosids - ps22
Eurosids - ps23
Malvids - ps24
Sap./Malv./Brassicales - ps25
Malvales/Brassicales - ps26
Brassicales - ps27
Cleomaceae/Brassicaceae - ps28
Brassicaceae - ps29
Camelineae - ps30
Arabidopsis - ps31
Arabidopsis_thaliana - ps32
p-value
1e-04
6e-04
3e-03
1e-02
7e-02
Log-odds
0.5
1.0
1.5
2.0
2.5
3.0
3.5
4.0

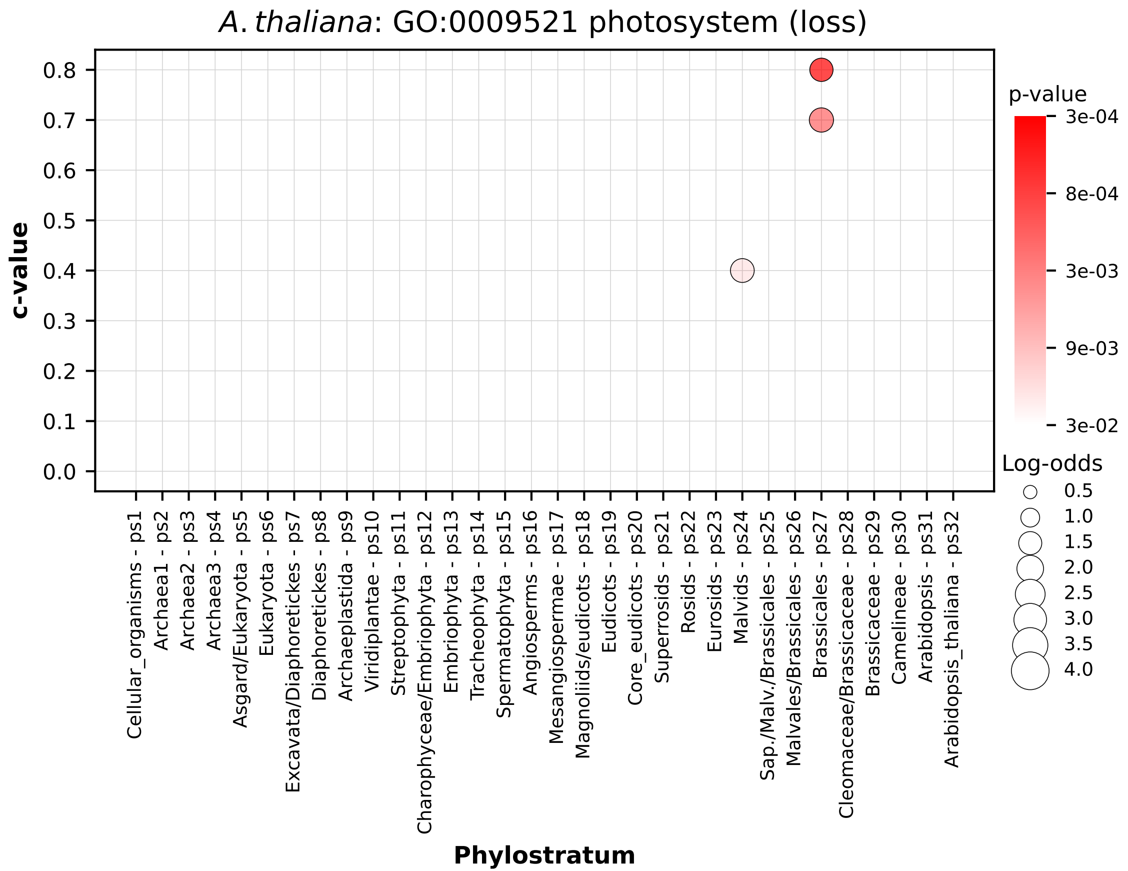
A. thaliana: GO:0009521 photosystem (loss)
c-value
0.0
0.1
0.2
0.3
0.4
0.5
0.6
0.7
0.8
p-value
3e-04
8e-04
3e-03
9e-03
3e-02
Log-odds
0.5
1.0
1.5
2.0
2.5
3.0
3.5
4.0
Cellular_organisms - ps1
Archaea1 - ps2
Archaea2 - ps3
Archaea3 - ps4
Asgard/Eukaryota - ps5
Eukaryota - ps6
Excavata/Diaphoretickes - ps7
Diaphoretickes - ps8
Archaeplastida - ps9
Viridiplantae - ps10
Streptophyta - ps11
Charophyceae/Embriophyta - ps12
Embriophyta - ps13
Tracheophyta - ps14
Spermatophyta - ps15
Angiosperms - ps16
Mesangiospermae - ps17
Magnoliids/eudicots - ps18
Eudicots - ps19
Core_eudicots - ps20
Superrosids - ps21
Rosids - ps22
Eurosids - ps23
Malvids - ps24
Sap./Malv./Brassicales - ps25
Malvales/Brassicales - ps26
Brassicales - ps27
Cleomaceae/Brassicaceae - ps28
Brassicaceae - ps29
Camelineae - ps30
Arabidopsis - ps31
Arabidopsis_thaliana - ps32
Phylostratum

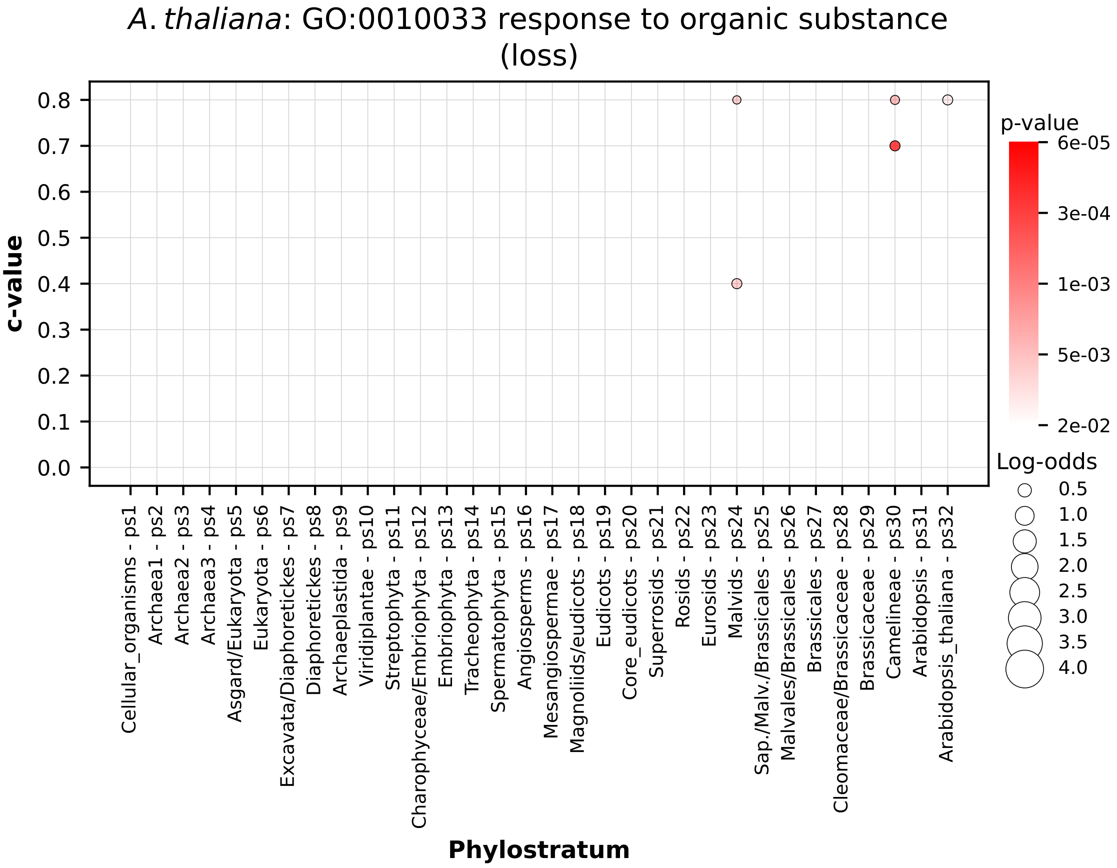
A. thaliana: GO:0010033 response to organic substance (loss)
c-value
0.8
0.7
0.6
0.5
0.4
0.3
0.2
0.1
0.0
p-value
6e-05
3e-04
1e-03
5e-03
2e-02
Log-odds
0.5
1.0
1.5
2.0
2.5
3.0
3.5
4.0
Cellular_organisms - ps1
Archaea1 - ps2
Archaea2 - ps3
Archaea3 - ps4
Asgard/Eukaryota - ps5
Eukaryota - ps6
Excavata/Diaphoretickes - ps7
Diaphoretickes - ps8
Archaeplastida - ps9
Viridiplantae - ps10
Streptophyta - ps11
Charophyceae/Embriophyta - ps12
Embriophyta - ps13
Tracheophyta - ps14
Spermatophyta - ps15
Angiosperms - ps16
Mesangiospermae - ps17
Magnoliids/eudicots - ps18
Eudicots - ps19
Core_eudicots - ps20
Superrosids - ps21
Rosids - ps22
Eurosids - ps23
Malvids - ps24
Sap./Malv./Brassicales - ps25
Malvales/Brassicales - ps26
Brassicales - ps27
Cleomaceae/Brassicaceae - ps28
Brassicaceae - ps29
Camelineae - ps30
Arabidopsis - ps31
Arabidopsis_thaliana - ps32
Phylostratum

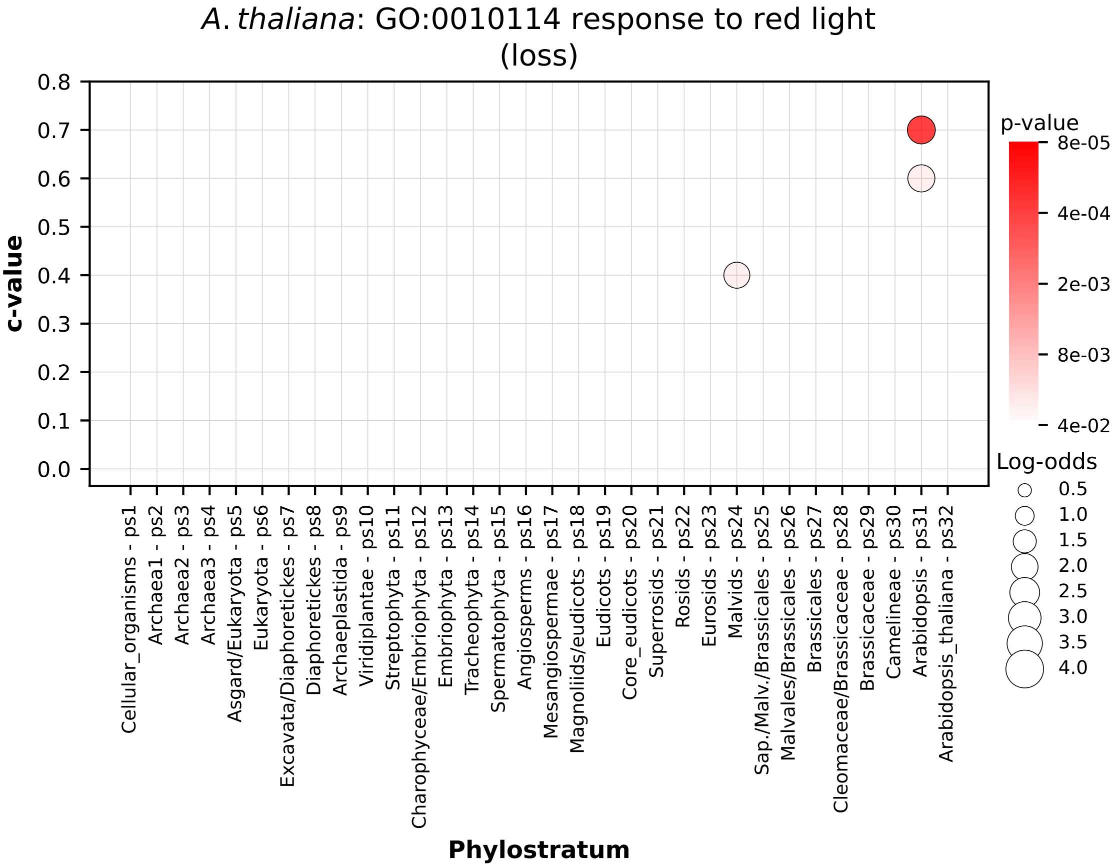

A. thaliana: GO:0010114 response to red light
(loss)
c-value
0.0
0.1
0.2
0.3
0.4
0.5
0.6
0.7
0.8
p-value
8e-05
4e-04
2e-03
8e-03
4e-02
Log-odds
0.5
1.0
1.5
2.0
2.5
3.0
3.5
4.0
Cellular_organisms - ps1
Archaea1 - ps2
Archaea2 - ps3
Archaea3 - ps4
Asgard/Eukaryota - ps5
Eukaryota - ps6
Excavata/Diaphoretickes - ps7
Diaphoretickes - ps8
Archaeplastida - ps9
Viridiplantae - ps10
Streptophyta - ps11
Charophyceae/Embriophyta - ps12
Embriophyta - ps13
Tracheophyta - ps14
Spermatophyta - ps15
Angiosperms - ps16
Mesangiospermae - ps17
Magnoliids/eudicots - ps18
Eudicots - ps19
Core_eudicots - ps20
Superrosids - ps21
Rosids - ps22
Eurosids - ps23
Malvids - ps24
Sap./Malv./Brassicales - ps25
Malvales/Brassicales - ps26
Brassicales - ps27
Cleomaceae/Brassicaceae - ps28
Brassicaceae - ps29
Camelineae - ps30
Arabidopsis - ps31
Arabidopsis_thaliana - ps32
Phylostratum

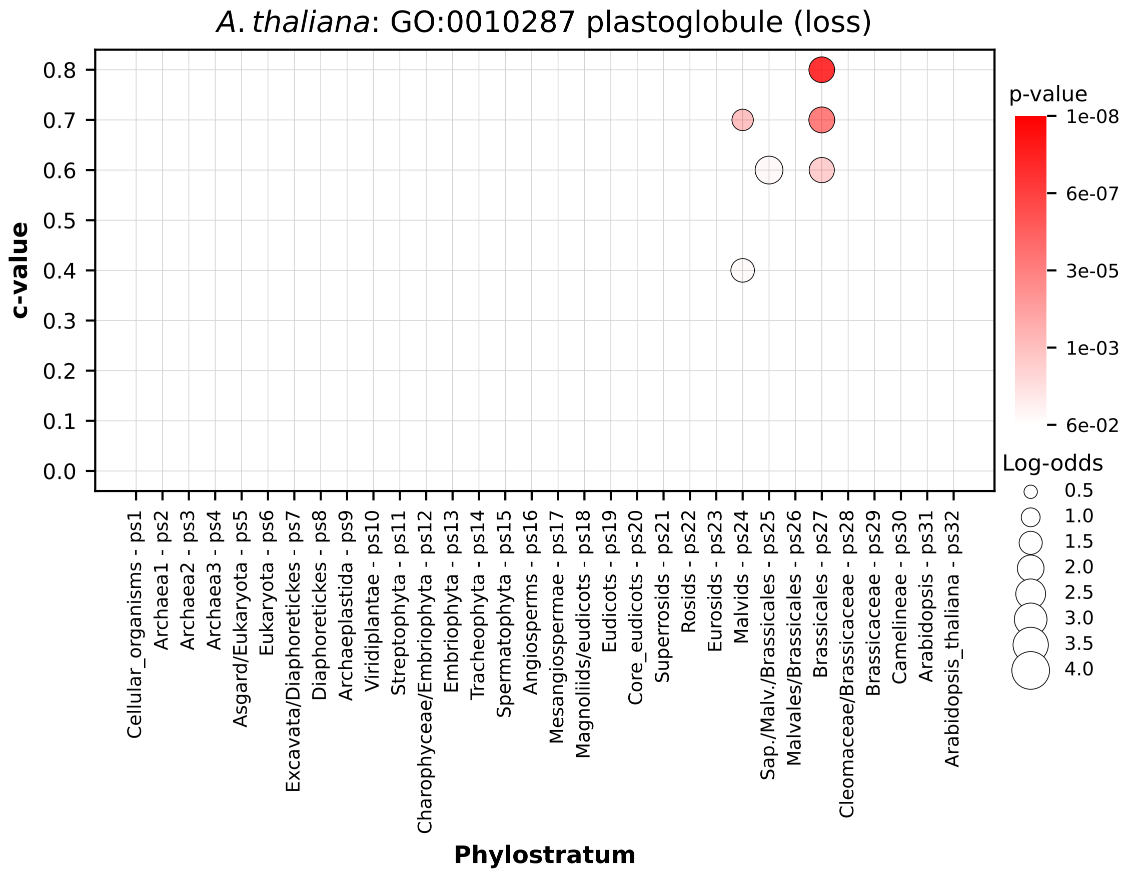

A. thaliana: GO:0010287 plastoglobule (loss)
c-value
0.8
0.7
0.6
0.5
0.4
0.3
0.2
0.1
0.0
p-value
1e-08
6e-07
3e-05
1e-03
6e-02
Log-odds
0.5
1.0
1.5
2.0
2.5
3.0
3.5
4.0
Cellular_organisms - ps1
Archaea1 - ps2
Archaea2 - ps3
Archaea3 - ps4
Asgard/Eukaryota - ps5
Eukaryota - ps6
Excavata/Diaphoretickes - ps7
Diaphoretickes - ps8
Archaeplastida - ps9
Viridiplantae - ps10
Streptophyta - ps11
Charophyceae/Embriophyta - ps12
Embriophyta - ps13
Tracheophyta - ps14
Spermatophyta - ps15
Angiosperms - ps16
Mesangiospermae - ps17
Magnoliids/eudicots - ps18
Eudicots - ps19
Core_eudicots - ps20
Superrosids - ps21
Rosids - ps22
Eurosids - ps23
Malvids - ps24
Sap./Malv./Brassicales - ps25
Malvales/Brassicales - ps26
Brassicales - ps27
Cleomaceae/Brassicaceae - ps28
Brassicaceae - ps29
Camelineae - ps30
Arabidopsis - ps31
Arabidopsis_thaliana - ps32
Phylostratum

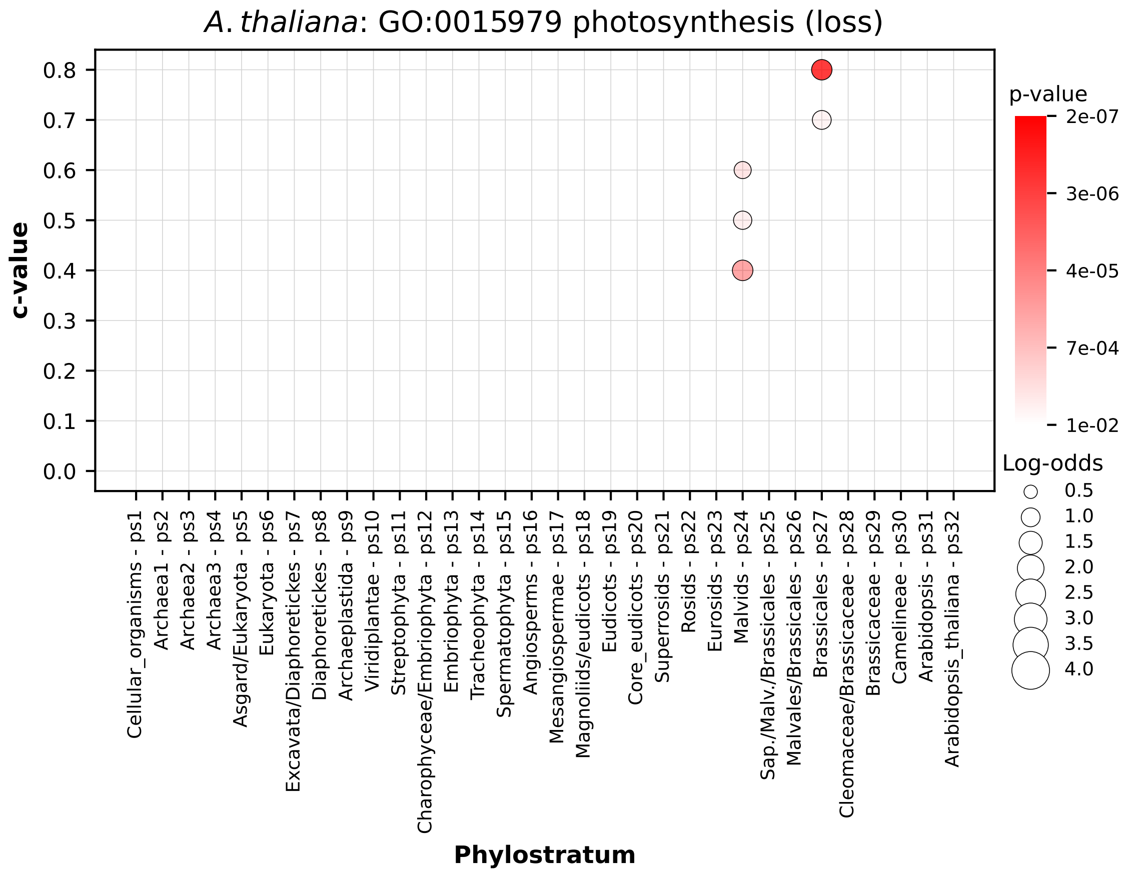

A. thaliana: GO:0015979 photosynthesis (loss)
c-value
0.8
0.7
0.6
0.5
0.4
0.3
0.2
0.1
0.0
p-value
2e-07
3e-06
4e-05
7e-04
1e-02
Log-odds
0.5
1.0
1.5
2.0
2.5
3.0
3.5
4.0
Cellular_organisms - ps1
Archaea1 - ps2
Archaea2 - ps3
Archaea3 - ps4
Asgard/Eukaryota - ps5
Eukaryota - ps6
Excavata/Diaphoretickes - ps7
Diaphoretickes - ps8
Archaeplastida - ps9
Viridiplantae - ps10
Streptophyta - ps11
Charophyceae/Embriophyta - ps12
Embriophyta - ps13
Tracheophyta - ps14
Spermatophyta - ps15
Angiosperms - ps16
Mesangiospermae - ps17
Magnoliids/eudicots - ps18
Eudicots - ps19
Core_eudicots - ps20
Superrosids - ps21
Rosids - ps22
Eurosids - ps23
Malvids - ps24
Sap./Malv./Brassicales - ps25
Malvales/Brassicales - ps26
Brassicales - ps27
Cleomaceae/Brassicaceae - ps28
Brassicaceae - ps29
Camelineae - ps30
Arabidopsis - ps31
Arabidopsis_thaliana - ps32
Phylostratum

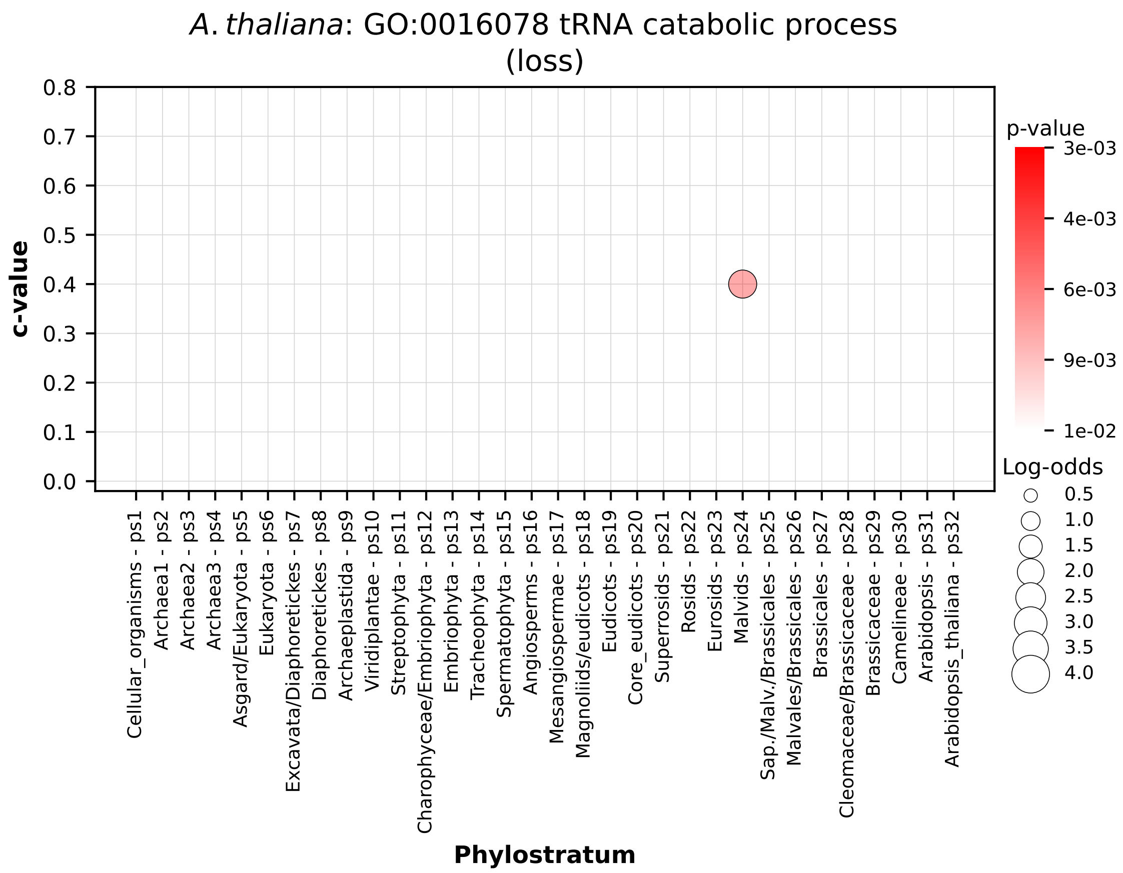

A. thaliana: GO:0016078 tRNA catabolic process
(loss)
c-value
0.8
0.7
0.6
0.5
0.4
0.3
0.2
0.1
0.0
Phylostratum
Cellular_organisms - ps1
Archaea1 - ps2
Archaea2 - ps3
Archaea3 - ps4
Asgard/Eukaryota - ps5
Eukaryota - ps6
Excavata/Diaphoretickes - ps7
Diaphoretickes - ps8
Archaeplastida - ps9
Viridiplantae - ps10
Streptophyta - ps11
Charophyceae/Embriophyta - ps12
Embriophyta - ps13
Tracheophyta - ps14
Spermatophyta - ps15
Angiosperms - ps16
Mesangiospermae - ps17
Magnoliids/eudicots - ps18
Eudicots - ps19
Core_eudicots - ps20
Superrosids - ps21
Rosids - ps22
Eurosids - ps23
Malvids - ps24
Sap./Malv./Brassicales - ps25
Malvales/Brassicales - ps26
Brassicales - ps27
Cleomaceae/Brassicaceae - ps28
Brassicaceae - ps29
Camelineae - ps30
Arabidopsis - ps31
Arabidopsis_thaliana - ps32
p-value
3e-03
4e-03
6e-03
9e-03
1e-02
Log-odds
0.5
1.0
1.5
2.0
2.5
3.0
3.5
4.0

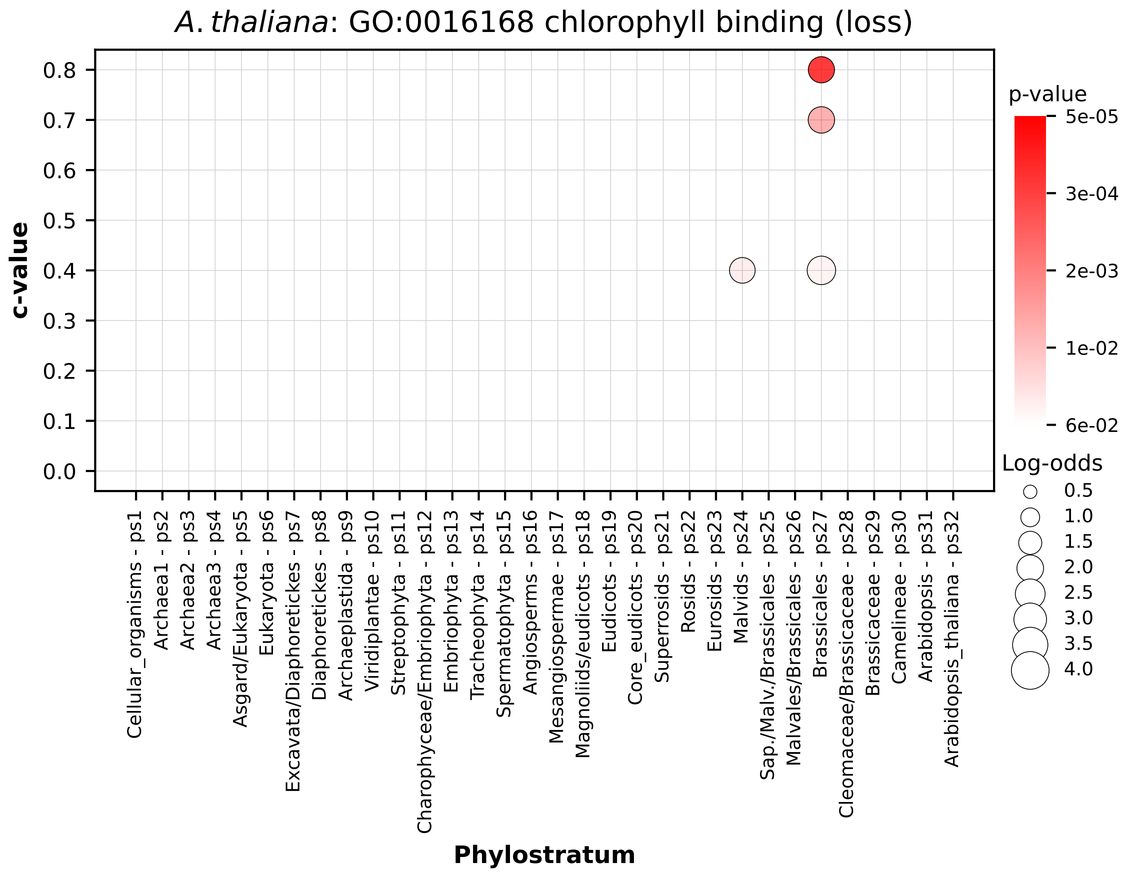

A. thaliana: GO:0016168 chlorophyll binding (loss)
c-value
0.8
0.7
0.6
0.5
0.4
0.3
0.2
0.1
0.0
p-value
5e-05
3e-04
2e-03
1e-02
6e-02
Log-odds
0.5
1.0
1.5
2.0
2.5
3.0
3.5
4.0
Cellular_organisms - ps1
Archaea1 - ps2
Archaea2 - ps3
Archaea3 - ps4
Asgard/Eukaryota - ps5
Eukaryota - ps6
Excavata/Diaphoretickes - ps7
Diaphoretickes - ps8
Archaeplastida - ps9
Viridiplantae - ps10
Streptophyta - ps11
Charophyceae/Embriophyta - ps12
Embriophyta - ps13
Tracheophyta - ps14
Spermatophyta - ps15
Angiosperms - ps16
Mesangiospermae - ps17
Magnoliids/eudicots - ps18
Eudicots - ps19
Core_eudicots - ps20
Superrosids - ps21
Rosids - ps22
Eurosids - ps23
Malvids - ps24
Sap./Malv./Brassicales - ps25
Malvales/Brassicales - ps26
Brassicales - ps27
Cleomaceae/Brassicaceae - ps28
Brassicaceae - ps29
Camelineae - ps30
Arabidopsis - ps31
Arabidopsis_thaliana - ps32
Phylostratum

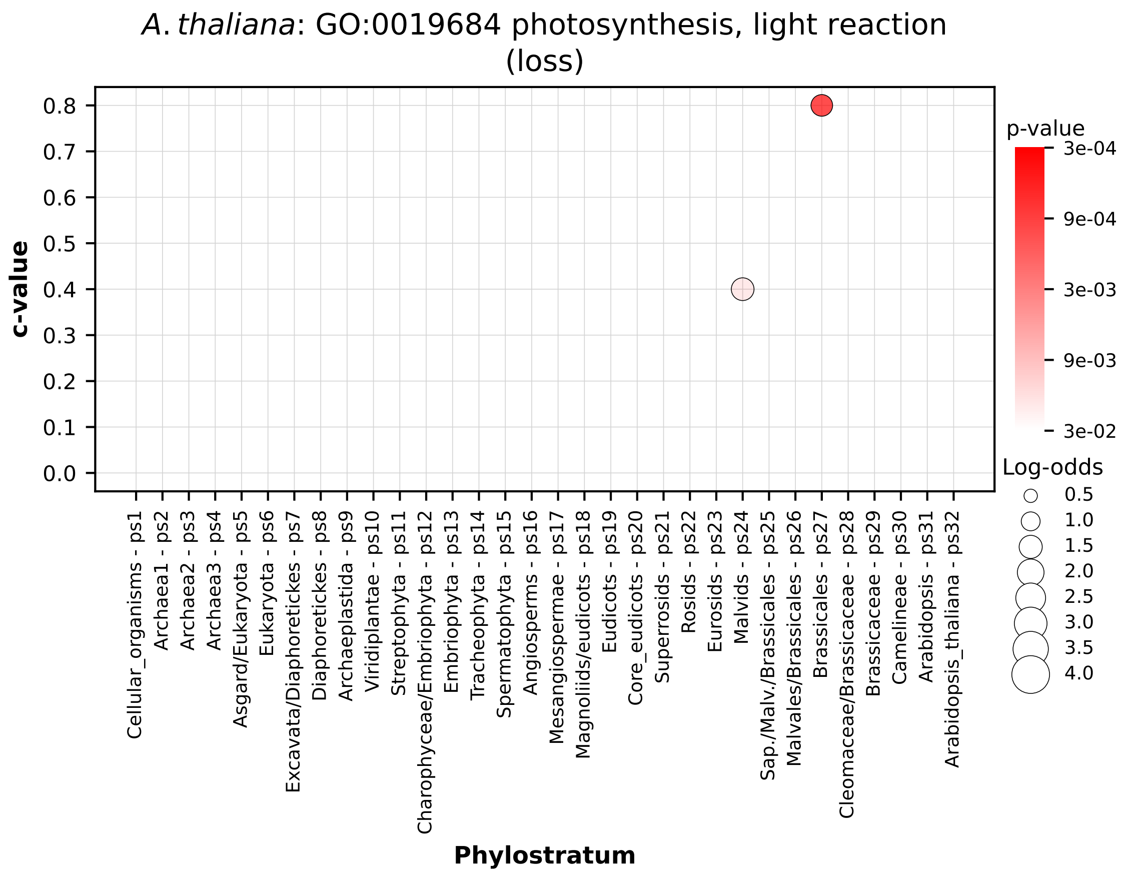

A. thaliana: GO:0019684 photosynthesis, light reaction
(loss)
c-value
0.0
0.1
0.2
0.3
0.4
0.5
0.6
0.7
0.8
p-value
3e-04
9e-04
3e-03
9e-03
3e-02
Log-odds
0.5
1.0
1.5
2.0
2.5
3.0
3.5
4.0
Cellular_organisms - ps1
Archaea1 - ps2
Archaea2 - ps3
Archaea3 - ps4
Asgard/Eukaryota - ps5
Eukaryota - ps6
Excavata/Diaphoretickes - ps7
Diaphoretickes - ps8
Archaeplastida - ps9
Viridiplantae - ps10
Streptophyta - ps11
Charophyceae/Embriophyta - ps12
Embriophyta - ps13
Tracheophyta - ps14
Spermatophyta - ps15
Angiosperms - ps16
Mesangiospermae - ps17
Magnoliids/eudicots - ps18
Eudicots - ps19
Core_eudicots - ps20
Superrosids - ps21
Rosids - ps22
Eurosids - ps23
Malvids - ps24
Sap./Malv./Brassicales - ps25
Malvales/Brassicales - ps26
Brassicales - ps27
Cleomaceae/Brassicaceae - ps28
Brassicaceae - ps29
Camelineae - ps30
Arabidopsis - ps31
Arabidopsis_thaliana - ps32
Phylostratum

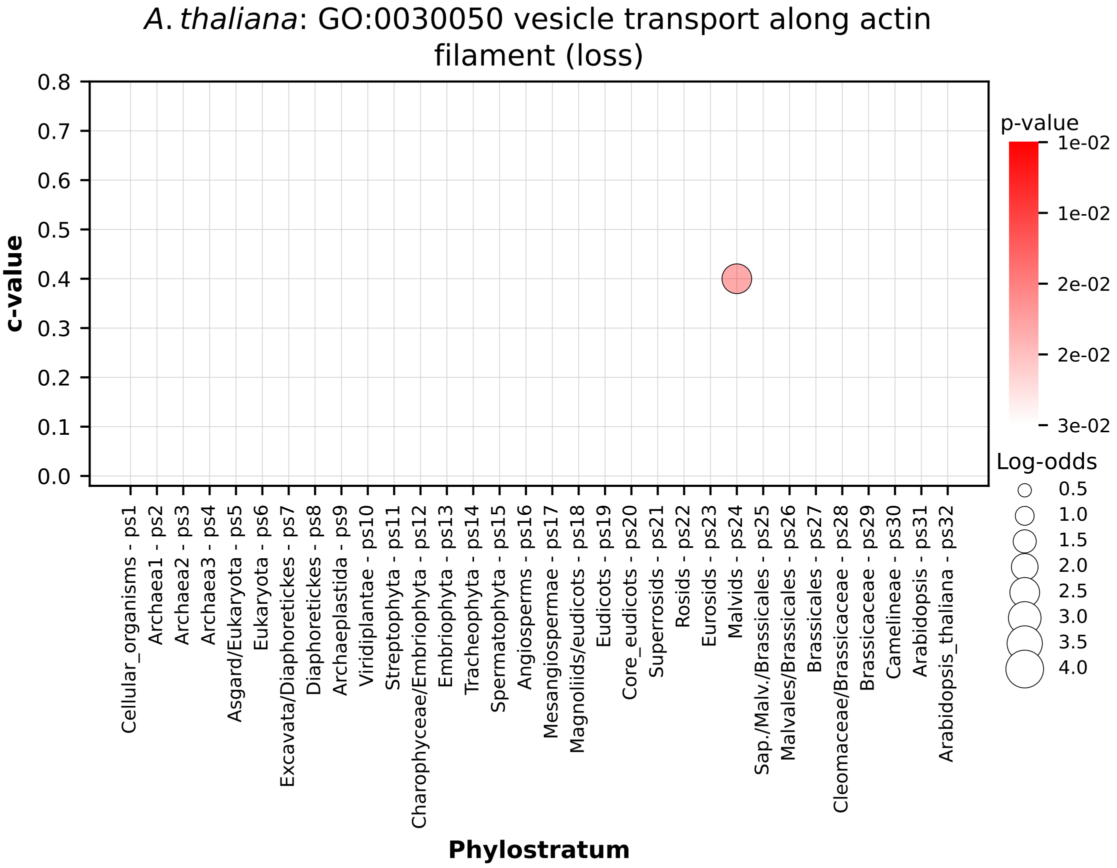

A. thaliana: GO:0030050 vesicle transport along actin filament (loss)
c-value
0.8
0.7
0.6
0.5
0.4
0.3
0.2
0.1
0.0
p-value
1e-02
1e-02
2e-02
2e-02
3e-02
Log-odds
0.5
1.0
1.5
2.0
2.5
3.0
3.5
4.0
Cellular_organisms - ps1
Archaea1 - ps2
Archaea2 - ps3
Archaea3 - ps4
Asgard/Eukaryota - ps5
Eukaryota - ps6
Excavata/Diaphoretickes - ps7
Diaphoretickes - ps8
Archaeplastida - ps9
Viridiplantae - ps10
Streptophyta - ps11
Charophyceae/Embriophyta - ps12
Embriophyta - ps13
Tracheophyta - ps14
Spermatophyta - ps15
Angiosperms - ps16
Mesangiospermae - ps17
Magnoliids/eudicots - ps18
Eudicots - ps19
Core_eudicots - ps20
Superrosids - ps21
Rosids - ps22
Eurosids - ps23
Malvids - ps24
Sap./Malv./Brassicales - ps25
Malvales/Brassicales - ps26
Brassicales - ps27
Cleomaceae/Brassicaceae - ps28
Brassicaceae - ps29
Camelineae - ps30
Arabidopsis - ps31
Arabidopsis_thaliana - ps32
Phylostratum

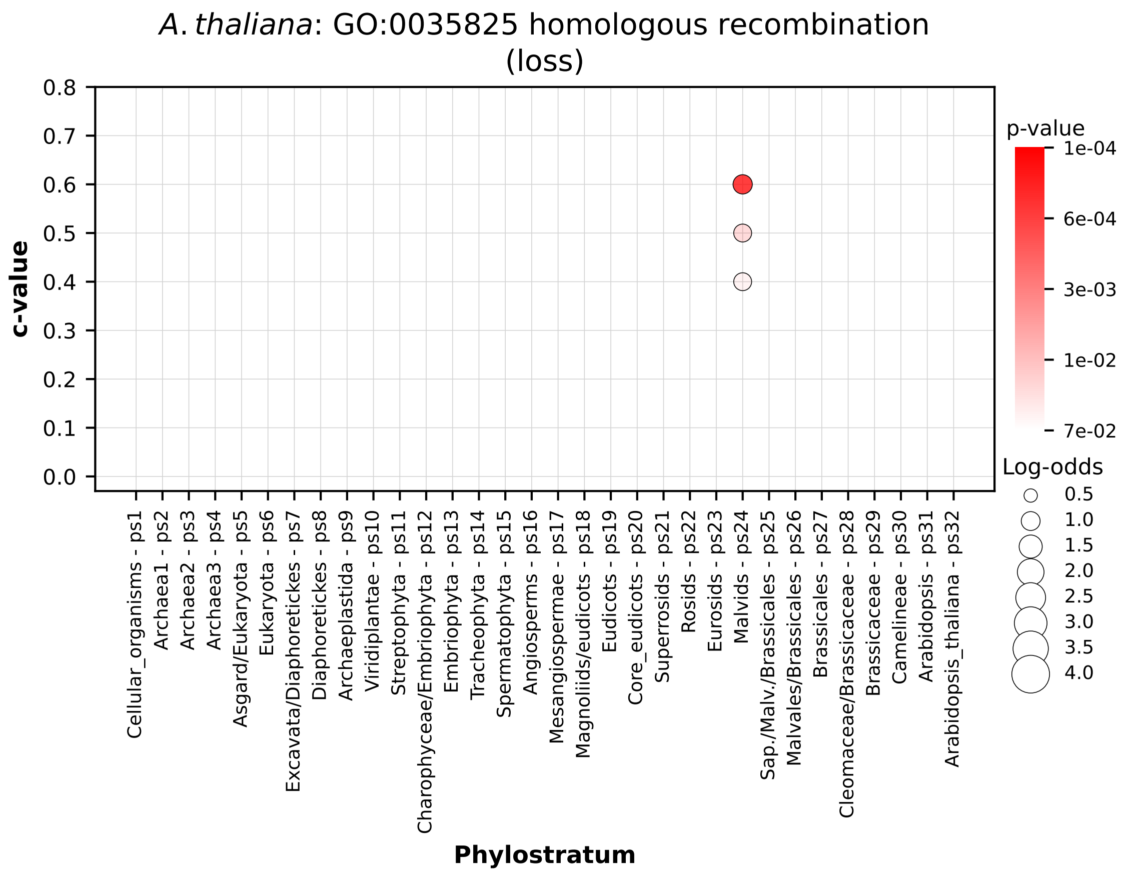

A. thaliana: GO:0035825 homologous recombination
(loss)
c-value
0.0
0.1
0.2
0.3
0.4
0.5
0.6
0.7
0.8
Phylostratum
Cellular_organisms - ps1
Archaea1 - ps2
Archaea2 - ps3
Archaea3 - ps4
Asgard/Eukaryota - ps5
Eukaryota - ps6
Excavata/Diaphoretickes - ps7
Diaphoretickes - ps8
Archaeplastida - ps9
Viridiplantae - ps10
Streptophyta - ps11
Charophyceae/Embriophyta - ps12
Embriophyta - ps13
Tracheophyta - ps14
Spermatophyta - ps15
Angiosperms - ps16
Mesangiospermae - ps17
Magnoliids/eudicots - ps18
Eudicots - ps19
Core_eudicots - ps20
Superrosids - ps21
Rosids - ps22
Eurosids - ps23
Malvids - ps24
Sap./Malv./Brassicales - ps25
Malvales/Brassicales - ps26
Brassicales - ps27
Cleomaceae/Brassicaceae - ps28
Brassicaceae - ps29
Camelineae - ps30
Arabidopsis - ps31
Arabidopsis_thaliana - ps32
p-value
1e-04
6e-04
3e-03
1e-02
7e-02
Log-odds
0.5
1.0
1.5
2.0
2.5
3.0
3.5
4.0

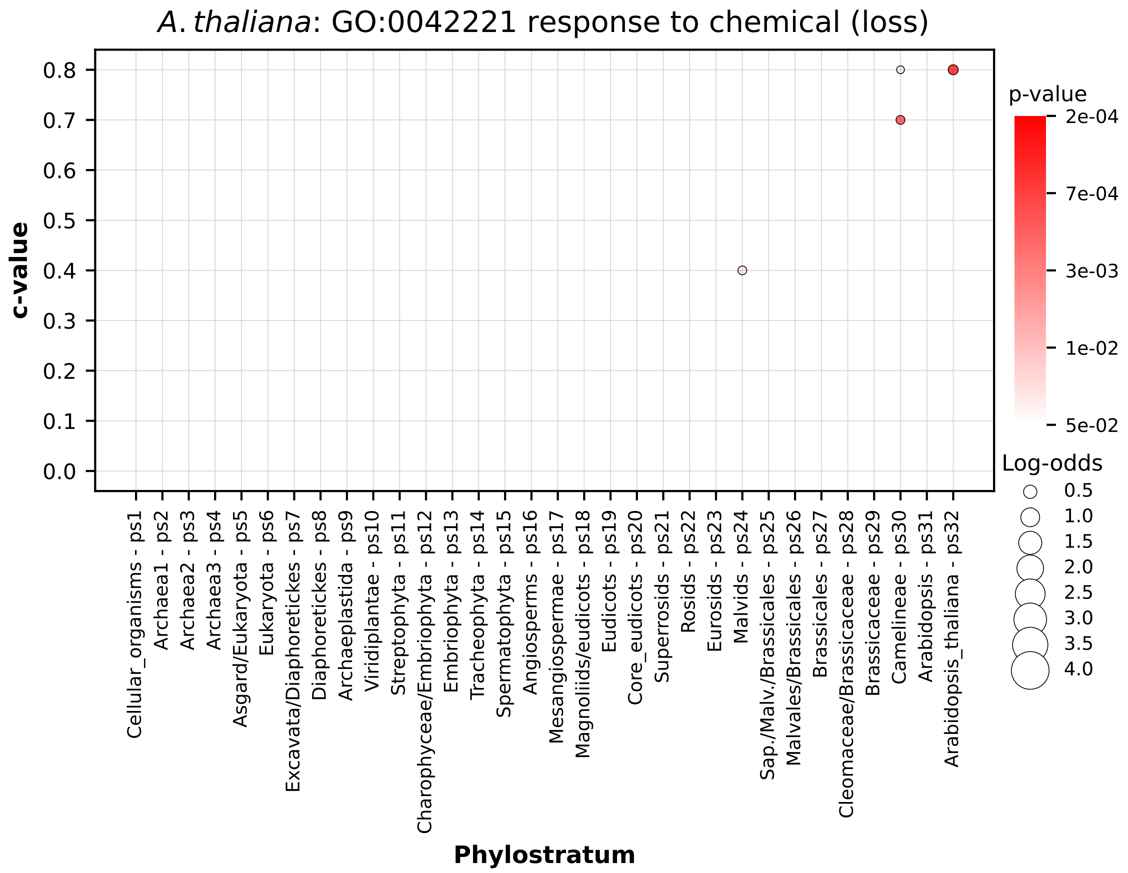

A. thaliana: GO:0042221 response to chemical (loss)
c-value
0.0
0.1
0.2
0.3
0.4
0.5
0.6
0.7
0.8
Phylostratum
Cellular_organisms - ps1
Archaea1 - ps2
Archaea2 - ps3
Archaea3 - ps4
Asgard/Eukaryota - ps5
Eukaryota - ps6
Excavata/Diaphoretickes - ps7
Diaphoretickes - ps8
Archaeplastida - ps9
Viridiplantae - ps10
Streptophyta - ps11
Charophyceae/Embriophyta - ps12
Embriophyta - ps13
Tracheophyta - ps14
Spermatophyta - ps15
Angiosperms - ps16
Mesangiospermae - ps17
Magnoliids/eudicots - ps18
Eudicots - ps19
Core_eudicots - ps20
Superrosids - ps21
Rosids - ps22
Eurosids - ps23
Malvids - ps24
Sap./Malv./Brassicales - ps25
Malvales/Brassicales - ps26
Brassicales - ps27
Cleomaceae/Brassicaceae - ps28
Brassicaceae - ps29
Camelineae - ps30
Arabidopsis - ps31
Arabidopsis_thaliana - ps32
p-value
2e-04
7e-04
3e-03
1e-02
5e-02
Log-odds
0.5
1.0
1.5
2.0
2.5
3.0
3.5
4.0

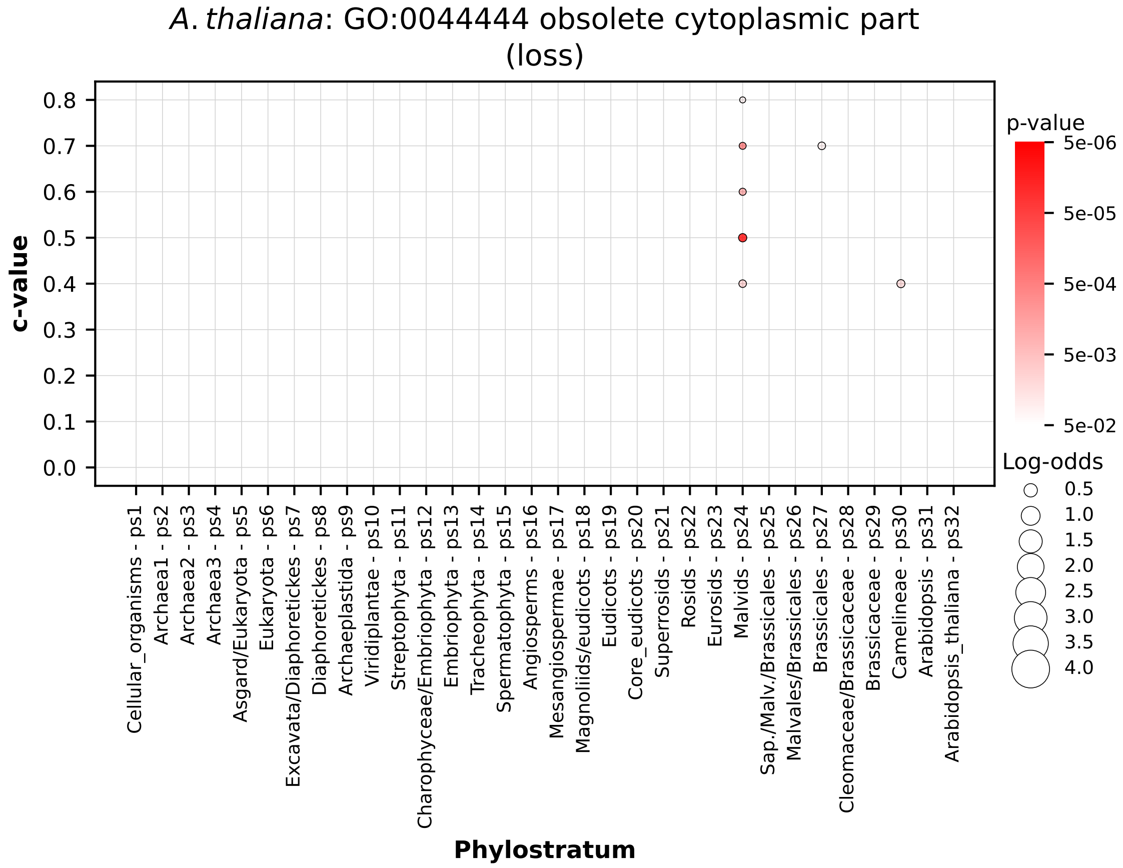

A. thaliana: GO:0044444 obsolete cytoplasmic part
(loss)
c-value
0.0
0.1
0.2
0.3
0.4
0.5
0.6
0.7
0.8
Phylostratum
Cellular_organisms - ps1
Archaea1 - ps2
Archaea2 - ps3
Archaea3 - ps4
Asgard/Eukaryota - ps5
Eukaryota - ps6
Excavata/Diaphoretickes - ps7
Diaphoretickes - ps8
Archaeplastida - ps9
Viridiplantae - ps10
Streptophyta - ps11
Charophyceae/Embriophyta - ps12
Embriophyta - ps13
Tracheophyta - ps14
Spermatophyta - ps15
Angiosperms - ps16
Mesangiospermae - ps17
Magnoliids/eudicots - ps18
Eudicots - ps19
Core_eudicots - ps20
Superrosids - ps21
Rosids - ps22
Eurosids - ps23
Malvids - ps24
Sap./Malv./Brassicales - ps25
Malvales/Brassicales - ps26
Brassicales - ps27
Cleomaceae/Brassicaceae - ps28
Brassicaceae - ps29
Camelineae - ps30
Arabidopsis - ps31
Arabidopsis_thaliana - ps32
p-value
5e-06
5e-05
5e-04
5e-03
5e-02
Log-odds
0.5
1.0
1.5
2.0
2.5
3.0
3.5
4.0

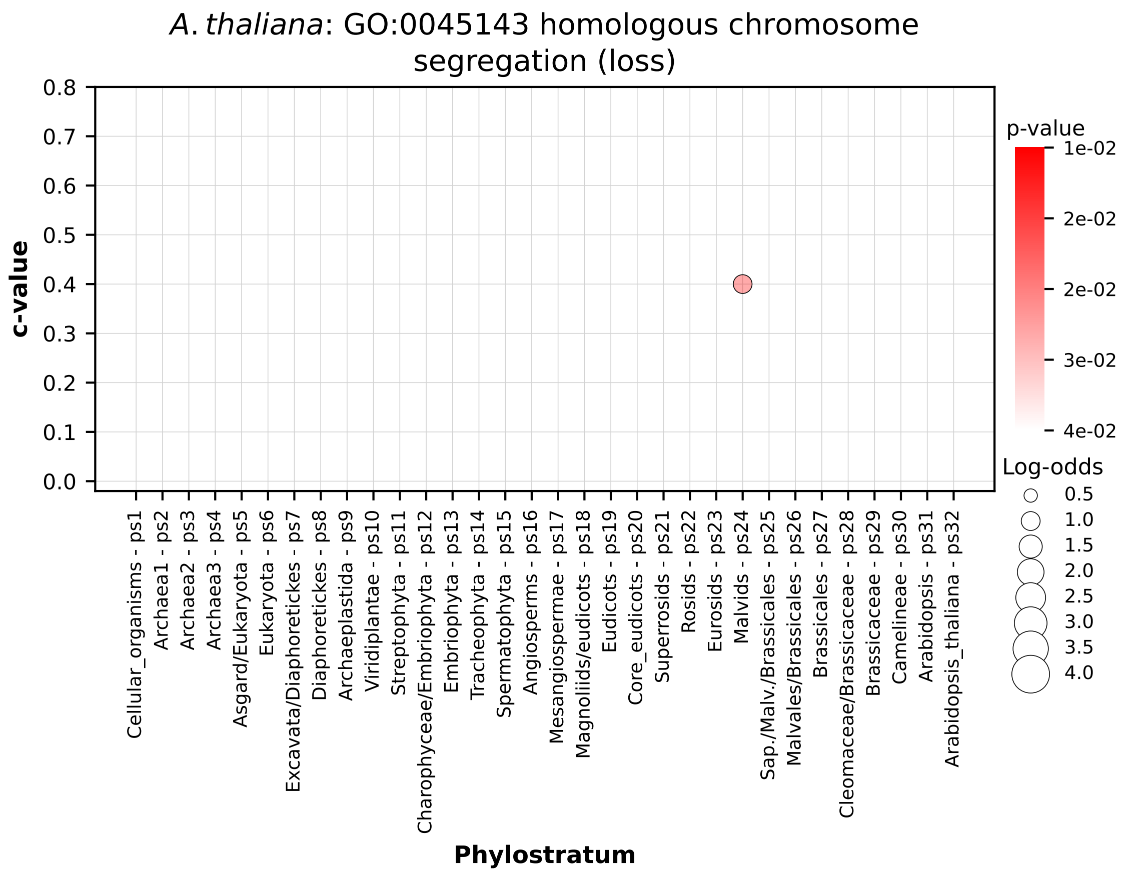

A. thaliana: GO:0045143 homologous chromosome segregation (loss)
c-value
0.8
0.7
0.6
0.5
0.4
0.3
0.2
0.1
0.0
p-value
1e-02
2e-02
2e-02
3e-02
4e-02
Log-odds
0.5
1.0
1.5
2.0
2.5
3.0
3.5
4.0
Cellular_organisms - ps1
Archaea1 - ps2
Archaea2 - ps3
Archaea3 - ps4
Asgard/Eukaryota - ps5
Eukaryota - ps6
Excavata/Diaphoretickes - ps7
Diaphoretickes - ps8
Archaeplastida - ps9
Viridiplantae - ps10
Streptophyta - ps11
Charophyceae/Embriophyta - ps12
Embriophyta - ps13
Tracheophyta - ps14
Spermatophyta - ps15
Angiosperms - ps16
Mesangiospermae - ps17
Magnoliids/eudicots - ps18
Eudicots - ps19
Core_eudicots - ps20
Superrosids - ps21
Rosids - ps22
Eurosids - ps23
Malvids - ps24
Sap./Malv./Brassicales - ps25
Malvales/Brassicales - ps26
Brassicales - ps27
Cleomaceae/Brassicaceae - ps28
Brassicaceae - ps29
Camelineae - ps30
Arabidopsis - ps31
Arabidopsis_thaliana - ps32
Phylostratum

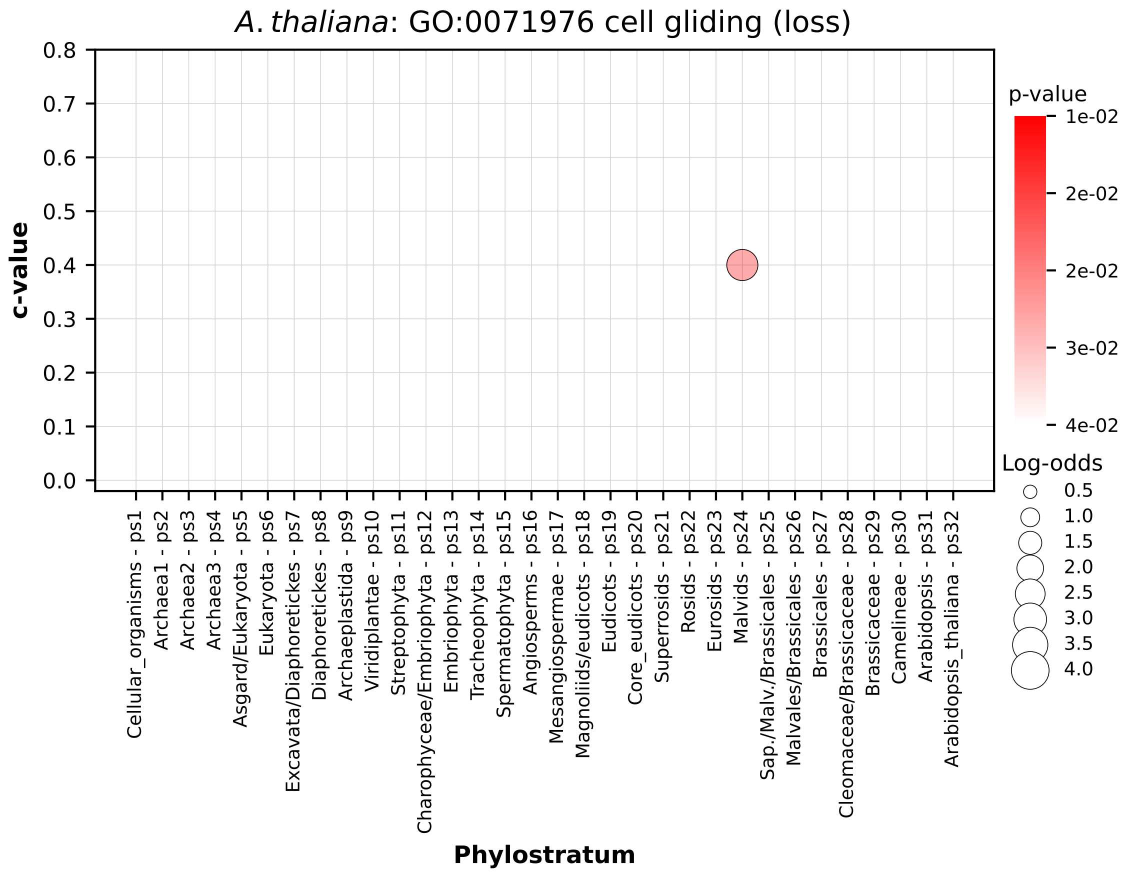

A. thaliana: GO:0071976 cell gliding (loss)
c-value
0.0
0.1
0.2
0.3
0.4
0.5
0.6
0.7
0.8
Phylostratum
Cellular_organisms - ps1
Archaea1 - ps2
Archaea2 - ps3
Archaea3 - ps4
Asgard/Eukaryota - ps5
Eukaryota - ps6
Excavata/Diaphoretickes - ps7
Diaphoretickes - ps8
Archaeplastida - ps9
Viridiplantae - ps10
Streptophyta - ps11
Charophyceae/Embriophyta - ps12
Embriophyta - ps13
Tracheophyta - ps14
Spermatophyta - ps15
Angiosperms - ps16
Mesangiospermae - ps17
Magnoliids/eudicots - ps18
Eudicots - ps19
Core_eudicots - ps20
Superrosids - ps21
Rosids - ps22
Eurosids - ps23
Malvids - ps24
Sap./Malv./Brassicales - ps25
Malvales/Brassicales - ps26
Brassicales - ps27
Cleomaceae/Brassicaceae - ps28
Brassicaceae - ps29
Camelineae - ps30
Arabidopsis - ps31
Arabidopsis_thaliana - ps32
p-value
1e-02
2e-02
2e-02
3e-02
4e-02
Log-odds
0.5
1.0
1.5
2.0
2.5
3.0
3.5
4.0

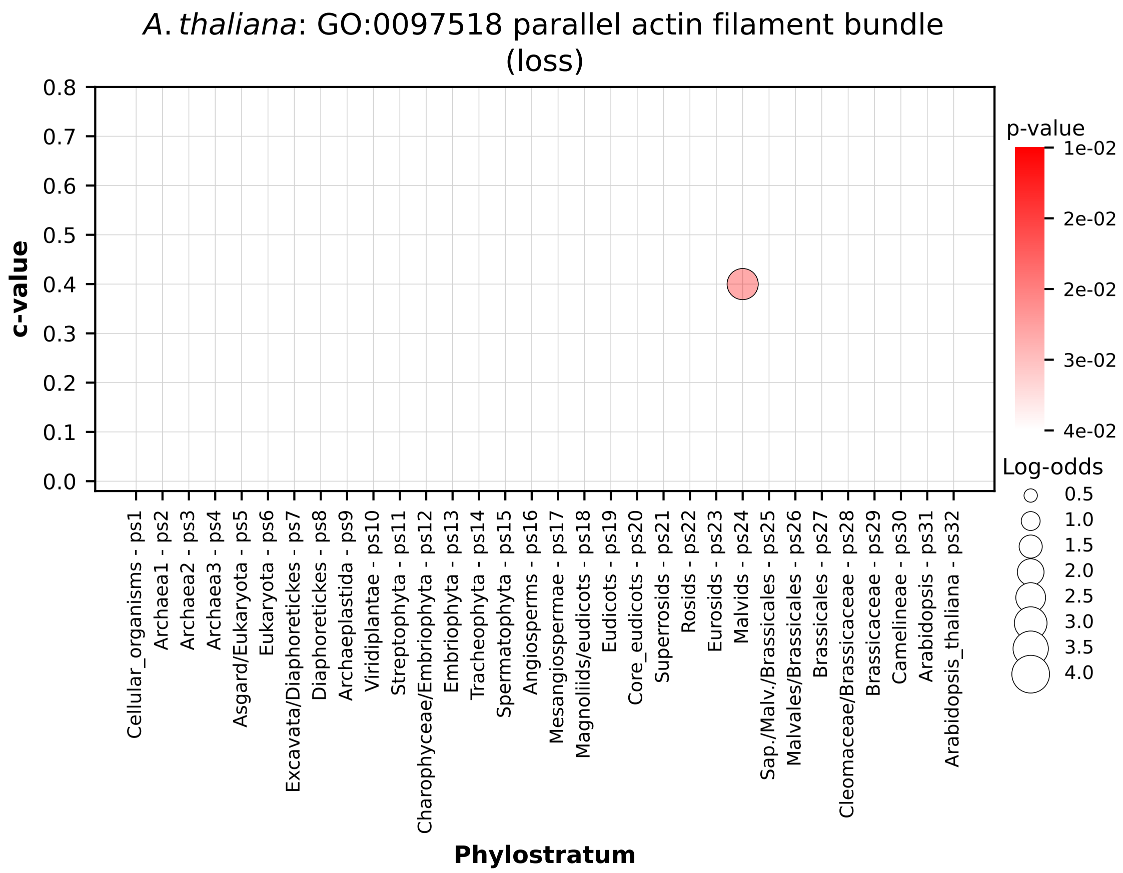
A. thaliana: GO:0097518 parallel actin filament bundle
(loss)
c-value
0.0
0.1
0.2
0.3
0.4
0.5
0.6
0.7
0.8
Phylostratum
Cellular_organisms - ps1
Archaea1 - ps2
Archaea2 - ps3
Archaea3 - ps4
Asgard/Eukaryota - ps5
Eukaryota - ps6
Excavata/Diaphoretickes - ps7
Diaphoretickes - ps8
Archaeplastida - ps9
Viridiplantae - ps10
Streptophyta - ps11
Charophyceae/Embriophyta - ps12
Embriophyta - ps13
Tracheophyta - ps14
Spermatophyta - ps15
Angiosperms - ps16
Mesangiospermae - ps17
Magnoliids/eudicots - ps18
Eudicots - ps19
Core_eudicots - ps20
Superrosids - ps21
Rosids - ps22
Eurosids - ps23
Malvids - ps24
Sap./Malv./Brassicales - ps25
Malvales/Brassicales - ps26
Brassicales - ps27
Cleomaceae/Brassicaceae - ps28
Brassicaceae - ps29
Camelineae - ps30
Arabidopsis - ps31
Arabidopsis_thaliana - ps32
p-value
1e-02
2e-02
2e-02
3e-02
4e-02
Log-odds
0.5
1.0
1.5
2.0
2.5
3.0
3.5
4.0

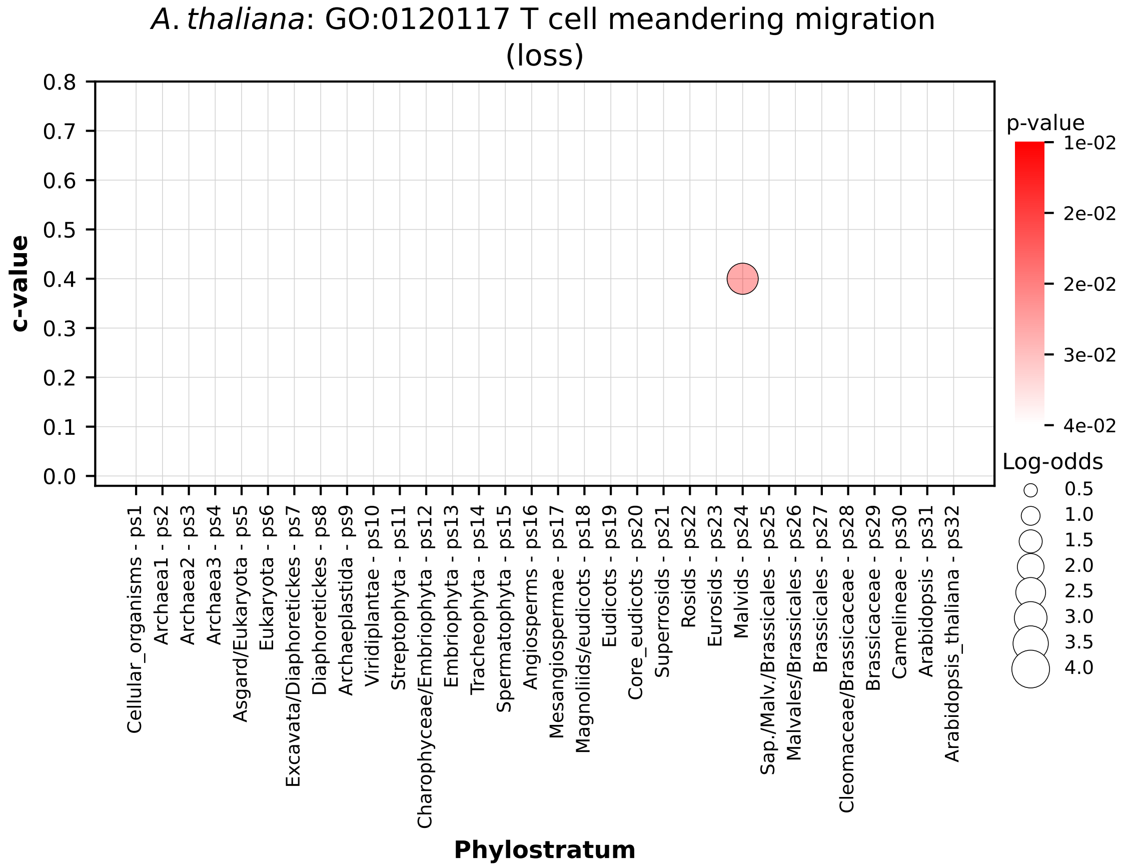

A. thaliana: GO:0120117 T cell meandering migration
(loss)
c-value
0.8
0.7
0.6
0.5
0.4
0.3
0.2
0.1
0.0
p-value
1e-02
2e-02
2e-02
3e-02
4e-02
Log-odds
0.5
1.0
1.5
2.0
2.5
3.0
3.5
4.0
Cellular_organisms - ps1
Archaea1 - ps2
Archaea2 - ps3
Archaea3 - ps4
Asgard/Eukaryota - ps5
Eukaryota - ps6
Excavata/Diaphoretickes - ps7
Diaphoretickes - ps8
Archaeplastida - ps9
Viridiplantae - ps10
Streptophyta - ps11
Charophyceae/Embriophyta - ps12
Embriophyta - ps13
Tracheophyta - ps14
Spermatophyta - ps15
Angiosperms - ps16
Mesangiospermae - ps17
Magnoliids/eudicots - ps18
Eudicots - ps19
Core_eudicots - ps20
Superrosids - ps21
Rosids - ps22
Eurosids - ps23
Malvids - ps24
Sap./Malv./Brassicales - ps25
Malvales/Brassicales - ps26
Brassicales - ps27
Cleomaceae/Brassicaceae - ps28
Brassicaceae - ps29
Camelineae - ps30
Arabidopsis - ps31
Arabidopsis_thaliana - ps32
Phylostratum

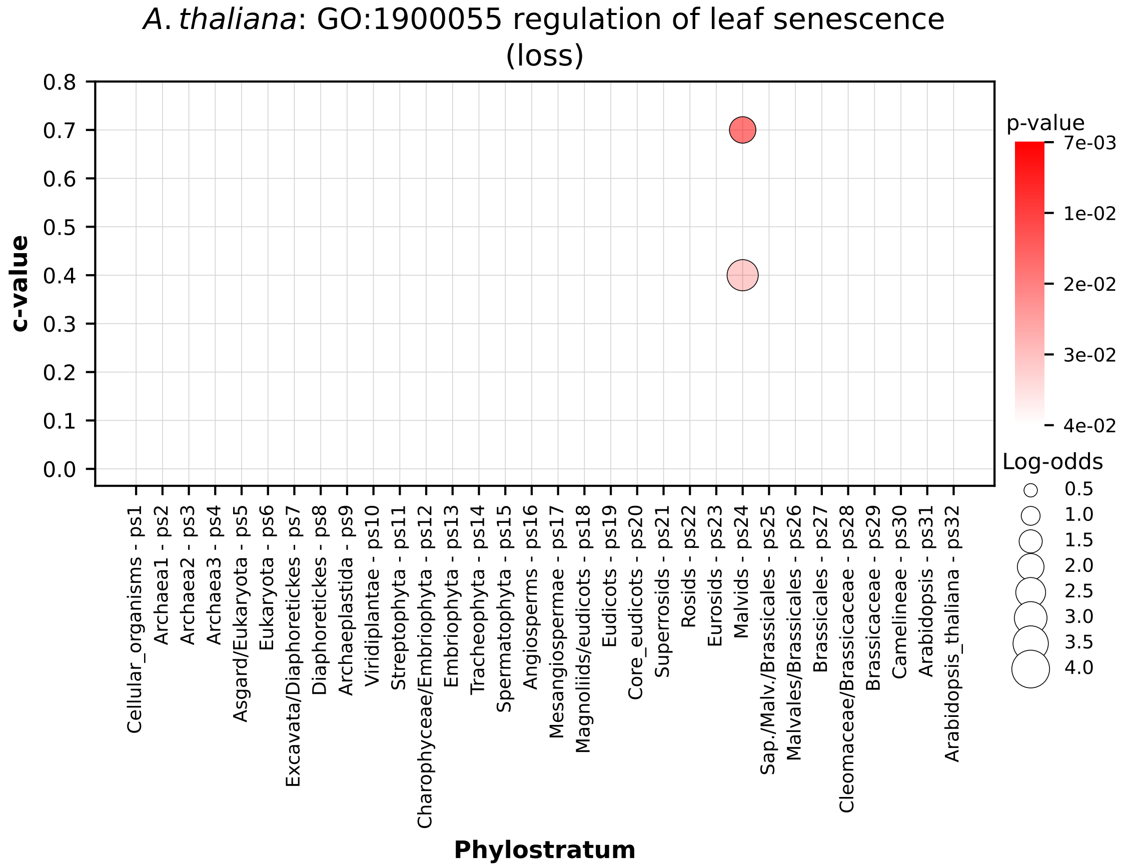

A. thaliana: GO:1900055 regulation of leaf senescence
(loss)
c-value
0.8
0.7
0.6
0.5
0.4
0.3
0.2
0.1
0.0
p-value
7e-03
1e-02
2e-02
3e-02
4e-02
Log-odds
0.5
1.0
1.5
2.0
2.5
3.0
3.5
4.0
Cellular_organisms - ps1
Archaea1 - ps2
Archaea2 - ps3
Archaea3 - ps4
Asgard/Eukaryota - ps5
Eukaryota - ps6
Excavata/Diaphoretickes - ps7
Diaphoretickes - ps8
Archaeplastida - ps9
Viridiplantae - ps10
Streptophyta - ps11
Charophyceae/Embriophyta - ps12
Embriophyta - ps13
Tracheophyta - ps14
Spermatophyta - ps15
Angiosperms - ps16
Mesangiospermae - ps17
Magnoliids/eudicots - ps18
Eudicots - ps19
Core_eudicots - ps20
Superrosids - ps21
Rosids - ps22
Eurosids - ps23
Malvids - ps24
Sap./Malv./Brassicales - ps25
Malvales/Brassicales - ps26
Brassicales - ps27
Cleomaceae/Brassicaceae - ps28
Brassicaceae - ps29
Camelineae - ps30
Arabidopsis - ps31
Arabidopsis_thaliana - ps32
Phylostratum

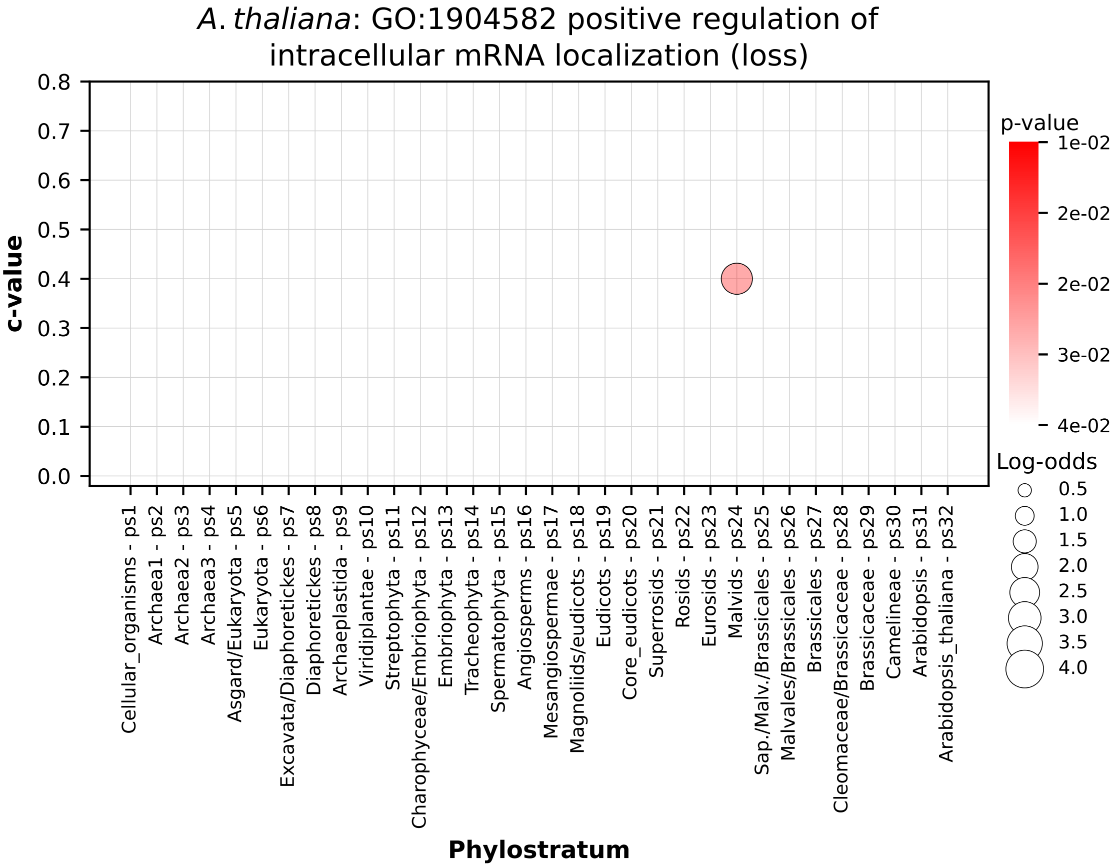

A. thaliana: GO:1904582 positive regulation of intracellular mRNA localization (loss)
c-value
0.8
0.7
0.6
0.5
0.4
0.3
0.2
0.1
0.0
p-value
1e-02
2e-02
2e-02
3e-02
4e-02
Log-odds
0.5
1.0
1.5
2.0
2.5
3.0
3.5
4.0
Cellular_organisms - ps1
Archaea1 - ps2
Archaea2 - ps3
Archaea3 - ps4
Asgard/Eukaryota - ps5
Eukaryota - ps6
Excavata/Diaphoretickes - ps7
Diaphoretickes - ps8
Archaeplastida - ps9
Viridiplantae - ps10
Streptophyta - ps11
Charophyceae/Embriophyta - ps12
Embriophyta - ps13
Tracheophyta - ps14
Spermatophyta - ps15
Angiosperms - ps16
Mesangiospermae - ps17
Magnoliids/eudicots - ps18
Eudicots - ps19
Core_eudicots - ps20
Superrosids - ps21
Rosids - ps22
Eurosids - ps23
Malvids - ps24
Sap./Malv./Brassicales - ps25
Malvales/Brassicales - ps26
Brassicales - ps27
Cleomaceae/Brassicaceae - ps28
Brassicaceae - ps29
Camelineae - ps30
Arabidopsis - ps31
Arabidopsis_thaliana - ps32
Phylostratum

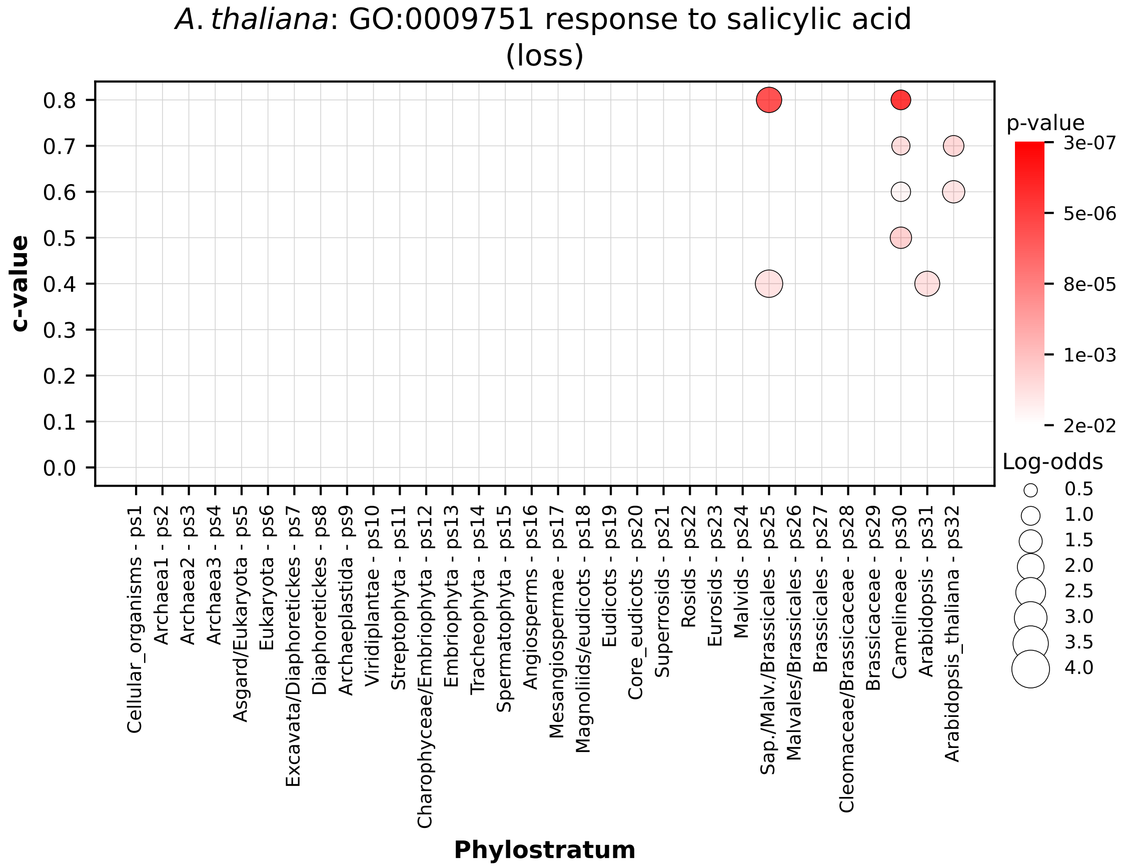
A. thaliana: GO:0009751 response to salicylic acid
(loss)
c-value
0.8
0.7
0.6
0.5
0.4
0.3
0.2
0.1
0.0
p-value
3e-07
5e-06
8e-05
1e-03
2e-02
Log-odds
0.5
1.0
1.5
2.0
2.5
3.0
3.5
4.0
Cellular_organisms - ps1
Archaea1 - ps2
Archaea2 - ps3
Archaea3 - ps4
Asgard/Eukaryota - ps5
Eukaryota - ps6
Excavata/Diaphoretickes - ps7
Diaphoretickes - ps8
Archaeplastida - ps9
Viridiplantae - ps10
Streptophyta - ps11
Charophyceae/Embriophyta - ps12
Embriophyta - ps13
Tracheophyta - ps14
Spermatophyta - ps15
Angiosperms - ps16
Mesangiospermae - ps17
Magnoliids/eudicots - ps18
Eudicots - ps19
Core_eudicots - ps20
Superrosids - ps21
Rosids - ps22
Eurosids - ps23
Malvids - ps24
Sap./Malv./Brassicales - ps25
Malvales/Brassicales - ps26
Brassicales - ps27
Cleomaceae/Brassicaceae - ps28
Brassicaceae - ps29
Camelineae - ps30
Arabidopsis - ps31
Arabidopsis_thaliana - ps32
Phylostratum

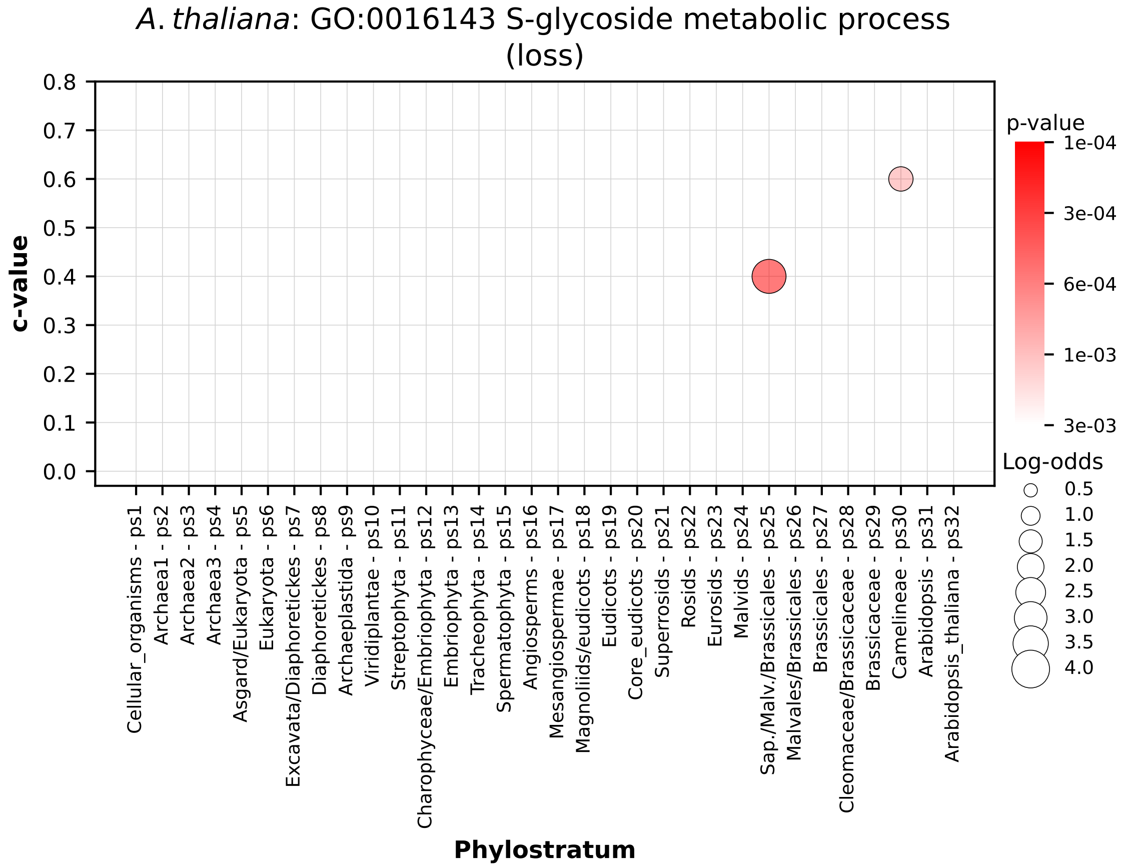

A. thaliana: GO:0016143 S-glycoside metabolic process (loss)
c-value
0.8
0.7
0.6
0.5
0.4
0.3
0.2
0.1
0.0
Phylostratum
Cellular_organisms - ps1
Archaea1 - ps2
Archaea2 - ps3
Archaea3 - ps4
Asgard/Eukaryota - ps5
Eukaryota - ps6
Excavata/Diaphoretickes - ps7
Diaphoretickes - ps8
Archaeplastida - ps9
Viridiplantae - ps10
Streptophyta - ps11
Charophyceae/Embriophyta - ps12
Embriophyta - ps13
Tracheophyta - ps14
Spermatophyta - ps15
Angiosperms - ps16
Mesangiospermae - ps17
Magnoliids/eudicots - ps18
Eudicots - ps19
Core_eudicots - ps20
Superrosids - ps21
Rosids - ps22
Eurosids - ps23
Malvids - ps24
Sap./Malv./Brassicales - ps25
Malvales/Brassicales - ps26
Brassicales - ps27
Cleomaceae/Brassicaceae - ps28
Brassicaceae - ps29
Camelineae - ps30
Arabidopsis - ps31
Arabidopsis_thaliana - ps32
p-value
1e-04
3e-04
6e-04
1e-03
3e-03
Log-odds
0.5
1.0
1.5
2.0
2.5
3.0
3.5
4.0

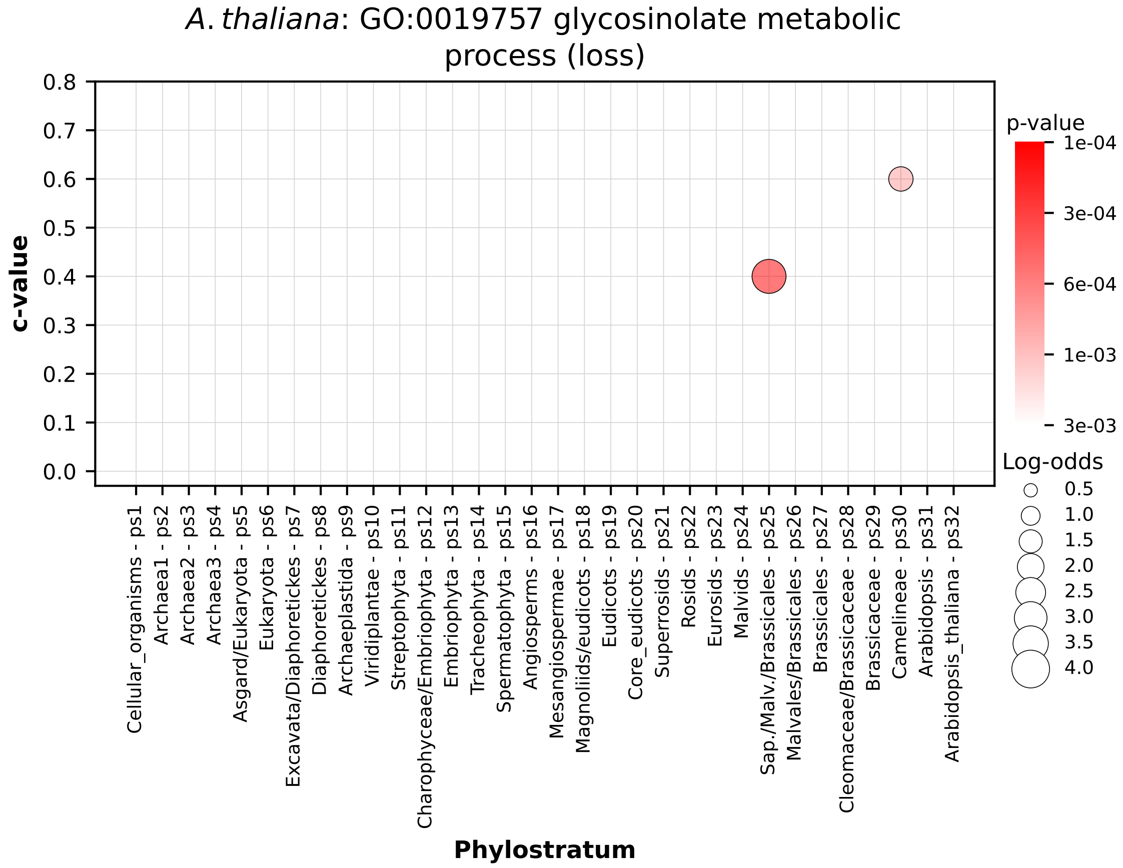
A. thaliana: GO:0019757 glycosinolate metabolic process (loss)
c-value
0.8
0.7
0.6
0.5
0.4
0.3
0.2
0.1
0.0
Phylostratum
Cellular_organisms - ps1
Archaea1 - ps2
Archaea2 - ps3
Archaea3 - ps4
Asgard/Eukaryota - ps5
Eukaryota - ps6
Excavata/Diaphoretickes - ps7
Diaphoretickes - ps8
Archaeplastida - ps9
Viridiplantae - ps10
Streptophyta - ps11
Charophyceae/Embriophyta - ps12
Embriophyta - ps13
Tracheophyta - ps14
Spermatophyta - ps15
Angiosperms - ps16
Mesangiospermae - ps17
Magnoliids/eudicots - ps18
Eudicots - ps19
Core_eudicots - ps20
Superrosids - ps21
Rosids - ps22
Eurosids - ps23
Malvids - ps24
Sap./Malv./Brassicales - ps25
Malvales/Brassicales - ps26
Brassicales - ps27
Cleomaceae/Brassicaceae - ps28
Brassicaceae - ps29
Camelineae - ps30
Arabidopsis - ps31
Arabidopsis_thaliana - ps32
p-value
1e-04
3e-04
6e-04
1e-03
3e-03
Log-odds
0.5
1.0
1.5
2.0
2.5
3.0
3.5
4.0

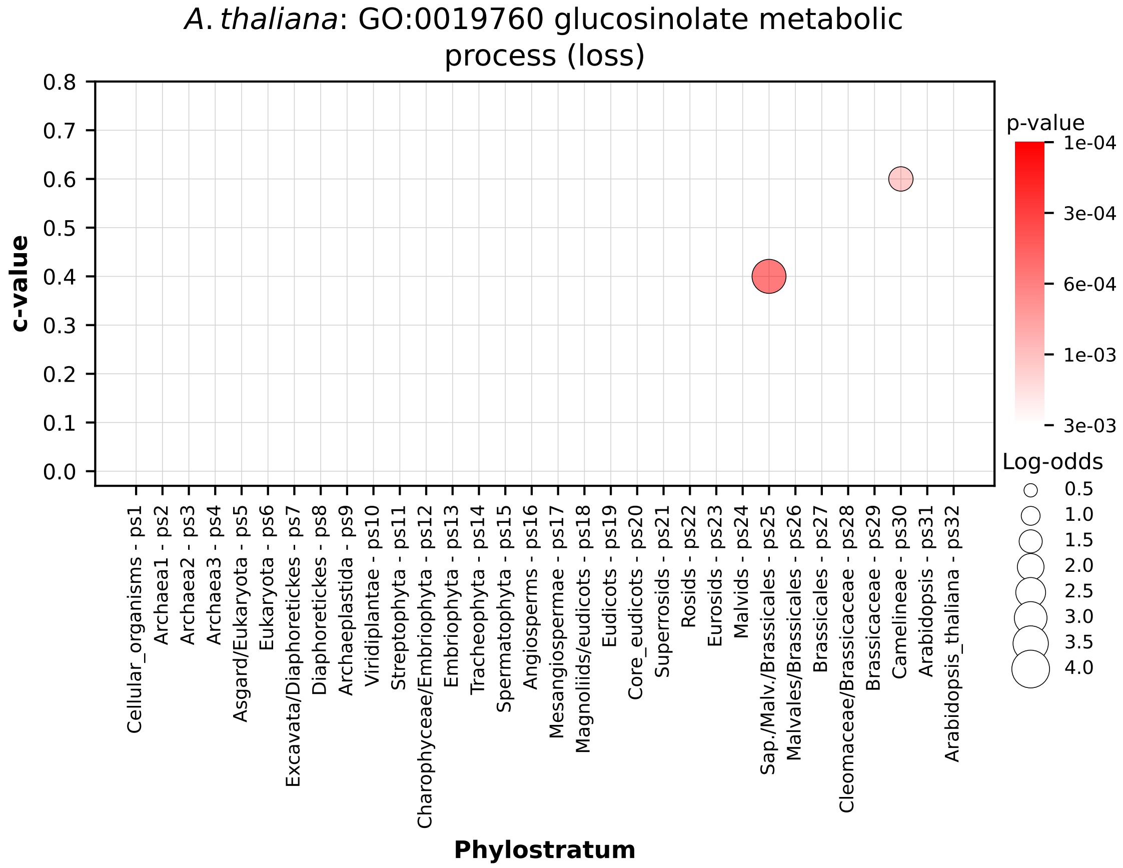

A. thaliana: GO:0019760 glucosinolate metabolic process (loss)
c-value
0.8
0.7
0.6
0.5
0.4
0.3
0.2
0.1
0.0
p-value
1e-04
3e-04
6e-04
1e-03
3e-03
Log-odds
0.5
1.0
1.5
2.0
2.5
3.0
3.5
4.0
Cellular_organisms - ps1
Archaea1 - ps2
Archaea2 - ps3
Archaea3 - ps4
Asgard/Eukaryota - ps5
Eukaryota - ps6
Excavata/Diaphoretickes - ps7
Diaphoretickes - ps8
Archaeplastida - ps9
Viridiplantae - ps10
Streptophyta - ps11
Charophyceae/Embriophyta - ps12
Embriophyta - ps13
Tracheophyta - ps14
Spermatophyta - ps15
Angiosperms - ps16
Mesangiospermae - ps17
Magnoliids/eudicots - ps18
Eudicots - ps19
Core_eudicots - ps20
Superrosids - ps21
Rosids - ps22
Eurosids - ps23
Malvids - ps24
Sap./Malv./Brassicales - ps25
Malvales/Brassicales - ps26
Brassicales - ps27
Cleomaceae/Brassicaceae - ps28
Brassicaceae - ps29
Camelineae - ps30
Arabidopsis - ps31
Arabidopsis_thaliana - ps32
Phylostratum

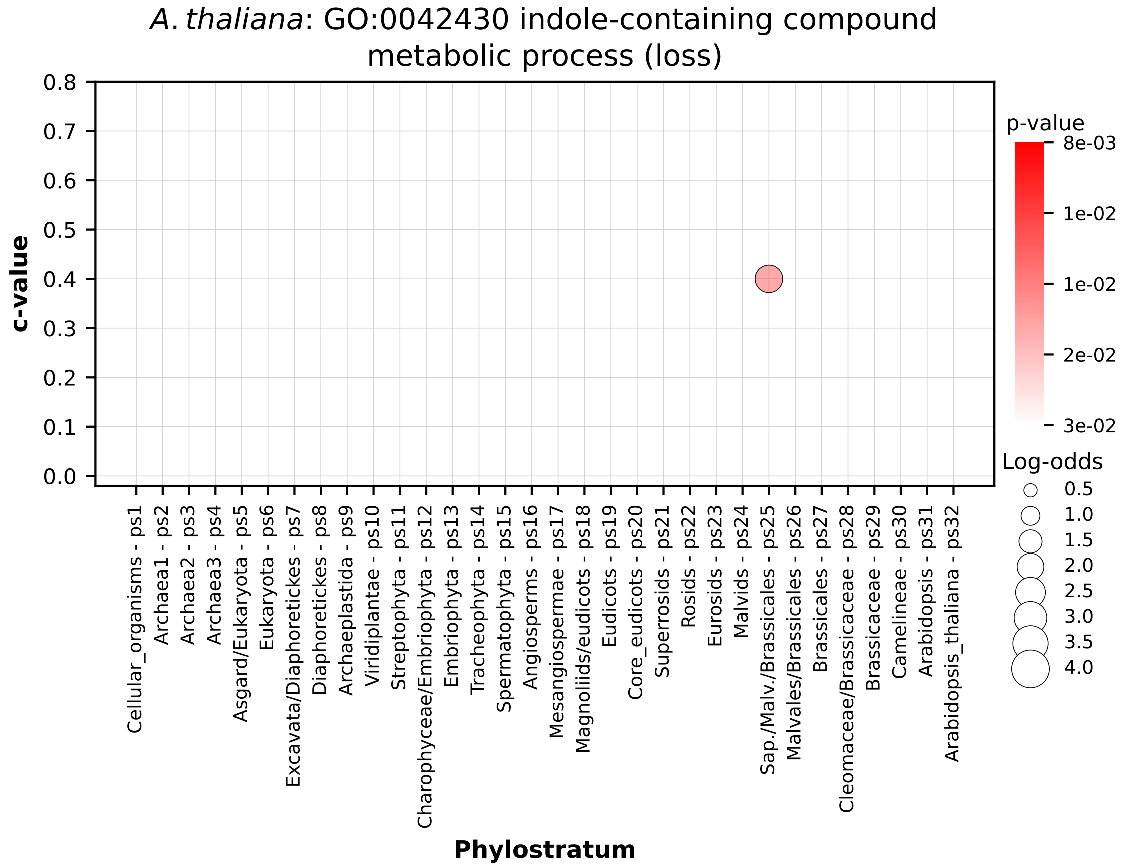
A. thaliana: GO:0042430 indole-containing compound metabolic process (loss)
c-value
0.8
0.7
0.6
0.5
0.4
0.3
0.2
0.1
0.0
p-value
8e-03
1e-02
1e-02
2e-02
3e-02
Log-odds
0.5
1.0
1.5
2.0
2.5
3.0
3.5
4.0
Cellular_organisms - ps1
Archaea1 - ps2
Archaea2 - ps3
Archaea3 - ps4
Asgard/Eukaryota - ps5
Eukaryota - ps6
Excavata/Diaphoretickes - ps7
Diaphoretickes - ps8
Archaeplastida - ps9
Viridiplantae - ps10
Streptophyta - ps11
Charophyceae/Embriophyta - ps12
Embriophyta - ps13
Tracheophyta - ps14
Spermatophyta - ps15
Angiosperms - ps16
Mesangiospermae - ps17
Magnoliids/eudicots - ps18
Eudicots - ps19
Core_eudicots - ps20
Superrosids - ps21
Rosids - ps22
Eurosids - ps23
Malvids - ps24
Sap./Malv./Brassicales - ps25
Malvales/Brassicales - ps26
Brassicales - ps27
Cleomaceae/Brassicaceae - ps28
Brassicaceae - ps29
Camelineae - ps30
Arabidopsis - ps31
Arabidopsis_thaliana - ps32
Phylostratum

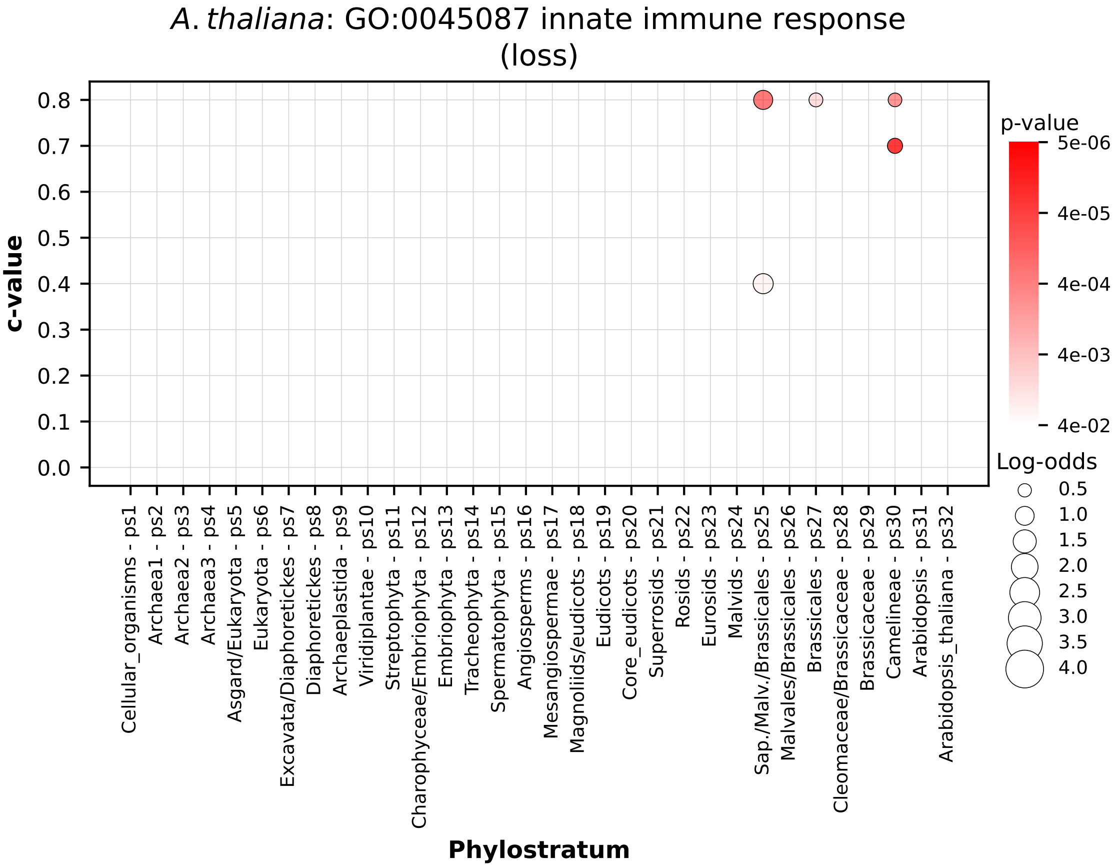

A. thaliana: GO:0045087 innate immune response
(loss)
c-value
0.8
0.7
0.6
0.5
0.4
0.3
0.2
0.1
0.0
p-value
5e-06
4e-05
4e-04
4e-03
4e-02
Log-odds
0.5
1.0
1.5
2.0
2.5
3.0
3.5
4.0
Cellular_organisms - ps1
Archaea1 - ps2
Archaea2 - ps3
Archaea3 - ps4
Asgard/Eukaryota - ps5
Eukaryota - ps6
Excavata/Diaphoretickes - ps7
Diaphoretickes - ps8
Archaeplastida - ps9
Viridiplantae - ps10
Streptophyta - ps11
Charophyceae/Embriophyta - ps12
Embriophyta - ps13
Tracheophyta - ps14
Spermatophyta - ps15
Angiosperms - ps16
Mesangiospermae - ps17
Magnoliids/eudicots - ps18
Eudicots - ps19
Core_eudicots - ps20
Superrosids - ps21
Rosids - ps22
Eurosids - ps23
Malvids - ps24
Sap./Malv./Brassicales - ps25
Malvales/Brassicales - ps26
Brassicales - ps27
Cleomaceae/Brassicaceae - ps28
Brassicaceae - ps29
Camelineae - ps30
Arabidopsis - ps31
Arabidopsis_thaliana - ps32
Phylostratum

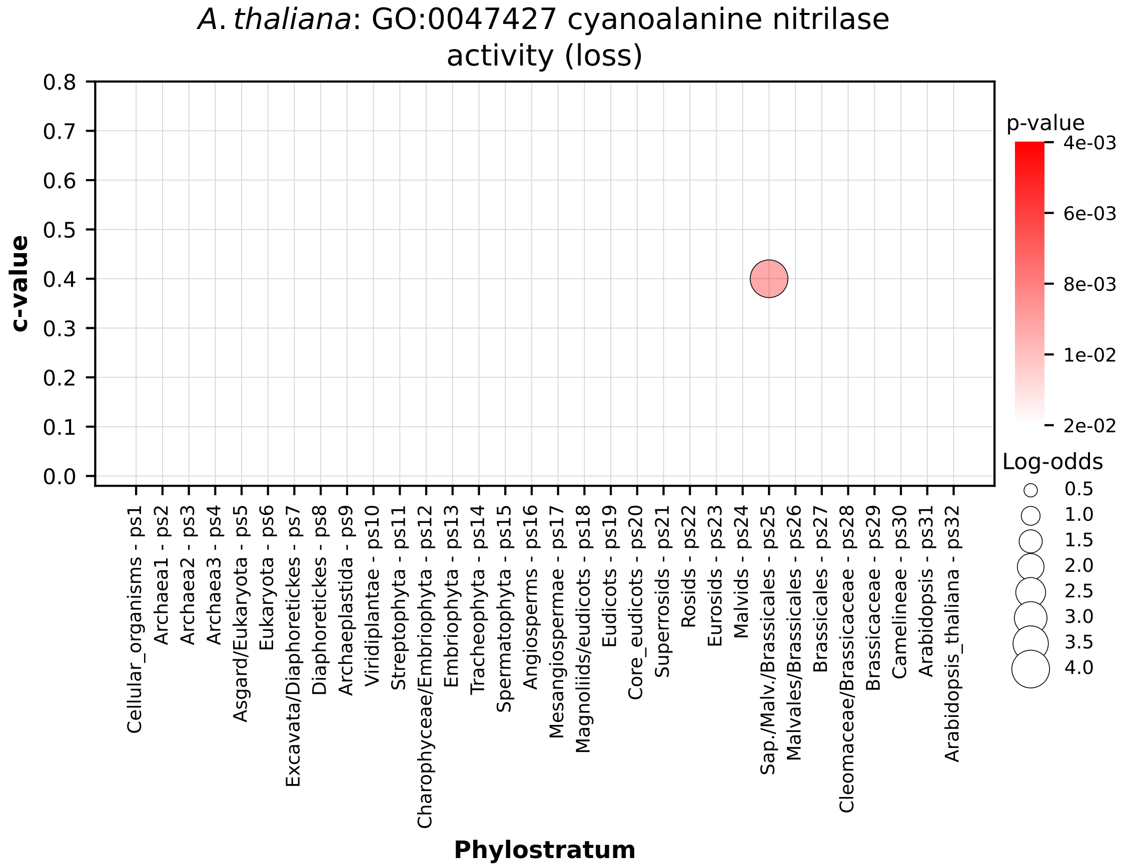

A. thaliana: GO:0047427 cyanoalanine nitrilase activity (loss)
c-value
0.8
0.7
0.6
0.5
0.4
0.3
0.2
0.1
0.0
Phylostratum
Cellular_organisms - ps1
Archaea1 - ps2
Archaea2 - ps3
Archaea3 - ps4
Asgard/Eukaryota - ps5
Eukaryota - ps6
Excavata/Diaphoretickes - ps7
Diaphoretickes - ps8
Archaeplastida - ps9
Viridiplantae - ps10
Streptophyta - ps11
Charophyceae/Embriophyta - ps12
Embriophyta - ps13
Tracheophyta - ps14
Spermatophyta - ps15
Angiosperms - ps16
Mesangiospermae - ps17
Magnoliids/eudicots - ps18
Eudicots - ps19
Core_eudicots - ps20
Superrosids - ps21
Rosids - ps22
Eurosids - ps23
Malvids - ps24
Sap./Malv./Brassicales - ps25
Malvales/Brassicales - ps26
Brassicales - ps27
Cleomaceae/Brassicaceae - ps28
Brassicaceae - ps29
Camelineae - ps30
Arabidopsis - ps31
Arabidopsis_thaliana - ps32
p-value
4e-03
6e-03
8e-03
1e-02
2e-02
Log-odds
0.5
1.0
1.5
2.0
2.5
3.0
3.5
4.0

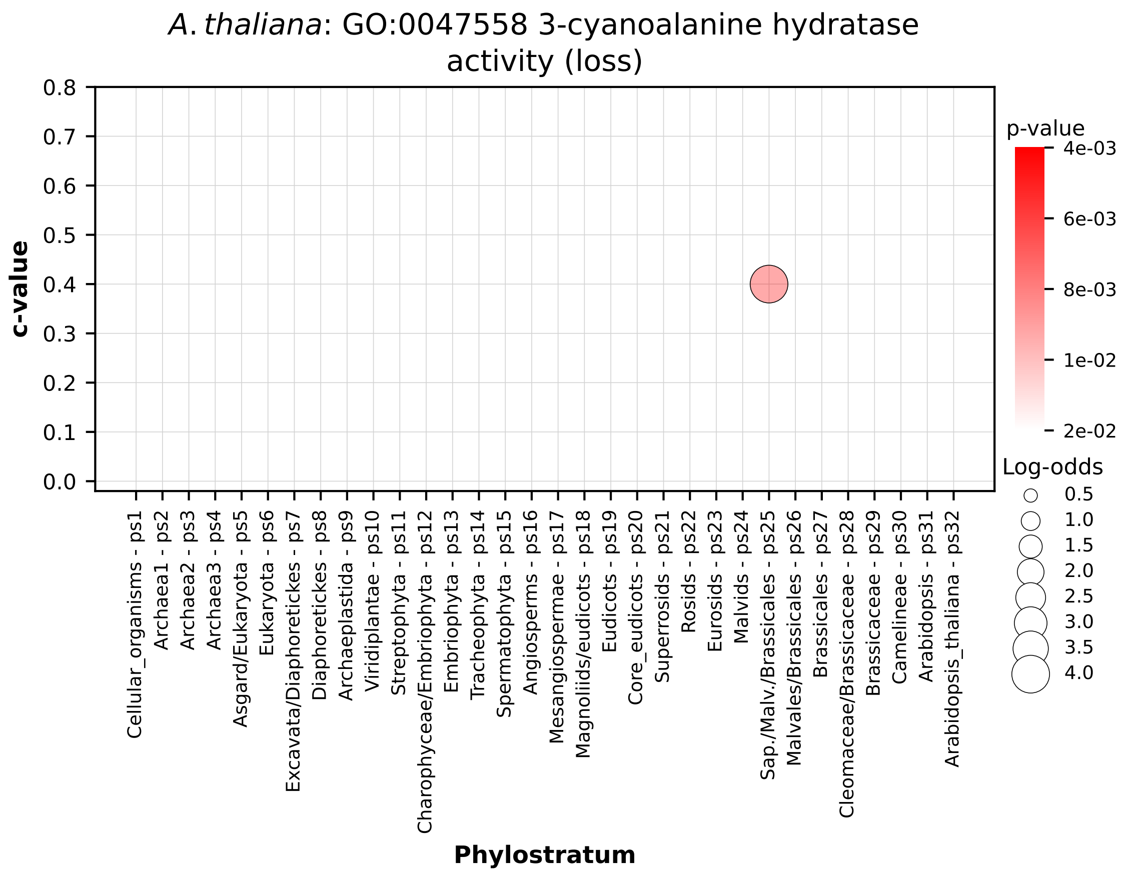

A. thaliana: GO:0047558 3-cyanoalanine hydratase activity (loss)
c-value
0.8
0.7
0.6
0.5
0.4
0.3
0.2
0.1
0.0
p-value
4e-03
6e-03
8e-03
1e-02
2e-02
Log-odds
0.5
1.0
1.5
2.0
2.5
3.0
3.5
4.0
Cellular_organisms - ps1
Archaea1 - ps2
Archaea2 - ps3
Archaea3 - ps4
Asgard/Eukaryota - ps5
Eukaryota - ps6
Excavata/Diaphoretickes - ps7
Diaphoretickes - ps8
Archaeplastida - ps9
Viridiplantae - ps10
Streptophyta - ps11
Charophyceae/Embriophyta - ps12
Embriophyta - ps13
Tracheophyta - ps14
Spermatophyta - ps15
Angiosperms - ps16
Mesangiospermae - ps17
Magnoliids/eudicots - ps18
Eudicots - ps19
Core_eudicots - ps20
Superrosids - ps21
Rosids - ps22
Eurosids - ps23
Malvids - ps24
Sap./Malv./Brassicales - ps25
Malvales/Brassicales - ps26
Brassicales - ps27
Cleomaceae/Brassicaceae - ps28
Brassicaceae - ps29
Camelineae - ps30
Arabidopsis - ps31
Arabidopsis_thaliana - ps32
Phylostratum

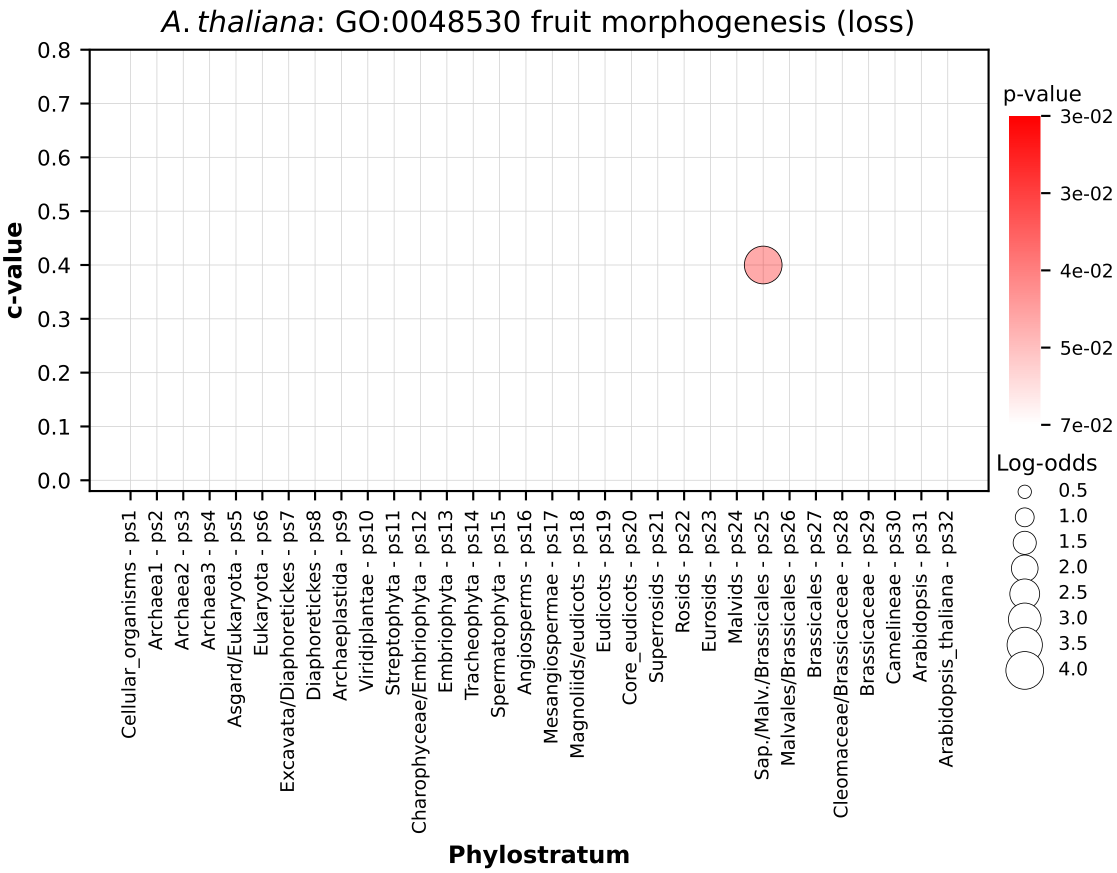

A. thaliana: GO:0048530 fruit morphogenesis (loss)
c-value
0.8
0.7
0.6
0.5
0.4
0.3
0.2
0.1
0.0
p-value
3e-02
3e-02
4e-02
5e-02
7e-02
Log-odds
0.5
1.0
1.5
2.0
2.5
3.0
3.5
4.0
Cellular_organisms - ps1
Archaea1 - ps2
Archaea2 - ps3
Archaea3 - ps4
Asgard/Eukaryota - ps5
Eukaryota - ps6
Excavata/Diaphoretickes - ps7
Diaphoretickes - ps8
Archaeplastida - ps9
Viridiplantae - ps10
Streptophyta - ps11
Charophyceae/Embriophyta - ps12
Embriophyta - ps13
Tracheophyta - ps14
Spermatophyta - ps15
Angiosperms - ps16
Mesangiospermae - ps17
Magnoliids/eudicots - ps18
Eudicots - ps19
Core_eudicots - ps20
Superrosids - ps21
Rosids - ps22
Eurosids - ps23
Malvids - ps24
Sap./Malv./Brassicales - ps25
Malvales/Brassicales - ps26
Brassicales - ps27
Cleomaceae/Brassicaceae - ps28
Brassicaceae - ps29
Camelineae - ps30
Arabidopsis - ps31
Arabidopsis_thaliana - ps32
Phylostratum

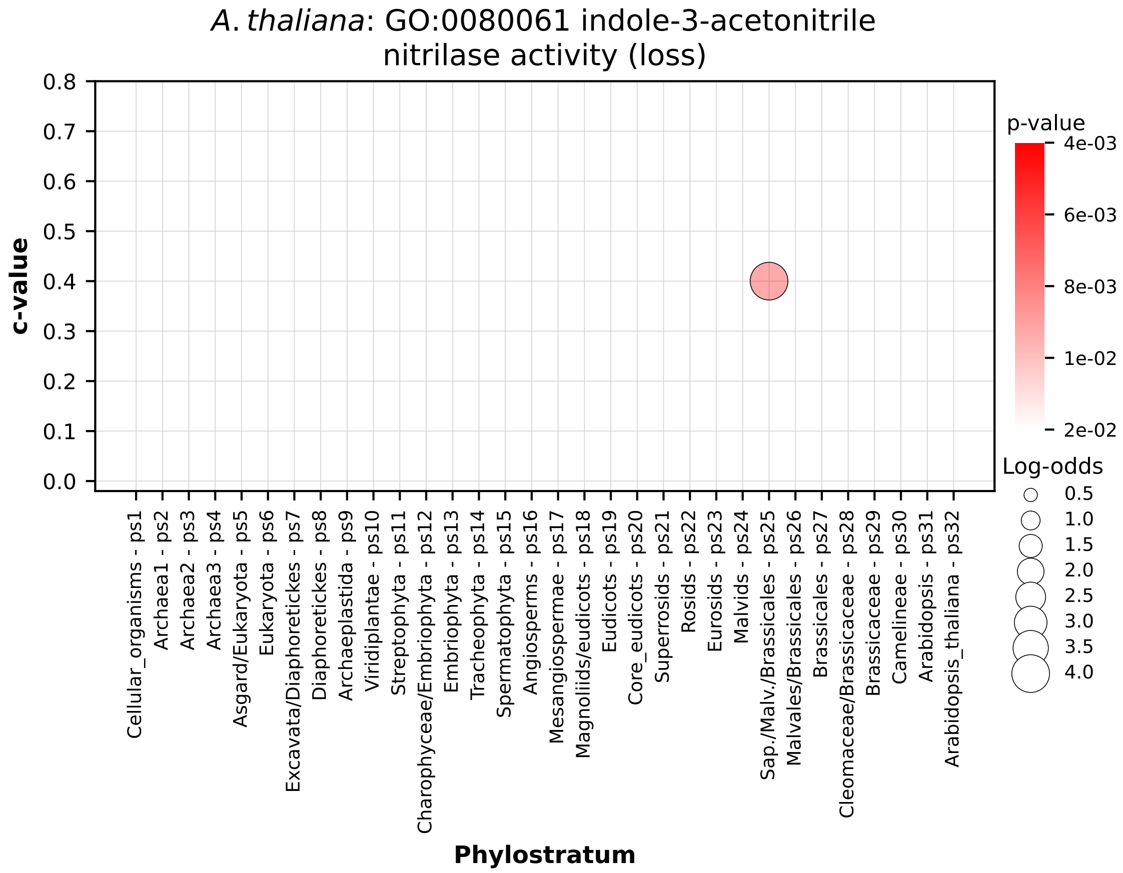
A. thaliana: GO:0080061 indole-3-acetonitrile nitrilase activity (loss)
c-value
0.0
0.1
0.2
0.3
0.4
0.5
0.6
0.7
0.8
p-value
4e-03
6e-03
8e-03
1e-02
2e-02
Log-odds
0.5
1.0
1.5
2.0
2.5
3.0
3.5
4.0
Cellular_organisms - ps1
Archaea1 - ps2
Archaea2 - ps3
Archaea3 - ps4
Asgard/Eukaryota - ps5
Eukaryota - ps6
Excavata/Diaphoretickes - ps7
Diaphoretickes - ps8
Archaeplastida - ps9
Viridiplantae - ps10
Streptophyta - ps11
Charophyceae/Embriophyta - ps12
Embriophyta - ps13
Tracheophyta - ps14
Spermatophyta - ps15
Angiosperms - ps16
Mesangiospermae - ps17
Magnoliids/eudicots - ps18
Eudicots - ps19
Core_eudicots - ps20
Superrosids - ps21
Rosids - ps22
Eurosids - ps23
Malvids - ps24
Sap./Malv./Brassicales - ps25
Malvales/Brassicales - ps26
Brassicales - ps27
Cleomaceae/Brassicaceae - ps28
Brassicaceae - ps29
Camelineae - ps30
Arabidopsis - ps31
Arabidopsis_thaliana - ps32
Phylostratum

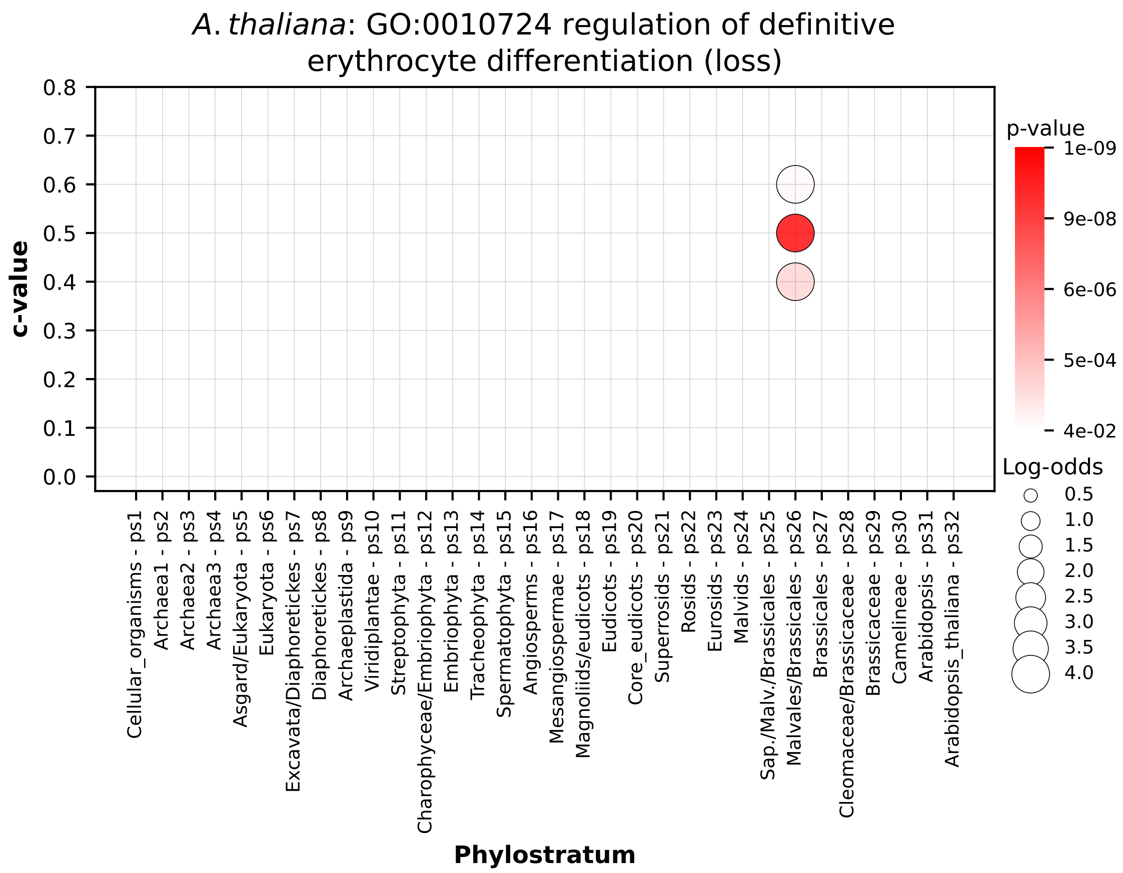

A. thaliana: GO:0010724 regulation of definitive erythrocyte differentiation (loss)
c-value
0.8
0.7
0.6
0.5
0.4
0.3
0.2
0.1
0.0
p-value
1e-09
9e-08
6e-06
5e-04
4e-02
Log-odds
0.5
1.0
1.5
2.0
2.5
3.0
3.5
4.0
Cellular_organisms - ps1
Archaea1 - ps2
Archaea2 - ps3
Archaea3 - ps4
Asgard/Eukaryota - ps5
Eukaryota - ps6
Excavata/Diaphoretickes - ps7
Diaphoretickes - ps8
Archaeplastida - ps9
Viridiplantae - ps10
Streptophyta - ps11
Charophyceae/Embriophyta - ps12
Embriophyta - ps13
Tracheophyta - ps14
Spermatophyta - ps15
Angiosperms - ps16
Mesangiospermae - ps17
Magnoliids/eudicots - ps18
Eudicots - ps19
Core_eudicots - ps20
Superrosids - ps21
Rosids - ps22
Eurosids - ps23
Malvids - ps24
Sap./Malv./Brassicales - ps25
Malvales/Brassicales - ps26
Brassicales - ps27
Cleomaceae/Brassicaceae - ps28
Brassicaceae - ps29
Camelineae - ps30
Arabidopsis - ps31
Arabidopsis_thaliana - ps32
Phylostratum

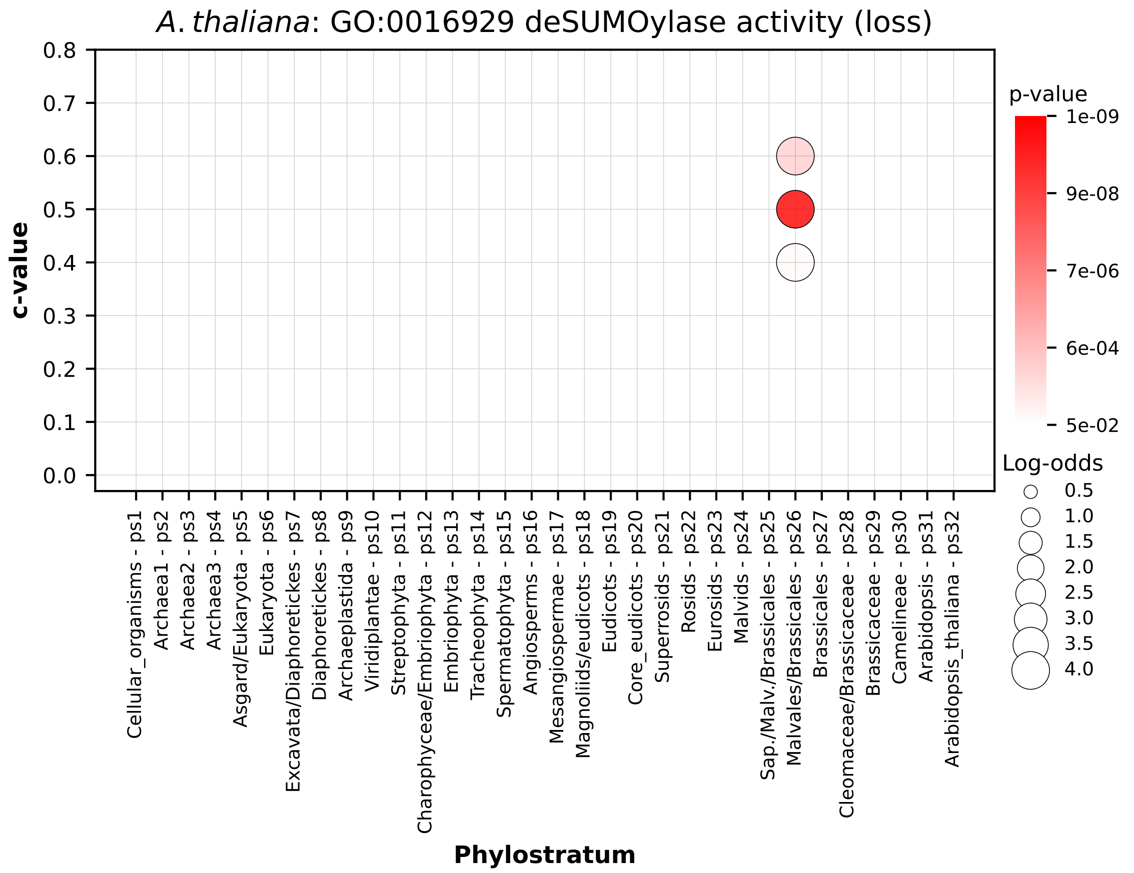

A. thaliana: GO:0016929 deSUMOylase activity (loss)
c-value
0.8
0.7
0.6
0.5
0.4
0.3
0.2
0.1
0.0
p-value
1e-09
9e-08
7e-06
6e-04
5e-02
Log-odds
0.5
1.0
1.5
2.0
2.5
3.0
3.5
4.0
Cellular_organisms - ps1
Archaea1 - ps2
Archaea2 - ps3
Archaea3 - ps4
Asgard/Eukaryota - ps5
Eukaryota - ps6
Excavata/Diaphoretickes - ps7
Diaphoretickes - ps8
Archaeplastida - ps9
Viridiplantae - ps10
Streptophyta - ps11
Charophyceae/Embriophyta - ps12
Embriophyta - ps13
Tracheophyta - ps14
Spermatophyta - ps15
Angiosperms - ps16
Mesangiospermae - ps17
Magnoliids/eudicots - ps18
Eudicots - ps19
Core_eudicots - ps20
Superrosids - ps21
Rosids - ps22
Eurosids - ps23
Malvids - ps24
Sap./Malv./Brassicales - ps25
Malvales/Brassicales - ps26
Brassicales - ps27
Cleomaceae/Brassicaceae - ps28
Brassicaceae - ps29
Camelineae - ps30
Arabidopsis - ps31
Arabidopsis_thaliana - ps32
Phylostratum

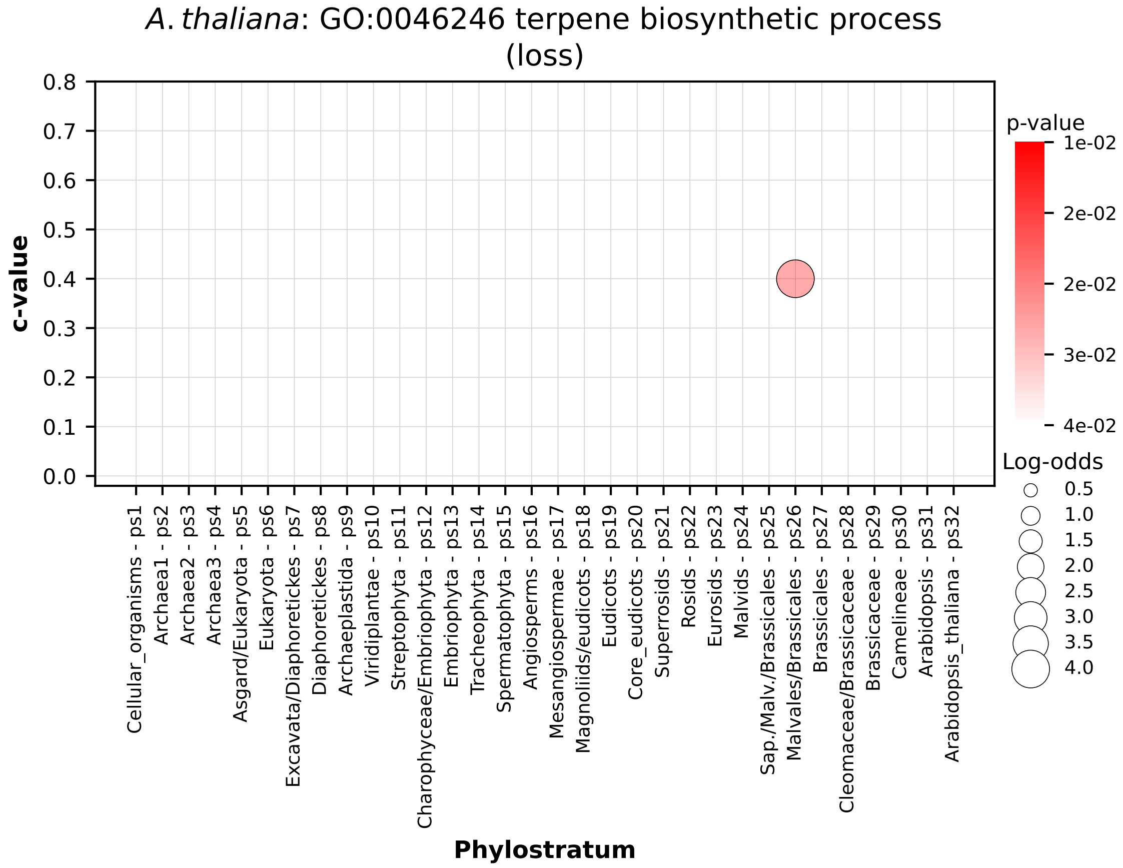

A. thaliana: GO:0046246 terpene biosynthetic process
(loss)
c-value
0.8
0.7
0.6
0.5
0.4
0.3
0.2
0.1
0.0
Phylostratum
Cellular_organisms - ps1
Archaea1 - ps2
Archaea2 - ps3
Archaea3 - ps4
Asgard/Eukaryota - ps5
Eukaryota - ps6
Excavata/Diaphoretickes - ps7
Diaphoretickes - ps8
Archaeplastida - ps9
Viridiplantae - ps10
Streptophyta - ps11
Charophyceae/Embriophyta - ps12
Embriophyta - ps13
Tracheophyta - ps14
Spermatophyta - ps15
Angiosperms - ps16
Mesangiospermae - ps17
Magnoliids/eudicots - ps18
Eudicots - ps19
Core_eudicots - ps20
Superrosids - ps21
Rosids - ps22
Eurosids - ps23
Malvids - ps24
Sap./Malv./Brassicales - ps25
Malvales/Brassicales - ps26
Brassicales - ps27
Cleomaceae/Brassicaceae - ps28
Brassicaceae - ps29
Camelineae - ps30
Arabidopsis - ps31
Arabidopsis_thaliana - ps32
p-value
1e-02
2e-02
2e-02
3e-02
4e-02
Log-odds
0.5
1.0
1.5
2.0
2.5
3.0
3.5
4.0

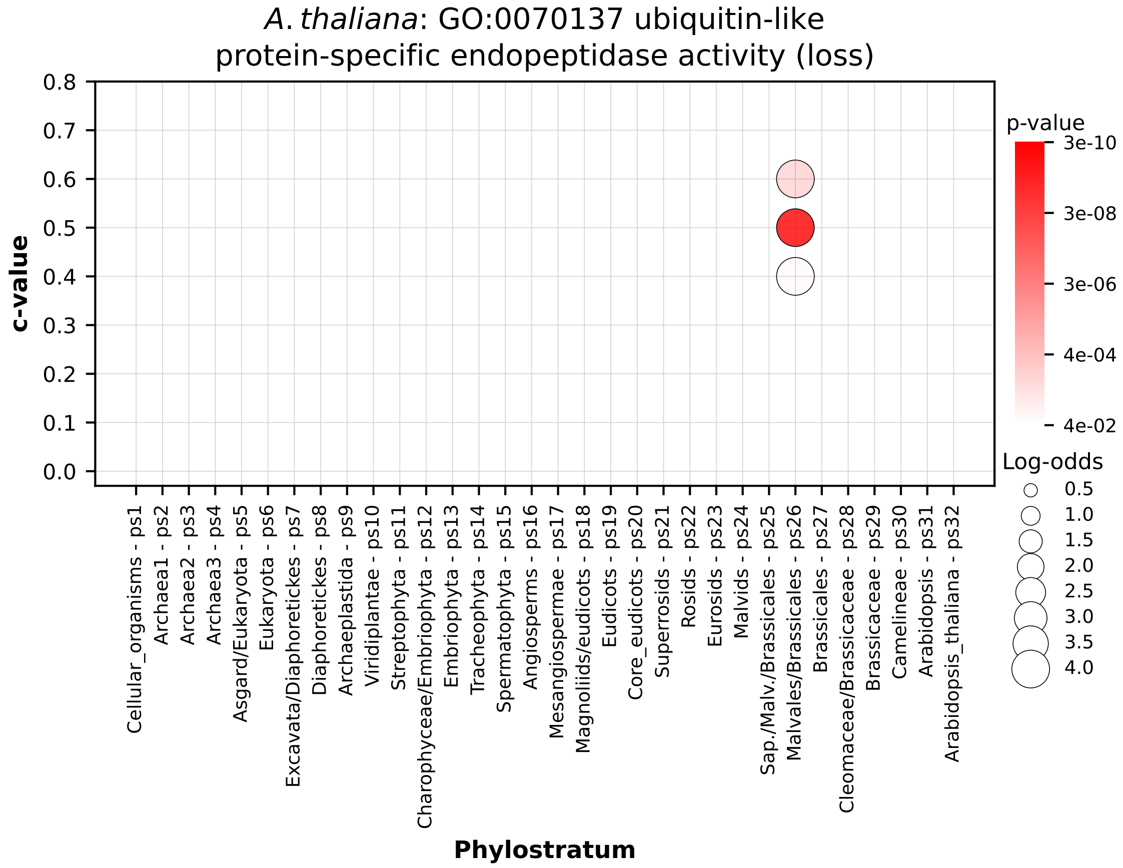

A. thaliana: GO:0070137 ubiquitin-like protein-specific endopeptidase activity (loss)
c-value
0.8
0.7
0.6
0.5
0.4
0.3
0.2
0.1
0.0
Phylostratum
Cellular_organisms - ps1
Archaea1 - ps2
Archaea2 - ps3
Archaea3 - ps4
Asgard/Eukaryota - ps5
Eukaryota - ps6
Excavata/Diaphoretickes - ps7
Diaphoretickes - ps8
Archaeplastida - ps9
Viridiplantae - ps10
Streptophyta - ps11
Charophyceae/Embriophyta - ps12
Embriophyta - ps13
Tracheophyta - ps14
Spermatophyta - ps15
Angiosperms - ps16
Mesangiospermae - ps17
Magnoliids/eudicots - ps18
Eudicots - ps19
Core_eudicots - ps20
Superrosids - ps21
Rosids - ps22
Eurosids - ps23
Malvids - ps24
Sap./Malv./Brassicales - ps25
Malvales/Brassicales - ps26
Brassicales - ps27
Cleomaceae/Brassicaceae - ps28
Brassicaceae - ps29
Camelineae - ps30
Arabidopsis - ps31
Arabidopsis_thaliana - ps32
p-value
3e-10
3e-08
3e-06
4e-04
4e-02
Log-odds
0.5
1.0
1.5
2.0
2.5
3.0
3.5
4.0

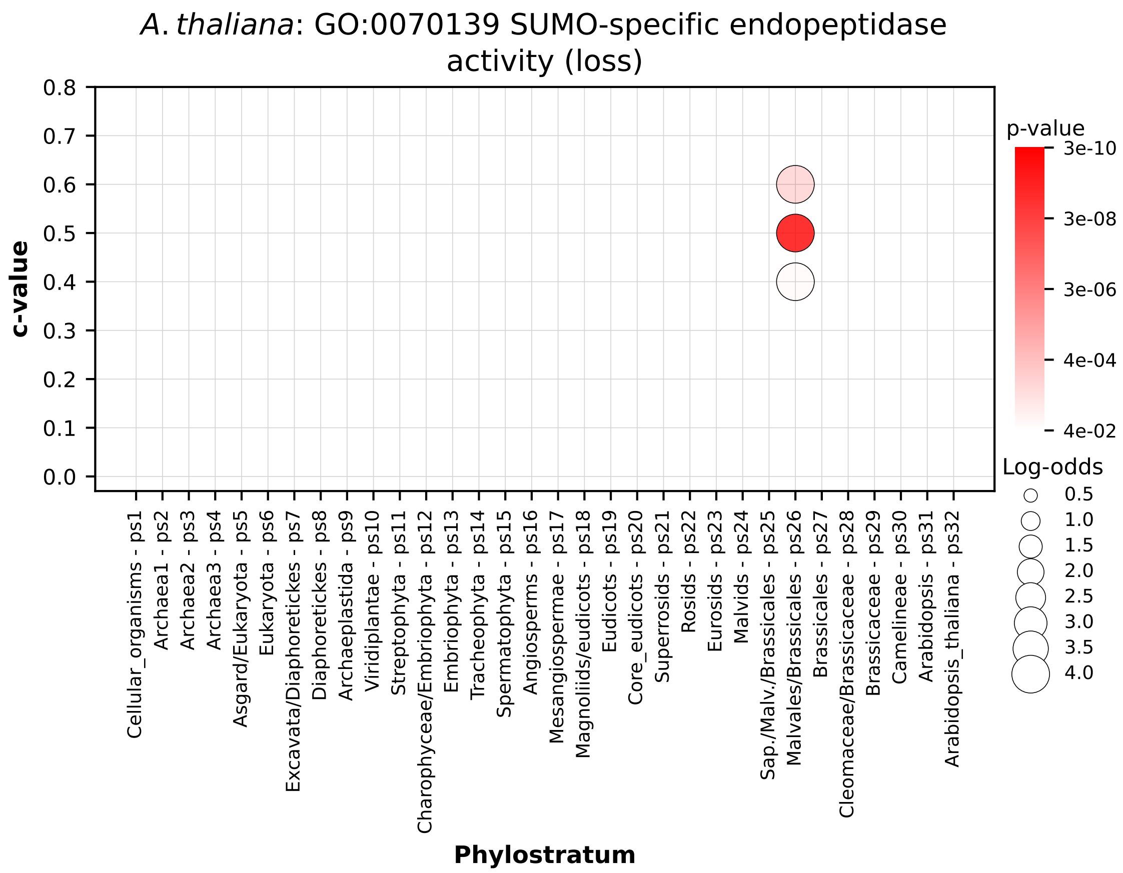

A. thaliana: GO:0070139 SUMO-specific endopeptidase activity (loss)
c-value
0.8
0.7
0.6
0.5
0.4
0.3
0.2
0.1
0.0
p-value
3e-10
3e-08
3e-06
4e-04
4e-02
Log-odds
0.5
1.0
1.5
2.0
2.5
3.0
3.5
4.0
Cellular_organisms - ps1
Archaea1 - ps2
Archaea2 - ps3
Archaea3 - ps4
Asgard/Eukaryota - ps5
Eukaryota - ps6
Excavata/Diaphoretickes - ps7
Diaphoretickes - ps8
Archaeplastida - ps9
Viridiplantae - ps10
Streptophyta - ps11
Charophyceae/Embriophyta - ps12
Embriophyta - ps13
Tracheophyta - ps14
Spermatophyta - ps15
Angiosperms - ps16
Mesangiospermae - ps17
Magnoliids/eudicots - ps18
Eudicots - ps19
Core_eudicots - ps20
Superrosids - ps21
Rosids - ps22
Eurosids - ps23
Malvids - ps24
Sap./Malv./Brassicales - ps25
Malvales/Brassicales - ps26
Brassicales - ps27
Cleomaceae/Brassicaceae - ps28
Brassicaceae - ps29
Camelineae - ps30
Arabidopsis - ps31
Arabidopsis_thaliana - ps32
Phylostratum

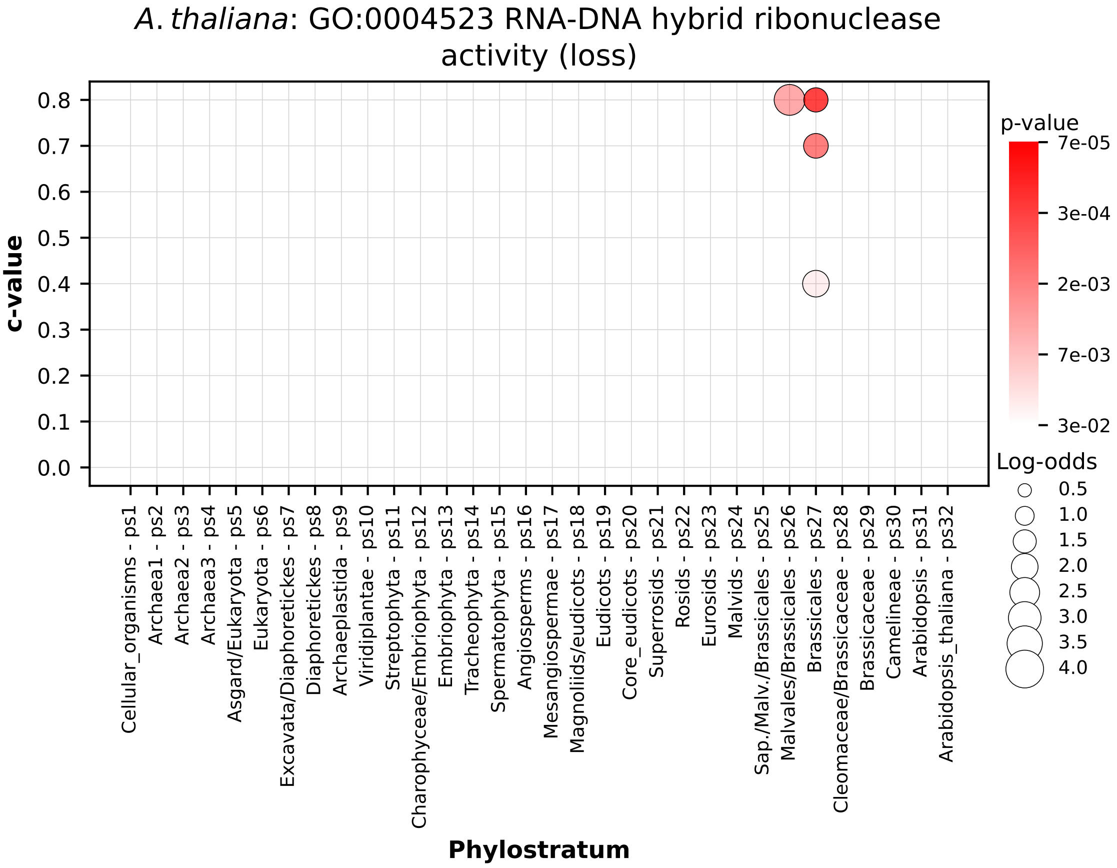
A. thaliana: GO:0004523 RNA-DNA hybrid ribonuclease activity (loss)
c-value
0.0
0.1
0.2
0.3
0.4
0.5
0.6
0.7
0.8
Phylostratum
Cellular_organisms - ps1
Archaea1 - ps2
Archaea2 - ps3
Archaea3 - ps4
Asgard/Eukaryota - ps5
Eukaryota - ps6
Excavata/Diaphoretickes - ps7
Diaphoretickes - ps8
Archaeplastida - ps9
Viridiplantae - ps10
Streptophyta - ps11
Charophyceae/Embriophyta - ps12
Embriophyta - ps13
Tracheophyta - ps14
Spermatophyta - ps15
Angiosperms - ps16
Mesangiospermae - ps17
Magnoliids/eudicots - ps18
Eudicots - ps19
Core_eudicots - ps20
Superrosids - ps21
Rosids - ps22
Eurosids - ps23
Malvids - ps24
Sap./Malv./Brassicales - ps25
Malvales/Brassicales - ps26
Brassicales - ps27
Cleomaceae/Brassicaceae - ps28
Brassicaceae - ps29
Camelineae - ps30
Arabidopsis - ps31
Arabidopsis_thaliana - ps32
p-value
7e-05
3e-04
2e-03
7e-03
3e-02
Log-odds
0.5
1.0
1.5
2.0
2.5
3.0
3.5
4.0

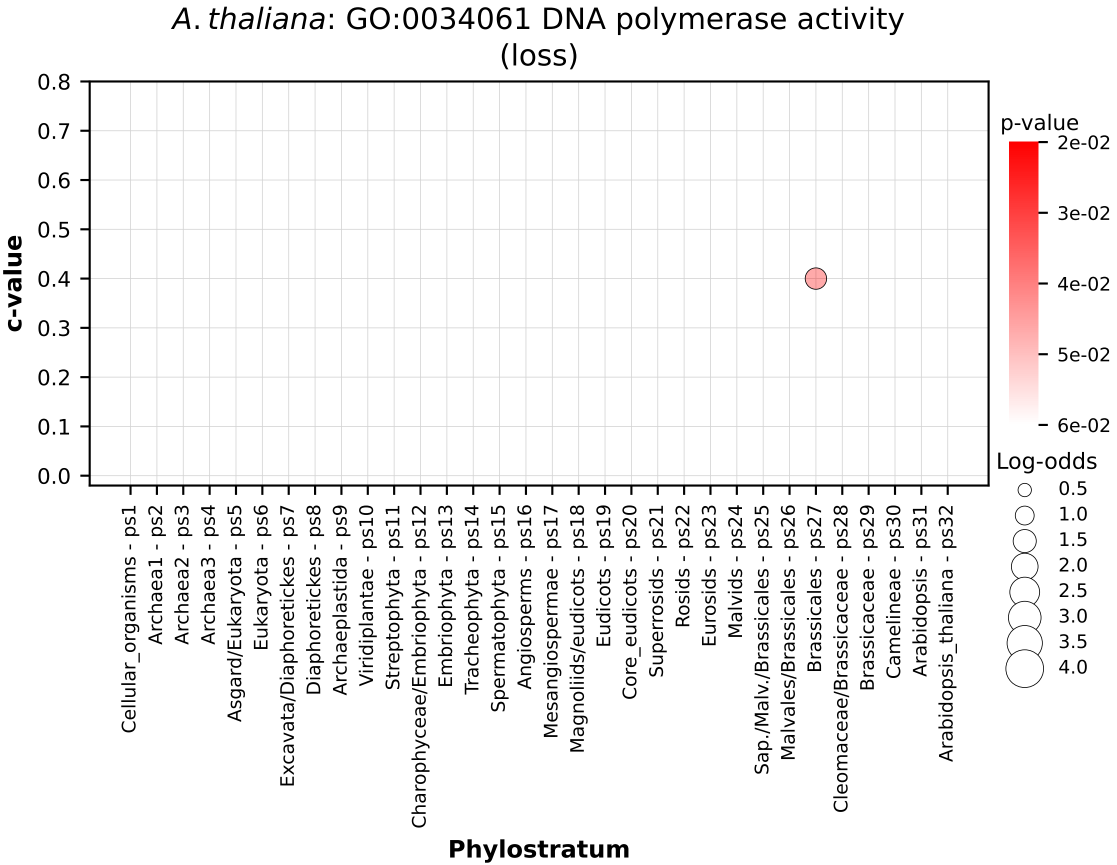
A. thaliana: GO:0034061 DNA polymerase activity
(loss)
c-value
0.8
0.7
0.6
0.5
0.4
0.3
0.2
0.1
0.0
p-value
2e-02
3e-02
4e-02
5e-02
6e-02
Log-odds
0.5
1.0
1.5
2.0
2.5
3.0
3.5
4.0
Cellular_organisms - ps1
Archaea1 - ps2
Archaea2 - ps3
Archaea3 - ps4
Asgard/Eukaryota - ps5
Eukaryota - ps6
Excavata/Diaphoretickes - ps7
Diaphoretickes - ps8
Archaeplastida - ps9
Viridiplantae - ps10
Streptophyta - ps11
Charophyceae/Embriophyta - ps12
Embriophyta - ps13
Tracheophyta - ps14
Spermatophyta - ps15
Angiosperms - ps16
Mesangiospermae - ps17
Magnoliids/eudicots - ps18
Eudicots - ps19
Core_eudicots - ps20
Superrosids - ps21
Rosids - ps22
Eurosids - ps23
Malvids - ps24
Sap./Malv./Brassicales - ps25
Malvales/Brassicales - ps26
Brassicales - ps27
Cleomaceae/Brassicaceae - ps28
Brassicaceae - ps29
Camelineae - ps30
Arabidopsis - ps31
Arabidopsis_thaliana - ps32
Phylostratum

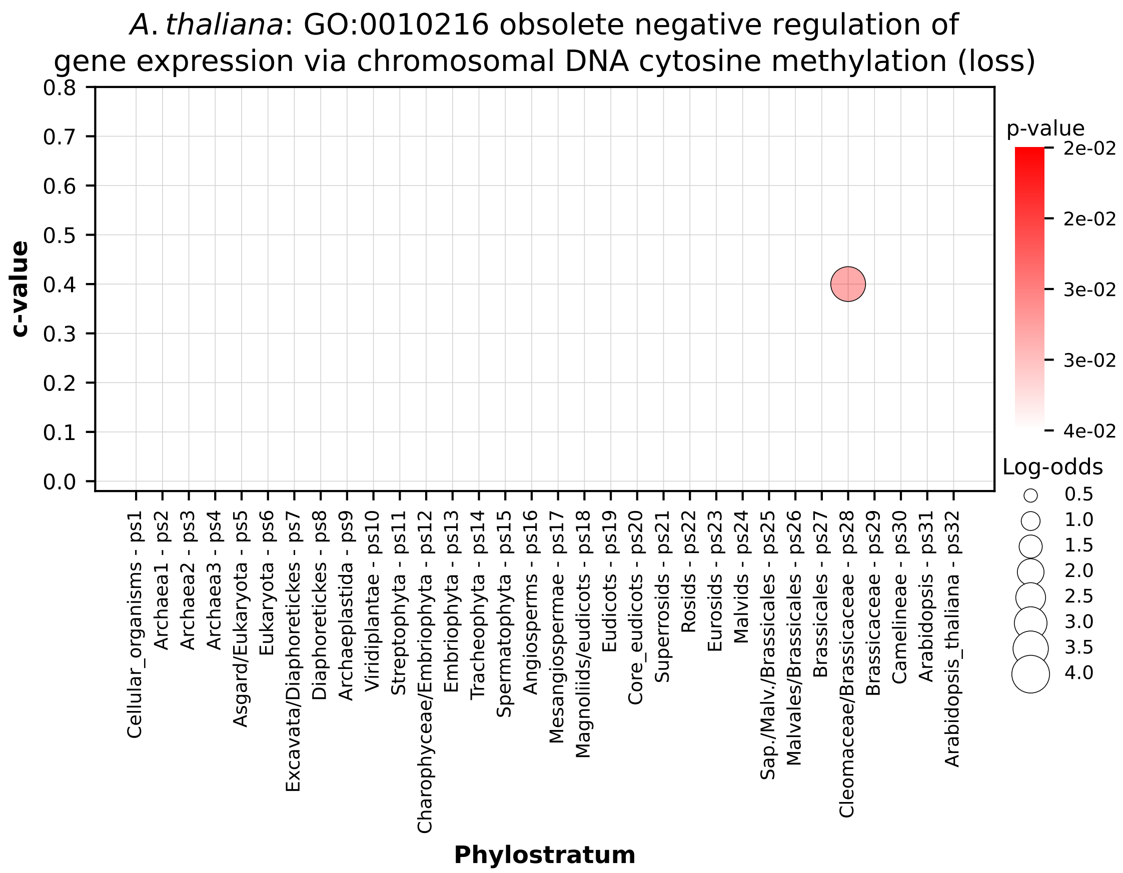

A. thaliana: GO:0010216 obsolete negative regulation of gene expression via chromosomal DNA cytosine methylation (loss)
c-value
0.8
0.7
0.6
0.5
0.4
0.3
0.2
0.1
0.0
p-value
2e-02
2e-02
3e-02
3e-02
4e-02
Log-odds
0.5
1.0
1.5
2.0
2.5
3.0
3.5
4.0
Cellular_organisms - ps1
Archaea1 - ps2
Archaea2 - ps3
Archaea3 - ps4
Asgard/Eukaryota - ps5
Eukaryota - ps6
Excavata/Diaphoretickes - ps7
Diaphoretickes - ps8
Archaeplastida - ps9
Viridiplantae - ps10
Streptophyta - ps11
Charophyceae/Embriophyta - ps12
Embriophyta - ps13
Tracheophyta - ps14
Spermatophyta - ps15
Angiosperms - ps16
Mesangiospermae - ps17
Magnoliids/eudicots - ps18
Eudicots - ps19
Core_eudicots - ps20
Superrosids - ps21
Rosids - ps22
Eurosids - ps23
Malvids - ps24
Sap./Malv./Brassicales - ps25
Malvales/Brassicales - ps26
Brassicales - ps27
Cleomaceae/Brassicaceae - ps28
Brassicaceae - ps29
Camelineae - ps30
Arabidopsis - ps31
Arabidopsis_thaliana - ps32
Phylostratum

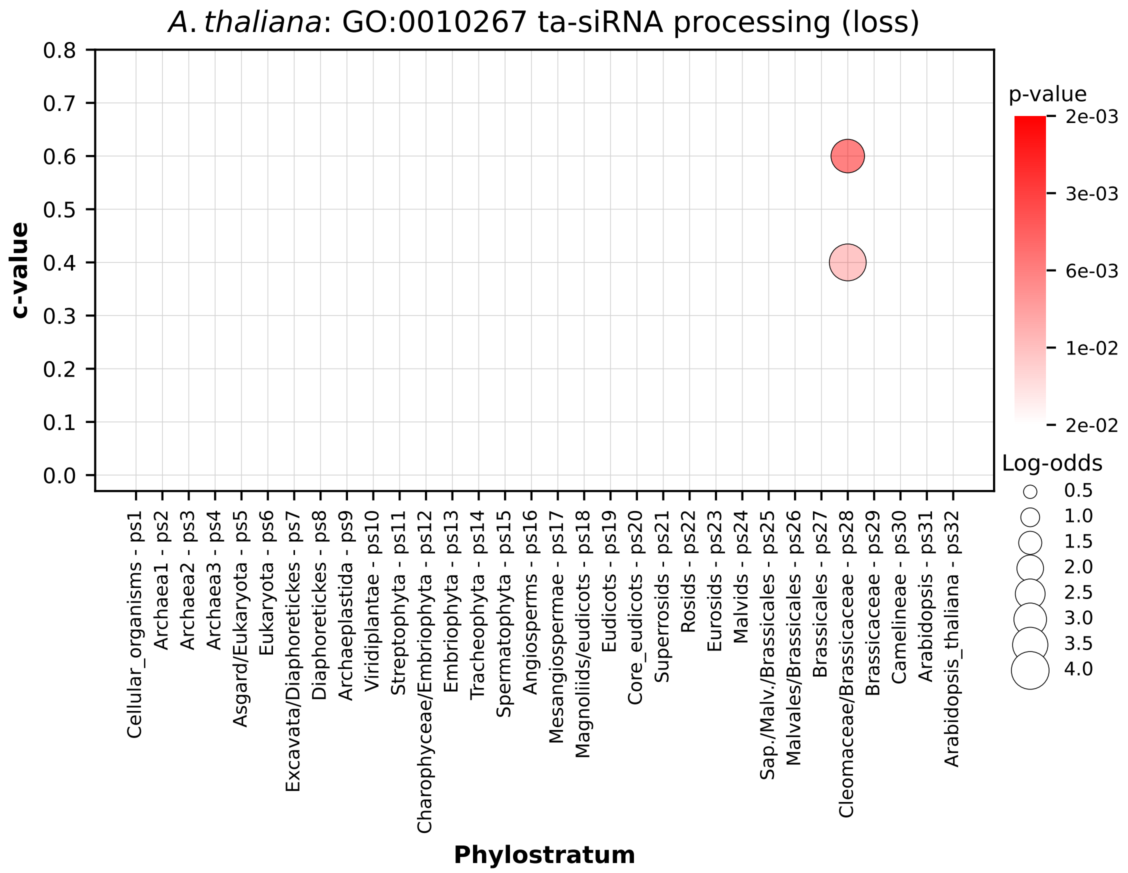

A. thaliana: GO:0010267 ta-siRNA processing (loss)
c-value
0.8
0.7
0.6
0.5
0.4
0.3
0.2
0.1
0.0
p-value
2e-03
3e-03
6e-03
1e-02
2e-02
Log-odds
0.5
1.0
1.5
2.0
2.5
3.0
3.5
4.0
Cellular_organisms - ps1
Archaea1 - ps2
Archaea2 - ps3
Archaea3 - ps4
Asgard/Eukaryota - ps5
Eukaryota - ps6
Excavata/Diaphoretickes - ps7
Diaphoretickes - ps8
Archaeplastida - ps9
Viridiplantae - ps10
Streptophyta - ps11
Charophyceae/Embriophyta - ps12
Embriophyta - ps13
Tracheophyta - ps14
Spermatophyta - ps15
Angiosperms - ps16
Mesangiospermae - ps17
Magnoliids/eudicots - ps18
Eudicots - ps19
Core_eudicots - ps20
Superrosids - ps21
Rosids - ps22
Eurosids - ps23
Malvids - ps24
Sap./Malv./Brassicales - ps25
Malvales/Brassicales - ps26
Brassicales - ps27
Cleomaceae/Brassicaceae - ps28
Brassicaceae - ps29
Camelineae - ps30
Arabidopsis - ps31
Arabidopsis_thaliana - ps32
Phylostratum

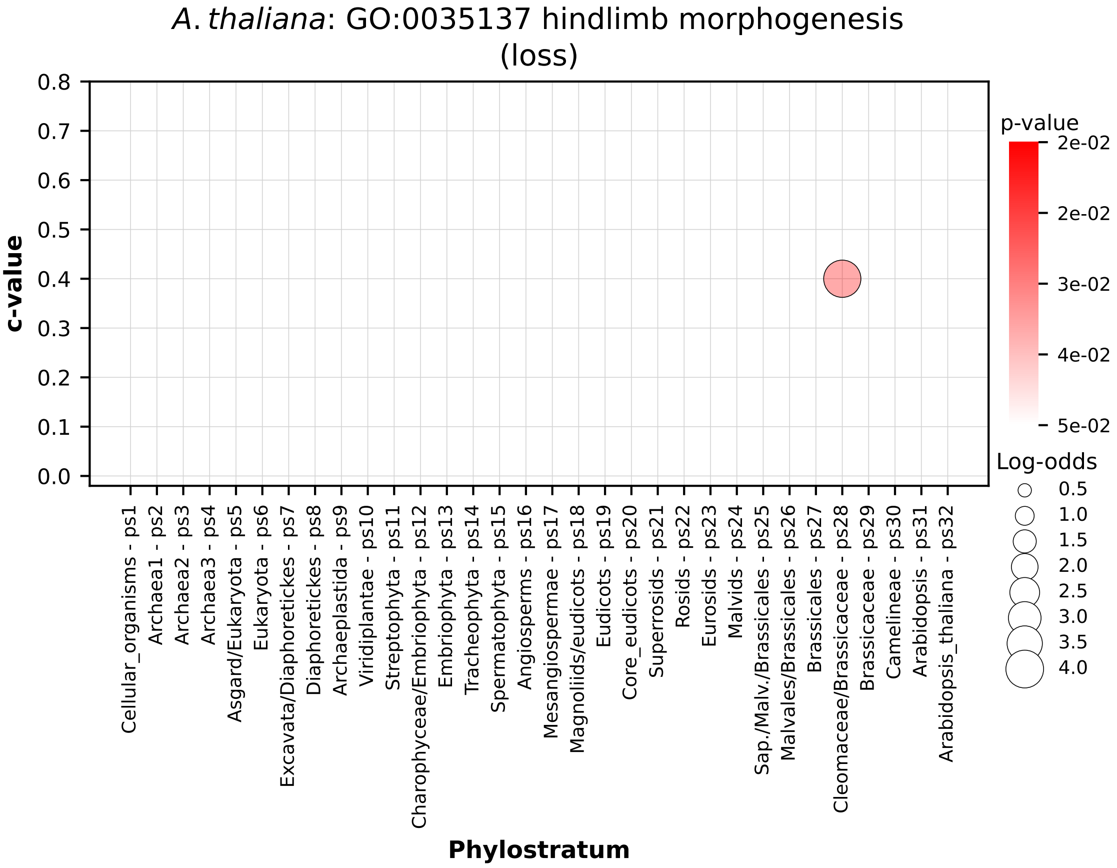

A. thaliana: GO:0035137 hindlimb morphogenesis
(loss)
c-value
0.8
0.7
0.6
0.5
0.4
0.3
0.2
0.1
0.0
p-value
2e-02
2e-02
3e-02
4e-02
5e-02
Log-odds
0.5
1.0
1.5
2.0
2.5
3.0
3.5
4.0
Cellular_organisms - ps1
Archaea1 - ps2
Archaea2 - ps3
Archaea3 - ps4
Asgard/Eukaryota - ps5
Eukaryota - ps6
Excavata/Diaphoretickes - ps7
Diaphoretickes - ps8
Archaeplastida - ps9
Viridiplantae - ps10
Streptophyta - ps11
Charophyceae/Embriophyta - ps12
Embriophyta - ps13
Tracheophyta - ps14
Spermatophyta - ps15
Angiosperms - ps16
Mesangiospermae - ps17
Magnoliids/eudicots - ps18
Eudicots - ps19
Core_eudicots - ps20
Superrosids - ps21
Rosids - ps22
Eurosids - ps23
Malvids - ps24
Sap./Malv./Brassicales - ps25
Malvales/Brassicales - ps26
Brassicales - ps27
Cleomaceae/Brassicaceae - ps28
Brassicaceae - ps29
Camelineae - ps30
Arabidopsis - ps31
Arabidopsis_thaliana - ps32
Phylostratum

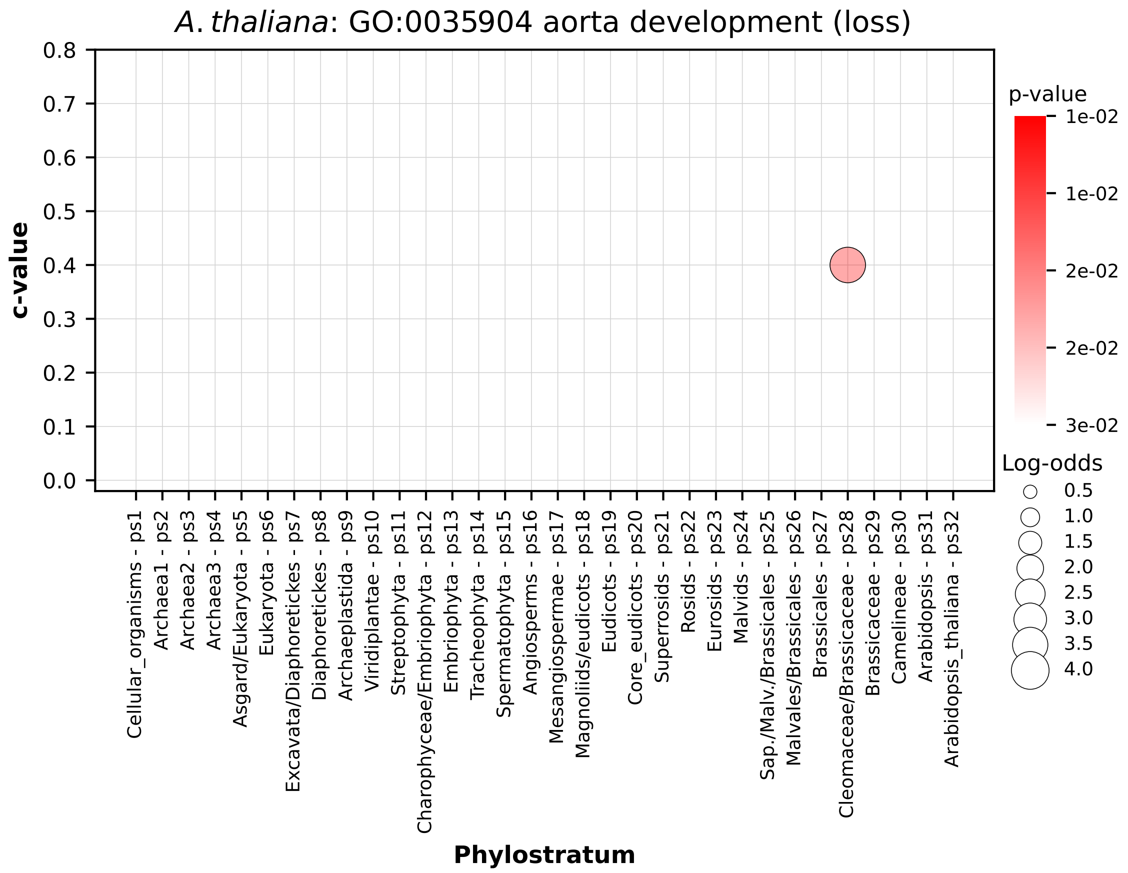

A. thaliana: GO:0035904 aorta development (loss)
c-value
0.0
0.1
0.2
0.3
0.4
0.5
0.6
0.7
0.8
p-value
1e-02
1e-02
2e-02
2e-02
3e-02
Log-odds
0.5
1.0
1.5
2.0
2.5
3.0
3.5
4.0
Cellular_organisms - ps1
Archaea1 - ps2
Archaea2 - ps3
Archaea3 - ps4
Asgard/Eukaryota - ps5
Eukaryota - ps6
Excavata/Diaphoretickes - ps7
Diaphoretickes - ps8
Archaeplastida - ps9
Viridiplantae - ps10
Streptophyta - ps11
Charophyceae/Embriophyta - ps12
Embriophyta - ps13
Tracheophyta - ps14
Spermatophyta - ps15
Angiosperms - ps16
Mesangiospermae - ps17
Magnoliids/eudicots - ps18
Eudicots - ps19
Core_eudicots - ps20
Superrosids - ps21
Rosids - ps22
Eurosids - ps23
Malvids - ps24
Sap./Malv./Brassicales - ps25
Malvales/Brassicales - ps26
Brassicales - ps27
Cleomaceae/Brassicaceae - ps28
Brassicaceae - ps29
Camelineae - ps30
Arabidopsis - ps31
Arabidopsis_thaliana - ps32
Phylostratum

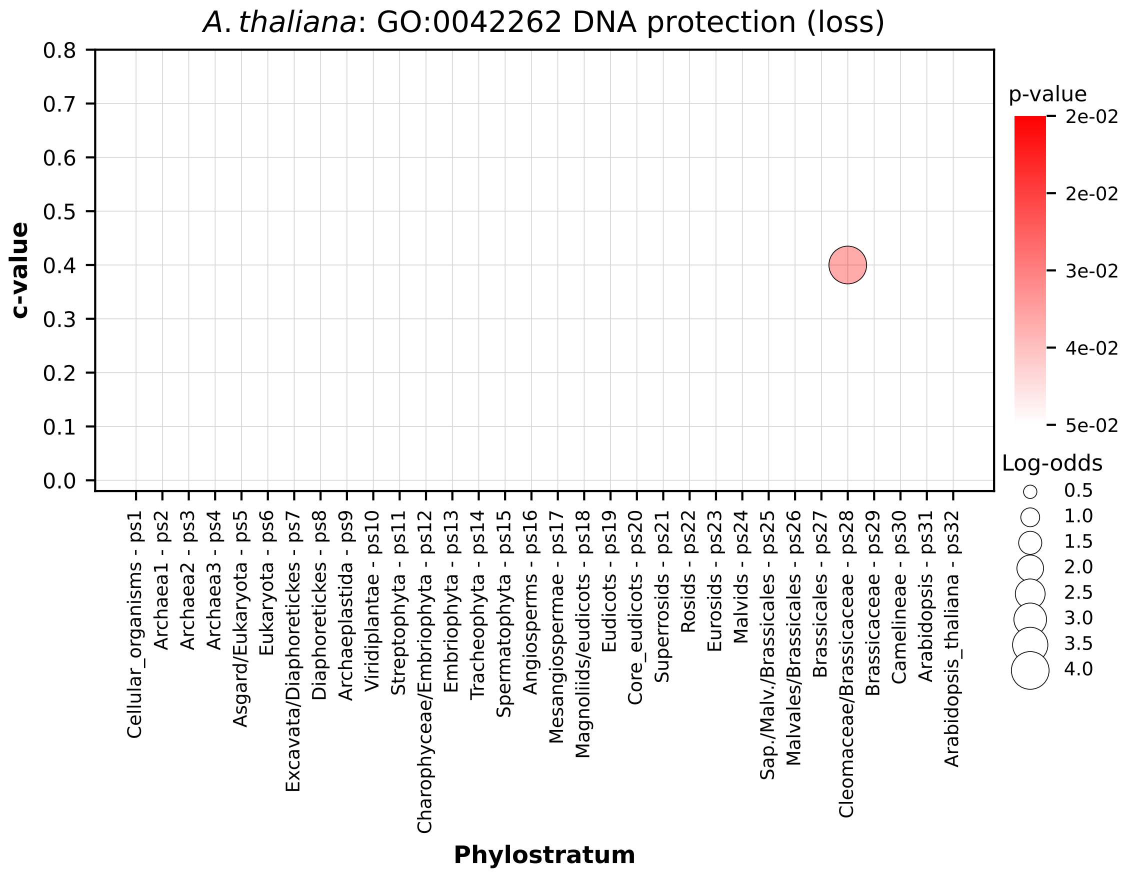

A. thaliana: GO:0042262 DNA protection (loss)
c-value
0.8
0.7
0.6
0.5
0.4
0.3
0.2
0.1
0.0
Phylostratum
Cellular_organisms - ps1
Archaea1 - ps2
Archaea2 - ps3
Archaea3 - ps4
Asgard/Eukaryota - ps5
Eukaryota - ps6
Excavata/Diaphoretickes - ps7
Diaphoretickes - ps8
Archaeplastida - ps9
Viridiplantae - ps10
Streptophyta - ps11
Charophyceae/Embriophyta - ps12
Embriophyta - ps13
Tracheophyta - ps14
Spermatophyta - ps15
Angiosperms - ps16
Mesangiospermae - ps17
Magnoliids/eudicots - ps18
Eudicots - ps19
Core_eudicots - ps20
Superrosids - ps21
Rosids - ps22
Eurosids - ps23
Malvids - ps24
Sap./Malv./Brassicales - ps25
Malvales/Brassicales - ps26
Brassicales - ps27
Cleomaceae/Brassicaceae - ps28
Brassicaceae - ps29
Camelineae - ps30
Arabidopsis - ps31
Arabidopsis_thaliana - ps32
p-value
2e-02
2e-02
3e-02
4e-02
5e-02
Log-odds
0.5
1.0
1.5
2.0
2.5
3.0
3.5
4.0

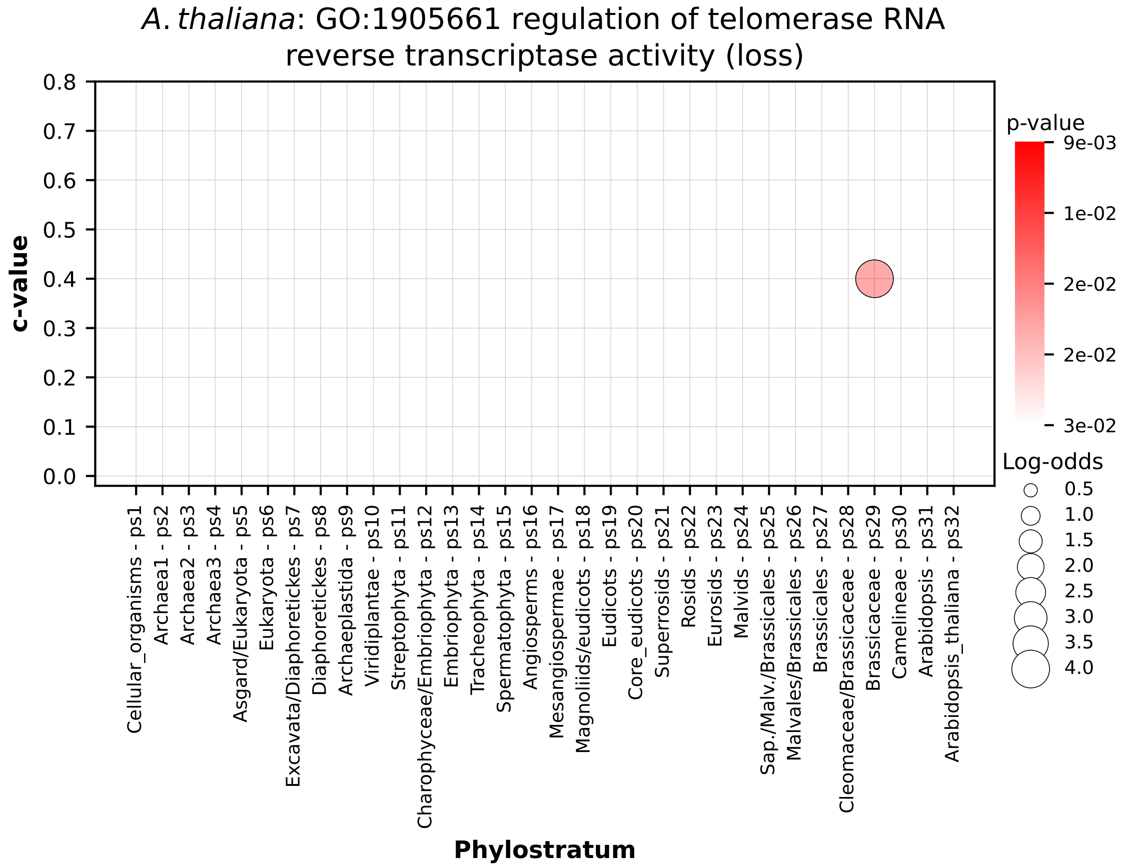

A. thaliana: GO:1905661 regulation of telomerase RNA reverse transcriptase activity (loss)
c-value
0.8
0.7
0.6
0.5
0.4
0.3
0.2
0.1
0.0
p-value
9e-03
1e-02
2e-02
2e-02
3e-02
Log-odds
0.5
1.0
1.5
2.0
2.5
3.0
3.5
4.0
Cellular_organisms - ps1
Archaea1 - ps2
Archaea2 - ps3
Archaea3 - ps4
Asgard/Eukaryota - ps5
Eukaryota - ps6
Excavata/Diaphoretickes - ps7
Diaphoretickes - ps8
Archaeplastida - ps9
Viridiplantae - ps10
Streptophyta - ps11
Charophyceae/Embriophyta - ps12
Embriophyta - ps13
Tracheophyta - ps14
Spermatophyta - ps15
Angiosperms - ps16
Mesangiospermae - ps17
Magnoliids/eudicots - ps18
Eudicots - ps19
Core_eudicots - ps20
Superrosids - ps21
Rosids - ps22
Eurosids - ps23
Malvids - ps24
Sap./Malv./Brassicales - ps25
Malvales/Brassicales - ps26
Brassicales - ps27
Cleomaceae/Brassicaceae - ps28
Brassicaceae - ps29
Camelineae - ps30
Arabidopsis - ps31
Arabidopsis_thaliana - ps32
Phylostratum

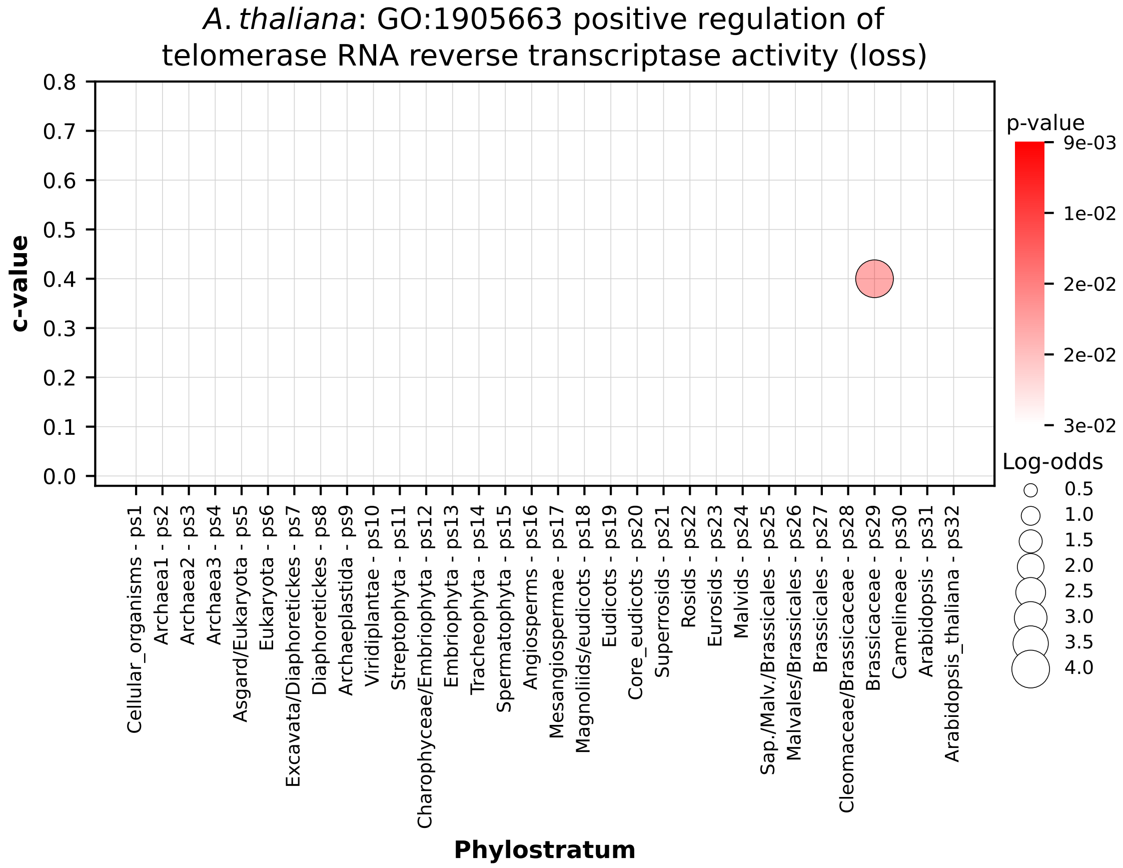

A. thaliana: GO:1905663 positive regulation of telomerase RNA reverse transcriptase activity (loss)
c-value
0.8
0.7
0.6
0.5
0.4
0.3
0.2
0.1
0.0
Phylostratum
Cellular_organisms - ps1
Archaea1 - ps2
Archaea2 - ps3
Archaea3 - ps4
Asgard/Eukaryota - ps5
Eukaryota - ps6
Excavata/Diaphoretickes - ps7
Diaphoretickes - ps8
Archaeplastida - ps9
Viridiplantae - ps10
Streptophyta - ps11
Charophyceae/Embriophyta - ps12
Embriophyta - ps13
Tracheophyta - ps14
Spermatophyta - ps15
Angiosperms - ps16
Mesangiospermae - ps17
Magnoliids/eudicots - ps18
Eudicots - ps19
Core_eudicots - ps20
Superrosids - ps21
Rosids - ps22
Eurosids - ps23
Malvids - ps24
Sap./Malv./Brassicales - ps25
Malvales/Brassicales - ps26
Brassicales - ps27
Cleomaceae/Brassicaceae - ps28
Brassicaceae - ps29
Camelineae - ps30
Arabidopsis - ps31
Arabidopsis_thaliana - ps32
p-value
9e-03
1e-02
2e-02
2e-02
3e-02
Log-odds
0.5
1.0
1.5
2.0
2.5
3.0
3.5
4.0

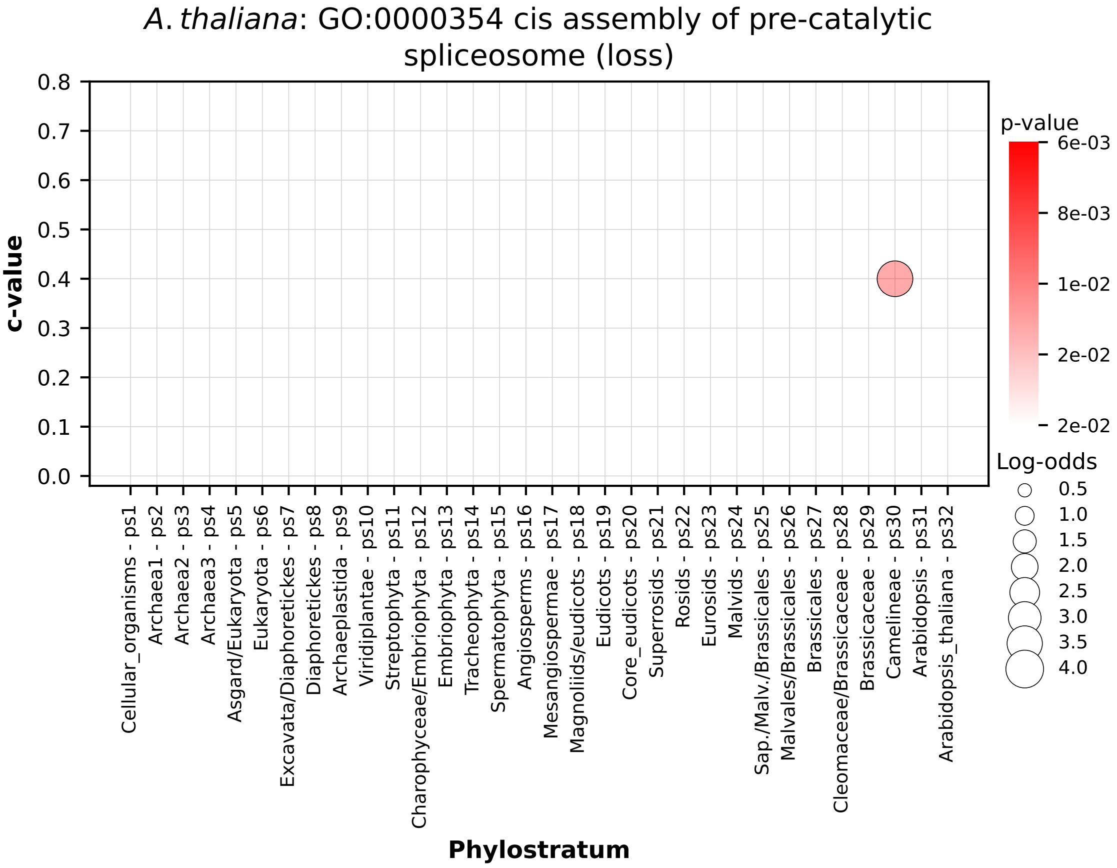
A. thaliana: GO:0000354 cis assembly of pre-catalytic spliceosome (loss)
c-value
0.8
0.7
0.6
0.5
0.4
0.3
0.2
0.1
0.0
Phylostratum
Cellular_organisms - ps1
Archaea1 - ps2
Archaea2 - ps3
Archaea3 - ps4
Asgard/Eukaryota - ps5
Eukaryota - ps6
Excavata/Diaphoretickes - ps7
Diaphoretickes - ps8
Archaeplastida - ps9
Viridiplantae - ps10
Streptophyta - ps11
Charophyceae/Embriophyta - ps12
Embriophyta - ps13
Tracheophyta - ps14
Spermatophyta - ps15
Angiosperms - ps16
Mesangiospermae - ps17
Magnoliids/eudicots - ps18
Eudicots - ps19
Core_eudicots - ps20
Superrosids - ps21
Rosids - ps22
Eurosids - ps23
Malvids - ps24
Sap./Malv./Brassicales - ps25
Malvales/Brassicales - ps26
Brassicales - ps27
Cleomaceae/Brassicaceae - ps28
Brassicaceae - ps29
Camelineae - ps30
Arabidopsis - ps31
Arabidopsis_thaliana - ps32
p-value
6e-03
8e-03
1e-02
2e-02
2e-02
Log-odds
0.5
1.0
1.5
2.0
2.5
3.0
3.5
4.0

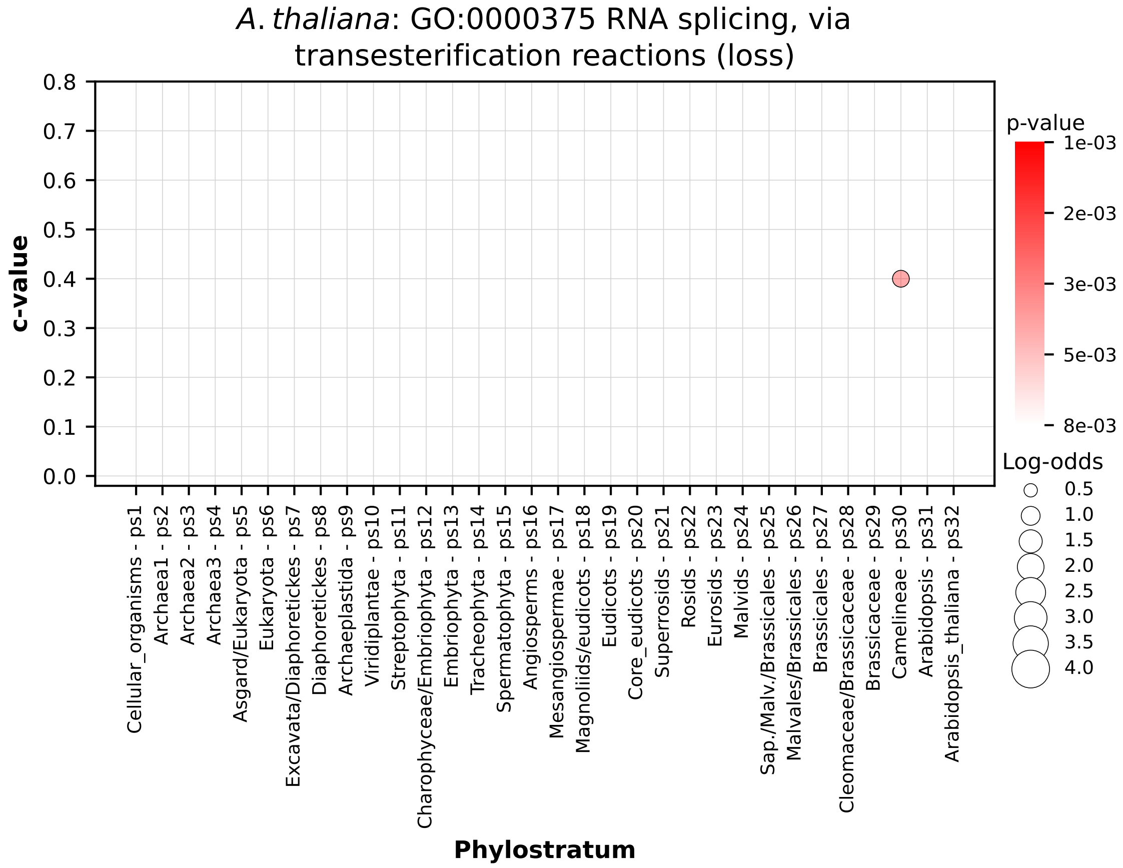

A. thaliana: GO:0000375 RNA splicing, via transesterification reactions (loss)
c-value
0.8
0.7
0.6
0.5
0.4
0.3
0.2
0.1
0.0
Phylostratum
Cellular_organisms - ps1
Archaea1 - ps2
Archaea2 - ps3
Archaea3 - ps4
Asgard/Eukaryota - ps5
Eukaryota - ps6
Excavata/Diaphoretickes - ps7
Diaphoretickes - ps8
Archaeplastida - ps9
Viridiplantae - ps10
Streptophyta - ps11
Charophyceae/Embriophyta - ps12
Embriophyta - ps13
Tracheophyta - ps14
Spermatophyta - ps15
Angiosperms - ps16
Mesangiospermae - ps17
Magnoliids/eudicots - ps18
Eudicots - ps19
Core_eudicots - ps20
Superrosids - ps21
Rosids - ps22
Eurosids - ps23
Malvids - ps24
Sap./Malv./Brassicales - ps25
Malvales/Brassicales - ps26
Brassicales - ps27
Cleomaceae/Brassicaceae - ps28
Brassicaceae - ps29
Camelineae - ps30
Arabidopsis - ps31
Arabidopsis_thaliana - ps32
p-value
1e-03
2e-03
3e-03
5e-03
8e-03
Log-odds
0.5
1.0
1.5
2.0
2.5
3.0
3.5
4.0

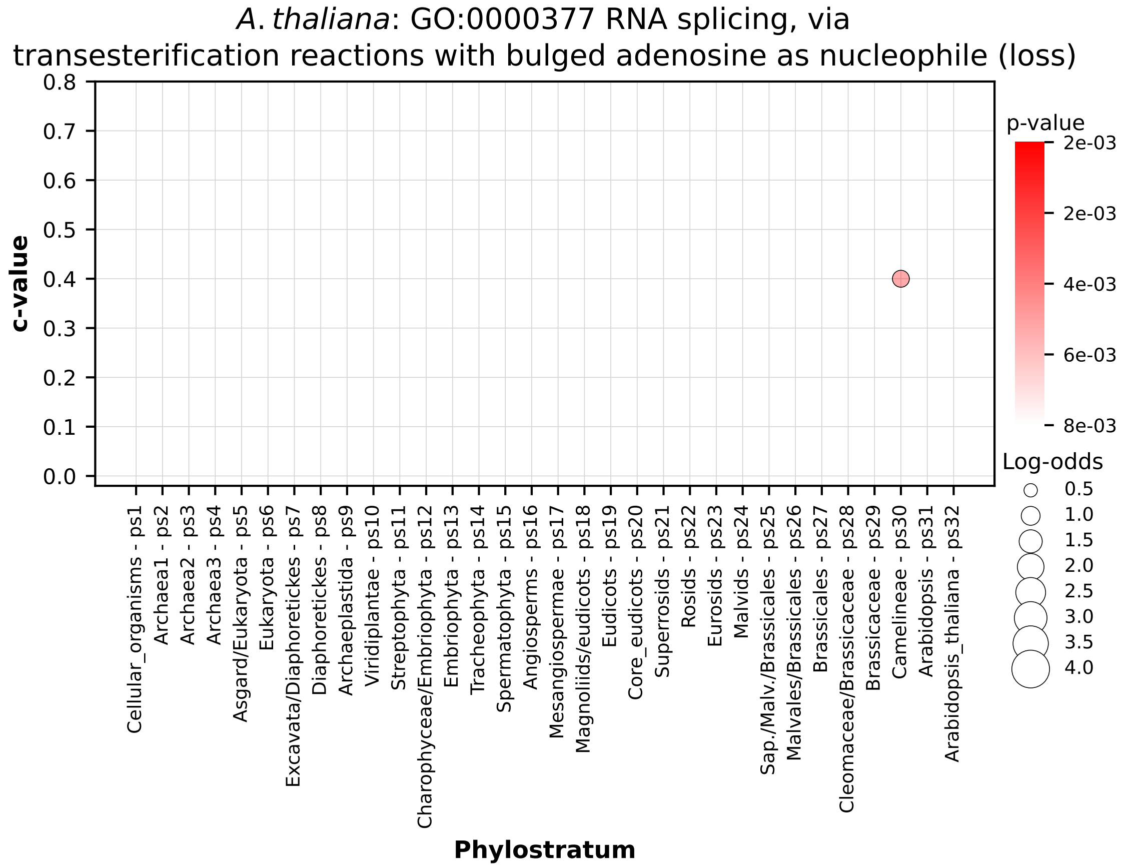

A. thaliana: GO:0000377 RNA splicing, via transesterification reactions with bulged adenosine as nucleophile (loss)
c-value
0.8
0.7
0.6
0.5
0.4
0.3
0.2
0.1
0.0
p-value
2e-03
2e-03
4e-03
6e-03
8e-03
Log-odds
0.5
1.0
1.5
2.0
2.5
3.0
3.5
4.0
Cellular_organisms - ps1
Archaea1 - ps2
Archaea2 - ps3
Archaea3 - ps4
Asgard/Eukaryota - ps5
Eukaryota - ps6
Excavata/Diaphoretickes - ps7
Diaphoretickes - ps8
Archaeplastida - ps9
Viridiplantae - ps10
Streptophyta - ps11
Charophyceae/Embriophyta - ps12
Embriophyta - ps13
Tracheophyta - ps14
Spermatophyta - ps15
Angiosperms - ps16
Mesangiospermae - ps17
Magnoliids/eudicots - ps18
Eudicots - ps19
Core_eudicots - ps20
Superrosids - ps21
Rosids - ps22
Eurosids - ps23
Malvids - ps24
Sap./Malv./Brassicales - ps25
Malvales/Brassicales - ps26
Brassicales - ps27
Cleomaceae/Brassicaceae - ps28
Brassicaceae - ps29
Camelineae - ps30
Arabidopsis - ps31
Arabidopsis_thaliana - ps32
Phylostratum

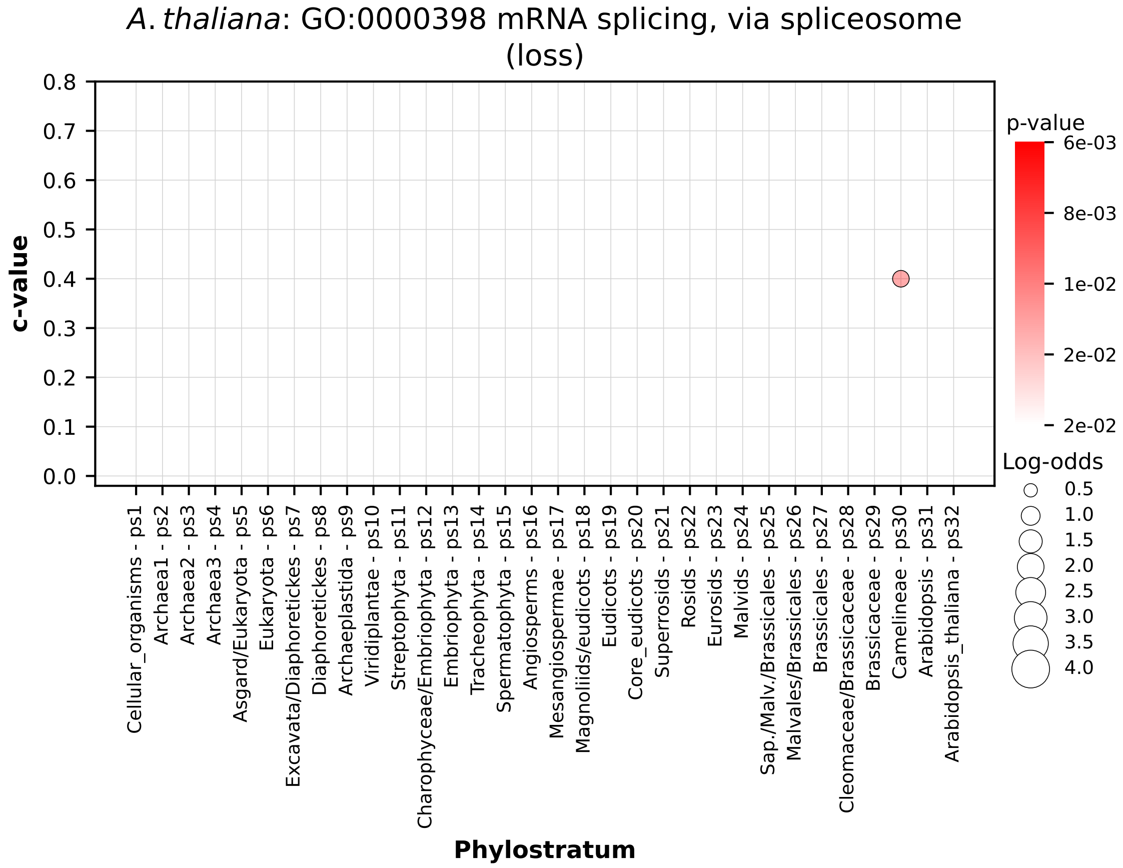

A. thaliana: GO:0000398 mRNA splicing, via spliceosome (loss)
c-value
0.8
0.7
0.6
0.5
0.4
0.3
0.2
0.1
0.0
p-value
6e-03
8e-03
1e-02
2e-02
2e-02
Log-odds
0.5
1.0
1.5
2.0
2.5
3.0
3.5
4.0
Cellular_organisms - ps1
Archaea1 - ps2
Archaea2 - ps3
Archaea3 - ps4
Asgard/Eukaryota - ps5
Eukaryota - ps6
Excavata/Diaphoretickes - ps7
Diaphoretickes - ps8
Archaeplastida - ps9
Viridiplantae - ps10
Streptophyta - ps11
Charophyceae/Embriophyta - ps12
Embriophyta - ps13
Tracheophyta - ps14
Spermatophyta - ps15
Angiosperms - ps16
Mesangiospermae - ps17
Magnoliids/eudicots - ps18
Eudicots - ps19
Core_eudicots - ps20
Superrosids - ps21
Rosids - ps22
Eurosids - ps23
Malvids - ps24
Sap./Malv./Brassicales - ps25
Malvales/Brassicales - ps26
Brassicales - ps27
Cleomaceae/Brassicaceae - ps28
Brassicaceae - ps29
Camelineae - ps30
Arabidopsis - ps31
Arabidopsis_thaliana - ps32
Phylostratum

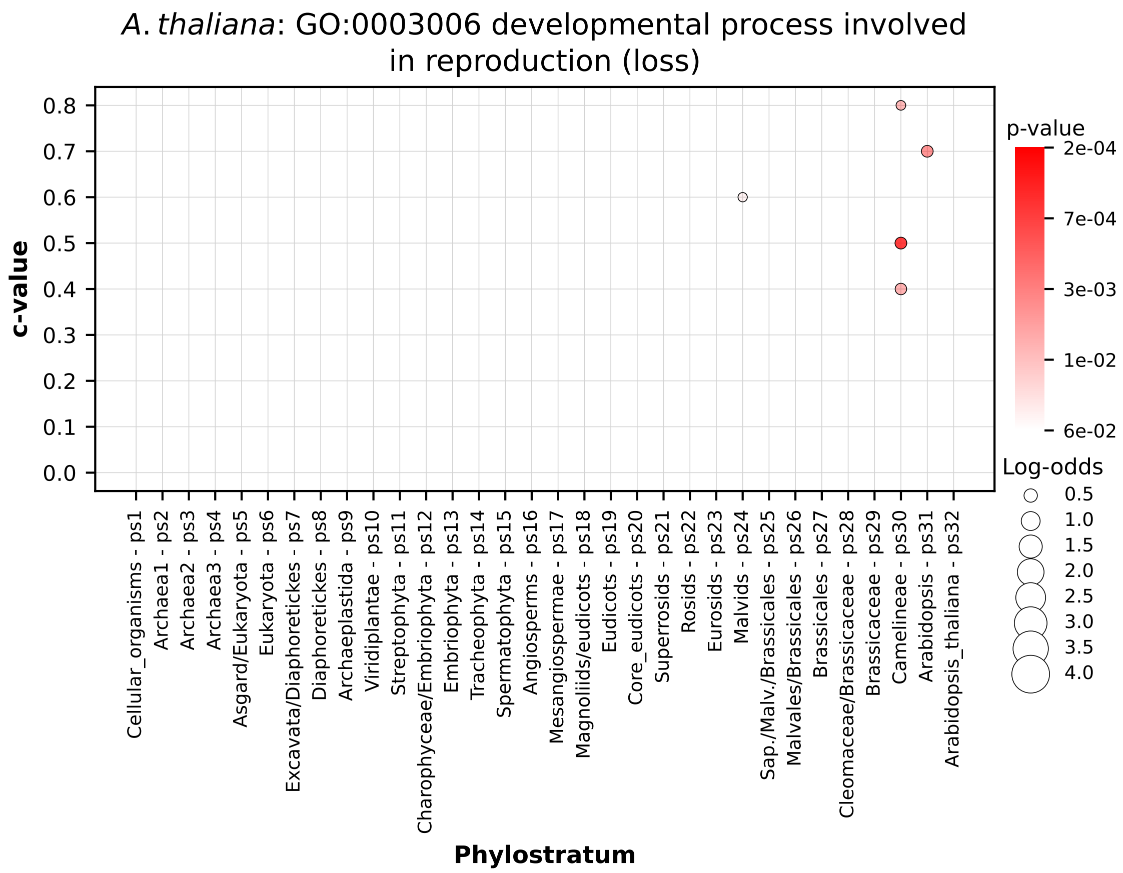

A. thaliana: GO:0003006 developmental process involved in reproduction (loss)
c-value
0.0
0.1
0.2
0.3
0.4
0.5
0.6
0.7
0.8
Phylostratum
Cellular_organisms - ps1
Archaea1 - ps2
Archaea2 - ps3
Archaea3 - ps4
Asgard/Eukaryota - ps5
Eukaryota - ps6
Excavata/Diaphoretickes - ps7
Diaphoretickes - ps8
Archaeplastida - ps9
Viridiplantae - ps10
Streptophyta - ps11
Charophyceae/Embriophyta - ps12
Embriophyta - ps13
Tracheophyta - ps14
Spermatophyta - ps15
Angiosperms - ps16
Mesangiospermae - ps17
Magnoliids/eudicots - ps18
Eudicots - ps19
Core_eudicots - ps20
Superrosids - ps21
Rosids - ps22
Eurosids - ps23
Malvids - ps24
Sap./Malv./Brassicales - ps25
Malvales/Brassicales - ps26
Brassicales - ps27
Cleomaceae/Brassicaceae - ps28
Brassicaceae - ps29
Camelineae - ps30
Arabidopsis - ps31
Arabidopsis_thaliana - ps32
p-value
2e-04
7e-04
3e-03
1e-02
6e-02
Log-odds
0.5
1.0
1.5
2.0
2.5
3.0
3.5
4.0

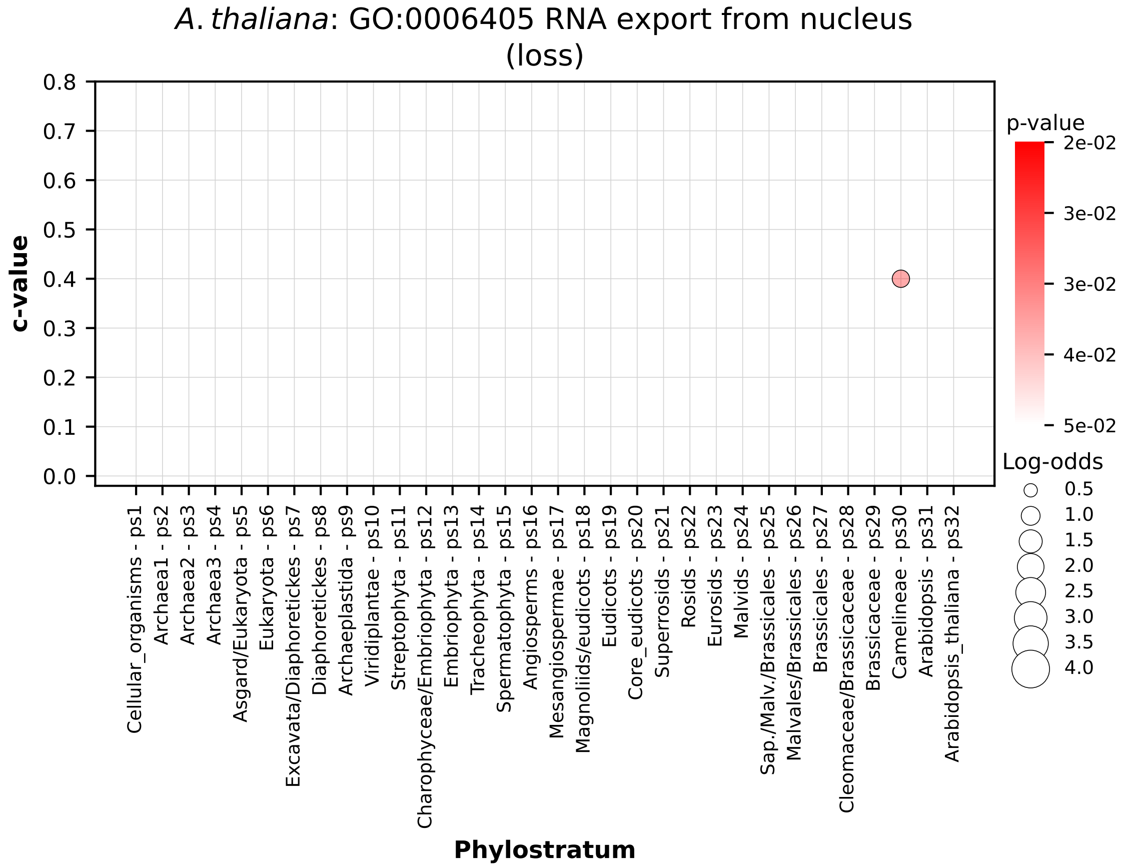

A. thaliana: GO:0006405 RNA export from nucleus
(loss)
c-value
0.8
0.7
0.6
0.5
0.4
0.3
0.2
0.1
0.0
Phylostratum
Cellular_organisms - ps1
Archaea1 - ps2
Archaea2 - ps3
Archaea3 - ps4
Asgard/Eukaryota - ps5
Eukaryota - ps6
Excavata/Diaphoretickes - ps7
Diaphoretickes - ps8
Archaeplastida - ps9
Viridiplantae - ps10
Streptophyta - ps11
Charophyceae/Embriophyta - ps12
Embriophyta - ps13
Tracheophyta - ps14
Spermatophyta - ps15
Angiosperms - ps16
Mesangiospermae - ps17
Magnoliids/eudicots - ps18
Eudicots - ps19
Core_eudicots - ps20
Superrosids - ps21
Rosids - ps22
Eurosids - ps23
Malvids - ps24
Sap./Malv./Brassicales - ps25
Malvales/Brassicales - ps26
Brassicales - ps27
Cleomaceae/Brassicaceae - ps28
Brassicaceae - ps29
Camelineae - ps30
Arabidopsis - ps31
Arabidopsis_thaliana - ps32
p-value
2e-02
3e-02
3e-02
4e-02
5e-02
Log-odds
0.5
1.0
1.5
2.0
2.5
3.0
3.5
4.0

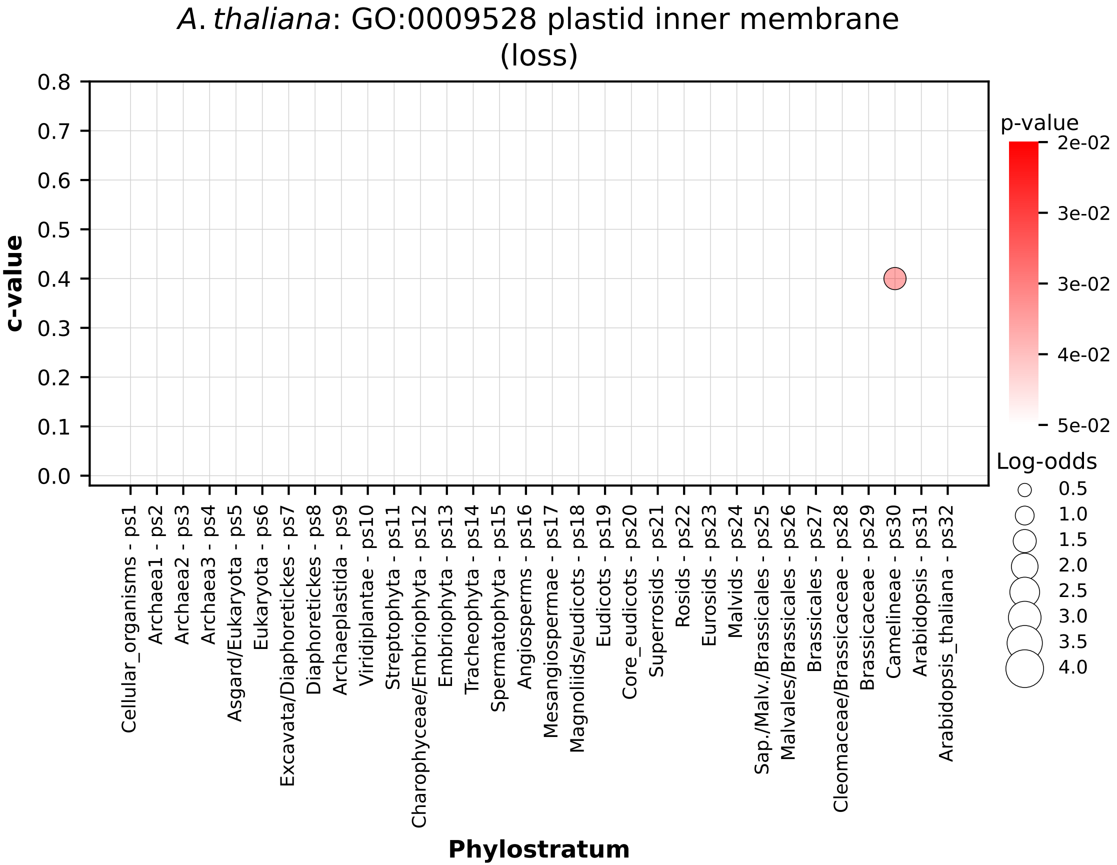

A. thaliana: GO:0009528 plastid inner membrane
(loss)
c-value
0.8
0.7
0.6
0.5
0.4
0.3
0.2
0.1
0.0
p-value
2e-02
3e-02
3e-02
4e-02
5e-02
Log-odds
0.5
1.0
1.5
2.0
2.5
3.0
3.5
4.0
Cellular_organisms - ps1
Archaea1 - ps2
Archaea2 - ps3
Archaea3 - ps4
Asgard/Eukaryota - ps5
Eukaryota - ps6
Excavata/Diaphoretickes - ps7
Diaphoretickes - ps8
Archaeplastida - ps9
Viridiplantae - ps10
Streptophyta - ps11
Charophyceae/Embriophyta - ps12
Embriophyta - ps13
Tracheophyta - ps14
Spermatophyta - ps15
Angiosperms - ps16
Mesangiospermae - ps17
Magnoliids/eudicots - ps18
Eudicots - ps19
Core_eudicots - ps20
Superrosids - ps21
Rosids - ps22
Eurosids - ps23
Malvids - ps24
Sap./Malv./Brassicales - ps25
Malvales/Brassicales - ps26
Brassicales - ps27
Cleomaceae/Brassicaceae - ps28
Brassicaceae - ps29
Camelineae - ps30
Arabidopsis - ps31
Arabidopsis_thaliana - ps32
Phylostratum

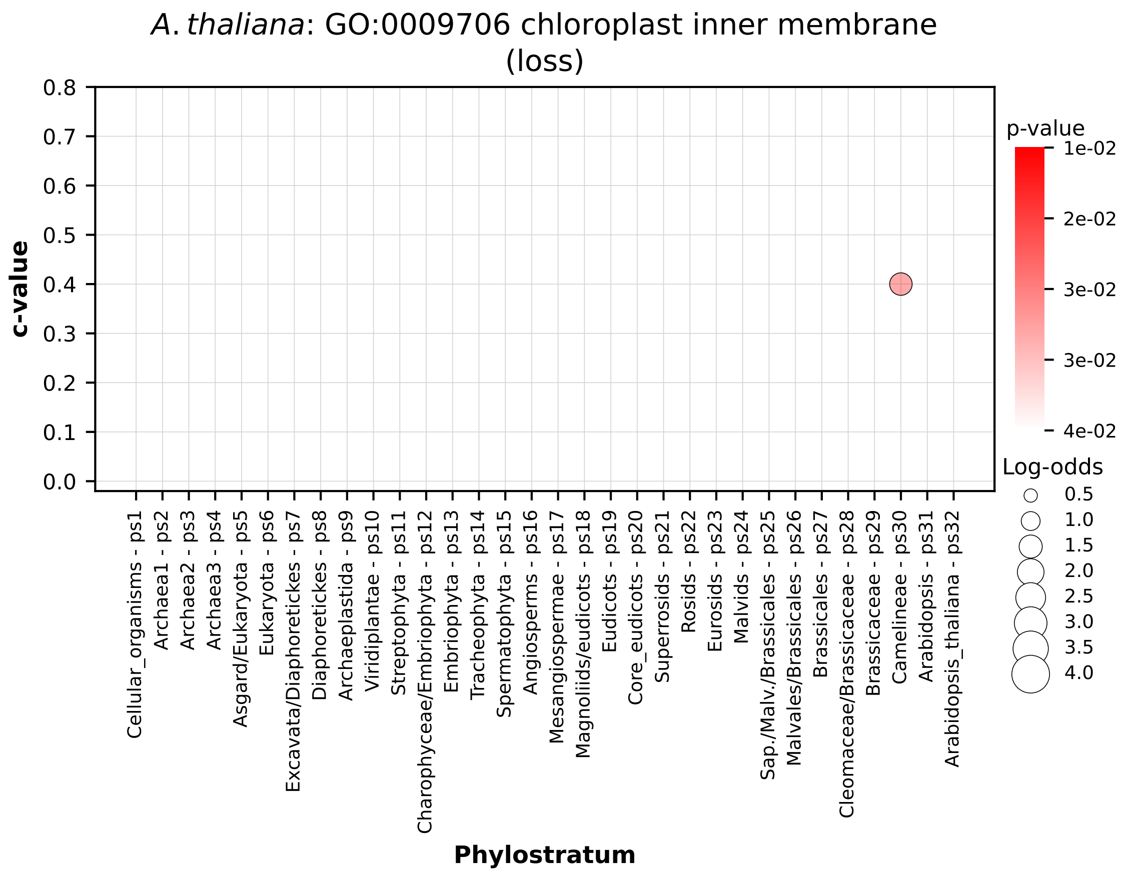

A. thaliana: GO:0009706 chloroplast inner membrane
(loss)
c-value
0.0
0.1
0.2
0.3
0.4
0.5
0.6
0.7
0.8
Phylostratum
Cellular_organisms - ps1
Archaea1 - ps2
Archaea2 - ps3
Archaea3 - ps4
Asgard/Eukaryota - ps5
Eukaryota - ps6
Excavata/Diaphoretickes - ps7
Diaphoretickes - ps8
Archaeplastida - ps9
Viridiplantae - ps10
Streptophyta - ps11
Charophyceae/Embriophyta - ps12
Embriophyta - ps13
Tracheophyta - ps14
Spermatophyta - ps15
Angiosperms - ps16
Mesangiospermae - ps17
Magnoliids/eudicots - ps18
Eudicots - ps19
Core_eudicots - ps20
Superrosids - ps21
Rosids - ps22
Eurosids - ps23
Malvids - ps24
Sap./Malv./Brassicales - ps25
Malvales/Brassicales - ps26
Brassicales - ps27
Cleomaceae/Brassicaceae - ps28
Brassicaceae - ps29
Camelineae - ps30
Arabidopsis - ps31
Arabidopsis_thaliana - ps32
p-value
1e-02
2e-02
3e-02
3e-02
4e-02
Log-odds
0.5
1.0
1.5
2.0
2.5
3.0
3.5
4.0

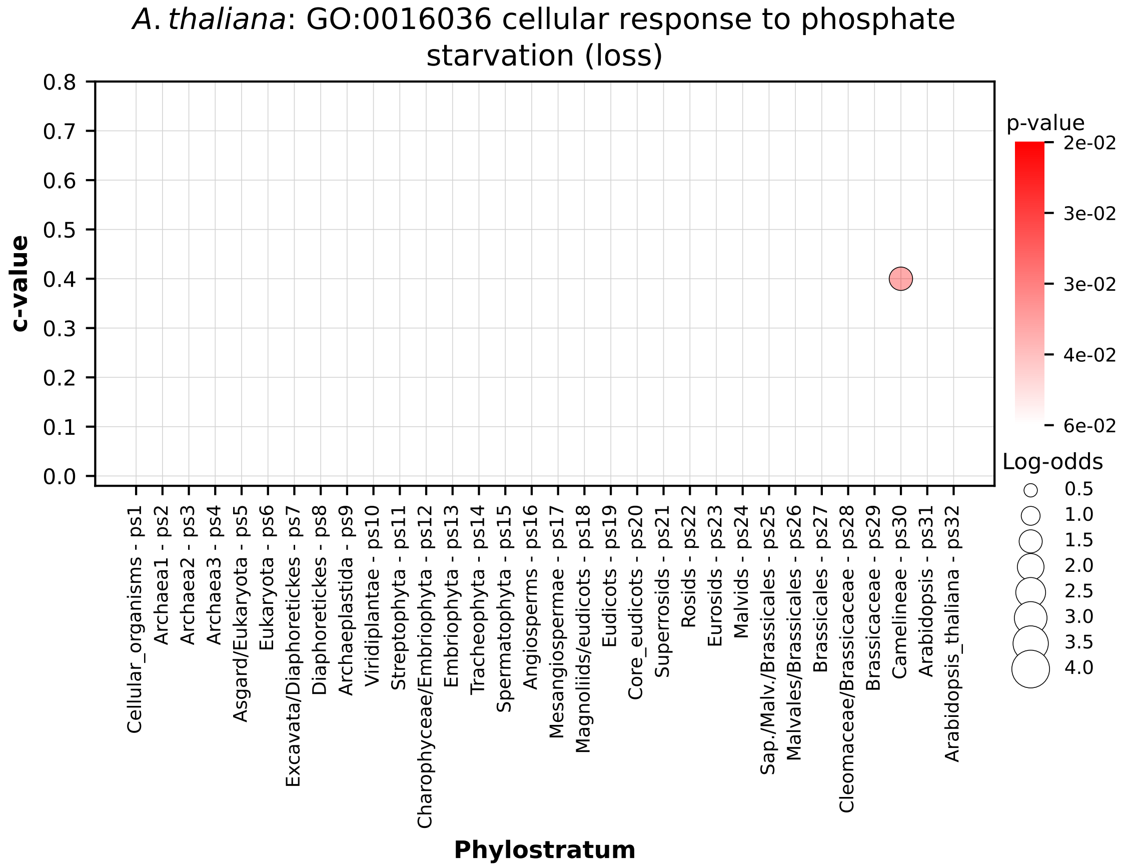

A. thaliana: GO:0016036 cellular response to phosphate starvation (loss)
c-value
0.8
0.7
0.6
0.5
0.4
0.3
0.2
0.1
0.0
Phylostratum
Cellular_organisms - ps1
Archaea1 - ps2
Archaea2 - ps3
Archaea3 - ps4
Asgard/Eukaryota - ps5
Eukaryota - ps6
Excavata/Diaphoretickes - ps7
Diaphoretickes - ps8
Archaeplastida - ps9
Viridiplantae - ps10
Streptophyta - ps11
Charophyceae/Embriophyta - ps12
Embriophyta - ps13
Tracheophyta - ps14
Spermatophyta - ps15
Angiosperms - ps16
Mesangiospermae - ps17
Magnoliids/eudicots - ps18
Eudicots - ps19
Core_eudicots - ps20
Superrosids - ps21
Rosids - ps22
Eurosids - ps23
Malvids - ps24
Sap./Malv./Brassicales - ps25
Malvales/Brassicales - ps26
Brassicales - ps27
Cleomaceae/Brassicaceae - ps28
Brassicaceae - ps29
Camelineae - ps30
Arabidopsis - ps31
Arabidopsis_thaliana - ps32
p-value
2e-02
3e-02
3e-02
4e-02
6e-02
Log-odds
0.5
1.0
1.5
2.0
2.5
3.0
3.5
4.0

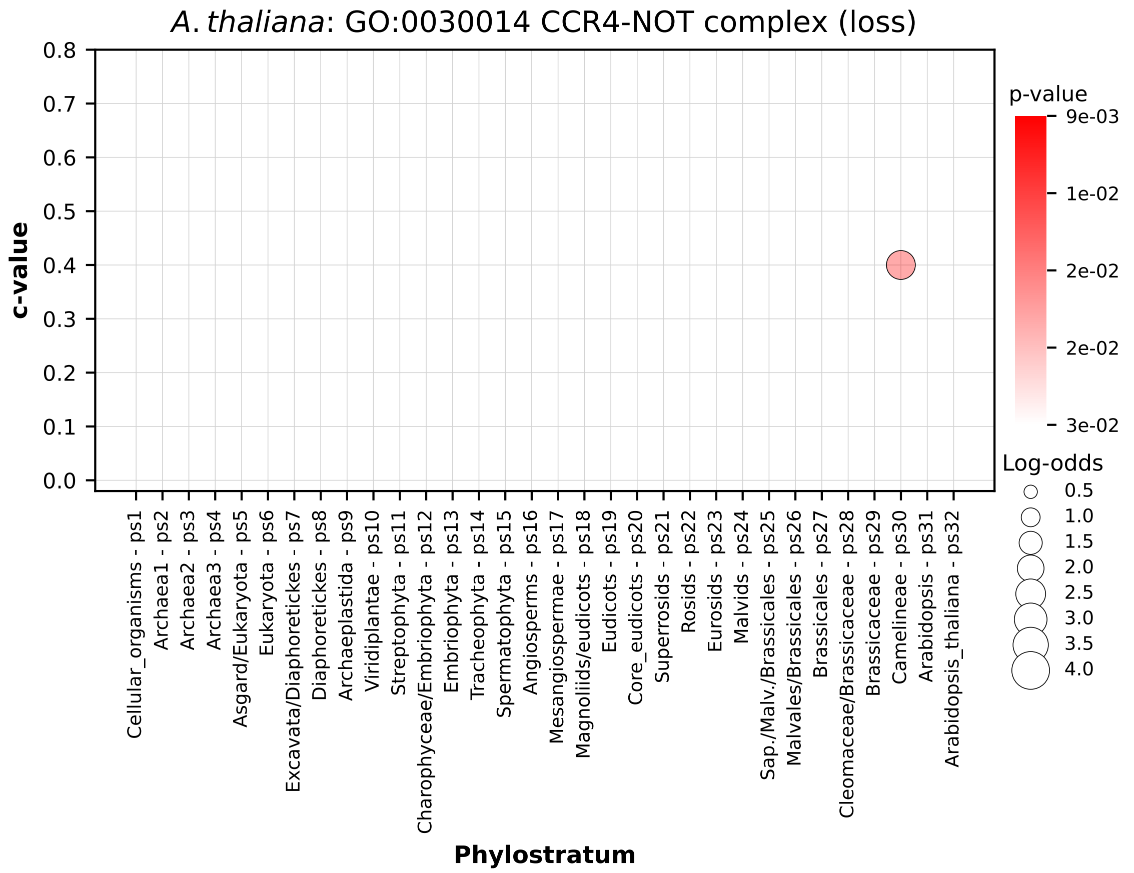

A. thaliana: GO:0030014 CCR4-NOT complex (loss)
c-value
0.0
0.1
0.2
0.3
0.4
0.5
0.6
0.7
0.8
Phylostratum
Cellular_organisms - ps1
Archaea1 - ps2
Archaea2 - ps3
Archaea3 - ps4
Asgard/Eukaryota - ps5
Eukaryota - ps6
Excavata/Diaphoretickes - ps7
Diaphoretickes - ps8
Archaeplastida - ps9
Viridiplantae - ps10
Streptophyta - ps11
Charophyceae/Embriophyta - ps12
Embriophyta - ps13
Tracheophyta - ps14
Spermatophyta - ps15
Angiosperms - ps16
Mesangiospermae - ps17
Magnoliids/eudicots - ps18
Eudicots - ps19
Core_eudicots - ps20
Superrosids - ps21
Rosids - ps22
Eurosids - ps23
Malvids - ps24
Sap./Malv./Brassicales - ps25
Malvales/Brassicales - ps26
Brassicales - ps27
Cleomaceae/Brassicaceae - ps28
Brassicaceae - ps29
Camelineae - ps30
Arabidopsis - ps31
Arabidopsis_thaliana - ps32
p-value
9e-03
1e-02
2e-02
2e-02
3e-02
Log-odds
0.5
1.0
1.5
2.0
2.5
3.0
3.5
4.0

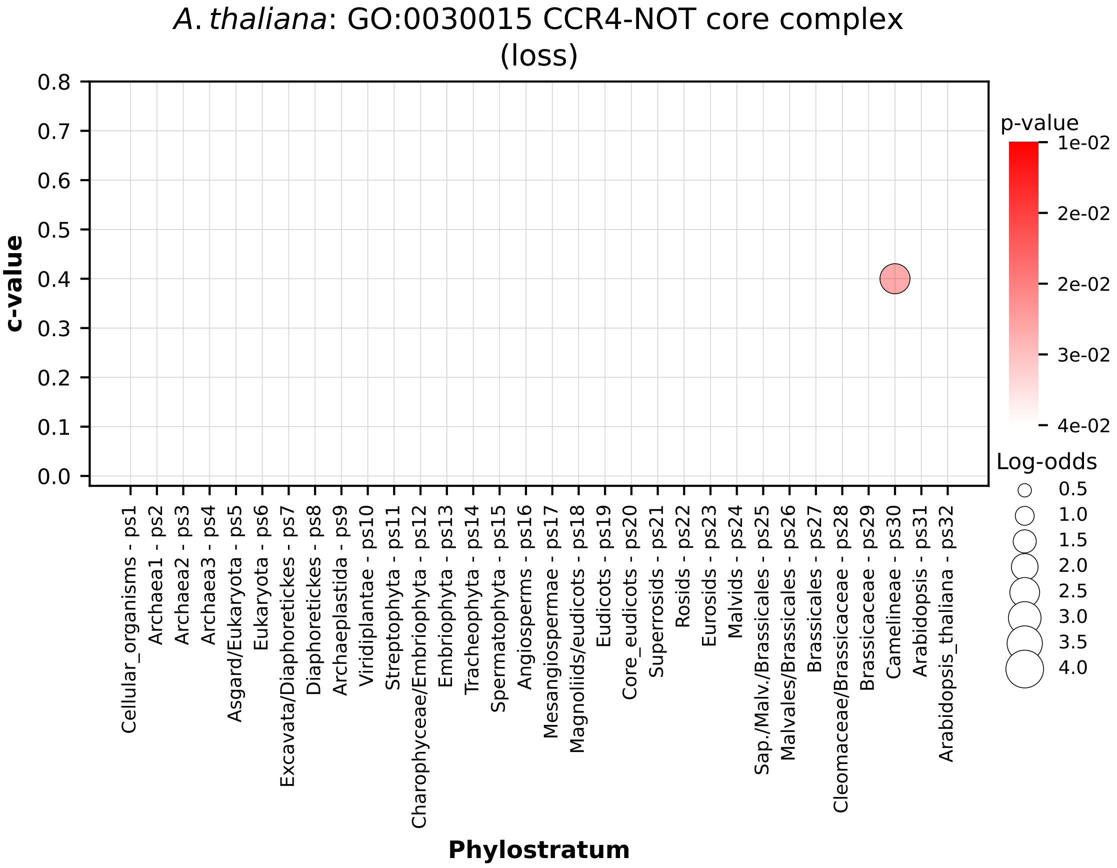

A. thaliana: GO:0030015 CCR4-NOT core complex
(loss)
c-value
0.8
0.7
0.6
0.5
0.4
0.3
0.2
0.1
0.0
p-value
1e-02
2e-02
2e-02
3e-02
4e-02
Log-odds
0.5
1.0
1.5
2.0
2.5
3.0
3.5
4.0
Cellular_organisms - ps1
Archaea1 - ps2
Archaea2 - ps3
Archaea3 - ps4
Asgard/Eukaryota - ps5
Eukaryota - ps6
Excavata/Diaphoretickes - ps7
Diaphoretickes - ps8
Archaeplastida - ps9
Viridiplantae - ps10
Streptophyta - ps11
Charophyceae/Embriophyta - ps12
Embriophyta - ps13
Tracheophyta - ps14
Spermatophyta - ps15
Angiosperms - ps16
Mesangiospermae - ps17
Magnoliids/eudicots - ps18
Eudicots - ps19
Core_eudicots - ps20
Superrosids - ps21
Rosids - ps22
Eurosids - ps23
Malvids - ps24
Sap./Malv./Brassicales - ps25
Malvales/Brassicales - ps26
Brassicales - ps27
Cleomaceae/Brassicaceae - ps28
Brassicaceae - ps29
Camelineae - ps30
Arabidopsis - ps31
Arabidopsis_thaliana - ps32
Phylostratum

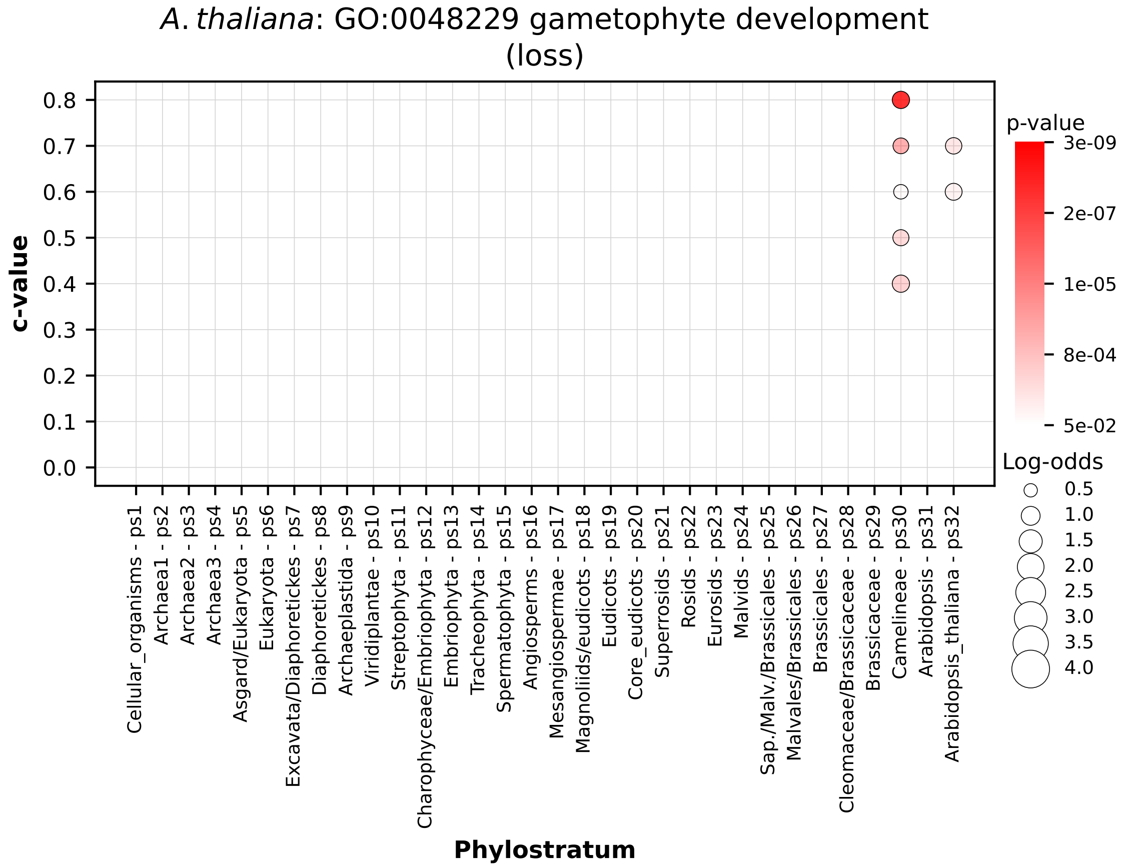

A. thaliana: GO:0048229 gametophyte development
(loss)
c-value
0.8
0.7
0.6
0.5
0.4
0.3
0.2
0.1
0.0
Phylostratum
Cellular_organisms - ps1
Archaea1 - ps2
Archaea2 - ps3
Archaea3 - ps4
Asgard/Eukaryota - ps5
Eukaryota - ps6
Excavata/Diaphoretickes - ps7
Diaphoretickes - ps8
Archaeplastida - ps9
Viridiplantae - ps10
Streptophyta - ps11
Charophyceae/Embriophyta - ps12
Embriophyta - ps13
Tracheophyta - ps14
Spermatophyta - ps15
Angiosperms - ps16
Mesangiospermae - ps17
Magnoliids/eudicots - ps18
Eudicots - ps19
Core_eudicots - ps20
Superrosids - ps21
Rosids - ps22
Eurosids - ps23
Malvids - ps24
Sap./Malv./Brassicales - ps25
Malvales/Brassicales - ps26
Brassicales - ps27
Cleomaceae/Brassicaceae - ps28
Brassicaceae - ps29
Camelineae - ps30
Arabidopsis - ps31
Arabidopsis_thaliana - ps32
p-value
3e-09
2e-07
1e-05
8e-04
5e-02
Log-odds
0.5
1.0
1.5
2.0
2.5
3.0
3.5
4.0

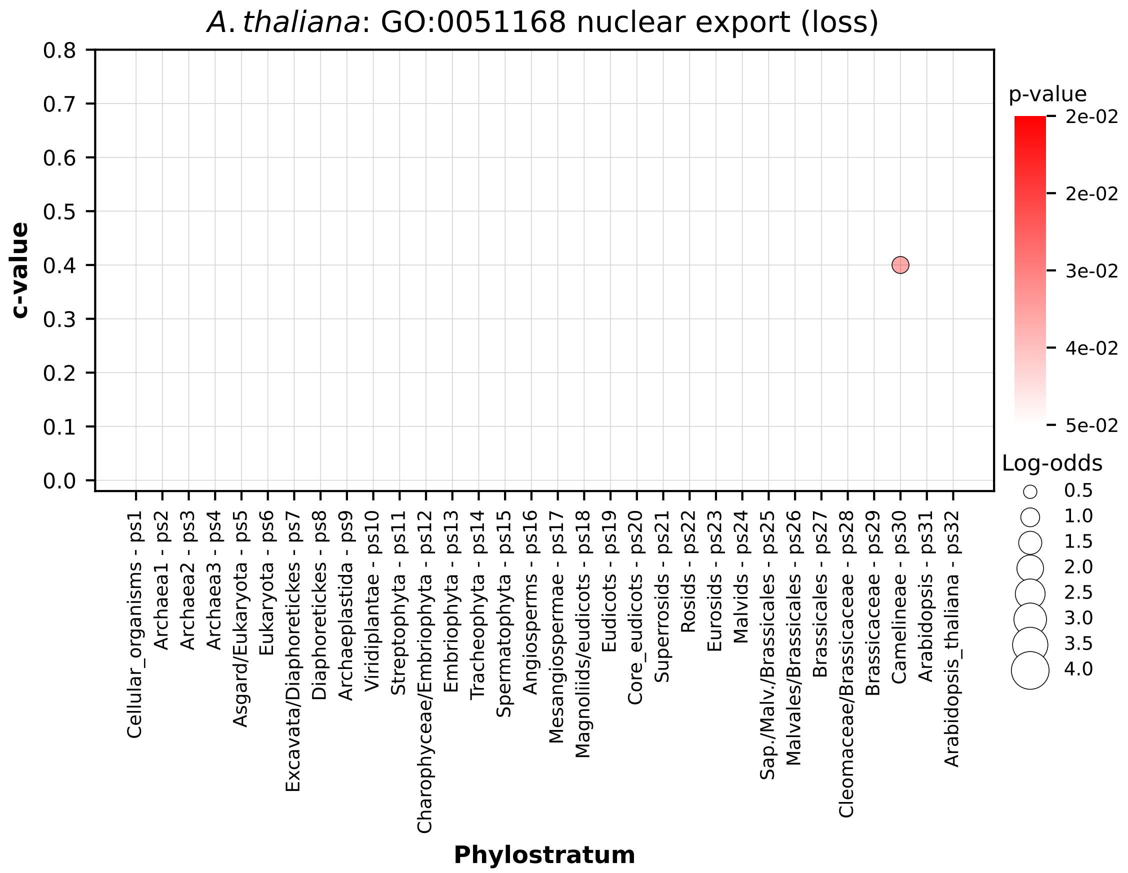

A. thaliana: GO:0051168 nuclear export (loss)
c-value
0.0
0.1
0.2
0.3
0.4
0.5
0.6
0.7
0.8
Phylostratum
Cellular_organisms - ps1
Archaea1 - ps2
Archaea2 - ps3
Archaea3 - ps4
Asgard/Eukaryota - ps5
Eukaryota - ps6
Excavata/Diaphoretickes - ps7
Diaphoretickes - ps8
Archaeplastida - ps9
Viridiplantae - ps10
Streptophyta - ps11
Charophyceae/Embriophyta - ps12
Embriophyta - ps13
Tracheophyta - ps14
Spermatophyta - ps15
Angiosperms - ps16
Mesangiospermae - ps17
Magnoliids/eudicots - ps18
Eudicots - ps19
Core_eudicots - ps20
Superrosids - ps21
Rosids - ps22
Eurosids - ps23
Malvids - ps24
Sap./Malv./Brassicales - ps25
Malvales/Brassicales - ps26
Brassicales - ps27
Cleomaceae/Brassicaceae - ps28
Brassicaceae - ps29
Camelineae - ps30
Arabidopsis - ps31
Arabidopsis_thaliana - ps32
p-value
2e-02
2e-02
3e-02
4e-02
5e-02
Log-odds
0.5
1.0
1.5
2.0
2.5
3.0
3.5
4.0

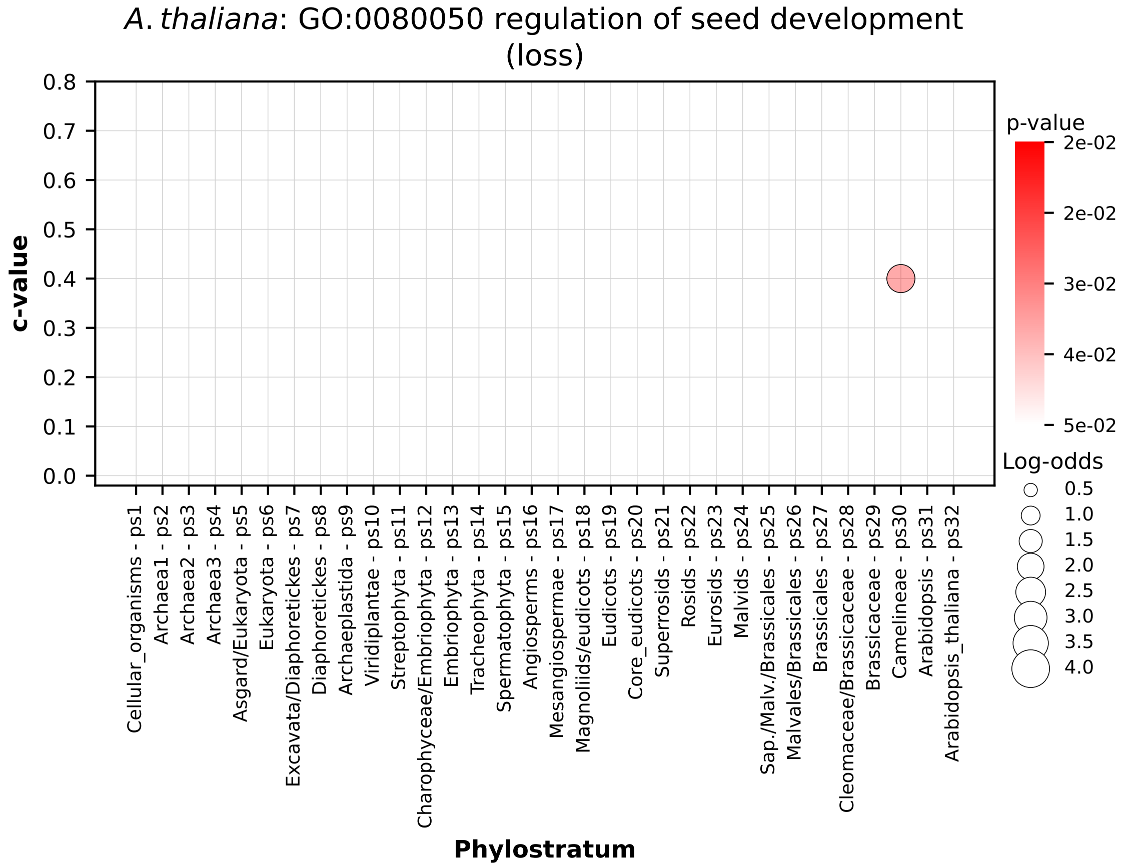

A. thaliana: GO:0080050 regulation of seed development (loss)
c-value
0.8
0.7
0.6
0.5
0.4
0.3
0.2
0.1
0.0
Phylostratum
Cellular_organisms - ps1
Archaea1 - ps2
Archaea2 - ps3
Archaea3 - ps4
Asgard/Eukaryota - ps5
Eukaryota - ps6
Excavata/Diaphoretickes - ps7
Diaphoretickes - ps8
Archaeplastida - ps9
Viridiplantae - ps10
Streptophyta - ps11
Charophyceae/Embriophyta - ps12
Embriophyta - ps13
Tracheophyta - ps14
Spermatophyta - ps15
Angiosperms - ps16
Mesangiospermae - ps17
Magnoliids/eudicots - ps18
Eudicots - ps19
Core_eudicots - ps20
Superrosids - ps21
Rosids - ps22
Eurosids - ps23
Malvids - ps24
Sap./Malv./Brassicales - ps25
Malvales/Brassicales - ps26
Brassicales - ps27
Cleomaceae/Brassicaceae - ps28
Brassicaceae - ps29
Camelineae - ps30
Arabidopsis - ps31
Arabidopsis_thaliana - ps32
p-value
2e-02
2e-02
3e-02
4e-02
5e-02
Log-odds
0.5
1.0
1.5
2.0
2.5
3.0
3.5
4.0

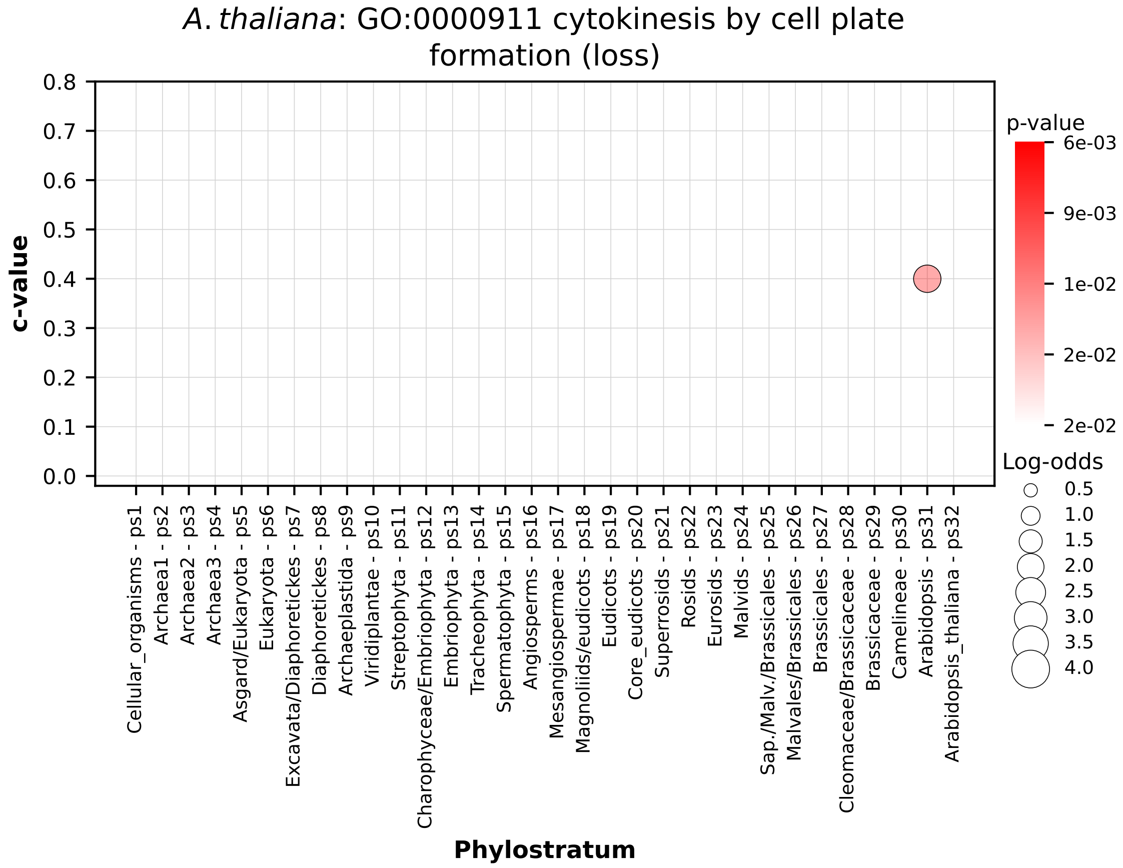

A. thaliana: GO:0000911 cytokinesis by cell plate formation (loss)
c-value
0.8
0.7
0.6
0.5
0.4
0.3
0.2
0.1
0.0
Phylostratum
Cellular_organisms - ps1
Archaea1 - ps2
Archaea2 - ps3
Archaea3 - ps4
Asgard/Eukaryota - ps5
Eukaryota - ps6
Excavata/Diaphoretickes - ps7
Diaphoretickes - ps8
Archaeplastida - ps9
Viridiplantae - ps10
Streptophyta - ps11
Charophyceae/Embriophyta - ps12
Embriophyta - ps13
Tracheophyta - ps14
Spermatophyta - ps15
Angiosperms - ps16
Mesangiospermae - ps17
Magnoliids/eudicots - ps18
Eudicots - ps19
Core_eudicots - ps20
Superrosids - ps21
Rosids - ps22
Eurosids - ps23
Malvids - ps24
Sap./Malv./Brassicales - ps25
Malvales/Brassicales - ps26
Brassicales - ps27
Cleomaceae/Brassicaceae - ps28
Brassicaceae - ps29
Camelineae - ps30
Arabidopsis - ps31
Arabidopsis_thaliana - ps32
p-value
6e-03
9e-03
1e-02
2e-02
2e-02
Log-odds
0.5
1.0
1.5
2.0
2.5
3.0
3.5
4.0

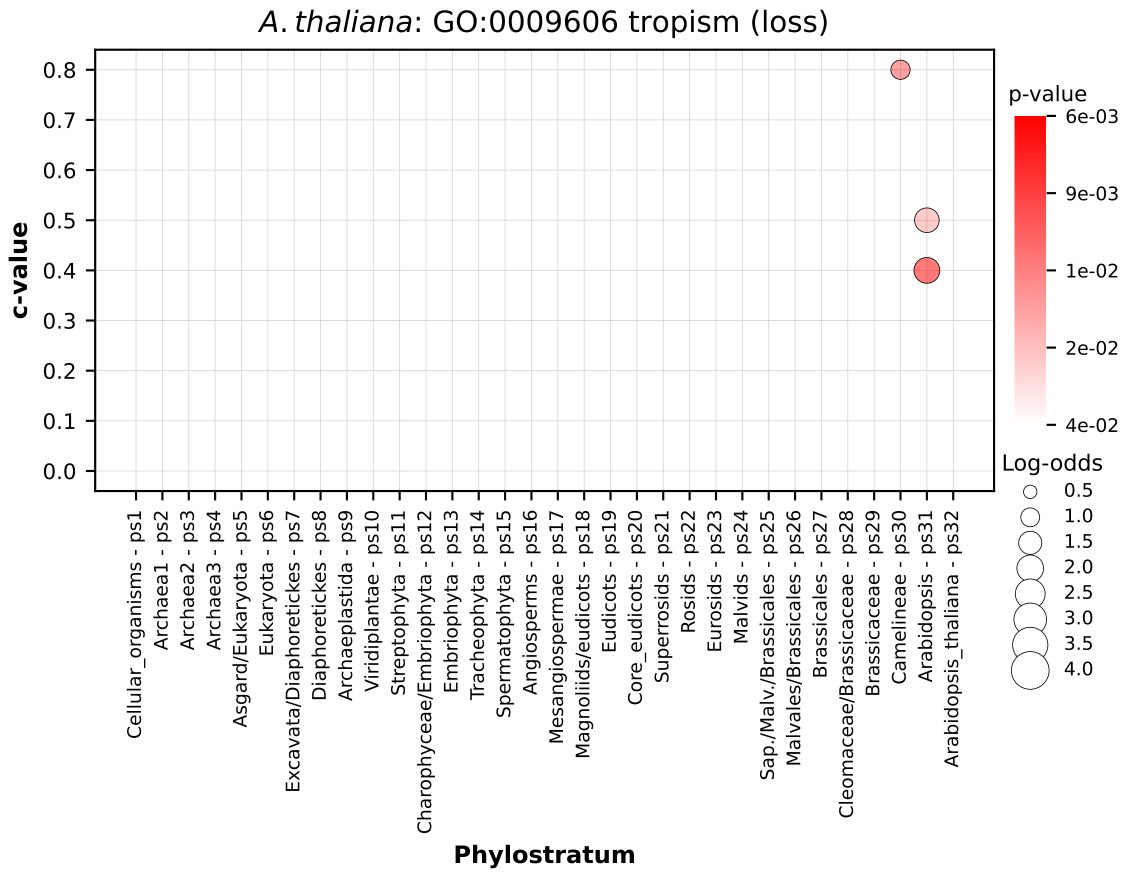
A. thaliana: GO:0009606 tropism (loss)
c-value
0.8
0.7
0.6
0.5
0.4
0.3
0.2
0.1
0.0
p-value
6e-03
9e-03
1e-02
2e-02
4e-02
Log-odds
0.5
1.0
1.5
2.0
2.5
3.0
3.5
4.0
Cellular_organisms - ps1
Archaea1 - ps2
Archaea2 - ps3
Archaea3 - ps4
Asgard/Eukaryota - ps5
Eukaryota - ps6
Excavata/Diaphoretickes - ps7
Diaphoretickes - ps8
Archaeplastida - ps9
Viridiplantae - ps10
Streptophyta - ps11
Charophyceae/Embriophyta - ps12
Embriophyta - ps13
Tracheophyta - ps14
Spermatophyta - ps15
Angiosperms - ps16
Mesangiospermae - ps17
Magnoliids/eudicots - ps18
Eudicots - ps19
Core_eudicots - ps20
Superrosids - ps21
Rosids - ps22
Eurosids - ps23
Malvids - ps24
Sap./Malv./Brassicales - ps25
Malvales/Brassicales - ps26
Brassicales - ps27
Cleomaceae/Brassicaceae - ps28
Brassicaceae - ps29
Camelineae - ps30
Arabidopsis - ps31
Arabidopsis_thaliana - ps32
Phylostratum

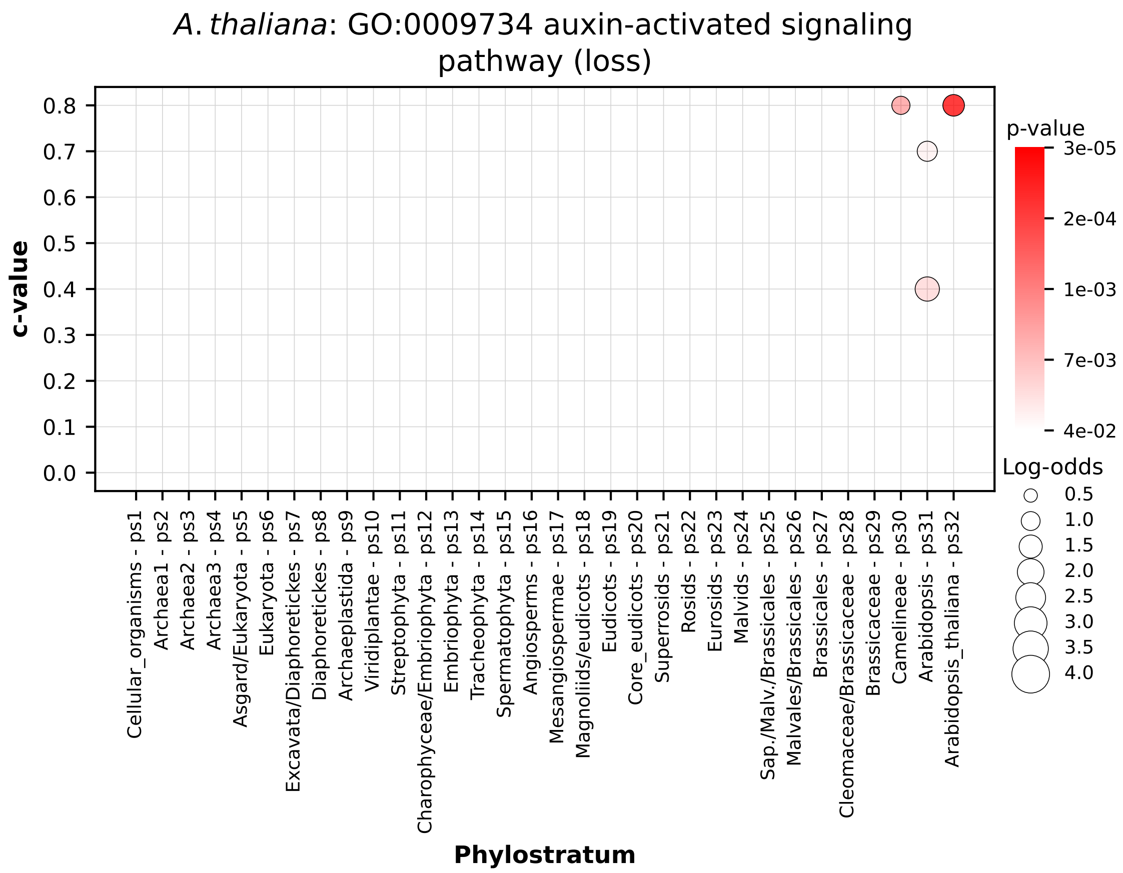
A. thaliana: GO:0009734 auxin-activated signaling pathway (loss)
c-value
0.0
0.1
0.2
0.3
0.4
0.5
0.6
0.7
0.8
Phylostratum
Cellular_organisms - ps1
Archaea1 - ps2
Archaea2 - ps3
Archaea3 - ps4
Asgard/Eukaryota - ps5
Eukaryota - ps6
Excavata/Diaphoretickes - ps7
Diaphoretickes - ps8
Archaeplastida - ps9
Viridiplantae - ps10
Streptophyta - ps11
Charophyceae/Embriophyta - ps12
Embriophyta - ps13
Tracheophyta - ps14
Spermatophyta - ps15
Angiosperms - ps16
Mesangiospermae - ps17
Magnoliids/eudicots - ps18
Eudicots - ps19
Core_eudicots - ps20
Superrosids - ps21
Rosids - ps22
Eurosids - ps23
Malvids - ps24
Sap./Malv./Brassicales - ps25
Malvales/Brassicales - ps26
Brassicales - ps27
Cleomaceae/Brassicaceae - ps28
Brassicaceae - ps29
Camelineae - ps30
Arabidopsis - ps31
Arabidopsis_thaliana - ps32
p-value
3e-05
2e-04
1e-03
7e-03
4e-02
Log-odds
0.5
1.0
1.5
2.0
2.5
3.0
3.5
4.0

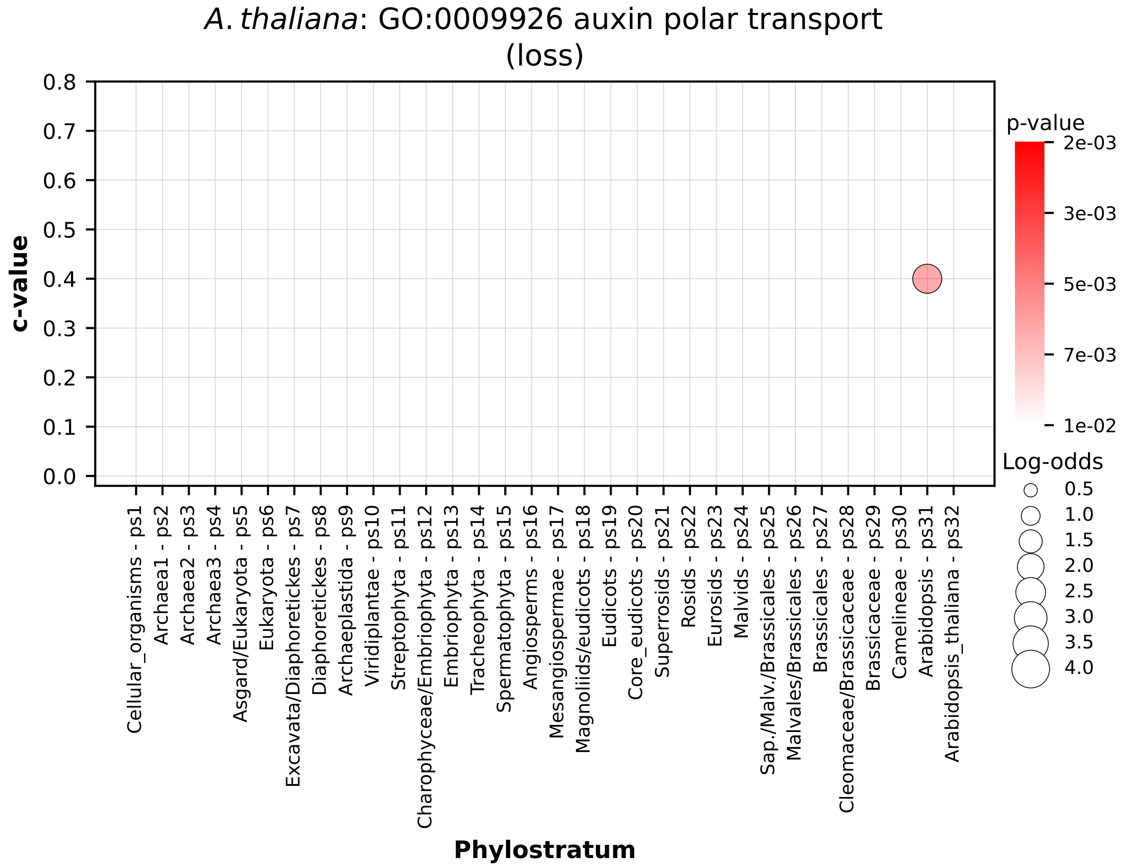

A. thaliana: GO:0009926 auxin polar transport
(loss)
c-value
0.8
0.7
0.6
0.5
0.4
0.3
0.2
0.1
0.0
p-value
2e-03
3e-03
5e-03
7e-03
1e-02
Log-odds
0.5
1.0
1.5
2.0
2.5
3.0
3.5
4.0
Cellular_organisms - ps1
Archaea1 - ps2
Archaea2 - ps3
Archaea3 - ps4
Asgard/Eukaryota - ps5
Eukaryota - ps6
Excavata/Diaphoretickes - ps7
Diaphoretickes - ps8
Archaeplastida - ps9
Viridiplantae - ps10
Streptophyta - ps11
Charophyceae/Embriophyta - ps12
Embriophyta - ps13
Tracheophyta - ps14
Spermatophyta - ps15
Angiosperms - ps16
Mesangiospermae - ps17
Magnoliids/eudicots - ps18
Eudicots - ps19
Core_eudicots - ps20
Superrosids - ps21
Rosids - ps22
Eurosids - ps23
Malvids - ps24
Sap./Malv./Brassicales - ps25
Malvales/Brassicales - ps26
Brassicales - ps27
Cleomaceae/Brassicaceae - ps28
Brassicaceae - ps29
Camelineae - ps30
Arabidopsis - ps31
Arabidopsis_thaliana - ps32
Phylostratum

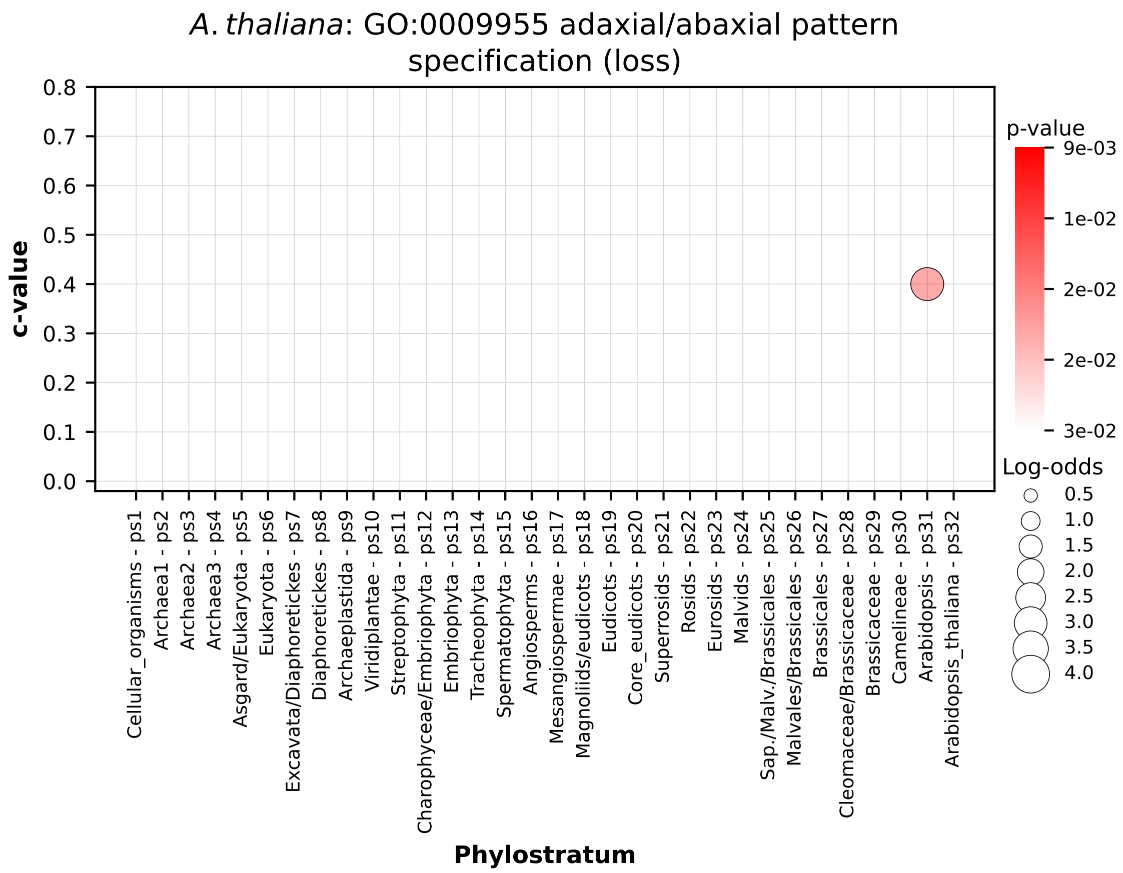

A. thaliana: GO:0009955 adaxial/abaxial pattern specification (loss)
c-value
0.8
0.7
0.6
0.5
0.4
0.3
0.2
0.1
0.0
Phylostratum
Cellular_organisms - ps1
Archaea1 - ps2
Archaea2 - ps3
Archaea3 - ps4
Asgard/Eukaryota - ps5
Eukaryota - ps6
Excavata/Diaphoretickes - ps7
Diaphoretickes - ps8
Archaeplastida - ps9
Viridiplantae - ps10
Streptophyta - ps11
Charophyceae/Embriophyta - ps12
Embriophyta - ps13
Tracheophyta - ps14
Spermatophyta - ps15
Angiosperms - ps16
Mesangiospermae - ps17
Magnoliids/eudicots - ps18
Eudicots - ps19
Core_eudicots - ps20
Superrosids - ps21
Rosids - ps22
Eurosids - ps23
Malvids - ps24
Sap./Malv./Brassicales - ps25
Malvales/Brassicales - ps26
Brassicales - ps27
Cleomaceae/Brassicaceae - ps28
Brassicaceae - ps29
Camelineae - ps30
Arabidopsis - ps31
Arabidopsis_thaliana - ps32
p-value
9e-03
1e-02
2e-02
2e-02
3e-02
Log-odds
0.5
1.0
1.5
2.0
2.5
3.0
3.5
4.0

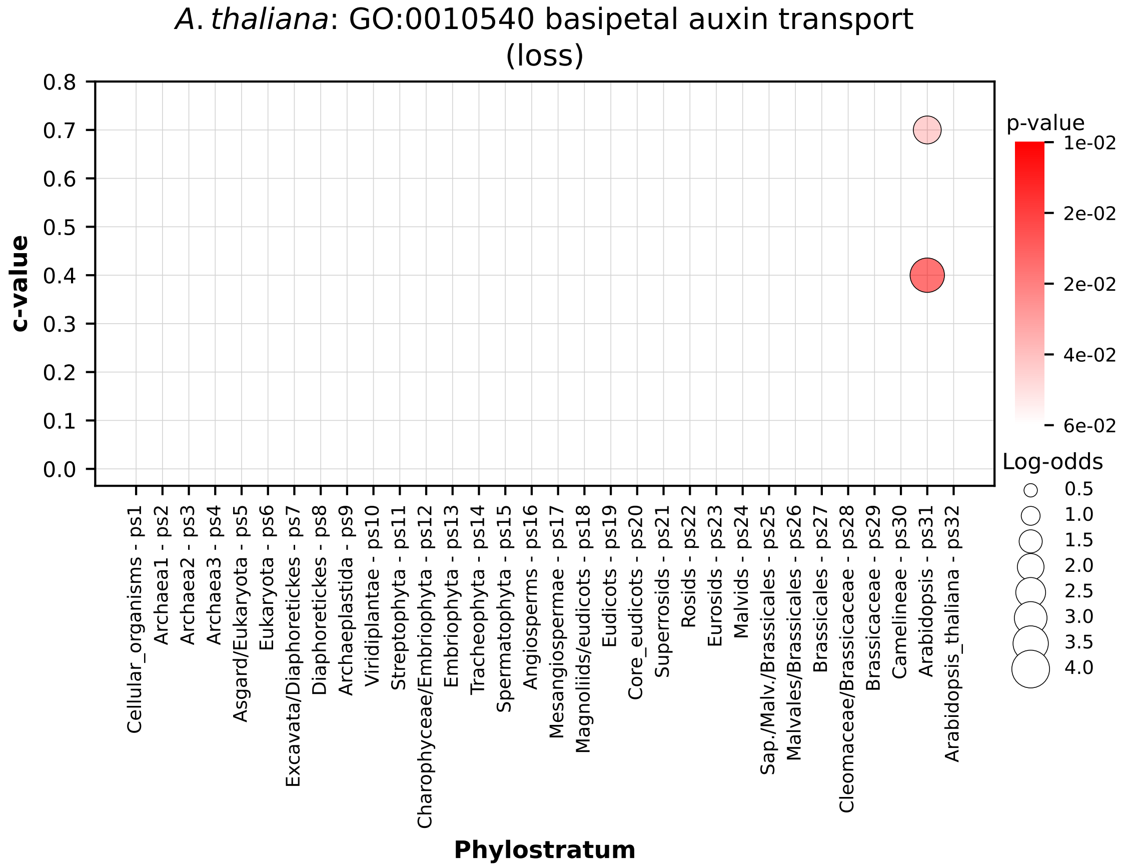

A. thaliana: GO:0010540 basipetal auxin transport
(loss)
c-value
0.8
0.7
0.6
0.5
0.4
0.3
0.2
0.1
0.0
Phylostratum
Cellular_organisms - ps1
Archaea1 - ps2
Archaea2 - ps3
Archaea3 - ps4
Asgard/Eukaryota - ps5
Eukaryota - ps6
Excavata/Diaphoretickes - ps7
Diaphoretickes - ps8
Archaeplastida - ps9
Viridiplantae - ps10
Streptophyta - ps11
Charophyceae/Embriophyta - ps12
Embriophyta - ps13
Tracheophyta - ps14
Spermatophyta - ps15
Angiosperms - ps16
Mesangiospermae - ps17
Magnoliids/eudicots - ps18
Eudicots - ps19
Core_eudicots - ps20
Superrosids - ps21
Rosids - ps22
Eurosids - ps23
Malvids - ps24
Sap./Malv./Brassicales - ps25
Malvales/Brassicales - ps26
Brassicales - ps27
Cleomaceae/Brassicaceae - ps28
Brassicaceae - ps29
Camelineae - ps30
Arabidopsis - ps31
Arabidopsis_thaliana - ps32
p-value
1e-02
2e-02
2e-02
4e-02
6e-02
Log-odds
0.5
1.0
1.5
2.0
2.5
3.0
3.5
4.0

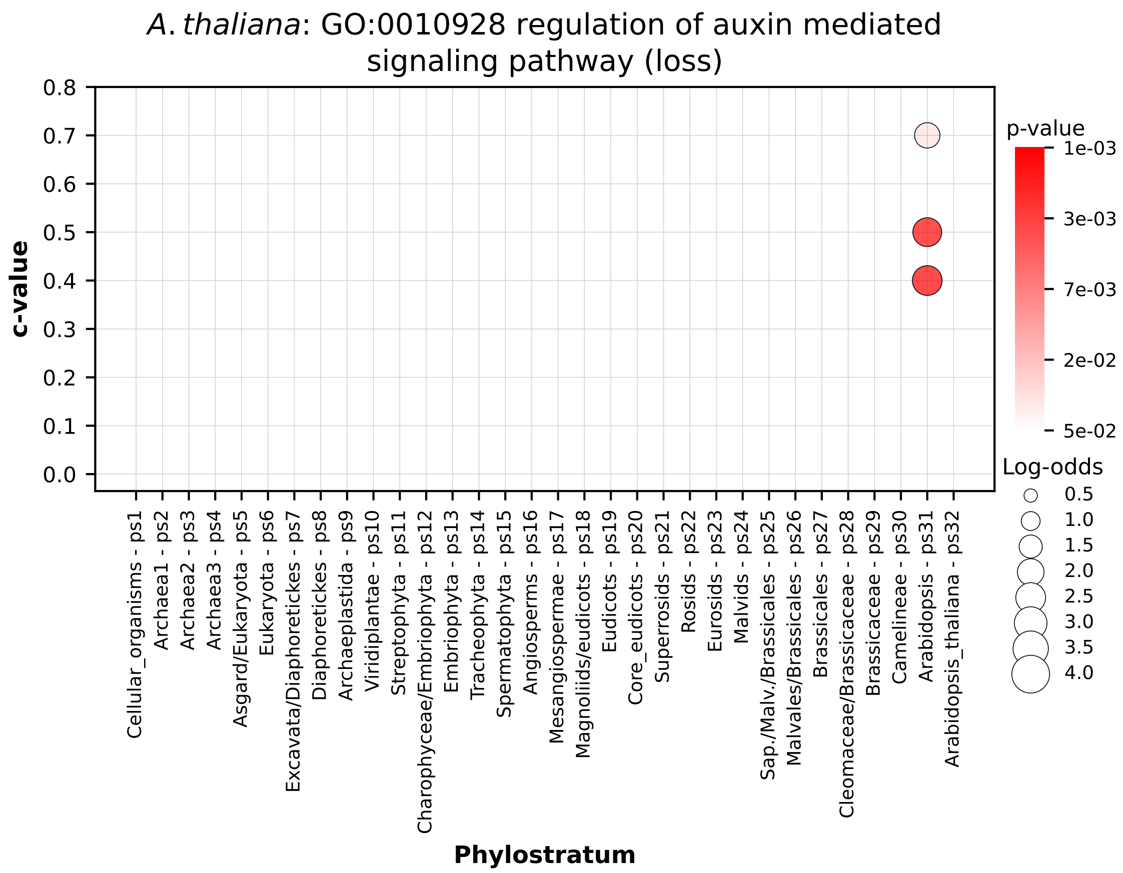

A. thaliana: GO:0010928 regulation of auxin mediated signaling pathway (loss)
c-value
0.8
0.7
0.6
0.5
0.4
0.3
0.2
0.1
0.0
p-value
1e-03
3e-03
7e-03
2e-02
5e-02
Log-odds
0.5
1.0
1.5
2.0
2.5
3.0
3.5
4.0
Cellular_organisms - ps1
Archaea1 - ps2
Archaea2 - ps3
Archaea3 - ps4
Asgard/Eukaryota - ps5
Eukaryota - ps6
Excavata/Diaphoretickes - ps7
Diaphoretickes - ps8
Archaeplastida - ps9
Viridiplantae - ps10
Streptophyta - ps11
Charophyceae/Embriophyta - ps12
Embriophyta - ps13
Tracheophyta - ps14
Spermatophyta - ps15
Angiosperms - ps16
Mesangiospermae - ps17
Magnoliids/eudicots - ps18
Eudicots - ps19
Core_eudicots - ps20
Superrosids - ps21
Rosids - ps22
Eurosids - ps23
Malvids - ps24
Sap./Malv./Brassicales - ps25
Malvales/Brassicales - ps26
Brassicales - ps27
Cleomaceae/Brassicaceae - ps28
Brassicaceae - ps29
Camelineae - ps30
Arabidopsis - ps31
Arabidopsis_thaliana - ps32
Phylostratum

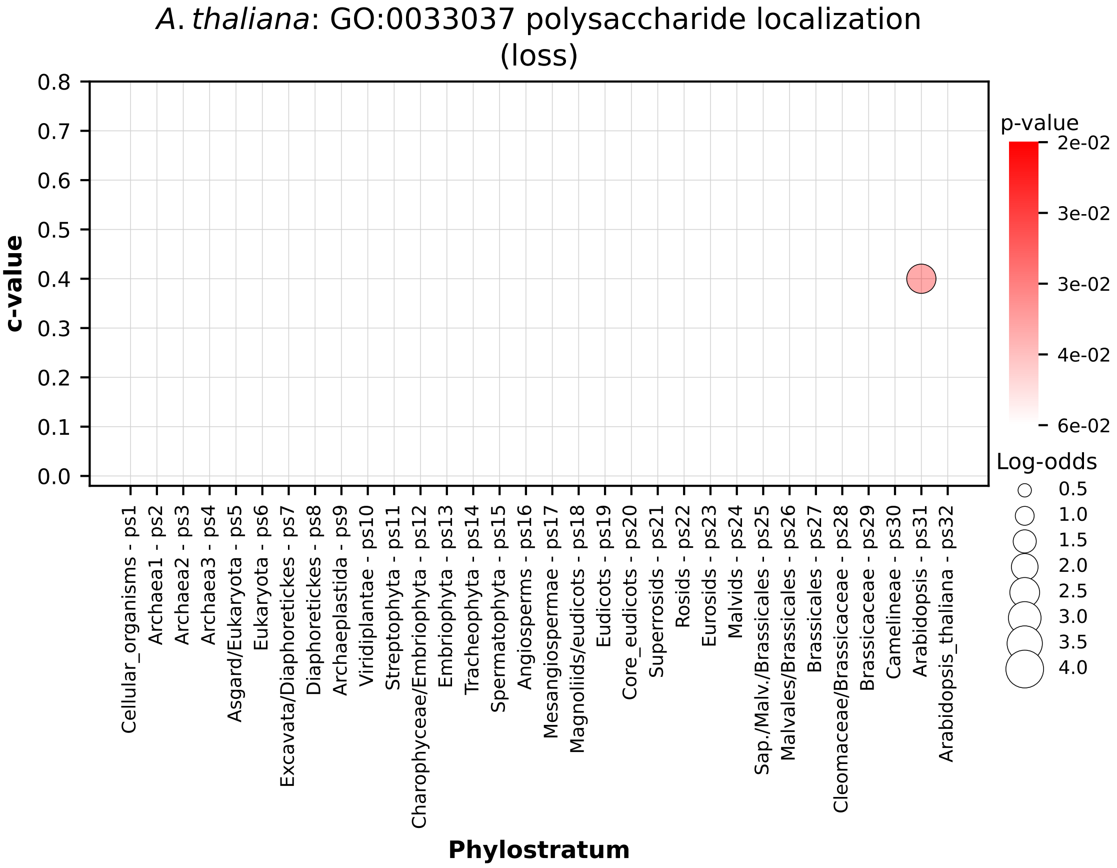
A. thaliana: GO:0033037 polysaccharide localization
(loss)
c-value
0.8
0.7
0.6
0.5
0.4
0.3
0.2
0.1
0.0
p-value
2e-02
3e-02
3e-02
4e-02
6e-02
Log-odds
0.5
1.0
1.5
2.0
2.5
3.0
3.5
4.0
Cellular_organisms - ps1
Archaea1 - ps2
Archaea2 - ps3
Archaea3 - ps4
Asgard/Eukaryota - ps5
Eukaryota - ps6
Excavata/Diaphoretickes - ps7
Diaphoretickes - ps8
Archaeplastida - ps9
Viridiplantae - ps10
Streptophyta - ps11
Charophyceae/Embriophyta - ps12
Embriophyta - ps13
Tracheophyta - ps14
Spermatophyta - ps15
Angiosperms - ps16
Mesangiospermae - ps17
Magnoliids/eudicots - ps18
Eudicots - ps19
Core_eudicots - ps20
Superrosids - ps21
Rosids - ps22
Eurosids - ps23
Malvids - ps24
Sap./Malv./Brassicales - ps25
Malvales/Brassicales - ps26
Brassicales - ps27
Cleomaceae/Brassicaceae - ps28
Brassicaceae - ps29
Camelineae - ps30
Arabidopsis - ps31
Arabidopsis_thaliana - ps32
Phylostratum

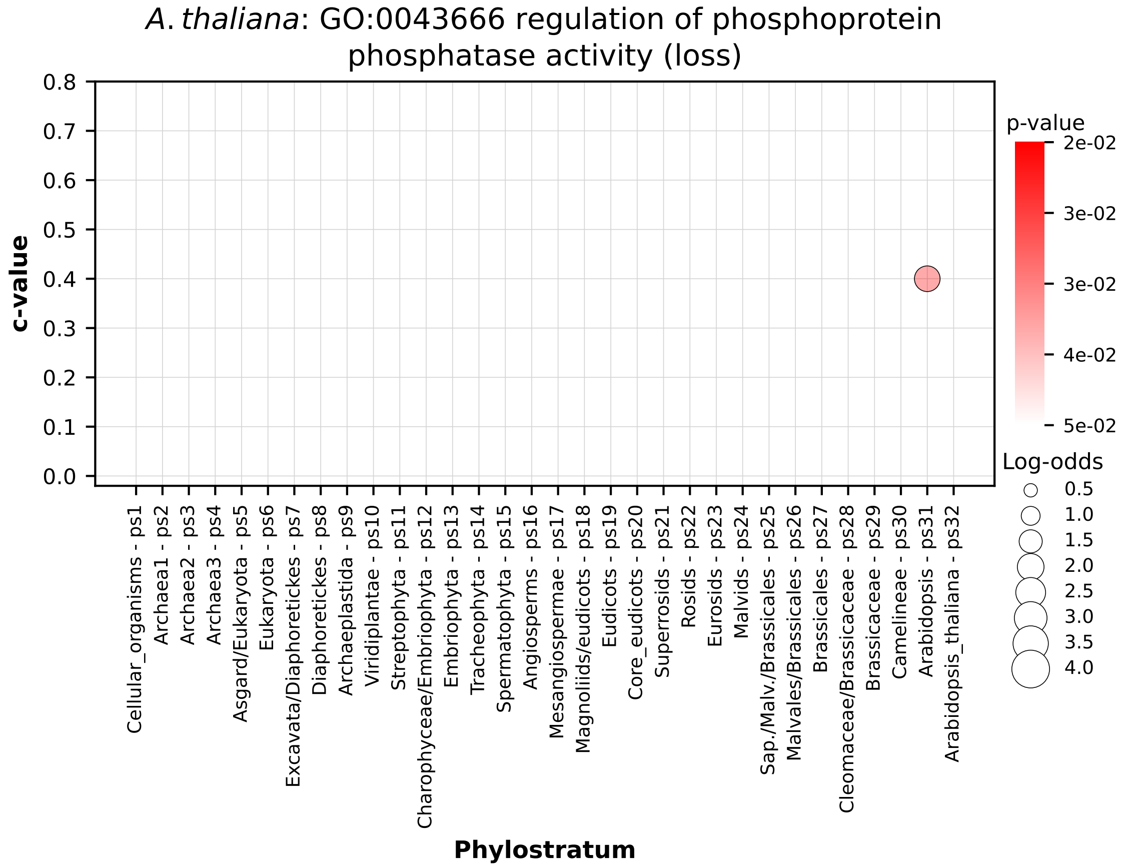
A. thaliana: GO:0043666 regulation of phosphoprotein phosphatase activity (loss)
c-value
0.8
0.7
0.6
0.5
0.4
0.3
0.2
0.1
0.0
Phylostratum
Cellular_organisms - ps1
Archaea1 - ps2
Archaea2 - ps3
Archaea3 - ps4
Asgard/Eukaryota - ps5
Eukaryota - ps6
Excavata/Diaphoretickes - ps7
Diaphoretickes - ps8
Archaeplastida - ps9
Viridiplantae - ps10
Streptophyta - ps11
Charophyceae/Embriophyta - ps12
Embriophyta - ps13
Tracheophyta - ps14
Spermatophyta - ps15
Angiosperms - ps16
Mesangiospermae - ps17
Magnoliids/eudicots - ps18
Eudicots - ps19
Core_eudicots - ps20
Superrosids - ps21
Rosids - ps22
Eurosids - ps23
Malvids - ps24
Sap./Malv./Brassicales - ps25
Malvales/Brassicales - ps26
Brassicales - ps27
Cleomaceae/Brassicaceae - ps28
Brassicaceae - ps29
Camelineae - ps30
Arabidopsis - ps31
Arabidopsis_thaliana - ps32
p-value
2e-02
3e-02
3e-02
4e-02
5e-02
Log-odds
0.5
1.0
1.5
2.0
2.5
3.0
3.5
4.0

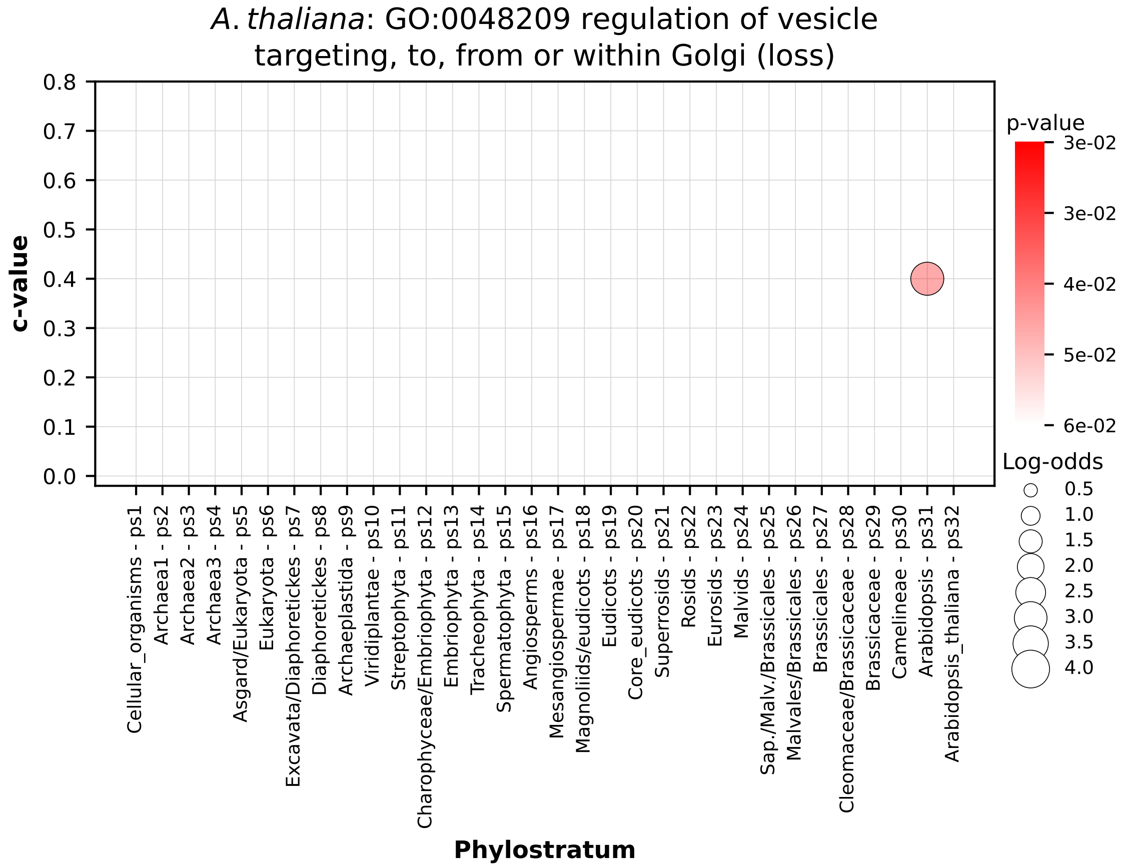

$A. thaliana$: GO:0048209 regulation of vesicle targeting, to, from or within Golgi (loss)
c-value
0.8
0.7
0.6
0.5
0.4
0.3
0.2
0.1
0.0
p-value
3e-02
3e-02
4e-02
5e-02
6e-02
Log-odds
0.5
1.0
1.5
2.0
2.5
3.0
3.5
4.0
Cellular_organisms - ps1
Archaea1 - ps2
Archaea2 - ps3
Archaea3 - ps4
Asgard/Eukaryota - ps5
Eukaryota - ps6
Excavata/Diaphoretickes - ps7
Diaphoretickes - ps8
Archaeplastida - ps9
Viridiplantae - ps10
Streptophyta - ps11
Charophyceae/Embriophyta - ps12
Embriophyta - ps13
Tracheophyta - ps14
Spermatophyta - ps15
Angiosperms - ps16
Mesangiospermae - ps17
Magnoliids/eudicots - ps18
Eudicots - ps19
Core_eudicots - ps20
Superrosids - ps21
Rosids - ps22
Eurosids - ps23
Malvids - ps24
Sap./Malv./Brassicales - ps25
Malvales/Brassicales - ps26
Brassicales - ps27
Cleomaceae/Brassicaceae - ps28
Brassicaceae - ps29
Camelineae - ps30
Arabidopsis - ps31
Arabidopsis_thaliana - ps32
Phylostratum

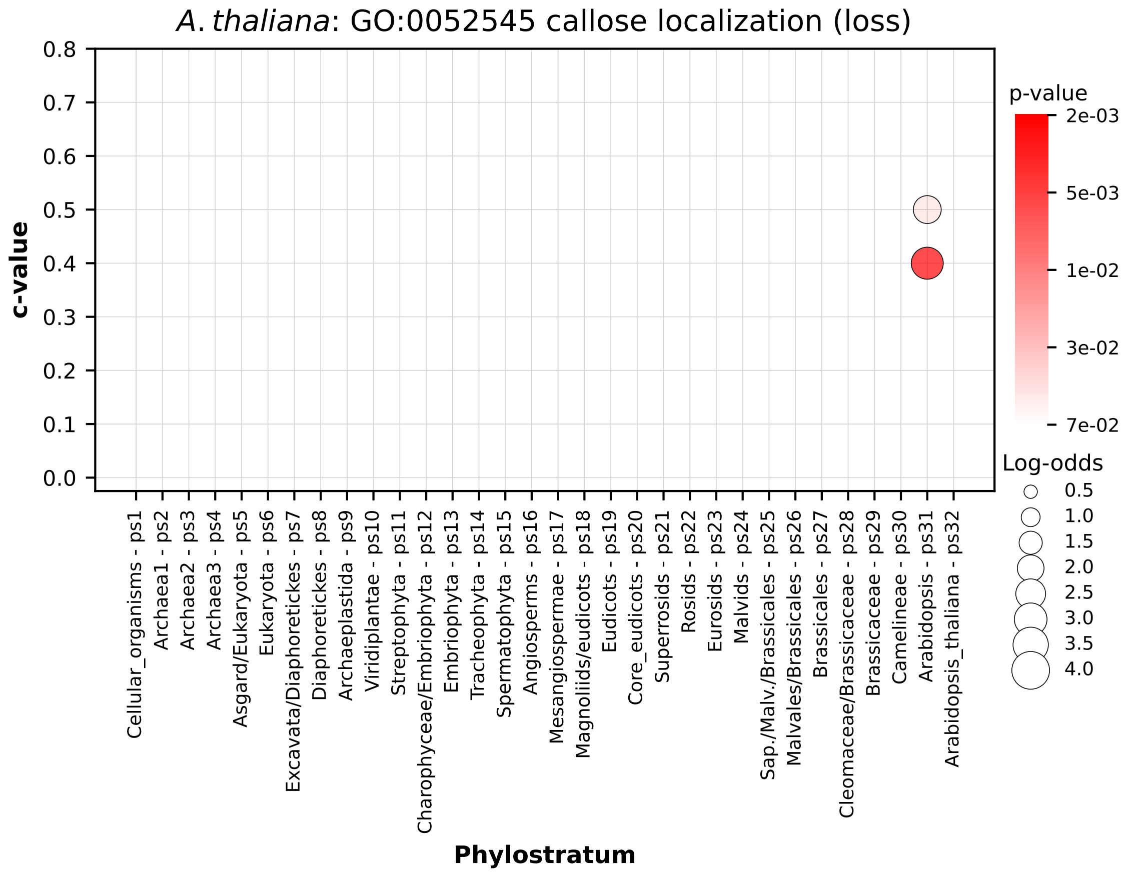

A. thaliana: GO:0052545 callose localization (loss)
c-value
0.0
0.1
0.2
0.3
0.4
0.5
0.6
0.7
0.8
Phylostratum
Cellular_organisms - ps1
Archaea1 - ps2
Archaea2 - ps3
Archaea3 - ps4
Asgard/Eukaryota - ps5
Eukaryota - ps6
Excavata/Diaphoretickes - ps7
Diaphoretickes - ps8
Archaeplastida - ps9
Viridiplantae - ps10
Streptophyta - ps11
Charophyceae/Embriophyta - ps12
Embriophyta - ps13
Tracheophyta - ps14
Spermatophyta - ps15
Angiosperms - ps16
Mesangiospermae - ps17
Magnoliids/eudicots - ps18
Eudicots - ps19
Core_eudicots - ps20
Superrosids - ps21
Rosids - ps22
Eurosids - ps23
Malvids - ps24
Sap./Malv./Brassicales - ps25
Malvales/Brassicales - ps26
Brassicales - ps27
Cleomaceae/Brassicaceae - ps28
Brassicaceae - ps29
Camelineae - ps30
Arabidopsis - ps31
Arabidopsis_thaliana - ps32
p-value
2e-03
5e-03
1e-02
3e-02
7e-02
Log-odds
0.5
1.0
1.5
2.0
2.5
3.0
3.5
4.0

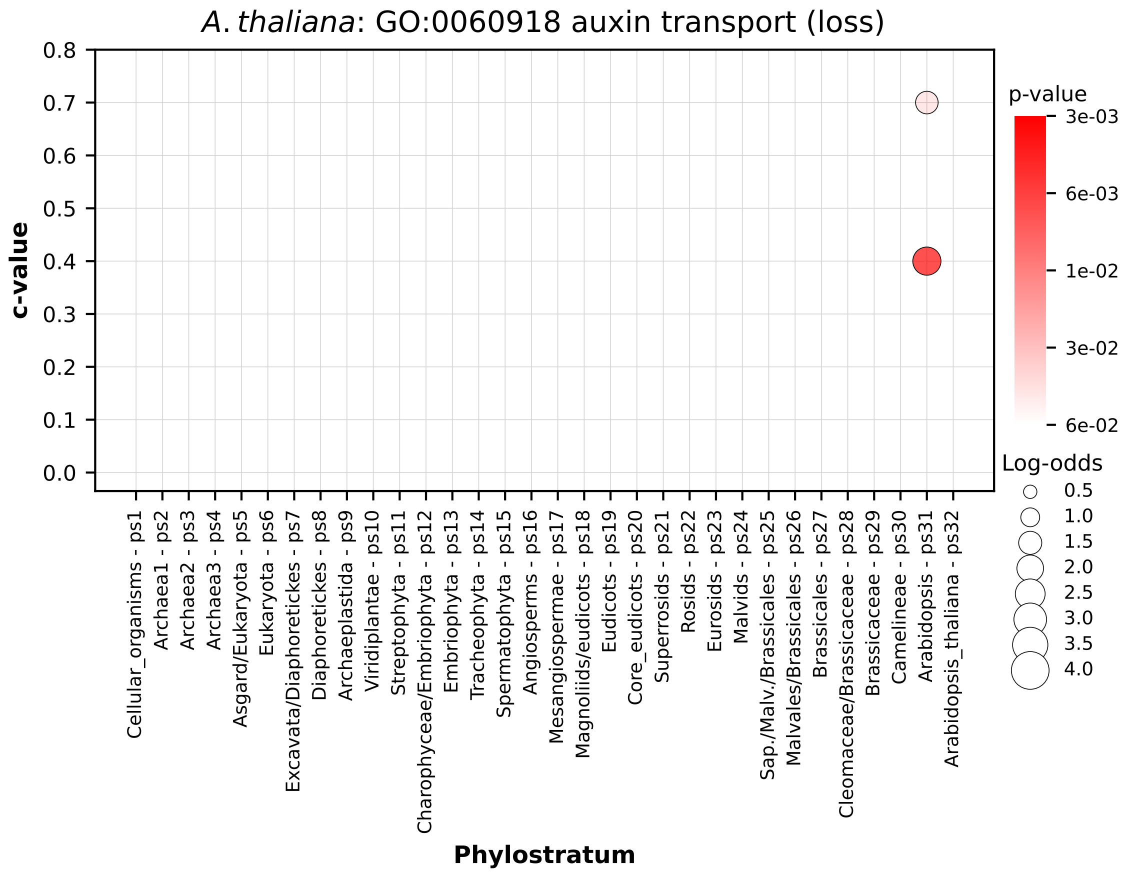

A. thaliana: GO:0060918 auxin transport (loss)
c-value
0.8
0.7
0.6
0.5
0.4
0.3
0.2
0.1
0.0
p-value
3e-03
6e-03
1e-02
3e-02
6e-02
Log-odds
0.5
1.0
1.5
2.0
2.5
3.0
3.5
4.0
Cellular_organisms - ps1
Archaea1 - ps2
Archaea2 - ps3
Archaea3 - ps4
Asgard/Eukaryota - ps5
Eukaryota - ps6
Excavata/Diaphoretickes - ps7
Diaphoretickes - ps8
Archaeplastida - ps9
Viridiplantae - ps10
Streptophyta - ps11
Charophyceae/Embriophyta - ps12
Embriophyta - ps13
Tracheophyta - ps14
Spermatophyta - ps15
Angiosperms - ps16
Mesangiospermae - ps17
Magnoliids/eudicots - ps18
Eudicots - ps19
Core_eudicots - ps20
Superrosids - ps21
Rosids - ps22
Eurosids - ps23
Malvids - ps24
Sap./Malv./Brassicales - ps25
Malvales/Brassicales - ps26
Brassicales - ps27
Cleomaceae/Brassicaceae - ps28
Brassicaceae - ps29
Camelineae - ps30
Arabidopsis - ps31
Arabidopsis_thaliana - ps32
Phylostratum

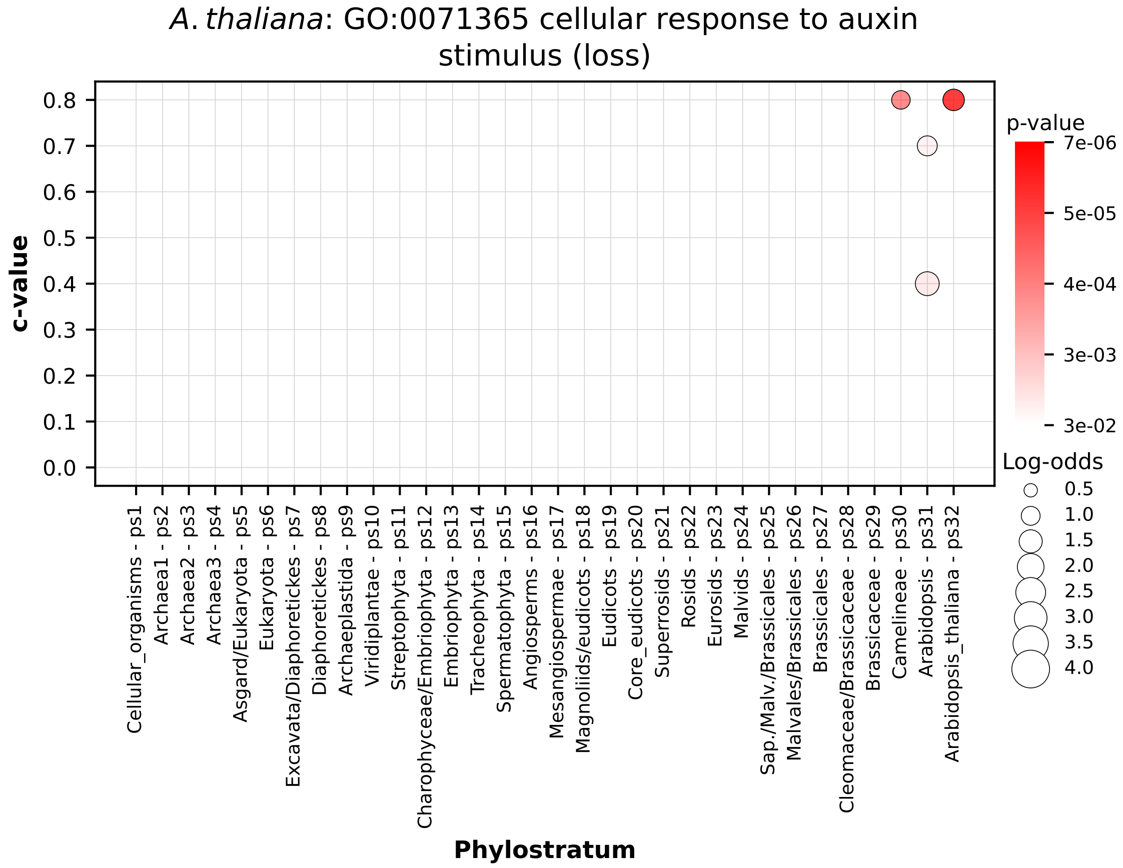

A. thaliana: GO:0071365 cellular response to auxin stimulus (loss)
c-value
0.0
0.1
0.2
0.3
0.4
0.5
0.6
0.7
0.8
Phylostratum
Cellular_organisms - ps1
Archaea1 - ps2
Archaea2 - ps3
Archaea3 - ps4
Asgard/Eukaryota - ps5
Eukaryota - ps6
Excavata/Diaphoretickes - ps7
Diaphoretickes - ps8
Archaeplastida - ps9
Viridiplantae - ps10
Streptophyta - ps11
Charophyceae/Embriophyta - ps12
Embriophyta - ps13
Tracheophyta - ps14
Spermatophyta - ps15
Angiosperms - ps16
Mesangiospermae - ps17
Magnoliids/eudicots - ps18
Eudicots - ps19
Core_eudicots - ps20
Superrosids - ps21
Rosids - ps22
Eurosids - ps23
Malvids - ps24
Sap./Malv./Brassicales - ps25
Malvales/Brassicales - ps26
Brassicales - ps27
Cleomaceae/Brassicaceae - ps28
Brassicaceae - ps29
Camelineae - ps30
Arabidopsis - ps31
Arabidopsis_thaliana - ps32
p-value
7e-06
5e-05
4e-04
3e-03
3e-02
Log-odds
0.5
1.0
1.5
2.0
2.5
3.0
3.5
4.0

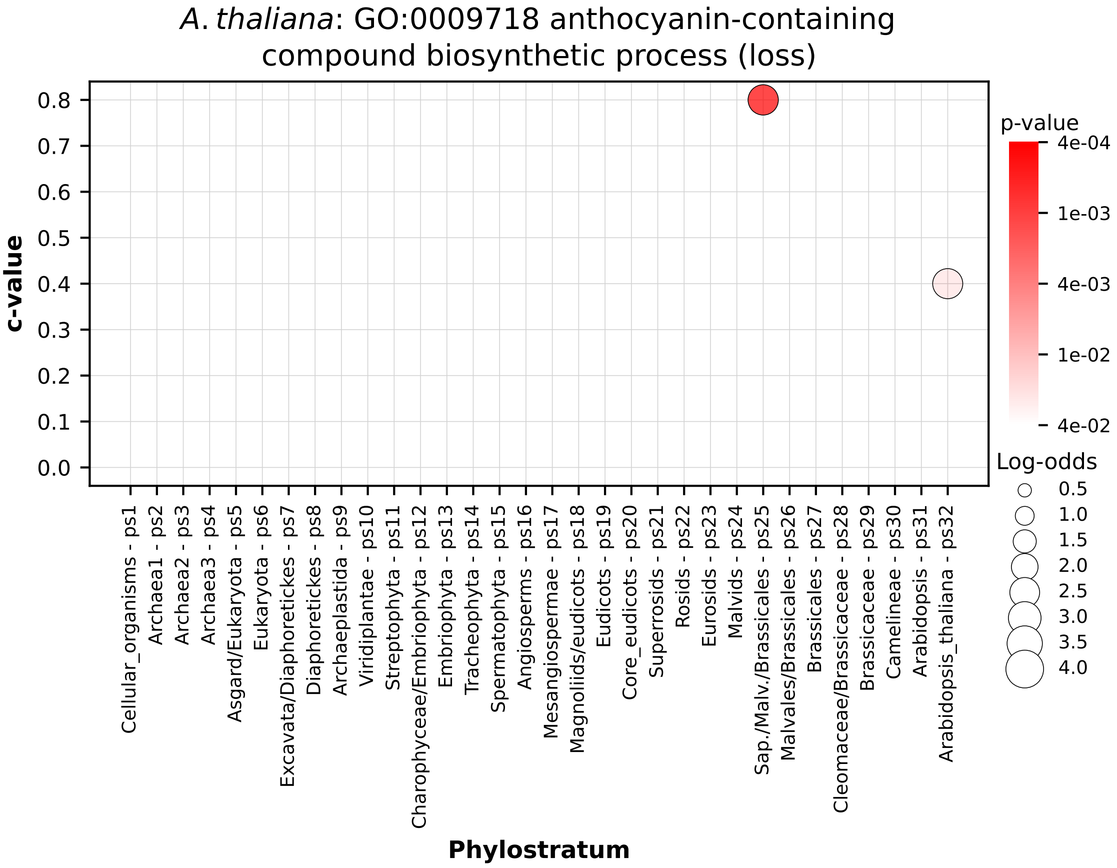

A. thaliana: GO:0009718 anthocyanin-containing compound biosynthetic process (loss)
c-value
0.0
0.1
0.2
0.3
0.4
0.5
0.6
0.7
0.8
p-value
4e-04
1e-03
4e-03
1e-02
4e-02
Log-odds
0.5
1.0
1.5
2.0
2.5
3.0
3.5
4.0
Cellular_organisms - ps1
Archaea1 - ps2
Archaea2 - ps3
Archaea3 - ps4
Asgard/Eukaryota - ps5
Eukaryota - ps6
Excavata/Diaphoretickes - ps7
Diaphoretickes - ps8
Archaeplastida - ps9
Viridiplantae - ps10
Streptophyta - ps11
Charophyceae/Embriophyta - ps12
Embriophyta - ps13
Tracheophyta - ps14
Spermatophyta - ps15
Angiosperms - ps16
Mesangiospermae - ps17
Magnoliids/eudicots - ps18
Eudicots - ps19
Core_eudicots - ps20
Superrosids - ps21
Rosids - ps22
Eurosids - ps23
Malvids - ps24
Sap./Malv./Brassicales - ps25
Malvales/Brassicales - ps26
Brassicales - ps27
Cleomaceae/Brassicaceae - ps28
Brassicaceae - ps29
Camelineae - ps30
Arabidopsis - ps31
Arabidopsis_thaliana - ps32
Phylostratum

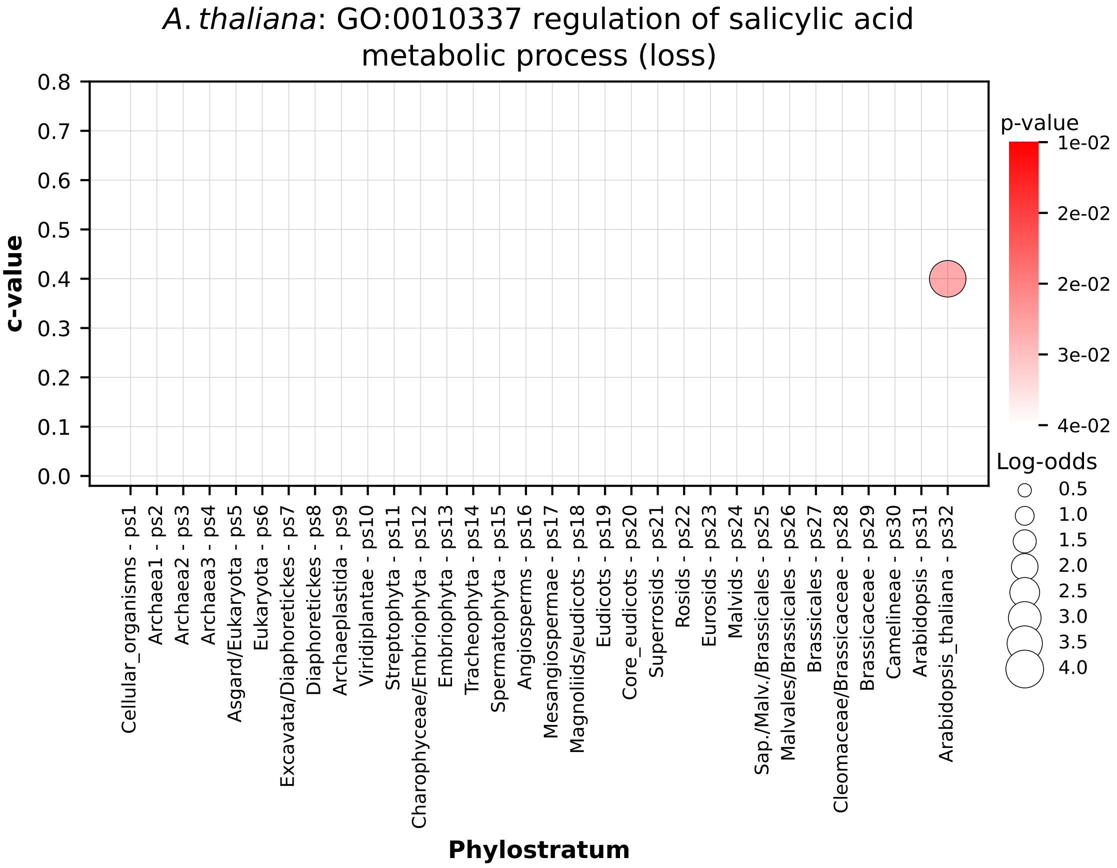

A. thaliana: GO:0010337 regulation of salicylic acid metabolic process (loss)
c-value
0.8
0.7
0.6
0.5
0.4
0.3
0.2
0.1
0.0
p-value
1e-02
2e-02
2e-02
3e-02
4e-02
Log-odds
0.5
1.0
1.5
2.0
2.5
3.0
3.5
4.0
Cellular_organisms - ps1
Archaea1 - ps2
Archaea2 - ps3
Archaea3 - ps4
Asgard/Eukaryota - ps5
Eukaryota - ps6
Excavata/Diaphoretickes - ps7
Diaphoretickes - ps8
Archaeplastida - ps9
Viridiplantae - ps10
Streptophyta - ps11
Charophyceae/Embriophyta - ps12
Embriophyta - ps13
Tracheophyta - ps14
Spermatophyta - ps15
Angiosperms - ps16
Mesangiospermae - ps17
Magnoliids/eudicots - ps18
Eudicots - ps19
Core_eudicots - ps20
Superrosids - ps21
Rosids - ps22
Eurosids - ps23
Malvids - ps24
Sap./Malv./Brassicales - ps25
Malvales/Brassicales - ps26
Brassicales - ps27
Cleomaceae/Brassicaceae - ps28
Brassicaceae - ps29
Camelineae - ps30
Arabidopsis - ps31
Arabidopsis_thaliana - ps32
Phylostratum

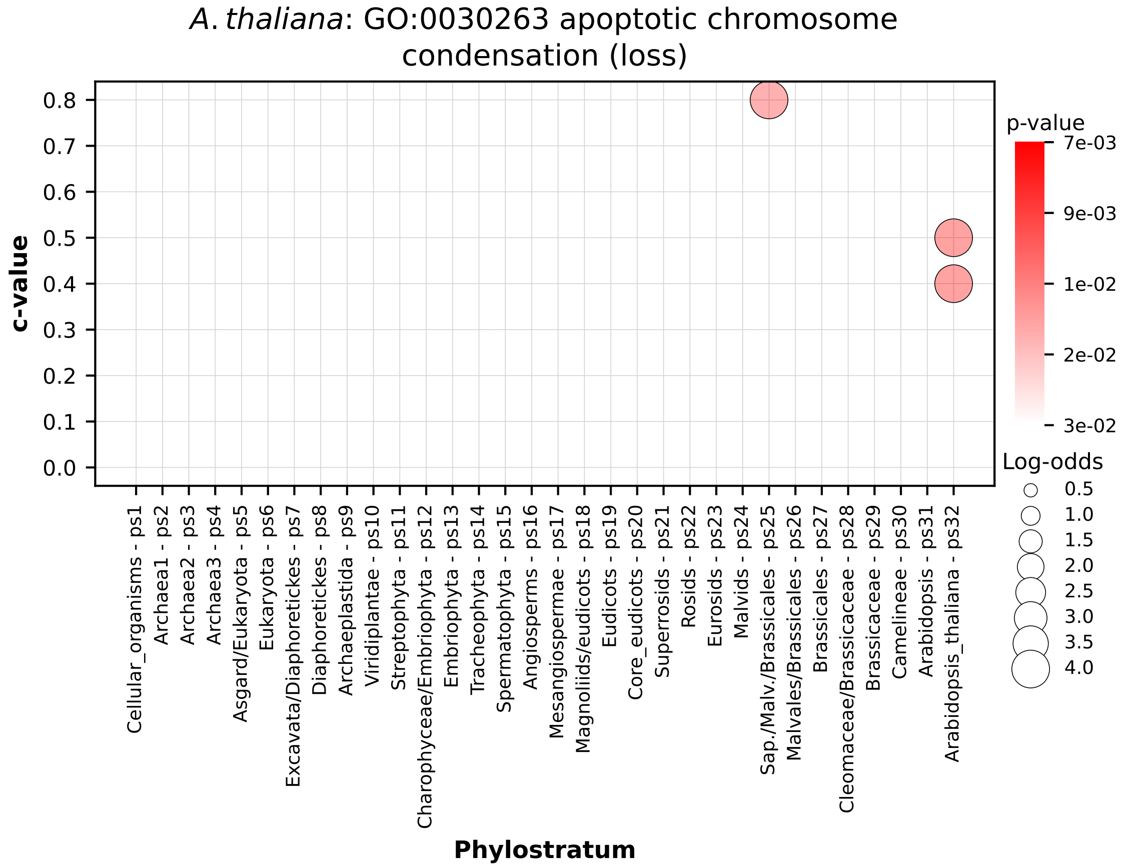
A. thaliana: GO:0030263 apoptotic chromosome condensation (loss)
c-value
0.0
0.1
0.2
0.3
0.4
0.5
0.6
0.7
0.8
Phylostratum
Cellular_organisms - ps1
Archaea1 - ps2
Archaea2 - ps3
Archaea3 - ps4
Asgard/Eukaryota - ps5
Eukaryota - ps6
Excavata/Diaphoretickes - ps7
Diaphoretickes - ps8
Archaeplastida - ps9
Viridiplantae - ps10
Streptophyta - ps11
Charophyceae/Embriophyta - ps12
Embriophyta - ps13
Tracheophyta - ps14
Spermatophyta - ps15
Angiosperms - ps16
Mesangiospermae - ps17
Magnoliids/eudicots - ps18
Eudicots - ps19
Core_eudicots - ps20
Superrosids - ps21
Rosids - ps22
Eurosids - ps23
Malvids - ps24
Sap./Malv./Brassicales - ps25
Malvales/Brassicales - ps26
Brassicales - ps27
Cleomaceae/Brassicaceae - ps28
Brassicaceae - ps29
Camelineae - ps30
Arabidopsis - ps31
Arabidopsis_thaliana - ps32
p-value
7e-03
9e-03
1e-02
2e-02
3e-02
Log-odds
0.5
1.0
1.5
2.0
2.5
3.0
3.5
4.0

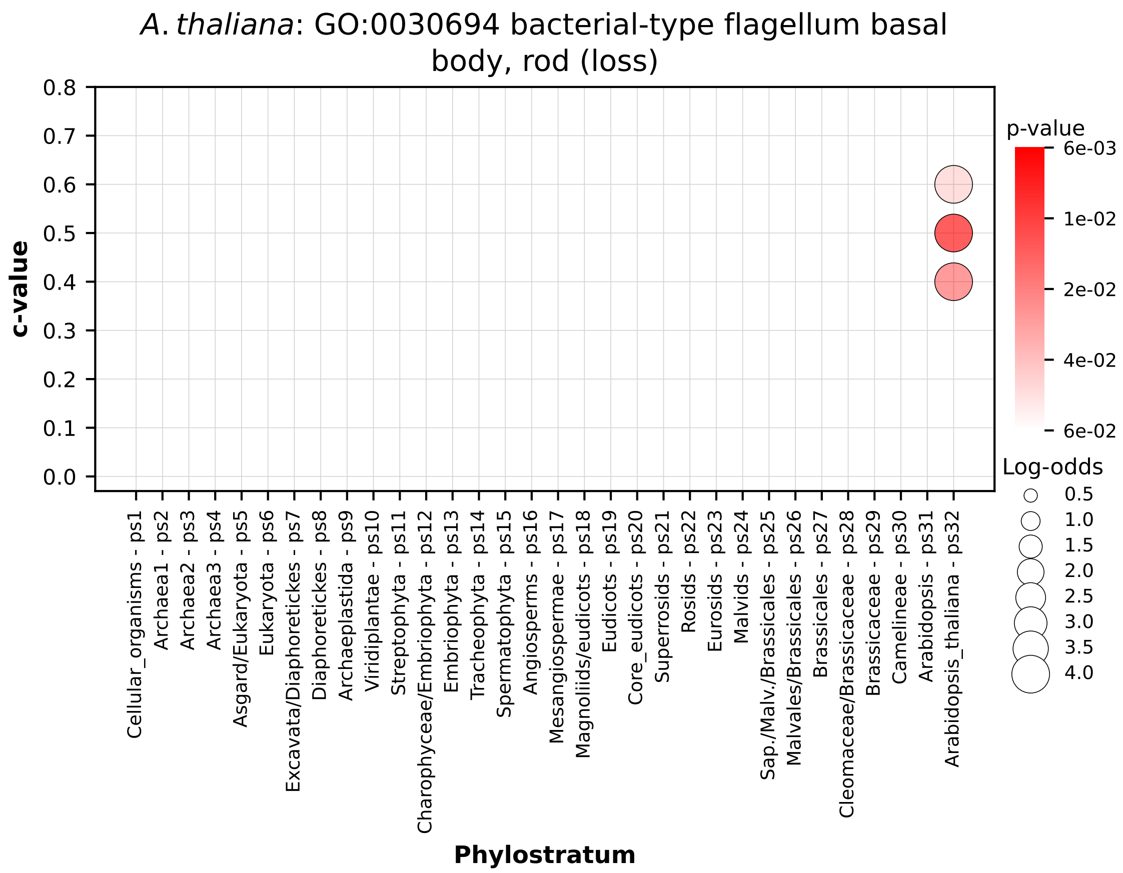
A. thaliana: GO:0030694 bacterial-type flagellum basal body, rod (loss)
c-value
0.8
0.7
0.6
0.5
0.4
0.3
0.2
0.1
0.0
p-value
6e-03
1e-02
2e-02
4e-02
6e-02
Log-odds
0.5
1.0
1.5
2.0
2.5
3.0
3.5
4.0
Cellular_organisms - ps1
Archaea1 - ps2
Archaea2 - ps3
Archaea3 - ps4
Asgard/Eukaryota - ps5
Eukaryota - ps6
Excavata/Diaphoretickes - ps7
Diaphoretickes - ps8
Archaeplastida - ps9
Viridiplantae - ps10
Streptophyta - ps11
Charophyceae/Embriophyta - ps12
Embriophyta - ps13
Tracheophyta - ps14
Spermatophyta - ps15
Angiosperms - ps16
Mesangiospermae - ps17
Magnoliids/eudicots - ps18
Eudicots - ps19
Core_eudicots - ps20
Superrosids - ps21
Rosids - ps22
Eurosids - ps23
Malvids - ps24
Sap./Malv./Brassicales - ps25
Malvales/Brassicales - ps26
Brassicales - ps27
Cleomaceae/Brassicaceae - ps28
Brassicaceae - ps29
Camelineae - ps30
Arabidopsis - ps31
Arabidopsis_thaliana - ps32
Phylostratum

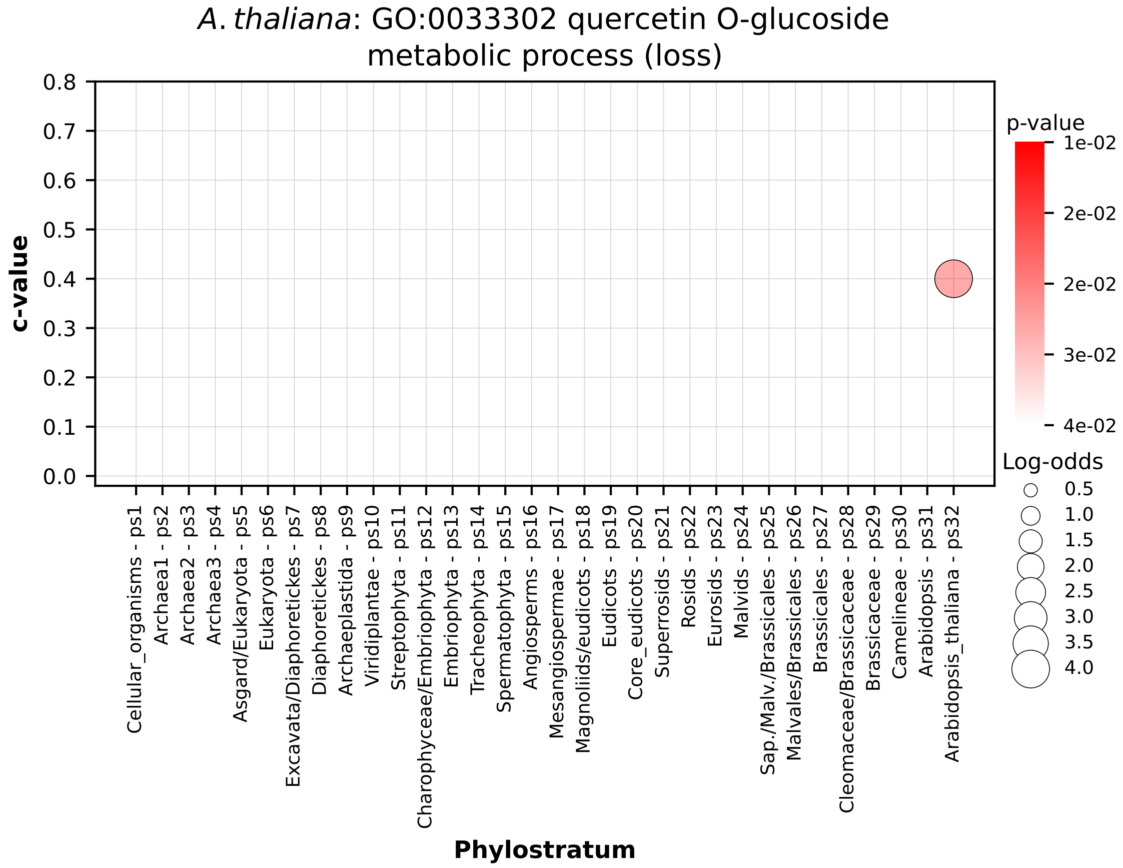
A. thaliana: GO:0033302 quercetin O-glucoside metabolic process (loss)
c-value
0.8
0.7
0.6
0.5
0.4
0.3
0.2
0.1
0.0
Phylostratum
Cellular_organisms - ps1
Archaea1 - ps2
Archaea2 - ps3
Archaea3 - ps4
Asgard/Eukaryota - ps5
Eukaryota - ps6
Excavata/Diaphoretickes - ps7
Diaphoretickes - ps8
Archaeplastida - ps9
Viridiplantae - ps10
Streptophyta - ps11
Charophyceae/Embriophyta - ps12
Embriophyta - ps13
Tracheophyta - ps14
Spermatophyta - ps15
Angiosperms - ps16
Mesangiospermae - ps17
Magnoliids/eudicots - ps18
Eudicots - ps19
Core_eudicots - ps20
Superrosids - ps21
Rosids - ps22
Eurosids - ps23
Malvids - ps24
Sap./Malv./Brassicales - ps25
Malvales/Brassicales - ps26
Brassicales - ps27
Cleomaceae/Brassicaceae - ps28
Brassicaceae - ps29
Camelineae - ps30
Arabidopsis - ps31
Arabidopsis_thaliana - ps32
p-value
1e-02
2e-02
2e-02
3e-02
4e-02
Log-odds
0.5
1.0
1.5
2.0
2.5
3.0
3.5
4.0

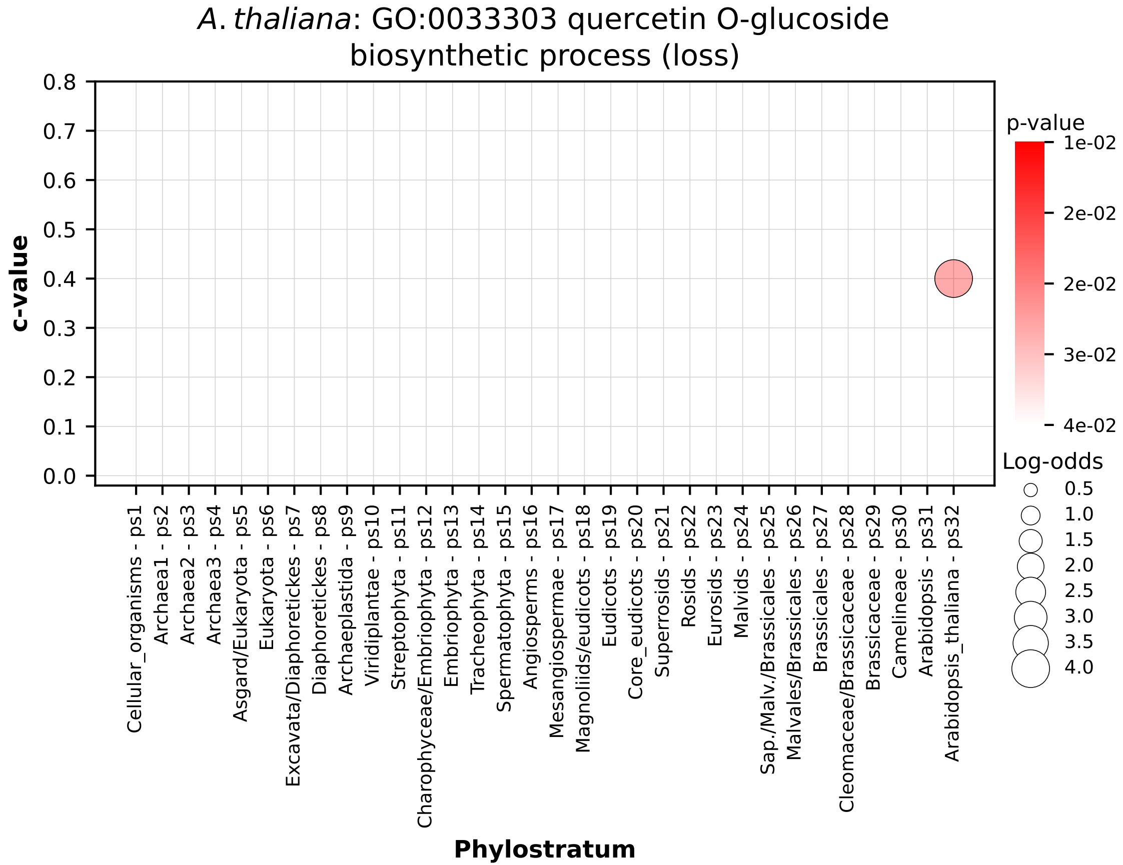

A. thaliana: GO:0033303 quercetin O-glucoside biosynthetic process (loss)
c-value
0.8
0.7
0.6
0.5
0.4
0.3
0.2
0.1
0.0
p-value
1e-02
2e-02
2e-02
3e-02
4e-02
Log-odds
0.5
1.0
1.5
2.0
2.5
3.0
3.5
4.0
Cellular_organisms - ps1
Archaea1 - ps2
Archaea2 - ps3
Archaea3 - ps4
Asgard/Eukaryota - ps5
Eukaryota - ps6
Excavata/Diaphoretickes - ps7
Diaphoretickes - ps8
Archaeplastida - ps9
Viridiplantae - ps10
Streptophyta - ps11
Charophyceae/Embriophyta - ps12
Embriophyta - ps13
Tracheophyta - ps14
Spermatophyta - ps15
Angiosperms - ps16
Mesangiospermae - ps17
Magnoliids/eudicots - ps18
Eudicots - ps19
Core_eudicots - ps20
Superrosids - ps21
Rosids - ps22
Eurosids - ps23
Malvids - ps24
Sap./Malv./Brassicales - ps25
Malvales/Brassicales - ps26
Brassicales - ps27
Cleomaceae/Brassicaceae - ps28
Brassicaceae - ps29
Camelineae - ps30
Arabidopsis - ps31
Arabidopsis_thaliana - ps32
Phylostratum

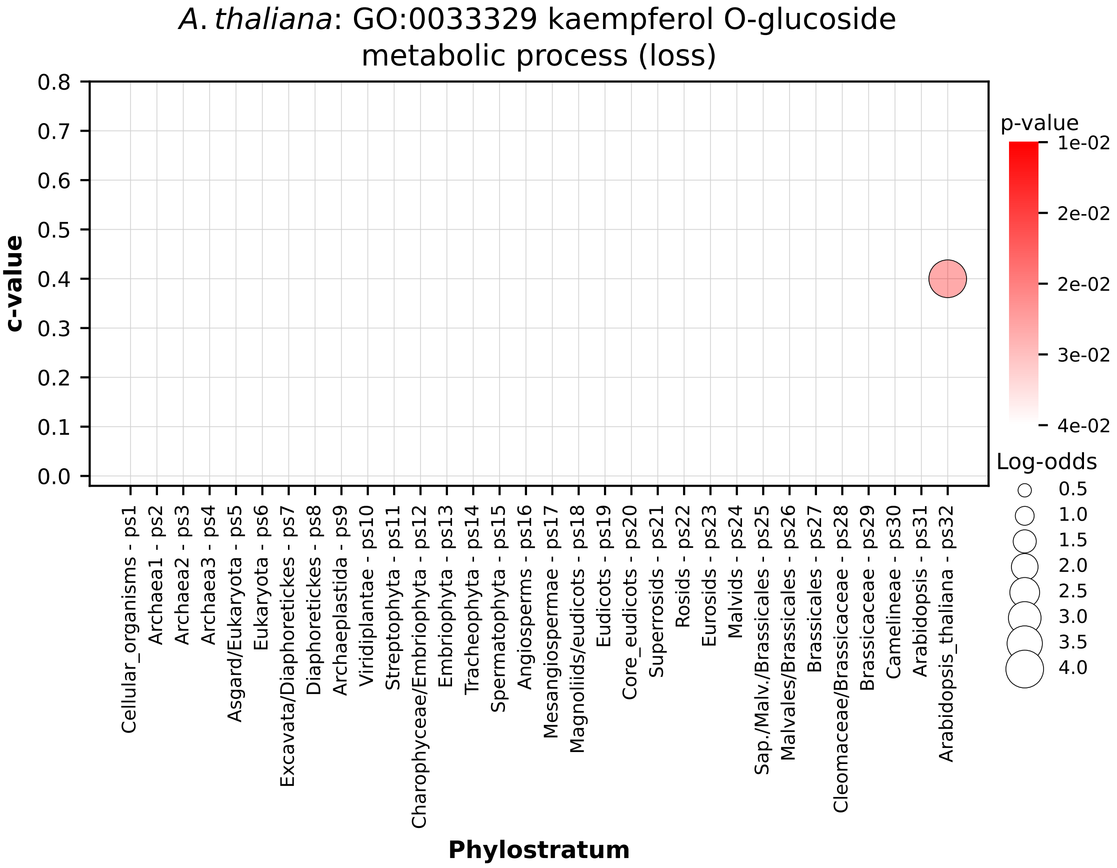

A. thaliana: GO:0033329 kaempferol O-glucoside metabolic process (loss)
c-value
0.8
0.7
0.6
0.5
0.4
0.3
0.2
0.1
0.0
Cellular_organisms - ps1
Archaea1 - ps2
Archaea2 - ps3
Archaea3 - ps4
Asgard/Eukaryota - ps5
Eukaryota - ps6
Excavata/Diaphoretickes - ps7
Diaphoretickes - ps8
Archaeplastida - ps9
Viridiplantae - ps10
Streptophyta - ps11
Charophyceae/Embriophyta - ps12
Embriophyta - ps13
Tracheophyta - ps14
Spermatophyta - ps15
Angiosperms - ps16
Mesangiospermae - ps17
Magnoliids/eudicots - ps18
Eudicots - ps19
Core_eudicots - ps20
Superrosids - ps21
Rosids - ps22
Eurosids - ps23
Malvids - ps24
Sap./Malv./Brassicales - ps25
Malvales/Brassicales - ps26
Brassicales - ps27
Cleomaceae/Brassicaceae - ps28
Brassicaceae - ps29
Camelineae - ps30
Arabidopsis - ps31
Arabidopsis_thaliana - ps32
Phylostratum
p-value
1e-02
2e-02
2e-02
3e-02
4e-02
Log-odds
0.5
1.0
1.5
2.0
2.5
3.0
3.5
4.0

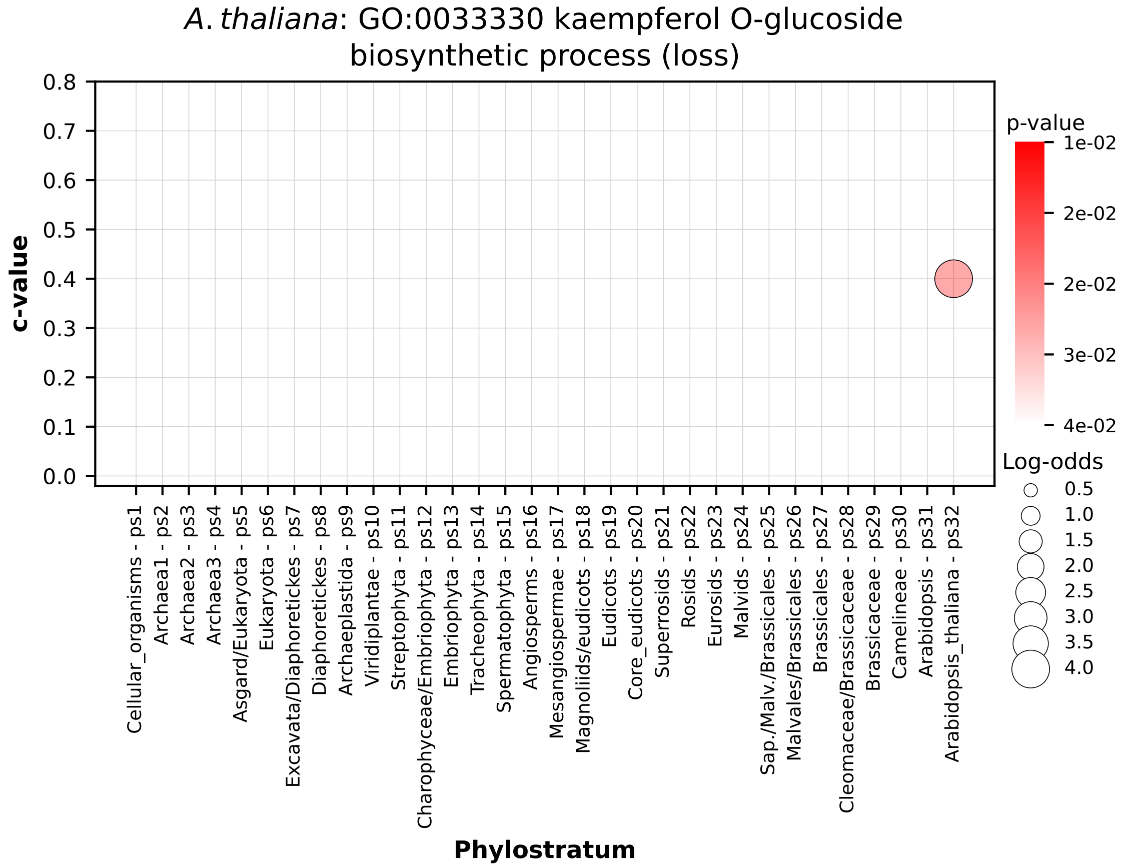

A. thaliana: GO:0033330 kaempferol O-glucoside biosynthetic process (loss)
c-value
0.8
0.7
0.6
0.5
0.4
0.3
0.2
0.1
0.0
p-value
1e-02
2e-02
2e-02
3e-02
4e-02
Log-odds
0.5
1.0
1.5
2.0
2.5
3.0
3.5
4.0
Cellular_organisms - ps1
Archaea1 - ps2
Archaea2 - ps3
Archaea3 - ps4
Asgard/Eukaryota - ps5
Eukaryota - ps6
Excavata/Diaphoretickes - ps7
Diaphoretickes - ps8
Archaeplastida - ps9
Viridiplantae - ps10
Streptophyta - ps11
Charophyceae/Embriophyta - ps12
Embriophyta - ps13
Tracheophyta - ps14
Spermatophyta - ps15
Angiosperms - ps16
Mesangiospermae - ps17
Magnoliids/eudicots - ps18
Eudicots - ps19
Core_eudicots - ps20
Superrosids - ps21
Rosids - ps22
Eurosids - ps23
Malvids - ps24
Sap./Malv./Brassicales - ps25
Malvales/Brassicales - ps26
Brassicales - ps27
Cleomaceae/Brassicaceae - ps28
Brassicaceae - ps29
Camelineae - ps30
Arabidopsis - ps31
Arabidopsis_thaliana - ps32
Phylostratum

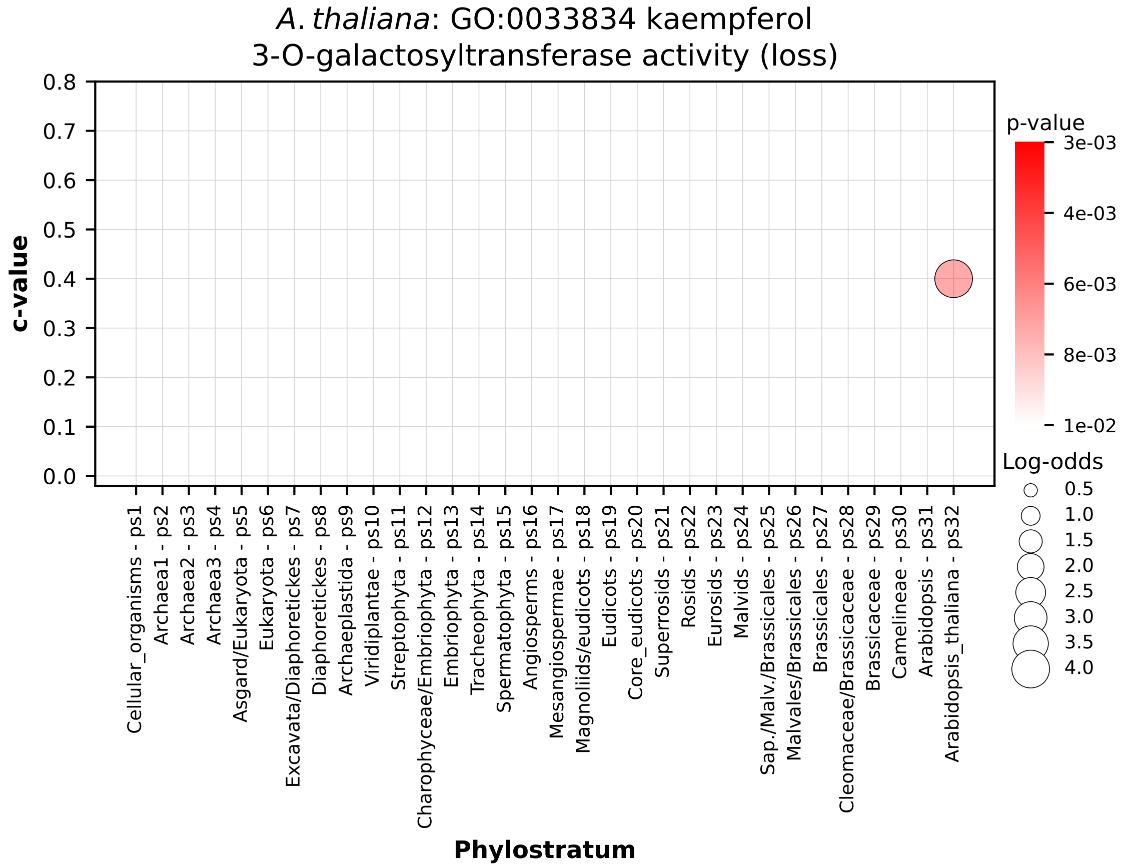

A. thaliana: GO:0033834 kaempferol 3-O-galactosyltransferase activity (loss)
c-value
0.8
0.7
0.6
0.5
0.4
0.3
0.2
0.1
0.0
Phylostratum
Cellular_organisms - ps1
Archaea1 - ps2
Archaea2 - ps3
Archaea3 - ps4
Asgard/Eukaryota - ps5
Eukaryota - ps6
Excavata/Diaphoretickes - ps7
Diaphoretickes - ps8
Archaeplastida - ps9
Viridiplantae - ps10
Streptophyta - ps11
Charophyceae/Embriophyta - ps12
Embriophyta - ps13
Tracheophyta - ps14
Spermatophyta - ps15
Angiosperms - ps16
Mesangiospermae - ps17
Magnoliids/eudicots - ps18
Eudicots - ps19
Core_eudicots - ps20
Superrosids - ps21
Rosids - ps22
Eurosids - ps23
Malvids - ps24
Sap./Malv./Brassicales - ps25
Malvales/Brassicales - ps26
Brassicales - ps27
Cleomaceae/Brassicaceae - ps28
Brassicaceae - ps29
Camelineae - ps30
Arabidopsis - ps31
Arabidopsis_thaliana - ps32
p-value
3e-03
4e-03
6e-03
8e-03
1e-02
Log-odds
0.5
1.0
1.5
2.0
2.5
3.0
3.5
4.0

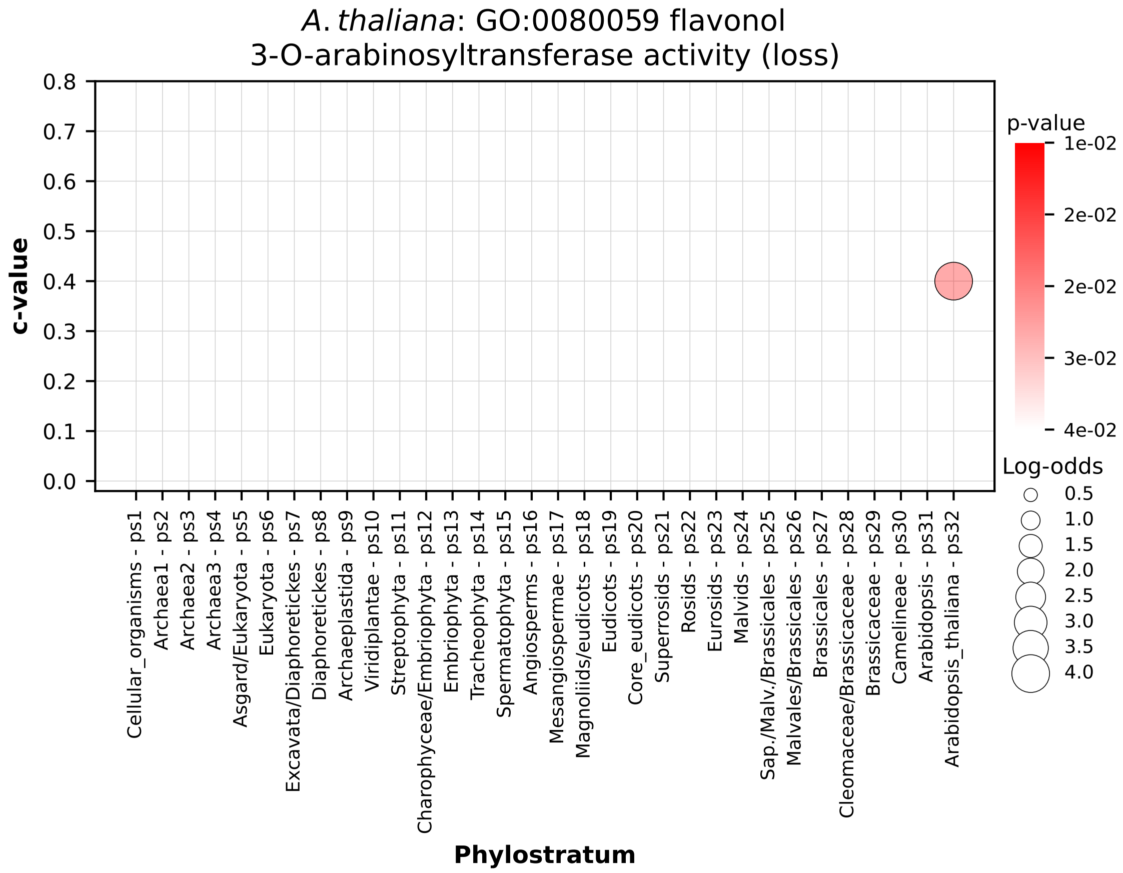

A. thaliana: GO:0080059 flavonol
3-O-arabinosyltransferase activity (loss)
c-value
0.0
0.1
0.2
0.3
0.4
0.5
0.6
0.7
0.8
Phylostratum
Cellular_organisms - ps1
Archaea1 - ps2
Archaea2 - ps3
Archaea3 - ps4
Asgard/Eukaryota - ps5
Eukaryota - ps6
Excavata/Diaphoretickes - ps7
Diaphoretickes - ps8
Archaeplastida - ps9
Viridiplantae - ps10
Streptophyta - ps11
Charophyceae/Embriophyta - ps12
Embriophyta - ps13
Tracheophyta - ps14
Spermatophyta - ps15
Angiosperms - ps16
Mesangiospermae - ps17
Magnoliids/eudicots - ps18
Eudicots - ps19
Core_eudicots - ps20
Superrosids - ps21
Rosids - ps22
Eurosids - ps23
Malvids - ps24
Sap./Malv./Brassicales - ps25
Malvales/Brassicales - ps26
Brassicales - ps27
Cleomaceae/Brassicaceae - ps28
Brassicaceae - ps29
Camelineae - ps30
Arabidopsis - ps31
Arabidopsis_thaliana - ps32
p-value
1e-02
2e-02
2e-02
3e-02
4e-02
Log-odds
0.5
1.0
1.5
2.0
2.5
3.0
3.5
4.0

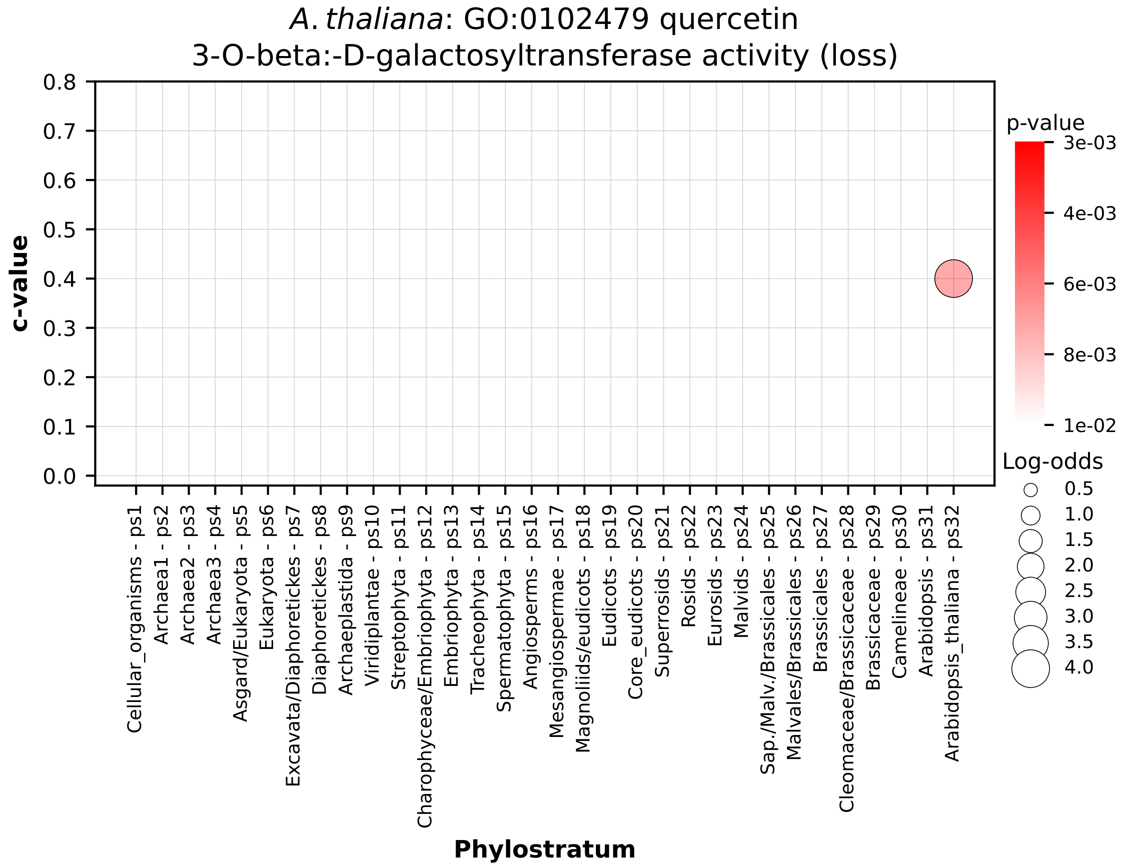

A. thaliana: GO:0102479 quercetin
3-O-beta:-D-galactosyltransferase activity (loss)
c-value
0.8
0.7
0.6
0.5
0.4
0.3
0.2
0.1
0.0
p-value
3e-03
4e-03
6e-03
8e-03
1e-02
Log-odds
0.5
1.0
1.5
2.0
2.5
3.0
3.5
4.0
Cellular_organisms - ps1
Archaea1 - ps2
Archaea2 - ps3
Archaea3 - ps4
Asgard/Eukaryota - ps5
Eukaryota - ps6
Excavata/Diaphoretickes - ps7
Diaphoretickes - ps8
Archaeplastida - ps9
Viridiplantae - ps10
Streptophyta - ps11
Charophyceae/Embriophyta - ps12
Embriophyta - ps13
Tracheophyta - ps14
Spermatophyta - ps15
Angiosperms - ps16
Mesangiospermae - ps17
Magnoliids/eudicots - ps18
Eudicots - ps19
Core_eudicots - ps20
Superrosids - ps21
Rosids - ps22
Eurosids - ps23
Malvids - ps24
Sap./Malv./Brassicales - ps25
Malvales/Brassicales - ps26
Brassicales - ps27
Cleomaceae/Brassicaceae - ps28
Brassicaceae - ps29
Camelineae - ps30
Arabidopsis - ps31
Arabidopsis_thaliana - ps32
Phylostratum

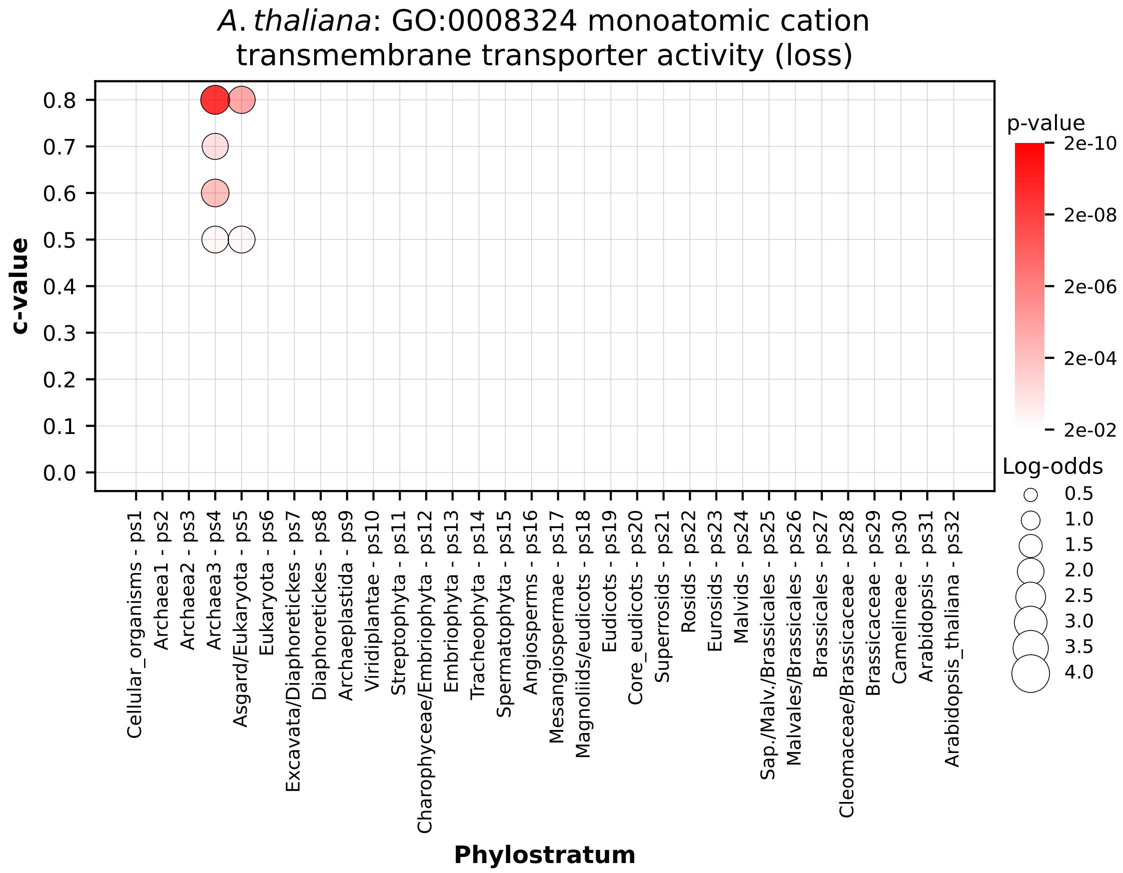

A. thaliana: GO:0008324 monoatomic cation transmembrane transporter activity (loss)
c-value
0.8
0.7
0.6
0.5
0.4
0.3
0.2
0.1
0.0
p-value
2e-10
2e-08
2e-06
2e-04
2e-02
Log-odds
0.5
1.0
1.5
2.0
2.5
3.0
3.5
4.0
Cellular_organisms - ps1
Archaea1 - ps2
Archaea2 - ps3
Archaea3 - ps4
Asgard/Eukaryota - ps5
Eukaryota - ps6
Excavata/Diaphoretickes - ps7
Diaphoretickes - ps8
Archaeplastida - ps9
Viridiplantae - ps10
Streptophyta - ps11
Charophyceae/Embriophyta - ps12
Embriophyta - ps13
Tracheophyta - ps14
Spermatophyta - ps15
Angiosperms - ps16
Mesangiospermae - ps17
Magnoliids/eudicots - ps18
Eudicots - ps19
Core_eudicots - ps20
Superrosids - ps21
Rosids - ps22
Eurosids - ps23
Malvids - ps24
Sap./Malv./Brassicales - ps25
Malvales/Brassicales - ps26
Brassicales - ps27
Cleomaceae/Brassicaceae - ps28
Brassicaceae - ps29
Camelineae - ps30
Arabidopsis - ps31
Arabidopsis_thaliana - ps32
Phylostratum

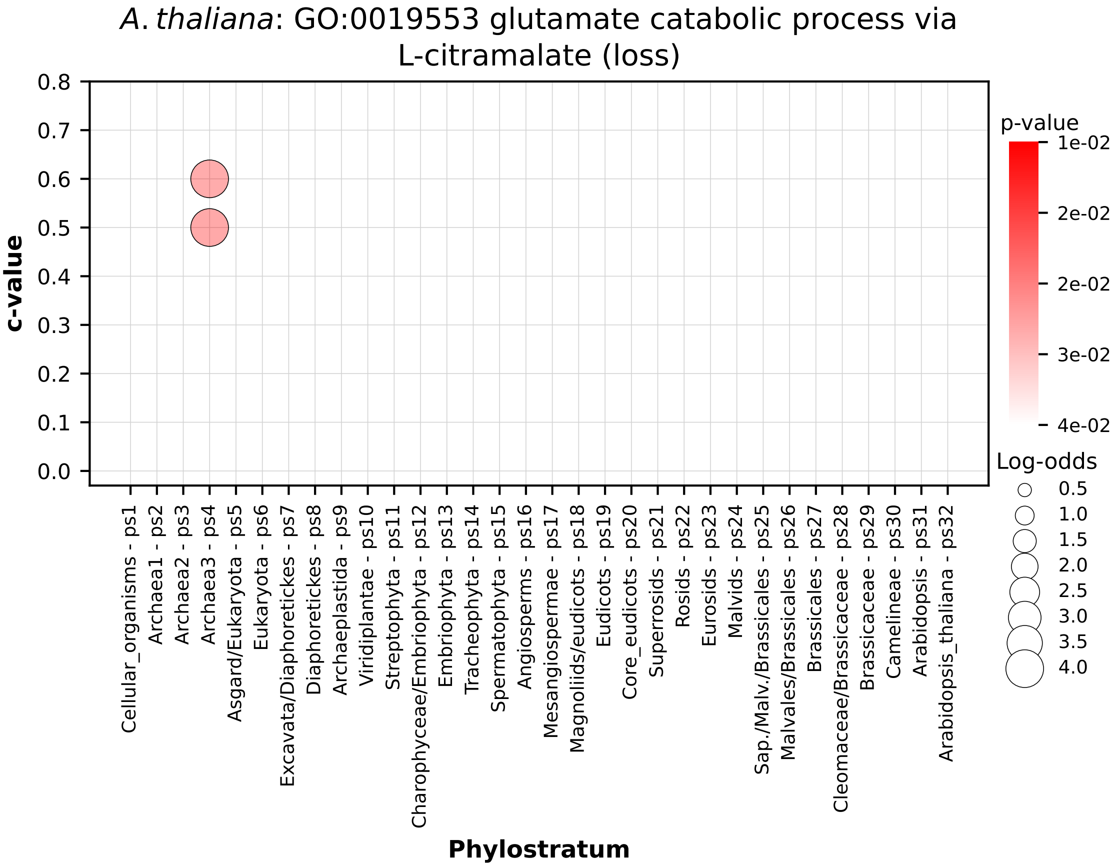
A. thaliana: GO:0019553 glutamate catabolic process via L-citramalate (loss)
c-value
0.0
0.1
0.2
0.3
0.4
0.5
0.6
0.7
0.8
Phylostratum
Cellular_organisms - ps1
Archaea1 - ps2
Archaea2 - ps3
Archaea3 - ps4
Asgard/Eukaryota - ps5
Eukaryota - ps6
Excavata/Diaphoretickes - ps7
Diaphoretickes - ps8
Archaeplastida - ps9
Viridiplantae - ps10
Streptophyta - ps11
Charophyceae/Embriophyta - ps12
Embriophyta - ps13
Tracheophyta - ps14
Spermatophyta - ps15
Angiosperms - ps16
Mesangiospermae - ps17
Magnoliids/eudicots - ps18
Eudicots - ps19
Core_eudicots - ps20
Superrosids - ps21
Rosids - ps22
Eurosids - ps23
Malvids - ps24
Sap./Malv./Brassicales - ps25
Malvales/Brassicales - ps26
Brassicales - ps27
Cleomaceae/Brassicaceae - ps28
Brassicaceae - ps29
Camelineae - ps30
Arabidopsis - ps31
Arabidopsis_thaliana - ps32
p-value
1e-02
2e-02
2e-02
3e-02
4e-02
Log-odds
0.5
1.0
1.5
2.0
2.5
3.0
3.5
4.0

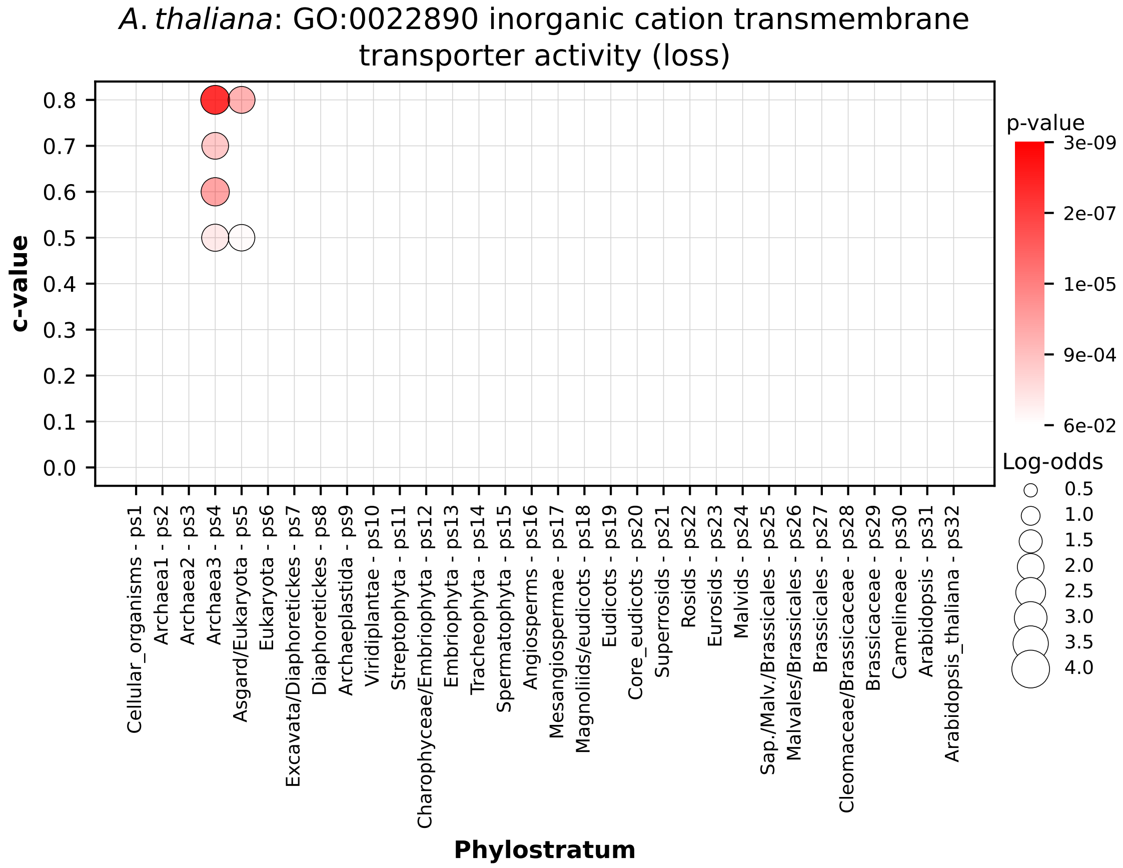
A. thaliana: GO:0022890 inorganic cation transmembrane transporter activity (loss)
c-value
0.8
0.7
0.6
0.5
0.4
0.3
0.2
0.1
0.0
p-value
3e-09
2e-07
1e-05
9e-04
6e-02
Log-odds
0.5
1.0
1.5
2.0
2.5
3.0
3.5
4.0
Cellular_organisms - ps1
Archaea1 - ps2
Archaea2 - ps3
Archaea3 - ps4
Asgard/Eukaryota - ps5
Eukaryota - ps6
Excavata/Diaphoretickes - ps7
Diaphoretickes - ps8
Archaeplastida - ps9
Viridiplantae - ps10
Streptophyta - ps11
Charophyceae/Embriophyta - ps12
Embriophyta - ps13
Tracheophyta - ps14
Spermatophyta - ps15
Angiosperms - ps16
Mesangiospermae - ps17
Magnoliids/eudicots - ps18
Eudicots - ps19
Core_eudicots - ps20
Superrosids - ps21
Rosids - ps22
Eurosids - ps23
Malvids - ps24
Sap./Malv./Brassicales - ps25
Malvales/Brassicales - ps26
Brassicales - ps27
Cleomaceae/Brassicaceae - ps28
Brassicaceae - ps29
Camelineae - ps30
Arabidopsis - ps31
Arabidopsis_thaliana - ps32
Phylostratum

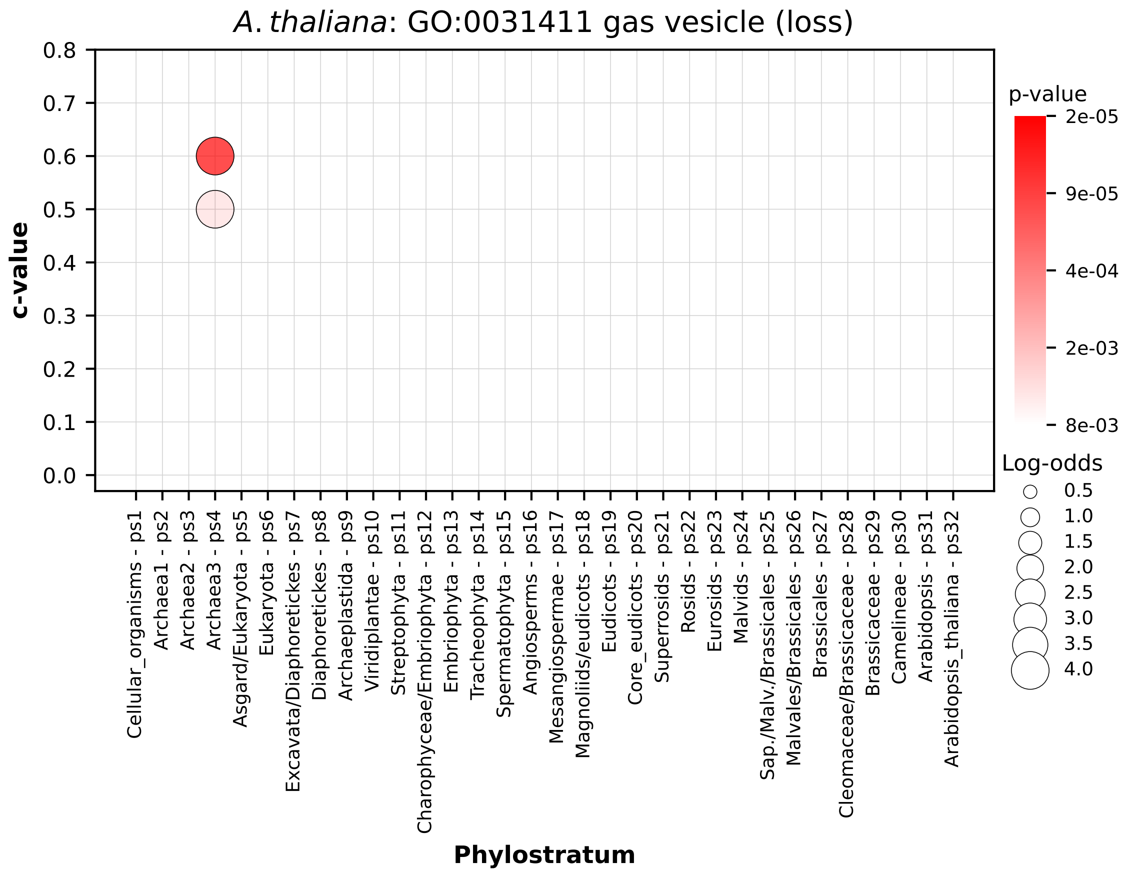

A. thaliana: GO:0031411 gas vesicle (loss)
c-value
0.8
0.7
0.6
0.5
0.4
0.3
0.2
0.1
0.0
p-value
2e-05
9e-05
4e-04
2e-03
8e-03
Log-odds
0.5
1.0
1.5
2.0
2.5
3.0
3.5
4.0
Cellular_organisms - ps1
Archaea1 - ps2
Archaea2 - ps3
Archaea3 - ps4
Asgard/Eukaryota - ps5
Eukaryota - ps6
Excavata/Diaphoretickes - ps7
Diaphoretickes - ps8
Archaeplastida - ps9
Viridiplantae - ps10
Streptophyta - ps11
Charophyceae/Embriophyta - ps12
Embriophyta - ps13
Tracheophyta - ps14
Spermatophyta - ps15
Angiosperms - ps16
Mesangiospermae - ps17
Magnoliids/eudicots - ps18
Eudicots - ps19
Core_eudicots - ps20
Superrosids - ps21
Rosids - ps22
Eurosids - ps23
Malvids - ps24
Sap./Malv./Brassicales - ps25
Malvales/Brassicales - ps26
Brassicales - ps27
Cleomaceae/Brassicaceae - ps28
Brassicaceae - ps29
Camelineae - ps30
Arabidopsis - ps31
Arabidopsis_thaliana - ps32
Phylostratum

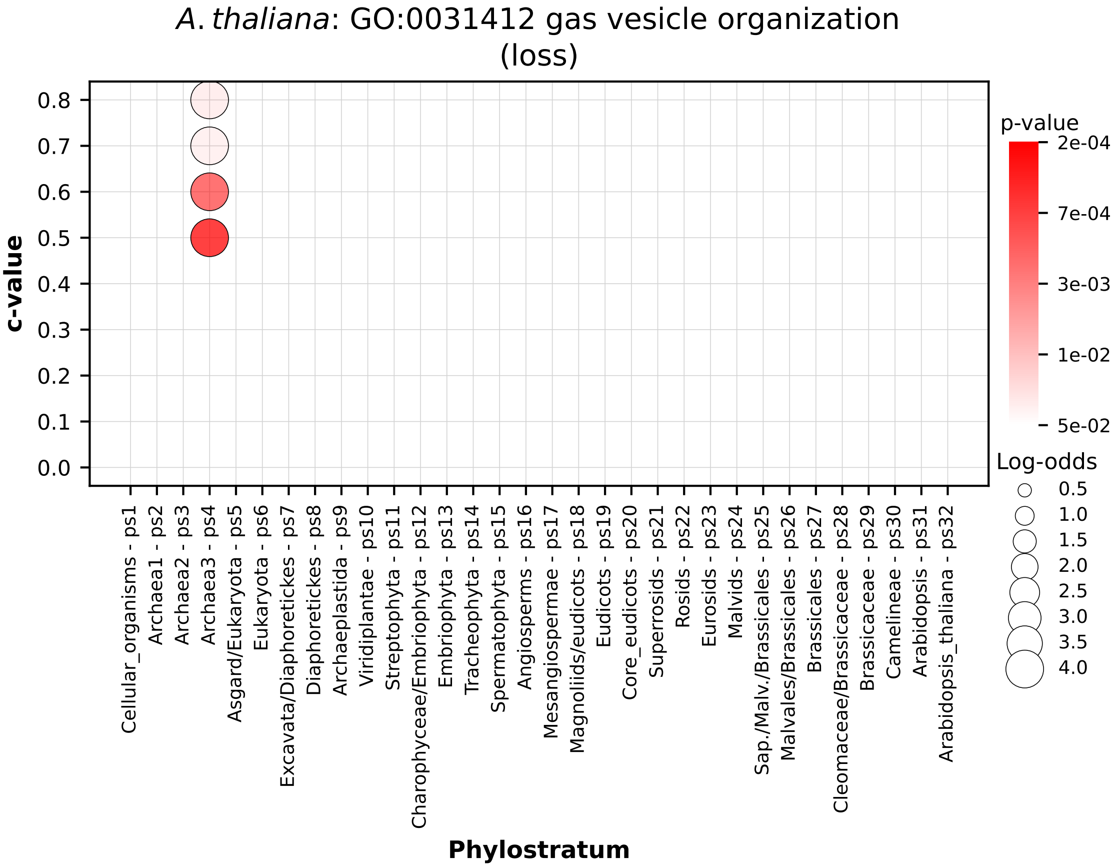

A. thaliana: GO:0031412 gas vesicle organization
(loss)
c-value
0.8
0.7
0.6
0.5
0.4
0.3
0.2
0.1
0.0
p-value
2e-04
7e-04
3e-03
1e-02
5e-02
Log-odds
0.5
1.0
1.5
2.0
2.5
3.0
3.5
4.0
Cellular_organisms - ps1
Archaea1 - ps2
Archaea2 - ps3
Archaea3 - ps4
Asgard/Eukaryota - ps5
Eukaryota - ps6
Excavata/Diaphoretickes - ps7
Diaphoretickes - ps8
Archaeplastida - ps9
Viridiplantae - ps10
Streptophyta - ps11
Charophyceae/Embriophyta - ps12
Embriophyta - ps13
Tracheophyta - ps14
Spermatophyta - ps15
Angiosperms - ps16
Mesangiospermae - ps17
Magnoliids/eudicots - ps18
Eudicots - ps19
Core_eudicots - ps20
Superrosids - ps21
Rosids - ps22
Eurosids - ps23
Malvids - ps24
Sap./Malv./Brassicales - ps25
Malvales/Brassicales - ps26
Brassicales - ps27
Cleomaceae/Brassicaceae - ps28
Brassicaceae - ps29
Camelineae - ps30
Arabidopsis - ps31
Arabidopsis_thaliana - ps32
Phylostratum

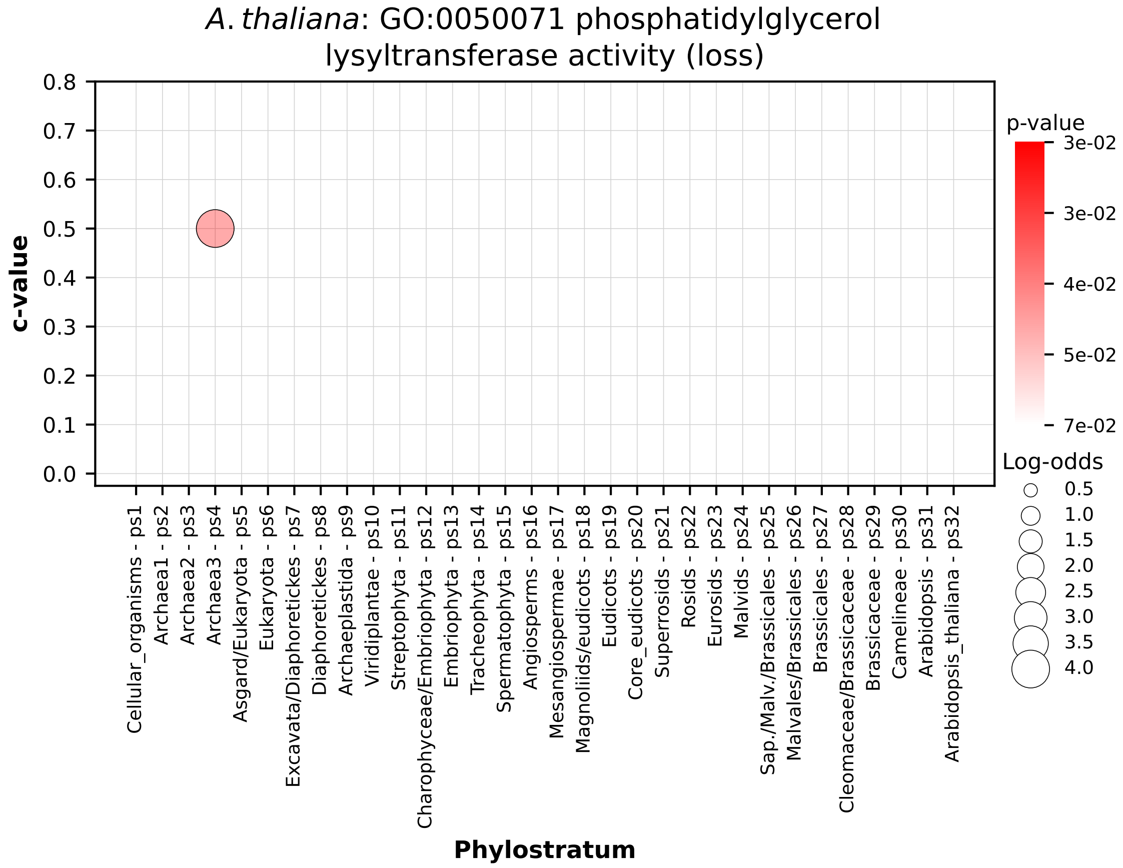

A. thaliana: GO:0050071 phosphatidylglycerol lysyltransferase activity (loss)
c-value
0.8
0.7
0.6
0.5
0.4
0.3
0.2
0.1
0.0
p-value
3e-02
3e-02
4e-02
5e-02
7e-02
Log-odds
0.5
1.0
1.5
2.0
2.5
3.0
3.5
4.0
Cellular_organisms - ps1
Archaea1 - ps2
Archaea2 - ps3
Archaea3 - ps4
Asgard/Eukaryota - ps5
Eukaryota - ps6
Excavata/Diaphoretickes - ps7
Diaphoretickes - ps8
Archaeplastida - ps9
Viridiplantae - ps10
Streptophyta - ps11
Charophyceae/Embriophyta - ps12
Embriophyta - ps13
Tracheophyta - ps14
Spermatophyta - ps15
Angiosperms - ps16
Mesangiospermae - ps17
Magnoliids/eudicots - ps18
Eudicots - ps19
Core_eudicots - ps20
Superrosids - ps21
Rosids - ps22
Eurosids - ps23
Malvids - ps24
Sap./Malv./Brassicales - ps25
Malvales/Brassicales - ps26
Brassicales - ps27
Cleomaceae/Brassicaceae - ps28
Brassicaceae - ps29
Camelineae - ps30
Arabidopsis - ps31
Arabidopsis_thaliana - ps32
Phylostratum

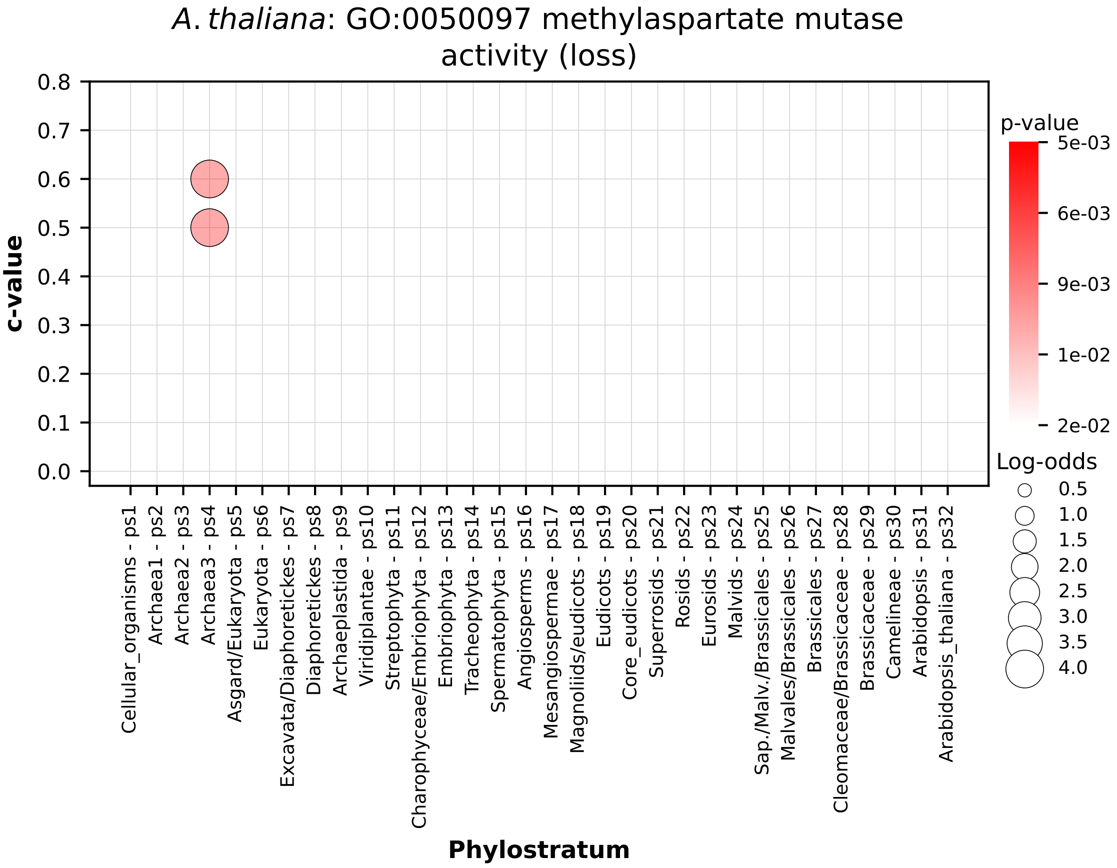

A. thaliana: GO:0050097 methylaspartate mutase activity (loss)
c-value
0.8
0.7
0.6
0.5
0.4
0.3
0.2
0.1
0.0
Phylostratum
Cellular_organisms - ps1
Archaea1 - ps2
Archaea2 - ps3
Archaea3 - ps4
Asgard/Eukaryota - ps5
Eukaryota - ps6
Excavata/Diaphoretickes - ps7
Diaphoretickes - ps8
Archaeplastida - ps9
Viridiplantae - ps10
Streptophyta - ps11
Charophyceae/Embriophyta - ps12
Embriophyta - ps13
Tracheophyta - ps14
Spermatophyta - ps15
Angiosperms - ps16
Mesangiospermae - ps17
Magnoliids/eudicots - ps18
Eudicots - ps19
Core_eudicots - ps20
Superrosids - ps21
Rosids - ps22
Eurosids - ps23
Malvids - ps24
Sap./Malv./Brassicales - ps25
Malvales/Brassicales - ps26
Brassicales - ps27
Cleomaceae/Brassicaceae - ps28
Brassicaceae - ps29
Camelineae - ps30
Arabidopsis - ps31
Arabidopsis_thaliana - ps32
p-value
5e-03
6e-03
9e-03
1e-02
2e-02
Log-odds
0.5
1.0
1.5
2.0
2.5
3.0
3.5
4.0

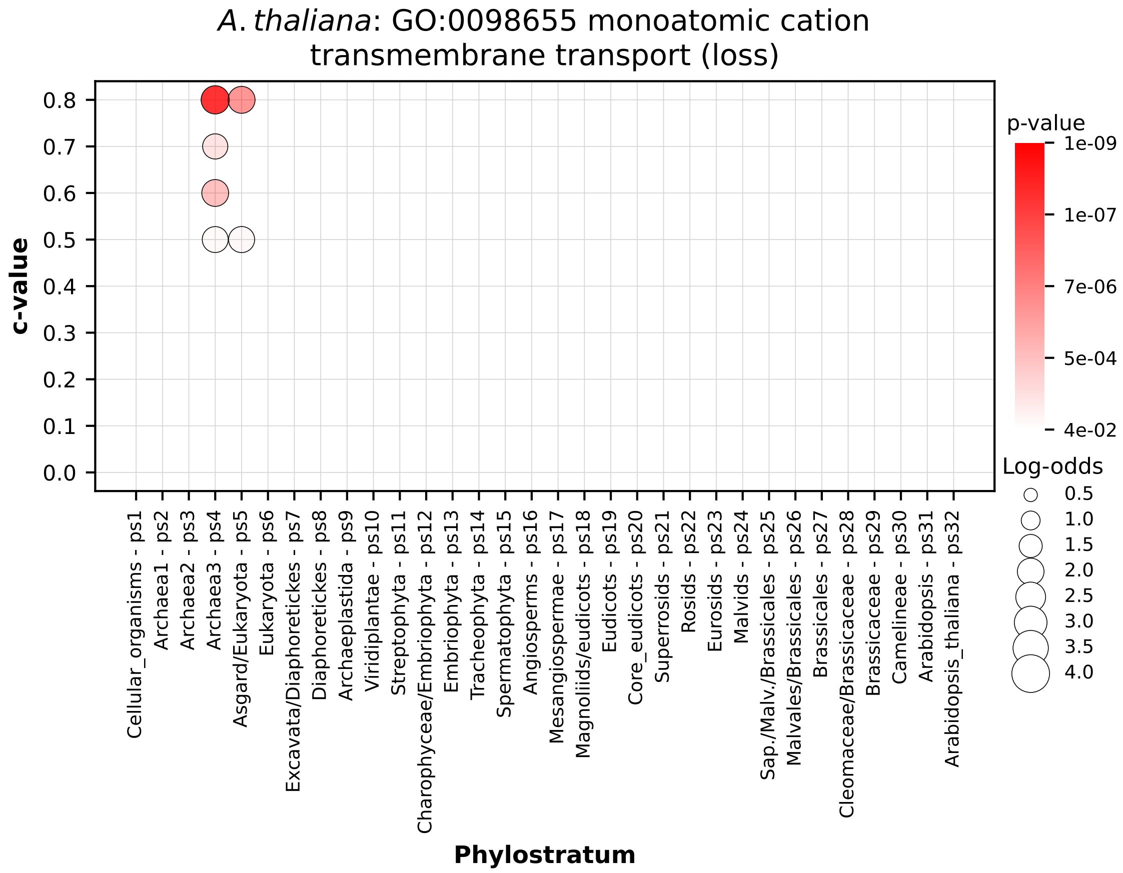

A. thaliana: GO:0098655 monoatomic cation transmembrane transport (loss)
c-value
0.8
0.7
0.6
0.5
0.4
0.3
0.2
0.1
0.0
Phylostratum
Cellular_organisms - ps1
Archaea1 - ps2
Archaea2 - ps3
Archaea3 - ps4
Asgard/Eukaryota - ps5
Eukaryota - ps6
Excavata/Diaphoretickes - ps7
Diaphoretickes - ps8
Archaeplastida - ps9
Viridiplantae - ps10
Streptophyta - ps11
Charophyceae/Embriophyta - ps12
Embriophyta - ps13
Tracheophyta - ps14
Spermatophyta - ps15
Angiosperms - ps16
Mesangiospermae - ps17
Magnoliids/eudicots - ps18
Eudicots - ps19
Core_eudicots - ps20
Superrosids - ps21
Rosids - ps22
Eurosids - ps23
Malvids - ps24
Sap./Malv./Brassicales - ps25
Malvales/Brassicales - ps26
Brassicales - ps27
Cleomaceae/Brassicaceae - ps28
Brassicaceae - ps29
Camelineae - ps30
Arabidopsis - ps31
Arabidopsis_thaliana - ps32
p-value
1e-09
1e-07
7e-06
5e-04
4e-02
Log-odds
0.5
1.0
1.5
2.0
2.5
3.0
3.5
4.0

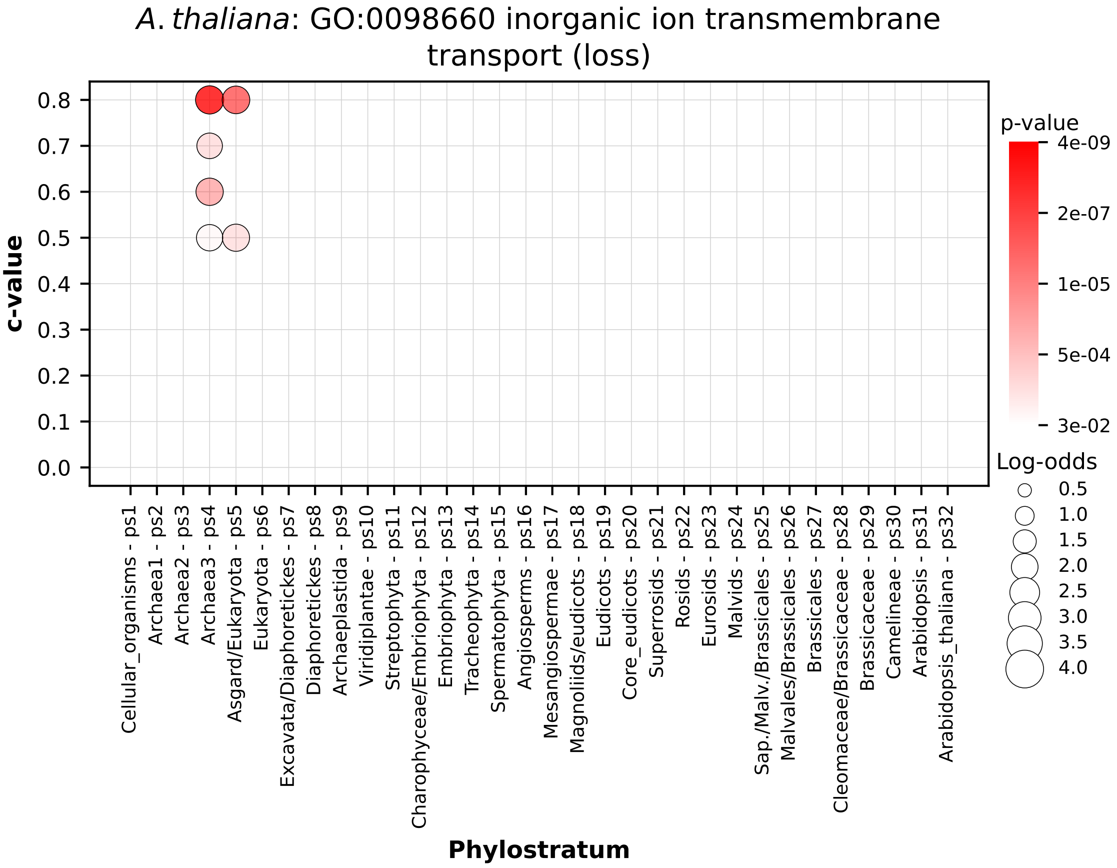

A. thaliana: GO:0098660 inorganic ion transmembrane transport (loss)
c-value
0.8
0.7
0.6
0.5
0.4
0.3
0.2
0.1
0.0
Phylostratum
Cellular_organisms - ps1
Archaea1 - ps2
Archaea2 - ps3
Archaea3 - ps4
Asgard/Eukaryota - ps5
Eukaryota - ps6
Excavata/Diaphoretickes - ps7
Diaphoretickes - ps8
Archaeplastida - ps9
Viridiplantae - ps10
Streptophyta - ps11
Charophyceae/Embriophyta - ps12
Embriophyta - ps13
Tracheophyta - ps14
Spermatophyta - ps15
Angiosperms - ps16
Mesangiospermae - ps17
Magnoliids/eudicots - ps18
Eudicots - ps19
Core_eudicots - ps20
Superrosids - ps21
Rosids - ps22
Eurosids - ps23
Malvids - ps24
Sap./Malv./Brassicales - ps25
Malvales/Brassicales - ps26
Brassicales - ps27
Cleomaceae/Brassicaceae - ps28
Brassicaceae - ps29
Camelineae - ps30
Arabidopsis - ps31
Arabidopsis_thaliana - ps32
p-value
4e-09
2e-07
1e-05
5e-04
3e-02
Log-odds
0.5
1.0
1.5
2.0
2.5
3.0
3.5
4.0

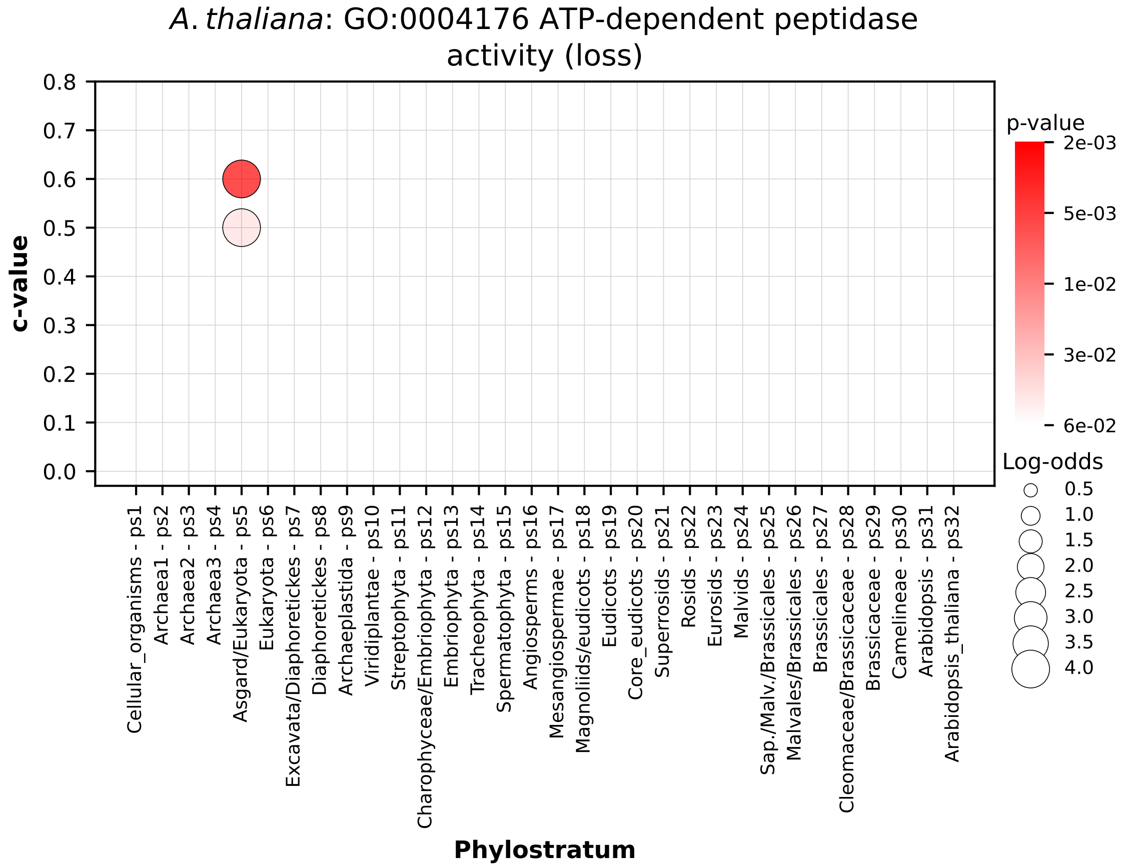

A. thaliana: GO:0004176 ATP-dependent peptidase activity (loss)
c-value
0.8
0.7
0.6
0.5
0.4
0.3
0.2
0.1
0.0
Cellular_organisms - ps1
Archaea1 - ps2
Archaea2 - ps3
Archaea3 - ps4
Asgard/Eukaryota - ps5
Eukaryota - ps6
Excavata/Diaphoretickes - ps7
Diaphoretickes - ps8
Archaeplastida - ps9
Viridiplantae - ps10
Streptophyta - ps11
Charophyceae/Embriophyta - ps12
Embriophyta - ps13
Tracheophyta - ps14
Spermatophyta - ps15
Angiosperms - ps16
Mesangiospermae - ps17
Magnoliids/eudicots - ps18
Eudicots - ps19
Core_eudicots - ps20
Superrosids - ps21
Rosids - ps22
Eurosids - ps23
Malvids - ps24
Sap./Malv./Brassicales - ps25
Malvales/Brassicales - ps26
Brassicales - ps27
Cleomaceae/Brassicaceae - ps28
Brassicaceae - ps29
Camelineae - ps30
Arabidopsis - ps31
Arabidopsis_thaliana - ps32
Phylostratum
p-value
2e-03
5e-03
1e-02
3e-02
6e-02
Log-odds
0.5
1.0
1.5
2.0
2.5
3.0
3.5
4.0

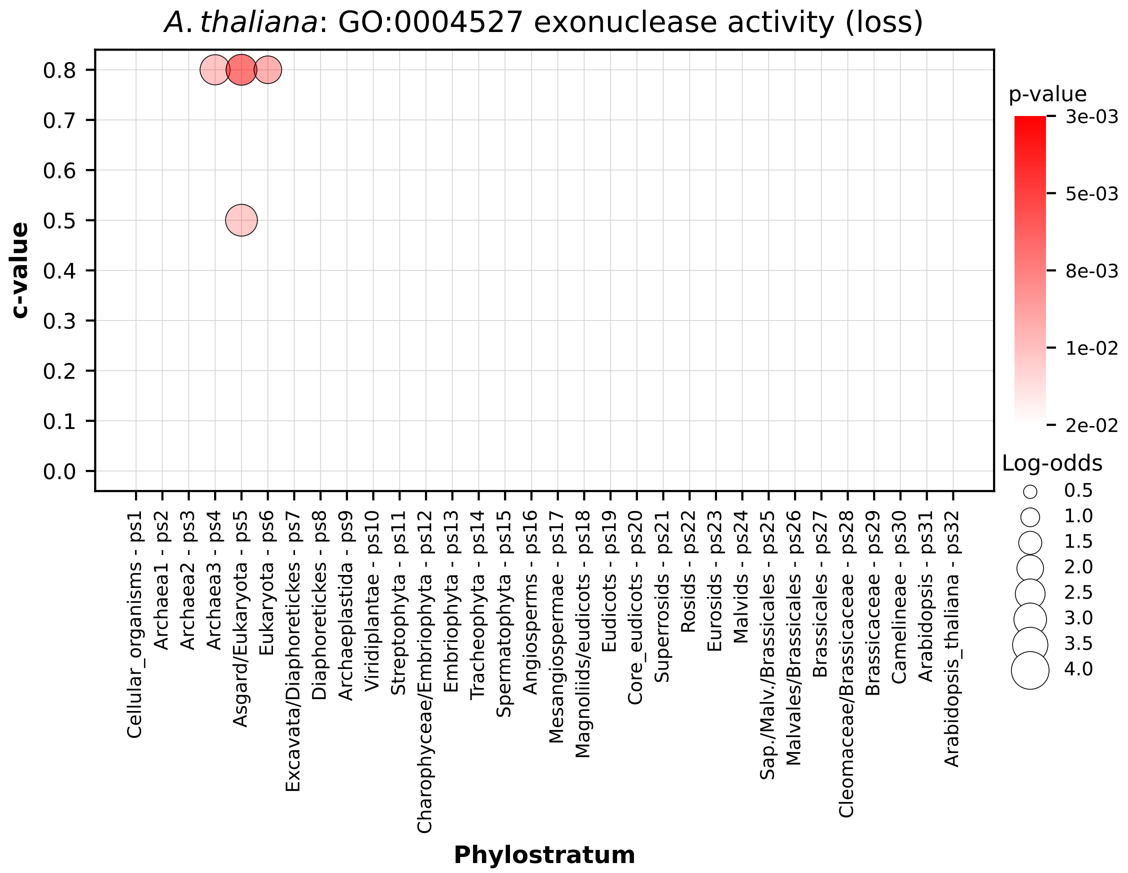

A. thaliana: GO:0004527 exonuclease activity (loss)
c-value
0.0
0.1
0.2
0.3
0.4
0.5
0.6
0.7
0.8
p-value
3e-03
5e-03
8e-03
1e-02
2e-02
Log-odds
0.5
1.0
1.5
2.0
2.5
3.0
3.5
4.0
Cellular_organisms - ps1
Archaea1 - ps2
Archaea2 - ps3
Archaea3 - ps4
Asgard/Eukaryota - ps5
Eukaryota - ps6
Excavata/Diaphoretickes - ps7
Diaphoretickes - ps8
Archaeplastida - ps9
Viridiplantae - ps10
Streptophyta - ps11
Charophyceae/Embriophyta - ps12
Embriophyta - ps13
Tracheophyta - ps14
Spermatophyta - ps15
Angiosperms - ps16
Mesangiospermae - ps17
Magnoliids/eudicots - ps18
Eudicots - ps19
Core_eudicots - ps20
Superrosids - ps21
Rosids - ps22
Eurosids - ps23
Malvids - ps24
Sap./Malv./Brassicales - ps25
Malvales/Brassicales - ps26
Brassicales - ps27
Cleomaceae/Brassicaceae - ps28
Brassicaceae - ps29
Camelineae - ps30
Arabidopsis - ps31
Arabidopsis_thaliana - ps32
Phylostratum

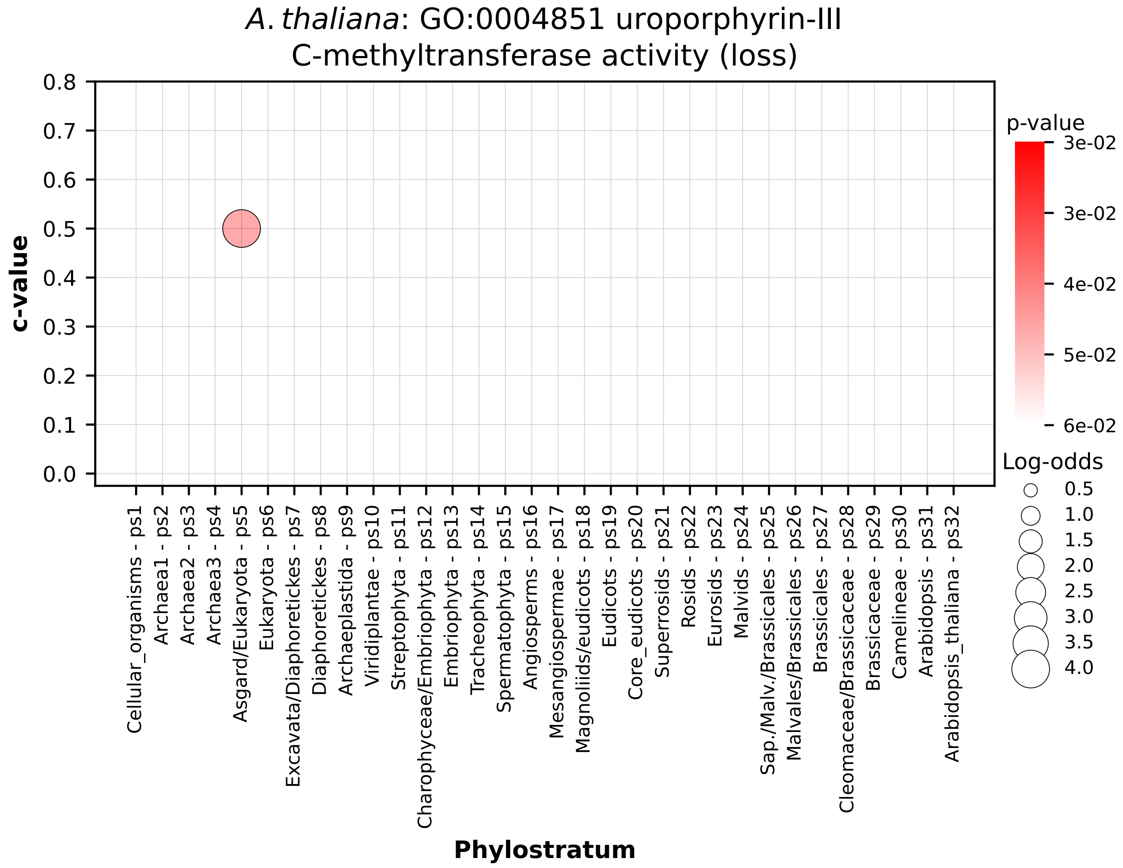

A. thaliana: GO:0004851 uroporphyrin-III C-methyltransferase activity (loss)
c-value
0.8
0.7
0.6
0.5
0.4
0.3
0.2
0.1
0.0
p-value
3e-02
3e-02
4e-02
5e-02
6e-02
Log-odds
0.5
1.0
1.5
2.0
2.5
3.0
3.5
4.0
Cellular_organisms - ps1
Archaea1 - ps2
Archaea2 - ps3
Archaea3 - ps4
Asgard/Eukaryota - ps5
Eukaryota - ps6
Excavata/Diaphoretickes - ps7
Diaphoretickes - ps8
Archaeplastida - ps9
Viridiplantae - ps10
Streptophyta - ps11
Charophyceae/Embriophyta - ps12
Embriophyta - ps13
Tracheophyta - ps14
Spermatophyta - ps15
Angiosperms - ps16
Mesangiospermae - ps17
Magnoliids/eudicots - ps18
Eudicots - ps19
Core_eudicots - ps20
Superrosids - ps21
Rosids - ps22
Eurosids - ps23
Malvids - ps24
Sap./Malv./Brassicales - ps25
Malvales/Brassicales - ps26
Brassicales - ps27
Cleomaceae/Brassicaceae - ps28
Brassicaceae - ps29
Camelineae - ps30
Arabidopsis - ps31
Arabidopsis_thaliana - ps32
Phylostratum

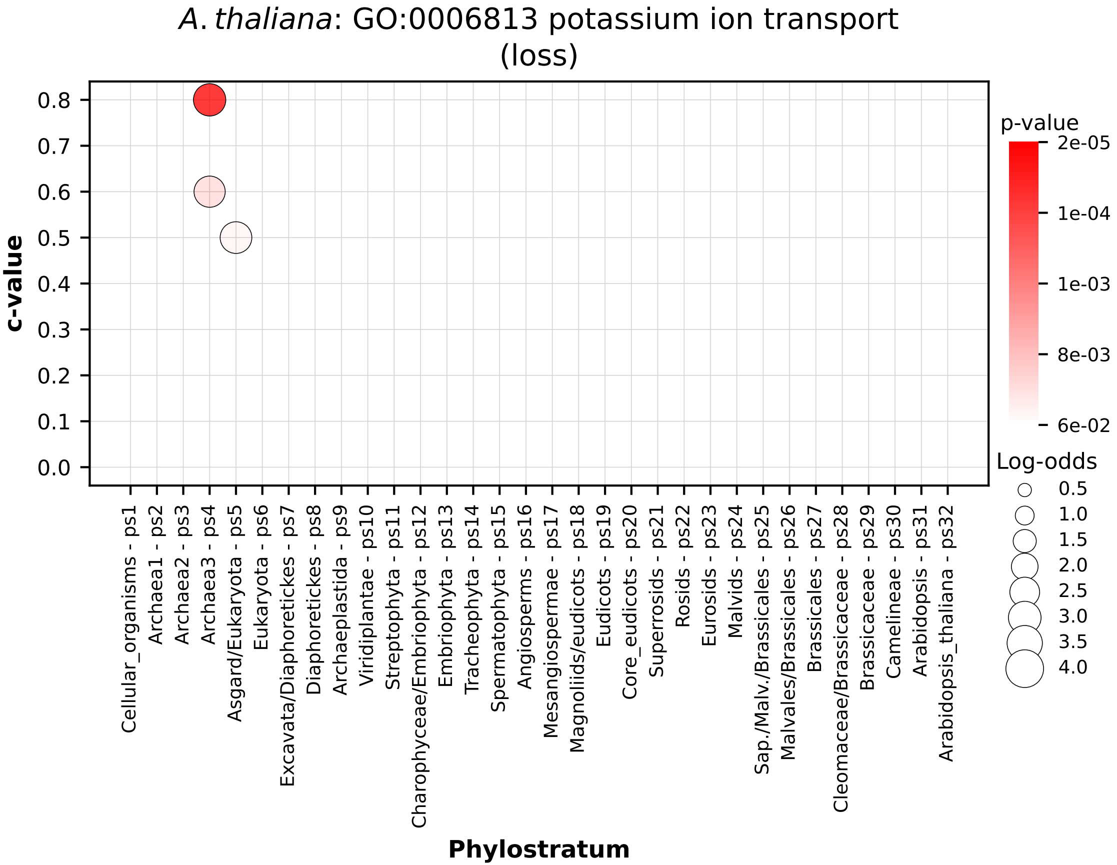

A. thaliana: GO:0006813 potassium ion transport
(loss)
c-value
0.0
0.1
0.2
0.3
0.4
0.5
0.6
0.7
0.8
p-value
2e-05
1e-04
1e-03
8e-03
6e-02
Log-odds
0.5
1.0
1.5
2.0
2.5
3.0
3.5
4.0
Cellular_organisms - ps1
Archaea1 - ps2
Archaea2 - ps3
Archaea3 - ps4
Asgard/Eukaryota - ps5
Eukaryota - ps6
Excavata/Diaphoretickes - ps7
Diaphoretickes - ps8
Archaeplastida - ps9
Viridiplantae - ps10
Streptophyta - ps11
Charophyceae/Embriophyta - ps12
Embriophyta - ps13
Tracheophyta - ps14
Spermatophyta - ps15
Angiosperms - ps16
Mesangiospermae - ps17
Magnoliids/eudicots - ps18
Eudicots - ps19
Core_eudicots - ps20
Superrosids - ps21
Rosids - ps22
Eurosids - ps23
Malvids - ps24
Sap./Malv./Brassicales - ps25
Malvales/Brassicales - ps26
Brassicales - ps27
Cleomaceae/Brassicaceae - ps28
Brassicaceae - ps29
Camelineae - ps30
Arabidopsis - ps31
Arabidopsis_thaliana - ps32
Phylostratum

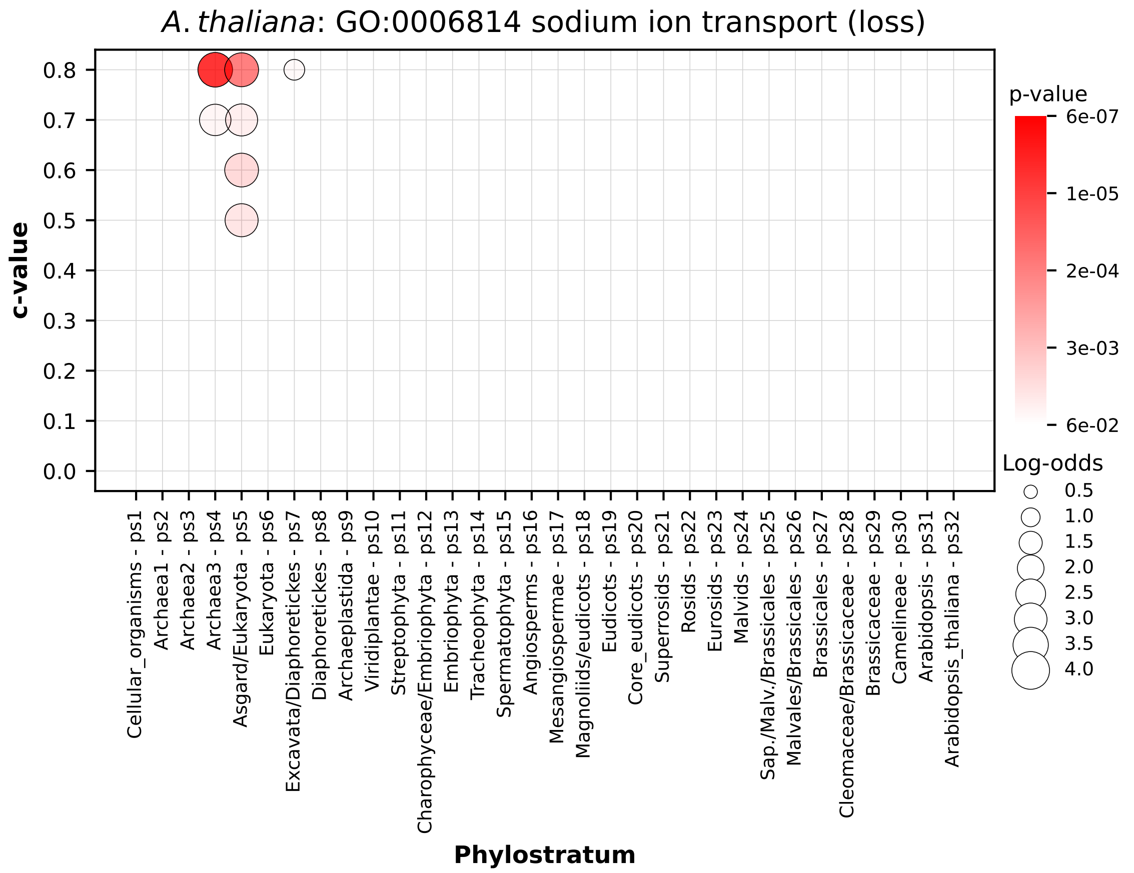
A. thaliana: GO:0006814 sodium ion transport (loss)
c-value
0.8
0.7
0.6
0.5
0.4
0.3
0.2
0.1
0.0
p-value
6e-07
1e-05
2e-04
3e-03
6e-02
Log-odds
0.5
1.0
1.5
2.0
2.5
3.0
3.5
4.0
Cellular_organisms - ps1
Archaea1 - ps2
Archaea2 - ps3
Archaea3 - ps4
Asgard/Eukaryota - ps5
Eukaryota - ps6
Excavata/Diaphoretickes - ps7
Diaphoretickes - ps8
Archaeplastida - ps9
Viridiplantae - ps10
Streptophyta - ps11
Charophyceae/Embriophyta - ps12
Embriophyta - ps13
Tracheophyta - ps14
Spermatophyta - ps15
Angiosperms - ps16
Mesangiospermae - ps17
Magnoliids/eudicots - ps18
Eudicots - ps19
Core_eudicots - ps20
Superrosids - ps21
Rosids - ps22
Eurosids - ps23
Malvids - ps24
Sap./Malv./Brassicales - ps25
Malvales/Brassicales - ps26
Brassicales - ps27
Cleomaceae/Brassicaceae - ps28
Brassicaceae - ps29
Camelineae - ps30
Arabidopsis - ps31
Arabidopsis_thaliana - ps32
Phylostratum

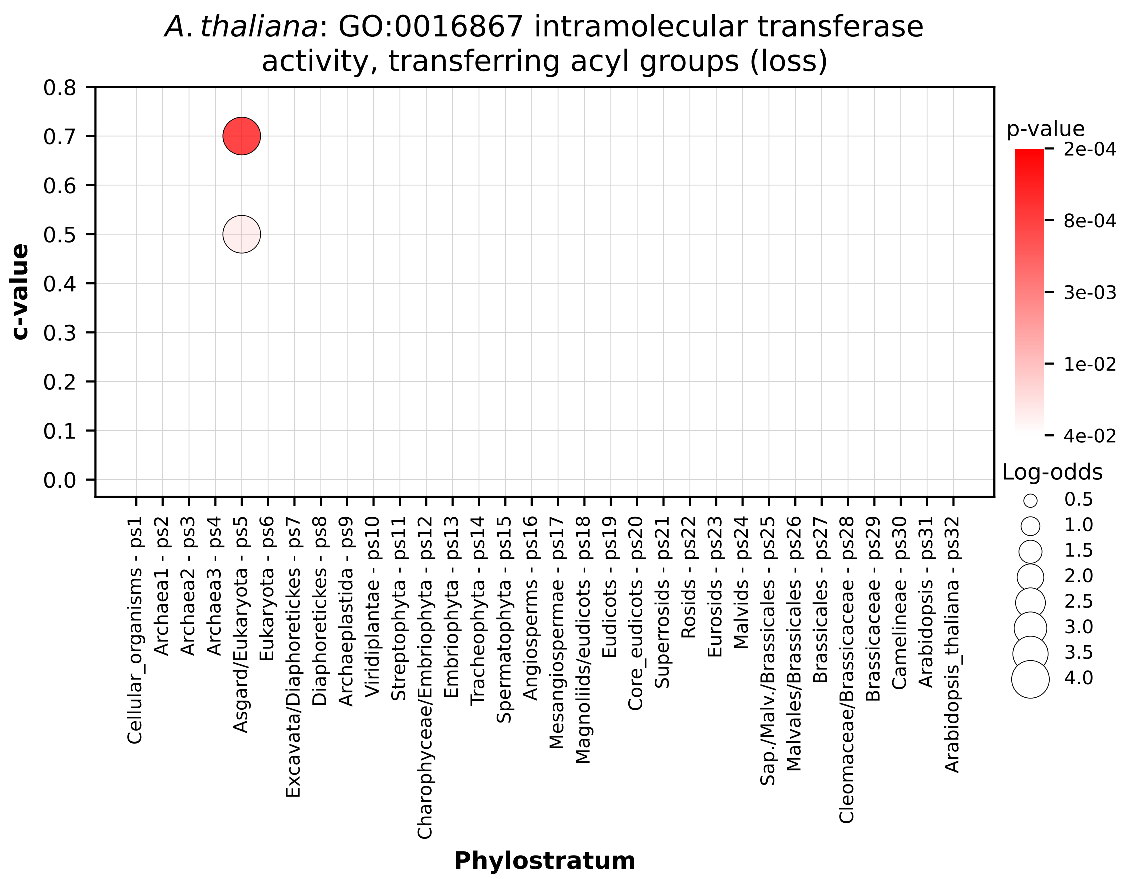

A. thaliana: GO:0016867 intramolecular transferase activity, transferring acyl groups (loss)
c-value
0.8
0.7
0.6
0.5
0.4
0.3
0.2
0.1
0.0
p-value
2e-04
8e-04
3e-03
1e-02
4e-02
Log-odds
0.5
1.0
1.5
2.0
2.5
3.0
3.5
4.0
Cellular_organisms - ps1
Archaea1 - ps2
Archaea2 - ps3
Archaea3 - ps4
Asgard/Eukaryota - ps5
Eukaryota - ps6
Excavata/Diaphoretickes - ps7
Diaphoretickes - ps8
Archaeplastida - ps9
Viridiplantae - ps10
Streptophyta - ps11
Charophyceae/Embriophyta - ps12
Embriophyta - ps13
Tracheophyta - ps14
Spermatophyta - ps15
Angiosperms - ps16
Mesangiospermae - ps17
Magnoliids/eudicots - ps18
Eudicots - ps19
Core_eudicots - ps20
Superrosids - ps21
Rosids - ps22
Eurosids - ps23
Malvids - ps24
Sap./Malv./Brassicales - ps25
Malvales/Brassicales - ps26
Brassicales - ps27
Cleomaceae/Brassicaceae - ps28
Brassicaceae - ps29
Camelineae - ps30
Arabidopsis - ps31
Arabidopsis_thaliana - ps32
Phylostratum

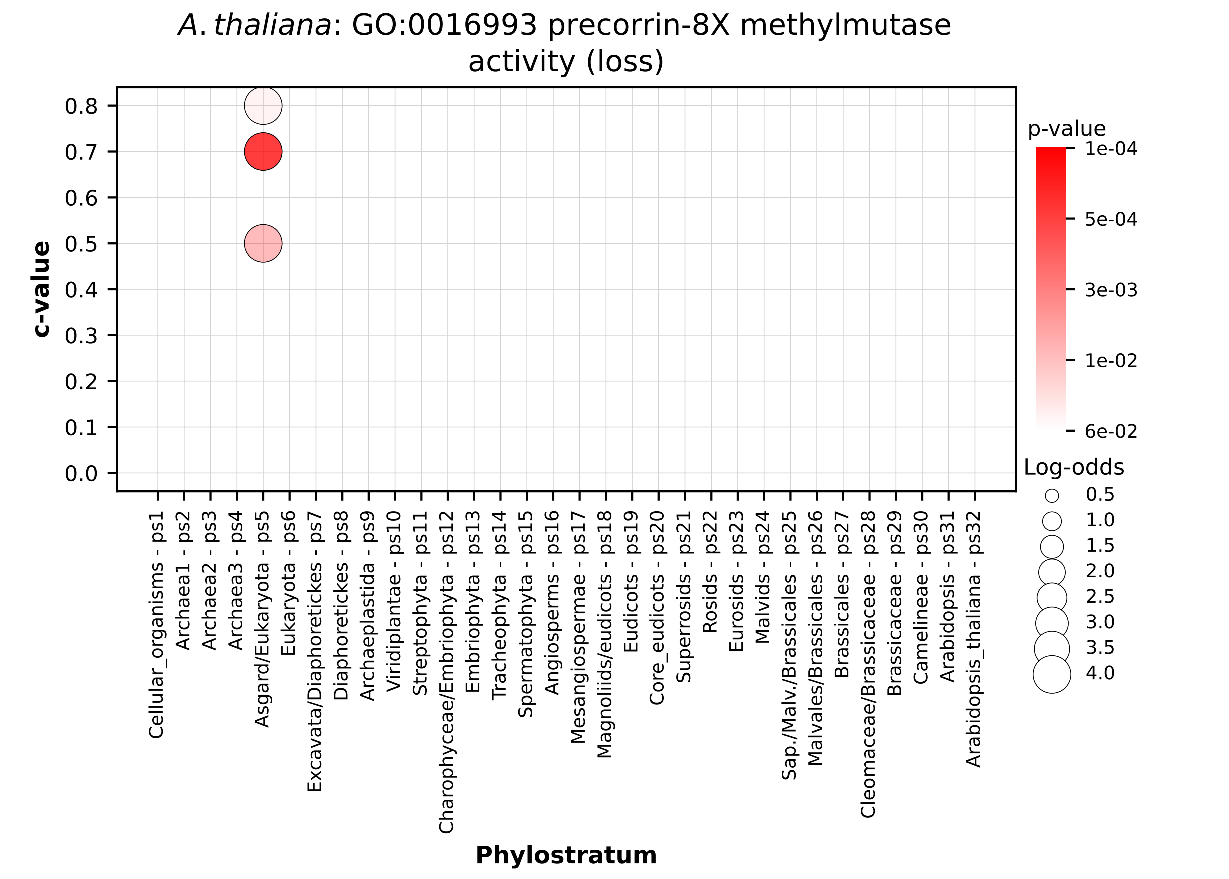

A. thaliana: GO:0016993 precorrin-8X methylmutase activity (loss)
c-value
0.8
0.7
0.6
0.5
0.4
0.3
0.2
0.1
0.0
Cellular_organisms - ps1
Archaea1 - ps2
Archaea2 - ps3
Archaea3 - ps4
Asgard/Eukaryota - ps5
Eukaryota - ps6
Excavata/Diaphoretickes - ps7
Diaphoretickes - ps8
Archaeplastida - ps9
Viridiplantae - ps10
Streptophyta - ps11
Charophyceae/Embriophyta - ps12
Embriophyta - ps13
Tracheophyta - ps14
Spermatophyta - ps15
Angiosperms - ps16
Mesangiospermae - ps17
Magnoliids/eudicots - ps18
Eudicots - ps19
Core_eudicots - ps20
Superrosids - ps21
Rosids - ps22
Eurosids - ps23
Malvids - ps24
Sap./Malv./Brassicales - ps25
Malvales/Brassicales - ps26
Brassicales - ps27
Cleomaceae/Brassicaceae - ps28
Brassicaceae - ps29
Camelineae - ps30
Arabidopsis - ps31
Arabidopsis_thaliana - ps32
Phylostratum
p-value
1e-04
5e-04
3e-03
1e-02
6e-02
Log-odds
0.5
1.0
1.5
2.0
2.5
3.0
3.5
4.0

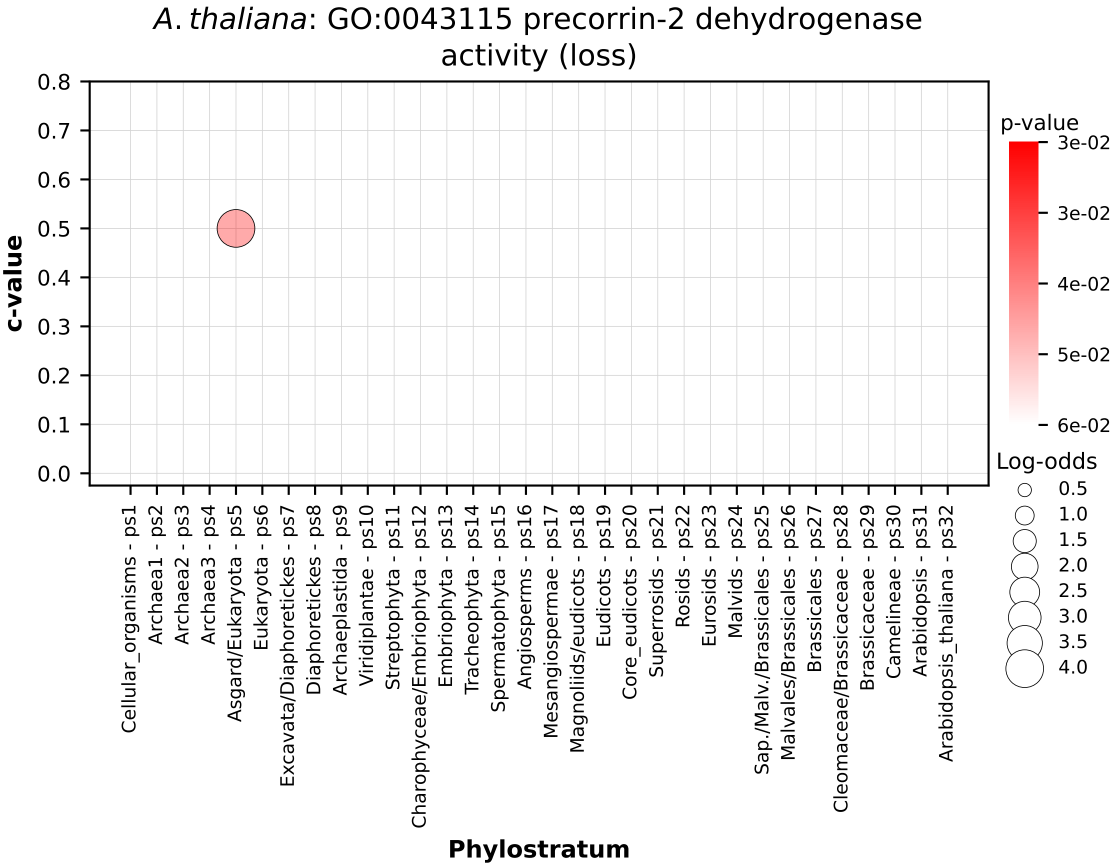
A. thaliana: GO:0043115 precorrin-2 dehydrogenase activity (loss)
c-value
0.8
0.7
0.6
0.5
0.4
0.3
0.2
0.1
0.0
Phylostratum
Cellular_organisms - ps1
Archaea1 - ps2
Archaea2 - ps3
Archaea3 - ps4
Asgard/Eukaryota - ps5
Eukaryota - ps6
Excavata/Diaphoretickes - ps7
Diaphoretickes - ps8
Archaeplastida - ps9
Viridiplantae - ps10
Streptophyta - ps11
Charophyceae/Embriophyta - ps12
Embriophyta - ps13
Tracheophyta - ps14
Spermatophyta - ps15
Angiosperms - ps16
Mesangiospermae - ps17
Magnoliids/eudicots - ps18
Eudicots - ps19
Core_eudicots - ps20
Superrosids - ps21
Rosids - ps22
Eurosids - ps23
Malvids - ps24
Sap./Malv./Brassicales - ps25
Malvales/Brassicales - ps26
Brassicales - ps27
Cleomaceae/Brassicaceae - ps28
Brassicaceae - ps29
Camelineae - ps30
Arabidopsis - ps31
Arabidopsis_thaliana - ps32
p-value
3e-02
3e-02
4e-02
5e-02
6e-02
Log-odds
0.5
1.0
1.5
2.0
2.5
3.0
3.5
4.0

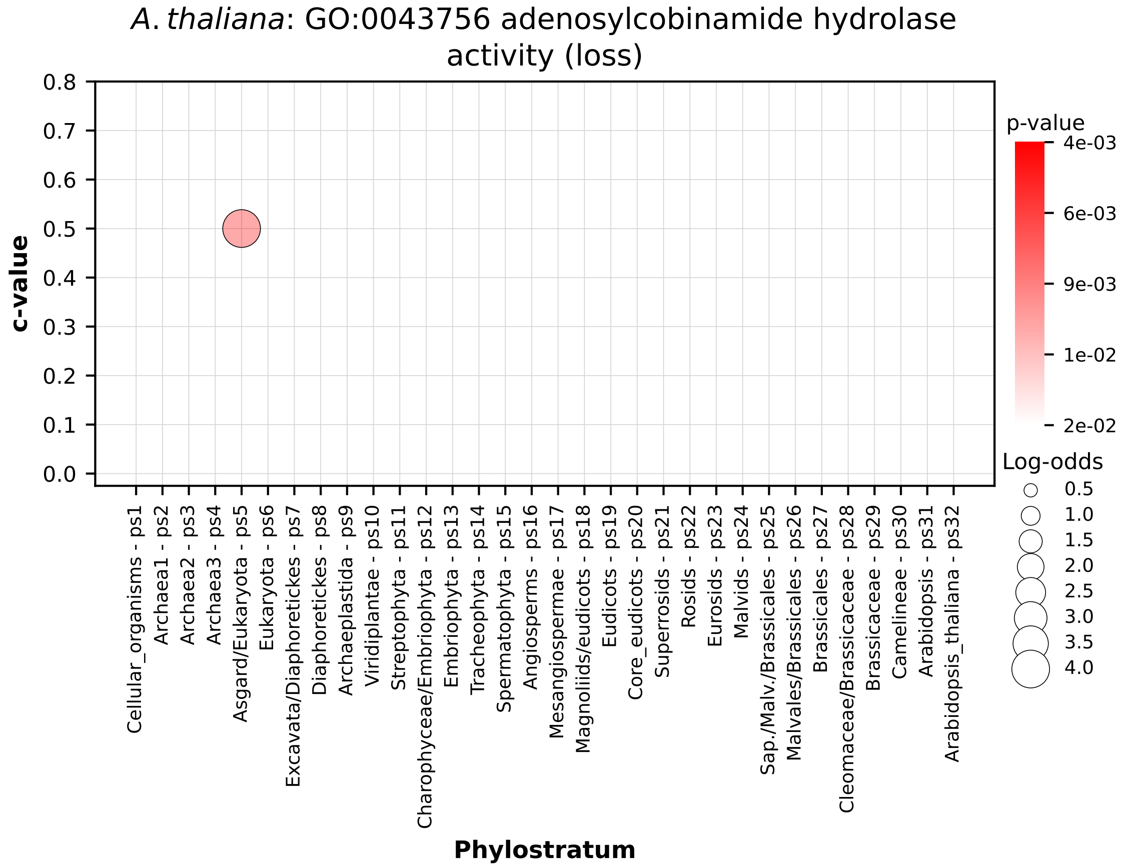

A. thaliana: GO:0043756 adenosylcobinamide hydrolase activity (loss)
c-value
0.8
0.7
0.6
0.5
0.4
0.3
0.2
0.1
0.0
p-value
4e-03
6e-03
9e-03
1e-02
2e-02
Log-odds
0.5
1.0
1.5
2.0
2.5
3.0
3.5
4.0
Cellular_organisms - ps1
Archaea1 - ps2
Archaea2 - ps3
Archaea3 - ps4
Asgard/Eukaryota - ps5
Eukaryota - ps6
Excavata/Diaphoretickes - ps7
Diaphoretickes - ps8
Archaeplastida - ps9
Viridiplantae - ps10
Streptophyta - ps11
Charophyceae/Embriophyta - ps12
Embriophyta - ps13
Tracheophyta - ps14
Spermatophyta - ps15
Angiosperms - ps16
Mesangiospermae - ps17
Magnoliids/eudicots - ps18
Eudicots - ps19
Core_eudicots - ps20
Superrosids - ps21
Rosids - ps22
Eurosids - ps23
Malvids - ps24
Sap./Malv./Brassicales - ps25
Malvales/Brassicales - ps26
Brassicales - ps27
Cleomaceae/Brassicaceae - ps28
Brassicaceae - ps29
Camelineae - ps30
Arabidopsis - ps31
Arabidopsis_thaliana - ps32
Phylostratum

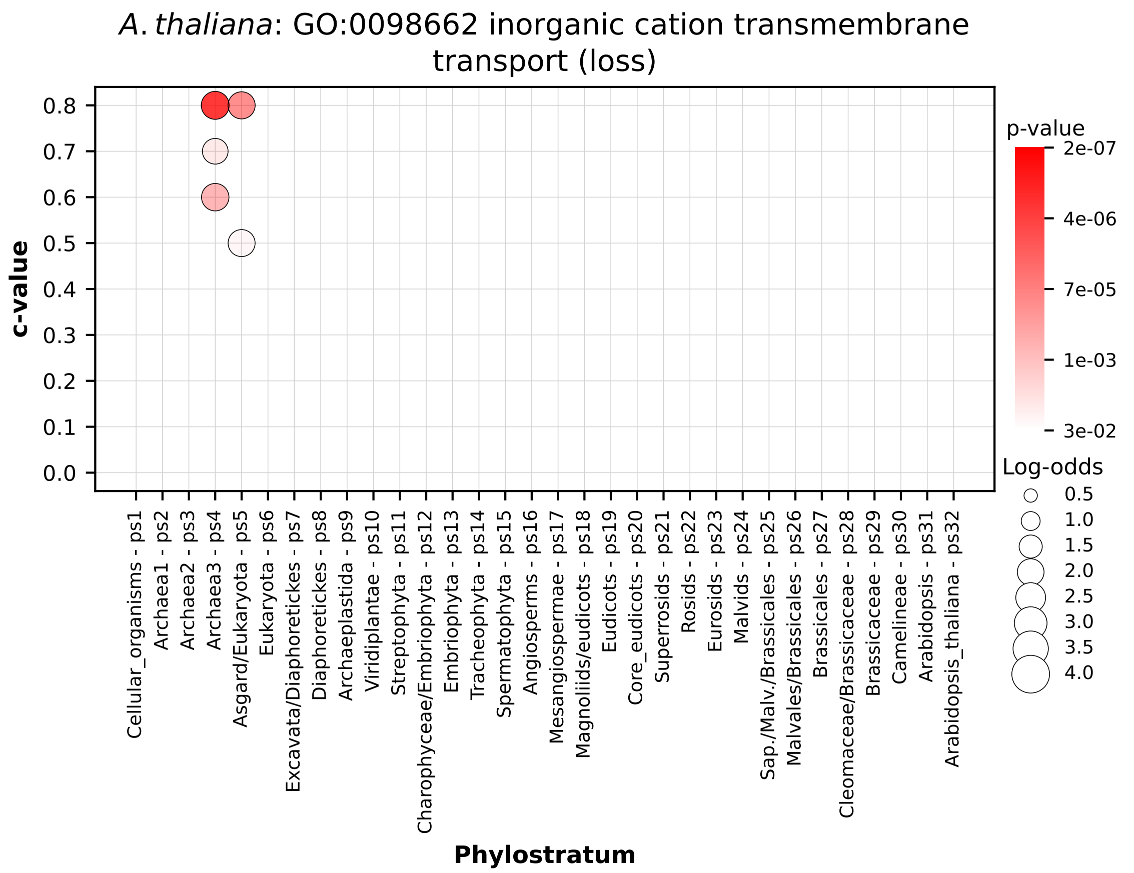

A. thaliana: GO:0098662 inorganic cation transmembrane transport (loss)
c-value
0.8
0.7
0.6
0.5
0.4
0.3
0.2
0.1
0.0
p-value
2e-07
4e-06
7e-05
1e-03
3e-02
Log-odds
0.5
1.0
1.5
2.0
2.5
3.0
3.5
4.0
Cellular_organisms - ps1
Archaea1 - ps2
Archaea2 - ps3
Archaea3 - ps4
Asgard/Eukaryota - ps5
Eukaryota - ps6
Excavata/Diaphoretickes - ps7
Diaphoretickes - ps8
Archaeplastida - ps9
Viridiplantae - ps10
Streptophyta - ps11
Charophyceae/Embriophyta - ps12
Embriophyta - ps13
Tracheophyta - ps14
Spermatophyta - ps15
Angiosperms - ps16
Mesangiospermae - ps17
Magnoliids/eudicots - ps18
Eudicots - ps19
Core_eudicots - ps20
Superrosids - ps21
Rosids - ps22
Eurosids - ps23
Malvids - ps24
Sap./Malv./Brassicales - ps25
Malvales/Brassicales - ps26
Brassicales - ps27
Cleomaceae/Brassicaceae - ps28
Brassicaceae - ps29
Camelineae - ps30
Arabidopsis - ps31
Arabidopsis_thaliana - ps32
Phylostratum

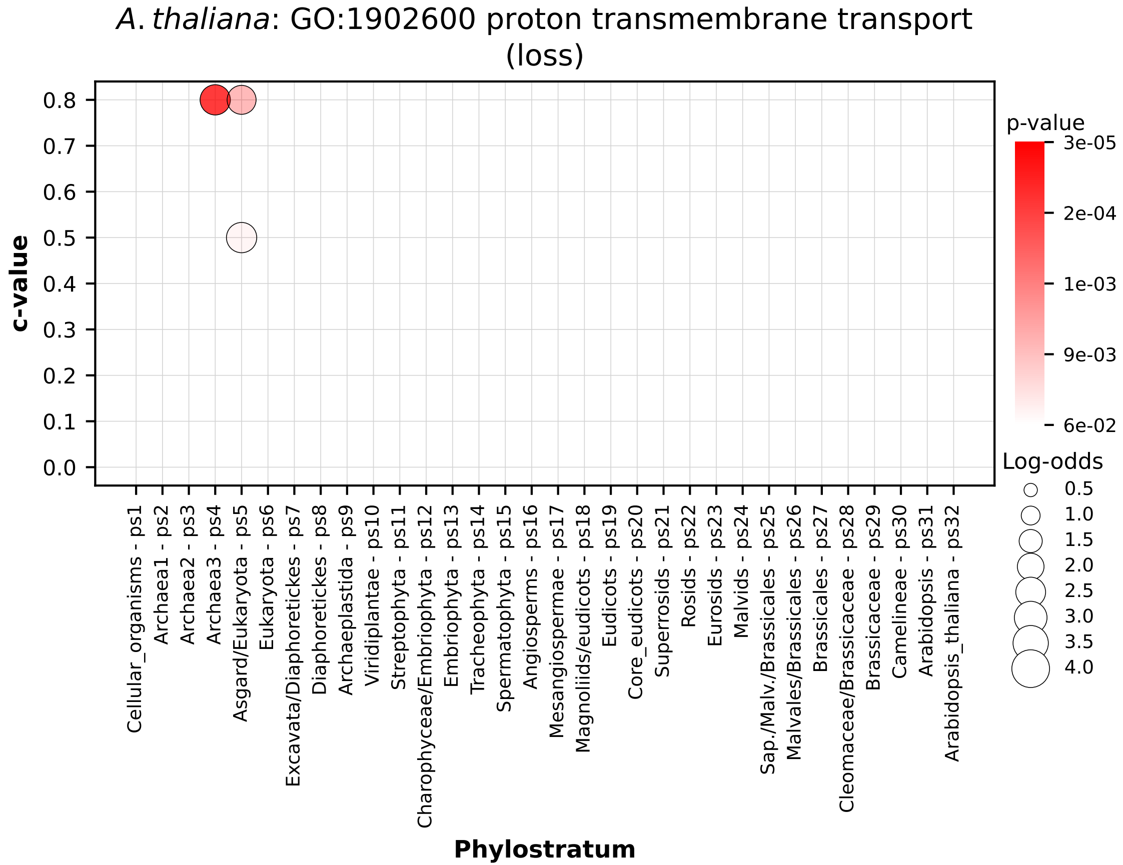

$A. thaliana$: GO:1902600 proton transmembrane transport (loss)
c-value
0.0
0.1
0.2
0.3
0.4
0.5
0.6
0.7
0.8
Phylostratum
Cellular_organisms - ps1
Archaea1 - ps2
Archaea2 - ps3
Archaea3 - ps4
Asgard/Eukaryota - ps5
Eukaryota - ps6
Excavata/Diaphoretickes - ps7
Diaphoretickes - ps8
Archaeplastida - ps9
Viridiplantae - ps10
Streptophyta - ps11
Charophyceae/Embriophyta - ps12
Embriophyta - ps13
Tracheophyta - ps14
Spermatophyta - ps15
Angiosperms - ps16
Mesangiospermae - ps17
Magnoliids/eudicots - ps18
Eudicots - ps19
Core_eudicots - ps20
Superrosids - ps21
Rosids - ps22
Eurosids - ps23
Malvids - ps24
Sap./Malv./Brassicales - ps25
Malvales/Brassicales - ps26
Brassicales - ps27
Cleomaceae/Brassicaceae - ps28
Brassicaceae - ps29
Camelineae - ps30
Arabidopsis - ps31
Arabidopsis_thaliana - ps32
p-value
3e-05
2e-04
1e-03
9e-03
6e-02
Log-odds
0.5
1.0
1.5
2.0
2.5
3.0
3.5
4.0

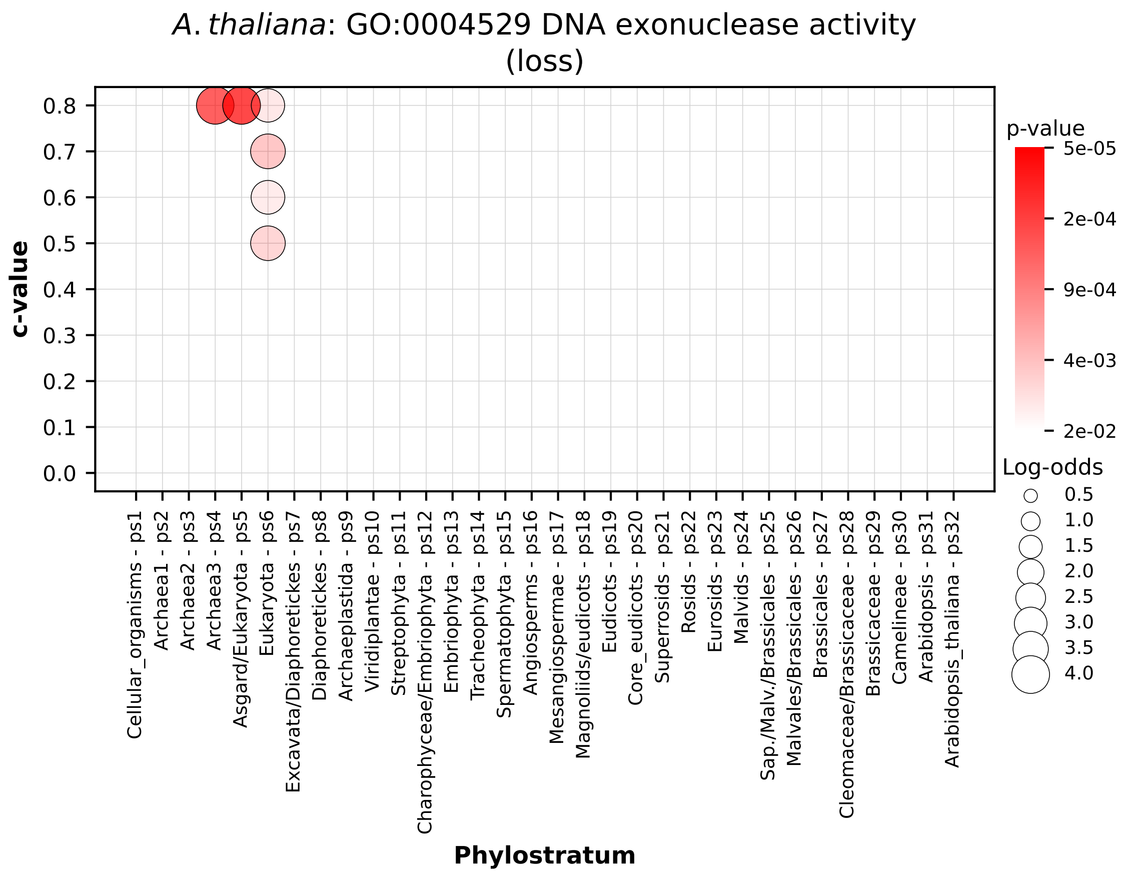

A. thaliana: GO:0004529 DNA exonuclease activity
(loss)
c-value
0.8
0.7
0.6
0.5
0.4
0.3
0.2
0.1
0.0
p-value
5e-05
2e-04
9e-04
4e-03
2e-02
Log-odds
0.5
1.0
1.5
2.0
2.5
3.0
3.5
4.0
Cellular_organisms - ps1
Archaea1 - ps2
Archaea2 - ps3
Archaea3 - ps4
Asgard/Eukaryota - ps5
Eukaryota - ps6
Excavata/Diaphoretickes - ps7
Diaphoretickes - ps8
Archaeplastida - ps9
Viridiplantae - ps10
Streptophyta - ps11
Charophyceae/Embriophyta - ps12
Embriophyta - ps13
Tracheophyta - ps14
Spermatophyta - ps15
Angiosperms - ps16
Mesangiospermae - ps17
Magnoliids/eudicots - ps18
Eudicots - ps19
Core_eudicots - ps20
Superrosids - ps21
Rosids - ps22
Eurosids - ps23
Malvids - ps24
Sap./Malv./Brassicales - ps25
Malvales/Brassicales - ps26
Brassicales - ps27
Cleomaceae/Brassicaceae - ps28
Brassicaceae - ps29
Camelineae - ps30
Arabidopsis - ps31
Arabidopsis_thaliana - ps32
Phylostratum

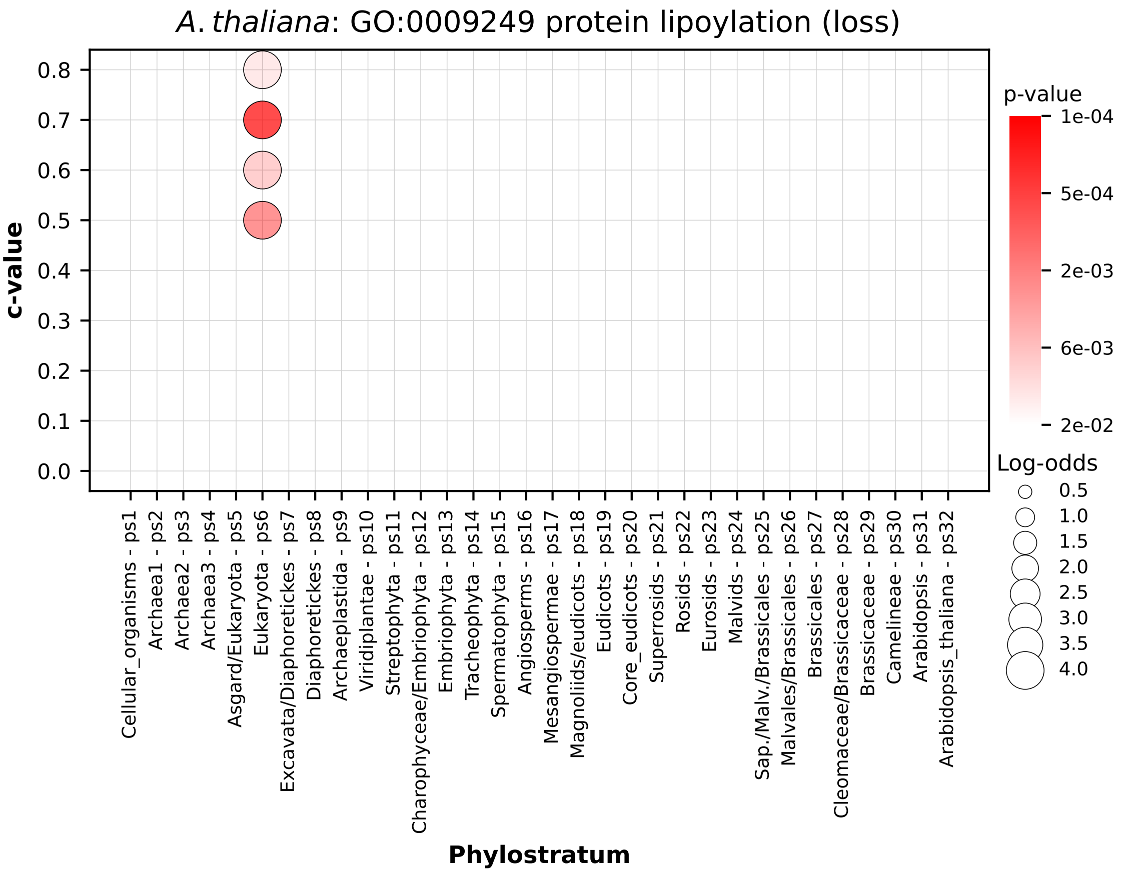

A. thaliana: GO:0009249 protein lipoylation (loss)
c-value
0.0
0.1
0.2
0.3
0.4
0.5
0.6
0.7
0.8
p-value
1e-04
5e-04
2e-03
6e-03
2e-02
Log-odds
0.5
1.0
1.5
2.0
2.5
3.0
3.5
4.0
Cellular_organisms - ps1
Archaea1 - ps2
Archaea2 - ps3
Archaea3 - ps4
Asgard/Eukaryota - ps5
Eukaryota - ps6
Excavata/Diaphoretickes - ps7
Diaphoretickes - ps8
Archaeplastida - ps9
Viridiplantae - ps10
Streptophyta - ps11
Charophyceae/Embriophyta - ps12
Embriophyta - ps13
Tracheophyta - ps14
Spermatophyta - ps15
Angiosperms - ps16
Mesangiospermae - ps17
Magnoliids/eudicots - ps18
Eudicots - ps19
Core_eudicots - ps20
Superrosids - ps21
Rosids - ps22
Eurosids - ps23
Malvids - ps24
Sap./Malv./Brassicales - ps25
Malvales/Brassicales - ps26
Brassicales - ps27
Cleomaceae/Brassicaceae - ps28
Brassicaceae - ps29
Camelineae - ps30
Arabidopsis - ps31
Arabidopsis_thaliana - ps32
Phylostratum

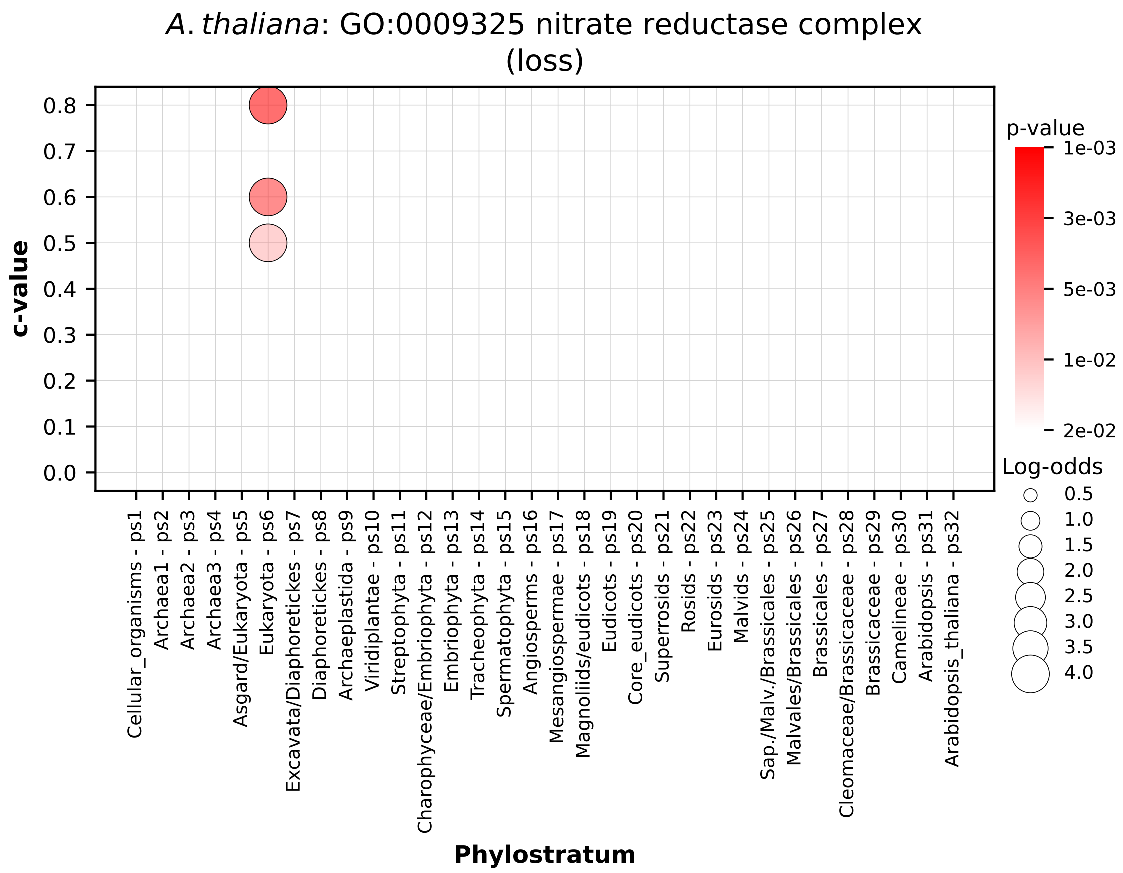

A. thaliana: GO:0009325 nitrate reductase complex
(loss)
c-value
0.0
0.1
0.2
0.3
0.4
0.5
0.6
0.7
0.8
Phylostratum
Cellular_organisms - ps1
Archaea1 - ps2
Archaea2 - ps3
Archaea3 - ps4
Asgard/Eukaryota - ps5
Eukaryota - ps6
Excavata/Diaphoretickes - ps7
Diaphoretickes - ps8
Archaeplastida - ps9
Viridiplantae - ps10
Streptophyta - ps11
Charophyceae/Embriophyta - ps12
Embriophyta - ps13
Tracheophyta - ps14
Spermatophyta - ps15
Angiosperms - ps16
Mesangiospermae - ps17
Magnoliids/eudicots - ps18
Eudicots - ps19
Core_eudicots - ps20
Superrosids - ps21
Rosids - ps22
Eurosids - ps23
Malvids - ps24
Sap./Malv./Brassicales - ps25
Malvales/Brassicales - ps26
Brassicales - ps27
Cleomaceae/Brassicaceae - ps28
Brassicaceae - ps29
Camelineae - ps30
Arabidopsis - ps31
Arabidopsis_thaliana - ps32
p-value
1e-03
3e-03
5e-03
1e-02
2e-02
Log-odds
0.5
1.0
1.5
2.0
2.5
3.0
3.5
4.0

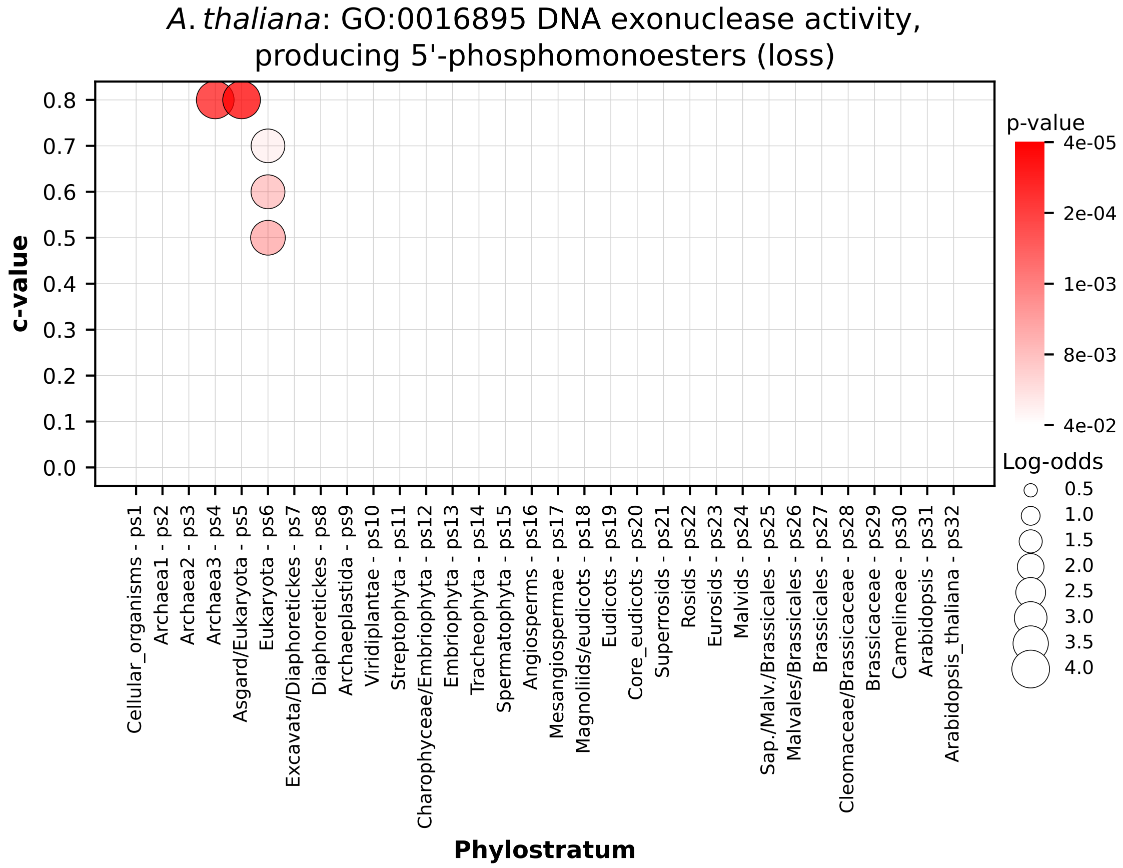

A. thaliana: GO:0016895 DNA exonuclease activity, producing 5'-phosphomonoesters (loss)
c-value
0.8
0.7
0.6
0.5
0.4
0.3
0.2
0.1
0.0
p-value
4e-05
2e-04
1e-03
8e-03
4e-02
Log-odds
0.5
1.0
1.5
2.0
2.5
3.0
3.5
4.0
Cellular_organisms - ps1
Archaea1 - ps2
Archaea2 - ps3
Archaea3 - ps4
Asgard/Eukaryota - ps5
Eukaryota - ps6
Excavata/Diaphoretickes - ps7
Diaphoretickes - ps8
Archaeplastida - ps9
Viridiplantae - ps10
Streptophyta - ps11
Charophyceae/Embriophyta - ps12
Embriophyta - ps13
Tracheophyta - ps14
Spermatophyta - ps15
Angiosperms - ps16
Mesangiospermae - ps17
Magnoliids/eudicots - ps18
Eudicots - ps19
Core_eudicots - ps20
Superrosids - ps21
Rosids - ps22
Eurosids - ps23
Malvids - ps24
Sap./Malv./Brassicales - ps25
Malvales/Brassicales - ps26
Brassicales - ps27
Cleomaceae/Brassicaceae - ps28
Brassicaceae - ps29
Camelineae - ps30
Arabidopsis - ps31
Arabidopsis_thaliana - ps32
Phylostratum

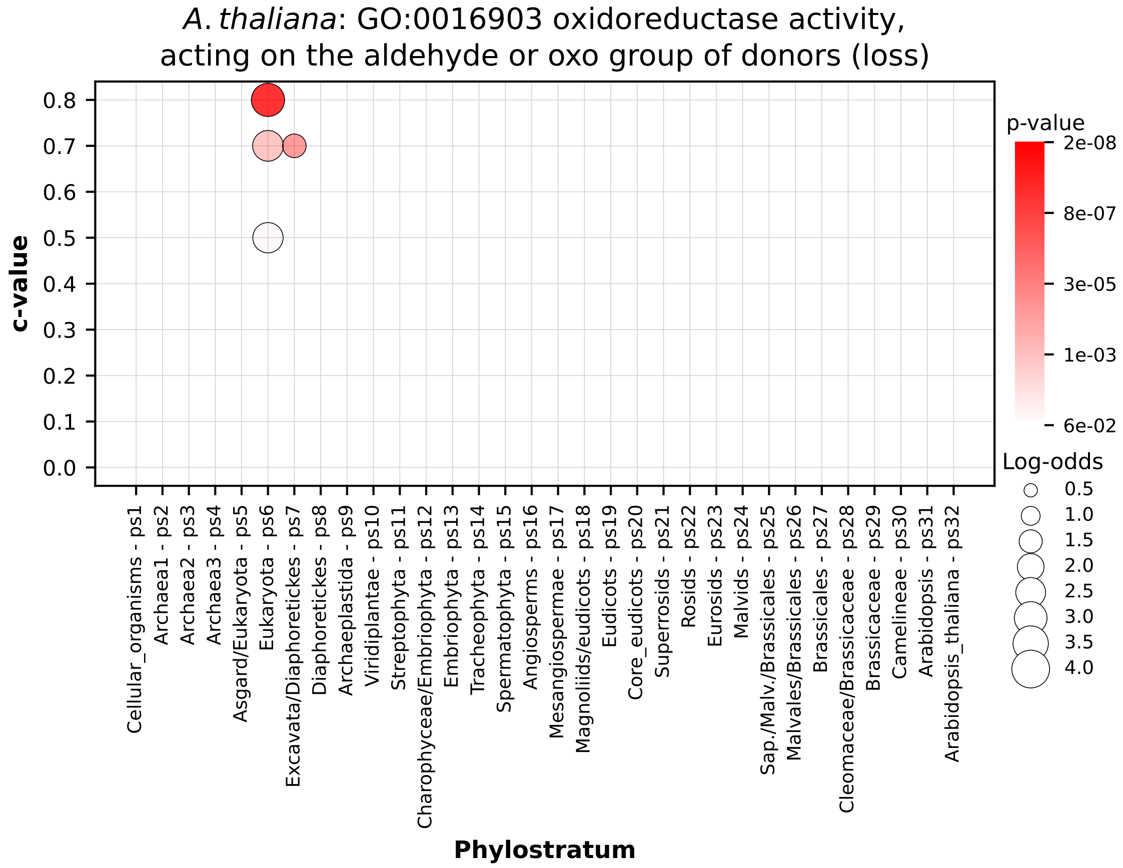

A. thaliana: GO:0016903 oxidoreductase activity, acting on the aldehyde or oxo group of donors (loss)
c-value
0.8
0.7
0.6
0.5
0.4
0.3
0.2
0.1
0.0
p-value
2e-08
8e-07
3e-05
1e-03
6e-02
Log-odds
0.5
1.0
1.5
2.0
2.5
3.0
3.5
4.0
Cellular_organisms - ps1
Archaea1 - ps2
Archaea2 - ps3
Archaea3 - ps4
Asgard/Eukaryota - ps5
Eukaryota - ps6
Excavata/Diaphoretickes - ps7
Diaphoretickes - ps8
Archaeplastida - ps9
Viridiplantae - ps10
Streptophyta - ps11
Charophyceae/Embriophyta - ps12
Embriophyta - ps13
Tracheophyta - ps14
Spermatophyta - ps15
Angiosperms - ps16
Mesangiospermae - ps17
Magnoliids/eudicots - ps18
Eudicots - ps19
Core_eudicots - ps20
Superrosids - ps21
Rosids - ps22
Eurosids - ps23
Malvids - ps24
Sap./Malv./Brassicales - ps25
Malvales/Brassicales - ps26
Brassicales - ps27
Cleomaceae/Brassicaceae - ps28
Brassicaceae - ps29
Camelineae - ps30
Arabidopsis - ps31
Arabidopsis_thaliana - ps32
Phylostratum

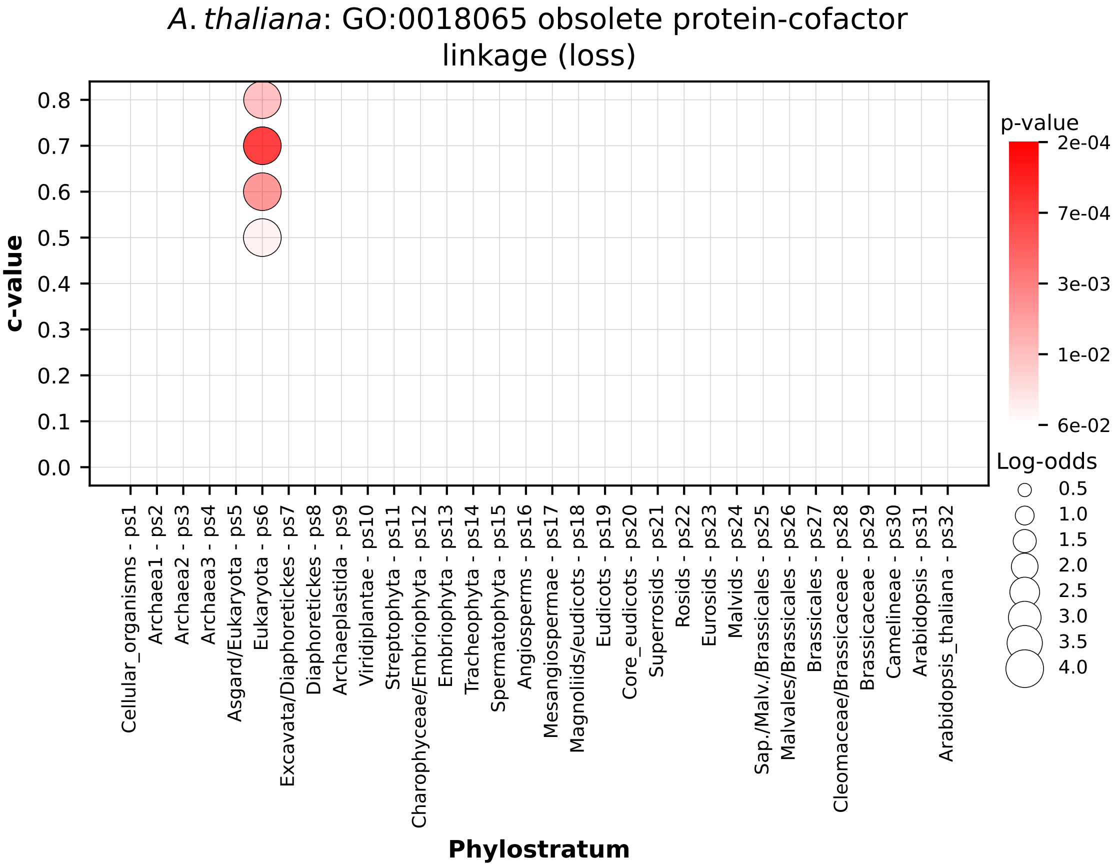

A. thaliana: GO:0018065 obsolete protein-cofactor linkage (loss)
c-value
0.0
0.1
0.2
0.3
0.4
0.5
0.6
0.7
0.8
p-value
2e-04
7e-04
3e-03
1e-02
6e-02
Log-odds
0.5
1.0
1.5
2.0
2.5
3.0
3.5
4.0
Cellular_organisms - ps1
Archaea1 - ps2
Archaea2 - ps3
Archaea3 - ps4
Asgard/Eukaryota - ps5
Eukaryota - ps6
Excavata/Diaphoretickes - ps7
Diaphoretickes - ps8
Archaeplastida - ps9
Viridiplantae - ps10
Streptophyta - ps11
Charophyceae/Embriophyta - ps12
Embriophyta - ps13
Tracheophyta - ps14
Spermatophyta - ps15
Angiosperms - ps16
Mesangiospermae - ps17
Magnoliids/eudicots - ps18
Eudicots - ps19
Core_eudicots - ps20
Superrosids - ps21
Rosids - ps22
Eurosids - ps23
Malvids - ps24
Sap./Malv./Brassicales - ps25
Malvales/Brassicales - ps26
Brassicales - ps27
Cleomaceae/Brassicaceae - ps28
Brassicaceae - ps29
Camelineae - ps30
Arabidopsis - ps31
Arabidopsis_thaliana - ps32
Phylostratum

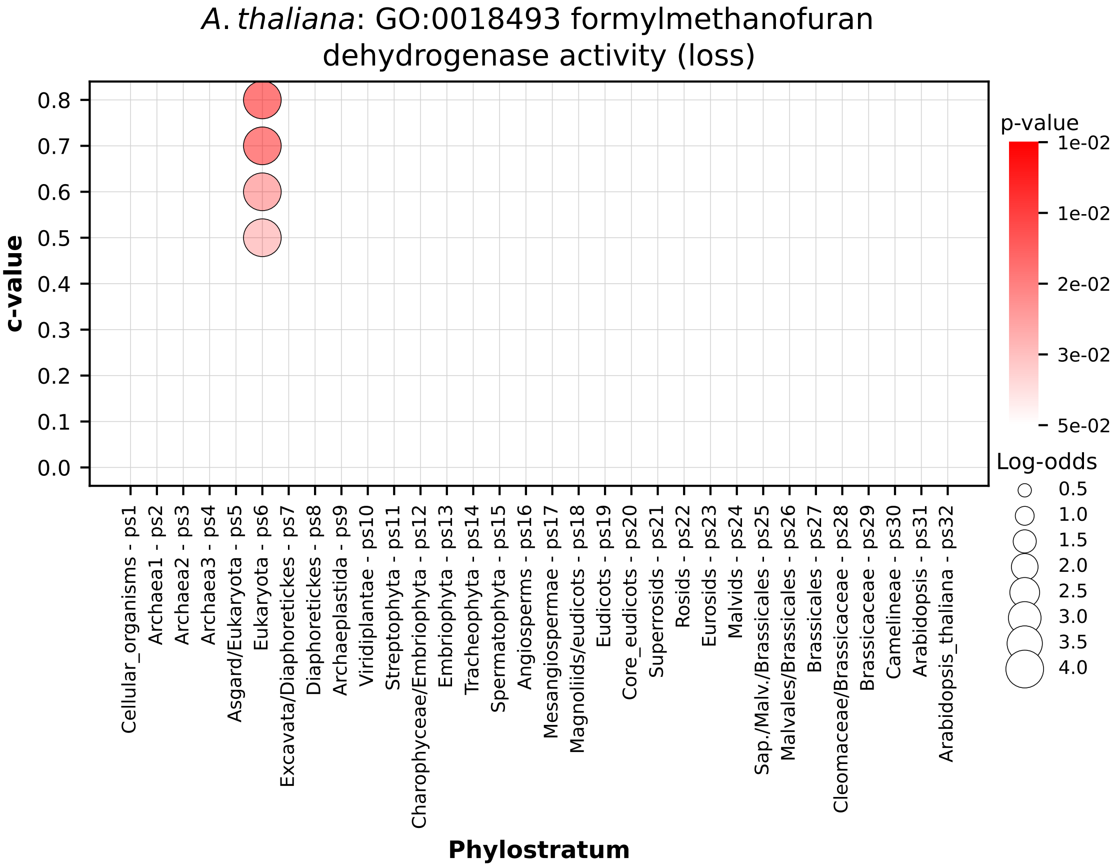

A. thaliana: GO:0018493 formylmethanofuran dehydrogenase activity (loss)
c-value
0.8
0.7
0.6
0.5
0.4
0.3
0.2
0.1
0.0
Phylostratum
Cellular_organisms - ps1
Archaea1 - ps2
Archaea2 - ps3
Archaea3 - ps4
Asgard/Eukaryota - ps5
Eukaryota - ps6
Excavata/Diaphoretickes - ps7
Diaphoretickes - ps8
Archaeplastida - ps9
Viridiplantae - ps10
Streptophyta - ps11
Charophyceae/Embriophyta - ps12
Embriophyta - ps13
Tracheophyta - ps14
Spermatophyta - ps15
Angiosperms - ps16
Mesangiospermae - ps17
Magnoliids/eudicots - ps18
Eudicots - ps19
Core_eudicots - ps20
Superrosids - ps21
Rosids - ps22
Eurosids - ps23
Malvids - ps24
Sap./Malv./Brassicales - ps25
Malvales/Brassicales - ps26
Brassicales - ps27
Cleomaceae/Brassicaceae - ps28
Brassicaceae - ps29
Camelineae - ps30
Arabidopsis - ps31
Arabidopsis_thaliana - ps32
p-value
1e-02
1e-02
2e-02
3e-02
5e-02
Log-odds
0.5
1.0
1.5
2.0
2.5
3.0
3.5
4.0

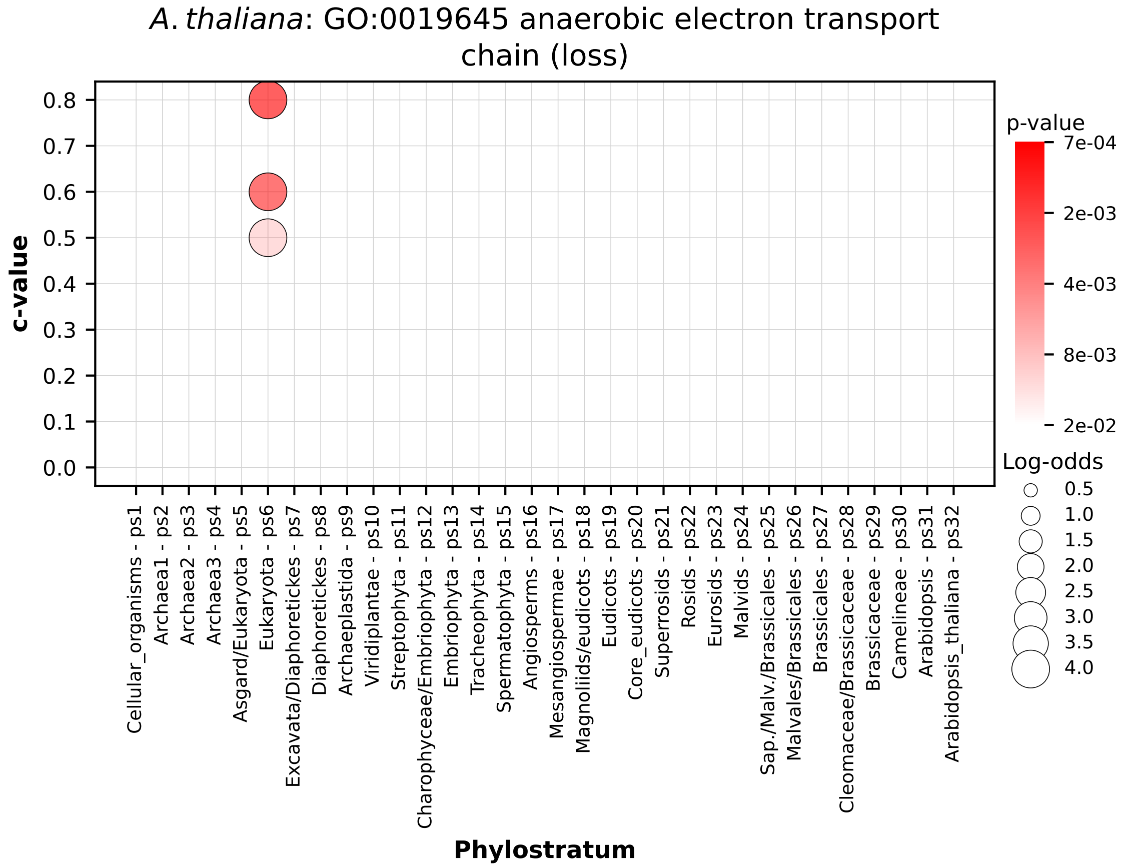
A. thaliana: GO:0019645 anaerobic electron transport chain (loss)
c-value
0.8
0.7
0.6
0.5
0.4
0.3
0.2
0.1
0.0
Phylostratum
Cellular_organisms - ps1
Archaea1 - ps2
Archaea2 - ps3
Archaea3 - ps4
Asgard/Eukaryota - ps5
Eukaryota - ps6
Excavata/Diaphoretickes - ps7
Diaphoretickes - ps8
Archaeplastida - ps9
Viridiplantae - ps10
Streptophyta - ps11
Charophyceae/Embriophyta - ps12
Embriophyta - ps13
Tracheophyta - ps14
Spermatophyta - ps15
Angiosperms - ps16
Mesangiospermae - ps17
Magnoliids/eudicots - ps18
Eudicots - ps19
Core_eudicots - ps20
Superrosids - ps21
Rosids - ps22
Eurosids - ps23
Malvids - ps24
Sap./Malv./Brassicales - ps25
Malvales/Brassicales - ps26
Brassicales - ps27
Cleomaceae/Brassicaceae - ps28
Brassicaceae - ps29
Camelineae - ps30
Arabidopsis - ps31
Arabidopsis_thaliana - ps32
p-value
7e-04
2e-03
4e-03
8e-03
2e-02
Log-odds
0.5
1.0
1.5
2.0
2.5
3.0
3.5
4.0

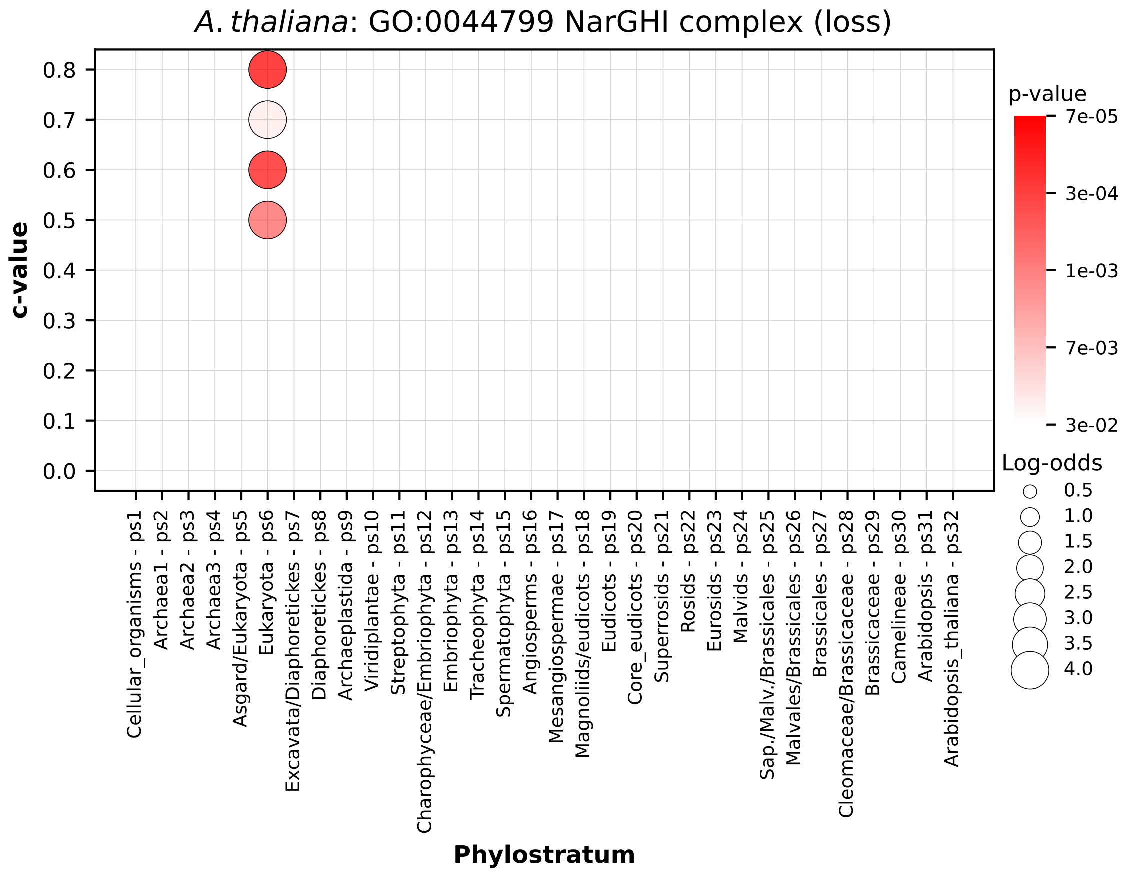
A. thaliana: GO:0044799 NarGHI complex (loss)
c-value
Phylostratum
p-value
7e-05
3e-04
1e-03
7e-03
3e-02
Log-odds
0.5
1.0
1.5
2.0
2.5
3.0
3.5
4.0
Cellular_organisms - ps1
Archaea1 - ps2
Archaea2 - ps3
Archaea3 - ps4
Asgard/Eukaryota - ps5
Eukaryota - ps6
Excavata/Diaphoretickes - ps7
Diaphoretickes - ps8
Archaeplastida - ps9
Viridiplantae - ps10
Streptophyta - ps11
Charophyceae/Embriophyta - ps12
Embriophyta - ps13
Tracheophyta - ps14
Spermatophyta - ps15
Angiosperms - ps16
Mesangiospermae - ps17
Magnoliids/eudicots - ps18
Eudicots - ps19
Core_eudicots - ps20
Superrosids - ps21
Rosids - ps22
Eurosids - ps23
Malvids - ps24
Sap./Malv./Brassicales - ps25
Malvales/Brassicales - ps26
Brassicales - ps27
Cleomaceae/Brassicaceae - ps28
Brassicaceae - ps29
Camelineae - ps30
Arabidopsis - ps31
Arabidopsis_thaliana - ps32

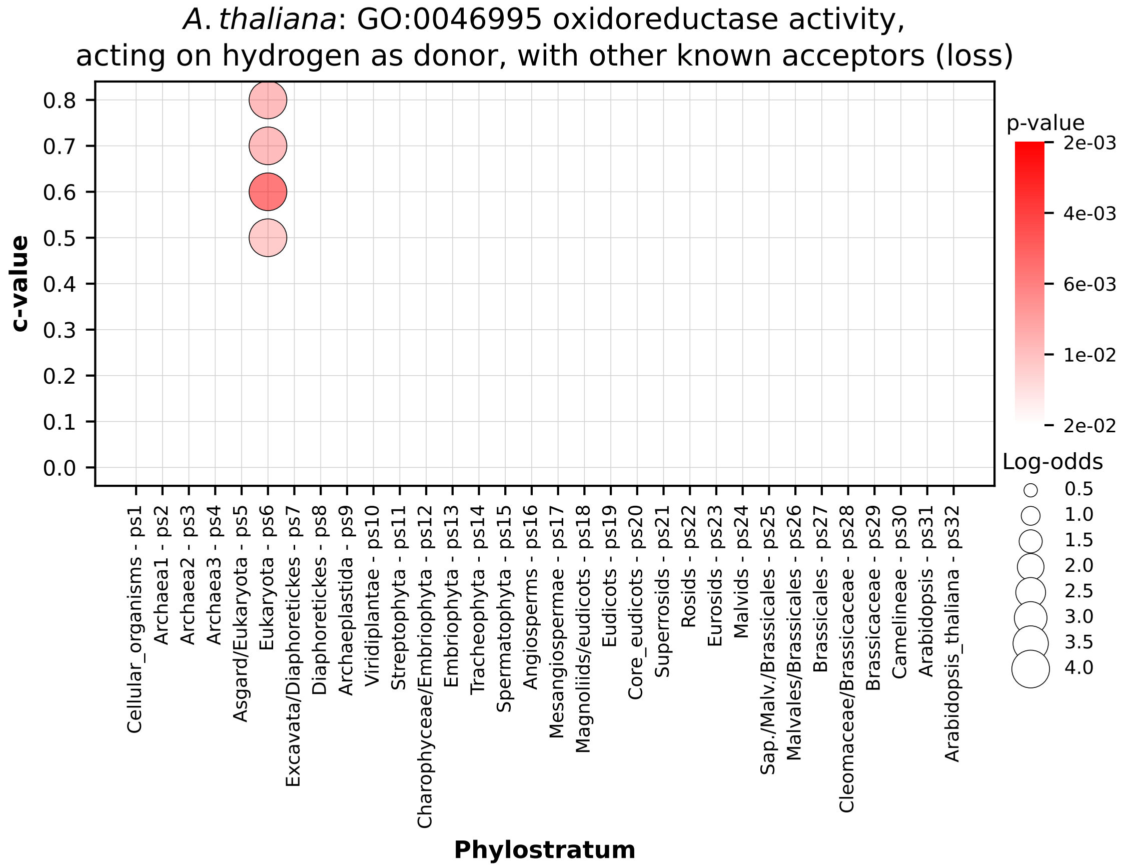

A. thaliana: GO:0046995 oxidoreductase activity, acting on hydrogen as donor, with other known acceptors (loss)
c-value
0.8
0.7
0.6
0.5
0.4
0.3
0.2
0.1
0.0
p-value
2e-03
4e-03
6e-03
1e-02
2e-02
Log-odds
0.5
1.0
1.5
2.0
2.5
3.0
3.5
4.0
Cellular_organisms - ps1
Archaea1 - ps2
Archaea2 - ps3
Archaea3 - ps4
Asgard/Eukaryota - ps5
Eukaryota - ps6
Excavata/Diaphoretickes - ps7
Diaphoretickes - ps8
Archaeplastida - ps9
Viridiplantae - ps10
Streptophyta - ps11
Charophyceae/Embriophyta - ps12
Embriophyta - ps13
Tracheophyta - ps14
Spermatophyta - ps15
Angiosperms - ps16
Mesangiospermae - ps17
Magnoliids/eudicots - ps18
Eudicots - ps19
Core_eudicots - ps20
Superrosids - ps21
Rosids - ps22
Eurosids - ps23
Malvids - ps24
Sap./Malv./Brassicales - ps25
Malvales/Brassicales - ps26
Brassicales - ps27
Cleomaceae/Brassicaceae - ps28
Brassicaceae - ps29
Camelineae - ps30
Arabidopsis - ps31
Arabidopsis_thaliana - ps32
Phylostratum

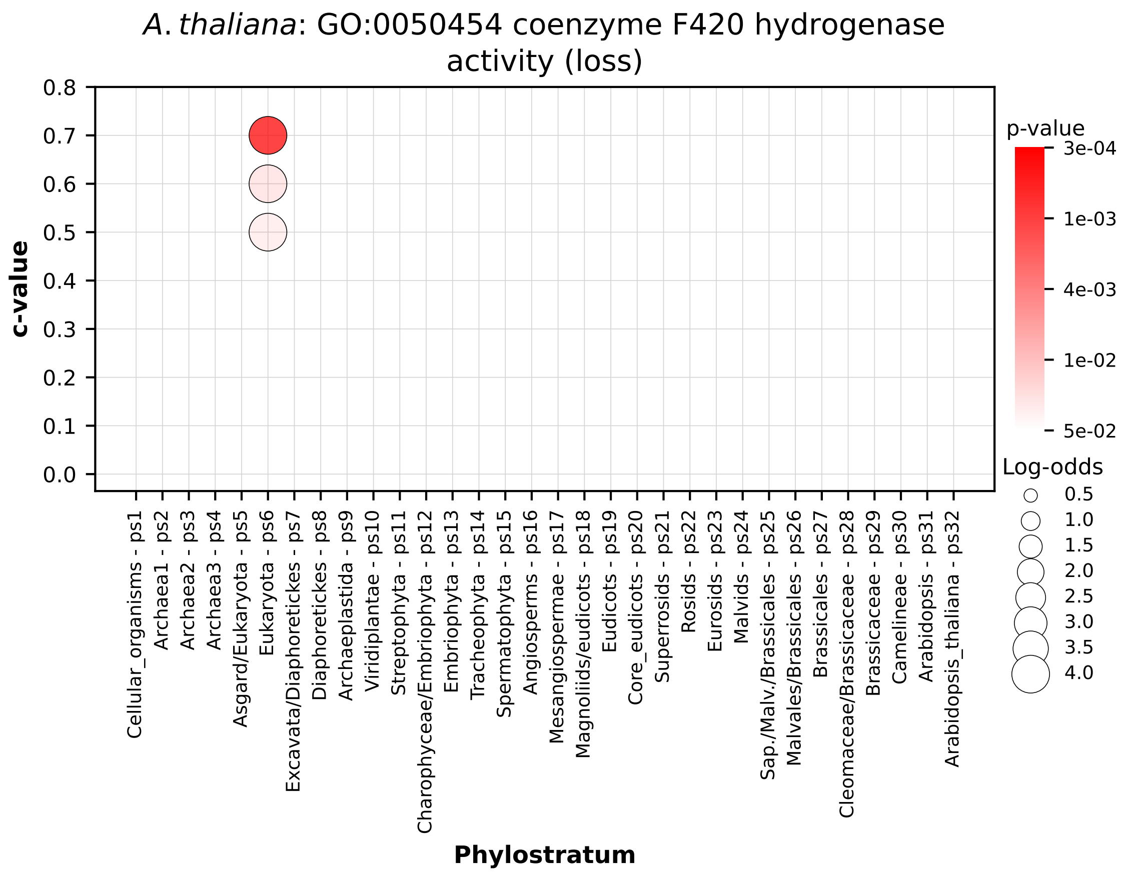

A. thaliana: GO:0050454 coenzyme F420 hydrogenase activity (loss)
c-value
0.8
0.7
0.6
0.5
0.4
0.3
0.2
0.1
0.0
p-value
3e-04
1e-03
4e-03
1e-02
5e-02
Log-odds
0.5
1.0
1.5
2.0
2.5
3.0
3.5
4.0
Cellular_organisms - ps1
Archaea1 - ps2
Archaea2 - ps3
Archaea3 - ps4
Asgard/Eukaryota - ps5
Eukaryota - ps6
Excavata/Diaphoretickes - ps7
Diaphoretickes - ps8
Archaeplastida - ps9
Viridiplantae - ps10
Streptophyta - ps11
Charophyceae/Embriophyta - ps12
Embriophyta - ps13
Tracheophyta - ps14
Spermatophyta - ps15
Angiosperms - ps16
Mesangiospermae - ps17
Magnoliids/eudicots - ps18
Eudicots - ps19
Core_eudicots - ps20
Superrosids - ps21
Rosids - ps22
Eurosids - ps23
Malvids - ps24
Sap./Malv./Brassicales - ps25
Malvales/Brassicales - ps26
Brassicales - ps27
Cleomaceae/Brassicaceae - ps28
Brassicaceae - ps29
Camelineae - ps30
Arabidopsis - ps31
Arabidopsis_thaliana - ps32
Phylostratum

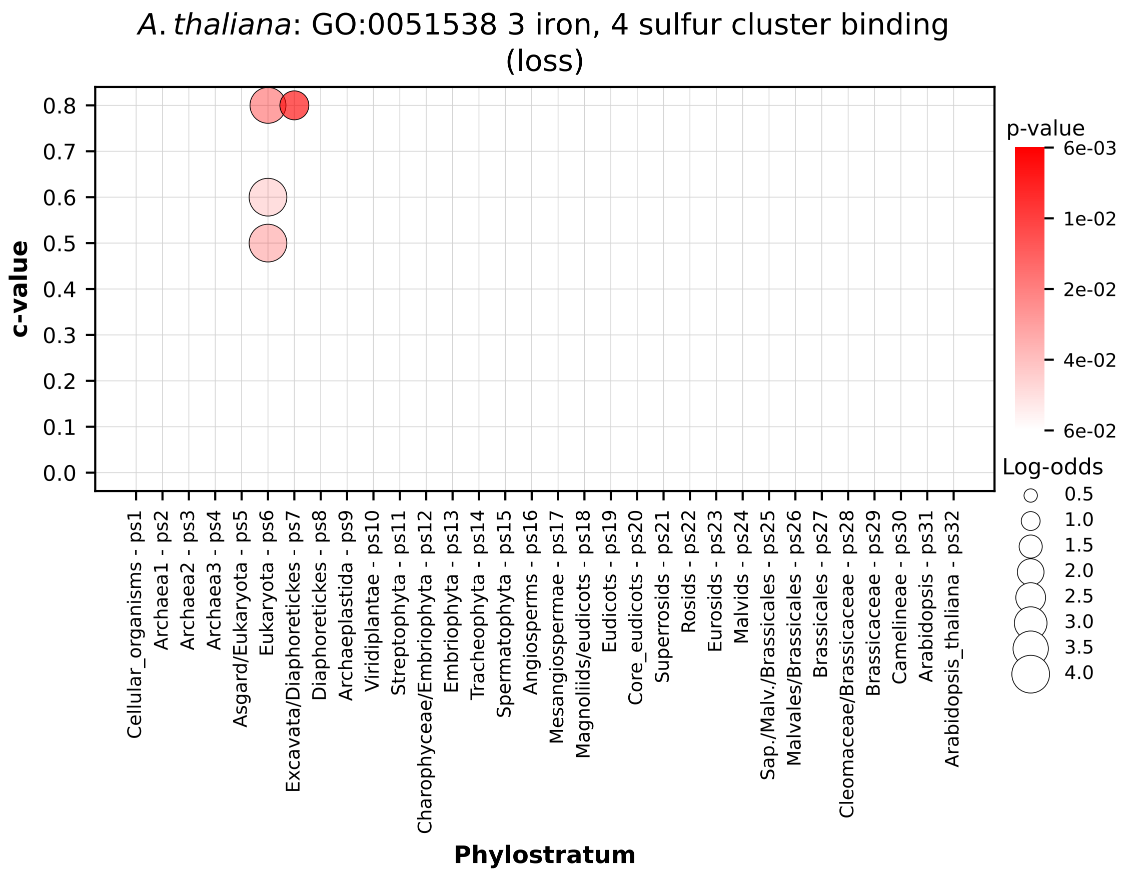

A. thaliana: GO:0051538 3 iron, 4 sulfur cluster binding
(loss)
c-value
0.0
0.1
0.2
0.3
0.4
0.5
0.6
0.7
0.8
p-value
6e-03
1e-02
2e-02
4e-02
6e-02
Log-odds
0.5
1.0
1.5
2.0
2.5
3.0
3.5
4.0
Cellular_organisms - ps1
Archaea1 - ps2
Archaea2 - ps3
Archaea3 - ps4
Asgard/Eukaryota - ps5
Eukaryota - ps6
Excavata/Diaphoretickes - ps7
Diaphoretickes - ps8
Archaeplastida - ps9
Viridiplantae - ps10
Streptophyta - ps11
Charophyceae/Embriophyta - ps12
Embriophyta - ps13
Tracheophyta - ps14
Spermatophyta - ps15
Angiosperms - ps16
Mesangiospermae - ps17
Magnoliids/eudicots - ps18
Eudicots - ps19
Core_eudicots - ps20
Superrosids - ps21
Rosids - ps22
Eurosids - ps23
Malvids - ps24
Sap./Malv./Brassicales - ps25
Malvales/Brassicales - ps26
Brassicales - ps27
Cleomaceae/Brassicaceae - ps28
Brassicaceae - ps29
Camelineae - ps30
Arabidopsis - ps31
Arabidopsis_thaliana - ps32
Phylostratum

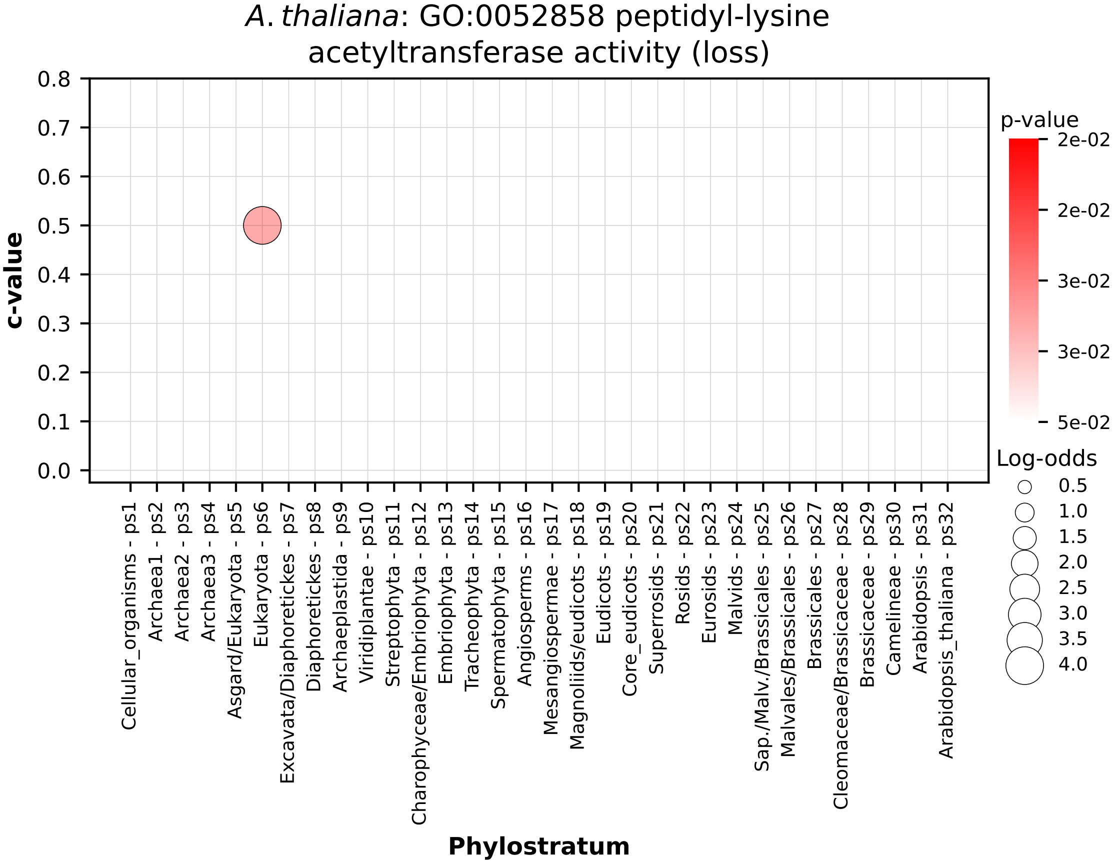

A. thaliana: GO:0052858 peptidyl-lysine acetyltransferase activity (loss)
c-value
0.8
0.7
0.6
0.5
0.4
0.3
0.2
0.1
0.0
p-value
2e-02
2e-02
3e-02
3e-02
5e-02
Log-odds
0.5
1.0
1.5
2.0
2.5
3.0
3.5
4.0
Cellular_organisms - ps1
Archaea1 - ps2
Archaea2 - ps3
Archaea3 - ps4
Asgard/Eukaryota - ps5
Eukaryota - ps6
Excavata/Diaphoretickes - ps7
Diaphoretickes - ps8
Archaeplastida - ps9
Viridiplantae - ps10
Streptophyta - ps11
Charophyceae/Embriophyta - ps12
Embriophyta - ps13
Tracheophyta - ps14
Spermatophyta - ps15
Angiosperms - ps16
Mesangiospermae - ps17
Magnoliids/eudicots - ps18
Eudicots - ps19
Core_eudicots - ps20
Superrosids - ps21
Rosids - ps22
Eurosids - ps23
Malvids - ps24
Sap./Malv./Brassicales - ps25
Malvales/Brassicales - ps26
Brassicales - ps27
Cleomaceae/Brassicaceae - ps28
Brassicaceae - ps29
Camelineae - ps30
Arabidopsis - ps31
Arabidopsis_thaliana - ps32
Phylostratum

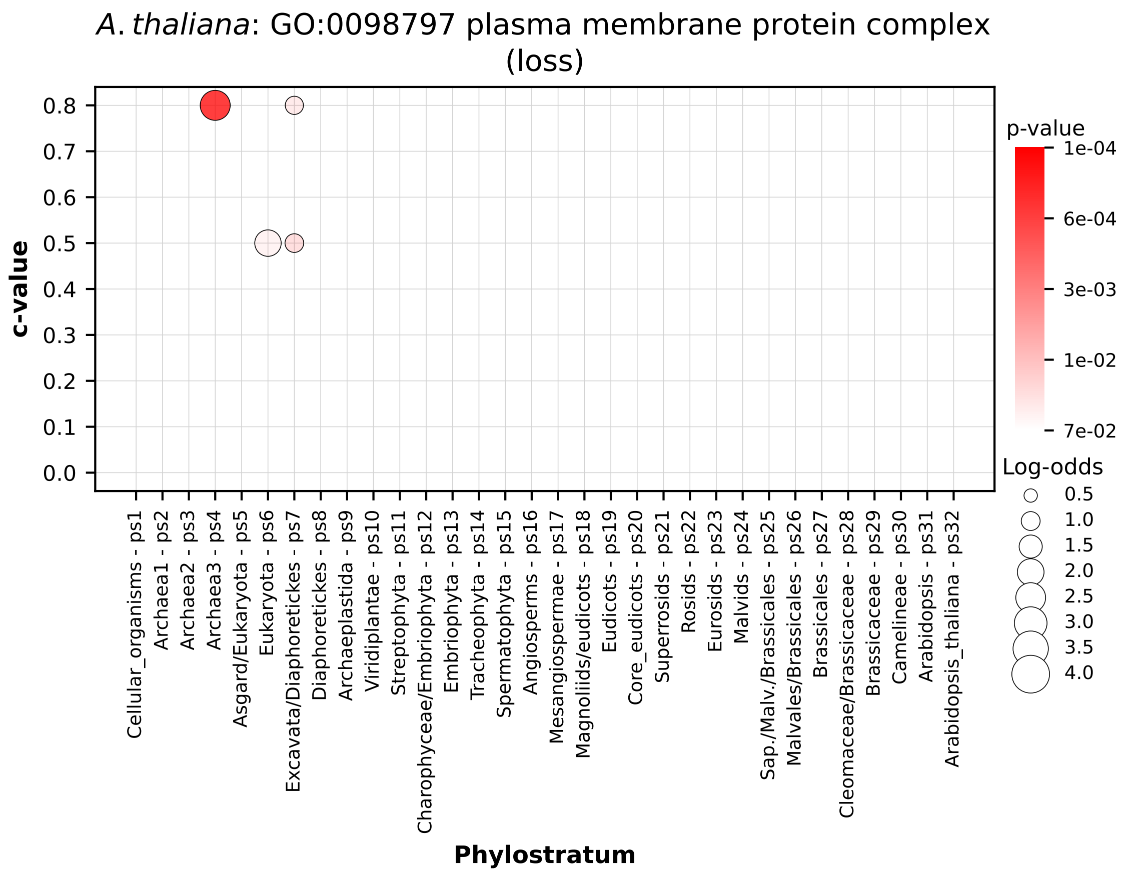

A. thaliana: GO:0098797 plasma membrane protein complex
(loss)
c-value
0.0
0.1
0.2
0.3
0.4
0.5
0.6
0.7
0.8
Phylostratum
Cellular_organisms - ps1
Archaea1 - ps2
Archaea2 - ps3
Archaea3 - ps4
Asgard/Eukaryota - ps5
Eukaryota - ps6
Excavata/Diaphoretickes - ps7
Diaphoretickes - ps8
Archaeplastida - ps9
Viridiplantae - ps10
Streptophyta - ps11
Charophyceae/Embriophyta - ps12
Embriophyta - ps13
Tracheophyta - ps14
Spermatophyta - ps15
Angiosperms - ps16
Mesangiospermae - ps17
Magnoliids/eudicots - ps18
Eudicots - ps19
Core_eudicots - ps20
Superrosids - ps21
Rosids - ps22
Eurosids - ps23
Malvids - ps24
Sap./Malv./Brassicales - ps25
Malvales/Brassicales - ps26
Brassicales - ps27
Cleomaceae/Brassicaceae - ps28
Brassicaceae - ps29
Camelineae - ps30
Arabidopsis - ps31
Arabidopsis_thaliana - ps32
p-value
1e-04
6e-04
3e-03
1e-02
7e-02
Log-odds
0.5
1.0
1.5
2.0
2.5
3.0
3.5
4.0

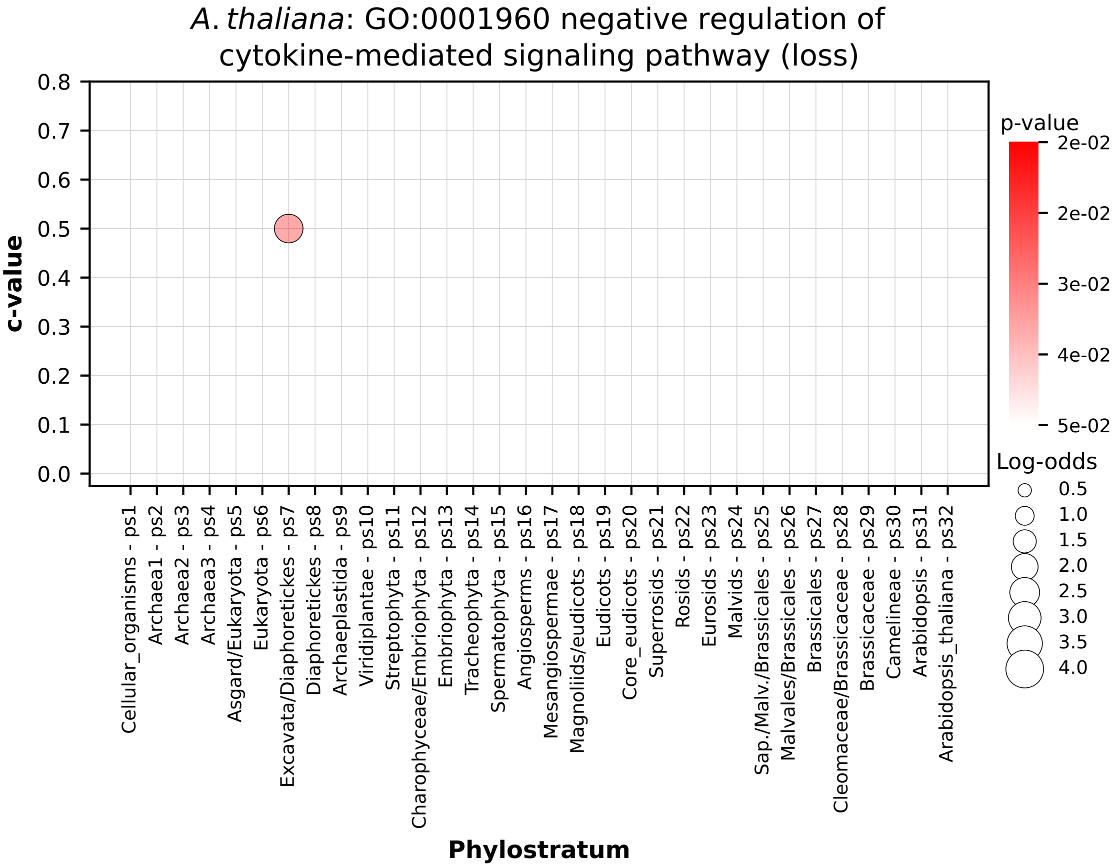

A. thaliana: GO:0001960 negative regulation of cytokine-mediated signaling pathway (loss)
c-value
0.8
0.7
0.6
0.5
0.4
0.3
0.2
0.1
0.0
p-value
2e-02
2e-02
3e-02
4e-02
5e-02
Log-odds
0.5
1.0
1.5
2.0
2.5
3.0
3.5
4.0
Cellular_organisms - ps1
Archaea1 - ps2
Archaea2 - ps3
Archaea3 - ps4
Asgard/Eukaryota - ps5
Eukaryota - ps6
Excavata/Diaphoretickes - ps7
Diaphoretickes - ps8
Archaeplastida - ps9
Viridiplantae - ps10
Streptophyta - ps11
Charophyceae/Embriophyta - ps12
Embriophyta - ps13
Tracheophyta - ps14
Spermatophyta - ps15
Angiosperms - ps16
Mesangiospermae - ps17
Magnoliids/eudicots - ps18
Eudicots - ps19
Core_eudicots - ps20
Superrosids - ps21
Rosids - ps22
Eurosids - ps23
Malvids - ps24
Sap./Malv./Brassicales - ps25
Malvales/Brassicales - ps26
Brassicales - ps27
Cleomaceae/Brassicaceae - ps28
Brassicaceae - ps29
Camelineae - ps30
Arabidopsis - ps31
Arabidopsis_thaliana - ps32
Phylostratum

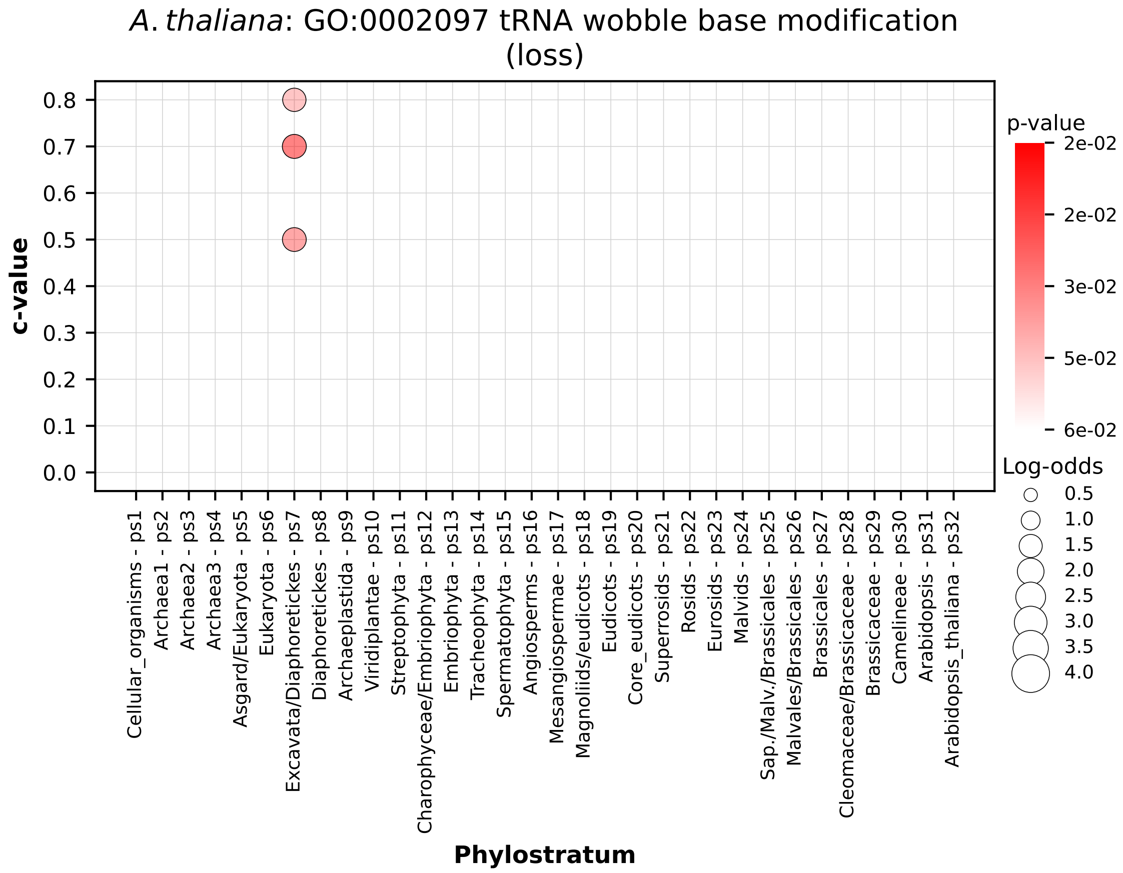

A. thaliana: GO:0002097 tRNA wobble base modification (loss)
c-value
0.8
0.7
0.6
0.5
0.4
0.3
0.2
0.1
0.0
p-value
2e-02
2e-02
3e-02
5e-02
6e-02
Log-odds
0.5
1.0
1.5
2.0
2.5
3.0
3.5
4.0
Cellular_organisms - ps1
Archaea1 - ps2
Archaea2 - ps3
Archaea3 - ps4
Asgard/Eukaryota - ps5
Eukaryota - ps6
Excavata/Diaphoretickes - ps7
Diaphoretickes - ps8
Archaeplastida - ps9
Viridiplantae - ps10
Streptophyta - ps11
Charophyceae/Embriophyta - ps12
Embriophyta - ps13
Tracheophyta - ps14
Spermatophyta - ps15
Angiosperms - ps16
Mesangiospermae - ps17
Magnoliids/eudicots - ps18
Eudicots - ps19
Core_eudicots - ps20
Superrosids - ps21
Rosids - ps22
Eurosids - ps23
Malvids - ps24
Sap./Malv./Brassicales - ps25
Malvales/Brassicales - ps26
Brassicales - ps27
Cleomaceae/Brassicaceae - ps28
Brassicaceae - ps29
Camelineae - ps30
Arabidopsis - ps31
Arabidopsis_thaliana - ps32
Phylostratum

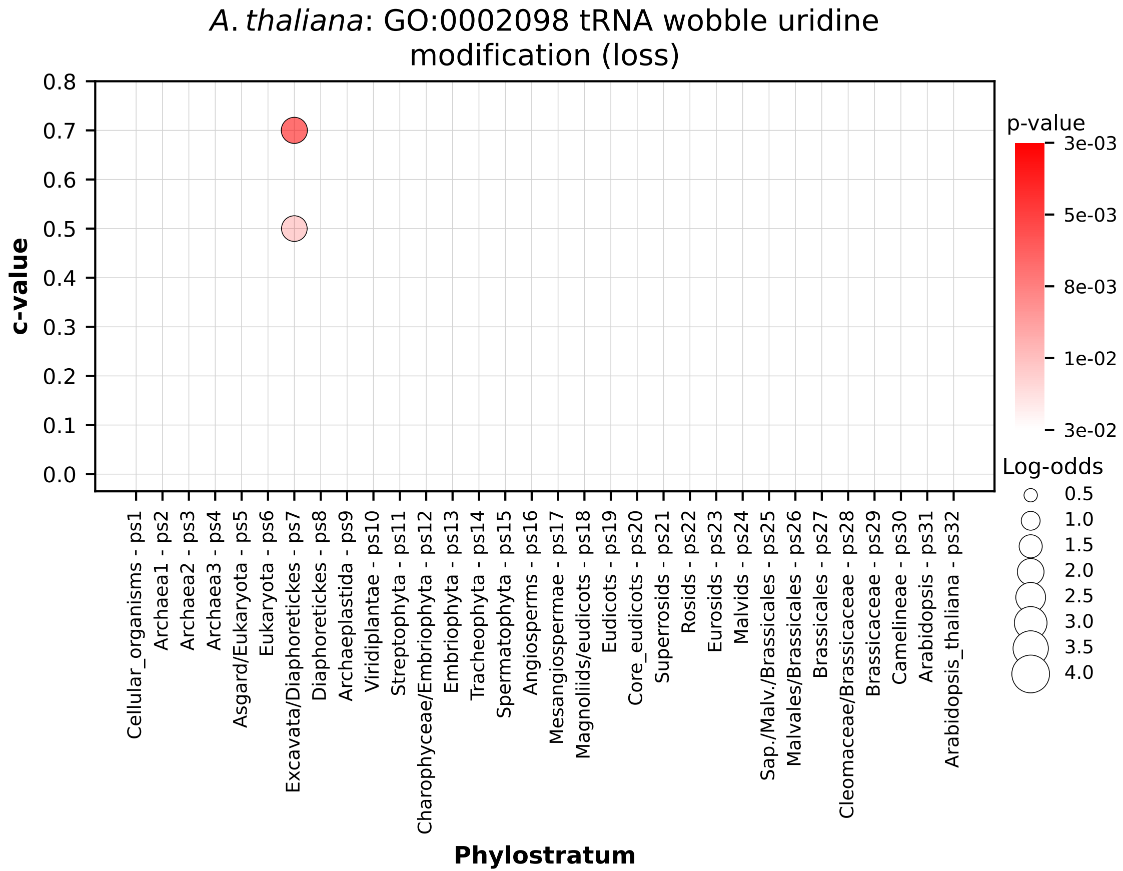

A. thaliana: GO:0002098 tRNA wobble uridine modification (loss)
c-value
0.8
0.7
0.6
0.5
0.4
0.3
0.2
0.1
0.0
p-value
3e-03
5e-03
8e-03
1e-02
3e-02
Log-odds
0.5
1.0
1.5
2.0
2.5
3.0
3.5
4.0
Cellular_organisms - ps1
Archaea1 - ps2
Archaea2 - ps3
Archaea3 - ps4
Asgard/Eukaryota - ps5
Eukaryota - ps6
Excavata/Diaphoretickes - ps7
Diaphoretickes - ps8
Archaeplastida - ps9
Viridiplantae - ps10
Streptophyta - ps11
Charophyceae/Embriophyta - ps12
Embriophyta - ps13
Tracheophyta - ps14
Spermatophyta - ps15
Angiosperms - ps16
Mesangiospermae - ps17
Magnoliids/eudicots - ps18
Eudicots - ps19
Core_eudicots - ps20
Superrosids - ps21
Rosids - ps22
Eurosids - ps23
Malvids - ps24
Sap./Malv./Brassicales - ps25
Malvales/Brassicales - ps26
Brassicales - ps27
Cleomaceae/Brassicaceae - ps28
Brassicaceae - ps29
Camelineae - ps30
Arabidopsis - ps31
Arabidopsis_thaliana - ps32
Phylostratum

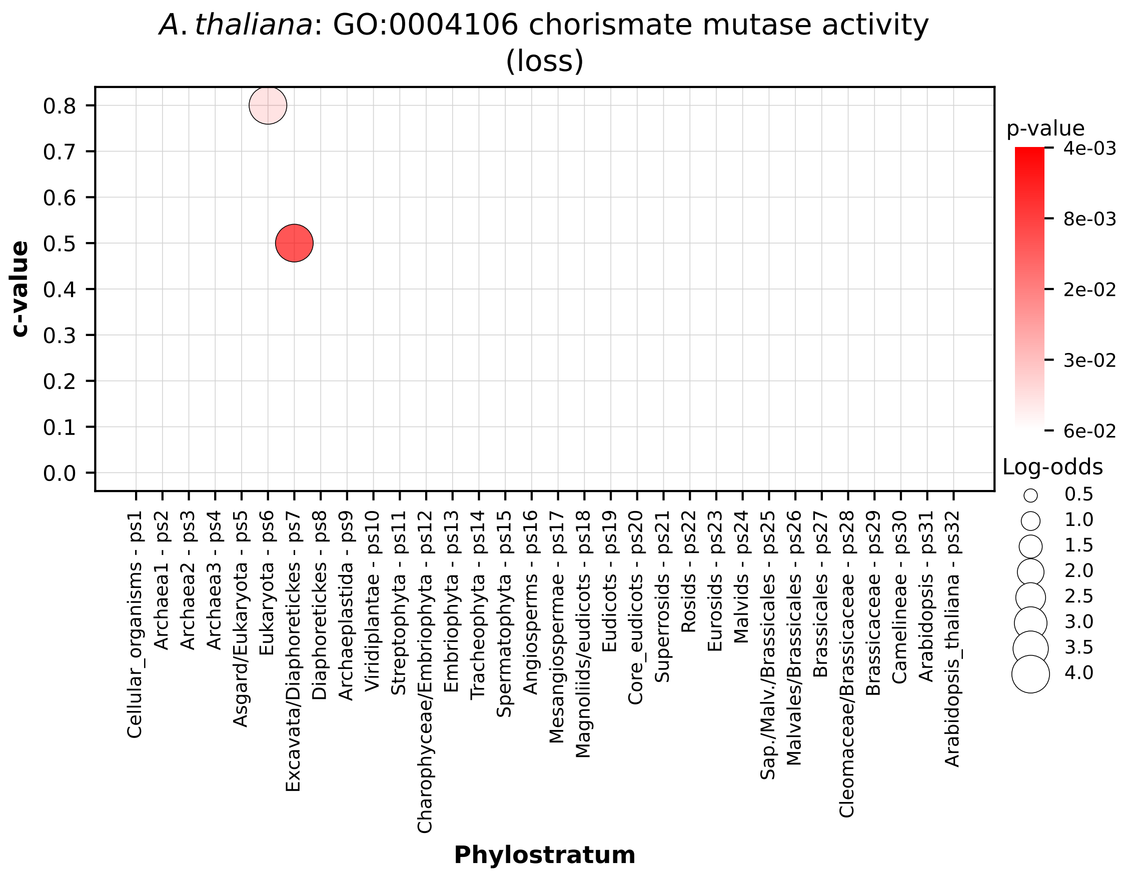

A. thaliana: GO:0004106 chorismate mutase activity
(loss)
c-value
0.8
0.7
0.6
0.5
0.4
0.3
0.2
0.1
0.0
Cellular_organisms - ps1
Archaea1 - ps2
Archaea2 - ps3
Archaea3 - ps4
Asgard/Eukaryota - ps5
Eukaryota - ps6
Excavata/Diaphoretickes - ps7
Diaphoretickes - ps8
Archaeplastida - ps9
Viridiplantae - ps10
Streptophyta - ps11
Charophyceae/Embriophyta - ps12
Embriophyta - ps13
Tracheophyta - ps14
Spermatophyta - ps15
Angiosperms - ps16
Mesangiospermae - ps17
Magnoliids/eudicots - ps18
Eudicots - ps19
Core_eudicots - ps20
Superrosids - ps21
Rosids - ps22
Eurosids - ps23
Malvids - ps24
Sap./Malv./Brassicales - ps25
Malvales/Brassicales - ps26
Brassicales - ps27
Cleomaceae/Brassicaceae - ps28
Brassicaceae - ps29
Camelineae - ps30
Arabidopsis - ps31
Arabidopsis_thaliana - ps32
Phylostratum
p-value
4e-03
8e-03
2e-02
3e-02
6e-02
Log-odds
0.5
1.0
1.5
2.0
2.5
3.0
3.5
4.0

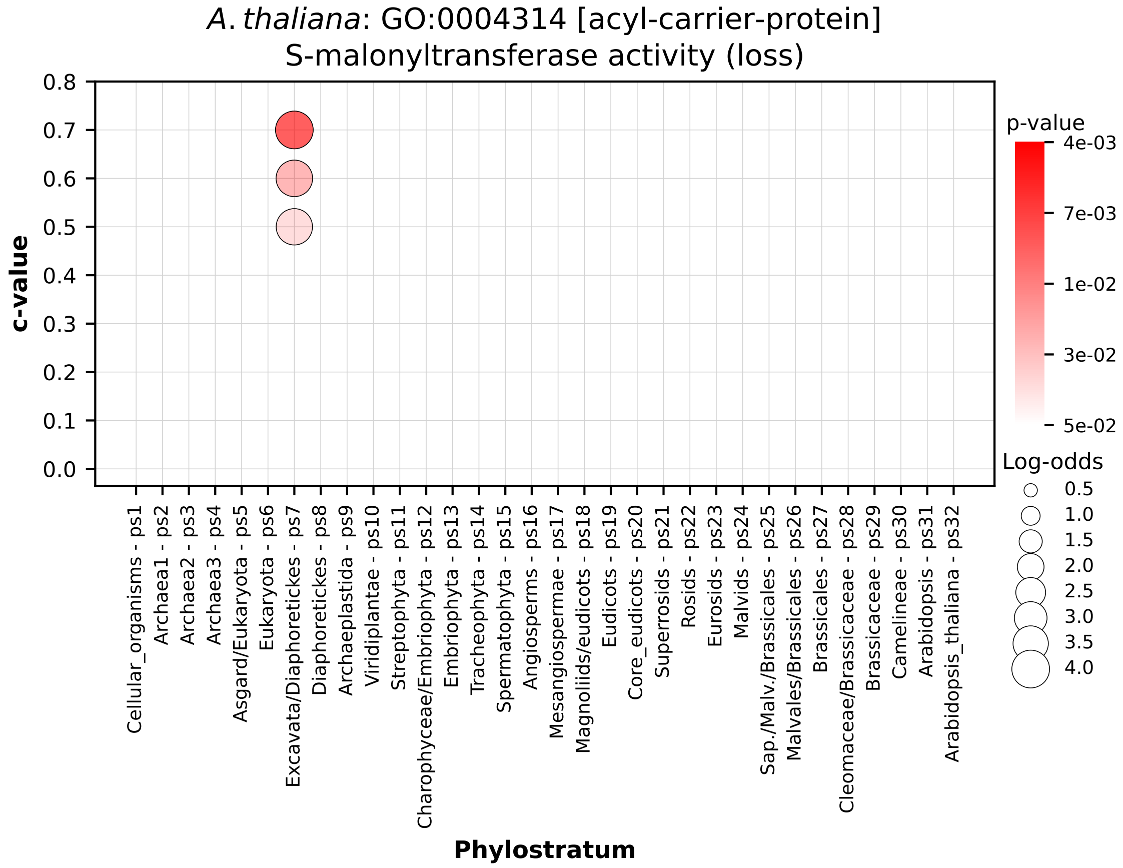

A. thaliana: GO:0004314 [acyl-carrier-protein]
S-malonyltransferase activity (loss)
c-value
0.8
0.7
0.6
0.5
0.4
0.3
0.2
0.1
0.0
Phylostratum
Cellular_organisms - ps1
Archaea1 - ps2
Archaea2 - ps3
Archaea3 - ps4
Asgard/Eukaryota - ps5
Eukaryota - ps6
Excavata/Diaphoretickes - ps7
Diaphoretickes - ps8
Archaeplastida - ps9
Viridiplantae - ps10
Streptophyta - ps11
Charophyceae/Embriophyta - ps12
Embriophyta - ps13
Tracheophyta - ps14
Spermatophyta - ps15
Angiosperms - ps16
Mesangiospermae - ps17
Magnoliids/eudicots - ps18
Eudicots - ps19
Core_eudicots - ps20
Superrosids - ps21
Rosids - ps22
Eurosids - ps23
Malvids - ps24
Sap./Malv./Brassicales - ps25
Malvales/Brassicales - ps26
Brassicales - ps27
Cleomaceae/Brassicaceae - ps28
Brassicaceae - ps29
Camelineae - ps30
Arabidopsis - ps31
Arabidopsis_thaliana - ps32
p-value
4e-03
7e-03
1e-02
3e-02
5e-02
Log-odds
0.5
1.0
1.5
2.0
2.5
3.0
3.5
4.0

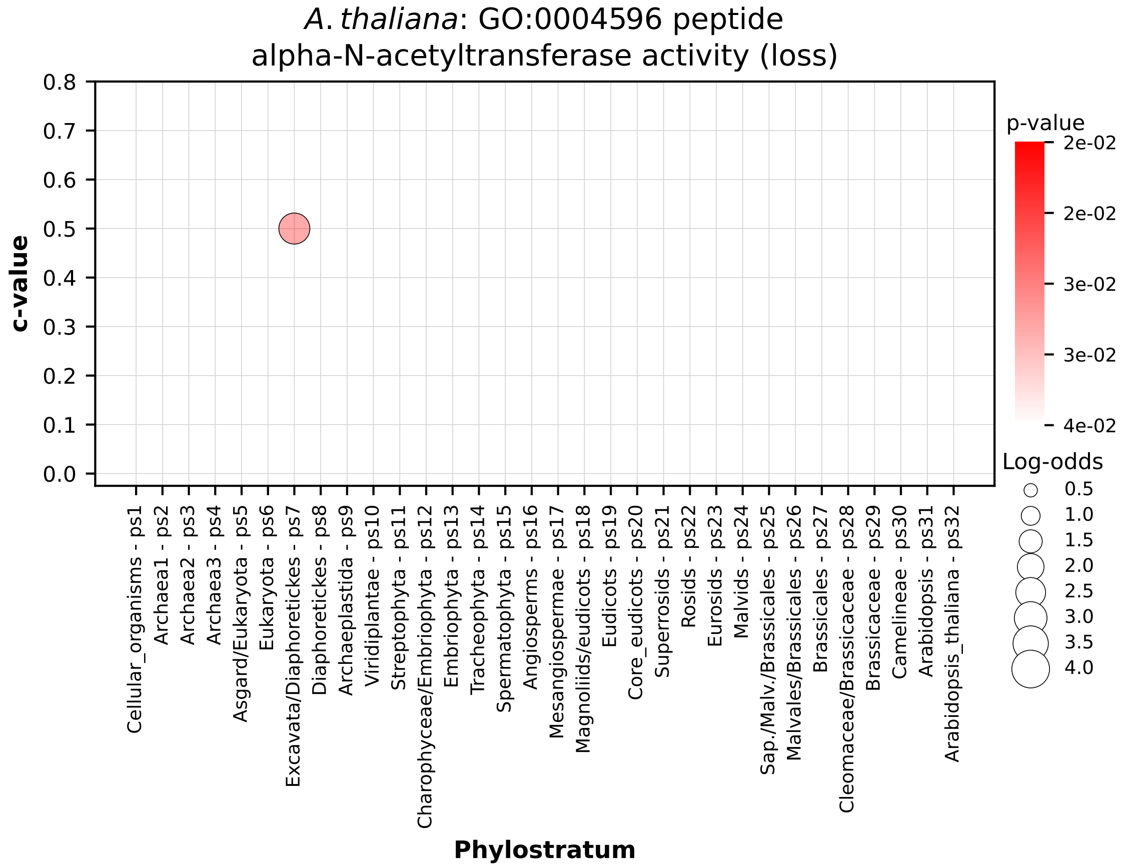
A. thaliana: GO:0004596 peptide alpha-N-acetyltransferase activity (loss)
c-value
0.8
0.7
0.6
0.5
0.4
0.3
0.2
0.1
0.0
p-value
2e-02
2e-02
3e-02
3e-02
4e-02
Log-odds
0.5
1.0
1.5
2.0
2.5
3.0
3.5
4.0
Cellular_organisms - ps1
Archaea1 - ps2
Archaea2 - ps3
Archaea3 - ps4
Asgard/Eukaryota - ps5
Eukaryota - ps6
Excavata/Diaphoretickes - ps7
Diaphoretickes - ps8
Archaeplastida - ps9
Viridiplantae - ps10
Streptophyta - ps11
Charophyceae/Embriophyta - ps12
Embriophyta - ps13
Tracheophyta - ps14
Spermatophyta - ps15
Angiosperms - ps16
Mesangiospermae - ps17
Magnoliids/eudicots - ps18
Eudicots - ps19
Core_eudicots - ps20
Superrosids - ps21
Rosids - ps22
Eurosids - ps23
Malvids - ps24
Sap./Malv./Brassicales - ps25
Malvales/Brassicales - ps26
Brassicales - ps27
Cleomaceae/Brassicaceae - ps28
Brassicaceae - ps29
Camelineae - ps30
Arabidopsis - ps31
Arabidopsis_thaliana - ps32
Phylostratum

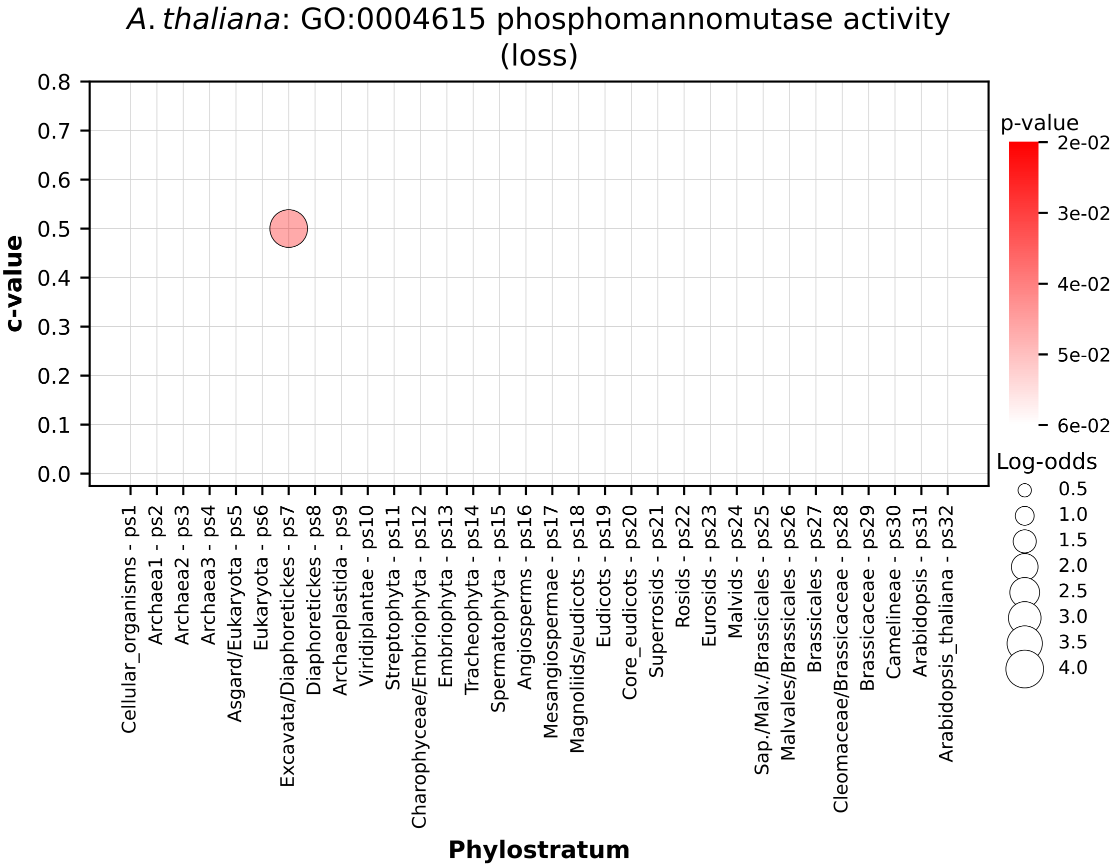

A. thaliana: GO:0004615 phosphomannomutase activity
(loss)
c-value
0.8
0.7
0.6
0.5
0.4
0.3
0.2
0.1
0.0
Phylostratum
Cellular_organisms - ps1
Archaea1 - ps2
Archaea2 - ps3
Archaea3 - ps4
Asgard/Eukaryota - ps5
Eukaryota - ps6
Excavata/Diaphoretickes - ps7
Diaphoretickes - ps8
Archaeplastida - ps9
Viridiplantae - ps10
Streptophyta - ps11
Charophyceae/Embriophyta - ps12
Embriophyta - ps13
Tracheophyta - ps14
Spermatophyta - ps15
Angiosperms - ps16
Mesangiospermae - ps17
Magnoliids/eudicots - ps18
Eudicots - ps19
Core_eudicots - ps20
Superrosids - ps21
Rosids - ps22
Eurosids - ps23
Malvids - ps24
Sap./Malv./Brassicales - ps25
Malvales/Brassicales - ps26
Brassicales - ps27
Cleomaceae/Brassicaceae - ps28
Brassicaceae - ps29
Camelineae - ps30
Arabidopsis - ps31
Arabidopsis_thaliana - ps32
p-value
2e-02
3e-02
4e-02
5e-02
6e-02
Log-odds
0.5
1.0
1.5
2.0
2.5
3.0
3.5
4.0

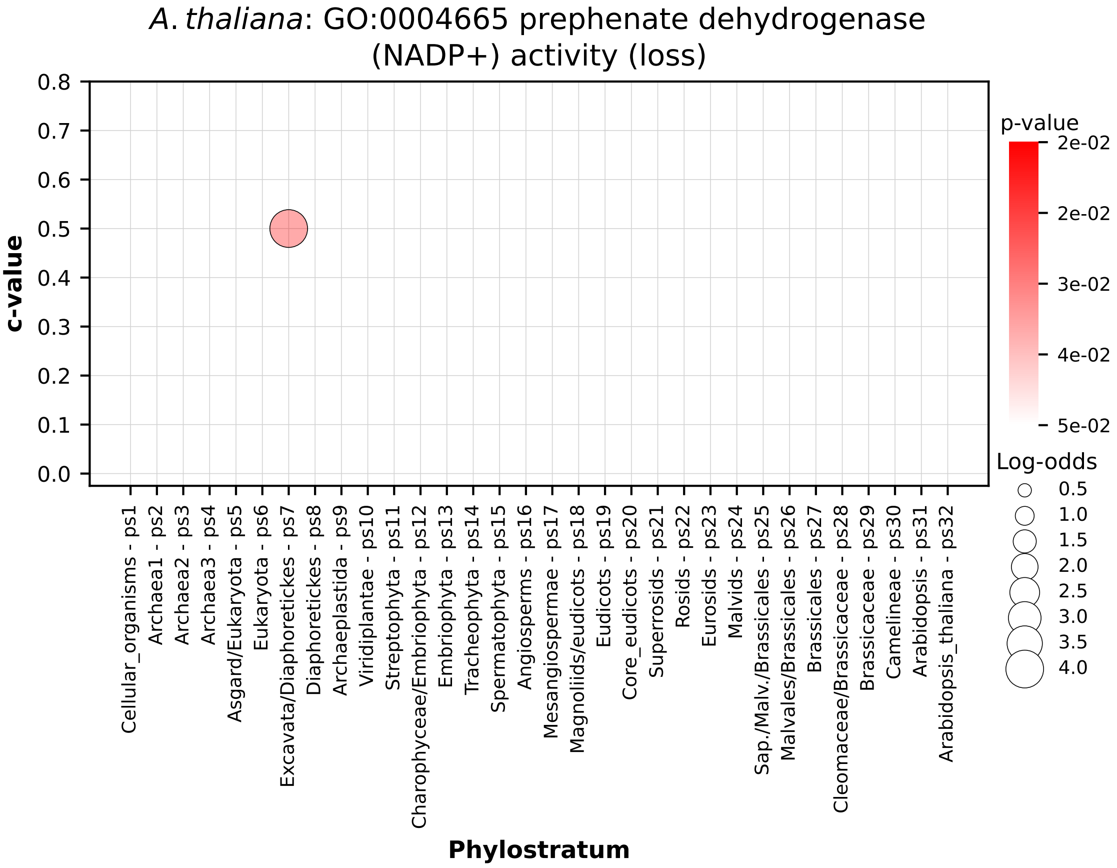
A. thaliana: GO:0004665 prephenate dehydrogenase (NADP+) activity (loss)
c-value
0.8
0.7
0.6
0.5
0.4
0.3
0.2
0.1
0.0
p-value
2e-02
2e-02
3e-02
4e-02
5e-02
Log-odds
0.5
1.0
1.5
2.0
2.5
3.0
3.5
4.0
Cellular_organisms - ps1
Archaea1 - ps2
Archaea2 - ps3
Archaea3 - ps4
Asgard/Eukaryota - ps5
Eukaryota - ps6
Excavata/Diaphoretickes - ps7
Diaphoretickes - ps8
Archaeplastida - ps9
Viridiplantae - ps10
Streptophyta - ps11
Charophyceae/Embriophyta - ps12
Embriophyta - ps13
Tracheophyta - ps14
Spermatophyta - ps15
Angiosperms - ps16
Mesangiospermae - ps17
Magnoliids/eudicots - ps18
Eudicots - ps19
Core_eudicots - ps20
Superrosids - ps21
Rosids - ps22
Eurosids - ps23
Malvids - ps24
Sap./Malv./Brassicales - ps25
Malvales/Brassicales - ps26
Brassicales - ps27
Cleomaceae/Brassicaceae - ps28
Brassicaceae - ps29
Camelineae - ps30
Arabidopsis - ps31
Arabidopsis_thaliana - ps32
Phylostratum

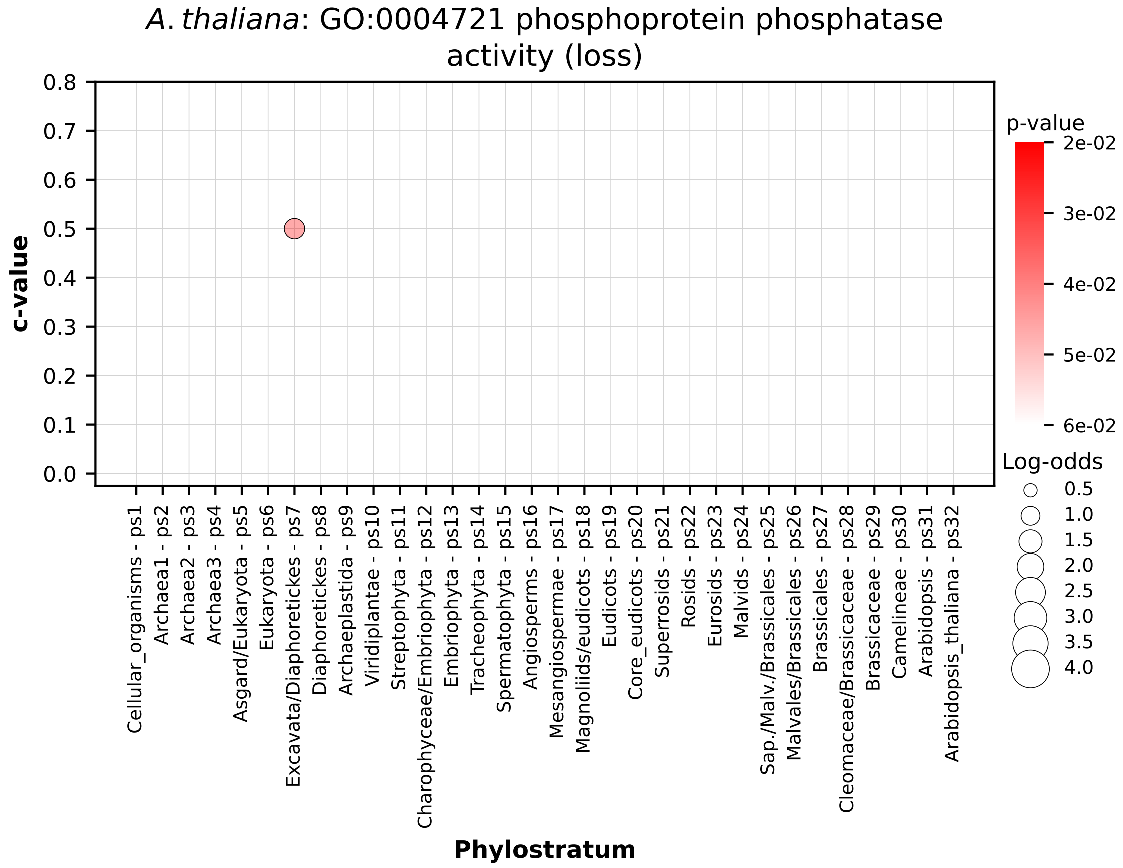

A. thaliana: GO:0004721 phosphoprotein phosphatase activity (loss)
c-value
0.8
0.7
0.6
0.5
0.4
0.3
0.2
0.1
0.0
Phylostratum
Cellular_organisms - ps1
Archaea1 - ps2
Archaea2 - ps3
Archaea3 - ps4
Asgard/Eukaryota - ps5
Eukaryota - ps6
Excavata/Diaphoretickes - ps7
Diaphoretickes - ps8
Archaeplastida - ps9
Viridiplantae - ps10
Streptophyta - ps11
Charophyceae/Embriophyta - ps12
Embriophyta - ps13
Tracheophyta - ps14
Spermatophyta - ps15
Angiosperms - ps16
Mesangiospermae - ps17
Magnoliids/eudicots - ps18
Eudicots - ps19
Core_eudicots - ps20
Superrosids - ps21
Rosids - ps22
Eurosids - ps23
Malvids - ps24
Sap./Malv./Brassicales - ps25
Malvales/Brassicales - ps26
Brassicales - ps27
Cleomaceae/Brassicaceae - ps28
Brassicaceae - ps29
Camelineae - ps30
Arabidopsis - ps31
Arabidopsis_thaliana - ps32
p-value
2e-02
3e-02
4e-02
5e-02
6e-02
Log-odds
0.5
1.0
1.5
2.0
2.5
3.0
3.5
4.0

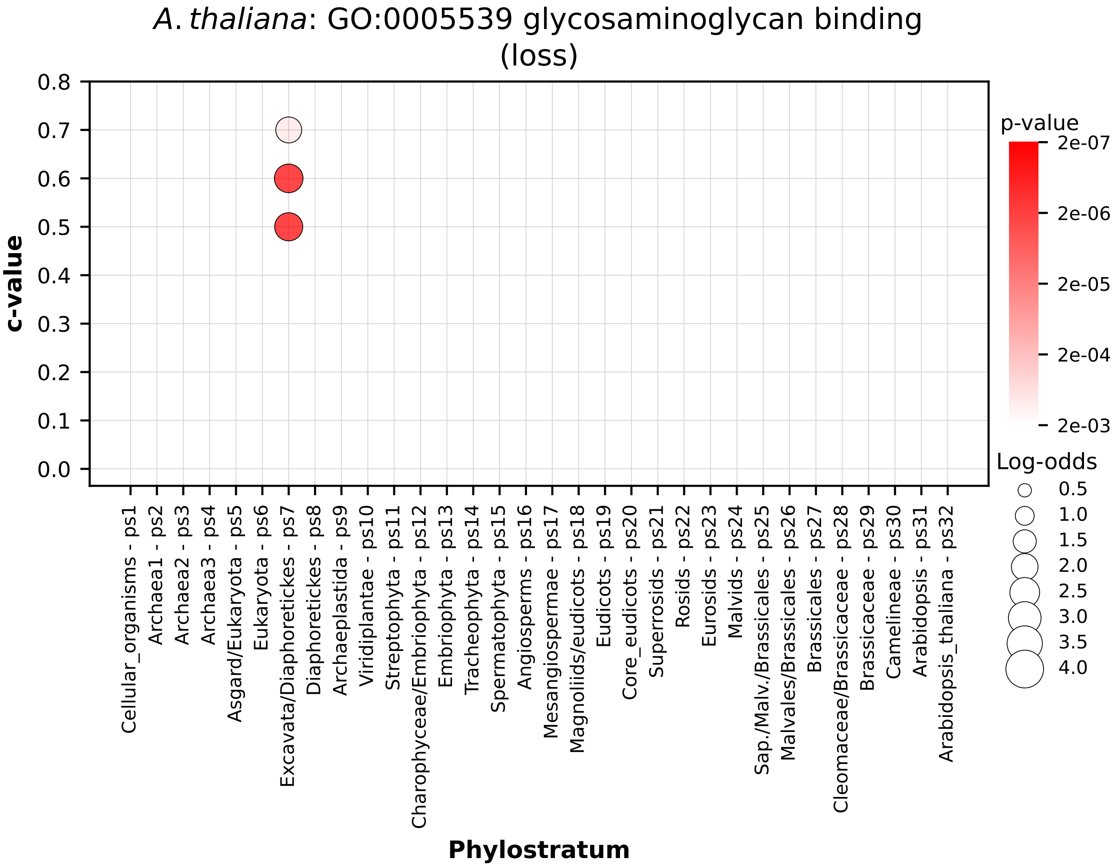

A. thaliana: GO:0005539 glycosaminoglycan binding (loss)
c-value
0.8
0.7
0.6
0.5
0.4
0.3
0.2
0.1
0.0
Phylostratum
Cellular_organisms - ps1
Archaea1 - ps2
Archaea2 - ps3
Archaea3 - ps4
Asgard/Eukaryota - ps5
Eukaryota - ps6
Excavata/Diaphoretickes - ps7
Diaphoretickes - ps8
Archaeplastida - ps9
Viridiplantae - ps10
Streptophyta - ps11
Charophyceae/Embriophyta - ps12
Embriophyta - ps13
Tracheophyta - ps14
Spermatophyta - ps15
Angiosperms - ps16
Mesangiospermae - ps17
Magnoliids/eudicots - ps18
Eudicots - ps19
Core_eudicots - ps20
Superrosids - ps21
Rosids - ps22
Eurosids - ps23
Malvids - ps24
Sap./Malv./Brassicales - ps25
Malvales/Brassicales - ps26
Brassicales - ps27
Cleomaceae/Brassicaceae - ps28
Brassicaceae - ps29
Camelineae - ps30
Arabidopsis - ps31
Arabidopsis_thaliana - ps32
p-value
2e-07
2e-06
2e-05
2e-04
2e-03
Log-odds
0.5
1.0
1.5
2.0
2.5
3.0
3.5
4.0

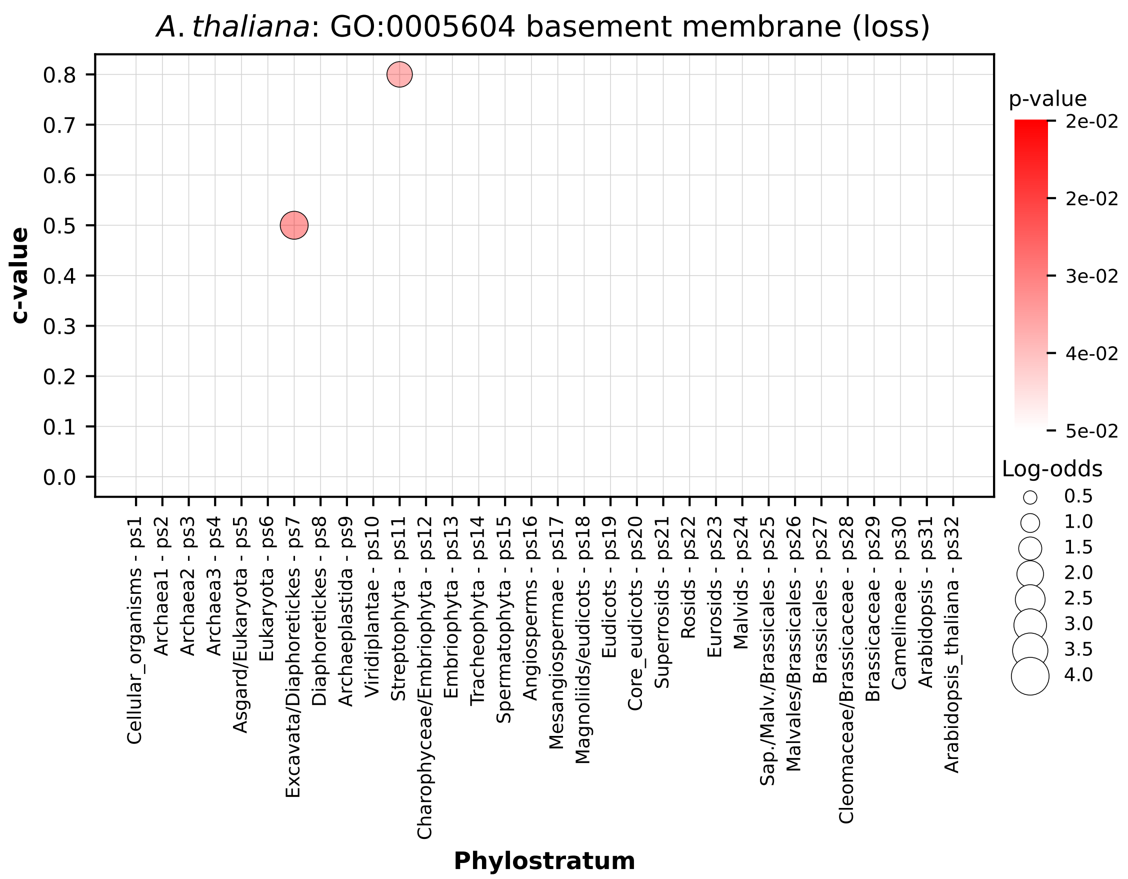

A. thaliana: GO:0005604 basement membrane (loss)
c-value
0.0
0.1
0.2
0.3
0.4
0.5
0.6
0.7
0.8
Phylostratum
Cellular_organisms - ps1
Archaea1 - ps2
Archaea2 - ps3
Archaea3 - ps4
Asgard/Eukaryota - ps5
Eukaryota - ps6
Excavata/Diaphoretickes - ps7
Diaphoretickes - ps8
Archaeplastida - ps9
Viridiplantae - ps10
Streptophyta - ps11
Charophyceae/Embriophyta - ps12
Embriophyta - ps13
Tracheophyta - ps14
Spermatophyta - ps15
Angiosperms - ps16
Mesangiospermae - ps17
Magnoliids/eudicots - ps18
Eudicots - ps19
Core_eudicots - ps20
Superrosids - ps21
Rosids - ps22
Eurosids - ps23
Malvids - ps24
Sap./Malv./Brassicales - ps25
Malvales/Brassicales - ps26
Brassicales - ps27
Cleomaceae/Brassicaceae - ps28
Brassicaceae - ps29
Camelineae - ps30
Arabidopsis - ps31
Arabidopsis_thaliana - ps32
p-value
2e-02
2e-02
3e-02
4e-02
5e-02
Log-odds
0.5
1.0
1.5
2.0
2.5
3.0
3.5
4.0

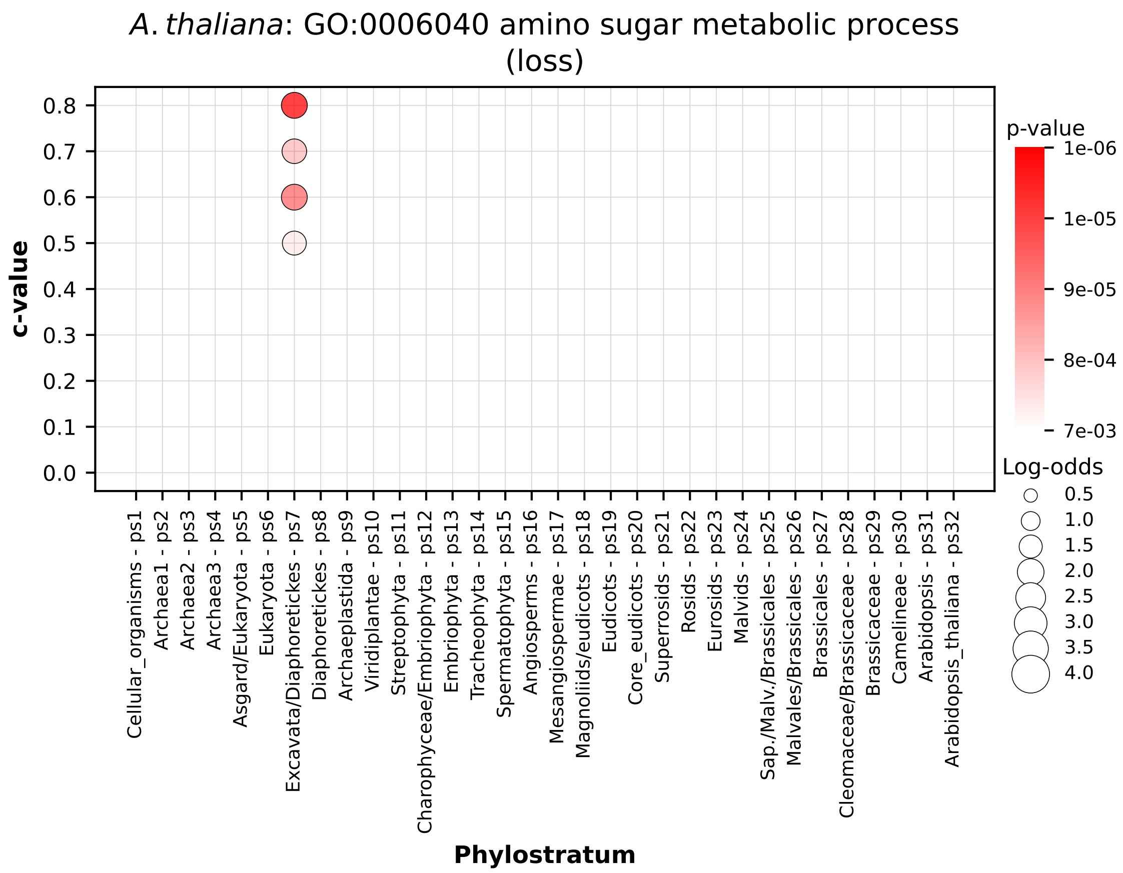

A. thaliana: GO:0006040 amino sugar metabolic process
(loss)
c-value
0.8
0.7
0.6
0.5
0.4
0.3
0.2
0.1
0.0
p-value
1e-06
1e-05
9e-05
8e-04
7e-03
Log-odds
0.5
1.0
1.5
2.0
2.5
3.0
3.5
4.0
Cellular_organisms - ps1
Archaea1 - ps2
Archaea2 - ps3
Archaea3 - ps4
Asgard/Eukaryota - ps5
Eukaryota - ps6
Excavata/Diaphoretickes - ps7
Diaphoretickes - ps8
Archaeplastida - ps9
Viridiplantae - ps10
Streptophyta - ps11
Charophyceae/Embriophyta - ps12
Embriophyta - ps13
Tracheophyta - ps14
Spermatophyta - ps15
Angiosperms - ps16
Mesangiospermae - ps17
Magnoliids/eudicots - ps18
Eudicots - ps19
Core_eudicots - ps20
Superrosids - ps21
Rosids - ps22
Eurosids - ps23
Malvids - ps24
Sap./Malv./Brassicales - ps25
Malvales/Brassicales - ps26
Brassicales - ps27
Cleomaceae/Brassicaceae - ps28
Brassicaceae - ps29
Camelineae - ps30
Arabidopsis - ps31
Arabidopsis_thaliana - ps32
Phylostratum

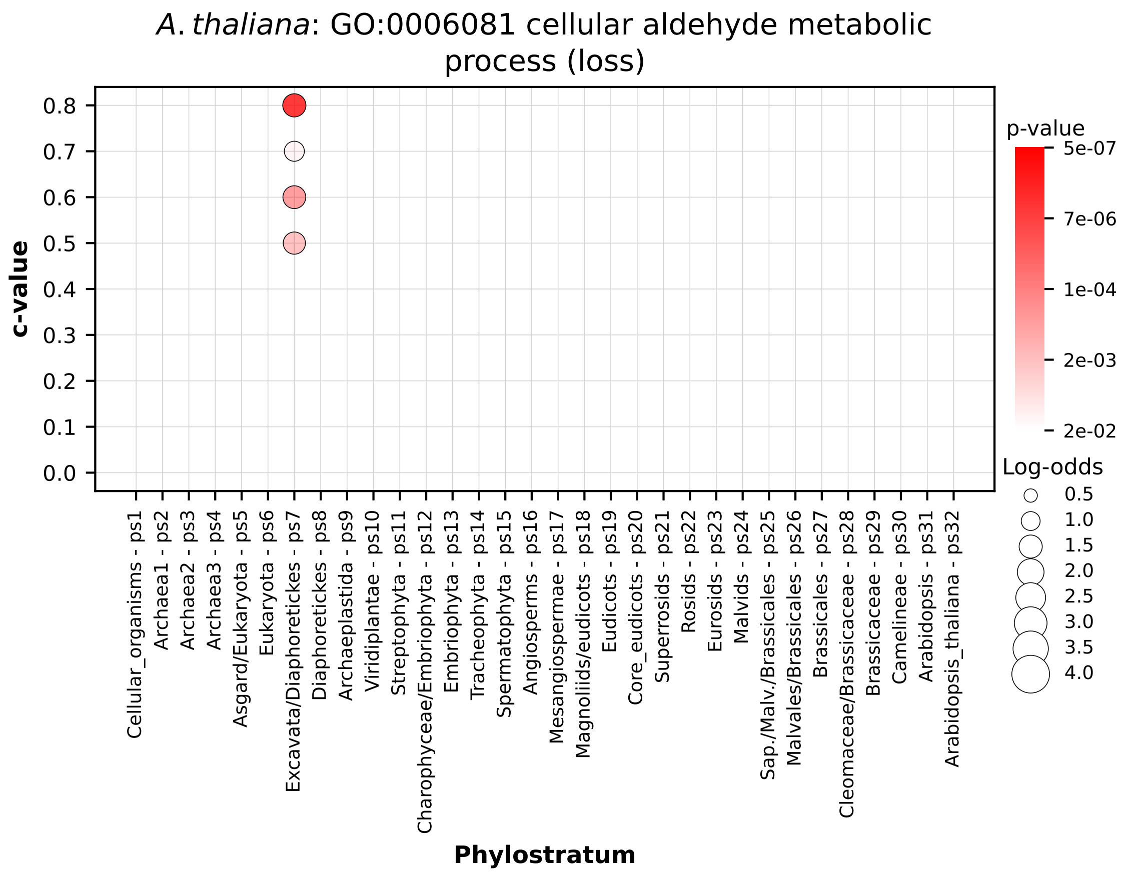
A. thaliana: GO:0006081 cellular aldehyde metabolic process (loss)
c-value
0.0
0.1
0.2
0.3
0.4
0.5
0.6
0.7
0.8
p-value
5e-07
7e-06
1e-04
2e-03
2e-02
Log-odds
0.5
1.0
1.5
2.0
2.5
3.0
3.5
4.0
Cellular_organisms - ps1
Archaea1 - ps2
Archaea2 - ps3
Archaea3 - ps4
Asgard/Eukaryota - ps5
Eukaryota - ps6
Excavata/Diaphoretickes - ps7
Diaphoretickes - ps8
Archaeplastida - ps9
Viridiplantae - ps10
Streptophyta - ps11
Charophyceae/Embriophyta - ps12
Embriophyta - ps13
Tracheophyta - ps14
Spermatophyta - ps15
Angiosperms - ps16
Mesangiospermae - ps17
Magnoliids/eudicots - ps18
Eudicots - ps19
Core_eudicots - ps20
Superrosids - ps21
Rosids - ps22
Eurosids - ps23
Malvids - ps24
Sap./Malv./Brassicales - ps25
Malvales/Brassicales - ps26
Brassicales - ps27
Cleomaceae/Brassicaceae - ps28
Brassicaceae - ps29
Camelineae - ps30
Arabidopsis - ps31
Arabidopsis_thaliana - ps32
Phylostratum

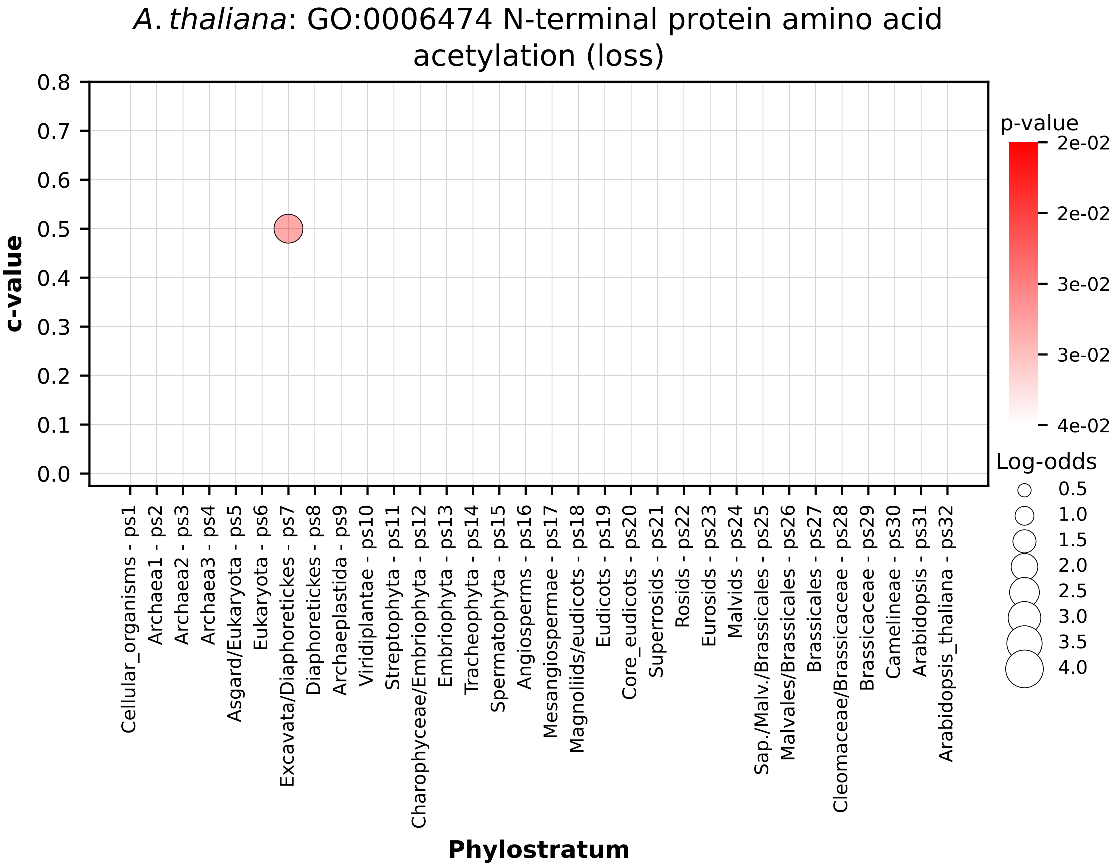

A. thaliana: GO:0006474 N-terminal protein amino acid acetylation (loss)
c-value
0.8
0.7
0.6
0.5
0.4
0.3
0.2
0.1
0.0
Phylostratum
Cellular_organisms - ps1
Archaea1 - ps2
Archaea2 - ps3
Archaea3 - ps4
Asgard/Eukaryota - ps5
Eukaryota - ps6
Excavata/Diaphoretickes - ps7
Diaphoretickes - ps8
Archaeplastida - ps9
Viridiplantae - ps10
Streptophyta - ps11
Charophyceae/Embriophyta - ps12
Embriophyta - ps13
Tracheophyta - ps14
Spermatophyta - ps15
Angiosperms - ps16
Mesangiospermae - ps17
Magnoliids/eudicots - ps18
Eudicots - ps19
Core_eudicots - ps20
Superrosids - ps21
Rosids - ps22
Eurosids - ps23
Malvids - ps24
Sap./Malv./Brassicales - ps25
Malvales/Brassicales - ps26
Brassicales - ps27
Cleomaceae/Brassicaceae - ps28
Brassicaceae - ps29
Camelineae - ps30
Arabidopsis - ps31
Arabidopsis_thaliana - ps32
p-value
2e-02
2e-02
3e-02
3e-02
4e-02
Log-odds
0.5
1.0
1.5
2.0
2.5
3.0
3.5
4.0

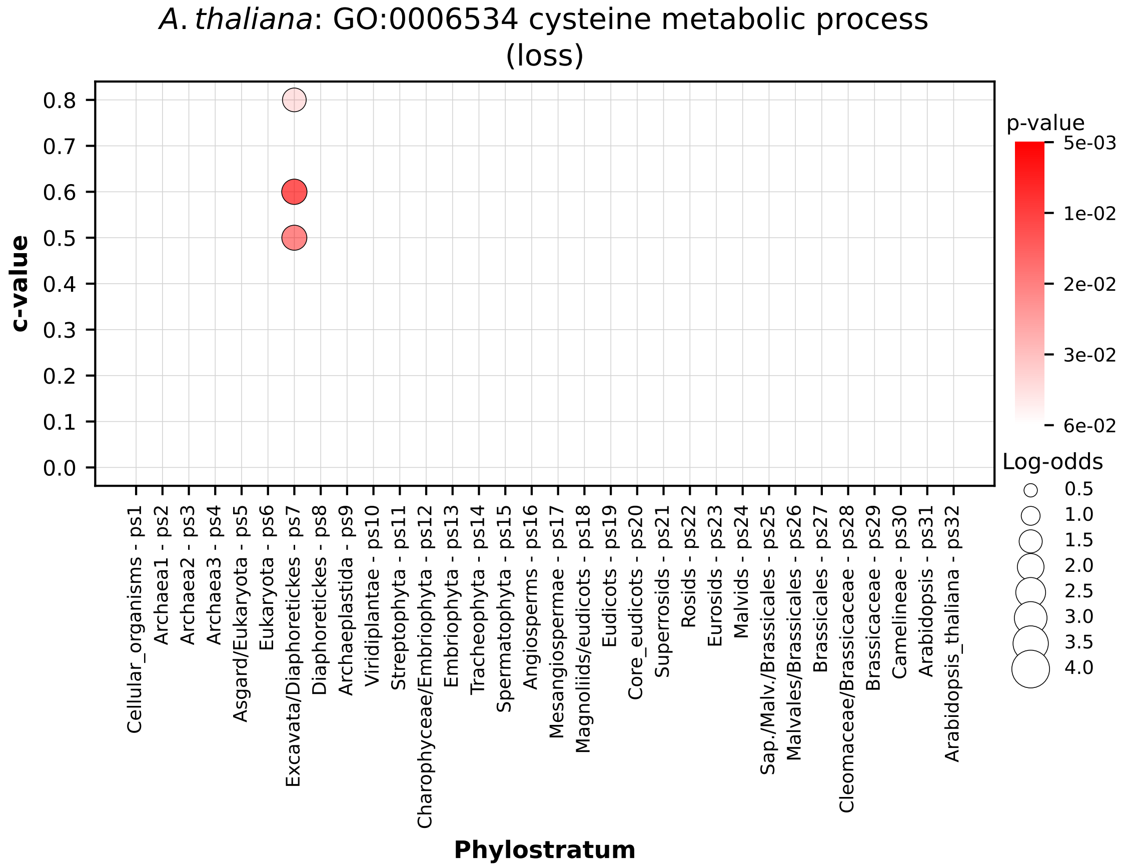

A. thaliana: GO:0006534 cysteine metabolic process
(loss)
c-value
0.0
0.1
0.2
0.3
0.4
0.5
0.6
0.7
0.8
Phylostratum
Cellular_organisms - ps1
Archaea1 - ps2
Archaea2 - ps3
Archaea3 - ps4
Asgard/Eukaryota - ps5
Eukaryota - ps6
Excavata/Diaphoretickes - ps7
Diaphoretickes - ps8
Archaeplastida - ps9
Viridiplantae - ps10
Streptophyta - ps11
Charophyceae/Embriophyta - ps12
Embriophyta - ps13
Tracheophyta - ps14
Spermatophyta - ps15
Angiosperms - ps16
Mesangiospermae - ps17
Magnoliids/eudicots - ps18
Eudicots - ps19
Core_eudicots - ps20
Superrosids - ps21
Rosids - ps22
Eurosids - ps23
Malvids - ps24
Sap./Malv./Brassicales - ps25
Malvales/Brassicales - ps26
Brassicales - ps27
Cleomaceae/Brassicaceae - ps28
Brassicaceae - ps29
Camelineae - ps30
Arabidopsis - ps31
Arabidopsis_thaliana - ps32
p-value
5e-03
1e-02
2e-02
3e-02
6e-02
Log-odds
0.5
1.0
1.5
2.0
2.5
3.0
3.5
4.0

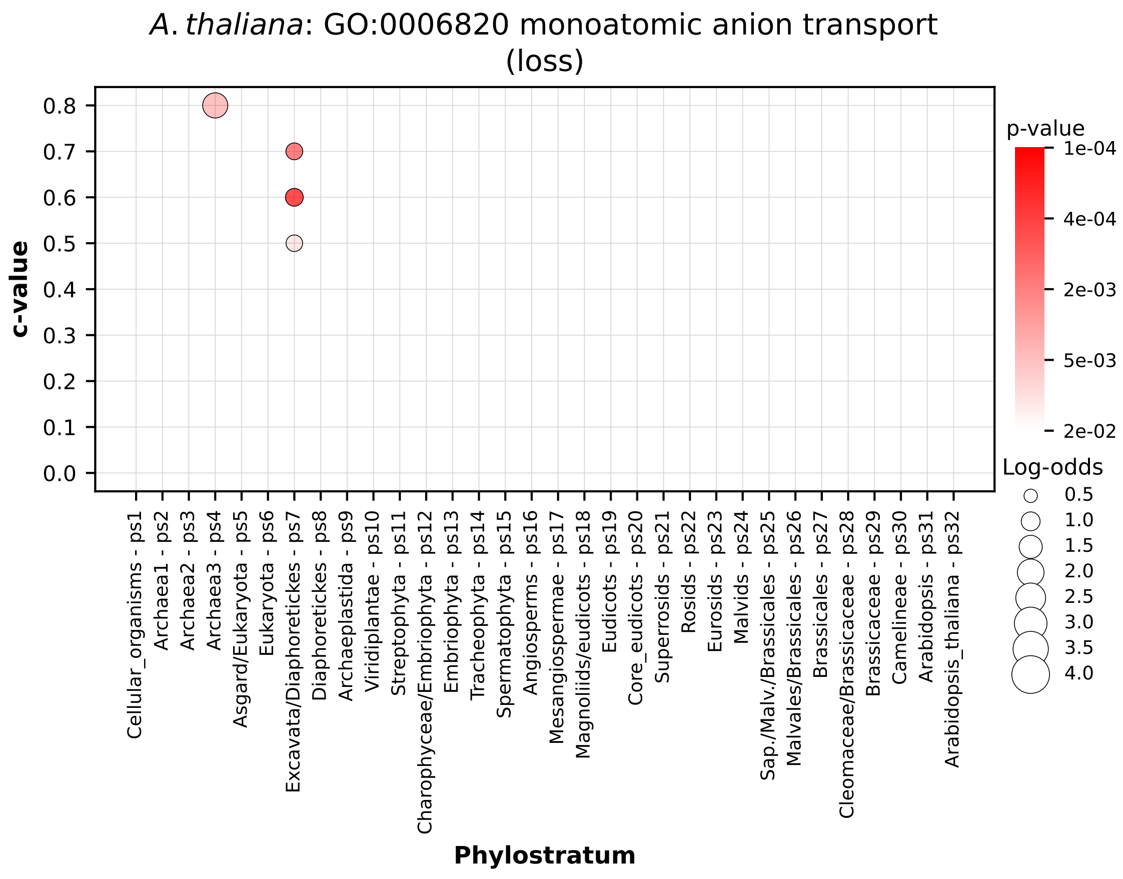

A. thaliana: GO:0006820 monoatomic anion transport
(loss)
c-value
0.0
0.1
0.2
0.3
0.4
0.5
0.6
0.7
0.8
Phylostratum
Cellular_organisms - ps1
Archaea1 - ps2
Archaea2 - ps3
Archaea3 - ps4
Asgard/Eukaryota - ps5
Eukaryota - ps6
Excavata/Diaphoretickes - ps7
Diaphoretickes - ps8
Archaeplastida - ps9
Viridiplantae - ps10
Streptophyta - ps11
Charophyceae/Embriophyta - ps12
Embriophyta - ps13
Tracheophyta - ps14
Spermatophyta - ps15
Angiosperms - ps16
Mesangiospermae - ps17
Magnoliids/eudicots - ps18
Eudicots - ps19
Core_eudicots - ps20
Superrosids - ps21
Rosids - ps22
Eurosids - ps23
Malvids - ps24
Sap./Malv./Brassicales - ps25
Malvales/Brassicales - ps26
Brassicales - ps27
Cleomaceae/Brassicaceae - ps28
Brassicaceae - ps29
Camelineae - ps30
Arabidopsis - ps31
Arabidopsis_thaliana - ps32
p-value
1e-04
4e-04
2e-03
5e-03
2e-02
Log-odds
0.5
1.0
1.5
2.0
2.5
3.0
3.5
4.0

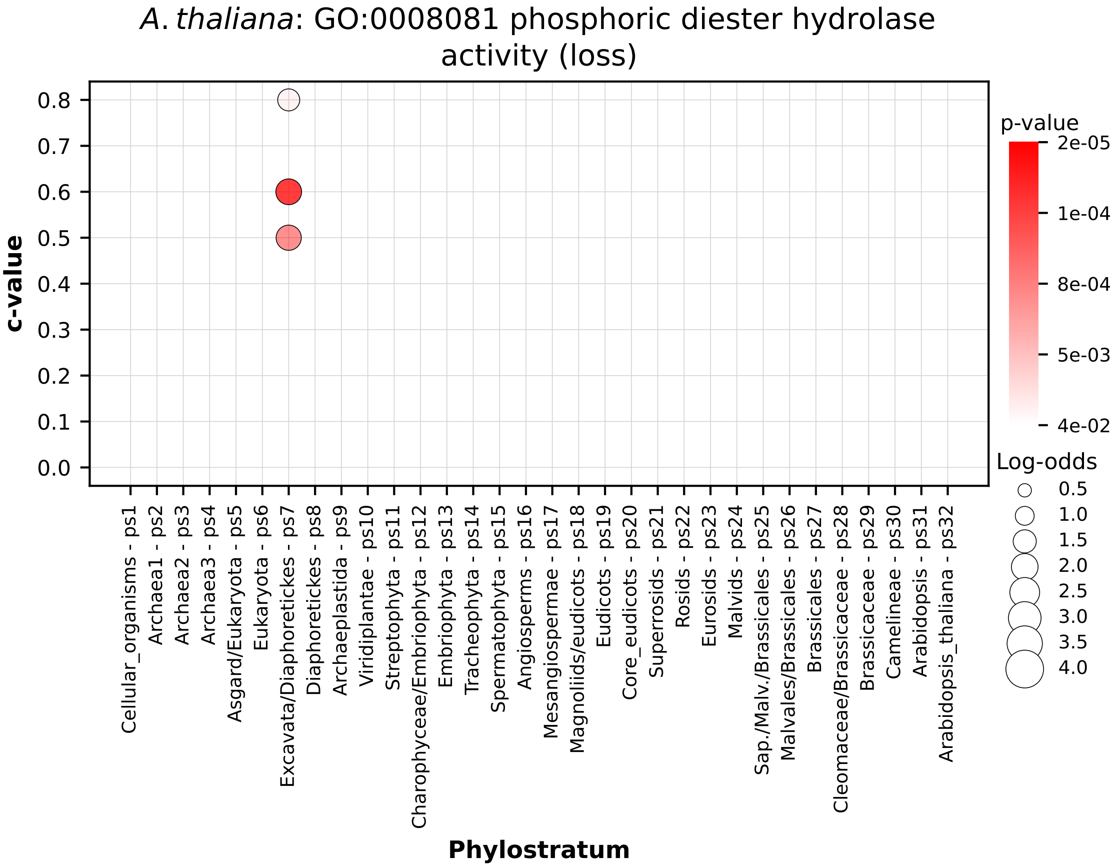

A. thaliana: GO:0008081 phosphoric diester hydrolase activity (loss)
c-value
0.0
0.1
0.2
0.3
0.4
0.5
0.6
0.7
0.8
p-value
2e-05
1e-04
8e-04
5e-03
4e-02
Log-odds
0.5
1.0
1.5
2.0
2.5
3.0
3.5
4.0
Cellular_organisms - ps1
Archaea1 - ps2
Archaea2 - ps3
Archaea3 - ps4
Asgard/Eukaryota - ps5
Eukaryota - ps6
Excavata/Diaphoretickes - ps7
Diaphoretickes - ps8
Archaeplastida - ps9
Viridiplantae - ps10
Streptophyta - ps11
Charophyceae/Embriophyta - ps12
Embriophyta - ps13
Tracheophyta - ps14
Spermatophyta - ps15
Angiosperms - ps16
Mesangiospermae - ps17
Magnoliids/eudicots - ps18
Eudicots - ps19
Core_eudicots - ps20
Superrosids - ps21
Rosids - ps22
Eurosids - ps23
Malvids - ps24
Sap./Malv./Brassicales - ps25
Malvales/Brassicales - ps26
Brassicales - ps27
Cleomaceae/Brassicaceae - ps28
Brassicaceae - ps29
Camelineae - ps30
Arabidopsis - ps31
Arabidopsis_thaliana - ps32
Phylostratum

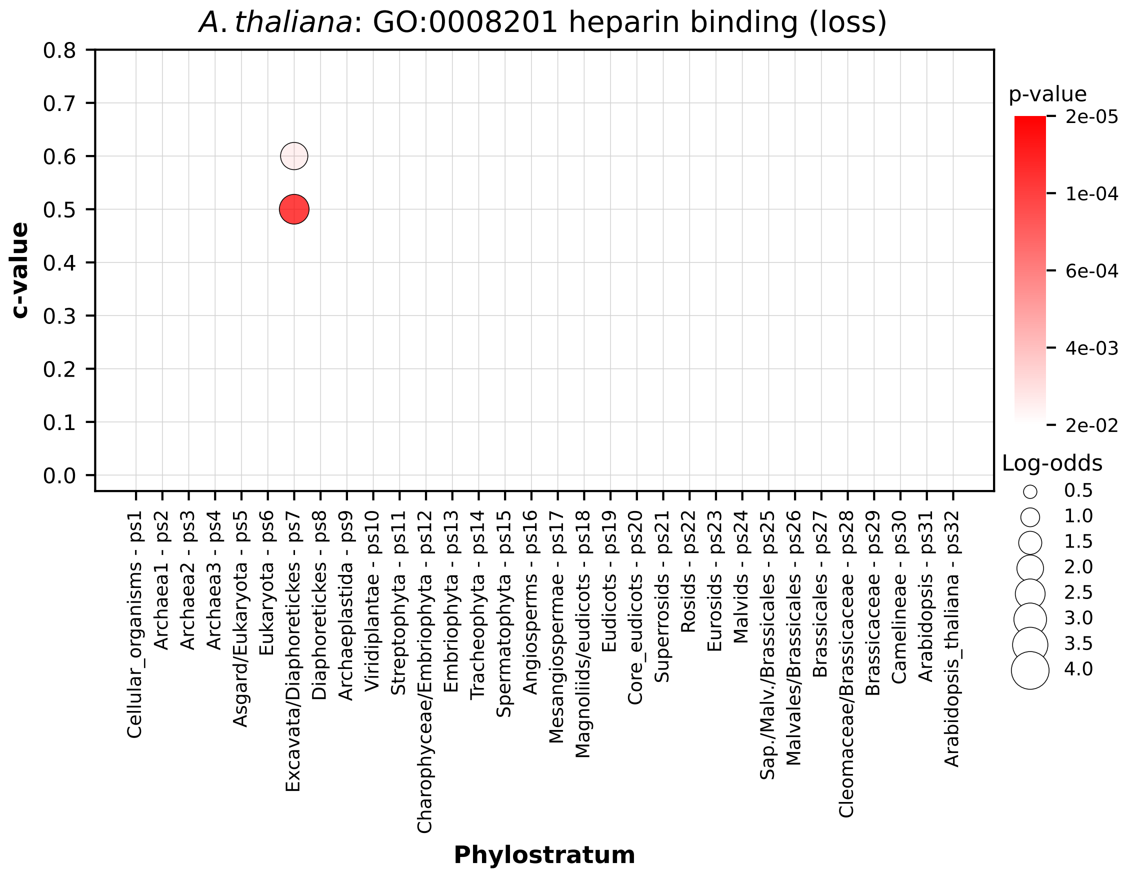

A. thaliana: GO:0008201 heparin binding (loss)
c-value
0.8
0.7
0.6
0.5
0.4
0.3
0.2
0.1
0.0
p-value
2e-05
1e-04
6e-04
4e-03
2e-02
Log-odds
0.5
1.0
1.5
2.0
2.5
3.0
3.5
4.0
Cellular_organisms - ps1
Archaea1 - ps2
Archaea2 - ps3
Archaea3 - ps4
Asgard/Eukaryota - ps5
Eukaryota - ps6
Excavata/Diaphoretickes - ps7
Diaphoretickes - ps8
Archaeplastida - ps9
Viridiplantae - ps10
Streptophyta - ps11
Charophyceae/Embriophyta - ps12
Embriophyta - ps13
Tracheophyta - ps14
Spermatophyta - ps15
Angiosperms - ps16
Mesangiospermae - ps17
Magnoliids/eudicots - ps18
Eudicots - ps19
Core_eudicots - ps20
Superrosids - ps21
Rosids - ps22
Eurosids - ps23
Malvids - ps24
Sap./Malv./Brassicales - ps25
Malvales/Brassicales - ps26
Brassicales - ps27
Cleomaceae/Brassicaceae - ps28
Brassicaceae - ps29
Camelineae - ps30
Arabidopsis - ps31
Arabidopsis_thaliana - ps32
Phylostratum

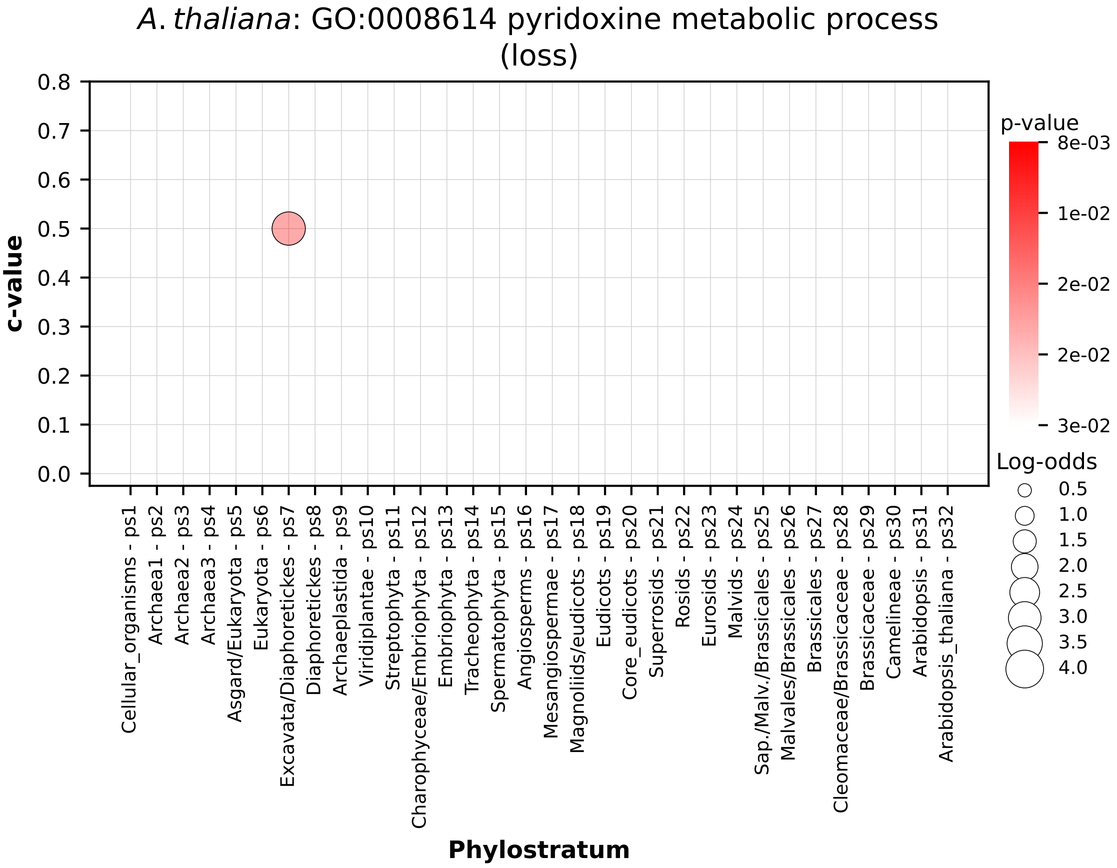

A. thaliana: GO:0008614 pyridoxine metabolic process (loss)
c-value
0.8
0.7
0.6
0.5
0.4
0.3
0.2
0.1
0.0
p-value
8e-03
1e-02
2e-02
2e-02
3e-02
Log-odds
0.5
1.0
1.5
2.0
2.5
3.0
3.5
4.0
Cellular_organisms - ps1
Archaea1 - ps2
Archaea2 - ps3
Archaea3 - ps4
Asgard/Eukaryota - ps5
Eukaryota - ps6
Excavata/Diaphoretickes - ps7
Diaphoretickes - ps8
Archaeplastida - ps9
Viridiplantae - ps10
Streptophyta - ps11
Charophyceae/Embriophyta - ps12
Embriophyta - ps13
Tracheophyta - ps14
Spermatophyta - ps15
Angiosperms - ps16
Mesangiospermae - ps17
Magnoliids/eudicots - ps18
Eudicots - ps19
Core_eudicots - ps20
Superrosids - ps21
Rosids - ps22
Eurosids - ps23
Malvids - ps24
Sap./Malv./Brassicales - ps25
Malvales/Brassicales - ps26
Brassicales - ps27
Cleomaceae/Brassicaceae - ps28
Brassicaceae - ps29
Camelineae - ps30
Arabidopsis - ps31
Arabidopsis_thaliana - ps32
Phylostratum

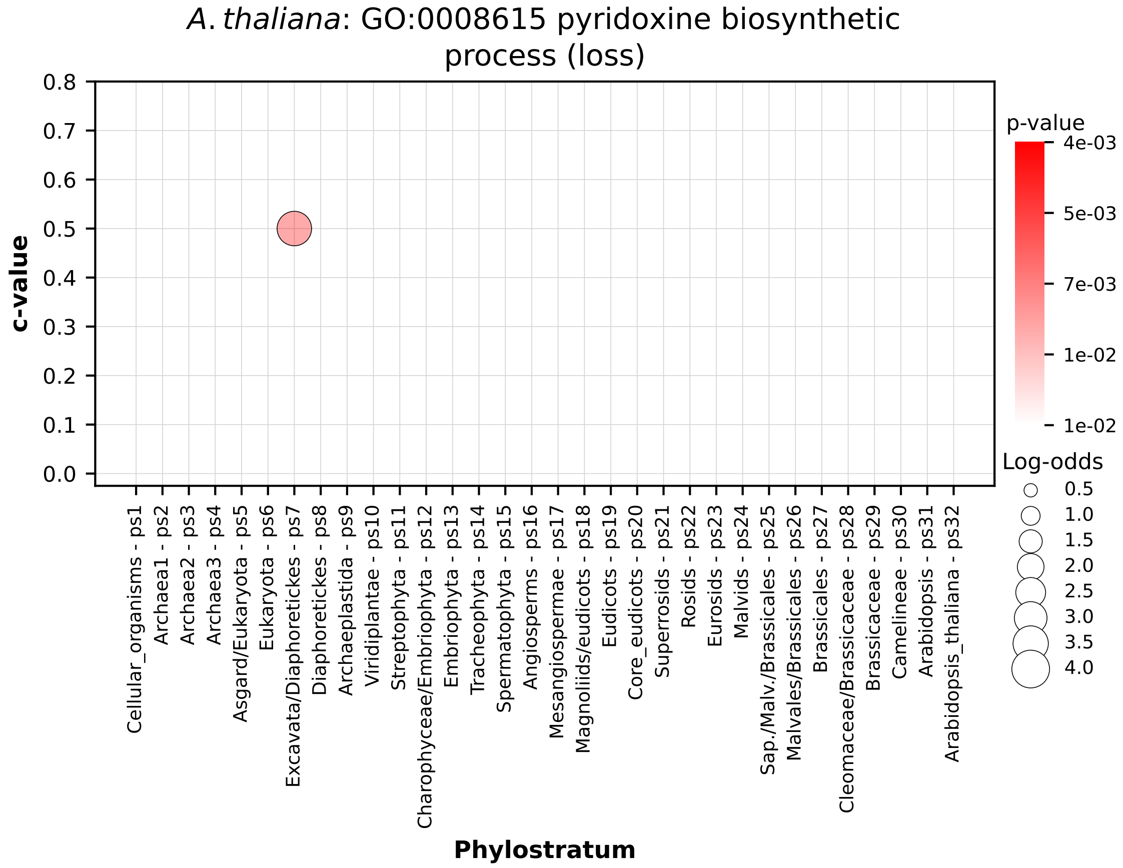

A. thaliana: GO:0008615 pyridoxine biosynthetic process (loss)
c-value
0.8
0.7
0.6
0.5
0.4
0.3
0.2
0.1
0.0
Phylostratum
Cellular_organisms - ps1
Archaea1 - ps2
Archaea2 - ps3
Archaea3 - ps4
Asgard/Eukaryota - ps5
Eukaryota - ps6
Excavata/Diaphoretickes - ps7
Diaphoretickes - ps8
Archaeplastida - ps9
Viridiplantae - ps10
Streptophyta - ps11
Charophyceae/Embriophyta - ps12
Embriophyta - ps13
Tracheophyta - ps14
Spermatophyta - ps15
Angiosperms - ps16
Mesangiospermae - ps17
Magnoliids/eudicots - ps18
Eudicots - ps19
Core_eudicots - ps20
Superrosids - ps21
Rosids - ps22
Eurosids - ps23
Malvids - ps24
Sap./Malv./Brassicales - ps25
Malvales/Brassicales - ps26
Brassicales - ps27
Cleomaceae/Brassicaceae - ps28
Brassicaceae - ps29
Camelineae - ps30
Arabidopsis - ps31
Arabidopsis_thaliana - ps32
p-value
4e-03
5e-03
7e-03
1e-02
1e-02
Log-odds
0.5
1.0
1.5
2.0
2.5
3.0
3.5
4.0

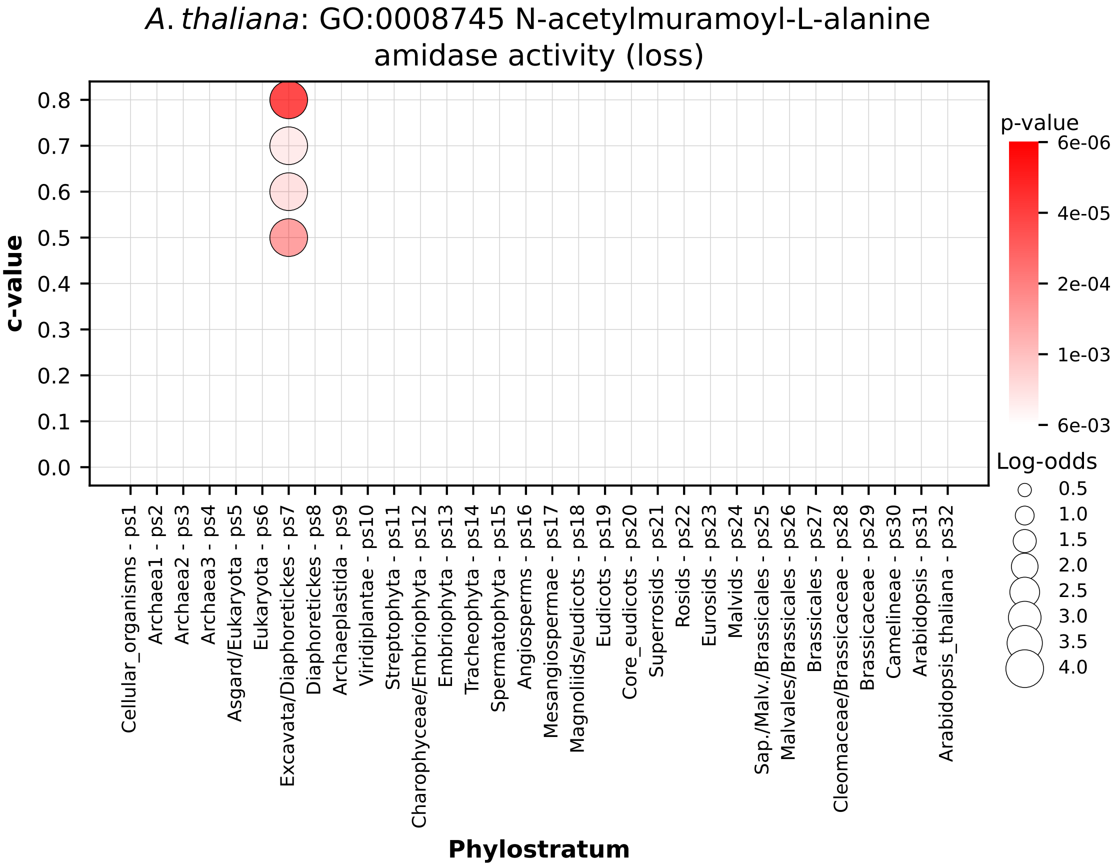

A. thaliana: GO:0008745 N-acetylmuramoyl-L-alanine amidase activity (loss)
c-value
0.8
0.7
0.6
0.5
0.4
0.3
0.2
0.1
0.0
Phylostratum
Cellular_organisms - ps1
Archaea1 - ps2
Archaea2 - ps3
Archaea3 - ps4
Asgard/Eukaryota - ps5
Eukaryota - ps6
Excavata/Diaphoretickes - ps7
Diaphoretickes - ps8
Archaeplastida - ps9
Viridiplantae - ps10
Streptophyta - ps11
Charophyceae/Embriophyta - ps12
Embriophyta - ps13
Tracheophyta - ps14
Spermatophyta - ps15
Angiosperms - ps16
Mesangiospermae - ps17
Magnoliids/eudicots - ps18
Eudicots - ps19
Core_eudicots - ps20
Superrosids - ps21
Rosids - ps22
Eurosids - ps23
Malvids - ps24
Sap./Malv./Brassicales - ps25
Malvales/Brassicales - ps26
Brassicales - ps27
Cleomaceae/Brassicaceae - ps28
Brassicaceae - ps29
Camelineae - ps30
Arabidopsis - ps31
Arabidopsis_thaliana - ps32
p-value
6e-06
4e-05
2e-04
1e-03
6e-03
Log-odds
0.5
1.0
1.5
2.0
2.5
3.0
3.5
4.0

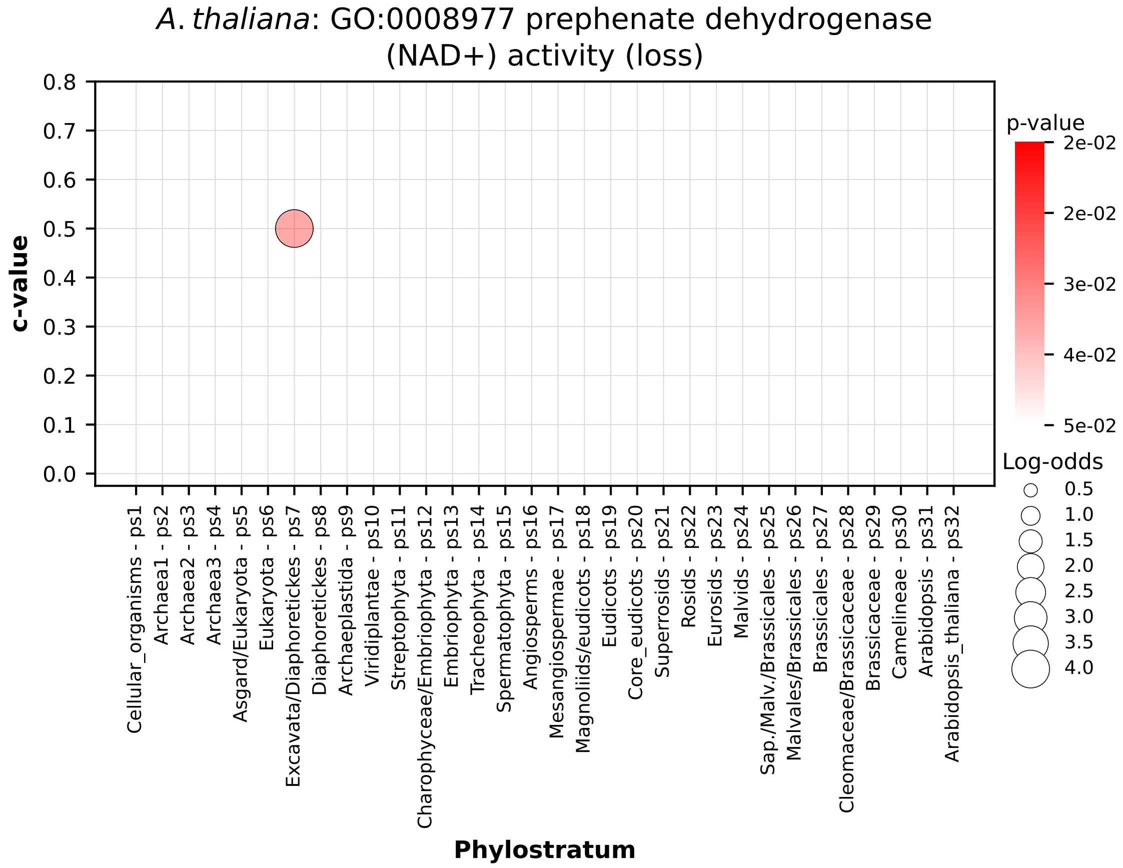
A. thaliana: GO:0008977 prephenate dehydrogenase (NAD+) activity (loss)
c-value
0.8
0.7
0.6
0.5
0.4
0.3
0.2
0.1
0.0
p-value
2e-02
2e-02
3e-02
4e-02
5e-02
Log-odds
0.5
1.0
1.5
2.0
2.5
3.0
3.5
4.0
Cellular_organisms - ps1
Archaea1 - ps2
Archaea2 - ps3
Archaea3 - ps4
Asgard/Eukaryota - ps5
Eukaryota - ps6
Excavata/Diaphoretickes - ps7
Diaphoretickes - ps8
Archaeplastida - ps9
Viridiplantae - ps10
Streptophyta - ps11
Charophyceae/Embriophyta - ps12
Embriophyta - ps13
Tracheophyta - ps14
Spermatophyta - ps15
Angiosperms - ps16
Mesangiospermae - ps17
Magnoliids/eudicots - ps18
Eudicots - ps19
Core_eudicots - ps20
Superrosids - ps21
Rosids - ps22
Eurosids - ps23
Malvids - ps24
Sap./Malv./Brassicales - ps25
Malvales/Brassicales - ps26
Brassicales - ps27
Cleomaceae/Brassicaceae - ps28
Brassicaceae - ps29
Camelineae - ps30
Arabidopsis - ps31
Arabidopsis_thaliana - ps32
Phylostratum

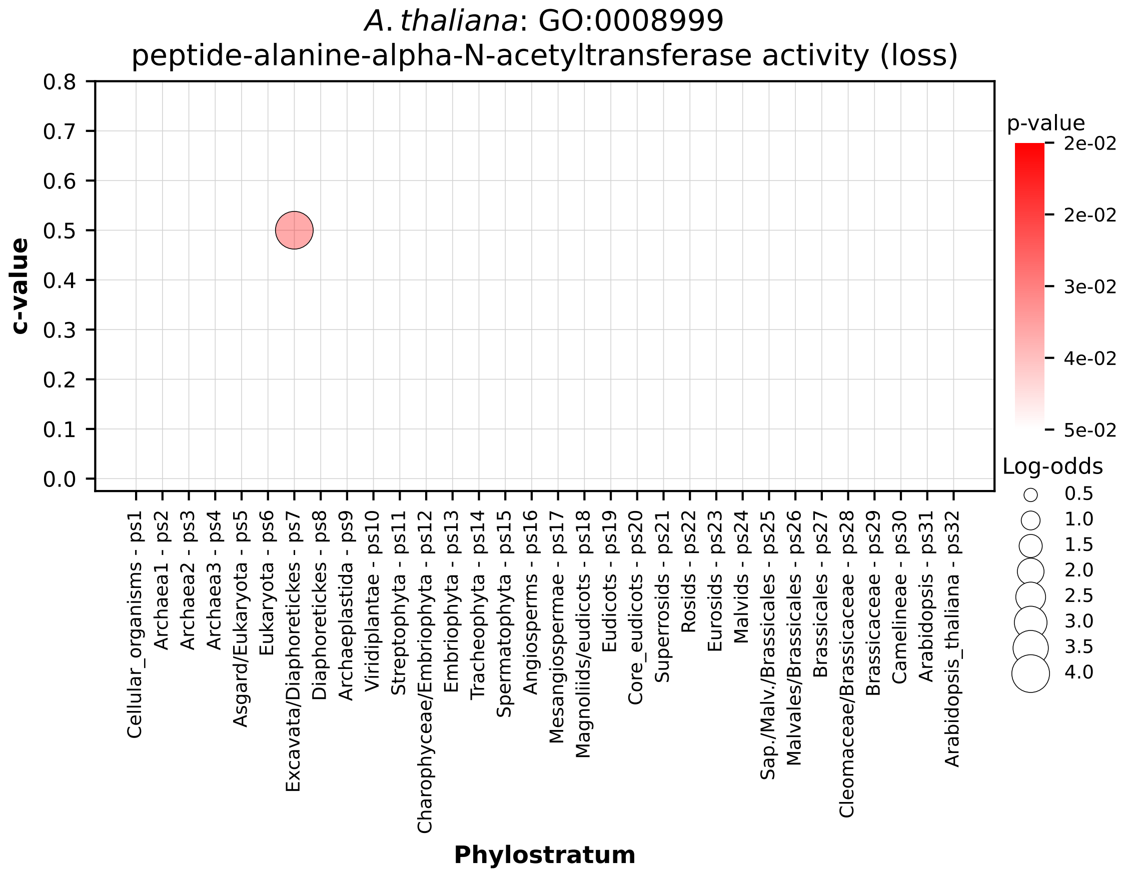

A. thaliana: GO:0008999
peptide-alanine-alpha-N-acetyltransferase activity (loss)
c-value
0.8
0.7
0.6
0.5
0.4
0.3
0.2
0.1
0.0
p-value
2e-02
2e-02
3e-02
4e-02
5e-02
Log-odds
0.5
1.0
1.5
2.0
2.5
3.0
3.5
4.0
Cellular_organisms - ps1
Archaea1 - ps2
Archaea2 - ps3
Archaea3 - ps4
Asgard/Eukaryota - ps5
Eukaryota - ps6
Excavata/Diaphoretickes - ps7
Diaphoretickes - ps8
Archaeplastida - ps9
Viridiplantae - ps10
Streptophyta - ps11
Charophyceae/Embriophyta - ps12
Embriophyta - ps13
Tracheophyta - ps14
Spermatophyta - ps15
Angiosperms - ps16
Mesangiospermae - ps17
Magnoliids/eudicots - ps18
Eudicots - ps19
Core_eudicots - ps20
Superrosids - ps21
Rosids - ps22
Eurosids - ps23
Malvids - ps24
Sap./Malv./Brassicales - ps25
Malvales/Brassicales - ps26
Brassicales - ps27
Cleomaceae/Brassicaceae - ps28
Brassicaceae - ps29
Camelineae - ps30
Arabidopsis - ps31
Arabidopsis_thaliana - ps32
Phylostratum

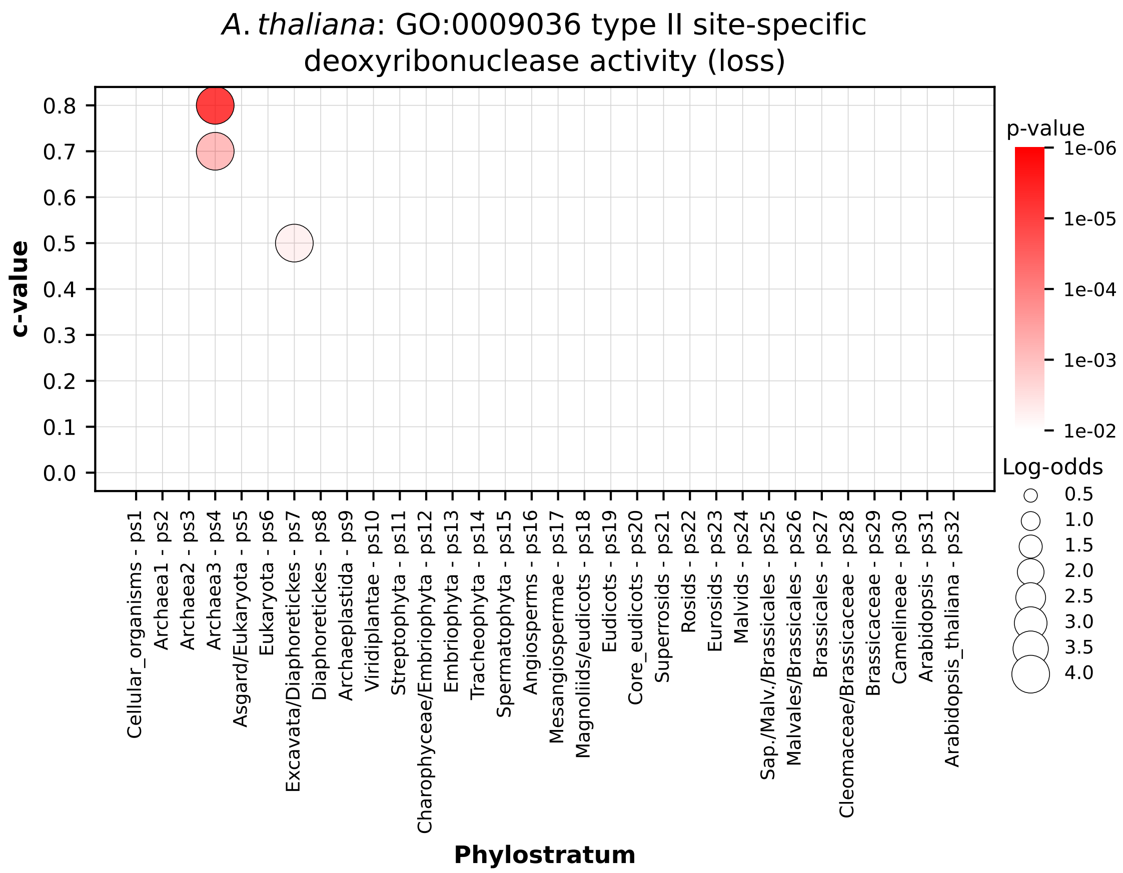
A. thaliana: GO:0009036 type II site-specific deoxyribonuclease activity (loss)
c-value
0.8
0.7
0.6
0.5
0.4
0.3
0.2
0.1
0.0
p-value
1e-06
1e-05
1e-04
1e-03
1e-02
Log-odds
0.5
1.0
1.5
2.0
2.5
3.0
3.5
4.0
Cellular_organisms - ps1
Archaea1 - ps2
Archaea2 - ps3
Archaea3 - ps4
Asgard/Eukaryota - ps5
Eukaryota - ps6
Excavata/Diaphoretickes - ps7
Diaphoretickes - ps8
Archaeplastida - ps9
Viridiplantae - ps10
Streptophyta - ps11
Charophyceae/Embriophyta - ps12
Embriophyta - ps13
Tracheophyta - ps14
Spermatophyta - ps15
Angiosperms - ps16
Mesangiospermae - ps17
Magnoliids/eudicots - ps18
Eudicots - ps19
Core_eudicots - ps20
Superrosids - ps21
Rosids - ps22
Eurosids - ps23
Malvids - ps24
Sap./Malv./Brassicales - ps25
Malvales/Brassicales - ps26
Brassicales - ps27
Cleomaceae/Brassicaceae - ps28
Brassicaceae - ps29
Camelineae - ps30
Arabidopsis - ps31
Arabidopsis_thaliana - ps32
Phylostratum

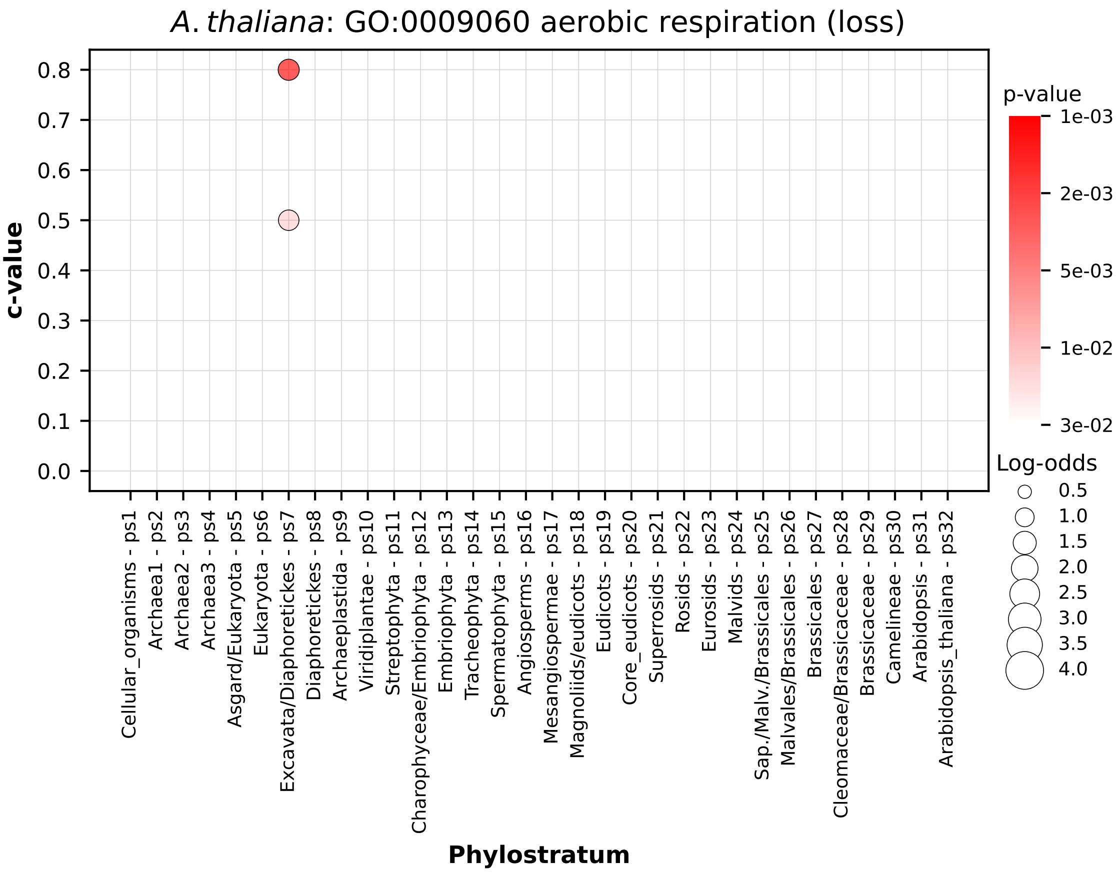

A. thaliana: GO:0009060 aerobic respiration (loss)
c-value
0.8
0.7
0.6
0.5
0.4
0.3
0.2
0.1
0.0
Cellular_organisms - ps1
Archaea1 - ps2
Archaea2 - ps3
Archaea3 - ps4
Asgard/Eukaryota - ps5
Eukaryota - ps6
Excavata/Diaphoretickes - ps7
Diaphoretickes - ps8
Archaeplastida - ps9
Viridiplantae - ps10
Streptophyta - ps11
Charophyceae/Embriophyta - ps12
Embriophyta - ps13
Tracheophyta - ps14
Spermatophyta - ps15
Angiosperms - ps16
Mesangiospermae - ps17
Magnoliids/eudicots - ps18
Eudicots - ps19
Core_eudicots - ps20
Superrosids - ps21
Rosids - ps22
Eurosids - ps23
Malvids - ps24
Sap./Malv./Brassicales - ps25
Malvales/Brassicales - ps26
Brassicales - ps27
Cleomaceae/Brassicaceae - ps28
Brassicaceae - ps29
Camelineae - ps30
Arabidopsis - ps31
Arabidopsis_thaliana - ps32
Phylostratum
p-value
1e-03
2e-03
5e-03
1e-02
3e-02
Log-odds
0.5
1.0
1.5
2.0
2.5
3.0
3.5
4.0

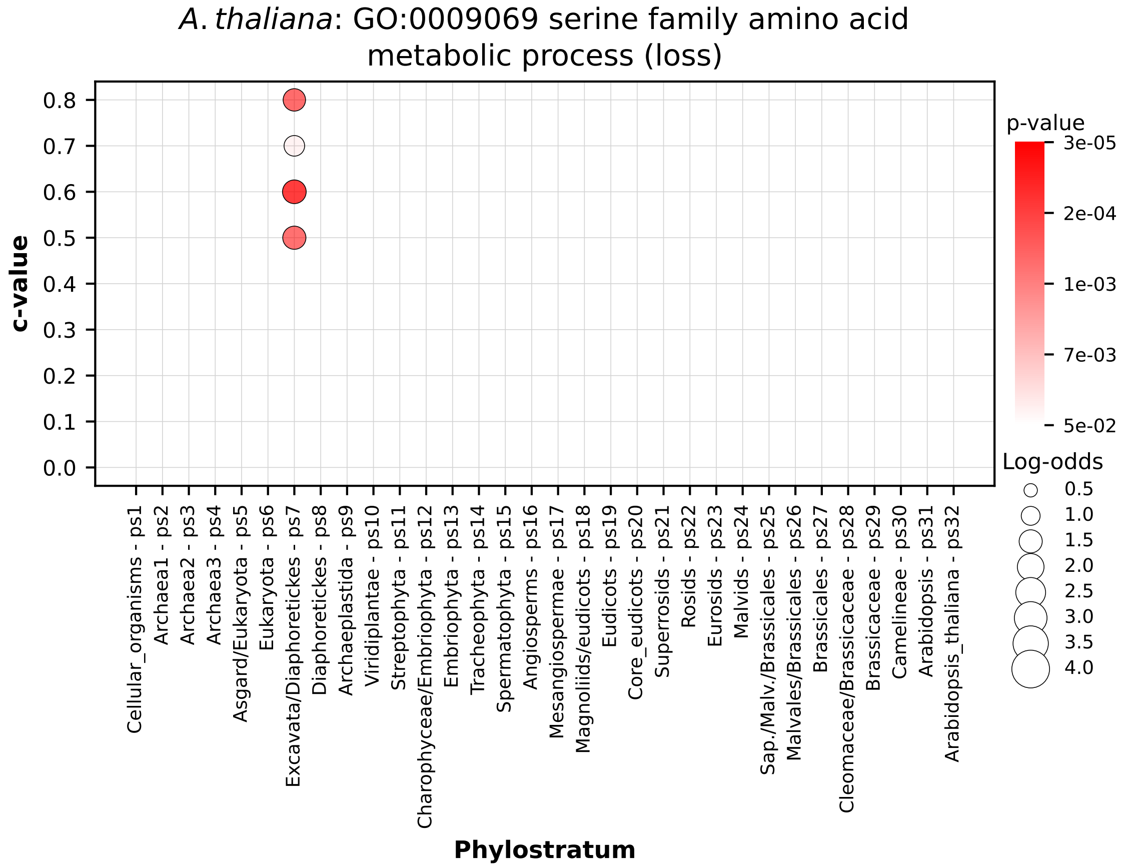

A. thaliana: GO:0009069 serine family amino acid metabolic process (loss)
c-value
0.0
0.1
0.2
0.3
0.4
0.5
0.6
0.7
0.8
Phylostratum
Cellular_organisms - ps1
Archaea1 - ps2
Archaea2 - ps3
Archaea3 - ps4
Asgard/Eukaryota - ps5
Eukaryota - ps6
Excavata/Diaphoretickes - ps7
Diaphoretickes - ps8
Archaeplastida - ps9
Viridiplantae - ps10
Streptophyta - ps11
Charophyceae/Embriophyta - ps12
Embriophyta - ps13
Tracheophyta - ps14
Spermatophyta - ps15
Angiosperms - ps16
Mesangiospermae - ps17
Magnoliids/eudicots - ps18
Eudicots - ps19
Core_eudicots - ps20
Superrosids - ps21
Rosids - ps22
Eurosids - ps23
Malvids - ps24
Sap./Malv./Brassicales - ps25
Malvales/Brassicales - ps26
Brassicales - ps27
Cleomaceae/Brassicaceae - ps28
Brassicaceae - ps29
Camelineae - ps30
Arabidopsis - ps31
Arabidopsis_thaliana - ps32
p-value
3e-05
2e-04
1e-03
7e-03
5e-02
Log-odds
0.5
1.0
1.5
2.0
2.5
3.0
3.5
4.0

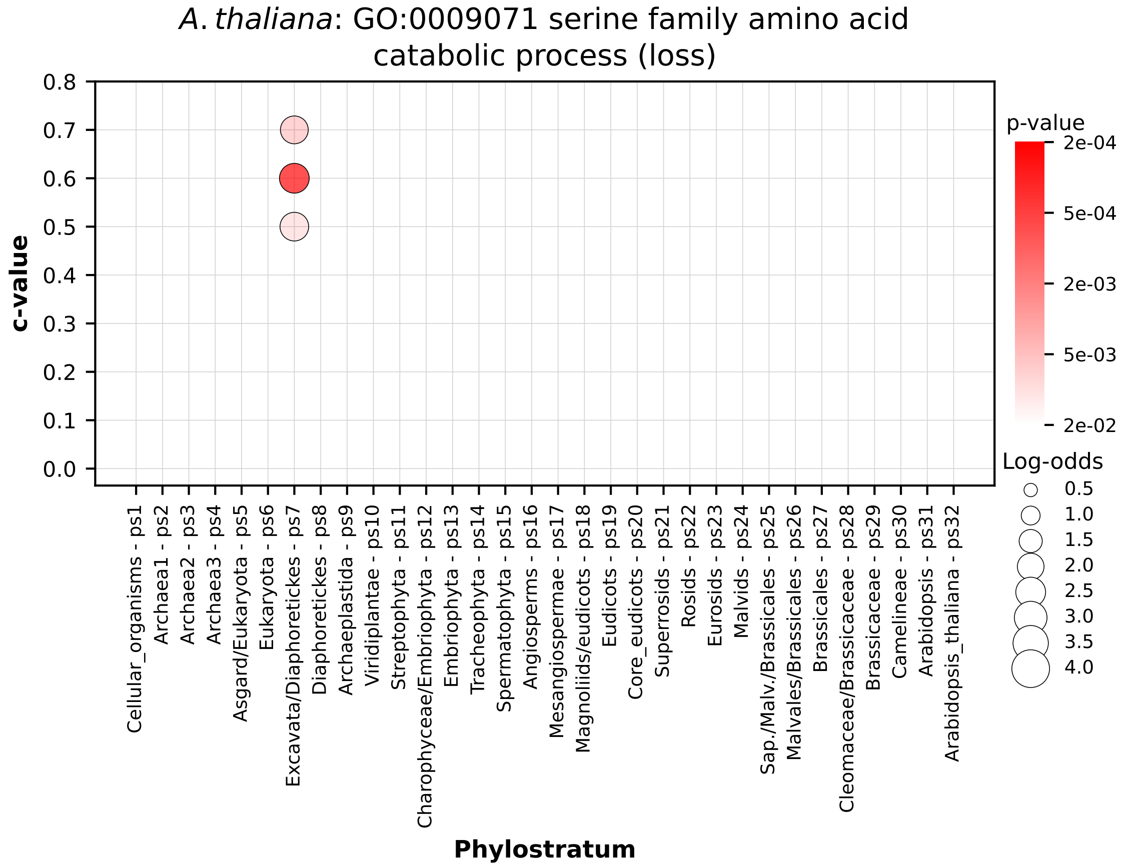

A. thaliana: GO:0009071 serine family amino acid catabolic process (loss)
c-value
0.8
0.7
0.6
0.5
0.4
0.3
0.2
0.1
0.0
p-value
2e-04
5e-04
2e-03
5e-03
2e-02
Log-odds
0.5
1.0
1.5
2.0
2.5
3.0
3.5
4.0
Cellular_organisms - ps1
Archaea1 - ps2
Archaea2 - ps3
Archaea3 - ps4
Asgard/Eukaryota - ps5
Eukaryota - ps6
Excavata/Diaphoretickes - ps7
Diaphoretickes - ps8
Archaeplastida - ps9
Viridiplantae - ps10
Streptophyta - ps11
Charophyceae/Embriophyta - ps12
Embriophyta - ps13
Tracheophyta - ps14
Spermatophyta - ps15
Angiosperms - ps16
Mesangiospermae - ps17
Magnoliids/eudicots - ps18
Eudicots - ps19
Core_eudicots - ps20
Superrosids - ps21
Rosids - ps22
Eurosids - ps23
Malvids - ps24
Sap./Malv./Brassicales - ps25
Malvales/Brassicales - ps26
Brassicales - ps27
Cleomaceae/Brassicaceae - ps28
Brassicaceae - ps29
Camelineae - ps30
Arabidopsis - ps31
Arabidopsis_thaliana - ps32
Phylostratum

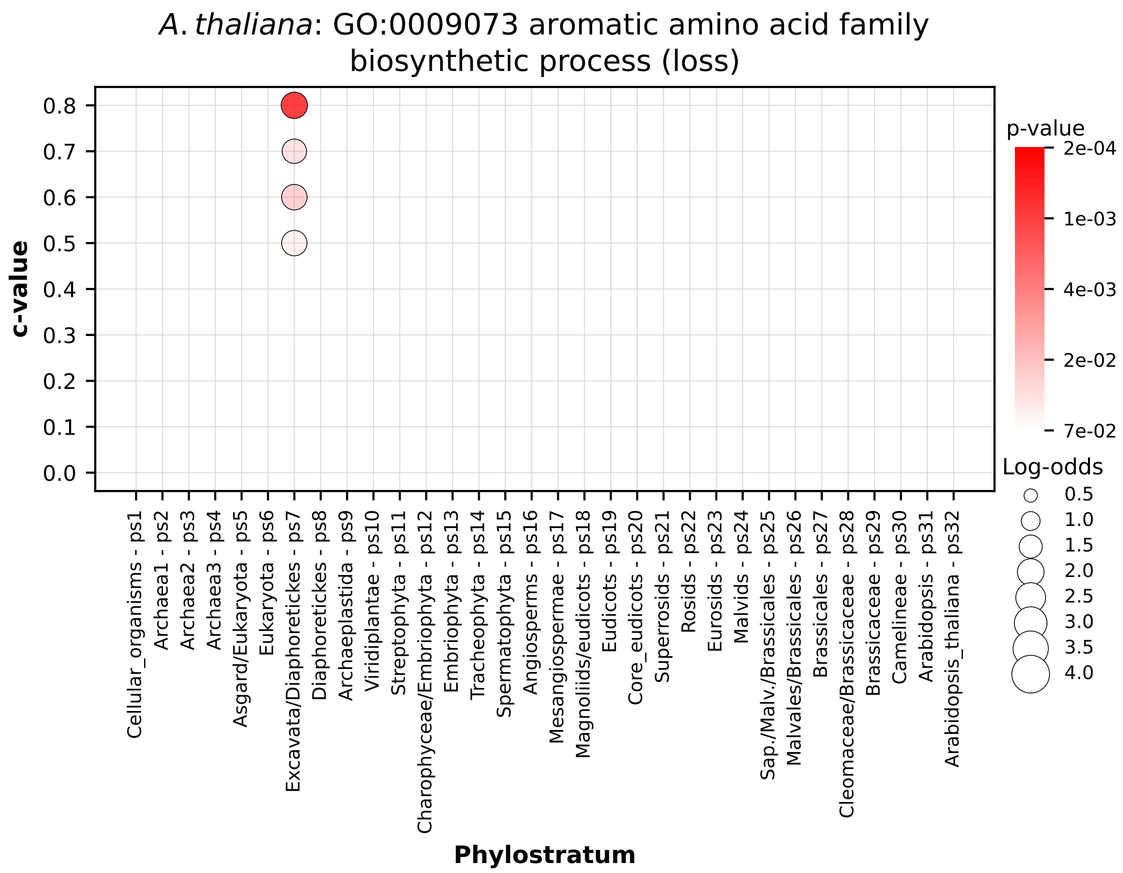

A. thaliana: GO:0009073 aromatic amino acid family biosynthetic process (loss)
c-value
0.8
0.7
0.6
0.5
0.4
0.3
0.2
0.1
0.0
p-value
2e-04
1e-03
4e-03
2e-02
7e-02
Log-odds
0.5
1.0
1.5
2.0
2.5
3.0
3.5
4.0
Cellular_organisms - ps1
Archaea1 - ps2
Archaea2 - ps3
Archaea3 - ps4
Asgard/Eukaryota - ps5
Eukaryota - ps6
Excavata/Diaphoretickes - ps7
Diaphoretickes - ps8
Archaeplastida - ps9
Viridiplantae - ps10
Streptophyta - ps11
Charophyceae/Embriophyta - ps12
Embriophyta - ps13
Tracheophyta - ps14
Spermatophyta - ps15
Angiosperms - ps16
Mesangiospermae - ps17
Magnoliids/eudicots - ps18
Eudicots - ps19
Core_eudicots - ps20
Superrosids - ps21
Rosids - ps22
Eurosids - ps23
Malvids - ps24
Sap./Malv./Brassicales - ps25
Malvales/Brassicales - ps26
Brassicales - ps27
Cleomaceae/Brassicaceae - ps28
Brassicaceae - ps29
Camelineae - ps30
Arabidopsis - ps31
Arabidopsis_thaliana - ps32
Phylostratum

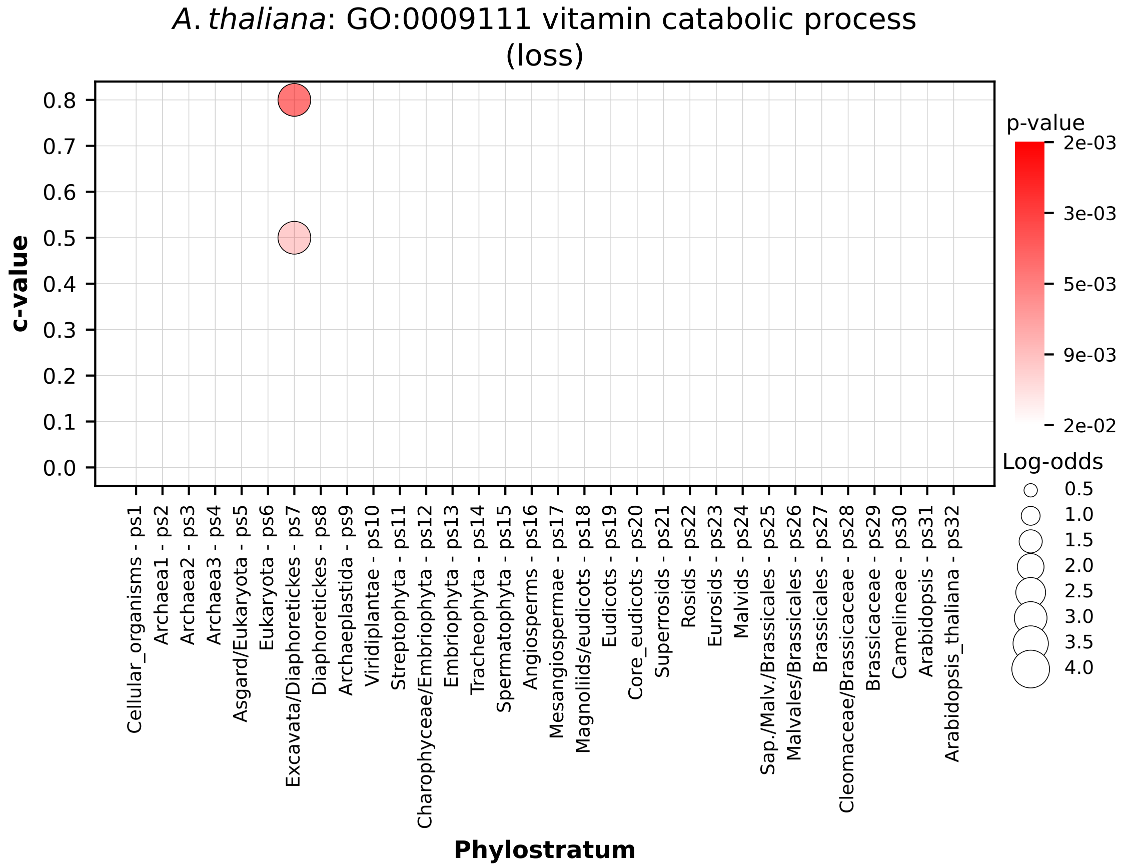

A. thaliana: GO:0009111 vitamin catabolic process
(loss)
c-value
0.8
0.7
0.6
0.5
0.4
0.3
0.2
0.1
0.0
Phylostratum
Cellular_organisms - ps1
Archaea1 - ps2
Archaea2 - ps3
Archaea3 - ps4
Asgard/Eukaryota - ps5
Eukaryota - ps6
Excavata/Diaphoretickes - ps7
Diaphoretickes - ps8
Archaeplastida - ps9
Viridiplantae - ps10
Streptophyta - ps11
Charophyceae/Embriophyta - ps12
Embriophyta - ps13
Tracheophyta - ps14
Spermatophyta - ps15
Angiosperms - ps16
Mesangiospermae - ps17
Magnoliids/eudicots - ps18
Eudicots - ps19
Core_eudicots - ps20
Superrosids - ps21
Rosids - ps22
Eurosids - ps23
Malvids - ps24
Sap./Malv./Brassicales - ps25
Malvales/Brassicales - ps26
Brassicales - ps27
Cleomaceae/Brassicaceae - ps28
Brassicaceae - ps29
Camelineae - ps30
Arabidopsis - ps31
Arabidopsis_thaliana - ps32
p-value
2e-03
3e-03
5e-03
9e-03
2e-02
Log-odds
0.5
1.0
1.5
2.0
2.5
3.0
3.5
4.0

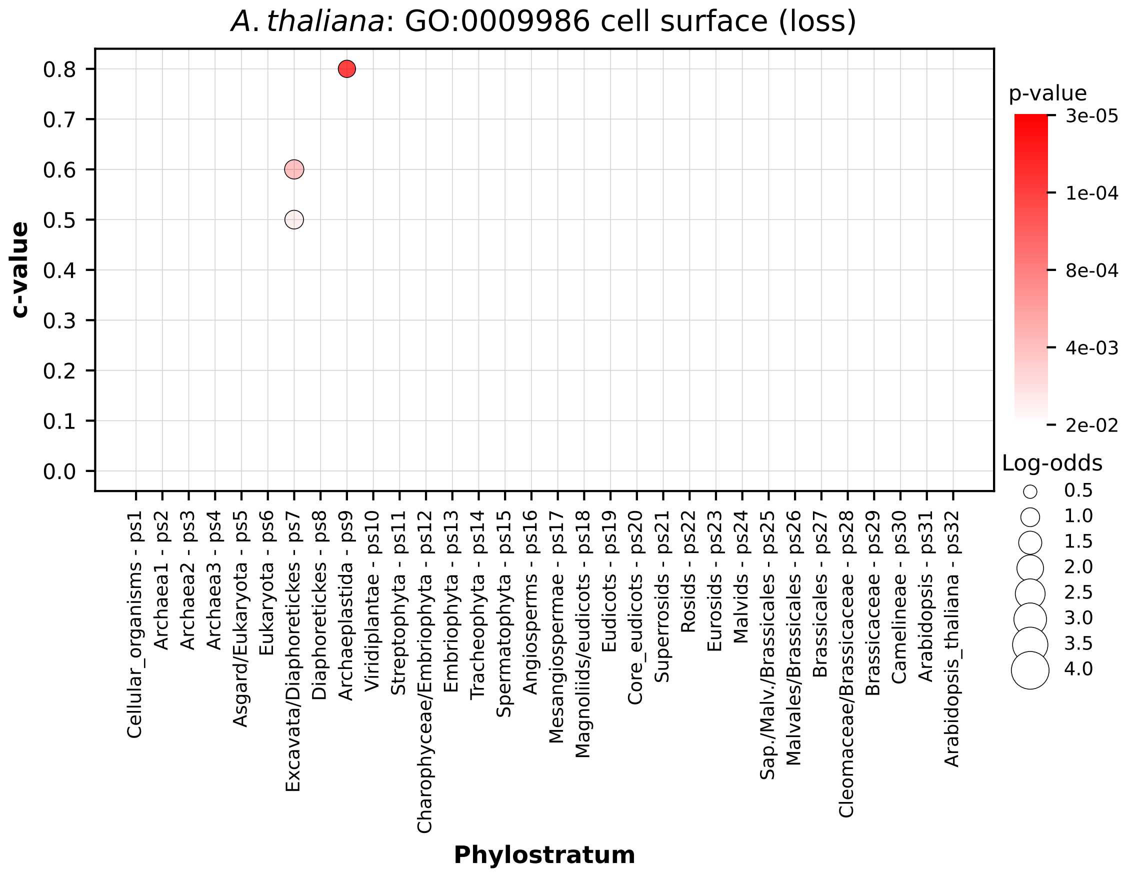

A. thaliana: GO:0009986 cell surface (loss)
c-value
0.8
0.7
0.6
0.5
0.4
0.3
0.2
0.1
0.0
p-value
3e-05
1e-04
8e-04
4e-03
2e-02
Log-odds
0.5
1.0
1.5
2.0
2.5
3.0
3.5
4.0
Cellular_organisms - ps1
Archaea1 - ps2
Archaea2 - ps3
Archaea3 - ps4
Asgard/Eukaryota - ps5
Eukaryota - ps6
Excavata/Diaphoretickes - ps7
Diaphoretickes - ps8
Archaeplastida - ps9
Viridiplantae - ps10
Streptophyta - ps11
Charophyceae/Embriophyta - ps12
Embriophyta - ps13
Tracheophyta - ps14
Spermatophyta - ps15
Angiosperms - ps16
Mesangiospermae - ps17
Magnoliids/eudicots - ps18
Eudicots - ps19
Core_eudicots - ps20
Superrosids - ps21
Rosids - ps22
Eurosids - ps23
Malvids - ps24
Sap./Malv./Brassicales - ps25
Malvales/Brassicales - ps26
Brassicales - ps27
Cleomaceae/Brassicaceae - ps28
Brassicaceae - ps29
Camelineae - ps30
Arabidopsis - ps31
Arabidopsis_thaliana - ps32
Phylostratum

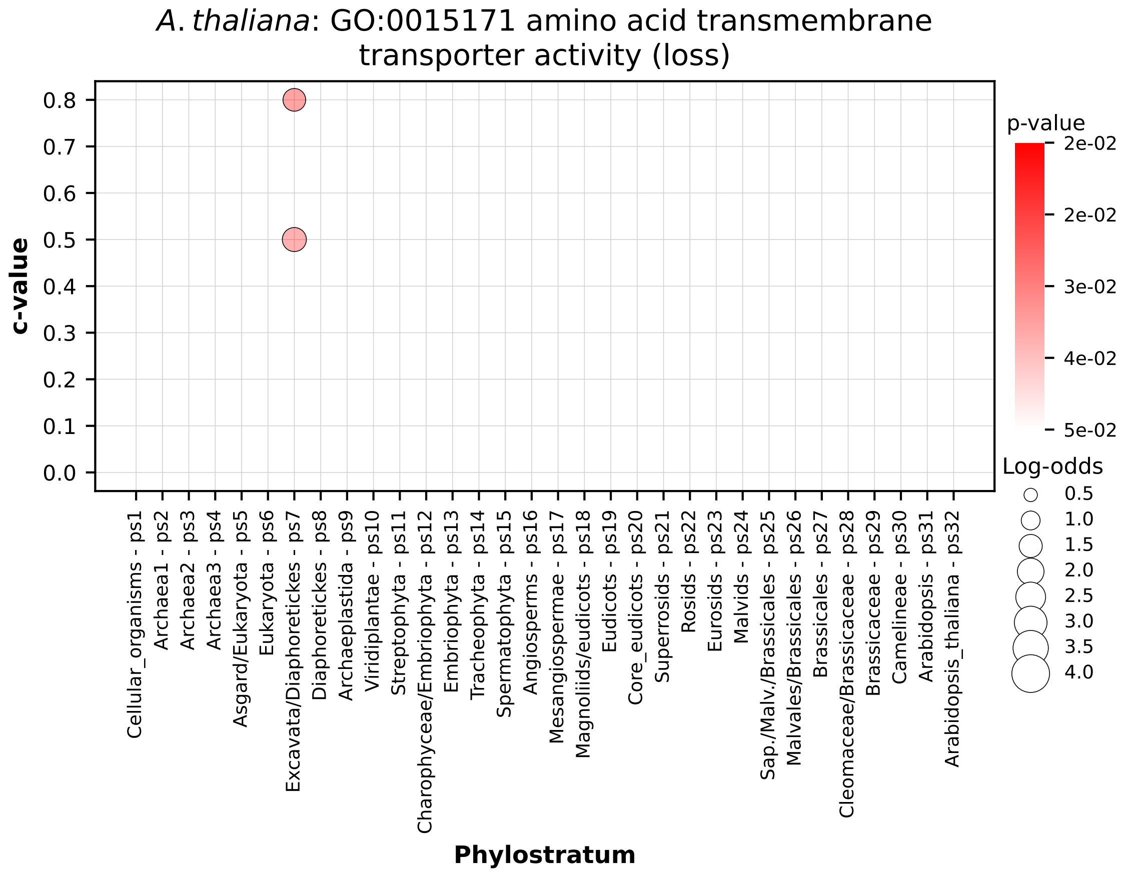

A. thaliana: GO:0015171 amino acid transmembrane transporter activity (loss)
c-value
0.8
0.7
0.6
0.5
0.4
0.3
0.2
0.1
0.0
Phylostratum
Cellular_organisms - ps1
Archaea1 - ps2
Archaea2 - ps3
Archaea3 - ps4
Asgard/Eukaryota - ps5
Eukaryota - ps6
Excavata/Diaphoretickes - ps7
Diaphoretickes - ps8
Archaeplastida - ps9
Viridiplantae - ps10
Streptophyta - ps11
Charophyceae/Embriophyta - ps12
Embriophyta - ps13
Tracheophyta - ps14
Spermatophyta - ps15
Angiosperms - ps16
Mesangiospermae - ps17
Magnoliids/eudicots - ps18
Eudicots - ps19
Core_eudicots - ps20
Superrosids - ps21
Rosids - ps22
Eurosids - ps23
Malvids - ps24
Sap./Malv./Brassicales - ps25
Malvales/Brassicales - ps26
Brassicales - ps27
Cleomaceae/Brassicaceae - ps28
Brassicaceae - ps29
Camelineae - ps30
Arabidopsis - ps31
Arabidopsis_thaliana - ps32
p-value
2e-02
2e-02
3e-02
4e-02
5e-02
Log-odds
0.5
1.0
1.5
2.0
2.5
3.0
3.5
4.0

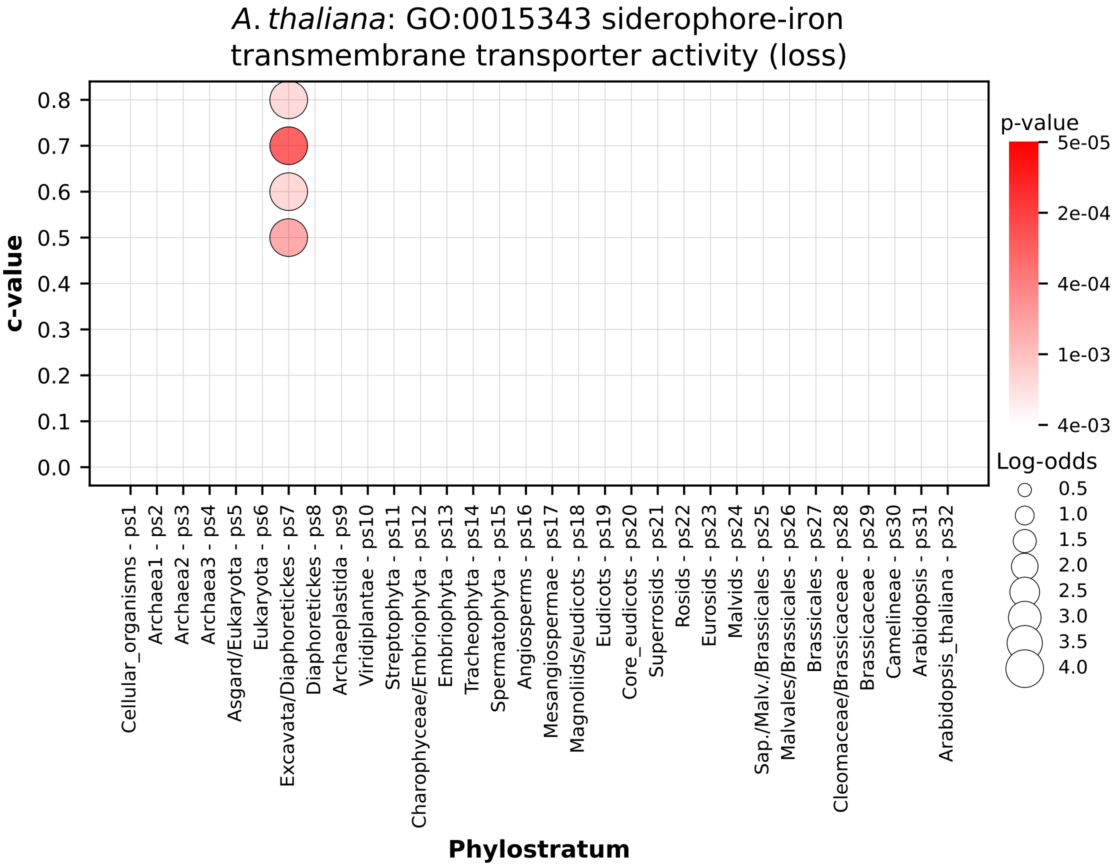

A. thaliana: GO:0015343 siderophore-iron transmembrane transporter activity (loss)
c-value
0.8
0.7
0.6
0.5
0.4
0.3
0.2
0.1
0.0
p-value
5e-05
2e-04
4e-04
1e-03
4e-03
Log-odds
0.5
1.0
1.5
2.0
2.5
3.0
3.5
4.0
Cellular_organisms - ps1
Archaea1 - ps2
Archaea2 - ps3
Archaea3 - ps4
Asgard/Eukaryota - ps5
Eukaryota - ps6
Excavata/Diaphoretickes - ps7
Diaphoretickes - ps8
Archaeplastida - ps9
Viridiplantae - ps10
Streptophyta - ps11
Charophyceae/Embriophyta - ps12
Embriophyta - ps13
Tracheophyta - ps14
Spermatophyta - ps15
Angiosperms - ps16
Mesangiospermae - ps17
Magnoliids/eudicots - ps18
Eudicots - ps19
Core_eudicots - ps20
Superrosids - ps21
Rosids - ps22
Eurosids - ps23
Malvids - ps24
Sap./Malv./Brassicales - ps25
Malvales/Brassicales - ps26
Brassicales - ps27
Cleomaceae/Brassicaceae - ps28
Brassicaceae - ps29
Camelineae - ps30
Arabidopsis - ps31
Arabidopsis_thaliana - ps32
Phylostratum

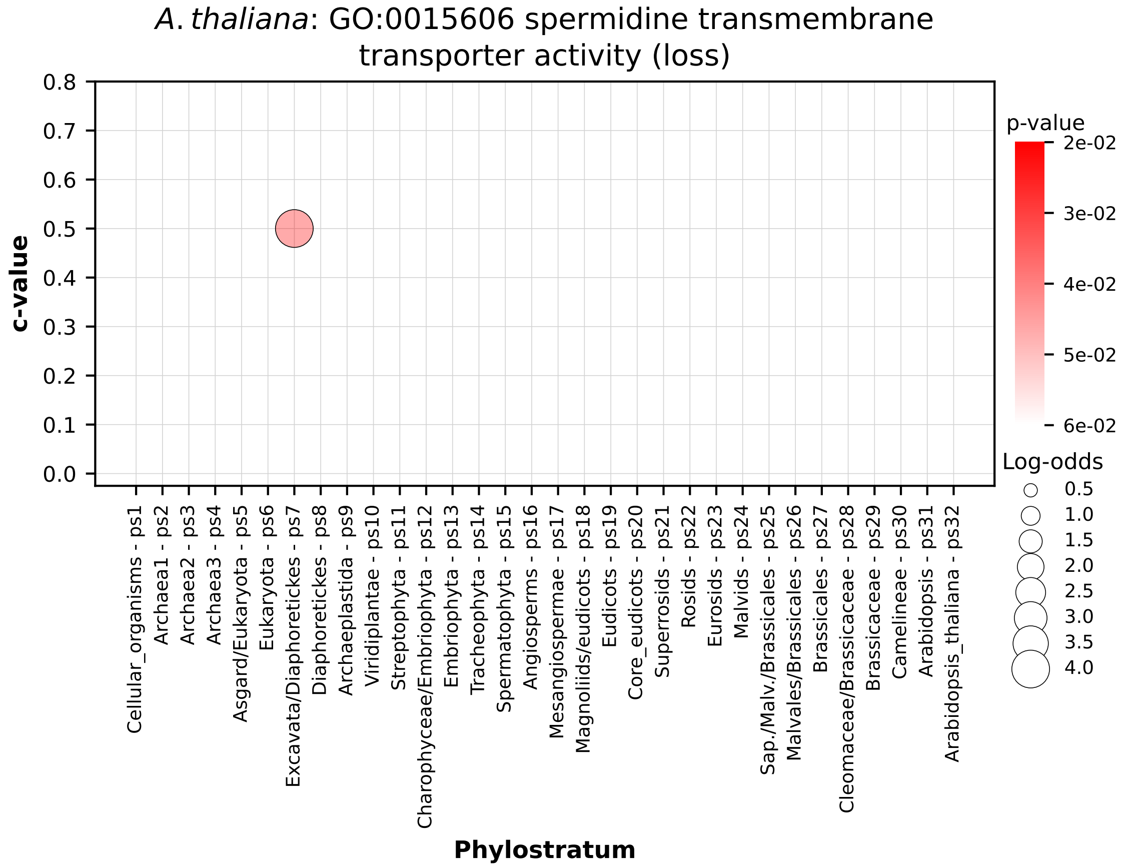
A. thaliana: GO:0015606 spermidine transmembrane transporter activity (loss)
c-value
0.8
0.7
0.6
0.5
0.4
0.3
0.2
0.1
0.0
Phylostratum
Cellular_organisms - ps1
Archaea1 - ps2
Archaea2 - ps3
Archaea3 - ps4
Asgard/Eukaryota - ps5
Eukaryota - ps6
Excavata/Diaphoretickes - ps7
Diaphoretickes - ps8
Archaeplastida - ps9
Viridiplantae - ps10
Streptophyta - ps11
Charophyceae/Embriophyta - ps12
Embriophyta - ps13
Tracheophyta - ps14
Spermatophyta - ps15
Angiosperms - ps16
Mesangiospermae - ps17
Magnoliids/eudicots - ps18
Eudicots - ps19
Core_eudicots - ps20
Superrosids - ps21
Rosids - ps22
Eurosids - ps23
Malvids - ps24
Sap./Malv./Brassicales - ps25
Malvales/Brassicales - ps26
Brassicales - ps27
Cleomaceae/Brassicaceae - ps28
Brassicaceae - ps29
Camelineae - ps30
Arabidopsis - ps31
Arabidopsis_thaliana - ps32
p-value
2e-02
3e-02
4e-02
5e-02
6e-02
Log-odds
0.5
1.0
1.5
2.0
2.5
3.0
3.5
4.0

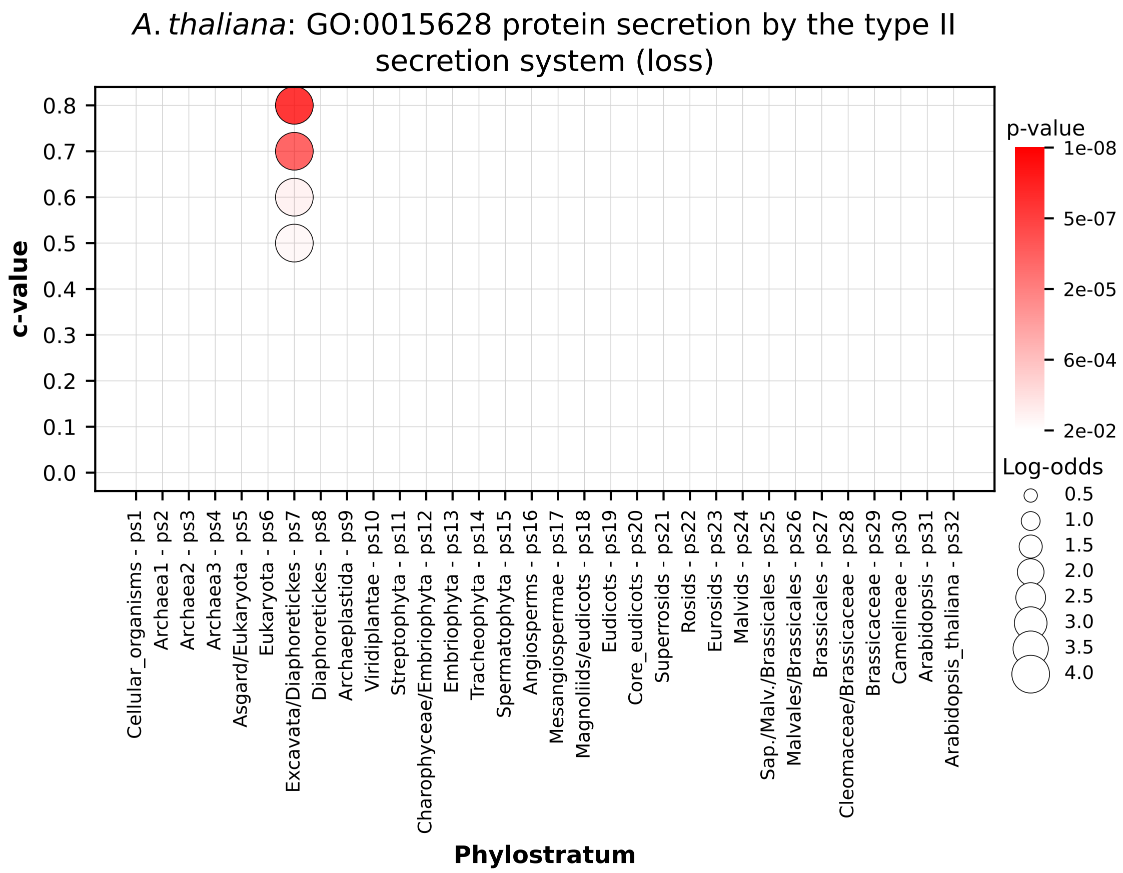
A. thaliana: GO:0015628 protein secretion by the type II secretion system (loss)
c-value
0.8
0.7
0.6
0.5
0.4
0.3
0.2
0.1
0.0
p-value
1e-08
5e-07
2e-05
6e-04
2e-02
Log-odds
0.5
1.0
1.5
2.0
2.5
3.0
3.5
4.0
Cellular_organisms - ps1
Archaea1 - ps2
Archaea2 - ps3
Archaea3 - ps4
Asgard/Eukaryota - ps5
Eukaryota - ps6
Excavata/Diaphoretickes - ps7
Diaphoretickes - ps8
Archaeplastida - ps9
Viridiplantae - ps10
Streptophyta - ps11
Charophyceae/Embriophyta - ps12
Embriophyta - ps13
Tracheophyta - ps14
Spermatophyta - ps15
Angiosperms - ps16
Mesangiospermae - ps17
Magnoliids/eudicots - ps18
Eudicots - ps19
Core_eudicots - ps20
Superrosids - ps21
Rosids - ps22
Eurosids - ps23
Malvids - ps24
Sap./Malv./Brassicales - ps25
Malvales/Brassicales - ps26
Brassicales - ps27
Cleomaceae/Brassicaceae - ps28
Brassicaceae - ps29
Camelineae - ps30
Arabidopsis - ps31
Arabidopsis_thaliana - ps32
Phylostratum

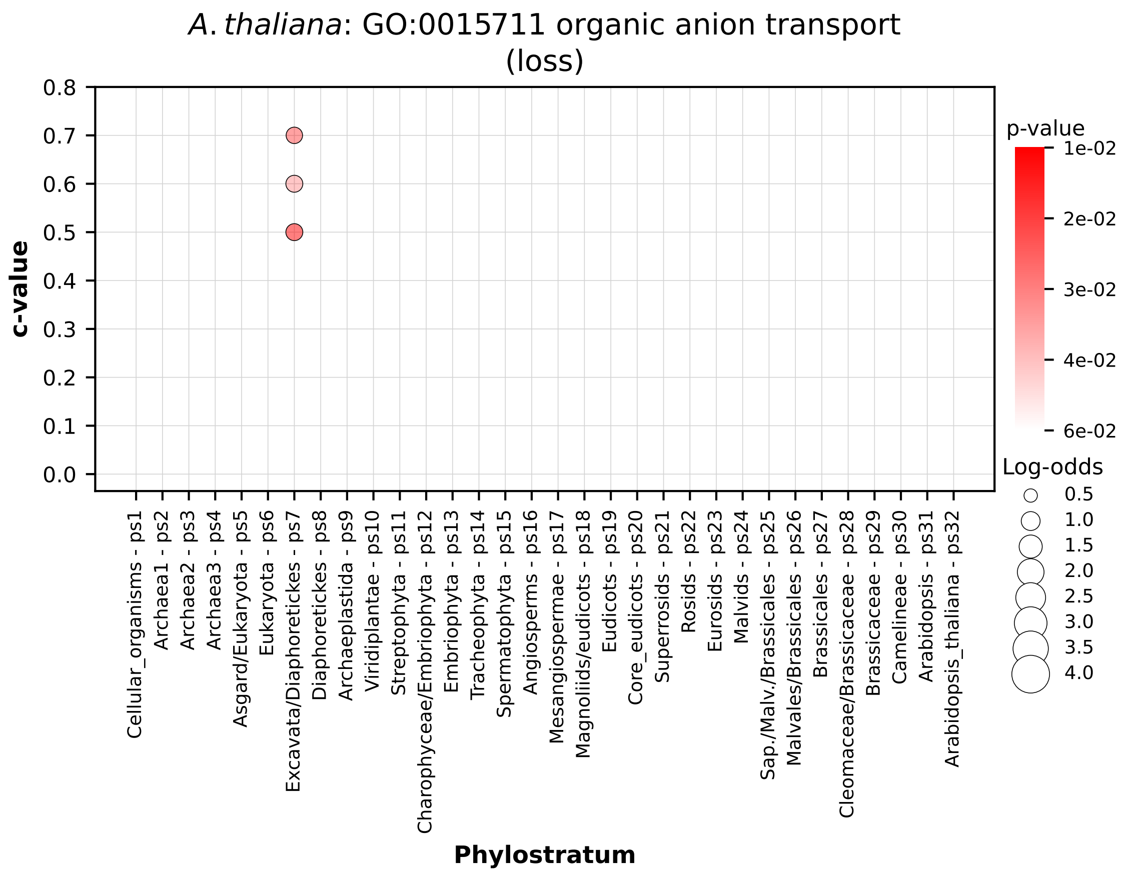
A. thaliana: GO:0015711 organic anion transport
(loss)
c-value
0.8
0.7
0.6
0.5
0.4
0.3
0.2
0.1
0.0
p-value
1e-02
2e-02
3e-02
4e-02
6e-02
Log-odds
0.5
1.0
1.5
2.0
2.5
3.0
3.5
4.0
Cellular_organisms - ps1
Archaea1 - ps2
Archaea2 - ps3
Archaea3 - ps4
Asgard/Eukaryota - ps5
Eukaryota - ps6
Excavata/Diaphoretickes - ps7
Diaphoretickes - ps8
Archaeplastida - ps9
Viridiplantae - ps10
Streptophyta - ps11
Charophyceae/Embriophyta - ps12
Embriophyta - ps13
Tracheophyta - ps14
Spermatophyta - ps15
Angiosperms - ps16
Mesangiospermae - ps17
Magnoliids/eudicots - ps18
Eudicots - ps19
Core_eudicots - ps20
Superrosids - ps21
Rosids - ps22
Eurosids - ps23
Malvids - ps24
Sap./Malv./Brassicales - ps25
Malvales/Brassicales - ps26
Brassicales - ps27
Cleomaceae/Brassicaceae - ps28
Brassicaceae - ps29
Camelineae - ps30
Arabidopsis - ps31
Arabidopsis_thaliana - ps32
Phylostratum

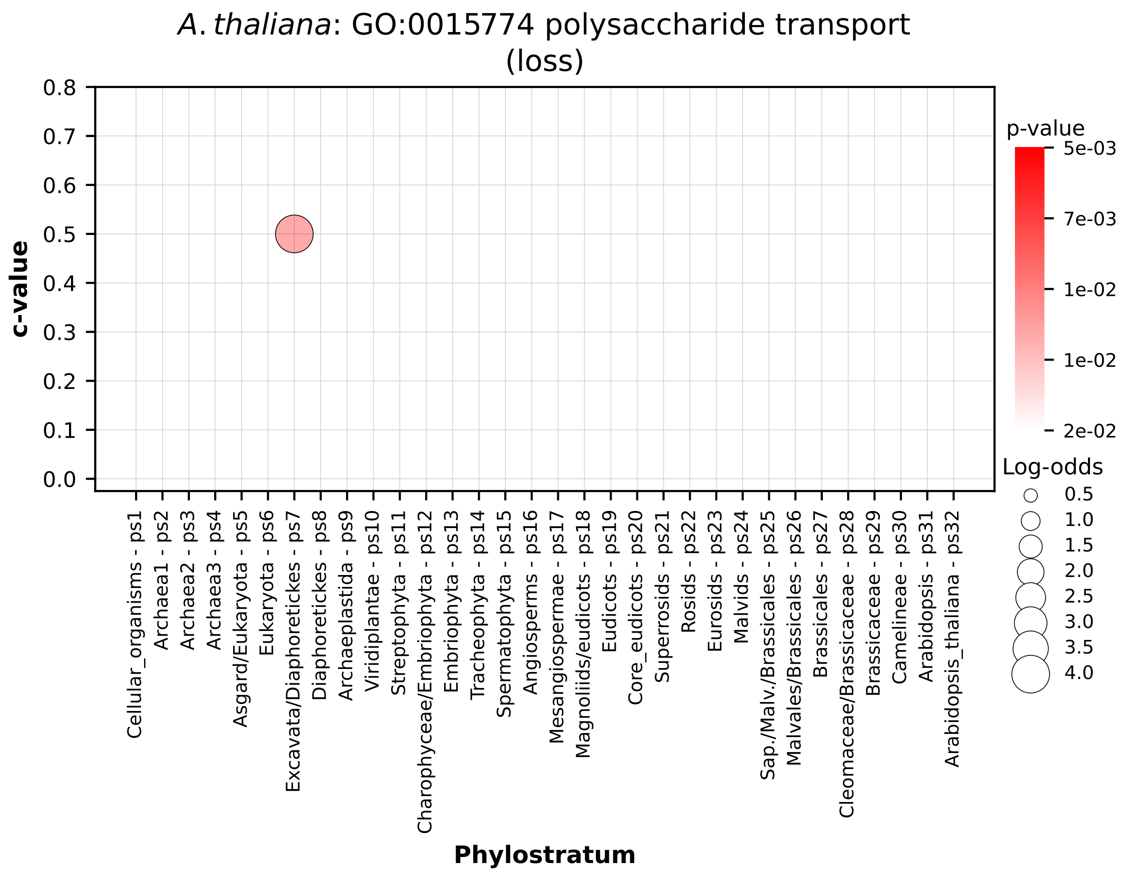

A. thaliana: GO:0015774 polysaccharide transport
(loss)
c-value
0.8
0.7
0.6
0.5
0.4
0.3
0.2
0.1
0.0
p-value
5e-03
7e-03
1e-02
1e-02
2e-02
Log-odds
0.5
1.0
1.5
2.0
2.5
3.0
3.5
4.0
Cellular_organisms - ps1
Archaea1 - ps2
Archaea2 - ps3
Archaea3 - ps4
Asgard/Eukaryota - ps5
Eukaryota - ps6
Excavata/Diaphoretickes - ps7
Diaphoretickes - ps8
Archaeplastida - ps9
Viridiplantae - ps10
Streptophyta - ps11
Charophyceae/Embriophyta - ps12
Embriophyta - ps13
Tracheophyta - ps14
Spermatophyta - ps15
Angiosperms - ps16
Mesangiospermae - ps17
Magnoliids/eudicots - ps18
Eudicots - ps19
Core_eudicots - ps20
Superrosids - ps21
Rosids - ps22
Eurosids - ps23
Malvids - ps24
Sap./Malv./Brassicales - ps25
Malvales/Brassicales - ps26
Brassicales - ps27
Cleomaceae/Brassicaceae - ps28
Brassicaceae - ps29
Camelineae - ps30
Arabidopsis - ps31
Arabidopsis_thaliana - ps32
Phylostratum

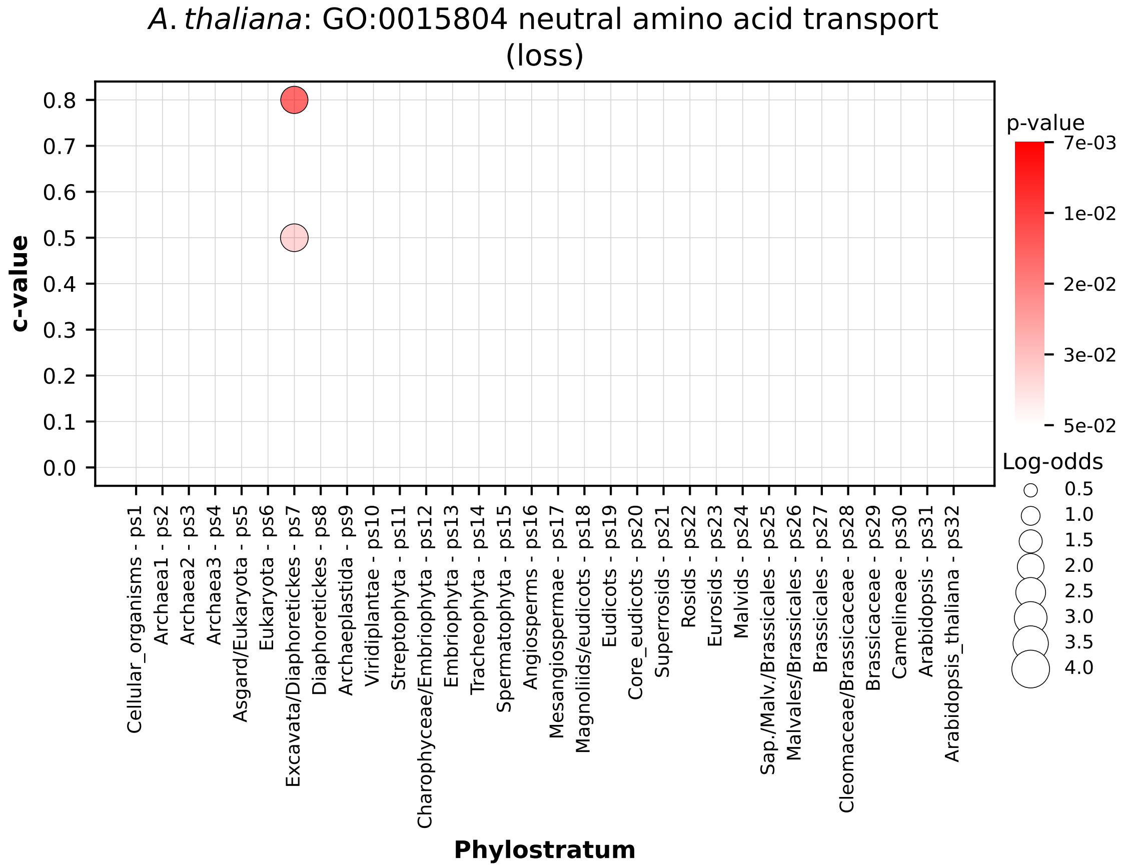

A. thaliana: GO:0015804 neutral amino acid transport
(loss)
c-value
0.8
0.7
0.6
0.5
0.4
0.3
0.2
0.1
0.0
p-value
7e-03
1e-02
2e-02
3e-02
5e-02
Log-odds
0.5
1.0
1.5
2.0
2.5
3.0
3.5
4.0
Cellular_organisms - ps1
Archaea1 - ps2
Archaea2 - ps3
Archaea3 - ps4
Asgard/Eukaryota - ps5
Eukaryota - ps6
Excavata/Diaphoretickes - ps7
Diaphoretickes - ps8
Archaeplastida - ps9
Viridiplantae - ps10
Streptophyta - ps11
Charophyceae/Embriophyta - ps12
Embriophyta - ps13
Tracheophyta - ps14
Spermatophyta - ps15
Angiosperms - ps16
Mesangiospermae - ps17
Magnoliids/eudicots - ps18
Eudicots - ps19
Core_eudicots - ps20
Superrosids - ps21
Rosids - ps22
Eurosids - ps23
Malvids - ps24
Sap./Malv./Brassicales - ps25
Malvales/Brassicales - ps26
Brassicales - ps27
Cleomaceae/Brassicaceae - ps28
Brassicaceae - ps29
Camelineae - ps30
Arabidopsis - ps31
Arabidopsis_thaliana - ps32
Phylostratum

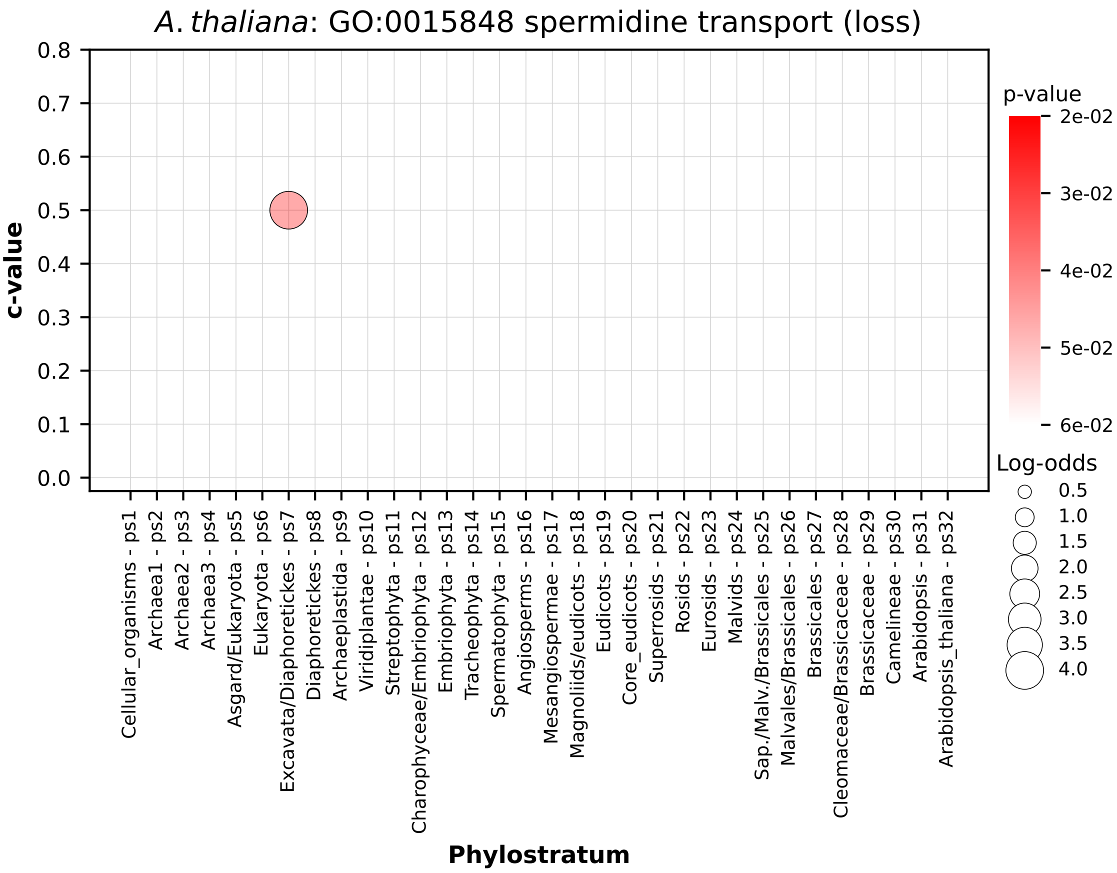
A. thaliana: GO:0015848 spermidine transport (loss)
c-value
0.8
0.7
0.6
0.5
0.4
0.3
0.2
0.1
0.0
Cellular_organisms - ps1
Archaea1 - ps2
Archaea2 - ps3
Archaea3 - ps4
Asgard/Eukaryota - ps5
Eukaryota - ps6
Excavata/Diaphoretickes - ps7
Diaphoretickes - ps8
Archaeplastida - ps9
Viridiplantae - ps10
Streptophyta - ps11
Charophyceae/Embriophyta - ps12
Embriophyta - ps13
Tracheophyta - ps14
Spermatophyta - ps15
Angiosperms - ps16
Mesangiospermae - ps17
Magnoliids/eudicots - ps18
Eudicots - ps19
Core_eudicots - ps20
Superrosids - ps21
Rosids - ps22
Eurosids - ps23
Malvids - ps24
Sap./Malv./Brassicales - ps25
Malvales/Brassicales - ps26
Brassicales - ps27
Cleomaceae/Brassicaceae - ps28
Brassicaceae - ps29
Camelineae - ps30
Arabidopsis - ps31
Arabidopsis_thaliana - ps32
Phylostratum
p-value
2e-02
3e-02
4e-02
5e-02
6e-02
Log-odds
0.5
1.0
1.5
2.0
2.5
3.0
3.5
4.0

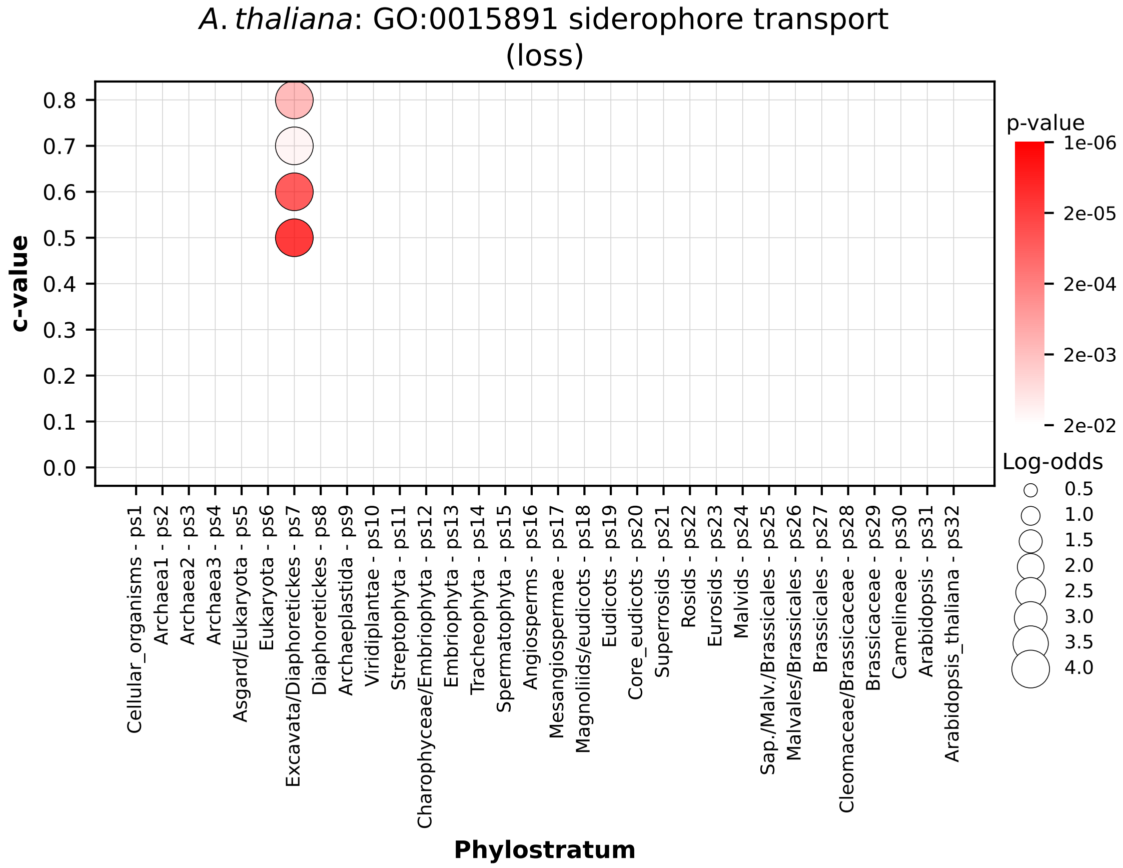

A. thaliana: GO:0015891 siderophore transport
(loss)
c-value
0.0
0.1
0.2
0.3
0.4
0.5
0.6
0.7
0.8
Phylostratum
Cellular_organisms - ps1
Archaea1 - ps2
Archaea2 - ps3
Archaea3 - ps4
Asgard/Eukaryota - ps5
Eukaryota - ps6
Excavata/Diaphoretickes - ps7
Diaphoretickes - ps8
Archaeplastida - ps9
Viridiplantae - ps10
Streptophyta - ps11
Charophyceae/Embriophyta - ps12
Embriophyta - ps13
Tracheophyta - ps14
Spermatophyta - ps15
Angiosperms - ps16
Mesangiospermae - ps17
Magnoliids/eudicots - ps18
Eudicots - ps19
Core_eudicots - ps20
Superrosids - ps21
Rosids - ps22
Eurosids - ps23
Malvids - ps24
Sap./Malv./Brassicales - ps25
Malvales/Brassicales - ps26
Brassicales - ps27
Cleomaceae/Brassicaceae - ps28
Brassicaceae - ps29
Camelineae - ps30
Arabidopsis - ps31
Arabidopsis_thaliana - ps32
p-value
1e-06
2e-05
2e-04
2e-03
2e-02
Log-odds
0.5
1.0
1.5
2.0
2.5
3.0
3.5
4.0

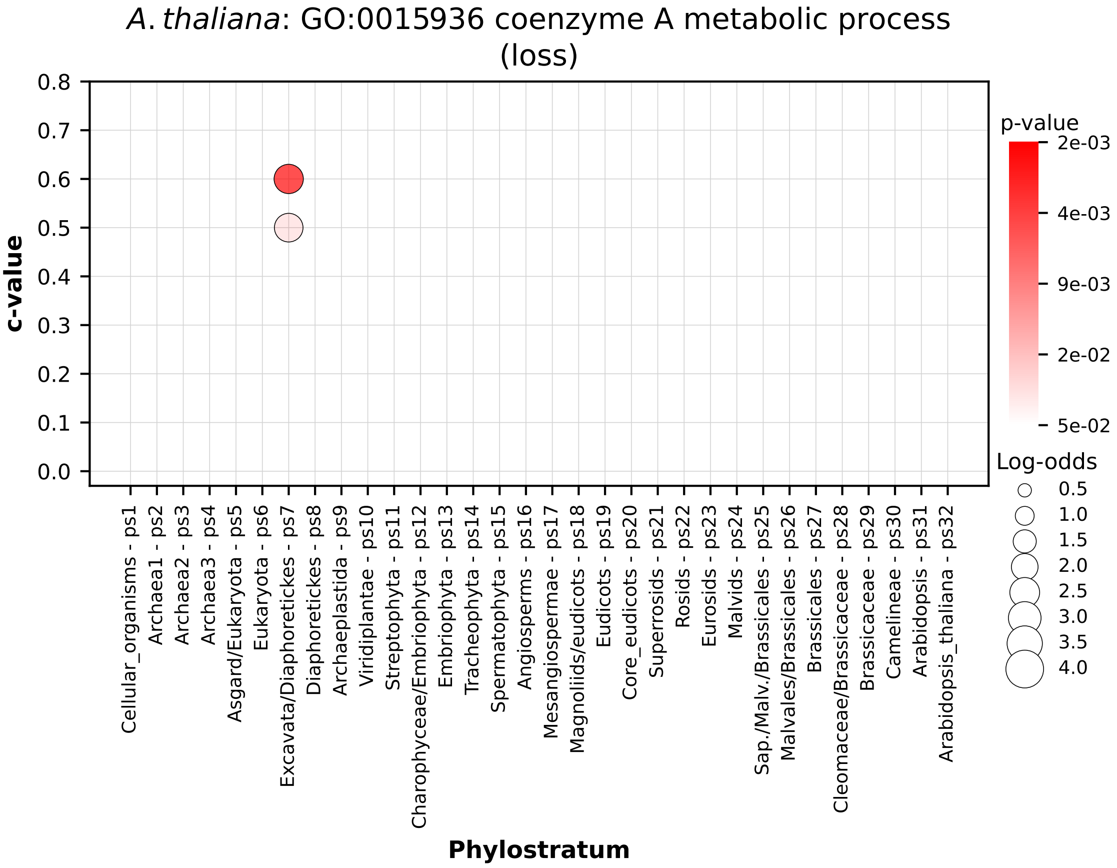

A. thaliana: GO:0015936 coenzyme A metabolic process (loss)
c-value
0.8
0.7
0.6
0.5
0.4
0.3
0.2
0.1
0.0
p-value
2e-03
4e-03
9e-03
2e-02
5e-02
Log-odds
0.5
1.0
1.5
2.0
2.5
3.0
3.5
4.0
Cellular_organisms - ps1
Archaea1 - ps2
Archaea2 - ps3
Archaea3 - ps4
Asgard/Eukaryota - ps5
Eukaryota - ps6
Excavata/Diaphoretickes - ps7
Diaphoretickes - ps8
Archaeplastida - ps9
Viridiplantae - ps10
Streptophyta - ps11
Charophyceae/Embriophyta - ps12
Embriophyta - ps13
Tracheophyta - ps14
Spermatophyta - ps15
Angiosperms - ps16
Mesangiospermae - ps17
Magnoliids/eudicots - ps18
Eudicots - ps19
Core_eudicots - ps20
Superrosids - ps21
Rosids - ps22
Eurosids - ps23
Malvids - ps24
Sap./Malv./Brassicales - ps25
Malvales/Brassicales - ps26
Brassicales - ps27
Cleomaceae/Brassicaceae - ps28
Brassicaceae - ps29
Camelineae - ps30
Arabidopsis - ps31
Arabidopsis_thaliana - ps32
Phylostratum

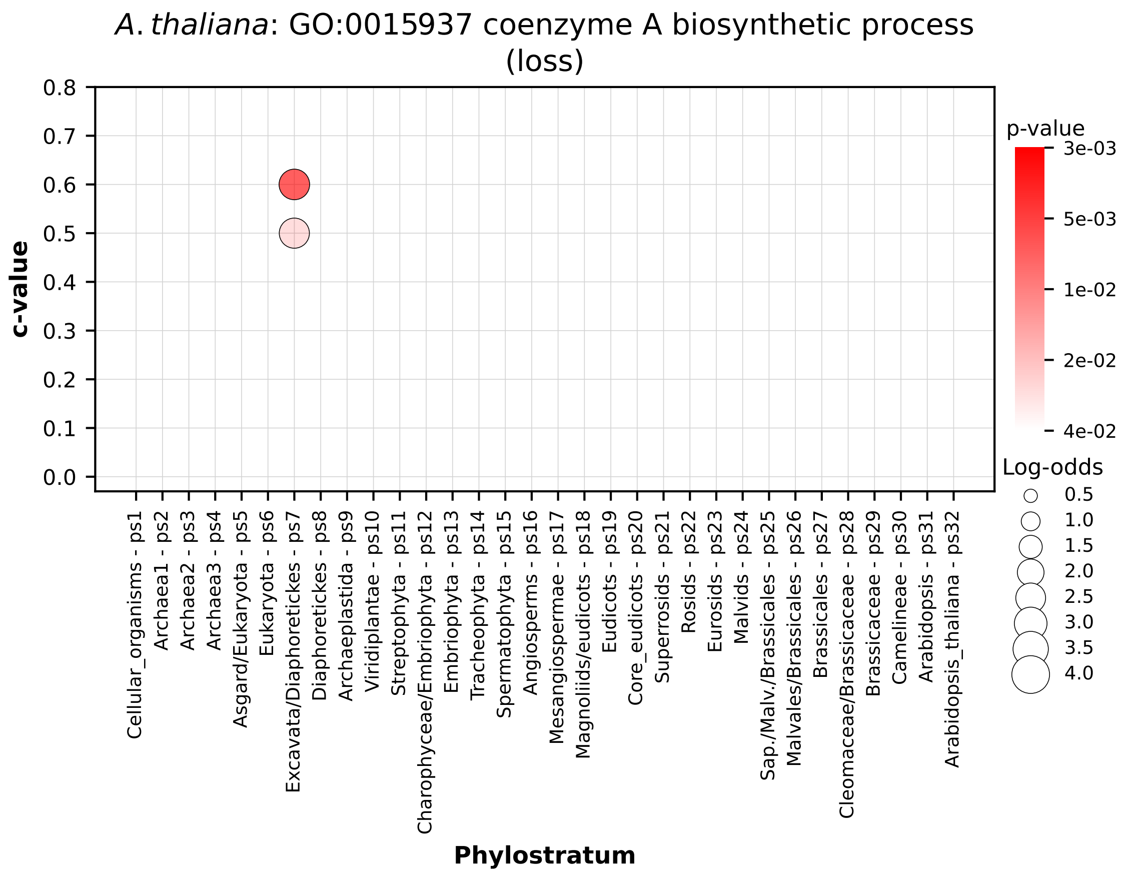

A. thaliana: GO:0015937 coenzyme A biosynthetic process
(loss)
c-value
0.8
0.7
0.6
0.5
0.4
0.3
0.2
0.1
0.0
Phylostratum
Cellular_organisms - ps1
Archaea1 - ps2
Archaea2 - ps3
Archaea3 - ps4
Asgard/Eukaryota - ps5
Eukaryota - ps6
Excavata/Diaphoretickes - ps7
Diaphoretickes - ps8
Archaeplastida - ps9
Viridiplantae - ps10
Streptophyta - ps11
Charophyceae/Embriophyta - ps12
Embriophyta - ps13
Tracheophyta - ps14
Spermatophyta - ps15
Angiosperms - ps16
Mesangiospermae - ps17
Magnoliids/eudicots - ps18
Eudicots - ps19
Core_eudicots - ps20
Superrosids - ps21
Rosids - ps22
Eurosids - ps23
Malvids - ps24
Sap./Malv./Brassicales - ps25
Malvales/Brassicales - ps26
Brassicales - ps27
Cleomaceae/Brassicaceae - ps28
Brassicaceae - ps29
Camelineae - ps30
Arabidopsis - ps31
Arabidopsis_thaliana - ps32
p-value
3e-03
5e-03
1e-02
2e-02
4e-02
Log-odds
0.5
1.0
1.5
2.0
2.5
3.0
3.5
4.0

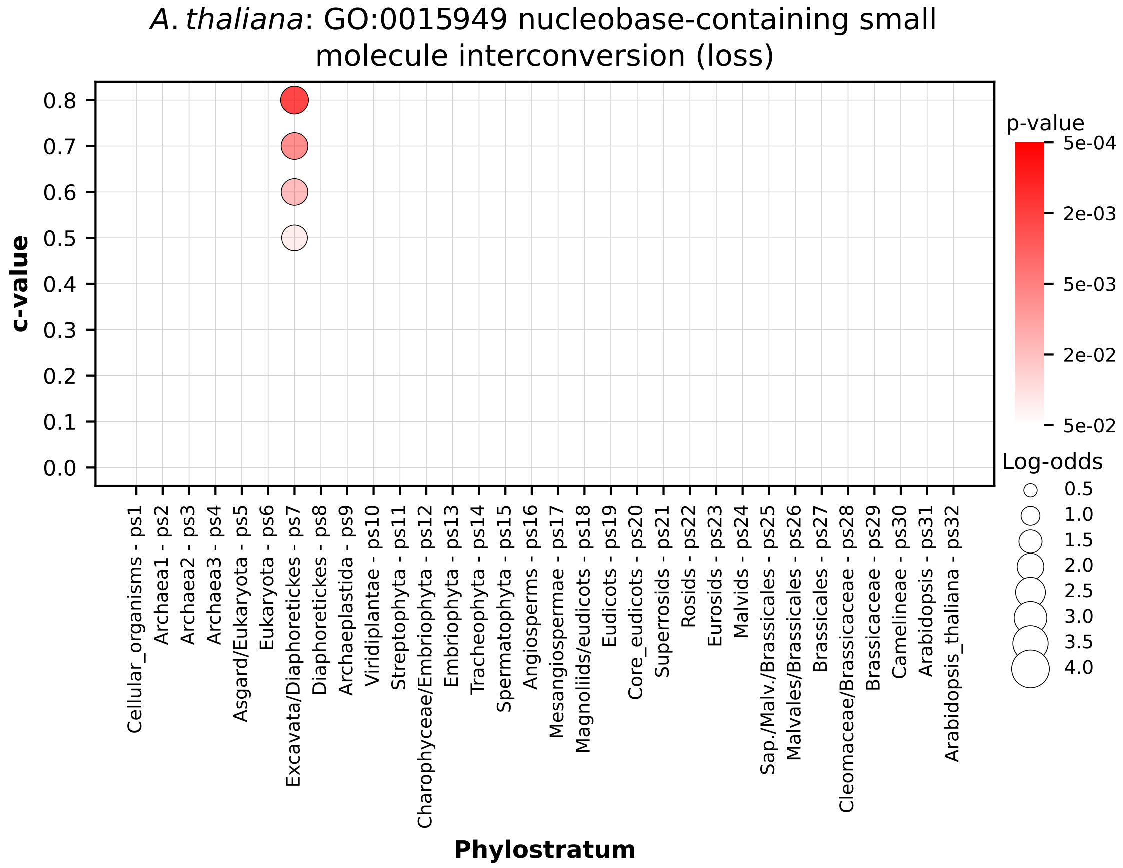

A. thaliana: GO:0015949 nucleobase-containing small molecule interconversion (loss)
c-value
0.0
0.1
0.2
0.3
0.4
0.5
0.6
0.7
0.8
p-value
5e-04
2e-03
5e-03
2e-02
5e-02
Log-odds
0.5
1.0
1.5
2.0
2.5
3.0
3.5
4.0
Cellular_organisms - ps1
Archaea1 - ps2
Archaea2 - ps3
Archaea3 - ps4
Asgard/Eukaryota - ps5
Eukaryota - ps6
Excavata/Diaphoretickes - ps7
Diaphoretickes - ps8
Archaeplastida - ps9
Viridiplantae - ps10
Streptophyta - ps11
Charophyceae/Embriophyta - ps12
Embriophyta - ps13
Tracheophyta - ps14
Spermatophyta - ps15
Angiosperms - ps16
Mesangiospermae - ps17
Magnoliids/eudicots - ps18
Eudicots - ps19
Core_eudicots - ps20
Superrosids - ps21
Rosids - ps22
Eurosids - ps23
Malvids - ps24
Sap./Malv./Brassicales - ps25
Malvales/Brassicales - ps26
Brassicales - ps27
Cleomaceae/Brassicaceae - ps28
Brassicaceae - ps29
Camelineae - ps30
Arabidopsis - ps31
Arabidopsis_thaliana - ps32
Phylostratum

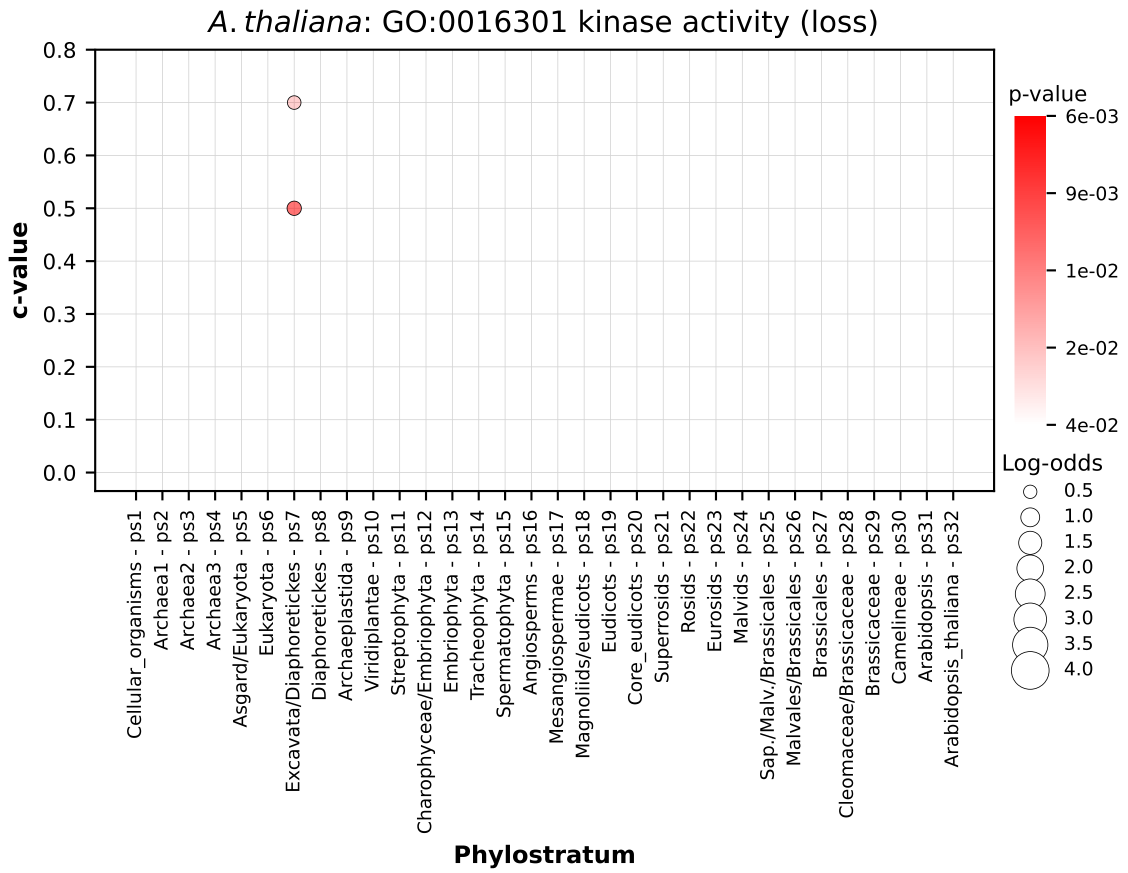
A. thaliana: GO:0016301 kinase activity (loss)
c-value
0.8
0.7
0.6
0.5
0.4
0.3
0.2
0.1
0.0
Phylostratum
Cellular_organisms - ps1
Archaea1 - ps2
Archaea2 - ps3
Archaea3 - ps4
Asgard/Eukaryota - ps5
Eukaryota - ps6
Excavata/Diaphoretickes - ps7
Diaphoretickes - ps8
Archaeplastida - ps9
Viridiplantae - ps10
Streptophyta - ps11
Charophyceae/Embriophyta - ps12
Embriophyta - ps13
Tracheophyta - ps14
Spermatophyta - ps15
Angiosperms - ps16
Mesangiospermae - ps17
Magnoliids/eudicots - ps18
Eudicots - ps19
Core_eudicots - ps20
Superrosids - ps21
Rosids - ps22
Eurosids - ps23
Malvids - ps24
Sap./Malv./Brassicales - ps25
Malvales/Brassicales - ps26
Brassicales - ps27
Cleomaceae/Brassicaceae - ps28
Brassicaceae - ps29
Camelineae - ps30
Arabidopsis - ps31
Arabidopsis_thaliana - ps32
p-value
6e-03
9e-03
1e-02
2e-02
4e-02
Log-odds
0.5
1.0
1.5
2.0
2.5
3.0
3.5
4.0

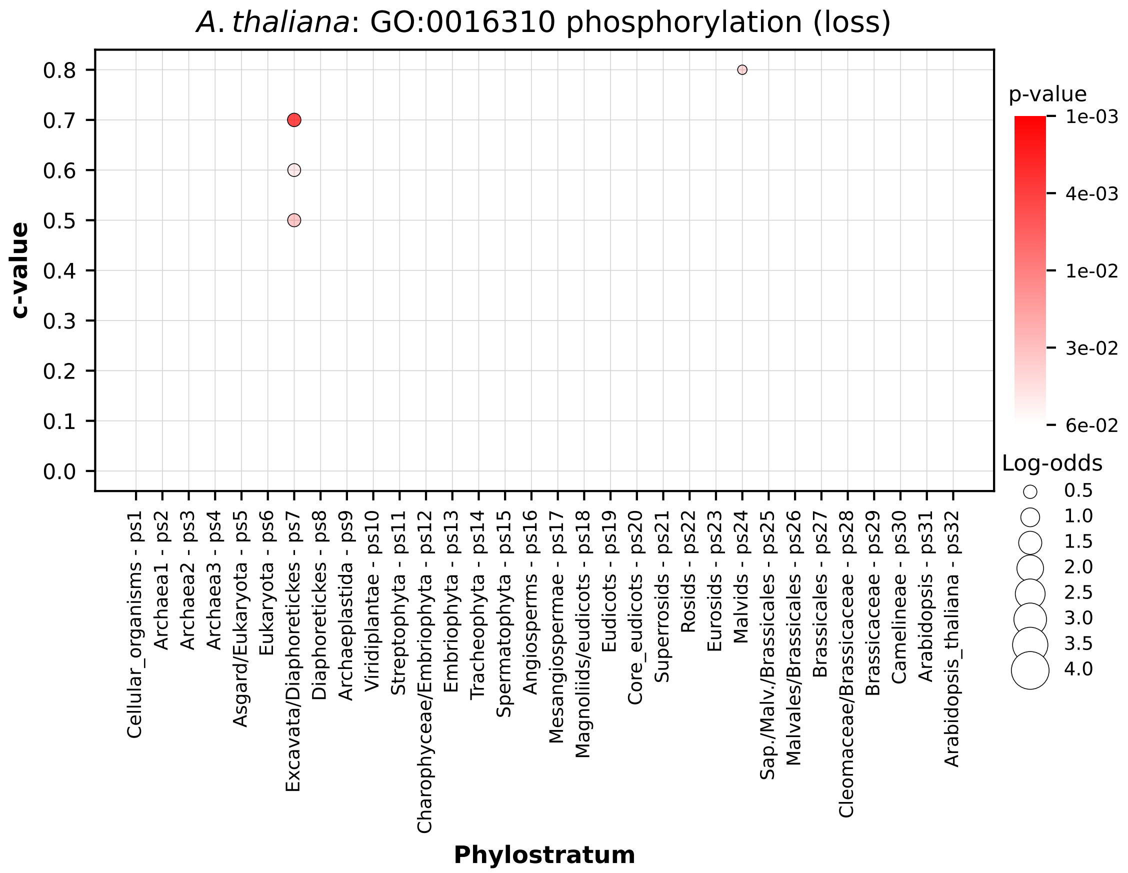

A. thaliana: GO:0016310 phosphorylation (loss)
c-value
0.8
0.7
0.6
0.5
0.4
0.3
0.2
0.1
0.0
Phylostratum
Cellular_organisms - ps1
Archaea1 - ps2
Archaea2 - ps3
Archaea3 - ps4
Asgard/Eukaryota - ps5
Eukaryota - ps6
Excavata/Diaphoretickes - ps7
Diaphoretickes - ps8
Archaeplastida - ps9
Viridiplantae - ps10
Streptophyta - ps11
Charophyceae/Embriophyta - ps12
Embriophyta - ps13
Tracheophyta - ps14
Spermatophyta - ps15
Angiosperms - ps16
Mesangiospermae - ps17
Magnoliids/eudicots - ps18
Eudicots - ps19
Core_eudicots - ps20
Superrosids - ps21
Rosids - ps22
Eurosids - ps23
Malvids - ps24
Sap./Malv./Brassicales - ps25
Malvales/Brassicales - ps26
Brassicales - ps27
Cleomaceae/Brassicaceae - ps28
Brassicaceae - ps29
Camelineae - ps30
Arabidopsis - ps31
Arabidopsis_thaliana - ps32
p-value
1e-03
4e-03
1e-02
3e-02
6e-02
Log-odds
0.5
1.0
1.5
2.0
2.5
3.0
3.5
4.0

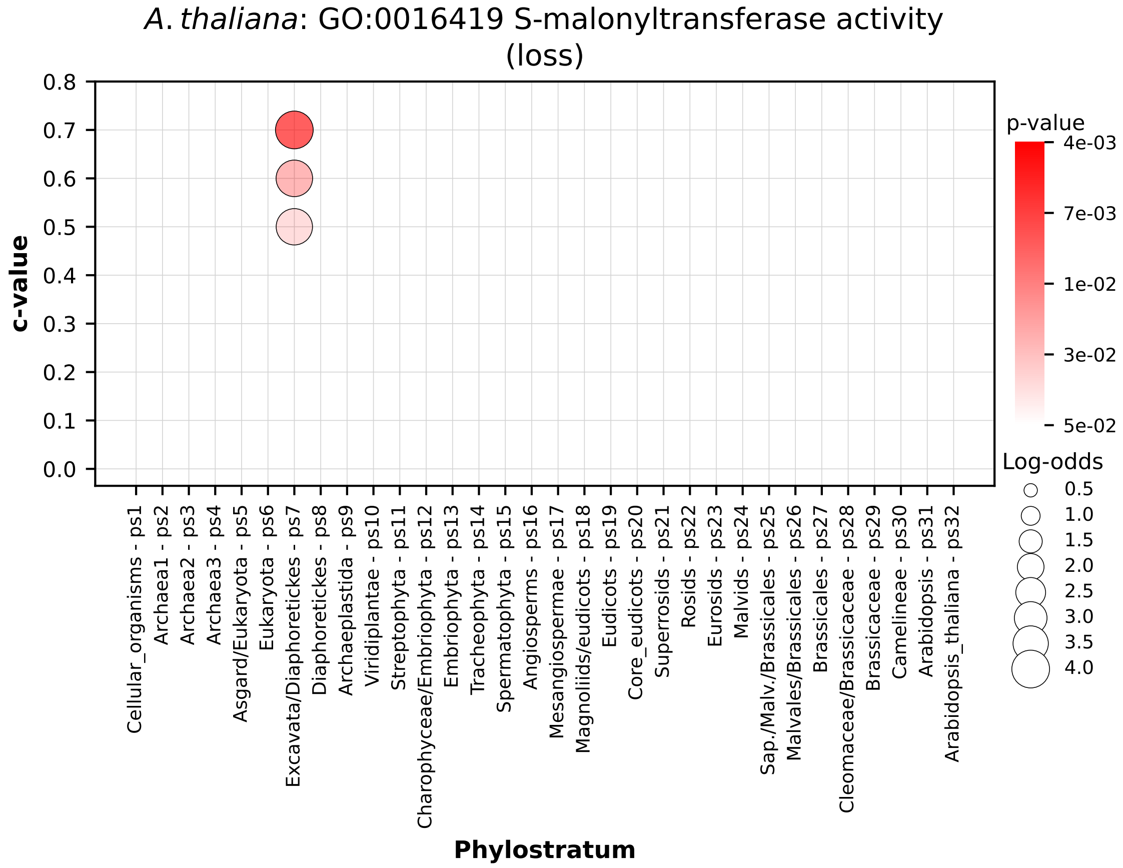

A. thaliana: GO:0016419 S-malonyltransferase activity
(loss)
c-value
0.8
0.7
0.6
0.5
0.4
0.3
0.2
0.1
0.0
Phylostratum
Cellular_organisms - ps1
Archaea1 - ps2
Archaea2 - ps3
Archaea3 - ps4
Asgard/Eukaryota - ps5
Eukaryota - ps6
Excavata/Diaphoretickes - ps7
Diaphoretickes - ps8
Archaeplastida - ps9
Viridiplantae - ps10
Streptophyta - ps11
Charophyceae/Embriophyta - ps12
Embriophyta - ps13
Tracheophyta - ps14
Spermatophyta - ps15
Angiosperms - ps16
Mesangiospermae - ps17
Magnoliids/eudicots - ps18
Eudicots - ps19
Core_eudicots - ps20
Superrosids - ps21
Rosids - ps22
Eurosids - ps23
Malvids - ps24
Sap./Malv./Brassicales - ps25
Malvales/Brassicales - ps26
Brassicales - ps27
Cleomaceae/Brassicaceae - ps28
Brassicaceae - ps29
Camelineae - ps30
Arabidopsis - ps31
Arabidopsis_thaliana - ps32
p-value
4e-03
7e-03
1e-02
3e-02
5e-02
Log-odds
0.5
1.0
1.5
2.0
2.5
3.0
3.5
4.0

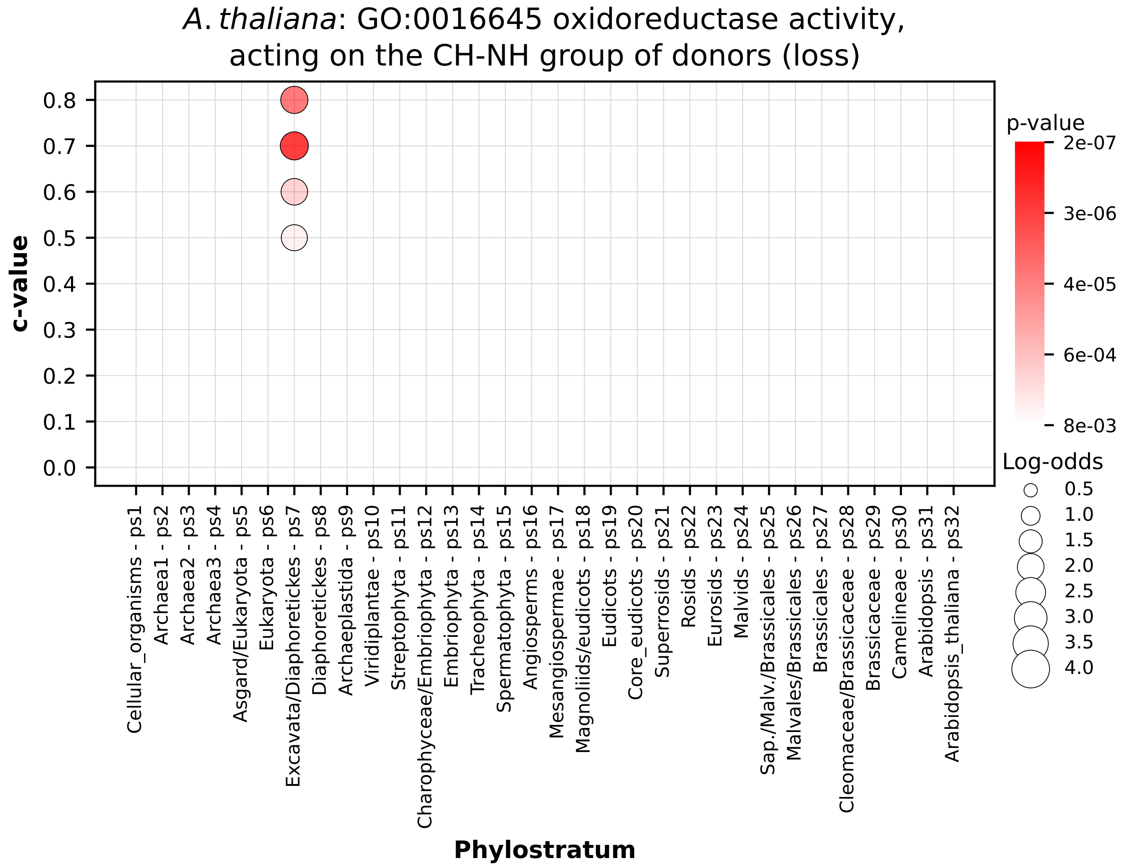

A. thaliana: GO:0016645 oxidoreductase activity,
acting on the CH-NH group of donors (loss)
c-value
0.0
0.1
0.2
0.3
0.4
0.5
0.6
0.7
0.8
Phylostratum
Cellular_organisms - ps1
Archaea1 - ps2
Archaea2 - ps3
Archaea3 - ps4
Asgard/Eukaryota - ps5
Eukaryota - ps6
Excavata/Diaphoretickes - ps7
Diaphoretickes - ps8
Archaeplastida - ps9
Viridiplantae - ps10
Streptophyta - ps11
Charophyceae/Embriophyta - ps12
Embriophyta - ps13
Tracheophyta - ps14
Spermatophyta - ps15
Angiosperms - ps16
Mesangiospermae - ps17
Magnoliids/eudicots - ps18
Eudicots - ps19
Core_eudicots - ps20
Superrosids - ps21
Rosids - ps22
Eurosids - ps23
Malvids - ps24
Sap./Malv./Brassicales - ps25
Malvales/Brassicales - ps26
Brassicales - ps27
Cleomaceae/Brassicaceae - ps28
Brassicaceae - ps29
Camelineae - ps30
Arabidopsis - ps31
Arabidopsis_thaliana - ps32
p-value
2e-07
3e-06
4e-05
6e-04
8e-03
Log-odds
0.5
1.0
1.5
2.0
2.5
3.0
3.5
4.0

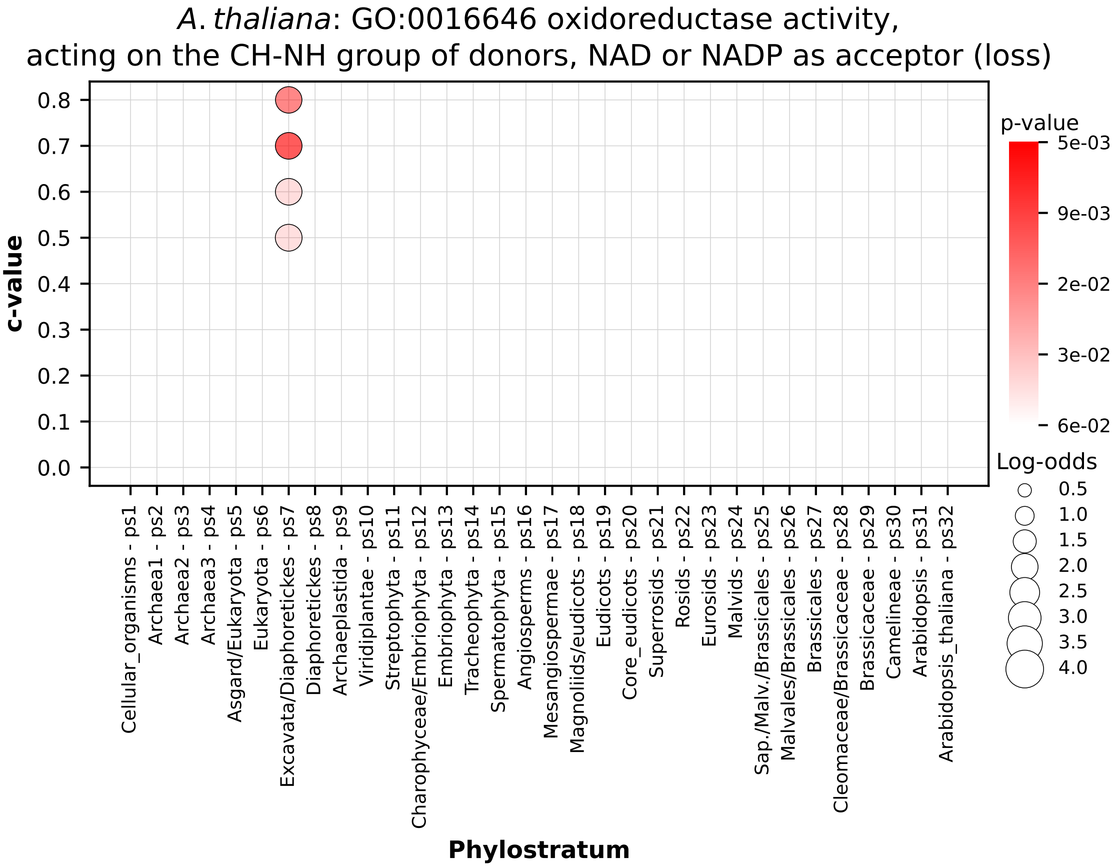
A. thaliana: GO:0016646 oxidoreductase activity,
acting on the CH-NH group of donors, NAD or NADP as acceptor (loss)
c-value
0.0
0.1
0.2
0.3
0.4
0.5
0.6
0.7
0.8
Phylostratum
Cellular_organisms - ps1
Archaea1 - ps2
Archaea2 - ps3
Archaea3 - ps4
Asgard/Eukaryota - ps5
Eukaryota - ps6
Excavata/Diaphoretickes - ps7
Diaphoretickes - ps8
Archaeplastida - ps9
Viridiplantae - ps10
Streptophyta - ps11
Charophyceae/Embriophyta - ps12
Embriophyta - ps13
Tracheophyta - ps14
Spermatophyta - ps15
Angiosperms - ps16
Mesangiospermae - ps17
Magnoliids/eudicots - ps18
Eudicots - ps19
Core_eudicots - ps20
Superrosids - ps21
Rosids - ps22
Eurosids - ps23
Malvids - ps24
Sap./Malv./Brassicales - ps25
Malvales/Brassicales - ps26
Brassicales - ps27
Cleomaceae/Brassicaceae - ps28
Brassicaceae - ps29
Camelineae - ps30
Arabidopsis - ps31
Arabidopsis_thaliana - ps32
p-value
5e-03
9e-03
2e-02
3e-02
6e-02
Log-odds
0.5
1.0
1.5
2.0
2.5
3.0
3.5
4.0

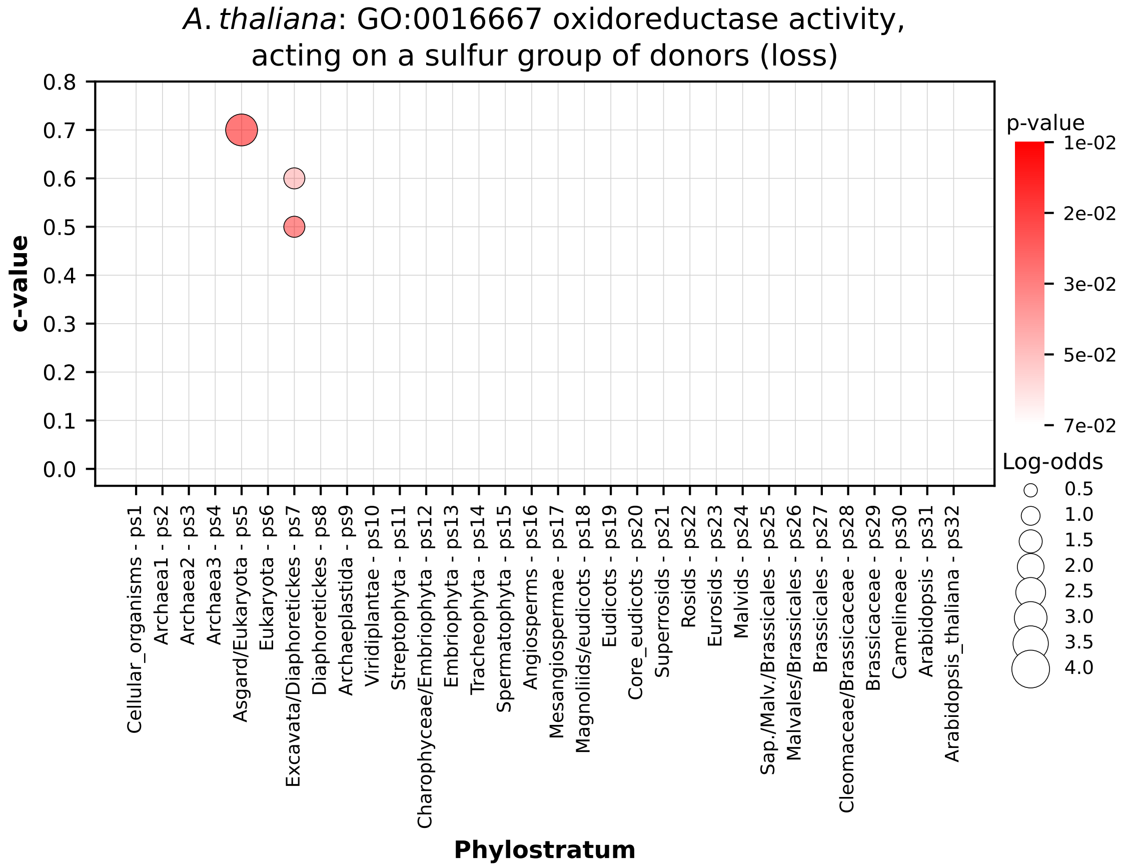

A. thaliana: GO:0016667 oxidoreductase activity, acting on a sulfur group of donors (loss)
c-value
0.8
0.7
0.6
0.5
0.4
0.3
0.2
0.1
0.0
Phylostratum
Cellular_organisms - ps1
Archaea1 - ps2
Archaea2 - ps3
Archaea3 - ps4
Asgard/Eukaryota - ps5
Eukaryota - ps6
Excavata/Diaphoretickes - ps7
Diaphoretickes - ps8
Archaeplastida - ps9
Viridiplantae - ps10
Streptophyta - ps11
Charophyceae/Embriophyta - ps12
Embriophyta - ps13
Tracheophyta - ps14
Spermatophyta - ps15
Angiosperms - ps16
Mesangiospermae - ps17
Magnoliids/eudicots - ps18
Eudicots - ps19
Core_eudicots - ps20
Superrosids - ps21
Rosids - ps22
Eurosids - ps23
Malvids - ps24
Sap./Malv./Brassicales - ps25
Malvales/Brassicales - ps26
Brassicales - ps27
Cleomaceae/Brassicaceae - ps28
Brassicaceae - ps29
Camelineae - ps30
Arabidopsis - ps31
Arabidopsis_thaliana - ps32
p-value
1e-02
2e-02
3e-02
5e-02
7e-02
Log-odds
0.5
1.0
1.5
2.0
2.5
3.0
3.5
4.0

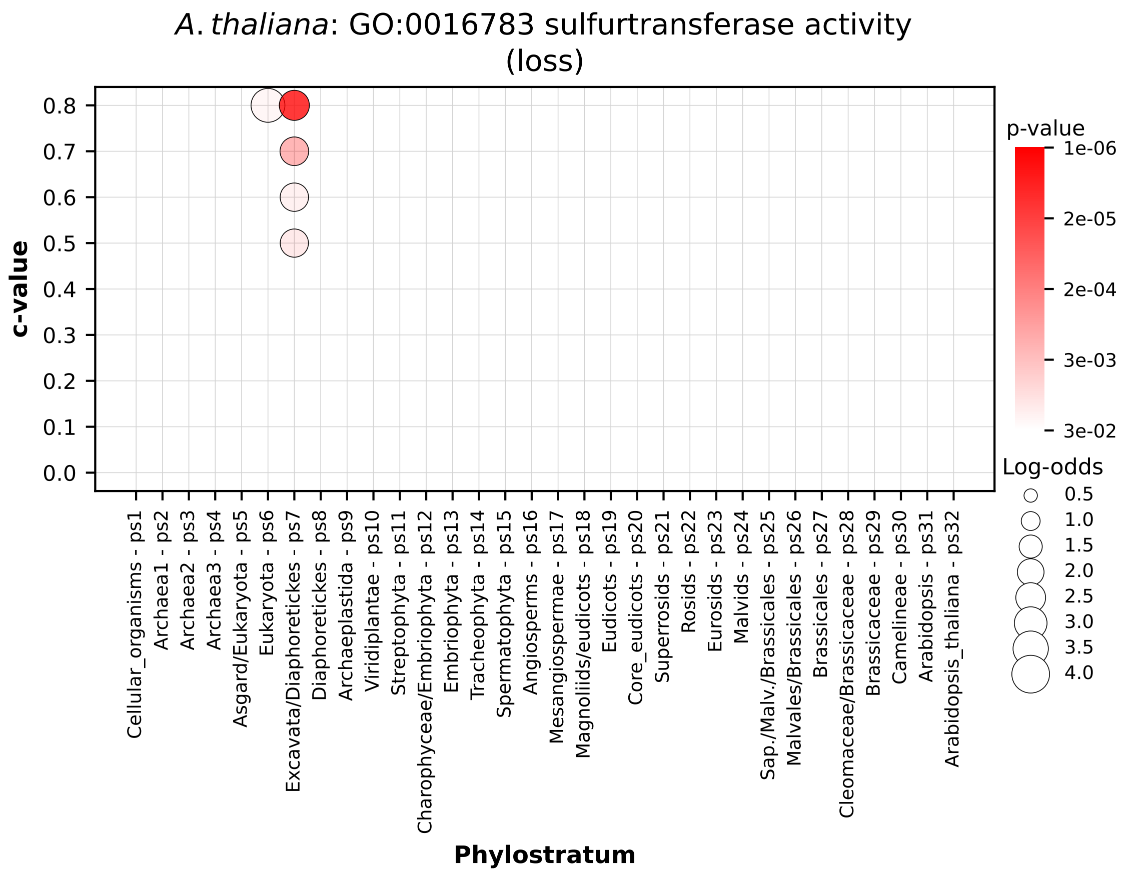

A. thaliana: GO:0016783 sulfurtransferase activity
(loss)
c-value
0.8
0.7
0.6
0.5
0.4
0.3
0.2
0.1
0.0
p-value
1e-06
2e-05
2e-04
3e-03
3e-02
Log-odds
0.5
1.0
1.5
2.0
2.5
3.0
3.5
4.0
Cellular_organisms - ps1
Archaea1 - ps2
Archaea2 - ps3
Archaea3 - ps4
Asgard/Eukaryota - ps5
Eukaryota - ps6
Excavata/Diaphoretickes - ps7
Diaphoretickes - ps8
Archaeplastida - ps9
Viridiplantae - ps10
Streptophyta - ps11
Charophyceae/Embriophyta - ps12
Embriophyta - ps13
Tracheophyta - ps14
Spermatophyta - ps15
Angiosperms - ps16
Mesangiospermae - ps17
Magnoliids/eudicots - ps18
Eudicots - ps19
Core_eudicots - ps20
Superrosids - ps21
Rosids - ps22
Eurosids - ps23
Malvids - ps24
Sap./Malv./Brassicales - ps25
Malvales/Brassicales - ps26
Brassicales - ps27
Cleomaceae/Brassicaceae - ps28
Brassicaceae - ps29
Camelineae - ps30
Arabidopsis - ps31
Arabidopsis_thaliana - ps32
Phylostratum

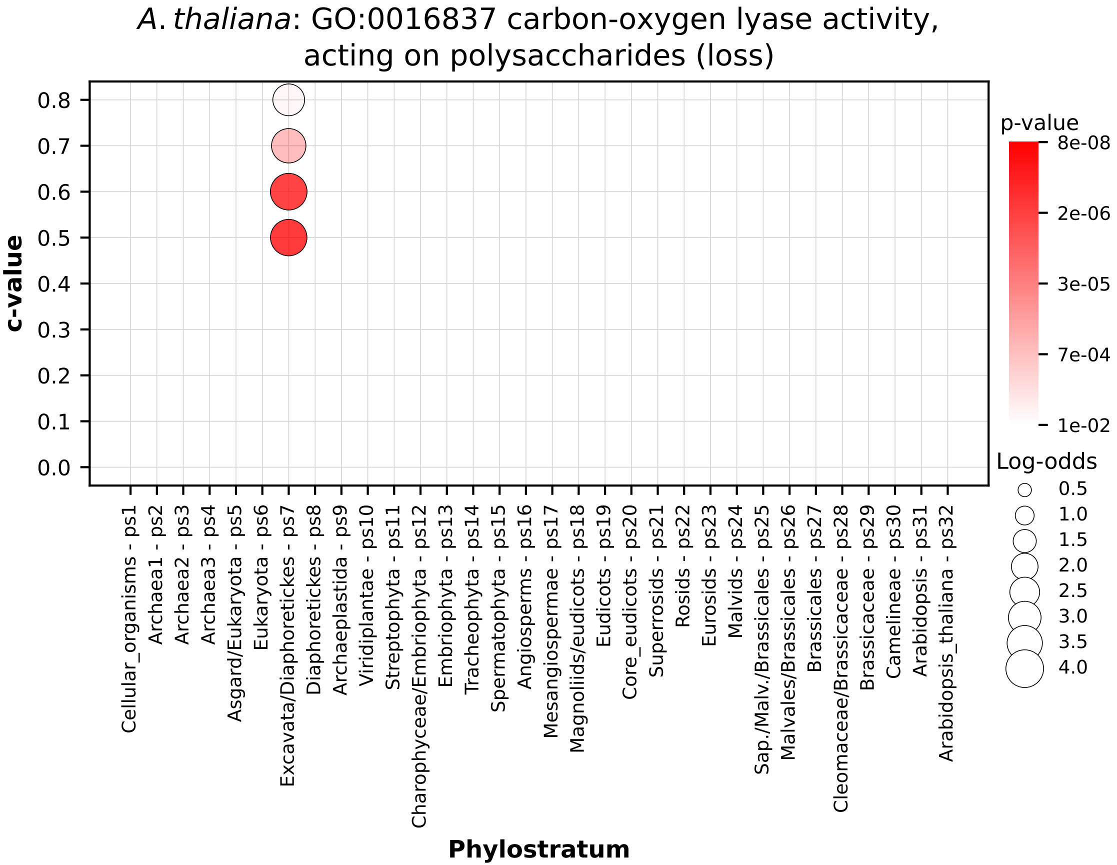

A. thaliana: GO:0016837 carbon-oxygen lyase activity, acting on polysaccharides (loss)
c-value
0.0
0.1
0.2
0.3
0.4
0.5
0.6
0.7
0.8
Phylostratum
Cellular_organisms - ps1
Archaea1 - ps2
Archaea2 - ps3
Archaea3 - ps4
Asgard/Eukaryota - ps5
Eukaryota - ps6
Excavata/Diaphoretickes - ps7
Diaphoretickes - ps8
Archaeplastida - ps9
Viridiplantae - ps10
Streptophyta - ps11
Charophyceae/Embriophyta - ps12
Embriophyta - ps13
Tracheophyta - ps14
Spermatophyta - ps15
Angiosperms - ps16
Mesangiospermae - ps17
Magnoliids/eudicots - ps18
Eudicots - ps19
Core_eudicots - ps20
Superrosids - ps21
Rosids - ps22
Eurosids - ps23
Malvids - ps24
Sap./Malv./Brassicales - ps25
Malvales/Brassicales - ps26
Brassicales - ps27
Cleomaceae/Brassicaceae - ps28
Brassicaceae - ps29
Camelineae - ps30
Arabidopsis - ps31
Arabidopsis_thaliana - ps32
p-value
8e-08
2e-06
3e-05
7e-04
1e-02
Log-odds
0.5
1.0
1.5
2.0
2.5
3.0
3.5
4.0

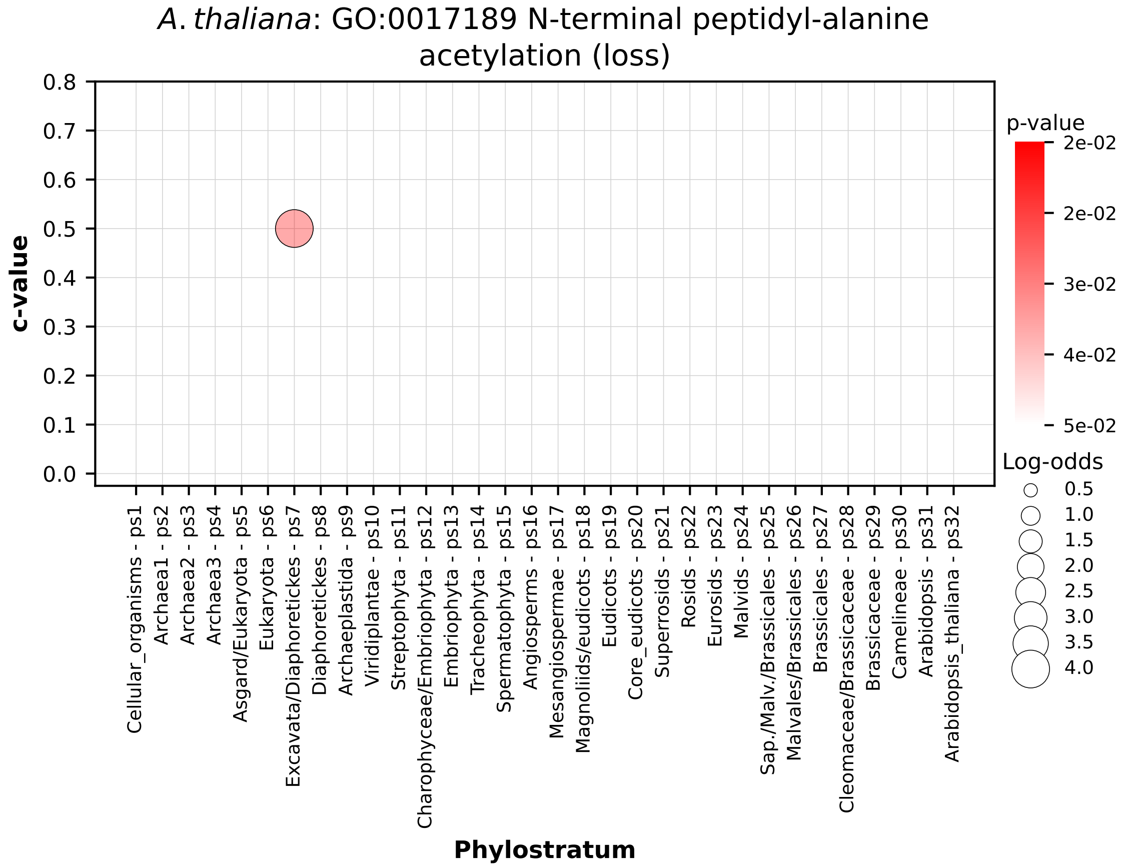

A. thaliana: GO:0017189 N-terminal peptidyl-alanine acetylation (loss)
c-value
0.8
0.7
0.6
0.5
0.4
0.3
0.2
0.1
0.0
Phylostratum
Cellular_organisms - ps1
Archaea1 - ps2
Archaea2 - ps3
Archaea3 - ps4
Asgard/Eukaryota - ps5
Eukaryota - ps6
Excavata/Diaphoretickes - ps7
Diaphoretickes - ps8
Archaeplastida - ps9
Viridiplantae - ps10
Streptophyta - ps11
Charophyceae/Embriophyta - ps12
Embriophyta - ps13
Tracheophyta - ps14
Spermatophyta - ps15
Angiosperms - ps16
Mesangiospermae - ps17
Magnoliids/eudicots - ps18
Eudicots - ps19
Core_eudicots - ps20
Superrosids - ps21
Rosids - ps22
Eurosids - ps23
Malvids - ps24
Sap./Malv./Brassicales - ps25
Malvales/Brassicales - ps26
Brassicales - ps27
Cleomaceae/Brassicaceae - ps28
Brassicaceae - ps29
Camelineae - ps30
Arabidopsis - ps31
Arabidopsis_thaliana - ps32
p-value
2e-02
2e-02
3e-02
4e-02
5e-02
Log-odds
0.5
1.0
1.5
2.0
2.5
3.0
3.5
4.0

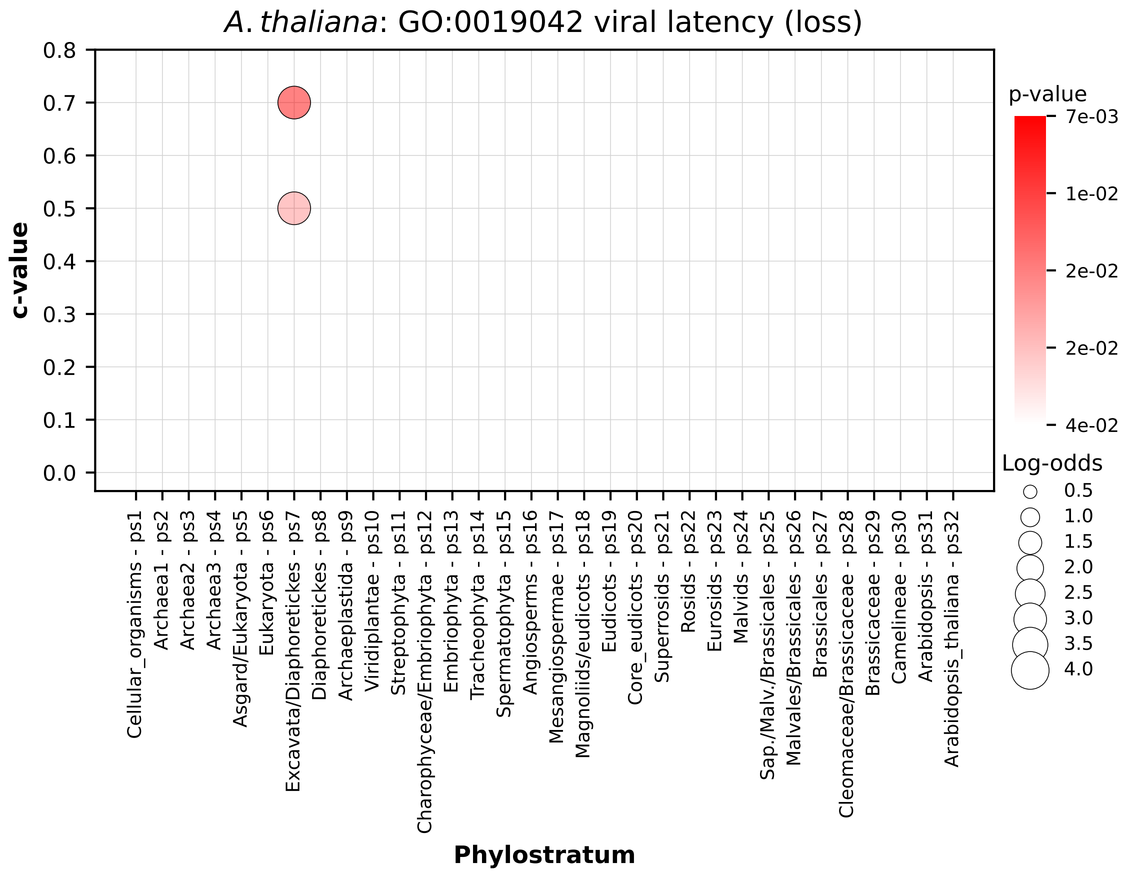
A. thaliana: GO:0019042 viral latency (loss)
c-value
0.0
0.1
0.2
0.3
0.4
0.5
0.6
0.7
0.8
Phylostratum
Cellular_organisms - ps1
Archaea1 - ps2
Archaea2 - ps3
Archaea3 - ps4
Asgard/Eukaryota - ps5
Eukaryota - ps6
Excavata/Diaphoretickes - ps7
Diaphoretickes - ps8
Archaeplastida - ps9
Viridiplantae - ps10
Streptophyta - ps11
Charophyceae/Embriophyta - ps12
Embriophyta - ps13
Tracheophyta - ps14
Spermatophyta - ps15
Angiosperms - ps16
Mesangiospermae - ps17
Magnoliids/eudicots - ps18
Eudicots - ps19
Core_eudicots - ps20
Superrosids - ps21
Rosids - ps22
Eurosids - ps23
Malvids - ps24
Sap./Malv./Brassicales - ps25
Malvales/Brassicales - ps26
Brassicales - ps27
Cleomaceae/Brassicaceae - ps28
Brassicaceae - ps29
Camelineae - ps30
Arabidopsis - ps31
Arabidopsis_thaliana - ps32
p-value
7e-03
1e-02
2e-02
2e-02
4e-02
Log-odds
0.5
1.0
1.5
2.0
2.5
3.0
3.5
4.0

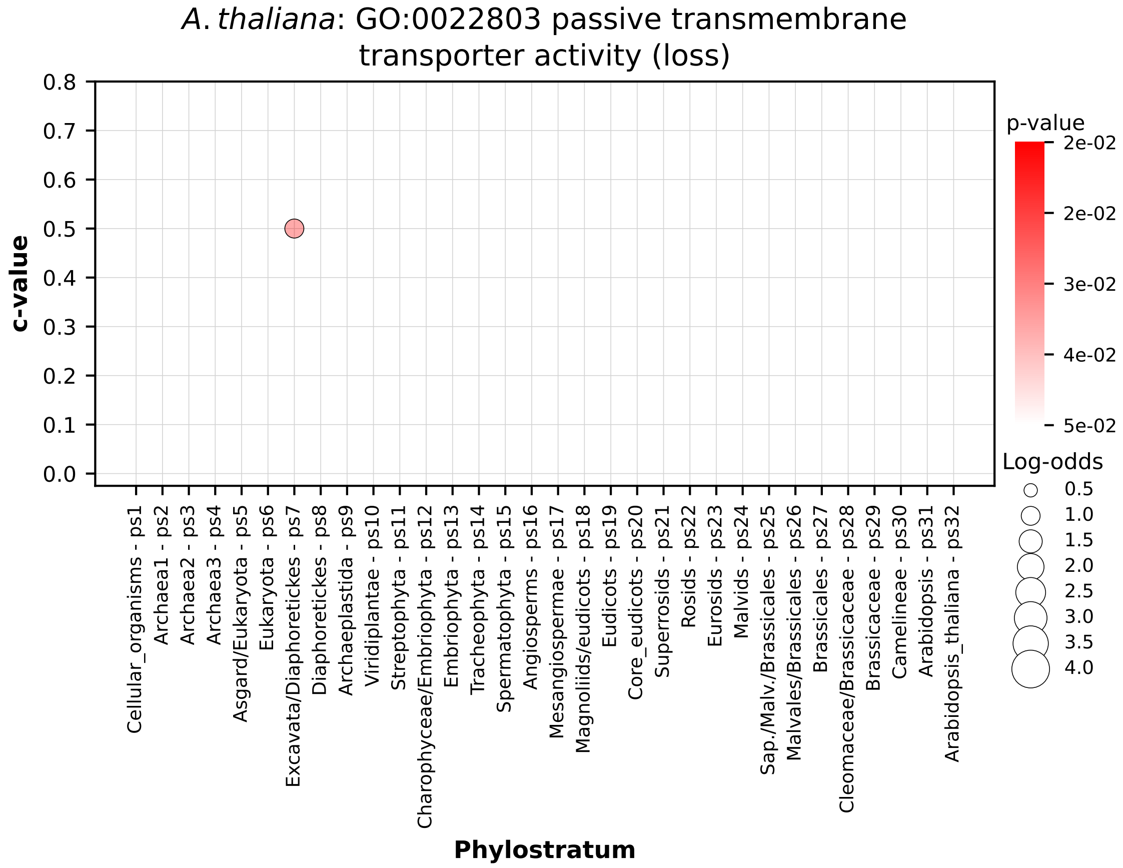

A. thaliana: GO:0022803 passive transmembrane transporter activity (loss)
c-value
0.0
0.1
0.2
0.3
0.4
0.5
0.6
0.7
0.8
Phylostratum
Cellular_organisms - ps1
Archaea1 - ps2
Archaea2 - ps3
Archaea3 - ps4
Asgard/Eukaryota - ps5
Eukaryota - ps6
Excavata/Diaphoretickes - ps7
Diaphoretickes - ps8
Archaeplastida - ps9
Viridiplantae - ps10
Streptophyta - ps11
Charophyceae/Embriophyta - ps12
Embriophyta - ps13
Tracheophyta - ps14
Spermatophyta - ps15
Angiosperms - ps16
Mesangiospermae - ps17
Magnoliids/eudicots - ps18
Eudicots - ps19
Core_eudicots - ps20
Superrosids - ps21
Rosids - ps22
Eurosids - ps23
Malvids - ps24
Sap./Malv./Brassicales - ps25
Malvales/Brassicales - ps26
Brassicales - ps27
Cleomaceae/Brassicaceae - ps28
Brassicaceae - ps29
Camelineae - ps30
Arabidopsis - ps31
Arabidopsis_thaliana - ps32
p-value
2e-02
2e-02
3e-02
4e-02
5e-02
Log-odds
0.5
1.0
1.5
2.0
2.5
3.0
3.5
4.0

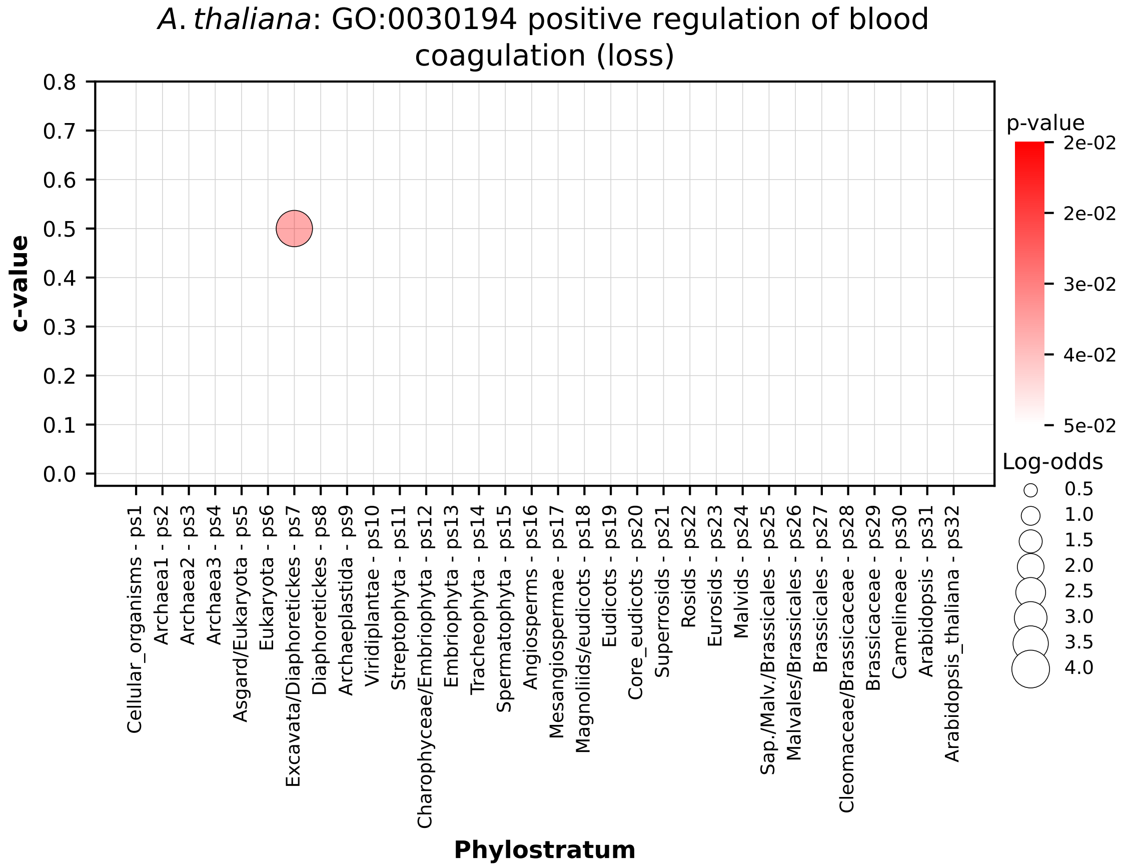

A. thaliana: GO:0030194 positive regulation of blood coagulation (loss)
c-value
0.8
0.7
0.6
0.5
0.4
0.3
0.2
0.1
0.0
Cellular_organisms - ps1
Archaea1 - ps2
Archaea2 - ps3
Archaea3 - ps4
Asgard/Eukaryota - ps5
Eukaryota - ps6
Excavata/Diaphoretickes - ps7
Diaphoretickes - ps8
Archaeplastida - ps9
Viridiplantae - ps10
Streptophyta - ps11
Charophyceae/Embriophyta - ps12
Embriophyta - ps13
Tracheophyta - ps14
Spermatophyta - ps15
Angiosperms - ps16
Mesangiospermae - ps17
Magnoliids/eudicots - ps18
Eudicots - ps19
Core_eudicots - ps20
Superrosids - ps21
Rosids - ps22
Eurosids - ps23
Malvids - ps24
Sap./Malv./Brassicales - ps25
Malvales/Brassicales - ps26
Brassicales - ps27
Cleomaceae/Brassicaceae - ps28
Brassicaceae - ps29
Camelineae - ps30
Arabidopsis - ps31
Arabidopsis_thaliana - ps32
Phylostratum
p-value
2e-02
2e-02
3e-02
4e-02
5e-02
Log-odds
0.5
1.0
1.5
2.0
2.5
3.0
3.5
4.0

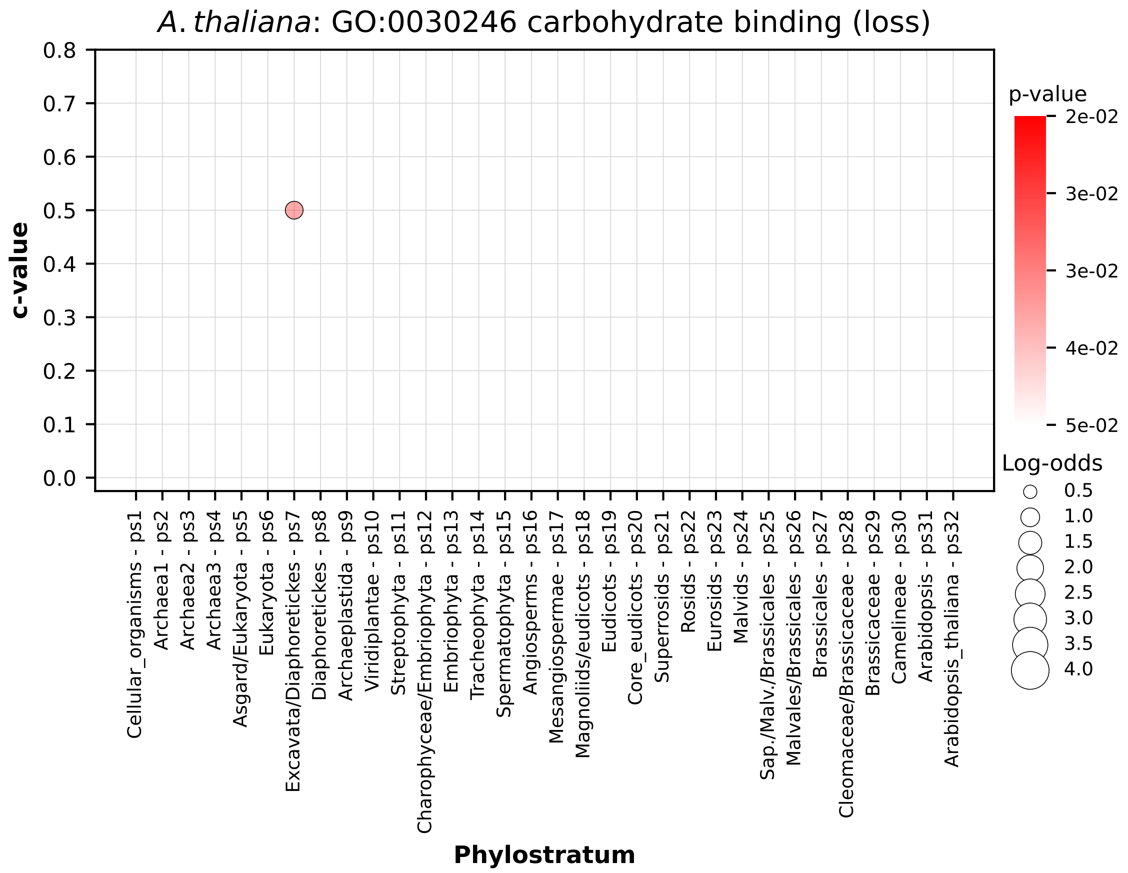
A. thaliana: GO:0030246 carbohydrate binding (loss)
c-value
0.8
0.7
0.6
0.5
0.4
0.3
0.2
0.1
0.0
p-value
2e-02
3e-02
3e-02
4e-02
5e-02
Log-odds
0.5
1.0
1.5
2.0
2.5
3.0
3.5
4.0
Cellular_organisms - ps1
Archaea1 - ps2
Archaea2 - ps3
Archaea3 - ps4
Asgard/Eukaryota - ps5
Eukaryota - ps6
Excavata/Diaphoretickes - ps7
Diaphoretickes - ps8
Archaeplastida - ps9
Viridiplantae - ps10
Streptophyta - ps11
Charophyceae/Embriophyta - ps12
Embriophyta - ps13
Tracheophyta - ps14
Spermatophyta - ps15
Angiosperms - ps16
Mesangiospermae - ps17
Magnoliids/eudicots - ps18
Eudicots - ps19
Core_eudicots - ps20
Superrosids - ps21
Rosids - ps22
Eurosids - ps23
Malvids - ps24
Sap./Malv./Brassicales - ps25
Malvales/Brassicales - ps26
Brassicales - ps27
Cleomaceae/Brassicaceae - ps28
Brassicaceae - ps29
Camelineae - ps30
Arabidopsis - ps31
Arabidopsis_thaliana - ps32
Phylostratum

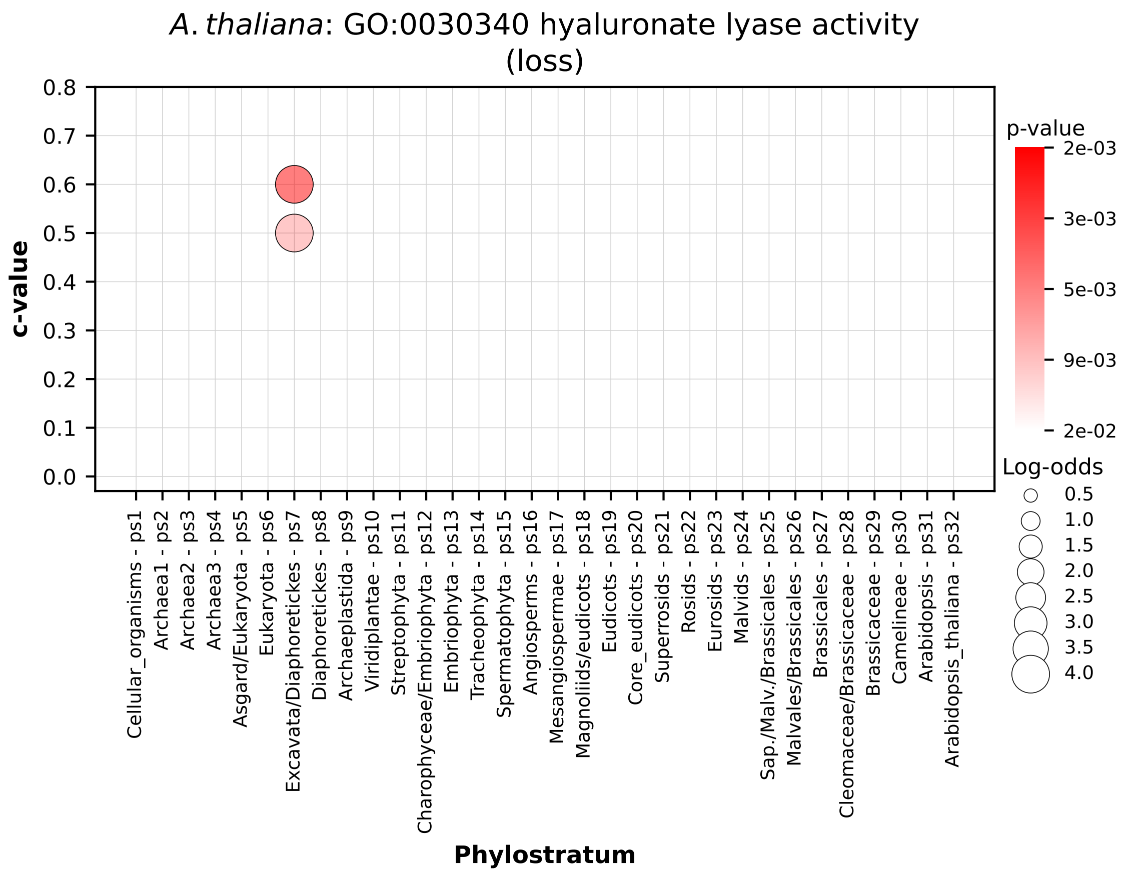
A. thaliana: GO:0030340 hyaluronate lyase activity (loss)
c-value
0.8
0.7
0.6
0.5
0.4
0.3
0.2
0.1
0.0
Phylostratum
Cellular_organisms - ps1
Archaea1 - ps2
Archaea2 - ps3
Archaea3 - ps4
Asgard/Eukaryota - ps5
Eukaryota - ps6
Excavata/Diaphoretickes - ps7
Diaphoretickes - ps8
Archaeplastida - ps9
Viridiplantae - ps10
Streptophyta - ps11
Charophyceae/Embriophyta - ps12
Embriophyta - ps13
Tracheophyta - ps14
Spermatophyta - ps15
Angiosperms - ps16
Mesangiospermae - ps17
Magnoliids/eudicots - ps18
Eudicots - ps19
Core_eudicots - ps20
Superrosids - ps21
Rosids - ps22
Eurosids - ps23
Malvids - ps24
Sap./Malv./Brassicales - ps25
Malvales/Brassicales - ps26
Brassicales - ps27
Cleomaceae/Brassicaceae - ps28
Brassicaceae - ps29
Camelineae - ps30
Arabidopsis - ps31
Arabidopsis_thaliana - ps32
p-value
2e-03
3e-03
5e-03
9e-03
2e-02
Log-odds
0.5
1.0
1.5
2.0
2.5
3.0
3.5
4.0

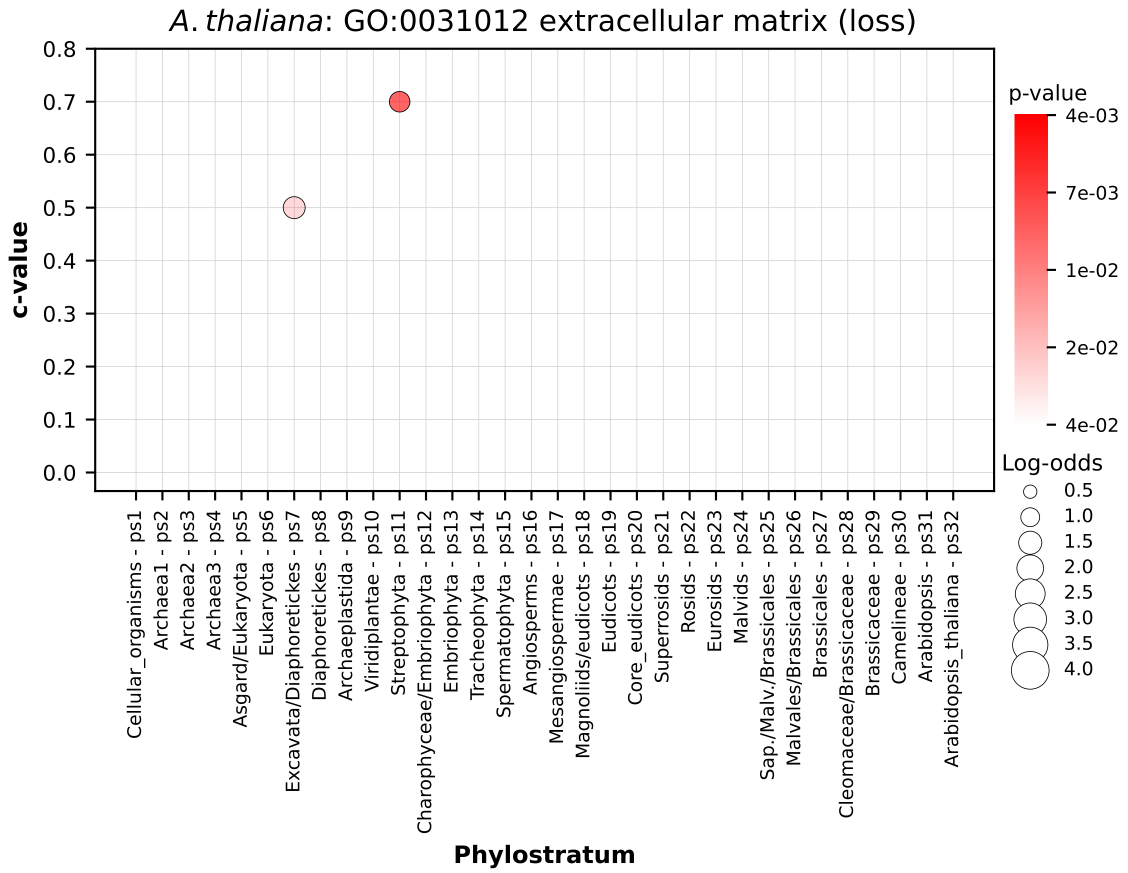

A. thaliana: GO:0031012 extracellular matrix (loss)
c-value
0.8
0.7
0.6
0.5
0.4
0.3
0.2
0.1
0.0
Phylostratum
Cellular_organisms - ps1
Archaea1 - ps2
Archaea2 - ps3
Archaea3 - ps4
Asgard/Eukaryota - ps5
Eukaryota - ps6
Excavata/Diaphoretickes - ps7
Diaphoretickes - ps8
Archaeplastida - ps9
Viridiplantae - ps10
Streptophyta - ps11
Charophyceae/Embriophyta - ps12
Embriophyta - ps13
Tracheophyta - ps14
Spermatophyta - ps15
Angiosperms - ps16
Mesangiospermae - ps17
Magnoliids/eudicots - ps18
Eudicots - ps19
Core_eudicots - ps20
Superrosids - ps21
Rosids - ps22
Eurosids - ps23
Malvids - ps24
Sap./Malv./Brassicales - ps25
Malvales/Brassicales - ps26
Brassicales - ps27
Cleomaceae/Brassicaceae - ps28
Brassicaceae - ps29
Camelineae - ps30
Arabidopsis - ps31
Arabidopsis_thaliana - ps32
p-value
4e-03
7e-03
1e-02
2e-02
4e-02
Log-odds
0.5
1.0
1.5
2.0
2.5
3.0
3.5
4.0

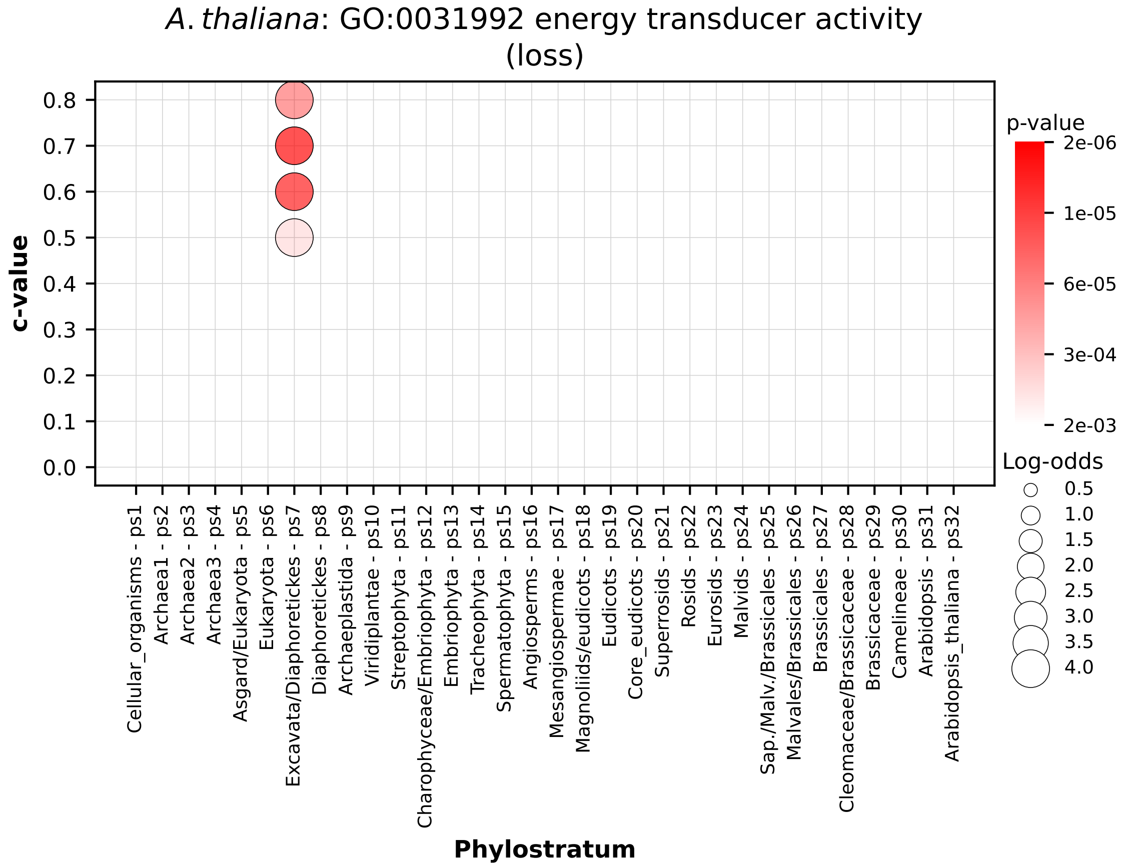
A. thaliana: GO:0031992 energy transducer activity
(loss)
c-value
0.8
0.7
0.6
0.5
0.4
0.3
0.2
0.1
0.0
Phylostratum
Cellular_organisms - ps1
Archaea1 - ps2
Archaea2 - ps3
Archaea3 - ps4
Asgard/Eukaryota - ps5
Eukaryota - ps6
Excavata/Diaphoretickes - ps7
Diaphoretickes - ps8
Archaeplastida - ps9
Viridiplantae - ps10
Streptophyta - ps11
Charophyceae/Embriophyta - ps12
Embriophyta - ps13
Tracheophyta - ps14
Spermatophyta - ps15
Angiosperms - ps16
Mesangiospermae - ps17
Magnoliids/eudicots - ps18
Eudicots - ps19
Core_eudicots - ps20
Superrosids - ps21
Rosids - ps22
Eurosids - ps23
Malvids - ps24
Sap./Malv./Brassicales - ps25
Malvales/Brassicales - ps26
Brassicales - ps27
Cleomaceae/Brassicaceae - ps28
Brassicaceae - ps29
Camelineae - ps30
Arabidopsis - ps31
Arabidopsis_thaliana - ps32
p-value
2e-06
1e-05
6e-05
3e-04
2e-03
Log-odds
0.5
1.0
1.5
2.0
2.5
3.0
3.5
4.0

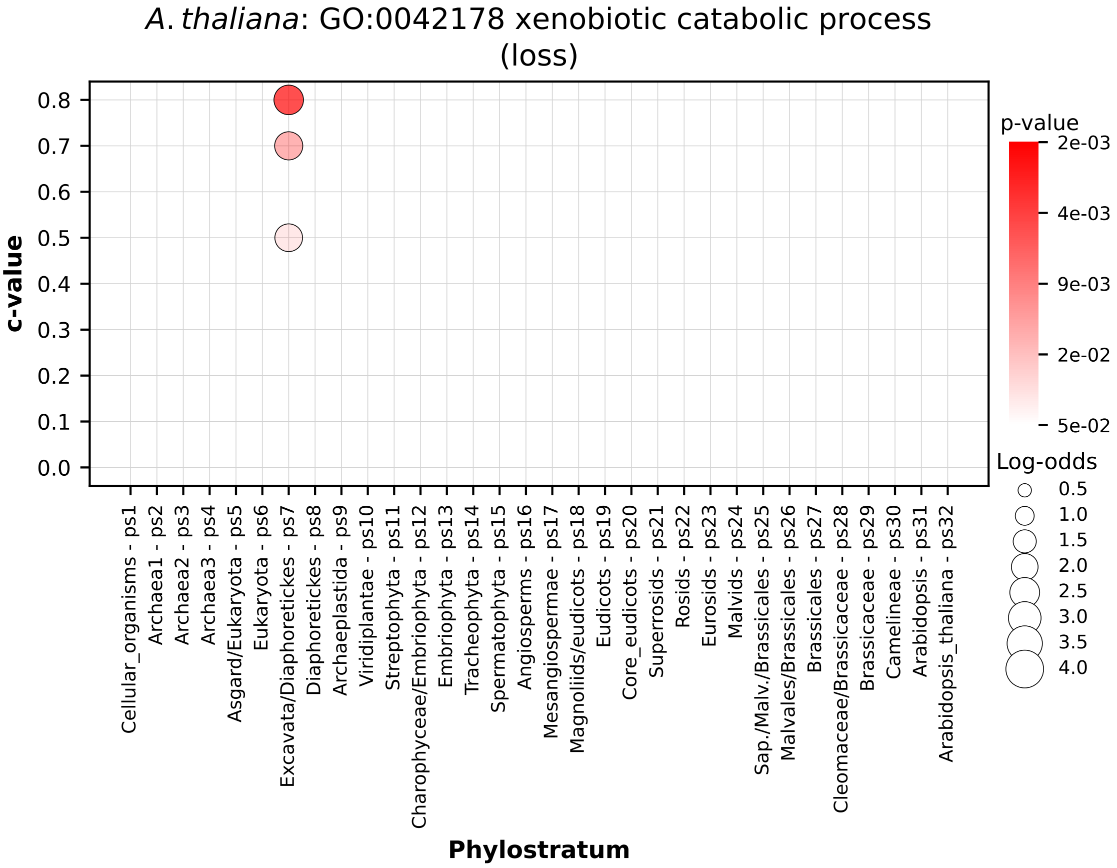

A. thaliana: GO:0042178 xenobiotic catabolic process (loss)
c-value
0.8
0.7
0.6
0.5
0.4
0.3
0.2
0.1
0.0
p-value
2e-03
4e-03
9e-03
2e-02
5e-02
Log-odds
0.5
1.0
1.5
2.0
2.5
3.0
3.5
4.0
Cellular_organisms - ps1
Archaea1 - ps2
Archaea2 - ps3
Archaea3 - ps4
Asgard/Eukaryota - ps5
Eukaryota - ps6
Excavata/Diaphoretickes - ps7
Diaphoretickes - ps8
Archaeplastida - ps9
Viridiplantae - ps10
Streptophyta - ps11
Charophyceae/Embriophyta - ps12
Embriophyta - ps13
Tracheophyta - ps14
Spermatophyta - ps15
Angiosperms - ps16
Mesangiospermae - ps17
Magnoliids/eudicots - ps18
Eudicots - ps19
Core_eudicots - ps20
Superrosids - ps21
Rosids - ps22
Eurosids - ps23
Malvids - ps24
Sap./Malv./Brassicales - ps25
Malvales/Brassicales - ps26
Brassicales - ps27
Cleomaceae/Brassicaceae - ps28
Brassicaceae - ps29
Camelineae - ps30
Arabidopsis - ps31
Arabidopsis_thaliana - ps32
Phylostratum

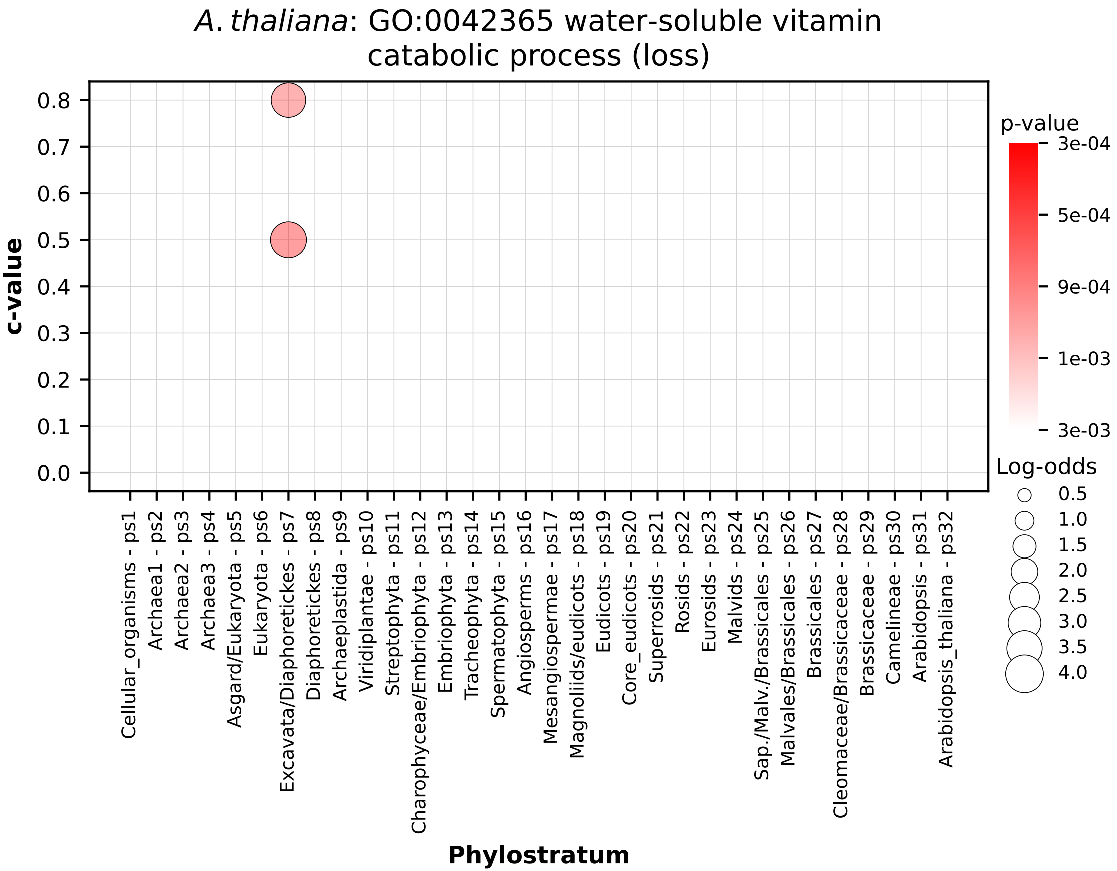

A. thaliana: GO:0042365 water-soluble vitamin catabolic process (loss)
c-value
0.8
0.7
0.6
0.5
0.4
0.3
0.2
0.1
0.0
Phylostratum
Cellular_organisms - ps1
Archaea1 - ps2
Archaea2 - ps3
Archaea3 - ps4
Asgard/Eukaryota - ps5
Eukaryota - ps6
Excavata/Diaphoretickes - ps7
Diaphoretickes - ps8
Archaeplastida - ps9
Viridiplantae - ps10
Streptophyta - ps11
Charophyceae/Embriophyta - ps12
Embriophyta - ps13
Tracheophyta - ps14
Spermatophyta - ps15
Angiosperms - ps16
Mesangiospermae - ps17
Magnoliids/eudicots - ps18
Eudicots - ps19
Core_eudicots - ps20
Superrosids - ps21
Rosids - ps22
Eurosids - ps23
Malvids - ps24
Sap./Malv./Brassicales - ps25
Malvales/Brassicales - ps26
Brassicales - ps27
Cleomaceae/Brassicaceae - ps28
Brassicaceae - ps29
Camelineae - ps30
Arabidopsis - ps31
Arabidopsis_thaliana - ps32
p-value
3e-04
5e-04
9e-04
1e-03
3e-03
Log-odds
0.5
1.0
1.5
2.0
2.5
3.0
3.5
4.0

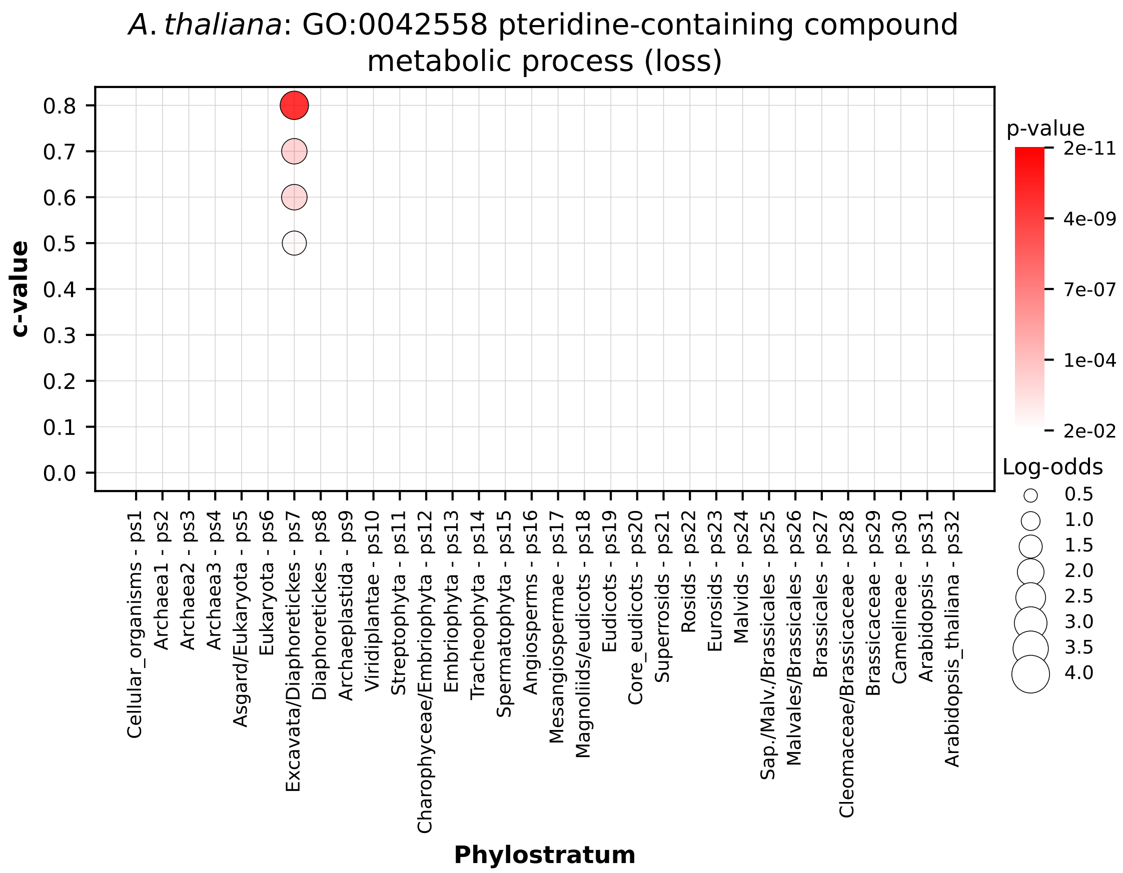

A. thaliana: GO:0042558 pteridine-containing compound metabolic process (loss)
c-value
0.8
0.7
0.6
0.5
0.4
0.3
0.2
0.1
0.0
p-value
2e-11
4e-09
7e-07
1e-04
2e-02
Log-odds
0.5
1.0
1.5
2.0
2.5
3.0
3.5
4.0
Cellular_organisms - ps1
Archaea1 - ps2
Archaea2 - ps3
Archaea3 - ps4
Asgard/Eukaryota - ps5
Eukaryota - ps6
Excavata/Diaphoretickes - ps7
Diaphoretickes - ps8
Archaeplastida - ps9
Viridiplantae - ps10
Streptophyta - ps11
Charophyceae/Embriophyta - ps12
Embriophyta - ps13
Tracheophyta - ps14
Spermatophyta - ps15
Angiosperms - ps16
Mesangiospermae - ps17
Magnoliids/eudicots - ps18
Eudicots - ps19
Core_eudicots - ps20
Superrosids - ps21
Rosids - ps22
Eurosids - ps23
Malvids - ps24
Sap./Malv./Brassicales - ps25
Malvales/Brassicales - ps26
Brassicales - ps27
Cleomaceae/Brassicaceae - ps28
Brassicaceae - ps29
Camelineae - ps30
Arabidopsis - ps31
Arabidopsis_thaliana - ps32
Phylostratum

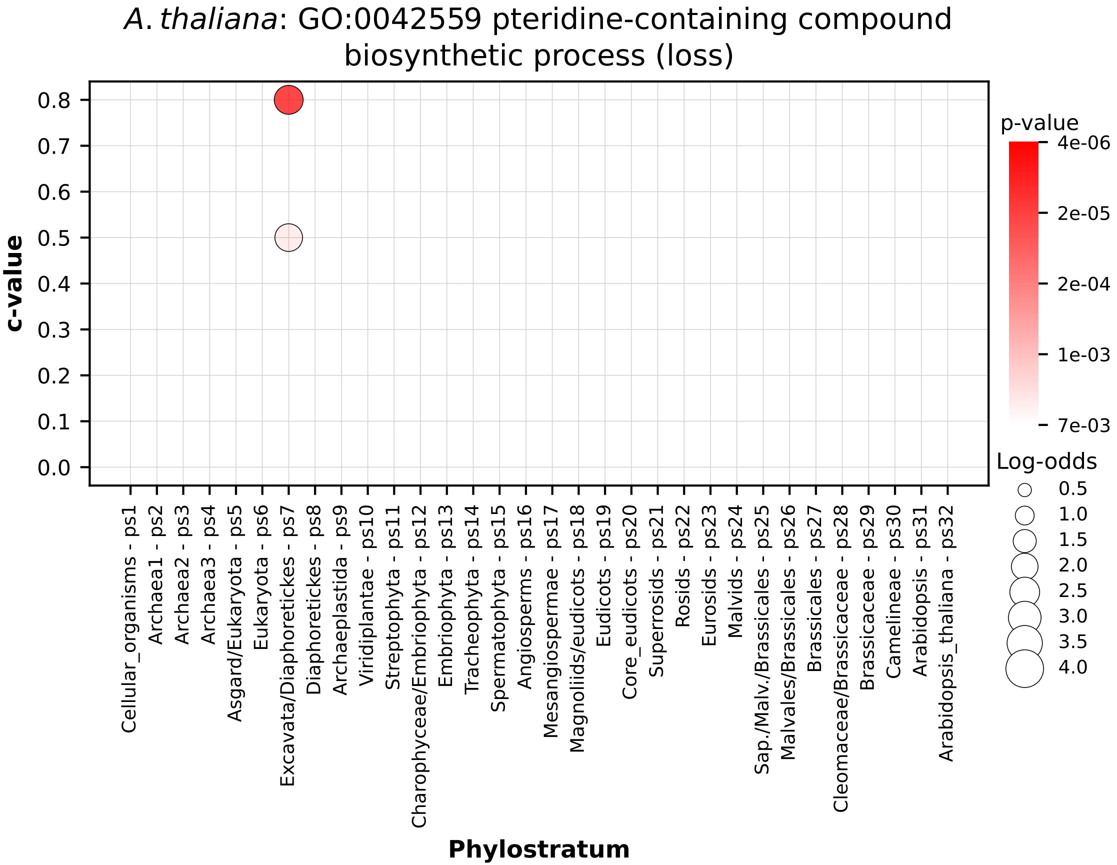

A. thaliana: GO:0042559 pteridine-containing compound biosynthetic process (loss)
c-value
0.0
0.1
0.2
0.3
0.4
0.5
0.6
0.7
0.8
Phylostratum
Cellular_organisms - ps1
Archaea1 - ps2
Archaea2 - ps3
Archaea3 - ps4
Asgard/Eukaryota - ps5
Eukaryota - ps6
Excavata/Diaphoretickes - ps7
Diaphoretickes - ps8
Archaeplastida - ps9
Viridiplantae - ps10
Streptophyta - ps11
Charophyceae/Embriophyta - ps12
Embriophyta - ps13
Tracheophyta - ps14
Spermatophyta - ps15
Angiosperms - ps16
Mesangiospermae - ps17
Magnoliids/eudicots - ps18
Eudicots - ps19
Core_eudicots - ps20
Superrosids - ps21
Rosids - ps22
Eurosids - ps23
Malvids - ps24
Sap./Malv./Brassicales - ps25
Malvales/Brassicales - ps26
Brassicales - ps27
Cleomaceae/Brassicaceae - ps28
Brassicaceae - ps29
Camelineae - ps30
Arabidopsis - ps31
Arabidopsis_thaliana - ps32
p-value
4e-06
2e-05
2e-04
1e-03
7e-03
Log-odds
0.5
1.0
1.5
2.0
2.5
3.0
3.5
4.0

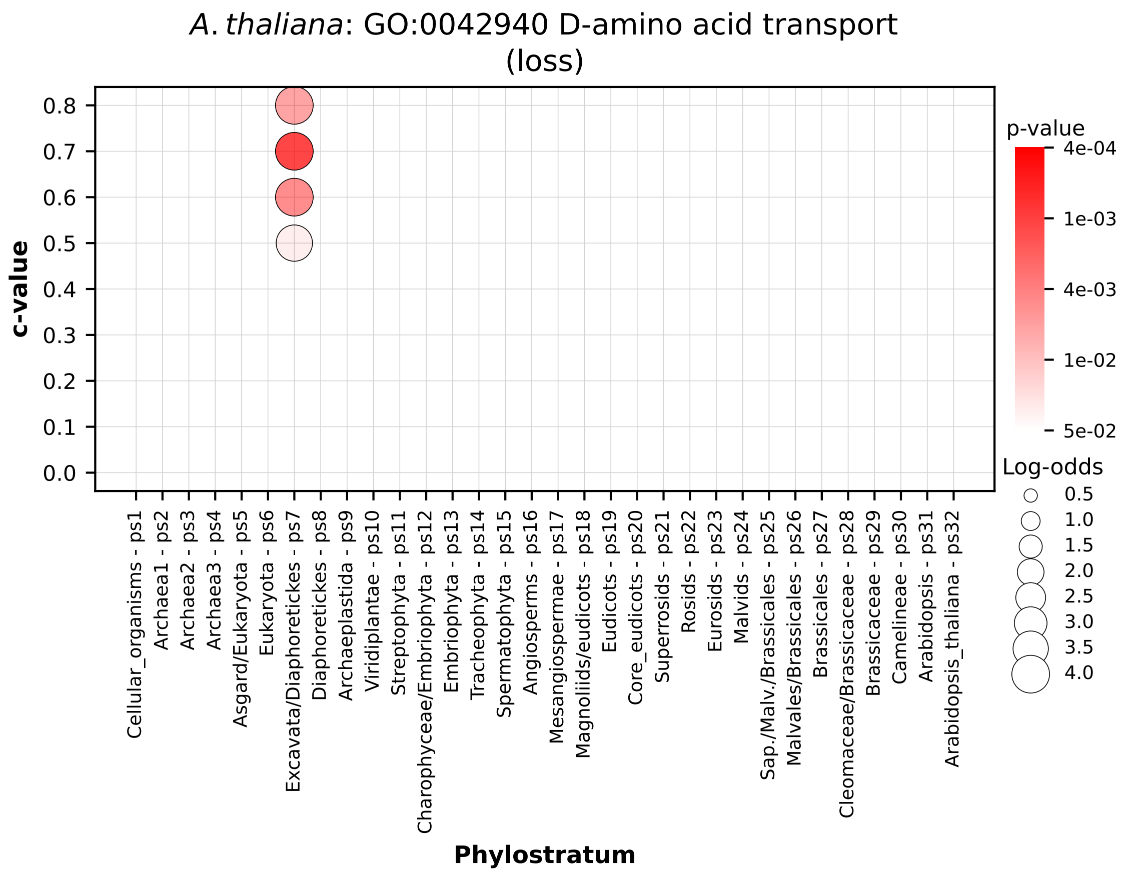

A. thaliana: GO:0042940 D-amino acid transport
(loss)
c-value
0.8
0.7
0.6
0.5
0.4
0.3
0.2
0.1
0.0
p-value
4e-04
1e-03
4e-03
1e-02
5e-02
Log-odds
0.5
1.0
1.5
2.0
2.5
3.0
3.5
4.0
Cellular_organisms - ps1
Archaea1 - ps2
Archaea2 - ps3
Archaea3 - ps4
Asgard/Eukaryota - ps5
Eukaryota - ps6
Excavata/Diaphoretickes - ps7
Diaphoretickes - ps8
Archaeplastida - ps9
Viridiplantae - ps10
Streptophyta - ps11
Charophyceae/Embriophyta - ps12
Embriophyta - ps13
Tracheophyta - ps14
Spermatophyta - ps15
Angiosperms - ps16
Mesangiospermae - ps17
Magnoliids/eudicots - ps18
Eudicots - ps19
Core_eudicots - ps20
Superrosids - ps21
Rosids - ps22
Eurosids - ps23
Malvids - ps24
Sap./Malv./Brassicales - ps25
Malvales/Brassicales - ps26
Brassicales - ps27
Cleomaceae/Brassicaceae - ps28
Brassicaceae - ps29
Camelineae - ps30
Arabidopsis - ps31
Arabidopsis_thaliana - ps32
Phylostratum

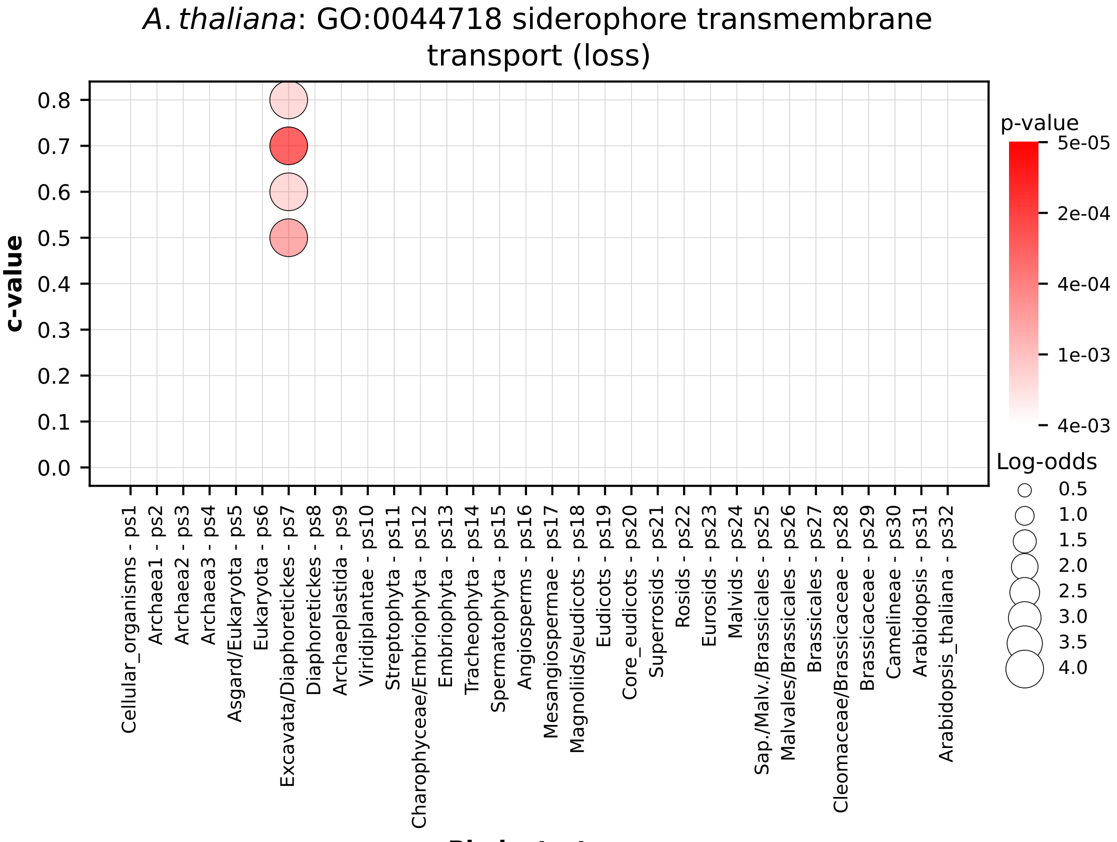

A. thaliana: GO:0044718 siderophore transmembrane transport (loss)
c-value
0.8
0.7
0.6
0.5
0.4
0.3
0.2
0.1
0.0
p-value
5e-05
2e-04
4e-04
1e-03
4e-03
Log-odds
0.5
1.0
1.5
2.0
2.5
3.0
3.5
4.0
Cellular_organisms - ps1
Archaea1 - ps2
Archaea2 - ps3
Archaea3 - ps4
Asgard/Eukaryota - ps5
Eukaryota - ps6
Excavata/Diaphoretickes - ps7
Diaphoretickes - ps8
Archaeplastida - ps9
Viridiplantae - ps10
Streptophyta - ps11
Charophyceae/Embriophyta - ps12
Embriophyta - ps13
Tracheophyta - ps14
Spermatophyta - ps15
Angiosperms - ps16
Mesangiospermae - ps17
Magnoliids/eudicots - ps18
Eudicots - ps19
Core_eudicots - ps20
Superrosids - ps21
Rosids - ps22
Eurosids - ps23
Malvids - ps24
Sap./Malv./Brassicales - ps25
Malvales/Brassicales - ps26
Brassicales - ps27
Cleomaceae/Brassicaceae - ps28
Brassicaceae - ps29
Camelineae - ps30
Arabidopsis - ps31
Arabidopsis_thaliana - ps32
Phylostratum

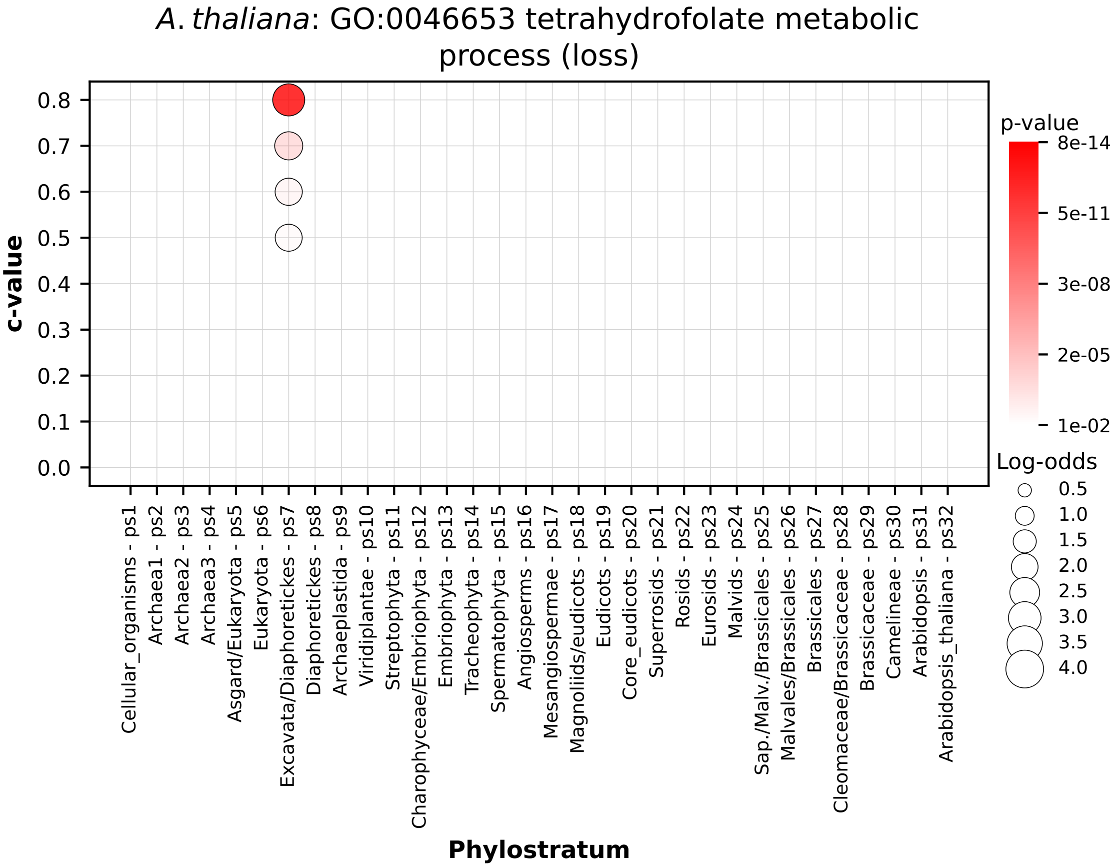

$A. thaliana$: GO:0046653 tetrahydrofolate metabolic process (loss)
c-value
0.8
0.7
0.6
0.5
0.4
0.3
0.2
0.1
0.0
Phylostratum
Cellular_organisms - ps1
Archaea1 - ps2
Archaea2 - ps3
Archaea3 - ps4
Asgard/Eukaryota - ps5
Eukaryota - ps6
Excavata/Diaphoretickes - ps7
Diaphoretickes - ps8
Archaeplastida - ps9
Viridiplantae - ps10
Streptophyta - ps11
Charophyceae/Embriophyta - ps12
Embriophyta - ps13
Tracheophyta - ps14
Spermatophyta - ps15
Angiosperms - ps16
Mesangiospermae - ps17
Magnoliids/eudicots - ps18
Eudicots - ps19
Core_eudicots - ps20
Superrosids - ps21
Rosids - ps22
Eurosids - ps23
Malvids - ps24
Sap./Malv./Brassicales - ps25
Malvales/Brassicales - ps26
Brassicales - ps27
Cleomaceae/Brassicaceae - ps28
Brassicaceae - ps29
Camelineae - ps30
Arabidopsis - ps31
Arabidopsis_thaliana - ps32
p-value
8e-14
5e-11
3e-08
2e-05
1e-02
Log-odds
0.5
1.0
1.5
2.0
2.5
3.0
3.5
4.0

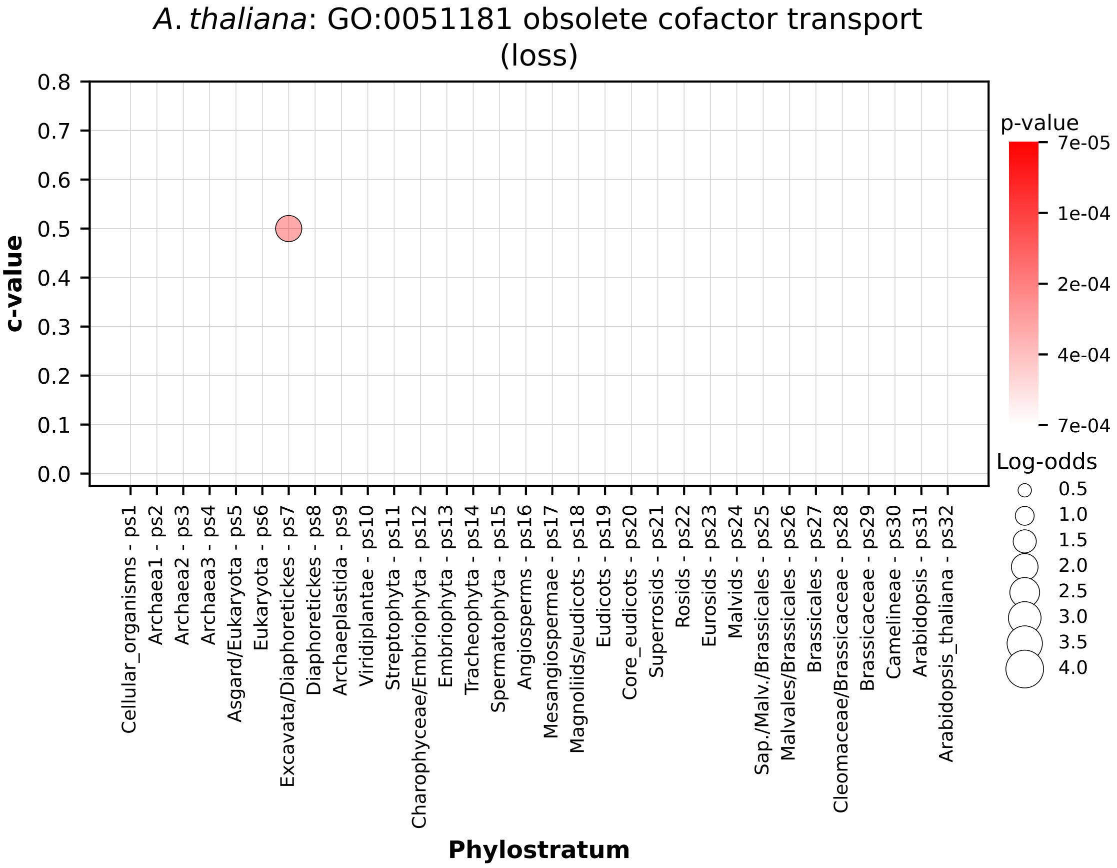

A. thaliana: GO:0051181 obsolete cofactor transport
(loss)
c-value
0.8
0.7
0.6
0.5
0.4
0.3
0.2
0.1
0.0
p-value
7e-05
1e-04
2e-04
4e-04
7e-04
Log-odds
0.5
1.0
1.5
2.0
2.5
3.0
3.5
4.0
Cellular_organisms - ps1
Archaea1 - ps2
Archaea2 - ps3
Archaea3 - ps4
Asgard/Eukaryota - ps5
Eukaryota - ps6
Excavata/Diaphoretickes - ps7
Diaphoretickes - ps8
Archaeplastida - ps9
Viridiplantae - ps10
Streptophyta - ps11
Charophyceae/Embriophyta - ps12
Embriophyta - ps13
Tracheophyta - ps14
Spermatophyta - ps15
Angiosperms - ps16
Mesangiospermae - ps17
Magnoliids/eudicots - ps18
Eudicots - ps19
Core_eudicots - ps20
Superrosids - ps21
Rosids - ps22
Eurosids - ps23
Malvids - ps24
Sap./Malv./Brassicales - ps25
Malvales/Brassicales - ps26
Brassicales - ps27
Cleomaceae/Brassicaceae - ps28
Brassicaceae - ps29
Camelineae - ps30
Arabidopsis - ps31
Arabidopsis_thaliana - ps32
Phylostratum

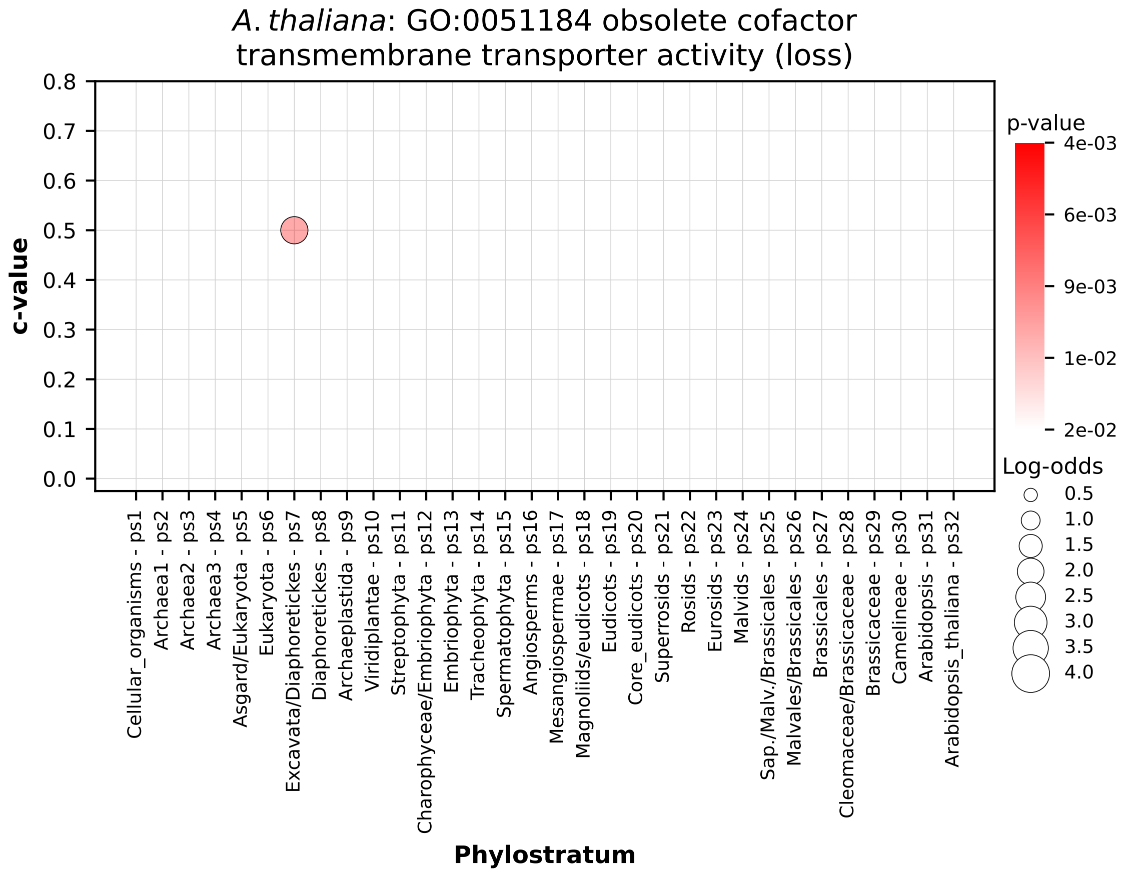

A. thaliana: GO:0051184 obsolete cofactor transmembrane transporter activity (loss)
c-value
0.8
0.7
0.6
0.5
0.4
0.3
0.2
0.1
0.0
p-value
4e-03
6e-03
9e-03
1e-02
2e-02
Log-odds
0.5
1.0
1.5
2.0
2.5
3.0
3.5
4.0
Cellular_organisms - ps1
Archaea1 - ps2
Archaea2 - ps3
Archaea3 - ps4
Asgard/Eukaryota - ps5
Eukaryota - ps6
Excavata/Diaphoretickes - ps7
Diaphoretickes - ps8
Archaeplastida - ps9
Viridiplantae - ps10
Streptophyta - ps11
Charophyceae/Embriophyta - ps12
Embriophyta - ps13
Tracheophyta - ps14
Spermatophyta - ps15
Angiosperms - ps16
Mesangiospermae - ps17
Magnoliids/eudicots - ps18
Eudicots - ps19
Core_eudicots - ps20
Superrosids - ps21
Rosids - ps22
Eurosids - ps23
Malvids - ps24
Sap./Malv./Brassicales - ps25
Malvales/Brassicales - ps26
Brassicales - ps27
Cleomaceae/Brassicaceae - ps28
Brassicaceae - ps29
Camelineae - ps30
Arabidopsis - ps31
Arabidopsis_thaliana - ps32
Phylostratum

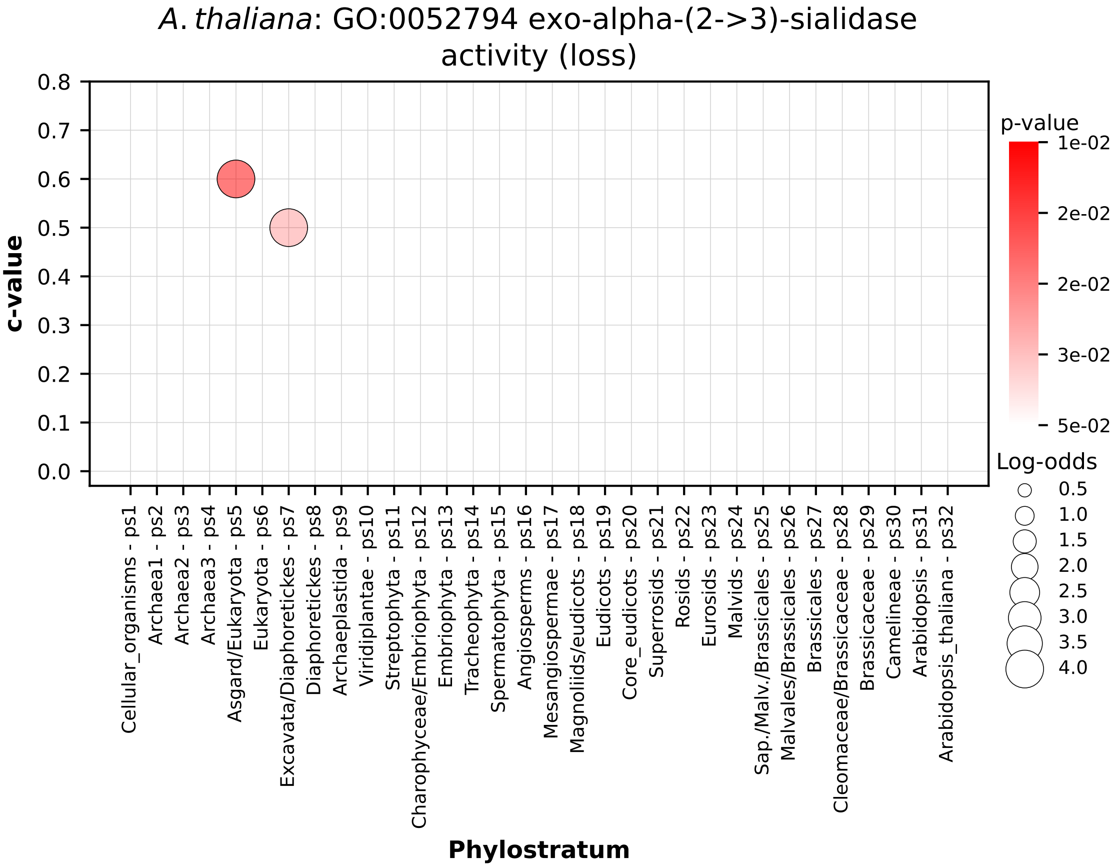

A. thaliana: GO:0052794 exo-alpha-(2->3)-sialidase activity (loss)
c-value
0.8
0.7
0.6
0.5
0.4
0.3
0.2
0.1
0.0
p-value
1e-02
2e-02
2e-02
3e-02
5e-02
Log-odds
0.5
1.0
1.5
2.0
2.5
3.0
3.5
4.0
Cellular_organisms - ps1
Archaea1 - ps2
Archaea2 - ps3
Archaea3 - ps4
Asgard/Eukaryota - ps5
Eukaryota - ps6
Excavata/Diaphoretickes - ps7
Diaphoretickes - ps8
Archaeplastida - ps9
Viridiplantae - ps10
Streptophyta - ps11
Charophyceae/Embriophyta - ps12
Embriophyta - ps13
Tracheophyta - ps14
Spermatophyta - ps15
Angiosperms - ps16
Mesangiospermae - ps17
Magnoliids/eudicots - ps18
Eudicots - ps19
Core_eudicots - ps20
Superrosids - ps21
Rosids - ps22
Eurosids - ps23
Malvids - ps24
Sap./Malv./Brassicales - ps25
Malvales/Brassicales - ps26
Brassicales - ps27
Cleomaceae/Brassicaceae - ps28
Brassicaceae - ps29
Camelineae - ps30
Arabidopsis - ps31
Arabidopsis_thaliana - ps32
Phylostratum

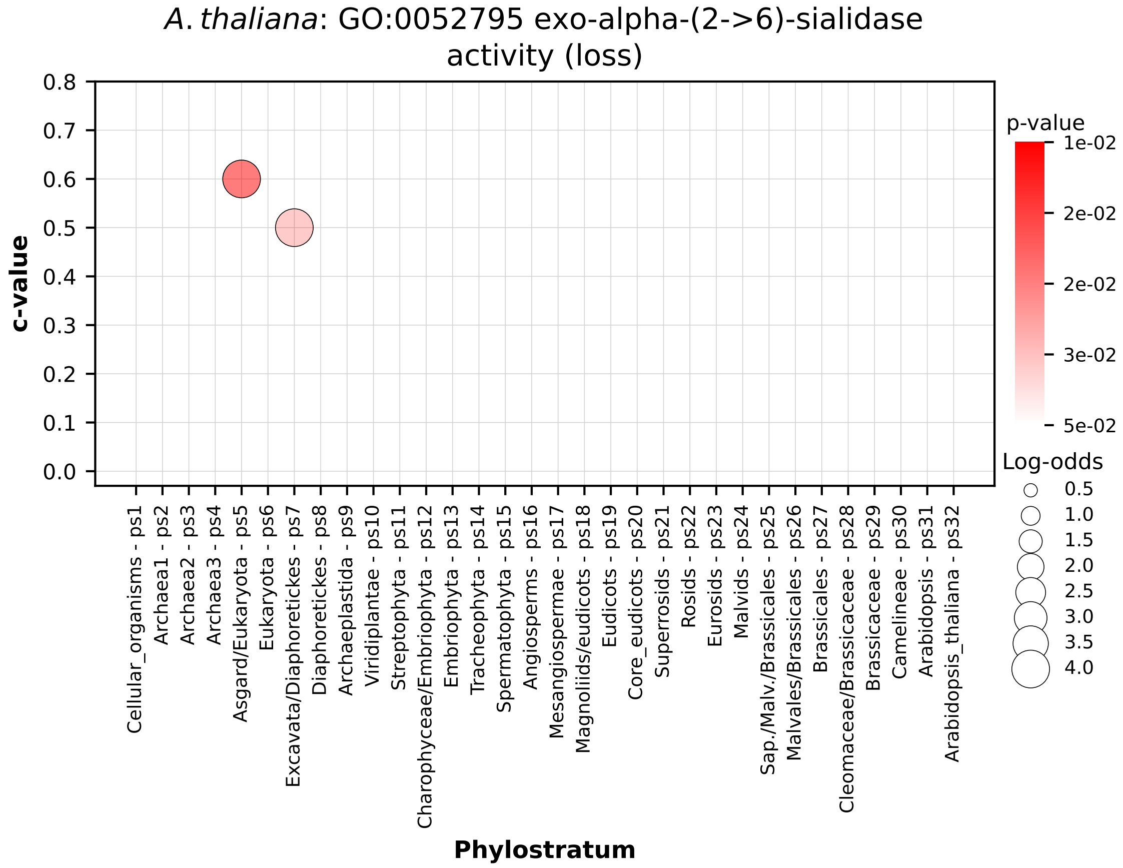
A. thaliana: GO:0052795 exo-alpha-(2->6)-sialidase activity (loss)
c-value
0.8
0.7
0.6
0.5
0.4
0.3
0.2
0.1
0.0
Phylostratum
Cellular_organisms - ps1
Archaea1 - ps2
Archaea2 - ps3
Archaea3 - ps4
Asgard/Eukaryota - ps5
Eukaryota - ps6
Excavata/Diaphoretickes - ps7
Diaphoretickes - ps8
Archaeplastida - ps9
Viridiplantae - ps10
Streptophyta - ps11
Charophyceae/Embriophyta - ps12
Embriophyta - ps13
Tracheophyta - ps14
Spermatophyta - ps15
Angiosperms - ps16
Mesangiospermae - ps17
Magnoliids/eudicots - ps18
Eudicots - ps19
Core_eudicots - ps20
Superrosids - ps21
Rosids - ps22
Eurosids - ps23
Malvids - ps24
Sap./Malv./Brassicales - ps25
Malvales/Brassicales - ps26
Brassicales - ps27
Cleomaceae/Brassicaceae - ps28
Brassicaceae - ps29
Camelineae - ps30
Arabidopsis - ps31
Arabidopsis_thaliana - ps32
p-value
1e-02
2e-02
2e-02
3e-02
5e-02
Log-odds
0.5
1.0
1.5
2.0
2.5
3.0
3.5
4.0

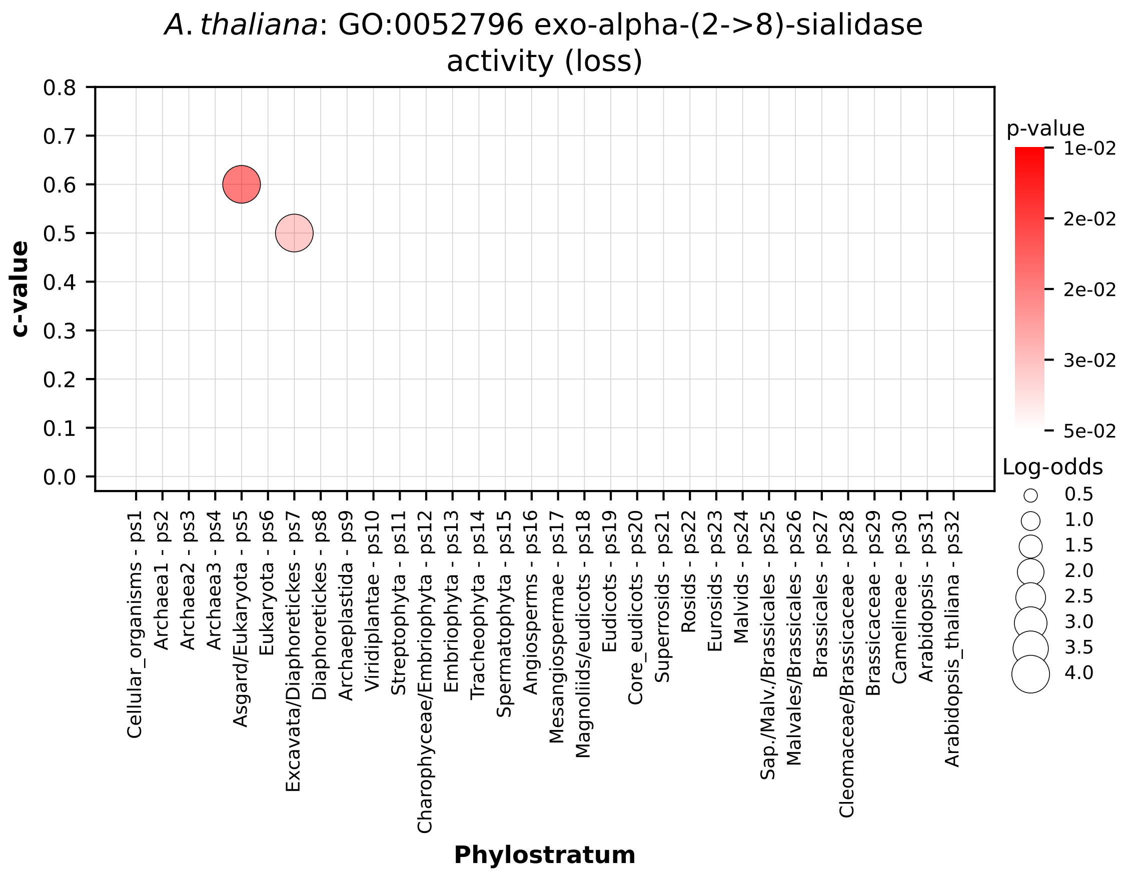

A. thaliana: GO:0052796 exo-alpha-(2->8)-sialidase activity (loss)
c-value
0.8
0.7
0.6
0.5
0.4
0.3
0.2
0.1
0.0
p-value
1e-02
2e-02
2e-02
3e-02
5e-02
Log-odds
0.5
1.0
1.5
2.0
2.5
3.0
3.5
4.0
Cellular_organisms - ps1
Archaea1 - ps2
Archaea2 - ps3
Archaea3 - ps4
Asgard/Eukaryota - ps5
Eukaryota - ps6
Excavata/Diaphoretickes - ps7
Diaphoretickes - ps8
Archaeplastida - ps9
Viridiplantae - ps10
Streptophyta - ps11
Charophyceae/Embriophyta - ps12
Embriophyta - ps13
Tracheophyta - ps14
Spermatophyta - ps15
Angiosperms - ps16
Mesangiospermae - ps17
Magnoliids/eudicots - ps18
Eudicots - ps19
Core_eudicots - ps20
Superrosids - ps21
Rosids - ps22
Eurosids - ps23
Malvids - ps24
Sap./Malv./Brassicales - ps25
Malvales/Brassicales - ps26
Brassicales - ps27
Cleomaceae/Brassicaceae - ps28
Brassicaceae - ps29
Camelineae - ps30
Arabidopsis - ps31
Arabidopsis_thaliana - ps32
Phylostratum

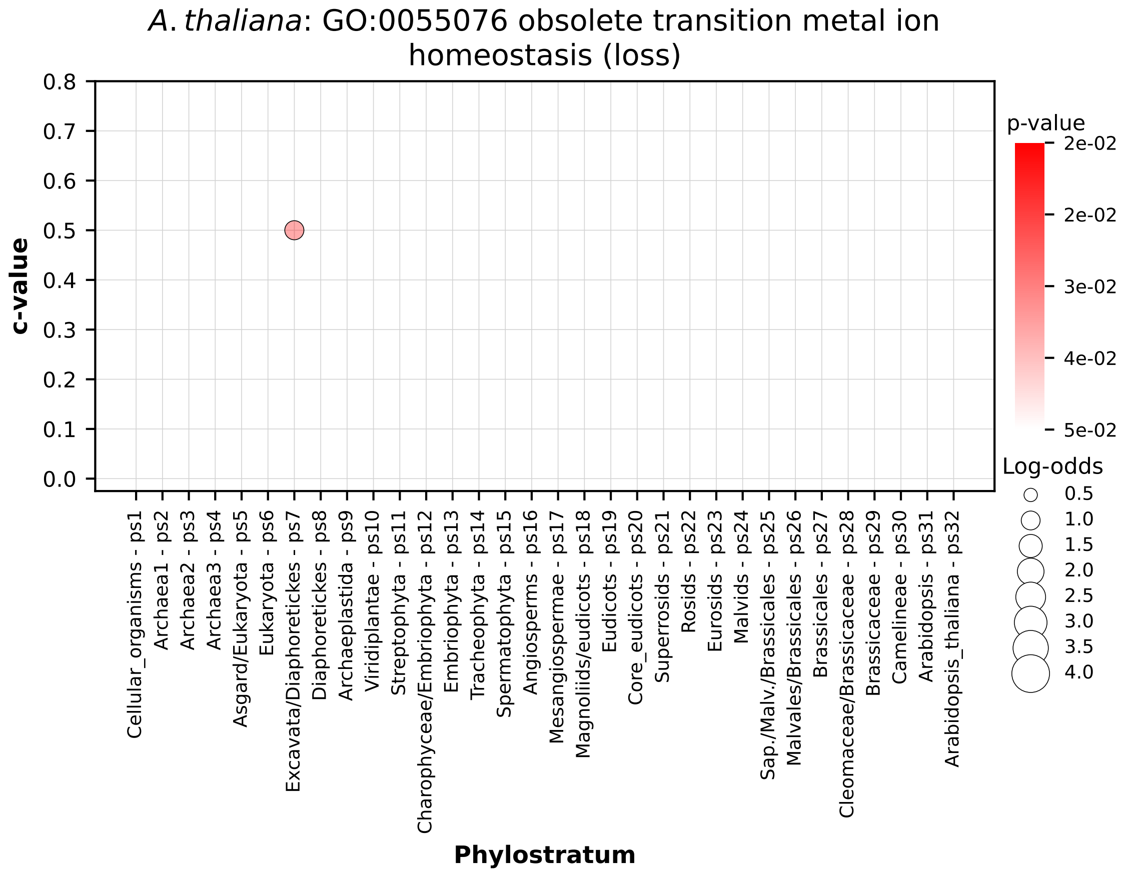

A. thaliana: GO:0055076 obsolete transition metal ion homeostasis (loss)
c-value
0.8
0.7
0.6
0.5
0.4
0.3
0.2
0.1
0.0
Phylostratum
Cellular_organisms - ps1
Archaea1 - ps2
Archaea2 - ps3
Archaea3 - ps4
Asgard/Eukaryota - ps5
Eukaryota - ps6
Excavata/Diaphoretickes - ps7
Diaphoretickes - ps8
Archaeplastida - ps9
Viridiplantae - ps10
Streptophyta - ps11
Charophyceae/Embriophyta - ps12
Embriophyta - ps13
Tracheophyta - ps14
Spermatophyta - ps15
Angiosperms - ps16
Mesangiospermae - ps17
Magnoliids/eudicots - ps18
Eudicots - ps19
Core_eudicots - ps20
Superrosids - ps21
Rosids - ps22
Eurosids - ps23
Malvids - ps24
Sap./Malv./Brassicales - ps25
Malvales/Brassicales - ps26
Brassicales - ps27
Cleomaceae/Brassicaceae - ps28
Brassicaceae - ps29
Camelineae - ps30
Arabidopsis - ps31
Arabidopsis_thaliana - ps32
p-value
2e-02
2e-02
3e-02
4e-02
5e-02
Log-odds
0.5
1.0
1.5
2.0
2.5
3.0
3.5
4.0

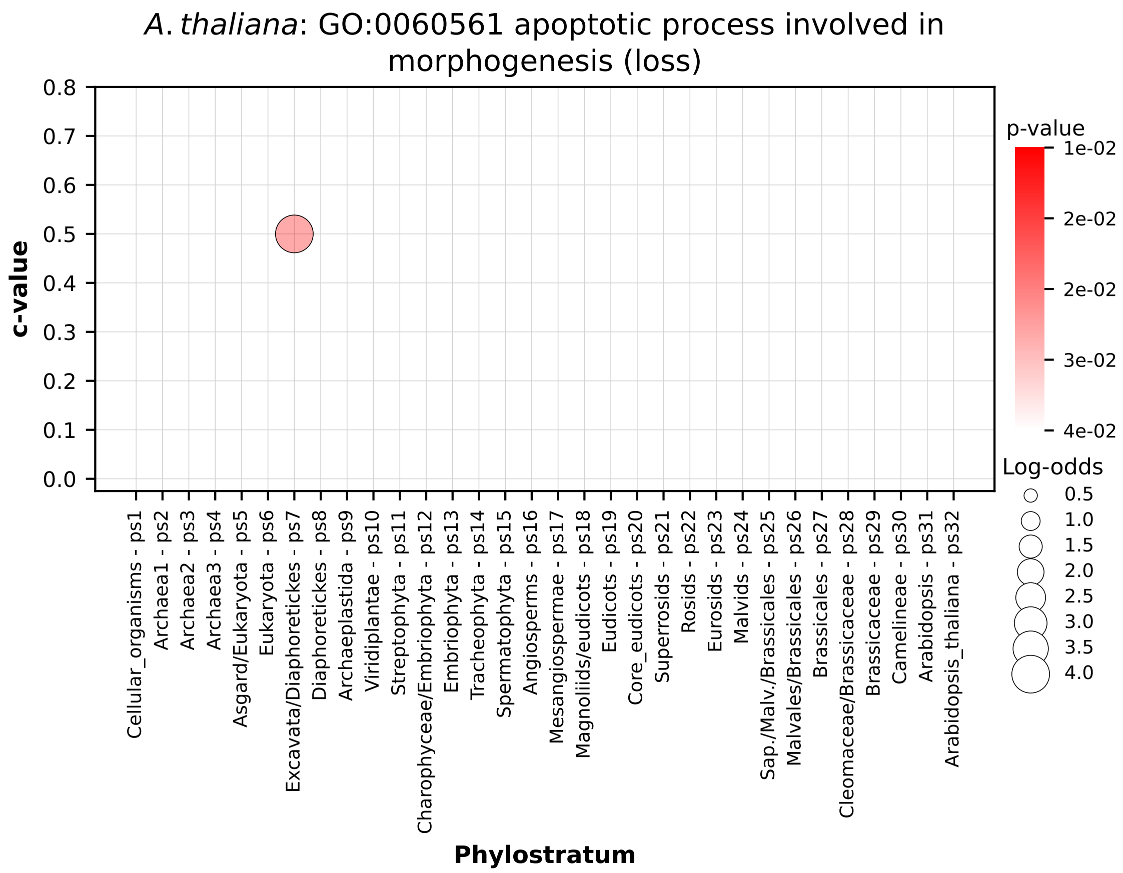

A. thaliana: GO:0060561 apoptotic process involved in morphogenesis (loss)
c-value
0.8
0.7
0.6
0.5
0.4
0.3
0.2
0.1
0.0
Phylostratum
Cellular_organisms - ps1
Archaea1 - ps2
Archaea2 - ps3
Archaea3 - ps4
Asgard/Eukaryota - ps5
Eukaryota - ps6
Excavata/Diaphoretickes - ps7
Diaphoretickes - ps8
Archaeplastida - ps9
Viridiplantae - ps10
Streptophyta - ps11
Charophyceae/Embriophyta - ps12
Embriophyta - ps13
Tracheophyta - ps14
Spermatophyta - ps15
Angiosperms - ps16
Mesangiospermae - ps17
Magnoliids/eudicots - ps18
Eudicots - ps19
Core_eudicots - ps20
Superrosids - ps21
Rosids - ps22
Eurosids - ps23
Malvids - ps24
Sap./Malv./Brassicales - ps25
Malvales/Brassicales - ps26
Brassicales - ps27
Cleomaceae/Brassicaceae - ps28
Brassicaceae - ps29
Camelineae - ps30
Arabidopsis - ps31
Arabidopsis_thaliana - ps32
p-value
1e-02
2e-02
2e-02
3e-02
4e-02
Log-odds
0.5
1.0
1.5
2.0
2.5
3.0
3.5
4.0

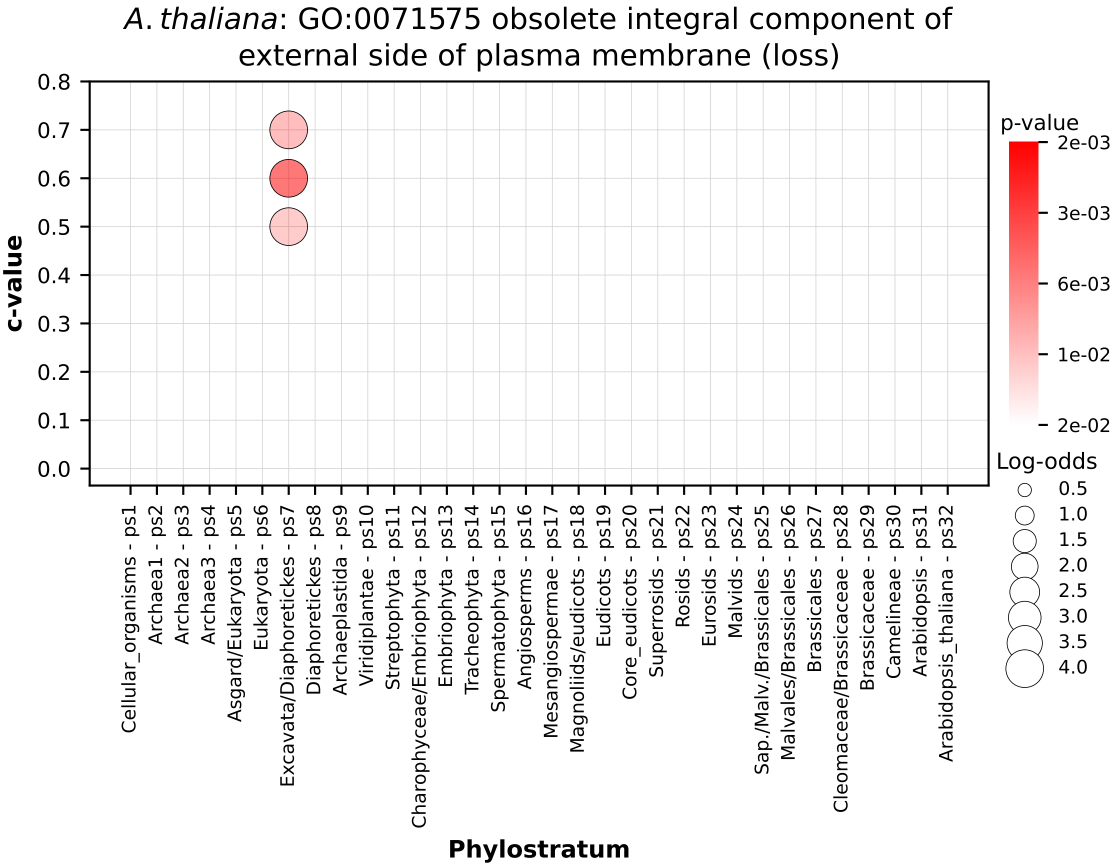

A. thaliana: GO:0071575 obsolete integral component of external side of plasma membrane (loss)
c-value
0.8
0.7
0.6
0.5
0.4
0.3
0.2
0.1
0.0
Phylostratum
Cellular_organisms - ps1
Archaea1 - ps2
Archaea2 - ps3
Archaea3 - ps4
Asgard/Eukaryota - ps5
Eukaryota - ps6
Excavata/Diaphoretickes - ps7
Diaphoretickes - ps8
Archaeplastida - ps9
Viridiplantae - ps10
Streptophyta - ps11
Charophyceae/Embriophyta - ps12
Embriophyta - ps13
Tracheophyta - ps14
Spermatophyta - ps15
Angiosperms - ps16
Mesangiospermae - ps17
Magnoliids/eudicots - ps18
Eudicots - ps19
Core_eudicots - ps20
Superrosids - ps21
Rosids - ps22
Eurosids - ps23
Malvids - ps24
Sap./Malv./Brassicales - ps25
Malvales/Brassicales - ps26
Brassicales - ps27
Cleomaceae/Brassicaceae - ps28
Brassicaceae - ps29
Camelineae - ps30
Arabidopsis - ps31
Arabidopsis_thaliana - ps32
p-value
2e-03
3e-03
6e-03
1e-02
2e-02
Log-odds
0.5
1.0
1.5
2.0
2.5
3.0
3.5
4.0

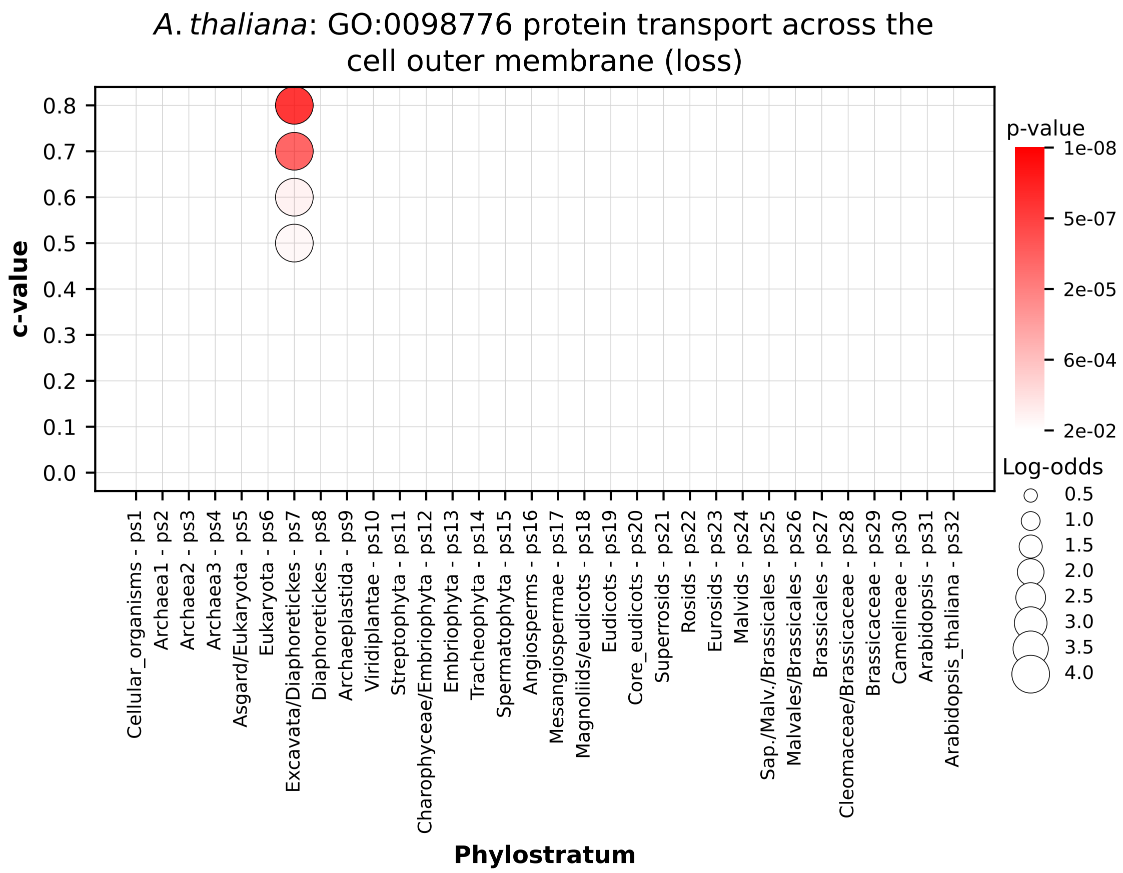

A. thaliana: GO:0098776 protein transport across the cell outer membrane (loss)
c-value
0.8
0.7
0.6
0.5
0.4
0.3
0.2
0.1
0.0
Phylostratum
Cellular_organisms - ps1
Archaea1 - ps2
Archaea2 - ps3
Archaea3 - ps4
Asgard/Eukaryota - ps5
Eukaryota - ps6
Excavata/Diaphoretickes - ps7
Diaphoretickes - ps8
Archaeplastida - ps9
Viridiplantae - ps10
Streptophyta - ps11
Charophyceae/Embriophyta - ps12
Embriophyta - ps13
Tracheophyta - ps14
Spermatophyta - ps15
Angiosperms - ps16
Mesangiospermae - ps17
Magnoliids/eudicots - ps18
Eudicots - ps19
Core_eudicots - ps20
Superrosids - ps21
Rosids - ps22
Eurosids - ps23
Malvids - ps24
Sap./Malv./Brassicales - ps25
Malvales/Brassicales - ps26
Brassicales - ps27
Cleomaceae/Brassicaceae - ps28
Brassicaceae - ps29
Camelineae - ps30
Arabidopsis - ps31
Arabidopsis_thaliana - ps32
p-value
1e-08
5e-07
2e-05
6e-04
2e-02
Log-odds
0.5
1.0
1.5
2.0
2.5
3.0
3.5
4.0

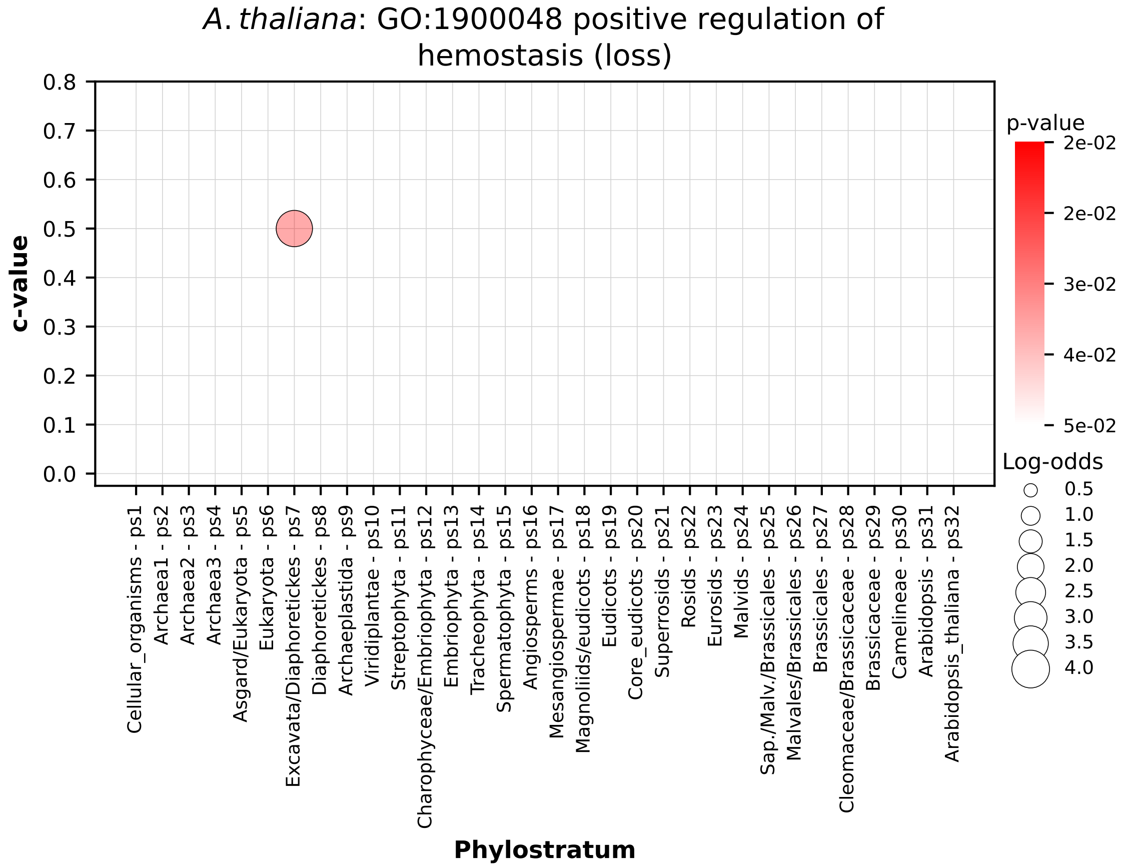
A. thaliana: GO:1900048 positive regulation of hemostasis (loss)
c-value
0.8
0.7
0.6
0.5
0.4
0.3
0.2
0.1
0.0
Phylostratum
Cellular_organisms - ps1
Archaea1 - ps2
Archaea2 - ps3
Archaea3 - ps4
Asgard/Eukaryota - ps5
Eukaryota - ps6
Excavata/Diaphoretickes - ps7
Diaphoretickes - ps8
Archaeplastida - ps9
Viridiplantae - ps10
Streptophyta - ps11
Charophyceae/Embriophyta - ps12
Embriophyta - ps13
Tracheophyta - ps14
Spermatophyta - ps15
Angiosperms - ps16
Mesangiospermae - ps17
Magnoliids/eudicots - ps18
Eudicots - ps19
Core_eudicots - ps20
Superrosids - ps21
Rosids - ps22
Eurosids - ps23
Malvids - ps24
Sap./Malv./Brassicales - ps25
Malvales/Brassicales - ps26
Brassicales - ps27
Cleomaceae/Brassicaceae - ps28
Brassicaceae - ps29
Camelineae - ps30
Arabidopsis - ps31
Arabidopsis_thaliana - ps32
p-value
2e-02
2e-02
3e-02
4e-02
5e-02
Log-odds
0.5
1.0
1.5
2.0
2.5
3.0
3.5
4.0

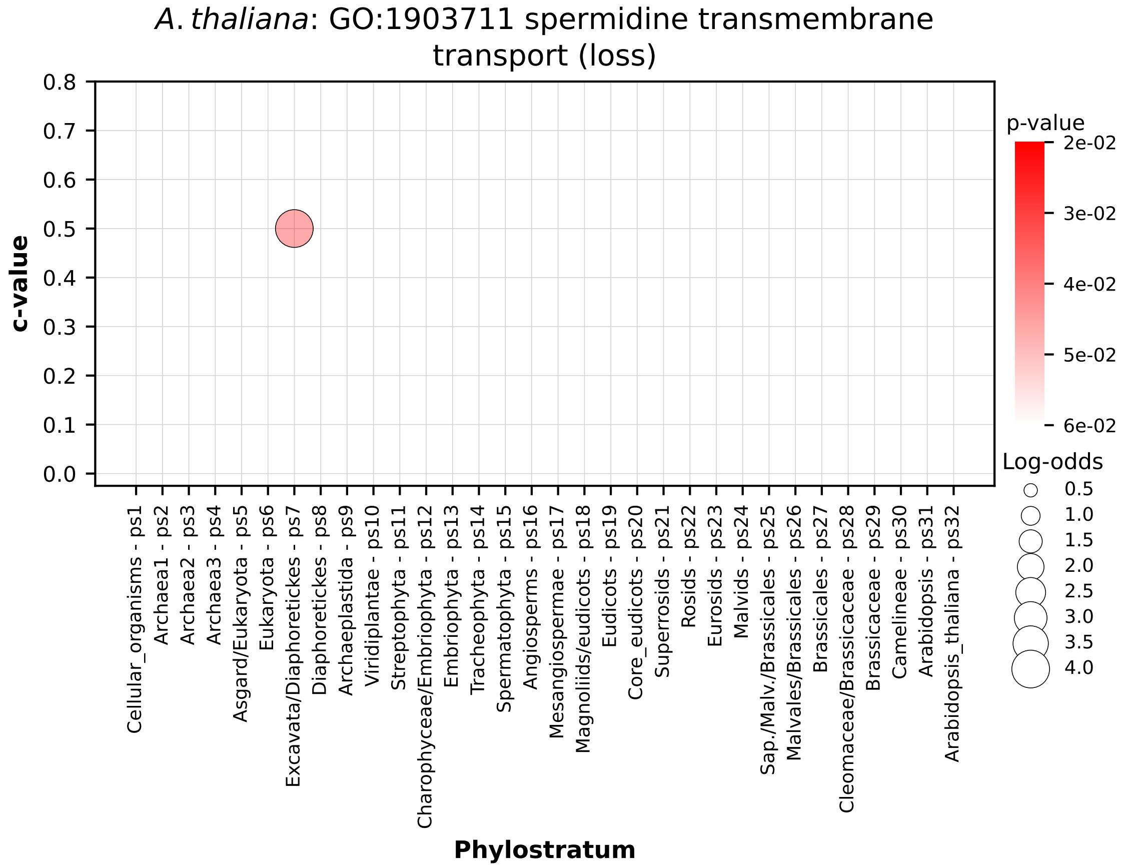

A. thaliana: GO:1903711 spermidine transmembrane transport (loss)
c-value
0.8
0.7
0.6
0.5
0.4
0.3
0.2
0.1
0.0
Phylostratum
Cellular_organisms - ps1
Archaea1 - ps2
Archaea2 - ps3
Archaea3 - ps4
Asgard/Eukaryota - ps5
Eukaryota - ps6
Excavata/Diaphoretickes - ps7
Diaphoretickes - ps8
Archaeplastida - ps9
Viridiplantae - ps10
Streptophyta - ps11
Charophyceae/Embriophyta - ps12
Embriophyta - ps13
Tracheophyta - ps14
Spermatophyta - ps15
Angiosperms - ps16
Mesangiospermae - ps17
Magnoliids/eudicots - ps18
Eudicots - ps19
Core_eudicots - ps20
Superrosids - ps21
Rosids - ps22
Eurosids - ps23
Malvids - ps24
Sap./Malv./Brassicales - ps25
Malvales/Brassicales - ps26
Brassicales - ps27
Cleomaceae/Brassicaceae - ps28
Brassicaceae - ps29
Camelineae - ps30
Arabidopsis - ps31
Arabidopsis_thaliana - ps32
p-value
2e-02
3e-02
4e-02
5e-02
6e-02
Log-odds
0.5
1.0
1.5
2.0
2.5
3.0
3.5
4.0

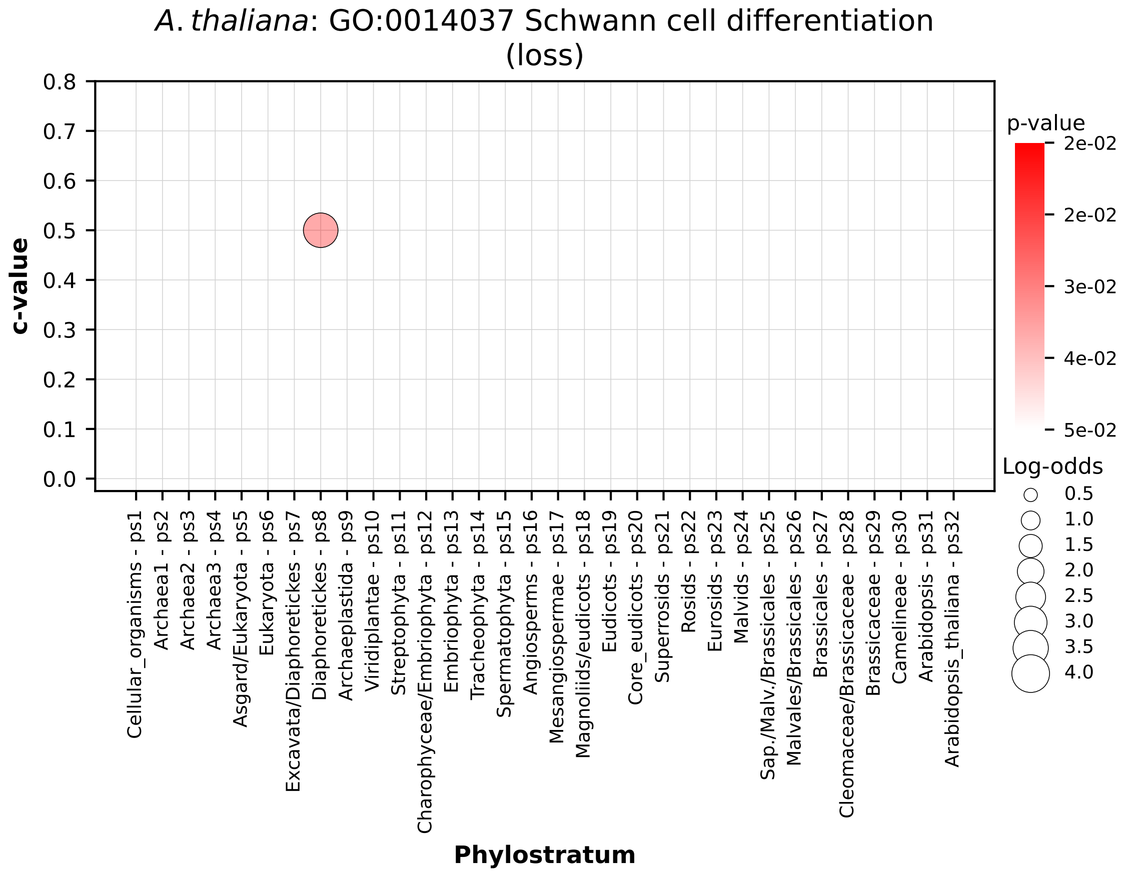

A. thaliana: GO:0014037 Schwann cell differentiation (loss)
c-value
0.0
0.1
0.2
0.3
0.4
0.5
0.6
0.7
0.8
Phylostratum
Cellular_organisms - ps1
Archaea1 - ps2
Archaea2 - ps3
Archaea3 - ps4
Asgard/Eukaryota - ps5
Eukaryota - ps6
Excavata/Diaphoretickes - ps7
Diaphoretickes - ps8
Archaeplastida - ps9
Viridiplantae - ps10
Streptophyta - ps11
Charophyceae/Embriophyta - ps12
Embriophyta - ps13
Tracheophyta - ps14
Spermatophyta - ps15
Angiosperms - ps16
Mesangiospermae - ps17
Magnoliids/eudicots - ps18
Eudicots - ps19
Core_eudicots - ps20
Superrosids - ps21
Rosids - ps22
Eurosids - ps23
Malvids - ps24
Sap./Malv./Brassicales - ps25
Malvales/Brassicales - ps26
Brassicales - ps27
Cleomaceae/Brassicaceae - ps28
Brassicaceae - ps29
Camelineae - ps30
Arabidopsis - ps31
Arabidopsis_thaliana - ps32
p-value
2e-02
2e-02
3e-02
4e-02
5e-02
Log-odds
0.5
1.0
1.5
2.0
2.5
3.0
3.5
4.0

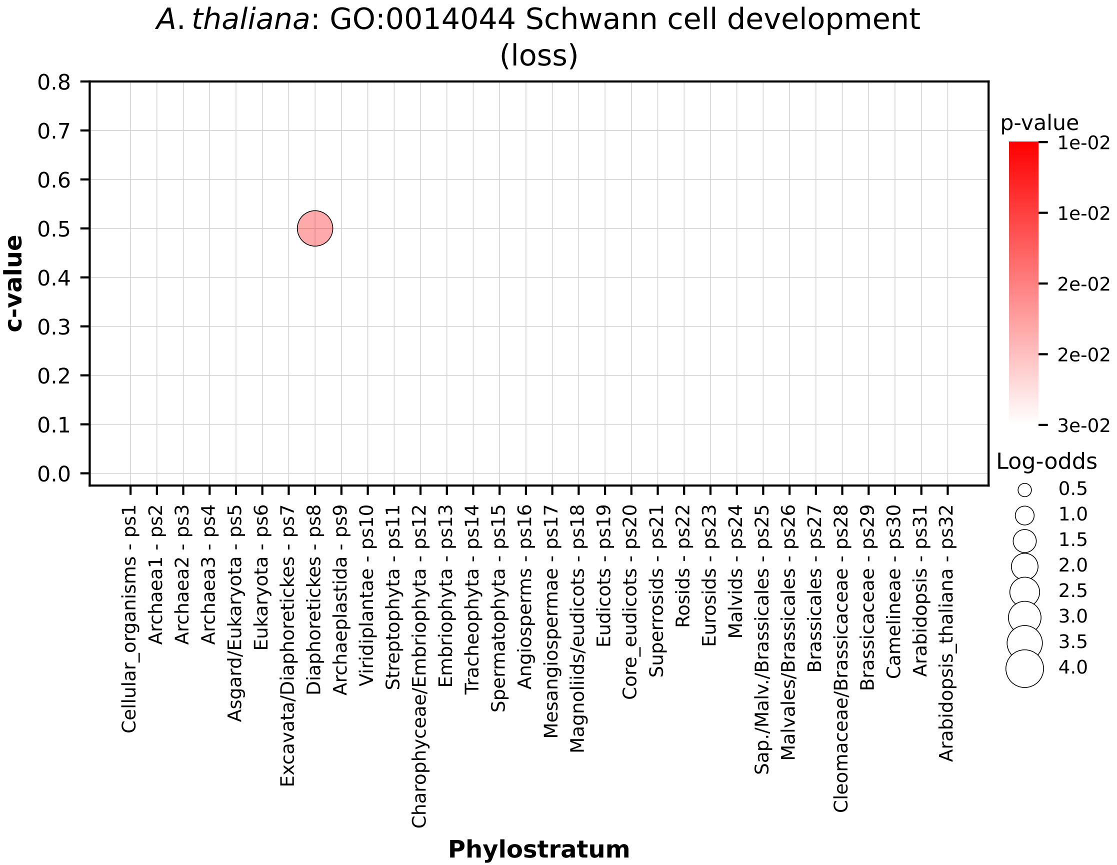

A. thaliana: GO:0014044 Schwann cell development (loss)
c-value
0.8
0.7
0.6
0.5
0.4
0.3
0.2
0.1
0.0
p-value
1e-02
1e-02
2e-02
2e-02
3e-02
Log-odds
0.5
1.0
1.5
2.0
2.5
3.0
3.5
4.0
Cellular_organisms - ps1
Archaea1 - ps2
Archaea2 - ps3
Archaea3 - ps4
Asgard/Eukaryota - ps5
Eukaryota - ps6
Excavata/Diaphoretickes - ps7
Diaphoretickes - ps8
Archaeplastida - ps9
Viridiplantae - ps10
Streptophyta - ps11
Charophyceae/Embriophyta - ps12
Embriophyta - ps13
Tracheophyta - ps14
Spermatophyta - ps15
Angiosperms - ps16
Mesangiospermae - ps17
Magnoliids/eudicots - ps18
Eudicots - ps19
Core_eudicots - ps20
Superrosids - ps21
Rosids - ps22
Eurosids - ps23
Malvids - ps24
Sap./Malv./Brassicales - ps25
Malvales/Brassicales - ps26
Brassicales - ps27
Cleomaceae/Brassicaceae - ps28
Brassicaceae - ps29
Camelineae - ps30
Arabidopsis - ps31
Arabidopsis_thaliana - ps32
Phylostratum

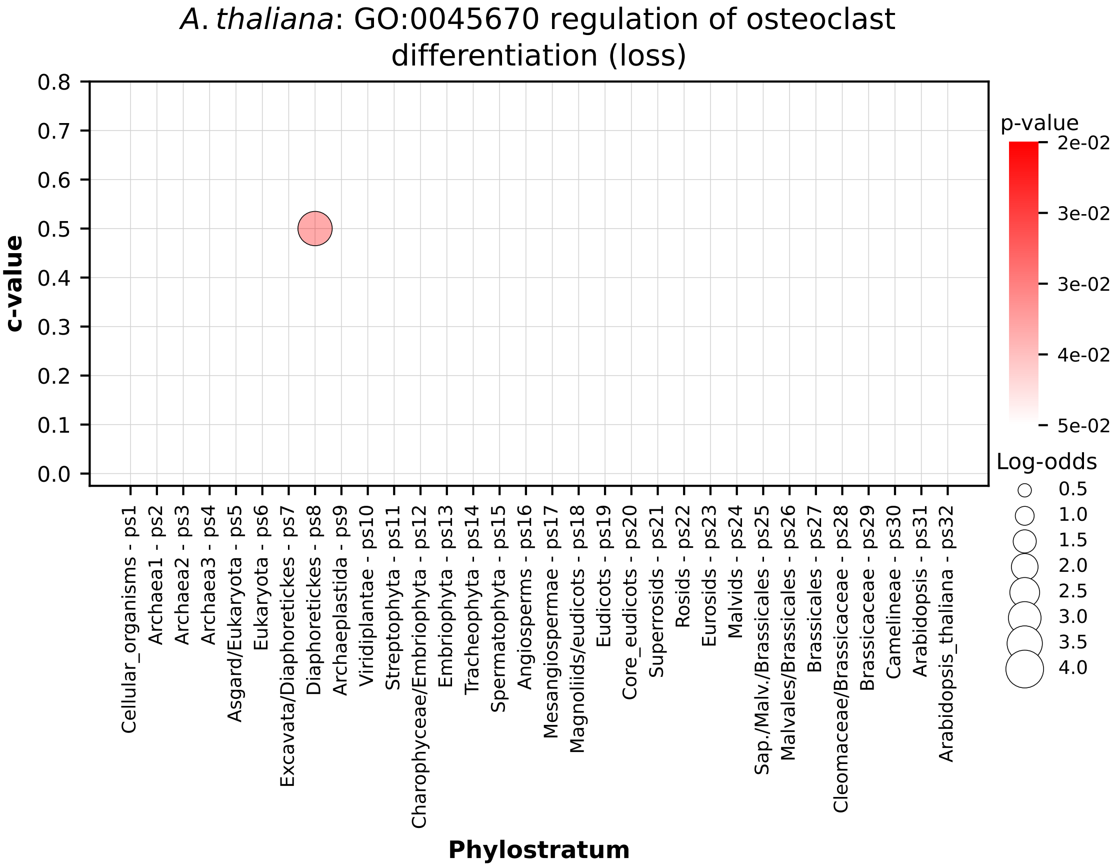

A. thaliana: GO:0045670 regulation of osteoclast differentiation (loss)
c-value
0.8
0.7
0.6
0.5
0.4
0.3
0.2
0.1
0.0
p-value
2e-02
3e-02
3e-02
4e-02
5e-02
Log-odds
0.5
1.0
1.5
2.0
2.5
3.0
3.5
4.0
Cellular_organisms - ps1
Archaea1 - ps2
Archaea2 - ps3
Archaea3 - ps4
Asgard/Eukaryota - ps5
Eukaryota - ps6
Excavata/Diaphoretickes - ps7
Diaphoretickes - ps8
Archaeplastida - ps9
Viridiplantae - ps10
Streptophyta - ps11
Charophyceae/Embriophyta - ps12
Embriophyta - ps13
Tracheophyta - ps14
Spermatophyta - ps15
Angiosperms - ps16
Mesangiospermae - ps17
Magnoliids/eudicots - ps18
Eudicots - ps19
Core_eudicots - ps20
Superrosids - ps21
Rosids - ps22
Eurosids - ps23
Malvids - ps24
Sap./Malv./Brassicales - ps25
Malvales/Brassicales - ps26
Brassicales - ps27
Cleomaceae/Brassicaceae - ps28
Brassicaceae - ps29
Camelineae - ps30
Arabidopsis - ps31
Arabidopsis_thaliana - ps32
Phylostratum

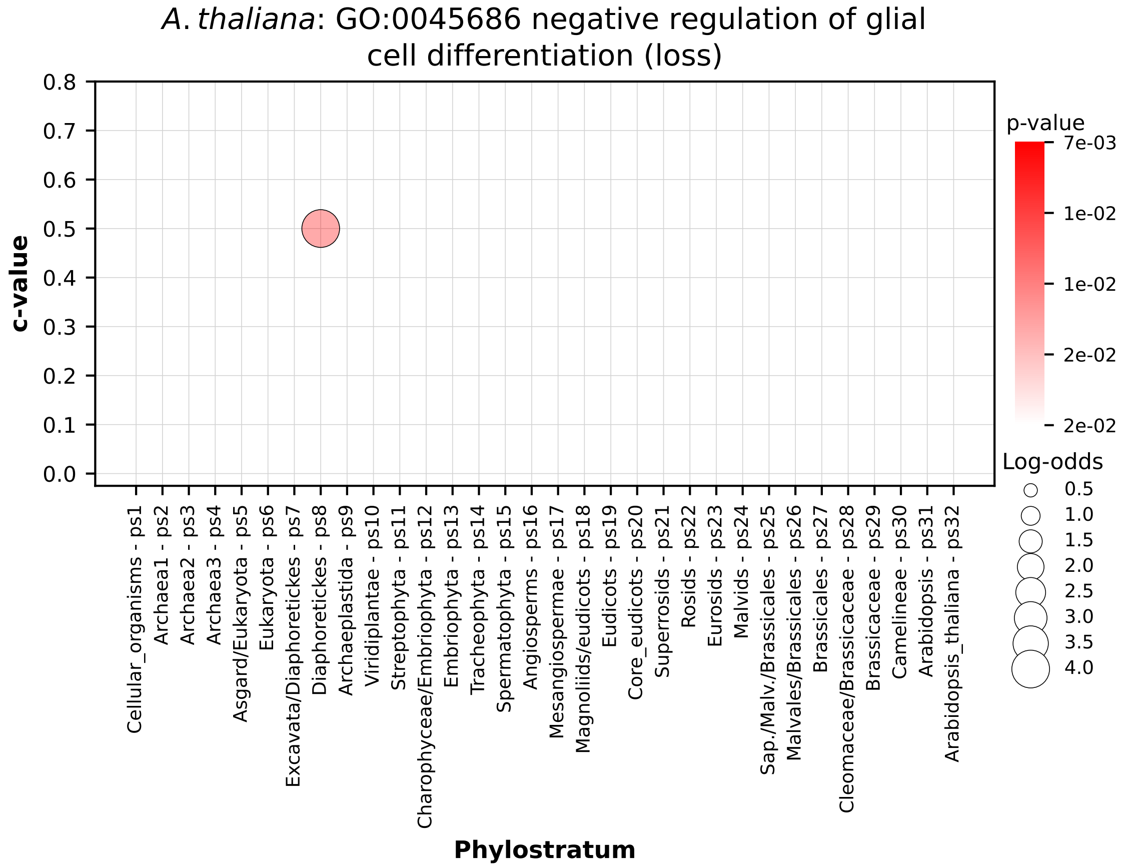

A. thaliana: GO:0045686 negative regulation of glial cell differentiation (loss)
c-value
0.8
0.7
0.6
0.5
0.4
0.3
0.2
0.1
0.0
Phylostratum
Cellular_organisms - ps1
Archaea1 - ps2
Archaea2 - ps3
Archaea3 - ps4
Asgard/Eukaryota - ps5
Eukaryota - ps6
Excavata/Diaphoretickes - ps7
Diaphoretickes - ps8
Archaeplastida - ps9
Viridiplantae - ps10
Streptophyta - ps11
Charophyceae/Embriophyta - ps12
Embriophyta - ps13
Tracheophyta - ps14
Spermatophyta - ps15
Angiosperms - ps16
Mesangiospermae - ps17
Magnoliids/eudicots - ps18
Eudicots - ps19
Core_eudicots - ps20
Superrosids - ps21
Rosids - ps22
Eurosids - ps23
Malvids - ps24
Sap./Malv./Brassicales - ps25
Malvales/Brassicales - ps26
Brassicales - ps27
Cleomaceae/Brassicaceae - ps28
Brassicaceae - ps29
Camelineae - ps30
Arabidopsis - ps31
Arabidopsis_thaliana - ps32
p-value
7e-03
1e-02
1e-02
2e-02
2e-02
Log-odds
0.5
1.0
1.5
2.0
2.5
3.0
3.5
4.0

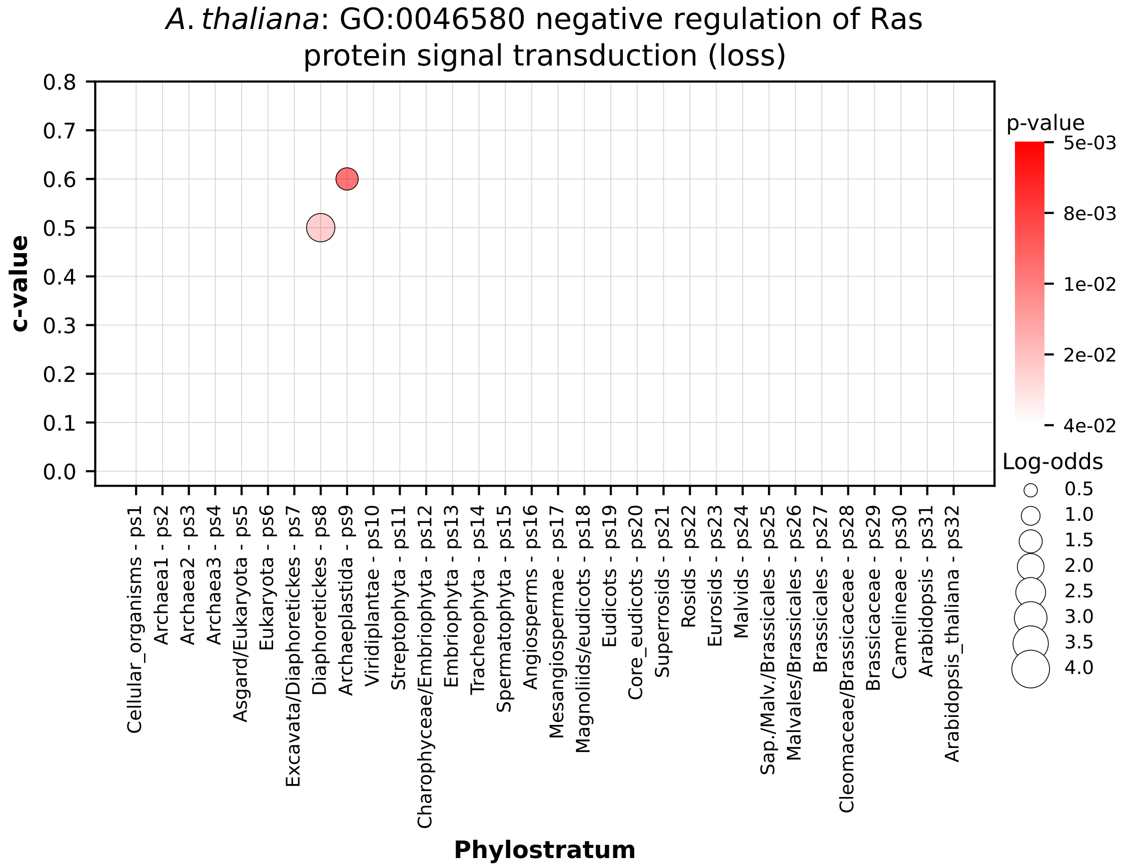

A. thaliana: GO:0046580 negative regulation of Ras protein signal transduction (loss)
c-value
0.0
0.1
0.2
0.3
0.4
0.5
0.6
0.7
0.8
Phylostratum
Cellular_organisms - ps1
Archaea1 - ps2
Archaea2 - ps3
Archaea3 - ps4
Asgard/Eukaryota - ps5
Eukaryota - ps6
Excavata/Diaphoretickes - ps7
Diaphoretickes - ps8
Archaeplastida - ps9
Viridiplantae - ps10
Streptophyta - ps11
Charophyceae/Embriophyta - ps12
Embriophyta - ps13
Tracheophyta - ps14
Spermatophyta - ps15
Angiosperms - ps16
Mesangiospermae - ps17
Magnoliids/eudicots - ps18
Eudicots - ps19
Core_eudicots - ps20
Superrosids - ps21
Rosids - ps22
Eurosids - ps23
Malvids - ps24
Sap./Malv./Brassicales - ps25
Malvales/Brassicales - ps26
Brassicales - ps27
Cleomaceae/Brassicaceae - ps28
Brassicaceae - ps29
Camelineae - ps30
Arabidopsis - ps31
Arabidopsis_thaliana - ps32
p-value
5e-03
8e-03
1e-02
2e-02
4e-02
Log-odds
0.5
1.0
1.5
2.0
2.5
3.0
3.5
4.0

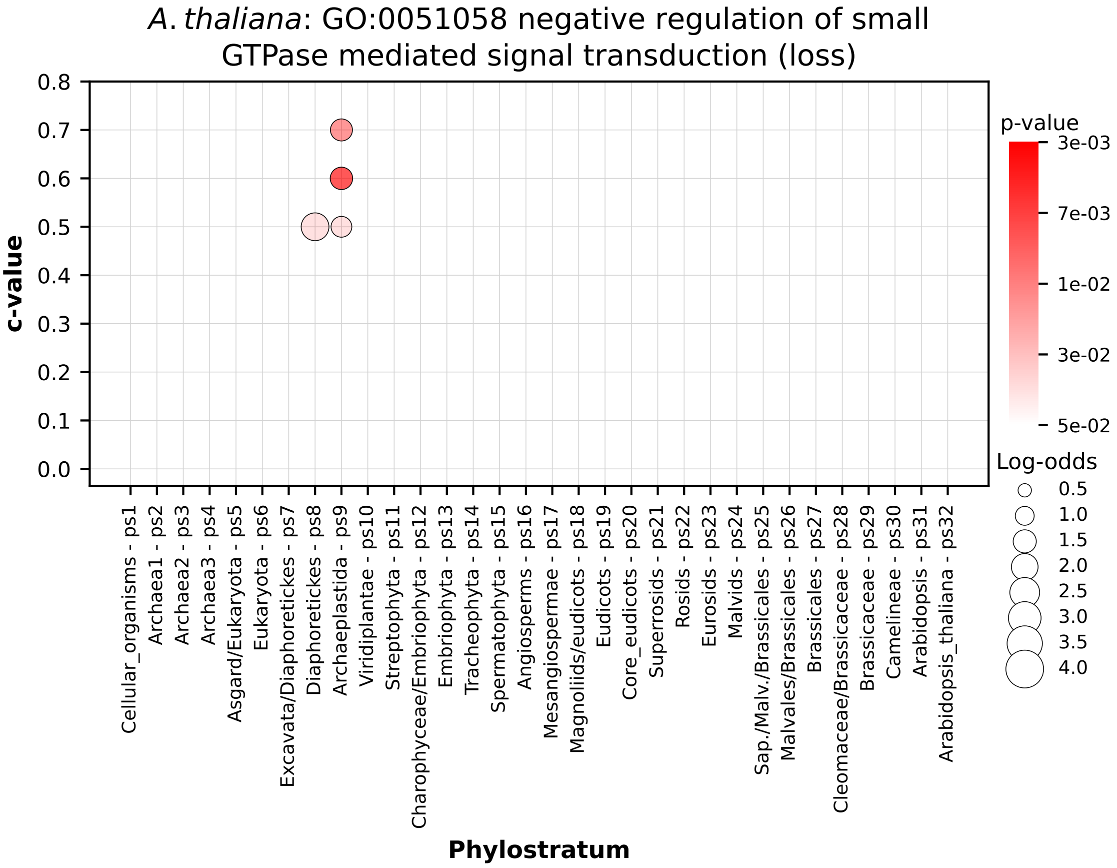
A. thaliana: GO:0051058 negative regulation of small GTPase mediated signal transduction (loss)
c-value
0.0
0.1
0.2
0.3
0.4
0.5
0.6
0.7
0.8
Phylostratum
Cellular_organisms - ps1
Archaea1 - ps2
Archaea2 - ps3
Archaea3 - ps4
Asgard/Eukaryota - ps5
Eukaryota - ps6
Excavata/Diaphoretickes - ps7
Diaphoretickes - ps8
Archaeplastida - ps9
Viridiplantae - ps10
Streptophyta - ps11
Charophyceae/Embriophyta - ps12
Embriophyta - ps13
Tracheophyta - ps14
Spermatophyta - ps15
Angiosperms - ps16
Mesangiospermae - ps17
Magnoliids/eudicots - ps18
Eudicots - ps19
Core_eudicots - ps20
Superrosids - ps21
Rosids - ps22
Eurosids - ps23
Malvids - ps24
Sap./Malv./Brassicales - ps25
Malvales/Brassicales - ps26
Brassicales - ps27
Cleomaceae/Brassicaceae - ps28
Brassicaceae - ps29
Camelineae - ps30
Arabidopsis - ps31
Arabidopsis_thaliana - ps32
p-value
3e-03
7e-03
1e-02
3e-02
5e-02
Log-odds
0.5
1.0
1.5
2.0
2.5
3.0
3.5
4.0

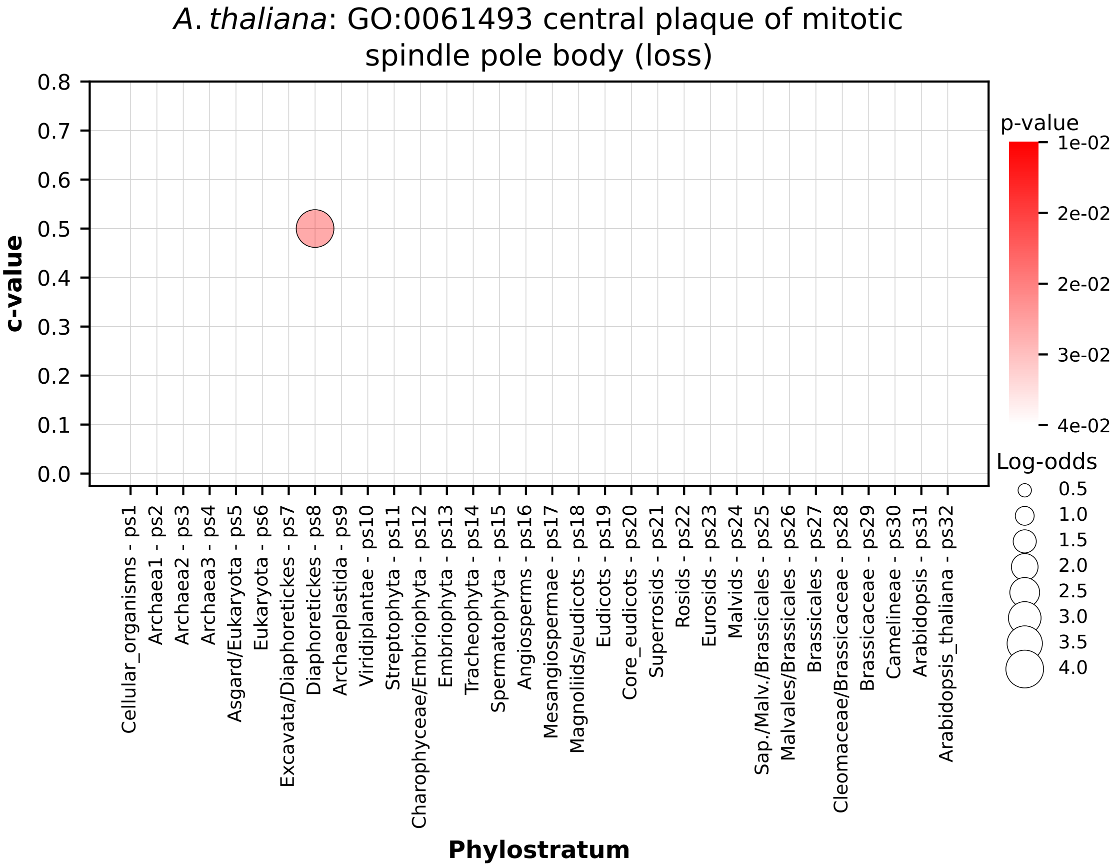

A. thaliana: GO:0061493 central plaque of mitotic spindle pole body (loss)
c-value
0.8
0.7
0.6
0.5
0.4
0.3
0.2
0.1
0.0
p-value
1e-02
2e-02
2e-02
3e-02
4e-02
Log-odds
0.5
1.0
1.5
2.0
2.5
3.0
3.5
4.0
Cellular_organisms - ps1
Archaea1 - ps2
Archaea2 - ps3
Archaea3 - ps4
Asgard/Eukaryota - ps5
Eukaryota - ps6
Excavata/Diaphoretickes - ps7
Diaphoretickes - ps8
Archaeplastida - ps9
Viridiplantae - ps10
Streptophyta - ps11
Charophyceae/Embriophyta - ps12
Embriophyta - ps13
Tracheophyta - ps14
Spermatophyta - ps15
Angiosperms - ps16
Mesangiospermae - ps17
Magnoliids/eudicots - ps18
Eudicots - ps19
Core_eudicots - ps20
Superrosids - ps21
Rosids - ps22
Eurosids - ps23
Malvids - ps24
Sap./Malv./Brassicales - ps25
Malvales/Brassicales - ps26
Brassicales - ps27
Cleomaceae/Brassicaceae - ps28
Brassicaceae - ps29
Camelineae - ps30
Arabidopsis - ps31
Arabidopsis_thaliana - ps32
Phylostratum

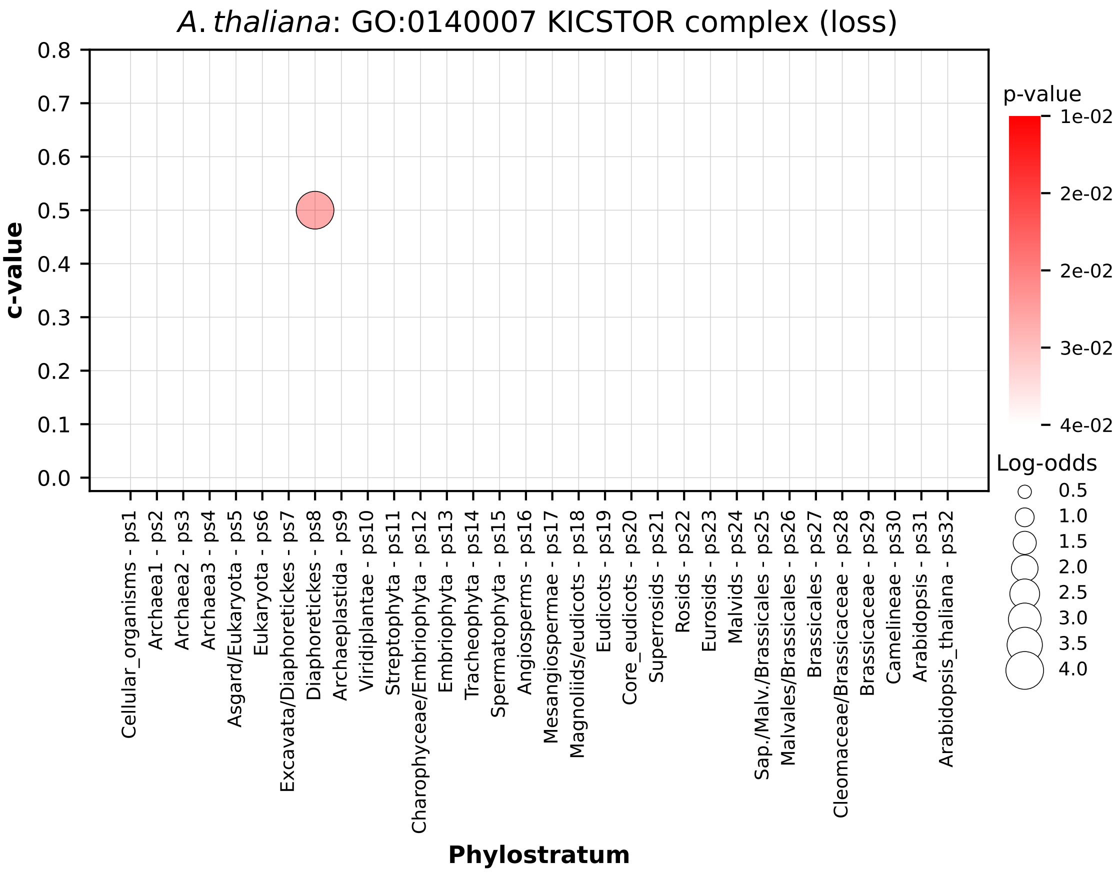

A. thaliana: GO:0140007 KICSTOR complex (loss)
c-value
0.8
0.7
0.6
0.5
0.4
0.3
0.2
0.1
0.0
p-value
1e-02
2e-02
2e-02
3e-02
4e-02
Log-odds
0.5
1.0
1.5
2.0
2.5
3.0
3.5
4.0
Cellular_organisms - ps1
Archaea1 - ps2
Archaea2 - ps3
Archaea3 - ps4
Asgard/Eukaryota - ps5
Eukaryota - ps6
Excavata/Diaphoretickes - ps7
Diaphoretickes - ps8
Archaeplastida - ps9
Viridiplantae - ps10
Streptophyta - ps11
Charophyceae/Embriophyta - ps12
Embriophyta - ps13
Tracheophyta - ps14
Spermatophyta - ps15
Angiosperms - ps16
Mesangiospermae - ps17
Magnoliids/eudicots - ps18
Eudicots - ps19
Core_eudicots - ps20
Superrosids - ps21
Rosids - ps22
Eurosids - ps23
Malvids - ps24
Sap./Malv./Brassicales - ps25
Malvales/Brassicales - ps26
Brassicales - ps27
Cleomaceae/Brassicaceae - ps28
Brassicaceae - ps29
Camelineae - ps30
Arabidopsis - ps31
Arabidopsis_thaliana - ps32
Phylostratum

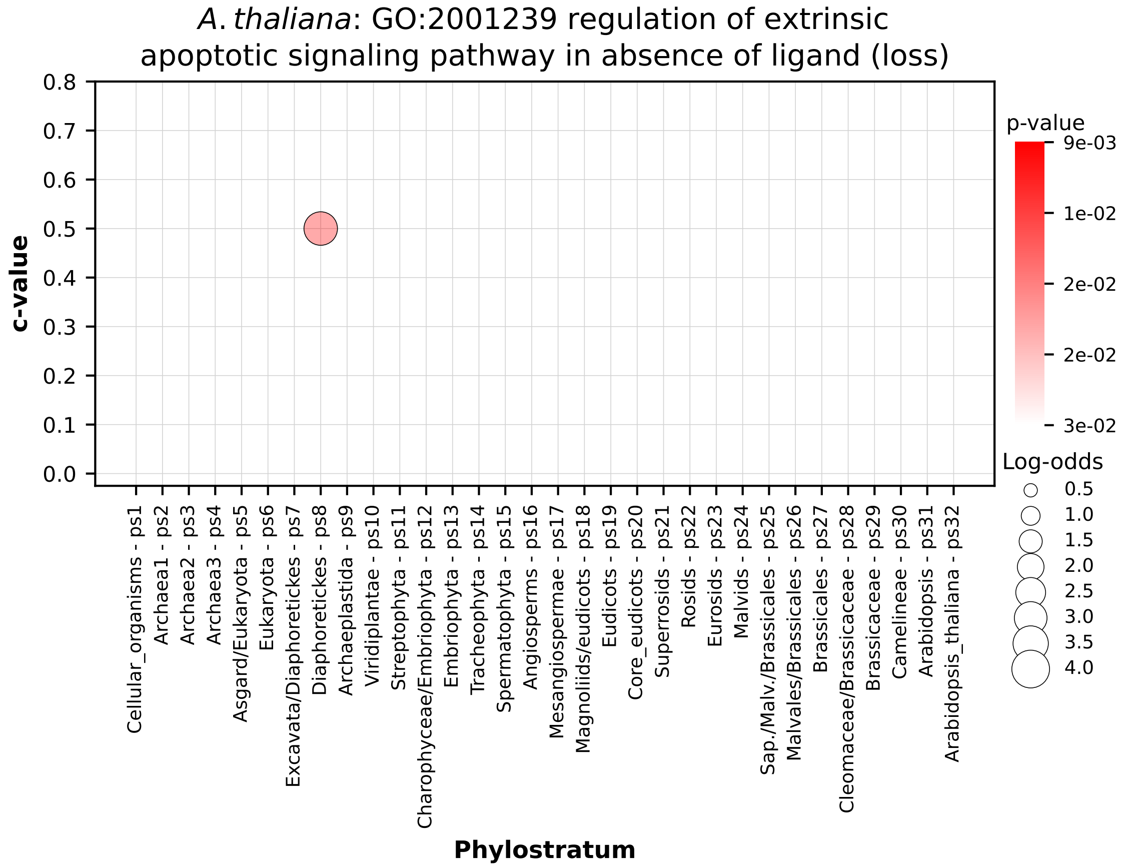

A. thaliana: GO:2001239 regulation of extrinsic apoptotic signaling pathway in absence of ligand (loss)
c-value
0.0
0.1
0.2
0.3
0.4
0.5
0.6
0.7
0.8
Phylostratum
Cellular_organisms - ps1
Archaea1 - ps2
Archaea2 - ps3
Archaea3 - ps4
Asgard/Eukaryota - ps5
Eukaryota - ps6
Excavata/Diaphoretickes - ps7
Diaphoretickes - ps8
Archaeplastida - ps9
Viridiplantae - ps10
Streptophyta - ps11
Charophyceae/Embriophyta - ps12
Embriophyta - ps13
Tracheophyta - ps14
Spermatophyta - ps15
Angiosperms - ps16
Mesangiospermae - ps17
Magnoliids/eudicots - ps18
Eudicots - ps19
Core_eudicots - ps20
Superrosids - ps21
Rosids - ps22
Eurosids - ps23
Malvids - ps24
Sap./Malv./Brassicales - ps25
Malvales/Brassicales - ps26
Brassicales - ps27
Cleomaceae/Brassicaceae - ps28
Brassicaceae - ps29
Camelineae - ps30
Arabidopsis - ps31
Arabidopsis_thaliana - ps32
p-value
9e-03
1e-02
2e-02
2e-02
3e-02
Log-odds
0.5
1.0
1.5
2.0
2.5
3.0
3.5
4.0

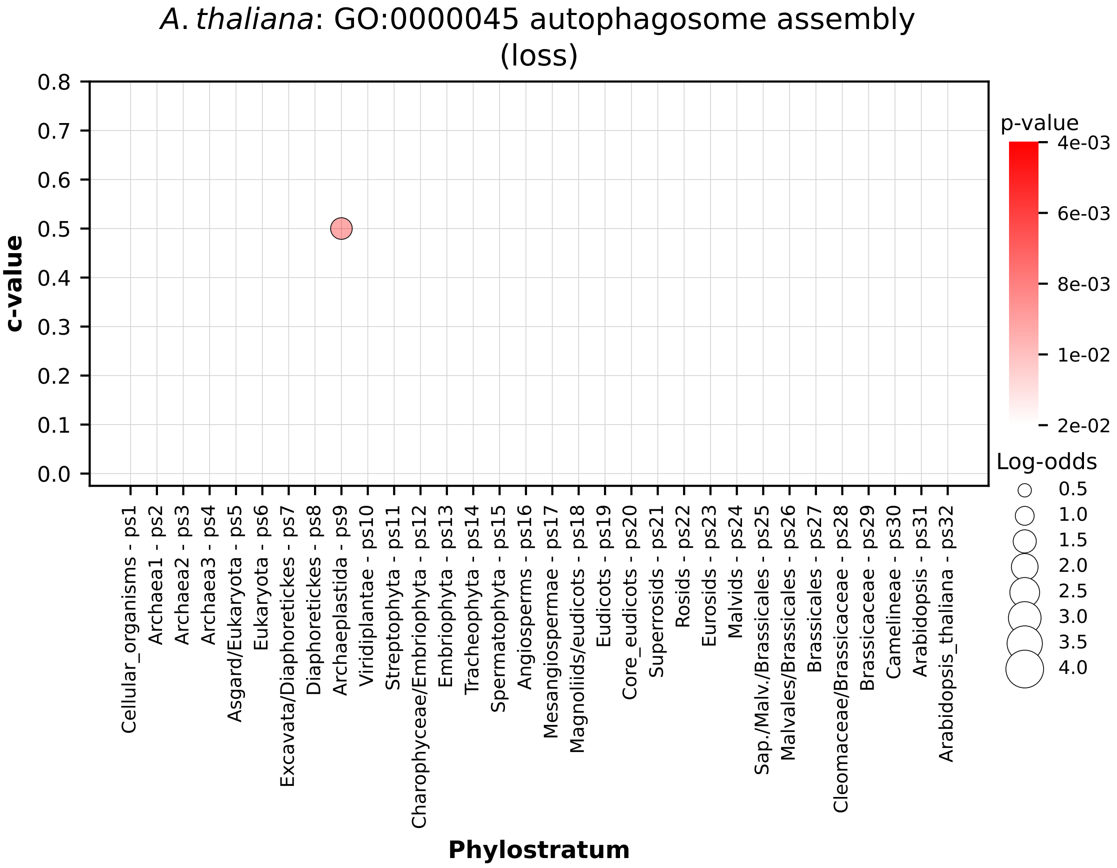
A. thaliana: GO:0000045 autophagosome assembly
(loss)
c-value
0.8
0.7
0.6
0.5
0.4
0.3
0.2
0.1
0.0
Phylostratum
Cellular_organisms - ps1
Archaea1 - ps2
Archaea2 - ps3
Archaea3 - ps4
Asgard/Eukaryota - ps5
Eukaryota - ps6
Excavata/Diaphoretickes - ps7
Diaphoretickes - ps8
Archaeplastida - ps9
Viridiplantae - ps10
Streptophyta - ps11
Charophyceae/Embriophyta - ps12
Embriophyta - ps13
Tracheophyta - ps14
Spermatophyta - ps15
Angiosperms - ps16
Mesangiospermae - ps17
Magnoliids/eudicots - ps18
Eudicots - ps19
Core_eudicots - ps20
Superrosids - ps21
Rosids - ps22
Eurosids - ps23
Malvids - ps24
Sap./Malv./Brassicales - ps25
Malvales/Brassicales - ps26
Brassicales - ps27
Cleomaceae/Brassicaceae - ps28
Brassicaceae - ps29
Camelineae - ps30
Arabidopsis - ps31
Arabidopsis_thaliana - ps32
p-value
4e-03
6e-03
8e-03
1e-02
2e-02
Log-odds
0.5
1.0
1.5
2.0
2.5
3.0
3.5
4.0

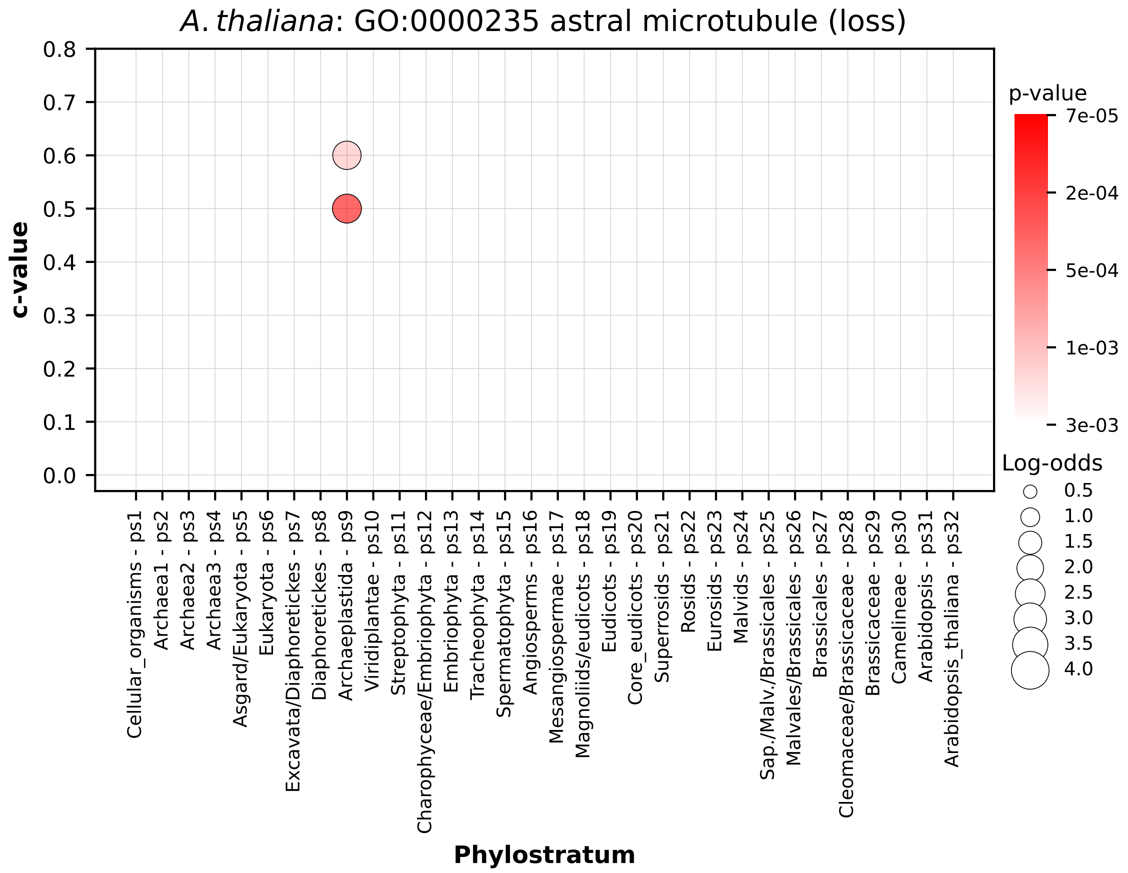

A. thaliana: GO:0000235 astral microtubule (loss)
c-value
0.8
0.7
0.6
0.5
0.4
0.3
0.2
0.1
0.0
Phylostratum
Cellular_organisms - ps1
Archaea1 - ps2
Archaea2 - ps3
Archaea3 - ps4
Asgard/Eukaryota - ps5
Eukaryota - ps6
Excavata/Diaphoretickes - ps7
Diaphoretickes - ps8
Archaeplastida - ps9
Viridiplantae - ps10
Streptophyta - ps11
Charophyceae/Embriophyta - ps12
Embriophyta - ps13
Tracheophyta - ps14
Spermatophyta - ps15
Angiosperms - ps16
Mesangiospermae - ps17
Magnoliids/eudicots - ps18
Eudicots - ps19
Core_eudicots - ps20
Superrosids - ps21
Rosids - ps22
Eurosids - ps23
Malvids - ps24
Sap./Malv./Brassicales - ps25
Malvales/Brassicales - ps26
Brassicales - ps27
Cleomaceae/Brassicaceae - ps28
Brassicaceae - ps29
Camelineae - ps30
Arabidopsis - ps31
Arabidopsis_thaliana - ps32
p-value
7e-05
2e-04
5e-04
1e-03
3e-03
Log-odds
0.5
1.0
1.5
2.0
2.5
3.0
3.5
4.0

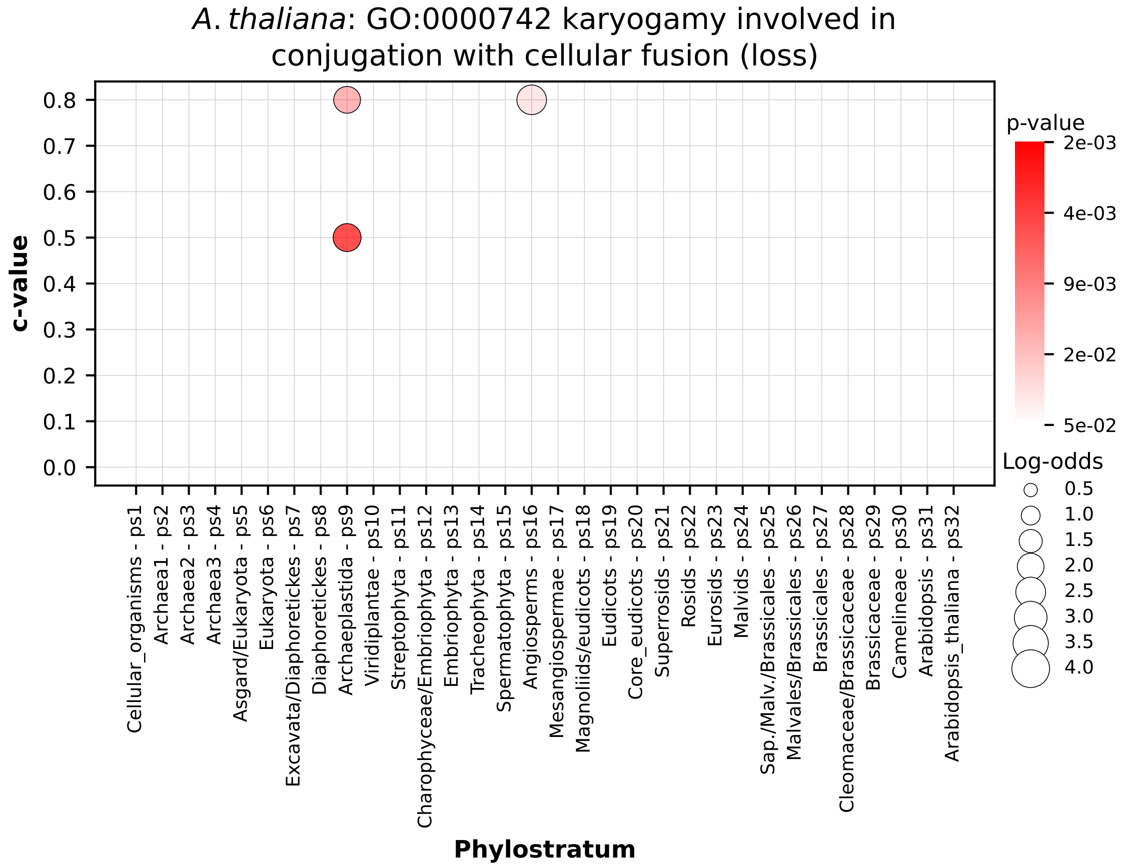
A. thaliana: GO:0000742 karyogamy involved in conjugation with cellular fusion (loss)
c-value
0.0
0.1
0.2
0.3
0.4
0.5
0.6
0.7
0.8
Phylostratum
Cellular_organisms - ps1
Archaea1 - ps2
Archaea2 - ps3
Archaea3 - ps4
Asgard/Eukaryota - ps5
Eukaryota - ps6
Excavata/Diaphoretickes - ps7
Diaphoretickes - ps8
Archaeplastida - ps9
Viridiplantae - ps10
Streptophyta - ps11
Charophyceae/Embriophyta - ps12
Embriophyta - ps13
Tracheophyta - ps14
Spermatophyta - ps15
Angiosperms - ps16
Mesangiospermae - ps17
Magnoliids/eudicots - ps18
Eudicots - ps19
Core_eudicots - ps20
Superrosids - ps21
Rosids - ps22
Eurosids - ps23
Malvids - ps24
Sap./Malv./Brassicales - ps25
Malvales/Brassicales - ps26
Brassicales - ps27
Cleomaceae/Brassicaceae - ps28
Brassicaceae - ps29
Camelineae - ps30
Arabidopsis - ps31
Arabidopsis_thaliana - ps32
p-value
2e-03
4e-03
9e-03
2e-02
5e-02
Log-odds
0.5
1.0
1.5
2.0
2.5
3.0
3.5
4.0

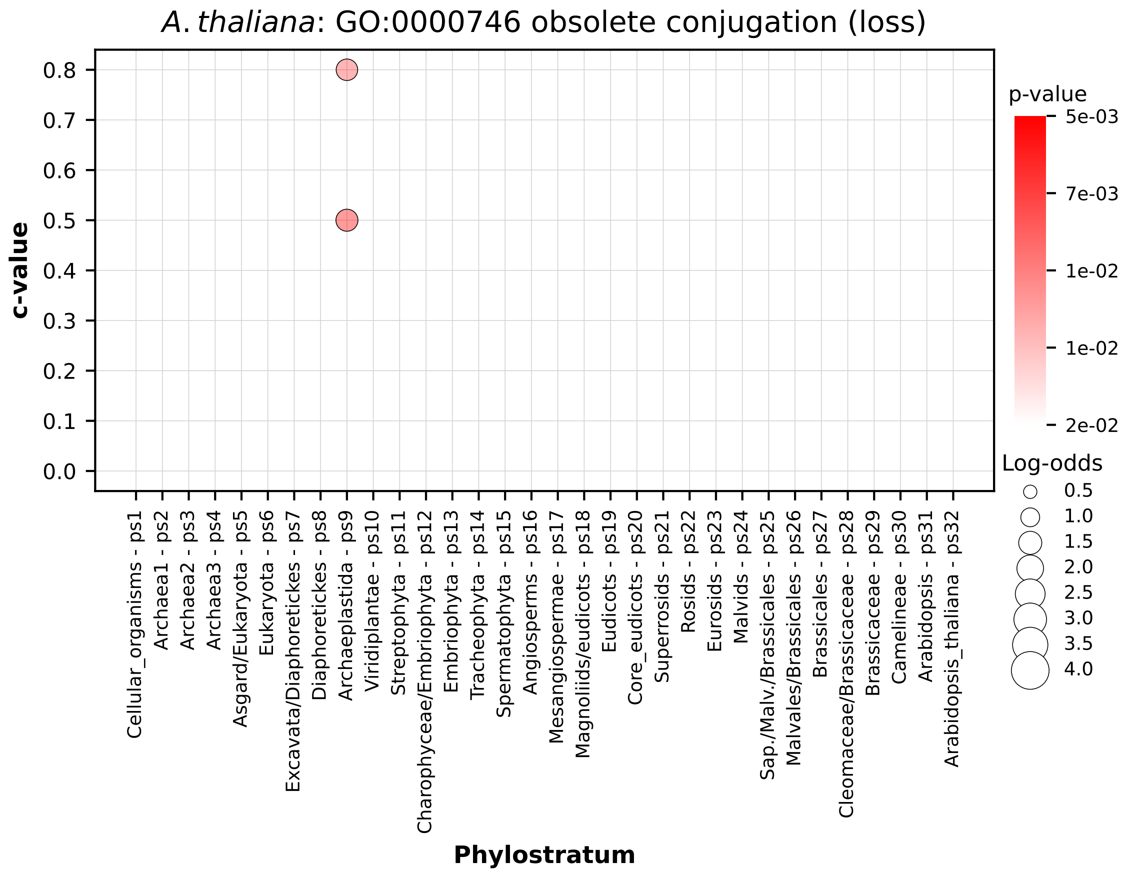

A. thaliana: GO:0000746 obsolete conjugation (loss)
c-value
0.8
0.7
0.6
0.5
0.4
0.3
0.2
0.1
0.0
p-value
5e-03
7e-03
1e-02
1e-02
2e-02
Log-odds
0.5
1.0
1.5
2.0
2.5
3.0
3.5
4.0
Cellular_organisms - ps1
Archaea1 - ps2
Archaea2 - ps3
Archaea3 - ps4
Asgard/Eukaryota - ps5
Eukaryota - ps6
Excavata/Diaphoretickes - ps7
Diaphoretickes - ps8
Archaeplastida - ps9
Viridiplantae - ps10
Streptophyta - ps11
Charophyceae/Embriophyta - ps12
Embriophyta - ps13
Tracheophyta - ps14
Spermatophyta - ps15
Angiosperms - ps16
Mesangiospermae - ps17
Magnoliids/eudicots - ps18
Eudicots - ps19
Core_eudicots - ps20
Superrosids - ps21
Rosids - ps22
Eurosids - ps23
Malvids - ps24
Sap./Malv./Brassicales - ps25
Malvales/Brassicales - ps26
Brassicales - ps27
Cleomaceae/Brassicaceae - ps28
Brassicaceae - ps29
Camelineae - ps30
Arabidopsis - ps31
Arabidopsis_thaliana - ps32
Phylostratum

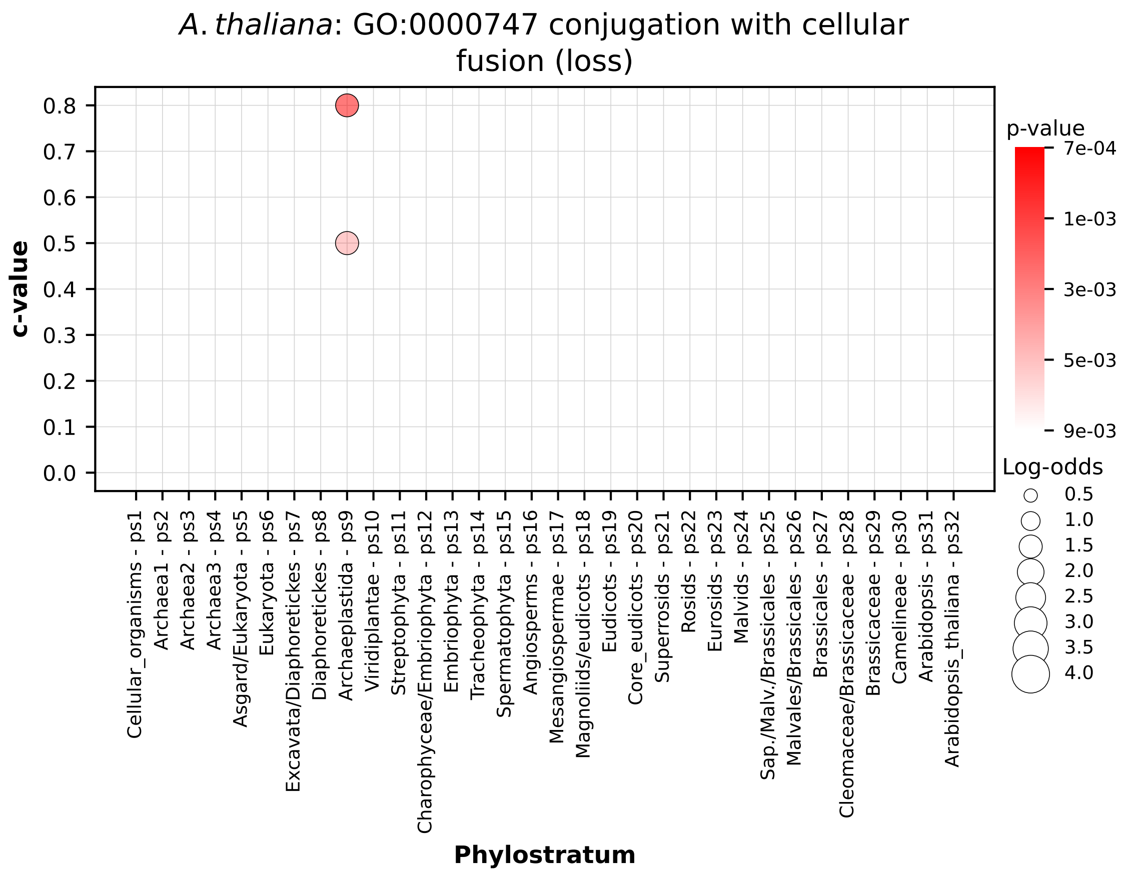

A. thaliana: GO:0000747 conjugation with cellular fusion (loss)
c-value
0.0
0.1
0.2
0.3
0.4
0.5
0.6
0.7
0.8
p-value
7e-04
1e-03
3e-03
5e-03
9e-03
Log-odds
0.5
1.0
1.5
2.0
2.5
3.0
3.5
4.0
Cellular_organisms - ps1
Archaea1 - ps2
Archaea2 - ps3
Archaea3 - ps4
Asgard/Eukaryota - ps5
Eukaryota - ps6
Excavata/Diaphoretickes - ps7
Diaphoretickes - ps8
Archaeplastida - ps9
Viridiplantae - ps10
Streptophyta - ps11
Charophyceae/Embriophyta - ps12
Embriophyta - ps13
Tracheophyta - ps14
Spermatophyta - ps15
Angiosperms - ps16
Mesangiospermae - ps17
Magnoliids/eudicots - ps18
Eudicots - ps19
Core_eudicots - ps20
Superrosids - ps21
Rosids - ps22
Eurosids - ps23
Malvids - ps24
Sap./Malv./Brassicales - ps25
Malvales/Brassicales - ps26
Brassicales - ps27
Cleomaceae/Brassicaceae - ps28
Brassicaceae - ps29
Camelineae - ps30
Arabidopsis - ps31
Arabidopsis_thaliana - ps32
Phylostratum

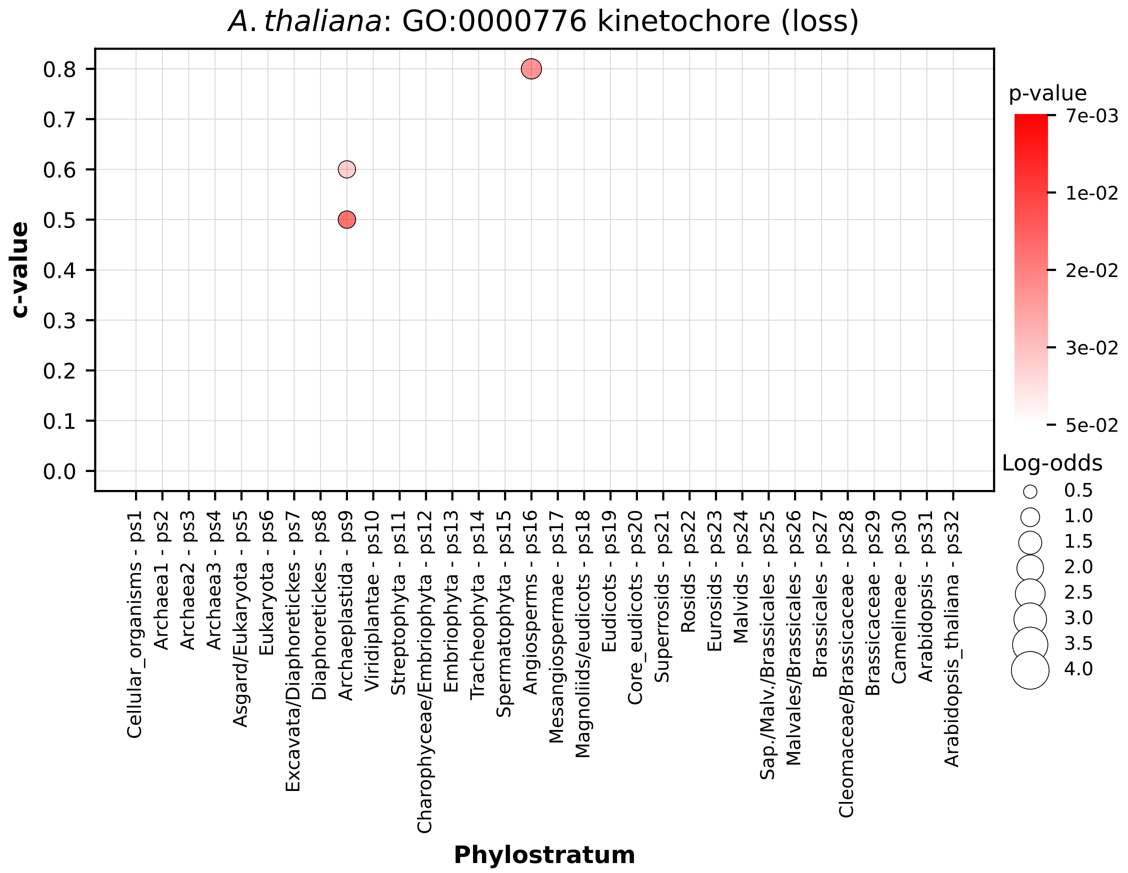
A. thaliana: GO:0000776 kinetochore (loss)
c-value
0.0
0.1
0.2
0.3
0.4
0.5
0.6
0.7
0.8
Phylostratum
Cellular_organisms - ps1
Archaea1 - ps2
Archaea2 - ps3
Archaea3 - ps4
Asgard/Eukaryota - ps5
Eukaryota - ps6
Excavata/Diaphoretickes - ps7
Diaphoretickes - ps8
Archaeplastida - ps9
Viridiplantae - ps10
Streptophyta - ps11
Charophyceae/Embriophyta - ps12
Embriophyta - ps13
Tracheophyta - ps14
Spermatophyta - ps15
Angiosperms - ps16
Mesangiospermae - ps17
Magnoliids/eudicots - ps18
Eudicots - ps19
Core_eudicots - ps20
Superrosids - ps21
Rosids - ps22
Eurosids - ps23
Malvids - ps24
Sap./Malv./Brassicales - ps25
Malvales/Brassicales - ps26
Brassicales - ps27
Cleomaceae/Brassicaceae - ps28
Brassicaceae - ps29
Camelineae - ps30
Arabidopsis - ps31
Arabidopsis_thaliana - ps32
p-value
7e-03
1e-02
2e-02
3e-02
5e-02
Log-odds
0.5
1.0
1.5
2.0
2.5
3.0
3.5
4.0

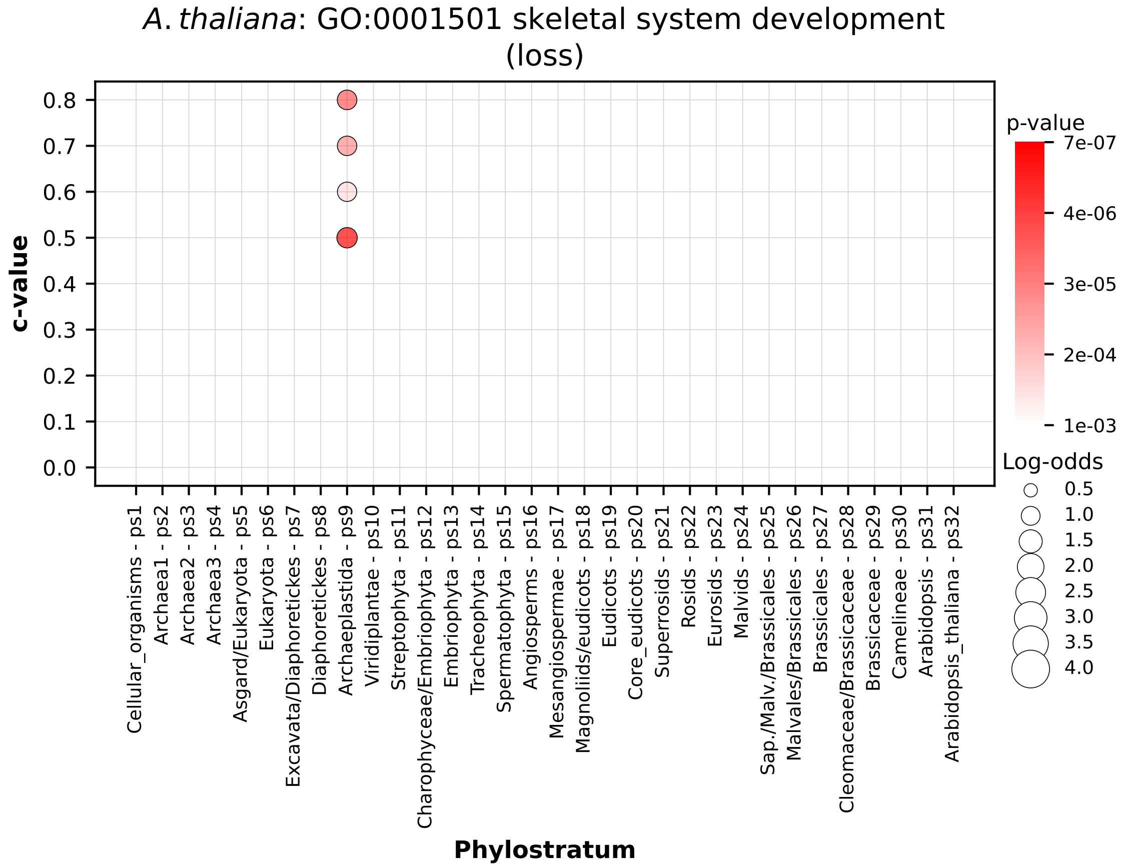

A. thaliana: GO:0001501 skeletal system development (loss)
c-value
0.0
0.1
0.2
0.3
0.4
0.5
0.6
0.7
0.8
Phylostratum
Cellular_organisms - ps1
Archaea1 - ps2
Archaea2 - ps3
Archaea3 - ps4
Asgard/Eukaryota - ps5
Eukaryota - ps6
Excavata/Diaphoretickes - ps7
Diaphoretickes - ps8
Archaeplastida - ps9
Viridiplantae - ps10
Streptophyta - ps11
Charophyceae/Embriophyta - ps12
Embriophyta - ps13
Tracheophyta - ps14
Spermatophyta - ps15
Angiosperms - ps16
Mesangiospermae - ps17
Magnoliids/eudicots - ps18
Eudicots - ps19
Core_eudicots - ps20
Superrosids - ps21
Rosids - ps22
Eurosids - ps23
Malvids - ps24
Sap./Malv./Brassicales - ps25
Malvales/Brassicales - ps26
Brassicales - ps27
Cleomaceae/Brassicaceae - ps28
Brassicaceae - ps29
Camelineae - ps30
Arabidopsis - ps31
Arabidopsis_thaliana - ps32
p-value
7e-07
4e-06
3e-05
2e-04
1e-03
Log-odds
0.5
1.0
1.5
2.0
2.5
3.0
3.5
4.0

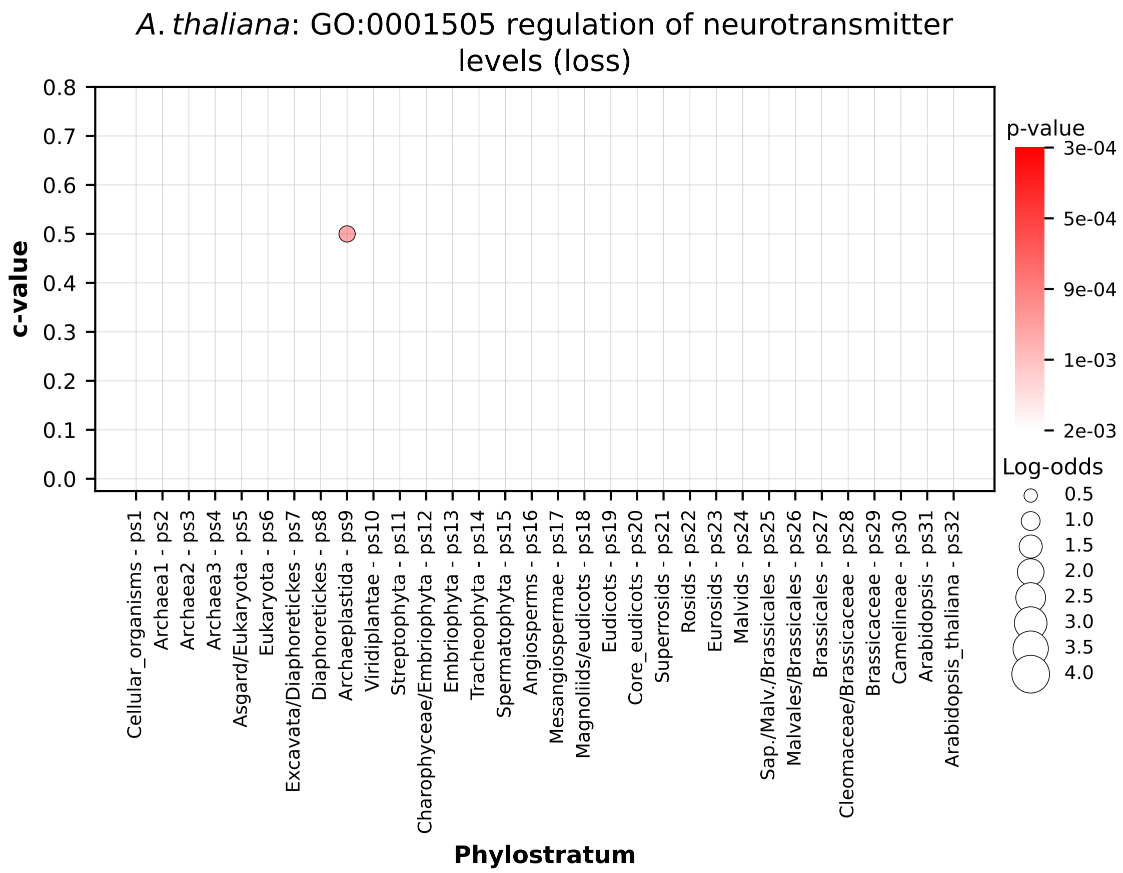
A. thaliana: GO:0001505 regulation of neurotransmitter levels (loss)
c-value
0.8
0.7
0.6
0.5
0.4
0.3
0.2
0.1
0.0
Phylostratum
Cellular_organisms - ps1
Archaea1 - ps2
Archaea2 - ps3
Archaea3 - ps4
Asgard/Eukaryota - ps5
Eukaryota - ps6
Excavata/Diaphoretickes - ps7
Diaphoretickes - ps8
Archaeplastida - ps9
Viridiplantae - ps10
Streptophyta - ps11
Charophyceae/Embriophyta - ps12
Embriophyta - ps13
Tracheophyta - ps14
Spermatophyta - ps15
Angiosperms - ps16
Mesangiospermae - ps17
Magnoliids/eudicots - ps18
Eudicots - ps19
Core_eudicots - ps20
Superrosids - ps21
Rosids - ps22
Eurosids - ps23
Malvids - ps24
Sap./Malv./Brassicales - ps25
Malvales/Brassicales - ps26
Brassicales - ps27
Cleomaceae/Brassicaceae - ps28
Brassicaceae - ps29
Camelineae - ps30
Arabidopsis - ps31
Arabidopsis_thaliana - ps32
p-value
3e-04
5e-04
9e-04
1e-03
2e-03
Log-odds
0.5
1.0
1.5
2.0
2.5
3.0
3.5
4.0

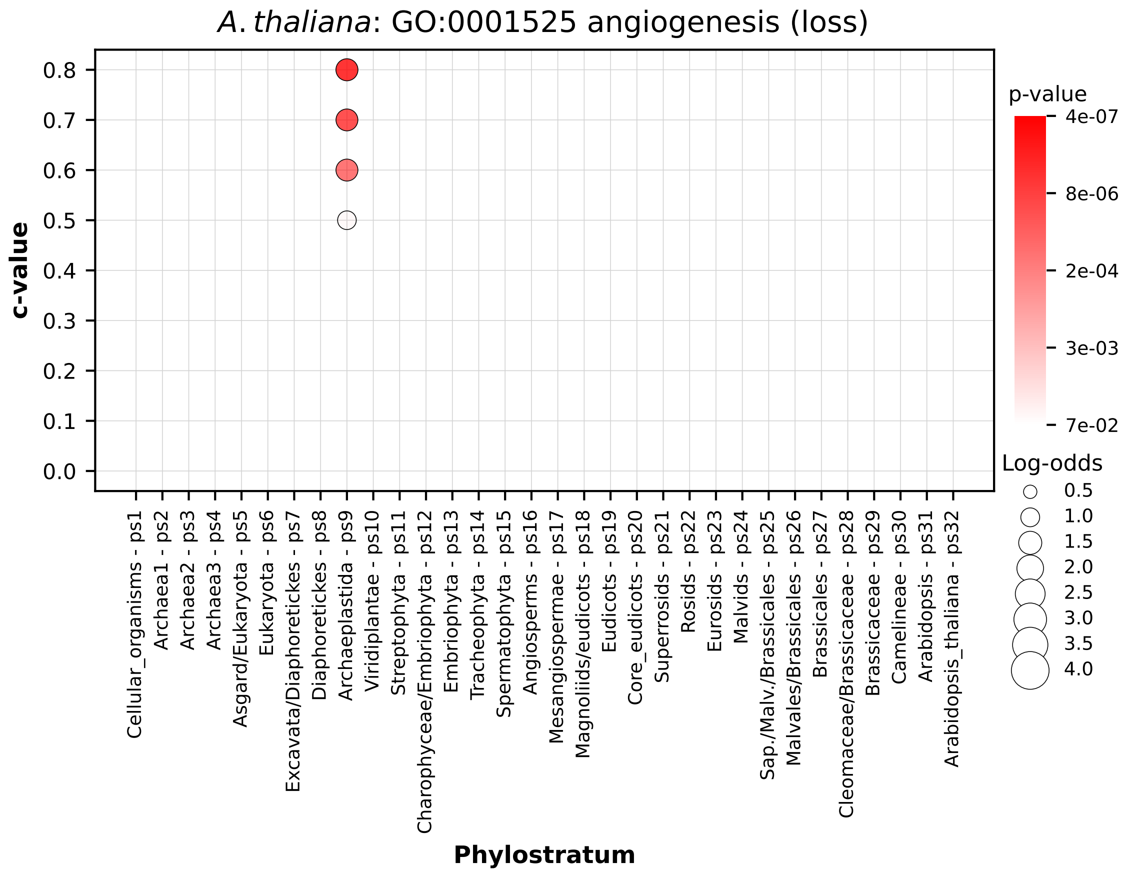

A. thaliana: GO:0001525 angiogenesis (loss)
c-value
0.0
0.1
0.2
0.3
0.4
0.5
0.6
0.7
0.8
p-value
4e-07
8e-06
2e-04
3e-03
7e-02
Log-odds
0.5
1.0
1.5
2.0
2.5
3.0
3.5
4.0
Cellular_organisms - ps1
Archaea1 - ps2
Archaea2 - ps3
Archaea3 - ps4
Asgard/Eukaryota - ps5
Eukaryota - ps6
Excavata/Diaphoretickes - ps7
Diaphoretickes - ps8
Archaeplastida - ps9
Viridiplantae - ps10
Streptophyta - ps11
Charophyceae/Embriophyta - ps12
Embriophyta - ps13
Tracheophyta - ps14
Spermatophyta - ps15
Angiosperms - ps16
Mesangiospermae - ps17
Magnoliids/eudicots - ps18
Eudicots - ps19
Core_eudicots - ps20
Superrosids - ps21
Rosids - ps22
Eurosids - ps23
Malvids - ps24
Sap./Malv./Brassicales - ps25
Malvales/Brassicales - ps26
Brassicales - ps27
Cleomaceae/Brassicaceae - ps28
Brassicaceae - ps29
Camelineae - ps30
Arabidopsis - ps31
Arabidopsis_thaliana - ps32
Phylostratum

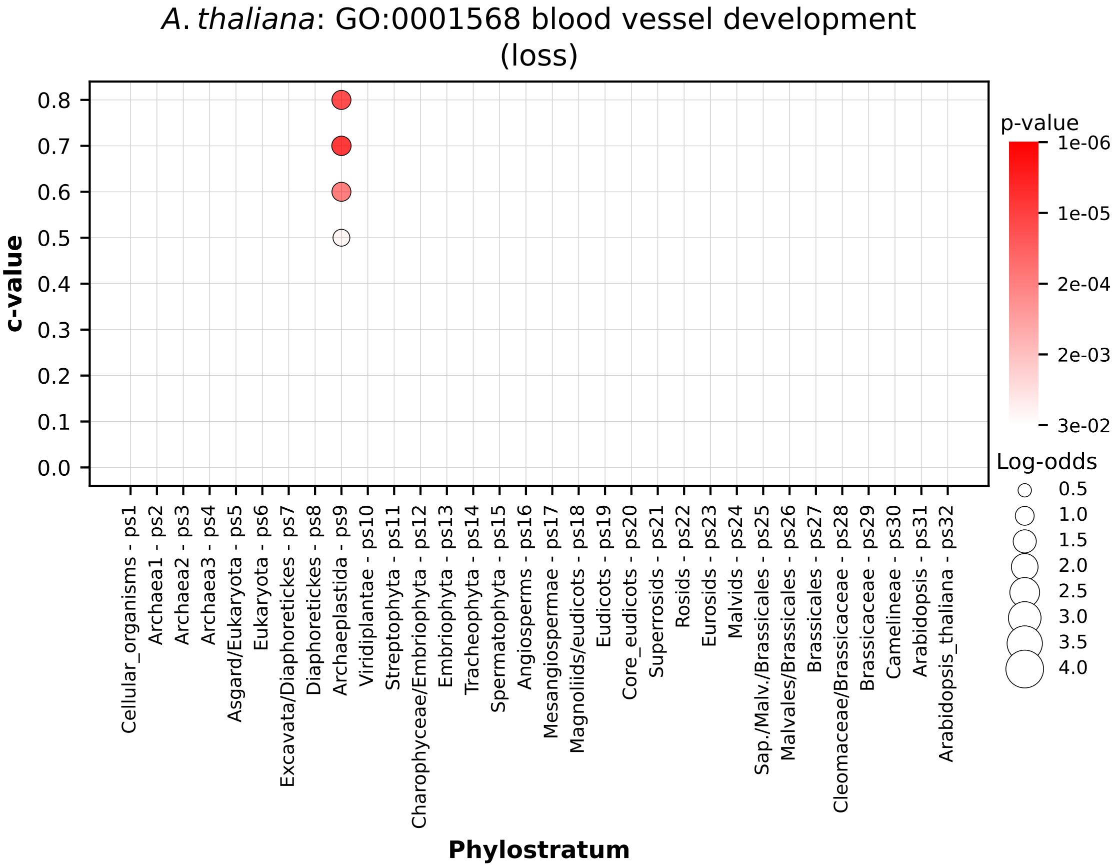

A. thaliana: GO:0001568 blood vessel development
(loss)
c-value
0.8
0.7
0.6
0.5
0.4
0.3
0.2
0.1
0.0
Phylostratum
Cellular_organisms - ps1
Archaea1 - ps2
Archaea2 - ps3
Archaea3 - ps4
Asgard/Eukaryota - ps5
Eukaryota - ps6
Excavata/Diaphoretickes - ps7
Diaphoretickes - ps8
Archaeplastida - ps9
Viridiplantae - ps10
Streptophyta - ps11
Charophyceae/Embriophyta - ps12
Embriophyta - ps13
Tracheophyta - ps14
Spermatophyta - ps15
Angiosperms - ps16
Mesangiospermae - ps17
Magnoliids/eudicots - ps18
Eudicots - ps19
Core_eudicots - ps20
Superrosids - ps21
Rosids - ps22
Eurosids - ps23
Malvids - ps24
Sap./Malv./Brassicales - ps25
Malvales/Brassicales - ps26
Brassicales - ps27
Cleomaceae/Brassicaceae - ps28
Brassicaceae - ps29
Camelineae - ps30
Arabidopsis - ps31
Arabidopsis_thaliana - ps32
p-value
1e-06
1e-05
2e-04
2e-03
3e-02
Log-odds
0.5
1.0
1.5
2.0
2.5
3.0
3.5
4.0

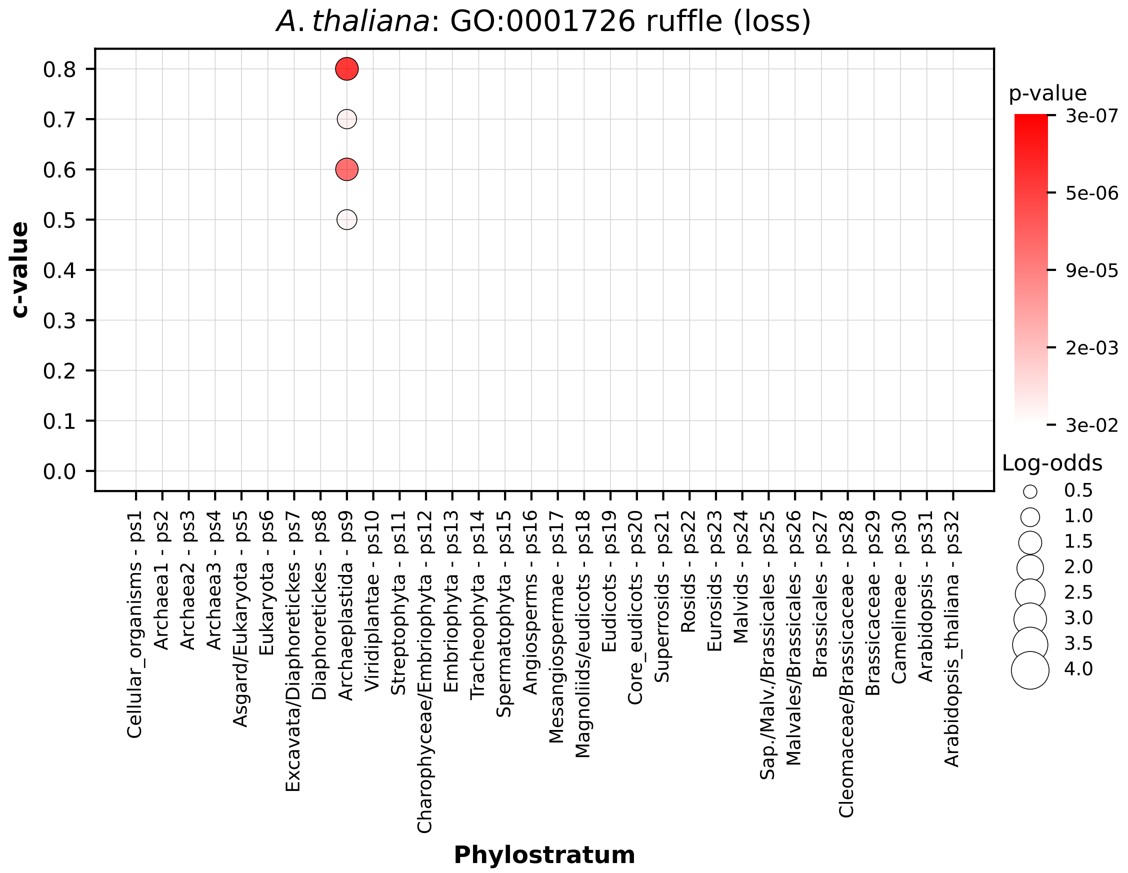

A. thaliana: GO:0001726 ruffle (loss)
c-value
0.0
0.1
0.2
0.3
0.4
0.5
0.6
0.7
0.8
Phylostratum
Cellular_organisms - ps1
Archaea1 - ps2
Archaea2 - ps3
Archaea3 - ps4
Asgard/Eukaryota - ps5
Eukaryota - ps6
Excavata/Diaphoretickes - ps7
Diaphoretickes - ps8
Archaeplastida - ps9
Viridiplantae - ps10
Streptophyta - ps11
Charophyceae/Embriophyta - ps12
Embriophyta - ps13
Tracheophyta - ps14
Spermatophyta - ps15
Angiosperms - ps16
Mesangiospermae - ps17
Magnoliids/eudicots - ps18
Eudicots - ps19
Core_eudicots - ps20
Superrosids - ps21
Rosids - ps22
Eurosids - ps23
Malvids - ps24
Sap./Malv./Brassicales - ps25
Malvales/Brassicales - ps26
Brassicales - ps27
Cleomaceae/Brassicaceae - ps28
Brassicaceae - ps29
Camelineae - ps30
Arabidopsis - ps31
Arabidopsis_thaliana - ps32
p-value
3e-07
5e-06
9e-05
2e-03
3e-02
Log-odds
0.5
1.0
1.5
2.0
2.5
3.0
3.5
4.0

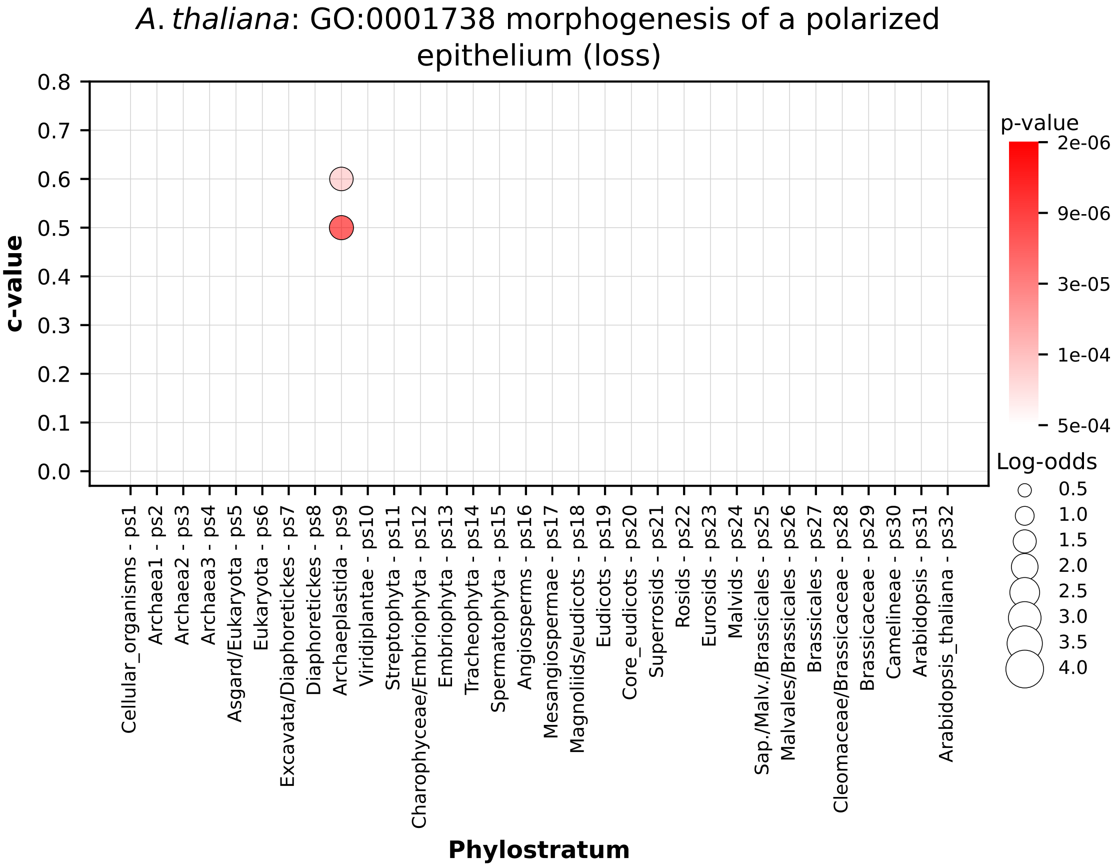

A. thaliana: GO:0001738 morphogenesis of a polarized epithelium (loss)
c-value
0.8
0.7
0.6
0.5
0.4
0.3
0.2
0.1
0.0
Phylostratum
Cellular_organisms - ps1
Archaea1 - ps2
Archaea2 - ps3
Archaea3 - ps4
Asgard/Eukaryota - ps5
Eukaryota - ps6
Excavata/Diaphoretickes - ps7
Diaphoretickes - ps8
Archaeplastida - ps9
Viridiplantae - ps10
Streptophyta - ps11
Charophyceae/Embriophyta - ps12
Embriophyta - ps13
Tracheophyta - ps14
Spermatophyta - ps15
Angiosperms - ps16
Mesangiospermae - ps17
Magnoliids/eudicots - ps18
Eudicots - ps19
Core_eudicots - ps20
Superrosids - ps21
Rosids - ps22
Eurosids - ps23
Malvids - ps24
Sap./Malv./Brassicales - ps25
Malvales/Brassicales - ps26
Brassicales - ps27
Cleomaceae/Brassicaceae - ps28
Brassicaceae - ps29
Camelineae - ps30
Arabidopsis - ps31
Arabidopsis_thaliana - ps32
p-value
2e-06
9e-06
3e-05
1e-04
5e-04
Log-odds
0.5
1.0
1.5
2.0
2.5
3.0
3.5
4.0

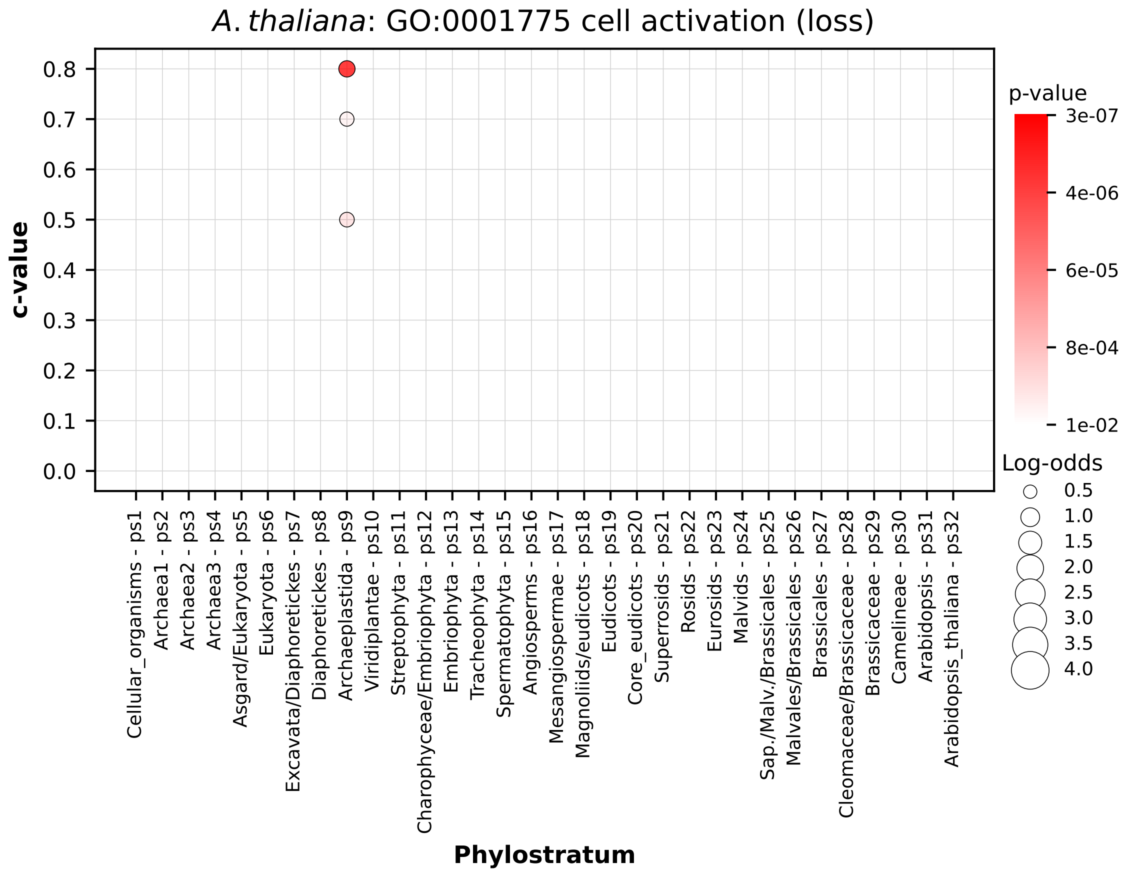

A. thaliana: GO:0001775 cell activation (loss)
c-value
0.8
0.7
0.6
0.5
0.4
0.3
0.2
0.1
0.0
Phylostratum
Cellular_organisms - ps1
Archaea1 - ps2
Archaea2 - ps3
Archaea3 - ps4
Asgard/Eukaryota - ps5
Eukaryota - ps6
Excavata/Diaphoretickes - ps7
Diaphoretickes - ps8
Archaeplastida - ps9
Viridiplantae - ps10
Streptophyta - ps11
Charophyceae/Embriophyta - ps12
Embriophyta - ps13
Tracheophyta - ps14
Spermatophyta - ps15
Angiosperms - ps16
Mesangiospermae - ps17
Magnoliids/eudicots - ps18
Eudicots - ps19
Core_eudicots - ps20
Superrosids - ps21
Rosids - ps22
Eurosids - ps23
Malvids - ps24
Sap./Malv./Brassicales - ps25
Malvales/Brassicales - ps26
Brassicales - ps27
Cleomaceae/Brassicaceae - ps28
Brassicaceae - ps29
Camelineae - ps30
Arabidopsis - ps31
Arabidopsis_thaliana - ps32
p-value
3e-07
4e-06
6e-05
8e-04
1e-02
Log-odds
0.5
1.0
1.5
2.0
2.5
3.0
3.5
4.0

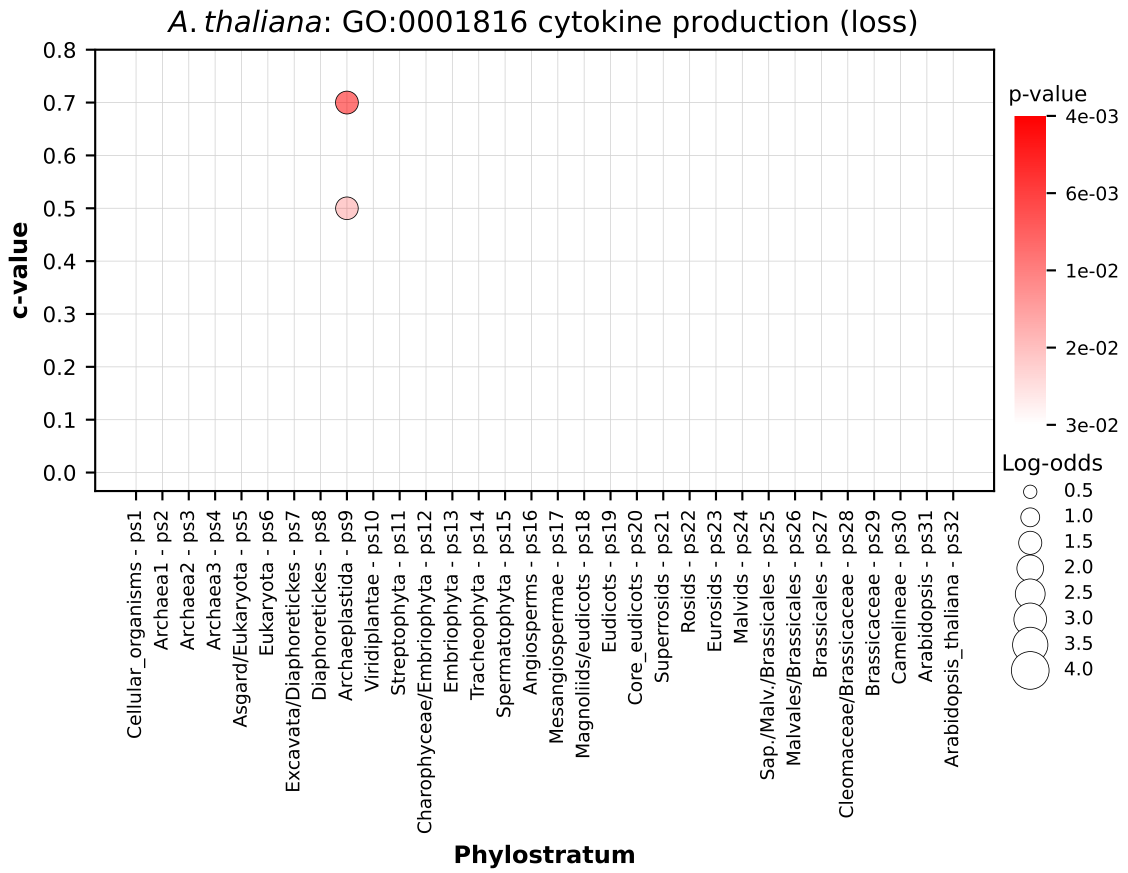

A. thaliana: GO:0001816 cytokine production (loss)
c-value
0.0
0.1
0.2
0.3
0.4
0.5
0.6
0.7
0.8
Phylostratum
Cellular_organisms - ps1
Archaea1 - ps2
Archaea2 - ps3
Archaea3 - ps4
Asgard/Eukaryota - ps5
Eukaryota - ps6
Excavata/Diaphoretickes - ps7
Diaphoretickes - ps8
Archaeplastida - ps9
Viridiplantae - ps10
Streptophyta - ps11
Charophyceae/Embriophyta - ps12
Embriophyta - ps13
Tracheophyta - ps14
Spermatophyta - ps15
Angiosperms - ps16
Mesangiospermae - ps17
Magnoliids/eudicots - ps18
Eudicots - ps19
Core_eudicots - ps20
Superrosids - ps21
Rosids - ps22
Eurosids - ps23
Malvids - ps24
Sap./Malv./Brassicales - ps25
Malvales/Brassicales - ps26
Brassicales - ps27
Cleomaceae/Brassicaceae - ps28
Brassicaceae - ps29
Camelineae - ps30
Arabidopsis - ps31
Arabidopsis_thaliana - ps32
p-value
4e-03
6e-03
1e-02
2e-02
3e-02
Log-odds
0.5
1.0
1.5
2.0
2.5
3.0
3.5
4.0

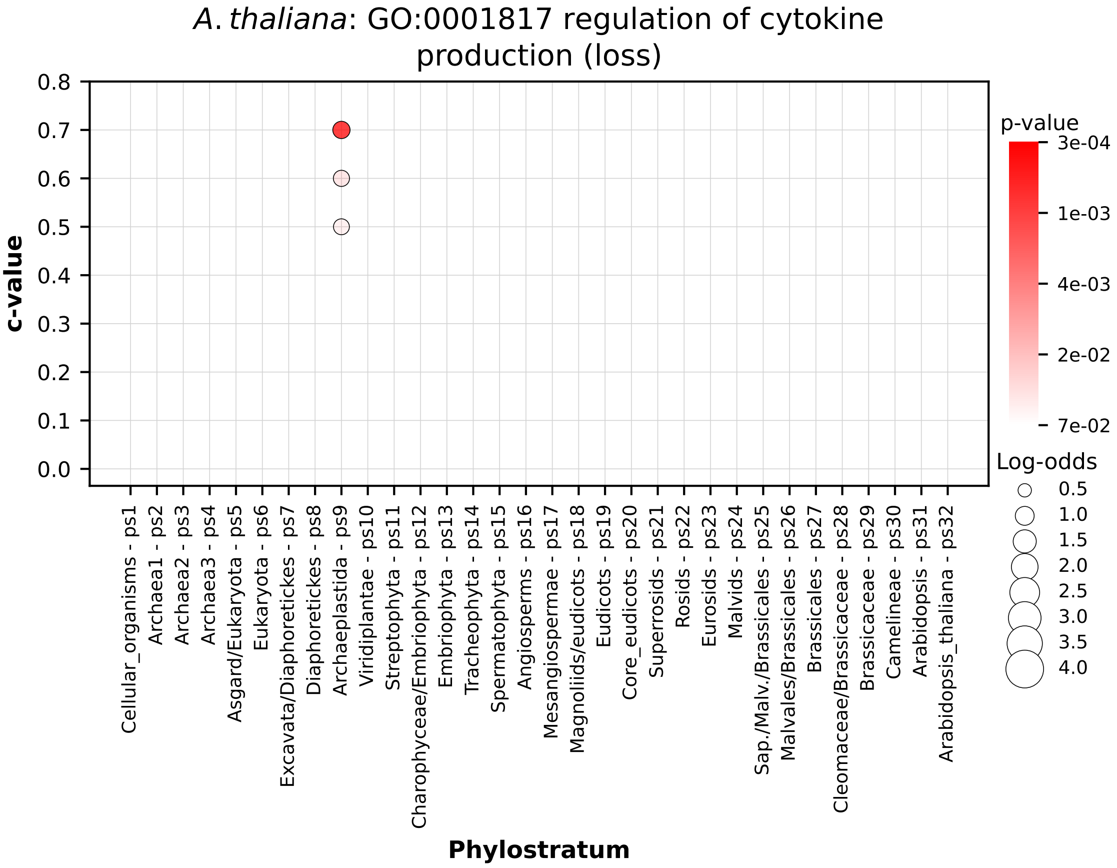
A. thaliana: GO:0001817 regulation of cytokine production (loss)
c-value
0.8
0.7
0.6
0.5
0.4
0.3
0.2
0.1
0.0
Phylostratum
Cellular_organisms - ps1
Archaea1 - ps2
Archaea2 - ps3
Archaea3 - ps4
Asgard/Eukaryota - ps5
Eukaryota - ps6
Excavata/Diaphoretickes - ps7
Diaphoretickes - ps8
Archaeplastida - ps9
Viridiplantae - ps10
Streptophyta - ps11
Charophyceae/Embriophyta - ps12
Embriophyta - ps13
Tracheophyta - ps14
Spermatophyta - ps15
Angiosperms - ps16
Mesangiospermae - ps17
Magnoliids/eudicots - ps18
Eudicots - ps19
Core_eudicots - ps20
Superrosids - ps21
Rosids - ps22
Eurosids - ps23
Malvids - ps24
Sap./Malv./Brassicales - ps25
Malvales/Brassicales - ps26
Brassicales - ps27
Cleomaceae/Brassicaceae - ps28
Brassicaceae - ps29
Camelineae - ps30
Arabidopsis - ps31
Arabidopsis_thaliana - ps32
p-value
3e-04
1e-03
4e-03
2e-02
7e-02
Log-odds
0.5
1.0
1.5
2.0
2.5
3.0
3.5
4.0

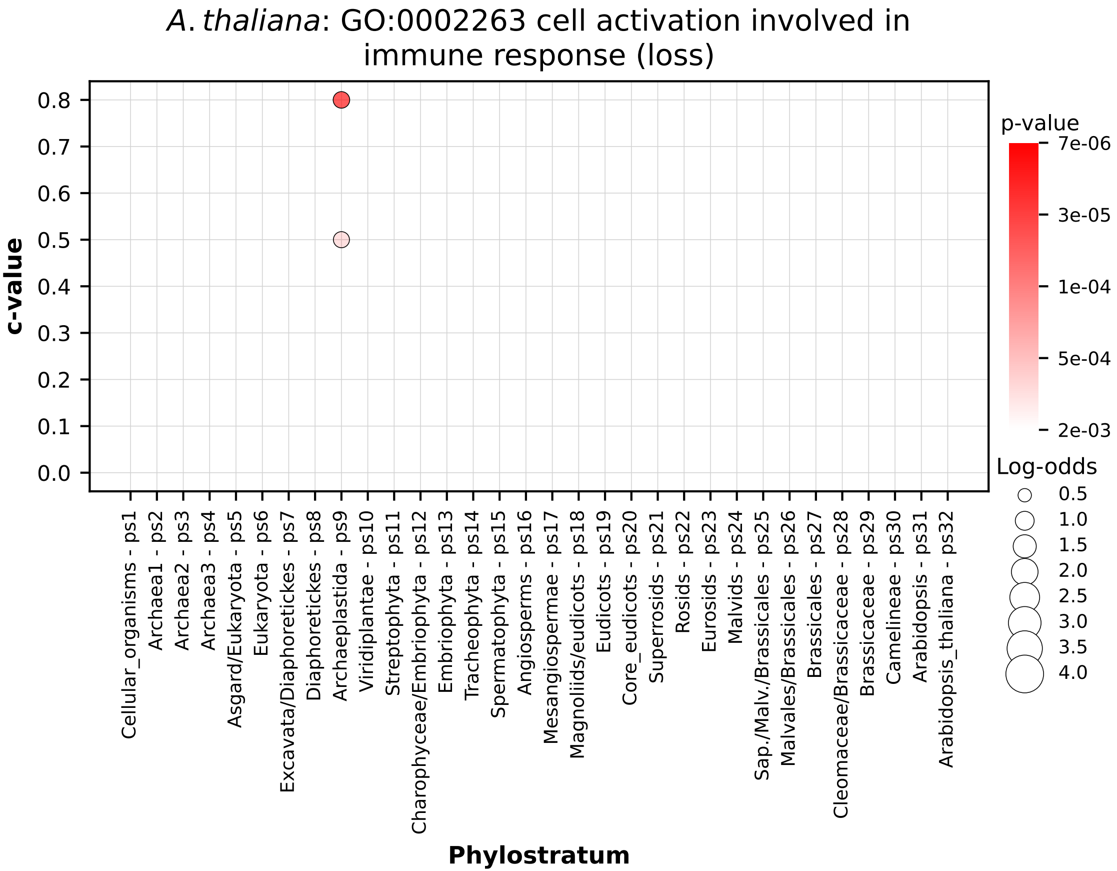

A. thaliana: GO:0002263 cell activation involved in immune response (loss)
c-value
0.0
0.1
0.2
0.3
0.4
0.5
0.6
0.7
0.8
Phylostratum
Cellular_organisms - ps1
Archaea1 - ps2
Archaea2 - ps3
Archaea3 - ps4
Asgard/Eukaryota - ps5
Eukaryota - ps6
Excavata/Diaphoretickes - ps7
Diaphoretickes - ps8
Archaeplastida - ps9
Viridiplantae - ps10
Streptophyta - ps11
Charophyceae/Embriophyta - ps12
Embriophyta - ps13
Tracheophyta - ps14
Spermatophyta - ps15
Angiosperms - ps16
Mesangiospermae - ps17
Magnoliids/eudicots - ps18
Eudicots - ps19
Core_eudicots - ps20
Superrosids - ps21
Rosids - ps22
Eurosids - ps23
Malvids - ps24
Sap./Malv./Brassicales - ps25
Malvales/Brassicales - ps26
Brassicales - ps27
Cleomaceae/Brassicaceae - ps28
Brassicaceae - ps29
Camelineae - ps30
Arabidopsis - ps31
Arabidopsis_thaliana - ps32
p-value
7e-06
3e-05
1e-04
5e-04
2e-03
Log-odds
0.5
1.0
1.5
2.0
2.5
3.0
3.5
4.0

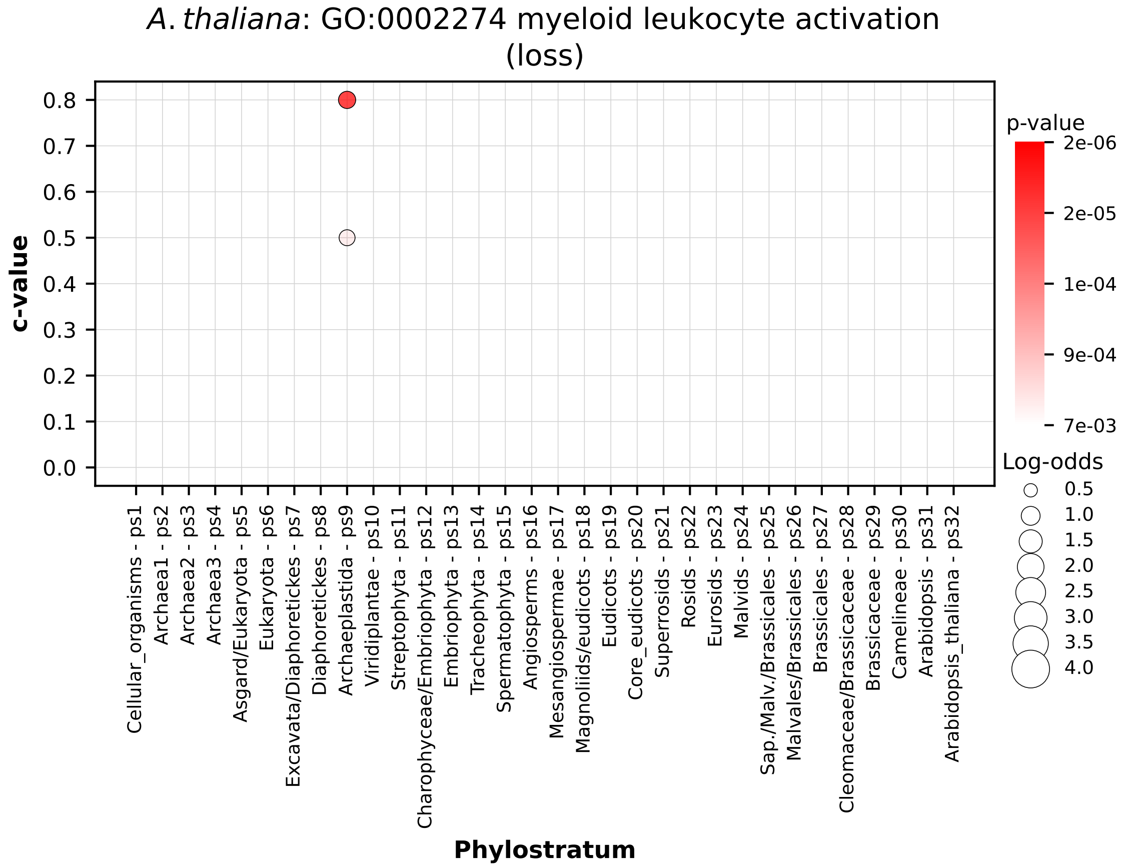

A. thaliana: GO:0002274 myeloid leukocyte activation
(loss)
c-value
0.0
0.1
0.2
0.3
0.4
0.5
0.6
0.7
0.8
p-value
2e-06
2e-05
1e-04
9e-04
7e-03
Log-odds
0.5
1.0
1.5
2.0
2.5
3.0
3.5
4.0
Cellular_organisms - ps1
Archaea1 - ps2
Archaea2 - ps3
Archaea3 - ps4
Asgard/Eukaryota - ps5
Eukaryota - ps6
Excavata/Diaphoretickes - ps7
Diaphoretickes - ps8
Archaeplastida - ps9
Viridiplantae - ps10
Streptophyta - ps11
Charophyceae/Embriophyta - ps12
Embriophyta - ps13
Tracheophyta - ps14
Spermatophyta - ps15
Angiosperms - ps16
Mesangiospermae - ps17
Magnoliids/eudicots - ps18
Eudicots - ps19
Core_eudicots - ps20
Superrosids - ps21
Rosids - ps22
Eurosids - ps23
Malvids - ps24
Sap./Malv./Brassicales - ps25
Malvales/Brassicales - ps26
Brassicales - ps27
Cleomaceae/Brassicaceae - ps28
Brassicaceae - ps29
Camelineae - ps30
Arabidopsis - ps31
Arabidopsis_thaliana - ps32
Phylostratum

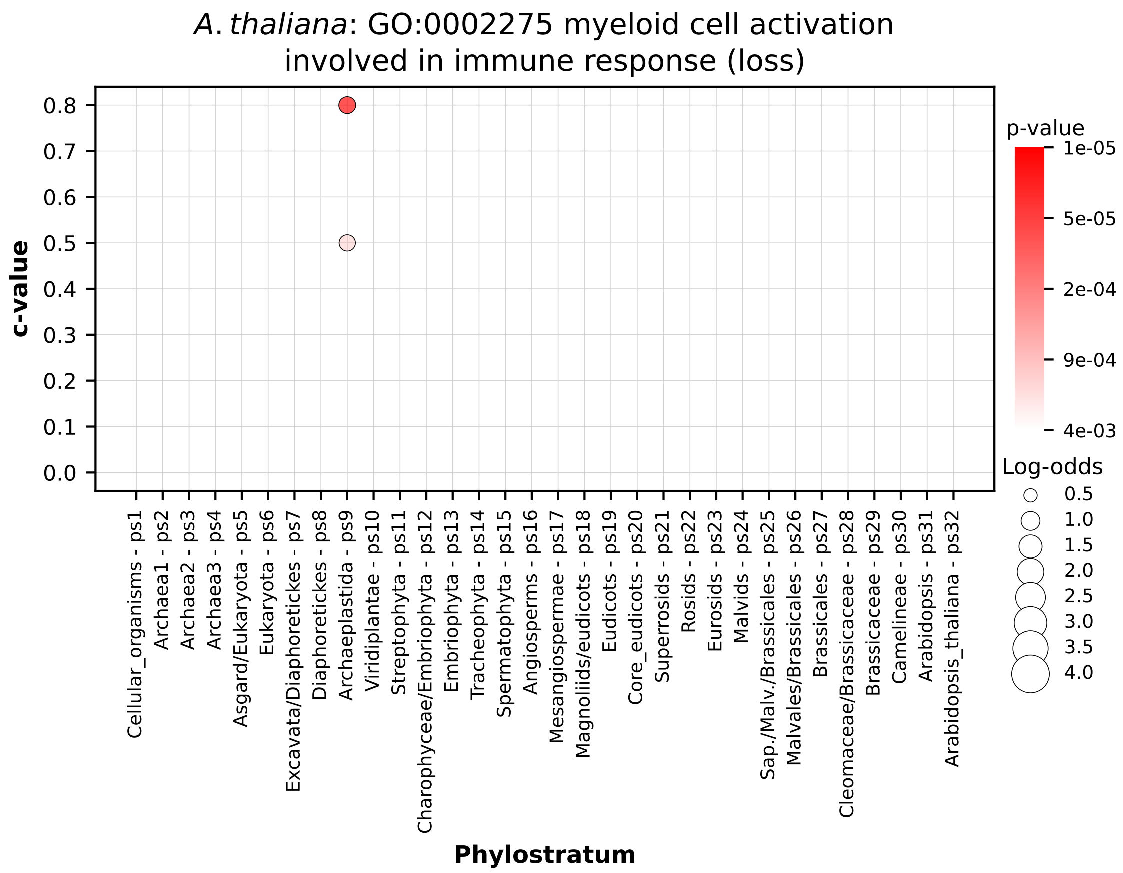

A. thaliana: GO:0002275 myeloid cell activation involved in immune response (loss)
c-value
0.0
0.1
0.2
0.3
0.4
0.5
0.6
0.7
0.8
Phylostratum
Cellular_organisms - ps1
Archaea1 - ps2
Archaea2 - ps3
Archaea3 - ps4
Asgard/Eukaryota - ps5
Eukaryota - ps6
Excavata/Diaphoretickes - ps7
Diaphoretickes - ps8
Archaeplastida - ps9
Viridiplantae - ps10
Streptophyta - ps11
Charophyceae/Embriophyta - ps12
Embriophyta - ps13
Tracheophyta - ps14
Spermatophyta - ps15
Angiosperms - ps16
Mesangiospermae - ps17
Magnoliids/eudicots - ps18
Eudicots - ps19
Core_eudicots - ps20
Superrosids - ps21
Rosids - ps22
Eurosids - ps23
Malvids - ps24
Sap./Malv./Brassicales - ps25
Malvales/Brassicales - ps26
Brassicales - ps27
Cleomaceae/Brassicaceae - ps28
Brassicaceae - ps29
Camelineae - ps30
Arabidopsis - ps31
Arabidopsis_thaliana - ps32
p-value
1e-05
5e-05
2e-04
9e-04
4e-03
Log-odds
0.5
1.0
1.5
2.0
2.5
3.0
3.5
4.0

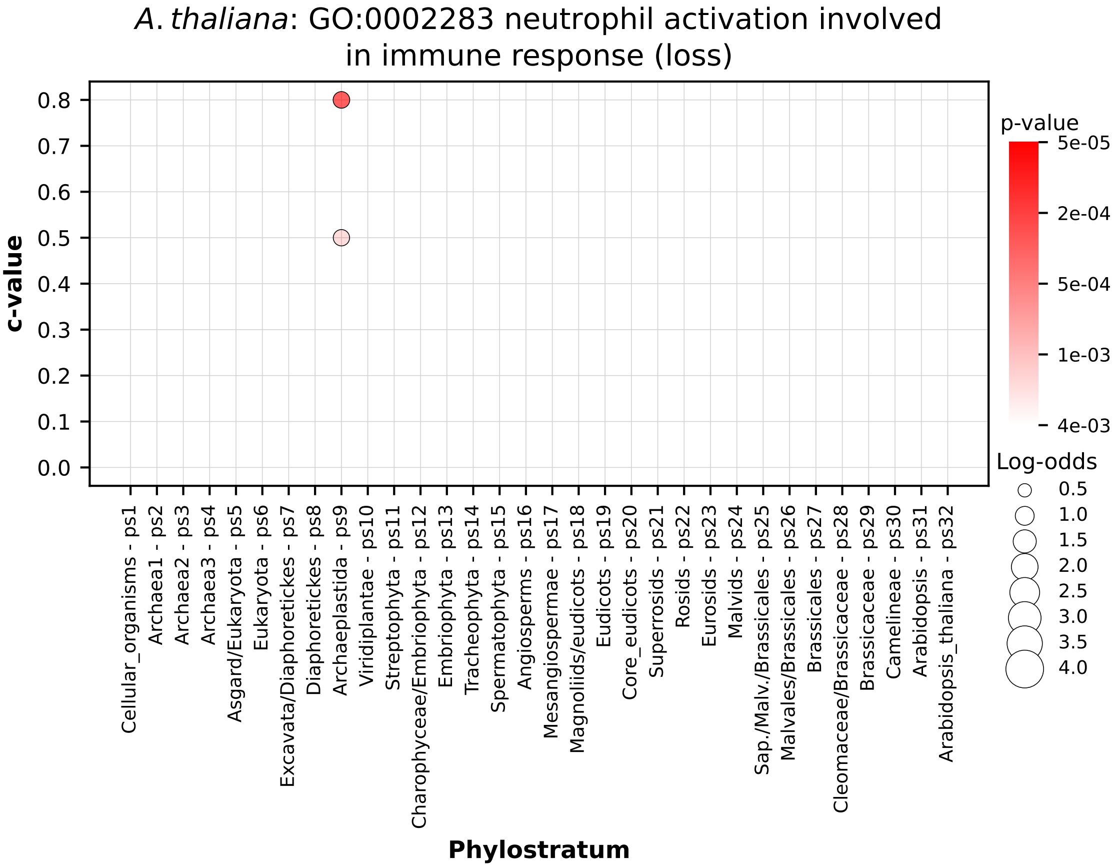

A. thaliana: GO:0002283 neutrophil activation involved in immune response (loss)
c-value
0.8
0.7
0.6
0.5
0.4
0.3
0.2
0.1
0.0
p-value
5e-05
2e-04
5e-04
1e-03
4e-03
Log-odds
0.5
1.0
1.5
2.0
2.5
3.0
3.5
4.0
Cellular_organisms - ps1
Archaea1 - ps2
Archaea2 - ps3
Archaea3 - ps4
Asgard/Eukaryota - ps5
Eukaryota - ps6
Excavata/Diaphoretickes - ps7
Diaphoretickes - ps8
Archaeplastida - ps9
Viridiplantae - ps10
Streptophyta - ps11
Charophyceae/Embriophyta - ps12
Embriophyta - ps13
Tracheophyta - ps14
Spermatophyta - ps15
Angiosperms - ps16
Mesangiospermae - ps17
Magnoliids/eudicots - ps18
Eudicots - ps19
Core_eudicots - ps20
Superrosids - ps21
Rosids - ps22
Eurosids - ps23
Malvids - ps24
Sap./Malv./Brassicales - ps25
Malvales/Brassicales - ps26
Brassicales - ps27
Cleomaceae/Brassicaceae - ps28
Brassicaceae - ps29
Camelineae - ps30
Arabidopsis - ps31
Arabidopsis_thaliana - ps32
Phylostratum

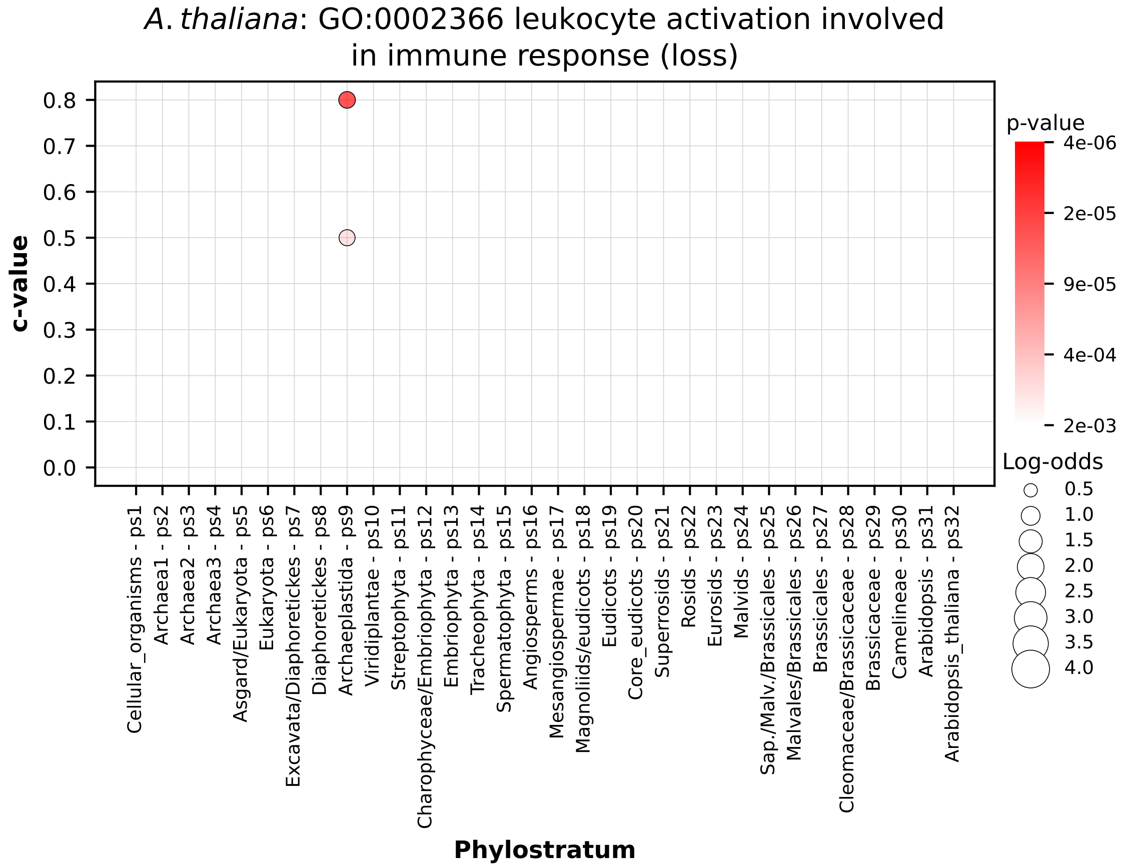

A. thaliana: GO:0002366 leukocyte activation involved in immune response (loss)
c-value
0.0
0.1
0.2
0.3
0.4
0.5
0.6
0.7
0.8
Phylostratum
Cellular_organisms - ps1
Archaea1 - ps2
Archaea2 - ps3
Archaea3 - ps4
Asgard/Eukaryota - ps5
Eukaryota - ps6
Excavata/Diaphoretickes - ps7
Diaphoretickes - ps8
Archaeplastida - ps9
Viridiplantae - ps10
Streptophyta - ps11
Charophyceae/Embriophyta - ps12
Embriophyta - ps13
Tracheophyta - ps14
Spermatophyta - ps15
Angiosperms - ps16
Mesangiospermae - ps17
Magnoliids/eudicots - ps18
Eudicots - ps19
Core_eudicots - ps20
Superrosids - ps21
Rosids - ps22
Eurosids - ps23
Malvids - ps24
Sap./Malv./Brassicales - ps25
Malvales/Brassicales - ps26
Brassicales - ps27
Cleomaceae/Brassicaceae - ps28
Brassicaceae - ps29
Camelineae - ps30
Arabidopsis - ps31
Arabidopsis_thaliana - ps32
p-value
4e-06
2e-05
9e-05
4e-04
2e-03
Log-odds
0.5
1.0
1.5
2.0
2.5
3.0
3.5
4.0

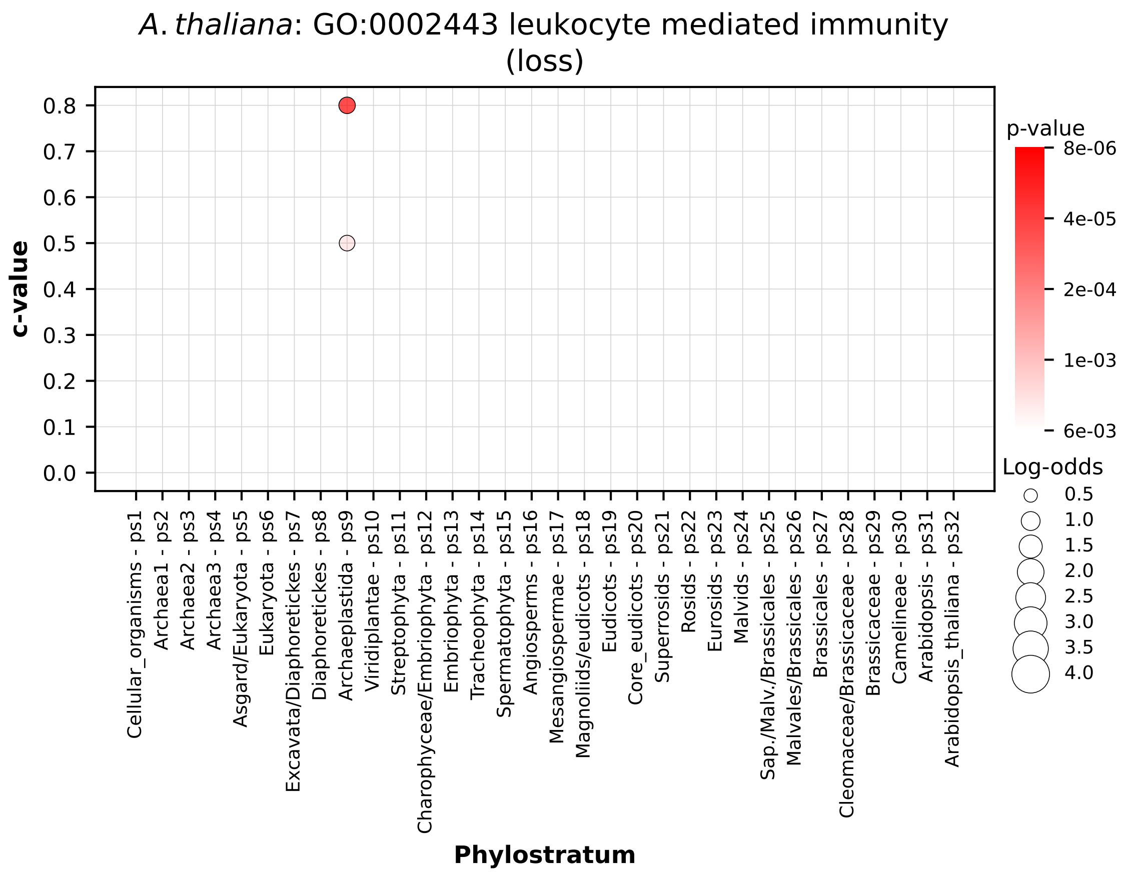

A. thaliana: GO:0002443 leukocyte mediated immunity
(loss)
c-value
0.8
0.7
0.6
0.5
0.4
0.3
0.2
0.1
0.0
p-value
8e-06
4e-05
2e-04
1e-03
6e-03
Log-odds
0.5
1.0
1.5
2.0
2.5
3.0
3.5
4.0
Cellular_organisms - ps1
Archaea1 - ps2
Archaea2 - ps3
Archaea3 - ps4
Asgard/Eukaryota - ps5
Eukaryota - ps6
Excavata/Diaphoretickes - ps7
Diaphoretickes - ps8
Archaeplastida - ps9
Viridiplantae - ps10
Streptophyta - ps11
Charophyceae/Embriophyta - ps12
Embriophyta - ps13
Tracheophyta - ps14
Spermatophyta - ps15
Angiosperms - ps16
Mesangiospermae - ps17
Magnoliids/eudicots - ps18
Eudicots - ps19
Core_eudicots - ps20
Superrosids - ps21
Rosids - ps22
Eurosids - ps23
Malvids - ps24
Sap./Malv./Brassicales - ps25
Malvales/Brassicales - ps26
Brassicales - ps27
Cleomaceae/Brassicaceae - ps28
Brassicaceae - ps29
Camelineae - ps30
Arabidopsis - ps31
Arabidopsis_thaliana - ps32
Phylostratum

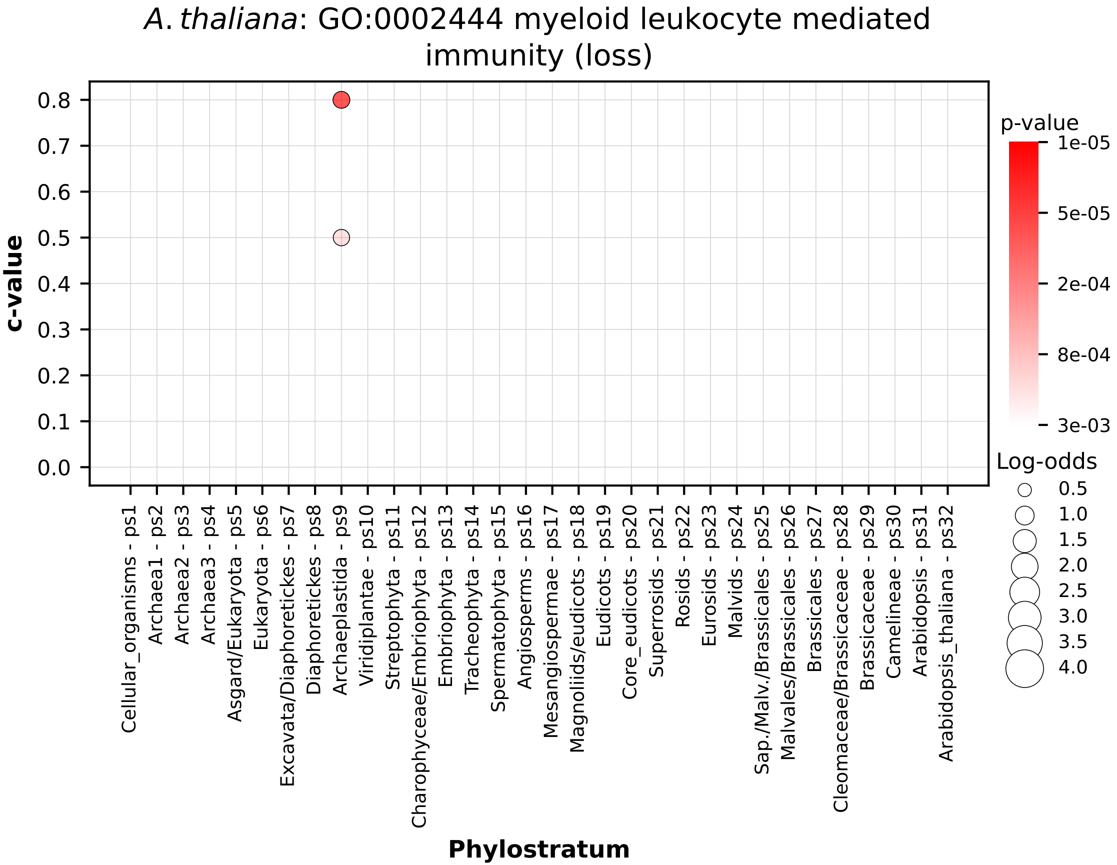

A. thaliana: GO:0002444 myeloid leukocyte mediated immunity (loss)
c-value
0.8
0.7
0.6
0.5
0.4
0.3
0.2
0.1
0.0
p-value
1e-05
5e-05
2e-04
8e-04
3e-03
Log-odds
0.5
1.0
1.5
2.0
2.5
3.0
3.5
4.0
Cellular_organisms - ps1
Archaea1 - ps2
Archaea2 - ps3
Archaea3 - ps4
Asgard/Eukaryota - ps5
Eukaryota - ps6
Excavata/Diaphoretickes - ps7
Diaphoretickes - ps8
Archaeplastida - ps9
Viridiplantae - ps10
Streptophyta - ps11
Charophyceae/Embriophyta - ps12
Embriophyta - ps13
Tracheophyta - ps14
Spermatophyta - ps15
Angiosperms - ps16
Mesangiospermae - ps17
Magnoliids/eudicots - ps18
Eudicots - ps19
Core_eudicots - ps20
Superrosids - ps21
Rosids - ps22
Eurosids - ps23
Malvids - ps24
Sap./Malv./Brassicales - ps25
Malvales/Brassicales - ps26
Brassicales - ps27
Cleomaceae/Brassicaceae - ps28
Brassicaceae - ps29
Camelineae - ps30
Arabidopsis - ps31
Arabidopsis_thaliana - ps32
Phylostratum

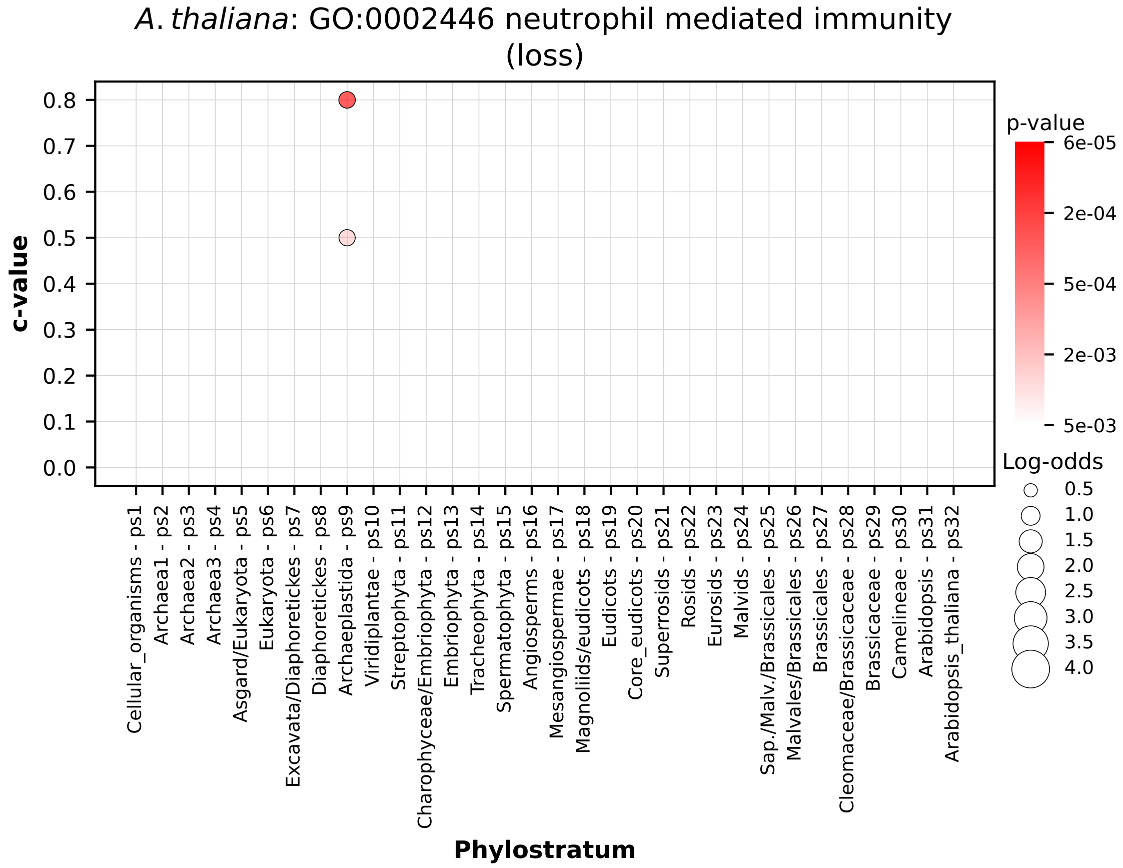

A. thaliana: GO:0002446 neutrophil mediated immunity (loss)
c-value
0.8
0.7
0.6
0.5
0.4
0.3
0.2
0.1
0.0
p-value
6e-05
2e-04
5e-04
2e-03
5e-03
Log-odds
0.5
1.0
1.5
2.0
2.5
3.0
3.5
4.0
Cellular_organisms - ps1
Archaea1 - ps2
Archaea2 - ps3
Archaea3 - ps4
Asgard/Eukaryota - ps5
Eukaryota - ps6
Excavata/Diaphoretickes - ps7
Diaphoretickes - ps8
Archaeplastida - ps9
Viridiplantae - ps10
Streptophyta - ps11
Charophyceae/Embriophyta - ps12
Embriophyta - ps13
Tracheophyta - ps14
Spermatophyta - ps15
Angiosperms - ps16
Mesangiospermae - ps17
Magnoliids/eudicots - ps18
Eudicots - ps19
Core_eudicots - ps20
Superrosids - ps21
Rosids - ps22
Eurosids - ps23
Malvids - ps24
Sap./Malv./Brassicales - ps25
Malvales/Brassicales - ps26
Brassicales - ps27
Cleomaceae/Brassicaceae - ps28
Brassicaceae - ps29
Camelineae - ps30
Arabidopsis - ps31
Arabidopsis_thaliana - ps32
Phylostratum

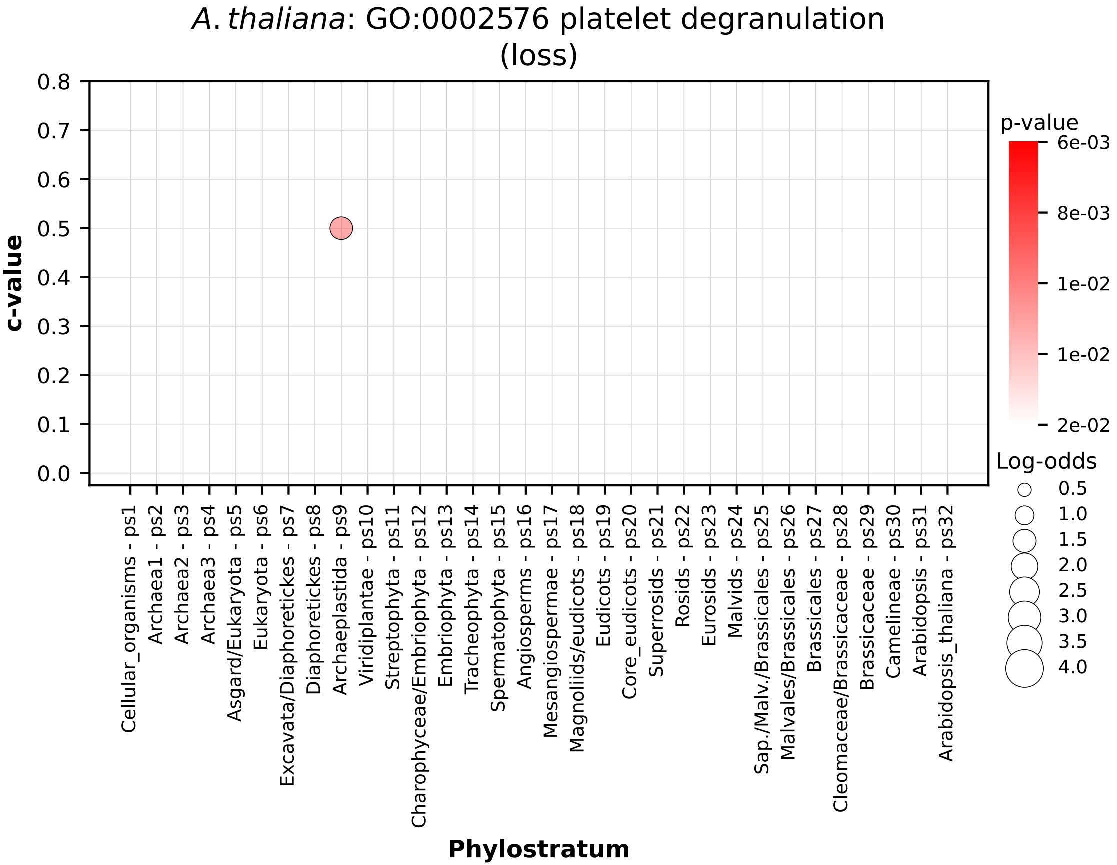
A. thaliana: GO:0002576 platelet degranulation
(loss)
c-value
0.8
0.7
0.6
0.5
0.4
0.3
0.2
0.1
0.0
p-value
6e-03
8e-03
1e-02
1e-02
2e-02
Log-odds
0.5
1.0
1.5
2.0
2.5
3.0
3.5
4.0
Cellular_organisms - ps1
Archaea1 - ps2
Archaea2 - ps3
Archaea3 - ps4
Asgard/Eukaryota - ps5
Eukaryota - ps6
Excavata/Diaphoretickes - ps7
Diaphoretickes - ps8
Archaeplastida - ps9
Viridiplantae - ps10
Streptophyta - ps11
Charophyceae/Embriophyta - ps12
Embriophyta - ps13
Tracheophyta - ps14
Spermatophyta - ps15
Angiosperms - ps16
Mesangiospermae - ps17
Magnoliids/eudicots - ps18
Eudicots - ps19
Core_eudicots - ps20
Superrosids - ps21
Rosids - ps22
Eurosids - ps23
Malvids - ps24
Sap./Malv./Brassicales - ps25
Malvales/Brassicales - ps26
Brassicales - ps27
Cleomaceae/Brassicaceae - ps28
Brassicaceae - ps29
Camelineae - ps30
Arabidopsis - ps31
Arabidopsis_thaliana - ps32
Phylostratum

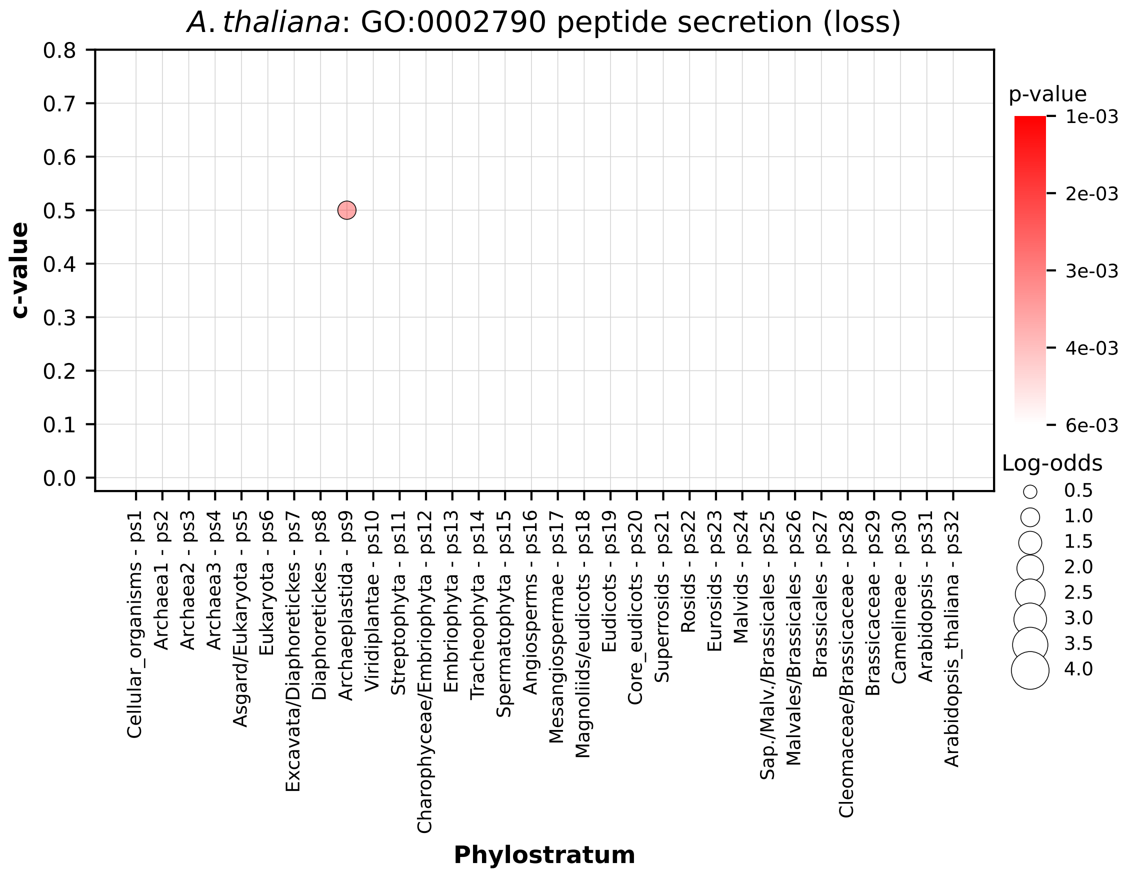

A. thaliana: GO:0002790 peptide secretion (loss)
c-value
0.8
0.7
0.6
0.5
0.4
0.3
0.2
0.1
0.0
p-value
1e-03
2e-03
3e-03
4e-03
6e-03
Log-odds
0.5
1.0
1.5
2.0
2.5
3.0
3.5
4.0
Cellular_organisms - ps1
Archaea1 - ps2
Archaea2 - ps3
Archaea3 - ps4
Asgard/Eukaryota - ps5
Eukaryota - ps6
Excavata/Diaphoretickes - ps7
Diaphoretickes - ps8
Archaeplastida - ps9
Viridiplantae - ps10
Streptophyta - ps11
Charophyceae/Embriophyta - ps12
Embriophyta - ps13
Tracheophyta - ps14
Spermatophyta - ps15
Angiosperms - ps16
Mesangiospermae - ps17
Magnoliids/eudicots - ps18
Eudicots - ps19
Core_eudicots - ps20
Superrosids - ps21
Rosids - ps22
Eurosids - ps23
Malvids - ps24
Sap./Malv./Brassicales - ps25
Malvales/Brassicales - ps26
Brassicales - ps27
Cleomaceae/Brassicaceae - ps28
Brassicaceae - ps29
Camelineae - ps30
Arabidopsis - ps31
Arabidopsis_thaliana - ps32
Phylostratum

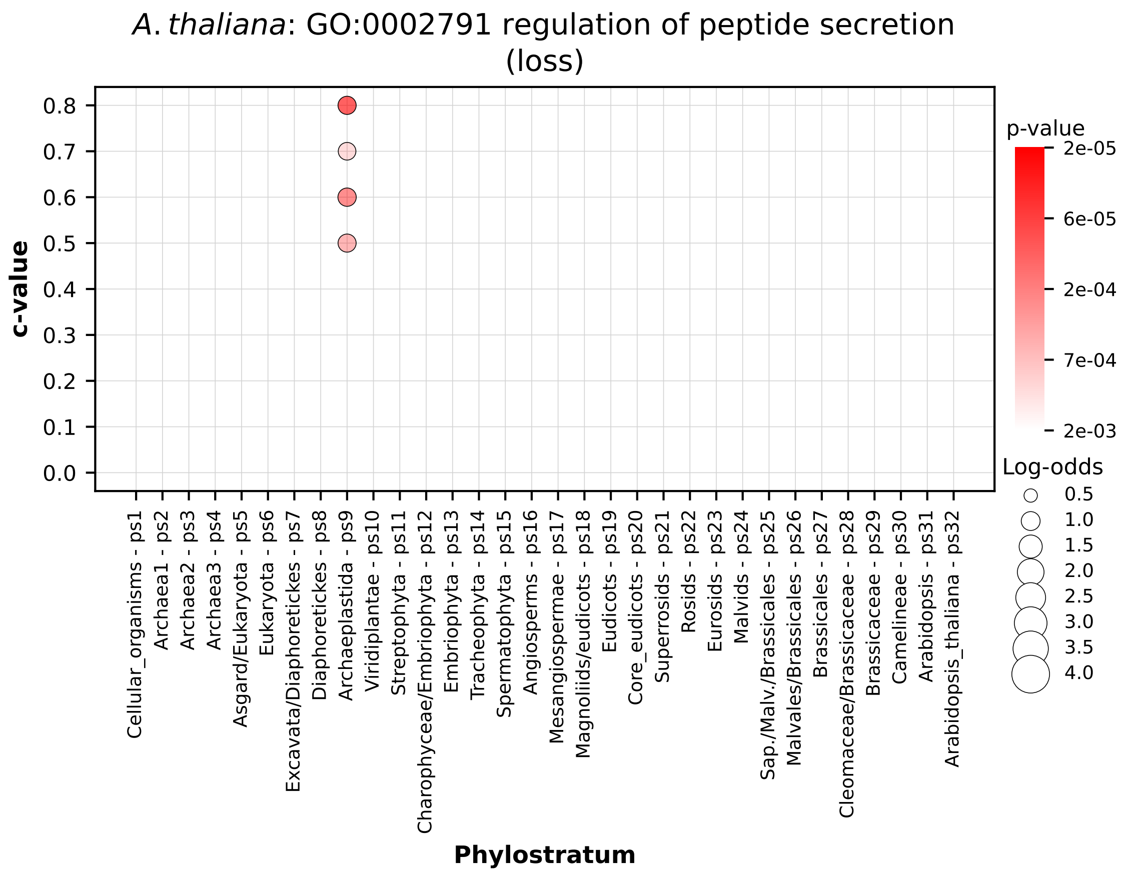

A. thaliana: GO:0002791 regulation of peptide secretion (loss)
c-value
0.8
0.7
0.6
0.5
0.4
0.3
0.2
0.1
0.0
p-value
2e-05
6e-05
2e-04
7e-04
2e-03
Log-odds
0.5
1.0
1.5
2.0
2.5
3.0
3.5
4.0
Cellular_organisms - ps1
Archaea1 - ps2
Archaea2 - ps3
Archaea3 - ps4
Asgard/Eukaryota - ps5
Eukaryota - ps6
Excavata/Diaphoretickes - ps7
Diaphoretickes - ps8
Archaeplastida - ps9
Viridiplantae - ps10
Streptophyta - ps11
Charophyceae/Embriophyta - ps12
Embriophyta - ps13
Tracheophyta - ps14
Spermatophyta - ps15
Angiosperms - ps16
Mesangiospermae - ps17
Magnoliids/eudicots - ps18
Eudicots - ps19
Core_eudicots - ps20
Superrosids - ps21
Rosids - ps22
Eurosids - ps23
Malvids - ps24
Sap./Malv./Brassicales - ps25
Malvales/Brassicales - ps26
Brassicales - ps27
Cleomaceae/Brassicaceae - ps28
Brassicaceae - ps29
Camelineae - ps30
Arabidopsis - ps31
Arabidopsis_thaliana - ps32
Phylostratum

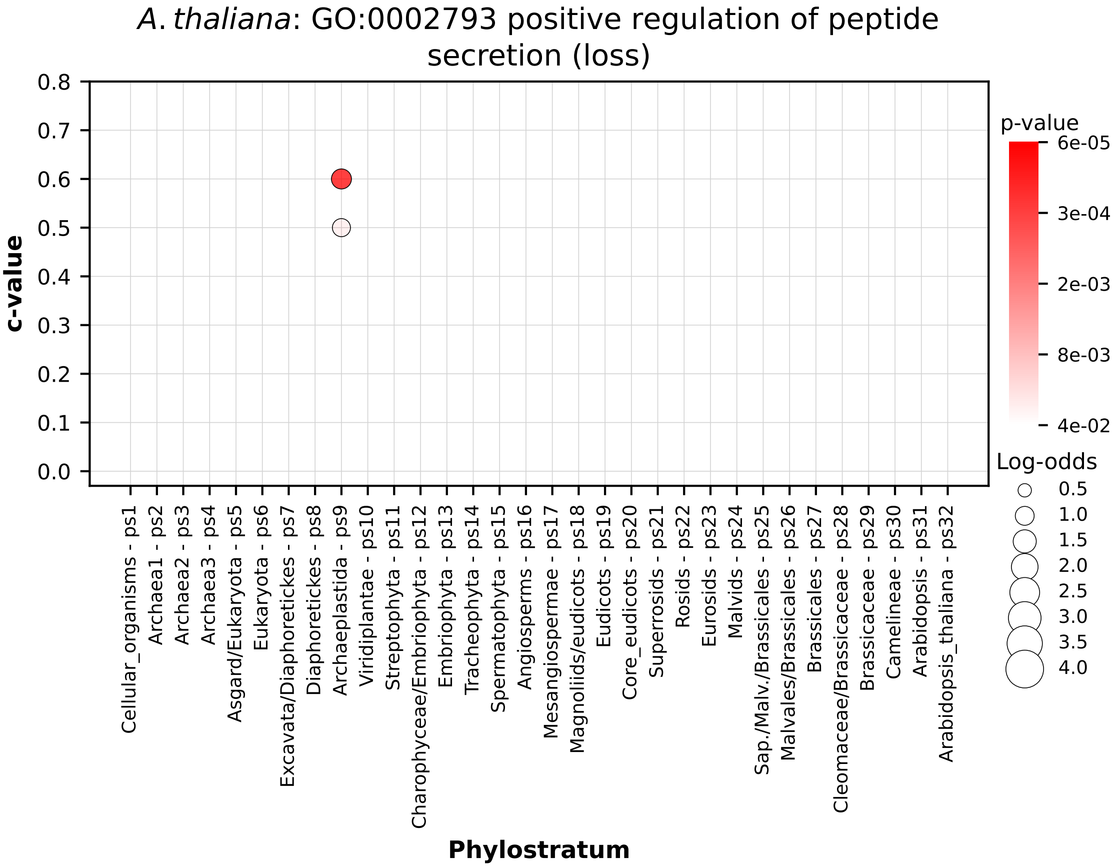
A. thaliana: GO:0002793 positive regulation of peptide secretion (loss)
c-value
0.0
0.1
0.2
0.3
0.4
0.5
0.6
0.7
0.8
p-value
6e-05
3e-04
2e-03
8e-03
4e-02
Log-odds
0.5
1.0
1.5
2.0
2.5
3.0
3.5
4.0
Cellular_organisms - ps1
Archaea1 - ps2
Archaea2 - ps3
Archaea3 - ps4
Asgard/Eukaryota - ps5
Eukaryota - ps6
Excavata/Diaphoretickes - ps7
Diaphoretickes - ps8
Archaeplastida - ps9
Viridiplantae - ps10
Streptophyta - ps11
Charophyceae/Embriophyta - ps12
Embriophyta - ps13
Tracheophyta - ps14
Spermatophyta - ps15
Angiosperms - ps16
Mesangiospermae - ps17
Magnoliids/eudicots - ps18
Eudicots - ps19
Core_eudicots - ps20
Superrosids - ps21
Rosids - ps22
Eurosids - ps23
Malvids - ps24
Sap./Malv./Brassicales - ps25
Malvales/Brassicales - ps26
Brassicales - ps27
Cleomaceae/Brassicaceae - ps28
Brassicaceae - ps29
Camelineae - ps30
Arabidopsis - ps31
Arabidopsis_thaliana - ps32
Phylostratum

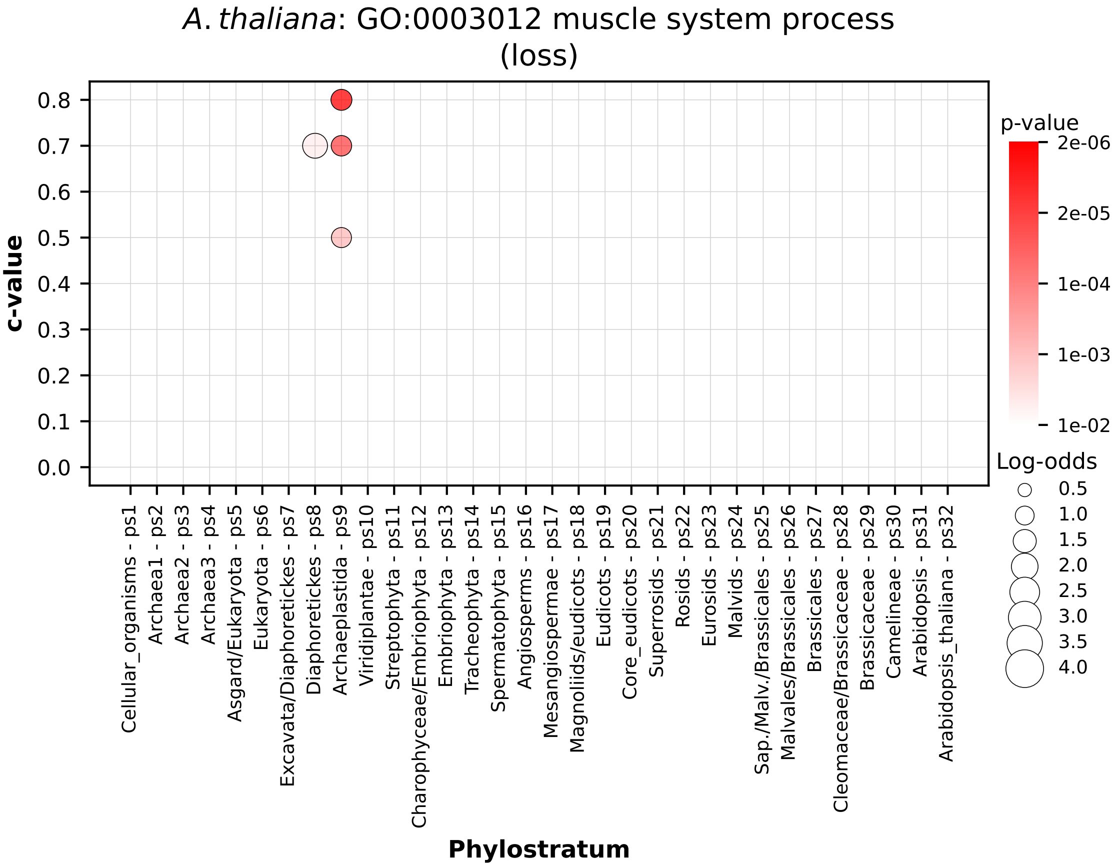

A. thaliana: GO:0003012 muscle system process
(loss)
c-value
0.8
0.7
0.6
0.5
0.4
0.3
0.2
0.1
0.0
p-value
2e-06
2e-05
1e-04
1e-03
1e-02
Log-odds
0.5
1.0
1.5
2.0
2.5
3.0
3.5
4.0
Cellular_organisms - ps1
Archaea1 - ps2
Archaea2 - ps3
Archaea3 - ps4
Asgard/Eukaryota - ps5
Eukaryota - ps6
Excavata/Diaphoretickes - ps7
Diaphoretickes - ps8
Archaeplastida - ps9
Viridiplantae - ps10
Streptophyta - ps11
Charophyceae/Embriophyta - ps12
Embriophyta - ps13
Tracheophyta - ps14
Spermatophyta - ps15
Angiosperms - ps16
Mesangiospermae - ps17
Magnoliids/eudicots - ps18
Eudicots - ps19
Core_eudicots - ps20
Superrosids - ps21
Rosids - ps22
Eurosids - ps23
Malvids - ps24
Sap./Malv./Brassicales - ps25
Malvales/Brassicales - ps26
Brassicales - ps27
Cleomaceae/Brassicaceae - ps28
Brassicaceae - ps29
Camelineae - ps30
Arabidopsis - ps31
Arabidopsis_thaliana - ps32
Phylostratum

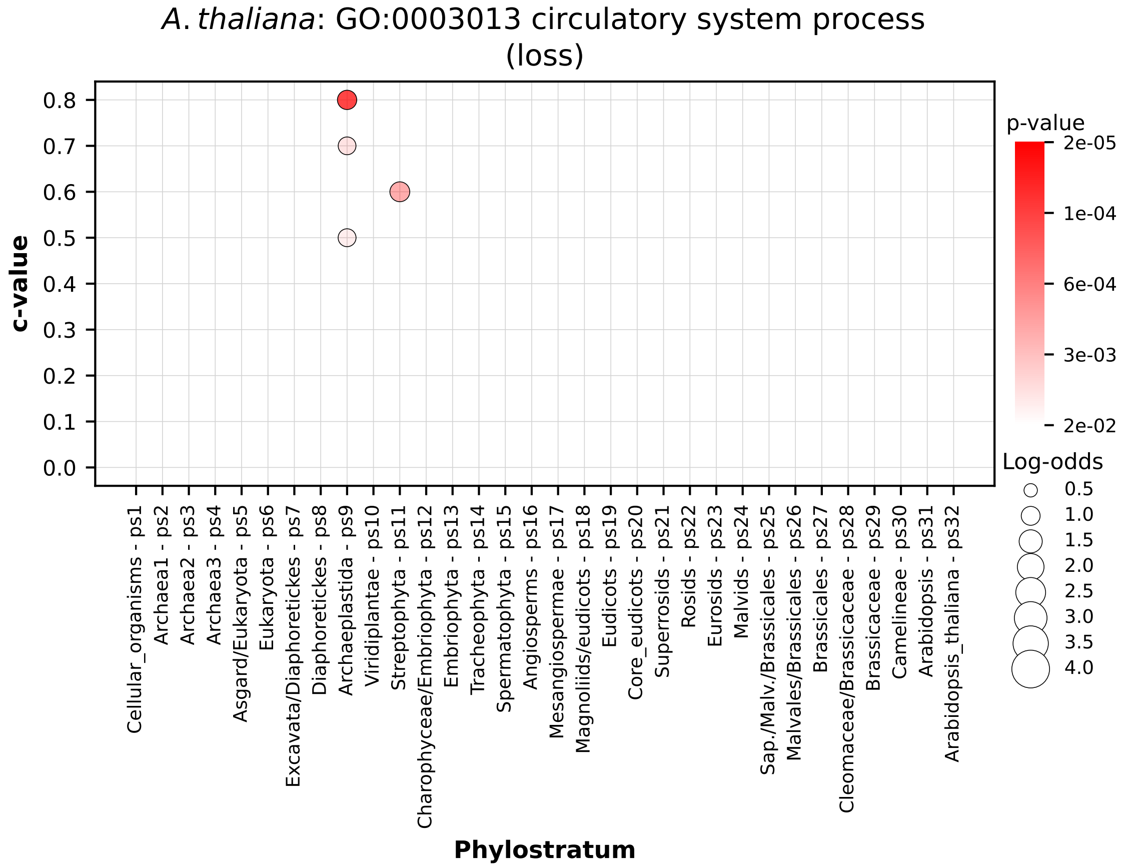

A. thaliana: GO:0003013 circulatory system process
(loss)
c-value
0.8
0.7
0.6
0.5
0.4
0.3
0.2
0.1
0.0
p-value
2e-05
1e-04
6e-04
3e-03
2e-02
Log-odds
0.5
1.0
1.5
2.0
2.5
3.0
3.5
4.0
Cellular_organisms - ps1
Archaea1 - ps2
Archaea2 - ps3
Archaea3 - ps4
Asgard/Eukaryota - ps5
Eukaryota - ps6
Excavata/Diaphoretickes - ps7
Diaphoretickes - ps8
Archaeplastida - ps9
Viridiplantae - ps10
Streptophyta - ps11
Charophyceae/Embriophyta - ps12
Embriophyta - ps13
Tracheophyta - ps14
Spermatophyta - ps15
Angiosperms - ps16
Mesangiospermae - ps17
Magnoliids/eudicots - ps18
Eudicots - ps19
Core_eudicots - ps20
Superrosids - ps21
Rosids - ps22
Eurosids - ps23
Malvids - ps24
Sap./Malv./Brassicales - ps25
Malvales/Brassicales - ps26
Brassicales - ps27
Cleomaceae/Brassicaceae - ps28
Brassicaceae - ps29
Camelineae - ps30
Arabidopsis - ps31
Arabidopsis_thaliana - ps32
Phylostratum

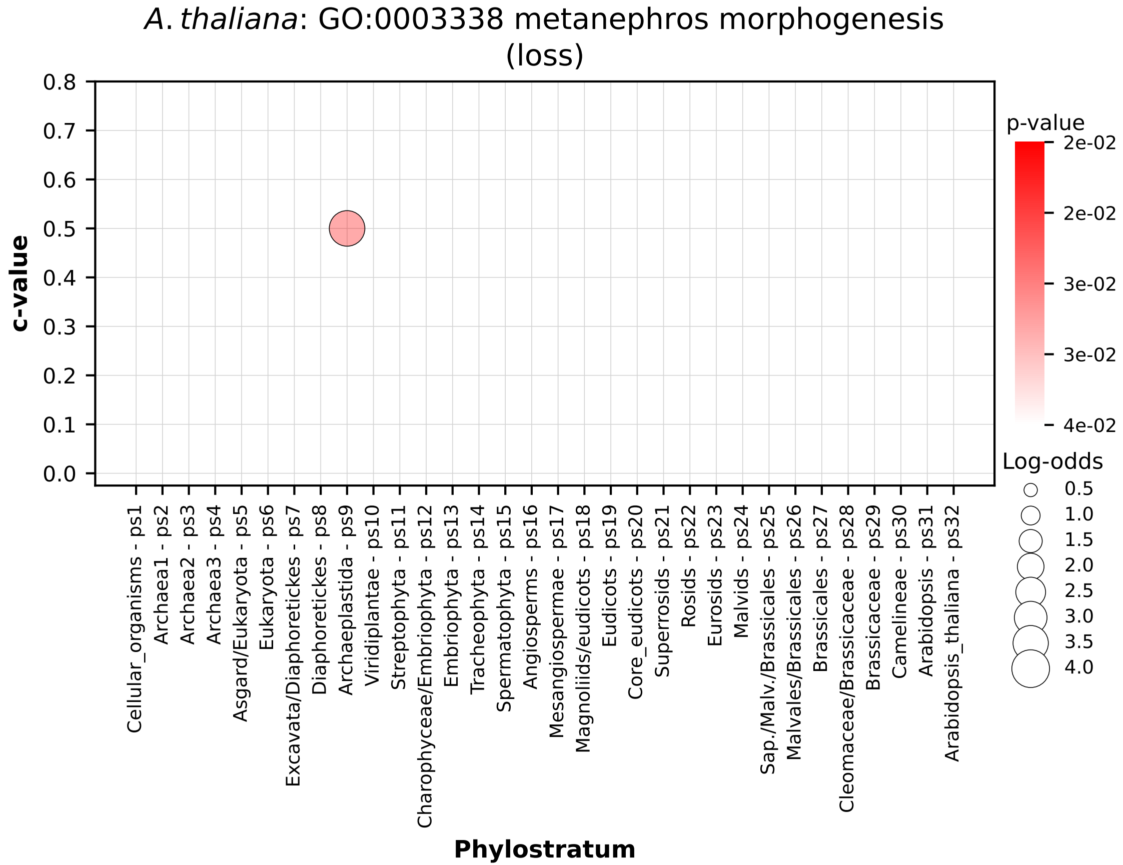

A. thaliana: GO:0003338 metanephros morphogenesis (loss)
c-value
0.8
0.7
0.6
0.5
0.4
0.3
0.2
0.1
0.0
Phylostratum
Cellular_organisms - ps1
Archaea1 - ps2
Archaea2 - ps3
Archaea3 - ps4
Asgard/Eukaryota - ps5
Eukaryota - ps6
Excavata/Diaphoretickes - ps7
Diaphoretickes - ps8
Archaeplastida - ps9
Viridiplantae - ps10
Streptophyta - ps11
Charophyceae/Embriophyta - ps12
Embriophyta - ps13
Tracheophyta - ps14
Spermatophyta - ps15
Angiosperms - ps16
Mesangiospermae - ps17
Magnoliids/eudicots - ps18
Eudicots - ps19
Core_eudicots - ps20
Superrosids - ps21
Rosids - ps22
Eurosids - ps23
Malvids - ps24
Sap./Malv./Brassicales - ps25
Malvales/Brassicales - ps26
Brassicales - ps27
Cleomaceae/Brassicaceae - ps28
Brassicaceae - ps29
Camelineae - ps30
Arabidopsis - ps31
Arabidopsis_thaliana - ps32
p-value
2e-02
2e-02
3e-02
3e-02
4e-02
Log-odds
0.5
1.0
1.5
2.0
2.5
3.0
3.5
4.0

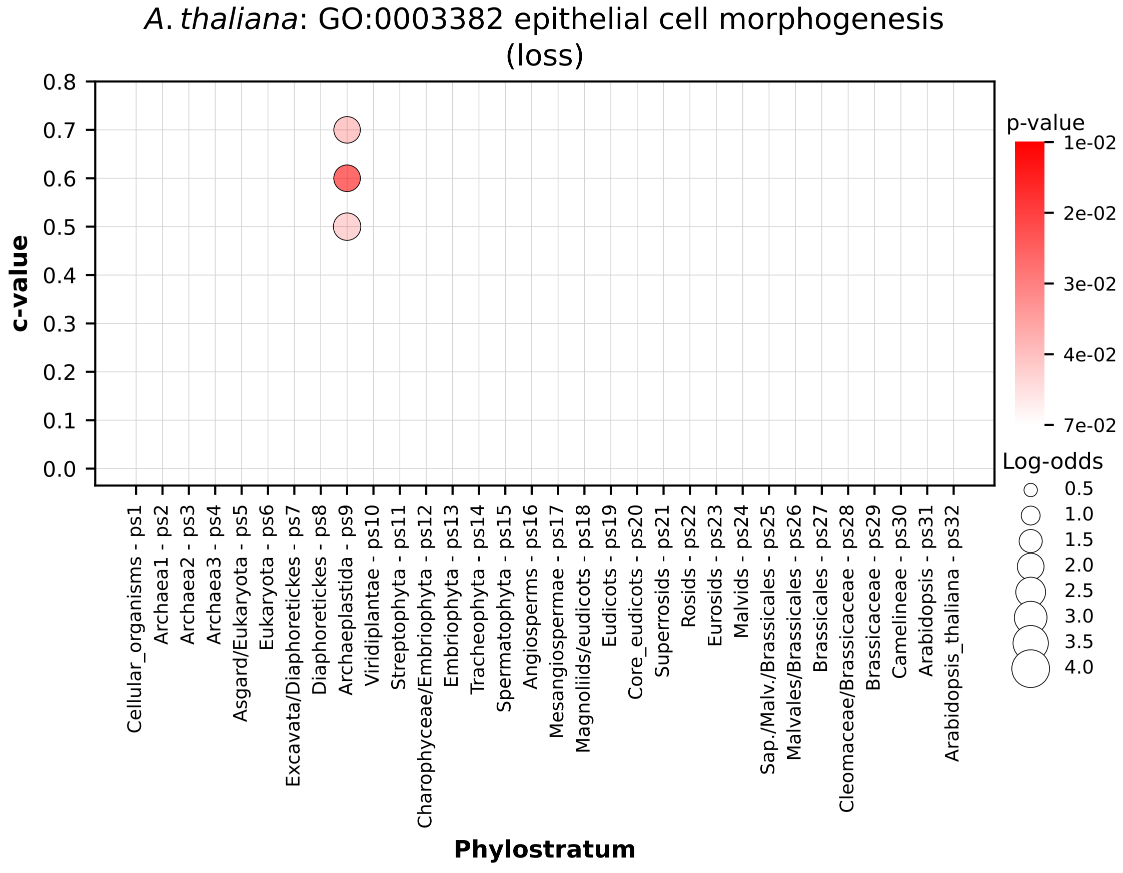

A. thaliana: GO:0003382 epithelial cell morphogenesis
(loss)
c-value
0.8
0.7
0.6
0.5
0.4
0.3
0.2
0.1
0.0
p-value
1e-02
2e-02
3e-02
4e-02
7e-02
Log-odds
0.5
1.0
1.5
2.0
2.5
3.0
3.5
4.0
Cellular_organisms - ps1
Archaea1 - ps2
Archaea2 - ps3
Archaea3 - ps4
Asgard/Eukaryota - ps5
Eukaryota - ps6
Excavata/Diaphoretickes - ps7
Diaphoretickes - ps8
Archaeplastida - ps9
Viridiplantae - ps10
Streptophyta - ps11
Charophyceae/Embriophyta - ps12
Embriophyta - ps13
Tracheophyta - ps14
Spermatophyta - ps15
Angiosperms - ps16
Mesangiospermae - ps17
Magnoliids/eudicots - ps18
Eudicots - ps19
Core_eudicots - ps20
Superrosids - ps21
Rosids - ps22
Eurosids - ps23
Malvids - ps24
Sap./Malv./Brassicales - ps25
Malvales/Brassicales - ps26
Brassicales - ps27
Cleomaceae/Brassicaceae - ps28
Brassicaceae - ps29
Camelineae - ps30
Arabidopsis - ps31
Arabidopsis_thaliana - ps32
Phylostratum

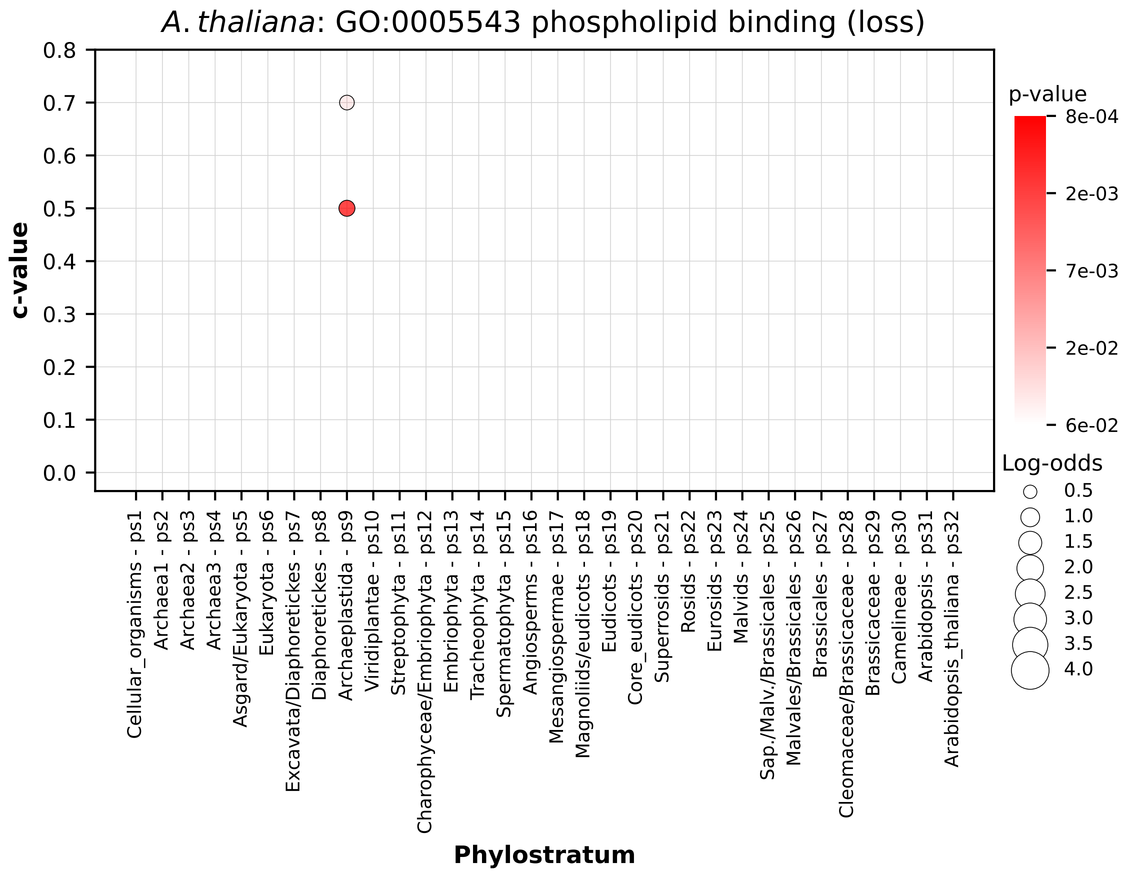

A. thaliana: GO:0005543 phospholipid binding (loss)
c-value
0.8
0.7
0.6
0.5
0.4
0.3
0.2
0.1
0.0
Phylostratum
Cellular_organisms - ps1
Archaea1 - ps2
Archaea2 - ps3
Archaea3 - ps4
Asgard/Eukaryota - ps5
Eukaryota - ps6
Excavata/Diaphoretickes - ps7
Diaphoretickes - ps8
Archaeplastida - ps9
Viridiplantae - ps10
Streptophyta - ps11
Charophyceae/Embriophyta - ps12
Embriophyta - ps13
Tracheophyta - ps14
Spermatophyta - ps15
Angiosperms - ps16
Mesangiospermae - ps17
Magnoliids/eudicots - ps18
Eudicots - ps19
Core_eudicots - ps20
Superrosids - ps21
Rosids - ps22
Eurosids - ps23
Malvids - ps24
Sap./Malv./Brassicales - ps25
Malvales/Brassicales - ps26
Brassicales - ps27
Cleomaceae/Brassicaceae - ps28
Brassicaceae - ps29
Camelineae - ps30
Arabidopsis - ps31
Arabidopsis_thaliana - ps32
p-value
8e-04
2e-03
7e-03
2e-02
6e-02
Log-odds
0.5
1.0
1.5
2.0
2.5
3.0
3.5
4.0

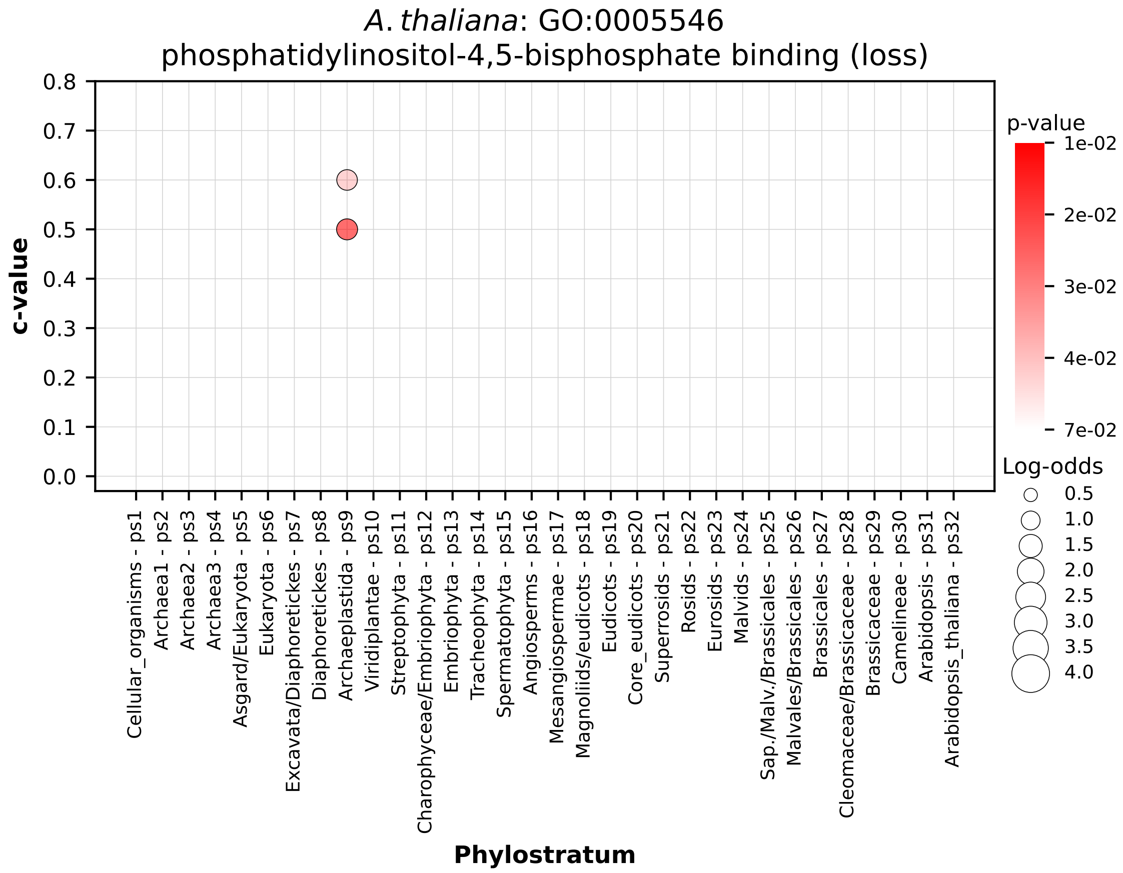

A. thaliana: GO:0005546
phosphatidylinositol-4,5-bisphosphate binding (loss)
c-value
0.8
0.7
0.6
0.5
0.4
0.3
0.2
0.1
0.0
Phylostratum
Cellular_organisms - ps1
Archaea1 - ps2
Archaea2 - ps3
Archaea3 - ps4
Asgard/Eukaryota - ps5
Eukaryota - ps6
Excavata/Diaphoretickes - ps7
Diaphoretickes - ps8
Archaeplastida - ps9
Viridiplantae - ps10
Streptophyta - ps11
Charophyceae/Embriophyta - ps12
Embriophyta - ps13
Tracheophyta - ps14
Spermatophyta - ps15
Angiosperms - ps16
Mesangiospermae - ps17
Magnoliids/eudicots - ps18
Eudicots - ps19
Core_eudicots - ps20
Superrosids - ps21
Rosids - ps22
Eurosids - ps23
Malvids - ps24
Sap./Malv./Brassicales - ps25
Malvales/Brassicales - ps26
Brassicales - ps27
Cleomaceae/Brassicaceae - ps28
Brassicaceae - ps29
Camelineae - ps30
Arabidopsis - ps31
Arabidopsis_thaliana - ps32
p-value
1e-02
2e-02
3e-02
4e-02
7e-02
Log-odds
0.5
1.0
1.5
2.0
2.5
3.0
3.5
4.0

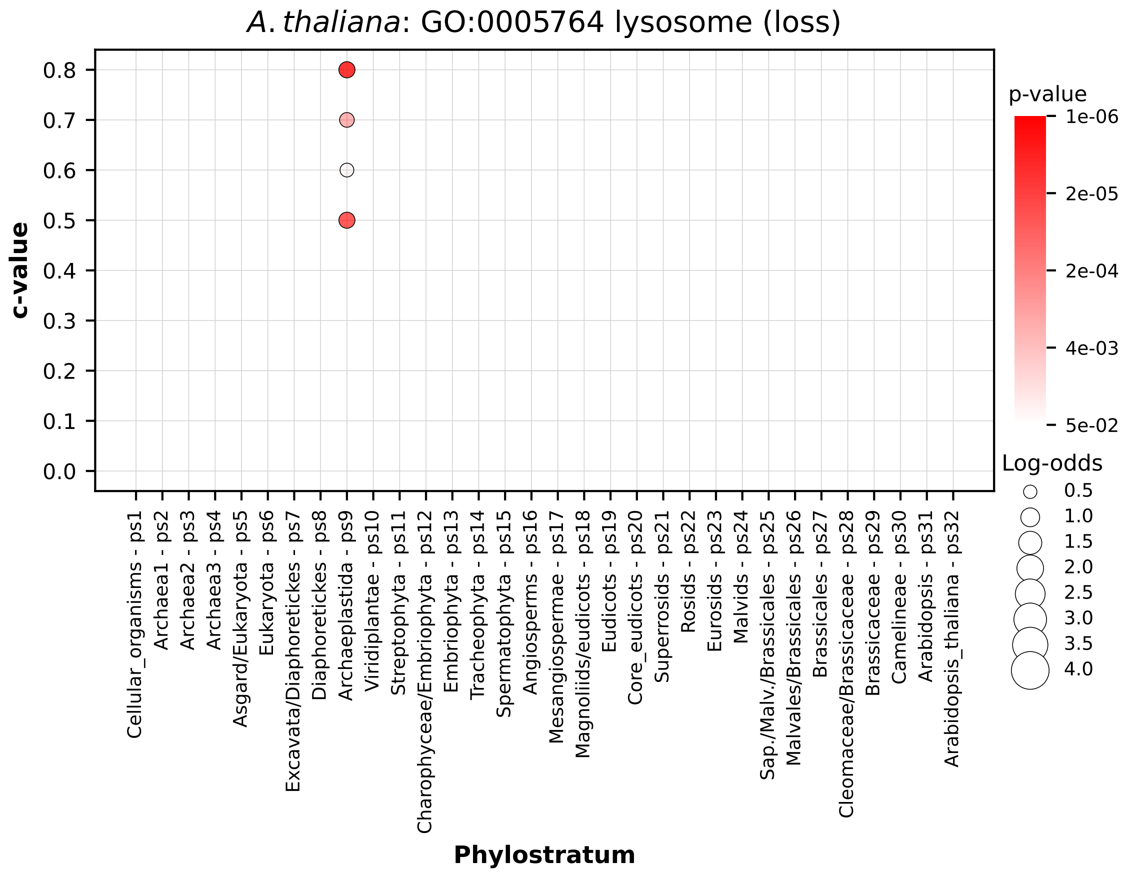

A. thaliana: GO:0005764 lysosome (loss)
c-value
0.0
0.1
0.2
0.3
0.4
0.5
0.6
0.7
0.8
p-value
1e-06
2e-05
2e-04
4e-03
5e-02
Log-odds
0.5
1.0
1.5
2.0
2.5
3.0
3.5
4.0
Cellular_organisms - ps1
Archaea1 - ps2
Archaea2 - ps3
Archaea3 - ps4
Asgard/Eukaryota - ps5
Eukaryota - ps6
Excavata/Diaphoretickes - ps7
Diaphoretickes - ps8
Archaeplastida - ps9
Viridiplantae - ps10
Streptophyta - ps11
Charophyceae/Embriophyta - ps12
Embriophyta - ps13
Tracheophyta - ps14
Spermatophyta - ps15
Angiosperms - ps16
Mesangiospermae - ps17
Magnoliids/eudicots - ps18
Eudicots - ps19
Core_eudicots - ps20
Superrosids - ps21
Rosids - ps22
Eurosids - ps23
Malvids - ps24
Sap./Malv./Brassicales - ps25
Malvales/Brassicales - ps26
Brassicales - ps27
Cleomaceae/Brassicaceae - ps28
Brassicaceae - ps29
Camelineae - ps30
Arabidopsis - ps31
Arabidopsis_thaliana - ps32
Phylostratum

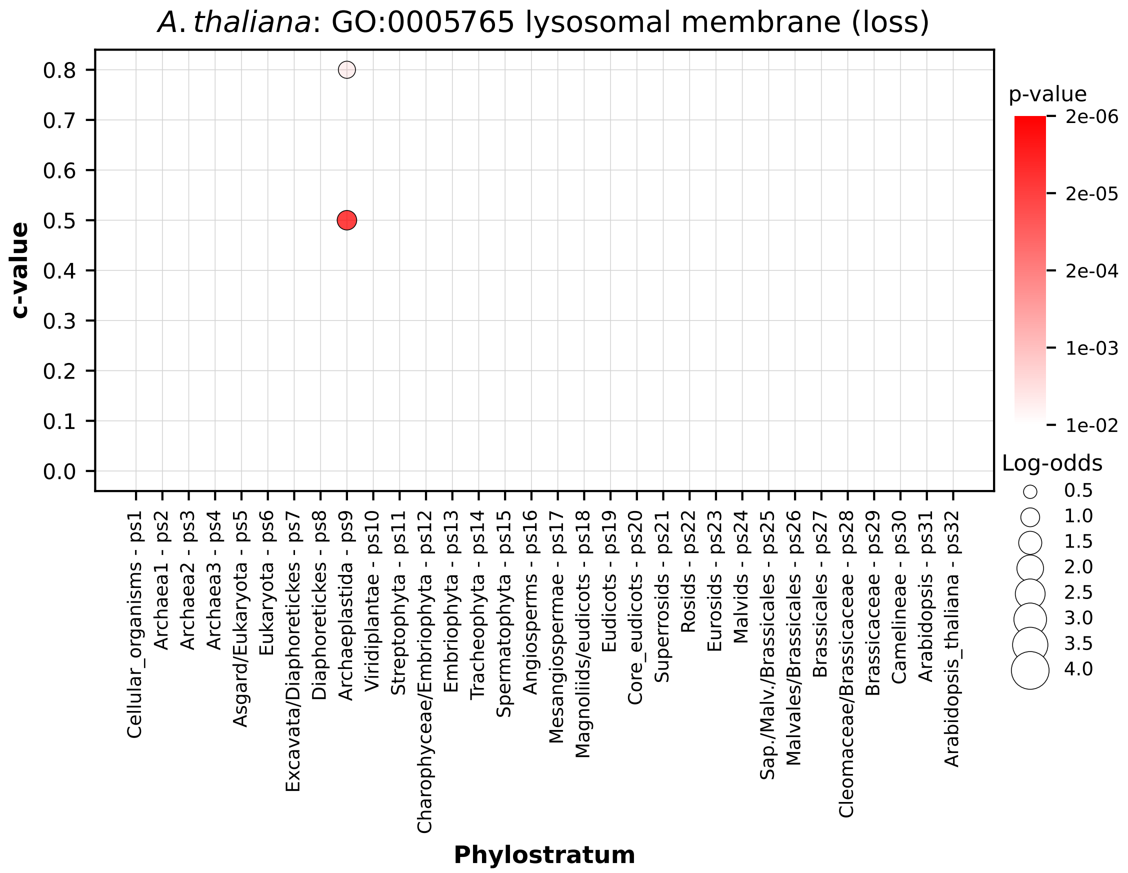

A. thaliana: GO:0005765 lysosomal membrane (loss)
c-value
0.0
0.1
0.2
0.3
0.4
0.5
0.6
0.7
0.8
Phylostratum
Cellular_organisms - ps1
Archaea1 - ps2
Archaea2 - ps3
Archaea3 - ps4
Asgard/Eukaryota - ps5
Eukaryota - ps6
Excavata/Diaphoretickes - ps7
Diaphoretickes - ps8
Archaeplastida - ps9
Viridiplantae - ps10
Streptophyta - ps11
Charophyceae/Embriophyta - ps12
Embriophyta - ps13
Tracheophyta - ps14
Spermatophyta - ps15
Angiosperms - ps16
Mesangiospermae - ps17
Magnoliids/eudicots - ps18
Eudicots - ps19
Core_eudicots - ps20
Superrosids - ps21
Rosids - ps22
Eurosids - ps23
Malvids - ps24
Sap./Malv./Brassicales - ps25
Malvales/Brassicales - ps26
Brassicales - ps27
Cleomaceae/Brassicaceae - ps28
Brassicaceae - ps29
Camelineae - ps30
Arabidopsis - ps31
Arabidopsis_thaliana - ps32
p-value
2e-06
2e-05
2e-04
1e-03
1e-02
Log-odds
0.5
1.0
1.5
2.0
2.5
3.0
3.5
4.0

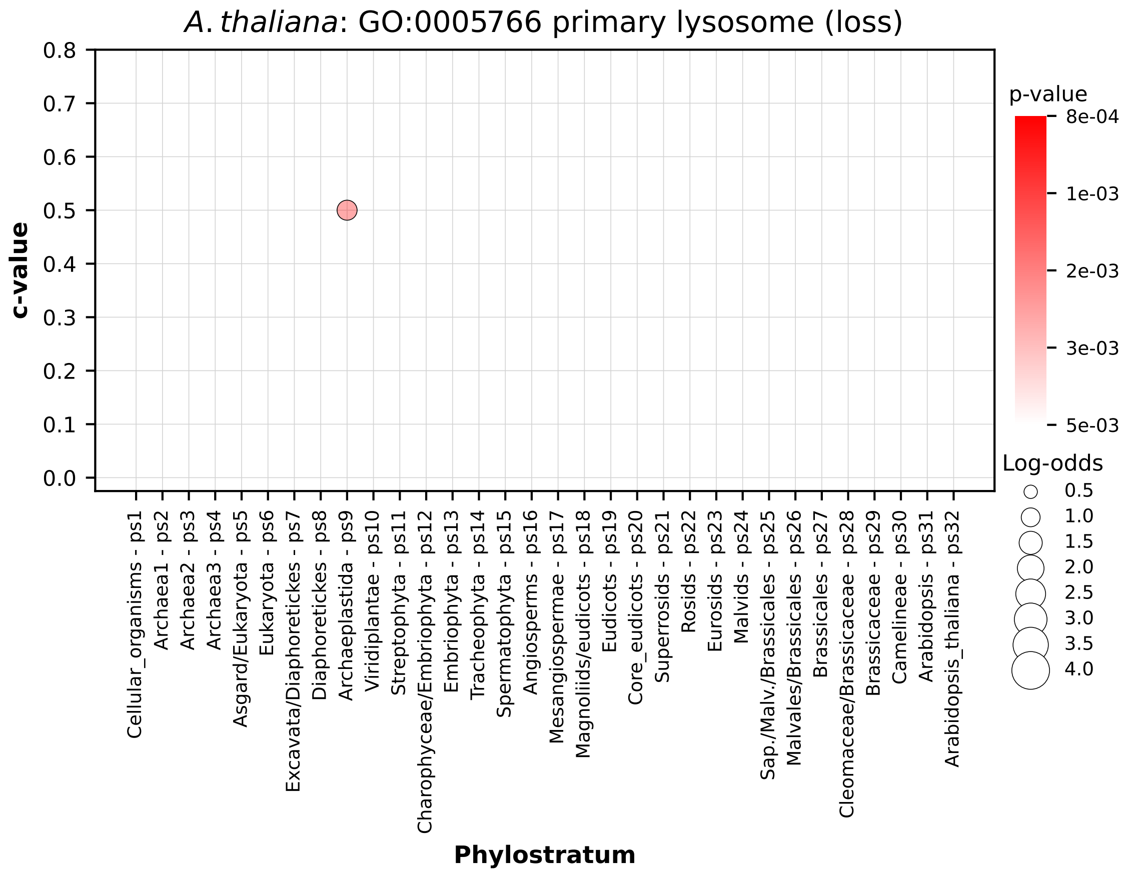
A. thaliana: GO:0005766 primary lysosome (loss)
c-value
0.8
0.7
0.6
0.5
0.4
0.3
0.2
0.1
0.0
Phylostratum
Cellular_organisms - ps1
Archaea1 - ps2
Archaea2 - ps3
Archaea3 - ps4
Asgard/Eukaryota - ps5
Eukaryota - ps6
Excavata/Diaphoretickes - ps7
Diaphoretickes - ps8
Archaeplastida - ps9
Viridiplantae - ps10
Streptophyta - ps11
Charophyceae/Embriophyta - ps12
Embriophyta - ps13
Tracheophyta - ps14
Spermatophyta - ps15
Angiosperms - ps16
Mesangiospermae - ps17
Magnoliids/eudicots - ps18
Eudicots - ps19
Core_eudicots - ps20
Superrosids - ps21
Rosids - ps22
Eurosids - ps23
Malvids - ps24
Sap./Malv./Brassicales - ps25
Malvales/Brassicales - ps26
Brassicales - ps27
Cleomaceae/Brassicaceae - ps28
Brassicaceae - ps29
Camelineae - ps30
Arabidopsis - ps31
Arabidopsis_thaliana - ps32
p-value
8e-04
1e-03
2e-03
3e-03
5e-03
Log-odds
0.5
1.0
1.5
2.0
2.5
3.0
3.5
4.0

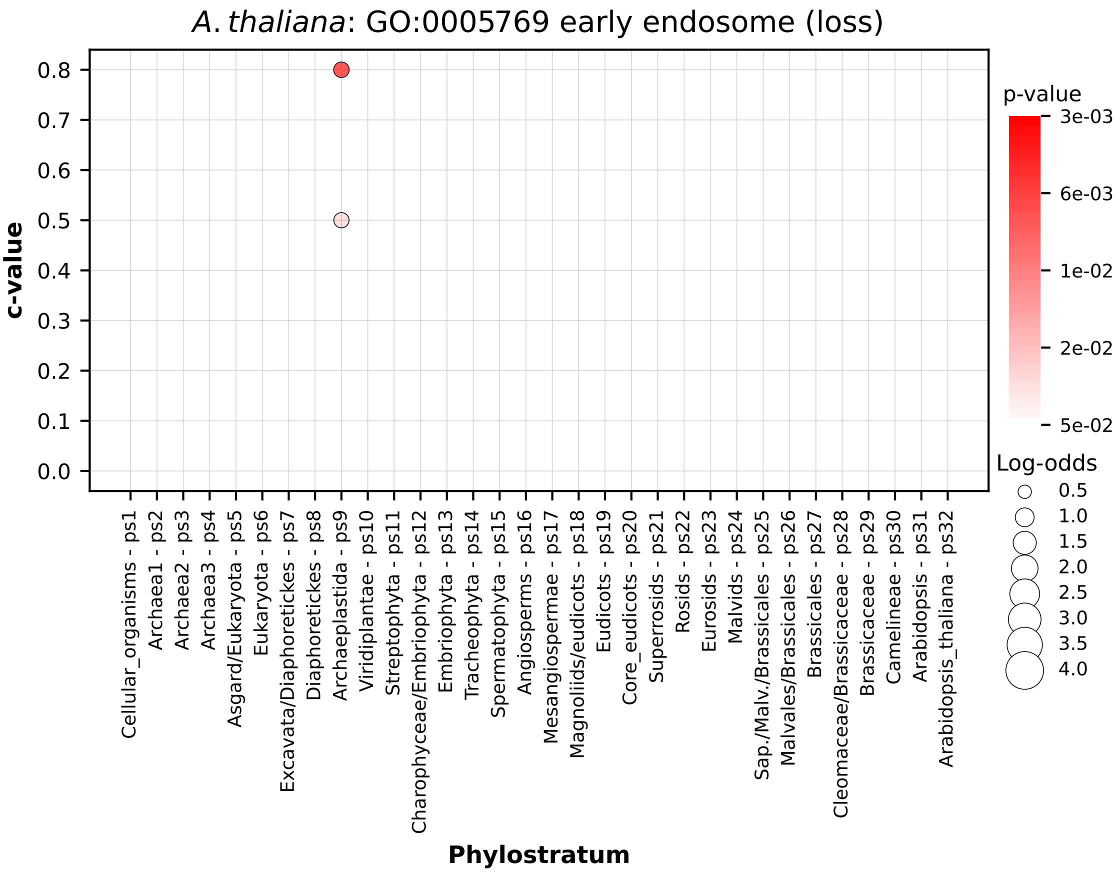

A. thaliana: GO:0005769 early endosome (loss)
c-value
0.8
0.7
0.6
0.5
0.4
0.3
0.2
0.1
0.0
p-value
3e-03
6e-03
1e-02
2e-02
5e-02
Log-odds
0.5
1.0
1.5
2.0
2.5
3.0
3.5
4.0
Cellular_organisms - ps1
Archaea1 - ps2
Archaea2 - ps3
Archaea3 - ps4
Asgard/Eukaryota - ps5
Eukaryota - ps6
Excavata/Diaphoretickes - ps7
Diaphoretickes - ps8
Archaeplastida - ps9
Viridiplantae - ps10
Streptophyta - ps11
Charophyceae/Embriophyta - ps12
Embriophyta - ps13
Tracheophyta - ps14
Spermatophyta - ps15
Angiosperms - ps16
Mesangiospermae - ps17
Magnoliids/eudicots - ps18
Eudicots - ps19
Core_eudicots - ps20
Superrosids - ps21
Rosids - ps22
Eurosids - ps23
Malvids - ps24
Sap./Malv./Brassicales - ps25
Malvales/Brassicales - ps26
Brassicales - ps27
Cleomaceae/Brassicaceae - ps28
Brassicaceae - ps29
Camelineae - ps30
Arabidopsis - ps31
Arabidopsis_thaliana - ps32
Phylostratum

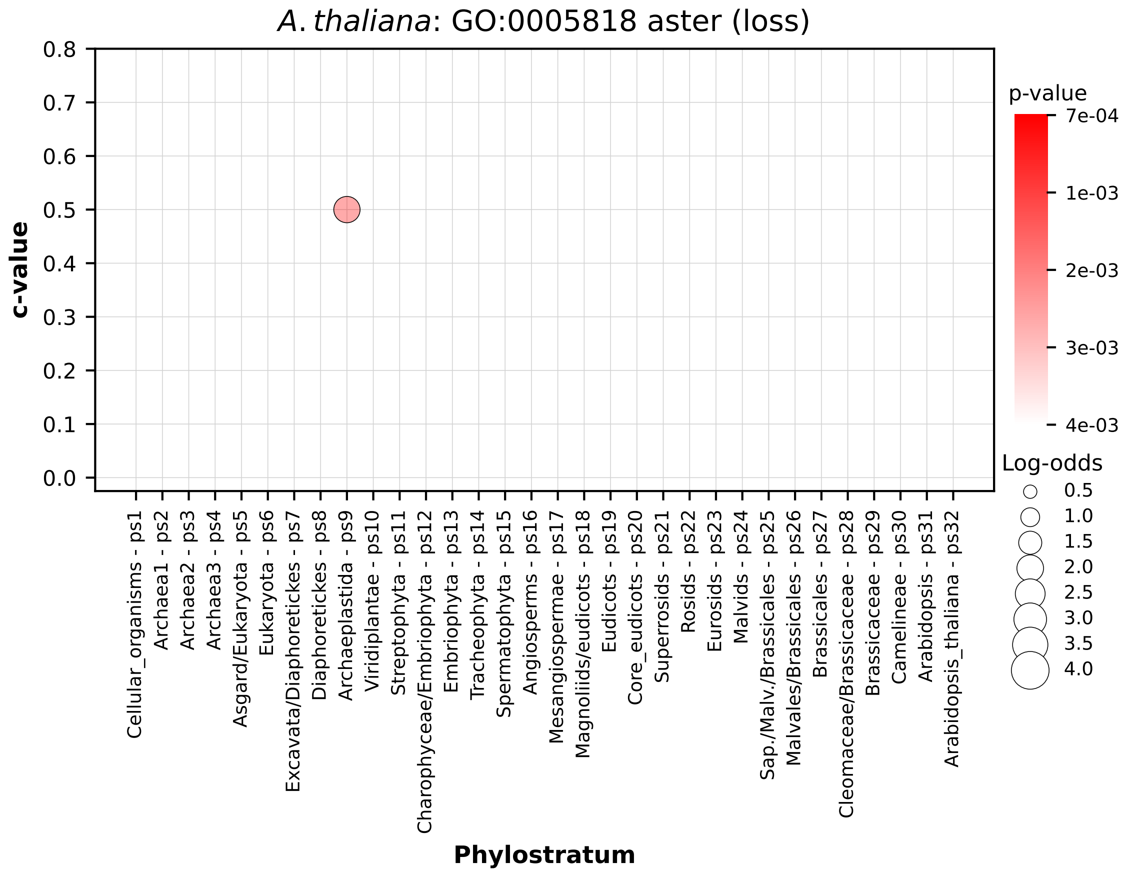

A. thaliana: GO:0005818 aster (loss)
c-value
0.0
0.1
0.2
0.3
0.4
0.5
0.6
0.7
0.8
Phylostratum
Cellular_organisms - ps1
Archaea1 - ps2
Archaea2 - ps3
Archaea3 - ps4
Asgard/Eukaryota - ps5
Eukaryota - ps6
Excavata/Diaphoretickes - ps7
Diaphoretickes - ps8
Archaeplastida - ps9
Viridiplantae - ps10
Streptophyta - ps11
Charophyceae/Embriophyta - ps12
Embriophyta - ps13
Tracheophyta - ps14
Spermatophyta - ps15
Angiosperms - ps16
Mesangiospermae - ps17
Magnoliids/eudicots - ps18
Eudicots - ps19
Core_eudicots - ps20
Superrosids - ps21
Rosids - ps22
Eurosids - ps23
Malvids - ps24
Sap./Malv./Brassicales - ps25
Malvales/Brassicales - ps26
Brassicales - ps27
Cleomaceae/Brassicaceae - ps28
Brassicaceae - ps29
Camelineae - ps30
Arabidopsis - ps31
Arabidopsis_thaliana - ps32
p-value
7e-04
1e-03
2e-03
3e-03
4e-03
Log-odds
0.5
1.0
1.5
2.0
2.5
3.0
3.5
4.0

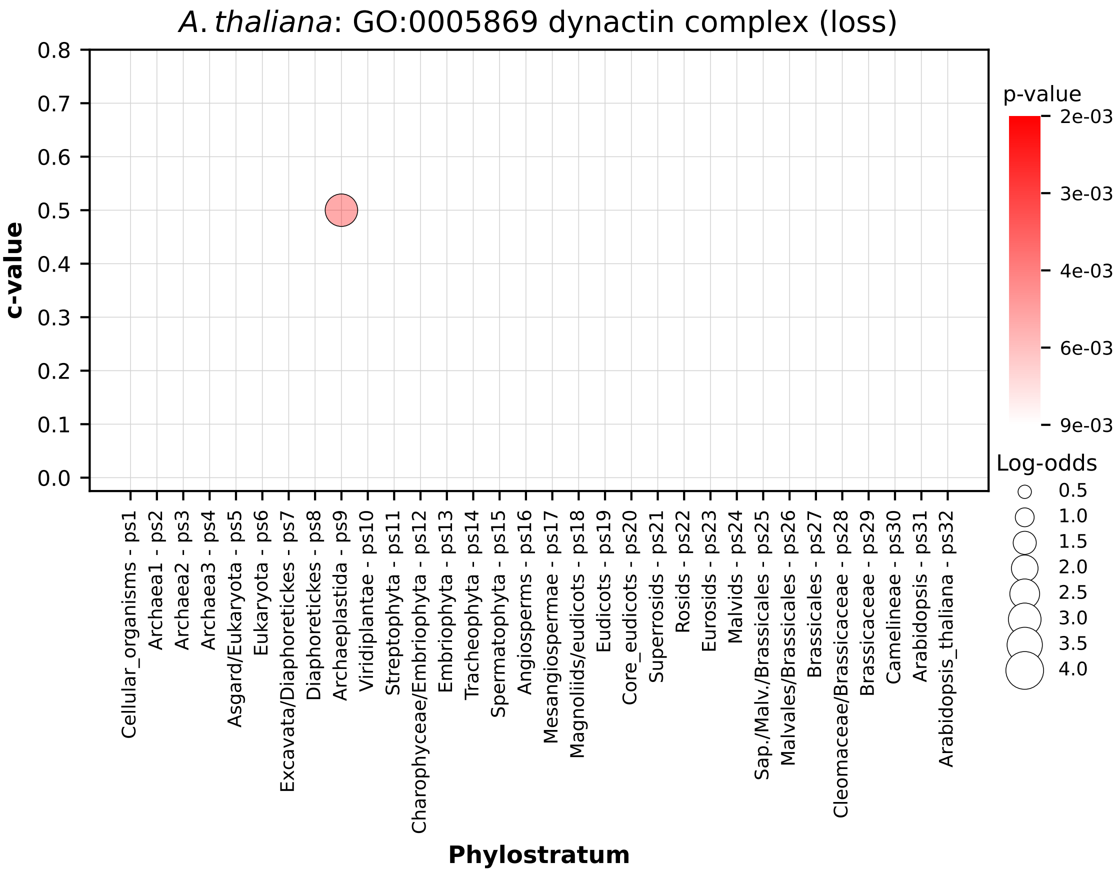

A. thaliana: GO:0005869 dynactin complex (loss)
c-value
0.0
0.1
0.2
0.3
0.4
0.5
0.6
0.7
0.8
Phylostratum
Cellular_organisms - ps1
Archaea1 - ps2
Archaea2 - ps3
Archaea3 - ps4
Asgard/Eukaryota - ps5
Eukaryota - ps6
Excavata/Diaphoretickes - ps7
Diaphoretickes - ps8
Archaeplastida - ps9
Viridiplantae - ps10
Streptophyta - ps11
Charophyceae/Embriophyta - ps12
Embriophyta - ps13
Tracheophyta - ps14
Spermatophyta - ps15
Angiosperms - ps16
Mesangiospermae - ps17
Magnoliids/eudicots - ps18
Eudicots - ps19
Core_eudicots - ps20
Superrosids - ps21
Rosids - ps22
Eurosids - ps23
Malvids - ps24
Sap./Malv./Brassicales - ps25
Malvales/Brassicales - ps26
Brassicales - ps27
Cleomaceae/Brassicaceae - ps28
Brassicaceae - ps29
Camelineae - ps30
Arabidopsis - ps31
Arabidopsis_thaliana - ps32
p-value
2e-03
3e-03
4e-03
6e-03
9e-03
Log-odds
0.5
1.0
1.5
2.0
2.5
3.0
3.5
4.0

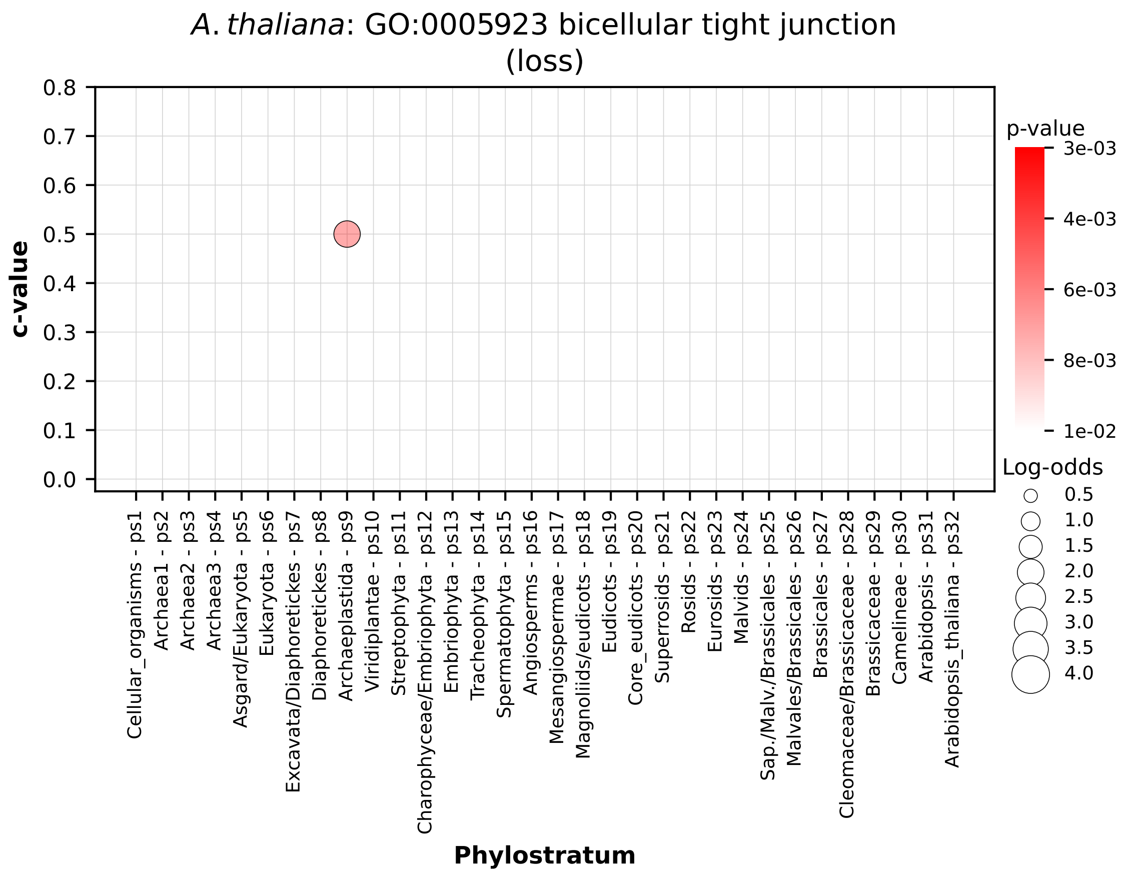
A. thaliana: GO:0005923 bicellular tight junction
(loss)
c-value
0.0
0.1
0.2
0.3
0.4
0.5
0.6
0.7
0.8
Phylostratum
Cellular_organisms - ps1
Archaea1 - ps2
Archaea2 - ps3
Archaea3 - ps4
Asgard/Eukaryota - ps5
Eukaryota - ps6
Excavata/Diaphoretickes - ps7
Diaphoretickes - ps8
Archaeplastida - ps9
Viridiplantae - ps10
Streptophyta - ps11
Charophyceae/Embriophyta - ps12
Embriophyta - ps13
Tracheophyta - ps14
Spermatophyta - ps15
Angiosperms - ps16
Mesangiospermae - ps17
Magnoliids/eudicots - ps18
Eudicots - ps19
Core_eudicots - ps20
Superrosids - ps21
Rosids - ps22
Eurosids - ps23
Malvids - ps24
Sap./Malv./Brassicales - ps25
Malvales/Brassicales - ps26
Brassicales - ps27
Cleomaceae/Brassicaceae - ps28
Brassicaceae - ps29
Camelineae - ps30
Arabidopsis - ps31
Arabidopsis_thaliana - ps32
p-value
3e-03
4e-03
6e-03
8e-03
1e-02
Log-odds
0.5
1.0
1.5
2.0
2.5
3.0
3.5
4.0

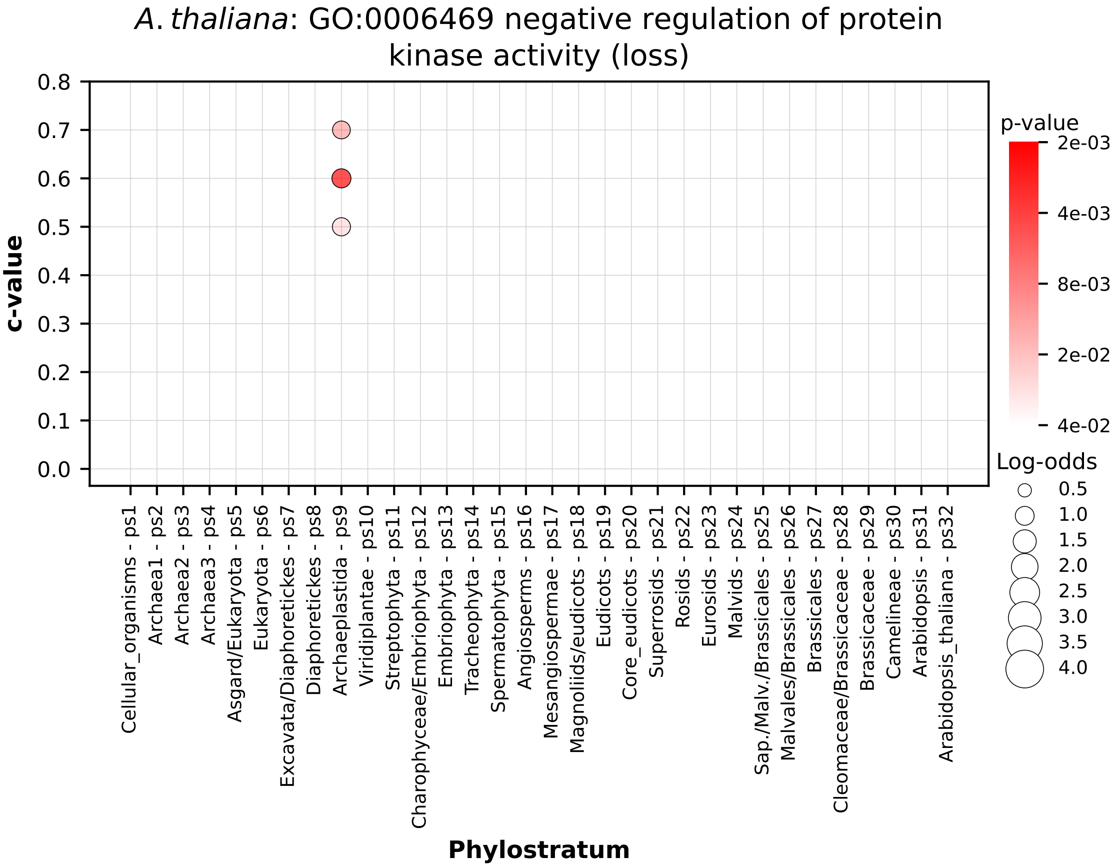

A. thaliana: GO:0006469 negative regulation of protein kinase activity (loss)
c-value
0.8
0.7
0.6
0.5
0.4
0.3
0.2
0.1
0.0
p-value
2e-03
4e-03
8e-03
2e-02
4e-02
Log-odds
0.5
1.0
1.5
2.0
2.5
3.0
3.5
4.0
Cellular_organisms - ps1
Archaea1 - ps2
Archaea2 - ps3
Archaea3 - ps4
Asgard/Eukaryota - ps5
Eukaryota - ps6
Excavata/Diaphoretickes - ps7
Diaphoretickes - ps8
Archaeplastida - ps9
Viridiplantae - ps10
Streptophyta - ps11
Charophyceae/Embriophyta - ps12
Embriophyta - ps13
Tracheophyta - ps14
Spermatophyta - ps15
Angiosperms - ps16
Mesangiospermae - ps17
Magnoliids/eudicots - ps18
Eudicots - ps19
Core_eudicots - ps20
Superrosids - ps21
Rosids - ps22
Eurosids - ps23
Malvids - ps24
Sap./Malv./Brassicales - ps25
Malvales/Brassicales - ps26
Brassicales - ps27
Cleomaceae/Brassicaceae - ps28
Brassicaceae - ps29
Camelineae - ps30
Arabidopsis - ps31
Arabidopsis_thaliana - ps32
Phylostratum

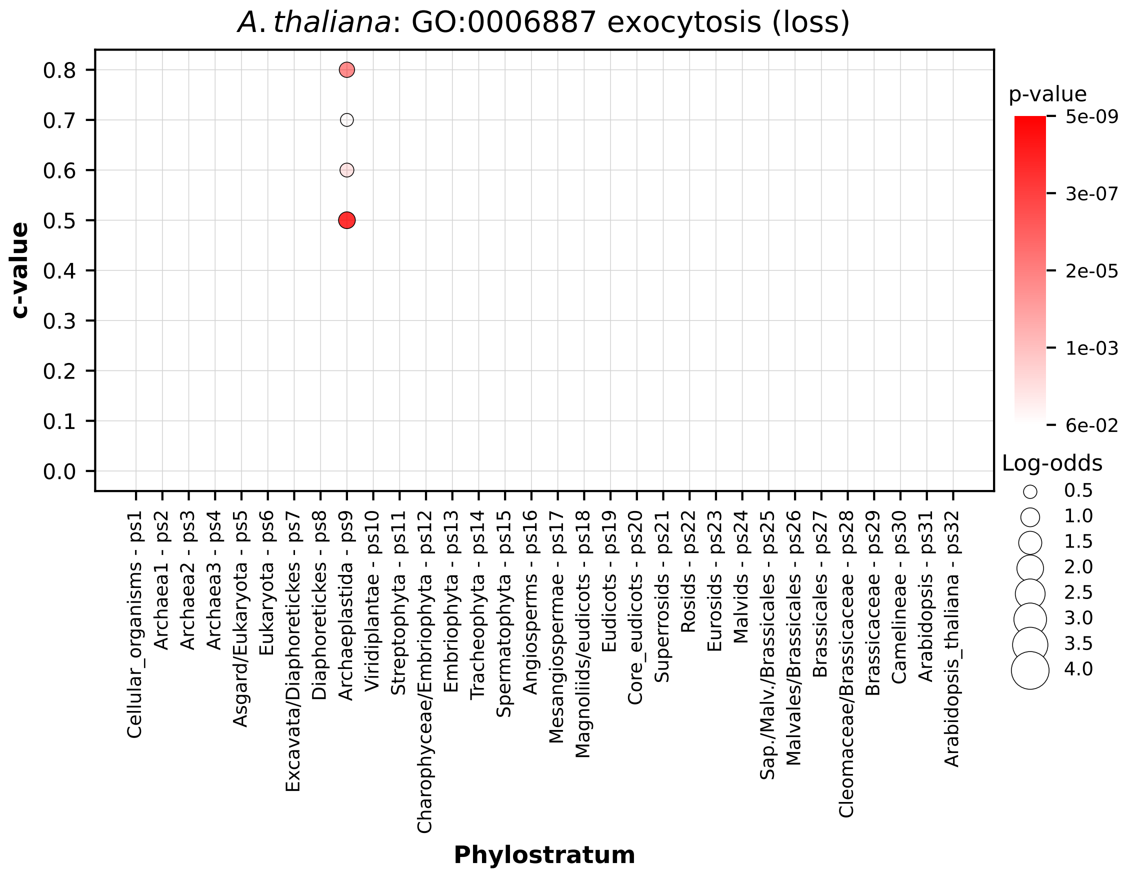

A. thaliana: GO:0006887 exocytosis (loss)
c-value
0.8
0.7
0.6
0.5
0.4
0.3
0.2
0.1
0.0
Phylostratum
Cellular_organisms - ps1
Archaea1 - ps2
Archaea2 - ps3
Archaea3 - ps4
Asgard/Eukaryota - ps5
Eukaryota - ps6
Excavata/Diaphoretickes - ps7
Diaphoretickes - ps8
Archaeplastida - ps9
Viridiplantae - ps10
Streptophyta - ps11
Charophyceae/Embriophyta - ps12
Embriophyta - ps13
Tracheophyta - ps14
Spermatophyta - ps15
Angiosperms - ps16
Mesangiospermae - ps17
Magnoliids/eudicots - ps18
Eudicots - ps19
Core_eudicots - ps20
Superrosids - ps21
Rosids - ps22
Eurosids - ps23
Malvids - ps24
Sap./Malv./Brassicales - ps25
Malvales/Brassicales - ps26
Brassicales - ps27
Cleomaceae/Brassicaceae - ps28
Brassicaceae - ps29
Camelineae - ps30
Arabidopsis - ps31
Arabidopsis_thaliana - ps32
p-value
5e-09
3e-07
2e-05
1e-03
6e-02
Log-odds
0.5
1.0
1.5
2.0
2.5
3.0
3.5
4.0

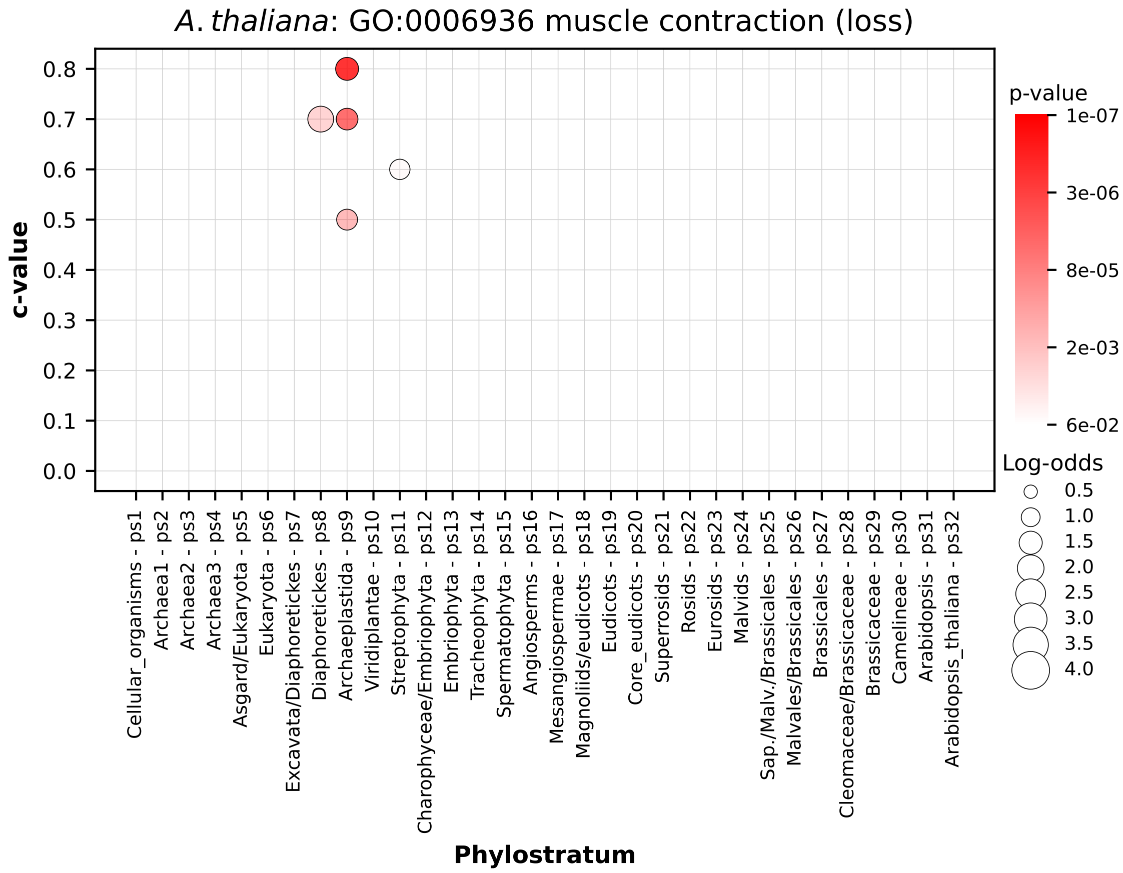

A. thaliana: GO:0006936 muscle contraction (loss)
c-value
0.0
0.1
0.2
0.3
0.4
0.5
0.6
0.7
0.8
Phylostratum
Cellular_organisms - ps1
Archaea1 - ps2
Archaea2 - ps3
Archaea3 - ps4
Asgard/Eukaryota - ps5
Eukaryota - ps6
Excavata/Diaphoretickes - ps7
Diaphoretickes - ps8
Archaeplastida - ps9
Viridiplantae - ps10
Streptophyta - ps11
Charophyceae/Embriophyta - ps12
Embriophyta - ps13
Tracheophyta - ps14
Spermatophyta - ps15
Angiosperms - ps16
Mesangiospermae - ps17
Magnoliids/eudicots - ps18
Eudicots - ps19
Core_eudicots - ps20
Superrosids - ps21
Rosids - ps22
Eurosids - ps23
Malvids - ps24
Sap./Malv./Brassicales - ps25
Malvales/Brassicales - ps26
Brassicales - ps27
Cleomaceae/Brassicaceae - ps28
Brassicaceae - ps29
Camelineae - ps30
Arabidopsis - ps31
Arabidopsis_thaliana - ps32
p-value
1e-07
3e-06
8e-05
2e-03
6e-02
Log-odds
0.5
1.0
1.5
2.0
2.5
3.0
3.5
4.0

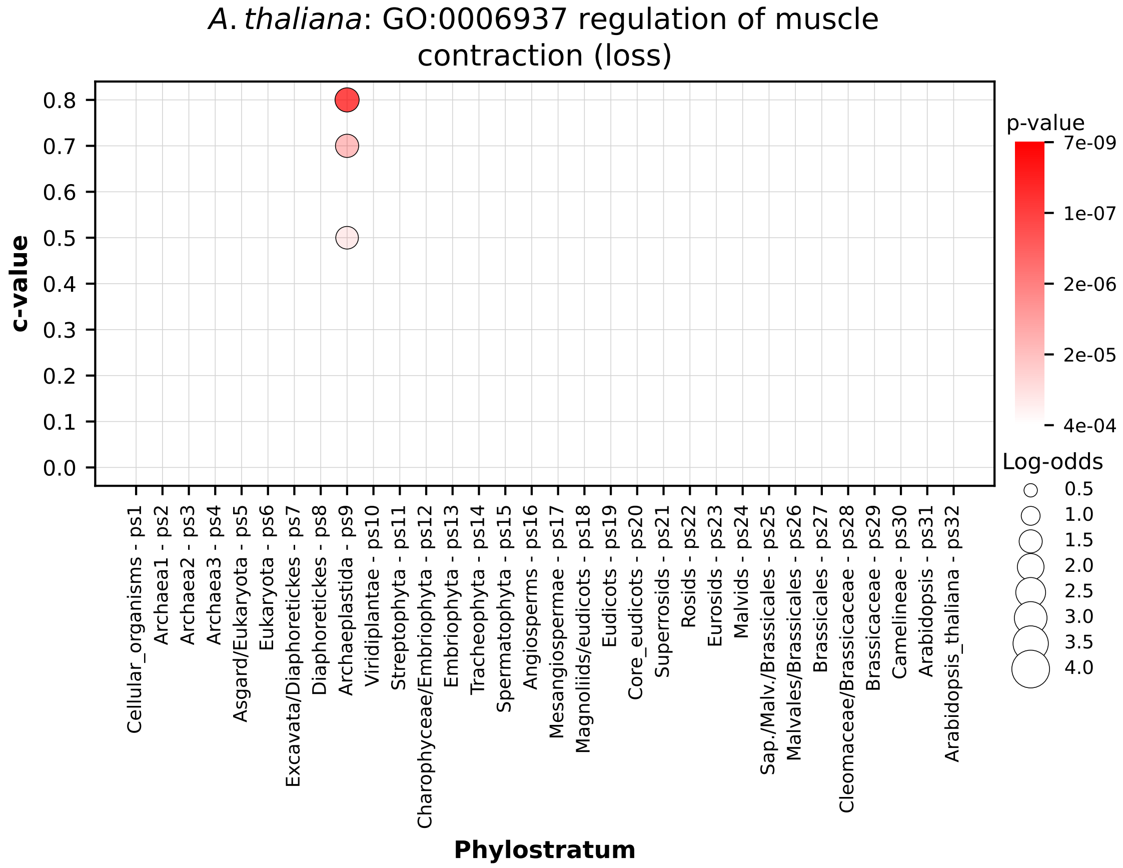

A. thaliana: GO:0006937 regulation of muscle contraction (loss)
c-value
0.8
0.7
0.6
0.5
0.4
0.3
0.2
0.1
0.0
p-value
7e-09
1e-07
2e-06
2e-05
4e-04
Log-odds
0.5
1.0
1.5
2.0
2.5
3.0
3.5
4.0
Cellular_organisms - ps1
Archaea1 - ps2
Archaea2 - ps3
Archaea3 - ps4
Asgard/Eukaryota - ps5
Eukaryota - ps6
Excavata/Diaphoretickes - ps7
Diaphoretickes - ps8
Archaeplastida - ps9
Viridiplantae - ps10
Streptophyta - ps11
Charophyceae/Embriophyta - ps12
Embriophyta - ps13
Tracheophyta - ps14
Spermatophyta - ps15
Angiosperms - ps16
Mesangiospermae - ps17
Magnoliids/eudicots - ps18
Eudicots - ps19
Core_eudicots - ps20
Superrosids - ps21
Rosids - ps22
Eurosids - ps23
Malvids - ps24
Sap./Malv./Brassicales - ps25
Malvales/Brassicales - ps26
Brassicales - ps27
Cleomaceae/Brassicaceae - ps28
Brassicaceae - ps29
Camelineae - ps30
Arabidopsis - ps31
Arabidopsis_thaliana - ps32
Phylostratum

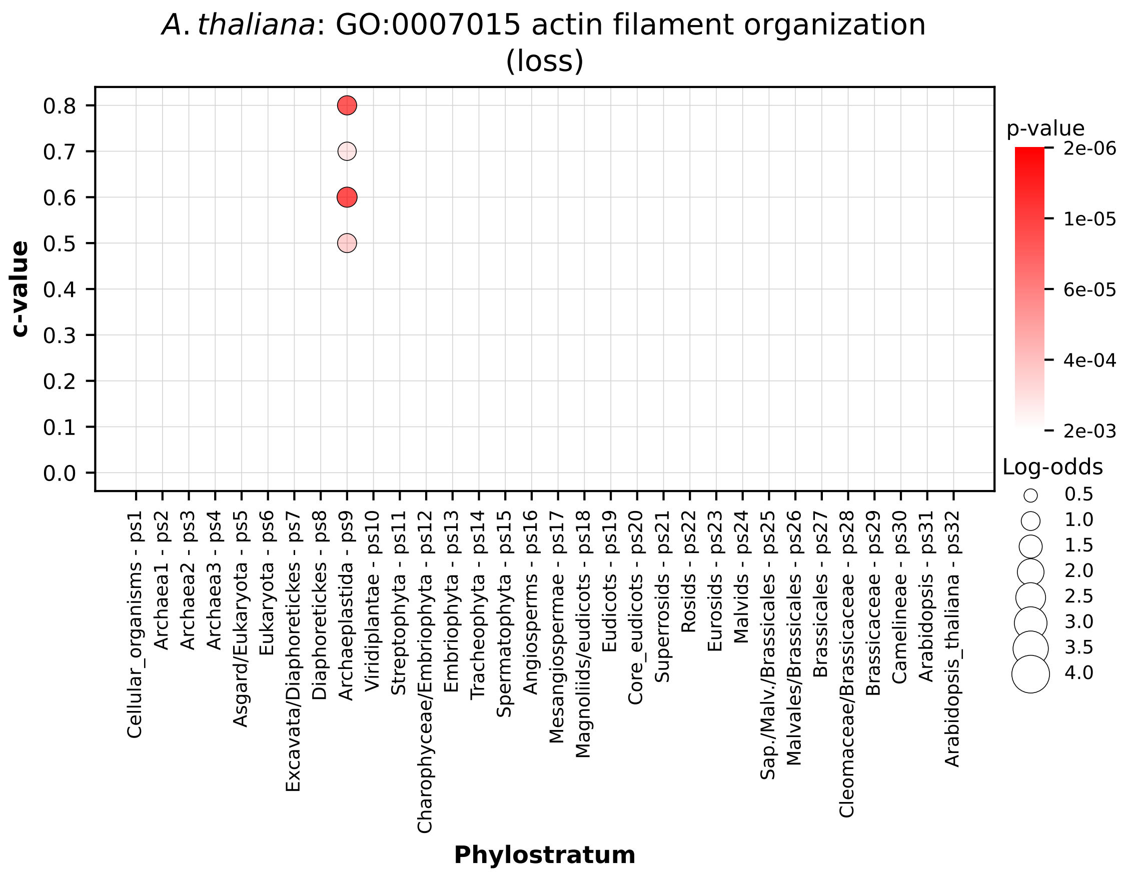

A. thaliana: GO:0007015 actin filament organization (loss)
c-value
0.8
0.7
0.6
0.5
0.4
0.3
0.2
0.1
0.0
Phylostratum
Cellular_organisms - ps1
Archaea1 - ps2
Archaea2 - ps3
Archaea3 - ps4
Asgard/Eukaryota - ps5
Eukaryota - ps6
Excavata/Diaphoretickes - ps7
Diaphoretickes - ps8
Archaeplastida - ps9
Viridiplantae - ps10
Streptophyta - ps11
Charophyceae/Embriophyta - ps12
Embriophyta - ps13
Tracheophyta - ps14
Spermatophyta - ps15
Angiosperms - ps16
Mesangiospermae - ps17
Magnoliids/eudicots - ps18
Eudicots - ps19
Core_eudicots - ps20
Superrosids - ps21
Rosids - ps22
Eurosids - ps23
Malvids - ps24
Sap./Malv./Brassicales - ps25
Malvales/Brassicales - ps26
Brassicales - ps27
Cleomaceae/Brassicaceae - ps28
Brassicaceae - ps29
Camelineae - ps30
Arabidopsis - ps31
Arabidopsis_thaliana - ps32
p-value
2e-06
1e-05
6e-05
4e-04
2e-03
Log-odds
0.5
1.0
1.5
2.0
2.5
3.0
3.5
4.0

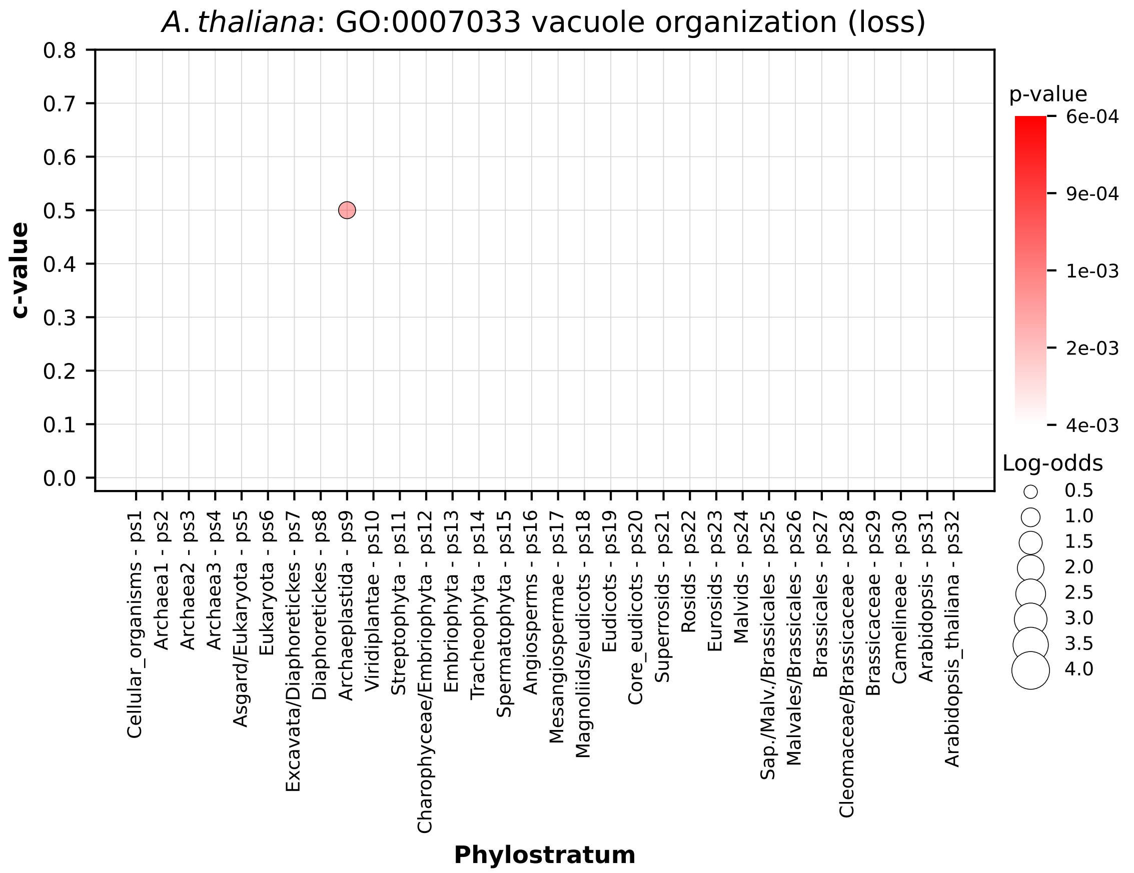

A. thaliana: GO:0007033 vacuole organization (loss)
c-value
0.8
0.7
0.6
0.5
0.4
0.3
0.2
0.1
0.0
Phylostratum
Cellular_organisms - ps1
Archaea1 - ps2
Archaea2 - ps3
Archaea3 - ps4
Asgard/Eukaryota - ps5
Eukaryota - ps6
Excavata/Diaphoretickes - ps7
Diaphoretickes - ps8
Archaeplastida - ps9
Viridiplantae - ps10
Streptophyta - ps11
Charophyceae/Embriophyta - ps12
Embriophyta - ps13
Tracheophyta - ps14
Spermatophyta - ps15
Angiosperms - ps16
Mesangiospermae - ps17
Magnoliids/eudicots - ps18
Eudicots - ps19
Core_eudicots - ps20
Superrosids - ps21
Rosids - ps22
Eurosids - ps23
Malvids - ps24
Sap./Malv./Brassicales - ps25
Malvales/Brassicales - ps26
Brassicales - ps27
Cleomaceae/Brassicaceae - ps28
Brassicaceae - ps29
Camelineae - ps30
Arabidopsis - ps31
Arabidopsis_thaliana - ps32
p-value
6e-04
9e-04
1e-03
2e-03
4e-03
Log-odds
0.5
1.0
1.5
2.0
2.5
3.0
3.5
4.0

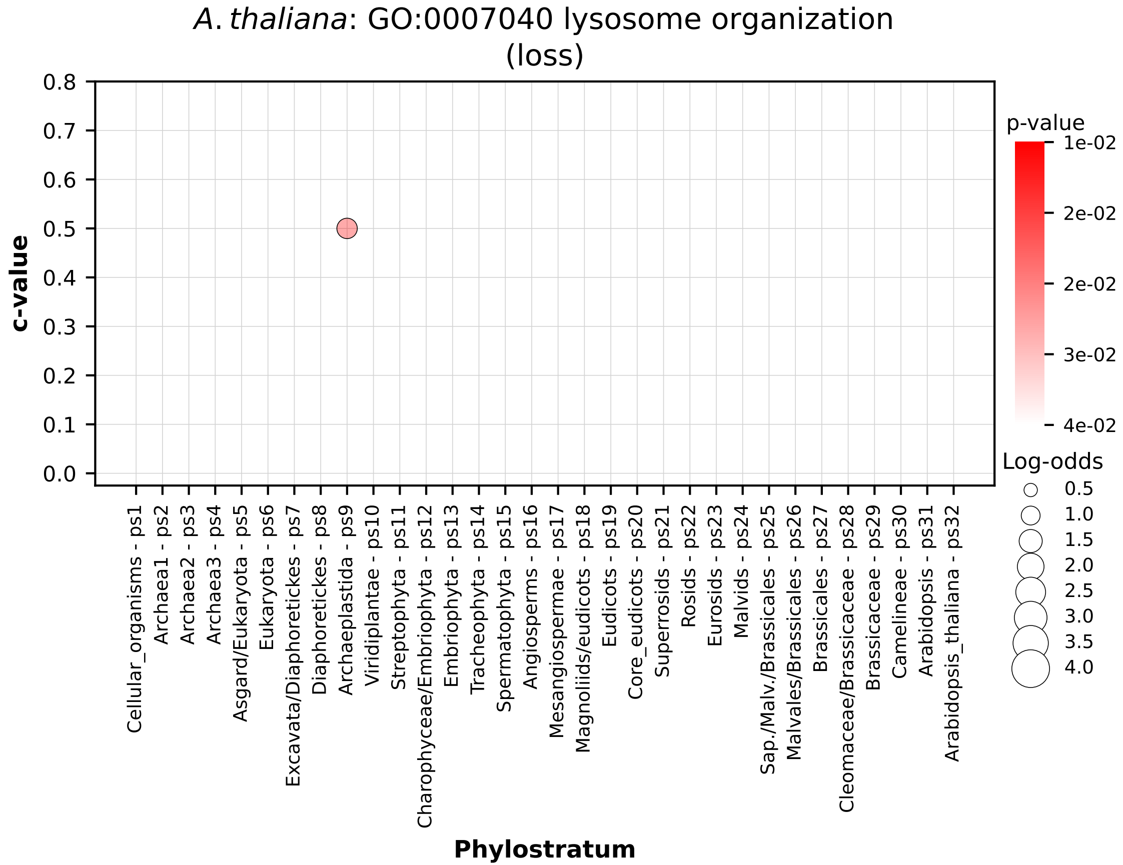

A. thaliana: GO:0007040 lysosome organization
(loss)
c-value
0.8
0.7
0.6
0.5
0.4
0.3
0.2
0.1
0.0
Phylostratum
Cellular_organisms - ps1
Archaea1 - ps2
Archaea2 - ps3
Archaea3 - ps4
Asgard/Eukaryota - ps5
Eukaryota - ps6
Excavata/Diaphoretickes - ps7
Diaphoretickes - ps8
Archaeplastida - ps9
Viridiplantae - ps10
Streptophyta - ps11
Charophyceae/Embriophyta - ps12
Embriophyta - ps13
Tracheophyta - ps14
Spermatophyta - ps15
Angiosperms - ps16
Mesangiospermae - ps17
Magnoliids/eudicots - ps18
Eudicots - ps19
Core_eudicots - ps20
Superrosids - ps21
Rosids - ps22
Eurosids - ps23
Malvids - ps24
Sap./Malv./Brassicales - ps25
Malvales/Brassicales - ps26
Brassicales - ps27
Cleomaceae/Brassicaceae - ps28
Brassicaceae - ps29
Camelineae - ps30
Arabidopsis - ps31
Arabidopsis_thaliana - ps32
p-value
1e-02
2e-02
2e-02
3e-02
4e-02
Log-odds
0.5
1.0
1.5
2.0
2.5
3.0
3.5
4.0

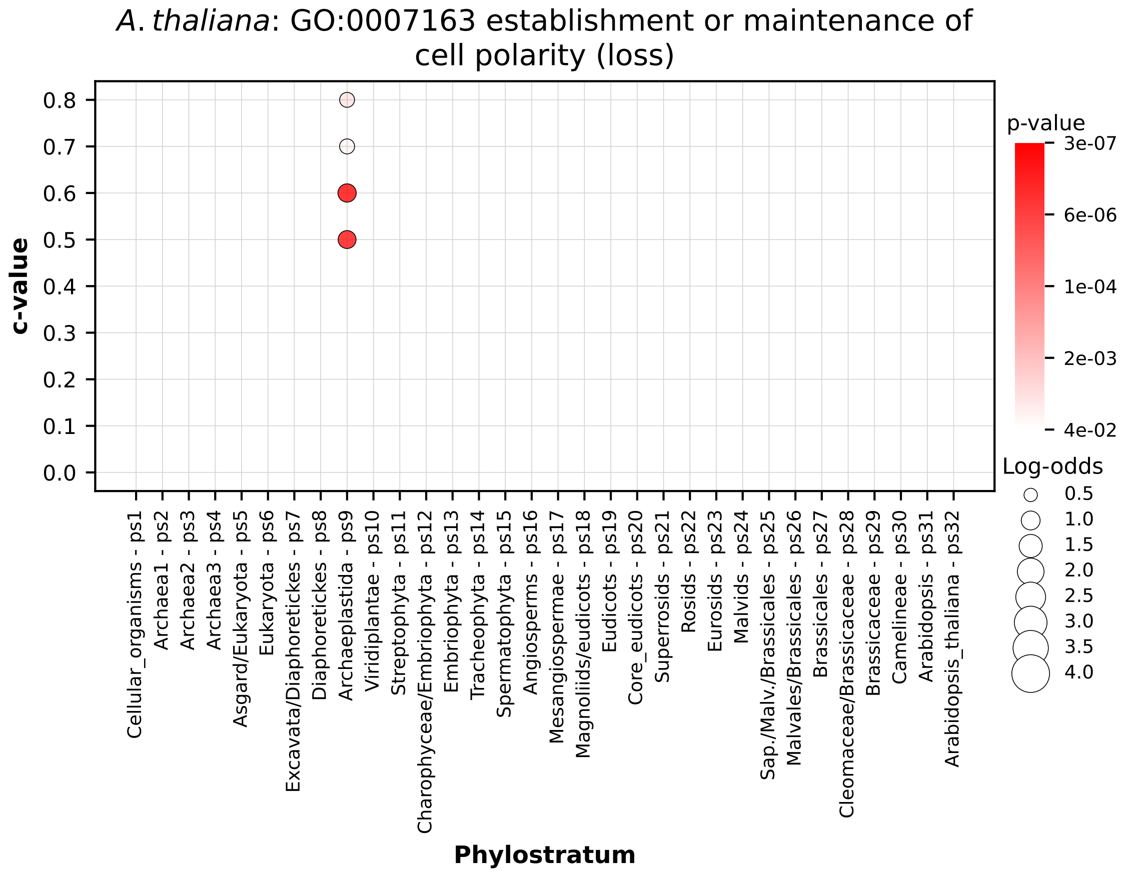
A. thaliana: GO:0007163 establishment or maintenance of cell polarity (loss)
c-value
0.8
0.7
0.6
0.5
0.4
0.3
0.2
0.1
0.0
Phylostratum
Cellular_organisms - ps1
Archaea1 - ps2
Archaea2 - ps3
Archaea3 - ps4
Asgard/Eukaryota - ps5
Eukaryota - ps6
Excavata/Diaphoretickes - ps7
Diaphoretickes - ps8
Archaeplastida - ps9
Viridiplantae - ps10
Streptophyta - ps11
Charophyceae/Embriophyta - ps12
Embriophyta - ps13
Tracheophyta - ps14
Spermatophyta - ps15
Angiosperms - ps16
Mesangiospermae - ps17
Magnoliids/eudicots - ps18
Eudicots - ps19
Core_eudicots - ps20
Superrosids - ps21
Rosids - ps22
Eurosids - ps23
Malvids - ps24
Sap./Malv./Brassicales - ps25
Malvales/Brassicales - ps26
Brassicales - ps27
Cleomaceae/Brassicaceae - ps28
Brassicaceae - ps29
Camelineae - ps30
Arabidopsis - ps31
Arabidopsis_thaliana - ps32
p-value
3e-07
6e-06
1e-04
2e-03
4e-02
Log-odds
0.5
1.0
1.5
2.0
2.5
3.0
3.5
4.0

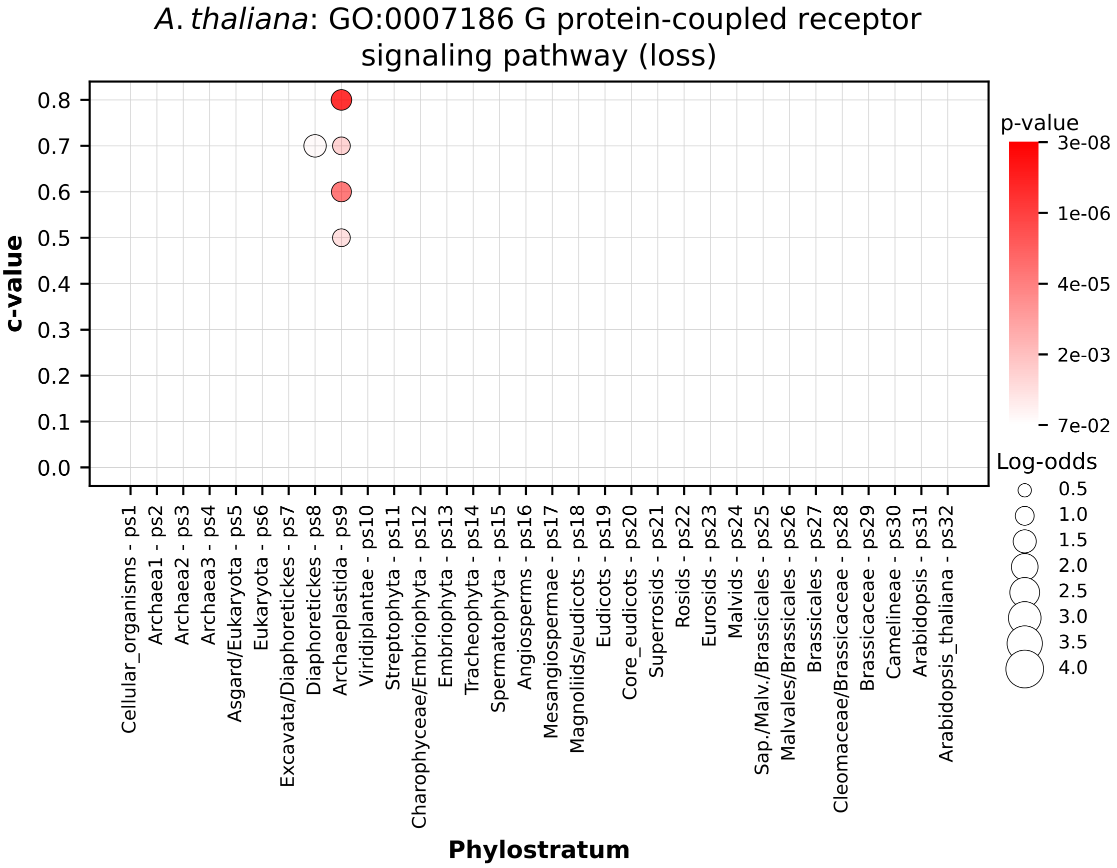
A. thaliana: GO:0007186 G protein-coupled receptor signaling pathway (loss)
c-value
0.0
0.1
0.2
0.3
0.4
0.5
0.6
0.7
0.8
Phylostratum
Cellular_organisms - ps1
Archaea1 - ps2
Archaea2 - ps3
Archaea3 - ps4
Asgard/Eukaryota - ps5
Eukaryota - ps6
Excavata/Diaphoretickes - ps7
Diaphoretickes - ps8
Archaeplastida - ps9
Viridiplantae - ps10
Streptophyta - ps11
Charophyceae/Embriophyta - ps12
Embriophyta - ps13
Tracheophyta - ps14
Spermatophyta - ps15
Angiosperms - ps16
Mesangiospermae - ps17
Magnoliids/eudicots - ps18
Eudicots - ps19
Core_eudicots - ps20
Superrosids - ps21
Rosids - ps22
Eurosids - ps23
Malvids - ps24
Sap./Malv./Brassicales - ps25
Malvales/Brassicales - ps26
Brassicales - ps27
Cleomaceae/Brassicaceae - ps28
Brassicaceae - ps29
Camelineae - ps30
Arabidopsis - ps31
Arabidopsis_thaliana - ps32
p-value
3e-08
1e-06
4e-05
2e-03
7e-02
Log-odds
0.5
1.0
1.5
2.0
2.5
3.0
3.5
4.0

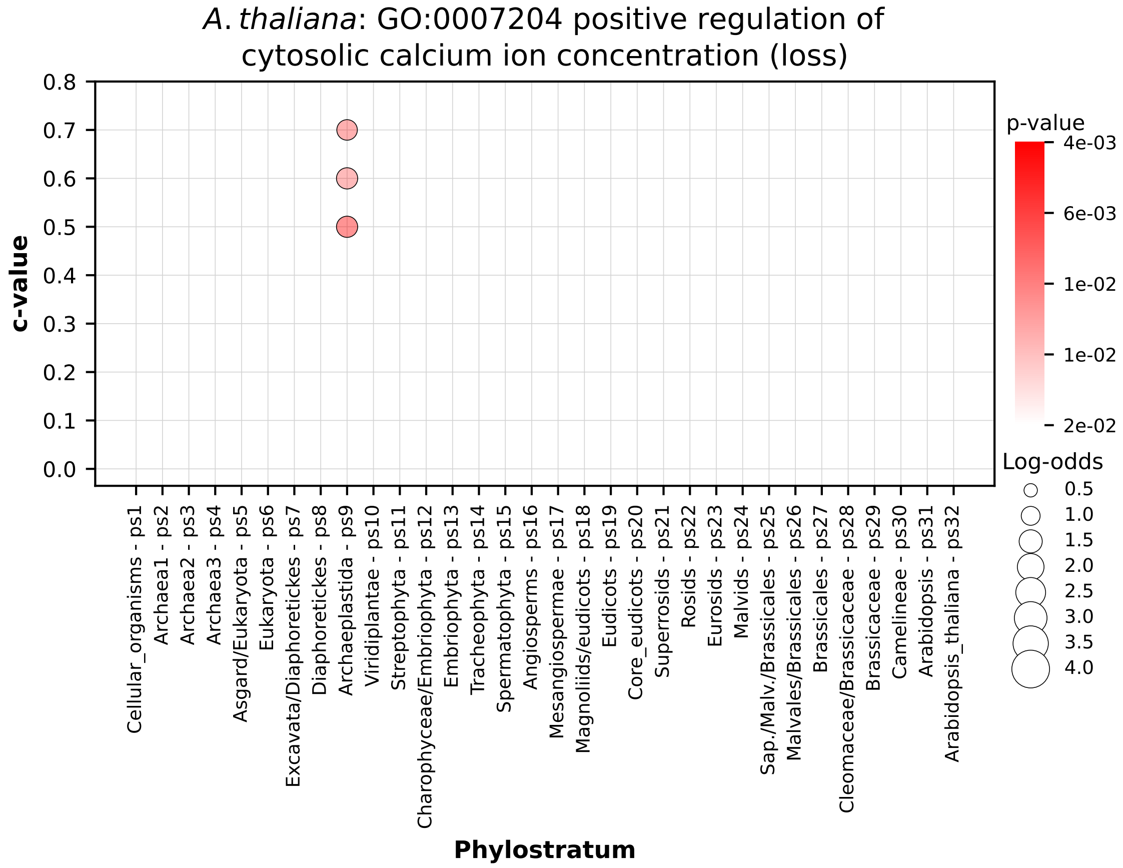

A. thaliana: GO:0007204 positive regulation of cytosolic calcium ion concentration (loss)
c-value
0.8
0.7
0.6
0.5
0.4
0.3
0.2
0.1
0.0
p-value
4e-03
6e-03
1e-02
1e-02
2e-02
Log-odds
0.5
1.0
1.5
2.0
2.5
3.0
3.5
4.0
Cellular_organisms - ps1
Archaea1 - ps2
Archaea2 - ps3
Archaea3 - ps4
Asgard/Eukaryota - ps5
Eukaryota - ps6
Excavata/Diaphoretickes - ps7
Diaphoretickes - ps8
Archaeplastida - ps9
Viridiplantae - ps10
Streptophyta - ps11
Charophyceae/Embriophyta - ps12
Embriophyta - ps13
Tracheophyta - ps14
Spermatophyta - ps15
Angiosperms - ps16
Mesangiospermae - ps17
Magnoliids/eudicots - ps18
Eudicots - ps19
Core_eudicots - ps20
Superrosids - ps21
Rosids - ps22
Eurosids - ps23
Malvids - ps24
Sap./Malv./Brassicales - ps25
Malvales/Brassicales - ps26
Brassicales - ps27
Cleomaceae/Brassicaceae - ps28
Brassicaceae - ps29
Camelineae - ps30
Arabidopsis - ps31
Arabidopsis_thaliana - ps32
Phylostratum

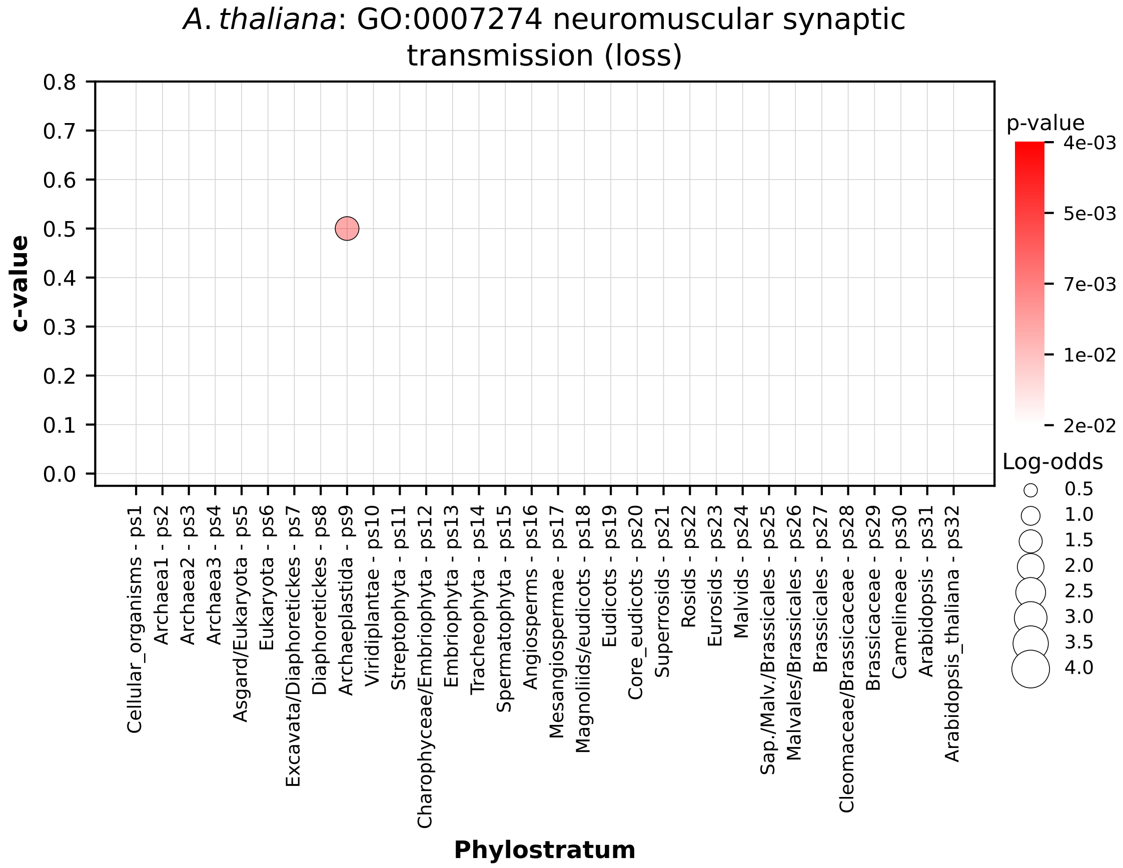
A. thaliana: GO:0007274 neuromuscular synaptic transmission (loss)
c-value
0.8
0.7
0.6
0.5
0.4
0.3
0.2
0.1
0.0
Phylostratum
Cellular_organisms - ps1
Archaea1 - ps2
Archaea2 - ps3
Archaea3 - ps4
Asgard/Eukaryota - ps5
Eukaryota - ps6
Excavata/Diaphoretickes - ps7
Diaphoretickes - ps8
Archaeplastida - ps9
Viridiplantae - ps10
Streptophyta - ps11
Charophyceae/Embriophyta - ps12
Embriophyta - ps13
Tracheophyta - ps14
Spermatophyta - ps15
Angiosperms - ps16
Mesangiospermae - ps17
Magnoliids/eudicots - ps18
Eudicots - ps19
Core_eudicots - ps20
Superrosids - ps21
Rosids - ps22
Eurosids - ps23
Malvids - ps24
Sap./Malv./Brassicales - ps25
Malvales/Brassicales - ps26
Brassicales - ps27
Cleomaceae/Brassicaceae - ps28
Brassicaceae - ps29
Camelineae - ps30
Arabidopsis - ps31
Arabidopsis_thaliana - ps32
p-value
4e-03
5e-03
7e-03
1e-02
2e-02
Log-odds
0.5
1.0
1.5
2.0
2.5
3.0
3.5
4.0

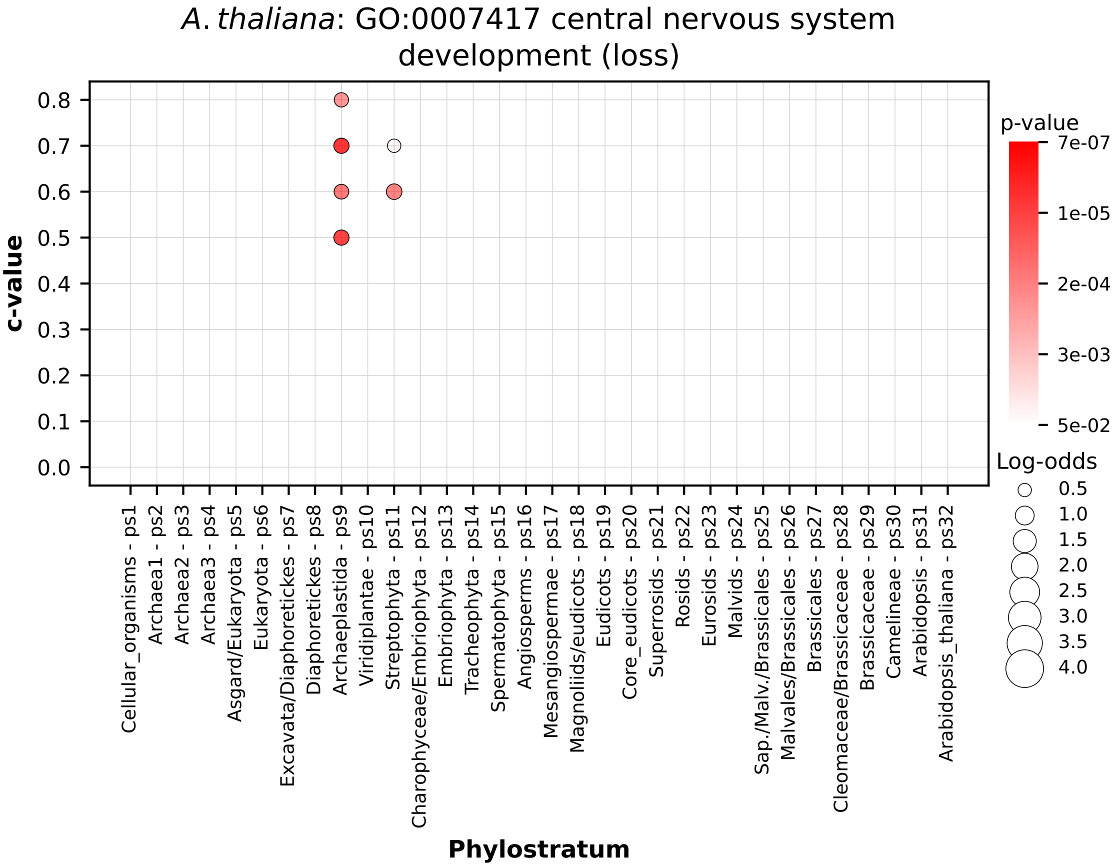

A. thaliana: GO:0007417 central nervous system development (loss)
c-value
0.8
0.7
0.6
0.5
0.4
0.3
0.2
0.1
0.0
p-value
7e-07
1e-05
2e-04
3e-03
5e-02
Log-odds
0.5
1.0
1.5
2.0
2.5
3.0
3.5
4.0
Cellular_organisms - ps1
Archaea1 - ps2
Archaea2 - ps3
Archaea3 - ps4
Asgard/Eukaryota - ps5
Eukaryota - ps6
Excavata/Diaphoretickes - ps7
Diaphoretickes - ps8
Archaeplastida - ps9
Viridiplantae - ps10
Streptophyta - ps11
Charophyceae/Embriophyta - ps12
Embriophyta - ps13
Tracheophyta - ps14
Spermatophyta - ps15
Angiosperms - ps16
Mesangiospermae - ps17
Magnoliids/eudicots - ps18
Eudicots - ps19
Core_eudicots - ps20
Superrosids - ps21
Rosids - ps22
Eurosids - ps23
Malvids - ps24
Sap./Malv./Brassicales - ps25
Malvales/Brassicales - ps26
Brassicales - ps27
Cleomaceae/Brassicaceae - ps28
Brassicaceae - ps29
Camelineae - ps30
Arabidopsis - ps31
Arabidopsis_thaliana - ps32
Phylostratum

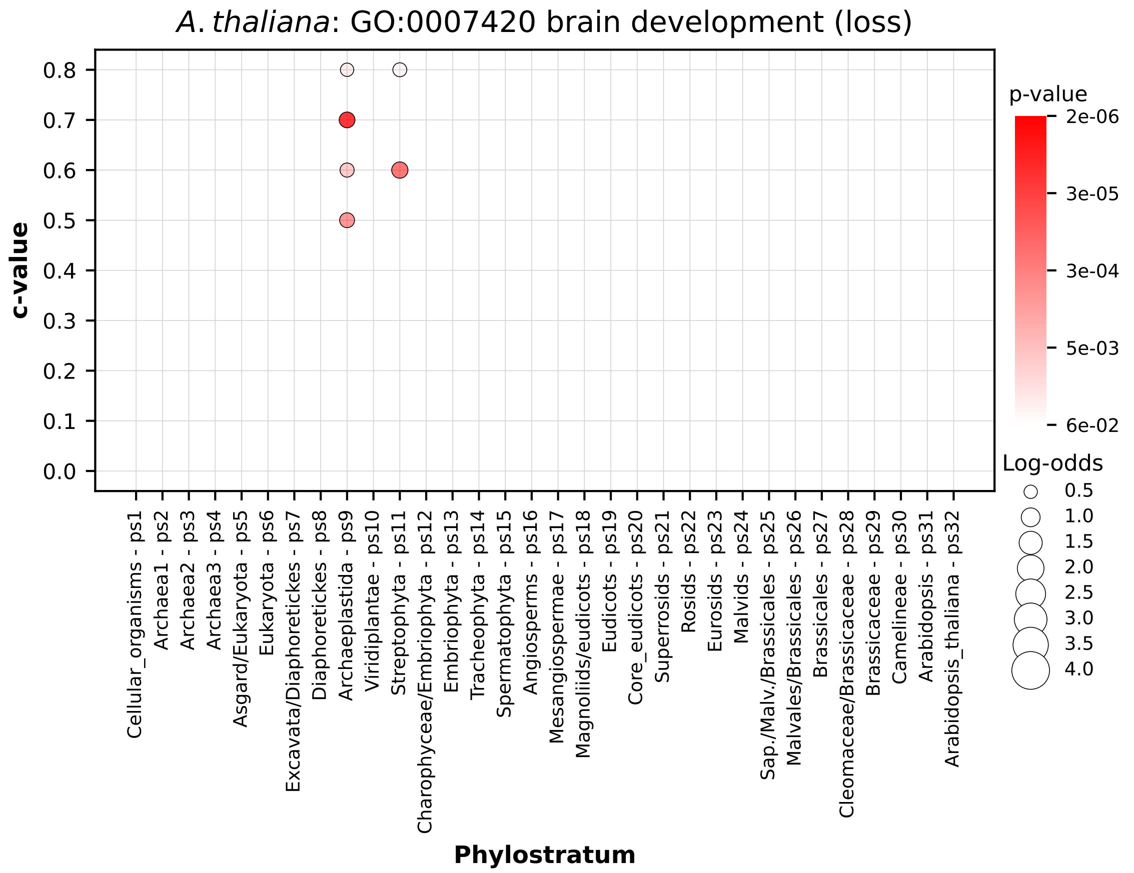

A. thaliana: GO:0007420 brain development (loss)
c-value
0.8
0.7
0.6
0.5
0.4
0.3
0.2
0.1
0.0
Phylostratum
Cellular_organisms - ps1
Archaea1 - ps2
Archaea2 - ps3
Archaea3 - ps4
Asgard/Eukaryota - ps5
Eukaryota - ps6
Excavata/Diaphoretickes - ps7
Diaphoretickes - ps8
Archaeplastida - ps9
Viridiplantae - ps10
Streptophyta - ps11
Charophyceae/Embriophyta - ps12
Embriophyta - ps13
Tracheophyta - ps14
Spermatophyta - ps15
Angiosperms - ps16
Mesangiospermae - ps17
Magnoliids/eudicots - ps18
Eudicots - ps19
Core_eudicots - ps20
Superrosids - ps21
Rosids - ps22
Eurosids - ps23
Malvids - ps24
Sap./Malv./Brassicales - ps25
Malvales/Brassicales - ps26
Brassicales - ps27
Cleomaceae/Brassicaceae - ps28
Brassicaceae - ps29
Camelineae - ps30
Arabidopsis - ps31
Arabidopsis_thaliana - ps32
p-value
2e-06
3e-05
3e-04
5e-03
6e-02
Log-odds
0.5
1.0
1.5
2.0
2.5
3.0
3.5
4.0

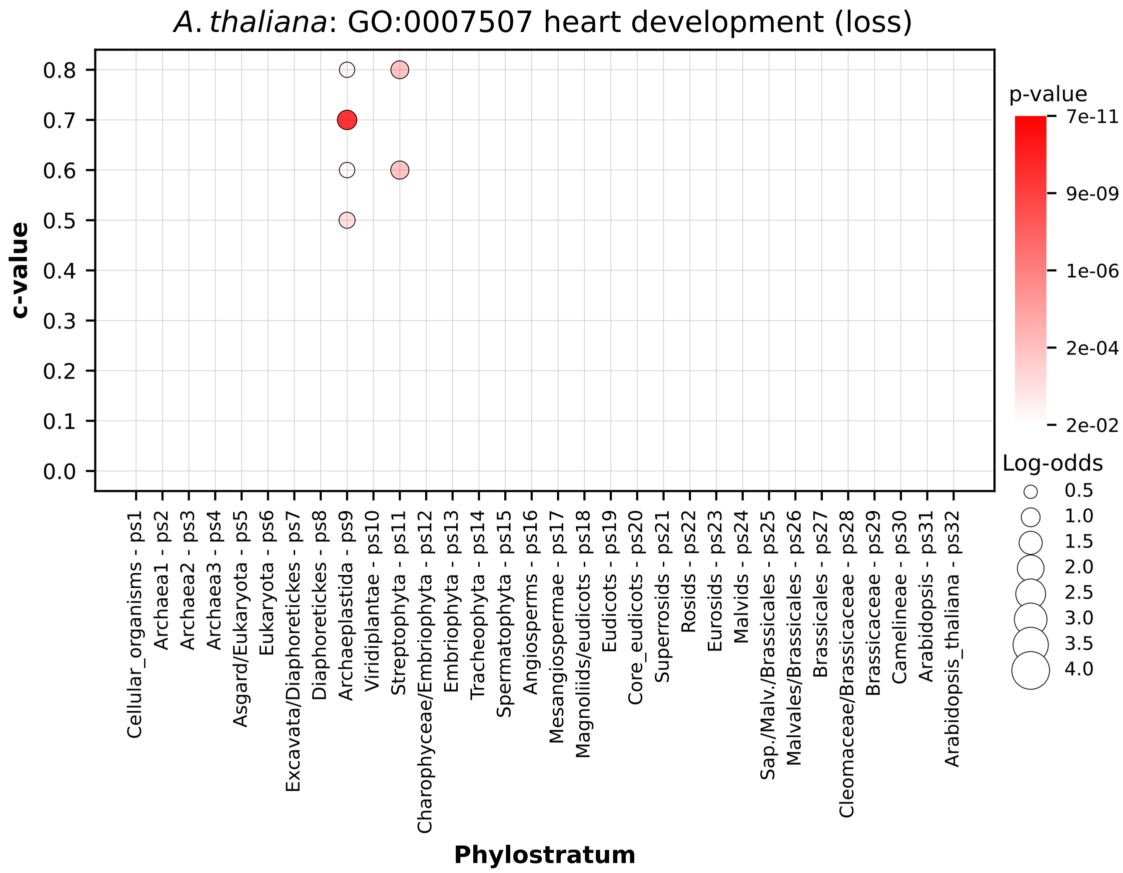

A. thaliana: GO:0007507 heart development (loss)
c-value
0.8
0.7
0.6
0.5
0.4
0.3
0.2
0.1
0.0
Phylostratum
Cellular_organisms - ps1
Archaea1 - ps2
Archaea2 - ps3
Archaea3 - ps4
Asgard/Eukaryota - ps5
Eukaryota - ps6
Excavata/Diaphoretickes - ps7
Diaphoretickes - ps8
Archaeplastida - ps9
Viridiplantae - ps10
Streptophyta - ps11
Charophyceae/Embriophyta - ps12
Embriophyta - ps13
Tracheophyta - ps14
Spermatophyta - ps15
Angiosperms - ps16
Mesangiospermae - ps17
Magnoliids/eudicots - ps18
Eudicots - ps19
Core_eudicots - ps20
Superrosids - ps21
Rosids - ps22
Eurosids - ps23
Malvids - ps24
Sap./Malv./Brassicales - ps25
Malvales/Brassicales - ps26
Brassicales - ps27
Cleomaceae/Brassicaceae - ps28
Brassicaceae - ps29
Camelineae - ps30
Arabidopsis - ps31
Arabidopsis_thaliana - ps32
p-value
7e-11
9e-09
1e-06
2e-04
2e-02
Log-odds
0.5
1.0
1.5
2.0
2.5
3.0
3.5
4.0

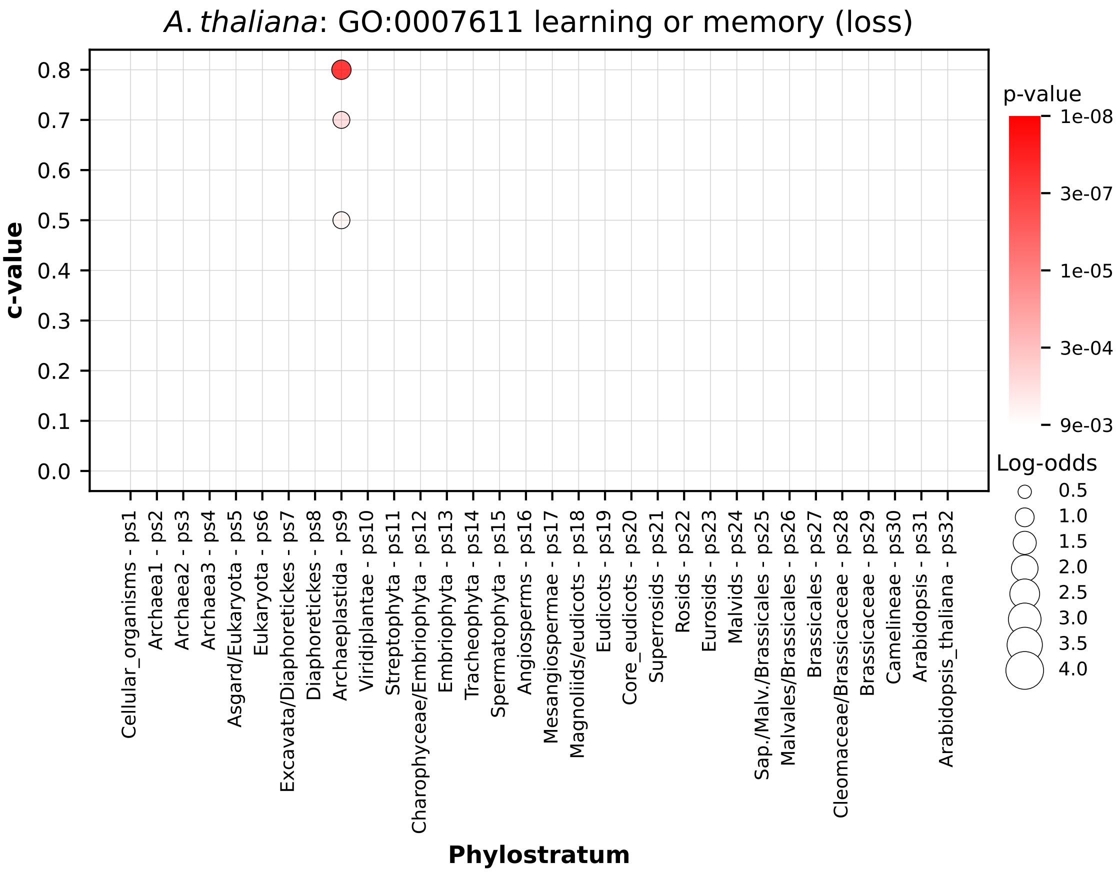

A. thaliana: GO:0007611 learning or memory (loss)
c-value
0.8
0.7
0.6
0.5
0.4
0.3
0.2
0.1
0.0
p-value
1e-08
3e-07
1e-05
3e-04
9e-03
Log-odds
0.5
1.0
1.5
2.0
2.5
3.0
3.5
4.0
Cellular_organisms - ps1
Archaea1 - ps2
Archaea2 - ps3
Archaea3 - ps4
Asgard/Eukaryota - ps5
Eukaryota - ps6
Excavata/Diaphoretickes - ps7
Diaphoretickes - ps8
Archaeplastida - ps9
Viridiplantae - ps10
Streptophyta - ps11
Charophyceae/Embriophyta - ps12
Embriophyta - ps13
Tracheophyta - ps14
Spermatophyta - ps15
Angiosperms - ps16
Mesangiospermae - ps17
Magnoliids/eudicots - ps18
Eudicots - ps19
Core_eudicots - ps20
Superrosids - ps21
Rosids - ps22
Eurosids - ps23
Malvids - ps24
Sap./Malv./Brassicales - ps25
Malvales/Brassicales - ps26
Brassicales - ps27
Cleomaceae/Brassicaceae - ps28
Brassicaceae - ps29
Camelineae - ps30
Arabidopsis - ps31
Arabidopsis_thaliana - ps32
Phylostratum

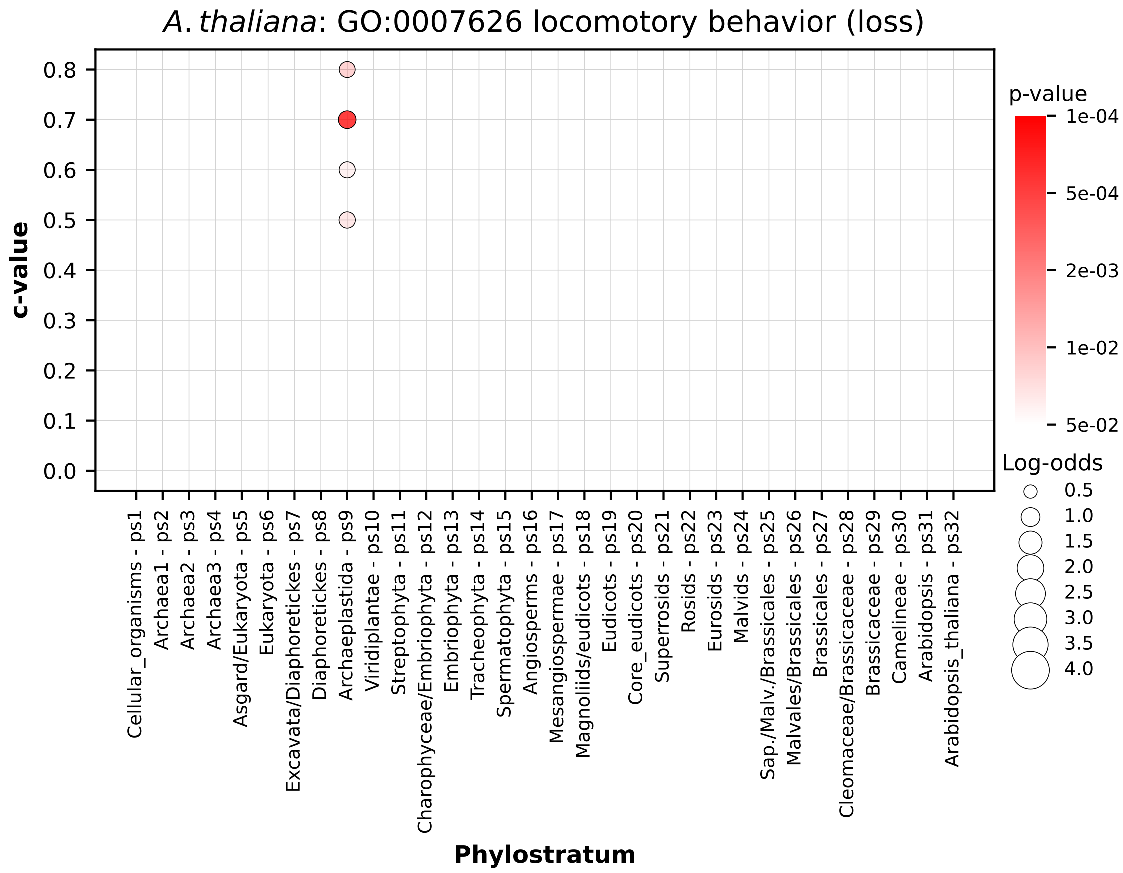

A. thaliana: GO:0007626 locomotory behavior (loss)
c-value
0.8
0.7
0.6
0.5
0.4
0.3
0.2
0.1
0.0
p-value
1e-04
5e-04
2e-03
1e-02
5e-02
Log-odds
0.5
1.0
1.5
2.0
2.5
3.0
3.5
4.0
Cellular_organisms - ps1
Archaea1 - ps2
Archaea2 - ps3
Archaea3 - ps4
Asgard/Eukaryota - ps5
Eukaryota - ps6
Excavata/Diaphoretickes - ps7
Diaphoretickes - ps8
Archaeplastida - ps9
Viridiplantae - ps10
Streptophyta - ps11
Charophyceae/Embriophyta - ps12
Embriophyta - ps13
Tracheophyta - ps14
Spermatophyta - ps15
Angiosperms - ps16
Mesangiospermae - ps17
Magnoliids/eudicots - ps18
Eudicots - ps19
Core_eudicots - ps20
Superrosids - ps21
Rosids - ps22
Eurosids - ps23
Malvids - ps24
Sap./Malv./Brassicales - ps25
Malvales/Brassicales - ps26
Brassicales - ps27
Cleomaceae/Brassicaceae - ps28
Brassicaceae - ps29
Camelineae - ps30
Arabidopsis - ps31
Arabidopsis_thaliana - ps32
Phylostratum

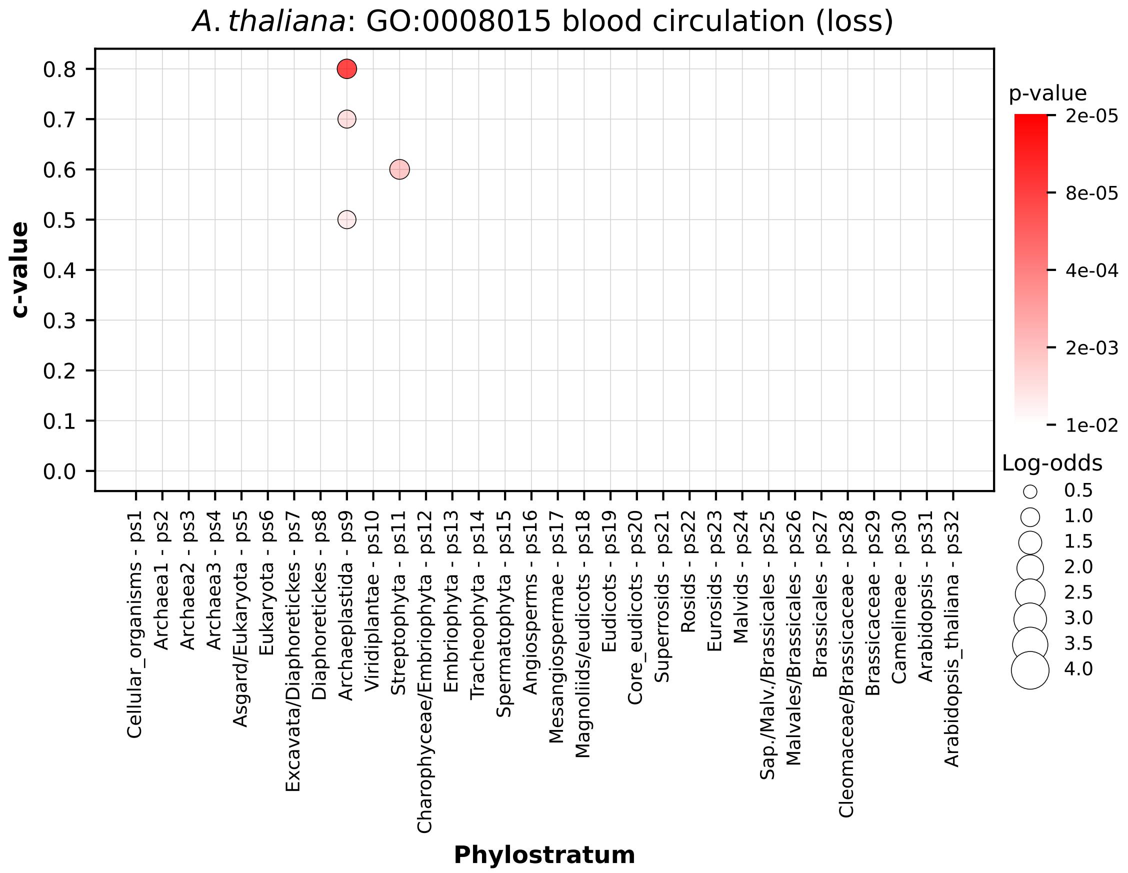

A. thaliana: GO:0008015 blood circulation (loss)
c-value
0.0
0.1
0.2
0.3
0.4
0.5
0.6
0.7
0.8
p-value
2e-05
8e-05
4e-04
2e-03
1e-02
Log-odds
0.5
1.0
1.5
2.0
2.5
3.0
3.5
4.0
Cellular_organisms - ps1
Archaea1 - ps2
Archaea2 - ps3
Archaea3 - ps4
Asgard/Eukaryota - ps5
Eukaryota - ps6
Excavata/Diaphoretickes - ps7
Diaphoretickes - ps8
Archaeplastida - ps9
Viridiplantae - ps10
Streptophyta - ps11
Charophyceae/Embriophyta - ps12
Embriophyta - ps13
Tracheophyta - ps14
Spermatophyta - ps15
Angiosperms - ps16
Mesangiospermae - ps17
Magnoliids/eudicots - ps18
Eudicots - ps19
Core_eudicots - ps20
Superrosids - ps21
Rosids - ps22
Eurosids - ps23
Malvids - ps24
Sap./Malv./Brassicales - ps25
Malvales/Brassicales - ps26
Brassicales - ps27
Cleomaceae/Brassicaceae - ps28
Brassicaceae - ps29
Camelineae - ps30
Arabidopsis - ps31
Arabidopsis_thaliana - ps32
Phylostratum

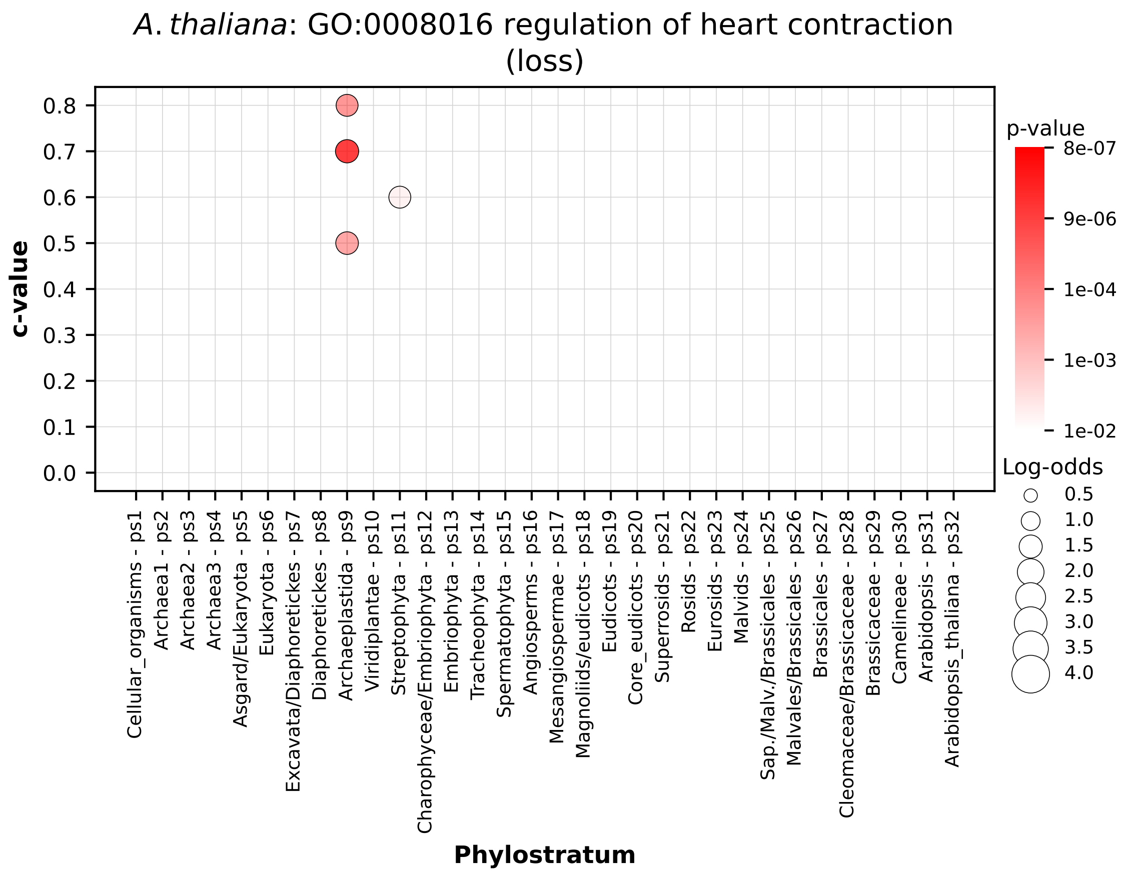
A. thaliana: GO:0008016 regulation of heart contraction
(loss)
c-value
0.8
0.7
0.6
0.5
0.4
0.3
0.2
0.1
0.0
p-value
8e-07
9e-06
1e-04
1e-03
1e-02
Log-odds
0.5
1.0
1.5
2.0
2.5
3.0
3.5
4.0
Cellular_organisms - ps1
Archaea1 - ps2
Archaea2 - ps3
Archaea3 - ps4
Asgard/Eukaryota - ps5
Eukaryota - ps6
Excavata/Diaphoretickes - ps7
Diaphoretickes - ps8
Archaeplastida - ps9
Viridiplantae - ps10
Streptophyta - ps11
Charophyceae/Embriophyta - ps12
Embriophyta - ps13
Tracheophyta - ps14
Spermatophyta - ps15
Angiosperms - ps16
Mesangiospermae - ps17
Magnoliids/eudicots - ps18
Eudicots - ps19
Core_eudicots - ps20
Superrosids - ps21
Rosids - ps22
Eurosids - ps23
Malvids - ps24
Sap./Malv./Brassicales - ps25
Malvales/Brassicales - ps26
Brassicales - ps27
Cleomaceae/Brassicaceae - ps28
Brassicaceae - ps29
Camelineae - ps30
Arabidopsis - ps31
Arabidopsis_thaliana - ps32
Phylostratum

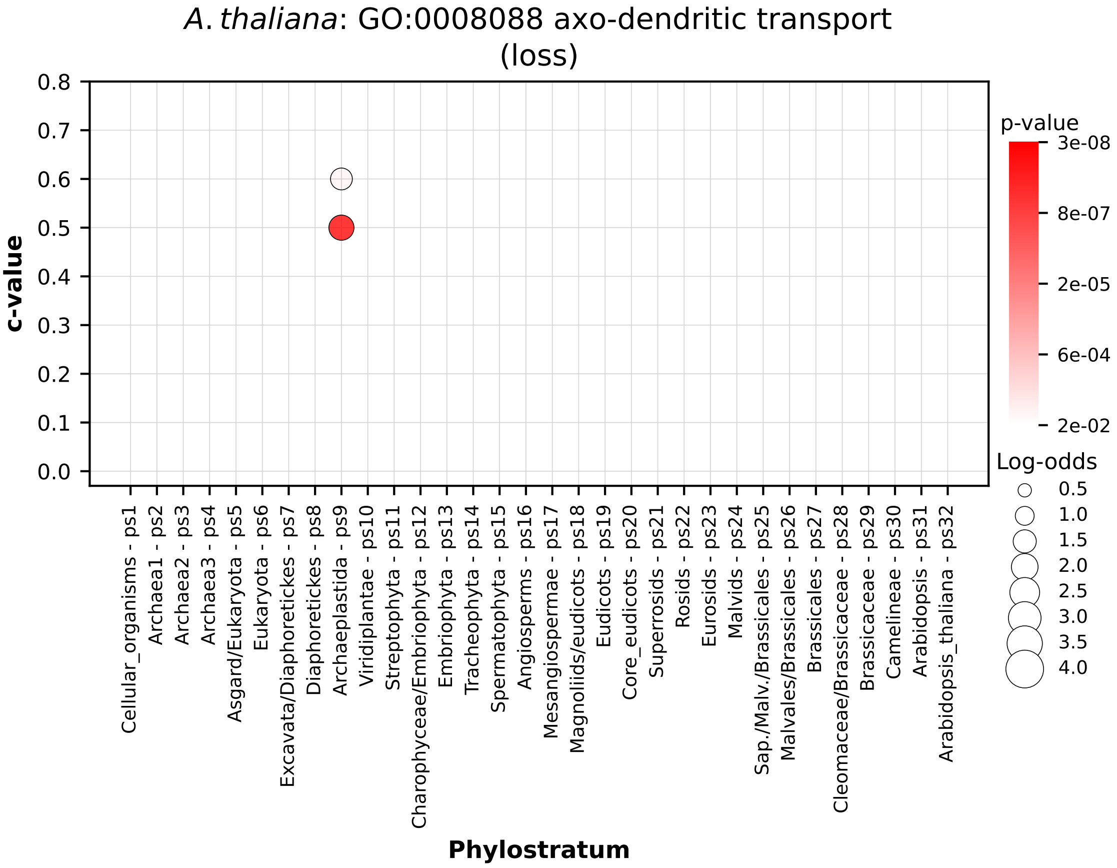
A. thaliana: GO:0008088 axo-dendritic transport
(loss)
c-value
0.8
0.7
0.6
0.5
0.4
0.3
0.2
0.1
0.0
Phylostratum
Cellular_organisms - ps1
Archaea1 - ps2
Archaea2 - ps3
Archaea3 - ps4
Asgard/Eukaryota - ps5
Eukaryota - ps6
Excavata/Diaphoretickes - ps7
Diaphoretickes - ps8
Archaeplastida - ps9
Viridiplantae - ps10
Streptophyta - ps11
Charophyceae/Embriophyta - ps12
Embriophyta - ps13
Tracheophyta - ps14
Spermatophyta - ps15
Angiosperms - ps16
Mesangiospermae - ps17
Magnoliids/eudicots - ps18
Eudicots - ps19
Core_eudicots - ps20
Superrosids - ps21
Rosids - ps22
Eurosids - ps23
Malvids - ps24
Sap./Malv./Brassicales - ps25
Malvales/Brassicales - ps26
Brassicales - ps27
Cleomaceae/Brassicaceae - ps28
Brassicaceae - ps29
Camelineae - ps30
Arabidopsis - ps31
Arabidopsis_thaliana - ps32
p-value
3e-08
8e-07
2e-05
6e-04
2e-02
Log-odds
0.5
1.0
1.5
2.0
2.5
3.0
3.5
4.0

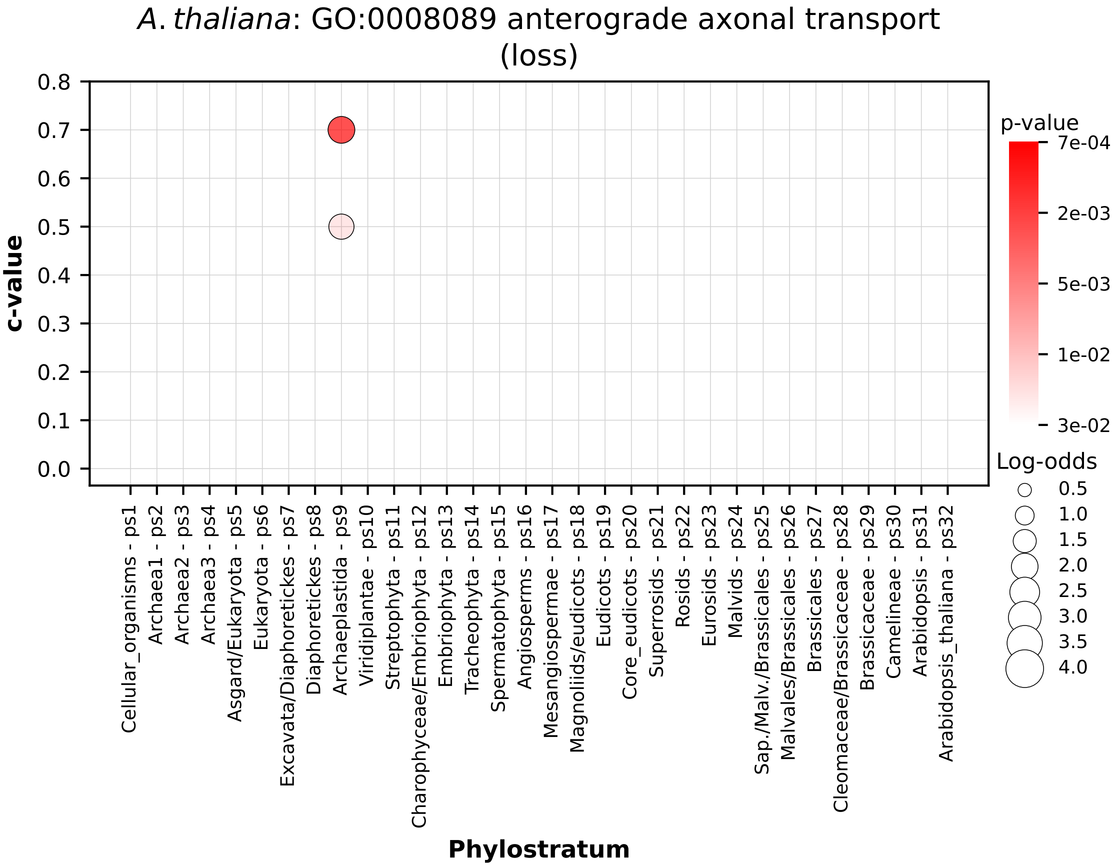

A. thaliana: GO:0008089 anterograde axonal transport
(loss)
c-value
0.8
0.7
0.6
0.5
0.4
0.3
0.2
0.1
0.0
Phylostratum
Cellular_organisms - ps1
Archaea1 - ps2
Archaea2 - ps3
Archaea3 - ps4
Asgard/Eukaryota - ps5
Eukaryota - ps6
Excavata/Diaphoretickes - ps7
Diaphoretickes - ps8
Archaeplastida - ps9
Viridiplantae - ps10
Streptophyta - ps11
Charophyceae/Embriophyta - ps12
Embriophyta - ps13
Tracheophyta - ps14
Spermatophyta - ps15
Angiosperms - ps16
Mesangiospermae - ps17
Magnoliids/eudicots - ps18
Eudicots - ps19
Core_eudicots - ps20
Superrosids - ps21
Rosids - ps22
Eurosids - ps23
Malvids - ps24
Sap./Malv./Brassicales - ps25
Malvales/Brassicales - ps26
Brassicales - ps27
Cleomaceae/Brassicaceae - ps28
Brassicaceae - ps29
Camelineae - ps30
Arabidopsis - ps31
Arabidopsis_thaliana - ps32
p-value
7e-04
2e-03
5e-03
1e-02
3e-02
Log-odds
0.5
1.0
1.5
2.0
2.5
3.0
3.5
4.0

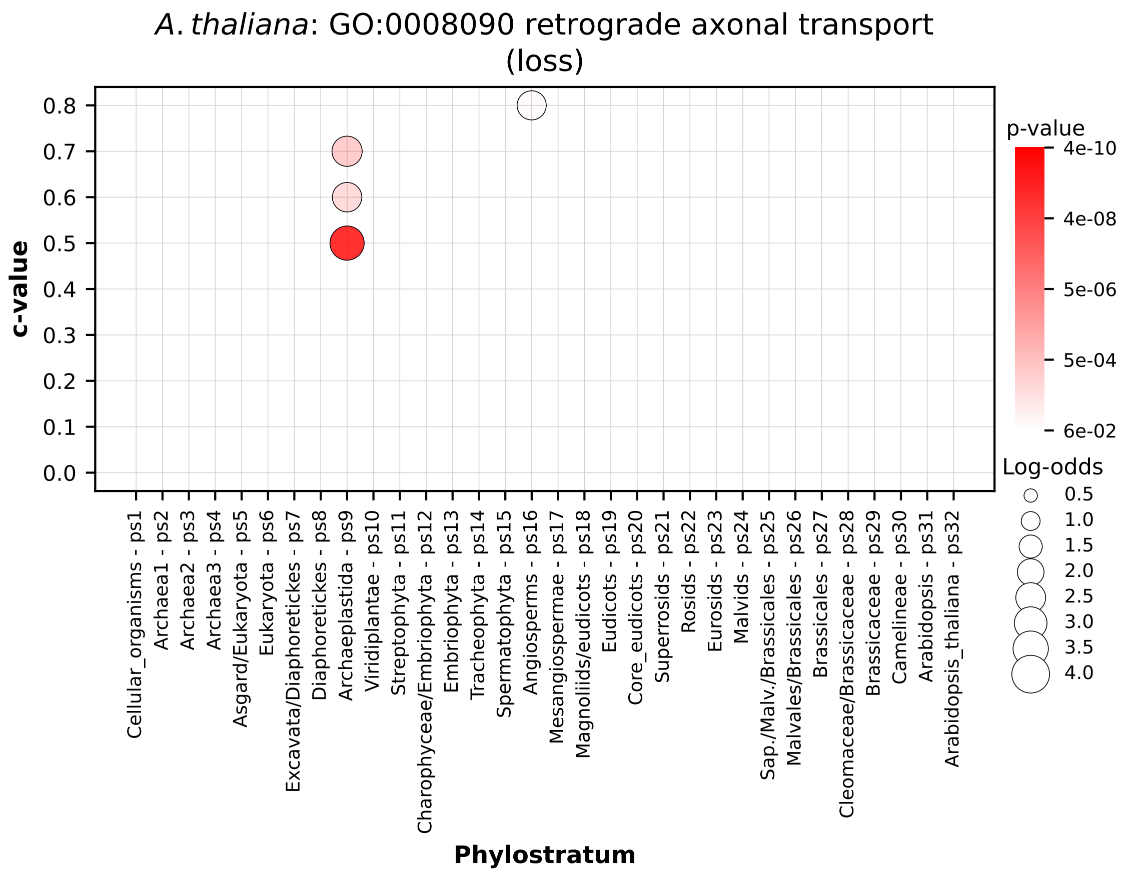

A. thaliana: GO:0008090 retrograde axonal transport
(loss)
c-value
0.0
0.1
0.2
0.3
0.4
0.5
0.6
0.7
0.8
Phylostratum
Cellular_organisms - ps1
Archaea1 - ps2
Archaea2 - ps3
Archaea3 - ps4
Asgard/Eukaryota - ps5
Eukaryota - ps6
Excavata/Diaphoretickes - ps7
Diaphoretickes - ps8
Archaeplastida - ps9
Viridiplantae - ps10
Streptophyta - ps11
Charophyceae/Embriophyta - ps12
Embriophyta - ps13
Tracheophyta - ps14
Spermatophyta - ps15
Angiosperms - ps16
Mesangiospermae - ps17
Magnoliids/eudicots - ps18
Eudicots - ps19
Core_eudicots - ps20
Superrosids - ps21
Rosids - ps22
Eurosids - ps23
Malvids - ps24
Sap./Malv./Brassicales - ps25
Malvales/Brassicales - ps26
Brassicales - ps27
Cleomaceae/Brassicaceae - ps28
Brassicaceae - ps29
Camelineae - ps30
Arabidopsis - ps31
Arabidopsis_thaliana - ps32
p-value
4e-10
4e-08
5e-06
5e-04
6e-02
Log-odds
0.5
1.0
1.5
2.0
2.5
3.0
3.5
4.0

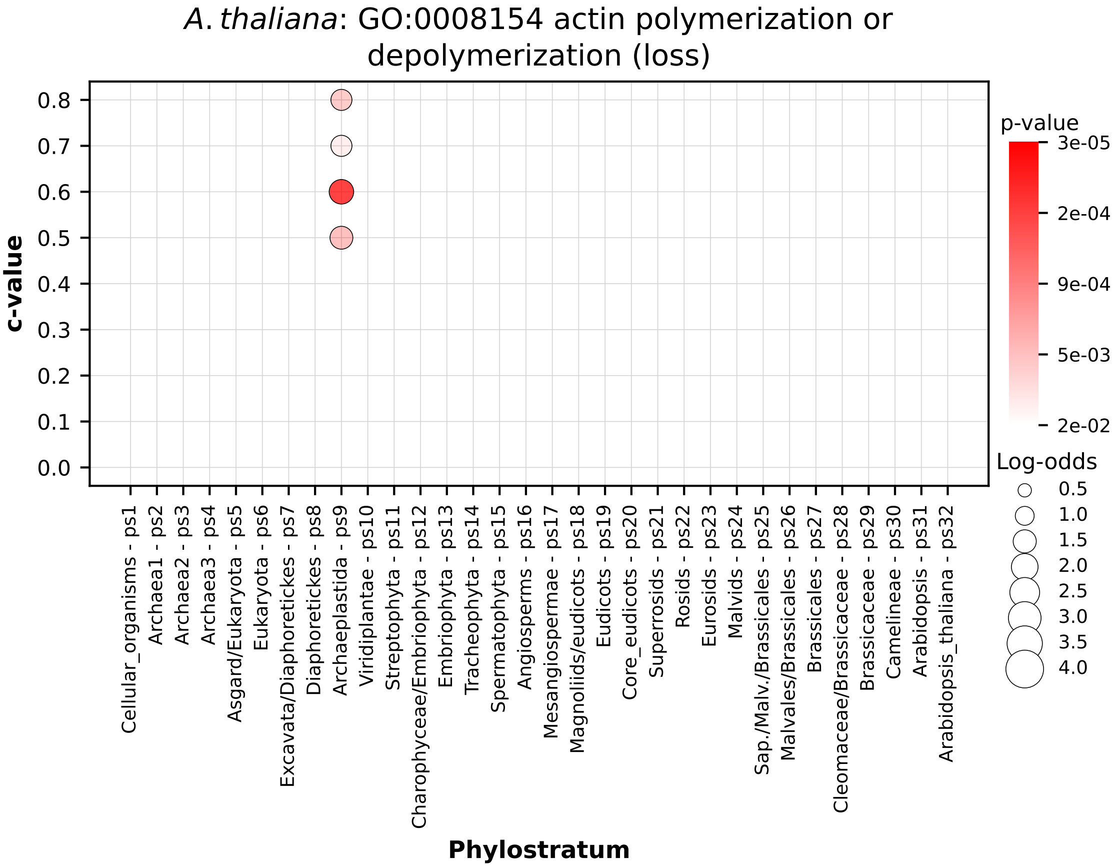

A. thaliana: GO:0008154 actin polymerization or depolymerization (loss)
c-value
0.8
0.7
0.6
0.5
0.4
0.3
0.2
0.1
0.0
Phylostratum
Cellular_organisms - ps1
Archaea1 - ps2
Archaea2 - ps3
Archaea3 - ps4
Asgard/Eukaryota - ps5
Eukaryota - ps6
Excavata/Diaphoretickes - ps7
Diaphoretickes - ps8
Archaeplastida - ps9
Viridiplantae - ps10
Streptophyta - ps11
Charophyceae/Embriophyta - ps12
Embriophyta - ps13
Tracheophyta - ps14
Spermatophyta - ps15
Angiosperms - ps16
Mesangiospermae - ps17
Magnoliids/eudicots - ps18
Eudicots - ps19
Core_eudicots - ps20
Superrosids - ps21
Rosids - ps22
Eurosids - ps23
Malvids - ps24
Sap./Malv./Brassicales - ps25
Malvales/Brassicales - ps26
Brassicales - ps27
Cleomaceae/Brassicaceae - ps28
Brassicaceae - ps29
Camelineae - ps30
Arabidopsis - ps31
Arabidopsis_thaliana - ps32
p-value
3e-05
2e-04
9e-04
5e-03
2e-02
Log-odds
0.5
1.0
1.5
2.0
2.5
3.0
3.5
4.0

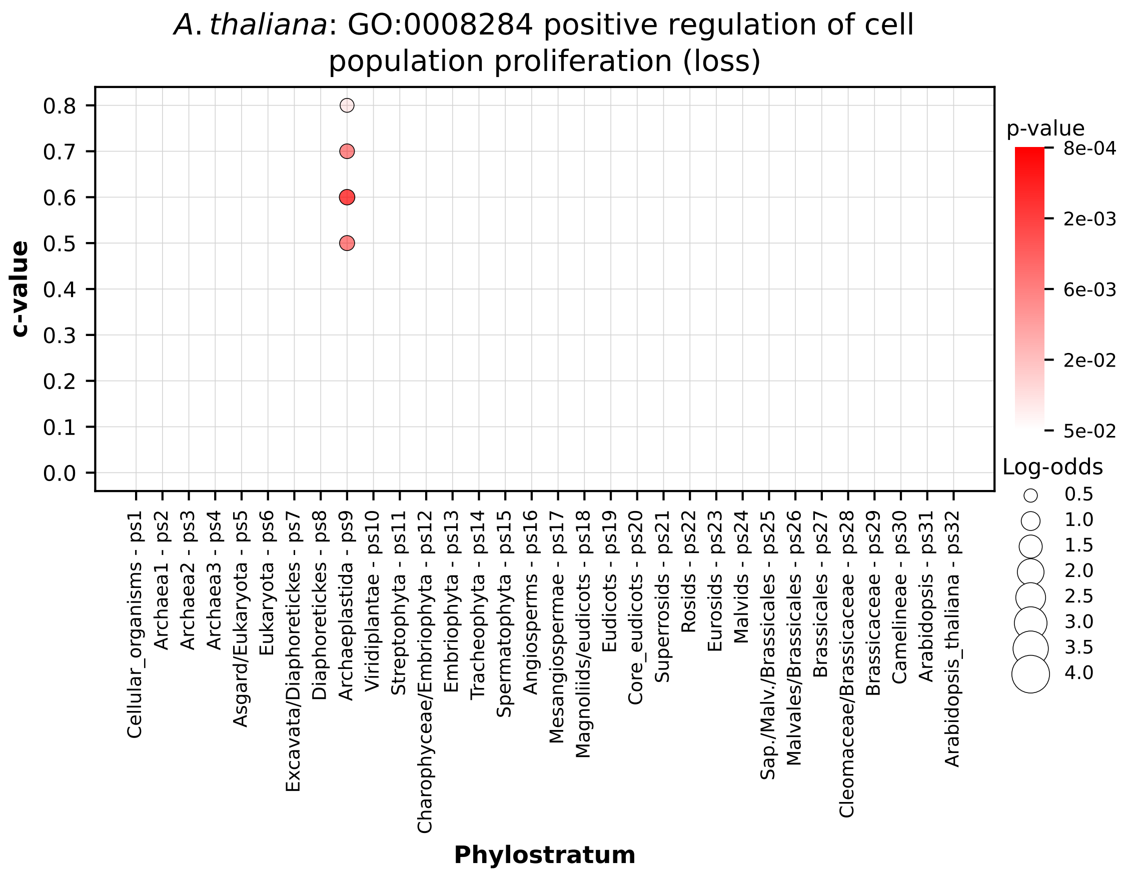

$A. thaliana$: GO:0008284 positive regulation of cell population proliferation (loss)
c-value
0.0
0.1
0.2
0.3
0.4
0.5
0.6
0.7
0.8
p-value
8e-04
2e-03
6e-03
2e-02
5e-02
Log-odds
0.5
1.0
1.5
2.0
2.5
3.0
3.5
4.0
Cellular_organisms - ps1
Archaea1 - ps2
Archaea2 - ps3
Archaea3 - ps4
Asgard/Eukaryota - ps5
Eukaryota - ps6
Excavata/Diaphoretickes - ps7
Diaphoretickes - ps8
Archaeplastida - ps9
Viridiplantae - ps10
Streptophyta - ps11
Charophyceae/Embriophyta - ps12
Embriophyta - ps13
Tracheophyta - ps14
Spermatophyta - ps15
Angiosperms - ps16
Mesangiospermae - ps17
Magnoliids/eudicots - ps18
Eudicots - ps19
Core_eudicots - ps20
Superrosids - ps21
Rosids - ps22
Eurosids - ps23
Malvids - ps24
Sap./Malv./Brassicales - ps25
Malvales/Brassicales - ps26
Brassicales - ps27
Cleomaceae/Brassicaceae - ps28
Brassicaceae - ps29
Camelineae - ps30
Arabidopsis - ps31
Arabidopsis_thaliana - ps32
Phylostratum

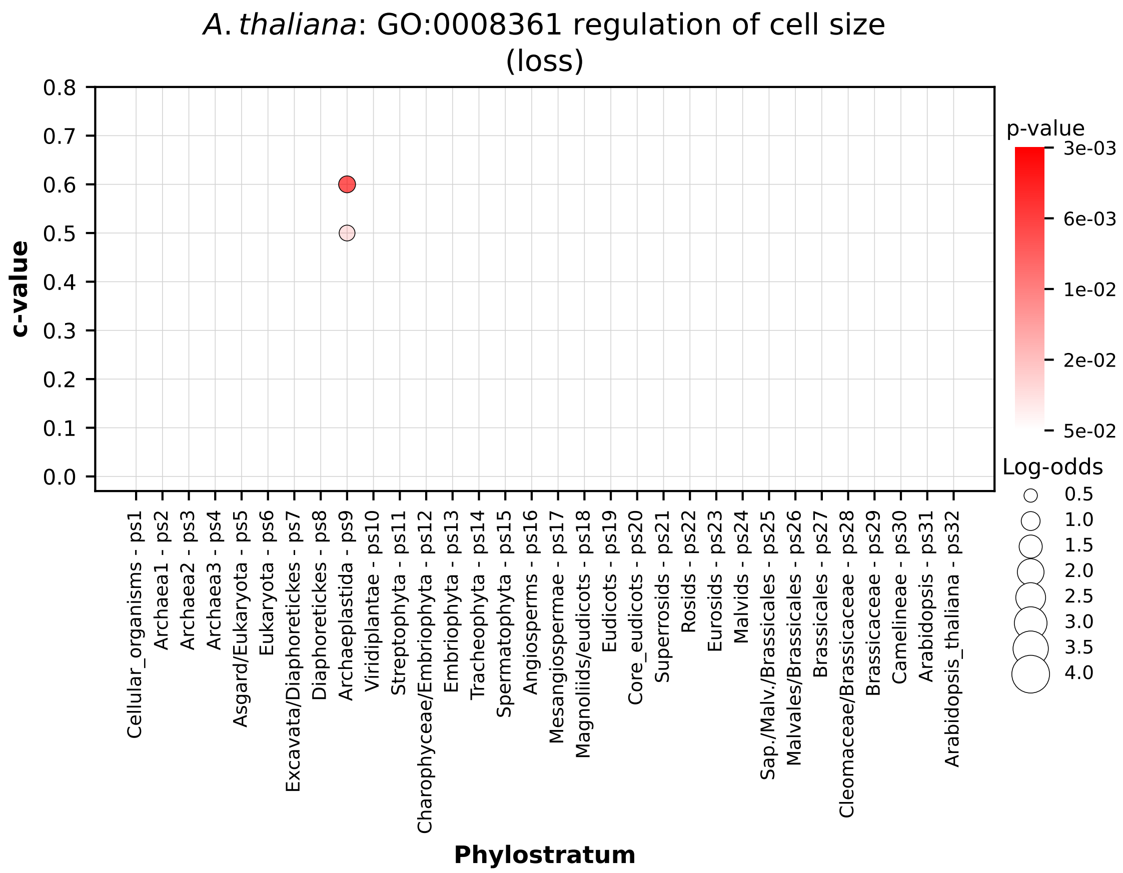

A. thaliana: GO:0008361 regulation of cell size
(loss)
c-value
0.8
0.7
0.6
0.5
0.4
0.3
0.2
0.1
0.0
Phylostratum
Cellular_organisms - ps1
Archaea1 - ps2
Archaea2 - ps3
Archaea3 - ps4
Asgard/Eukaryota - ps5
Eukaryota - ps6
Excavata/Diaphoretickes - ps7
Diaphoretickes - ps8
Archaeplastida - ps9
Viridiplantae - ps10
Streptophyta - ps11
Charophyceae/Embriophyta - ps12
Embriophyta - ps13
Tracheophyta - ps14
Spermatophyta - ps15
Angiosperms - ps16
Mesangiospermae - ps17
Magnoliids/eudicots - ps18
Eudicots - ps19
Core_eudicots - ps20
Superrosids - ps21
Rosids - ps22
Eurosids - ps23
Malvids - ps24
Sap./Malv./Brassicales - ps25
Malvales/Brassicales - ps26
Brassicales - ps27
Cleomaceae/Brassicaceae - ps28
Brassicaceae - ps29
Camelineae - ps30
Arabidopsis - ps31
Arabidopsis_thaliana - ps32
p-value
3e-03
6e-03
1e-02
2e-02
5e-02
Log-odds
0.5
1.0
1.5
2.0
2.5
3.0
3.5
4.0

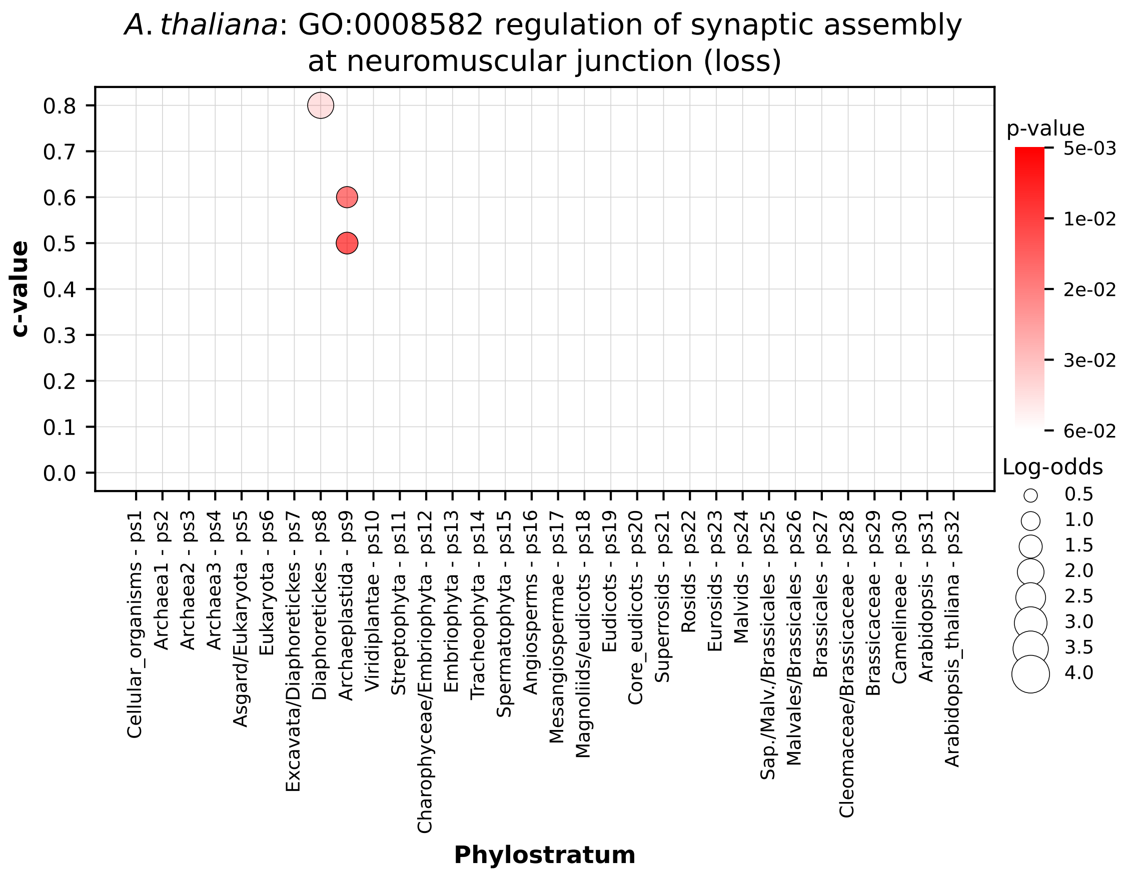

A. thaliana: GO:0008582 regulation of synaptic assembly at neuromuscular junction (loss)
c-value
0.8
0.7
0.6
0.5
0.4
0.3
0.2
0.1
0.0
p-value
5e-03
1e-02
2e-02
3e-02
6e-02
Log-odds
0.5
1.0
1.5
2.0
2.5
3.0
3.5
4.0
Cellular_organisms - ps1
Archaea1 - ps2
Archaea2 - ps3
Archaea3 - ps4
Asgard/Eukaryota - ps5
Eukaryota - ps6
Excavata/Diaphoretickes - ps7
Diaphoretickes - ps8
Archaeplastida - ps9
Viridiplantae - ps10
Streptophyta - ps11
Charophyceae/Embriophyta - ps12
Embriophyta - ps13
Tracheophyta - ps14
Spermatophyta - ps15
Angiosperms - ps16
Mesangiospermae - ps17
Magnoliids/eudicots - ps18
Eudicots - ps19
Core_eudicots - ps20
Superrosids - ps21
Rosids - ps22
Eurosids - ps23
Malvids - ps24
Sap./Malv./Brassicales - ps25
Malvales/Brassicales - ps26
Brassicales - ps27
Cleomaceae/Brassicaceae - ps28
Brassicaceae - ps29
Camelineae - ps30
Arabidopsis - ps31
Arabidopsis_thaliana - ps32
Phylostratum

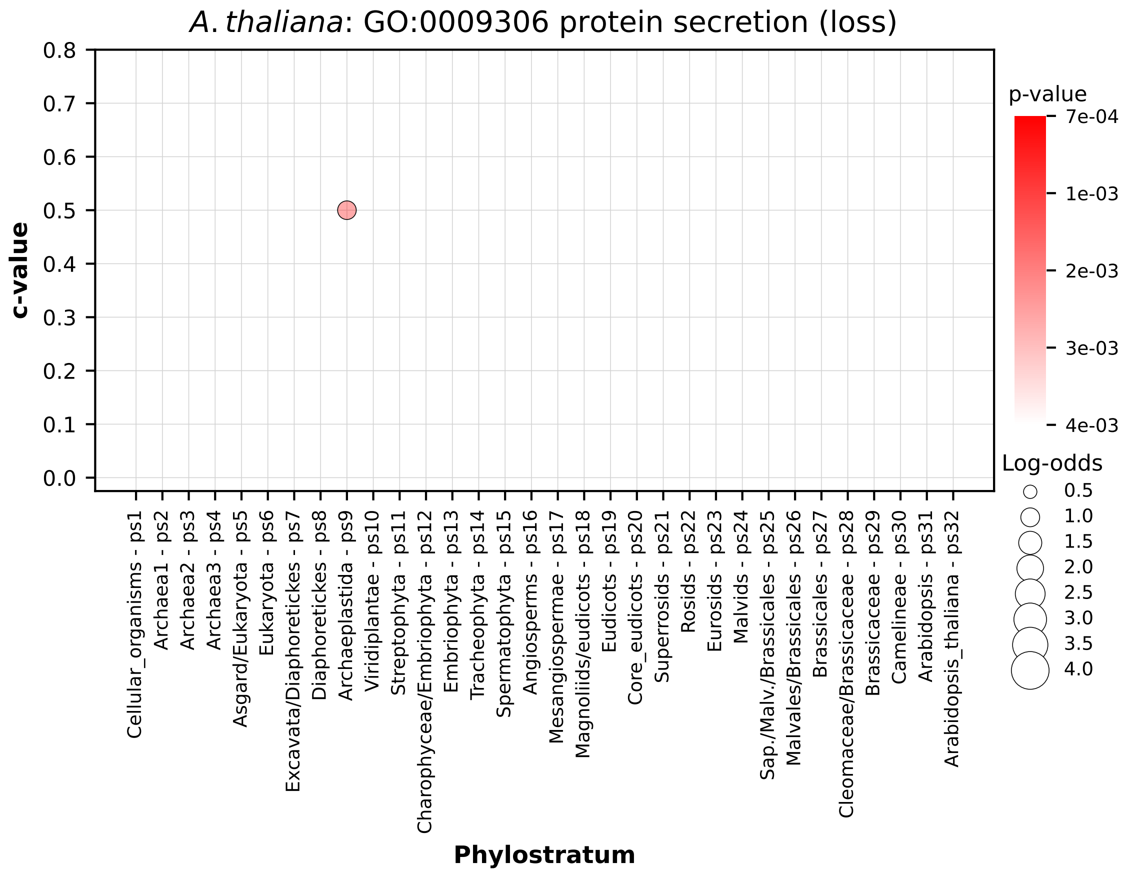

A. thaliana: GO:0009306 protein secretion (loss)
c-value
0.0
0.1
0.2
0.3
0.4
0.5
0.6
0.7
0.8
Phylostratum
Cellular_organisms - ps1
Archaea1 - ps2
Archaea2 - ps3
Archaea3 - ps4
Asgard/Eukaryota - ps5
Eukaryota - ps6
Excavata/Diaphoretickes - ps7
Diaphoretickes - ps8
Archaeplastida - ps9
Viridiplantae - ps10
Streptophyta - ps11
Charophyceae/Embriophyta - ps12
Embriophyta - ps13
Tracheophyta - ps14
Spermatophyta - ps15
Angiosperms - ps16
Mesangiospermae - ps17
Magnoliids/eudicots - ps18
Eudicots - ps19
Core_eudicots - ps20
Superrosids - ps21
Rosids - ps22
Eurosids - ps23
Malvids - ps24
Sap./Malv./Brassicales - ps25
Malvales/Brassicales - ps26
Brassicales - ps27
Cleomaceae/Brassicaceae - ps28
Brassicaceae - ps29
Camelineae - ps30
Arabidopsis - ps31
Arabidopsis_thaliana - ps32
p-value
7e-04
1e-03
2e-03
3e-03
4e-03
Log-odds
0.5
1.0
1.5
2.0
2.5
3.0
3.5
4.0

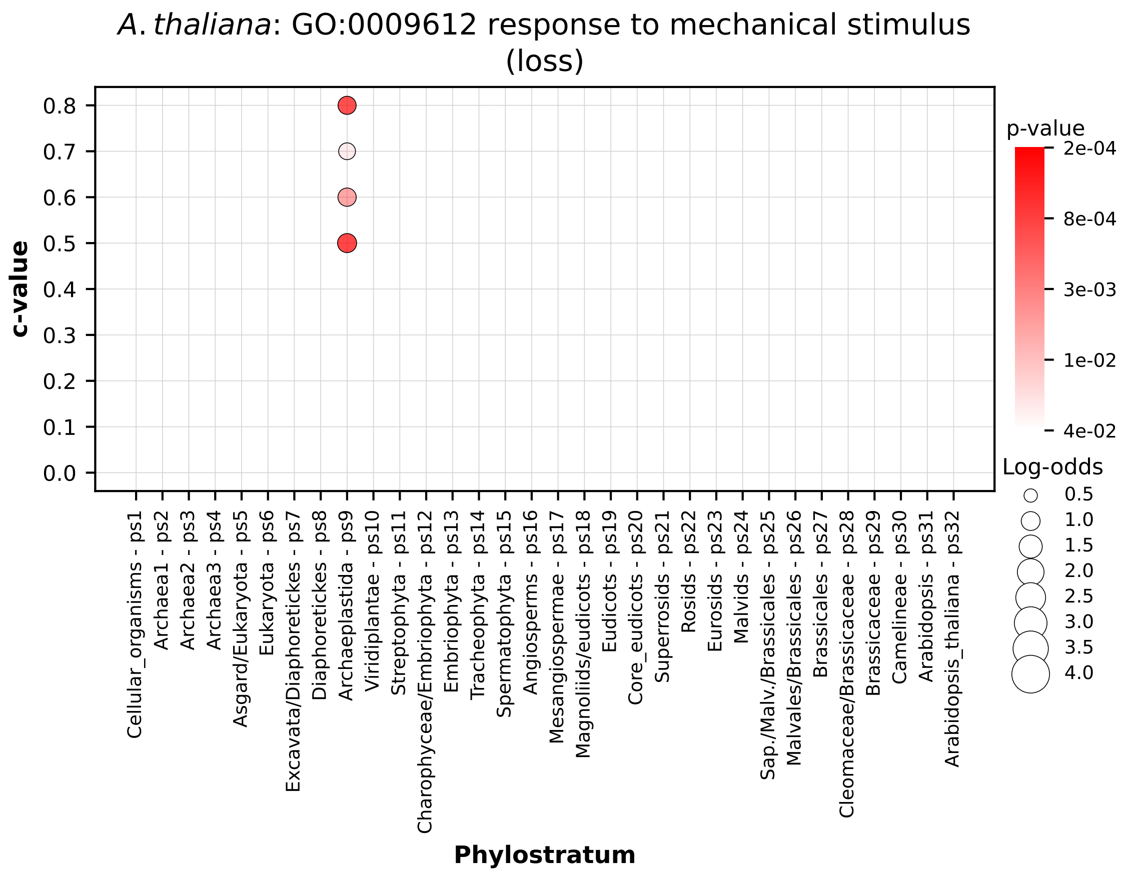

A. thaliana: GO:0009612 response to mechanical stimulus
(loss)
c-value
0.0
0.1
0.2
0.3
0.4
0.5
0.6
0.7
0.8
p-value
2e-04
8e-04
3e-03
1e-02
4e-02
Log-odds
0.5
1.0
1.5
2.0
2.5
3.0
3.5
4.0
Cellular_organisms - ps1
Archaea1 - ps2
Archaea2 - ps3
Archaea3 - ps4
Asgard/Eukaryota - ps5
Eukaryota - ps6
Excavata/Diaphoretickes - ps7
Diaphoretickes - ps8
Archaeplastida - ps9
Viridiplantae - ps10
Streptophyta - ps11
Charophyceae/Embriophyta - ps12
Embriophyta - ps13
Tracheophyta - ps14
Spermatophyta - ps15
Angiosperms - ps16
Mesangiospermae - ps17
Magnoliids/eudicots - ps18
Eudicots - ps19
Core_eudicots - ps20
Superrosids - ps21
Rosids - ps22
Eurosids - ps23
Malvids - ps24
Sap./Malv./Brassicales - ps25
Malvales/Brassicales - ps26
Brassicales - ps27
Cleomaceae/Brassicaceae - ps28
Brassicaceae - ps29
Camelineae - ps30
Arabidopsis - ps31
Arabidopsis_thaliana - ps32
Phylostratum

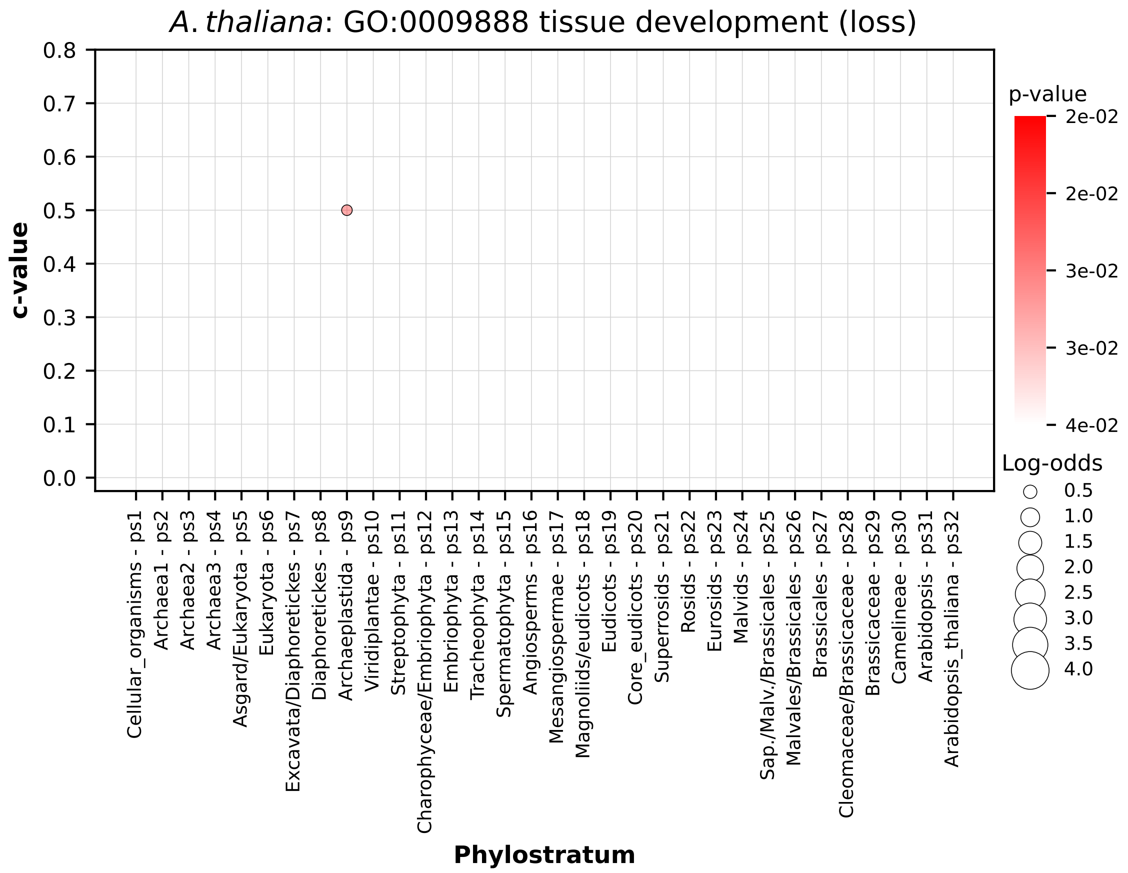

A. thaliana: GO:0009888 tissue development (loss)
c-value
0.8
0.7
0.6
0.5
0.4
0.3
0.2
0.1
0.0
p-value
2e-02
2e-02
3e-02
3e-02
4e-02
Log-odds
0.5
1.0
1.5
2.0
2.5
3.0
3.5
4.0
Cellular_organisms - ps1
Archaea1 - ps2
Archaea2 - ps3
Archaea3 - ps4
Asgard/Eukaryota - ps5
Eukaryota - ps6
Excavata/Diaphoretickes - ps7
Diaphoretickes - ps8
Archaeplastida - ps9
Viridiplantae - ps10
Streptophyta - ps11
Charophyceae/Embriophyta - ps12
Embriophyta - ps13
Tracheophyta - ps14
Spermatophyta - ps15
Angiosperms - ps16
Mesangiospermae - ps17
Magnoliids/eudicots - ps18
Eudicots - ps19
Core_eudicots - ps20
Superrosids - ps21
Rosids - ps22
Eurosids - ps23
Malvids - ps24
Sap./Malv./Brassicales - ps25
Malvales/Brassicales - ps26
Brassicales - ps27
Cleomaceae/Brassicaceae - ps28
Brassicaceae - ps29
Camelineae - ps30
Arabidopsis - ps31
Arabidopsis_thaliana - ps32
Phylostratum

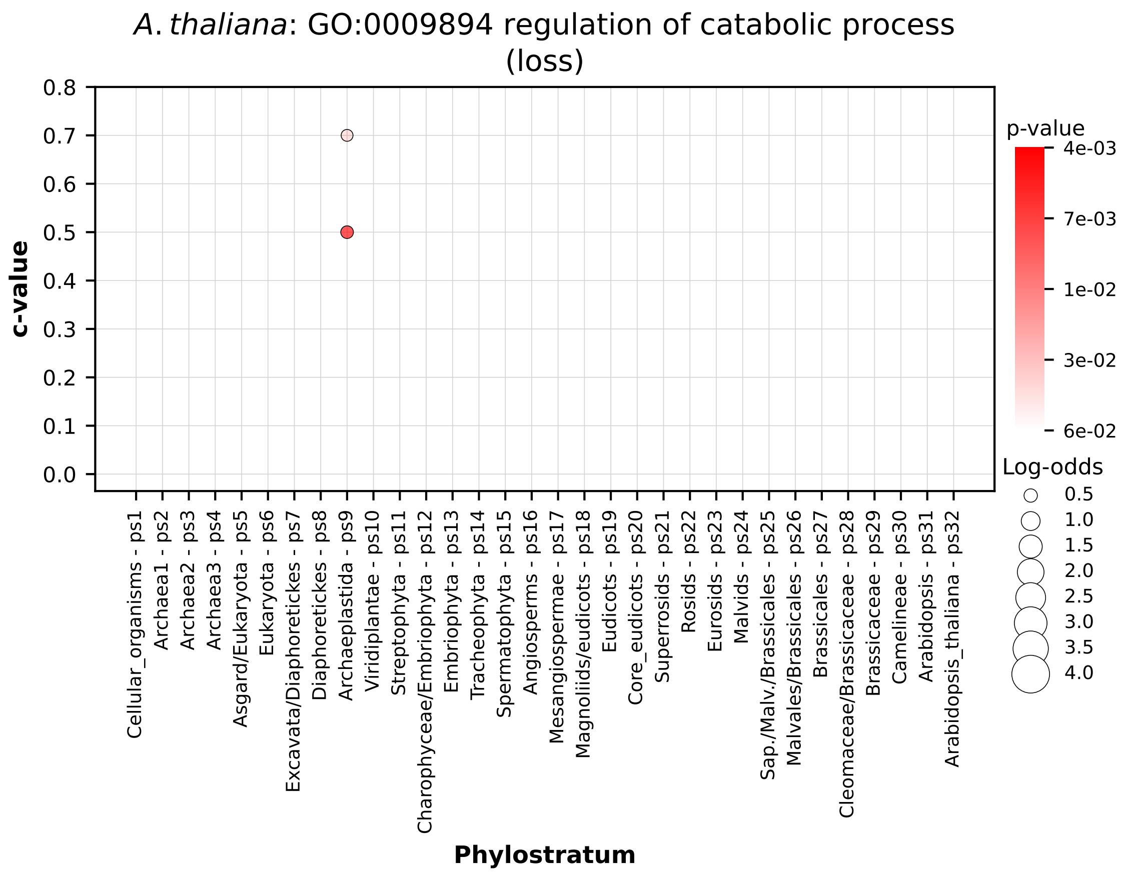
A. thaliana: GO:0009894 regulation of catabolic process
(loss)
c-value
0.8
0.7
0.6
0.5
0.4
0.3
0.2
0.1
0.0
Phylostratum
Cellular_organisms - ps1
Archaea1 - ps2
Archaea2 - ps3
Archaea3 - ps4
Asgard/Eukaryota - ps5
Eukaryota - ps6
Excavata/Diaphoretickes - ps7
Diaphoretickes - ps8
Archaeplastida - ps9
Viridiplantae - ps10
Streptophyta - ps11
Charophyceae/Embriophyta - ps12
Embriophyta - ps13
Tracheophyta - ps14
Spermatophyta - ps15
Angiosperms - ps16
Mesangiospermae - ps17
Magnoliids/eudicots - ps18
Eudicots - ps19
Core_eudicots - ps20
Superrosids - ps21
Rosids - ps22
Eurosids - ps23
Malvids - ps24
Sap./Malv./Brassicales - ps25
Malvales/Brassicales - ps26
Brassicales - ps27
Cleomaceae/Brassicaceae - ps28
Brassicaceae - ps29
Camelineae - ps30
Arabidopsis - ps31
Arabidopsis_thaliana - ps32
p-value
4e-03
7e-03
1e-02
3e-02
6e-02
Log-odds
0.5
1.0
1.5
2.0
2.5
3.0
3.5
4.0

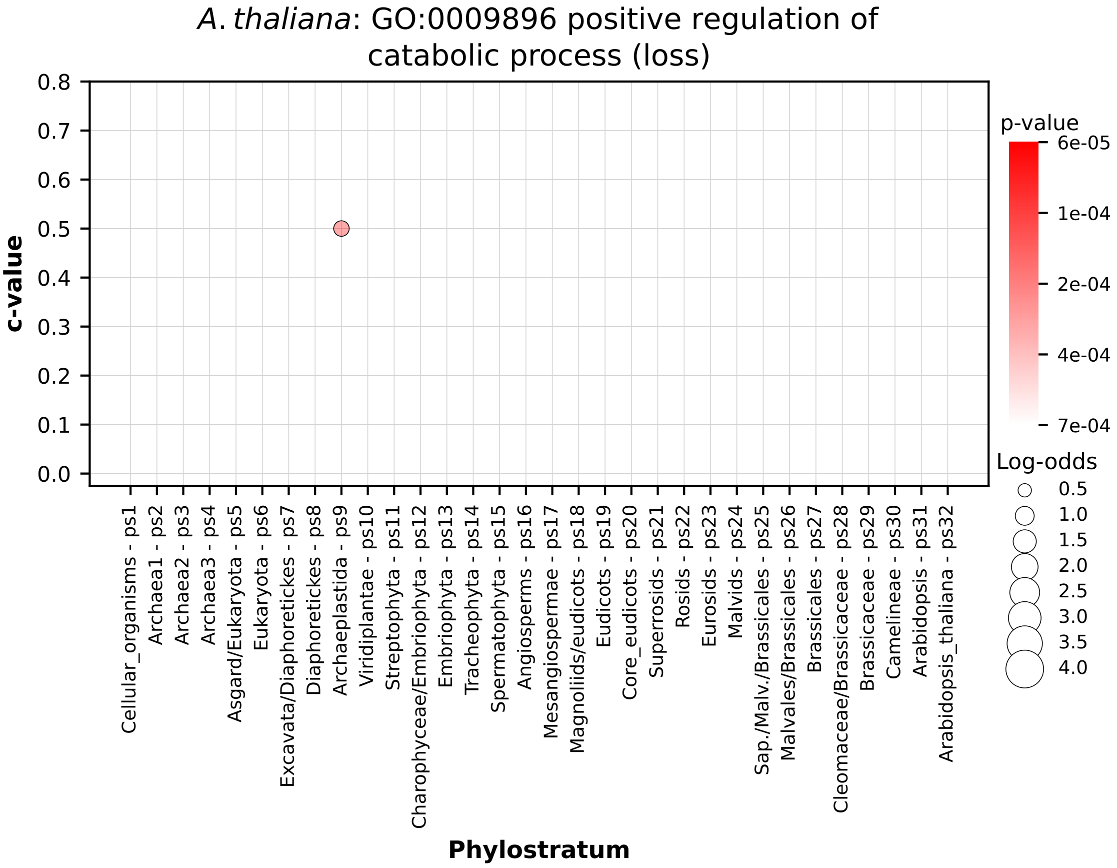

A. thaliana: GO:0009896 positive regulation of catabolic process (loss)
c-value
0.8
0.7
0.6
0.5
0.4
0.3
0.2
0.1
0.0
Phylostratum
Cellular_organisms - ps1
Archaea1 - ps2
Archaea2 - ps3
Archaea3 - ps4
Asgard/Eukaryota - ps5
Eukaryota - ps6
Excavata/Diaphoretickes - ps7
Diaphoretickes - ps8
Archaeplastida - ps9
Viridiplantae - ps10
Streptophyta - ps11
Charophyceae/Embriophyta - ps12
Embriophyta - ps13
Tracheophyta - ps14
Spermatophyta - ps15
Angiosperms - ps16
Mesangiospermae - ps17
Magnoliids/eudicots - ps18
Eudicots - ps19
Core_eudicots - ps20
Superrosids - ps21
Rosids - ps22
Eurosids - ps23
Malvids - ps24
Sap./Malv./Brassicales - ps25
Malvales/Brassicales - ps26
Brassicales - ps27
Cleomaceae/Brassicaceae - ps28
Brassicaceae - ps29
Camelineae - ps30
Arabidopsis - ps31
Arabidopsis_thaliana - ps32
p-value
6e-05
1e-04
2e-04
4e-04
7e-04
Log-odds
0.5
1.0
1.5
2.0
2.5
3.0
3.5
4.0

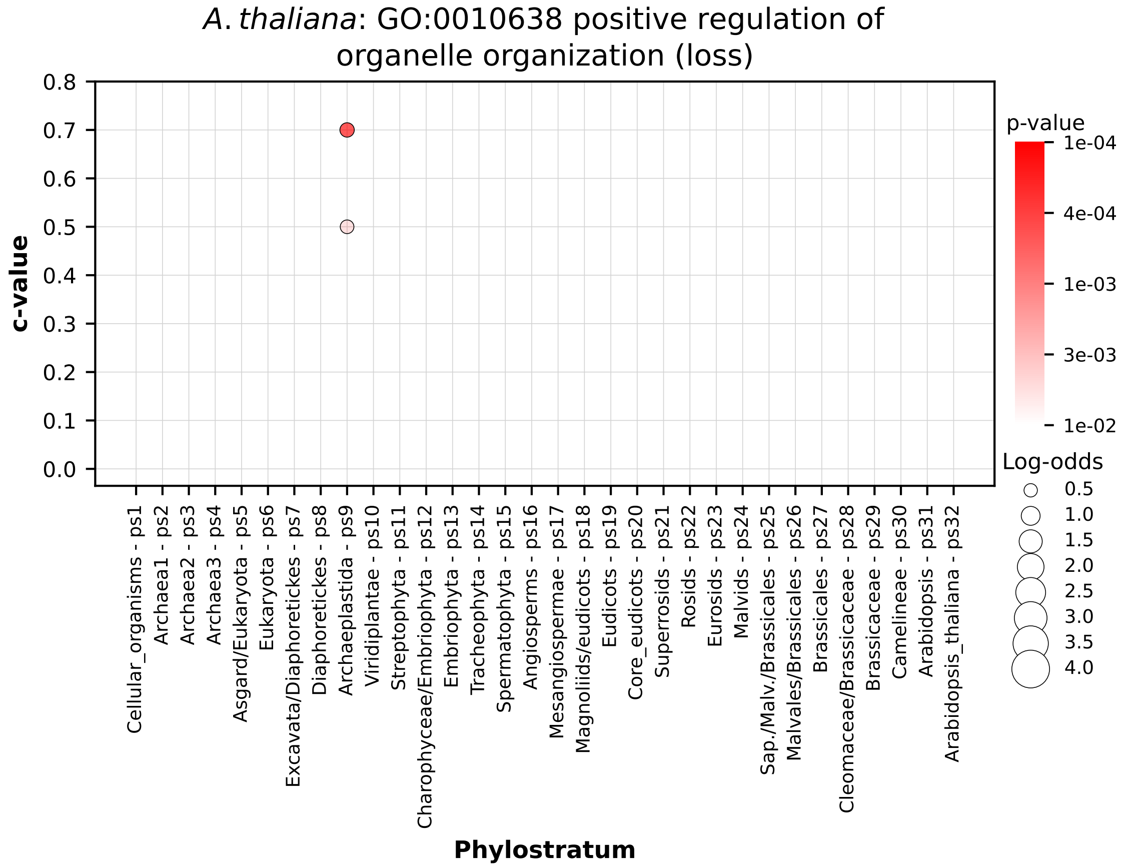
A. thaliana: GO:0010638 positive regulation of organelle organization (loss)
c-value
0.8
0.7
0.6
0.5
0.4
0.3
0.2
0.1
0.0
p-value
1e-04
4e-04
1e-03
3e-03
1e-02
Log-odds
0.5
1.0
1.5
2.0
2.5
3.0
3.5
4.0
Cellular_organisms - ps1
Archaea1 - ps2
Archaea2 - ps3
Archaea3 - ps4
Asgard/Eukaryota - ps5
Eukaryota - ps6
Excavata/Diaphoretickes - ps7
Diaphoretickes - ps8
Archaeplastida - ps9
Viridiplantae - ps10
Streptophyta - ps11
Charophyceae/Embriophyta - ps12
Embriophyta - ps13
Tracheophyta - ps14
Spermatophyta - ps15
Angiosperms - ps16
Mesangiospermae - ps17
Magnoliids/eudicots - ps18
Eudicots - ps19
Core_eudicots - ps20
Superrosids - ps21
Rosids - ps22
Eurosids - ps23
Malvids - ps24
Sap./Malv./Brassicales - ps25
Malvales/Brassicales - ps26
Brassicales - ps27
Cleomaceae/Brassicaceae - ps28
Brassicaceae - ps29
Camelineae - ps30
Arabidopsis - ps31
Arabidopsis_thaliana - ps32
Phylostratum

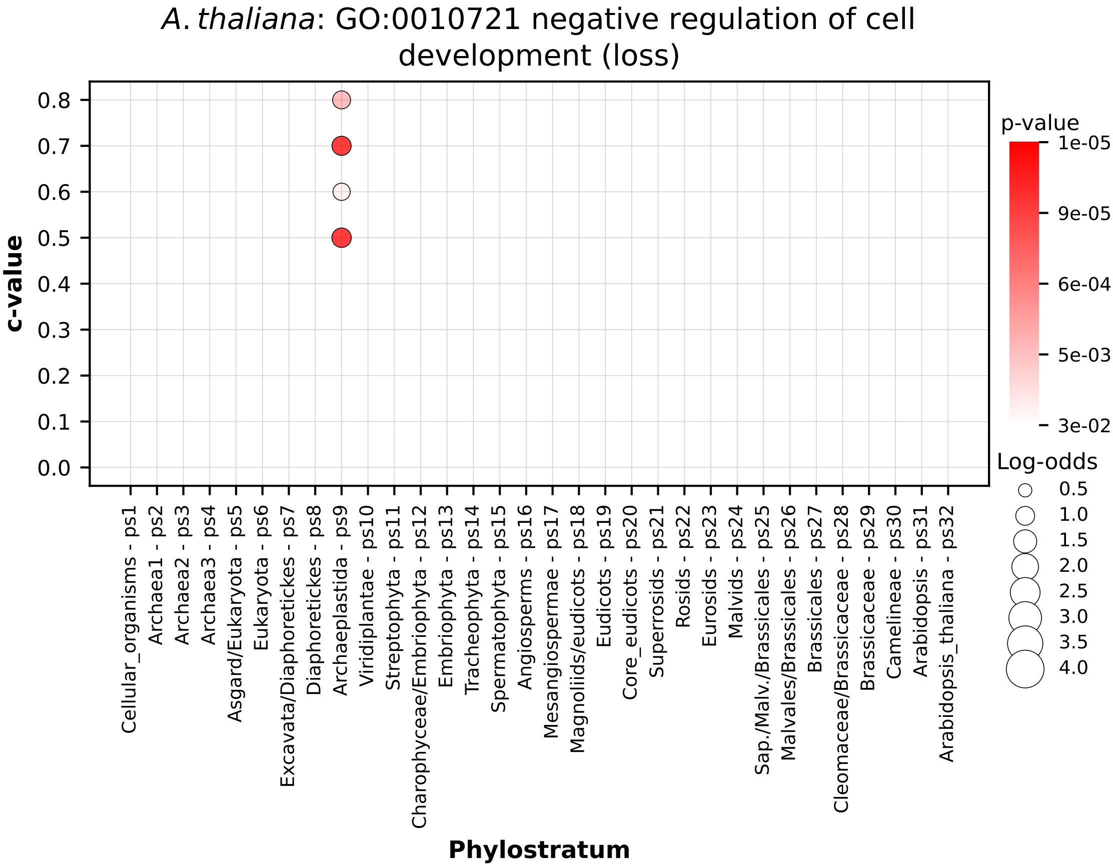
A. thaliana: GO:0010721 negative regulation of cell development (loss)
c-value
0.8
0.7
0.6
0.5
0.4
0.3
0.2
0.1
0.0
p-value
1e-05
9e-05
6e-04
5e-03
3e-02
Log-odds
0.5
1.0
1.5
2.0
2.5
3.0
3.5
4.0
Cellular_organisms - ps1
Archaea1 - ps2
Archaea2 - ps3
Archaea3 - ps4
Asgard/Eukaryota - ps5
Eukaryota - ps6
Excavata/Diaphoretickes - ps7
Diaphoretickes - ps8
Archaeplastida - ps9
Viridiplantae - ps10
Streptophyta - ps11
Charophyceae/Embriophyta - ps12
Embriophyta - ps13
Tracheophyta - ps14
Spermatophyta - ps15
Angiosperms - ps16
Mesangiospermae - ps17
Magnoliids/eudicots - ps18
Eudicots - ps19
Core_eudicots - ps20
Superrosids - ps21
Rosids - ps22
Eurosids - ps23
Malvids - ps24
Sap./Malv./Brassicales - ps25
Malvales/Brassicales - ps26
Brassicales - ps27
Cleomaceae/Brassicaceae - ps28
Brassicaceae - ps29
Camelineae - ps30
Arabidopsis - ps31
Arabidopsis_thaliana - ps32
Phylostratum

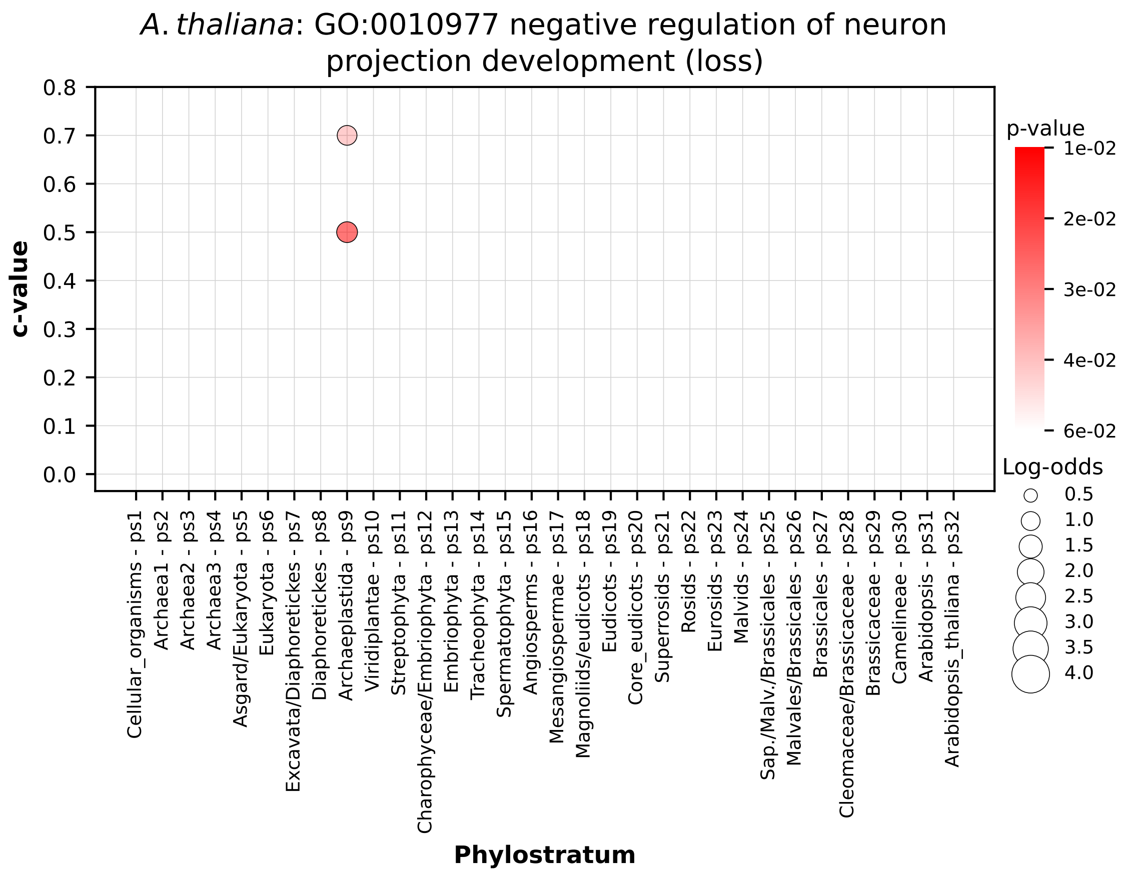

A. thaliana: GO:0010977 negative regulation of neuron projection development (loss)
c-value
0.8
0.7
0.6
0.5
0.4
0.3
0.2
0.1
0.0
p-value
1e-02
2e-02
3e-02
4e-02
6e-02
Log-odds
0.5
1.0
1.5
2.0
2.5
3.0
3.5
4.0
Cellular_organisms - ps1
Archaea1 - ps2
Archaea2 - ps3
Archaea3 - ps4
Asgard/Eukaryota - ps5
Eukaryota - ps6
Excavata/Diaphoretickes - ps7
Diaphoretickes - ps8
Archaeplastida - ps9
Viridiplantae - ps10
Streptophyta - ps11
Charophyceae/Embriophyta - ps12
Embriophyta - ps13
Tracheophyta - ps14
Spermatophyta - ps15
Angiosperms - ps16
Mesangiospermae - ps17
Magnoliids/eudicots - ps18
Eudicots - ps19
Core_eudicots - ps20
Superrosids - ps21
Rosids - ps22
Eurosids - ps23
Malvids - ps24
Sap./Malv./Brassicales - ps25
Malvales/Brassicales - ps26
Brassicales - ps27
Cleomaceae/Brassicaceae - ps28
Brassicaceae - ps29
Camelineae - ps30
Arabidopsis - ps31
Arabidopsis_thaliana - ps32
Phylostratum

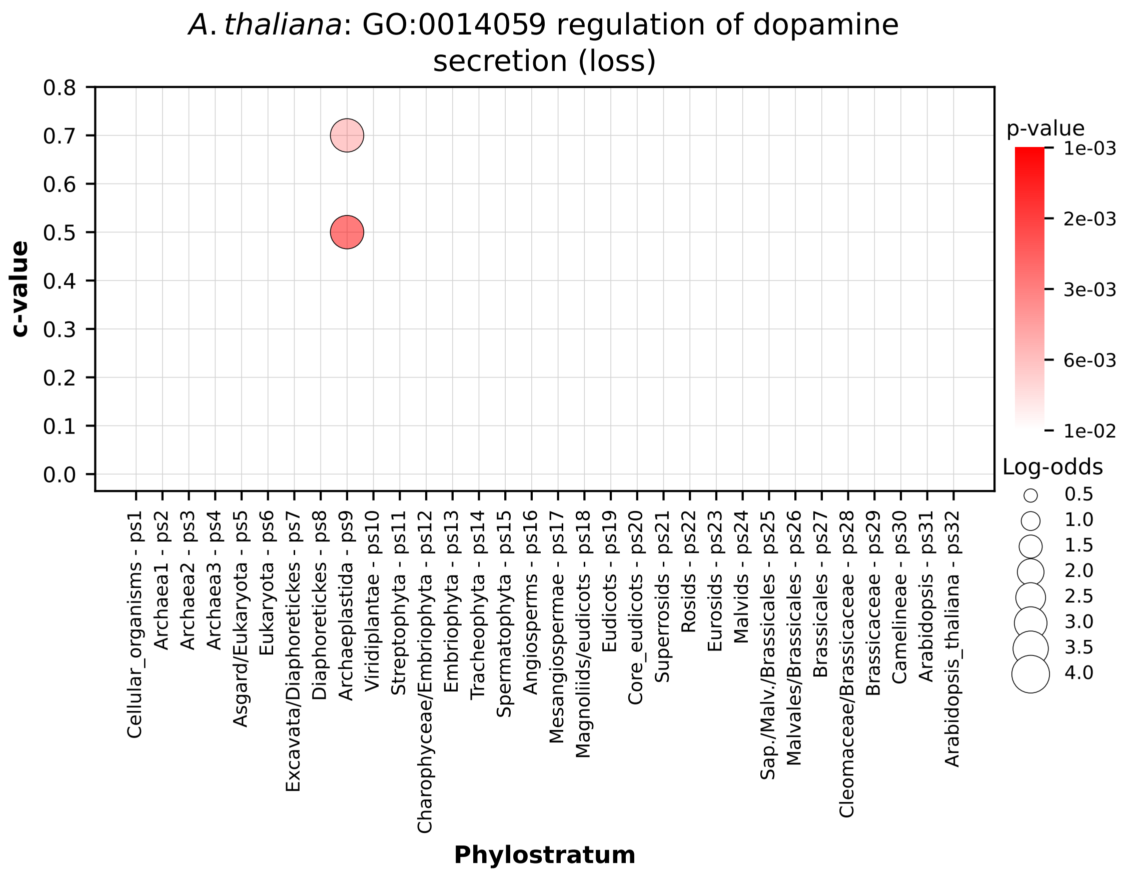
A. thaliana: GO:0014059 regulation of dopamine secretion (loss)
c-value
0.0
0.1
0.2
0.3
0.4
0.5
0.6
0.7
0.8
p-value
1e-03
2e-03
3e-03
6e-03
1e-02
Log-odds
0.5
1.0
1.5
2.0
2.5
3.0
3.5
4.0
Cellular_organisms - ps1
Archaea1 - ps2
Archaea2 - ps3
Archaea3 - ps4
Asgard/Eukaryota - ps5
Eukaryota - ps6
Excavata/Diaphoretickes - ps7
Diaphoretickes - ps8
Archaeplastida - ps9
Viridiplantae - ps10
Streptophyta - ps11
Charophyceae/Embriophyta - ps12
Embriophyta - ps13
Tracheophyta - ps14
Spermatophyta - ps15
Angiosperms - ps16
Mesangiospermae - ps17
Magnoliids/eudicots - ps18
Eudicots - ps19
Core_eudicots - ps20
Superrosids - ps21
Rosids - ps22
Eurosids - ps23
Malvids - ps24
Sap./Malv./Brassicales - ps25
Malvales/Brassicales - ps26
Brassicales - ps27
Cleomaceae/Brassicaceae - ps28
Brassicaceae - ps29
Camelineae - ps30
Arabidopsis - ps31
Arabidopsis_thaliana - ps32
Phylostratum

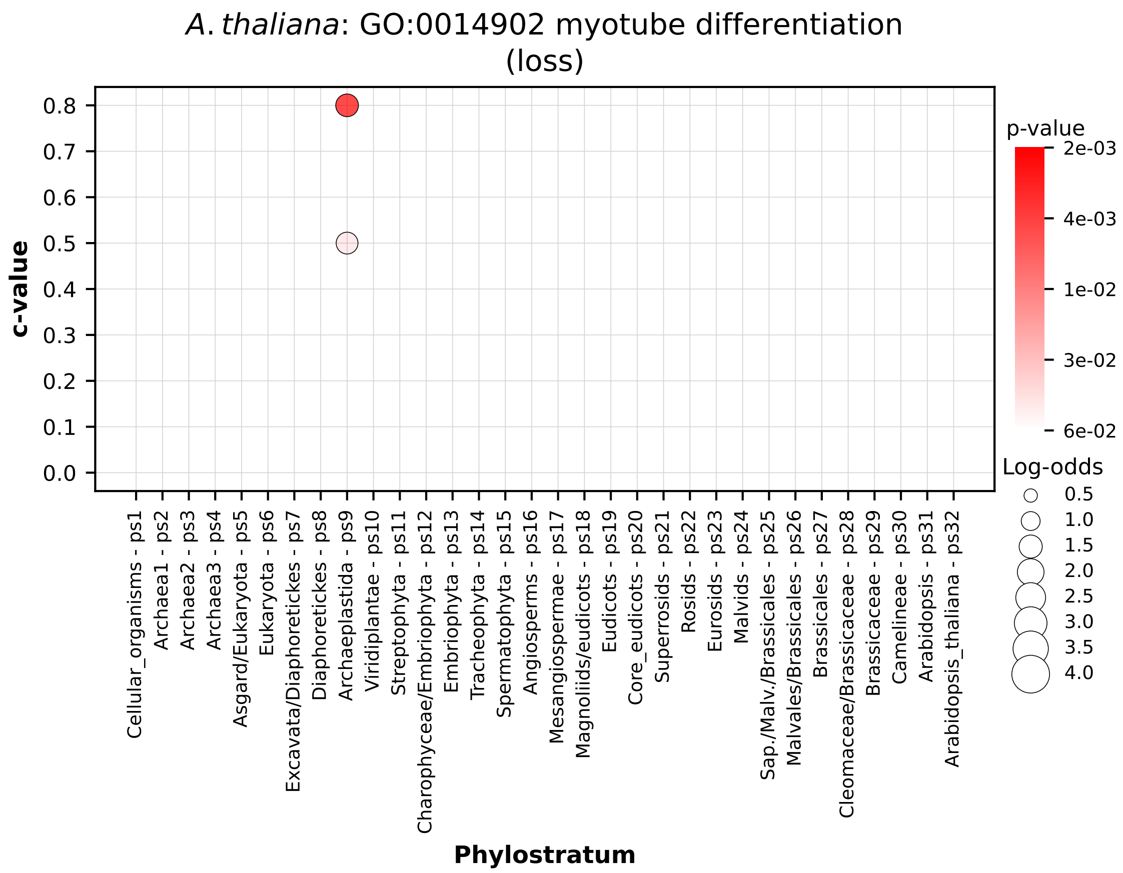

A. thaliana: GO:0014902 myotube differentiation
(loss)
c-value
0.0
0.1
0.2
0.3
0.4
0.5
0.6
0.7
0.8
p-value
2e-03
4e-03
1e-02
3e-02
6e-02
Log-odds
0.5
1.0
1.5
2.0
2.5
3.0
3.5
4.0
Cellular_organisms - ps1
Archaea1 - ps2
Archaea2 - ps3
Archaea3 - ps4
Asgard/Eukaryota - ps5
Eukaryota - ps6
Excavata/Diaphoretickes - ps7
Diaphoretickes - ps8
Archaeplastida - ps9
Viridiplantae - ps10
Streptophyta - ps11
Charophyceae/Embriophyta - ps12
Embriophyta - ps13
Tracheophyta - ps14
Spermatophyta - ps15
Angiosperms - ps16
Mesangiospermae - ps17
Magnoliids/eudicots - ps18
Eudicots - ps19
Core_eudicots - ps20
Superrosids - ps21
Rosids - ps22
Eurosids - ps23
Malvids - ps24
Sap./Malv./Brassicales - ps25
Malvales/Brassicales - ps26
Brassicales - ps27
Cleomaceae/Brassicaceae - ps28
Brassicaceae - ps29
Camelineae - ps30
Arabidopsis - ps31
Arabidopsis_thaliana - ps32
Phylostratum

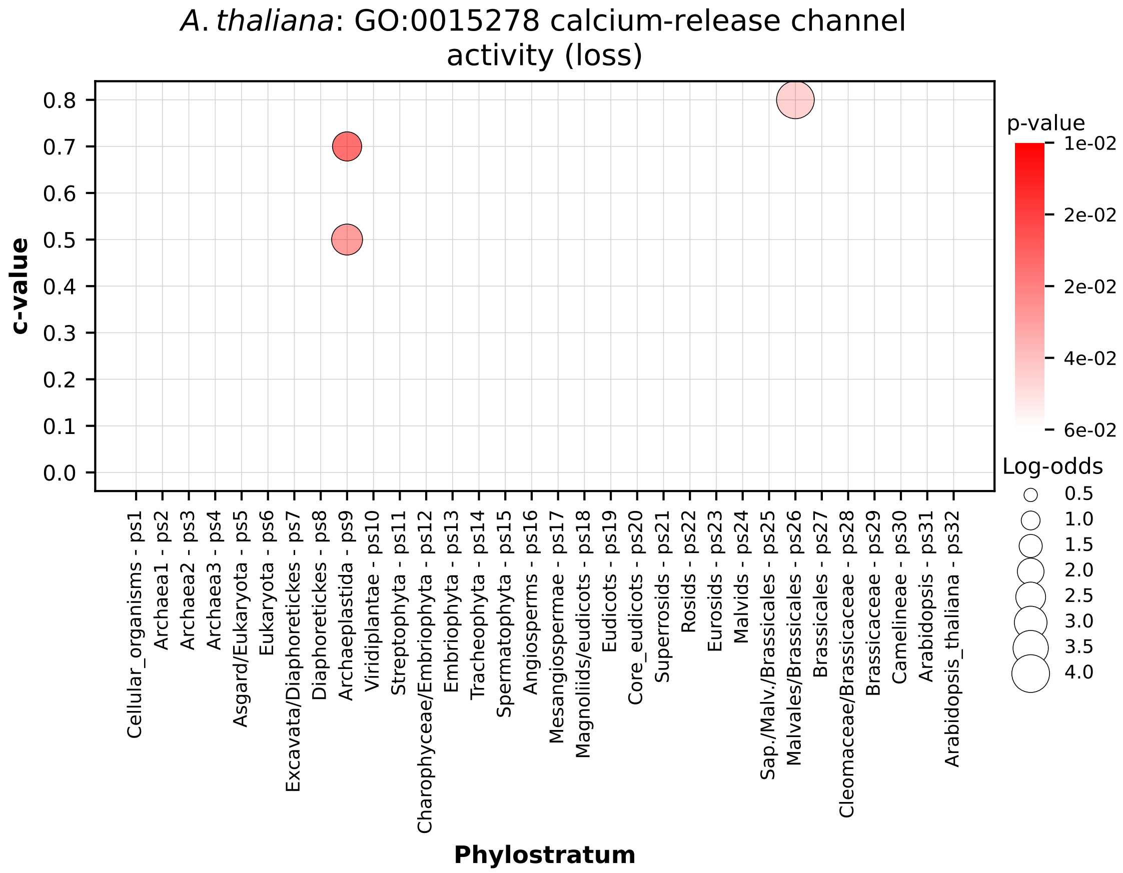
A. thaliana: GO:0015278 calcium-release channel activity (loss)
c-value
0.8
0.7
0.6
0.5
0.4
0.3
0.2
0.1
0.0
p-value
1e-02
2e-02
2e-02
4e-02
6e-02
Log-odds
0.5
1.0
1.5
2.0
2.5
3.0
3.5
4.0
Cellular_organisms - ps1
Archaea1 - ps2
Archaea2 - ps3
Archaea3 - ps4
Asgard/Eukaryota - ps5
Eukaryota - ps6
Excavata/Diaphoretickes - ps7
Diaphoretickes - ps8
Archaeplastida - ps9
Viridiplantae - ps10
Streptophyta - ps11
Charophyceae/Embriophyta - ps12
Embriophyta - ps13
Tracheophyta - ps14
Spermatophyta - ps15
Angiosperms - ps16
Mesangiospermae - ps17
Magnoliids/eudicots - ps18
Eudicots - ps19
Core_eudicots - ps20
Superrosids - ps21
Rosids - ps22
Eurosids - ps23
Malvids - ps24
Sap./Malv./Brassicales - ps25
Malvales/Brassicales - ps26
Brassicales - ps27
Cleomaceae/Brassicaceae - ps28
Brassicaceae - ps29
Camelineae - ps30
Arabidopsis - ps31
Arabidopsis_thaliana - ps32
Phylostratum

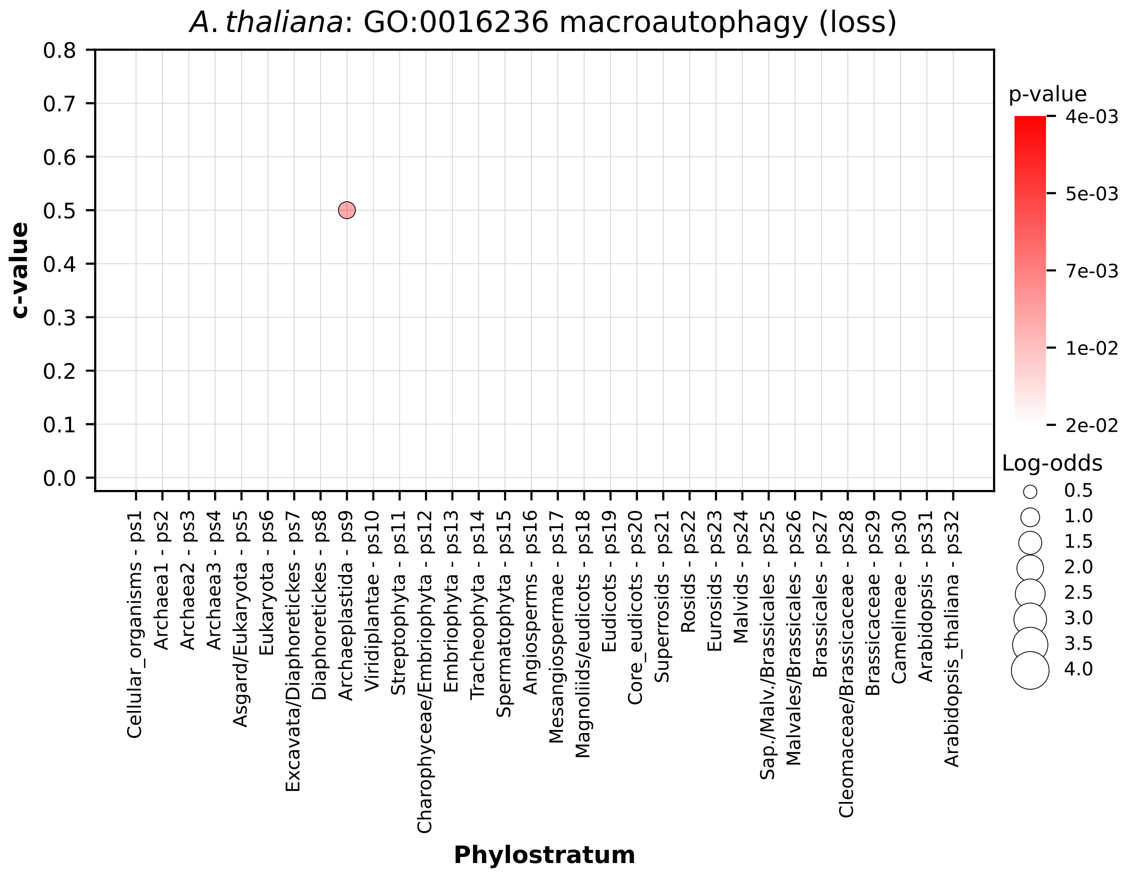

A. thaliana: GO:0016236 macroautophagy (loss)
c-value
0.8
0.7
0.6
0.5
0.4
0.3
0.2
0.1
0.0
p-value
4e-03
5e-03
7e-03
1e-02
2e-02
Log-odds
0.5
1.0
1.5
2.0
2.5
3.0
3.5
4.0
Cellular_organisms - ps1
Archaea1 - ps2
Archaea2 - ps3
Archaea3 - ps4
Asgard/Eukaryota - ps5
Eukaryota - ps6
Excavata/Diaphoretickes - ps7
Diaphoretickes - ps8
Archaeplastida - ps9
Viridiplantae - ps10
Streptophyta - ps11
Charophyceae/Embriophyta - ps12
Embriophyta - ps13
Tracheophyta - ps14
Spermatophyta - ps15
Angiosperms - ps16
Mesangiospermae - ps17
Magnoliids/eudicots - ps18
Eudicots - ps19
Core_eudicots - ps20
Superrosids - ps21
Rosids - ps22
Eurosids - ps23
Malvids - ps24
Sap./Malv./Brassicales - ps25
Malvales/Brassicales - ps26
Brassicales - ps27
Cleomaceae/Brassicaceae - ps28
Brassicaceae - ps29
Camelineae - ps30
Arabidopsis - ps31
Arabidopsis_thaliana - ps32
Phylostratum

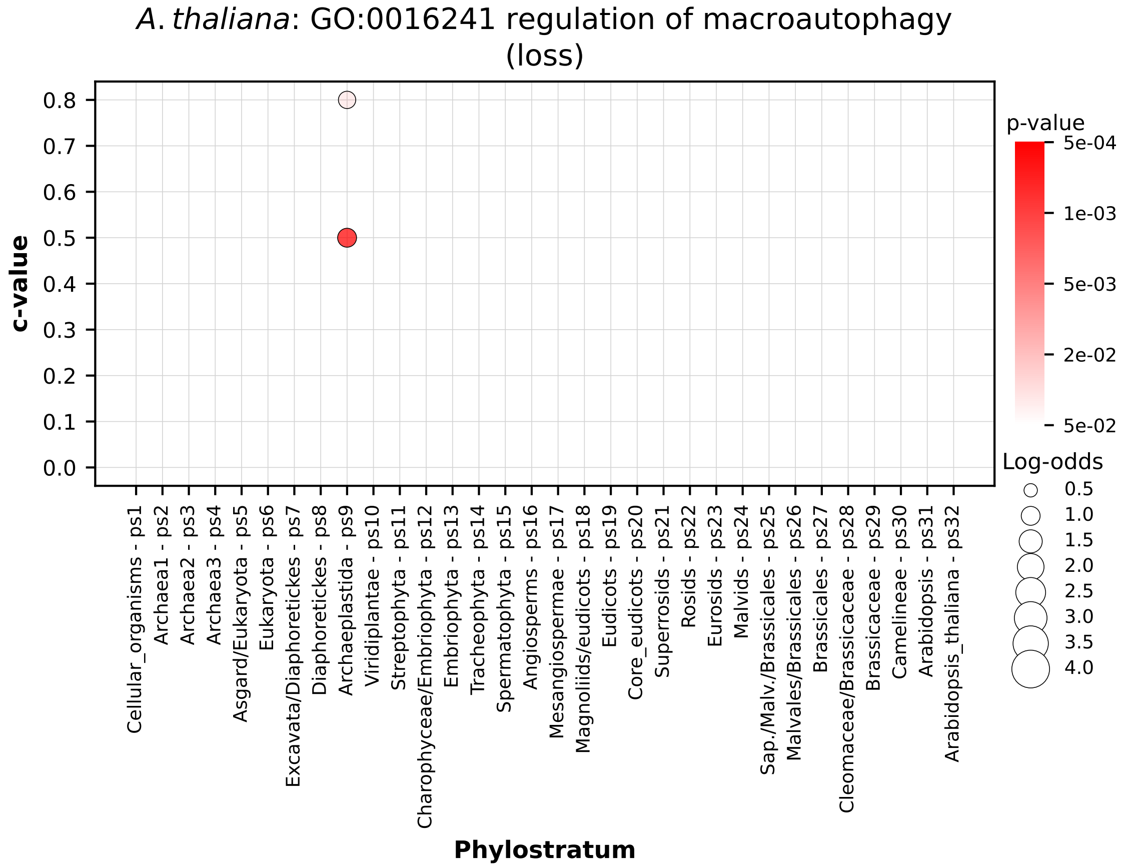
A. thaliana: GO:0016241 regulation of macroautophagy
(loss)
c-value
0.8
0.7
0.6
0.5
0.4
0.3
0.2
0.1
0.0
Phylostratum
Cellular_organisms - ps1
Archaea1 - ps2
Archaea2 - ps3
Archaea3 - ps4
Asgard/Eukaryota - ps5
Eukaryota - ps6
Excavata/Diaphoretickes - ps7
Diaphoretickes - ps8
Archaeplastida - ps9
Viridiplantae - ps10
Streptophyta - ps11
Charophyceae/Embriophyta - ps12
Embriophyta - ps13
Tracheophyta - ps14
Spermatophyta - ps15
Angiosperms - ps16
Mesangiospermae - ps17
Magnoliids/eudicots - ps18
Eudicots - ps19
Core_eudicots - ps20
Superrosids - ps21
Rosids - ps22
Eurosids - ps23
Malvids - ps24
Sap./Malv./Brassicales - ps25
Malvales/Brassicales - ps26
Brassicales - ps27
Cleomaceae/Brassicaceae - ps28
Brassicaceae - ps29
Camelineae - ps30
Arabidopsis - ps31
Arabidopsis_thaliana - ps32
p-value
5e-04
1e-03
5e-03
2e-02
5e-02
Log-odds
0.5
1.0
1.5
2.0
2.5
3.0
3.5
4.0

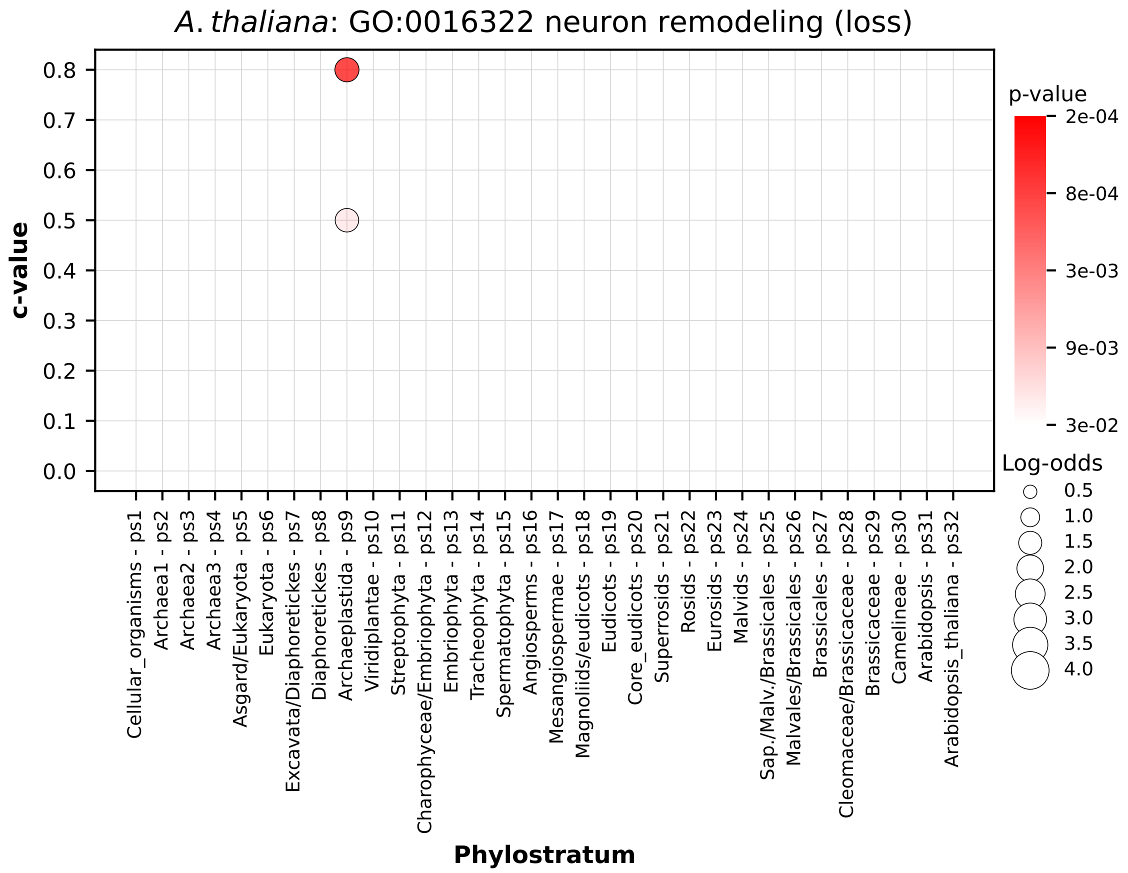

A. thaliana: GO:0016322 neuron remodeling (loss)
c-value
0.0
0.1
0.2
0.3
0.4
0.5
0.6
0.7
0.8
p-value
2e-04
8e-04
3e-03
9e-03
3e-02
Log-odds
0.5
1.0
1.5
2.0
2.5
3.0
3.5
4.0
Cellular_organisms - ps1
Archaea1 - ps2
Archaea2 - ps3
Archaea3 - ps4
Asgard/Eukaryota - ps5
Eukaryota - ps6
Excavata/Diaphoretickes - ps7
Diaphoretickes - ps8
Archaeplastida - ps9
Viridiplantae - ps10
Streptophyta - ps11
Charophyceae/Embriophyta - ps12
Embriophyta - ps13
Tracheophyta - ps14
Spermatophyta - ps15
Angiosperms - ps16
Mesangiospermae - ps17
Magnoliids/eudicots - ps18
Eudicots - ps19
Core_eudicots - ps20
Superrosids - ps21
Rosids - ps22
Eurosids - ps23
Malvids - ps24
Sap./Malv./Brassicales - ps25
Malvales/Brassicales - ps26
Brassicales - ps27
Cleomaceae/Brassicaceae - ps28
Brassicaceae - ps29
Camelineae - ps30
Arabidopsis - ps31
Arabidopsis_thaliana - ps32
Phylostratum

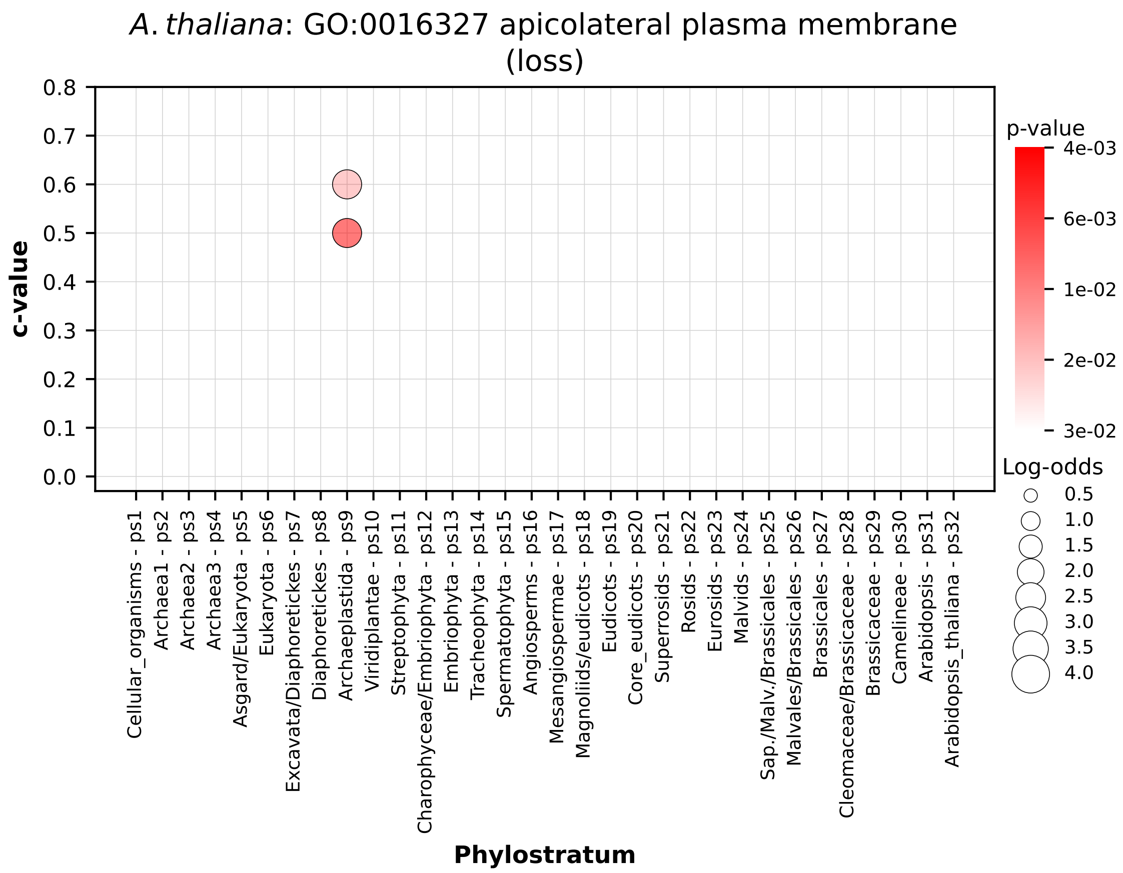

A. thaliana: GO:0016327 apicolateral plasma membrane
(loss)
c-value
0.8
0.7
0.6
0.5
0.4
0.3
0.2
0.1
0.0
p-value
4e-03
6e-03
1e-02
2e-02
3e-02
Log-odds
0.5
1.0
1.5
2.0
2.5
3.0
3.5
4.0
Cellular_organisms - ps1
Archaea1 - ps2
Archaea2 - ps3
Archaea3 - ps4
Asgard/Eukaryota - ps5
Eukaryota - ps6
Excavata/Diaphoretickes - ps7
Diaphoretickes - ps8
Archaeplastida - ps9
Viridiplantae - ps10
Streptophyta - ps11
Charophyceae/Embriophyta - ps12
Embriophyta - ps13
Tracheophyta - ps14
Spermatophyta - ps15
Angiosperms - ps16
Mesangiospermae - ps17
Magnoliids/eudicots - ps18
Eudicots - ps19
Core_eudicots - ps20
Superrosids - ps21
Rosids - ps22
Eurosids - ps23
Malvids - ps24
Sap./Malv./Brassicales - ps25
Malvales/Brassicales - ps26
Brassicales - ps27
Cleomaceae/Brassicaceae - ps28
Brassicaceae - ps29
Camelineae - ps30
Arabidopsis - ps31
Arabidopsis_thaliana - ps32
Phylostratum

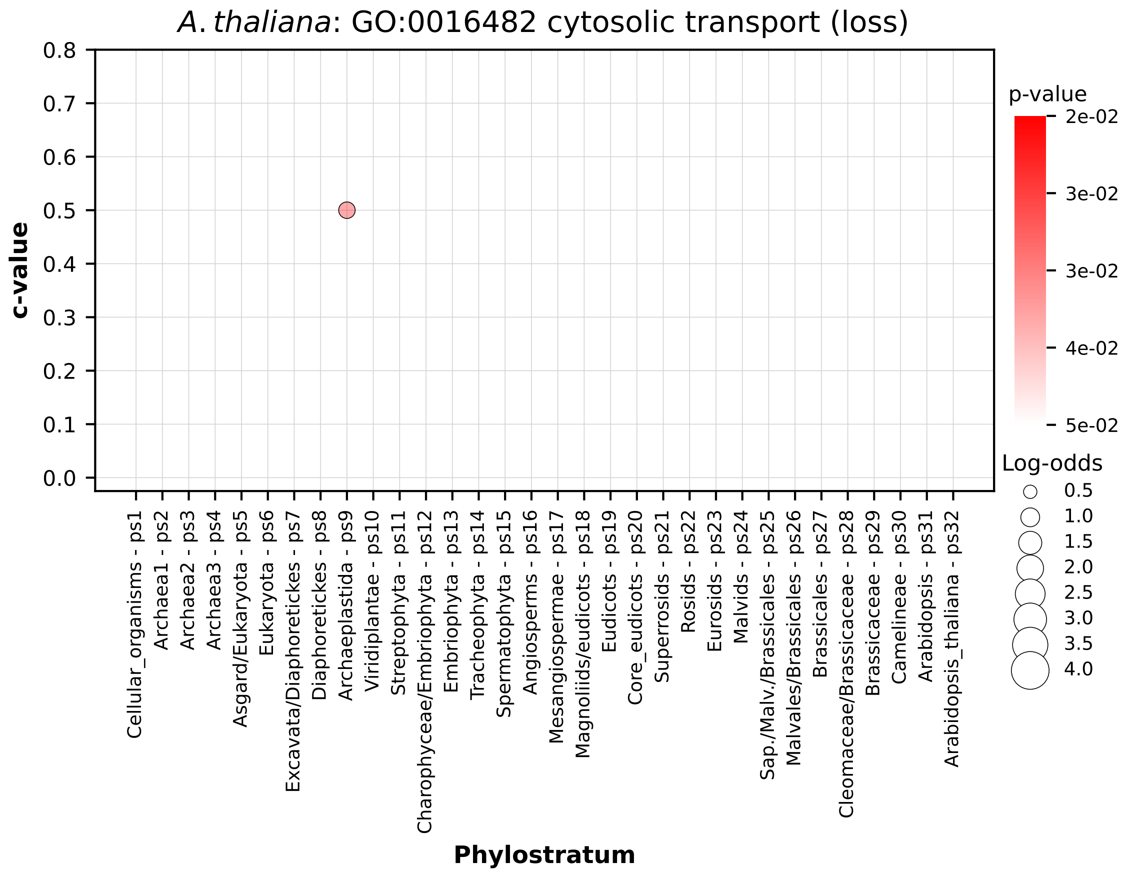
A. thaliana: GO:0016482 cytosolic transport (loss)
c-value
0.0
0.1
0.2
0.3
0.4
0.5
0.6
0.7
0.8
Phylostratum
Cellular_organisms - ps1
Archaea1 - ps2
Archaea2 - ps3
Archaea3 - ps4
Asgard/Eukaryota - ps5
Eukaryota - ps6
Excavata/Diaphoretickes - ps7
Diaphoretickes - ps8
Archaeplastida - ps9
Viridiplantae - ps10
Streptophyta - ps11
Charophyceae/Embriophyta - ps12
Embriophyta - ps13
Tracheophyta - ps14
Spermatophyta - ps15
Angiosperms - ps16
Mesangiospermae - ps17
Magnoliids/eudicots - ps18
Eudicots - ps19
Core_eudicots - ps20
Superrosids - ps21
Rosids - ps22
Eurosids - ps23
Malvids - ps24
Sap./Malv./Brassicales - ps25
Malvales/Brassicales - ps26
Brassicales - ps27
Cleomaceae/Brassicaceae - ps28
Brassicaceae - ps29
Camelineae - ps30
Arabidopsis - ps31
Arabidopsis_thaliana - ps32
p-value
2e-02
3e-02
3e-02
4e-02
5e-02
Log-odds
0.5
1.0
1.5
2.0
2.5
3.0
3.5
4.0

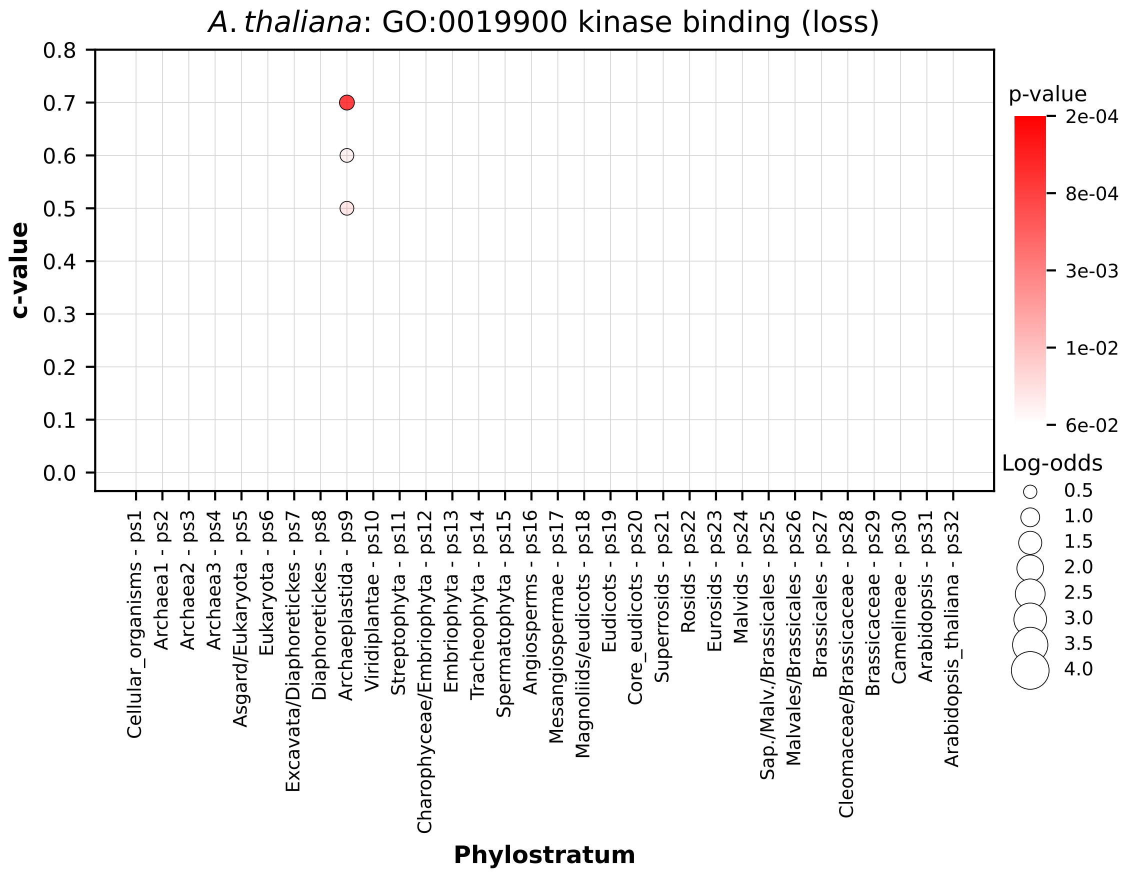

A. thaliana: GO:0019900 kinase binding (loss)
c-value
0.8
0.7
0.6
0.5
0.4
0.3
0.2
0.1
0.0
Cellular_organisms - ps1
Archaea1 - ps2
Archaea2 - ps3
Archaea3 - ps4
Asgard/Eukaryota - ps5
Eukaryota - ps6
Excavata/Diaphoretickes - ps7
Diaphoretickes - ps8
Archaeplastida - ps9
Viridiplantae - ps10
Streptophyta - ps11
Charophyceae/Embriophyta - ps12
Embriophyta - ps13
Tracheophyta - ps14
Spermatophyta - ps15
Angiosperms - ps16
Mesangiospermae - ps17
Magnoliids/eudicots - ps18
Eudicots - ps19
Core_eudicots - ps20
Superrosids - ps21
Rosids - ps22
Eurosids - ps23
Malvids - ps24
Sap./Malv./Brassicales - ps25
Malvales/Brassicales - ps26
Brassicales - ps27
Cleomaceae/Brassicaceae - ps28
Brassicaceae - ps29
Camelineae - ps30
Arabidopsis - ps31
Arabidopsis_thaliana - ps32
Phylostratum
p-value
2e-04
8e-04
3e-03
1e-02
6e-02
Log-odds
0.5
1.0
1.5
2.0
2.5
3.0
3.5
4.0

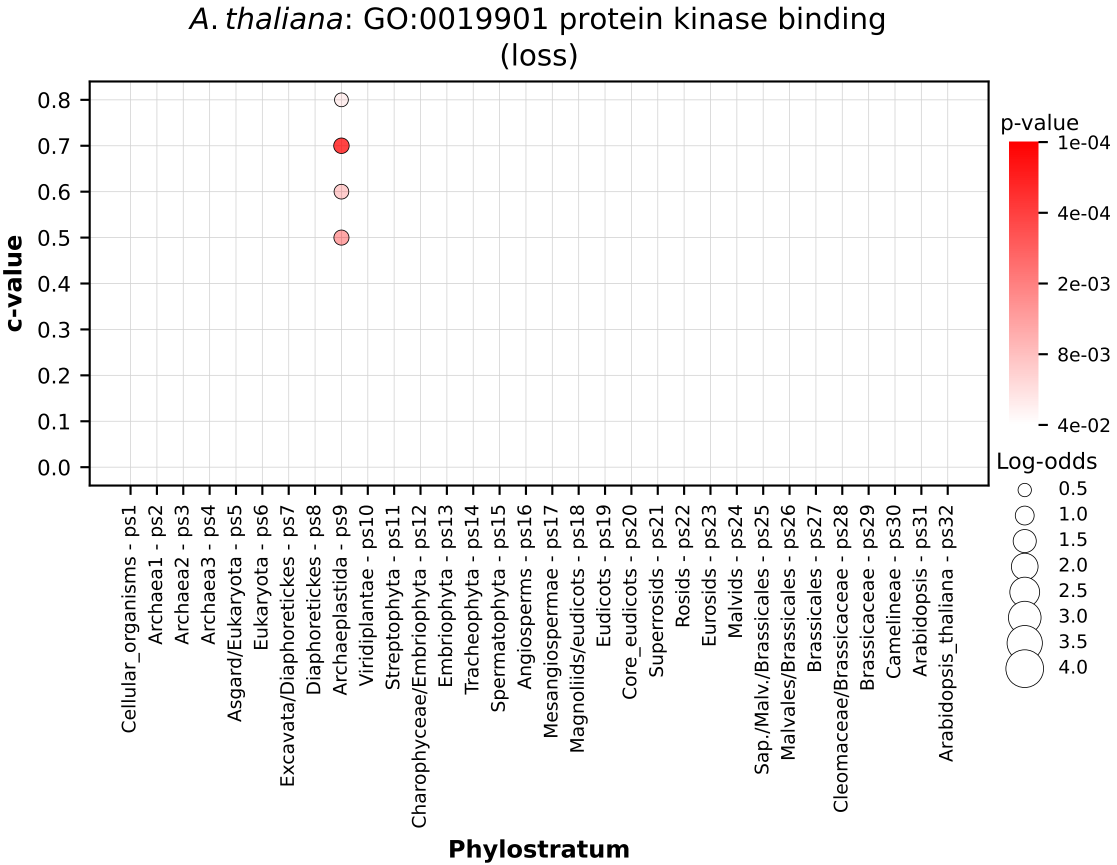
A. thaliana: GO:0019901 protein kinase binding
(loss)
c-value
0.0
0.1
0.2
0.3
0.4
0.5
0.6
0.7
0.8
p-value
1e-04
4e-04
2e-03
8e-03
4e-02
Log-odds
0.5
1.0
1.5
2.0
2.5
3.0
3.5
4.0
Cellular_organisms - ps1
Archaea1 - ps2
Archaea2 - ps3
Archaea3 - ps4
Asgard/Eukaryota - ps5
Eukaryota - ps6
Excavata/Diaphoretickes - ps7
Diaphoretickes - ps8
Archaeplastida - ps9
Viridiplantae - ps10
Streptophyta - ps11
Charophyceae/Embriophyta - ps12
Embriophyta - ps13
Tracheophyta - ps14
Spermatophyta - ps15
Angiosperms - ps16
Mesangiospermae - ps17
Magnoliids/eudicots - ps18
Eudicots - ps19
Core_eudicots - ps20
Superrosids - ps21
Rosids - ps22
Eurosids - ps23
Malvids - ps24
Sap./Malv./Brassicales - ps25
Malvales/Brassicales - ps26
Brassicales - ps27
Cleomaceae/Brassicaceae - ps28
Brassicaceae - ps29
Camelineae - ps30
Arabidopsis - ps31
Arabidopsis_thaliana - ps32
Phylostratum

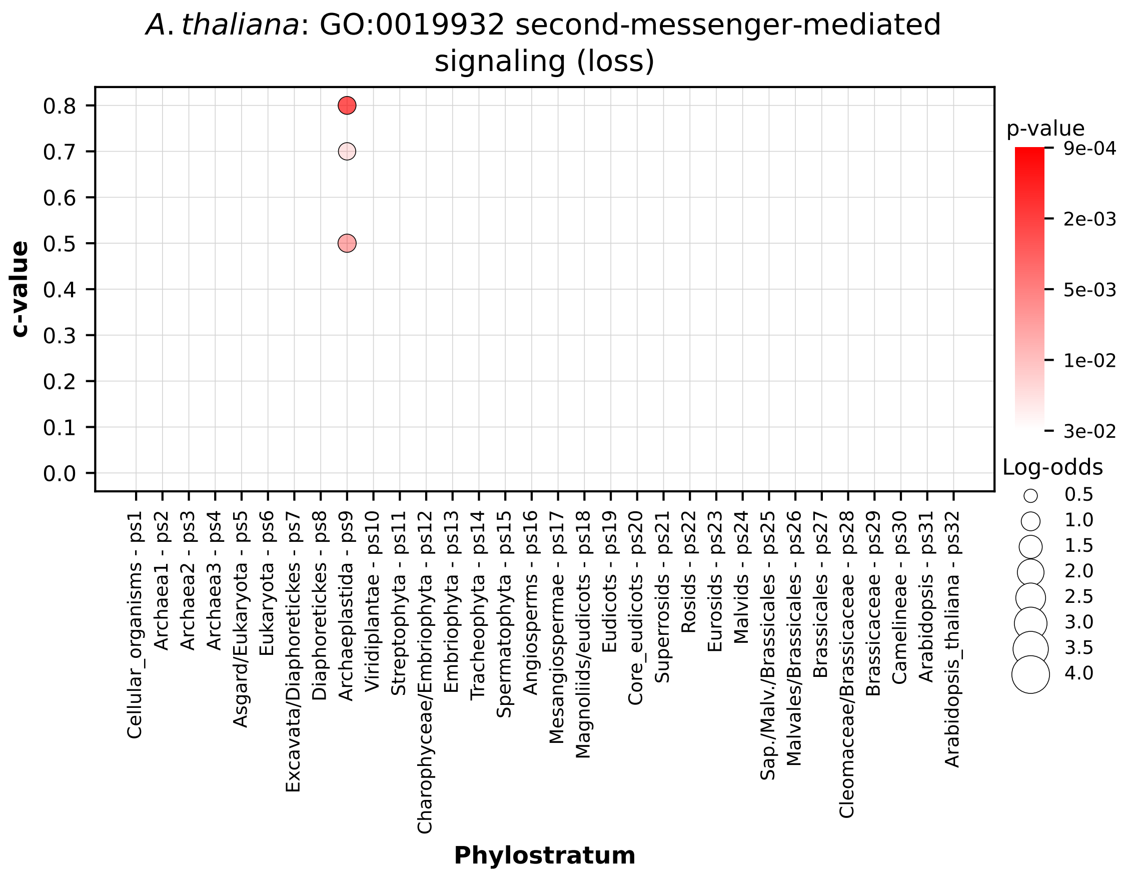

A. thaliana: GO:0019932 second-messenger-mediated signaling (loss)
c-value
0.8
0.7
0.6
0.5
0.4
0.3
0.2
0.1
0.0
p-value
9e-04
2e-03
5e-03
1e-02
3e-02
Log-odds
0.5
1.0
1.5
2.0
2.5
3.0
3.5
4.0
Cellular_organisms - ps1
Archaea1 - ps2
Archaea2 - ps3
Archaea3 - ps4
Asgard/Eukaryota - ps5
Eukaryota - ps6
Excavata/Diaphoretickes - ps7
Diaphoretickes - ps8
Archaeplastida - ps9
Viridiplantae - ps10
Streptophyta - ps11
Charophyceae/Embriophyta - ps12
Embriophyta - ps13
Tracheophyta - ps14
Spermatophyta - ps15
Angiosperms - ps16
Mesangiospermae - ps17
Magnoliids/eudicots - ps18
Eudicots - ps19
Core_eudicots - ps20
Superrosids - ps21
Rosids - ps22
Eurosids - ps23
Malvids - ps24
Sap./Malv./Brassicales - ps25
Malvales/Brassicales - ps26
Brassicales - ps27
Cleomaceae/Brassicaceae - ps28
Brassicaceae - ps29
Camelineae - ps30
Arabidopsis - ps31
Arabidopsis_thaliana - ps32
Phylostratum

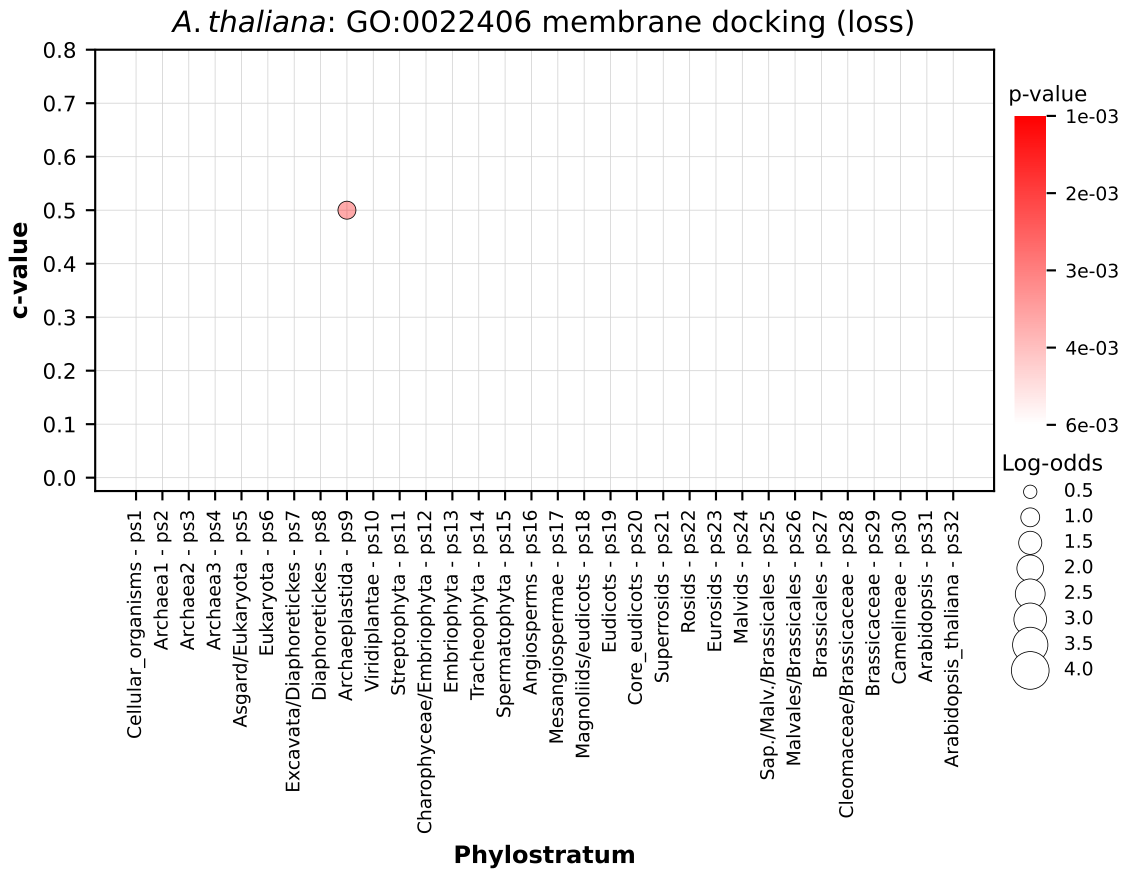

A. thaliana: GO:0022406 membrane docking (loss)
c-value
0.8
0.7
0.6
0.5
0.4
0.3
0.2
0.1
0.0
p-value
1e-03
2e-03
3e-03
4e-03
6e-03
Log-odds
0.5
1.0
1.5
2.0
2.5
3.0
3.5
4.0
Cellular_organisms - ps1
Archaea1 - ps2
Archaea2 - ps3
Archaea3 - ps4
Asgard/Eukaryota - ps5
Eukaryota - ps6
Excavata/Diaphoretickes - ps7
Diaphoretickes - ps8
Archaeplastida - ps9
Viridiplantae - ps10
Streptophyta - ps11
Charophyceae/Embriophyta - ps12
Embriophyta - ps13
Tracheophyta - ps14
Spermatophyta - ps15
Angiosperms - ps16
Mesangiospermae - ps17
Magnoliids/eudicots - ps18
Eudicots - ps19
Core_eudicots - ps20
Superrosids - ps21
Rosids - ps22
Eurosids - ps23
Malvids - ps24
Sap./Malv./Brassicales - ps25
Malvales/Brassicales - ps26
Brassicales - ps27
Cleomaceae/Brassicaceae - ps28
Brassicaceae - ps29
Camelineae - ps30
Arabidopsis - ps31
Arabidopsis_thaliana - ps32
Phylostratum

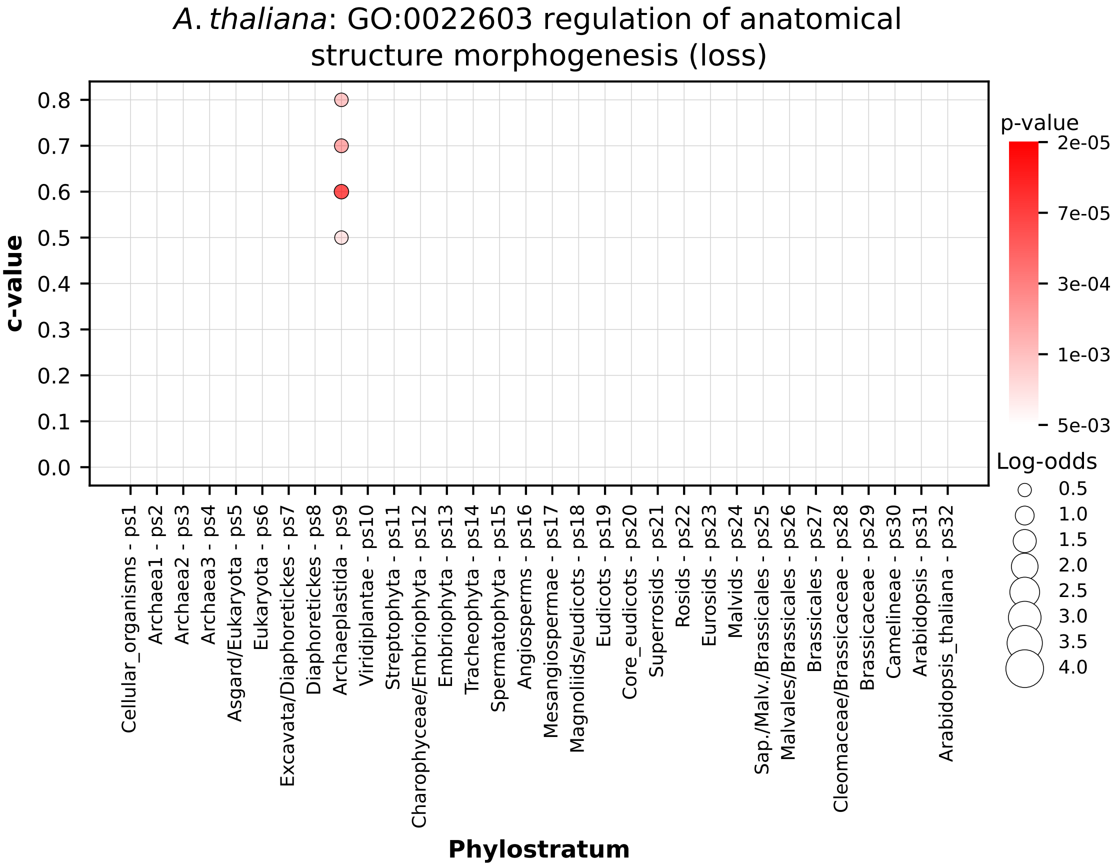

A. thaliana: GO:0022603 regulation of anatomical structure morphogenesis (loss)
c-value
0.0
0.1
0.2
0.3
0.4
0.5
0.6
0.7
0.8
p-value
2e-05
7e-05
3e-04
1e-03
5e-03
Log-odds
0.5
1.0
1.5
2.0
2.5
3.0
3.5
4.0
Cellular_organisms - ps1
Archaea1 - ps2
Archaea2 - ps3
Archaea3 - ps4
Asgard/Eukaryota - ps5
Eukaryota - ps6
Excavata/Diaphoretickes - ps7
Diaphoretickes - ps8
Archaeplastida - ps9
Viridiplantae - ps10
Streptophyta - ps11
Charophyceae/Embriophyta - ps12
Embriophyta - ps13
Tracheophyta - ps14
Spermatophyta - ps15
Angiosperms - ps16
Mesangiospermae - ps17
Magnoliids/eudicots - ps18
Eudicots - ps19
Core_eudicots - ps20
Superrosids - ps21
Rosids - ps22
Eurosids - ps23
Malvids - ps24
Sap./Malv./Brassicales - ps25
Malvales/Brassicales - ps26
Brassicales - ps27
Cleomaceae/Brassicaceae - ps28
Brassicaceae - ps29
Camelineae - ps30
Arabidopsis - ps31
Arabidopsis_thaliana - ps32
Phylostratum

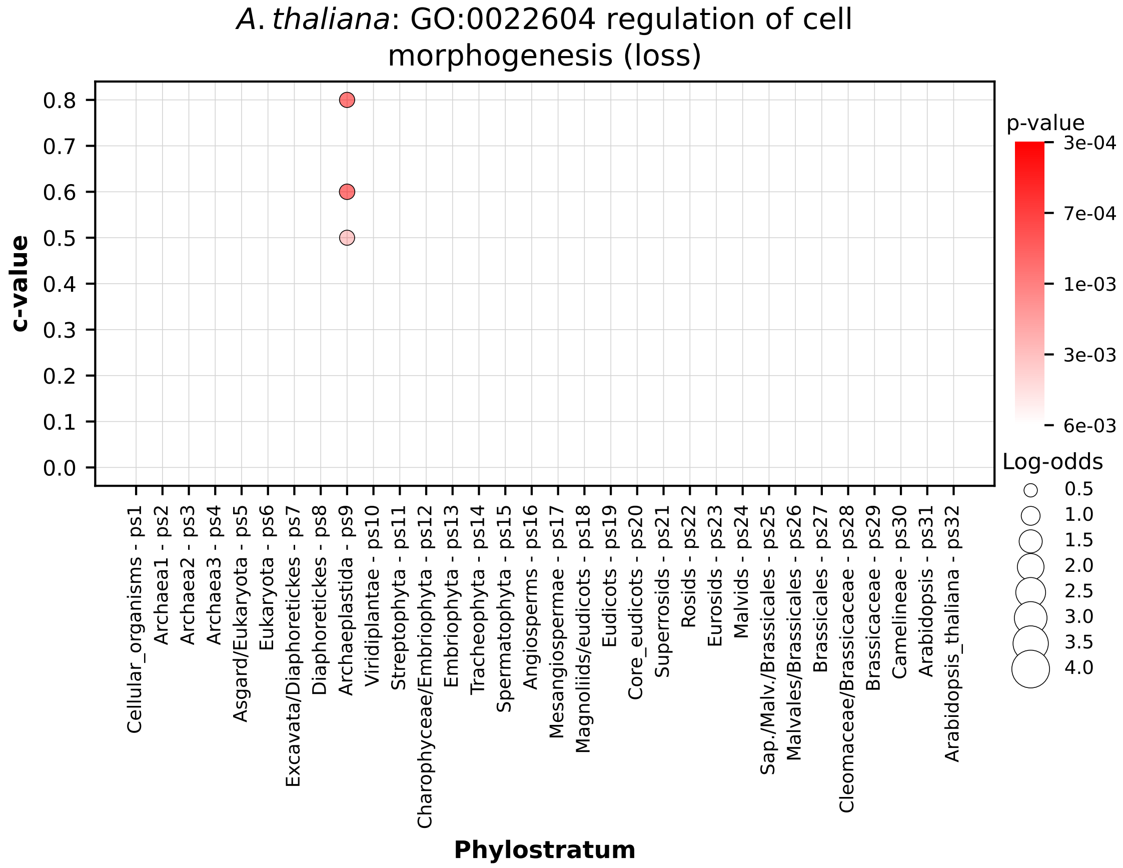

A. thaliana: GO:0022604 regulation of cell morphogenesis (loss)
c-value
0.0
0.1
0.2
0.3
0.4
0.5
0.6
0.7
0.8
Phylostratum
Cellular_organisms - ps1
Archaea1 - ps2
Archaea2 - ps3
Archaea3 - ps4
Asgard/Eukaryota - ps5
Eukaryota - ps6
Excavata/Diaphoretickes - ps7
Diaphoretickes - ps8
Archaeplastida - ps9
Viridiplantae - ps10
Streptophyta - ps11
Charophyceae/Embriophyta - ps12
Embriophyta - ps13
Tracheophyta - ps14
Spermatophyta - ps15
Angiosperms - ps16
Mesangiospermae - ps17
Magnoliids/eudicots - ps18
Eudicots - ps19
Core_eudicots - ps20
Superrosids - ps21
Rosids - ps22
Eurosids - ps23
Malvids - ps24
Sap./Malv./Brassicales - ps25
Malvales/Brassicales - ps26
Brassicales - ps27
Cleomaceae/Brassicaceae - ps28
Brassicaceae - ps29
Camelineae - ps30
Arabidopsis - ps31
Arabidopsis_thaliana - ps32
p-value
3e-04
7e-04
1e-03
3e-03
6e-03
Log-odds
0.5
1.0
1.5
2.0
2.5
3.0
3.5
4.0

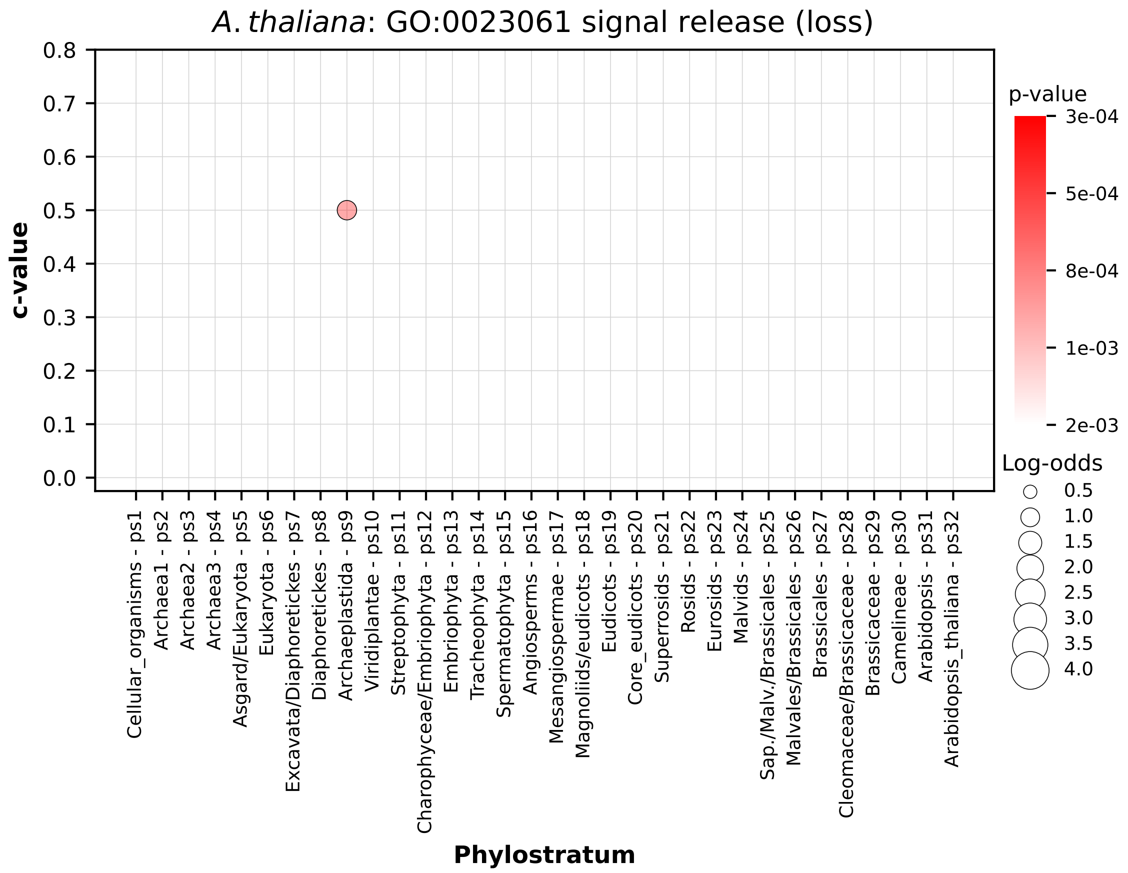
A. thaliana: GO:0023061 signal release (loss)
c-value
0.8
0.7
0.6
0.5
0.4
0.3
0.2
0.1
0.0
p-value
3e-04
5e-04
8e-04
1e-03
2e-03
Log-odds
0.5
1.0
1.5
2.0
2.5
3.0
3.5
4.0
Cellular_organisms - ps1
Archaea1 - ps2
Archaea2 - ps3
Archaea3 - ps4
Asgard/Eukaryota - ps5
Eukaryota - ps6
Excavata/Diaphoretickes - ps7
Diaphoretickes - ps8
Archaeplastida - ps9
Viridiplantae - ps10
Streptophyta - ps11
Charophyceae/Embriophyta - ps12
Embriophyta - ps13
Tracheophyta - ps14
Spermatophyta - ps15
Angiosperms - ps16
Mesangiospermae - ps17
Magnoliids/eudicots - ps18
Eudicots - ps19
Core_eudicots - ps20
Superrosids - ps21
Rosids - ps22
Eurosids - ps23
Malvids - ps24
Sap./Malv./Brassicales - ps25
Malvales/Brassicales - ps26
Brassicales - ps27
Cleomaceae/Brassicaceae - ps28
Brassicaceae - ps29
Camelineae - ps30
Arabidopsis - ps31
Arabidopsis_thaliana - ps32
Phylostratum

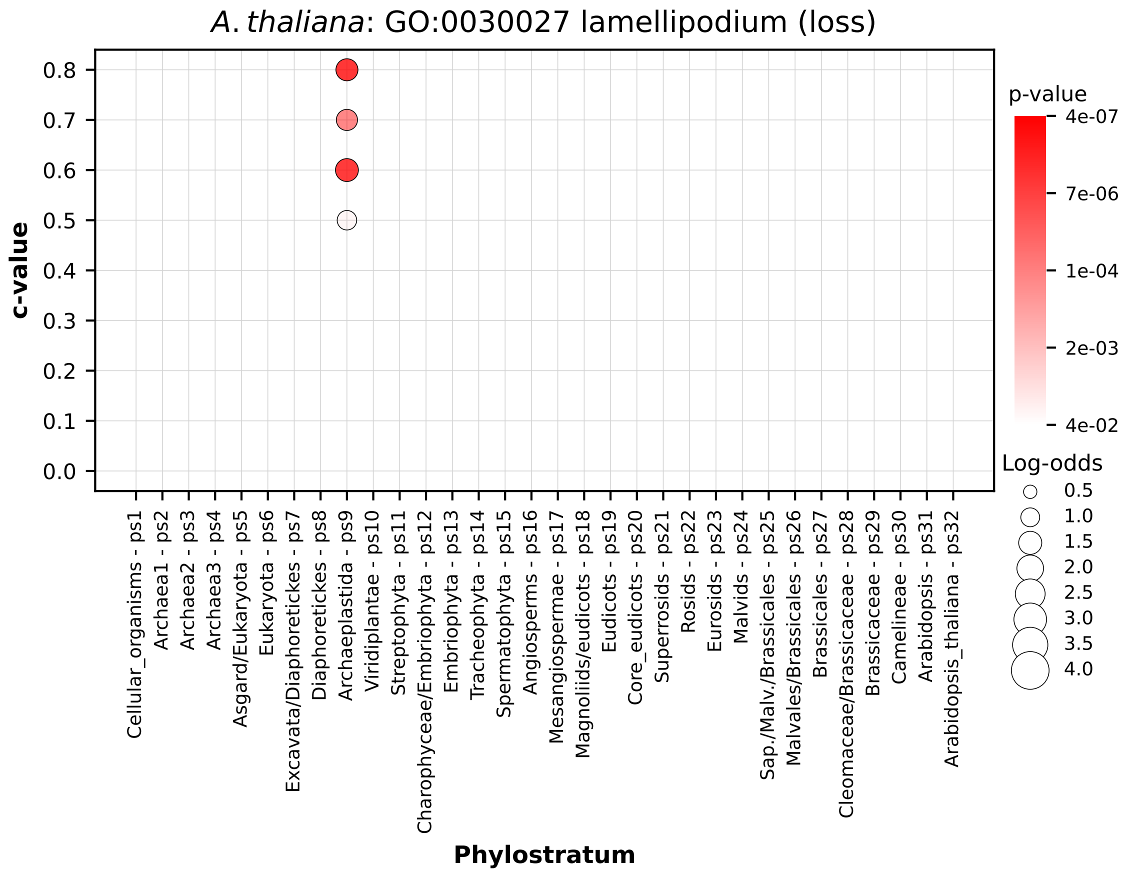

A. thaliana: GO:0030027 lamellipodium (loss)
c-value
0.8
0.7
0.6
0.5
0.4
0.3
0.2
0.1
0.0
p-value
4e-07
7e-06
1e-04
2e-03
4e-02
Log-odds
0.5
1.0
1.5
2.0
2.5
3.0
3.5
4.0
Cellular_organisms - ps1
Archaea1 - ps2
Archaea2 - ps3
Archaea3 - ps4
Asgard/Eukaryota - ps5
Eukaryota - ps6
Excavata/Diaphoretickes - ps7
Diaphoretickes - ps8
Archaeplastida - ps9
Viridiplantae - ps10
Streptophyta - ps11
Charophyceae/Embriophyta - ps12
Embriophyta - ps13
Tracheophyta - ps14
Spermatophyta - ps15
Angiosperms - ps16
Mesangiospermae - ps17
Magnoliids/eudicots - ps18
Eudicots - ps19
Core_eudicots - ps20
Superrosids - ps21
Rosids - ps22
Eurosids - ps23
Malvids - ps24
Sap./Malv./Brassicales - ps25
Malvales/Brassicales - ps26
Brassicales - ps27
Cleomaceae/Brassicaceae - ps28
Brassicaceae - ps29
Camelineae - ps30
Arabidopsis - ps31
Arabidopsis_thaliana - ps32
Phylostratum

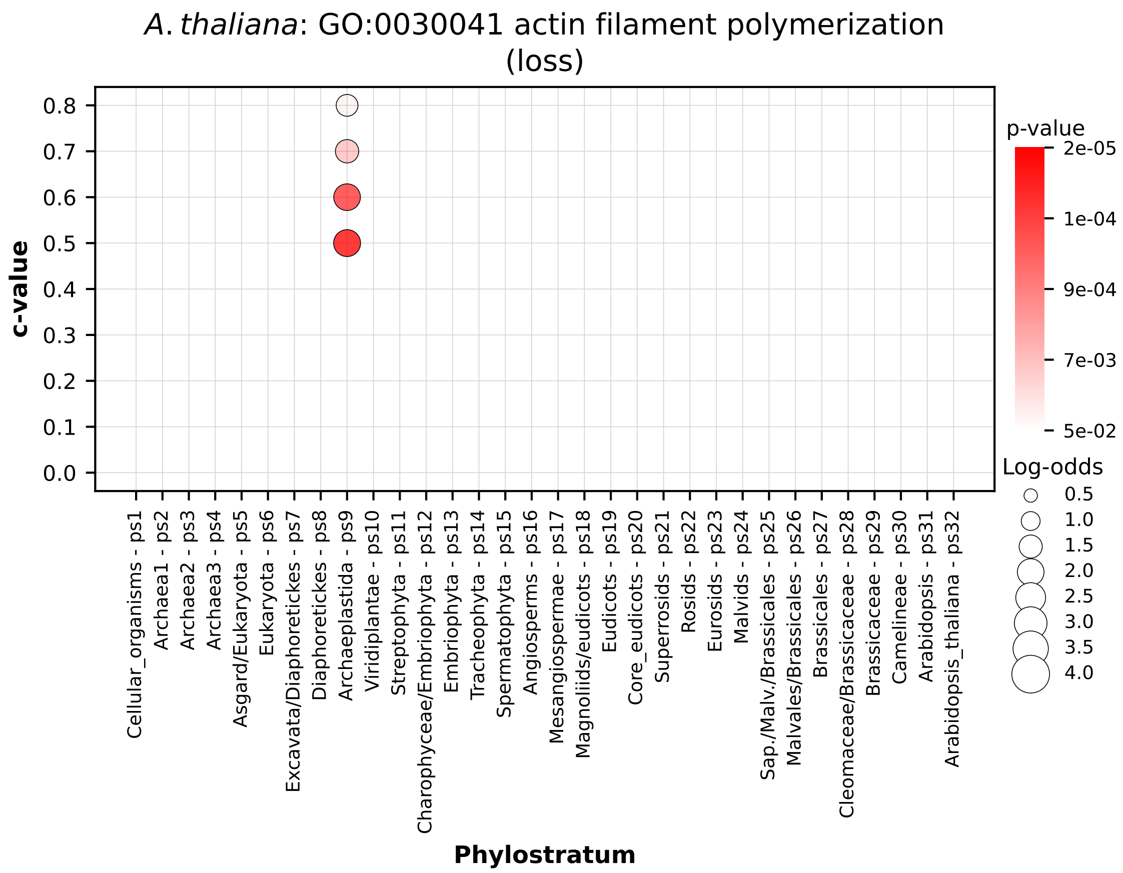

A. thaliana: GO:0030041 actin filament polymerization
(loss)
c-value
0.0
0.1
0.2
0.3
0.4
0.5
0.6
0.7
0.8
p-value
2e-05
1e-04
9e-04
7e-03
5e-02
Log-odds
0.5
1.0
1.5
2.0
2.5
3.0
3.5
4.0
Cellular_organisms - ps1
Archaea1 - ps2
Archaea2 - ps3
Archaea3 - ps4
Asgard/Eukaryota - ps5
Eukaryota - ps6
Excavata/Diaphoretickes - ps7
Diaphoretickes - ps8
Archaeplastida - ps9
Viridiplantae - ps10
Streptophyta - ps11
Charophyceae/Embriophyta - ps12
Embriophyta - ps13
Tracheophyta - ps14
Spermatophyta - ps15
Angiosperms - ps16
Mesangiospermae - ps17
Magnoliids/eudicots - ps18
Eudicots - ps19
Core_eudicots - ps20
Superrosids - ps21
Rosids - ps22
Eurosids - ps23
Malvids - ps24
Sap./Malv./Brassicales - ps25
Malvales/Brassicales - ps26
Brassicales - ps27
Cleomaceae/Brassicaceae - ps28
Brassicaceae - ps29
Camelineae - ps30
Arabidopsis - ps31
Arabidopsis_thaliana - ps32
Phylostratum

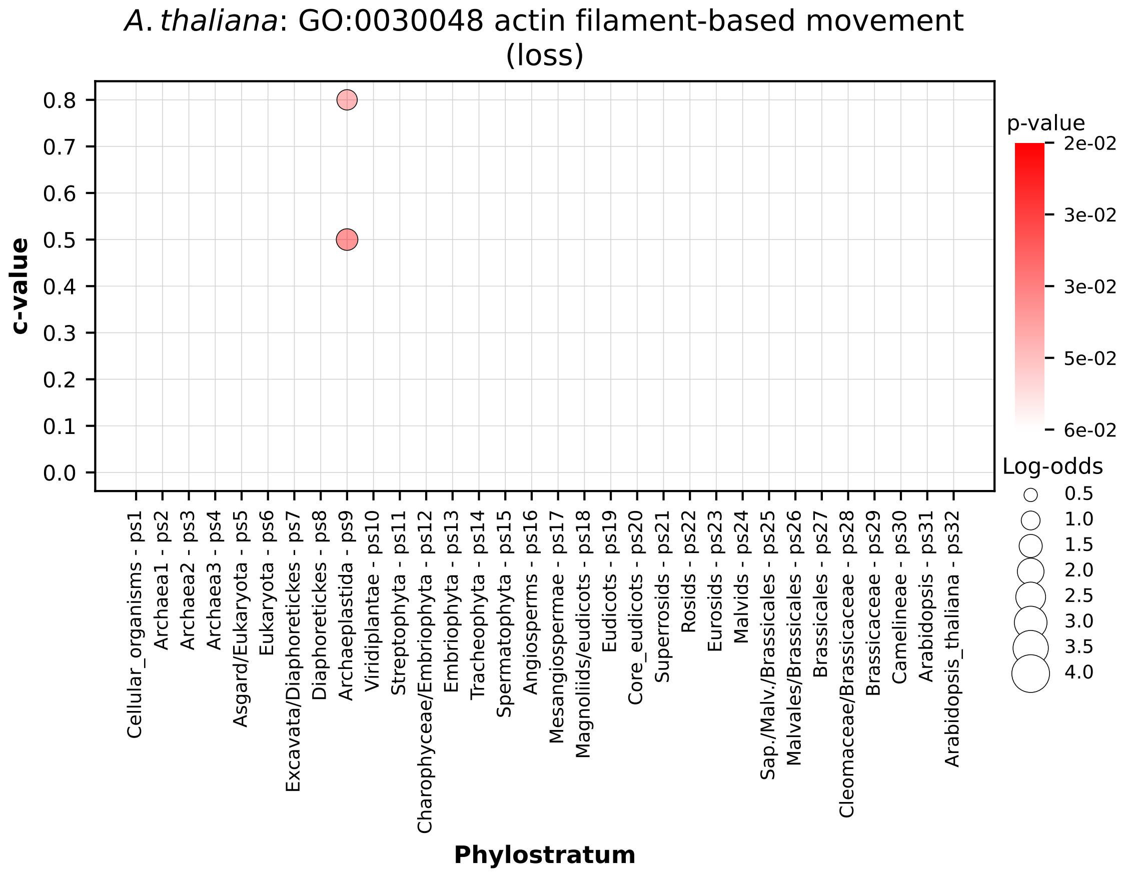

A. thaliana: GO:0030048 actin filament-based movement (loss)
c-value
0.0
0.1
0.2
0.3
0.4
0.5
0.6
0.7
0.8
Phylostratum
Cellular_organisms - ps1
Archaea1 - ps2
Archaea2 - ps3
Archaea3 - ps4
Asgard/Eukaryota - ps5
Eukaryota - ps6
Excavata/Diaphoretickes - ps7
Diaphoretickes - ps8
Archaeplastida - ps9
Viridiplantae - ps10
Streptophyta - ps11
Charophyceae/Embriophyta - ps12
Embriophyta - ps13
Tracheophyta - ps14
Spermatophyta - ps15
Angiosperms - ps16
Mesangiospermae - ps17
Magnoliids/eudicots - ps18
Eudicots - ps19
Core_eudicots - ps20
Superrosids - ps21
Rosids - ps22
Eurosids - ps23
Malvids - ps24
Sap./Malv./Brassicales - ps25
Malvales/Brassicales - ps26
Brassicales - ps27
Cleomaceae/Brassicaceae - ps28
Brassicaceae - ps29
Camelineae - ps30
Arabidopsis - ps31
Arabidopsis_thaliana - ps32
p-value
2e-02
3e-02
3e-02
5e-02
6e-02
Log-odds
0.5
1.0
1.5
2.0
2.5
3.0
3.5
4.0

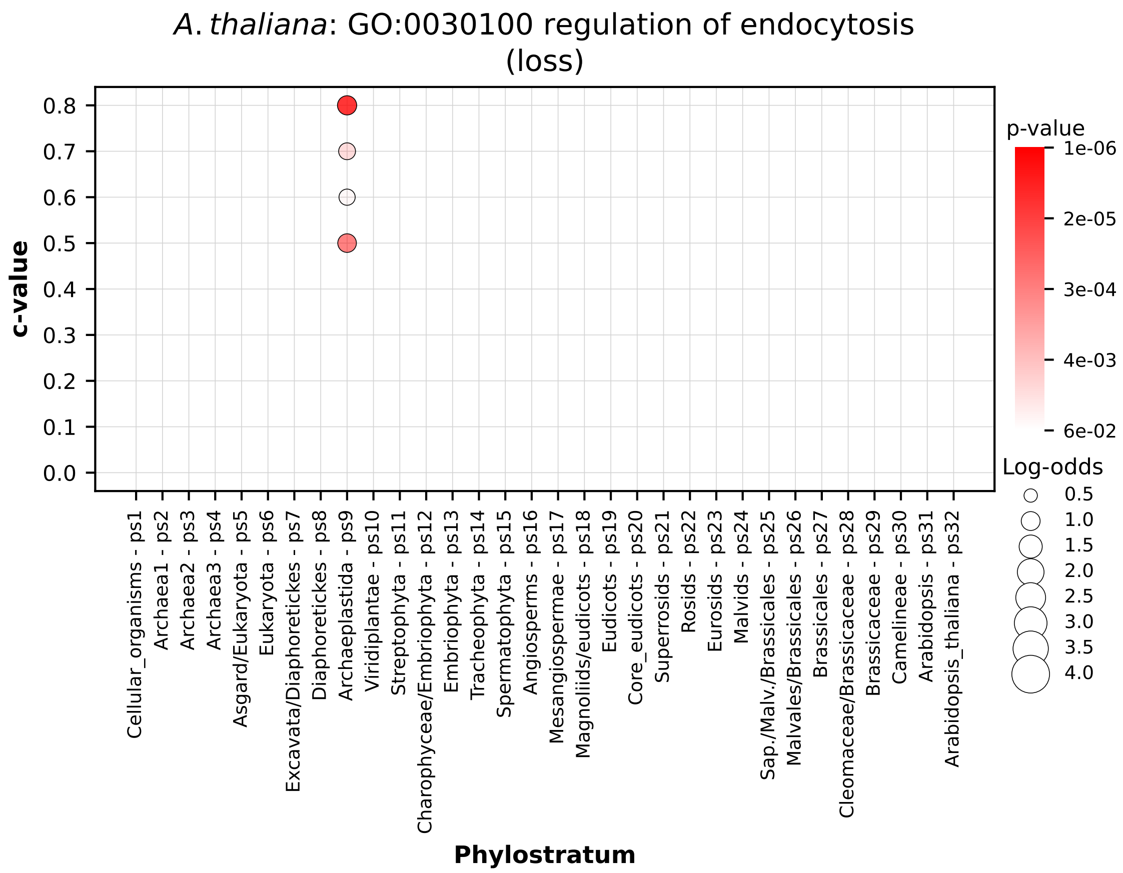

A. thaliana: GO:0030100 regulation of endocytosis
(loss)
c-value
0.8
0.7
0.6
0.5
0.4
0.3
0.2
0.1
0.0
Phylostratum
Cellular_organisms - ps1
Archaea1 - ps2
Archaea2 - ps3
Archaea3 - ps4
Asgard/Eukaryota - ps5
Eukaryota - ps6
Excavata/Diaphoretickes - ps7
Diaphoretickes - ps8
Archaeplastida - ps9
Viridiplantae - ps10
Streptophyta - ps11
Charophyceae/Embriophyta - ps12
Embriophyta - ps13
Tracheophyta - ps14
Spermatophyta - ps15
Angiosperms - ps16
Mesangiospermae - ps17
Magnoliids/eudicots - ps18
Eudicots - ps19
Core_eudicots - ps20
Superrosids - ps21
Rosids - ps22
Eurosids - ps23
Malvids - ps24
Sap./Malv./Brassicales - ps25
Malvales/Brassicales - ps26
Brassicales - ps27
Cleomaceae/Brassicaceae - ps28
Brassicaceae - ps29
Camelineae - ps30
Arabidopsis - ps31
Arabidopsis_thaliana - ps32
p-value
1e-06
2e-05
3e-04
4e-03
6e-02
Log-odds
0.5
1.0
1.5
2.0
2.5
3.0
3.5
4.0

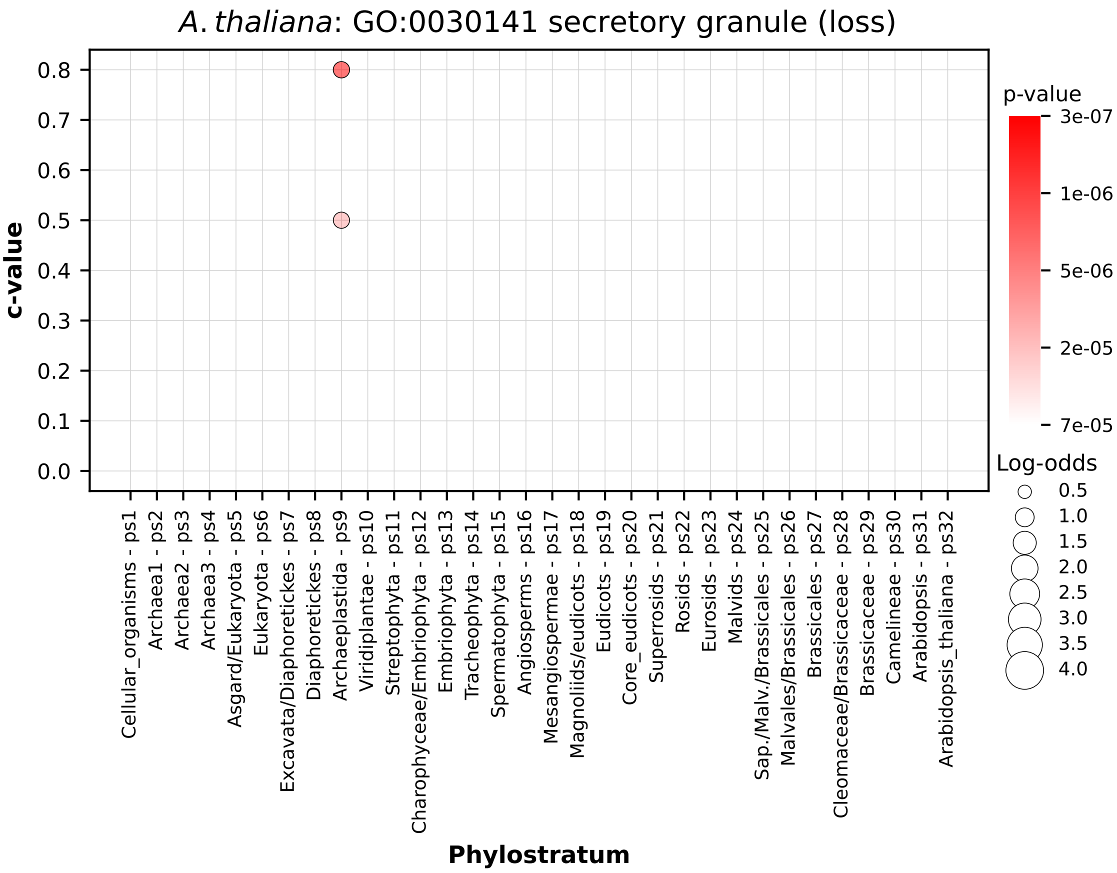
A. thaliana: GO:0030141 secretory granule (loss)
c-value
0.0
0.1
0.2
0.3
0.4
0.5
0.6
0.7
0.8
Phylostratum
Cellular_organisms - ps1
Archaea1 - ps2
Archaea2 - ps3
Archaea3 - ps4
Asgard/Eukaryota - ps5
Eukaryota - ps6
Excavata/Diaphoretickes - ps7
Diaphoretickes - ps8
Archaeplastida - ps9
Viridiplantae - ps10
Streptophyta - ps11
Charophyceae/Embriophyta - ps12
Embriophyta - ps13
Tracheophyta - ps14
Spermatophyta - ps15
Angiosperms - ps16
Mesangiospermae - ps17
Magnoliids/eudicots - ps18
Eudicots - ps19
Core_eudicots - ps20
Superrosids - ps21
Rosids - ps22
Eurosids - ps23
Malvids - ps24
Sap./Malv./Brassicales - ps25
Malvales/Brassicales - ps26
Brassicales - ps27
Cleomaceae/Brassicaceae - ps28
Brassicaceae - ps29
Camelineae - ps30
Arabidopsis - ps31
Arabidopsis_thaliana - ps32
p-value
3e-07
1e-06
5e-06
2e-05
7e-05
Log-odds
0.5
1.0
1.5
2.0
2.5
3.0
3.5
4.0

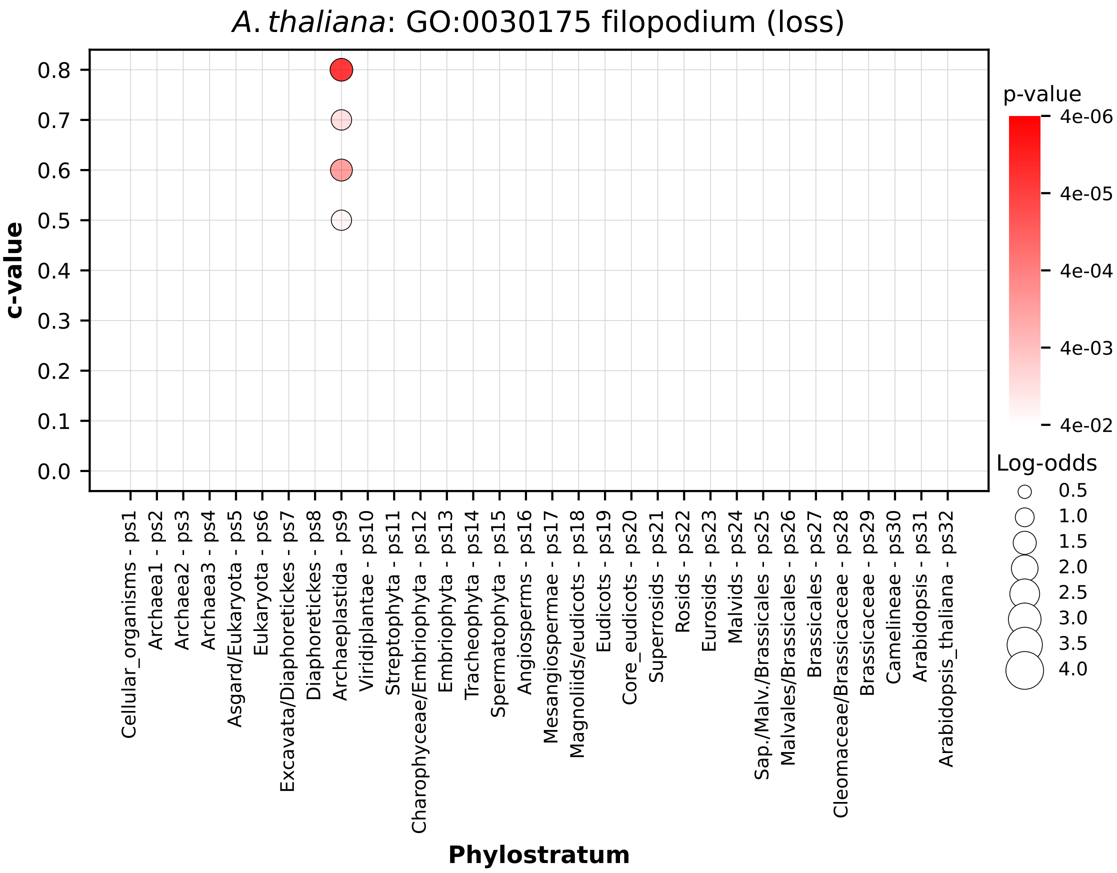

A. thaliana: GO:0030175 filopodium (loss)
c-value
0.8
0.7
0.6
0.5
0.4
0.3
0.2
0.1
0.0
p-value
4e-06
4e-05
4e-04
4e-03
4e-02
Log-odds
0.5
1.0
1.5
2.0
2.5
3.0
3.5
4.0
Cellular_organisms - ps1
Archaea1 - ps2
Archaea2 - ps3
Archaea3 - ps4
Asgard/Eukaryota - ps5
Eukaryota - ps6
Excavata/Diaphoretickes - ps7
Diaphoretickes - ps8
Archaeplastida - ps9
Viridiplantae - ps10
Streptophyta - ps11
Charophyceae/Embriophyta - ps12
Embriophyta - ps13
Tracheophyta - ps14
Spermatophyta - ps15
Angiosperms - ps16
Mesangiospermae - ps17
Magnoliids/eudicots - ps18
Eudicots - ps19
Core_eudicots - ps20
Superrosids - ps21
Rosids - ps22
Eurosids - ps23
Malvids - ps24
Sap./Malv./Brassicales - ps25
Malvales/Brassicales - ps26
Brassicales - ps27
Cleomaceae/Brassicaceae - ps28
Brassicaceae - ps29
Camelineae - ps30
Arabidopsis - ps31
Arabidopsis_thaliana - ps32
Phylostratum

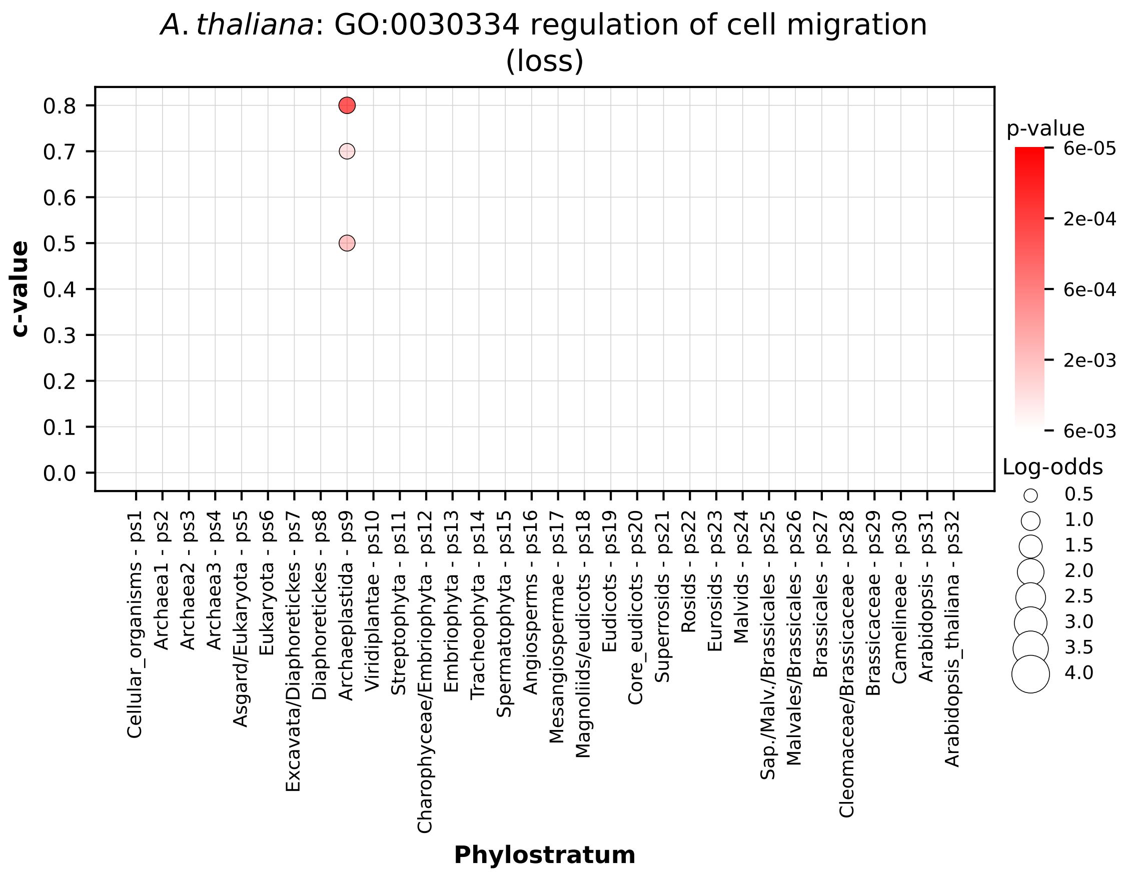

A. thaliana: GO:0030334 regulation of cell migration (loss)
c-value
0.0
0.1
0.2
0.3
0.4
0.5
0.6
0.7
0.8
Phylostratum
Cellular_organisms - ps1
Archaea1 - ps2
Archaea2 - ps3
Archaea3 - ps4
Asgard/Eukaryota - ps5
Eukaryota - ps6
Excavata/Diaphoretickes - ps7
Diaphoretickes - ps8
Archaeplastida - ps9
Viridiplantae - ps10
Streptophyta - ps11
Charophyceae/Embriophyta - ps12
Embriophyta - ps13
Tracheophyta - ps14
Spermatophyta - ps15
Angiosperms - ps16
Mesangiospermae - ps17
Magnoliids/eudicots - ps18
Eudicots - ps19
Core_eudicots - ps20
Superrosids - ps21
Rosids - ps22
Eurosids - ps23
Malvids - ps24
Sap./Malv./Brassicales - ps25
Malvales/Brassicales - ps26
Brassicales - ps27
Cleomaceae/Brassicaceae - ps28
Brassicaceae - ps29
Camelineae - ps30
Arabidopsis - ps31
Arabidopsis_thaliana - ps32
p-value
6e-05
2e-04
6e-04
2e-03
6e-03
Log-odds
0.5
1.0
1.5
2.0
2.5
3.0
3.5
4.0

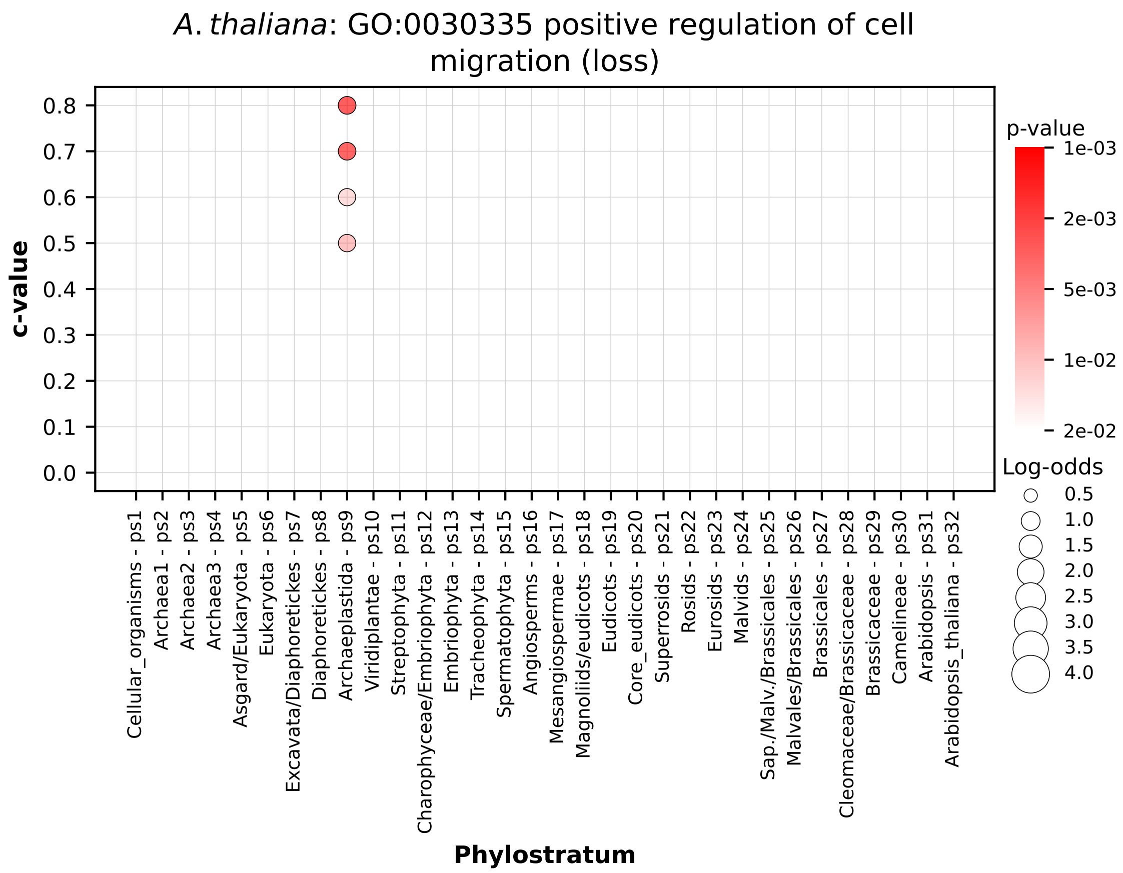

A. thaliana: GO:0030335 positive regulation of cell migration (loss)
c-value
0.8
0.7
0.6
0.5
0.4
0.3
0.2
0.1
0.0
p-value
1e-03
2e-03
5e-03
1e-02
2e-02
Log-odds
0.5
1.0
1.5
2.0
2.5
3.0
3.5
4.0
Cellular_organisms - ps1
Archaea1 - ps2
Archaea2 - ps3
Archaea3 - ps4
Asgard/Eukaryota - ps5
Eukaryota - ps6
Excavata/Diaphoretickes - ps7
Diaphoretickes - ps8
Archaeplastida - ps9
Viridiplantae - ps10
Streptophyta - ps11
Charophyceae/Embriophyta - ps12
Embriophyta - ps13
Tracheophyta - ps14
Spermatophyta - ps15
Angiosperms - ps16
Mesangiospermae - ps17
Magnoliids/eudicots - ps18
Eudicots - ps19
Core_eudicots - ps20
Superrosids - ps21
Rosids - ps22
Eurosids - ps23
Malvids - ps24
Sap./Malv./Brassicales - ps25
Malvales/Brassicales - ps26
Brassicales - ps27
Cleomaceae/Brassicaceae - ps28
Brassicaceae - ps29
Camelineae - ps30
Arabidopsis - ps31
Arabidopsis_thaliana - ps32
Phylostratum

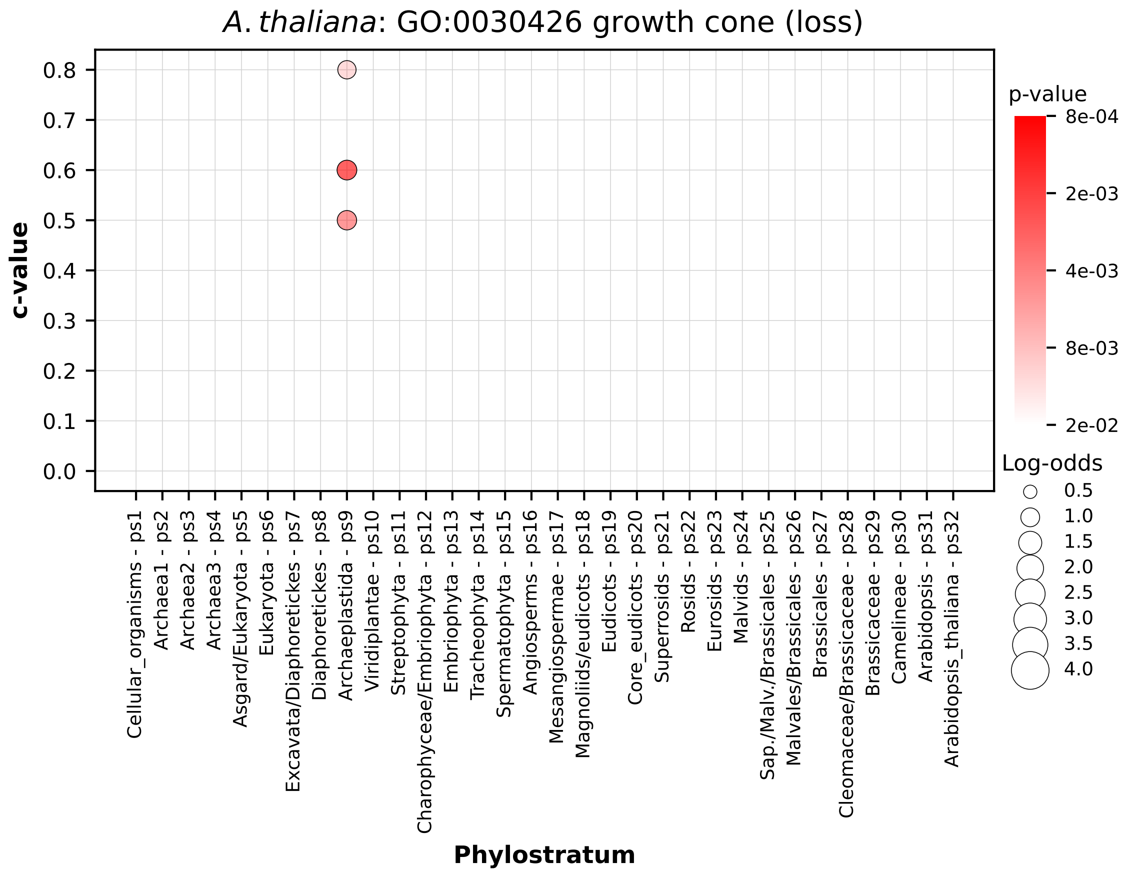
A. thaliana: GO:0030426 growth cone (loss)
c-value
0.0
0.1
0.2
0.3
0.4
0.5
0.6
0.7
0.8
p-value
8e-04
2e-03
4e-03
8e-03
2e-02
Log-odds
0.5
1.0
1.5
2.0
2.5
3.0
3.5
4.0
Cellular_organisms - ps1
Archaea1 - ps2
Archaea2 - ps3
Archaea3 - ps4
Asgard/Eukaryota - ps5
Eukaryota - ps6
Excavata/Diaphoretickes - ps7
Diaphoretickes - ps8
Archaeplastida - ps9
Viridiplantae - ps10
Streptophyta - ps11
Charophyceae/Embriophyta - ps12
Embriophyta - ps13
Tracheophyta - ps14
Spermatophyta - ps15
Angiosperms - ps16
Mesangiospermae - ps17
Magnoliids/eudicots - ps18
Eudicots - ps19
Core_eudicots - ps20
Superrosids - ps21
Rosids - ps22
Eurosids - ps23
Malvids - ps24
Sap./Malv./Brassicales - ps25
Malvales/Brassicales - ps26
Brassicales - ps27
Cleomaceae/Brassicaceae - ps28
Brassicaceae - ps29
Camelineae - ps30
Arabidopsis - ps31
Arabidopsis_thaliana - ps32
Phylostratum

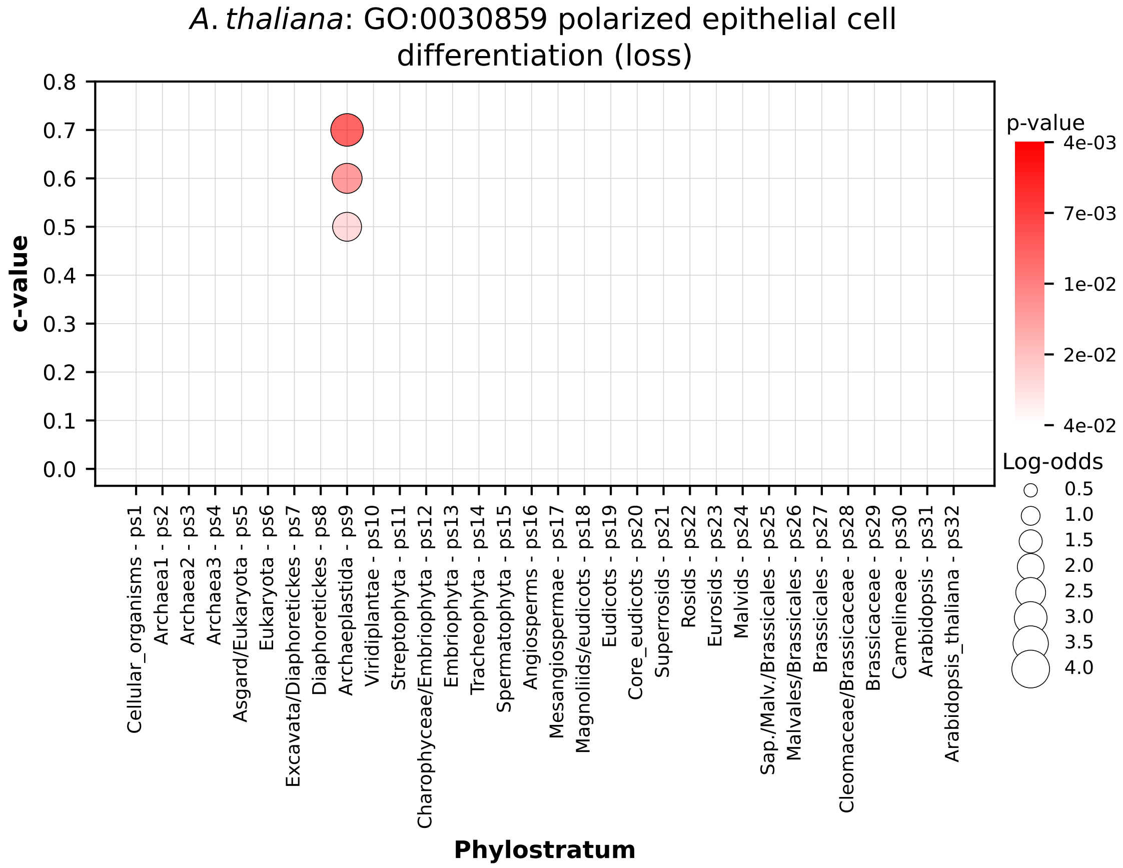

A. thaliana: GO:0030859 polarized epithelial cell differentiation (loss)
c-value
0.8
0.7
0.6
0.5
0.4
0.3
0.2
0.1
0.0
p-value
4e-03
7e-03
1e-02
2e-02
4e-02
Log-odds
0.5
1.0
1.5
2.0
2.5
3.0
3.5
4.0
Cellular_organisms - ps1
Archaea1 - ps2
Archaea2 - ps3
Archaea3 - ps4
Asgard/Eukaryota - ps5
Eukaryota - ps6
Excavata/Diaphoretickes - ps7
Diaphoretickes - ps8
Archaeplastida - ps9
Viridiplantae - ps10
Streptophyta - ps11
Charophyceae/Embriophyta - ps12
Embriophyta - ps13
Tracheophyta - ps14
Spermatophyta - ps15
Angiosperms - ps16
Mesangiospermae - ps17
Magnoliids/eudicots - ps18
Eudicots - ps19
Core_eudicots - ps20
Superrosids - ps21
Rosids - ps22
Eurosids - ps23
Malvids - ps24
Sap./Malv./Brassicales - ps25
Malvales/Brassicales - ps26
Brassicales - ps27
Cleomaceae/Brassicaceae - ps28
Brassicaceae - ps29
Camelineae - ps30
Arabidopsis - ps31
Arabidopsis_thaliana - ps32
Phylostratum

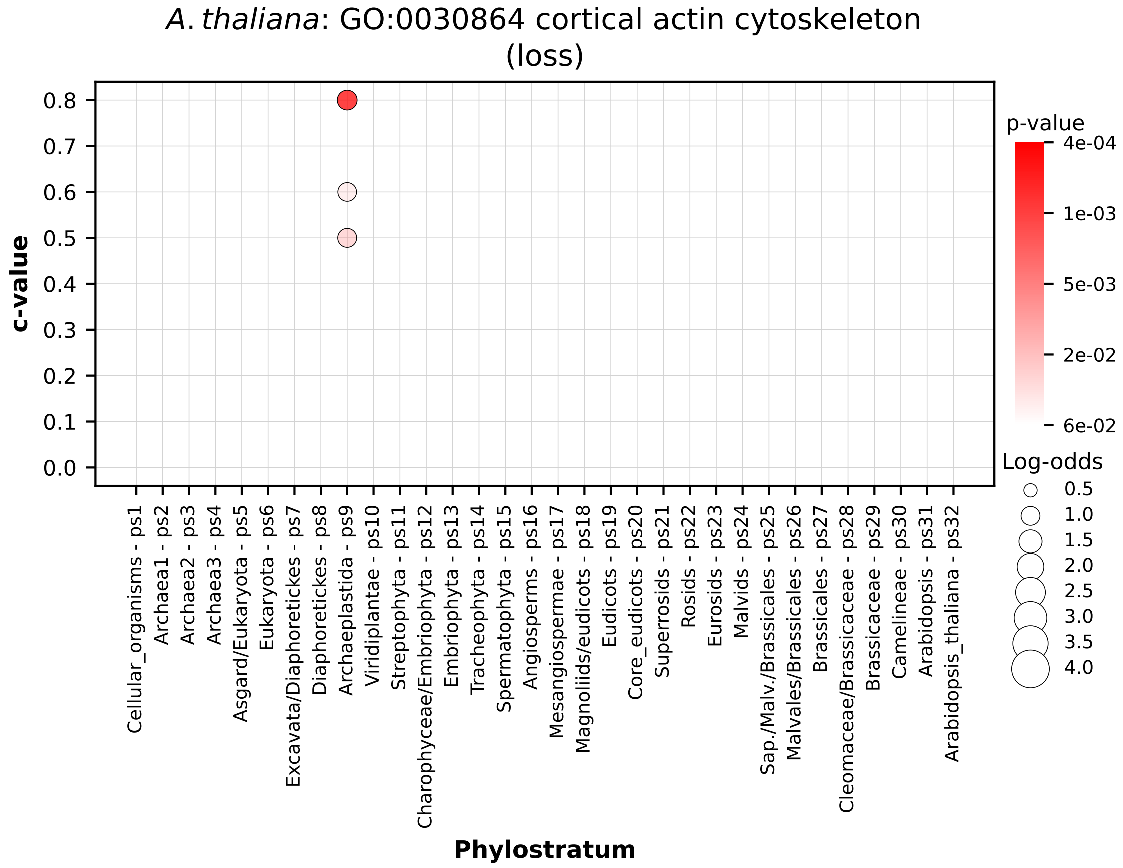
A. thaliana: GO:0030864 cortical actin cytoskeleton
(loss)
c-value
0.0
0.1
0.2
0.3
0.4
0.5
0.6
0.7
0.8
Phylostratum
Cellular_organisms - ps1
Archaea1 - ps2
Archaea2 - ps3
Archaea3 - ps4
Asgard/Eukaryota - ps5
Eukaryota - ps6
Excavata/Diaphoretickes - ps7
Diaphoretickes - ps8
Archaeplastida - ps9
Viridiplantae - ps10
Streptophyta - ps11
Charophyceae/Embriophyta - ps12
Embriophyta - ps13
Tracheophyta - ps14
Spermatophyta - ps15
Angiosperms - ps16
Mesangiospermae - ps17
Magnoliids/eudicots - ps18
Eudicots - ps19
Core_eudicots - ps20
Superrosids - ps21
Rosids - ps22
Eurosids - ps23
Malvids - ps24
Sap./Malv./Brassicales - ps25
Malvales/Brassicales - ps26
Brassicales - ps27
Cleomaceae/Brassicaceae - ps28
Brassicaceae - ps29
Camelineae - ps30
Arabidopsis - ps31
Arabidopsis_thaliana - ps32
p-value
4e-04
1e-03
5e-03
2e-02
6e-02
Log-odds
0.5
1.0
1.5
2.0
2.5
3.0
3.5
4.0

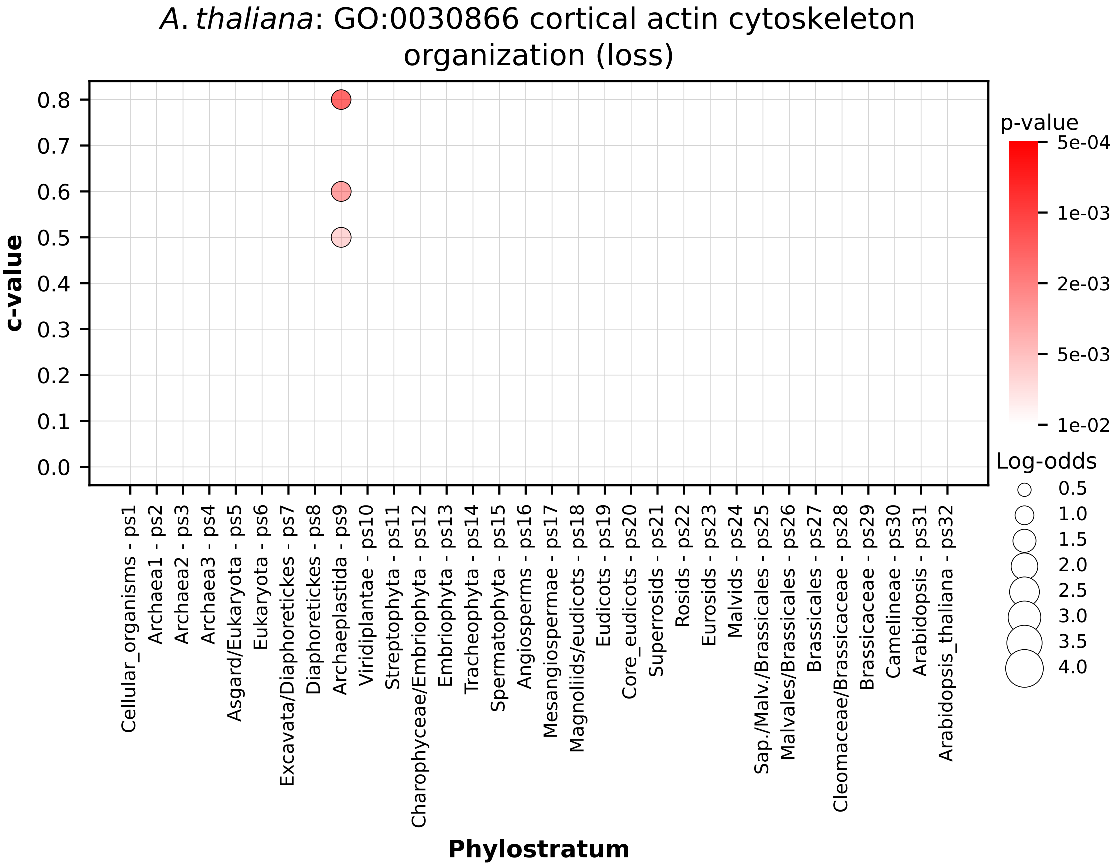

A. thaliana: GO:0030866 cortical actin cytoskeleton organization (loss)
c-value
0.0
0.1
0.2
0.3
0.4
0.5
0.6
0.7
0.8
p-value
5e-04
1e-03
2e-03
5e-03
1e-02
Log-odds
0.5
1.0
1.5
2.0
2.5
3.0
3.5
4.0
Cellular_organisms - ps1
Archaea1 - ps2
Archaea2 - ps3
Archaea3 - ps4
Asgard/Eukaryota - ps5
Eukaryota - ps6
Excavata/Diaphoretickes - ps7
Diaphoretickes - ps8
Archaeplastida - ps9
Viridiplantae - ps10
Streptophyta - ps11
Charophyceae/Embriophyta - ps12
Embriophyta - ps13
Tracheophyta - ps14
Spermatophyta - ps15
Angiosperms - ps16
Mesangiospermae - ps17
Magnoliids/eudicots - ps18
Eudicots - ps19
Core_eudicots - ps20
Superrosids - ps21
Rosids - ps22
Eurosids - ps23
Malvids - ps24
Sap./Malv./Brassicales - ps25
Malvales/Brassicales - ps26
Brassicales - ps27
Cleomaceae/Brassicaceae - ps28
Brassicaceae - ps29
Camelineae - ps30
Arabidopsis - ps31
Arabidopsis_thaliana - ps32
Phylostratum

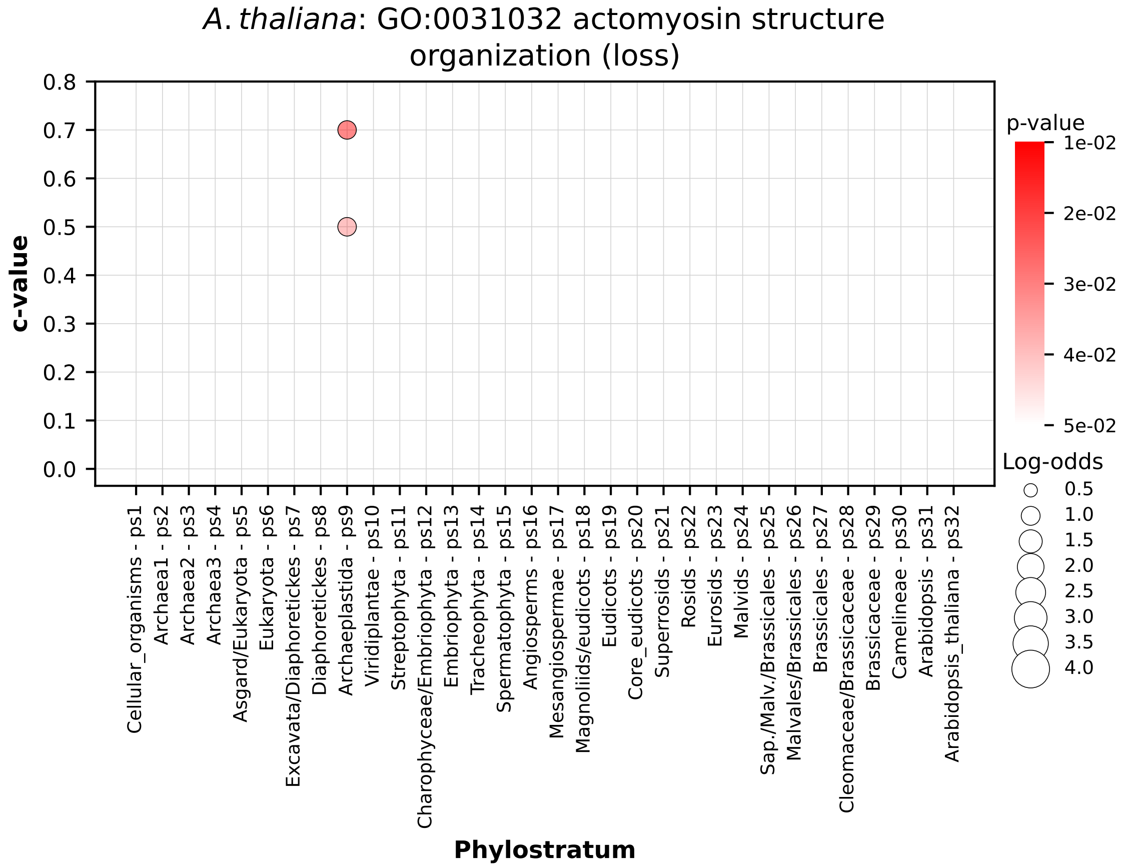
A. thaliana: GO:0031032 actomyosin structure organization (loss)
c-value
0.8
0.7
0.6
0.5
0.4
0.3
0.2
0.1
0.0
Phylostratum
Cellular_organisms - ps1
Archaea1 - ps2
Archaea2 - ps3
Archaea3 - ps4
Asgard/Eukaryota - ps5
Eukaryota - ps6
Excavata/Diaphoretickes - ps7
Diaphoretickes - ps8
Archaeplastida - ps9
Viridiplantae - ps10
Streptophyta - ps11
Charophyceae/Embriophyta - ps12
Embriophyta - ps13
Tracheophyta - ps14
Spermatophyta - ps15
Angiosperms - ps16
Mesangiospermae - ps17
Magnoliids/eudicots - ps18
Eudicots - ps19
Core_eudicots - ps20
Superrosids - ps21
Rosids - ps22
Eurosids - ps23
Malvids - ps24
Sap./Malv./Brassicales - ps25
Malvales/Brassicales - ps26
Brassicales - ps27
Cleomaceae/Brassicaceae - ps28
Brassicaceae - ps29
Camelineae - ps30
Arabidopsis - ps31
Arabidopsis_thaliana - ps32
p-value
1e-02
2e-02
3e-02
4e-02
5e-02
Log-odds
0.5
1.0
1.5
2.0
2.5
3.0
3.5
4.0

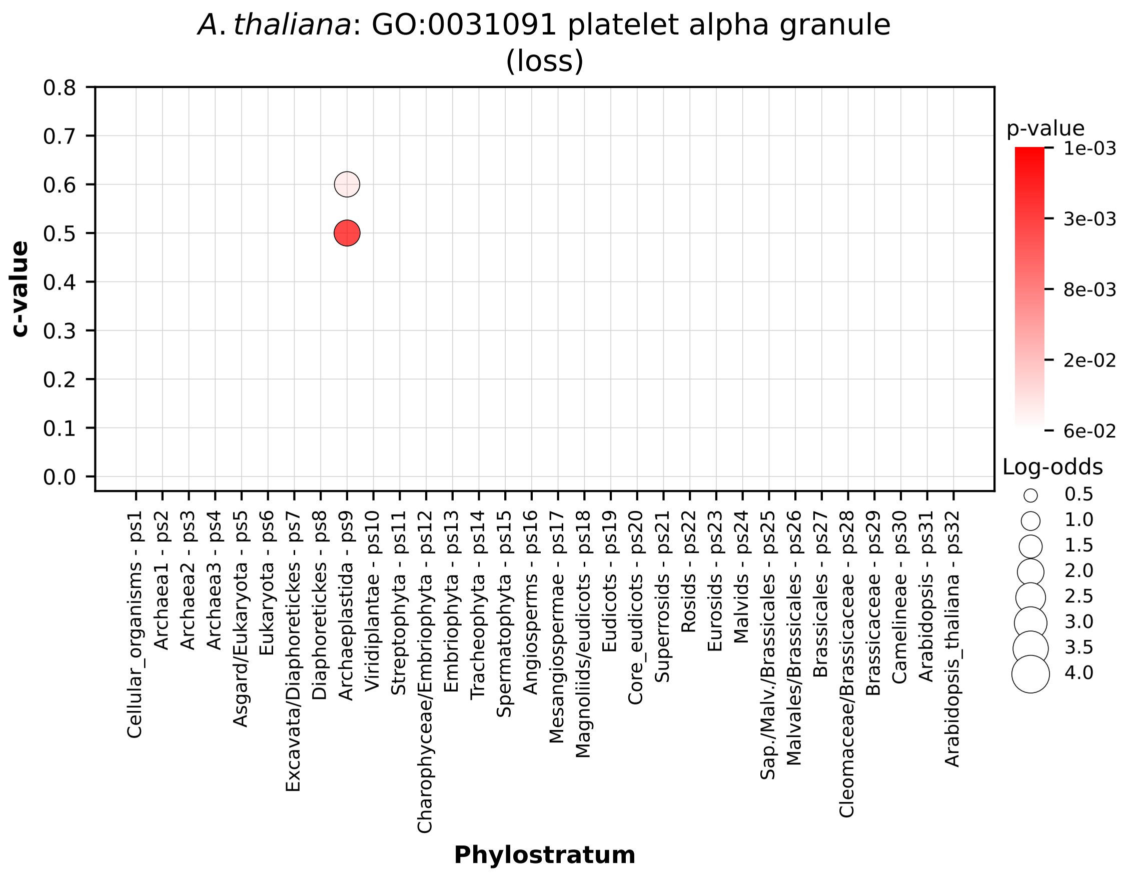

A. thaliana: GO:0031091 platelet alpha granule
(loss)
c-value
0.0
0.1
0.2
0.3
0.4
0.5
0.6
0.7
0.8
Phylostratum
Cellular_organisms - ps1
Archaea1 - ps2
Archaea2 - ps3
Archaea3 - ps4
Asgard/Eukaryota - ps5
Eukaryota - ps6
Excavata/Diaphoretickes - ps7
Diaphoretickes - ps8
Archaeplastida - ps9
Viridiplantae - ps10
Streptophyta - ps11
Charophyceae/Embriophyta - ps12
Embriophyta - ps13
Tracheophyta - ps14
Spermatophyta - ps15
Angiosperms - ps16
Mesangiospermae - ps17
Magnoliids/eudicots - ps18
Eudicots - ps19
Core_eudicots - ps20
Superrosids - ps21
Rosids - ps22
Eurosids - ps23
Malvids - ps24
Sap./Malv./Brassicales - ps25
Malvales/Brassicales - ps26
Brassicales - ps27
Cleomaceae/Brassicaceae - ps28
Brassicaceae - ps29
Camelineae - ps30
Arabidopsis - ps31
Arabidopsis_thaliana - ps32
p-value
1e-03
3e-03
8e-03
2e-02
6e-02
Log-odds
0.5
1.0
1.5
2.0
2.5
3.0
3.5
4.0

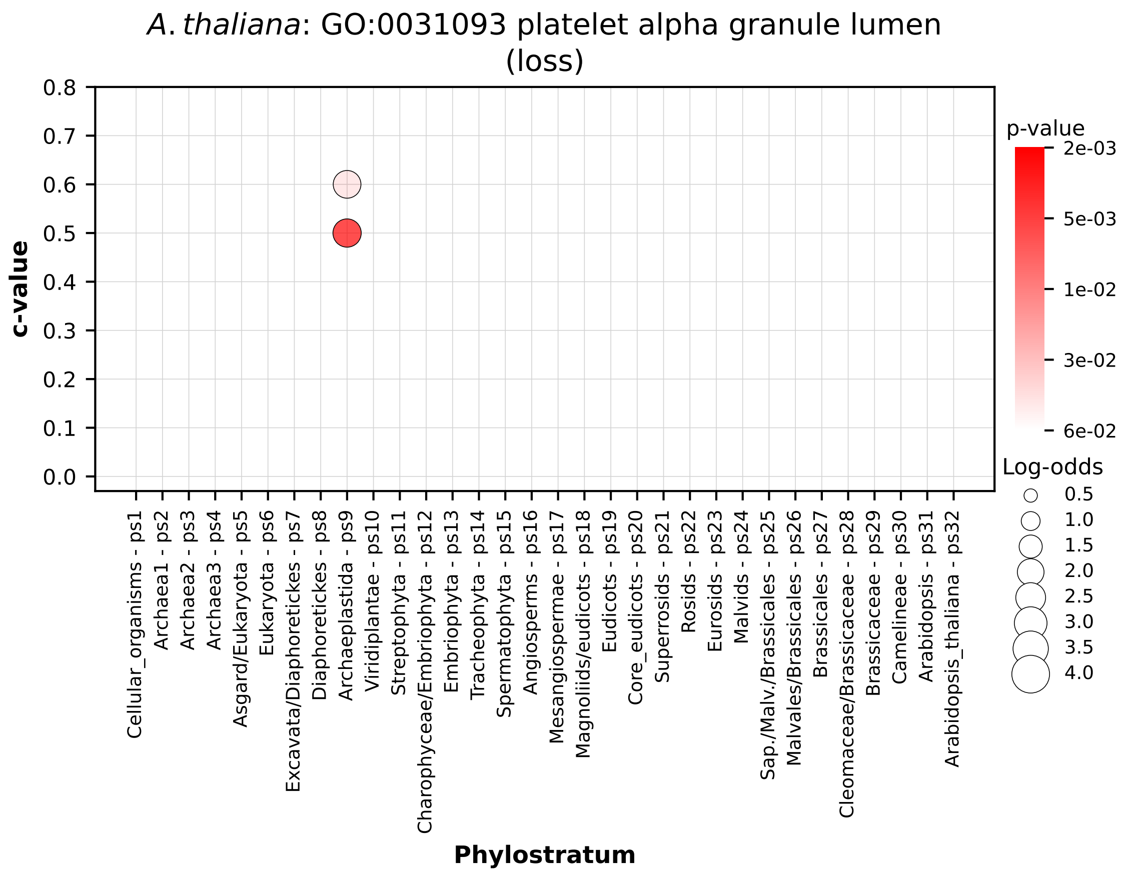

A. thaliana: GO:0031093 platelet alpha granule lumen (loss)
c-value
0.8
0.7
0.6
0.5
0.4
0.3
0.2
0.1
0.0
p-value
2e-03
5e-03
1e-02
3e-02
6e-02
Log-odds
0.5
1.0
1.5
2.0
2.5
3.0
3.5
4.0
Cellular_organisms - ps1
Archaea1 - ps2
Archaea2 - ps3
Archaea3 - ps4
Asgard/Eukaryota - ps5
Eukaryota - ps6
Excavata/Diaphoretickes - ps7
Diaphoretickes - ps8
Archaeplastida - ps9
Viridiplantae - ps10
Streptophyta - ps11
Charophyceae/Embriophyta - ps12
Embriophyta - ps13
Tracheophyta - ps14
Spermatophyta - ps15
Angiosperms - ps16
Mesangiospermae - ps17
Magnoliids/eudicots - ps18
Eudicots - ps19
Core_eudicots - ps20
Superrosids - ps21
Rosids - ps22
Eurosids - ps23
Malvids - ps24
Sap./Malv./Brassicales - ps25
Malvales/Brassicales - ps26
Brassicales - ps27
Cleomaceae/Brassicaceae - ps28
Brassicaceae - ps29
Camelineae - ps30
Arabidopsis - ps31
Arabidopsis_thaliana - ps32
Phylostratum

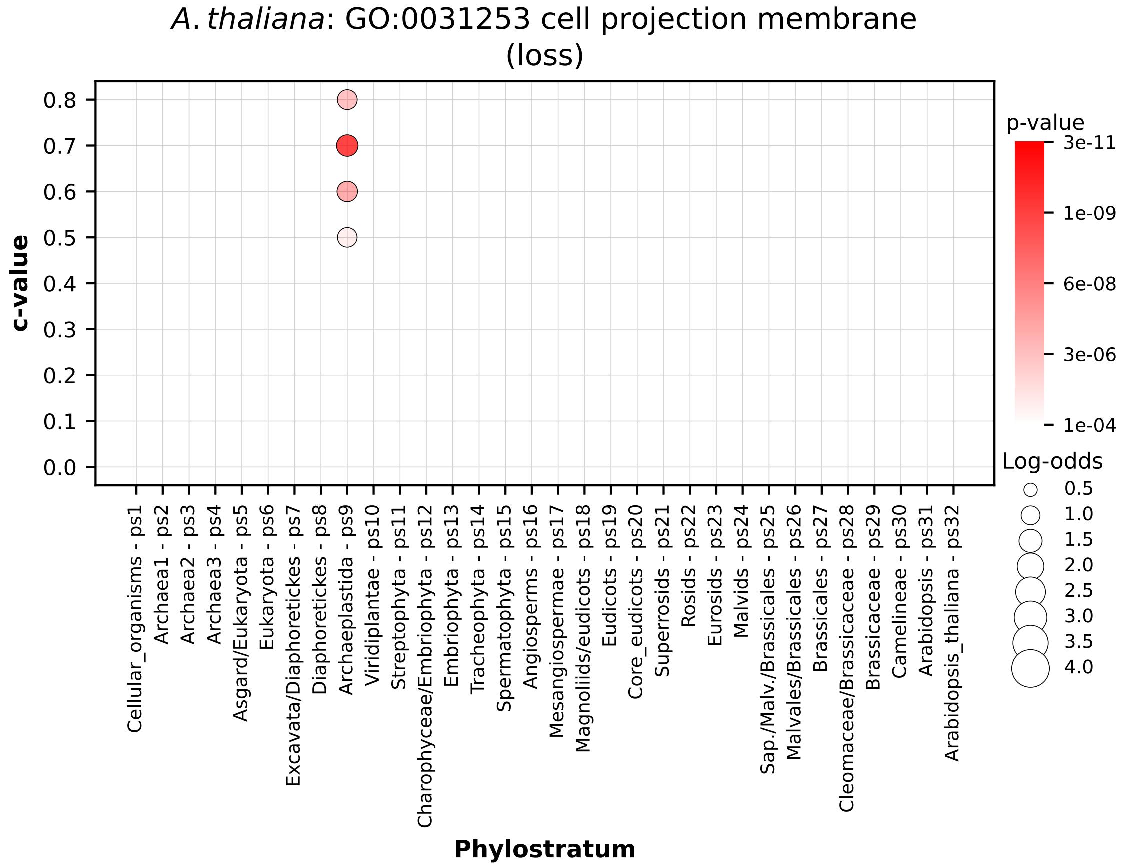

A. thaliana: GO:0031253 cell projection membrane (loss)
c-value
0.8
0.7
0.6
0.5
0.4
0.3
0.2
0.1
0.0
Phylostratum
Cellular_organisms - ps1
Archaea1 - ps2
Archaea2 - ps3
Archaea3 - ps4
Asgard/Eukaryota - ps5
Eukaryota - ps6
Excavata/Diaphoretickes - ps7
Diaphoretickes - ps8
Archaeplastida - ps9
Viridiplantae - ps10
Streptophyta - ps11
Charophyceae/Embriophyta - ps12
Embriophyta - ps13
Tracheophyta - ps14
Spermatophyta - ps15
Angiosperms - ps16
Mesangiospermae - ps17
Magnoliids/eudicots - ps18
Eudicots - ps19
Core_eudicots - ps20
Superrosids - ps21
Rosids - ps22
Eurosids - ps23
Malvids - ps24
Sap./Malv./Brassicales - ps25
Malvales/Brassicales - ps26
Brassicales - ps27
Cleomaceae/Brassicaceae - ps28
Brassicaceae - ps29
Camelineae - ps30
Arabidopsis - ps31
Arabidopsis_thaliana - ps32
p-value
3e-11
1e-09
6e-08
3e-06
1e-04
Log-odds
0.5
1.0
1.5
2.0
2.5
3.0
3.5
4.0

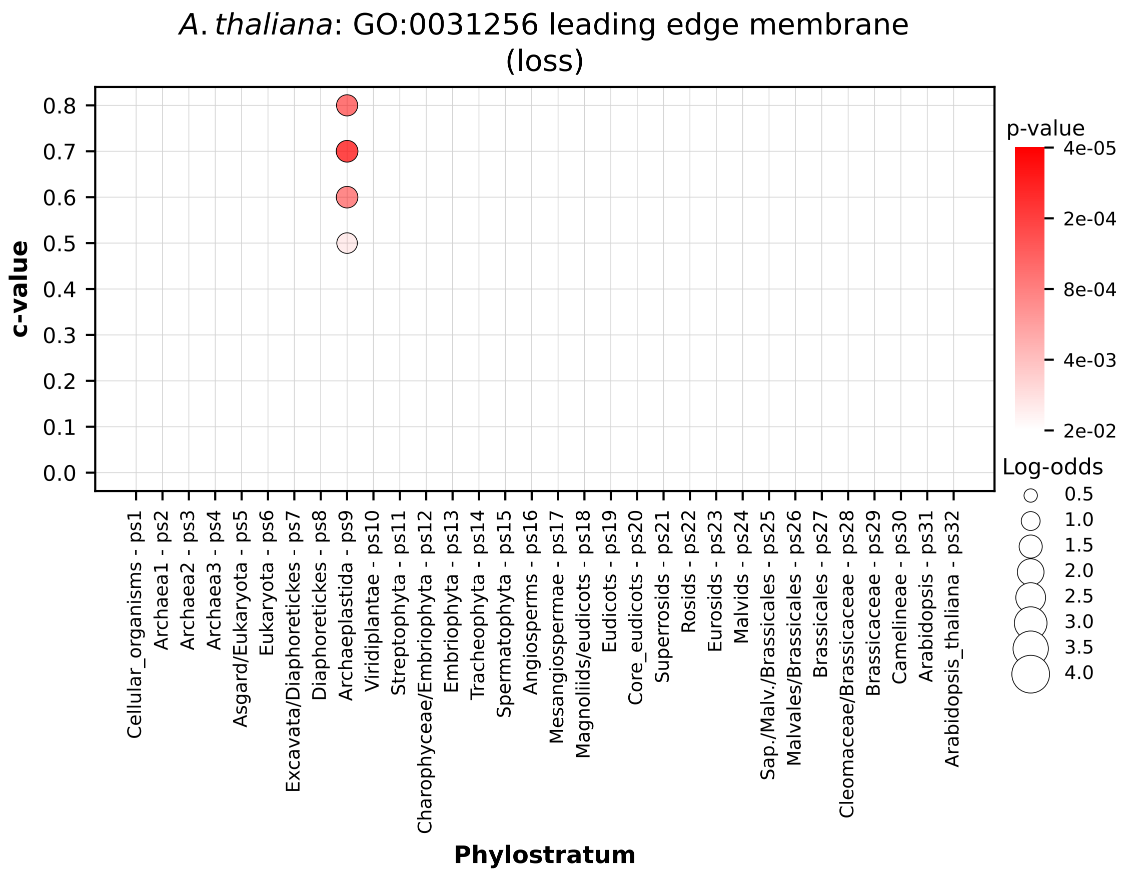

A. thaliana: GO:0031256 leading edge membrane
(loss)
c-value
0.8
0.7
0.6
0.5
0.4
0.3
0.2
0.1
0.0
p-value
4e-05
2e-04
8e-04
4e-03
2e-02
Log-odds
0.5
1.0
1.5
2.0
2.5
3.0
3.5
4.0
Cellular_organisms - ps1
Archaea1 - ps2
Archaea2 - ps3
Archaea3 - ps4
Asgard/Eukaryota - ps5
Eukaryota - ps6
Excavata/Diaphoretickes - ps7
Diaphoretickes - ps8
Archaeplastida - ps9
Viridiplantae - ps10
Streptophyta - ps11
Charophyceae/Embriophyta - ps12
Embriophyta - ps13
Tracheophyta - ps14
Spermatophyta - ps15
Angiosperms - ps16
Mesangiospermae - ps17
Magnoliids/eudicots - ps18
Eudicots - ps19
Core_eudicots - ps20
Superrosids - ps21
Rosids - ps22
Eurosids - ps23
Malvids - ps24
Sap./Malv./Brassicales - ps25
Malvales/Brassicales - ps26
Brassicales - ps27
Cleomaceae/Brassicaceae - ps28
Brassicaceae - ps29
Camelineae - ps30
Arabidopsis - ps31
Arabidopsis_thaliana - ps32
Phylostratum

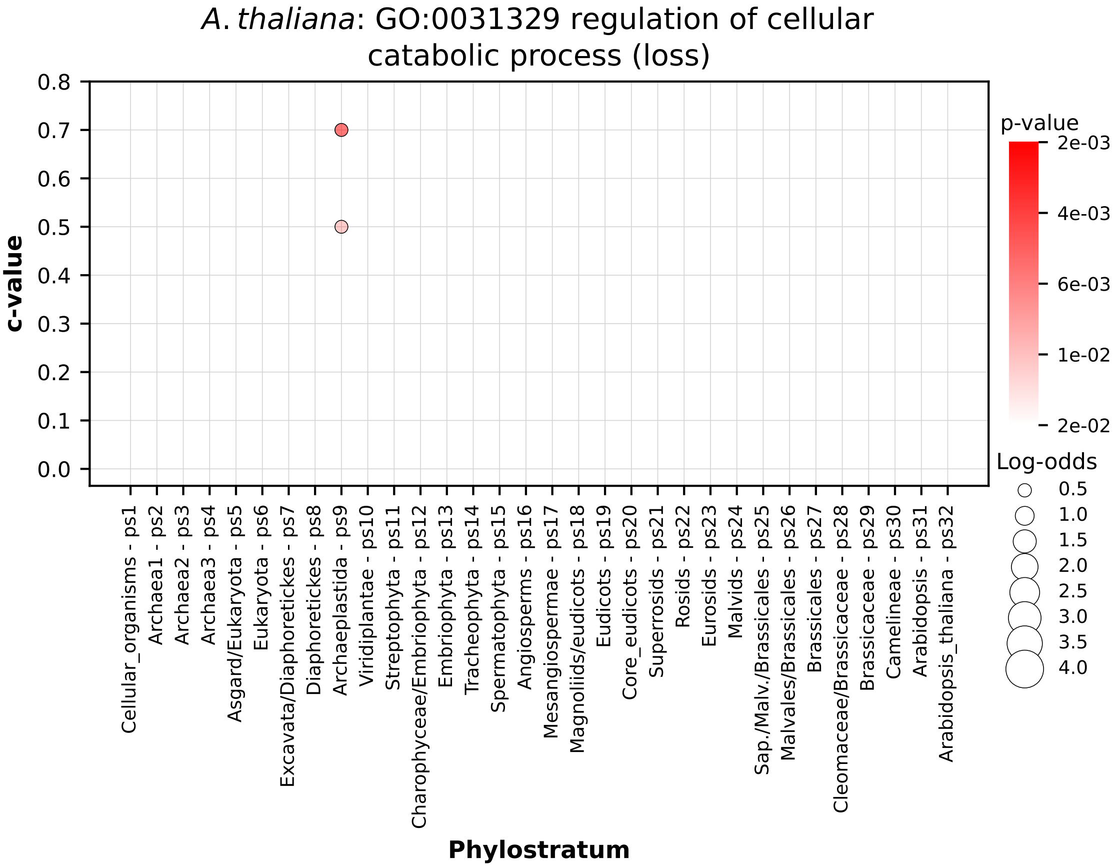

A. thaliana: GO:0031329 regulation of cellular catabolic process (loss)
c-value
0.0
0.1
0.2
0.3
0.4
0.5
0.6
0.7
0.8
Phylostratum
Cellular_organisms - ps1
Archaea1 - ps2
Archaea2 - ps3
Archaea3 - ps4
Asgard/Eukaryota - ps5
Eukaryota - ps6
Excavata/Diaphoretickes - ps7
Diaphoretickes - ps8
Archaeplastida - ps9
Viridiplantae - ps10
Streptophyta - ps11
Charophyceae/Embriophyta - ps12
Embriophyta - ps13
Tracheophyta - ps14
Spermatophyta - ps15
Angiosperms - ps16
Mesangiospermae - ps17
Magnoliids/eudicots - ps18
Eudicots - ps19
Core_eudicots - ps20
Superrosids - ps21
Rosids - ps22
Eurosids - ps23
Malvids - ps24
Sap./Malv./Brassicales - ps25
Malvales/Brassicales - ps26
Brassicales - ps27
Cleomaceae/Brassicaceae - ps28
Brassicaceae - ps29
Camelineae - ps30
Arabidopsis - ps31
Arabidopsis_thaliana - ps32
p-value
2e-03
4e-03
6e-03
1e-02
2e-02
Log-odds
0.5
1.0
1.5
2.0
2.5
3.0
3.5
4.0

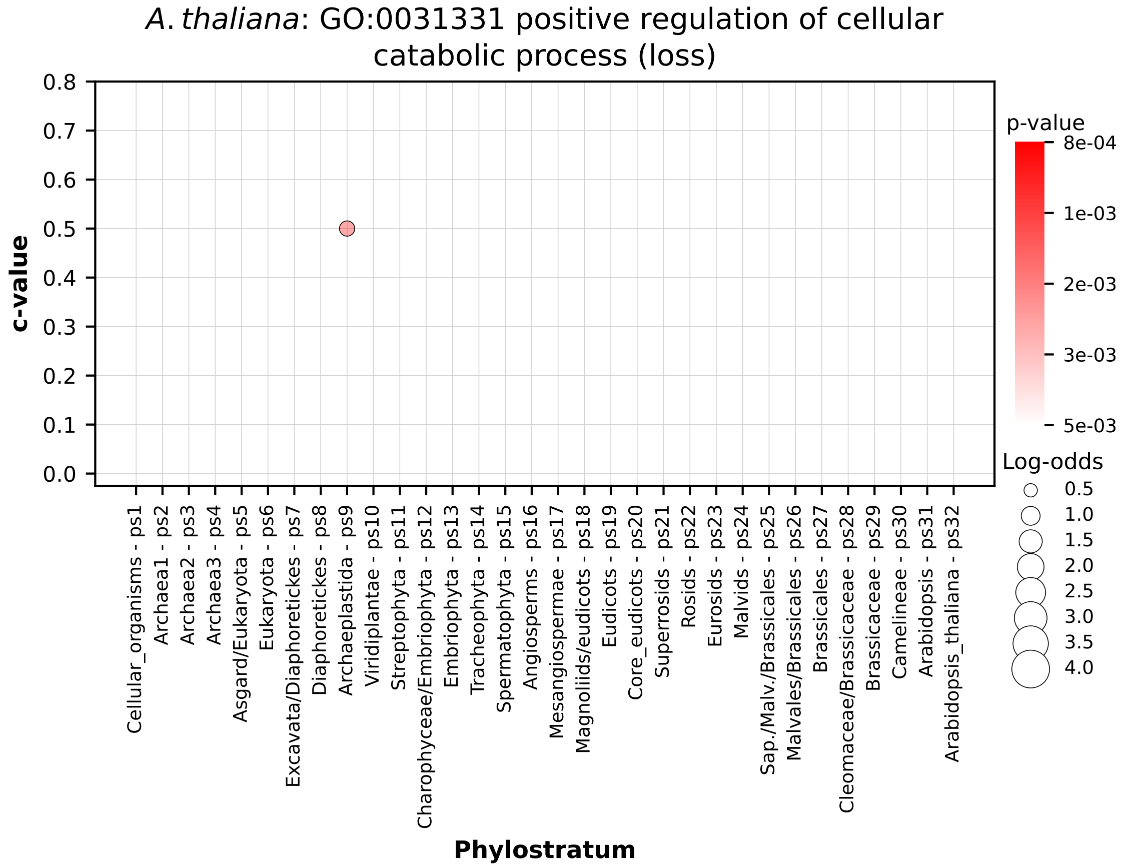

A. thaliana: GO:0031331 positive regulation of cellular catabolic process (loss)
c-value
0.8
0.7
0.6
0.5
0.4
0.3
0.2
0.1
0.0
Phylostratum
Cellular_organisms - ps1
Archaea1 - ps2
Archaea2 - ps3
Archaea3 - ps4
Asgard/Eukaryota - ps5
Eukaryota - ps6
Excavata/Diaphoretickes - ps7
Diaphoretickes - ps8
Archaeplastida - ps9
Viridiplantae - ps10
Streptophyta - ps11
Charophyceae/Embriophyta - ps12
Embriophyta - ps13
Tracheophyta - ps14
Spermatophyta - ps15
Angiosperms - ps16
Mesangiospermae - ps17
Magnoliids/eudicots - ps18
Eudicots - ps19
Core_eudicots - ps20
Superrosids - ps21
Rosids - ps22
Eurosids - ps23
Malvids - ps24
Sap./Malv./Brassicales - ps25
Malvales/Brassicales - ps26
Brassicales - ps27
Cleomaceae/Brassicaceae - ps28
Brassicaceae - ps29
Camelineae - ps30
Arabidopsis - ps31
Arabidopsis_thaliana - ps32
p-value
8e-04
1e-03
2e-03
3e-03
5e-03
Log-odds
0.5
1.0
1.5
2.0
2.5
3.0
3.5
4.0

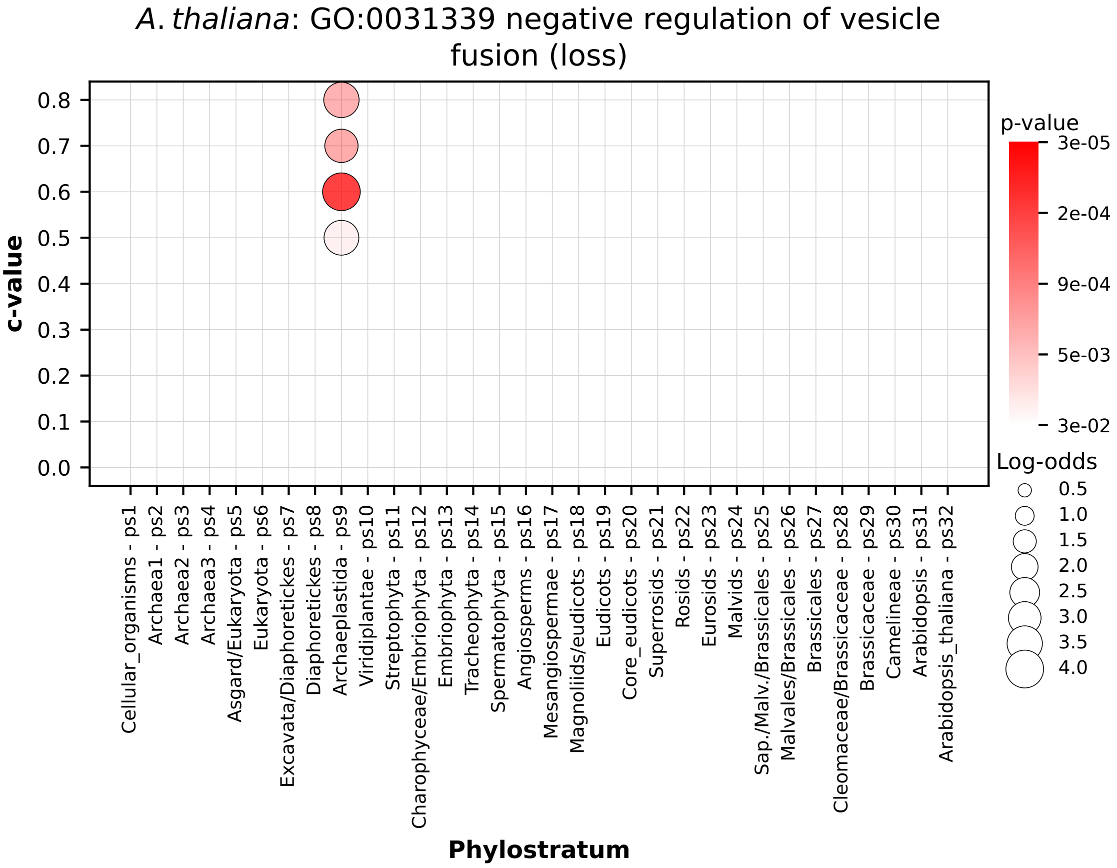

A. thaliana: GO:0031339 negative regulation of vesicle fusion (loss)
c-value
0.8
0.7
0.6
0.5
0.4
0.3
0.2
0.1
0.0
Phylostratum
Cellular_organisms - ps1
Archaea1 - ps2
Archaea2 - ps3
Archaea3 - ps4
Asgard/Eukaryota - ps5
Eukaryota - ps6
Excavata/Diaphoretickes - ps7
Diaphoretickes - ps8
Archaeplastida - ps9
Viridiplantae - ps10
Streptophyta - ps11
Charophyceae/Embriophyta - ps12
Embriophyta - ps13
Tracheophyta - ps14
Spermatophyta - ps15
Angiosperms - ps16
Mesangiospermae - ps17
Magnoliids/eudicots - ps18
Eudicots - ps19
Core_eudicots - ps20
Superrosids - ps21
Rosids - ps22
Eurosids - ps23
Malvids - ps24
Sap./Malv./Brassicales - ps25
Malvales/Brassicales - ps26
Brassicales - ps27
Cleomaceae/Brassicaceae - ps28
Brassicaceae - ps29
Camelineae - ps30
Arabidopsis - ps31
Arabidopsis_thaliana - ps32
p-value
3e-05
2e-04
9e-04
5e-03
3e-02
Log-odds
0.5
1.0
1.5
2.0
2.5
3.0
3.5
4.0

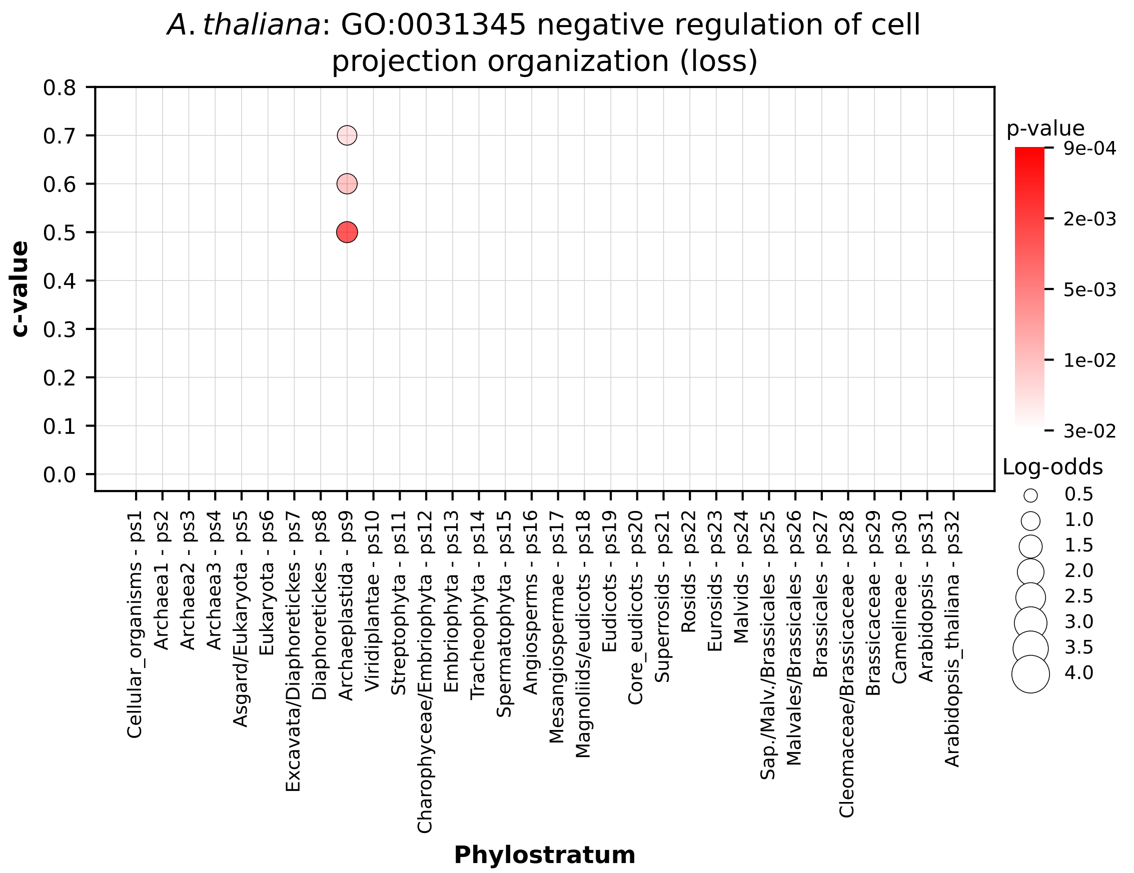

A. thaliana: GO:0031345 negative regulation of cell projection organization (loss)
c-value
0.8
0.7
0.6
0.5
0.4
0.3
0.2
0.1
0.0
Phylostratum
Cellular_organisms - ps1
Archaea1 - ps2
Archaea2 - ps3
Archaea3 - ps4
Asgard/Eukaryota - ps5
Eukaryota - ps6
Excavata/Diaphoretickes - ps7
Diaphoretickes - ps8
Archaeplastida - ps9
Viridiplantae - ps10
Streptophyta - ps11
Charophyceae/Embriophyta - ps12
Embriophyta - ps13
Tracheophyta - ps14
Spermatophyta - ps15
Angiosperms - ps16
Mesangiospermae - ps17
Magnoliids/eudicots - ps18
Eudicots - ps19
Core_eudicots - ps20
Superrosids - ps21
Rosids - ps22
Eurosids - ps23
Malvids - ps24
Sap./Malv./Brassicales - ps25
Malvales/Brassicales - ps26
Brassicales - ps27
Cleomaceae/Brassicaceae - ps28
Brassicaceae - ps29
Camelineae - ps30
Arabidopsis - ps31
Arabidopsis_thaliana - ps32
p-value
9e-04
2e-03
5e-03
1e-02
3e-02
Log-odds
0.5
1.0
1.5
2.0
2.5
3.0
3.5
4.0

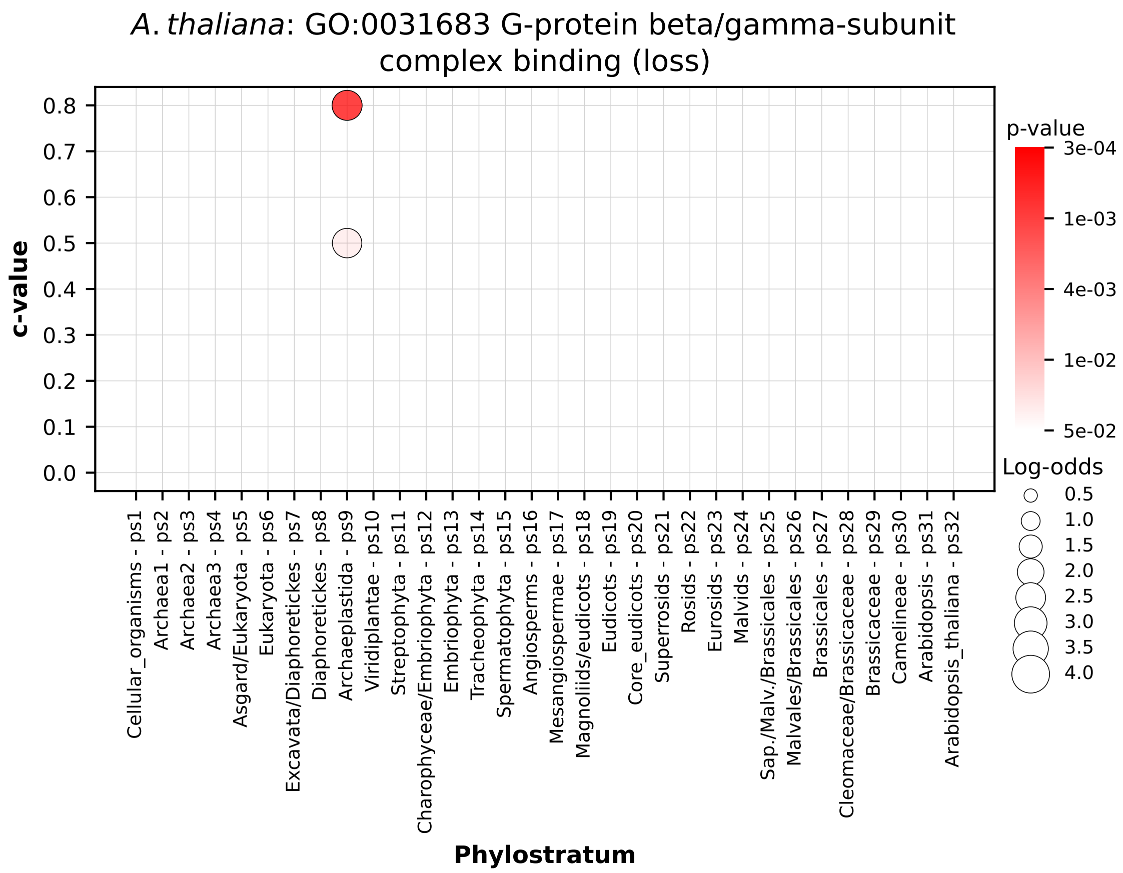

A. thaliana: GO:0031683 G-protein beta/gamma-subunit complex binding (loss)
c-value
0.0
0.1
0.2
0.3
0.4
0.5
0.6
0.7
0.8
p-value
3e-04
1e-03
4e-03
1e-02
5e-02
Log-odds
0.5
1.0
1.5
2.0
2.5
3.0
3.5
4.0
Cellular_organisms - ps1
Archaea1 - ps2
Archaea2 - ps3
Archaea3 - ps4
Asgard/Eukaryota - ps5
Eukaryota - ps6
Excavata/Diaphoretickes - ps7
Diaphoretickes - ps8
Archaeplastida - ps9
Viridiplantae - ps10
Streptophyta - ps11
Charophyceae/Embriophyta - ps12
Embriophyta - ps13
Tracheophyta - ps14
Spermatophyta - ps15
Angiosperms - ps16
Mesangiospermae - ps17
Magnoliids/eudicots - ps18
Eudicots - ps19
Core_eudicots - ps20
Superrosids - ps21
Rosids - ps22
Eurosids - ps23
Malvids - ps24
Sap./Malv./Brassicales - ps25
Malvales/Brassicales - ps26
Brassicales - ps27
Cleomaceae/Brassicaceae - ps28
Brassicaceae - ps29
Camelineae - ps30
Arabidopsis - ps31
Arabidopsis_thaliana - ps32
Phylostratum

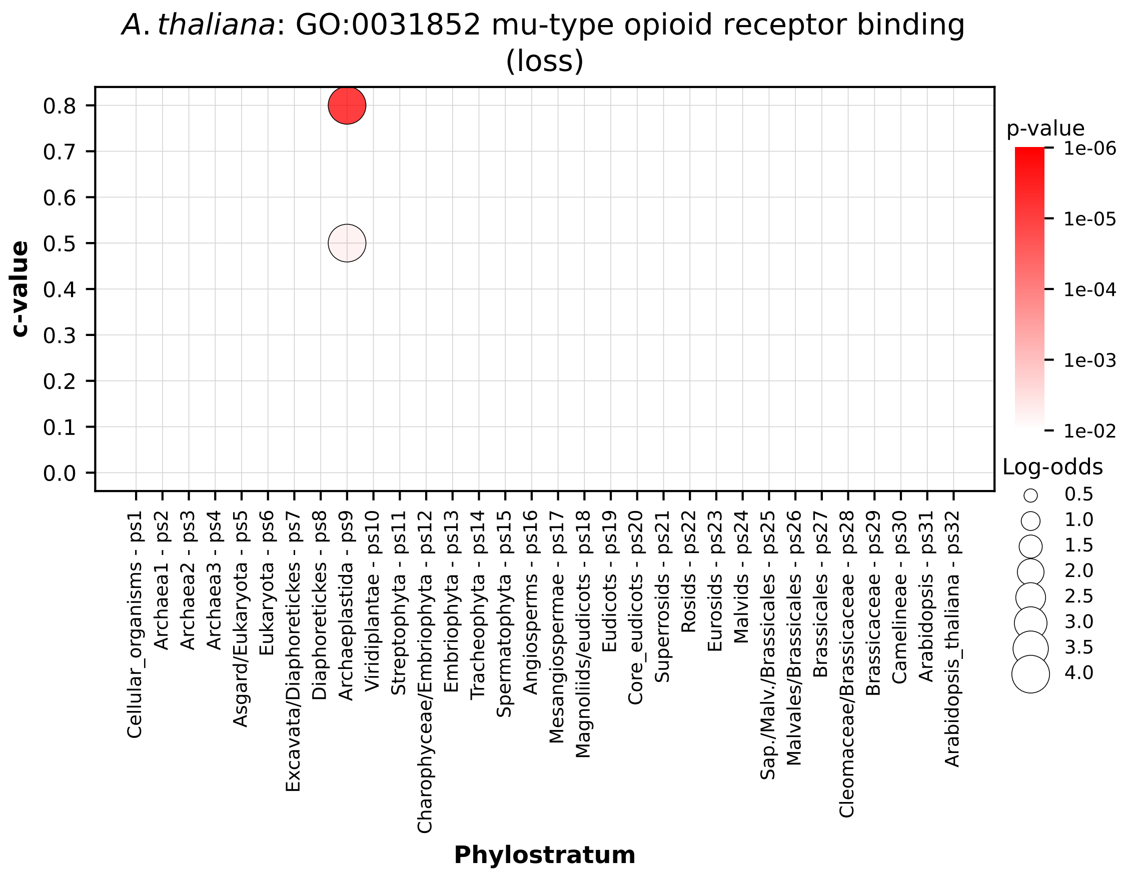

A. thaliana: GO:0031852 mu-type opioid receptor binding (loss)
c-value
0.0
0.1
0.2
0.3
0.4
0.5
0.6
0.7
0.8
p-value
1e-06
1e-05
1e-04
1e-03
1e-02
Log-odds
0.5
1.0
1.5
2.0
2.5
3.0
3.5
4.0
Cellular_organisms - ps1
Archaea1 - ps2
Archaea2 - ps3
Archaea3 - ps4
Asgard/Eukaryota - ps5
Eukaryota - ps6
Excavata/Diaphoretickes - ps7
Diaphoretickes - ps8
Archaeplastida - ps9
Viridiplantae - ps10
Streptophyta - ps11
Charophyceae/Embriophyta - ps12
Embriophyta - ps13
Tracheophyta - ps14
Spermatophyta - ps15
Angiosperms - ps16
Mesangiospermae - ps17
Magnoliids/eudicots - ps18
Eudicots - ps19
Core_eudicots - ps20
Superrosids - ps21
Rosids - ps22
Eurosids - ps23
Malvids - ps24
Sap./Malv./Brassicales - ps25
Malvales/Brassicales - ps26
Brassicales - ps27
Cleomaceae/Brassicaceae - ps28
Brassicaceae - ps29
Camelineae - ps30
Arabidopsis - ps31
Arabidopsis_thaliana - ps32
Phylostratum

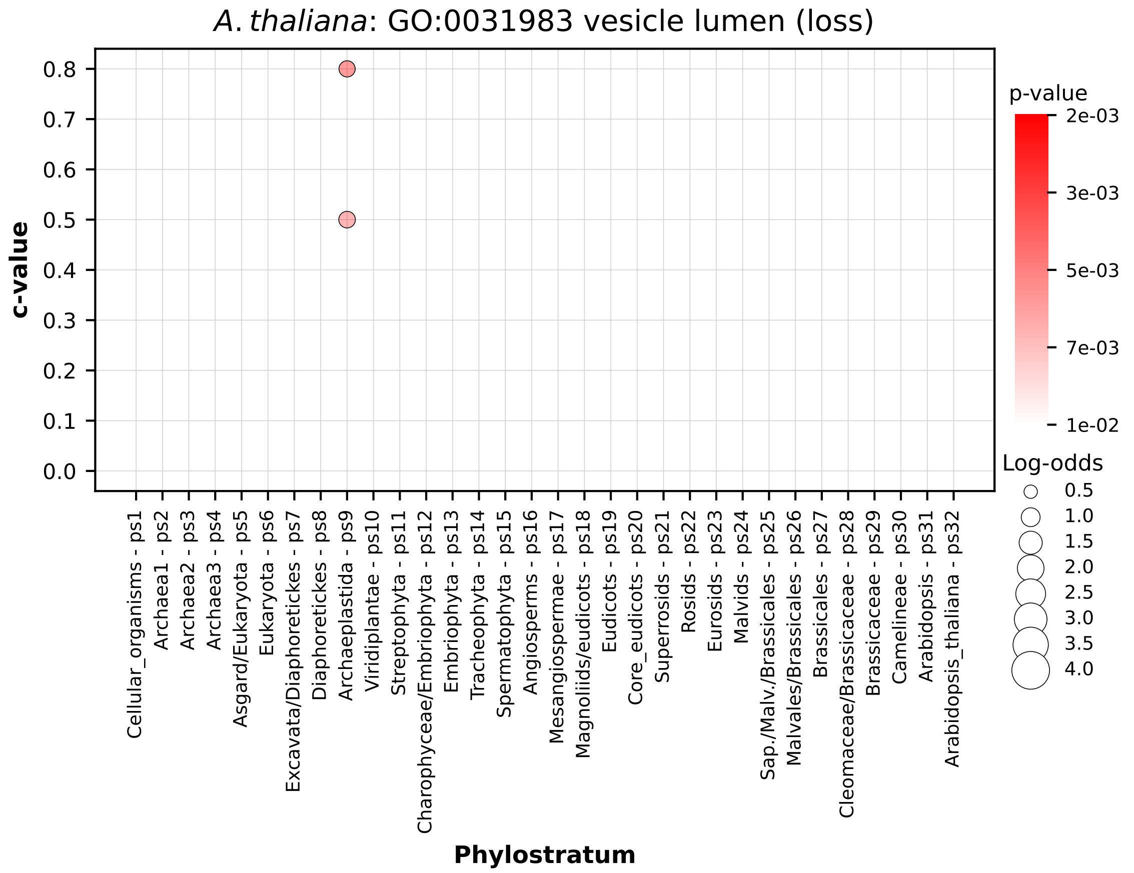

A. thaliana: GO:0031983 vesicle lumen (loss)
c-value
0.0
0.1
0.2
0.3
0.4
0.5
0.6
0.7
0.8
Phylostratum
Cellular_organisms - ps1
Archaea1 - ps2
Archaea2 - ps3
Archaea3 - ps4
Asgard/Eukaryota - ps5
Eukaryota - ps6
Excavata/Diaphoretickes - ps7
Diaphoretickes - ps8
Archaeplastida - ps9
Viridiplantae - ps10
Streptophyta - ps11
Charophyceae/Embriophyta - ps12
Embriophyta - ps13
Tracheophyta - ps14
Spermatophyta - ps15
Angiosperms - ps16
Mesangiospermae - ps17
Magnoliids/eudicots - ps18
Eudicots - ps19
Core_eudicots - ps20
Superrosids - ps21
Rosids - ps22
Eurosids - ps23
Malvids - ps24
Sap./Malv./Brassicales - ps25
Malvales/Brassicales - ps26
Brassicales - ps27
Cleomaceae/Brassicaceae - ps28
Brassicaceae - ps29
Camelineae - ps30
Arabidopsis - ps31
Arabidopsis_thaliana - ps32
p-value
2e-03
3e-03
5e-03
7e-03
1e-02
Log-odds
0.5
1.0
1.5
2.0
2.5
3.0
3.5
4.0

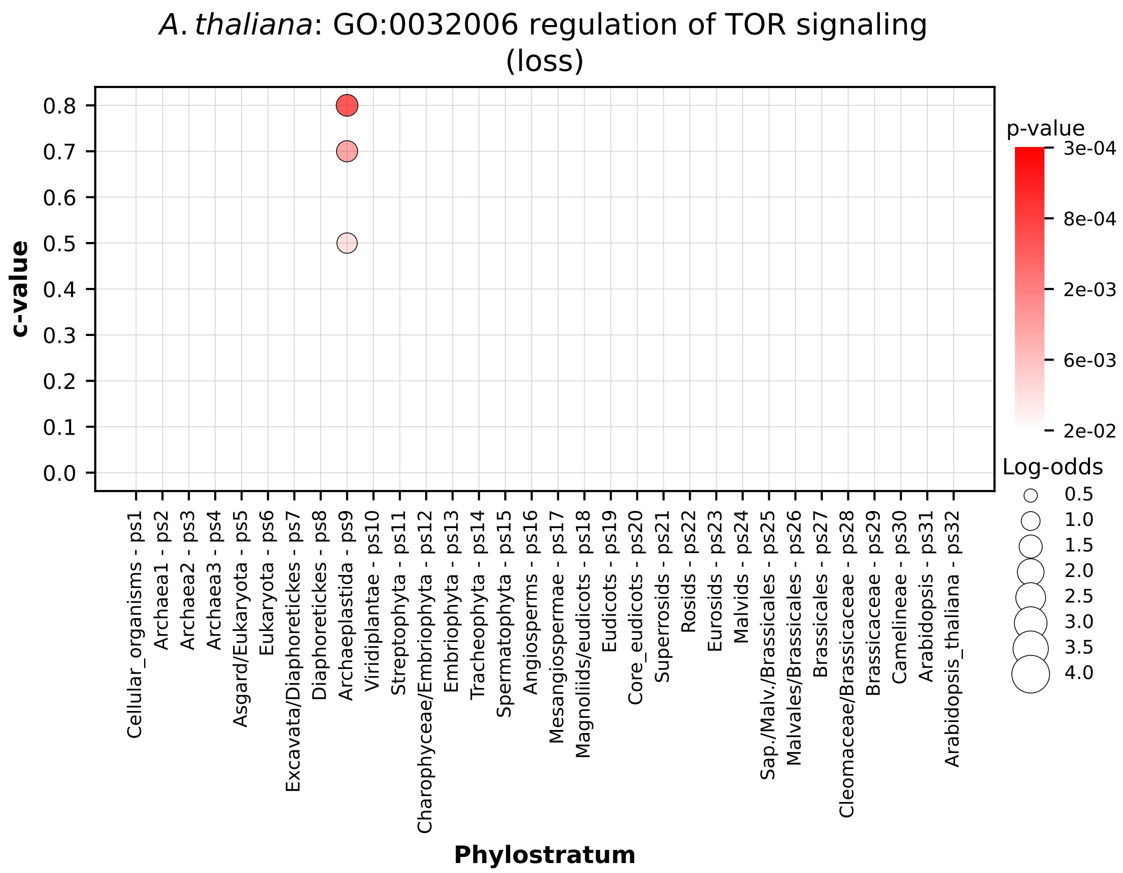
A. thaliana: GO:0032006 regulation of TOR signaling
(loss)
c-value
0.8
0.7
0.6
0.5
0.4
0.3
0.2
0.1
0.0
p-value
3e-04
8e-04
2e-03
6e-03
2e-02
Log-odds
0.5
1.0
1.5
2.0
2.5
3.0
3.5
4.0
Cellular_organisms - ps1
Archaea1 - ps2
Archaea2 - ps3
Archaea3 - ps4
Asgard/Eukaryota - ps5
Eukaryota - ps6
Excavata/Diaphoretickes - ps7
Diaphoretickes - ps8
Archaeplastida - ps9
Viridiplantae - ps10
Streptophyta - ps11
Charophyceae/Embriophyta - ps12
Embriophyta - ps13
Tracheophyta - ps14
Spermatophyta - ps15
Angiosperms - ps16
Mesangiospermae - ps17
Magnoliids/eudicots - ps18
Eudicots - ps19
Core_eudicots - ps20
Superrosids - ps21
Rosids - ps22
Eurosids - ps23
Malvids - ps24
Sap./Malv./Brassicales - ps25
Malvales/Brassicales - ps26
Brassicales - ps27
Cleomaceae/Brassicaceae - ps28
Brassicaceae - ps29
Camelineae - ps30
Arabidopsis - ps31
Arabidopsis_thaliana - ps32
Phylostratum

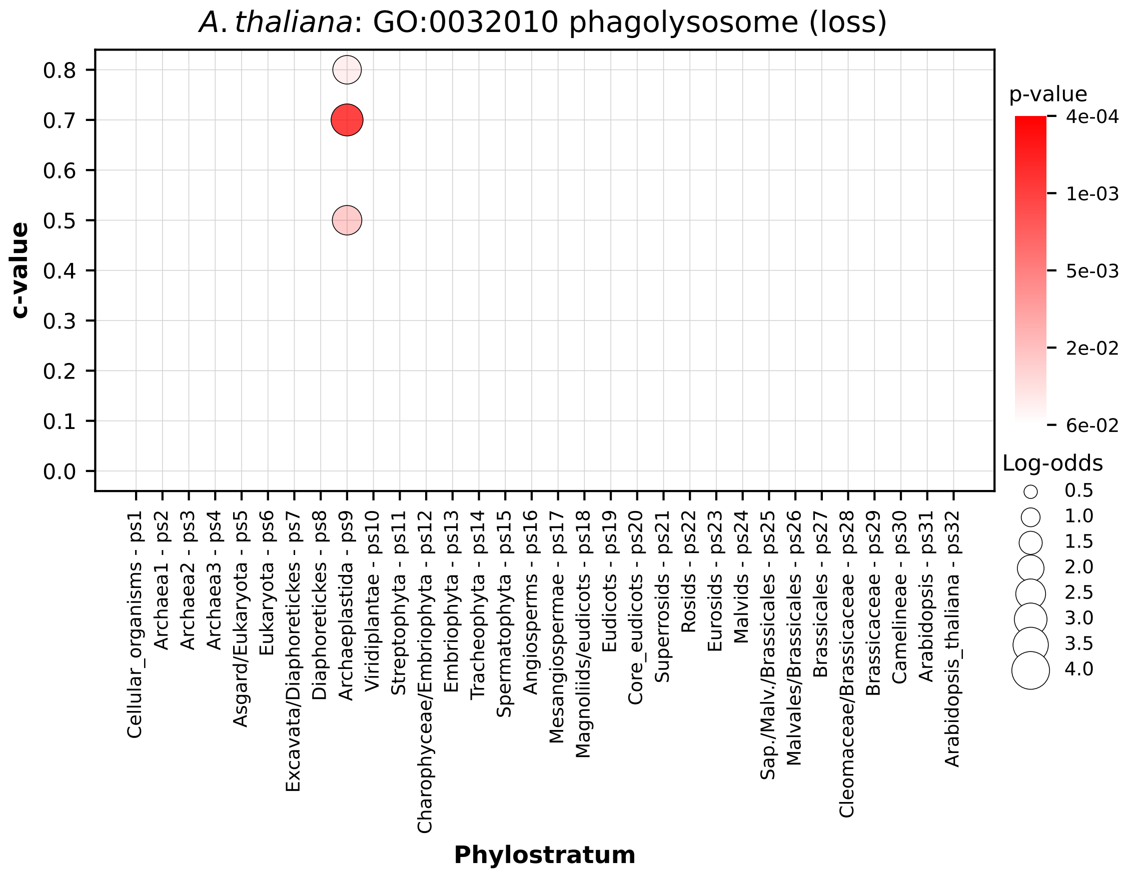

A. thaliana: GO:0032010 phagolysosome (loss)
c-value
0.0
0.1
0.2
0.3
0.4
0.5
0.6
0.7
0.8
Phylostratum
Cellular_organisms - ps1
Archaea1 - ps2
Archaea2 - ps3
Archaea3 - ps4
Asgard/Eukaryota - ps5
Eukaryota - ps6
Excavata/Diaphoretickes - ps7
Diaphoretickes - ps8
Archaeplastida - ps9
Viridiplantae - ps10
Streptophyta - ps11
Charophyceae/Embriophyta - ps12
Embriophyta - ps13
Tracheophyta - ps14
Spermatophyta - ps15
Angiosperms - ps16
Mesangiospermae - ps17
Magnoliids/eudicots - ps18
Eudicots - ps19
Core_eudicots - ps20
Superrosids - ps21
Rosids - ps22
Eurosids - ps23
Malvids - ps24
Sap./Malv./Brassicales - ps25
Malvales/Brassicales - ps26
Brassicales - ps27
Cleomaceae/Brassicaceae - ps28
Brassicaceae - ps29
Camelineae - ps30
Arabidopsis - ps31
Arabidopsis_thaliana - ps32
p-value
4e-04
1e-03
5e-03
2e-02
6e-02
Log-odds
0.5
1.0
1.5
2.0
2.5
3.0
3.5
4.0

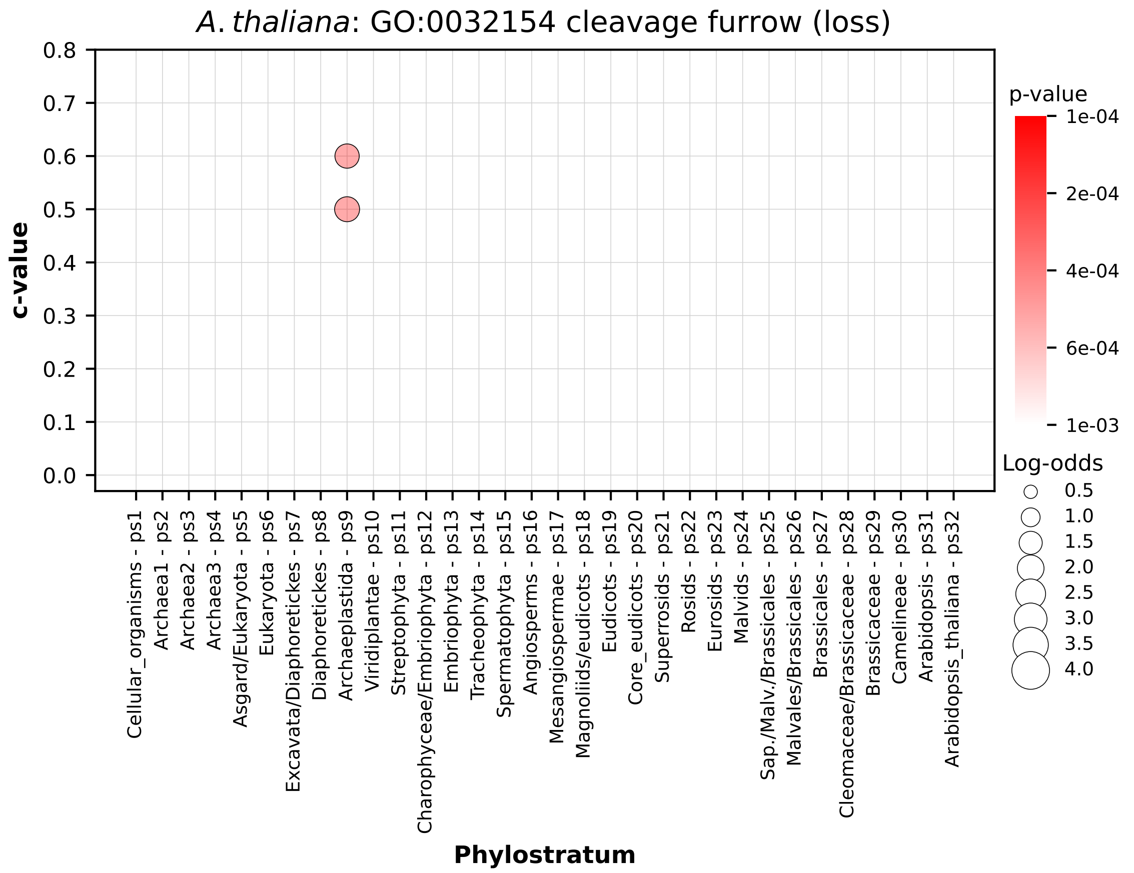

A. thaliana: GO:0032154 cleavage furrow (loss)
c-value
0.8
0.7
0.6
0.5
0.4
0.3
0.2
0.1
0.0
Phylostratum
Cellular_organisms - ps1
Archaea1 - ps2
Archaea2 - ps3
Archaea3 - ps4
Asgard/Eukaryota - ps5
Eukaryota - ps6
Excavata/Diaphoretickes - ps7
Diaphoretickes - ps8
Archaeplastida - ps9
Viridiplantae - ps10
Streptophyta - ps11
Charophyceae/Embriophyta - ps12
Embriophyta - ps13
Tracheophyta - ps14
Spermatophyta - ps15
Angiosperms - ps16
Mesangiospermae - ps17
Magnoliids/eudicots - ps18
Eudicots - ps19
Core_eudicots - ps20
Superrosids - ps21
Rosids - ps22
Eurosids - ps23
Malvids - ps24
Sap./Malv./Brassicales - ps25
Malvales/Brassicales - ps26
Brassicales - ps27
Cleomaceae/Brassicaceae - ps28
Brassicaceae - ps29
Camelineae - ps30
Arabidopsis - ps31
Arabidopsis_thaliana - ps32
p-value
1e-04
2e-04
4e-04
6e-04
1e-03
Log-odds
0.5
1.0
1.5
2.0
2.5
3.0
3.5
4.0

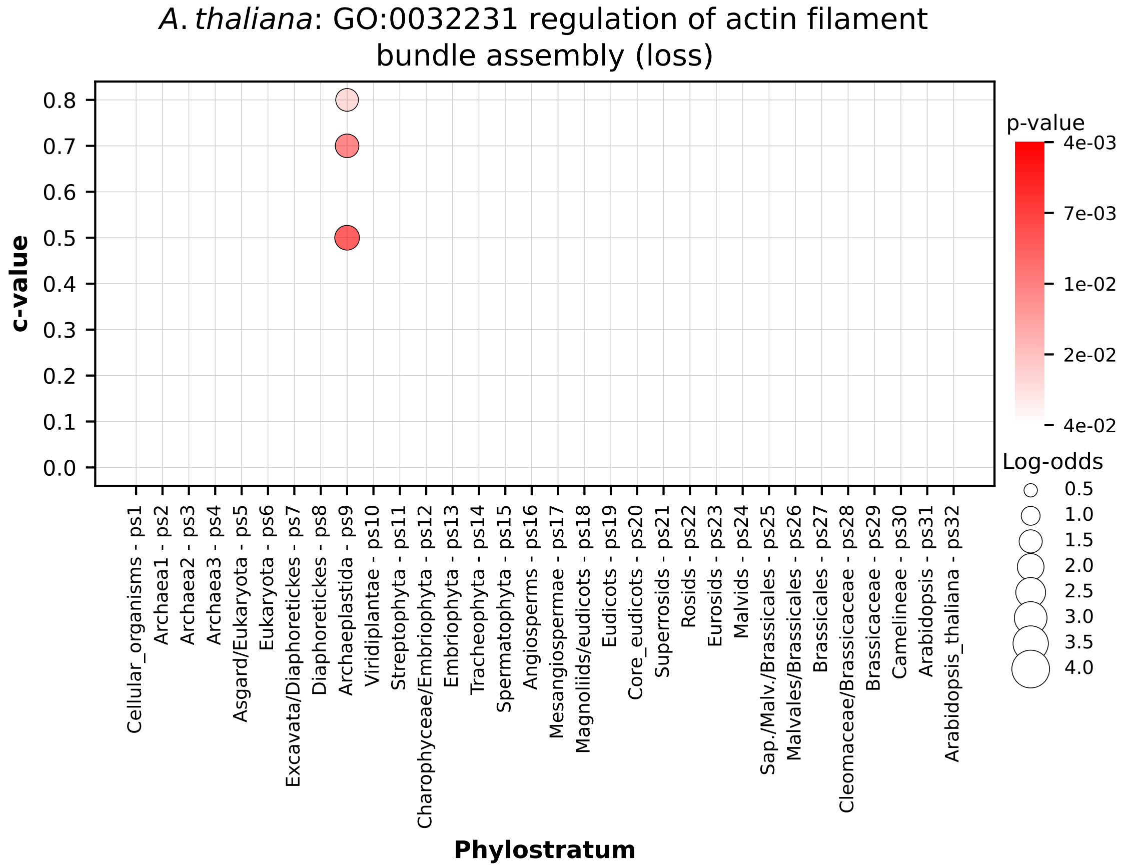

A. thaliana: GO:0032231 regulation of actin filament bundle assembly (loss)
c-value
0.8
0.7
0.6
0.5
0.4
0.3
0.2
0.1
0.0
Phylostratum
Cellular_organisms - ps1
Archaea1 - ps2
Archaea2 - ps3
Archaea3 - ps4
Asgard/Eukaryota - ps5
Eukaryota - ps6
Excavata/Diaphoretickes - ps7
Diaphoretickes - ps8
Archaeplastida - ps9
Viridiplantae - ps10
Streptophyta - ps11
Charophyceae/Embriophyta - ps12
Embriophyta - ps13
Tracheophyta - ps14
Spermatophyta - ps15
Angiosperms - ps16
Mesangiospermae - ps17
Magnoliids/eudicots - ps18
Eudicots - ps19
Core_eudicots - ps20
Superrosids - ps21
Rosids - ps22
Eurosids - ps23
Malvids - ps24
Sap./Malv./Brassicales - ps25
Malvales/Brassicales - ps26
Brassicales - ps27
Cleomaceae/Brassicaceae - ps28
Brassicaceae - ps29
Camelineae - ps30
Arabidopsis - ps31
Arabidopsis_thaliana - ps32
p-value
4e-03
7e-03
1e-02
2e-02
4e-02
Log-odds
0.5
1.0
1.5
2.0
2.5
3.0
3.5
4.0

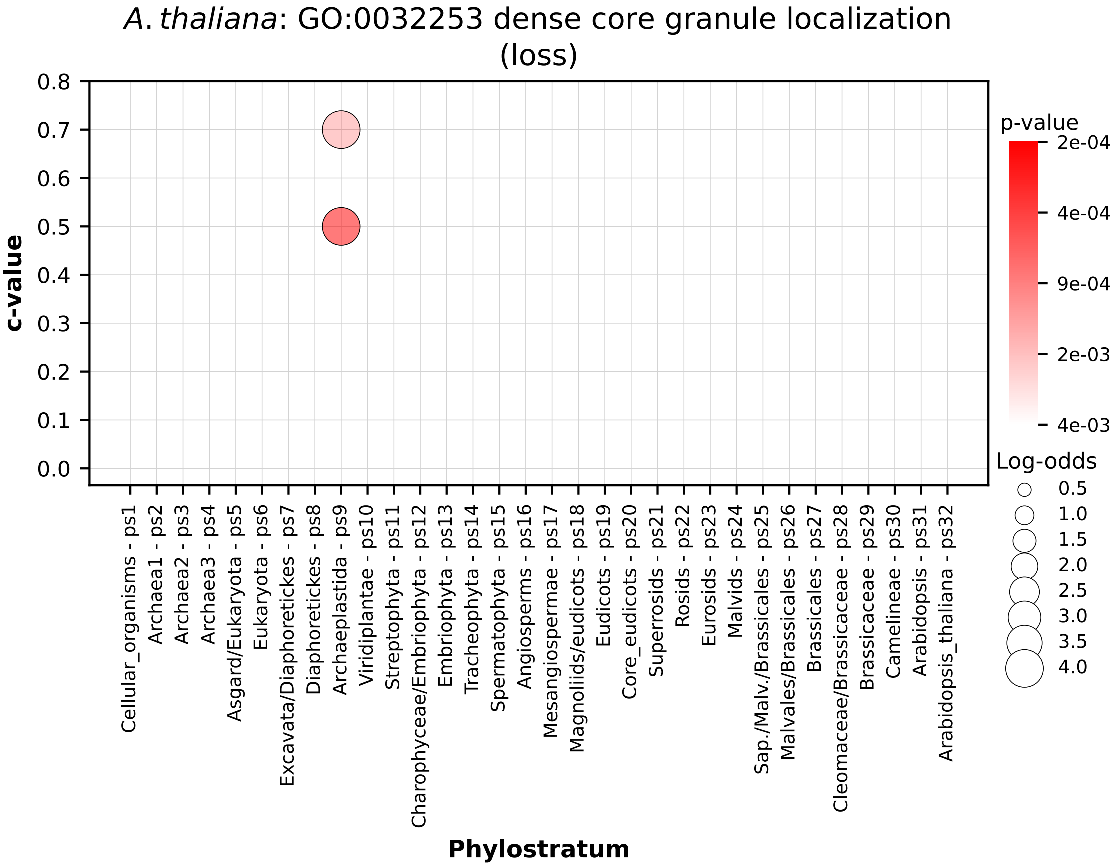

A. thaliana: GO:0032253 dense core granule localization
(loss)
c-value
0.8
0.7
0.6
0.5
0.4
0.3
0.2
0.1
0.0
p-value
2e-04
4e-04
9e-04
2e-03
4e-03
Log-odds
0.5
1.0
1.5
2.0
2.5
3.0
3.5
4.0
Cellular_organisms - ps1
Archaea1 - ps2
Archaea2 - ps3
Archaea3 - ps4
Asgard/Eukaryota - ps5
Eukaryota - ps6
Excavata/Diaphoretickes - ps7
Diaphoretickes - ps8
Archaeplastida - ps9
Viridiplantae - ps10
Streptophyta - ps11
Charophyceae/Embriophyta - ps12
Embriophyta - ps13
Tracheophyta - ps14
Spermatophyta - ps15
Angiosperms - ps16
Mesangiospermae - ps17
Magnoliids/eudicots - ps18
Eudicots - ps19
Core_eudicots - ps20
Superrosids - ps21
Rosids - ps22
Eurosids - ps23
Malvids - ps24
Sap./Malv./Brassicales - ps25
Malvales/Brassicales - ps26
Brassicales - ps27
Cleomaceae/Brassicaceae - ps28
Brassicaceae - ps29
Camelineae - ps30
Arabidopsis - ps31
Arabidopsis_thaliana - ps32
Phylostratum

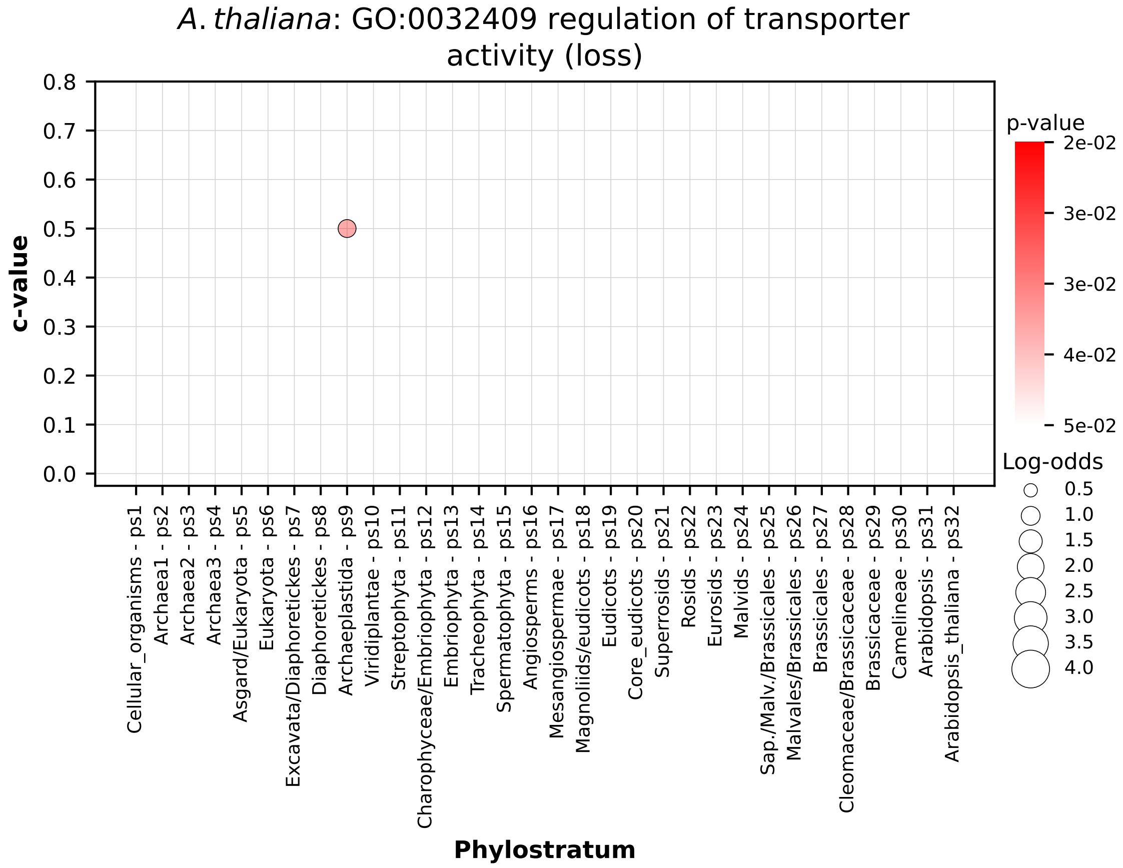

$A. thaliana$: GO:0032409 regulation of transporter activity (loss)
c-value
0.8
0.7
0.6
0.5
0.4
0.3
0.2
0.1
0.0
Phylostratum
Cellular_organisms - ps1
Archaea1 - ps2
Archaea2 - ps3
Archaea3 - ps4
Asgard/Eukaryota - ps5
Eukaryota - ps6
Excavata/Diaphoretickes - ps7
Diaphoretickes - ps8
Archaeplastida - ps9
Viridiplantae - ps10
Streptophyta - ps11
Charophyceae/Embriophyta - ps12
Embriophyta - ps13
Tracheophyta - ps14
Spermatophyta - ps15
Angiosperms - ps16
Mesangiospermae - ps17
Magnoliids/eudicots - ps18
Eudicots - ps19
Core_eudicots - ps20
Superrosids - ps21
Rosids - ps22
Eurosids - ps23
Malvids - ps24
Sap./Malv./Brassicales - ps25
Malvales/Brassicales - ps26
Brassicales - ps27
Cleomaceae/Brassicaceae - ps28
Brassicaceae - ps29
Camelineae - ps30
Arabidopsis - ps31
Arabidopsis_thaliana - ps32
p-value
2e-02
3e-02
3e-02
4e-02
5e-02
Log-odds
0.5
1.0
1.5
2.0
2.5
3.0
3.5
4.0

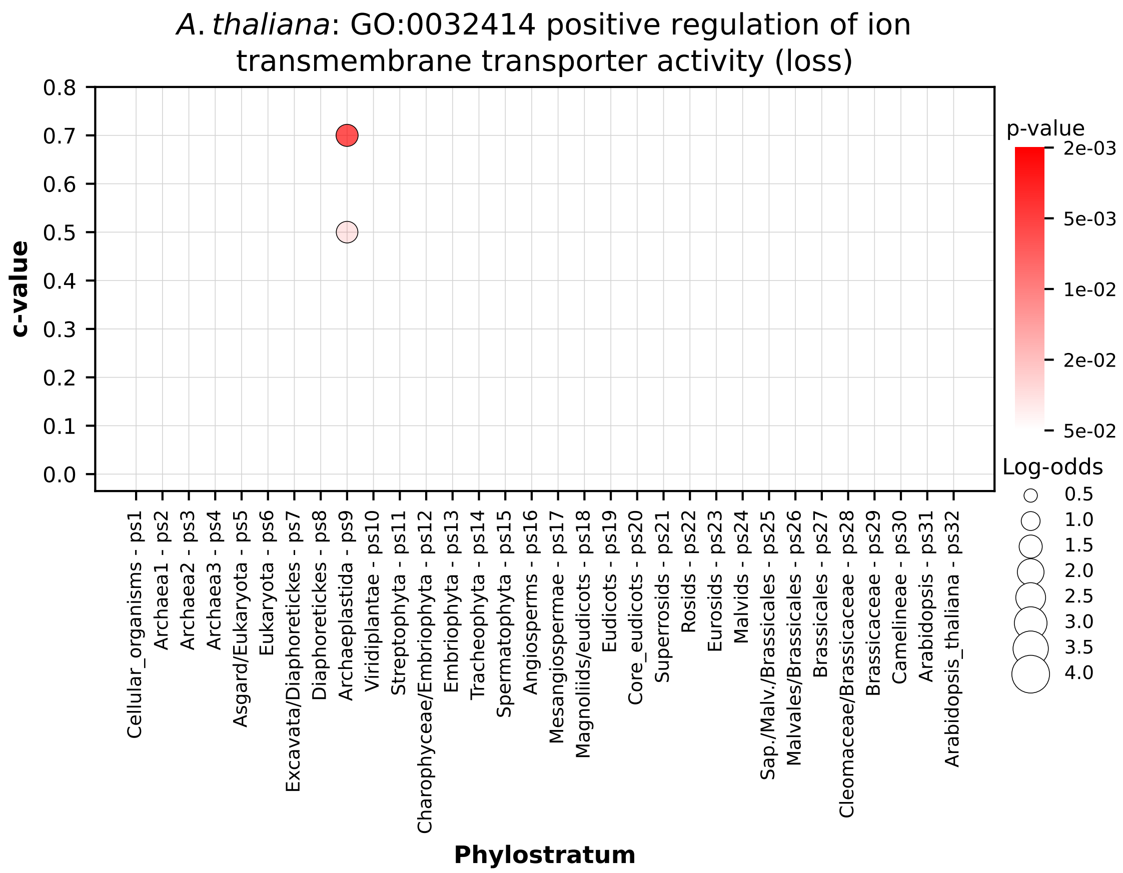
A. thaliana: GO:0032414 positive regulation of ion transmembrane transporter activity (loss)
c-value
0.8
0.7
0.6
0.5
0.4
0.3
0.2
0.1
0.0
p-value
2e-03
5e-03
1e-02
2e-02
5e-02
Log-odds
0.5
1.0
1.5
2.0
2.5
3.0
3.5
4.0
Cellular_organisms - ps1
Archaea1 - ps2
Archaea2 - ps3
Archaea3 - ps4
Asgard/Eukaryota - ps5
Eukaryota - ps6
Excavata/Diaphoretickes - ps7
Diaphoretickes - ps8
Archaeplastida - ps9
Viridiplantae - ps10
Streptophyta - ps11
Charophyceae/Embriophyta - ps12
Embriophyta - ps13
Tracheophyta - ps14
Spermatophyta - ps15
Angiosperms - ps16
Mesangiospermae - ps17
Magnoliids/eudicots - ps18
Eudicots - ps19
Core_eudicots - ps20
Superrosids - ps21
Rosids - ps22
Eurosids - ps23
Malvids - ps24
Sap./Malv./Brassicales - ps25
Malvales/Brassicales - ps26
Brassicales - ps27
Cleomaceae/Brassicaceae - ps28
Brassicaceae - ps29
Camelineae - ps30
Arabidopsis - ps31
Arabidopsis_thaliana - ps32
Phylostratum

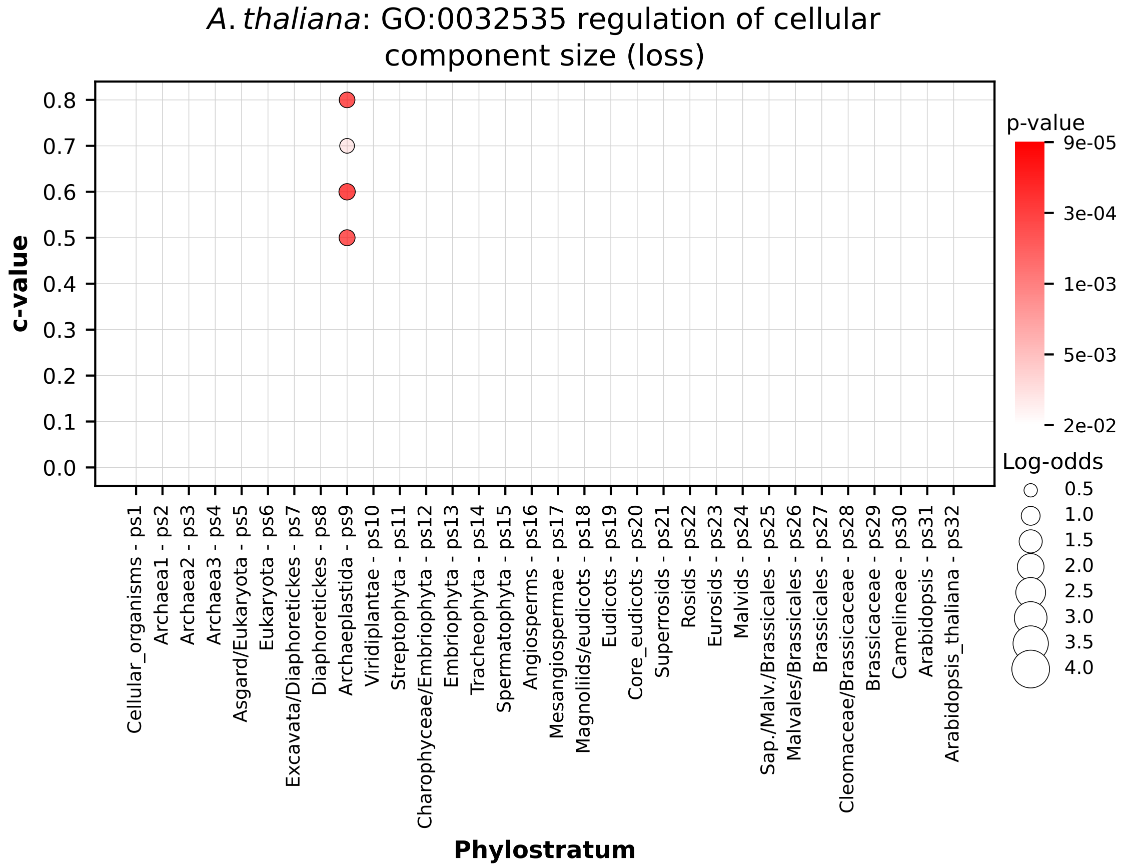

A. thaliana: GO:0032535 regulation of cellular component size (loss)
c-value
0.8
0.7
0.6
0.5
0.4
0.3
0.2
0.1
0.0
p-value
9e-05
3e-04
1e-03
5e-03
2e-02
Log-odds
0.5
1.0
1.5
2.0
2.5
3.0
3.5
4.0
Cellular_organisms - ps1
Archaea1 - ps2
Archaea2 - ps3
Archaea3 - ps4
Asgard/Eukaryota - ps5
Eukaryota - ps6
Excavata/Diaphoretickes - ps7
Diaphoretickes - ps8
Archaeplastida - ps9
Viridiplantae - ps10
Streptophyta - ps11
Charophyceae/Embriophyta - ps12
Embriophyta - ps13
Tracheophyta - ps14
Spermatophyta - ps15
Angiosperms - ps16
Mesangiospermae - ps17
Magnoliids/eudicots - ps18
Eudicots - ps19
Core_eudicots - ps20
Superrosids - ps21
Rosids - ps22
Eurosids - ps23
Malvids - ps24
Sap./Malv./Brassicales - ps25
Malvales/Brassicales - ps26
Brassicales - ps27
Cleomaceae/Brassicaceae - ps28
Brassicaceae - ps29
Camelineae - ps30
Arabidopsis - ps31
Arabidopsis_thaliana - ps32
Phylostratum

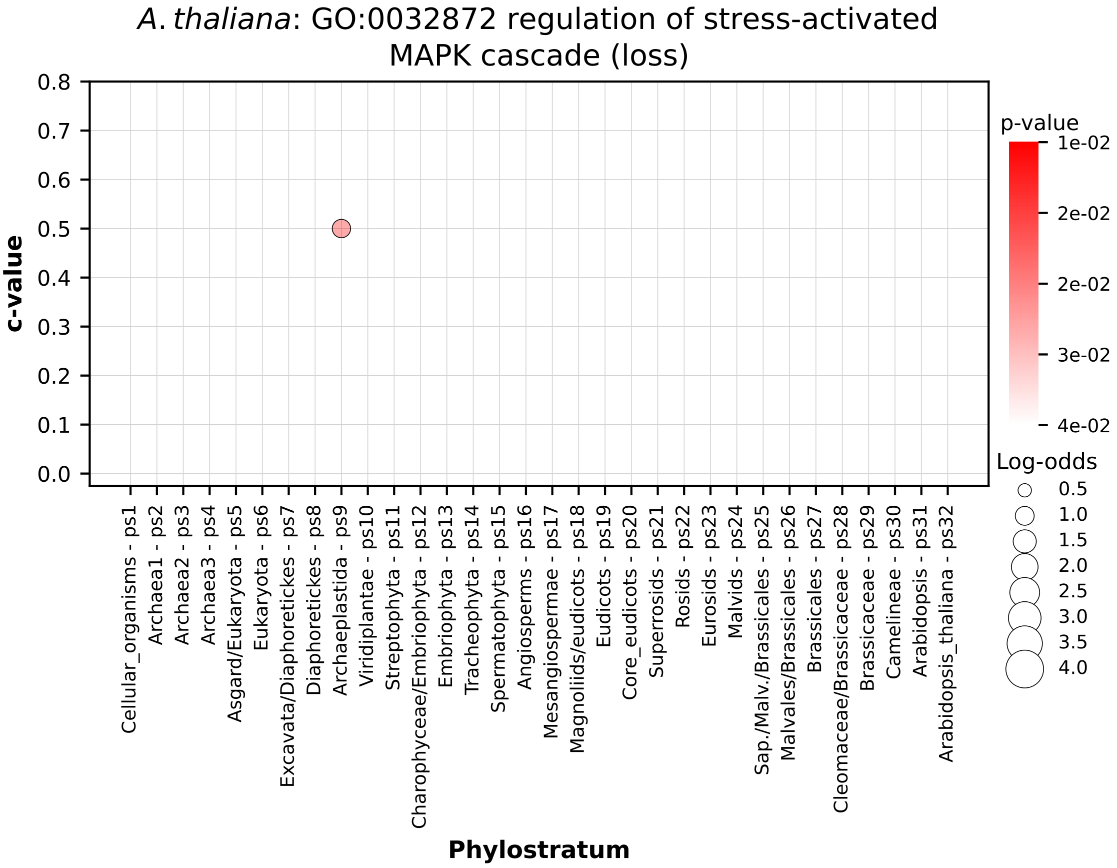

A. thaliana: GO:0032872 regulation of stress-activated MAPK cascade (loss)
c-value
0.8
0.7
0.6
0.5
0.4
0.3
0.2
0.1
0.0
p-value
1e-02
2e-02
2e-02
3e-02
4e-02
Log-odds
0.5
1.0
1.5
2.0
2.5
3.0
3.5
4.0
Cellular_organisms - ps1
Archaea1 - ps2
Archaea2 - ps3
Archaea3 - ps4
Asgard/Eukaryota - ps5
Eukaryota - ps6
Excavata/Diaphoretickes - ps7
Diaphoretickes - ps8
Archaeplastida - ps9
Viridiplantae - ps10
Streptophyta - ps11
Charophyceae/Embriophyta - ps12
Embriophyta - ps13
Tracheophyta - ps14
Spermatophyta - ps15
Angiosperms - ps16
Mesangiospermae - ps17
Magnoliids/eudicots - ps18
Eudicots - ps19
Core_eudicots - ps20
Superrosids - ps21
Rosids - ps22
Eurosids - ps23
Malvids - ps24
Sap./Malv./Brassicales - ps25
Malvales/Brassicales - ps26
Brassicales - ps27
Cleomaceae/Brassicaceae - ps28
Brassicaceae - ps29
Camelineae - ps30
Arabidopsis - ps31
Arabidopsis_thaliana - ps32
Phylostratum

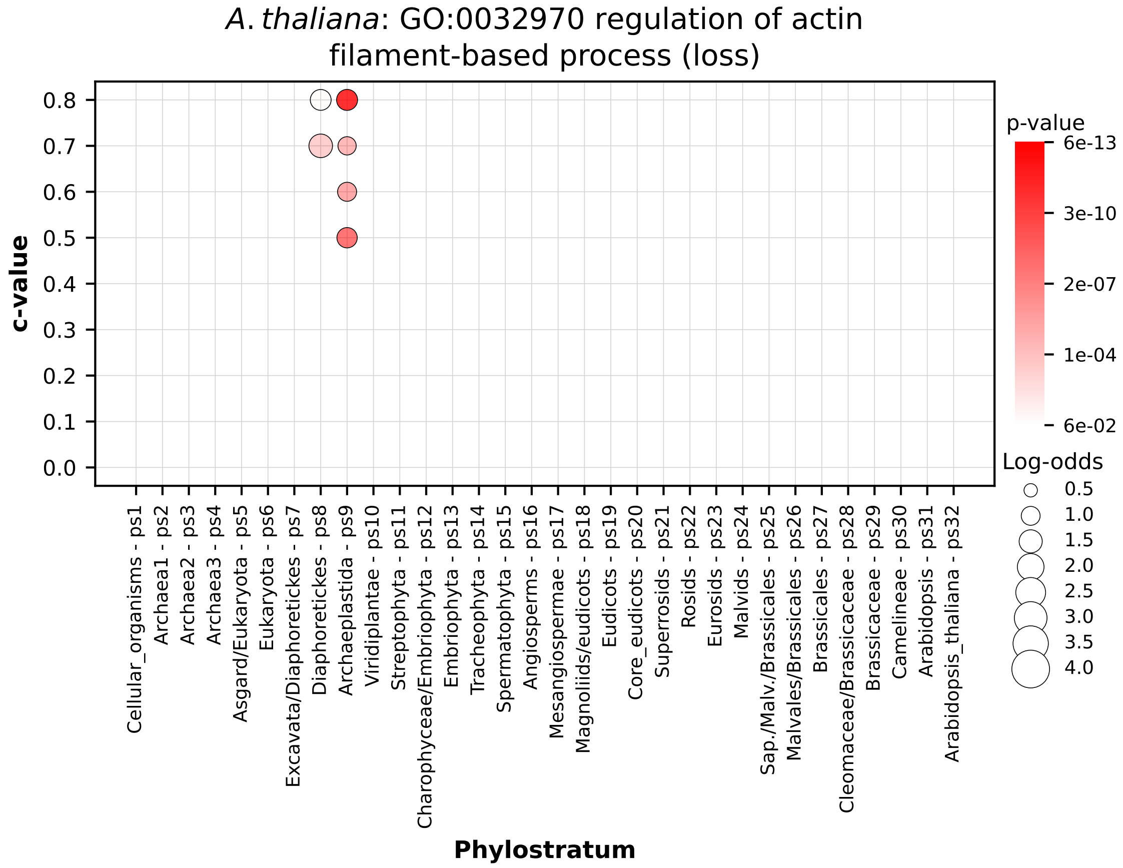

A. thaliana: GO:0032970 regulation of actin filament-based process (loss)
c-value
0.8
0.7
0.6
0.5
0.4
0.3
0.2
0.1
0.0
p-value
6e-13
3e-10
2e-07
1e-04
6e-02
Log-odds
0.5
1.0
1.5
2.0
2.5
3.0
3.5
4.0
Cellular_organisms - ps1
Archaea1 - ps2
Archaea2 - ps3
Archaea3 - ps4
Asgard/Eukaryota - ps5
Eukaryota - ps6
Excavata/Diaphoretickes - ps7
Diaphoretickes - ps8
Archaeplastida - ps9
Viridiplantae - ps10
Streptophyta - ps11
Charophyceae/Embriophyta - ps12
Embriophyta - ps13
Tracheophyta - ps14
Spermatophyta - ps15
Angiosperms - ps16
Mesangiospermae - ps17
Magnoliids/eudicots - ps18
Eudicots - ps19
Core_eudicots - ps20
Superrosids - ps21
Rosids - ps22
Eurosids - ps23
Malvids - ps24
Sap./Malv./Brassicales - ps25
Malvales/Brassicales - ps26
Brassicales - ps27
Cleomaceae/Brassicaceae - ps28
Brassicaceae - ps29
Camelineae - ps30
Arabidopsis - ps31
Arabidopsis_thaliana - ps32
Phylostratum

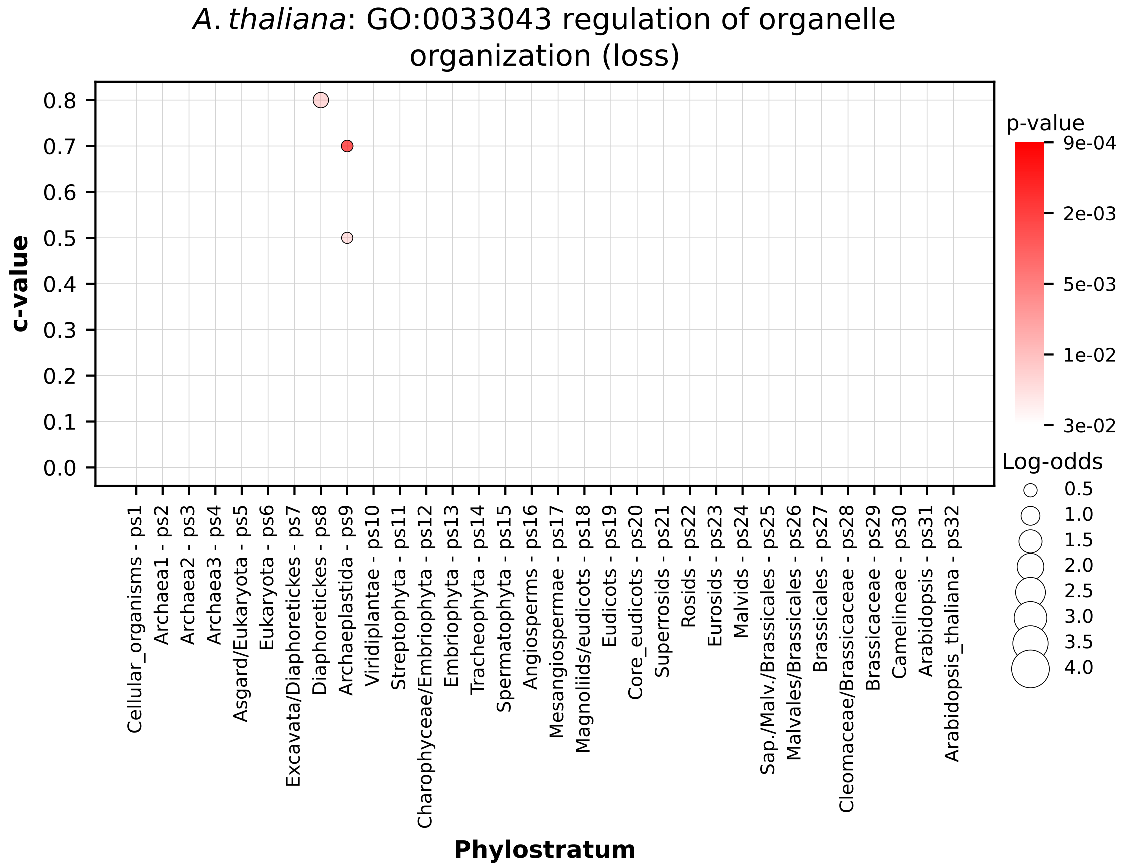

A. thaliana: GO:0033043 regulation of organelle organization (loss)
c-value
0.0
0.1
0.2
0.3
0.4
0.5
0.6
0.7
0.8
Phylostratum
Cellular_organisms - ps1
Archaea1 - ps2
Archaea2 - ps3
Archaea3 - ps4
Asgard/Eukaryota - ps5
Eukaryota - ps6
Excavata/Diaphoretickes - ps7
Diaphoretickes - ps8
Archaeplastida - ps9
Viridiplantae - ps10
Streptophyta - ps11
Charophyceae/Embriophyta - ps12
Embriophyta - ps13
Tracheophyta - ps14
Spermatophyta - ps15
Angiosperms - ps16
Mesangiospermae - ps17
Magnoliids/eudicots - ps18
Eudicots - ps19
Core_eudicots - ps20
Superrosids - ps21
Rosids - ps22
Eurosids - ps23
Malvids - ps24
Sap./Malv./Brassicales - ps25
Malvales/Brassicales - ps26
Brassicales - ps27
Cleomaceae/Brassicaceae - ps28
Brassicaceae - ps29
Camelineae - ps30
Arabidopsis - ps31
Arabidopsis_thaliana - ps32
p-value
9e-04
2e-03
5e-03
1e-02
3e-02
Log-odds
0.5
1.0
1.5
2.0
2.5
3.0
3.5
4.0

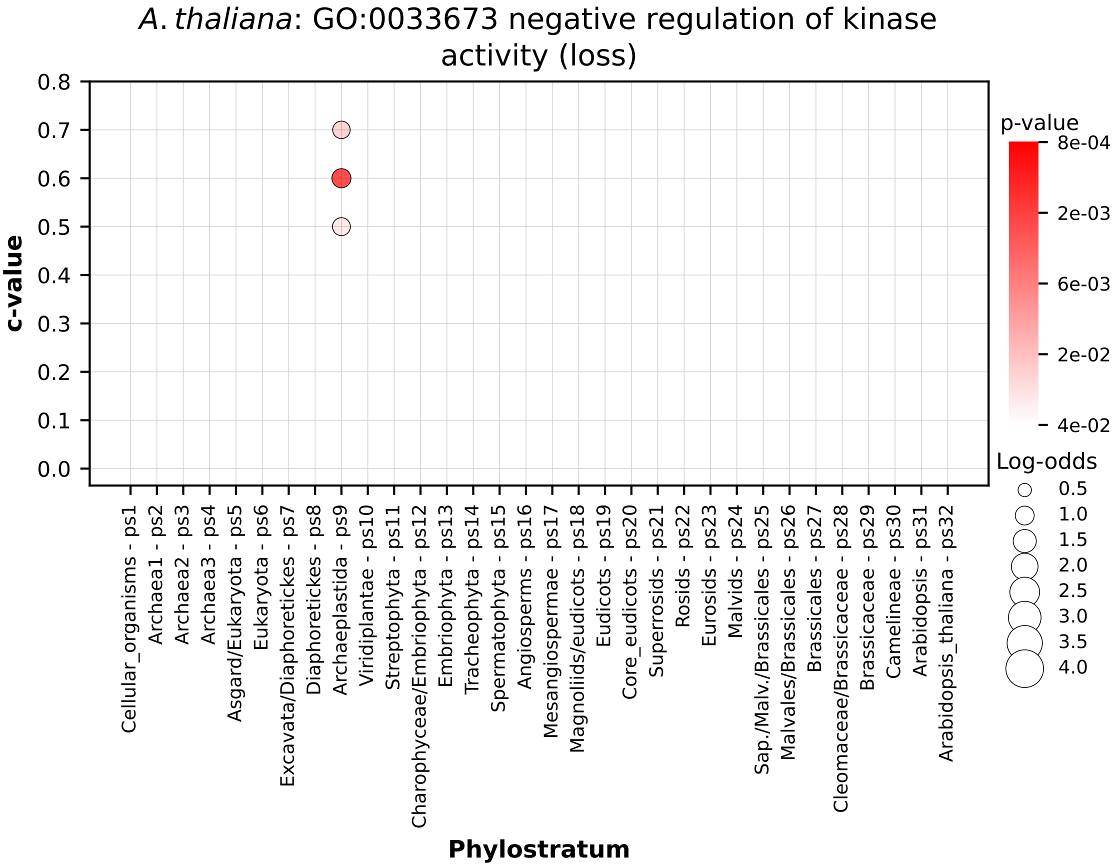

A. thaliana: GO:0033673 negative regulation of kinase activity (loss)
c-value
0.8
0.7
0.6
0.5
0.4
0.3
0.2
0.1
0.0
p-value
8e-04
2e-03
6e-03
2e-02
4e-02
Log-odds
0.5
1.0
1.5
2.0
2.5
3.0
3.5
4.0
Cellular_organisms - ps1
Archaea1 - ps2
Archaea2 - ps3
Archaea3 - ps4
Asgard/Eukaryota - ps5
Eukaryota - ps6
Excavata/Diaphoretickes - ps7
Diaphoretickes - ps8
Archaeplastida - ps9
Viridiplantae - ps10
Streptophyta - ps11
Charophyceae/Embriophyta - ps12
Embriophyta - ps13
Tracheophyta - ps14
Spermatophyta - ps15
Angiosperms - ps16
Mesangiospermae - ps17
Magnoliids/eudicots - ps18
Eudicots - ps19
Core_eudicots - ps20
Superrosids - ps21
Rosids - ps22
Eurosids - ps23
Malvids - ps24
Sap./Malv./Brassicales - ps25
Malvales/Brassicales - ps26
Brassicales - ps27
Cleomaceae/Brassicaceae - ps28
Brassicaceae - ps29
Camelineae - ps30
Arabidopsis - ps31
Arabidopsis_thaliana - ps32
Phylostratum

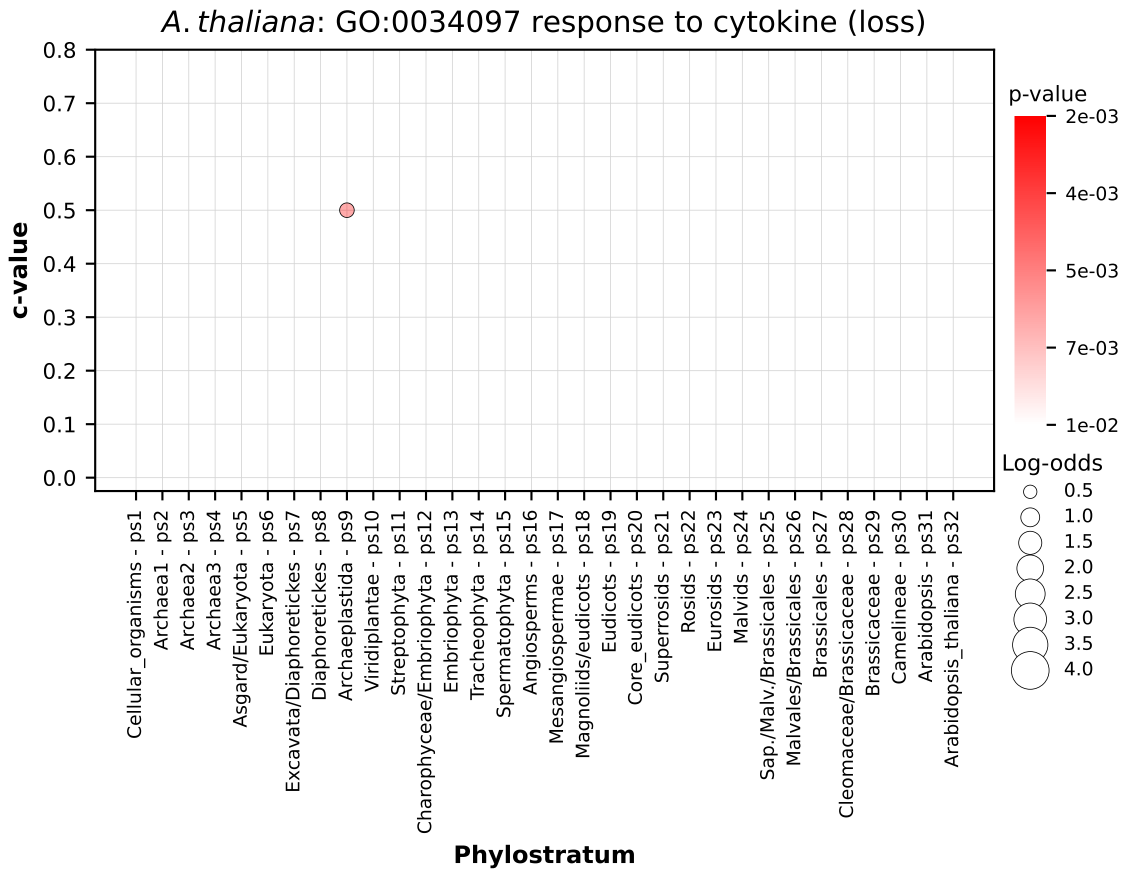
A. thaliana: GO:0034097 response to cytokine (loss)
c-value
0.8
0.7
0.6
0.5
0.4
0.3
0.2
0.1
0.0
Phylostratum
Cellular_organisms - ps1
Archaea1 - ps2
Archaea2 - ps3
Archaea3 - ps4
Asgard/Eukaryota - ps5
Eukaryota - ps6
Excavata/Diaphoretickes - ps7
Diaphoretickes - ps8
Archaeplastida - ps9
Viridiplantae - ps10
Streptophyta - ps11
Charophyceae/Embriophyta - ps12
Embriophyta - ps13
Tracheophyta - ps14
Spermatophyta - ps15
Angiosperms - ps16
Mesangiospermae - ps17
Magnoliids/eudicots - ps18
Eudicots - ps19
Core_eudicots - ps20
Superrosids - ps21
Rosids - ps22
Eurosids - ps23
Malvids - ps24
Sap./Malv./Brassicales - ps25
Malvales/Brassicales - ps26
Brassicales - ps27
Cleomaceae/Brassicaceae - ps28
Brassicaceae - ps29
Camelineae - ps30
Arabidopsis - ps31
Arabidopsis_thaliana - ps32
p-value
2e-03
4e-03
5e-03
7e-03
1e-02
Log-odds
0.5
1.0
1.5
2.0
2.5
3.0
3.5
4.0

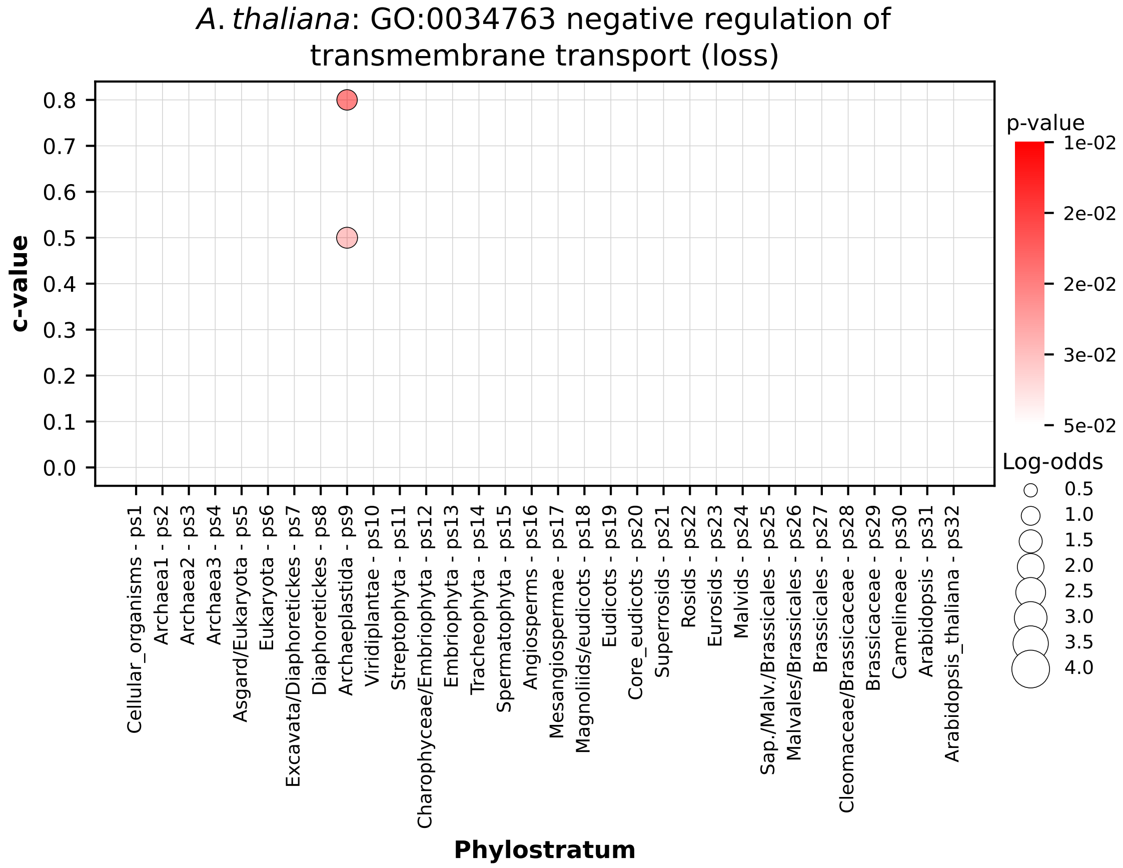
A. thaliana: GO:0034763 negative regulation of transmembrane transport (loss)
c-value
0.0
0.1
0.2
0.3
0.4
0.5
0.6
0.7
0.8
Phylostratum
Cellular_organisms - ps1
Archaea1 - ps2
Archaea2 - ps3
Archaea3 - ps4
Asgard/Eukaryota - ps5
Eukaryota - ps6
Excavata/Diaphoretickes - ps7
Diaphoretickes - ps8
Archaeplastida - ps9
Viridiplantae - ps10
Streptophyta - ps11
Charophyceae/Embriophyta - ps12
Embriophyta - ps13
Tracheophyta - ps14
Spermatophyta - ps15
Angiosperms - ps16
Mesangiospermae - ps17
Magnoliids/eudicots - ps18
Eudicots - ps19
Core_eudicots - ps20
Superrosids - ps21
Rosids - ps22
Eurosids - ps23
Malvids - ps24
Sap./Malv./Brassicales - ps25
Malvales/Brassicales - ps26
Brassicales - ps27
Cleomaceae/Brassicaceae - ps28
Brassicaceae - ps29
Camelineae - ps30
Arabidopsis - ps31
Arabidopsis_thaliana - ps32
p-value
1e-02
2e-02
2e-02
3e-02
5e-02
Log-odds
0.5
1.0
1.5
2.0
2.5
3.0
3.5
4.0

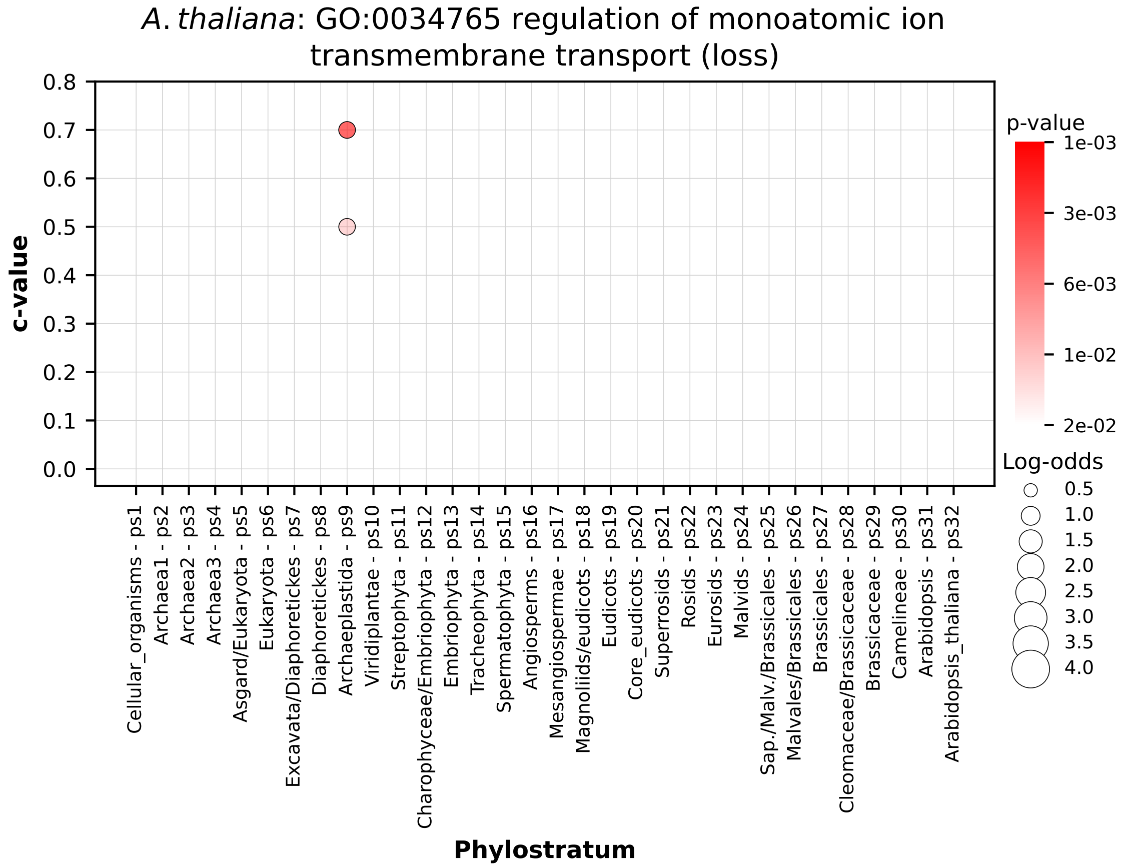

A. thaliana: GO:0034765 regulation of monoatomic ion transmembrane transport (loss)
c-value
0.8
0.7
0.6
0.5
0.4
0.3
0.2
0.1
0.0
p-value
1e-03
3e-03
6e-03
1e-02
2e-02
Log-odds
0.5
1.0
1.5
2.0
2.5
3.0
3.5
4.0
Cellular_organisms - ps1
Archaea1 - ps2
Archaea2 - ps3
Archaea3 - ps4
Asgard/Eukaryota - ps5
Eukaryota - ps6
Excavata/Diaphoretickes - ps7
Diaphoretickes - ps8
Archaeplastida - ps9
Viridiplantae - ps10
Streptophyta - ps11
Charophyceae/Embriophyta - ps12
Embriophyta - ps13
Tracheophyta - ps14
Spermatophyta - ps15
Angiosperms - ps16
Mesangiospermae - ps17
Magnoliids/eudicots - ps18
Eudicots - ps19
Core_eudicots - ps20
Superrosids - ps21
Rosids - ps22
Eurosids - ps23
Malvids - ps24
Sap./Malv./Brassicales - ps25
Malvales/Brassicales - ps26
Brassicales - ps27
Cleomaceae/Brassicaceae - ps28
Brassicaceae - ps29
Camelineae - ps30
Arabidopsis - ps31
Arabidopsis_thaliana - ps32
Phylostratum

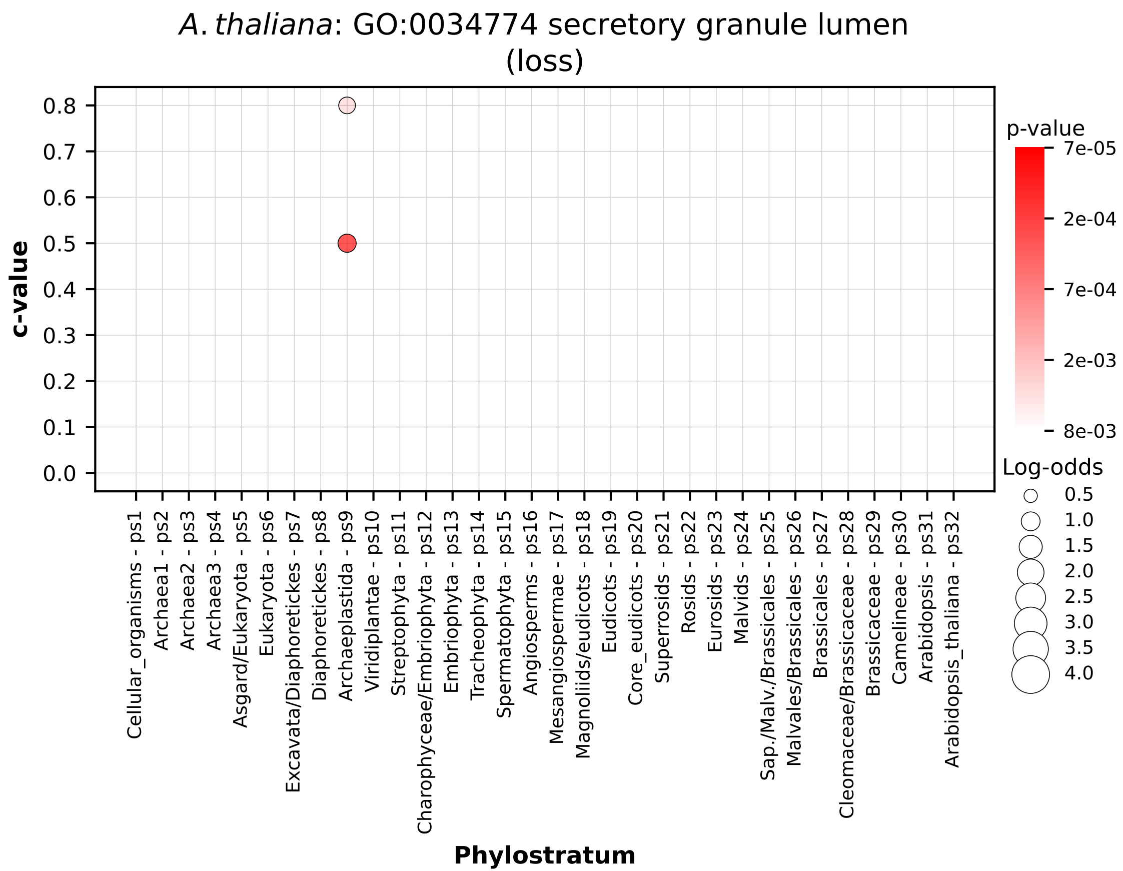

A. thaliana: GO:0034774 secretory granule lumen
(loss)
c-value
0.0
0.1
0.2
0.3
0.4
0.5
0.6
0.7
0.8
Phylostratum
Cellular_organisms - ps1
Archaea1 - ps2
Archaea2 - ps3
Archaea3 - ps4
Asgard/Eukaryota - ps5
Eukaryota - ps6
Excavata/Diaphoretickes - ps7
Diaphoretickes - ps8
Archaeplastida - ps9
Viridiplantae - ps10
Streptophyta - ps11
Charophyceae/Embriophyta - ps12
Embriophyta - ps13
Tracheophyta - ps14
Spermatophyta - ps15
Angiosperms - ps16
Mesangiospermae - ps17
Magnoliids/eudicots - ps18
Eudicots - ps19
Core_eudicots - ps20
Superrosids - ps21
Rosids - ps22
Eurosids - ps23
Malvids - ps24
Sap./Malv./Brassicales - ps25
Malvales/Brassicales - ps26
Brassicales - ps27
Cleomaceae/Brassicaceae - ps28
Brassicaceae - ps29
Camelineae - ps30
Arabidopsis - ps31
Arabidopsis_thaliana - ps32
p-value
7e-05
2e-04
7e-04
2e-03
8e-03
Log-odds
0.5
1.0
1.5
2.0
2.5
3.0
3.5
4.0

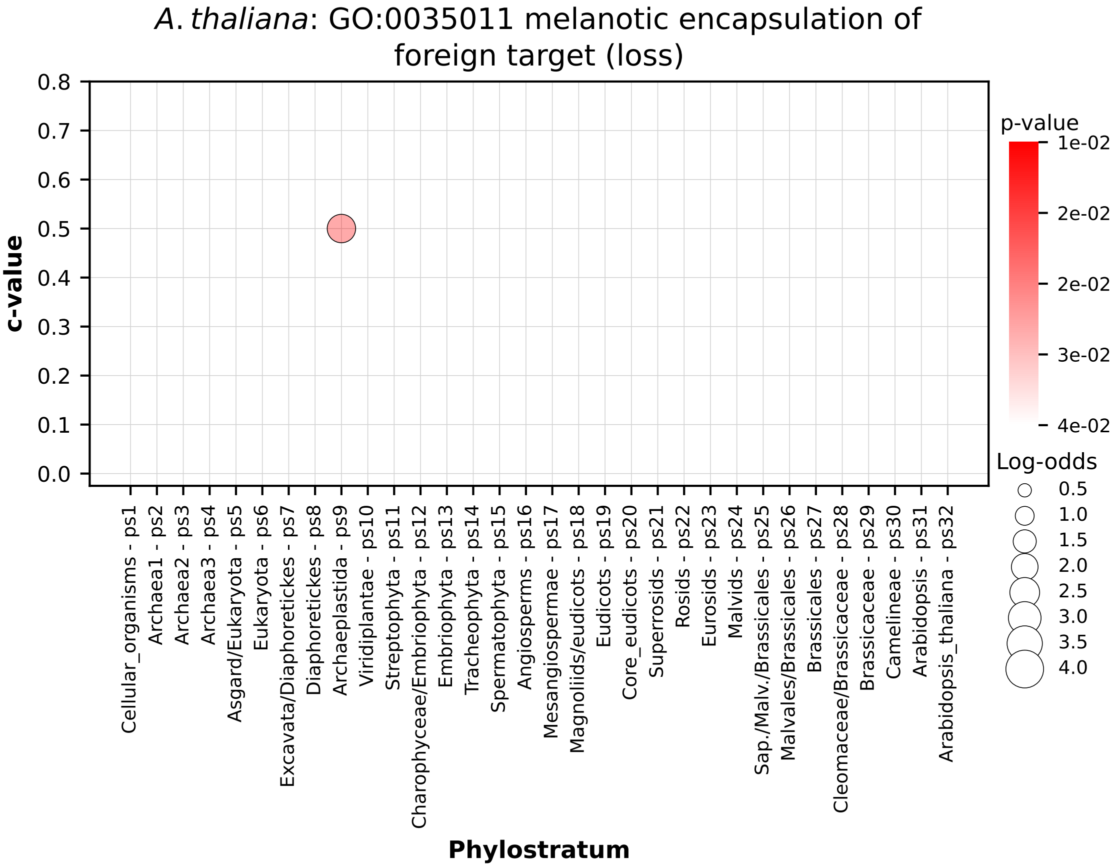

A. thaliana: GO:0035011 melanotic encapsulation of foreign target (loss)
c-value
0.8
0.7
0.6
0.5
0.4
0.3
0.2
0.1
0.0
Phylostratum
Cellular_organisms - ps1
Archaea1 - ps2
Archaea2 - ps3
Archaea3 - ps4
Asgard/Eukaryota - ps5
Eukaryota - ps6
Excavata/Diaphoretickes - ps7
Diaphoretickes - ps8
Archaeplastida - ps9
Viridiplantae - ps10
Streptophyta - ps11
Charophyceae/Embriophyta - ps12
Embriophyta - ps13
Tracheophyta - ps14
Spermatophyta - ps15
Angiosperms - ps16
Mesangiospermae - ps17
Magnoliids/eudicots - ps18
Eudicots - ps19
Core_eudicots - ps20
Superrosids - ps21
Rosids - ps22
Eurosids - ps23
Malvids - ps24
Sap./Malv./Brassicales - ps25
Malvales/Brassicales - ps26
Brassicales - ps27
Cleomaceae/Brassicaceae - ps28
Brassicaceae - ps29
Camelineae - ps30
Arabidopsis - ps31
Arabidopsis_thaliana - ps32
p-value
1e-02
2e-02
2e-02
3e-02
4e-02
Log-odds
0.5
1.0
1.5
2.0
2.5
3.0
3.5
4.0

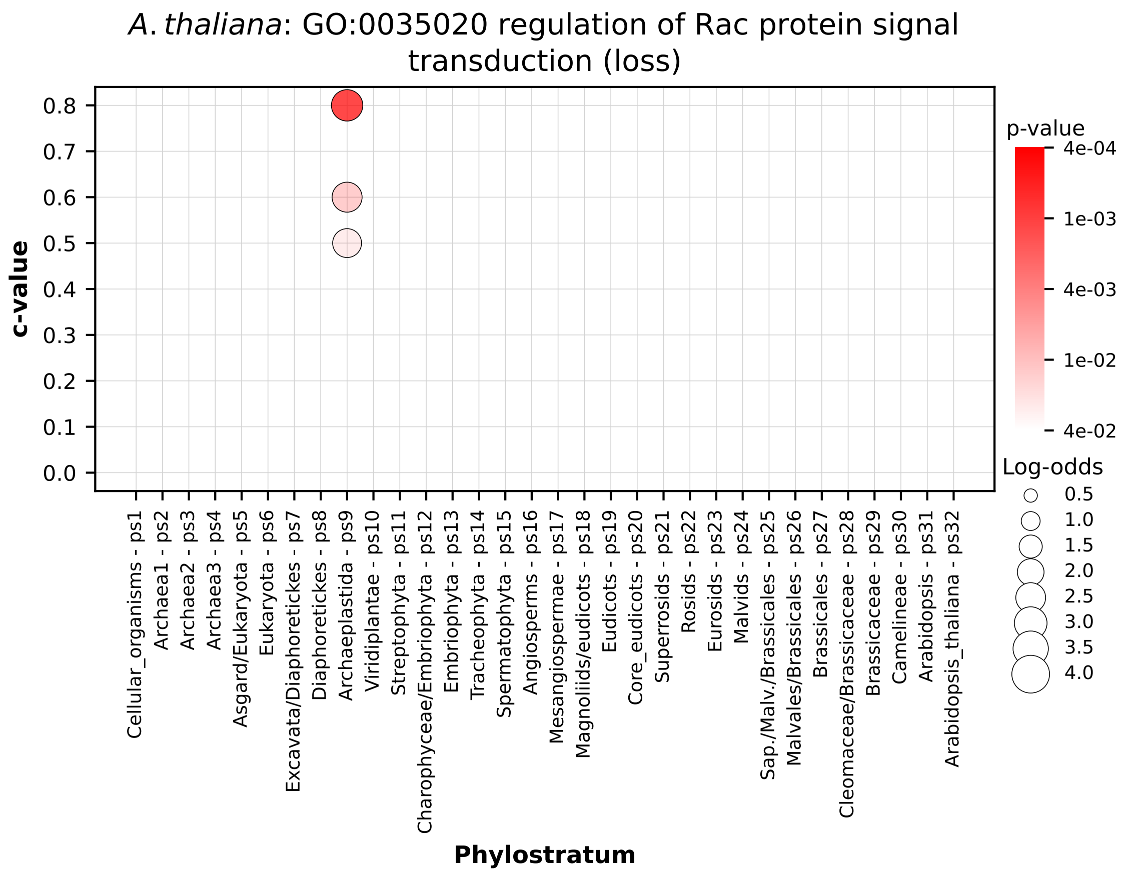

A. thaliana: GO:0035020 regulation of Rac protein signal transduction (loss)
c-value
0.0
0.1
0.2
0.3
0.4
0.5
0.6
0.7
0.8
p-value
4e-04
1e-03
4e-03
1e-02
4e-02
Log-odds
0.5
1.0
1.5
2.0
2.5
3.0
3.5
4.0
Cellular_organisms - ps1
Archaea1 - ps2
Archaea2 - ps3
Archaea3 - ps4
Asgard/Eukaryota - ps5
Eukaryota - ps6
Excavata/Diaphoretickes - ps7
Diaphoretickes - ps8
Archaeplastida - ps9
Viridiplantae - ps10
Streptophyta - ps11
Charophyceae/Embriophyta - ps12
Embriophyta - ps13
Tracheophyta - ps14
Spermatophyta - ps15
Angiosperms - ps16
Mesangiospermae - ps17
Magnoliids/eudicots - ps18
Eudicots - ps19
Core_eudicots - ps20
Superrosids - ps21
Rosids - ps22
Eurosids - ps23
Malvids - ps24
Sap./Malv./Brassicales - ps25
Malvales/Brassicales - ps26
Brassicales - ps27
Cleomaceae/Brassicaceae - ps28
Brassicaceae - ps29
Camelineae - ps30
Arabidopsis - ps31
Arabidopsis_thaliana - ps32
Phylostratum

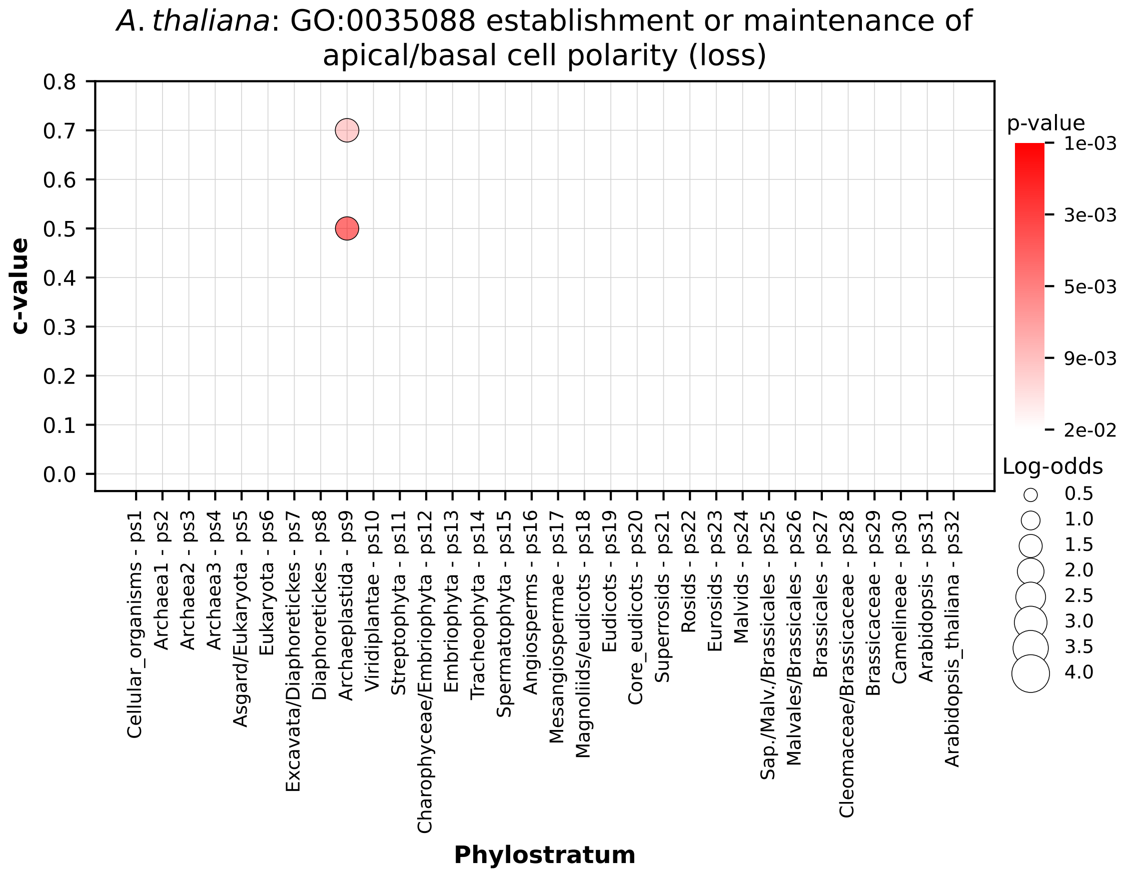

A. thaliana: GO:0035088 establishment or maintenance of apical/basal cell polarity (loss)
c-value
0.0
0.1
0.2
0.3
0.4
0.5
0.6
0.7
0.8
Phylostratum
Cellular_organisms - ps1
Archaea1 - ps2
Archaea2 - ps3
Archaea3 - ps4
Asgard/Eukaryota - ps5
Eukaryota - ps6
Excavata/Diaphoretickes - ps7
Diaphoretickes - ps8
Archaeplastida - ps9
Viridiplantae - ps10
Streptophyta - ps11
Charophyceae/Embriophyta - ps12
Embriophyta - ps13
Tracheophyta - ps14
Spermatophyta - ps15
Angiosperms - ps16
Mesangiospermae - ps17
Magnoliids/eudicots - ps18
Eudicots - ps19
Core_eudicots - ps20
Superrosids - ps21
Rosids - ps22
Eurosids - ps23
Malvids - ps24
Sap./Malv./Brassicales - ps25
Malvales/Brassicales - ps26
Brassicales - ps27
Cleomaceae/Brassicaceae - ps28
Brassicaceae - ps29
Camelineae - ps30
Arabidopsis - ps31
Arabidopsis_thaliana - ps32
p-value
1e-03
3e-03
5e-03
9e-03
2e-02
Log-odds
0.5
1.0
1.5
2.0
2.5
3.0
3.5
4.0

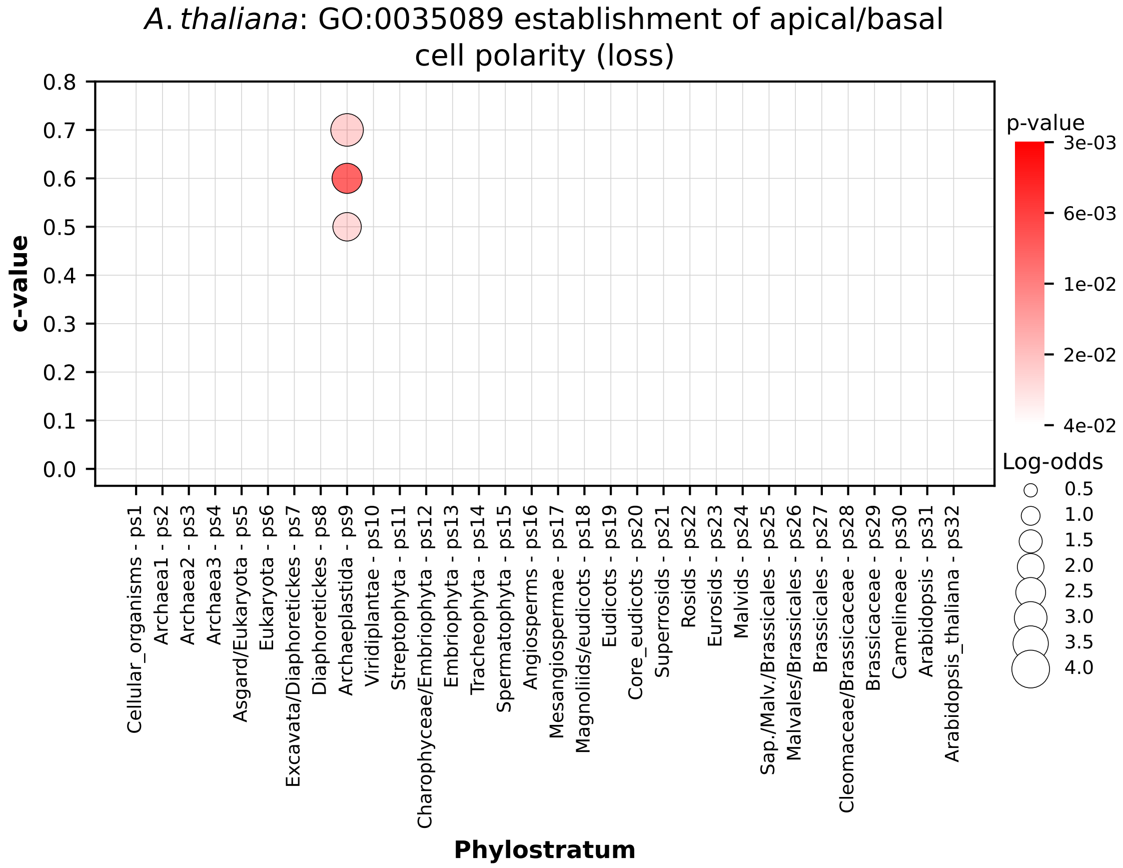

A. thaliana: GO:0035089 establishment of apical/basal cell polarity (loss)
c-value
0.8
0.7
0.6
0.5
0.4
0.3
0.2
0.1
0.0
p-value
3e-03
6e-03
1e-02
2e-02
4e-02
Log-odds
0.5
1.0
1.5
2.0
2.5
3.0
3.5
4.0
Cellular_organisms - ps1
Archaea1 - ps2
Archaea2 - ps3
Archaea3 - ps4
Asgard/Eukaryota - ps5
Eukaryota - ps6
Excavata/Diaphoretickes - ps7
Diaphoretickes - ps8
Archaeplastida - ps9
Viridiplantae - ps10
Streptophyta - ps11
Charophyceae/Embriophyta - ps12
Embriophyta - ps13
Tracheophyta - ps14
Spermatophyta - ps15
Angiosperms - ps16
Mesangiospermae - ps17
Magnoliids/eudicots - ps18
Eudicots - ps19
Core_eudicots - ps20
Superrosids - ps21
Rosids - ps22
Eurosids - ps23
Malvids - ps24
Sap./Malv./Brassicales - ps25
Malvales/Brassicales - ps26
Brassicales - ps27
Cleomaceae/Brassicaceae - ps28
Brassicaceae - ps29
Camelineae - ps30
Arabidopsis - ps31
Arabidopsis_thaliana - ps32
Phylostratum

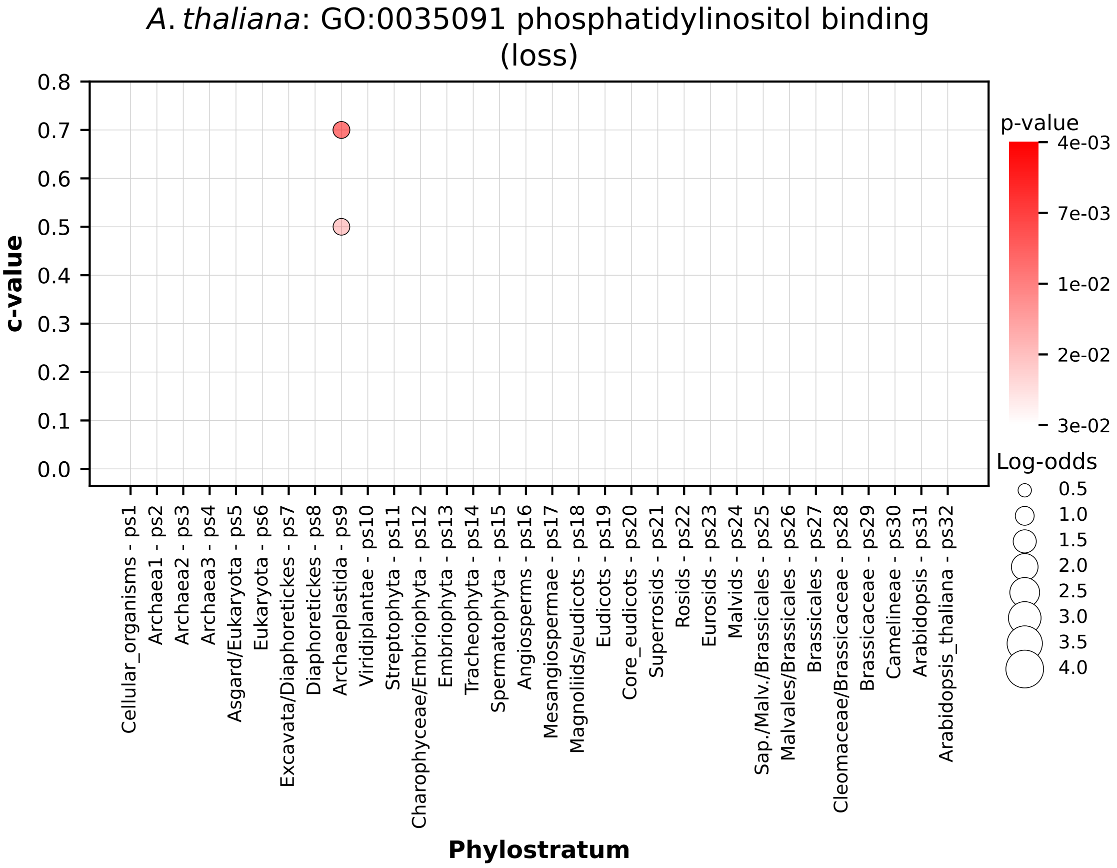

A. thaliana: GO:0035091 phosphatidylinositol binding
(loss)
c-value
0.8
0.7
0.6
0.5
0.4
0.3
0.2
0.1
0.0
Phylostratum
Cellular_organisms - ps1
Archaea1 - ps2
Archaea2 - ps3
Archaea3 - ps4
Asgard/Eukaryota - ps5
Eukaryota - ps6
Excavata/Diaphoretickes - ps7
Diaphoretickes - ps8
Archaeplastida - ps9
Viridiplantae - ps10
Streptophyta - ps11
Charophyceae/Embriophyta - ps12
Embriophyta - ps13
Tracheophyta - ps14
Spermatophyta - ps15
Angiosperms - ps16
Mesangiospermae - ps17
Magnoliids/eudicots - ps18
Eudicots - ps19
Core_eudicots - ps20
Superrosids - ps21
Rosids - ps22
Eurosids - ps23
Malvids - ps24
Sap./Malv./Brassicales - ps25
Malvales/Brassicales - ps26
Brassicales - ps27
Cleomaceae/Brassicaceae - ps28
Brassicaceae - ps29
Camelineae - ps30
Arabidopsis - ps31
Arabidopsis_thaliana - ps32
p-value
4e-03
7e-03
1e-02
2e-02
3e-02
Log-odds
0.5
1.0
1.5
2.0
2.5
3.0
3.5
4.0

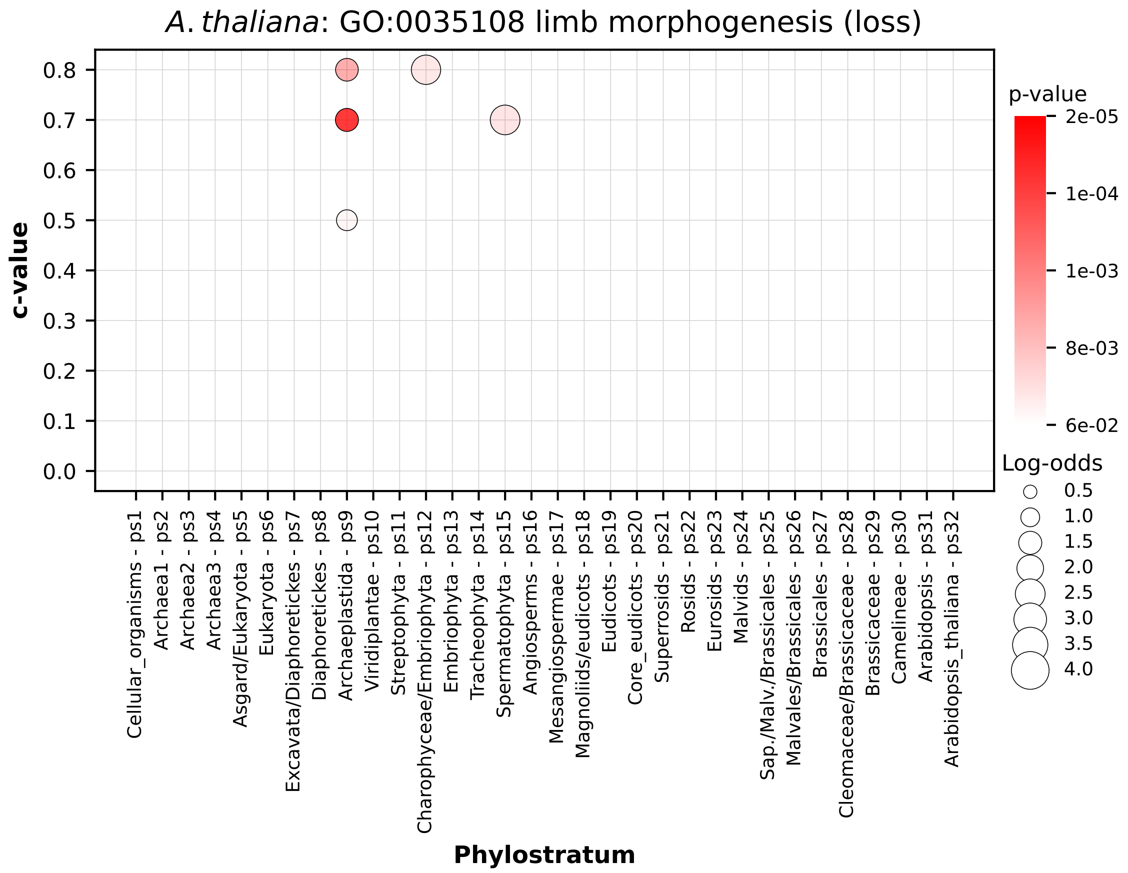

A. thaliana: GO:0035108 limb morphogenesis (loss)
c-value
0.0
0.1
0.2
0.3
0.4
0.5
0.6
0.7
0.8
Phylostratum
Cellular_organisms - ps1
Archaea1 - ps2
Archaea2 - ps3
Archaea3 - ps4
Asgard/Eukaryota - ps5
Eukaryota - ps6
Excavata/Diaphoretickes - ps7
Diaphoretickes - ps8
Archaeplastida - ps9
Viridiplantae - ps10
Streptophyta - ps11
Charophyceae/Embriophyta - ps12
Embriophyta - ps13
Tracheophyta - ps14
Spermatophyta - ps15
Angiosperms - ps16
Mesangiospermae - ps17
Magnoliids/eudicots - ps18
Eudicots - ps19
Core_eudicots - ps20
Superrosids - ps21
Rosids - ps22
Eurosids - ps23
Malvids - ps24
Sap./Malv./Brassicales - ps25
Malvales/Brassicales - ps26
Brassicales - ps27
Cleomaceae/Brassicaceae - ps28
Brassicaceae - ps29
Camelineae - ps30
Arabidopsis - ps31
Arabidopsis_thaliana - ps32
p-value
2e-05
1e-04
1e-03
8e-03
6e-02
Log-odds
0.5
1.0
1.5
2.0
2.5
3.0
3.5
4.0

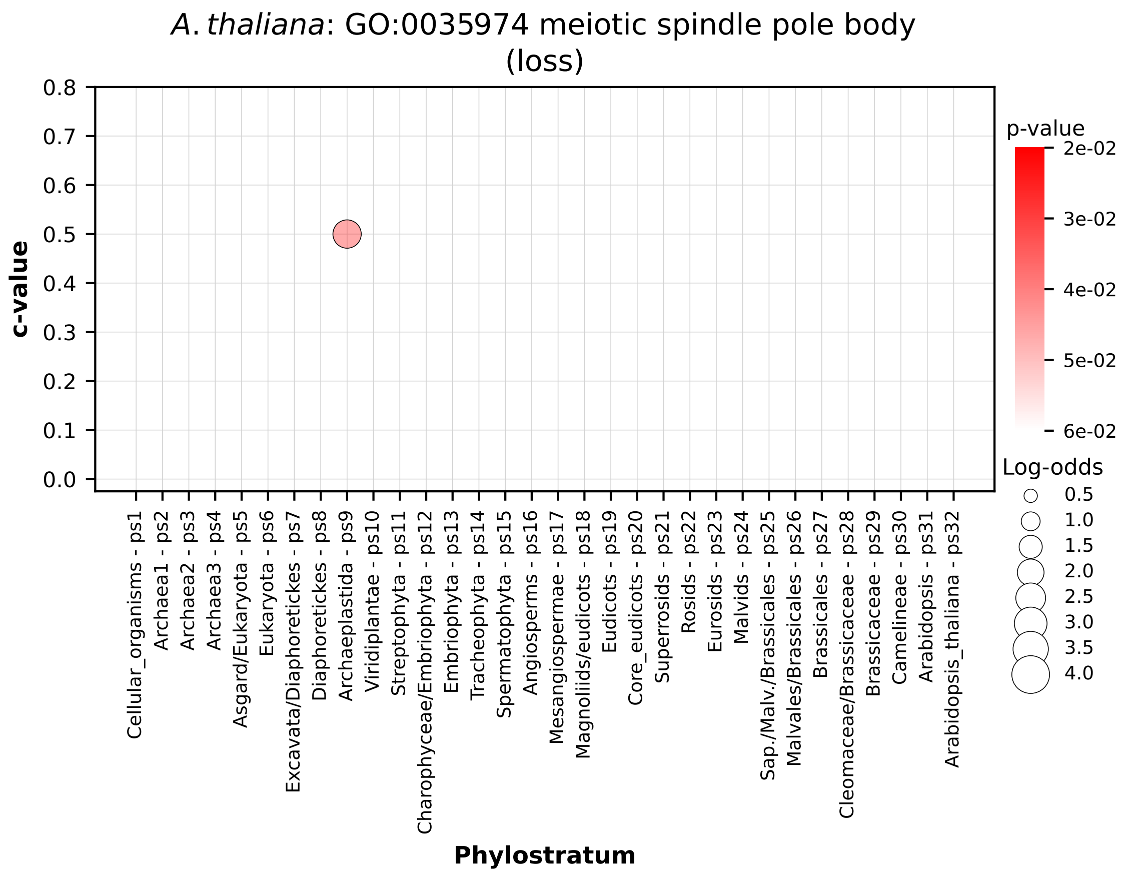

A. thaliana: GO:0035974 meiotic spindle pole body
(loss)
c-value
0.8
0.7
0.6
0.5
0.4
0.3
0.2
0.1
0.0
p-value
2e-02
3e-02
4e-02
5e-02
6e-02
Log-odds
0.5
1.0
1.5
2.0
2.5
3.0
3.5
4.0
Cellular_organisms - ps1
Archaea1 - ps2
Archaea2 - ps3
Archaea3 - ps4
Asgard/Eukaryota - ps5
Eukaryota - ps6
Excavata/Diaphoretickes - ps7
Diaphoretickes - ps8
Archaeplastida - ps9
Viridiplantae - ps10
Streptophyta - ps11
Charophyceae/Embriophyta - ps12
Embriophyta - ps13
Tracheophyta - ps14
Spermatophyta - ps15
Angiosperms - ps16
Mesangiospermae - ps17
Magnoliids/eudicots - ps18
Eudicots - ps19
Core_eudicots - ps20
Superrosids - ps21
Rosids - ps22
Eurosids - ps23
Malvids - ps24
Sap./Malv./Brassicales - ps25
Malvales/Brassicales - ps26
Brassicales - ps27
Cleomaceae/Brassicaceae - ps28
Brassicaceae - ps29
Camelineae - ps30
Arabidopsis - ps31
Arabidopsis_thaliana - ps32
Phylostratum

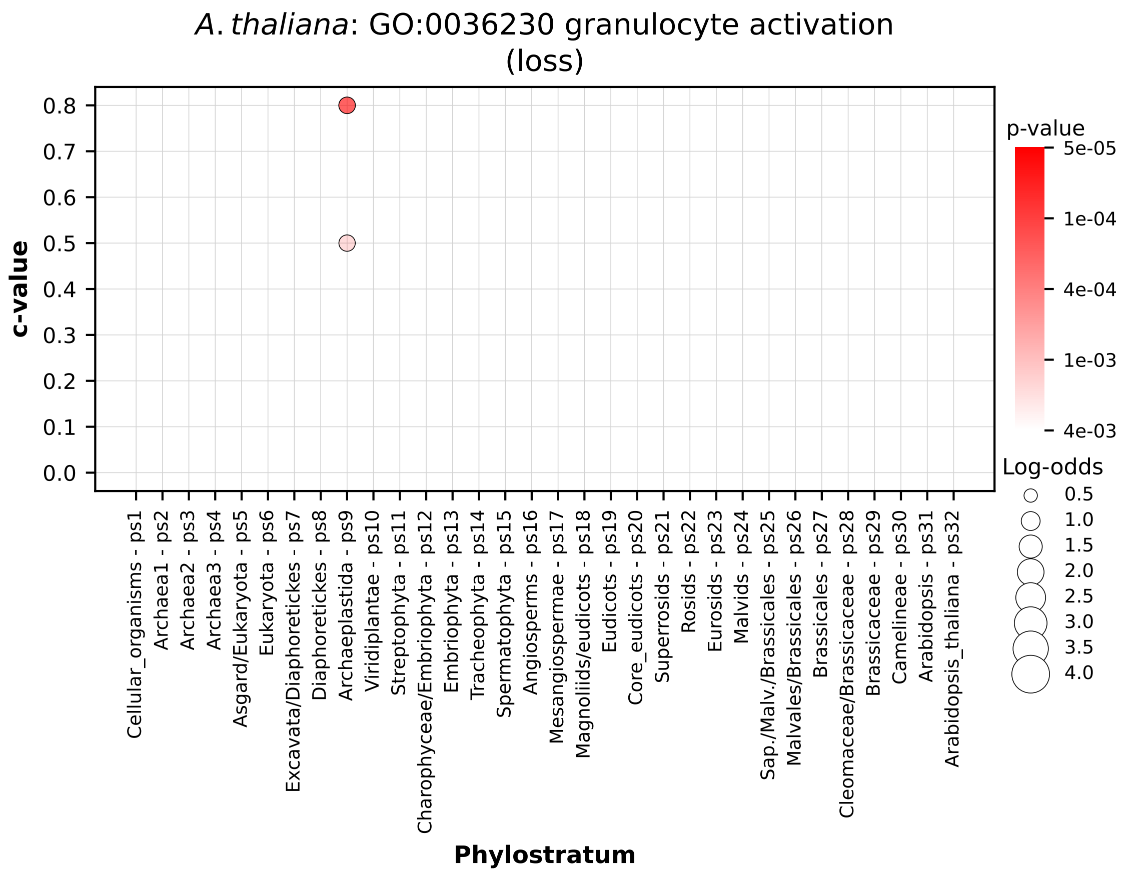

A. thaliana: GO:0036230 granulocyte activation
(loss)
c-value
0.0
0.1
0.2
0.3
0.4
0.5
0.6
0.7
0.8
p-value
5e-05
1e-04
4e-04
1e-03
4e-03
Log-odds
0.5
1.0
1.5
2.0
2.5
3.0
3.5
4.0
Cellular_organisms - ps1
Archaea1 - ps2
Archaea2 - ps3
Archaea3 - ps4
Asgard/Eukaryota - ps5
Eukaryota - ps6
Excavata/Diaphoretickes - ps7
Diaphoretickes - ps8
Archaeplastida - ps9
Viridiplantae - ps10
Streptophyta - ps11
Charophyceae/Embriophyta - ps12
Embriophyta - ps13
Tracheophyta - ps14
Spermatophyta - ps15
Angiosperms - ps16
Mesangiospermae - ps17
Magnoliids/eudicots - ps18
Eudicots - ps19
Core_eudicots - ps20
Superrosids - ps21
Rosids - ps22
Eurosids - ps23
Malvids - ps24
Sap./Malv./Brassicales - ps25
Malvales/Brassicales - ps26
Brassicales - ps27
Cleomaceae/Brassicaceae - ps28
Brassicaceae - ps29
Camelineae - ps30
Arabidopsis - ps31
Arabidopsis_thaliana - ps32
Phylostratum

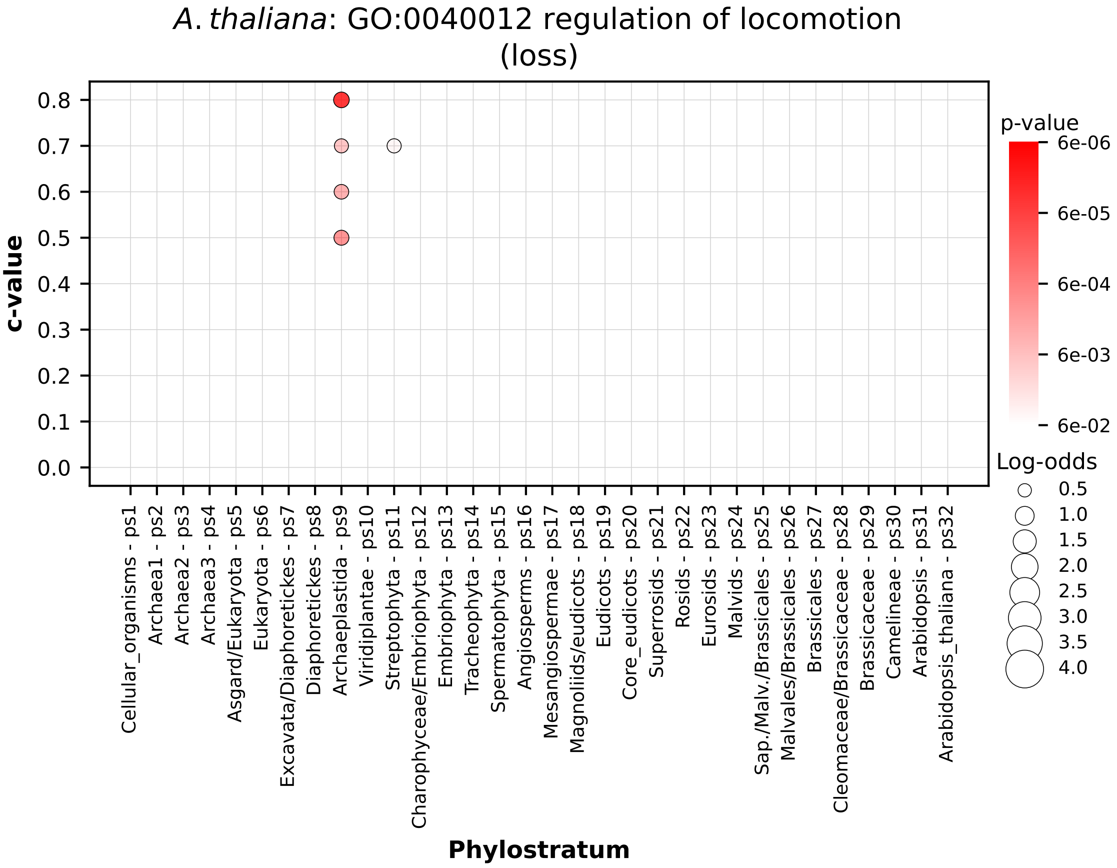

A. thaliana: GO:0040012 regulation of locomotion
(loss)
c-value
0.8
0.7
0.6
0.5
0.4
0.3
0.2
0.1
0.0
p-value
6e-06
6e-05
6e-04
6e-03
6e-02
Log-odds
0.5
1.0
1.5
2.0
2.5
3.0
3.5
4.0
Cellular_organisms - ps1
Archaea1 - ps2
Archaea2 - ps3
Archaea3 - ps4
Asgard/Eukaryota - ps5
Eukaryota - ps6
Excavata/Diaphoretickes - ps7
Diaphoretickes - ps8
Archaeplastida - ps9
Viridiplantae - ps10
Streptophyta - ps11
Charophyceae/Embriophyta - ps12
Embriophyta - ps13
Tracheophyta - ps14
Spermatophyta - ps15
Angiosperms - ps16
Mesangiospermae - ps17
Magnoliids/eudicots - ps18
Eudicots - ps19
Core_eudicots - ps20
Superrosids - ps21
Rosids - ps22
Eurosids - ps23
Malvids - ps24
Sap./Malv./Brassicales - ps25
Malvales/Brassicales - ps26
Brassicales - ps27
Cleomaceae/Brassicaceae - ps28
Brassicaceae - ps29
Camelineae - ps30
Arabidopsis - ps31
Arabidopsis_thaliana - ps32
Phylostratum

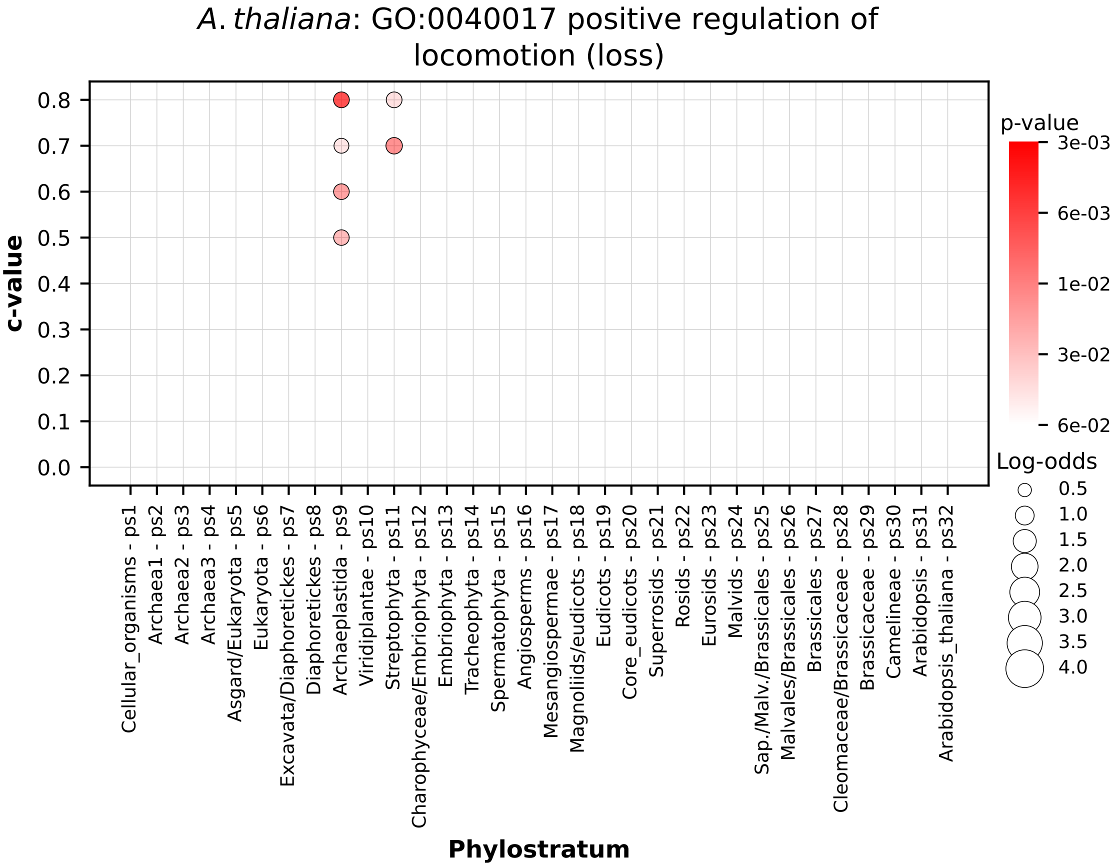

A. thaliana: GO:0040017 positive regulation of locomotion (loss)
c-value
0.0
0.1
0.2
0.3
0.4
0.5
0.6
0.7
0.8
Phylostratum
Cellular_organisms - ps1
Archaea1 - ps2
Archaea2 - ps3
Archaea3 - ps4
Asgard/Eukaryota - ps5
Eukaryota - ps6
Excavata/Diaphoretickes - ps7
Diaphoretickes - ps8
Archaeplastida - ps9
Viridiplantae - ps10
Streptophyta - ps11
Charophyceae/Embriophyta - ps12
Embriophyta - ps13
Tracheophyta - ps14
Spermatophyta - ps15
Angiosperms - ps16
Mesangiospermae - ps17
Magnoliids/eudicots - ps18
Eudicots - ps19
Core_eudicots - ps20
Superrosids - ps21
Rosids - ps22
Eurosids - ps23
Malvids - ps24
Sap./Malv./Brassicales - ps25
Malvales/Brassicales - ps26
Brassicales - ps27
Cleomaceae/Brassicaceae - ps28
Brassicaceae - ps29
Camelineae - ps30
Arabidopsis - ps31
Arabidopsis_thaliana - ps32
p-value
3e-03
6e-03
1e-02
3e-02
6e-02
Log-odds
0.5
1.0
1.5
2.0
2.5
3.0
3.5
4.0

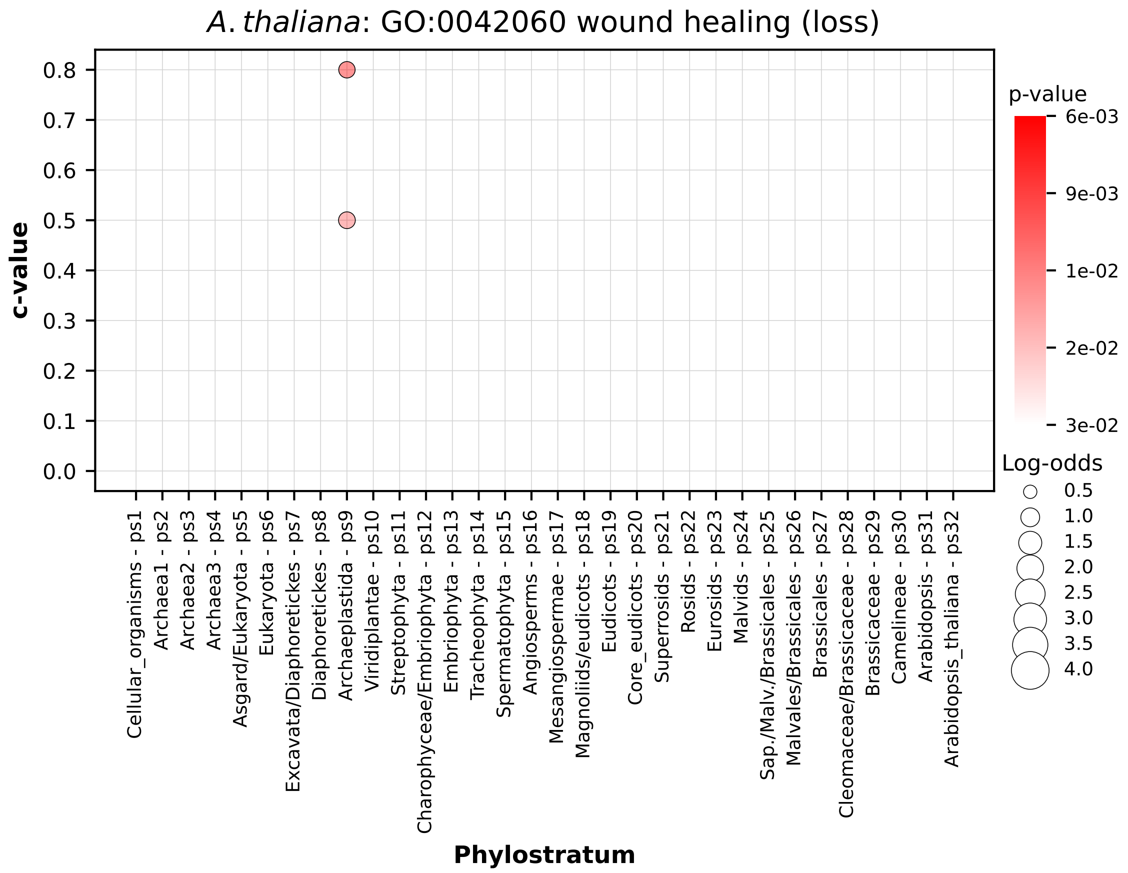
A. thaliana: GO:0042060 wound healing (loss)
c-value
0.8
0.7
0.6
0.5
0.4
0.3
0.2
0.1
0.0
p-value
6e-03
9e-03
1e-02
2e-02
3e-02
Log-odds
0.5
1.0
1.5
2.0
2.5
3.0
3.5
4.0
Cellular_organisms - ps1
Archaea1 - ps2
Archaea2 - ps3
Archaea3 - ps4
Asgard/Eukaryota - ps5
Eukaryota - ps6
Excavata/Diaphoretickes - ps7
Diaphoretickes - ps8
Archaeplastida - ps9
Viridiplantae - ps10
Streptophyta - ps11
Charophyceae/Embriophyta - ps12
Embriophyta - ps13
Tracheophyta - ps14
Spermatophyta - ps15
Angiosperms - ps16
Mesangiospermae - ps17
Magnoliids/eudicots - ps18
Eudicots - ps19
Core_eudicots - ps20
Superrosids - ps21
Rosids - ps22
Eurosids - ps23
Malvids - ps24
Sap./Malv./Brassicales - ps25
Malvales/Brassicales - ps26
Brassicales - ps27
Cleomaceae/Brassicaceae - ps28
Brassicaceae - ps29
Camelineae - ps30
Arabidopsis - ps31
Arabidopsis_thaliana - ps32
Phylostratum

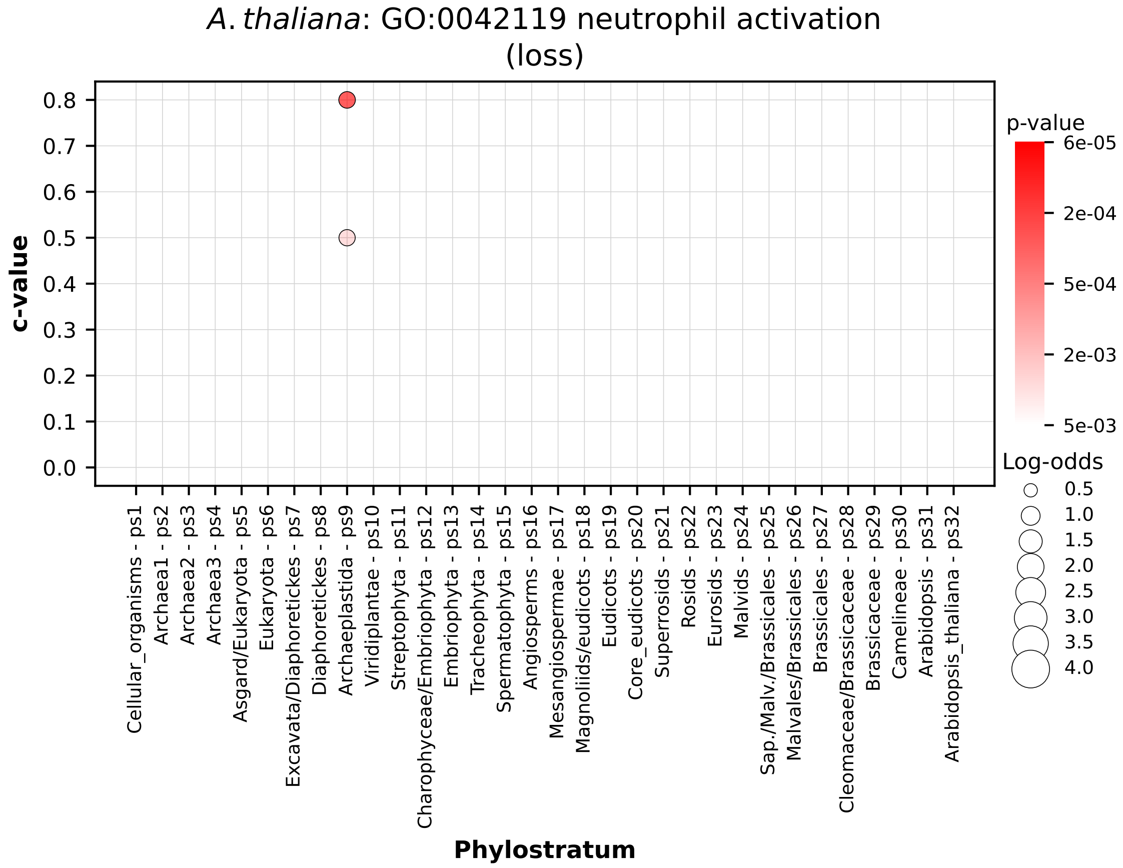

A. thaliana: GO:0042119 neutrophil activation (loss)
c-value
0.8
0.7
0.6
0.5
0.4
0.3
0.2
0.1
0.0
p-value
6e-05
2e-04
5e-04
2e-03
5e-03
Log-odds
0.5
1.0
1.5
2.0
2.5
3.0
3.5
4.0
Cellular_organisms - ps1
Archaea1 - ps2
Archaea2 - ps3
Archaea3 - ps4
Asgard/Eukaryota - ps5
Eukaryota - ps6
Excavata/Diaphoretickes - ps7
Diaphoretickes - ps8
Archaeplastida - ps9
Viridiplantae - ps10
Streptophyta - ps11
Charophyceae/Embriophyta - ps12
Embriophyta - ps13
Tracheophyta - ps14
Spermatophyta - ps15
Angiosperms - ps16
Mesangiospermae - ps17
Magnoliids/eudicots - ps18
Eudicots - ps19
Core_eudicots - ps20
Superrosids - ps21
Rosids - ps22
Eurosids - ps23
Malvids - ps24
Sap./Malv./Brassicales - ps25
Malvales/Brassicales - ps26
Brassicales - ps27
Cleomaceae/Brassicaceae - ps28
Brassicaceae - ps29
Camelineae - ps30
Arabidopsis - ps31
Arabidopsis_thaliana - ps32
Phylostratum

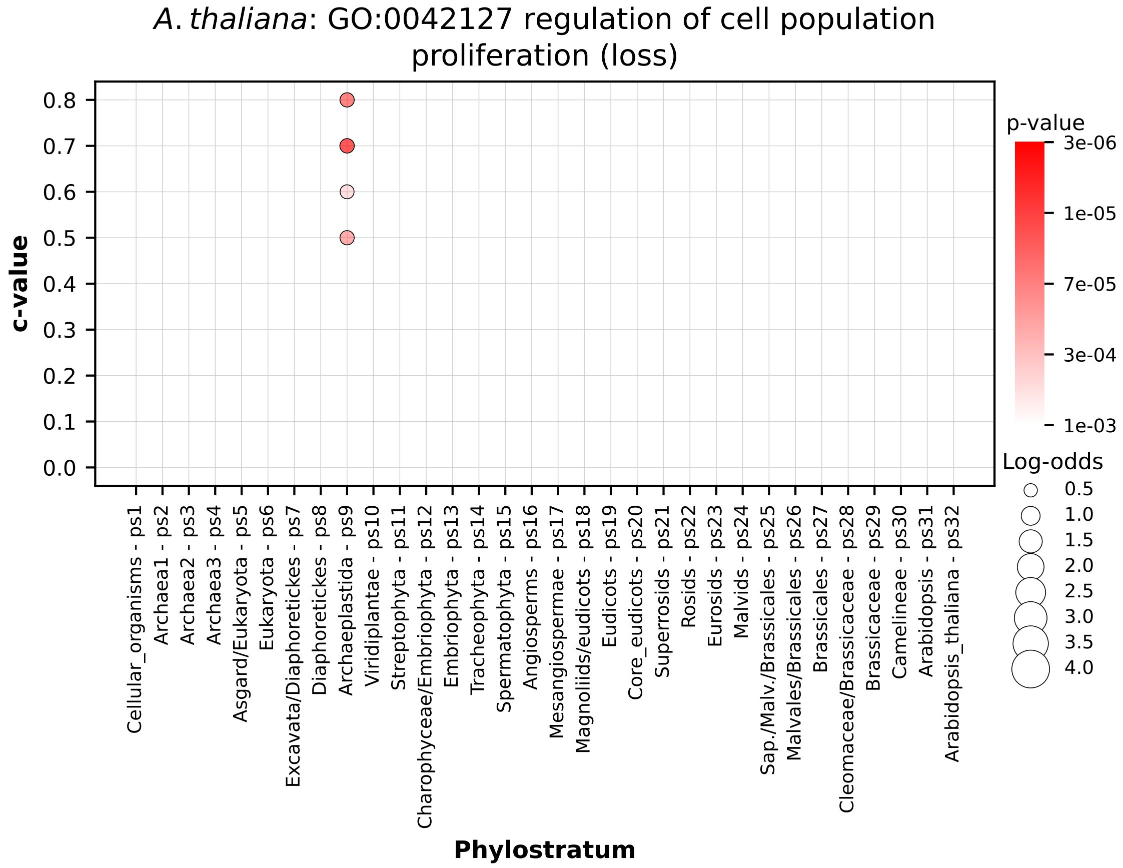

A. thaliana: GO:0042127 regulation of cell population proliferation (loss)
c-value
0.8
0.7
0.6
0.5
0.4
0.3
0.2
0.1
0.0
Phylostratum
Cellular_organisms - ps1
Archaea1 - ps2
Archaea2 - ps3
Archaea3 - ps4
Asgard/Eukaryota - ps5
Eukaryota - ps6
Excavata/Diaphoretickes - ps7
Diaphoretickes - ps8
Archaeplastida - ps9
Viridiplantae - ps10
Streptophyta - ps11
Charophyceae/Embriophyta - ps12
Embriophyta - ps13
Tracheophyta - ps14
Spermatophyta - ps15
Angiosperms - ps16
Mesangiospermae - ps17
Magnoliids/eudicots - ps18
Eudicots - ps19
Core_eudicots - ps20
Superrosids - ps21
Rosids - ps22
Eurosids - ps23
Malvids - ps24
Sap./Malv./Brassicales - ps25
Malvales/Brassicales - ps26
Brassicales - ps27
Cleomaceae/Brassicaceae - ps28
Brassicaceae - ps29
Camelineae - ps30
Arabidopsis - ps31
Arabidopsis_thaliana - ps32
p-value
3e-06
1e-05
7e-05
3e-04
1e-03
Log-odds
0.5
1.0
1.5
2.0
2.5
3.0
3.5
4.0

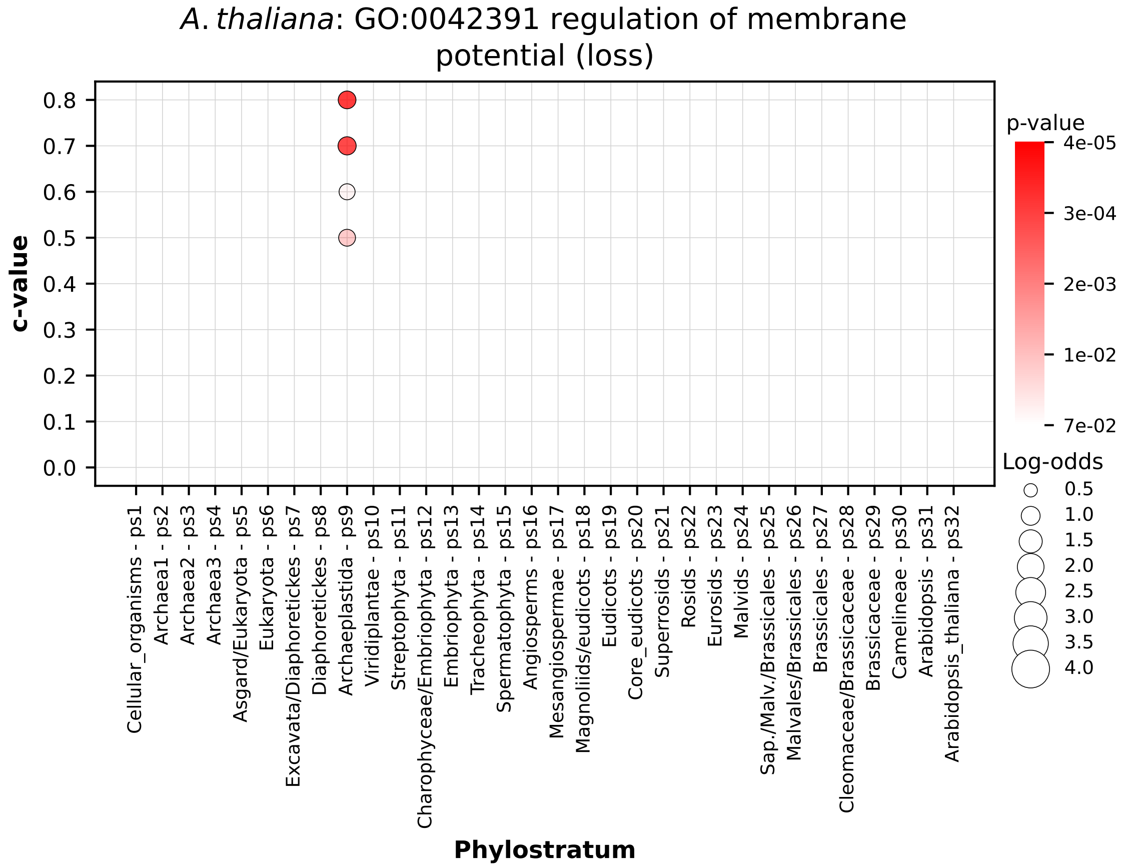

A. thaliana: GO:0042391 regulation of membrane potential (loss)
c-value
0.8
0.7
0.6
0.5
0.4
0.3
0.2
0.1
0.0
p-value
4e-05
3e-04
2e-03
1e-02
7e-02
Log-odds
0.5
1.0
1.5
2.0
2.5
3.0
3.5
4.0
Cellular_organisms - ps1
Archaea1 - ps2
Archaea2 - ps3
Archaea3 - ps4
Asgard/Eukaryota - ps5
Eukaryota - ps6
Excavata/Diaphoretickes - ps7
Diaphoretickes - ps8
Archaeplastida - ps9
Viridiplantae - ps10
Streptophyta - ps11
Charophyceae/Embriophyta - ps12
Embriophyta - ps13
Tracheophyta - ps14
Spermatophyta - ps15
Angiosperms - ps16
Mesangiospermae - ps17
Magnoliids/eudicots - ps18
Eudicots - ps19
Core_eudicots - ps20
Superrosids - ps21
Rosids - ps22
Eurosids - ps23
Malvids - ps24
Sap./Malv./Brassicales - ps25
Malvales/Brassicales - ps26
Brassicales - ps27
Cleomaceae/Brassicaceae - ps28
Brassicaceae - ps29
Camelineae - ps30
Arabidopsis - ps31
Arabidopsis_thaliana - ps32
Phylostratum

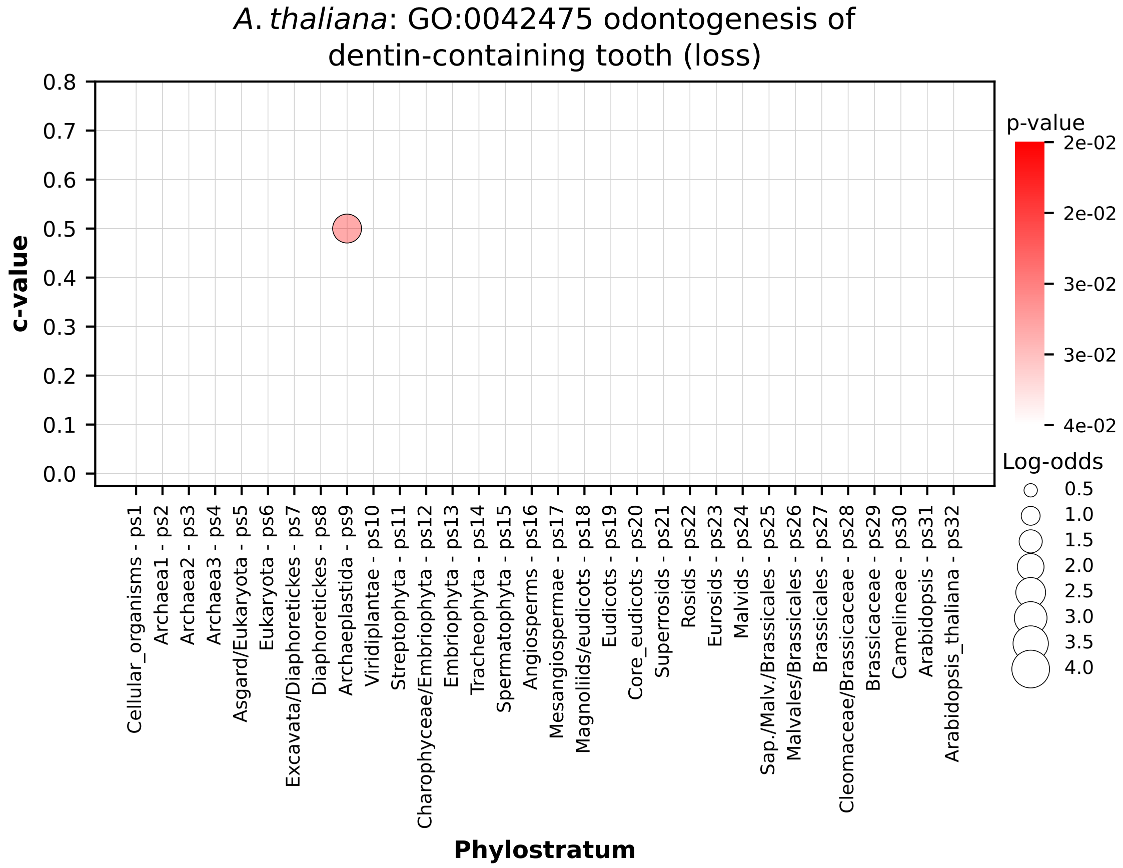

A. thaliana: GO:0042475 odontogenesis of dentin-containing tooth (loss)
c-value
0.8
0.7
0.6
0.5
0.4
0.3
0.2
0.1
0.0
p-value
2e-02
2e-02
3e-02
3e-02
4e-02
Log-odds
0.5
1.0
1.5
2.0
2.5
3.0
3.5
4.0
Cellular_organisms - ps1
Archaea1 - ps2
Archaea2 - ps3
Archaea3 - ps4
Asgard/Eukaryota - ps5
Eukaryota - ps6
Excavata/Diaphoretickes - ps7
Diaphoretickes - ps8
Archaeplastida - ps9
Viridiplantae - ps10
Streptophyta - ps11
Charophyceae/Embriophyta - ps12
Embriophyta - ps13
Tracheophyta - ps14
Spermatophyta - ps15
Angiosperms - ps16
Mesangiospermae - ps17
Magnoliids/eudicots - ps18
Eudicots - ps19
Core_eudicots - ps20
Superrosids - ps21
Rosids - ps22
Eurosids - ps23
Malvids - ps24
Sap./Malv./Brassicales - ps25
Malvales/Brassicales - ps26
Brassicales - ps27
Cleomaceae/Brassicaceae - ps28
Brassicaceae - ps29
Camelineae - ps30
Arabidopsis - ps31
Arabidopsis_thaliana - ps32
Phylostratum

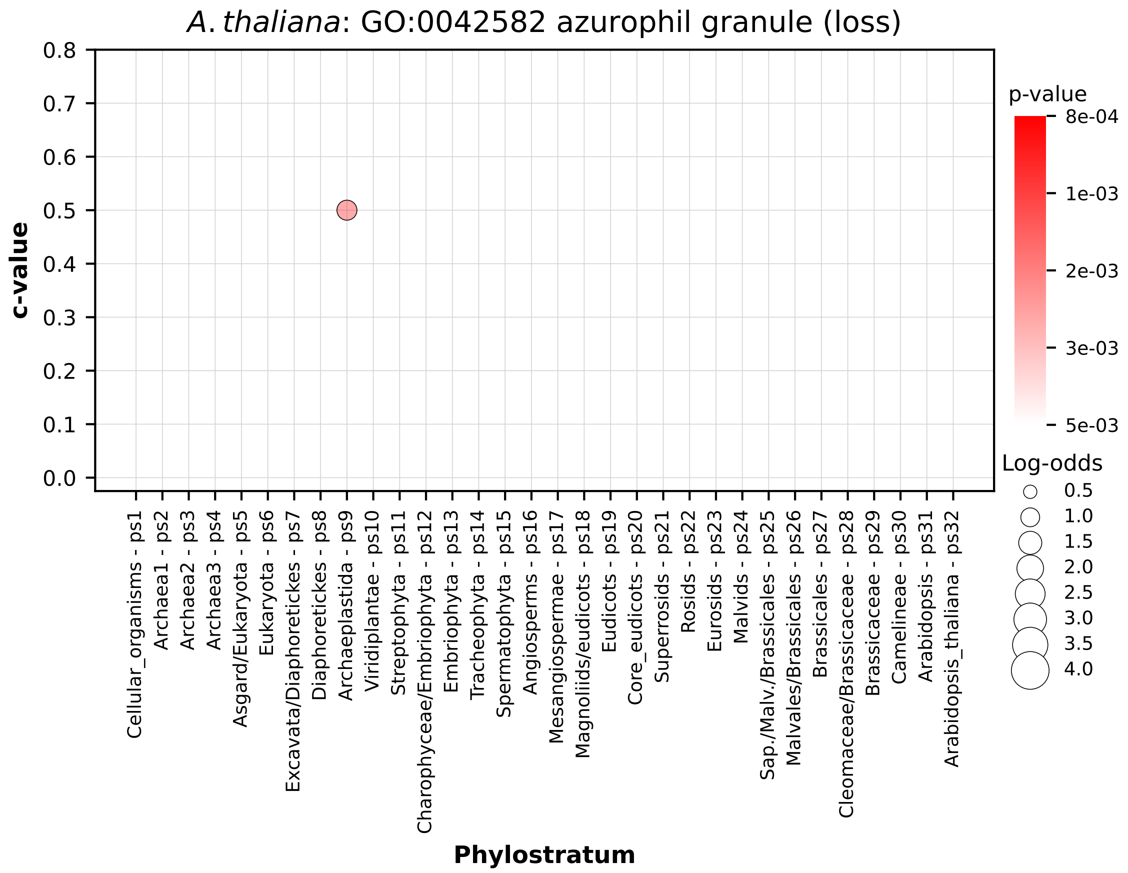

A. thaliana: GO:0042582 azurophil granule (loss)
c-value
0.8
0.7
0.6
0.5
0.4
0.3
0.2
0.1
0.0
Phylostratum
Cellular_organisms - ps1
Archaea1 - ps2
Archaea2 - ps3
Archaea3 - ps4
Asgard/Eukaryota - ps5
Eukaryota - ps6
Excavata/Diaphoretickes - ps7
Diaphoretickes - ps8
Archaeplastida - ps9
Viridiplantae - ps10
Streptophyta - ps11
Charophyceae/Embriophyta - ps12
Embriophyta - ps13
Tracheophyta - ps14
Spermatophyta - ps15
Angiosperms - ps16
Mesangiospermae - ps17
Magnoliids/eudicots - ps18
Eudicots - ps19
Core_eudicots - ps20
Superrosids - ps21
Rosids - ps22
Eurosids - ps23
Malvids - ps24
Sap./Malv./Brassicales - ps25
Malvales/Brassicales - ps26
Brassicales - ps27
Cleomaceae/Brassicaceae - ps28
Brassicaceae - ps29
Camelineae - ps30
Arabidopsis - ps31
Arabidopsis_thaliana - ps32
p-value
8e-04
1e-03
2e-03
3e-03
5e-03
Log-odds
0.5
1.0
1.5
2.0
2.5
3.0
3.5
4.0

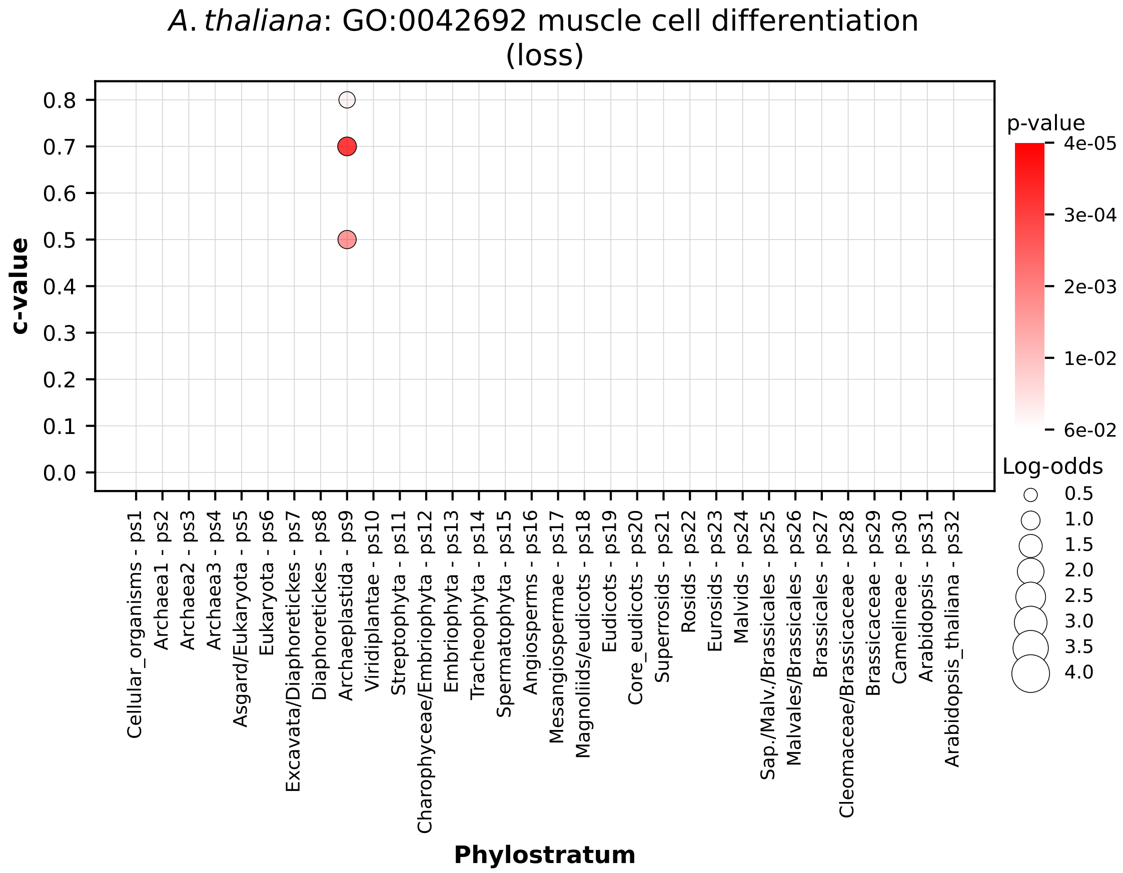

A. thaliana: GO:0042692 muscle cell differentiation (loss)
c-value
0.8
0.7
0.6
0.5
0.4
0.3
0.2
0.1
0.0
p-value
4e-05
3e-04
2e-03
1e-02
6e-02
Log-odds
0.5
1.0
1.5
2.0
2.5
3.0
3.5
4.0
Cellular_organisms - ps1
Archaea1 - ps2
Archaea2 - ps3
Archaea3 - ps4
Asgard/Eukaryota - ps5
Eukaryota - ps6
Excavata/Diaphoretickes - ps7
Diaphoretickes - ps8
Archaeplastida - ps9
Viridiplantae - ps10
Streptophyta - ps11
Charophyceae/Embriophyta - ps12
Embriophyta - ps13
Tracheophyta - ps14
Spermatophyta - ps15
Angiosperms - ps16
Mesangiospermae - ps17
Magnoliids/eudicots - ps18
Eudicots - ps19
Core_eudicots - ps20
Superrosids - ps21
Rosids - ps22
Eurosids - ps23
Malvids - ps24
Sap./Malv./Brassicales - ps25
Malvales/Brassicales - ps26
Brassicales - ps27
Cleomaceae/Brassicaceae - ps28
Brassicaceae - ps29
Camelineae - ps30
Arabidopsis - ps31
Arabidopsis_thaliana - ps32
Phylostratum

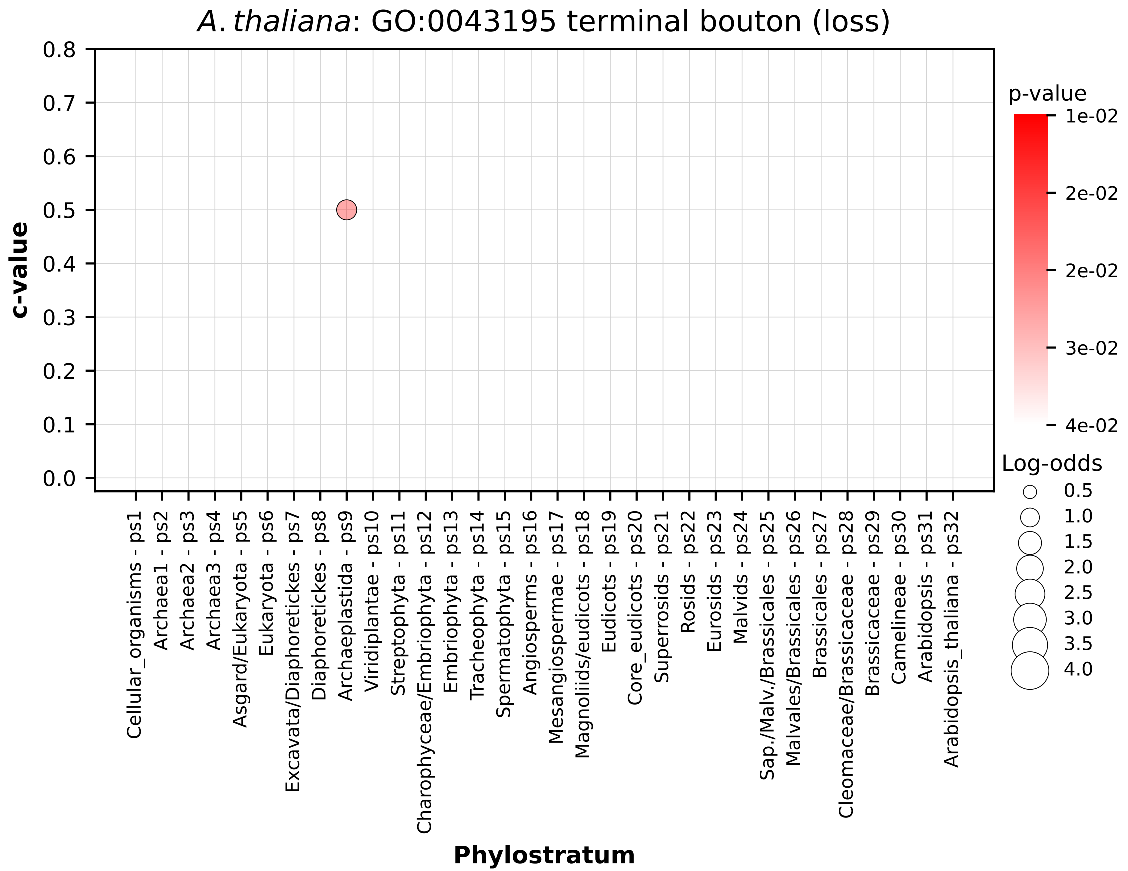
A. thaliana: GO:0043195 terminal bouton (loss)
c-value
0.8
0.7
0.6
0.5
0.4
0.3
0.2
0.1
0.0
Phylostratum
Cellular_organisms - ps1
Archaea1 - ps2
Archaea2 - ps3
Archaea3 - ps4
Asgard/Eukaryota - ps5
Eukaryota - ps6
Excavata/Diaphoretickes - ps7
Diaphoretickes - ps8
Archaeplastida - ps9
Viridiplantae - ps10
Streptophyta - ps11
Charophyceae/Embriophyta - ps12
Embriophyta - ps13
Tracheophyta - ps14
Spermatophyta - ps15
Angiosperms - ps16
Mesangiospermae - ps17
Magnoliids/eudicots - ps18
Eudicots - ps19
Core_eudicots - ps20
Superrosids - ps21
Rosids - ps22
Eurosids - ps23
Malvids - ps24
Sap./Malv./Brassicales - ps25
Malvales/Brassicales - ps26
Brassicales - ps27
Cleomaceae/Brassicaceae - ps28
Brassicaceae - ps29
Camelineae - ps30
Arabidopsis - ps31
Arabidopsis_thaliana - ps32
p-value
1e-02
2e-02
2e-02
3e-02
4e-02
Log-odds
0.5
1.0
1.5
2.0
2.5
3.0
3.5
4.0

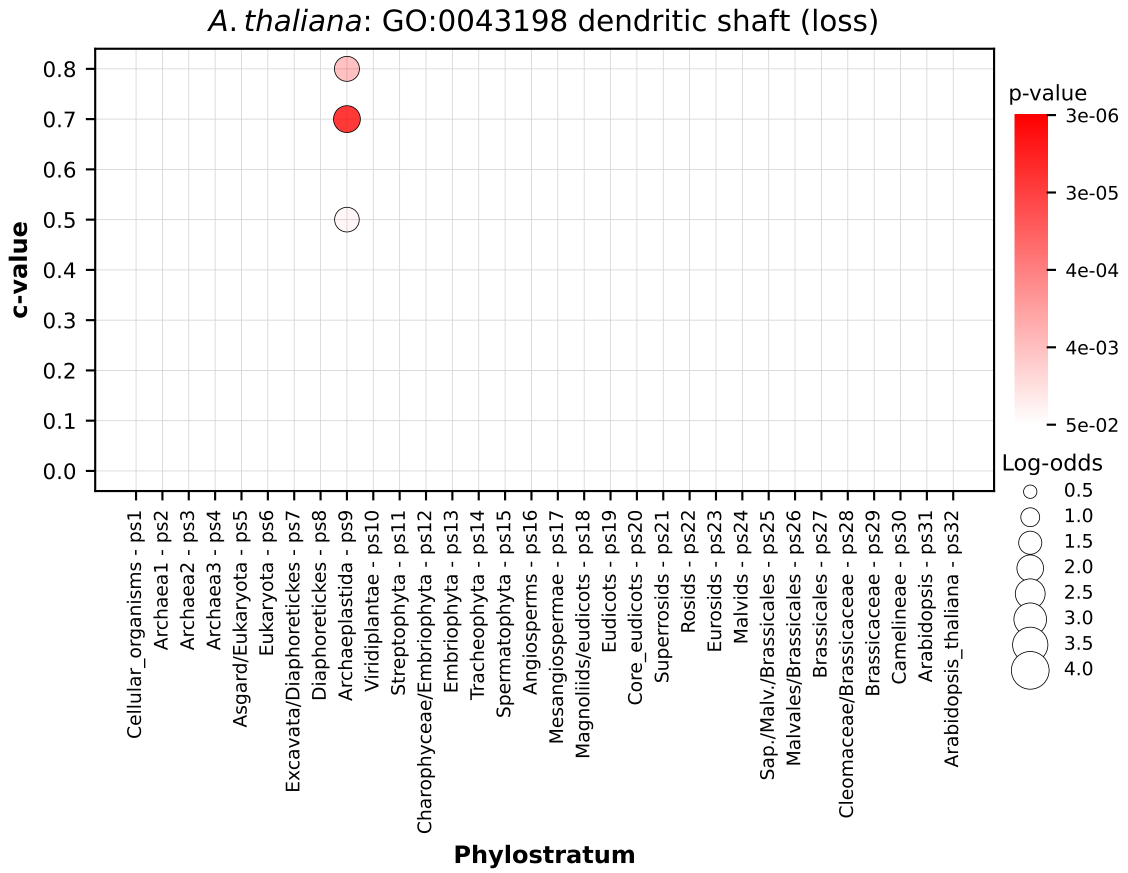

A. thaliana: GO:0043198 dendritic shaft (loss)
c-value
0.8
0.7
0.6
0.5
0.4
0.3
0.2
0.1
0.0
p-value
3e-06
3e-05
4e-04
4e-03
5e-02
Log-odds
0.5
1.0
1.5
2.0
2.5
3.0
3.5
4.0
Cellular_organisms - ps1
Archaea1 - ps2
Archaea2 - ps3
Archaea3 - ps4
Asgard/Eukaryota - ps5
Eukaryota - ps6
Excavata/Diaphoretickes - ps7
Diaphoretickes - ps8
Archaeplastida - ps9
Viridiplantae - ps10
Streptophyta - ps11
Charophyceae/Embriophyta - ps12
Embriophyta - ps13
Tracheophyta - ps14
Spermatophyta - ps15
Angiosperms - ps16
Mesangiospermae - ps17
Magnoliids/eudicots - ps18
Eudicots - ps19
Core_eudicots - ps20
Superrosids - ps21
Rosids - ps22
Eurosids - ps23
Malvids - ps24
Sap./Malv./Brassicales - ps25
Malvales/Brassicales - ps26
Brassicales - ps27
Cleomaceae/Brassicaceae - ps28
Brassicaceae - ps29
Camelineae - ps30
Arabidopsis - ps31
Arabidopsis_thaliana - ps32
Phylostratum

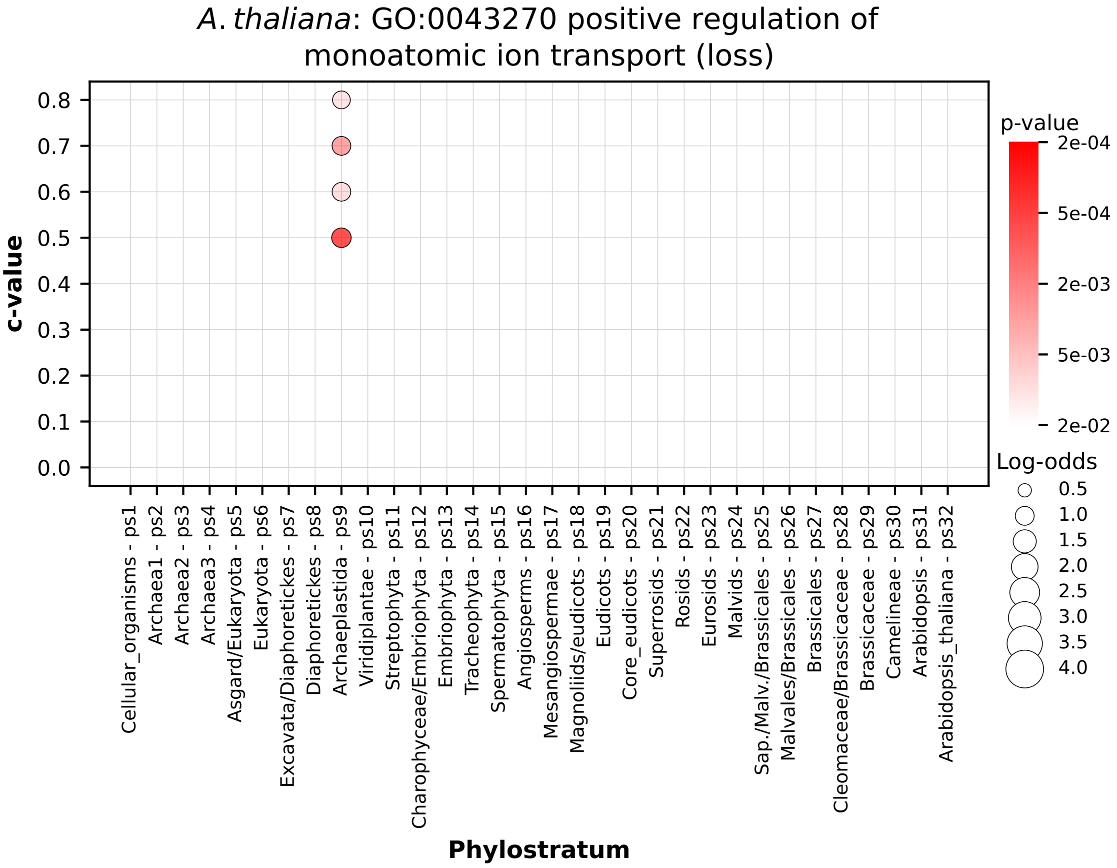

A. thaliana: GO:0043270 positive regulation of monoatomic ion transport (loss)
c-value
0.8
0.7
0.6
0.5
0.4
0.3
0.2
0.1
0.0
p-value
2e-04
5e-04
2e-03
5e-03
2e-02
Log-odds
0.5
1.0
1.5
2.0
2.5
3.0
3.5
4.0
Cellular_organisms - ps1
Archaea1 - ps2
Archaea2 - ps3
Archaea3 - ps4
Asgard/Eukaryota - ps5
Eukaryota - ps6
Excavata/Diaphoretickes - ps7
Diaphoretickes - ps8
Archaeplastida - ps9
Viridiplantae - ps10
Streptophyta - ps11
Charophyceae/Embriophyta - ps12
Embriophyta - ps13
Tracheophyta - ps14
Spermatophyta - ps15
Angiosperms - ps16
Mesangiospermae - ps17
Magnoliids/eudicots - ps18
Eudicots - ps19
Core_eudicots - ps20
Superrosids - ps21
Rosids - ps22
Eurosids - ps23
Malvids - ps24
Sap./Malv./Brassicales - ps25
Malvales/Brassicales - ps26
Brassicales - ps27
Cleomaceae/Brassicaceae - ps28
Brassicaceae - ps29
Camelineae - ps30
Arabidopsis - ps31
Arabidopsis_thaliana - ps32
Phylostratum

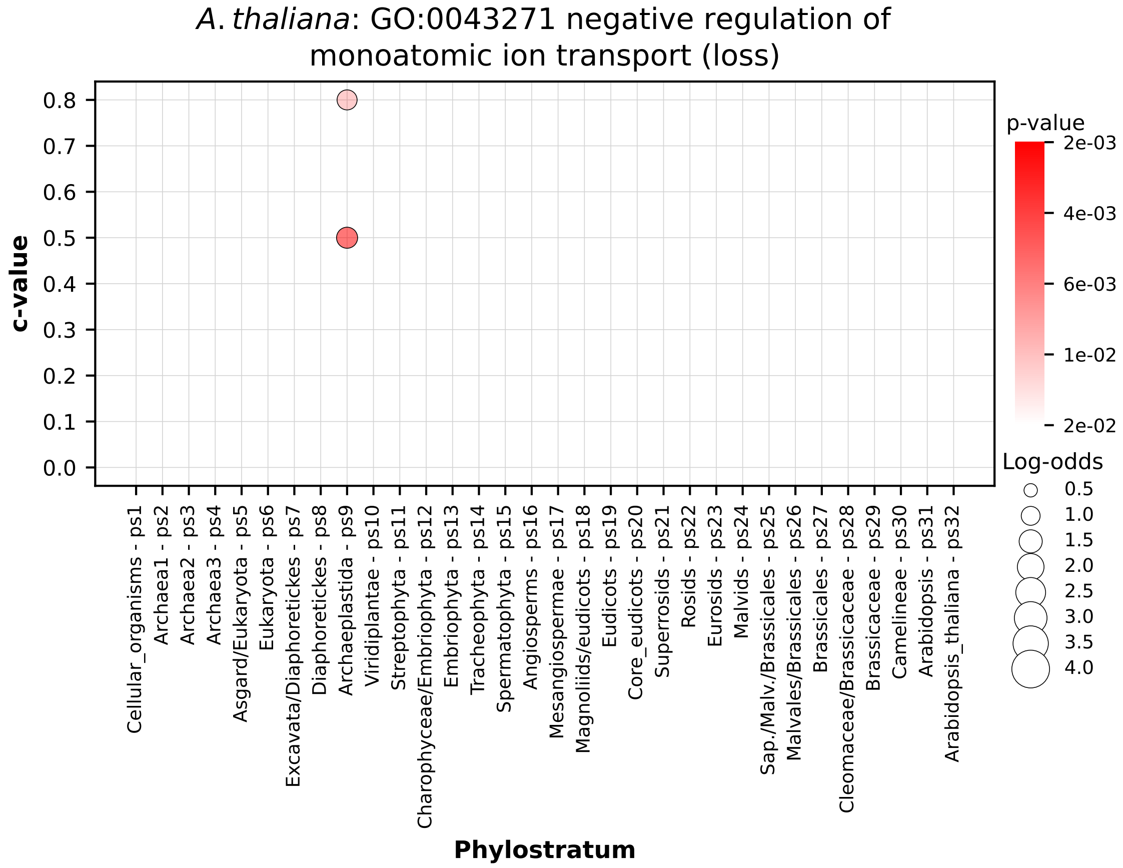

A. thaliana: GO:0043271 negative regulation of monoatomic ion transport (loss)
c-value
0.8
0.7
0.6
0.5
0.4
0.3
0.2
0.1
0.0
p-value
2e-03
4e-03
6e-03
1e-02
2e-02
Log-odds
0.5
1.0
1.5
2.0
2.5
3.0
3.5
4.0
Cellular_organisms - ps1
Archaea1 - ps2
Archaea2 - ps3
Archaea3 - ps4
Asgard/Eukaryota - ps5
Eukaryota - ps6
Excavata/Diaphoretickes - ps7
Diaphoretickes - ps8
Archaeplastida - ps9
Viridiplantae - ps10
Streptophyta - ps11
Charophyceae/Embriophyta - ps12
Embriophyta - ps13
Tracheophyta - ps14
Spermatophyta - ps15
Angiosperms - ps16
Mesangiospermae - ps17
Magnoliids/eudicots - ps18
Eudicots - ps19
Core_eudicots - ps20
Superrosids - ps21
Rosids - ps22
Eurosids - ps23
Malvids - ps24
Sap./Malv./Brassicales - ps25
Malvales/Brassicales - ps26
Brassicales - ps27
Cleomaceae/Brassicaceae - ps28
Brassicaceae - ps29
Camelineae - ps30
Arabidopsis - ps31
Arabidopsis_thaliana - ps32
Phylostratum

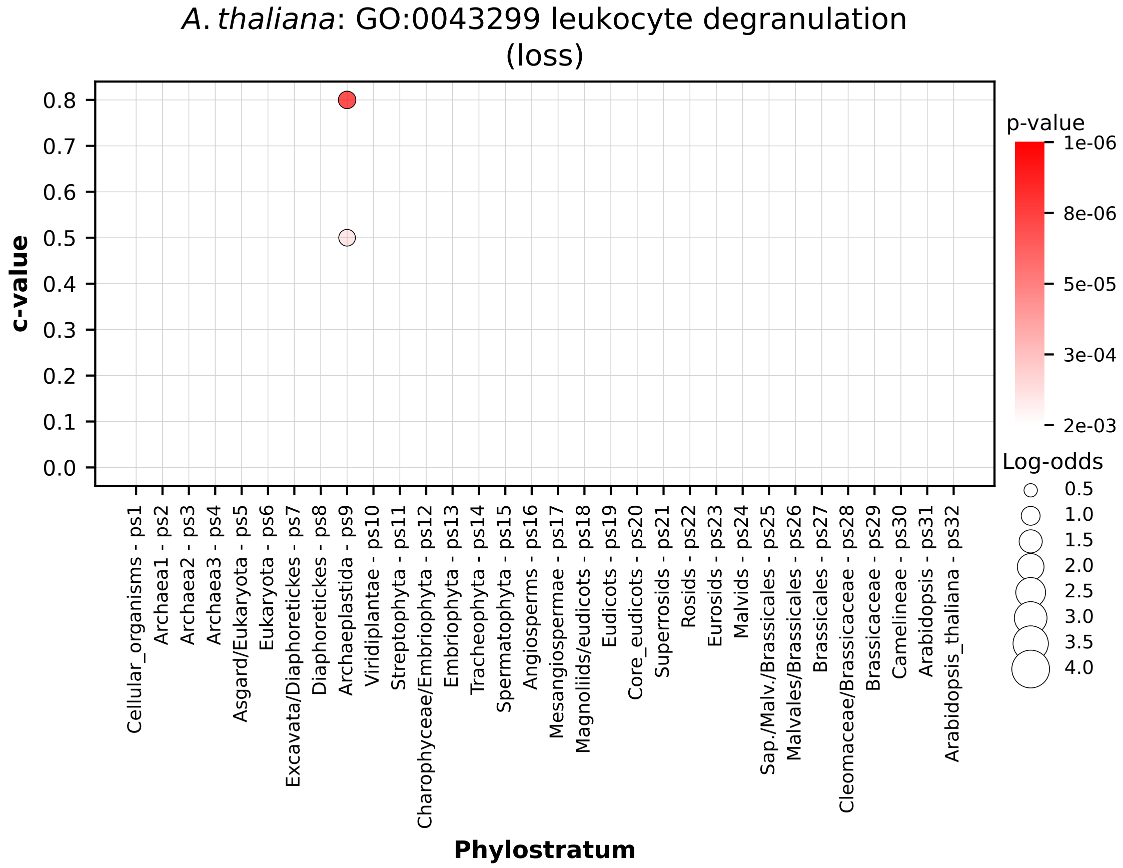

A. thaliana: GO:0043299 leukocyte degranulation
(loss)
c-value
0.0
0.1
0.2
0.3
0.4
0.5
0.6
0.7
0.8
Phylostratum
Cellular_organisms - ps1
Archaea1 - ps2
Archaea2 - ps3
Archaea3 - ps4
Asgard/Eukaryota - ps5
Eukaryota - ps6
Excavata/Diaphoretickes - ps7
Diaphoretickes - ps8
Archaeplastida - ps9
Viridiplantae - ps10
Streptophyta - ps11
Charophyceae/Embriophyta - ps12
Embriophyta - ps13
Tracheophyta - ps14
Spermatophyta - ps15
Angiosperms - ps16
Mesangiospermae - ps17
Magnoliids/eudicots - ps18
Eudicots - ps19
Core_eudicots - ps20
Superrosids - ps21
Rosids - ps22
Eurosids - ps23
Malvids - ps24
Sap./Malv./Brassicales - ps25
Malvales/Brassicales - ps26
Brassicales - ps27
Cleomaceae/Brassicaceae - ps28
Brassicaceae - ps29
Camelineae - ps30
Arabidopsis - ps31
Arabidopsis_thaliana - ps32
p-value
1e-06
8e-06
5e-05
3e-04
2e-03
Log-odds
0.5
1.0
1.5
2.0
2.5
3.0
3.5
4.0

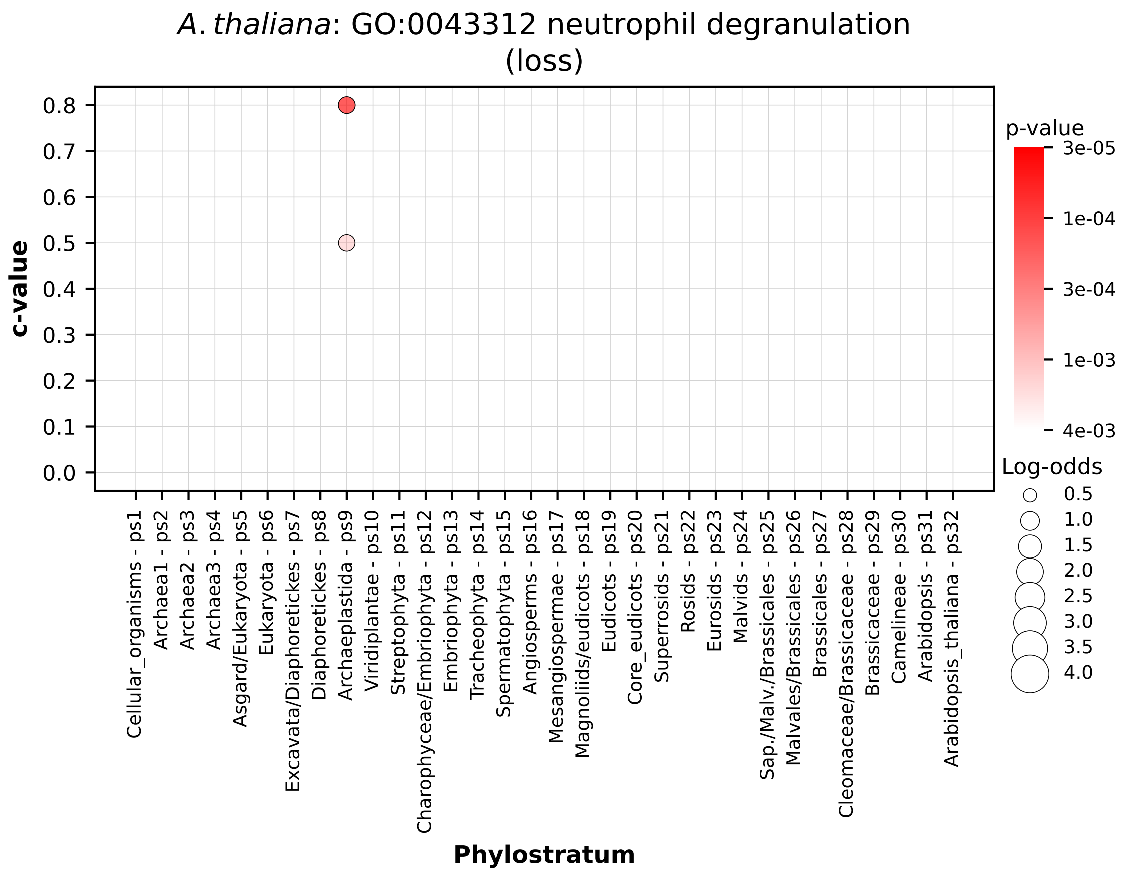

A. thaliana: GO:0043312 neutrophil degranulation
(loss)
c-value
0.8
0.7
0.6
0.5
0.4
0.3
0.2
0.1
0.0
p-value
3e-05
1e-04
3e-04
1e-03
4e-03
Log-odds
0.5
1.0
1.5
2.0
2.5
3.0
3.5
4.0
Cellular_organisms - ps1
Archaea1 - ps2
Archaea2 - ps3
Archaea3 - ps4
Asgard/Eukaryota - ps5
Eukaryota - ps6
Excavata/Diaphoretickes - ps7
Diaphoretickes - ps8
Archaeplastida - ps9
Viridiplantae - ps10
Streptophyta - ps11
Charophyceae/Embriophyta - ps12
Embriophyta - ps13
Tracheophyta - ps14
Spermatophyta - ps15
Angiosperms - ps16
Mesangiospermae - ps17
Magnoliids/eudicots - ps18
Eudicots - ps19
Core_eudicots - ps20
Superrosids - ps21
Rosids - ps22
Eurosids - ps23
Malvids - ps24
Sap./Malv./Brassicales - ps25
Malvales/Brassicales - ps26
Brassicales - ps27
Cleomaceae/Brassicaceae - ps28
Brassicaceae - ps29
Camelineae - ps30
Arabidopsis - ps31
Arabidopsis_thaliana - ps32
Phylostratum

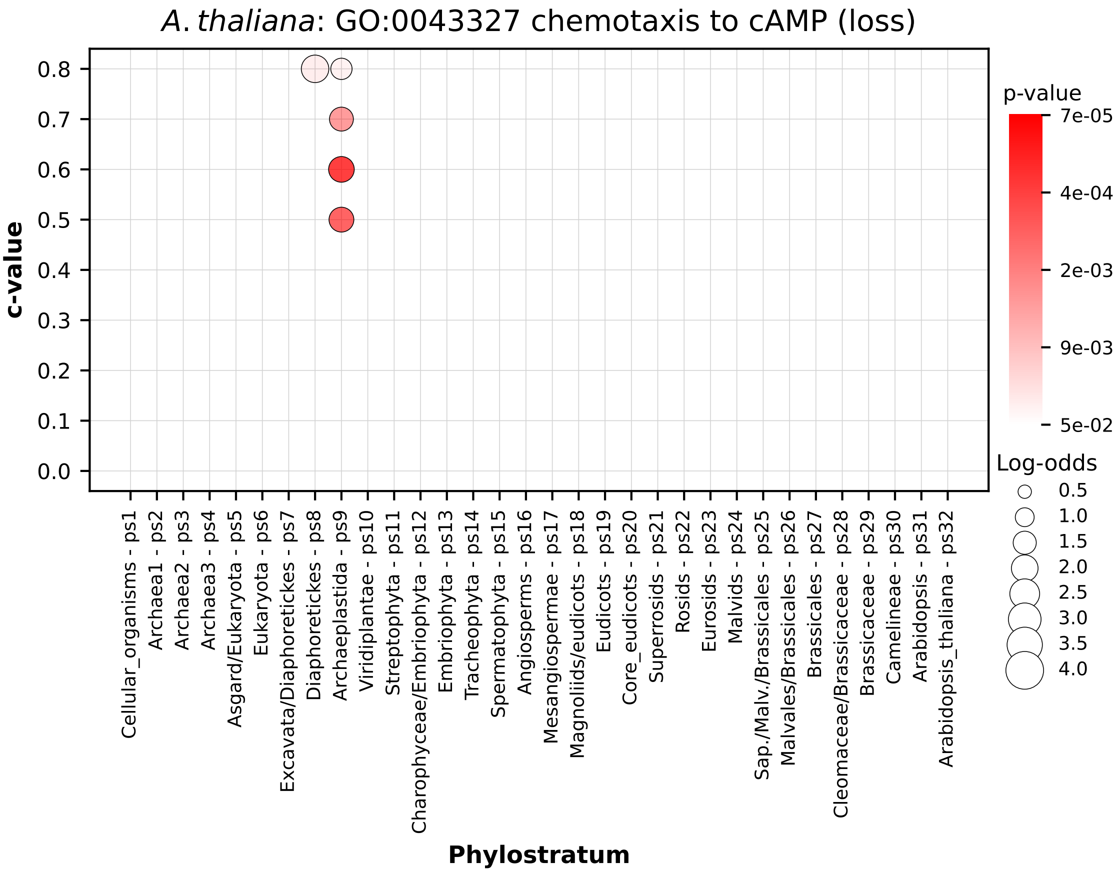

A. thaliana: GO:0043327 chemotaxis to cAMP (loss)
c-value
0.0
0.1
0.2
0.3
0.4
0.5
0.6
0.7
0.8
Phylostratum
Cellular_organisms - ps1
Archaea1 - ps2
Archaea2 - ps3
Archaea3 - ps4
Asgard/Eukaryota - ps5
Eukaryota - ps6
Excavata/Diaphoretickes - ps7
Diaphoretickes - ps8
Archaeplastida - ps9
Viridiplantae - ps10
Streptophyta - ps11
Charophyceae/Embriophyta - ps12
Embriophyta - ps13
Tracheophyta - ps14
Spermatophyta - ps15
Angiosperms - ps16
Mesangiospermae - ps17
Magnoliids/eudicots - ps18
Eudicots - ps19
Core_eudicots - ps20
Superrosids - ps21
Rosids - ps22
Eurosids - ps23
Malvids - ps24
Sap./Malv./Brassicales - ps25
Malvales/Brassicales - ps26
Brassicales - ps27
Cleomaceae/Brassicaceae - ps28
Brassicaceae - ps29
Camelineae - ps30
Arabidopsis - ps31
Arabidopsis_thaliana - ps32
p-value
7e-05
4e-04
2e-03
9e-03
5e-02
Log-odds
0.5
1.0
1.5
2.0
2.5
3.0
3.5
4.0

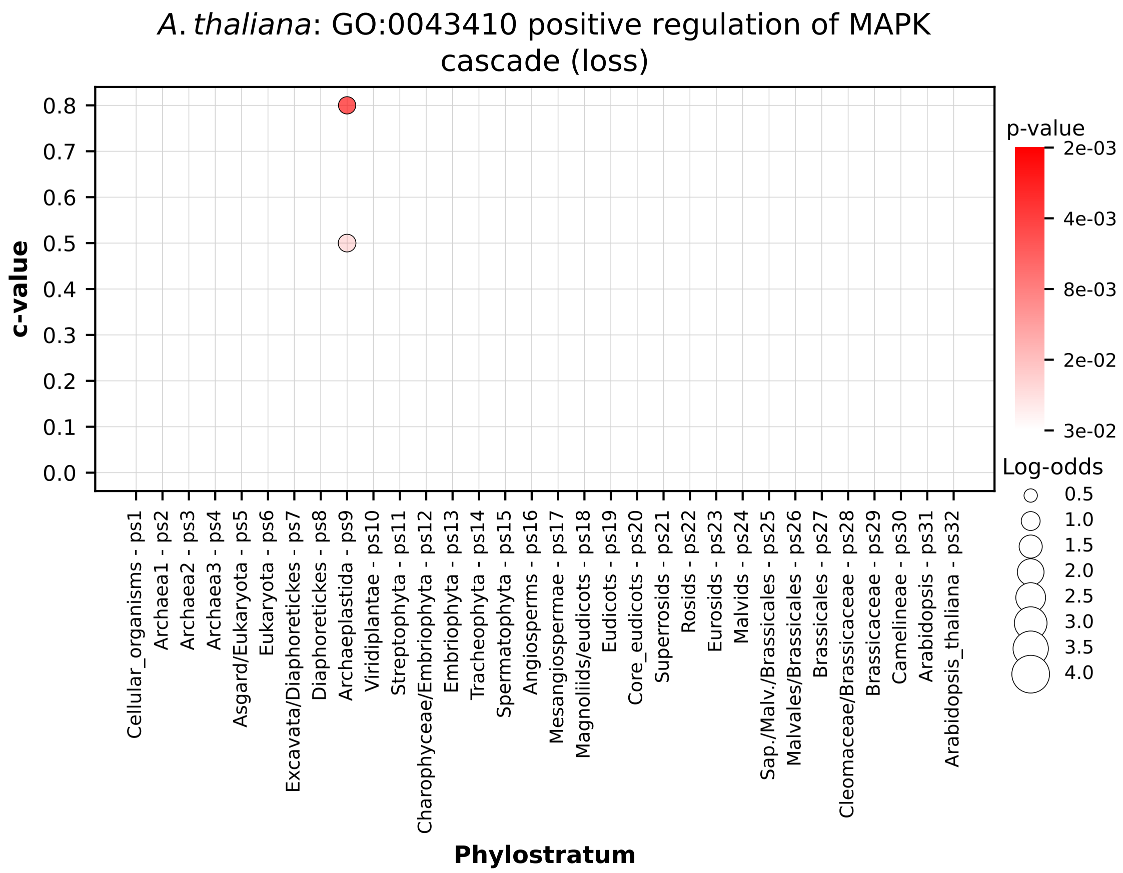

A. thaliana: GO:0043410 positive regulation of MAPK cascade (loss)
c-value
0.0
0.1
0.2
0.3
0.4
0.5
0.6
0.7
0.8
p-value
2e-03
4e-03
8e-03
2e-02
3e-02
Log-odds
0.5
1.0
1.5
2.0
2.5
3.0
3.5
4.0
Cellular_organisms - ps1
Archaea1 - ps2
Archaea2 - ps3
Archaea3 - ps4
Asgard/Eukaryota - ps5
Eukaryota - ps6
Excavata/Diaphoretickes - ps7
Diaphoretickes - ps8
Archaeplastida - ps9
Viridiplantae - ps10
Streptophyta - ps11
Charophyceae/Embriophyta - ps12
Embriophyta - ps13
Tracheophyta - ps14
Spermatophyta - ps15
Angiosperms - ps16
Mesangiospermae - ps17
Magnoliids/eudicots - ps18
Eudicots - ps19
Core_eudicots - ps20
Superrosids - ps21
Rosids - ps22
Eurosids - ps23
Malvids - ps24
Sap./Malv./Brassicales - ps25
Malvales/Brassicales - ps26
Brassicales - ps27
Cleomaceae/Brassicaceae - ps28
Brassicaceae - ps29
Camelineae - ps30
Arabidopsis - ps31
Arabidopsis_thaliana - ps32
Phylostratum

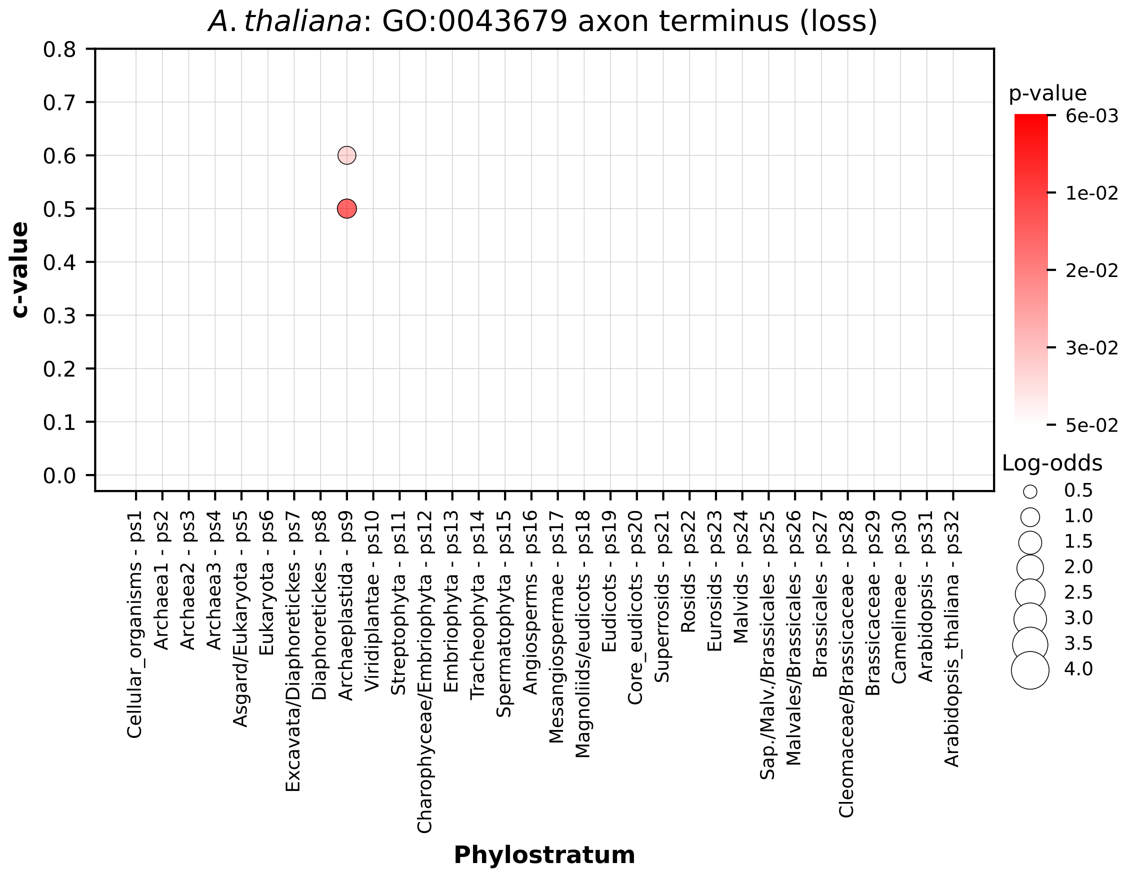
A. thaliana: GO:0043679 axon terminus (loss)
c-value
0.0
0.1
0.2
0.3
0.4
0.5
0.6
0.7
0.8
Phylostratum
Cellular_organisms - ps1
Archaea1 - ps2
Archaea2 - ps3
Archaea3 - ps4
Asgard/Eukaryota - ps5
Eukaryota - ps6
Excavata/Diaphoretickes - ps7
Diaphoretickes - ps8
Archaeplastida - ps9
Viridiplantae - ps10
Streptophyta - ps11
Charophyceae/Embriophyta - ps12
Embriophyta - ps13
Tracheophyta - ps14
Spermatophyta - ps15
Angiosperms - ps16
Mesangiospermae - ps17
Magnoliids/eudicots - ps18
Eudicots - ps19
Core_eudicots - ps20
Superrosids - ps21
Rosids - ps22
Eurosids - ps23
Malvids - ps24
Sap./Malv./Brassicales - ps25
Malvales/Brassicales - ps26
Brassicales - ps27
Cleomaceae/Brassicaceae - ps28
Brassicaceae - ps29
Camelineae - ps30
Arabidopsis - ps31
Arabidopsis_thaliana - ps32
p-value
6e-03
1e-02
2e-02
3e-02
5e-02
Log-odds
0.5
1.0
1.5
2.0
2.5
3.0
3.5
4.0

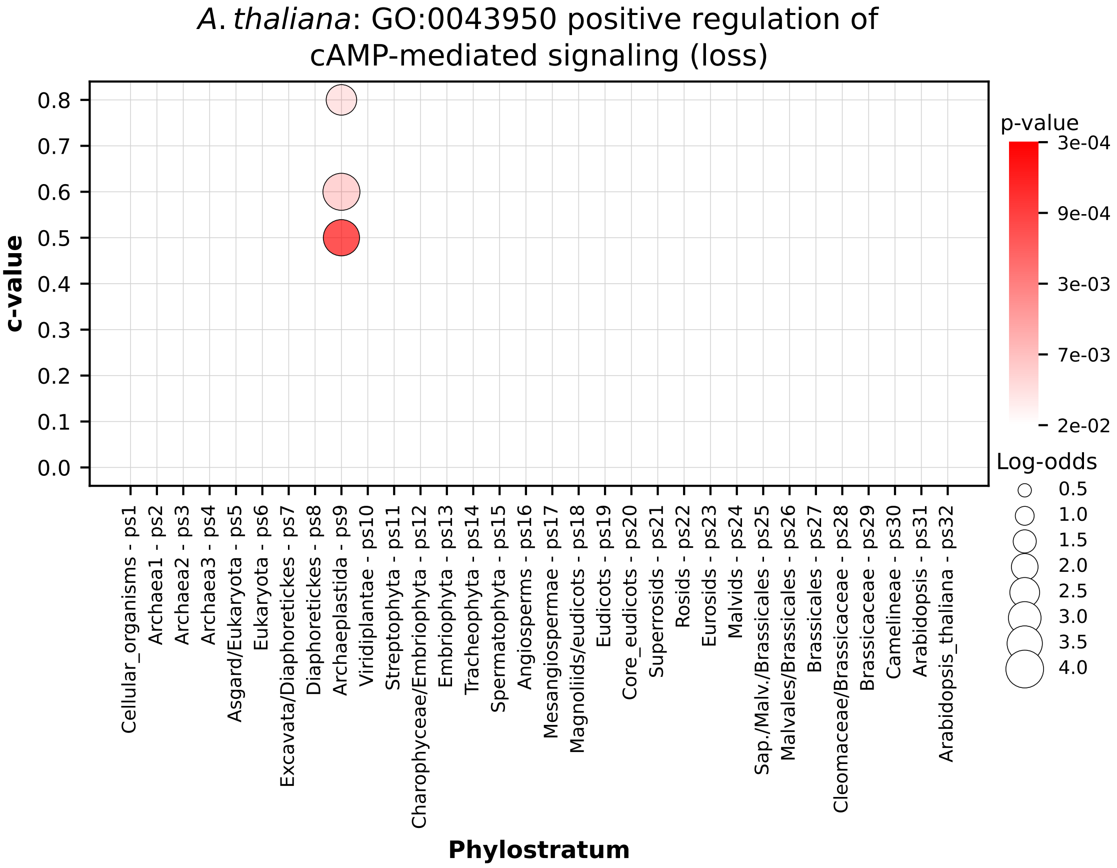
A. thaliana: GO:0043950 positive regulation of cAMP-mediated signaling (loss)
c-value
0.8
0.7
0.6
0.5
0.4
0.3
0.2
0.1
0.0
Phylostratum
Cellular_organisms - ps1
Archaea1 - ps2
Archaea2 - ps3
Archaea3 - ps4
Asgard/Eukaryota - ps5
Eukaryota - ps6
Excavata/Diaphoretickes - ps7
Diaphoretickes - ps8
Archaeplastida - ps9
Viridiplantae - ps10
Streptophyta - ps11
Charophyceae/Embriophyta - ps12
Embriophyta - ps13
Tracheophyta - ps14
Spermatophyta - ps15
Angiosperms - ps16
Mesangiospermae - ps17
Magnoliids/eudicots - ps18
Eudicots - ps19
Core_eudicots - ps20
Superrosids - ps21
Rosids - ps22
Eurosids - ps23
Malvids - ps24
Sap./Malv./Brassicales - ps25
Malvales/Brassicales - ps26
Brassicales - ps27
Cleomaceae/Brassicaceae - ps28
Brassicaceae - ps29
Camelineae - ps30
Arabidopsis - ps31
Arabidopsis_thaliana - ps32
p-value
3e-04
9e-04
3e-03
7e-03
2e-02
Log-odds
0.5
1.0
1.5
2.0
2.5
3.0
3.5
4.0

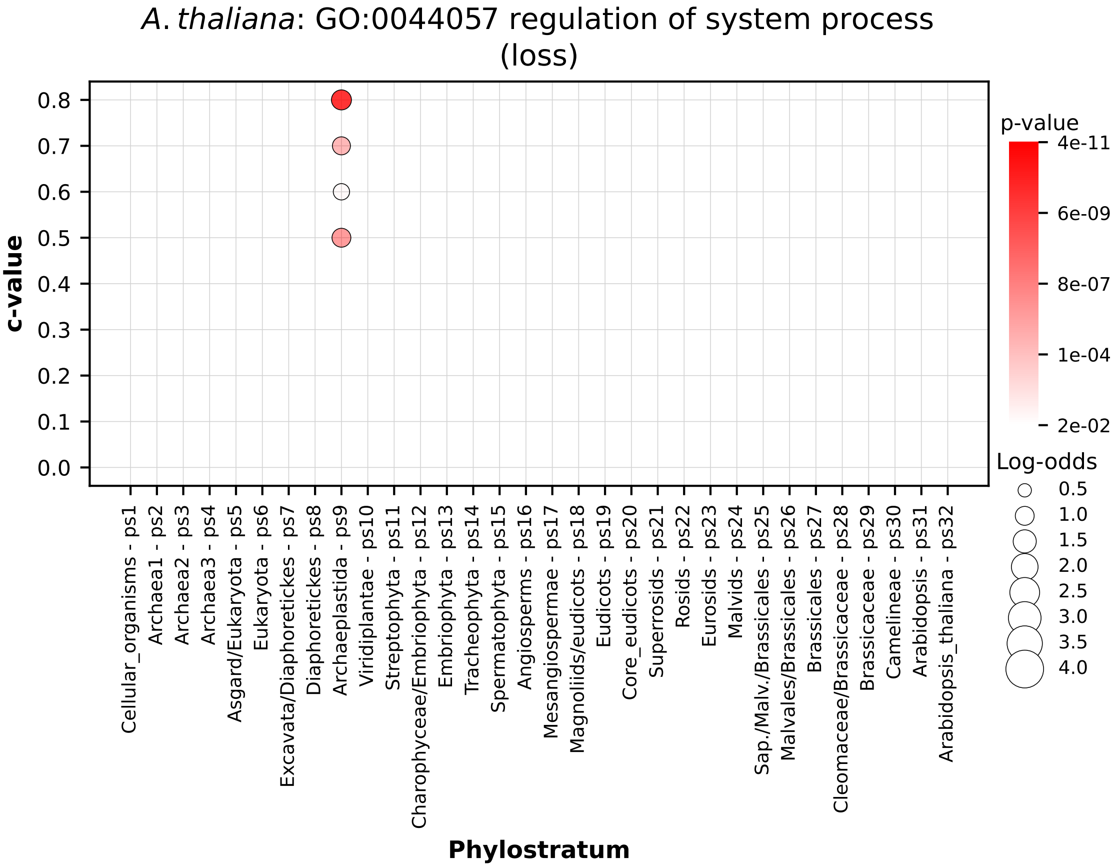

A. thaliana: GO:0044057 regulation of system process (loss)
c-value
0.8
0.7
0.6
0.5
0.4
0.3
0.2
0.1
0.0
p-value
4e-11
6e-09
8e-07
1e-04
2e-02
Log-odds
0.5
1.0
1.5
2.0
2.5
3.0
3.5
4.0
Cellular_organisms - ps1
Archaea1 - ps2
Archaea2 - ps3
Archaea3 - ps4
Asgard/Eukaryota - ps5
Eukaryota - ps6
Excavata/Diaphoretickes - ps7
Diaphoretickes - ps8
Archaeplastida - ps9
Viridiplantae - ps10
Streptophyta - ps11
Charophyceae/Embriophyta - ps12
Embriophyta - ps13
Tracheophyta - ps14
Spermatophyta - ps15
Angiosperms - ps16
Mesangiospermae - ps17
Magnoliids/eudicots - ps18
Eudicots - ps19
Core_eudicots - ps20
Superrosids - ps21
Rosids - ps22
Eurosids - ps23
Malvids - ps24
Sap./Malv./Brassicales - ps25
Malvales/Brassicales - ps26
Brassicales - ps27
Cleomaceae/Brassicaceae - ps28
Brassicaceae - ps29
Camelineae - ps30
Arabidopsis - ps31
Arabidopsis_thaliana - ps32
Phylostratum

A. thaliana: GO:0044090 positive regulation of vacuole organization (loss)

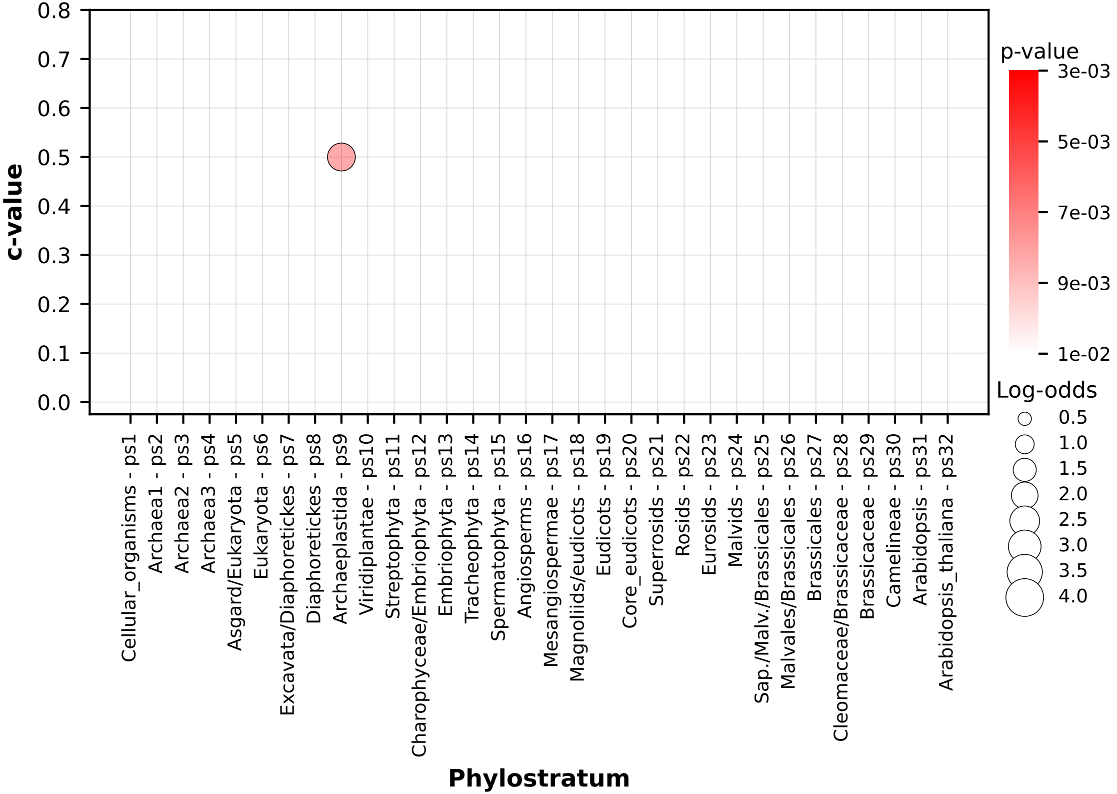

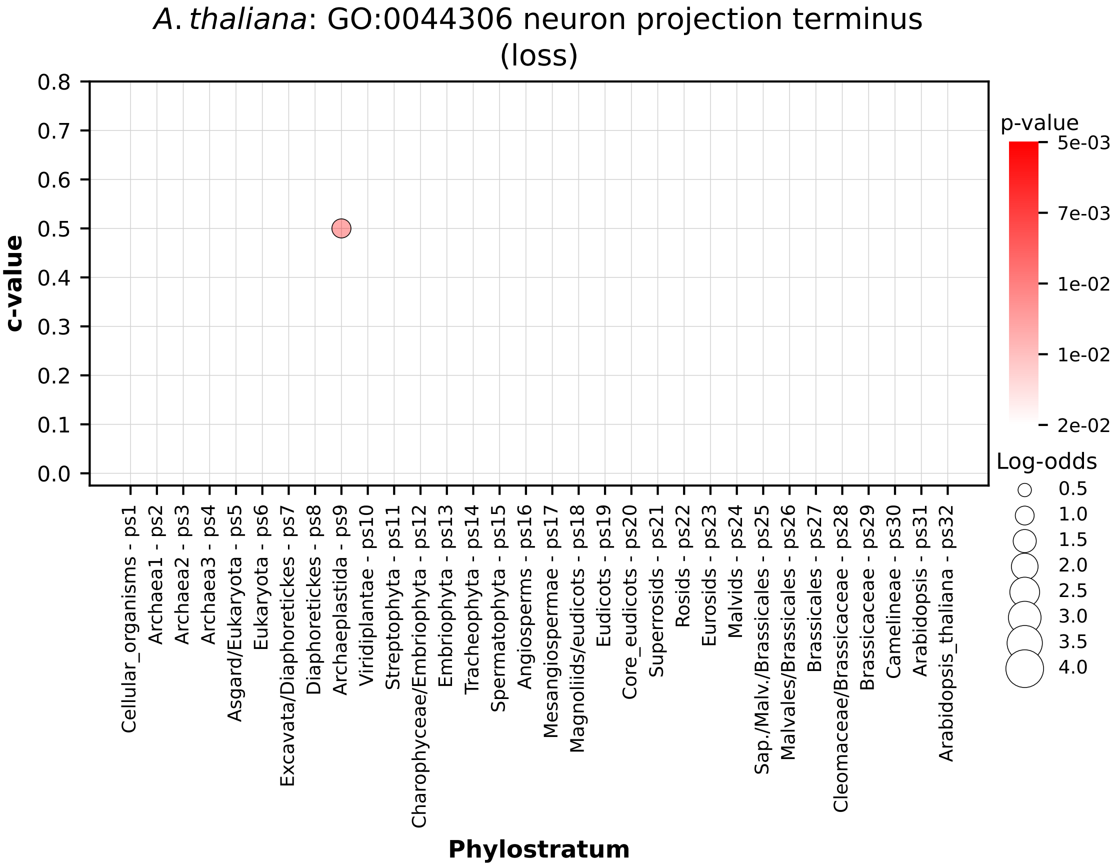

A. thaliana: GO:0044306 neuron projection terminus
(loss)
c-value
0.8
0.7
0.6
0.5
0.4
0.3
0.2
0.1
0.0
p-value
5e-03
7e-03
1e-02
1e-02
2e-02
Log-odds
0.5
1.0
1.5
2.0
2.5
3.0
3.5
4.0
Cellular_organisms - ps1
Archaea1 - ps2
Archaea2 - ps3
Archaea3 - ps4
Asgard/Eukaryota - ps5
Eukaryota - ps6
Excavata/Diaphoretickes - ps7
Diaphoretickes - ps8
Archaeplastida - ps9
Viridiplantae - ps10
Streptophyta - ps11
Charophyceae/Embriophyta - ps12
Embriophyta - ps13
Tracheophyta - ps14
Spermatophyta - ps15
Angiosperms - ps16
Mesangiospermae - ps17
Magnoliids/eudicots - ps18
Eudicots - ps19
Core_eudicots - ps20
Superrosids - ps21
Rosids - ps22
Eurosids - ps23
Malvids - ps24
Sap./Malv./Brassicales - ps25
Malvales/Brassicales - ps26
Brassicales - ps27
Cleomaceae/Brassicaceae - ps28
Brassicaceae - ps29
Camelineae - ps30
Arabidopsis - ps31
Arabidopsis_thaliana - ps32
Phylostratum

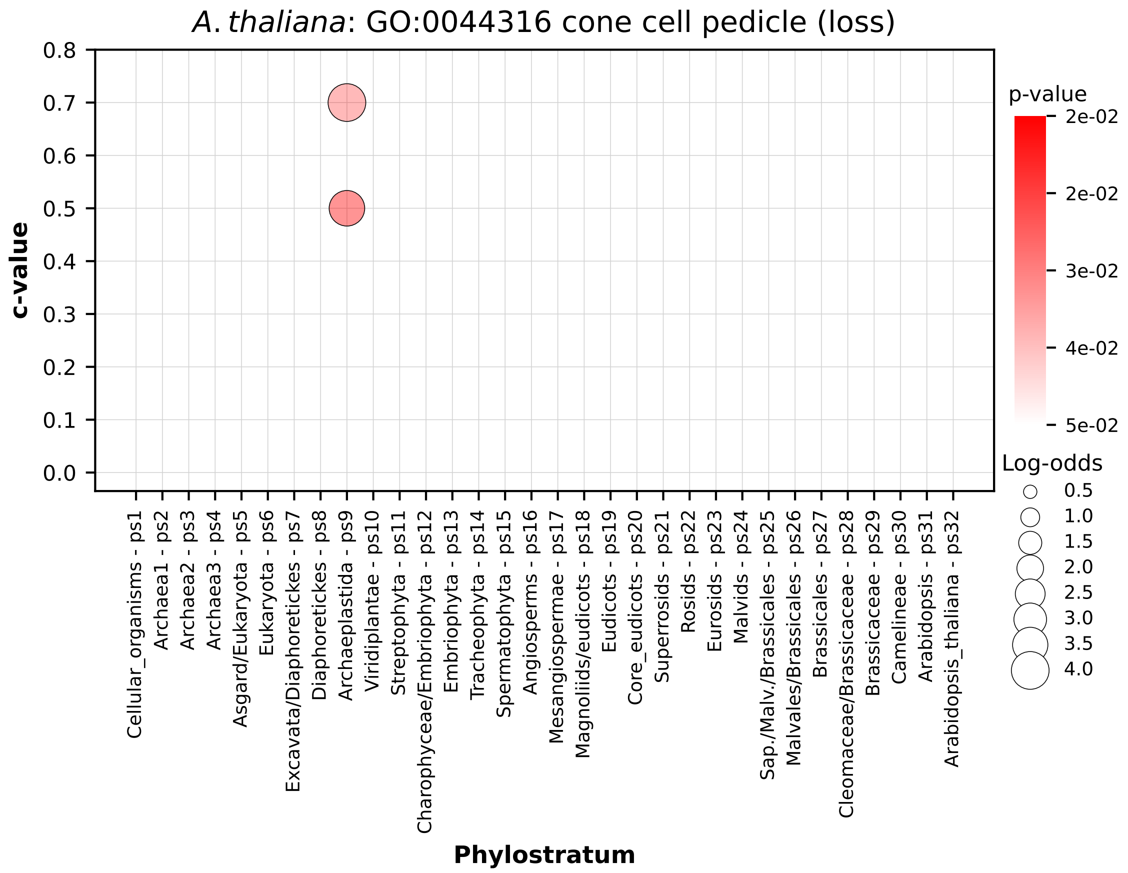
A. thaliana: GO:0044316 cone cell pedicle (loss)
c-value
0.8
0.7
0.6
0.5
0.4
0.3
0.2
0.1
0.0
p-value
2e-02
2e-02
3e-02
4e-02
5e-02
Log-odds
0.5
1.0
1.5
2.0
2.5
3.0
3.5
4.0
Cellular_organisms - ps1
Archaea1 - ps2
Archaea2 - ps3
Archaea3 - ps4
Asgard/Eukaryota - ps5
Eukaryota - ps6
Excavata/Diaphoretickes - ps7
Diaphoretickes - ps8
Archaeplastida - ps9
Viridiplantae - ps10
Streptophyta - ps11
Charophyceae/Embriophyta - ps12
Embriophyta - ps13
Tracheophyta - ps14
Spermatophyta - ps15
Angiosperms - ps16
Mesangiospermae - ps17
Magnoliids/eudicots - ps18
Eudicots - ps19
Core_eudicots - ps20
Superrosids - ps21
Rosids - ps22
Eurosids - ps23
Malvids - ps24
Sap./Malv./Brassicales - ps25
Malvales/Brassicales - ps26
Brassicales - ps27
Cleomaceae/Brassicaceae - ps28
Brassicaceae - ps29
Camelineae - ps30
Arabidopsis - ps31
Arabidopsis_thaliana - ps32
Phylostratum

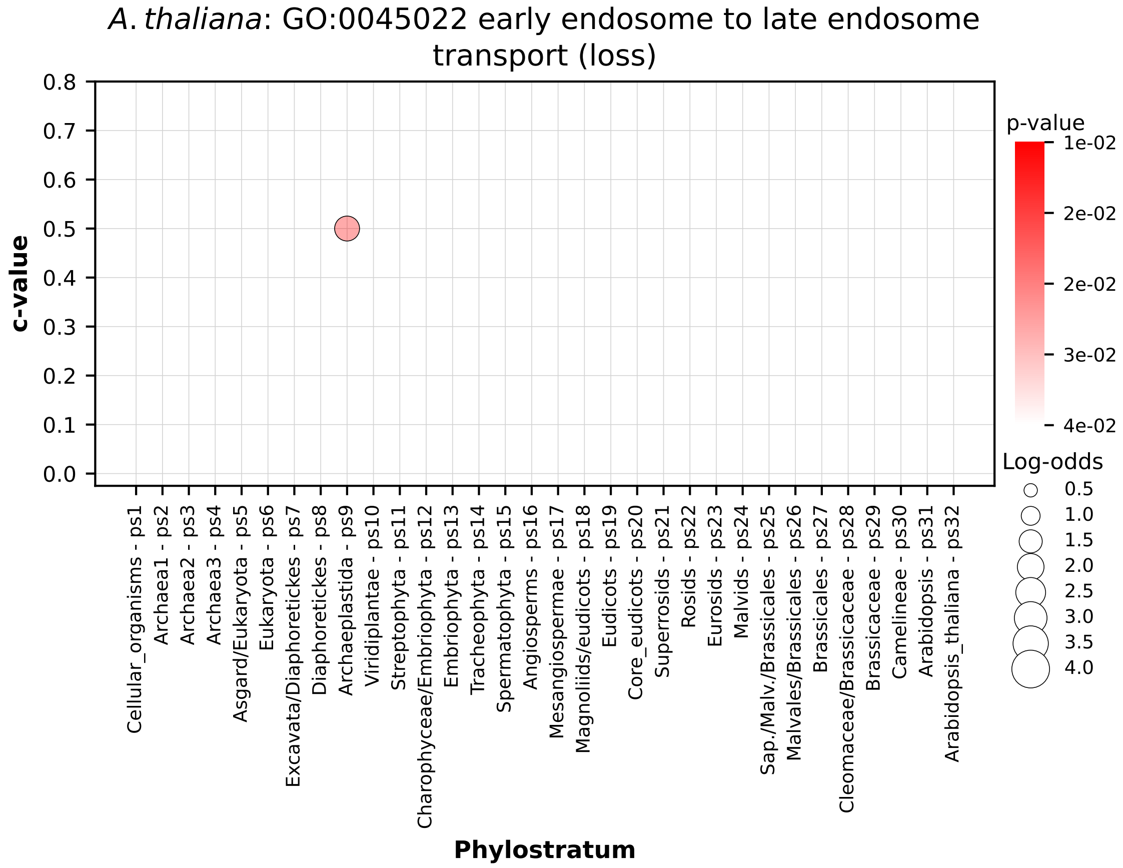

A. thaliana: GO:0045022 early endosome to late endosome transport (loss)
c-value
0.8
0.7
0.6
0.5
0.4
0.3
0.2
0.1
0.0
Phylostratum
Cellular_organisms - ps1
Archaea1 - ps2
Archaea2 - ps3
Archaea3 - ps4
Asgard/Eukaryota - ps5
Eukaryota - ps6
Excavata/Diaphoretickes - ps7
Diaphoretickes - ps8
Archaeplastida - ps9
Viridiplantae - ps10
Streptophyta - ps11
Charophyceae/Embriophyta - ps12
Embriophyta - ps13
Tracheophyta - ps14
Spermatophyta - ps15
Angiosperms - ps16
Mesangiospermae - ps17
Magnoliids/eudicots - ps18
Eudicots - ps19
Core_eudicots - ps20
Superrosids - ps21
Rosids - ps22
Eurosids - ps23
Malvids - ps24
Sap./Malv./Brassicales - ps25
Malvales/Brassicales - ps26
Brassicales - ps27
Cleomaceae/Brassicaceae - ps28
Brassicaceae - ps29
Camelineae - ps30
Arabidopsis - ps31
Arabidopsis_thaliana - ps32
p-value
1e-02
2e-02
2e-02
3e-02
4e-02
Log-odds
0.5
1.0
1.5
2.0
2.5
3.0
3.5
4.0

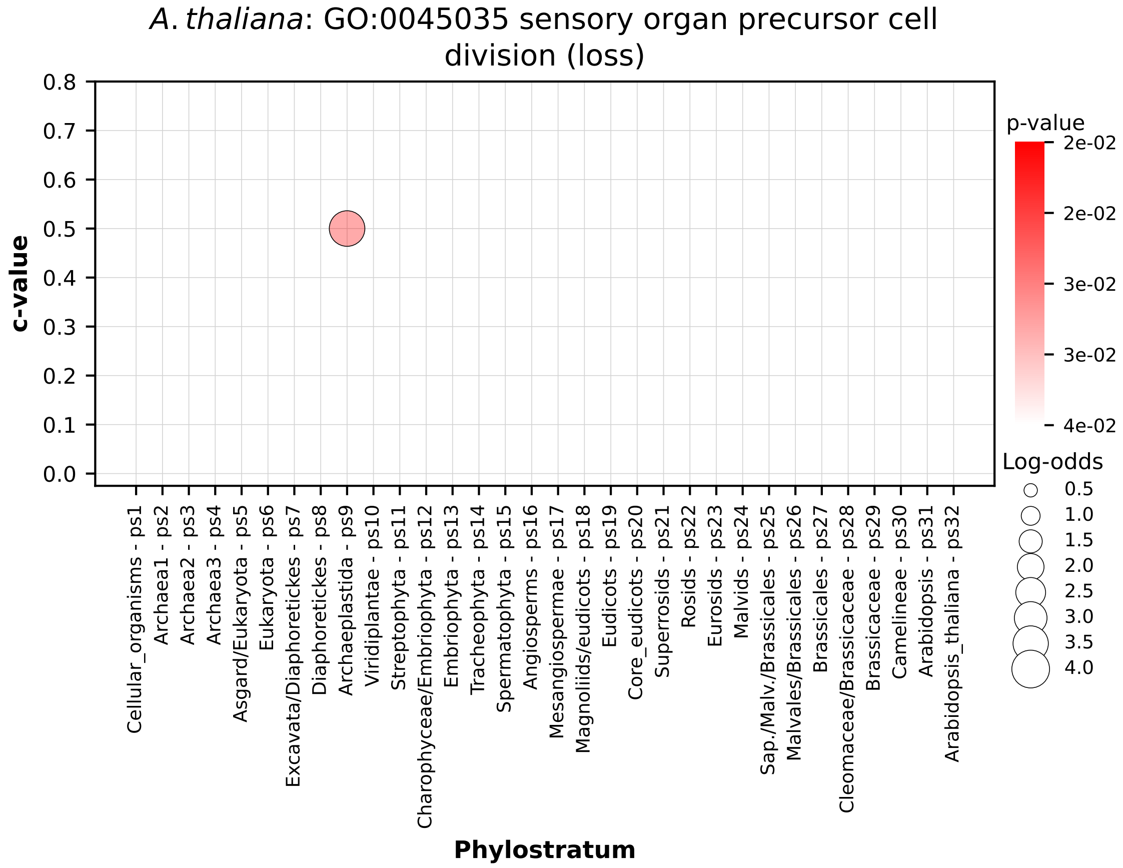
A. thaliana: GO:0045035 sensory organ precursor cell division (loss)
c-value
0.8
0.7
0.6
0.5
0.4
0.3
0.2
0.1
0.0
Phylostratum
Cellular_organisms - ps1
Archaea1 - ps2
Archaea2 - ps3
Archaea3 - ps4
Asgard/Eukaryota - ps5
Eukaryota - ps6
Excavata/Diaphoretickes - ps7
Diaphoretickes - ps8
Archaeplastida - ps9
Viridiplantae - ps10
Streptophyta - ps11
Charophyceae/Embriophyta - ps12
Embriophyta - ps13
Tracheophyta - ps14
Spermatophyta - ps15
Angiosperms - ps16
Mesangiospermae - ps17
Magnoliids/eudicots - ps18
Eudicots - ps19
Core_eudicots - ps20
Superrosids - ps21
Rosids - ps22
Eurosids - ps23
Malvids - ps24
Sap./Malv./Brassicales - ps25
Malvales/Brassicales - ps26
Brassicales - ps27
Cleomaceae/Brassicaceae - ps28
Brassicaceae - ps29
Camelineae - ps30
Arabidopsis - ps31
Arabidopsis_thaliana - ps32
p-value
2e-02
2e-02
3e-02
3e-02
4e-02
Log-odds
0.5
1.0
1.5
2.0
2.5
3.0
3.5
4.0

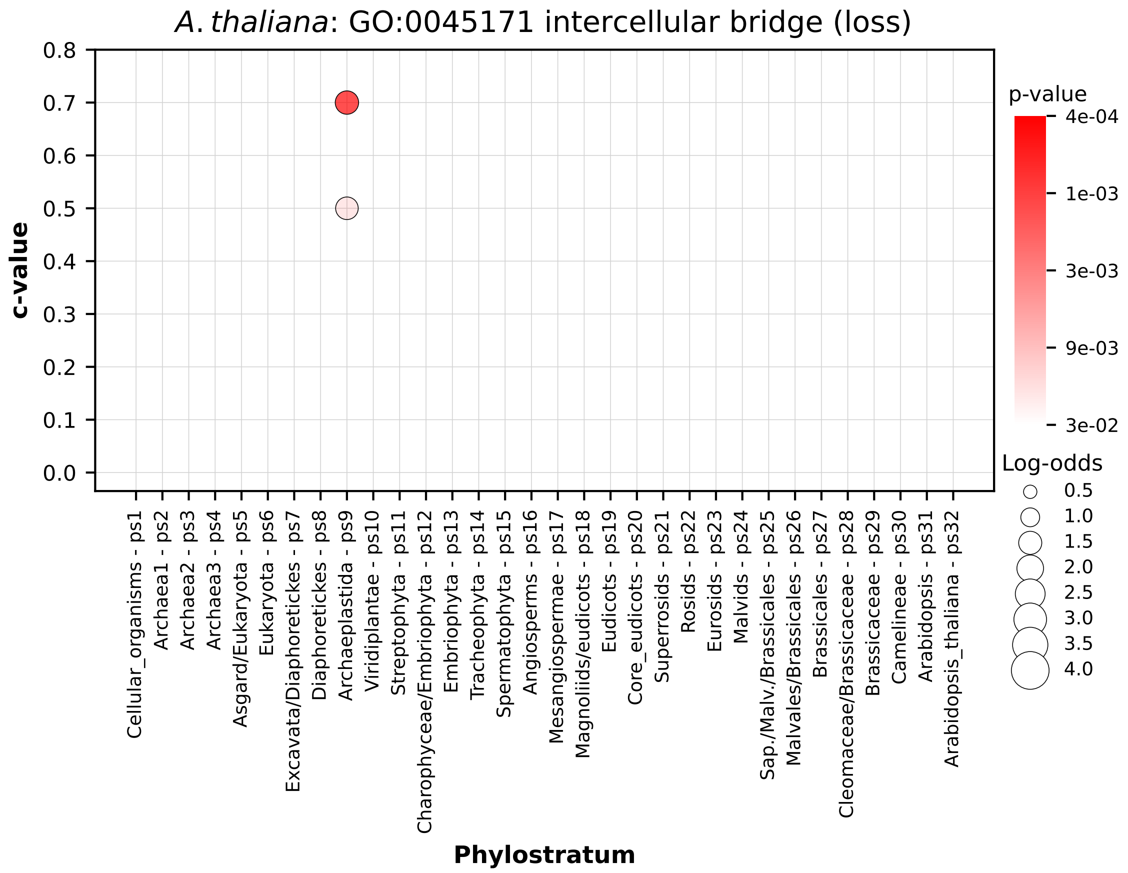

A. thaliana: GO:0045171 intercellular bridge (loss)
c-value
0.8
0.7
0.6
0.5
0.4
0.3
0.2
0.1
0.0
Phylostratum
Cellular_organisms - ps1
Archaea1 - ps2
Archaea2 - ps3
Archaea3 - ps4
Asgard/Eukaryota - ps5
Eukaryota - ps6
Excavata/Diaphoretickes - ps7
Diaphoretickes - ps8
Archaeplastida - ps9
Viridiplantae - ps10
Streptophyta - ps11
Charophyceae/Embriophyta - ps12
Embriophyta - ps13
Tracheophyta - ps14
Spermatophyta - ps15
Angiosperms - ps16
Mesangiospermae - ps17
Magnoliids/eudicots - ps18
Eudicots - ps19
Core_eudicots - ps20
Superrosids - ps21
Rosids - ps22
Eurosids - ps23
Malvids - ps24
Sap./Malv./Brassicales - ps25
Malvales/Brassicales - ps26
Brassicales - ps27
Cleomaceae/Brassicaceae - ps28
Brassicaceae - ps29
Camelineae - ps30
Arabidopsis - ps31
Arabidopsis_thaliana - ps32
p-value
4e-04
1e-03
3e-03
9e-03
3e-02
Log-odds
0.5
1.0
1.5
2.0
2.5
3.0
3.5
4.0

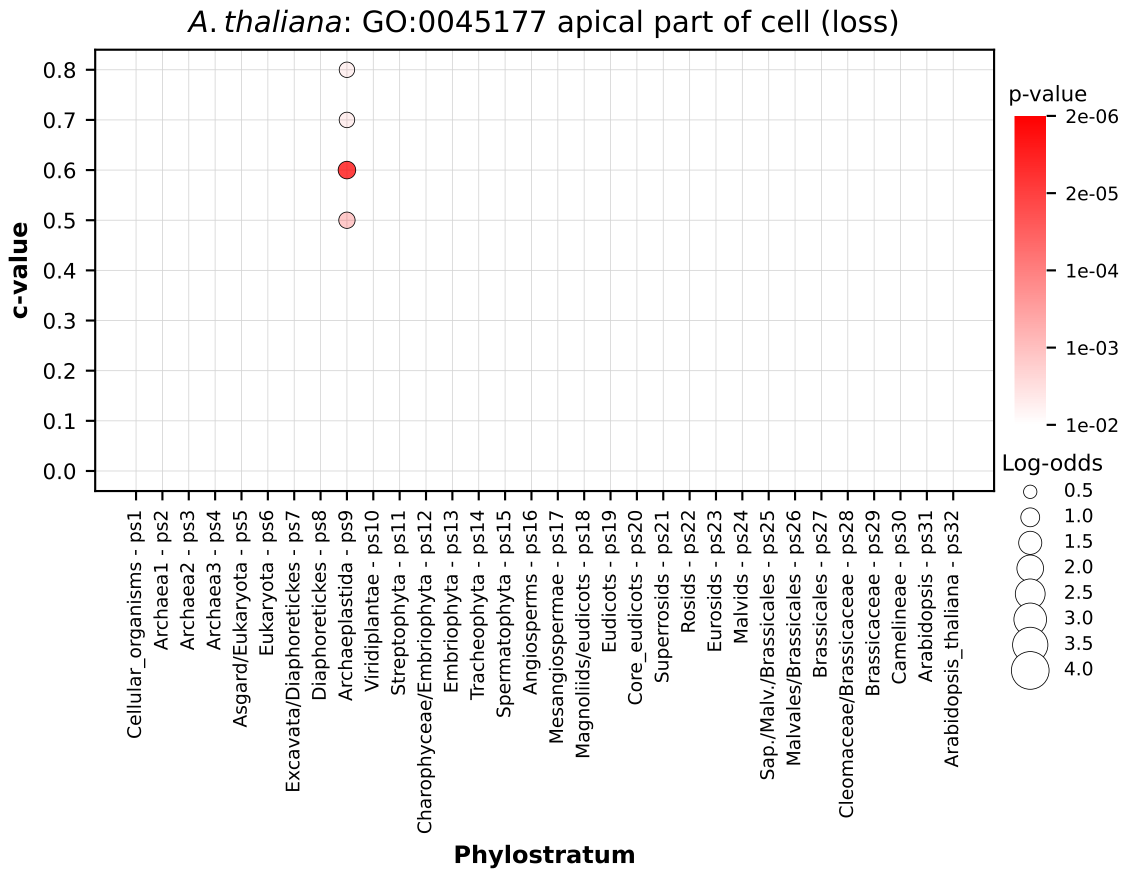
A. thaliana: GO:0045177 apical part of cell (loss)
c-value
0.8
0.7
0.6
0.5
0.4
0.3
0.2
0.1
0.0
p-value
2e-06
2e-05
1e-04
1e-03
1e-02
Log-odds
0.5
1.0
1.5
2.0
2.5
3.0
3.5
4.0
Cellular_organisms - ps1
Archaea1 - ps2
Archaea2 - ps3
Archaea3 - ps4
Asgard/Eukaryota - ps5
Eukaryota - ps6
Excavata/Diaphoretickes - ps7
Diaphoretickes - ps8
Archaeplastida - ps9
Viridiplantae - ps10
Streptophyta - ps11
Charophyceae/Embriophyta - ps12
Embriophyta - ps13
Tracheophyta - ps14
Spermatophyta - ps15
Angiosperms - ps16
Mesangiospermae - ps17
Magnoliids/eudicots - ps18
Eudicots - ps19
Core_eudicots - ps20
Superrosids - ps21
Rosids - ps22
Eurosids - ps23
Malvids - ps24
Sap./Malv./Brassicales - ps25
Malvales/Brassicales - ps26
Brassicales - ps27
Cleomaceae/Brassicaceae - ps28
Brassicaceae - ps29
Camelineae - ps30
Arabidopsis - ps31
Arabidopsis_thaliana - ps32
Phylostratum

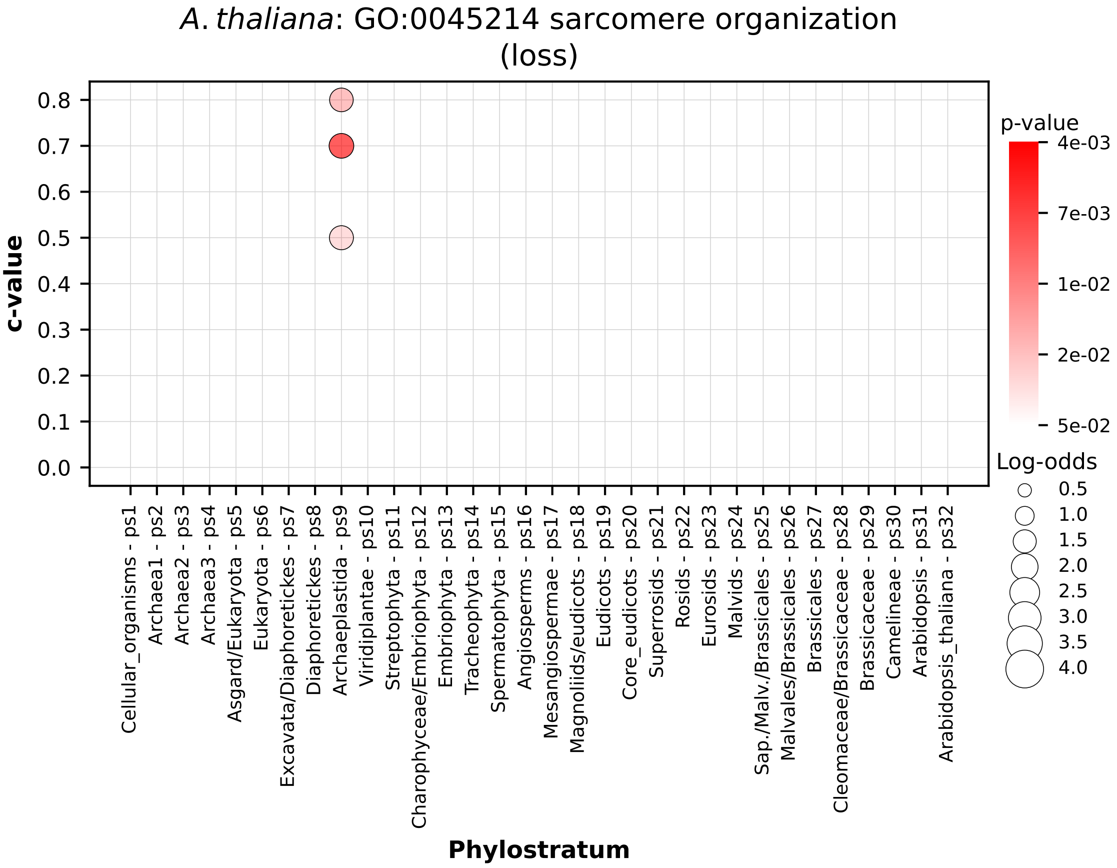

A. thaliana: GO:0045214 sarcomere organization
(loss)
c-value
0.8
0.7
0.6
0.5
0.4
0.3
0.2
0.1
0.0
Phylostratum
Cellular_organisms - ps1
Archaea1 - ps2
Archaea2 - ps3
Archaea3 - ps4
Asgard/Eukaryota - ps5
Eukaryota - ps6
Excavata/Diaphoretickes - ps7
Diaphoretickes - ps8
Archaeplastida - ps9
Viridiplantae - ps10
Streptophyta - ps11
Charophyceae/Embriophyta - ps12
Embriophyta - ps13
Tracheophyta - ps14
Spermatophyta - ps15
Angiosperms - ps16
Mesangiospermae - ps17
Magnoliids/eudicots - ps18
Eudicots - ps19
Core_eudicots - ps20
Superrosids - ps21
Rosids - ps22
Eurosids - ps23
Malvids - ps24
Sap./Malv./Brassicales - ps25
Malvales/Brassicales - ps26
Brassicales - ps27
Cleomaceae/Brassicaceae - ps28
Brassicaceae - ps29
Camelineae - ps30
Arabidopsis - ps31
Arabidopsis_thaliana - ps32
p-value
4e-03
7e-03
1e-02
2e-02
5e-02
Log-odds
0.5
1.0
1.5
2.0
2.5
3.0
3.5
4.0

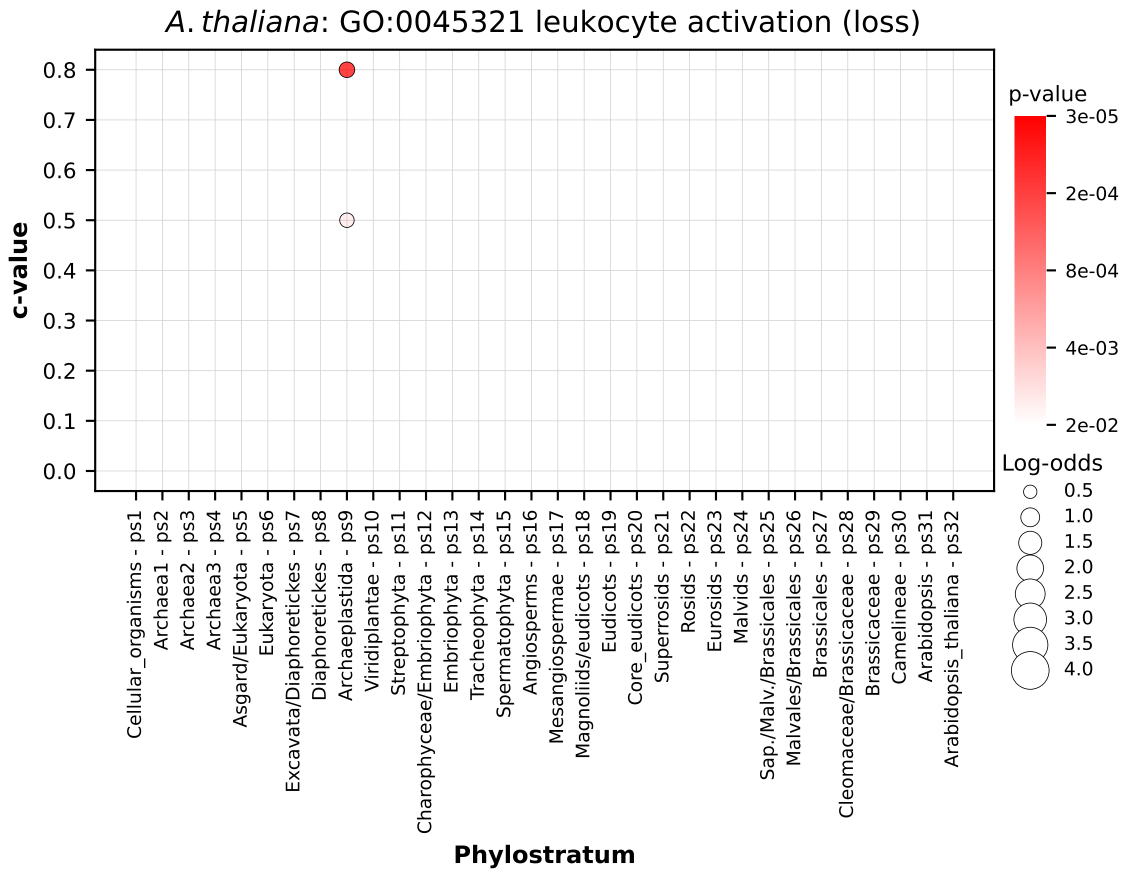

A. thaliana: GO:0045321 leukocyte activation (loss)
c-value
0.0
0.1
0.2
0.3
0.4
0.5
0.6
0.7
0.8
Phylostratum
Cellular_organisms - ps1
Archaea1 - ps2
Archaea2 - ps3
Archaea3 - ps4
Asgard/Eukaryota - ps5
Eukaryota - ps6
Excavata/Diaphoretickes - ps7
Diaphoretickes - ps8
Archaeplastida - ps9
Viridiplantae - ps10
Streptophyta - ps11
Charophyceae/Embriophyta - ps12
Embriophyta - ps13
Tracheophyta - ps14
Spermatophyta - ps15
Angiosperms - ps16
Mesangiospermae - ps17
Magnoliids/eudicots - ps18
Eudicots - ps19
Core_eudicots - ps20
Superrosids - ps21
Rosids - ps22
Eurosids - ps23
Malvids - ps24
Sap./Malv./Brassicales - ps25
Malvales/Brassicales - ps26
Brassicales - ps27
Cleomaceae/Brassicaceae - ps28
Brassicaceae - ps29
Camelineae - ps30
Arabidopsis - ps31
Arabidopsis_thaliana - ps32
p-value
3e-05
2e-04
8e-04
4e-03
2e-02
Log-odds
0.5
1.0
1.5
2.0
2.5
3.0
3.5
4.0

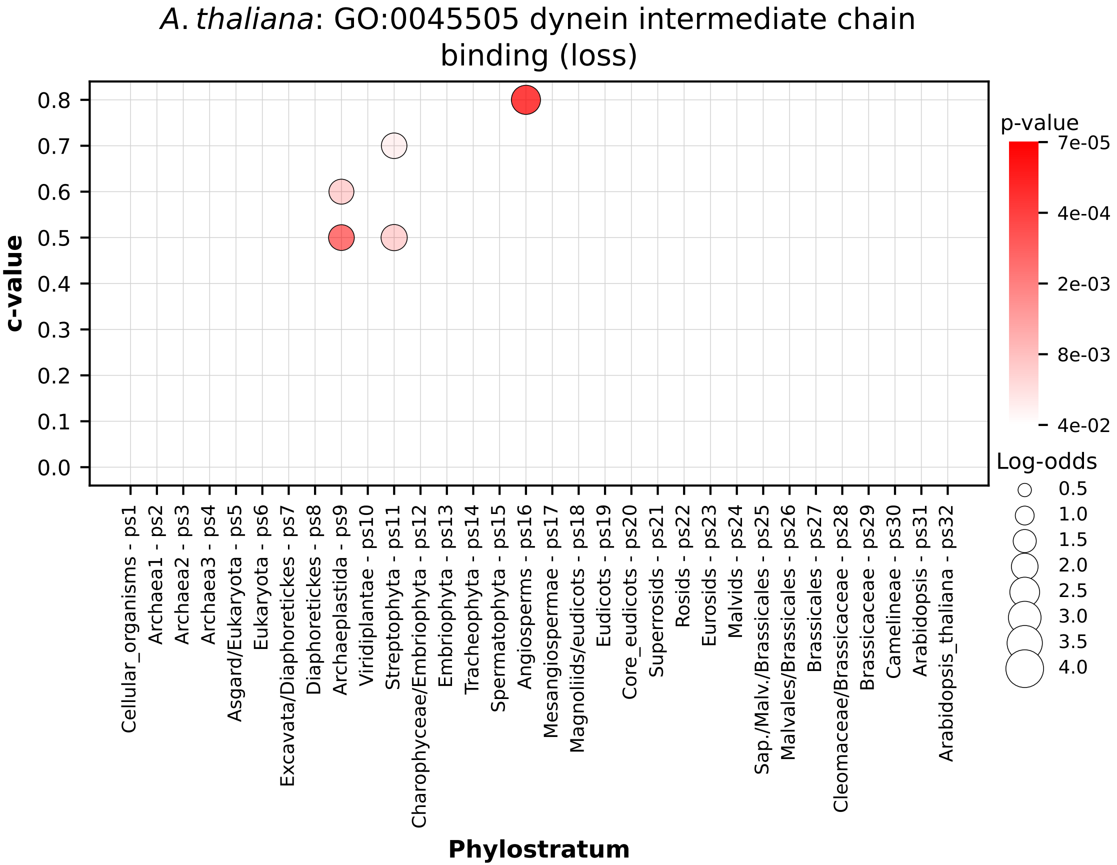

A. thaliana: GO:0045505 dynein intermediate chain binding (loss)
c-value
0.0
0.1
0.2
0.3
0.4
0.5
0.6
0.7
0.8
p-value
7e-05
4e-04
2e-03
8e-03
4e-02
Log-odds
0.5
1.0
1.5
2.0
2.5
3.0
3.5
4.0
Cellular_organisms - ps1
Archaea1 - ps2
Archaea2 - ps3
Archaea3 - ps4
Asgard/Eukaryota - ps5
Eukaryota - ps6
Excavata/Diaphoretickes - ps7
Diaphoretickes - ps8
Archaeplastida - ps9
Viridiplantae - ps10
Streptophyta - ps11
Charophyceae/Embriophyta - ps12
Embriophyta - ps13
Tracheophyta - ps14
Spermatophyta - ps15
Angiosperms - ps16
Mesangiospermae - ps17
Magnoliids/eudicots - ps18
Eudicots - ps19
Core_eudicots - ps20
Superrosids - ps21
Rosids - ps22
Eurosids - ps23
Malvids - ps24
Sap./Malv./Brassicales - ps25
Malvales/Brassicales - ps26
Brassicales - ps27
Cleomaceae/Brassicaceae - ps28
Brassicaceae - ps29
Camelineae - ps30
Arabidopsis - ps31
Arabidopsis_thaliana - ps32
Phylostratum

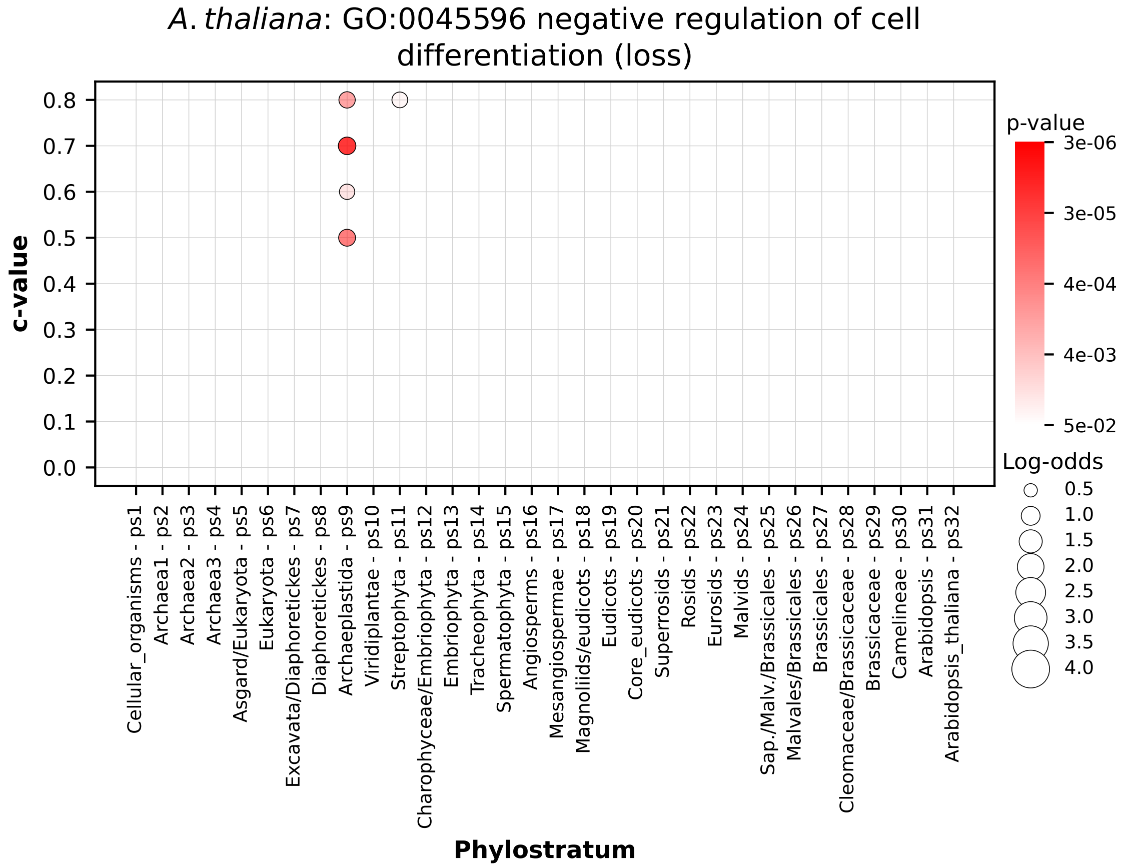

A. thaliana: GO:0045596 negative regulation of cell differentiation (loss)
c-value
0.8
0.7
0.6
0.5
0.4
0.3
0.2
0.1
0.0
p-value
3e-06
3e-05
4e-04
4e-03
5e-02
Log-odds
0.5
1.0
1.5
2.0
2.5
3.0
3.5
4.0
Cellular_organisms - ps1
Archaea1 - ps2
Archaea2 - ps3
Archaea3 - ps4
Asgard/Eukaryota - ps5
Eukaryota - ps6
Excavata/Diaphoretickes - ps7
Diaphoretickes - ps8
Archaeplastida - ps9
Viridiplantae - ps10
Streptophyta - ps11
Charophyceae/Embriophyta - ps12
Embriophyta - ps13
Tracheophyta - ps14
Spermatophyta - ps15
Angiosperms - ps16
Mesangiospermae - ps17
Magnoliids/eudicots - ps18
Eudicots - ps19
Core_eudicots - ps20
Superrosids - ps21
Rosids - ps22
Eurosids - ps23
Malvids - ps24
Sap./Malv./Brassicales - ps25
Malvales/Brassicales - ps26
Brassicales - ps27
Cleomaceae/Brassicaceae - ps28
Brassicaceae - ps29
Camelineae - ps30
Arabidopsis - ps31
Arabidopsis_thaliana - ps32
Phylostratum

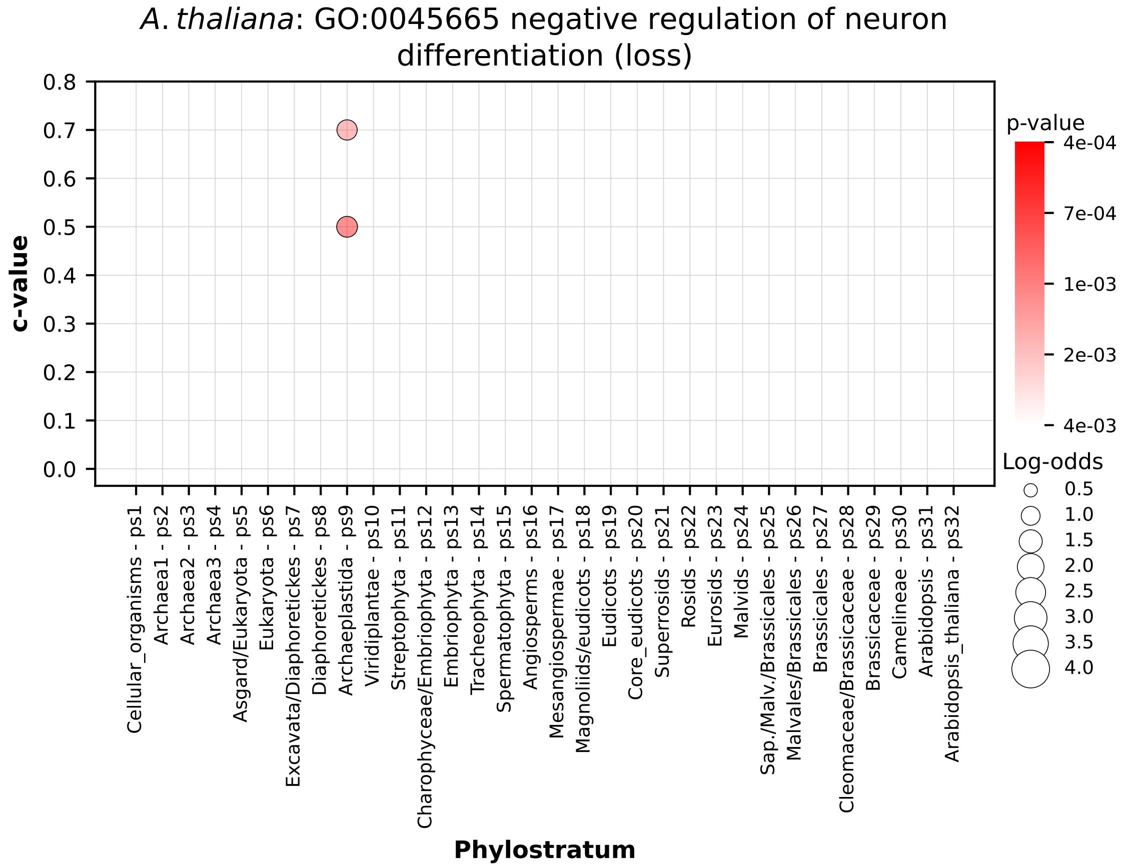
A. thaliana: GO:0045665 negative regulation of neuron differentiation (loss)
c-value
0.8
0.7
0.6
0.5
0.4
0.3
0.2
0.1
0.0
Phylostratum
Cellular_organisms - ps1
Archaea1 - ps2
Archaea2 - ps3
Archaea3 - ps4
Asgard/Eukaryota - ps5
Eukaryota - ps6
Excavata/Diaphoretickes - ps7
Diaphoretickes - ps8
Archaeplastida - ps9
Viridiplantae - ps10
Streptophyta - ps11
Charophyceae/Embriophyta - ps12
Embriophyta - ps13
Tracheophyta - ps14
Spermatophyta - ps15
Angiosperms - ps16
Mesangiospermae - ps17
Magnoliids/eudicots - ps18
Eudicots - ps19
Core_eudicots - ps20
Superrosids - ps21
Rosids - ps22
Eurosids - ps23
Malvids - ps24
Sap./Malv./Brassicales - ps25
Malvales/Brassicales - ps26
Brassicales - ps27
Cleomaceae/Brassicaceae - ps28
Brassicaceae - ps29
Camelineae - ps30
Arabidopsis - ps31
Arabidopsis_thaliana - ps32
p-value
4e-04
7e-04
1e-03
2e-03
4e-03
Log-odds
0.5
1.0
1.5
2.0
2.5
3.0
3.5
4.0

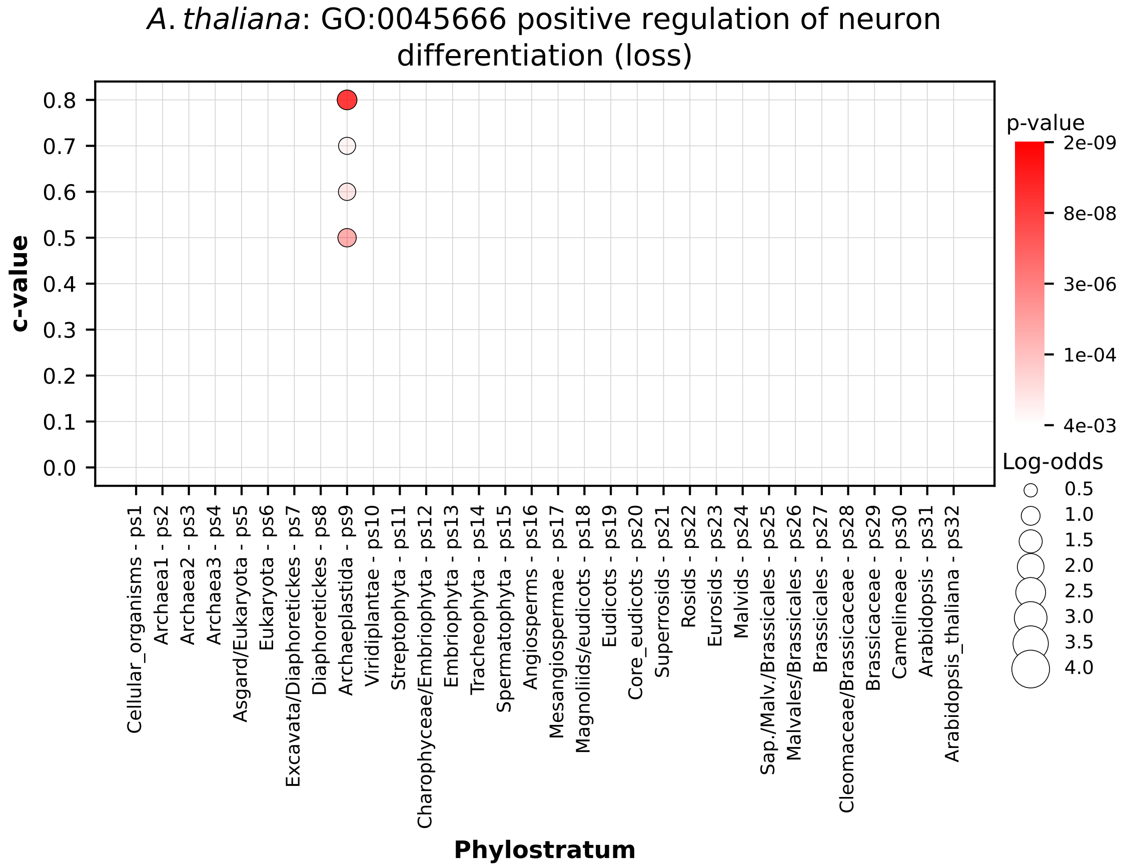
A. thaliana: GO:0045666 positive regulation of neuron differentiation (loss)
c-value
0.8
0.7
0.6
0.5
0.4
0.3
0.2
0.1
0.0
p-value
2e-09
8e-08
3e-06
1e-04
4e-03
Log-odds
0.5
1.0
1.5
2.0
2.5
3.0
3.5
4.0
Cellular_organisms - ps1
Archaea1 - ps2
Archaea2 - ps3
Archaea3 - ps4
Asgard/Eukaryota - ps5
Eukaryota - ps6
Excavata/Diaphoretickes - ps7
Diaphoretickes - ps8
Archaeplastida - ps9
Viridiplantae - ps10
Streptophyta - ps11
Charophyceae/Embriophyta - ps12
Embriophyta - ps13
Tracheophyta - ps14
Spermatophyta - ps15
Angiosperms - ps16
Mesangiospermae - ps17
Magnoliids/eudicots - ps18
Eudicots - ps19
Core_eudicots - ps20
Superrosids - ps21
Rosids - ps22
Eurosids - ps23
Malvids - ps24
Sap./Malv./Brassicales - ps25
Malvales/Brassicales - ps26
Brassicales - ps27
Cleomaceae/Brassicaceae - ps28
Brassicaceae - ps29
Camelineae - ps30
Arabidopsis - ps31
Arabidopsis_thaliana - ps32
Phylostratum

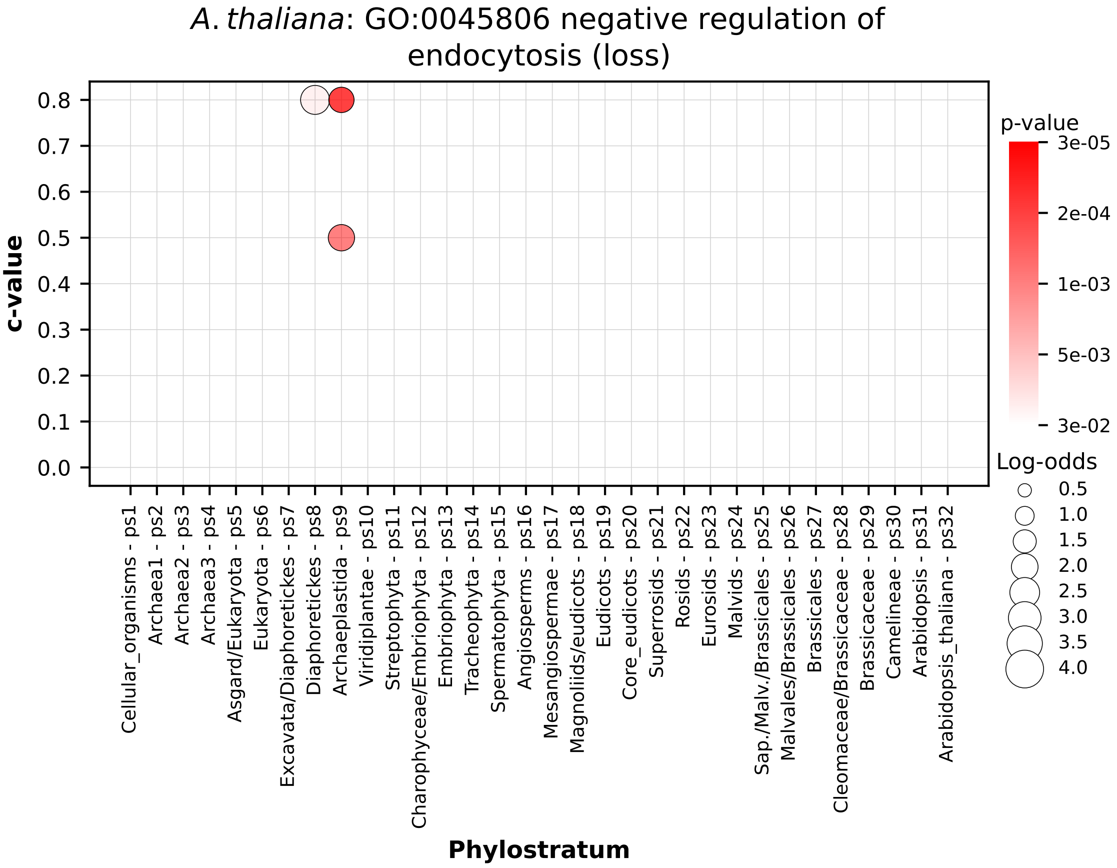

A. thaliana: GO:0045806 negative regulation of endocytosis (loss)
c-value
0.0
0.1
0.2
0.3
0.4
0.5
0.6
0.7
0.8
Phylostratum
Cellular_organisms - ps1
Archaea1 - ps2
Archaea2 - ps3
Archaea3 - ps4
Asgard/Eukaryota - ps5
Eukaryota - ps6
Excavata/Diaphoretickes - ps7
Diaphoretickes - ps8
Archaeplastida - ps9
Viridiplantae - ps10
Streptophyta - ps11
Charophyceae/Embriophyta - ps12
Embriophyta - ps13
Tracheophyta - ps14
Spermatophyta - ps15
Angiosperms - ps16
Mesangiospermae - ps17
Magnoliids/eudicots - ps18
Eudicots - ps19
Core_eudicots - ps20
Superrosids - ps21
Rosids - ps22
Eurosids - ps23
Malvids - ps24
Sap./Malv./Brassicales - ps25
Malvales/Brassicales - ps26
Brassicales - ps27
Cleomaceae/Brassicaceae - ps28
Brassicaceae - ps29
Camelineae - ps30
Arabidopsis - ps31
Arabidopsis_thaliana - ps32
p-value
3e-05
2e-04
1e-03
5e-03
3e-02
Log-odds
0.5
1.0
1.5
2.0
2.5
3.0
3.5
4.0

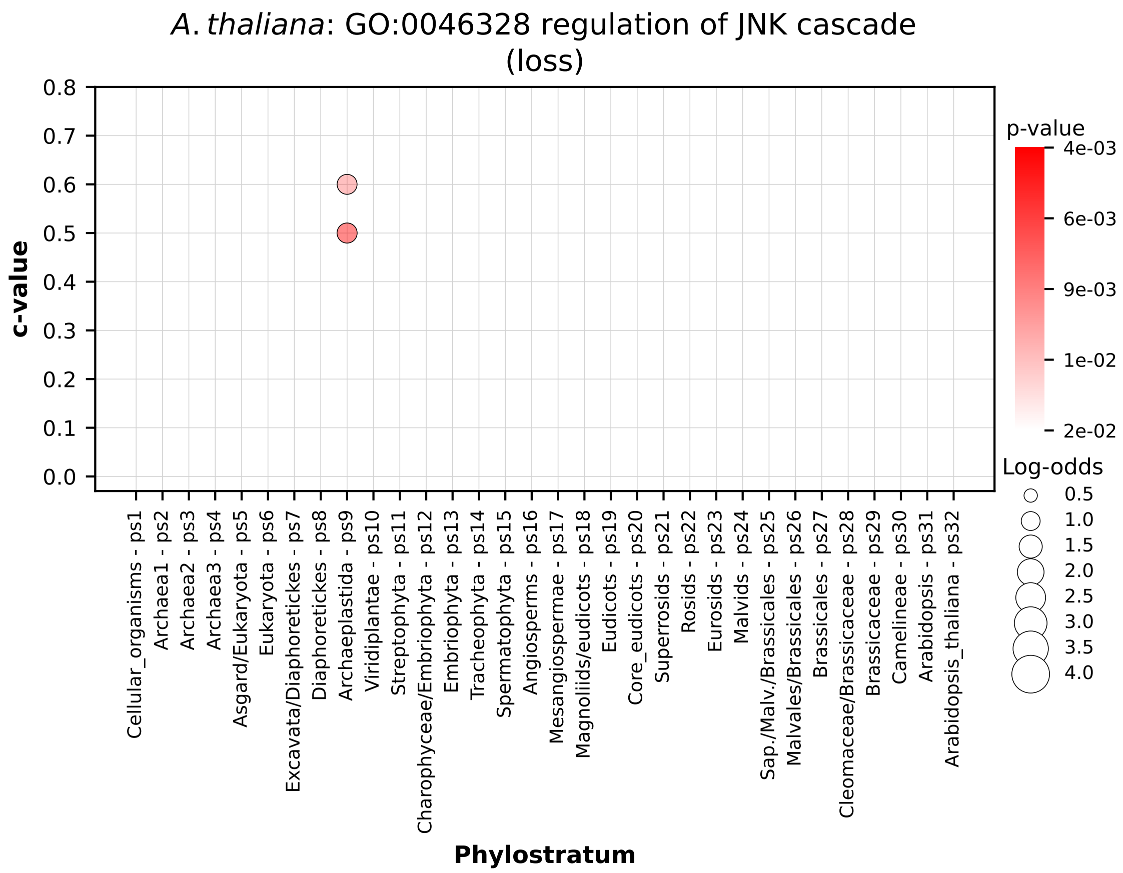

A. thaliana: GO:0046328 regulation of JNK cascade
(loss)
c-value
0.8
0.7
0.6
0.5
0.4
0.3
0.2
0.1
0.0
p-value
4e-03
6e-03
9e-03
1e-02
2e-02
Log-odds
0.5
1.0
1.5
2.0
2.5
3.0
3.5
4.0
Cellular_organisms - ps1
Archaea1 - ps2
Archaea2 - ps3
Archaea3 - ps4
Asgard/Eukaryota - ps5
Eukaryota - ps6
Excavata/Diaphoretickes - ps7
Diaphoretickes - ps8
Archaeplastida - ps9
Viridiplantae - ps10
Streptophyta - ps11
Charophyceae/Embriophyta - ps12
Embriophyta - ps13
Tracheophyta - ps14
Spermatophyta - ps15
Angiosperms - ps16
Mesangiospermae - ps17
Magnoliids/eudicots - ps18
Eudicots - ps19
Core_eudicots - ps20
Superrosids - ps21
Rosids - ps22
Eurosids - ps23
Malvids - ps24
Sap./Malv./Brassicales - ps25
Malvales/Brassicales - ps26
Brassicales - ps27
Cleomaceae/Brassicaceae - ps28
Brassicaceae - ps29
Camelineae - ps30
Arabidopsis - ps31
Arabidopsis_thaliana - ps32
Phylostratum

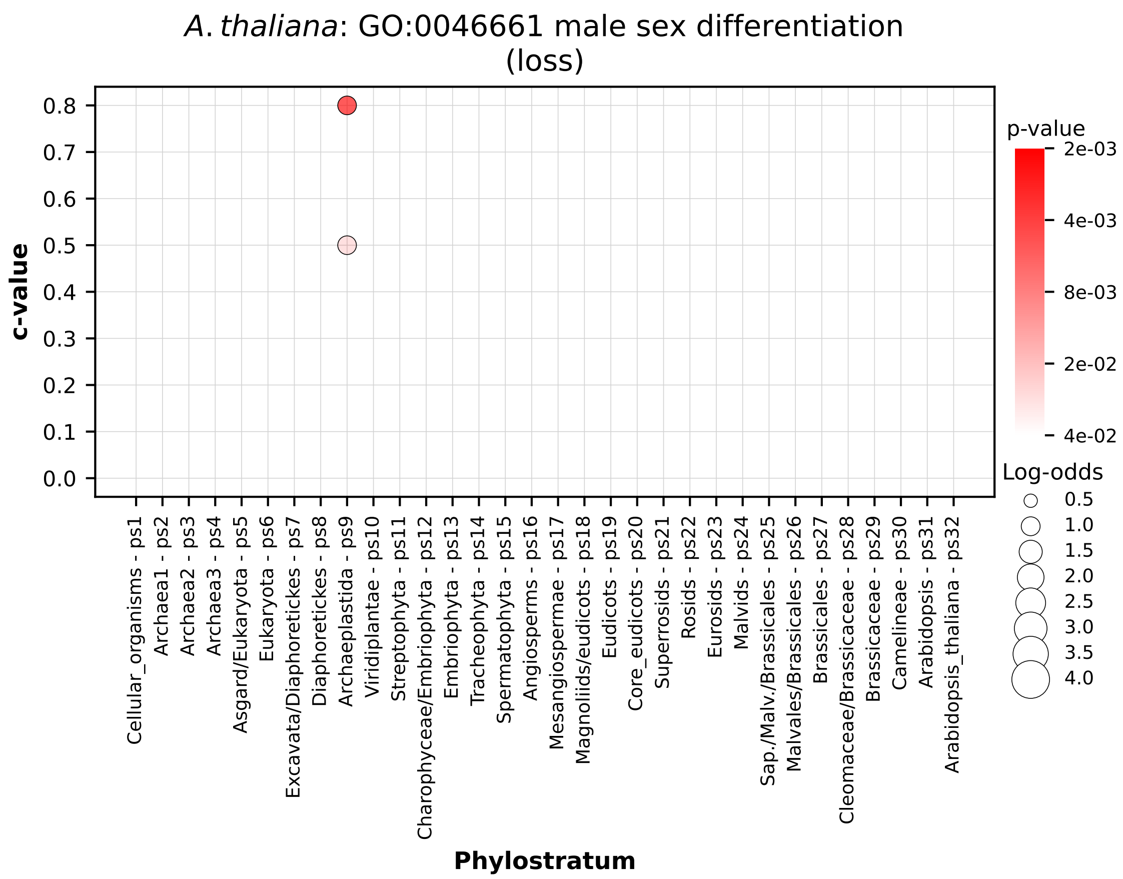

A. thaliana: GO:0046661 male sex differentiation
(loss)
c-value
0.8
0.7
0.6
0.5
0.4
0.3
0.2
0.1
0.0
p-value
2e-03
4e-03
8e-03
2e-02
4e-02
Log-odds
0.5
1.0
1.5
2.0
2.5
3.0
3.5
4.0
Cellular_organisms - ps1
Archaea1 - ps2
Archaea2 - ps3
Archaea3 - ps4
Asgard/Eukaryota - ps5
Eukaryota - ps6
Excavata/Diaphoretickes - ps7
Diaphoretickes - ps8
Archaeplastida - ps9
Viridiplantae - ps10
Streptophyta - ps11
Charophyceae/Embriophyta - ps12
Embriophyta - ps13
Tracheophyta - ps14
Spermatophyta - ps15
Angiosperms - ps16
Mesangiospermae - ps17
Magnoliids/eudicots - ps18
Eudicots - ps19
Core_eudicots - ps20
Superrosids - ps21
Rosids - ps22
Eurosids - ps23
Malvids - ps24
Sap./Malv./Brassicales - ps25
Malvales/Brassicales - ps26
Brassicales - ps27
Cleomaceae/Brassicaceae - ps28
Brassicaceae - ps29
Camelineae - ps30
Arabidopsis - ps31
Arabidopsis_thaliana - ps32
Phylostratum

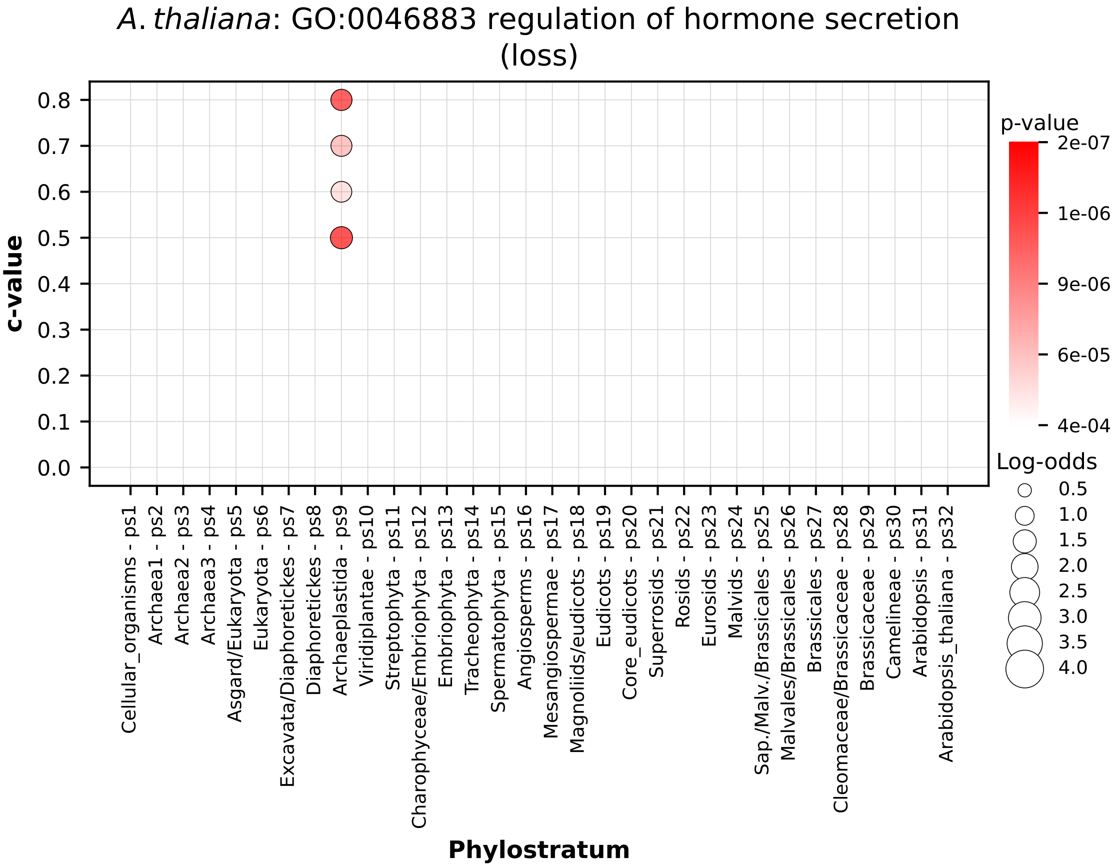

A. thaliana: GO:0046883 regulation of hormone secretion (loss)
c-value
0.0
0.1
0.2
0.3
0.4
0.5
0.6
0.7
0.8
Phylostratum
Cellular_organisms - ps1
Archaea1 - ps2
Archaea2 - ps3
Archaea3 - ps4
Asgard/Eukaryota - ps5
Eukaryota - ps6
Excavata/Diaphoretickes - ps7
Diaphoretickes - ps8
Archaeplastida - ps9
Viridiplantae - ps10
Streptophyta - ps11
Charophyceae/Embriophyta - ps12
Embriophyta - ps13
Tracheophyta - ps14
Spermatophyta - ps15
Angiosperms - ps16
Mesangiospermae - ps17
Magnoliids/eudicots - ps18
Eudicots - ps19
Core_eudicots - ps20
Superrosids - ps21
Rosids - ps22
Eurosids - ps23
Malvids - ps24
Sap./Malv./Brassicales - ps25
Malvales/Brassicales - ps26
Brassicales - ps27
Cleomaceae/Brassicaceae - ps28
Brassicaceae - ps29
Camelineae - ps30
Arabidopsis - ps31
Arabidopsis_thaliana - ps32
p-value
2e-07
1e-06
9e-06
6e-05
4e-04
Log-odds
0.5
1.0
1.5
2.0
2.5
3.0
3.5
4.0

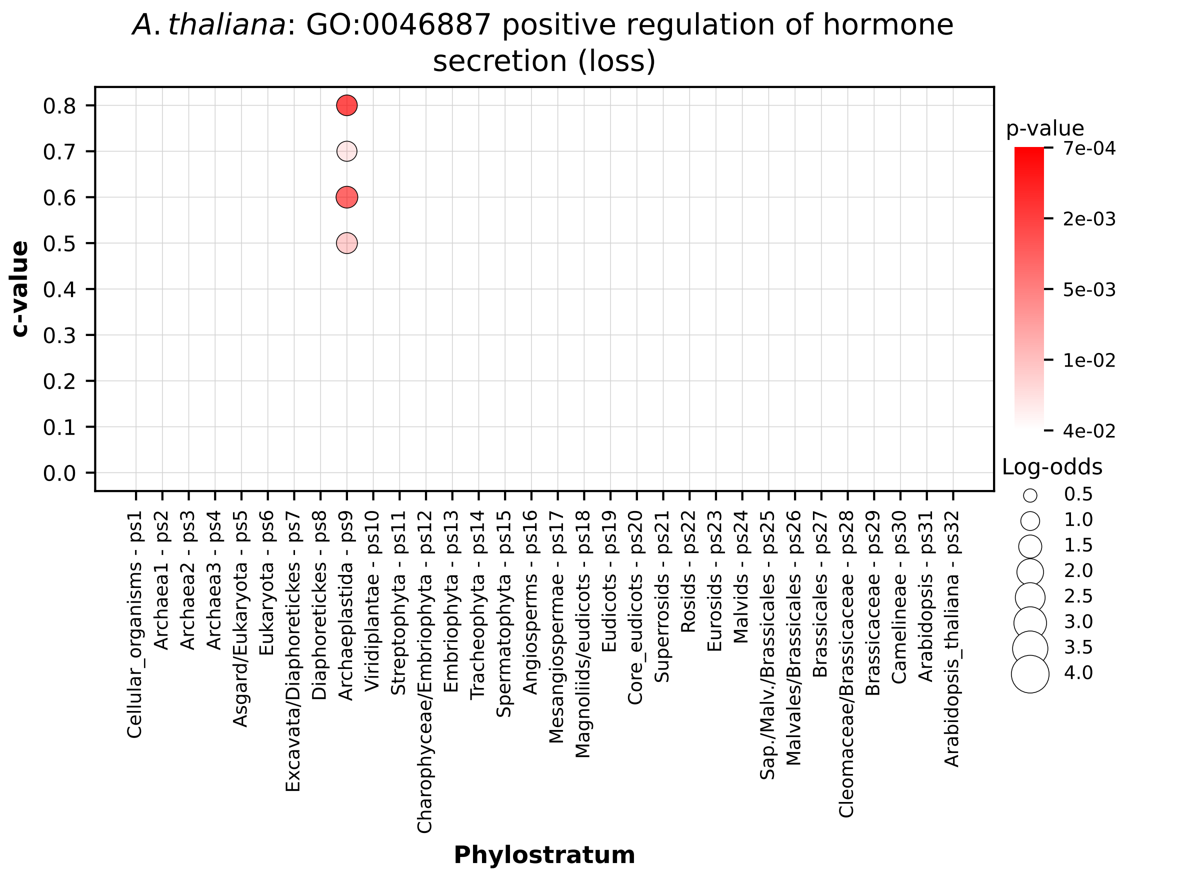

$A. thaliana$: GO:0046887 positive regulation of hormone secretion (loss)
c-value
0.8
0.7
0.6
0.5
0.4
0.3
0.2
0.1
0.0
p-value
7e-04
2e-03
5e-03
1e-02
4e-02
Log-odds
0.5
1.0
1.5
2.0
2.5
3.0
3.5
4.0
Cellular_organisms - ps1
Archaea1 - ps2
Archaea2 - ps3
Archaea3 - ps4
Asgard/Eukaryota - ps5
Eukaryota - ps6
Excavata/Diaphoretickes - ps7
Diaphoretickes - ps8
Archaeplastida - ps9
Viridiplantae - ps10
Streptophyta - ps11
Charophyceae/Embriophyta - ps12
Embriophyta - ps13
Tracheophyta - ps14
Spermatophyta - ps15
Angiosperms - ps16
Mesangiospermae - ps17
Magnoliids/eudicots - ps18
Eudicots - ps19
Core_eudicots - ps20
Superrosids - ps21
Rosids - ps22
Eurosids - ps23
Malvids - ps24
Sap./Malv./Brassicales - ps25
Malvales/Brassicales - ps26
Brassicales - ps27
Cleomaceae/Brassicaceae - ps28
Brassicaceae - ps29
Camelineae - ps30
Arabidopsis - ps31
Arabidopsis_thaliana - ps32
Phylostratum

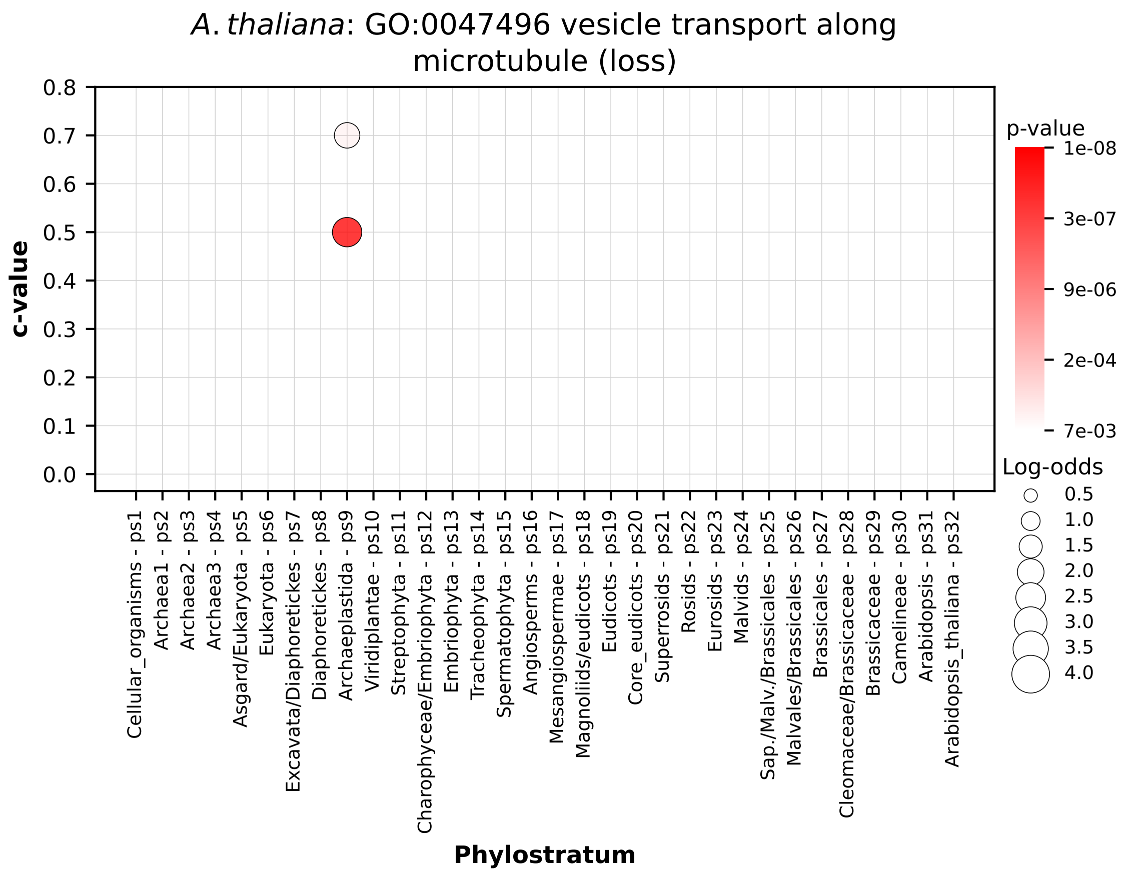

A. thaliana: GO:0047496 vesicle transport along microtubule (loss)
c-value
0.8
0.7
0.6
0.5
0.4
0.3
0.2
0.1
0.0
p-value
1e-08
3e-07
9e-06
2e-04
7e-03
Log-odds
0.5
1.0
1.5
2.0
2.5
3.0
3.5
4.0
Cellular_organisms - ps1
Archaea1 - ps2
Archaea2 - ps3
Archaea3 - ps4
Asgard/Eukaryota - ps5
Eukaryota - ps6
Excavata/Diaphoretickes - ps7
Diaphoretickes - ps8
Archaeplastida - ps9
Viridiplantae - ps10
Streptophyta - ps11
Charophyceae/Embriophyta - ps12
Embriophyta - ps13
Tracheophyta - ps14
Spermatophyta - ps15
Angiosperms - ps16
Mesangiospermae - ps17
Magnoliids/eudicots - ps18
Eudicots - ps19
Core_eudicots - ps20
Superrosids - ps21
Rosids - ps22
Eurosids - ps23
Malvids - ps24
Sap./Malv./Brassicales - ps25
Malvales/Brassicales - ps26
Brassicales - ps27
Cleomaceae/Brassicaceae - ps28
Brassicaceae - ps29
Camelineae - ps30
Arabidopsis - ps31
Arabidopsis_thaliana - ps32
Phylostratum

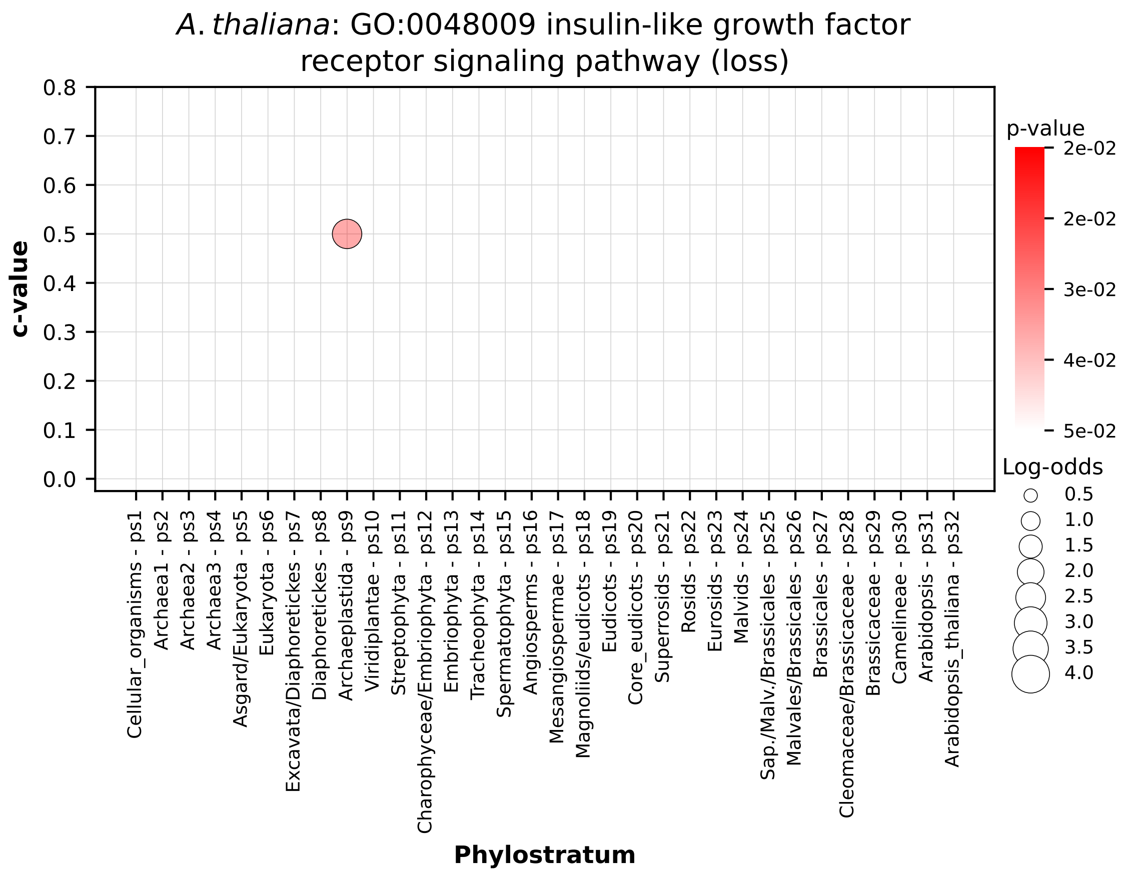

A. thaliana: GO:0048009 insulin-like growth factor receptor signaling pathway (loss)
c-value
0.8
0.7
0.6
0.5
0.4
0.3
0.2
0.1
0.0
Phylostratum
Cellular_organisms - ps1
Archaea1 - ps2
Archaea2 - ps3
Archaea3 - ps4
Asgard/Eukaryota - ps5
Eukaryota - ps6
Excavata/Diaphoretickes - ps7
Diaphoretickes - ps8
Archaeplastida - ps9
Viridiplantae - ps10
Streptophyta - ps11
Charophyceae/Embriophyta - ps12
Embriophyta - ps13
Tracheophyta - ps14
Spermatophyta - ps15
Angiosperms - ps16
Mesangiospermae - ps17
Magnoliids/eudicots - ps18
Eudicots - ps19
Core_eudicots - ps20
Superrosids - ps21
Rosids - ps22
Eurosids - ps23
Malvids - ps24
Sap./Malv./Brassicales - ps25
Malvales/Brassicales - ps26
Brassicales - ps27
Cleomaceae/Brassicaceae - ps28
Brassicaceae - ps29
Camelineae - ps30
Arabidopsis - ps31
Arabidopsis_thaliana - ps32
p-value
2e-02
2e-02
3e-02
4e-02
5e-02
Log-odds
0.5
1.0
1.5
2.0
2.5
3.0
3.5
4.0

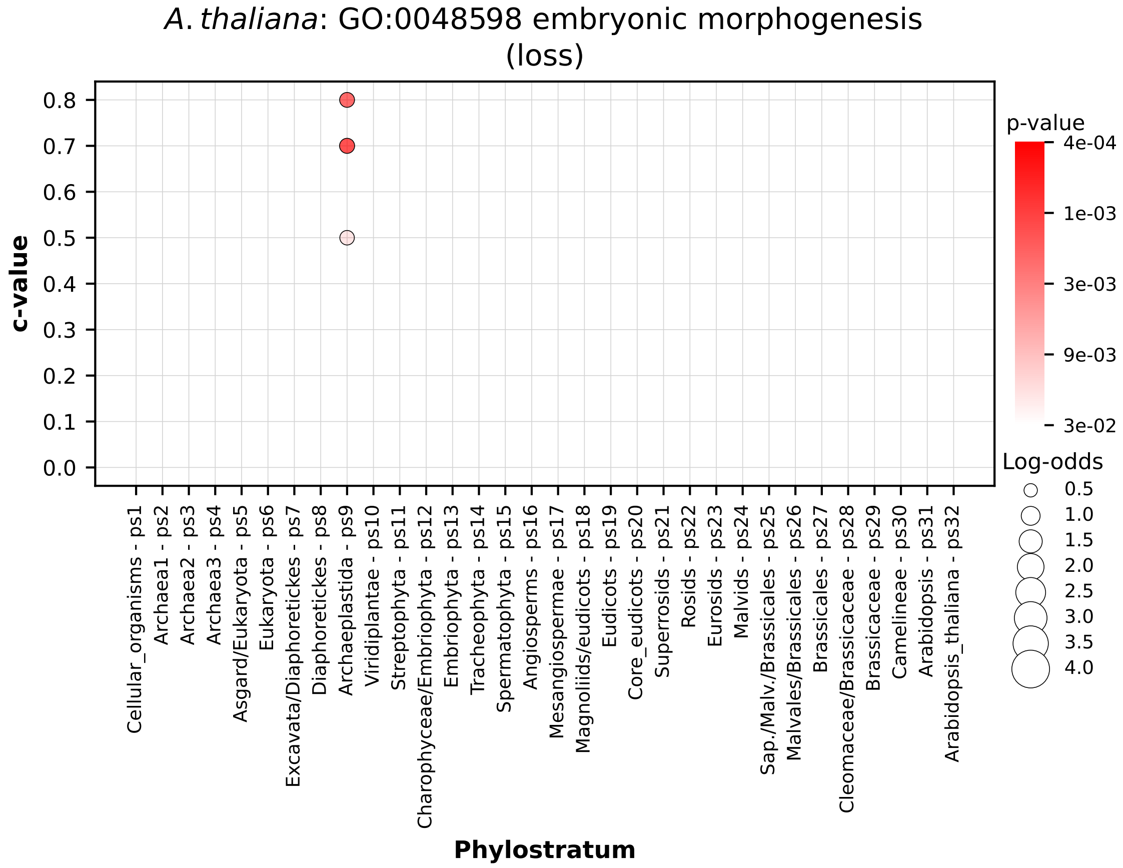

A. thaliana: GO:0048598 embryonic morphogenesis
(loss)
c-value
0.0
0.1
0.2
0.3
0.4
0.5
0.6
0.7
0.8
Phylostratum
Cellular_organisms - ps1
Archaea1 - ps2
Archaea2 - ps3
Archaea3 - ps4
Asgard/Eukaryota - ps5
Eukaryota - ps6
Excavata/Diaphoretickes - ps7
Diaphoretickes - ps8
Archaeplastida - ps9
Viridiplantae - ps10
Streptophyta - ps11
Charophyceae/Embriophyta - ps12
Embriophyta - ps13
Tracheophyta - ps14
Spermatophyta - ps15
Angiosperms - ps16
Mesangiospermae - ps17
Magnoliids/eudicots - ps18
Eudicots - ps19
Core_eudicots - ps20
Superrosids - ps21
Rosids - ps22
Eurosids - ps23
Malvids - ps24
Sap./Malv./Brassicales - ps25
Malvales/Brassicales - ps26
Brassicales - ps27
Cleomaceae/Brassicaceae - ps28
Brassicaceae - ps29
Camelineae - ps30
Arabidopsis - ps31
Arabidopsis_thaliana - ps32
p-value
4e-04
1e-03
3e-03
9e-03
3e-02
Log-odds
0.5
1.0
1.5
2.0
2.5
3.0
3.5
4.0

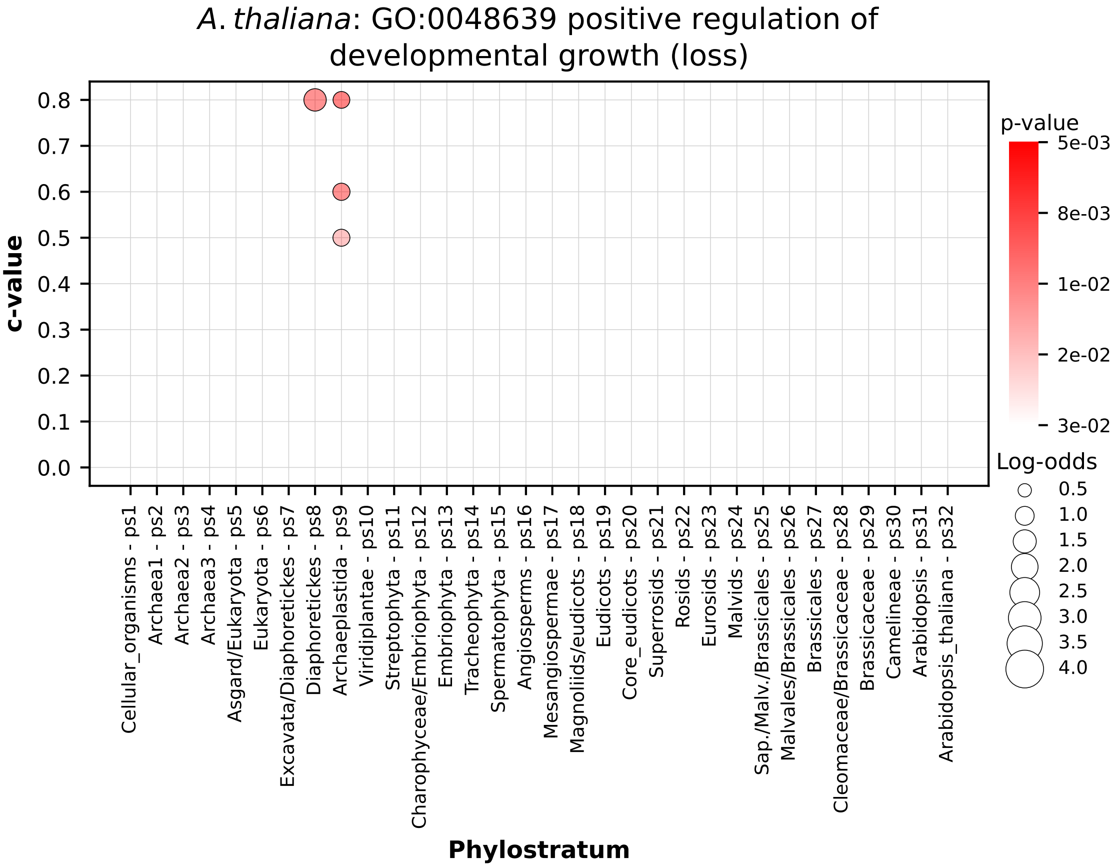
A. thaliana: GO:0048639 positive regulation of developmental growth (loss)
c-value
0.0
0.1
0.2
0.3
0.4
0.5
0.6
0.7
0.8
p-value
5e-03
8e-03
1e-02
2e-02
3e-02
Log-odds
0.5
1.0
1.5
2.0
2.5
3.0
3.5
4.0
Cellular_organisms - ps1
Archaea1 - ps2
Archaea2 - ps3
Archaea3 - ps4
Asgard/Eukaryota - ps5
Eukaryota - ps6
Excavata/Diaphoretickes - ps7
Diaphoretickes - ps8
Archaeplastida - ps9
Viridiplantae - ps10
Streptophyta - ps11
Charophyceae/Embriophyta - ps12
Embriophyta - ps13
Tracheophyta - ps14
Spermatophyta - ps15
Angiosperms - ps16
Mesangiospermae - ps17
Magnoliids/eudicots - ps18
Eudicots - ps19
Core_eudicots - ps20
Superrosids - ps21
Rosids - ps22
Eurosids - ps23
Malvids - ps24
Sap./Malv./Brassicales - ps25
Malvales/Brassicales - ps26
Brassicales - ps27
Cleomaceae/Brassicaceae - ps28
Brassicaceae - ps29
Camelineae - ps30
Arabidopsis - ps31
Arabidopsis_thaliana - ps32
Phylostratum

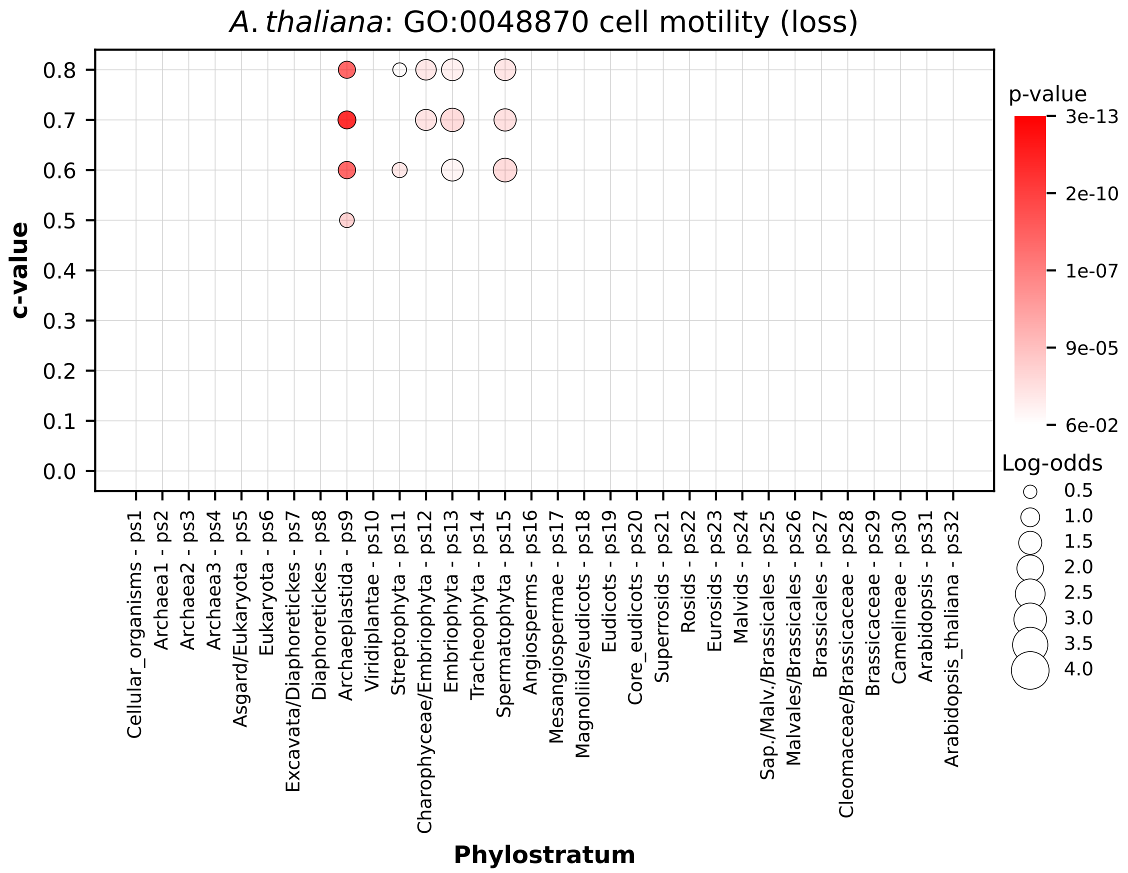

A. thaliana: GO:0048870 cell motility (loss)
c-value
0.8
0.7
0.6
0.5
0.4
0.3
0.2
0.1
0.0
p-value
3e-13
2e-10
1e-07
9e-05
6e-02
Log-odds
0.5
1.0
1.5
2.0
2.5
3.0
3.5
4.0
Cellular_organisms - ps1
Archaea1 - ps2
Archaea2 - ps3
Archaea3 - ps4
Asgard/Eukaryota - ps5
Eukaryota - ps6
Excavata/Diaphoretickes - ps7
Diaphoretickes - ps8
Archaeplastida - ps9
Viridiplantae - ps10
Streptophyta - ps11
Charophyceae/Embriophyta - ps12
Embriophyta - ps13
Tracheophyta - ps14
Spermatophyta - ps15
Angiosperms - ps16
Mesangiospermae - ps17
Magnoliids/eudicots - ps18
Eudicots - ps19
Core_eudicots - ps20
Superrosids - ps21
Rosids - ps22
Eurosids - ps23
Malvids - ps24
Sap./Malv./Brassicales - ps25
Malvales/Brassicales - ps26
Brassicales - ps27
Cleomaceae/Brassicaceae - ps28
Brassicaceae - ps29
Camelineae - ps30
Arabidopsis - ps31
Arabidopsis_thaliana - ps32
Phylostratum

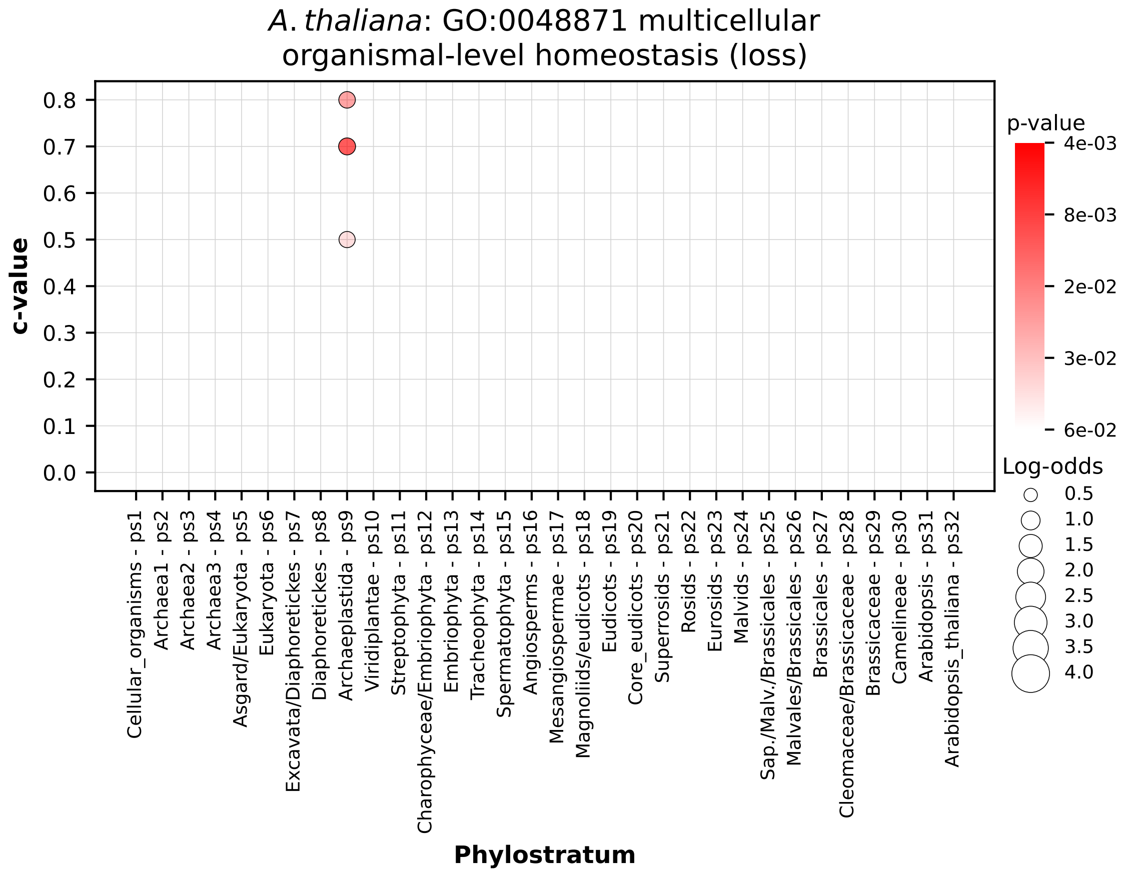

A. thaliana: GO:0048871 multicellular organismal-level homeostasis (loss)
c-value
0.0
0.1
0.2
0.3
0.4
0.5
0.6
0.7
0.8
p-value
4e-03
8e-03
2e-02
3e-02
6e-02
Log-odds
0.5
1.0
1.5
2.0
2.5
3.0
3.5
4.0
Cellular_organisms - ps1
Archaea1 - ps2
Archaea2 - ps3
Archaea3 - ps4
Asgard/Eukaryota - ps5
Eukaryota - ps6
Excavata/Diaphoretickes - ps7
Diaphoretickes - ps8
Archaeplastida - ps9
Viridiplantae - ps10
Streptophyta - ps11
Charophyceae/Embriophyta - ps12
Embriophyta - ps13
Tracheophyta - ps14
Spermatophyta - ps15
Angiosperms - ps16
Mesangiospermae - ps17
Magnoliids/eudicots - ps18
Eudicots - ps19
Core_eudicots - ps20
Superrosids - ps21
Rosids - ps22
Eurosids - ps23
Malvids - ps24
Sap./Malv./Brassicales - ps25
Malvales/Brassicales - ps26
Brassicales - ps27
Cleomaceae/Brassicaceae - ps28
Brassicaceae - ps29
Camelineae - ps30
Arabidopsis - ps31
Arabidopsis_thaliana - ps32
Phylostratum

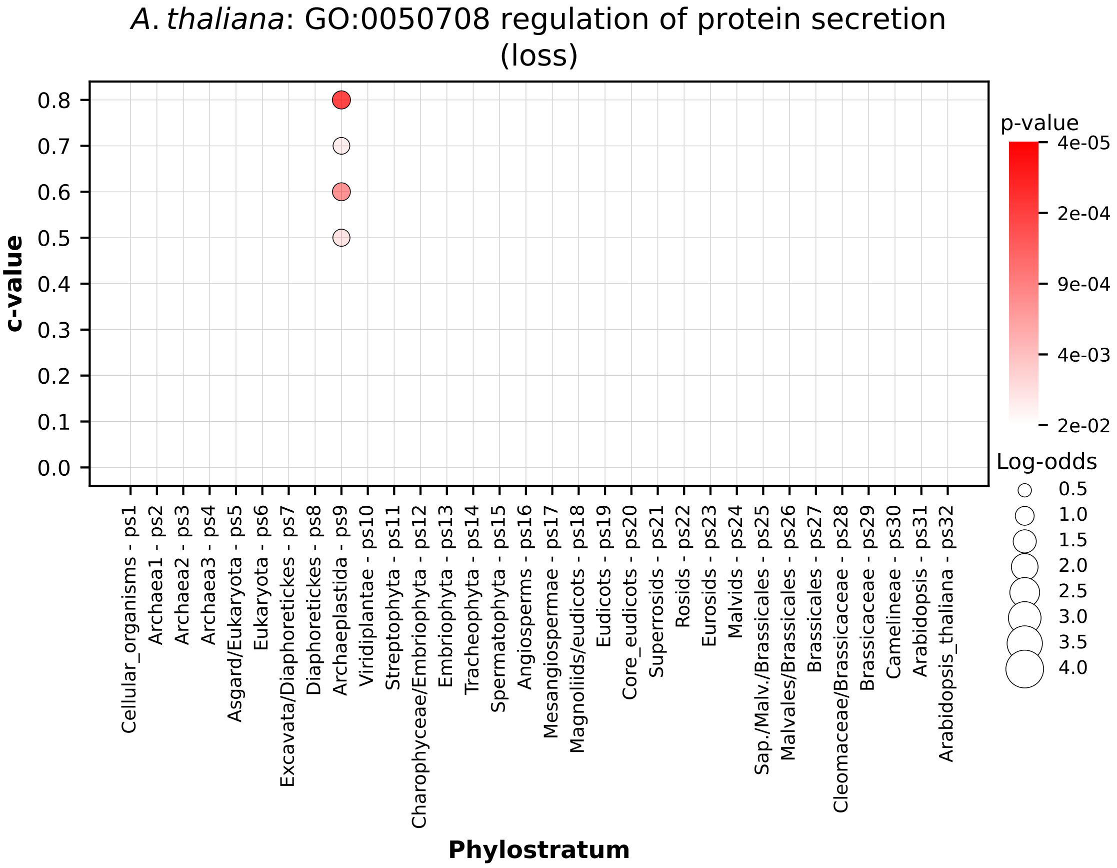

A. thaliana: GO:0050708 regulation of protein secretion
(loss)
c-value
0.0
0.1
0.2
0.3
0.4
0.5
0.6
0.7
0.8
p-value
4e-05
2e-04
9e-04
4e-03
2e-02
Log-odds
0.5
1.0
1.5
2.0
2.5
3.0
3.5
4.0
Cellular_organisms - ps1
Archaea1 - ps2
Archaea2 - ps3
Archaea3 - ps4
Asgard/Eukaryota - ps5
Eukaryota - ps6
Excavata/Diaphoretickes - ps7
Diaphoretickes - ps8
Archaeplastida - ps9
Viridiplantae - ps10
Streptophyta - ps11
Charophyceae/Embriophyta - ps12
Embriophyta - ps13
Tracheophyta - ps14
Spermatophyta - ps15
Angiosperms - ps16
Mesangiospermae - ps17
Magnoliids/eudicots - ps18
Eudicots - ps19
Core_eudicots - ps20
Superrosids - ps21
Rosids - ps22
Eurosids - ps23
Malvids - ps24
Sap./Malv./Brassicales - ps25
Malvales/Brassicales - ps26
Brassicales - ps27
Cleomaceae/Brassicaceae - ps28
Brassicaceae - ps29
Camelineae - ps30
Arabidopsis - ps31
Arabidopsis_thaliana - ps32
Phylostratum

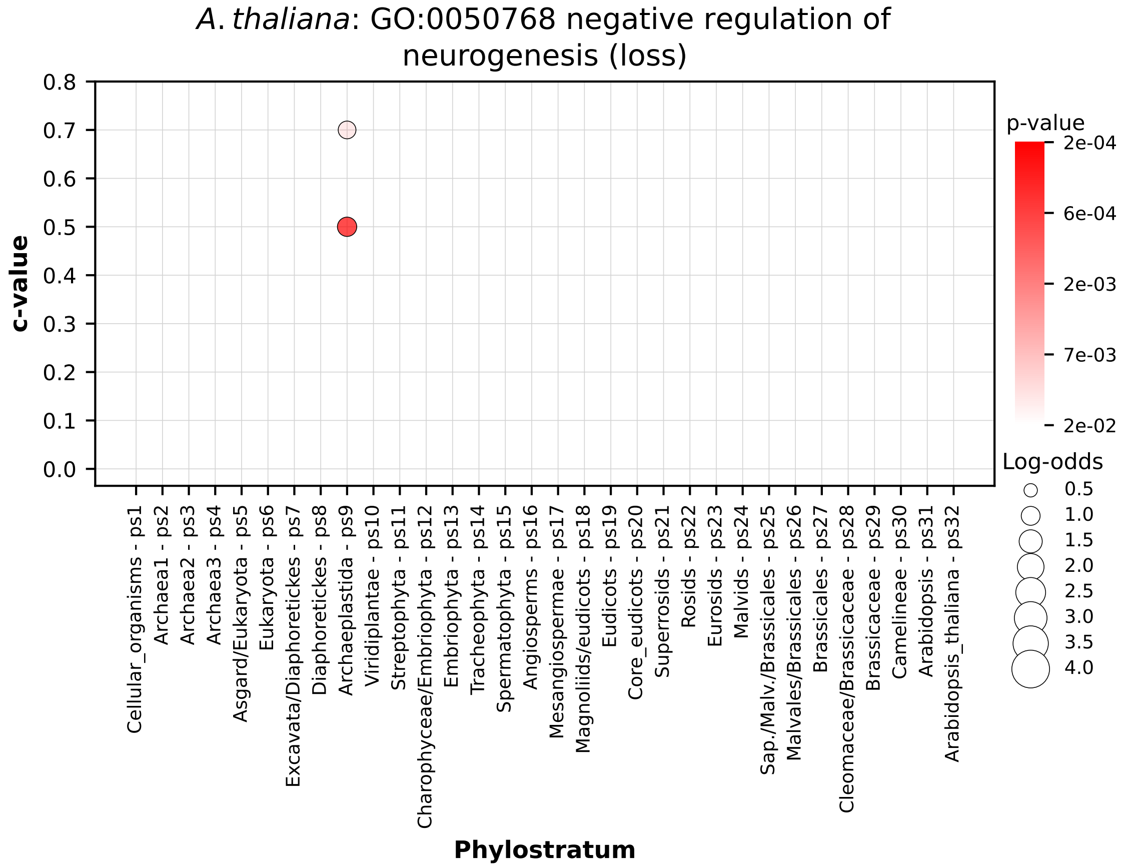

A. thaliana: GO:0050768 negative regulation of neurogenesis (loss)
c-value
0.8
0.7
0.6
0.5
0.4
0.3
0.2
0.1
0.0
Phylostratum
Cellular_organisms - ps1
Archaea1 - ps2
Archaea2 - ps3
Archaea3 - ps4
Asgard/Eukaryota - ps5
Eukaryota - ps6
Excavata/Diaphoretickes - ps7
Diaphoretickes - ps8
Archaeplastida - ps9
Viridiplantae - ps10
Streptophyta - ps11
Charophyceae/Embriophyta - ps12
Embriophyta - ps13
Tracheophyta - ps14
Spermatophyta - ps15
Angiosperms - ps16
Mesangiospermae - ps17
Magnoliids/eudicots - ps18
Eudicots - ps19
Core_eudicots - ps20
Superrosids - ps21
Rosids - ps22
Eurosids - ps23
Malvids - ps24
Sap./Malv./Brassicales - ps25
Malvales/Brassicales - ps26
Brassicales - ps27
Cleomaceae/Brassicaceae - ps28
Brassicaceae - ps29
Camelineae - ps30
Arabidopsis - ps31
Arabidopsis_thaliana - ps32
p-value
2e-04
6e-04
2e-03
7e-03
2e-02
Log-odds
0.5
1.0
1.5
2.0
2.5
3.0
3.5
4.0

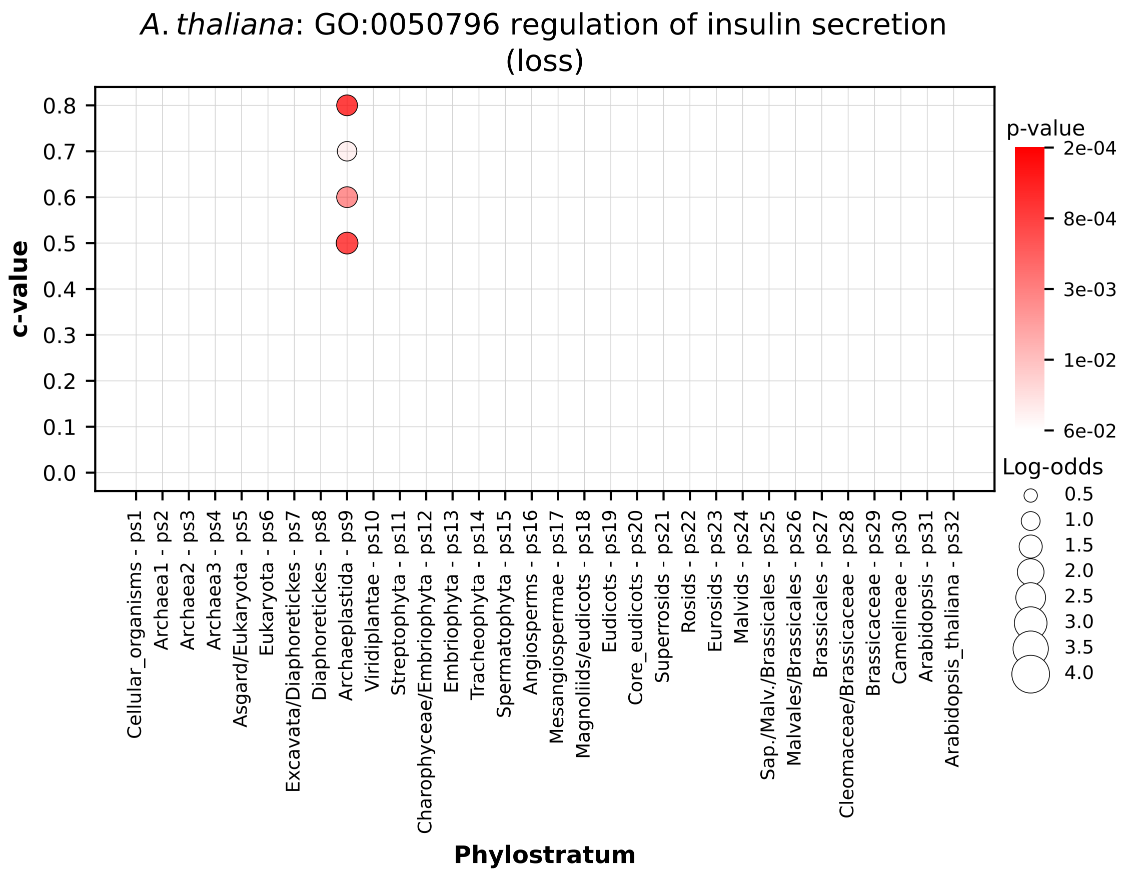

A. thaliana: GO:0050796 regulation of insulin secretion
(loss)
c-value
0.8
0.7
0.6
0.5
0.4
0.3
0.2
0.1
0.0
p-value
2e-04
8e-04
3e-03
1e-02
6e-02
Log-odds
0.5
1.0
1.5
2.0
2.5
3.0
3.5
4.0
Cellular_organisms - ps1
Archaea1 - ps2
Archaea2 - ps3
Archaea3 - ps4
Asgard/Eukaryota - ps5
Eukaryota - ps6
Excavata/Diaphoretickes - ps7
Diaphoretickes - ps8
Archaeplastida - ps9
Viridiplantae - ps10
Streptophyta - ps11
Charophyceae/Embriophyta - ps12
Embriophyta - ps13
Tracheophyta - ps14
Spermatophyta - ps15
Angiosperms - ps16
Mesangiospermae - ps17
Magnoliids/eudicots - ps18
Eudicots - ps19
Core_eudicots - ps20
Superrosids - ps21
Rosids - ps22
Eurosids - ps23
Malvids - ps24
Sap./Malv./Brassicales - ps25
Malvales/Brassicales - ps26
Brassicales - ps27
Cleomaceae/Brassicaceae - ps28
Brassicaceae - ps29
Camelineae - ps30
Arabidopsis - ps31
Arabidopsis_thaliana - ps32
Phylostratum

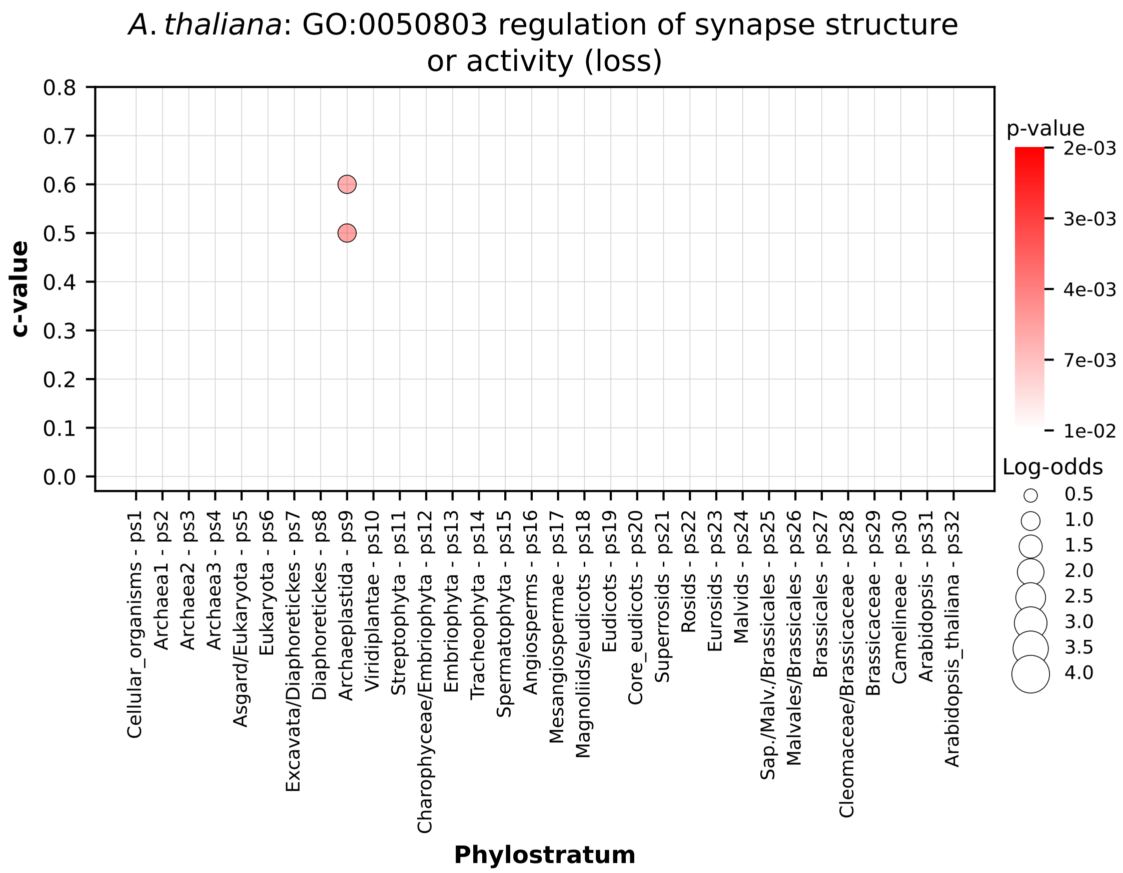

A. thaliana: GO:0050803 regulation of synapse structure or activity (loss)
c-value
0.8
0.7
0.6
0.5
0.4
0.3
0.2
0.1
0.0
p-value
2e-03
3e-03
4e-03
7e-03
1e-02
Log-odds
0.5
1.0
1.5
2.0
2.5
3.0
3.5
4.0
Cellular_organisms - ps1
Archaea1 - ps2
Archaea2 - ps3
Archaea3 - ps4
Asgard/Eukaryota - ps5
Eukaryota - ps6
Excavata/Diaphoretickes - ps7
Diaphoretickes - ps8
Archaeplastida - ps9
Viridiplantae - ps10
Streptophyta - ps11
Charophyceae/Embriophyta - ps12
Embriophyta - ps13
Tracheophyta - ps14
Spermatophyta - ps15
Angiosperms - ps16
Mesangiospermae - ps17
Magnoliids/eudicots - ps18
Eudicots - ps19
Core_eudicots - ps20
Superrosids - ps21
Rosids - ps22
Eurosids - ps23
Malvids - ps24
Sap./Malv./Brassicales - ps25
Malvales/Brassicales - ps26
Brassicales - ps27
Cleomaceae/Brassicaceae - ps28
Brassicaceae - ps29
Camelineae - ps30
Arabidopsis - ps31
Arabidopsis_thaliana - ps32
Phylostratum

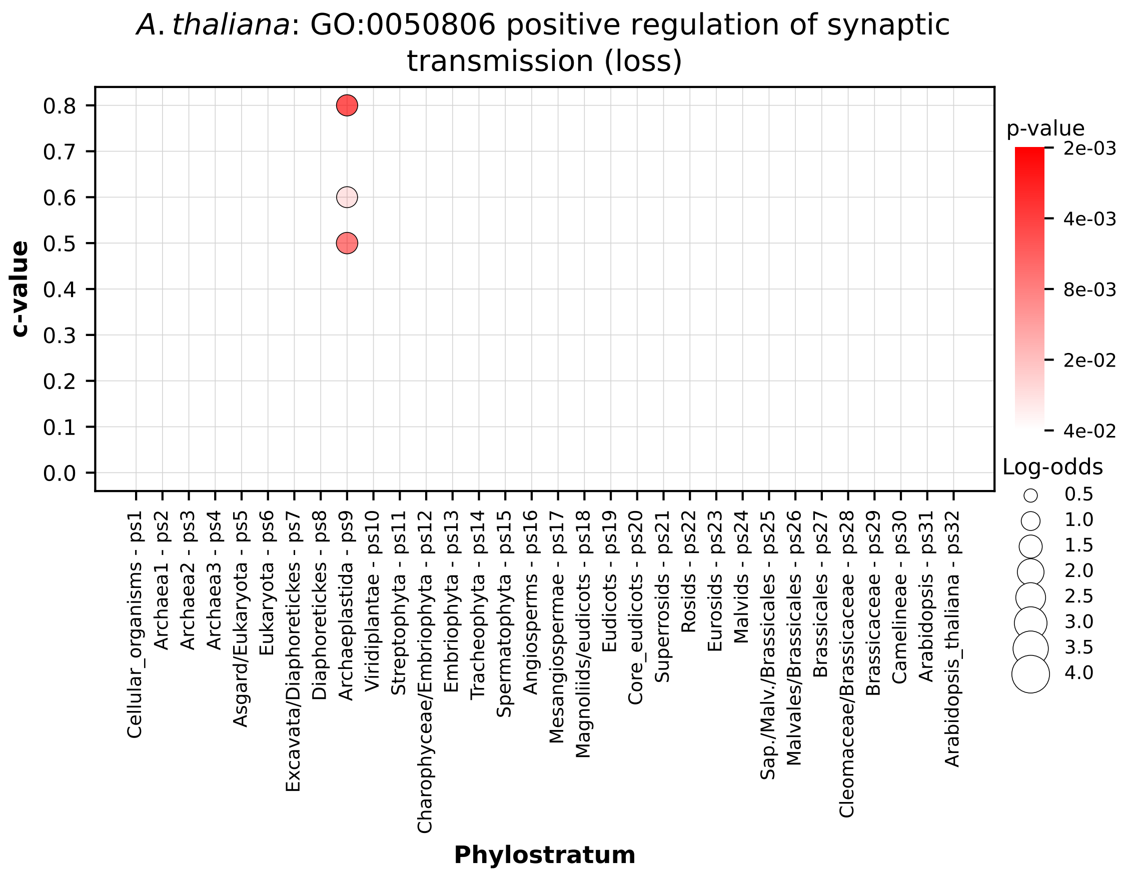

A. thaliana: GO:0050806 positive regulation of synaptic transmission (loss)
c-value
0.8
0.7
0.6
0.5
0.4
0.3
0.2
0.1
0.0
p-value
2e-03
4e-03
8e-03
2e-02
4e-02
Log-odds
0.5
1.0
1.5
2.0
2.5
3.0
3.5
4.0
Cellular_organisms - ps1
Archaea1 - ps2
Archaea2 - ps3
Archaea3 - ps4
Asgard/Eukaryota - ps5
Eukaryota - ps6
Excavata/Diaphoretickes - ps7
Diaphoretickes - ps8
Archaeplastida - ps9
Viridiplantae - ps10
Streptophyta - ps11
Charophyceae/Embriophyta - ps12
Embriophyta - ps13
Tracheophyta - ps14
Spermatophyta - ps15
Angiosperms - ps16
Mesangiospermae - ps17
Magnoliids/eudicots - ps18
Eudicots - ps19
Core_eudicots - ps20
Superrosids - ps21
Rosids - ps22
Eurosids - ps23
Malvids - ps24
Sap./Malv./Brassicales - ps25
Malvales/Brassicales - ps26
Brassicales - ps27
Cleomaceae/Brassicaceae - ps28
Brassicaceae - ps29
Camelineae - ps30
Arabidopsis - ps31
Arabidopsis_thaliana - ps32
Phylostratum

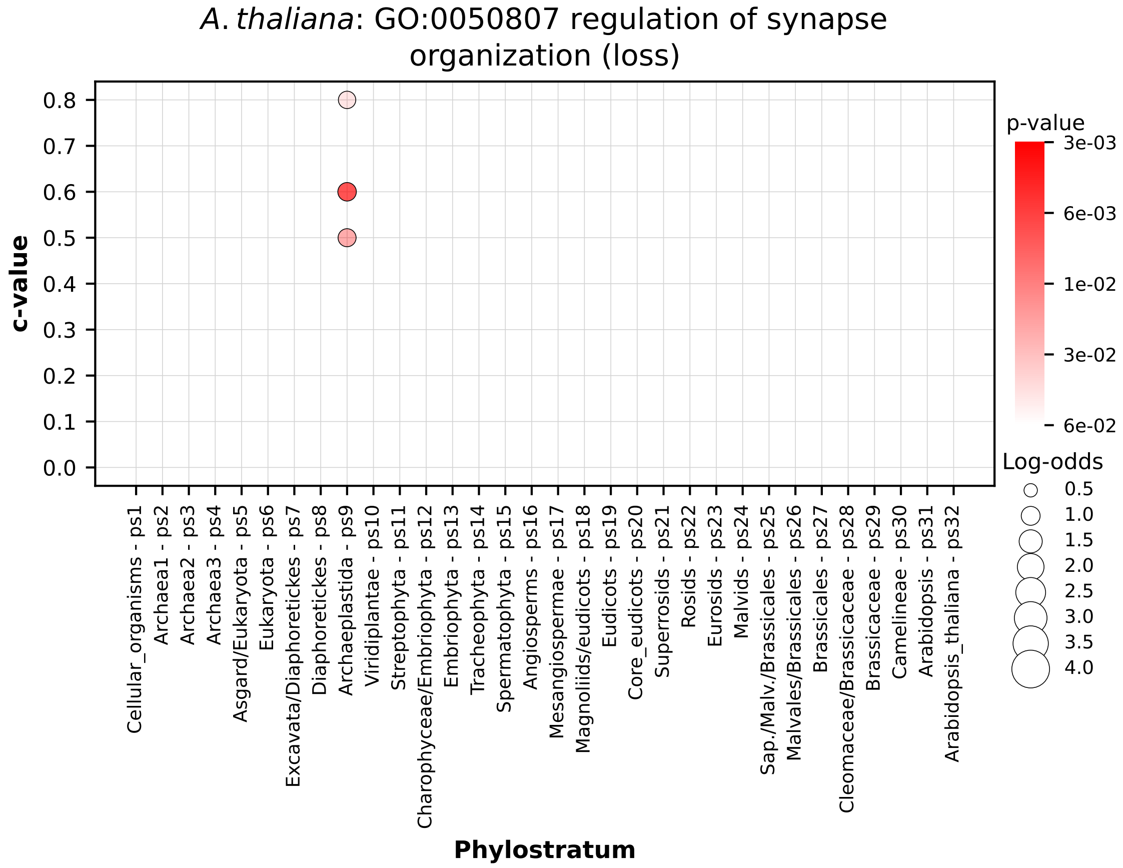

A. thaliana: GO:0050807 regulation of synapse organization (loss)
c-value
0.8
0.7
0.6
0.5
0.4
0.3
0.2
0.1
0.0
p-value
3e-03
6e-03
1e-02
3e-02
6e-02
Log-odds
0.5
1.0
1.5
2.0
2.5
3.0
3.5
4.0
Cellular_organisms - ps1
Archaea1 - ps2
Archaea2 - ps3
Archaea3 - ps4
Asgard/Eukaryota - ps5
Eukaryota - ps6
Excavata/Diaphoretickes - ps7
Diaphoretickes - ps8
Archaeplastida - ps9
Viridiplantae - ps10
Streptophyta - ps11
Charophyceae/Embriophyta - ps12
Embriophyta - ps13
Tracheophyta - ps14
Spermatophyta - ps15
Angiosperms - ps16
Mesangiospermae - ps17
Magnoliids/eudicots - ps18
Eudicots - ps19
Core_eudicots - ps20
Superrosids - ps21
Rosids - ps22
Eurosids - ps23
Malvids - ps24
Sap./Malv./Brassicales - ps25
Malvales/Brassicales - ps26
Brassicales - ps27
Cleomaceae/Brassicaceae - ps28
Brassicaceae - ps29
Camelineae - ps30
Arabidopsis - ps31
Arabidopsis_thaliana - ps32
Phylostratum

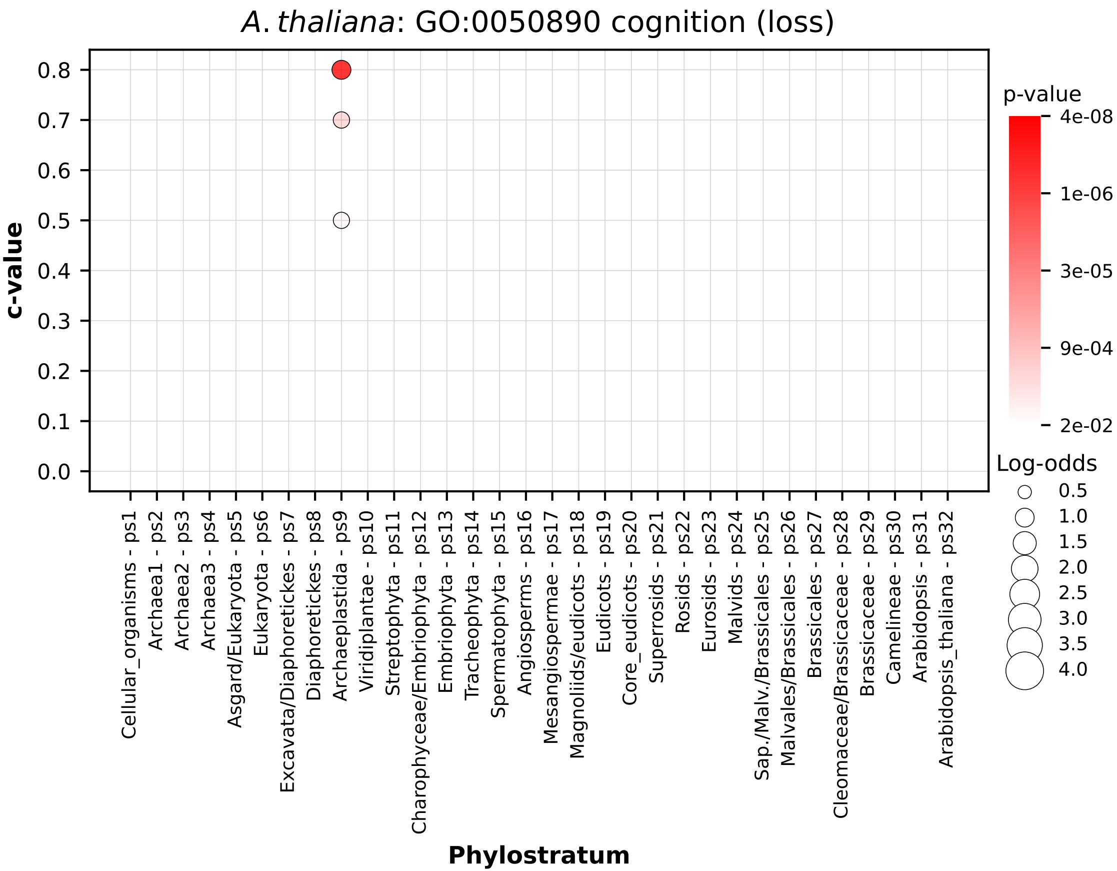

A. thaliana: GO:0050890 cognition (loss)
c-value
0.0
0.1
0.2
0.3
0.4
0.5
0.6
0.7
0.8
Phylostratum
Cellular_organisms - ps1
Archaea1 - ps2
Archaea2 - ps3
Archaea3 - ps4
Asgard/Eukaryota - ps5
Eukaryota - ps6
Excavata/Diaphoretickes - ps7
Diaphoretickes - ps8
Archaeplastida - ps9
Viridiplantae - ps10
Streptophyta - ps11
Charophyceae/Embriophyta - ps12
Embriophyta - ps13
Tracheophyta - ps14
Spermatophyta - ps15
Angiosperms - ps16
Mesangiospermae - ps17
Magnoliids/eudicots - ps18
Eudicots - ps19
Core_eudicots - ps20
Superrosids - ps21
Rosids - ps22
Eurosids - ps23
Malvids - ps24
Sap./Malv./Brassicales - ps25
Malvales/Brassicales - ps26
Brassicales - ps27
Cleomaceae/Brassicaceae - ps28
Brassicaceae - ps29
Camelineae - ps30
Arabidopsis - ps31
Arabidopsis_thaliana - ps32
p-value
4e-08
1e-06
3e-05
9e-04
2e-02
Log-odds
0.5
1.0
1.5
2.0
2.5
3.0
3.5
4.0

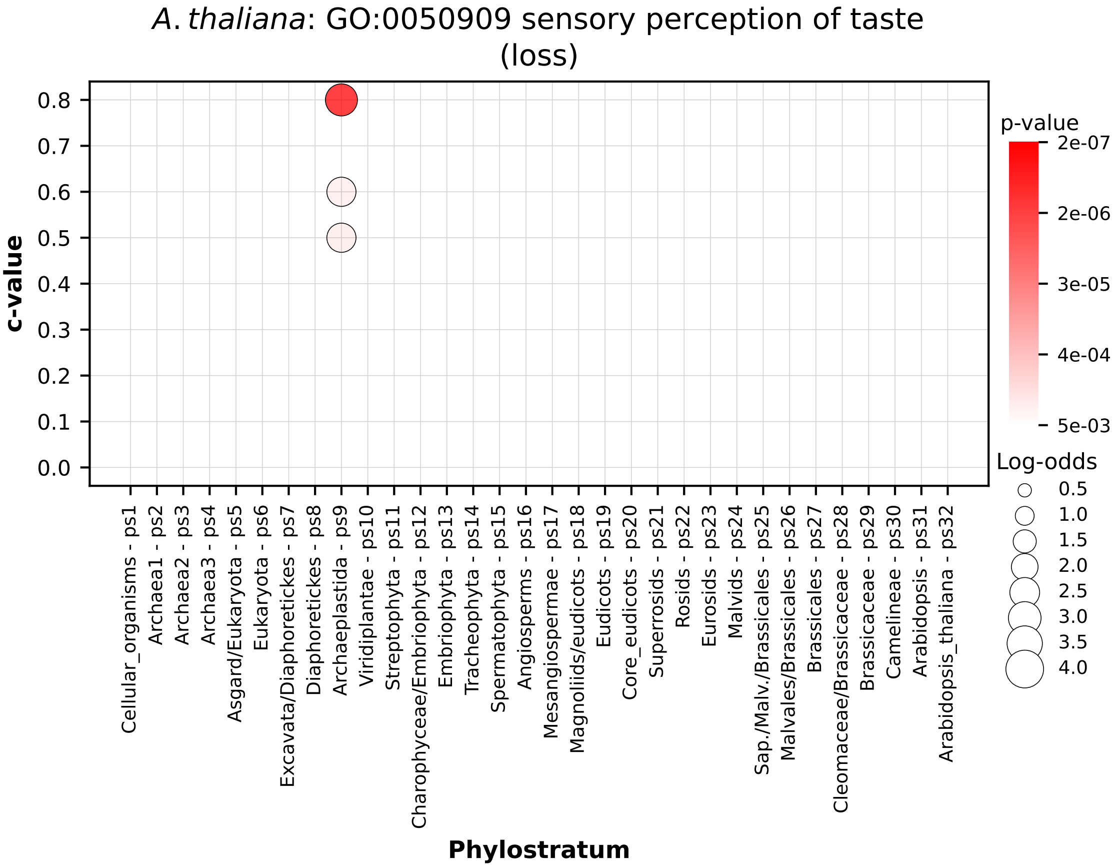

A. thaliana: GO:0050909 sensory perception of taste
(loss)
c-value
0.0
0.1
0.2
0.3
0.4
0.5
0.6
0.7
0.8
p-value
2e-07
2e-06
3e-05
4e-04
5e-03
Log-odds
0.5
1.0
1.5
2.0
2.5
3.0
3.5
4.0
Cellular_organisms - ps1
Archaea1 - ps2
Archaea2 - ps3
Archaea3 - ps4
Asgard/Eukaryota - ps5
Eukaryota - ps6
Excavata/Diaphoretickes - ps7
Diaphoretickes - ps8
Archaeplastida - ps9
Viridiplantae - ps10
Streptophyta - ps11
Charophyceae/Embriophyta - ps12
Embriophyta - ps13
Tracheophyta - ps14
Spermatophyta - ps15
Angiosperms - ps16
Mesangiospermae - ps17
Magnoliids/eudicots - ps18
Eudicots - ps19
Core_eudicots - ps20
Superrosids - ps21
Rosids - ps22
Eurosids - ps23
Malvids - ps24
Sap./Malv./Brassicales - ps25
Malvales/Brassicales - ps26
Brassicales - ps27
Cleomaceae/Brassicaceae - ps28
Brassicaceae - ps29
Camelineae - ps30
Arabidopsis - ps31
Arabidopsis_thaliana - ps32
Phylostratum

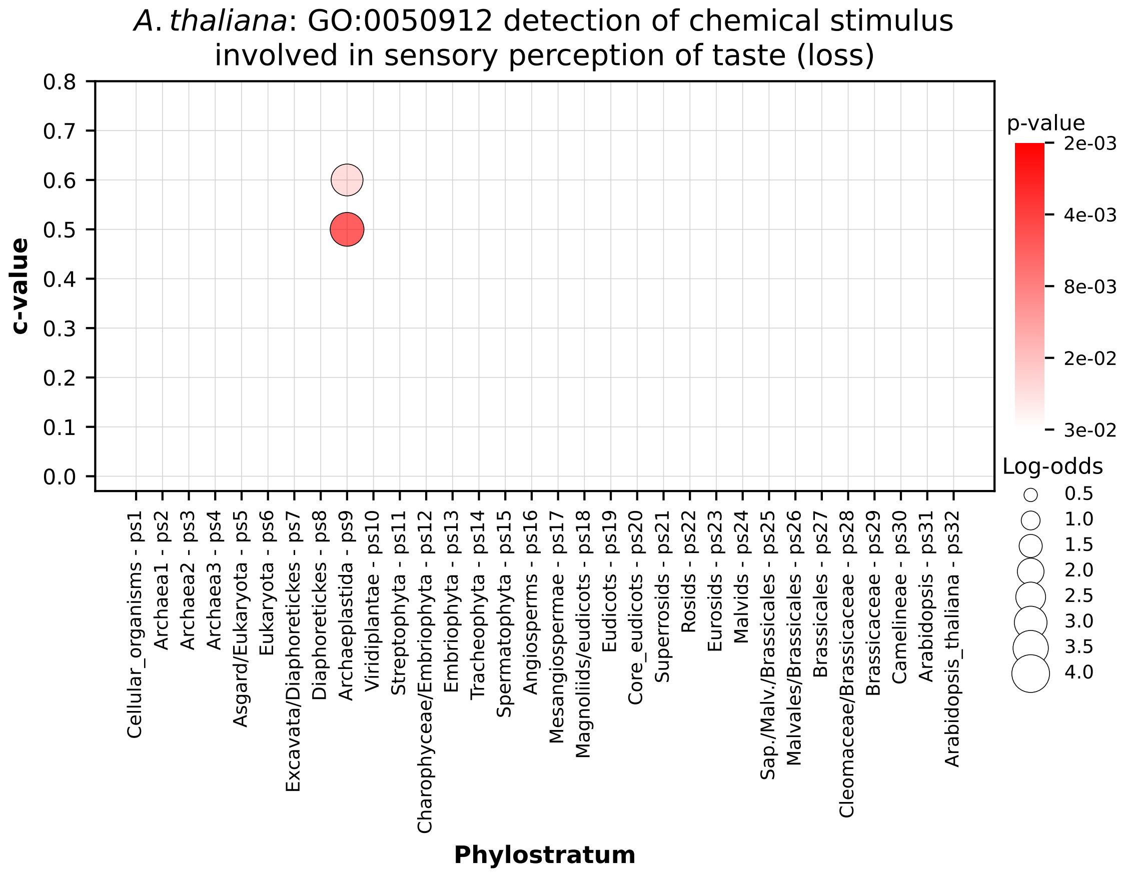

A. thaliana: GO:0050912 detection of chemical stimulus involved in sensory perception of taste (loss)
c-value
0.8
0.7
0.6
0.5
0.4
0.3
0.2
0.1
0.0
p-value
2e-03
4e-03
8e-03
2e-02
3e-02
Log-odds
0.5
1.0
1.5
2.0
2.5
3.0
3.5
4.0
Cellular_organisms - ps1
Archaea1 - ps2
Archaea2 - ps3
Archaea3 - ps4
Asgard/Eukaryota - ps5
Eukaryota - ps6
Excavata/Diaphoretickes - ps7
Diaphoretickes - ps8
Archaeplastida - ps9
Viridiplantae - ps10
Streptophyta - ps11
Charophyceae/Embriophyta - ps12
Embriophyta - ps13
Tracheophyta - ps14
Spermatophyta - ps15
Angiosperms - ps16
Mesangiospermae - ps17
Magnoliids/eudicots - ps18
Eudicots - ps19
Core_eudicots - ps20
Superrosids - ps21
Rosids - ps22
Eurosids - ps23
Malvids - ps24
Sap./Malv./Brassicales - ps25
Malvales/Brassicales - ps26
Brassicales - ps27
Cleomaceae/Brassicaceae - ps28
Brassicaceae - ps29
Camelineae - ps30
Arabidopsis - ps31
Arabidopsis_thaliana - ps32
Phylostratum

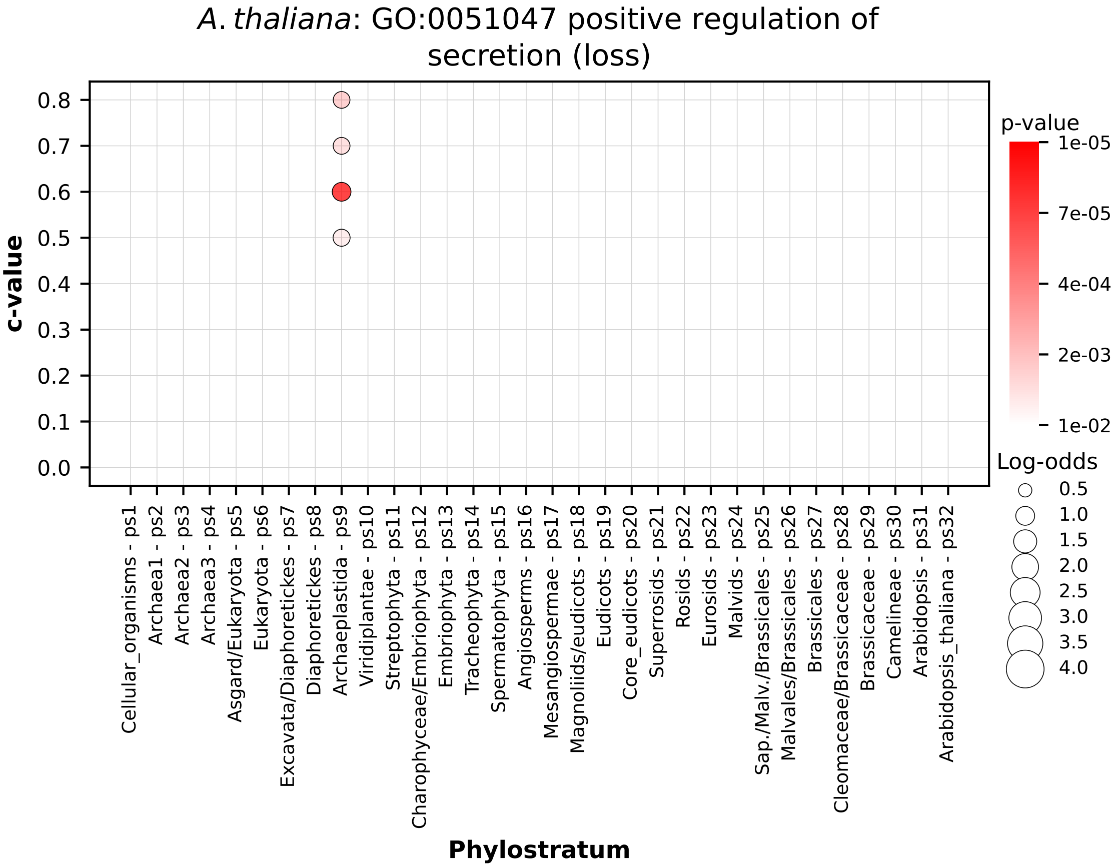

A. thaliana: GO:0051047 positive regulation of secretion (loss)
c-value
0.8
0.7
0.6
0.5
0.4
0.3
0.2
0.1
0.0
p-value
1e-05
7e-05
4e-04
2e-03
1e-02
Log-odds
0.5
1.0
1.5
2.0
2.5
3.0
3.5
4.0
Cellular_organisms - ps1
Archaea1 - ps2
Archaea2 - ps3
Archaea3 - ps4
Asgard/Eukaryota - ps5
Eukaryota - ps6
Excavata/Diaphoretickes - ps7
Diaphoretickes - ps8
Archaeplastida - ps9
Viridiplantae - ps10
Streptophyta - ps11
Charophyceae/Embriophyta - ps12
Embriophyta - ps13
Tracheophyta - ps14
Spermatophyta - ps15
Angiosperms - ps16
Mesangiospermae - ps17
Magnoliids/eudicots - ps18
Eudicots - ps19
Core_eudicots - ps20
Superrosids - ps21
Rosids - ps22
Eurosids - ps23
Malvids - ps24
Sap./Malv./Brassicales - ps25
Malvales/Brassicales - ps26
Brassicales - ps27
Cleomaceae/Brassicaceae - ps28
Brassicaceae - ps29
Camelineae - ps30
Arabidopsis - ps31
Arabidopsis_thaliana - ps32
Phylostratum

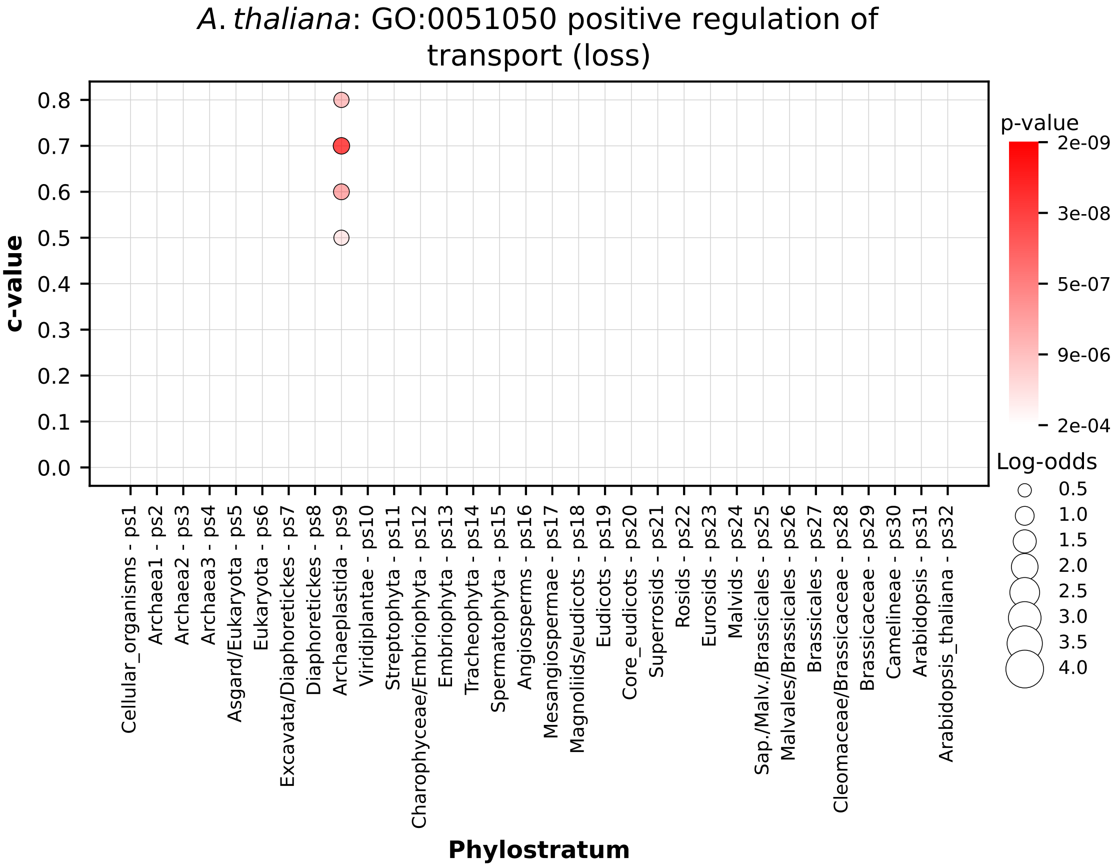
A. thaliana: GO:0051050 positive regulation of transport (loss)
c-value
0.8
0.7
0.6
0.5
0.4
0.3
0.2
0.1
0.0
Phylostratum
Cellular_organisms - ps1
Archaea1 - ps2
Archaea2 - ps3
Archaea3 - ps4
Asgard/Eukaryota - ps5
Eukaryota - ps6
Excavata/Diaphoretickes - ps7
Diaphoretickes - ps8
Archaeplastida - ps9
Viridiplantae - ps10
Streptophyta - ps11
Charophyceae/Embriophyta - ps12
Embriophyta - ps13
Tracheophyta - ps14
Spermatophyta - ps15
Angiosperms - ps16
Mesangiospermae - ps17
Magnoliids/eudicots - ps18
Eudicots - ps19
Core_eudicots - ps20
Superrosids - ps21
Rosids - ps22
Eurosids - ps23
Malvids - ps24
Sap./Malv./Brassicales - ps25
Malvales/Brassicales - ps26
Brassicales - ps27
Cleomaceae/Brassicaceae - ps28
Brassicaceae - ps29
Camelineae - ps30
Arabidopsis - ps31
Arabidopsis_thaliana - ps32
p-value
2e-09
3e-08
5e-07
9e-06
2e-04
Log-odds
0.5
1.0
1.5
2.0
2.5
3.0
3.5
4.0

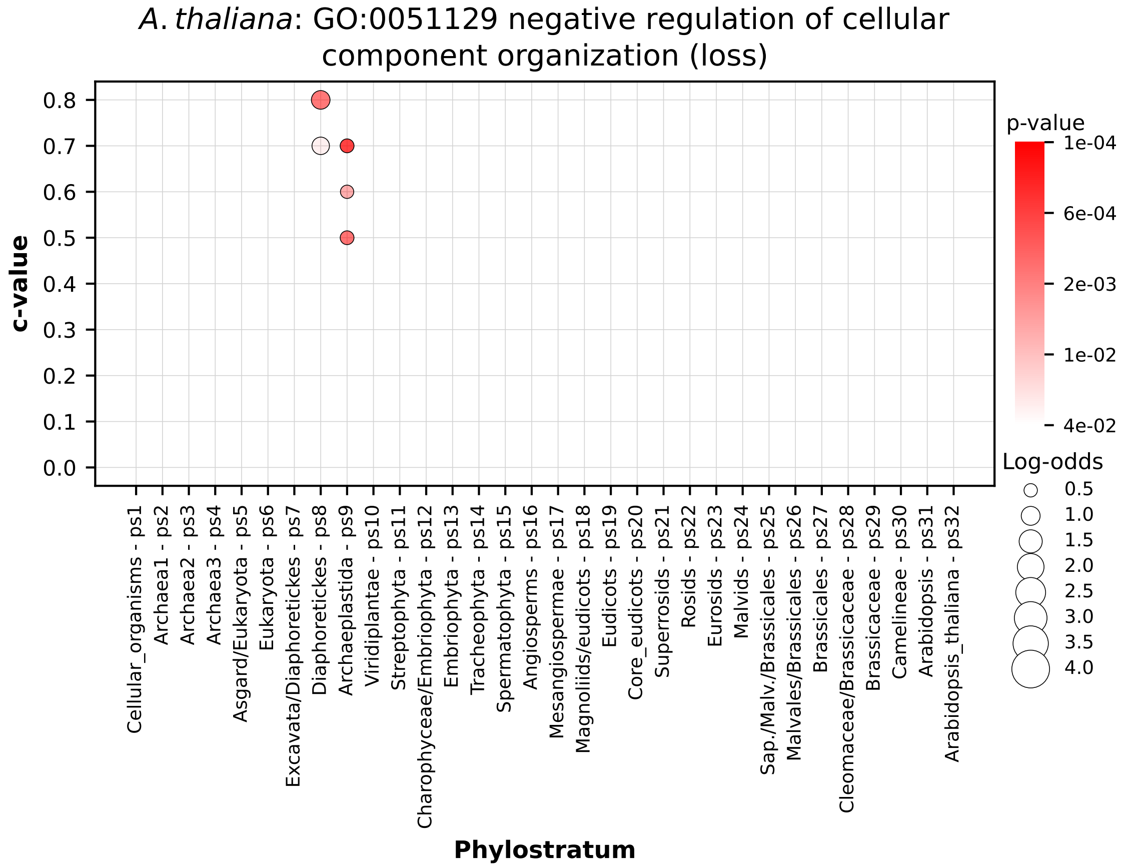

A. thaliana: GO:0051129 negative regulation of cellular component organization (loss)
c-value
0.8
0.7
0.6
0.5
0.4
0.3
0.2
0.1
0.0
p-value
1e-04
6e-04
2e-03
1e-02
4e-02
Log-odds
0.5
1.0
1.5
2.0
2.5
3.0
3.5
4.0
Cellular_organisms - ps1
Archaea1 - ps2
Archaea2 - ps3
Archaea3 - ps4
Asgard/Eukaryota - ps5
Eukaryota - ps6
Excavata/Diaphoretickes - ps7
Diaphoretickes - ps8
Archaeplastida - ps9
Viridiplantae - ps10
Streptophyta - ps11
Charophyceae/Embriophyta - ps12
Embriophyta - ps13
Tracheophyta - ps14
Spermatophyta - ps15
Angiosperms - ps16
Mesangiospermae - ps17
Magnoliids/eudicots - ps18
Eudicots - ps19
Core_eudicots - ps20
Superrosids - ps21
Rosids - ps22
Eurosids - ps23
Malvids - ps24
Sap./Malv./Brassicales - ps25
Malvales/Brassicales - ps26
Brassicales - ps27
Cleomaceae/Brassicaceae - ps28
Brassicaceae - ps29
Camelineae - ps30
Arabidopsis - ps31
Arabidopsis_thaliana - ps32
Phylostratum

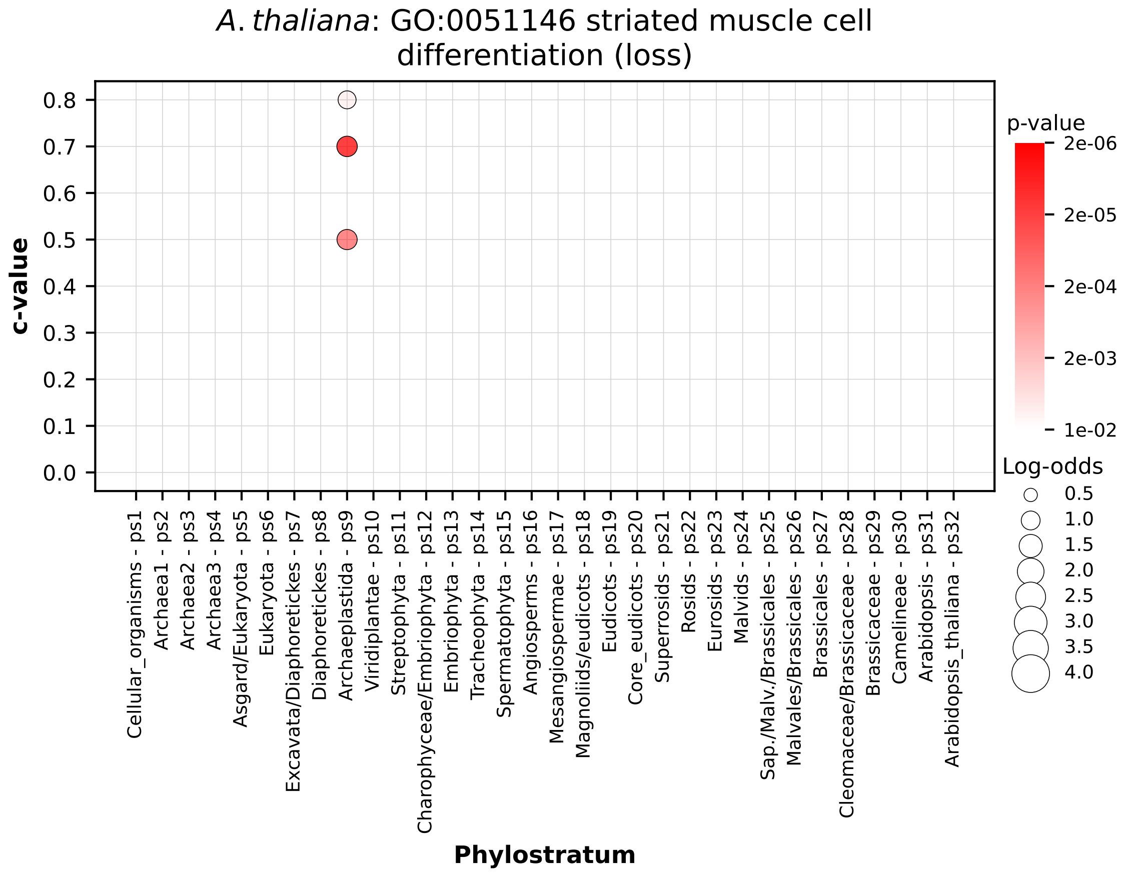
A. thaliana: GO:0051146 striated muscle cell differentiation (loss)
c-value
0.8
0.7
0.6
0.5
0.4
0.3
0.2
0.1
0.0
Phylostratum
Cellular_organisms - ps1
Archaea1 - ps2
Archaea2 - ps3
Archaea3 - ps4
Asgard/Eukaryota - ps5
Eukaryota - ps6
Excavata/Diaphoretickes - ps7
Diaphoretickes - ps8
Archaeplastida - ps9
Viridiplantae - ps10
Streptophyta - ps11
Charophyceae/Embriophyta - ps12
Embriophyta - ps13
Tracheophyta - ps14
Spermatophyta - ps15
Angiosperms - ps16
Mesangiospermae - ps17
Magnoliids/eudicots - ps18
Eudicots - ps19
Core_eudicots - ps20
Superrosids - ps21
Rosids - ps22
Eurosids - ps23
Malvids - ps24
Sap./Malv./Brassicales - ps25
Malvales/Brassicales - ps26
Brassicales - ps27
Cleomaceae/Brassicaceae - ps28
Brassicaceae - ps29
Camelineae - ps30
Arabidopsis - ps31
Arabidopsis_thaliana - ps32
p-value
2e-06
2e-05
2e-04
2e-03
1e-02
Log-odds
0.5
1.0
1.5
2.0
2.5
3.0
3.5
4.0

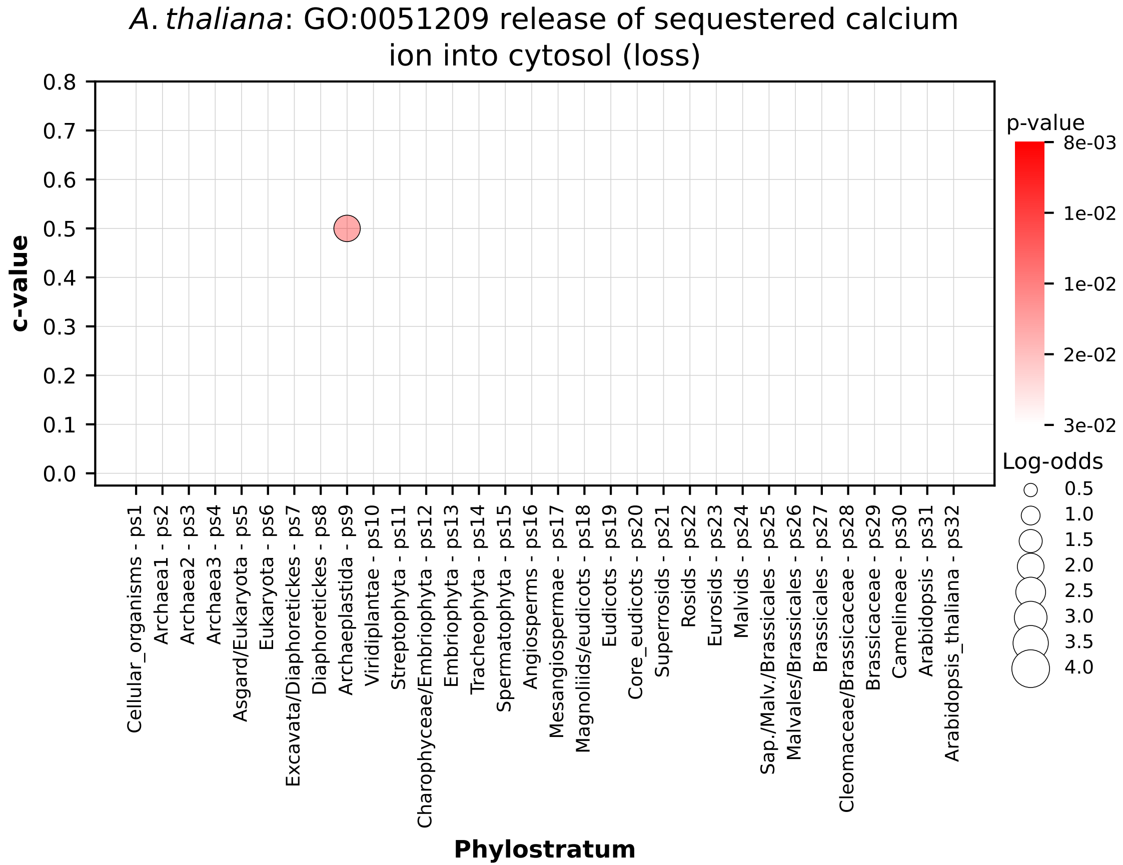

A. thaliana: GO:0051209 release of sequestered calcium ion into cytosol (loss)
c-value
0.8
0.7
0.6
0.5
0.4
0.3
0.2
0.1
0.0
Phylostratum
Cellular_organisms - ps1
Archaea1 - ps2
Archaea2 - ps3
Archaea3 - ps4
Asgard/Eukaryota - ps5
Eukaryota - ps6
Excavata/Diaphoretickes - ps7
Diaphoretickes - ps8
Archaeplastida - ps9
Viridiplantae - ps10
Streptophyta - ps11
Charophyceae/Embriophyta - ps12
Embriophyta - ps13
Tracheophyta - ps14
Spermatophyta - ps15
Angiosperms - ps16
Mesangiospermae - ps17
Magnoliids/eudicots - ps18
Eudicots - ps19
Core_eudicots - ps20
Superrosids - ps21
Rosids - ps22
Eurosids - ps23
Malvids - ps24
Sap./Malv./Brassicales - ps25
Malvales/Brassicales - ps26
Brassicales - ps27
Cleomaceae/Brassicaceae - ps28
Brassicaceae - ps29
Camelineae - ps30
Arabidopsis - ps31
Arabidopsis_thaliana - ps32
p-value
8e-03
1e-02
1e-02
2e-02
3e-02
Log-odds
0.5
1.0
1.5
2.0
2.5
3.0
3.5
4.0

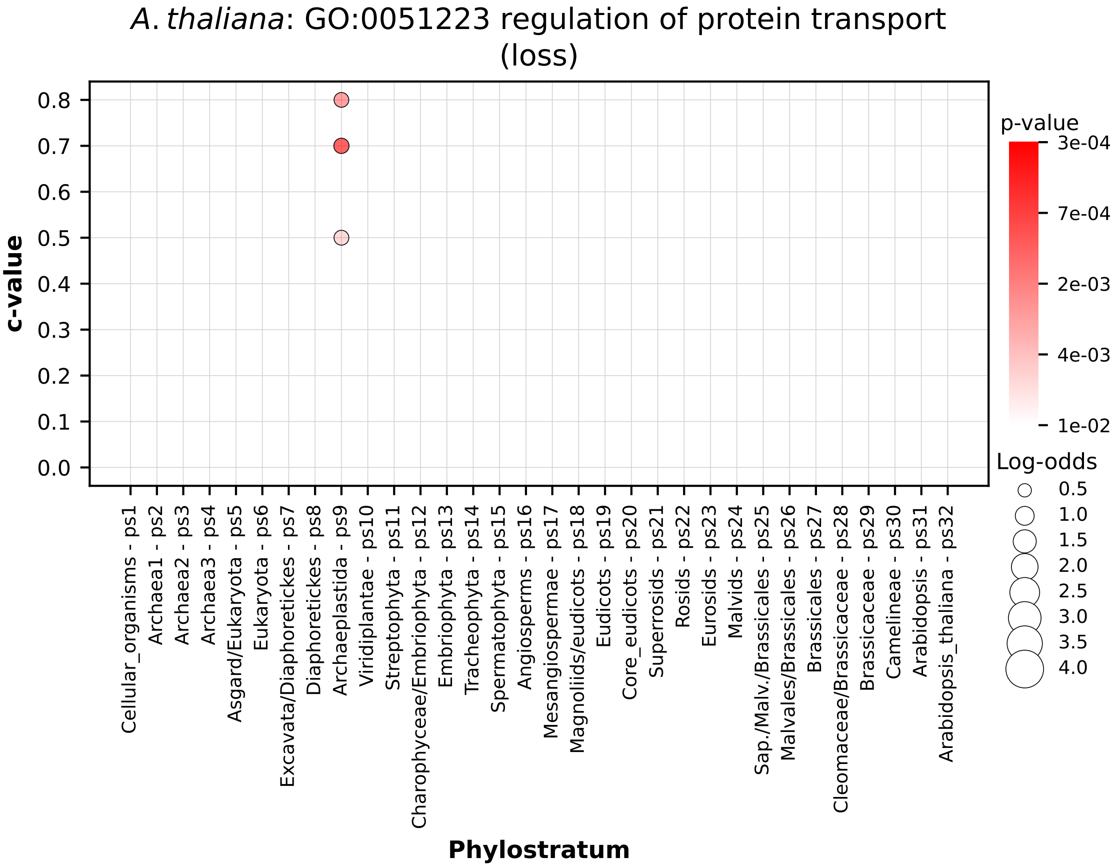

A. thaliana: GO:0051223 regulation of protein transport
(loss)
c-value
0.8
0.7
0.6
0.5
0.4
0.3
0.2
0.1
0.0
p-value
3e-04
7e-04
2e-03
4e-03
1e-02
Log-odds
0.5
1.0
1.5
2.0
2.5
3.0
3.5
4.0
Cellular_organisms - ps1
Archaea1 - ps2
Archaea2 - ps3
Archaea3 - ps4
Asgard/Eukaryota - ps5
Eukaryota - ps6
Excavata/Diaphoretickes - ps7
Diaphoretickes - ps8
Archaeplastida - ps9
Viridiplantae - ps10
Streptophyta - ps11
Charophyceae/Embriophyta - ps12
Embriophyta - ps13
Tracheophyta - ps14
Spermatophyta - ps15
Angiosperms - ps16
Mesangiospermae - ps17
Magnoliids/eudicots - ps18
Eudicots - ps19
Core_eudicots - ps20
Superrosids - ps21
Rosids - ps22
Eurosids - ps23
Malvids - ps24
Sap./Malv./Brassicales - ps25
Malvales/Brassicales - ps26
Brassicales - ps27
Cleomaceae/Brassicaceae - ps28
Brassicaceae - ps29
Camelineae - ps30
Arabidopsis - ps31
Arabidopsis_thaliana - ps32
Phylostratum

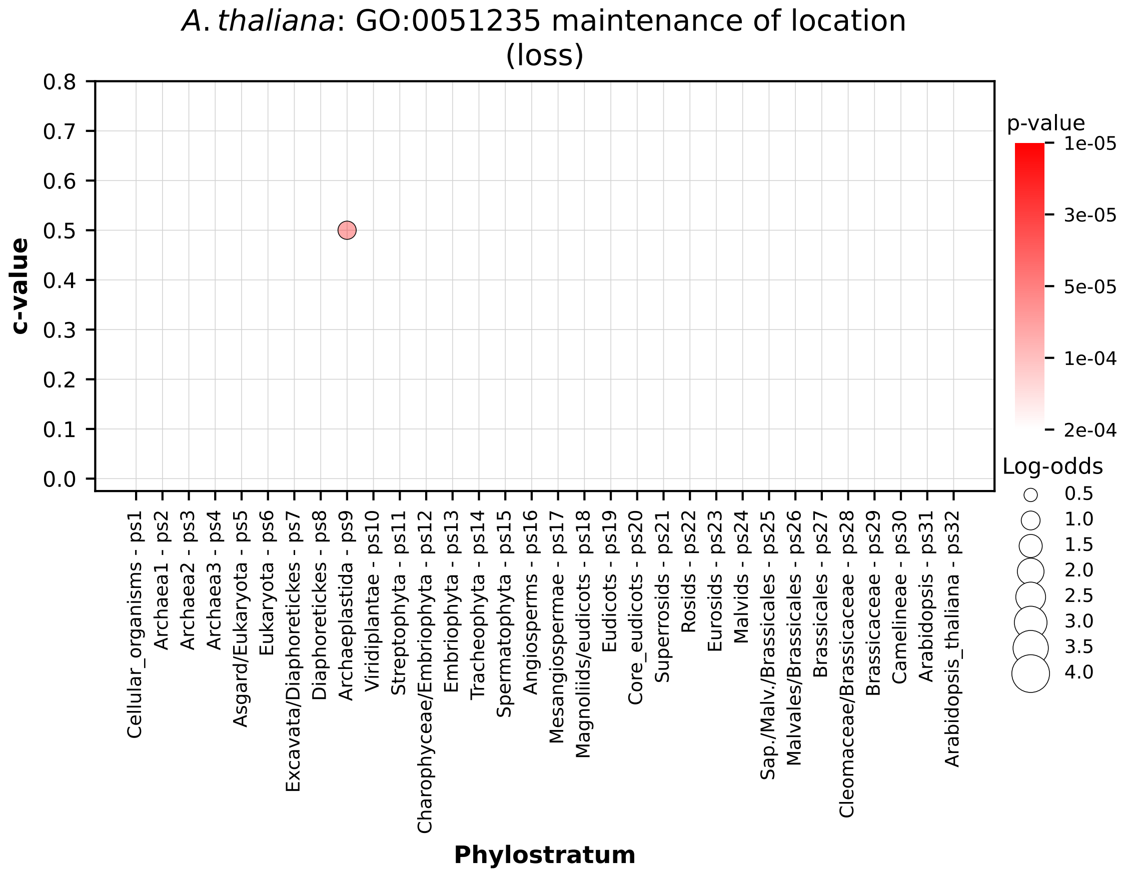

A. thaliana: GO:0051235 maintenance of location
(loss)
c-value
0.0
0.1
0.2
0.3
0.4
0.5
0.6
0.7
0.8
Phylostratum
Cellular_organisms - ps1
Archaea1 - ps2
Archaea2 - ps3
Archaea3 - ps4
Asgard/Eukaryota - ps5
Eukaryota - ps6
Excavata/Diaphoretickes - ps7
Diaphoretickes - ps8
Archaeplastida - ps9
Viridiplantae - ps10
Streptophyta - ps11
Charophyceae/Embriophyta - ps12
Embriophyta - ps13
Tracheophyta - ps14
Spermatophyta - ps15
Angiosperms - ps16
Mesangiospermae - ps17
Magnoliids/eudicots - ps18
Eudicots - ps19
Core_eudicots - ps20
Superrosids - ps21
Rosids - ps22
Eurosids - ps23
Malvids - ps24
Sap./Malv./Brassicales - ps25
Malvales/Brassicales - ps26
Brassicales - ps27
Cleomaceae/Brassicaceae - ps28
Brassicaceae - ps29
Camelineae - ps30
Arabidopsis - ps31
Arabidopsis_thaliana - ps32
p-value
1e-05
3e-05
5e-05
1e-04
2e-04
Log-odds
0.5
1.0
1.5
2.0
2.5
3.0
3.5
4.0

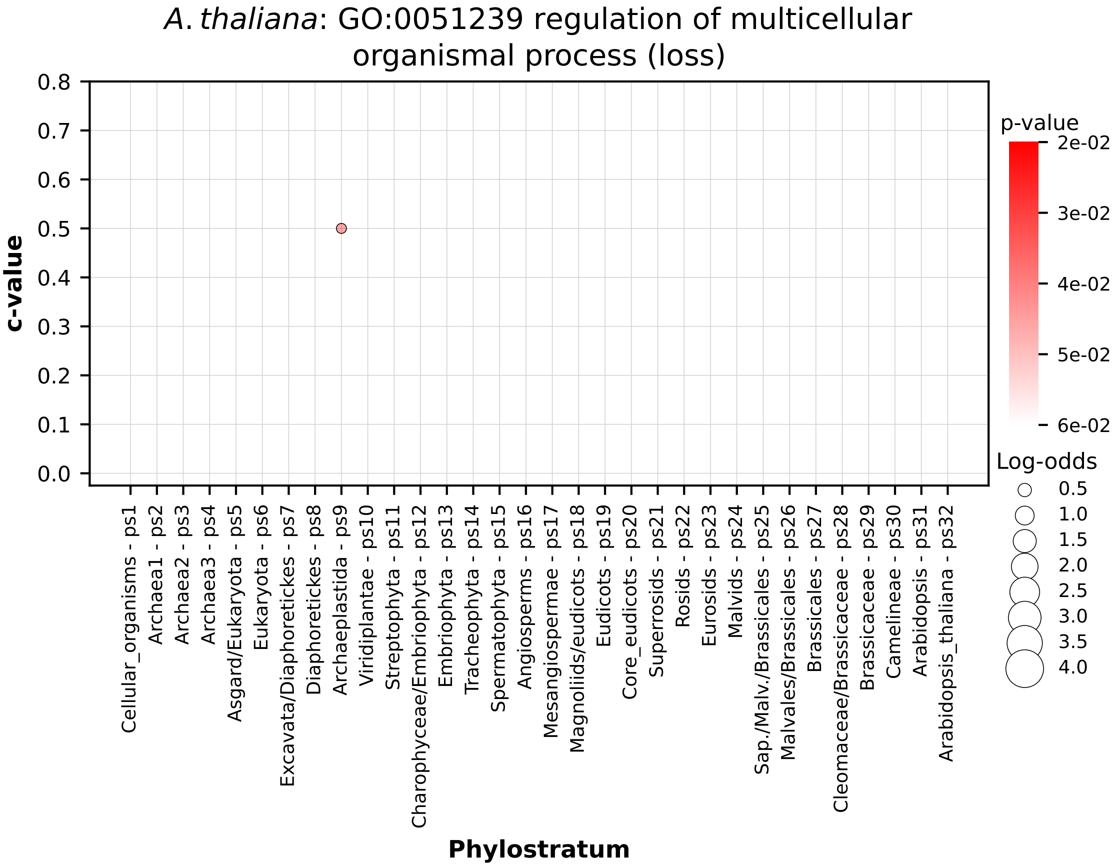
A. thaliana: GO:0051239 regulation of multicellular organismal process (loss)
c-value
0.8
0.7
0.6
0.5
0.4
0.3
0.2
0.1
0.0
Phylostratum
Cellular_organisms - ps1
Archaea1 - ps2
Archaea2 - ps3
Archaea3 - ps4
Asgard/Eukaryota - ps5
Eukaryota - ps6
Excavata/Diaphoretickes - ps7
Diaphoretickes - ps8
Archaeplastida - ps9
Viridiplantae - ps10
Streptophyta - ps11
Charophyceae/Embriophyta - ps12
Embriophyta - ps13
Tracheophyta - ps14
Spermatophyta - ps15
Angiosperms - ps16
Mesangiospermae - ps17
Magnoliids/eudicots - ps18
Eudicots - ps19
Core_eudicots - ps20
Superrosids - ps21
Rosids - ps22
Eurosids - ps23
Malvids - ps24
Sap./Malv./Brassicales - ps25
Malvales/Brassicales - ps26
Brassicales - ps27
Cleomaceae/Brassicaceae - ps28
Brassicaceae - ps29
Camelineae - ps30
Arabidopsis - ps31
Arabidopsis_thaliana - ps32
p-value
2e-02
3e-02
4e-02
5e-02
6e-02
Log-odds
0.5
1.0
1.5
2.0
2.5
3.0
3.5
4.0

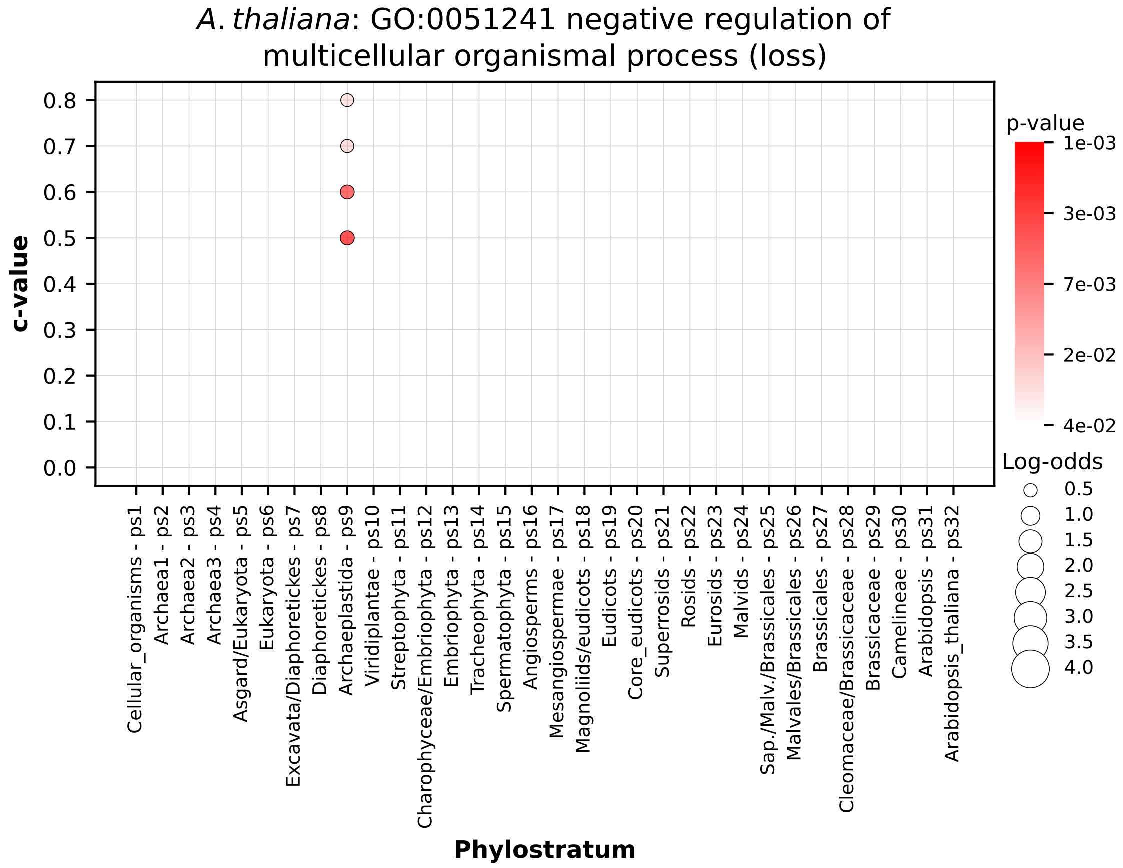

A. thaliana: GO:0051241 negative regulation of multicellular organismal process (loss)
c-value
0.0
0.1
0.2
0.3
0.4
0.5
0.6
0.7
0.8
Phylostratum
Cellular_organisms - ps1
Archaea1 - ps2
Archaea2 - ps3
Archaea3 - ps4
Asgard/Eukaryota - ps5
Eukaryota - ps6
Excavata/Diaphoretickes - ps7
Diaphoretickes - ps8
Archaeplastida - ps9
Viridiplantae - ps10
Streptophyta - ps11
Charophyceae/Embriophyta - ps12
Embriophyta - ps13
Tracheophyta - ps14
Spermatophyta - ps15
Angiosperms - ps16
Mesangiospermae - ps17
Magnoliids/eudicots - ps18
Eudicots - ps19
Core_eudicots - ps20
Superrosids - ps21
Rosids - ps22
Eurosids - ps23
Malvids - ps24
Sap./Malv./Brassicales - ps25
Malvales/Brassicales - ps26
Brassicales - ps27
Cleomaceae/Brassicaceae - ps28
Brassicaceae - ps29
Camelineae - ps30
Arabidopsis - ps31
Arabidopsis_thaliana - ps32
p-value
1e-03
3e-03
7e-03
2e-02
4e-02
Log-odds
0.5
1.0
1.5
2.0
2.5
3.0
3.5
4.0

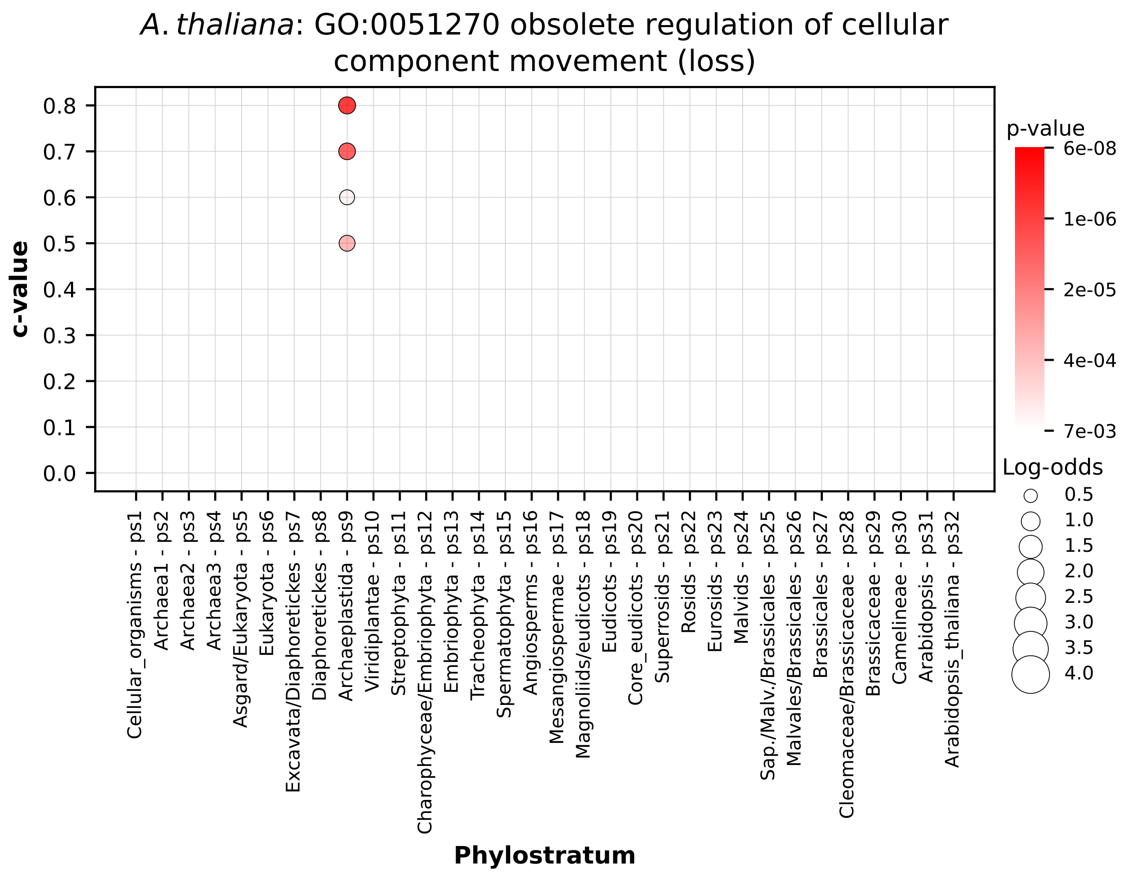

A. thaliana: GO:0051270 obsolete regulation of cellular component movement (loss)
c-value
0.0
0.1
0.2
0.3
0.4
0.5
0.6
0.7
0.8
p-value
6e-08
1e-06
2e-05
4e-04
7e-03
Log-odds
0.5
1.0
1.5
2.0
2.5
3.0
3.5
4.0
Cellular_organisms - ps1
Archaea1 - ps2
Archaea2 - ps3
Archaea3 - ps4
Asgard/Eukaryota - ps5
Eukaryota - ps6
Excavata/Diaphoretickes - ps7
Diaphoretickes - ps8
Archaeplastida - ps9
Viridiplantae - ps10
Streptophyta - ps11
Charophyceae/Embriophyta - ps12
Embriophyta - ps13
Tracheophyta - ps14
Spermatophyta - ps15
Angiosperms - ps16
Mesangiospermae - ps17
Magnoliids/eudicots - ps18
Eudicots - ps19
Core_eudicots - ps20
Superrosids - ps21
Rosids - ps22
Eurosids - ps23
Malvids - ps24
Sap./Malv./Brassicales - ps25
Malvales/Brassicales - ps26
Brassicales - ps27
Cleomaceae/Brassicaceae - ps28
Brassicaceae - ps29
Camelineae - ps30
Arabidopsis - ps31
Arabidopsis_thaliana - ps32
Phylostratum

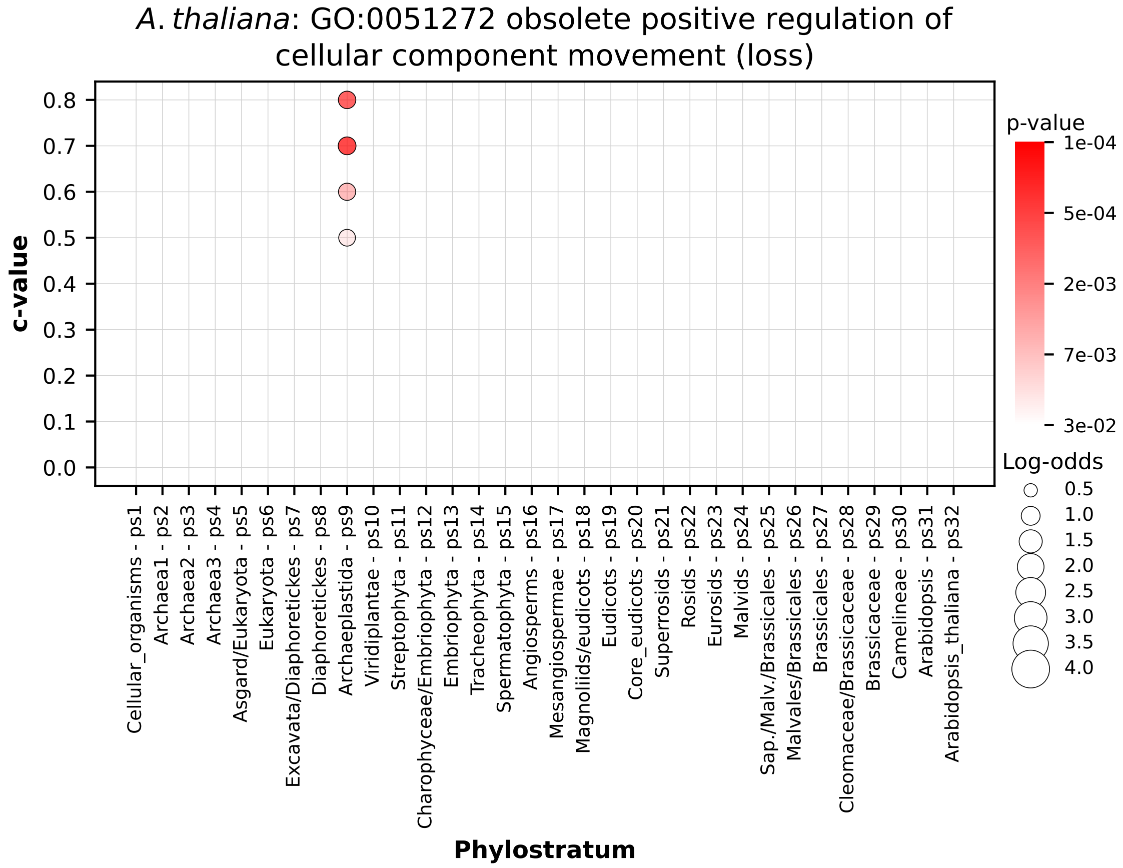

A. thaliana: GO:0051272 obsolete positive regulation of cellular component movement (loss)
c-value
0.0
0.1
0.2
0.3
0.4
0.5
0.6
0.7
0.8
p-value
1e-04
5e-04
2e-03
7e-03
3e-02
Log-odds
0.5
1.0
1.5
2.0
2.5
3.0
3.5
4.0
Cellular_organisms - ps1
Archaea1 - ps2
Archaea2 - ps3
Archaea3 - ps4
Asgard/Eukaryota - ps5
Eukaryota - ps6
Excavata/Diaphoretickes - ps7
Diaphoretickes - ps8
Archaeplastida - ps9
Viridiplantae - ps10
Streptophyta - ps11
Charophyceae/Embriophyta - ps12
Embriophyta - ps13
Tracheophyta - ps14
Spermatophyta - ps15
Angiosperms - ps16
Mesangiospermae - ps17
Magnoliids/eudicots - ps18
Eudicots - ps19
Core_eudicots - ps20
Superrosids - ps21
Rosids - ps22
Eurosids - ps23
Malvids - ps24
Sap./Malv./Brassicales - ps25
Malvales/Brassicales - ps26
Brassicales - ps27
Cleomaceae/Brassicaceae - ps28
Brassicaceae - ps29
Camelineae - ps30
Arabidopsis - ps31
Arabidopsis_thaliana - ps32
Phylostratum

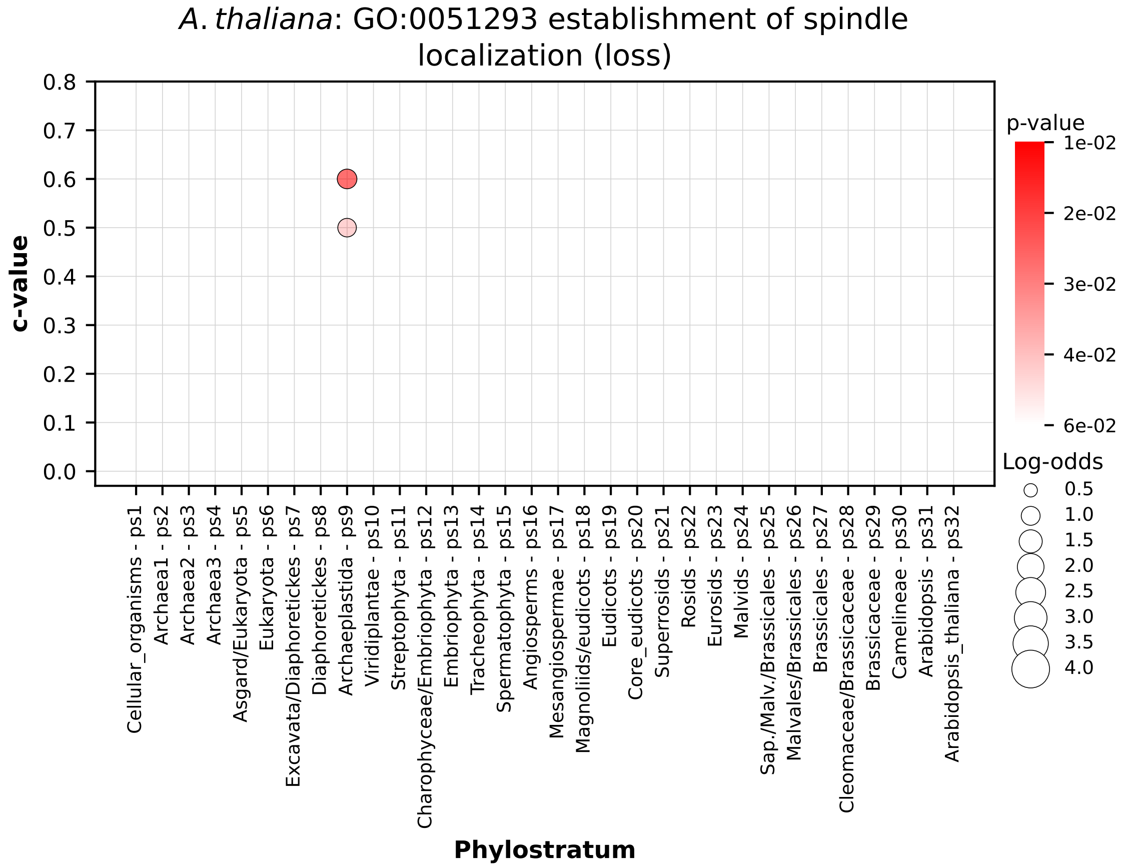

A. thaliana: GO:0051293 establishment of spindle localization (loss)
c-value
0.8
0.7
0.6
0.5
0.4
0.3
0.2
0.1
0.0
Phylostratum
Cellular_organisms - ps1
Archaea1 - ps2
Archaea2 - ps3
Archaea3 - ps4
Asgard/Eukaryota - ps5
Eukaryota - ps6
Excavata/Diaphoretickes - ps7
Diaphoretickes - ps8
Archaeplastida - ps9
Viridiplantae - ps10
Streptophyta - ps11
Charophyceae/Embriophyta - ps12
Embriophyta - ps13
Tracheophyta - ps14
Spermatophyta - ps15
Angiosperms - ps16
Mesangiospermae - ps17
Magnoliids/eudicots - ps18
Eudicots - ps19
Core_eudicots - ps20
Superrosids - ps21
Rosids - ps22
Eurosids - ps23
Malvids - ps24
Sap./Malv./Brassicales - ps25
Malvales/Brassicales - ps26
Brassicales - ps27
Cleomaceae/Brassicaceae - ps28
Brassicaceae - ps29
Camelineae - ps30
Arabidopsis - ps31
Arabidopsis_thaliana - ps32
p-value
1e-02
2e-02
3e-02
4e-02
6e-02
Log-odds
0.5
1.0
1.5
2.0
2.5
3.0
3.5
4.0

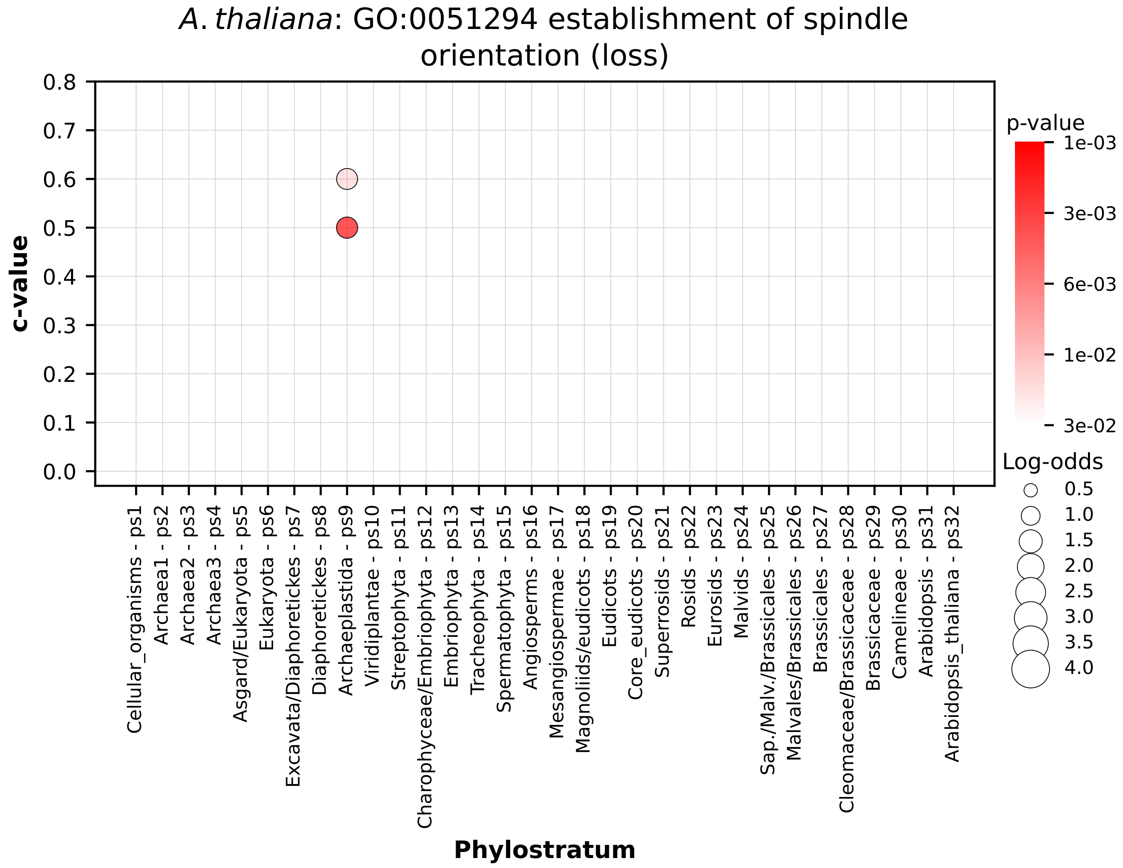

A. thaliana: GO:0051294 establishment of spindle orientation (loss)
c-value
0.8
0.7
0.6
0.5
0.4
0.3
0.2
0.1
0.0
p-value
1e-03
3e-03
6e-03
1e-02
3e-02
Log-odds
0.5
1.0
1.5
2.0
2.5
3.0
3.5
4.0
Cellular_organisms - ps1
Archaea1 - ps2
Archaea2 - ps3
Archaea3 - ps4
Asgard/Eukaryota - ps5
Eukaryota - ps6
Excavata/Diaphoretickes - ps7
Diaphoretickes - ps8
Archaeplastida - ps9
Viridiplantae - ps10
Streptophyta - ps11
Charophyceae/Embriophyta - ps12
Embriophyta - ps13
Tracheophyta - ps14
Spermatophyta - ps15
Angiosperms - ps16
Mesangiospermae - ps17
Magnoliids/eudicots - ps18
Eudicots - ps19
Core_eudicots - ps20
Superrosids - ps21
Rosids - ps22
Eurosids - ps23
Malvids - ps24
Sap./Malv./Brassicales - ps25
Malvales/Brassicales - ps26
Brassicales - ps27
Cleomaceae/Brassicaceae - ps28
Brassicaceae - ps29
Camelineae - ps30
Arabidopsis - ps31
Arabidopsis_thaliana - ps32
Phylostratum

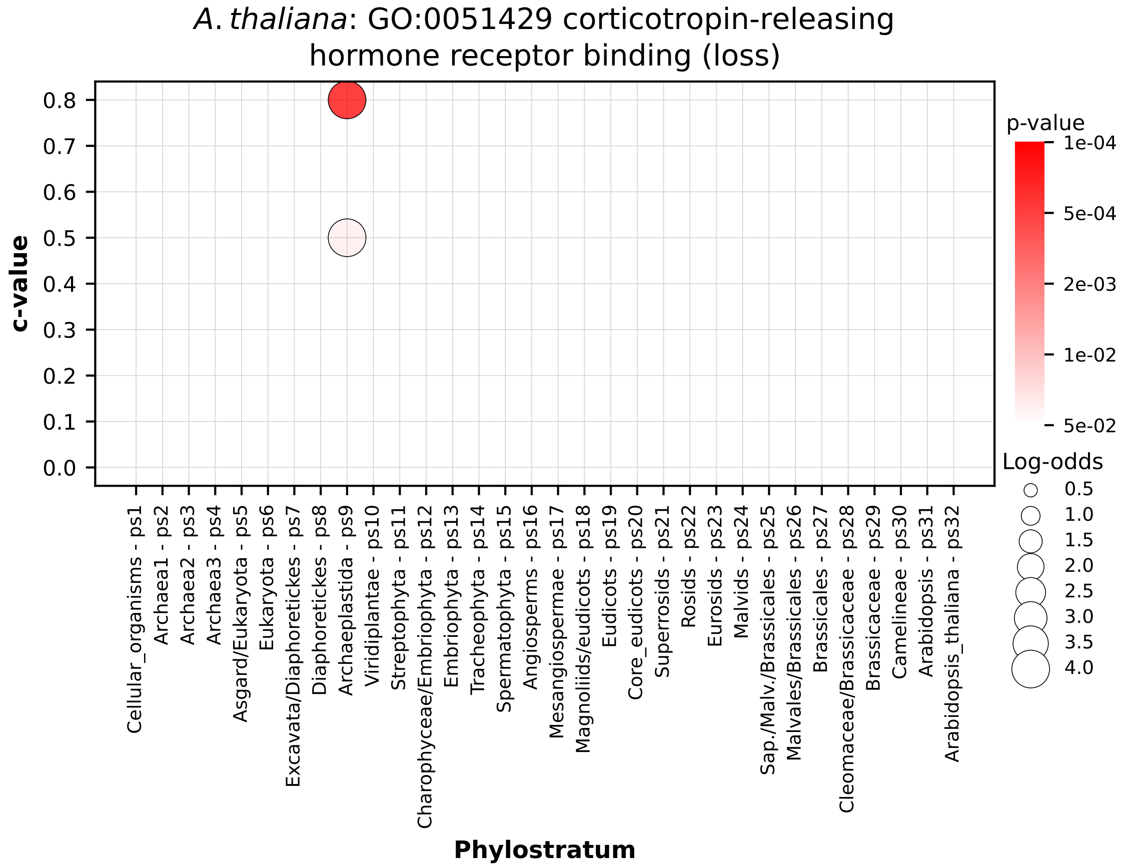

A. thaliana: GO:0051429 corticotropin-releasing hormone receptor binding (loss)
c-value
0.0
0.1
0.2
0.3
0.4
0.5
0.6
0.7
0.8
Phylostratum
Cellular_organisms - ps1
Archaea1 - ps2
Archaea2 - ps3
Archaea3 - ps4
Asgard/Eukaryota - ps5
Eukaryota - ps6
Excavata/Diaphoretickes - ps7
Diaphoretickes - ps8
Archaeplastida - ps9
Viridiplantae - ps10
Streptophyta - ps11
Charophyceae/Embriophyta - ps12
Embriophyta - ps13
Tracheophyta - ps14
Spermatophyta - ps15
Angiosperms - ps16
Mesangiospermae - ps17
Magnoliids/eudicots - ps18
Eudicots - ps19
Core_eudicots - ps20
Superrosids - ps21
Rosids - ps22
Eurosids - ps23
Malvids - ps24
Sap./Malv./Brassicales - ps25
Malvales/Brassicales - ps26
Brassicales - ps27
Cleomaceae/Brassicaceae - ps28
Brassicaceae - ps29
Camelineae - ps30
Arabidopsis - ps31
Arabidopsis_thaliana - ps32
p-value
1e-04
5e-04
2e-03
1e-02
5e-02
Log-odds
0.5
1.0
1.5
2.0
2.5
3.0
3.5
4.0

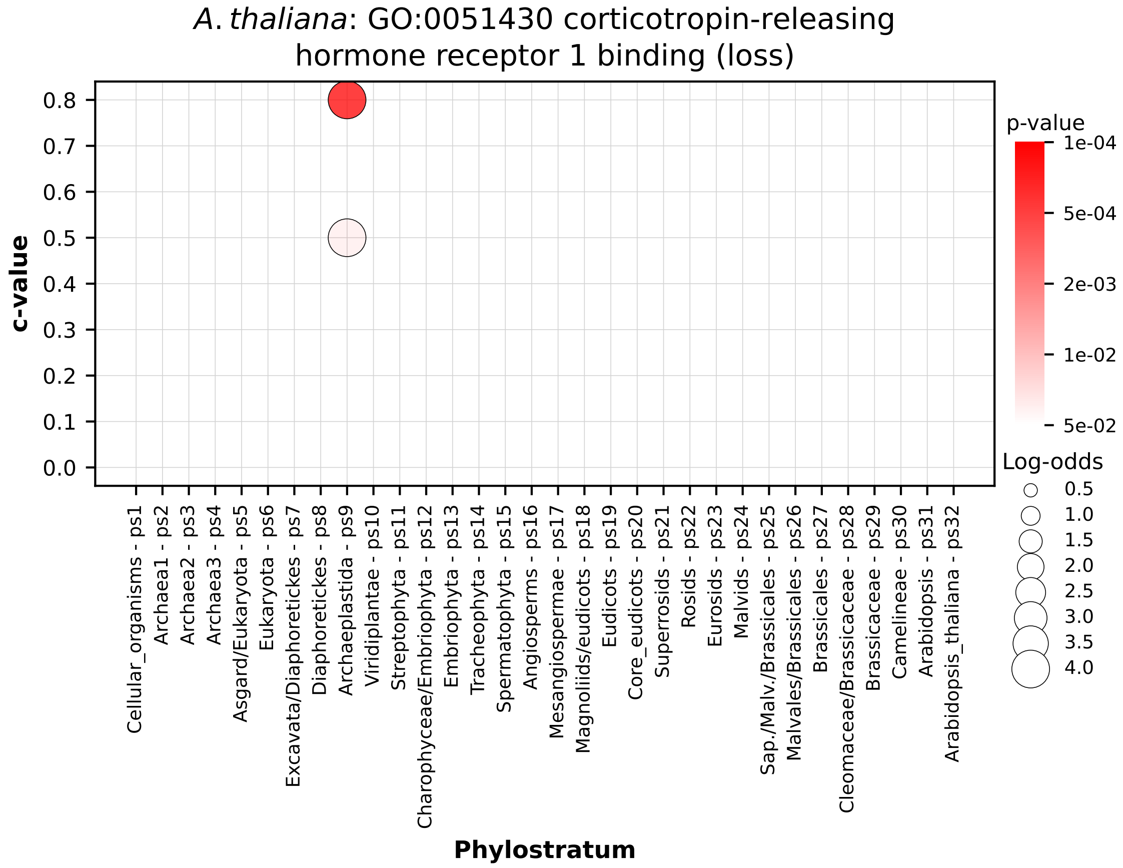

A. thaliana: GO:0051430 corticotropin-releasing hormone receptor 1 binding (loss)
c-value
0.0
0.1
0.2
0.3
0.4
0.5
0.6
0.7
0.8
p-value
1e-04
5e-04
2e-03
1e-02
5e-02
Log-odds
0.5
1.0
1.5
2.0
2.5
3.0
3.5
4.0
Cellular_organisms - ps1
Archaea1 - ps2
Archaea2 - ps3
Archaea3 - ps4
Asgard/Eukaryota - ps5
Eukaryota - ps6
Excavata/Diaphoretickes - ps7
Diaphoretickes - ps8
Archaeplastida - ps9
Viridiplantae - ps10
Streptophyta - ps11
Charophyceae/Embriophyta - ps12
Embriophyta - ps13
Tracheophyta - ps14
Spermatophyta - ps15
Angiosperms - ps16
Mesangiospermae - ps17
Magnoliids/eudicots - ps18
Eudicots - ps19
Core_eudicots - ps20
Superrosids - ps21
Rosids - ps22
Eurosids - ps23
Malvids - ps24
Sap./Malv./Brassicales - ps25
Malvales/Brassicales - ps26
Brassicales - ps27
Cleomaceae/Brassicaceae - ps28
Brassicaceae - ps29
Camelineae - ps30
Arabidopsis - ps31
Arabidopsis_thaliana - ps32
Phylostratum

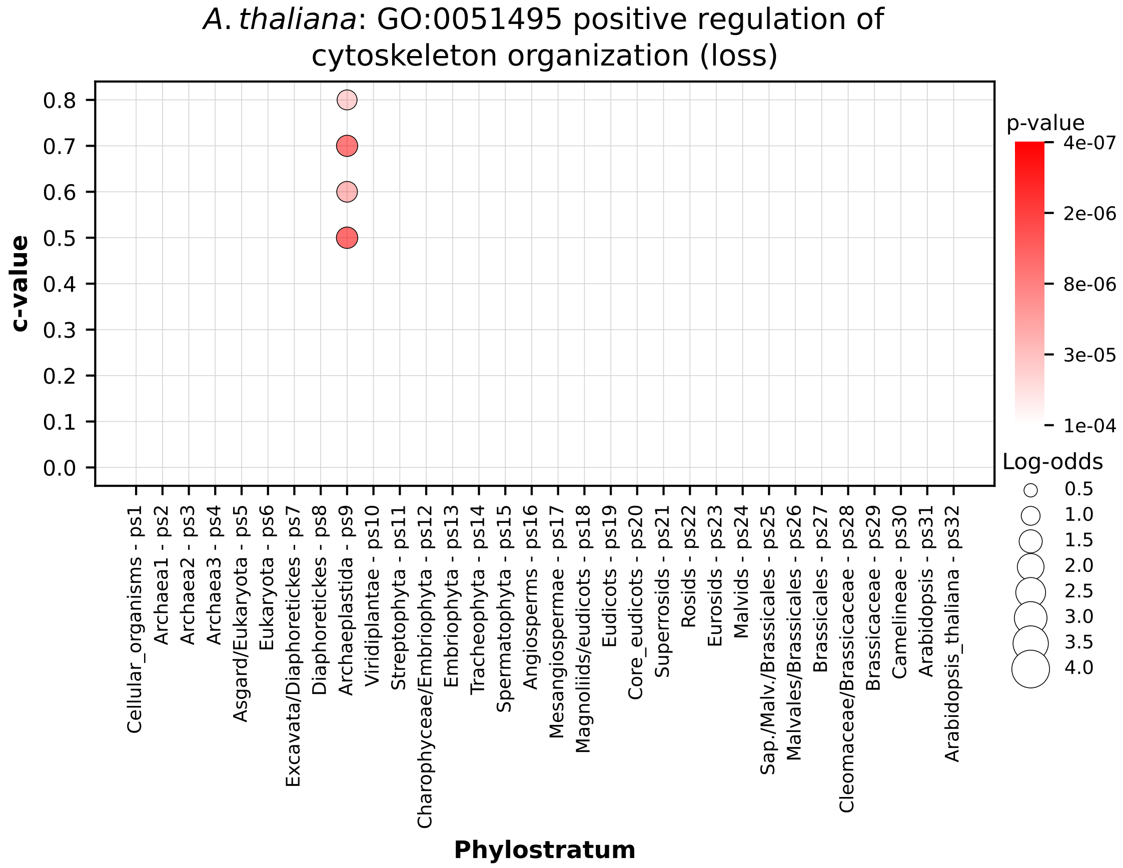

A. thaliana: GO:0051495 positive regulation of cytoskeleton organization (loss)
c-value
0.8
0.7
0.6
0.5
0.4
0.3
0.2
0.1
0.0
p-value
4e-07
2e-06
8e-06
3e-05
1e-04
Log-odds
0.5
1.0
1.5
2.0
2.5
3.0
3.5
4.0
Cellular_organisms - ps1
Archaea1 - ps2
Archaea2 - ps3
Archaea3 - ps4
Asgard/Eukaryota - ps5
Eukaryota - ps6
Excavata/Diaphoretickes - ps7
Diaphoretickes - ps8
Archaeplastida - ps9
Viridiplantae - ps10
Streptophyta - ps11
Charophyceae/Embriophyta - ps12
Embriophyta - ps13
Tracheophyta - ps14
Spermatophyta - ps15
Angiosperms - ps16
Mesangiospermae - ps17
Magnoliids/eudicots - ps18
Eudicots - ps19
Core_eudicots - ps20
Superrosids - ps21
Rosids - ps22
Eurosids - ps23
Malvids - ps24
Sap./Malv./Brassicales - ps25
Malvales/Brassicales - ps26
Brassicales - ps27
Cleomaceae/Brassicaceae - ps28
Brassicaceae - ps29
Camelineae - ps30
Arabidopsis - ps31
Arabidopsis_thaliana - ps32
Phylostratum

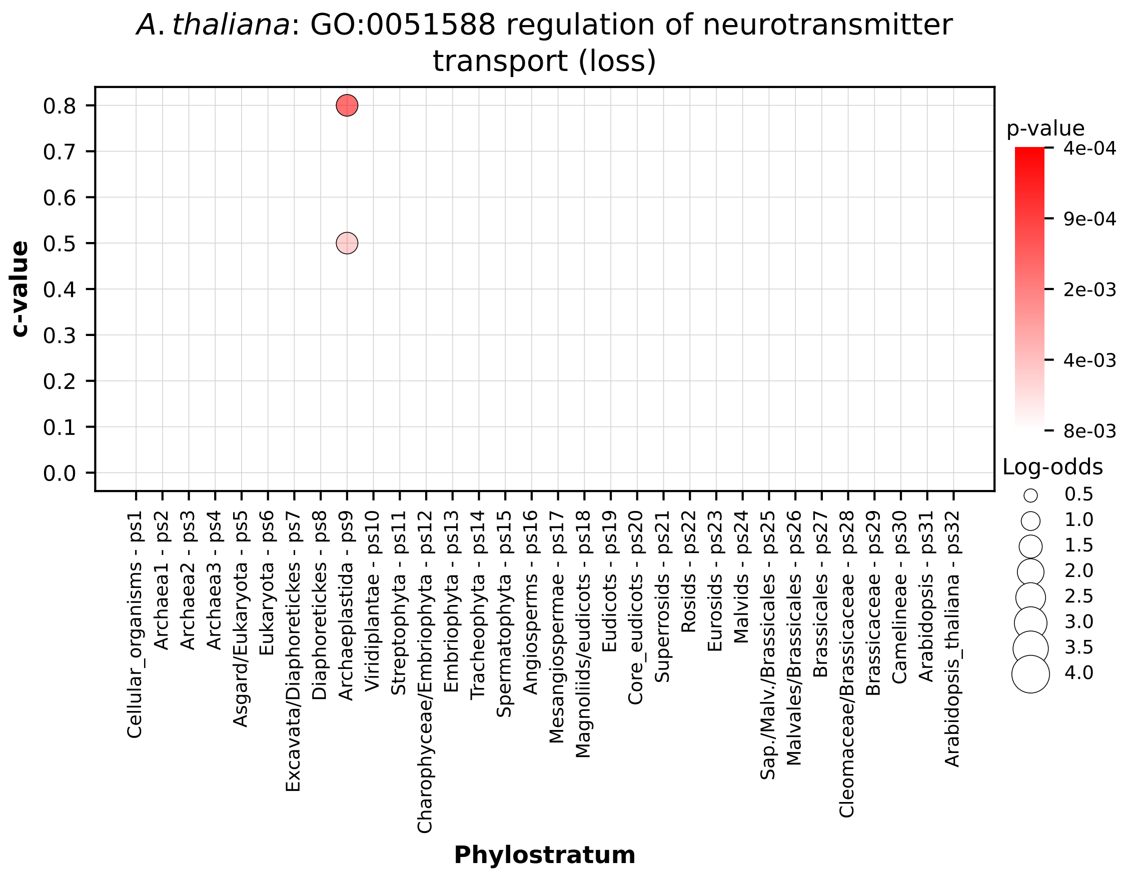

A. thaliana: GO:0051588 regulation of neurotransmitter transport (loss)
c-value
0.8
0.7
0.6
0.5
0.4
0.3
0.2
0.1
0.0
Phylostratum
Cellular_organisms - ps1
Archaea1 - ps2
Archaea2 - ps3
Archaea3 - ps4
Asgard/Eukaryota - ps5
Eukaryota - ps6
Excavata/Diaphoretickes - ps7
Diaphoretickes - ps8
Archaeplastida - ps9
Viridiplantae - ps10
Streptophyta - ps11
Charophyceae/Embriophyta - ps12
Embriophyta - ps13
Tracheophyta - ps14
Spermatophyta - ps15
Angiosperms - ps16
Mesangiospermae - ps17
Magnoliids/eudicots - ps18
Eudicots - ps19
Core_eudicots - ps20
Superrosids - ps21
Rosids - ps22
Eurosids - ps23
Malvids - ps24
Sap./Malv./Brassicales - ps25
Malvales/Brassicales - ps26
Brassicales - ps27
Cleomaceae/Brassicaceae - ps28
Brassicaceae - ps29
Camelineae - ps30
Arabidopsis - ps31
Arabidopsis_thaliana - ps32
p-value
4e-04
9e-04
2e-03
4e-03
8e-03
Log-odds
0.5
1.0
1.5
2.0
2.5
3.0
3.5
4.0

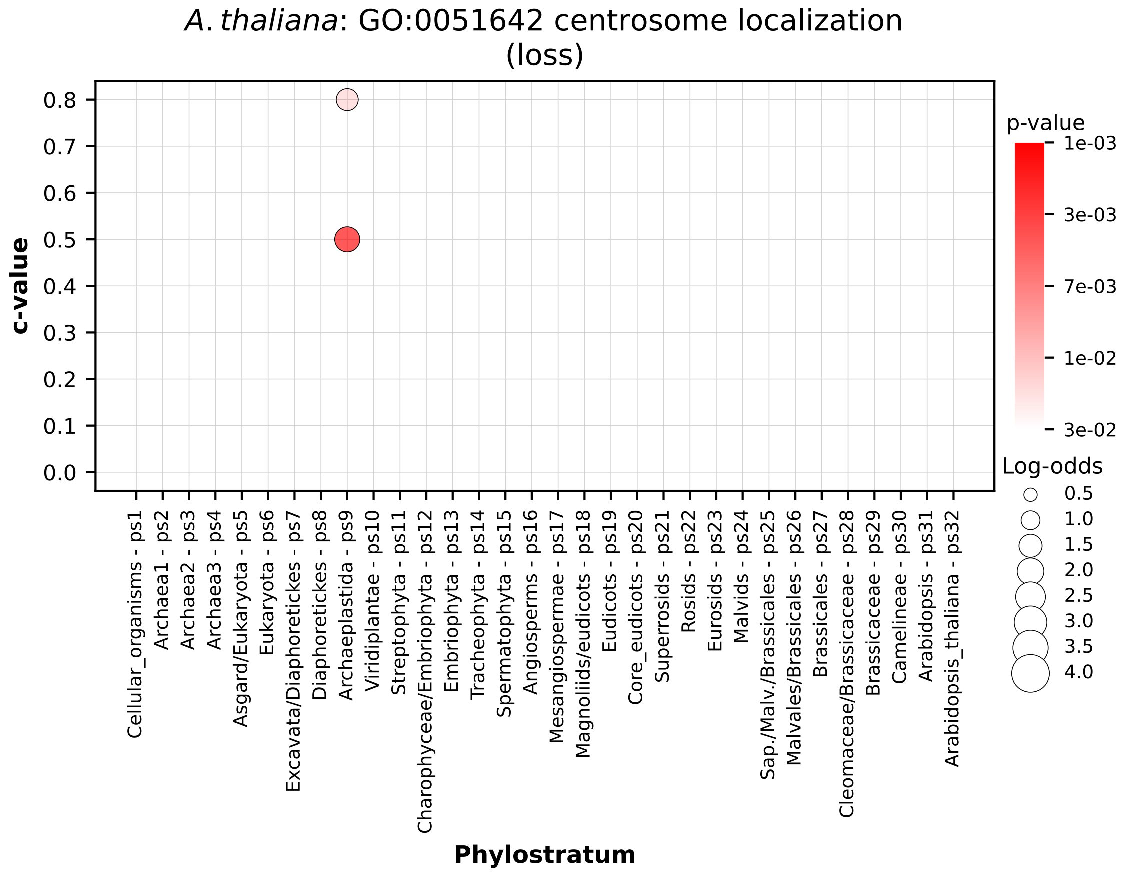
A. thaliana: GO:0051642 centrosome localization
(loss)
c-value
0.8
0.7
0.6
0.5
0.4
0.3
0.2
0.1
0.0
Phylostratum
Cellular_organisms - ps1
Archaea1 - ps2
Archaea2 - ps3
Archaea3 - ps4
Asgard/Eukaryota - ps5
Eukaryota - ps6
Excavata/Diaphoretickes - ps7
Diaphoretickes - ps8
Archaeplastida - ps9
Viridiplantae - ps10
Streptophyta - ps11
Charophyceae/Embriophyta - ps12
Embriophyta - ps13
Tracheophyta - ps14
Spermatophyta - ps15
Angiosperms - ps16
Mesangiospermae - ps17
Magnoliids/eudicots - ps18
Eudicots - ps19
Core_eudicots - ps20
Superrosids - ps21
Rosids - ps22
Eurosids - ps23
Malvids - ps24
Sap./Malv./Brassicales - ps25
Malvales/Brassicales - ps26
Brassicales - ps27
Cleomaceae/Brassicaceae - ps28
Brassicaceae - ps29
Camelineae - ps30
Arabidopsis - ps31
Arabidopsis_thaliana - ps32
p-value
1e-03
3e-03
7e-03
1e-02
3e-02
Log-odds
0.5
1.0
1.5
2.0
2.5
3.0
3.5
4.0

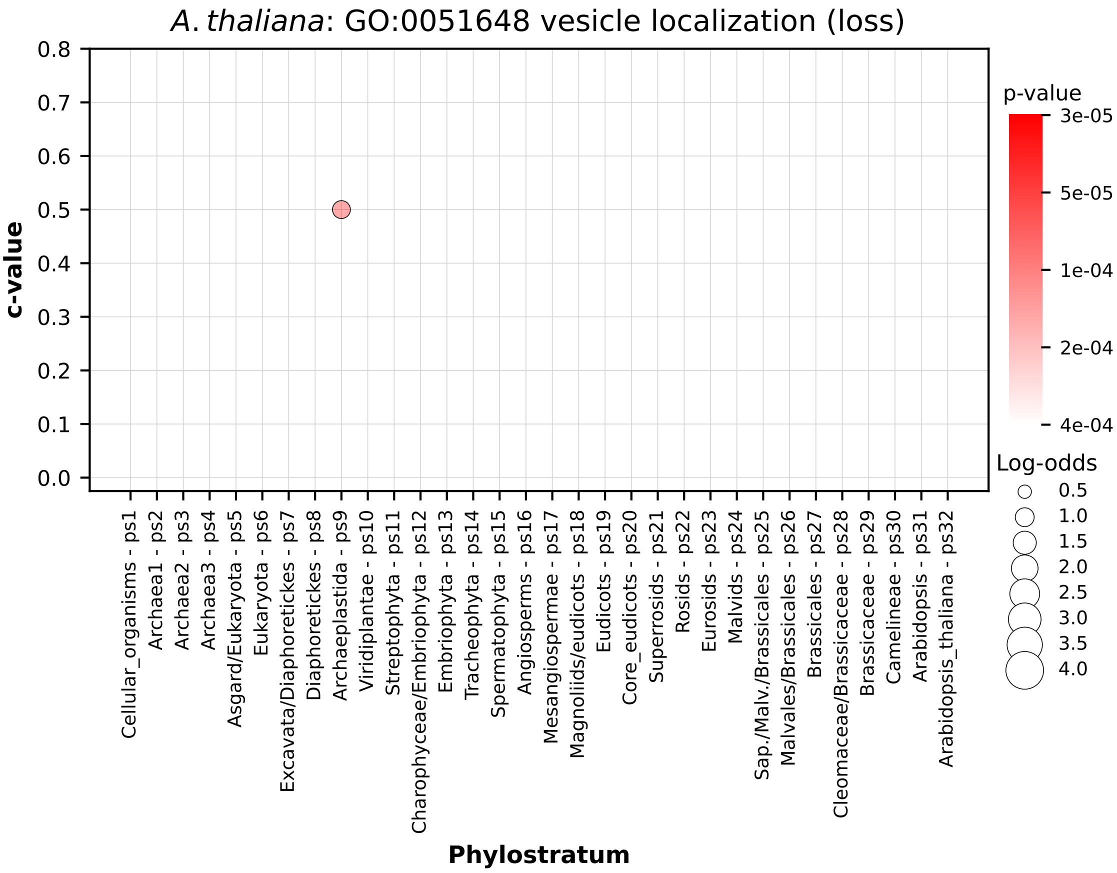

A. thaliana: GO:0051648 vesicle localization (loss)
c-value
0.0
0.1
0.2
0.3
0.4
0.5
0.6
0.7
0.8
p-value
3e-05
5e-05
1e-04
2e-04
4e-04
Log-odds
0.5
1.0
1.5
2.0
2.5
3.0
3.5
4.0
Cellular_organisms - ps1
Archaea1 - ps2
Archaea2 - ps3
Archaea3 - ps4
Asgard/Eukaryota - ps5
Eukaryota - ps6
Excavata/Diaphoretickes - ps7
Diaphoretickes - ps8
Archaeplastida - ps9
Viridiplantae - ps10
Streptophyta - ps11
Charophyceae/Embriophyta - ps12
Embriophyta - ps13
Tracheophyta - ps14
Spermatophyta - ps15
Angiosperms - ps16
Mesangiospermae - ps17
Magnoliids/eudicots - ps18
Eudicots - ps19
Core_eudicots - ps20
Superrosids - ps21
Rosids - ps22
Eurosids - ps23
Malvids - ps24
Sap./Malv./Brassicales - ps25
Malvales/Brassicales - ps26
Brassicales - ps27
Cleomaceae/Brassicaceae - ps28
Brassicaceae - ps29
Camelineae - ps30
Arabidopsis - ps31
Arabidopsis_thaliana - ps32
Phylostratum

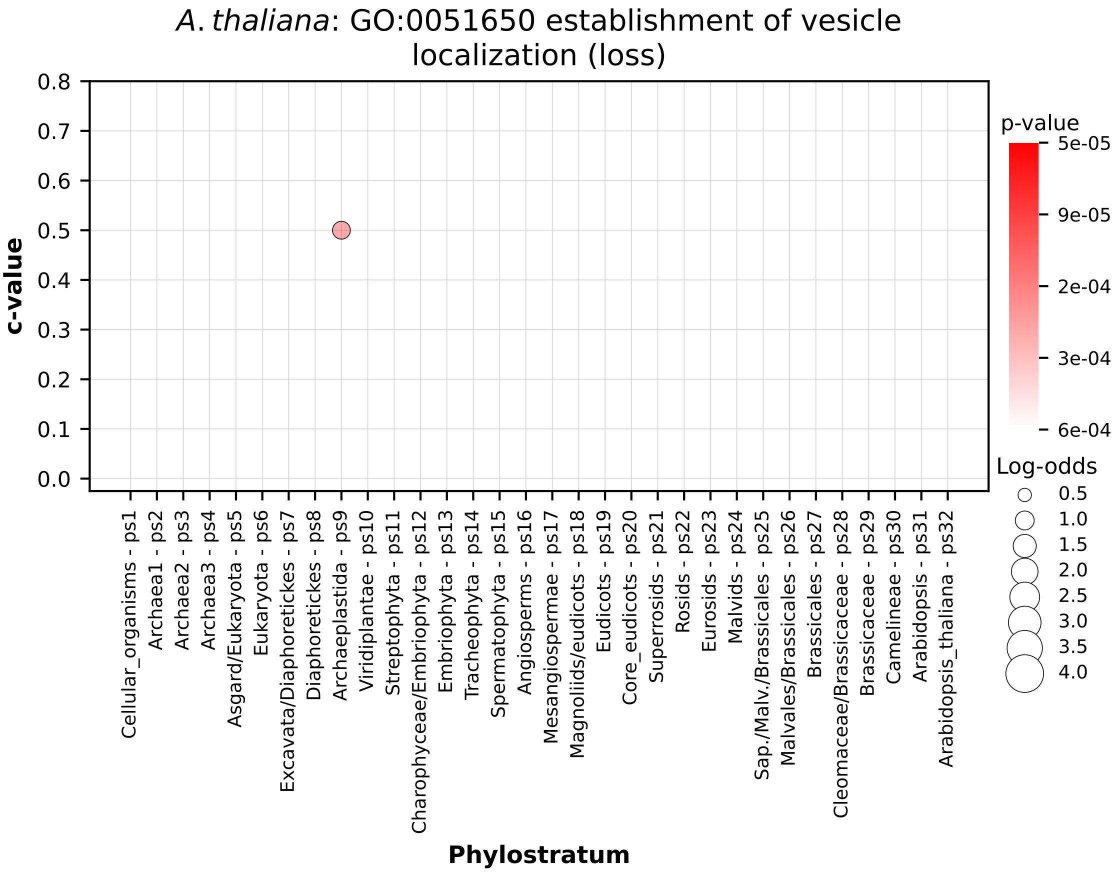

A. thaliana: GO:0051650 establishment of vesicle localization (loss)
c-value
0.0
0.1
0.2
0.3
0.4
0.5
0.6
0.7
0.8
Phylostratum
Cellular_organisms - ps1
Archaea1 - ps2
Archaea2 - ps3
Archaea3 - ps4
Asgard/Eukaryota - ps5
Eukaryota - ps6
Excavata/Diaphoretickes - ps7
Diaphoretickes - ps8
Archaeplastida - ps9
Viridiplantae - ps10
Streptophyta - ps11
Charophyceae/Embriophyta - ps12
Embriophyta - ps13
Tracheophyta - ps14
Spermatophyta - ps15
Angiosperms - ps16
Mesangiospermae - ps17
Magnoliids/eudicots - ps18
Eudicots - ps19
Core_eudicots - ps20
Superrosids - ps21
Rosids - ps22
Eurosids - ps23
Malvids - ps24
Sap./Malv./Brassicales - ps25
Malvales/Brassicales - ps26
Brassicales - ps27
Cleomaceae/Brassicaceae - ps28
Brassicaceae - ps29
Camelineae - ps30
Arabidopsis - ps31
Arabidopsis_thaliana - ps32
p-value
5e-05
9e-05
2e-04
3e-04
6e-04
Log-odds
0.5
1.0
1.5
2.0
2.5
3.0
3.5
4.0

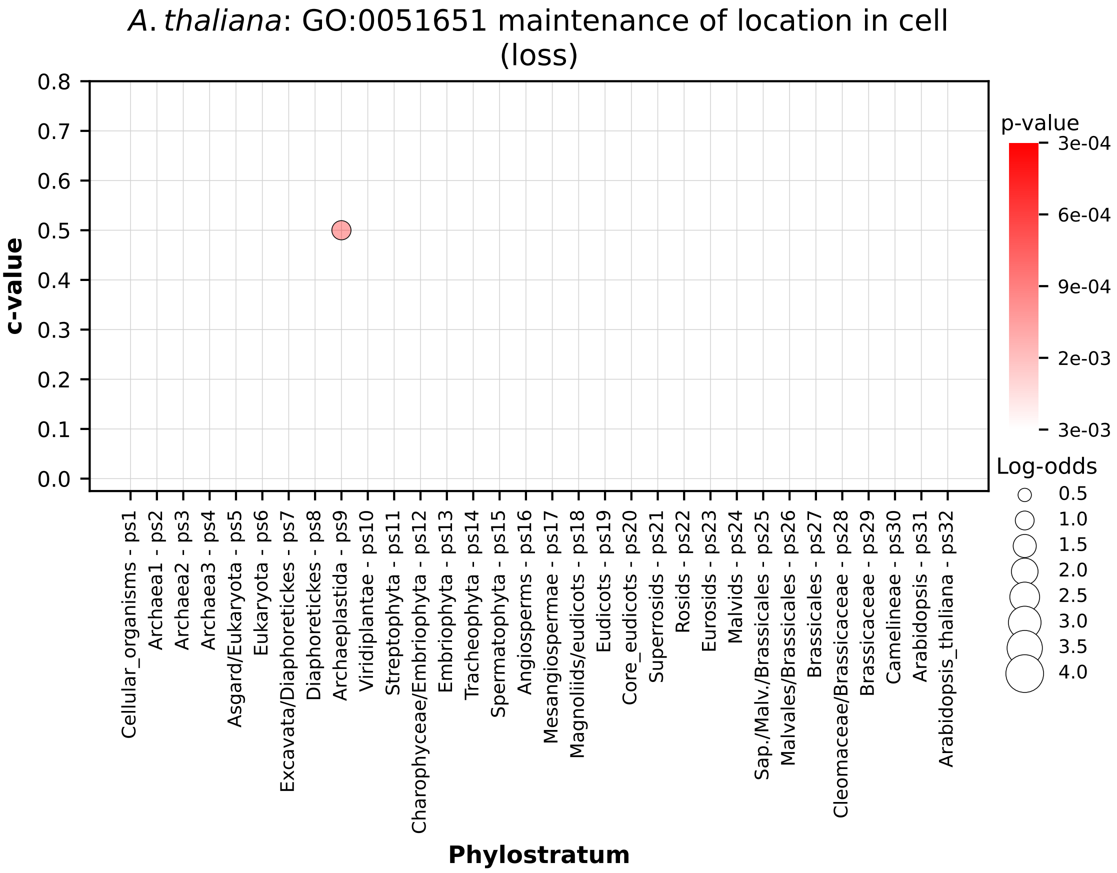
A. thaliana: GO:0051651 maintenance of location in cell (loss)
c-value
0.8
0.7
0.6
0.5
0.4
0.3
0.2
0.1
0.0
Phylostratum
Cellular_organisms - ps1
Archaea1 - ps2
Archaea2 - ps3
Archaea3 - ps4
Asgard/Eukaryota - ps5
Eukaryota - ps6
Excavata/Diaphoretickes - ps7
Diaphoretickes - ps8
Archaeplastida - ps9
Viridiplantae - ps10
Streptophyta - ps11
Charophyceae/Embriophyta - ps12
Embriophyta - ps13
Tracheophyta - ps14
Spermatophyta - ps15
Angiosperms - ps16
Mesangiospermae - ps17
Magnoliids/eudicots - ps18
Eudicots - ps19
Core_eudicots - ps20
Superrosids - ps21
Rosids - ps22
Eurosids - ps23
Malvids - ps24
Sap./Malv./Brassicales - ps25
Malvales/Brassicales - ps26
Brassicales - ps27
Cleomaceae/Brassicaceae - ps28
Brassicaceae - ps29
Camelineae - ps30
Arabidopsis - ps31
Arabidopsis_thaliana - ps32
p-value
3e-04
6e-04
9e-04
2e-03
3e-03
Log-odds
0.5
1.0
1.5
2.0
2.5
3.0
3.5
4.0

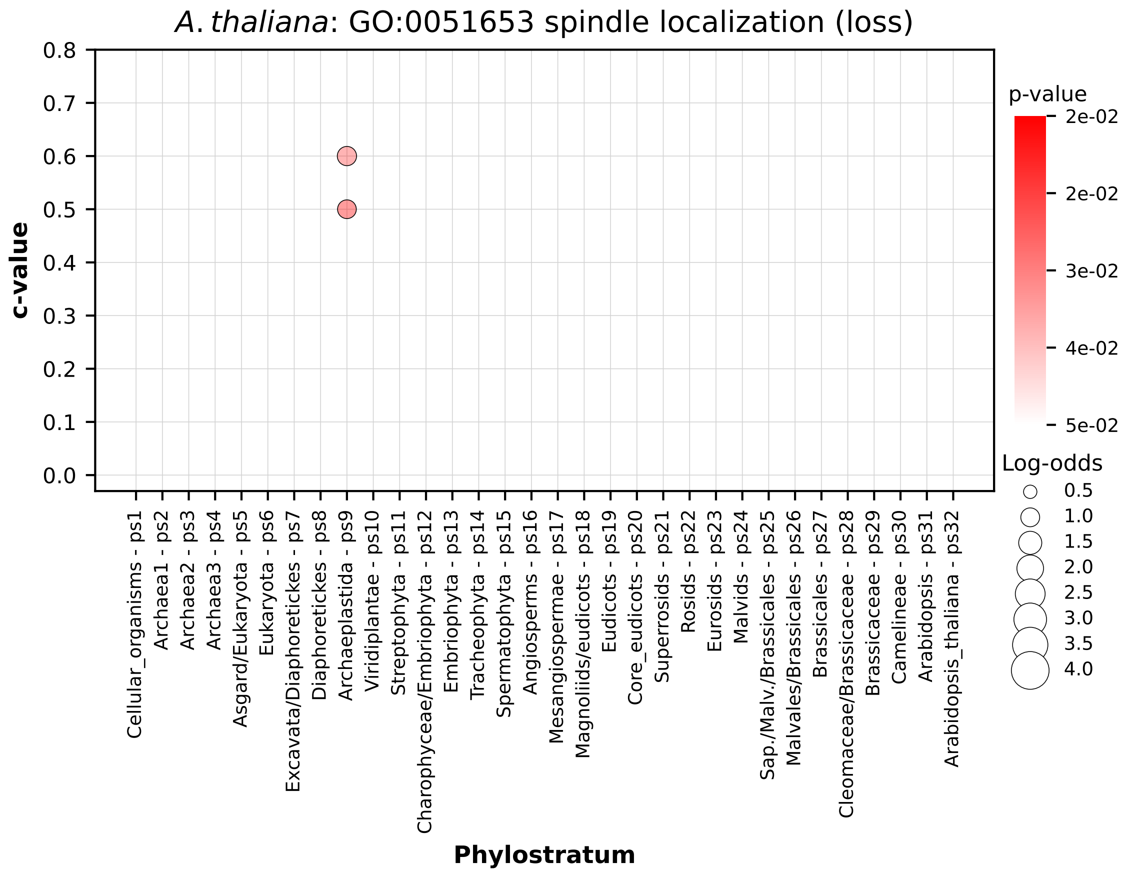

A. thaliana: GO:0051653 spindle localization (loss)
c-value
0.8
0.7
0.6
0.5
0.4
0.3
0.2
0.1
0.0
Phylostratum
Cellular_organisms - ps1
Archaea1 - ps2
Archaea2 - ps3
Archaea3 - ps4
Asgard/Eukaryota - ps5
Eukaryota - ps6
Excavata/Diaphoretickes - ps7
Diaphoretickes - ps8
Archaeplastida - ps9
Viridiplantae - ps10
Streptophyta - ps11
Charophyceae/Embriophyta - ps12
Embriophyta - ps13
Tracheophyta - ps14
Spermatophyta - ps15
Angiosperms - ps16
Mesangiospermae - ps17
Magnoliids/eudicots - ps18
Eudicots - ps19
Core_eudicots - ps20
Superrosids - ps21
Rosids - ps22
Eurosids - ps23
Malvids - ps24
Sap./Malv./Brassicales - ps25
Malvales/Brassicales - ps26
Brassicales - ps27
Cleomaceae/Brassicaceae - ps28
Brassicaceae - ps29
Camelineae - ps30
Arabidopsis - ps31
Arabidopsis_thaliana - ps32
p-value
2e-02
2e-02
3e-02
4e-02
5e-02
Log-odds
0.5
1.0
1.5
2.0
2.5
3.0
3.5
4.0

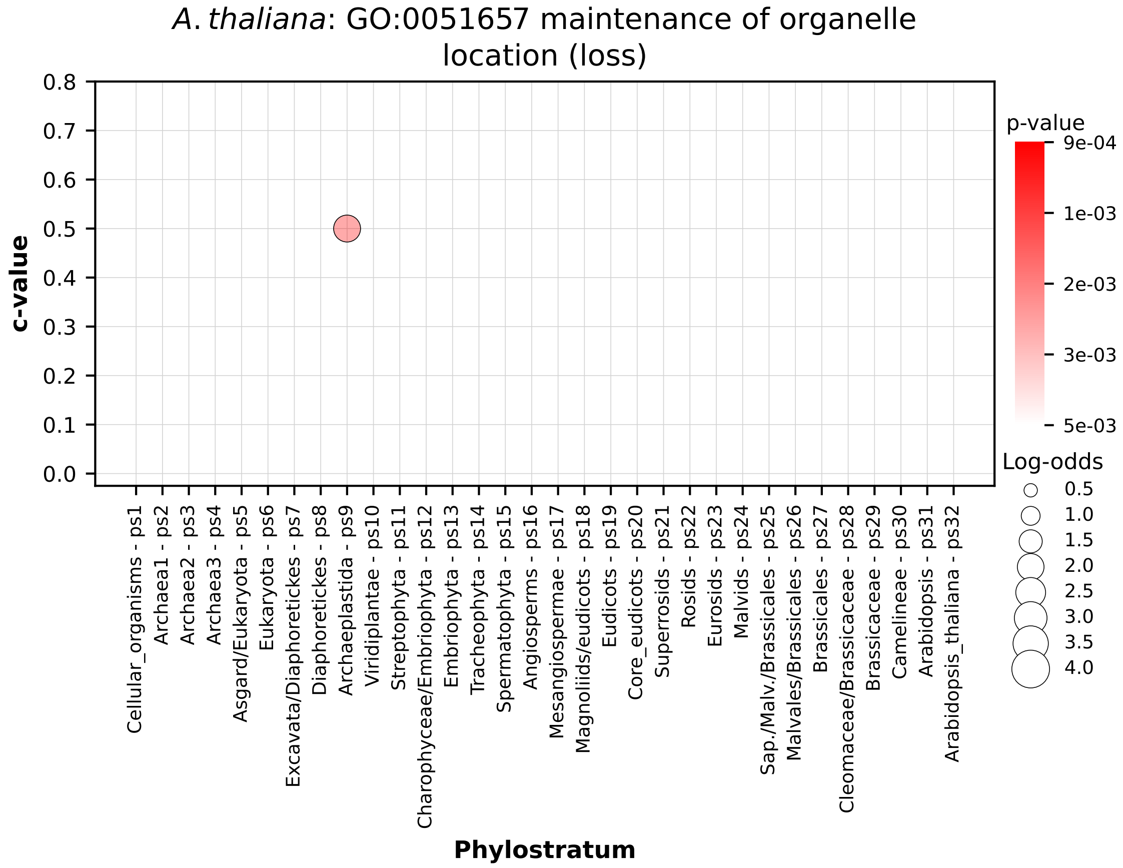
A. thaliana: GO:0051657 maintenance of organelle location (loss)
c-value
0.8
0.7
0.6
0.5
0.4
0.3
0.2
0.1
0.0
p-value
9e-04
1e-03
2e-03
3e-03
5e-03
Log-odds
0.5
1.0
1.5
2.0
2.5
3.0
3.5
4.0
Cellular_organisms - ps1
Archaea1 - ps2
Archaea2 - ps3
Archaea3 - ps4
Asgard/Eukaryota - ps5
Eukaryota - ps6
Excavata/Diaphoretickes - ps7
Diaphoretickes - ps8
Archaeplastida - ps9
Viridiplantae - ps10
Streptophyta - ps11
Charophyceae/Embriophyta - ps12
Embriophyta - ps13
Tracheophyta - ps14
Spermatophyta - ps15
Angiosperms - ps16
Mesangiospermae - ps17
Magnoliids/eudicots - ps18
Eudicots - ps19
Core_eudicots - ps20
Superrosids - ps21
Rosids - ps22
Eurosids - ps23
Malvids - ps24
Sap./Malv./Brassicales - ps25
Malvales/Brassicales - ps26
Brassicales - ps27
Cleomaceae/Brassicaceae - ps28
Brassicaceae - ps29
Camelineae - ps30
Arabidopsis - ps31
Arabidopsis_thaliana - ps32
Phylostratum

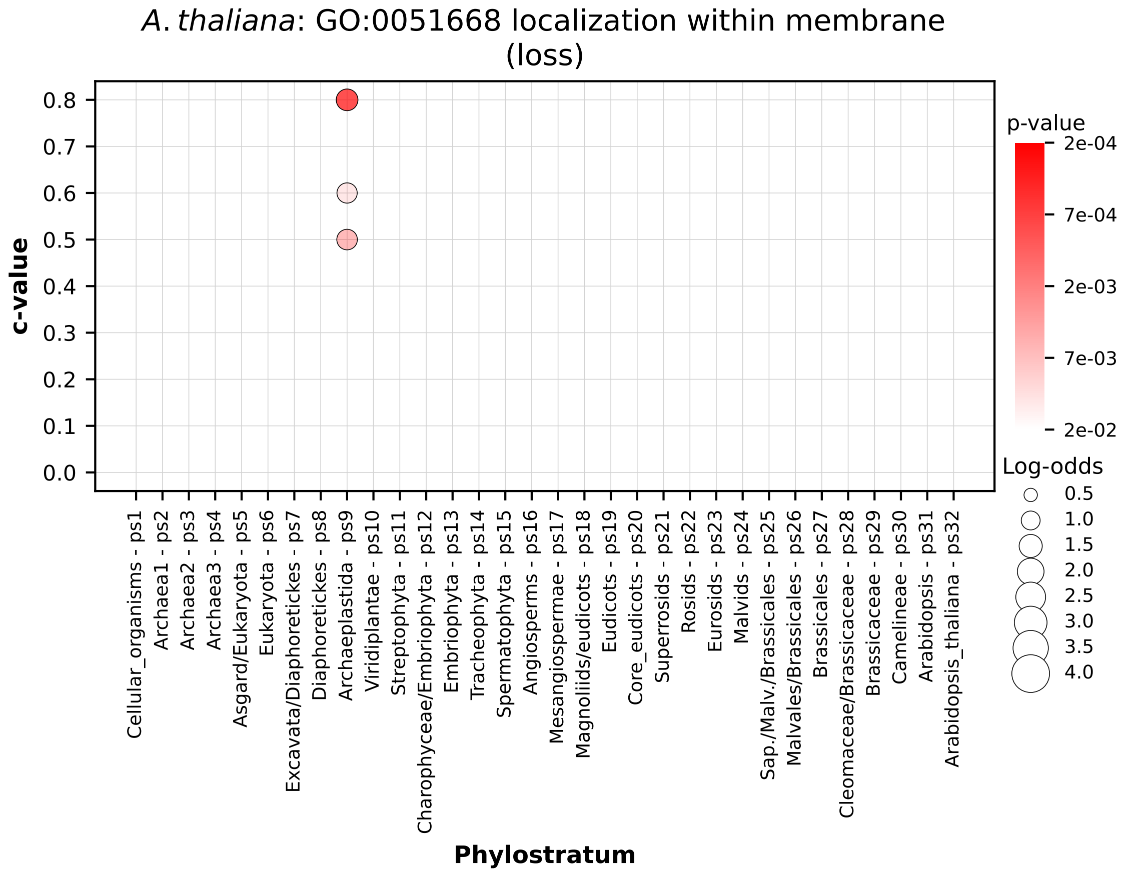

A. thaliana: GO:0051668 localization within membrane (loss)
c-value
0.8
0.7
0.6
0.5
0.4
0.3
0.2
0.1
0.0
p-value
2e-04
7e-04
2e-03
7e-03
2e-02
Log-odds
0.5
1.0
1.5
2.0
2.5
3.0
3.5
4.0
Cellular_organisms - ps1
Archaea1 - ps2
Archaea2 - ps3
Archaea3 - ps4
Asgard/Eukaryota - ps5
Eukaryota - ps6
Excavata/Diaphoretickes - ps7
Diaphoretickes - ps8
Archaeplastida - ps9
Viridiplantae - ps10
Streptophyta - ps11
Charophyceae/Embriophyta - ps12
Embriophyta - ps13
Tracheophyta - ps14
Spermatophyta - ps15
Angiosperms - ps16
Mesangiospermae - ps17
Magnoliids/eudicots - ps18
Eudicots - ps19
Core_eudicots - ps20
Superrosids - ps21
Rosids - ps22
Eurosids - ps23
Malvids - ps24
Sap./Malv./Brassicales - ps25
Malvales/Brassicales - ps26
Brassicales - ps27
Cleomaceae/Brassicaceae - ps28
Brassicaceae - ps29
Camelineae - ps30
Arabidopsis - ps31
Arabidopsis_thaliana - ps32
Phylostratum

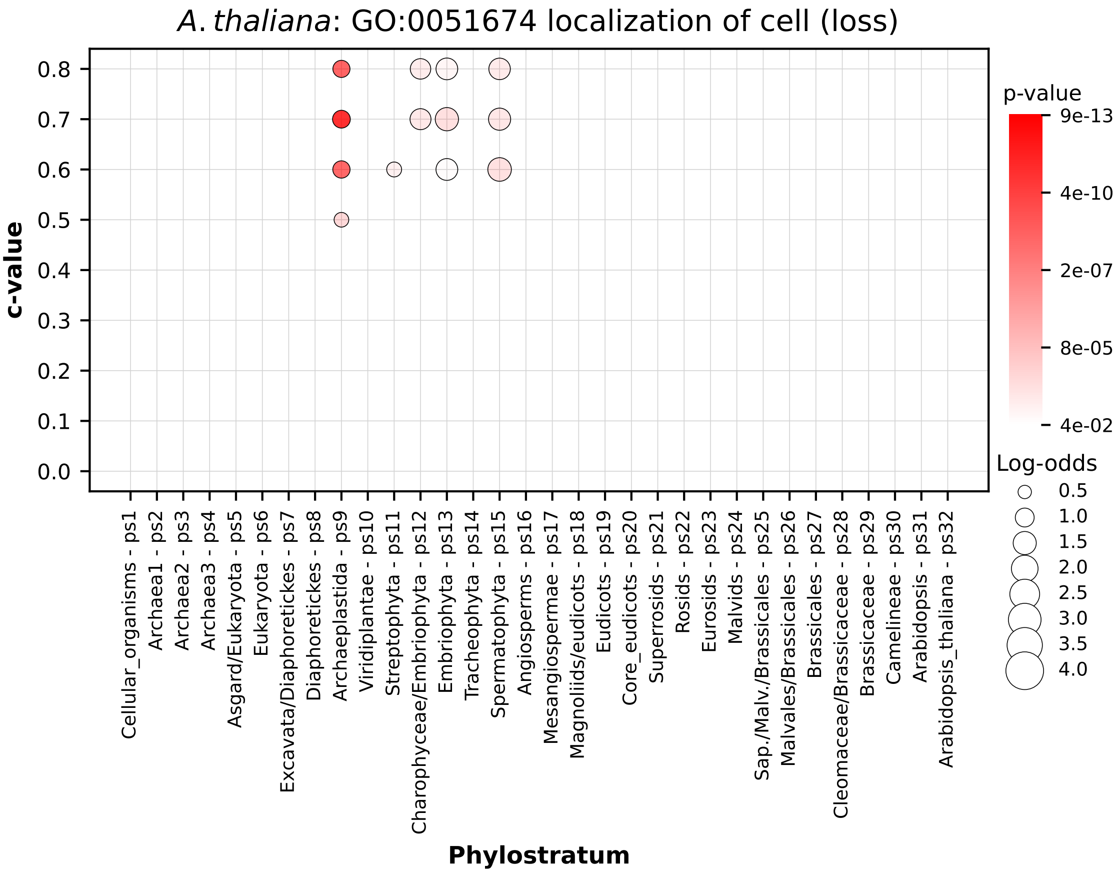

A. thaliana: GO:0051674 localization of cell (loss)
c-value
0.8
0.7
0.6
0.5
0.4
0.3
0.2
0.1
0.0
p-value
9e-13
4e-10
2e-07
8e-05
4e-02
Log-odds
0.5
1.0
1.5
2.0
2.5
3.0
3.5
4.0
Cellular_organisms - ps1
Archaea1 - ps2
Archaea2 - ps3
Archaea3 - ps4
Asgard/Eukaryota - ps5
Eukaryota - ps6
Excavata/Diaphoretickes - ps7
Diaphoretickes - ps8
Archaeplastida - ps9
Viridiplantae - ps10
Streptophyta - ps11
Charophyceae/Embriophyta - ps12
Embriophyta - ps13
Tracheophyta - ps14
Spermatophyta - ps15
Angiosperms - ps16
Mesangiospermae - ps17
Magnoliids/eudicots - ps18
Eudicots - ps19
Core_eudicots - ps20
Superrosids - ps21
Rosids - ps22
Eurosids - ps23
Malvids - ps24
Sap./Malv./Brassicales - ps25
Malvales/Brassicales - ps26
Brassicales - ps27
Cleomaceae/Brassicaceae - ps28
Brassicaceae - ps29
Camelineae - ps30
Arabidopsis - ps31
Arabidopsis_thaliana - ps32
Phylostratum

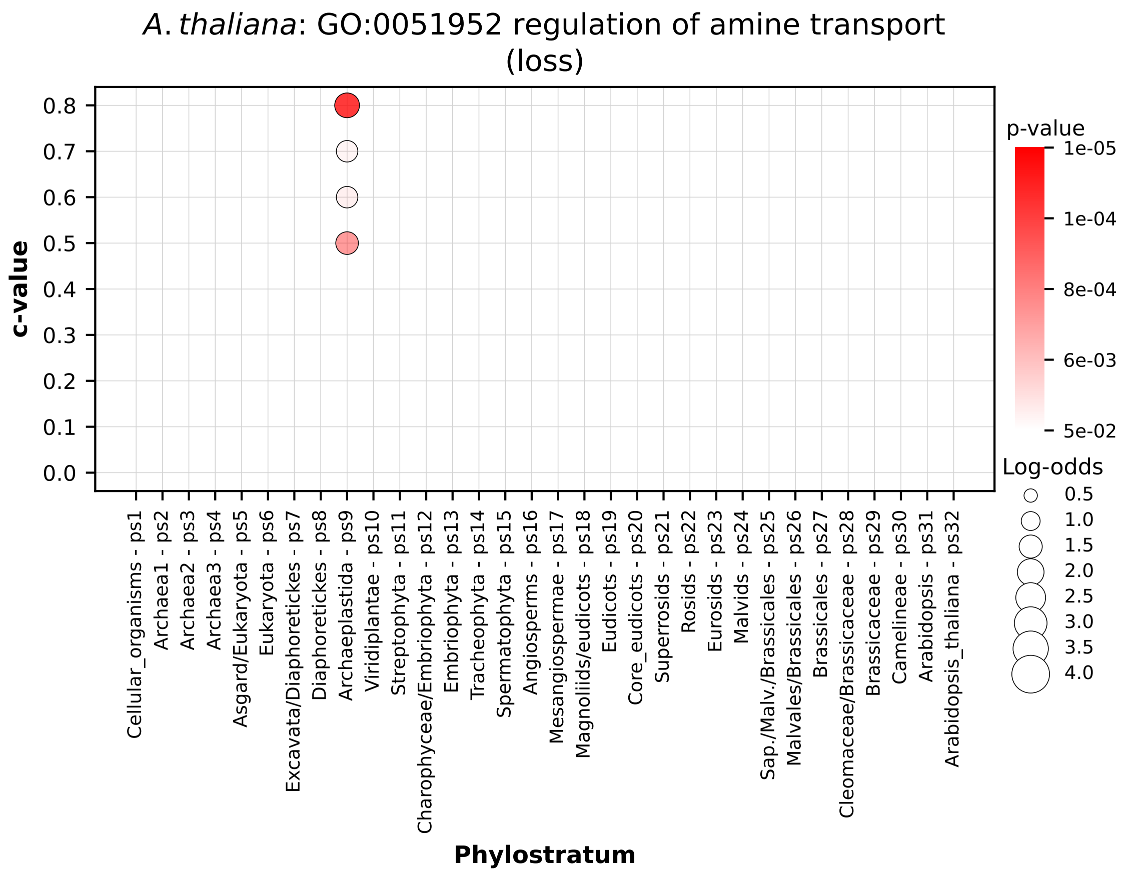

A. thaliana: GO:0051952 regulation of amine transport (loss)
c-value
0.8
0.7
0.6
0.5
0.4
0.3
0.2
0.1
0.0
p-value
1e-05
1e-04
8e-04
6e-03
5e-02
Log-odds
0.5
1.0
1.5
2.0
2.5
3.0
3.5
4.0
Cellular_organisms - ps1
Archaea1 - ps2
Archaea2 - ps3
Archaea3 - ps4
Asgard/Eukaryota - ps5
Eukaryota - ps6
Excavata/Diaphoretickes - ps7
Diaphoretickes - ps8
Archaeplastida - ps9
Viridiplantae - ps10
Streptophyta - ps11
Charophyceae/Embriophyta - ps12
Embriophyta - ps13
Tracheophyta - ps14
Spermatophyta - ps15
Angiosperms - ps16
Mesangiospermae - ps17
Magnoliids/eudicots - ps18
Eudicots - ps19
Core_eudicots - ps20
Superrosids - ps21
Rosids - ps22
Eurosids - ps23
Malvids - ps24
Sap./Malv./Brassicales - ps25
Malvales/Brassicales - ps26
Brassicales - ps27
Cleomaceae/Brassicaceae - ps28
Brassicaceae - ps29
Camelineae - ps30
Arabidopsis - ps31
Arabidopsis_thaliana - ps32
Phylostratum

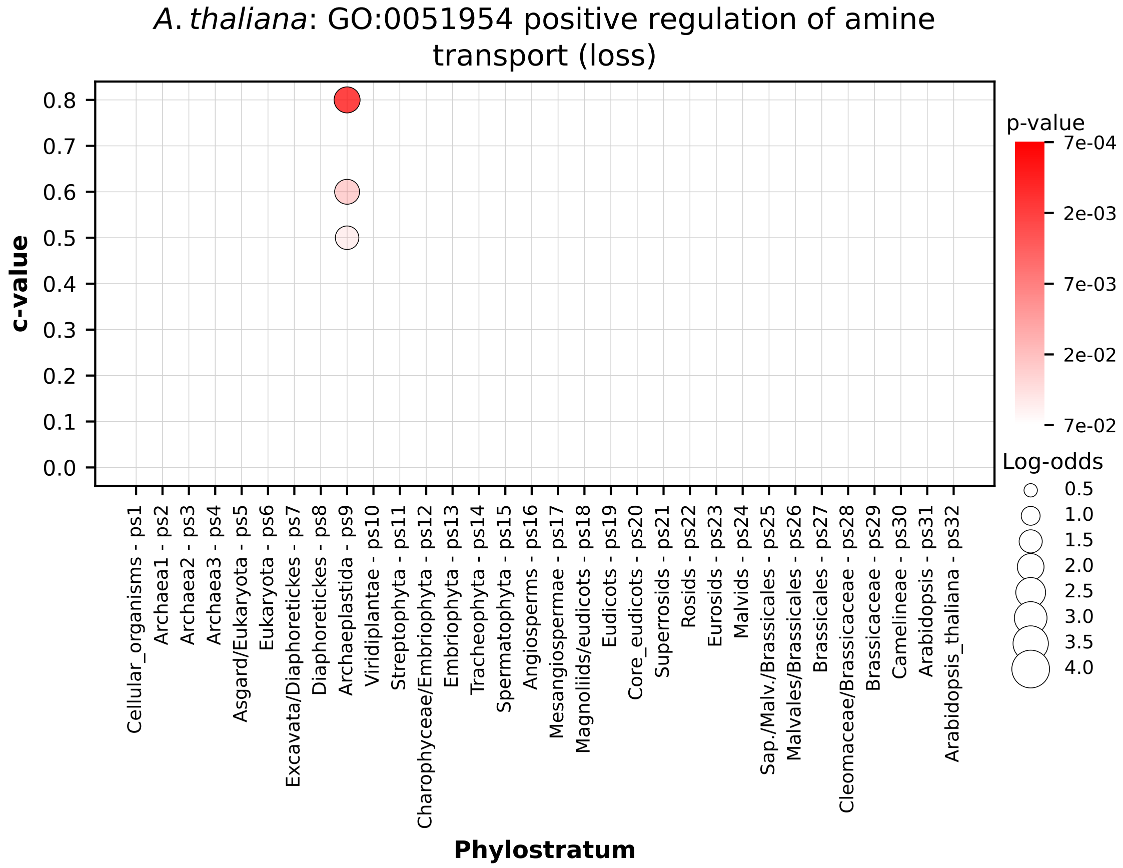

A. thaliana: GO:0051954 positive regulation of amine transport (loss)
c-value
0.0
0.1
0.2
0.3
0.4
0.5
0.6
0.7
0.8
p-value
7e-04
2e-03
7e-03
2e-02
7e-02
Log-odds
0.5
1.0
1.5
2.0
2.5
3.0
3.5
4.0
Cellular_organisms - ps1
Archaea1 - ps2
Archaea2 - ps3
Archaea3 - ps4
Asgard/Eukaryota - ps5
Eukaryota - ps6
Excavata/Diaphoretickes - ps7
Diaphoretickes - ps8
Archaeplastida - ps9
Viridiplantae - ps10
Streptophyta - ps11
Charophyceae/Embriophyta - ps12
Embriophyta - ps13
Tracheophyta - ps14
Spermatophyta - ps15
Angiosperms - ps16
Mesangiospermae - ps17
Magnoliids/eudicots - ps18
Eudicots - ps19
Core_eudicots - ps20
Superrosids - ps21
Rosids - ps22
Eurosids - ps23
Malvids - ps24
Sap./Malv./Brassicales - ps25
Malvales/Brassicales - ps26
Brassicales - ps27
Cleomaceae/Brassicaceae - ps28
Brassicaceae - ps29
Camelineae - ps30
Arabidopsis - ps31
Arabidopsis_thaliana - ps32
Phylostratum

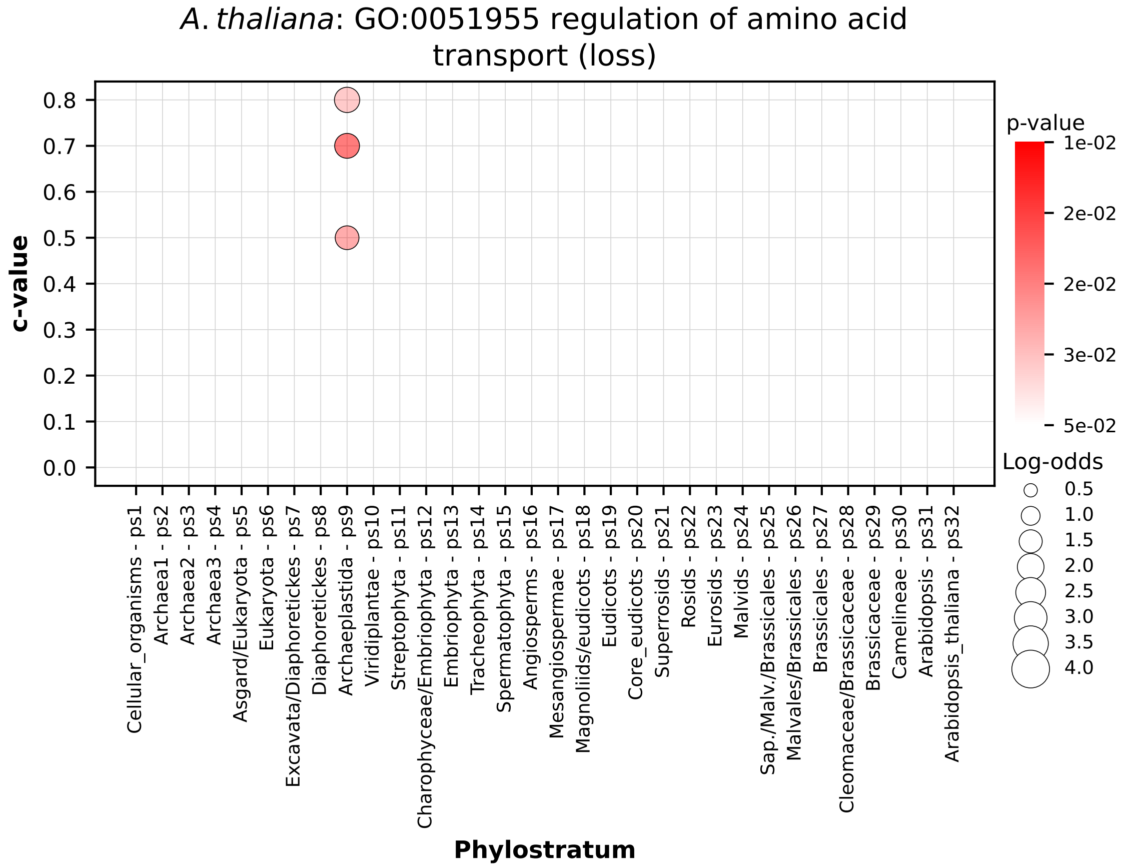

A. thaliana: GO:0051955 regulation of amino acid transport (loss)
c-value
0.0
0.1
0.2
0.3
0.4
0.5
0.6
0.7
0.8
p-value
1e-02
2e-02
2e-02
3e-02
5e-02
Log-odds
0.5
1.0
1.5
2.0
2.5
3.0
3.5
4.0
Cellular_organisms - ps1
Archaea1 - ps2
Archaea2 - ps3
Archaea3 - ps4
Asgard/Eukaryota - ps5
Eukaryota - ps6
Excavata/Diaphoretickes - ps7
Diaphoretickes - ps8
Archaeplastida - ps9
Viridiplantae - ps10
Streptophyta - ps11
Charophyceae/Embriophyta - ps12
Embriophyta - ps13
Tracheophyta - ps14
Spermatophyta - ps15
Angiosperms - ps16
Mesangiospermae - ps17
Magnoliids/eudicots - ps18
Eudicots - ps19
Core_eudicots - ps20
Superrosids - ps21
Rosids - ps22
Eurosids - ps23
Malvids - ps24
Sap./Malv./Brassicales - ps25
Malvales/Brassicales - ps26
Brassicales - ps27
Cleomaceae/Brassicaceae - ps28
Brassicaceae - ps29
Camelineae - ps30
Arabidopsis - ps31
Arabidopsis_thaliana - ps32
Phylostratum

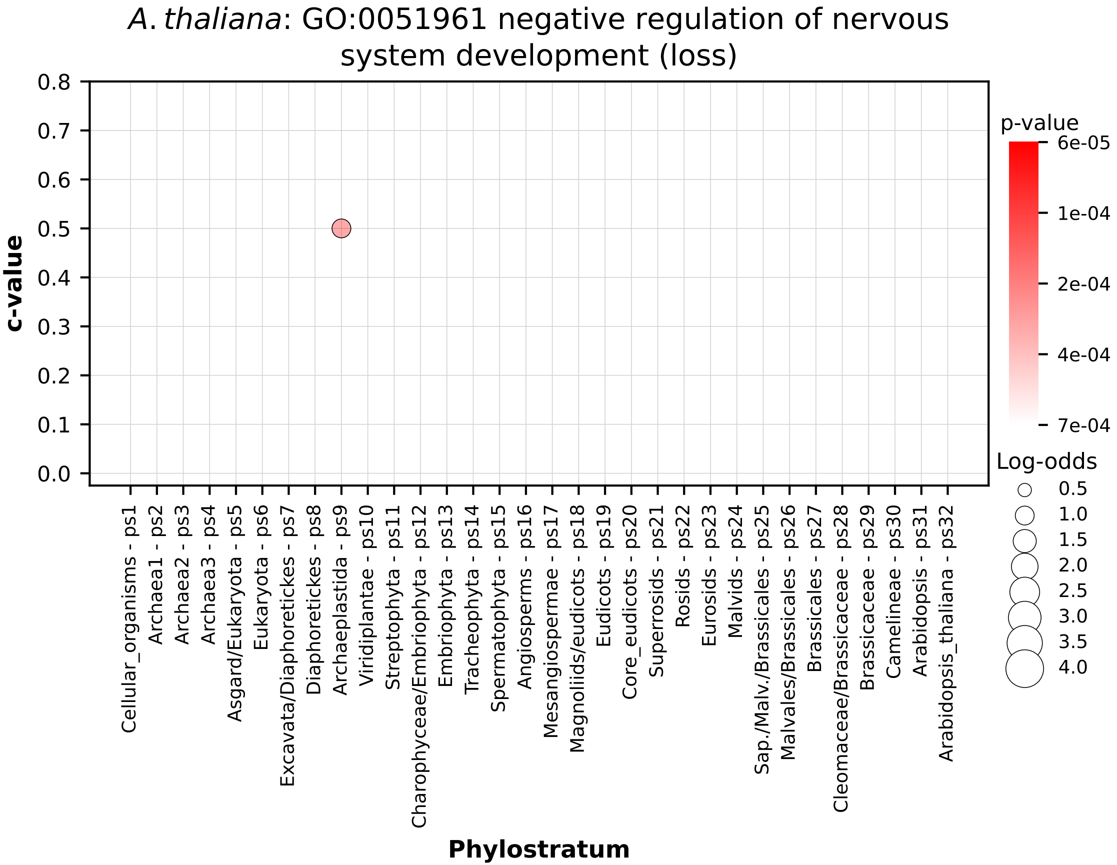

A. thaliana: GO:0051961 negative regulation of nervous system development (loss)
c-value
0.8
0.7
0.6
0.5
0.4
0.3
0.2
0.1
0.0
Phylostratum
Cellular_organisms - ps1
Archaea1 - ps2
Archaea2 - ps3
Archaea3 - ps4
Asgard/Eukaryota - ps5
Eukaryota - ps6
Excavata/Diaphoretickes - ps7
Diaphoretickes - ps8
Archaeplastida - ps9
Viridiplantae - ps10
Streptophyta - ps11
Charophyceae/Embriophyta - ps12
Embriophyta - ps13
Tracheophyta - ps14
Spermatophyta - ps15
Angiosperms - ps16
Mesangiospermae - ps17
Magnoliids/eudicots - ps18
Eudicots - ps19
Core_eudicots - ps20
Superrosids - ps21
Rosids - ps22
Eurosids - ps23
Malvids - ps24
Sap./Malv./Brassicales - ps25
Malvales/Brassicales - ps26
Brassicales - ps27
Cleomaceae/Brassicaceae - ps28
Brassicaceae - ps29
Camelineae - ps30
Arabidopsis - ps31
Arabidopsis_thaliana - ps32
p-value
6e-05
1e-04
2e-04
4e-04
7e-04
Log-odds
0.5
1.0
1.5
2.0
2.5
3.0
3.5
4.0

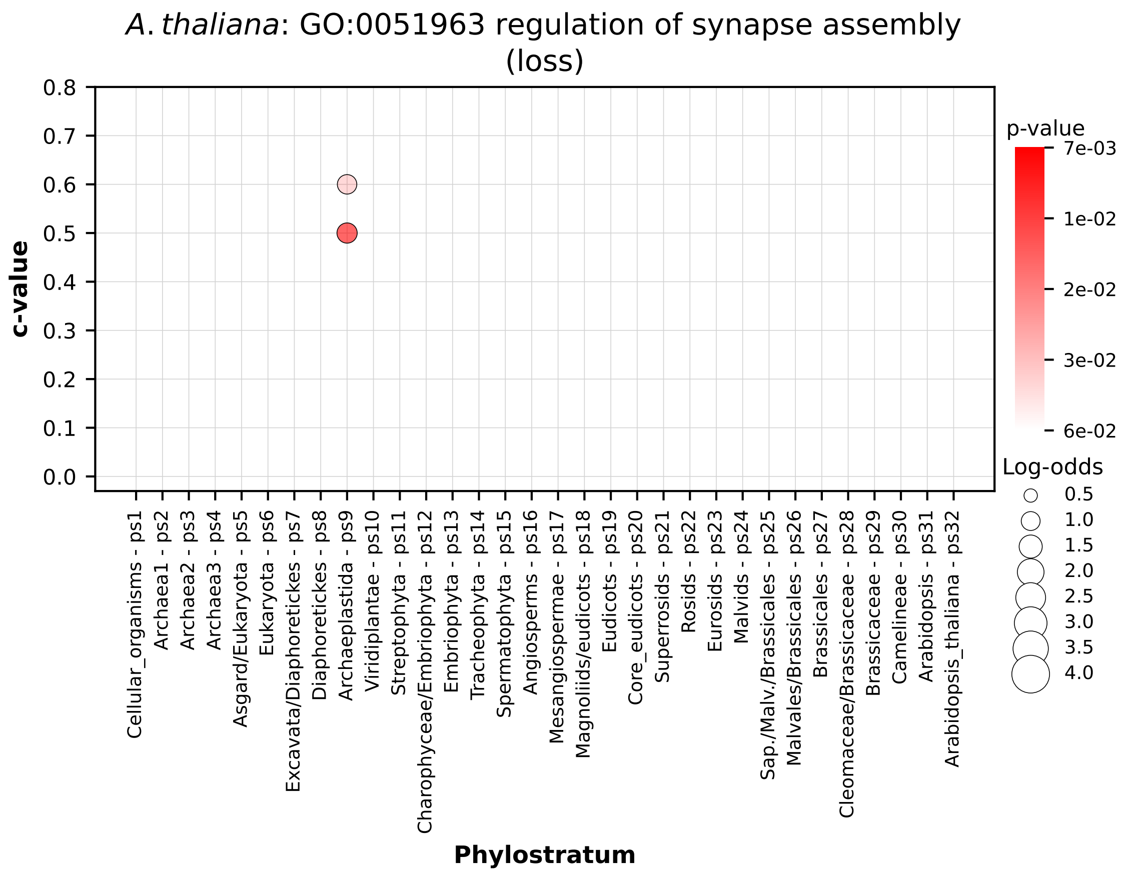
A. thaliana: GO:0051963 regulation of synapse assembly (loss)
c-value
0.0
0.1
0.2
0.3
0.4
0.5
0.6
0.7
0.8
Phylostratum
Cellular_organisms - ps1
Archaea1 - ps2
Archaea2 - ps3
Archaea3 - ps4
Asgard/Eukaryota - ps5
Eukaryota - ps6
Excavata/Diaphoretickes - ps7
Diaphoretickes - ps8
Archaeplastida - ps9
Viridiplantae - ps10
Streptophyta - ps11
Charophyceae/Embriophyta - ps12
Embriophyta - ps13
Tracheophyta - ps14
Spermatophyta - ps15
Angiosperms - ps16
Mesangiospermae - ps17
Magnoliids/eudicots - ps18
Eudicots - ps19
Core_eudicots - ps20
Superrosids - ps21
Rosids - ps22
Eurosids - ps23
Malvids - ps24
Sap./Malv./Brassicales - ps25
Malvales/Brassicales - ps26
Brassicales - ps27
Cleomaceae/Brassicaceae - ps28
Brassicaceae - ps29
Camelineae - ps30
Arabidopsis - ps31
Arabidopsis_thaliana - ps32
p-value
7e-03
1e-02
2e-02
3e-02
6e-02
Log-odds
0.5
1.0
1.5
2.0
2.5
3.0
3.5
4.0

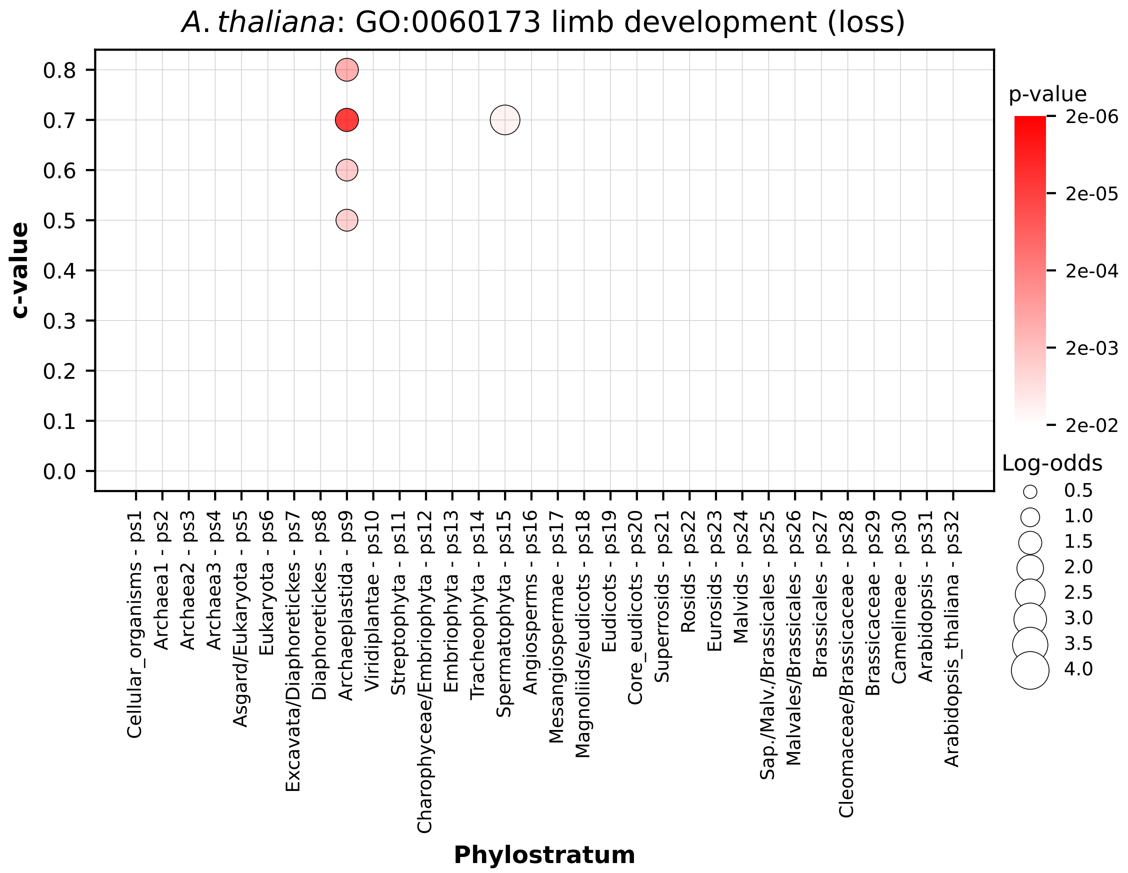
A. thaliana: GO:0060173 limb development (loss)
c-value
0.8
0.7
0.6
0.5
0.4
0.3
0.2
0.1
0.0
p-value
2e-06
2e-05
2e-04
2e-03
2e-02
Log-odds
0.5
1.0
1.5
2.0
2.5
3.0
3.5
4.0
Cellular_organisms - ps1
Archaea1 - ps2
Archaea2 - ps3
Archaea3 - ps4
Asgard/Eukaryota - ps5
Eukaryota - ps6
Excavata/Diaphoretickes - ps7
Diaphoretickes - ps8
Archaeplastida - ps9
Viridiplantae - ps10
Streptophyta - ps11
Charophyceae/Embriophyta - ps12
Embriophyta - ps13
Tracheophyta - ps14
Spermatophyta - ps15
Angiosperms - ps16
Mesangiospermae - ps17
Magnoliids/eudicots - ps18
Eudicots - ps19
Core_eudicots - ps20
Superrosids - ps21
Rosids - ps22
Eurosids - ps23
Malvids - ps24
Sap./Malv./Brassicales - ps25
Malvales/Brassicales - ps26
Brassicales - ps27
Cleomaceae/Brassicaceae - ps28
Brassicaceae - ps29
Camelineae - ps30
Arabidopsis - ps31
Arabidopsis_thaliana - ps32
Phylostratum

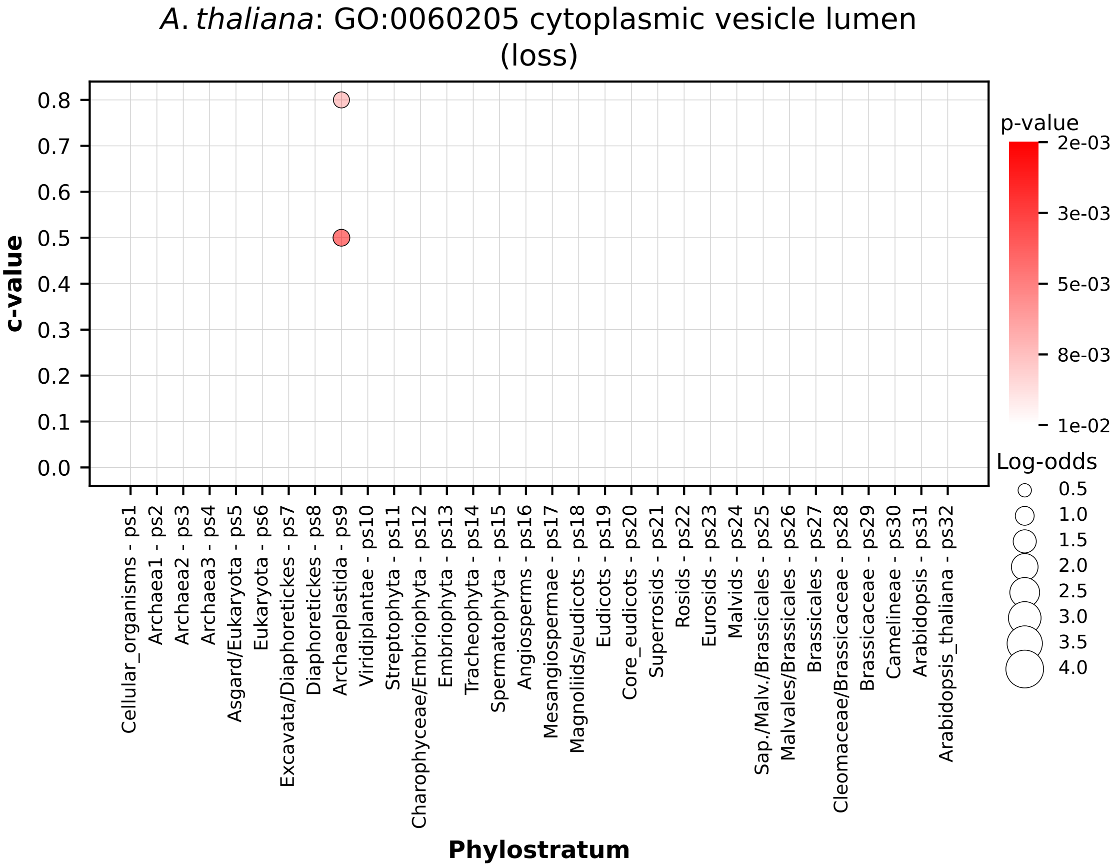

A. thaliana: GO:0060205 cytoplasmic vesicle lumen
(loss)
c-value
0.8
0.7
0.6
0.5
0.4
0.3
0.2
0.1
0.0
p-value
2e-03
3e-03
5e-03
8e-03
1e-02
Log-odds
0.5
1.0
1.5
2.0
2.5
3.0
3.5
4.0
Cellular_organisms - ps1
Archaea1 - ps2
Archaea2 - ps3
Archaea3 - ps4
Asgard/Eukaryota - ps5
Eukaryota - ps6
Excavata/Diaphoretickes - ps7
Diaphoretickes - ps8
Archaeplastida - ps9
Viridiplantae - ps10
Streptophyta - ps11
Charophyceae/Embriophyta - ps12
Embriophyta - ps13
Tracheophyta - ps14
Spermatophyta - ps15
Angiosperms - ps16
Mesangiospermae - ps17
Magnoliids/eudicots - ps18
Eudicots - ps19
Core_eudicots - ps20
Superrosids - ps21
Rosids - ps22
Eurosids - ps23
Malvids - ps24
Sap./Malv./Brassicales - ps25
Malvales/Brassicales - ps26
Brassicales - ps27
Cleomaceae/Brassicaceae - ps28
Brassicaceae - ps29
Camelineae - ps30
Arabidopsis - ps31
Arabidopsis_thaliana - ps32
Phylostratum

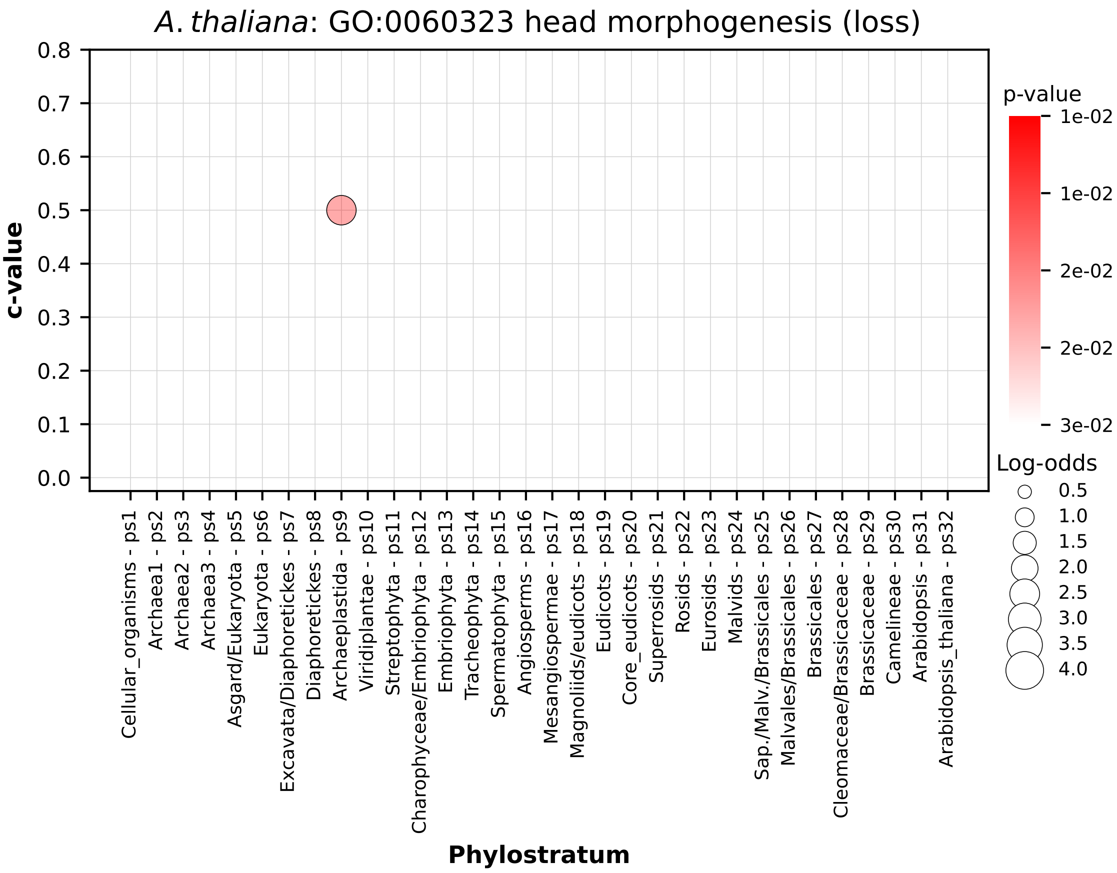

A. thaliana: GO:0060323 head morphogenesis (loss)
c-value
0.8
0.7
0.6
0.5
0.4
0.3
0.2
0.1
0.0
p-value
1e-02
1e-02
2e-02
2e-02
3e-02
Log-odds
0.5
1.0
1.5
2.0
2.5
3.0
3.5
4.0
Cellular_organisms - ps1
Archaea1 - ps2
Archaea2 - ps3
Archaea3 - ps4
Asgard/Eukaryota - ps5
Eukaryota - ps6
Excavata/Diaphoretickes - ps7
Diaphoretickes - ps8
Archaeplastida - ps9
Viridiplantae - ps10
Streptophyta - ps11
Charophyceae/Embriophyta - ps12
Embriophyta - ps13
Tracheophyta - ps14
Spermatophyta - ps15
Angiosperms - ps16
Mesangiospermae - ps17
Magnoliids/eudicots - ps18
Eudicots - ps19
Core_eudicots - ps20
Superrosids - ps21
Rosids - ps22
Eurosids - ps23
Malvids - ps24
Sap./Malv./Brassicales - ps25
Malvales/Brassicales - ps26
Brassicales - ps27
Cleomaceae/Brassicaceae - ps28
Brassicaceae - ps29
Camelineae - ps30
Arabidopsis - ps31
Arabidopsis_thaliana - ps32
Phylostratum

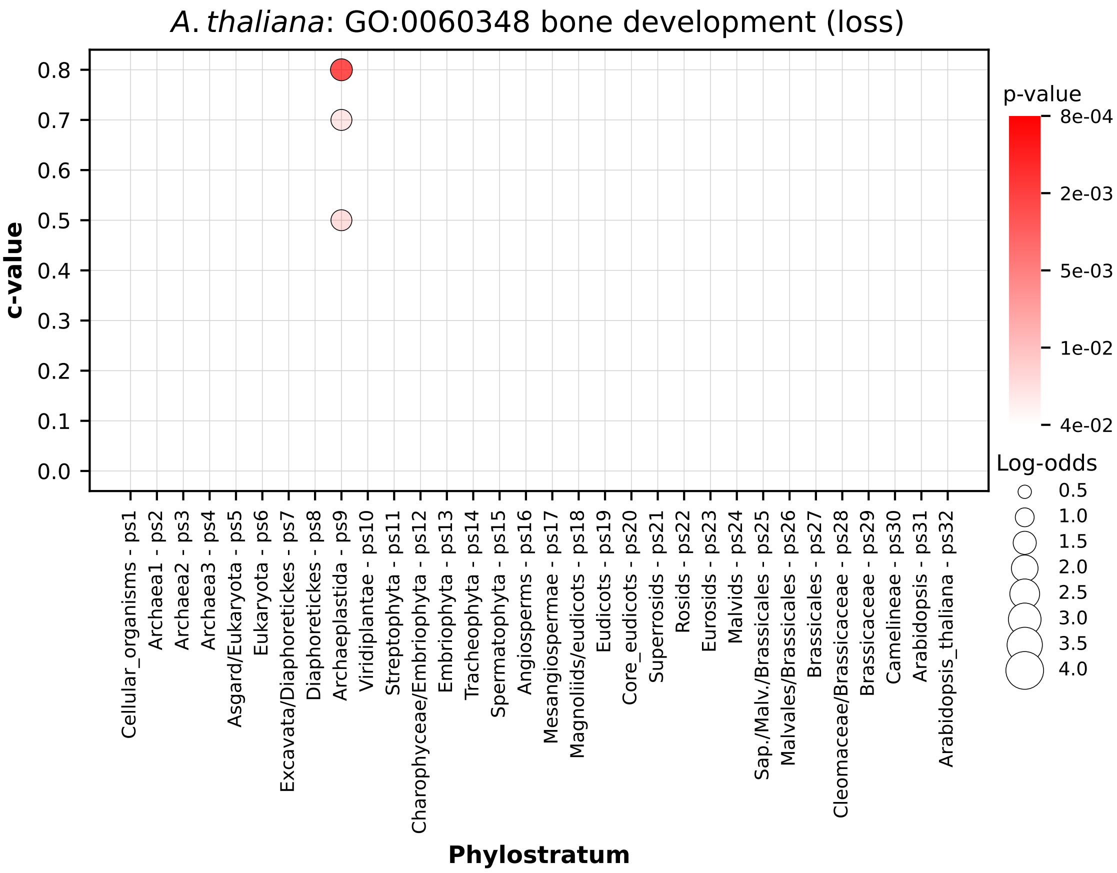

A. thaliana: GO:0060348 bone development (loss)
c-value
0.8
0.7
0.6
0.5
0.4
0.3
0.2
0.1
0.0
p-value
8e-04
2e-03
5e-03
1e-02
4e-02
Log-odds
0.5
1.0
1.5
2.0
2.5
3.0
3.5
4.0
Cellular_organisms - ps1
Archaea1 - ps2
Archaea2 - ps3
Archaea3 - ps4
Asgard/Eukaryota - ps5
Eukaryota - ps6
Excavata/Diaphoretickes - ps7
Diaphoretickes - ps8
Archaeplastida - ps9
Viridiplantae - ps10
Streptophyta - ps11
Charophyceae/Embriophyta - ps12
Embriophyta - ps13
Tracheophyta - ps14
Spermatophyta - ps15
Angiosperms - ps16
Mesangiospermae - ps17
Magnoliids/eudicots - ps18
Eudicots - ps19
Core_eudicots - ps20
Superrosids - ps21
Rosids - ps22
Eurosids - ps23
Malvids - ps24
Sap./Malv./Brassicales - ps25
Malvales/Brassicales - ps26
Brassicales - ps27
Cleomaceae/Brassicaceae - ps28
Brassicaceae - ps29
Camelineae - ps30
Arabidopsis - ps31
Arabidopsis_thaliana - ps32
Phylostratum

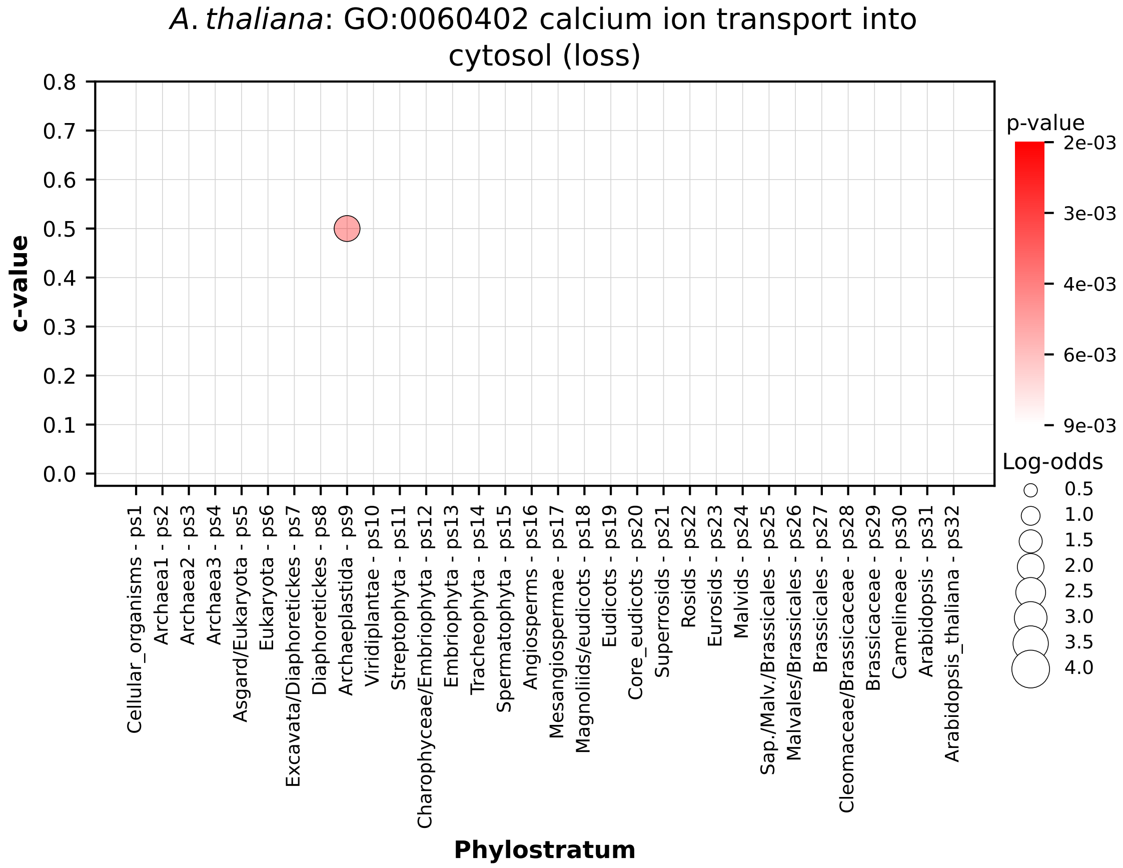
A. thaliana: GO:0060402 calcium ion transport into cytosol (loss)
c-value
0.0
0.1
0.2
0.3
0.4
0.5
0.6
0.7
0.8
Phylostratum
Cellular_organisms - ps1
Archaea1 - ps2
Archaea2 - ps3
Archaea3 - ps4
Asgard/Eukaryota - ps5
Eukaryota - ps6
Excavata/Diaphoretickes - ps7
Diaphoretickes - ps8
Archaeplastida - ps9
Viridiplantae - ps10
Streptophyta - ps11
Charophyceae/Embriophyta - ps12
Embriophyta - ps13
Tracheophyta - ps14
Spermatophyta - ps15
Angiosperms - ps16
Mesangiospermae - ps17
Magnoliids/eudicots - ps18
Eudicots - ps19
Core_eudicots - ps20
Superrosids - ps21
Rosids - ps22
Eurosids - ps23
Malvids - ps24
Sap./Malv./Brassicales - ps25
Malvales/Brassicales - ps26
Brassicales - ps27
Cleomaceae/Brassicaceae - ps28
Brassicaceae - ps29
Camelineae - ps30
Arabidopsis - ps31
Arabidopsis_thaliana - ps32
p-value
2e-03
3e-03
4e-03
6e-03
9e-03
Log-odds
0.5
1.0
1.5
2.0
2.5
3.0
3.5
4.0

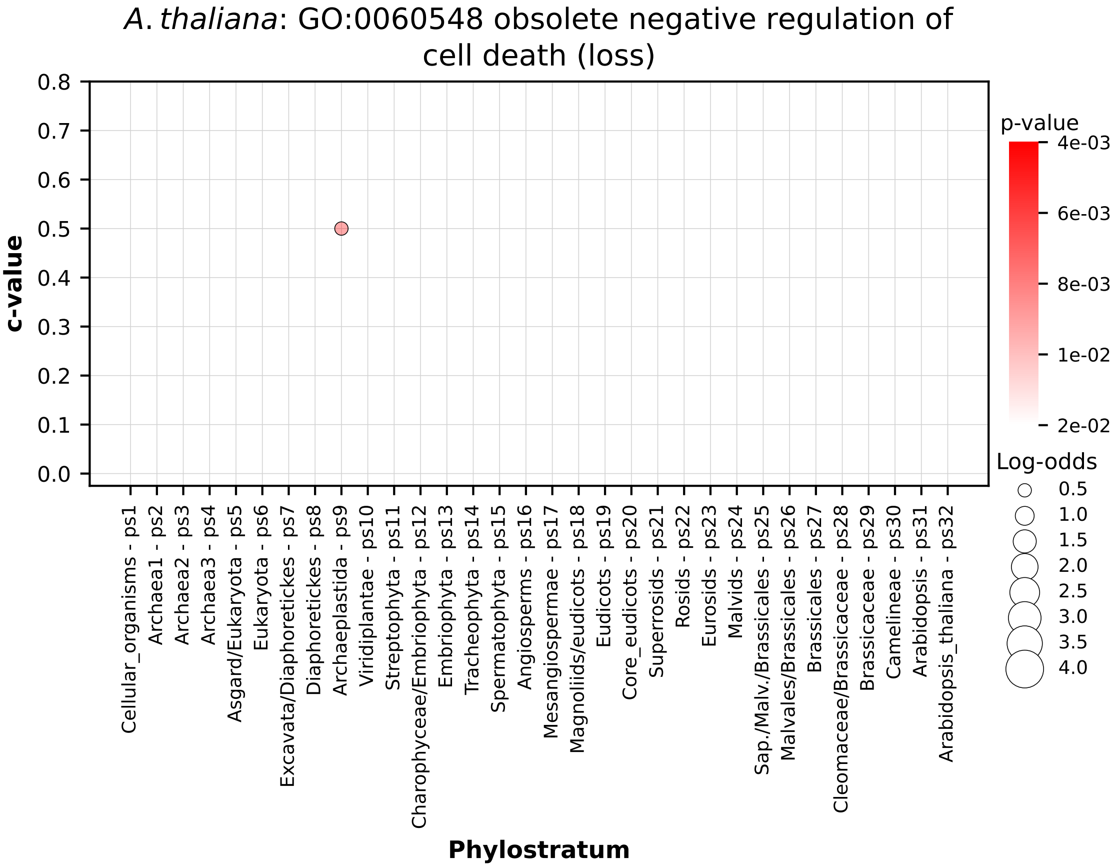

A. thaliana: GO:0060548 obsolete negative regulation of cell death (loss)
c-value
0.8
0.7
0.6
0.5
0.4
0.3
0.2
0.1
0.0
Phylostratum
Cellular_organisms - ps1
Archaea1 - ps2
Archaea2 - ps3
Archaea3 - ps4
Asgard/Eukaryota - ps5
Eukaryota - ps6
Excavata/Diaphoretickes - ps7
Diaphoretickes - ps8
Archaeplastida - ps9
Viridiplantae - ps10
Streptophyta - ps11
Charophyceae/Embriophyta - ps12
Embriophyta - ps13
Tracheophyta - ps14
Spermatophyta - ps15
Angiosperms - ps16
Mesangiospermae - ps17
Magnoliids/eudicots - ps18
Eudicots - ps19
Core_eudicots - ps20
Superrosids - ps21
Rosids - ps22
Eurosids - ps23
Malvids - ps24
Sap./Malv./Brassicales - ps25
Malvales/Brassicales - ps26
Brassicales - ps27
Cleomaceae/Brassicaceae - ps28
Brassicaceae - ps29
Camelineae - ps30
Arabidopsis - ps31
Arabidopsis_thaliana - ps32
p-value
4e-03
6e-03
8e-03
1e-02
2e-02
Log-odds
0.5
1.0
1.5
2.0
2.5
3.0
3.5
4.0

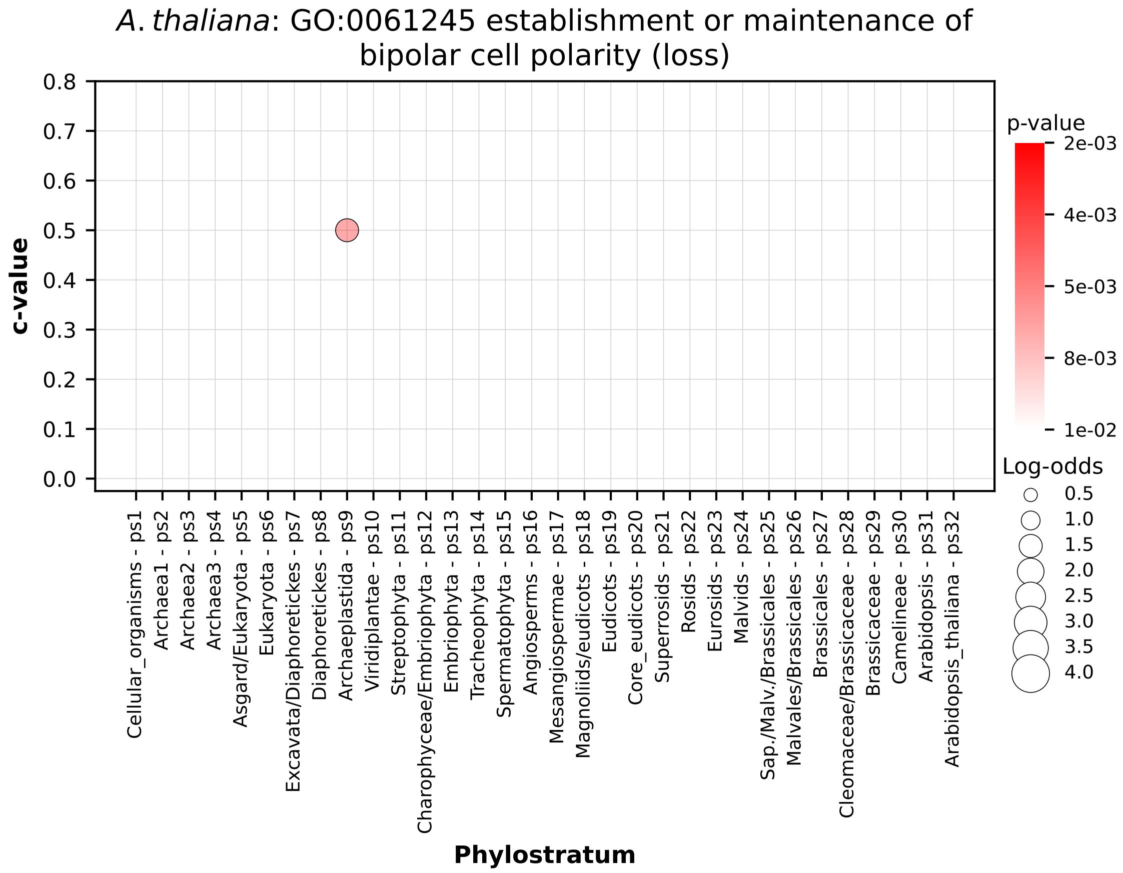

A. thaliana: GO:0061245 establishment or maintenance of bipolar cell polarity (loss)
c-value
0.8
0.7
0.6
0.5
0.4
0.3
0.2
0.1
0.0
p-value
2e-03
4e-03
5e-03
8e-03
1e-02
Log-odds
0.5
1.0
1.5
2.0
2.5
3.0
3.5
4.0
Cellular_organisms - ps1
Archaea1 - ps2
Archaea2 - ps3
Archaea3 - ps4
Asgard/Eukaryota - ps5
Eukaryota - ps6
Excavata/Diaphoretickes - ps7
Diaphoretickes - ps8
Archaeplastida - ps9
Viridiplantae - ps10
Streptophyta - ps11
Charophyceae/Embriophyta - ps12
Embriophyta - ps13
Tracheophyta - ps14
Spermatophyta - ps15
Angiosperms - ps16
Mesangiospermae - ps17
Magnoliids/eudicots - ps18
Eudicots - ps19
Core_eudicots - ps20
Superrosids - ps21
Rosids - ps22
Eurosids - ps23
Malvids - ps24
Sap./Malv./Brassicales - ps25
Malvales/Brassicales - ps26
Brassicales - ps27
Cleomaceae/Brassicaceae - ps28
Brassicaceae - ps29
Camelineae - ps30
Arabidopsis - ps31
Arabidopsis_thaliana - ps32
Phylostratum

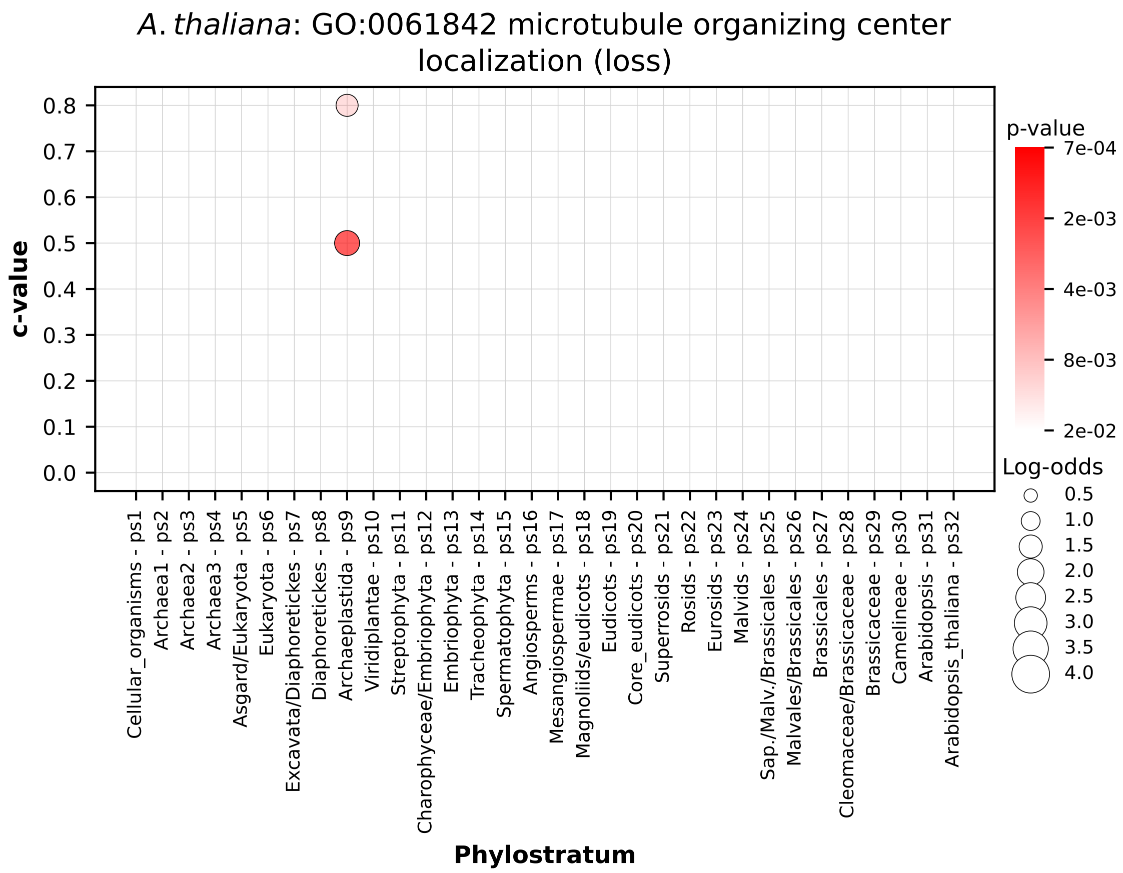

A. thaliana: GO:0061842 microtubule organizing center localization (loss)
c-value
0.0
0.1
0.2
0.3
0.4
0.5
0.6
0.7
0.8
p-value
7e-04
2e-03
4e-03
8e-03
2e-02
Log-odds
0.5
1.0
1.5
2.0
2.5
3.0
3.5
4.0
Cellular_organisms - ps1
Archaea1 - ps2
Archaea2 - ps3
Archaea3 - ps4
Asgard/Eukaryota - ps5
Eukaryota - ps6
Excavata/Diaphoretickes - ps7
Diaphoretickes - ps8
Archaeplastida - ps9
Viridiplantae - ps10
Streptophyta - ps11
Charophyceae/Embriophyta - ps12
Embriophyta - ps13
Tracheophyta - ps14
Spermatophyta - ps15
Angiosperms - ps16
Mesangiospermae - ps17
Magnoliids/eudicots - ps18
Eudicots - ps19
Core_eudicots - ps20
Superrosids - ps21
Rosids - ps22
Eurosids - ps23
Malvids - ps24
Sap./Malv./Brassicales - ps25
Malvales/Brassicales - ps26
Brassicales - ps27
Cleomaceae/Brassicaceae - ps28
Brassicaceae - ps29
Camelineae - ps30
Arabidopsis - ps31
Arabidopsis_thaliana - ps32
Phylostratum

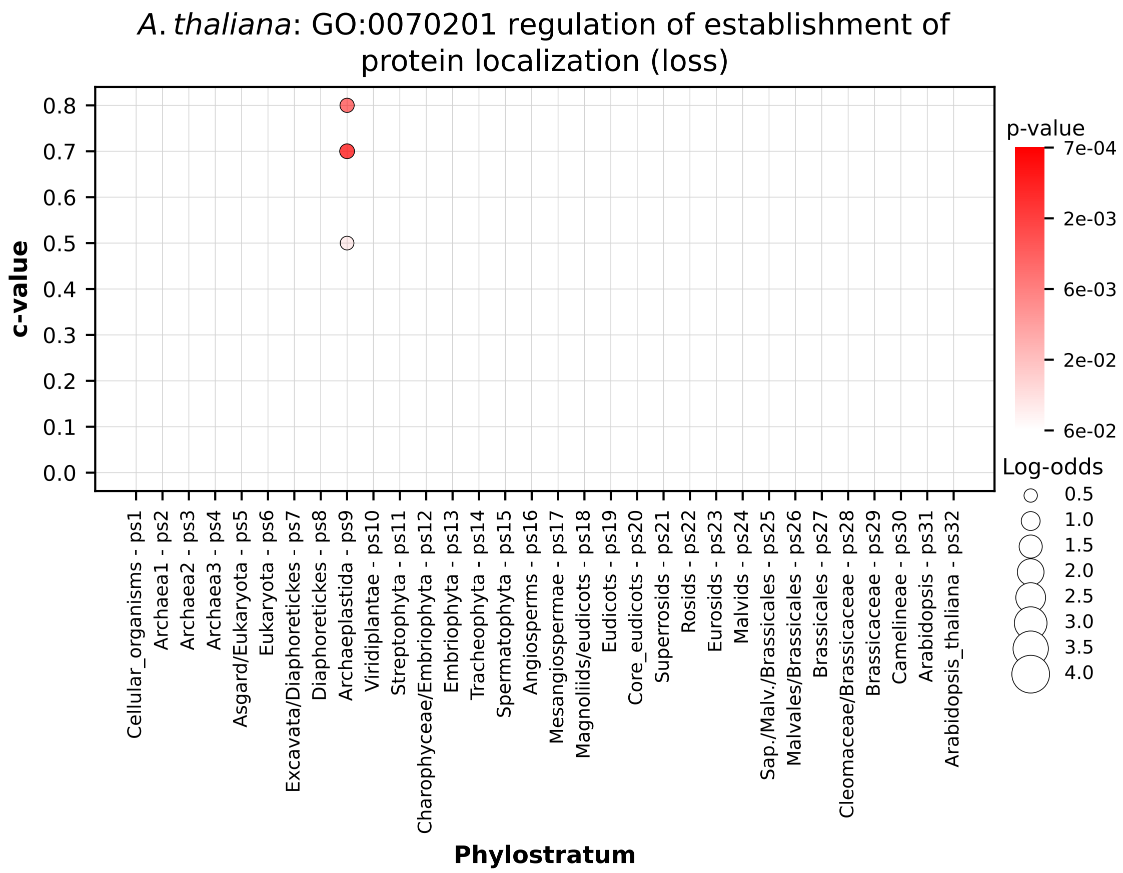

A. thaliana: GO:0070201 regulation of establishment of protein localization (loss)
c-value
0.8
0.7
0.6
0.5
0.4
0.3
0.2
0.1
0.0
p-value
7e-04
2e-03
6e-03
2e-02
6e-02
Log-odds
0.5
1.0
1.5
2.0
2.5
3.0
3.5
4.0
Cellular_organisms - ps1
Archaea1 - ps2
Archaea2 - ps3
Archaea3 - ps4
Asgard/Eukaryota - ps5
Eukaryota - ps6
Excavata/Diaphoretickes - ps7
Diaphoretickes - ps8
Archaeplastida - ps9
Viridiplantae - ps10
Streptophyta - ps11
Charophyceae/Embriophyta - ps12
Embriophyta - ps13
Tracheophyta - ps14
Spermatophyta - ps15
Angiosperms - ps16
Mesangiospermae - ps17
Magnoliids/eudicots - ps18
Eudicots - ps19
Core_eudicots - ps20
Superrosids - ps21
Rosids - ps22
Eurosids - ps23
Malvids - ps24
Sap./Malv./Brassicales - ps25
Malvales/Brassicales - ps26
Brassicales - ps27
Cleomaceae/Brassicaceae - ps28
Brassicaceae - ps29
Camelineae - ps30
Arabidopsis - ps31
Arabidopsis_thaliana - ps32
Phylostratum

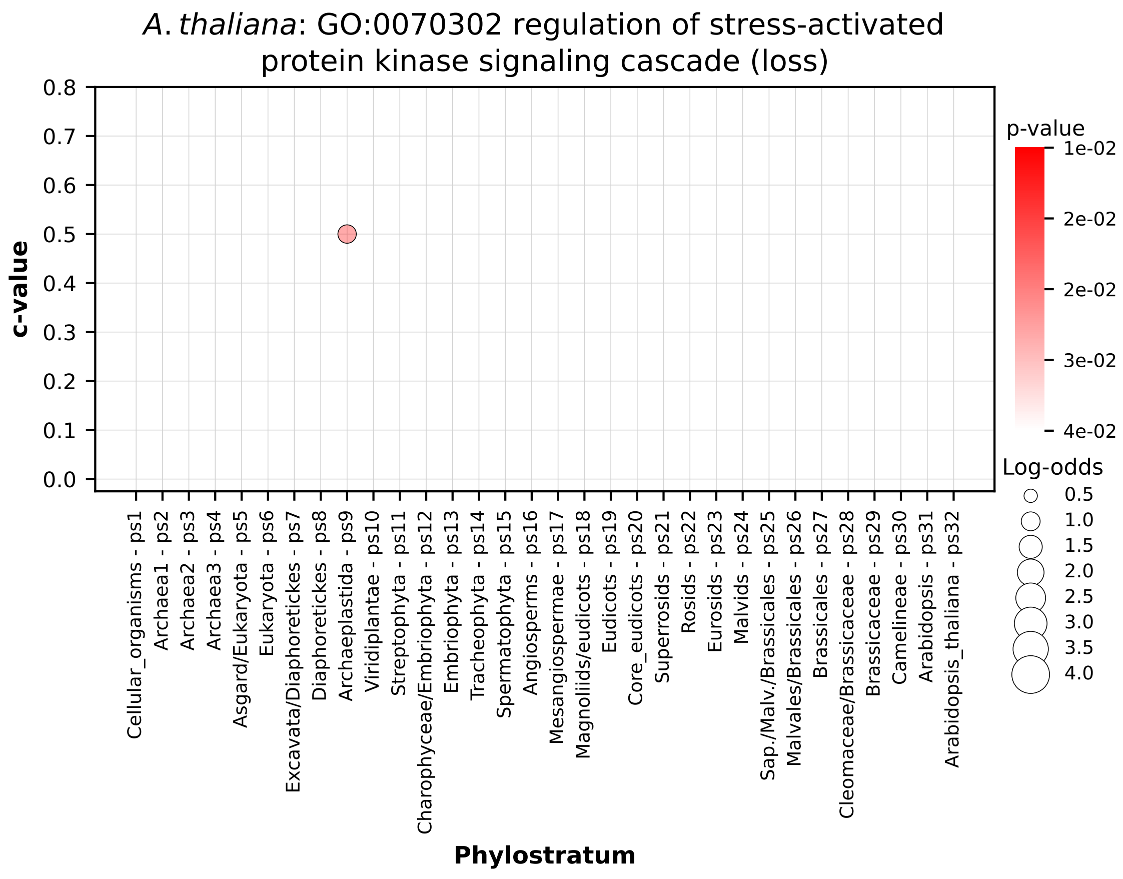
A. thaliana: GO:0070302 regulation of stress-activated protein kinase signaling cascade (loss)
c-value
0.0
0.1
0.2
0.3
0.4
0.5
0.6
0.7
0.8
Phylostratum
Cellular_organisms - ps1
Archaea1 - ps2
Archaea2 - ps3
Archaea3 - ps4
Asgard/Eukaryota - ps5
Eukaryota - ps6
Excavata/Diaphoretickes - ps7
Diaphoretickes - ps8
Archaeplastida - ps9
Viridiplantae - ps10
Streptophyta - ps11
Charophyceae/Embriophyta - ps12
Embriophyta - ps13
Tracheophyta - ps14
Spermatophyta - ps15
Angiosperms - ps16
Mesangiospermae - ps17
Magnoliids/eudicots - ps18
Eudicots - ps19
Core_eudicots - ps20
Superrosids - ps21
Rosids - ps22
Eurosids - ps23
Malvids - ps24
Sap./Malv./Brassicales - ps25
Malvales/Brassicales - ps26
Brassicales - ps27
Cleomaceae/Brassicaceae - ps28
Brassicaceae - ps29
Camelineae - ps30
Arabidopsis - ps31
Arabidopsis_thaliana - ps32
p-value
1e-02
2e-02
2e-02
3e-02
4e-02
Log-odds
0.5
1.0
1.5
2.0
2.5
3.0
3.5
4.0

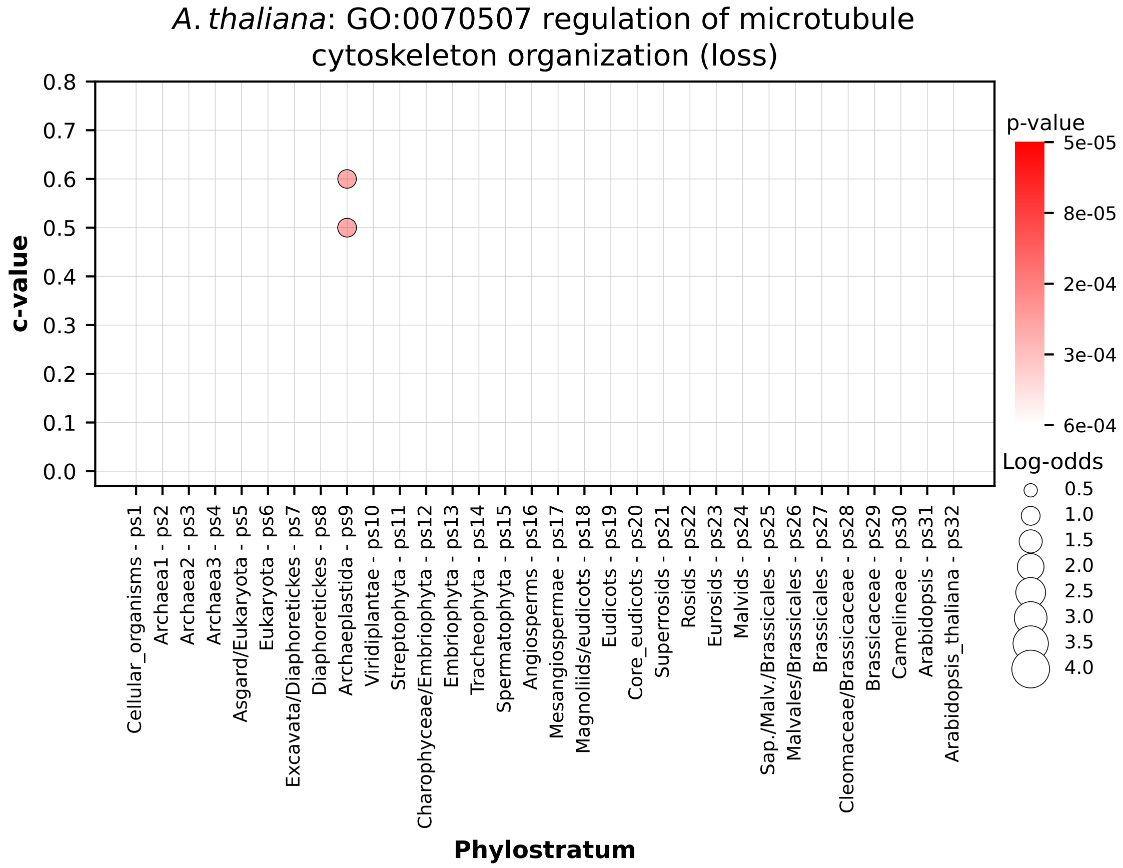

A. thaliana: GO:0070507 regulation of microtubule cytoskeleton organization (loss)
c-value
0.0
0.1
0.2
0.3
0.4
0.5
0.6
0.7
0.8
Phylostratum
Cellular_organisms - ps1
Archaea1 - ps2
Archaea2 - ps3
Archaea3 - ps4
Asgard/Eukaryota - ps5
Eukaryota - ps6
Excavata/Diaphoretickes - ps7
Diaphoretickes - ps8
Archaeplastida - ps9
Viridiplantae - ps10
Streptophyta - ps11
Charophyceae/Embriophyta - ps12
Embriophyta - ps13
Tracheophyta - ps14
Spermatophyta - ps15
Angiosperms - ps16
Mesangiospermae - ps17
Magnoliids/eudicots - ps18
Eudicots - ps19
Core_eudicots - ps20
Superrosids - ps21
Rosids - ps22
Eurosids - ps23
Malvids - ps24
Sap./Malv./Brassicales - ps25
Malvales/Brassicales - ps26
Brassicales - ps27
Cleomaceae/Brassicaceae - ps28
Brassicaceae - ps29
Camelineae - ps30
Arabidopsis - ps31
Arabidopsis_thaliana - ps32
p-value
5e-05
8e-05
2e-04
3e-04
6e-04
Log-odds
0.5
1.0
1.5
2.0
2.5
3.0
3.5
4.0

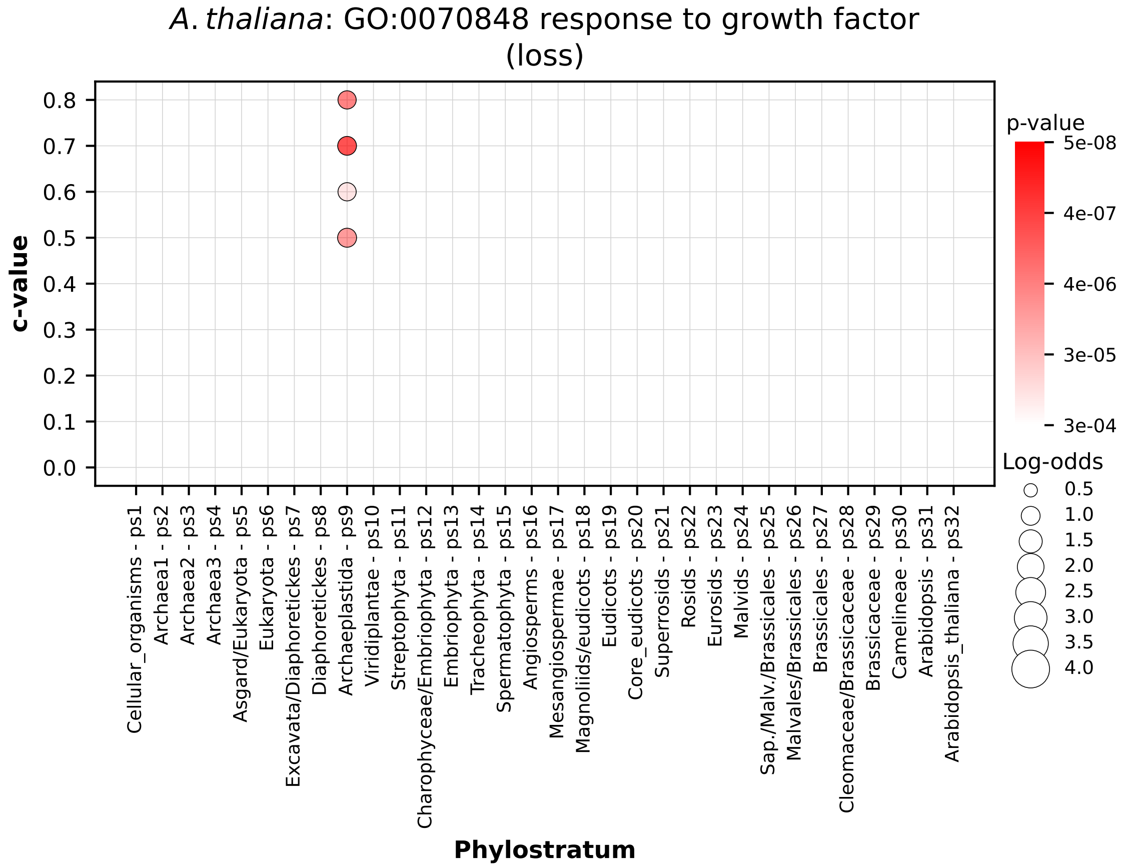
A. thaliana: GO:0070848 response to growth factor
(loss)
c-value
0.8
0.7
0.6
0.5
0.4
0.3
0.2
0.1
0.0
p-value
5e-08
4e-07
4e-06
3e-05
3e-04
Log-odds
0.5
1.0
1.5
2.0
2.5
3.0
3.5
4.0
Cellular_organisms - ps1
Archaea1 - ps2
Archaea2 - ps3
Archaea3 - ps4
Asgard/Eukaryota - ps5
Eukaryota - ps6
Excavata/Diaphoretickes - ps7
Diaphoretickes - ps8
Archaeplastida - ps9
Viridiplantae - ps10
Streptophyta - ps11
Charophyceae/Embriophyta - ps12
Embriophyta - ps13
Tracheophyta - ps14
Spermatophyta - ps15
Angiosperms - ps16
Mesangiospermae - ps17
Magnoliids/eudicots - ps18
Eudicots - ps19
Core_eudicots - ps20
Superrosids - ps21
Rosids - ps22
Eurosids - ps23
Malvids - ps24
Sap./Malv./Brassicales - ps25
Malvales/Brassicales - ps26
Brassicales - ps27
Cleomaceae/Brassicaceae - ps28
Brassicaceae - ps29
Camelineae - ps30
Arabidopsis - ps31
Arabidopsis_thaliana - ps32
Phylostratum

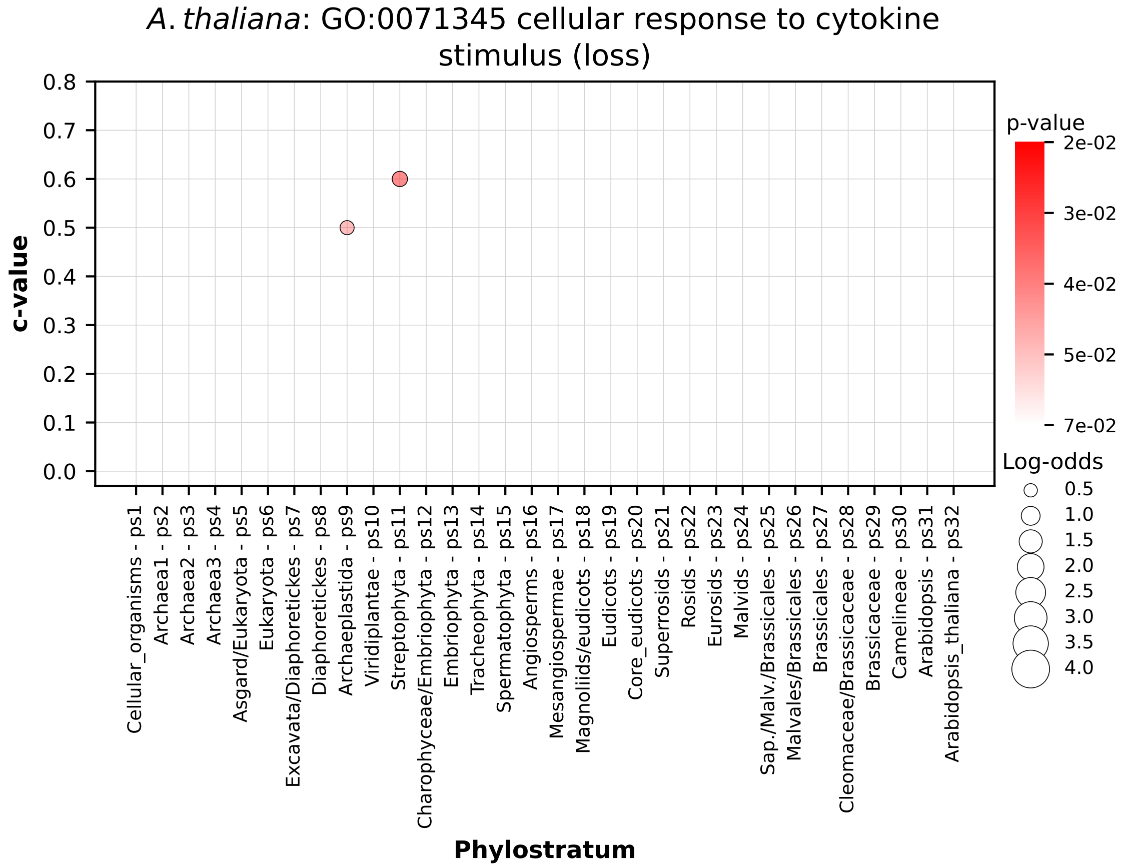

A. thaliana: GO:0071345 cellular response to cytokine stimulus (loss)
c-value
0.8
0.7
0.6
0.5
0.4
0.3
0.2
0.1
0.0
Phylostratum
Cellular_organisms - ps1
Archaea1 - ps2
Archaea2 - ps3
Archaea3 - ps4
Asgard/Eukaryota - ps5
Eukaryota - ps6
Excavata/Diaphoretickes - ps7
Diaphoretickes - ps8
Archaeplastida - ps9
Viridiplantae - ps10
Streptophyta - ps11
Charophyceae/Embriophyta - ps12
Embriophyta - ps13
Tracheophyta - ps14
Spermatophyta - ps15
Angiosperms - ps16
Mesangiospermae - ps17
Magnoliids/eudicots - ps18
Eudicots - ps19
Core_eudicots - ps20
Superrosids - ps21
Rosids - ps22
Eurosids - ps23
Malvids - ps24
Sap./Malv./Brassicales - ps25
Malvales/Brassicales - ps26
Brassicales - ps27
Cleomaceae/Brassicaceae - ps28
Brassicaceae - ps29
Camelineae - ps30
Arabidopsis - ps31
Arabidopsis_thaliana - ps32
p-value
2e-02
3e-02
4e-02
5e-02
7e-02
Log-odds
0.5
1.0
1.5
2.0
2.5
3.0
3.5
4.0

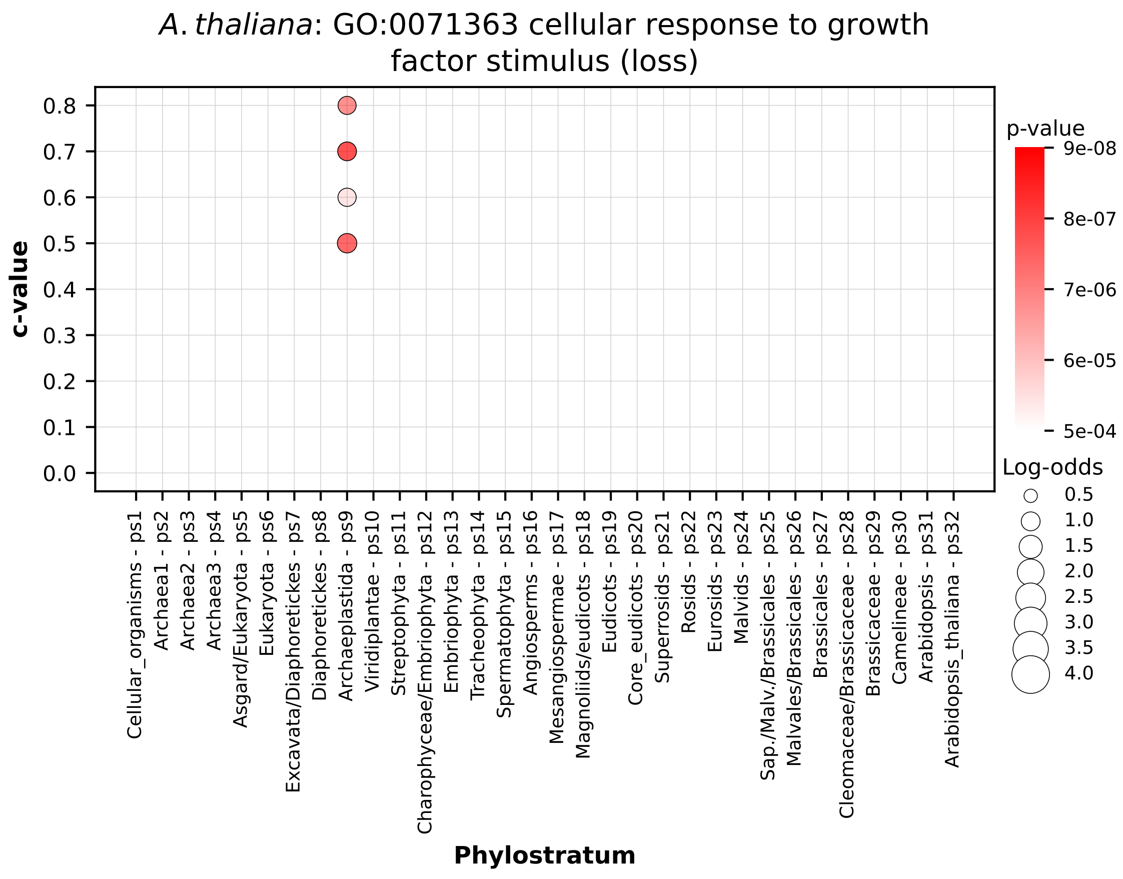

A. thaliana: GO:0071363 cellular response to growth factor stimulus (loss)
c-value
0.8
0.7
0.6
0.5
0.4
0.3
0.2
0.1
0.0
p-value
9e-08
8e-07
7e-06
6e-05
5e-04
Log-odds
0.5
1.0
1.5
2.0
2.5
3.0
3.5
4.0
Cellular_organisms - ps1
Archaea1 - ps2
Archaea2 - ps3
Archaea3 - ps4
Asgard/Eukaryota - ps5
Eukaryota - ps6
Excavata/Diaphoretickes - ps7
Diaphoretickes - ps8
Archaeplastida - ps9
Viridiplantae - ps10
Streptophyta - ps11
Charophyceae/Embriophyta - ps12
Embriophyta - ps13
Tracheophyta - ps14
Spermatophyta - ps15
Angiosperms - ps16
Mesangiospermae - ps17
Magnoliids/eudicots - ps18
Eudicots - ps19
Core_eudicots - ps20
Superrosids - ps21
Rosids - ps22
Eurosids - ps23
Malvids - ps24
Sap./Malv./Brassicales - ps25
Malvales/Brassicales - ps26
Brassicales - ps27
Cleomaceae/Brassicaceae - ps28
Brassicaceae - ps29
Camelineae - ps30
Arabidopsis - ps31
Arabidopsis_thaliana - ps32
Phylostratum

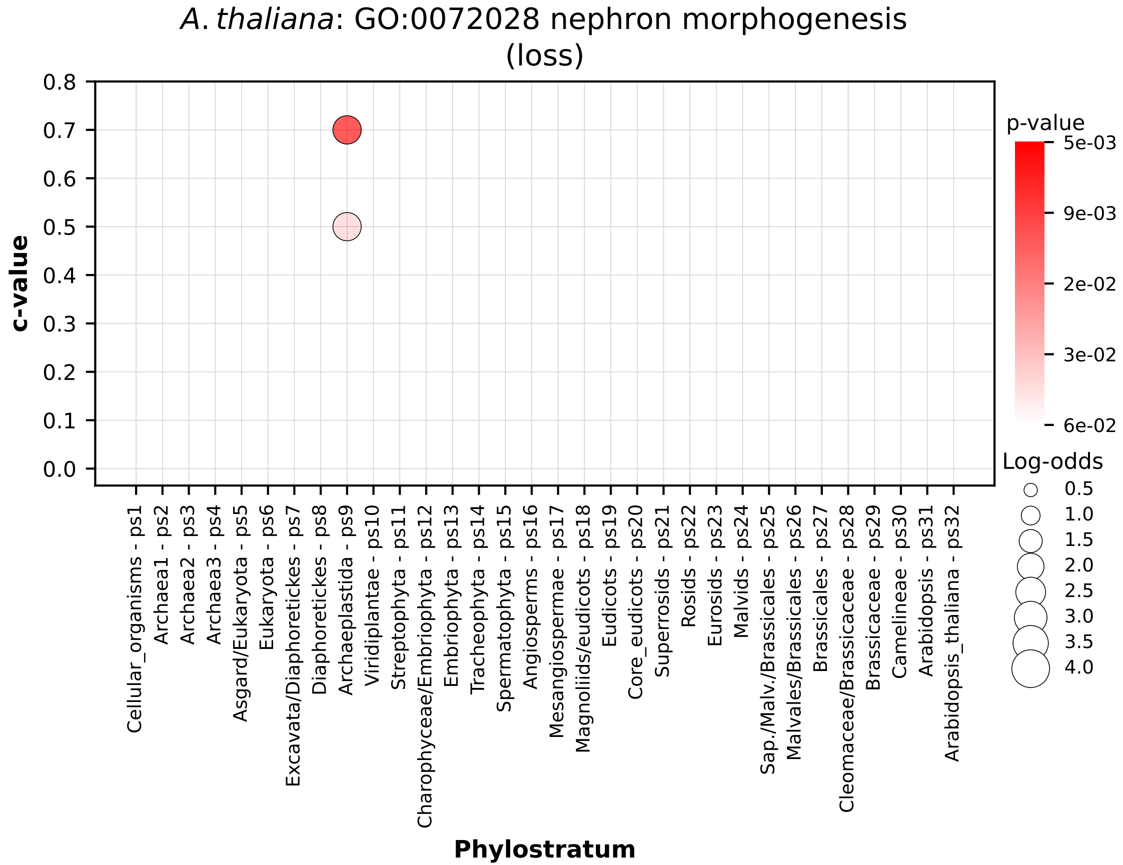

A. thaliana: GO:0072028 nephron morphogenesis
(loss)
c-value
0.8
0.7
0.6
0.5
0.4
0.3
0.2
0.1
0.0
p-value
5e-03
9e-03
2e-02
3e-02
6e-02
Log-odds
0.5
1.0
1.5
2.0
2.5
3.0
3.5
4.0
Cellular_organisms - ps1
Archaea1 - ps2
Archaea2 - ps3
Archaea3 - ps4
Asgard/Eukaryota - ps5
Eukaryota - ps6
Excavata/Diaphoretickes - ps7
Diaphoretickes - ps8
Archaeplastida - ps9
Viridiplantae - ps10
Streptophyta - ps11
Charophyceae/Embriophyta - ps12
Embriophyta - ps13
Tracheophyta - ps14
Spermatophyta - ps15
Angiosperms - ps16
Mesangiospermae - ps17
Magnoliids/eudicots - ps18
Eudicots - ps19
Core_eudicots - ps20
Superrosids - ps21
Rosids - ps22
Eurosids - ps23
Malvids - ps24
Sap./Malv./Brassicales - ps25
Malvales/Brassicales - ps26
Brassicales - ps27
Cleomaceae/Brassicaceae - ps28
Brassicaceae - ps29
Camelineae - ps30
Arabidopsis - ps31
Arabidopsis_thaliana - ps32
Phylostratum

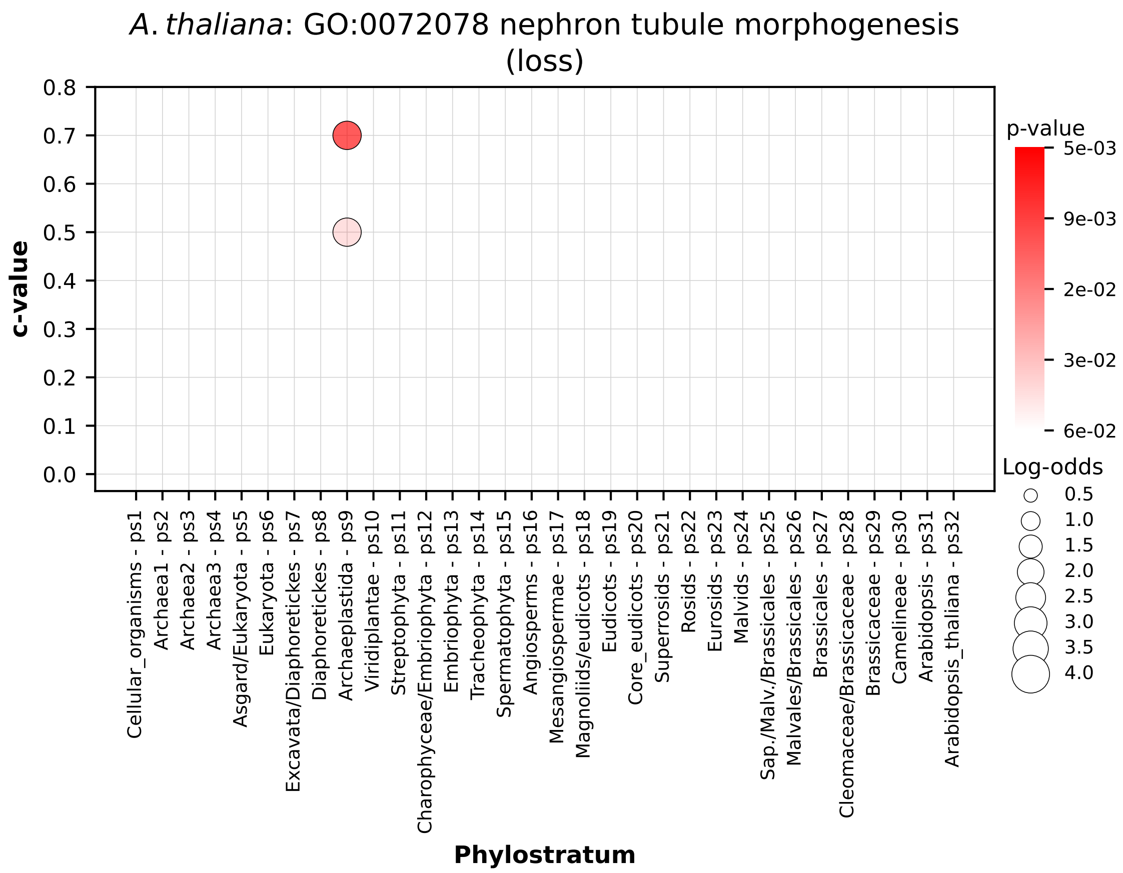
A. thaliana: GO:0072078 nephron tubule morphogenesis
(loss)
c-value
0.8
0.7
0.6
0.5
0.4
0.3
0.2
0.1
0.0
p-value
5e-03
9e-03
2e-02
3e-02
6e-02
Log-odds
0.5
1.0
1.5
2.0
2.5
3.0
3.5
4.0
Cellular_organisms - ps1
Archaea1 - ps2
Archaea2 - ps3
Archaea3 - ps4
Asgard/Eukaryota - ps5
Eukaryota - ps6
Excavata/Diaphoretickes - ps7
Diaphoretickes - ps8
Archaeplastida - ps9
Viridiplantae - ps10
Streptophyta - ps11
Charophyceae/Embriophyta - ps12
Embriophyta - ps13
Tracheophyta - ps14
Spermatophyta - ps15
Angiosperms - ps16
Mesangiospermae - ps17
Magnoliids/eudicots - ps18
Eudicots - ps19
Core_eudicots - ps20
Superrosids - ps21
Rosids - ps22
Eurosids - ps23
Malvids - ps24
Sap./Malv./Brassicales - ps25
Malvales/Brassicales - ps26
Brassicales - ps27
Cleomaceae/Brassicaceae - ps28
Brassicaceae - ps29
Camelineae - ps30
Arabidopsis - ps31
Arabidopsis_thaliana - ps32
Phylostratum

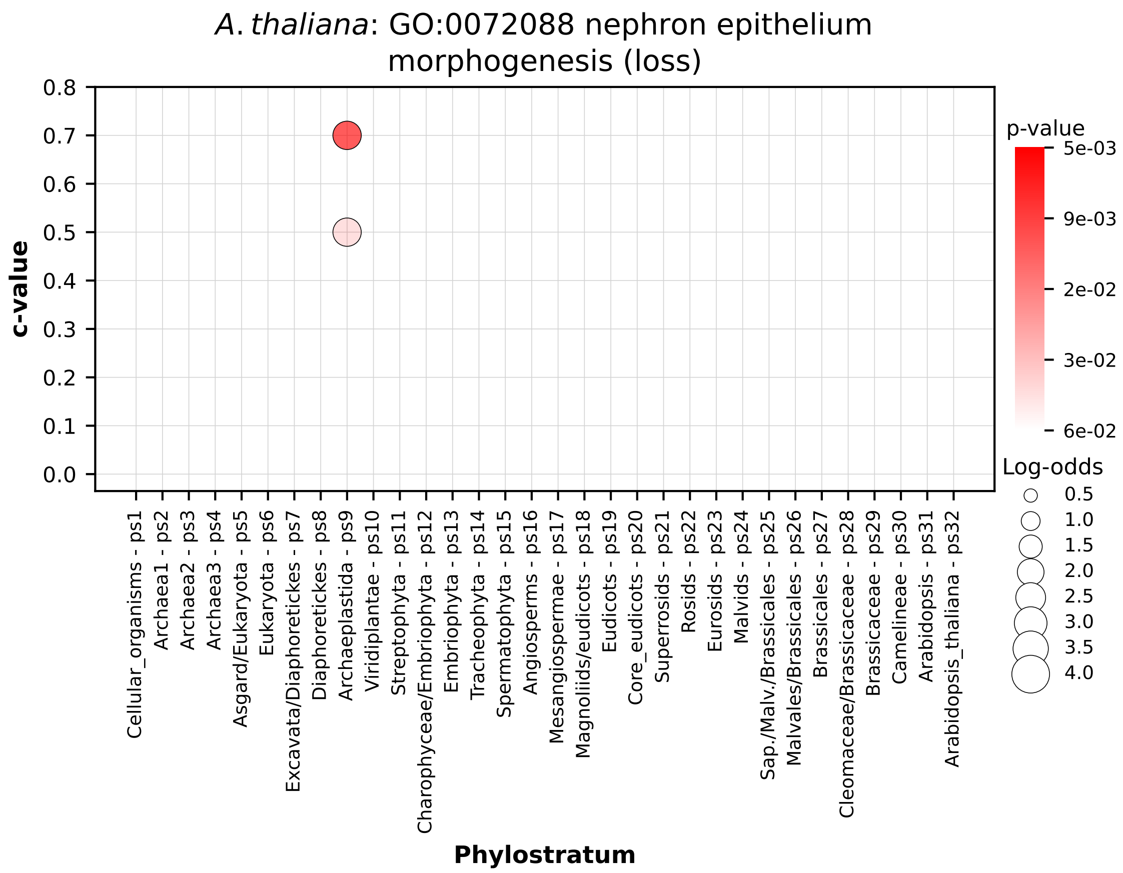

A. thaliana: GO:0072088 nephron epithelium morphogenesis (loss)
c-value
0.8
0.7
0.6
0.5
0.4
0.3
0.2
0.1
0.0
p-value
5e-03
9e-03
2e-02
3e-02
6e-02
Log-odds
0.5
1.0
1.5
2.0
2.5
3.0
3.5
4.0
Cellular_organisms - ps1
Archaea1 - ps2
Archaea2 - ps3
Archaea3 - ps4
Asgard/Eukaryota - ps5
Eukaryota - ps6
Excavata/Diaphoretickes - ps7
Diaphoretickes - ps8
Archaeplastida - ps9
Viridiplantae - ps10
Streptophyta - ps11
Charophyceae/Embriophyta - ps12
Embriophyta - ps13
Tracheophyta - ps14
Spermatophyta - ps15
Angiosperms - ps16
Mesangiospermae - ps17
Magnoliids/eudicots - ps18
Eudicots - ps19
Core_eudicots - ps20
Superrosids - ps21
Rosids - ps22
Eurosids - ps23
Malvids - ps24
Sap./Malv./Brassicales - ps25
Malvales/Brassicales - ps26
Brassicales - ps27
Cleomaceae/Brassicaceae - ps28
Brassicaceae - ps29
Camelineae - ps30
Arabidopsis - ps31
Arabidopsis_thaliana - ps32
Phylostratum

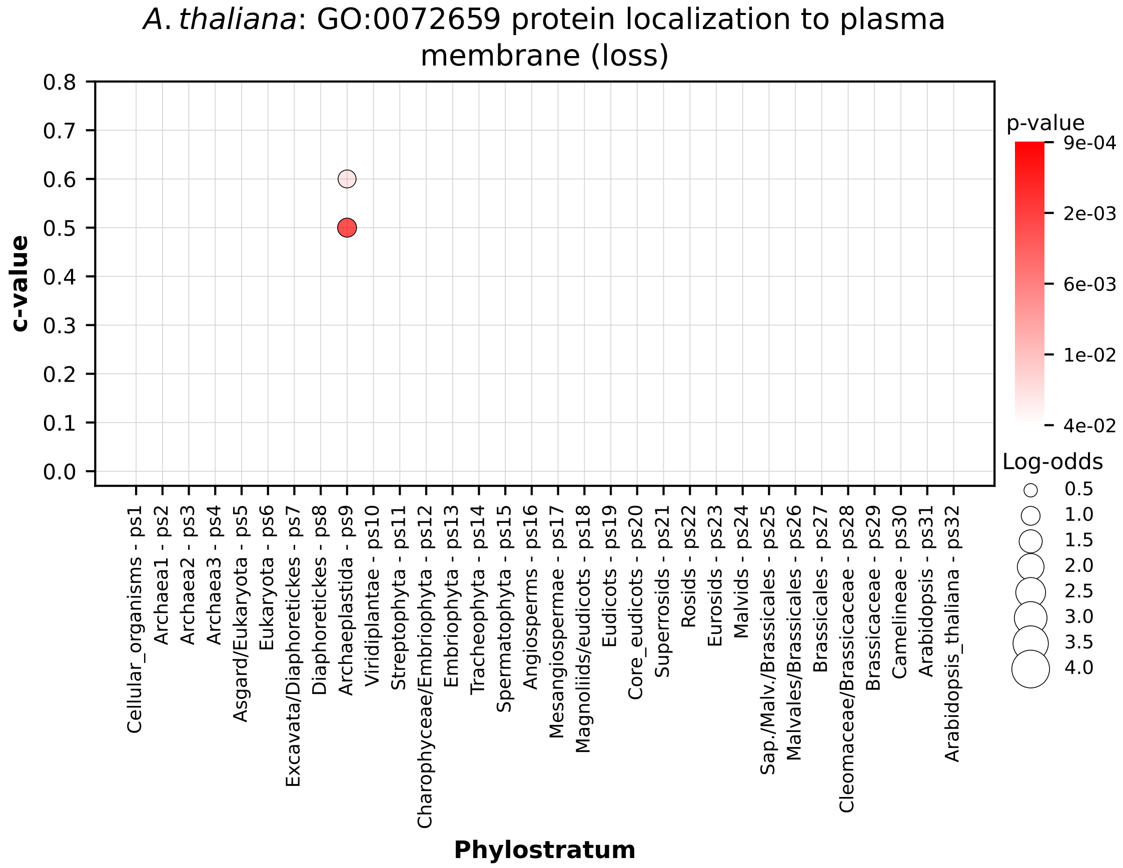

A. thaliana: GO:0072659 protein localization to plasma membrane (loss)
c-value
0.8
0.7
0.6
0.5
0.4
0.3
0.2
0.1
0.0
p-value
9e-04
2e-03
6e-03
1e-02
4e-02
Log-odds
0.5
1.0
1.5
2.0
2.5
3.0
3.5
4.0
Cellular_organisms - ps1
Archaea1 - ps2
Archaea2 - ps3
Archaea3 - ps4
Asgard/Eukaryota - ps5
Eukaryota - ps6
Excavata/Diaphoretickes - ps7
Diaphoretickes - ps8
Archaeplastida - ps9
Viridiplantae - ps10
Streptophyta - ps11
Charophyceae/Embriophyta - ps12
Embriophyta - ps13
Tracheophyta - ps14
Spermatophyta - ps15
Angiosperms - ps16
Mesangiospermae - ps17
Magnoliids/eudicots - ps18
Eudicots - ps19
Core_eudicots - ps20
Superrosids - ps21
Rosids - ps22
Eurosids - ps23
Malvids - ps24
Sap./Malv./Brassicales - ps25
Malvales/Brassicales - ps26
Brassicales - ps27
Cleomaceae/Brassicaceae - ps28
Brassicaceae - ps29
Camelineae - ps30
Arabidopsis - ps31
Arabidopsis_thaliana - ps32
Phylostratum

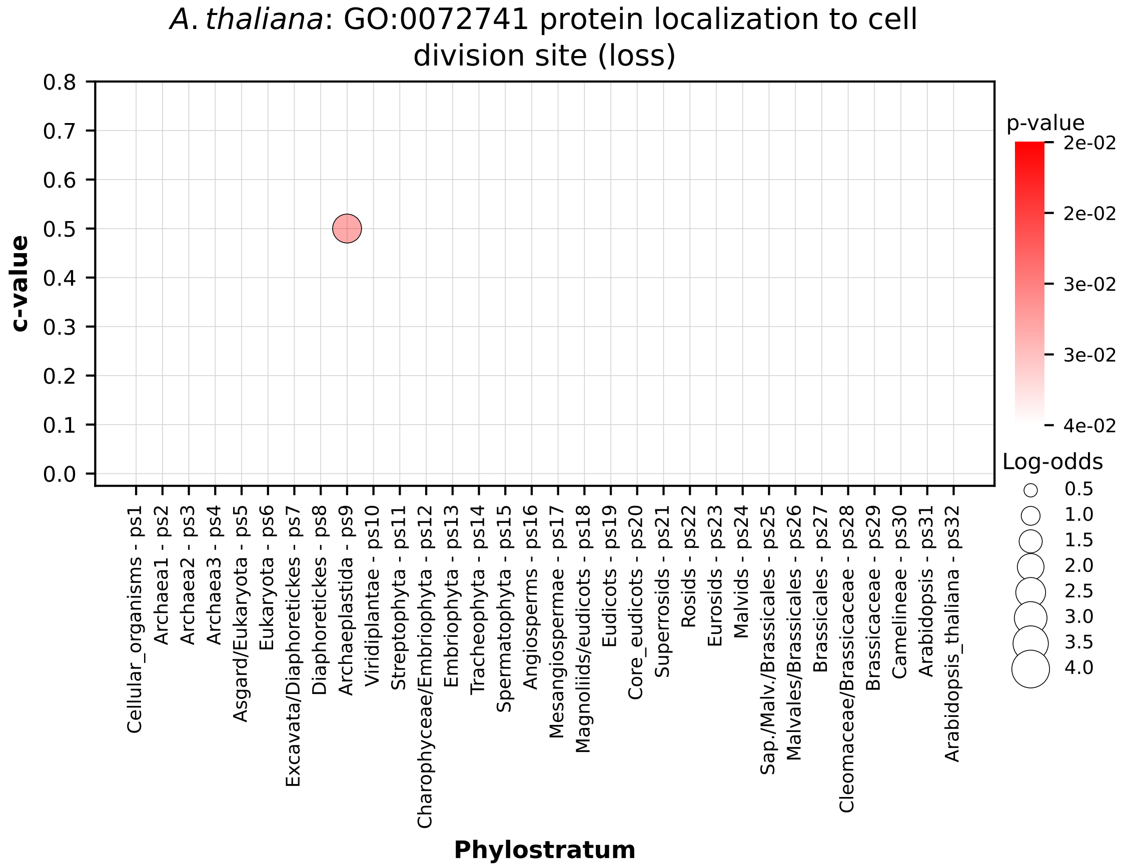

A. thaliana: GO:0072741 protein localization to cell division site (loss)
c-value
0.8
0.7
0.6
0.5
0.4
0.3
0.2
0.1
0.0
Phylostratum
Cellular_organisms - ps1
Archaea1 - ps2
Archaea2 - ps3
Archaea3 - ps4
Asgard/Eukaryota - ps5
Eukaryota - ps6
Excavata/Diaphoretickes - ps7
Diaphoretickes - ps8
Archaeplastida - ps9
Viridiplantae - ps10
Streptophyta - ps11
Charophyceae/Embriophyta - ps12
Embriophyta - ps13
Tracheophyta - ps14
Spermatophyta - ps15
Angiosperms - ps16
Mesangiospermae - ps17
Magnoliids/eudicots - ps18
Eudicots - ps19
Core_eudicots - ps20
Superrosids - ps21
Rosids - ps22
Eurosids - ps23
Malvids - ps24
Sap./Malv./Brassicales - ps25
Malvales/Brassicales - ps26
Brassicales - ps27
Cleomaceae/Brassicaceae - ps28
Brassicaceae - ps29
Camelineae - ps30
Arabidopsis - ps31
Arabidopsis_thaliana - ps32
p-value
2e-02
2e-02
3e-02
3e-02
4e-02
Log-odds
0.5
1.0
1.5
2.0
2.5
3.0
3.5
4.0

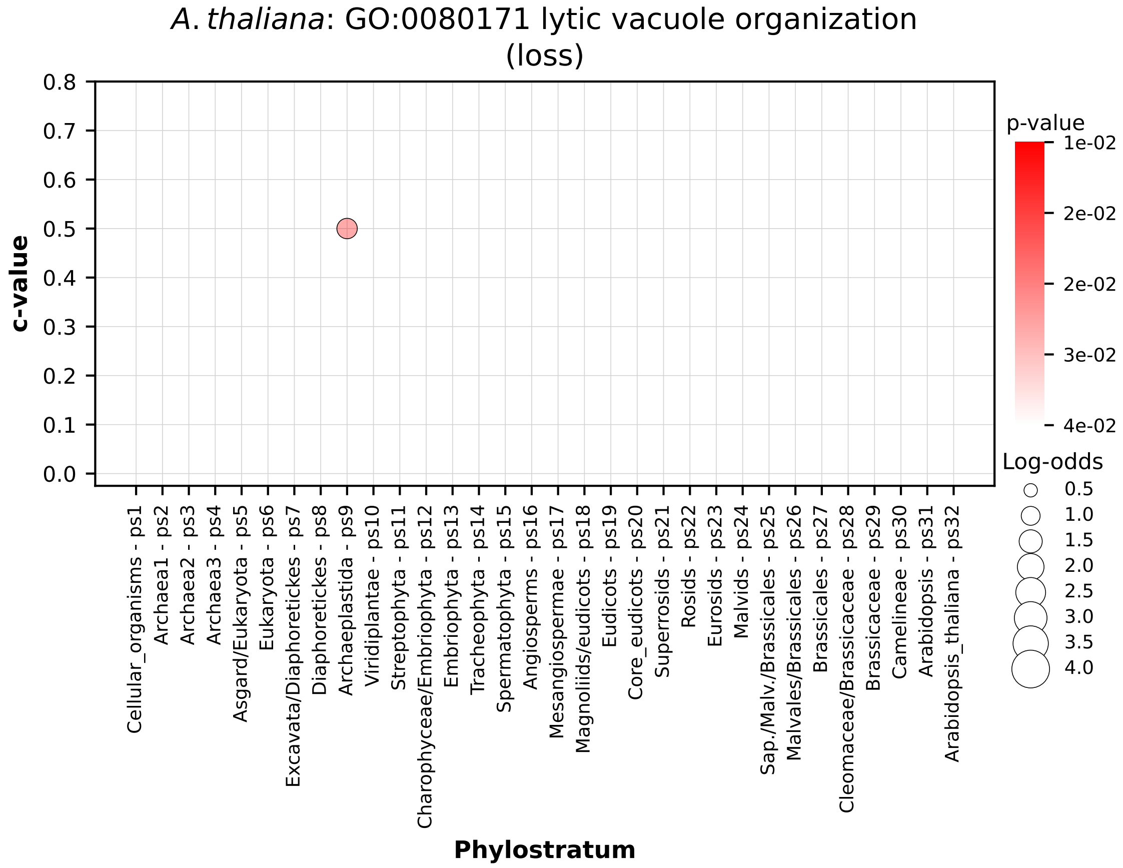

A. thaliana: GO:0080171 lytic vacuole organization
(loss)
c-value
0.8
0.7
0.6
0.5
0.4
0.3
0.2
0.1
0.0
Phylostratum
Cellular_organisms - ps1
Archaea1 - ps2
Archaea2 - ps3
Archaea3 - ps4
Asgard/Eukaryota - ps5
Eukaryota - ps6
Excavata/Diaphoretickes - ps7
Diaphoretickes - ps8
Archaeplastida - ps9
Viridiplantae - ps10
Streptophyta - ps11
Charophyceae/Embriophyta - ps12
Embriophyta - ps13
Tracheophyta - ps14
Spermatophyta - ps15
Angiosperms - ps16
Mesangiospermae - ps17
Magnoliids/eudicots - ps18
Eudicots - ps19
Core_eudicots - ps20
Superrosids - ps21
Rosids - ps22
Eurosids - ps23
Malvids - ps24
Sap./Malv./Brassicales - ps25
Malvales/Brassicales - ps26
Brassicales - ps27
Cleomaceae/Brassicaceae - ps28
Brassicaceae - ps29
Camelineae - ps30
Arabidopsis - ps31
Arabidopsis_thaliana - ps32
p-value
1e-02
2e-02
2e-02
3e-02
4e-02
Log-odds
0.5
1.0
1.5
2.0
2.5
3.0
3.5
4.0

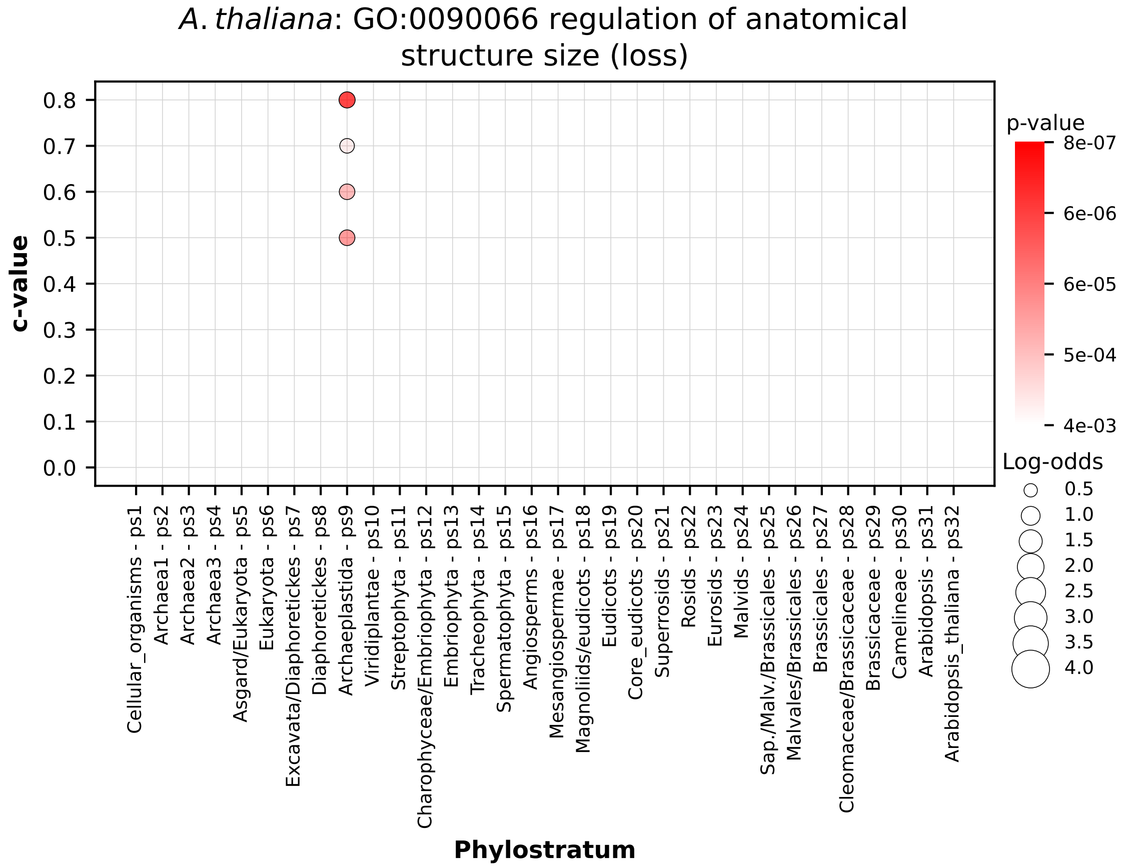

A. thaliana: GO:0090066 regulation of anatomical structure size (loss)
c-value
0.8
0.7
0.6
0.5
0.4
0.3
0.2
0.1
0.0
p-value
8e-07
6e-06
6e-05
5e-04
4e-03
Log-odds
0.5
1.0
1.5
2.0
2.5
3.0
3.5
4.0
Cellular_organisms - ps1
Archaea1 - ps2
Archaea2 - ps3
Archaea3 - ps4
Asgard/Eukaryota - ps5
Eukaryota - ps6
Excavata/Diaphoretickes - ps7
Diaphoretickes - ps8
Archaeplastida - ps9
Viridiplantae - ps10
Streptophyta - ps11
Charophyceae/Embriophyta - ps12
Embriophyta - ps13
Tracheophyta - ps14
Spermatophyta - ps15
Angiosperms - ps16
Mesangiospermae - ps17
Magnoliids/eudicots - ps18
Eudicots - ps19
Core_eudicots - ps20
Superrosids - ps21
Rosids - ps22
Eurosids - ps23
Malvids - ps24
Sap./Malv./Brassicales - ps25
Malvales/Brassicales - ps26
Brassicales - ps27
Cleomaceae/Brassicaceae - ps28
Brassicaceae - ps29
Camelineae - ps30
Arabidopsis - ps31
Arabidopsis_thaliana - ps32
Phylostratum

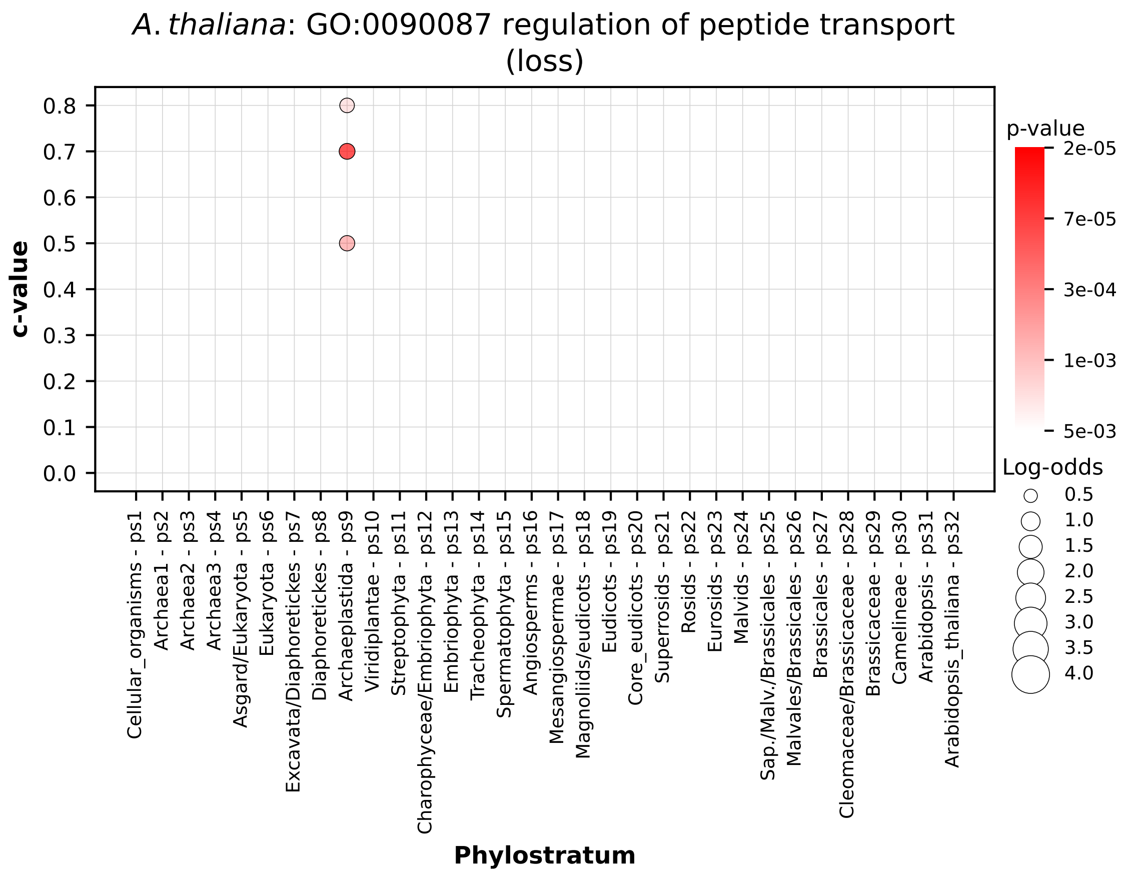
A. thaliana: GO:0090087 regulation of peptide transport
(loss)
c-value
0.8
0.7
0.6
0.5
0.4
0.3
0.2
0.1
0.0
p-value
2e-05
7e-05
3e-04
1e-03
5e-03
Log-odds
0.5
1.0
1.5
2.0
2.5
3.0
3.5
4.0
Cellular_organisms - ps1
Archaea1 - ps2
Archaea2 - ps3
Archaea3 - ps4
Asgard/Eukaryota - ps5
Eukaryota - ps6
Excavata/Diaphoretickes - ps7
Diaphoretickes - ps8
Archaeplastida - ps9
Viridiplantae - ps10
Streptophyta - ps11
Charophyceae/Embriophyta - ps12
Embriophyta - ps13
Tracheophyta - ps14
Spermatophyta - ps15
Angiosperms - ps16
Mesangiospermae - ps17
Magnoliids/eudicots - ps18
Eudicots - ps19
Core_eudicots - ps20
Superrosids - ps21
Rosids - ps22
Eurosids - ps23
Malvids - ps24
Sap./Malv./Brassicales - ps25
Malvales/Brassicales - ps26
Brassicales - ps27
Cleomaceae/Brassicaceae - ps28
Brassicaceae - ps29
Camelineae - ps30
Arabidopsis - ps31
Arabidopsis_thaliana - ps32
Phylostratum

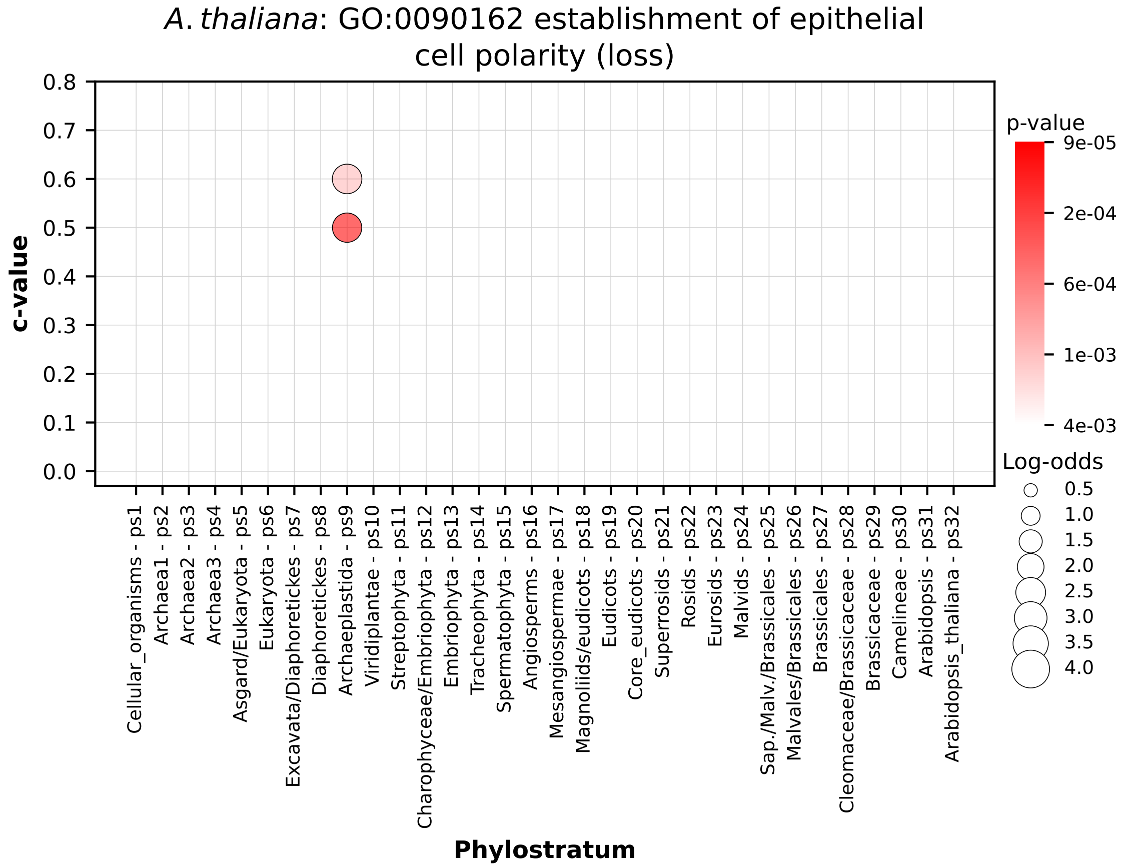

A. thaliana: GO:0090162 establishment of epithelial cell polarity (loss)
c-value
0.8
0.7
0.6
0.5
0.4
0.3
0.2
0.1
0.0
Phylostratum
Cellular_organisms - ps1
Archaea1 - ps2
Archaea2 - ps3
Archaea3 - ps4
Asgard/Eukaryota - ps5
Eukaryota - ps6
Excavata/Diaphoretickes - ps7
Diaphoretickes - ps8
Archaeplastida - ps9
Viridiplantae - ps10
Streptophyta - ps11
Charophyceae/Embriophyta - ps12
Embriophyta - ps13
Tracheophyta - ps14
Spermatophyta - ps15
Angiosperms - ps16
Mesangiospermae - ps17
Magnoliids/eudicots - ps18
Eudicots - ps19
Core_eudicots - ps20
Superrosids - ps21
Rosids - ps22
Eurosids - ps23
Malvids - ps24
Sap./Malv./Brassicales - ps25
Malvales/Brassicales - ps26
Brassicales - ps27
Cleomaceae/Brassicaceae - ps28
Brassicaceae - ps29
Camelineae - ps30
Arabidopsis - ps31
Arabidopsis_thaliana - ps32
p-value
9e-05
2e-04
6e-04
1e-03
4e-03
Log-odds
0.5
1.0
1.5
2.0
2.5
3.0
3.5
4.0

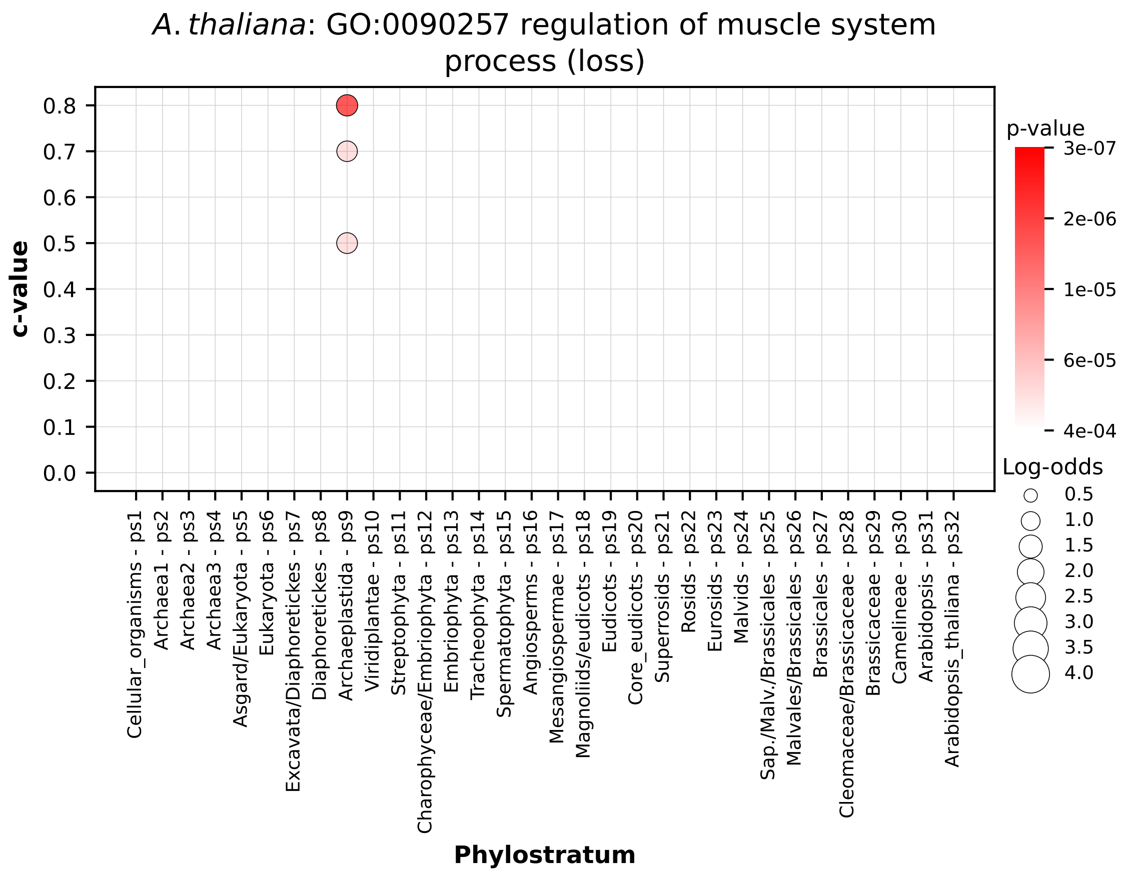

A. thaliana: GO:0090257 regulation of muscle system process (loss)
c-value
0.0
0.1
0.2
0.3
0.4
0.5
0.6
0.7
0.8
p-value
3e-07
2e-06
1e-05
6e-05
4e-04
Log-odds
0.5
1.0
1.5
2.0
2.5
3.0
3.5
4.0
Cellular_organisms - ps1
Archaea1 - ps2
Archaea2 - ps3
Archaea3 - ps4
Asgard/Eukaryota - ps5
Eukaryota - ps6
Excavata/Diaphoretickes - ps7
Diaphoretickes - ps8
Archaeplastida - ps9
Viridiplantae - ps10
Streptophyta - ps11
Charophyceae/Embriophyta - ps12
Embriophyta - ps13
Tracheophyta - ps14
Spermatophyta - ps15
Angiosperms - ps16
Mesangiospermae - ps17
Magnoliids/eudicots - ps18
Eudicots - ps19
Core_eudicots - ps20
Superrosids - ps21
Rosids - ps22
Eurosids - ps23
Malvids - ps24
Sap./Malv./Brassicales - ps25
Malvales/Brassicales - ps26
Brassicales - ps27
Cleomaceae/Brassicaceae - ps28
Brassicaceae - ps29
Camelineae - ps30
Arabidopsis - ps31
Arabidopsis_thaliana - ps32
Phylostratum

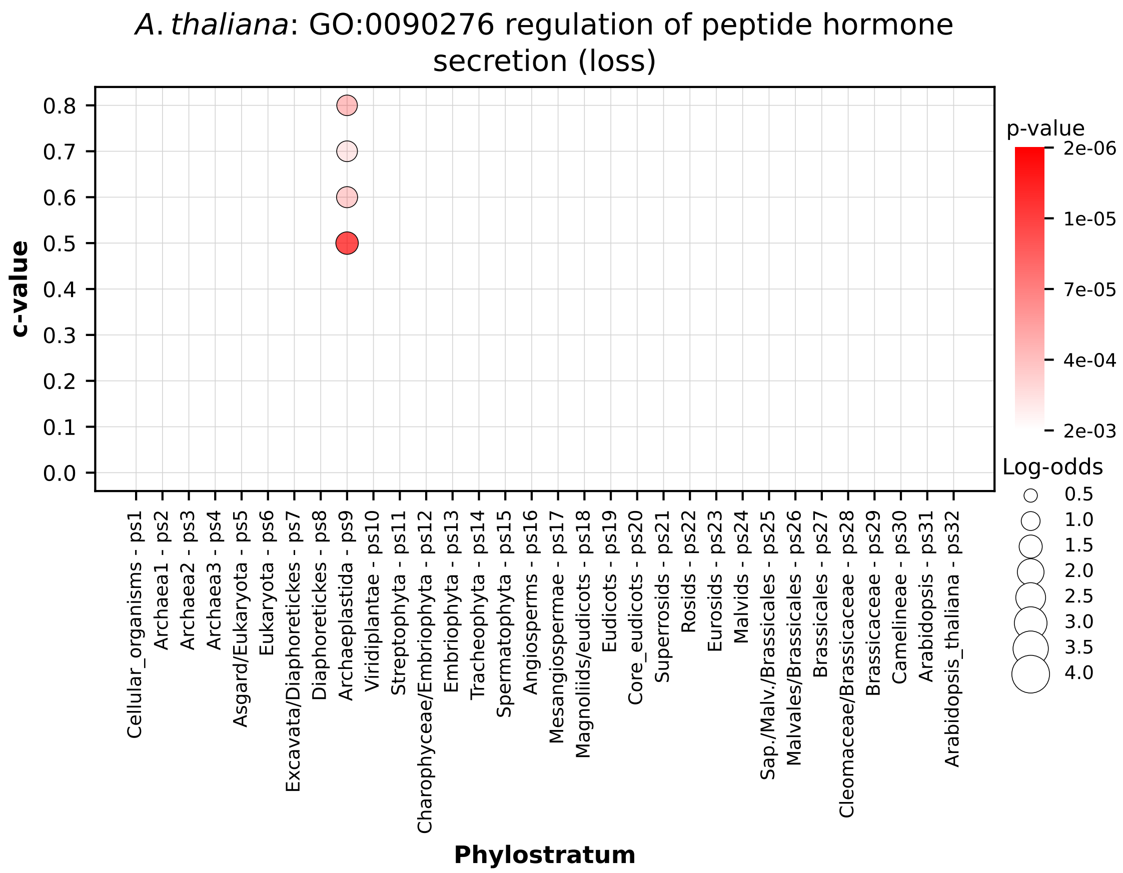
A. thaliana: GO:0090276 regulation of peptide hormone secretion (loss)
c-value
0.0
0.1
0.2
0.3
0.4
0.5
0.6
0.7
0.8
Phylostratum
Cellular_organisms - ps1
Archaea1 - ps2
Archaea2 - ps3
Archaea3 - ps4
Asgard/Eukaryota - ps5
Eukaryota - ps6
Excavata/Diaphoretickes - ps7
Diaphoretickes - ps8
Archaeplastida - ps9
Viridiplantae - ps10
Streptophyta - ps11
Charophyceae/Embriophyta - ps12
Embriophyta - ps13
Tracheophyta - ps14
Spermatophyta - ps15
Angiosperms - ps16
Mesangiospermae - ps17
Magnoliids/eudicots - ps18
Eudicots - ps19
Core_eudicots - ps20
Superrosids - ps21
Rosids - ps22
Eurosids - ps23
Malvids - ps24
Sap./Malv./Brassicales - ps25
Malvales/Brassicales - ps26
Brassicales - ps27
Cleomaceae/Brassicaceae - ps28
Brassicaceae - ps29
Camelineae - ps30
Arabidopsis - ps31
Arabidopsis_thaliana - ps32
p-value
2e-06
1e-05
7e-05
4e-04
2e-03
Log-odds
0.5
1.0
1.5
2.0
2.5
3.0
3.5
4.0

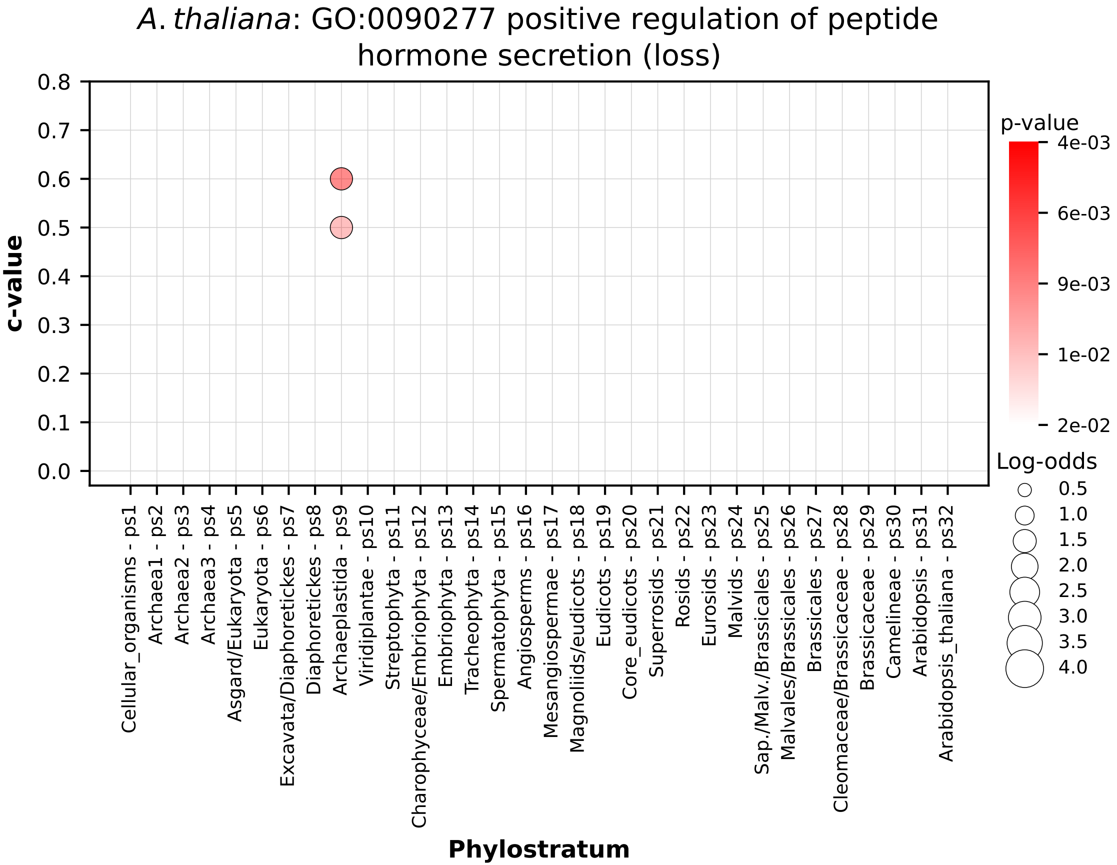
A. thaliana: GO:0090277 positive regulation of peptide hormone secretion (loss)
c-value
0.8
0.7
0.6
0.5
0.4
0.3
0.2
0.1
0.0
p-value
4e-03
6e-03
9e-03
1e-02
2e-02
Log-odds
0.5
1.0
1.5
2.0
2.5
3.0
3.5
4.0
Cellular_organisms - ps1
Archaea1 - ps2
Archaea2 - ps3
Archaea3 - ps4
Asgard/Eukaryota - ps5
Eukaryota - ps6
Excavata/Diaphoretickes - ps7
Diaphoretickes - ps8
Archaeplastida - ps9
Viridiplantae - ps10
Streptophyta - ps11
Charophyceae/Embriophyta - ps12
Embriophyta - ps13
Tracheophyta - ps14
Spermatophyta - ps15
Angiosperms - ps16
Mesangiospermae - ps17
Magnoliids/eudicots - ps18
Eudicots - ps19
Core_eudicots - ps20
Superrosids - ps21
Rosids - ps22
Eurosids - ps23
Malvids - ps24
Sap./Malv./Brassicales - ps25
Malvales/Brassicales - ps26
Brassicales - ps27
Cleomaceae/Brassicaceae - ps28
Brassicaceae - ps29
Camelineae - ps30
Arabidopsis - ps31
Arabidopsis_thaliana - ps32
Phylostratum

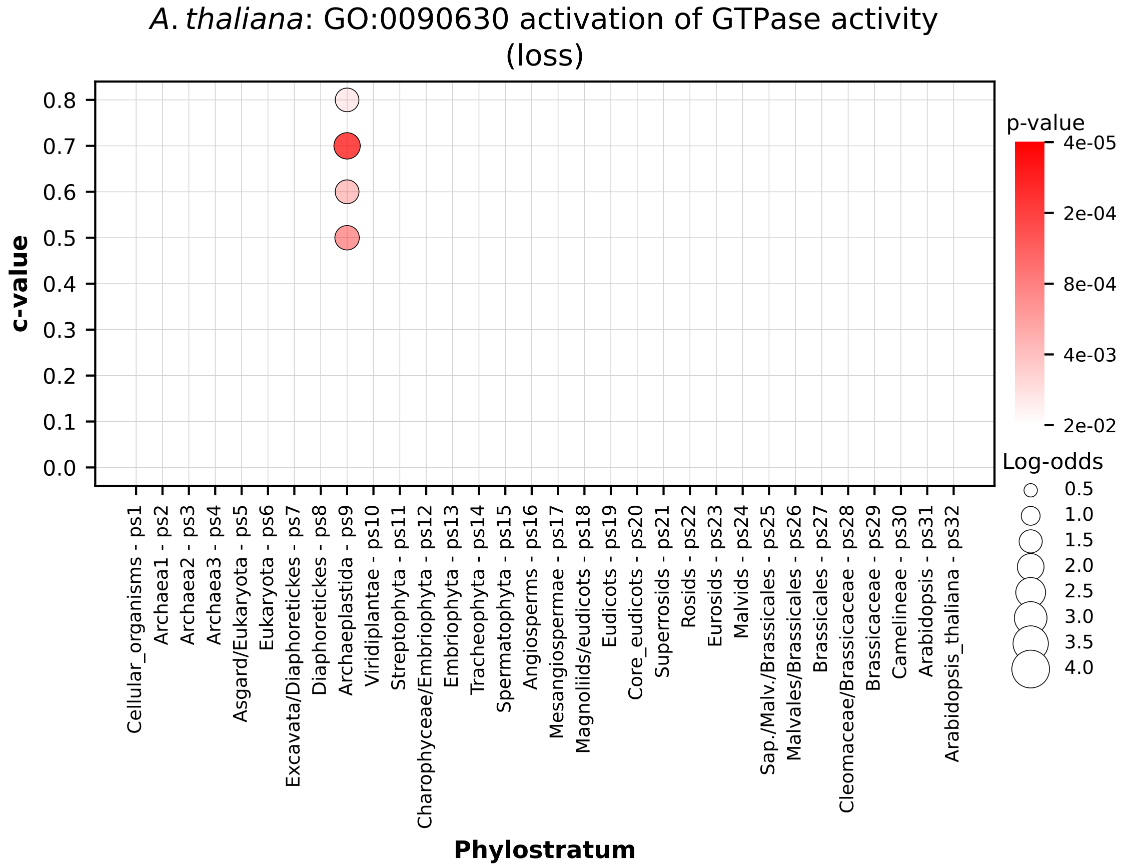

A. thaliana: GO:0090630 activation of GTPase activity
(loss)
c-value
0.8
0.7
0.6
0.5
0.4
0.3
0.2
0.1
0.0
p-value
4e-05
2e-04
8e-04
4e-03
2e-02
Log-odds
0.5
1.0
1.5
2.0
2.5
3.0
3.5
4.0
Cellular_organisms - ps1
Archaea1 - ps2
Archaea2 - ps3
Archaea3 - ps4
Asgard/Eukaryota - ps5
Eukaryota - ps6
Excavata/Diaphoretickes - ps7
Diaphoretickes - ps8
Archaeplastida - ps9
Viridiplantae - ps10
Streptophyta - ps11
Charophyceae/Embriophyta - ps12
Embriophyta - ps13
Tracheophyta - ps14
Spermatophyta - ps15
Angiosperms - ps16
Mesangiospermae - ps17
Magnoliids/eudicots - ps18
Eudicots - ps19
Core_eudicots - ps20
Superrosids - ps21
Rosids - ps22
Eurosids - ps23
Malvids - ps24
Sap./Malv./Brassicales - ps25
Malvales/Brassicales - ps26
Brassicales - ps27
Cleomaceae/Brassicaceae - ps28
Brassicaceae - ps29
Camelineae - ps30
Arabidopsis - ps31
Arabidopsis_thaliana - ps32
Phylostratum

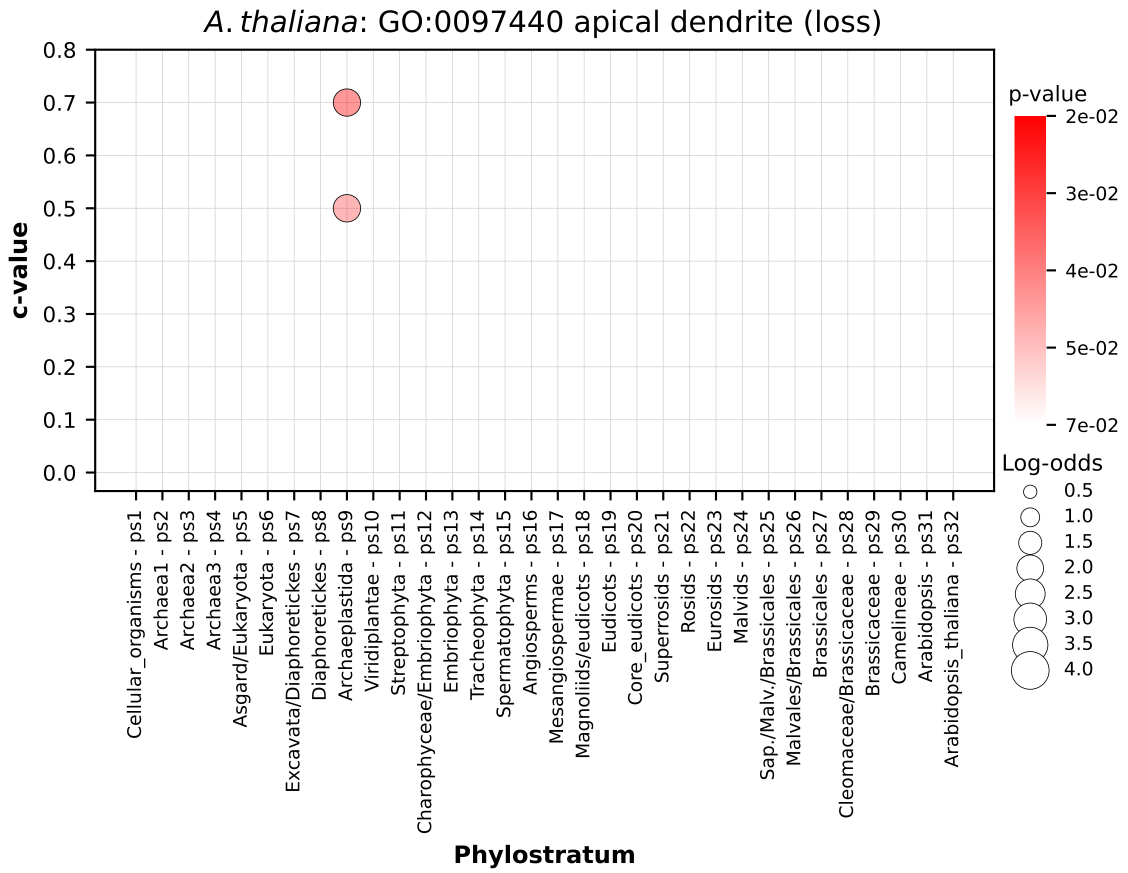

A. thaliana: GO:0097440 apical dendrite (loss)
c-value
0.0
0.1
0.2
0.3
0.4
0.5
0.6
0.7
0.8
p-value
2e-02
3e-02
4e-02
5e-02
7e-02
Log-odds
0.5
1.0
1.5
2.0
2.5
3.0
3.5
4.0
Cellular_organisms - ps1
Archaea1 - ps2
Archaea2 - ps3
Archaea3 - ps4
Asgard/Eukaryota - ps5
Eukaryota - ps6
Excavata/Diaphoretickes - ps7
Diaphoretickes - ps8
Archaeplastida - ps9
Viridiplantae - ps10
Streptophyta - ps11
Charophyceae/Embriophyta - ps12
Embriophyta - ps13
Tracheophyta - ps14
Spermatophyta - ps15
Angiosperms - ps16
Mesangiospermae - ps17
Magnoliids/eudicots - ps18
Eudicots - ps19
Core_eudicots - ps20
Superrosids - ps21
Rosids - ps22
Eurosids - ps23
Malvids - ps24
Sap./Malv./Brassicales - ps25
Malvales/Brassicales - ps26
Brassicales - ps27
Cleomaceae/Brassicaceae - ps28
Brassicaceae - ps29
Camelineae - ps30
Arabidopsis - ps31
Arabidopsis_thaliana - ps32
Phylostratum

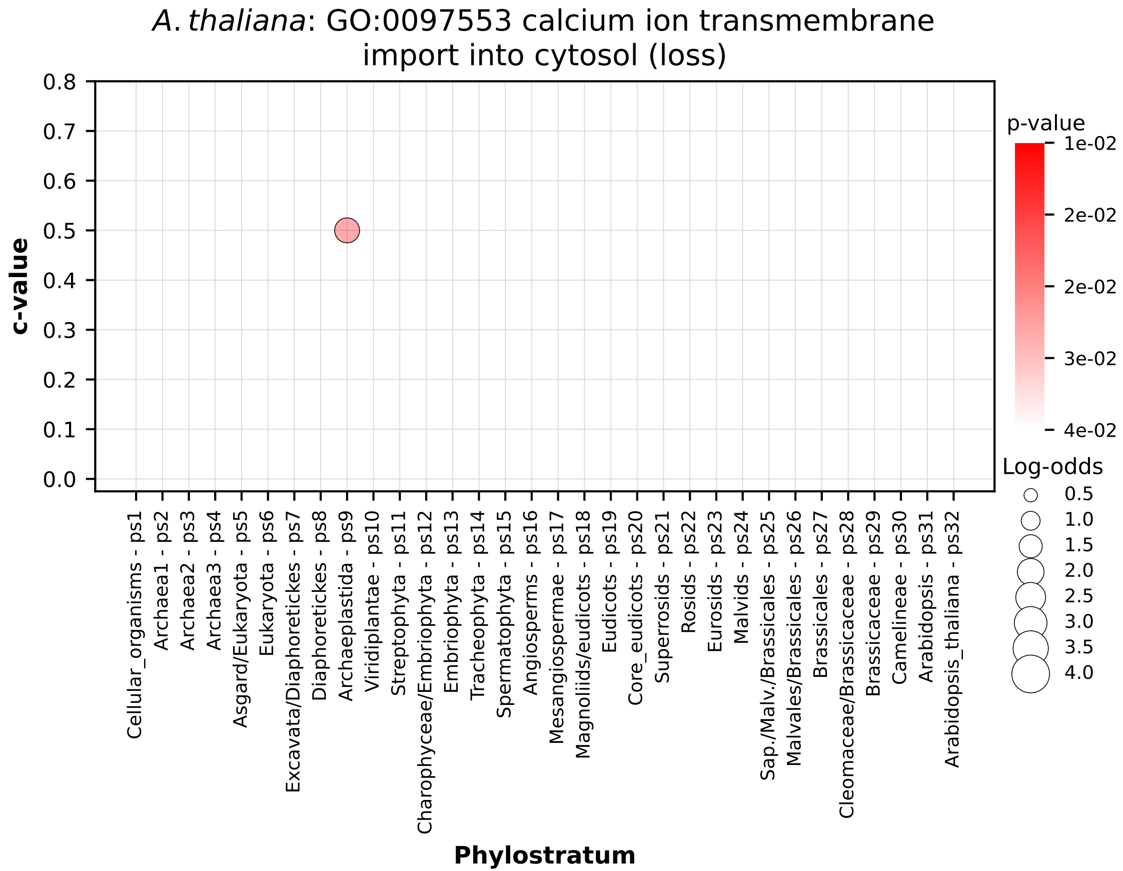

A. thaliana: GO:0097553 calcium ion transmembrane import into cytosol (loss)
c-value
0.0
0.1
0.2
0.3
0.4
0.5
0.6
0.7
0.8
Phylostratum
Cellular_organisms - ps1
Archaea1 - ps2
Archaea2 - ps3
Archaea3 - ps4
Asgard/Eukaryota - ps5
Eukaryota - ps6
Excavata/Diaphoretickes - ps7
Diaphoretickes - ps8
Archaeplastida - ps9
Viridiplantae - ps10
Streptophyta - ps11
Charophyceae/Embriophyta - ps12
Embriophyta - ps13
Tracheophyta - ps14
Spermatophyta - ps15
Angiosperms - ps16
Mesangiospermae - ps17
Magnoliids/eudicots - ps18
Eudicots - ps19
Core_eudicots - ps20
Superrosids - ps21
Rosids - ps22
Eurosids - ps23
Malvids - ps24
Sap./Malv./Brassicales - ps25
Malvales/Brassicales - ps26
Brassicales - ps27
Cleomaceae/Brassicaceae - ps28
Brassicaceae - ps29
Camelineae - ps30
Arabidopsis - ps31
Arabidopsis_thaliana - ps32
p-value
1e-02
2e-02
2e-02
3e-02
4e-02
Log-odds
0.5
1.0
1.5
2.0
2.5
3.0
3.5
4.0

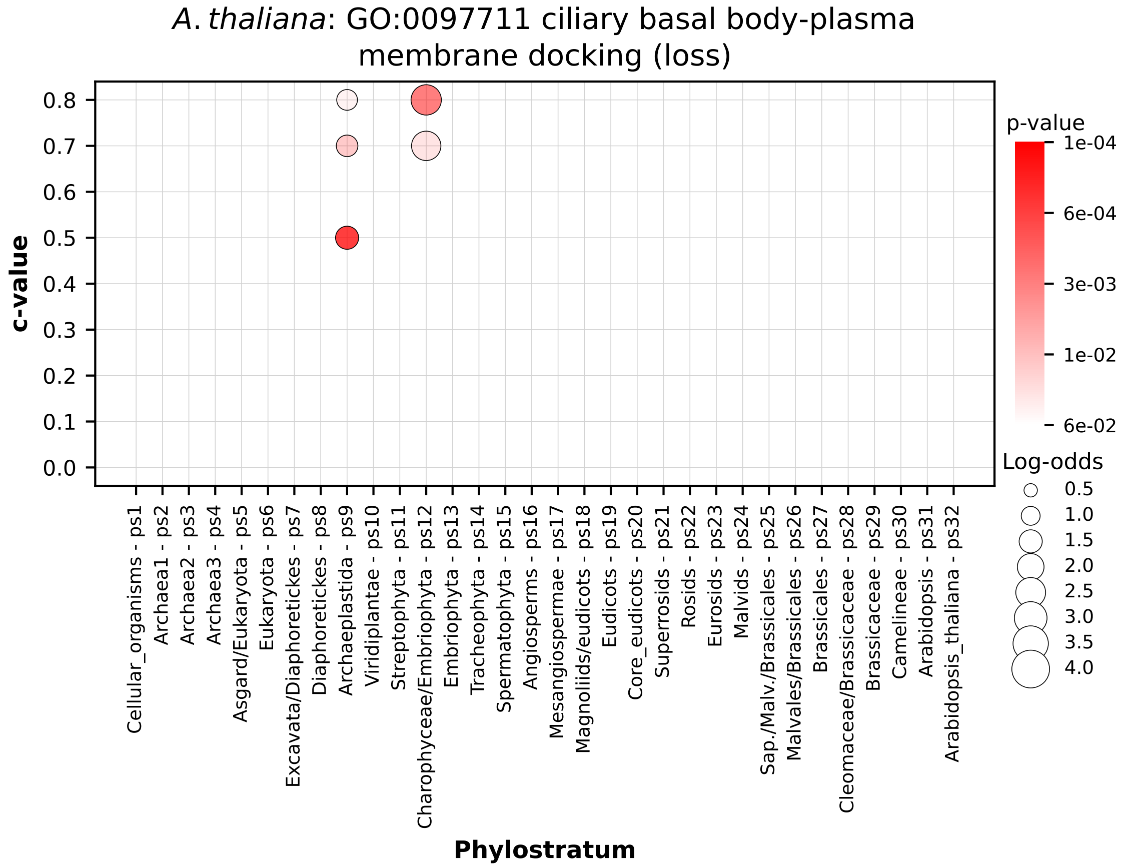

A. thaliana: GO:0097711 ciliary basal body-plasma membrane docking (loss)
c-value
0.8
0.7
0.6
0.5
0.4
0.3
0.2
0.1
0.0
p-value
1e-04
6e-04
3e-03
1e-02
6e-02
Log-odds
0.5
1.0
1.5
2.0
2.5
3.0
3.5
4.0
Cellular_organisms - ps1
Archaea1 - ps2
Archaea2 - ps3
Archaea3 - ps4
Asgard/Eukaryota - ps5
Eukaryota - ps6
Excavata/Diaphoretickes - ps7
Diaphoretickes - ps8
Archaeplastida - ps9
Viridiplantae - ps10
Streptophyta - ps11
Charophyceae/Embriophyta - ps12
Embriophyta - ps13
Tracheophyta - ps14
Spermatophyta - ps15
Angiosperms - ps16
Mesangiospermae - ps17
Magnoliids/eudicots - ps18
Eudicots - ps19
Core_eudicots - ps20
Superrosids - ps21
Rosids - ps22
Eurosids - ps23
Malvids - ps24
Sap./Malv./Brassicales - ps25
Malvales/Brassicales - ps26
Brassicales - ps27
Cleomaceae/Brassicaceae - ps28
Brassicaceae - ps29
Camelineae - ps30
Arabidopsis - ps31
Arabidopsis_thaliana - ps32
Phylostratum

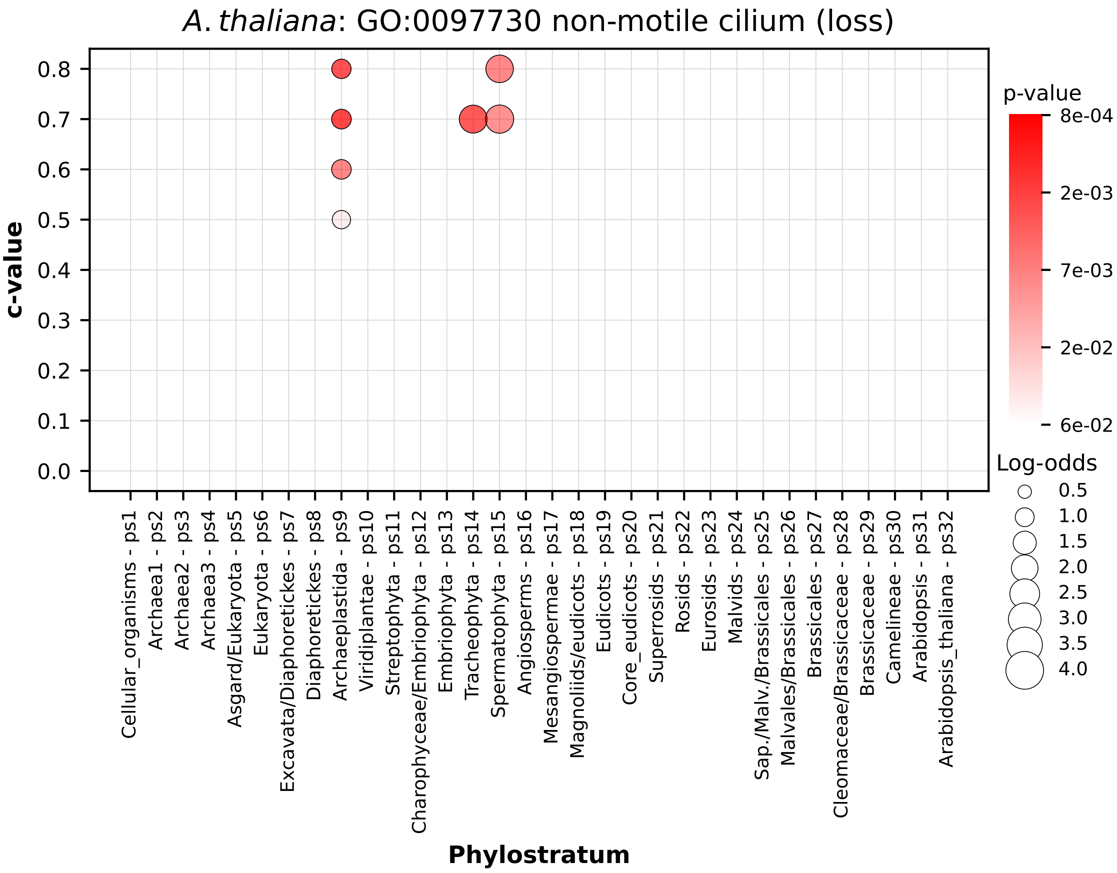

A. thaliana: GO:0097730 non-motile cilium (loss)
c-value
0.8
0.7
0.6
0.5
0.4
0.3
0.2
0.1
0.0
p-value
8e-04
2e-03
7e-03
2e-02
6e-02
Log-odds
0.5
1.0
1.5
2.0
2.5
3.0
3.5
4.0
Cellular_organisms - ps1
Archaea1 - ps2
Archaea2 - ps3
Archaea3 - ps4
Asgard/Eukaryota - ps5
Eukaryota - ps6
Excavata/Diaphoretickes - ps7
Diaphoretickes - ps8
Archaeplastida - ps9
Viridiplantae - ps10
Streptophyta - ps11
Charophyceae/Embriophyta - ps12
Embriophyta - ps13
Tracheophyta - ps14
Spermatophyta - ps15
Angiosperms - ps16
Mesangiospermae - ps17
Magnoliids/eudicots - ps18
Eudicots - ps19
Core_eudicots - ps20
Superrosids - ps21
Rosids - ps22
Eurosids - ps23
Malvids - ps24
Sap./Malv./Brassicales - ps25
Malvales/Brassicales - ps26
Brassicales - ps27
Cleomaceae/Brassicaceae - ps28
Brassicaceae - ps29
Camelineae - ps30
Arabidopsis - ps31
Arabidopsis_thaliana - ps32
Phylostratum

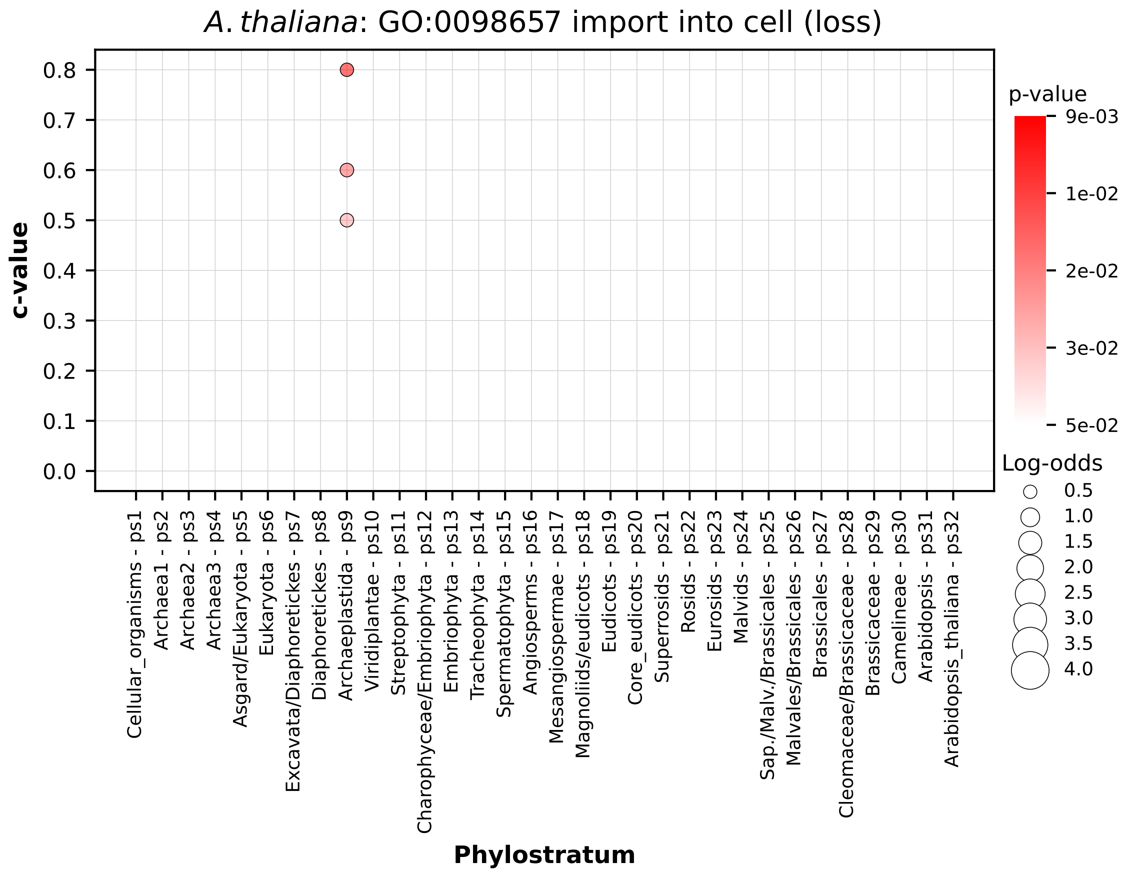

A. thaliana: GO:0098657 import into cell (loss)
c-value
0.0
0.1
0.2
0.3
0.4
0.5
0.6
0.7
0.8
Phylostratum
Cellular_organisms - ps1
Archaea1 - ps2
Archaea2 - ps3
Archaea3 - ps4
Asgard/Eukaryota - ps5
Eukaryota - ps6
Excavata/Diaphoretickes - ps7
Diaphoretickes - ps8
Archaeplastida - ps9
Viridiplantae - ps10
Streptophyta - ps11
Charophyceae/Embriophyta - ps12
Embriophyta - ps13
Tracheophyta - ps14
Spermatophyta - ps15
Angiosperms - ps16
Mesangiospermae - ps17
Magnoliids/eudicots - ps18
Eudicots - ps19
Core_eudicots - ps20
Superrosids - ps21
Rosids - ps22
Eurosids - ps23
Malvids - ps24
Sap./Malv./Brassicales - ps25
Malvales/Brassicales - ps26
Brassicales - ps27
Cleomaceae/Brassicaceae - ps28
Brassicaceae - ps29
Camelineae - ps30
Arabidopsis - ps31
Arabidopsis_thaliana - ps32
p-value
9e-03
1e-02
2e-02
3e-02
5e-02
Log-odds
0.5
1.0
1.5
2.0
2.5
3.0
3.5
4.0

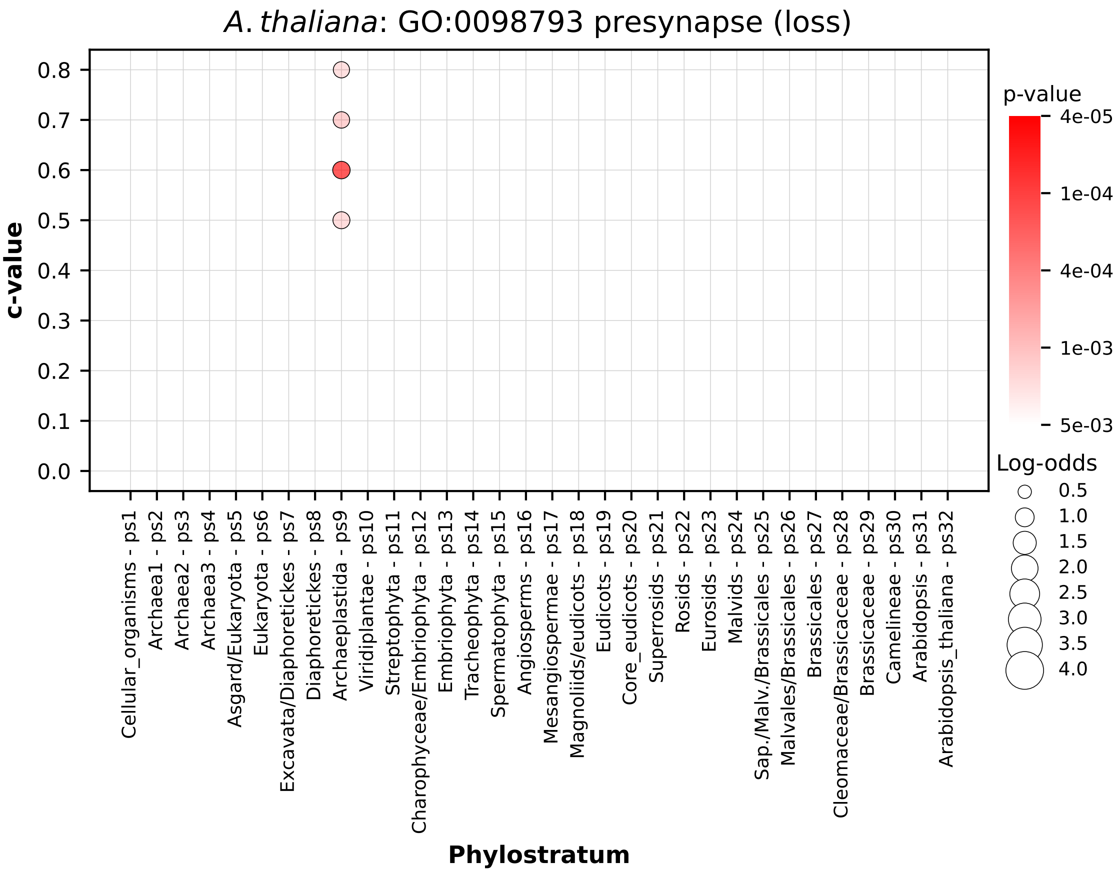

A. thaliana: GO:0098793 presynapse (loss)
c-value
0.8
0.7
0.6
0.5
0.4
0.3
0.2
0.1
0.0
p-value
4e-05
1e-04
4e-04
1e-03
5e-03
Log-odds
0.5
1.0
1.5
2.0
2.5
3.0
3.5
4.0
Cellular_organisms - ps1
Archaea1 - ps2
Archaea2 - ps3
Archaea3 - ps4
Asgard/Eukaryota - ps5
Eukaryota - ps6
Excavata/Diaphoretickes - ps7
Diaphoretickes - ps8
Archaeplastida - ps9
Viridiplantae - ps10
Streptophyta - ps11
Charophyceae/Embriophyta - ps12
Embriophyta - ps13
Tracheophyta - ps14
Spermatophyta - ps15
Angiosperms - ps16
Mesangiospermae - ps17
Magnoliids/eudicots - ps18
Eudicots - ps19
Core_eudicots - ps20
Superrosids - ps21
Rosids - ps22
Eurosids - ps23
Malvids - ps24
Sap./Malv./Brassicales - ps25
Malvales/Brassicales - ps26
Brassicales - ps27
Cleomaceae/Brassicaceae - ps28
Brassicaceae - ps29
Camelineae - ps30
Arabidopsis - ps31
Arabidopsis_thaliana - ps32
Phylostratum

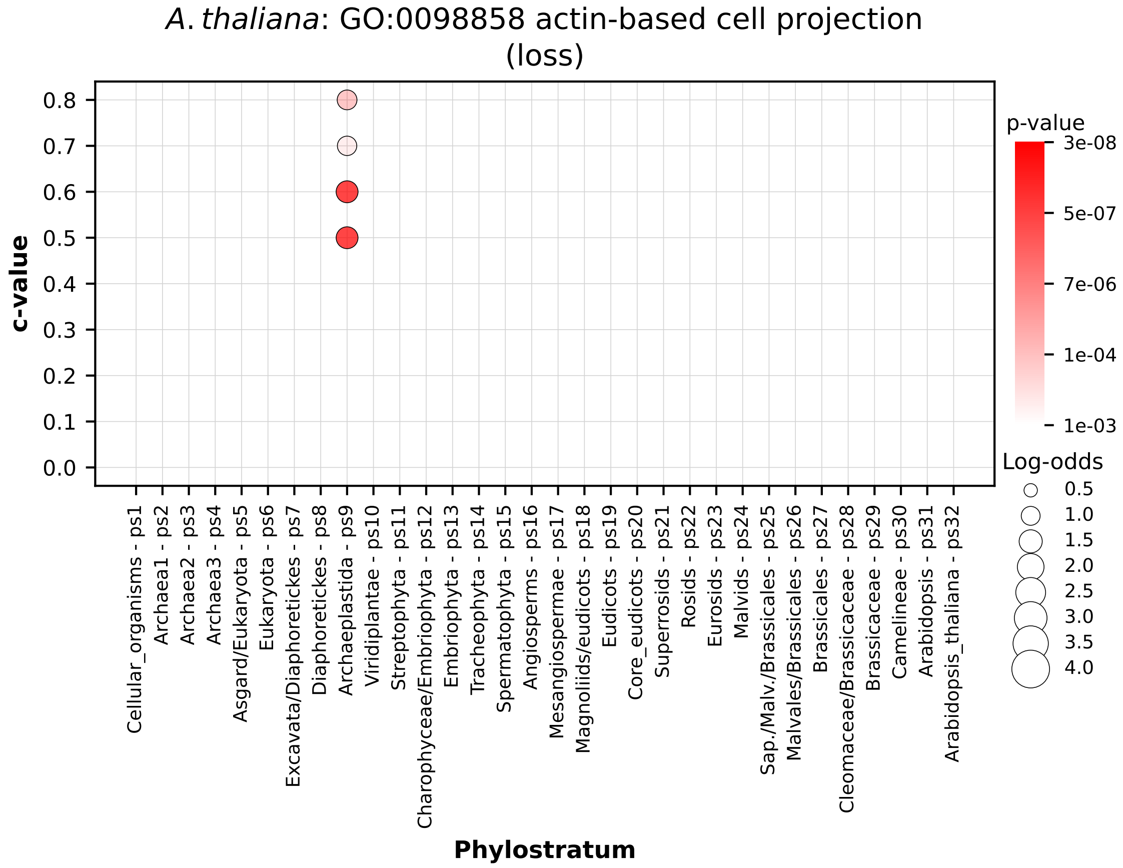

A. thaliana: GO:0098858 actin-based cell projection
(loss)
c-value
0.8
0.7
0.6
0.5
0.4
0.3
0.2
0.1
0.0
p-value
3e-08
5e-07
7e-06
1e-04
1e-03
Log-odds
0.5
1.0
1.5
2.0
2.5
3.0
3.5
4.0
Cellular_organisms - ps1
Archaea1 - ps2
Archaea2 - ps3
Archaea3 - ps4
Asgard/Eukaryota - ps5
Eukaryota - ps6
Excavata/Diaphoretickes - ps7
Diaphoretickes - ps8
Archaeplastida - ps9
Viridiplantae - ps10
Streptophyta - ps11
Charophyceae/Embriophyta - ps12
Embriophyta - ps13
Tracheophyta - ps14
Spermatophyta - ps15
Angiosperms - ps16
Mesangiospermae - ps17
Magnoliids/eudicots - ps18
Eudicots - ps19
Core_eudicots - ps20
Superrosids - ps21
Rosids - ps22
Eurosids - ps23
Malvids - ps24
Sap./Malv./Brassicales - ps25
Malvales/Brassicales - ps26
Brassicales - ps27
Cleomaceae/Brassicaceae - ps28
Brassicaceae - ps29
Camelineae - ps30
Arabidopsis - ps31
Arabidopsis_thaliana - ps32
Phylostratum

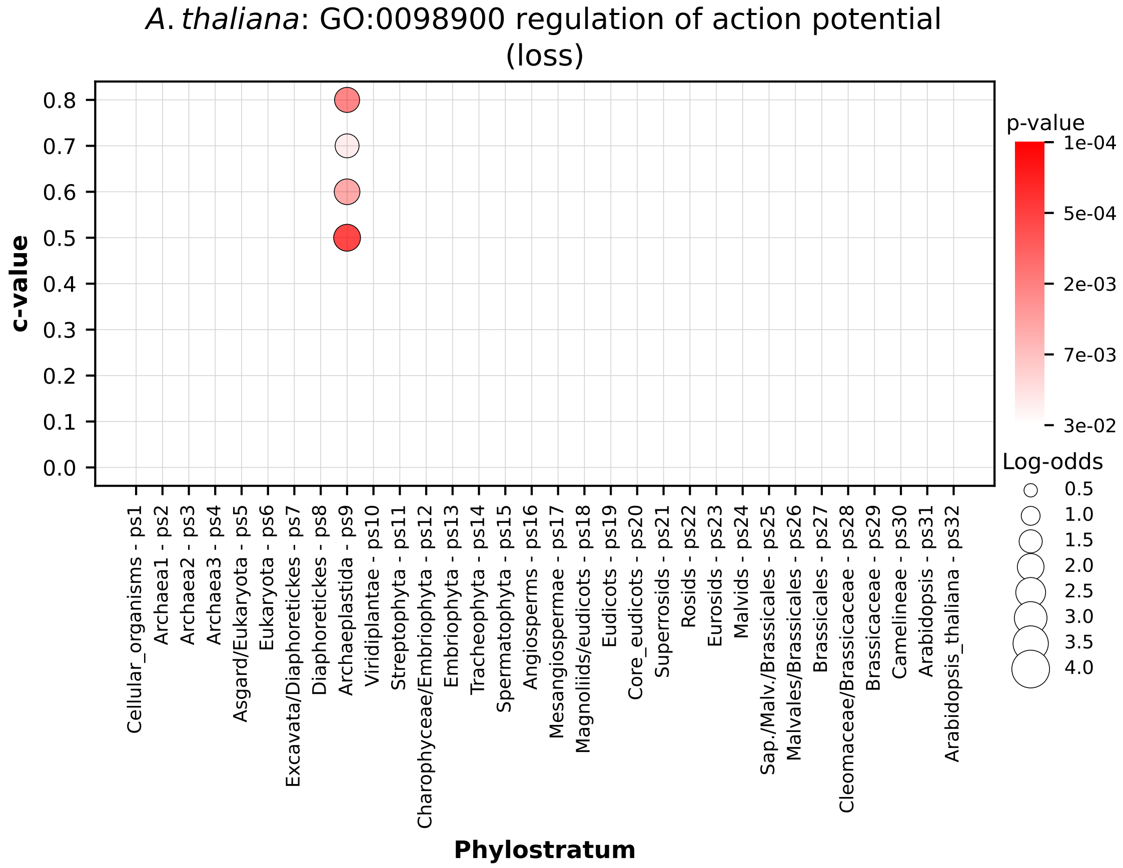

A. thaliana: GO:0098900 regulation of action potential
(loss)
c-value
0.8
0.7
0.6
0.5
0.4
0.3
0.2
0.1
0.0
p-value
1e-04
5e-04
2e-03
7e-03
3e-02
Log-odds
0.5
1.0
1.5
2.0
2.5
3.0
3.5
4.0
Cellular_organisms - ps1
Archaea1 - ps2
Archaea2 - ps3
Archaea3 - ps4
Asgard/Eukaryota - ps5
Eukaryota - ps6
Excavata/Diaphoretickes - ps7
Diaphoretickes - ps8
Archaeplastida - ps9
Viridiplantae - ps10
Streptophyta - ps11
Charophyceae/Embriophyta - ps12
Embriophyta - ps13
Tracheophyta - ps14
Spermatophyta - ps15
Angiosperms - ps16
Mesangiospermae - ps17
Magnoliids/eudicots - ps18
Eudicots - ps19
Core_eudicots - ps20
Superrosids - ps21
Rosids - ps22
Eurosids - ps23
Malvids - ps24
Sap./Malv./Brassicales - ps25
Malvales/Brassicales - ps26
Brassicales - ps27
Cleomaceae/Brassicaceae - ps28
Brassicaceae - ps29
Camelineae - ps30
Arabidopsis - ps31
Arabidopsis_thaliana - ps32
Phylostratum

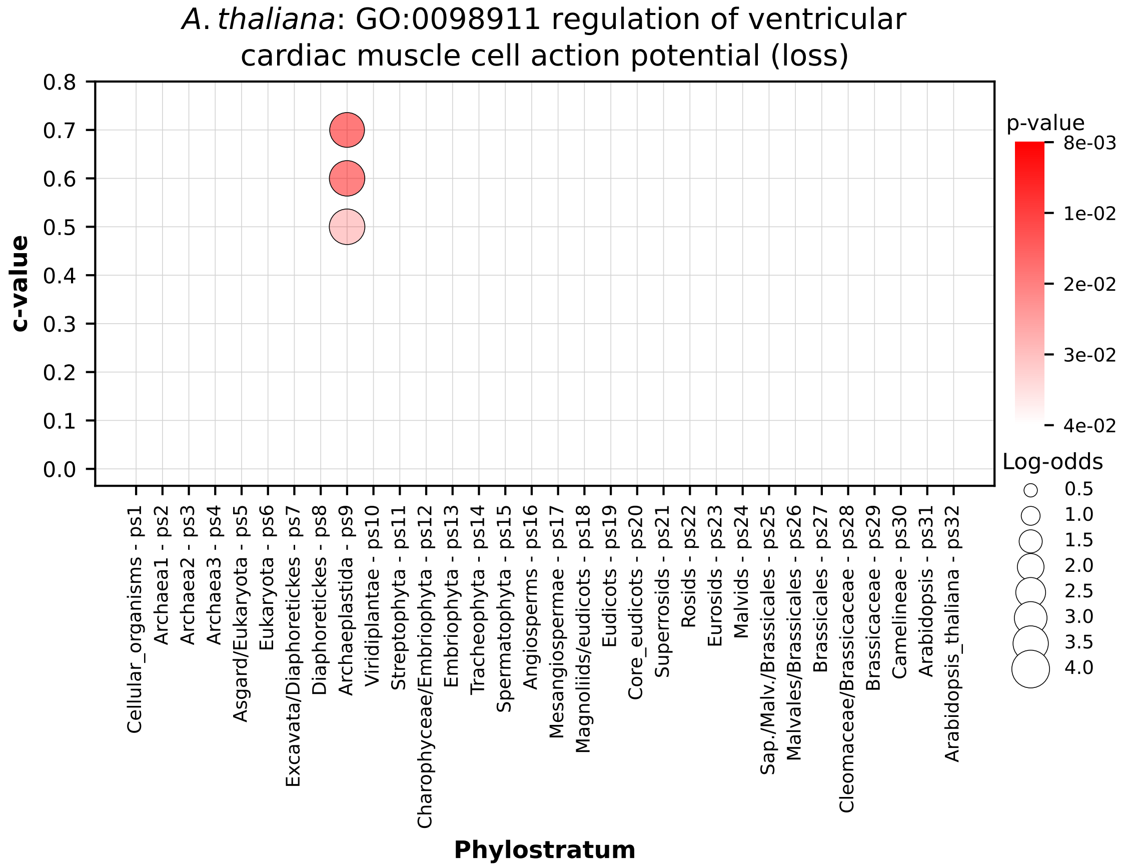

A. thaliana: GO:0098911 regulation of ventricular cardiac muscle cell action potential (loss)
c-value
0.8
0.7
0.6
0.5
0.4
0.3
0.2
0.1
0.0
Phylostratum
Cellular_organisms - ps1
Archaea1 - ps2
Archaea2 - ps3
Archaea3 - ps4
Asgard/Eukaryota - ps5
Eukaryota - ps6
Excavata/Diaphoretickes - ps7
Diaphoretickes - ps8
Archaeplastida - ps9
Viridiplantae - ps10
Streptophyta - ps11
Charophyceae/Embriophyta - ps12
Embriophyta - ps13
Tracheophyta - ps14
Spermatophyta - ps15
Angiosperms - ps16
Mesangiospermae - ps17
Magnoliids/eudicots - ps18
Eudicots - ps19
Core_eudicots - ps20
Superrosids - ps21
Rosids - ps22
Eurosids - ps23
Malvids - ps24
Sap./Malv./Brassicales - ps25
Malvales/Brassicales - ps26
Brassicales - ps27
Cleomaceae/Brassicaceae - ps28
Brassicaceae - ps29
Camelineae - ps30
Arabidopsis - ps31
Arabidopsis_thaliana - ps32
p-value
8e-03
1e-02
2e-02
3e-02
4e-02
Log-odds
0.5
1.0
1.5
2.0
2.5
3.0
3.5
4.0

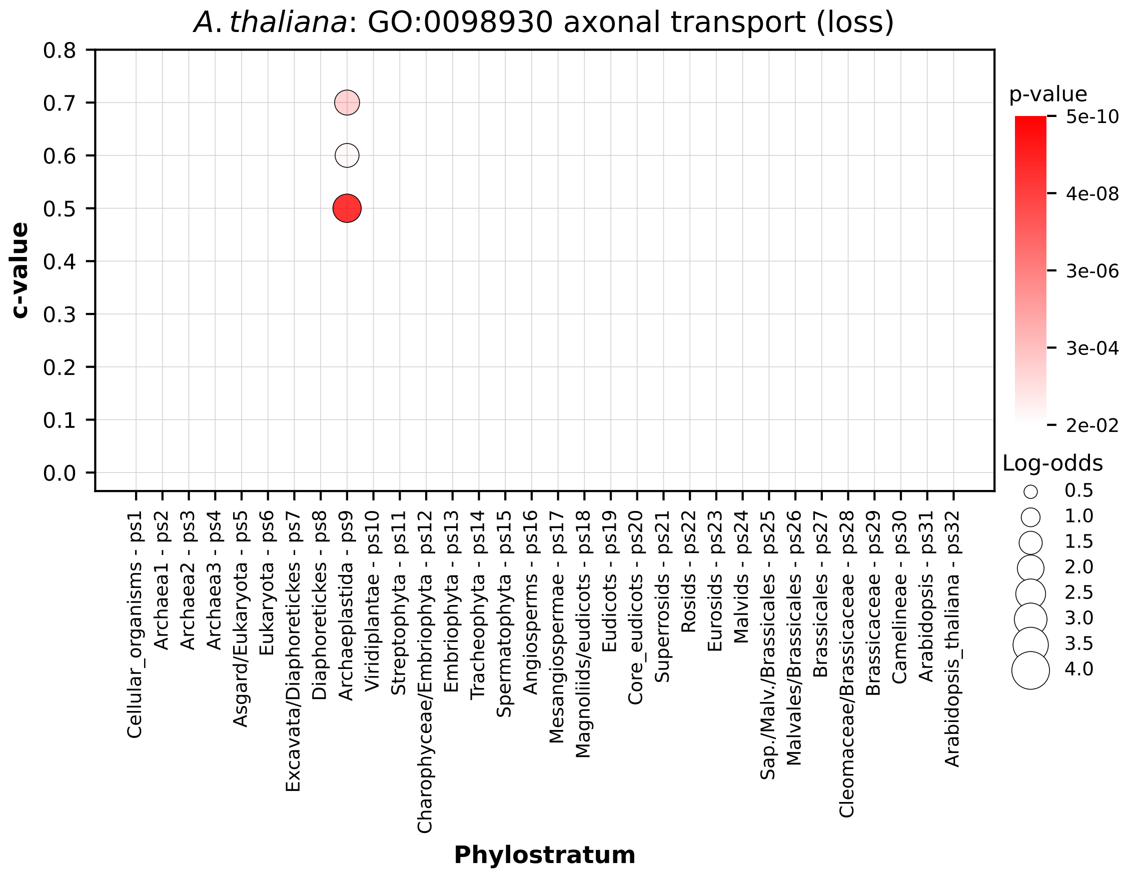

A. thaliana: GO:0098930 axonal transport (loss)
c-value
0.0
0.1
0.2
0.3
0.4
0.5
0.6
0.7
0.8
Phylostratum
Cellular_organisms - ps1
Archaea1 - ps2
Archaea2 - ps3
Archaea3 - ps4
Asgard/Eukaryota - ps5
Eukaryota - ps6
Excavata/Diaphoretickes - ps7
Diaphoretickes - ps8
Archaeplastida - ps9
Viridiplantae - ps10
Streptophyta - ps11
Charophyceae/Embriophyta - ps12
Embriophyta - ps13
Tracheophyta - ps14
Spermatophyta - ps15
Angiosperms - ps16
Mesangiospermae - ps17
Magnoliids/eudicots - ps18
Eudicots - ps19
Core_eudicots - ps20
Superrosids - ps21
Rosids - ps22
Eurosids - ps23
Malvids - ps24
Sap./Malv./Brassicales - ps25
Malvales/Brassicales - ps26
Brassicales - ps27
Cleomaceae/Brassicaceae - ps28
Brassicaceae - ps29
Camelineae - ps30
Arabidopsis - ps31
Arabidopsis_thaliana - ps32
p-value
5e-10
4e-08
3e-06
3e-04
2e-02
Log-odds
0.5
1.0
1.5
2.0
2.5
3.0
3.5
4.0

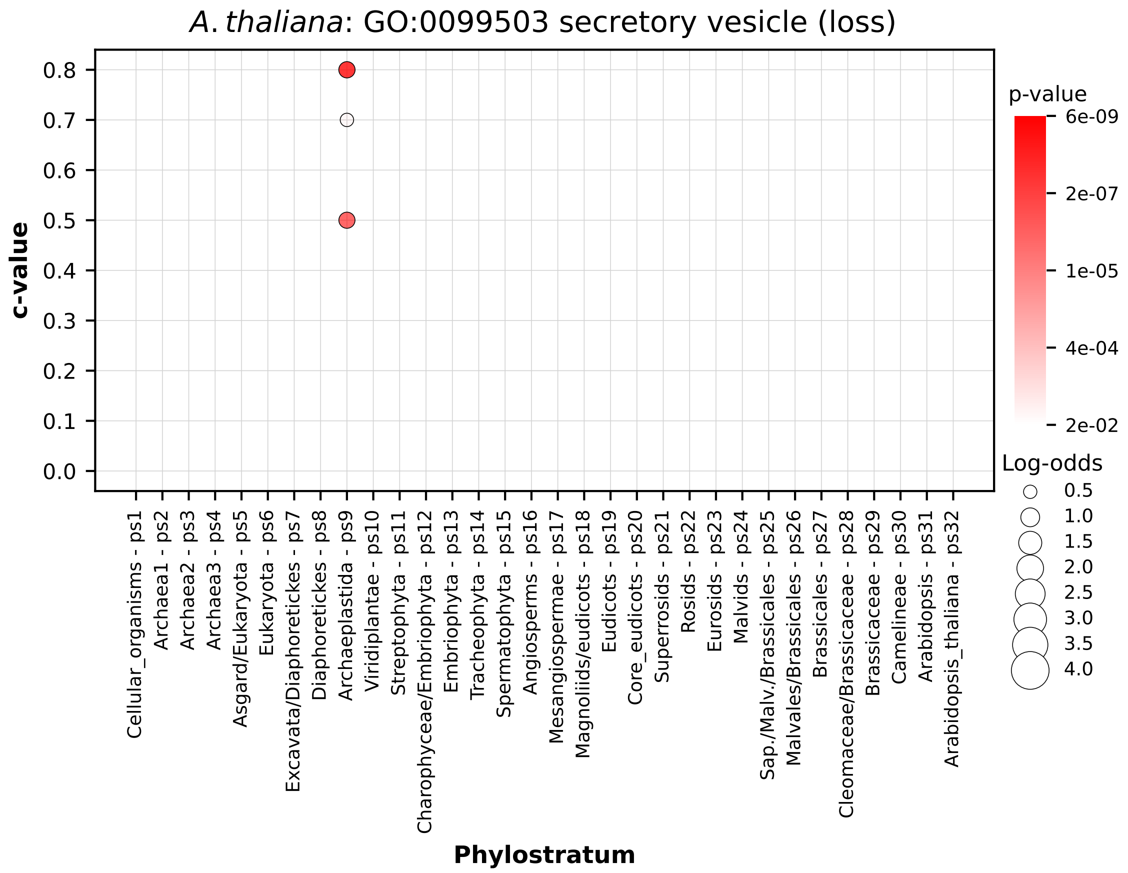
A. thaliana: GO:0099503 secretory vesicle (loss)
c-value
0.8
0.7
0.6
0.5
0.4
0.3
0.2
0.1
0.0
p-value
6e-09
2e-07
1e-05
4e-04
2e-02
Log-odds
0.5
1.0
1.5
2.0
2.5
3.0
3.5
4.0
Cellular_organisms - ps1
Archaea1 - ps2
Archaea2 - ps3
Archaea3 - ps4
Asgard/Eukaryota - ps5
Eukaryota - ps6
Excavata/Diaphoretickes - ps7
Diaphoretickes - ps8
Archaeplastida - ps9
Viridiplantae - ps10
Streptophyta - ps11
Charophyceae/Embriophyta - ps12
Embriophyta - ps13
Tracheophyta - ps14
Spermatophyta - ps15
Angiosperms - ps16
Mesangiospermae - ps17
Magnoliids/eudicots - ps18
Eudicots - ps19
Core_eudicots - ps20
Superrosids - ps21
Rosids - ps22
Eurosids - ps23
Malvids - ps24
Sap./Malv./Brassicales - ps25
Malvales/Brassicales - ps26
Brassicales - ps27
Cleomaceae/Brassicaceae - ps28
Brassicaceae - ps29
Camelineae - ps30
Arabidopsis - ps31
Arabidopsis_thaliana - ps32
Phylostratum

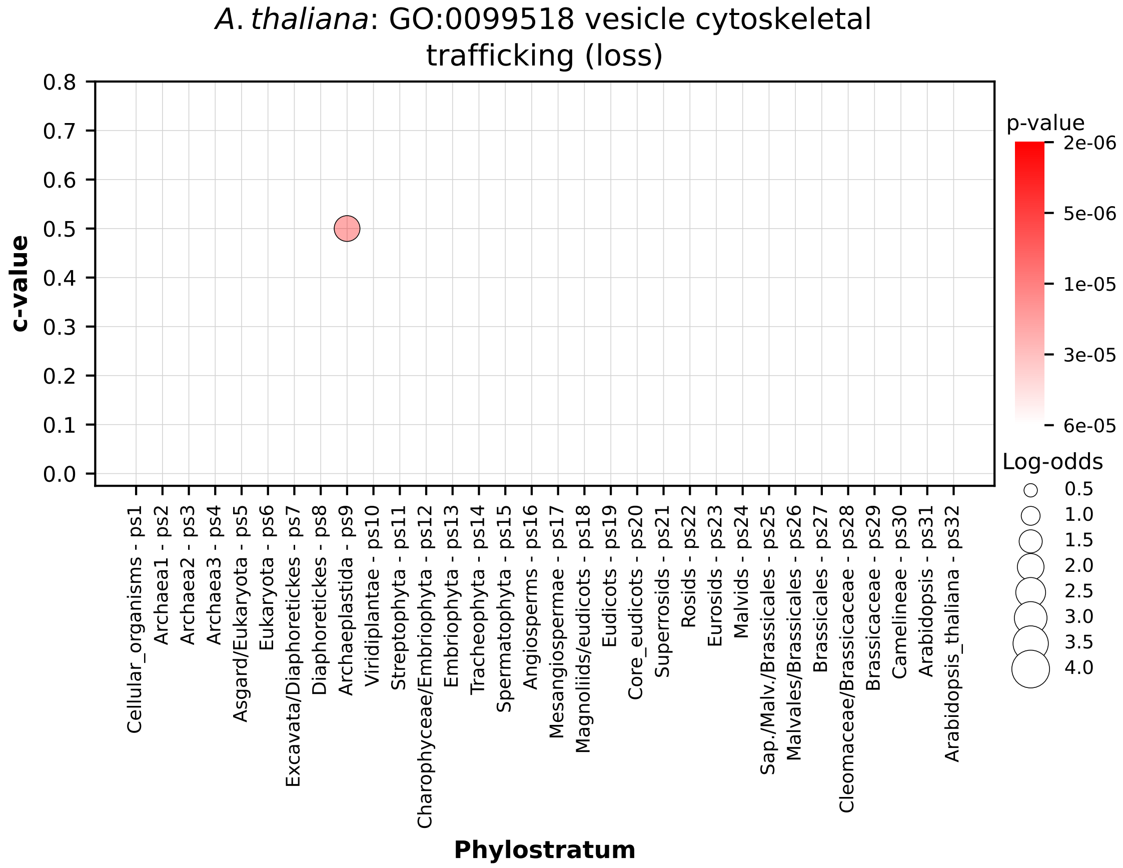
A. thaliana: GO:0099518 vesicle cytoskeletal trafficking (loss)
c-value
0.8
0.7
0.6
0.5
0.4
0.3
0.2
0.1
0.0
Phylostratum
Cellular_organisms - ps1
Archaea1 - ps2
Archaea2 - ps3
Archaea3 - ps4
Asgard/Eukaryota - ps5
Eukaryota - ps6
Excavata/Diaphoretickes - ps7
Diaphoretickes - ps8
Archaeplastida - ps9
Viridiplantae - ps10
Streptophyta - ps11
Charophyceae/Embriophyta - ps12
Embriophyta - ps13
Tracheophyta - ps14
Spermatophyta - ps15
Angiosperms - ps16
Mesangiospermae - ps17
Magnoliids/eudicots - ps18
Eudicots - ps19
Core_eudicots - ps20
Superrosids - ps21
Rosids - ps22
Eurosids - ps23
Malvids - ps24
Sap./Malv./Brassicales - ps25
Malvales/Brassicales - ps26
Brassicales - ps27
Cleomaceae/Brassicaceae - ps28
Brassicaceae - ps29
Camelineae - ps30
Arabidopsis - ps31
Arabidopsis_thaliana - ps32
p-value
2e-06
5e-06
1e-05
3e-05
6e-05
Log-odds
0.5
1.0
1.5
2.0
2.5
3.0
3.5
4.0

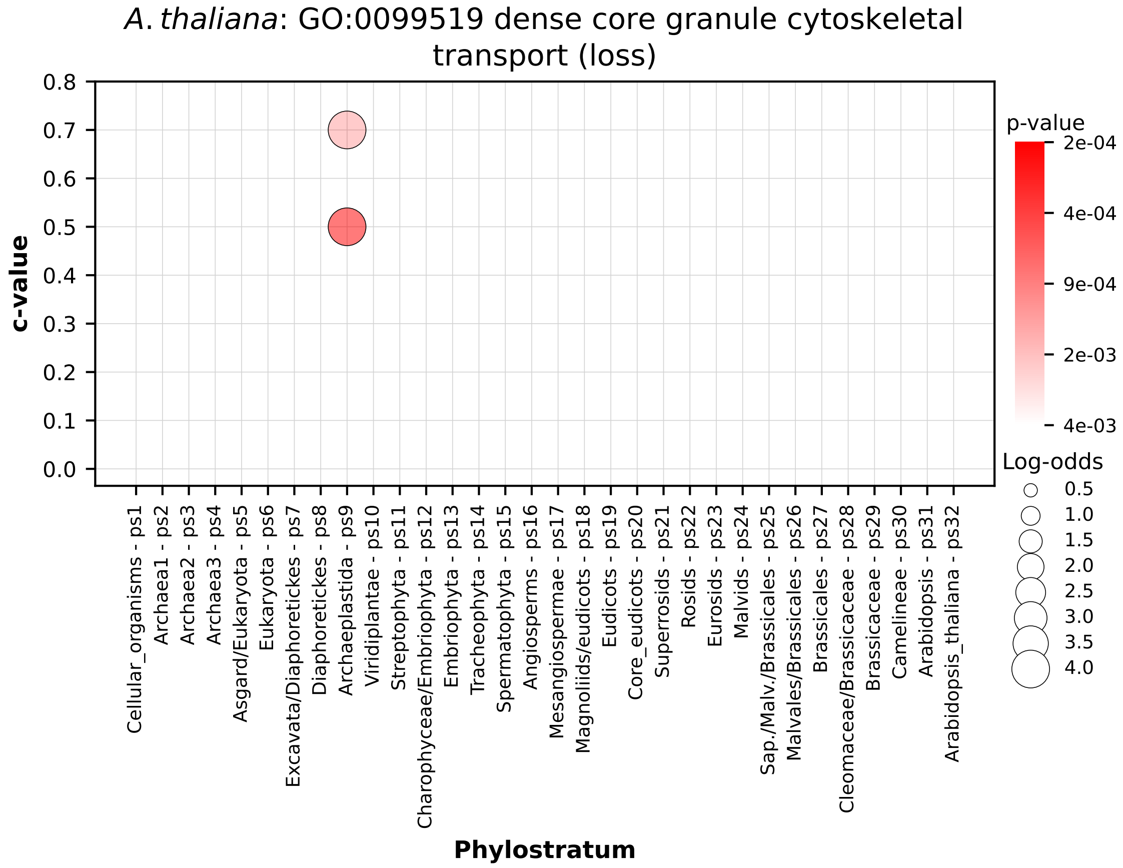
A. thaliana: GO:0099519 dense core granule cytoskeletal transport (loss)
c-value
0.8
0.7
0.6
0.5
0.4
0.3
0.2
0.1
0.0
p-value
2e-04
4e-04
9e-04
2e-03
4e-03
Log-odds
0.5
1.0
1.5
2.0
2.5
3.0
3.5
4.0
Cellular_organisms - ps1
Archaea1 - ps2
Archaea2 - ps3
Archaea3 - ps4
Asgard/Eukaryota - ps5
Eukaryota - ps6
Excavata/Diaphoretickes - ps7
Diaphoretickes - ps8
Archaeplastida - ps9
Viridiplantae - ps10
Streptophyta - ps11
Charophyceae/Embriophyta - ps12
Embriophyta - ps13
Tracheophyta - ps14
Spermatophyta - ps15
Angiosperms - ps16
Mesangiospermae - ps17
Magnoliids/eudicots - ps18
Eudicots - ps19
Core_eudicots - ps20
Superrosids - ps21
Rosids - ps22
Eurosids - ps23
Malvids - ps24
Sap./Malv./Brassicales - ps25
Malvales/Brassicales - ps26
Brassicales - ps27
Cleomaceae/Brassicaceae - ps28
Brassicaceae - ps29
Camelineae - ps30
Arabidopsis - ps31
Arabidopsis_thaliana - ps32
Phylostratum

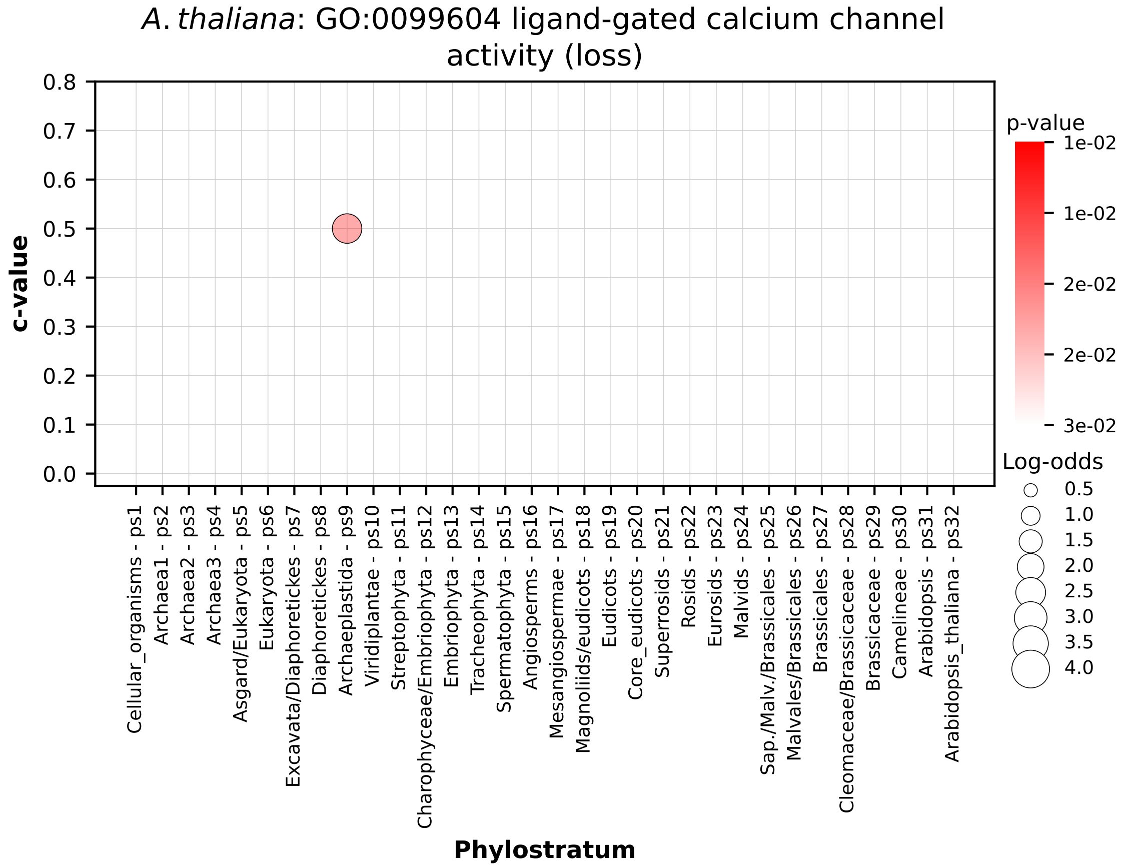

A. thaliana: GO:0099604 ligand-gated calcium channel activity (loss)
c-value
0.8
0.7
0.6
0.5
0.4
0.3
0.2
0.1
0.0
Phylostratum
Cellular_organisms - ps1
Archaea1 - ps2
Archaea2 - ps3
Archaea3 - ps4
Asgard/Eukaryota - ps5
Eukaryota - ps6
Excavata/Diaphoretickes - ps7
Diaphoretickes - ps8
Archaeplastida - ps9
Viridiplantae - ps10
Streptophyta - ps11
Charophyceae/Embriophyta - ps12
Embriophyta - ps13
Tracheophyta - ps14
Spermatophyta - ps15
Angiosperms - ps16
Mesangiospermae - ps17
Magnoliids/eudicots - ps18
Eudicots - ps19
Core_eudicots - ps20
Superrosids - ps21
Rosids - ps22
Eurosids - ps23
Malvids - ps24
Sap./Malv./Brassicales - ps25
Malvales/Brassicales - ps26
Brassicales - ps27
Cleomaceae/Brassicaceae - ps28
Brassicaceae - ps29
Camelineae - ps30
Arabidopsis - ps31
Arabidopsis_thaliana - ps32
p-value
1e-02
1e-02
2e-02
2e-02
3e-02
Log-odds
0.5
1.0
1.5
2.0
2.5
3.0
3.5
4.0

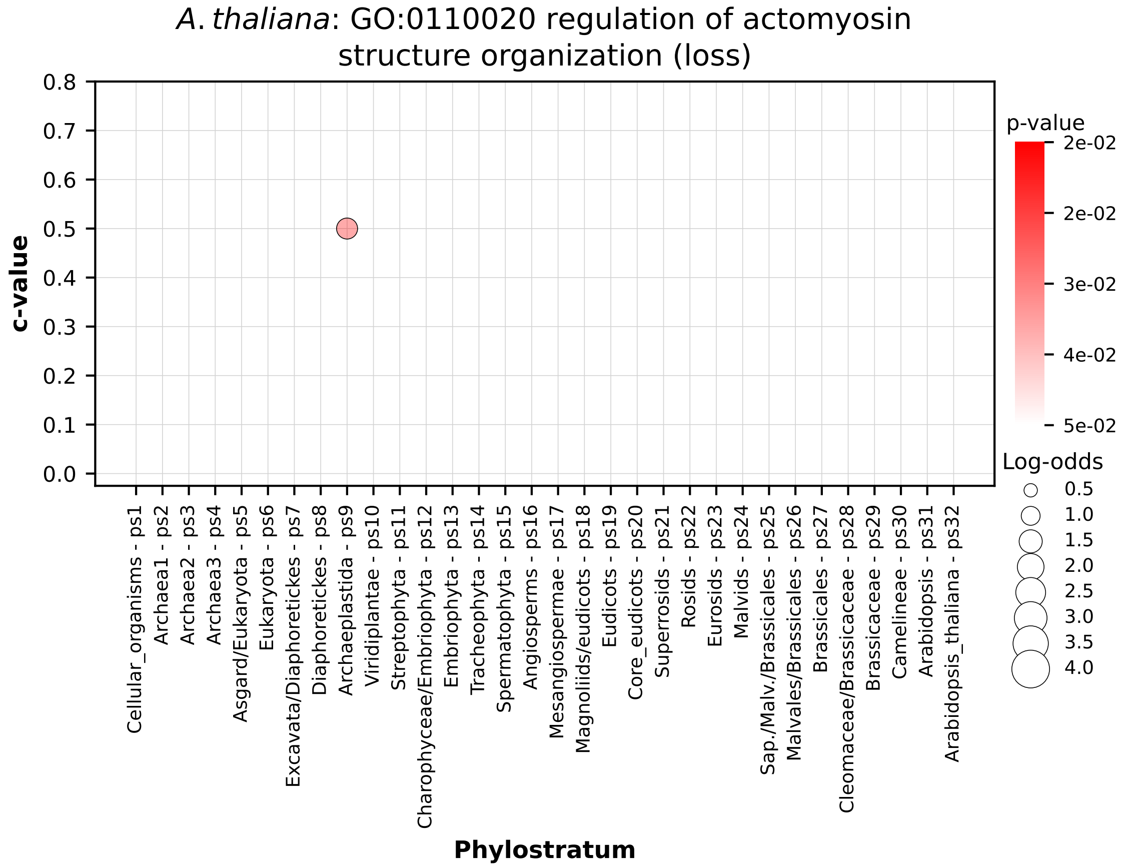

A. thaliana: GO:0110020 regulation of actomyosin structure organization (loss)
c-value
0.8
0.7
0.6
0.5
0.4
0.3
0.2
0.1
0.0
Phylostratum
Cellular_organisms - ps1
Archaea1 - ps2
Archaea2 - ps3
Archaea3 - ps4
Asgard/Eukaryota - ps5
Eukaryota - ps6
Excavata/Diaphoretickes - ps7
Diaphoretickes - ps8
Archaeplastida - ps9
Viridiplantae - ps10
Streptophyta - ps11
Charophyceae/Embriophyta - ps12
Embriophyta - ps13
Tracheophyta - ps14
Spermatophyta - ps15
Angiosperms - ps16
Mesangiospermae - ps17
Magnoliids/eudicots - ps18
Eudicots - ps19
Core_eudicots - ps20
Superrosids - ps21
Rosids - ps22
Eurosids - ps23
Malvids - ps24
Sap./Malv./Brassicales - ps25
Malvales/Brassicales - ps26
Brassicales - ps27
Cleomaceae/Brassicaceae - ps28
Brassicaceae - ps29
Camelineae - ps30
Arabidopsis - ps31
Arabidopsis_thaliana - ps32
p-value
2e-02
2e-02
3e-02
4e-02
5e-02
Log-odds
0.5
1.0
1.5
2.0
2.5
3.0
3.5
4.0

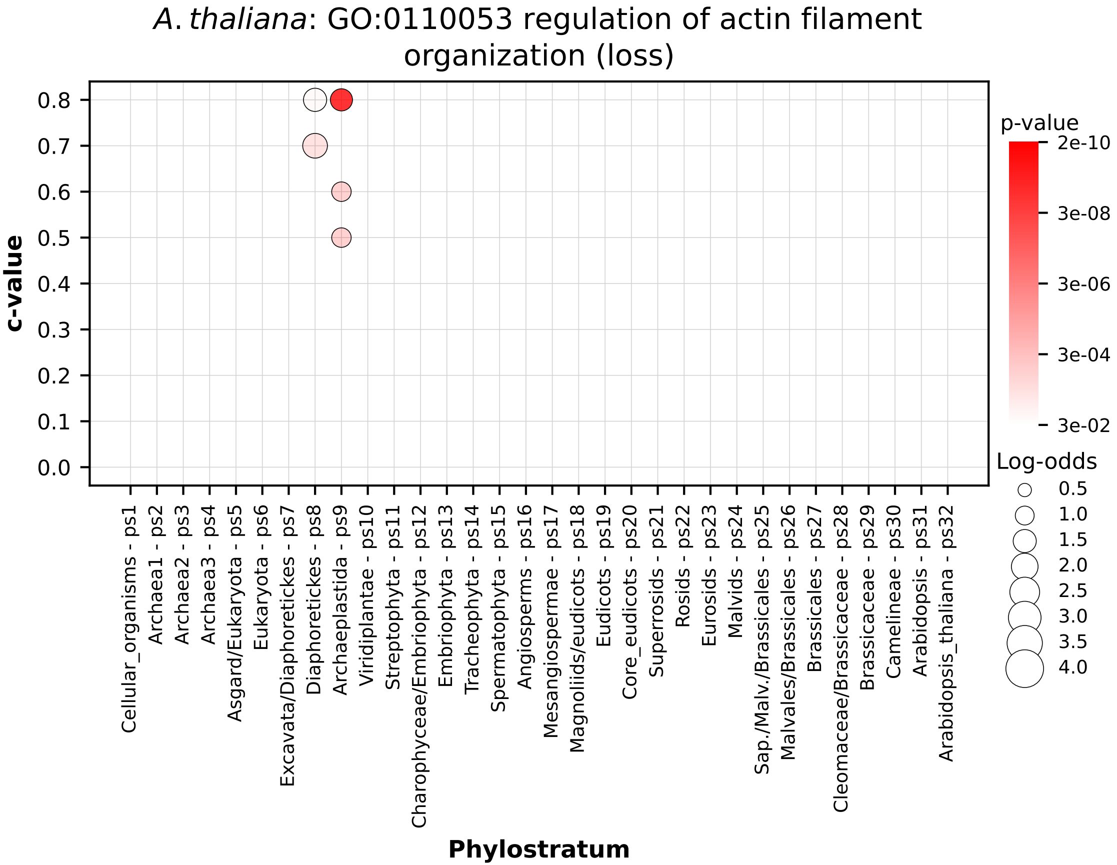

A. thaliana: GO:0110053 regulation of actin filament organization (loss)
c-value
0.8
0.7
0.6
0.5
0.4
0.3
0.2
0.1
0.0
p-value
2e-10
3e-08
3e-06
3e-04
3e-02
Log-odds
0.5
1.0
1.5
2.0
2.5
3.0
3.5
4.0
Cellular_organisms - ps1
Archaea1 - ps2
Archaea2 - ps3
Archaea3 - ps4
Asgard/Eukaryota - ps5
Eukaryota - ps6
Excavata/Diaphoretickes - ps7
Diaphoretickes - ps8
Archaeplastida - ps9
Viridiplantae - ps10
Streptophyta - ps11
Charophyceae/Embriophyta - ps12
Embriophyta - ps13
Tracheophyta - ps14
Spermatophyta - ps15
Angiosperms - ps16
Mesangiospermae - ps17
Magnoliids/eudicots - ps18
Eudicots - ps19
Core_eudicots - ps20
Superrosids - ps21
Rosids - ps22
Eurosids - ps23
Malvids - ps24
Sap./Malv./Brassicales - ps25
Malvales/Brassicales - ps26
Brassicales - ps27
Cleomaceae/Brassicaceae - ps28
Brassicaceae - ps29
Camelineae - ps30
Arabidopsis - ps31
Arabidopsis_thaliana - ps32
Phylostratum

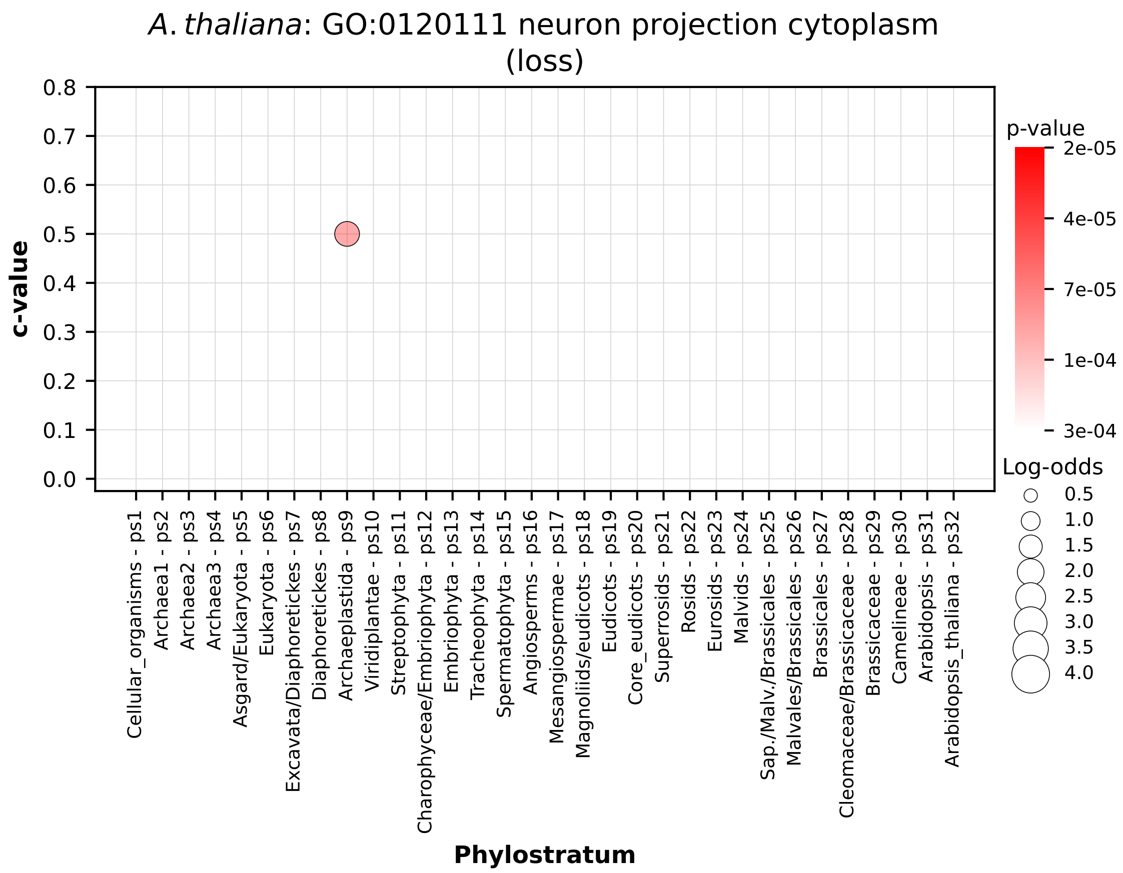

A. thaliana: GO:0120111 neuron projection cytoplasm
(loss)
c-value
0.0
0.1
0.2
0.3
0.4
0.5
0.6
0.7
0.8
Phylostratum
Cellular_organisms - ps1
Archaea1 - ps2
Archaea2 - ps3
Archaea3 - ps4
Asgard/Eukaryota - ps5
Eukaryota - ps6
Excavata/Diaphoretickes - ps7
Diaphoretickes - ps8
Archaeplastida - ps9
Viridiplantae - ps10
Streptophyta - ps11
Charophyceae/Embriophyta - ps12
Embriophyta - ps13
Tracheophyta - ps14
Spermatophyta - ps15
Angiosperms - ps16
Mesangiospermae - ps17
Magnoliids/eudicots - ps18
Eudicots - ps19
Core_eudicots - ps20
Superrosids - ps21
Rosids - ps22
Eurosids - ps23
Malvids - ps24
Sap./Malv./Brassicales - ps25
Malvales/Brassicales - ps26
Brassicales - ps27
Cleomaceae/Brassicaceae - ps28
Brassicaceae - ps29
Camelineae - ps30
Arabidopsis - ps31
Arabidopsis_thaliana - ps32
p-value
2e-05
4e-05
7e-05
1e-04
3e-04
Log-odds
0.5
1.0
1.5
2.0
2.5
3.0
3.5
4.0

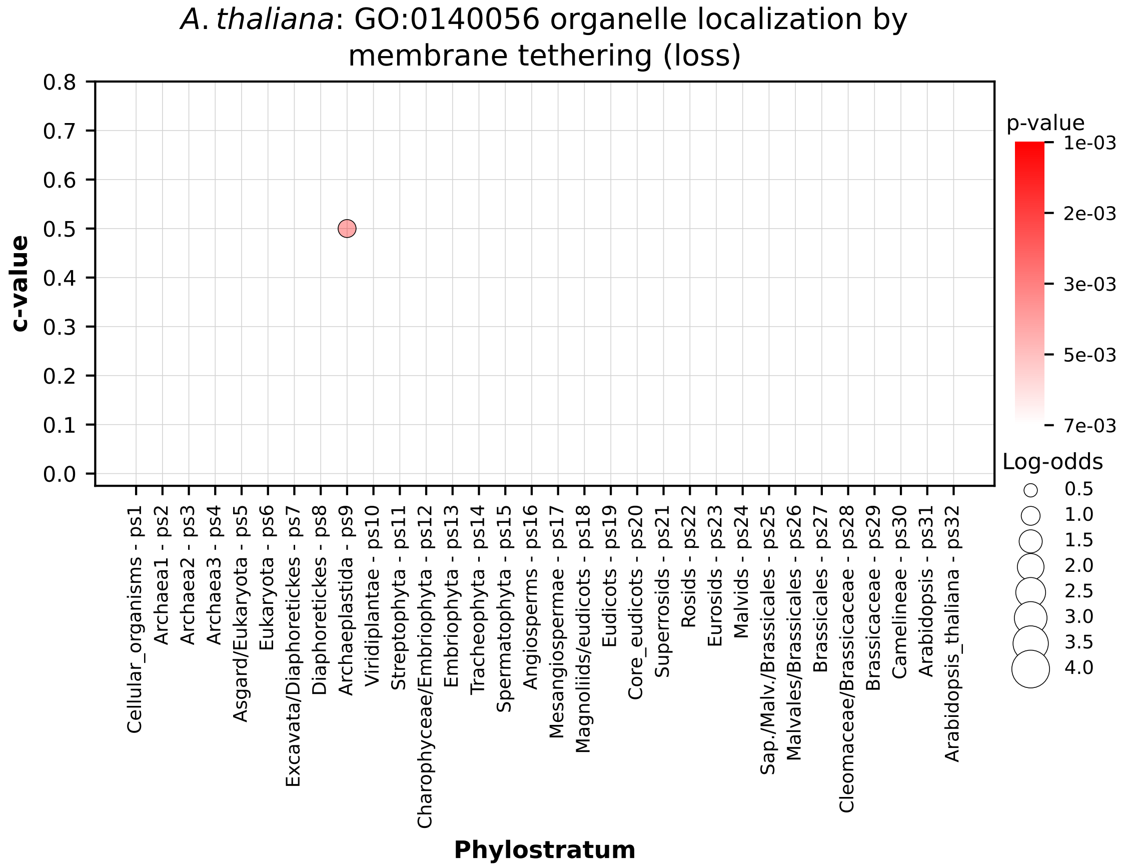

A. thaliana: GO:0140056 organelle localization by membrane tethering (loss)
c-value
0.8
0.7
0.6
0.5
0.4
0.3
0.2
0.1
0.0
p-value
1e-03
2e-03
3e-03
5e-03
7e-03
Log-odds
0.5
1.0
1.5
2.0
2.5
3.0
3.5
4.0
Cellular_organisms - ps1
Archaea1 - ps2
Archaea2 - ps3
Archaea3 - ps4
Asgard/Eukaryota - ps5
Eukaryota - ps6
Excavata/Diaphoretickes - ps7
Diaphoretickes - ps8
Archaeplastida - ps9
Viridiplantae - ps10
Streptophyta - ps11
Charophyceae/Embriophyta - ps12
Embriophyta - ps13
Tracheophyta - ps14
Spermatophyta - ps15
Angiosperms - ps16
Mesangiospermae - ps17
Magnoliids/eudicots - ps18
Eudicots - ps19
Core_eudicots - ps20
Superrosids - ps21
Rosids - ps22
Eurosids - ps23
Malvids - ps24
Sap./Malv./Brassicales - ps25
Malvales/Brassicales - ps26
Brassicales - ps27
Cleomaceae/Brassicaceae - ps28
Brassicaceae - ps29
Camelineae - ps30
Arabidopsis - ps31
Arabidopsis_thaliana - ps32
Phylostratum

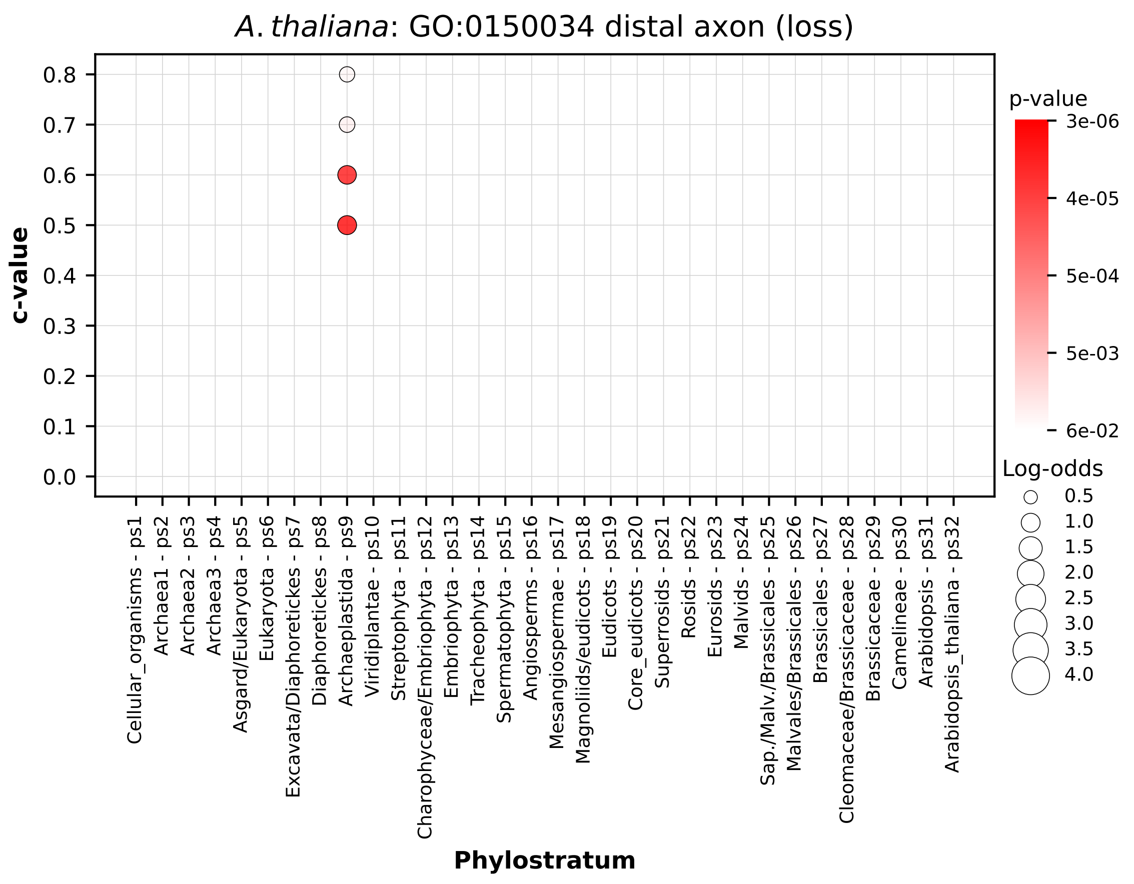

A. thaliana: GO:0150034 distal axon (loss)
c-value
0.8
0.7
0.6
0.5
0.4
0.3
0.2
0.1
0.0
p-value
3e-06
4e-05
5e-04
5e-03
6e-02
Log-odds
0.5
1.0
1.5
2.0
2.5
3.0
3.5
4.0
Cellular_organisms - ps1
Archaea1 - ps2
Archaea2 - ps3
Archaea3 - ps4
Asgard/Eukaryota - ps5
Eukaryota - ps6
Excavata/Diaphoretickes - ps7
Diaphoretickes - ps8
Archaeplastida - ps9
Viridiplantae - ps10
Streptophyta - ps11
Charophyceae/Embriophyta - ps12
Embriophyta - ps13
Tracheophyta - ps14
Spermatophyta - ps15
Angiosperms - ps16
Mesangiospermae - ps17
Magnoliids/eudicots - ps18
Eudicots - ps19
Core_eudicots - ps20
Superrosids - ps21
Rosids - ps22
Eurosids - ps23
Malvids - ps24
Sap./Malv./Brassicales - ps25
Malvales/Brassicales - ps26
Brassicales - ps27
Cleomaceae/Brassicaceae - ps28
Brassicaceae - ps29
Camelineae - ps30
Arabidopsis - ps31
Arabidopsis_thaliana - ps32
Phylostratum

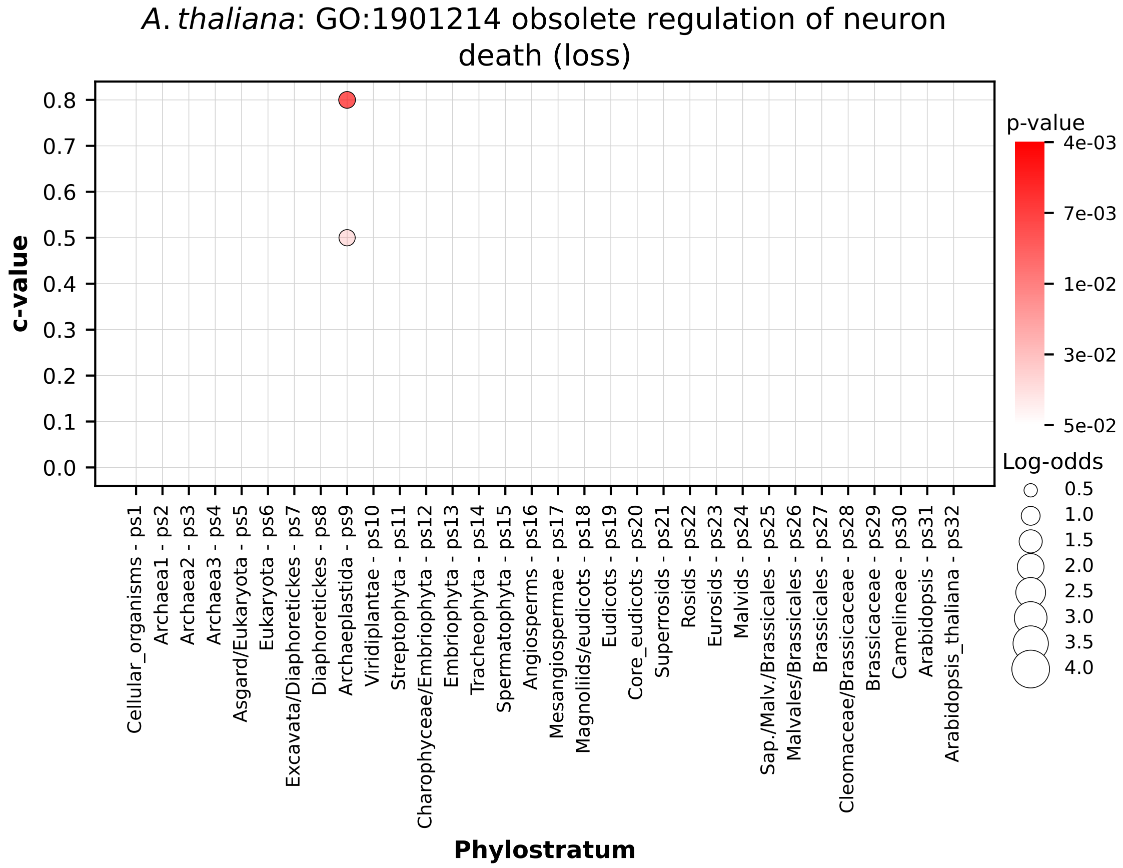

A. thaliana: GO:1901214 obsolete regulation of neuron death (loss)
c-value
0.8
0.7
0.6
0.5
0.4
0.3
0.2
0.1
0.0
Phylostratum
Cellular_organisms - ps1
Archaea1 - ps2
Archaea2 - ps3
Archaea3 - ps4
Asgard/Eukaryota - ps5
Eukaryota - ps6
Excavata/Diaphoretickes - ps7
Diaphoretickes - ps8
Archaeplastida - ps9
Viridiplantae - ps10
Streptophyta - ps11
Charophyceae/Embriophyta - ps12
Embriophyta - ps13
Tracheophyta - ps14
Spermatophyta - ps15
Angiosperms - ps16
Mesangiospermae - ps17
Magnoliids/eudicots - ps18
Eudicots - ps19
Core_eudicots - ps20
Superrosids - ps21
Rosids - ps22
Eurosids - ps23
Malvids - ps24
Sap./Malv./Brassicales - ps25
Malvales/Brassicales - ps26
Brassicales - ps27
Cleomaceae/Brassicaceae - ps28
Brassicaceae - ps29
Camelineae - ps30
Arabidopsis - ps31
Arabidopsis_thaliana - ps32
p-value
4e-03
7e-03
1e-02
3e-02
5e-02
Log-odds
0.5
1.0
1.5
2.0
2.5
3.0
3.5
4.0

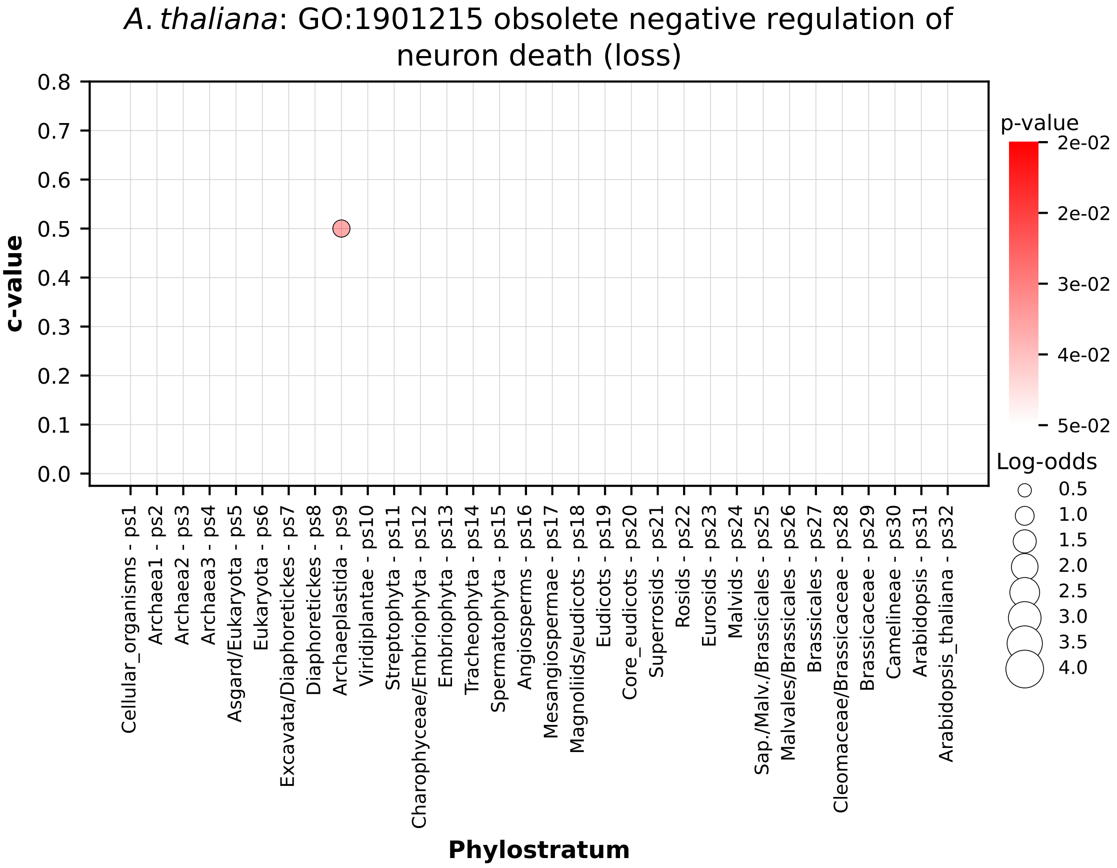

A. thaliana: GO:1901215 obsolete negative regulation of neuron death (loss)
c-value
0.8
0.7
0.6
0.5
0.4
0.3
0.2
0.1
0.0
Phylostratum
Cellular_organisms - ps1
Archaea1 - ps2
Archaea2 - ps3
Archaea3 - ps4
Asgard/Eukaryota - ps5
Eukaryota - ps6
Excavata/Diaphoretickes - ps7
Diaphoretickes - ps8
Archaeplastida - ps9
Viridiplantae - ps10
Streptophyta - ps11
Charophyceae/Embriophyta - ps12
Embriophyta - ps13
Tracheophyta - ps14
Spermatophyta - ps15
Angiosperms - ps16
Mesangiospermae - ps17
Magnoliids/eudicots - ps18
Eudicots - ps19
Core_eudicots - ps20
Superrosids - ps21
Rosids - ps22
Eurosids - ps23
Malvids - ps24
Sap./Malv./Brassicales - ps25
Malvales/Brassicales - ps26
Brassicales - ps27
Cleomaceae/Brassicaceae - ps28
Brassicaceae - ps29
Camelineae - ps30
Arabidopsis - ps31
Arabidopsis_thaliana - ps32
p-value
2e-02
2e-02
3e-02
4e-02
5e-02
Log-odds
0.5
1.0
1.5
2.0
2.5
3.0
3.5
4.0

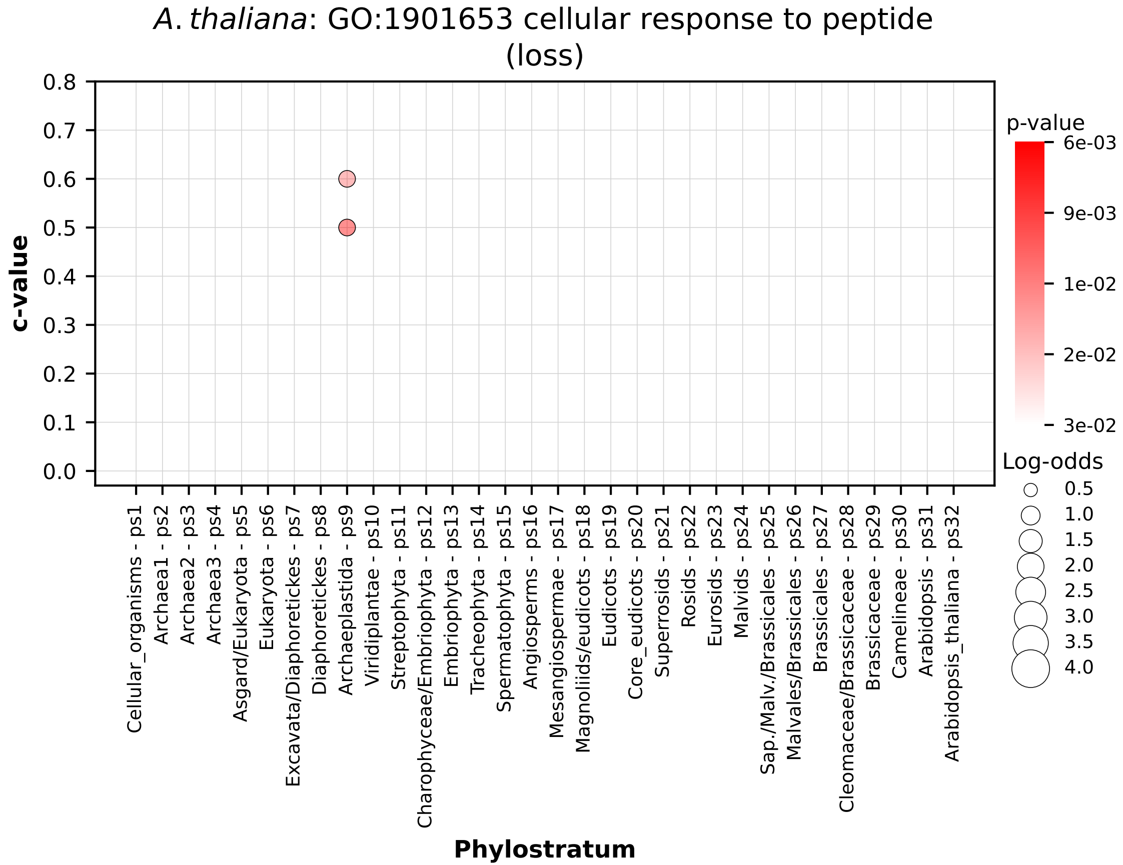

A. thaliana: GO:1901653 cellular response to peptide
(loss)
c-value
0.8
0.7
0.6
0.5
0.4
0.3
0.2
0.1
0.0
Phylostratum
Cellular_organisms - ps1
Archaea1 - ps2
Archaea2 - ps3
Archaea3 - ps4
Asgard/Eukaryota - ps5
Eukaryota - ps6
Excavata/Diaphoretickes - ps7
Diaphoretickes - ps8
Archaeplastida - ps9
Viridiplantae - ps10
Streptophyta - ps11
Charophyceae/Embriophyta - ps12
Embriophyta - ps13
Tracheophyta - ps14
Spermatophyta - ps15
Angiosperms - ps16
Mesangiospermae - ps17
Magnoliids/eudicots - ps18
Eudicots - ps19
Core_eudicots - ps20
Superrosids - ps21
Rosids - ps22
Eurosids - ps23
Malvids - ps24
Sap./Malv./Brassicales - ps25
Malvales/Brassicales - ps26
Brassicales - ps27
Cleomaceae/Brassicaceae - ps28
Brassicaceae - ps29
Camelineae - ps30
Arabidopsis - ps31
Arabidopsis_thaliana - ps32
p-value
6e-03
9e-03
1e-02
2e-02
3e-02
Log-odds
0.5
1.0
1.5
2.0
2.5
3.0
3.5
4.0

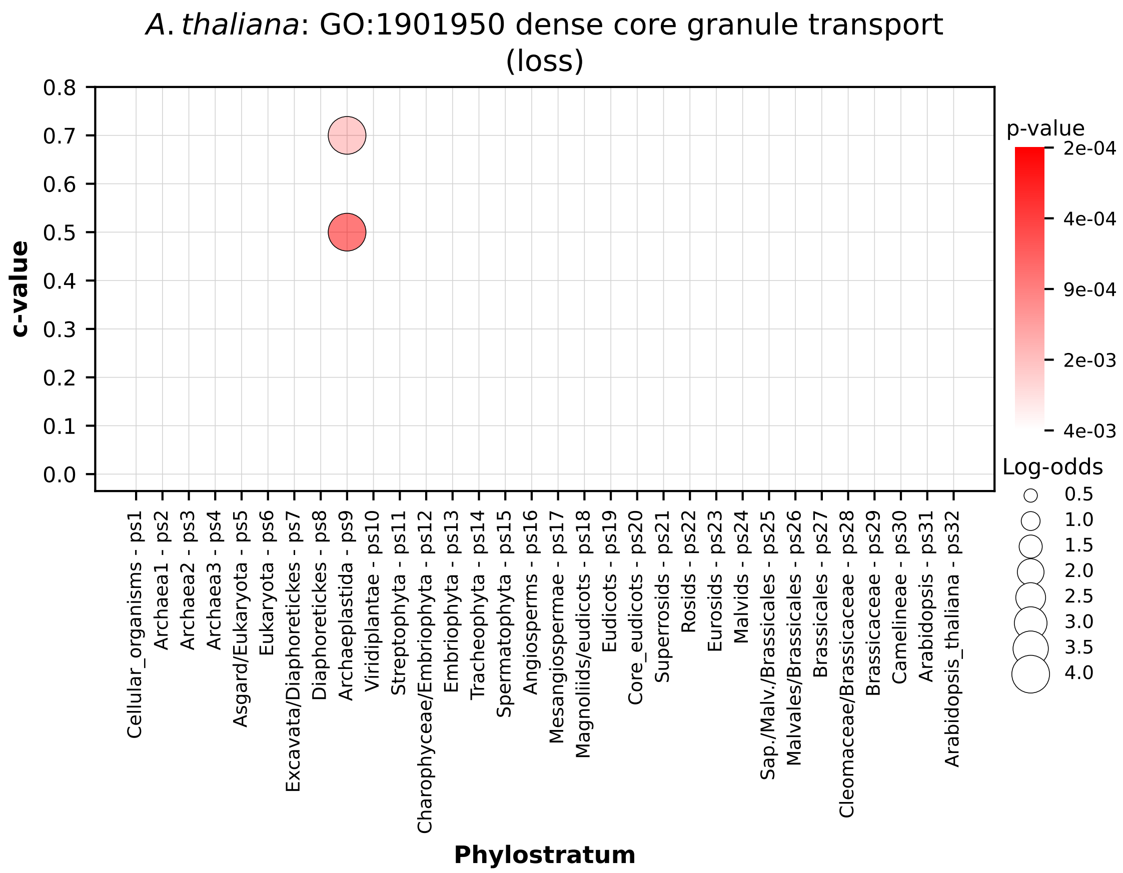
A. thaliana: GO:1901950 dense core granule transport
(loss)
c-value
0.8
0.7
0.6
0.5
0.4
0.3
0.2
0.1
0.0
p-value
2e-04
4e-04
9e-04
2e-03
4e-03
Log-odds
0.5
1.0
1.5
2.0
2.5
3.0
3.5
4.0
Cellular_organisms - ps1
Archaea1 - ps2
Archaea2 - ps3
Archaea3 - ps4
Asgard/Eukaryota - ps5
Eukaryota - ps6
Excavata/Diaphoretickes - ps7
Diaphoretickes - ps8
Archaeplastida - ps9
Viridiplantae - ps10
Streptophyta - ps11
Charophyceae/Embriophyta - ps12
Embriophyta - ps13
Tracheophyta - ps14
Spermatophyta - ps15
Angiosperms - ps16
Mesangiospermae - ps17
Magnoliids/eudicots - ps18
Eudicots - ps19
Core_eudicots - ps20
Superrosids - ps21
Rosids - ps22
Eurosids - ps23
Malvids - ps24
Sap./Malv./Brassicales - ps25
Malvales/Brassicales - ps26
Brassicales - ps27
Cleomaceae/Brassicaceae - ps28
Brassicaceae - ps29
Camelineae - ps30
Arabidopsis - ps31
Arabidopsis_thaliana - ps32
Phylostratum

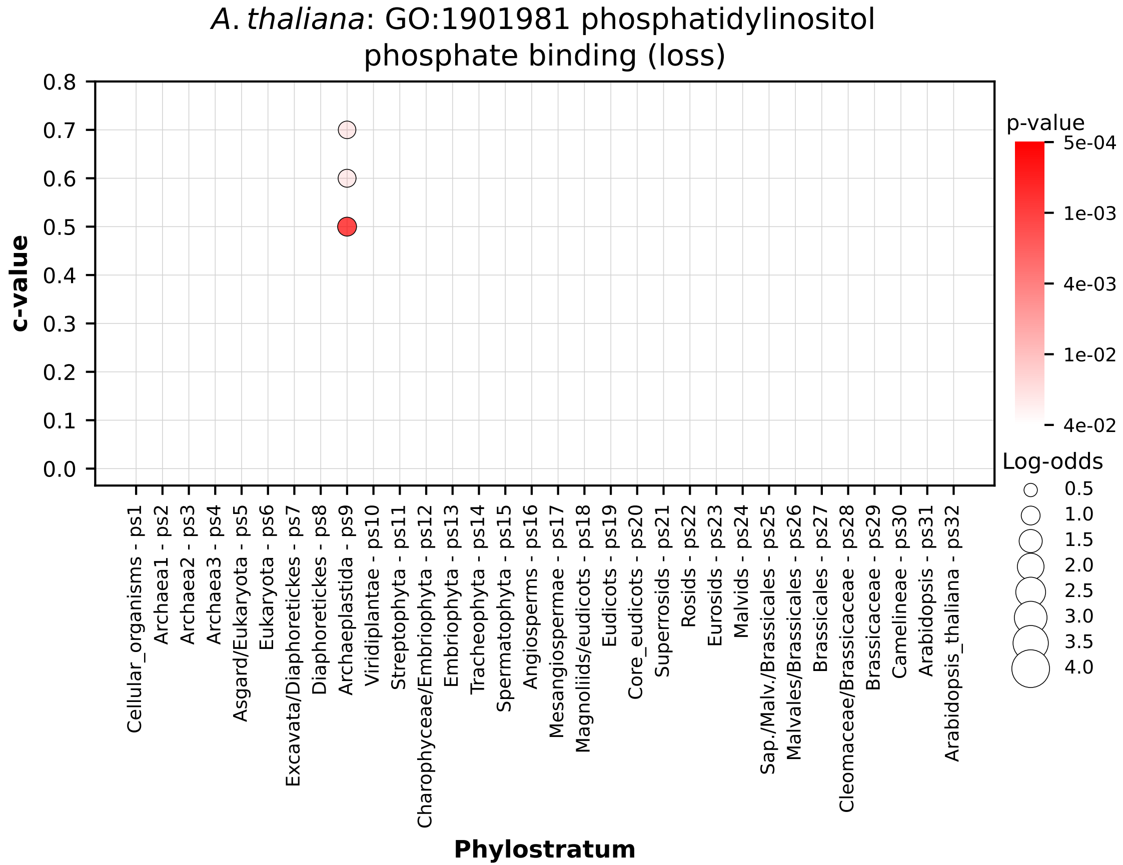

A. thaliana: GO:1901981 phosphatidylinositol phosphate binding (loss)
c-value
0.0
0.1
0.2
0.3
0.4
0.5
0.6
0.7
0.8
Phylostratum
Cellular_organisms - ps1
Archaea1 - ps2
Archaea2 - ps3
Archaea3 - ps4
Asgard/Eukaryota - ps5
Eukaryota - ps6
Excavata/Diaphoretickes - ps7
Diaphoretickes - ps8
Archaeplastida - ps9
Viridiplantae - ps10
Streptophyta - ps11
Charophyceae/Embriophyta - ps12
Embriophyta - ps13
Tracheophyta - ps14
Spermatophyta - ps15
Angiosperms - ps16
Mesangiospermae - ps17
Magnoliids/eudicots - ps18
Eudicots - ps19
Core_eudicots - ps20
Superrosids - ps21
Rosids - ps22
Eurosids - ps23
Malvids - ps24
Sap./Malv./Brassicales - ps25
Malvales/Brassicales - ps26
Brassicales - ps27
Cleomaceae/Brassicaceae - ps28
Brassicaceae - ps29
Camelineae - ps30
Arabidopsis - ps31
Arabidopsis_thaliana - ps32
p-value
5e-04
1e-03
4e-03
1e-02
4e-02
Log-odds
0.5
1.0
1.5
2.0
2.5
3.0
3.5
4.0

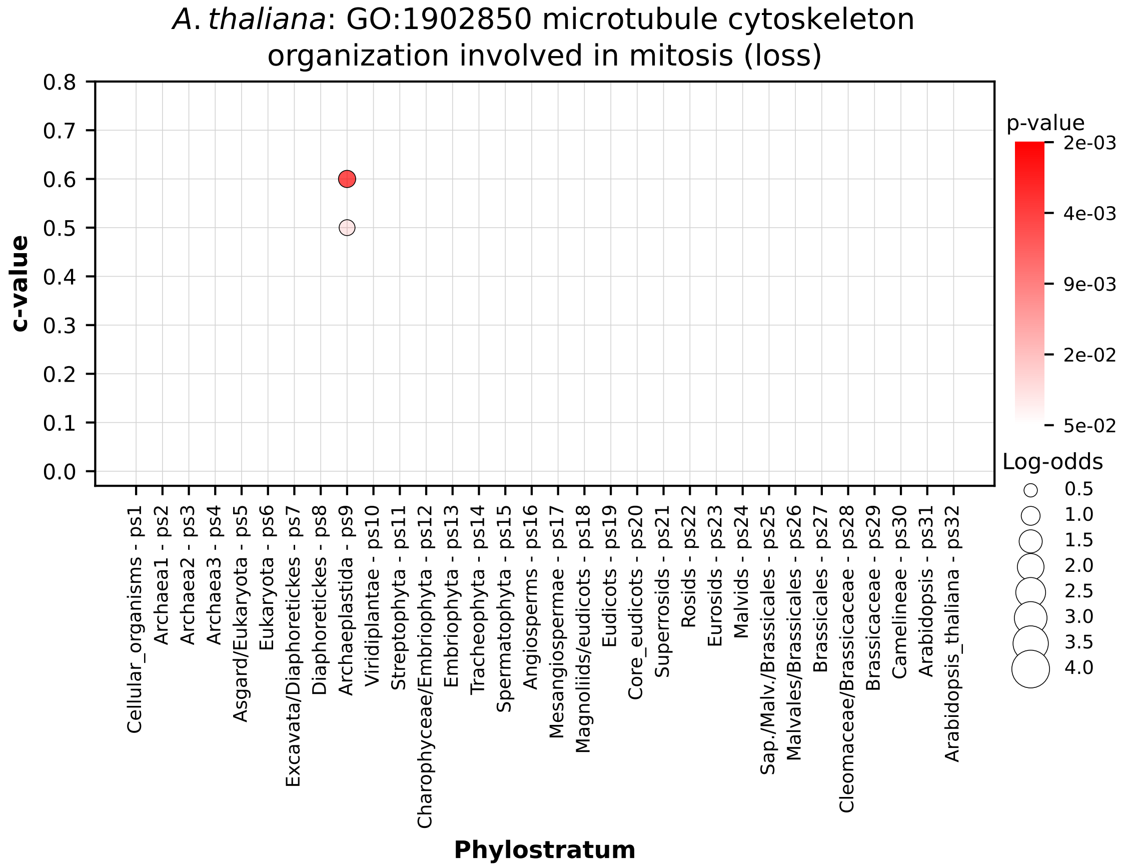

A. thaliana: GO:1902850 microtubule cytoskeleton organization involved in mitosis (loss)
c-value
0.8
0.7
0.6
0.5
0.4
0.3
0.2
0.1
0.0
Phylostratum
Cellular_organisms - ps1
Archaea1 - ps2
Archaea2 - ps3
Archaea3 - ps4
Asgard/Eukaryota - ps5
Eukaryota - ps6
Excavata/Diaphoretickes - ps7
Diaphoretickes - ps8
Archaeplastida - ps9
Viridiplantae - ps10
Streptophyta - ps11
Charophyceae/Embriophyta - ps12
Embriophyta - ps13
Tracheophyta - ps14
Spermatophyta - ps15
Angiosperms - ps16
Mesangiospermae - ps17
Magnoliids/eudicots - ps18
Eudicots - ps19
Core_eudicots - ps20
Superrosids - ps21
Rosids - ps22
Eurosids - ps23
Malvids - ps24
Sap./Malv./Brassicales - ps25
Malvales/Brassicales - ps26
Brassicales - ps27
Cleomaceae/Brassicaceae - ps28
Brassicaceae - ps29
Camelineae - ps30
Arabidopsis - ps31
Arabidopsis_thaliana - ps32
p-value
2e-03
4e-03
9e-03
2e-02
5e-02
Log-odds
0.5
1.0
1.5
2.0
2.5
3.0
3.5
4.0

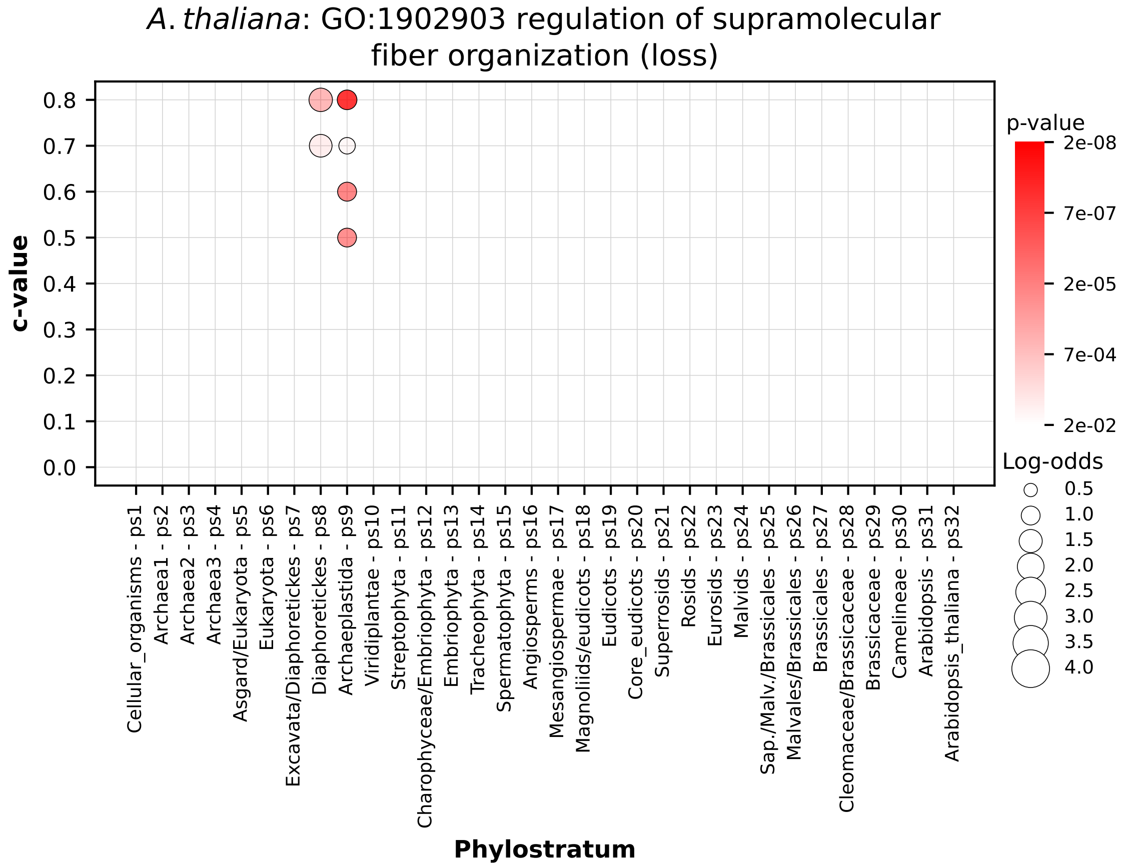

A. thaliana: GO:1902903 regulation of supramolecular fiber organization (loss)
c-value
0.0
0.1
0.2
0.3
0.4
0.5
0.6
0.7
0.8
Phylostratum
Cellular_organisms - ps1
Archaea1 - ps2
Archaea2 - ps3
Archaea3 - ps4
Asgard/Eukaryota - ps5
Eukaryota - ps6
Excavata/Diaphoretickes - ps7
Diaphoretickes - ps8
Archaeplastida - ps9
Viridiplantae - ps10
Streptophyta - ps11
Charophyceae/Embriophyta - ps12
Embriophyta - ps13
Tracheophyta - ps14
Spermatophyta - ps15
Angiosperms - ps16
Mesangiospermae - ps17
Magnoliids/eudicots - ps18
Eudicots - ps19
Core_eudicots - ps20
Superrosids - ps21
Rosids - ps22
Eurosids - ps23
Malvids - ps24
Sap./Malv./Brassicales - ps25
Malvales/Brassicales - ps26
Brassicales - ps27
Cleomaceae/Brassicaceae - ps28
Brassicaceae - ps29
Camelineae - ps30
Arabidopsis - ps31
Arabidopsis_thaliana - ps32
p-value
2e-08
7e-07
2e-05
7e-04
2e-02
Log-odds
0.5
1.0
1.5
2.0
2.5
3.0
3.5
4.0

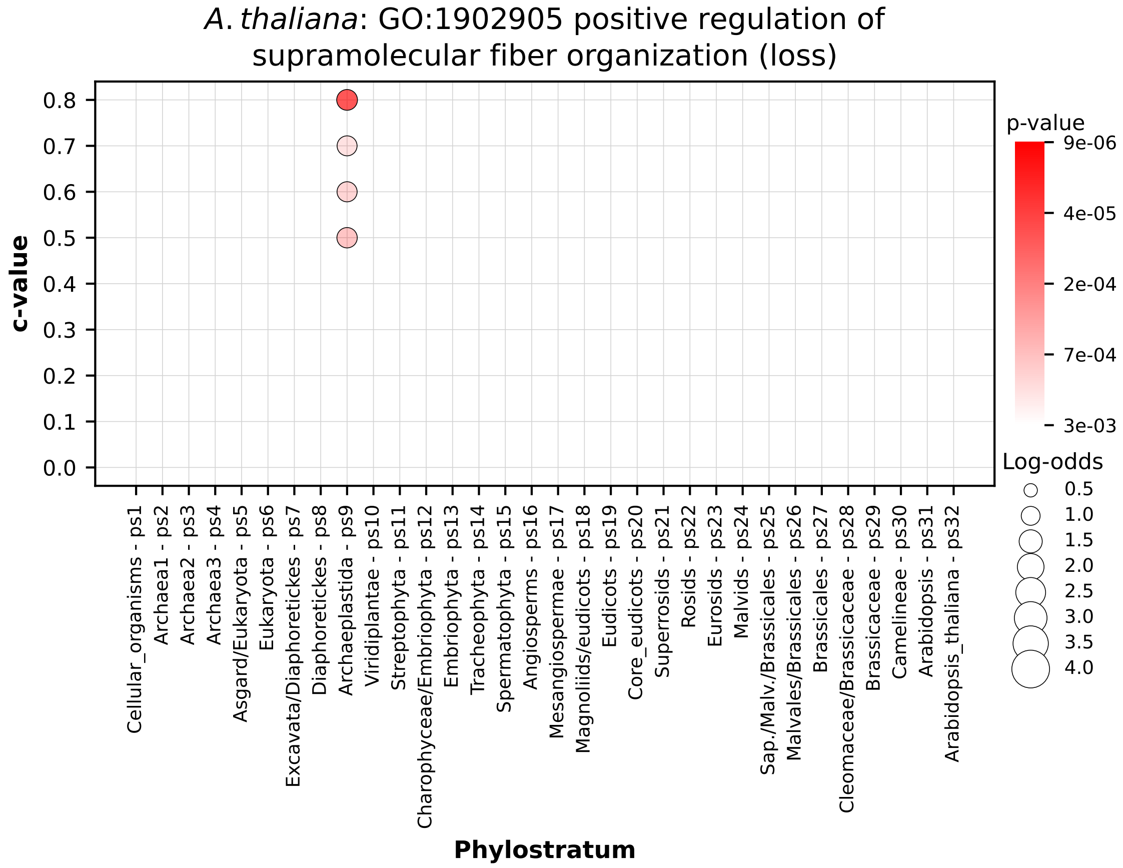

A. thaliana: GO:1902905 positive regulation of supramolecular fiber organization (loss)
c-value
0.8
0.7
0.6
0.5
0.4
0.3
0.2
0.1
0.0
Phylostratum
Cellular_organisms - ps1
Archaea1 - ps2
Archaea2 - ps3
Archaea3 - ps4
Asgard/Eukaryota - ps5
Eukaryota - ps6
Excavata/Diaphoretickes - ps7
Diaphoretickes - ps8
Archaeplastida - ps9
Viridiplantae - ps10
Streptophyta - ps11
Charophyceae/Embriophyta - ps12
Embriophyta - ps13
Tracheophyta - ps14
Spermatophyta - ps15
Angiosperms - ps16
Mesangiospermae - ps17
Magnoliids/eudicots - ps18
Eudicots - ps19
Core_eudicots - ps20
Superrosids - ps21
Rosids - ps22
Eurosids - ps23
Malvids - ps24
Sap./Malv./Brassicales - ps25
Malvales/Brassicales - ps26
Brassicales - ps27
Cleomaceae/Brassicaceae - ps28
Brassicaceae - ps29
Camelineae - ps30
Arabidopsis - ps31
Arabidopsis_thaliana - ps32
p-value
9e-06
4e-05
2e-04
7e-04
3e-03
Log-odds
0.5
1.0
1.5
2.0
2.5
3.0
3.5
4.0

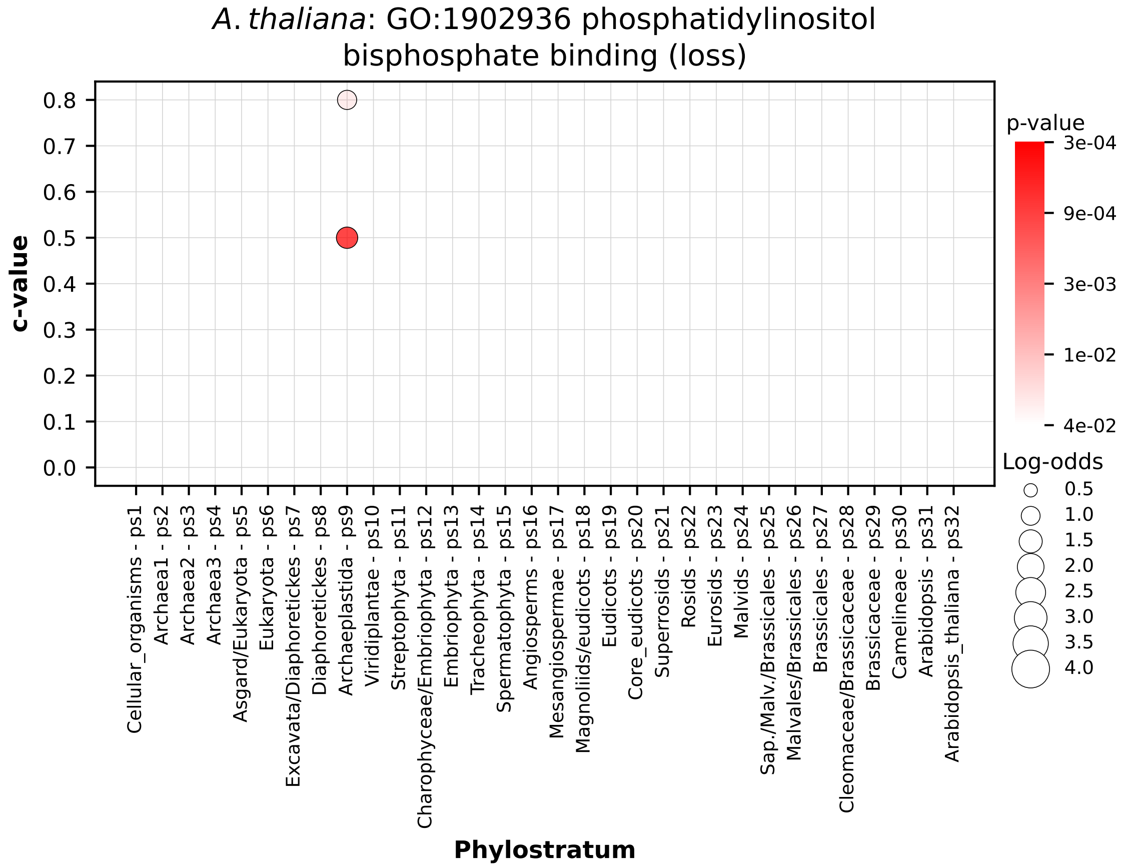

A. thaliana: GO:1902936 phosphatidylinositol bisphosphate binding (loss)
c-value
0.0
0.1
0.2
0.3
0.4
0.5
0.6
0.7
0.8
Phylostratum
Cellular_organisms - ps1
Archaea1 - ps2
Archaea2 - ps3
Archaea3 - ps4
Asgard/Eukaryota - ps5
Eukaryota - ps6
Excavata/Diaphoretickes - ps7
Diaphoretickes - ps8
Archaeplastida - ps9
Viridiplantae - ps10
Streptophyta - ps11
Charophyceae/Embriophyta - ps12
Embriophyta - ps13
Tracheophyta - ps14
Spermatophyta - ps15
Angiosperms - ps16
Mesangiospermae - ps17
Magnoliids/eudicots - ps18
Eudicots - ps19
Core_eudicots - ps20
Superrosids - ps21
Rosids - ps22
Eurosids - ps23
Malvids - ps24
Sap./Malv./Brassicales - ps25
Malvales/Brassicales - ps26
Brassicales - ps27
Cleomaceae/Brassicaceae - ps28
Brassicaceae - ps29
Camelineae - ps30
Arabidopsis - ps31
Arabidopsis_thaliana - ps32
p-value
3e-04
9e-04
3e-03
1e-02
4e-02
Log-odds
0.5
1.0
1.5
2.0
2.5
3.0
3.5
4.0

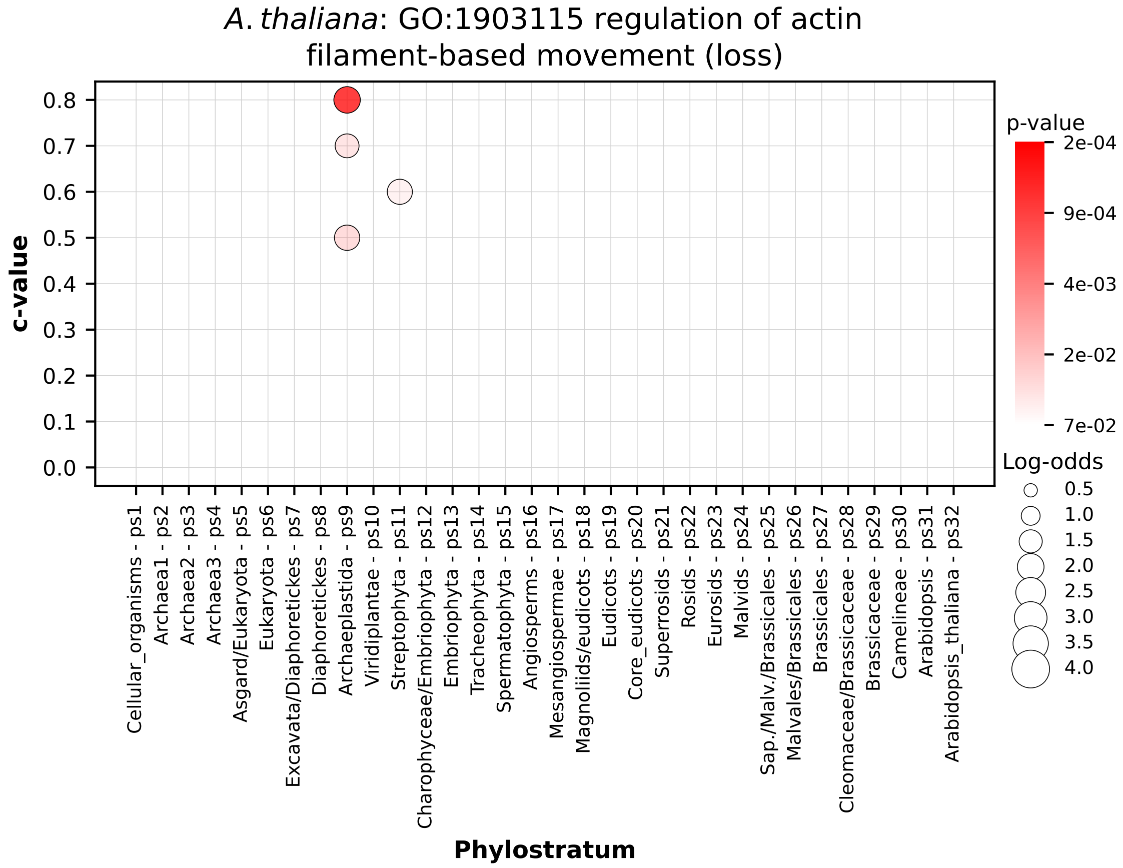

A. thaliana: GO:1903115 regulation of actin filament-based movement (loss)
c-value
0.8
0.7
0.6
0.5
0.4
0.3
0.2
0.1
0.0
Phylostratum
Cellular_organisms - ps1
Archaea1 - ps2
Archaea2 - ps3
Archaea3 - ps4
Asgard/Eukaryota - ps5
Eukaryota - ps6
Excavata/Diaphoretickes - ps7
Diaphoretickes - ps8
Archaeplastida - ps9
Viridiplantae - ps10
Streptophyta - ps11
Charophyceae/Embriophyta - ps12
Embriophyta - ps13
Tracheophyta - ps14
Spermatophyta - ps15
Angiosperms - ps16
Mesangiospermae - ps17
Magnoliids/eudicots - ps18
Eudicots - ps19
Core_eudicots - ps20
Superrosids - ps21
Rosids - ps22
Eurosids - ps23
Malvids - ps24
Sap./Malv./Brassicales - ps25
Malvales/Brassicales - ps26
Brassicales - ps27
Cleomaceae/Brassicaceae - ps28
Brassicaceae - ps29
Camelineae - ps30
Arabidopsis - ps31
Arabidopsis_thaliana - ps32
p-value
2e-04
9e-04
4e-03
2e-02
7e-02
Log-odds
0.5
1.0
1.5
2.0
2.5
3.0
3.5
4.0

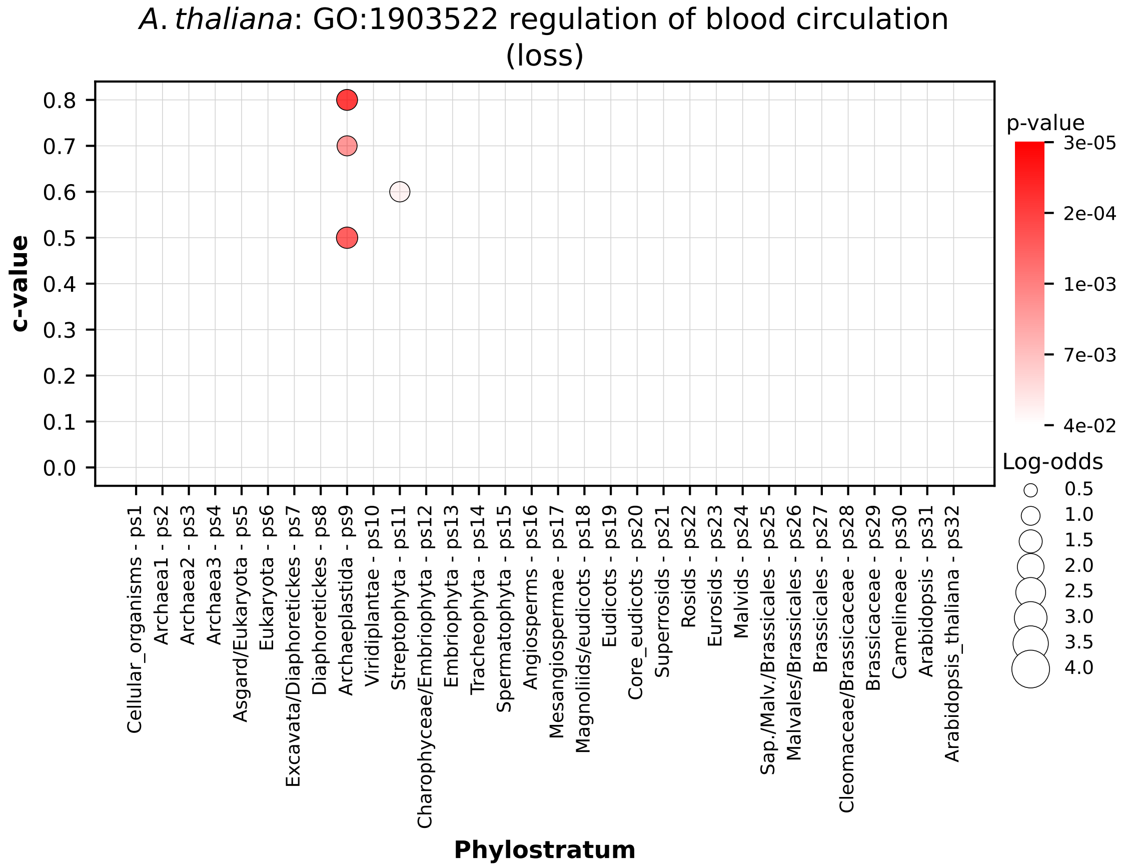
A. thaliana: GO:1903522 regulation of blood circulation (loss)
c-value
0.0
0.1
0.2
0.3
0.4
0.5
0.6
0.7
0.8
Phylostratum
Cellular_organisms - ps1
Archaea1 - ps2
Archaea2 - ps3
Archaea3 - ps4
Asgard/Eukaryota - ps5
Eukaryota - ps6
Excavata/Diaphoretickes - ps7
Diaphoretickes - ps8
Archaeplastida - ps9
Viridiplantae - ps10
Streptophyta - ps11
Charophyceae/Embriophyta - ps12
Embriophyta - ps13
Tracheophyta - ps14
Spermatophyta - ps15
Angiosperms - ps16
Mesangiospermae - ps17
Magnoliids/eudicots - ps18
Eudicots - ps19
Core_eudicots - ps20
Superrosids - ps21
Rosids - ps22
Eurosids - ps23
Malvids - ps24
Sap./Malv./Brassicales - ps25
Malvales/Brassicales - ps26
Brassicales - ps27
Cleomaceae/Brassicaceae - ps28
Brassicaceae - ps29
Camelineae - ps30
Arabidopsis - ps31
Arabidopsis_thaliana - ps32
p-value
3e-05
2e-04
1e-03
7e-03
4e-02
Log-odds
0.5
1.0
1.5
2.0
2.5
3.0
3.5
4.0

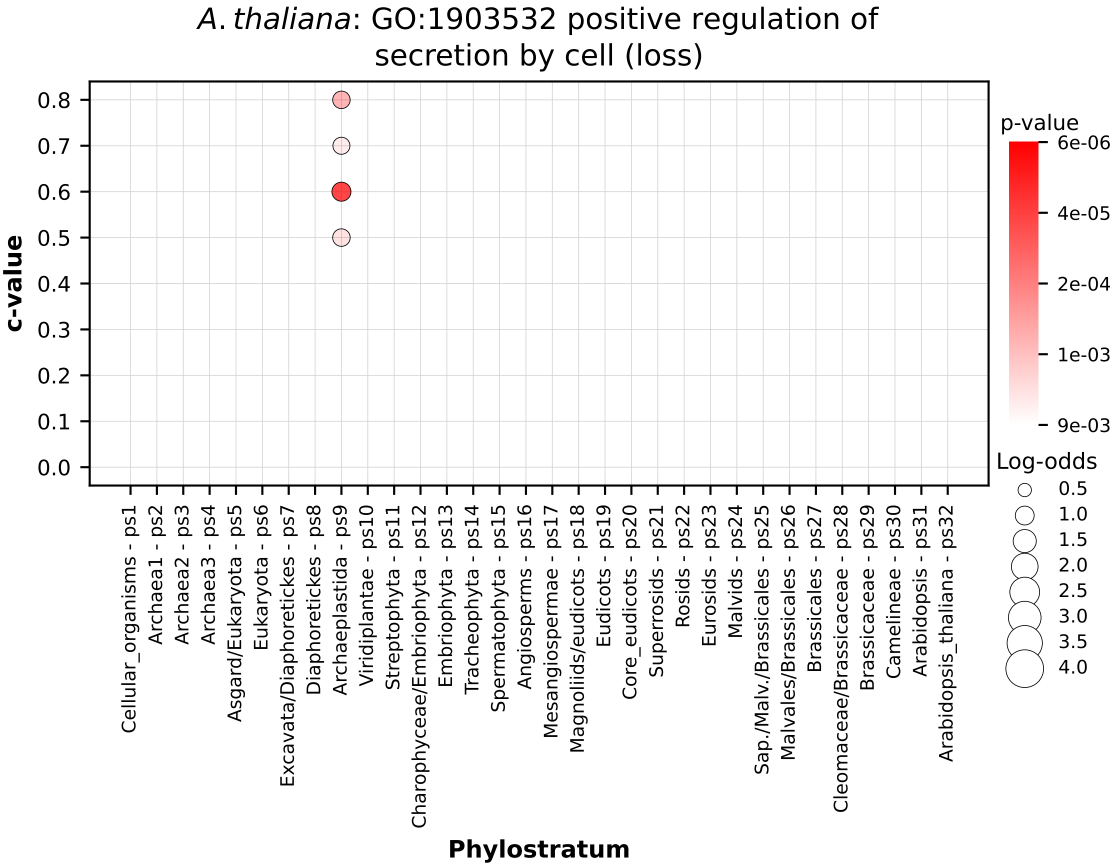

A. thaliana: GO:1903532 positive regulation of secretion by cell (loss)
c-value
0.8
0.7
0.6
0.5
0.4
0.3
0.2
0.1
0.0
p-value
6e-06
4e-05
2e-04
1e-03
9e-03
Log-odds
0.5
1.0
1.5
2.0
2.5
3.0
3.5
4.0
Cellular_organisms - ps1
Archaea1 - ps2
Archaea2 - ps3
Archaea3 - ps4
Asgard/Eukaryota - ps5
Eukaryota - ps6
Excavata/Diaphoretickes - ps7
Diaphoretickes - ps8
Archaeplastida - ps9
Viridiplantae - ps10
Streptophyta - ps11
Charophyceae/Embriophyta - ps12
Embriophyta - ps13
Tracheophyta - ps14
Spermatophyta - ps15
Angiosperms - ps16
Mesangiospermae - ps17
Magnoliids/eudicots - ps18
Eudicots - ps19
Core_eudicots - ps20
Superrosids - ps21
Rosids - ps22
Eurosids - ps23
Malvids - ps24
Sap./Malv./Brassicales - ps25
Malvales/Brassicales - ps26
Brassicales - ps27
Cleomaceae/Brassicaceae - ps28
Brassicaceae - ps29
Camelineae - ps30
Arabidopsis - ps31
Arabidopsis_thaliana - ps32
Phylostratum

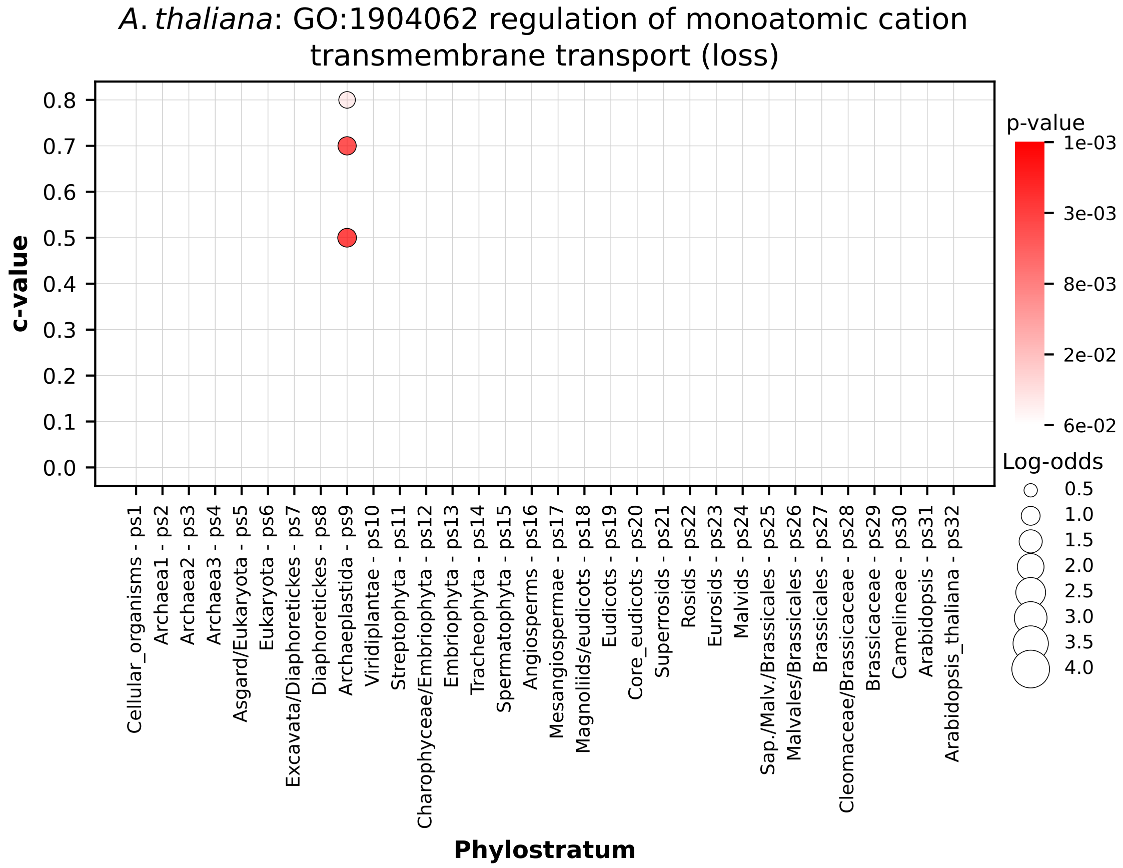

A. thaliana: GO:1904062 regulation of monoatomic cation transmembrane transport (loss)
c-value
0.0
0.1
0.2
0.3
0.4
0.5
0.6
0.7
0.8
p-value
1e-03
3e-03
8e-03
2e-02
6e-02
Log-odds
0.5
1.0
1.5
2.0
2.5
3.0
3.5
4.0
Cellular_organisms - ps1
Archaea1 - ps2
Archaea2 - ps3
Archaea3 - ps4
Asgard/Eukaryota - ps5
Eukaryota - ps6
Excavata/Diaphoretickes - ps7
Diaphoretickes - ps8
Archaeplastida - ps9
Viridiplantae - ps10
Streptophyta - ps11
Charophyceae/Embriophyta - ps12
Embriophyta - ps13
Tracheophyta - ps14
Spermatophyta - ps15
Angiosperms - ps16
Mesangiospermae - ps17
Magnoliids/eudicots - ps18
Eudicots - ps19
Core_eudicots - ps20
Superrosids - ps21
Rosids - ps22
Eurosids - ps23
Malvids - ps24
Sap./Malv./Brassicales - ps25
Malvales/Brassicales - ps26
Brassicales - ps27
Cleomaceae/Brassicaceae - ps28
Brassicaceae - ps29
Camelineae - ps30
Arabidopsis - ps31
Arabidopsis_thaliana - ps32
Phylostratum

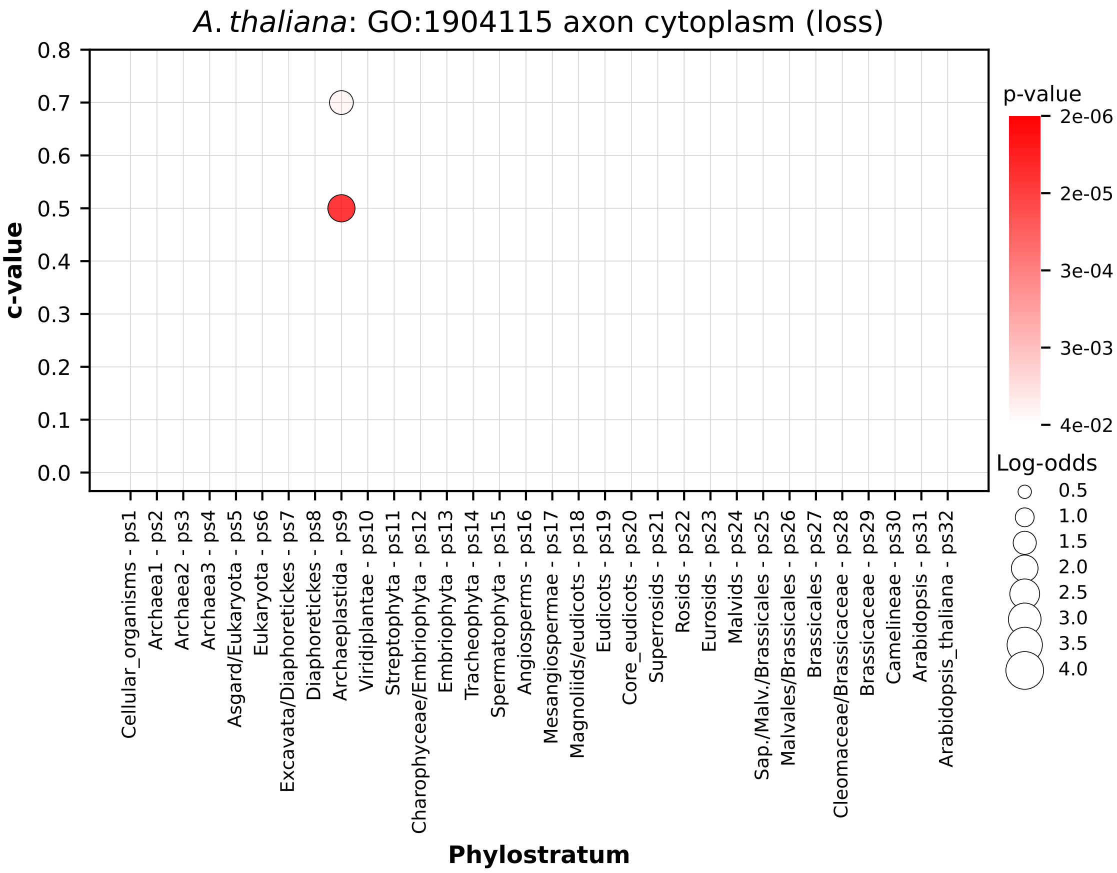
A. thaliana: GO:1904115 axon cytoplasm (loss)
c-value
0.0
0.1
0.2
0.3
0.4
0.5
0.6
0.7
0.8
Phylostratum
Cellular_organisms - ps1
Archaea1 - ps2
Archaea2 - ps3
Archaea3 - ps4
Asgard/Eukaryota - ps5
Eukaryota - ps6
Excavata/Diaphoretickes - ps7
Diaphoretickes - ps8
Archaeplastida - ps9
Viridiplantae - ps10
Streptophyta - ps11
Charophyceae/Embriophyta - ps12
Embriophyta - ps13
Tracheophyta - ps14
Spermatophyta - ps15
Angiosperms - ps16
Mesangiospermae - ps17
Magnoliids/eudicots - ps18
Eudicots - ps19
Core_eudicots - ps20
Superrosids - ps21
Rosids - ps22
Eurosids - ps23
Malvids - ps24
Sap./Malv./Brassicales - ps25
Malvales/Brassicales - ps26
Brassicales - ps27
Cleomaceae/Brassicaceae - ps28
Brassicaceae - ps29
Camelineae - ps30
Arabidopsis - ps31
Arabidopsis_thaliana - ps32
p-value
2e-06
2e-05
3e-04
3e-03
4e-02
Log-odds
0.5
1.0
1.5
2.0
2.5
3.0
3.5
4.0

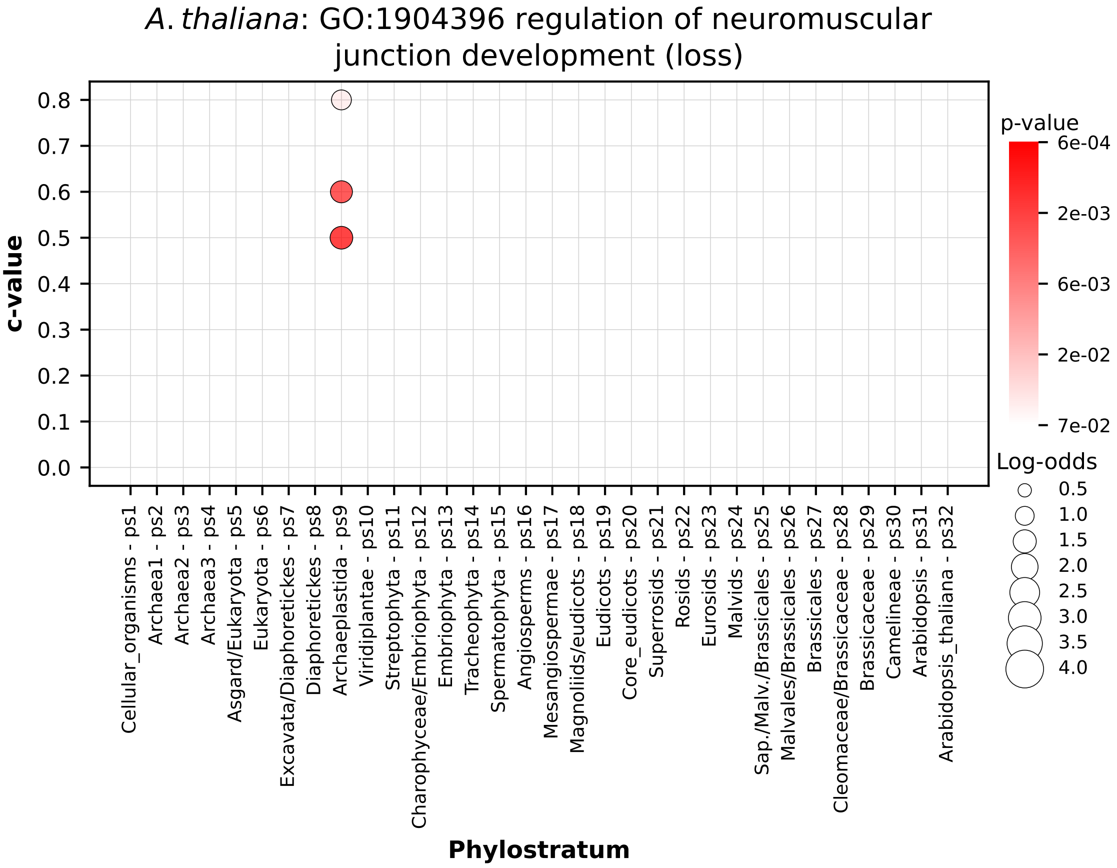

A. thaliana: GO:1904396 regulation of neuromuscular junction development (loss)
c-value
0.8
0.7
0.6
0.5
0.4
0.3
0.2
0.1
0.0
p-value
6e-04
2e-03
6e-03
2e-02
7e-02
Log-odds
0.5
1.0
1.5
2.0
2.5
3.0
3.5
4.0
Cellular_organisms - ps1
Archaea1 - ps2
Archaea2 - ps3
Archaea3 - ps4
Asgard/Eukaryota - ps5
Eukaryota - ps6
Excavata/Diaphoretickes - ps7
Diaphoretickes - ps8
Archaeplastida - ps9
Viridiplantae - ps10
Streptophyta - ps11
Charophyceae/Embriophyta - ps12
Embriophyta - ps13
Tracheophyta - ps14
Spermatophyta - ps15
Angiosperms - ps16
Mesangiospermae - ps17
Magnoliids/eudicots - ps18
Eudicots - ps19
Core_eudicots - ps20
Superrosids - ps21
Rosids - ps22
Eurosids - ps23
Malvids - ps24
Sap./Malv./Brassicales - ps25
Malvales/Brassicales - ps26
Brassicales - ps27
Cleomaceae/Brassicaceae - ps28
Brassicaceae - ps29
Camelineae - ps30
Arabidopsis - ps31
Arabidopsis_thaliana - ps32
Phylostratum

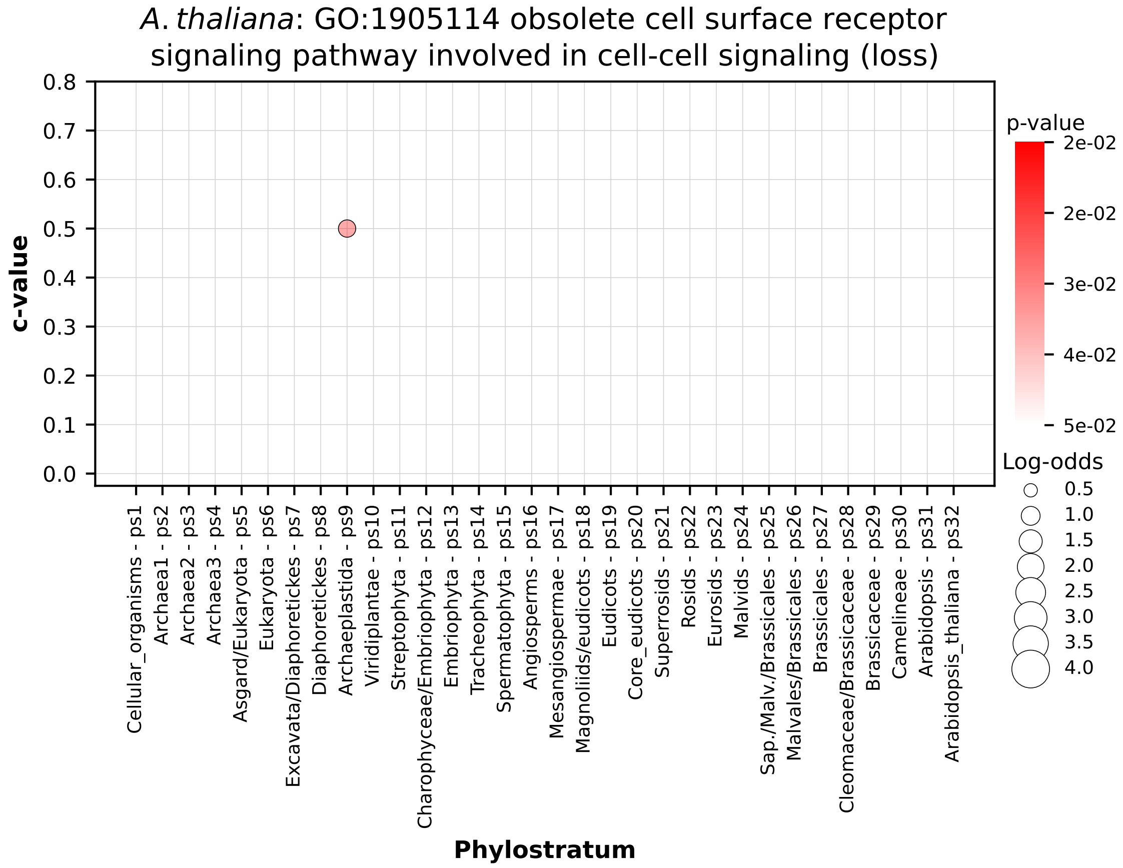
A. thaliana: GO:1905114 obsolete cell surface receptor signaling pathway involved in cell-cell signaling (loss)
c-value
0.8
0.7
0.6
0.5
0.4
0.3
0.2
0.1
0.0
p-value
2e-02
2e-02
3e-02
4e-02
5e-02
Log-odds
0.5
1.0
1.5
2.0
2.5
3.0
3.5
4.0
Cellular_organisms - ps1
Archaea1 - ps2
Archaea2 - ps3
Archaea3 - ps4
Asgard/Eukaryota - ps5
Eukaryota - ps6
Excavata/Diaphoretickes - ps7
Diaphoretickes - ps8
Archaeplastida - ps9
Viridiplantae - ps10
Streptophyta - ps11
Charophyceae/Embriophyta - ps12
Embriophyta - ps13
Tracheophyta - ps14
Spermatophyta - ps15
Angiosperms - ps16
Mesangiospermae - ps17
Magnoliids/eudicots - ps18
Eudicots - ps19
Core_eudicots - ps20
Superrosids - ps21
Rosids - ps22
Eurosids - ps23
Malvids - ps24
Sap./Malv./Brassicales - ps25
Malvales/Brassicales - ps26
Brassicales - ps27
Cleomaceae/Brassicaceae - ps28
Brassicaceae - ps29
Camelineae - ps30
Arabidopsis - ps31
Arabidopsis_thaliana - ps32
Phylostratum

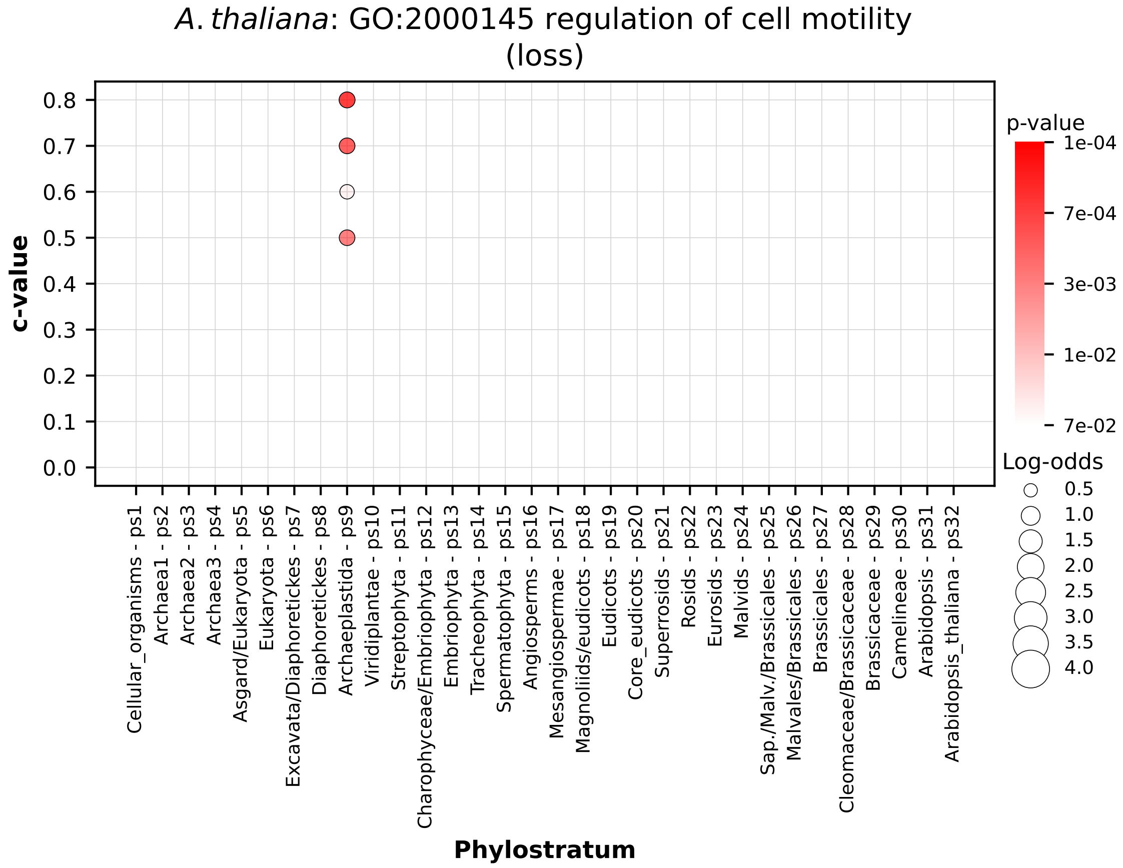

A. thaliana: GO:2000145 regulation of cell motility
(loss)
c-value
0.8
0.7
0.6
0.5
0.4
0.3
0.2
0.1
0.0
p-value
1e-04
7e-04
3e-03
1e-02
7e-02
Log-odds
0.5
1.0
1.5
2.0
2.5
3.0
3.5
4.0
Cellular_organisms - ps1
Archaea1 - ps2
Archaea2 - ps3
Archaea3 - ps4
Asgard/Eukaryota - ps5
Eukaryota - ps6
Excavata/Diaphoretickes - ps7
Diaphoretickes - ps8
Archaeplastida - ps9
Viridiplantae - ps10
Streptophyta - ps11
Charophyceae/Embriophyta - ps12
Embriophyta - ps13
Tracheophyta - ps14
Spermatophyta - ps15
Angiosperms - ps16
Mesangiospermae - ps17
Magnoliids/eudicots - ps18
Eudicots - ps19
Core_eudicots - ps20
Superrosids - ps21
Rosids - ps22
Eurosids - ps23
Malvids - ps24
Sap./Malv./Brassicales - ps25
Malvales/Brassicales - ps26
Brassicales - ps27
Cleomaceae/Brassicaceae - ps28
Brassicaceae - ps29
Camelineae - ps30
Arabidopsis - ps31
Arabidopsis_thaliana - ps32
Phylostratum

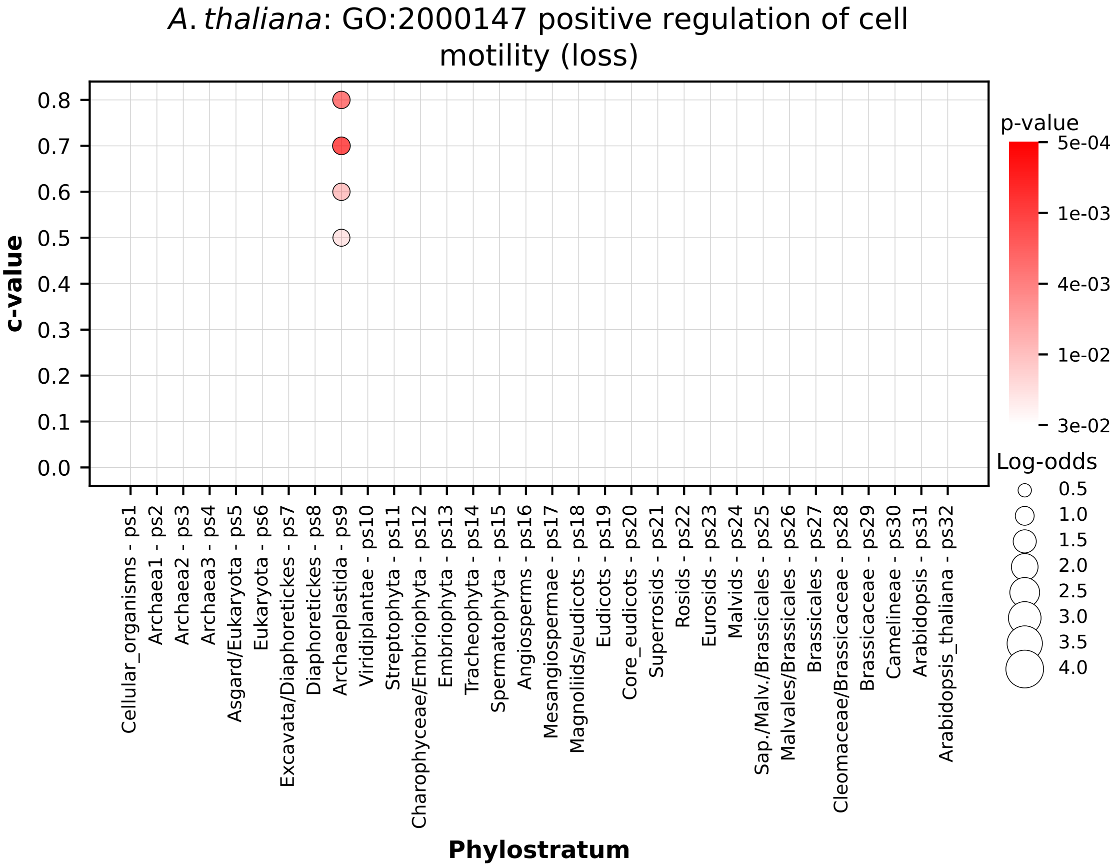

A. thaliana: GO:2000147 positive regulation of cell motility (loss)
c-value
0.0
0.1
0.2
0.3
0.4
0.5
0.6
0.7
0.8
p-value
5e-04
1e-03
4e-03
1e-02
3e-02
Log-odds
0.5
1.0
1.5
2.0
2.5
3.0
3.5
4.0
Cellular_organisms - ps1
Archaea1 - ps2
Archaea2 - ps3
Archaea3 - ps4
Asgard/Eukaryota - ps5
Eukaryota - ps6
Excavata/Diaphoretickes - ps7
Diaphoretickes - ps8
Archaeplastida - ps9
Viridiplantae - ps10
Streptophyta - ps11
Charophyceae/Embriophyta - ps12
Embriophyta - ps13
Tracheophyta - ps14
Spermatophyta - ps15
Angiosperms - ps16
Mesangiospermae - ps17
Magnoliids/eudicots - ps18
Eudicots - ps19
Core_eudicots - ps20
Superrosids - ps21
Rosids - ps22
Eurosids - ps23
Malvids - ps24
Sap./Malv./Brassicales - ps25
Malvales/Brassicales - ps26
Brassicales - ps27
Cleomaceae/Brassicaceae - ps28
Brassicaceae - ps29
Camelineae - ps30
Arabidopsis - ps31
Arabidopsis_thaliana - ps32
Phylostratum

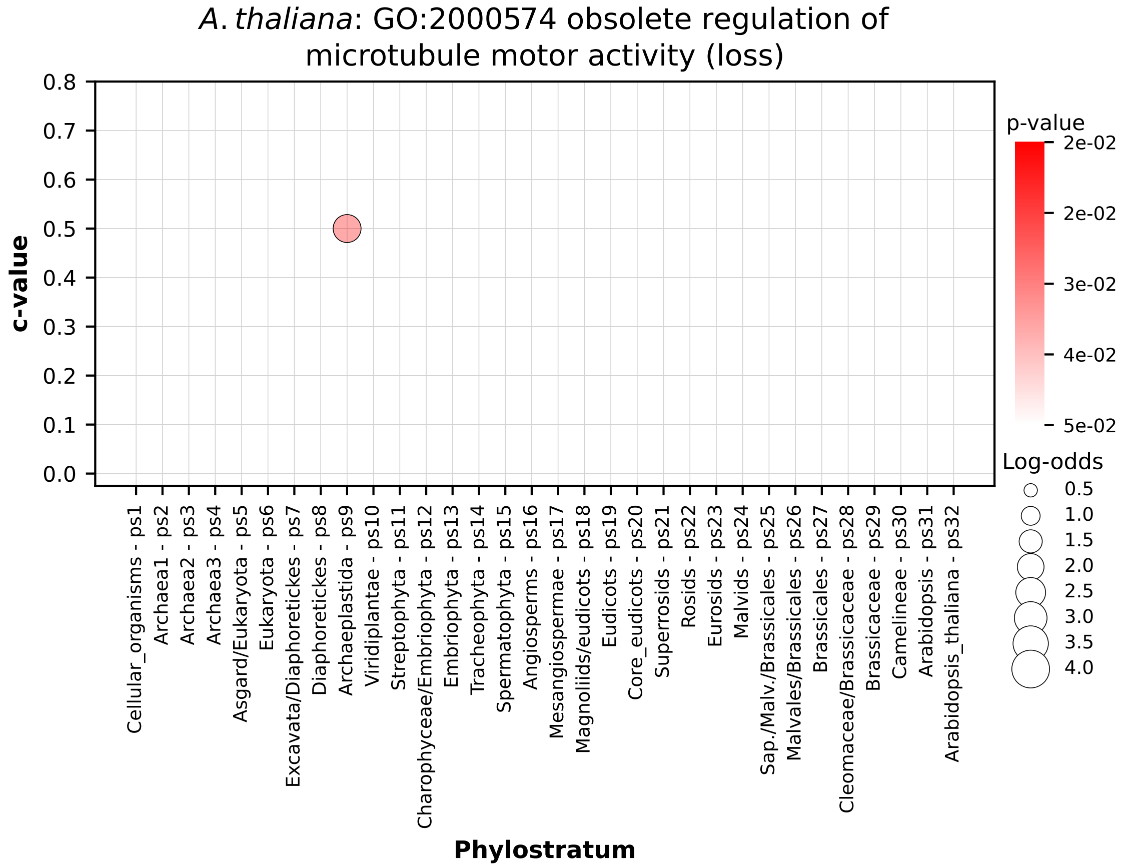
A. thaliana: GO:2000574 obsolete regulation of microtubule motor activity (loss)
c-value
0.8
0.7
0.6
0.5
0.4
0.3
0.2
0.1
0.0
p-value
2e-02
2e-02
3e-02
4e-02
5e-02
Log-odds
0.5
1.0
1.5
2.0
2.5
3.0
3.5
4.0
Cellular_organisms - ps1
Archaea1 - ps2
Archaea2 - ps3
Archaea3 - ps4
Asgard/Eukaryota - ps5
Eukaryota - ps6
Excavata/Diaphoretickes - ps7
Diaphoretickes - ps8
Archaeplastida - ps9
Viridiplantae - ps10
Streptophyta - ps11
Charophyceae/Embriophyta - ps12
Embriophyta - ps13
Tracheophyta - ps14
Spermatophyta - ps15
Angiosperms - ps16
Mesangiospermae - ps17
Magnoliids/eudicots - ps18
Eudicots - ps19
Core_eudicots - ps20
Superrosids - ps21
Rosids - ps22
Eurosids - ps23
Malvids - ps24
Sap./Malv./Brassicales - ps25
Malvales/Brassicales - ps26
Brassicales - ps27
Cleomaceae/Brassicaceae - ps28
Brassicaceae - ps29
Camelineae - ps30
Arabidopsis - ps31
Arabidopsis_thaliana - ps32
Phylostratum

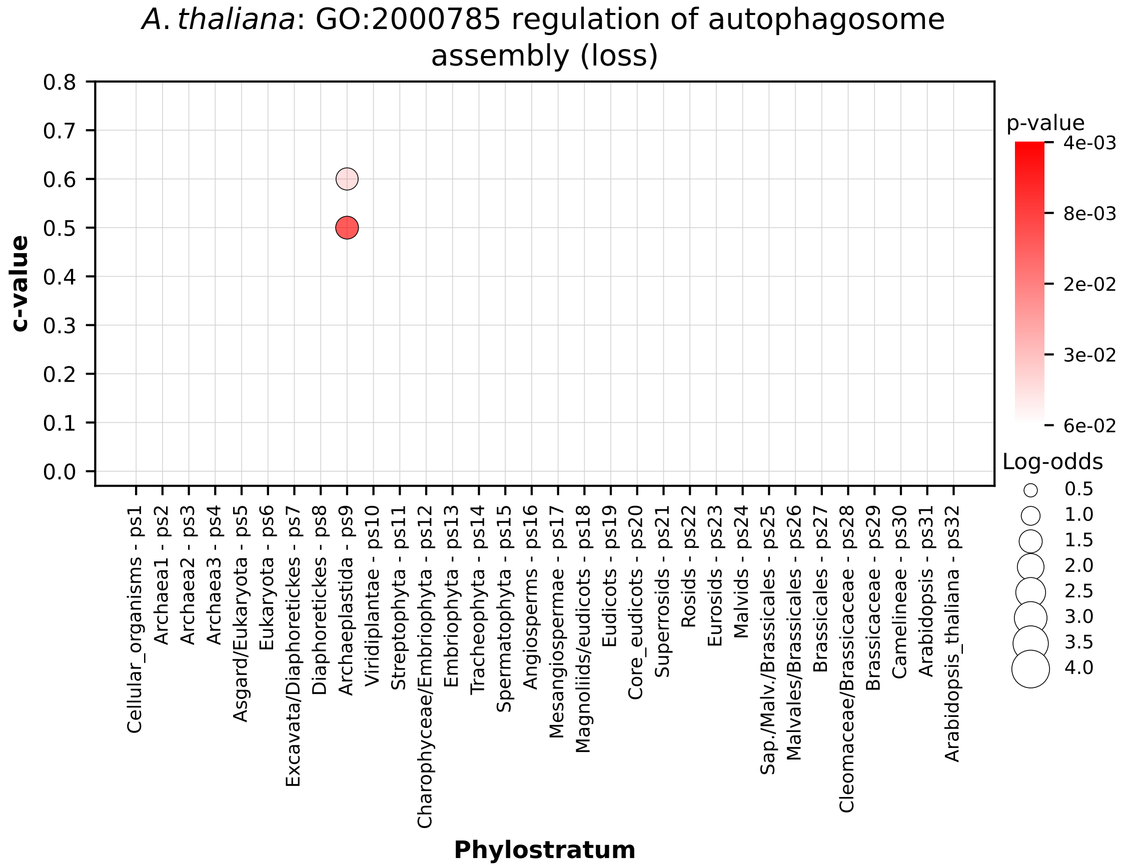

A. thaliana: GO:2000785 regulation of autophagosome assembly (loss)
c-value
0.0
0.1
0.2
0.3
0.4
0.5
0.6
0.7
0.8
Phylostratum
Cellular_organisms - ps1
Archaea1 - ps2
Archaea2 - ps3
Archaea3 - ps4
Asgard/Eukaryota - ps5
Eukaryota - ps6
Excavata/Diaphoretickes - ps7
Diaphoretickes - ps8
Archaeplastida - ps9
Viridiplantae - ps10
Streptophyta - ps11
Charophyceae/Embriophyta - ps12
Embriophyta - ps13
Tracheophyta - ps14
Spermatophyta - ps15
Angiosperms - ps16
Mesangiospermae - ps17
Magnoliids/eudicots - ps18
Eudicots - ps19
Core_eudicots - ps20
Superrosids - ps21
Rosids - ps22
Eurosids - ps23
Malvids - ps24
Sap./Malv./Brassicales - ps25
Malvales/Brassicales - ps26
Brassicales - ps27
Cleomaceae/Brassicaceae - ps28
Brassicaceae - ps29
Camelineae - ps30
Arabidopsis - ps31
Arabidopsis_thaliana - ps32
p-value
4e-03
8e-03
2e-02
3e-02
6e-02
Log-odds
0.5
1.0
1.5
2.0
2.5
3.0
3.5
4.0

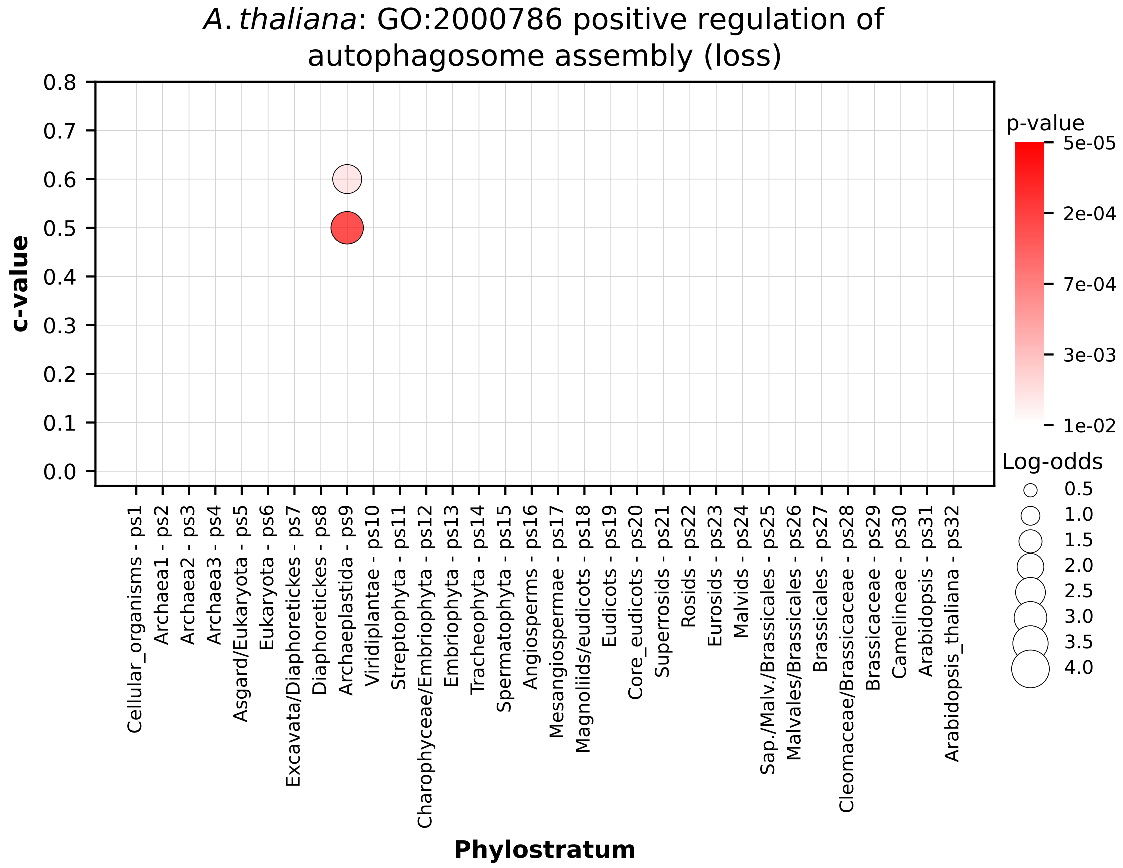
A. thaliana: GO:2000786 positive regulation of autophagosome assembly (loss)
c-value
0.8
0.7
0.6
0.5
0.4
0.3
0.2
0.1
0.0
Phylostratum
Cellular_organisms - ps1
Archaea1 - ps2
Archaea2 - ps3
Archaea3 - ps4
Asgard/Eukaryota - ps5
Eukaryota - ps6
Excavata/Diaphoretickes - ps7
Diaphoretickes - ps8
Archaeplastida - ps9
Viridiplantae - ps10
Streptophyta - ps11
Charophyceae/Embriophyta - ps12
Embriophyta - ps13
Tracheophyta - ps14
Spermatophyta - ps15
Angiosperms - ps16
Mesangiospermae - ps17
Magnoliids/eudicots - ps18
Eudicots - ps19
Core_eudicots - ps20
Superrosids - ps21
Rosids - ps22
Eurosids - ps23
Malvids - ps24
Sap./Malv./Brassicales - ps25
Malvales/Brassicales - ps26
Brassicales - ps27
Cleomaceae/Brassicaceae - ps28
Brassicaceae - ps29
Camelineae - ps30
Arabidopsis - ps31
Arabidopsis_thaliana - ps32
p-value
5e-05
2e-04
7e-04
3e-03
1e-02
Log-odds
0.5
1.0
1.5
2.0
2.5
3.0
3.5
4.0

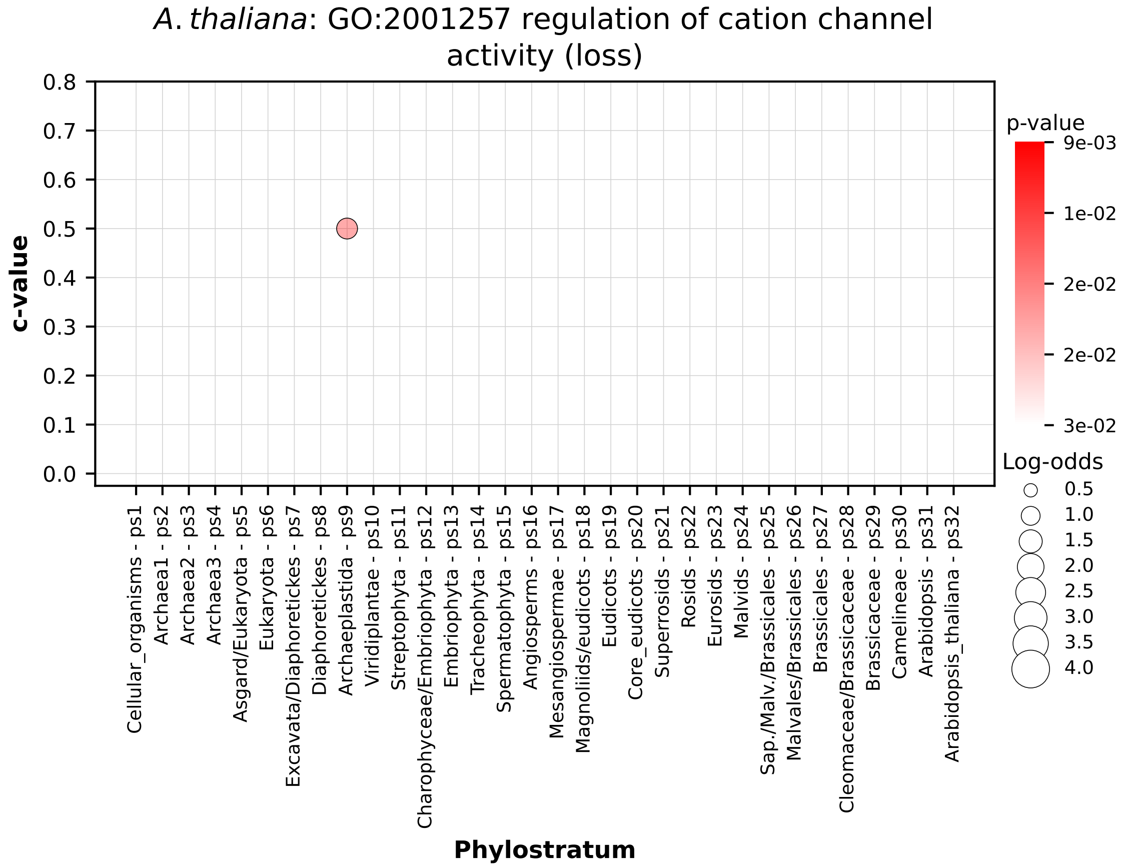

A. thaliana: GO:2001257 regulation of cation channel activity (loss)
c-value
0.0
0.1
0.2
0.3
0.4
0.5
0.6
0.7
0.8
Phylostratum
Cellular_organisms - ps1
Archaea1 - ps2
Archaea2 - ps3
Archaea3 - ps4
Asgard/Eukaryota - ps5
Eukaryota - ps6
Excavata/Diaphoretickes - ps7
Diaphoretickes - ps8
Archaeplastida - ps9
Viridiplantae - ps10
Streptophyta - ps11
Charophyceae/Embriophyta - ps12
Embriophyta - ps13
Tracheophyta - ps14
Spermatophyta - ps15
Angiosperms - ps16
Mesangiospermae - ps17
Magnoliids/eudicots - ps18
Eudicots - ps19
Core_eudicots - ps20
Superrosids - ps21
Rosids - ps22
Eurosids - ps23
Malvids - ps24
Sap./Malv./Brassicales - ps25
Malvales/Brassicales - ps26
Brassicales - ps27
Cleomaceae/Brassicaceae - ps28
Brassicaceae - ps29
Camelineae - ps30
Arabidopsis - ps31
Arabidopsis_thaliana - ps32
p-value
9e-03
1e-02
2e-02
2e-02
3e-02
Log-odds
0.5
1.0
1.5
2.0
2.5
3.0
3.5
4.0

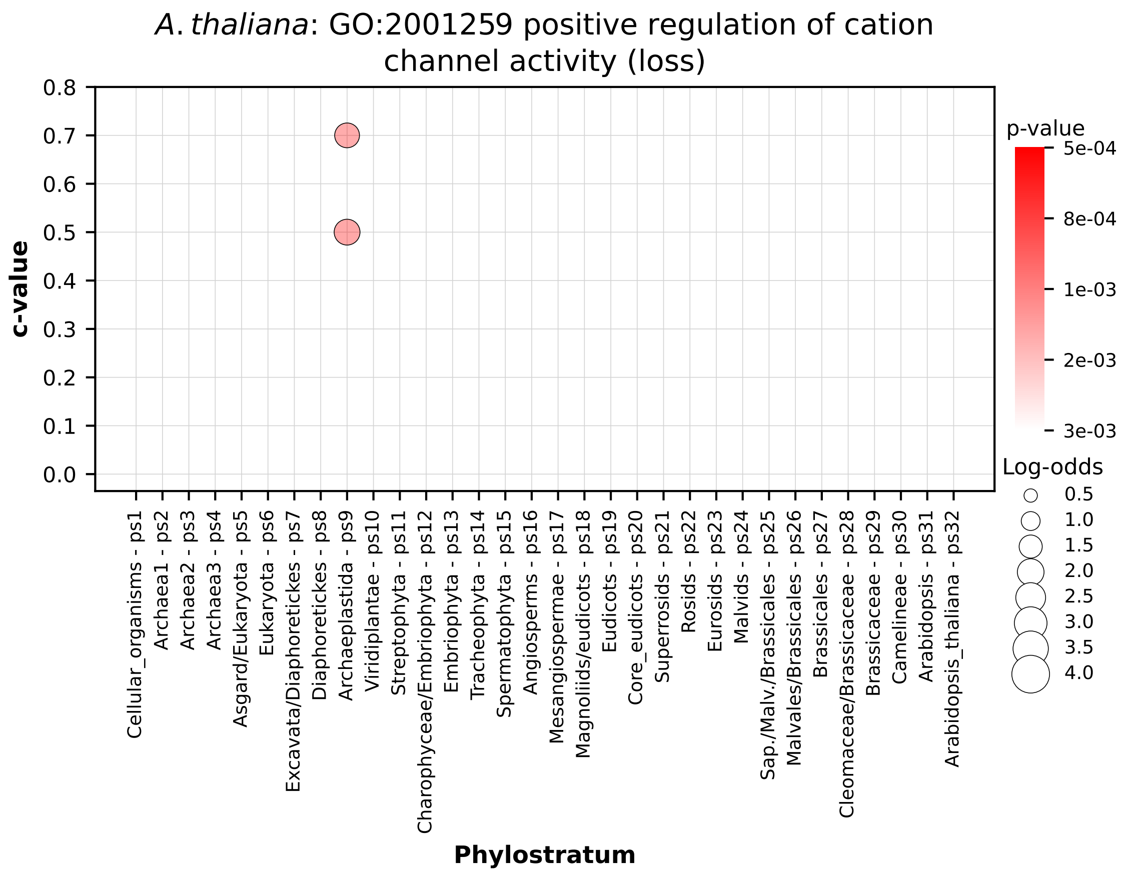

A. thaliana: GO:2001259 positive regulation of cation channel activity (loss)
c-value
0.8
0.7
0.6
0.5
0.4
0.3
0.2
0.1
0.0
Phylostratum
Cellular_organisms - ps1
Archaea1 - ps2
Archaea2 - ps3
Archaea3 - ps4
Asgard/Eukaryota - ps5
Eukaryota - ps6
Excavata/Diaphoretickes - ps7
Diaphoretickes - ps8
Archaeplastida - ps9
Viridiplantae - ps10
Streptophyta - ps11
Charophyceae/Embriophyta - ps12
Embriophyta - ps13
Tracheophyta - ps14
Spermatophyta - ps15
Angiosperms - ps16
Mesangiospermae - ps17
Magnoliids/eudicots - ps18
Eudicots - ps19
Core_eudicots - ps20
Superrosids - ps21
Rosids - ps22
Eurosids - ps23
Malvids - ps24
Sap./Malv./Brassicales - ps25
Malvales/Brassicales - ps26
Brassicales - ps27
Cleomaceae/Brassicaceae - ps28
Brassicaceae - ps29
Camelineae - ps30
Arabidopsis - ps31
Arabidopsis_thaliana - ps32
p-value
5e-04
8e-04
1e-03
2e-03
3e-03
Log-odds
0.5
1.0
1.5
2.0
2.5
3.0
3.5
4.0

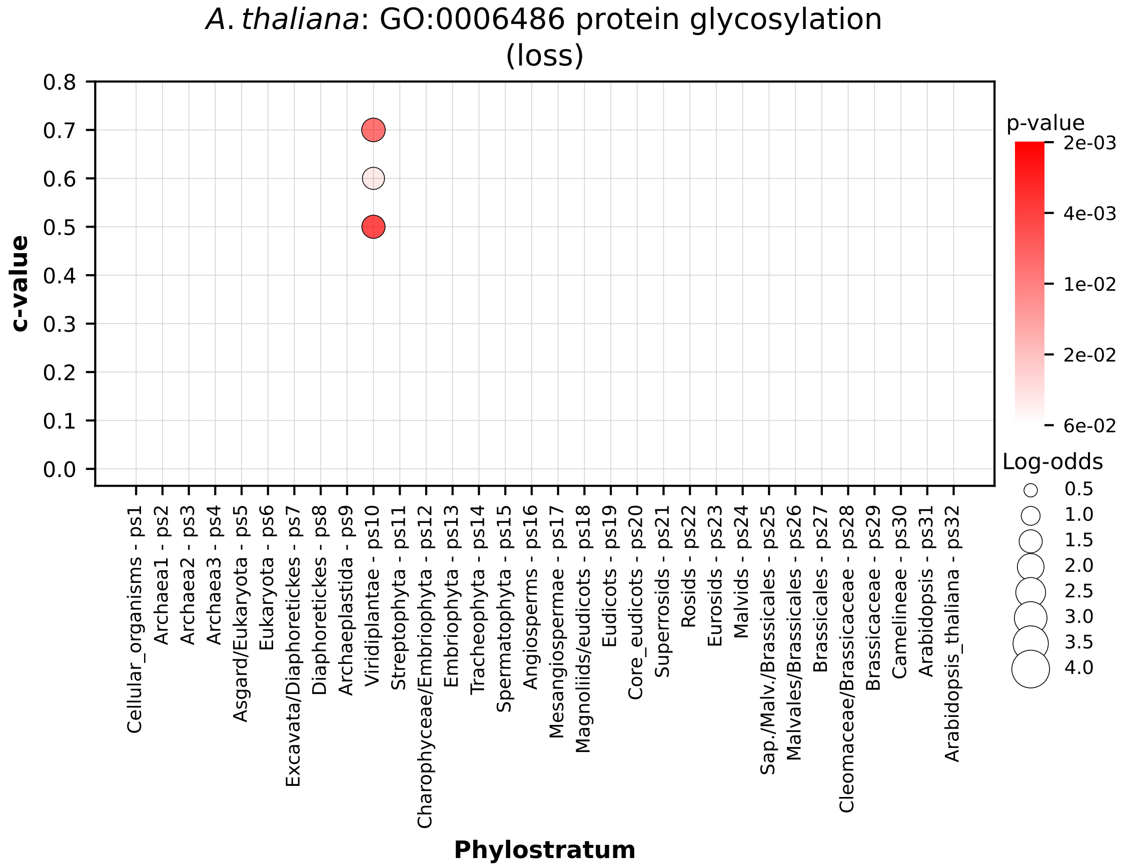
A. thaliana: GO:0006486 protein glycosylation
(loss)
c-value
0.8
0.7
0.6
0.5
0.4
0.3
0.2
0.1
0.0
p-value
2e-03
4e-03
1e-02
2e-02
6e-02
Log-odds
0.5
1.0
1.5
2.0
2.5
3.0
3.5
4.0
Cellular_organisms - ps1
Archaea1 - ps2
Archaea2 - ps3
Archaea3 - ps4
Asgard/Eukaryota - ps5
Eukaryota - ps6
Excavata/Diaphoretickes - ps7
Diaphoretickes - ps8
Archaeplastida - ps9
Viridiplantae - ps10
Streptophyta - ps11
Charophyceae/Embriophyta - ps12
Embriophyta - ps13
Tracheophyta - ps14
Spermatophyta - ps15
Angiosperms - ps16
Mesangiospermae - ps17
Magnoliids/eudicots - ps18
Eudicots - ps19
Core_eudicots - ps20
Superrosids - ps21
Rosids - ps22
Eurosids - ps23
Malvids - ps24
Sap./Malv./Brassicales - ps25
Malvales/Brassicales - ps26
Brassicales - ps27
Cleomaceae/Brassicaceae - ps28
Brassicaceae - ps29
Camelineae - ps30
Arabidopsis - ps31
Arabidopsis_thaliana - ps32
Phylostratum

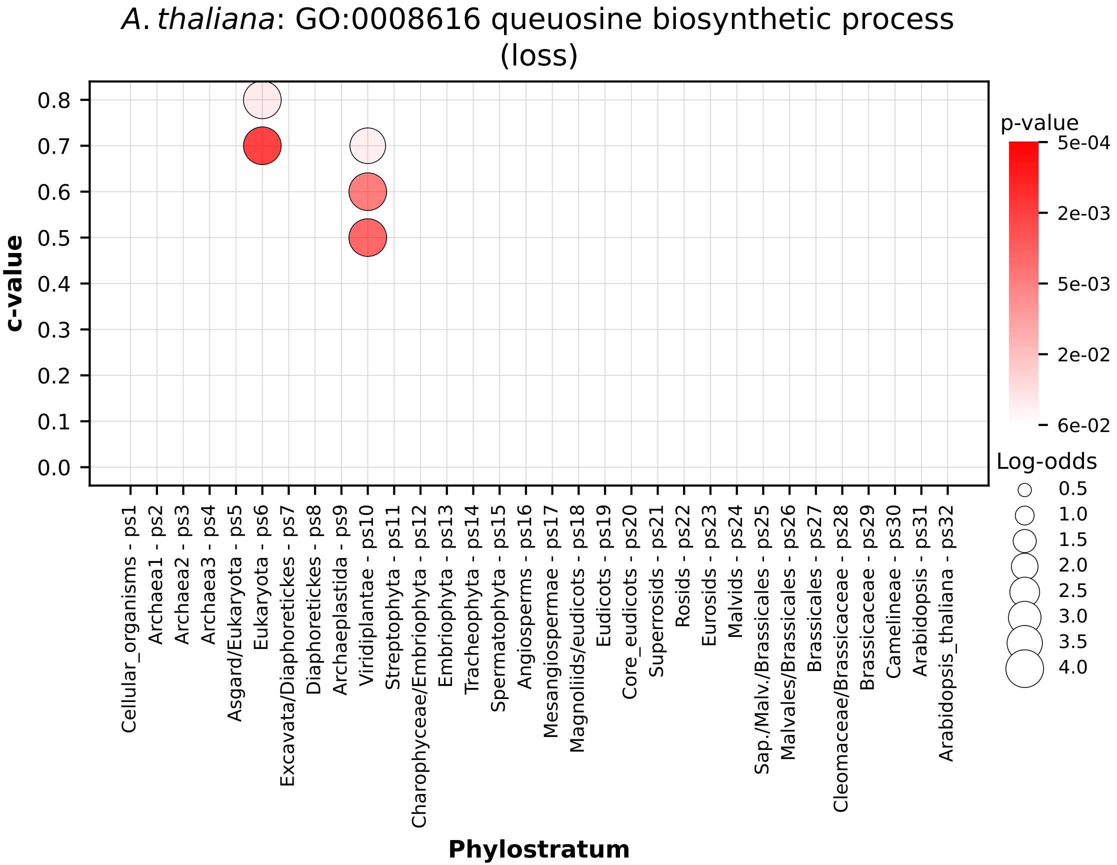

A. thaliana: GO:0008616 queuosine biosynthetic process (loss)
c-value
0.8
0.7
0.6
0.5
0.4
0.3
0.2
0.1
0.0
Phylostratum
Cellular_organisms - ps1
Archaea1 - ps2
Archaea2 - ps3
Archaea3 - ps4
Asgard/Eukaryota - ps5
Eukaryota - ps6
Excavata/Diaphoretickes - ps7
Diaphoretickes - ps8
Archaeplastida - ps9
Viridiplantae - ps10
Streptophyta - ps11
Charophyceae/Embriophyta - ps12
Embriophyta - ps13
Tracheophyta - ps14
Spermatophyta - ps15
Angiosperms - ps16
Mesangiospermae - ps17
Magnoliids/eudicots - ps18
Eudicots - ps19
Core_eudicots - ps20
Superrosids - ps21
Rosids - ps22
Eurosids - ps23
Malvids - ps24
Sap./Malv./Brassicales - ps25
Malvales/Brassicales - ps26
Brassicales - ps27
Cleomaceae/Brassicaceae - ps28
Brassicaceae - ps29
Camelineae - ps30
Arabidopsis - ps31
Arabidopsis_thaliana - ps32
p-value
5e-04
2e-03
5e-03
2e-02
6e-02
Log-odds
0.5
1.0
1.5
2.0
2.5
3.0
3.5
4.0

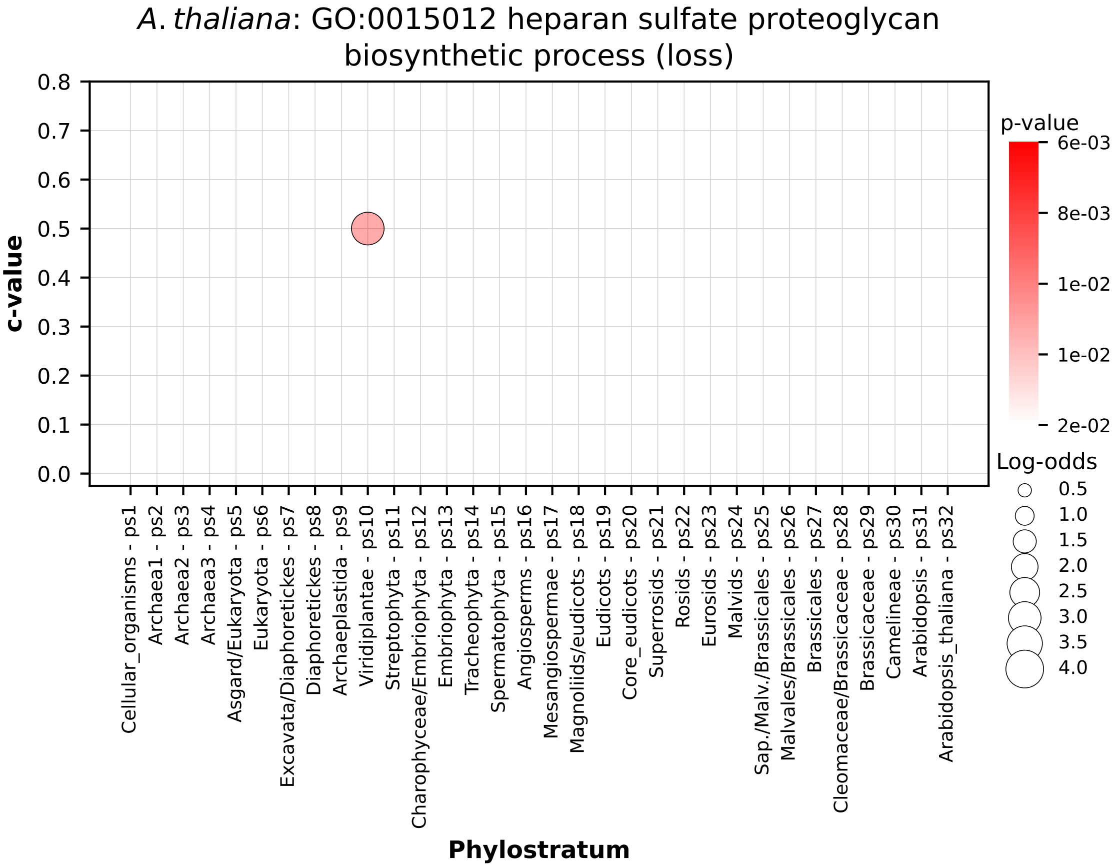

A. thaliana: GO:0015012 heparan sulfate proteoglycan biosynthetic process (loss)
c-value
0.0
0.1
0.2
0.3
0.4
0.5
0.6
0.7
0.8
Phylostratum
Cellular_organisms - ps1
Archaea1 - ps2
Archaea2 - ps3
Archaea3 - ps4
Asgard/Eukaryota - ps5
Eukaryota - ps6
Excavata/Diaphoretickes - ps7
Diaphoretickes - ps8
Archaeplastida - ps9
Viridiplantae - ps10
Streptophyta - ps11
Charophyceae/Embriophyta - ps12
Embriophyta - ps13
Tracheophyta - ps14
Spermatophyta - ps15
Angiosperms - ps16
Mesangiospermae - ps17
Magnoliids/eudicots - ps18
Eudicots - ps19
Core_eudicots - ps20
Superrosids - ps21
Rosids - ps22
Eurosids - ps23
Malvids - ps24
Sap./Malv./Brassicales - ps25
Malvales/Brassicales - ps26
Brassicales - ps27
Cleomaceae/Brassicaceae - ps28
Brassicaceae - ps29
Camelineae - ps30
Arabidopsis - ps31
Arabidopsis_thaliana - ps32
p-value
6e-03
8e-03
1e-02
1e-02
2e-02
Log-odds
0.5
1.0
1.5
2.0
2.5
3.0
3.5
4.0

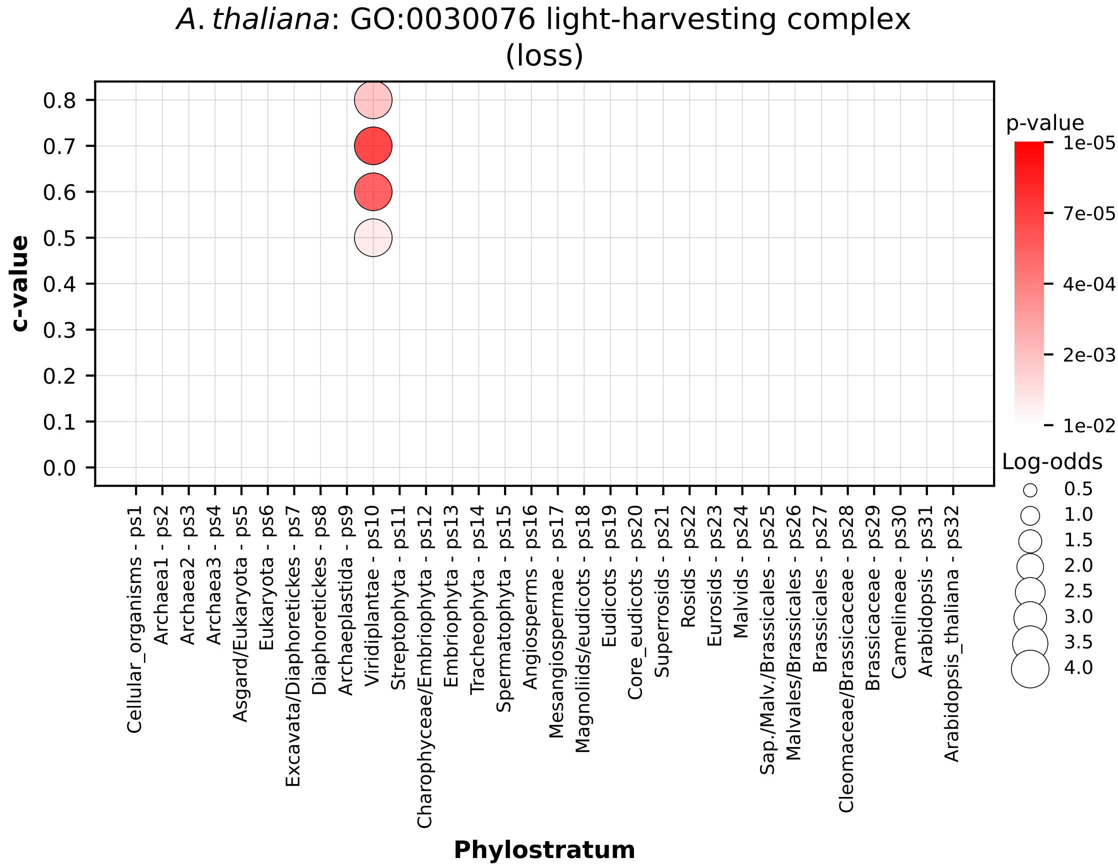

A. thaliana: GO:0030076 light-harvesting complex
(loss)
c-value
0.8
0.7
0.6
0.5
0.4
0.3
0.2
0.1
0.0
p-value
1e-05
7e-05
4e-04
2e-03
1e-02
Log-odds
0.5
1.0
1.5
2.0
2.5
3.0
3.5
4.0
Cellular_organisms - ps1
Archaea1 - ps2
Archaea2 - ps3
Archaea3 - ps4
Asgard/Eukaryota - ps5
Eukaryota - ps6
Excavata/Diaphoretickes - ps7
Diaphoretickes - ps8
Archaeplastida - ps9
Viridiplantae - ps10
Streptophyta - ps11
Charophyceae/Embriophyta - ps12
Embriophyta - ps13
Tracheophyta - ps14
Spermatophyta - ps15
Angiosperms - ps16
Mesangiospermae - ps17
Magnoliids/eudicots - ps18
Eudicots - ps19
Core_eudicots - ps20
Superrosids - ps21
Rosids - ps22
Eurosids - ps23
Malvids - ps24
Sap./Malv./Brassicales - ps25
Malvales/Brassicales - ps26
Brassicales - ps27
Cleomaceae/Brassicaceae - ps28
Brassicaceae - ps29
Camelineae - ps30
Arabidopsis - ps31
Arabidopsis_thaliana - ps32
Phylostratum

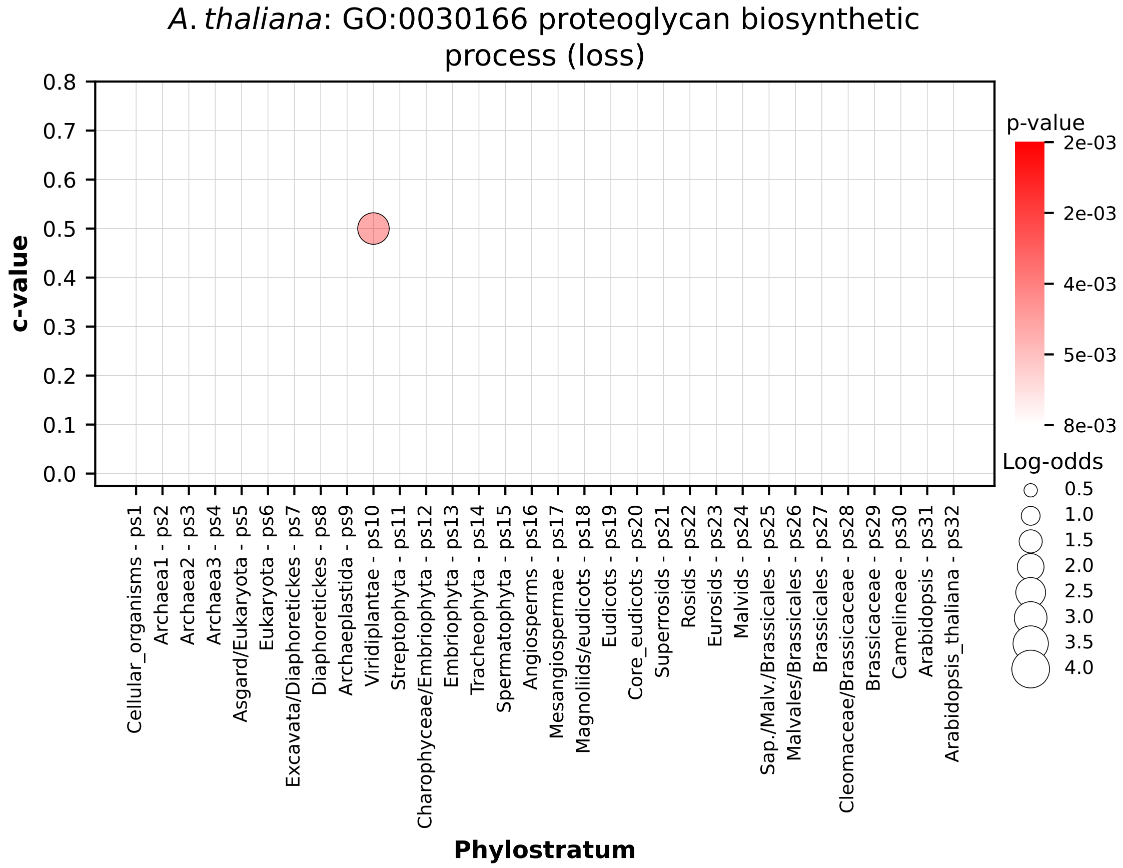

A. thaliana: GO:0030166 proteoglycan biosynthetic process (loss)
c-value
0.8
0.7
0.6
0.5
0.4
0.3
0.2
0.1
0.0
Phylostratum
Cellular_organisms - ps1
Archaea1 - ps2
Archaea2 - ps3
Archaea3 - ps4
Asgard/Eukaryota - ps5
Eukaryota - ps6
Excavata/Diaphoretickes - ps7
Diaphoretickes - ps8
Archaeplastida - ps9
Viridiplantae - ps10
Streptophyta - ps11
Charophyceae/Embriophyta - ps12
Embriophyta - ps13
Tracheophyta - ps14
Spermatophyta - ps15
Angiosperms - ps16
Mesangiospermae - ps17
Magnoliids/eudicots - ps18
Eudicots - ps19
Core_eudicots - ps20
Superrosids - ps21
Rosids - ps22
Eurosids - ps23
Malvids - ps24
Sap./Malv./Brassicales - ps25
Malvales/Brassicales - ps26
Brassicales - ps27
Cleomaceae/Brassicaceae - ps28
Brassicaceae - ps29
Camelineae - ps30
Arabidopsis - ps31
Arabidopsis_thaliana - ps32
p-value
2e-03
2e-03
4e-03
5e-03
8e-03
Log-odds
0.5
1.0
1.5
2.0
2.5
3.0
3.5
4.0

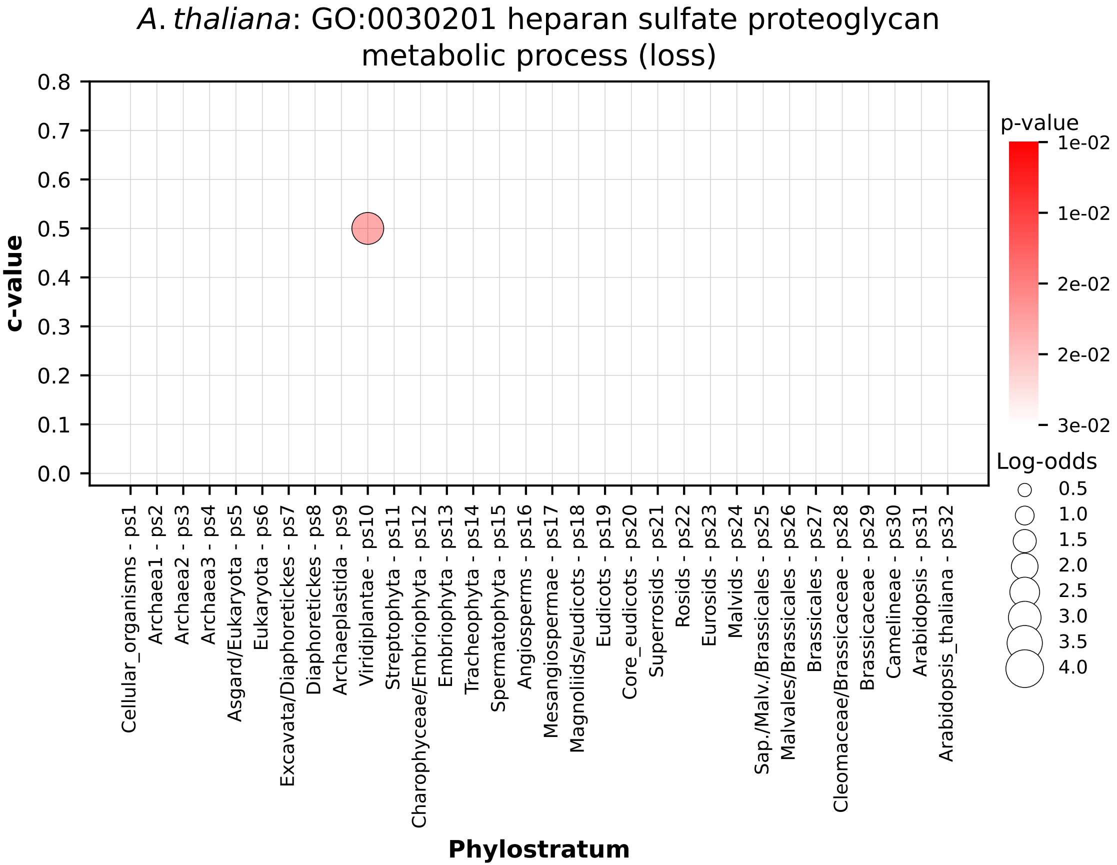

A. thaliana: GO:0030201 heparan sulfate proteoglycan metabolic process (loss)
c-value
0.8
0.7
0.6
0.5
0.4
0.3
0.2
0.1
0.0
p-value
1e-02
1e-02
2e-02
2e-02
3e-02
Log-odds
0.5
1.0
1.5
2.0
2.5
3.0
3.5
4.0
Cellular_organisms - ps1
Archaea1 - ps2
Archaea2 - ps3
Archaea3 - ps4
Asgard/Eukaryota - ps5
Eukaryota - ps6
Excavata/Diaphoretickes - ps7
Diaphoretickes - ps8
Archaeplastida - ps9
Viridiplantae - ps10
Streptophyta - ps11
Charophyceae/Embriophyta - ps12
Embriophyta - ps13
Tracheophyta - ps14
Spermatophyta - ps15
Angiosperms - ps16
Mesangiospermae - ps17
Magnoliids/eudicots - ps18
Eudicots - ps19
Core_eudicots - ps20
Superrosids - ps21
Rosids - ps22
Eurosids - ps23
Malvids - ps24
Sap./Malv./Brassicales - ps25
Malvales/Brassicales - ps26
Brassicales - ps27
Cleomaceae/Brassicaceae - ps28
Brassicaceae - ps29
Camelineae - ps30
Arabidopsis - ps31
Arabidopsis_thaliana - ps32
Phylostratum

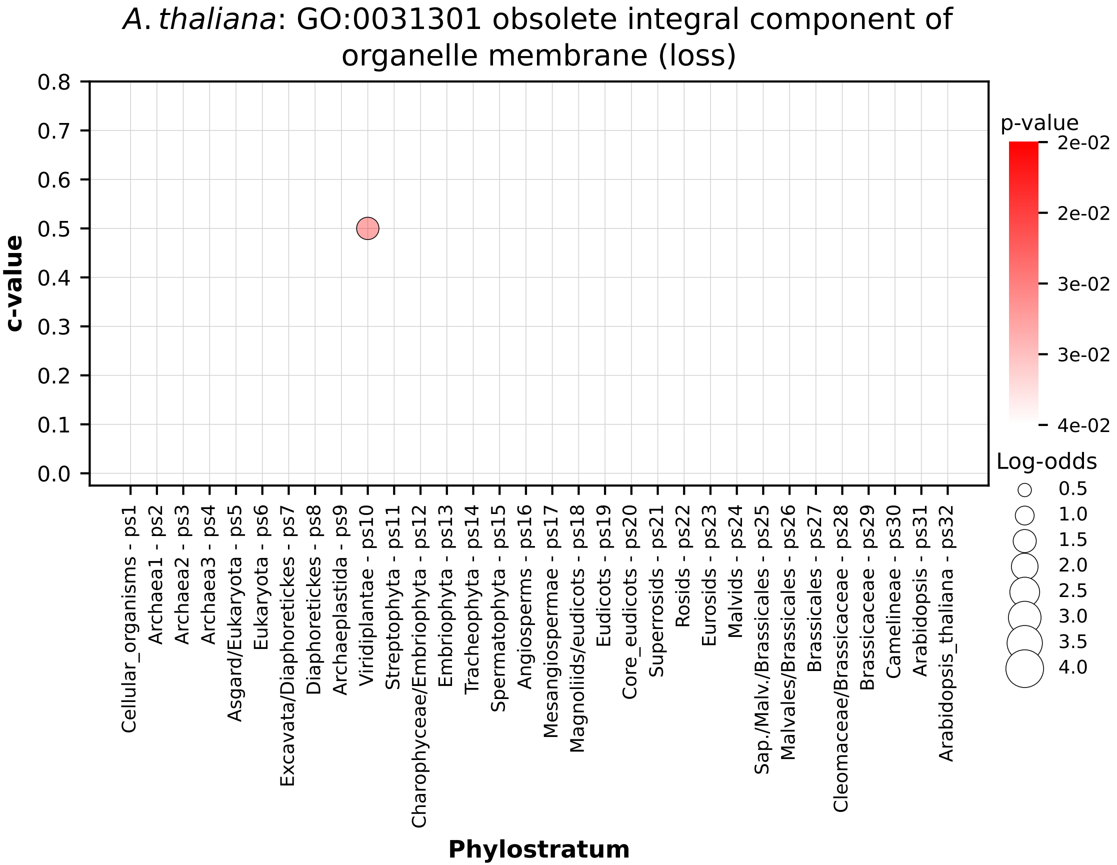

A. thaliana: GO:0031301 obsolete integral component of organelle membrane (loss)
c-value
0.8
0.7
0.6
0.5
0.4
0.3
0.2
0.1
0.0
p-value
2e-02
2e-02
3e-02
3e-02
4e-02
Log-odds
0.5
1.0
1.5
2.0
2.5
3.0
3.5
4.0
Cellular_organisms - ps1
Archaea1 - ps2
Archaea2 - ps3
Archaea3 - ps4
Asgard/Eukaryota - ps5
Eukaryota - ps6
Excavata/Diaphoretickes - ps7
Diaphoretickes - ps8
Archaeplastida - ps9
Viridiplantae - ps10
Streptophyta - ps11
Charophyceae/Embriophyta - ps12
Embriophyta - ps13
Tracheophyta - ps14
Spermatophyta - ps15
Angiosperms - ps16
Mesangiospermae - ps17
Magnoliids/eudicots - ps18
Eudicots - ps19
Core_eudicots - ps20
Superrosids - ps21
Rosids - ps22
Eurosids - ps23
Malvids - ps24
Sap./Malv./Brassicales - ps25
Malvales/Brassicales - ps26
Brassicales - ps27
Cleomaceae/Brassicaceae - ps28
Brassicaceae - ps29
Camelineae - ps30
Arabidopsis - ps31
Arabidopsis_thaliana - ps32
Phylostratum

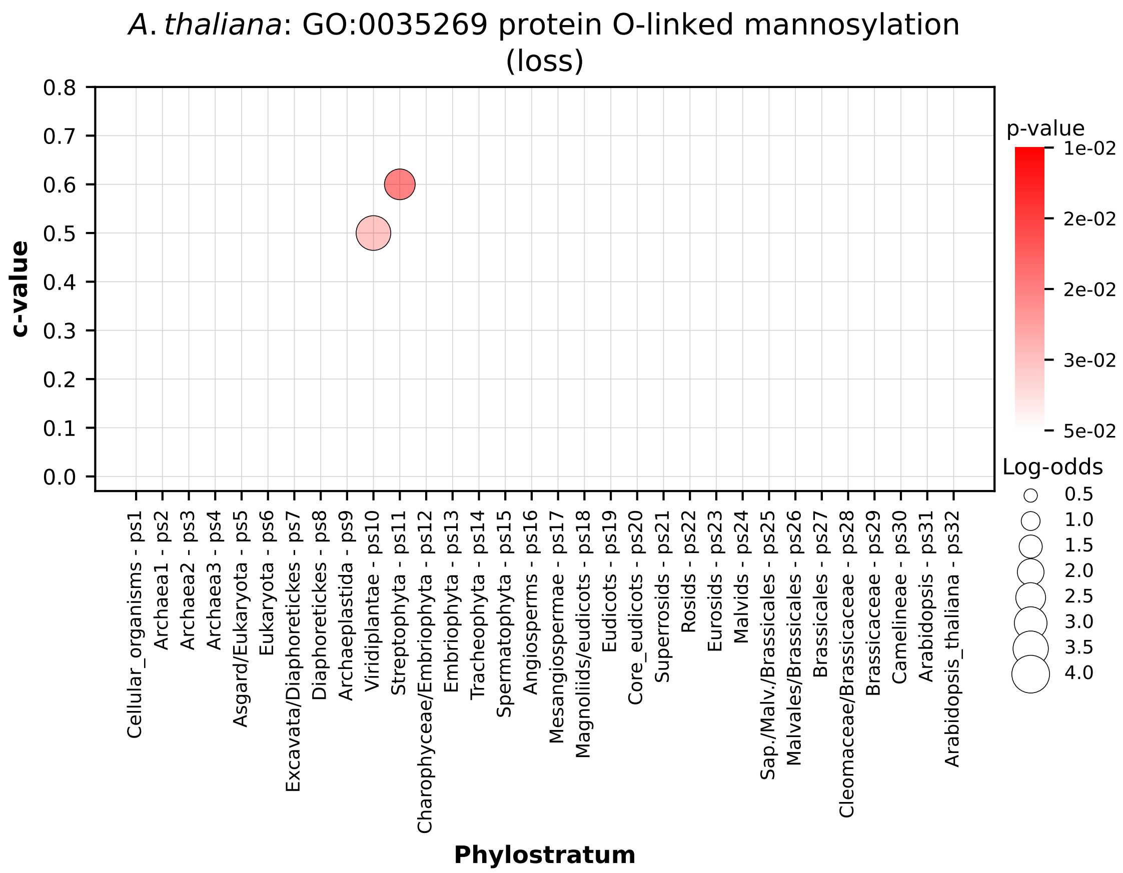

A. thaliana: GO:0035269 protein O-linked mannosylation (loss)
c-value
0.0
0.1
0.2
0.3
0.4
0.5
0.6
0.7
0.8
Phylostratum
Cellular_organisms - ps1
Archaea1 - ps2
Archaea2 - ps3
Archaea3 - ps4
Asgard/Eukaryota - ps5
Eukaryota - ps6
Excavata/Diaphoretickes - ps7
Diaphoretickes - ps8
Archaeplastida - ps9
Viridiplantae - ps10
Streptophyta - ps11
Charophyceae/Embriophyta - ps12
Embriophyta - ps13
Tracheophyta - ps14
Spermatophyta - ps15
Angiosperms - ps16
Mesangiospermae - ps17
Magnoliids/eudicots - ps18
Eudicots - ps19
Core_eudicots - ps20
Superrosids - ps21
Rosids - ps22
Eurosids - ps23
Malvids - ps24
Sap./Malv./Brassicales - ps25
Malvales/Brassicales - ps26
Brassicales - ps27
Cleomaceae/Brassicaceae - ps28
Brassicaceae - ps29
Camelineae - ps30
Arabidopsis - ps31
Arabidopsis_thaliana - ps32
p-value
1e-02
2e-02
2e-02
3e-02
5e-02
Log-odds
0.5
1.0
1.5
2.0
2.5
3.0
3.5
4.0

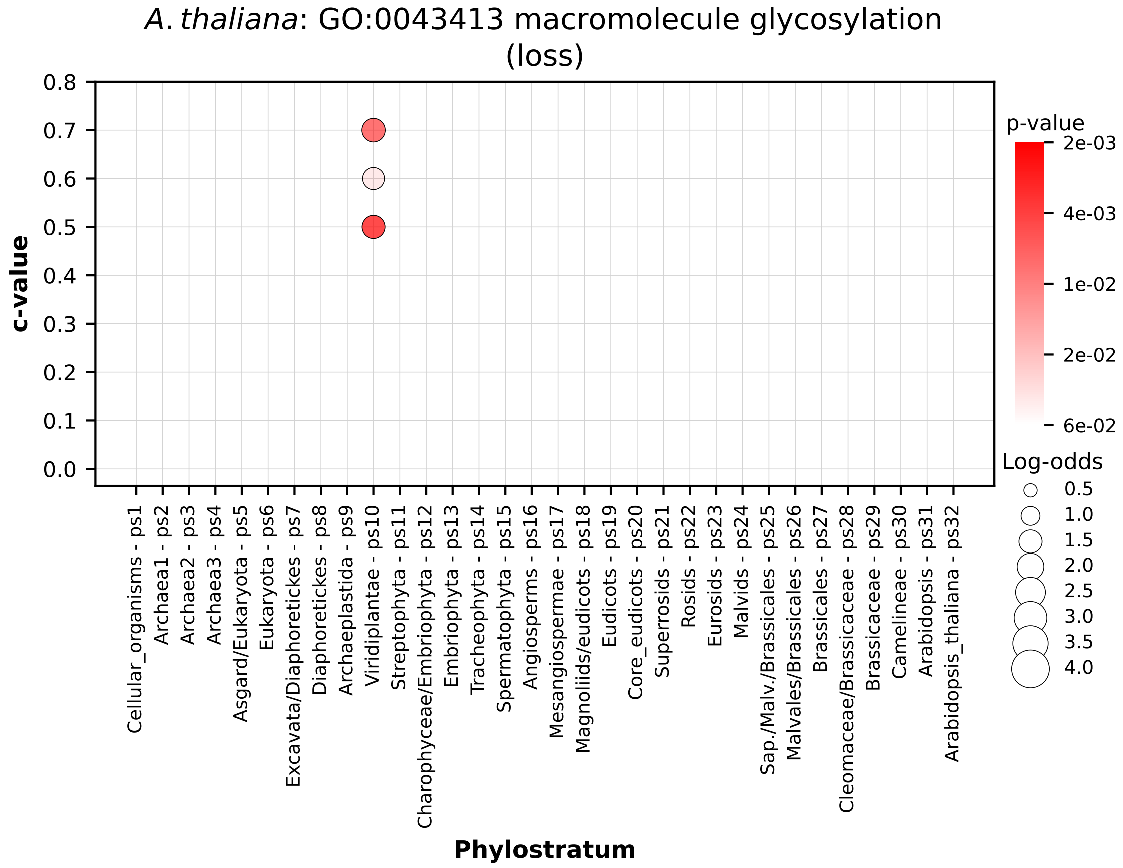

A. thaliana: GO:0043413 macromolecule glycosylation
(loss)
c-value
0.8
0.7
0.6
0.5
0.4
0.3
0.2
0.1
0.0
p-value
2e-03
4e-03
1e-02
2e-02
6e-02
Log-odds
0.5
1.0
1.5
2.0
2.5
3.0
3.5
4.0
Cellular_organisms - ps1
Archaea1 - ps2
Archaea2 - ps3
Archaea3 - ps4
Asgard/Eukaryota - ps5
Eukaryota - ps6
Excavata/Diaphoretickes - ps7
Diaphoretickes - ps8
Archaeplastida - ps9
Viridiplantae - ps10
Streptophyta - ps11
Charophyceae/Embriophyta - ps12
Embriophyta - ps13
Tracheophyta - ps14
Spermatophyta - ps15
Angiosperms - ps16
Mesangiospermae - ps17
Magnoliids/eudicots - ps18
Eudicots - ps19
Core_eudicots - ps20
Superrosids - ps21
Rosids - ps22
Eurosids - ps23
Malvids - ps24
Sap./Malv./Brassicales - ps25
Malvales/Brassicales - ps26
Brassicales - ps27
Cleomaceae/Brassicaceae - ps28
Brassicaceae - ps29
Camelineae - ps30
Arabidopsis - ps31
Arabidopsis_thaliana - ps32
Phylostratum

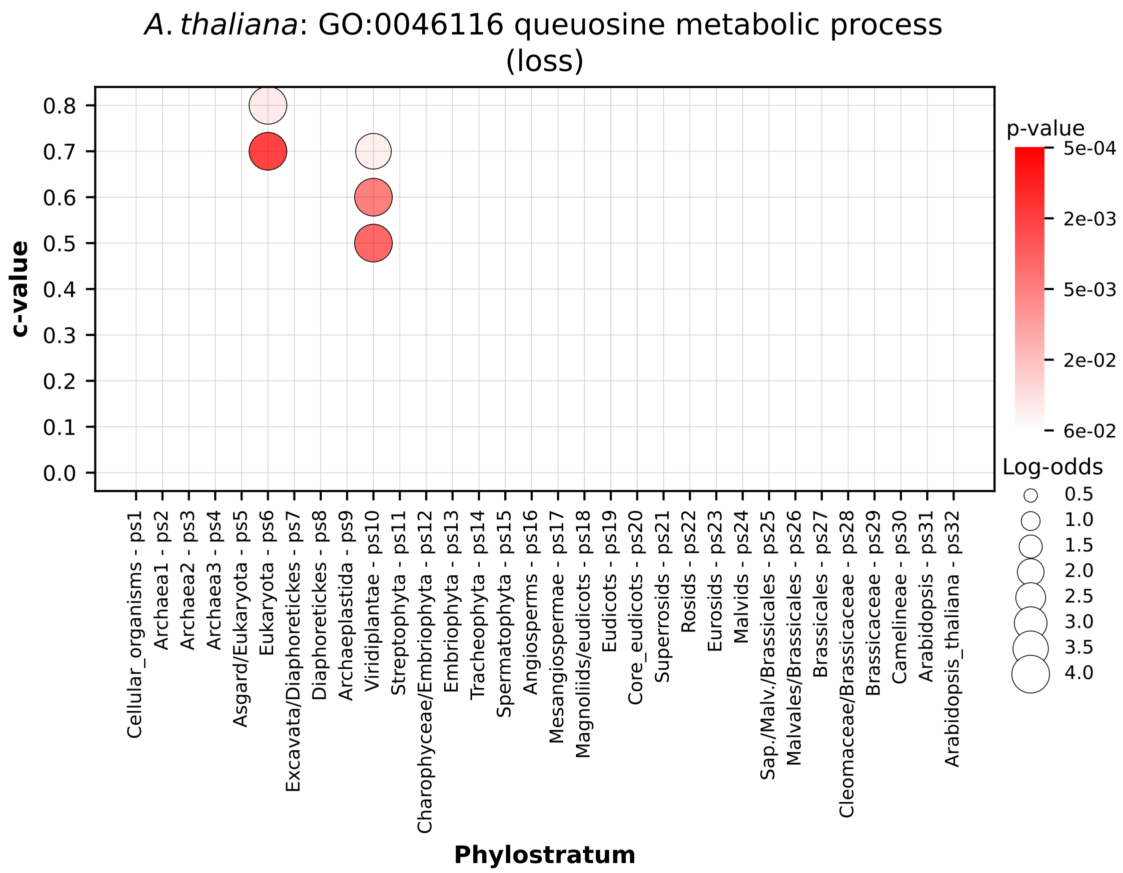
A. thaliana: GO:0046116 queuosine metabolic process (loss)
c-value
0.8
0.7
0.6
0.5
0.4
0.3
0.2
0.1
0.0
Phylostratum
Cellular_organisms - ps1
Archaea1 - ps2
Archaea2 - ps3
Archaea3 - ps4
Asgard/Eukaryota - ps5
Eukaryota - ps6
Excavata/Diaphoretickes - ps7
Diaphoretickes - ps8
Archaeplastida - ps9
Viridiplantae - ps10
Streptophyta - ps11
Charophyceae/Embriophyta - ps12
Embriophyta - ps13
Tracheophyta - ps14
Spermatophyta - ps15
Angiosperms - ps16
Mesangiospermae - ps17
Magnoliids/eudicots - ps18
Eudicots - ps19
Core_eudicots - ps20
Superrosids - ps21
Rosids - ps22
Eurosids - ps23
Malvids - ps24
Sap./Malv./Brassicales - ps25
Malvales/Brassicales - ps26
Brassicales - ps27
Cleomaceae/Brassicaceae - ps28
Brassicaceae - ps29
Camelineae - ps30
Arabidopsis - ps31
Arabidopsis_thaliana - ps32
p-value
5e-04
2e-03
5e-03
2e-02
6e-02
Log-odds
0.5
1.0
1.5
2.0
2.5
3.0
3.5
4.0

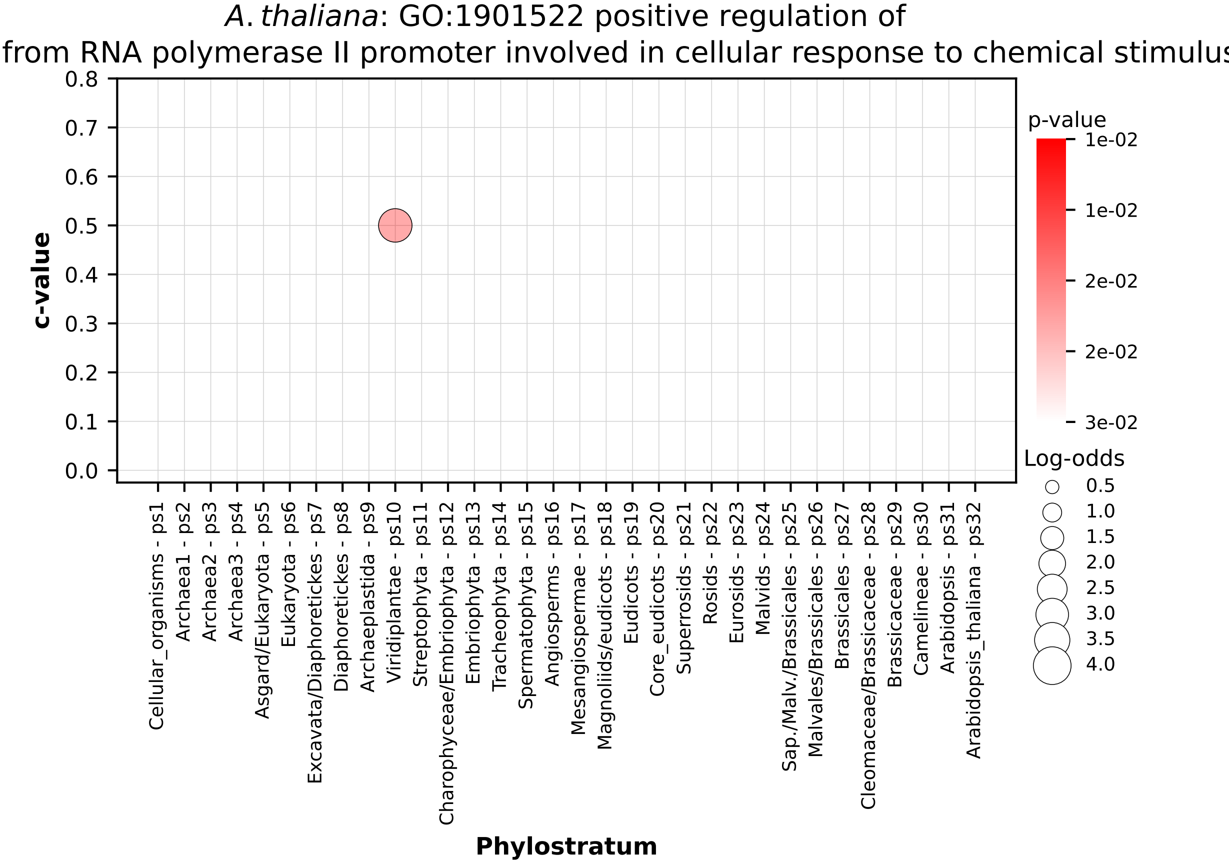

A. thaliana: GO:1901522 positive regulation of
from RNA polymerase II promoter involved in cellular response to chemical stimulus
c-value
0.8
0.7
0.6
0.5
0.4
0.3
0.2
0.1
0.0
p-value
1e-02
1e-02
2e-02
2e-02
3e-02
Log-odds
0.5
1.0
1.5
2.0
2.5
3.0
3.5
4.0
Cellular_organisms - ps1
Archaea1 - ps2
Archaea2 - ps3
Archaea3 - ps4
Asgard/Eukaryota - ps5
Eukaryota - ps6
Excavata/Diaphoretickes - ps7
Diaphoretickes - ps8
Archaeplastida - ps9
Viridiplantae - ps10
Streptophyta - ps11
Charophyceae/Embriophyta - ps12
Embriophyta - ps13
Tracheophyta - ps14
Spermatophyta - ps15
Angiosperms - ps16
Mesangiospermae - ps17
Magnoliids/eudicots - ps18
Eudicots - ps19
Core_eudicots - ps20
Superrosids - ps21
Rosids - ps22
Eurosids - ps23
Malvids - ps24
Sap./Malv./Brassicales - ps25
Malvales/Brassicales - ps26
Brassicales - ps27
Cleomaceae/Brassicaceae - ps28
Brassicaceae - ps29
Camelineae - ps30
Arabidopsis - ps31
Arabidopsis_thaliana - ps32
Phylostratum

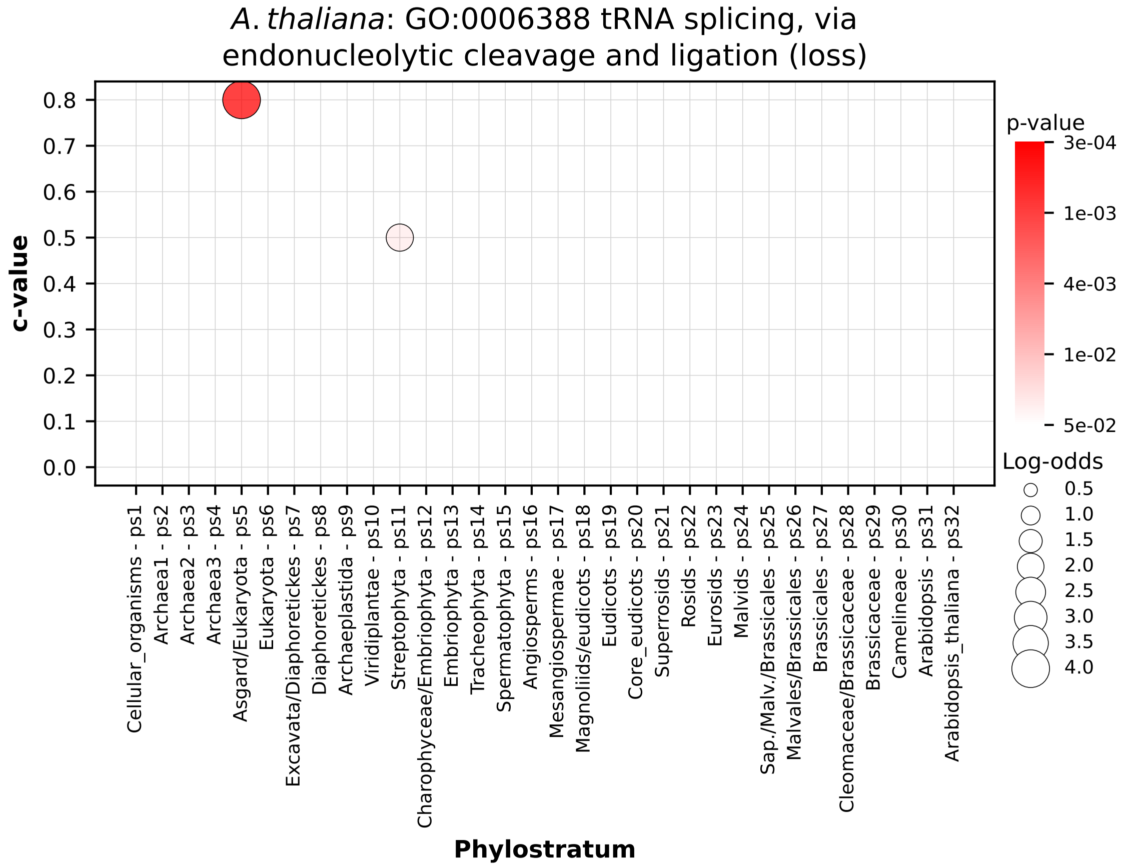

A. thaliana: GO:0006388 tRNA splicing, via endonucleolytic cleavage and ligation (loss)
c-value
0.8
0.7
0.6
0.5
0.4
0.3
0.2
0.1
0.0
Phylostratum
Cellular_organisms - ps1
Archaea1 - ps2
Archaea2 - ps3
Archaea3 - ps4
Asgard/Eukaryota - ps5
Eukaryota - ps6
Excavata/Diaphoretickes - ps7
Diaphoretickes - ps8
Archaeplastida - ps9
Viridiplantae - ps10
Streptophyta - ps11
Charophyceae/Embriophyta - ps12
Embriophyta - ps13
Tracheophyta - ps14
Spermatophyta - ps15
Angiosperms - ps16
Mesangiospermae - ps17
Magnoliids/eudicots - ps18
Eudicots - ps19
Core_eudicots - ps20
Superrosids - ps21
Rosids - ps22
Eurosids - ps23
Malvids - ps24
Sap./Malv./Brassicales - ps25
Malvales/Brassicales - ps26
Brassicales - ps27
Cleomaceae/Brassicaceae - ps28
Brassicaceae - ps29
Camelineae - ps30
Arabidopsis - ps31
Arabidopsis_thaliana - ps32
p-value
3e-04
1e-03
4e-03
1e-02
5e-02
Log-odds
0.5
1.0
1.5
2.0
2.5
3.0
3.5
4.0

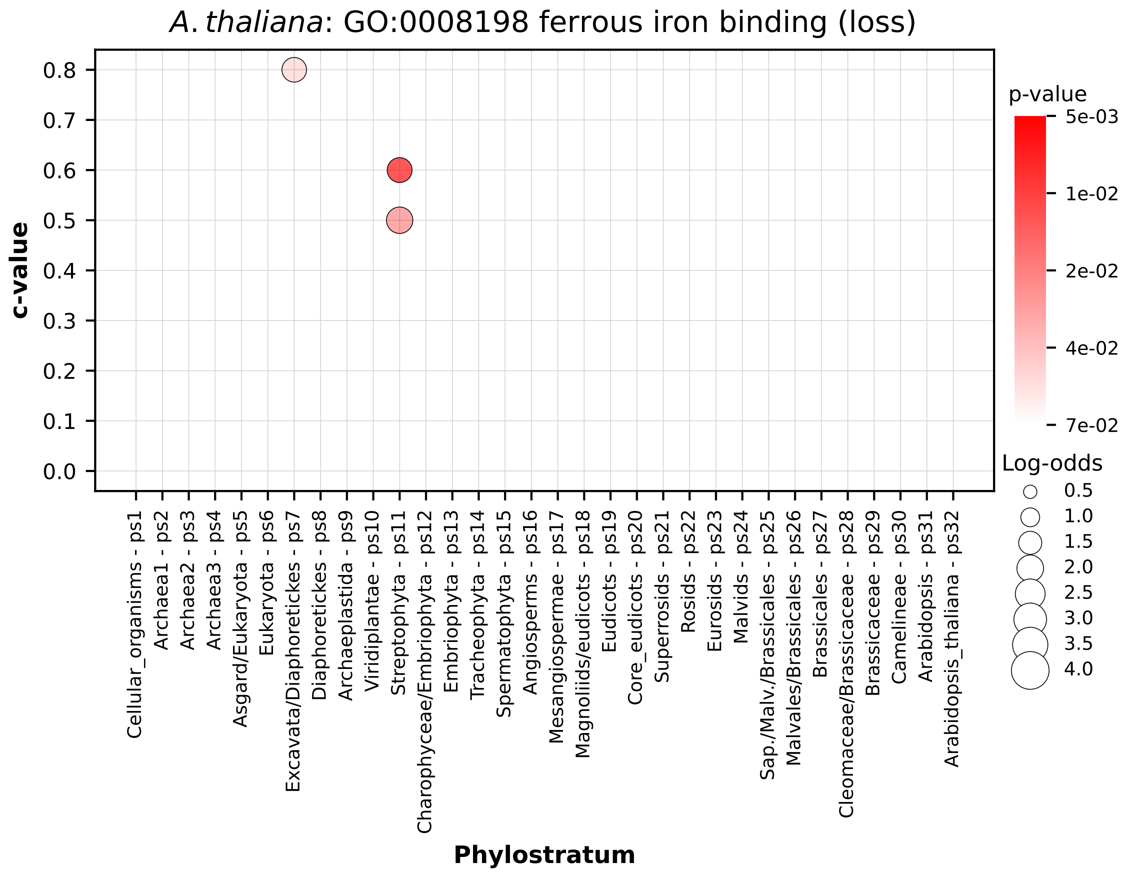

A. thaliana: GO:0008198 ferrous iron binding (loss)
c-value
0.0
0.1
0.2
0.3
0.4
0.5
0.6
0.7
0.8
p-value
5e-03
1e-02
2e-02
4e-02
7e-02
Log-odds
0.5
1.0
1.5
2.0
2.5
3.0
3.5
4.0
Cellular_organisms - ps1
Archaea1 - ps2
Archaea2 - ps3
Archaea3 - ps4
Asgard/Eukaryota - ps5
Eukaryota - ps6
Excavata/Diaphoretickes - ps7
Diaphoretickes - ps8
Archaeplastida - ps9
Viridiplantae - ps10
Streptophyta - ps11
Charophyceae/Embriophyta - ps12
Embriophyta - ps13
Tracheophyta - ps14
Spermatophyta - ps15
Angiosperms - ps16
Mesangiospermae - ps17
Magnoliids/eudicots - ps18
Eudicots - ps19
Core_eudicots - ps20
Superrosids - ps21
Rosids - ps22
Eurosids - ps23
Malvids - ps24
Sap./Malv./Brassicales - ps25
Malvales/Brassicales - ps26
Brassicales - ps27
Cleomaceae/Brassicaceae - ps28
Brassicaceae - ps29
Camelineae - ps30
Arabidopsis - ps31
Arabidopsis_thaliana - ps32
Phylostratum

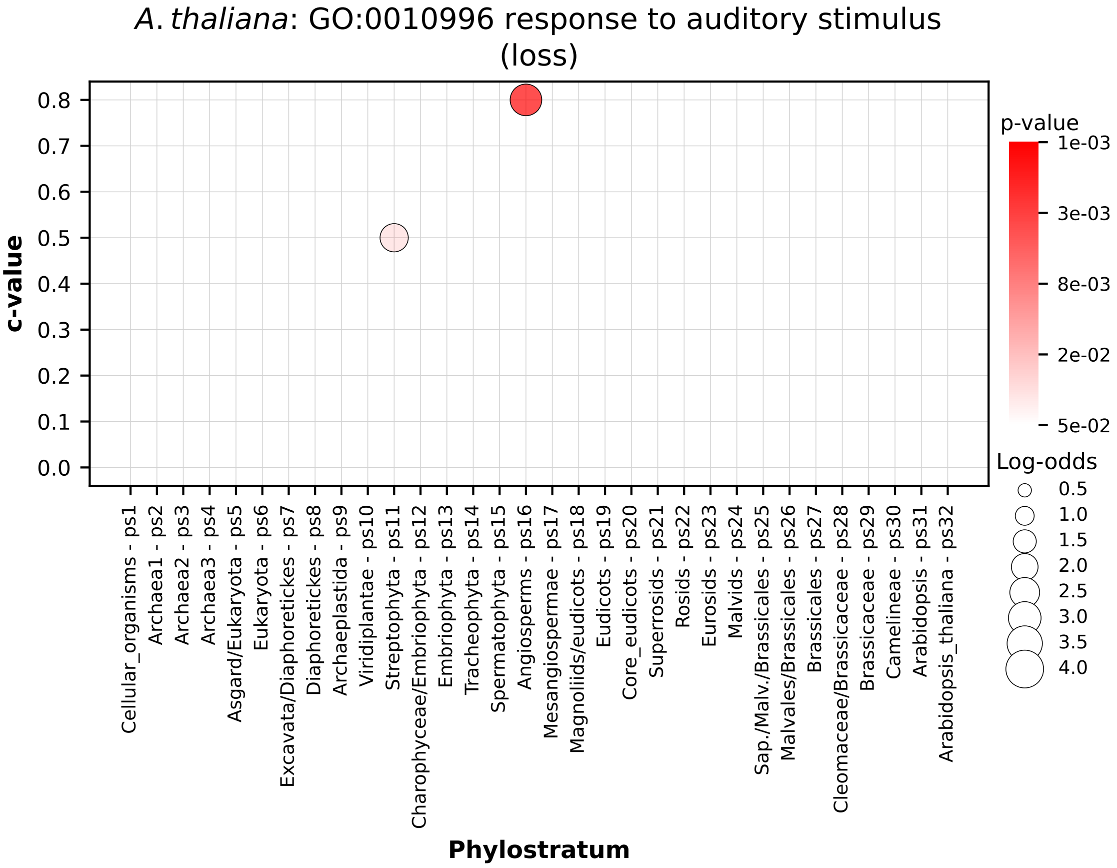
A. thaliana: GO:0010996 response to auditory stimulus
(loss)
c-value
0.0
0.1
0.2
0.3
0.4
0.5
0.6
0.7
0.8
Phylostratum
Cellular_organisms - ps1
Archaea1 - ps2
Archaea2 - ps3
Archaea3 - ps4
Asgard/Eukaryota - ps5
Eukaryota - ps6
Excavata/Diaphoretickes - ps7
Diaphoretickes - ps8
Archaeplastida - ps9
Viridiplantae - ps10
Streptophyta - ps11
Charophyceae/Embriophyta - ps12
Embriophyta - ps13
Tracheophyta - ps14
Spermatophyta - ps15
Angiosperms - ps16
Mesangiospermae - ps17
Magnoliids/eudicots - ps18
Eudicots - ps19
Core_eudicots - ps20
Superrosids - ps21
Rosids - ps22
Eurosids - ps23
Malvids - ps24
Sap./Malv./Brassicales - ps25
Malvales/Brassicales - ps26
Brassicales - ps27
Cleomaceae/Brassicaceae - ps28
Brassicaceae - ps29
Camelineae - ps30
Arabidopsis - ps31
Arabidopsis_thaliana - ps32
p-value
1e-03
3e-03
8e-03
2e-02
5e-02
Log-odds
0.5
1.0
1.5
2.0
2.5
3.0
3.5
4.0

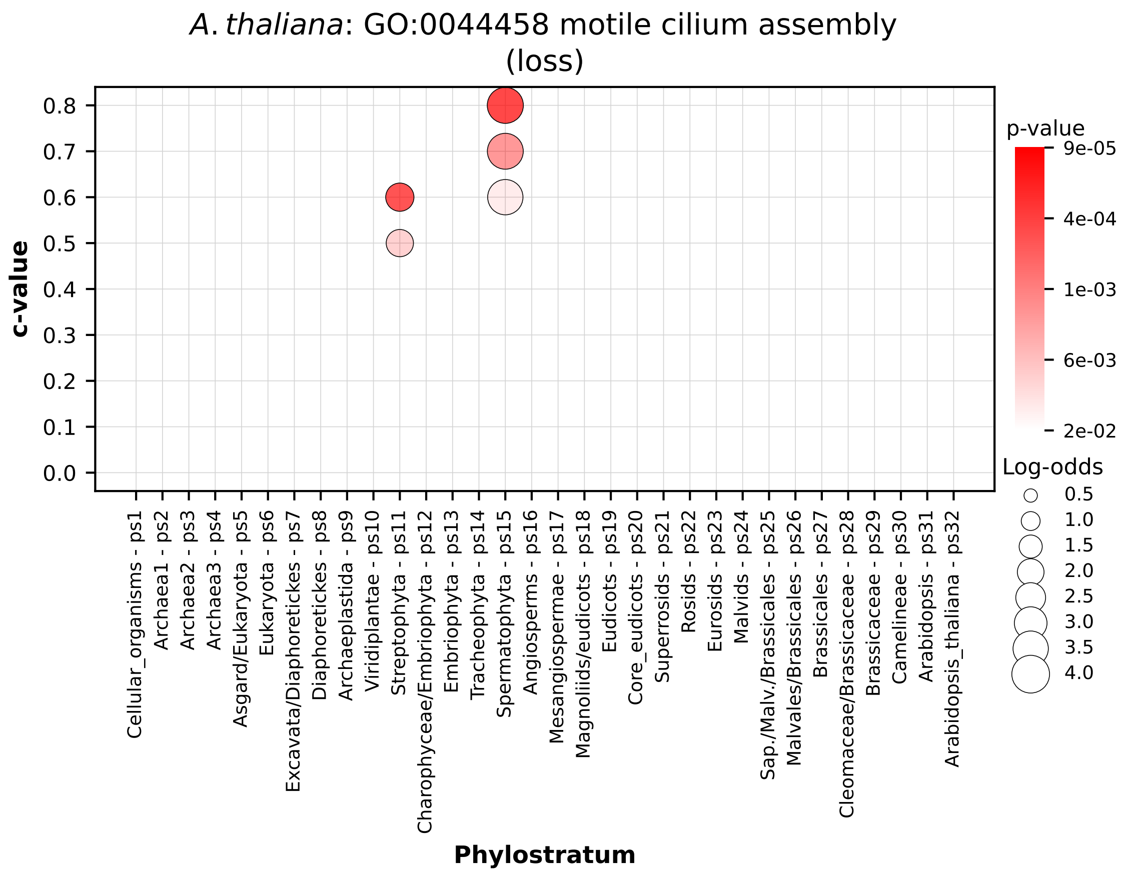

A. thaliana: GO:0044458 motile cilium assembly
(loss)
c-value
0.0
0.1
0.2
0.3
0.4
0.5
0.6
0.7
0.8
p-value
9e-05
4e-04
1e-03
6e-03
2e-02
Log-odds
0.5
1.0
1.5
2.0
2.5
3.0
3.5
4.0
Cellular_organisms - ps1
Archaea1 - ps2
Archaea2 - ps3
Archaea3 - ps4
Asgard/Eukaryota - ps5
Eukaryota - ps6
Excavata/Diaphoretickes - ps7
Diaphoretickes - ps8
Archaeplastida - ps9
Viridiplantae - ps10
Streptophyta - ps11
Charophyceae/Embriophyta - ps12
Embriophyta - ps13
Tracheophyta - ps14
Spermatophyta - ps15
Angiosperms - ps16
Mesangiospermae - ps17
Magnoliids/eudicots - ps18
Eudicots - ps19
Core_eudicots - ps20
Superrosids - ps21
Rosids - ps22
Eurosids - ps23
Malvids - ps24
Sap./Malv./Brassicales - ps25
Malvales/Brassicales - ps26
Brassicales - ps27
Cleomaceae/Brassicaceae - ps28
Brassicaceae - ps29
Camelineae - ps30
Arabidopsis - ps31
Arabidopsis_thaliana - ps32
Phylostratum

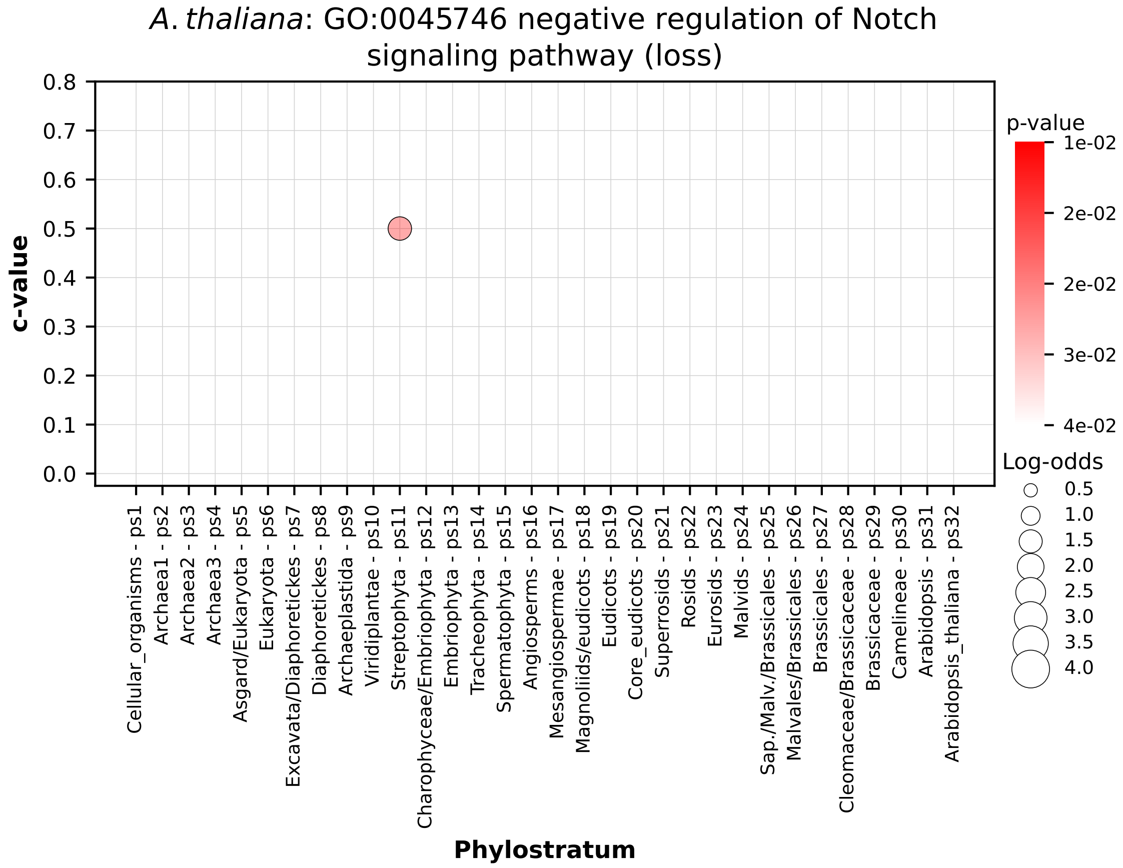

A. thaliana: GO:0045746 negative regulation of Notch signaling pathway (loss)
c-value
0.8
0.7
0.6
0.5
0.4
0.3
0.2
0.1
0.0
p-value
1e-02
2e-02
2e-02
3e-02
4e-02
Log-odds
0.5
1.0
1.5
2.0
2.5
3.0
3.5
4.0
Cellular_organisms - ps1
Archaea1 - ps2
Archaea2 - ps3
Archaea3 - ps4
Asgard/Eukaryota - ps5
Eukaryota - ps6
Excavata/Diaphoretickes - ps7
Diaphoretickes - ps8
Archaeplastida - ps9
Viridiplantae - ps10
Streptophyta - ps11
Charophyceae/Embriophyta - ps12
Embriophyta - ps13
Tracheophyta - ps14
Spermatophyta - ps15
Angiosperms - ps16
Mesangiospermae - ps17
Magnoliids/eudicots - ps18
Eudicots - ps19
Core_eudicots - ps20
Superrosids - ps21
Rosids - ps22
Eurosids - ps23
Malvids - ps24
Sap./Malv./Brassicales - ps25
Malvales/Brassicales - ps26
Brassicales - ps27
Cleomaceae/Brassicaceae - ps28
Brassicaceae - ps29
Camelineae - ps30
Arabidopsis - ps31
Arabidopsis_thaliana - ps32
Phylostratum

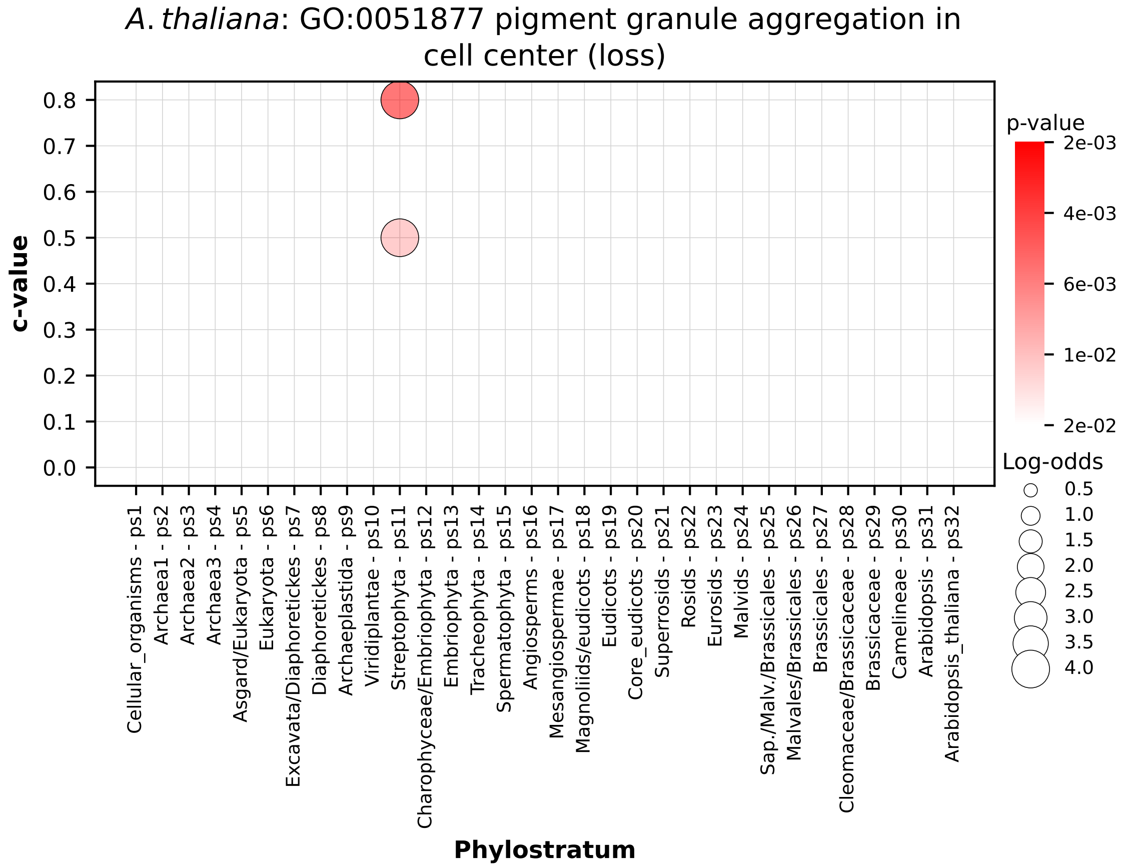

A. thaliana: GO:0051877 pigment granule aggregation in cell center (loss)
c-value
0.0
0.1
0.2
0.3
0.4
0.5
0.6
0.7
0.8
Phylostratum
Cellular_organisms - ps1
Archaea1 - ps2
Archaea2 - ps3
Archaea3 - ps4
Asgard/Eukaryota - ps5
Eukaryota - ps6
Excavata/Diaphoretickes - ps7
Diaphoretickes - ps8
Archaeplastida - ps9
Viridiplantae - ps10
Streptophyta - ps11
Charophyceae/Embriophyta - ps12
Embriophyta - ps13
Tracheophyta - ps14
Spermatophyta - ps15
Angiosperms - ps16
Mesangiospermae - ps17
Magnoliids/eudicots - ps18
Eudicots - ps19
Core_eudicots - ps20
Superrosids - ps21
Rosids - ps22
Eurosids - ps23
Malvids - ps24
Sap./Malv./Brassicales - ps25
Malvales/Brassicales - ps26
Brassicales - ps27
Cleomaceae/Brassicaceae - ps28
Brassicaceae - ps29
Camelineae - ps30
Arabidopsis - ps31
Arabidopsis_thaliana - ps32
p-value
2e-03
4e-03
6e-03
1e-02
2e-02
Log-odds
0.5
1.0
1.5
2.0
2.5
3.0
3.5
4.0

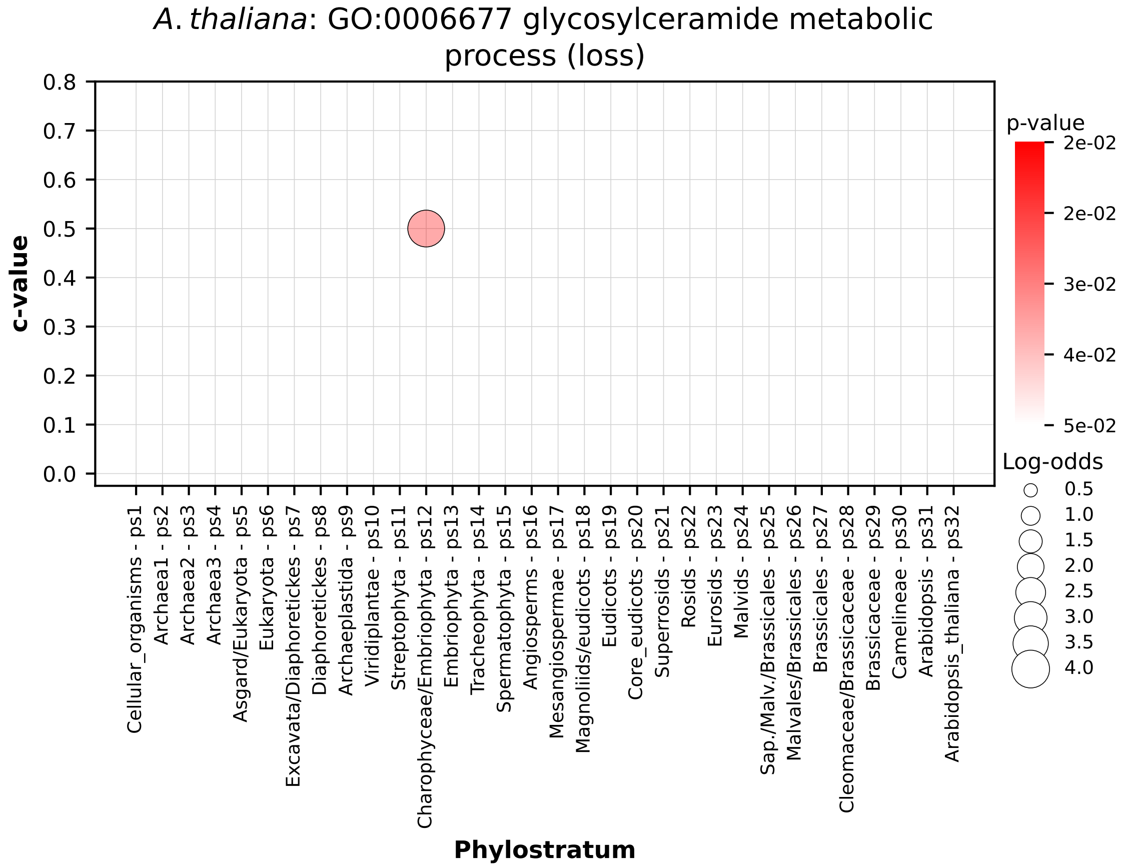

A. thaliana: GO:0006677 glycosylceramide metabolic process (loss)
c-value
0.8
0.7
0.6
0.5
0.4
0.3
0.2
0.1
0.0
p-value
2e-02
2e-02
3e-02
4e-02
5e-02
Log-odds
0.5
1.0
1.5
2.0
2.5
3.0
3.5
4.0
Cellular_organisms - ps1
Archaea1 - ps2
Archaea2 - ps3
Archaea3 - ps4
Asgard/Eukaryota - ps5
Eukaryota - ps6
Excavata/Diaphoretickes - ps7
Diaphoretickes - ps8
Archaeplastida - ps9
Viridiplantae - ps10
Streptophyta - ps11
Charophyceae/Embriophyta - ps12
Embriophyta - ps13
Tracheophyta - ps14
Spermatophyta - ps15
Angiosperms - ps16
Mesangiospermae - ps17
Magnoliids/eudicots - ps18
Eudicots - ps19
Core_eudicots - ps20
Superrosids - ps21
Rosids - ps22
Eurosids - ps23
Malvids - ps24
Sap./Malv./Brassicales - ps25
Malvales/Brassicales - ps26
Brassicales - ps27
Cleomaceae/Brassicaceae - ps28
Brassicaceae - ps29
Camelineae - ps30
Arabidopsis - ps31
Arabidopsis_thaliana - ps32
Phylostratum

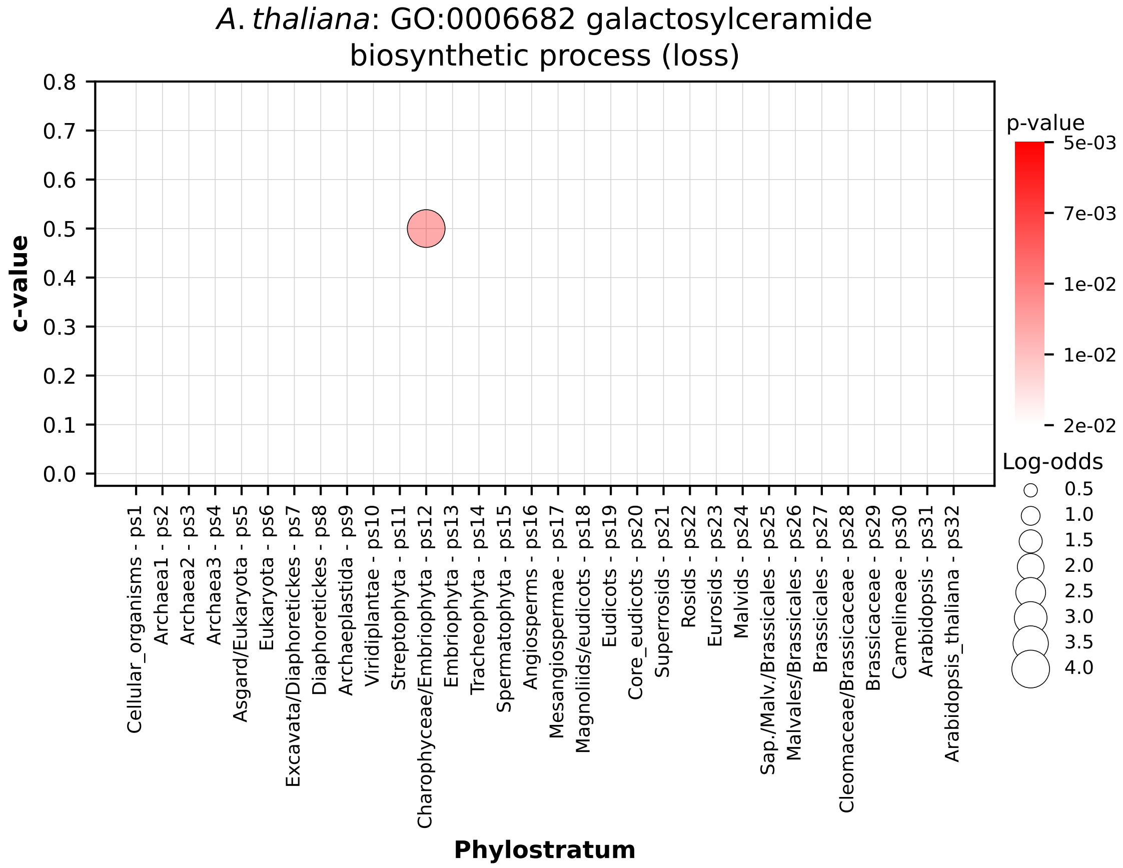

A. thaliana: GO:0006682 galactosylceramide biosynthetic process (loss)
c-value
0.8
0.7
0.6
0.5
0.4
0.3
0.2
0.1
0.0
p-value
5e-03
7e-03
1e-02
1e-02
2e-02
Log-odds
0.5
1.0
1.5
2.0
2.5
3.0
3.5
4.0
Cellular_organisms - ps1
Archaea1 - ps2
Archaea2 - ps3
Archaea3 - ps4
Asgard/Eukaryota - ps5
Eukaryota - ps6
Excavata/Diaphoretickes - ps7
Diaphoretickes - ps8
Archaeplastida - ps9
Viridiplantae - ps10
Streptophyta - ps11
Charophyceae/Embriophyta - ps12
Embriophyta - ps13
Tracheophyta - ps14
Spermatophyta - ps15
Angiosperms - ps16
Mesangiospermae - ps17
Magnoliids/eudicots - ps18
Eudicots - ps19
Core_eudicots - ps20
Superrosids - ps21
Rosids - ps22
Eurosids - ps23
Malvids - ps24
Sap./Malv./Brassicales - ps25
Malvales/Brassicales - ps26
Brassicales - ps27
Cleomaceae/Brassicaceae - ps28
Brassicaceae - ps29
Camelineae - ps30
Arabidopsis - ps31
Arabidopsis_thaliana - ps32
Phylostratum

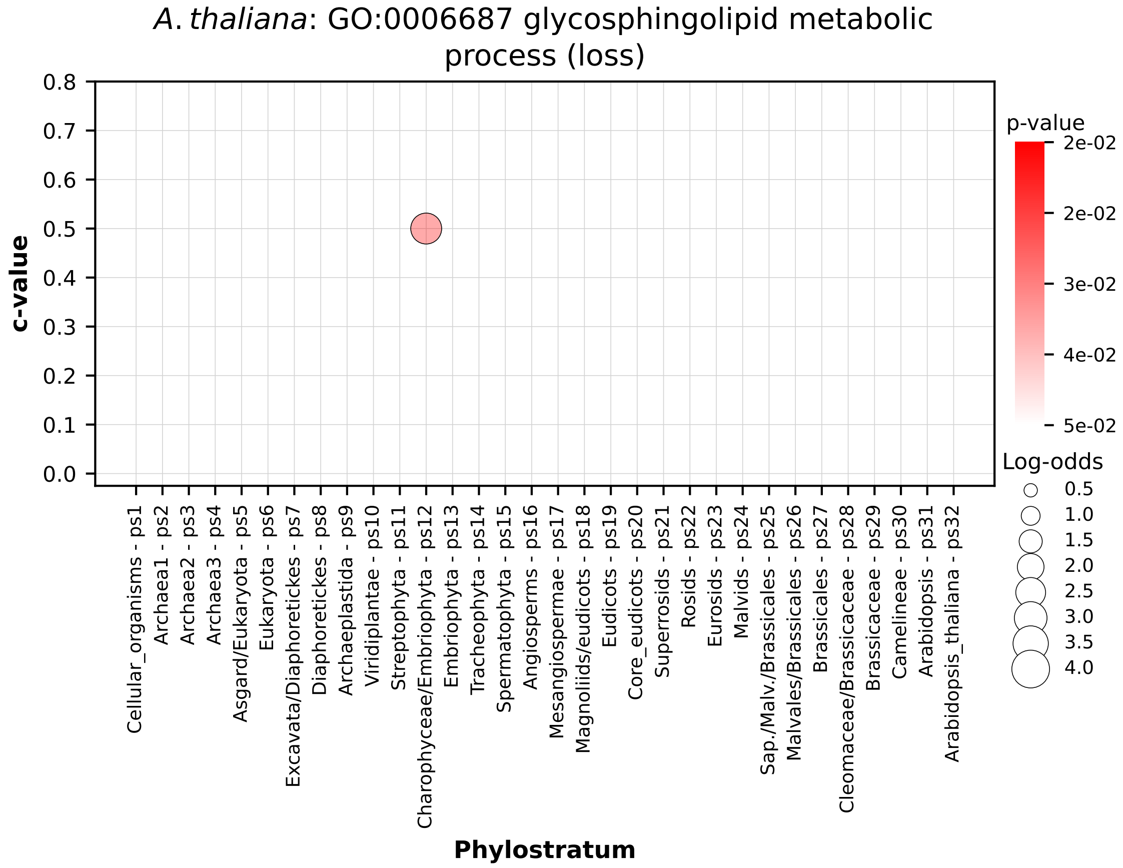

A. thaliana: GO:0006687 glycosphingolipid metabolic process (loss)
c-value
0.8
0.7
0.6
0.5
0.4
0.3
0.2
0.1
0.0
p-value
2e-02
2e-02
3e-02
4e-02
5e-02
Log-odds
0.5
1.0
1.5
2.0
2.5
3.0
3.5
4.0
Cellular_organisms - ps1
Archaea1 - ps2
Archaea2 - ps3
Archaea3 - ps4
Asgard/Eukaryota - ps5
Eukaryota - ps6
Excavata/Diaphoretickes - ps7
Diaphoretickes - ps8
Archaeplastida - ps9
Viridiplantae - ps10
Streptophyta - ps11
Charophyceae/Embriophyta - ps12
Embriophyta - ps13
Tracheophyta - ps14
Spermatophyta - ps15
Angiosperms - ps16
Mesangiospermae - ps17
Magnoliids/eudicots - ps18
Eudicots - ps19
Core_eudicots - ps20
Superrosids - ps21
Rosids - ps22
Eurosids - ps23
Malvids - ps24
Sap./Malv./Brassicales - ps25
Malvales/Brassicales - ps26
Brassicales - ps27
Cleomaceae/Brassicaceae - ps28
Brassicaceae - ps29
Camelineae - ps30
Arabidopsis - ps31
Arabidopsis_thaliana - ps32
Phylostratum

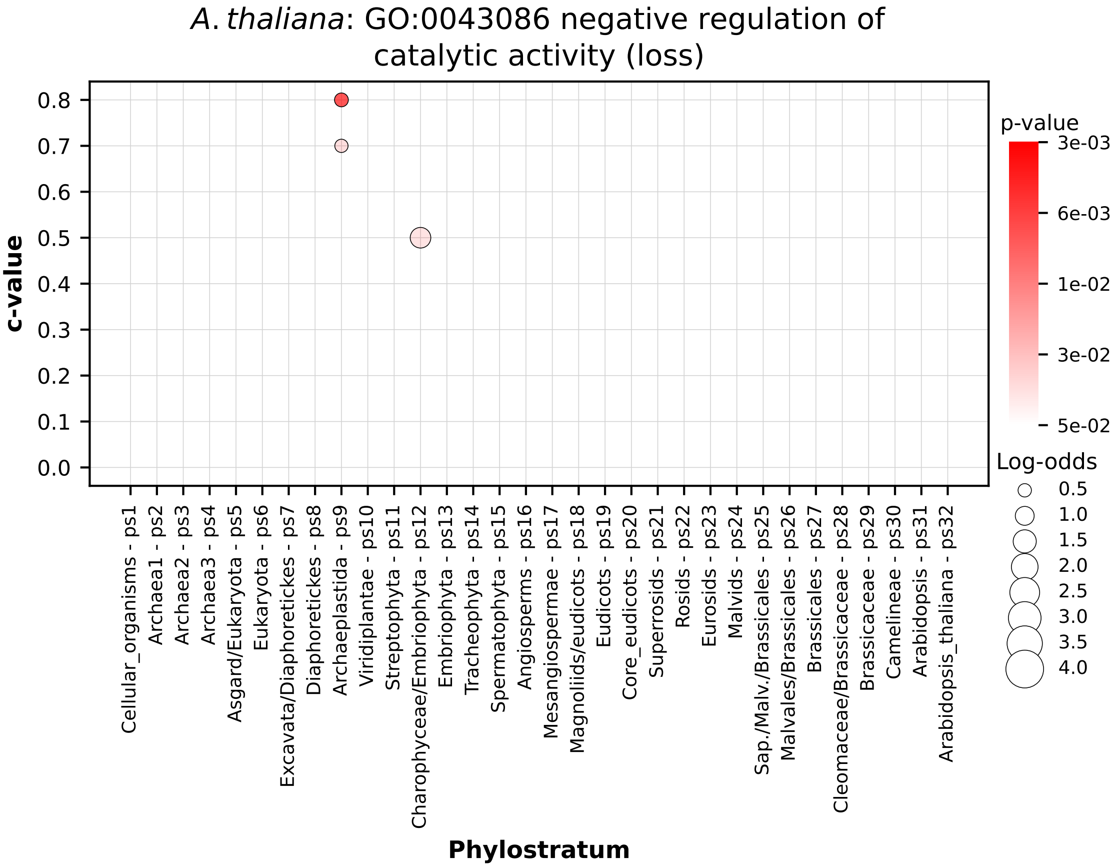

A. thaliana: GO:0043086 negative regulation of catalytic activity (loss)
c-value
Phylostratum
p-value
3e-03
6e-03
1e-02
3e-02
5e-02
Log-odds
0.5
1.0
1.5
2.0
2.5
3.0
3.5
4.0
Cellular_organisms - ps1
Archaea1 - ps2
Archaea2 - ps3
Archaea3 - ps4
Asgard/Eukaryota - ps5
Eukaryota - ps6
Excavata/Diaphoretickes - ps7
Diaphoretickes - ps8
Archaeplastida - ps9
Viridiplantae - ps10
Streptophyta - ps11
Charophyceae/Embriophyta - ps12
Embriophyta - ps13
Tracheophyta - ps14
Spermatophyta - ps15
Angiosperms - ps16
Mesangiospermae - ps17
Magnoliids/eudicots - ps18
Eudicots - ps19
Core_eudicots - ps20
Superrosids - ps21
Rosids - ps22
Eurosids - ps23
Malvids - ps24
Sap./Malv./Brassicales - ps25
Malvales/Brassicales - ps26
Brassicales - ps27
Cleomaceae/Brassicaceae - ps28
Brassicaceae - ps29
Camelineae - ps30
Arabidopsis - ps31
Arabidopsis_thaliana - ps32

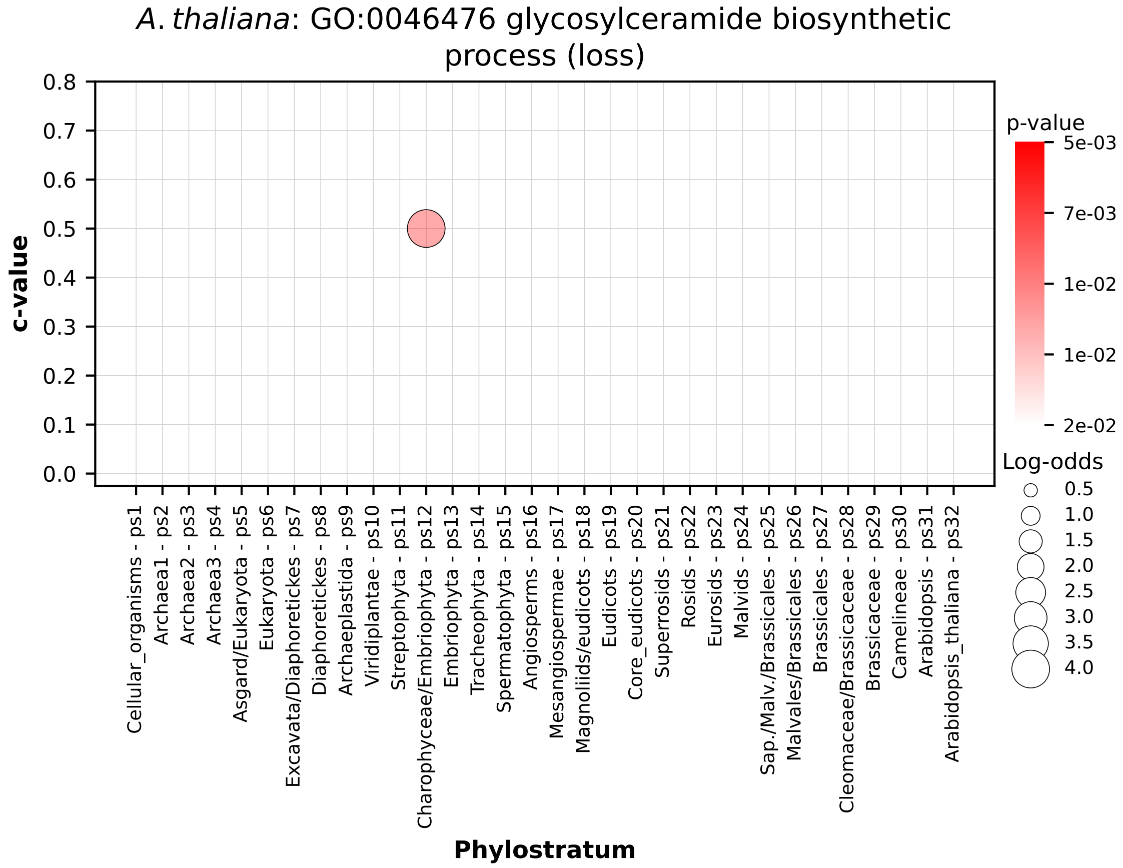

A. thaliana: GO:0046476 glycosylceramide biosynthetic process (loss)
c-value
0.8
0.7
0.6
0.5
0.4
0.3
0.2
0.1
0.0
Phylostratum
Cellular_organisms - ps1
Archaea1 - ps2
Archaea2 - ps3
Archaea3 - ps4
Asgard/Eukaryota - ps5
Eukaryota - ps6
Excavata/Diaphoretickes - ps7
Diaphoretickes - ps8
Archaeplastida - ps9
Viridiplantae - ps10
Streptophyta - ps11
Charophyceae/Embriophyta - ps12
Embriophyta - ps13
Tracheophyta - ps14
Spermatophyta - ps15
Angiosperms - ps16
Mesangiospermae - ps17
Magnoliids/eudicots - ps18
Eudicots - ps19
Core_eudicots - ps20
Superrosids - ps21
Rosids - ps22
Eurosids - ps23
Malvids - ps24
Sap./Malv./Brassicales - ps25
Malvales/Brassicales - ps26
Brassicales - ps27
Cleomaceae/Brassicaceae - ps28
Brassicaceae - ps29
Camelineae - ps30
Arabidopsis - ps31
Arabidopsis_thaliana - ps32
p-value
5e-03
7e-03
1e-02
1e-02
2e-02
Log-odds
0.5
1.0
1.5
2.0
2.5
3.0
3.5
4.0

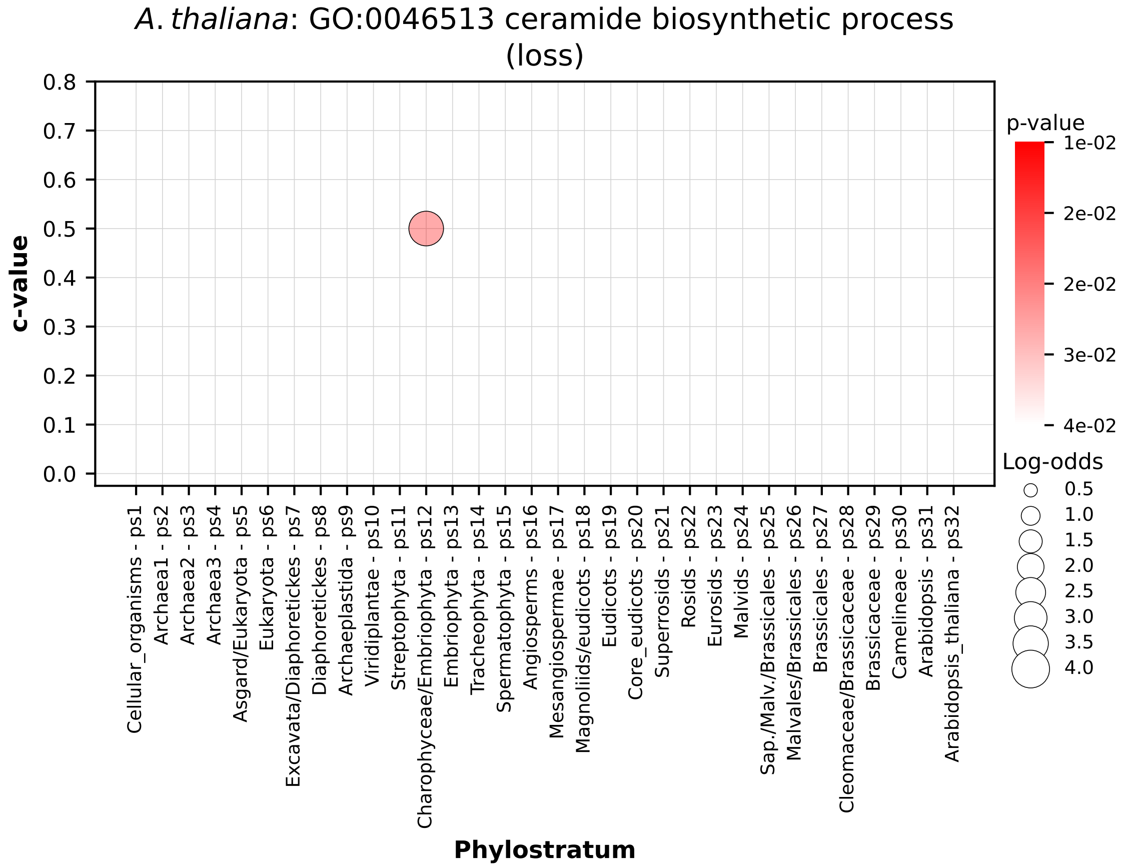

A. thaliana: GO:0046513 ceramide biosynthetic process
(loss)
c-value
0.8
0.7
0.6
0.5
0.4
0.3
0.2
0.1
0.0
Phylostratum
Cellular_organisms - ps1
Archaea1 - ps2
Archaea2 - ps3
Archaea3 - ps4
Asgard/Eukaryota - ps5
Eukaryota - ps6
Excavata/Diaphoretickes - ps7
Diaphoretickes - ps8
Archaeplastida - ps9
Viridiplantae - ps10
Streptophyta - ps11
Charophyceae/Embriophyta - ps12
Embriophyta - ps13
Tracheophyta - ps14
Spermatophyta - ps15
Angiosperms - ps16
Mesangiospermae - ps17
Magnoliids/eudicots - ps18
Eudicots - ps19
Core_eudicots - ps20
Superrosids - ps21
Rosids - ps22
Eurosids - ps23
Malvids - ps24
Sap./Malv./Brassicales - ps25
Malvales/Brassicales - ps26
Brassicales - ps27
Cleomaceae/Brassicaceae - ps28
Brassicaceae - ps29
Camelineae - ps30
Arabidopsis - ps31
Arabidopsis_thaliana - ps32
p-value
1e-02
2e-02
2e-02
3e-02
4e-02
Log-odds
0.5
1.0
1.5
2.0
2.5
3.0
3.5
4.0

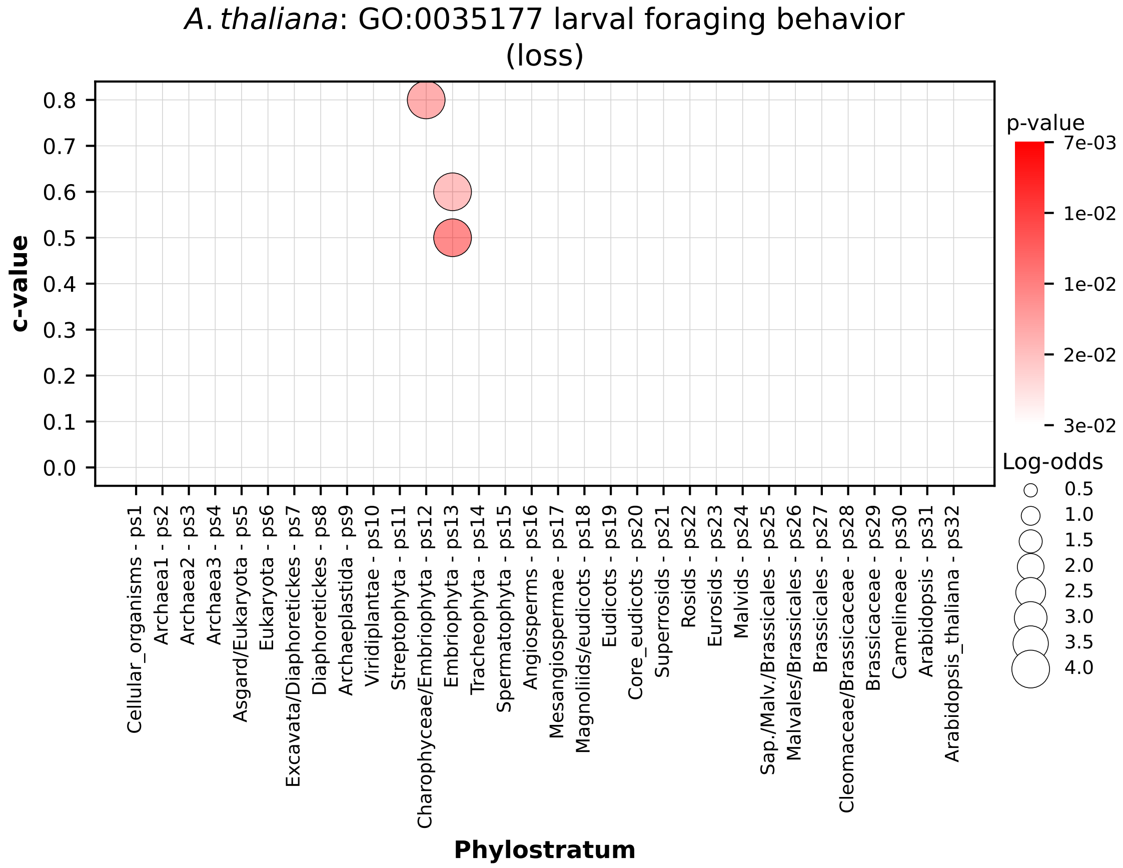

A. thaliana: GO:0035177 larval foraging behavior
(loss)
c-value
0.8
0.7
0.6
0.5
0.4
0.3
0.2
0.1
0.0
Phylostratum
Cellular_organisms - ps1
Archaea1 - ps2
Archaea2 - ps3
Archaea3 - ps4
Asgard/Eukaryota - ps5
Eukaryota - ps6
Excavata/Diaphoretickes - ps7
Diaphoretickes - ps8
Archaeplastida - ps9
Viridiplantae - ps10
Streptophyta - ps11
Charophyceae/Embriophyta - ps12
Embriophyta - ps13
Tracheophyta - ps14
Spermatophyta - ps15
Angiosperms - ps16
Mesangiospermae - ps17
Magnoliids/eudicots - ps18
Eudicots - ps19
Core_eudicots - ps20
Superrosids - ps21
Rosids - ps22
Eurosids - ps23
Malvids - ps24
Sap./Malv./Brassicales - ps25
Malvales/Brassicales - ps26
Brassicales - ps27
Cleomaceae/Brassicaceae - ps28
Brassicaceae - ps29
Camelineae - ps30
Arabidopsis - ps31
Arabidopsis_thaliana - ps32
p-value
7e-03
1e-02
1e-02
2e-02
3e-02
Log-odds
0.5
1.0
1.5
2.0
2.5
3.0
3.5
4.0

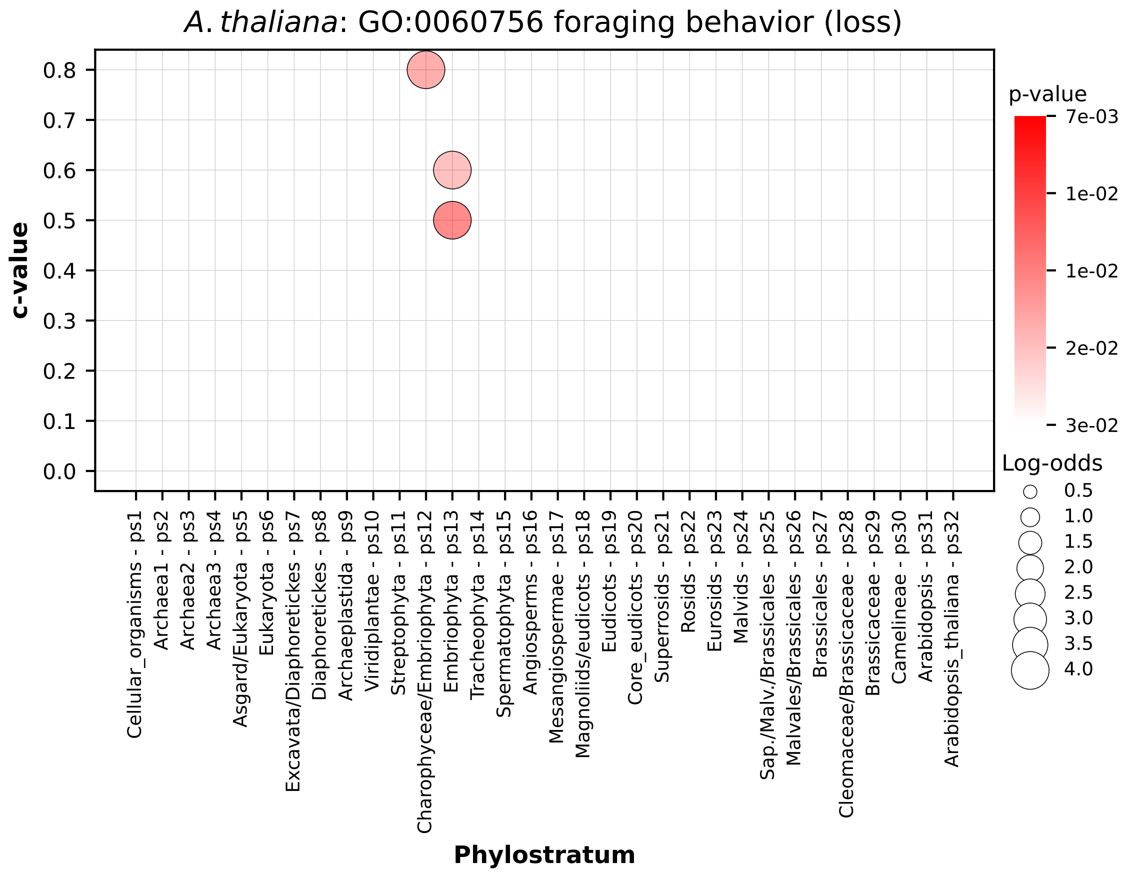

A. thaliana: GO:0060756 foraging behavior (loss)
c-value
0.0
0.1
0.2
0.3
0.4
0.5
0.6
0.7
0.8
Phylostratum
Cellular_organisms - ps1
Archaea1 - ps2
Archaea2 - ps3
Archaea3 - ps4
Asgard/Eukaryota - ps5
Eukaryota - ps6
Excavata/Diaphoretickes - ps7
Diaphoretickes - ps8
Archaeplastida - ps9
Viridiplantae - ps10
Streptophyta - ps11
Charophyceae/Embriophyta - ps12
Embriophyta - ps13
Tracheophyta - ps14
Spermatophyta - ps15
Angiosperms - ps16
Mesangiospermae - ps17
Magnoliids/eudicots - ps18
Eudicots - ps19
Core_eudicots - ps20
Superrosids - ps21
Rosids - ps22
Eurosids - ps23
Malvids - ps24
Sap./Malv./Brassicales - ps25
Malvales/Brassicales - ps26
Brassicales - ps27
Cleomaceae/Brassicaceae - ps28
Brassicaceae - ps29
Camelineae - ps30
Arabidopsis - ps31
Arabidopsis_thaliana - ps32
p-value
7e-03
1e-02
1e-02
2e-02
3e-02
Log-odds
0.5
1.0
1.5
2.0
2.5
3.0
3.5
4.0

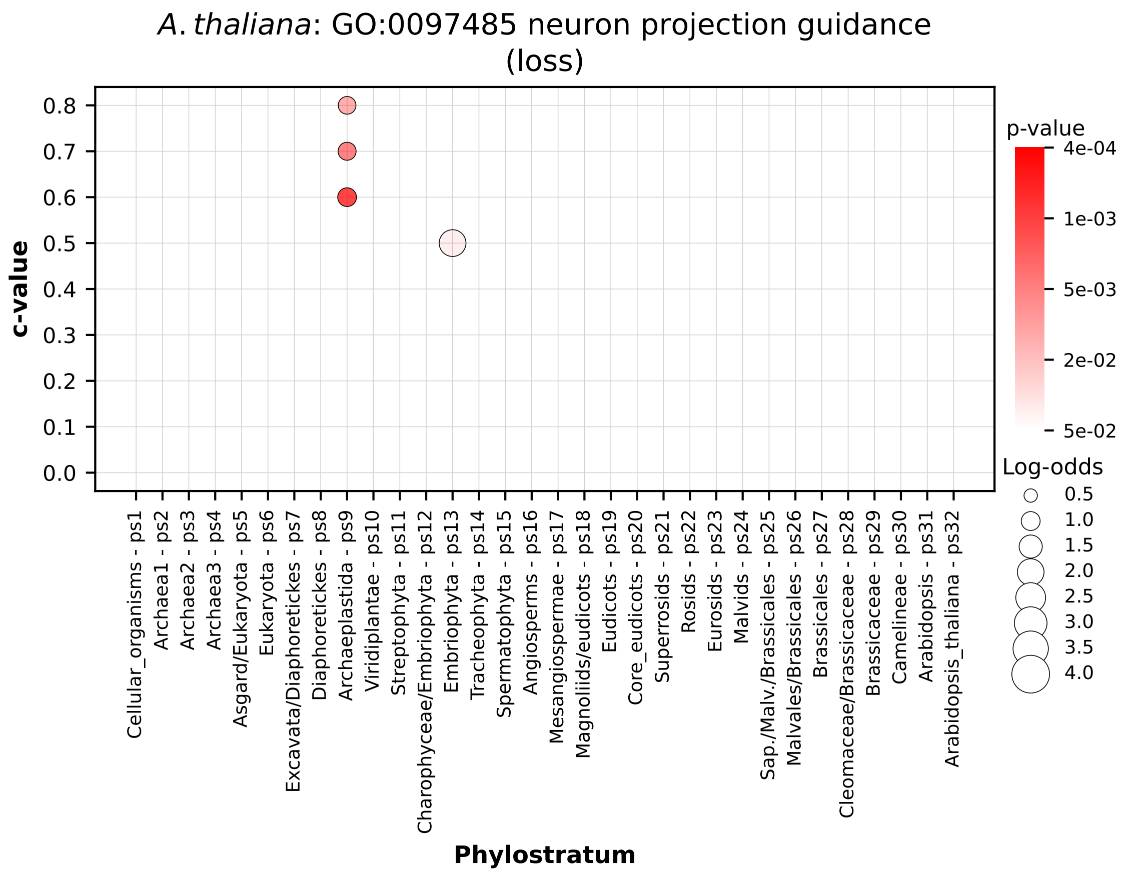

A. thaliana: GO:0097485 neuron projection guidance
(loss)
c-value
0.8
0.7
0.6
0.5
0.4
0.3
0.2
0.1
0.0
p-value
4e-04
1e-03
5e-03
2e-02
5e-02
Log-odds
0.5
1.0
1.5
2.0
2.5
3.0
3.5
4.0
Cellular_organisms - ps1
Archaea1 - ps2
Archaea2 - ps3
Archaea3 - ps4
Asgard/Eukaryota - ps5
Eukaryota - ps6
Excavata/Diaphoretickes - ps7
Diaphoretickes - ps8
Archaeplastida - ps9
Viridiplantae - ps10
Streptophyta - ps11
Charophyceae/Embriophyta - ps12
Embriophyta - ps13
Tracheophyta - ps14
Spermatophyta - ps15
Angiosperms - ps16
Mesangiospermae - ps17
Magnoliids/eudicots - ps18
Eudicots - ps19
Core_eudicots - ps20
Superrosids - ps21
Rosids - ps22
Eurosids - ps23
Malvids - ps24
Sap./Malv./Brassicales - ps25
Malvales/Brassicales - ps26
Brassicales - ps27
Cleomaceae/Brassicaceae - ps28
Brassicaceae - ps29
Camelineae - ps30
Arabidopsis - ps31
Arabidopsis_thaliana - ps32
Phylostratum

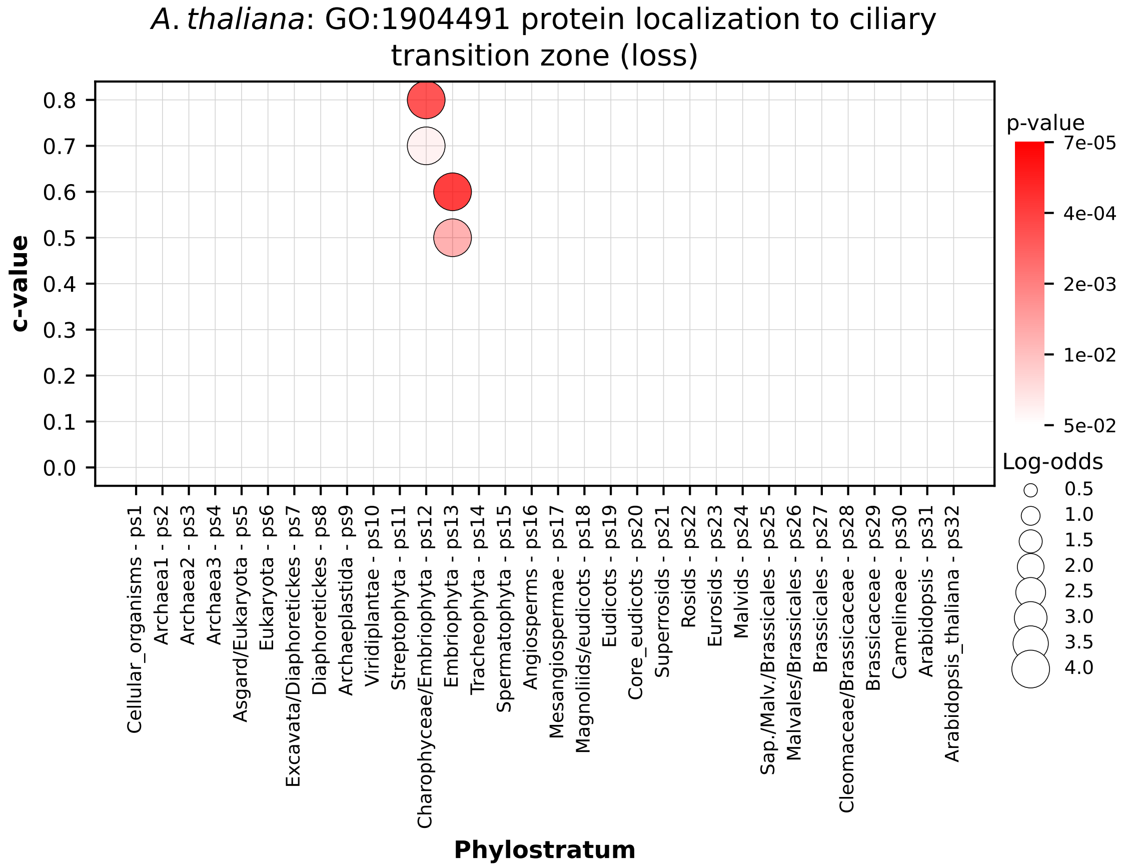
A. thaliana: GO:1904491 protein localization to ciliary transition zone (loss)
c-value
0.0
0.1
0.2
0.3
0.4
0.5
0.6
0.7
0.8
Cellular_organisms - ps1
Archaea1 - ps2
Archaea2 - ps3
Archaea3 - ps4
Asgard/Eukaryota - ps5
Eukaryota - ps6
Excavata/Diaphoretickes - ps7
Diaphoretickes - ps8
Archaeplastida - ps9
Viridiplantae - ps10
Streptophyta - ps11
Charophyceae/Embriophyta - ps12
Embriophyta - ps13
Tracheophyta - ps14
Spermatophyta - ps15
Angiosperms - ps16
Mesangiospermae - ps17
Magnoliids/eudicots - ps18
Eudicots - ps19
Core_eudicots - ps20
Superrosids - ps21
Rosids - ps22
Eurosids - ps23
Malvids - ps24
Sap./Malv./Brassicales - ps25
Malvales/Brassicales - ps26
Brassicales - ps27
Cleomaceae/Brassicaceae - ps28
Brassicaceae - ps29
Camelineae - ps30
Arabidopsis - ps31
Arabidopsis_thaliana - ps32
Phylostratum
p-value
7e-05
4e-04
2e-03
1e-02
5e-02
Log-odds
0.5
1.0
1.5
2.0
2.5
3.0
3.5
4.0

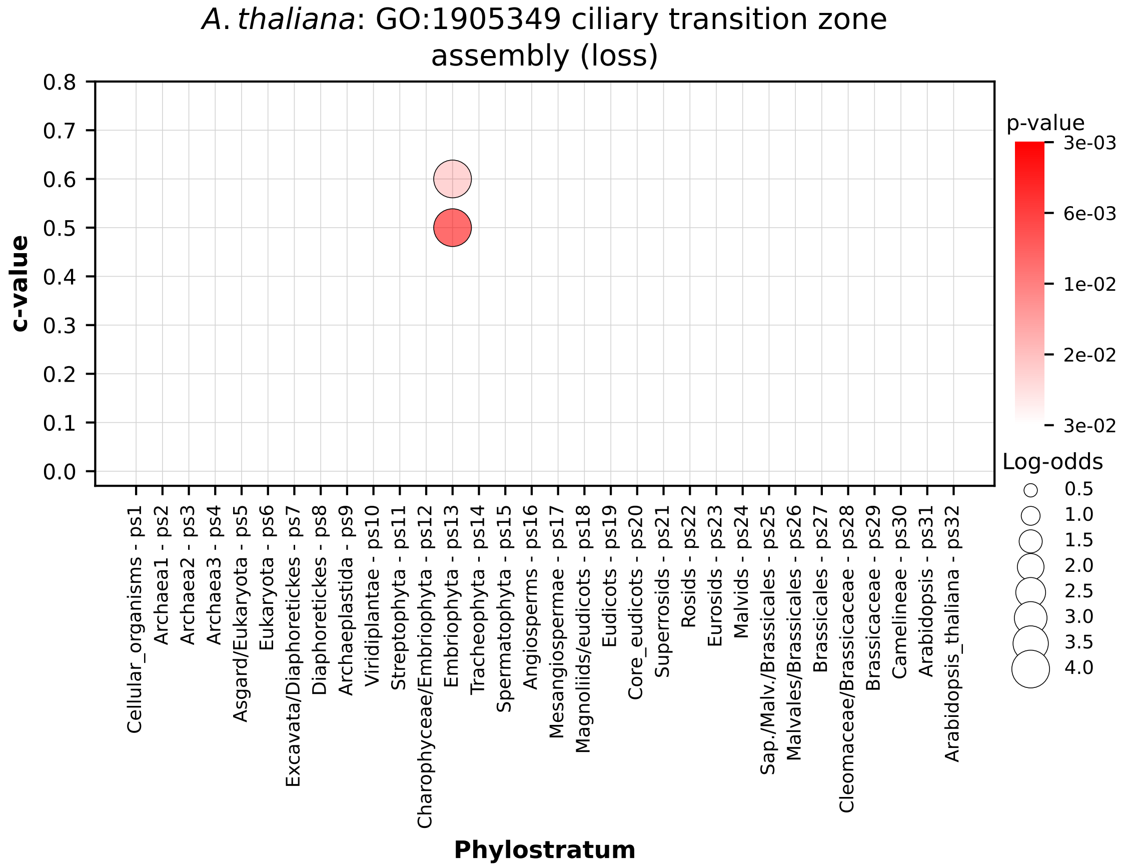

A. thaliana: GO:1905349 ciliary transition zone assembly (loss)
c-value
0.8
0.7
0.6
0.5
0.4
0.3
0.2
0.1
0.0
p-value
3e-03
6e-03
1e-02
2e-02
3e-02
Log-odds
0.5
1.0
1.5
2.0
2.5
3.0
3.5
4.0
Cellular_organisms - ps1
Archaea1 - ps2
Archaea2 - ps3
Archaea3 - ps4
Asgard/Eukaryota - ps5
Eukaryota - ps6
Excavata/Diaphoretickes - ps7
Diaphoretickes - ps8
Archaeplastida - ps9
Viridiplantae - ps10
Streptophyta - ps11
Charophyceae/Embriophyta - ps12
Embriophyta - ps13
Tracheophyta - ps14
Spermatophyta - ps15
Angiosperms - ps16
Mesangiospermae - ps17
Magnoliids/eudicots - ps18
Eudicots - ps19
Core_eudicots - ps20
Superrosids - ps21
Rosids - ps22
Eurosids - ps23
Malvids - ps24
Sap./Malv./Brassicales - ps25
Malvales/Brassicales - ps26
Brassicales - ps27
Cleomaceae/Brassicaceae - ps28
Brassicaceae - ps29
Camelineae - ps30
Arabidopsis - ps31
Arabidopsis_thaliana - ps32
Phylostratum

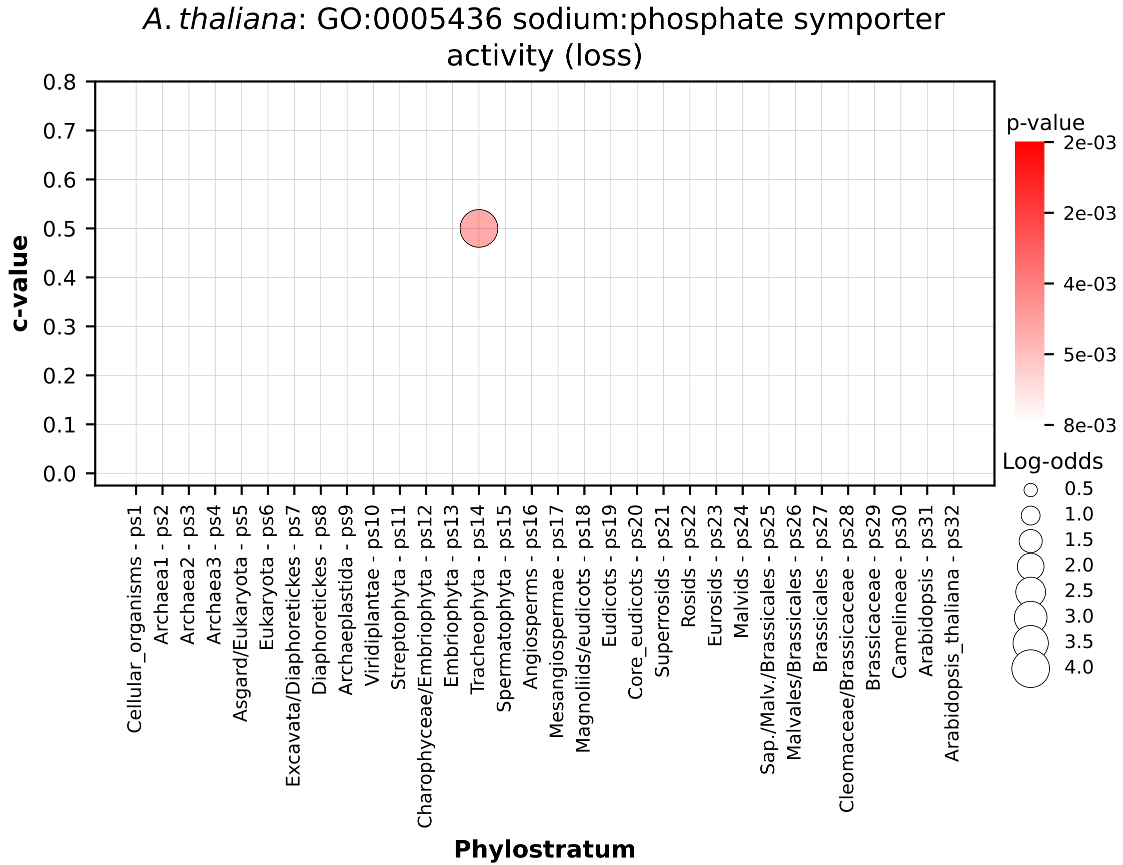

A. thaliana: GO:0005436 sodium:phosphate symporter activity (loss)
c-value
0.8
0.7
0.6
0.5
0.4
0.3
0.2
0.1
0.0
Phylostratum
Cellular_organisms - ps1
Archaea1 - ps2
Archaea2 - ps3
Archaea3 - ps4
Asgard/Eukaryota - ps5
Eukaryota - ps6
Excavata/Diaphoretickes - ps7
Diaphoretickes - ps8
Archaeplastida - ps9
Viridiplantae - ps10
Streptophyta - ps11
Charophyceae/Embriophyta - ps12
Embriophyta - ps13
Tracheophyta - ps14
Spermatophyta - ps15
Angiosperms - ps16
Mesangiospermae - ps17
Magnoliids/eudicots - ps18
Eudicots - ps19
Core_eudicots - ps20
Superrosids - ps21
Rosids - ps22
Eurosids - ps23
Malvids - ps24
Sap./Malv./Brassicales - ps25
Malvales/Brassicales - ps26
Brassicales - ps27
Cleomaceae/Brassicaceae - ps28
Brassicaceae - ps29
Camelineae - ps30
Arabidopsis - ps31
Arabidopsis_thaliana - ps32
p-value
2e-03
2e-03
4e-03
5e-03
8e-03
Log-odds
0.5
1.0
1.5
2.0
2.5
3.0
3.5
4.0

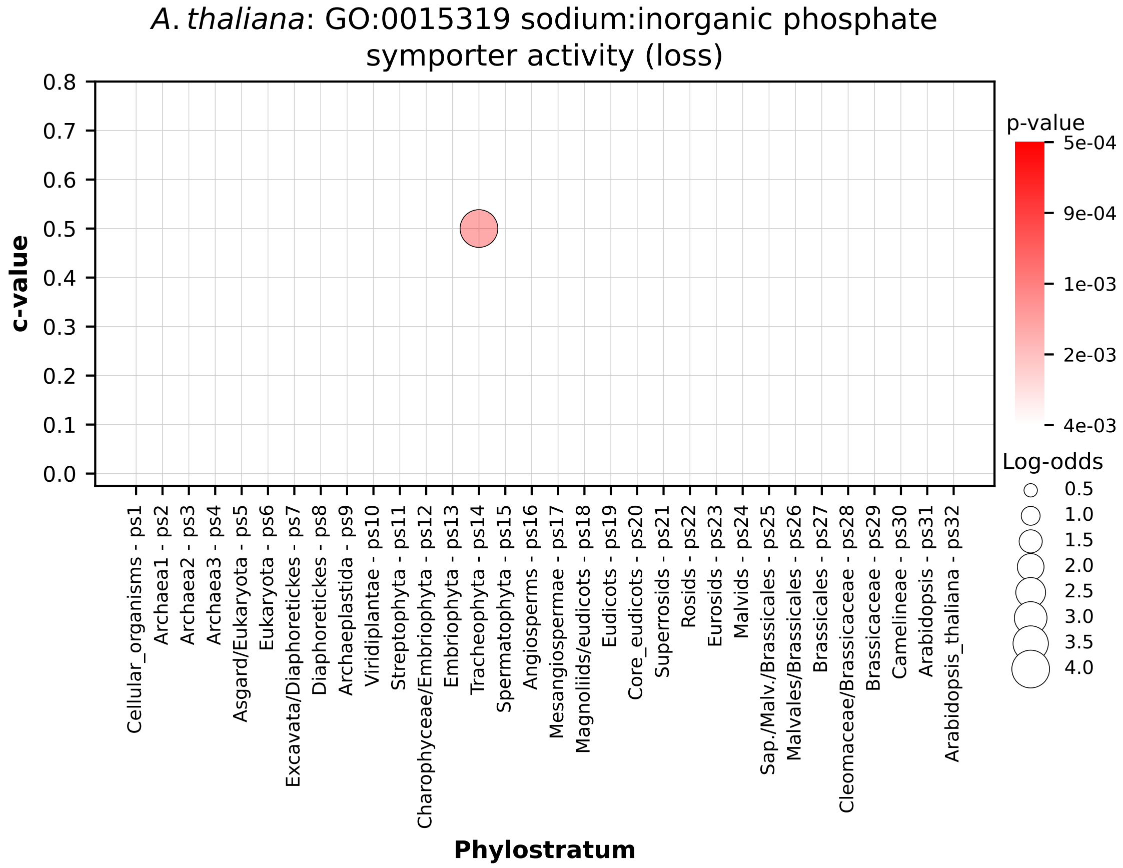
A. thaliana: GO:0015319 sodium:inorganic phosphate symporter activity (loss)
c-value
0.8
0.7
0.6
0.5
0.4
0.3
0.2
0.1
0.0
Phylostratum
Cellular_organisms - ps1
Archaea1 - ps2
Archaea2 - ps3
Archaea3 - ps4
Asgard/Eukaryota - ps5
Eukaryota - ps6
Excavata/Diaphoretickes - ps7
Diaphoretickes - ps8
Archaeplastida - ps9
Viridiplantae - ps10
Streptophyta - ps11
Charophyceae/Embriophyta - ps12
Embriophyta - ps13
Tracheophyta - ps14
Spermatophyta - ps15
Angiosperms - ps16
Mesangiospermae - ps17
Magnoliids/eudicots - ps18
Eudicots - ps19
Core_eudicots - ps20
Superrosids - ps21
Rosids - ps22
Eurosids - ps23
Malvids - ps24
Sap./Malv./Brassicales - ps25
Malvales/Brassicales - ps26
Brassicales - ps27
Cleomaceae/Brassicaceae - ps28
Brassicaceae - ps29
Camelineae - ps30
Arabidopsis - ps31
Arabidopsis_thaliana - ps32
p-value
5e-04
9e-04
1e-03
2e-03
4e-03
Log-odds
0.5
1.0
1.5
2.0
2.5
3.0
3.5
4.0

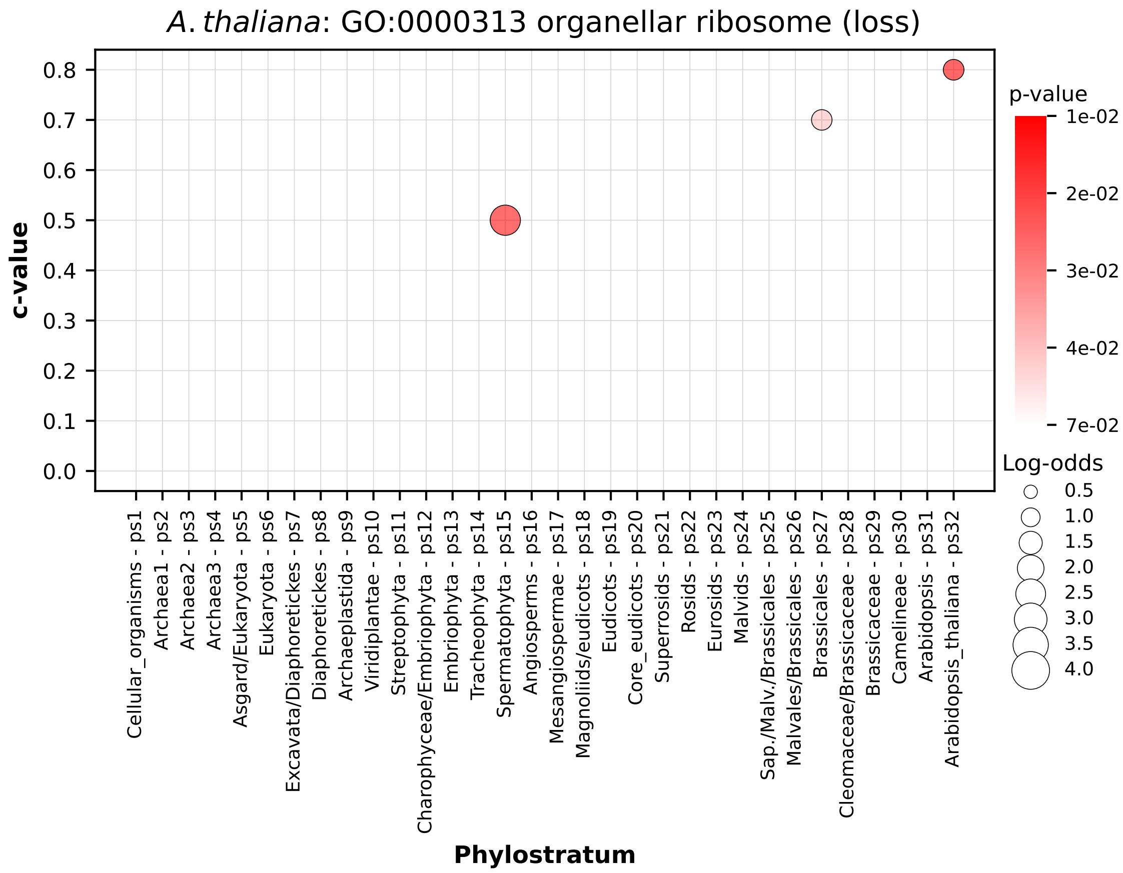

A. thaliana: GO:0000313 organellar ribosome (loss)
c-value
0.0
0.1
0.2
0.3
0.4
0.5
0.6
0.7
0.8
Phylostratum
Cellular_organisms - ps1
Archaea1 - ps2
Archaea2 - ps3
Archaea3 - ps4
Asgard/Eukaryota - ps5
Eukaryota - ps6
Excavata/Diaphoretickes - ps7
Diaphoretickes - ps8
Archaeplastida - ps9
Viridiplantae - ps10
Streptophyta - ps11
Charophyceae/Embriophyta - ps12
Embriophyta - ps13
Tracheophyta - ps14
Spermatophyta - ps15
Angiosperms - ps16
Mesangiospermae - ps17
Magnoliids/eudicots - ps18
Eudicots - ps19
Core_eudicots - ps20
Superrosids - ps21
Rosids - ps22
Eurosids - ps23
Malvids - ps24
Sap./Malv./Brassicales - ps25
Malvales/Brassicales - ps26
Brassicales - ps27
Cleomaceae/Brassicaceae - ps28
Brassicaceae - ps29
Camelineae - ps30
Arabidopsis - ps31
Arabidopsis_thaliana - ps32
p-value
1e-02
2e-02
3e-02
4e-02
7e-02
Log-odds
0.5
1.0
1.5
2.0
2.5
3.0
3.5
4.0

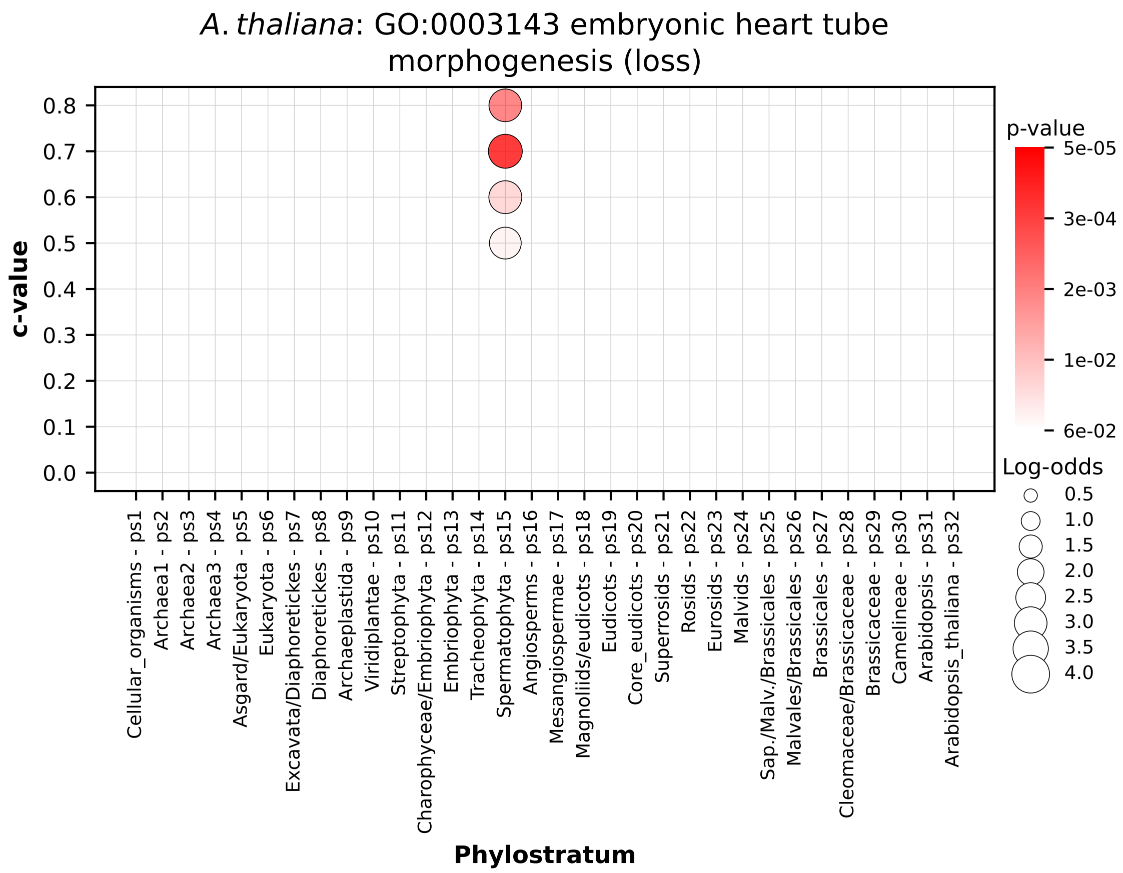

A. thaliana: GO:0003143 embryonic heart tube morphogenesis (loss)
c-value
0.8
0.7
0.6
0.5
0.4
0.3
0.2
0.1
0.0
p-value
5e-05
3e-04
2e-03
1e-02
6e-02
Log-odds
0.5
1.0
1.5
2.0
2.5
3.0
3.5
4.0
Cellular_organisms - ps1
Archaea1 - ps2
Archaea2 - ps3
Archaea3 - ps4
Asgard/Eukaryota - ps5
Eukaryota - ps6
Excavata/Diaphoretickes - ps7
Diaphoretickes - ps8
Archaeplastida - ps9
Viridiplantae - ps10
Streptophyta - ps11
Charophyceae/Embriophyta - ps12
Embriophyta - ps13
Tracheophyta - ps14
Spermatophyta - ps15
Angiosperms - ps16
Mesangiospermae - ps17
Magnoliids/eudicots - ps18
Eudicots - ps19
Core_eudicots - ps20
Superrosids - ps21
Rosids - ps22
Eurosids - ps23
Malvids - ps24
Sap./Malv./Brassicales - ps25
Malvales/Brassicales - ps26
Brassicales - ps27
Cleomaceae/Brassicaceae - ps28
Brassicaceae - ps29
Camelineae - ps30
Arabidopsis - ps31
Arabidopsis_thaliana - ps32
Phylostratum

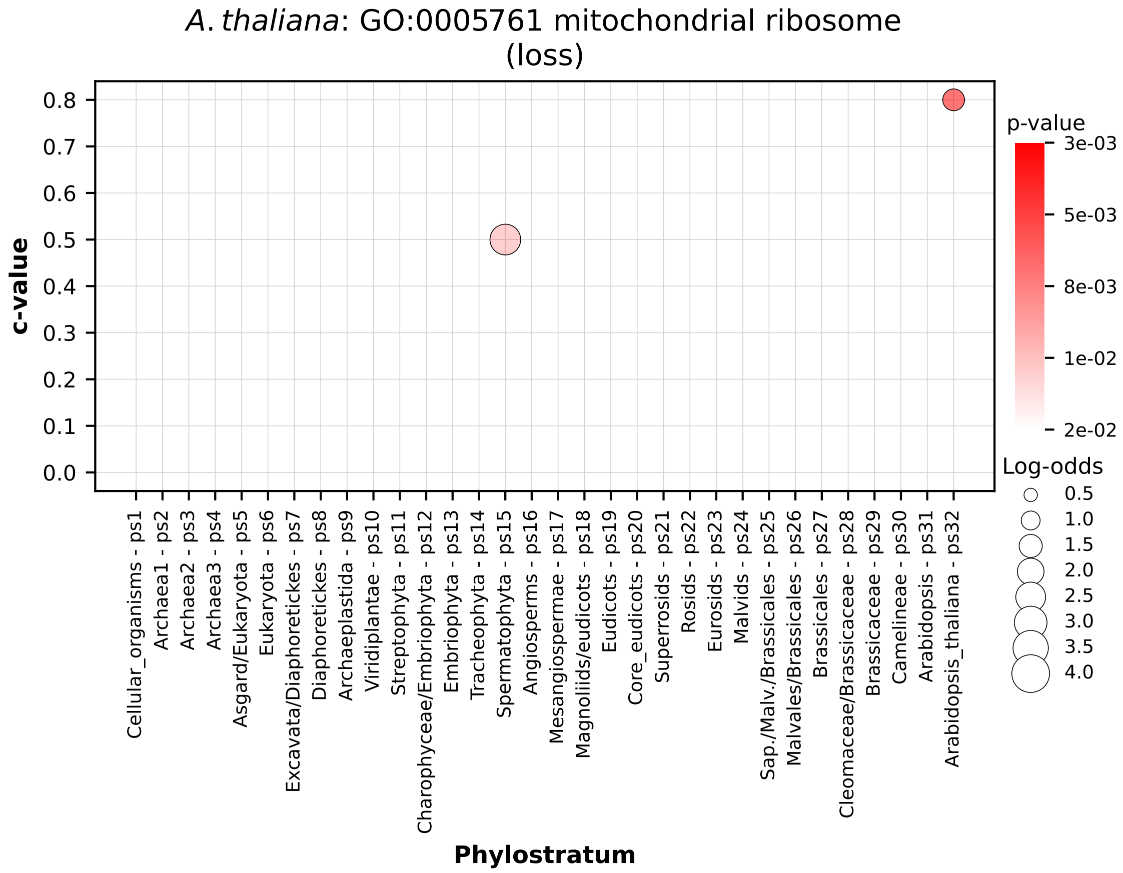
A. thaliana: GO:0005761 mitochondrial ribosome
(loss)
c-value
0.8
0.7
0.6
0.5
0.4
0.3
0.2
0.1
0.0
Phylostratum
Cellular_organisms - ps1
Archaea1 - ps2
Archaea2 - ps3
Archaea3 - ps4
Asgard/Eukaryota - ps5
Eukaryota - ps6
Excavata/Diaphoretickes - ps7
Diaphoretickes - ps8
Archaeplastida - ps9
Viridiplantae - ps10
Streptophyta - ps11
Charophyceae/Embriophyta - ps12
Embriophyta - ps13
Tracheophyta - ps14
Spermatophyta - ps15
Angiosperms - ps16
Mesangiospermae - ps17
Magnoliids/eudicots - ps18
Eudicots - ps19
Core_eudicots - ps20
Superrosids - ps21
Rosids - ps22
Eurosids - ps23
Malvids - ps24
Sap./Malv./Brassicales - ps25
Malvales/Brassicales - ps26
Brassicales - ps27
Cleomaceae/Brassicaceae - ps28
Brassicaceae - ps29
Camelineae - ps30
Arabidopsis - ps31
Arabidopsis_thaliana - ps32
p-value
3e-03
5e-03
8e-03
1e-02
2e-02
Log-odds
0.5
1.0
1.5
2.0
2.5
3.0
3.5
4.0

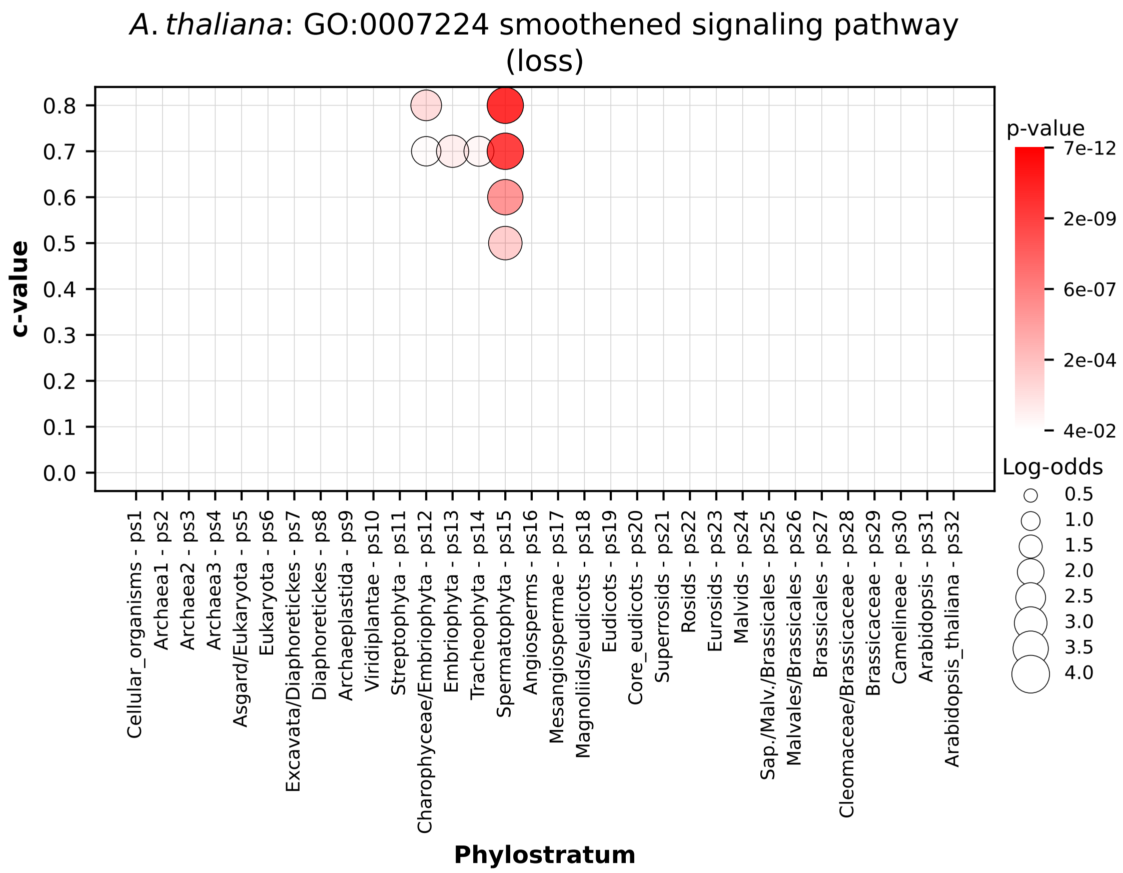

A. thaliana: GO:0007224 smoothened signaling pathway (loss)
c-value
0.0
0.1
0.2
0.3
0.4
0.5
0.6
0.7
0.8
Phylostratum
Cellular_organisms - ps1
Archaea1 - ps2
Archaea2 - ps3
Archaea3 - ps4
Asgard/Eukaryota - ps5
Eukaryota - ps6
Excavata/Diaphoretickes - ps7
Diaphoretickes - ps8
Archaeplastida - ps9
Viridiplantae - ps10
Streptophyta - ps11
Charophyceae/Embriophyta - ps12
Embriophyta - ps13
Tracheophyta - ps14
Spermatophyta - ps15
Angiosperms - ps16
Mesangiospermae - ps17
Magnoliids/eudicots - ps18
Eudicots - ps19
Core_eudicots - ps20
Superrosids - ps21
Rosids - ps22
Eurosids - ps23
Malvids - ps24
Sap./Malv./Brassicales - ps25
Malvales/Brassicales - ps26
Brassicales - ps27
Cleomaceae/Brassicaceae - ps28
Brassicaceae - ps29
Camelineae - ps30
Arabidopsis - ps31
Arabidopsis_thaliana - ps32
p-value
7e-12
2e-09
6e-07
2e-04
4e-02
Log-odds
0.5
1.0
1.5
2.0
2.5
3.0
3.5
4.0

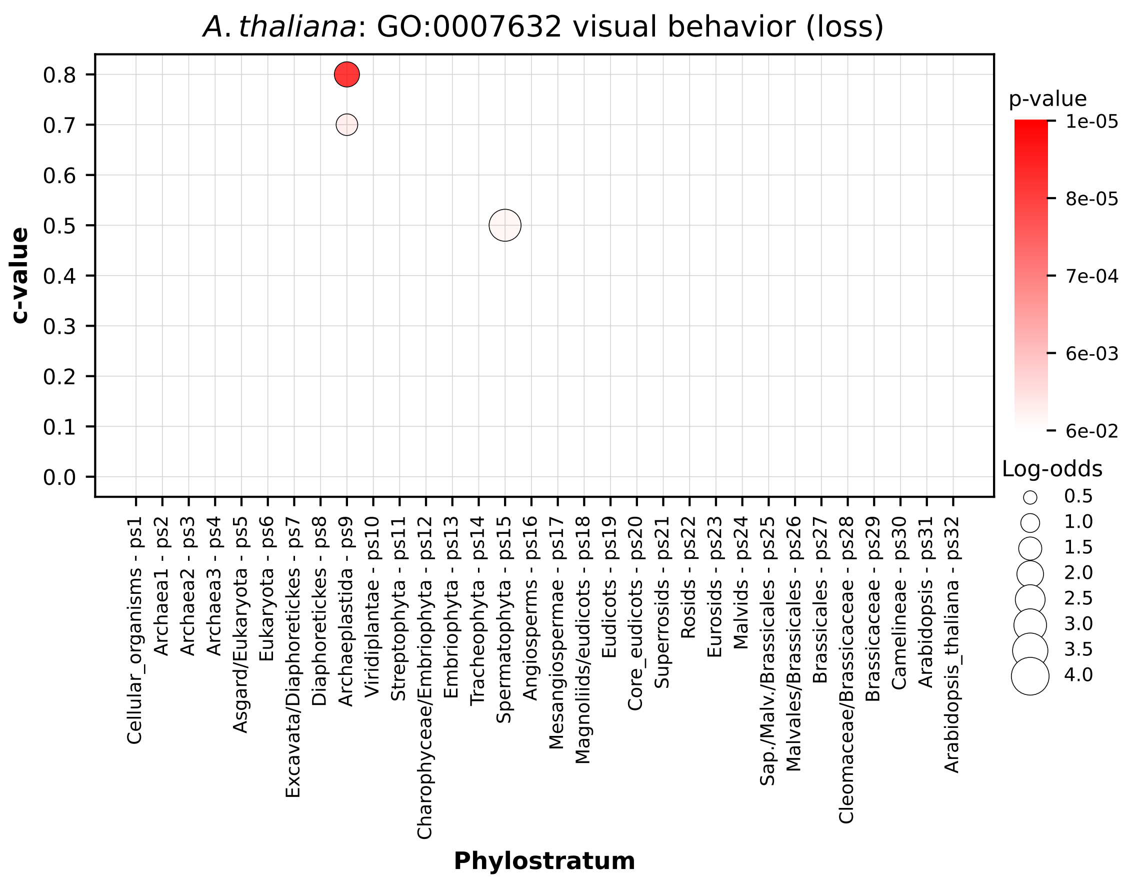

A. thaliana: GO:0007632 visual behavior (loss)
c-value
0.0
0.1
0.2
0.3
0.4
0.5
0.6
0.7
0.8
Phylostratum
Cellular_organisms - ps1
Archaea1 - ps2
Archaea2 - ps3
Archaea3 - ps4
Asgard/Eukaryota - ps5
Eukaryota - ps6
Excavata/Diaphoretickes - ps7
Diaphoretickes - ps8
Archaeplastida - ps9
Viridiplantae - ps10
Streptophyta - ps11
Charophyceae/Embriophyta - ps12
Embriophyta - ps13
Tracheophyta - ps14
Spermatophyta - ps15
Angiosperms - ps16
Mesangiospermae - ps17
Magnoliids/eudicots - ps18
Eudicots - ps19
Core_eudicots - ps20
Superrosids - ps21
Rosids - ps22
Eurosids - ps23
Malvids - ps24
Sap./Malv./Brassicales - ps25
Malvales/Brassicales - ps26
Brassicales - ps27
Cleomaceae/Brassicaceae - ps28
Brassicaceae - ps29
Camelineae - ps30
Arabidopsis - ps31
Arabidopsis_thaliana - ps32
p-value
1e-05
8e-05
7e-04
6e-03
6e-02
Log-odds
0.5
1.0
1.5
2.0
2.5
3.0
3.5
4.0

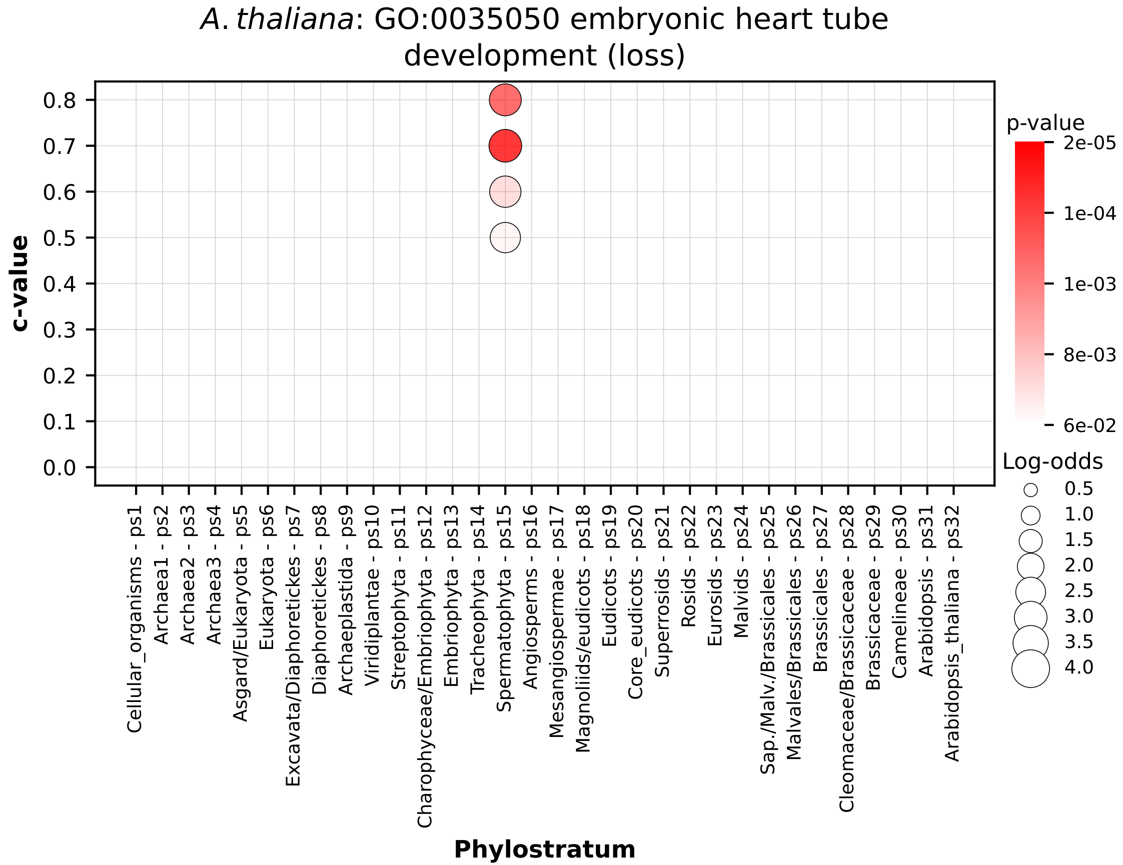

A. thaliana: GO:0035050 embryonic heart tube development (loss)
c-value
0.8
0.7
0.6
0.5
0.4
0.3
0.2
0.1
0.0
p-value
2e-05
1e-04
1e-03
8e-03
6e-02
Log-odds
0.5
1.0
1.5
2.0
2.5
3.0
3.5
4.0
Cellular_organisms - ps1
Archaea1 - ps2
Archaea2 - ps3
Archaea3 - ps4
Asgard/Eukaryota - ps5
Eukaryota - ps6
Excavata/Diaphoretickes - ps7
Diaphoretickes - ps8
Archaeplastida - ps9
Viridiplantae - ps10
Streptophyta - ps11
Charophyceae/Embriophyta - ps12
Embriophyta - ps13
Tracheophyta - ps14
Spermatophyta - ps15
Angiosperms - ps16
Mesangiospermae - ps17
Magnoliids/eudicots - ps18
Eudicots - ps19
Core_eudicots - ps20
Superrosids - ps21
Rosids - ps22
Eurosids - ps23
Malvids - ps24
Sap./Malv./Brassicales - ps25
Malvales/Brassicales - ps26
Brassicales - ps27
Cleomaceae/Brassicaceae - ps28
Brassicaceae - ps29
Camelineae - ps30
Arabidopsis - ps31
Arabidopsis_thaliana - ps32
Phylostratum

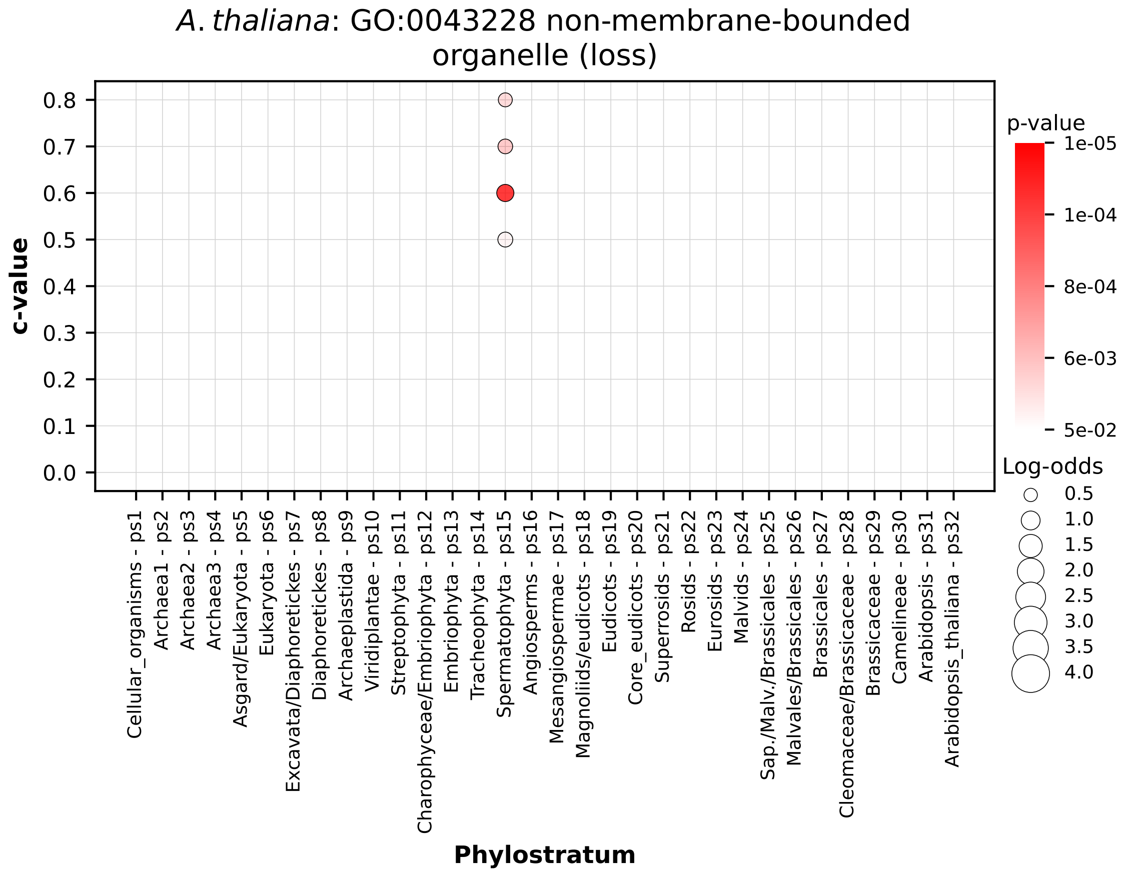

A. thaliana: GO:0043228 non-membrane-bounded organelle (loss)
c-value
0.8
0.7
0.6
0.5
0.4
0.3
0.2
0.1
0.0
p-value
1e-05
1e-04
8e-04
6e-03
5e-02
Log-odds
0.5
1.0
1.5
2.0
2.5
3.0
3.5
4.0
Cellular_organisms - ps1
Archaea1 - ps2
Archaea2 - ps3
Archaea3 - ps4
Asgard/Eukaryota - ps5
Eukaryota - ps6
Excavata/Diaphoretickes - ps7
Diaphoretickes - ps8
Archaeplastida - ps9
Viridiplantae - ps10
Streptophyta - ps11
Charophyceae/Embriophyta - ps12
Embriophyta - ps13
Tracheophyta - ps14
Spermatophyta - ps15
Angiosperms - ps16
Mesangiospermae - ps17
Magnoliids/eudicots - ps18
Eudicots - ps19
Core_eudicots - ps20
Superrosids - ps21
Rosids - ps22
Eurosids - ps23
Malvids - ps24
Sap./Malv./Brassicales - ps25
Malvales/Brassicales - ps26
Brassicales - ps27
Cleomaceae/Brassicaceae - ps28
Brassicaceae - ps29
Camelineae - ps30
Arabidopsis - ps31
Arabidopsis_thaliana - ps32
Phylostratum

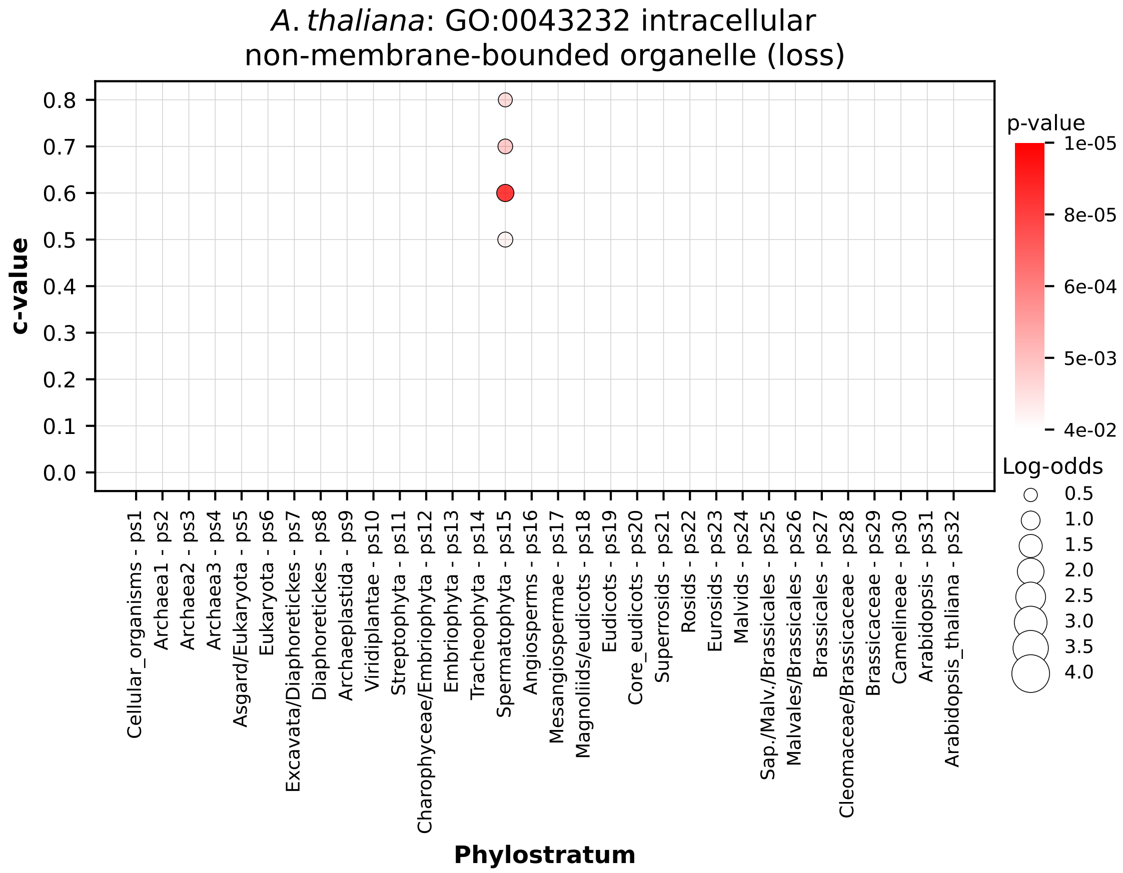

A. thaliana: GO:0043232 intracellular
non-membrane-bounded organelle (loss)
c-value
0.8
0.7
0.6
0.5
0.4
0.3
0.2
0.1
0.0
p-value
1e-05
8e-05
6e-04
5e-03
4e-02
Log-odds
0.5
1.0
1.5
2.0
2.5
3.0
3.5
4.0
Cellular_organisms - ps1
Archaea1 - ps2
Archaea2 - ps3
Archaea3 - ps4
Asgard/Eukaryota - ps5
Eukaryota - ps6
Excavata/Diaphoretickes - ps7
Diaphoretickes - ps8
Archaeplastida - ps9
Viridiplantae - ps10
Streptophyta - ps11
Charophyceae/Embriophyta - ps12
Embriophyta - ps13
Tracheophyta - ps14
Spermatophyta - ps15
Angiosperms - ps16
Mesangiospermae - ps17
Magnoliids/eudicots - ps18
Eudicots - ps19
Core_eudicots - ps20
Superrosids - ps21
Rosids - ps22
Eurosids - ps23
Malvids - ps24
Sap./Malv./Brassicales - ps25
Malvales/Brassicales - ps26
Brassicales - ps27
Cleomaceae/Brassicaceae - ps28
Brassicaceae - ps29
Camelineae - ps30
Arabidopsis - ps31
Arabidopsis_thaliana - ps32
Phylostratum

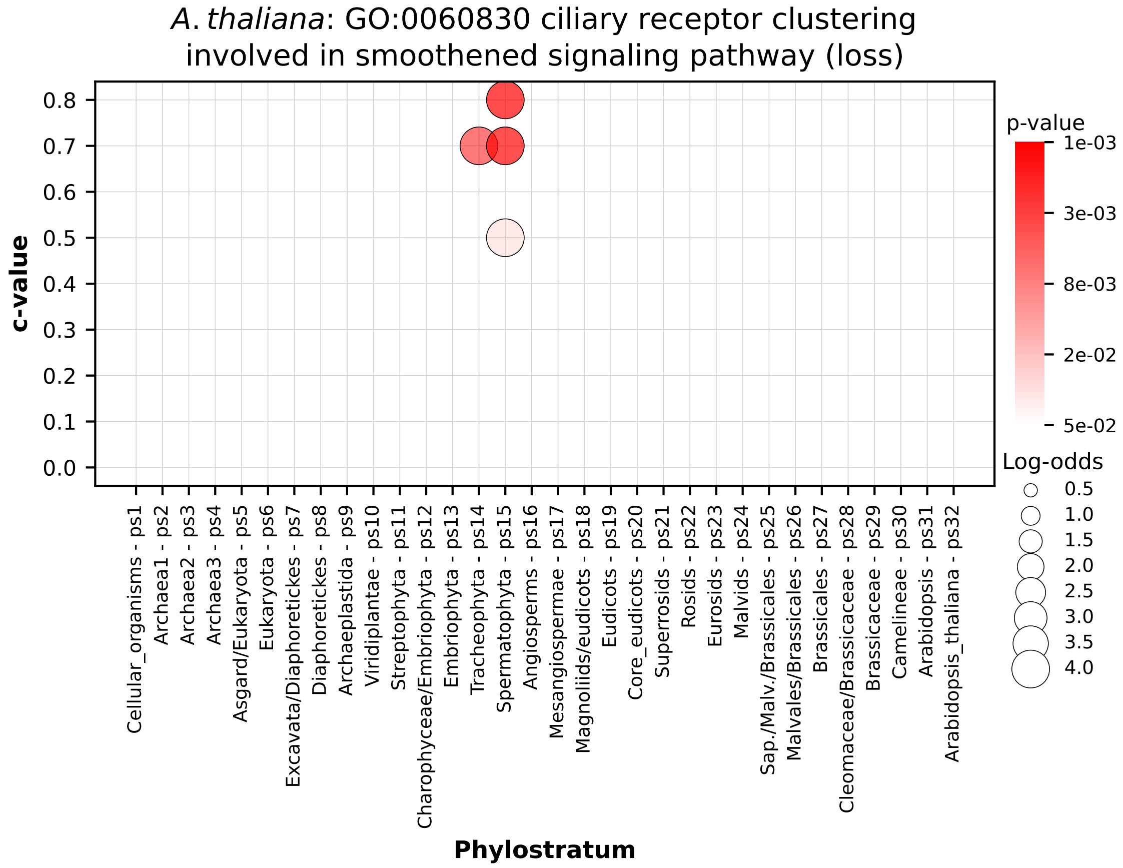

A. thaliana: GO:0060830 ciliary receptor clustering involved in smoothened signaling pathway (loss)
c-value
0.8
0.7
0.6
0.5
0.4
0.3
0.2
0.1
0.0
p-value
1e-03
3e-03
8e-03
2e-02
5e-02
Log-odds
0.5
1.0
1.5
2.0
2.5
3.0
3.5
4.0
Cellular_organisms - ps1
Archaea1 - ps2
Archaea2 - ps3
Archaea3 - ps4
Asgard/Eukaryota - ps5
Eukaryota - ps6
Excavata/Diaphoretickes - ps7
Diaphoretickes - ps8
Archaeplastida - ps9
Viridiplantae - ps10
Streptophyta - ps11
Charophyceae/Embriophyta - ps12
Embriophyta - ps13
Tracheophyta - ps14
Spermatophyta - ps15
Angiosperms - ps16
Mesangiospermae - ps17
Magnoliids/eudicots - ps18
Eudicots - ps19
Core_eudicots - ps20
Superrosids - ps21
Rosids - ps22
Eurosids - ps23
Malvids - ps24
Sap./Malv./Brassicales - ps25
Malvales/Brassicales - ps26
Brassicales - ps27
Cleomaceae/Brassicaceae - ps28
Brassicaceae - ps29
Camelineae - ps30
Arabidopsis - ps31
Arabidopsis_thaliana - ps32
Phylostratum

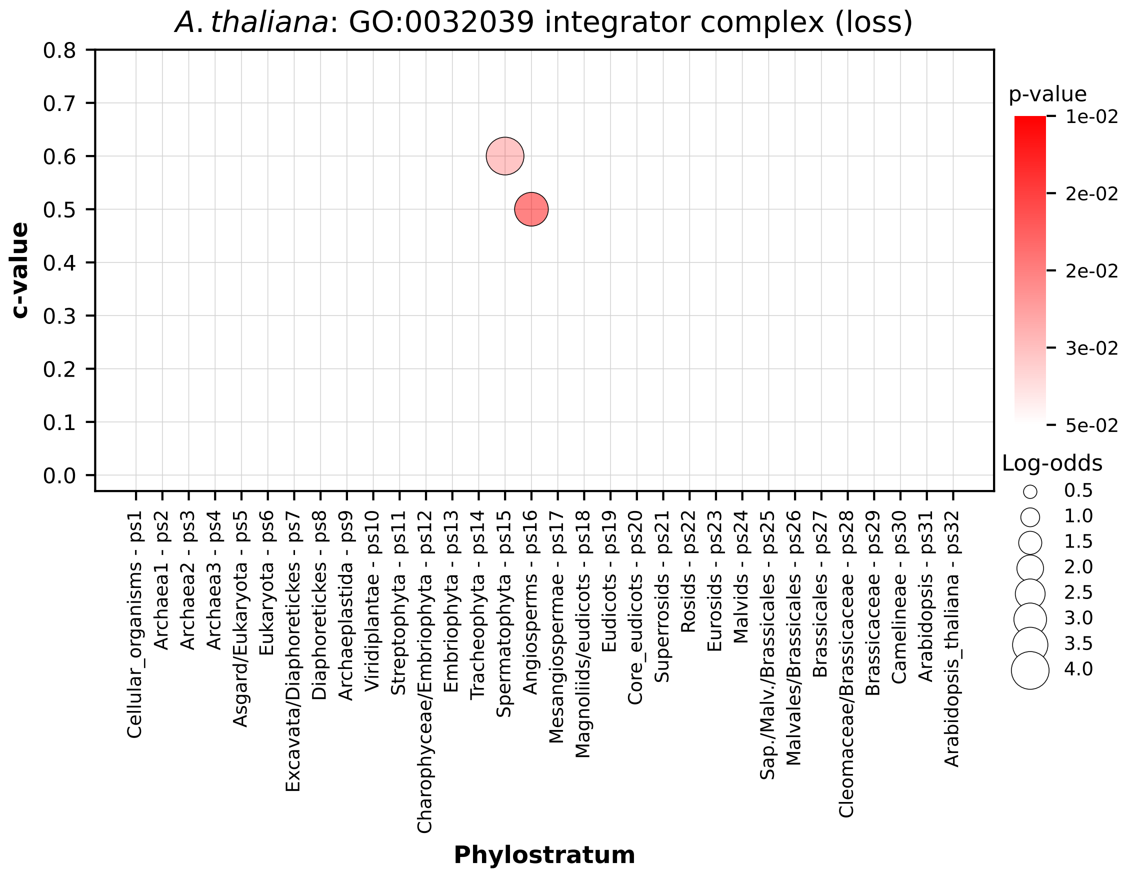
A. thaliana: GO:0032039 integrator complex (loss)
c-value
0.0
0.1
0.2
0.3
0.4
0.5
0.6
0.7
0.8
Phylostratum
Cellular_organisms - ps1
Archaea1 - ps2
Archaea2 - ps3
Archaea3 - ps4
Asgard/Eukaryota - ps5
Eukaryota - ps6
Excavata/Diaphoretickes - ps7
Diaphoretickes - ps8
Archaeplastida - ps9
Viridiplantae - ps10
Streptophyta - ps11
Charophyceae/Embriophyta - ps12
Embriophyta - ps13
Tracheophyta - ps14
Spermatophyta - ps15
Angiosperms - ps16
Mesangiospermae - ps17
Magnoliids/eudicots - ps18
Eudicots - ps19
Core_eudicots - ps20
Superrosids - ps21
Rosids - ps22
Eurosids - ps23
Malvids - ps24
Sap./Malv./Brassicales - ps25
Malvales/Brassicales - ps26
Brassicales - ps27
Cleomaceae/Brassicaceae - ps28
Brassicaceae - ps29
Camelineae - ps30
Arabidopsis - ps31
Arabidopsis_thaliana - ps32
p-value
1e-02
2e-02
2e-02
3e-02
5e-02
Log-odds
0.5
1.0
1.5
2.0
2.5
3.0
3.5
4.0

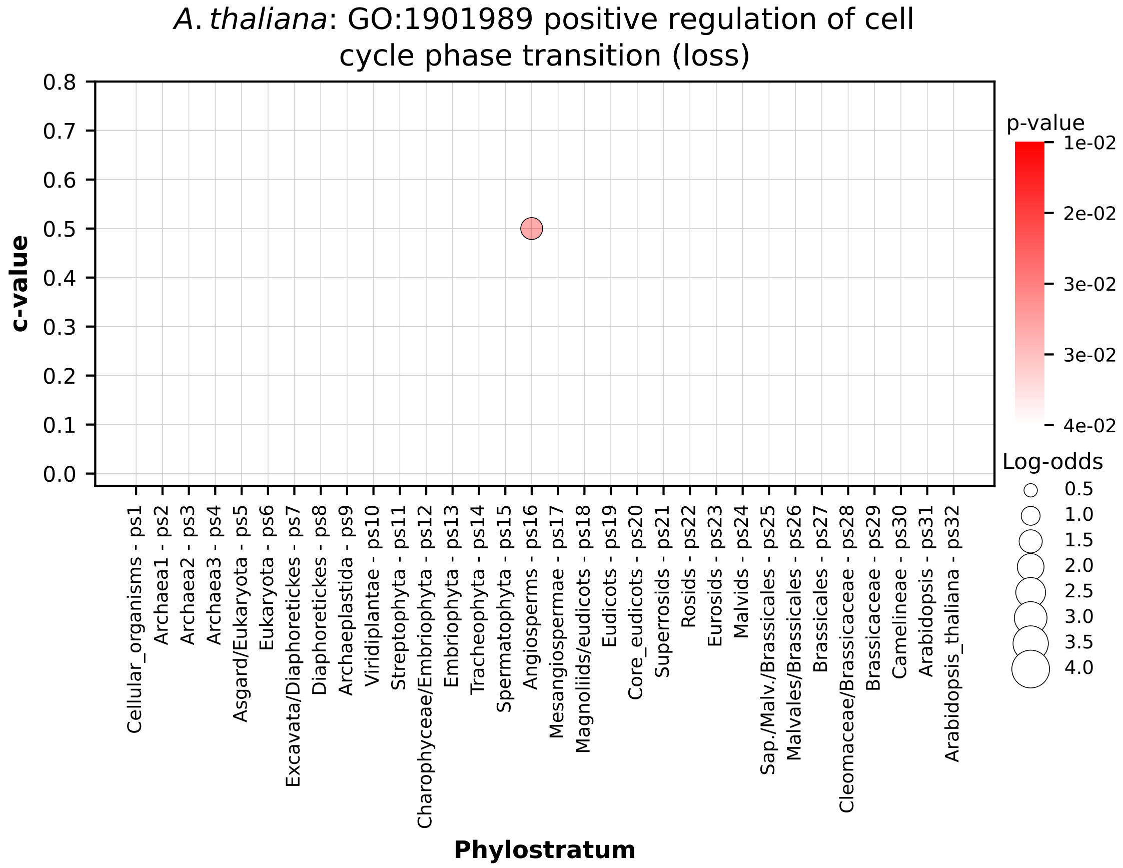

A. thaliana: GO:1901989 positive regulation of cell cycle phase transition (loss)
c-value
0.8
0.7
0.6
0.5
0.4
0.3
0.2
0.1
0.0
p-value
1e-02
2e-02
3e-02
3e-02
4e-02
Log-odds
0.5
1.0
1.5
2.0
2.5
3.0
3.5
4.0
Cellular_organisms - ps1
Archaea1 - ps2
Archaea2 - ps3
Archaea3 - ps4
Asgard/Eukaryota - ps5
Eukaryota - ps6
Excavata/Diaphoretickes - ps7
Diaphoretickes - ps8
Archaeplastida - ps9
Viridiplantae - ps10
Streptophyta - ps11
Charophyceae/Embriophyta - ps12
Embriophyta - ps13
Tracheophyta - ps14
Spermatophyta - ps15
Angiosperms - ps16
Mesangiospermae - ps17
Magnoliids/eudicots - ps18
Eudicots - ps19
Core_eudicots - ps20
Superrosids - ps21
Rosids - ps22
Eurosids - ps23
Malvids - ps24
Sap./Malv./Brassicales - ps25
Malvales/Brassicales - ps26
Brassicales - ps27
Cleomaceae/Brassicaceae - ps28
Brassicaceae - ps29
Camelineae - ps30
Arabidopsis - ps31
Arabidopsis_thaliana - ps32
Phylostratum

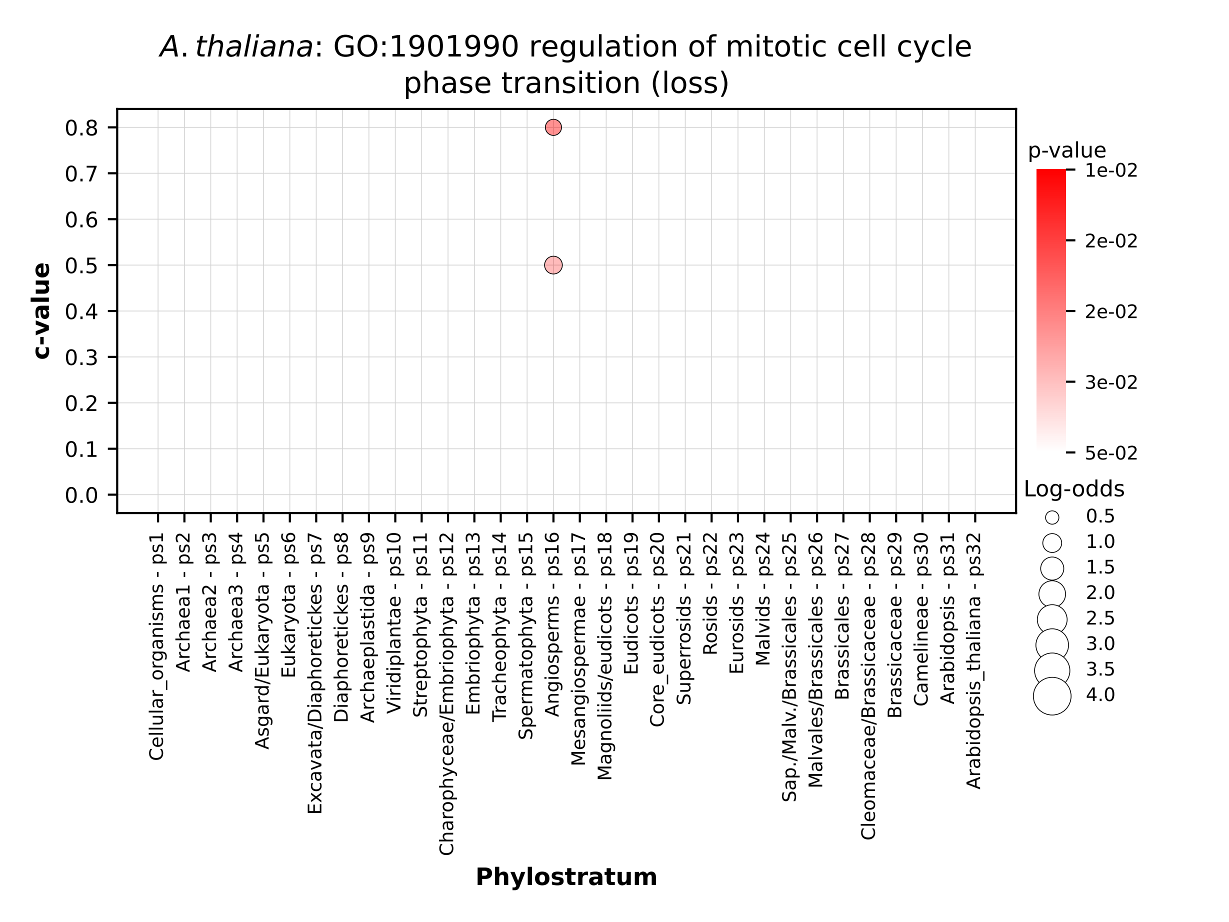

A. thaliana: GO:1901990 regulation of mitotic cell cycle phase transition (loss)
c-value
0.8
0.7
0.6
0.5
0.4
0.3
0.2
0.1
0.0
Phylostratum
Cellular_organisms - ps1
Archaea1 - ps2
Archaea2 - ps3
Archaea3 - ps4
Asgard/Eukaryota - ps5
Eukaryota - ps6
Excavata/Diaphoretickes - ps7
Diaphoretickes - ps8
Archaeplastida - ps9
Viridiplantae - ps10
Streptophyta - ps11
Charophyceae/Embriophyta - ps12
Embriophyta - ps13
Tracheophyta - ps14
Spermatophyta - ps15
Angiosperms - ps16
Mesangiospermae - ps17
Magnoliids/eudicots - ps18
Eudicots - ps19
Core_eudicots - ps20
Superrosids - ps21
Rosids - ps22
Eurosids - ps23
Malvids - ps24
Sap./Malv./Brassicales - ps25
Malvales/Brassicales - ps26
Brassicales - ps27
Cleomaceae/Brassicaceae - ps28
Brassicaceae - ps29
Camelineae - ps30
Arabidopsis - ps31
Arabidopsis_thaliana - ps32
p-value
1e-02
2e-02
2e-02
3e-02
5e-02
Log-odds
0.5
1.0
1.5
2.0
2.5
3.0
3.5
4.0

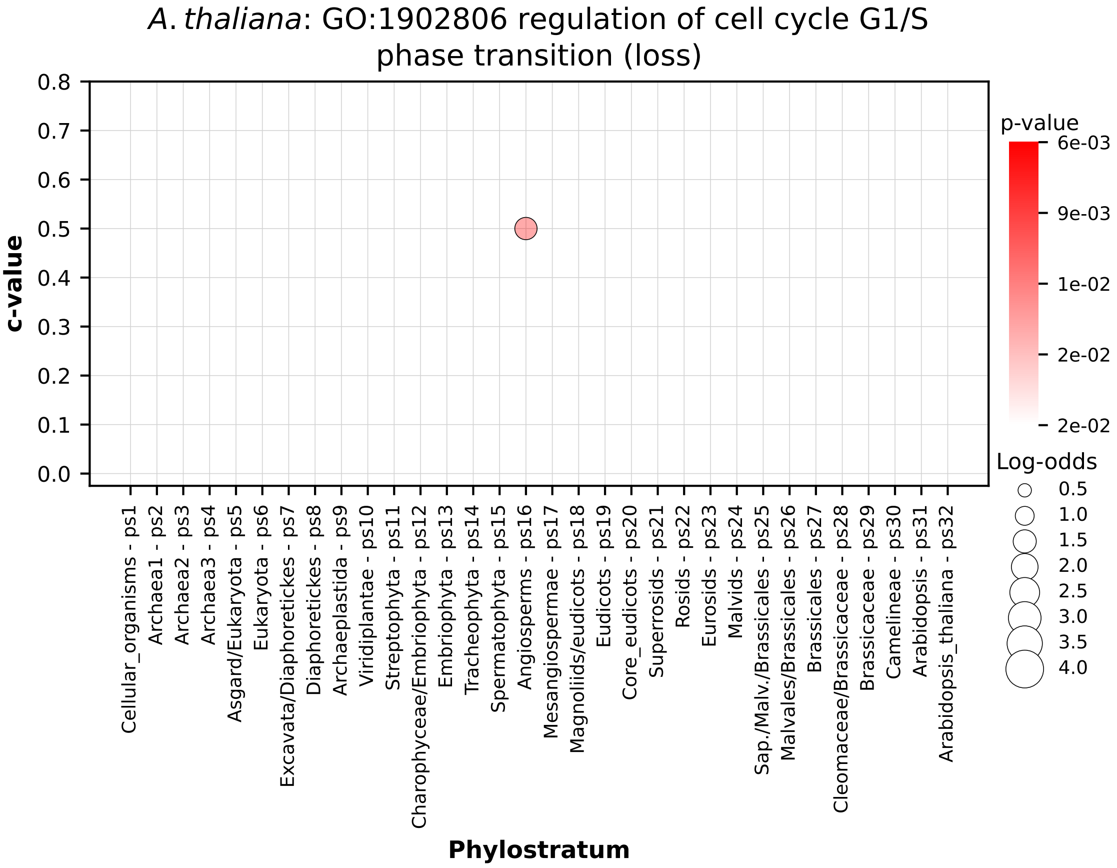
A. thaliana: GO:1902806 regulation of cell cycle G1/S phase transition (loss)
c-value
0.0
0.1
0.2
0.3
0.4
0.5
0.6
0.7
0.8
Phylostratum
Cellular_organisms - ps1
Archaea1 - ps2
Archaea2 - ps3
Archaea3 - ps4
Asgard/Eukaryota - ps5
Eukaryota - ps6
Excavata/Diaphoretickes - ps7
Diaphoretickes - ps8
Archaeplastida - ps9
Viridiplantae - ps10
Streptophyta - ps11
Charophyceae/Embriophyta - ps12
Embriophyta - ps13
Tracheophyta - ps14
Spermatophyta - ps15
Angiosperms - ps16
Mesangiospermae - ps17
Magnoliids/eudicots - ps18
Eudicots - ps19
Core_eudicots - ps20
Superrosids - ps21
Rosids - ps22
Eurosids - ps23
Malvids - ps24
Sap./Malv./Brassicales - ps25
Malvales/Brassicales - ps26
Brassicales - ps27
Cleomaceae/Brassicaceae - ps28
Brassicaceae - ps29
Camelineae - ps30
Arabidopsis - ps31
Arabidopsis_thaliana - ps32
p-value
6e-03
9e-03
1e-02
2e-02
2e-02
Log-odds
0.5
1.0
1.5
2.0
2.5
3.0
3.5
4.0

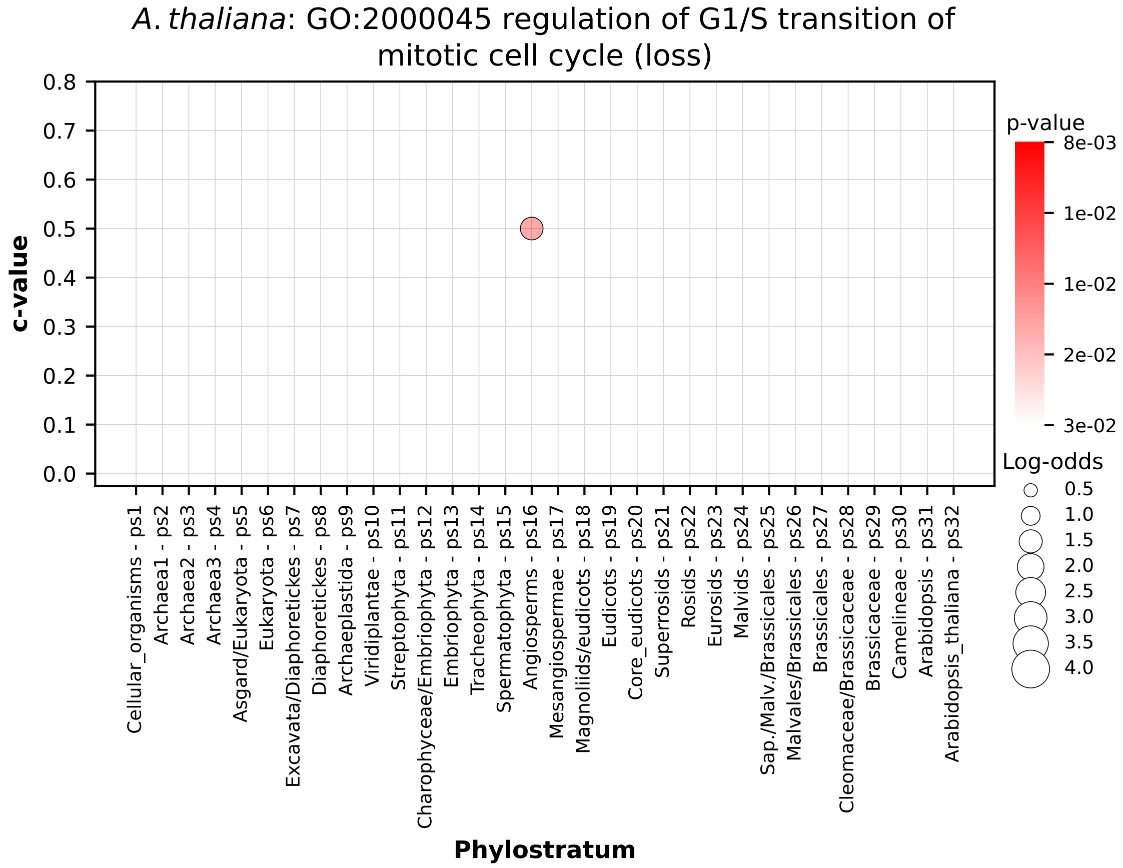

A. thaliana: GO:2000045 regulation of G1/S transition of mitotic cell cycle (loss)
c-value
0.8
0.7
0.6
0.5
0.4
0.3
0.2
0.1
0.0
Phylostratum
Cellular_organisms - ps1
Archaea1 - ps2
Archaea2 - ps3
Archaea3 - ps4
Asgard/Eukaryota - ps5
Eukaryota - ps6
Excavata/Diaphoretickes - ps7
Diaphoretickes - ps8
Archaeplastida - ps9
Viridiplantae - ps10
Streptophyta - ps11
Charophyceae/Embriophyta - ps12
Embriophyta - ps13
Tracheophyta - ps14
Spermatophyta - ps15
Angiosperms - ps16
Mesangiospermae - ps17
Magnoliids/eudicots - ps18
Eudicots - ps19
Core_eudicots - ps20
Superrosids - ps21
Rosids - ps22
Eurosids - ps23
Malvids - ps24
Sap./Malv./Brassicales - ps25
Malvales/Brassicales - ps26
Brassicales - ps27
Cleomaceae/Brassicaceae - ps28
Brassicaceae - ps29
Camelineae - ps30
Arabidopsis - ps31
Arabidopsis_thaliana - ps32
p-value
8e-03
1e-02
1e-02
2e-02
3e-02
Log-odds
0.5
1.0
1.5
2.0
2.5
3.0
3.5
4.0

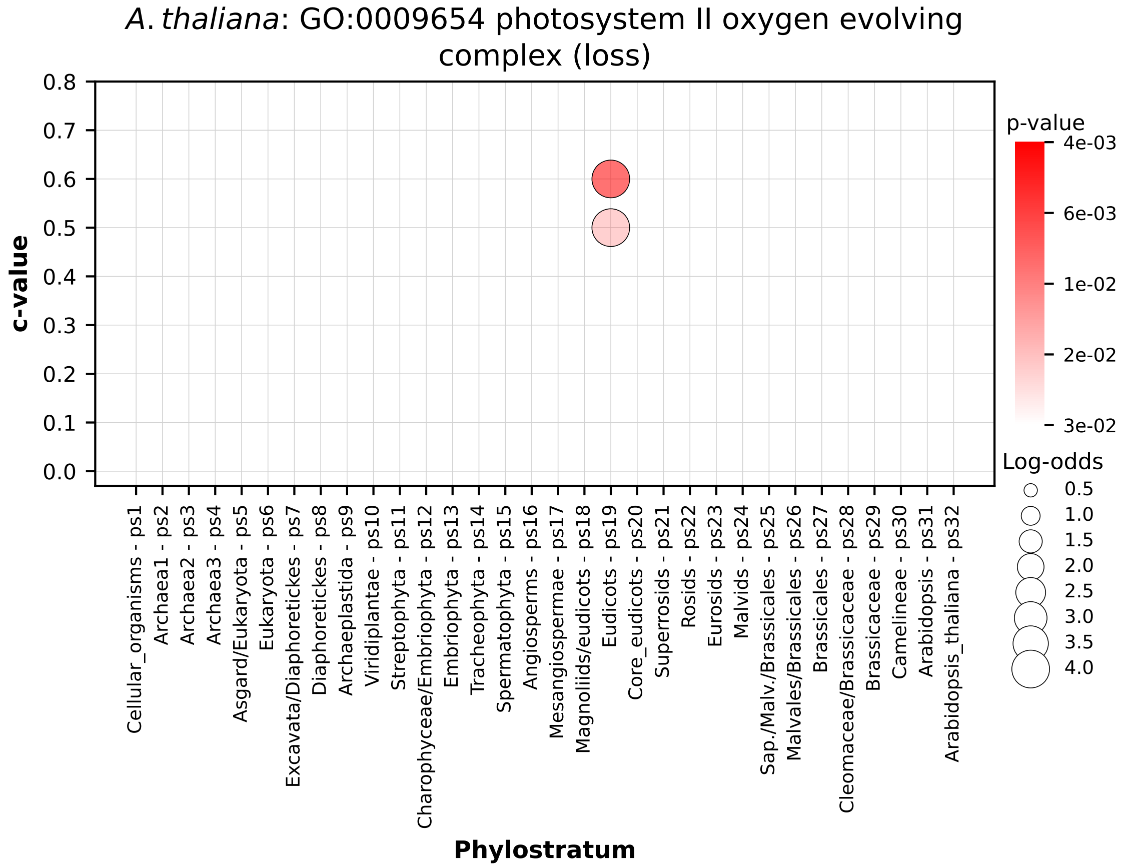

A. thaliana: GO:0009654 photosystem II oxygen evolving complex (loss)
c-value
0.8
0.7
0.6
0.5
0.4
0.3
0.2
0.1
0.0
Phylostratum
Cellular_organisms - ps1
Archaea1 - ps2
Archaea2 - ps3
Archaea3 - ps4
Asgard/Eukaryota - ps5
Eukaryota - ps6
Excavata/Diaphoretickes - ps7
Diaphoretickes - ps8
Archaeplastida - ps9
Viridiplantae - ps10
Streptophyta - ps11
Charophyceae/Embriophyta - ps12
Embriophyta - ps13
Tracheophyta - ps14
Spermatophyta - ps15
Angiosperms - ps16
Mesangiospermae - ps17
Magnoliids/eudicots - ps18
Eudicots - ps19
Core_eudicots - ps20
Superrosids - ps21
Rosids - ps22
Eurosids - ps23
Malvids - ps24
Sap./Malv./Brassicales - ps25
Malvales/Brassicales - ps26
Brassicales - ps27
Cleomaceae/Brassicaceae - ps28
Brassicaceae - ps29
Camelineae - ps30
Arabidopsis - ps31
Arabidopsis_thaliana - ps32
p-value
4e-03
6e-03
1e-02
2e-02
3e-02
Log-odds
0.5
1.0
1.5
2.0
2.5
3.0
3.5
4.0

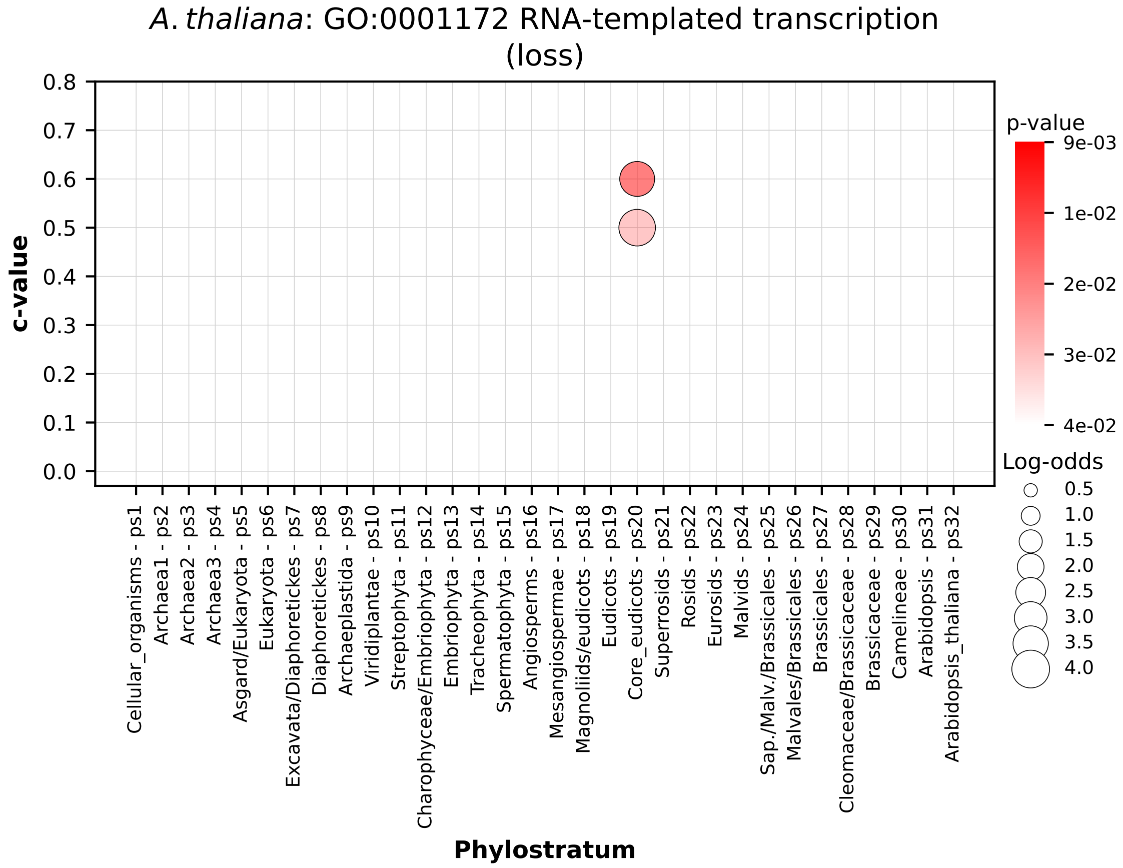

A. thaliana: GO:0001172 RNA-templated transcription
(loss)
c-value
0.8
0.7
0.6
0.5
0.4
0.3
0.2
0.1
0.0
p-value
9e-03
1e-02
2e-02
3e-02
4e-02
Log-odds
0.5
1.0
1.5
2.0
2.5
3.0
3.5
4.0
Cellular_organisms - ps1
Archaea1 - ps2
Archaea2 - ps3
Archaea3 - ps4
Asgard/Eukaryota - ps5
Eukaryota - ps6
Excavata/Diaphoretickes - ps7
Diaphoretickes - ps8
Archaeplastida - ps9
Viridiplantae - ps10
Streptophyta - ps11
Charophyceae/Embriophyta - ps12
Embriophyta - ps13
Tracheophyta - ps14
Spermatophyta - ps15
Angiosperms - ps16
Mesangiospermae - ps17
Magnoliids/eudicots - ps18
Eudicots - ps19
Core_eudicots - ps20
Superrosids - ps21
Rosids - ps22
Eurosids - ps23
Malvids - ps24
Sap./Malv./Brassicales - ps25
Malvales/Brassicales - ps26
Brassicales - ps27
Cleomaceae/Brassicaceae - ps28
Brassicaceae - ps29
Camelineae - ps30
Arabidopsis - ps31
Arabidopsis_thaliana - ps32
Phylostratum

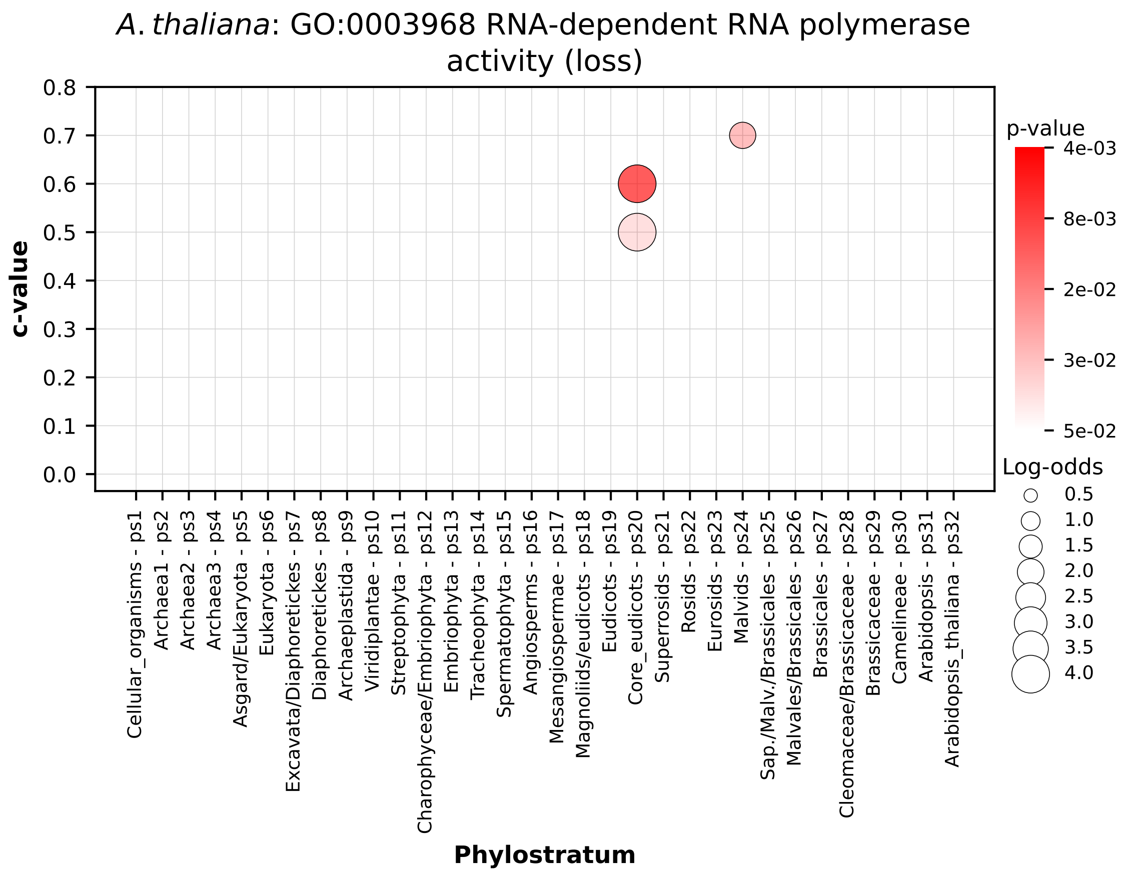

A. thaliana: GO:0003968 RNA-dependent RNA polymerase activity (loss)
c-value
0.8
0.7
0.6
0.5
0.4
0.3
0.2
0.1
0.0
Phylostratum
Cellular_organisms - ps1
Archaea1 - ps2
Archaea2 - ps3
Archaea3 - ps4
Asgard/Eukaryota - ps5
Eukaryota - ps6
Excavata/Diaphoretickes - ps7
Diaphoretickes - ps8
Archaeplastida - ps9
Viridiplantae - ps10
Streptophyta - ps11
Charophyceae/Embriophyta - ps12
Embriophyta - ps13
Tracheophyta - ps14
Spermatophyta - ps15
Angiosperms - ps16
Mesangiospermae - ps17
Magnoliids/eudicots - ps18
Eudicots - ps19
Core_eudicots - ps20
Superrosids - ps21
Rosids - ps22
Eurosids - ps23
Malvids - ps24
Sap./Malv./Brassicales - ps25
Malvales/Brassicales - ps26
Brassicales - ps27
Cleomaceae/Brassicaceae - ps28
Brassicaceae - ps29
Camelineae - ps30
Arabidopsis - ps31
Arabidopsis_thaliana - ps32
p-value
4e-03
8e-03
2e-02
3e-02
5e-02
Log-odds
0.5
1.0
1.5
2.0
2.5
3.0
3.5
4.0

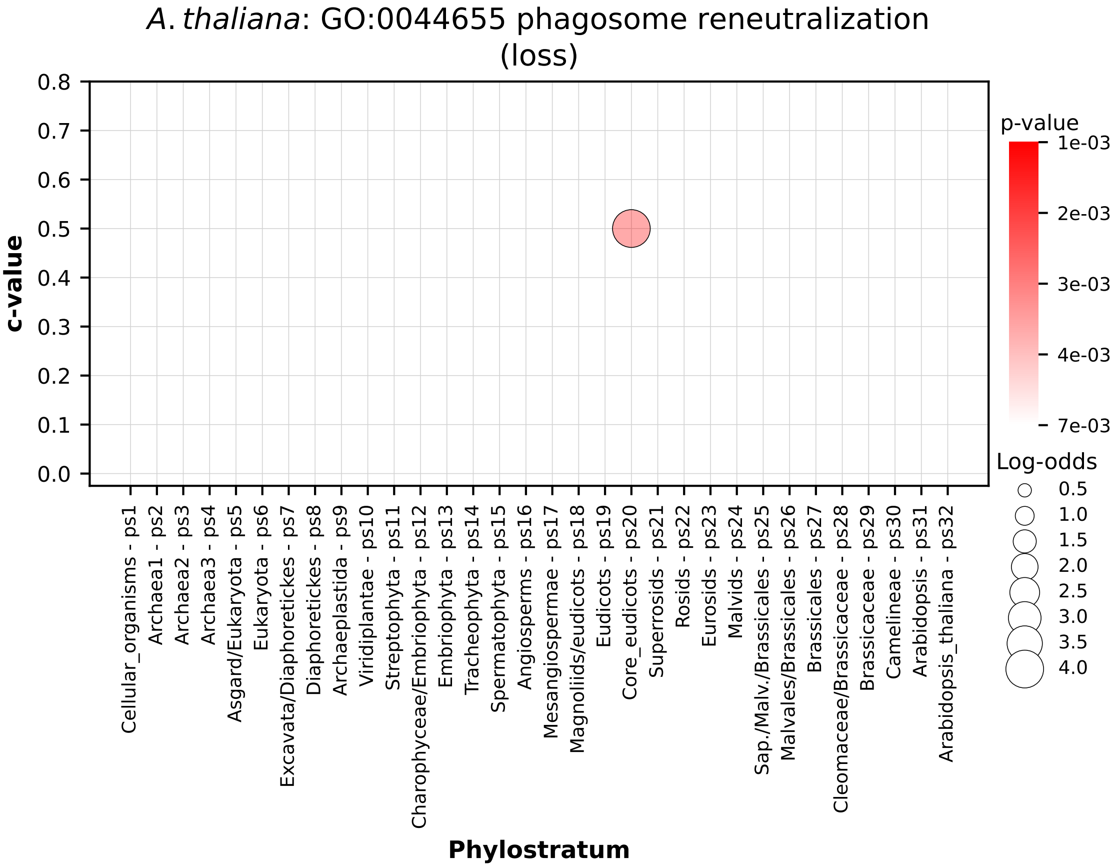

A. thaliana: GO:0044655 phagosome reneutralization
(loss)
c-value
0.8
0.7
0.6
0.5
0.4
0.3
0.2
0.1
0.0
p-value
1e-03
2e-03
3e-03
4e-03
7e-03
Log-odds
0.5
1.0
1.5
2.0
2.5
3.0
3.5
4.0
Cellular_organisms - ps1
Archaea1 - ps2
Archaea2 - ps3
Archaea3 - ps4
Asgard/Eukaryota - ps5
Eukaryota - ps6
Excavata/Diaphoretickes - ps7
Diaphoretickes - ps8
Archaeplastida - ps9
Viridiplantae - ps10
Streptophyta - ps11
Charophyceae/Embriophyta - ps12
Embriophyta - ps13
Tracheophyta - ps14
Spermatophyta - ps15
Angiosperms - ps16
Mesangiospermae - ps17
Magnoliids/eudicots - ps18
Eudicots - ps19
Core_eudicots - ps20
Superrosids - ps21
Rosids - ps22
Eurosids - ps23
Malvids - ps24
Sap./Malv./Brassicales - ps25
Malvales/Brassicales - ps26
Brassicales - ps27
Cleomaceae/Brassicaceae - ps28
Brassicaceae - ps29
Camelineae - ps30
Arabidopsis - ps31
Arabidopsis_thaliana - ps32
Phylostratum

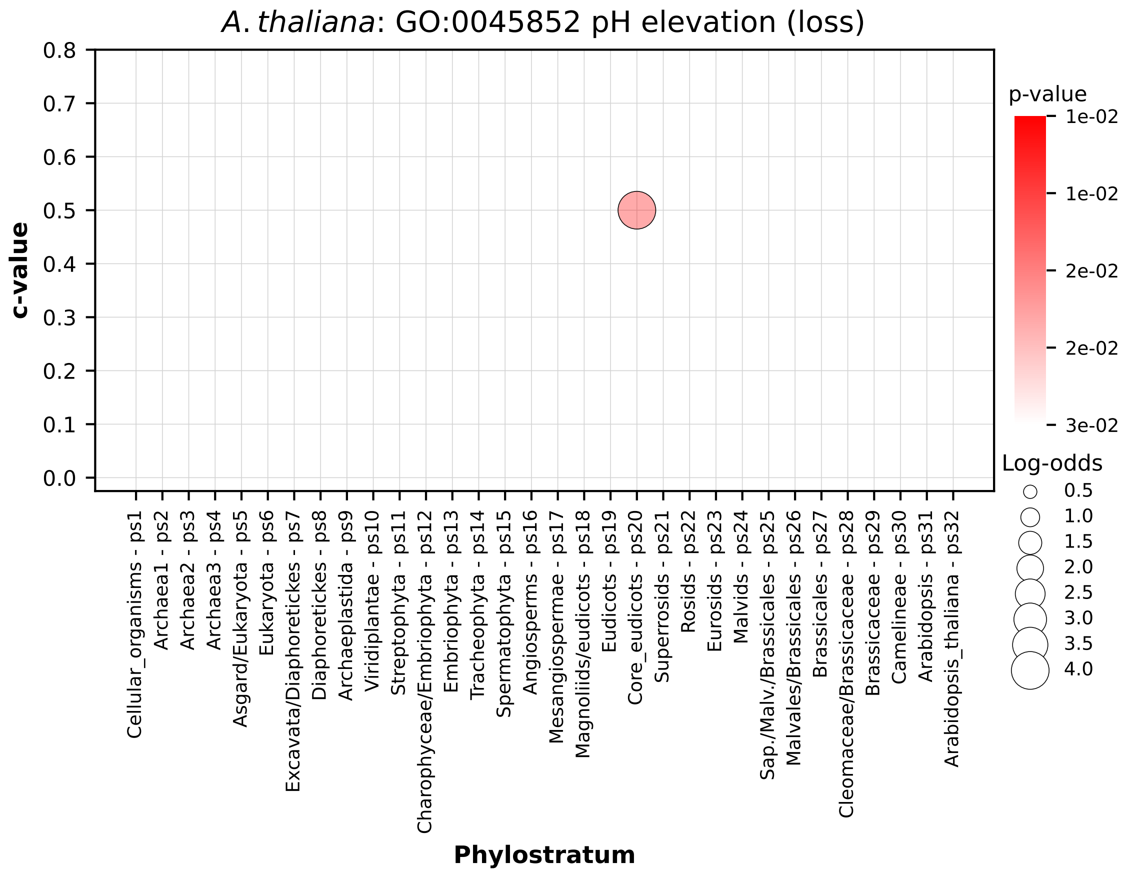

A. thaliana: GO:0045852 pH elevation (loss)
c-value
0.8
0.7
0.6
0.5
0.4
0.3
0.2
0.1
0.0
Phylostratum
Cellular_organisms - ps1
Archaea1 - ps2
Archaea2 - ps3
Archaea3 - ps4
Asgard/Eukaryota - ps5
Eukaryota - ps6
Excavata/Diaphoretickes - ps7
Diaphoretickes - ps8
Archaeplastida - ps9
Viridiplantae - ps10
Streptophyta - ps11
Charophyceae/Embriophyta - ps12
Embriophyta - ps13
Tracheophyta - ps14
Spermatophyta - ps15
Angiosperms - ps16
Mesangiospermae - ps17
Magnoliids/eudicots - ps18
Eudicots - ps19
Core_eudicots - ps20
Superrosids - ps21
Rosids - ps22
Eurosids - ps23
Malvids - ps24
Sap./Malv./Brassicales - ps25
Malvales/Brassicales - ps26
Brassicales - ps27
Cleomaceae/Brassicaceae - ps28
Brassicaceae - ps29
Camelineae - ps30
Arabidopsis - ps31
Arabidopsis_thaliana - ps32
p-value
1e-02
1e-02
2e-02
2e-02
3e-02
Log-odds
0.5
1.0
1.5
2.0
2.5
3.0
3.5
4.0

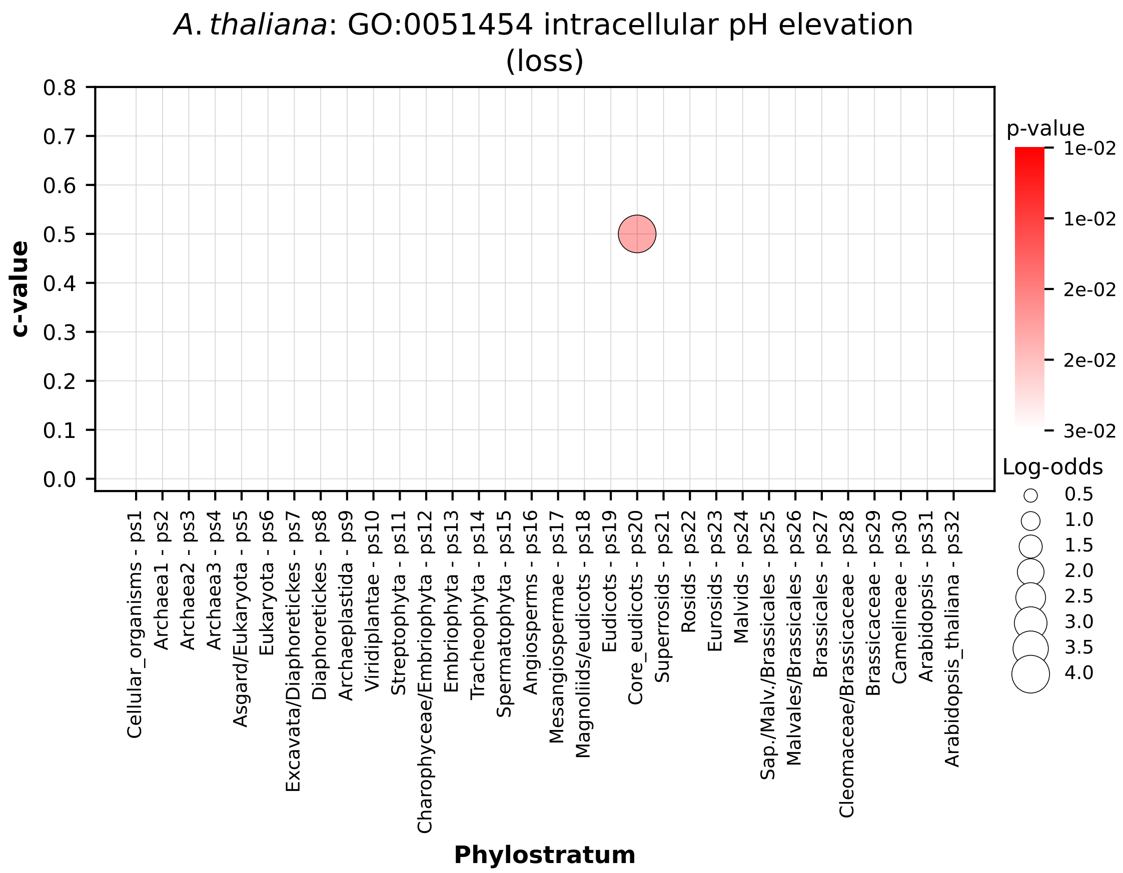

A. thaliana: GO:0051454 intracellular pH elevation
(loss)
c-value
0.8
0.7
0.6
0.5
0.4
0.3
0.2
0.1
0.0
p-value
1e-02
1e-02
2e-02
2e-02
3e-02
Log-odds
0.5
1.0
1.5
2.0
2.5
3.0
3.5
4.0
Cellular_organisms - ps1
Archaea1 - ps2
Archaea2 - ps3
Archaea3 - ps4
Asgard/Eukaryota - ps5
Eukaryota - ps6
Excavata/Diaphoretickes - ps7
Diaphoretickes - ps8
Archaeplastida - ps9
Viridiplantae - ps10
Streptophyta - ps11
Charophyceae/Embriophyta - ps12
Embriophyta - ps13
Tracheophyta - ps14
Spermatophyta - ps15
Angiosperms - ps16
Mesangiospermae - ps17
Magnoliids/eudicots - ps18
Eudicots - ps19
Core_eudicots - ps20
Superrosids - ps21
Rosids - ps22
Eurosids - ps23
Malvids - ps24
Sap./Malv./Brassicales - ps25
Malvales/Brassicales - ps26
Brassicales - ps27
Cleomaceae/Brassicaceae - ps28
Brassicaceae - ps29
Camelineae - ps30
Arabidopsis - ps31
Arabidopsis_thaliana - ps32
Phylostratum

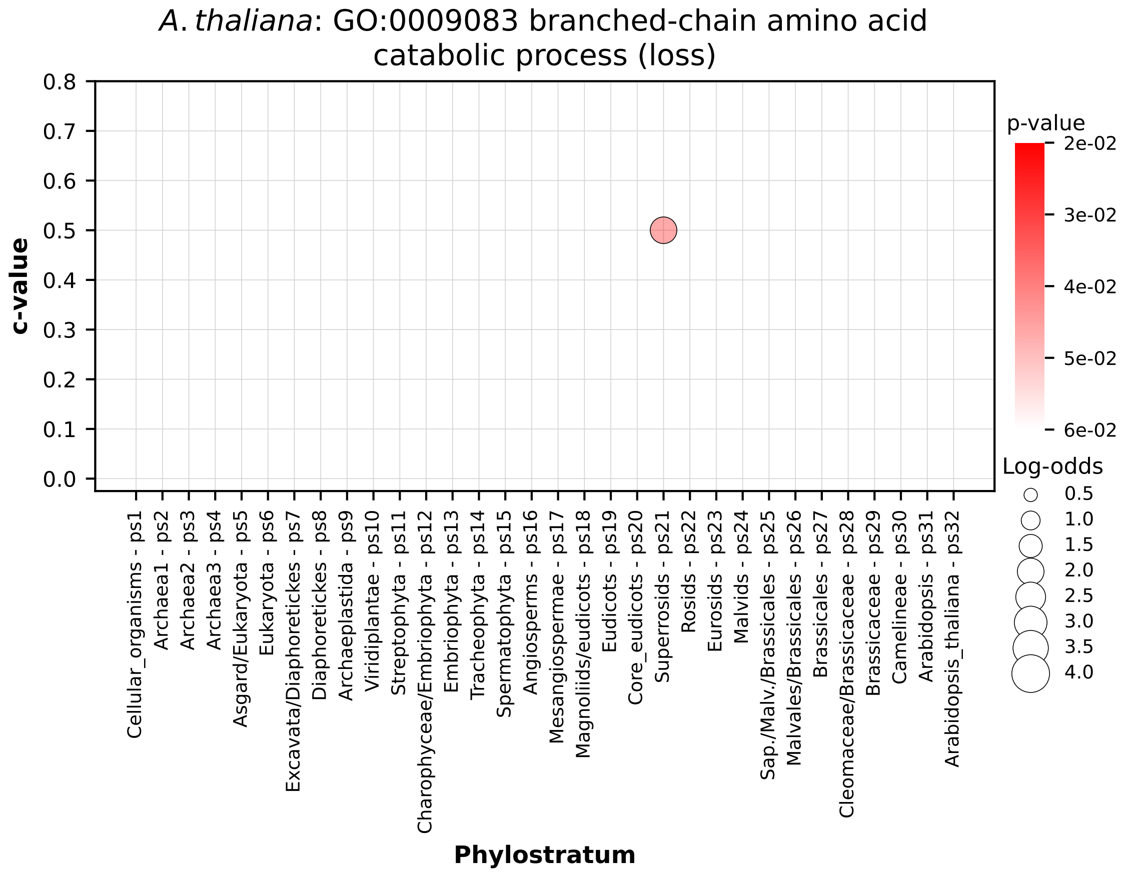

A. thaliana: GO:0009083 branched-chain amino acid catabolic process (loss)
c-value
0.8
0.7
0.6
0.5
0.4
0.3
0.2
0.1
0.0
Phylostratum
Cellular_organisms - ps1
Archaea1 - ps2
Archaea2 - ps3
Archaea3 - ps4
Asgard/Eukaryota - ps5
Eukaryota - ps6
Excavata/Diaphoretickes - ps7
Diaphoretickes - ps8
Archaeplastida - ps9
Viridiplantae - ps10
Streptophyta - ps11
Charophyceae/Embriophyta - ps12
Embriophyta - ps13
Tracheophyta - ps14
Spermatophyta - ps15
Angiosperms - ps16
Mesangiospermae - ps17
Magnoliids/eudicots - ps18
Eudicots - ps19
Core_eudicots - ps20
Superrosids - ps21
Rosids - ps22
Eurosids - ps23
Malvids - ps24
Sap./Malv./Brassicales - ps25
Malvales/Brassicales - ps26
Brassicales - ps27
Cleomaceae/Brassicaceae - ps28
Brassicaceae - ps29
Camelineae - ps30
Arabidopsis - ps31
Arabidopsis_thaliana - ps32
p-value
2e-02
3e-02
4e-02
5e-02
6e-02
Log-odds
0.5
1.0
1.5
2.0
2.5
3.0
3.5
4.0

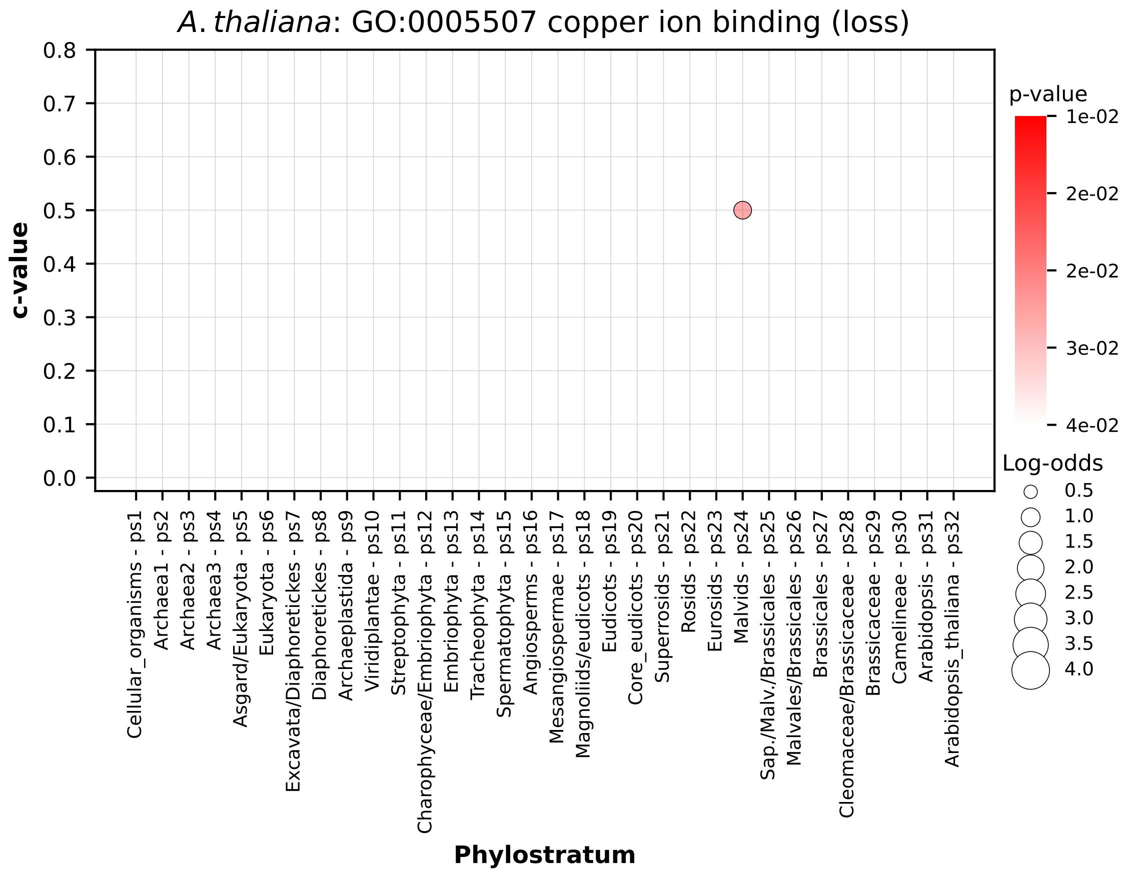

A. thaliana: GO:0005507 copper ion binding (loss)
c-value
0.0
0.1
0.2
0.3
0.4
0.5
0.6
0.7
0.8
p-value
1e-02
2e-02
2e-02
3e-02
4e-02
Log-odds
0.5
1.0
1.5
2.0
2.5
3.0
3.5
4.0
Cellular_organisms - ps1
Archaea1 - ps2
Archaea2 - ps3
Archaea3 - ps4
Asgard/Eukaryota - ps5
Eukaryota - ps6
Excavata/Diaphoretickes - ps7
Diaphoretickes - ps8
Archaeplastida - ps9
Viridiplantae - ps10
Streptophyta - ps11
Charophyceae/Embriophyta - ps12
Embriophyta - ps13
Tracheophyta - ps14
Spermatophyta - ps15
Angiosperms - ps16
Mesangiospermae - ps17
Magnoliids/eudicots - ps18
Eudicots - ps19
Core_eudicots - ps20
Superrosids - ps21
Rosids - ps22
Eurosids - ps23
Malvids - ps24
Sap./Malv./Brassicales - ps25
Malvales/Brassicales - ps26
Brassicales - ps27
Cleomaceae/Brassicaceae - ps28
Brassicaceae - ps29
Camelineae - ps30
Arabidopsis - ps31
Arabidopsis_thaliana - ps32
Phylostratum

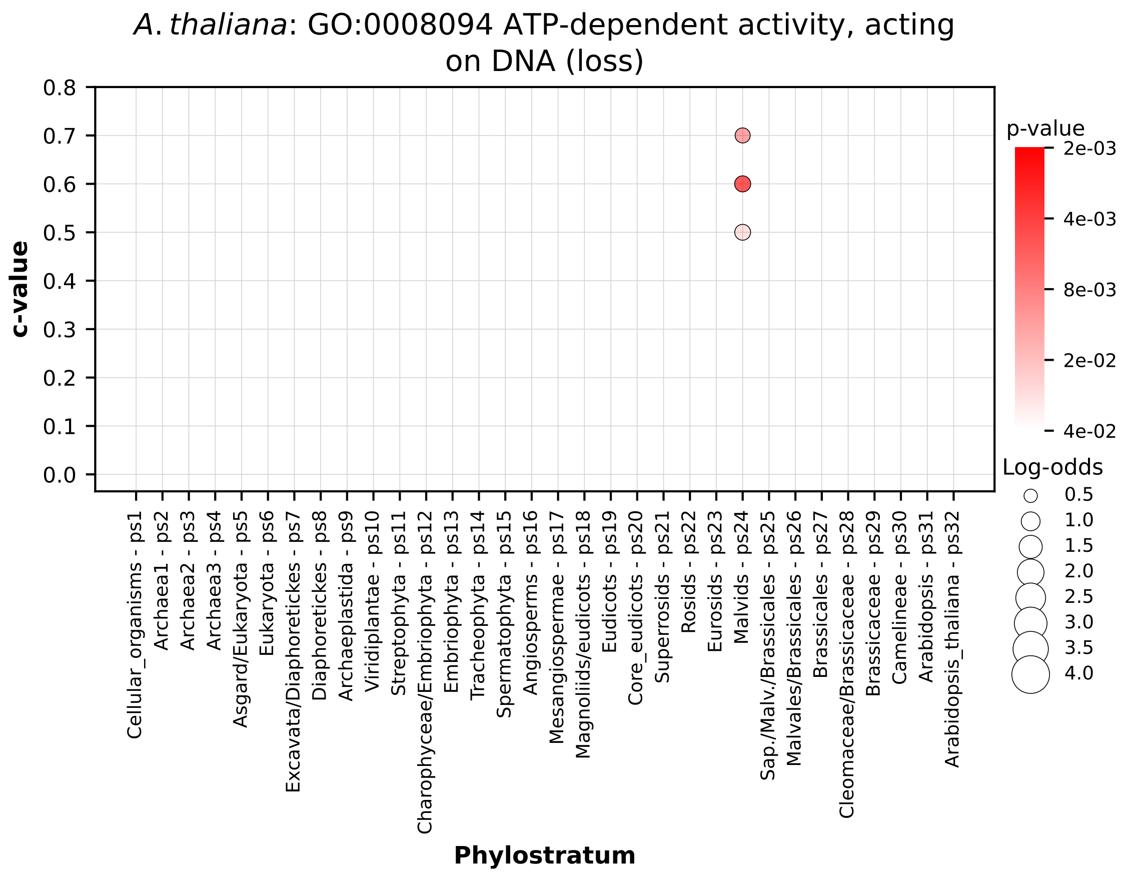

A. thaliana: GO:0008094 ATP-dependent activity, acting on DNA (loss)
c-value
0.8
0.7
0.6
0.5
0.4
0.3
0.2
0.1
0.0
p-value
2e-03
4e-03
8e-03
2e-02
4e-02
Log-odds
0.5
1.0
1.5
2.0
2.5
3.0
3.5
4.0
Cellular_organisms - ps1
Archaea1 - ps2
Archaea2 - ps3
Archaea3 - ps4
Asgard/Eukaryota - ps5
Eukaryota - ps6
Excavata/Diaphoretickes - ps7
Diaphoretickes - ps8
Archaeplastida - ps9
Viridiplantae - ps10
Streptophyta - ps11
Charophyceae/Embriophyta - ps12
Embriophyta - ps13
Tracheophyta - ps14
Spermatophyta - ps15
Angiosperms - ps16
Mesangiospermae - ps17
Magnoliids/eudicots - ps18
Eudicots - ps19
Core_eudicots - ps20
Superrosids - ps21
Rosids - ps22
Eurosids - ps23
Malvids - ps24
Sap./Malv./Brassicales - ps25
Malvales/Brassicales - ps26
Brassicales - ps27
Cleomaceae/Brassicaceae - ps28
Brassicaceae - ps29
Camelineae - ps30
Arabidopsis - ps31
Arabidopsis_thaliana - ps32
Phylostratum

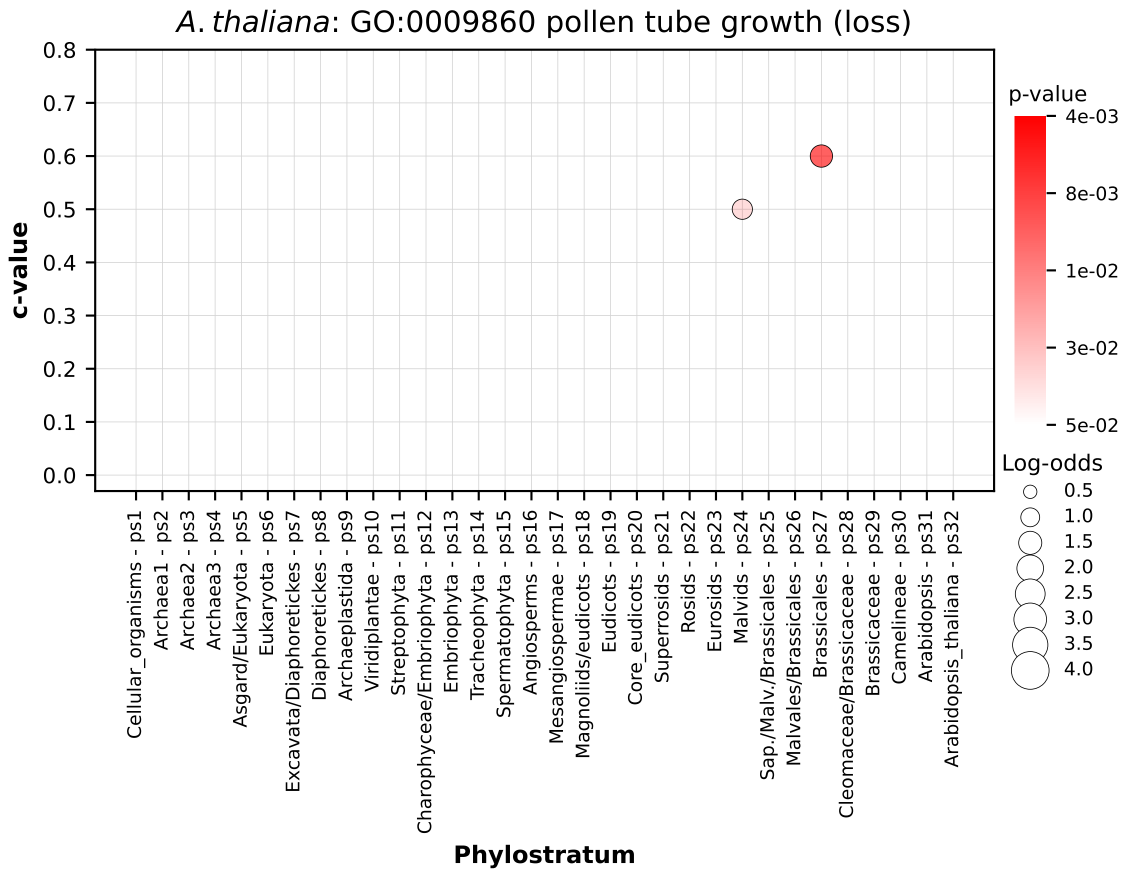

A. thaliana: GO:0009860 pollen tube growth (loss)
c-value
0.0
0.1
0.2
0.3
0.4
0.5
0.6
0.7
0.8
Phylostratum
Cellular_organisms - ps1
Archaea1 - ps2
Archaea2 - ps3
Archaea3 - ps4
Asgard/Eukaryota - ps5
Eukaryota - ps6
Excavata/Diaphoretickes - ps7
Diaphoretickes - ps8
Archaeplastida - ps9
Viridiplantae - ps10
Streptophyta - ps11
Charophyceae/Embriophyta - ps12
Embriophyta - ps13
Tracheophyta - ps14
Spermatophyta - ps15
Angiosperms - ps16
Mesangiospermae - ps17
Magnoliids/eudicots - ps18
Eudicots - ps19
Core_eudicots - ps20
Superrosids - ps21
Rosids - ps22
Eurosids - ps23
Malvids - ps24
Sap./Malv./Brassicales - ps25
Malvales/Brassicales - ps26
Brassicales - ps27
Cleomaceae/Brassicaceae - ps28
Brassicaceae - ps29
Camelineae - ps30
Arabidopsis - ps31
Arabidopsis_thaliana - ps32
p-value
4e-03
8e-03
1e-02
3e-02
5e-02
Log-odds
0.5
1.0
1.5
2.0
2.5
3.0
3.5
4.0

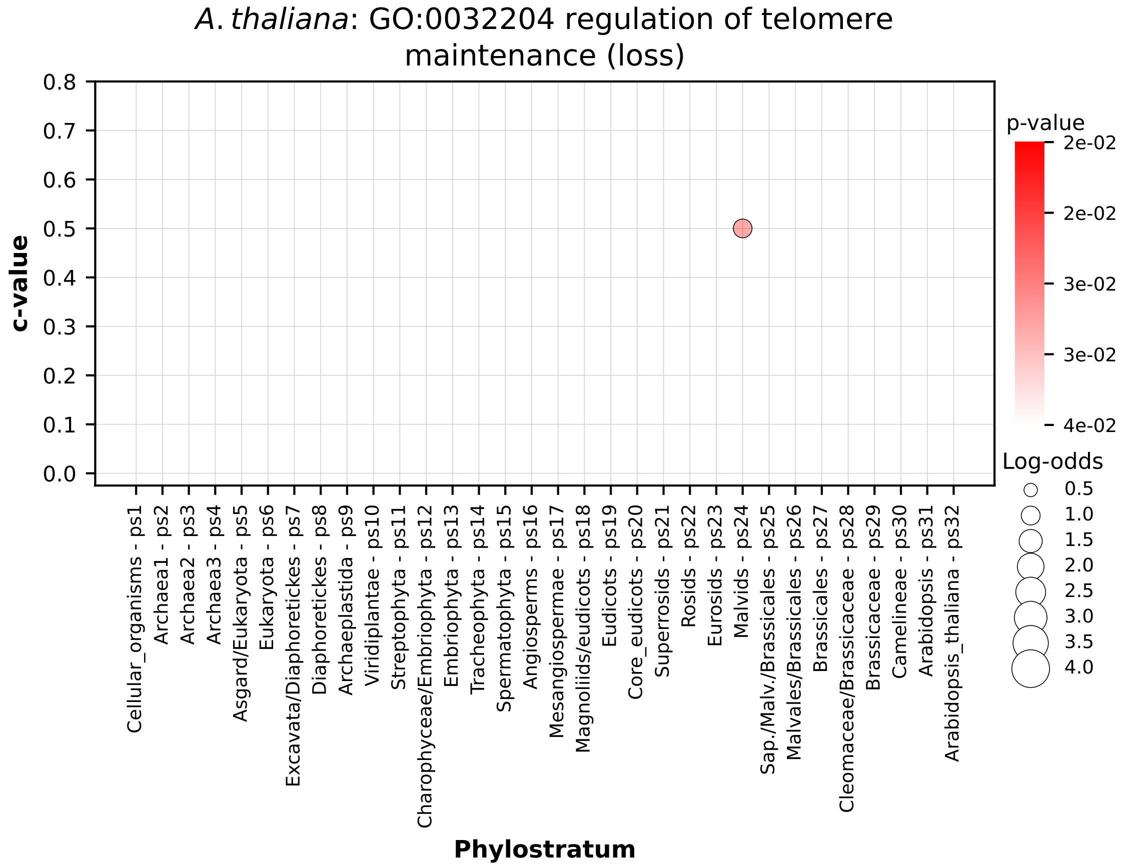

A. thaliana: GO:0032204 regulation of telomere maintenance (loss)
c-value
0.8
0.7
0.6
0.5
0.4
0.3
0.2
0.1
0.0
Phylostratum
Cellular_organisms - ps1
Archaea1 - ps2
Archaea2 - ps3
Archaea3 - ps4
Asgard/Eukaryota - ps5
Eukaryota - ps6
Excavata/Diaphoretickes - ps7
Diaphoretickes - ps8
Archaeplastida - ps9
Viridiplantae - ps10
Streptophyta - ps11
Charophyceae/Embriophyta - ps12
Embriophyta - ps13
Tracheophyta - ps14
Spermatophyta - ps15
Angiosperms - ps16
Mesangiospermae - ps17
Magnoliids/eudicots - ps18
Eudicots - ps19
Core_eudicots - ps20
Superrosids - ps21
Rosids - ps22
Eurosids - ps23
Malvids - ps24
Sap./Malv./Brassicales - ps25
Malvales/Brassicales - ps26
Brassicales - ps27
Cleomaceae/Brassicaceae - ps28
Brassicaceae - ps29
Camelineae - ps30
Arabidopsis - ps31
Arabidopsis_thaliana - ps32
p-value
2e-02
2e-02
3e-02
3e-02
4e-02
Log-odds
0.5
1.0
1.5
2.0
2.5
3.0
3.5
4.0

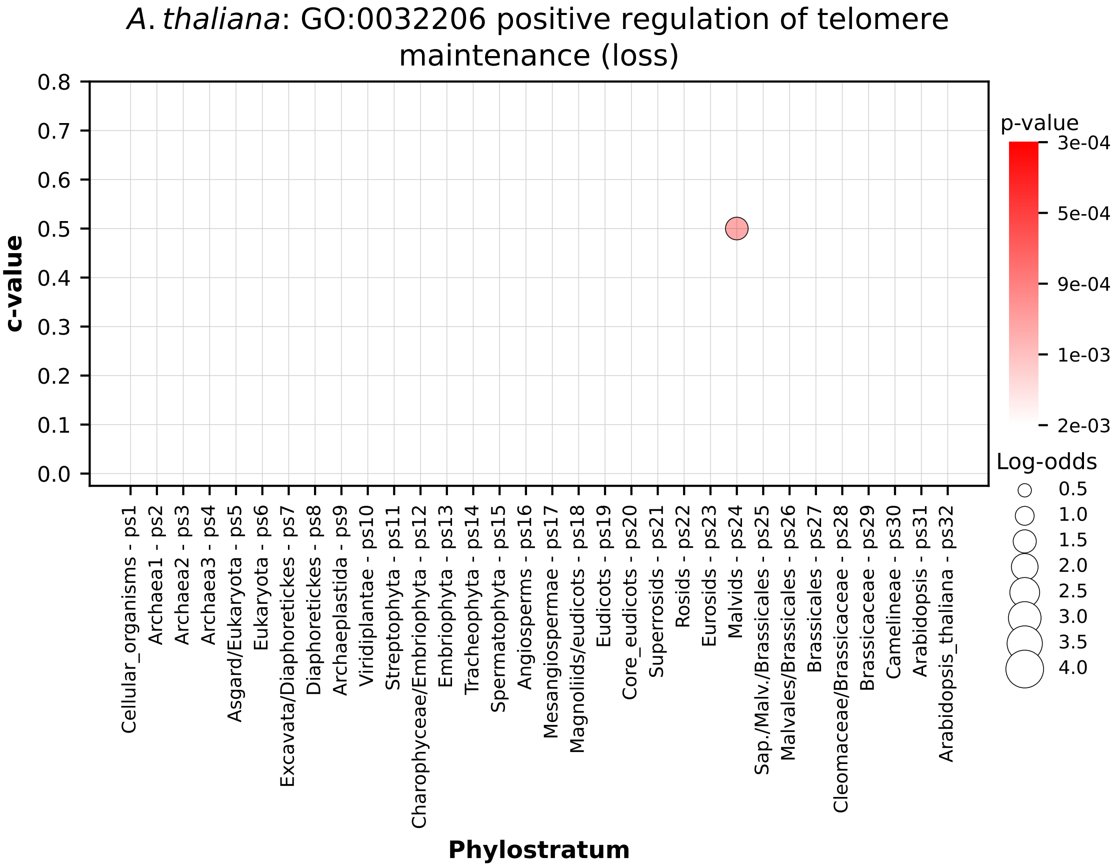

A. thaliana: GO:0032206 positive regulation of telomere maintenance (loss)
c-value
0.8
0.7
0.6
0.5
0.4
0.3
0.2
0.1
0.0
Phylostratum
Cellular_organisms - ps1
Archaea1 - ps2
Archaea2 - ps3
Archaea3 - ps4
Asgard/Eukaryota - ps5
Eukaryota - ps6
Excavata/Diaphoretickes - ps7
Diaphoretickes - ps8
Archaeplastida - ps9
Viridiplantae - ps10
Streptophyta - ps11
Charophyceae/Embriophyta - ps12
Embriophyta - ps13
Tracheophyta - ps14
Spermatophyta - ps15
Angiosperms - ps16
Mesangiospermae - ps17
Magnoliids/eudicots - ps18
Eudicots - ps19
Core_eudicots - ps20
Superrosids - ps21
Rosids - ps22
Eurosids - ps23
Malvids - ps24
Sap./Malv./Brassicales - ps25
Malvales/Brassicales - ps26
Brassicales - ps27
Cleomaceae/Brassicaceae - ps28
Brassicaceae - ps29
Camelineae - ps30
Arabidopsis - ps31
Arabidopsis_thaliana - ps32
p-value
3e-04
5e-04
9e-04
1e-03
2e-03
Log-odds
0.5
1.0
1.5
2.0
2.5
3.0
3.5
4.0

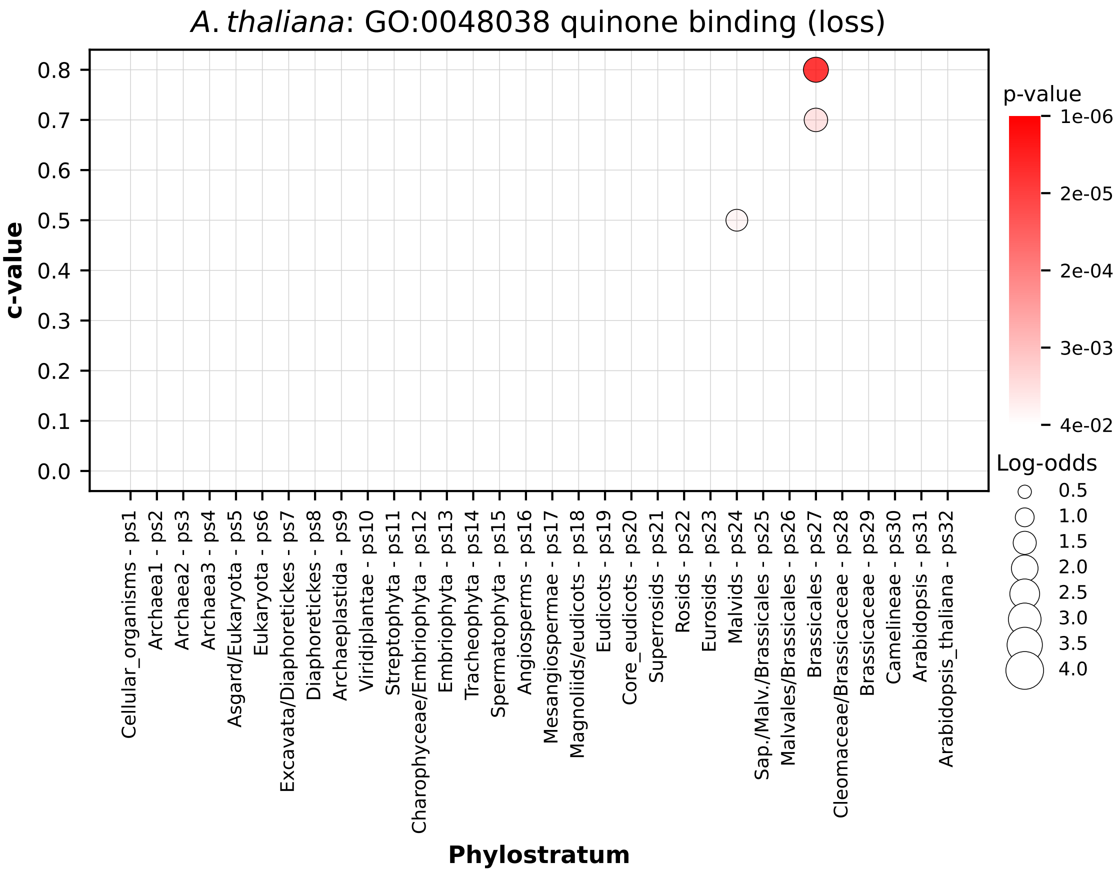
A. thaliana: GO:0048038 quinone binding (loss)
c-value
0.0
0.1
0.2
0.3
0.4
0.5
0.6
0.7
0.8
Phylostratum
Cellular_organisms - ps1
Archaea1 - ps2
Archaea2 - ps3
Archaea3 - ps4
Asgard/Eukaryota - ps5
Eukaryota - ps6
Excavata/Diaphoretickes - ps7
Diaphoretickes - ps8
Archaeplastida - ps9
Viridiplantae - ps10
Streptophyta - ps11
Charophyceae/Embriophyta - ps12
Embriophyta - ps13
Tracheophyta - ps14
Spermatophyta - ps15
Angiosperms - ps16
Mesangiospermae - ps17
Magnoliids/eudicots - ps18
Eudicots - ps19
Core_eudicots - ps20
Superrosids - ps21
Rosids - ps22
Eurosids - ps23
Malvids - ps24
Sap./Malv./Brassicales - ps25
Malvales/Brassicales - ps26
Brassicales - ps27
Cleomaceae/Brassicaceae - ps28
Brassicaceae - ps29
Camelineae - ps30
Arabidopsis - ps31
Arabidopsis_thaliana - ps32
p-value
1e-06
2e-05
2e-04
3e-03
4e-02
Log-odds
0.5
1.0
1.5
2.0
2.5
3.0
3.5
4.0

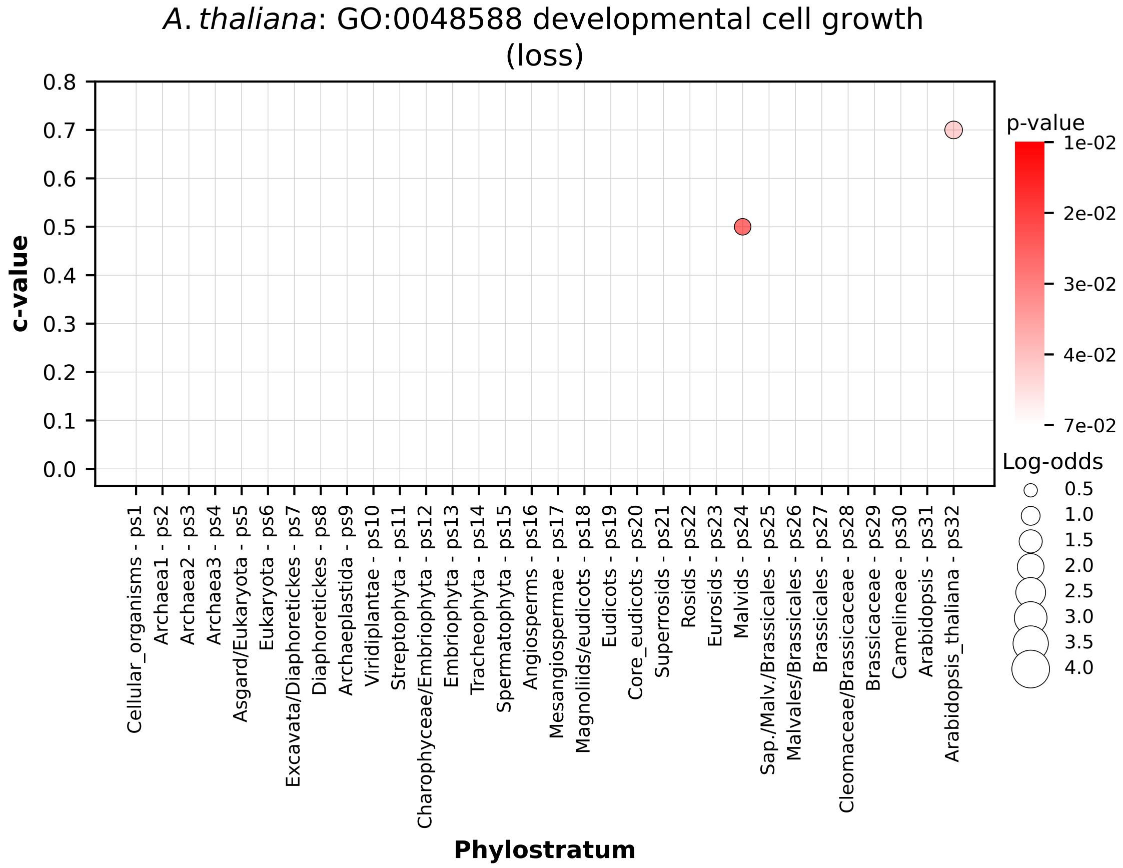

A. thaliana: GO:0048588 developmental cell growth
(loss)
c-value
0.8
0.7
0.6
0.5
0.4
0.3
0.2
0.1
0.0
Phylostratum
Cellular_organisms - ps1
Archaea1 - ps2
Archaea2 - ps3
Archaea3 - ps4
Asgard/Eukaryota - ps5
Eukaryota - ps6
Excavata/Diaphoretickes - ps7
Diaphoretickes - ps8
Archaeplastida - ps9
Viridiplantae - ps10
Streptophyta - ps11
Charophyceae/Embriophyta - ps12
Embriophyta - ps13
Tracheophyta - ps14
Spermatophyta - ps15
Angiosperms - ps16
Mesangiospermae - ps17
Magnoliids/eudicots - ps18
Eudicots - ps19
Core_eudicots - ps20
Superrosids - ps21
Rosids - ps22
Eurosids - ps23
Malvids - ps24
Sap./Malv./Brassicales - ps25
Malvales/Brassicales - ps26
Brassicales - ps27
Cleomaceae/Brassicaceae - ps28
Brassicaceae - ps29
Camelineae - ps30
Arabidopsis - ps31
Arabidopsis_thaliana - ps32
p-value
1e-02
2e-02
3e-02
4e-02
7e-02
Log-odds
0.5
1.0
1.5
2.0
2.5
3.0
3.5
4.0

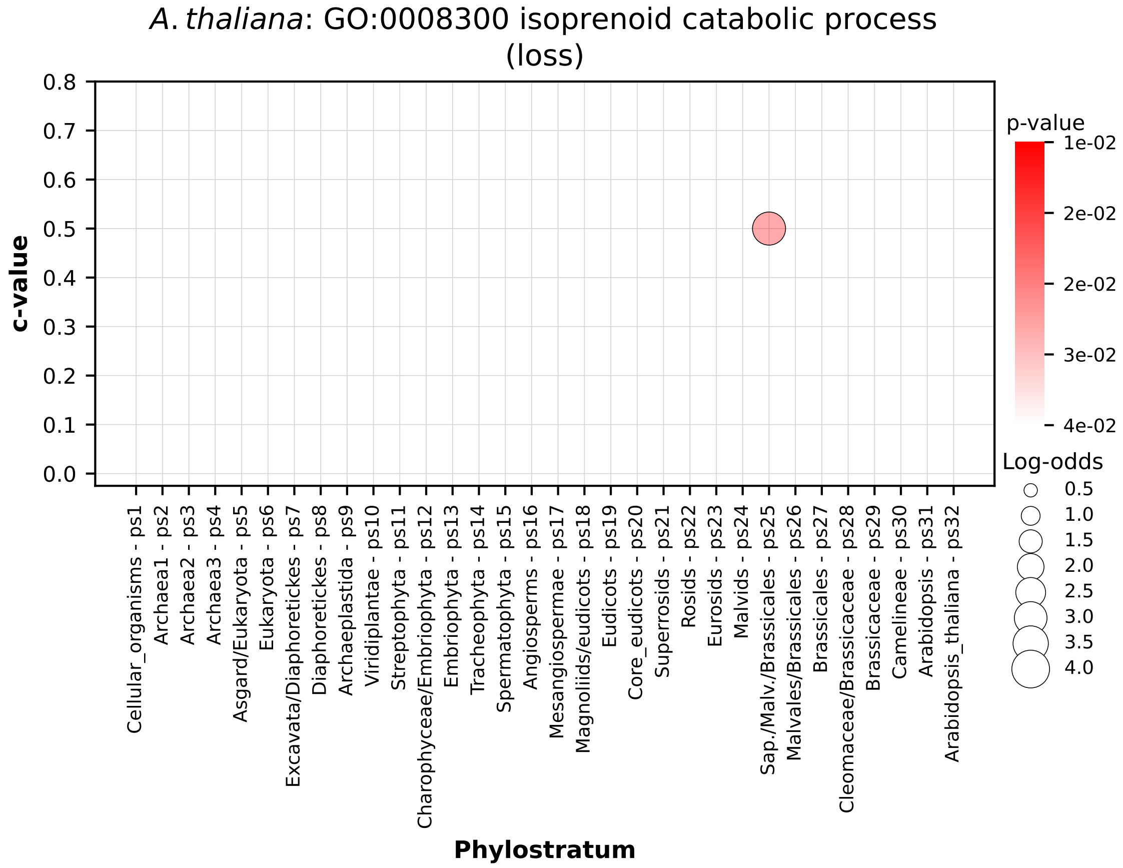

A. thaliana: GO:0008300 isoprenoid catabolic process
(loss)
c-value
0.8
0.7
0.6
0.5
0.4
0.3
0.2
0.1
0.0
p-value
1e-02
2e-02
2e-02
3e-02
4e-02
Log-odds
0.5
1.0
1.5
2.0
2.5
3.0
3.5
4.0
Cellular_organisms - ps1
Archaea1 - ps2
Archaea2 - ps3
Archaea3 - ps4
Asgard/Eukaryota - ps5
Eukaryota - ps6
Excavata/Diaphoretickes - ps7
Diaphoretickes - ps8
Archaeplastida - ps9
Viridiplantae - ps10
Streptophyta - ps11
Charophyceae/Embriophyta - ps12
Embriophyta - ps13
Tracheophyta - ps14
Spermatophyta - ps15
Angiosperms - ps16
Mesangiospermae - ps17
Magnoliids/eudicots - ps18
Eudicots - ps19
Core_eudicots - ps20
Superrosids - ps21
Rosids - ps22
Eurosids - ps23
Malvids - ps24
Sap./Malv./Brassicales - ps25
Malvales/Brassicales - ps26
Brassicales - ps27
Cleomaceae/Brassicaceae - ps28
Brassicaceae - ps29
Camelineae - ps30
Arabidopsis - ps31
Arabidopsis_thaliana - ps32
Phylostratum

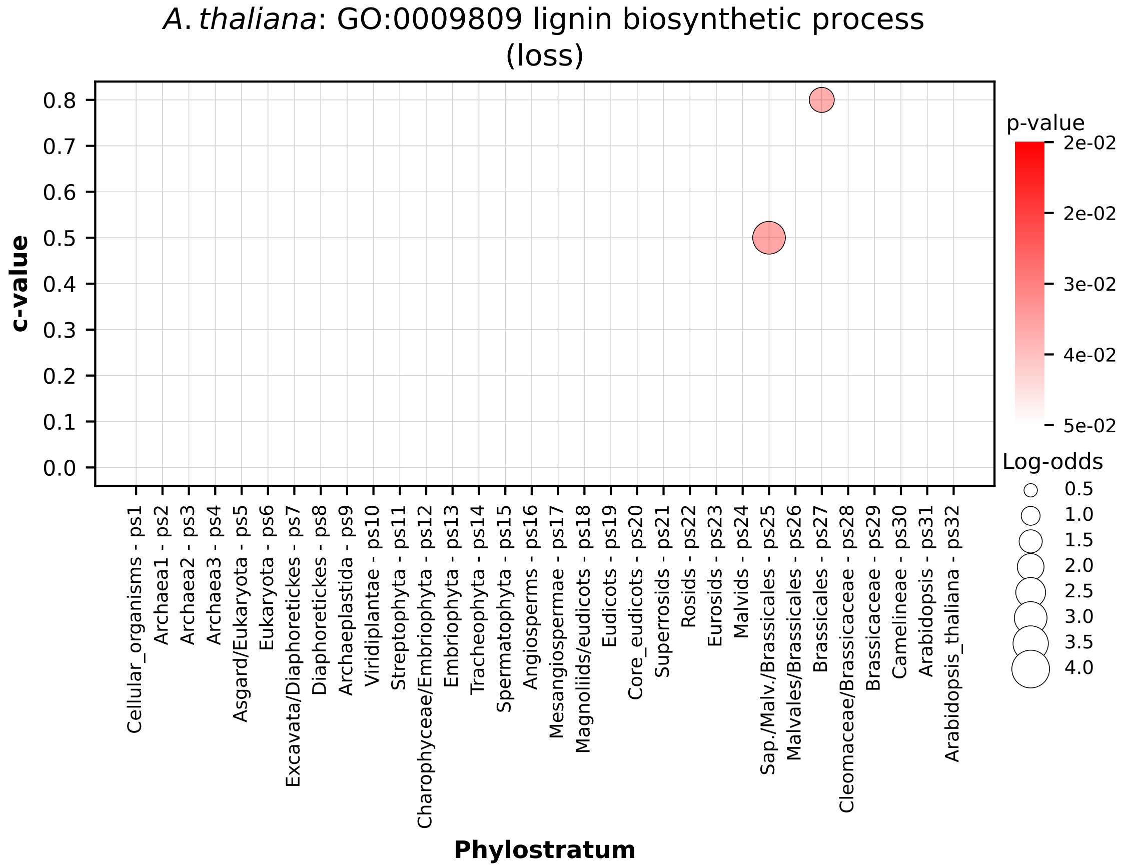
A. thaliana: GO:0009809 lignin biosynthetic process
(loss)
c-value
0.8
0.7
0.6
0.5
0.4
0.3
0.2
0.1
0.0
Phylostratum
Cellular_organisms - ps1
Archaea1 - ps2
Archaea2 - ps3
Archaea3 - ps4
Asgard/Eukaryota - ps5
Eukaryota - ps6
Excavata/Diaphoretickes - ps7
Diaphoretickes - ps8
Archaeplastida - ps9
Viridiplantae - ps10
Streptophyta - ps11
Charophyceae/Embriophyta - ps12
Embriophyta - ps13
Tracheophyta - ps14
Spermatophyta - ps15
Angiosperms - ps16
Mesangiospermae - ps17
Magnoliids/eudicots - ps18
Eudicots - ps19
Core_eudicots - ps20
Superrosids - ps21
Rosids - ps22
Eurosids - ps23
Malvids - ps24
Sap./Malv./Brassicales - ps25
Malvales/Brassicales - ps26
Brassicales - ps27
Cleomaceae/Brassicaceae - ps28
Brassicaceae - ps29
Camelineae - ps30
Arabidopsis - ps31
Arabidopsis_thaliana - ps32
p-value
2e-02
2e-02
3e-02
4e-02
5e-02
Log-odds
0.5
1.0
1.5
2.0
2.5
3.0
3.5
4.0

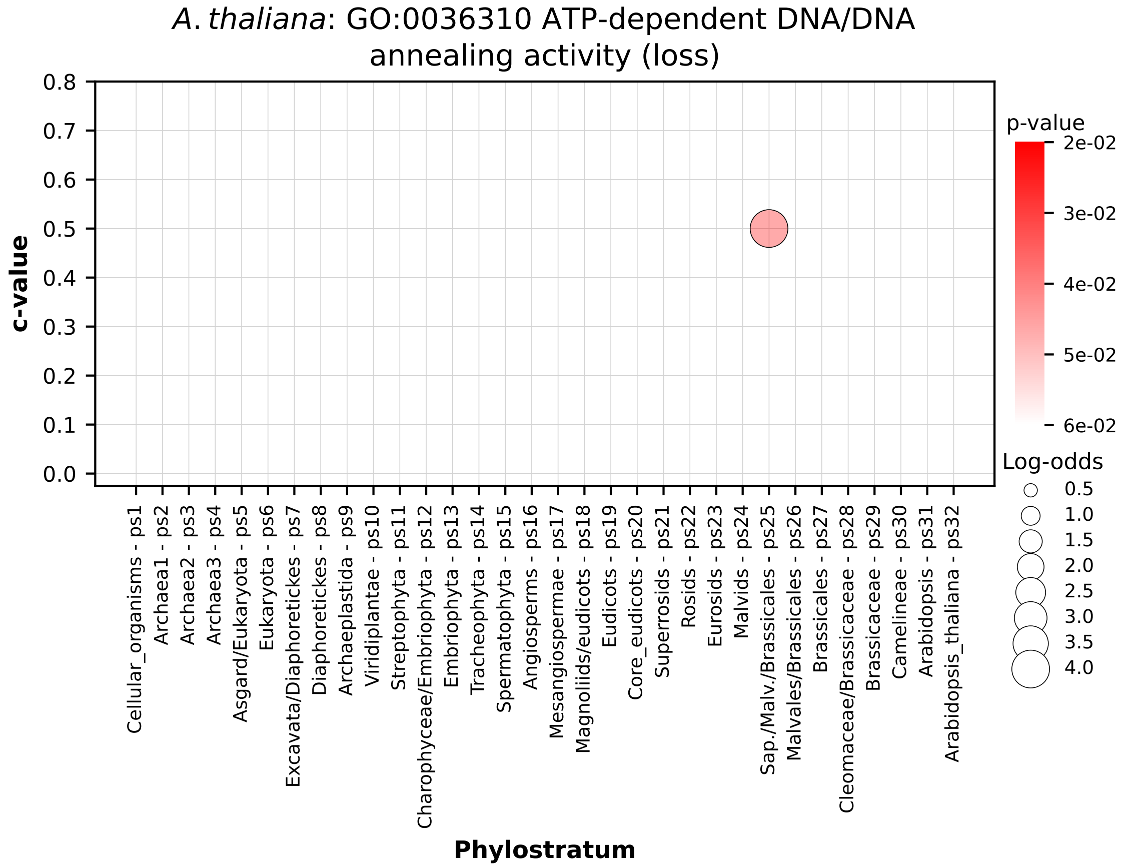

A. thaliana: GO:0036310 ATP-dependent DNA/DNA annealing activity (loss)
c-value
0.0
0.1
0.2
0.3
0.4
0.5
0.6
0.7
0.8
Phylostratum
Cellular_organisms - ps1
Archaea1 - ps2
Archaea2 - ps3
Archaea3 - ps4
Asgard/Eukaryota - ps5
Eukaryota - ps6
Excavata/Diaphoretickes - ps7
Diaphoretickes - ps8
Archaeplastida - ps9
Viridiplantae - ps10
Streptophyta - ps11
Charophyceae/Embriophyta - ps12
Embriophyta - ps13
Tracheophyta - ps14
Spermatophyta - ps15
Angiosperms - ps16
Mesangiospermae - ps17
Magnoliids/eudicots - ps18
Eudicots - ps19
Core_eudicots - ps20
Superrosids - ps21
Rosids - ps22
Eurosids - ps23
Malvids - ps24
Sap./Malv./Brassicales - ps25
Malvales/Brassicales - ps26
Brassicales - ps27
Cleomaceae/Brassicaceae - ps28
Brassicaceae - ps29
Camelineae - ps30
Arabidopsis - ps31
Arabidopsis_thaliana - ps32
p-value
2e-02
3e-02
4e-02
5e-02
6e-02
Log-odds
0.5
1.0
1.5
2.0
2.5
3.0
3.5
4.0

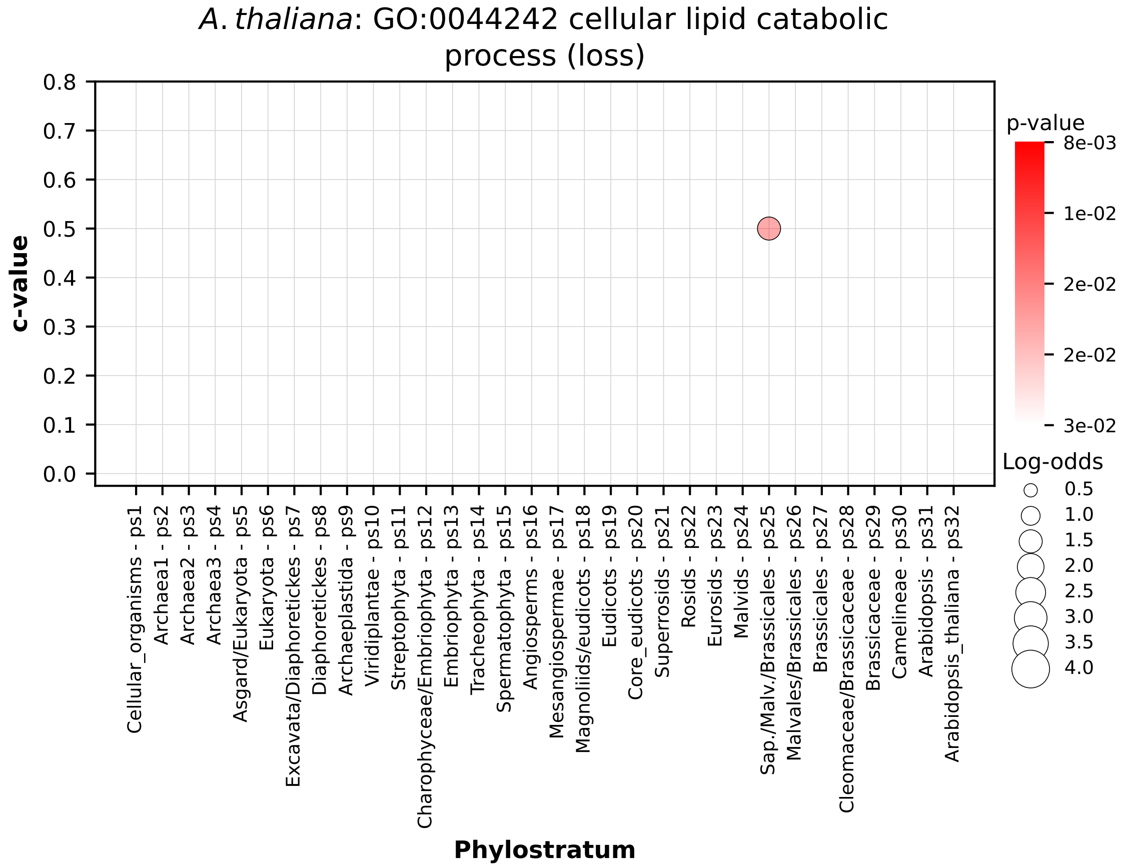

A. thaliana: GO:0044242 cellular lipid catabolic process (loss)
c-value
0.8
0.7
0.6
0.5
0.4
0.3
0.2
0.1
0.0
Phylostratum
Cellular_organisms - ps1
Archaea1 - ps2
Archaea2 - ps3
Archaea3 - ps4
Asgard/Eukaryota - ps5
Eukaryota - ps6
Excavata/Diaphoretickes - ps7
Diaphoretickes - ps8
Archaeplastida - ps9
Viridiplantae - ps10
Streptophyta - ps11
Charophyceae/Embriophyta - ps12
Embriophyta - ps13
Tracheophyta - ps14
Spermatophyta - ps15
Angiosperms - ps16
Mesangiospermae - ps17
Magnoliids/eudicots - ps18
Eudicots - ps19
Core_eudicots - ps20
Superrosids - ps21
Rosids - ps22
Eurosids - ps23
Malvids - ps24
Sap./Malv./Brassicales - ps25
Malvales/Brassicales - ps26
Brassicales - ps27
Cleomaceae/Brassicaceae - ps28
Brassicaceae - ps29
Camelineae - ps30
Arabidopsis - ps31
Arabidopsis_thaliana - ps32
p-value
8e-03
1e-02
2e-02
2e-02
3e-02
Log-odds
0.5
1.0
1.5
2.0
2.5
3.0
3.5
4.0

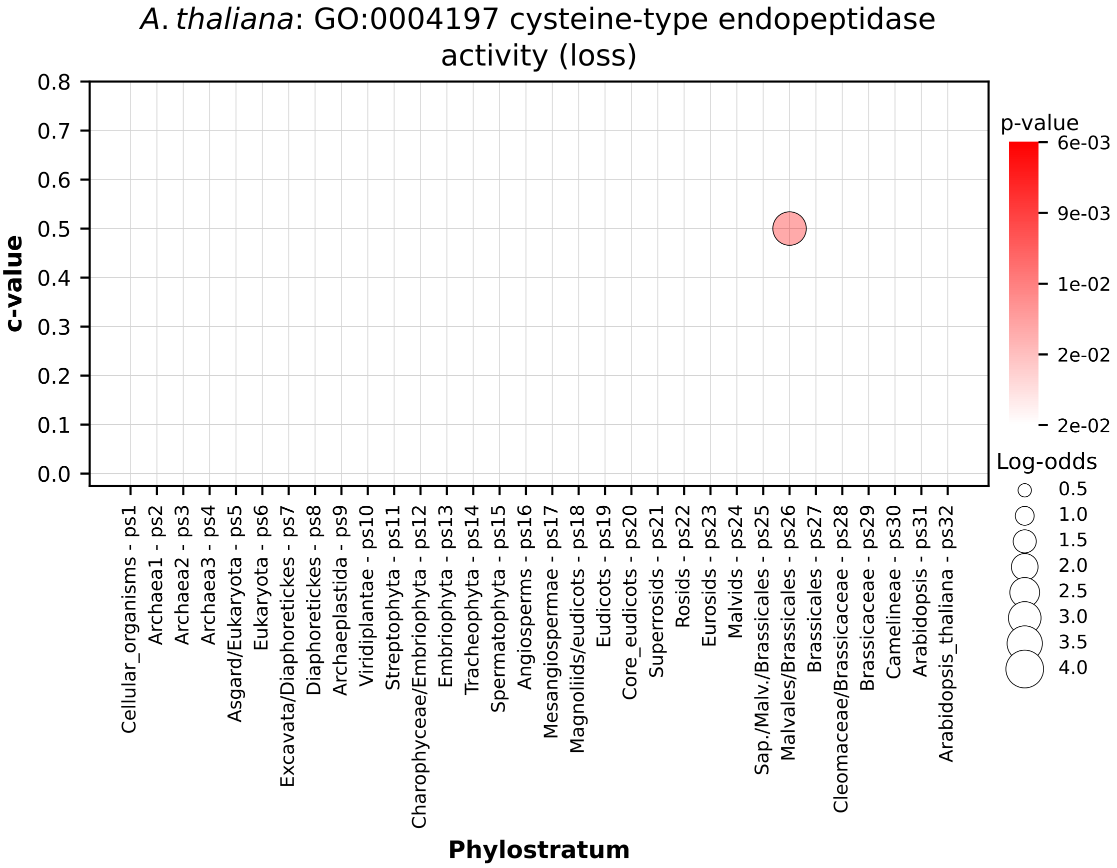
A. thaliana: GO:0004197 cysteine-type endopeptidase activity (loss)
c-value
0.0
0.1
0.2
0.3
0.4
0.5
0.6
0.7
0.8
Phylostratum
Cellular_organisms - ps1
Archaea1 - ps2
Archaea2 - ps3
Archaea3 - ps4
Asgard/Eukaryota - ps5
Eukaryota - ps6
Excavata/Diaphoretickes - ps7
Diaphoretickes - ps8
Archaeplastida - ps9
Viridiplantae - ps10
Streptophyta - ps11
Charophyceae/Embriophyta - ps12
Embriophyta - ps13
Tracheophyta - ps14
Spermatophyta - ps15
Angiosperms - ps16
Mesangiospermae - ps17
Magnoliids/eudicots - ps18
Eudicots - ps19
Core_eudicots - ps20
Superrosids - ps21
Rosids - ps22
Eurosids - ps23
Malvids - ps24
Sap./Malv./Brassicales - ps25
Malvales/Brassicales - ps26
Brassicales - ps27
Cleomaceae/Brassicaceae - ps28
Brassicaceae - ps29
Camelineae - ps30
Arabidopsis - ps31
Arabidopsis_thaliana - ps32
p-value
6e-03
9e-03
1e-02
2e-02
2e-02
Log-odds
0.5
1.0
1.5
2.0
2.5
3.0
3.5
4.0

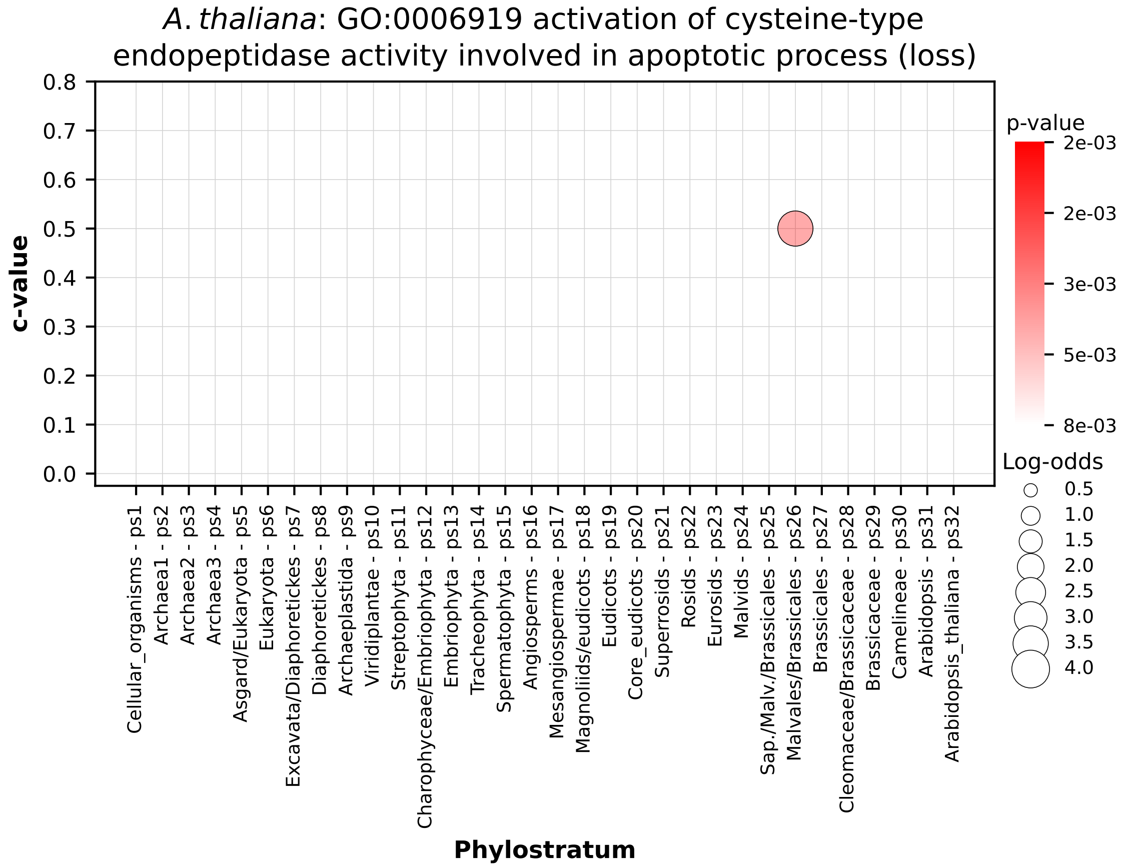

A. thaliana: GO:0006919 activation of cysteine-type endopeptidase activity involved in apoptotic process (loss)
c-value
0.8
0.7
0.6
0.5
0.4
0.3
0.2
0.1
0.0
p-value
2e-03
2e-03
3e-03
5e-03
8e-03
Log-odds
0.5
1.0
1.5
2.0
2.5
3.0
3.5
4.0
Cellular_organisms - ps1
Archaea1 - ps2
Archaea2 - ps3
Archaea3 - ps4
Asgard/Eukaryota - ps5
Eukaryota - ps6
Excavata/Diaphoretickes - ps7
Diaphoretickes - ps8
Archaeplastida - ps9
Viridiplantae - ps10
Streptophyta - ps11
Charophyceae/Embriophyta - ps12
Embriophyta - ps13
Tracheophyta - ps14
Spermatophyta - ps15
Angiosperms - ps16
Mesangiospermae - ps17
Magnoliids/eudicots - ps18
Eudicots - ps19
Core_eudicots - ps20
Superrosids - ps21
Rosids - ps22
Eurosids - ps23
Malvids - ps24
Sap./Malv./Brassicales - ps25
Malvales/Brassicales - ps26
Brassicales - ps27
Cleomaceae/Brassicaceae - ps28
Brassicaceae - ps29
Camelineae - ps30
Arabidopsis - ps31
Arabidopsis_thaliana - ps32
Phylostratum

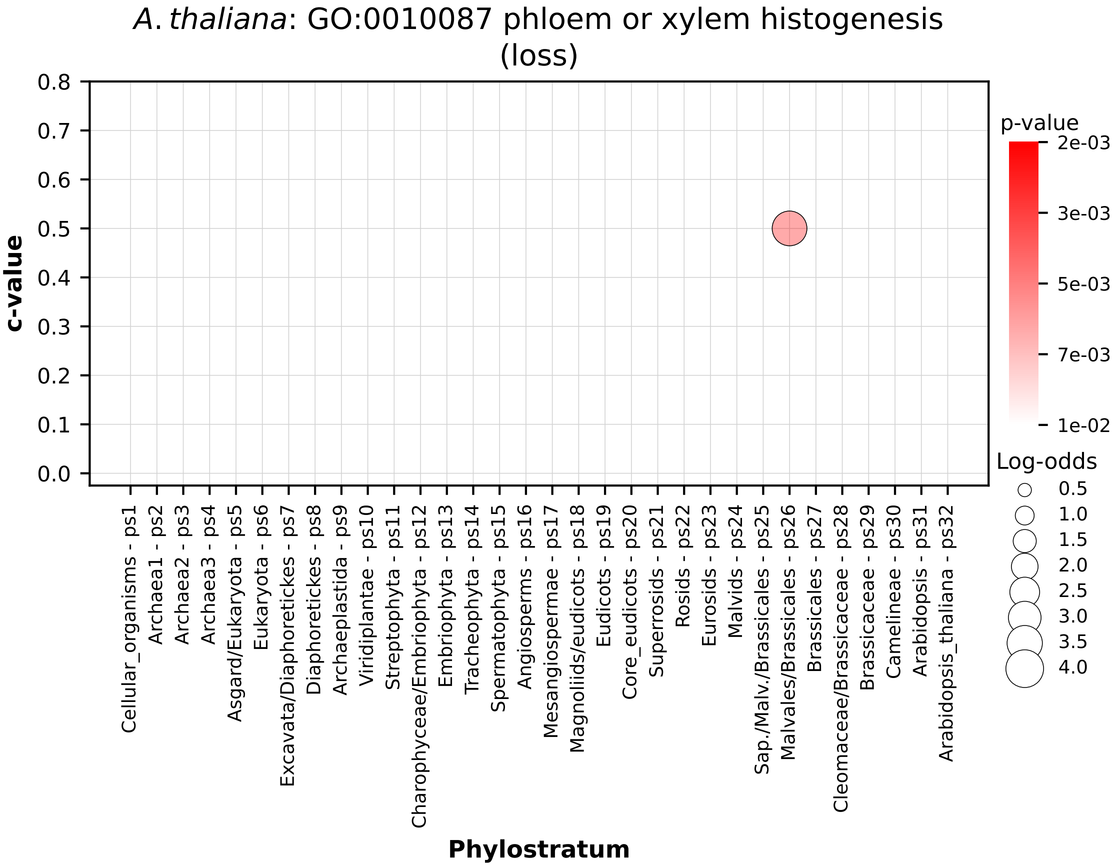
A. thaliana: GO:0010087 phloem or xylem histogenesis
(loss)
c-value
0.8
0.7
0.6
0.5
0.4
0.3
0.2
0.1
0.0
p-value
2e-03
3e-03
5e-03
7e-03
1e-02
Log-odds
0.5
1.0
1.5
2.0
2.5
3.0
3.5
4.0
Cellular_organisms - ps1
Archaea1 - ps2
Archaea2 - ps3
Archaea3 - ps4
Asgard/Eukaryota - ps5
Eukaryota - ps6
Excavata/Diaphoretickes - ps7
Diaphoretickes - ps8
Archaeplastida - ps9
Viridiplantae - ps10
Streptophyta - ps11
Charophyceae/Embriophyta - ps12
Embriophyta - ps13
Tracheophyta - ps14
Spermatophyta - ps15
Angiosperms - ps16
Mesangiospermae - ps17
Magnoliids/eudicots - ps18
Eudicots - ps19
Core_eudicots - ps20
Superrosids - ps21
Rosids - ps22
Eurosids - ps23
Malvids - ps24
Sap./Malv./Brassicales - ps25
Malvales/Brassicales - ps26
Brassicales - ps27
Cleomaceae/Brassicaceae - ps28
Brassicaceae - ps29
Camelineae - ps30
Arabidopsis - ps31
Arabidopsis_thaliana - ps32
Phylostratum

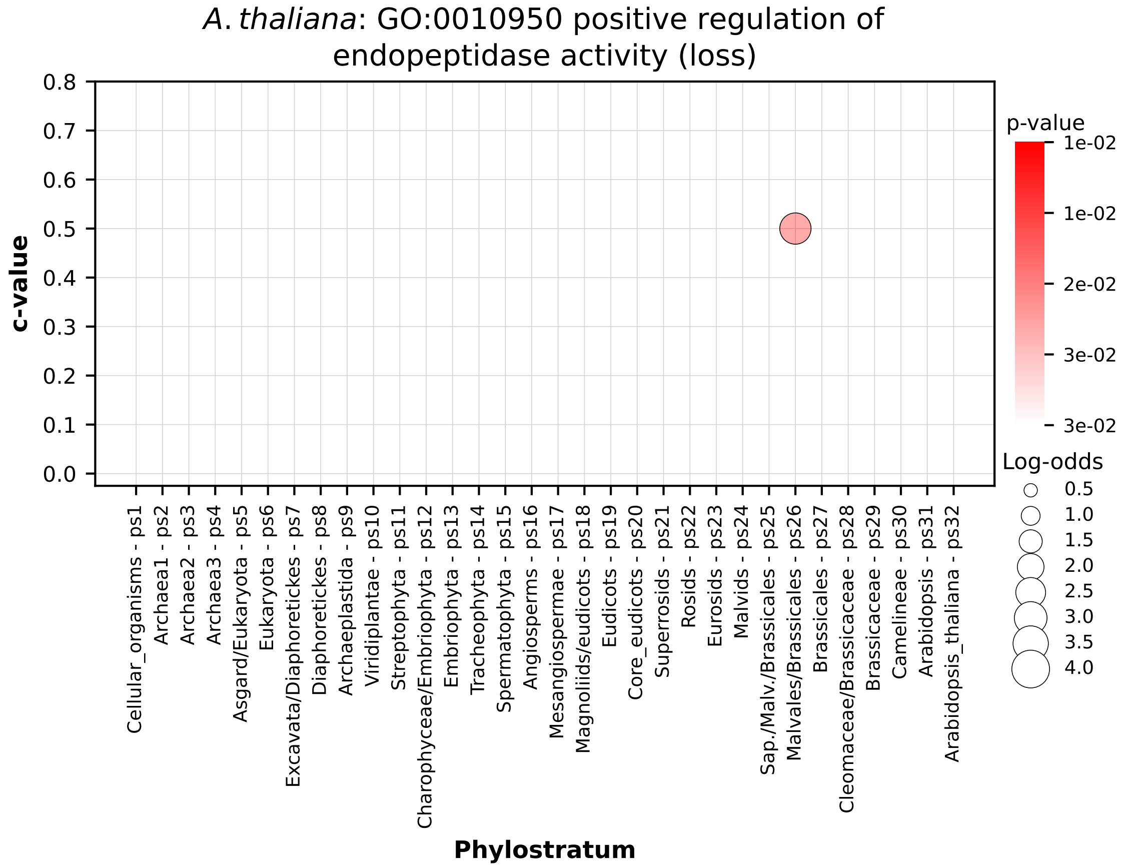

A. thaliana: GO:0010950 positive regulation of endopeptidase activity (loss)
c-value
0.8
0.7
0.6
0.5
0.4
0.3
0.2
0.1
0.0
Phylostratum
Cellular_organisms - ps1
Archaea1 - ps2
Archaea2 - ps3
Archaea3 - ps4
Asgard/Eukaryota - ps5
Eukaryota - ps6
Excavata/Diaphoretickes - ps7
Diaphoretickes - ps8
Archaeplastida - ps9
Viridiplantae - ps10
Streptophyta - ps11
Charophyceae/Embriophyta - ps12
Embriophyta - ps13
Tracheophyta - ps14
Spermatophyta - ps15
Angiosperms - ps16
Mesangiospermae - ps17
Magnoliids/eudicots - ps18
Eudicots - ps19
Core_eudicots - ps20
Superrosids - ps21
Rosids - ps22
Eurosids - ps23
Malvids - ps24
Sap./Malv./Brassicales - ps25
Malvales/Brassicales - ps26
Brassicales - ps27
Cleomaceae/Brassicaceae - ps28
Brassicaceae - ps29
Camelineae - ps30
Arabidopsis - ps31
Arabidopsis_thaliana - ps32
p-value
1e-02
1e-02
2e-02
3e-02
3e-02
Log-odds
0.5
1.0
1.5
2.0
2.5
3.0
3.5
4.0

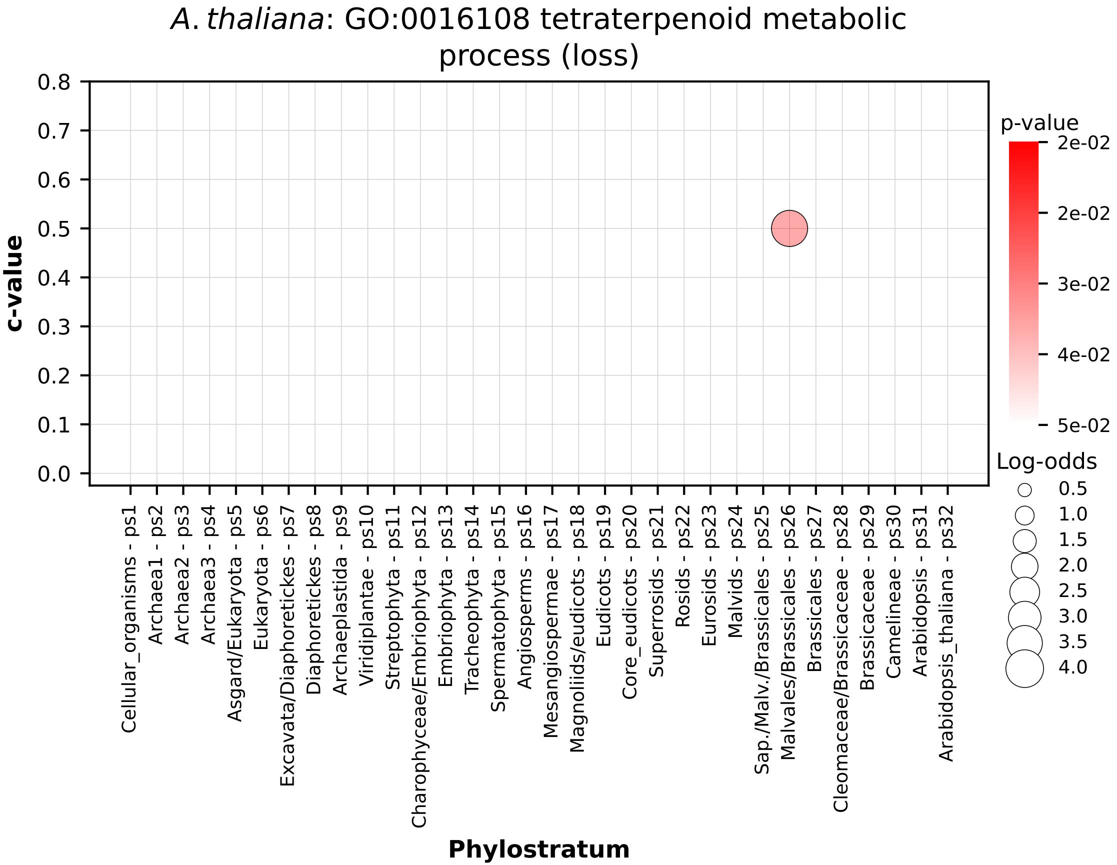

A. thaliana: GO:0016108 tetraterpenoid metabolic process (loss)
c-value
0.8
0.7
0.6
0.5
0.4
0.3
0.2
0.1
0.0
p-value
2e-02
2e-02
3e-02
4e-02
5e-02
Log-odds
0.5
1.0
1.5
2.0
2.5
3.0
3.5
4.0
Cellular_organisms - ps1
Archaea1 - ps2
Archaea2 - ps3
Archaea3 - ps4
Asgard/Eukaryota - ps5
Eukaryota - ps6
Excavata/Diaphoretickes - ps7
Diaphoretickes - ps8
Archaeplastida - ps9
Viridiplantae - ps10
Streptophyta - ps11
Charophyceae/Embriophyta - ps12
Embriophyta - ps13
Tracheophyta - ps14
Spermatophyta - ps15
Angiosperms - ps16
Mesangiospermae - ps17
Magnoliids/eudicots - ps18
Eudicots - ps19
Core_eudicots - ps20
Superrosids - ps21
Rosids - ps22
Eurosids - ps23
Malvids - ps24
Sap./Malv./Brassicales - ps25
Malvales/Brassicales - ps26
Brassicales - ps27
Cleomaceae/Brassicaceae - ps28
Brassicaceae - ps29
Camelineae - ps30
Arabidopsis - ps31
Arabidopsis_thaliana - ps32
Phylostratum

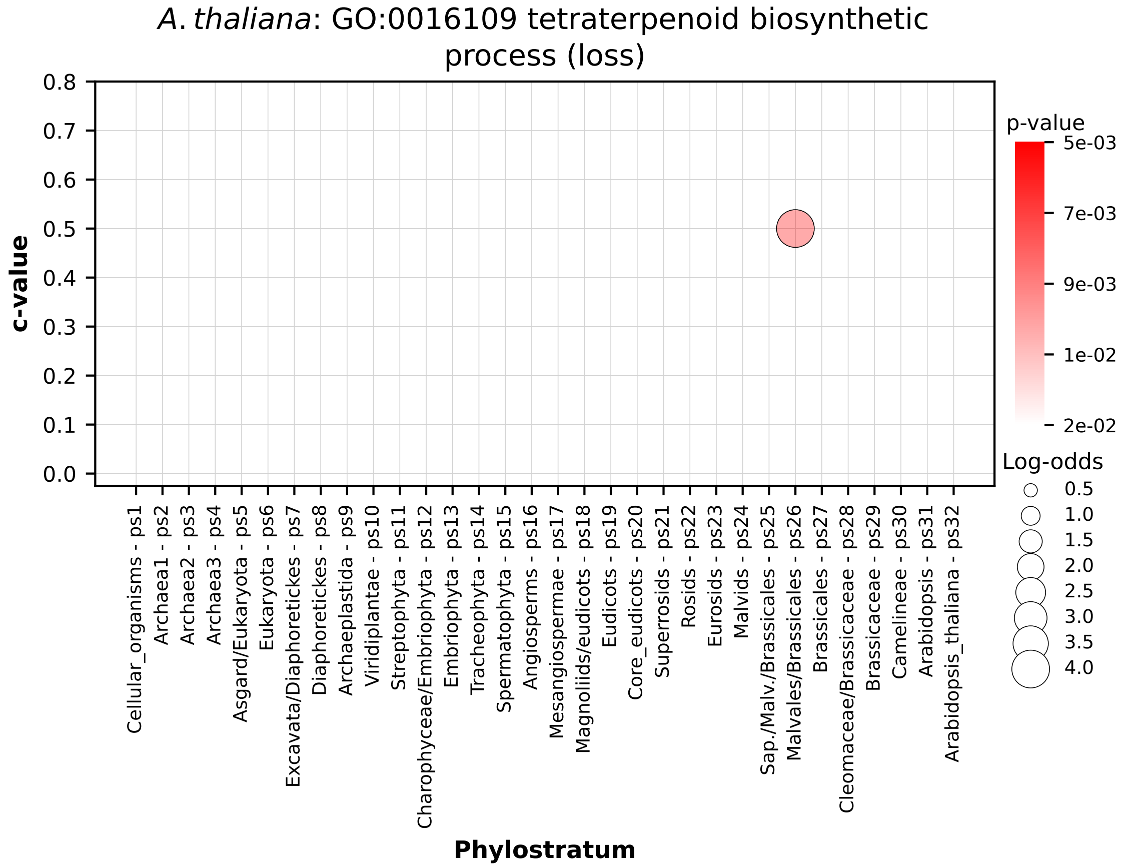

A. thaliana: GO:0016109 tetraterpenoid biosynthetic process (loss)
c-value
0.8
0.7
0.6
0.5
0.4
0.3
0.2
0.1
0.0
Phylostratum
Cellular_organisms - ps1
Archaea1 - ps2
Archaea2 - ps3
Archaea3 - ps4
Asgard/Eukaryota - ps5
Eukaryota - ps6
Excavata/Diaphoretickes - ps7
Diaphoretickes - ps8
Archaeplastida - ps9
Viridiplantae - ps10
Streptophyta - ps11
Charophyceae/Embriophyta - ps12
Embriophyta - ps13
Tracheophyta - ps14
Spermatophyta - ps15
Angiosperms - ps16
Mesangiospermae - ps17
Magnoliids/eudicots - ps18
Eudicots - ps19
Core_eudicots - ps20
Superrosids - ps21
Rosids - ps22
Eurosids - ps23
Malvids - ps24
Sap./Malv./Brassicales - ps25
Malvales/Brassicales - ps26
Brassicales - ps27
Cleomaceae/Brassicaceae - ps28
Brassicaceae - ps29
Camelineae - ps30
Arabidopsis - ps31
Arabidopsis_thaliana - ps32
p-value
5e-03
7e-03
9e-03
1e-02
2e-02
Log-odds
0.5
1.0
1.5
2.0
2.5
3.0
3.5
4.0

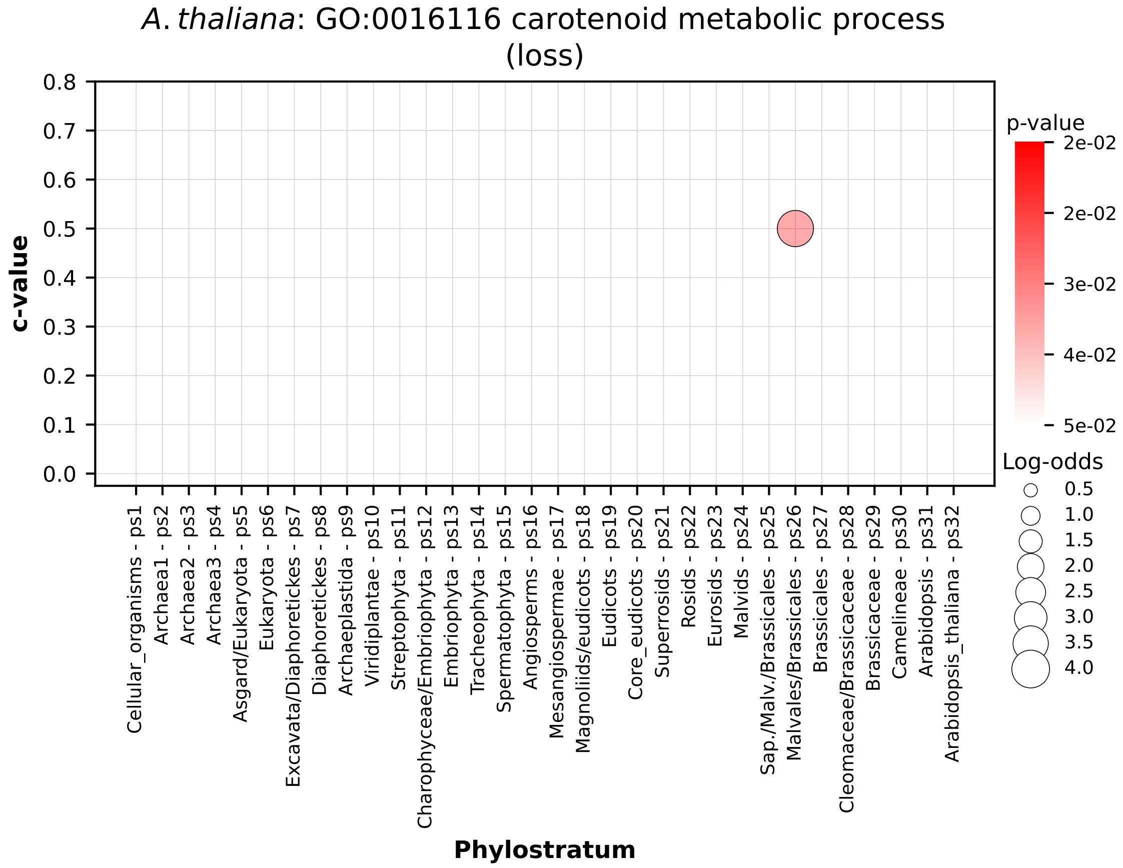

A. thaliana: GO:0016116 carotenoid metabolic process
(loss)
c-value
0.8
0.7
0.6
0.5
0.4
0.3
0.2
0.1
0.0
p-value
2e-02
2e-02
3e-02
4e-02
5e-02
Log-odds
0.5
1.0
1.5
2.0
2.5
3.0
3.5
4.0
Cellular_organisms - ps1
Archaea1 - ps2
Archaea2 - ps3
Archaea3 - ps4
Asgard/Eukaryota - ps5
Eukaryota - ps6
Excavata/Diaphoretickes - ps7
Diaphoretickes - ps8
Archaeplastida - ps9
Viridiplantae - ps10
Streptophyta - ps11
Charophyceae/Embriophyta - ps12
Embriophyta - ps13
Tracheophyta - ps14
Spermatophyta - ps15
Angiosperms - ps16
Mesangiospermae - ps17
Magnoliids/eudicots - ps18
Eudicots - ps19
Core_eudicots - ps20
Superrosids - ps21
Rosids - ps22
Eurosids - ps23
Malvids - ps24
Sap./Malv./Brassicales - ps25
Malvales/Brassicales - ps26
Brassicales - ps27
Cleomaceae/Brassicaceae - ps28
Brassicaceae - ps29
Camelineae - ps30
Arabidopsis - ps31
Arabidopsis_thaliana - ps32
Phylostratum

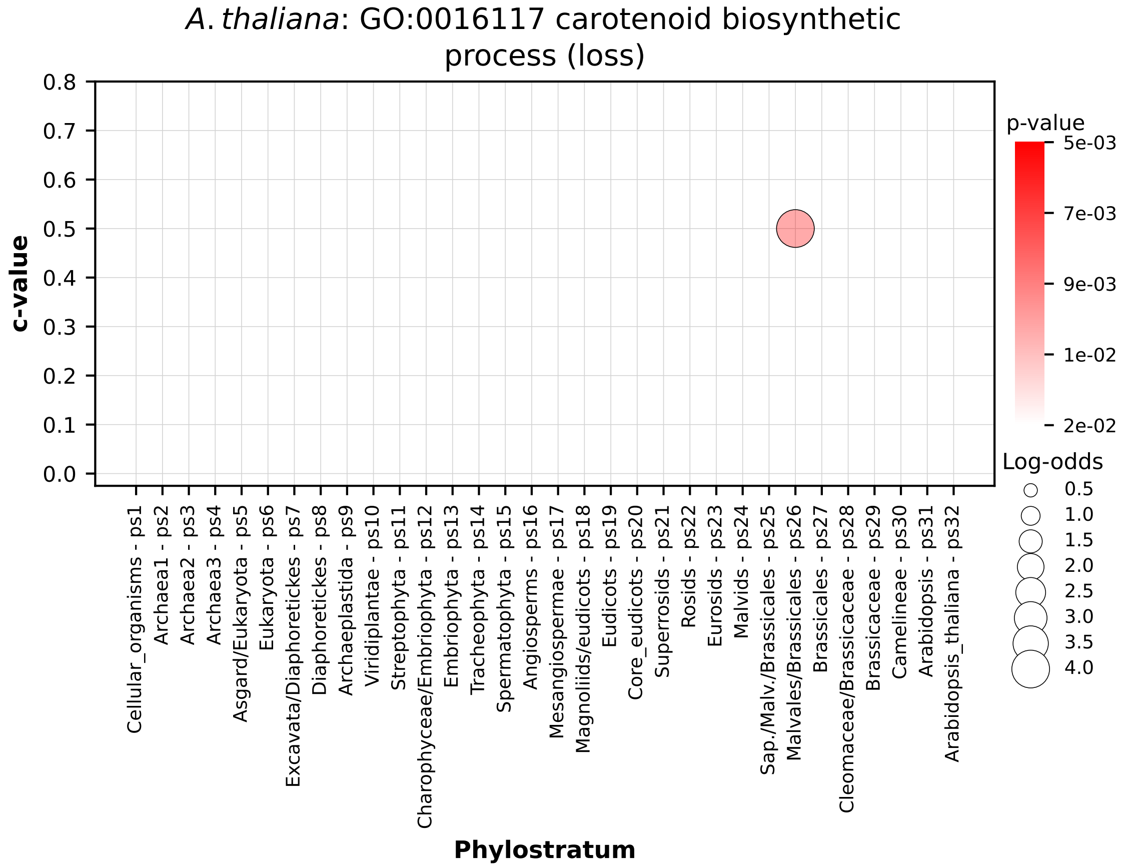

A. thaliana: GO:0016117 carotenoid biosynthetic process (loss)
c-value
0.8
0.7
0.6
0.5
0.4
0.3
0.2
0.1
0.0
p-value
5e-03
7e-03
9e-03
1e-02
2e-02
Log-odds
0.5
1.0
1.5
2.0
2.5
3.0
3.5
4.0
Cellular_organisms - ps1
Archaea1 - ps2
Archaea2 - ps3
Archaea3 - ps4
Asgard/Eukaryota - ps5
Eukaryota - ps6
Excavata/Diaphoretickes - ps7
Diaphoretickes - ps8
Archaeplastida - ps9
Viridiplantae - ps10
Streptophyta - ps11
Charophyceae/Embriophyta - ps12
Embriophyta - ps13
Tracheophyta - ps14
Spermatophyta - ps15
Angiosperms - ps16
Mesangiospermae - ps17
Magnoliids/eudicots - ps18
Eudicots - ps19
Core_eudicots - ps20
Superrosids - ps21
Rosids - ps22
Eurosids - ps23
Malvids - ps24
Sap./Malv./Brassicales - ps25
Malvales/Brassicales - ps26
Brassicales - ps27
Cleomaceae/Brassicaceae - ps28
Brassicaceae - ps29
Camelineae - ps30
Arabidopsis - ps31
Arabidopsis_thaliana - ps32
Phylostratum

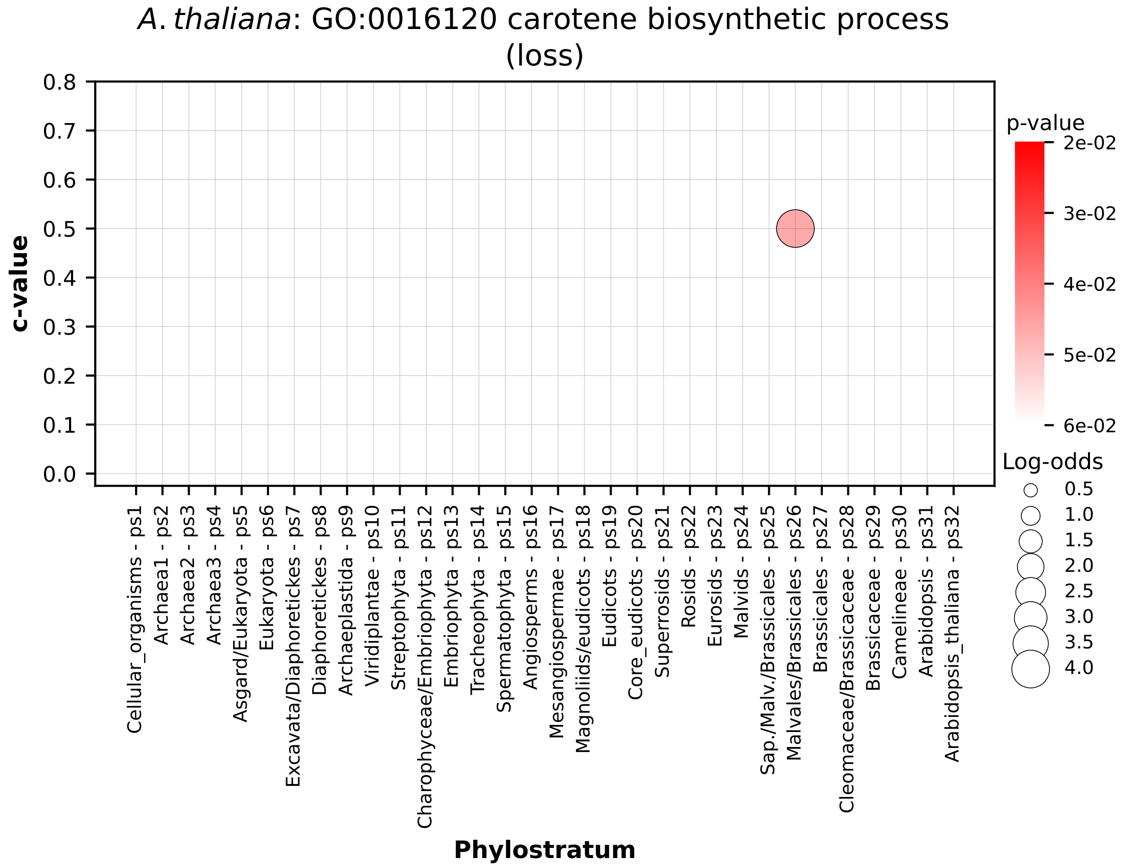

A. thaliana: GO:0016120 carotene biosynthetic process
(loss)
c-value
0.8
0.7
0.6
0.5
0.4
0.3
0.2
0.1
0.0
p-value
2e-02
3e-02
4e-02
5e-02
6e-02
Log-odds
0.5
1.0
1.5
2.0
2.5
3.0
3.5
4.0
Cellular_organisms - ps1
Archaea1 - ps2
Archaea2 - ps3
Archaea3 - ps4
Asgard/Eukaryota - ps5
Eukaryota - ps6
Excavata/Diaphoretickes - ps7
Diaphoretickes - ps8
Archaeplastida - ps9
Viridiplantae - ps10
Streptophyta - ps11
Charophyceae/Embriophyta - ps12
Embriophyta - ps13
Tracheophyta - ps14
Spermatophyta - ps15
Angiosperms - ps16
Mesangiospermae - ps17
Magnoliids/eudicots - ps18
Eudicots - ps19
Core_eudicots - ps20
Superrosids - ps21
Rosids - ps22
Eurosids - ps23
Malvids - ps24
Sap./Malv./Brassicales - ps25
Malvales/Brassicales - ps26
Brassicales - ps27
Cleomaceae/Brassicaceae - ps28
Brassicaceae - ps29
Camelineae - ps30
Arabidopsis - ps31
Arabidopsis_thaliana - ps32
Phylostratum

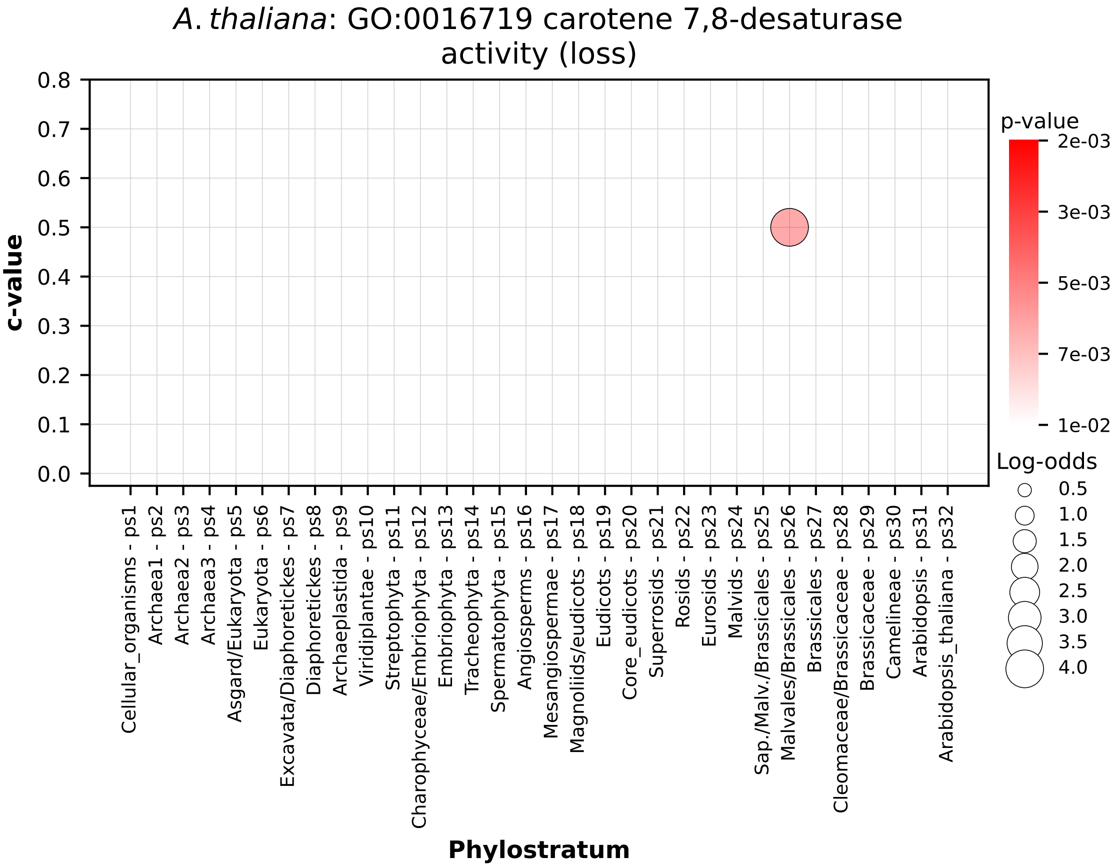

A. thaliana: GO:0016719 carotene 7,8-desaturase activity (loss)
c-value
0.8
0.7
0.6
0.5
0.4
0.3
0.2
0.1
0.0
p-value
2e-03
3e-03
5e-03
7e-03
1e-02
Log-odds
0.5
1.0
1.5
2.0
2.5
3.0
3.5
4.0
Cellular_organisms - ps1
Archaea1 - ps2
Archaea2 - ps3
Archaea3 - ps4
Asgard/Eukaryota - ps5
Eukaryota - ps6
Excavata/Diaphoretickes - ps7
Diaphoretickes - ps8
Archaeplastida - ps9
Viridiplantae - ps10
Streptophyta - ps11
Charophyceae/Embriophyta - ps12
Embriophyta - ps13
Tracheophyta - ps14
Spermatophyta - ps15
Angiosperms - ps16
Mesangiospermae - ps17
Magnoliids/eudicots - ps18
Eudicots - ps19
Core_eudicots - ps20
Superrosids - ps21
Rosids - ps22
Eurosids - ps23
Malvids - ps24
Sap./Malv./Brassicales - ps25
Malvales/Brassicales - ps26
Brassicales - ps27
Cleomaceae/Brassicaceae - ps28
Brassicaceae - ps29
Camelineae - ps30
Arabidopsis - ps31
Arabidopsis_thaliana - ps32
Phylostratum

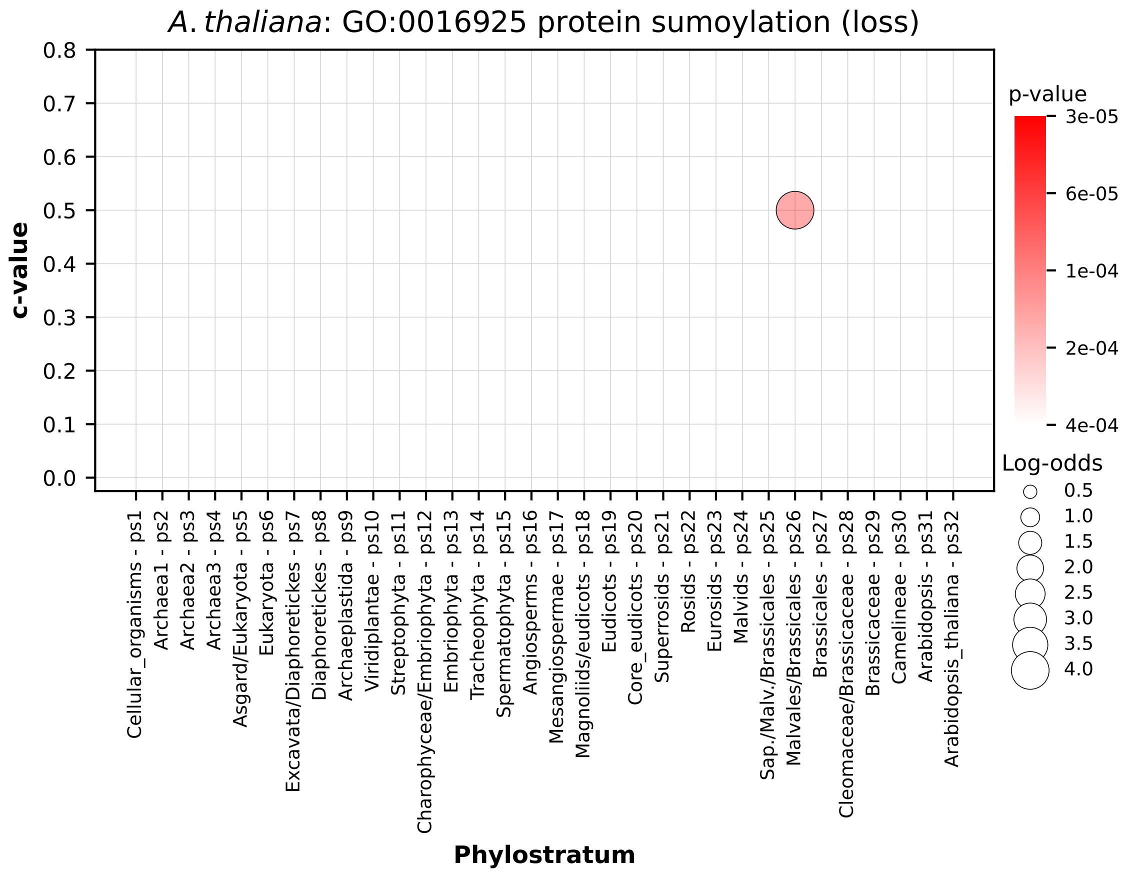

A. thaliana: GO:0016925 protein sumoylation (loss)
c-value
0.0
0.1
0.2
0.3
0.4
0.5
0.6
0.7
0.8
Phylostratum
Cellular_organisms - ps1
Archaea1 - ps2
Archaea2 - ps3
Archaea3 - ps4
Asgard/Eukaryota - ps5
Eukaryota - ps6
Excavata/Diaphoretickes - ps7
Diaphoretickes - ps8
Archaeplastida - ps9
Viridiplantae - ps10
Streptophyta - ps11
Charophyceae/Embriophyta - ps12
Embriophyta - ps13
Tracheophyta - ps14
Spermatophyta - ps15
Angiosperms - ps16
Mesangiospermae - ps17
Magnoliids/eudicots - ps18
Eudicots - ps19
Core_eudicots - ps20
Superrosids - ps21
Rosids - ps22
Eurosids - ps23
Malvids - ps24
Sap./Malv./Brassicales - ps25
Malvales/Brassicales - ps26
Brassicales - ps27
Cleomaceae/Brassicaceae - ps28
Brassicaceae - ps29
Camelineae - ps30
Arabidopsis - ps31
Arabidopsis_thaliana - ps32
p-value
3e-05
6e-05
1e-04
2e-04
4e-04
Log-odds
0.5
1.0
1.5
2.0
2.5
3.0
3.5
4.0

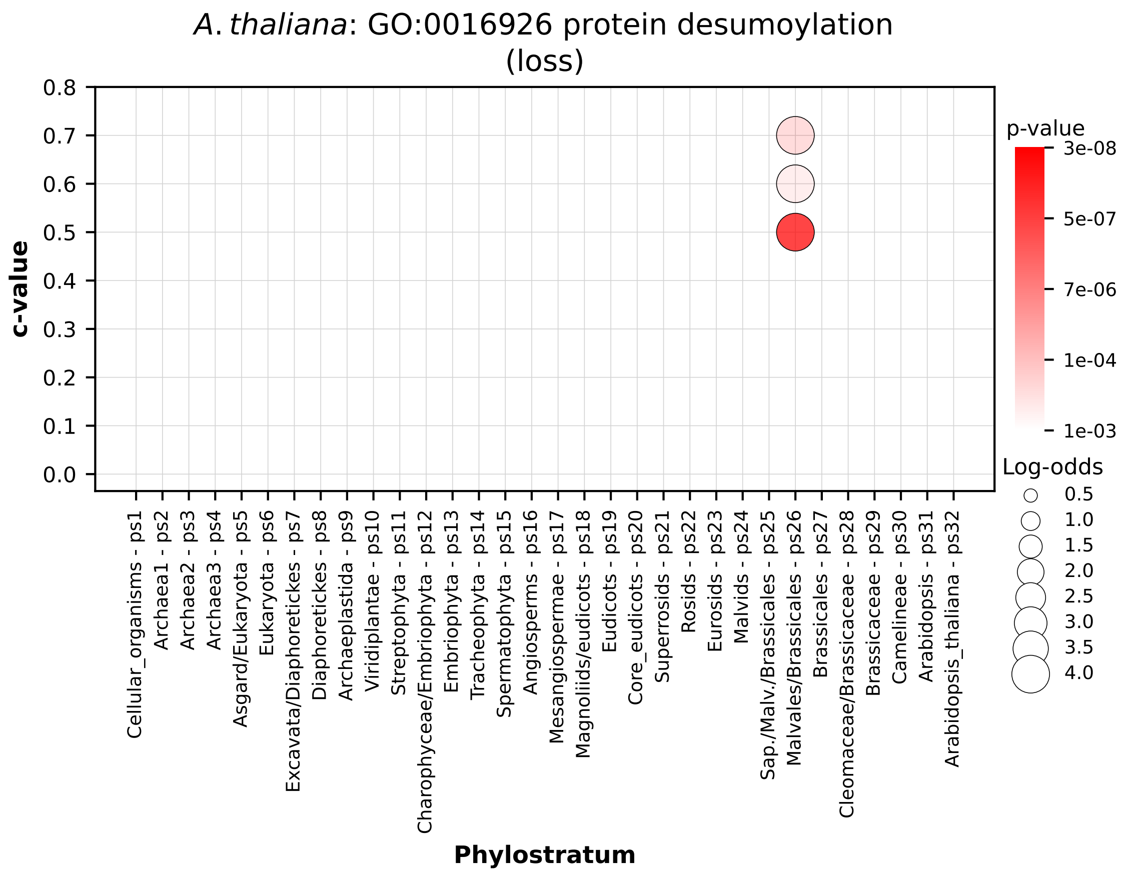
A. thaliana: GO:0016926 protein desumoylation
(loss)
c-value
0.0
0.1
0.2
0.3
0.4
0.5
0.6
0.7
0.8
Phylostratum
Cellular_organisms - ps1
Archaea1 - ps2
Archaea2 - ps3
Archaea3 - ps4
Asgard/Eukaryota - ps5
Eukaryota - ps6
Excavata/Diaphoretickes - ps7
Diaphoretickes - ps8
Archaeplastida - ps9
Viridiplantae - ps10
Streptophyta - ps11
Charophyceae/Embriophyta - ps12
Embriophyta - ps13
Tracheophyta - ps14
Spermatophyta - ps15
Angiosperms - ps16
Mesangiospermae - ps17
Magnoliids/eudicots - ps18
Eudicots - ps19
Core_eudicots - ps20
Superrosids - ps21
Rosids - ps22
Eurosids - ps23
Malvids - ps24
Sap./Malv./Brassicales - ps25
Malvales/Brassicales - ps26
Brassicales - ps27
Cleomaceae/Brassicaceae - ps28
Brassicaceae - ps29
Camelineae - ps30
Arabidopsis - ps31
Arabidopsis_thaliana - ps32
p-value
3e-08
5e-07
7e-06
1e-04
1e-03
Log-odds
0.5
1.0
1.5
2.0
2.5
3.0
3.5
4.0

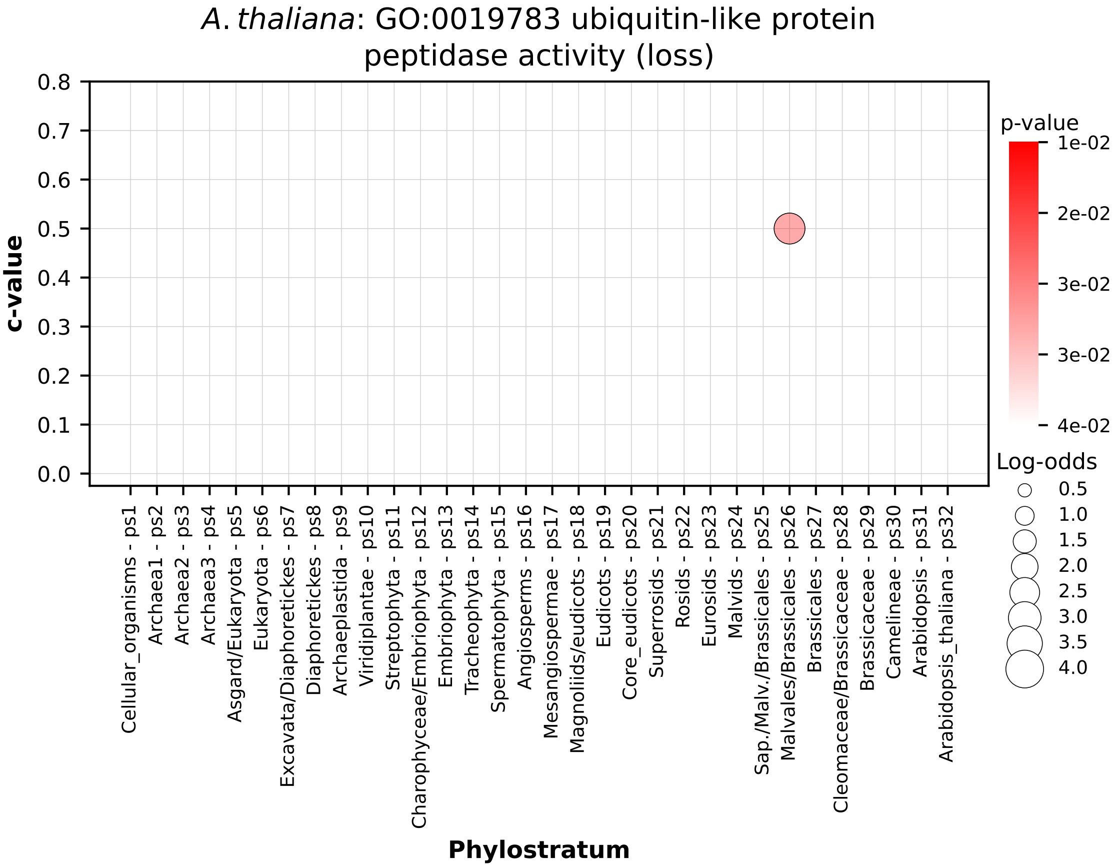
A. thaliana: GO:0019783 ubiquitin-like protein peptidase activity (loss)
c-value
0.8
0.7
0.6
0.5
0.4
0.3
0.2
0.1
0.0
p-value
1e-02
2e-02
3e-02
3e-02
4e-02
Log-odds
0.5
1.0
1.5
2.0
2.5
3.0
3.5
4.0
Cellular_organisms - ps1
Archaea1 - ps2
Archaea2 - ps3
Archaea3 - ps4
Asgard/Eukaryota - ps5
Eukaryota - ps6
Excavata/Diaphoretickes - ps7
Diaphoretickes - ps8
Archaeplastida - ps9
Viridiplantae - ps10
Streptophyta - ps11
Charophyceae/Embriophyta - ps12
Embriophyta - ps13
Tracheophyta - ps14
Spermatophyta - ps15
Angiosperms - ps16
Mesangiospermae - ps17
Magnoliids/eudicots - ps18
Eudicots - ps19
Core_eudicots - ps20
Superrosids - ps21
Rosids - ps22
Eurosids - ps23
Malvids - ps24
Sap./Malv./Brassicales - ps25
Malvales/Brassicales - ps26
Brassicales - ps27
Cleomaceae/Brassicaceae - ps28
Brassicaceae - ps29
Camelineae - ps30
Arabidopsis - ps31
Arabidopsis_thaliana - ps32
Phylostratum

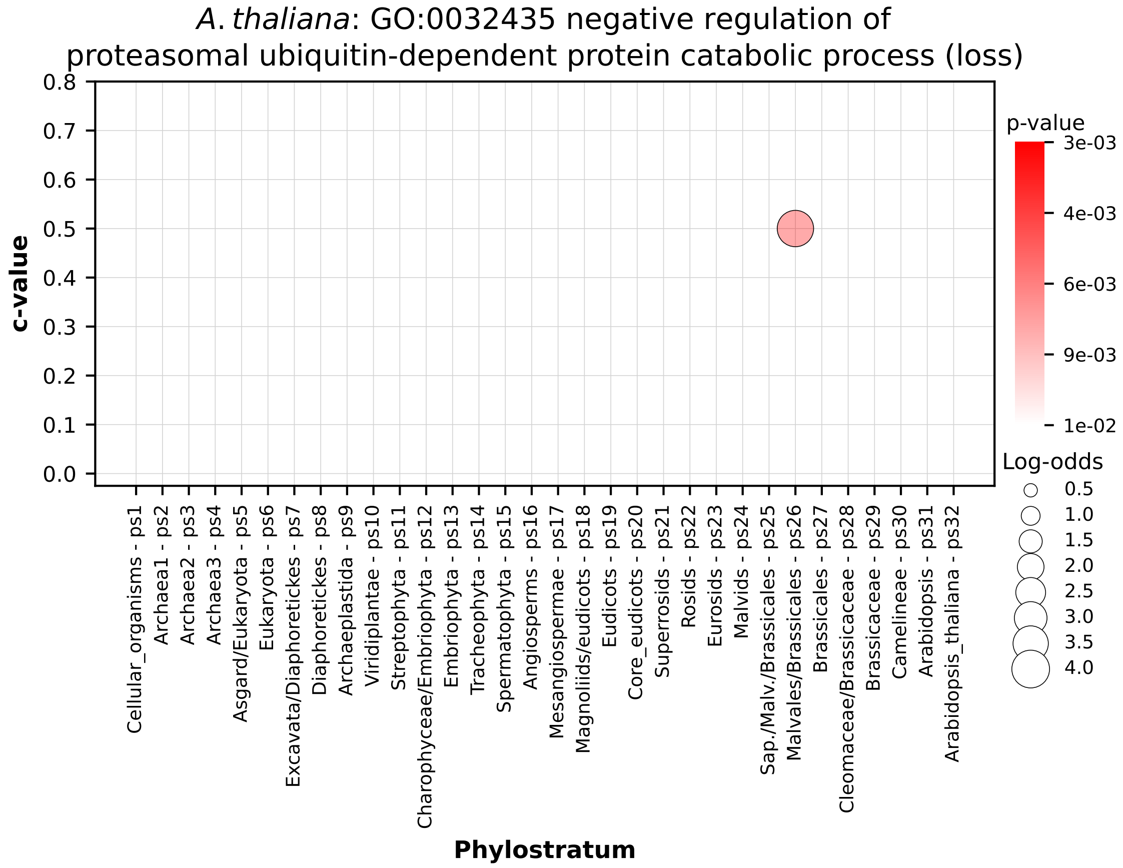
A. thaliana: GO:0032435 negative regulation of proteasomal ubiquitin-dependent protein catabolic process (loss)
c-value
0.8
0.7
0.6
0.5
0.4
0.3
0.2
0.1
0.0
p-value
3e-03
4e-03
6e-03
9e-03
1e-02
Log-odds
0.5
1.0
1.5
2.0
2.5
3.0
3.5
4.0
Cellular_organisms - ps1
Archaea1 - ps2
Archaea2 - ps3
Archaea3 - ps4
Asgard/Eukaryota - ps5
Eukaryota - ps6
Excavata/Diaphoretickes - ps7
Diaphoretickes - ps8
Archaeplastida - ps9
Viridiplantae - ps10
Streptophyta - ps11
Charophyceae/Embriophyta - ps12
Embriophyta - ps13
Tracheophyta - ps14
Spermatophyta - ps15
Angiosperms - ps16
Mesangiospermae - ps17
Magnoliids/eudicots - ps18
Eudicots - ps19
Core_eudicots - ps20
Superrosids - ps21
Rosids - ps22
Eurosids - ps23
Malvids - ps24
Sap./Malv./Brassicales - ps25
Malvales/Brassicales - ps26
Brassicales - ps27
Cleomaceae/Brassicaceae - ps28
Brassicaceae - ps29
Camelineae - ps30
Arabidopsis - ps31
Arabidopsis_thaliana - ps32
Phylostratum

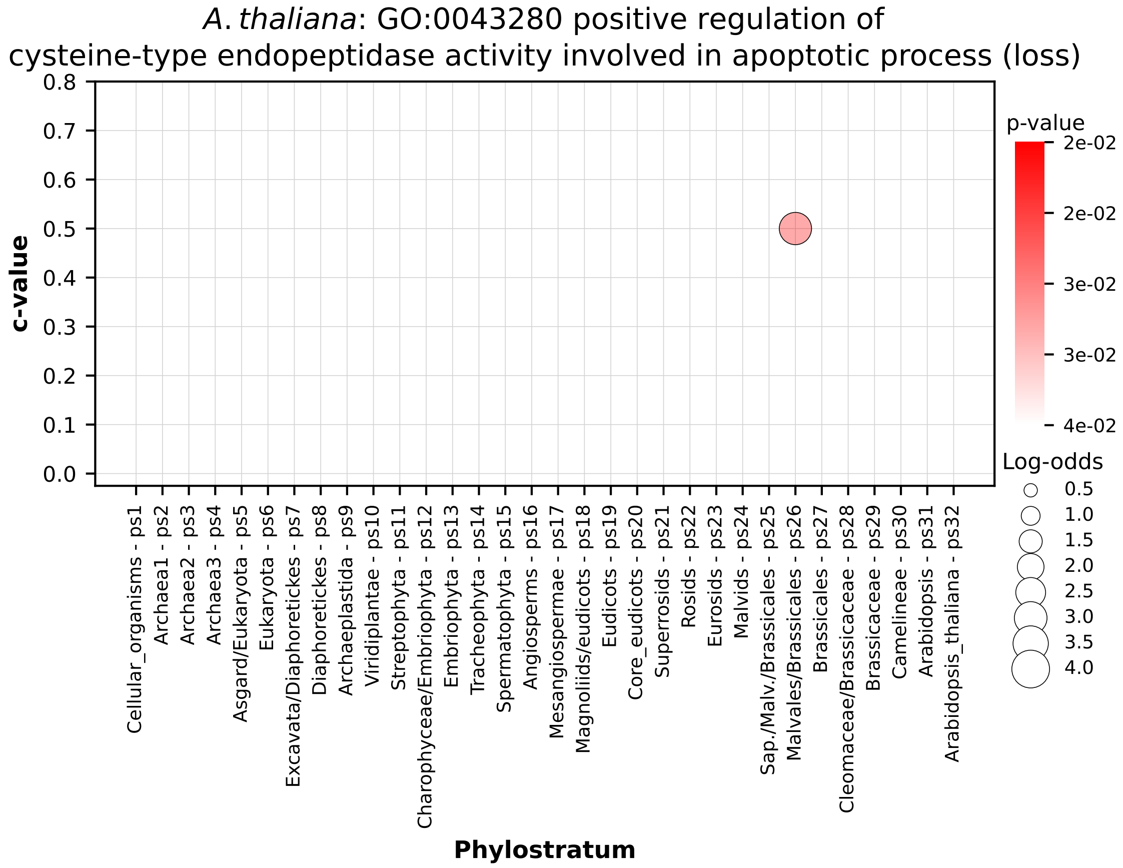

A. thaliana: GO:0043280 positive regulation of
cysteine-type endopeptidase activity involved in apoptotic process (loss)
c-value
0.8
0.7
0.6
0.5
0.4
0.3
0.2
0.1
0.0
p-value
2e-02
2e-02
3e-02
3e-02
4e-02
Log-odds
0.5
1.0
1.5
2.0
2.5
3.0
3.5
4.0
Cellular_organisms - ps1
Archaea1 - ps2
Archaea2 - ps3
Archaea3 - ps4
Asgard/Eukaryota - ps5
Eukaryota - ps6
Excavata/Diaphoretickes - ps7
Diaphoretickes - ps8
Archaeplastida - ps9
Viridiplantae - ps10
Streptophyta - ps11
Charophyceae/Embriophyta - ps12
Embriophyta - ps13
Tracheophyta - ps14
Spermatophyta - ps15
Angiosperms - ps16
Mesangiospermae - ps17
Magnoliids/eudicots - ps18
Eudicots - ps19
Core_eudicots - ps20
Superrosids - ps21
Rosids - ps22
Eurosids - ps23
Malvids - ps24
Sap./Malv./Brassicales - ps25
Malvales/Brassicales - ps26
Brassicales - ps27
Cleomaceae/Brassicaceae - ps28
Brassicaceae - ps29
Camelineae - ps30
Arabidopsis - ps31
Arabidopsis_thaliana - ps32
Phylostratum

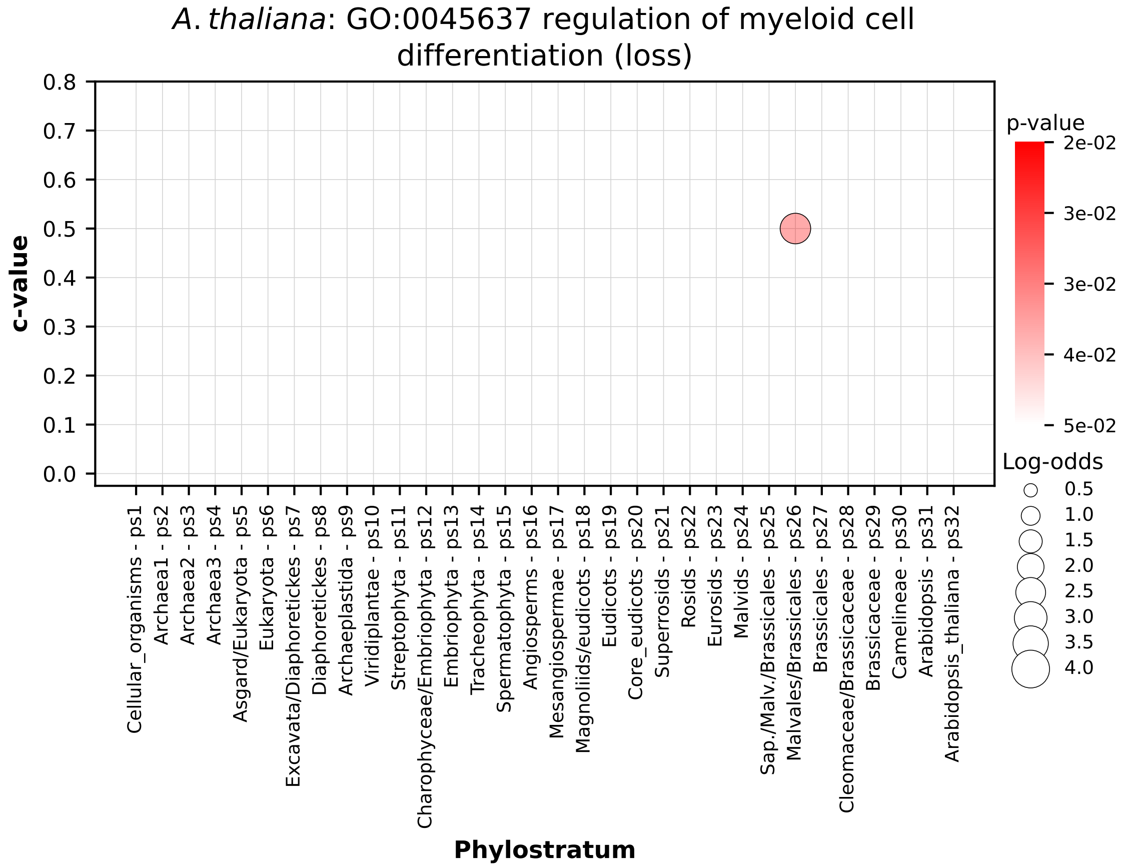

A. thaliana: GO:0045637 regulation of myeloid cell differentiation (loss)
c-value
0.8
0.7
0.6
0.5
0.4
0.3
0.2
0.1
0.0
Phylostratum
Cellular_organisms - ps1
Archaea1 - ps2
Archaea2 - ps3
Archaea3 - ps4
Asgard/Eukaryota - ps5
Eukaryota - ps6
Excavata/Diaphoretickes - ps7
Diaphoretickes - ps8
Archaeplastida - ps9
Viridiplantae - ps10
Streptophyta - ps11
Charophyceae/Embriophyta - ps12
Embriophyta - ps13
Tracheophyta - ps14
Spermatophyta - ps15
Angiosperms - ps16
Mesangiospermae - ps17
Magnoliids/eudicots - ps18
Eudicots - ps19
Core_eudicots - ps20
Superrosids - ps21
Rosids - ps22
Eurosids - ps23
Malvids - ps24
Sap./Malv./Brassicales - ps25
Malvales/Brassicales - ps26
Brassicales - ps27
Cleomaceae/Brassicaceae - ps28
Brassicaceae - ps29
Camelineae - ps30
Arabidopsis - ps31
Arabidopsis_thaliana - ps32
p-value
2e-02
3e-02
3e-02
4e-02
5e-02
Log-odds
0.5
1.0
1.5
2.0
2.5
3.0
3.5
4.0

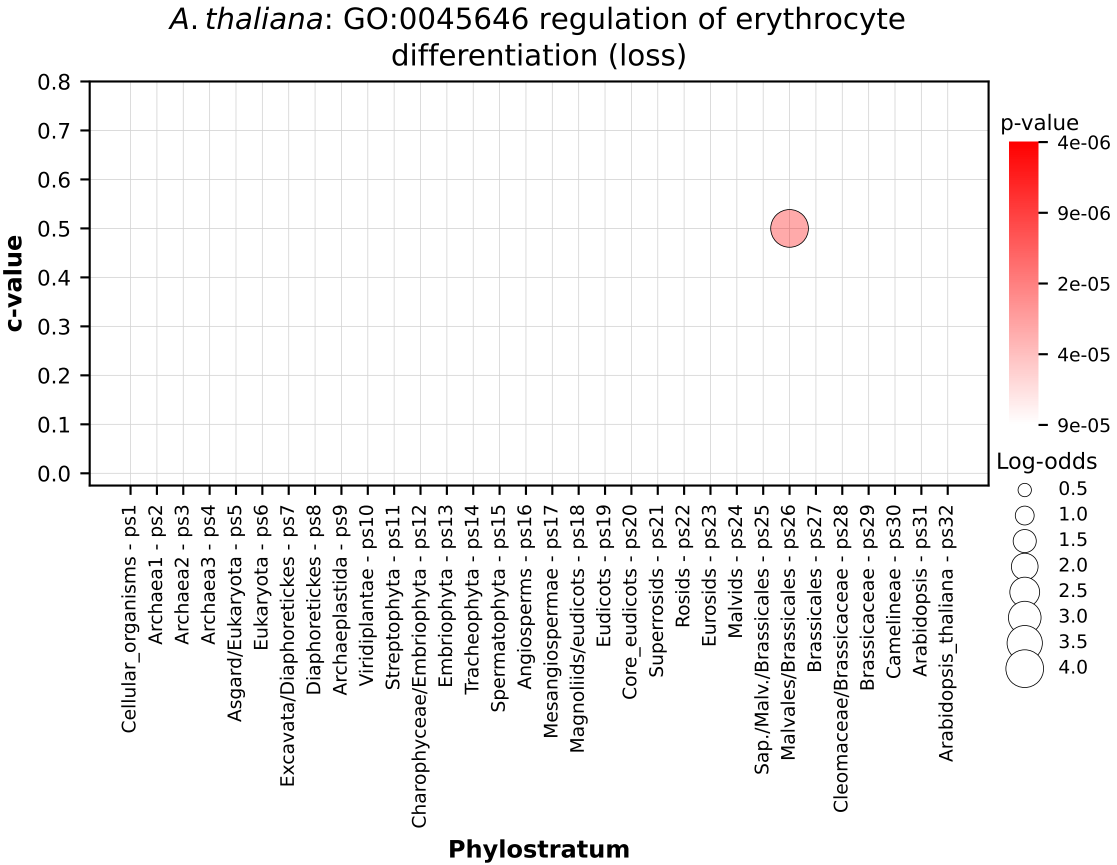

A. thaliana: GO:0045646 regulation of erythrocyte differentiation (loss)
c-value
0.8
0.7
0.6
0.5
0.4
0.3
0.2
0.1
0.0
p-value
4e-06
9e-06
2e-05
4e-05
9e-05
Log-odds
0.5
1.0
1.5
2.0
2.5
3.0
3.5
4.0
Cellular_organisms - ps1
Archaea1 - ps2
Archaea2 - ps3
Archaea3 - ps4
Asgard/Eukaryota - ps5
Eukaryota - ps6
Excavata/Diaphoretickes - ps7
Diaphoretickes - ps8
Archaeplastida - ps9
Viridiplantae - ps10
Streptophyta - ps11
Charophyceae/Embriophyta - ps12
Embriophyta - ps13
Tracheophyta - ps14
Spermatophyta - ps15
Angiosperms - ps16
Mesangiospermae - ps17
Magnoliids/eudicots - ps18
Eudicots - ps19
Core_eudicots - ps20
Superrosids - ps21
Rosids - ps22
Eurosids - ps23
Malvids - ps24
Sap./Malv./Brassicales - ps25
Malvales/Brassicales - ps26
Brassicales - ps27
Cleomaceae/Brassicaceae - ps28
Brassicaceae - ps29
Camelineae - ps30
Arabidopsis - ps31
Arabidopsis_thaliana - ps32
Phylostratum

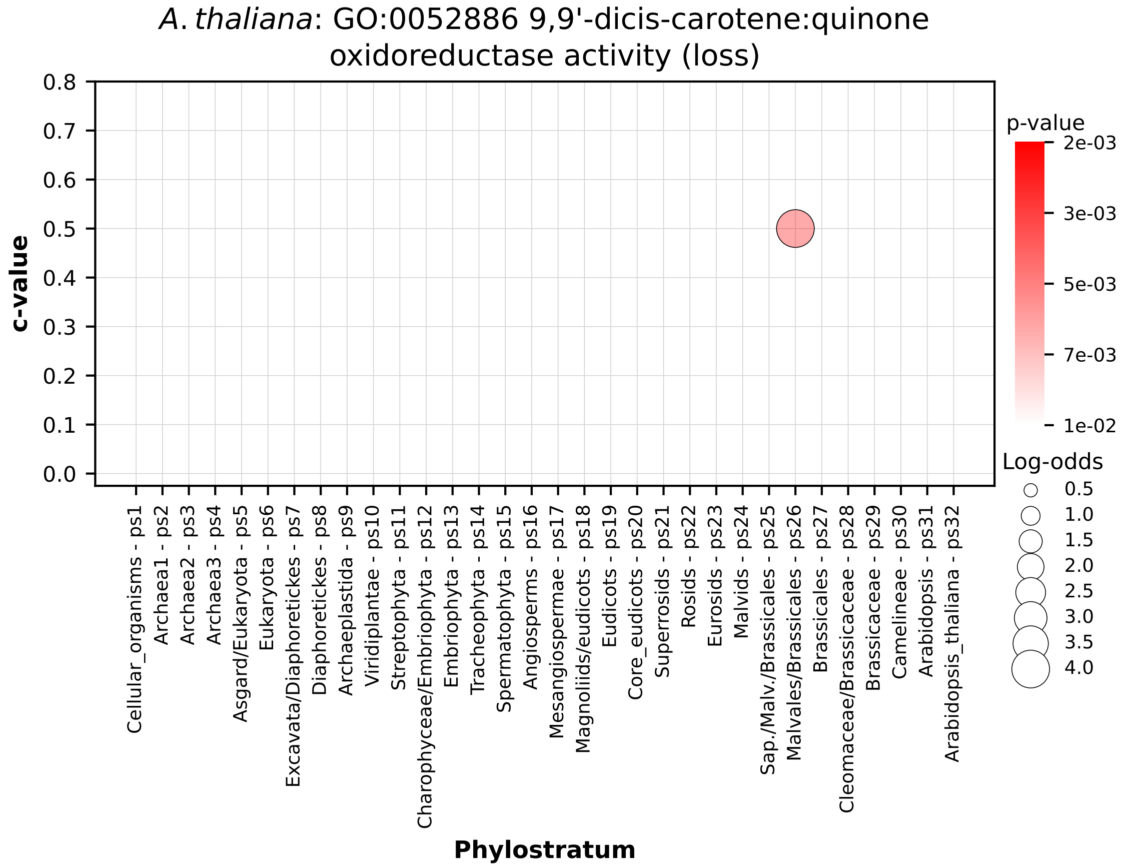

A. thaliana: GO:0052886 9,9'-dicis-carotene:quinone oxidoreductase activity (loss)
c-value
0.8
0.7
0.6
0.5
0.4
0.3
0.2
0.1
0.0
Phylostratum
Cellular_organisms - ps1
Archaea1 - ps2
Archaea2 - ps3
Archaea3 - ps4
Asgard/Eukaryota - ps5
Eukaryota - ps6
Excavata/Diaphoretickes - ps7
Diaphoretickes - ps8
Archaeplastida - ps9
Viridiplantae - ps10
Streptophyta - ps11
Charophyceae/Embriophyta - ps12
Embriophyta - ps13
Tracheophyta - ps14
Spermatophyta - ps15
Angiosperms - ps16
Mesangiospermae - ps17
Magnoliids/eudicots - ps18
Eudicots - ps19
Core_eudicots - ps20
Superrosids - ps21
Rosids - ps22
Eurosids - ps23
Malvids - ps24
Sap./Malv./Brassicales - ps25
Malvales/Brassicales - ps26
Brassicales - ps27
Cleomaceae/Brassicaceae - ps28
Brassicaceae - ps29
Camelineae - ps30
Arabidopsis - ps31
Arabidopsis_thaliana - ps32
p-value
2e-03
3e-03
5e-03
7e-03
1e-02
Log-odds
0.5
1.0
1.5
2.0
2.5
3.0
3.5
4.0

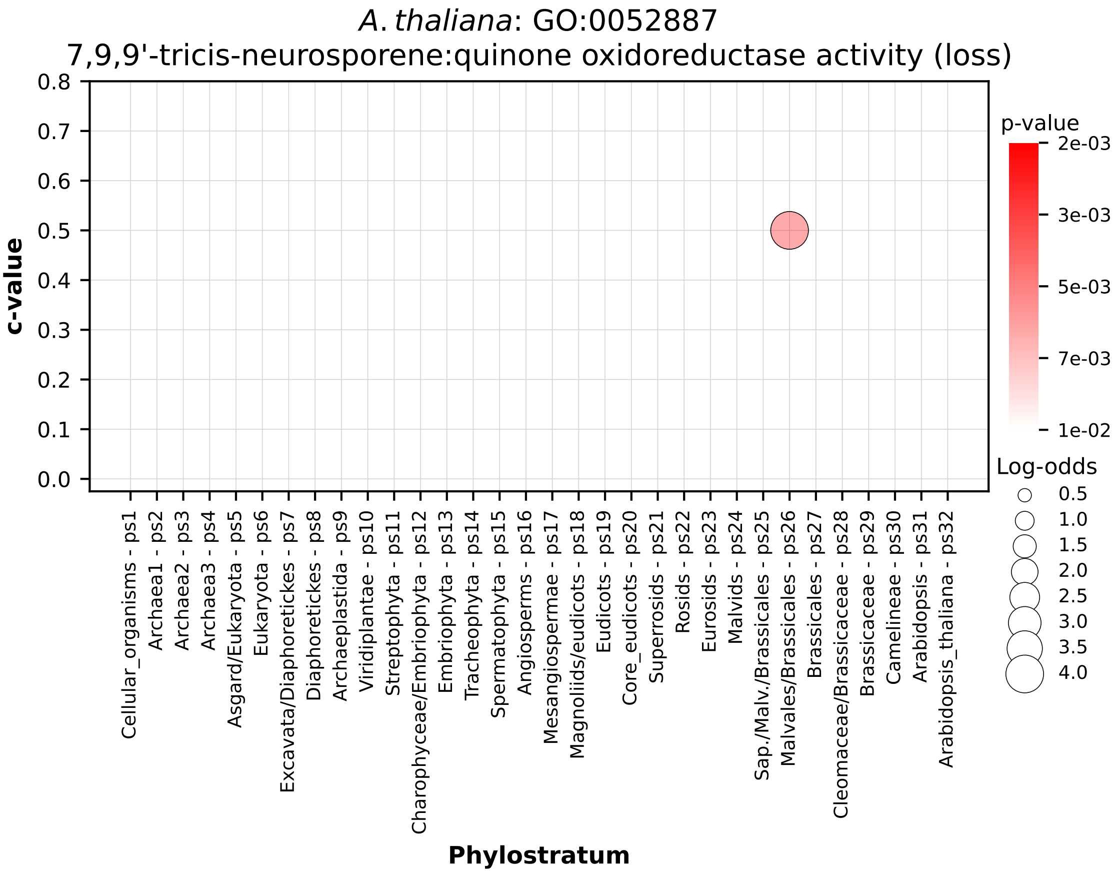

A. thaliana: GO:0052887
7,9,9'-tricis-neurosporene:quinone oxidoreductase activity (loss)
c-value
0.8
0.7
0.6
0.5
0.4
0.3
0.2
0.1
0.0
p-value
2e-03
3e-03
5e-03
7e-03
1e-02
Log-odds
0.5
1.0
1.5
2.0
2.5
3.0
3.5
4.0
Cellular_organisms - ps1
Archaea1 - ps2
Archaea2 - ps3
Archaea3 - ps4
Asgard/Eukaryota - ps5
Eukaryota - ps6
Excavata/Diaphoretickes - ps7
Diaphoretickes - ps8
Archaeplastida - ps9
Viridiplantae - ps10
Streptophyta - ps11
Charophyceae/Embriophyta - ps12
Embriophyta - ps13
Tracheophyta - ps14
Spermatophyta - ps15
Angiosperms - ps16
Mesangiospermae - ps17
Magnoliids/eudicots - ps18
Eudicots - ps19
Core_eudicots - ps20
Superrosids - ps21
Rosids - ps22
Eurosids - ps23
Malvids - ps24
Sap./Malv./Brassicales - ps25
Malvales/Brassicales - ps26
Brassicales - ps27
Cleomaceae/Brassicaceae - ps28
Brassicaceae - ps29
Camelineae - ps30
Arabidopsis - ps31
Arabidopsis_thaliana - ps32
Phylostratum

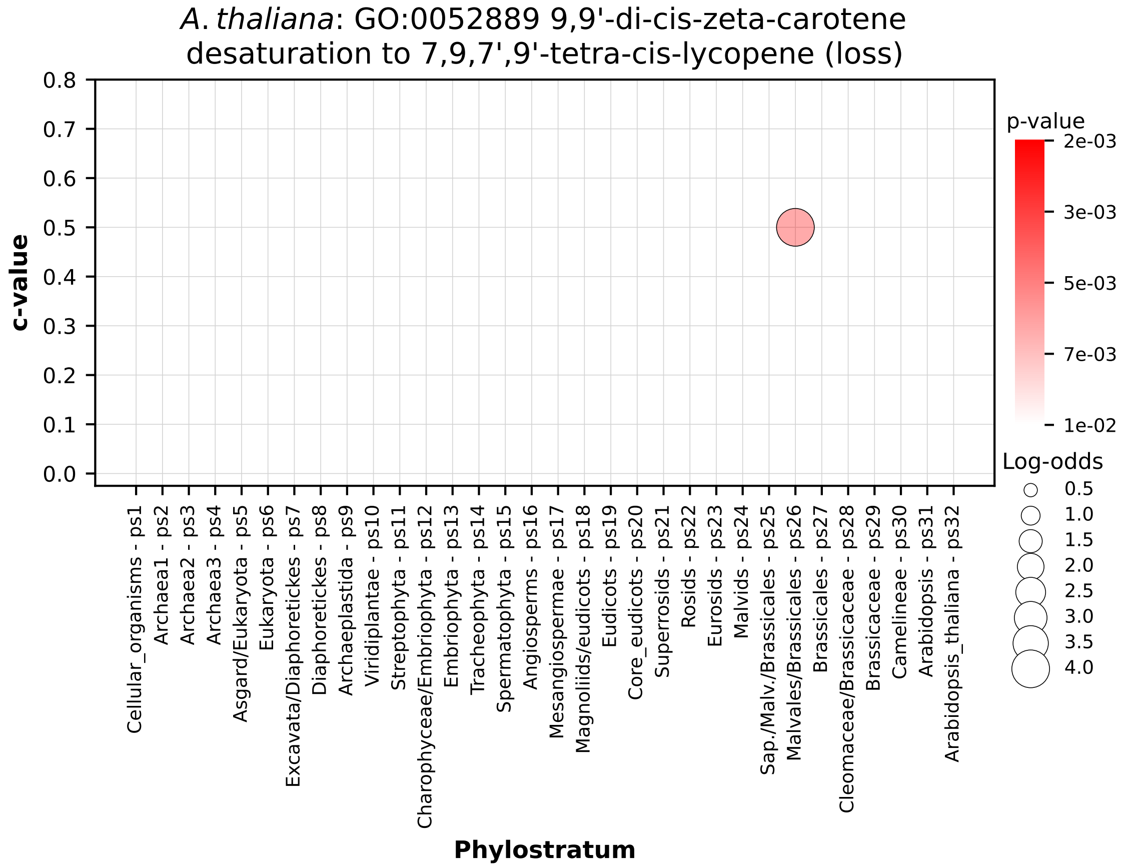

A. thaliana: GO:0052889 9,9'-di-cis-zeta-carotene desaturation to 7,9,7',9'-tetra-cis-lycopene (loss)
c-value
0.0
0.1
0.2
0.3
0.4
0.5
0.6
0.7
0.8
Phylostratum
Cellular_organisms - ps1
Archaea1 - ps2
Archaea2 - ps3
Archaea3 - ps4
Asgard/Eukaryota - ps5
Eukaryota - ps6
Excavata/Diaphoretickes - ps7
Diaphoretickes - ps8
Archaeplastida - ps9
Viridiplantae - ps10
Streptophyta - ps11
Charophyceae/Embriophyta - ps12
Embriophyta - ps13
Tracheophyta - ps14
Spermatophyta - ps15
Angiosperms - ps16
Mesangiospermae - ps17
Magnoliids/eudicots - ps18
Eudicots - ps19
Core_eudicots - ps20
Superrosids - ps21
Rosids - ps22
Eurosids - ps23
Malvids - ps24
Sap./Malv./Brassicales - ps25
Malvales/Brassicales - ps26
Brassicales - ps27
Cleomaceae/Brassicaceae - ps28
Brassicaceae - ps29
Camelineae - ps30
Arabidopsis - ps31
Arabidopsis_thaliana - ps32
p-value
2e-03
3e-03
5e-03
7e-03
1e-02
Log-odds
0.5
1.0
1.5
2.0
2.5
3.0
3.5
4.0

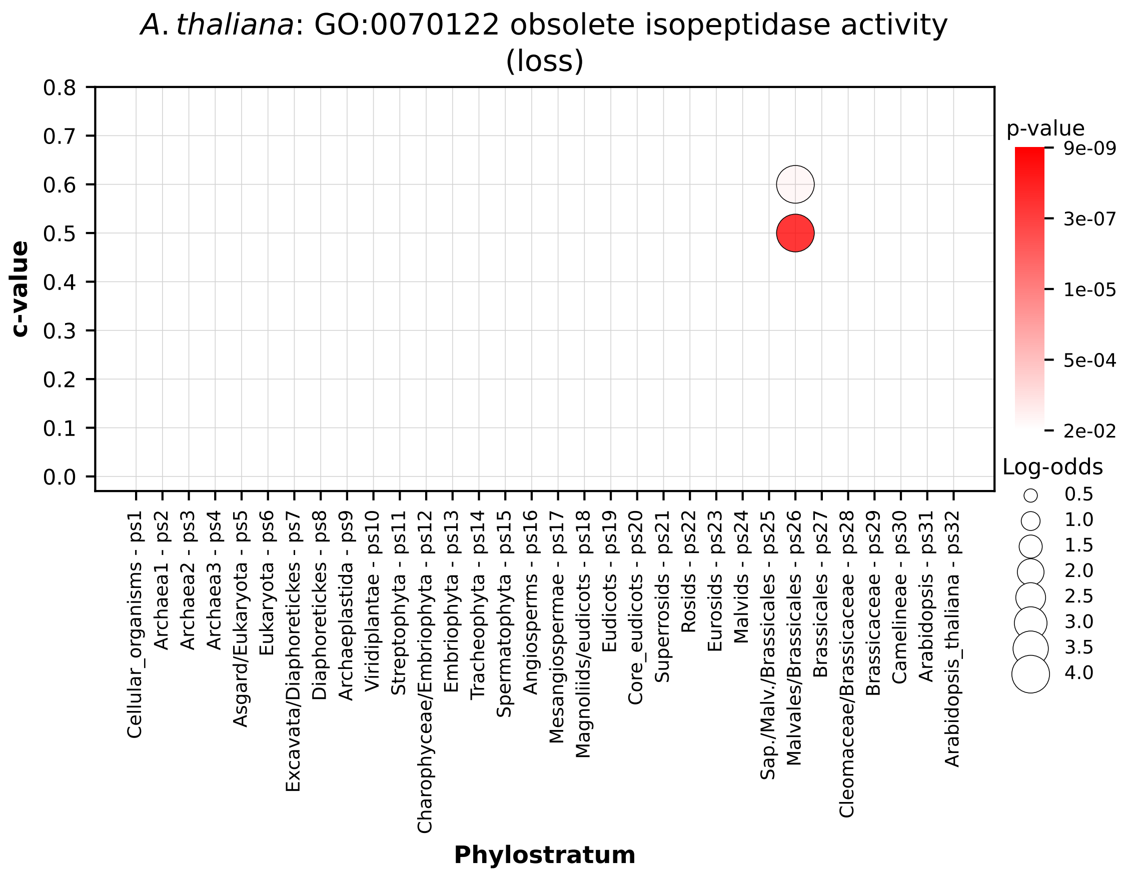
A. thaliana: GO:0070122 obsolete isopeptidase activity (loss)
c-value
0.8
0.7
0.6
0.5
0.4
0.3
0.2
0.1
0.0
p-value
9e-09
3e-07
1e-05
5e-04
2e-02
Log-odds
0.5
1.0
1.5
2.0
2.5
3.0
3.5
4.0
Cellular_organisms - ps1
Archaea1 - ps2
Archaea2 - ps3
Archaea3 - ps4
Asgard/Eukaryota - ps5
Eukaryota - ps6
Excavata/Diaphoretickes - ps7
Diaphoretickes - ps8
Archaeplastida - ps9
Viridiplantae - ps10
Streptophyta - ps11
Charophyceae/Embriophyta - ps12
Embriophyta - ps13
Tracheophyta - ps14
Spermatophyta - ps15
Angiosperms - ps16
Mesangiospermae - ps17
Magnoliids/eudicots - ps18
Eudicots - ps19
Core_eudicots - ps20
Superrosids - ps21
Rosids - ps22
Eurosids - ps23
Malvids - ps24
Sap./Malv./Brassicales - ps25
Malvales/Brassicales - ps26
Brassicales - ps27
Cleomaceae/Brassicaceae - ps28
Brassicaceae - ps29
Camelineae - ps30
Arabidopsis - ps31
Arabidopsis_thaliana - ps32
Phylostratum

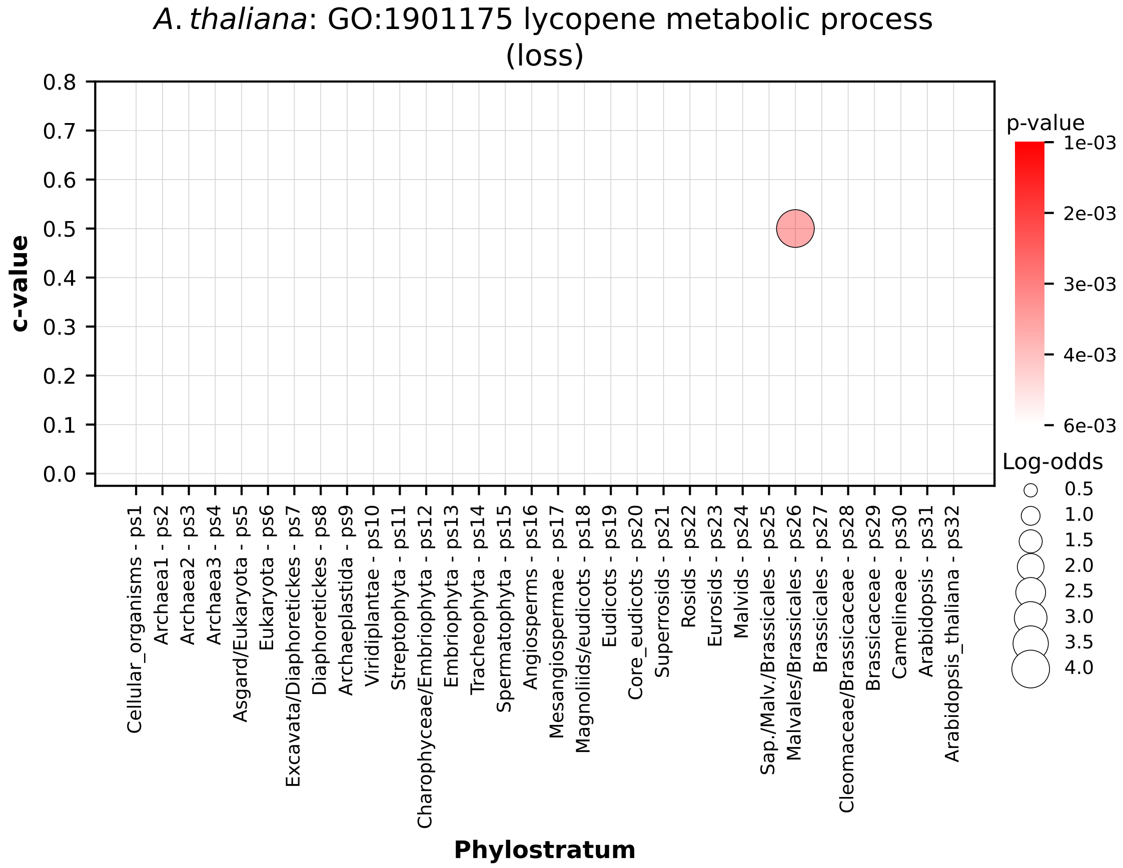

A. thaliana: GO:1901175 lycopene metabolic process
(loss)
c-value
0.8
0.7
0.6
0.5
0.4
0.3
0.2
0.1
0.0
p-value
1e-03
2e-03
3e-03
4e-03
6e-03
Log-odds
0.5
1.0
1.5
2.0
2.5
3.0
3.5
4.0
Cellular_organisms - ps1
Archaea1 - ps2
Archaea2 - ps3
Archaea3 - ps4
Asgard/Eukaryota - ps5
Eukaryota - ps6
Excavata/Diaphoretickes - ps7
Diaphoretickes - ps8
Archaeplastida - ps9
Viridiplantae - ps10
Streptophyta - ps11
Charophyceae/Embriophyta - ps12
Embriophyta - ps13
Tracheophyta - ps14
Spermatophyta - ps15
Angiosperms - ps16
Mesangiospermae - ps17
Magnoliids/eudicots - ps18
Eudicots - ps19
Core_eudicots - ps20
Superrosids - ps21
Rosids - ps22
Eurosids - ps23
Malvids - ps24
Sap./Malv./Brassicales - ps25
Malvales/Brassicales - ps26
Brassicales - ps27
Cleomaceae/Brassicaceae - ps28
Brassicaceae - ps29
Camelineae - ps30
Arabidopsis - ps31
Arabidopsis_thaliana - ps32
Phylostratum

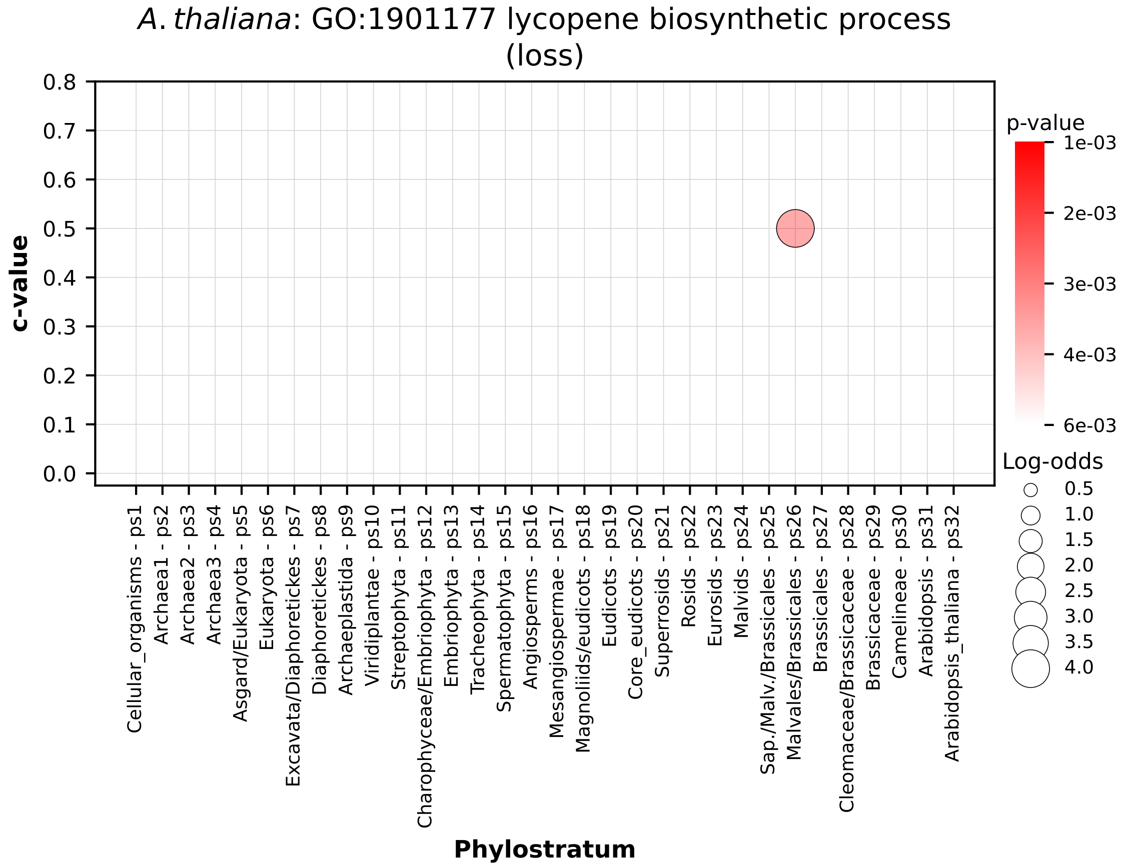

A. thaliana: GO:1901177 lycopene biosynthetic process
(loss)
c-value
0.8
0.7
0.6
0.5
0.4
0.3
0.2
0.1
0.0
p-value
1e-03
2e-03
3e-03
4e-03
6e-03
Log-odds
0.5
1.0
1.5
2.0
2.5
3.0
3.5
4.0
Cellular_organisms - ps1
Archaea1 - ps2
Archaea2 - ps3
Archaea3 - ps4
Asgard/Eukaryota - ps5
Eukaryota - ps6
Excavata/Diaphoretickes - ps7
Diaphoretickes - ps8
Archaeplastida - ps9
Viridiplantae - ps10
Streptophyta - ps11
Charophyceae/Embriophyta - ps12
Embriophyta - ps13
Tracheophyta - ps14
Spermatophyta - ps15
Angiosperms - ps16
Mesangiospermae - ps17
Magnoliids/eudicots - ps18
Eudicots - ps19
Core_eudicots - ps20
Superrosids - ps21
Rosids - ps22
Eurosids - ps23
Malvids - ps24
Sap./Malv./Brassicales - ps25
Malvales/Brassicales - ps26
Brassicales - ps27
Cleomaceae/Brassicaceae - ps28
Brassicaceae - ps29
Camelineae - ps30
Arabidopsis - ps31
Arabidopsis_thaliana - ps32
Phylostratum

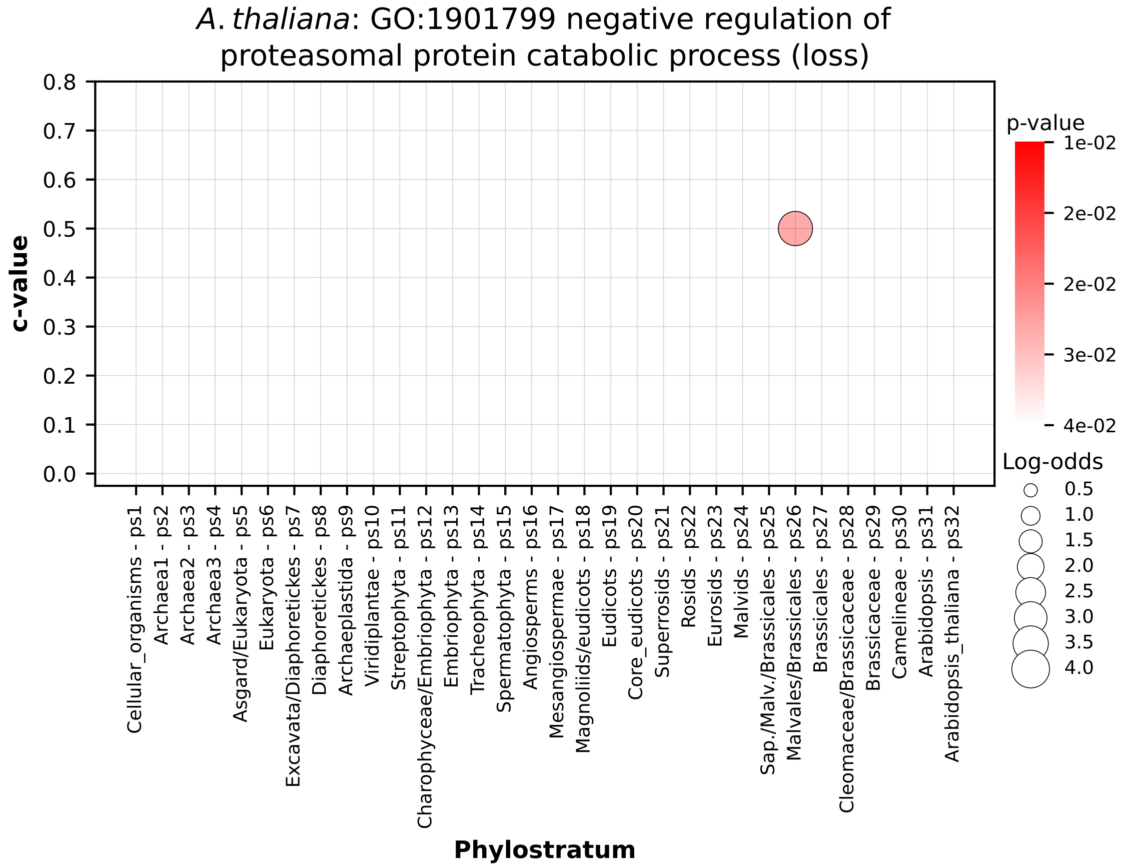

A. thaliana: GO:1901799 negative regulation of proteasomal protein catabolic process (loss)
c-value
0.8
0.7
0.6
0.5
0.4
0.3
0.2
0.1
0.0
p-value
1e-02
2e-02
2e-02
3e-02
4e-02
Log-odds
0.5
1.0
1.5
2.0
2.5
3.0
3.5
4.0
Cellular_organisms - ps1
Archaea1 - ps2
Archaea2 - ps3
Archaea3 - ps4
Asgard/Eukaryota - ps5
Eukaryota - ps6
Excavata/Diaphoretickes - ps7
Diaphoretickes - ps8
Archaeplastida - ps9
Viridiplantae - ps10
Streptophyta - ps11
Charophyceae/Embriophyta - ps12
Embriophyta - ps13
Tracheophyta - ps14
Spermatophyta - ps15
Angiosperms - ps16
Mesangiospermae - ps17
Magnoliids/eudicots - ps18
Eudicots - ps19
Core_eudicots - ps20
Superrosids - ps21
Rosids - ps22
Eurosids - ps23
Malvids - ps24
Sap./Malv./Brassicales - ps25
Malvales/Brassicales - ps26
Brassicales - ps27
Cleomaceae/Brassicaceae - ps28
Brassicaceae - ps29
Camelineae - ps30
Arabidopsis - ps31
Arabidopsis_thaliana - ps32
Phylostratum

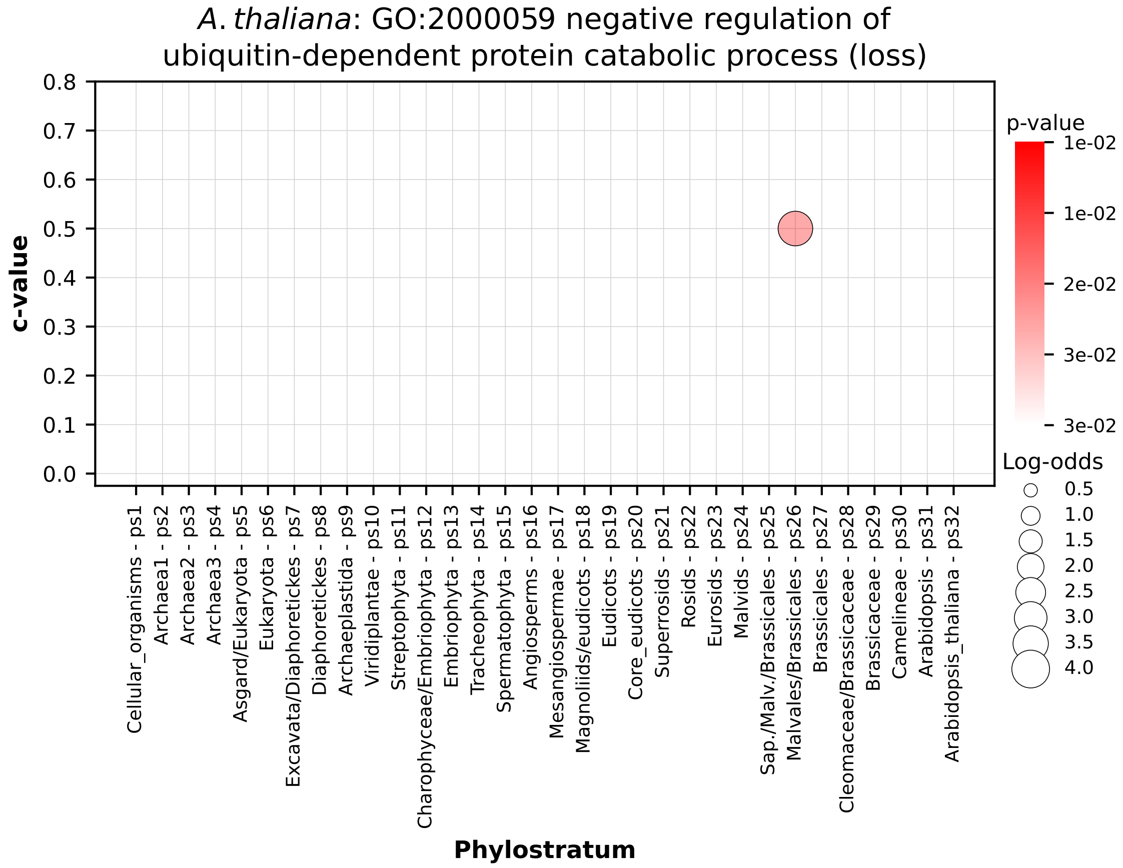

A. thaliana: GO:2000059 negative regulation of ubiquitin-dependent protein catabolic process (loss)
c-value
0.8
0.7
0.6
0.5
0.4
0.3
0.2
0.1
0.0
p-value
1e-02
1e-02
2e-02
3e-02
3e-02
Log-odds
0.5
1.0
1.5
2.0
2.5
3.0
3.5
4.0
Cellular_organisms - ps1
Archaea1 - ps2
Archaea2 - ps3
Archaea3 - ps4
Asgard/Eukaryota - ps5
Eukaryota - ps6
Excavata/Diaphoretickes - ps7
Diaphoretickes - ps8
Archaeplastida - ps9
Viridiplantae - ps10
Streptophyta - ps11
Charophyceae/Embriophyta - ps12
Embriophyta - ps13
Tracheophyta - ps14
Spermatophyta - ps15
Angiosperms - ps16
Mesangiospermae - ps17
Magnoliids/eudicots - ps18
Eudicots - ps19
Core_eudicots - ps20
Superrosids - ps21
Rosids - ps22
Eurosids - ps23
Malvids - ps24
Sap./Malv./Brassicales - ps25
Malvales/Brassicales - ps26
Brassicales - ps27
Cleomaceae/Brassicaceae - ps28
Brassicaceae - ps29
Camelineae - ps30
Arabidopsis - ps31
Arabidopsis_thaliana - ps32
Phylostratum

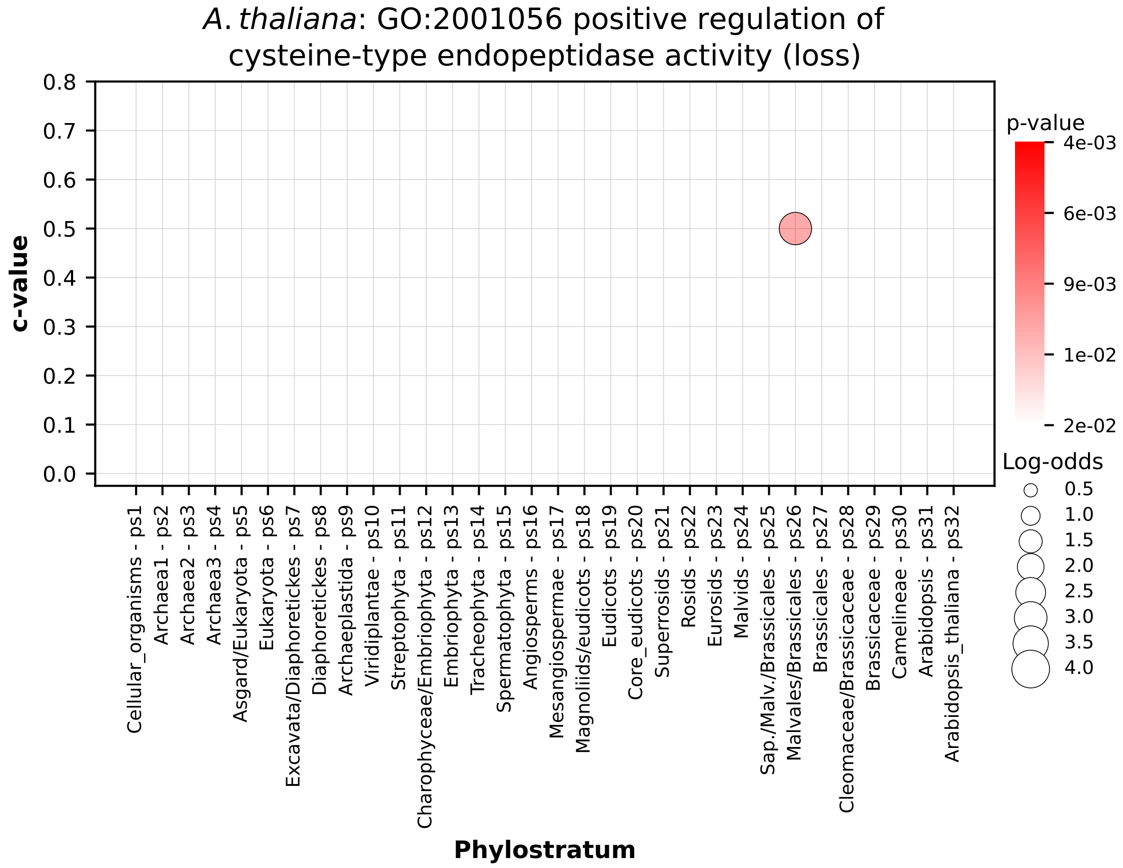

A. thaliana: GO:2001056 positive regulation of cysteine-type endopeptidase activity (loss)
c-value
0.8
0.7
0.6
0.5
0.4
0.3
0.2
0.1
0.0
Phylostratum
Cellular_organisms - ps1
Archaea1 - ps2
Archaea2 - ps3
Archaea3 - ps4
Asgard/Eukaryota - ps5
Eukaryota - ps6
Excavata/Diaphoretickes - ps7
Diaphoretickes - ps8
Archaeplastida - ps9
Viridiplantae - ps10
Streptophyta - ps11
Charophyceae/Embriophyta - ps12
Embriophyta - ps13
Tracheophyta - ps14
Spermatophyta - ps15
Angiosperms - ps16
Mesangiospermae - ps17
Magnoliids/eudicots - ps18
Eudicots - ps19
Core_eudicots - ps20
Superrosids - ps21
Rosids - ps22
Eurosids - ps23
Malvids - ps24
Sap./Malv./Brassicales - ps25
Malvales/Brassicales - ps26
Brassicales - ps27
Cleomaceae/Brassicaceae - ps28
Brassicaceae - ps29
Camelineae - ps30
Arabidopsis - ps31
Arabidopsis_thaliana - ps32
p-value
4e-03
6e-03
9e-03
1e-02
2e-02
Log-odds
0.5
1.0
1.5
2.0
2.5
3.0
3.5
4.0

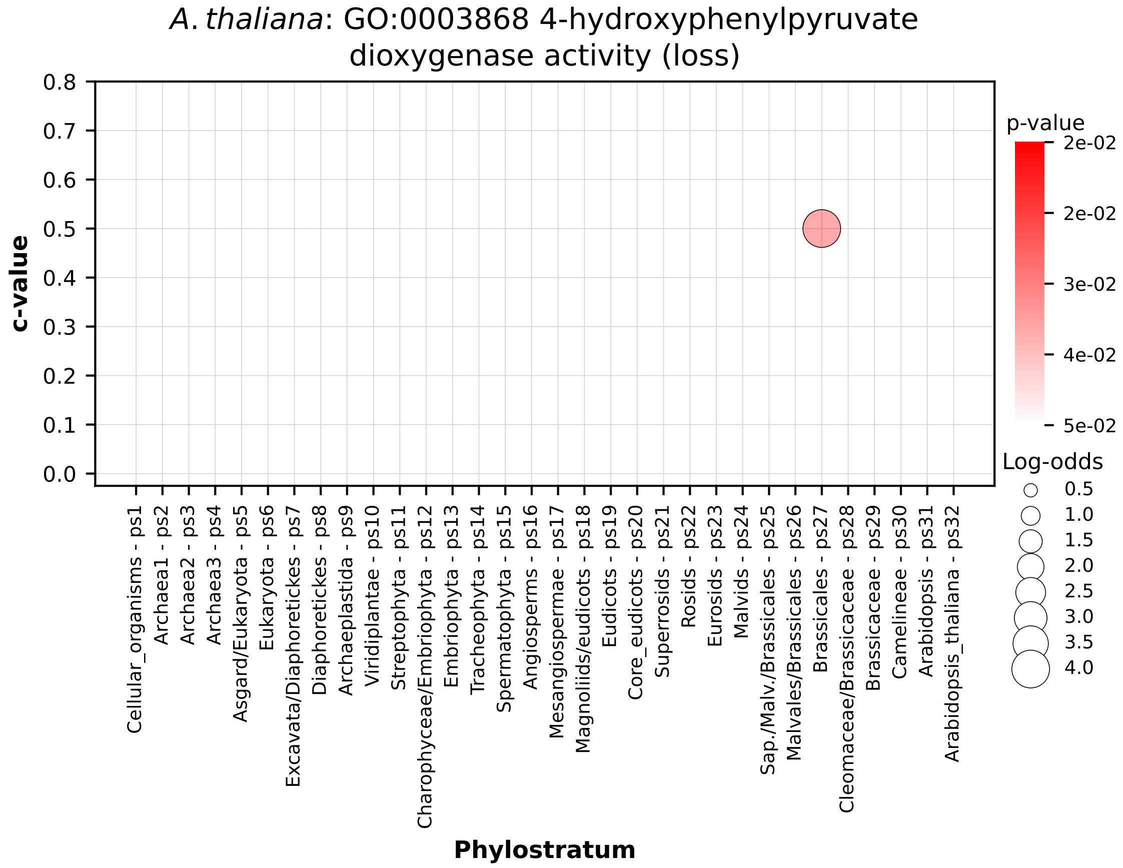
A. thaliana: GO:0003868 4-hydroxyphenylpyruvate dioxygenase activity (loss)
c-value
0.8
0.7
0.6
0.5
0.4
0.3
0.2
0.1
0.0
Phylostratum
Cellular_organisms - ps1
Archaea1 - ps2
Archaea2 - ps3
Archaea3 - ps4
Asgard/Eukaryota - ps5
Eukaryota - ps6
Excavata/Diaphoretickes - ps7
Diaphoretickes - ps8
Archaeplastida - ps9
Viridiplantae - ps10
Streptophyta - ps11
Charophyceae/Embriophyta - ps12
Embriophyta - ps13
Tracheophyta - ps14
Spermatophyta - ps15
Angiosperms - ps16
Mesangiospermae - ps17
Magnoliids/eudicots - ps18
Eudicots - ps19
Core_eudicots - ps20
Superrosids - ps21
Rosids - ps22
Eurosids - ps23
Malvids - ps24
Sap./Malv./Brassicales - ps25
Malvales/Brassicales - ps26
Brassicales - ps27
Cleomaceae/Brassicaceae - ps28
Brassicaceae - ps29
Camelineae - ps30
Arabidopsis - ps31
Arabidopsis_thaliana - ps32
p-value
2e-02
2e-02
3e-02
4e-02
5e-02
Log-odds
0.5
1.0
1.5
2.0
2.5
3.0
3.5
4.0

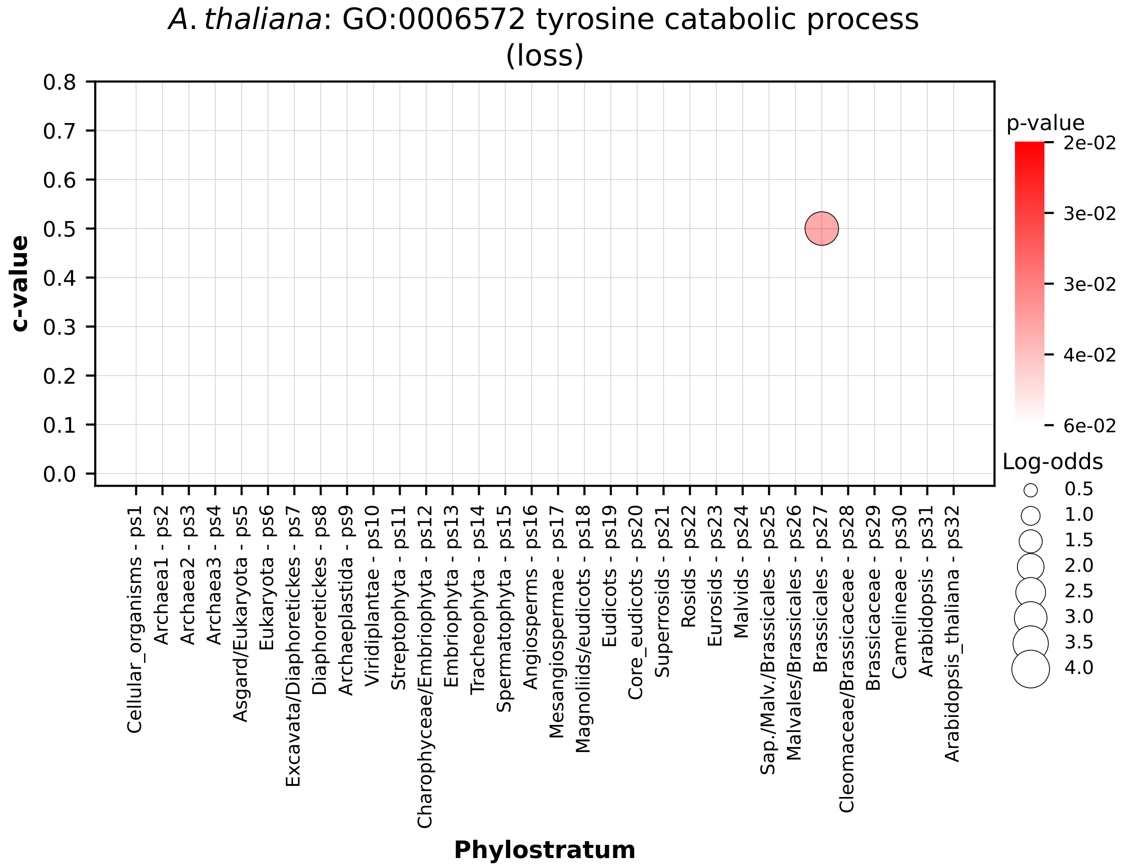

A. thaliana: GO:0006572 tyrosine catabolic process
(loss)
c-value
0.8
0.7
0.6
0.5
0.4
0.3
0.2
0.1
0.0
p-value
2e-02
3e-02
3e-02
4e-02
6e-02
Log-odds
0.5
1.0
1.5
2.0
2.5
3.0
3.5
4.0
Cellular_organisms - ps1
Archaea1 - ps2
Archaea2 - ps3
Archaea3 - ps4
Asgard/Eukaryota - ps5
Eukaryota - ps6
Excavata/Diaphoretickes - ps7
Diaphoretickes - ps8
Archaeplastida - ps9
Viridiplantae - ps10
Streptophyta - ps11
Charophyceae/Embriophyta - ps12
Embriophyta - ps13
Tracheophyta - ps14
Spermatophyta - ps15
Angiosperms - ps16
Mesangiospermae - ps17
Magnoliids/eudicots - ps18
Eudicots - ps19
Core_eudicots - ps20
Superrosids - ps21
Rosids - ps22
Eurosids - ps23
Malvids - ps24
Sap./Malv./Brassicales - ps25
Malvales/Brassicales - ps26
Brassicales - ps27
Cleomaceae/Brassicaceae - ps28
Brassicaceae - ps29
Camelineae - ps30
Arabidopsis - ps31
Arabidopsis_thaliana - ps32
Phylostratum

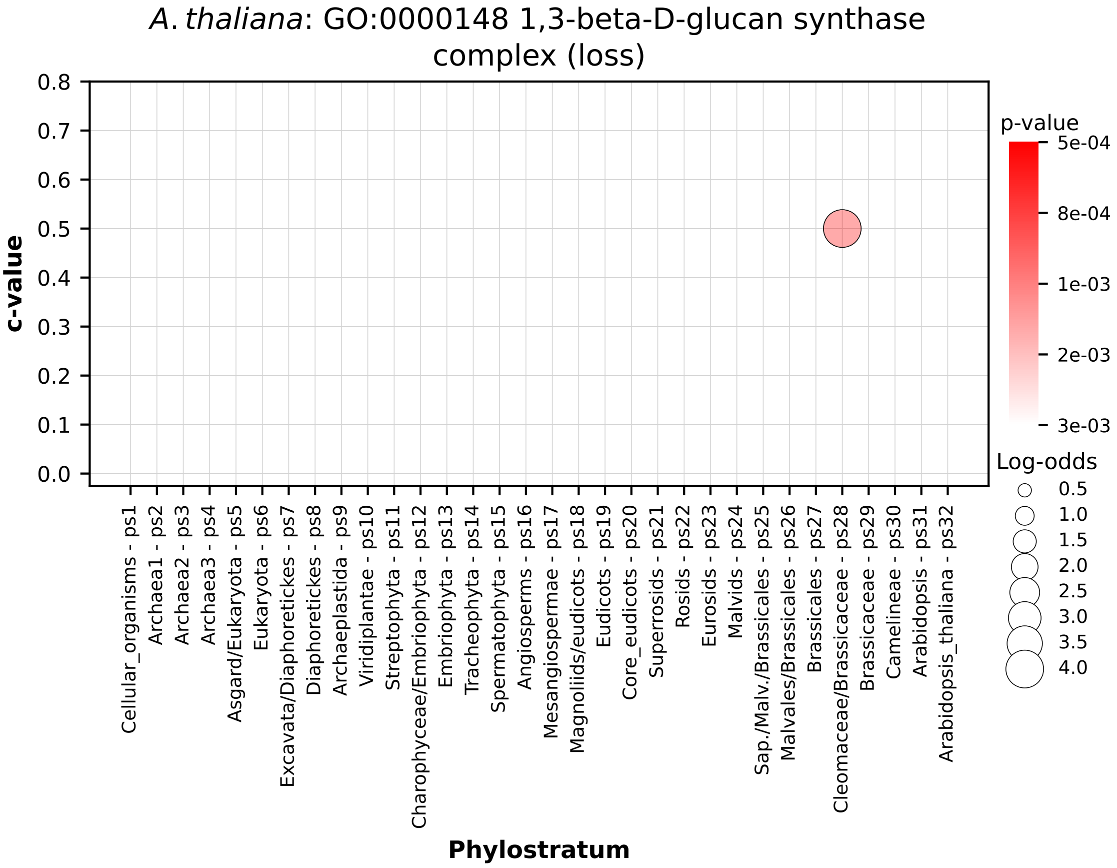

A. thaliana: GO:0000148 1,3-beta-D-glucan synthase complex (loss)
c-value
0.8
0.7
0.6
0.5
0.4
0.3
0.2
0.1
0.0
Cellular_organisms - ps1
Archaea1 - ps2
Archaea2 - ps3
Archaea3 - ps4
Asgard/Eukaryota - ps5
Eukaryota - ps6
Excavata/Diaphoretickes - ps7
Diaphoretickes - ps8
Archaeplastida - ps9
Viridiplantae - ps10
Streptophyta - ps11
Charophyceae/Embriophyta - ps12
Embriophyta - ps13
Tracheophyta - ps14
Spermatophyta - ps15
Angiosperms - ps16
Mesangiospermae - ps17
Magnoliids/eudicots - ps18
Eudicots - ps19
Core_eudicots - ps20
Superrosids - ps21
Rosids - ps22
Eurosids - ps23
Malvids - ps24
Sap./Malv./Brassicales - ps25
Malvales/Brassicales - ps26
Brassicales - ps27
Cleomaceae/Brassicaceae - ps28
Brassicaceae - ps29
Camelineae - ps30
Arabidopsis - ps31
Arabidopsis_thaliana - ps32
Phylostratum
p-value
5e-04
8e-04
1e-03
2e-03
3e-03
Log-odds
0.5
1.0
1.5
2.0
2.5
3.0
3.5
4.0

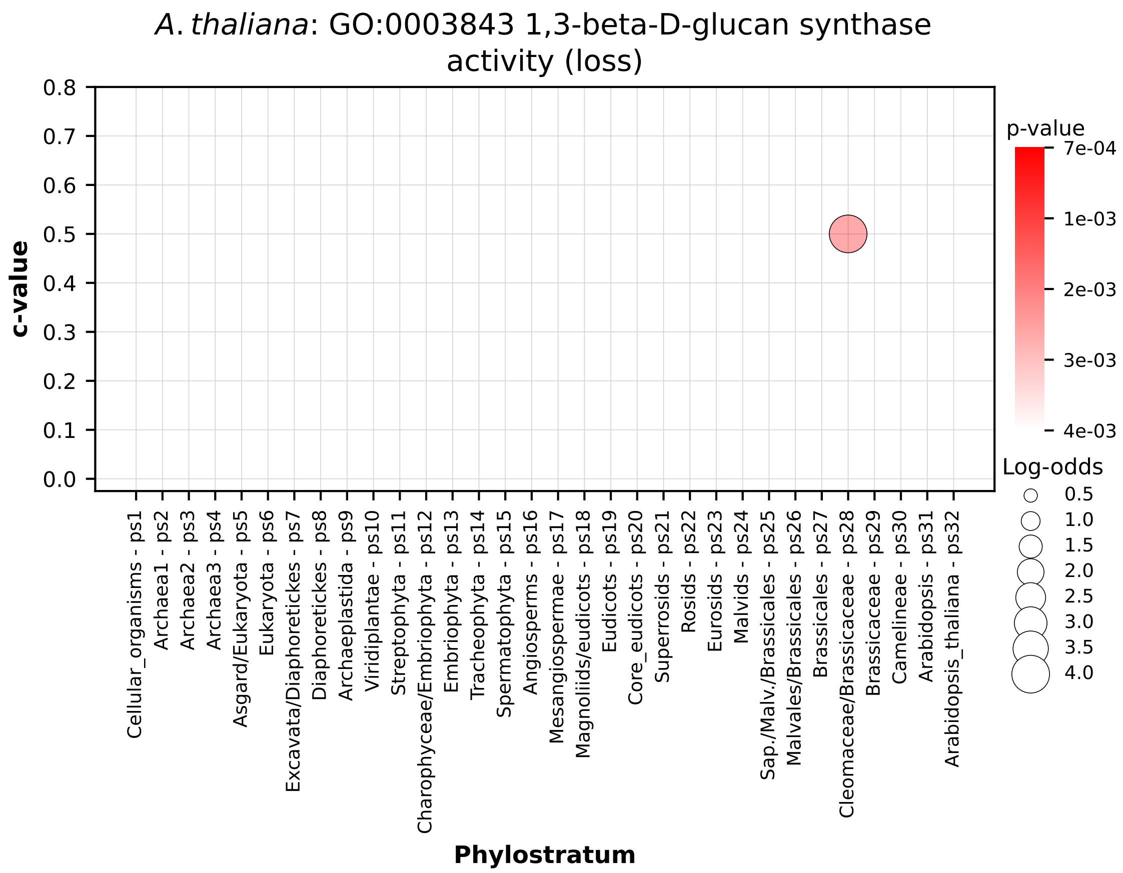

A. thaliana: GO:0003843 1,3-beta-D-glucan synthase activity (loss)
c-value
0.0
0.1
0.2
0.3
0.4
0.5
0.6
0.7
0.8
p-value
7e-04
1e-03
2e-03
3e-03
4e-03
Log-odds
0.5
1.0
1.5
2.0
2.5
3.0
3.5
4.0
Cellular_organisms - ps1
Archaea1 - ps2
Archaea2 - ps3
Archaea3 - ps4
Asgard/Eukaryota - ps5
Eukaryota - ps6
Excavata/Diaphoretickes - ps7
Diaphoretickes - ps8
Archaeplastida - ps9
Viridiplantae - ps10
Streptophyta - ps11
Charophyceae/Embriophyta - ps12
Embriophyta - ps13
Tracheophyta - ps14
Spermatophyta - ps15
Angiosperms - ps16
Mesangiospermae - ps17
Magnoliids/eudicots - ps18
Eudicots - ps19
Core_eudicots - ps20
Superrosids - ps21
Rosids - ps22
Eurosids - ps23
Malvids - ps24
Sap./Malv./Brassicales - ps25
Malvales/Brassicales - ps26
Brassicales - ps27
Cleomaceae/Brassicaceae - ps28
Brassicaceae - ps29
Camelineae - ps30
Arabidopsis - ps31
Arabidopsis_thaliana - ps32
Phylostratum

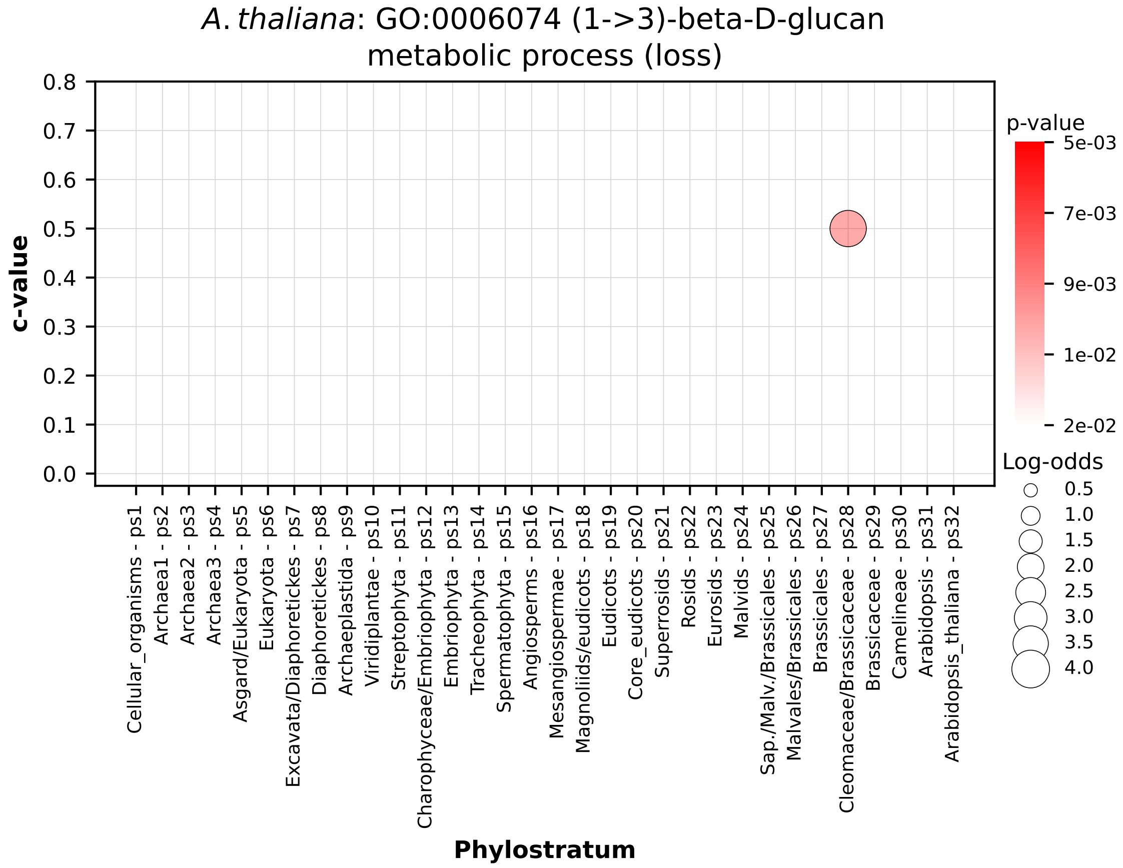

$A. thaliana$: GO:0006074 (1->3)-beta-D-glucan metabolic process (loss)
c-value
0.0
0.1
0.2
0.3
0.4
0.5
0.6
0.7
0.8
Phylostratum
Cellular_organisms - ps1
Archaea1 - ps2
Archaea2 - ps3
Archaea3 - ps4
Asgard/Eukaryota - ps5
Eukaryota - ps6
Excavata/Diaphoretickes - ps7
Diaphoretickes - ps8
Archaeplastida - ps9
Viridiplantae - ps10
Streptophyta - ps11
Charophyceae/Embriophyta - ps12
Embriophyta - ps13
Tracheophyta - ps14
Spermatophyta - ps15
Angiosperms - ps16
Mesangiospermae - ps17
Magnoliids/eudicots - ps18
Eudicots - ps19
Core_eudicots - ps20
Superrosids - ps21
Rosids - ps22
Eurosids - ps23
Malvids - ps24
Sap./Malv./Brassicales - ps25
Malvales/Brassicales - ps26
Brassicales - ps27
Cleomaceae/Brassicaceae - ps28
Brassicaceae - ps29
Camelineae - ps30
Arabidopsis - ps31
Arabidopsis_thaliana - ps32
p-value
5e-03
7e-03
9e-03
1e-02
2e-02
Log-odds
0.5
1.0
1.5
2.0
2.5
3.0
3.5
4.0

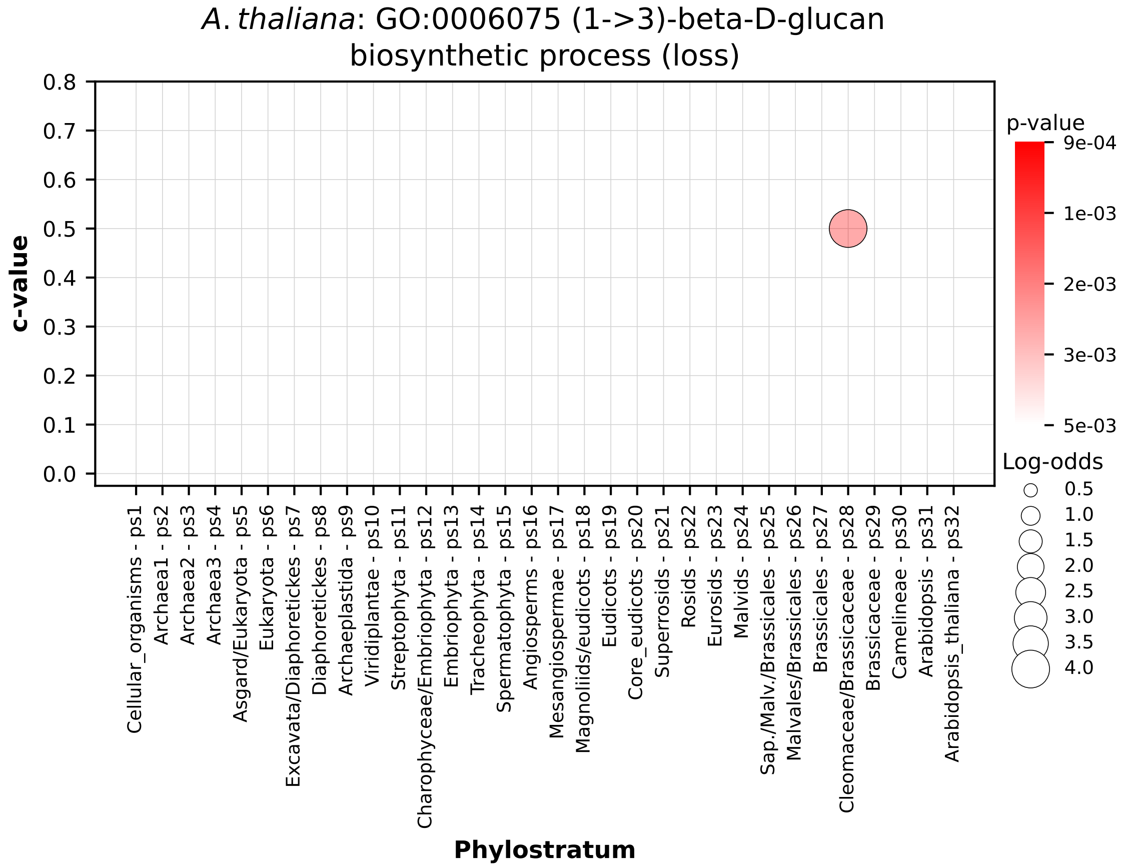

A. thaliana: GO:0006075 (1->3)-beta-D-glucan biosynthetic process (loss)
c-value
0.0
0.1
0.2
0.3
0.4
0.5
0.6
0.7
0.8
Phylostratum
Cellular_organisms - ps1
Archaea1 - ps2
Archaea2 - ps3
Archaea3 - ps4
Asgard/Eukaryota - ps5
Eukaryota - ps6
Excavata/Diaphoretickes - ps7
Diaphoretickes - ps8
Archaeplastida - ps9
Viridiplantae - ps10
Streptophyta - ps11
Charophyceae/Embriophyta - ps12
Embriophyta - ps13
Tracheophyta - ps14
Spermatophyta - ps15
Angiosperms - ps16
Mesangiospermae - ps17
Magnoliids/eudicots - ps18
Eudicots - ps19
Core_eudicots - ps20
Superrosids - ps21
Rosids - ps22
Eurosids - ps23
Malvids - ps24
Sap./Malv./Brassicales - ps25
Malvales/Brassicales - ps26
Brassicales - ps27
Cleomaceae/Brassicaceae - ps28
Brassicaceae - ps29
Camelineae - ps30
Arabidopsis - ps31
Arabidopsis_thaliana - ps32
p-value
9e-04
1e-03
2e-03
3e-03
5e-03
Log-odds
0.5
1.0
1.5
2.0
2.5
3.0
3.5
4.0

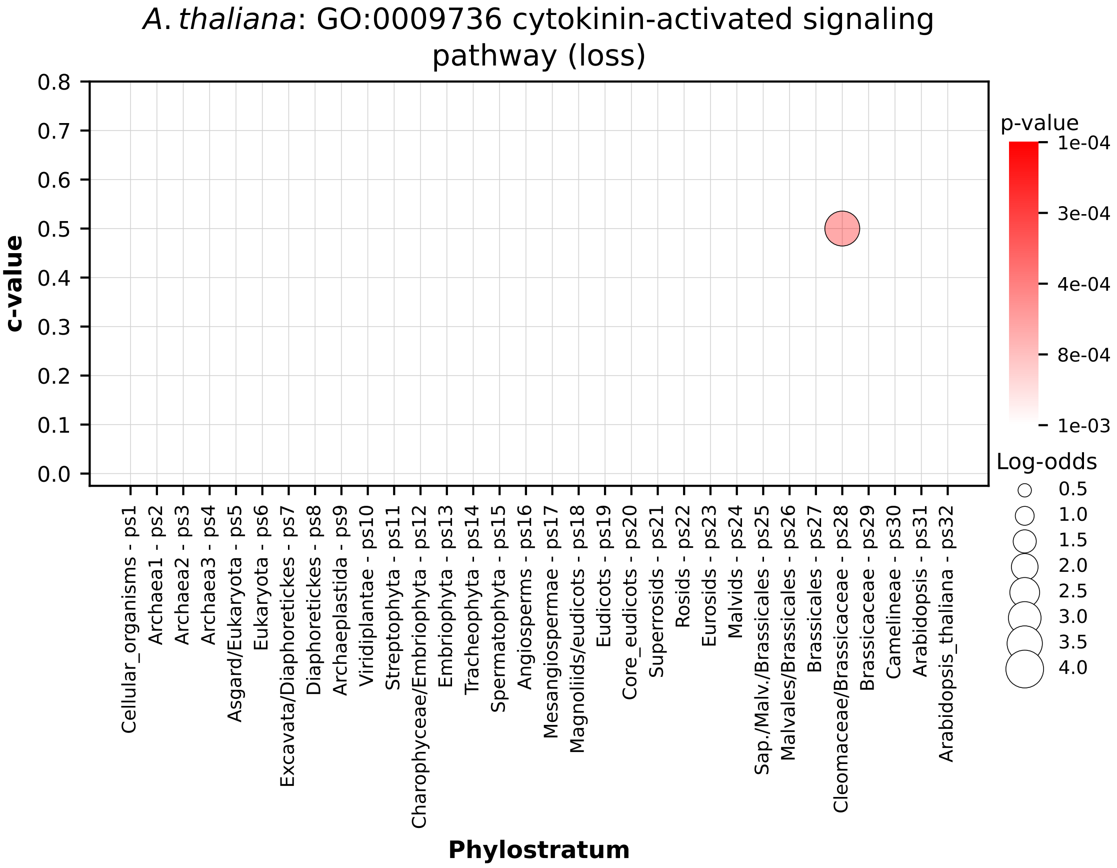

A. thaliana: GO:0009736 cytokinin-activated signaling pathway (loss)
c-value
0.8
0.7
0.6
0.5
0.4
0.3
0.2
0.1
0.0
p-value
1e-04
3e-04
4e-04
8e-04
1e-03
Log-odds
0.5
1.0
1.5
2.0
2.5
3.0
3.5
4.0
Cellular_organisms - ps1
Archaea1 - ps2
Archaea2 - ps3
Archaea3 - ps4
Asgard/Eukaryota - ps5
Eukaryota - ps6
Excavata/Diaphoretickes - ps7
Diaphoretickes - ps8
Archaeplastida - ps9
Viridiplantae - ps10
Streptophyta - ps11
Charophyceae/Embriophyta - ps12
Embriophyta - ps13
Tracheophyta - ps14
Spermatophyta - ps15
Angiosperms - ps16
Mesangiospermae - ps17
Magnoliids/eudicots - ps18
Eudicots - ps19
Core_eudicots - ps20
Superrosids - ps21
Rosids - ps22
Eurosids - ps23
Malvids - ps24
Sap./Malv./Brassicales - ps25
Malvales/Brassicales - ps26
Brassicales - ps27
Cleomaceae/Brassicaceae - ps28
Brassicaceae - ps29
Camelineae - ps30
Arabidopsis - ps31
Arabidopsis_thaliana - ps32
Phylostratum

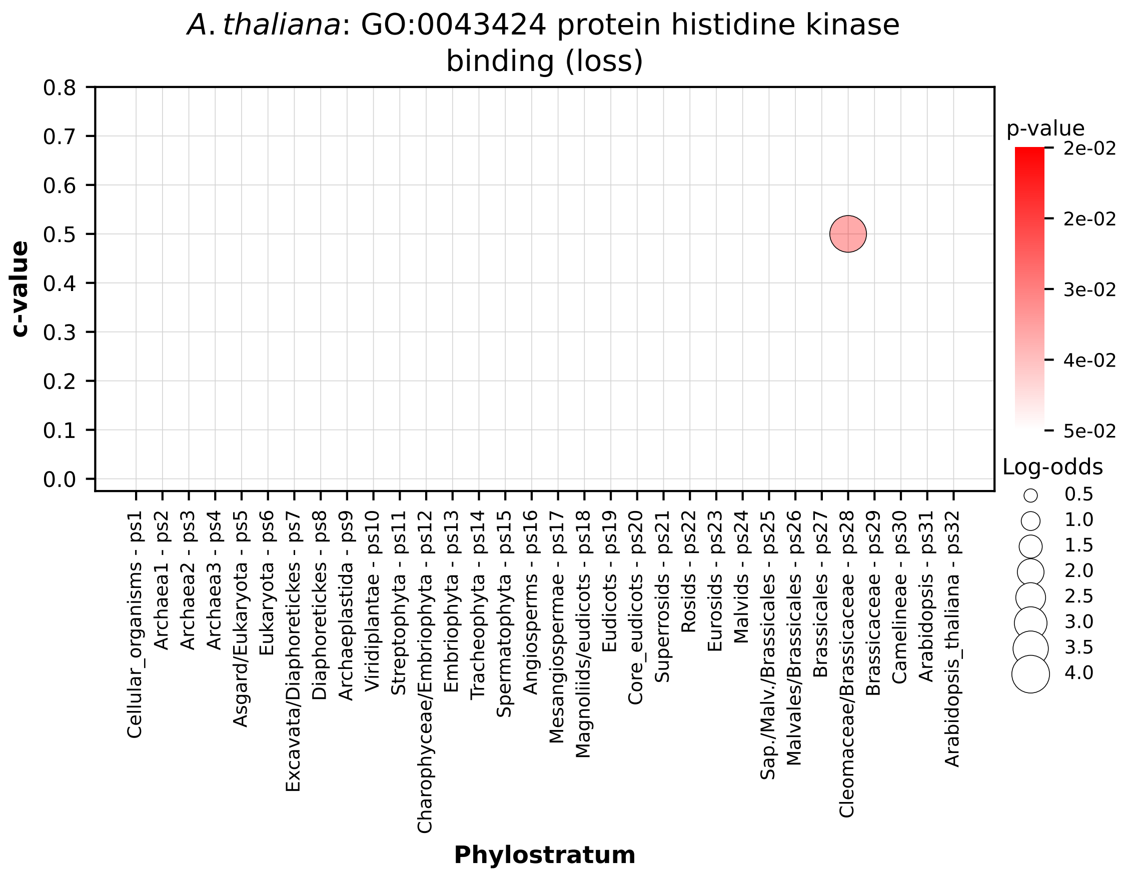

A. thaliana: GO:0043424 protein histidine kinase binding (loss)
c-value
0.0
0.1
0.2
0.3
0.4
0.5
0.6
0.7
0.8
Phylostratum
Cellular_organisms - ps1
Archaea1 - ps2
Archaea2 - ps3
Archaea3 - ps4
Asgard/Eukaryota - ps5
Eukaryota - ps6
Excavata/Diaphoretickes - ps7
Diaphoretickes - ps8
Archaeplastida - ps9
Viridiplantae - ps10
Streptophyta - ps11
Charophyceae/Embriophyta - ps12
Embriophyta - ps13
Tracheophyta - ps14
Spermatophyta - ps15
Angiosperms - ps16
Mesangiospermae - ps17
Magnoliids/eudicots - ps18
Eudicots - ps19
Core_eudicots - ps20
Superrosids - ps21
Rosids - ps22
Eurosids - ps23
Malvids - ps24
Sap./Malv./Brassicales - ps25
Malvales/Brassicales - ps26
Brassicales - ps27
Cleomaceae/Brassicaceae - ps28
Brassicaceae - ps29
Camelineae - ps30
Arabidopsis - ps31
Arabidopsis_thaliana - ps32
p-value
2e-02
2e-02
3e-02
4e-02
5e-02
Log-odds
0.5
1.0
1.5
2.0
2.5
3.0
3.5
4.0

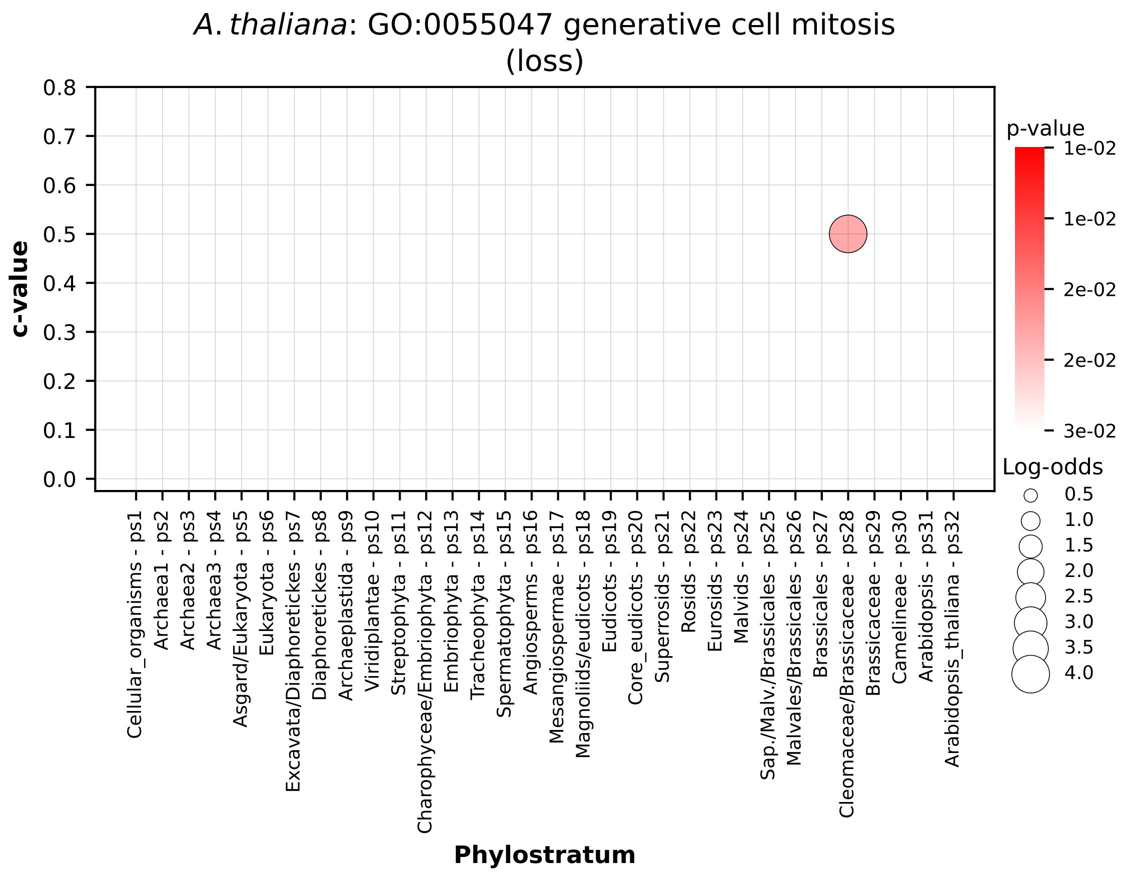

A. thaliana: GO:0055047 generative cell mitosis
(loss)
c-value
0.8
0.7
0.6
0.5
0.4
0.3
0.2
0.1
0.0
p-value
1e-02
1e-02
2e-02
2e-02
3e-02
Log-odds
0.5
1.0
1.5
2.0
2.5
3.0
3.5
4.0
Cellular_organisms - ps1
Archaea1 - ps2
Archaea2 - ps3
Archaea3 - ps4
Asgard/Eukaryota - ps5
Eukaryota - ps6
Excavata/Diaphoretickes - ps7
Diaphoretickes - ps8
Archaeplastida - ps9
Viridiplantae - ps10
Streptophyta - ps11
Charophyceae/Embriophyta - ps12
Embriophyta - ps13
Tracheophyta - ps14
Spermatophyta - ps15
Angiosperms - ps16
Mesangiospermae - ps17
Magnoliids/eudicots - ps18
Eudicots - ps19
Core_eudicots - ps20
Superrosids - ps21
Rosids - ps22
Eurosids - ps23
Malvids - ps24
Sap./Malv./Brassicales - ps25
Malvales/Brassicales - ps26
Brassicales - ps27
Cleomaceae/Brassicaceae - ps28
Brassicaceae - ps29
Camelineae - ps30
Arabidopsis - ps31
Arabidopsis_thaliana - ps32
Phylostratum

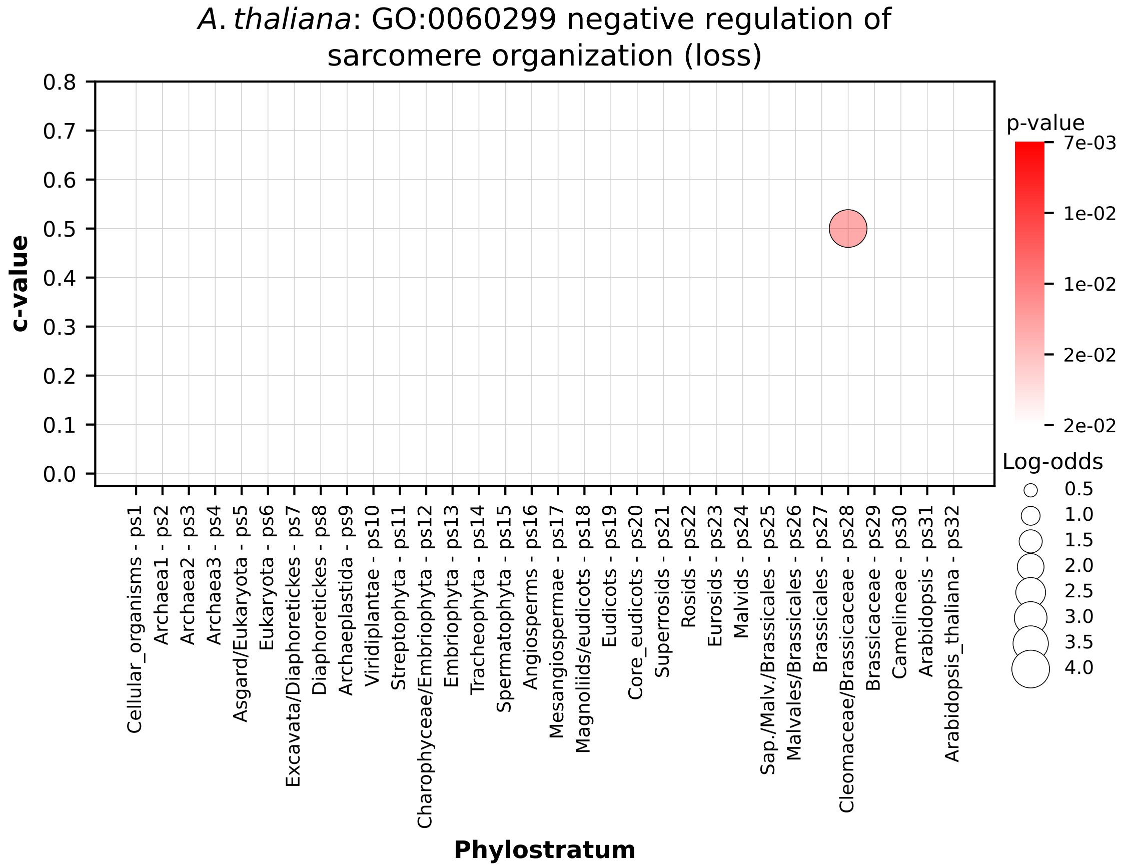

A. thaliana: GO:0060299 negative regulation of sarcomere organization (loss)
c-value
0.0
0.1
0.2
0.3
0.4
0.5
0.6
0.7
0.8
Phylostratum
Cellular_organisms - ps1
Archaea1 - ps2
Archaea2 - ps3
Archaea3 - ps4
Asgard/Eukaryota - ps5
Eukaryota - ps6
Excavata/Diaphoretickes - ps7
Diaphoretickes - ps8
Archaeplastida - ps9
Viridiplantae - ps10
Streptophyta - ps11
Charophyceae/Embriophyta - ps12
Embriophyta - ps13
Tracheophyta - ps14
Spermatophyta - ps15
Angiosperms - ps16
Mesangiospermae - ps17
Magnoliids/eudicots - ps18
Eudicots - ps19
Core_eudicots - ps20
Superrosids - ps21
Rosids - ps22
Eurosids - ps23
Malvids - ps24
Sap./Malv./Brassicales - ps25
Malvales/Brassicales - ps26
Brassicales - ps27
Cleomaceae/Brassicaceae - ps28
Brassicaceae - ps29
Camelineae - ps30
Arabidopsis - ps31
Arabidopsis_thaliana - ps32
p-value
7e-03
1e-02
1e-02
2e-02
2e-02
Log-odds
0.5
1.0
1.5
2.0
2.5
3.0
3.5
4.0

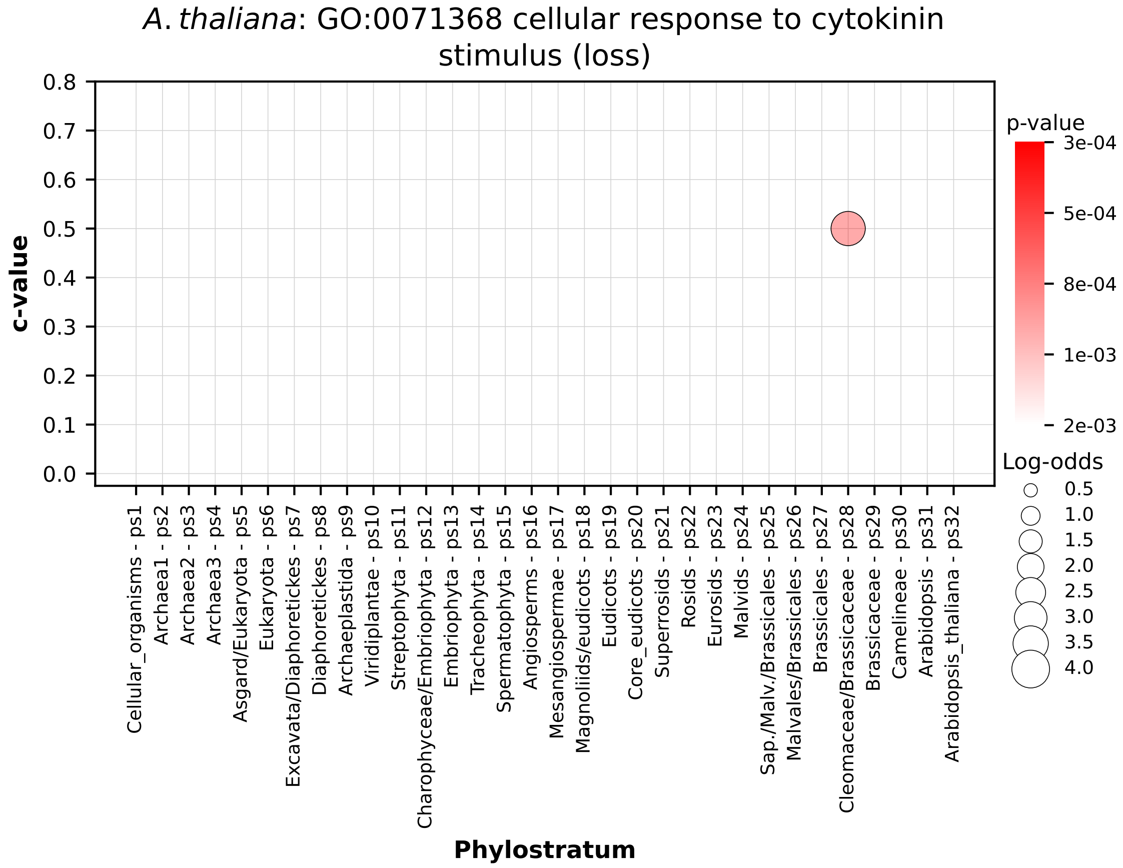

A. thaliana: GO:0071368 cellular response to cytokinin stimulus (loss)
c-value
0.8
0.7
0.6
0.5
0.4
0.3
0.2
0.1
0.0
Phylostratum
Cellular_organisms - ps1
Archaea1 - ps2
Archaea2 - ps3
Archaea3 - ps4
Asgard/Eukaryota - ps5
Eukaryota - ps6
Excavata/Diaphoretickes - ps7
Diaphoretickes - ps8
Archaeplastida - ps9
Viridiplantae - ps10
Streptophyta - ps11
Charophyceae/Embriophyta - ps12
Embriophyta - ps13
Tracheophyta - ps14
Spermatophyta - ps15
Angiosperms - ps16
Mesangiospermae - ps17
Magnoliids/eudicots - ps18
Eudicots - ps19
Core_eudicots - ps20
Superrosids - ps21
Rosids - ps22
Eurosids - ps23
Malvids - ps24
Sap./Malv./Brassicales - ps25
Malvales/Brassicales - ps26
Brassicales - ps27
Cleomaceae/Brassicaceae - ps28
Brassicaceae - ps29
Camelineae - ps30
Arabidopsis - ps31
Arabidopsis_thaliana - ps32
p-value
3e-04
5e-04
8e-04
1e-03
2e-03
Log-odds
0.5
1.0
1.5
2.0
2.5
3.0
3.5
4.0

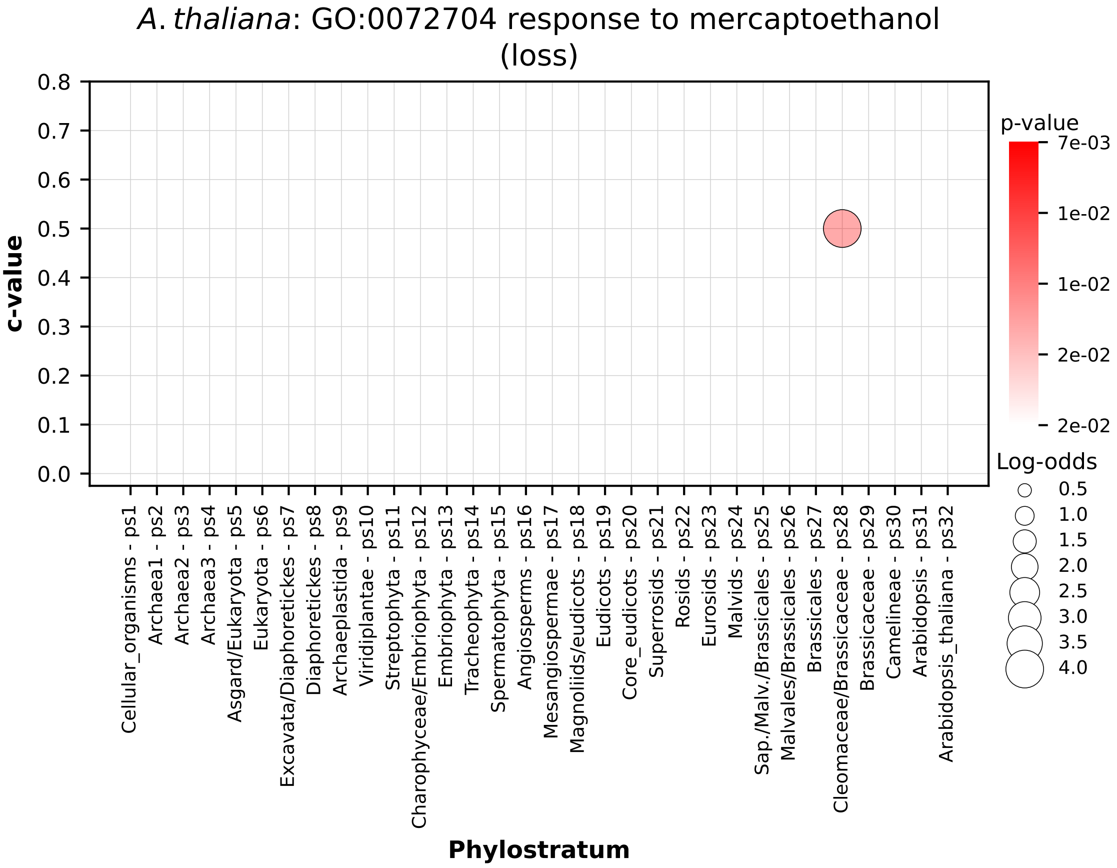

A. thaliana: GO:0072704 response to mercaptoethanol (loss)
c-value
0.8
0.7
0.6
0.5
0.4
0.3
0.2
0.1
0.0
p-value
7e-03
1e-02
1e-02
2e-02
2e-02
Log-odds
0.5
1.0
1.5
2.0
2.5
3.0
3.5
4.0
Cellular_organisms - ps1
Archaea1 - ps2
Archaea2 - ps3
Archaea3 - ps4
Asgard/Eukaryota - ps5
Eukaryota - ps6
Excavata/Diaphoretickes - ps7
Diaphoretickes - ps8
Archaeplastida - ps9
Viridiplantae - ps10
Streptophyta - ps11
Charophyceae/Embriophyta - ps12
Embriophyta - ps13
Tracheophyta - ps14
Spermatophyta - ps15
Angiosperms - ps16
Mesangiospermae - ps17
Magnoliids/eudicots - ps18
Eudicots - ps19
Core_eudicots - ps20
Superrosids - ps21
Rosids - ps22
Eurosids - ps23
Malvids - ps24
Sap./Malv./Brassicales - ps25
Malvales/Brassicales - ps26
Brassicales - ps27
Cleomaceae/Brassicaceae - ps28
Brassicaceae - ps29
Camelineae - ps30
Arabidopsis - ps31
Arabidopsis_thaliana - ps32
Phylostratum

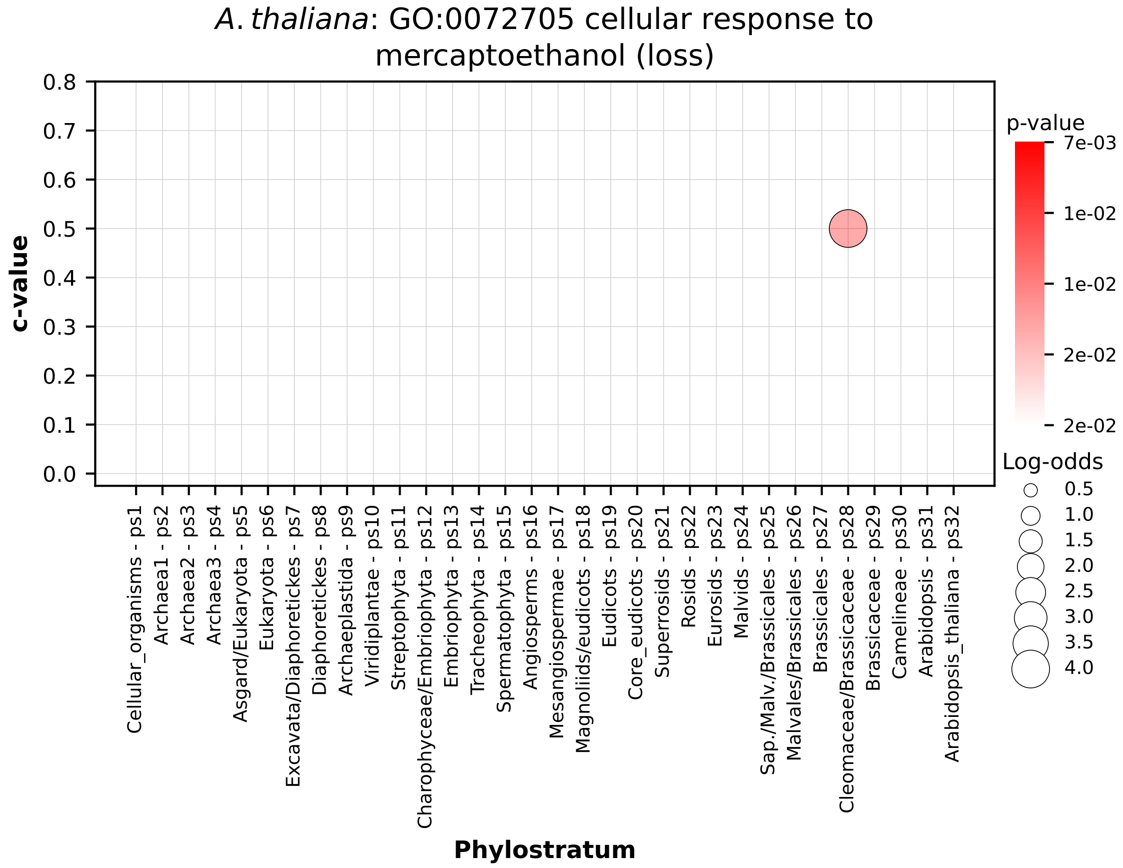

A. thaliana: GO:0072705 cellular response to mercaptoethanol (loss)
c-value
0.8
0.7
0.6
0.5
0.4
0.3
0.2
0.1
0.0
Phylostratum
Cellular_organisms - ps1
Archaea1 - ps2
Archaea2 - ps3
Archaea3 - ps4
Asgard/Eukaryota - ps5
Eukaryota - ps6
Excavata/Diaphoretickes - ps7
Diaphoretickes - ps8
Archaeplastida - ps9
Viridiplantae - ps10
Streptophyta - ps11
Charophyceae/Embriophyta - ps12
Embriophyta - ps13
Tracheophyta - ps14
Spermatophyta - ps15
Angiosperms - ps16
Mesangiospermae - ps17
Magnoliids/eudicots - ps18
Eudicots - ps19
Core_eudicots - ps20
Superrosids - ps21
Rosids - ps22
Eurosids - ps23
Malvids - ps24
Sap./Malv./Brassicales - ps25
Malvales/Brassicales - ps26
Brassicales - ps27
Cleomaceae/Brassicaceae - ps28
Brassicaceae - ps29
Camelineae - ps30
Arabidopsis - ps31
Arabidopsis_thaliana - ps32
p-value
7e-03
1e-02
1e-02
2e-02
2e-02
Log-odds
0.5
1.0
1.5
2.0
2.5
3.0
3.5
4.0

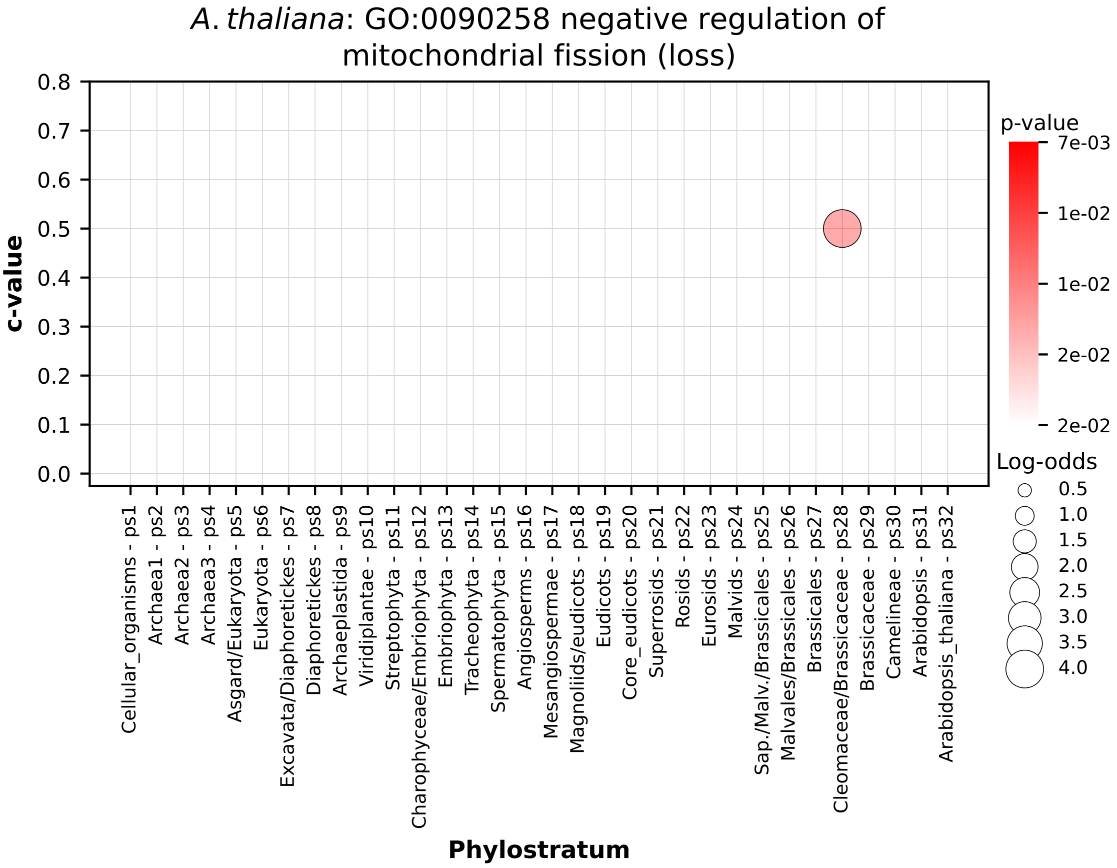

A. thaliana: GO:0090258 negative regulation of mitochondrial fission (loss)
c-value
0.8
0.7
0.6
0.5
0.4
0.3
0.2
0.1
0.0
p-value
7e-03
1e-02
1e-02
2e-02
2e-02
Log-odds
0.5
1.0
1.5
2.0
2.5
3.0
3.5
4.0
Cellular_organisms - ps1
Archaea1 - ps2
Archaea2 - ps3
Archaea3 - ps4
Asgard/Eukaryota - ps5
Eukaryota - ps6
Excavata/Diaphoretickes - ps7
Diaphoretickes - ps8
Archaeplastida - ps9
Viridiplantae - ps10
Streptophyta - ps11
Charophyceae/Embriophyta - ps12
Embriophyta - ps13
Tracheophyta - ps14
Spermatophyta - ps15
Angiosperms - ps16
Mesangiospermae - ps17
Magnoliids/eudicots - ps18
Eudicots - ps19
Core_eudicots - ps20
Superrosids - ps21
Rosids - ps22
Eurosids - ps23
Malvids - ps24
Sap./Malv./Brassicales - ps25
Malvales/Brassicales - ps26
Brassicales - ps27
Cleomaceae/Brassicaceae - ps28
Brassicaceae - ps29
Camelineae - ps30
Arabidopsis - ps31
Arabidopsis_thaliana - ps32
Phylostratum

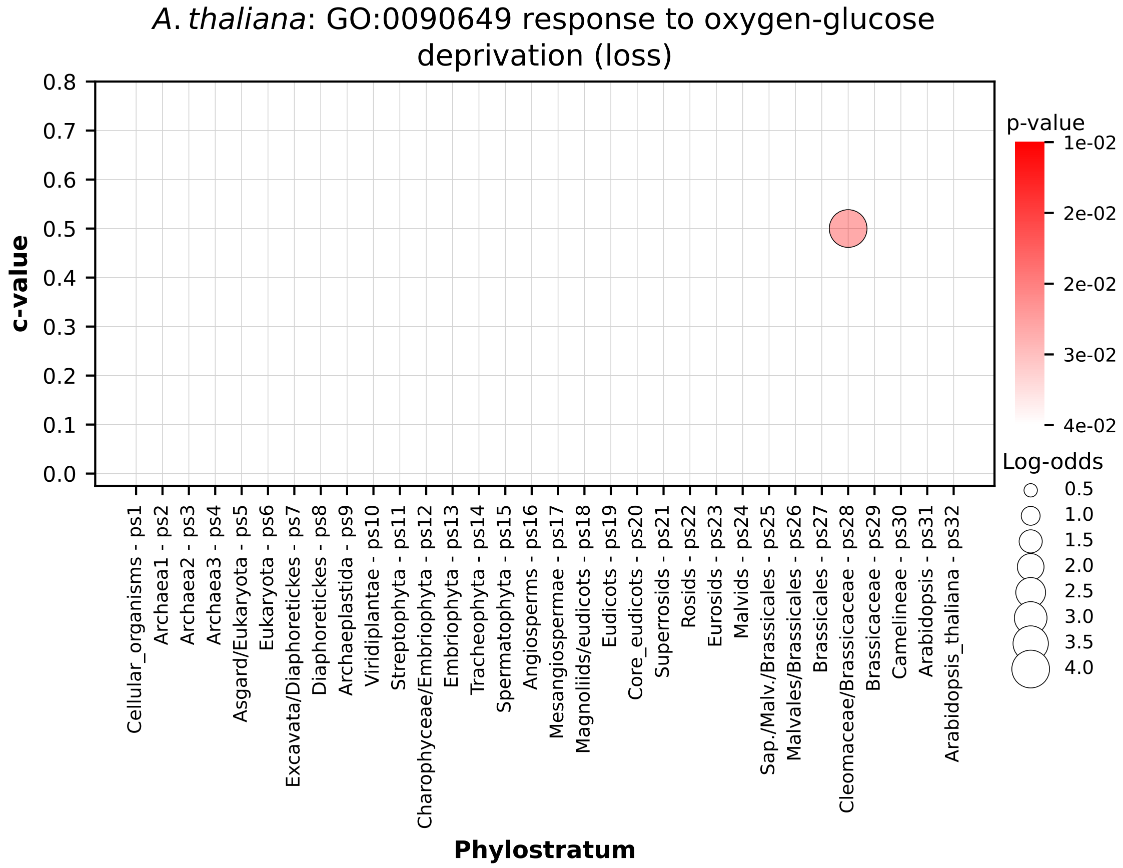

A. thaliana: GO:0090649 response to oxygen-glucose deprivation (loss)
c-value
0.8
0.7
0.6
0.5
0.4
0.3
0.2
0.1
0.0
p-value
1e-02
2e-02
2e-02
3e-02
4e-02
Log-odds
0.5
1.0
1.5
2.0
2.5
3.0
3.5
4.0
Cellular_organisms - ps1
Archaea1 - ps2
Archaea2 - ps3
Archaea3 - ps4
Asgard/Eukaryota - ps5
Eukaryota - ps6
Excavata/Diaphoretickes - ps7
Diaphoretickes - ps8
Archaeplastida - ps9
Viridiplantae - ps10
Streptophyta - ps11
Charophyceae/Embriophyta - ps12
Embriophyta - ps13
Tracheophyta - ps14
Spermatophyta - ps15
Angiosperms - ps16
Mesangiospermae - ps17
Magnoliids/eudicots - ps18
Eudicots - ps19
Core_eudicots - ps20
Superrosids - ps21
Rosids - ps22
Eurosids - ps23
Malvids - ps24
Sap./Malv./Brassicales - ps25
Malvales/Brassicales - ps26
Brassicales - ps27
Cleomaceae/Brassicaceae - ps28
Brassicaceae - ps29
Camelineae - ps30
Arabidopsis - ps31
Arabidopsis_thaliana - ps32
Phylostratum

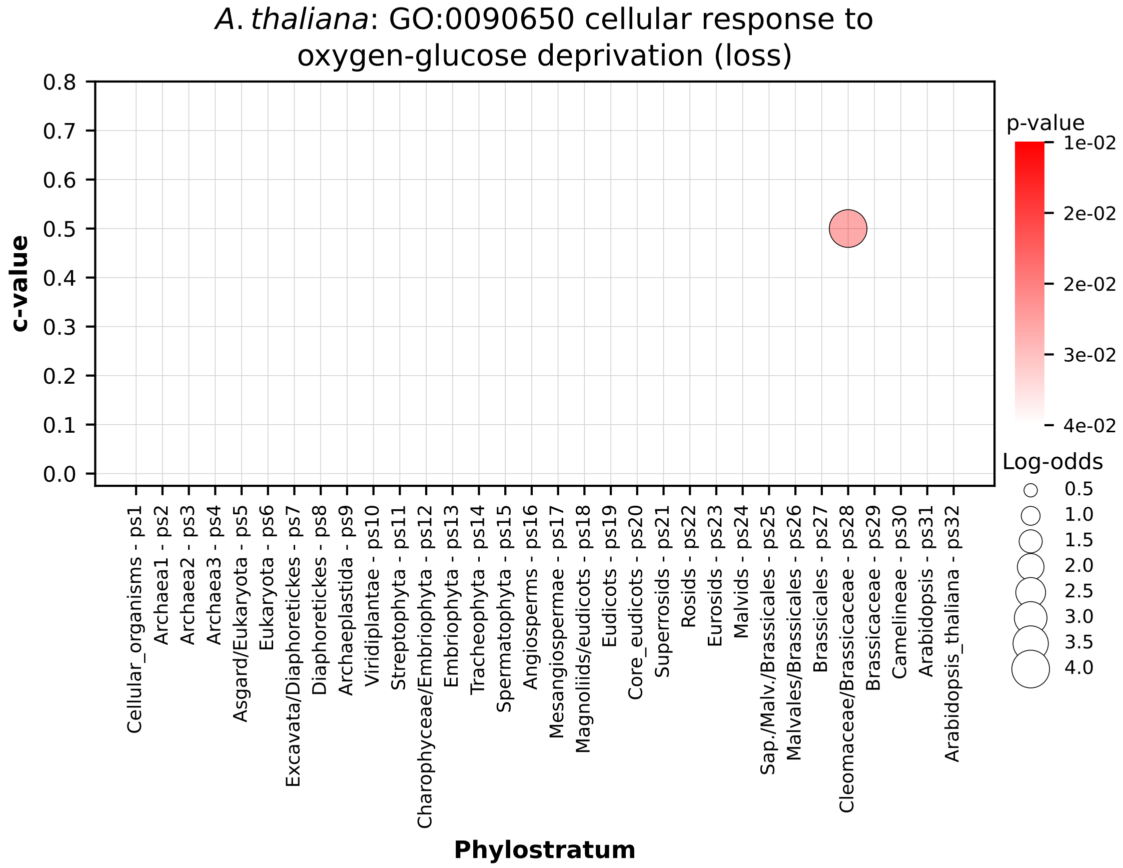

A. thaliana: GO:0090650 cellular response to oxygen-glucose deprivation (loss)
c-value
0.8
0.7
0.6
0.5
0.4
0.3
0.2
0.1
0.0
p-value
1e-02
2e-02
2e-02
3e-02
4e-02
Log-odds
0.5
1.0
1.5
2.0
2.5
3.0
3.5
4.0
Cellular_organisms - ps1
Archaea1 - ps2
Archaea2 - ps3
Archaea3 - ps4
Asgard/Eukaryota - ps5
Eukaryota - ps6
Excavata/Diaphoretickes - ps7
Diaphoretickes - ps8
Archaeplastida - ps9
Viridiplantae - ps10
Streptophyta - ps11
Charophyceae/Embriophyta - ps12
Embriophyta - ps13
Tracheophyta - ps14
Spermatophyta - ps15
Angiosperms - ps16
Mesangiospermae - ps17
Magnoliids/eudicots - ps18
Eudicots - ps19
Core_eudicots - ps20
Superrosids - ps21
Rosids - ps22
Eurosids - ps23
Malvids - ps24
Sap./Malv./Brassicales - ps25
Malvales/Brassicales - ps26
Brassicales - ps27
Cleomaceae/Brassicaceae - ps28
Brassicaceae - ps29
Camelineae - ps30
Arabidopsis - ps31
Arabidopsis_thaliana - ps32
Phylostratum

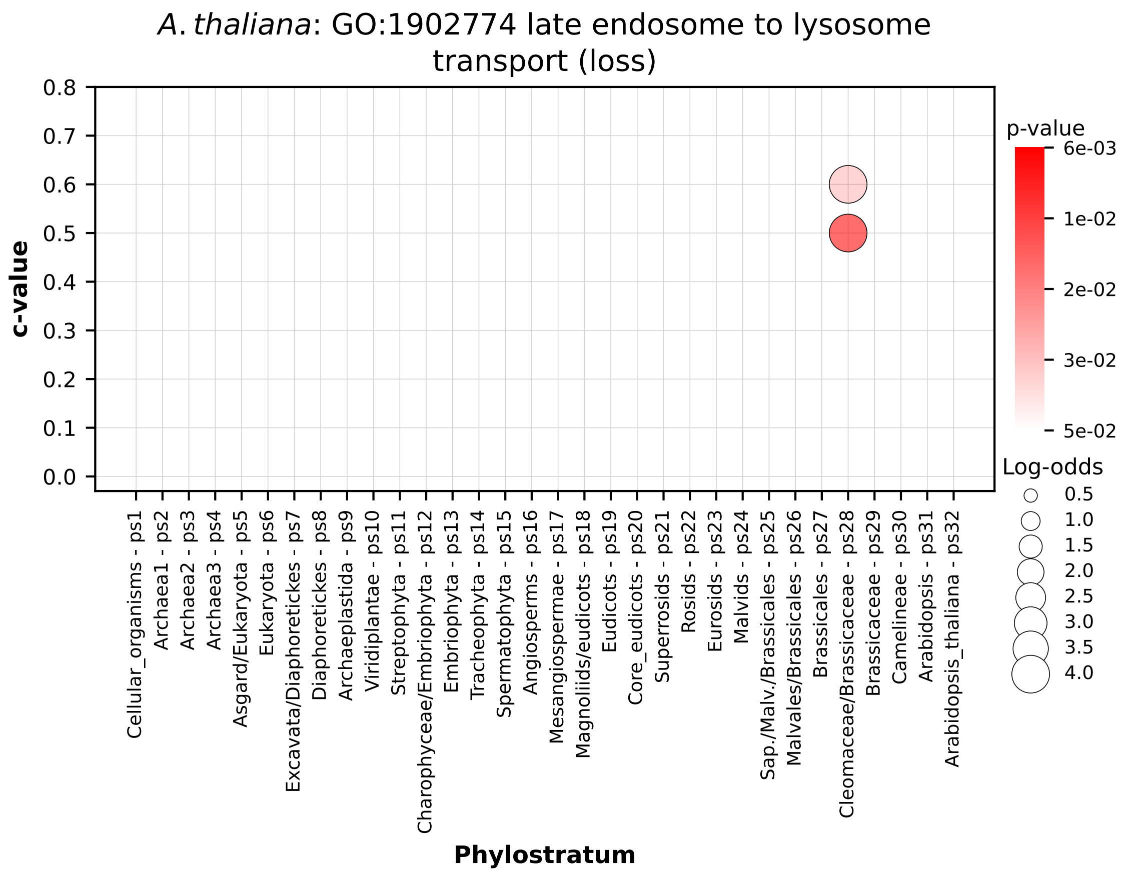

A. thaliana: GO:1902774 late endosome to lysosome transport (loss)
c-value
0.8
0.7
0.6
0.5
0.4
0.3
0.2
0.1
0.0
p-value
6e-03
1e-02
2e-02
3e-02
5e-02
Log-odds
0.5
1.0
1.5
2.0
2.5
3.0
3.5
4.0
Cellular_organisms - ps1
Archaea1 - ps2
Archaea2 - ps3
Archaea3 - ps4
Asgard/Eukaryota - ps5
Eukaryota - ps6
Excavata/Diaphoretickes - ps7
Diaphoretickes - ps8
Archaeplastida - ps9
Viridiplantae - ps10
Streptophyta - ps11
Charophyceae/Embriophyta - ps12
Embriophyta - ps13
Tracheophyta - ps14
Spermatophyta - ps15
Angiosperms - ps16
Mesangiospermae - ps17
Magnoliids/eudicots - ps18
Eudicots - ps19
Core_eudicots - ps20
Superrosids - ps21
Rosids - ps22
Eurosids - ps23
Malvids - ps24
Sap./Malv./Brassicales - ps25
Malvales/Brassicales - ps26
Brassicales - ps27
Cleomaceae/Brassicaceae - ps28
Brassicaceae - ps29
Camelineae - ps30
Arabidopsis - ps31
Arabidopsis_thaliana - ps32
Phylostratum

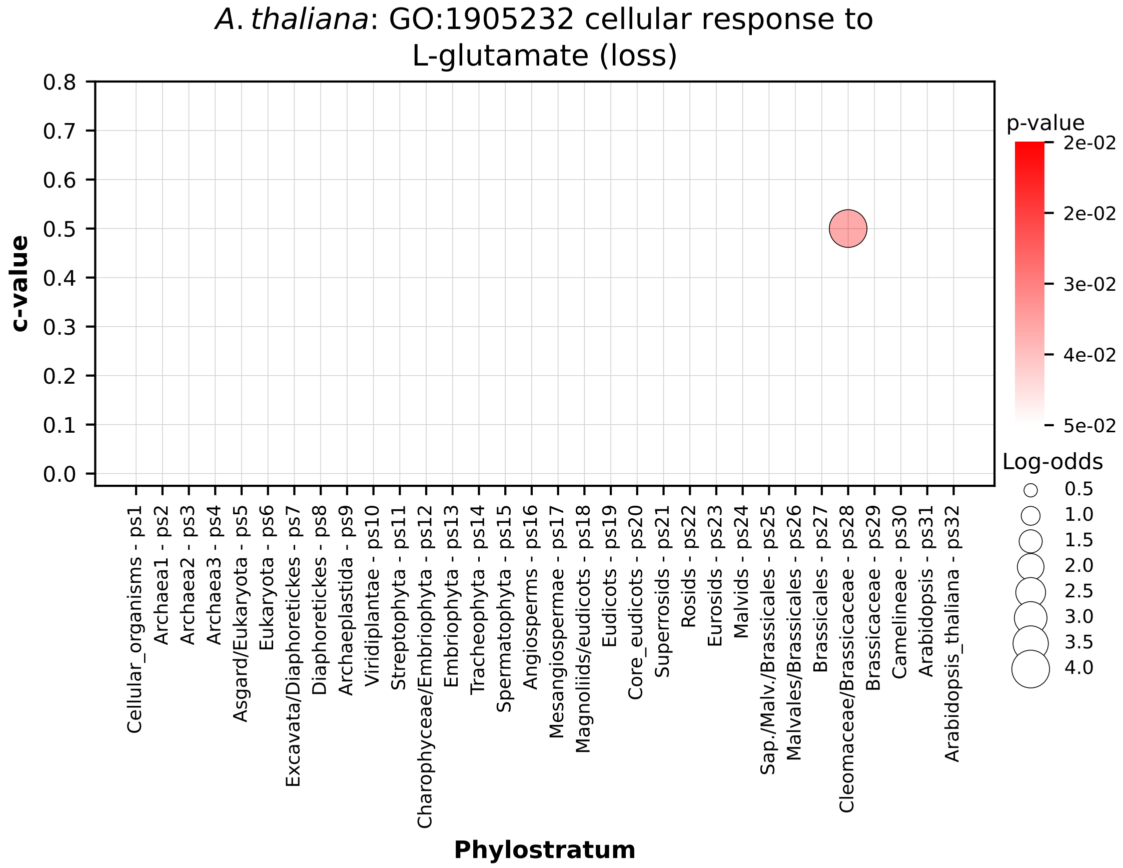

A. thaliana: GO:1905232 cellular response to L-glutamate (loss)
c-value
0.8
0.7
0.6
0.5
0.4
0.3
0.2
0.1
0.0
Phylostratum
Cellular_organisms - ps1
Archaea1 - ps2
Archaea2 - ps3
Archaea3 - ps4
Asgard/Eukaryota - ps5
Eukaryota - ps6
Excavata/Diaphoretickes - ps7
Diaphoretickes - ps8
Archaeplastida - ps9
Viridiplantae - ps10
Streptophyta - ps11
Charophyceae/Embriophyta - ps12
Embriophyta - ps13
Tracheophyta - ps14
Spermatophyta - ps15
Angiosperms - ps16
Mesangiospermae - ps17
Magnoliids/eudicots - ps18
Eudicots - ps19
Core_eudicots - ps20
Superrosids - ps21
Rosids - ps22
Eurosids - ps23
Malvids - ps24
Sap./Malv./Brassicales - ps25
Malvales/Brassicales - ps26
Brassicales - ps27
Cleomaceae/Brassicaceae - ps28
Brassicaceae - ps29
Camelineae - ps30
Arabidopsis - ps31
Arabidopsis_thaliana - ps32
p-value
2e-02
2e-02
3e-02
4e-02
5e-02
Log-odds
0.5
1.0
1.5
2.0
2.5
3.0
3.5
4.0

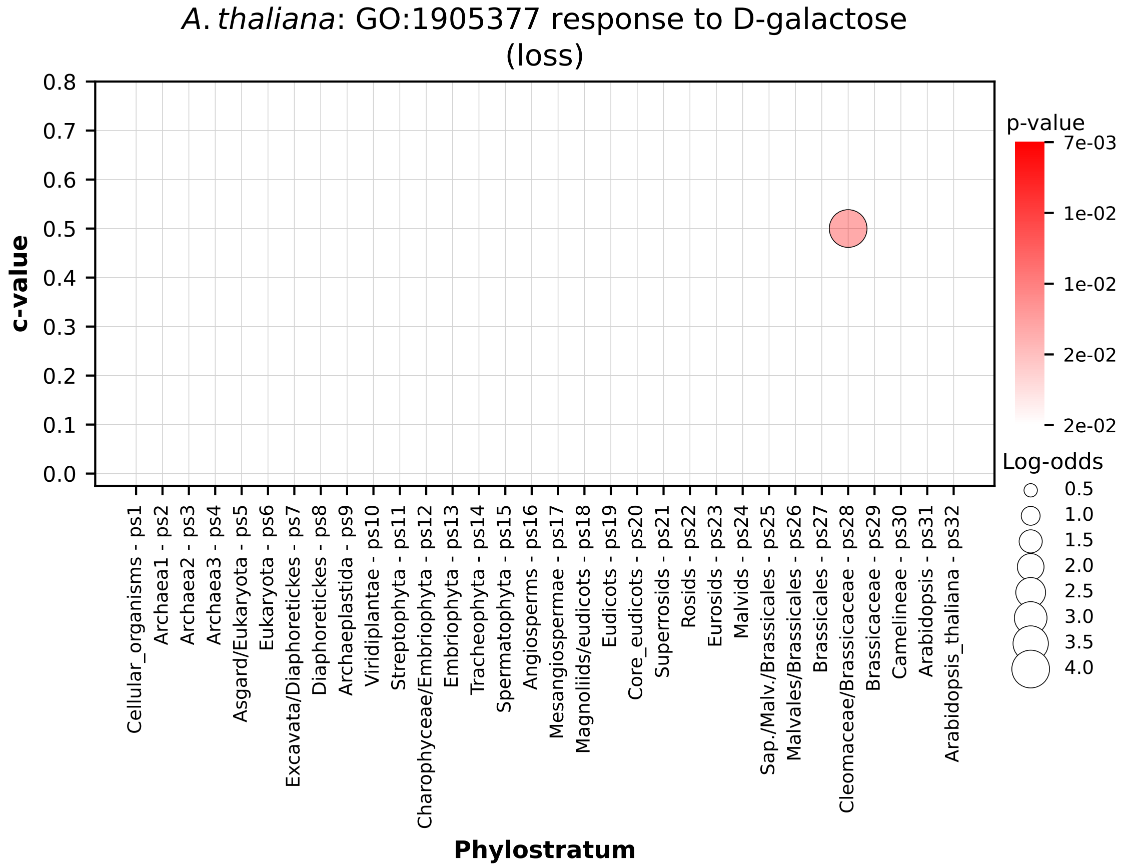

A. thaliana: GO:1905377 response to D-galactose (loss)
c-value
0.8
0.7
0.6
0.5
0.4
0.3
0.2
0.1
0.0
p-value
7e-03
1e-02
1e-02
2e-02
2e-02
Log-odds
0.5
1.0
1.5
2.0
2.5
3.0
3.5
4.0
Cellular_organisms - ps1
Archaea1 - ps2
Archaea2 - ps3
Archaea3 - ps4
Asgard/Eukaryota - ps5
Eukaryota - ps6
Excavata/Diaphoretickes - ps7
Diaphoretickes - ps8
Archaeplastida - ps9
Viridiplantae - ps10
Streptophyta - ps11
Charophyceae/Embriophyta - ps12
Embriophyta - ps13
Tracheophyta - ps14
Spermatophyta - ps15
Angiosperms - ps16
Mesangiospermae - ps17
Magnoliids/eudicots - ps18
Eudicots - ps19
Core_eudicots - ps20
Superrosids - ps21
Rosids - ps22
Eurosids - ps23
Malvids - ps24
Sap./Malv./Brassicales - ps25
Malvales/Brassicales - ps26
Brassicales - ps27
Cleomaceae/Brassicaceae - ps28
Brassicaceae - ps29
Camelineae - ps30
Arabidopsis - ps31
Arabidopsis_thaliana - ps32
Phylostratum

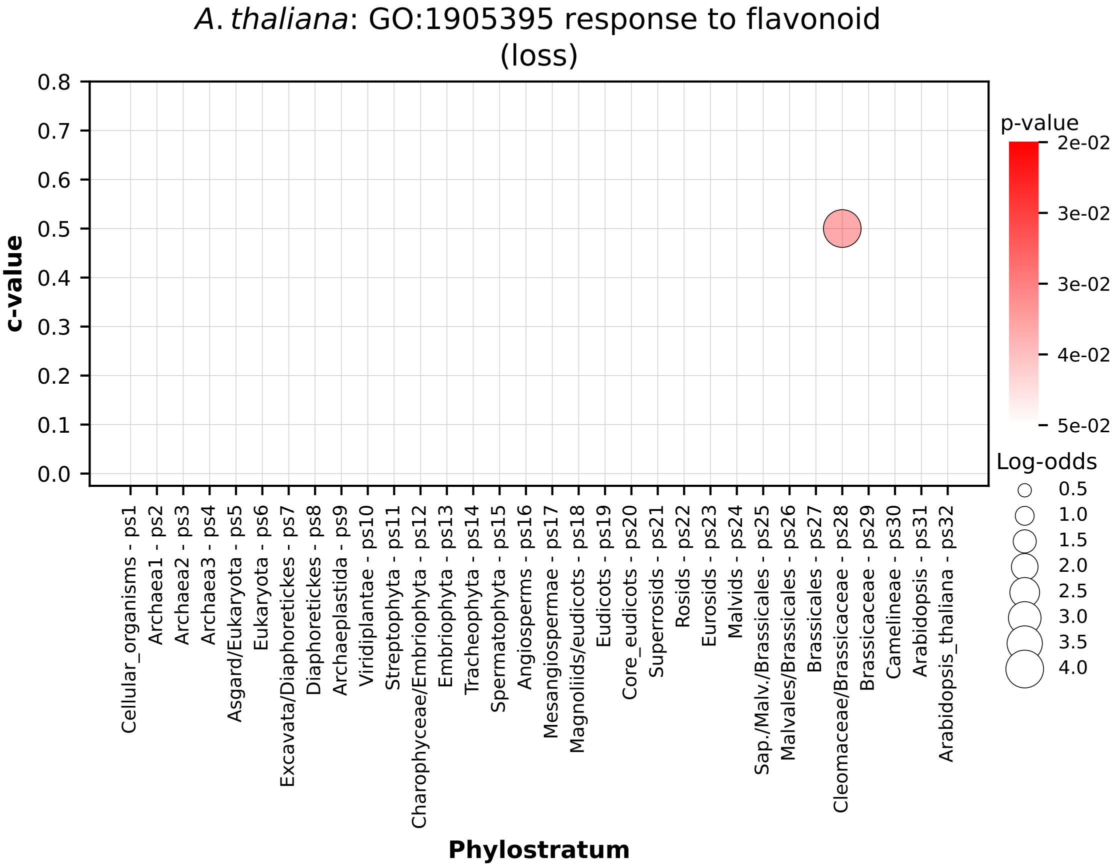

A. thaliana: GO:1905395 response to flavonoid
(loss)
c-value
0.8
0.7
0.6
0.5
0.4
0.3
0.2
0.1
0.0
p-value
2e-02
3e-02
3e-02
4e-02
5e-02
Log-odds
0.5
1.0
1.5
2.0
2.5
3.0
3.5
4.0
Cellular_organisms - ps1
Archaea1 - ps2
Archaea2 - ps3
Archaea3 - ps4
Asgard/Eukaryota - ps5
Eukaryota - ps6
Excavata/Diaphoretickes - ps7
Diaphoretickes - ps8
Archaeplastida - ps9
Viridiplantae - ps10
Streptophyta - ps11
Charophyceae/Embriophyta - ps12
Embriophyta - ps13
Tracheophyta - ps14
Spermatophyta - ps15
Angiosperms - ps16
Mesangiospermae - ps17
Magnoliids/eudicots - ps18
Eudicots - ps19
Core_eudicots - ps20
Superrosids - ps21
Rosids - ps22
Eurosids - ps23
Malvids - ps24
Sap./Malv./Brassicales - ps25
Malvales/Brassicales - ps26
Brassicales - ps27
Cleomaceae/Brassicaceae - ps28
Brassicaceae - ps29
Camelineae - ps30
Arabidopsis - ps31
Arabidopsis_thaliana - ps32
Phylostratum

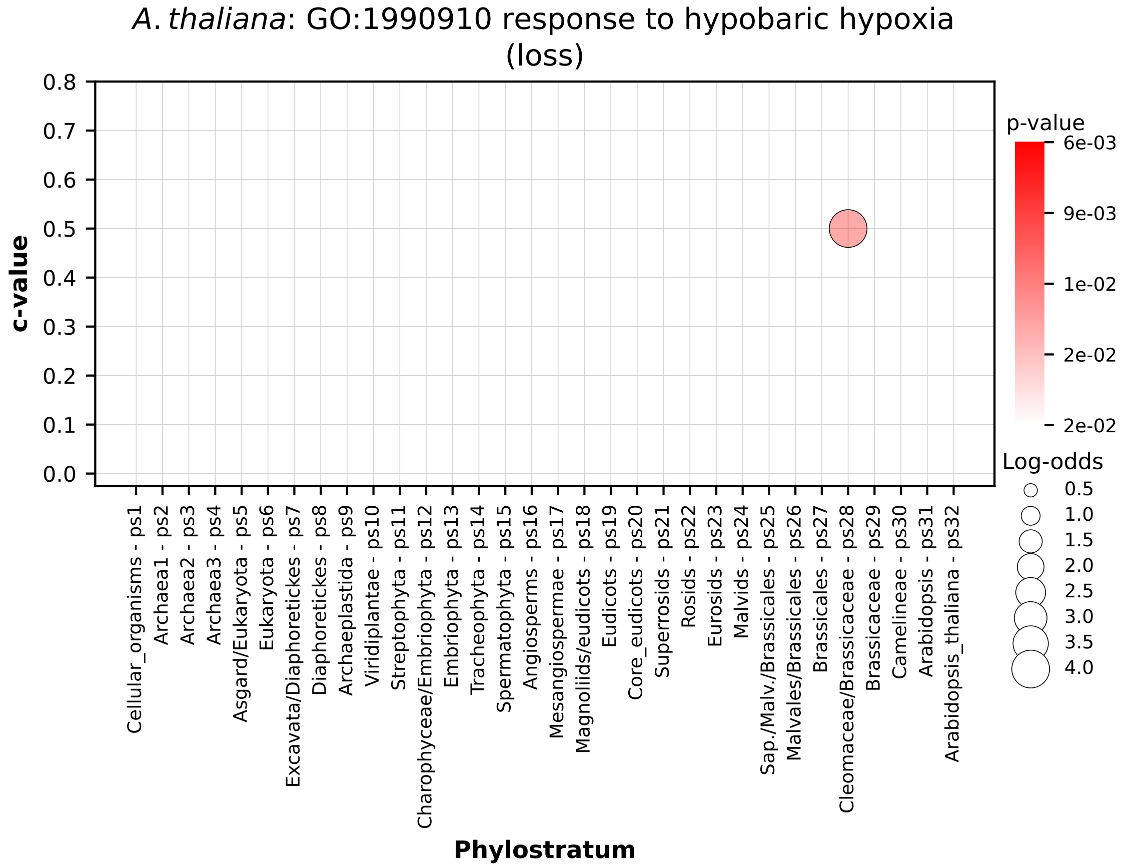

A. thaliana: GO:1990910 response to hypobaric hypoxia
(loss)
c-value
0.8
0.7
0.6
0.5
0.4
0.3
0.2
0.1
0.0
Phylostratum
Cellular_organisms - ps1
Archaea1 - ps2
Archaea2 - ps3
Archaea3 - ps4
Asgard/Eukaryota - ps5
Eukaryota - ps6
Excavata/Diaphoretickes - ps7
Diaphoretickes - ps8
Archaeplastida - ps9
Viridiplantae - ps10
Streptophyta - ps11
Charophyceae/Embriophyta - ps12
Embriophyta - ps13
Tracheophyta - ps14
Spermatophyta - ps15
Angiosperms - ps16
Mesangiospermae - ps17
Magnoliids/eudicots - ps18
Eudicots - ps19
Core_eudicots - ps20
Superrosids - ps21
Rosids - ps22
Eurosids - ps23
Malvids - ps24
Sap./Malv./Brassicales - ps25
Malvales/Brassicales - ps26
Brassicales - ps27
Cleomaceae/Brassicaceae - ps28
Brassicaceae - ps29
Camelineae - ps30
Arabidopsis - ps31
Arabidopsis_thaliana - ps32
p-value
6e-03
9e-03
1e-02
2e-02
2e-02
Log-odds
0.5
1.0
1.5
2.0
2.5
3.0
3.5
4.0

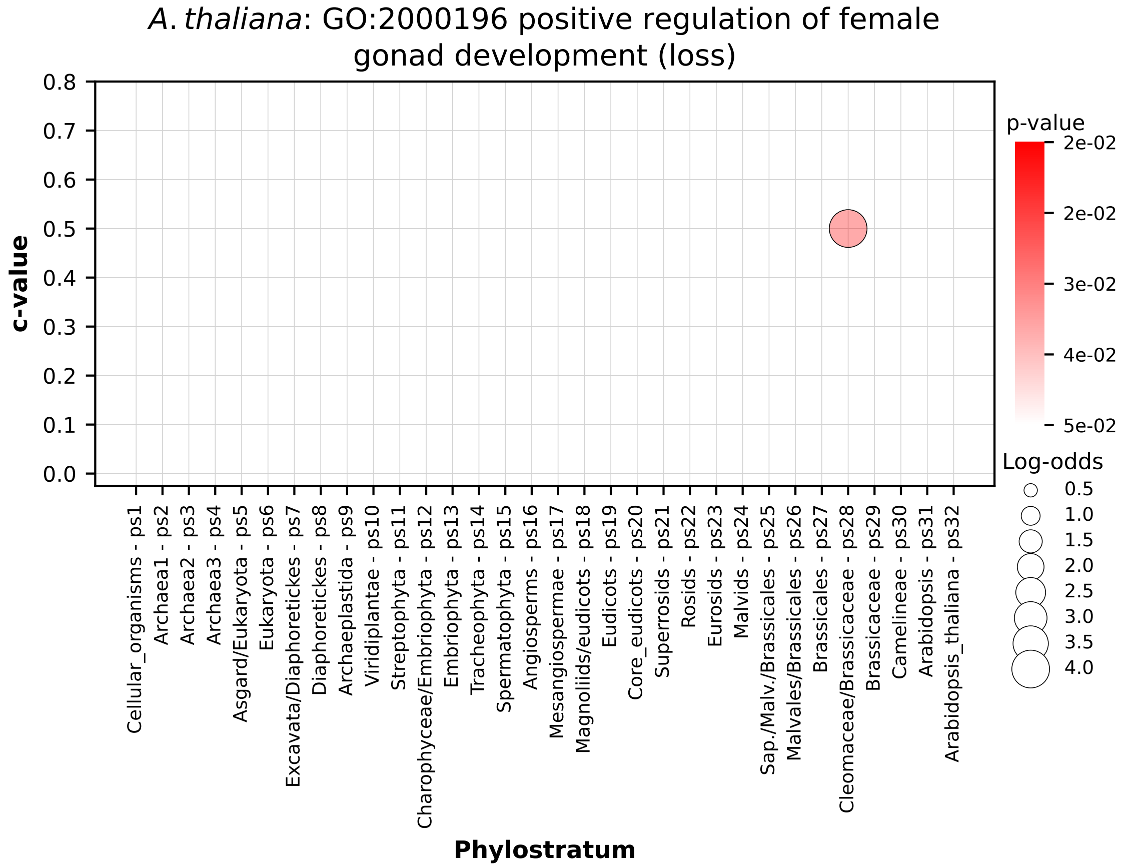

A. thaliana: GO:2000196 positive regulation of female gonad development (loss)
c-value
0.8
0.7
0.6
0.5
0.4
0.3
0.2
0.1
0.0
p-value
2e-02
2e-02
3e-02
4e-02
5e-02
Log-odds
0.5
1.0
1.5
2.0
2.5
3.0
3.5
4.0
Cellular_organisms - ps1
Archaea1 - ps2
Archaea2 - ps3
Archaea3 - ps4
Asgard/Eukaryota - ps5
Eukaryota - ps6
Excavata/Diaphoretickes - ps7
Diaphoretickes - ps8
Archaeplastida - ps9
Viridiplantae - ps10
Streptophyta - ps11
Charophyceae/Embriophyta - ps12
Embriophyta - ps13
Tracheophyta - ps14
Spermatophyta - ps15
Angiosperms - ps16
Mesangiospermae - ps17
Magnoliids/eudicots - ps18
Eudicots - ps19
Core_eudicots - ps20
Superrosids - ps21
Rosids - ps22
Eurosids - ps23
Malvids - ps24
Sap./Malv./Brassicales - ps25
Malvales/Brassicales - ps26
Brassicales - ps27
Cleomaceae/Brassicaceae - ps28
Brassicaceae - ps29
Camelineae - ps30
Arabidopsis - ps31
Arabidopsis_thaliana - ps32
Phylostratum

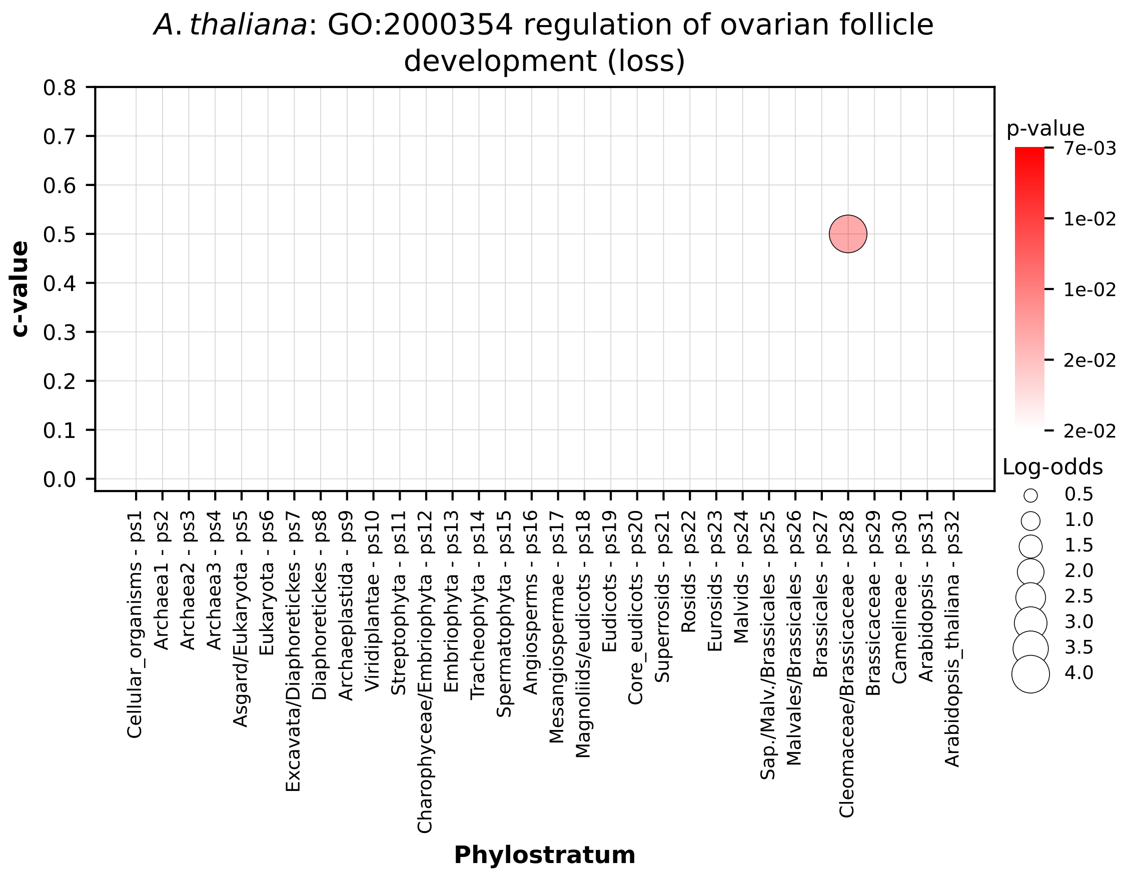

A. thaliana: GO:2000354 regulation of ovarian follicle development (loss)
c-value
0.8
0.7
0.6
0.5
0.4
0.3
0.2
0.1
0.0
Phylostratum
Cellular_organisms - ps1
Archaea1 - ps2
Archaea2 - ps3
Archaea3 - ps4
Asgard/Eukaryota - ps5
Eukaryota - ps6
Excavata/Diaphoretickes - ps7
Diaphoretickes - ps8
Archaeplastida - ps9
Viridiplantae - ps10
Streptophyta - ps11
Charophyceae/Embriophyta - ps12
Embriophyta - ps13
Tracheophyta - ps14
Spermatophyta - ps15
Angiosperms - ps16
Mesangiospermae - ps17
Magnoliids/eudicots - ps18
Eudicots - ps19
Core_eudicots - ps20
Superrosids - ps21
Rosids - ps22
Eurosids - ps23
Malvids - ps24
Sap./Malv./Brassicales - ps25
Malvales/Brassicales - ps26
Brassicales - ps27
Cleomaceae/Brassicaceae - ps28
Brassicaceae - ps29
Camelineae - ps30
Arabidopsis - ps31
Arabidopsis_thaliana - ps32
p-value
7e-03
1e-02
1e-02
2e-02
2e-02
Log-odds
0.5
1.0
1.5
2.0
2.5
3.0
3.5
4.0

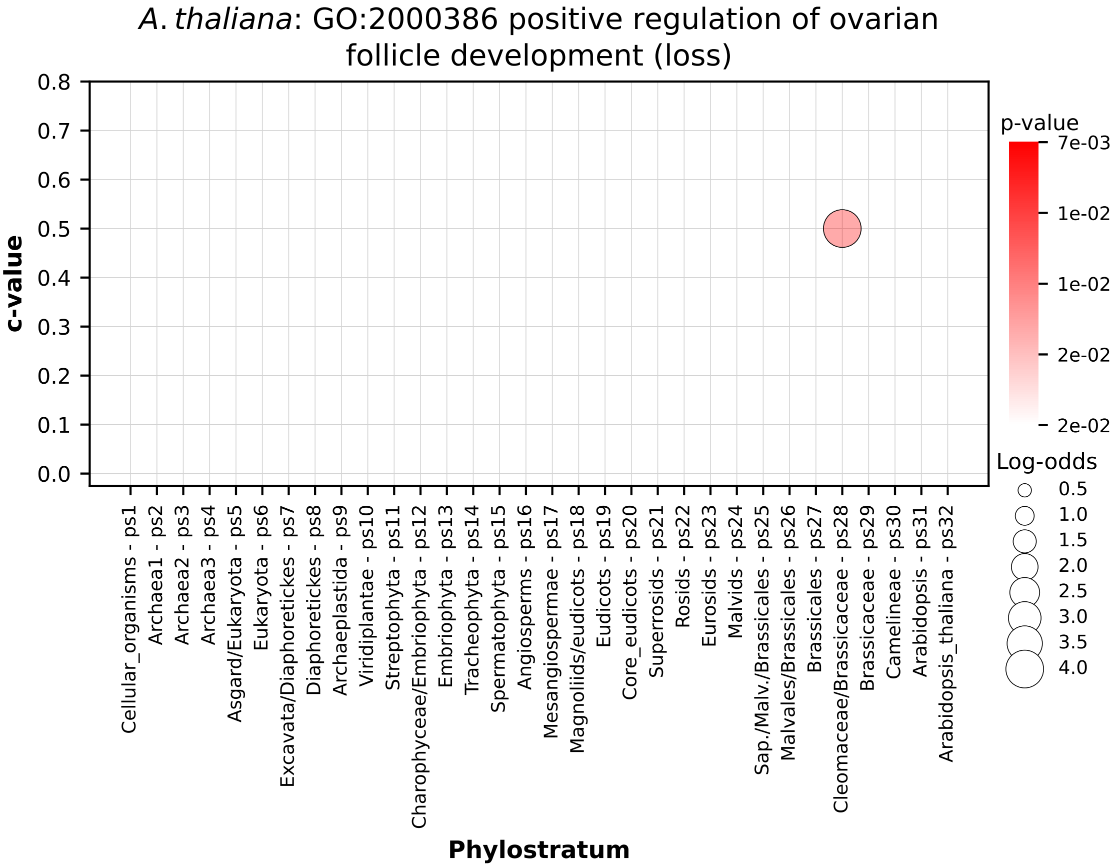
A. thaliana: GO:2000386 positive regulation of ovarian follicle development (loss)
c-value
0.8
0.7
0.6
0.5
0.4
0.3
0.2
0.1
0.0
Phylostratum
Cellular_organisms - ps1
Archaea1 - ps2
Archaea2 - ps3
Archaea3 - ps4
Asgard/Eukaryota - ps5
Eukaryota - ps6
Excavata/Diaphoretickes - ps7
Diaphoretickes - ps8
Archaeplastida - ps9
Viridiplantae - ps10
Streptophyta - ps11
Charophyceae/Embriophyta - ps12
Embriophyta - ps13
Tracheophyta - ps14
Spermatophyta - ps15
Angiosperms - ps16
Mesangiospermae - ps17
Magnoliids/eudicots - ps18
Eudicots - ps19
Core_eudicots - ps20
Superrosids - ps21
Rosids - ps22
Eurosids - ps23
Malvids - ps24
Sap./Malv./Brassicales - ps25
Malvales/Brassicales - ps26
Brassicales - ps27
Cleomaceae/Brassicaceae - ps28
Brassicaceae - ps29
Camelineae - ps30
Arabidopsis - ps31
Arabidopsis_thaliana - ps32
p-value
7e-03
1e-02
1e-02
2e-02
2e-02
Log-odds
0.5
1.0
1.5
2.0
2.5
3.0
3.5
4.0

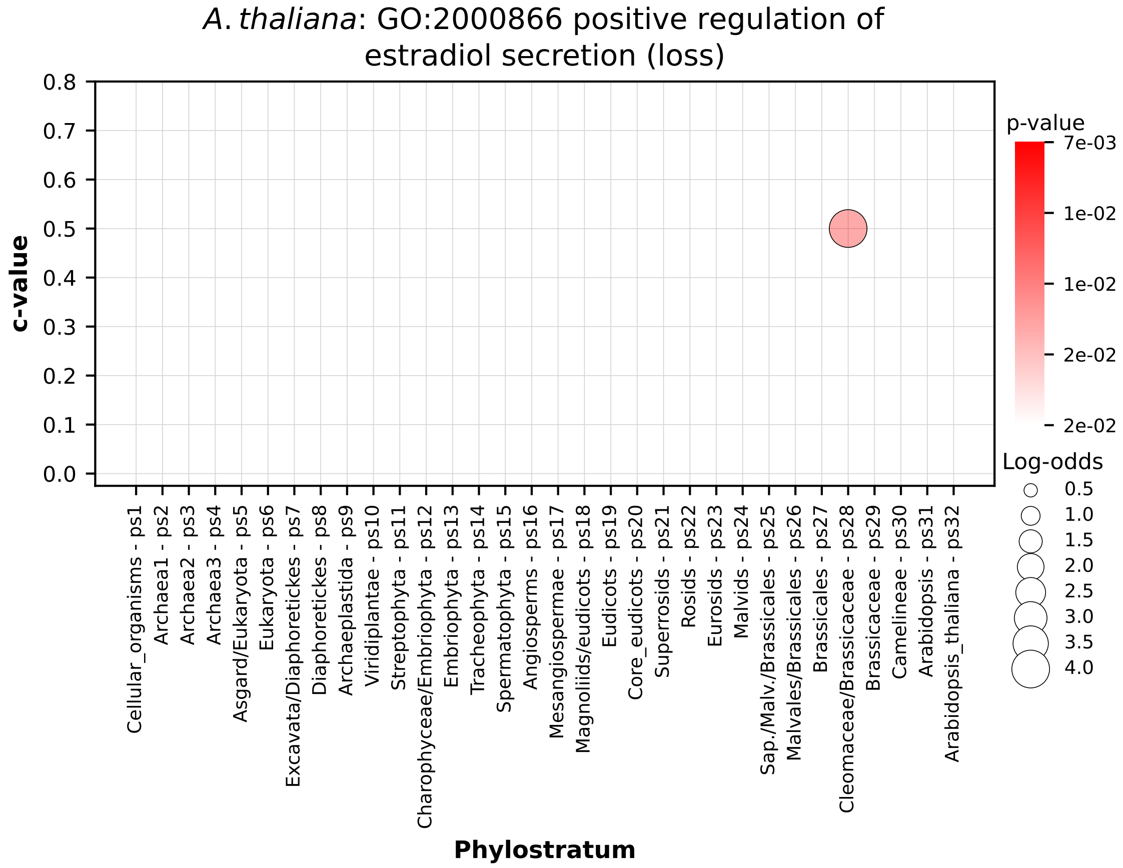

A. thaliana: GO:2000866 positive regulation of estradiol secretion (loss)
c-value
0.8
0.7
0.6
0.5
0.4
0.3
0.2
0.1
0.0
p-value
7e-03
1e-02
1e-02
2e-02
2e-02
Log-odds
0.5
1.0
1.5
2.0
2.5
3.0
3.5
4.0
Cellular_organisms - ps1
Archaea1 - ps2
Archaea2 - ps3
Archaea3 - ps4
Asgard/Eukaryota - ps5
Eukaryota - ps6
Excavata/Diaphoretickes - ps7
Diaphoretickes - ps8
Archaeplastida - ps9
Viridiplantae - ps10
Streptophyta - ps11
Charophyceae/Embriophyta - ps12
Embriophyta - ps13
Tracheophyta - ps14
Spermatophyta - ps15
Angiosperms - ps16
Mesangiospermae - ps17
Magnoliids/eudicots - ps18
Eudicots - ps19
Core_eudicots - ps20
Superrosids - ps21
Rosids - ps22
Eurosids - ps23
Malvids - ps24
Sap./Malv./Brassicales - ps25
Malvales/Brassicales - ps26
Brassicales - ps27
Cleomaceae/Brassicaceae - ps28
Brassicaceae - ps29
Camelineae - ps30
Arabidopsis - ps31
Arabidopsis_thaliana - ps32
Phylostratum

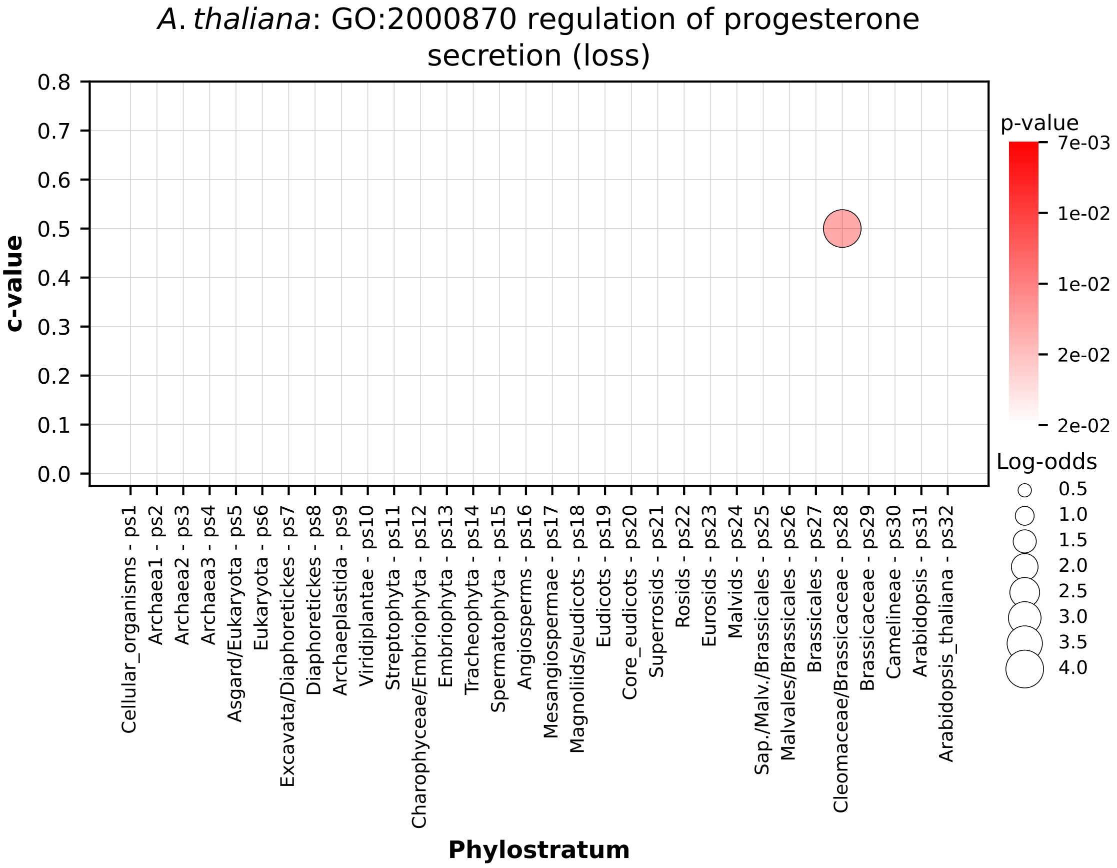
A. thaliana: GO:2000870 regulation of progesterone secretion (loss)
c-value
0.0
0.1
0.2
0.3
0.4
0.5
0.6
0.7
0.8
p-value
7e-03
1e-02
1e-02
2e-02
2e-02
Log-odds
0.5
1.0
1.5
2.0
2.5
3.0
3.5
4.0
Cellular_organisms - ps1
Archaea1 - ps2
Archaea2 - ps3
Archaea3 - ps4
Asgard/Eukaryota - ps5
Eukaryota - ps6
Excavata/Diaphoretickes - ps7
Diaphoretickes - ps8
Archaeplastida - ps9
Viridiplantae - ps10
Streptophyta - ps11
Charophyceae/Embriophyta - ps12
Embriophyta - ps13
Tracheophyta - ps14
Spermatophyta - ps15
Angiosperms - ps16
Mesangiospermae - ps17
Magnoliids/eudicots - ps18
Eudicots - ps19
Core_eudicots - ps20
Superrosids - ps21
Rosids - ps22
Eurosids - ps23
Malvids - ps24
Sap./Malv./Brassicales - ps25
Malvales/Brassicales - ps26
Brassicales - ps27
Cleomaceae/Brassicaceae - ps28
Brassicaceae - ps29
Camelineae - ps30
Arabidopsis - ps31
Arabidopsis_thaliana - ps32
Phylostratum

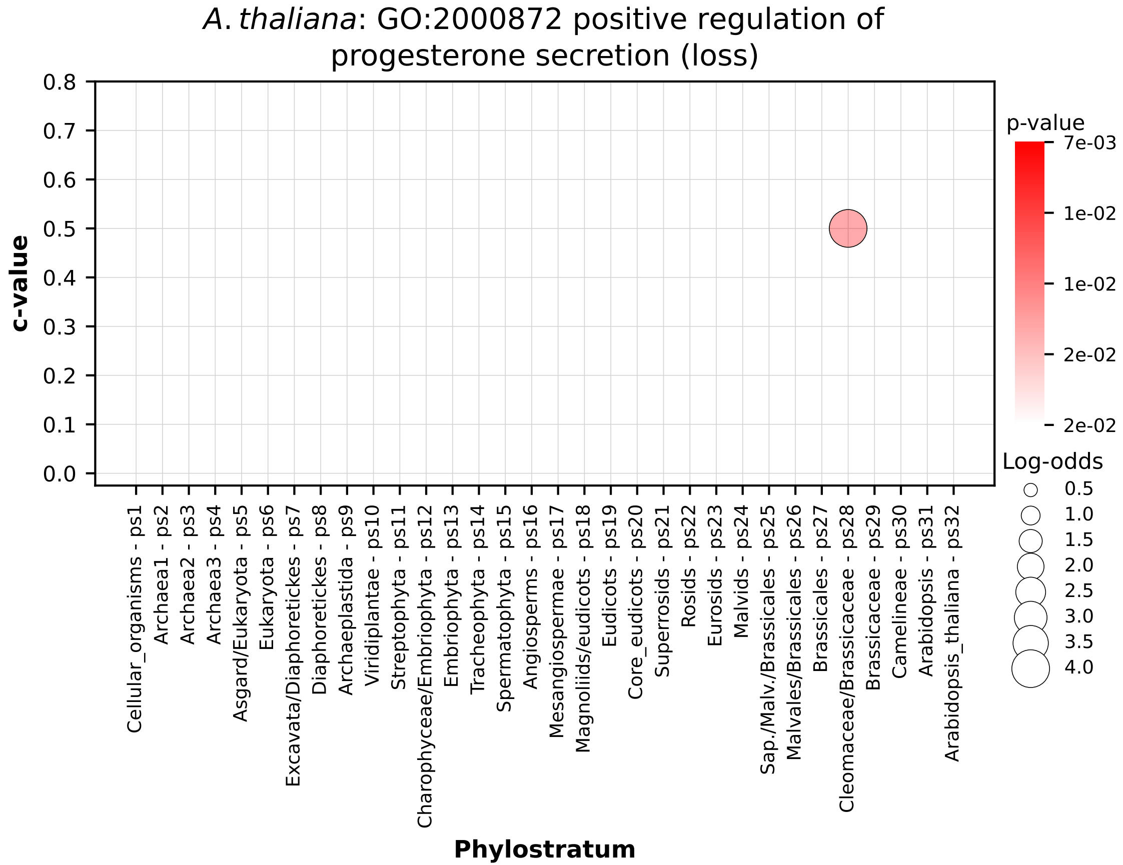

A. thaliana: GO:2000872 positive regulation of progesterone secretion (loss)
c-value
0.8
0.7
0.6
0.5
0.4
0.3
0.2
0.1
0.0
Phylostratum
Cellular_organisms - ps1
Archaea1 - ps2
Archaea2 - ps3
Archaea3 - ps4
Asgard/Eukaryota - ps5
Eukaryota - ps6
Excavata/Diaphoretickes - ps7
Diaphoretickes - ps8
Archaeplastida - ps9
Viridiplantae - ps10
Streptophyta - ps11
Charophyceae/Embriophyta - ps12
Embriophyta - ps13
Tracheophyta - ps14
Spermatophyta - ps15
Angiosperms - ps16
Mesangiospermae - ps17
Magnoliids/eudicots - ps18
Eudicots - ps19
Core_eudicots - ps20
Superrosids - ps21
Rosids - ps22
Eurosids - ps23
Malvids - ps24
Sap./Malv./Brassicales - ps25
Malvales/Brassicales - ps26
Brassicales - ps27
Cleomaceae/Brassicaceae - ps28
Brassicaceae - ps29
Camelineae - ps30
Arabidopsis - ps31
Arabidopsis_thaliana - ps32
p-value
7e-03
1e-02
1e-02
2e-02
2e-02
Log-odds
0.5
1.0
1.5
2.0
2.5
3.0
3.5
4.0

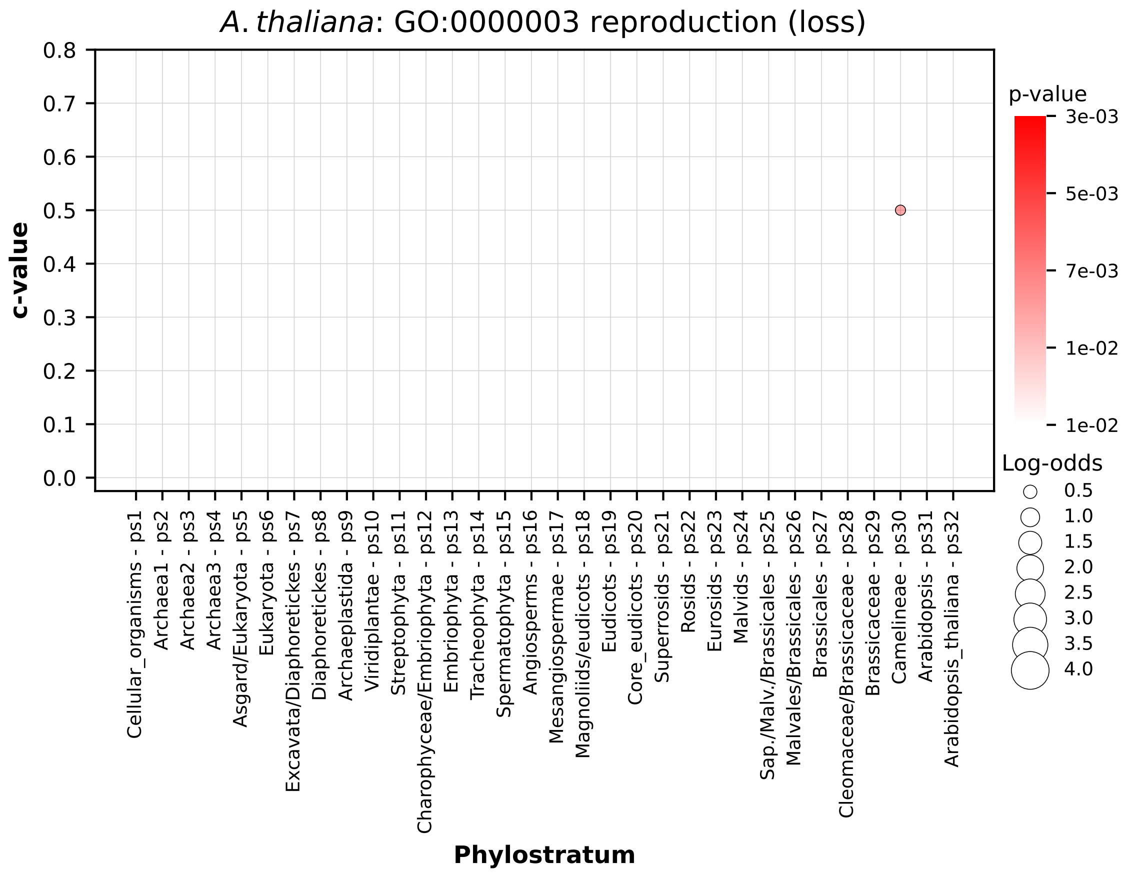

A. thaliana: GO:0000003 reproduction (loss)
c-value
0.8
0.7
0.6
0.5
0.4
0.3
0.2
0.1
0.0
p-value
3e-03
5e-03
7e-03
1e-02
1e-02
Log-odds
0.5
1.0
1.5
2.0
2.5
3.0
3.5
4.0
Cellular_organisms - ps1
Archaea1 - ps2
Archaea2 - ps3
Archaea3 - ps4
Asgard/Eukaryota - ps5
Eukaryota - ps6
Excavata/Diaphoretickes - ps7
Diaphoretickes - ps8
Archaeplastida - ps9
Viridiplantae - ps10
Streptophyta - ps11
Charophyceae/Embriophyta - ps12
Embriophyta - ps13
Tracheophyta - ps14
Spermatophyta - ps15
Angiosperms - ps16
Mesangiospermae - ps17
Magnoliids/eudicots - ps18
Eudicots - ps19
Core_eudicots - ps20
Superrosids - ps21
Rosids - ps22
Eurosids - ps23
Malvids - ps24
Sap./Malv./Brassicales - ps25
Malvales/Brassicales - ps26
Brassicales - ps27
Cleomaceae/Brassicaceae - ps28
Brassicaceae - ps29
Camelineae - ps30
Arabidopsis - ps31
Arabidopsis_thaliana - ps32
Phylostratum

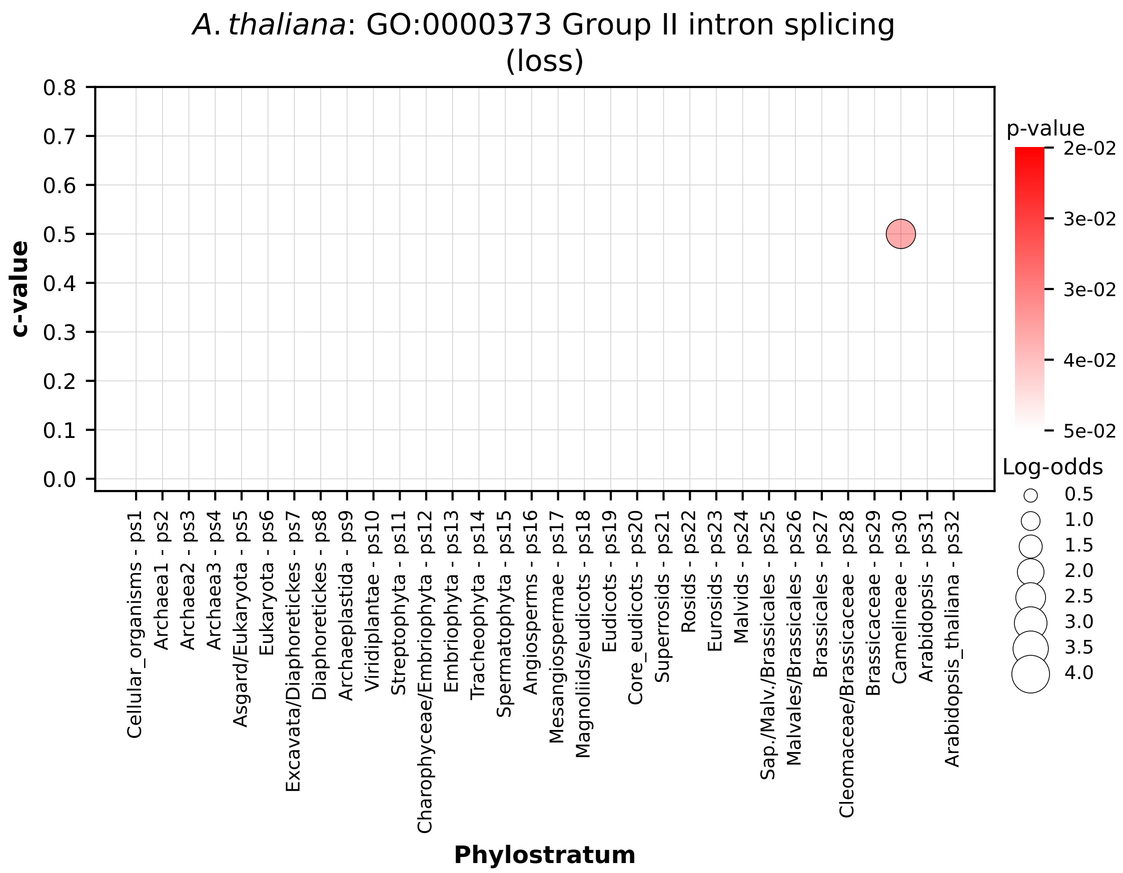

A. thaliana: GO:0000373 Group II intron splicing
(loss)
c-value
0.8
0.7
0.6
0.5
0.4
0.3
0.2
0.1
0.0
p-value
2e-02
3e-02
3e-02
4e-02
5e-02
Log-odds
0.5
1.0
1.5
2.0
2.5
3.0
3.5
4.0
Cellular_organisms - ps1
Archaea1 - ps2
Archaea2 - ps3
Archaea3 - ps4
Asgard/Eukaryota - ps5
Eukaryota - ps6
Excavata/Diaphoretickes - ps7
Diaphoretickes - ps8
Archaeplastida - ps9
Viridiplantae - ps10
Streptophyta - ps11
Charophyceae/Embriophyta - ps12
Embriophyta - ps13
Tracheophyta - ps14
Spermatophyta - ps15
Angiosperms - ps16
Mesangiospermae - ps17
Magnoliids/eudicots - ps18
Eudicots - ps19
Core_eudicots - ps20
Superrosids - ps21
Rosids - ps22
Eurosids - ps23
Malvids - ps24
Sap./Malv./Brassicales - ps25
Malvales/Brassicales - ps26
Brassicales - ps27
Cleomaceae/Brassicaceae - ps28
Brassicaceae - ps29
Camelineae - ps30
Arabidopsis - ps31
Arabidopsis_thaliana - ps32
Phylostratum

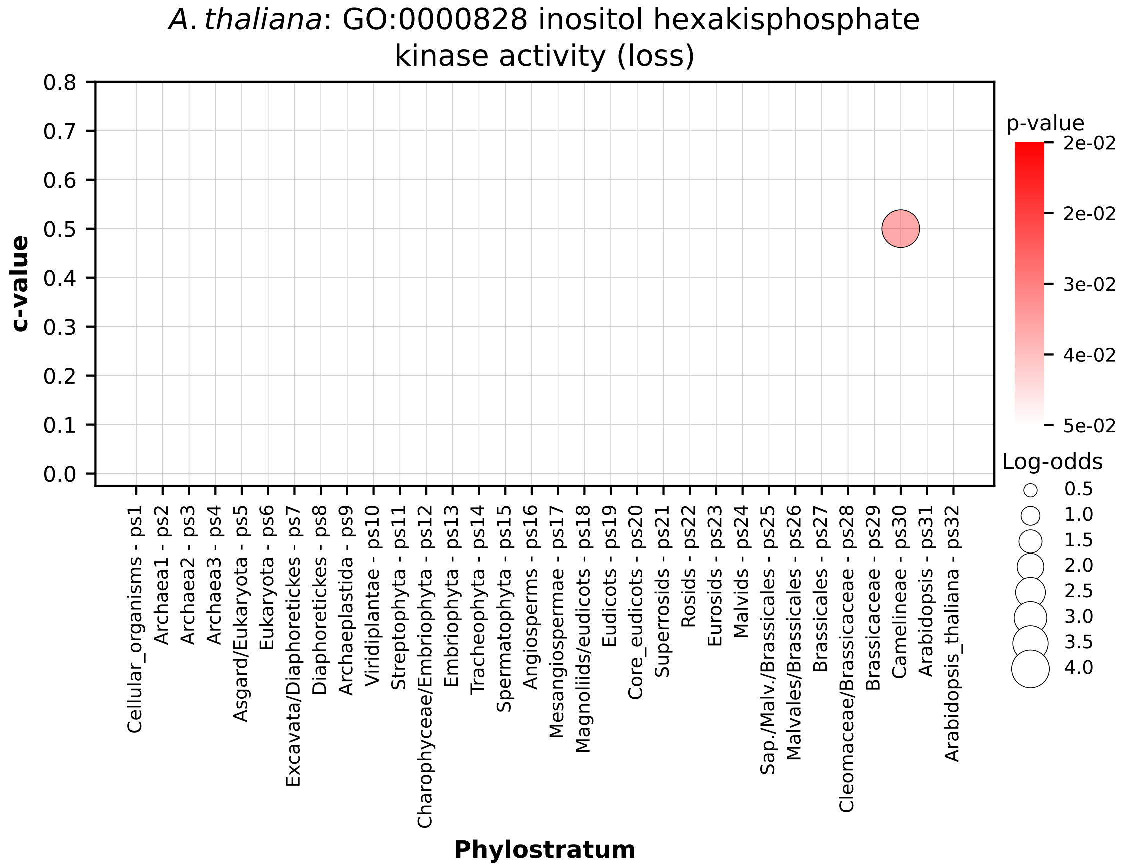

A. thaliana: GO:0000828 inositol hexakisphosphate kinase activity (loss)
c-value
0.8
0.7
0.6
0.5
0.4
0.3
0.2
0.1
0.0
Phylostratum
Cellular_organisms - ps1
Archaea1 - ps2
Archaea2 - ps3
Archaea3 - ps4
Asgard/Eukaryota - ps5
Eukaryota - ps6
Excavata/Diaphoretickes - ps7
Diaphoretickes - ps8
Archaeplastida - ps9
Viridiplantae - ps10
Streptophyta - ps11
Charophyceae/Embriophyta - ps12
Embriophyta - ps13
Tracheophyta - ps14
Spermatophyta - ps15
Angiosperms - ps16
Mesangiospermae - ps17
Magnoliids/eudicots - ps18
Eudicots - ps19
Core_eudicots - ps20
Superrosids - ps21
Rosids - ps22
Eurosids - ps23
Malvids - ps24
Sap./Malv./Brassicales - ps25
Malvales/Brassicales - ps26
Brassicales - ps27
Cleomaceae/Brassicaceae - ps28
Brassicaceae - ps29
Camelineae - ps30
Arabidopsis - ps31
Arabidopsis_thaliana - ps32
p-value
2e-02
2e-02
3e-02
4e-02
5e-02
Log-odds
0.5
1.0
1.5
2.0
2.5
3.0
3.5
4.0

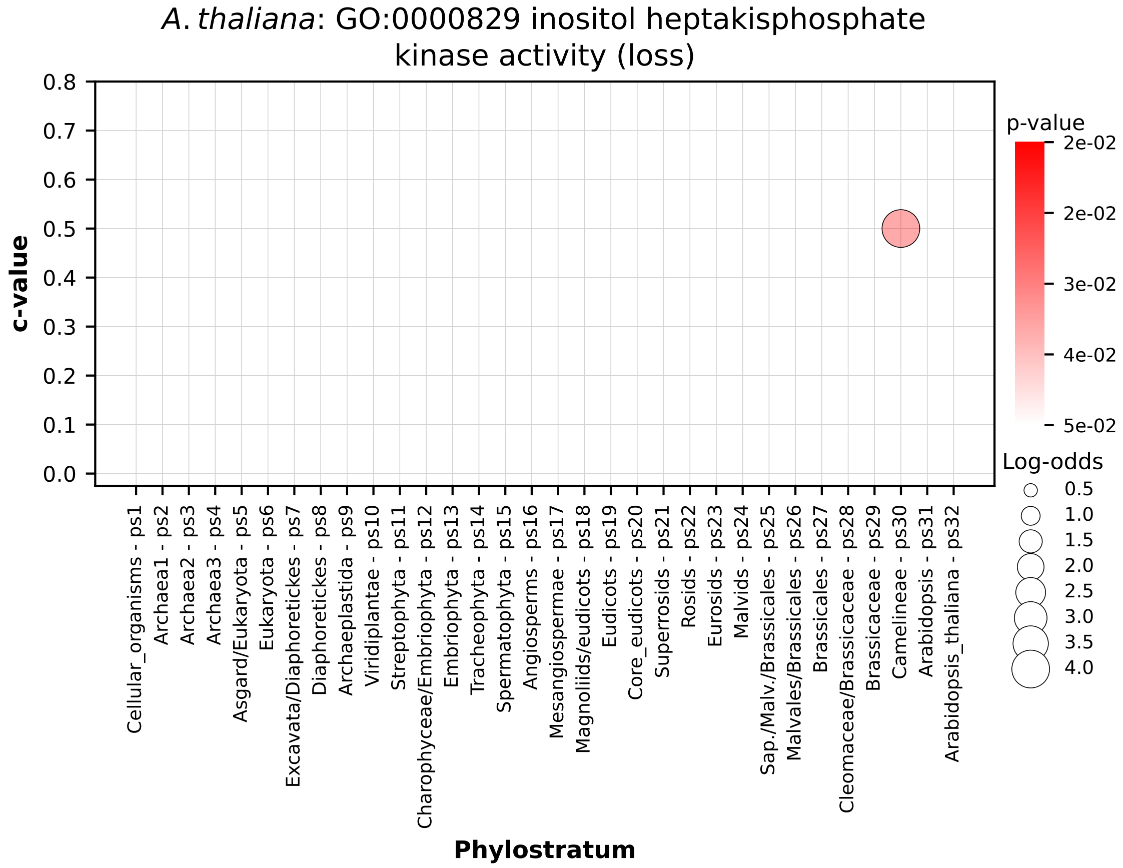

A. thaliana: GO:0000829 inositol heptakisphosphate kinase activity (loss)
c-value
0.8
0.7
0.6
0.5
0.4
0.3
0.2
0.1
0.0
p-value
2e-02
2e-02
3e-02
4e-02
5e-02
Log-odds
0.5
1.0
1.5
2.0
2.5
3.0
3.5
4.0
Cellular_organisms - ps1
Archaea1 - ps2
Archaea2 - ps3
Archaea3 - ps4
Asgard/Eukaryota - ps5
Eukaryota - ps6
Excavata/Diaphoretickes - ps7
Diaphoretickes - ps8
Archaeplastida - ps9
Viridiplantae - ps10
Streptophyta - ps11
Charophyceae/Embriophyta - ps12
Embriophyta - ps13
Tracheophyta - ps14
Spermatophyta - ps15
Angiosperms - ps16
Mesangiospermae - ps17
Magnoliids/eudicots - ps18
Eudicots - ps19
Core_eudicots - ps20
Superrosids - ps21
Rosids - ps22
Eurosids - ps23
Malvids - ps24
Sap./Malv./Brassicales - ps25
Malvales/Brassicales - ps26
Brassicales - ps27
Cleomaceae/Brassicaceae - ps28
Brassicaceae - ps29
Camelineae - ps30
Arabidopsis - ps31
Arabidopsis_thaliana - ps32
Phylostratum

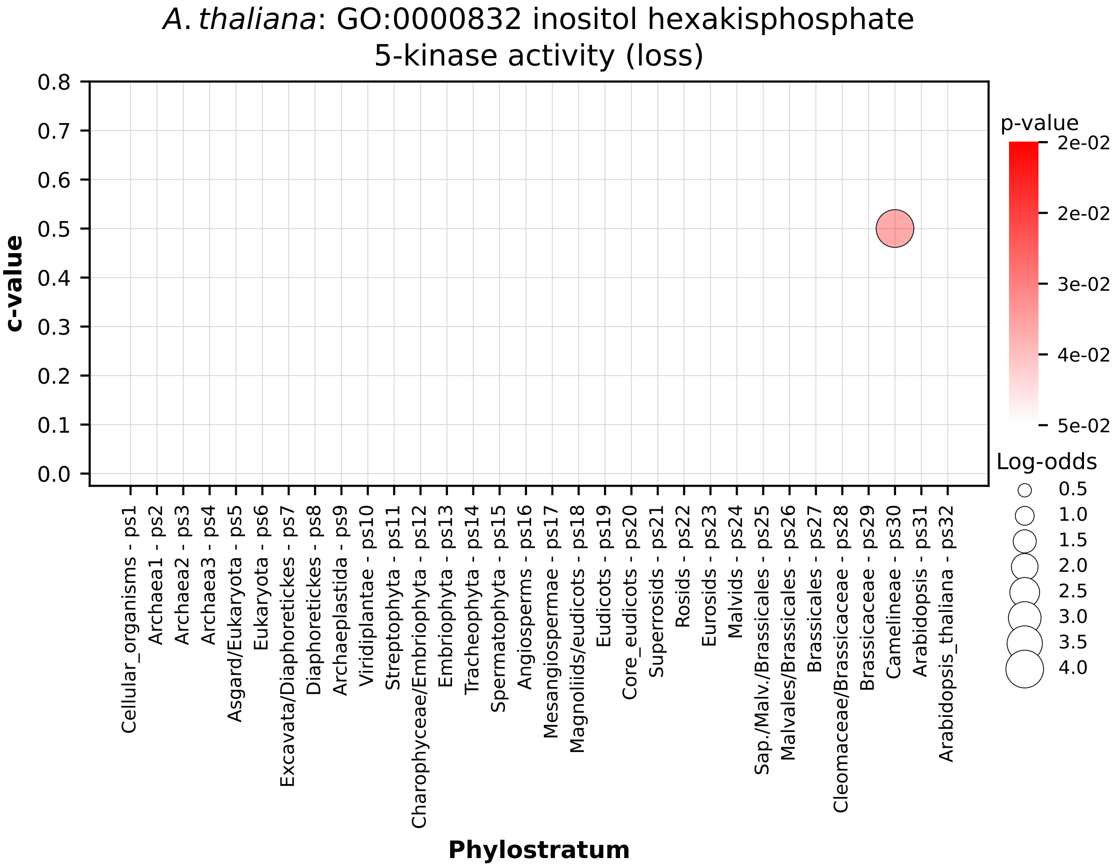

A. thaliana: GO:0000832 inositol hexakisphosphate 5-kinase activity (loss)
c-value
0.8
0.7
0.6
0.5
0.4
0.3
0.2
0.1
0.0
p-value
2e-02
2e-02
3e-02
4e-02
5e-02
Log-odds
0.5
1.0
1.5
2.0
2.5
3.0
3.5
4.0
Cellular_organisms - ps1
Archaea1 - ps2
Archaea2 - ps3
Archaea3 - ps4
Asgard/Eukaryota - ps5
Eukaryota - ps6
Excavata/Diaphoretickes - ps7
Diaphoretickes - ps8
Archaeplastida - ps9
Viridiplantae - ps10
Streptophyta - ps11
Charophyceae/Embriophyta - ps12
Embriophyta - ps13
Tracheophyta - ps14
Spermatophyta - ps15
Angiosperms - ps16
Mesangiospermae - ps17
Magnoliids/eudicots - ps18
Eudicots - ps19
Core_eudicots - ps20
Superrosids - ps21
Rosids - ps22
Eurosids - ps23
Malvids - ps24
Sap./Malv./Brassicales - ps25
Malvales/Brassicales - ps26
Brassicales - ps27
Cleomaceae/Brassicaceae - ps28
Brassicaceae - ps29
Camelineae - ps30
Arabidopsis - ps31
Arabidopsis_thaliana - ps32
Phylostratum

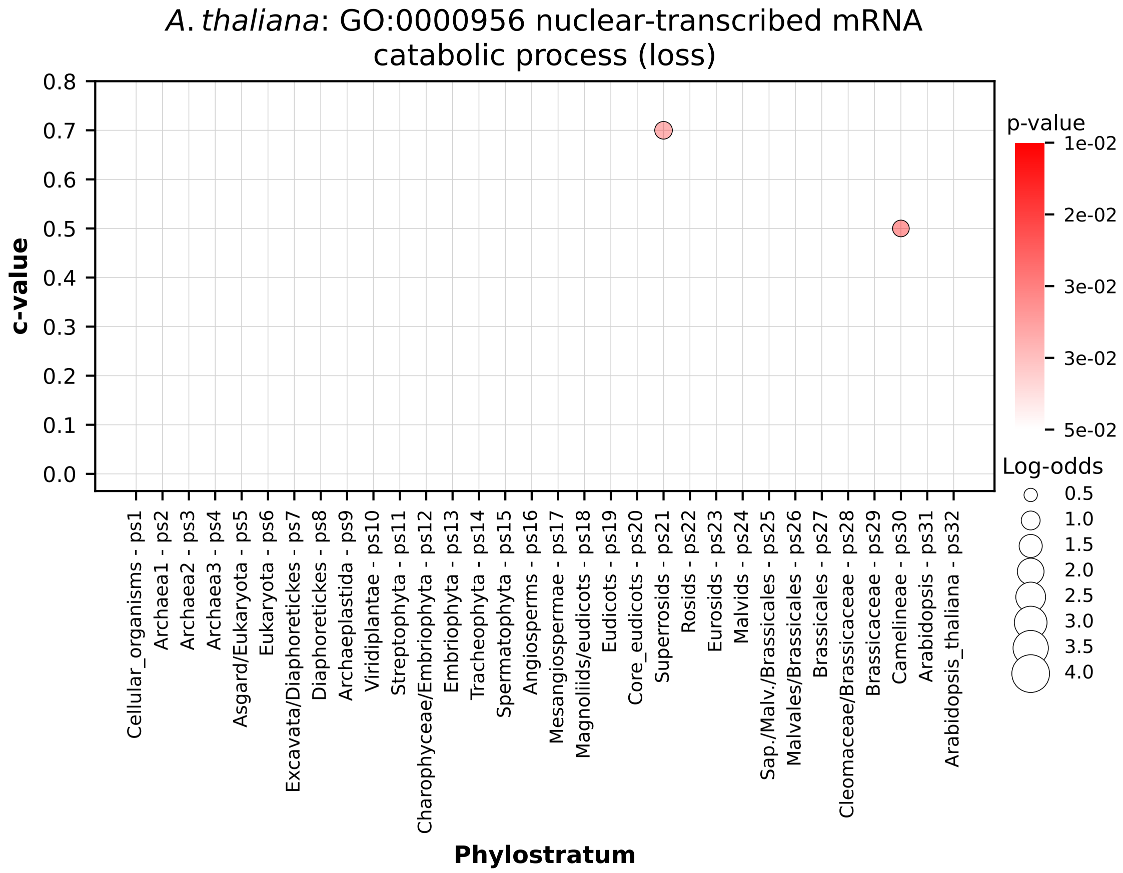

A. thaliana: GO:0000956 nuclear-transcribed mRNA catabolic process (loss)
c-value
0.0
0.1
0.2
0.3
0.4
0.5
0.6
0.7
0.8
Phylostratum
Cellular_organisms - ps1
Archaea1 - ps2
Archaea2 - ps3
Archaea3 - ps4
Asgard/Eukaryota - ps5
Eukaryota - ps6
Excavata/Diaphoretickes - ps7
Diaphoretickes - ps8
Archaeplastida - ps9
Viridiplantae - ps10
Streptophyta - ps11
Charophyceae/Embriophyta - ps12
Embriophyta - ps13
Tracheophyta - ps14
Spermatophyta - ps15
Angiosperms - ps16
Mesangiospermae - ps17
Magnoliids/eudicots - ps18
Eudicots - ps19
Core_eudicots - ps20
Superrosids - ps21
Rosids - ps22
Eurosids - ps23
Malvids - ps24
Sap./Malv./Brassicales - ps25
Malvales/Brassicales - ps26
Brassicales - ps27
Cleomaceae/Brassicaceae - ps28
Brassicaceae - ps29
Camelineae - ps30
Arabidopsis - ps31
Arabidopsis_thaliana - ps32
p-value
1e-02
2e-02
3e-02
3e-02
5e-02
Log-odds
0.5
1.0
1.5
2.0
2.5
3.0
3.5
4.0

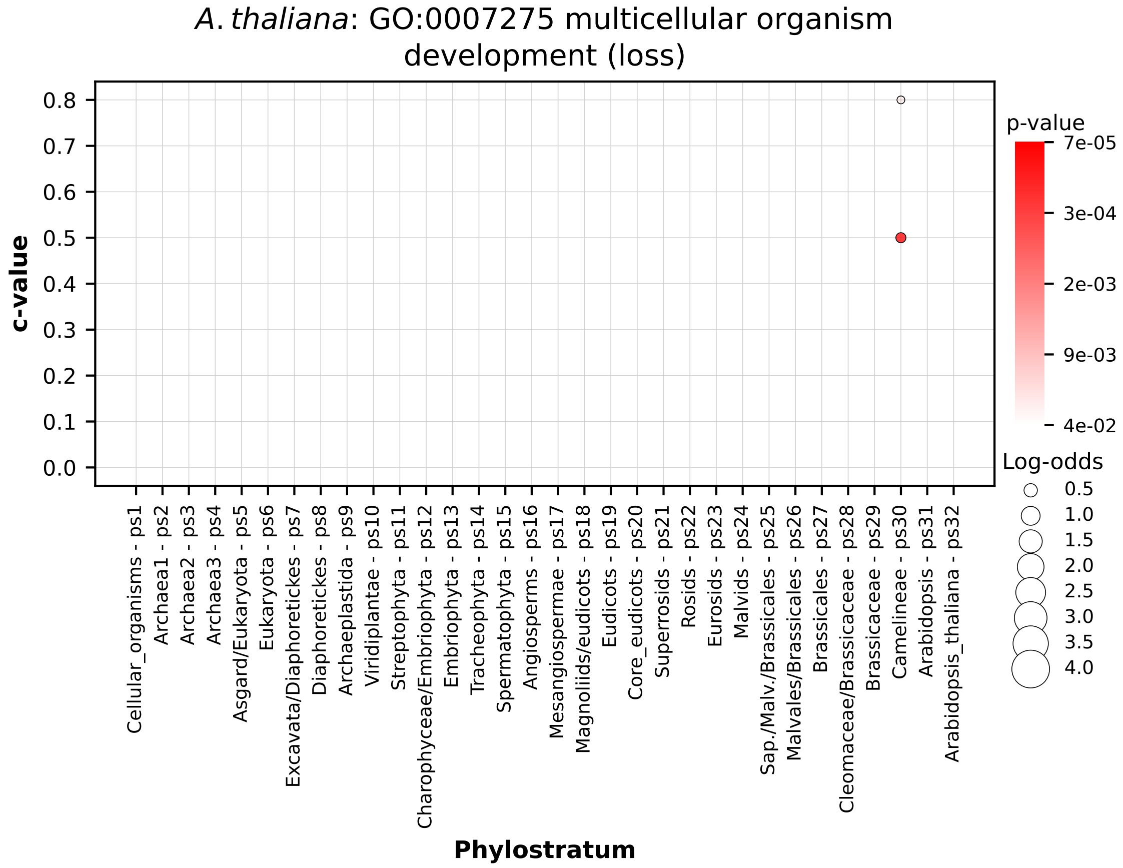

$A. thaliana$: GO:0007275 multicellular organism development (loss)
c-value
0.0
0.1
0.2
0.3
0.4
0.5
0.6
0.7
0.8
Phylostratum
Cellular_organisms - ps1
Archaea1 - ps2
Archaea2 - ps3
Archaea3 - ps4
Asgard/Eukaryota - ps5
Eukaryota - ps6
Excavata/Diaphoretickes - ps7
Diaphoretickes - ps8
Archaeplastida - ps9
Viridiplantae - ps10
Streptophyta - ps11
Charophyceae/Embriophyta - ps12
Embriophyta - ps13
Tracheophyta - ps14
Spermatophyta - ps15
Angiosperms - ps16
Mesangiospermae - ps17
Magnoliids/eudicots - ps18
Eudicots - ps19
Core_eudicots - ps20
Superrosids - ps21
Rosids - ps22
Eurosids - ps23
Malvids - ps24
Sap./Malv./Brassicales - ps25
Malvales/Brassicales - ps26
Brassicales - ps27
Cleomaceae/Brassicaceae - ps28
Brassicaceae - ps29
Camelineae - ps30
Arabidopsis - ps31
Arabidopsis_thaliana - ps32
p-value
7e-05
3e-04
2e-03
9e-03
4e-02
Log-odds
0.5
1.0
1.5
2.0
2.5
3.0
3.5
4.0

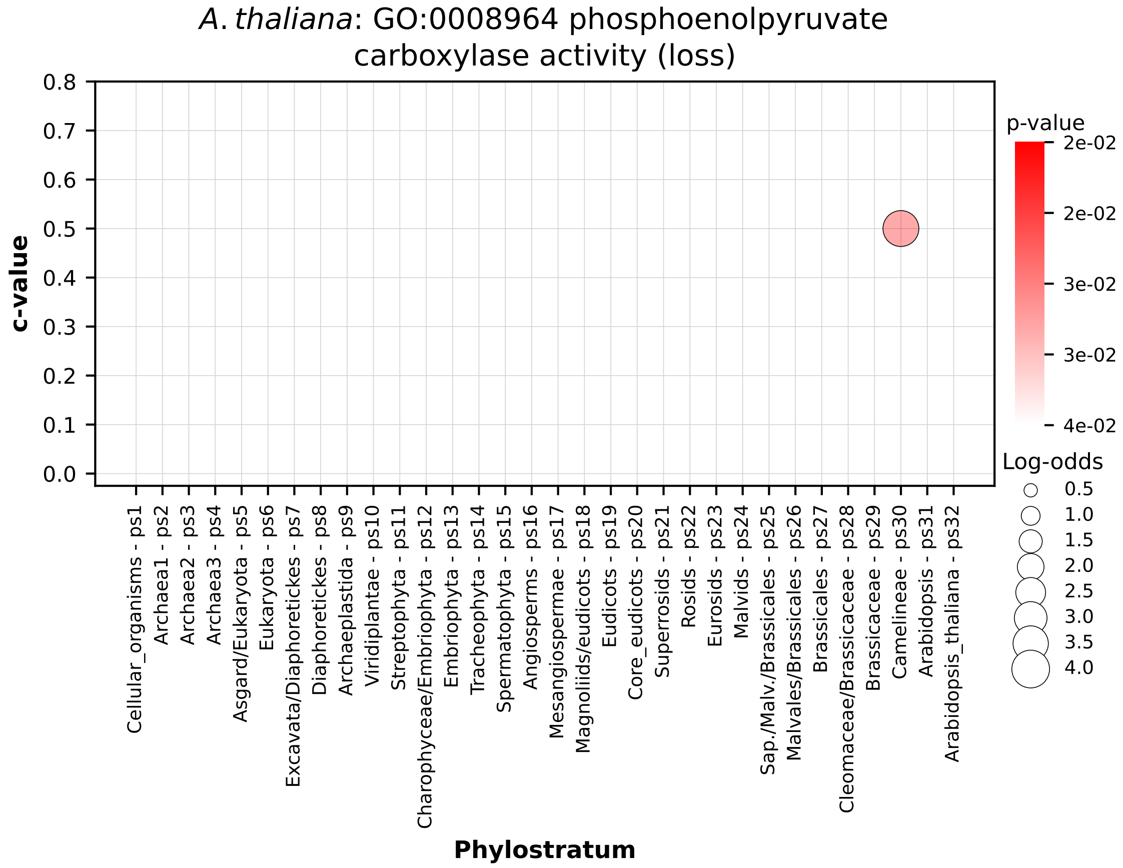

A. thaliana: GO:0008964 phosphoenolpyruvate carboxylase activity (loss)
c-value
0.8
0.7
0.6
0.5
0.4
0.3
0.2
0.1
0.0
Phylostratum
Cellular_organisms - ps1
Archaea1 - ps2
Archaea2 - ps3
Archaea3 - ps4
Asgard/Eukaryota - ps5
Eukaryota - ps6
Excavata/Diaphoretickes - ps7
Diaphoretickes - ps8
Archaeplastida - ps9
Viridiplantae - ps10
Streptophyta - ps11
Charophyceae/Embriophyta - ps12
Embriophyta - ps13
Tracheophyta - ps14
Spermatophyta - ps15
Angiosperms - ps16
Mesangiospermae - ps17
Magnoliids/eudicots - ps18
Eudicots - ps19
Core_eudicots - ps20
Superrosids - ps21
Rosids - ps22
Eurosids - ps23
Malvids - ps24
Sap./Malv./Brassicales - ps25
Malvales/Brassicales - ps26
Brassicales - ps27
Cleomaceae/Brassicaceae - ps28
Brassicaceae - ps29
Camelineae - ps30
Arabidopsis - ps31
Arabidopsis_thaliana - ps32
p-value
2e-02
2e-02
3e-02
3e-02
4e-02
Log-odds
0.5
1.0
1.5
2.0
2.5
3.0
3.5
4.0

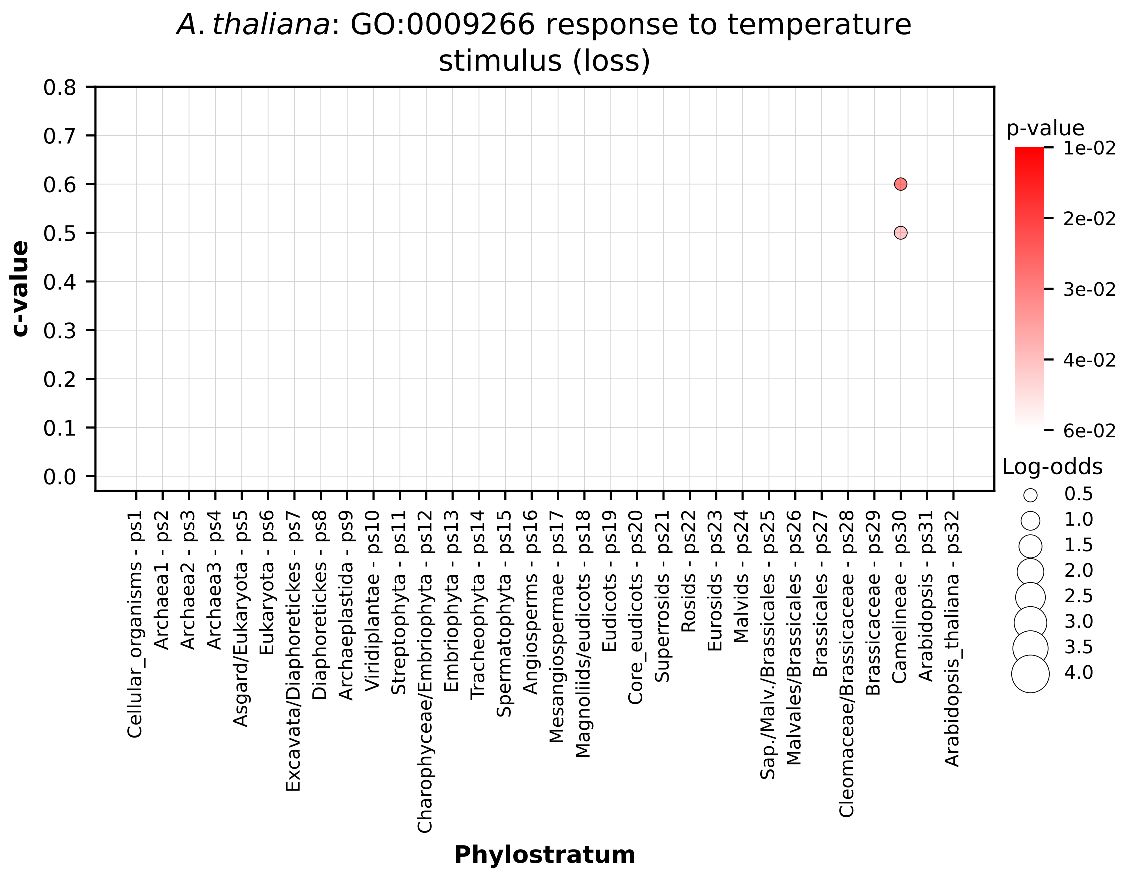

A. thaliana: GO:0009266 response to temperature stimulus (loss)
c-value
0.8
0.7
0.6
0.5
0.4
0.3
0.2
0.1
0.0
Phylostratum
Cellular_organisms - ps1
Archaea1 - ps2
Archaea2 - ps3
Archaea3 - ps4
Asgard/Eukaryota - ps5
Eukaryota - ps6
Excavata/Diaphoretickes - ps7
Diaphoretickes - ps8
Archaeplastida - ps9
Viridiplantae - ps10
Streptophyta - ps11
Charophyceae/Embriophyta - ps12
Embriophyta - ps13
Tracheophyta - ps14
Spermatophyta - ps15
Angiosperms - ps16
Mesangiospermae - ps17
Magnoliids/eudicots - ps18
Eudicots - ps19
Core_eudicots - ps20
Superrosids - ps21
Rosids - ps22
Eurosids - ps23
Malvids - ps24
Sap./Malv./Brassicales - ps25
Malvales/Brassicales - ps26
Brassicales - ps27
Cleomaceae/Brassicaceae - ps28
Brassicaceae - ps29
Camelineae - ps30
Arabidopsis - ps31
Arabidopsis_thaliana - ps32
p-value
1e-02
2e-02
3e-02
4e-02
6e-02
Log-odds
0.5
1.0
1.5
2.0
2.5
3.0
3.5
4.0

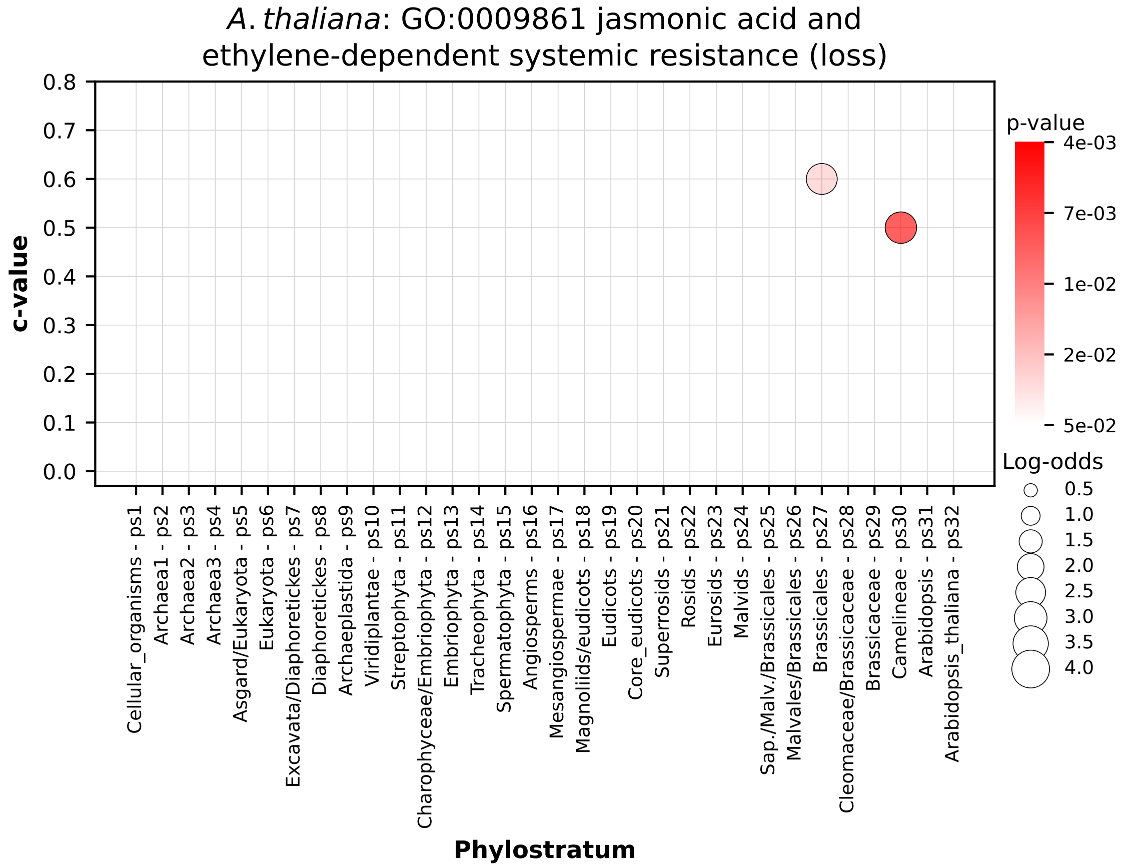

A. thaliana: GO:0009861 jasmonic acid and ethylene-dependent systemic resistance (loss)
c-value
0.8
0.7
0.6
0.5
0.4
0.3
0.2
0.1
0.0
p-value
4e-03
7e-03
1e-02
2e-02
5e-02
Log-odds
0.5
1.0
1.5
2.0
2.5
3.0
3.5
4.0
Cellular_organisms - ps1
Archaea1 - ps2
Archaea2 - ps3
Archaea3 - ps4
Asgard/Eukaryota - ps5
Eukaryota - ps6
Excavata/Diaphoretickes - ps7
Diaphoretickes - ps8
Archaeplastida - ps9
Viridiplantae - ps10
Streptophyta - ps11
Charophyceae/Embriophyta - ps12
Embriophyta - ps13
Tracheophyta - ps14
Spermatophyta - ps15
Angiosperms - ps16
Mesangiospermae - ps17
Magnoliids/eudicots - ps18
Eudicots - ps19
Core_eudicots - ps20
Superrosids - ps21
Rosids - ps22
Eurosids - ps23
Malvids - ps24
Sap./Malv./Brassicales - ps25
Malvales/Brassicales - ps26
Brassicales - ps27
Cleomaceae/Brassicaceae - ps28
Brassicaceae - ps29
Camelineae - ps30
Arabidopsis - ps31
Arabidopsis_thaliana - ps32
Phylostratum

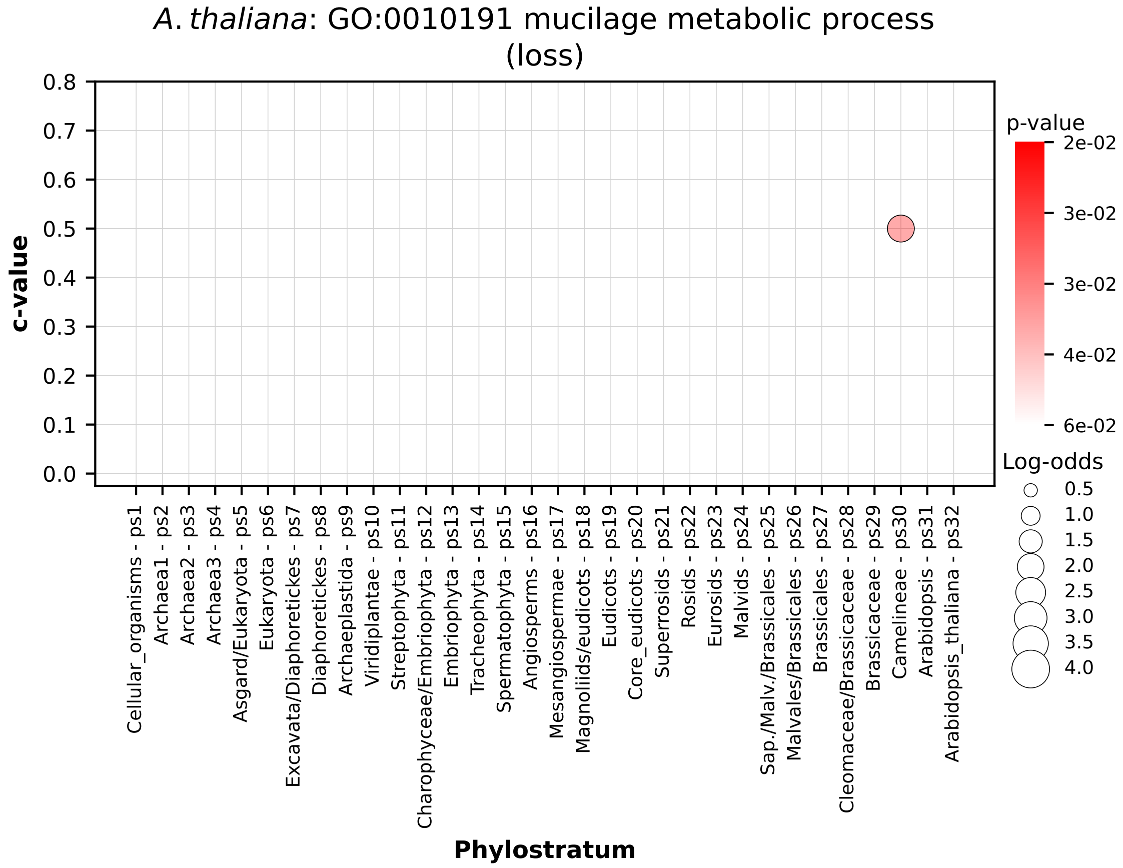

A. thaliana: GO:0010191 mucilage metabolic process
(loss)
c-value
0.8
0.7
0.6
0.5
0.4
0.3
0.2
0.1
0.0
p-value
2e-02
3e-02
3e-02
4e-02
6e-02
Log-odds
0.5
1.0
1.5
2.0
2.5
3.0
3.5
4.0
Cellular_organisms - ps1
Archaea1 - ps2
Archaea2 - ps3
Archaea3 - ps4
Asgard/Eukaryota - ps5
Eukaryota - ps6
Excavata/Diaphoretickes - ps7
Diaphoretickes - ps8
Archaeplastida - ps9
Viridiplantae - ps10
Streptophyta - ps11
Charophyceae/Embriophyta - ps12
Embriophyta - ps13
Tracheophyta - ps14
Spermatophyta - ps15
Angiosperms - ps16
Mesangiospermae - ps17
Magnoliids/eudicots - ps18
Eudicots - ps19
Core_eudicots - ps20
Superrosids - ps21
Rosids - ps22
Eurosids - ps23
Malvids - ps24
Sap./Malv./Brassicales - ps25
Malvales/Brassicales - ps26
Brassicales - ps27
Cleomaceae/Brassicaceae - ps28
Brassicaceae - ps29
Camelineae - ps30
Arabidopsis - ps31
Arabidopsis_thaliana - ps32
Phylostratum

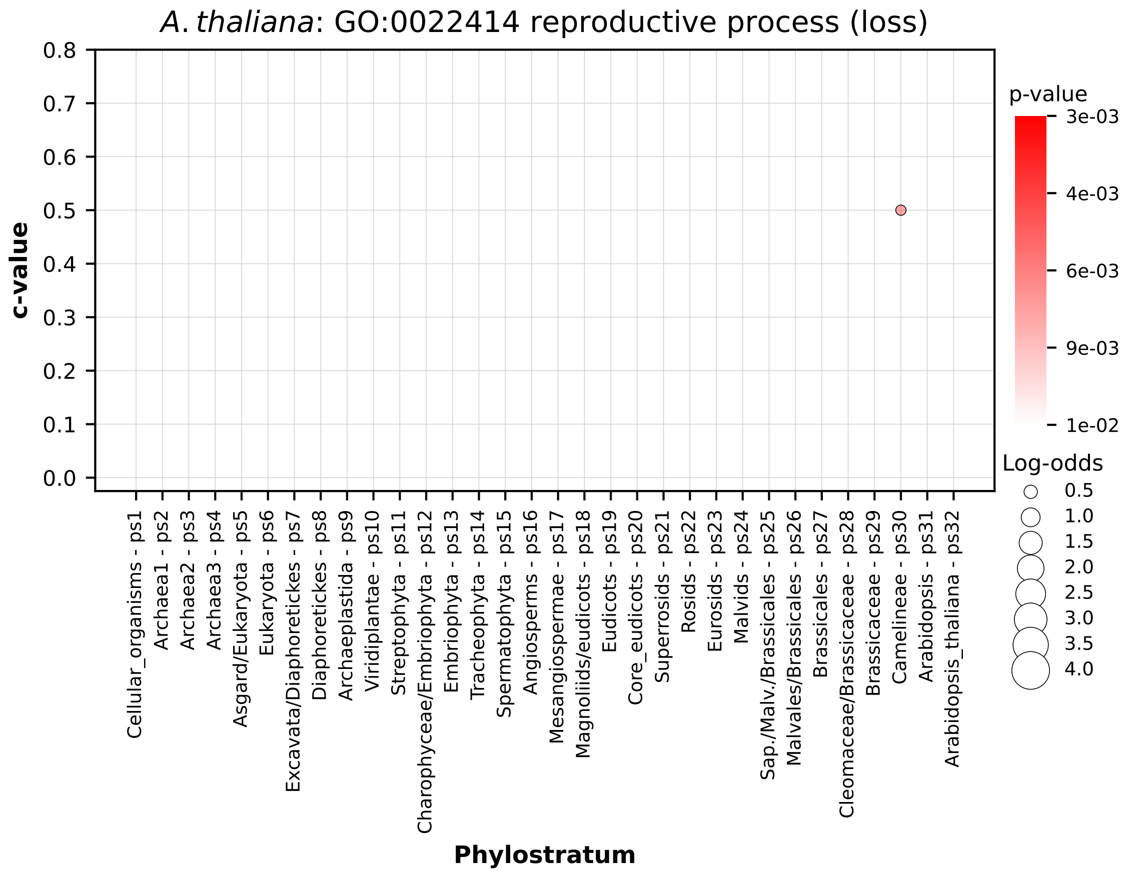

A. thaliana: GO:0022414 reproductive process (loss)
c-value
0.0
0.1
0.2
0.3
0.4
0.5
0.6
0.7
0.8
p-value
3e-03
4e-03
6e-03
9e-03
1e-02
Log-odds
0.5
1.0
1.5
2.0
2.5
3.0
3.5
4.0
Cellular_organisms - ps1
Archaea1 - ps2
Archaea2 - ps3
Archaea3 - ps4
Asgard/Eukaryota - ps5
Eukaryota - ps6
Excavata/Diaphoretickes - ps7
Diaphoretickes - ps8
Archaeplastida - ps9
Viridiplantae - ps10
Streptophyta - ps11
Charophyceae/Embriophyta - ps12
Embriophyta - ps13
Tracheophyta - ps14
Spermatophyta - ps15
Angiosperms - ps16
Mesangiospermae - ps17
Magnoliids/eudicots - ps18
Eudicots - ps19
Core_eudicots - ps20
Superrosids - ps21
Rosids - ps22
Eurosids - ps23
Malvids - ps24
Sap./Malv./Brassicales - ps25
Malvales/Brassicales - ps26
Brassicales - ps27
Cleomaceae/Brassicaceae - ps28
Brassicaceae - ps29
Camelineae - ps30
Arabidopsis - ps31
Arabidopsis_thaliana - ps32
Phylostratum

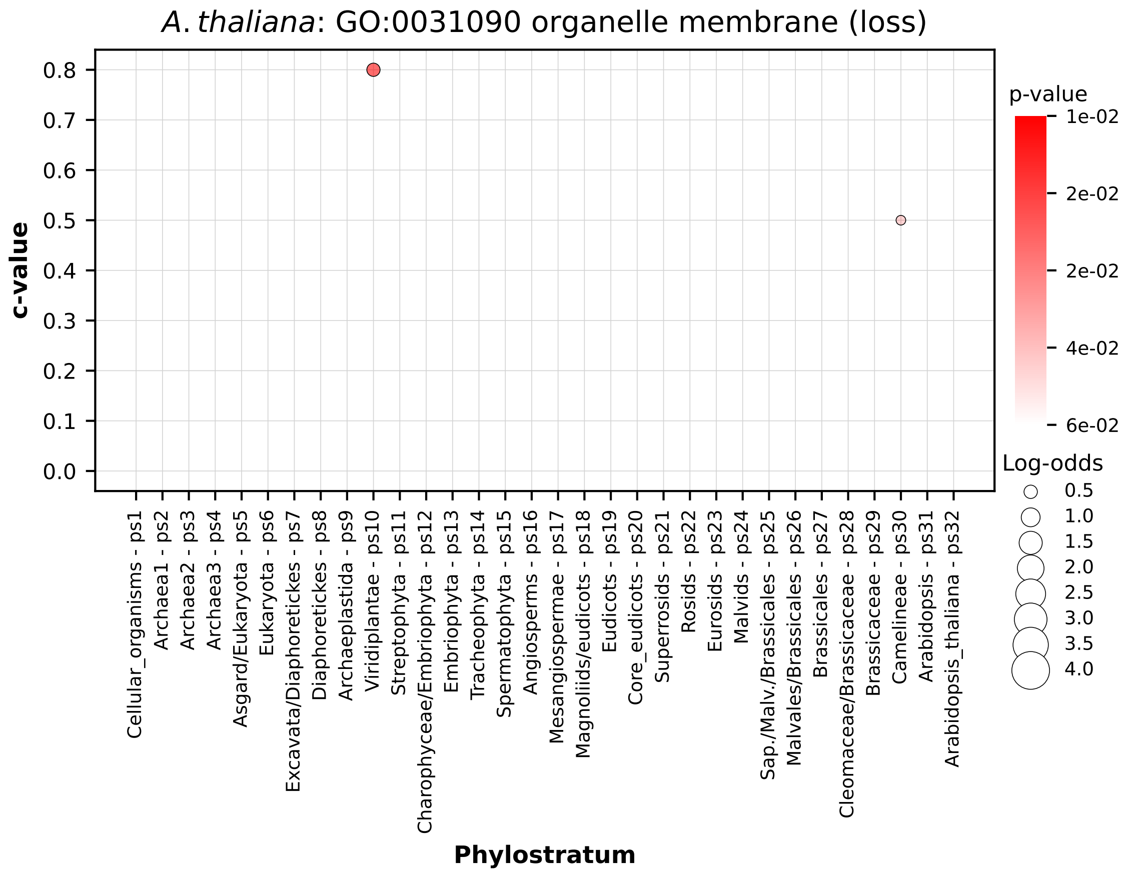

A. thaliana: GO:0031090 organelle membrane (loss)
c-value
0.0
0.1
0.2
0.3
0.4
0.5
0.6
0.7
0.8
Phylostratum
Cellular_organisms - ps1
Archaea1 - ps2
Archaea2 - ps3
Archaea3 - ps4
Asgard/Eukaryota - ps5
Eukaryota - ps6
Excavata/Diaphoretickes - ps7
Diaphoretickes - ps8
Archaeplastida - ps9
Viridiplantae - ps10
Streptophyta - ps11
Charophyceae/Embriophyta - ps12
Embriophyta - ps13
Tracheophyta - ps14
Spermatophyta - ps15
Angiosperms - ps16
Mesangiospermae - ps17
Magnoliids/eudicots - ps18
Eudicots - ps19
Core_eudicots - ps20
Superrosids - ps21
Rosids - ps22
Eurosids - ps23
Malvids - ps24
Sap./Malv./Brassicales - ps25
Malvales/Brassicales - ps26
Brassicales - ps27
Cleomaceae/Brassicaceae - ps28
Brassicaceae - ps29
Camelineae - ps30
Arabidopsis - ps31
Arabidopsis_thaliana - ps32
p-value
1e-02
2e-02
2e-02
4e-02
6e-02
Log-odds
0.5
1.0
1.5
2.0
2.5
3.0
3.5
4.0

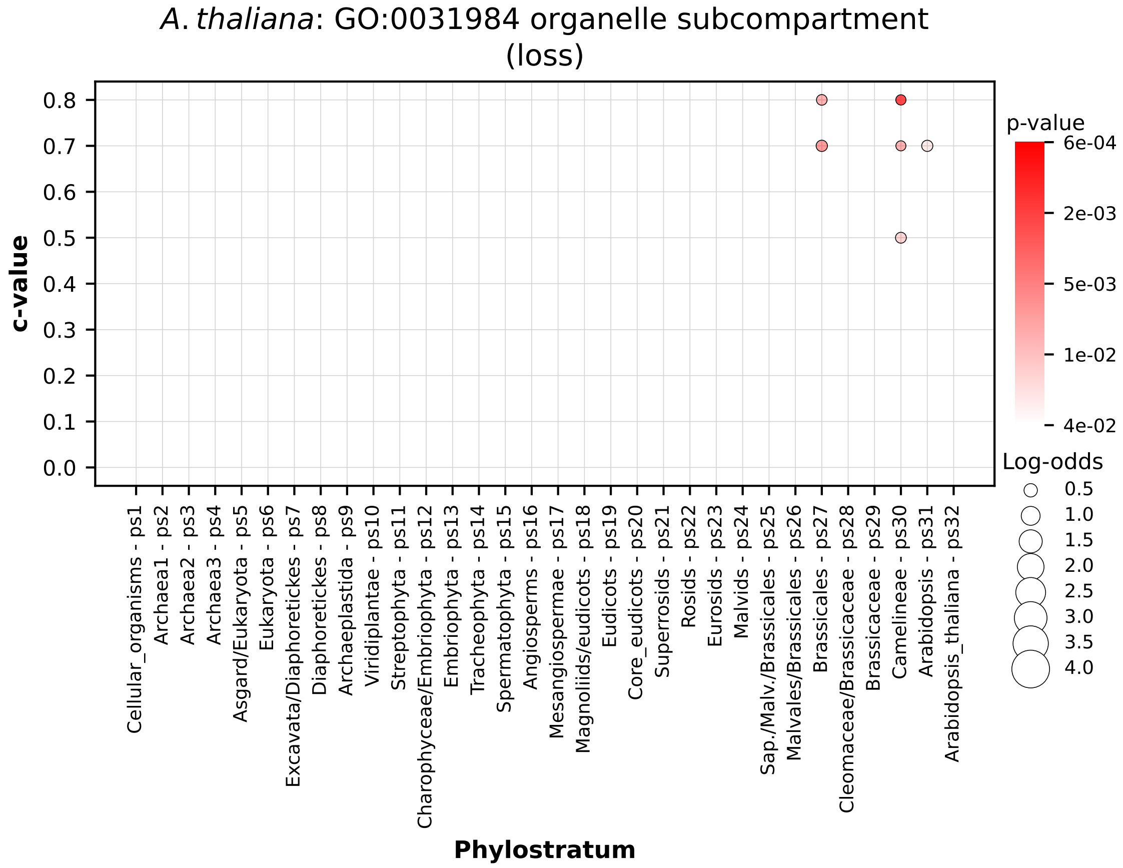

A. thaliana: GO:0031984 organelle subcompartment
(loss)
c-value
0.0
0.1
0.2
0.3
0.4
0.5
0.6
0.7
0.8
Phylostratum
Cellular_organisms - ps1
Archaea1 - ps2
Archaea2 - ps3
Archaea3 - ps4
Asgard/Eukaryota - ps5
Eukaryota - ps6
Excavata/Diaphoretickes - ps7
Diaphoretickes - ps8
Archaeplastida - ps9
Viridiplantae - ps10
Streptophyta - ps11
Charophyceae/Embriophyta - ps12
Embriophyta - ps13
Tracheophyta - ps14
Spermatophyta - ps15
Angiosperms - ps16
Mesangiospermae - ps17
Magnoliids/eudicots - ps18
Eudicots - ps19
Core_eudicots - ps20
Superrosids - ps21
Rosids - ps22
Eurosids - ps23
Malvids - ps24
Sap./Malv./Brassicales - ps25
Malvales/Brassicales - ps26
Brassicales - ps27
Cleomaceae/Brassicaceae - ps28
Brassicaceae - ps29
Camelineae - ps30
Arabidopsis - ps31
Arabidopsis_thaliana - ps32
p-value
6e-04
2e-03
5e-03
1e-02
4e-02
Log-odds
0.5
1.0
1.5
2.0
2.5
3.0
3.5
4.0

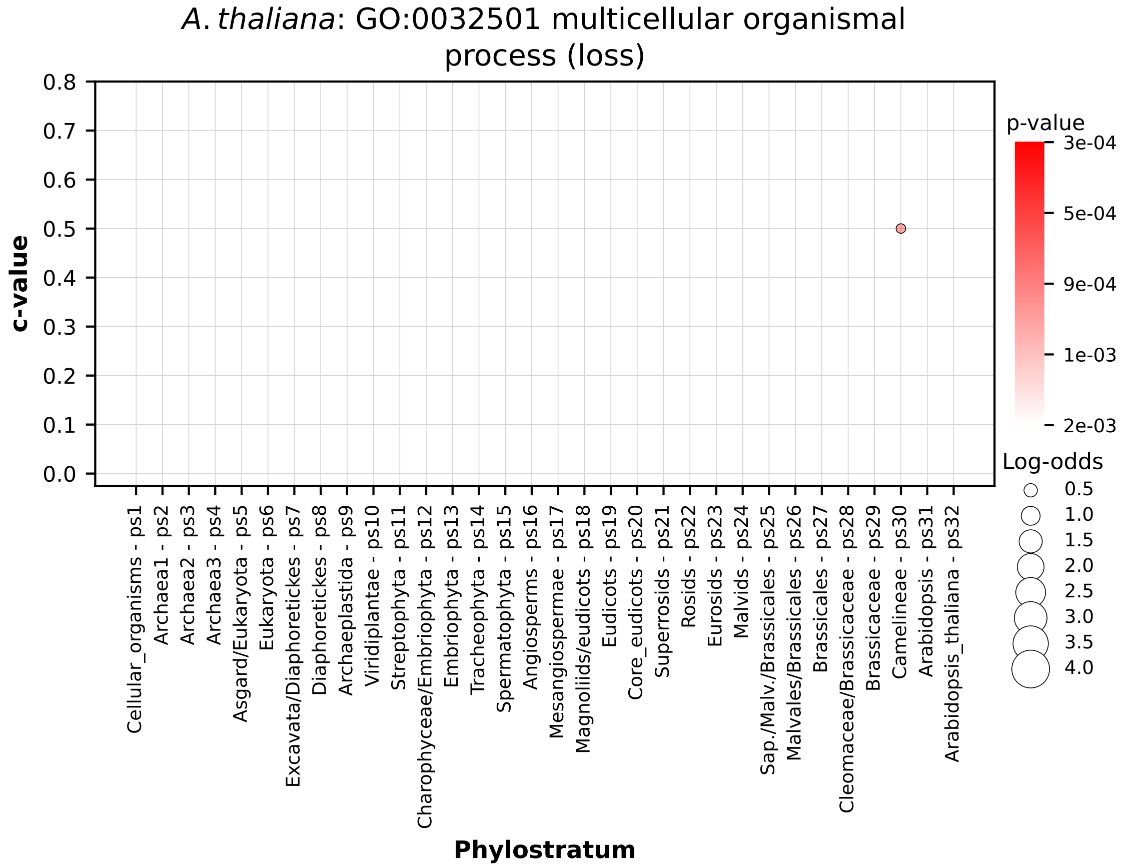

A. thaliana: GO:0032501 multicellular organismal process (loss)
c-value
0.8
0.7
0.6
0.5
0.4
0.3
0.2
0.1
0.0
p-value
3e-04
5e-04
9e-04
1e-03
2e-03
Log-odds
0.5
1.0
1.5
2.0
2.5
3.0
3.5
4.0
Cellular_organisms - ps1
Archaea1 - ps2
Archaea2 - ps3
Archaea3 - ps4
Asgard/Eukaryota - ps5
Eukaryota - ps6
Excavata/Diaphoretickes - ps7
Diaphoretickes - ps8
Archaeplastida - ps9
Viridiplantae - ps10
Streptophyta - ps11
Charophyceae/Embriophyta - ps12
Embriophyta - ps13
Tracheophyta - ps14
Spermatophyta - ps15
Angiosperms - ps16
Mesangiospermae - ps17
Magnoliids/eudicots - ps18
Eudicots - ps19
Core_eudicots - ps20
Superrosids - ps21
Rosids - ps22
Eurosids - ps23
Malvids - ps24
Sap./Malv./Brassicales - ps25
Malvales/Brassicales - ps26
Brassicales - ps27
Cleomaceae/Brassicaceae - ps28
Brassicaceae - ps29
Camelineae - ps30
Arabidopsis - ps31
Arabidopsis_thaliana - ps32
Phylostratum

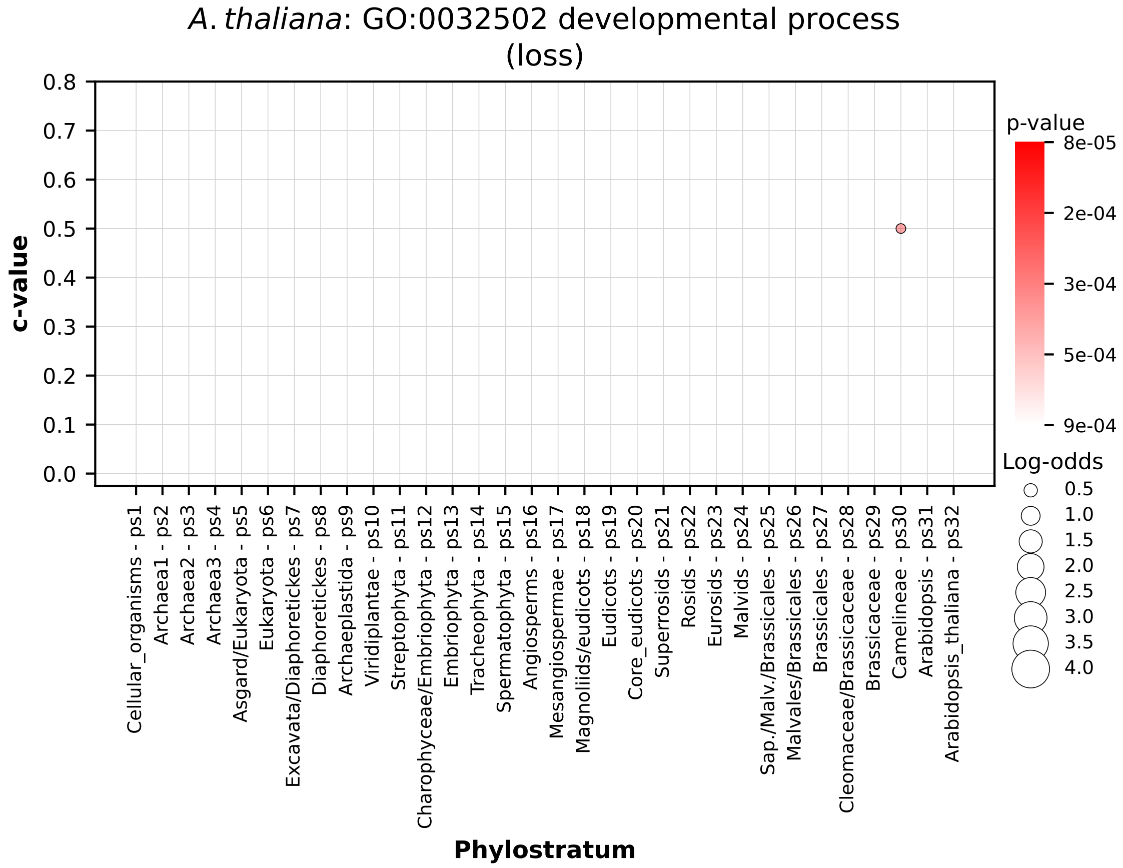

A. thaliana: GO:0032502 developmental process
(loss)
c-value
0.8
0.7
0.6
0.5
0.4
0.3
0.2
0.1
0.0
Phylostratum
Cellular_organisms - ps1
Archaea1 - ps2
Archaea2 - ps3
Archaea3 - ps4
Asgard/Eukaryota - ps5
Eukaryota - ps6
Excavata/Diaphoretickes - ps7
Diaphoretickes - ps8
Archaeplastida - ps9
Viridiplantae - ps10
Streptophyta - ps11
Charophyceae/Embriophyta - ps12
Embriophyta - ps13
Tracheophyta - ps14
Spermatophyta - ps15
Angiosperms - ps16
Mesangiospermae - ps17
Magnoliids/eudicots - ps18
Eudicots - ps19
Core_eudicots - ps20
Superrosids - ps21
Rosids - ps22
Eurosids - ps23
Malvids - ps24
Sap./Malv./Brassicales - ps25
Malvales/Brassicales - ps26
Brassicales - ps27
Cleomaceae/Brassicaceae - ps28
Brassicaceae - ps29
Camelineae - ps30
Arabidopsis - ps31
Arabidopsis_thaliana - ps32
p-value
8e-05
2e-04
3e-04
5e-04
9e-04
Log-odds
0.5
1.0
1.5
2.0
2.5
3.0
3.5
4.0

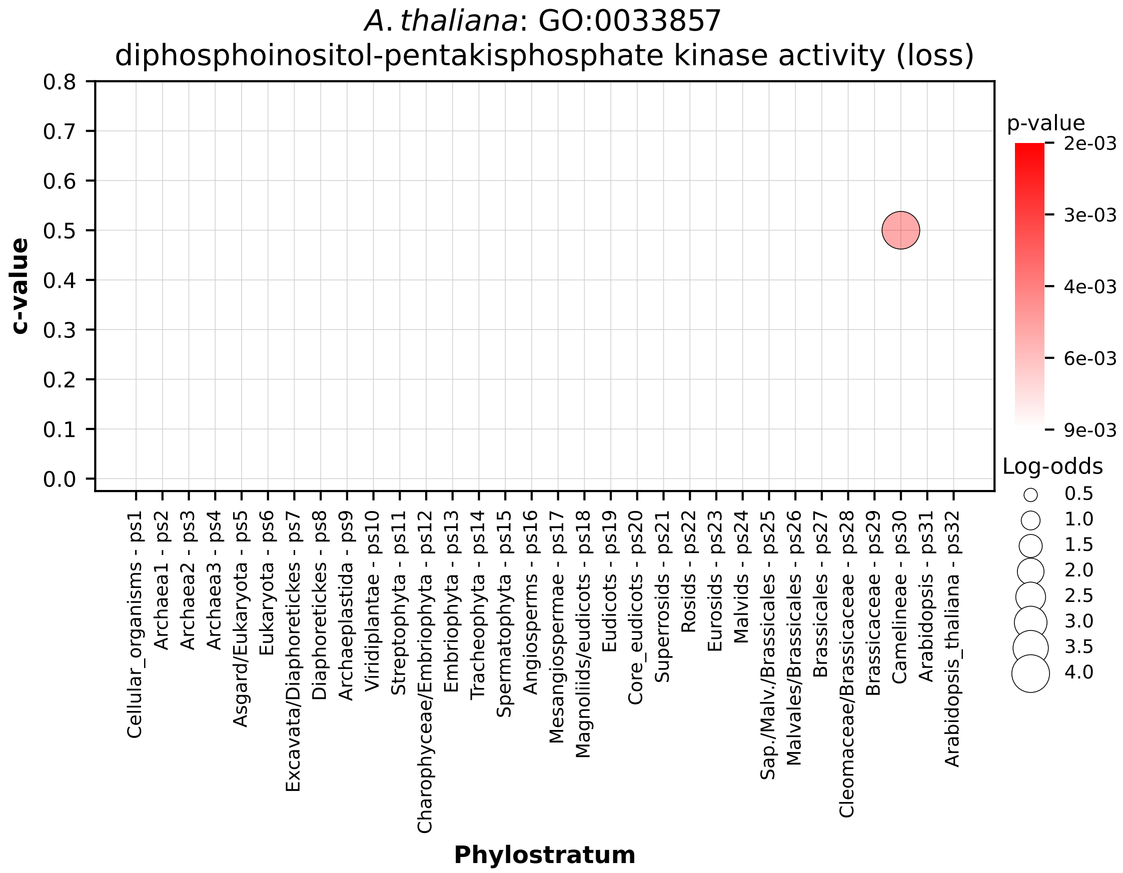

A. thaliana: GO:0033857
diphosphoinositol-pentakisphosphate kinase activity (loss)
c-value
0.8
0.7
0.6
0.5
0.4
0.3
0.2
0.1
0.0
p-value
2e-03
3e-03
4e-03
6e-03
9e-03
Log-odds
0.5
1.0
1.5
2.0
2.5
3.0
3.5
4.0
Cellular_organisms - ps1
Archaea1 - ps2
Archaea2 - ps3
Archaea3 - ps4
Asgard/Eukaryota - ps5
Eukaryota - ps6
Excavata/Diaphoretickes - ps7
Diaphoretickes - ps8
Archaeplastida - ps9
Viridiplantae - ps10
Streptophyta - ps11
Charophyceae/Embriophyta - ps12
Embriophyta - ps13
Tracheophyta - ps14
Spermatophyta - ps15
Angiosperms - ps16
Mesangiospermae - ps17
Magnoliids/eudicots - ps18
Eudicots - ps19
Core_eudicots - ps20
Superrosids - ps21
Rosids - ps22
Eurosids - ps23
Malvids - ps24
Sap./Malv./Brassicales - ps25
Malvales/Brassicales - ps26
Brassicales - ps27
Cleomaceae/Brassicaceae - ps28
Brassicaceae - ps29
Camelineae - ps30
Arabidopsis - ps31
Arabidopsis_thaliana - ps32
Phylostratum

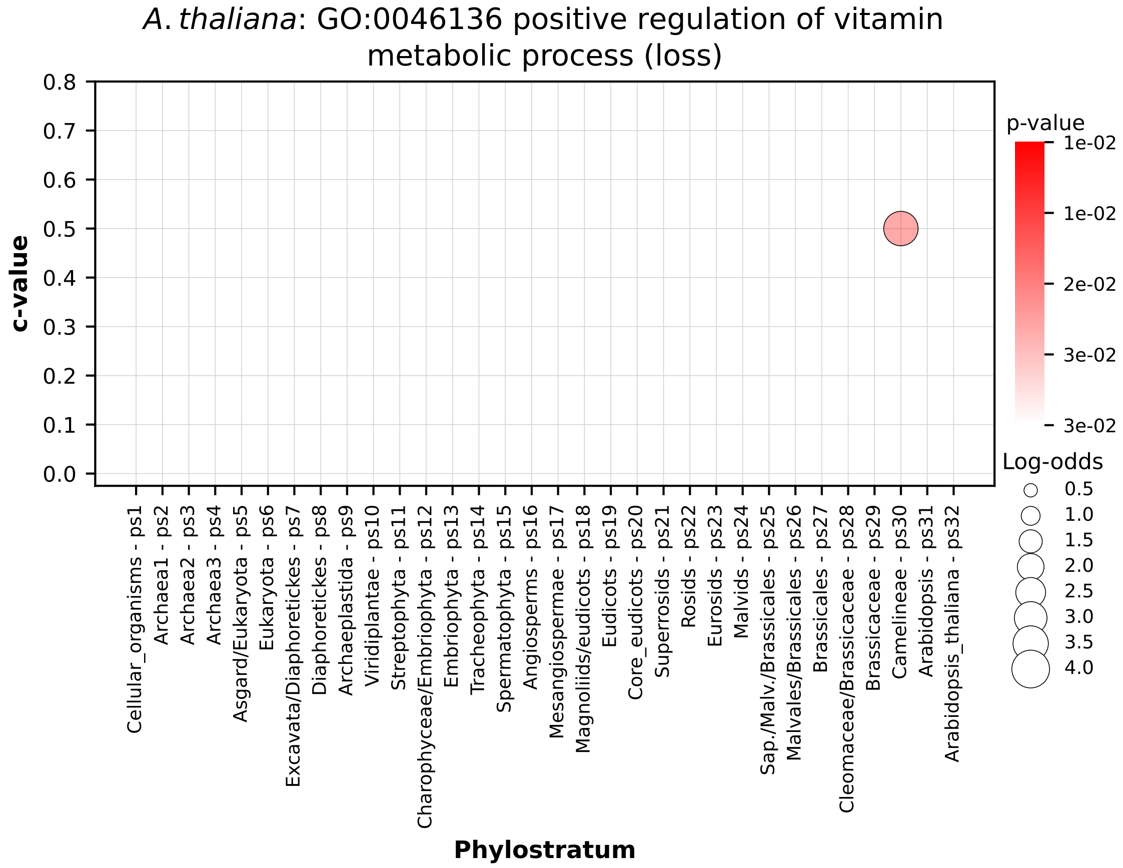

A. thaliana: GO:0046136 positive regulation of vitamin metabolic process (loss)
c-value
0.8
0.7
0.6
0.5
0.4
0.3
0.2
0.1
0.0
p-value
1e-02
1e-02
2e-02
3e-02
3e-02
Log-odds
0.5
1.0
1.5
2.0
2.5
3.0
3.5
4.0
Cellular_organisms - ps1
Archaea1 - ps2
Archaea2 - ps3
Archaea3 - ps4
Asgard/Eukaryota - ps5
Eukaryota - ps6
Excavata/Diaphoretickes - ps7
Diaphoretickes - ps8
Archaeplastida - ps9
Viridiplantae - ps10
Streptophyta - ps11
Charophyceae/Embriophyta - ps12
Embriophyta - ps13
Tracheophyta - ps14
Spermatophyta - ps15
Angiosperms - ps16
Mesangiospermae - ps17
Magnoliids/eudicots - ps18
Eudicots - ps19
Core_eudicots - ps20
Superrosids - ps21
Rosids - ps22
Eurosids - ps23
Malvids - ps24
Sap./Malv./Brassicales - ps25
Malvales/Brassicales - ps26
Brassicales - ps27
Cleomaceae/Brassicaceae - ps28
Brassicaceae - ps29
Camelineae - ps30
Arabidopsis - ps31
Arabidopsis_thaliana - ps32
Phylostratum

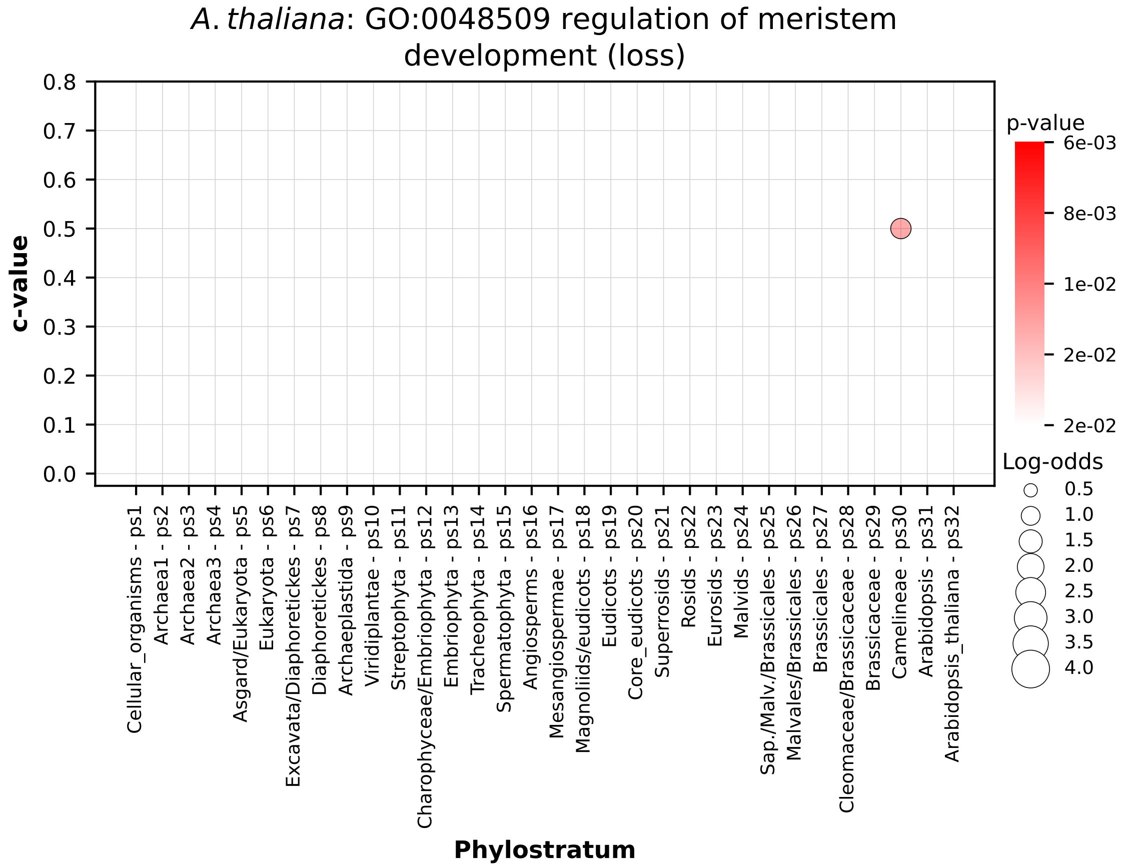

A. thaliana: GO:0048509 regulation of meristem development (loss)
c-value
0.8
0.7
0.6
0.5
0.4
0.3
0.2
0.1
0.0
p-value
6e-03
8e-03
1e-02
2e-02
2e-02
Log-odds
0.5
1.0
1.5
2.0
2.5
3.0
3.5
4.0
Cellular_organisms - ps1
Archaea1 - ps2
Archaea2 - ps3
Archaea3 - ps4
Asgard/Eukaryota - ps5
Eukaryota - ps6
Excavata/Diaphoretickes - ps7
Diaphoretickes - ps8
Archaeplastida - ps9
Viridiplantae - ps10
Streptophyta - ps11
Charophyceae/Embriophyta - ps12
Embriophyta - ps13
Tracheophyta - ps14
Spermatophyta - ps15
Angiosperms - ps16
Mesangiospermae - ps17
Magnoliids/eudicots - ps18
Eudicots - ps19
Core_eudicots - ps20
Superrosids - ps21
Rosids - ps22
Eurosids - ps23
Malvids - ps24
Sap./Malv./Brassicales - ps25
Malvales/Brassicales - ps26
Brassicales - ps27
Cleomaceae/Brassicaceae - ps28
Brassicaceae - ps29
Camelineae - ps30
Arabidopsis - ps31
Arabidopsis_thaliana - ps32
Phylostratum

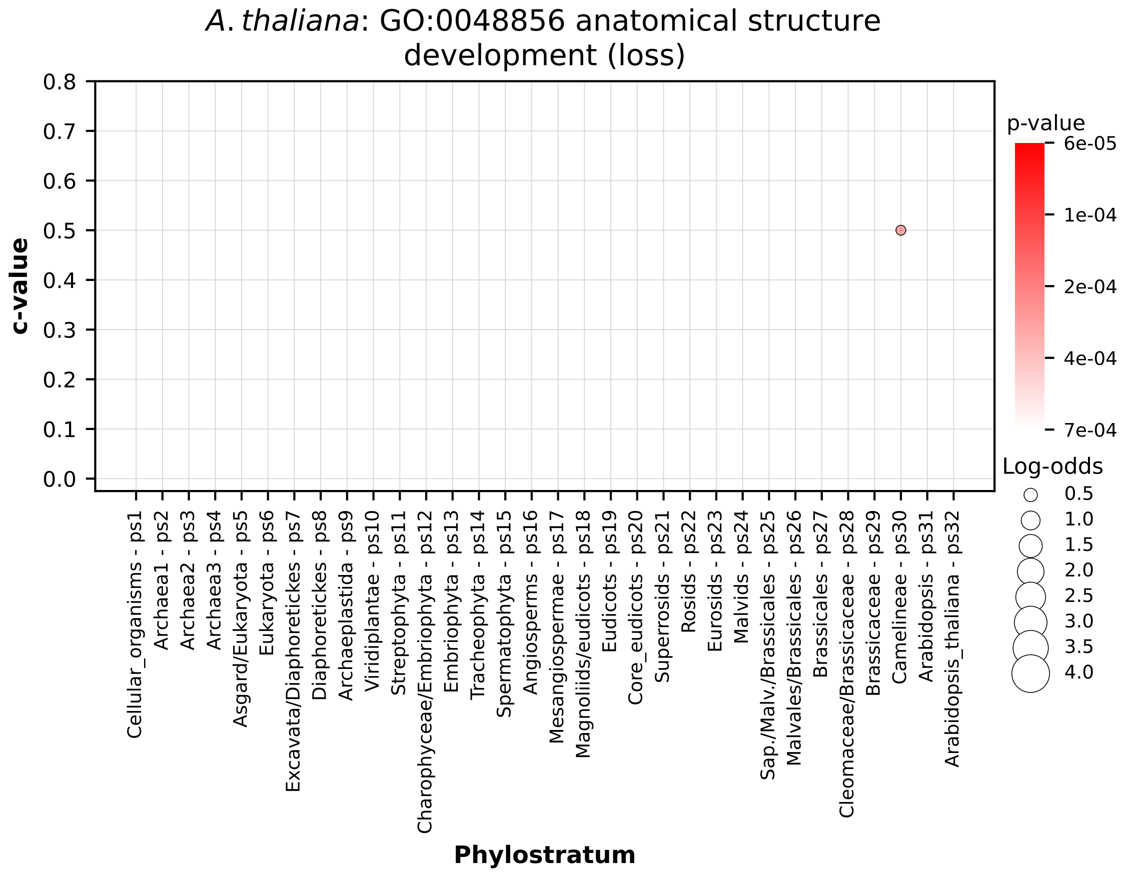

A. thaliana: GO:0048856 anatomical structure development (loss)
c-value
0.0
0.1
0.2
0.3
0.4
0.5
0.6
0.7
0.8
Phylostratum
Cellular_organisms - ps1
Archaea1 - ps2
Archaea2 - ps3
Archaea3 - ps4
Asgard/Eukaryota - ps5
Eukaryota - ps6
Excavata/Diaphoretickes - ps7
Diaphoretickes - ps8
Archaeplastida - ps9
Viridiplantae - ps10
Streptophyta - ps11
Charophyceae/Embriophyta - ps12
Embriophyta - ps13
Tracheophyta - ps14
Spermatophyta - ps15
Angiosperms - ps16
Mesangiospermae - ps17
Magnoliids/eudicots - ps18
Eudicots - ps19
Core_eudicots - ps20
Superrosids - ps21
Rosids - ps22
Eurosids - ps23
Malvids - ps24
Sap./Malv./Brassicales - ps25
Malvales/Brassicales - ps26
Brassicales - ps27
Cleomaceae/Brassicaceae - ps28
Brassicaceae - ps29
Camelineae - ps30
Arabidopsis - ps31
Arabidopsis_thaliana - ps32
p-value
6e-05
1e-04
2e-04
4e-04
7e-04
Log-odds
0.5
1.0
1.5
2.0
2.5
3.0
3.5
4.0

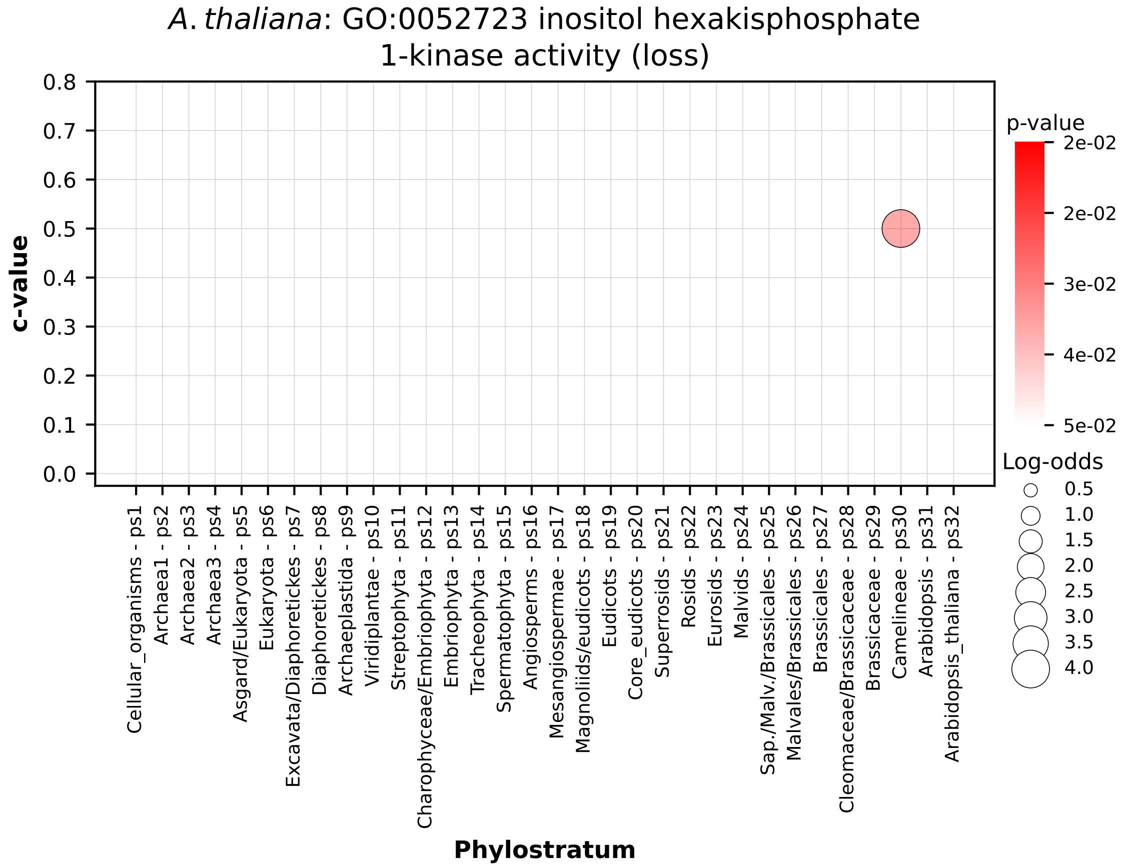

A. thaliana: GO:0052723 inositol hexakisphosphate 1-kinase activity (loss)
c-value
0.8
0.7
0.6
0.5
0.4
0.3
0.2
0.1
0.0
p-value
2e-02
2e-02
3e-02
4e-02
5e-02
Log-odds
0.5
1.0
1.5
2.0
2.5
3.0
3.5
4.0
Cellular_organisms - ps1
Archaea1 - ps2
Archaea2 - ps3
Archaea3 - ps4
Asgard/Eukaryota - ps5
Eukaryota - ps6
Excavata/Diaphoretickes - ps7
Diaphoretickes - ps8
Archaeplastida - ps9
Viridiplantae - ps10
Streptophyta - ps11
Charophyceae/Embriophyta - ps12
Embriophyta - ps13
Tracheophyta - ps14
Spermatophyta - ps15
Angiosperms - ps16
Mesangiospermae - ps17
Magnoliids/eudicots - ps18
Eudicots - ps19
Core_eudicots - ps20
Superrosids - ps21
Rosids - ps22
Eurosids - ps23
Malvids - ps24
Sap./Malv./Brassicales - ps25
Malvales/Brassicales - ps26
Brassicales - ps27
Cleomaceae/Brassicaceae - ps28
Brassicaceae - ps29
Camelineae - ps30
Arabidopsis - ps31
Arabidopsis_thaliana - ps32
Phylostratum

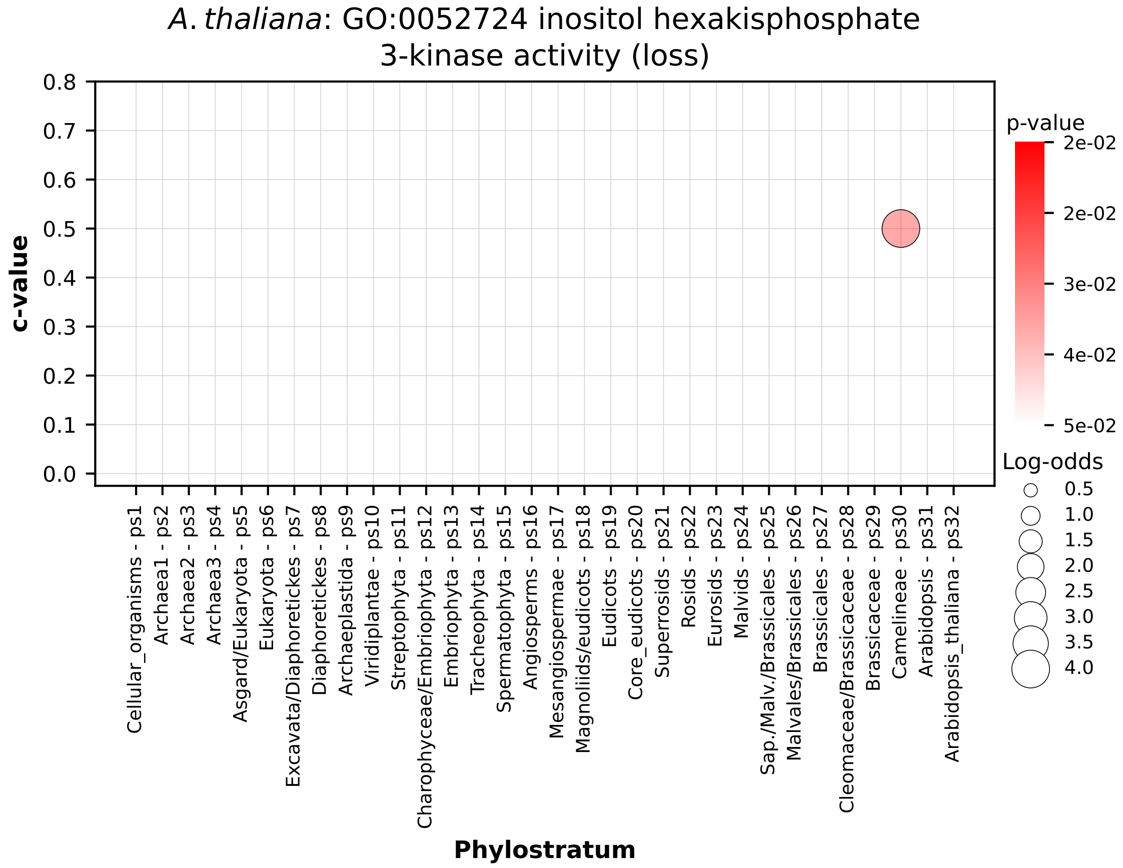

A. thaliana: GO:0052724 inositol hexakisphosphate 3-kinase activity (loss)
c-value
0.8
0.7
0.6
0.5
0.4
0.3
0.2
0.1
0.0
p-value
2e-02
2e-02
3e-02
4e-02
5e-02
Log-odds
0.5
1.0
1.5
2.0
2.5
3.0
3.5
4.0
Cellular_organisms - ps1
Archaea1 - ps2
Archaea2 - ps3
Archaea3 - ps4
Asgard/Eukaryota - ps5
Eukaryota - ps6
Excavata/Diaphoretickes - ps7
Diaphoretickes - ps8
Archaeplastida - ps9
Viridiplantae - ps10
Streptophyta - ps11
Charophyceae/Embriophyta - ps12
Embriophyta - ps13
Tracheophyta - ps14
Spermatophyta - ps15
Angiosperms - ps16
Mesangiospermae - ps17
Magnoliids/eudicots - ps18
Eudicots - ps19
Core_eudicots - ps20
Superrosids - ps21
Rosids - ps22
Eurosids - ps23
Malvids - ps24
Sap./Malv./Brassicales - ps25
Malvales/Brassicales - ps26
Brassicales - ps27
Cleomaceae/Brassicaceae - ps28
Brassicaceae - ps29
Camelineae - ps30
Arabidopsis - ps31
Arabidopsis_thaliana - ps32
Phylostratum

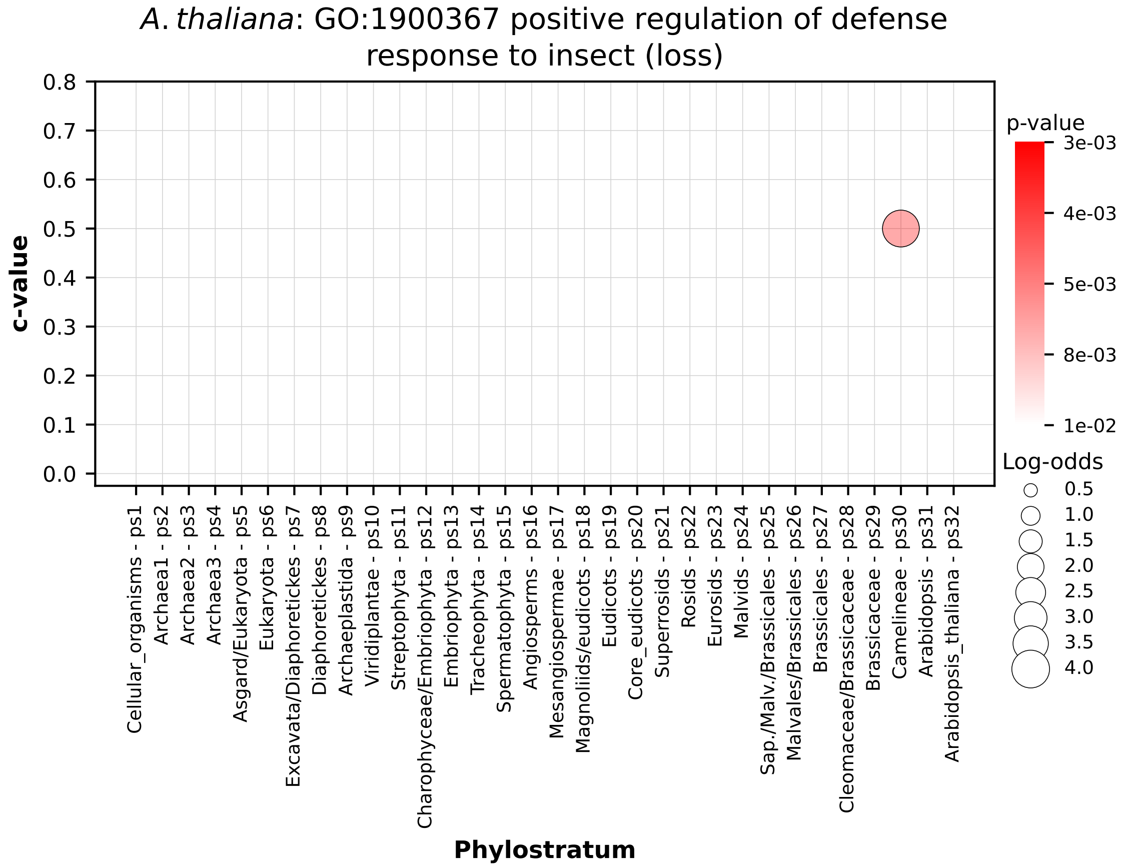

A. thaliana: GO:1900367 positive regulation of defense response to insect (loss)
c-value
0.8
0.7
0.6
0.5
0.4
0.3
0.2
0.1
0.0
Cellular_organisms - ps1
Archaea1 - ps2
Archaea2 - ps3
Archaea3 - ps4
Asgard/Eukaryota - ps5
Eukaryota - ps6
Excavata/Diaphoretickes - ps7
Diaphoretickes - ps8
Archaeplastida - ps9
Viridiplantae - ps10
Streptophyta - ps11
Charophyceae/Embriophyta - ps12
Embriophyta - ps13
Tracheophyta - ps14
Spermatophyta - ps15
Angiosperms - ps16
Mesangiospermae - ps17
Magnoliids/eudicots - ps18
Eudicots - ps19
Core_eudicots - ps20
Superrosids - ps21
Rosids - ps22
Eurosids - ps23
Malvids - ps24
Sap./Malv./Brassicales - ps25
Malvales/Brassicales - ps26
Brassicales - ps27
Cleomaceae/Brassicaceae - ps28
Brassicaceae - ps29
Camelineae - ps30
Arabidopsis - ps31
Arabidopsis_thaliana - ps32
Phylostratum
p-value
3e-03
4e-03
5e-03
8e-03
1e-02
Log-odds
0.5
1.0
1.5
2.0
2.5
3.0
3.5
4.0

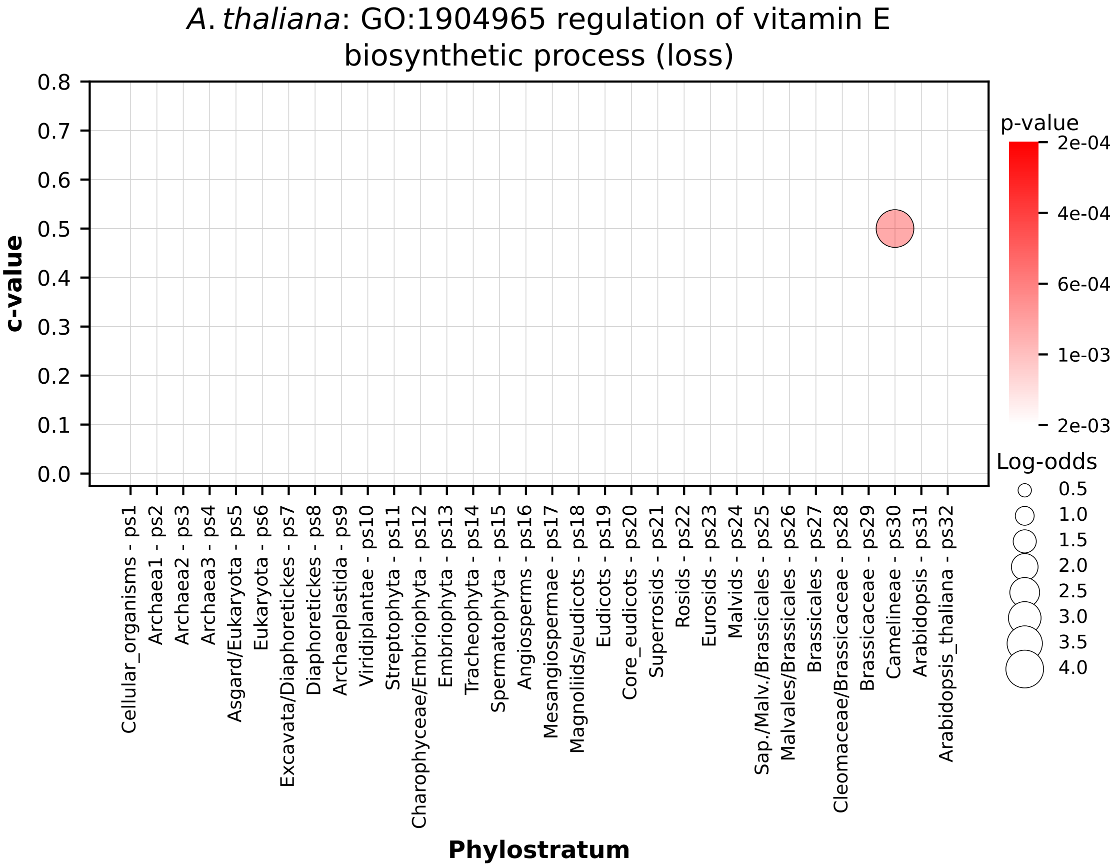

A. thaliana: GO:1904965 regulation of vitamin E biosynthetic process (loss)
c-value
0.8
0.7
0.6
0.5
0.4
0.3
0.2
0.1
0.0
p-value
2e-04
4e-04
6e-04
1e-03
2e-03
Log-odds
0.5
1.0
1.5
2.0
2.5
3.0
3.5
4.0
Cellular_organisms - ps1
Archaea1 - ps2
Archaea2 - ps3
Archaea3 - ps4
Asgard/Eukaryota - ps5
Eukaryota - ps6
Excavata/Diaphoretickes - ps7
Diaphoretickes - ps8
Archaeplastida - ps9
Viridiplantae - ps10
Streptophyta - ps11
Charophyceae/Embriophyta - ps12
Embriophyta - ps13
Tracheophyta - ps14
Spermatophyta - ps15
Angiosperms - ps16
Mesangiospermae - ps17
Magnoliids/eudicots - ps18
Eudicots - ps19
Core_eudicots - ps20
Superrosids - ps21
Rosids - ps22
Eurosids - ps23
Malvids - ps24
Sap./Malv./Brassicales - ps25
Malvales/Brassicales - ps26
Brassicales - ps27
Cleomaceae/Brassicaceae - ps28
Brassicaceae - ps29
Camelineae - ps30
Arabidopsis - ps31
Arabidopsis_thaliana - ps32
Phylostratum

A. thaliana: GO:1904966 positive regulation of vitamin E biosynthetic process (loss)

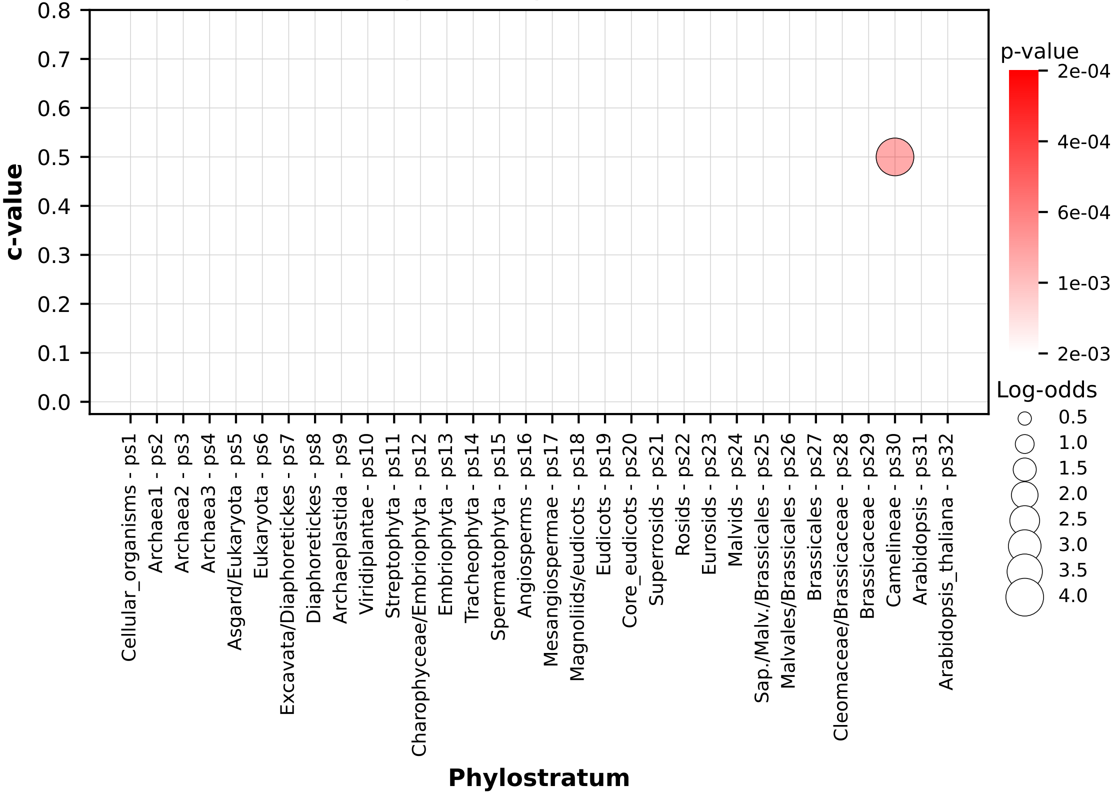

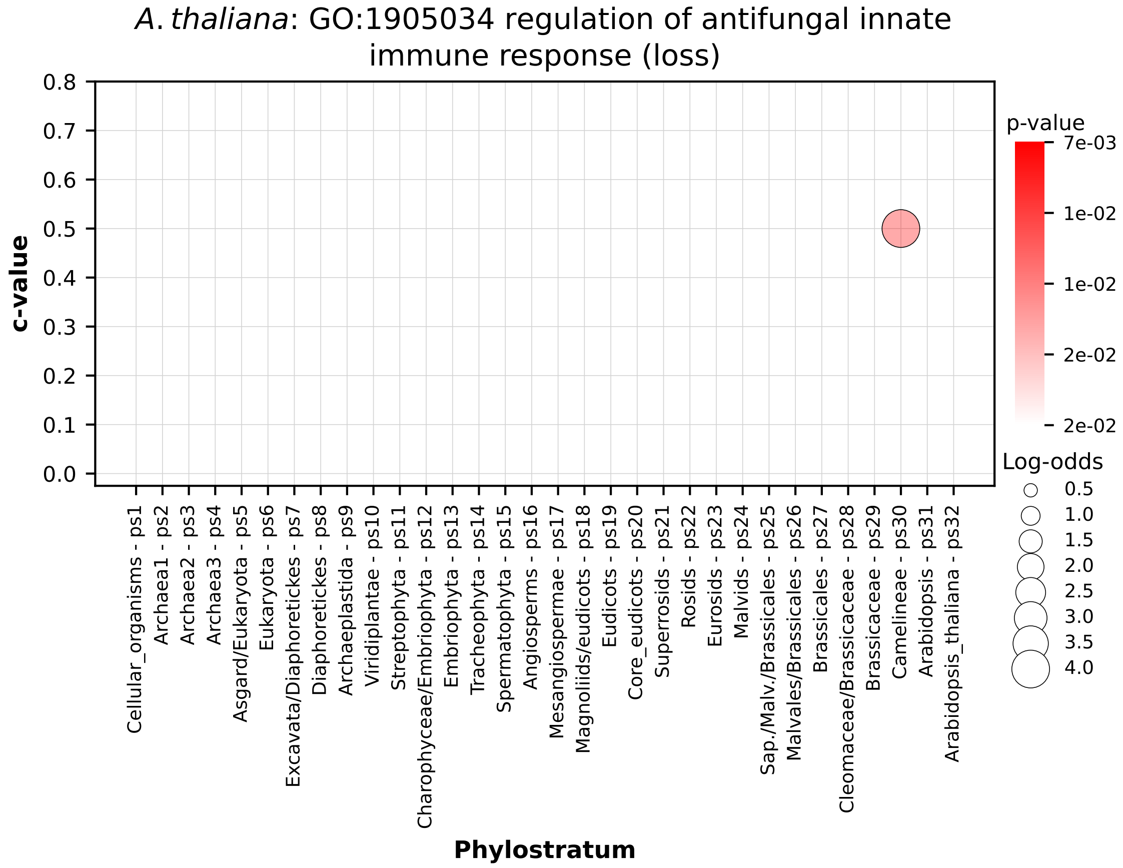

A. thaliana: GO:1905034 regulation of antifungal innate immune response (loss)
c-value
0.8
0.7
0.6
0.5
0.4
0.3
0.2
0.1
0.0
Phylostratum
Cellular_organisms - ps1
Archaea1 - ps2
Archaea2 - ps3
Archaea3 - ps4
Asgard/Eukaryota - ps5
Eukaryota - ps6
Excavata/Diaphoretickes - ps7
Diaphoretickes - ps8
Archaeplastida - ps9
Viridiplantae - ps10
Streptophyta - ps11
Charophyceae/Embriophyta - ps12
Embriophyta - ps13
Tracheophyta - ps14
Spermatophyta - ps15
Angiosperms - ps16
Mesangiospermae - ps17
Magnoliids/eudicots - ps18
Eudicots - ps19
Core_eudicots - ps20
Superrosids - ps21
Rosids - ps22
Eurosids - ps23
Malvids - ps24
Sap./Malv./Brassicales - ps25
Malvales/Brassicales - ps26
Brassicales - ps27
Cleomaceae/Brassicaceae - ps28
Brassicaceae - ps29
Camelineae - ps30
Arabidopsis - ps31
Arabidopsis_thaliana - ps32
p-value
7e-03
1e-02
1e-02
2e-02
2e-02
Log-odds
0.5
1.0
1.5
2.0
2.5
3.0
3.5
4.0

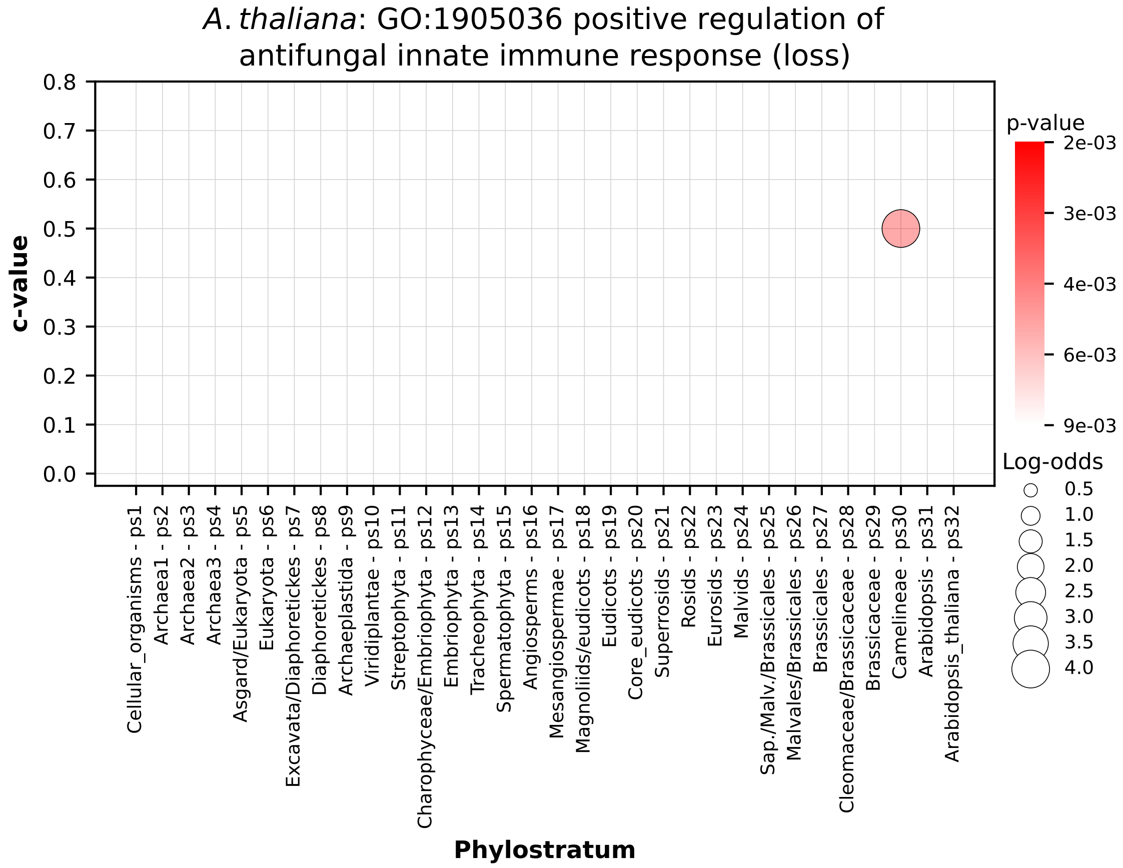

A. thaliana: GO:1905036 positive regulation of antifungal innate immune response (loss)
c-value
0.8
0.7
0.6
0.5
0.4
0.3
0.2
0.1
0.0
p-value
2e-03
3e-03
4e-03
6e-03
9e-03
Log-odds
0.5
1.0
1.5
2.0
2.5
3.0
3.5
4.0
Cellular_organisms - ps1
Archaea1 - ps2
Archaea2 - ps3
Archaea3 - ps4
Asgard/Eukaryota - ps5
Eukaryota - ps6
Excavata/Diaphoretickes - ps7
Diaphoretickes - ps8
Archaeplastida - ps9
Viridiplantae - ps10
Streptophyta - ps11
Charophyceae/Embriophyta - ps12
Embriophyta - ps13
Tracheophyta - ps14
Spermatophyta - ps15
Angiosperms - ps16
Mesangiospermae - ps17
Magnoliids/eudicots - ps18
Eudicots - ps19
Core_eudicots - ps20
Superrosids - ps21
Rosids - ps22
Eurosids - ps23
Malvids - ps24
Sap./Malv./Brassicales - ps25
Malvales/Brassicales - ps26
Brassicales - ps27
Cleomaceae/Brassicaceae - ps28
Brassicaceae - ps29
Camelineae - ps30
Arabidopsis - ps31
Arabidopsis_thaliana - ps32
Phylostratum

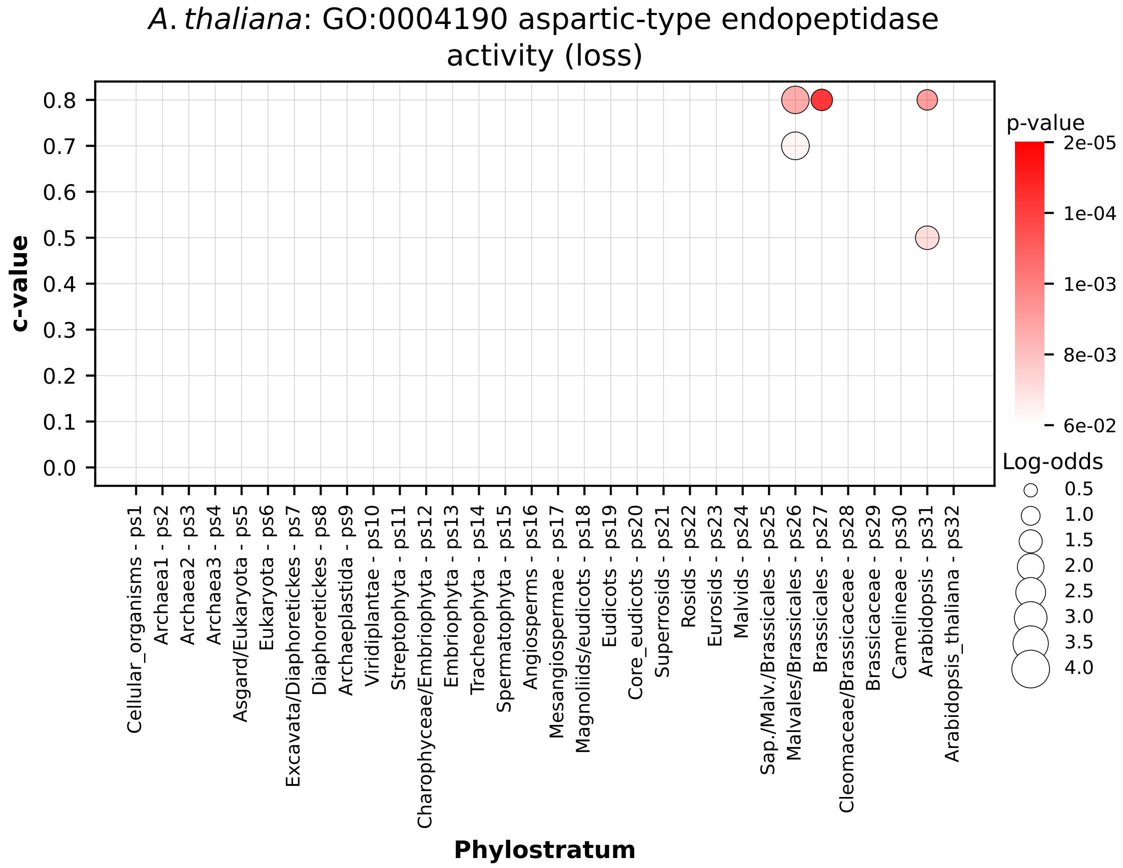
A. thaliana: GO:0004190 aspartic-type endopeptidase activity (loss)
c-value
0.8
0.7
0.6
0.5
0.4
0.3
0.2
0.1
0.0
p-value
2e-05
1e-04
1e-03
8e-03
6e-02
Log-odds
0.5
1.0
1.5
2.0
2.5
3.0
3.5
4.0
Cellular_organisms - ps1
Archaea1 - ps2
Archaea2 - ps3
Archaea3 - ps4
Asgard/Eukaryota - ps5
Eukaryota - ps6
Excavata/Diaphoretickes - ps7
Diaphoretickes - ps8
Archaeplastida - ps9
Viridiplantae - ps10
Streptophyta - ps11
Charophyceae/Embriophyta - ps12
Embriophyta - ps13
Tracheophyta - ps14
Spermatophyta - ps15
Angiosperms - ps16
Mesangiospermae - ps17
Magnoliids/eudicots - ps18
Eudicots - ps19
Core_eudicots - ps20
Superrosids - ps21
Rosids - ps22
Eurosids - ps23
Malvids - ps24
Sap./Malv./Brassicales - ps25
Malvales/Brassicales - ps26
Brassicales - ps27
Cleomaceae/Brassicaceae - ps28
Brassicaceae - ps29
Camelineae - ps30
Arabidopsis - ps31
Arabidopsis_thaliana - ps32
Phylostratum

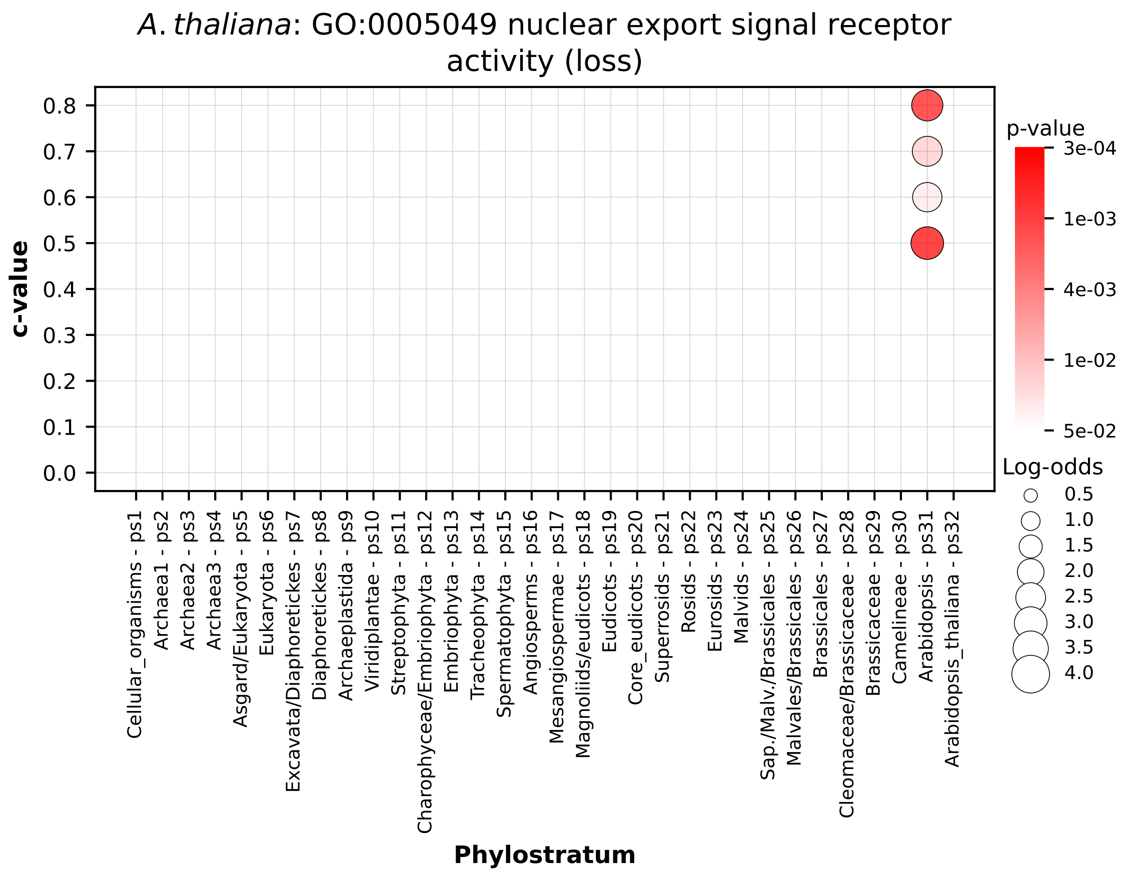

A. thaliana: GO:0005049 nuclear export signal receptor activity (loss)
c-value
0.8
0.7
0.6
0.5
0.4
0.3
0.2
0.1
0.0
Phylostratum
Cellular_organisms - ps1
Archaea1 - ps2
Archaea2 - ps3
Archaea3 - ps4
Asgard/Eukaryota - ps5
Eukaryota - ps6
Excavata/Diaphoretickes - ps7
Diaphoretickes - ps8
Archaeplastida - ps9
Viridiplantae - ps10
Streptophyta - ps11
Charophyceae/Embriophyta - ps12
Embriophyta - ps13
Tracheophyta - ps14
Spermatophyta - ps15
Angiosperms - ps16
Mesangiospermae - ps17
Magnoliids/eudicots - ps18
Eudicots - ps19
Core_eudicots - ps20
Superrosids - ps21
Rosids - ps22
Eurosids - ps23
Malvids - ps24
Sap./Malv./Brassicales - ps25
Malvales/Brassicales - ps26
Brassicales - ps27
Cleomaceae/Brassicaceae - ps28
Brassicaceae - ps29
Camelineae - ps30
Arabidopsis - ps31
Arabidopsis_thaliana - ps32
p-value
3e-04
1e-03
4e-03
1e-02
5e-02
Log-odds
0.5
1.0
1.5
2.0
2.5
3.0
3.5
4.0

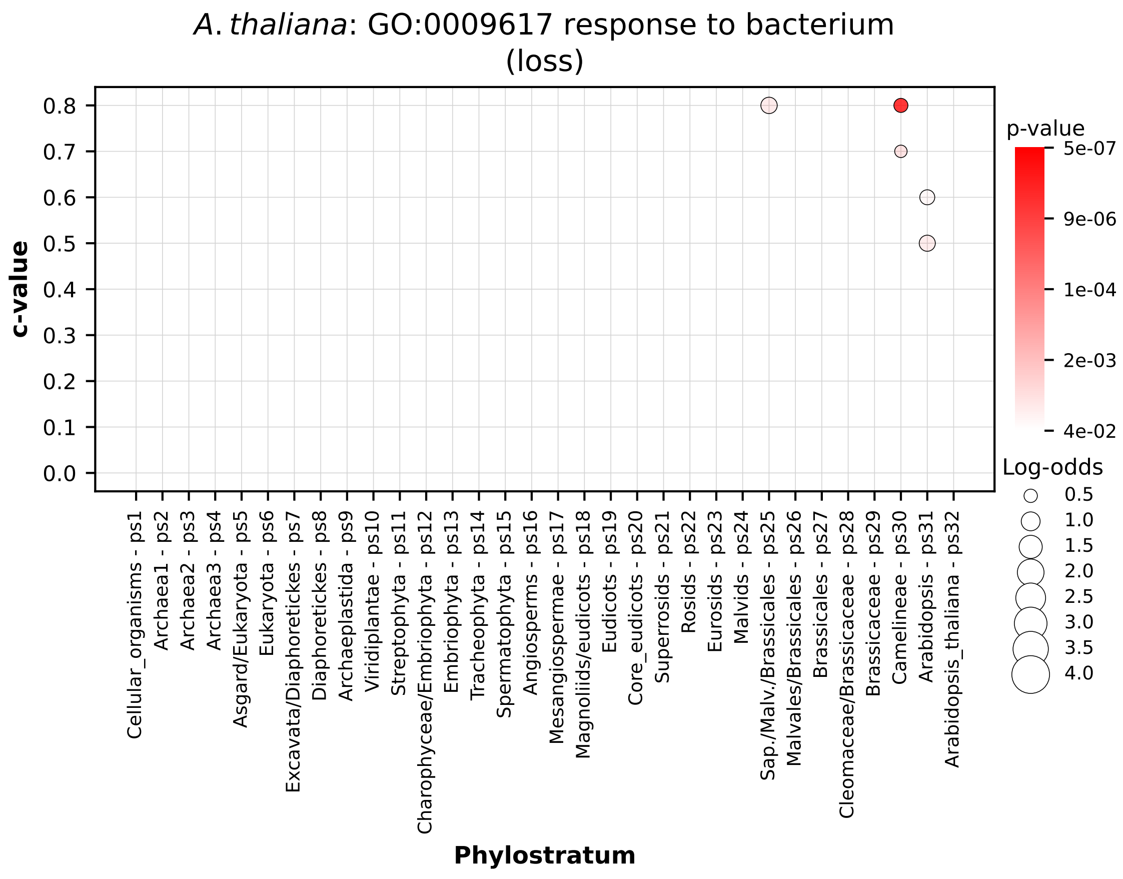

A. thaliana: GO:0009617 response to bacterium
(loss)
c-value
0.8
0.7
0.6
0.5
0.4
0.3
0.2
0.1
0.0
p-value
5e-07
9e-06
1e-04
2e-03
4e-02
Log-odds
0.5
1.0
1.5
2.0
2.5
3.0
3.5
4.0
Cellular_organisms - ps1
Archaea1 - ps2
Archaea2 - ps3
Archaea3 - ps4
Asgard/Eukaryota - ps5
Eukaryota - ps6
Excavata/Diaphoretickes - ps7
Diaphoretickes - ps8
Archaeplastida - ps9
Viridiplantae - ps10
Streptophyta - ps11
Charophyceae/Embriophyta - ps12
Embriophyta - ps13
Tracheophyta - ps14
Spermatophyta - ps15
Angiosperms - ps16
Mesangiospermae - ps17
Magnoliids/eudicots - ps18
Eudicots - ps19
Core_eudicots - ps20
Superrosids - ps21
Rosids - ps22
Eurosids - ps23
Malvids - ps24
Sap./Malv./Brassicales - ps25
Malvales/Brassicales - ps26
Brassicales - ps27
Cleomaceae/Brassicaceae - ps28
Brassicaceae - ps29
Camelineae - ps30
Arabidopsis - ps31
Arabidopsis_thaliana - ps32
Phylostratum

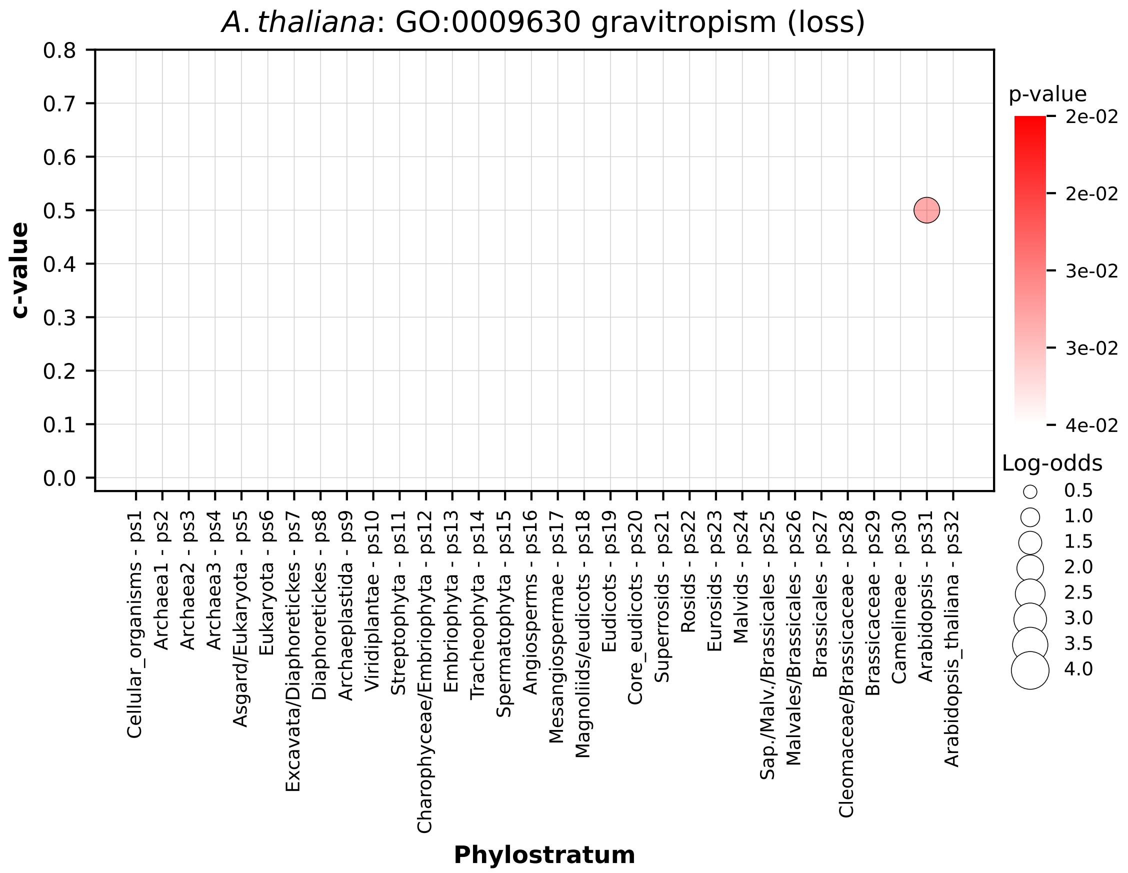
A. thaliana: GO:0009630 gravitropism (loss)
c-value
0.0
0.1
0.2
0.3
0.4
0.5
0.6
0.7
0.8
Phylostratum
Cellular_organisms - ps1
Archaea1 - ps2
Archaea2 - ps3
Archaea3 - ps4
Asgard/Eukaryota - ps5
Eukaryota - ps6
Excavata/Diaphoretickes - ps7
Diaphoretickes - ps8
Archaeplastida - ps9
Viridiplantae - ps10
Streptophyta - ps11
Charophyceae/Embriophyta - ps12
Embriophyta - ps13
Tracheophyta - ps14
Spermatophyta - ps15
Angiosperms - ps16
Mesangiospermae - ps17
Magnoliids/eudicots - ps18
Eudicots - ps19
Core_eudicots - ps20
Superrosids - ps21
Rosids - ps22
Eurosids - ps23
Malvids - ps24
Sap./Malv./Brassicales - ps25
Malvales/Brassicales - ps26
Brassicales - ps27
Cleomaceae/Brassicaceae - ps28
Brassicaceae - ps29
Camelineae - ps30
Arabidopsis - ps31
Arabidopsis_thaliana - ps32
p-value
2e-02
2e-02
3e-02
3e-02
4e-02
Log-odds
0.5
1.0
1.5
2.0
2.5
3.0
3.5
4.0

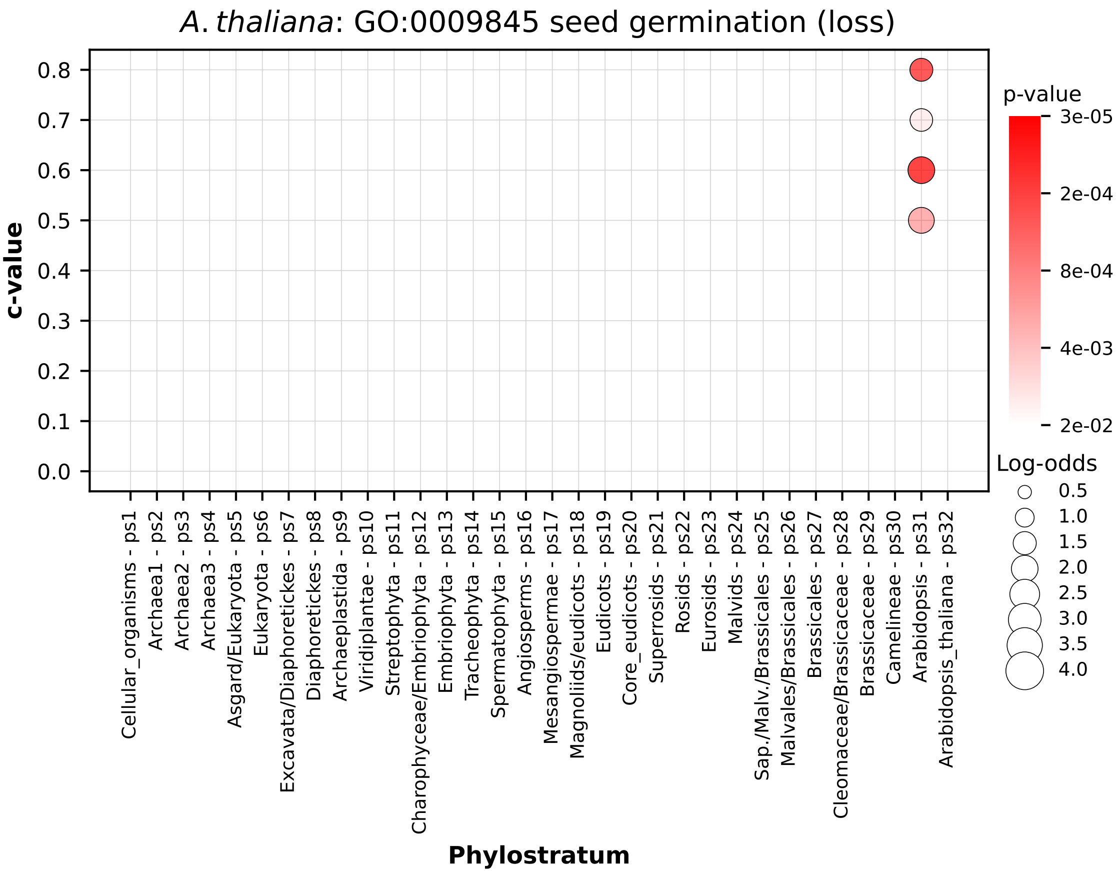

A. thaliana: GO:0009845 seed germination (loss)
c-value
0.8
0.7
0.6
0.5
0.4
0.3
0.2
0.1
0.0
p-value
3e-05
2e-04
8e-04
4e-03
2e-02
Log-odds
0.5
1.0
1.5
2.0
2.5
3.0
3.5
4.0
Cellular_organisms - ps1
Archaea1 - ps2
Archaea2 - ps3
Archaea3 - ps4
Asgard/Eukaryota - ps5
Eukaryota - ps6
Excavata/Diaphoretickes - ps7
Diaphoretickes - ps8
Archaeplastida - ps9
Viridiplantae - ps10
Streptophyta - ps11
Charophyceae/Embriophyta - ps12
Embriophyta - ps13
Tracheophyta - ps14
Spermatophyta - ps15
Angiosperms - ps16
Mesangiospermae - ps17
Magnoliids/eudicots - ps18
Eudicots - ps19
Core_eudicots - ps20
Superrosids - ps21
Rosids - ps22
Eurosids - ps23
Malvids - ps24
Sap./Malv./Brassicales - ps25
Malvales/Brassicales - ps26
Brassicales - ps27
Cleomaceae/Brassicaceae - ps28
Brassicaceae - ps29
Camelineae - ps30
Arabidopsis - ps31
Arabidopsis_thaliana - ps32
Phylostratum

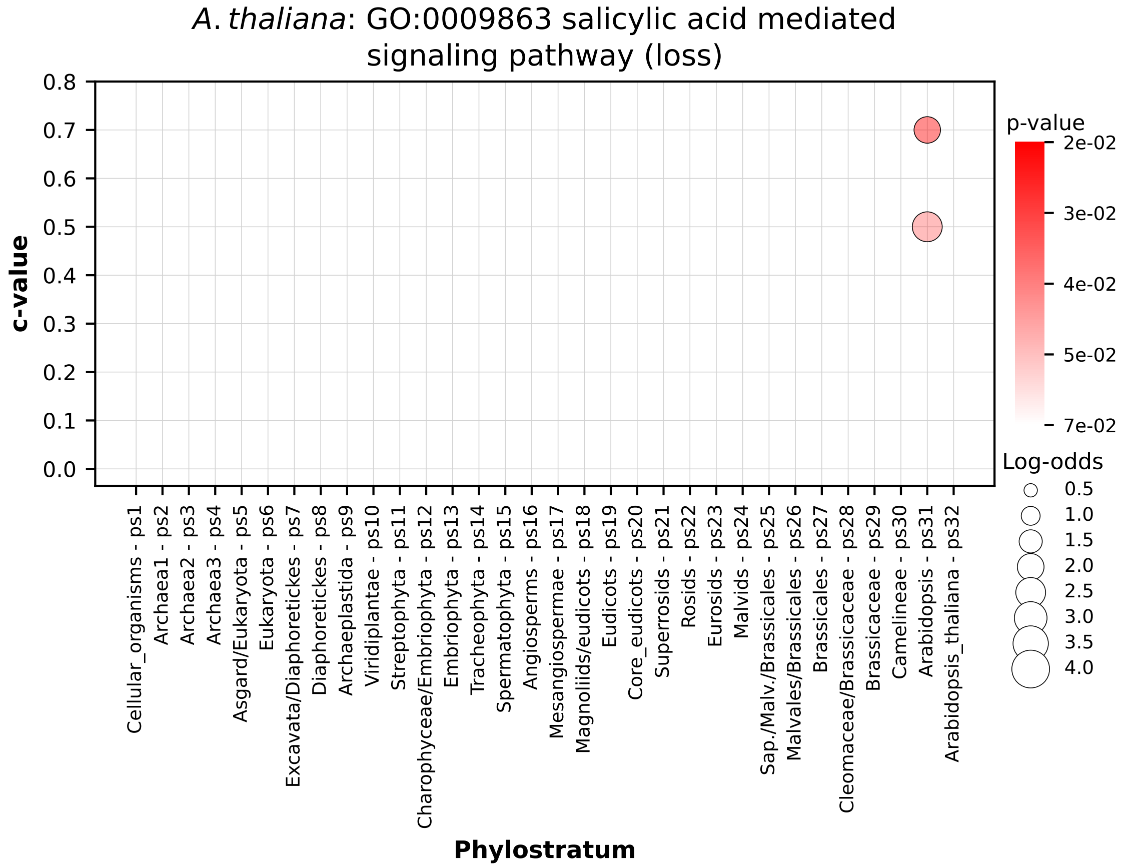
A. thaliana: GO:0009863 salicylic acid mediated signaling pathway (loss)
c-value
0.8
0.7
0.6
0.5
0.4
0.3
0.2
0.1
0.0
p-value
2e-02
3e-02
4e-02
5e-02
7e-02
Log-odds
0.5
1.0
1.5
2.0
2.5
3.0
3.5
4.0
Cellular_organisms - ps1
Archaea1 - ps2
Archaea2 - ps3
Archaea3 - ps4
Asgard/Eukaryota - ps5
Eukaryota - ps6
Excavata/Diaphoretickes - ps7
Diaphoretickes - ps8
Archaeplastida - ps9
Viridiplantae - ps10
Streptophyta - ps11
Charophyceae/Embriophyta - ps12
Embriophyta - ps13
Tracheophyta - ps14
Spermatophyta - ps15
Angiosperms - ps16
Mesangiospermae - ps17
Magnoliids/eudicots - ps18
Eudicots - ps19
Core_eudicots - ps20
Superrosids - ps21
Rosids - ps22
Eurosids - ps23
Malvids - ps24
Sap./Malv./Brassicales - ps25
Malvales/Brassicales - ps26
Brassicales - ps27
Cleomaceae/Brassicaceae - ps28
Brassicaceae - ps29
Camelineae - ps30
Arabidopsis - ps31
Arabidopsis_thaliana - ps32
Phylostratum

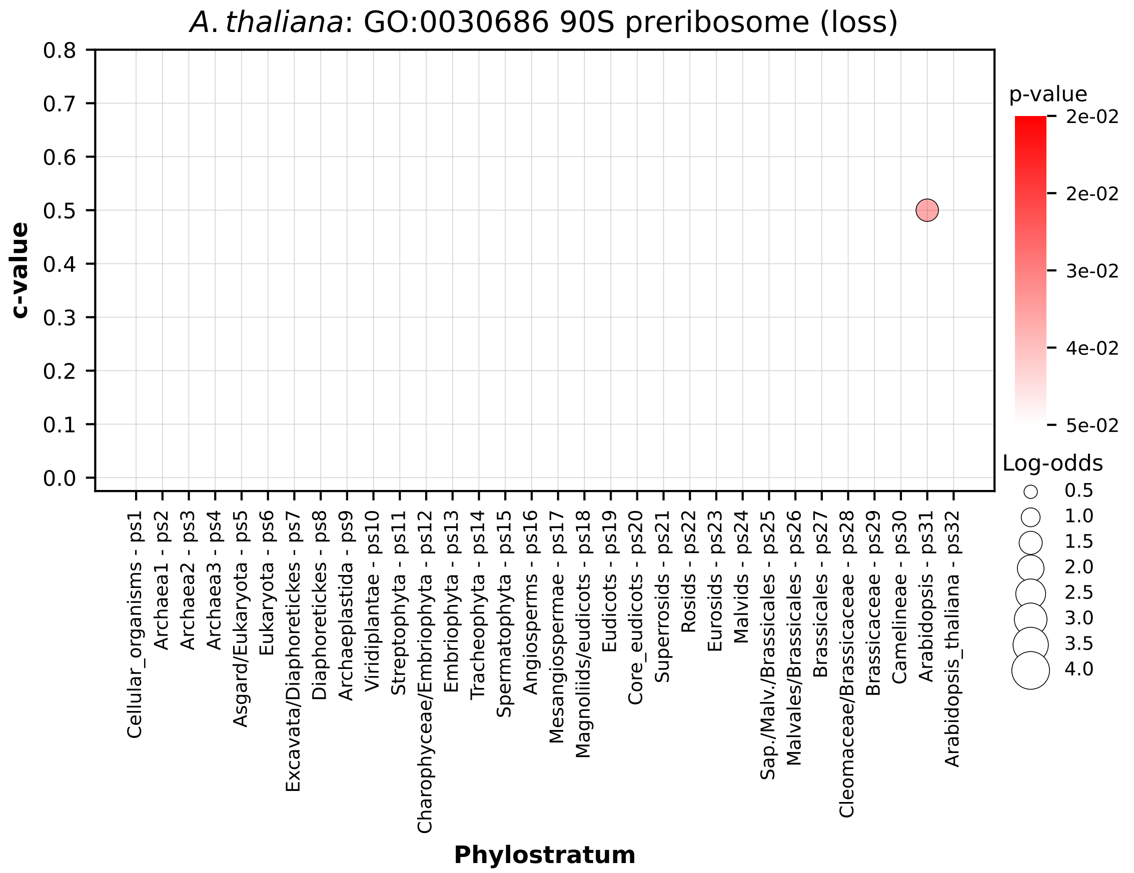

A. thaliana: GO:0030686 90S preribosome (loss)
c-value
0.8
0.7
0.6
0.5
0.4
0.3
0.2
0.1
0.0
p-value
2e-02
2e-02
3e-02
4e-02
5e-02
Log-odds
0.5
1.0
1.5
2.0
2.5
3.0
3.5
4.0
Cellular_organisms - ps1
Archaea1 - ps2
Archaea2 - ps3
Archaea3 - ps4
Asgard/Eukaryota - ps5
Eukaryota - ps6
Excavata/Diaphoretickes - ps7
Diaphoretickes - ps8
Archaeplastida - ps9
Viridiplantae - ps10
Streptophyta - ps11
Charophyceae/Embriophyta - ps12
Embriophyta - ps13
Tracheophyta - ps14
Spermatophyta - ps15
Angiosperms - ps16
Mesangiospermae - ps17
Magnoliids/eudicots - ps18
Eudicots - ps19
Core_eudicots - ps20
Superrosids - ps21
Rosids - ps22
Eurosids - ps23
Malvids - ps24
Sap./Malv./Brassicales - ps25
Malvales/Brassicales - ps26
Brassicales - ps27
Cleomaceae/Brassicaceae - ps28
Brassicaceae - ps29
Camelineae - ps30
Arabidopsis - ps31
Arabidopsis_thaliana - ps32
Phylostratum

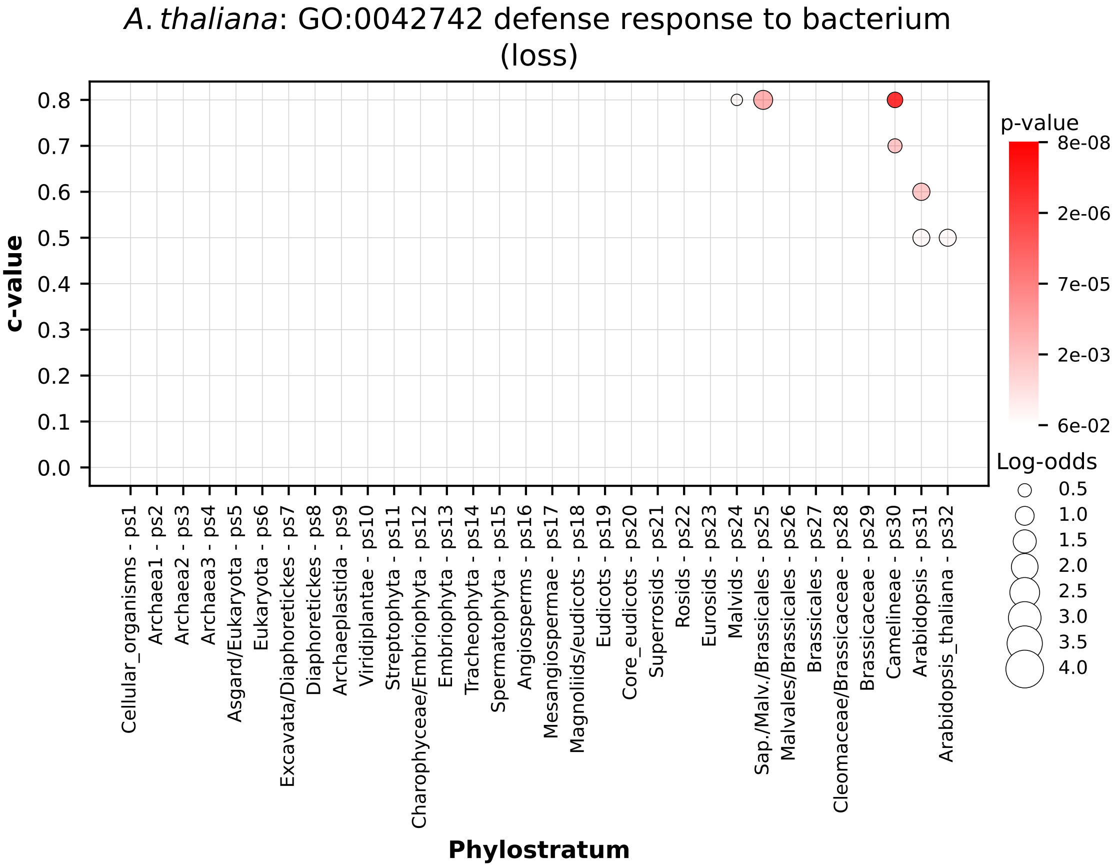

A. thaliana: GO:0042742 defense response to bacterium
(loss)
c-value
0.8
0.7
0.6
0.5
0.4
0.3
0.2
0.1
0.0
p-value
8e-08
2e-06
7e-05
2e-03
6e-02
Log-odds
0.5
1.0
1.5
2.0
2.5
3.0
3.5
4.0
Cellular_organisms - ps1
Archaea1 - ps2
Archaea2 - ps3
Archaea3 - ps4
Asgard/Eukaryota - ps5
Eukaryota - ps6
Excavata/Diaphoretickes - ps7
Diaphoretickes - ps8
Archaeplastida - ps9
Viridiplantae - ps10
Streptophyta - ps11
Charophyceae/Embriophyta - ps12
Embriophyta - ps13
Tracheophyta - ps14
Spermatophyta - ps15
Angiosperms - ps16
Mesangiospermae - ps17
Magnoliids/eudicots - ps18
Eudicots - ps19
Core_eudicots - ps20
Superrosids - ps21
Rosids - ps22
Eurosids - ps23
Malvids - ps24
Sap./Malv./Brassicales - ps25
Malvales/Brassicales - ps26
Brassicales - ps27
Cleomaceae/Brassicaceae - ps28
Brassicaceae - ps29
Camelineae - ps30
Arabidopsis - ps31
Arabidopsis_thaliana - ps32
Phylostratum

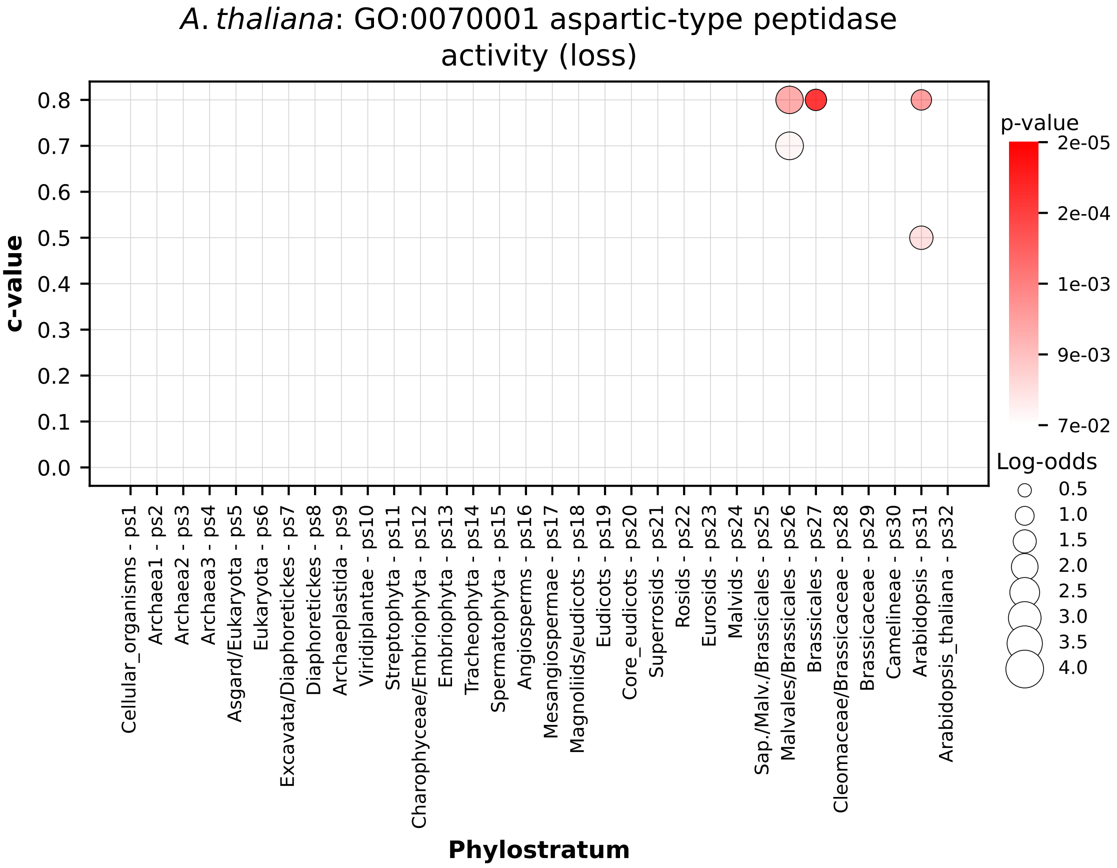

A. thaliana: GO:0070001 aspartic-type peptidase activity (loss)
c-value
0.0
0.1
0.2
0.3
0.4
0.5
0.6
0.7
0.8
p-value
2e-05
2e-04
1e-03
9e-03
7e-02
Log-odds
0.5
1.0
1.5
2.0
2.5
3.0
3.5
4.0
Cellular_organisms - ps1
Archaea1 - ps2
Archaea2 - ps3
Archaea3 - ps4
Asgard/Eukaryota - ps5
Eukaryota - ps6
Excavata/Diaphoretickes - ps7
Diaphoretickes - ps8
Archaeplastida - ps9
Viridiplantae - ps10
Streptophyta - ps11
Charophyceae/Embriophyta - ps12
Embriophyta - ps13
Tracheophyta - ps14
Spermatophyta - ps15
Angiosperms - ps16
Mesangiospermae - ps17
Magnoliids/eudicots - ps18
Eudicots - ps19
Core_eudicots - ps20
Superrosids - ps21
Rosids - ps22
Eurosids - ps23
Malvids - ps24
Sap./Malv./Brassicales - ps25
Malvales/Brassicales - ps26
Brassicales - ps27
Cleomaceae/Brassicaceae - ps28
Brassicaceae - ps29
Camelineae - ps30
Arabidopsis - ps31
Arabidopsis_thaliana - ps32
Phylostratum

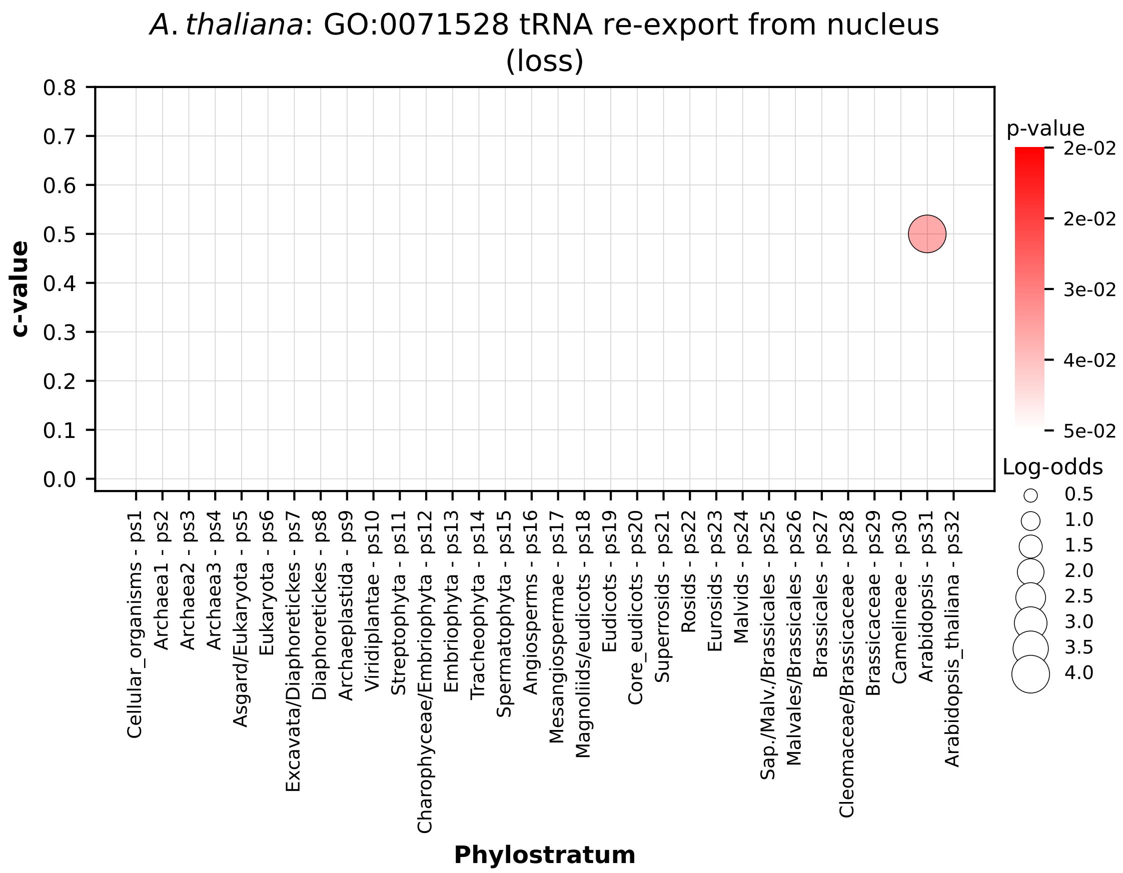

A. thaliana: GO:0071528 tRNA re-export from nucleus
(loss)
c-value
0.8
0.7
0.6
0.5
0.4
0.3
0.2
0.1
0.0
p-value
2e-02
2e-02
3e-02
4e-02
5e-02
Log-odds
0.5
1.0
1.5
2.0
2.5
3.0
3.5
4.0
Cellular_organisms - ps1
Archaea1 - ps2
Archaea2 - ps3
Archaea3 - ps4
Asgard/Eukaryota - ps5
Eukaryota - ps6
Excavata/Diaphoretickes - ps7
Diaphoretickes - ps8
Archaeplastida - ps9
Viridiplantae - ps10
Streptophyta - ps11
Charophyceae/Embriophyta - ps12
Embriophyta - ps13
Tracheophyta - ps14
Spermatophyta - ps15
Angiosperms - ps16
Mesangiospermae - ps17
Magnoliids/eudicots - ps18
Eudicots - ps19
Core_eudicots - ps20
Superrosids - ps21
Rosids - ps22
Eurosids - ps23
Malvids - ps24
Sap./Malv./Brassicales - ps25
Malvales/Brassicales - ps26
Brassicales - ps27
Cleomaceae/Brassicaceae - ps28
Brassicaceae - ps29
Camelineae - ps30
Arabidopsis - ps31
Arabidopsis_thaliana - ps32
Phylostratum

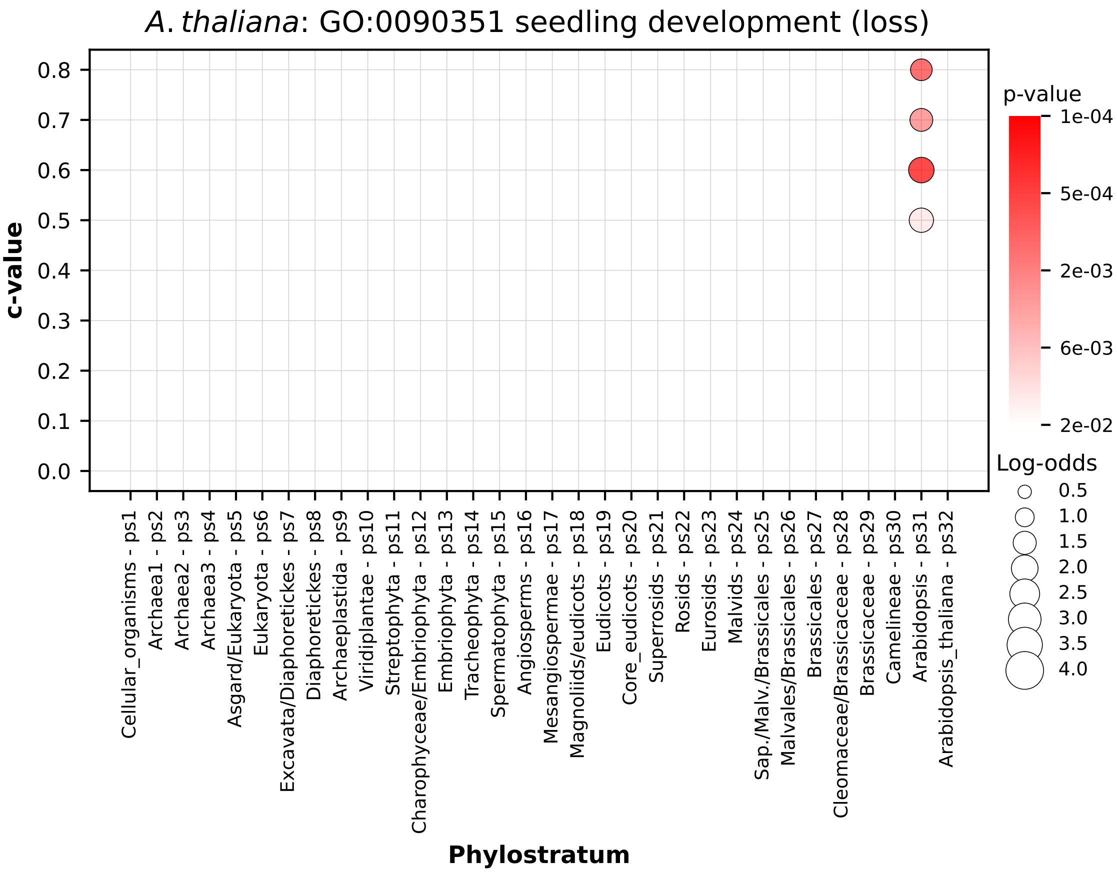
A. thaliana: GO:0090351 seedling development (loss)
c-value
0.0
0.1
0.2
0.3
0.4
0.5
0.6
0.7
0.8
Phylostratum
Cellular_organisms - ps1
Archaea1 - ps2
Archaea2 - ps3
Archaea3 - ps4
Asgard/Eukaryota - ps5
Eukaryota - ps6
Excavata/Diaphoretickes - ps7
Diaphoretickes - ps8
Archaeplastida - ps9
Viridiplantae - ps10
Streptophyta - ps11
Charophyceae/Embriophyta - ps12
Embriophyta - ps13
Tracheophyta - ps14
Spermatophyta - ps15
Angiosperms - ps16
Mesangiospermae - ps17
Magnoliids/eudicots - ps18
Eudicots - ps19
Core_eudicots - ps20
Superrosids - ps21
Rosids - ps22
Eurosids - ps23
Malvids - ps24
Sap./Malv./Brassicales - ps25
Malvales/Brassicales - ps26
Brassicales - ps27
Cleomaceae/Brassicaceae - ps28
Brassicaceae - ps29
Camelineae - ps30
Arabidopsis - ps31
Arabidopsis_thaliana - ps32
p-value
1e-04
5e-04
2e-03
6e-03
2e-02
Log-odds
0.5
1.0
1.5
2.0
2.5
3.0
3.5
4.0

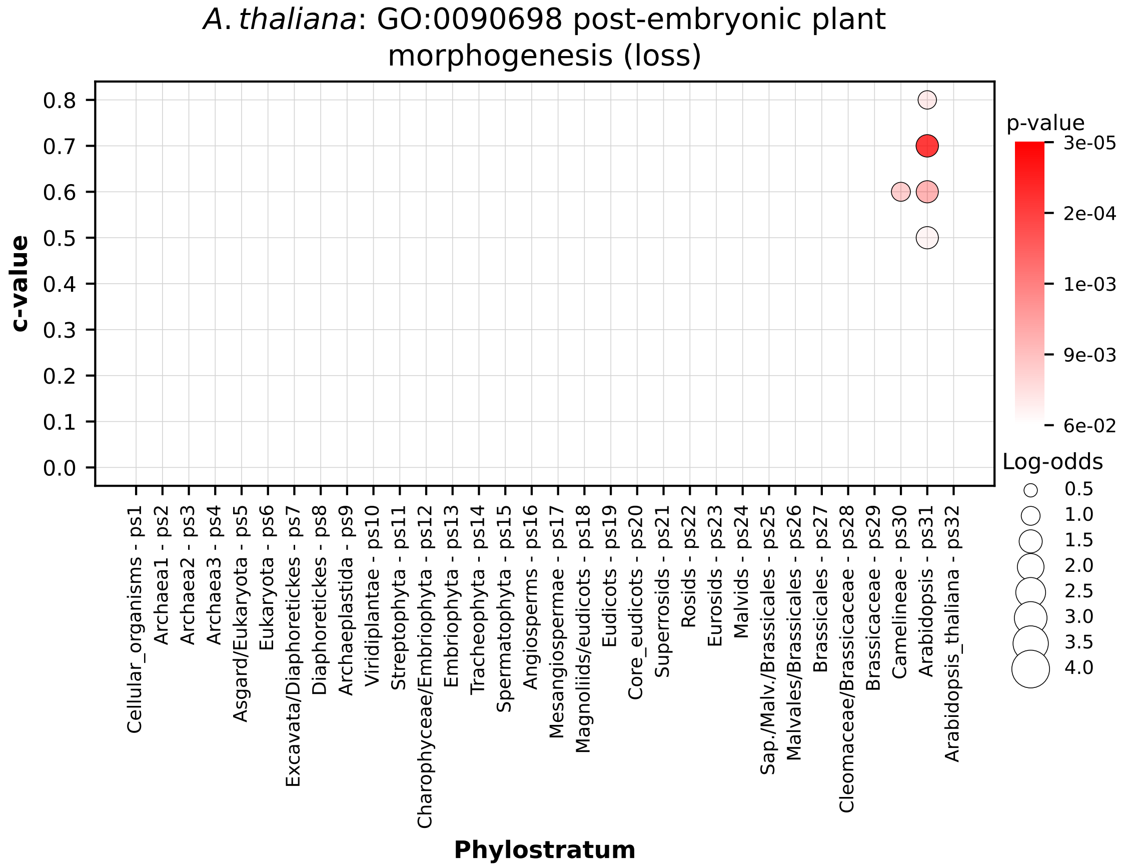

A. thaliana: GO:0090698 post-embryonic plant morphogenesis (loss)
c-value
0.8
0.7
0.6
0.5
0.4
0.3
0.2
0.1
0.0
Phylostratum
Cellular_organisms - ps1
Archaea1 - ps2
Archaea2 - ps3
Archaea3 - ps4
Asgard/Eukaryota - ps5
Eukaryota - ps6
Excavata/Diaphoretickes - ps7
Diaphoretickes - ps8
Archaeplastida - ps9
Viridiplantae - ps10
Streptophyta - ps11
Charophyceae/Embriophyta - ps12
Embriophyta - ps13
Tracheophyta - ps14
Spermatophyta - ps15
Angiosperms - ps16
Mesangiospermae - ps17
Magnoliids/eudicots - ps18
Eudicots - ps19
Core_eudicots - ps20
Superrosids - ps21
Rosids - ps22
Eurosids - ps23
Malvids - ps24
Sap./Malv./Brassicales - ps25
Malvales/Brassicales - ps26
Brassicales - ps27
Cleomaceae/Brassicaceae - ps28
Brassicaceae - ps29
Camelineae - ps30
Arabidopsis - ps31
Arabidopsis_thaliana - ps32
p-value
3e-05
2e-04
1e-03
9e-03
6e-02
Log-odds
0.5
1.0
1.5
2.0
2.5
3.0
3.5
4.0

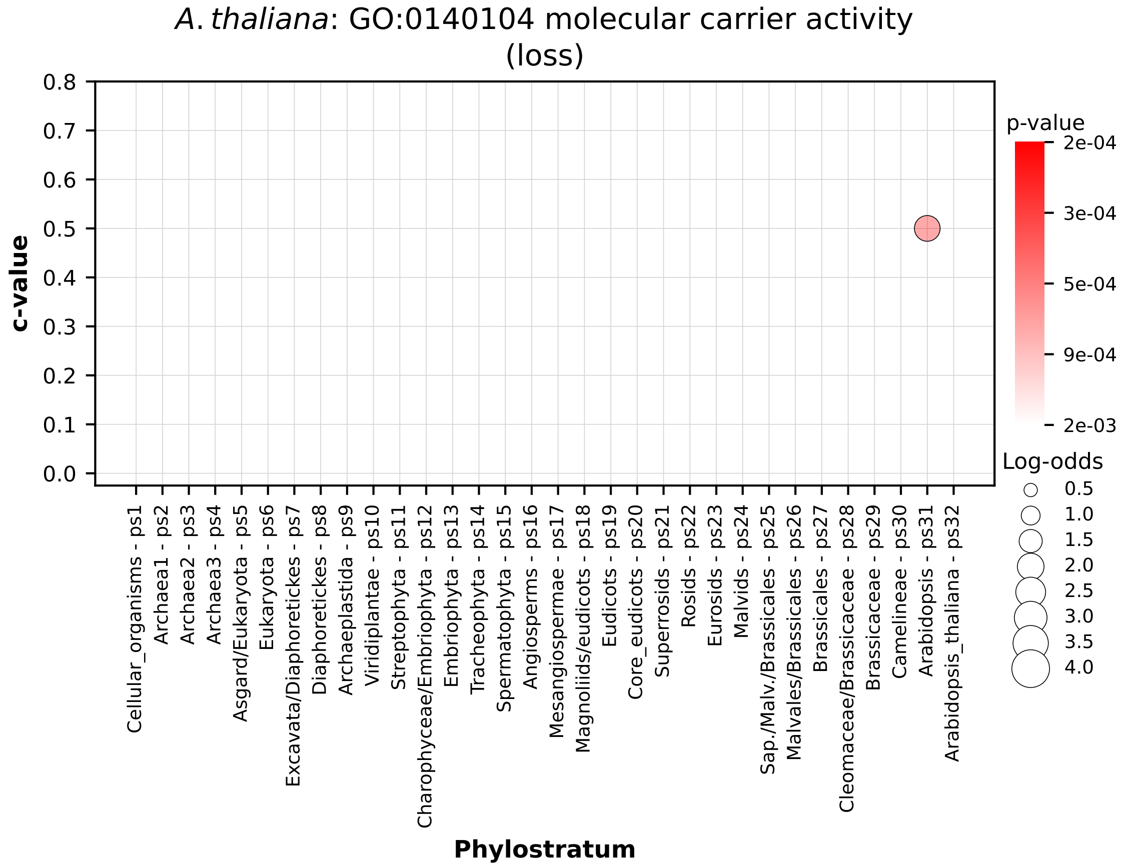

A. thaliana: GO:0140104 molecular carrier activity
(loss)
c-value
0.0
0.1
0.2
0.3
0.4
0.5
0.6
0.7
0.8
p-value
2e-04
3e-04
5e-04
9e-04
2e-03
Log-odds
0.5
1.0
1.5
2.0
2.5
3.0
3.5
4.0
Cellular_organisms - ps1
Archaea1 - ps2
Archaea2 - ps3
Archaea3 - ps4
Asgard/Eukaryota - ps5
Eukaryota - ps6
Excavata/Diaphoretickes - ps7
Diaphoretickes - ps8
Archaeplastida - ps9
Viridiplantae - ps10
Streptophyta - ps11
Charophyceae/Embriophyta - ps12
Embriophyta - ps13
Tracheophyta - ps14
Spermatophyta - ps15
Angiosperms - ps16
Mesangiospermae - ps17
Magnoliids/eudicots - ps18
Eudicots - ps19
Core_eudicots - ps20
Superrosids - ps21
Rosids - ps22
Eurosids - ps23
Malvids - ps24
Sap./Malv./Brassicales - ps25
Malvales/Brassicales - ps26
Brassicales - ps27
Cleomaceae/Brassicaceae - ps28
Brassicaceae - ps29
Camelineae - ps30
Arabidopsis - ps31
Arabidopsis_thaliana - ps32
Phylostratum

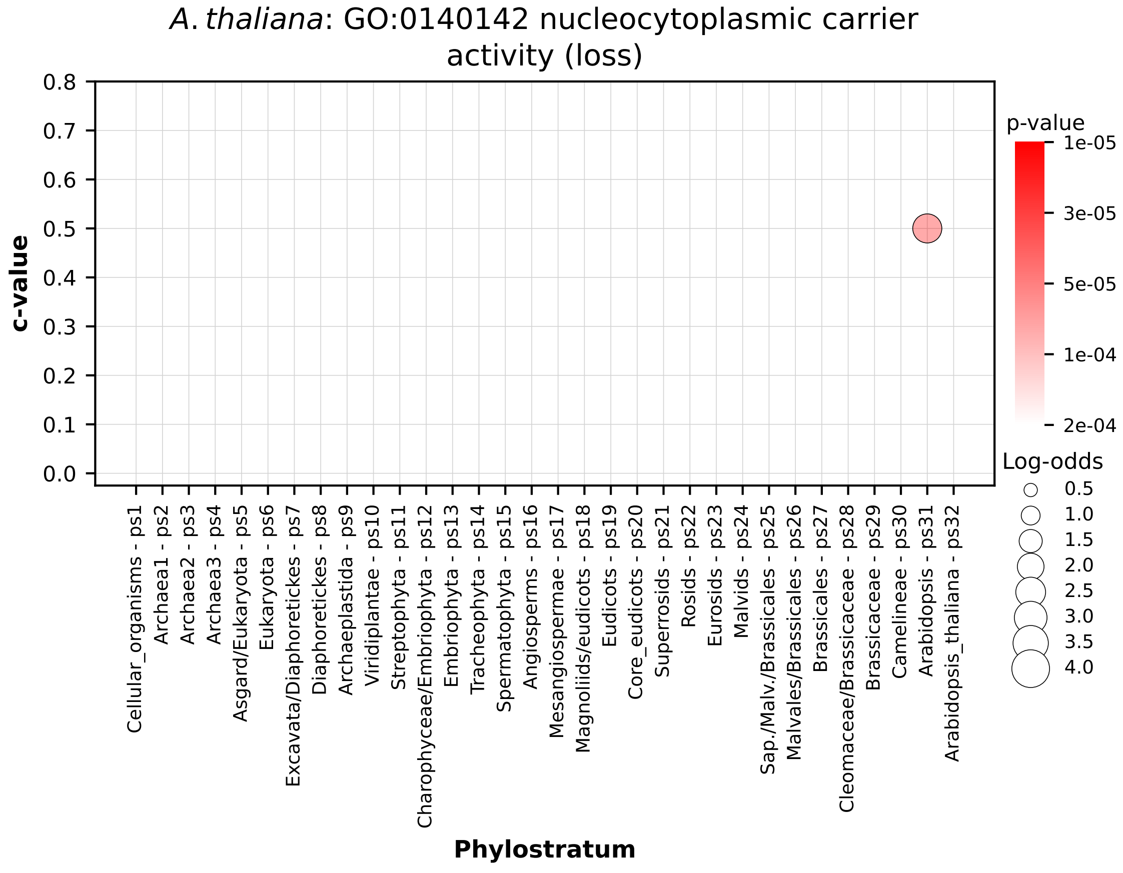
A. thaliana: GO:0140142 nucleocytoplasmic carrier activity (loss)
c-value
0.8
0.7
0.6
0.5
0.4
0.3
0.2
0.1
0.0
p-value
1e-05
3e-05
5e-05
1e-04
2e-04
Log-odds
0.5
1.0
1.5
2.0
2.5
3.0
3.5
4.0
Cellular_organisms - ps1
Archaea1 - ps2
Archaea2 - ps3
Archaea3 - ps4
Asgard/Eukaryota - ps5
Eukaryota - ps6
Excavata/Diaphoretickes - ps7
Diaphoretickes - ps8
Archaeplastida - ps9
Viridiplantae - ps10
Streptophyta - ps11
Charophyceae/Embriophyta - ps12
Embriophyta - ps13
Tracheophyta - ps14
Spermatophyta - ps15
Angiosperms - ps16
Mesangiospermae - ps17
Magnoliids/eudicots - ps18
Eudicots - ps19
Core_eudicots - ps20
Superrosids - ps21
Rosids - ps22
Eurosids - ps23
Malvids - ps24
Sap./Malv./Brassicales - ps25
Malvales/Brassicales - ps26
Brassicales - ps27
Cleomaceae/Brassicaceae - ps28
Brassicaceae - ps29
Camelineae - ps30
Arabidopsis - ps31
Arabidopsis_thaliana - ps32
Phylostratum

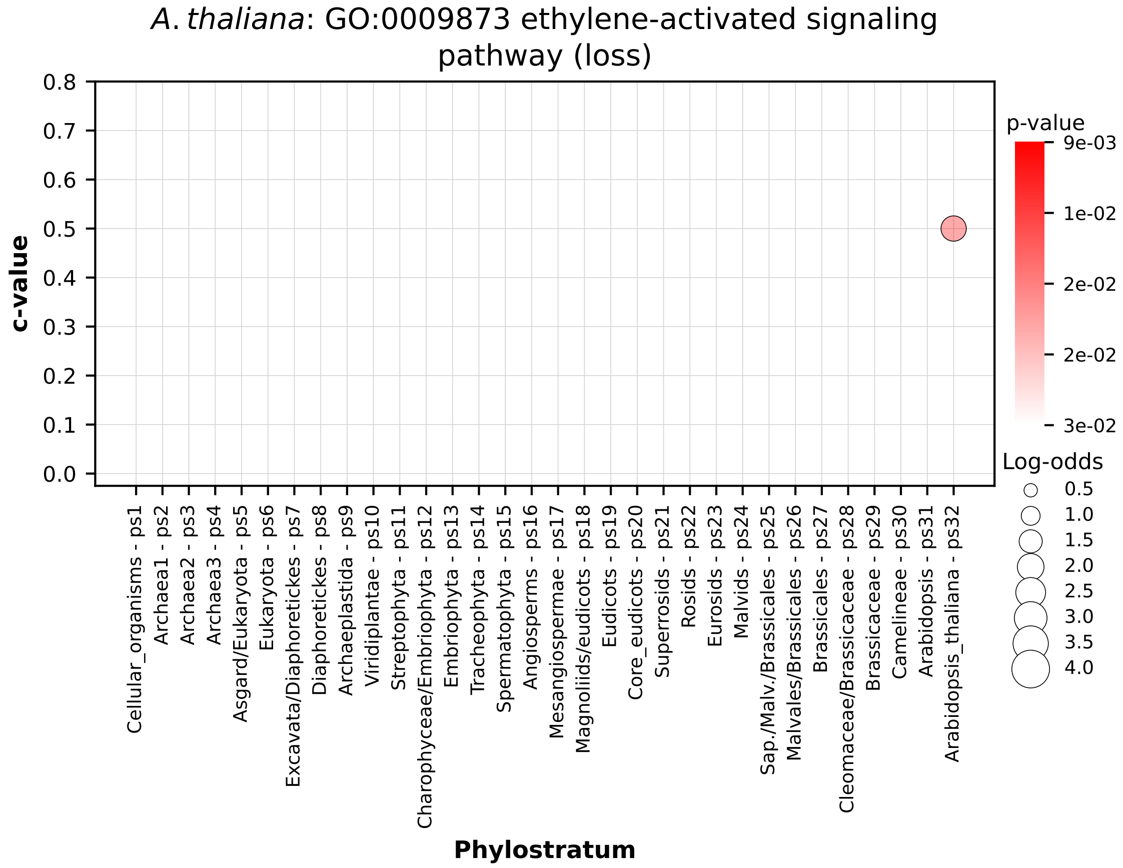
A. thaliana: GO:0009873 ethylene-activated signaling pathway (loss)
c-value
0.0
0.1
0.2
0.3
0.4
0.5
0.6
0.7
0.8
Phylostratum
Cellular_organisms - ps1
Archaea1 - ps2
Archaea2 - ps3
Archaea3 - ps4
Asgard/Eukaryota - ps5
Eukaryota - ps6
Excavata/Diaphoretickes - ps7
Diaphoretickes - ps8
Archaeplastida - ps9
Viridiplantae - ps10
Streptophyta - ps11
Charophyceae/Embriophyta - ps12
Embriophyta - ps13
Tracheophyta - ps14
Spermatophyta - ps15
Angiosperms - ps16
Mesangiospermae - ps17
Magnoliids/eudicots - ps18
Eudicots - ps19
Core_eudicots - ps20
Superrosids - ps21
Rosids - ps22
Eurosids - ps23
Malvids - ps24
Sap./Malv./Brassicales - ps25
Malvales/Brassicales - ps26
Brassicales - ps27
Cleomaceae/Brassicaceae - ps28
Brassicaceae - ps29
Camelineae - ps30
Arabidopsis - ps31
Arabidopsis_thaliana - ps32
p-value
9e-03
1e-02
2e-02
2e-02
3e-02
Log-odds
0.5
1.0
1.5
2.0
2.5
3.0
3.5
4.0

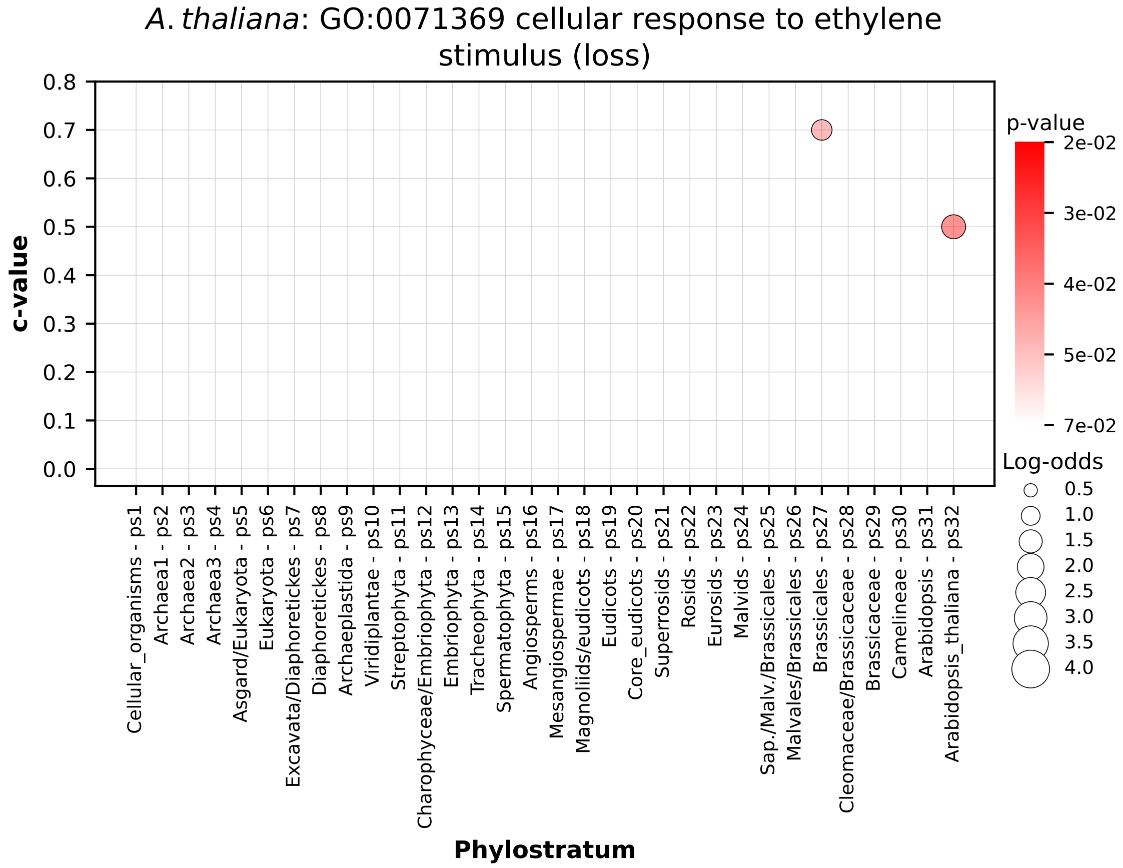

A. thaliana: GO:0071369 cellular response to ethylene stimulus (loss)
c-value
0.8
0.7
0.6
0.5
0.4
0.3
0.2
0.1
0.0
p-value
2e-02
3e-02
4e-02
5e-02
7e-02
Log-odds
0.5
1.0
1.5
2.0
2.5
3.0
3.5
4.0
Cellular_organisms - ps1
Archaea1 - ps2
Archaea2 - ps3
Archaea3 - ps4
Asgard/Eukaryota - ps5
Eukaryota - ps6
Excavata/Diaphoretickes - ps7
Diaphoretickes - ps8
Archaeplastida - ps9
Viridiplantae - ps10
Streptophyta - ps11
Charophyceae/Embriophyta - ps12
Embriophyta - ps13
Tracheophyta - ps14
Spermatophyta - ps15
Angiosperms - ps16
Mesangiospermae - ps17
Magnoliids/eudicots - ps18
Eudicots - ps19
Core_eudicots - ps20
Superrosids - ps21
Rosids - ps22
Eurosids - ps23
Malvids - ps24
Sap./Malv./Brassicales - ps25
Malvales/Brassicales - ps26
Brassicales - ps27
Cleomaceae/Brassicaceae - ps28
Brassicaceae - ps29
Camelineae - ps30
Arabidopsis - ps31
Arabidopsis_thaliana - ps32
Phylostratum

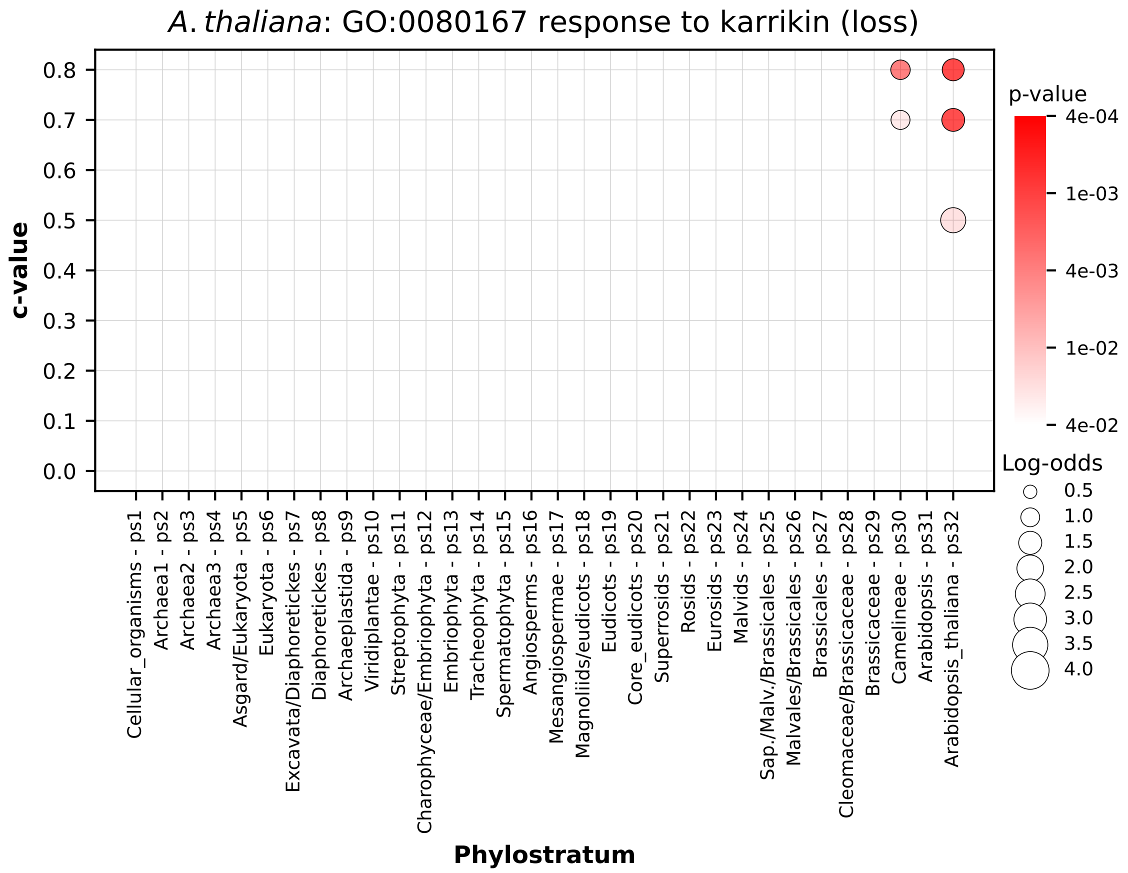
A. thaliana: GO:0080167 response to karrikin (loss)
c-value
0.8
0.7
0.6
0.5
0.4
0.3
0.2
0.1
0.0
Phylostratum
Cellular_organisms - ps1
Archaea1 - ps2
Archaea2 - ps3
Archaea3 - ps4
Asgard/Eukaryota - ps5
Eukaryota - ps6
Excavata/Diaphoretickes - ps7
Diaphoretickes - ps8
Archaeplastida - ps9
Viridiplantae - ps10
Streptophyta - ps11
Charophyceae/Embriophyta - ps12
Embriophyta - ps13
Tracheophyta - ps14
Spermatophyta - ps15
Angiosperms - ps16
Mesangiospermae - ps17
Magnoliids/eudicots - ps18
Eudicots - ps19
Core_eudicots - ps20
Superrosids - ps21
Rosids - ps22
Eurosids - ps23
Malvids - ps24
Sap./Malv./Brassicales - ps25
Malvales/Brassicales - ps26
Brassicales - ps27
Cleomaceae/Brassicaceae - ps28
Brassicaceae - ps29
Camelineae - ps30
Arabidopsis - ps31
Arabidopsis_thaliana - ps32
p-value
4e-04
1e-03
4e-03
1e-02
4e-02
Log-odds
0.5
1.0
1.5
2.0
2.5
3.0
3.5
4.0

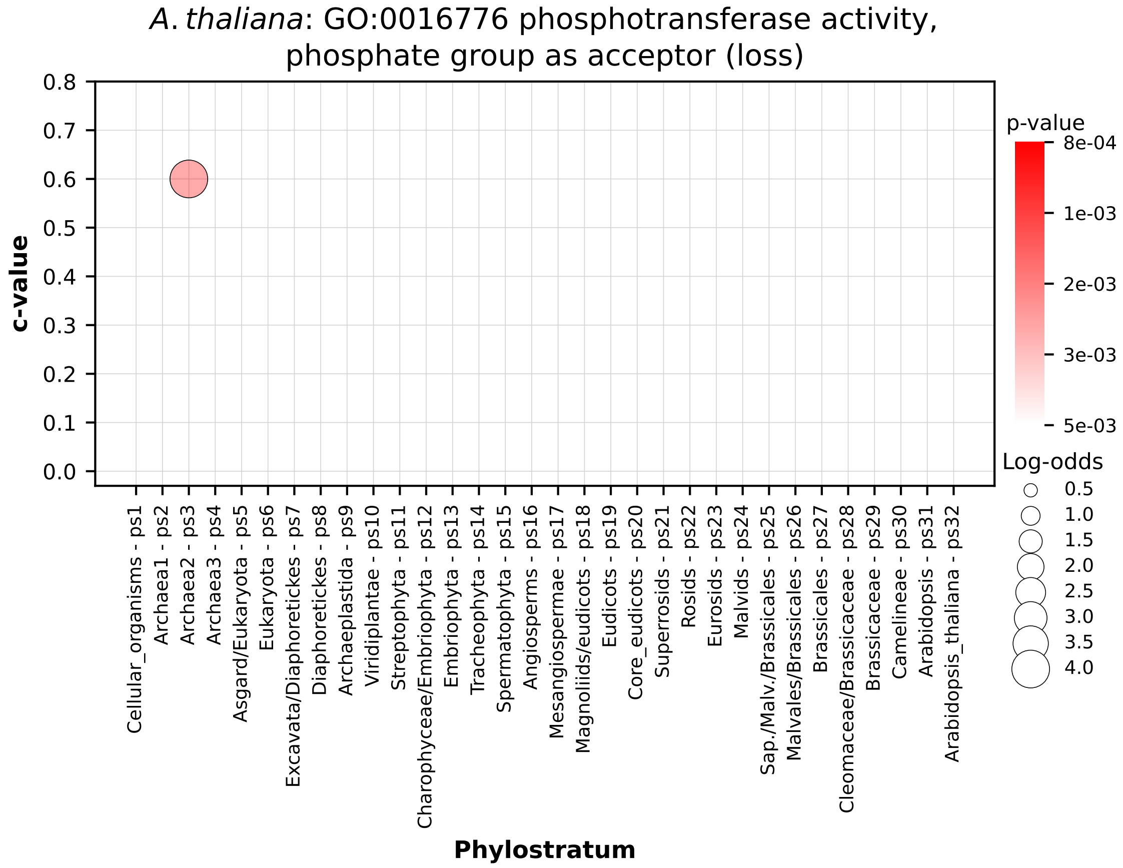

A. thaliana: GO:0016776 phosphotransferase activity, phosphate group as acceptor (loss)
c-value
0.8
0.7
0.6
0.5
0.4
0.3
0.2
0.1
0.0
Cellular_organisms - ps1
Archaea1 - ps2
Archaea2 - ps3
Archaea3 - ps4
Asgard/Eukaryota - ps5
Eukaryota - ps6
Excavata/Diaphoretickes - ps7
Diaphoretickes - ps8
Archaeplastida - ps9
Viridiplantae - ps10
Streptophyta - ps11
Charophyceae/Embriophyta - ps12
Embriophyta - ps13
Tracheophyta - ps14
Spermatophyta - ps15
Angiosperms - ps16
Mesangiospermae - ps17
Magnoliids/eudicots - ps18
Eudicots - ps19
Core_eudicots - ps20
Superrosids - ps21
Rosids - ps22
Eurosids - ps23
Malvids - ps24
Sap./Malv./Brassicales - ps25
Malvales/Brassicales - ps26
Brassicales - ps27
Cleomaceae/Brassicaceae - ps28
Brassicaceae - ps29
Camelineae - ps30
Arabidopsis - ps31
Arabidopsis_thaliana - ps32
Phylostratum
p-value
8e-04
1e-03
2e-03
3e-03
5e-03
Log-odds
0.5
1.0
1.5
2.0
2.5
3.0
3.5
4.0

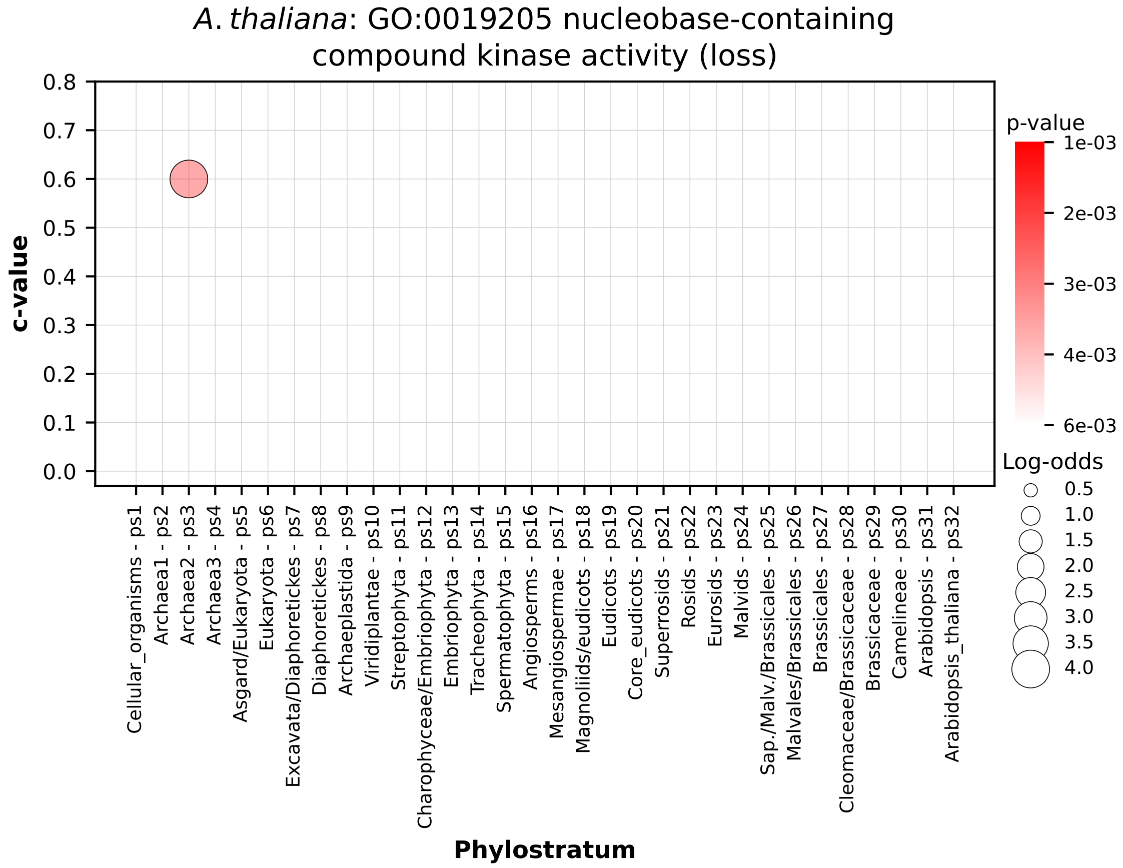
A. thaliana: GO:0019205 nucleobase-containing compound kinase activity (loss)
c-value
0.8
0.7
0.6
0.5
0.4
0.3
0.2
0.1
0.0
p-value
1e-03
2e-03
3e-03
4e-03
6e-03
Log-odds
0.5
1.0
1.5
2.0
2.5
3.0
3.5
4.0
Cellular_organisms - ps1
Archaea1 - ps2
Archaea2 - ps3
Archaea3 - ps4
Asgard/Eukaryota - ps5
Eukaryota - ps6
Excavata/Diaphoretickes - ps7
Diaphoretickes - ps8
Archaeplastida - ps9
Viridiplantae - ps10
Streptophyta - ps11
Charophyceae/Embriophyta - ps12
Embriophyta - ps13
Tracheophyta - ps14
Spermatophyta - ps15
Angiosperms - ps16
Mesangiospermae - ps17
Magnoliids/eudicots - ps18
Eudicots - ps19
Core_eudicots - ps20
Superrosids - ps21
Rosids - ps22
Eurosids - ps23
Malvids - ps24
Sap./Malv./Brassicales - ps25
Malvales/Brassicales - ps26
Brassicales - ps27
Cleomaceae/Brassicaceae - ps28
Brassicaceae - ps29
Camelineae - ps30
Arabidopsis - ps31
Arabidopsis_thaliana - ps32
Phylostratum

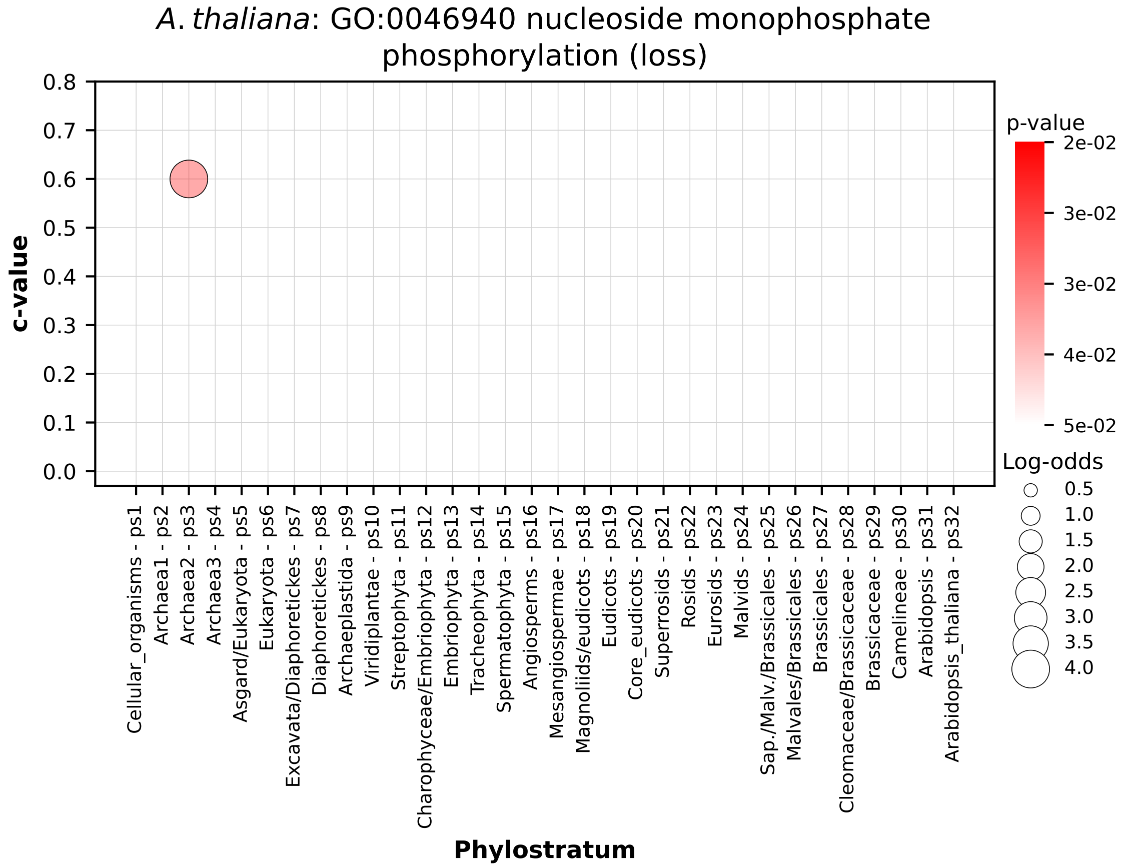

A. thaliana: GO:0046940 nucleoside monophosphate phosphorylation (loss)
c-value
0.8
0.7
0.6
0.5
0.4
0.3
0.2
0.1
0.0
Phylostratum
Cellular_organisms - ps1
Archaea1 - ps2
Archaea2 - ps3
Archaea3 - ps4
Asgard/Eukaryota - ps5
Eukaryota - ps6
Excavata/Diaphoretickes - ps7
Diaphoretickes - ps8
Archaeplastida - ps9
Viridiplantae - ps10
Streptophyta - ps11
Charophyceae/Embriophyta - ps12
Embriophyta - ps13
Tracheophyta - ps14
Spermatophyta - ps15
Angiosperms - ps16
Mesangiospermae - ps17
Magnoliids/eudicots - ps18
Eudicots - ps19
Core_eudicots - ps20
Superrosids - ps21
Rosids - ps22
Eurosids - ps23
Malvids - ps24
Sap./Malv./Brassicales - ps25
Malvales/Brassicales - ps26
Brassicales - ps27
Cleomaceae/Brassicaceae - ps28
Brassicaceae - ps29
Camelineae - ps30
Arabidopsis - ps31
Arabidopsis_thaliana - ps32
p-value
2e-02
3e-02
3e-02
4e-02
5e-02
Log-odds
0.5
1.0
1.5
2.0
2.5
3.0
3.5
4.0

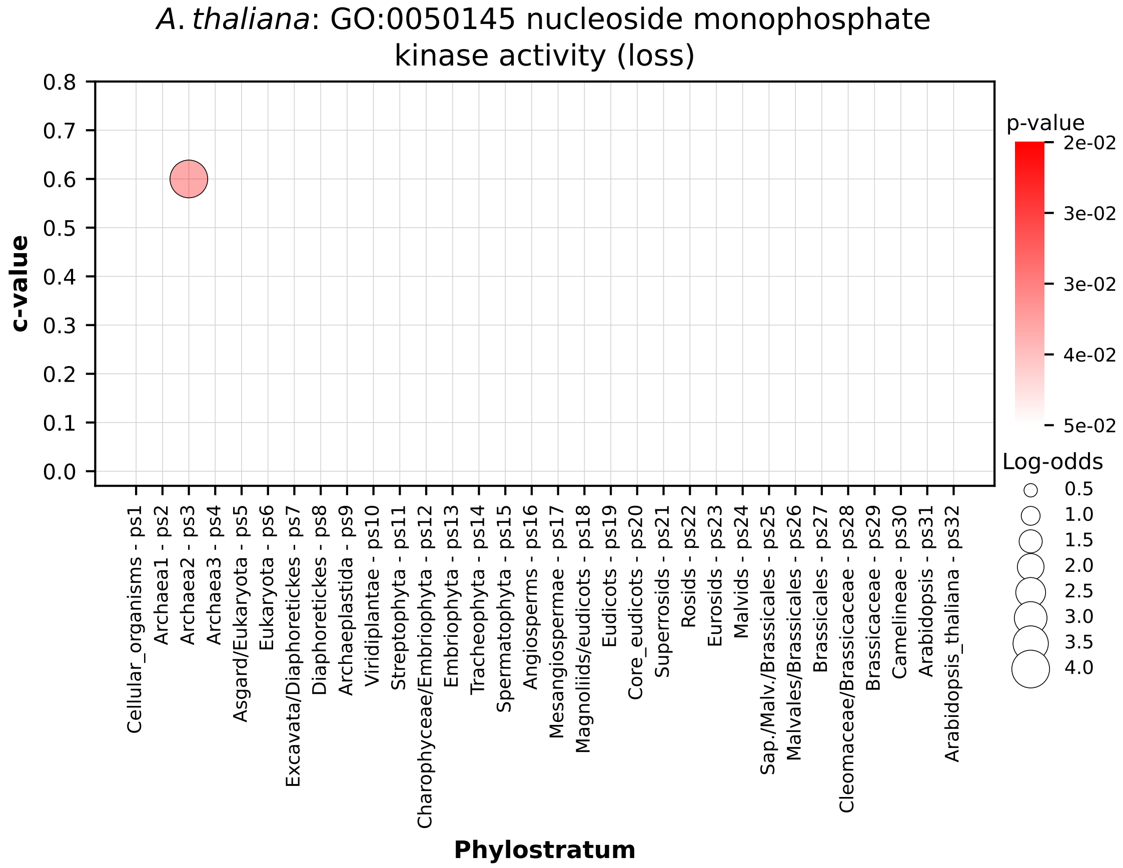

A. thaliana: GO:0050145 nucleoside monophosphate kinase activity (loss)
c-value
0.8
0.7
0.6
0.5
0.4
0.3
0.2
0.1
0.0
Phylostratum
Cellular_organisms - ps1
Archaea1 - ps2
Archaea2 - ps3
Archaea3 - ps4
Asgard/Eukaryota - ps5
Eukaryota - ps6
Excavata/Diaphoretickes - ps7
Diaphoretickes - ps8
Archaeplastida - ps9
Viridiplantae - ps10
Streptophyta - ps11
Charophyceae/Embriophyta - ps12
Embriophyta - ps13
Tracheophyta - ps14
Spermatophyta - ps15
Angiosperms - ps16
Mesangiospermae - ps17
Magnoliids/eudicots - ps18
Eudicots - ps19
Core_eudicots - ps20
Superrosids - ps21
Rosids - ps22
Eurosids - ps23
Malvids - ps24
Sap./Malv./Brassicales - ps25
Malvales/Brassicales - ps26
Brassicales - ps27
Cleomaceae/Brassicaceae - ps28
Brassicaceae - ps29
Camelineae - ps30
Arabidopsis - ps31
Arabidopsis_thaliana - ps32
p-value
2e-02
3e-02
3e-02
4e-02
5e-02
Log-odds
0.5
1.0
1.5
2.0
2.5
3.0
3.5
4.0

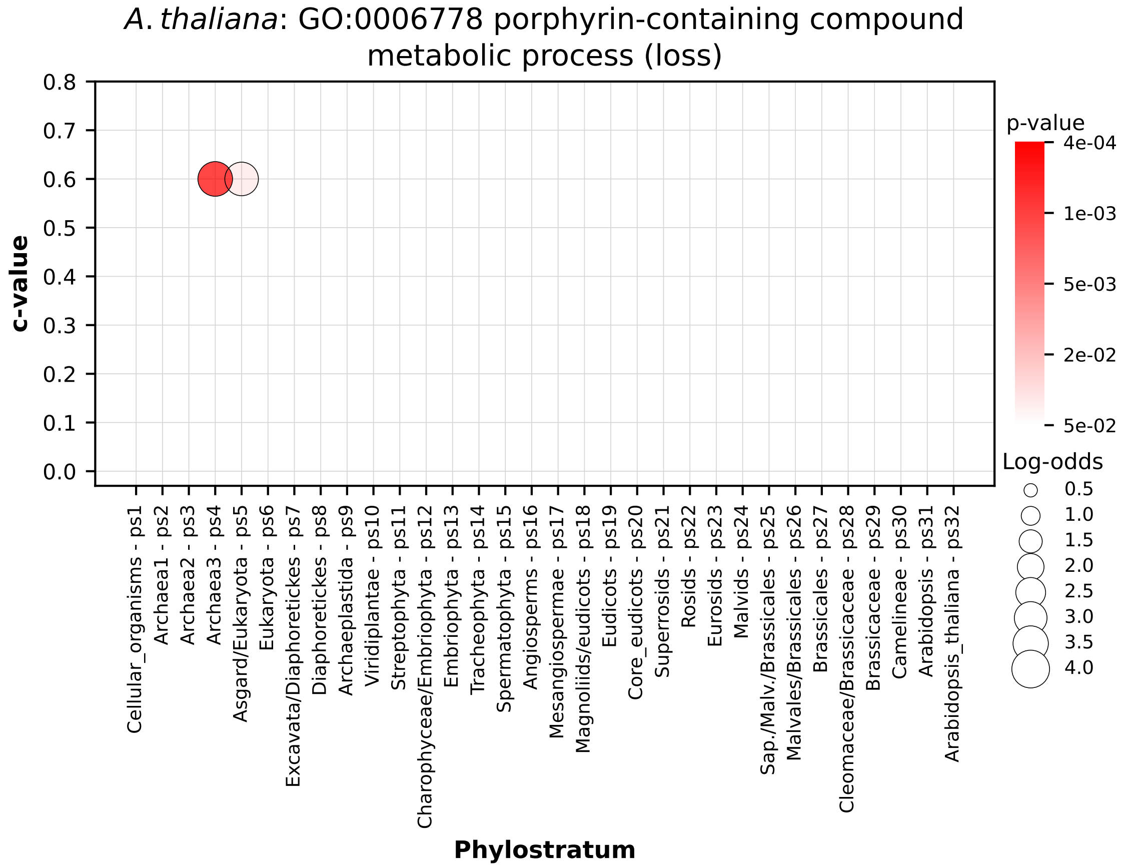
A. thaliana: GO:0006778 porphyrin-containing compound metabolic process (loss)
c-value
0.8
0.7
0.6
0.5
0.4
0.3
0.2
0.1
0.0
Phylostratum
Cellular_organisms - ps1
Archaea1 - ps2
Archaea2 - ps3
Archaea3 - ps4
Asgard/Eukaryota - ps5
Eukaryota - ps6
Excavata/Diaphoretickes - ps7
Diaphoretickes - ps8
Archaeplastida - ps9
Viridiplantae - ps10
Streptophyta - ps11
Charophyceae/Embriophyta - ps12
Embriophyta - ps13
Tracheophyta - ps14
Spermatophyta - ps15
Angiosperms - ps16
Mesangiospermae - ps17
Magnoliids/eudicots - ps18
Eudicots - ps19
Core_eudicots - ps20
Superrosids - ps21
Rosids - ps22
Eurosids - ps23
Malvids - ps24
Sap./Malv./Brassicales - ps25
Malvales/Brassicales - ps26
Brassicales - ps27
Cleomaceae/Brassicaceae - ps28
Brassicaceae - ps29
Camelineae - ps30
Arabidopsis - ps31
Arabidopsis_thaliana - ps32
p-value
4e-04
1e-03
5e-03
2e-02
5e-02
Log-odds
0.5
1.0
1.5
2.0
2.5
3.0
3.5
4.0

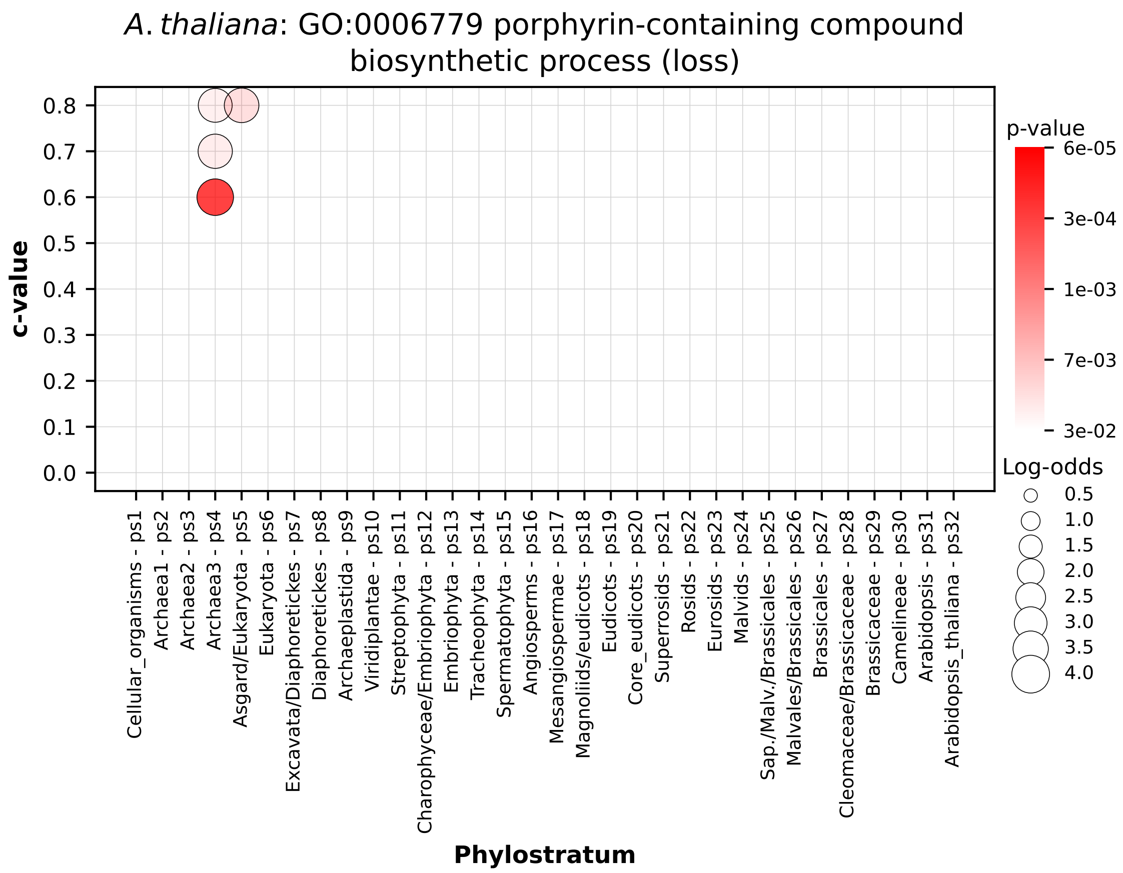

A. thaliana: GO:0006779 porphyrin-containing compound biosynthetic process (loss)
c-value
0.8
0.7
0.6
0.5
0.4
0.3
0.2
0.1
0.0
p-value
6e-05
3e-04
1e-03
7e-03
3e-02
Log-odds
0.5
1.0
1.5
2.0
2.5
3.0
3.5
4.0
Cellular_organisms - ps1
Archaea1 - ps2
Archaea2 - ps3
Archaea3 - ps4
Asgard/Eukaryota - ps5
Eukaryota - ps6
Excavata/Diaphoretickes - ps7
Diaphoretickes - ps8
Archaeplastida - ps9
Viridiplantae - ps10
Streptophyta - ps11
Charophyceae/Embriophyta - ps12
Embriophyta - ps13
Tracheophyta - ps14
Spermatophyta - ps15
Angiosperms - ps16
Mesangiospermae - ps17
Magnoliids/eudicots - ps18
Eudicots - ps19
Core_eudicots - ps20
Superrosids - ps21
Rosids - ps22
Eurosids - ps23
Malvids - ps24
Sap./Malv./Brassicales - ps25
Malvales/Brassicales - ps26
Brassicales - ps27
Cleomaceae/Brassicaceae - ps28
Brassicaceae - ps29
Camelineae - ps30
Arabidopsis - ps31
Arabidopsis_thaliana - ps32
Phylostratum

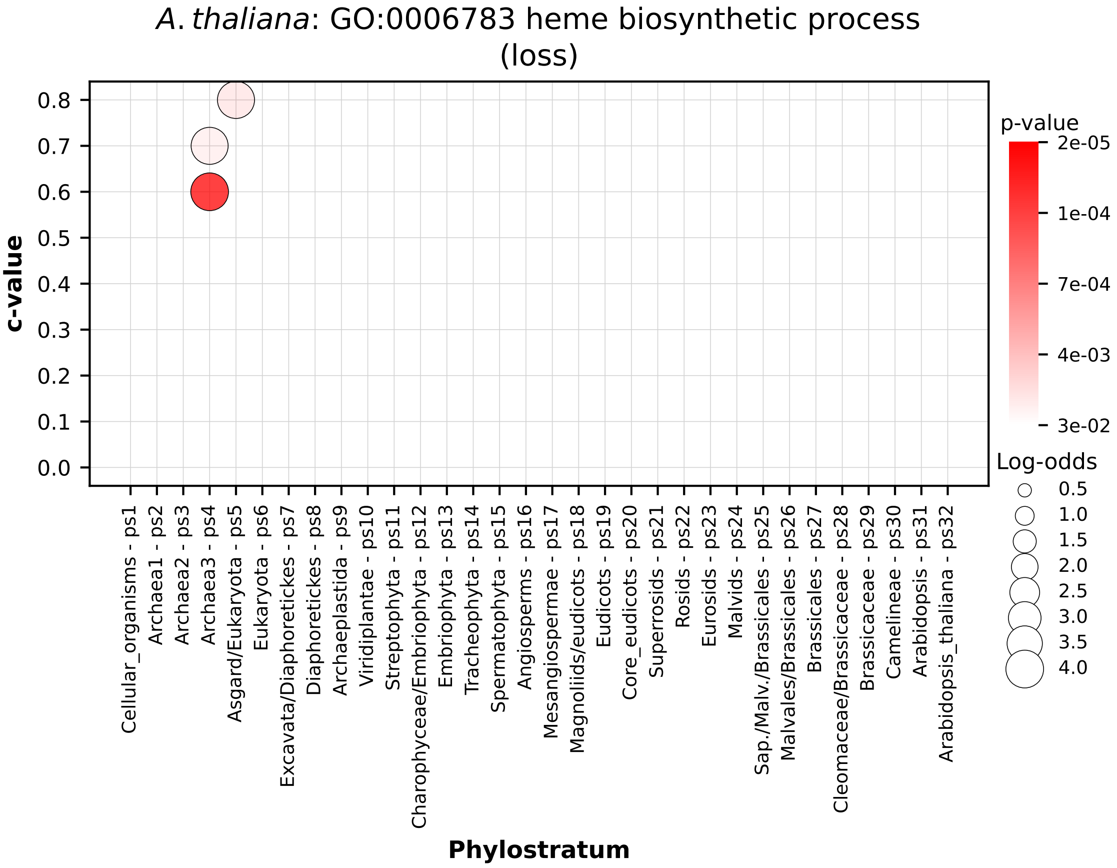

A. thaliana: GO:0006783 heme biosynthetic process
(loss)
c-value
0.8
0.7
0.6
0.5
0.4
0.3
0.2
0.1
0.0
p-value
2e-05
1e-04
7e-04
4e-03
3e-02
Log-odds
0.5
1.0
1.5
2.0
2.5
3.0
3.5
4.0
Cellular_organisms - ps1
Archaea1 - ps2
Archaea2 - ps3
Archaea3 - ps4
Asgard/Eukaryota - ps5
Eukaryota - ps6
Excavata/Diaphoretickes - ps7
Diaphoretickes - ps8
Archaeplastida - ps9
Viridiplantae - ps10
Streptophyta - ps11
Charophyceae/Embriophyta - ps12
Embriophyta - ps13
Tracheophyta - ps14
Spermatophyta - ps15
Angiosperms - ps16
Mesangiospermae - ps17
Magnoliids/eudicots - ps18
Eudicots - ps19
Core_eudicots - ps20
Superrosids - ps21
Rosids - ps22
Eurosids - ps23
Malvids - ps24
Sap./Malv./Brassicales - ps25
Malvales/Brassicales - ps26
Brassicales - ps27
Cleomaceae/Brassicaceae - ps28
Brassicaceae - ps29
Camelineae - ps30
Arabidopsis - ps31
Arabidopsis_thaliana - ps32
Phylostratum

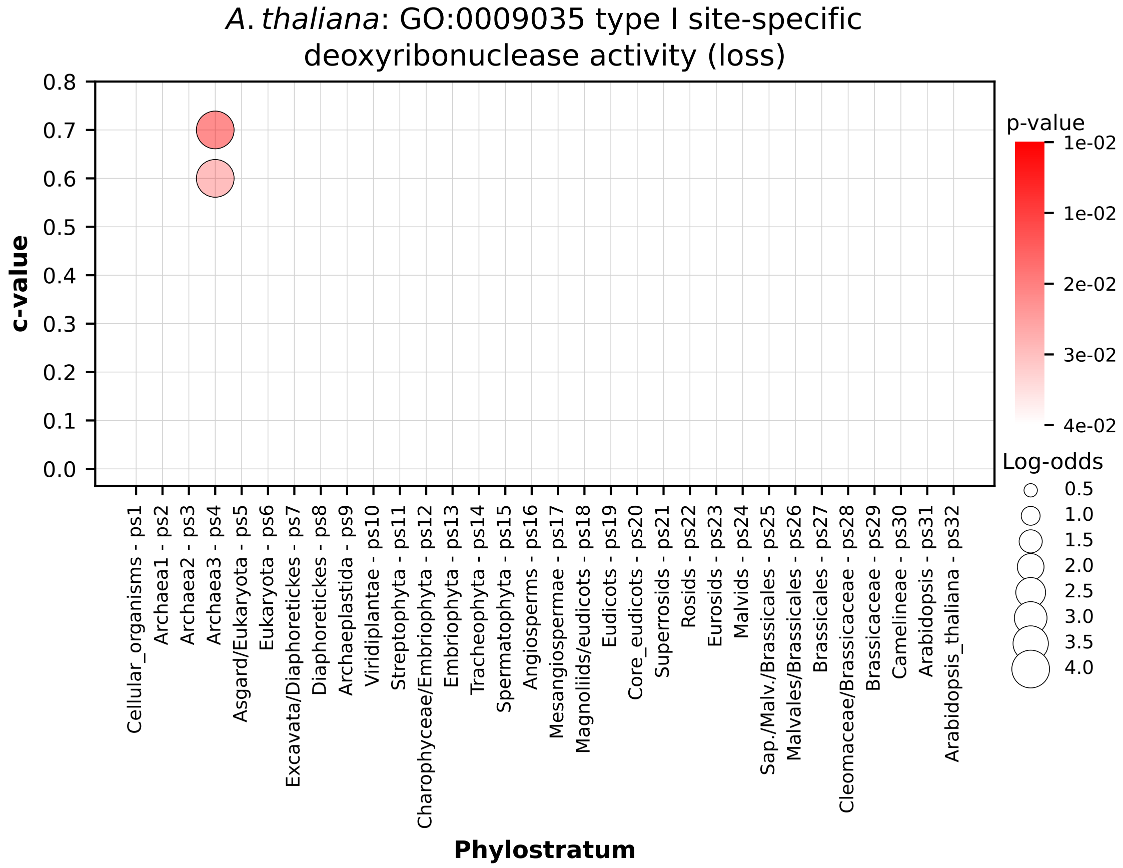
A. thaliana: GO:0009035 type I site-specific deoxyribonuclease activity (loss)
c-value
0.8
0.7
0.6
0.5
0.4
0.3
0.2
0.1
0.0
Phylostratum
Cellular_organisms - ps1
Archaea1 - ps2
Archaea2 - ps3
Archaea3 - ps4
Asgard/Eukaryota - ps5
Eukaryota - ps6
Excavata/Diaphoretickes - ps7
Diaphoretickes - ps8
Archaeplastida - ps9
Viridiplantae - ps10
Streptophyta - ps11
Charophyceae/Embriophyta - ps12
Embriophyta - ps13
Tracheophyta - ps14
Spermatophyta - ps15
Angiosperms - ps16
Mesangiospermae - ps17
Magnoliids/eudicots - ps18
Eudicots - ps19
Core_eudicots - ps20
Superrosids - ps21
Rosids - ps22
Eurosids - ps23
Malvids - ps24
Sap./Malv./Brassicales - ps25
Malvales/Brassicales - ps26
Brassicales - ps27
Cleomaceae/Brassicaceae - ps28
Brassicaceae - ps29
Camelineae - ps30
Arabidopsis - ps31
Arabidopsis_thaliana - ps32
p-value
1e-02
1e-02
2e-02
3e-02
4e-02
Log-odds
0.5
1.0
1.5
2.0
2.5
3.0
3.5
4.0

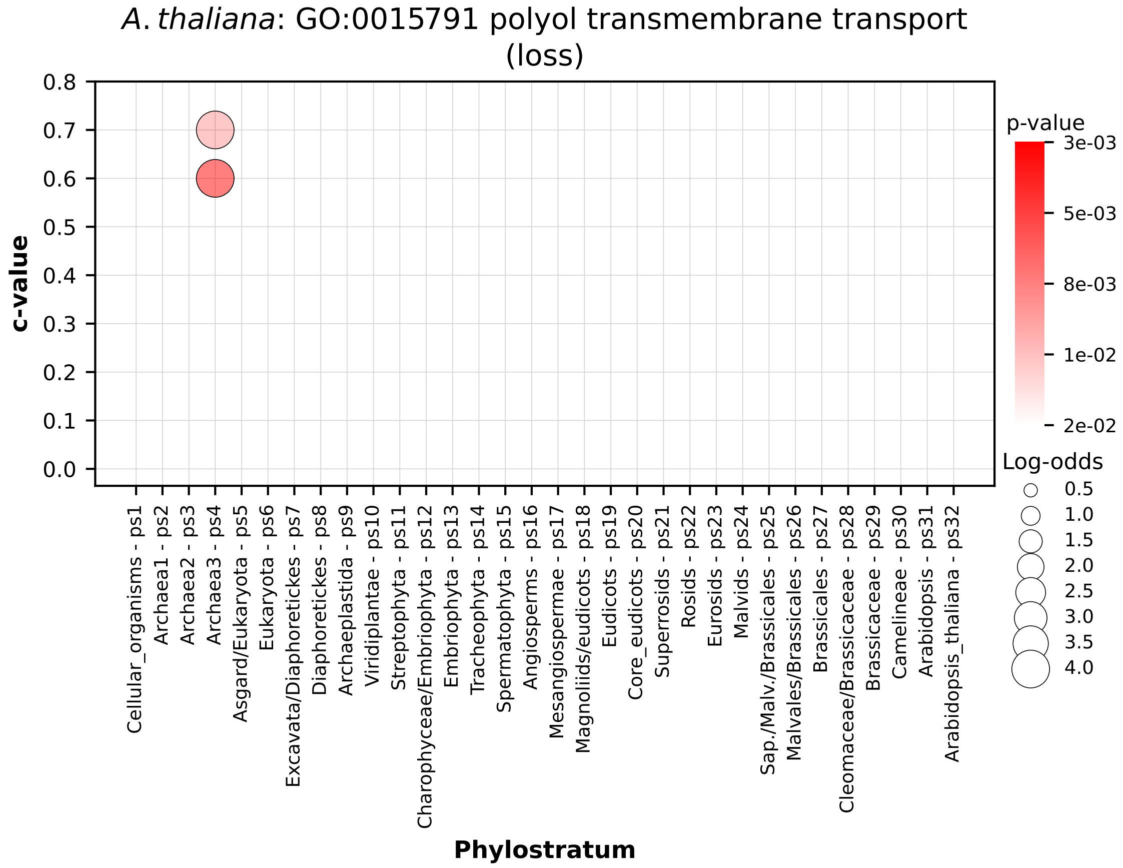

A. thaliana: GO:0015791 polyol transmembrane transport
(loss)
c-value
0.8
0.7
0.6
0.5
0.4
0.3
0.2
0.1
0.0
Phylostratum
Cellular_organisms - ps1
Archaea1 - ps2
Archaea2 - ps3
Archaea3 - ps4
Asgard/Eukaryota - ps5
Eukaryota - ps6
Excavata/Diaphoretickes - ps7
Diaphoretickes - ps8
Archaeplastida - ps9
Viridiplantae - ps10
Streptophyta - ps11
Charophyceae/Embriophyta - ps12
Embriophyta - ps13
Tracheophyta - ps14
Spermatophyta - ps15
Angiosperms - ps16
Mesangiospermae - ps17
Magnoliids/eudicots - ps18
Eudicots - ps19
Core_eudicots - ps20
Superrosids - ps21
Rosids - ps22
Eurosids - ps23
Malvids - ps24
Sap./Malv./Brassicales - ps25
Malvales/Brassicales - ps26
Brassicales - ps27
Cleomaceae/Brassicaceae - ps28
Brassicaceae - ps29
Camelineae - ps30
Arabidopsis - ps31
Arabidopsis_thaliana - ps32
p-value
3e-03
5e-03
8e-03
1e-02
2e-02
Log-odds
0.5
1.0
1.5
2.0
2.5
3.0
3.5
4.0

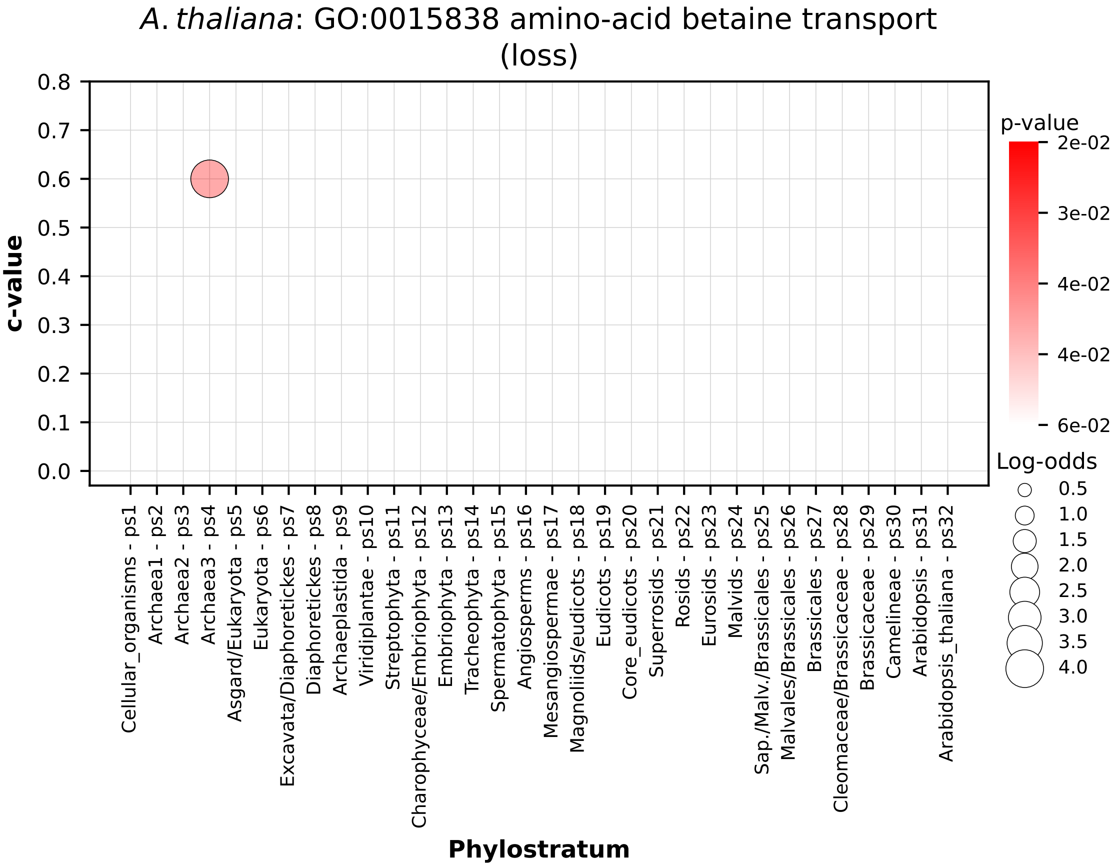
A. thaliana: GO:0015838 amino-acid betaine transport
(loss)
c-value
0.8
0.7
0.6
0.5
0.4
0.3
0.2
0.1
0.0
Phylostratum
Cellular_organisms - ps1
Archaea1 - ps2
Archaea2 - ps3
Archaea3 - ps4
Asgard/Eukaryota - ps5
Eukaryota - ps6
Excavata/Diaphoretickes - ps7
Diaphoretickes - ps8
Archaeplastida - ps9
Viridiplantae - ps10
Streptophyta - ps11
Charophyceae/Embriophyta - ps12
Embriophyta - ps13
Tracheophyta - ps14
Spermatophyta - ps15
Angiosperms - ps16
Mesangiospermae - ps17
Magnoliids/eudicots - ps18
Eudicots - ps19
Core_eudicots - ps20
Superrosids - ps21
Rosids - ps22
Eurosids - ps23
Malvids - ps24
Sap./Malv./Brassicales - ps25
Malvales/Brassicales - ps26
Brassicales - ps27
Cleomaceae/Brassicaceae - ps28
Brassicaceae - ps29
Camelineae - ps30
Arabidopsis - ps31
Arabidopsis_thaliana - ps32
p-value
2e-02
3e-02
4e-02
4e-02
6e-02
Log-odds
0.5
1.0
1.5
2.0
2.5
3.0
3.5
4.0

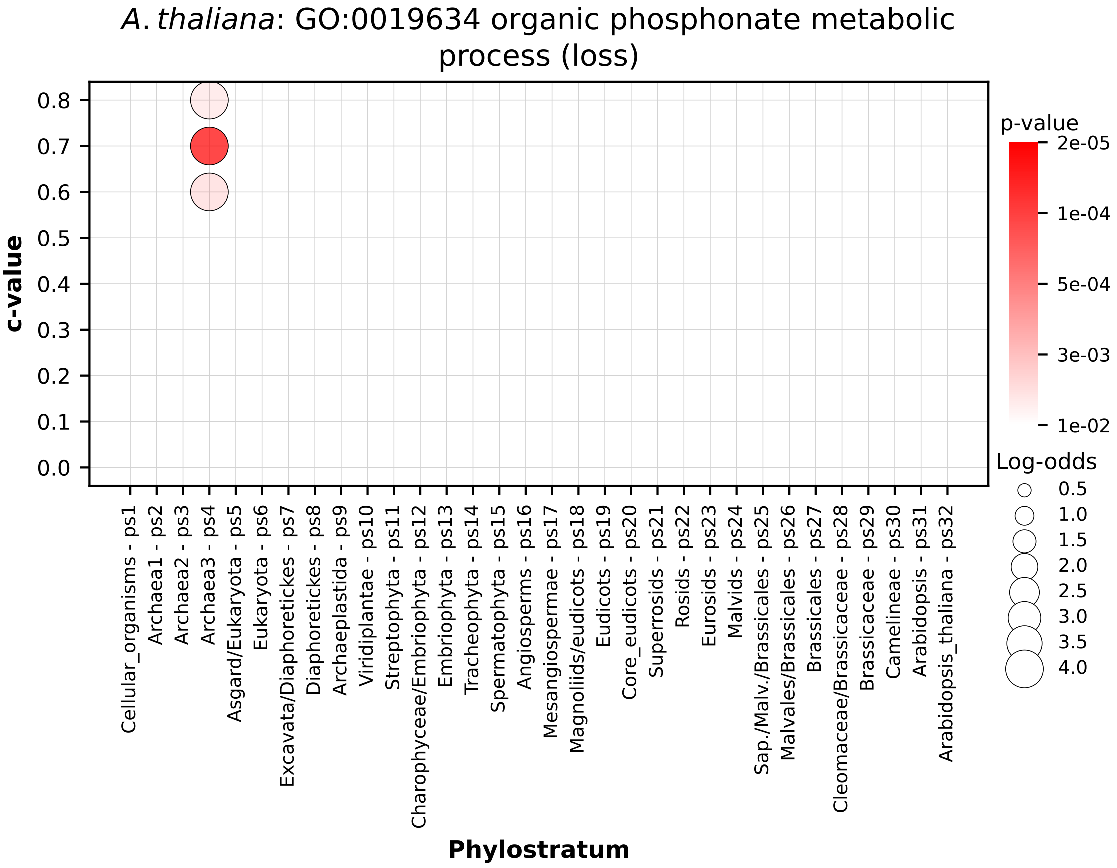

A. thaliana: GO:0019634 organic phosphonate metabolic process (loss)
c-value
0.0
0.1
0.2
0.3
0.4
0.5
0.6
0.7
0.8
Phylostratum
Cellular_organisms - ps1
Archaea1 - ps2
Archaea2 - ps3
Archaea3 - ps4
Asgard/Eukaryota - ps5
Eukaryota - ps6
Excavata/Diaphoretickes - ps7
Diaphoretickes - ps8
Archaeplastida - ps9
Viridiplantae - ps10
Streptophyta - ps11
Charophyceae/Embriophyta - ps12
Embriophyta - ps13
Tracheophyta - ps14
Spermatophyta - ps15
Angiosperms - ps16
Mesangiospermae - ps17
Magnoliids/eudicots - ps18
Eudicots - ps19
Core_eudicots - ps20
Superrosids - ps21
Rosids - ps22
Eurosids - ps23
Malvids - ps24
Sap./Malv./Brassicales - ps25
Malvales/Brassicales - ps26
Brassicales - ps27
Cleomaceae/Brassicaceae - ps28
Brassicaceae - ps29
Camelineae - ps30
Arabidopsis - ps31
Arabidopsis_thaliana - ps32
p-value
2e-05
1e-04
5e-04
3e-03
1e-02
Log-odds
0.5
1.0
1.5
2.0
2.5
3.0
3.5
4.0

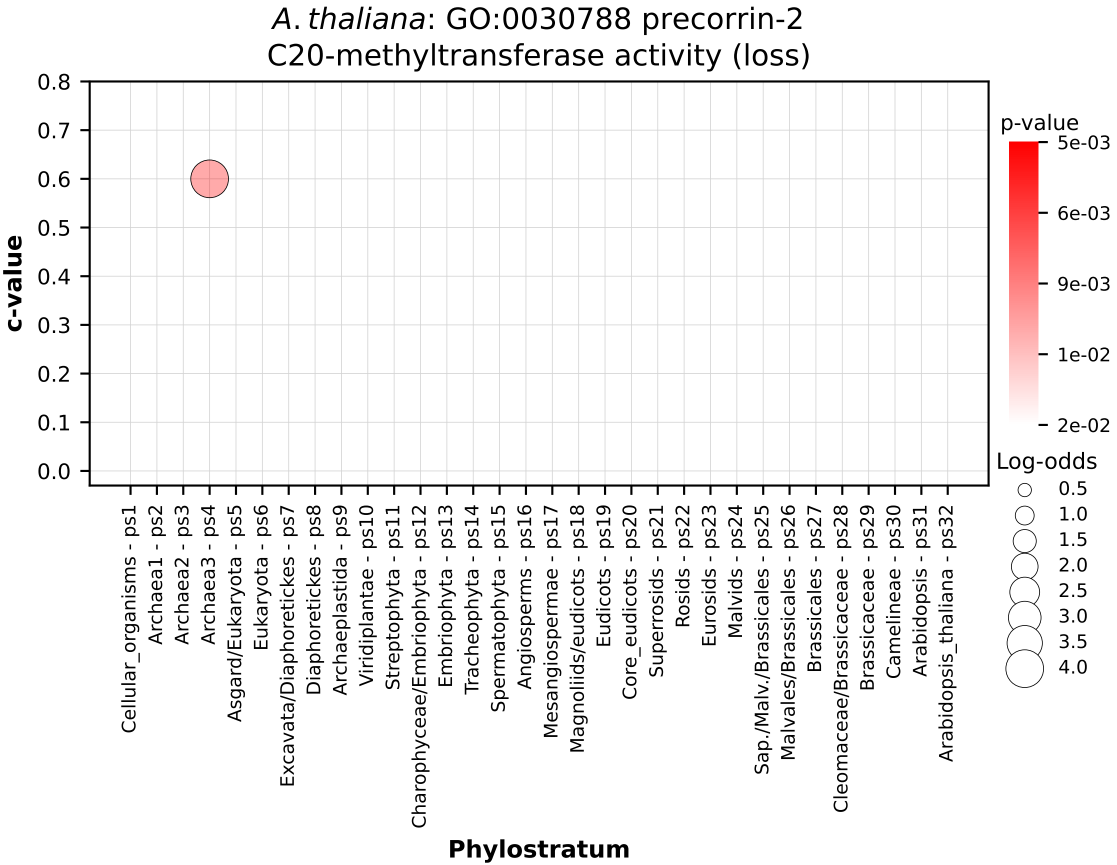

A. thaliana: GO:0030788 precorrin-2
C20-methyltransferase activity (loss)
c-value
0.8
0.7
0.6
0.5
0.4
0.3
0.2
0.1
0.0
p-value
5e-03
6e-03
9e-03
1e-02
2e-02
Log-odds
0.5
1.0
1.5
2.0
2.5
3.0
3.5
4.0
Cellular_organisms - ps1
Archaea1 - ps2
Archaea2 - ps3
Archaea3 - ps4
Asgard/Eukaryota - ps5
Eukaryota - ps6
Excavata/Diaphoretickes - ps7
Diaphoretickes - ps8
Archaeplastida - ps9
Viridiplantae - ps10
Streptophyta - ps11
Charophyceae/Embriophyta - ps12
Embriophyta - ps13
Tracheophyta - ps14
Spermatophyta - ps15
Angiosperms - ps16
Mesangiospermae - ps17
Magnoliids/eudicots - ps18
Eudicots - ps19
Core_eudicots - ps20
Superrosids - ps21
Rosids - ps22
Eurosids - ps23
Malvids - ps24
Sap./Malv./Brassicales - ps25
Malvales/Brassicales - ps26
Brassicales - ps27
Cleomaceae/Brassicaceae - ps28
Brassicaceae - ps29
Camelineae - ps30
Arabidopsis - ps31
Arabidopsis_thaliana - ps32
Phylostratum

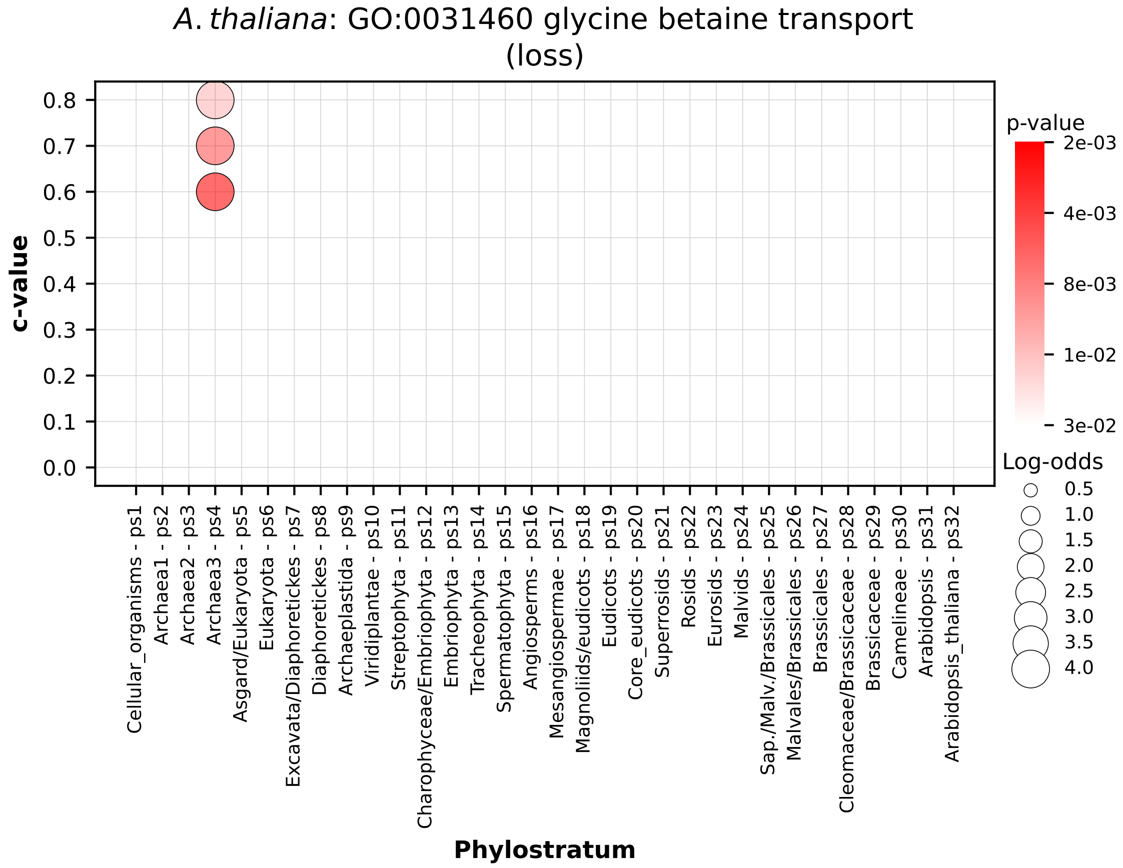

A. thaliana: GO:0031460 glycine betaine transport (loss)
c-value
0.8
0.7
0.6
0.5
0.4
0.3
0.2
0.1
0.0
Phylostratum
Cellular_organisms - ps1
Archaea1 - ps2
Archaea2 - ps3
Archaea3 - ps4
Asgard/Eukaryota - ps5
Eukaryota - ps6
Excavata/Diaphoretickes - ps7
Diaphoretickes - ps8
Archaeplastida - ps9
Viridiplantae - ps10
Streptophyta - ps11
Charophyceae/Embriophyta - ps12
Embriophyta - ps13
Tracheophyta - ps14
Spermatophyta - ps15
Angiosperms - ps16
Mesangiospermae - ps17
Magnoliids/eudicots - ps18
Eudicots - ps19
Core_eudicots - ps20
Superrosids - ps21
Rosids - ps22
Eurosids - ps23
Malvids - ps24
Sap./Malv./Brassicales - ps25
Malvales/Brassicales - ps26
Brassicales - ps27
Cleomaceae/Brassicaceae - ps28
Brassicaceae - ps29
Camelineae - ps30
Arabidopsis - ps31
Arabidopsis_thaliana - ps32
p-value
2e-03
4e-03
8e-03
1e-02
3e-02
Log-odds
0.5
1.0
1.5
2.0
2.5
3.0
3.5
4.0

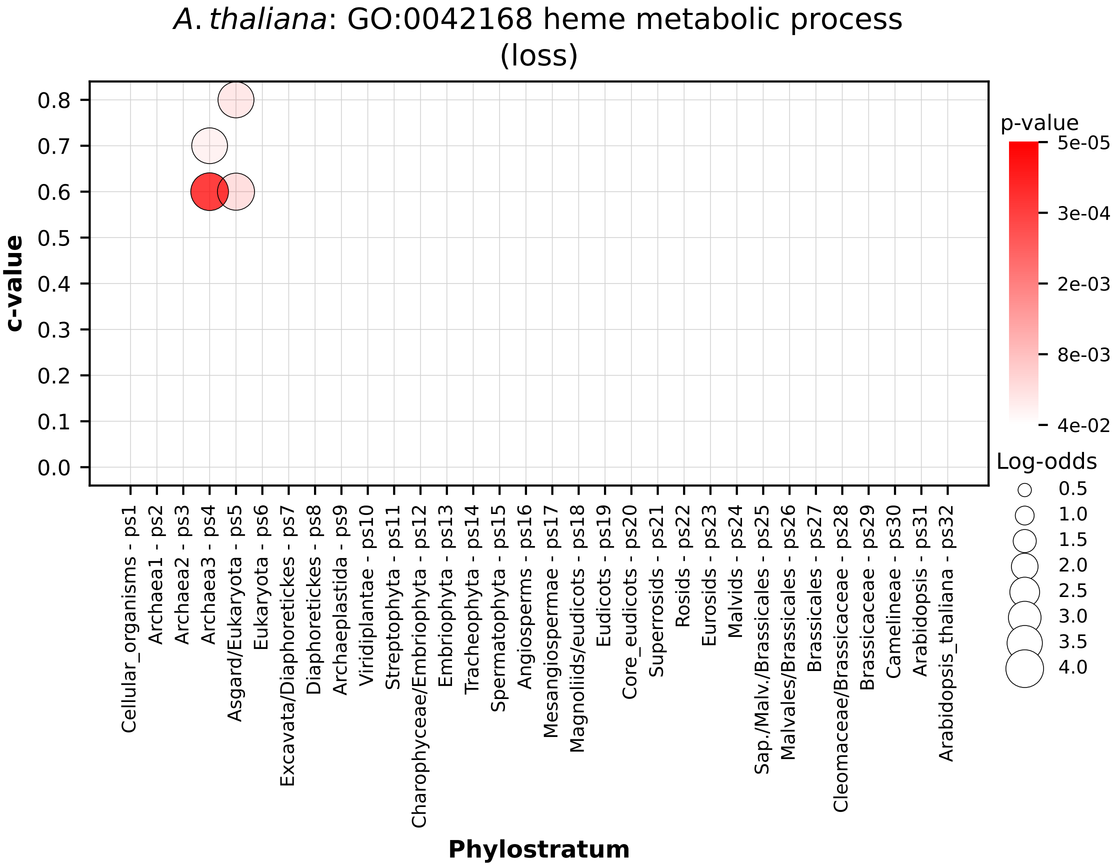

A. thaliana: GO:0042168 heme metabolic process
(loss)
c-value
0.0
0.1
0.2
0.3
0.4
0.5
0.6
0.7
0.8
Phylostratum
Cellular_organisms - ps1
Archaea1 - ps2
Archaea2 - ps3
Archaea3 - ps4
Asgard/Eukaryota - ps5
Eukaryota - ps6
Excavata/Diaphoretickes - ps7
Diaphoretickes - ps8
Archaeplastida - ps9
Viridiplantae - ps10
Streptophyta - ps11
Charophyceae/Embriophyta - ps12
Embriophyta - ps13
Tracheophyta - ps14
Spermatophyta - ps15
Angiosperms - ps16
Mesangiospermae - ps17
Magnoliids/eudicots - ps18
Eudicots - ps19
Core_eudicots - ps20
Superrosids - ps21
Rosids - ps22
Eurosids - ps23
Malvids - ps24
Sap./Malv./Brassicales - ps25
Malvales/Brassicales - ps26
Brassicales - ps27
Cleomaceae/Brassicaceae - ps28
Brassicaceae - ps29
Camelineae - ps30
Arabidopsis - ps31
Arabidopsis_thaliana - ps32
p-value
5e-05
3e-04
2e-03
8e-03
4e-02
Log-odds
0.5
1.0
1.5
2.0
2.5
3.0
3.5
4.0

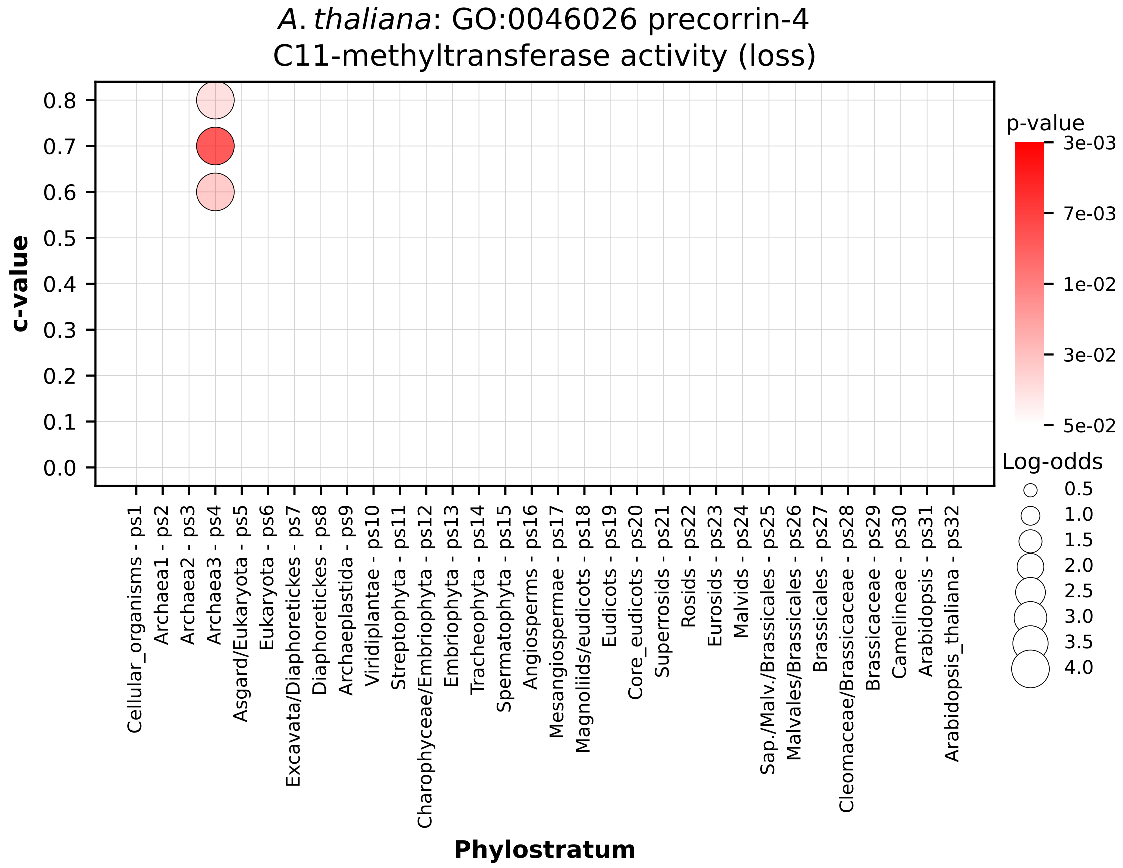

A. thaliana: GO:0046026 precorrin-4
C11-methyltransferase activity (loss)
c-value
0.8
0.7
0.6
0.5
0.4
0.3
0.2
0.1
0.0
p-value
3e-03
7e-03
1e-02
3e-02
5e-02
Log-odds
0.5
1.0
1.5
2.0
2.5
3.0
3.5
4.0
Cellular_organisms - ps1
Archaea1 - ps2
Archaea2 - ps3
Archaea3 - ps4
Asgard/Eukaryota - ps5
Eukaryota - ps6
Excavata/Diaphoretickes - ps7
Diaphoretickes - ps8
Archaeplastida - ps9
Viridiplantae - ps10
Streptophyta - ps11
Charophyceae/Embriophyta - ps12
Embriophyta - ps13
Tracheophyta - ps14
Spermatophyta - ps15
Angiosperms - ps16
Mesangiospermae - ps17
Magnoliids/eudicots - ps18
Eudicots - ps19
Core_eudicots - ps20
Superrosids - ps21
Rosids - ps22
Eurosids - ps23
Malvids - ps24
Sap./Malv./Brassicales - ps25
Malvales/Brassicales - ps26
Brassicales - ps27
Cleomaceae/Brassicaceae - ps28
Brassicaceae - ps29
Camelineae - ps30
Arabidopsis - ps31
Arabidopsis_thaliana - ps32
Phylostratum

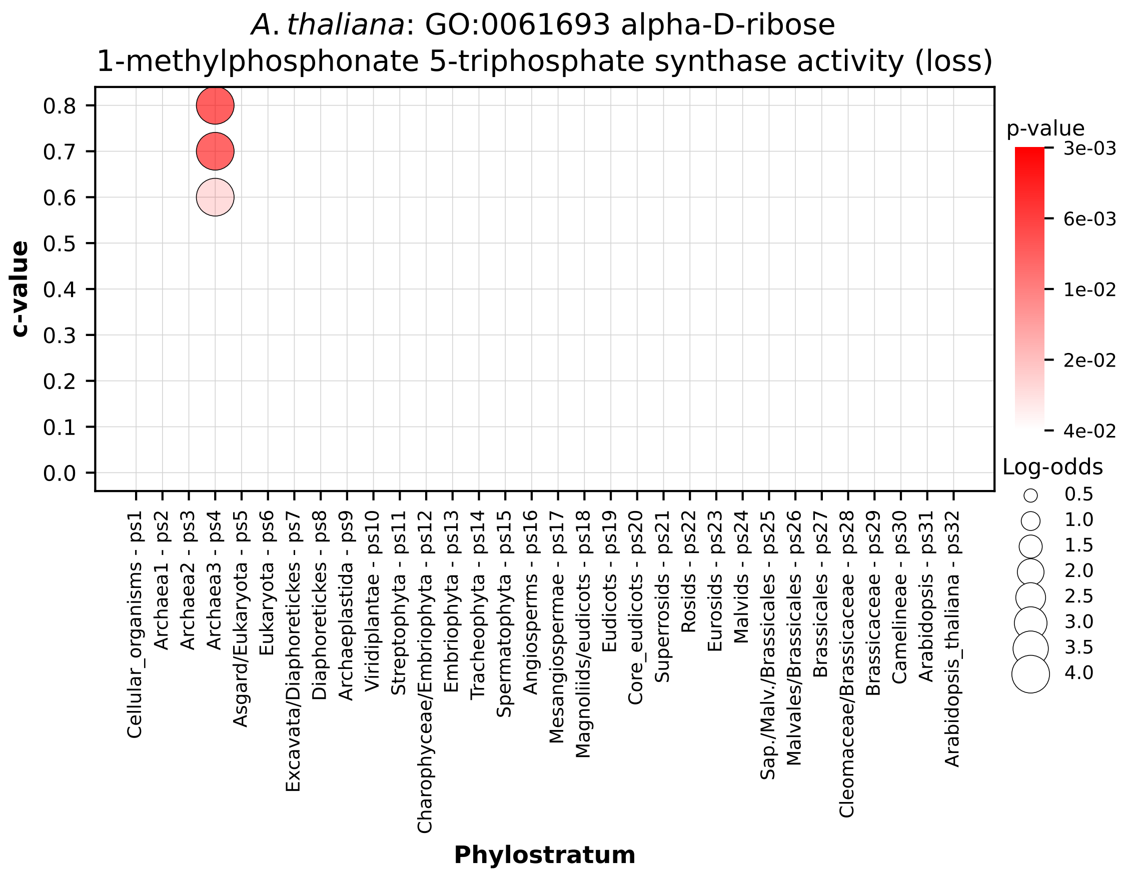

A. thaliana: GO:0061693 alpha-D-ribose 1-methylphosphonate 5-triphosphate synthase activity (loss)
c-value
0.8
0.7
0.6
0.5
0.4
0.3
0.2
0.1
0.0
p-value
3e-03
6e-03
1e-02
2e-02
4e-02
Log-odds
0.5
1.0
1.5
2.0
2.5
3.0
3.5
4.0
Cellular_organisms - ps1
Archaea1 - ps2
Archaea2 - ps3
Archaea3 - ps4
Asgard/Eukaryota - ps5
Eukaryota - ps6
Excavata/Diaphoretickes - ps7
Diaphoretickes - ps8
Archaeplastida - ps9
Viridiplantae - ps10
Streptophyta - ps11
Charophyceae/Embriophyta - ps12
Embriophyta - ps13
Tracheophyta - ps14
Spermatophyta - ps15
Angiosperms - ps16
Mesangiospermae - ps17
Magnoliids/eudicots - ps18
Eudicots - ps19
Core_eudicots - ps20
Superrosids - ps21
Rosids - ps22
Eurosids - ps23
Malvids - ps24
Sap./Malv./Brassicales - ps25
Malvales/Brassicales - ps26
Brassicales - ps27
Cleomaceae/Brassicaceae - ps28
Brassicaceae - ps29
Camelineae - ps30
Arabidopsis - ps31
Arabidopsis_thaliana - ps32
Phylostratum

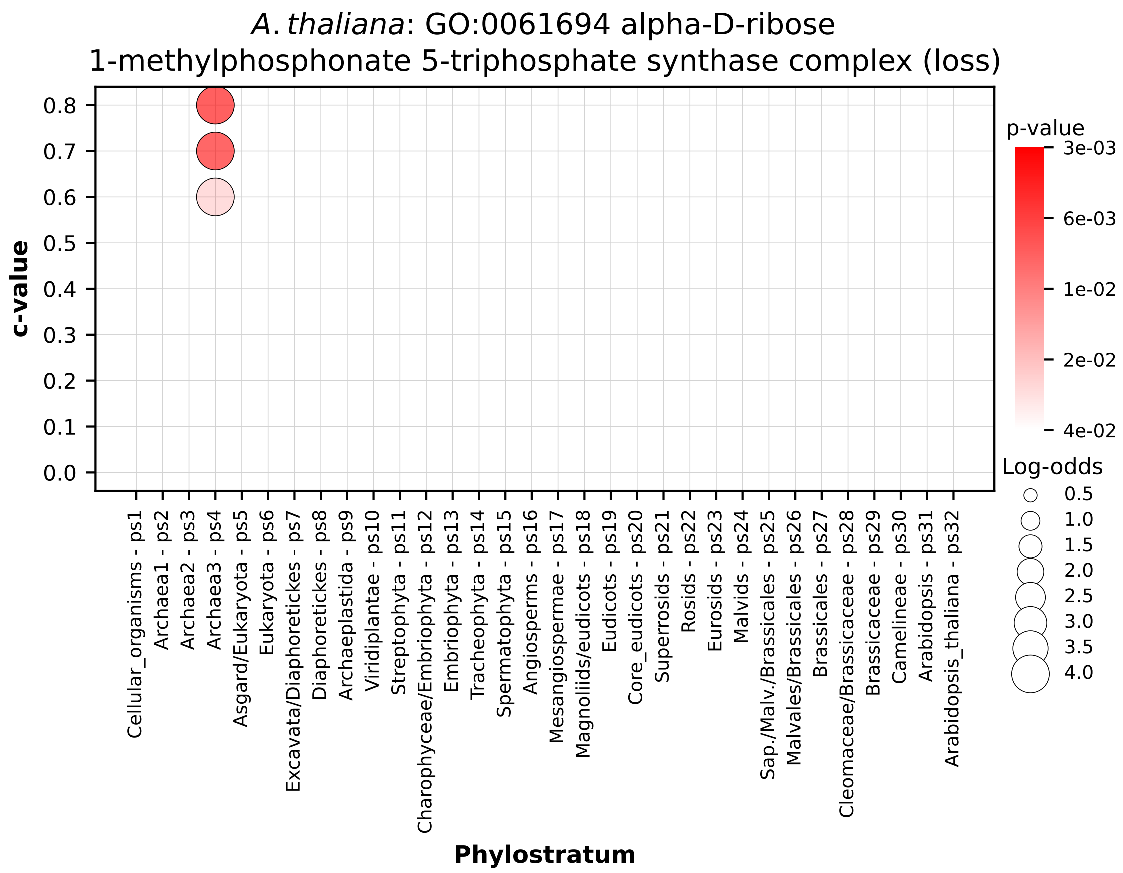

$A. thaliana$: GO:0061694 alpha-D-ribose
1-methylphosphonate 5-triphosphate synthase complex (loss)
c-value
0.0
0.1
0.2
0.3
0.4
0.5
0.6
0.7
0.8
Phylostratum
Cellular_organisms - ps1
Archaea1 - ps2
Archaea2 - ps3
Archaea3 - ps4
Asgard/Eukaryota - ps5
Eukaryota - ps6
Excavata/Diaphoretickes - ps7
Diaphoretickes - ps8
Archaeplastida - ps9
Viridiplantae - ps10
Streptophyta - ps11
Charophyceae/Embriophyta - ps12
Embriophyta - ps13
Tracheophyta - ps14
Spermatophyta - ps15
Angiosperms - ps16
Mesangiospermae - ps17
Magnoliids/eudicots - ps18
Eudicots - ps19
Core_eudicots - ps20
Superrosids - ps21
Rosids - ps22
Eurosids - ps23
Malvids - ps24
Sap./Malv./Brassicales - ps25
Malvales/Brassicales - ps26
Brassicales - ps27
Cleomaceae/Brassicaceae - ps28
Brassicaceae - ps29
Camelineae - ps30
Arabidopsis - ps31
Arabidopsis_thaliana - ps32
p-value
3e-03
6e-03
1e-02
2e-02
4e-02
Log-odds
0.5
1.0
1.5
2.0
2.5
3.0
3.5
4.0

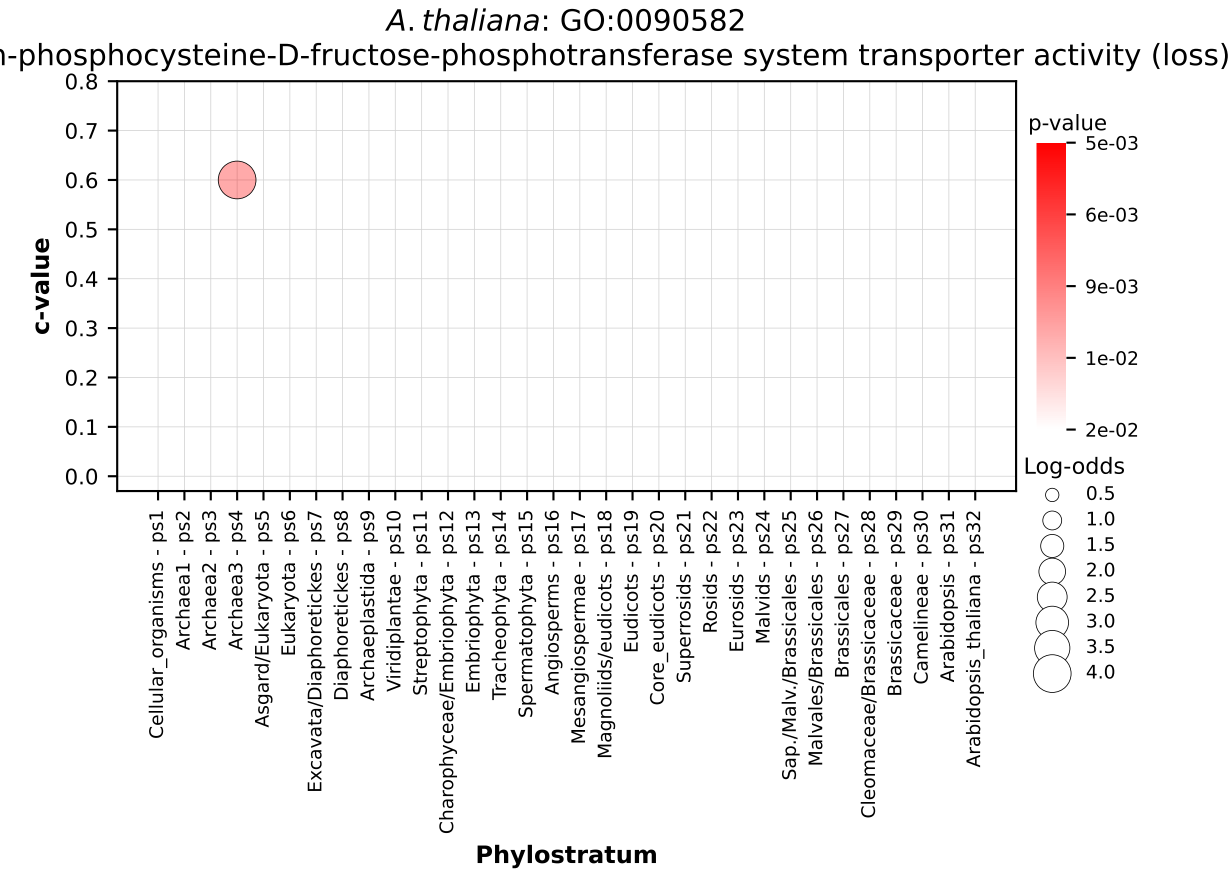
A. thaliana: GO:0090582
n-phosphocysteine-D-fructose-phosphotransferase system transporter activity (loss)
c-value
0.8
0.7
0.6
0.5
0.4
0.3
0.2
0.1
0.0
p-value
5e-03
6e-03
9e-03
1e-02
2e-02
Log-odds
0.5
1.0
1.5
2.0
2.5
3.0
3.5
4.0
Cellular_organisms - ps1
Archaea1 - ps2
Archaea2 - ps3
Archaea3 - ps4
Asgard/Eukaryota - ps5
Eukaryota - ps6
Excavata/Diaphoretickes - ps7
Diaphoretickes - ps8
Archaeplastida - ps9
Viridiplantae - ps10
Streptophyta - ps11
Charophyceae/Embriophyta - ps12
Embriophyta - ps13
Tracheophyta - ps14
Spermatophyta - ps15
Angiosperms - ps16
Mesangiospermae - ps17
Magnoliids/eudicots - ps18
Eudicots - ps19
Core_eudicots - ps20
Superrosids - ps21
Rosids - ps22
Eurosids - ps23
Malvids - ps24
Sap./Malv./Brassicales - ps25
Malvales/Brassicales - ps26
Brassicales - ps27
Cleomaceae/Brassicaceae - ps28
Brassicaceae - ps29
Camelineae - ps30
Arabidopsis - ps31
Arabidopsis_thaliana - ps32
Phylostratum

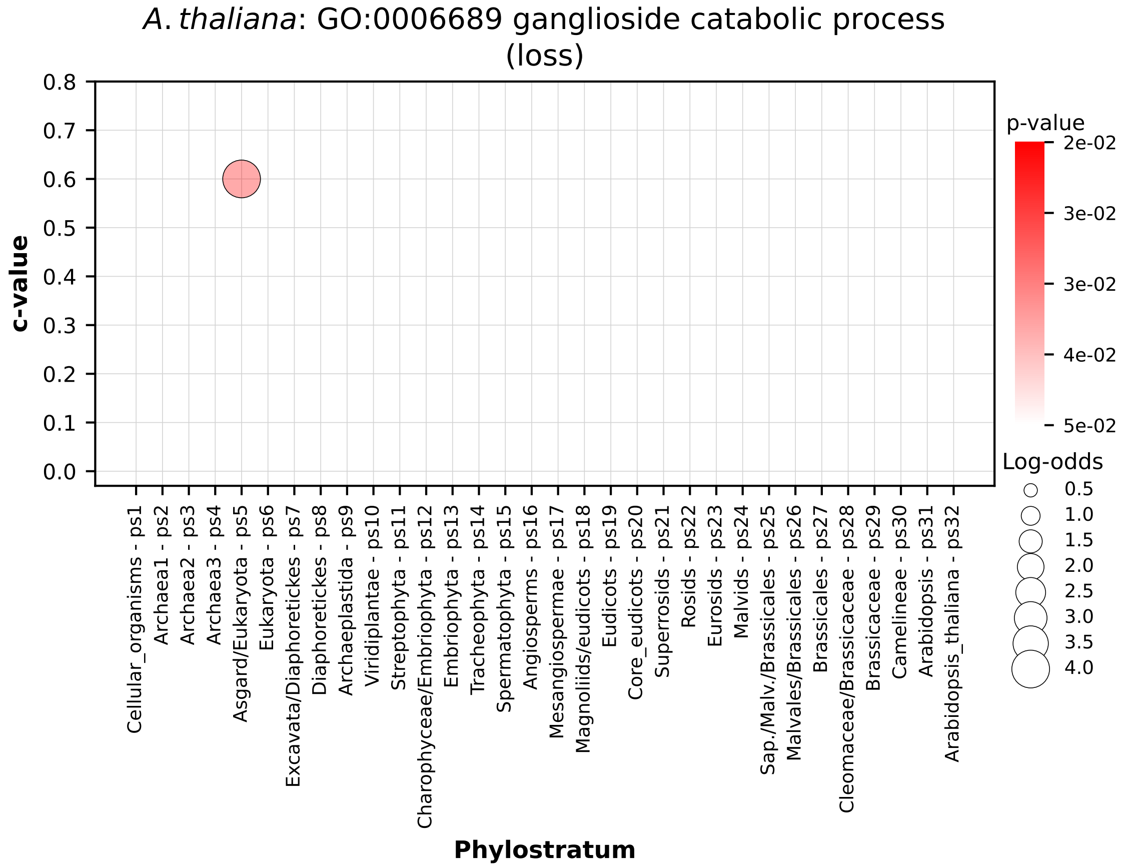

A. thaliana: GO:0006689 ganglioside catabolic process
(loss)
c-value
0.8
0.7
0.6
0.5
0.4
0.3
0.2
0.1
0.0
p-value
2e-02
3e-02
3e-02
4e-02
5e-02
Log-odds
0.5
1.0
1.5
2.0
2.5
3.0
3.5
4.0
Cellular_organisms - ps1
Archaea1 - ps2
Archaea2 - ps3
Archaea3 - ps4
Asgard/Eukaryota - ps5
Eukaryota - ps6
Excavata/Diaphoretickes - ps7
Diaphoretickes - ps8
Archaeplastida - ps9
Viridiplantae - ps10
Streptophyta - ps11
Charophyceae/Embriophyta - ps12
Embriophyta - ps13
Tracheophyta - ps14
Spermatophyta - ps15
Angiosperms - ps16
Mesangiospermae - ps17
Magnoliids/eudicots - ps18
Eudicots - ps19
Core_eudicots - ps20
Superrosids - ps21
Rosids - ps22
Eurosids - ps23
Malvids - ps24
Sap./Malv./Brassicales - ps25
Malvales/Brassicales - ps26
Brassicales - ps27
Cleomaceae/Brassicaceae - ps28
Brassicaceae - ps29
Camelineae - ps30
Arabidopsis - ps31
Arabidopsis_thaliana - ps32
Phylostratum

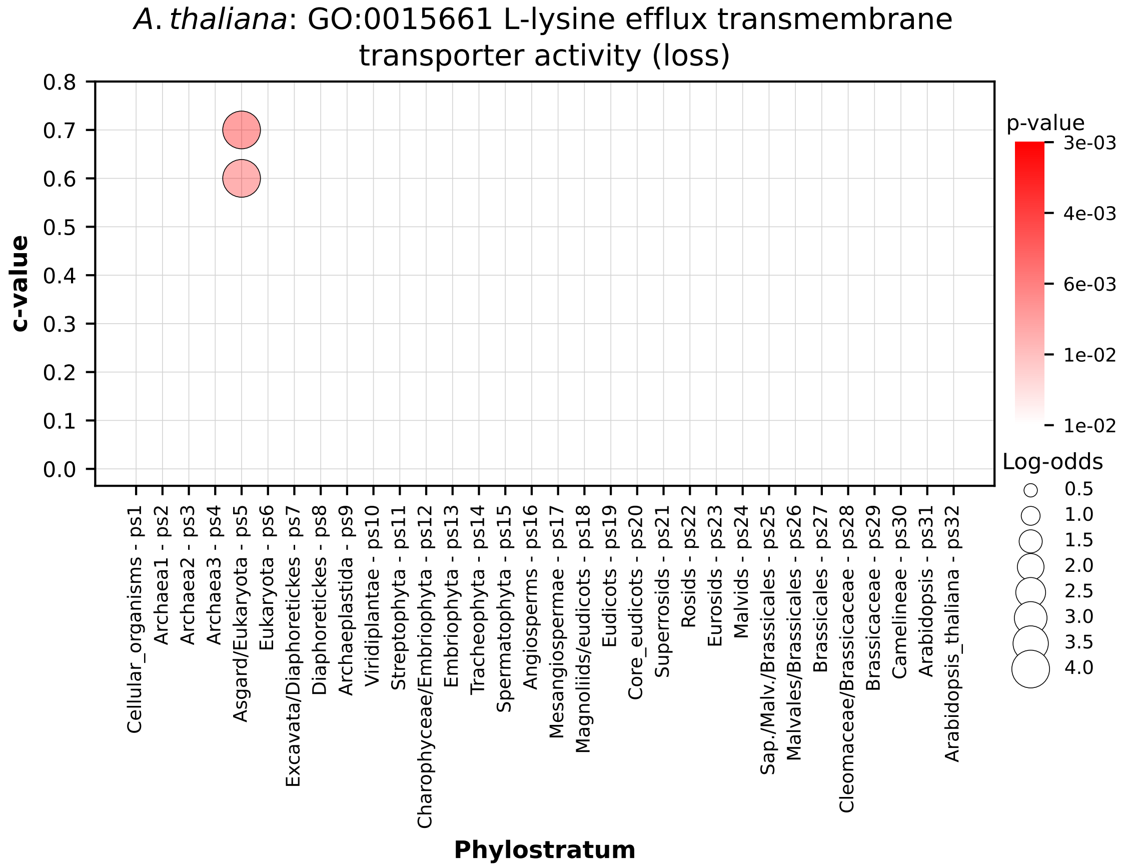

A. thaliana: GO:0015661 L-lysine efflux transmembrane transporter activity (loss)
c-value
0.8
0.7
0.6
0.5
0.4
0.3
0.2
0.1
0.0
p-value
3e-03
4e-03
6e-03
1e-02
1e-02
Log-odds
0.5
1.0
1.5
2.0
2.5
3.0
3.5
4.0
Cellular_organisms - ps1
Archaea1 - ps2
Archaea2 - ps3
Archaea3 - ps4
Asgard/Eukaryota - ps5
Eukaryota - ps6
Excavata/Diaphoretickes - ps7
Diaphoretickes - ps8
Archaeplastida - ps9
Viridiplantae - ps10
Streptophyta - ps11
Charophyceae/Embriophyta - ps12
Embriophyta - ps13
Tracheophyta - ps14
Spermatophyta - ps15
Angiosperms - ps16
Mesangiospermae - ps17
Magnoliids/eudicots - ps18
Eudicots - ps19
Core_eudicots - ps20
Superrosids - ps21
Rosids - ps22
Eurosids - ps23
Malvids - ps24
Sap./Malv./Brassicales - ps25
Malvales/Brassicales - ps26
Brassicales - ps27
Cleomaceae/Brassicaceae - ps28
Brassicaceae - ps29
Camelineae - ps30
Arabidopsis - ps31
Arabidopsis_thaliana - ps32
Phylostratum

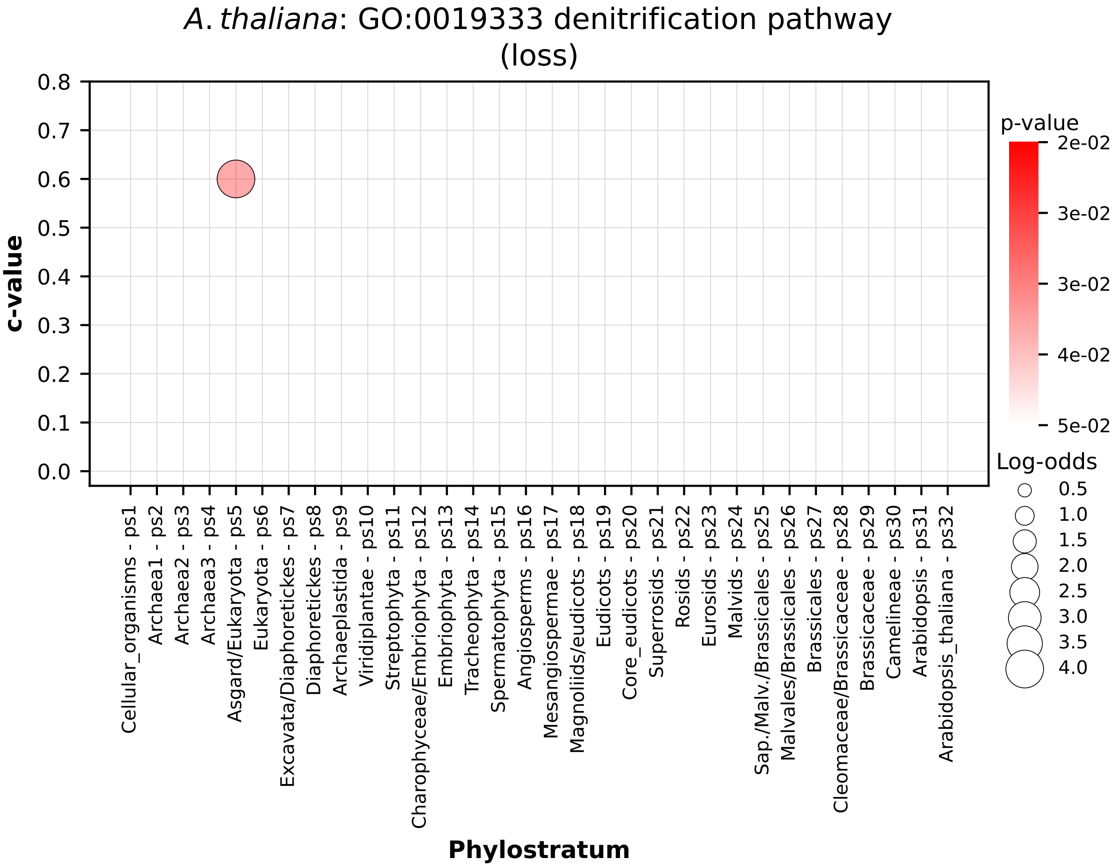

A. thaliana: GO:0019333 denitrification pathway
(loss)
c-value
0.8
0.7
0.6
0.5
0.4
0.3
0.2
0.1
0.0
p-value
2e-02
3e-02
3e-02
4e-02
5e-02
Log-odds
0.5
1.0
1.5
2.0
2.5
3.0
3.5
4.0
Cellular_organisms - ps1
Archaea1 - ps2
Archaea2 - ps3
Archaea3 - ps4
Asgard/Eukaryota - ps5
Eukaryota - ps6
Excavata/Diaphoretickes - ps7
Diaphoretickes - ps8
Archaeplastida - ps9
Viridiplantae - ps10
Streptophyta - ps11
Charophyceae/Embriophyta - ps12
Embriophyta - ps13
Tracheophyta - ps14
Spermatophyta - ps15
Angiosperms - ps16
Mesangiospermae - ps17
Magnoliids/eudicots - ps18
Eudicots - ps19
Core_eudicots - ps20
Superrosids - ps21
Rosids - ps22
Eurosids - ps23
Malvids - ps24
Sap./Malv./Brassicales - ps25
Malvales/Brassicales - ps26
Brassicales - ps27
Cleomaceae/Brassicaceae - ps28
Brassicaceae - ps29
Camelineae - ps30
Arabidopsis - ps31
Arabidopsis_thaliana - ps32
Phylostratum

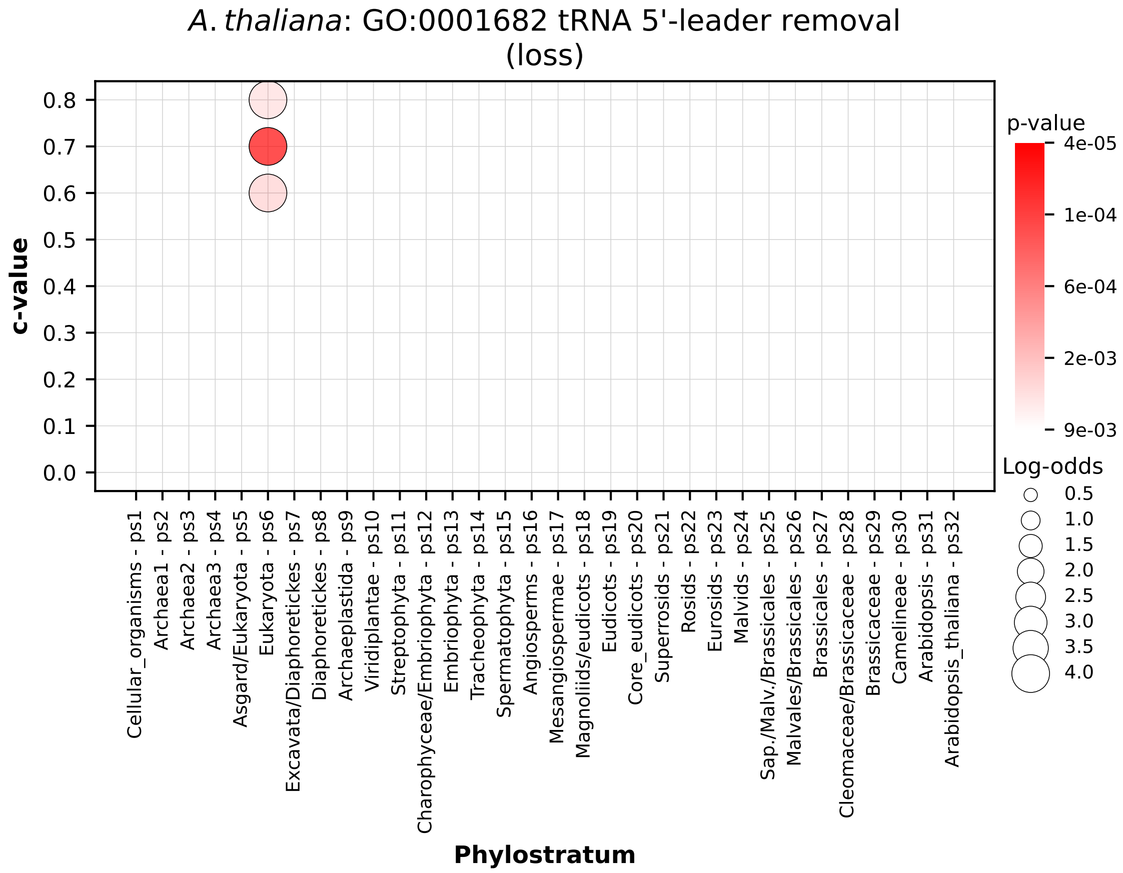

A. thaliana: GO:0001682 tRNA 5'-leader removal
(loss)
c-value
0.8
0.7
0.6
0.5
0.4
0.3
0.2
0.1
0.0
p-value
4e-05
1e-04
6e-04
2e-03
9e-03
Log-odds
0.5
1.0
1.5
2.0
2.5
3.0
3.5
4.0
Cellular_organisms - ps1
Archaea1 - ps2
Archaea2 - ps3
Archaea3 - ps4
Asgard/Eukaryota - ps5
Eukaryota - ps6
Excavata/Diaphoretickes - ps7
Diaphoretickes - ps8
Archaeplastida - ps9
Viridiplantae - ps10
Streptophyta - ps11
Charophyceae/Embriophyta - ps12
Embriophyta - ps13
Tracheophyta - ps14
Spermatophyta - ps15
Angiosperms - ps16
Mesangiospermae - ps17
Magnoliids/eudicots - ps18
Eudicots - ps19
Core_eudicots - ps20
Superrosids - ps21
Rosids - ps22
Eurosids - ps23
Malvids - ps24
Sap./Malv./Brassicales - ps25
Malvales/Brassicales - ps26
Brassicales - ps27
Cleomaceae/Brassicaceae - ps28
Brassicaceae - ps29
Camelineae - ps30
Arabidopsis - ps31
Arabidopsis_thaliana - ps32
Phylostratum

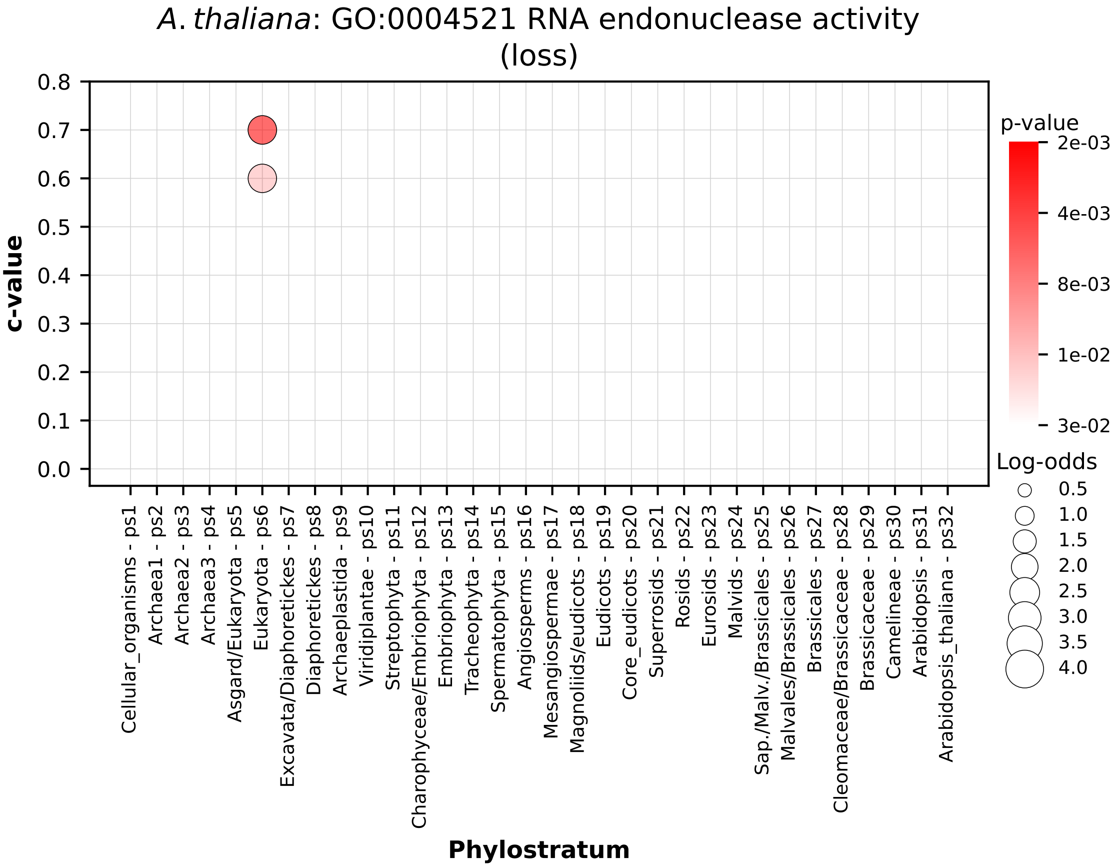

A. thaliana: GO:0004521 RNA endonuclease activity
(loss)
c-value
0.8
0.7
0.6
0.5
0.4
0.3
0.2
0.1
0.0
Phylostratum
Cellular_organisms - ps1
Archaea1 - ps2
Archaea2 - ps3
Archaea3 - ps4
Asgard/Eukaryota - ps5
Eukaryota - ps6
Excavata/Diaphoretickes - ps7
Diaphoretickes - ps8
Archaeplastida - ps9
Viridiplantae - ps10
Streptophyta - ps11
Charophyceae/Embriophyta - ps12
Embriophyta - ps13
Tracheophyta - ps14
Spermatophyta - ps15
Angiosperms - ps16
Mesangiospermae - ps17
Magnoliids/eudicots - ps18
Eudicots - ps19
Core_eudicots - ps20
Superrosids - ps21
Rosids - ps22
Eurosids - ps23
Malvids - ps24
Sap./Malv./Brassicales - ps25
Malvales/Brassicales - ps26
Brassicales - ps27
Cleomaceae/Brassicaceae - ps28
Brassicaceae - ps29
Camelineae - ps30
Arabidopsis - ps31
Arabidopsis_thaliana - ps32
p-value
2e-03
4e-03
8e-03
1e-02
3e-02
Log-odds
0.5
1.0
1.5
2.0
2.5
3.0
3.5
4.0

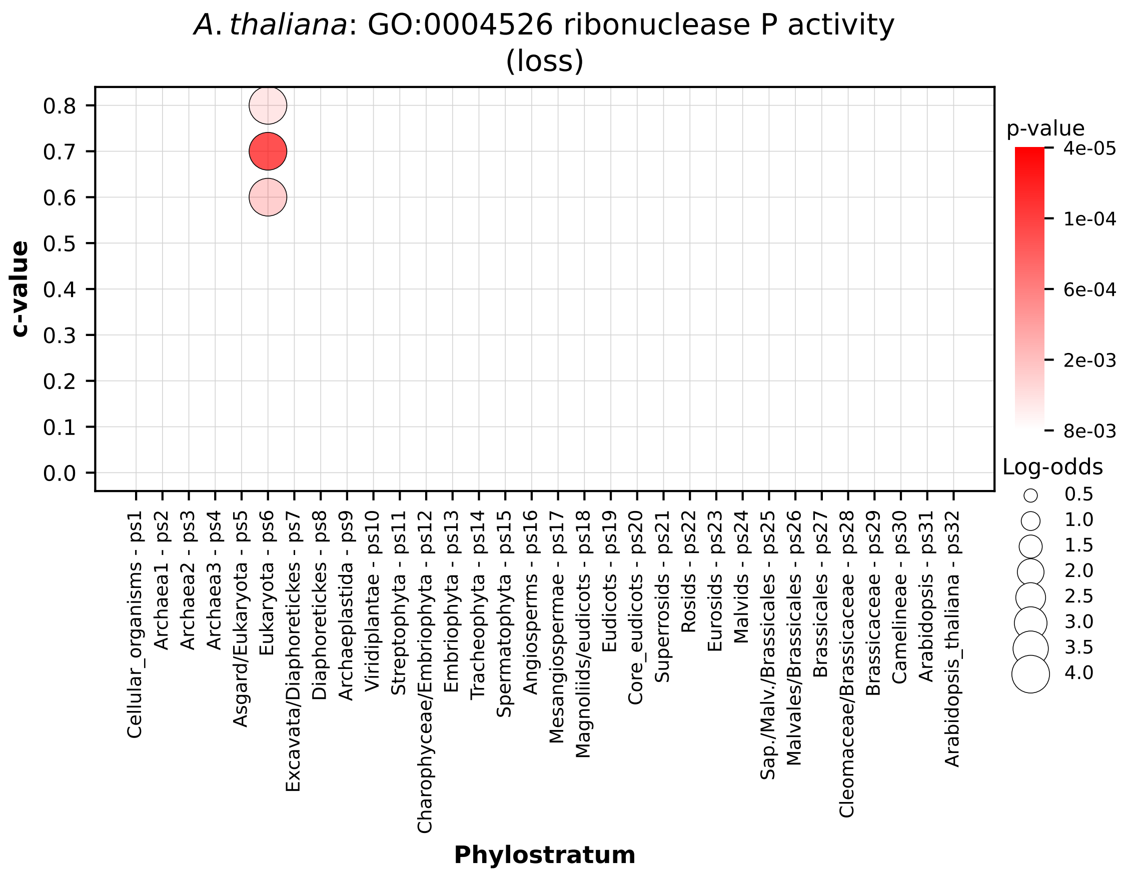

A. thaliana: GO:0004526 ribonuclease P activity
(loss)
c-value
0.8
0.7
0.6
0.5
0.4
0.3
0.2
0.1
0.0
p-value
4e-05
1e-04
6e-04
2e-03
8e-03
Log-odds
0.5
1.0
1.5
2.0
2.5
3.0
3.5
4.0
Cellular_organisms - ps1
Archaea1 - ps2
Archaea2 - ps3
Archaea3 - ps4
Asgard/Eukaryota - ps5
Eukaryota - ps6
Excavata/Diaphoretickes - ps7
Diaphoretickes - ps8
Archaeplastida - ps9
Viridiplantae - ps10
Streptophyta - ps11
Charophyceae/Embriophyta - ps12
Embriophyta - ps13
Tracheophyta - ps14
Spermatophyta - ps15
Angiosperms - ps16
Mesangiospermae - ps17
Magnoliids/eudicots - ps18
Eudicots - ps19
Core_eudicots - ps20
Superrosids - ps21
Rosids - ps22
Eurosids - ps23
Malvids - ps24
Sap./Malv./Brassicales - ps25
Malvales/Brassicales - ps26
Brassicales - ps27
Cleomaceae/Brassicaceae - ps28
Brassicaceae - ps29
Camelineae - ps30
Arabidopsis - ps31
Arabidopsis_thaliana - ps32
Phylostratum

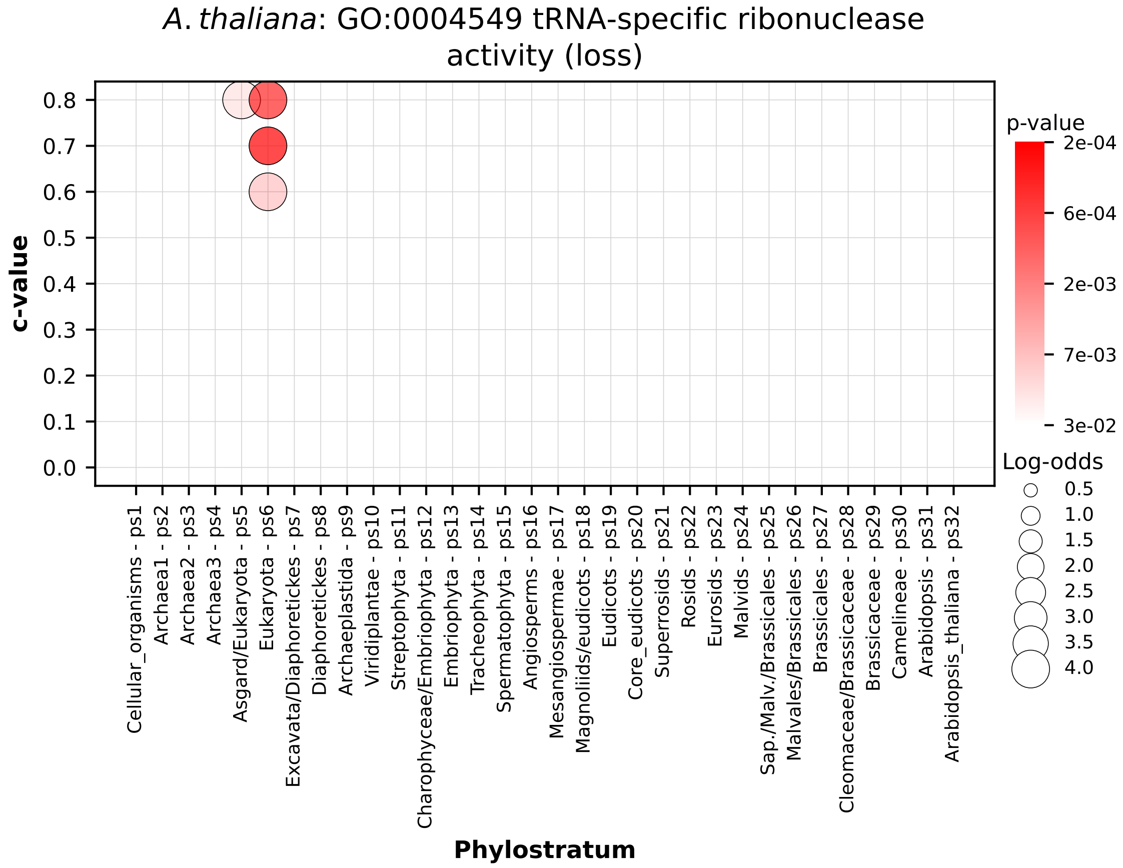

A. thaliana: GO:0004549 tRNA-specific ribonuclease activity (loss)
c-value
0.8
0.7
0.6
0.5
0.4
0.3
0.2
0.1
0.0
p-value
2e-04
6e-04
2e-03
7e-03
3e-02
Log-odds
0.5
1.0
1.5
2.0
2.5
3.0
3.5
4.0
Cellular_organisms - ps1
Archaea1 - ps2
Archaea2 - ps3
Archaea3 - ps4
Asgard/Eukaryota - ps5
Eukaryota - ps6
Excavata/Diaphoretickes - ps7
Diaphoretickes - ps8
Archaeplastida - ps9
Viridiplantae - ps10
Streptophyta - ps11
Charophyceae/Embriophyta - ps12
Embriophyta - ps13
Tracheophyta - ps14
Spermatophyta - ps15
Angiosperms - ps16
Mesangiospermae - ps17
Magnoliids/eudicots - ps18
Eudicots - ps19
Core_eudicots - ps20
Superrosids - ps21
Rosids - ps22
Eurosids - ps23
Malvids - ps24
Sap./Malv./Brassicales - ps25
Malvales/Brassicales - ps26
Brassicales - ps27
Cleomaceae/Brassicaceae - ps28
Brassicaceae - ps29
Camelineae - ps30
Arabidopsis - ps31
Arabidopsis_thaliana - ps32
Phylostratum

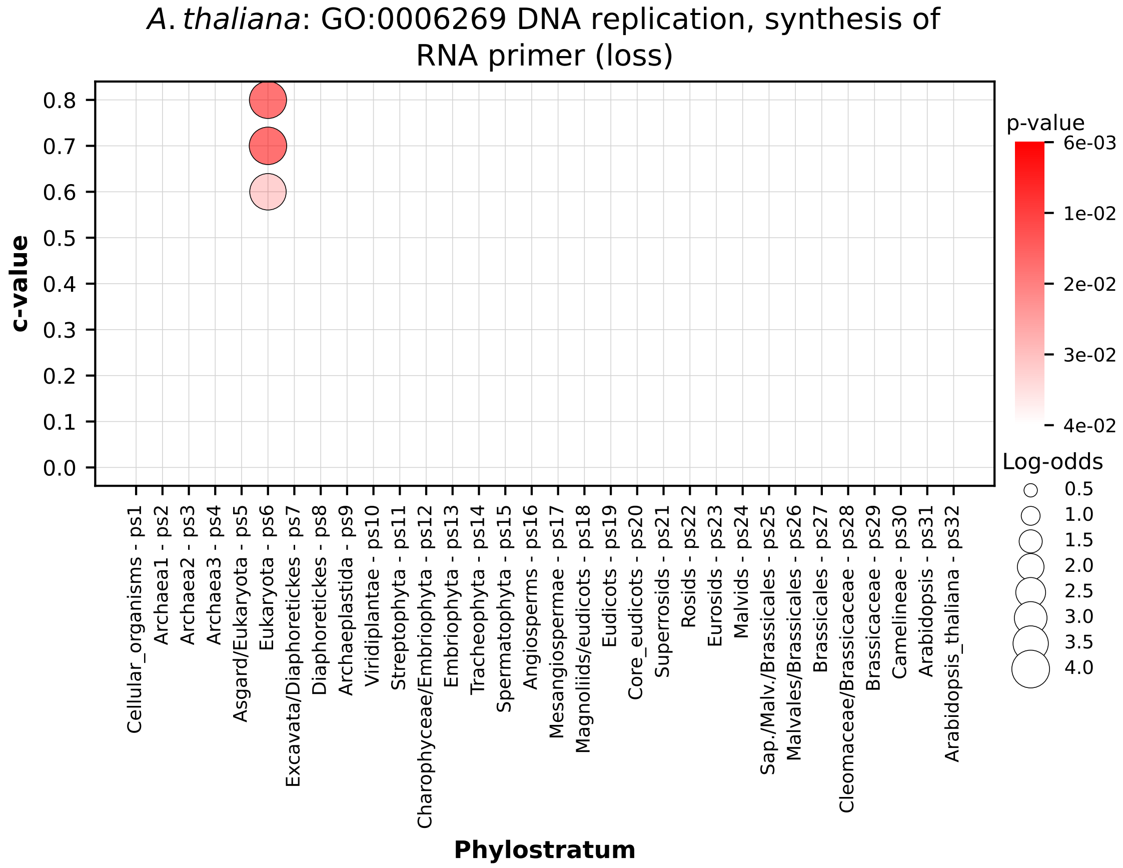

A. thaliana: GO:0006269 DNA replication, synthesis of RNA primer (loss)
c-value
0.8
0.7
0.6
0.5
0.4
0.3
0.2
0.1
0.0
p-value
6e-03
1e-02
2e-02
3e-02
4e-02
Log-odds
0.5
1.0
1.5
2.0
2.5
3.0
3.5
4.0
Cellular_organisms - ps1
Archaea1 - ps2
Archaea2 - ps3
Archaea3 - ps4
Asgard/Eukaryota - ps5
Eukaryota - ps6
Excavata/Diaphoretickes - ps7
Diaphoretickes - ps8
Archaeplastida - ps9
Viridiplantae - ps10
Streptophyta - ps11
Charophyceae/Embriophyta - ps12
Embriophyta - ps13
Tracheophyta - ps14
Spermatophyta - ps15
Angiosperms - ps16
Mesangiospermae - ps17
Magnoliids/eudicots - ps18
Eudicots - ps19
Core_eudicots - ps20
Superrosids - ps21
Rosids - ps22
Eurosids - ps23
Malvids - ps24
Sap./Malv./Brassicales - ps25
Malvales/Brassicales - ps26
Brassicales - ps27
Cleomaceae/Brassicaceae - ps28
Brassicaceae - ps29
Camelineae - ps30
Arabidopsis - ps31
Arabidopsis_thaliana - ps32
Phylostratum

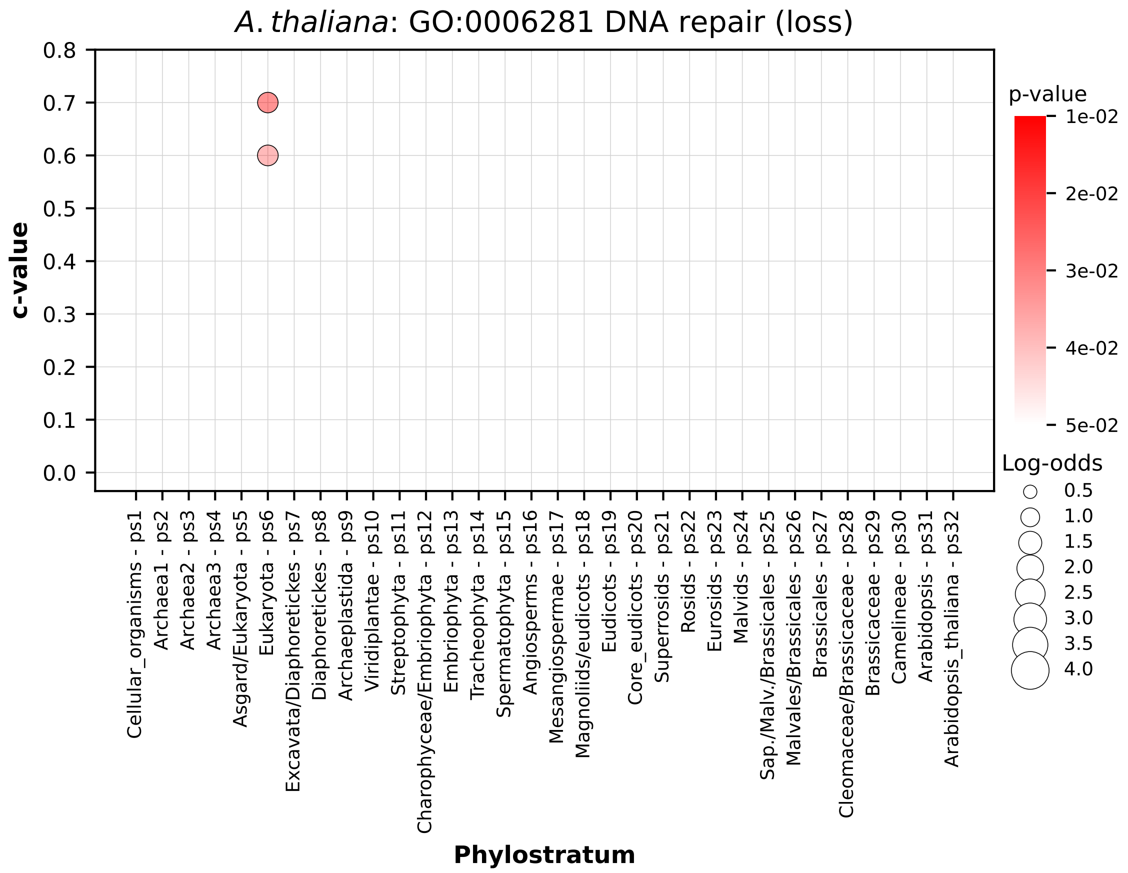

A. thaliana: GO:0006281 DNA repair (loss)
c-value
0.8
0.7
0.6
0.5
0.4
0.3
0.2
0.1
0.0
p-value
1e-02
2e-02
3e-02
4e-02
5e-02
Log-odds
0.5
1.0
1.5
2.0
2.5
3.0
3.5
4.0
Cellular_organisms - ps1
Archaea1 - ps2
Archaea2 - ps3
Archaea3 - ps4
Asgard/Eukaryota - ps5
Eukaryota - ps6
Excavata/Diaphoretickes - ps7
Diaphoretickes - ps8
Archaeplastida - ps9
Viridiplantae - ps10
Streptophyta - ps11
Charophyceae/Embriophyta - ps12
Embriophyta - ps13
Tracheophyta - ps14
Spermatophyta - ps15
Angiosperms - ps16
Mesangiospermae - ps17
Magnoliids/eudicots - ps18
Eudicots - ps19
Core_eudicots - ps20
Superrosids - ps21
Rosids - ps22
Eurosids - ps23
Malvids - ps24
Sap./Malv./Brassicales - ps25
Malvales/Brassicales - ps26
Brassicales - ps27
Cleomaceae/Brassicaceae - ps28
Brassicaceae - ps29
Camelineae - ps30
Arabidopsis - ps31
Arabidopsis_thaliana - ps32
Phylostratum

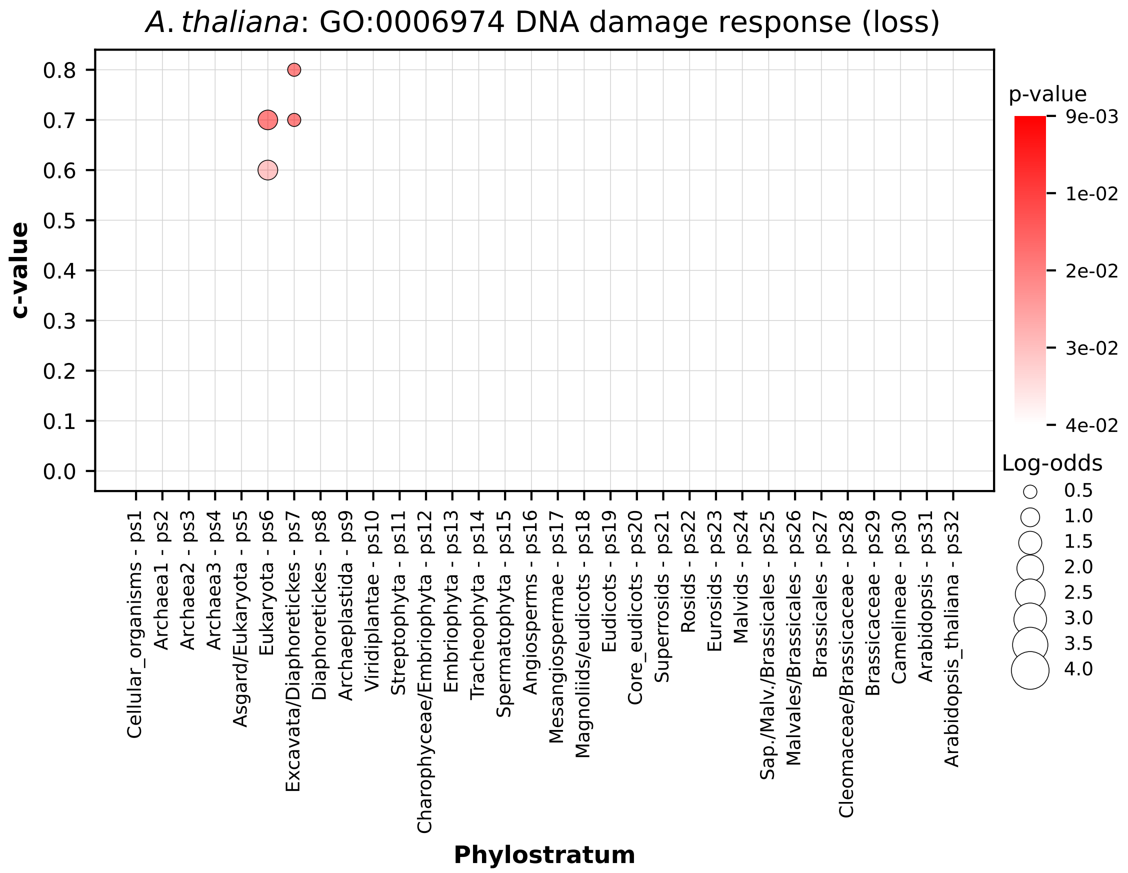

A. thaliana: GO:0006974 DNA damage response (loss)
c-value
0.0
0.1
0.2
0.3
0.4
0.5
0.6
0.7
0.8
Phylostratum
Cellular_organisms - ps1
Archaea1 - ps2
Archaea2 - ps3
Archaea3 - ps4
Asgard/Eukaryota - ps5
Eukaryota - ps6
Excavata/Diaphoretickes - ps7
Diaphoretickes - ps8
Archaeplastida - ps9
Viridiplantae - ps10
Streptophyta - ps11
Charophyceae/Embriophyta - ps12
Embriophyta - ps13
Tracheophyta - ps14
Spermatophyta - ps15
Angiosperms - ps16
Mesangiospermae - ps17
Magnoliids/eudicots - ps18
Eudicots - ps19
Core_eudicots - ps20
Superrosids - ps21
Rosids - ps22
Eurosids - ps23
Malvids - ps24
Sap./Malv./Brassicales - ps25
Malvales/Brassicales - ps26
Brassicales - ps27
Cleomaceae/Brassicaceae - ps28
Brassicaceae - ps29
Camelineae - ps30
Arabidopsis - ps31
Arabidopsis_thaliana - ps32
p-value
9e-03
1e-02
2e-02
3e-02
4e-02
Log-odds
0.5
1.0
1.5
2.0
2.5
3.0
3.5
4.0

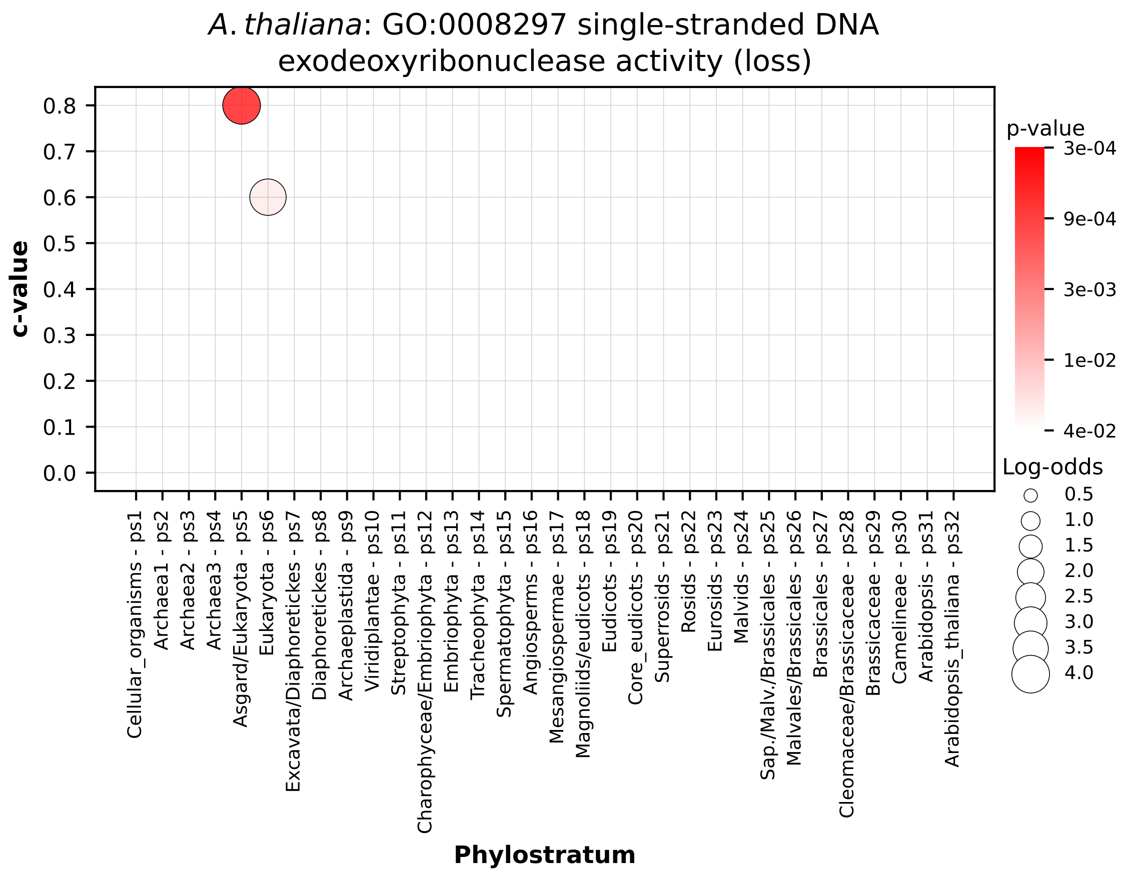

A. thaliana: GO:0008297 single-stranded DNA exodeoxyribonuclease activity (loss)
c-value
0.8
0.7
0.6
0.5
0.4
0.3
0.2
0.1
0.0
p-value
3e-04
9e-04
3e-03
1e-02
4e-02
Log-odds
0.5
1.0
1.5
2.0
2.5
3.0
3.5
4.0
Cellular_organisms - ps1
Archaea1 - ps2
Archaea2 - ps3
Archaea3 - ps4
Asgard/Eukaryota - ps5
Eukaryota - ps6
Excavata/Diaphoretickes - ps7
Diaphoretickes - ps8
Archaeplastida - ps9
Viridiplantae - ps10
Streptophyta - ps11
Charophyceae/Embriophyta - ps12
Embriophyta - ps13
Tracheophyta - ps14
Spermatophyta - ps15
Angiosperms - ps16
Mesangiospermae - ps17
Magnoliids/eudicots - ps18
Eudicots - ps19
Core_eudicots - ps20
Superrosids - ps21
Rosids - ps22
Eurosids - ps23
Malvids - ps24
Sap./Malv./Brassicales - ps25
Malvales/Brassicales - ps26
Brassicales - ps27
Cleomaceae/Brassicaceae - ps28
Brassicaceae - ps29
Camelineae - ps30
Arabidopsis - ps31
Arabidopsis_thaliana - ps32
Phylostratum

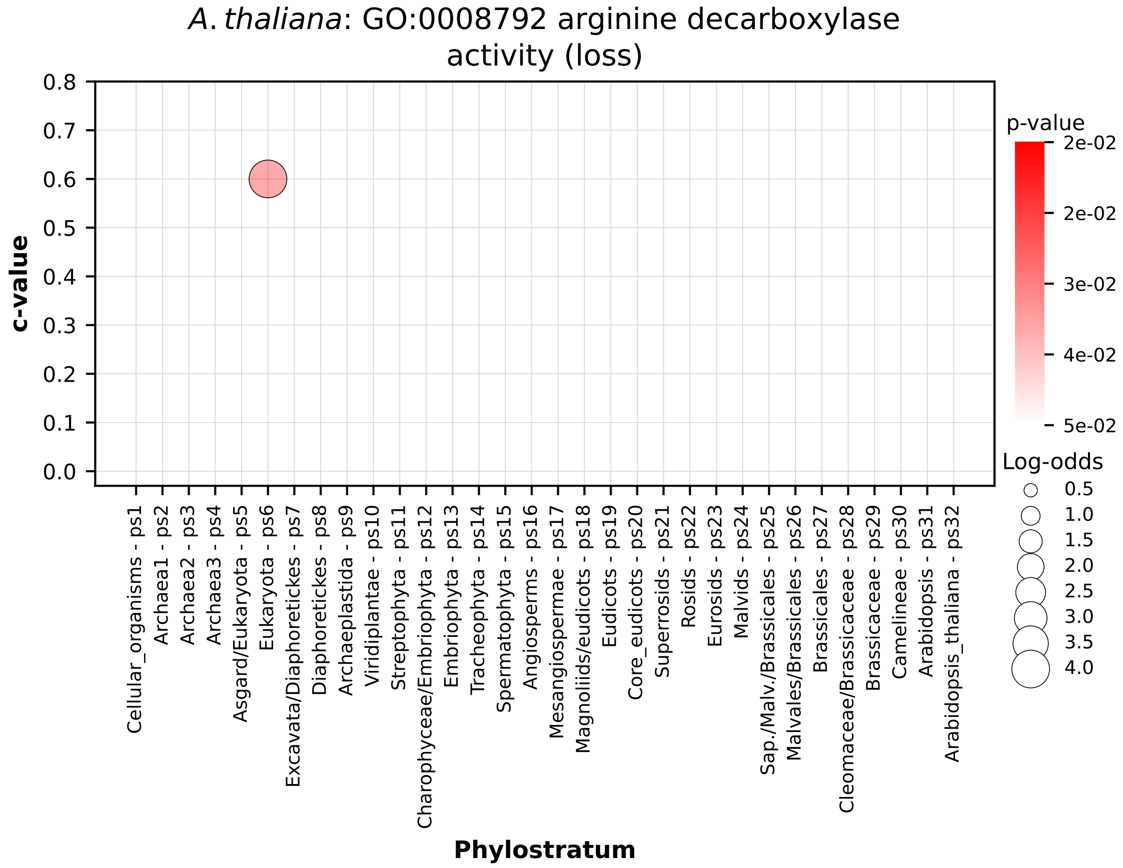

A. thaliana: GO:0008792 arginine decarboxylase activity (loss)
c-value
0.8
0.7
0.6
0.5
0.4
0.3
0.2
0.1
0.0
Phylostratum
Cellular_organisms - ps1
Archaea1 - ps2
Archaea2 - ps3
Archaea3 - ps4
Asgard/Eukaryota - ps5
Eukaryota - ps6
Excavata/Diaphoretickes - ps7
Diaphoretickes - ps8
Archaeplastida - ps9
Viridiplantae - ps10
Streptophyta - ps11
Charophyceae/Embriophyta - ps12
Embriophyta - ps13
Tracheophyta - ps14
Spermatophyta - ps15
Angiosperms - ps16
Mesangiospermae - ps17
Magnoliids/eudicots - ps18
Eudicots - ps19
Core_eudicots - ps20
Superrosids - ps21
Rosids - ps22
Eurosids - ps23
Malvids - ps24
Sap./Malv./Brassicales - ps25
Malvales/Brassicales - ps26
Brassicales - ps27
Cleomaceae/Brassicaceae - ps28
Brassicaceae - ps29
Camelineae - ps30
Arabidopsis - ps31
Arabidopsis_thaliana - ps32
p-value
2e-02
2e-02
3e-02
4e-02
5e-02
Log-odds
0.5
1.0
1.5
2.0
2.5
3.0
3.5
4.0

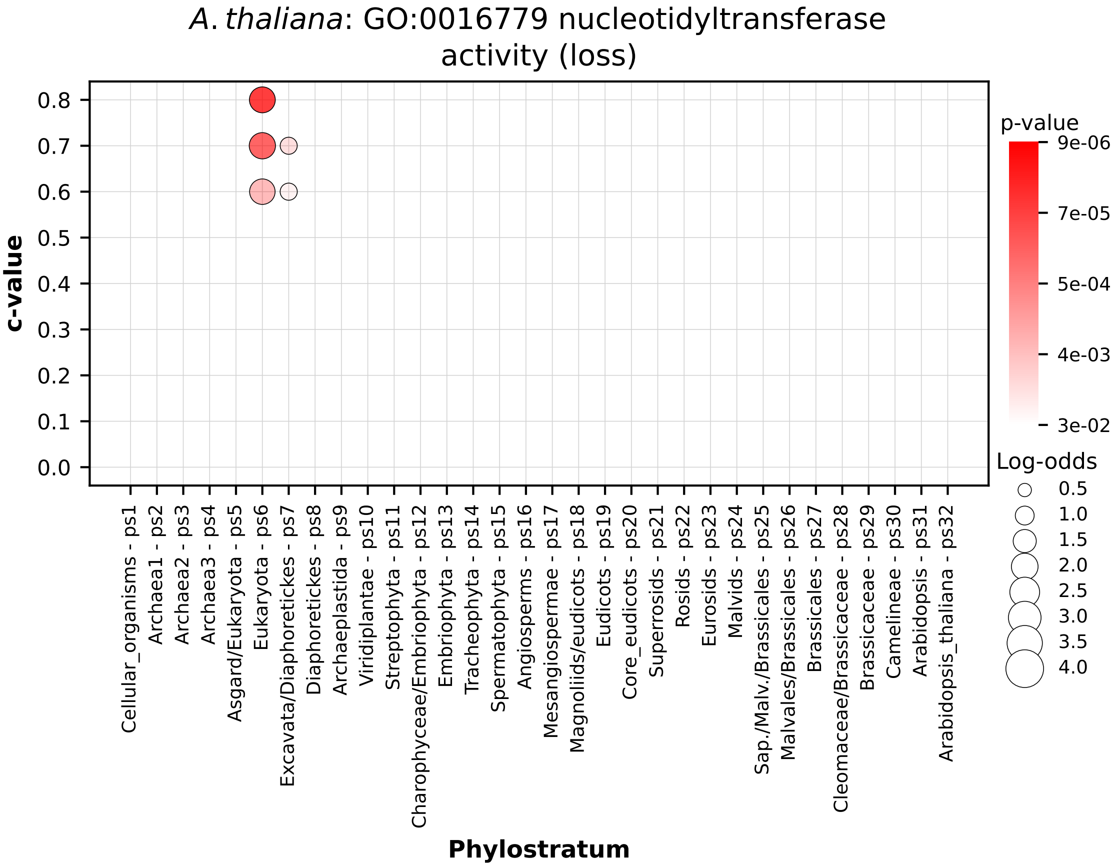

A. thaliana: GO:0016779 nucleotidyltransferase activity (loss)
c-value
0.0
0.1
0.2
0.3
0.4
0.5
0.6
0.7
0.8
p-value
9e-06
7e-05
5e-04
4e-03
3e-02
Log-odds
0.5
1.0
1.5
2.0
2.5
3.0
3.5
4.0
Cellular_organisms - ps1
Archaea1 - ps2
Archaea2 - ps3
Archaea3 - ps4
Asgard/Eukaryota - ps5
Eukaryota - ps6
Excavata/Diaphoretickes - ps7
Diaphoretickes - ps8
Archaeplastida - ps9
Viridiplantae - ps10
Streptophyta - ps11
Charophyceae/Embriophyta - ps12
Embriophyta - ps13
Tracheophyta - ps14
Spermatophyta - ps15
Angiosperms - ps16
Mesangiospermae - ps17
Magnoliids/eudicots - ps18
Eudicots - ps19
Core_eudicots - ps20
Superrosids - ps21
Rosids - ps22
Eurosids - ps23
Malvids - ps24
Sap./Malv./Brassicales - ps25
Malvales/Brassicales - ps26
Brassicales - ps27
Cleomaceae/Brassicaceae - ps28
Brassicaceae - ps29
Camelineae - ps30
Arabidopsis - ps31
Arabidopsis_thaliana - ps32
Phylostratum

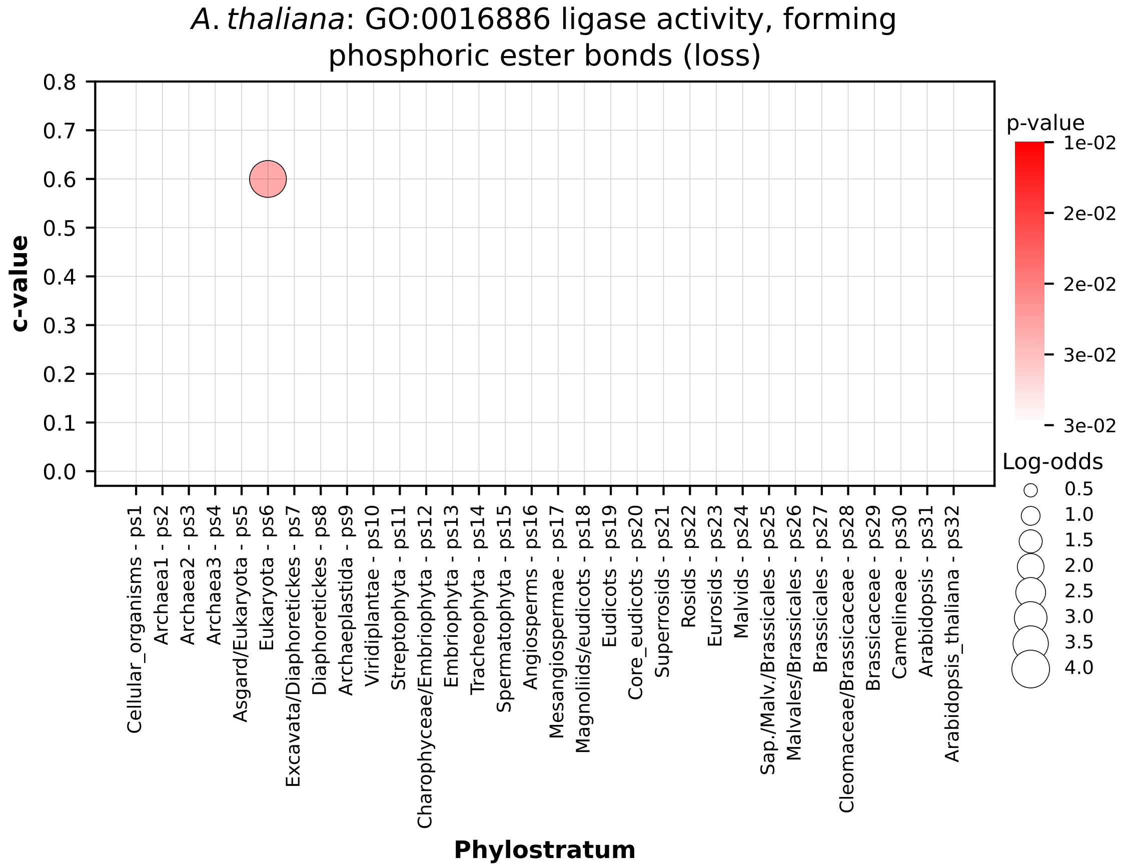

A. thaliana: GO:0016886 ligase activity, forming phosphoric ester bonds (loss)
c-value
0.8
0.7
0.6
0.5
0.4
0.3
0.2
0.1
0.0
p-value
1e-02
2e-02
2e-02
3e-02
3e-02
Log-odds
0.5
1.0
1.5
2.0
2.5
3.0
3.5
4.0
Cellular_organisms - ps1
Archaea1 - ps2
Archaea2 - ps3
Archaea3 - ps4
Asgard/Eukaryota - ps5
Eukaryota - ps6
Excavata/Diaphoretickes - ps7
Diaphoretickes - ps8
Archaeplastida - ps9
Viridiplantae - ps10
Streptophyta - ps11
Charophyceae/Embriophyta - ps12
Embriophyta - ps13
Tracheophyta - ps14
Spermatophyta - ps15
Angiosperms - ps16
Mesangiospermae - ps17
Magnoliids/eudicots - ps18
Eudicots - ps19
Core_eudicots - ps20
Superrosids - ps21
Rosids - ps22
Eurosids - ps23
Malvids - ps24
Sap./Malv./Brassicales - ps25
Malvales/Brassicales - ps26
Brassicales - ps27
Cleomaceae/Brassicaceae - ps28
Brassicaceae - ps29
Camelineae - ps30
Arabidopsis - ps31
Arabidopsis_thaliana - ps32
Phylostratum

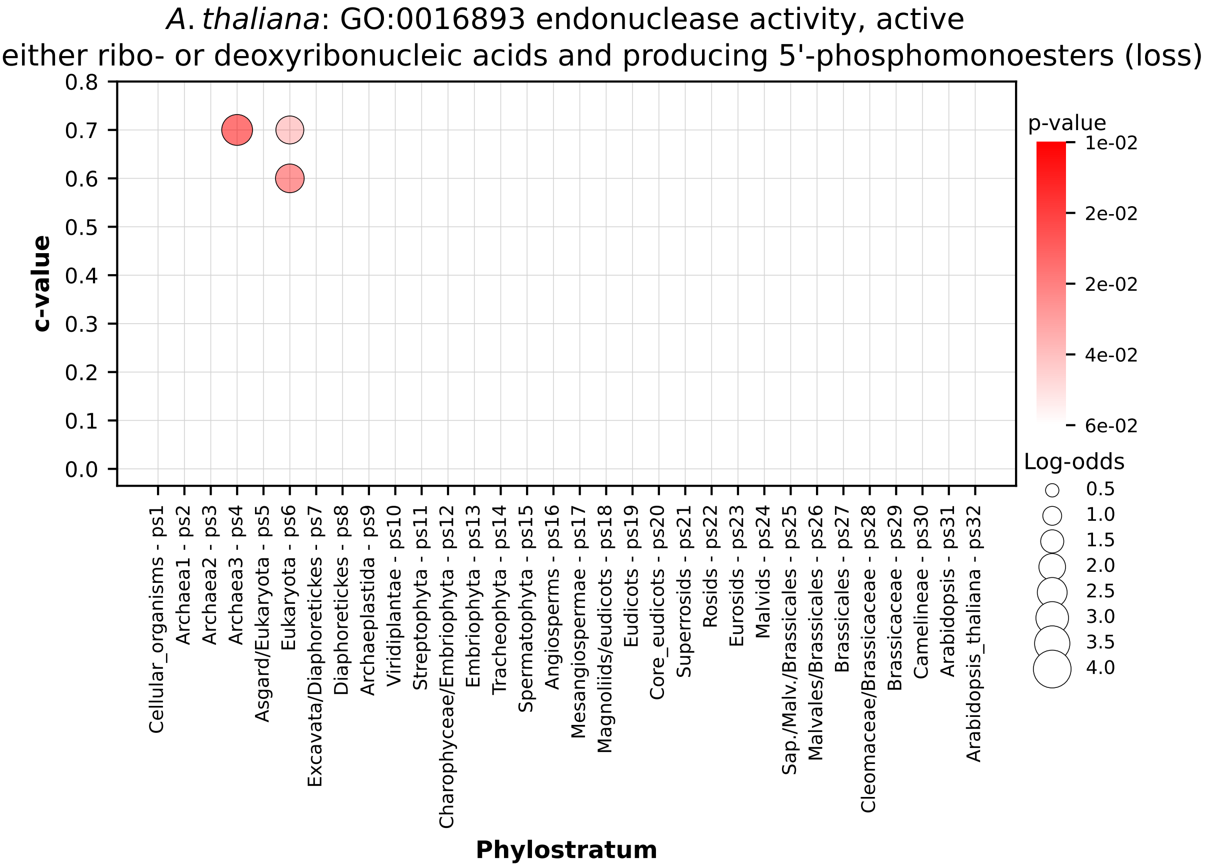

A. thaliana: GO:0016893 endonuclease activity, active
either ribo- or deoxyribonucleic acids and producing 5'-phosphomonoesters (loss)
c-value
0.8
0.7
0.6
0.5
0.4
0.3
0.2
0.1
0.0
p-value
1e-02
2e-02
2e-02
4e-02
6e-02
Log-odds
0.5
1.0
1.5
2.0
2.5
3.0
3.5
4.0
Cellular_organisms - ps1
Archaea1 - ps2
Archaea2 - ps3
Archaea3 - ps4
Asgard/Eukaryota - ps5
Eukaryota - ps6
Excavata/Diaphoretickes - ps7
Diaphoretickes - ps8
Archaeplastida - ps9
Viridiplantae - ps10
Streptophyta - ps11
Charophyceae/Embriophyta - ps12
Embriophyta - ps13
Tracheophyta - ps14
Spermatophyta - ps15
Angiosperms - ps16
Mesangiospermae - ps17
Magnoliids/eudicots - ps18
Eudicots - ps19
Core_eudicots - ps20
Superrosids - ps21
Rosids - ps22
Eurosids - ps23
Malvids - ps24
Sap./Malv./Brassicales - ps25
Malvales/Brassicales - ps26
Brassicales - ps27
Cleomaceae/Brassicaceae - ps28
Brassicaceae - ps29
Camelineae - ps30
Arabidopsis - ps31
Arabidopsis_thaliana - ps32
Phylostratum

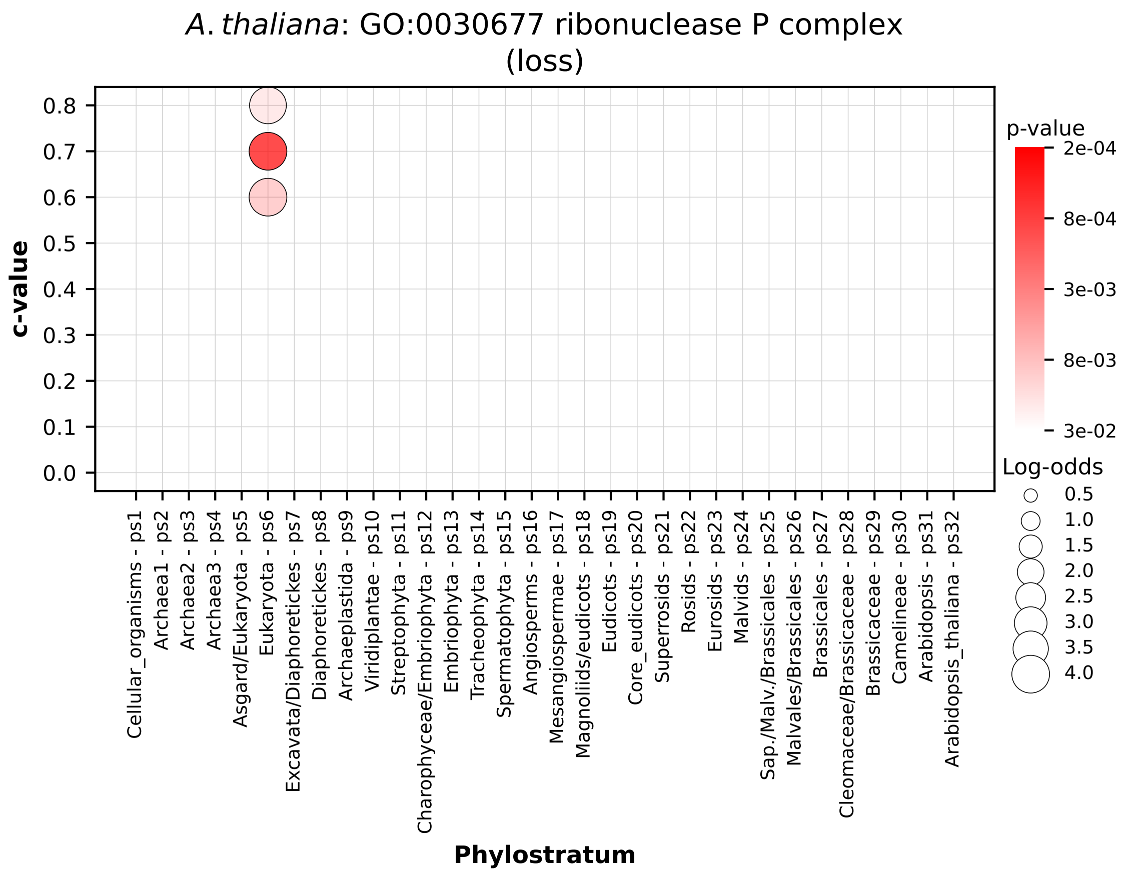
A. thaliana: GO:0030677 ribonuclease P complex
(loss)
c-value
0.0
0.1
0.2
0.3
0.4
0.5
0.6
0.7
0.8
p-value
2e-04
8e-04
3e-03
8e-03
3e-02
Log-odds
0.5
1.0
1.5
2.0
2.5
3.0
3.5
4.0
Cellular_organisms - ps1
Archaea1 - ps2
Archaea2 - ps3
Archaea3 - ps4
Asgard/Eukaryota - ps5
Eukaryota - ps6
Excavata/Diaphoretickes - ps7
Diaphoretickes - ps8
Archaeplastida - ps9
Viridiplantae - ps10
Streptophyta - ps11
Charophyceae/Embriophyta - ps12
Embriophyta - ps13
Tracheophyta - ps14
Spermatophyta - ps15
Angiosperms - ps16
Mesangiospermae - ps17
Magnoliids/eudicots - ps18
Eudicots - ps19
Core_eudicots - ps20
Superrosids - ps21
Rosids - ps22
Eurosids - ps23
Malvids - ps24
Sap./Malv./Brassicales - ps25
Malvales/Brassicales - ps26
Brassicales - ps27
Cleomaceae/Brassicaceae - ps28
Brassicaceae - ps29
Camelineae - ps30
Arabidopsis - ps31
Arabidopsis_thaliana - ps32
Phylostratum

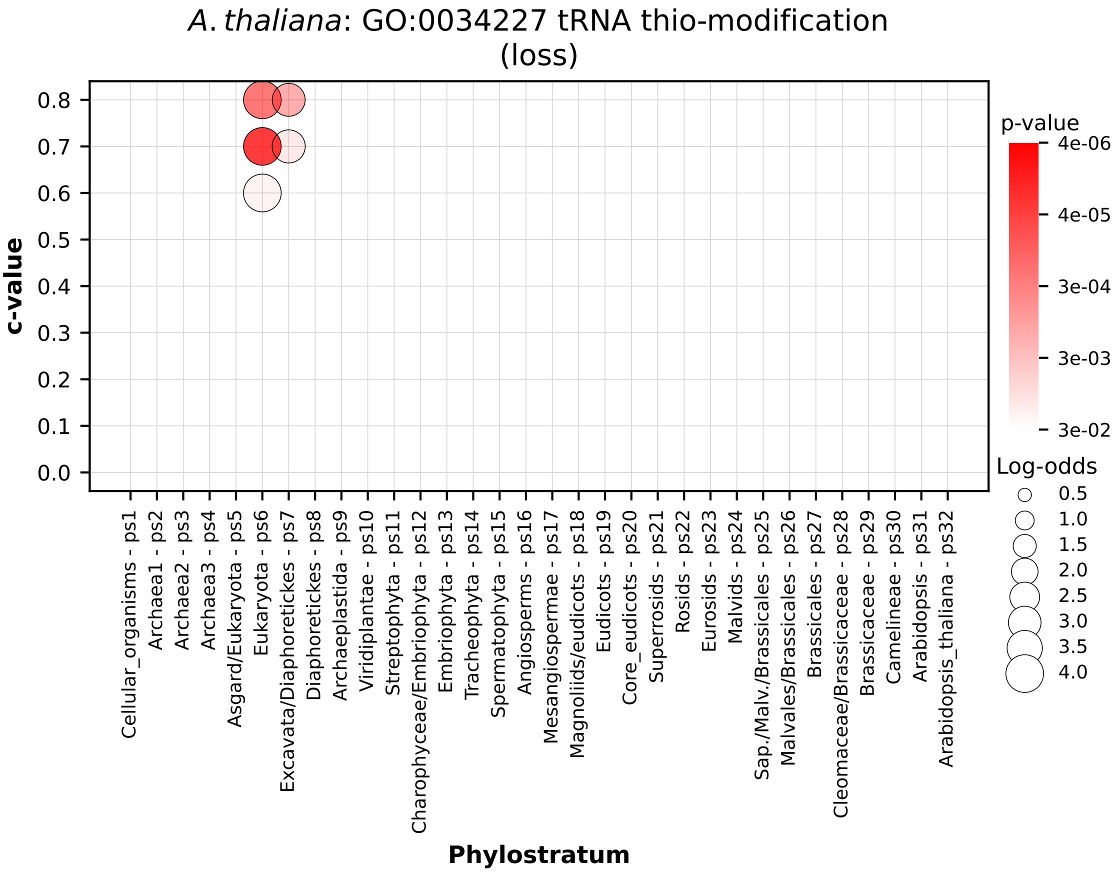

A. thaliana: GO:0034227 tRNA thio-modification
(loss)
c-value
0.0
0.1
0.2
0.3
0.4
0.5
0.6
0.7
0.8
Phylostratum
Cellular_organisms - ps1
Archaea1 - ps2
Archaea2 - ps3
Archaea3 - ps4
Asgard/Eukaryota - ps5
Eukaryota - ps6
Excavata/Diaphoretickes - ps7
Diaphoretickes - ps8
Archaeplastida - ps9
Viridiplantae - ps10
Streptophyta - ps11
Charophyceae/Embriophyta - ps12
Embriophyta - ps13
Tracheophyta - ps14
Spermatophyta - ps15
Angiosperms - ps16
Mesangiospermae - ps17
Magnoliids/eudicots - ps18
Eudicots - ps19
Core_eudicots - ps20
Superrosids - ps21
Rosids - ps22
Eurosids - ps23
Malvids - ps24
Sap./Malv./Brassicales - ps25
Malvales/Brassicales - ps26
Brassicales - ps27
Cleomaceae/Brassicaceae - ps28
Brassicaceae - ps29
Camelineae - ps30
Arabidopsis - ps31
Arabidopsis_thaliana - ps32
p-value
4e-06
4e-05
3e-04
3e-03
3e-02
Log-odds
0.5
1.0
1.5
2.0
2.5
3.0
3.5
4.0

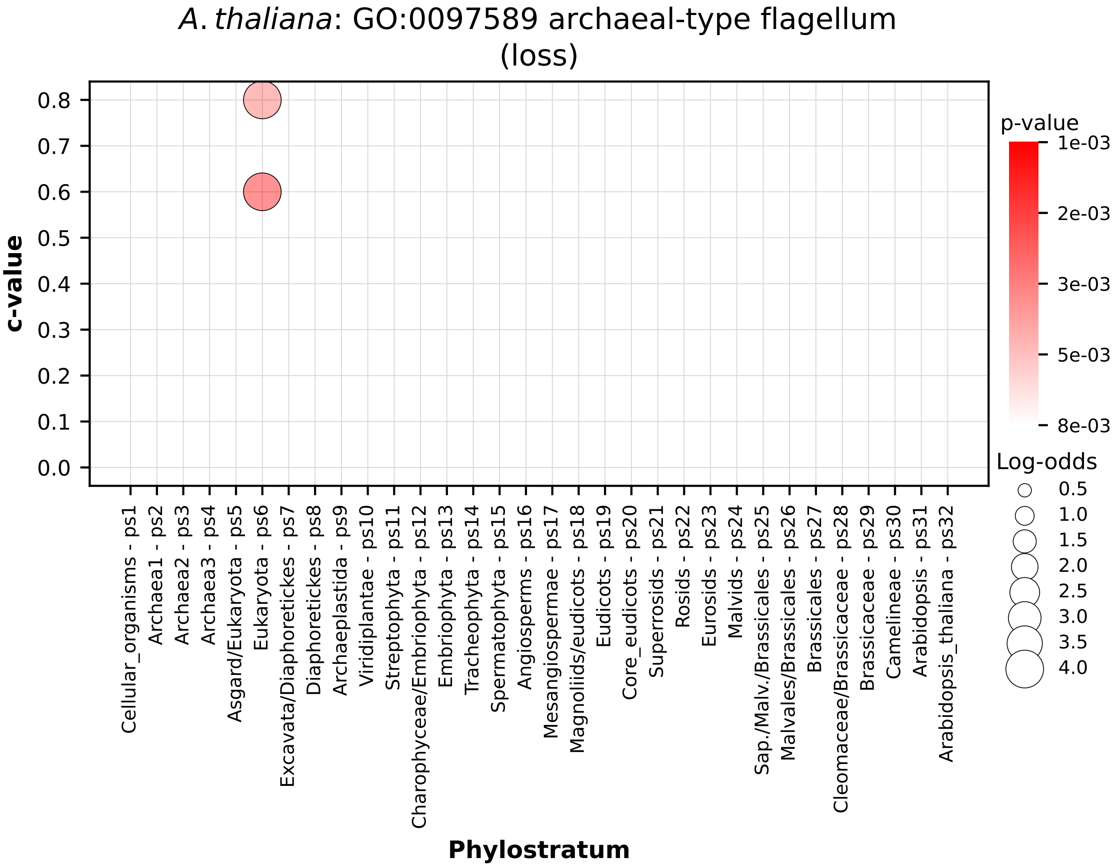

A. thaliana: GO:0097589 archaeal-type flagellum
(loss)
c-value
0.0
0.1
0.2
0.3
0.4
0.5
0.6
0.7
0.8
p-value
1e-03
2e-03
3e-03
5e-03
8e-03
Log-odds
0.5
1.0
1.5
2.0
2.5
3.0
3.5
4.0
Cellular_organisms - ps1
Archaea1 - ps2
Archaea2 - ps3
Archaea3 - ps4
Asgard/Eukaryota - ps5
Eukaryota - ps6
Excavata/Diaphoretickes - ps7
Diaphoretickes - ps8
Archaeplastida - ps9
Viridiplantae - ps10
Streptophyta - ps11
Charophyceae/Embriophyta - ps12
Embriophyta - ps13
Tracheophyta - ps14
Spermatophyta - ps15
Angiosperms - ps16
Mesangiospermae - ps17
Magnoliids/eudicots - ps18
Eudicots - ps19
Core_eudicots - ps20
Superrosids - ps21
Rosids - ps22
Eurosids - ps23
Malvids - ps24
Sap./Malv./Brassicales - ps25
Malvales/Brassicales - ps26
Brassicales - ps27
Cleomaceae/Brassicaceae - ps28
Brassicaceae - ps29
Camelineae - ps30
Arabidopsis - ps31
Arabidopsis_thaliana - ps32
Phylostratum

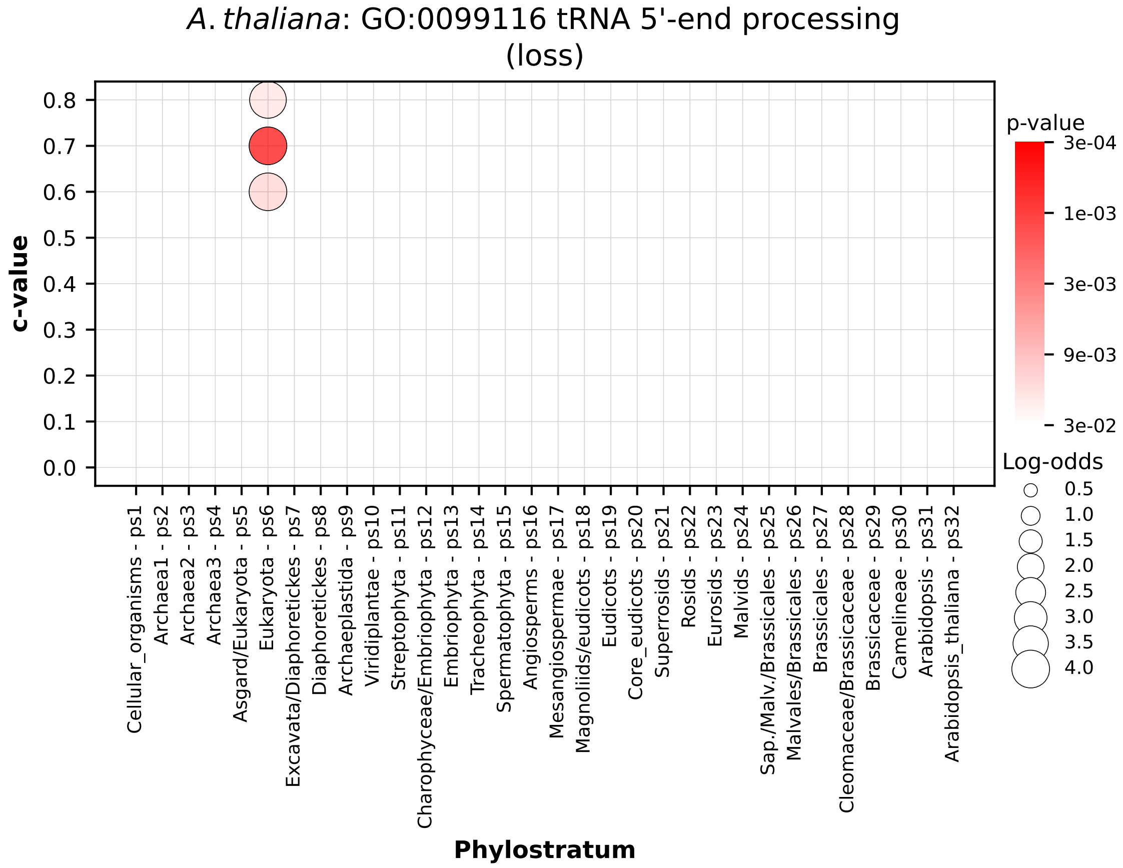

A. thaliana: GO:0099116 tRNA 5'-end processing
(loss)
c-value
0.8
0.7
0.6
0.5
0.4
0.3
0.2
0.1
0.0
p-value
3e-04
1e-03
3e-03
9e-03
3e-02
Log-odds
0.5
1.0
1.5
2.0
2.5
3.0
3.5
4.0
Cellular_organisms - ps1
Archaea1 - ps2
Archaea2 - ps3
Archaea3 - ps4
Asgard/Eukaryota - ps5
Eukaryota - ps6
Excavata/Diaphoretickes - ps7
Diaphoretickes - ps8
Archaeplastida - ps9
Viridiplantae - ps10
Streptophyta - ps11
Charophyceae/Embriophyta - ps12
Embriophyta - ps13
Tracheophyta - ps14
Spermatophyta - ps15
Angiosperms - ps16
Mesangiospermae - ps17
Magnoliids/eudicots - ps18
Eudicots - ps19
Core_eudicots - ps20
Superrosids - ps21
Rosids - ps22
Eurosids - ps23
Malvids - ps24
Sap./Malv./Brassicales - ps25
Malvales/Brassicales - ps26
Brassicales - ps27
Cleomaceae/Brassicaceae - ps28
Brassicaceae - ps29
Camelineae - ps30
Arabidopsis - ps31
Arabidopsis_thaliana - ps32
Phylostratum

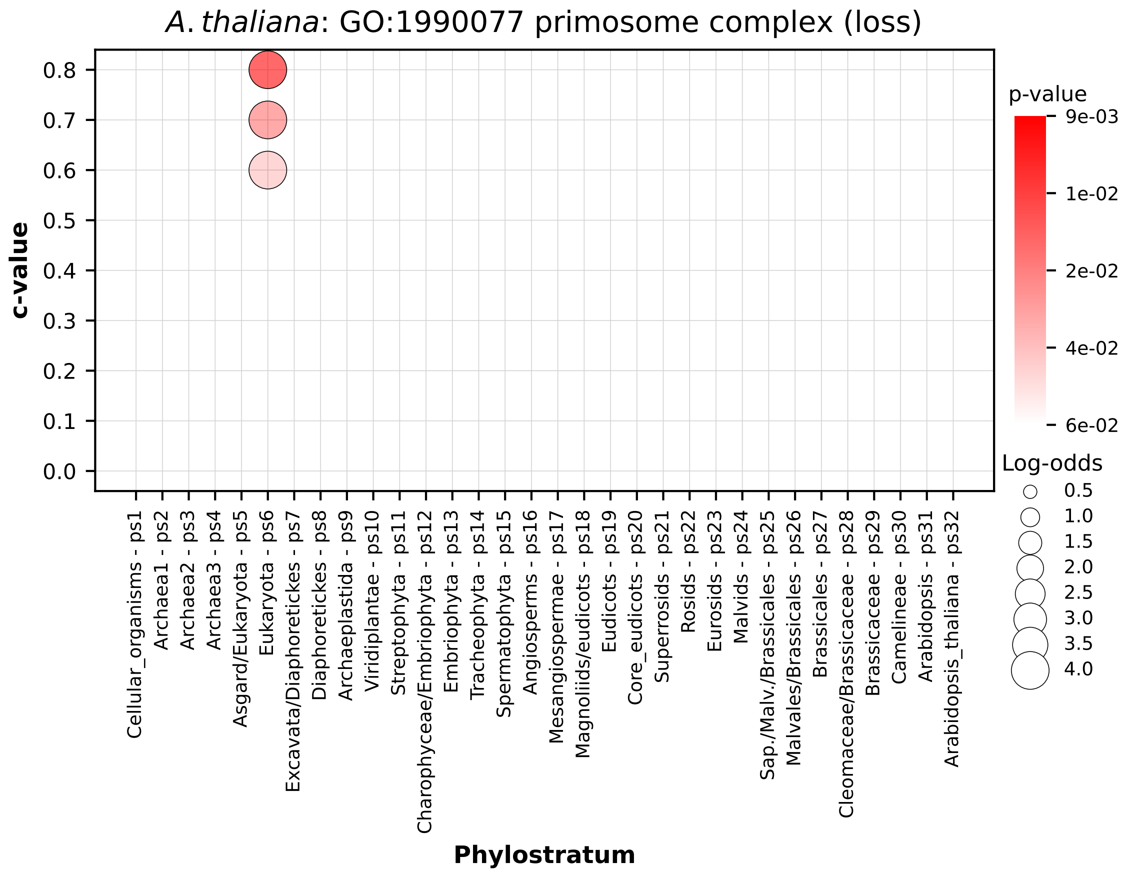

A. thaliana: GO:1990077 primosome complex (loss)
c-value
0.8
0.7
0.6
0.5
0.4
0.3
0.2
0.1
0.0
Phylostratum
Cellular_organisms - ps1
Archaea1 - ps2
Archaea2 - ps3
Archaea3 - ps4
Asgard/Eukaryota - ps5
Eukaryota - ps6
Excavata/Diaphoretickes - ps7
Diaphoretickes - ps8
Archaeplastida - ps9
Viridiplantae - ps10
Streptophyta - ps11
Charophyceae/Embriophyta - ps12
Embriophyta - ps13
Tracheophyta - ps14
Spermatophyta - ps15
Angiosperms - ps16
Mesangiospermae - ps17
Magnoliids/eudicots - ps18
Eudicots - ps19
Core_eudicots - ps20
Superrosids - ps21
Rosids - ps22
Eurosids - ps23
Malvids - ps24
Sap./Malv./Brassicales - ps25
Malvales/Brassicales - ps26
Brassicales - ps27
Cleomaceae/Brassicaceae - ps28
Brassicaceae - ps29
Camelineae - ps30
Arabidopsis - ps31
Arabidopsis_thaliana - ps32
p-value
9e-03
1e-02
2e-02
4e-02
6e-02
Log-odds
0.5
1.0
1.5
2.0
2.5
3.0
3.5
4.0

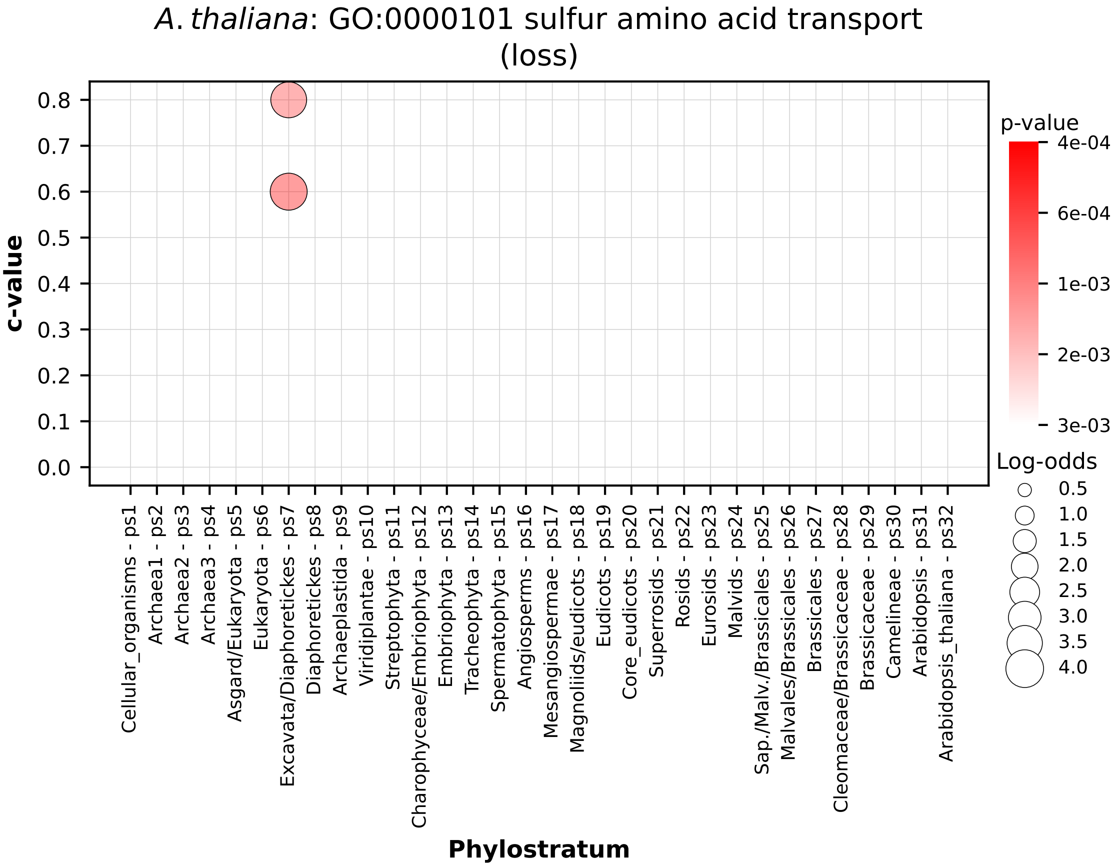

A. thaliana: GO:0000101 sulfur amino acid transport
(loss)
c-value
0.0
0.1
0.2
0.3
0.4
0.5
0.6
0.7
0.8
Phylostratum
Cellular_organisms - ps1
Archaea1 - ps2
Archaea2 - ps3
Archaea3 - ps4
Asgard/Eukaryota - ps5
Eukaryota - ps6
Excavata/Diaphoretickes - ps7
Diaphoretickes - ps8
Archaeplastida - ps9
Viridiplantae - ps10
Streptophyta - ps11
Charophyceae/Embriophyta - ps12
Embriophyta - ps13
Tracheophyta - ps14
Spermatophyta - ps15
Angiosperms - ps16
Mesangiospermae - ps17
Magnoliids/eudicots - ps18
Eudicots - ps19
Core_eudicots - ps20
Superrosids - ps21
Rosids - ps22
Eurosids - ps23
Malvids - ps24
Sap./Malv./Brassicales - ps25
Malvales/Brassicales - ps26
Brassicales - ps27
Cleomaceae/Brassicaceae - ps28
Brassicaceae - ps29
Camelineae - ps30
Arabidopsis - ps31
Arabidopsis_thaliana - ps32
p-value
4e-04
6e-04
1e-03
2e-03
3e-03
Log-odds
0.5
1.0
1.5
2.0
2.5
3.0
3.5
4.0

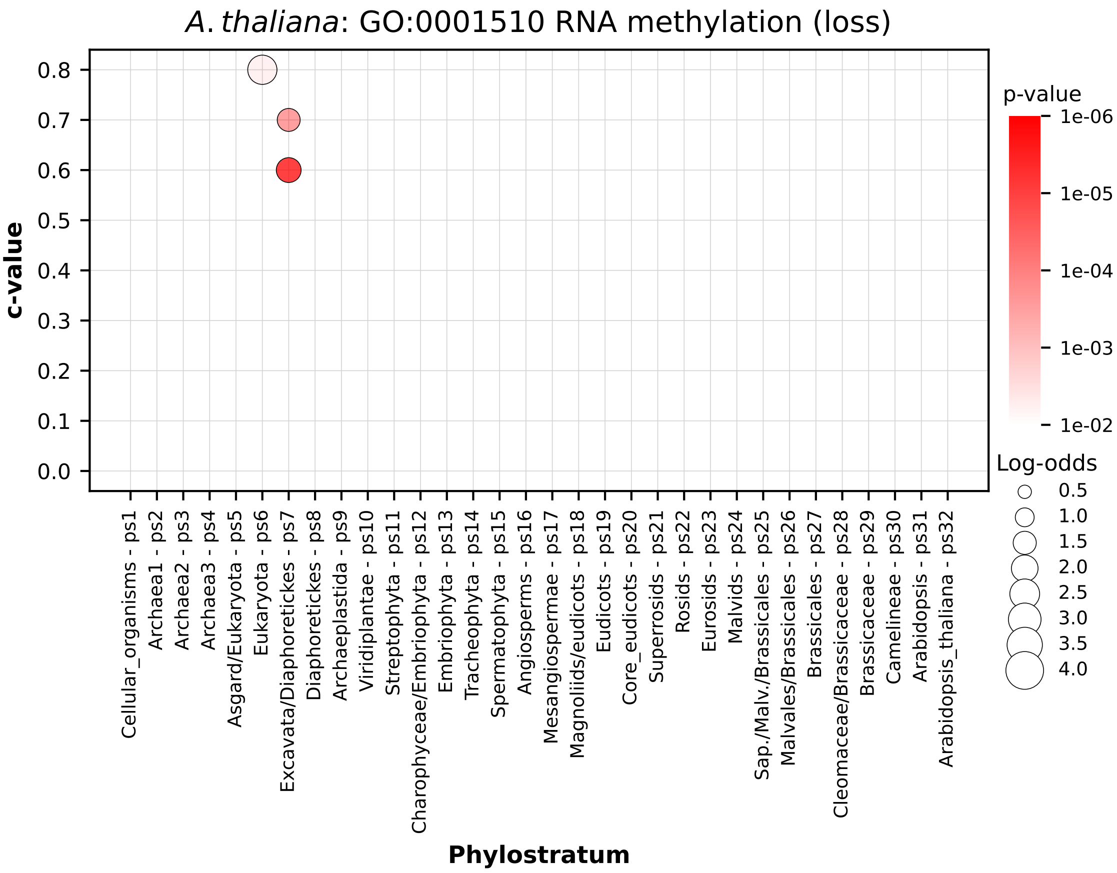

A. thaliana: GO:0001510 RNA methylation (loss)
c-value
0.8
0.7
0.6
0.5
0.4
0.3
0.2
0.1
0.0
p-value
1e-06
1e-05
1e-04
1e-03
1e-02
Log-odds
0.5
1.0
1.5
2.0
2.5
3.0
3.5
4.0
Cellular_organisms - ps1
Archaea1 - ps2
Archaea2 - ps3
Archaea3 - ps4
Asgard/Eukaryota - ps5
Eukaryota - ps6
Excavata/Diaphoretickes - ps7
Diaphoretickes - ps8
Archaeplastida - ps9
Viridiplantae - ps10
Streptophyta - ps11
Charophyceae/Embriophyta - ps12
Embriophyta - ps13
Tracheophyta - ps14
Spermatophyta - ps15
Angiosperms - ps16
Mesangiospermae - ps17
Magnoliids/eudicots - ps18
Eudicots - ps19
Core_eudicots - ps20
Superrosids - ps21
Rosids - ps22
Eurosids - ps23
Malvids - ps24
Sap./Malv./Brassicales - ps25
Malvales/Brassicales - ps26
Brassicales - ps27
Cleomaceae/Brassicaceae - ps28
Brassicaceae - ps29
Camelineae - ps30
Arabidopsis - ps31
Arabidopsis_thaliana - ps32
Phylostratum

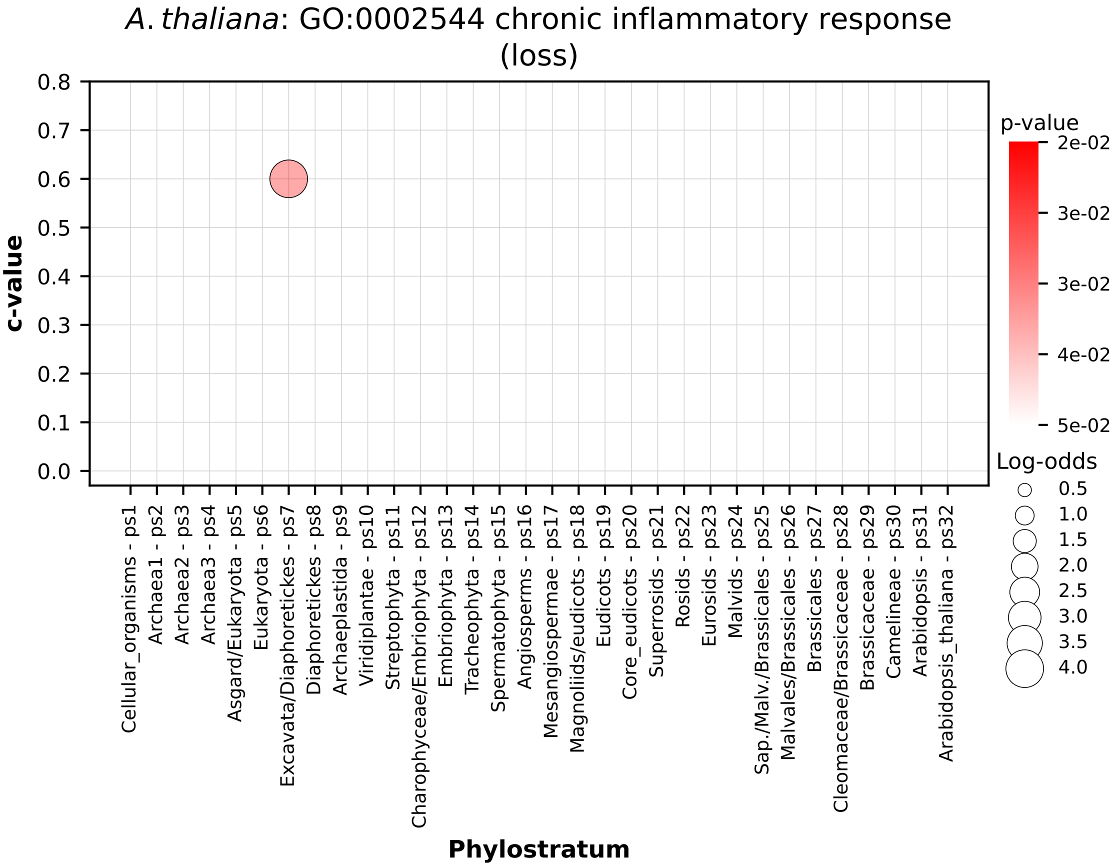

A. thaliana: GO:0002544 chronic inflammatory response (loss)
c-value
0.8
0.7
0.6
0.5
0.4
0.3
0.2
0.1
0.0
Phylostratum
Cellular_organisms - ps1
Archaea1 - ps2
Archaea2 - ps3
Archaea3 - ps4
Asgard/Eukaryota - ps5
Eukaryota - ps6
Excavata/Diaphoretickes - ps7
Diaphoretickes - ps8
Archaeplastida - ps9
Viridiplantae - ps10
Streptophyta - ps11
Charophyceae/Embriophyta - ps12
Embriophyta - ps13
Tracheophyta - ps14
Spermatophyta - ps15
Angiosperms - ps16
Mesangiospermae - ps17
Magnoliids/eudicots - ps18
Eudicots - ps19
Core_eudicots - ps20
Superrosids - ps21
Rosids - ps22
Eurosids - ps23
Malvids - ps24
Sap./Malv./Brassicales - ps25
Malvales/Brassicales - ps26
Brassicales - ps27
Cleomaceae/Brassicaceae - ps28
Brassicaceae - ps29
Camelineae - ps30
Arabidopsis - ps31
Arabidopsis_thaliana - ps32
p-value
2e-02
3e-02
3e-02
4e-02
5e-02
Log-odds
0.5
1.0
1.5
2.0
2.5
3.0
3.5
4.0

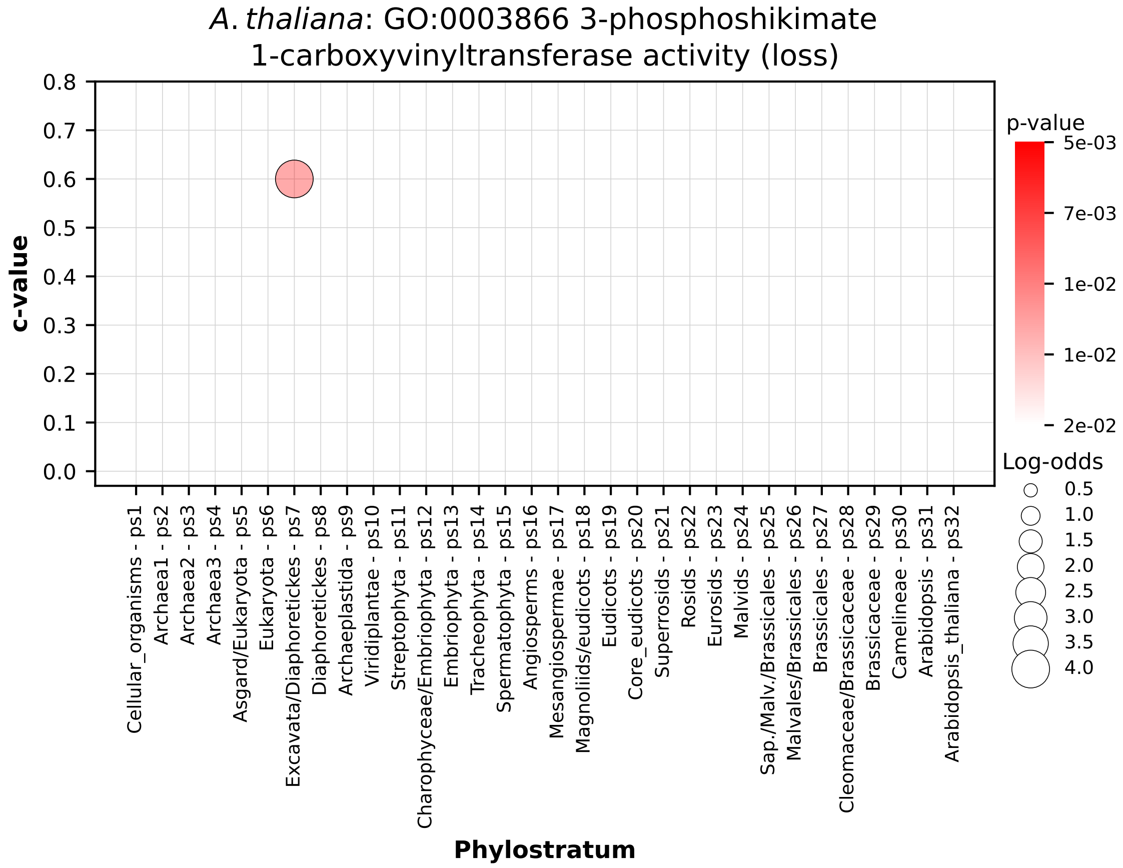

A. thaliana: GO:0003866 3-phosphoshikimate 1-carboxyvinyltransferase activity (loss)
c-value
0.8
0.7
0.6
0.5
0.4
0.3
0.2
0.1
0.0
p-value
5e-03
7e-03
1e-02
1e-02
2e-02
Log-odds
0.5
1.0
1.5
2.0
2.5
3.0
3.5
4.0
Cellular_organisms - ps1
Archaea1 - ps2
Archaea2 - ps3
Archaea3 - ps4
Asgard/Eukaryota - ps5
Eukaryota - ps6
Excavata/Diaphoretickes - ps7
Diaphoretickes - ps8
Archaeplastida - ps9
Viridiplantae - ps10
Streptophyta - ps11
Charophyceae/Embriophyta - ps12
Embriophyta - ps13
Tracheophyta - ps14
Spermatophyta - ps15
Angiosperms - ps16
Mesangiospermae - ps17
Magnoliids/eudicots - ps18
Eudicots - ps19
Core_eudicots - ps20
Superrosids - ps21
Rosids - ps22
Eurosids - ps23
Malvids - ps24
Sap./Malv./Brassicales - ps25
Malvales/Brassicales - ps26
Brassicales - ps27
Cleomaceae/Brassicaceae - ps28
Brassicaceae - ps29
Camelineae - ps30
Arabidopsis - ps31
Arabidopsis_thaliana - ps32
Phylostratum

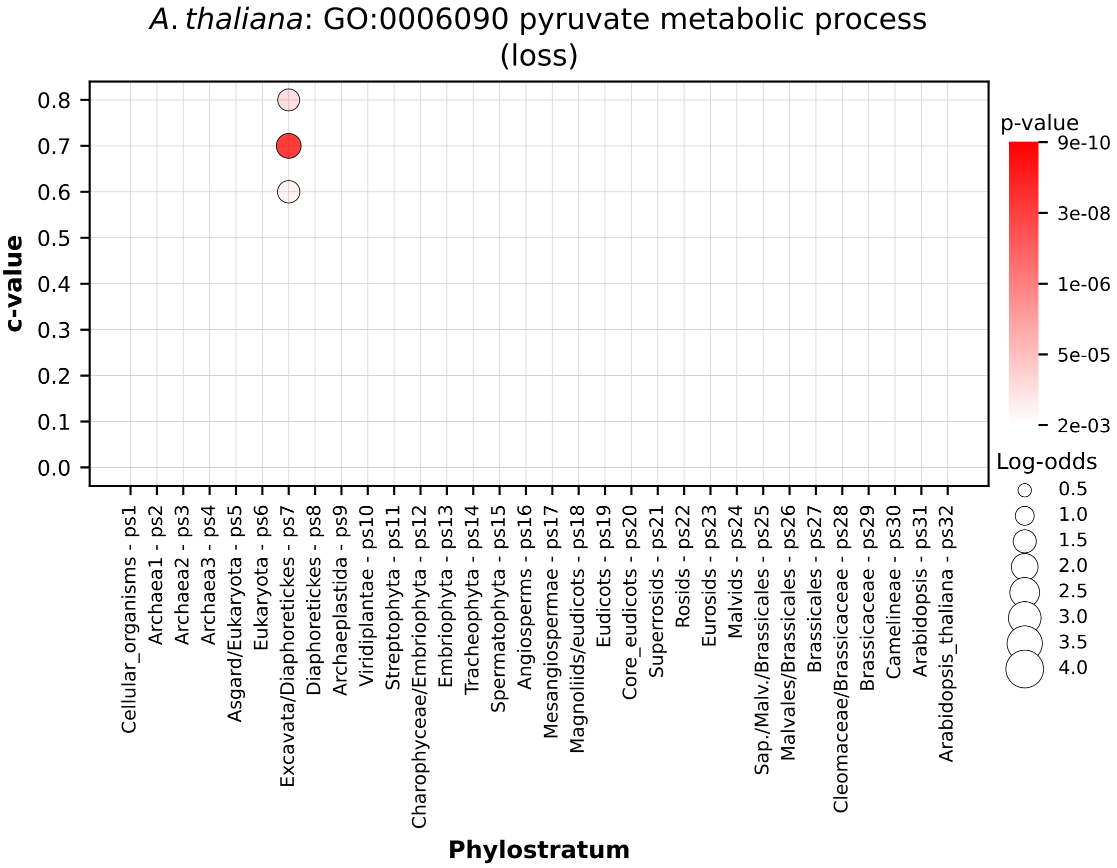
A. thaliana: GO:0006090 pyruvate metabolic process
(loss)
c-value
0.0
0.1
0.2
0.3
0.4
0.5
0.6
0.7
0.8
p-value
9e-10
3e-08
1e-06
5e-05
2e-03
Log-odds
0.5
1.0
1.5
2.0
2.5
3.0
3.5
4.0
Cellular_organisms - ps1
Archaea1 - ps2
Archaea2 - ps3
Archaea3 - ps4
Asgard/Eukaryota - ps5
Eukaryota - ps6
Excavata/Diaphoretickes - ps7
Diaphoretickes - ps8
Archaeplastida - ps9
Viridiplantae - ps10
Streptophyta - ps11
Charophyceae/Embriophyta - ps12
Embriophyta - ps13
Tracheophyta - ps14
Spermatophyta - ps15
Angiosperms - ps16
Mesangiospermae - ps17
Magnoliids/eudicots - ps18
Eudicots - ps19
Core_eudicots - ps20
Superrosids - ps21
Rosids - ps22
Eurosids - ps23
Malvids - ps24
Sap./Malv./Brassicales - ps25
Malvales/Brassicales - ps26
Brassicales - ps27
Cleomaceae/Brassicaceae - ps28
Brassicaceae - ps29
Camelineae - ps30
Arabidopsis - ps31
Arabidopsis_thaliana - ps32
Phylostratum

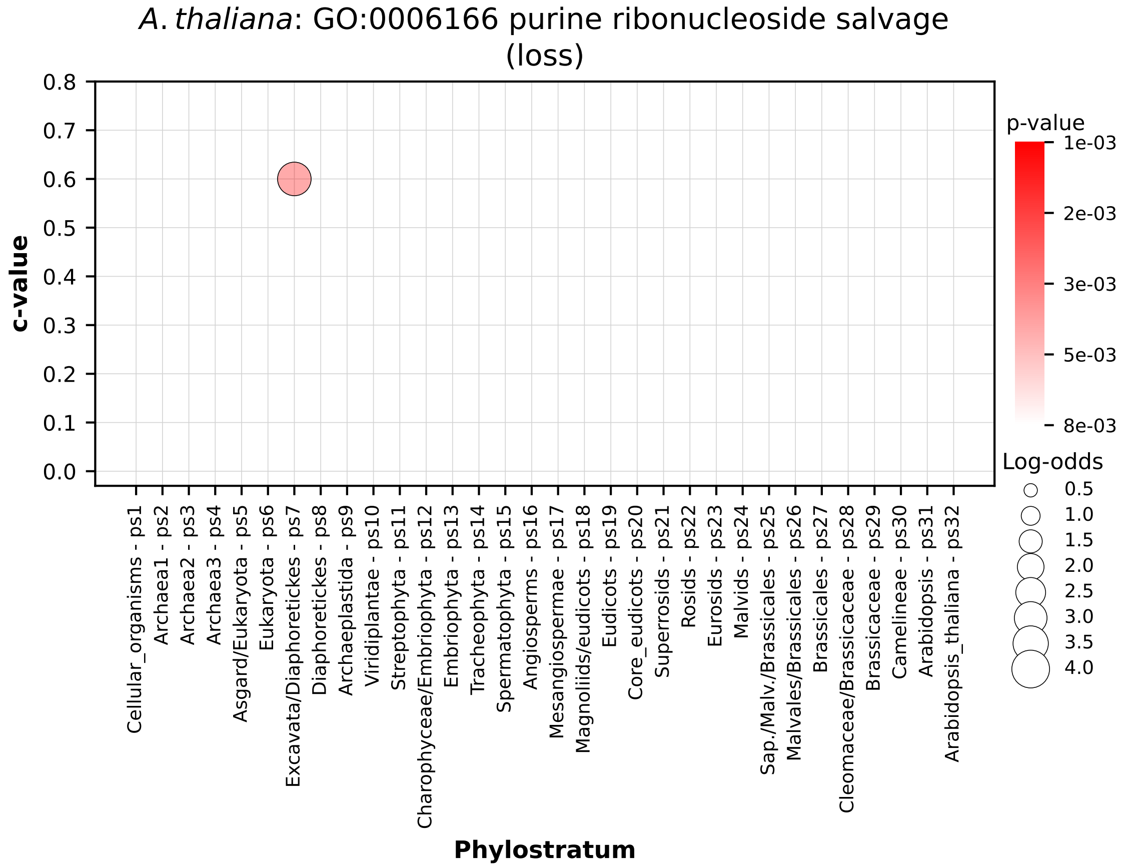
A. thaliana: GO:0006166 purine ribonucleoside salvage
(loss)
c-value
0.8
0.7
0.6
0.5
0.4
0.3
0.2
0.1
0.0
p-value
1e-03
2e-03
3e-03
5e-03
8e-03
Log-odds
0.5
1.0
1.5
2.0
2.5
3.0
3.5
4.0
Cellular_organisms - ps1
Archaea1 - ps2
Archaea2 - ps3
Archaea3 - ps4
Asgard/Eukaryota - ps5
Eukaryota - ps6
Excavata/Diaphoretickes - ps7
Diaphoretickes - ps8
Archaeplastida - ps9
Viridiplantae - ps10
Streptophyta - ps11
Charophyceae/Embriophyta - ps12
Embriophyta - ps13
Tracheophyta - ps14
Spermatophyta - ps15
Angiosperms - ps16
Mesangiospermae - ps17
Magnoliids/eudicots - ps18
Eudicots - ps19
Core_eudicots - ps20
Superrosids - ps21
Rosids - ps22
Eurosids - ps23
Malvids - ps24
Sap./Malv./Brassicales - ps25
Malvales/Brassicales - ps26
Brassicales - ps27
Cleomaceae/Brassicaceae - ps28
Brassicaceae - ps29
Camelineae - ps30
Arabidopsis - ps31
Arabidopsis_thaliana - ps32
Phylostratum

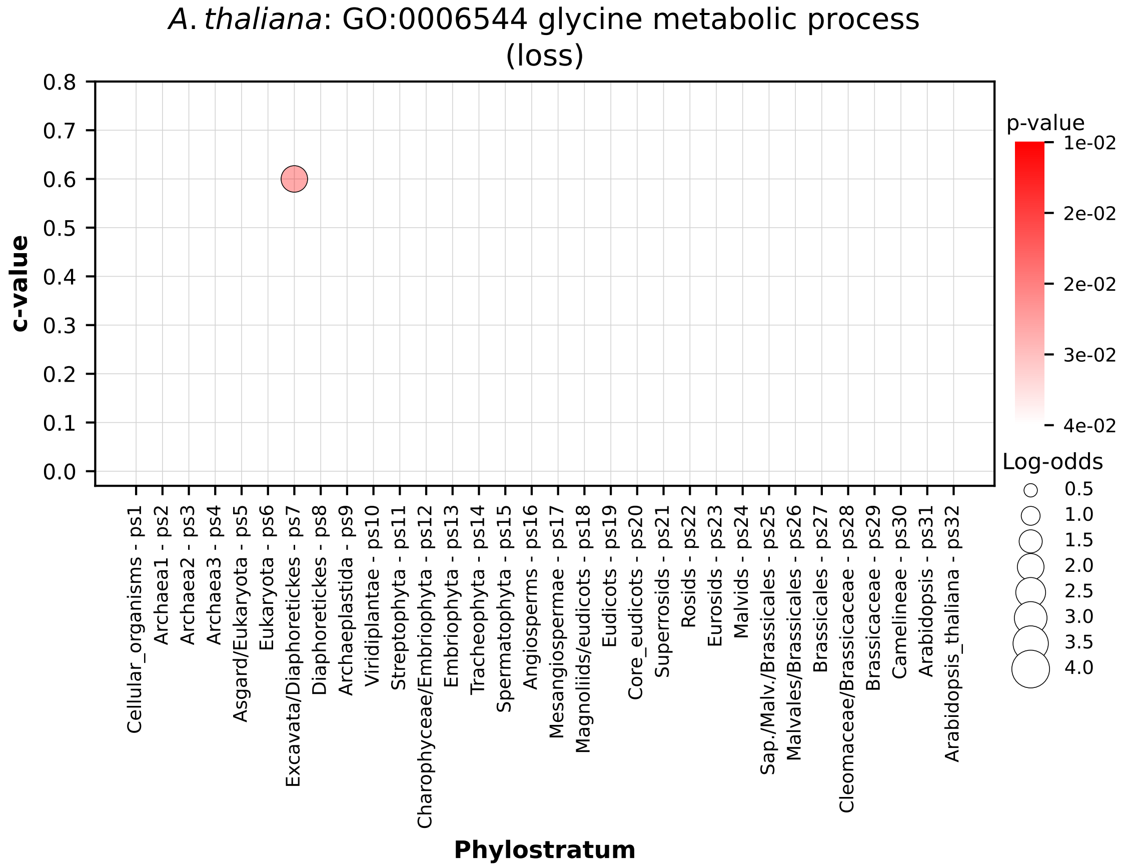

A. thaliana: GO:0006544 glycine metabolic process
(loss)
c-value
0.8
0.7
0.6
0.5
0.4
0.3
0.2
0.1
0.0
p-value
1e-02
2e-02
2e-02
3e-02
4e-02
Log-odds
0.5
1.0
1.5
2.0
2.5
3.0
3.5
4.0
Cellular_organisms - ps1
Archaea1 - ps2
Archaea2 - ps3
Archaea3 - ps4
Asgard/Eukaryota - ps5
Eukaryota - ps6
Excavata/Diaphoretickes - ps7
Diaphoretickes - ps8
Archaeplastida - ps9
Viridiplantae - ps10
Streptophyta - ps11
Charophyceae/Embriophyta - ps12
Embriophyta - ps13
Tracheophyta - ps14
Spermatophyta - ps15
Angiosperms - ps16
Mesangiospermae - ps17
Magnoliids/eudicots - ps18
Eudicots - ps19
Core_eudicots - ps20
Superrosids - ps21
Rosids - ps22
Eurosids - ps23
Malvids - ps24
Sap./Malv./Brassicales - ps25
Malvales/Brassicales - ps26
Brassicales - ps27
Cleomaceae/Brassicaceae - ps28
Brassicaceae - ps29
Camelineae - ps30
Arabidopsis - ps31
Arabidopsis_thaliana - ps32
Phylostratum

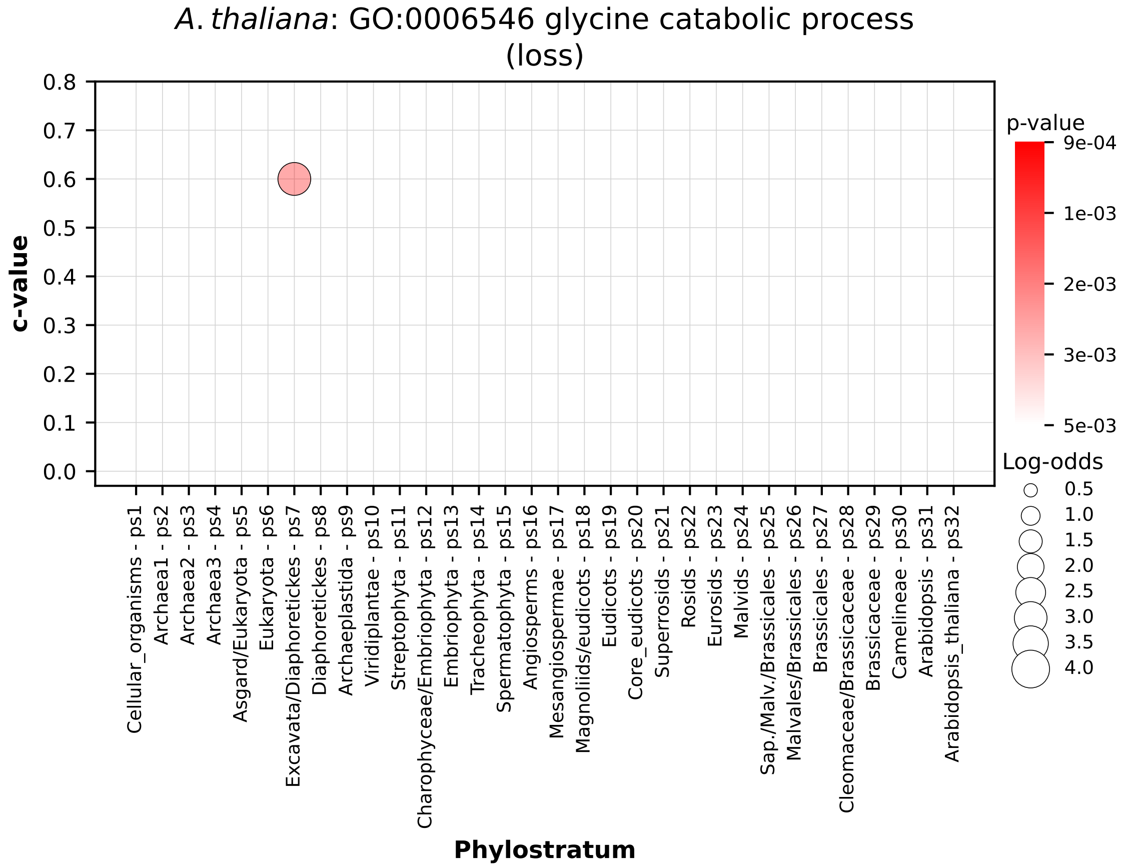

A. thaliana: GO:0006546 glycine catabolic process
(loss)
c-value
0.8
0.7
0.6
0.5
0.4
0.3
0.2
0.1
0.0
Phylostratum
Cellular_organisms - ps1
Archaea1 - ps2
Archaea2 - ps3
Archaea3 - ps4
Asgard/Eukaryota - ps5
Eukaryota - ps6
Excavata/Diaphoretickes - ps7
Diaphoretickes - ps8
Archaeplastida - ps9
Viridiplantae - ps10
Streptophyta - ps11
Charophyceae/Embriophyta - ps12
Embriophyta - ps13
Tracheophyta - ps14
Spermatophyta - ps15
Angiosperms - ps16
Mesangiospermae - ps17
Magnoliids/eudicots - ps18
Eudicots - ps19
Core_eudicots - ps20
Superrosids - ps21
Rosids - ps22
Eurosids - ps23
Malvids - ps24
Sap./Malv./Brassicales - ps25
Malvales/Brassicales - ps26
Brassicales - ps27
Cleomaceae/Brassicaceae - ps28
Brassicaceae - ps29
Camelineae - ps30
Arabidopsis - ps31
Arabidopsis_thaliana - ps32
p-value
9e-04
1e-03
2e-03
3e-03
5e-03
Log-odds
0.5
1.0
1.5
2.0
2.5
3.0
3.5
4.0

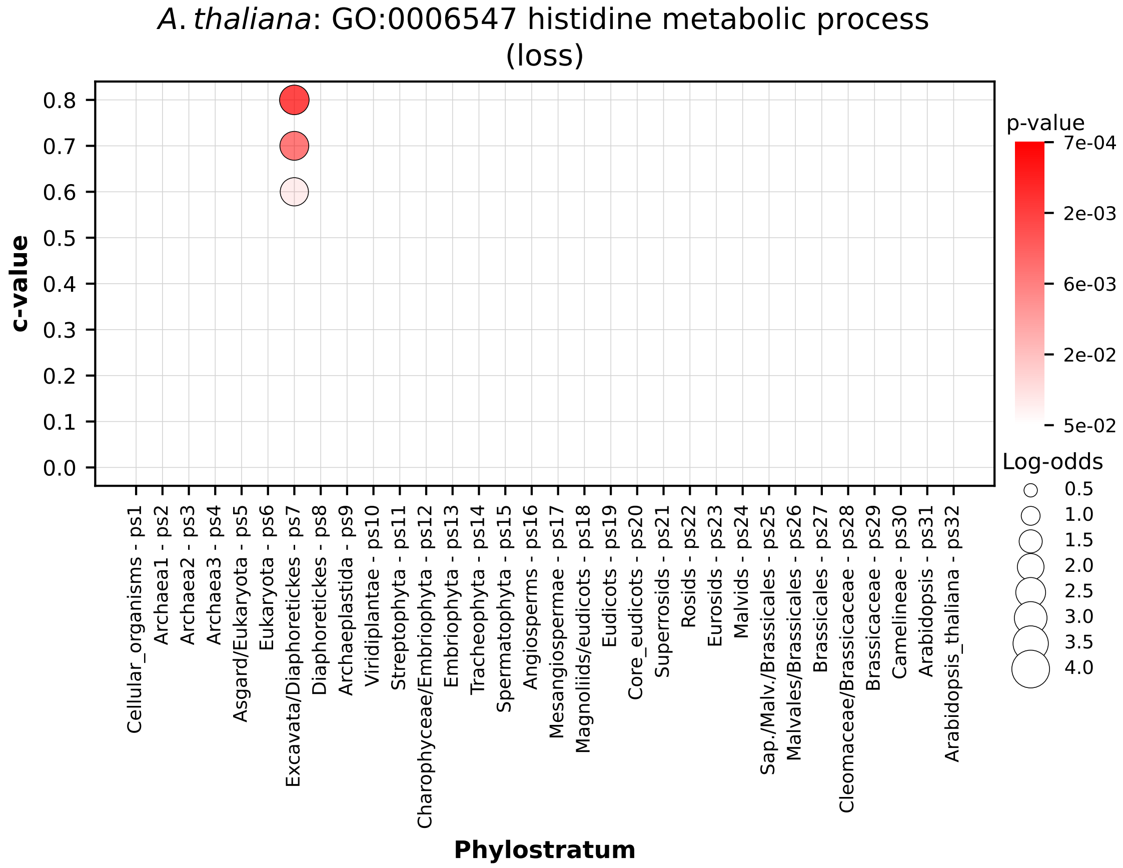

A. thaliana: GO:0006547 histidine metabolic process
(loss)
c-value
0.0
0.1
0.2
0.3
0.4
0.5
0.6
0.7
0.8
Phylostratum
Cellular_organisms - ps1
Archaea1 - ps2
Archaea2 - ps3
Archaea3 - ps4
Asgard/Eukaryota - ps5
Eukaryota - ps6
Excavata/Diaphoretickes - ps7
Diaphoretickes - ps8
Archaeplastida - ps9
Viridiplantae - ps10
Streptophyta - ps11
Charophyceae/Embriophyta - ps12
Embriophyta - ps13
Tracheophyta - ps14
Spermatophyta - ps15
Angiosperms - ps16
Mesangiospermae - ps17
Magnoliids/eudicots - ps18
Eudicots - ps19
Core_eudicots - ps20
Superrosids - ps21
Rosids - ps22
Eurosids - ps23
Malvids - ps24
Sap./Malv./Brassicales - ps25
Malvales/Brassicales - ps26
Brassicales - ps27
Cleomaceae/Brassicaceae - ps28
Brassicaceae - ps29
Camelineae - ps30
Arabidopsis - ps31
Arabidopsis_thaliana - ps32
p-value
7e-04
2e-03
6e-03
2e-02
5e-02
Log-odds
0.5
1.0
1.5
2.0
2.5
3.0
3.5
4.0

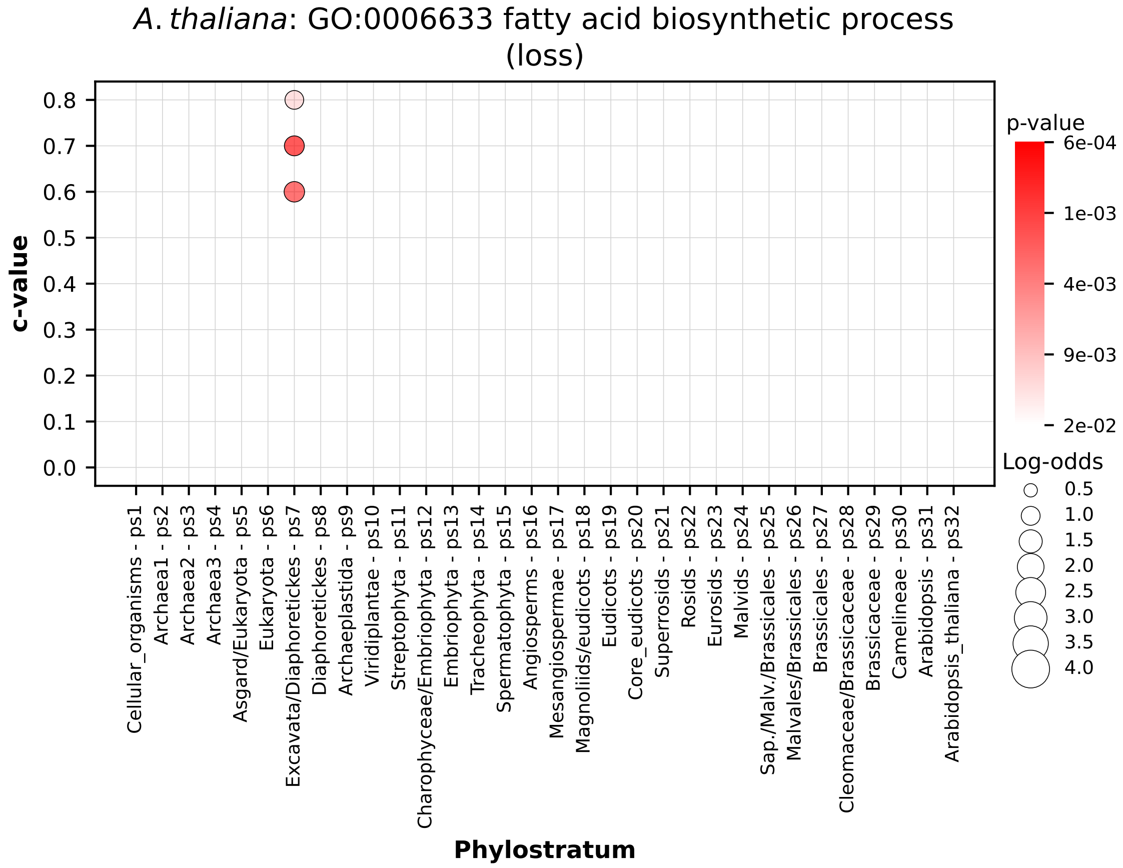

A. thaliana: GO:0006633 fatty acid biosynthetic process (loss)
c-value
0.8
0.7
0.6
0.5
0.4
0.3
0.2
0.1
0.0
p-value
6e-04
1e-03
4e-03
9e-03
2e-02
Log-odds
0.5
1.0
1.5
2.0
2.5
3.0
3.5
4.0
Cellular_organisms - ps1
Archaea1 - ps2
Archaea2 - ps3
Archaea3 - ps4
Asgard/Eukaryota - ps5
Eukaryota - ps6
Excavata/Diaphoretickes - ps7
Diaphoretickes - ps8
Archaeplastida - ps9
Viridiplantae - ps10
Streptophyta - ps11
Charophyceae/Embriophyta - ps12
Embriophyta - ps13
Tracheophyta - ps14
Spermatophyta - ps15
Angiosperms - ps16
Mesangiospermae - ps17
Magnoliids/eudicots - ps18
Eudicots - ps19
Core_eudicots - ps20
Superrosids - ps21
Rosids - ps22
Eurosids - ps23
Malvids - ps24
Sap./Malv./Brassicales - ps25
Malvales/Brassicales - ps26
Brassicales - ps27
Cleomaceae/Brassicaceae - ps28
Brassicaceae - ps29
Camelineae - ps30
Arabidopsis - ps31
Arabidopsis_thaliana - ps32
Phylostratum

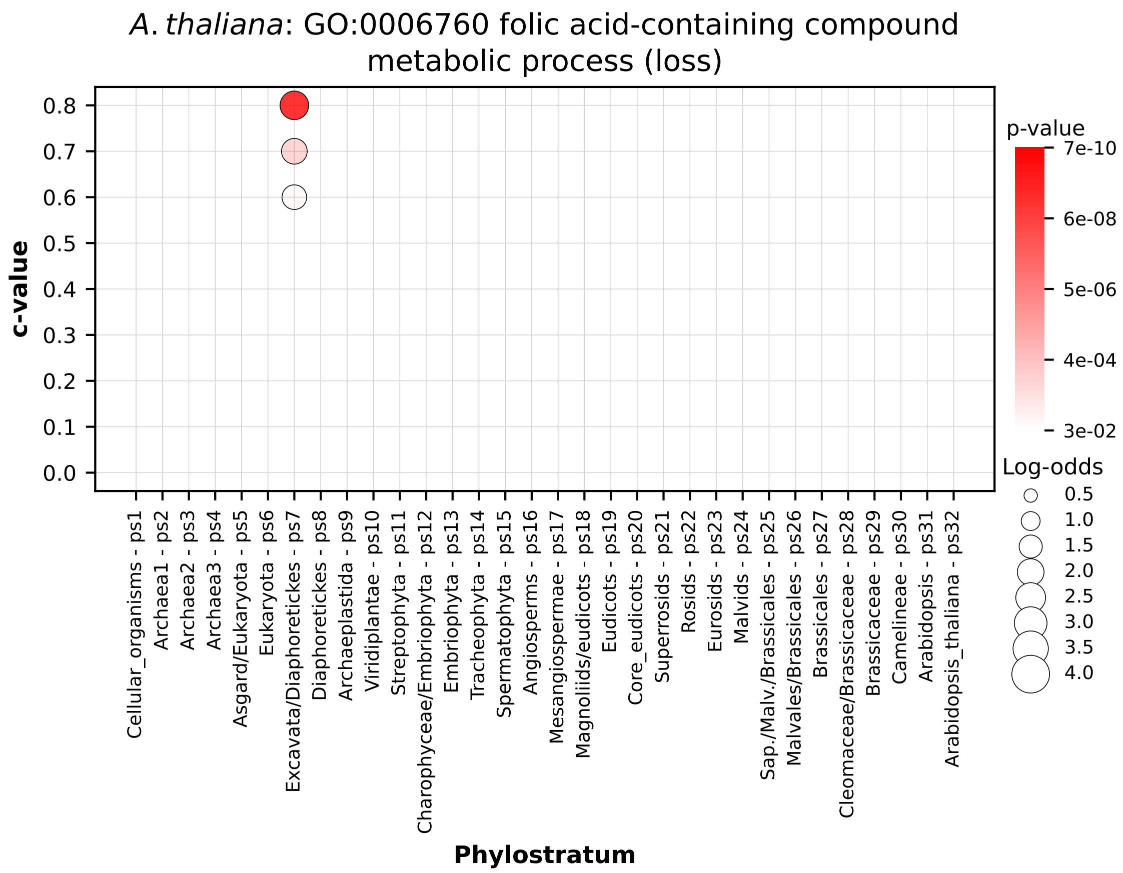

A. thaliana: GO:0006760 folic acid-containing compound metabolic process (loss)
c-value
0.0
0.1
0.2
0.3
0.4
0.5
0.6
0.7
0.8
Phylostratum
Cellular_organisms - ps1
Archaea1 - ps2
Archaea2 - ps3
Archaea3 - ps4
Asgard/Eukaryota - ps5
Eukaryota - ps6
Excavata/Diaphoretickes - ps7
Diaphoretickes - ps8
Archaeplastida - ps9
Viridiplantae - ps10
Streptophyta - ps11
Charophyceae/Embriophyta - ps12
Embriophyta - ps13
Tracheophyta - ps14
Spermatophyta - ps15
Angiosperms - ps16
Mesangiospermae - ps17
Magnoliids/eudicots - ps18
Eudicots - ps19
Core_eudicots - ps20
Superrosids - ps21
Rosids - ps22
Eurosids - ps23
Malvids - ps24
Sap./Malv./Brassicales - ps25
Malvales/Brassicales - ps26
Brassicales - ps27
Cleomaceae/Brassicaceae - ps28
Brassicaceae - ps29
Camelineae - ps30
Arabidopsis - ps31
Arabidopsis_thaliana - ps32
p-value
7e-10
6e-08
5e-06
4e-04
3e-02
Log-odds
0.5
1.0
1.5
2.0
2.5
3.0
3.5
4.0

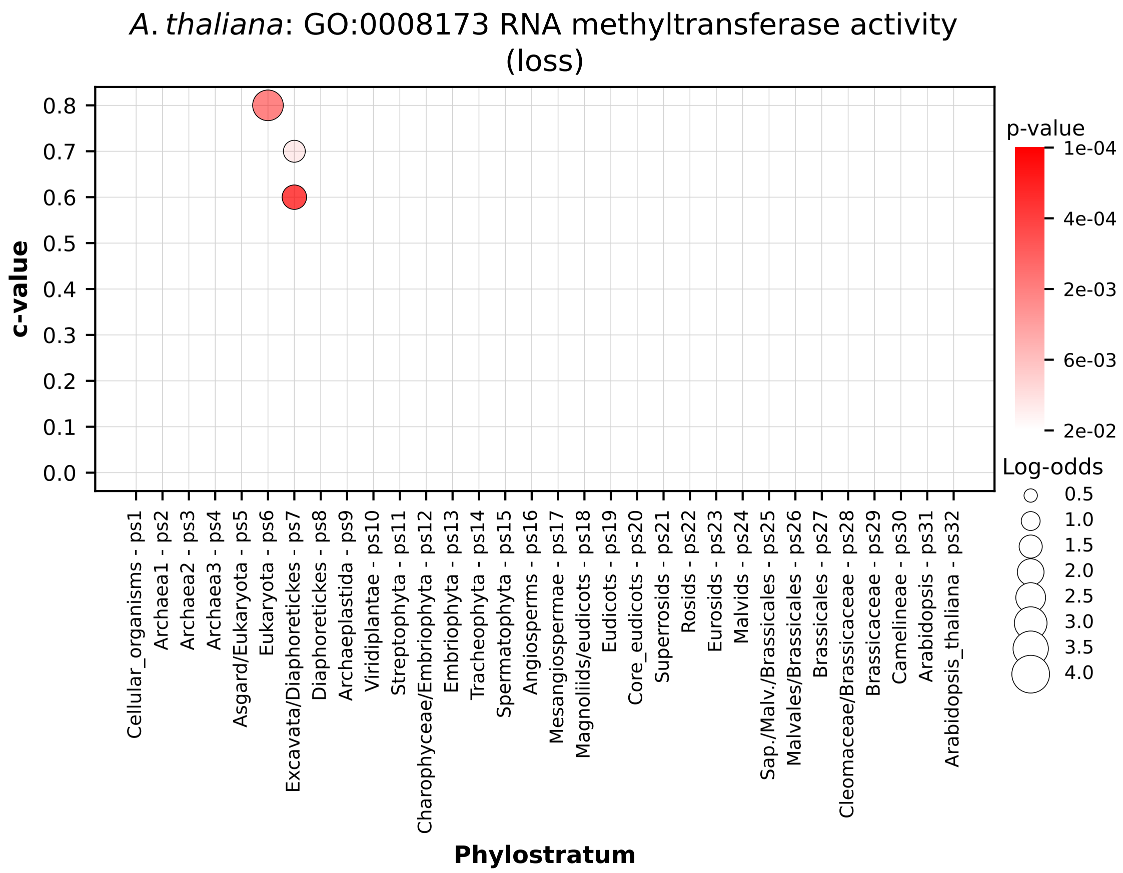

A. thaliana: GO:0008173 RNA methyltransferase activity
(loss)
c-value
0.0
0.1
0.2
0.3
0.4
0.5
0.6
0.7
0.8
Phylostratum
Cellular_organisms - ps1
Archaea1 - ps2
Archaea2 - ps3
Archaea3 - ps4
Asgard/Eukaryota - ps5
Eukaryota - ps6
Excavata/Diaphoretickes - ps7
Diaphoretickes - ps8
Archaeplastida - ps9
Viridiplantae - ps10
Streptophyta - ps11
Charophyceae/Embriophyta - ps12
Embriophyta - ps13
Tracheophyta - ps14
Spermatophyta - ps15
Angiosperms - ps16
Mesangiospermae - ps17
Magnoliids/eudicots - ps18
Eudicots - ps19
Core_eudicots - ps20
Superrosids - ps21
Rosids - ps22
Eurosids - ps23
Malvids - ps24
Sap./Malv./Brassicales - ps25
Malvales/Brassicales - ps26
Brassicales - ps27
Cleomaceae/Brassicaceae - ps28
Brassicaceae - ps29
Camelineae - ps30
Arabidopsis - ps31
Arabidopsis_thaliana - ps32
p-value
1e-04
4e-04
2e-03
6e-03
2e-02
Log-odds
0.5
1.0
1.5
2.0
2.5
3.0
3.5
4.0

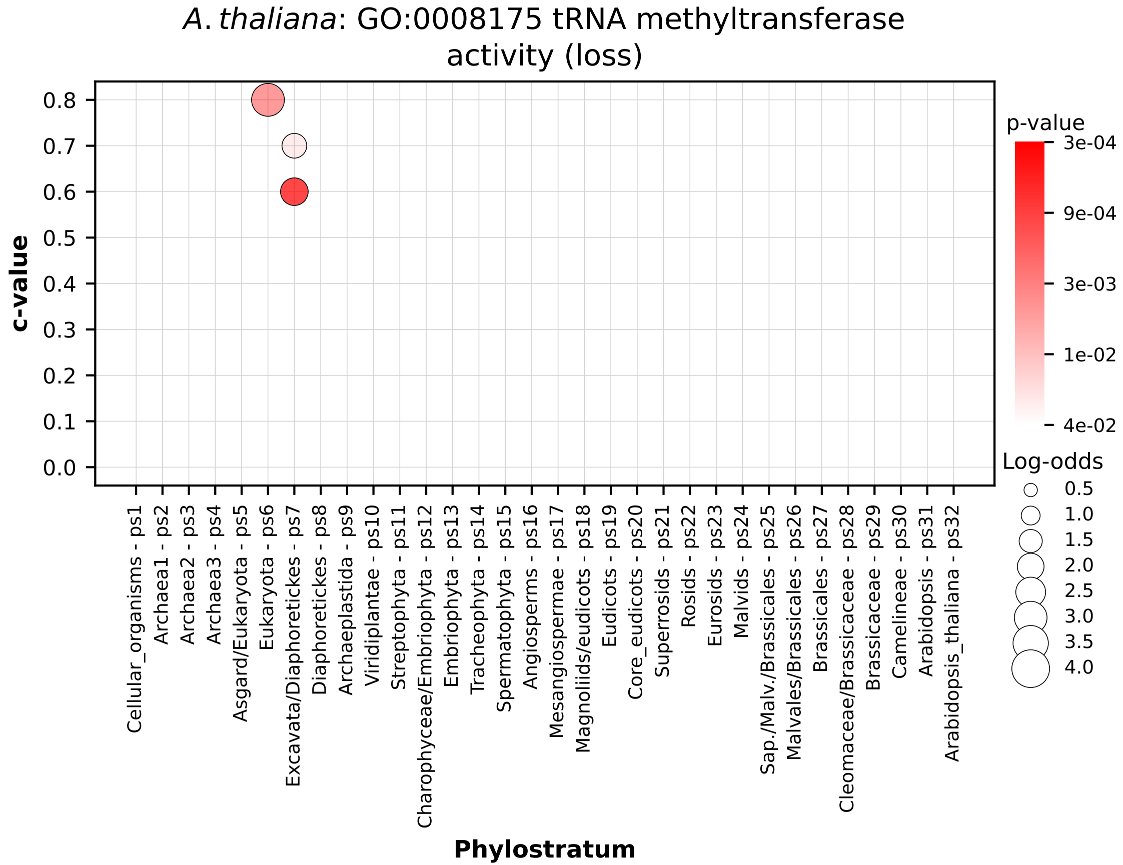

A. thaliana: GO:0008175 tRNA methyltransferase activity (loss)
c-value
0.0
0.1
0.2
0.3
0.4
0.5
0.6
0.7
0.8
Phylostratum
Cellular_organisms - ps1
Archaea1 - ps2
Archaea2 - ps3
Archaea3 - ps4
Asgard/Eukaryota - ps5
Eukaryota - ps6
Excavata/Diaphoretickes - ps7
Diaphoretickes - ps8
Archaeplastida - ps9
Viridiplantae - ps10
Streptophyta - ps11
Charophyceae/Embriophyta - ps12
Embriophyta - ps13
Tracheophyta - ps14
Spermatophyta - ps15
Angiosperms - ps16
Mesangiospermae - ps17
Magnoliids/eudicots - ps18
Eudicots - ps19
Core_eudicots - ps20
Superrosids - ps21
Rosids - ps22
Eurosids - ps23
Malvids - ps24
Sap./Malv./Brassicales - ps25
Malvales/Brassicales - ps26
Brassicales - ps27
Cleomaceae/Brassicaceae - ps28
Brassicaceae - ps29
Camelineae - ps30
Arabidopsis - ps31
Arabidopsis_thaliana - ps32
p-value
3e-04
9e-04
3e-03
1e-02
4e-02
Log-odds
0.5
1.0
1.5
2.0
2.5
3.0
3.5
4.0

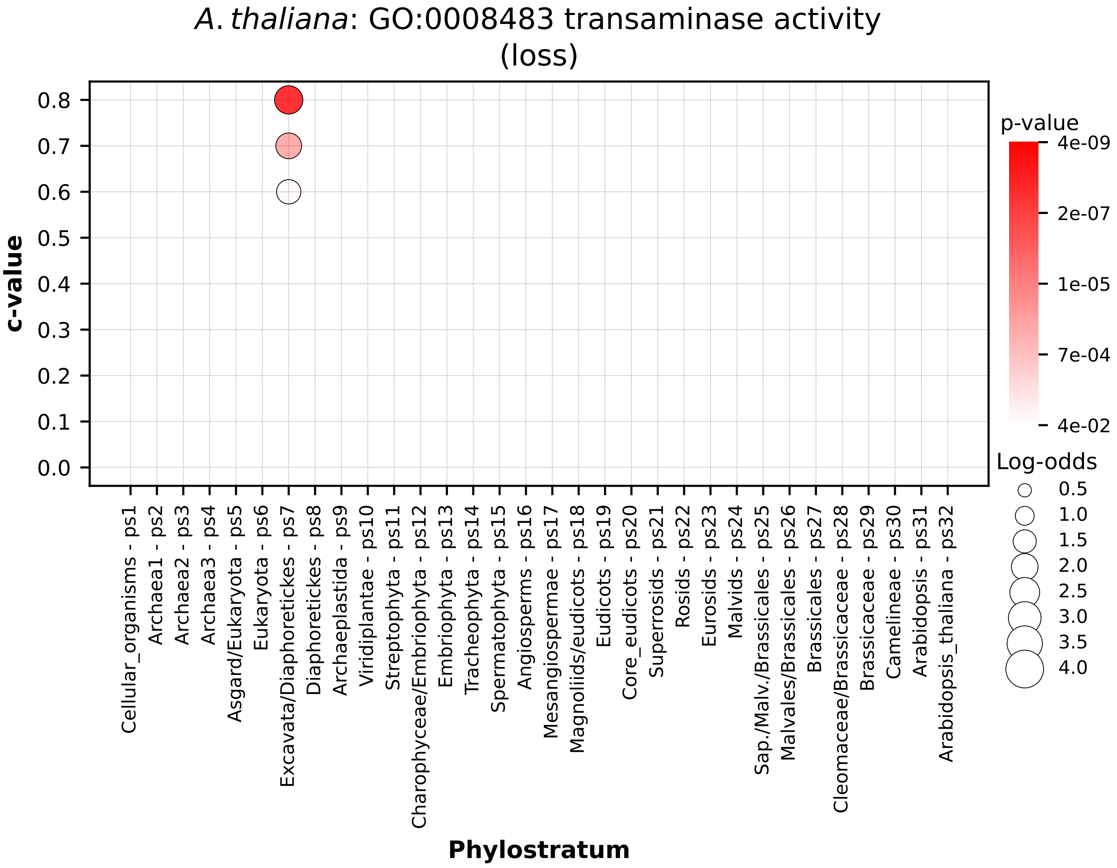

A. thaliana: GO:0008483 transaminase activity
(loss)
c-value
0.0
0.1
0.2
0.3
0.4
0.5
0.6
0.7
0.8
Phylostratum
Cellular_organisms - ps1
Archaea1 - ps2
Archaea2 - ps3
Archaea3 - ps4
Asgard/Eukaryota - ps5
Eukaryota - ps6
Excavata/Diaphoretickes - ps7
Diaphoretickes - ps8
Archaeplastida - ps9
Viridiplantae - ps10
Streptophyta - ps11
Charophyceae/Embriophyta - ps12
Embriophyta - ps13
Tracheophyta - ps14
Spermatophyta - ps15
Angiosperms - ps16
Mesangiospermae - ps17
Magnoliids/eudicots - ps18
Eudicots - ps19
Core_eudicots - ps20
Superrosids - ps21
Rosids - ps22
Eurosids - ps23
Malvids - ps24
Sap./Malv./Brassicales - ps25
Malvales/Brassicales - ps26
Brassicales - ps27
Cleomaceae/Brassicaceae - ps28
Brassicaceae - ps29
Camelineae - ps30
Arabidopsis - ps31
Arabidopsis_thaliana - ps32
p-value
4e-09
2e-07
1e-05
7e-04
4e-02
Log-odds
0.5
1.0
1.5
2.0
2.5
3.0
3.5
4.0

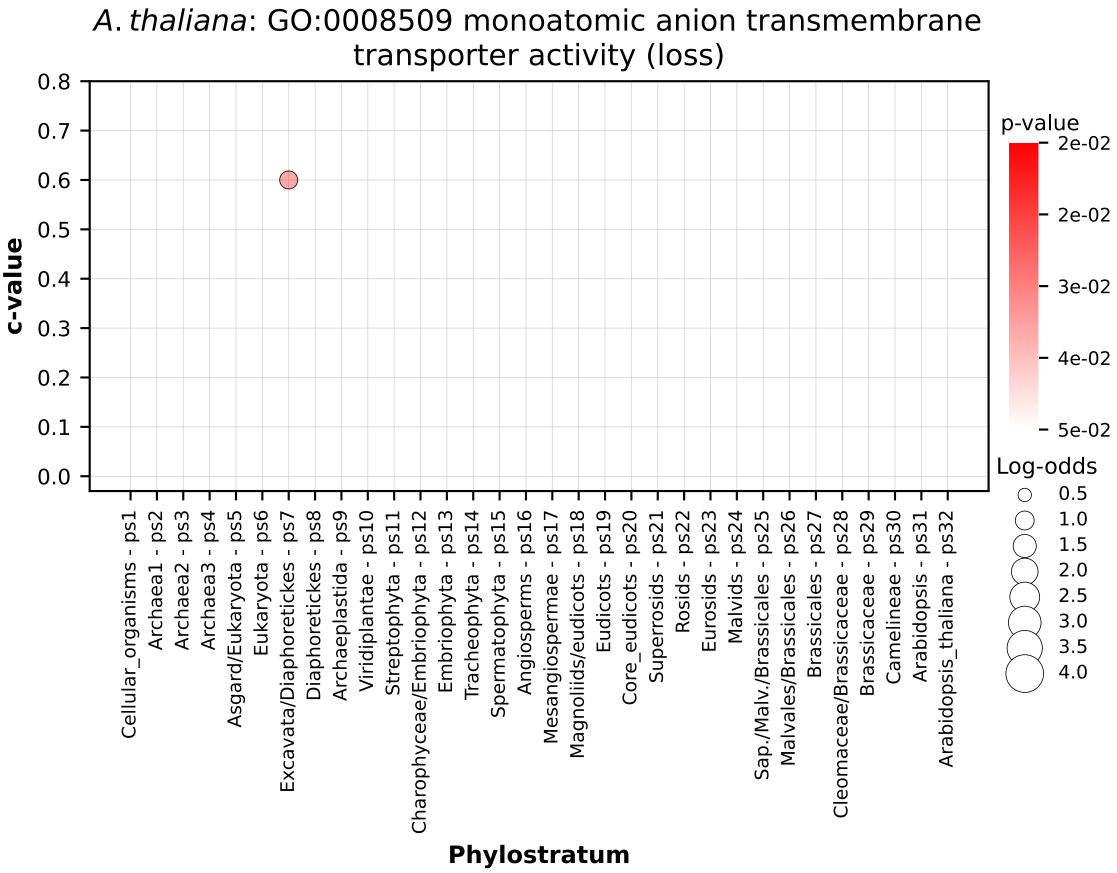

A. thaliana: GO:0008509 monoatomic anion transmembrane transporter activity (loss)
c-value
0.8
0.7
0.6
0.5
0.4
0.3
0.2
0.1
0.0
p-value
2e-02
2e-02
3e-02
4e-02
5e-02
Log-odds
0.5
1.0
1.5
2.0
2.5
3.0
3.5
4.0
Cellular_organisms - ps1
Archaea1 - ps2
Archaea2 - ps3
Archaea3 - ps4
Asgard/Eukaryota - ps5
Eukaryota - ps6
Excavata/Diaphoretickes - ps7
Diaphoretickes - ps8
Archaeplastida - ps9
Viridiplantae - ps10
Streptophyta - ps11
Charophyceae/Embriophyta - ps12
Embriophyta - ps13
Tracheophyta - ps14
Spermatophyta - ps15
Angiosperms - ps16
Mesangiospermae - ps17
Magnoliids/eudicots - ps18
Eudicots - ps19
Core_eudicots - ps20
Superrosids - ps21
Rosids - ps22
Eurosids - ps23
Malvids - ps24
Sap./Malv./Brassicales - ps25
Malvales/Brassicales - ps26
Brassicales - ps27
Cleomaceae/Brassicaceae - ps28
Brassicaceae - ps29
Camelineae - ps30
Arabidopsis - ps31
Arabidopsis_thaliana - ps32
Phylostratum

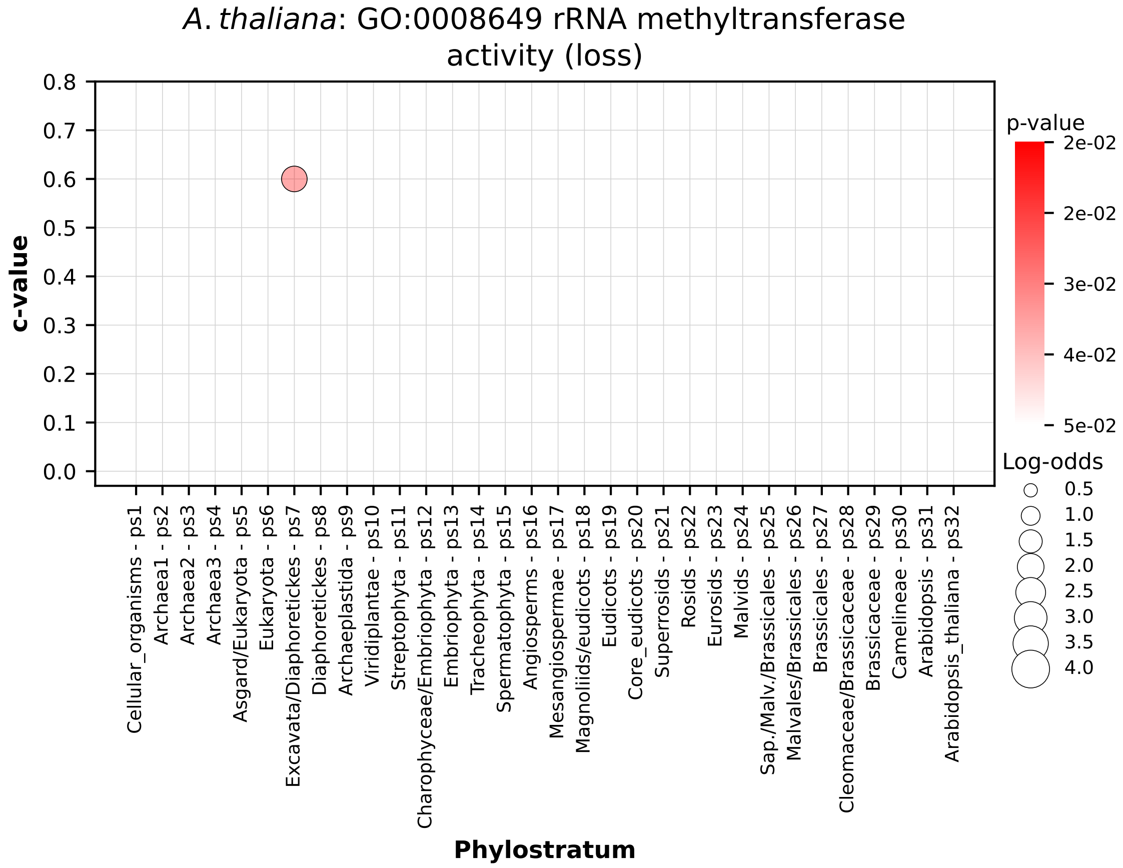
A. thaliana: GO:0008649 rRNA methyltransferase activity (loss)
c-value
0.0
0.1
0.2
0.3
0.4
0.5
0.6
0.7
0.8
Phylostratum
Cellular_organisms - ps1
Archaea1 - ps2
Archaea2 - ps3
Archaea3 - ps4
Asgard/Eukaryota - ps5
Eukaryota - ps6
Excavata/Diaphoretickes - ps7
Diaphoretickes - ps8
Archaeplastida - ps9
Viridiplantae - ps10
Streptophyta - ps11
Charophyceae/Embriophyta - ps12
Embriophyta - ps13
Tracheophyta - ps14
Spermatophyta - ps15
Angiosperms - ps16
Mesangiospermae - ps17
Magnoliids/eudicots - ps18
Eudicots - ps19
Core_eudicots - ps20
Superrosids - ps21
Rosids - ps22
Eurosids - ps23
Malvids - ps24
Sap./Malv./Brassicales - ps25
Malvales/Brassicales - ps26
Brassicales - ps27
Cleomaceae/Brassicaceae - ps28
Brassicaceae - ps29
Camelineae - ps30
Arabidopsis - ps31
Arabidopsis_thaliana - ps32
p-value
2e-02
2e-02
3e-02
4e-02
5e-02
Log-odds
0.5
1.0
1.5
2.0
2.5
3.0
3.5
4.0

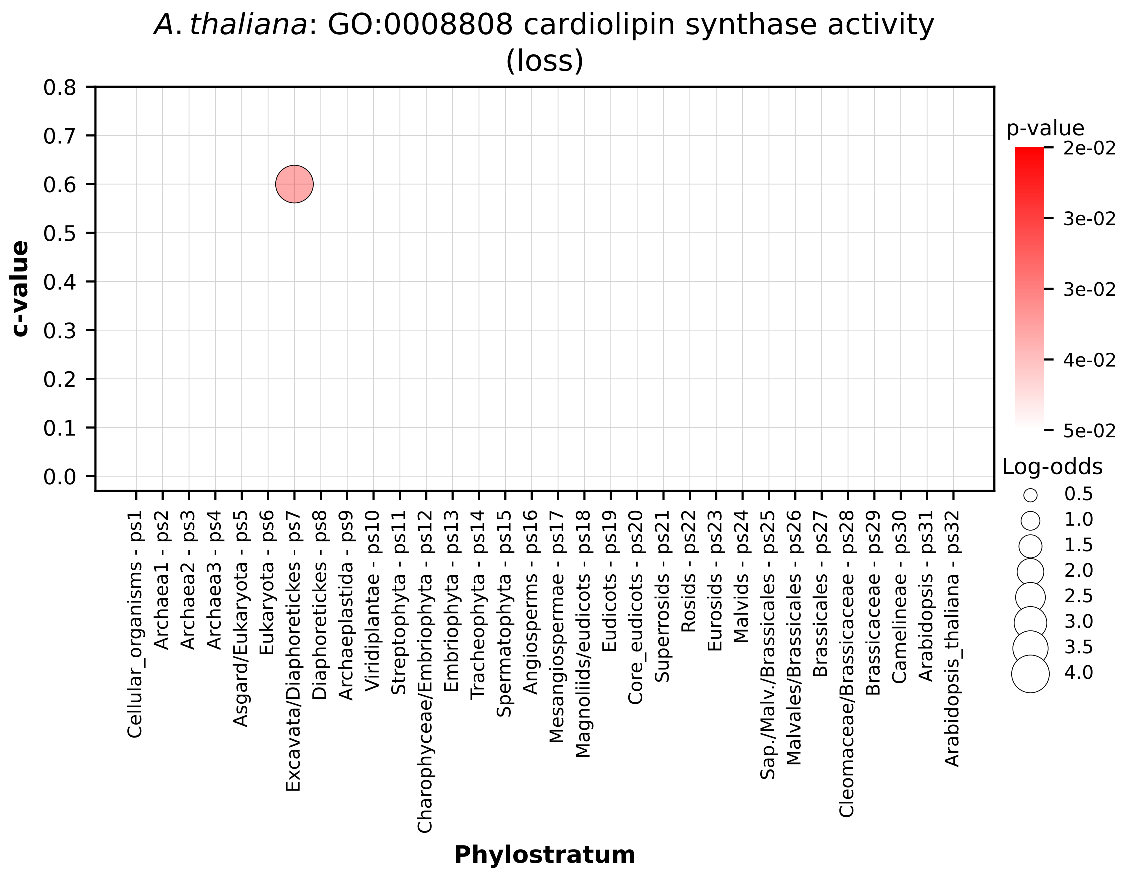

A. thaliana: GO:0008808 cardiolipin synthase activity
(loss)
c-value
0.8
0.7
0.6
0.5
0.4
0.3
0.2
0.1
0.0
p-value
2e-02
3e-02
3e-02
4e-02
5e-02
Log-odds
0.5
1.0
1.5
2.0
2.5
3.0
3.5
4.0
Cellular_organisms - ps1
Archaea1 - ps2
Archaea2 - ps3
Archaea3 - ps4
Asgard/Eukaryota - ps5
Eukaryota - ps6
Excavata/Diaphoretickes - ps7
Diaphoretickes - ps8
Archaeplastida - ps9
Viridiplantae - ps10
Streptophyta - ps11
Charophyceae/Embriophyta - ps12
Embriophyta - ps13
Tracheophyta - ps14
Spermatophyta - ps15
Angiosperms - ps16
Mesangiospermae - ps17
Magnoliids/eudicots - ps18
Eudicots - ps19
Core_eudicots - ps20
Superrosids - ps21
Rosids - ps22
Eurosids - ps23
Malvids - ps24
Sap./Malv./Brassicales - ps25
Malvales/Brassicales - ps26
Brassicales - ps27
Cleomaceae/Brassicaceae - ps28
Brassicaceae - ps29
Camelineae - ps30
Arabidopsis - ps31
Arabidopsis_thaliana - ps32
Phylostratum

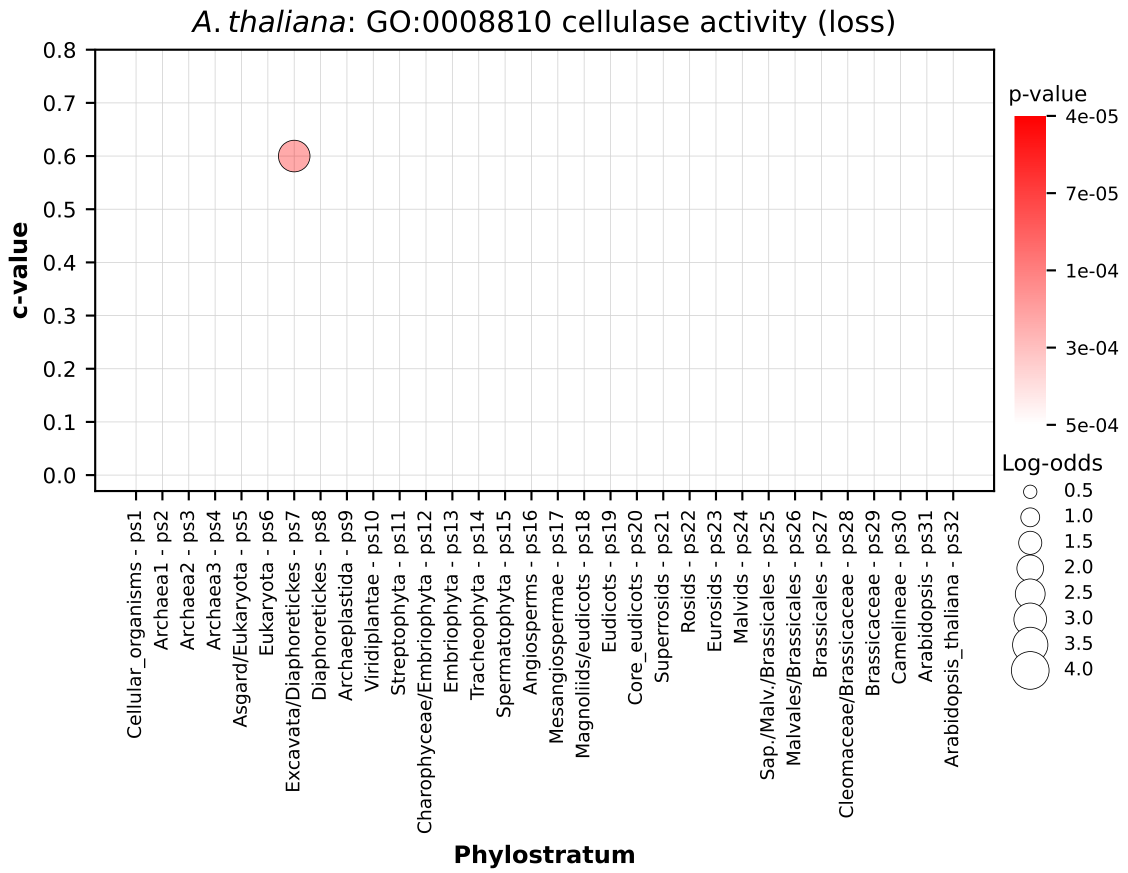
A. thaliana: GO:0008810 cellulase activity (loss)
c-value
0.8
0.7
0.6
0.5
0.4
0.3
0.2
0.1
0.0
p-value
4e-05
7e-05
1e-04
3e-04
5e-04
Log-odds
0.5
1.0
1.5
2.0
2.5
3.0
3.5
4.0
Cellular_organisms - ps1
Archaea1 - ps2
Archaea2 - ps3
Archaea3 - ps4
Asgard/Eukaryota - ps5
Eukaryota - ps6
Excavata/Diaphoretickes - ps7
Diaphoretickes - ps8
Archaeplastida - ps9
Viridiplantae - ps10
Streptophyta - ps11
Charophyceae/Embriophyta - ps12
Embriophyta - ps13
Tracheophyta - ps14
Spermatophyta - ps15
Angiosperms - ps16
Mesangiospermae - ps17
Magnoliids/eudicots - ps18
Eudicots - ps19
Core_eudicots - ps20
Superrosids - ps21
Rosids - ps22
Eurosids - ps23
Malvids - ps24
Sap./Malv./Brassicales - ps25
Malvales/Brassicales - ps26
Brassicales - ps27
Cleomaceae/Brassicaceae - ps28
Brassicaceae - ps29
Camelineae - ps30
Arabidopsis - ps31
Arabidopsis_thaliana - ps32
Phylostratum

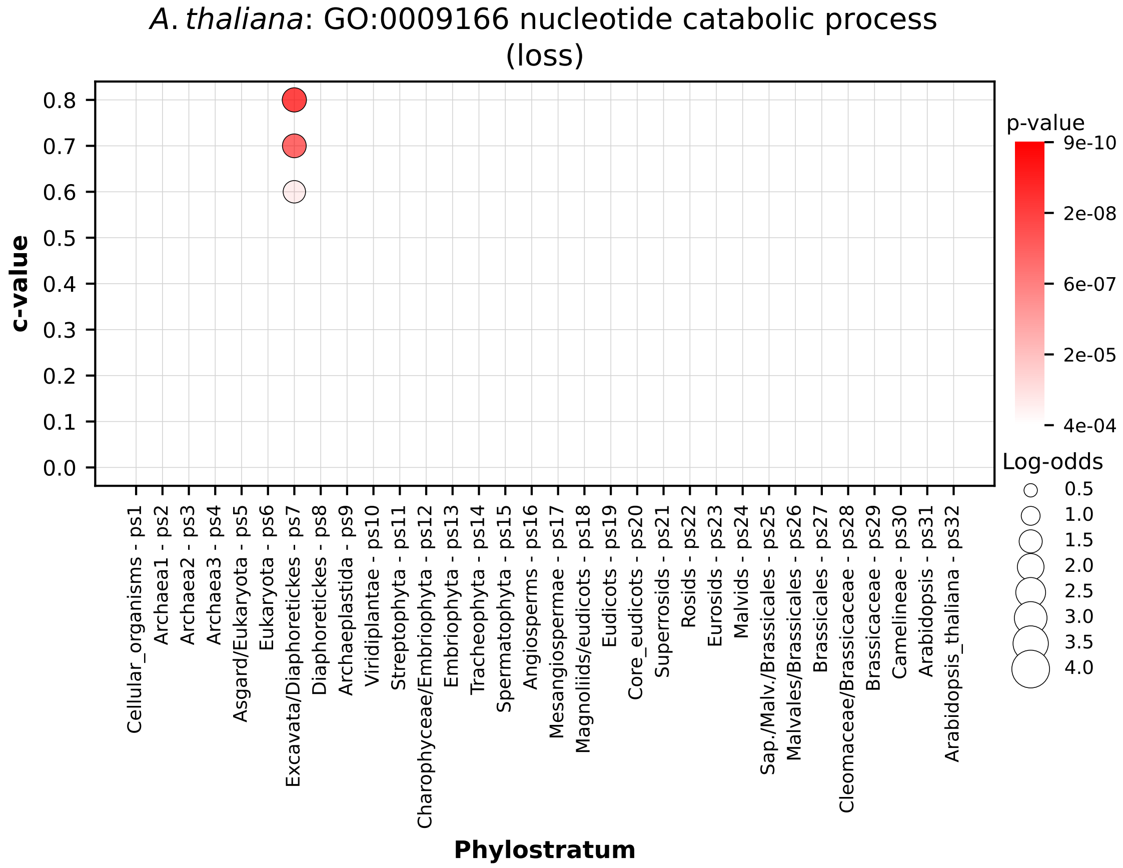
A. thaliana: GO:0009166 nucleotide catabolic process
(loss)
c-value
0.0
0.1
0.2
0.3
0.4
0.5
0.6
0.7
0.8
Phylostratum
Cellular_organisms - ps1
Archaea1 - ps2
Archaea2 - ps3
Archaea3 - ps4
Asgard/Eukaryota - ps5
Eukaryota - ps6
Excavata/Diaphoretickes - ps7
Diaphoretickes - ps8
Archaeplastida - ps9
Viridiplantae - ps10
Streptophyta - ps11
Charophyceae/Embriophyta - ps12
Embriophyta - ps13
Tracheophyta - ps14
Spermatophyta - ps15
Angiosperms - ps16
Mesangiospermae - ps17
Magnoliids/eudicots - ps18
Eudicots - ps19
Core_eudicots - ps20
Superrosids - ps21
Rosids - ps22
Eurosids - ps23
Malvids - ps24
Sap./Malv./Brassicales - ps25
Malvales/Brassicales - ps26
Brassicales - ps27
Cleomaceae/Brassicaceae - ps28
Brassicaceae - ps29
Camelineae - ps30
Arabidopsis - ps31
Arabidopsis_thaliana - ps32
p-value
9e-10
2e-08
6e-07
2e-05
4e-04
Log-odds
0.5
1.0
1.5
2.0
2.5
3.0
3.5
4.0

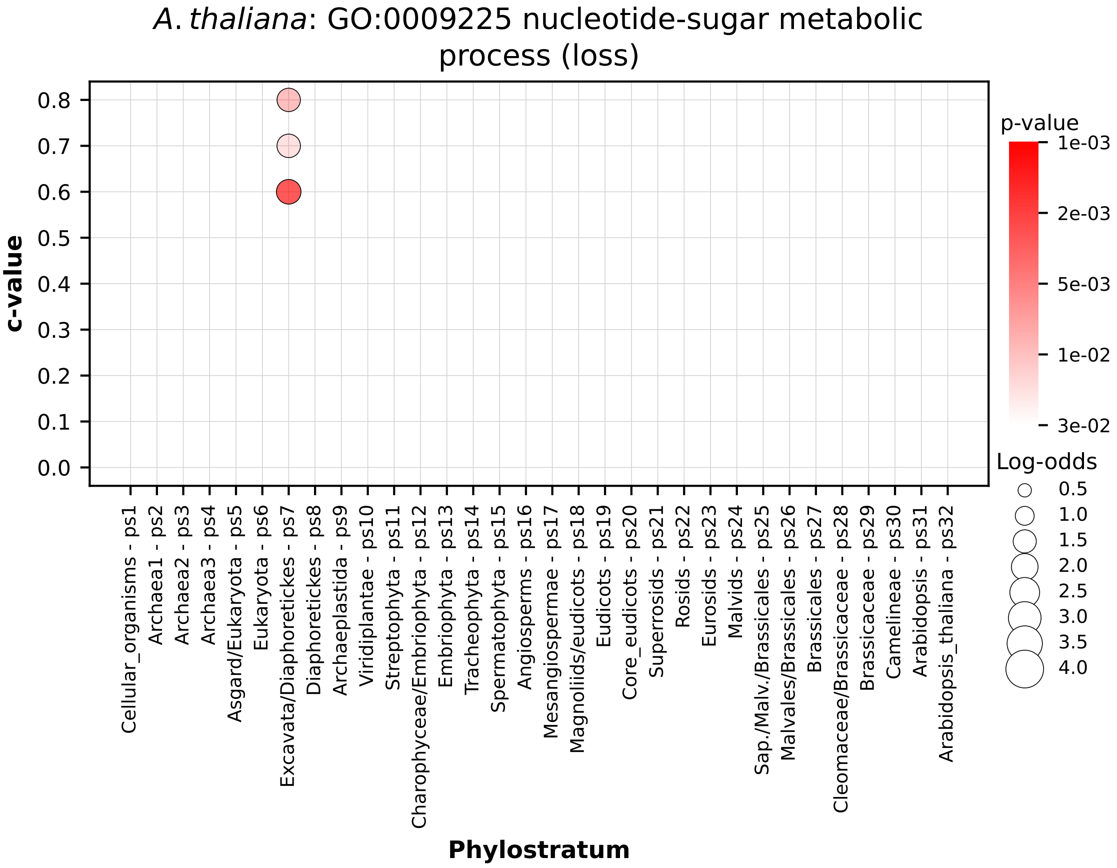
A. thaliana: GO:0009225 nucleotide-sugar metabolic process (loss)
c-value
0.8
0.7
0.6
0.5
0.4
0.3
0.2
0.1
0.0
p-value
1e-03
2e-03
5e-03
1e-02
3e-02
Log-odds
0.5
1.0
1.5
2.0
2.5
3.0
3.5
4.0
Cellular_organisms - ps1
Archaea1 - ps2
Archaea2 - ps3
Archaea3 - ps4
Asgard/Eukaryota - ps5
Eukaryota - ps6
Excavata/Diaphoretickes - ps7
Diaphoretickes - ps8
Archaeplastida - ps9
Viridiplantae - ps10
Streptophyta - ps11
Charophyceae/Embriophyta - ps12
Embriophyta - ps13
Tracheophyta - ps14
Spermatophyta - ps15
Angiosperms - ps16
Mesangiospermae - ps17
Magnoliids/eudicots - ps18
Eudicots - ps19
Core_eudicots - ps20
Superrosids - ps21
Rosids - ps22
Eurosids - ps23
Malvids - ps24
Sap./Malv./Brassicales - ps25
Malvales/Brassicales - ps26
Brassicales - ps27
Cleomaceae/Brassicaceae - ps28
Brassicaceae - ps29
Camelineae - ps30
Arabidopsis - ps31
Arabidopsis_thaliana - ps32
Phylostratum

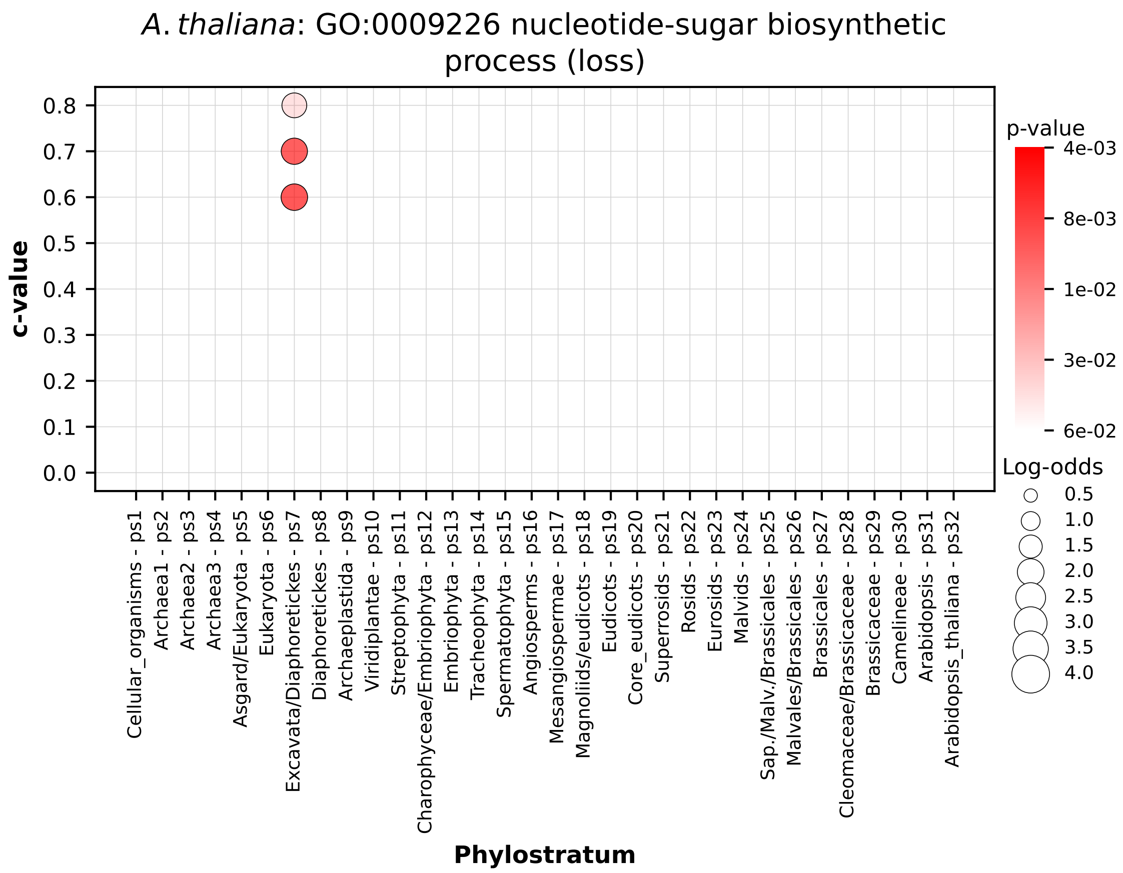
A. thaliana: GO:0009226 nucleotide-sugar biosynthetic process (loss)
c-value
0.8
0.7
0.6
0.5
0.4
0.3
0.2
0.1
0.0
p-value
4e-03
8e-03
1e-02
3e-02
6e-02
Log-odds
0.5
1.0
1.5
2.0
2.5
3.0
3.5
4.0
Cellular_organisms - ps1
Archaea1 - ps2
Archaea2 - ps3
Archaea3 - ps4
Asgard/Eukaryota - ps5
Eukaryota - ps6
Excavata/Diaphoretickes - ps7
Diaphoretickes - ps8
Archaeplastida - ps9
Viridiplantae - ps10
Streptophyta - ps11
Charophyceae/Embriophyta - ps12
Embriophyta - ps13
Tracheophyta - ps14
Spermatophyta - ps15
Angiosperms - ps16
Mesangiospermae - ps17
Magnoliids/eudicots - ps18
Eudicots - ps19
Core_eudicots - ps20
Superrosids - ps21
Rosids - ps22
Eurosids - ps23
Malvids - ps24
Sap./Malv./Brassicales - ps25
Malvales/Brassicales - ps26
Brassicales - ps27
Cleomaceae/Brassicaceae - ps28
Brassicaceae - ps29
Camelineae - ps30
Arabidopsis - ps31
Arabidopsis_thaliana - ps32
Phylostratum

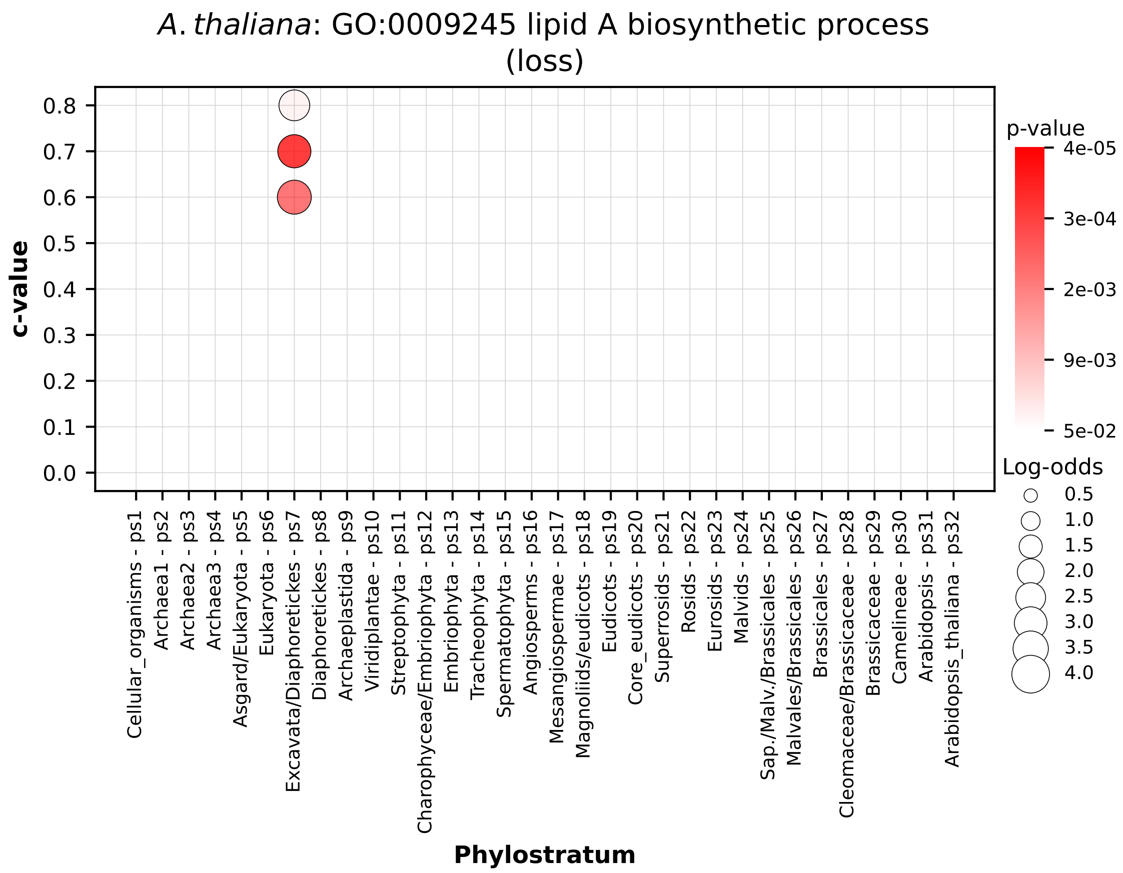

A. thaliana: GO:0009245 lipid A biosynthetic process
(loss)
c-value
0.0
0.1
0.2
0.3
0.4
0.5
0.6
0.7
0.8
Phylostratum
Cellular_organisms - ps1
Archaea1 - ps2
Archaea2 - ps3
Archaea3 - ps4
Asgard/Eukaryota - ps5
Eukaryota - ps6
Excavata/Diaphoretickes - ps7
Diaphoretickes - ps8
Archaeplastida - ps9
Viridiplantae - ps10
Streptophyta - ps11
Charophyceae/Embriophyta - ps12
Embriophyta - ps13
Tracheophyta - ps14
Spermatophyta - ps15
Angiosperms - ps16
Mesangiospermae - ps17
Magnoliids/eudicots - ps18
Eudicots - ps19
Core_eudicots - ps20
Superrosids - ps21
Rosids - ps22
Eurosids - ps23
Malvids - ps24
Sap./Malv./Brassicales - ps25
Malvales/Brassicales - ps26
Brassicales - ps27
Cleomaceae/Brassicaceae - ps28
Brassicaceae - ps29
Camelineae - ps30
Arabidopsis - ps31
Arabidopsis_thaliana - ps32
p-value
4e-05
3e-04
2e-03
9e-03
5e-02
Log-odds
0.5
1.0
1.5
2.0
2.5
3.0
3.5
4.0

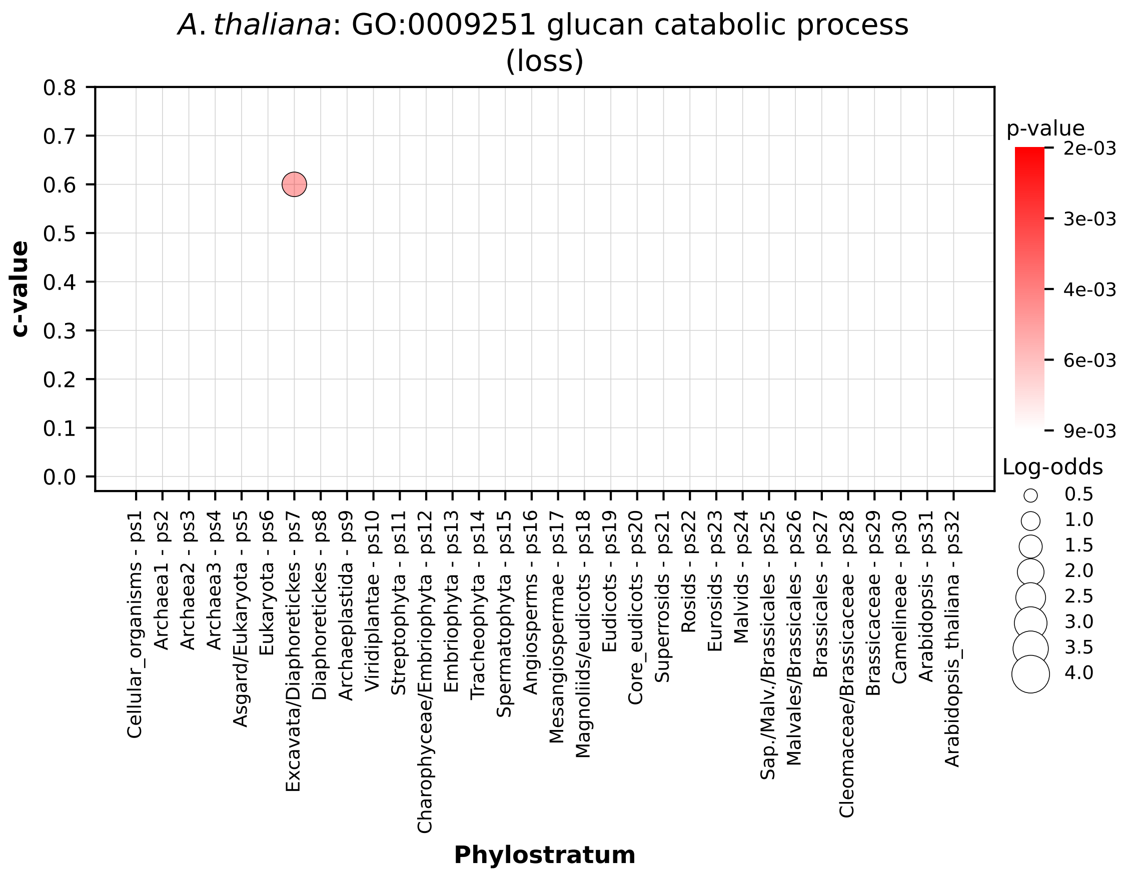

A. thaliana: GO:0009251 glucan catabolic process
(loss)
c-value
0.8
0.7
0.6
0.5
0.4
0.3
0.2
0.1
0.0
p-value
2e-03
3e-03
4e-03
6e-03
9e-03
Log-odds
0.5
1.0
1.5
2.0
2.5
3.0
3.5
4.0
Cellular_organisms - ps1
Archaea1 - ps2
Archaea2 - ps3
Archaea3 - ps4
Asgard/Eukaryota - ps5
Eukaryota - ps6
Excavata/Diaphoretickes - ps7
Diaphoretickes - ps8
Archaeplastida - ps9
Viridiplantae - ps10
Streptophyta - ps11
Charophyceae/Embriophyta - ps12
Embriophyta - ps13
Tracheophyta - ps14
Spermatophyta - ps15
Angiosperms - ps16
Mesangiospermae - ps17
Magnoliids/eudicots - ps18
Eudicots - ps19
Core_eudicots - ps20
Superrosids - ps21
Rosids - ps22
Eurosids - ps23
Malvids - ps24
Sap./Malv./Brassicales - ps25
Malvales/Brassicales - ps26
Brassicales - ps27
Cleomaceae/Brassicaceae - ps28
Brassicaceae - ps29
Camelineae - ps30
Arabidopsis - ps31
Arabidopsis_thaliana - ps32
Phylostratum

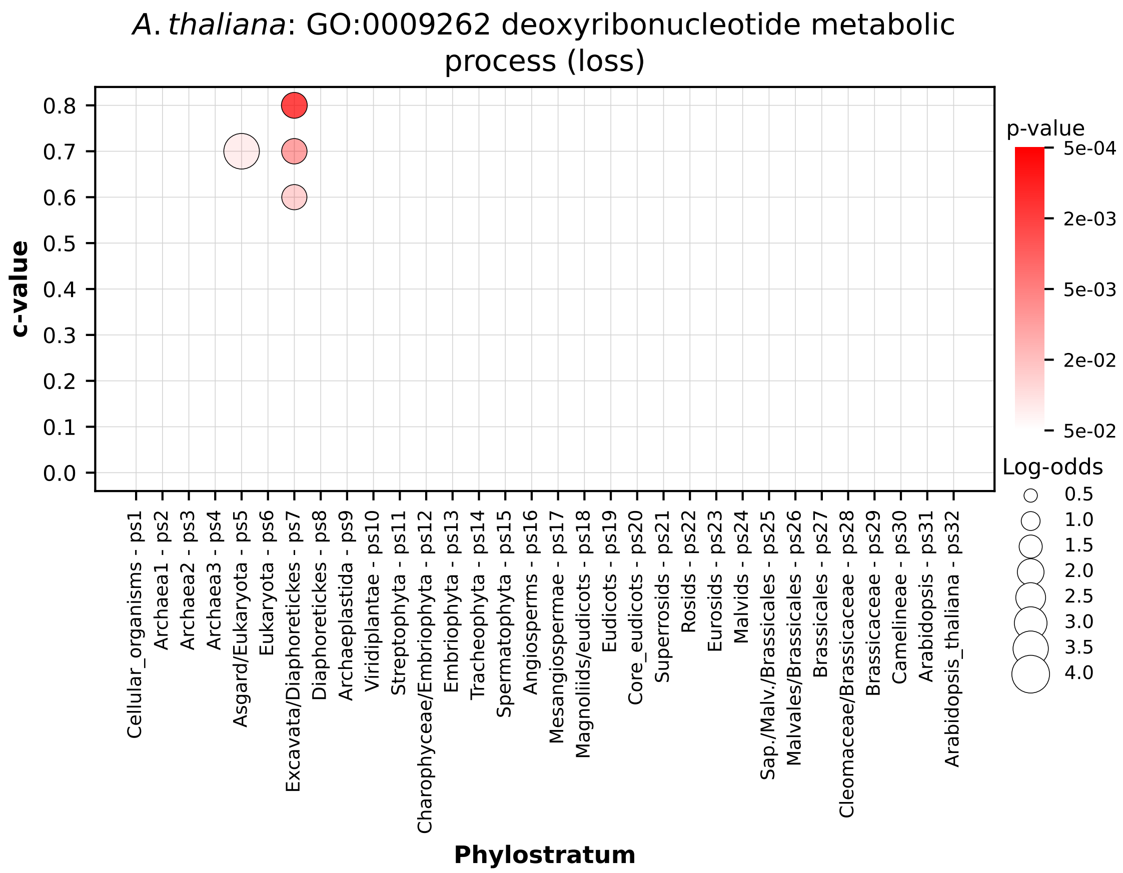

A. thaliana: GO:0009262 deoxyribonucleotide metabolic process (loss)
c-value
0.8
0.7
0.6
0.5
0.4
0.3
0.2
0.1
0.0
p-value
5e-04
2e-03
5e-03
2e-02
5e-02
Log-odds
0.5
1.0
1.5
2.0
2.5
3.0
3.5
4.0
Cellular_organisms - ps1
Archaea1 - ps2
Archaea2 - ps3
Archaea3 - ps4
Asgard/Eukaryota - ps5
Eukaryota - ps6
Excavata/Diaphoretickes - ps7
Diaphoretickes - ps8
Archaeplastida - ps9
Viridiplantae - ps10
Streptophyta - ps11
Charophyceae/Embriophyta - ps12
Embriophyta - ps13
Tracheophyta - ps14
Spermatophyta - ps15
Angiosperms - ps16
Mesangiospermae - ps17
Magnoliids/eudicots - ps18
Eudicots - ps19
Core_eudicots - ps20
Superrosids - ps21
Rosids - ps22
Eurosids - ps23
Malvids - ps24
Sap./Malv./Brassicales - ps25
Malvales/Brassicales - ps26
Brassicales - ps27
Cleomaceae/Brassicaceae - ps28
Brassicaceae - ps29
Camelineae - ps30
Arabidopsis - ps31
Arabidopsis_thaliana - ps32
Phylostratum

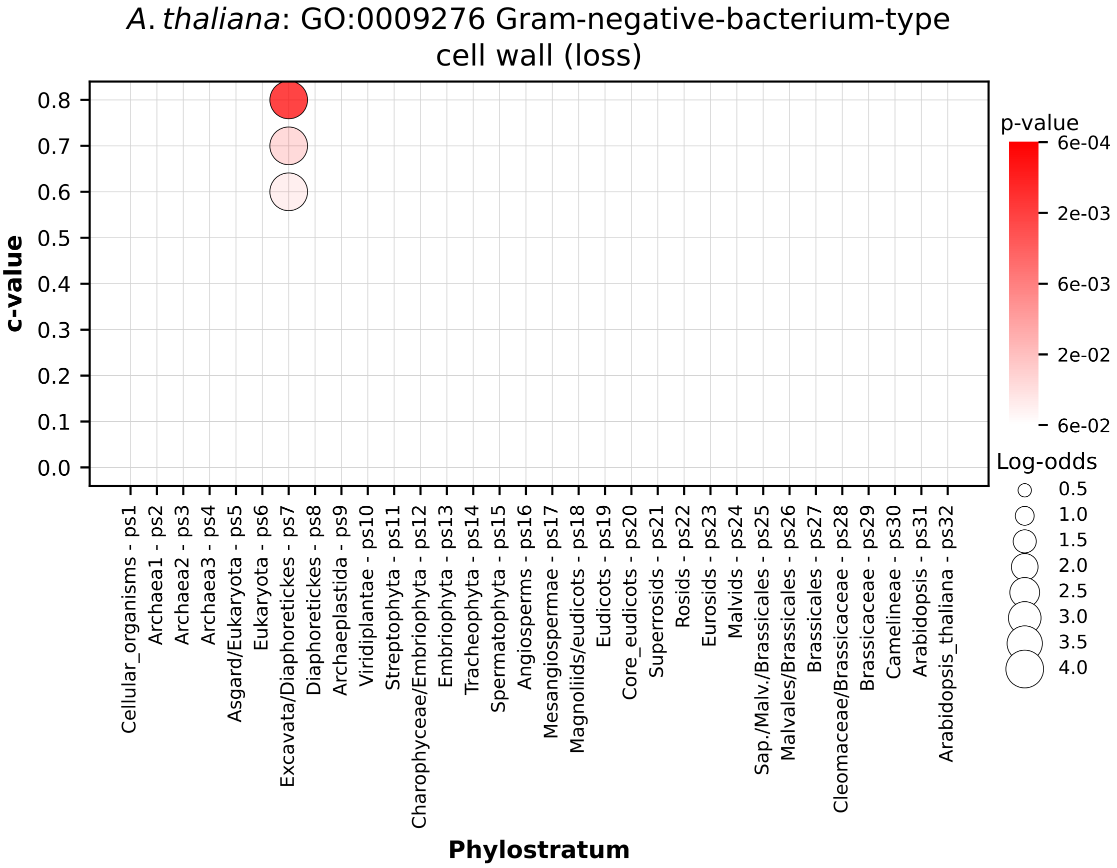

A. thaliana: GO:0009276 Gram-negative-bacterium-type cell wall (loss)
c-value
0.8
0.7
0.6
0.5
0.4
0.3
0.2
0.1
0.0
p-value
6e-04
2e-03
6e-03
2e-02
6e-02
Log-odds
0.5
1.0
1.5
2.0
2.5
3.0
3.5
4.0
Cellular_organisms - ps1
Archaea1 - ps2
Archaea2 - ps3
Archaea3 - ps4
Asgard/Eukaryota - ps5
Eukaryota - ps6
Excavata/Diaphoretickes - ps7
Diaphoretickes - ps8
Archaeplastida - ps9
Viridiplantae - ps10
Streptophyta - ps11
Charophyceae/Embriophyta - ps12
Embriophyta - ps13
Tracheophyta - ps14
Spermatophyta - ps15
Angiosperms - ps16
Mesangiospermae - ps17
Magnoliids/eudicots - ps18
Eudicots - ps19
Core_eudicots - ps20
Superrosids - ps21
Rosids - ps22
Eurosids - ps23
Malvids - ps24
Sap./Malv./Brassicales - ps25
Malvales/Brassicales - ps26
Brassicales - ps27
Cleomaceae/Brassicaceae - ps28
Brassicaceae - ps29
Camelineae - ps30
Arabidopsis - ps31
Arabidopsis_thaliana - ps32
Phylostratum

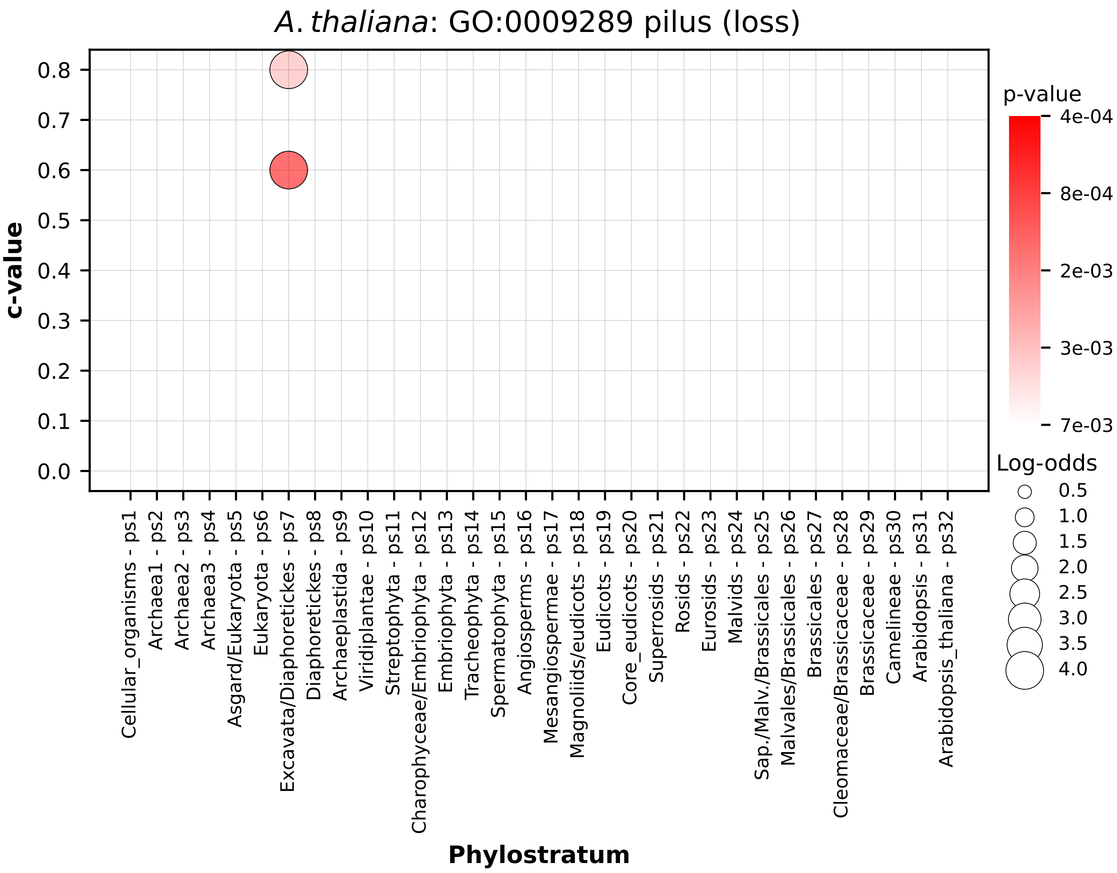

A. thaliana: GO:0009289 pilus (loss)
c-value
0.8
0.7
0.6
0.5
0.4
0.3
0.2
0.1
0.0
p-value
4e-04
8e-04
2e-03
3e-03
7e-03
Log-odds
0.5
1.0
1.5
2.0
2.5
3.0
3.5
4.0
Cellular_organisms - ps1
Archaea1 - ps2
Archaea2 - ps3
Archaea3 - ps4
Asgard/Eukaryota - ps5
Eukaryota - ps6
Excavata/Diaphoretickes - ps7
Diaphoretickes - ps8
Archaeplastida - ps9
Viridiplantae - ps10
Streptophyta - ps11
Charophyceae/Embriophyta - ps12
Embriophyta - ps13
Tracheophyta - ps14
Spermatophyta - ps15
Angiosperms - ps16
Mesangiospermae - ps17
Magnoliids/eudicots - ps18
Eudicots - ps19
Core_eudicots - ps20
Superrosids - ps21
Rosids - ps22
Eurosids - ps23
Malvids - ps24
Sap./Malv./Brassicales - ps25
Malvales/Brassicales - ps26
Brassicales - ps27
Cleomaceae/Brassicaceae - ps28
Brassicaceae - ps29
Camelineae - ps30
Arabidopsis - ps31
Arabidopsis_thaliana - ps32
Phylostratum

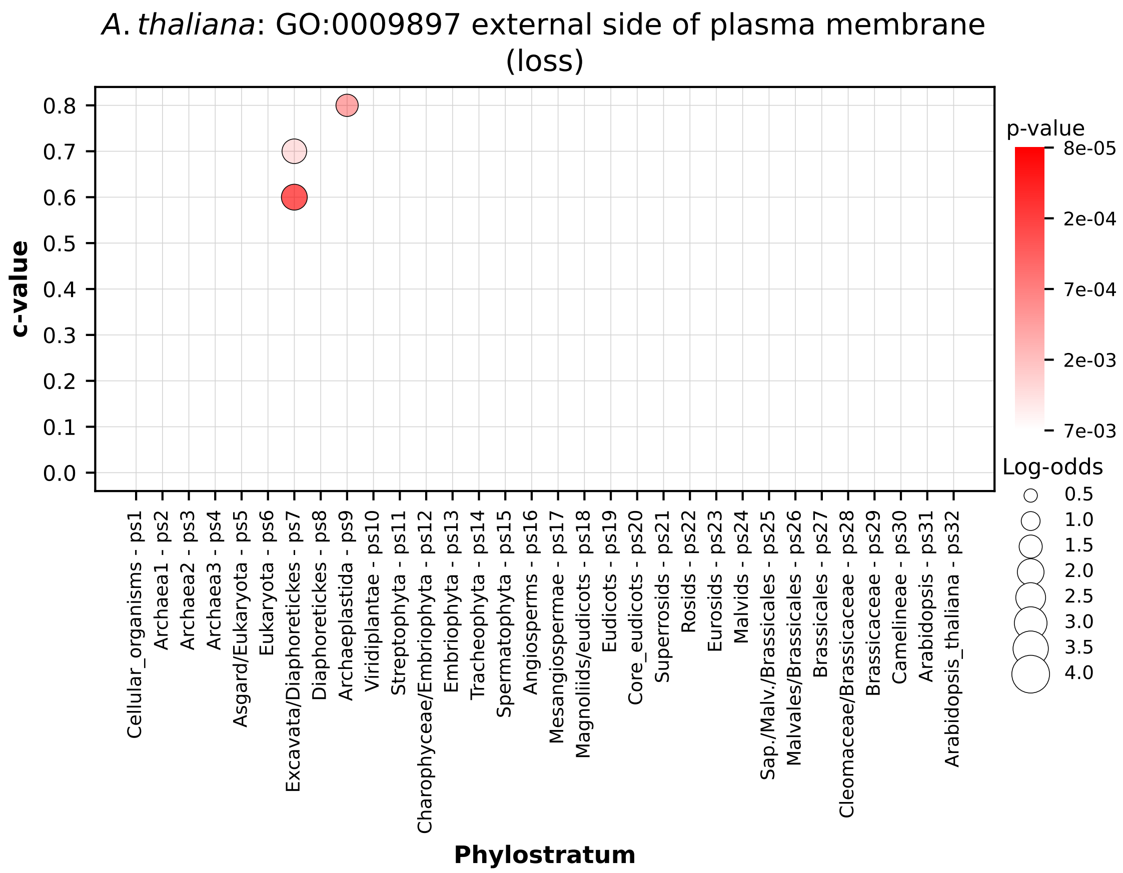

A. thaliana: GO:0009897 external side of plasma membrane
(loss)
c-value
0.0
0.1
0.2
0.3
0.4
0.5
0.6
0.7
0.8
p-value
8e-05
2e-04
7e-04
2e-03
7e-03
Log-odds
0.5
1.0
1.5
2.0
2.5
3.0
3.5
4.0
Cellular_organisms - ps1
Archaea1 - ps2
Archaea2 - ps3
Archaea3 - ps4
Asgard/Eukaryota - ps5
Eukaryota - ps6
Excavata/Diaphoretickes - ps7
Diaphoretickes - ps8
Archaeplastida - ps9
Viridiplantae - ps10
Streptophyta - ps11
Charophyceae/Embriophyta - ps12
Embriophyta - ps13
Tracheophyta - ps14
Spermatophyta - ps15
Angiosperms - ps16
Mesangiospermae - ps17
Magnoliids/eudicots - ps18
Eudicots - ps19
Core_eudicots - ps20
Superrosids - ps21
Rosids - ps22
Eurosids - ps23
Malvids - ps24
Sap./Malv./Brassicales - ps25
Malvales/Brassicales - ps26
Brassicales - ps27
Cleomaceae/Brassicaceae - ps28
Brassicaceae - ps29
Camelineae - ps30
Arabidopsis - ps31
Arabidopsis_thaliana - ps32
Phylostratum

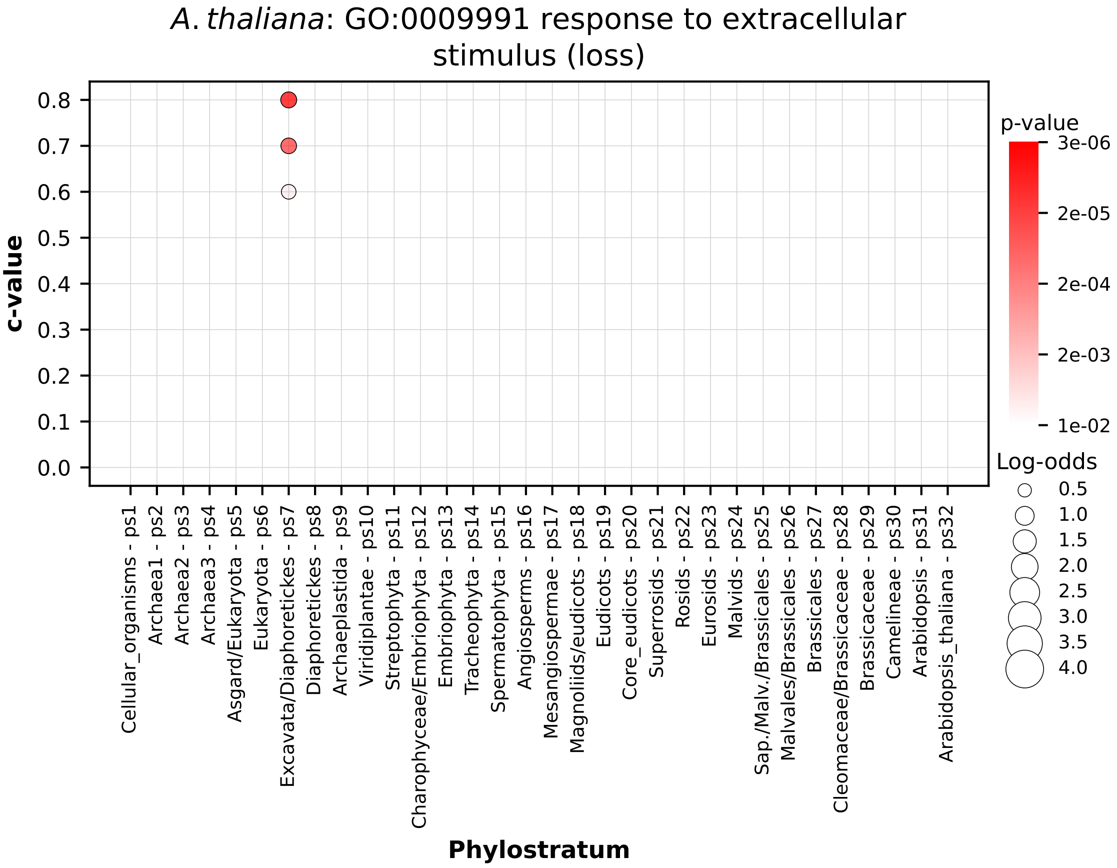

A. thaliana: GO:0009991 response to extracellular stimulus (loss)
c-value
0.8
0.7
0.6
0.5
0.4
0.3
0.2
0.1
0.0
Phylostratum
Cellular_organisms - ps1
Archaea1 - ps2
Archaea2 - ps3
Archaea3 - ps4
Asgard/Eukaryota - ps5
Eukaryota - ps6
Excavata/Diaphoretickes - ps7
Diaphoretickes - ps8
Archaeplastida - ps9
Viridiplantae - ps10
Streptophyta - ps11
Charophyceae/Embriophyta - ps12
Embriophyta - ps13
Tracheophyta - ps14
Spermatophyta - ps15
Angiosperms - ps16
Mesangiospermae - ps17
Magnoliids/eudicots - ps18
Eudicots - ps19
Core_eudicots - ps20
Superrosids - ps21
Rosids - ps22
Eurosids - ps23
Malvids - ps24
Sap./Malv./Brassicales - ps25
Malvales/Brassicales - ps26
Brassicales - ps27
Cleomaceae/Brassicaceae - ps28
Brassicaceae - ps29
Camelineae - ps30
Arabidopsis - ps31
Arabidopsis_thaliana - ps32
p-value
3e-06
2e-05
2e-04
2e-03
1e-02
Log-odds
0.5
1.0
1.5
2.0
2.5
3.0
3.5
4.0

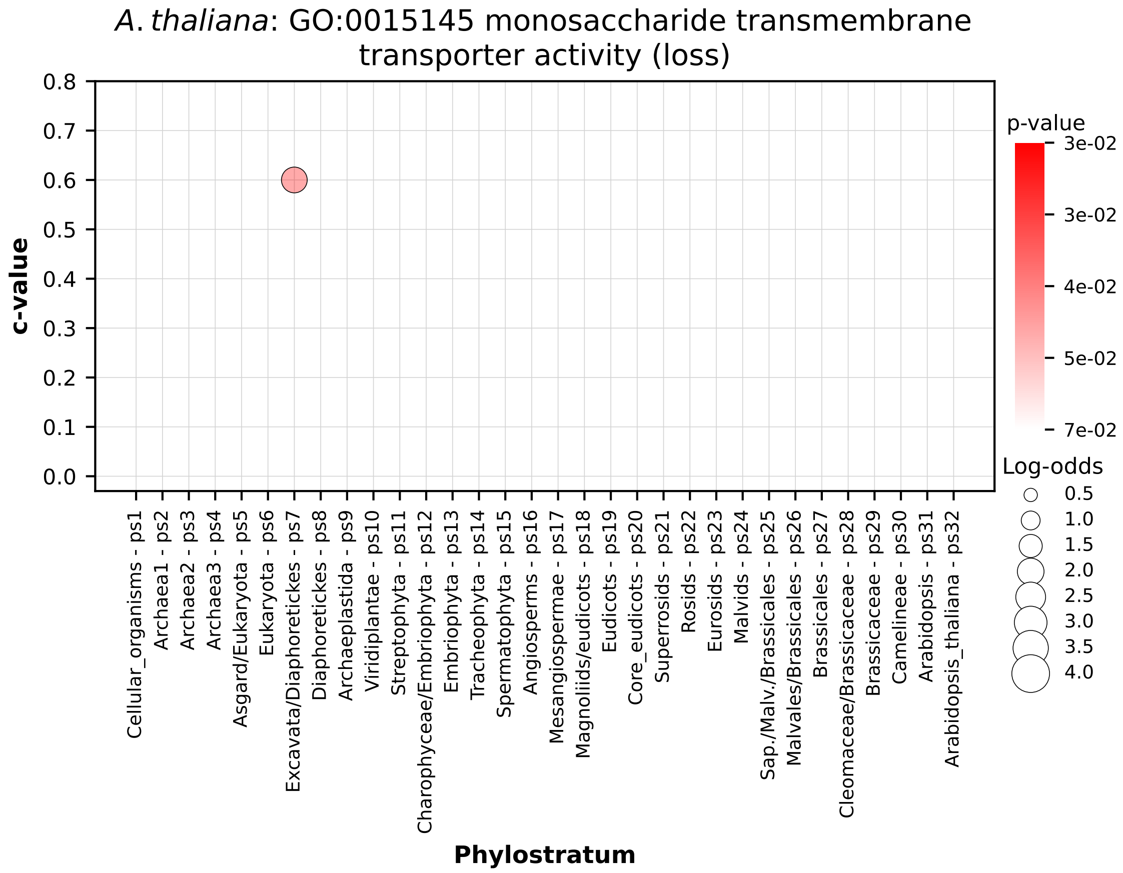

$A. thaliana$: GO:0015145 monosaccharide transmembrane transporter activity (loss)
c-value
0.8
0.7
0.6
0.5
0.4
0.3
0.2
0.1
0.0
Phylostratum
Cellular_organisms - ps1
Archaea1 - ps2
Archaea2 - ps3
Archaea3 - ps4
Asgard/Eukaryota - ps5
Eukaryota - ps6
Excavata/Diaphoretickes - ps7
Diaphoretickes - ps8
Archaeplastida - ps9
Viridiplantae - ps10
Streptophyta - ps11
Charophyceae/Embriophyta - ps12
Embriophyta - ps13
Tracheophyta - ps14
Spermatophyta - ps15
Angiosperms - ps16
Mesangiospermae - ps17
Magnoliids/eudicots - ps18
Eudicots - ps19
Core_eudicots - ps20
Superrosids - ps21
Rosids - ps22
Eurosids - ps23
Malvids - ps24
Sap./Malv./Brassicales - ps25
Malvales/Brassicales - ps26
Brassicales - ps27
Cleomaceae/Brassicaceae - ps28
Brassicaceae - ps29
Camelineae - ps30
Arabidopsis - ps31
Arabidopsis_thaliana - ps32
p-value
3e-02
3e-02
4e-02
5e-02
7e-02
Log-odds
0.5
1.0
1.5
2.0
2.5
3.0
3.5
4.0

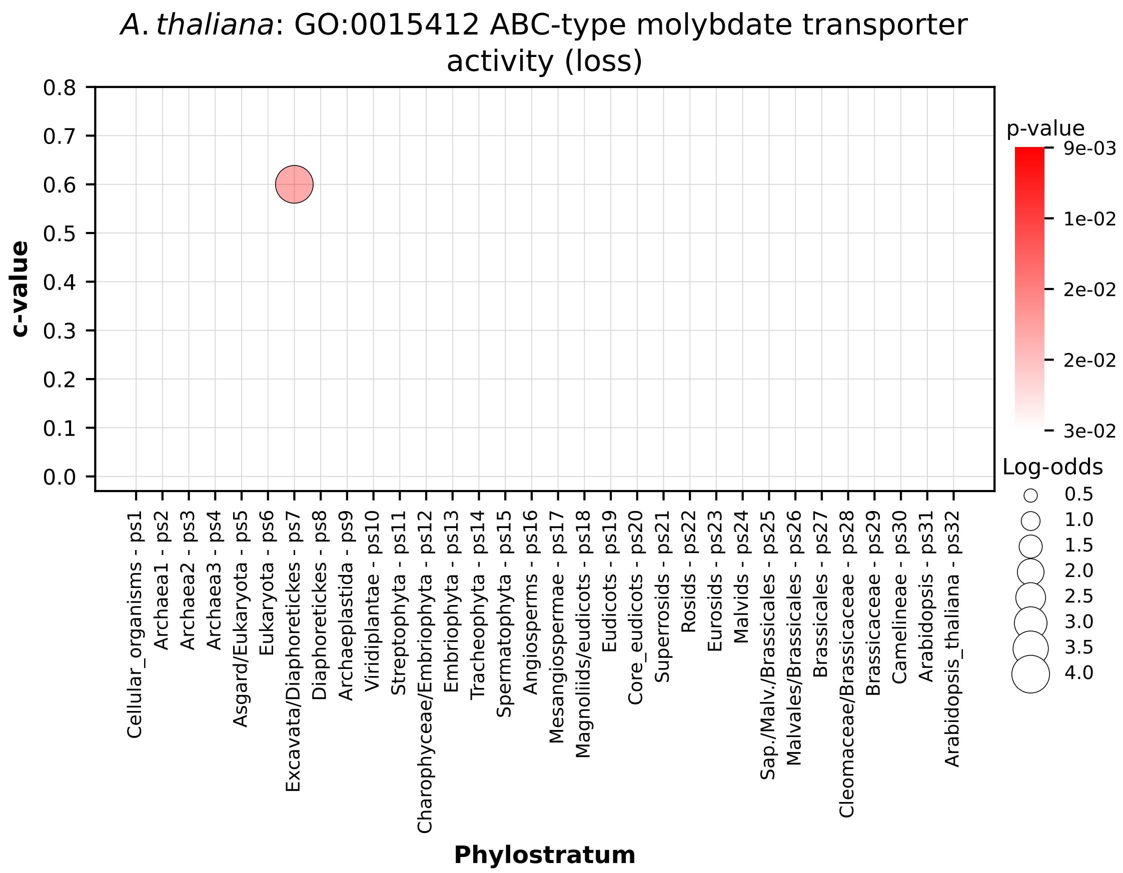
A. thaliana: GO:0015412 ABC-type molybdate transporter activity (loss)
c-value
0.8
0.7
0.6
0.5
0.4
0.3
0.2
0.1
0.0
Phylostratum
Cellular_organisms - ps1
Archaea1 - ps2
Archaea2 - ps3
Archaea3 - ps4
Asgard/Eukaryota - ps5
Eukaryota - ps6
Excavata/Diaphoretickes - ps7
Diaphoretickes - ps8
Archaeplastida - ps9
Viridiplantae - ps10
Streptophyta - ps11
Charophyceae/Embriophyta - ps12
Embriophyta - ps13
Tracheophyta - ps14
Spermatophyta - ps15
Angiosperms - ps16
Mesangiospermae - ps17
Magnoliids/eudicots - ps18
Eudicots - ps19
Core_eudicots - ps20
Superrosids - ps21
Rosids - ps22
Eurosids - ps23
Malvids - ps24
Sap./Malv./Brassicales - ps25
Malvales/Brassicales - ps26
Brassicales - ps27
Cleomaceae/Brassicaceae - ps28
Brassicaceae - ps29
Camelineae - ps30
Arabidopsis - ps31
Arabidopsis_thaliana - ps32
p-value
9e-03
1e-02
2e-02
2e-02
3e-02
Log-odds
0.5
1.0
1.5
2.0
2.5
3.0
3.5
4.0

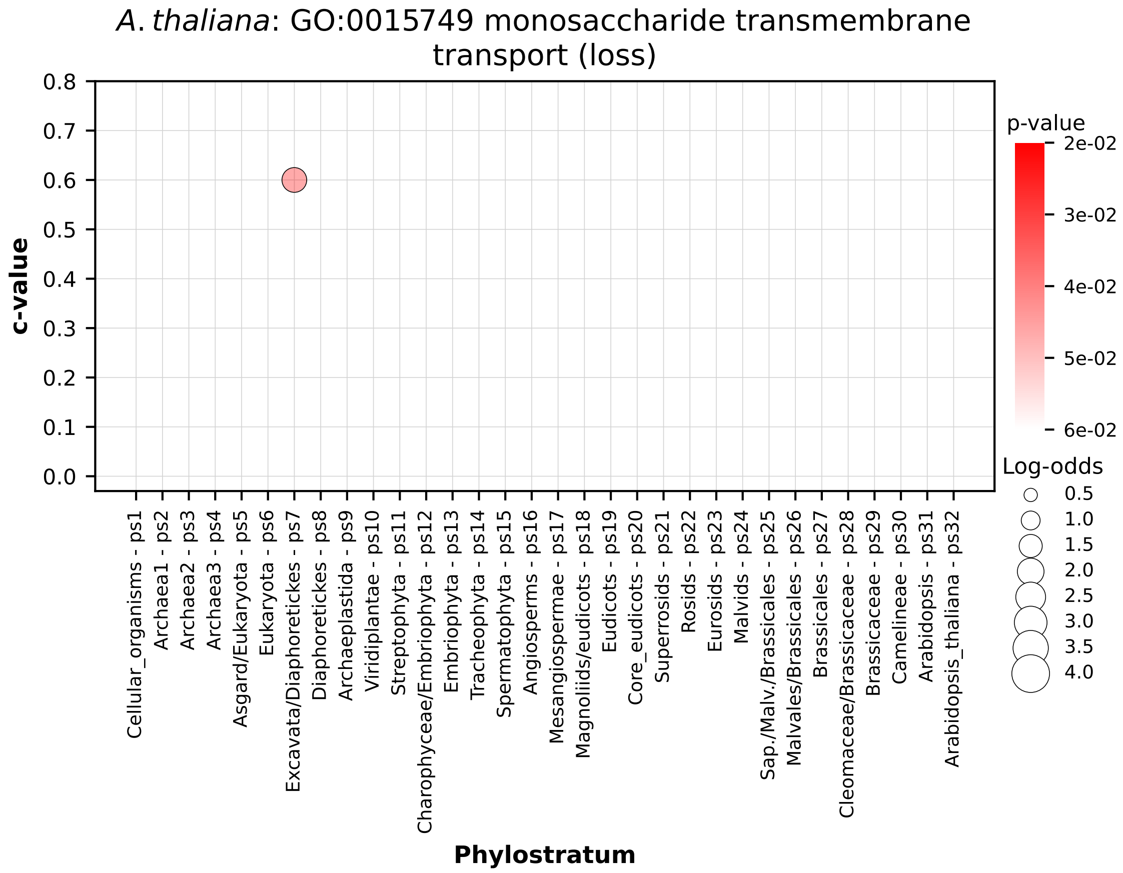

A. thaliana: GO:0015749 monosaccharide transmembrane transport (loss)
c-value
0.8
0.7
0.6
0.5
0.4
0.3
0.2
0.1
0.0
p-value
2e-02
3e-02
4e-02
5e-02
6e-02
Log-odds
0.5
1.0
1.5
2.0
2.5
3.0
3.5
4.0
Cellular_organisms - ps1
Archaea1 - ps2
Archaea2 - ps3
Archaea3 - ps4
Asgard/Eukaryota - ps5
Eukaryota - ps6
Excavata/Diaphoretickes - ps7
Diaphoretickes - ps8
Archaeplastida - ps9
Viridiplantae - ps10
Streptophyta - ps11
Charophyceae/Embriophyta - ps12
Embriophyta - ps13
Tracheophyta - ps14
Spermatophyta - ps15
Angiosperms - ps16
Mesangiospermae - ps17
Magnoliids/eudicots - ps18
Eudicots - ps19
Core_eudicots - ps20
Superrosids - ps21
Rosids - ps22
Eurosids - ps23
Malvids - ps24
Sap./Malv./Brassicales - ps25
Malvales/Brassicales - ps26
Brassicales - ps27
Cleomaceae/Brassicaceae - ps28
Brassicaceae - ps29
Camelineae - ps30
Arabidopsis - ps31
Arabidopsis_thaliana - ps32
Phylostratum

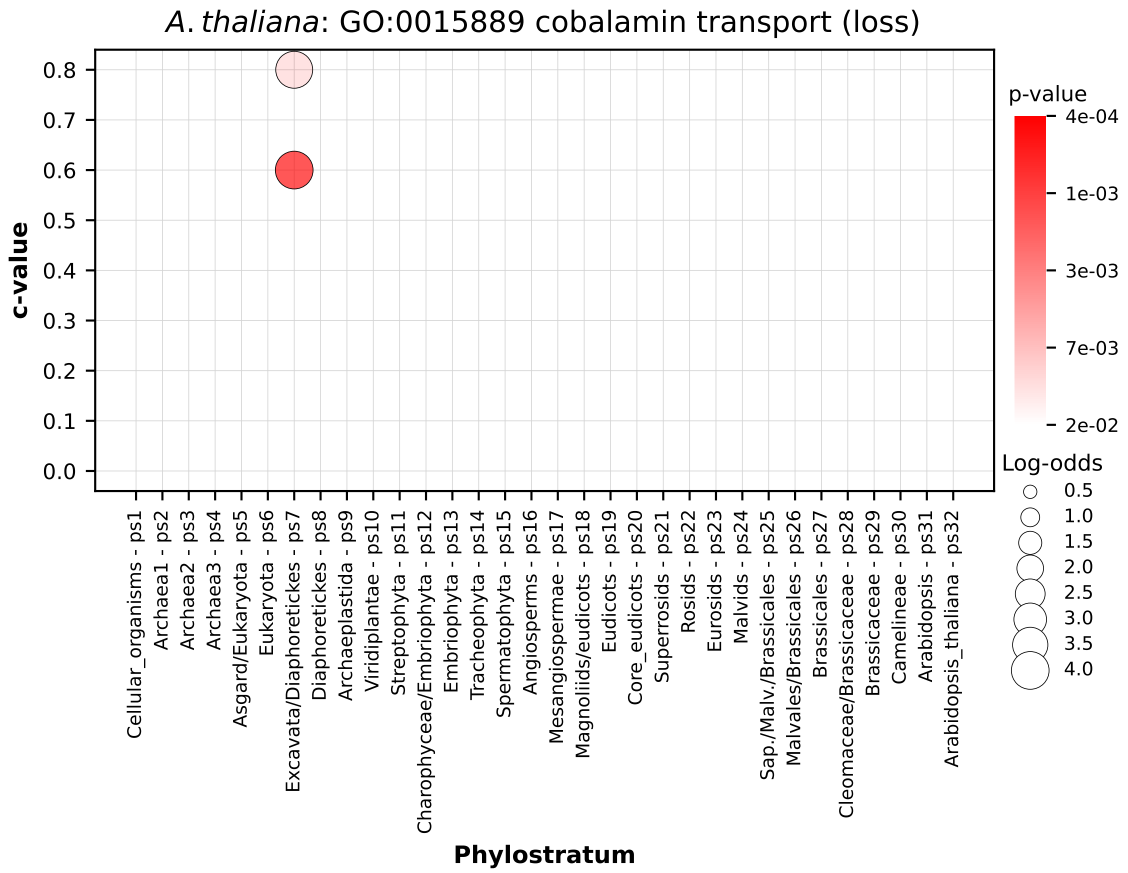

A. thaliana: GO:0015889 cobalamin transport (loss)
c-value
0.0
0.1
0.2
0.3
0.4
0.5
0.6
0.7
0.8
Phylostratum
Cellular_organisms - ps1
Archaea1 - ps2
Archaea2 - ps3
Archaea3 - ps4
Asgard/Eukaryota - ps5
Eukaryota - ps6
Excavata/Diaphoretickes - ps7
Diaphoretickes - ps8
Archaeplastida - ps9
Viridiplantae - ps10
Streptophyta - ps11
Charophyceae/Embriophyta - ps12
Embriophyta - ps13
Tracheophyta - ps14
Spermatophyta - ps15
Angiosperms - ps16
Mesangiospermae - ps17
Magnoliids/eudicots - ps18
Eudicots - ps19
Core_eudicots - ps20
Superrosids - ps21
Rosids - ps22
Eurosids - ps23
Malvids - ps24
Sap./Malv./Brassicales - ps25
Malvales/Brassicales - ps26
Brassicales - ps27
Cleomaceae/Brassicaceae - ps28
Brassicaceae - ps29
Camelineae - ps30
Arabidopsis - ps31
Arabidopsis_thaliana - ps32
p-value
4e-04
1e-03
3e-03
7e-03
2e-02
Log-odds
0.5
1.0
1.5
2.0
2.5
3.0
3.5
4.0

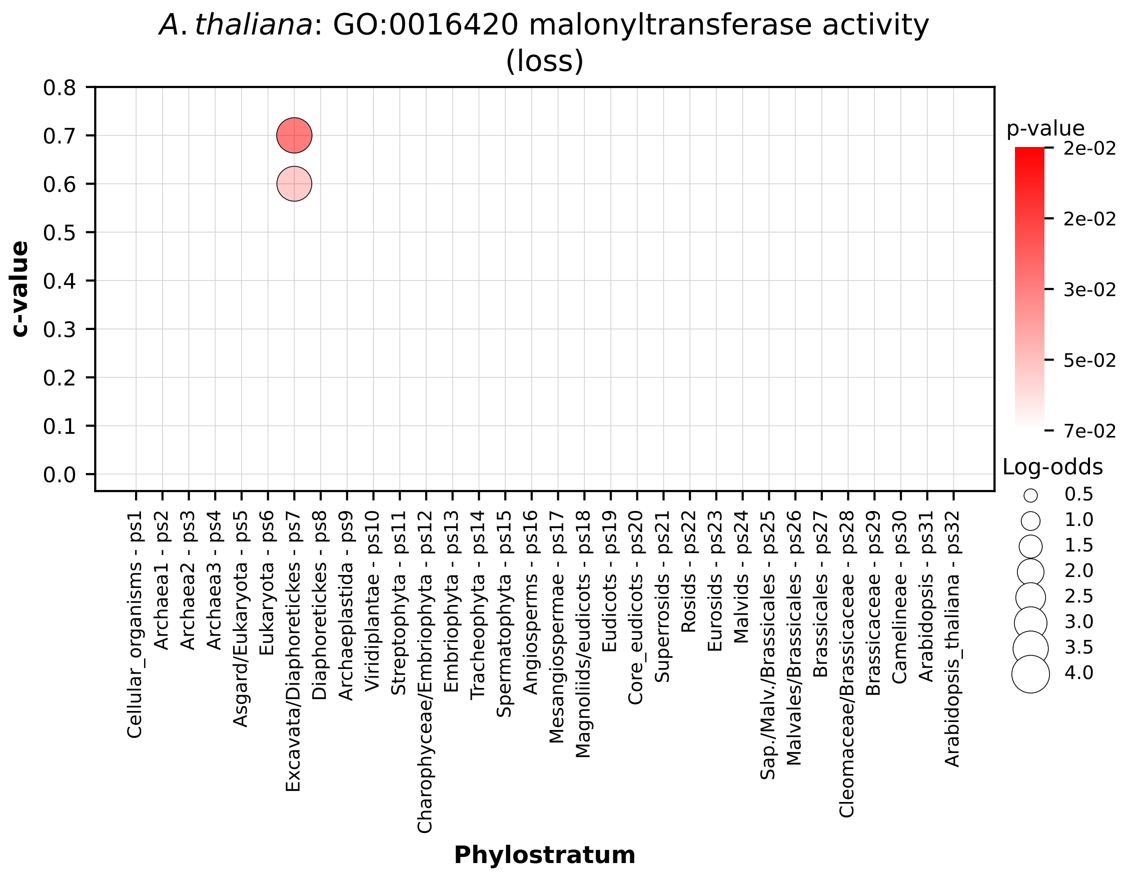

A. thaliana: GO:0016420 malonyltransferase activity (loss)
c-value
0.0
0.1
0.2
0.3
0.4
0.5
0.6
0.7
0.8
Phylostratum
Cellular_organisms - ps1
Archaea1 - ps2
Archaea2 - ps3
Archaea3 - ps4
Asgard/Eukaryota - ps5
Eukaryota - ps6
Excavata/Diaphoretickes - ps7
Diaphoretickes - ps8
Archaeplastida - ps9
Viridiplantae - ps10
Streptophyta - ps11
Charophyceae/Embriophyta - ps12
Embriophyta - ps13
Tracheophyta - ps14
Spermatophyta - ps15
Angiosperms - ps16
Mesangiospermae - ps17
Magnoliids/eudicots - ps18
Eudicots - ps19
Core_eudicots - ps20
Superrosids - ps21
Rosids - ps22
Eurosids - ps23
Malvids - ps24
Sap./Malv./Brassicales - ps25
Malvales/Brassicales - ps26
Brassicales - ps27
Cleomaceae/Brassicaceae - ps28
Brassicaceae - ps29
Camelineae - ps30
Arabidopsis - ps31
Arabidopsis_thaliana - ps32
p-value
2e-02
2e-02
3e-02
5e-02
7e-02
Log-odds
0.5
1.0
1.5
2.0
2.5
3.0
3.5
4.0

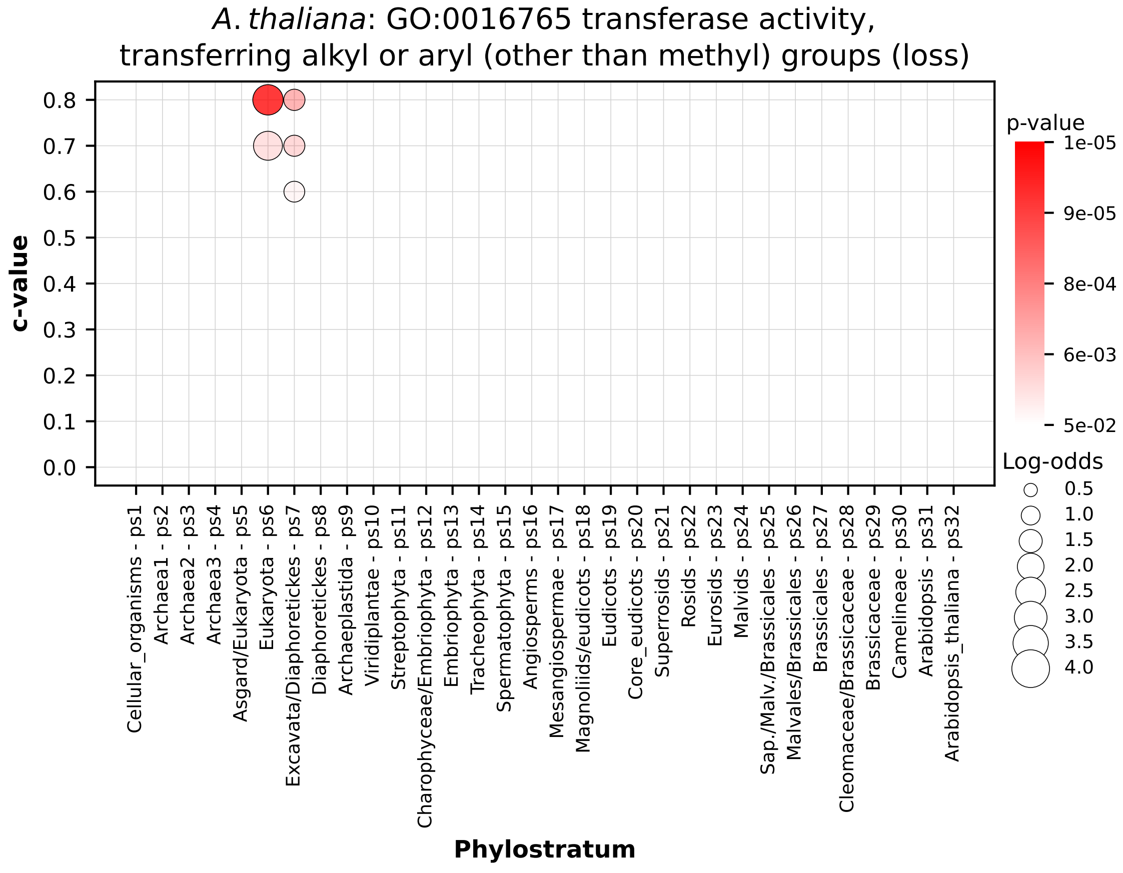

A. thaliana: GO:0016765 transferase activity, transferring alkyl or aryl (other than methyl) groups (loss)
c-value
0.8
0.7
0.6
0.5
0.4
0.3
0.2
0.1
0.0
p-value
1e-05
9e-05
8e-04
6e-03
5e-02
Log-odds
0.5
1.0
1.5
2.0
2.5
3.0
3.5
4.0
Cellular_organisms - ps1
Archaea1 - ps2
Archaea2 - ps3
Archaea3 - ps4
Asgard/Eukaryota - ps5
Eukaryota - ps6
Excavata/Diaphoretickes - ps7
Diaphoretickes - ps8
Archaeplastida - ps9
Viridiplantae - ps10
Streptophyta - ps11
Charophyceae/Embriophyta - ps12
Embriophyta - ps13
Tracheophyta - ps14
Spermatophyta - ps15
Angiosperms - ps16
Mesangiospermae - ps17
Magnoliids/eudicots - ps18
Eudicots - ps19
Core_eudicots - ps20
Superrosids - ps21
Rosids - ps22
Eurosids - ps23
Malvids - ps24
Sap./Malv./Brassicales - ps25
Malvales/Brassicales - ps26
Brassicales - ps27
Cleomaceae/Brassicaceae - ps28
Brassicaceae - ps29
Camelineae - ps30
Arabidopsis - ps31
Arabidopsis_thaliana - ps32
Phylostratum

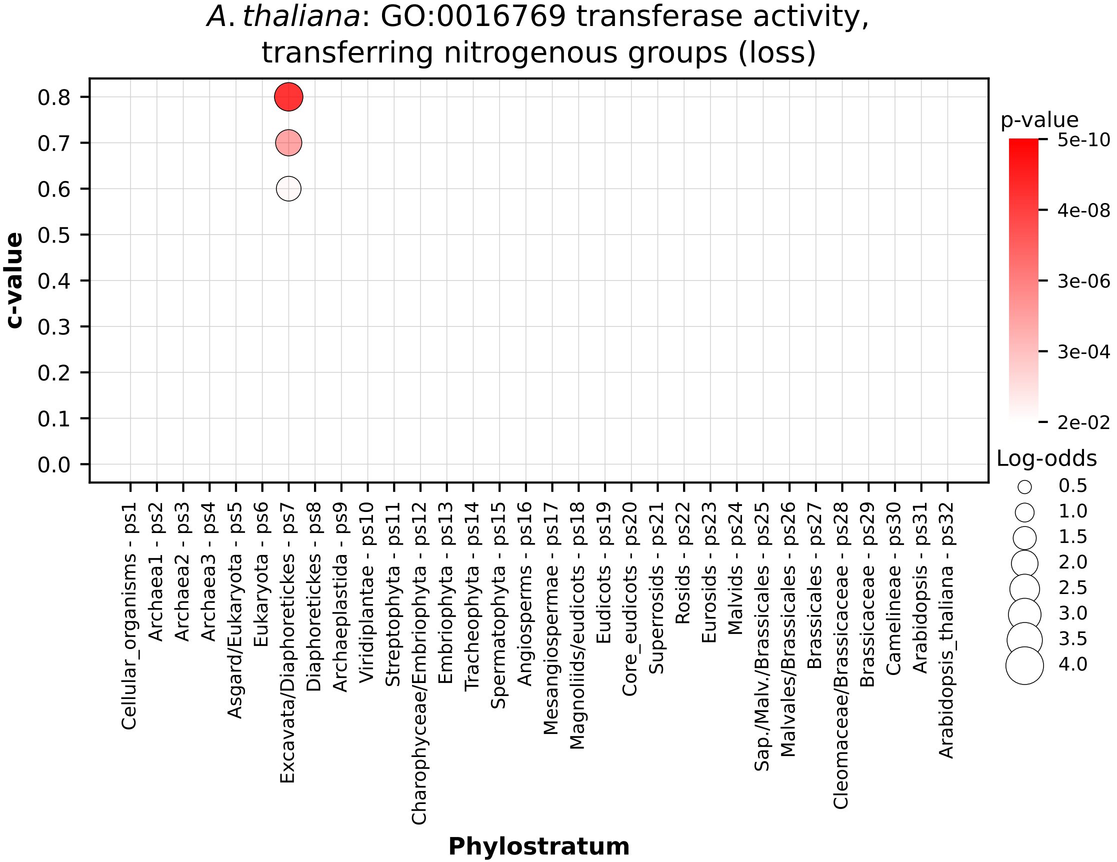
A. thaliana: GO:0016769 transferase activity, transferring nitrogenous groups (loss)
c-value
0.8
0.7
0.6
0.5
0.4
0.3
0.2
0.1
0.0
Phylostratum
Cellular_organisms - ps1
Archaea1 - ps2
Archaea2 - ps3
Archaea3 - ps4
Asgard/Eukaryota - ps5
Eukaryota - ps6
Excavata/Diaphoretickes - ps7
Diaphoretickes - ps8
Archaeplastida - ps9
Viridiplantae - ps10
Streptophyta - ps11
Charophyceae/Embriophyta - ps12
Embriophyta - ps13
Tracheophyta - ps14
Spermatophyta - ps15
Angiosperms - ps16
Mesangiospermae - ps17
Magnoliids/eudicots - ps18
Eudicots - ps19
Core_eudicots - ps20
Superrosids - ps21
Rosids - ps22
Eurosids - ps23
Malvids - ps24
Sap./Malv./Brassicales - ps25
Malvales/Brassicales - ps26
Brassicales - ps27
Cleomaceae/Brassicaceae - ps28
Brassicaceae - ps29
Camelineae - ps30
Arabidopsis - ps31
Arabidopsis_thaliana - ps32
p-value
5e-10
4e-08
3e-06
3e-04
2e-02
Log-odds
0.5
1.0
1.5
2.0
2.5
3.0
3.5
4.0

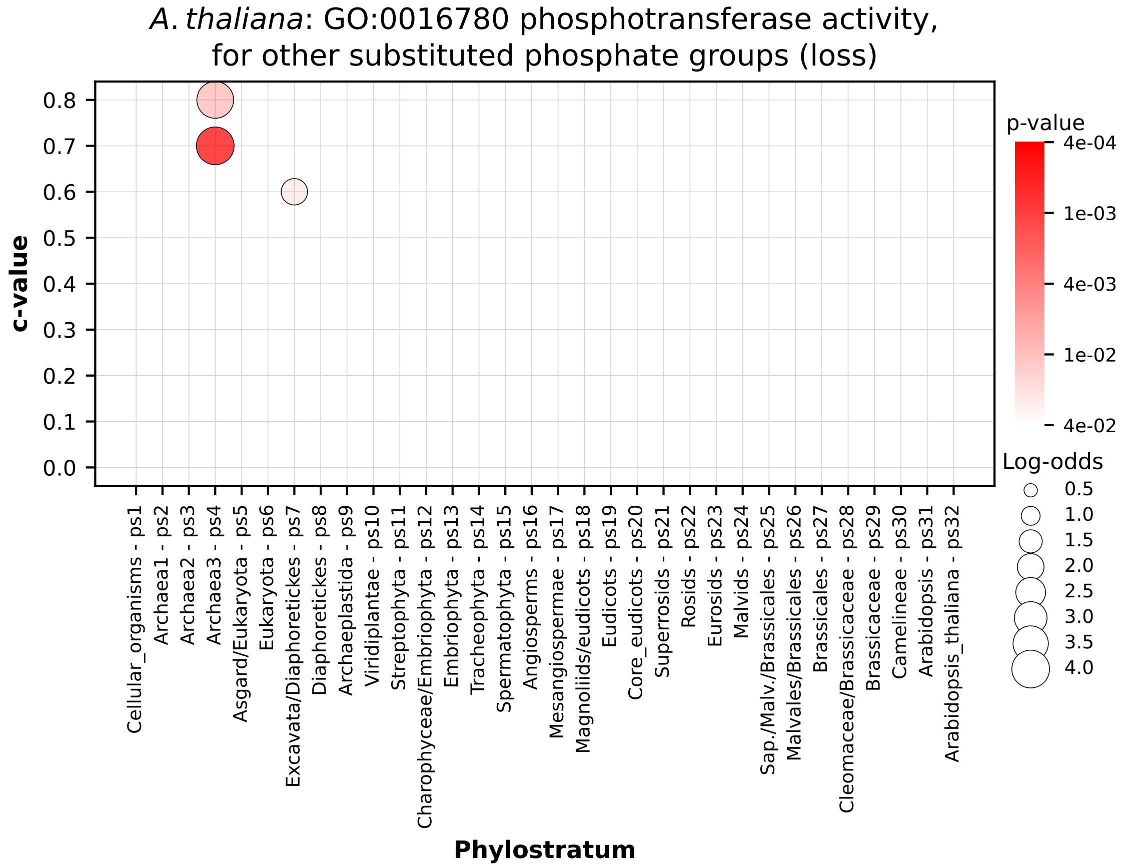

A. thaliana: GO:0016780 phosphotransferase activity, for other substituted phosphate groups (loss)
c-value
0.8
0.7
0.6
0.5
0.4
0.3
0.2
0.1
0.0
p-value
4e-04
1e-03
4e-03
1e-02
4e-02
Log-odds
0.5
1.0
1.5
2.0
2.5
3.0
3.5
4.0
Cellular_organisms - ps1
Archaea1 - ps2
Archaea2 - ps3
Archaea3 - ps4
Asgard/Eukaryota - ps5
Eukaryota - ps6
Excavata/Diaphoretickes - ps7
Diaphoretickes - ps8
Archaeplastida - ps9
Viridiplantae - ps10
Streptophyta - ps11
Charophyceae/Embriophyta - ps12
Embriophyta - ps13
Tracheophyta - ps14
Spermatophyta - ps15
Angiosperms - ps16
Mesangiospermae - ps17
Magnoliids/eudicots - ps18
Eudicots - ps19
Core_eudicots - ps20
Superrosids - ps21
Rosids - ps22
Eurosids - ps23
Malvids - ps24
Sap./Malv./Brassicales - ps25
Malvales/Brassicales - ps26
Brassicales - ps27
Cleomaceae/Brassicaceae - ps28
Brassicaceae - ps29
Camelineae - ps30
Arabidopsis - ps31
Arabidopsis_thaliana - ps32
Phylostratum

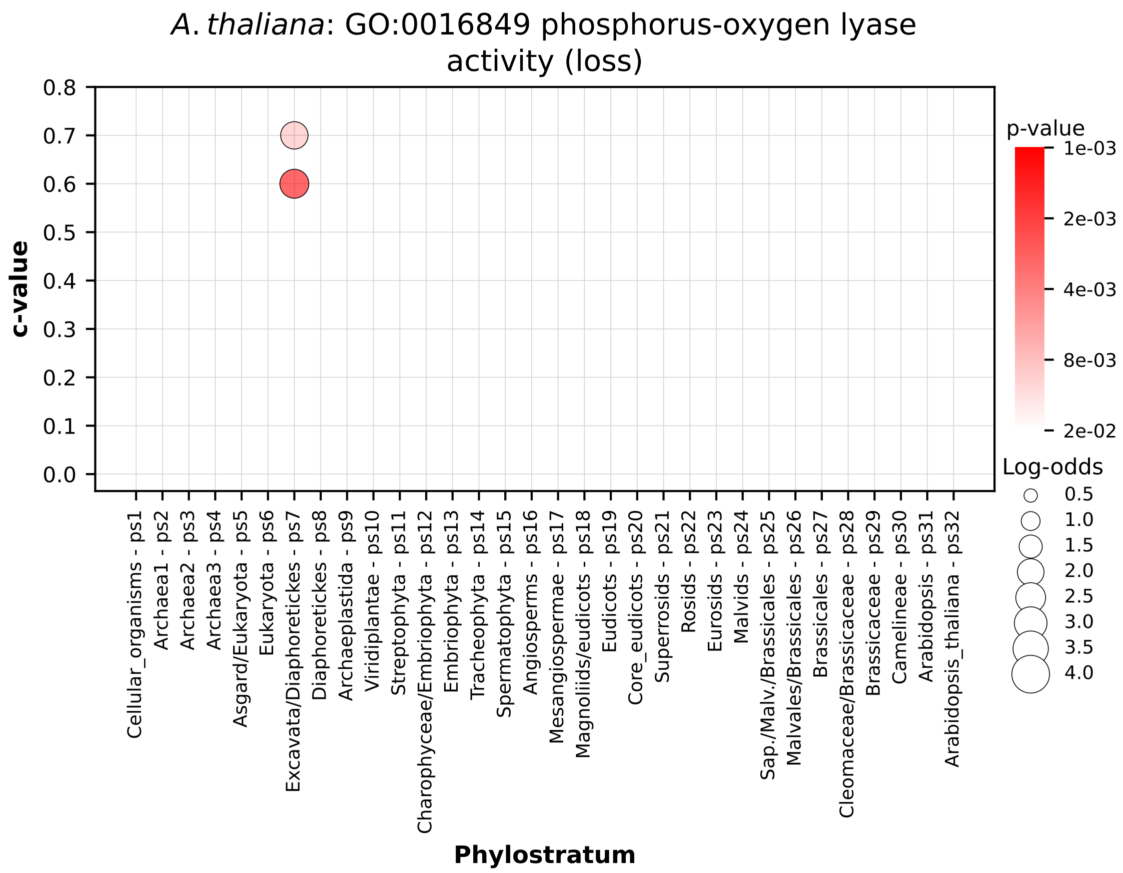

A. thaliana: GO:0016849 phosphorus-oxygen lyase activity (loss)
c-value
0.8
0.7
0.6
0.5
0.4
0.3
0.2
0.1
0.0
p-value
1e-03
2e-03
4e-03
8e-03
2e-02
Log-odds
0.5
1.0
1.5
2.0
2.5
3.0
3.5
4.0
Cellular_organisms - ps1
Archaea1 - ps2
Archaea2 - ps3
Archaea3 - ps4
Asgard/Eukaryota - ps5
Eukaryota - ps6
Excavata/Diaphoretickes - ps7
Diaphoretickes - ps8
Archaeplastida - ps9
Viridiplantae - ps10
Streptophyta - ps11
Charophyceae/Embriophyta - ps12
Embriophyta - ps13
Tracheophyta - ps14
Spermatophyta - ps15
Angiosperms - ps16
Mesangiospermae - ps17
Magnoliids/eudicots - ps18
Eudicots - ps19
Core_eudicots - ps20
Superrosids - ps21
Rosids - ps22
Eurosids - ps23
Malvids - ps24
Sap./Malv./Brassicales - ps25
Malvales/Brassicales - ps26
Brassicales - ps27
Cleomaceae/Brassicaceae - ps28
Brassicaceae - ps29
Camelineae - ps30
Arabidopsis - ps31
Arabidopsis_thaliana - ps32
Phylostratum

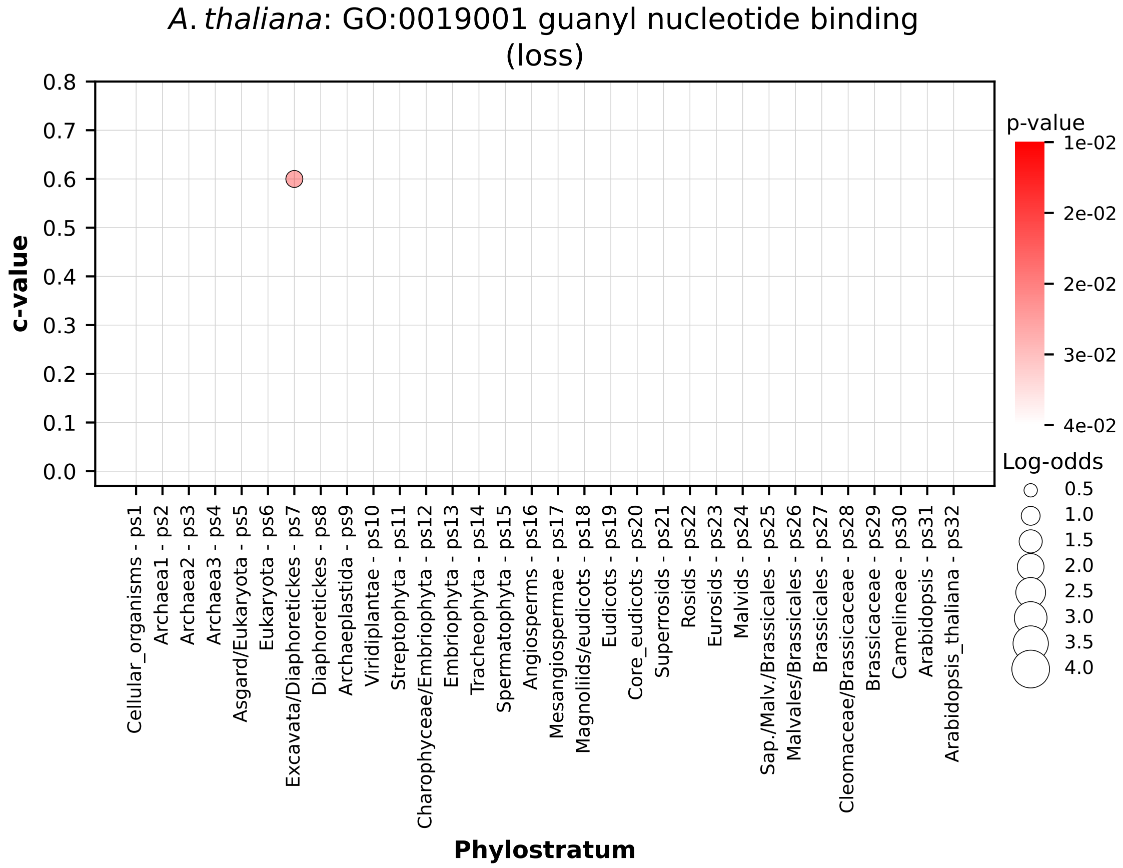
A. thaliana: GO:0019001 guanyl nucleotide binding
(loss)
c-value
0.8
0.7
0.6
0.5
0.4
0.3
0.2
0.1
0.0
Phylostratum
Cellular_organisms - ps1
Archaea1 - ps2
Archaea2 - ps3
Archaea3 - ps4
Asgard/Eukaryota - ps5
Eukaryota - ps6
Excavata/Diaphoretickes - ps7
Diaphoretickes - ps8
Archaeplastida - ps9
Viridiplantae - ps10
Streptophyta - ps11
Charophyceae/Embriophyta - ps12
Embriophyta - ps13
Tracheophyta - ps14
Spermatophyta - ps15
Angiosperms - ps16
Mesangiospermae - ps17
Magnoliids/eudicots - ps18
Eudicots - ps19
Core_eudicots - ps20
Superrosids - ps21
Rosids - ps22
Eurosids - ps23
Malvids - ps24
Sap./Malv./Brassicales - ps25
Malvales/Brassicales - ps26
Brassicales - ps27
Cleomaceae/Brassicaceae - ps28
Brassicaceae - ps29
Camelineae - ps30
Arabidopsis - ps31
Arabidopsis_thaliana - ps32
p-value
1e-02
2e-02
2e-02
3e-02
4e-02
Log-odds
0.5
1.0
1.5
2.0
2.5
3.0
3.5
4.0

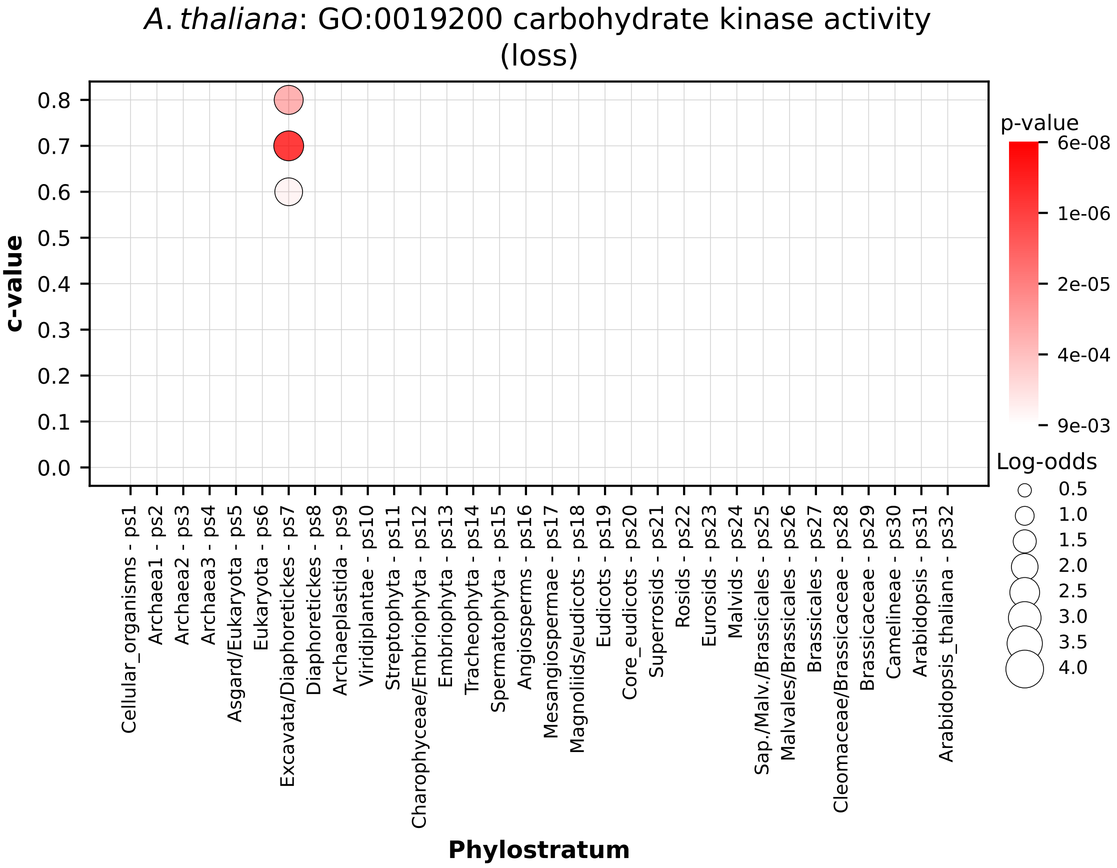

A. thaliana: GO:0019200 carbohydrate kinase activity
(loss)
c-value
0.0
0.1
0.2
0.3
0.4
0.5
0.6
0.7
0.8
p-value
6e-08
1e-06
2e-05
4e-04
9e-03
Log-odds
0.5
1.0
1.5
2.0
2.5
3.0
3.5
4.0
Cellular_organisms - ps1
Archaea1 - ps2
Archaea2 - ps3
Archaea3 - ps4
Asgard/Eukaryota - ps5
Eukaryota - ps6
Excavata/Diaphoretickes - ps7
Diaphoretickes - ps8
Archaeplastida - ps9
Viridiplantae - ps10
Streptophyta - ps11
Charophyceae/Embriophyta - ps12
Embriophyta - ps13
Tracheophyta - ps14
Spermatophyta - ps15
Angiosperms - ps16
Mesangiospermae - ps17
Magnoliids/eudicots - ps18
Eudicots - ps19
Core_eudicots - ps20
Superrosids - ps21
Rosids - ps22
Eurosids - ps23
Malvids - ps24
Sap./Malv./Brassicales - ps25
Malvales/Brassicales - ps26
Brassicales - ps27
Cleomaceae/Brassicaceae - ps28
Brassicaceae - ps29
Camelineae - ps30
Arabidopsis - ps31
Arabidopsis_thaliana - ps32
Phylostratum

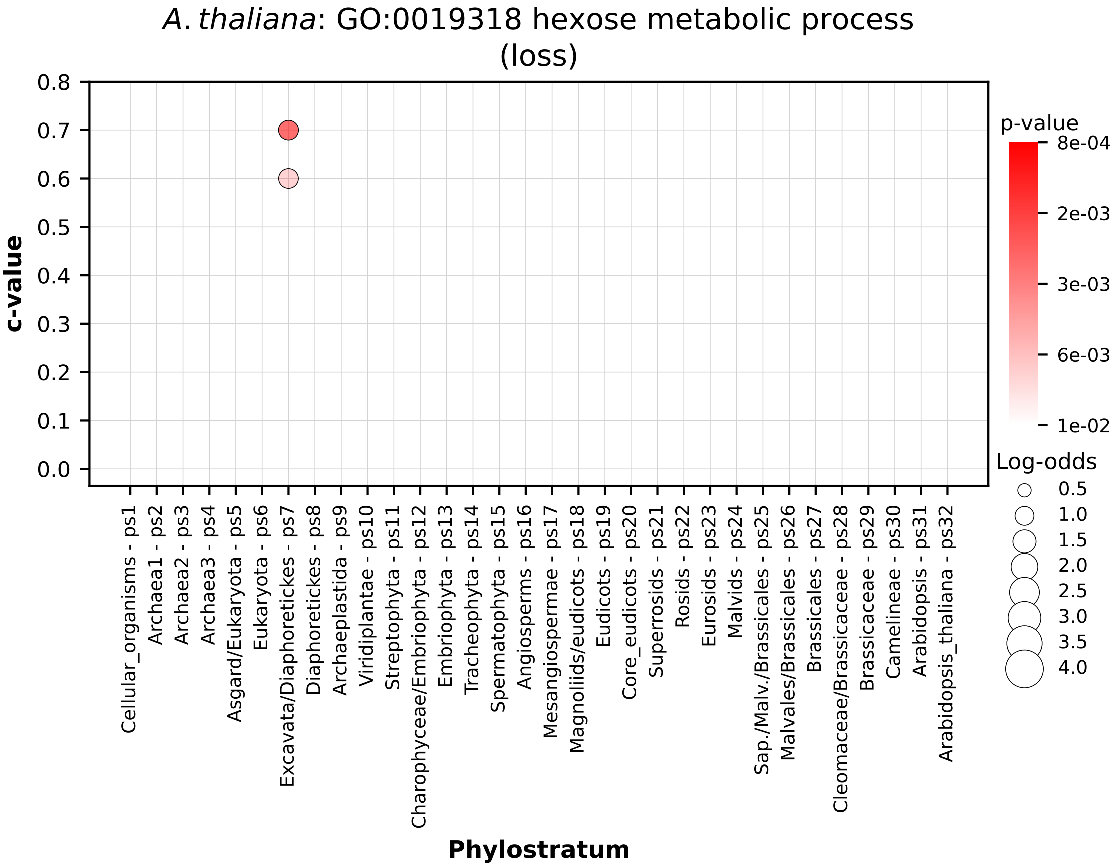

A. thaliana: GO:0019318 hexose metabolic process
(loss)
c-value
0.8
0.7
0.6
0.5
0.4
0.3
0.2
0.1
0.0
p-value
8e-04
2e-03
3e-03
6e-03
1e-02
Log-odds
0.5
1.0
1.5
2.0
2.5
3.0
3.5
4.0
Cellular_organisms - ps1
Archaea1 - ps2
Archaea2 - ps3
Archaea3 - ps4
Asgard/Eukaryota - ps5
Eukaryota - ps6
Excavata/Diaphoretickes - ps7
Diaphoretickes - ps8
Archaeplastida - ps9
Viridiplantae - ps10
Streptophyta - ps11
Charophyceae/Embriophyta - ps12
Embriophyta - ps13
Tracheophyta - ps14
Spermatophyta - ps15
Angiosperms - ps16
Mesangiospermae - ps17
Magnoliids/eudicots - ps18
Eudicots - ps19
Core_eudicots - ps20
Superrosids - ps21
Rosids - ps22
Eurosids - ps23
Malvids - ps24
Sap./Malv./Brassicales - ps25
Malvales/Brassicales - ps26
Brassicales - ps27
Cleomaceae/Brassicaceae - ps28
Brassicaceae - ps29
Camelineae - ps30
Arabidopsis - ps31
Arabidopsis_thaliana - ps32
Phylostratum

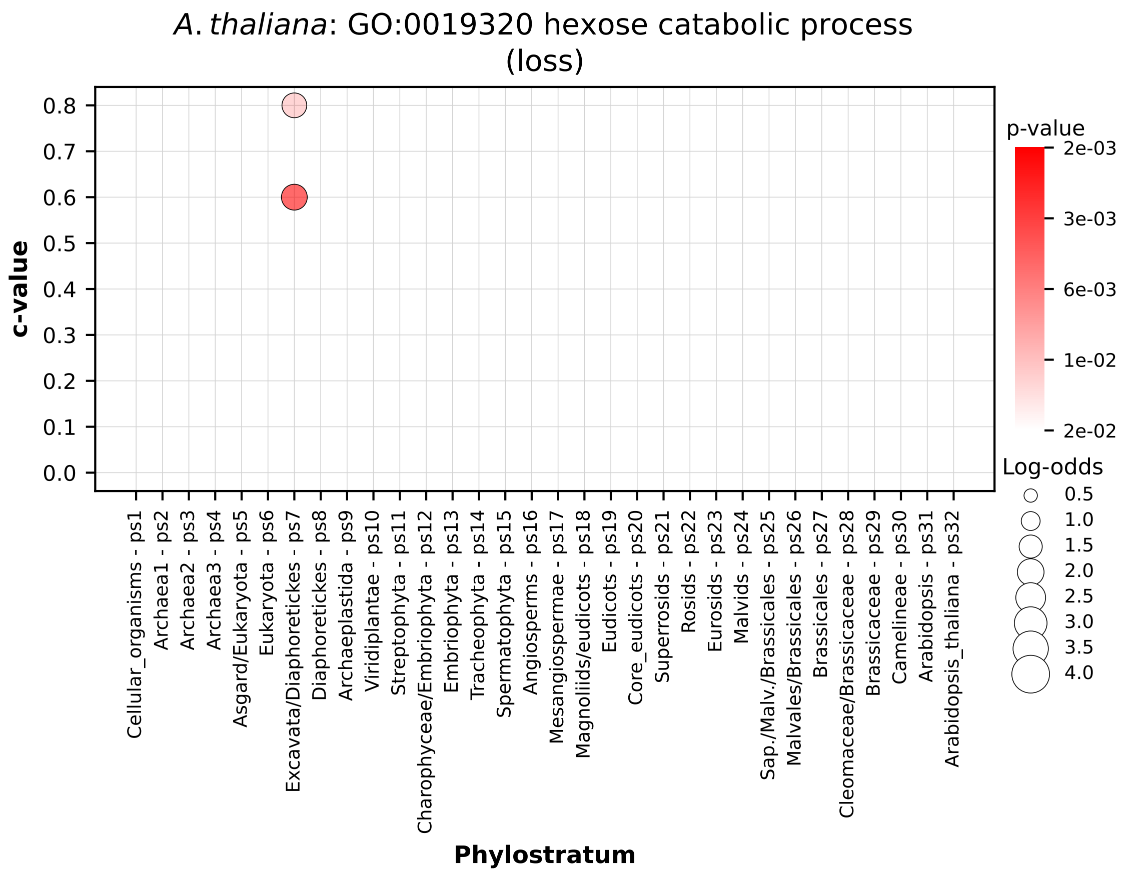

A. thaliana: GO:0019320 hexose catabolic process
(loss)
c-value
0.0
0.1
0.2
0.3
0.4
0.5
0.6
0.7
0.8
Phylostratum
Cellular_organisms - ps1
Archaea1 - ps2
Archaea2 - ps3
Archaea3 - ps4
Asgard/Eukaryota - ps5
Eukaryota - ps6
Excavata/Diaphoretickes - ps7
Diaphoretickes - ps8
Archaeplastida - ps9
Viridiplantae - ps10
Streptophyta - ps11
Charophyceae/Embriophyta - ps12
Embriophyta - ps13
Tracheophyta - ps14
Spermatophyta - ps15
Angiosperms - ps16
Mesangiospermae - ps17
Magnoliids/eudicots - ps18
Eudicots - ps19
Core_eudicots - ps20
Superrosids - ps21
Rosids - ps22
Eurosids - ps23
Malvids - ps24
Sap./Malv./Brassicales - ps25
Malvales/Brassicales - ps26
Brassicales - ps27
Cleomaceae/Brassicaceae - ps28
Brassicaceae - ps29
Camelineae - ps30
Arabidopsis - ps31
Arabidopsis_thaliana - ps32
p-value
2e-03
3e-03
6e-03
1e-02
2e-02
Log-odds
0.5
1.0
1.5
2.0
2.5
3.0
3.5
4.0

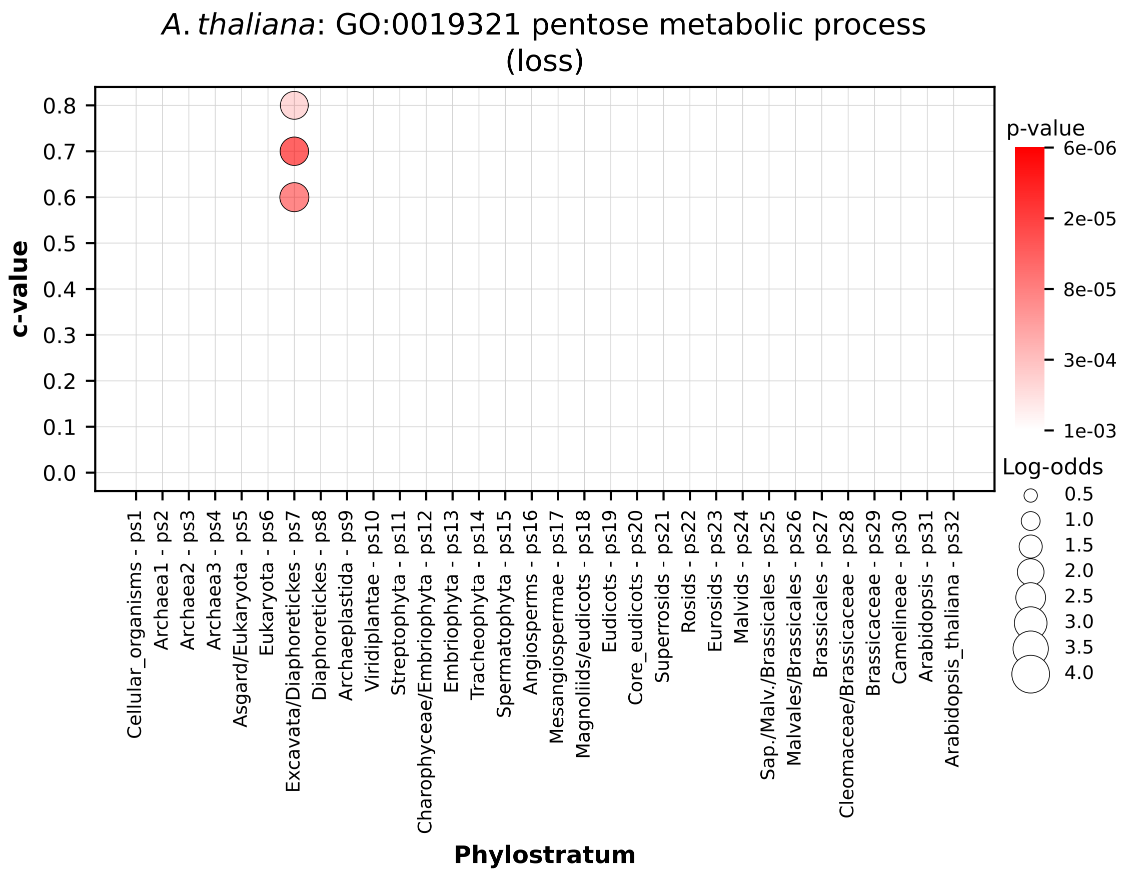

A. thaliana: GO:0019321 pentose metabolic process
(loss)
c-value
0.0
0.1
0.2
0.3
0.4
0.5
0.6
0.7
0.8
Phylostratum
Cellular_organisms - ps1
Archaea1 - ps2
Archaea2 - ps3
Archaea3 - ps4
Asgard/Eukaryota - ps5
Eukaryota - ps6
Excavata/Diaphoretickes - ps7
Diaphoretickes - ps8
Archaeplastida - ps9
Viridiplantae - ps10
Streptophyta - ps11
Charophyceae/Embriophyta - ps12
Embriophyta - ps13
Tracheophyta - ps14
Spermatophyta - ps15
Angiosperms - ps16
Mesangiospermae - ps17
Magnoliids/eudicots - ps18
Eudicots - ps19
Core_eudicots - ps20
Superrosids - ps21
Rosids - ps22
Eurosids - ps23
Malvids - ps24
Sap./Malv./Brassicales - ps25
Malvales/Brassicales - ps26
Brassicales - ps27
Cleomaceae/Brassicaceae - ps28
Brassicaceae - ps29
Camelineae - ps30
Arabidopsis - ps31
Arabidopsis_thaliana - ps32
p-value
6e-06
2e-05
8e-05
3e-04
1e-03
Log-odds
0.5
1.0
1.5
2.0
2.5
3.0
3.5
4.0

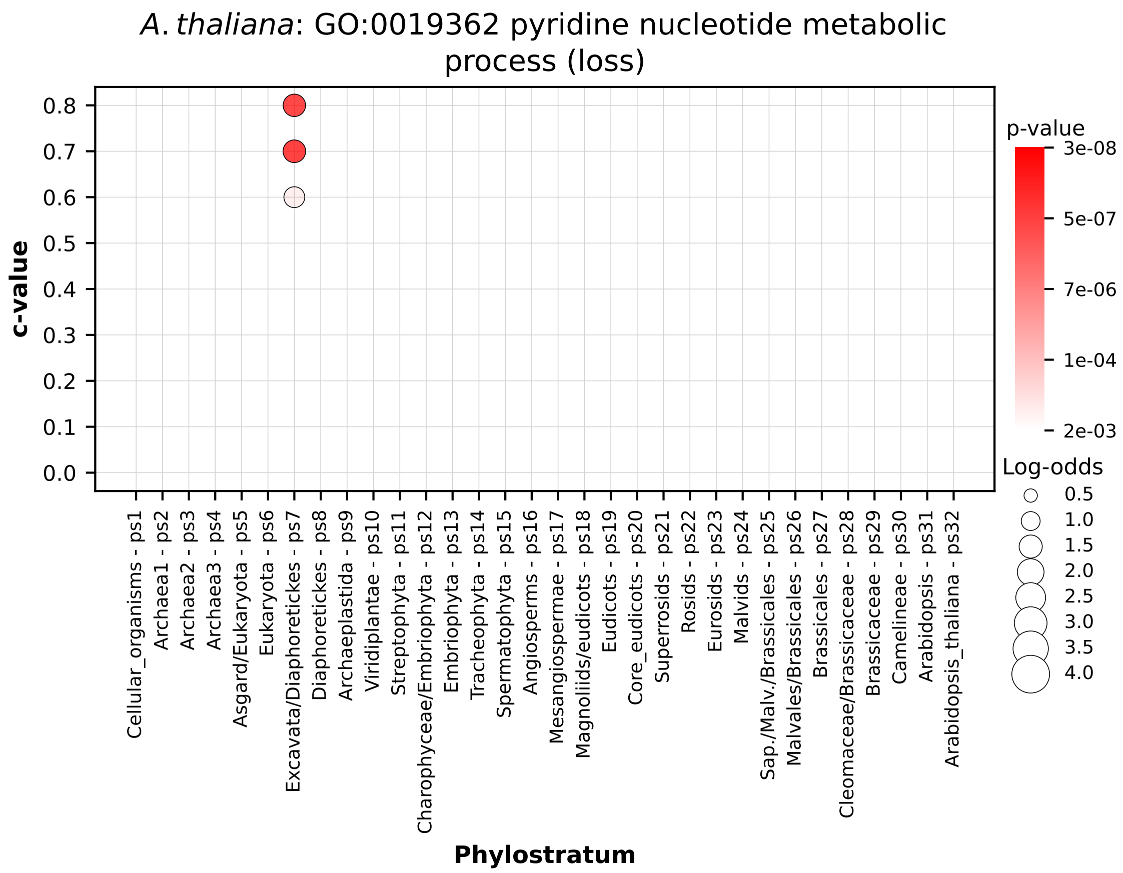

A. thaliana: GO:0019362 pyridine nucleotide metabolic process (loss)
c-value
0.8
0.7
0.6
0.5
0.4
0.3
0.2
0.1
0.0
p-value
3e-08
5e-07
7e-06
1e-04
2e-03
Log-odds
0.5
1.0
1.5
2.0
2.5
3.0
3.5
4.0
Cellular_organisms - ps1
Archaea1 - ps2
Archaea2 - ps3
Archaea3 - ps4
Asgard/Eukaryota - ps5
Eukaryota - ps6
Excavata/Diaphoretickes - ps7
Diaphoretickes - ps8
Archaeplastida - ps9
Viridiplantae - ps10
Streptophyta - ps11
Charophyceae/Embriophyta - ps12
Embriophyta - ps13
Tracheophyta - ps14
Spermatophyta - ps15
Angiosperms - ps16
Mesangiospermae - ps17
Magnoliids/eudicots - ps18
Eudicots - ps19
Core_eudicots - ps20
Superrosids - ps21
Rosids - ps22
Eurosids - ps23
Malvids - ps24
Sap./Malv./Brassicales - ps25
Malvales/Brassicales - ps26
Brassicales - ps27
Cleomaceae/Brassicaceae - ps28
Brassicaceae - ps29
Camelineae - ps30
Arabidopsis - ps31
Arabidopsis_thaliana - ps32
Phylostratum

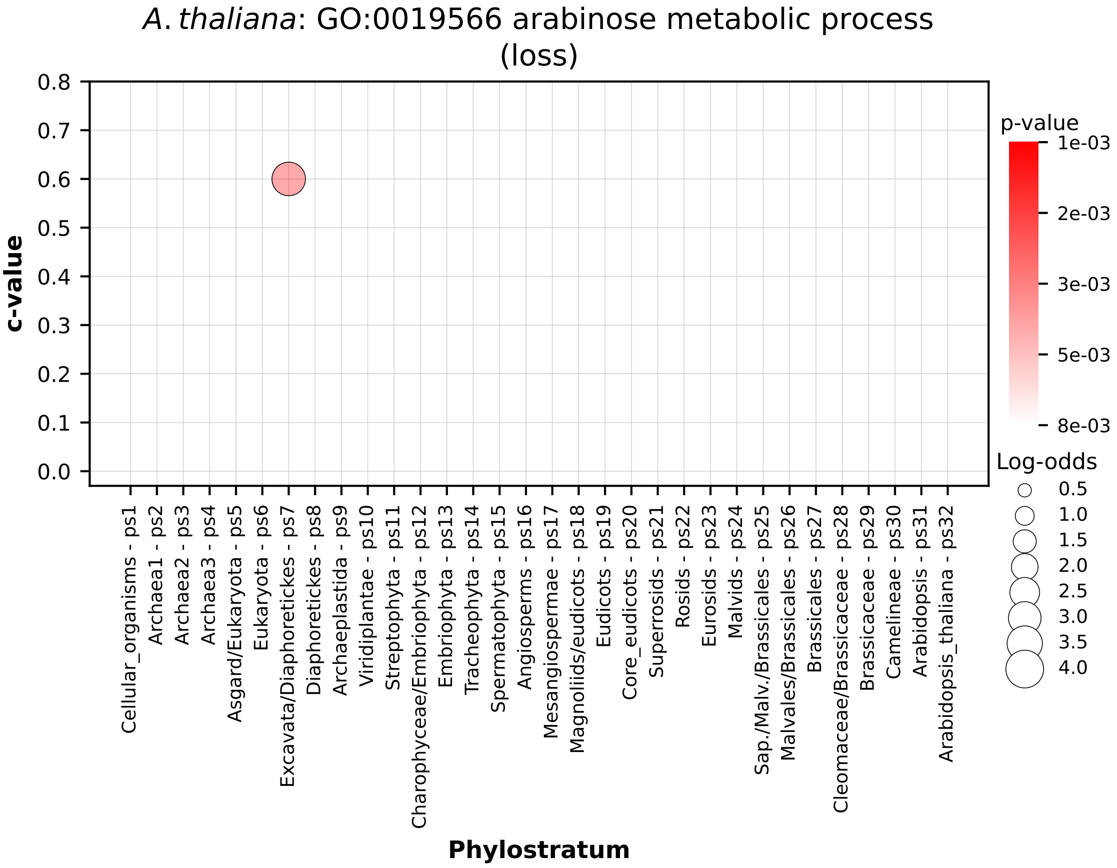

A. thaliana: GO:0019566 arabinose metabolic process
(loss)
c-value
0.8
0.7
0.6
0.5
0.4
0.3
0.2
0.1
0.0
Phylostratum
Cellular_organisms - ps1
Archaea1 - ps2
Archaea2 - ps3
Archaea3 - ps4
Asgard/Eukaryota - ps5
Eukaryota - ps6
Excavata/Diaphoretickes - ps7
Diaphoretickes - ps8
Archaeplastida - ps9
Viridiplantae - ps10
Streptophyta - ps11
Charophyceae/Embriophyta - ps12
Embriophyta - ps13
Tracheophyta - ps14
Spermatophyta - ps15
Angiosperms - ps16
Mesangiospermae - ps17
Magnoliids/eudicots - ps18
Eudicots - ps19
Core_eudicots - ps20
Superrosids - ps21
Rosids - ps22
Eurosids - ps23
Malvids - ps24
Sap./Malv./Brassicales - ps25
Malvales/Brassicales - ps26
Brassicales - ps27
Cleomaceae/Brassicaceae - ps28
Brassicaceae - ps29
Camelineae - ps30
Arabidopsis - ps31
Arabidopsis_thaliana - ps32
p-value
1e-03
2e-03
3e-03
5e-03
8e-03
Log-odds
0.5
1.0
1.5
2.0
2.5
3.0
3.5
4.0

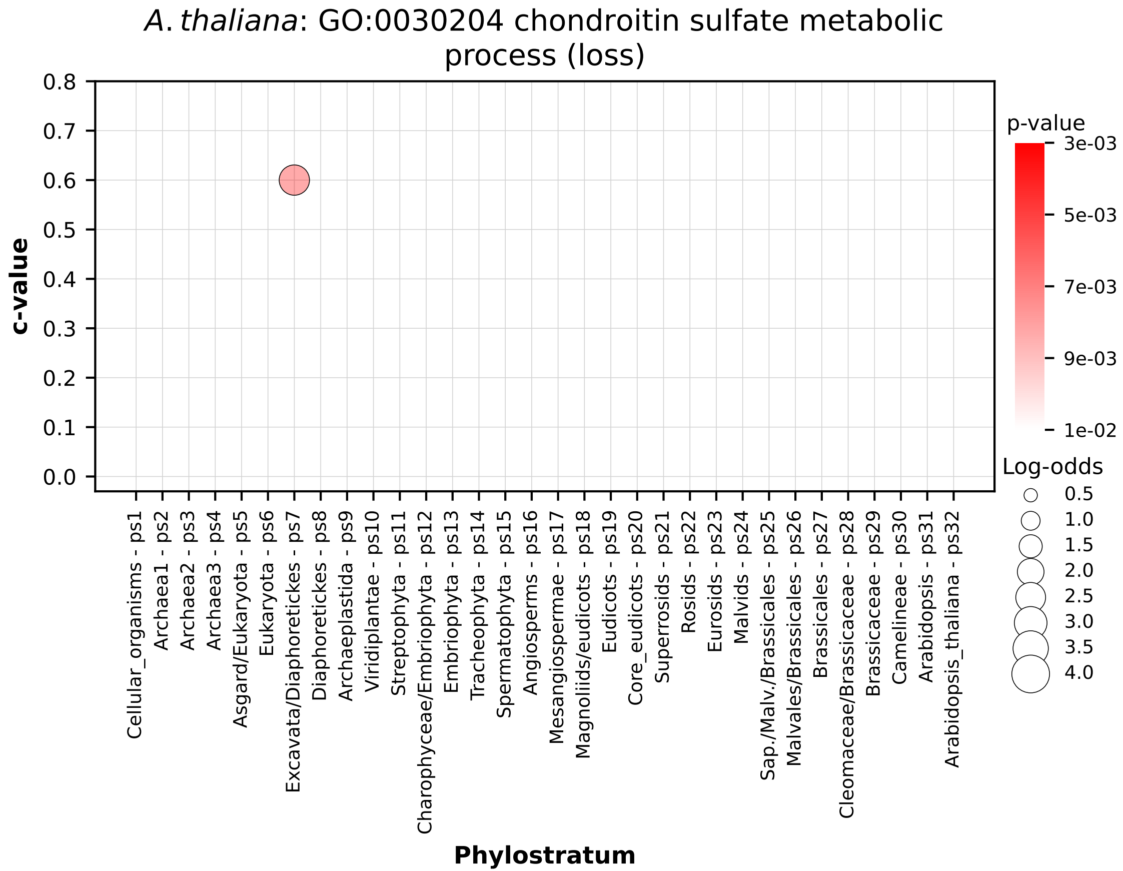

A. thaliana: GO:0030204 chondroitin sulfate metabolic process (loss)
c-value
0.8
0.7
0.6
0.5
0.4
0.3
0.2
0.1
0.0
p-value
3e-03
5e-03
7e-03
9e-03
1e-02
Log-odds
0.5
1.0
1.5
2.0
2.5
3.0
3.5
4.0
Cellular_organisms - ps1
Archaea1 - ps2
Archaea2 - ps3
Archaea3 - ps4
Asgard/Eukaryota - ps5
Eukaryota - ps6
Excavata/Diaphoretickes - ps7
Diaphoretickes - ps8
Archaeplastida - ps9
Viridiplantae - ps10
Streptophyta - ps11
Charophyceae/Embriophyta - ps12
Embriophyta - ps13
Tracheophyta - ps14
Spermatophyta - ps15
Angiosperms - ps16
Mesangiospermae - ps17
Magnoliids/eudicots - ps18
Eudicots - ps19
Core_eudicots - ps20
Superrosids - ps21
Rosids - ps22
Eurosids - ps23
Malvids - ps24
Sap./Malv./Brassicales - ps25
Malvales/Brassicales - ps26
Brassicales - ps27
Cleomaceae/Brassicaceae - ps28
Brassicaceae - ps29
Camelineae - ps30
Arabidopsis - ps31
Arabidopsis_thaliana - ps32
Phylostratum

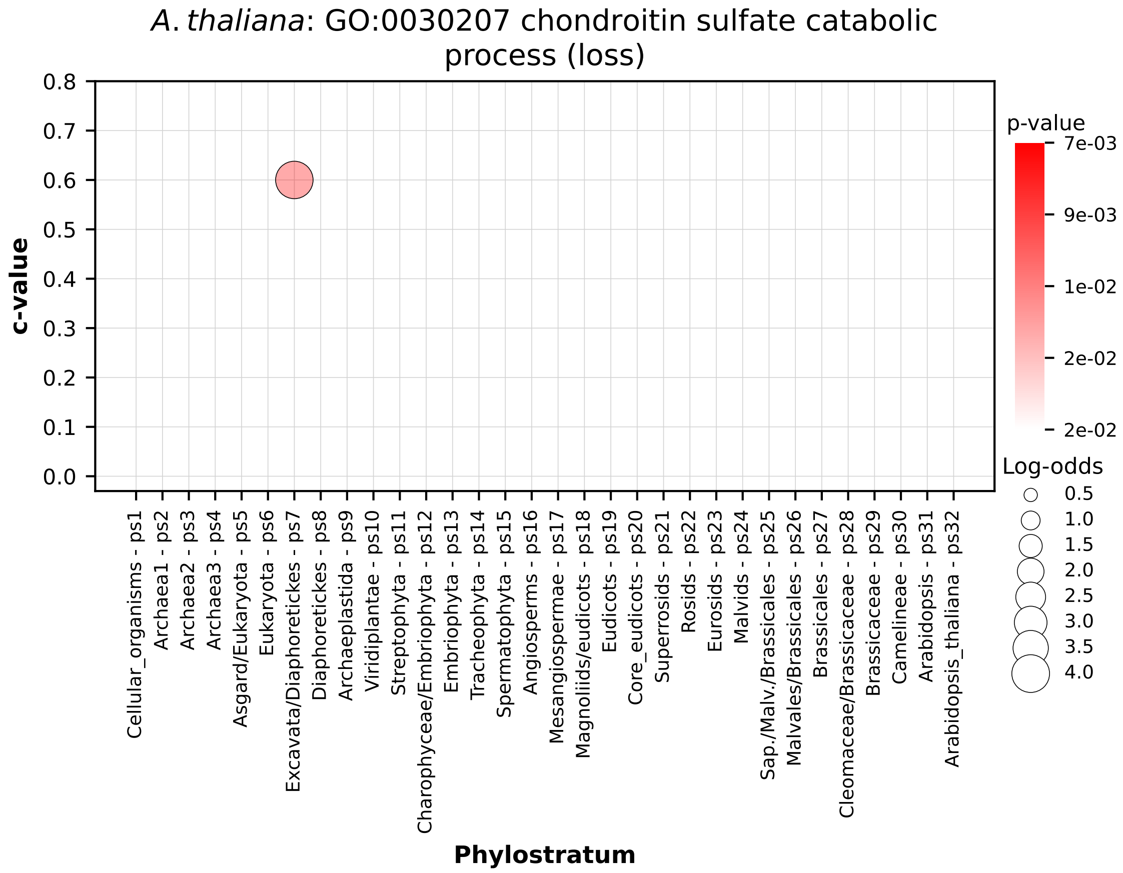

A. thaliana: GO:0030207 chondroitin sulfate catabolic process (loss)
c-value
0.8
0.7
0.6
0.5
0.4
0.3
0.2
0.1
0.0
Phylostratum
Cellular_organisms - ps1
Archaea1 - ps2
Archaea2 - ps3
Archaea3 - ps4
Asgard/Eukaryota - ps5
Eukaryota - ps6
Excavata/Diaphoretickes - ps7
Diaphoretickes - ps8
Archaeplastida - ps9
Viridiplantae - ps10
Streptophyta - ps11
Charophyceae/Embriophyta - ps12
Embriophyta - ps13
Tracheophyta - ps14
Spermatophyta - ps15
Angiosperms - ps16
Mesangiospermae - ps17
Magnoliids/eudicots - ps18
Eudicots - ps19
Core_eudicots - ps20
Superrosids - ps21
Rosids - ps22
Eurosids - ps23
Malvids - ps24
Sap./Malv./Brassicales - ps25
Malvales/Brassicales - ps26
Brassicales - ps27
Cleomaceae/Brassicaceae - ps28
Brassicaceae - ps29
Camelineae - ps30
Arabidopsis - ps31
Arabidopsis_thaliana - ps32
p-value
7e-03
9e-03
1e-02
2e-02
2e-02
Log-odds
0.5
1.0
1.5
2.0
2.5
3.0
3.5
4.0

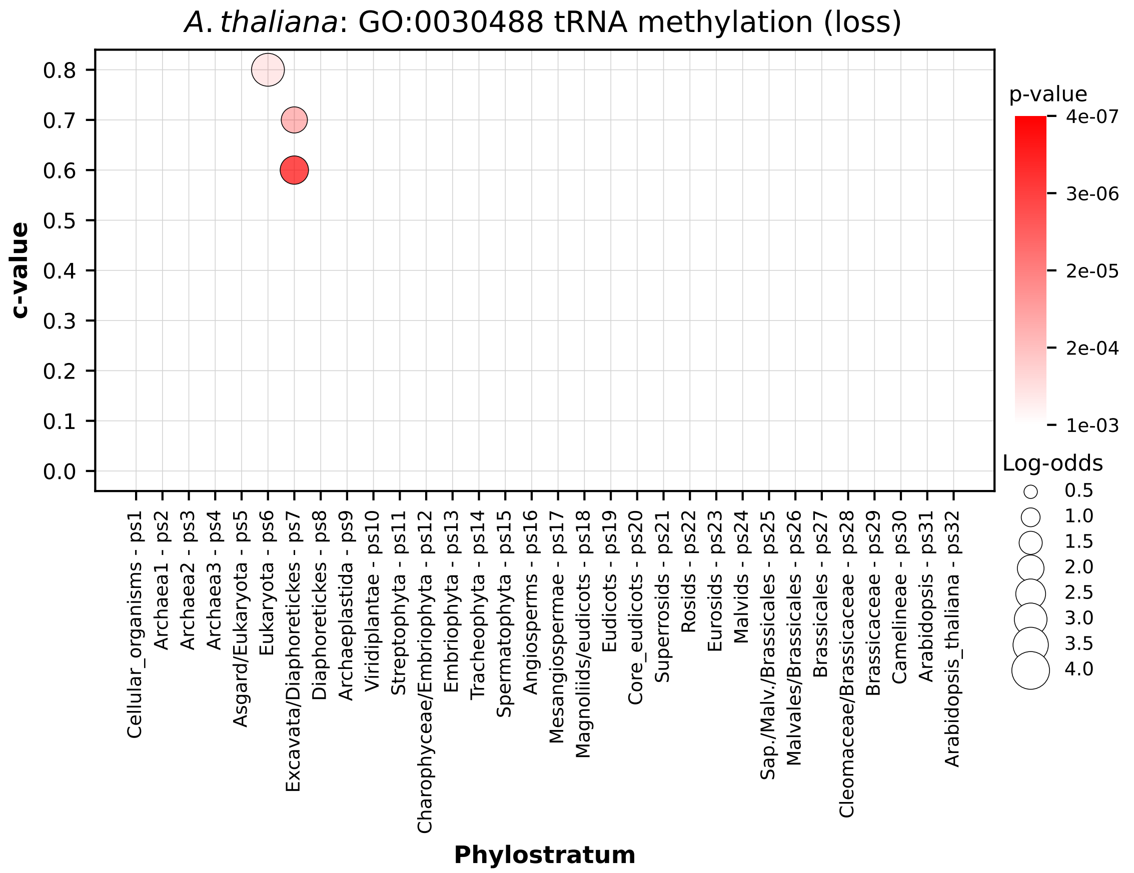

A. thaliana: GO:0030488 tRNA methylation (loss)
c-value
0.0
0.1
0.2
0.3
0.4
0.5
0.6
0.7
0.8
Phylostratum
Cellular_organisms - ps1
Archaea1 - ps2
Archaea2 - ps3
Archaea3 - ps4
Asgard/Eukaryota - ps5
Eukaryota - ps6
Excavata/Diaphoretickes - ps7
Diaphoretickes - ps8
Archaeplastida - ps9
Viridiplantae - ps10
Streptophyta - ps11
Charophyceae/Embriophyta - ps12
Embriophyta - ps13
Tracheophyta - ps14
Spermatophyta - ps15
Angiosperms - ps16
Mesangiospermae - ps17
Magnoliids/eudicots - ps18
Eudicots - ps19
Core_eudicots - ps20
Superrosids - ps21
Rosids - ps22
Eurosids - ps23
Malvids - ps24
Sap./Malv./Brassicales - ps25
Malvales/Brassicales - ps26
Brassicales - ps27
Cleomaceae/Brassicaceae - ps28
Brassicaceae - ps29
Camelineae - ps30
Arabidopsis - ps31
Arabidopsis_thaliana - ps32
p-value
4e-07
3e-06
2e-05
2e-04
1e-03
Log-odds
0.5
1.0
1.5
2.0
2.5
3.0
3.5
4.0

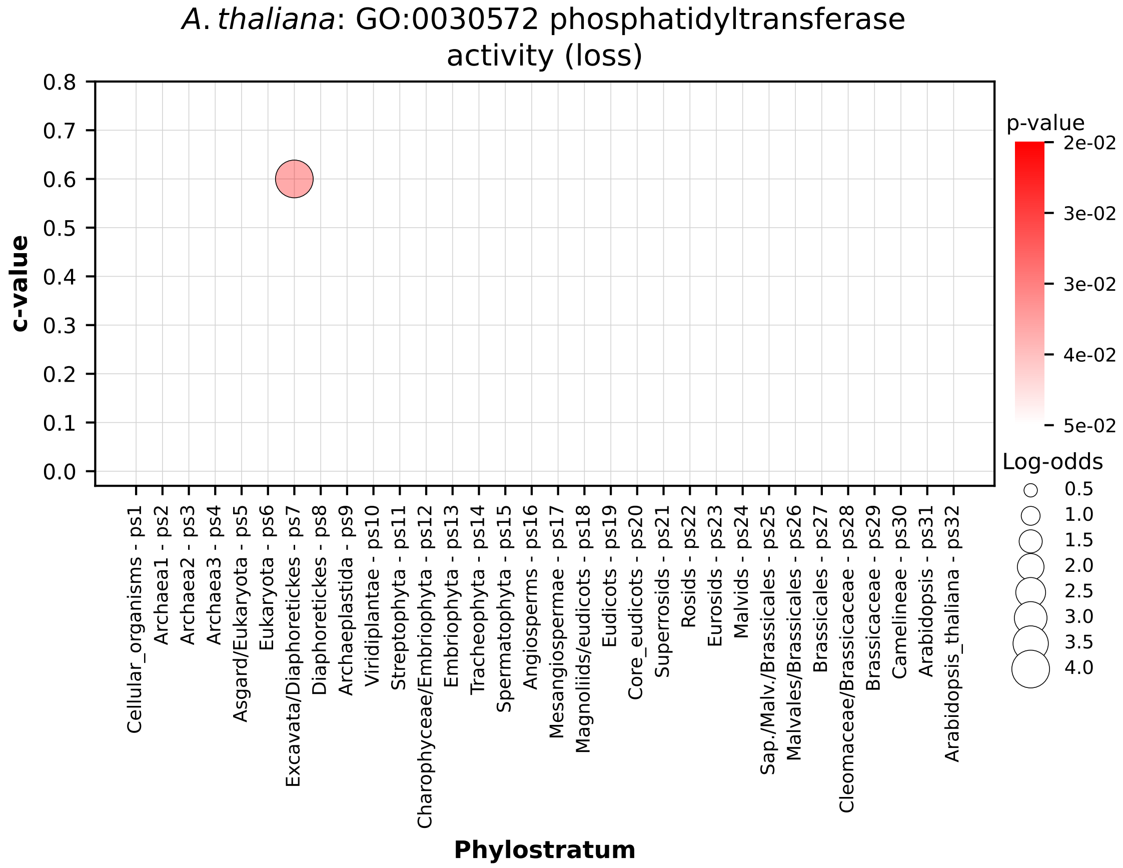

A. thaliana: GO:0030572 phosphatidyltransferase activity (loss)
c-value
0.8
0.7
0.6
0.5
0.4
0.3
0.2
0.1
0.0
p-value
2e-02
3e-02
3e-02
4e-02
5e-02
Log-odds
0.5
1.0
1.5
2.0
2.5
3.0
3.5
4.0
Cellular_organisms - ps1
Archaea1 - ps2
Archaea2 - ps3
Archaea3 - ps4
Asgard/Eukaryota - ps5
Eukaryota - ps6
Excavata/Diaphoretickes - ps7
Diaphoretickes - ps8
Archaeplastida - ps9
Viridiplantae - ps10
Streptophyta - ps11
Charophyceae/Embriophyta - ps12
Embriophyta - ps13
Tracheophyta - ps14
Spermatophyta - ps15
Angiosperms - ps16
Mesangiospermae - ps17
Magnoliids/eudicots - ps18
Eudicots - ps19
Core_eudicots - ps20
Superrosids - ps21
Rosids - ps22
Eurosids - ps23
Malvids - ps24
Sap./Malv./Brassicales - ps25
Malvales/Brassicales - ps26
Brassicales - ps27
Cleomaceae/Brassicaceae - ps28
Brassicaceae - ps29
Camelineae - ps30
Arabidopsis - ps31
Arabidopsis_thaliana - ps32
Phylostratum

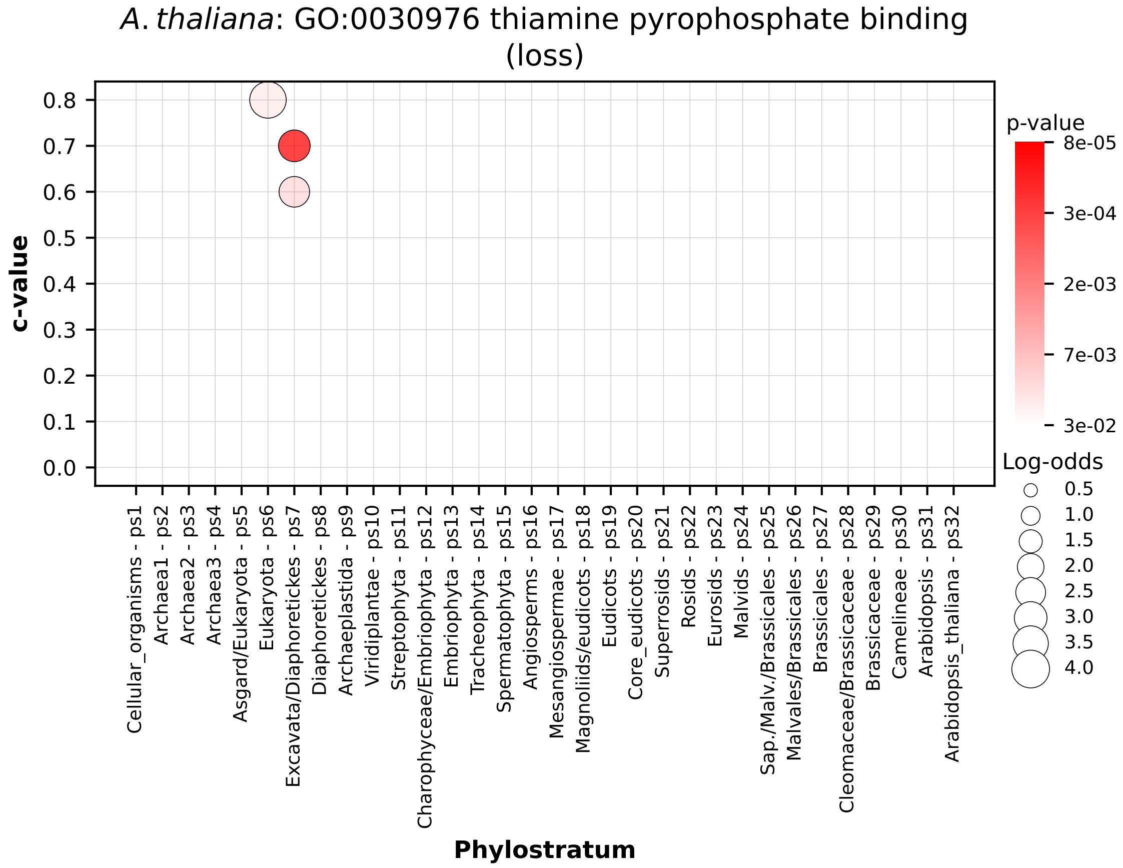

A. thaliana: GO:0030976 thiamine pyrophosphate binding
(loss)
c-value
0.0
0.1
0.2
0.3
0.4
0.5
0.6
0.7
0.8
Phylostratum
Cellular_organisms - ps1
Archaea1 - ps2
Archaea2 - ps3
Archaea3 - ps4
Asgard/Eukaryota - ps5
Eukaryota - ps6
Excavata/Diaphoretickes - ps7
Diaphoretickes - ps8
Archaeplastida - ps9
Viridiplantae - ps10
Streptophyta - ps11
Charophyceae/Embriophyta - ps12
Embriophyta - ps13
Tracheophyta - ps14
Spermatophyta - ps15
Angiosperms - ps16
Mesangiospermae - ps17
Magnoliids/eudicots - ps18
Eudicots - ps19
Core_eudicots - ps20
Superrosids - ps21
Rosids - ps22
Eurosids - ps23
Malvids - ps24
Sap./Malv./Brassicales - ps25
Malvales/Brassicales - ps26
Brassicales - ps27
Cleomaceae/Brassicaceae - ps28
Brassicaceae - ps29
Camelineae - ps30
Arabidopsis - ps31
Arabidopsis_thaliana - ps32
p-value
8e-05
3e-04
2e-03
7e-03
3e-02
Log-odds
0.5
1.0
1.5
2.0
2.5
3.0
3.5
4.0

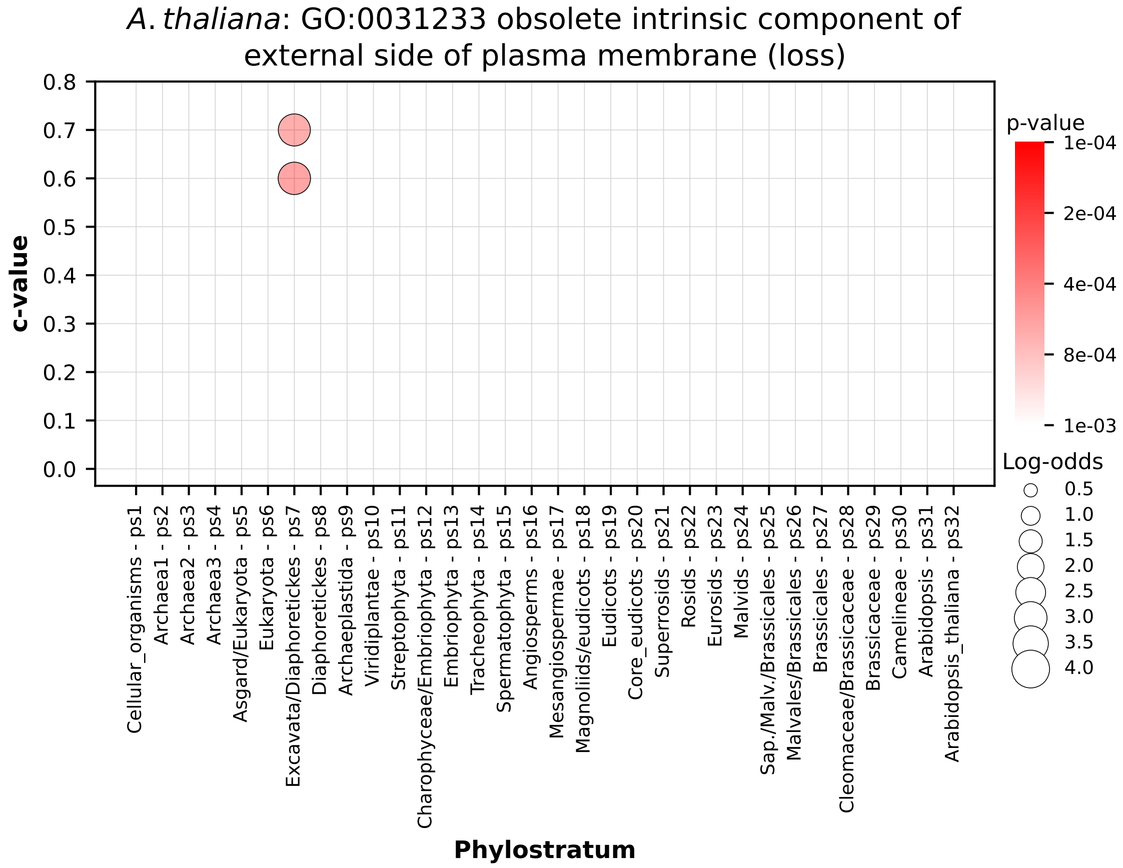
A. thaliana: GO:0031233 obsolete intrinsic component of external side of plasma membrane (loss)
c-value
0.0
0.1
0.2
0.3
0.4
0.5
0.6
0.7
0.8
Phylostratum
Cellular_organisms - ps1
Archaea1 - ps2
Archaea2 - ps3
Archaea3 - ps4
Asgard/Eukaryota - ps5
Eukaryota - ps6
Excavata/Diaphoretickes - ps7
Diaphoretickes - ps8
Archaeplastida - ps9
Viridiplantae - ps10
Streptophyta - ps11
Charophyceae/Embriophyta - ps12
Embriophyta - ps13
Tracheophyta - ps14
Spermatophyta - ps15
Angiosperms - ps16
Mesangiospermae - ps17
Magnoliids/eudicots - ps18
Eudicots - ps19
Core_eudicots - ps20
Superrosids - ps21
Rosids - ps22
Eurosids - ps23
Malvids - ps24
Sap./Malv./Brassicales - ps25
Malvales/Brassicales - ps26
Brassicales - ps27
Cleomaceae/Brassicaceae - ps28
Brassicaceae - ps29
Camelineae - ps30
Arabidopsis - ps31
Arabidopsis_thaliana - ps32
p-value
1e-04
2e-04
4e-04
8e-04
1e-03
Log-odds
0.5
1.0
1.5
2.0
2.5
3.0
3.5
4.0

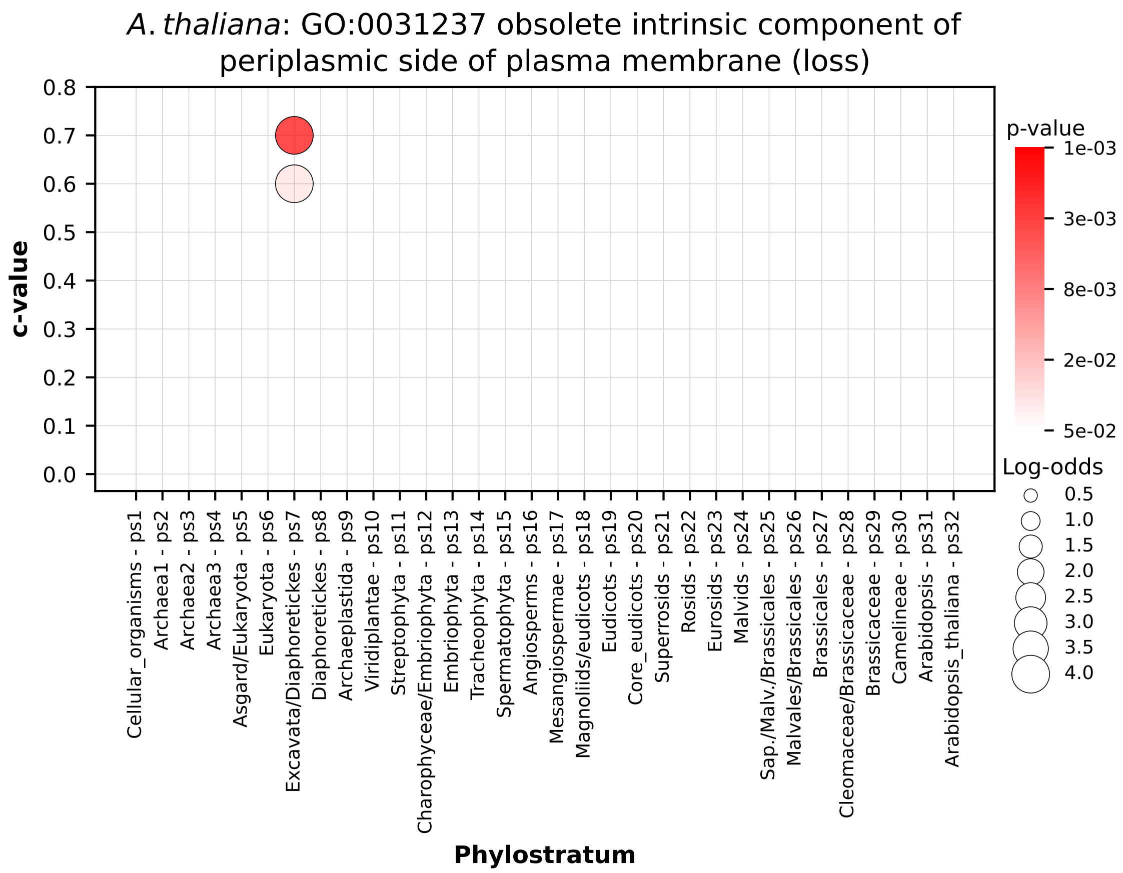
A. thaliana: GO:0031237 obsolete intrinsic component of periplasmic side of plasma membrane (loss)
c-value
0.8
0.7
0.6
0.5
0.4
0.3
0.2
0.1
0.0
p-value
1e-03
3e-03
8e-03
2e-02
5e-02
Log-odds
0.5
1.0
1.5
2.0
2.5
3.0
3.5
4.0
Cellular_organisms - ps1
Archaea1 - ps2
Archaea2 - ps3
Archaea3 - ps4
Asgard/Eukaryota - ps5
Eukaryota - ps6
Excavata/Diaphoretickes - ps7
Diaphoretickes - ps8
Archaeplastida - ps9
Viridiplantae - ps10
Streptophyta - ps11
Charophyceae/Embriophyta - ps12
Embriophyta - ps13
Tracheophyta - ps14
Spermatophyta - ps15
Angiosperms - ps16
Mesangiospermae - ps17
Magnoliids/eudicots - ps18
Eudicots - ps19
Core_eudicots - ps20
Superrosids - ps21
Rosids - ps22
Eurosids - ps23
Malvids - ps24
Sap./Malv./Brassicales - ps25
Malvales/Brassicales - ps26
Brassicales - ps27
Cleomaceae/Brassicaceae - ps28
Brassicaceae - ps29
Camelineae - ps30
Arabidopsis - ps31
Arabidopsis_thaliana - ps32
Phylostratum

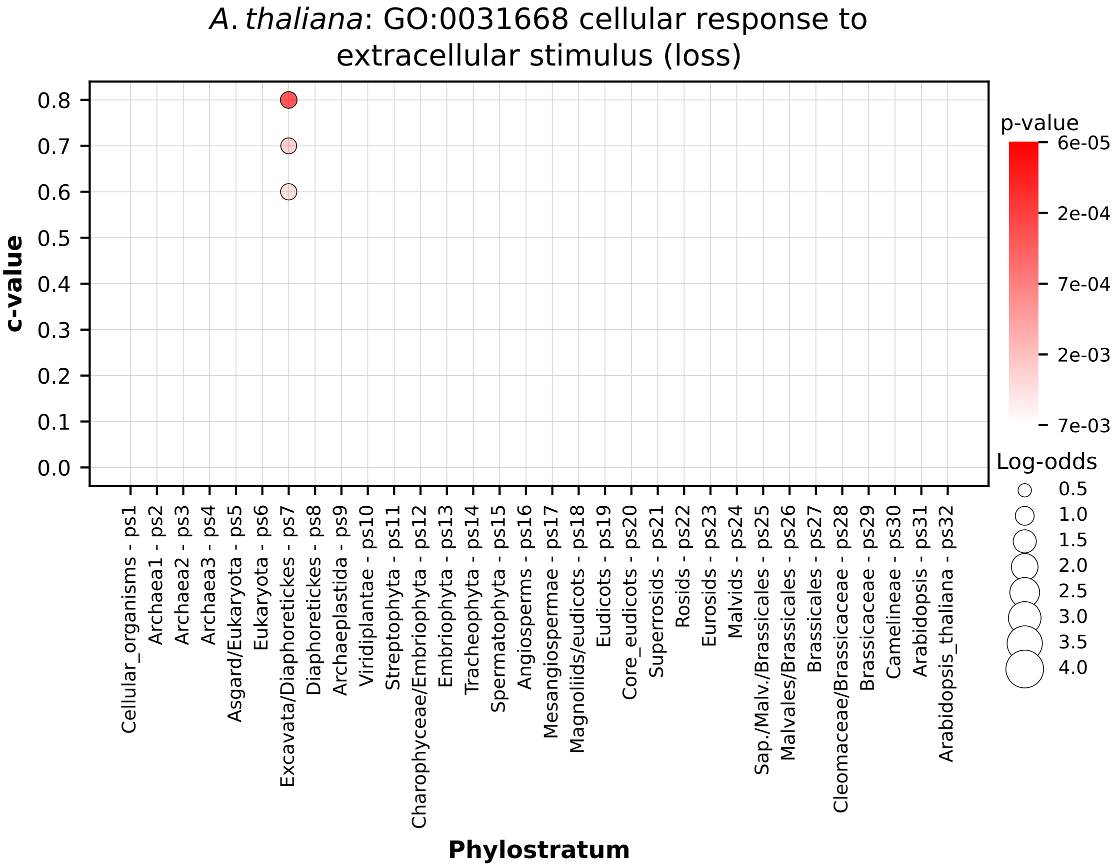

A. thaliana: GO:0031668 cellular response to extracellular stimulus (loss)
c-value
0.8
0.7
0.6
0.5
0.4
0.3
0.2
0.1
0.0
Phylostratum
Cellular_organisms - ps1
Archaea1 - ps2
Archaea2 - ps3
Archaea3 - ps4
Asgard/Eukaryota - ps5
Eukaryota - ps6
Excavata/Diaphoretickes - ps7
Diaphoretickes - ps8
Archaeplastida - ps9
Viridiplantae - ps10
Streptophyta - ps11
Charophyceae/Embriophyta - ps12
Embriophyta - ps13
Tracheophyta - ps14
Spermatophyta - ps15
Angiosperms - ps16
Mesangiospermae - ps17
Magnoliids/eudicots - ps18
Eudicots - ps19
Core_eudicots - ps20
Superrosids - ps21
Rosids - ps22
Eurosids - ps23
Malvids - ps24
Sap./Malv./Brassicales - ps25
Malvales/Brassicales - ps26
Brassicales - ps27
Cleomaceae/Brassicaceae - ps28
Brassicaceae - ps29
Camelineae - ps30
Arabidopsis - ps31
Arabidopsis_thaliana - ps32
p-value
6e-05
2e-04
7e-04
2e-03
7e-03
Log-odds
0.5
1.0
1.5
2.0
2.5
3.0
3.5
4.0

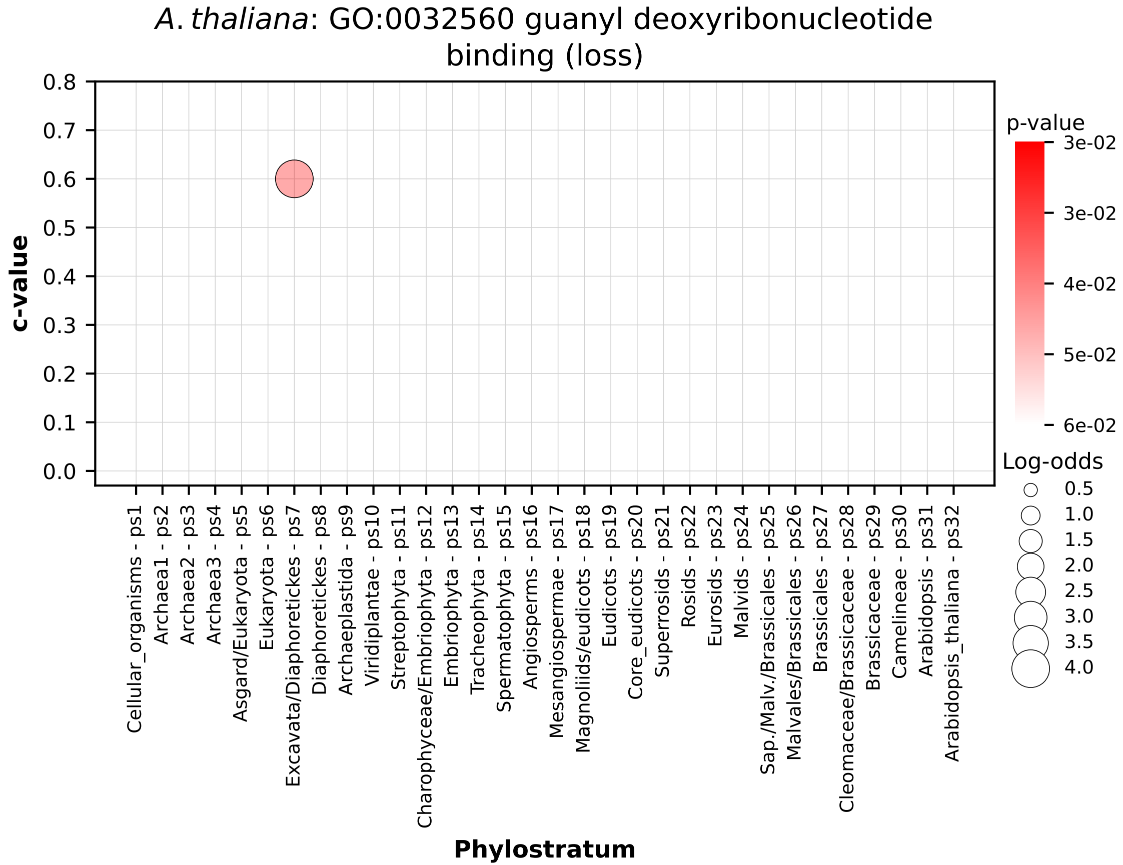

A. thaliana: GO:0032560 guanyl deoxyribonucleotide binding (loss)
c-value
0.8
0.7
0.6
0.5
0.4
0.3
0.2
0.1
0.0
Phylostratum
Cellular_organisms - ps1
Archaea1 - ps2
Archaea2 - ps3
Archaea3 - ps4
Asgard/Eukaryota - ps5
Eukaryota - ps6
Excavata/Diaphoretickes - ps7
Diaphoretickes - ps8
Archaeplastida - ps9
Viridiplantae - ps10
Streptophyta - ps11
Charophyceae/Embriophyta - ps12
Embriophyta - ps13
Tracheophyta - ps14
Spermatophyta - ps15
Angiosperms - ps16
Mesangiospermae - ps17
Magnoliids/eudicots - ps18
Eudicots - ps19
Core_eudicots - ps20
Superrosids - ps21
Rosids - ps22
Eurosids - ps23
Malvids - ps24
Sap./Malv./Brassicales - ps25
Malvales/Brassicales - ps26
Brassicales - ps27
Cleomaceae/Brassicaceae - ps28
Brassicaceae - ps29
Camelineae - ps30
Arabidopsis - ps31
Arabidopsis_thaliana - ps32
p-value
3e-02
3e-02
4e-02
5e-02
6e-02
Log-odds
0.5
1.0
1.5
2.0
2.5
3.0
3.5
4.0

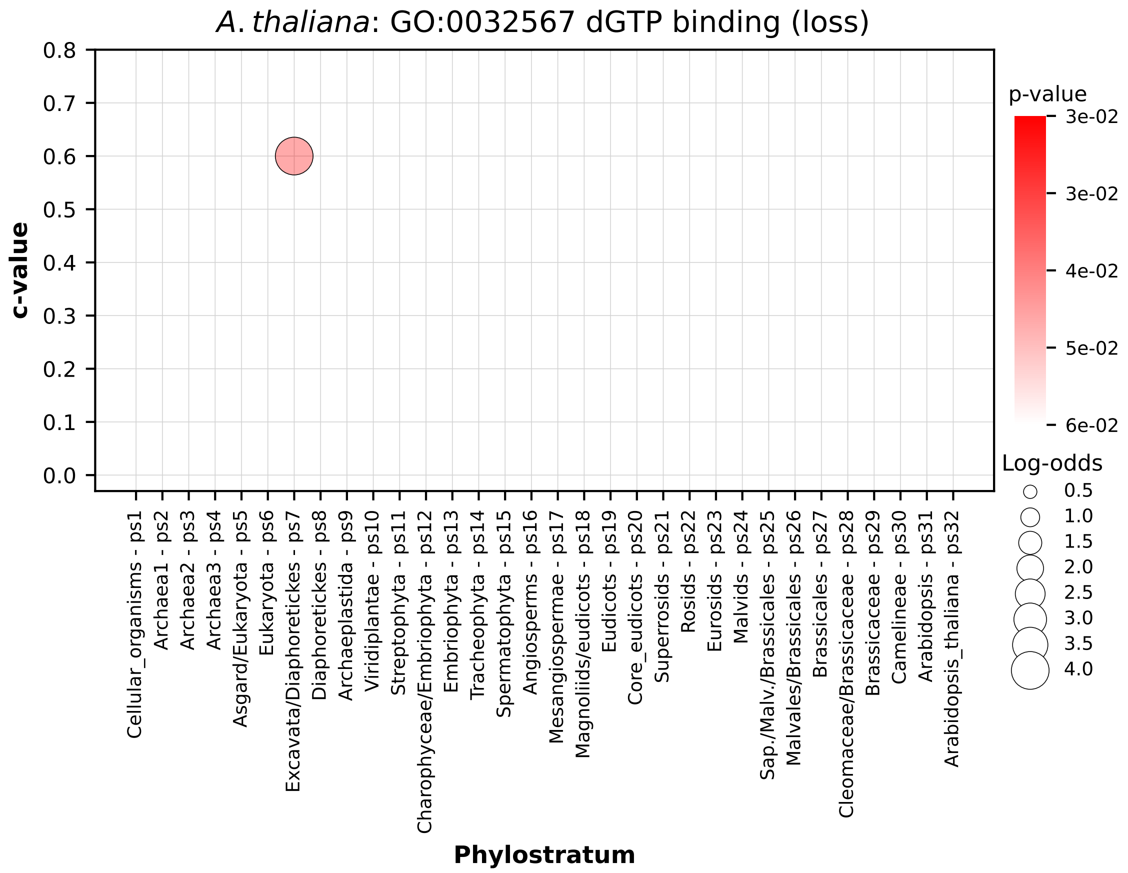

A. thaliana: GO:0032567 dGTP binding (loss)
c-value
0.8
0.7
0.6
0.5
0.4
0.3
0.2
0.1
0.0
p-value
3e-02
3e-02
4e-02
5e-02
6e-02
Log-odds
0.5
1.0
1.5
2.0
2.5
3.0
3.5
4.0
Cellular_organisms - ps1
Archaea1 - ps2
Archaea2 - ps3
Archaea3 - ps4
Asgard/Eukaryota - ps5
Eukaryota - ps6
Excavata/Diaphoretickes - ps7
Diaphoretickes - ps8
Archaeplastida - ps9
Viridiplantae - ps10
Streptophyta - ps11
Charophyceae/Embriophyta - ps12
Embriophyta - ps13
Tracheophyta - ps14
Spermatophyta - ps15
Angiosperms - ps16
Mesangiospermae - ps17
Magnoliids/eudicots - ps18
Eudicots - ps19
Core_eudicots - ps20
Superrosids - ps21
Rosids - ps22
Eurosids - ps23
Malvids - ps24
Sap./Malv./Brassicales - ps25
Malvales/Brassicales - ps26
Brassicales - ps27
Cleomaceae/Brassicaceae - ps28
Brassicaceae - ps29
Camelineae - ps30
Arabidopsis - ps31
Arabidopsis_thaliana - ps32
Phylostratum

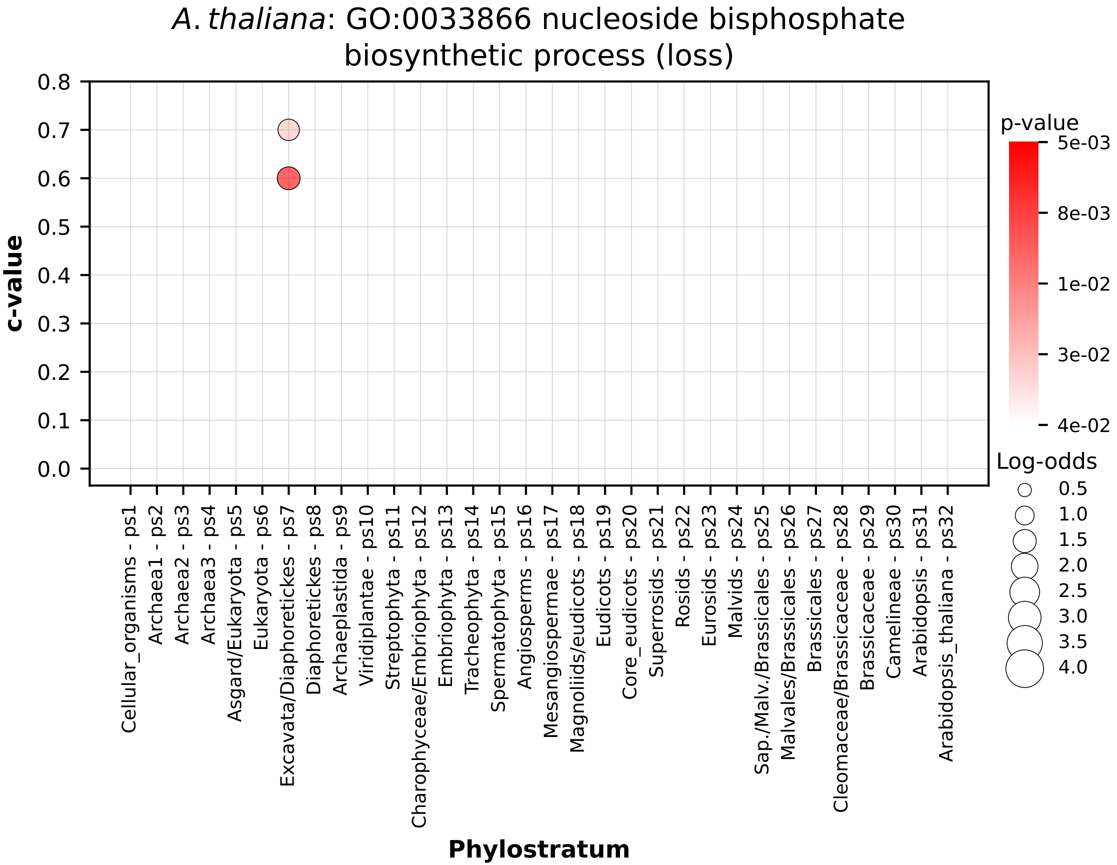

A. thaliana: GO:0033866 nucleoside bisphosphate biosynthetic process (loss)
c-value
0.8
0.7
0.6
0.5
0.4
0.3
0.2
0.1
0.0
Phylostratum
Cellular_organisms - ps1
Archaea1 - ps2
Archaea2 - ps3
Archaea3 - ps4
Asgard/Eukaryota - ps5
Eukaryota - ps6
Excavata/Diaphoretickes - ps7
Diaphoretickes - ps8
Archaeplastida - ps9
Viridiplantae - ps10
Streptophyta - ps11
Charophyceae/Embriophyta - ps12
Embriophyta - ps13
Tracheophyta - ps14
Spermatophyta - ps15
Angiosperms - ps16
Mesangiospermae - ps17
Magnoliids/eudicots - ps18
Eudicots - ps19
Core_eudicots - ps20
Superrosids - ps21
Rosids - ps22
Eurosids - ps23
Malvids - ps24
Sap./Malv./Brassicales - ps25
Malvales/Brassicales - ps26
Brassicales - ps27
Cleomaceae/Brassicaceae - ps28
Brassicaceae - ps29
Camelineae - ps30
Arabidopsis - ps31
Arabidopsis_thaliana - ps32
p-value
5e-03
8e-03
1e-02
3e-02
4e-02
Log-odds
0.5
1.0
1.5
2.0
2.5
3.0
3.5
4.0

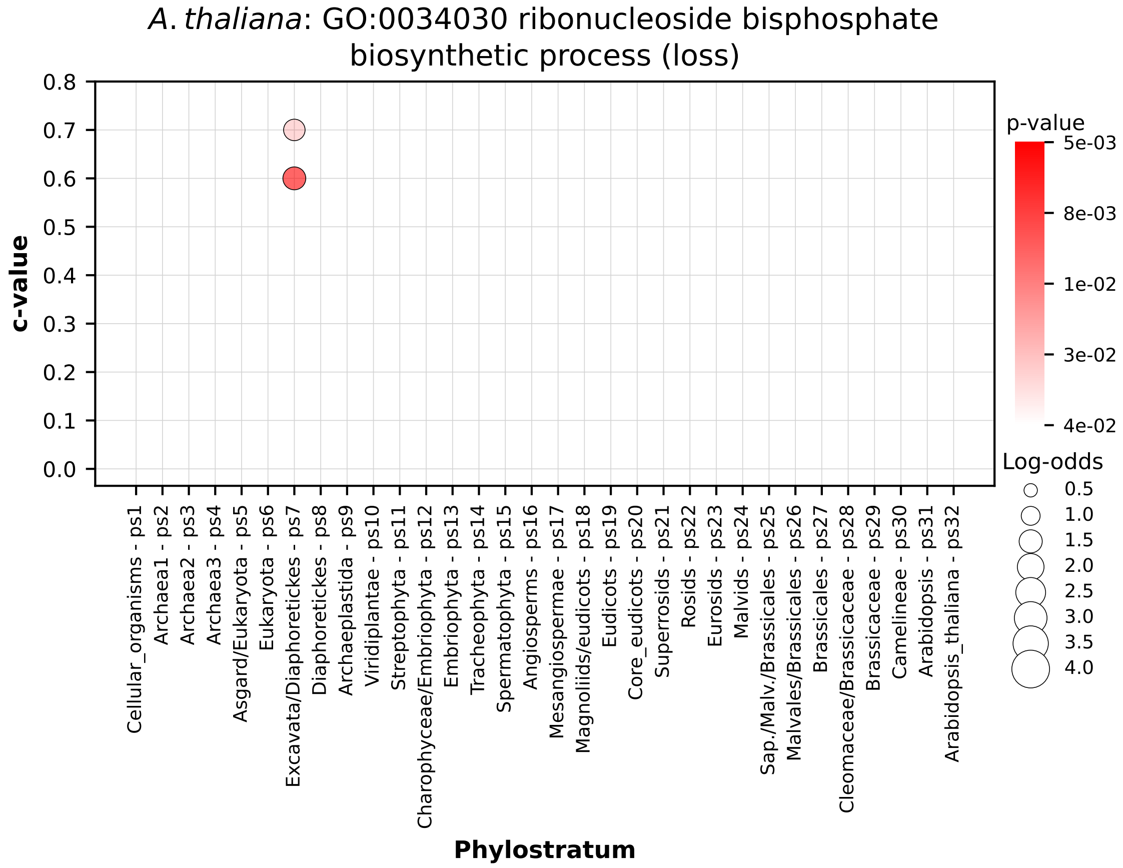

A. thaliana: GO:0034030 ribonucleoside bisphosphate biosynthetic process (loss)
c-value
0.8
0.7
0.6
0.5
0.4
0.3
0.2
0.1
0.0
p-value
5e-03
8e-03
1e-02
3e-02
4e-02
Log-odds
0.5
1.0
1.5
2.0
2.5
3.0
3.5
4.0
Cellular_organisms - ps1
Archaea1 - ps2
Archaea2 - ps3
Archaea3 - ps4
Asgard/Eukaryota - ps5
Eukaryota - ps6
Excavata/Diaphoretickes - ps7
Diaphoretickes - ps8
Archaeplastida - ps9
Viridiplantae - ps10
Streptophyta - ps11
Charophyceae/Embriophyta - ps12
Embriophyta - ps13
Tracheophyta - ps14
Spermatophyta - ps15
Angiosperms - ps16
Mesangiospermae - ps17
Magnoliids/eudicots - ps18
Eudicots - ps19
Core_eudicots - ps20
Superrosids - ps21
Rosids - ps22
Eurosids - ps23
Malvids - ps24
Sap./Malv./Brassicales - ps25
Malvales/Brassicales - ps26
Brassicales - ps27
Cleomaceae/Brassicaceae - ps28
Brassicaceae - ps29
Camelineae - ps30
Arabidopsis - ps31
Arabidopsis_thaliana - ps32
Phylostratum

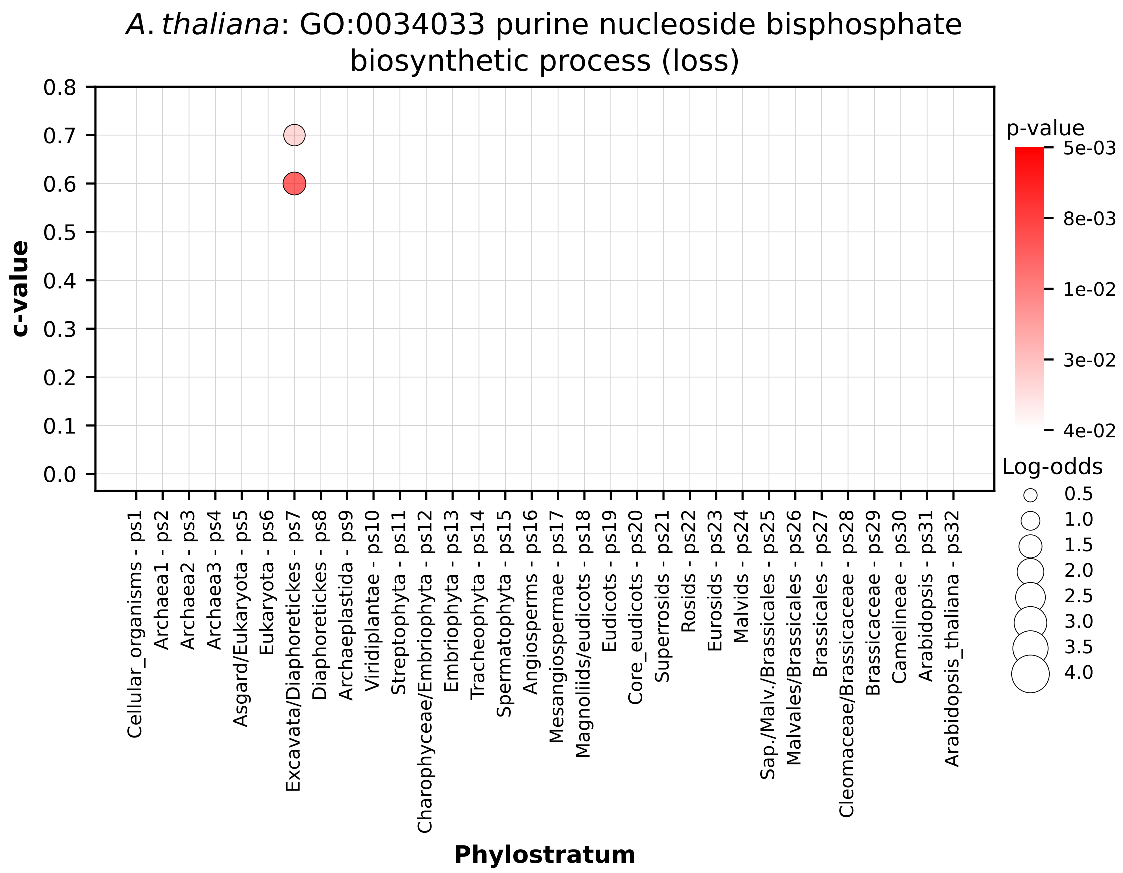

A. thaliana: GO:0034033 purine nucleoside bisphosphate biosynthetic process (loss)
c-value
0.8
0.7
0.6
0.5
0.4
0.3
0.2
0.1
0.0
p-value
5e-03
8e-03
1e-02
3e-02
4e-02
Log-odds
0.5
1.0
1.5
2.0
2.5
3.0
3.5
4.0
Cellular_organisms - ps1
Archaea1 - ps2
Archaea2 - ps3
Archaea3 - ps4
Asgard/Eukaryota - ps5
Eukaryota - ps6
Excavata/Diaphoretickes - ps7
Diaphoretickes - ps8
Archaeplastida - ps9
Viridiplantae - ps10
Streptophyta - ps11
Charophyceae/Embriophyta - ps12
Embriophyta - ps13
Tracheophyta - ps14
Spermatophyta - ps15
Angiosperms - ps16
Mesangiospermae - ps17
Magnoliids/eudicots - ps18
Eudicots - ps19
Core_eudicots - ps20
Superrosids - ps21
Rosids - ps22
Eurosids - ps23
Malvids - ps24
Sap./Malv./Brassicales - ps25
Malvales/Brassicales - ps26
Brassicales - ps27
Cleomaceae/Brassicaceae - ps28
Brassicaceae - ps29
Camelineae - ps30
Arabidopsis - ps31
Arabidopsis_thaliana - ps32
Phylostratum

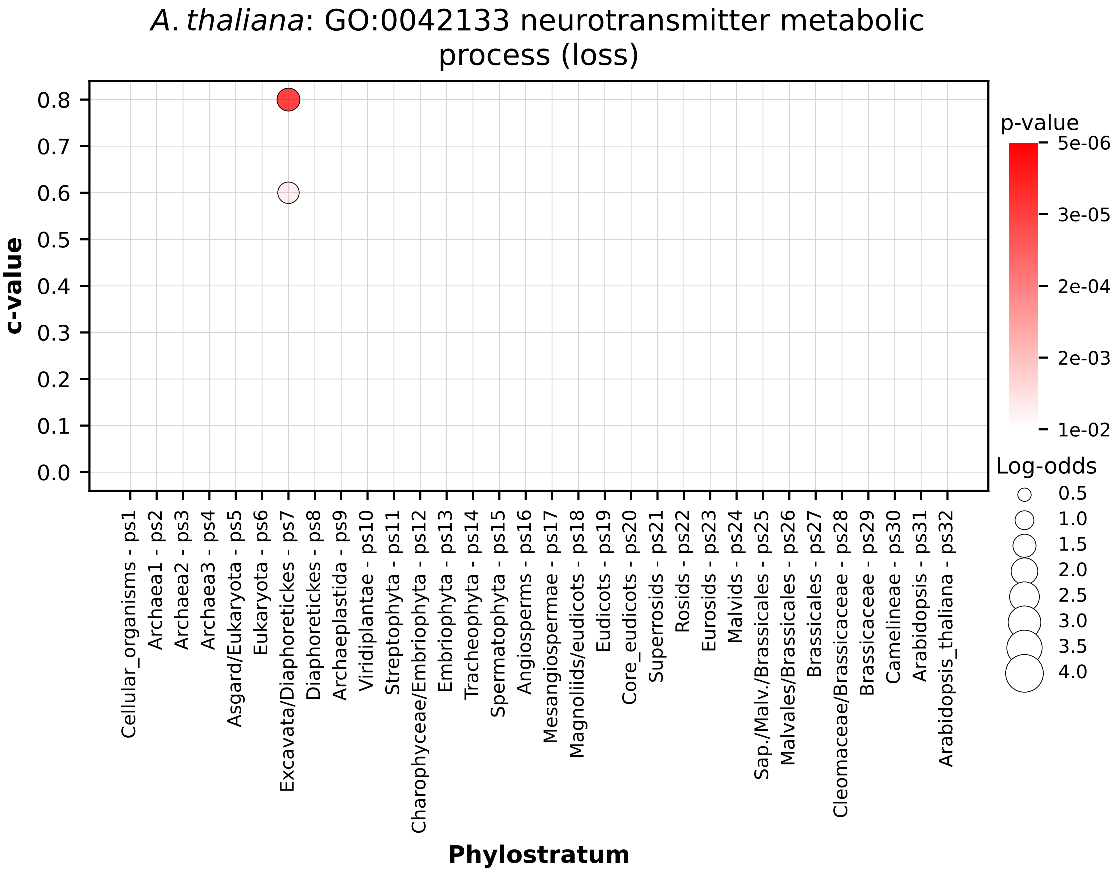

A. thaliana: GO:0042133 neurotransmitter metabolic process (loss)
c-value
0.0
0.1
0.2
0.3
0.4
0.5
0.6
0.7
0.8
Phylostratum
Cellular_organisms - ps1
Archaea1 - ps2
Archaea2 - ps3
Archaea3 - ps4
Asgard/Eukaryota - ps5
Eukaryota - ps6
Excavata/Diaphoretickes - ps7
Diaphoretickes - ps8
Archaeplastida - ps9
Viridiplantae - ps10
Streptophyta - ps11
Charophyceae/Embriophyta - ps12
Embriophyta - ps13
Tracheophyta - ps14
Spermatophyta - ps15
Angiosperms - ps16
Mesangiospermae - ps17
Magnoliids/eudicots - ps18
Eudicots - ps19
Core_eudicots - ps20
Superrosids - ps21
Rosids - ps22
Eurosids - ps23
Malvids - ps24
Sap./Malv./Brassicales - ps25
Malvales/Brassicales - ps26
Brassicales - ps27
Cleomaceae/Brassicaceae - ps28
Brassicaceae - ps29
Camelineae - ps30
Arabidopsis - ps31
Arabidopsis_thaliana - ps32
p-value
5e-06
3e-05
2e-04
2e-03
1e-02
Log-odds
0.5
1.0
1.5
2.0
2.5
3.0
3.5
4.0

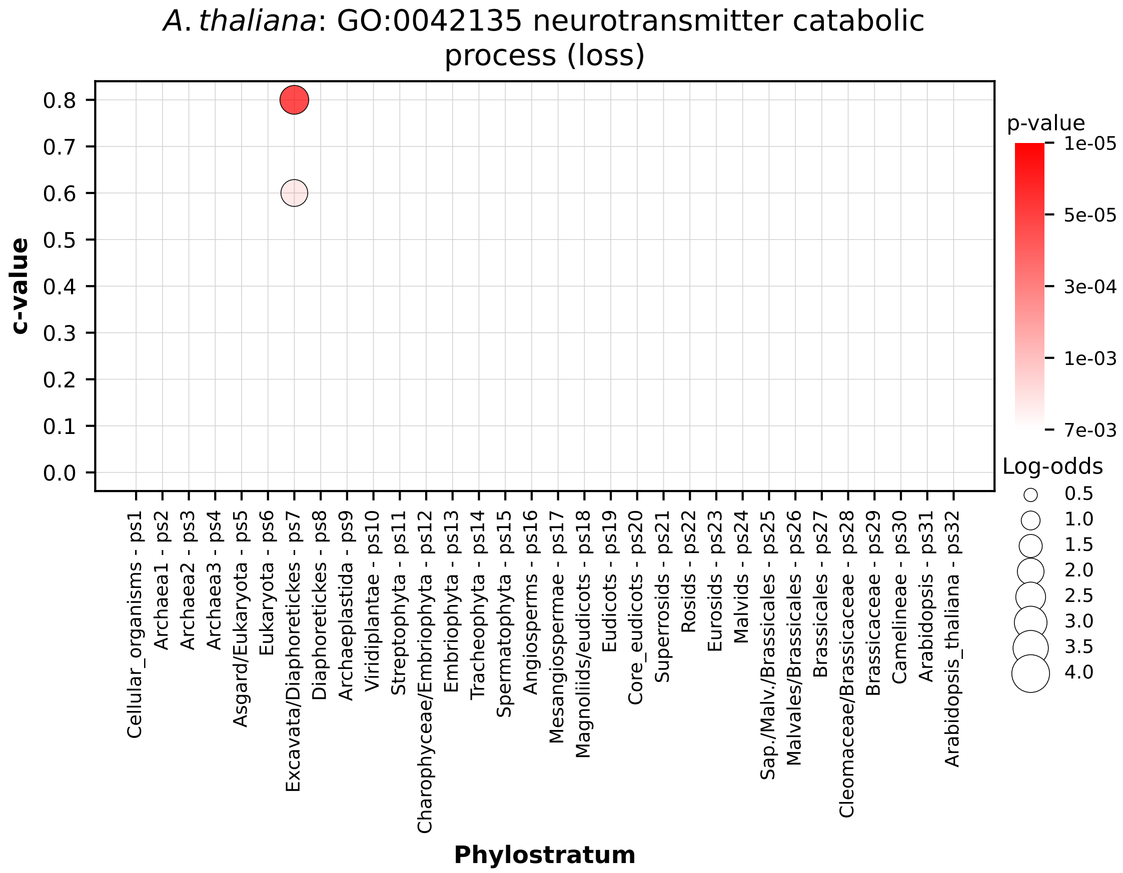

A. thaliana: GO:0042135 neurotransmitter catabolic process (loss)
c-value
0.0
0.1
0.2
0.3
0.4
0.5
0.6
0.7
0.8
Phylostratum
Cellular_organisms - ps1
Archaea1 - ps2
Archaea2 - ps3
Archaea3 - ps4
Asgard/Eukaryota - ps5
Eukaryota - ps6
Excavata/Diaphoretickes - ps7
Diaphoretickes - ps8
Archaeplastida - ps9
Viridiplantae - ps10
Streptophyta - ps11
Charophyceae/Embriophyta - ps12
Embriophyta - ps13
Tracheophyta - ps14
Spermatophyta - ps15
Angiosperms - ps16
Mesangiospermae - ps17
Magnoliids/eudicots - ps18
Eudicots - ps19
Core_eudicots - ps20
Superrosids - ps21
Rosids - ps22
Eurosids - ps23
Malvids - ps24
Sap./Malv./Brassicales - ps25
Malvales/Brassicales - ps26
Brassicales - ps27
Cleomaceae/Brassicaceae - ps28
Brassicaceae - ps29
Camelineae - ps30
Arabidopsis - ps31
Arabidopsis_thaliana - ps32
p-value
1e-05
5e-05
3e-04
1e-03
7e-03
Log-odds
0.5
1.0
1.5
2.0
2.5
3.0
3.5
4.0

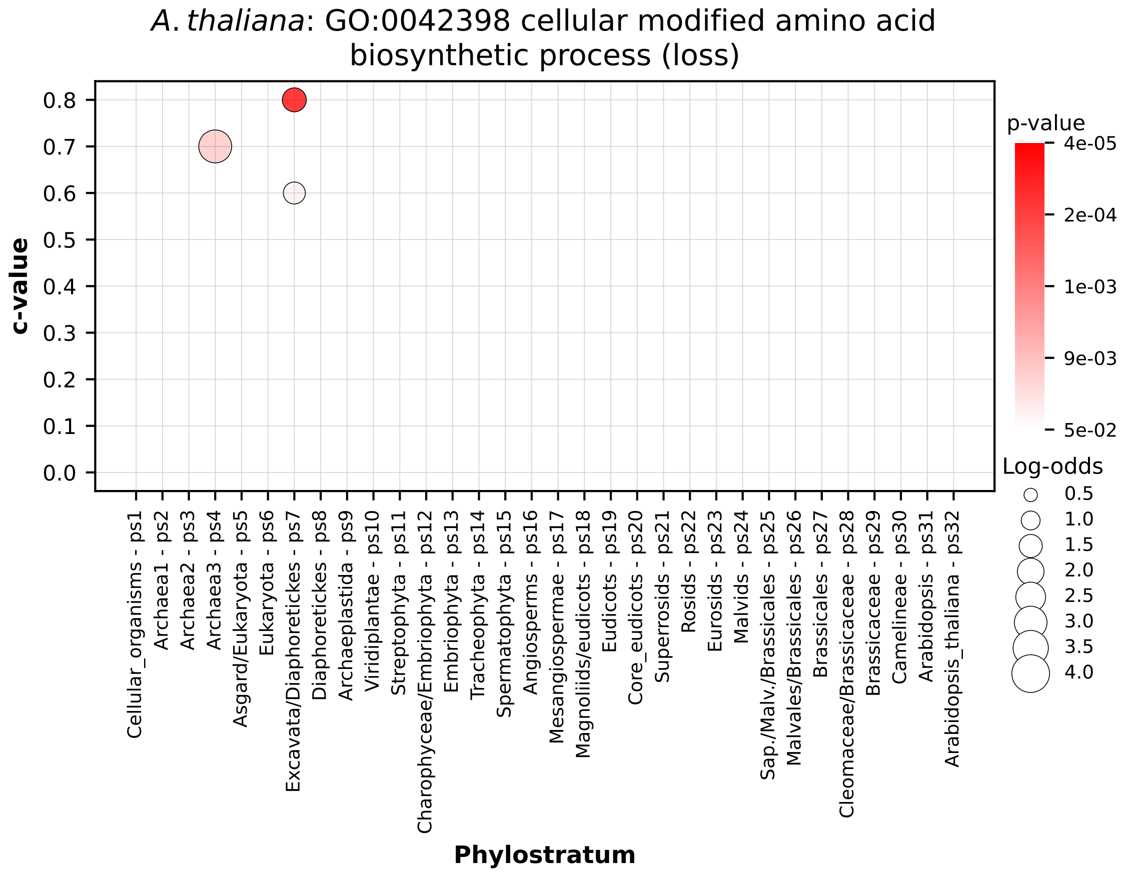

A. thaliana: GO:0042398 cellular modified amino acid biosynthetic process (loss)
c-value
0.0
0.1
0.2
0.3
0.4
0.5
0.6
0.7
0.8
p-value
4e-05
2e-04
1e-03
9e-03
5e-02
Log-odds
0.5
1.0
1.5
2.0
2.5
3.0
3.5
4.0
Cellular_organisms - ps1
Archaea1 - ps2
Archaea2 - ps3
Archaea3 - ps4
Asgard/Eukaryota - ps5
Eukaryota - ps6
Excavata/Diaphoretickes - ps7
Diaphoretickes - ps8
Archaeplastida - ps9
Viridiplantae - ps10
Streptophyta - ps11
Charophyceae/Embriophyta - ps12
Embriophyta - ps13
Tracheophyta - ps14
Spermatophyta - ps15
Angiosperms - ps16
Mesangiospermae - ps17
Magnoliids/eudicots - ps18
Eudicots - ps19
Core_eudicots - ps20
Superrosids - ps21
Rosids - ps22
Eurosids - ps23
Malvids - ps24
Sap./Malv./Brassicales - ps25
Malvales/Brassicales - ps26
Brassicales - ps27
Cleomaceae/Brassicaceae - ps28
Brassicaceae - ps29
Camelineae - ps30
Arabidopsis - ps31
Arabidopsis_thaliana - ps32
Phylostratum

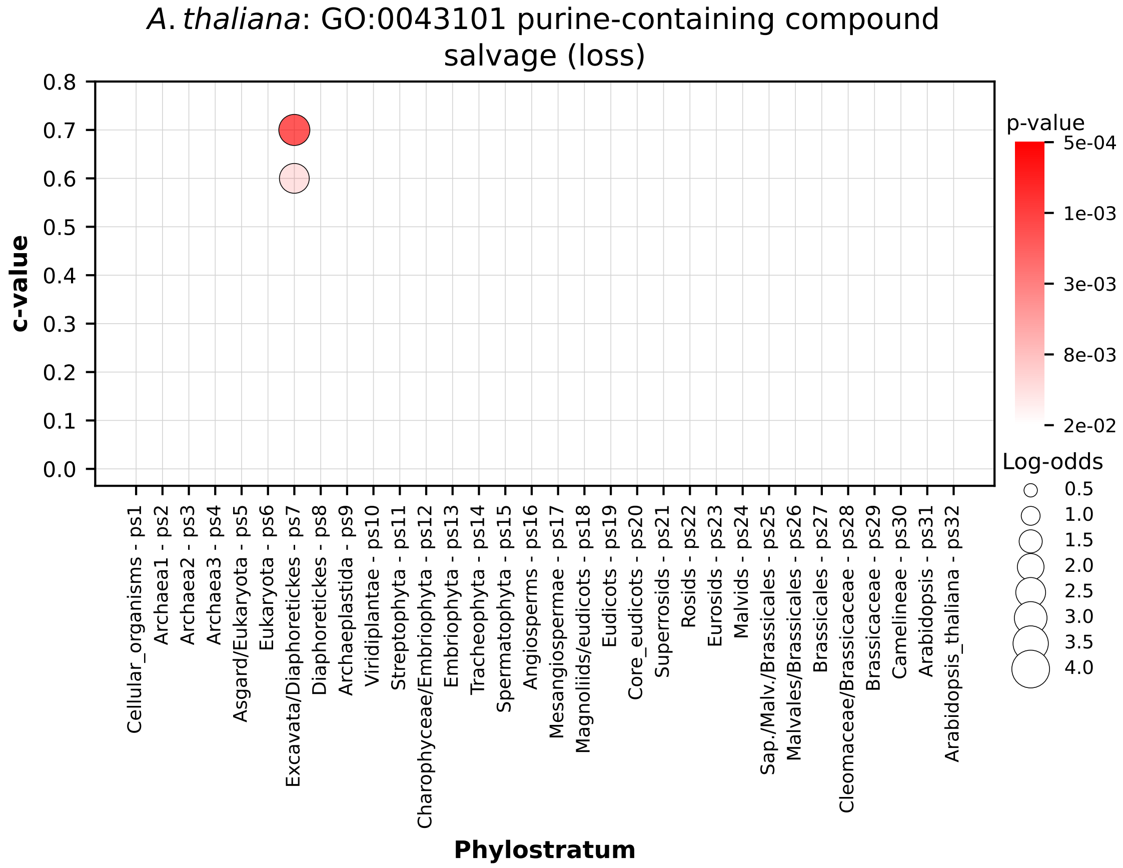

A. thaliana: GO:0043101 purine-containing compound salvage (loss)
c-value
0.8
0.7
0.6
0.5
0.4
0.3
0.2
0.1
0.0
p-value
5e-04
1e-03
3e-03
8e-03
2e-02
Log-odds
0.5
1.0
1.5
2.0
2.5
3.0
3.5
4.0
Cellular_organisms - ps1
Archaea1 - ps2
Archaea2 - ps3
Archaea3 - ps4
Asgard/Eukaryota - ps5
Eukaryota - ps6
Excavata/Diaphoretickes - ps7
Diaphoretickes - ps8
Archaeplastida - ps9
Viridiplantae - ps10
Streptophyta - ps11
Charophyceae/Embriophyta - ps12
Embriophyta - ps13
Tracheophyta - ps14
Spermatophyta - ps15
Angiosperms - ps16
Mesangiospermae - ps17
Magnoliids/eudicots - ps18
Eudicots - ps19
Core_eudicots - ps20
Superrosids - ps21
Rosids - ps22
Eurosids - ps23
Malvids - ps24
Sap./Malv./Brassicales - ps25
Malvales/Brassicales - ps26
Brassicales - ps27
Cleomaceae/Brassicaceae - ps28
Brassicaceae - ps29
Camelineae - ps30
Arabidopsis - ps31
Arabidopsis_thaliana - ps32
Phylostratum

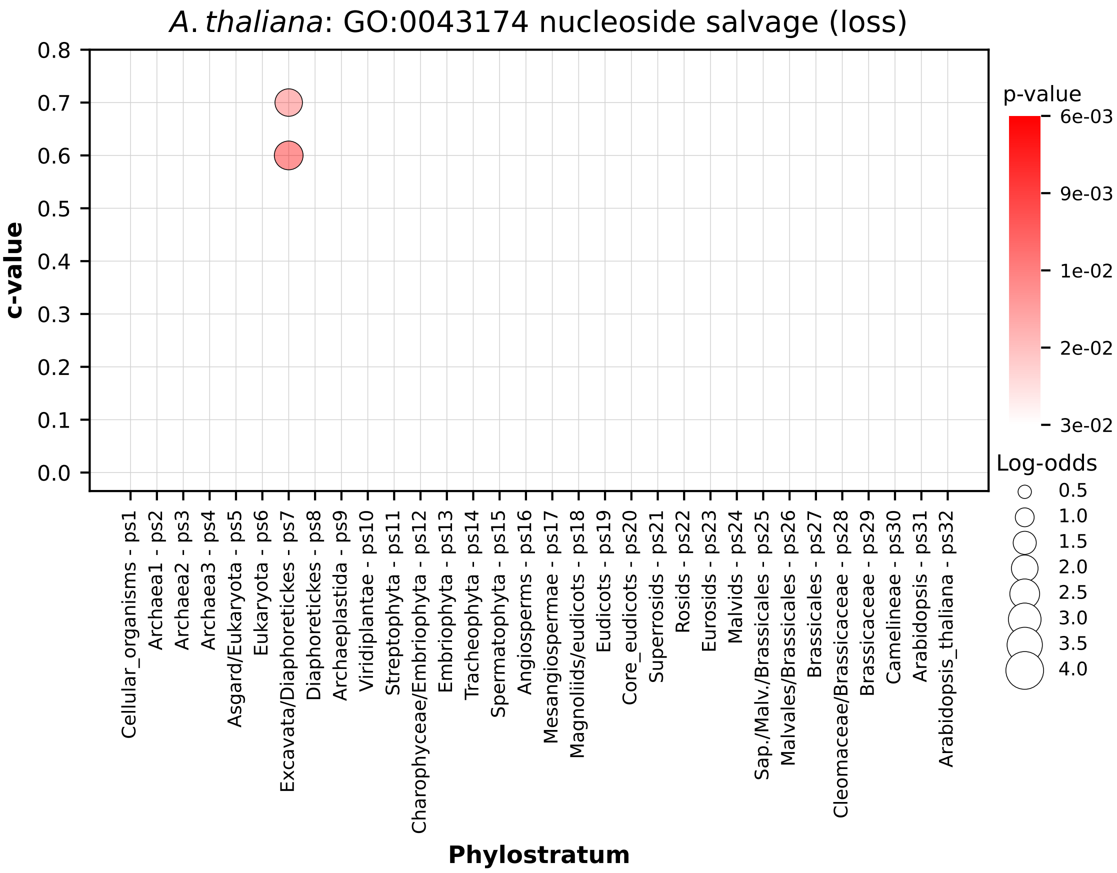

A. thaliana: GO:0043174 nucleoside salvage (loss)
c-value
0.0
0.1
0.2
0.3
0.4
0.5
0.6
0.7
0.8
Phylostratum
Cellular_organisms - ps1
Archaea1 - ps2
Archaea2 - ps3
Archaea3 - ps4
Asgard/Eukaryota - ps5
Eukaryota - ps6
Excavata/Diaphoretickes - ps7
Diaphoretickes - ps8
Archaeplastida - ps9
Viridiplantae - ps10
Streptophyta - ps11
Charophyceae/Embriophyta - ps12
Embriophyta - ps13
Tracheophyta - ps14
Spermatophyta - ps15
Angiosperms - ps16
Mesangiospermae - ps17
Magnoliids/eudicots - ps18
Eudicots - ps19
Core_eudicots - ps20
Superrosids - ps21
Rosids - ps22
Eurosids - ps23
Malvids - ps24
Sap./Malv./Brassicales - ps25
Malvales/Brassicales - ps26
Brassicales - ps27
Cleomaceae/Brassicaceae - ps28
Brassicaceae - ps29
Camelineae - ps30
Arabidopsis - ps31
Arabidopsis_thaliana - ps32
p-value
6e-03
9e-03
1e-02
2e-02
3e-02
Log-odds
0.5
1.0
1.5
2.0
2.5
3.0
3.5
4.0

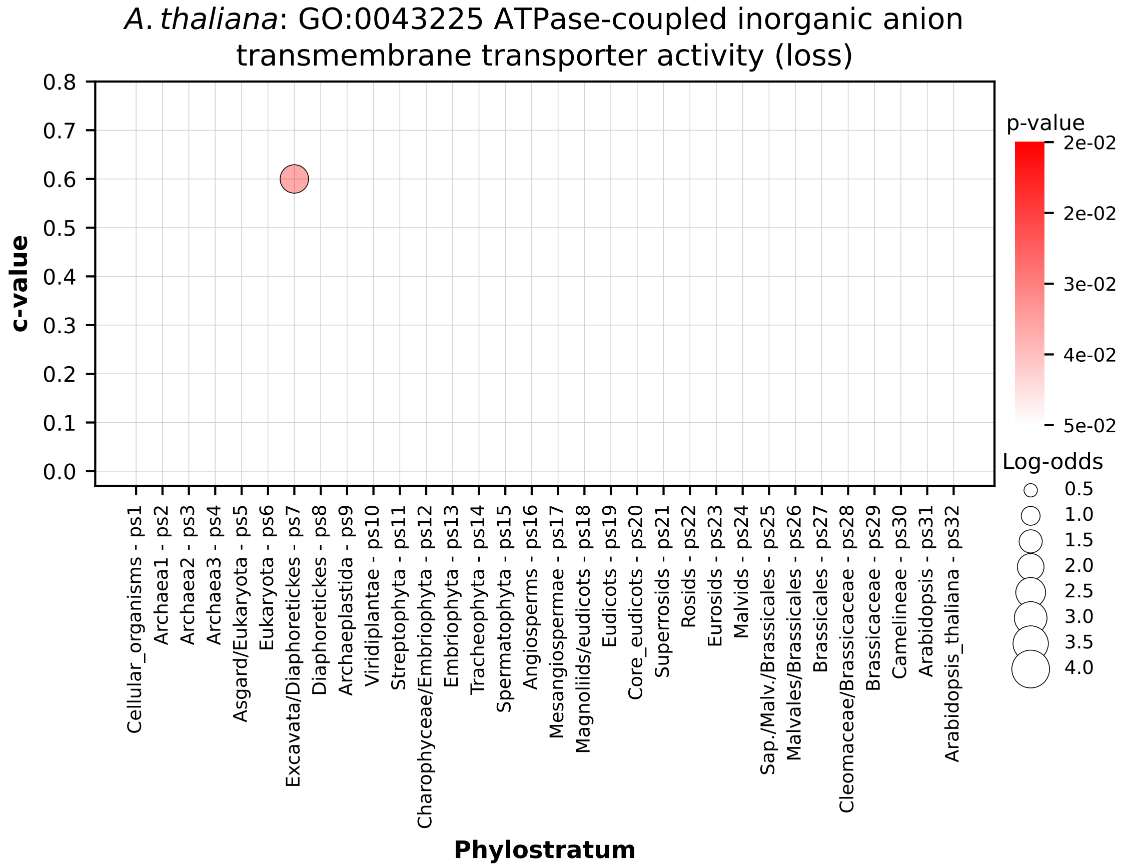

A. thaliana: GO:0043225 ATPase-coupled inorganic anion transmembrane transporter activity (loss)
c-value
0.8
0.7
0.6
0.5
0.4
0.3
0.2
0.1
0.0
Phylostratum
Cellular_organisms - ps1
Archaea1 - ps2
Archaea2 - ps3
Archaea3 - ps4
Asgard/Eukaryota - ps5
Eukaryota - ps6
Excavata/Diaphoretickes - ps7
Diaphoretickes - ps8
Archaeplastida - ps9
Viridiplantae - ps10
Streptophyta - ps11
Charophyceae/Embriophyta - ps12
Embriophyta - ps13
Tracheophyta - ps14
Spermatophyta - ps15
Angiosperms - ps16
Mesangiospermae - ps17
Magnoliids/eudicots - ps18
Eudicots - ps19
Core_eudicots - ps20
Superrosids - ps21
Rosids - ps22
Eurosids - ps23
Malvids - ps24
Sap./Malv./Brassicales - ps25
Malvales/Brassicales - ps26
Brassicales - ps27
Cleomaceae/Brassicaceae - ps28
Brassicaceae - ps29
Camelineae - ps30
Arabidopsis - ps31
Arabidopsis_thaliana - ps32
p-value
2e-02
2e-02
3e-02
4e-02
5e-02
Log-odds
0.5
1.0
1.5
2.0
2.5
3.0
3.5
4.0

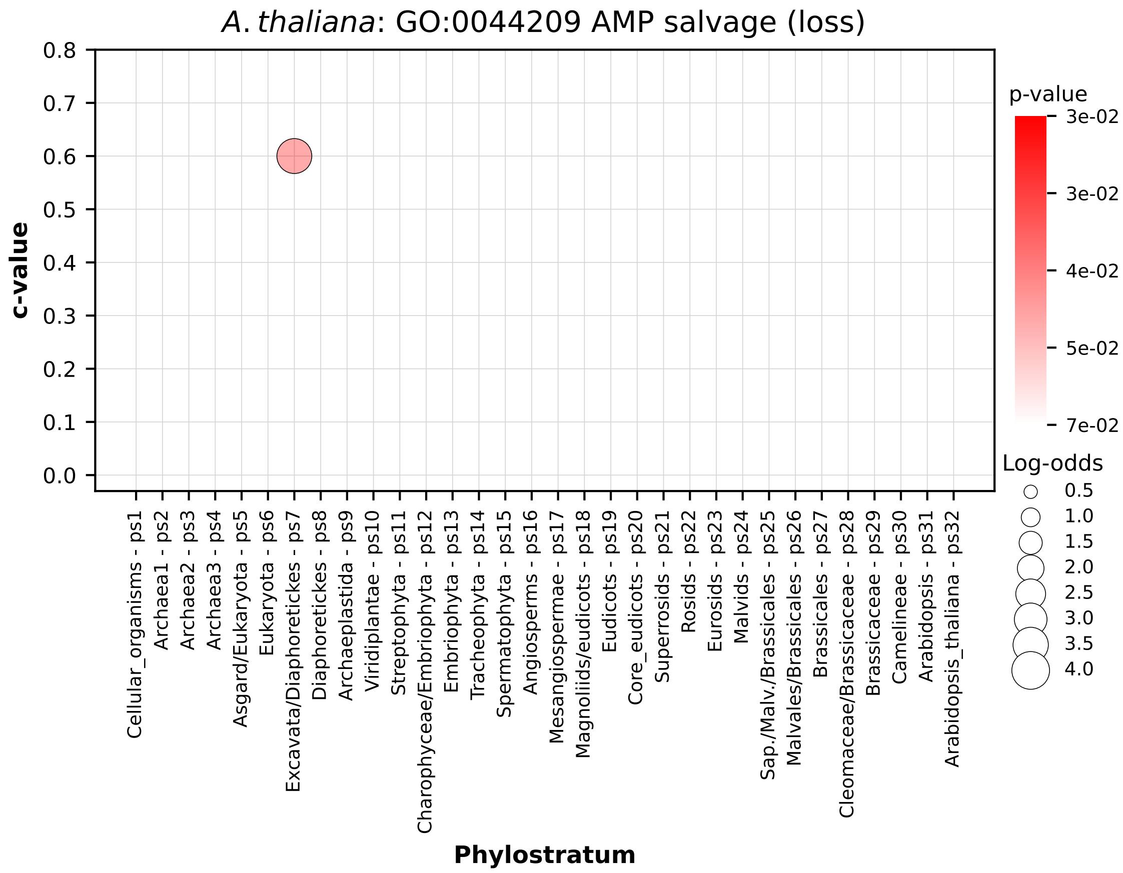
A. thaliana: GO:0044209 AMP salvage (loss)
c-value
0.8
0.7
0.6
0.5
0.4
0.3
0.2
0.1
0.0
Phylostratum
Cellular_organisms - ps1
Archaea1 - ps2
Archaea2 - ps3
Archaea3 - ps4
Asgard/Eukaryota - ps5
Eukaryota - ps6
Excavata/Diaphoretickes - ps7
Diaphoretickes - ps8
Archaeplastida - ps9
Viridiplantae - ps10
Streptophyta - ps11
Charophyceae/Embriophyta - ps12
Embriophyta - ps13
Tracheophyta - ps14
Spermatophyta - ps15
Angiosperms - ps16
Mesangiospermae - ps17
Magnoliids/eudicots - ps18
Eudicots - ps19
Core_eudicots - ps20
Superrosids - ps21
Rosids - ps22
Eurosids - ps23
Malvids - ps24
Sap./Malv./Brassicales - ps25
Malvales/Brassicales - ps26
Brassicales - ps27
Cleomaceae/Brassicaceae - ps28
Brassicaceae - ps29
Camelineae - ps30
Arabidopsis - ps31
Arabidopsis_thaliana - ps32
p-value
3e-02
3e-02
4e-02
5e-02
7e-02
Log-odds
0.5
1.0
1.5
2.0
2.5
3.0
3.5
4.0

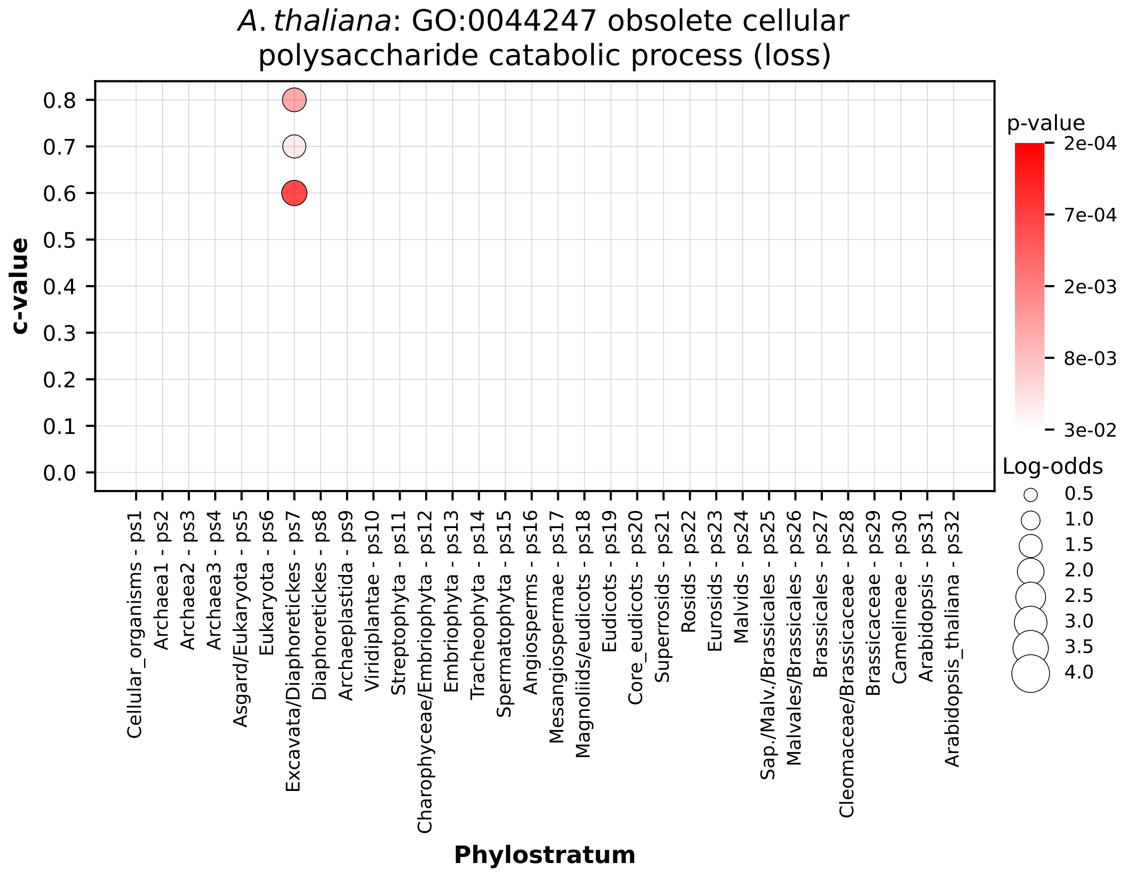

A. thaliana: GO:0044247 obsolete cellular polysaccharide catabolic process (loss)
c-value
0.0
0.1
0.2
0.3
0.4
0.5
0.6
0.7
0.8
Phylostratum
Cellular_organisms - ps1
Archaea1 - ps2
Archaea2 - ps3
Archaea3 - ps4
Asgard/Eukaryota - ps5
Eukaryota - ps6
Excavata/Diaphoretickes - ps7
Diaphoretickes - ps8
Archaeplastida - ps9
Viridiplantae - ps10
Streptophyta - ps11
Charophyceae/Embriophyta - ps12
Embriophyta - ps13
Tracheophyta - ps14
Spermatophyta - ps15
Angiosperms - ps16
Mesangiospermae - ps17
Magnoliids/eudicots - ps18
Eudicots - ps19
Core_eudicots - ps20
Superrosids - ps21
Rosids - ps22
Eurosids - ps23
Malvids - ps24
Sap./Malv./Brassicales - ps25
Malvales/Brassicales - ps26
Brassicales - ps27
Cleomaceae/Brassicaceae - ps28
Brassicaceae - ps29
Camelineae - ps30
Arabidopsis - ps31
Arabidopsis_thaliana - ps32
p-value
2e-04
7e-04
2e-03
8e-03
3e-02
Log-odds
0.5
1.0
1.5
2.0
2.5
3.0
3.5
4.0

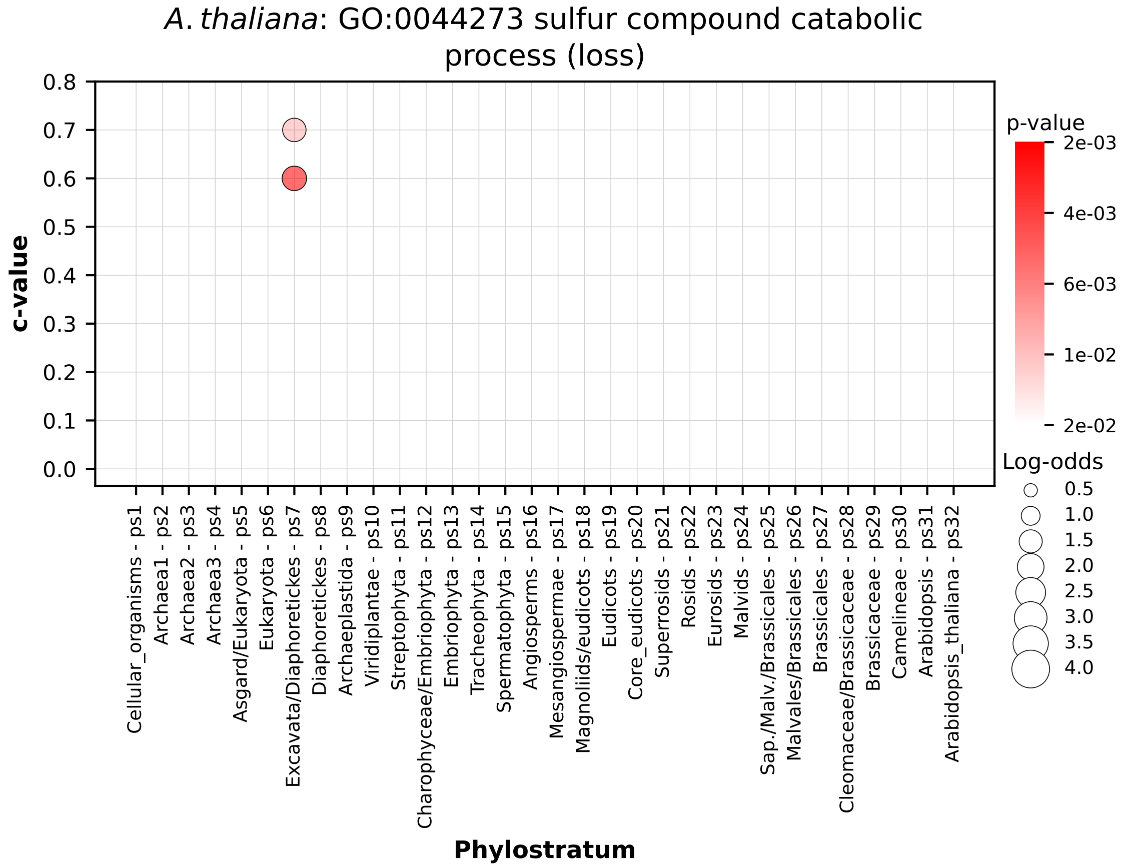
A. thaliana: GO:0044273 sulfur compound catabolic process (loss)
c-value
0.8
0.7
0.6
0.5
0.4
0.3
0.2
0.1
0.0
p-value
2e-03
4e-03
6e-03
1e-02
2e-02
Log-odds
0.5
1.0
1.5
2.0
2.5
3.0
3.5
4.0
Cellular_organisms - ps1
Archaea1 - ps2
Archaea2 - ps3
Archaea3 - ps4
Asgard/Eukaryota - ps5
Eukaryota - ps6
Excavata/Diaphoretickes - ps7
Diaphoretickes - ps8
Archaeplastida - ps9
Viridiplantae - ps10
Streptophyta - ps11
Charophyceae/Embriophyta - ps12
Embriophyta - ps13
Tracheophyta - ps14
Spermatophyta - ps15
Angiosperms - ps16
Mesangiospermae - ps17
Magnoliids/eudicots - ps18
Eudicots - ps19
Core_eudicots - ps20
Superrosids - ps21
Rosids - ps22
Eurosids - ps23
Malvids - ps24
Sap./Malv./Brassicales - ps25
Malvales/Brassicales - ps26
Brassicales - ps27
Cleomaceae/Brassicaceae - ps28
Brassicaceae - ps29
Camelineae - ps30
Arabidopsis - ps31
Arabidopsis_thaliana - ps32
Phylostratum

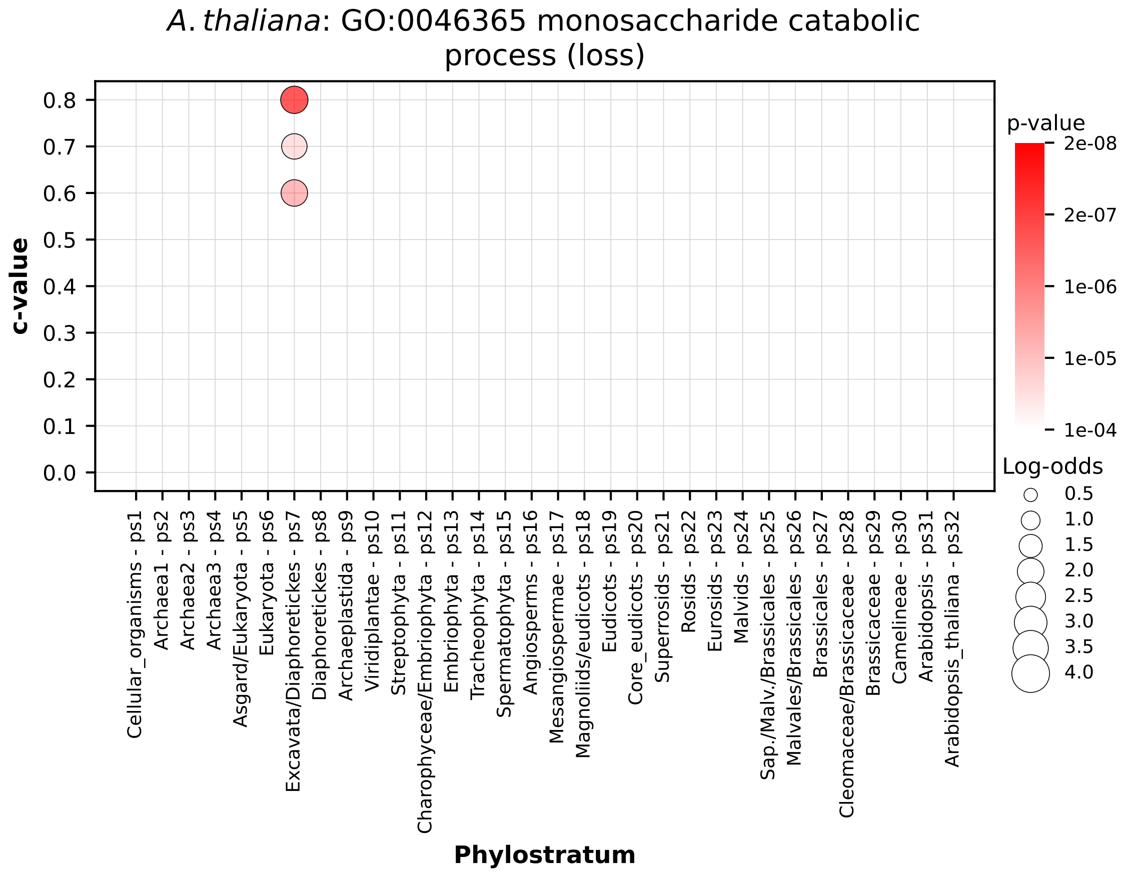
A. thaliana: GO:0046365 monosaccharide catabolic process (loss)
c-value
0.8
0.7
0.6
0.5
0.4
0.3
0.2
0.1
0.0
p-value
2e-08
2e-07
1e-06
1e-05
1e-04
Log-odds
0.5
1.0
1.5
2.0
2.5
3.0
3.5
4.0
Cellular_organisms - ps1
Archaea1 - ps2
Archaea2 - ps3
Archaea3 - ps4
Asgard/Eukaryota - ps5
Eukaryota - ps6
Excavata/Diaphoretickes - ps7
Diaphoretickes - ps8
Archaeplastida - ps9
Viridiplantae - ps10
Streptophyta - ps11
Charophyceae/Embriophyta - ps12
Embriophyta - ps13
Tracheophyta - ps14
Spermatophyta - ps15
Angiosperms - ps16
Mesangiospermae - ps17
Magnoliids/eudicots - ps18
Eudicots - ps19
Core_eudicots - ps20
Superrosids - ps21
Rosids - ps22
Eurosids - ps23
Malvids - ps24
Sap./Malv./Brassicales - ps25
Malvales/Brassicales - ps26
Brassicales - ps27
Cleomaceae/Brassicaceae - ps28
Brassicaceae - ps29
Camelineae - ps30
Arabidopsis - ps31
Arabidopsis_thaliana - ps32
Phylostratum

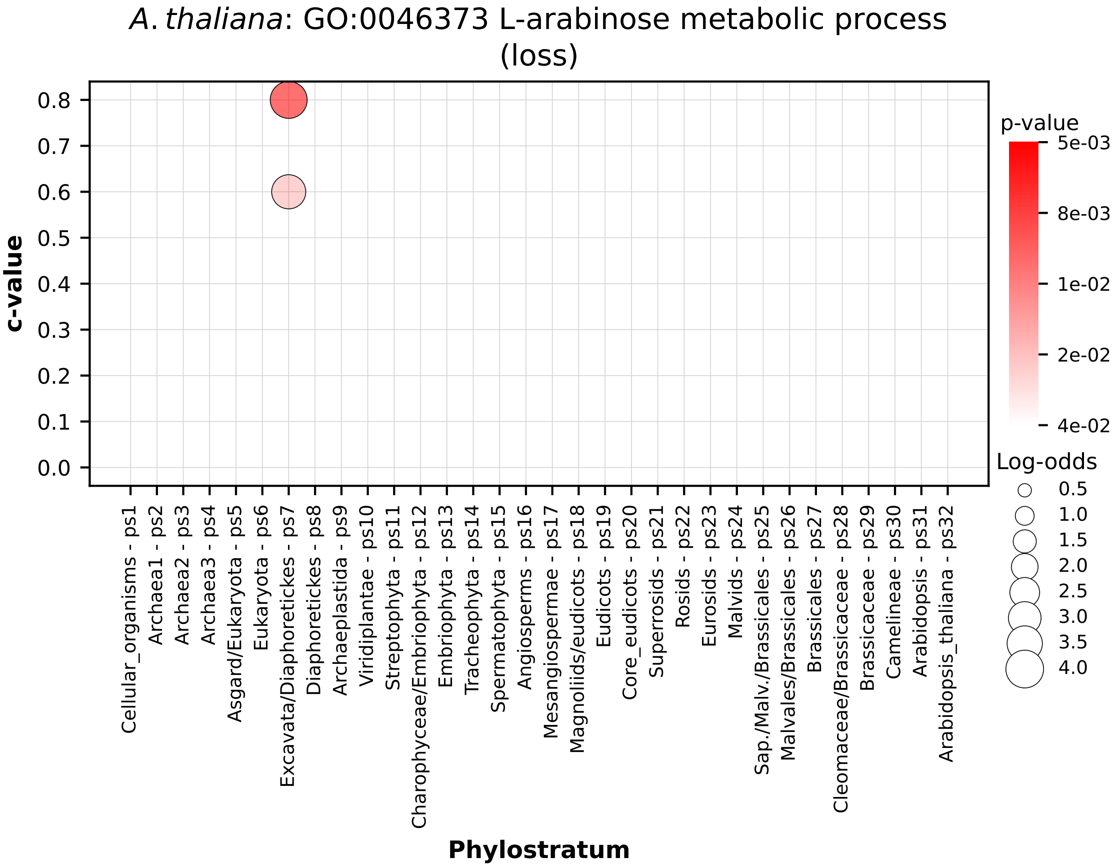

A. thaliana: GO:0046373 L-arabinose metabolic process
(loss)
c-value
0.0
0.1
0.2
0.3
0.4
0.5
0.6
0.7
0.8
Phylostratum
Cellular_organisms - ps1
Archaea1 - ps2
Archaea2 - ps3
Archaea3 - ps4
Asgard/Eukaryota - ps5
Eukaryota - ps6
Excavata/Diaphoretickes - ps7
Diaphoretickes - ps8
Archaeplastida - ps9
Viridiplantae - ps10
Streptophyta - ps11
Charophyceae/Embriophyta - ps12
Embriophyta - ps13
Tracheophyta - ps14
Spermatophyta - ps15
Angiosperms - ps16
Mesangiospermae - ps17
Magnoliids/eudicots - ps18
Eudicots - ps19
Core_eudicots - ps20
Superrosids - ps21
Rosids - ps22
Eurosids - ps23
Malvids - ps24
Sap./Malv./Brassicales - ps25
Malvales/Brassicales - ps26
Brassicales - ps27
Cleomaceae/Brassicaceae - ps28
Brassicaceae - ps29
Camelineae - ps30
Arabidopsis - ps31
Arabidopsis_thaliana - ps32
p-value
5e-03
8e-03
1e-02
2e-02
4e-02
Log-odds
0.5
1.0
1.5
2.0
2.5
3.0
3.5
4.0

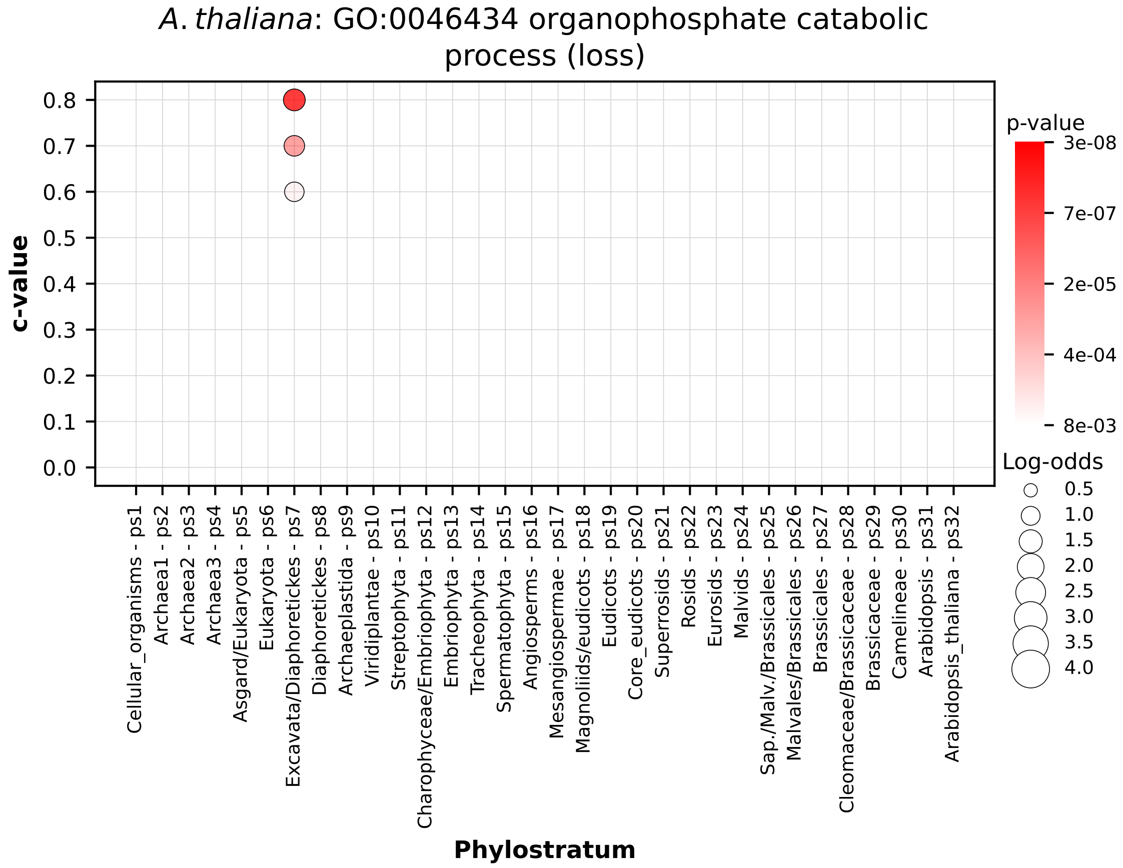

A. thaliana: GO:0046434 organophosphate catabolic process (loss)
c-value
0.0
0.1
0.2
0.3
0.4
0.5
0.6
0.7
0.8
Phylostratum
Cellular_organisms - ps1
Archaea1 - ps2
Archaea2 - ps3
Archaea3 - ps4
Asgard/Eukaryota - ps5
Eukaryota - ps6
Excavata/Diaphoretickes - ps7
Diaphoretickes - ps8
Archaeplastida - ps9
Viridiplantae - ps10
Streptophyta - ps11
Charophyceae/Embriophyta - ps12
Embriophyta - ps13
Tracheophyta - ps14
Spermatophyta - ps15
Angiosperms - ps16
Mesangiospermae - ps17
Magnoliids/eudicots - ps18
Eudicots - ps19
Core_eudicots - ps20
Superrosids - ps21
Rosids - ps22
Eurosids - ps23
Malvids - ps24
Sap./Malv./Brassicales - ps25
Malvales/Brassicales - ps26
Brassicales - ps27
Cleomaceae/Brassicaceae - ps28
Brassicaceae - ps29
Camelineae - ps30
Arabidopsis - ps31
Arabidopsis_thaliana - ps32
p-value
3e-08
7e-07
2e-05
4e-04
8e-03
Log-odds
0.5
1.0
1.5
2.0
2.5
3.0
3.5
4.0

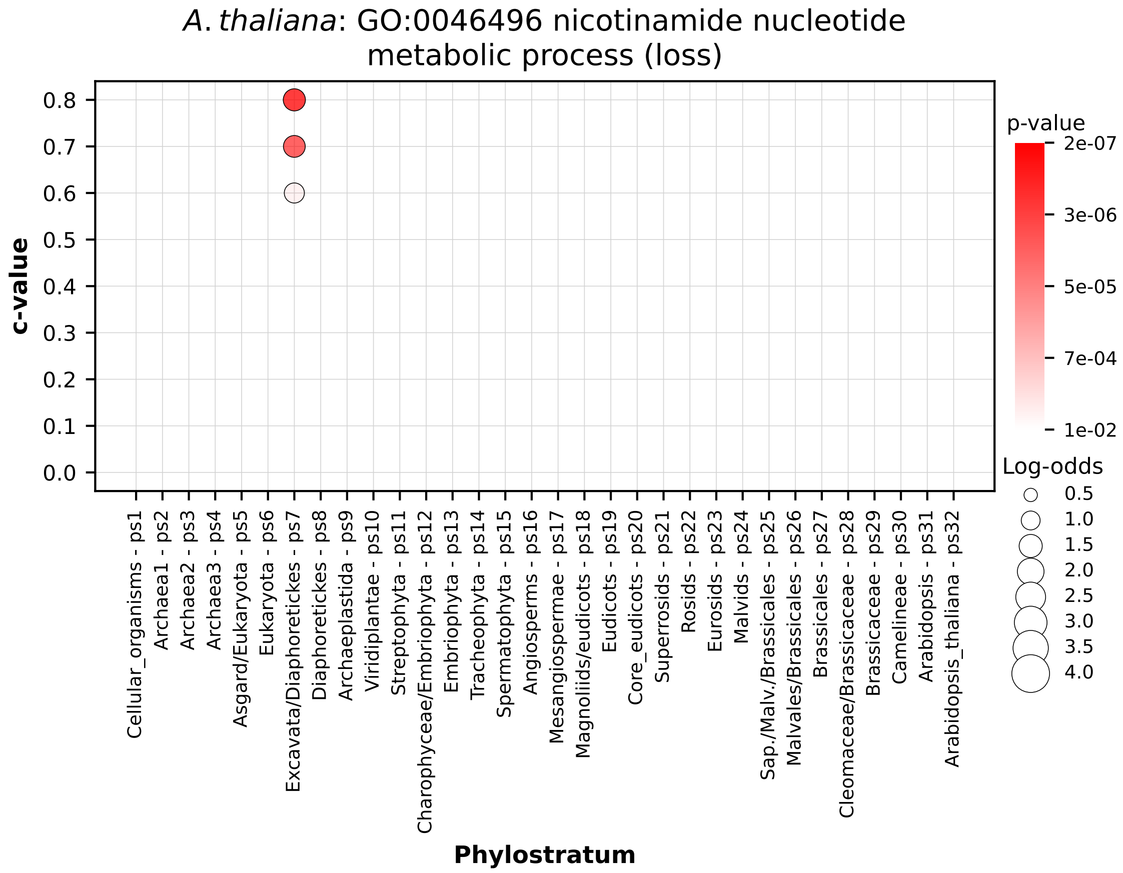

A. thaliana: GO:0046496 nicotinamide nucleotide metabolic process (loss)
c-value
0.0
0.1
0.2
0.3
0.4
0.5
0.6
0.7
0.8
p-value
2e-07
3e-06
5e-05
7e-04
1e-02
Log-odds
0.5
1.0
1.5
2.0
2.5
3.0
3.5
4.0
Cellular_organisms - ps1
Archaea1 - ps2
Archaea2 - ps3
Archaea3 - ps4
Asgard/Eukaryota - ps5
Eukaryota - ps6
Excavata/Diaphoretickes - ps7
Diaphoretickes - ps8
Archaeplastida - ps9
Viridiplantae - ps10
Streptophyta - ps11
Charophyceae/Embriophyta - ps12
Embriophyta - ps13
Tracheophyta - ps14
Spermatophyta - ps15
Angiosperms - ps16
Mesangiospermae - ps17
Magnoliids/eudicots - ps18
Eudicots - ps19
Core_eudicots - ps20
Superrosids - ps21
Rosids - ps22
Eurosids - ps23
Malvids - ps24
Sap./Malv./Brassicales - ps25
Malvales/Brassicales - ps26
Brassicales - ps27
Cleomaceae/Brassicaceae - ps28
Brassicaceae - ps29
Camelineae - ps30
Arabidopsis - ps31
Arabidopsis_thaliana - ps32
Phylostratum

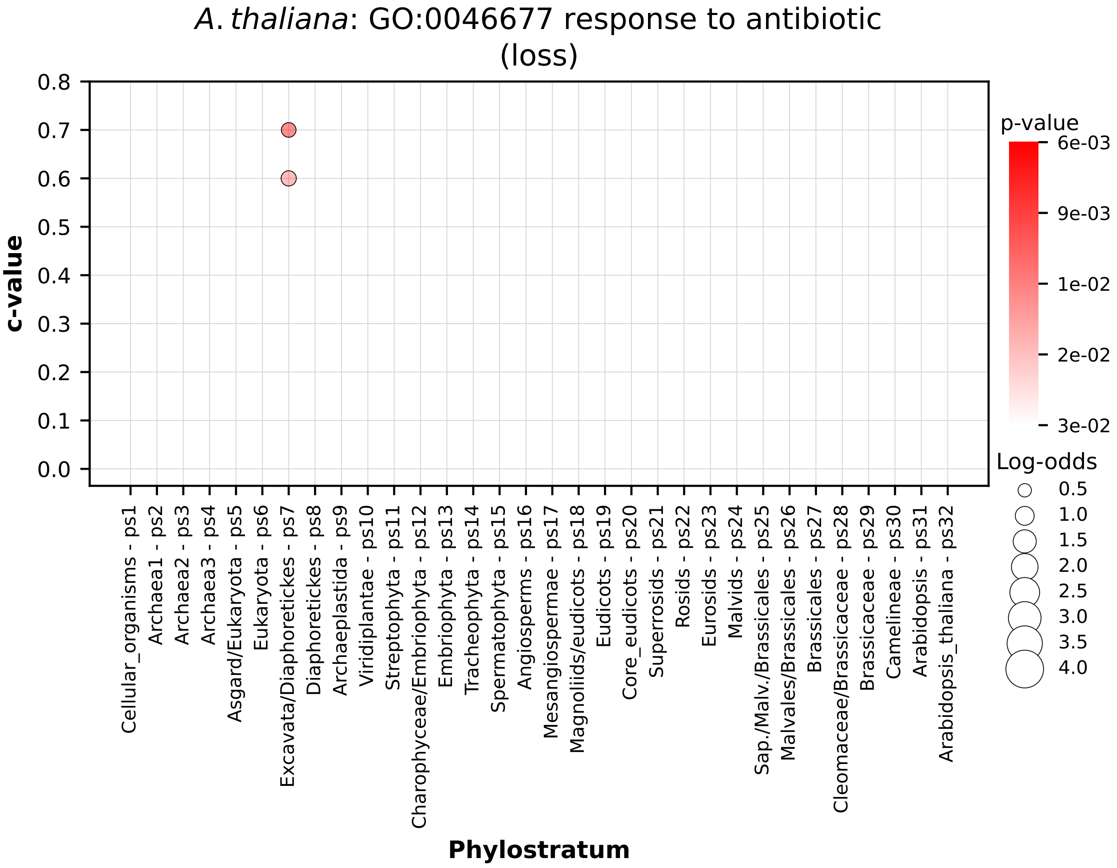

A. thaliana: GO:0046677 response to antibiotic
(loss)
c-value
0.8
0.7
0.6
0.5
0.4
0.3
0.2
0.1
0.0
Phylostratum
Cellular_organisms - ps1
Archaea1 - ps2
Archaea2 - ps3
Archaea3 - ps4
Asgard/Eukaryota - ps5
Eukaryota - ps6
Excavata/Diaphoretickes - ps7
Diaphoretickes - ps8
Archaeplastida - ps9
Viridiplantae - ps10
Streptophyta - ps11
Charophyceae/Embriophyta - ps12
Embriophyta - ps13
Tracheophyta - ps14
Spermatophyta - ps15
Angiosperms - ps16
Mesangiospermae - ps17
Magnoliids/eudicots - ps18
Eudicots - ps19
Core_eudicots - ps20
Superrosids - ps21
Rosids - ps22
Eurosids - ps23
Malvids - ps24
Sap./Malv./Brassicales - ps25
Malvales/Brassicales - ps26
Brassicales - ps27
Cleomaceae/Brassicaceae - ps28
Brassicaceae - ps29
Camelineae - ps30
Arabidopsis - ps31
Arabidopsis_thaliana - ps32
p-value
6e-03
9e-03
1e-02
2e-02
3e-02
Log-odds
0.5
1.0
1.5
2.0
2.5
3.0
3.5
4.0

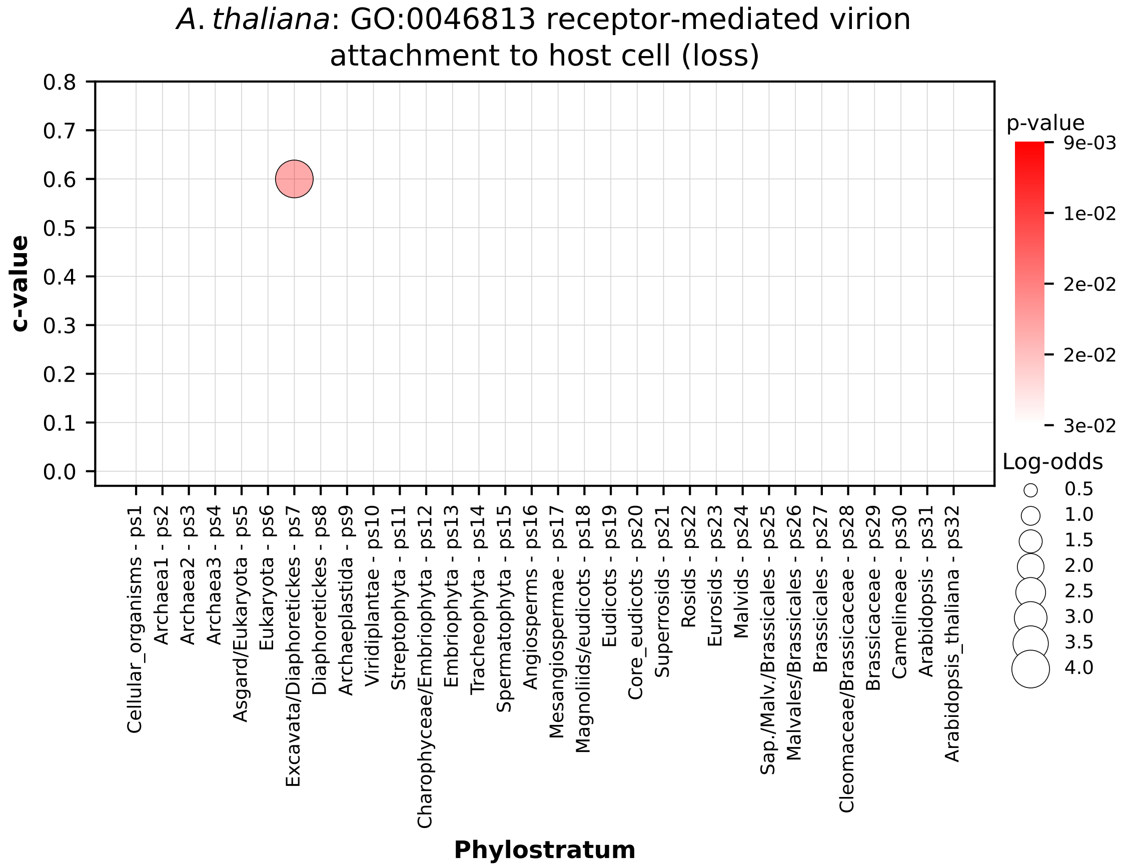

A. thaliana: GO:0046813 receptor-mediated virion attachment to host cell (loss)
c-value
0.8
0.7
0.6
0.5
0.4
0.3
0.2
0.1
0.0
p-value
9e-03
1e-02
2e-02
2e-02
3e-02
Log-odds
0.5
1.0
1.5
2.0
2.5
3.0
3.5
4.0
Cellular_organisms - ps1
Archaea1 - ps2
Archaea2 - ps3
Archaea3 - ps4
Asgard/Eukaryota - ps5
Eukaryota - ps6
Excavata/Diaphoretickes - ps7
Diaphoretickes - ps8
Archaeplastida - ps9
Viridiplantae - ps10
Streptophyta - ps11
Charophyceae/Embriophyta - ps12
Embriophyta - ps13
Tracheophyta - ps14
Spermatophyta - ps15
Angiosperms - ps16
Mesangiospermae - ps17
Magnoliids/eudicots - ps18
Eudicots - ps19
Core_eudicots - ps20
Superrosids - ps21
Rosids - ps22
Eurosids - ps23
Malvids - ps24
Sap./Malv./Brassicales - ps25
Malvales/Brassicales - ps26
Brassicales - ps27
Cleomaceae/Brassicaceae - ps28
Brassicaceae - ps29
Camelineae - ps30
Arabidopsis - ps31
Arabidopsis_thaliana - ps32
Phylostratum

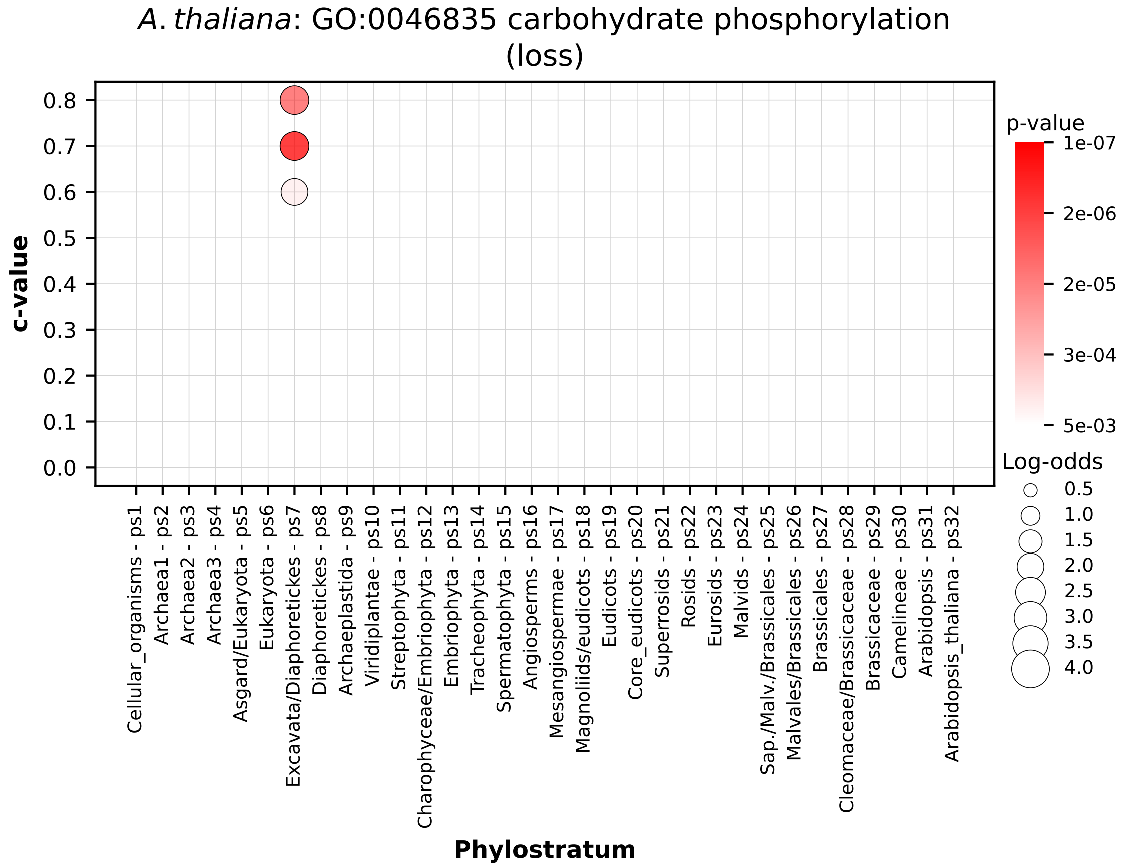

A. thaliana: GO:0046835 carbohydrate phosphorylation
(loss)
c-value
0.8
0.7
0.6
0.5
0.4
0.3
0.2
0.1
0.0
Phylostratum
Cellular_organisms - ps1
Archaea1 - ps2
Archaea2 - ps3
Archaea3 - ps4
Asgard/Eukaryota - ps5
Eukaryota - ps6
Excavata/Diaphoretickes - ps7
Diaphoretickes - ps8
Archaeplastida - ps9
Viridiplantae - ps10
Streptophyta - ps11
Charophyceae/Embriophyta - ps12
Embriophyta - ps13
Tracheophyta - ps14
Spermatophyta - ps15
Angiosperms - ps16
Mesangiospermae - ps17
Magnoliids/eudicots - ps18
Eudicots - ps19
Core_eudicots - ps20
Superrosids - ps21
Rosids - ps22
Eurosids - ps23
Malvids - ps24
Sap./Malv./Brassicales - ps25
Malvales/Brassicales - ps26
Brassicales - ps27
Cleomaceae/Brassicaceae - ps28
Brassicaceae - ps29
Camelineae - ps30
Arabidopsis - ps31
Arabidopsis_thaliana - ps32
p-value
1e-07
2e-06
2e-05
3e-04
5e-03
Log-odds
0.5
1.0
1.5
2.0
2.5
3.0
3.5
4.0

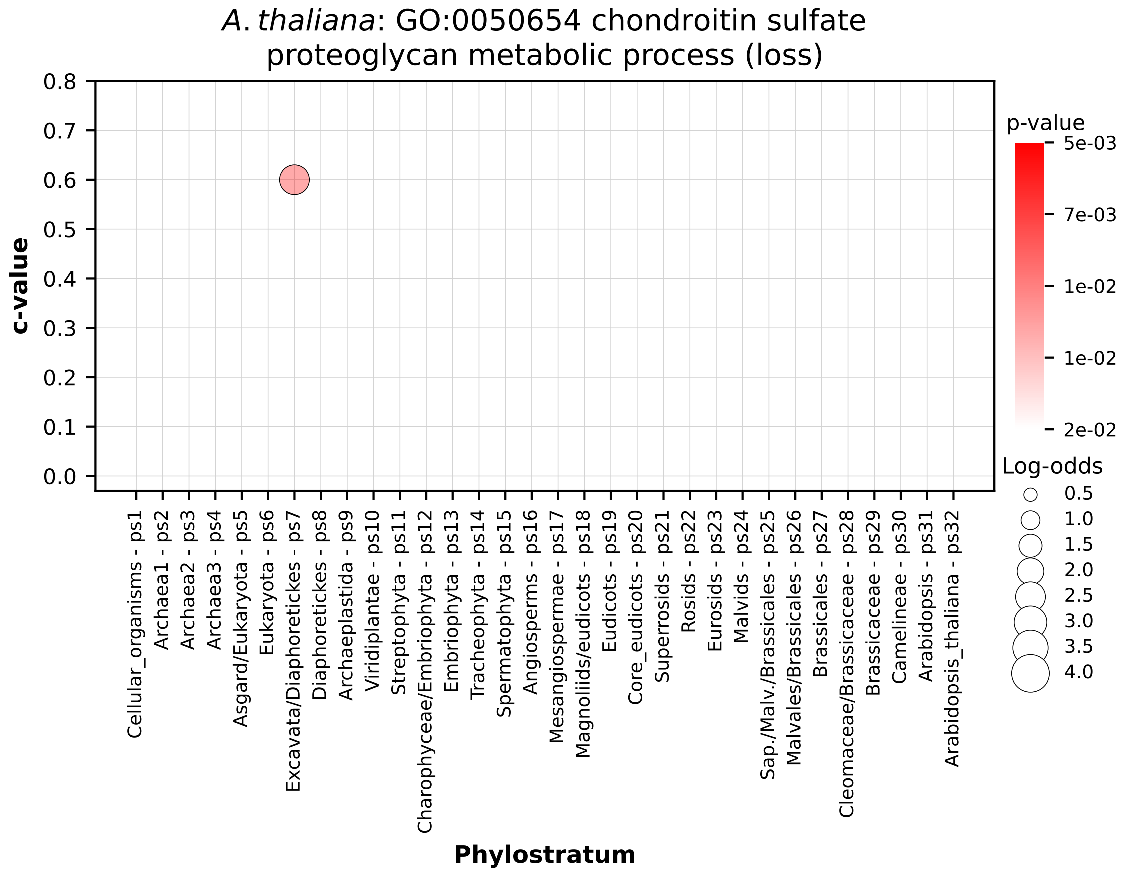
A. thaliana: GO:0050654 chondroitin sulfate proteoglycan metabolic process (loss)
c-value
0.8
0.7
0.6
0.5
0.4
0.3
0.2
0.1
0.0
p-value
5e-03
7e-03
1e-02
1e-02
2e-02
Log-odds
0.5
1.0
1.5
2.0
2.5
3.0
3.5
4.0
Cellular_organisms - ps1
Archaea1 - ps2
Archaea2 - ps3
Archaea3 - ps4
Asgard/Eukaryota - ps5
Eukaryota - ps6
Excavata/Diaphoretickes - ps7
Diaphoretickes - ps8
Archaeplastida - ps9
Viridiplantae - ps10
Streptophyta - ps11
Charophyceae/Embriophyta - ps12
Embriophyta - ps13
Tracheophyta - ps14
Spermatophyta - ps15
Angiosperms - ps16
Mesangiospermae - ps17
Magnoliids/eudicots - ps18
Eudicots - ps19
Core_eudicots - ps20
Superrosids - ps21
Rosids - ps22
Eurosids - ps23
Malvids - ps24
Sap./Malv./Brassicales - ps25
Malvales/Brassicales - ps26
Brassicales - ps27
Cleomaceae/Brassicaceae - ps28
Brassicaceae - ps29
Camelineae - ps30
Arabidopsis - ps31
Arabidopsis_thaliana - ps32
Phylostratum

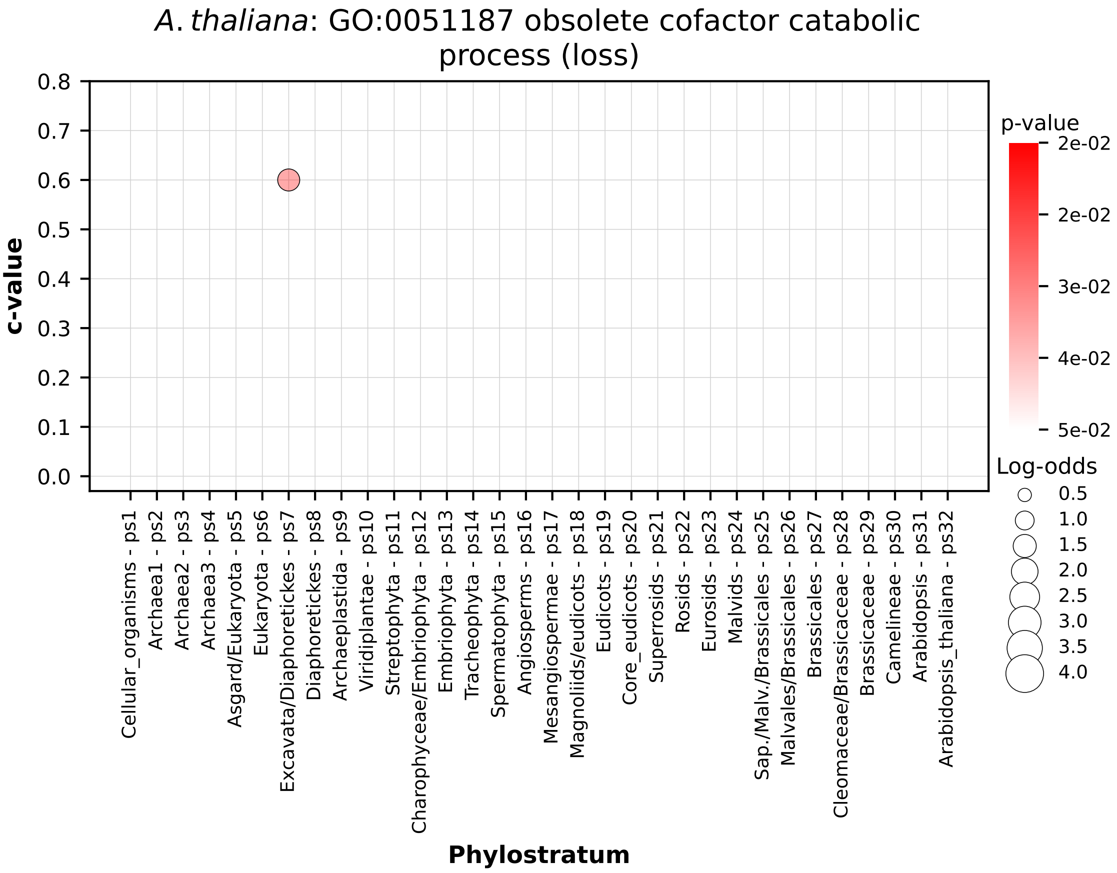

A. thaliana: GO:0051187 obsolete cofactor catabolic process (loss)
c-value
0.8
0.7
0.6
0.5
0.4
0.3
0.2
0.1
0.0
Phylostratum
Cellular_organisms - ps1
Archaea1 - ps2
Archaea2 - ps3
Archaea3 - ps4
Asgard/Eukaryota - ps5
Eukaryota - ps6
Excavata/Diaphoretickes - ps7
Diaphoretickes - ps8
Archaeplastida - ps9
Viridiplantae - ps10
Streptophyta - ps11
Charophyceae/Embriophyta - ps12
Embriophyta - ps13
Tracheophyta - ps14
Spermatophyta - ps15
Angiosperms - ps16
Mesangiospermae - ps17
Magnoliids/eudicots - ps18
Eudicots - ps19
Core_eudicots - ps20
Superrosids - ps21
Rosids - ps22
Eurosids - ps23
Malvids - ps24
Sap./Malv./Brassicales - ps25
Malvales/Brassicales - ps26
Brassicales - ps27
Cleomaceae/Brassicaceae - ps28
Brassicaceae - ps29
Camelineae - ps30
Arabidopsis - ps31
Arabidopsis_thaliana - ps32
p-value
2e-02
2e-02
3e-02
4e-02
5e-02
Log-odds
0.5
1.0
1.5
2.0
2.5
3.0
3.5
4.0

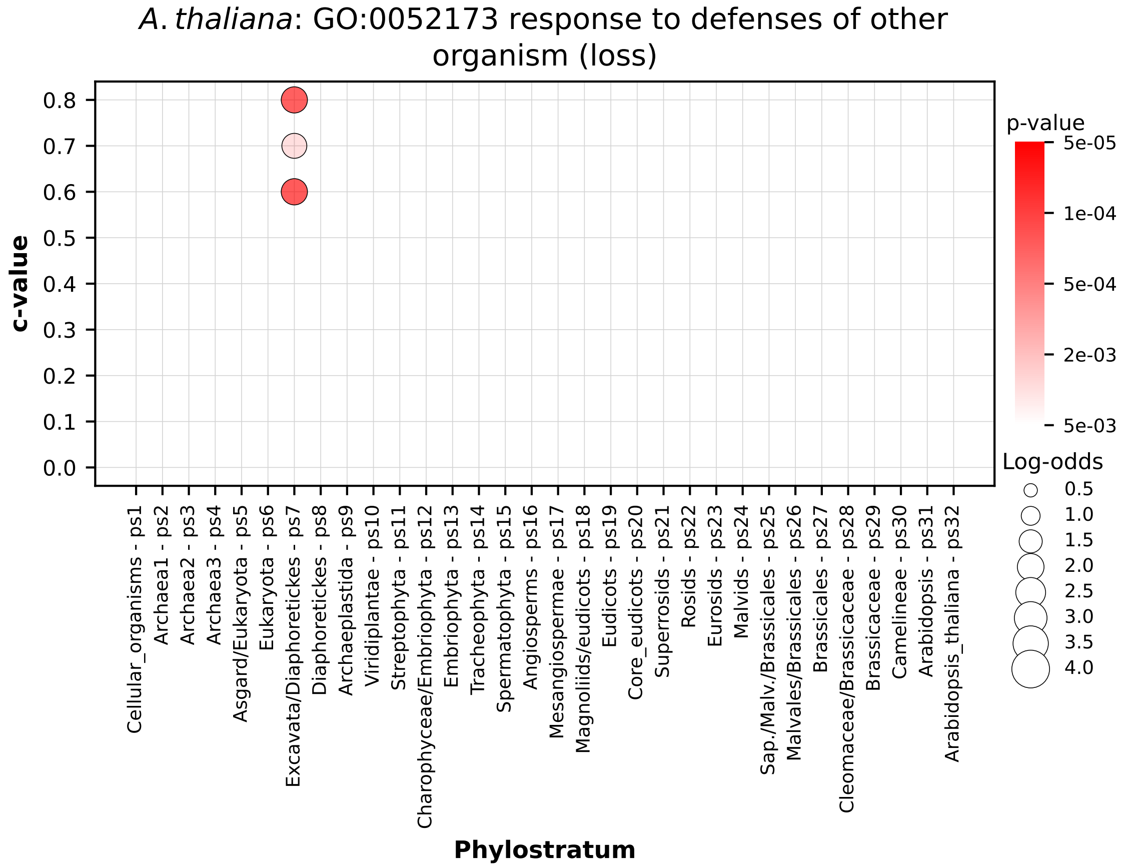
A. thaliana: GO:0052173 response to defenses of other organism (loss)
c-value
0.0
0.1
0.2
0.3
0.4
0.5
0.6
0.7
0.8
Phylostratum
Cellular_organisms - ps1
Archaea1 - ps2
Archaea2 - ps3
Archaea3 - ps4
Asgard/Eukaryota - ps5
Eukaryota - ps6
Excavata/Diaphoretickes - ps7
Diaphoretickes - ps8
Archaeplastida - ps9
Viridiplantae - ps10
Streptophyta - ps11
Charophyceae/Embriophyta - ps12
Embriophyta - ps13
Tracheophyta - ps14
Spermatophyta - ps15
Angiosperms - ps16
Mesangiospermae - ps17
Magnoliids/eudicots - ps18
Eudicots - ps19
Core_eudicots - ps20
Superrosids - ps21
Rosids - ps22
Eurosids - ps23
Malvids - ps24
Sap./Malv./Brassicales - ps25
Malvales/Brassicales - ps26
Brassicales - ps27
Cleomaceae/Brassicaceae - ps28
Brassicaceae - ps29
Camelineae - ps30
Arabidopsis - ps31
Arabidopsis_thaliana - ps32
p-value
5e-05
1e-04
5e-04
2e-03
5e-03
Log-odds
0.5
1.0
1.5
2.0
2.5
3.0
3.5
4.0

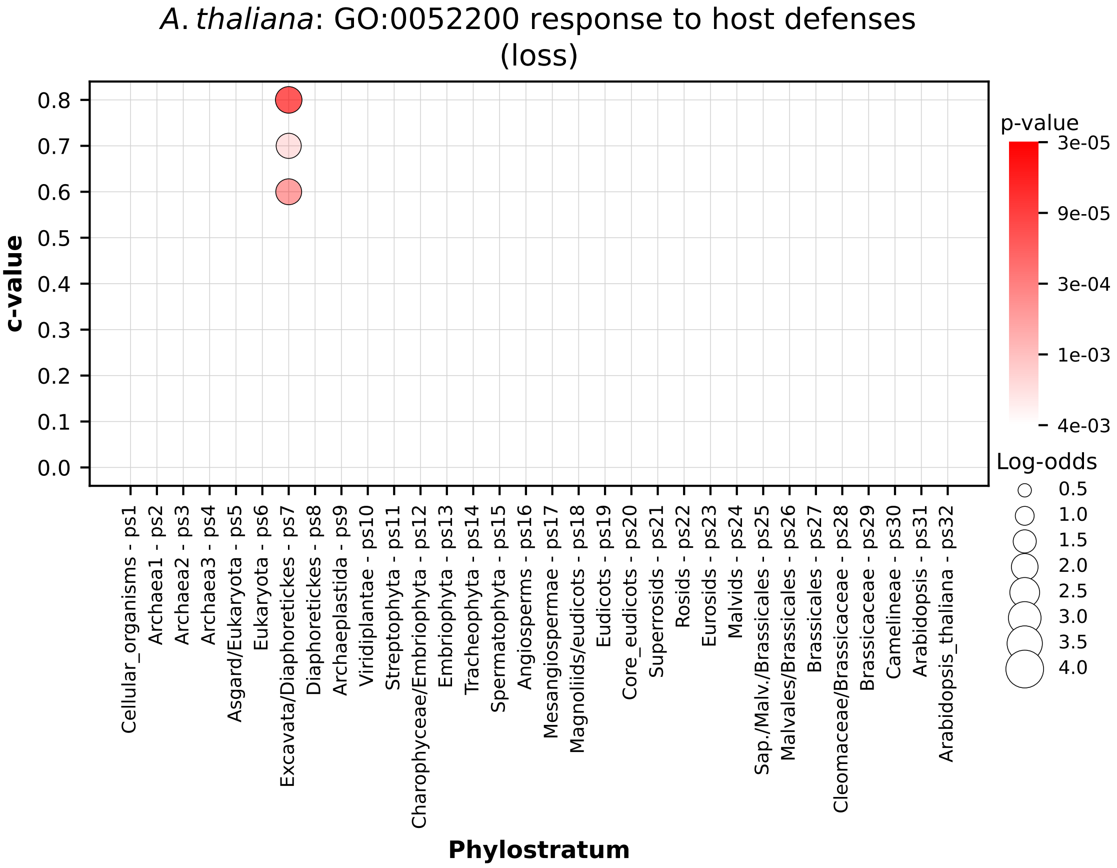

A. thaliana: GO:0052200 response to host defenses
(loss)
c-value
0.8
0.7
0.6
0.5
0.4
0.3
0.2
0.1
0.0
Phylostratum
Cellular_organisms - ps1
Archaea1 - ps2
Archaea2 - ps3
Archaea3 - ps4
Asgard/Eukaryota - ps5
Eukaryota - ps6
Excavata/Diaphoretickes - ps7
Diaphoretickes - ps8
Archaeplastida - ps9
Viridiplantae - ps10
Streptophyta - ps11
Charophyceae/Embriophyta - ps12
Embriophyta - ps13
Tracheophyta - ps14
Spermatophyta - ps15
Angiosperms - ps16
Mesangiospermae - ps17
Magnoliids/eudicots - ps18
Eudicots - ps19
Core_eudicots - ps20
Superrosids - ps21
Rosids - ps22
Eurosids - ps23
Malvids - ps24
Sap./Malv./Brassicales - ps25
Malvales/Brassicales - ps26
Brassicales - ps27
Cleomaceae/Brassicaceae - ps28
Brassicaceae - ps29
Camelineae - ps30
Arabidopsis - ps31
Arabidopsis_thaliana - ps32
p-value
3e-05
9e-05
3e-04
1e-03
4e-03
Log-odds
0.5
1.0
1.5
2.0
2.5
3.0
3.5
4.0

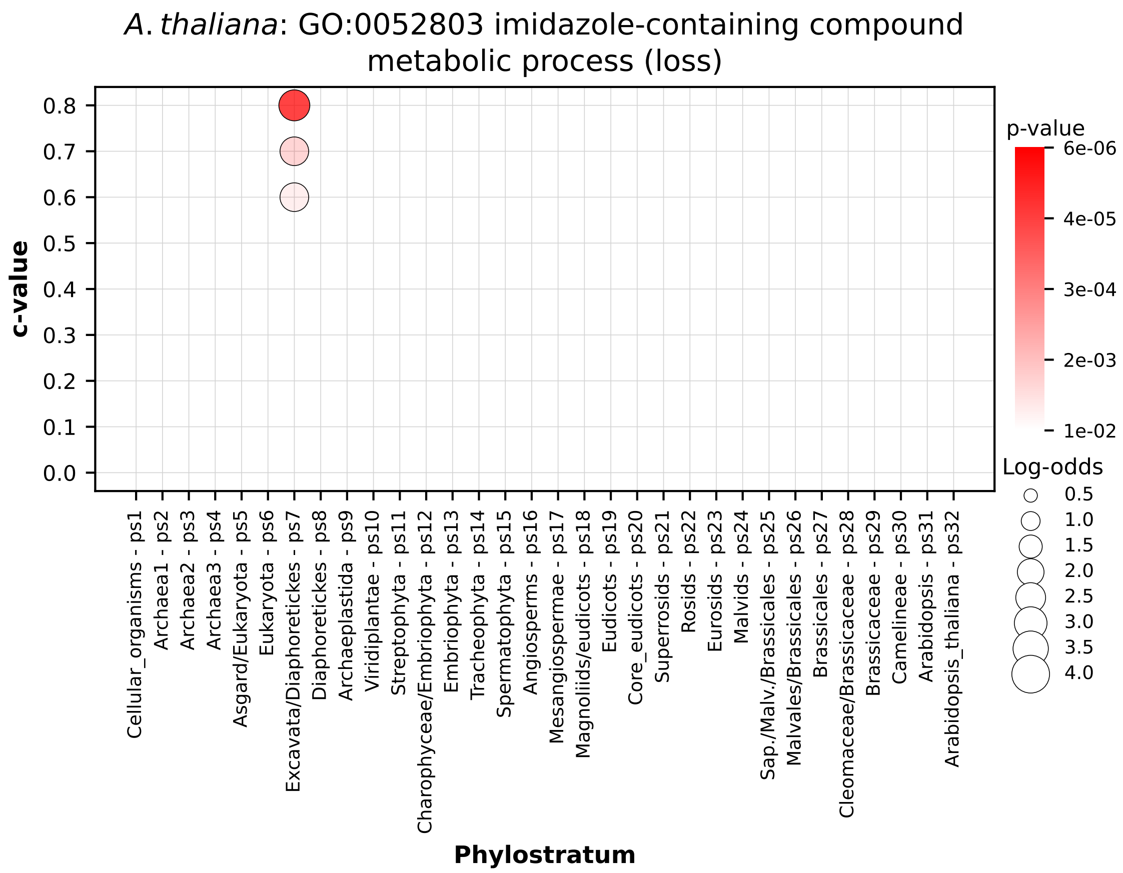

A. thaliana: GO:0052803 imidazole-containing compound metabolic process (loss)
c-value
0.0
0.1
0.2
0.3
0.4
0.5
0.6
0.7
0.8
p-value
6e-06
4e-05
3e-04
2e-03
1e-02
Log-odds
0.5
1.0
1.5
2.0
2.5
3.0
3.5
4.0
Cellular_organisms - ps1
Archaea1 - ps2
Archaea2 - ps3
Archaea3 - ps4
Asgard/Eukaryota - ps5
Eukaryota - ps6
Excavata/Diaphoretickes - ps7
Diaphoretickes - ps8
Archaeplastida - ps9
Viridiplantae - ps10
Streptophyta - ps11
Charophyceae/Embriophyta - ps12
Embriophyta - ps13
Tracheophyta - ps14
Spermatophyta - ps15
Angiosperms - ps16
Mesangiospermae - ps17
Magnoliids/eudicots - ps18
Eudicots - ps19
Core_eudicots - ps20
Superrosids - ps21
Rosids - ps22
Eurosids - ps23
Malvids - ps24
Sap./Malv./Brassicales - ps25
Malvales/Brassicales - ps26
Brassicales - ps27
Cleomaceae/Brassicaceae - ps28
Brassicaceae - ps29
Camelineae - ps30
Arabidopsis - ps31
Arabidopsis_thaliana - ps32
Phylostratum

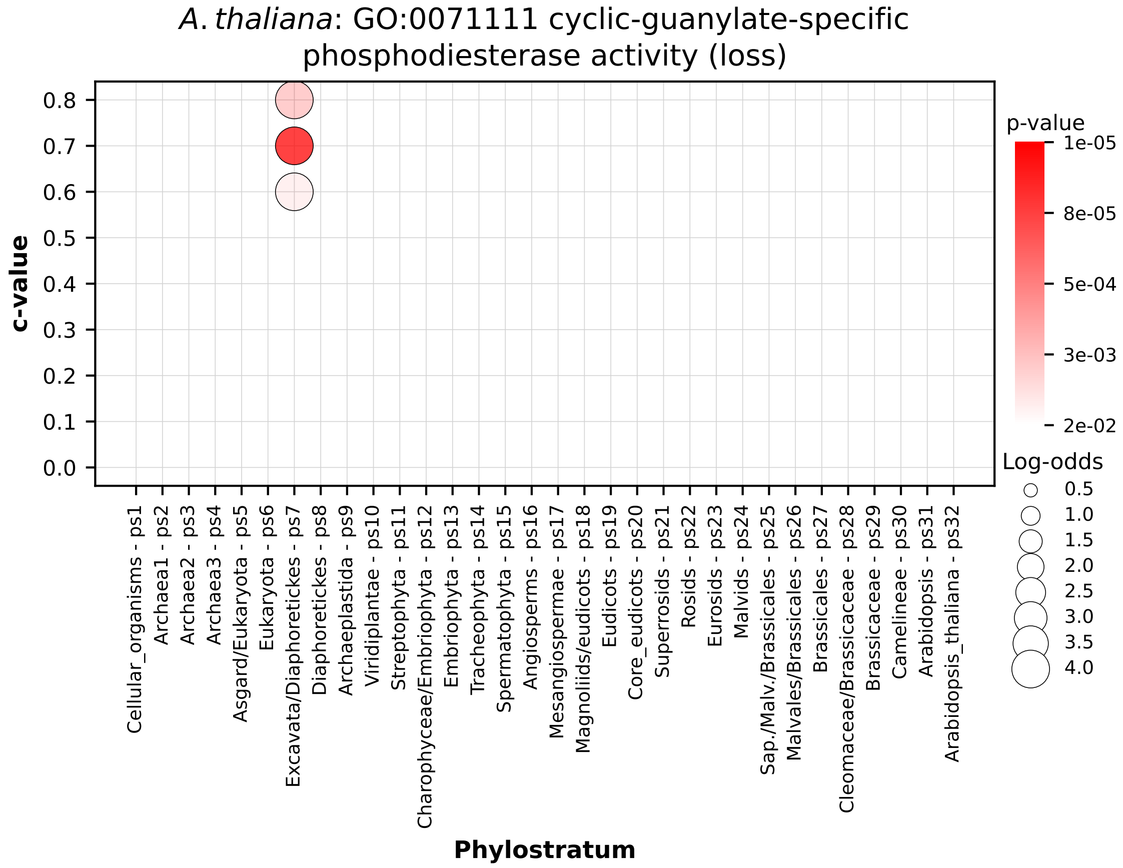
A. thaliana: GO:0071111 cyclic-guanylate-specific phosphodiesterase activity (loss)
c-value
0.0
0.1
0.2
0.3
0.4
0.5
0.6
0.7
0.8
Phylostratum
Cellular_organisms - ps1
Archaea1 - ps2
Archaea2 - ps3
Archaea3 - ps4
Asgard/Eukaryota - ps5
Eukaryota - ps6
Excavata/Diaphoretickes - ps7
Diaphoretickes - ps8
Archaeplastida - ps9
Viridiplantae - ps10
Streptophyta - ps11
Charophyceae/Embriophyta - ps12
Embriophyta - ps13
Tracheophyta - ps14
Spermatophyta - ps15
Angiosperms - ps16
Mesangiospermae - ps17
Magnoliids/eudicots - ps18
Eudicots - ps19
Core_eudicots - ps20
Superrosids - ps21
Rosids - ps22
Eurosids - ps23
Malvids - ps24
Sap./Malv./Brassicales - ps25
Malvales/Brassicales - ps26
Brassicales - ps27
Cleomaceae/Brassicaceae - ps28
Brassicaceae - ps29
Camelineae - ps30
Arabidopsis - ps31
Arabidopsis_thaliana - ps32
p-value
1e-05
8e-05
5e-04
3e-03
2e-02
Log-odds
0.5
1.0
1.5
2.0
2.5
3.0
3.5
4.0

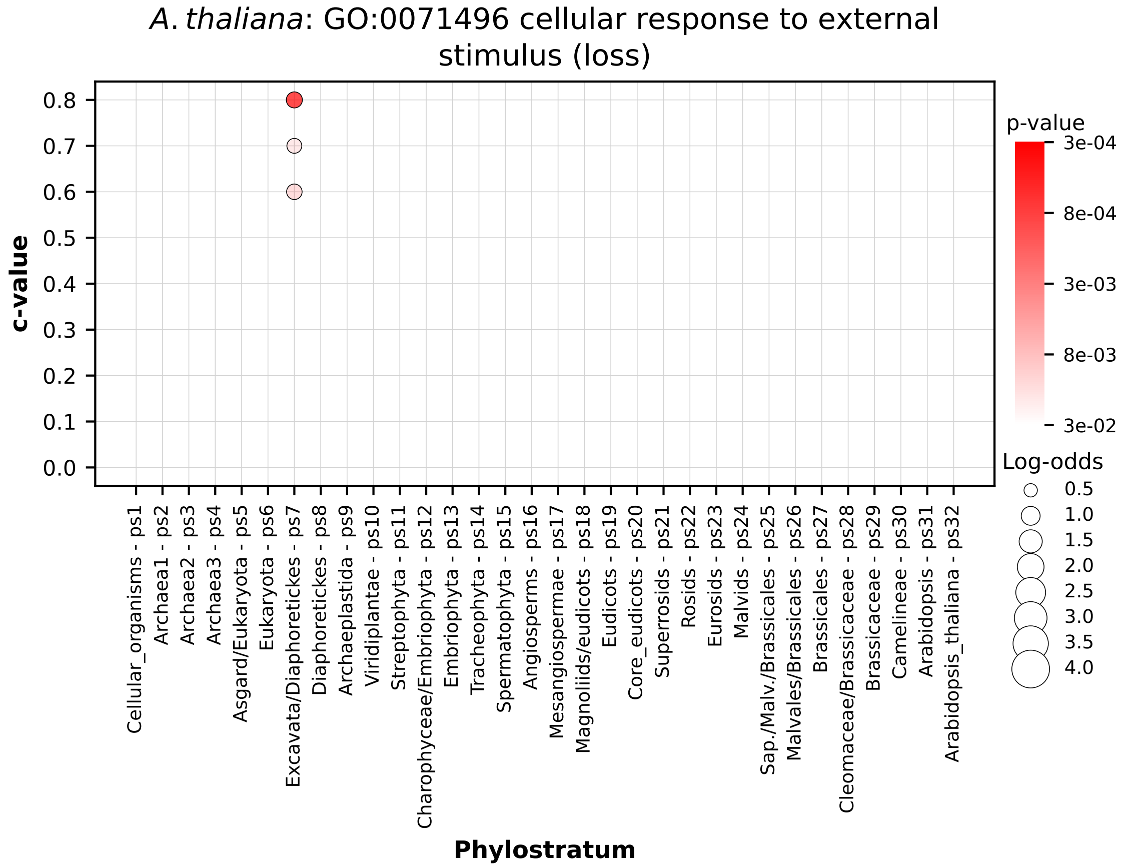

A. thaliana: GO:0071496 cellular response to external stimulus (loss)
c-value
0.0
0.1
0.2
0.3
0.4
0.5
0.6
0.7
0.8
Phylostratum
Cellular_organisms - ps1
Archaea1 - ps2
Archaea2 - ps3
Archaea3 - ps4
Asgard/Eukaryota - ps5
Eukaryota - ps6
Excavata/Diaphoretickes - ps7
Diaphoretickes - ps8
Archaeplastida - ps9
Viridiplantae - ps10
Streptophyta - ps11
Charophyceae/Embriophyta - ps12
Embriophyta - ps13
Tracheophyta - ps14
Spermatophyta - ps15
Angiosperms - ps16
Mesangiospermae - ps17
Magnoliids/eudicots - ps18
Eudicots - ps19
Core_eudicots - ps20
Superrosids - ps21
Rosids - ps22
Eurosids - ps23
Malvids - ps24
Sap./Malv./Brassicales - ps25
Malvales/Brassicales - ps26
Brassicales - ps27
Cleomaceae/Brassicaceae - ps28
Brassicaceae - ps29
Camelineae - ps30
Arabidopsis - ps31
Arabidopsis_thaliana - ps32
p-value
3e-04
8e-04
3e-03
8e-03
3e-02
Log-odds
0.5
1.0
1.5
2.0
2.5
3.0
3.5
4.0

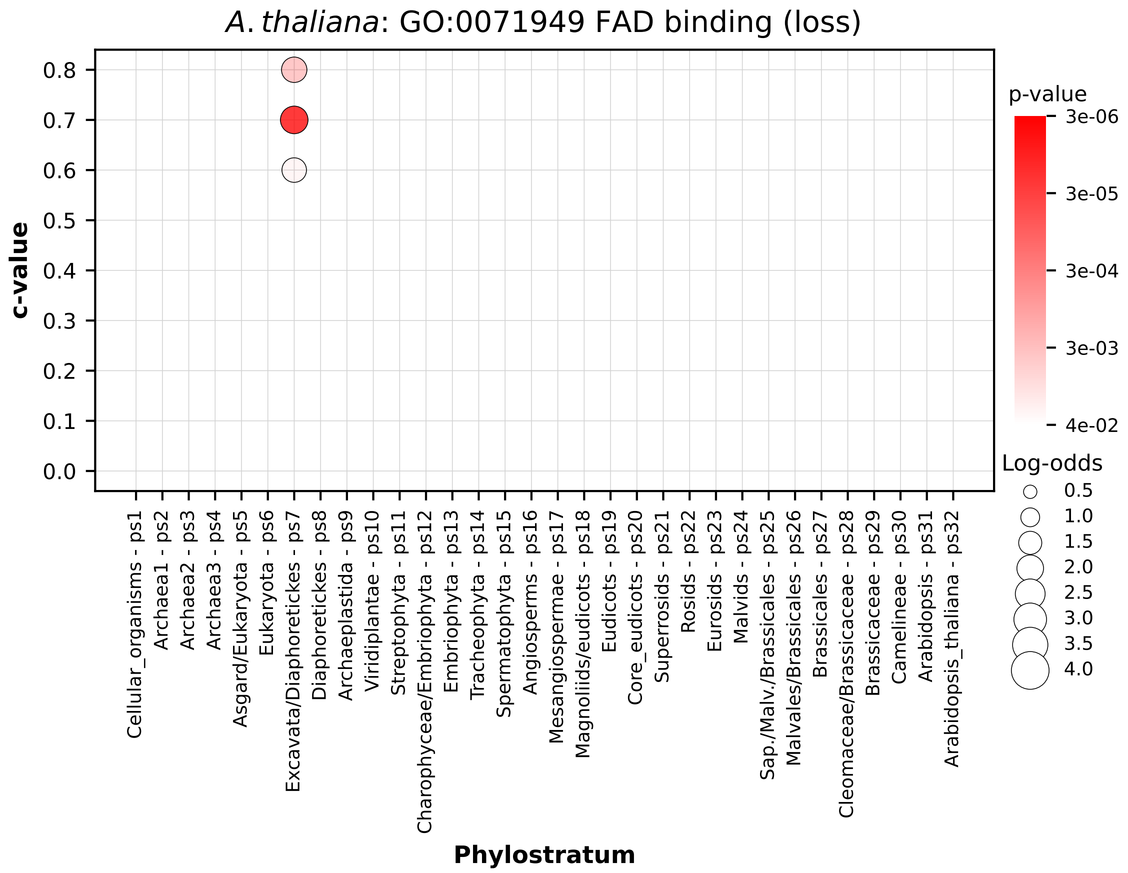

A. thaliana: GO:0071949 FAD binding (loss)
c-value
0.0
0.1
0.2
0.3
0.4
0.5
0.6
0.7
0.8
Phylostratum
Cellular_organisms - ps1
Archaea1 - ps2
Archaea2 - ps3
Archaea3 - ps4
Asgard/Eukaryota - ps5
Eukaryota - ps6
Excavata/Diaphoretickes - ps7
Diaphoretickes - ps8
Archaeplastida - ps9
Viridiplantae - ps10
Streptophyta - ps11
Charophyceae/Embriophyta - ps12
Embriophyta - ps13
Tracheophyta - ps14
Spermatophyta - ps15
Angiosperms - ps16
Mesangiospermae - ps17
Magnoliids/eudicots - ps18
Eudicots - ps19
Core_eudicots - ps20
Superrosids - ps21
Rosids - ps22
Eurosids - ps23
Malvids - ps24
Sap./Malv./Brassicales - ps25
Malvales/Brassicales - ps26
Brassicales - ps27
Cleomaceae/Brassicaceae - ps28
Brassicaceae - ps29
Camelineae - ps30
Arabidopsis - ps31
Arabidopsis_thaliana - ps32
p-value
3e-06
3e-05
3e-04
3e-03
4e-02
Log-odds
0.5
1.0
1.5
2.0
2.5
3.0
3.5
4.0

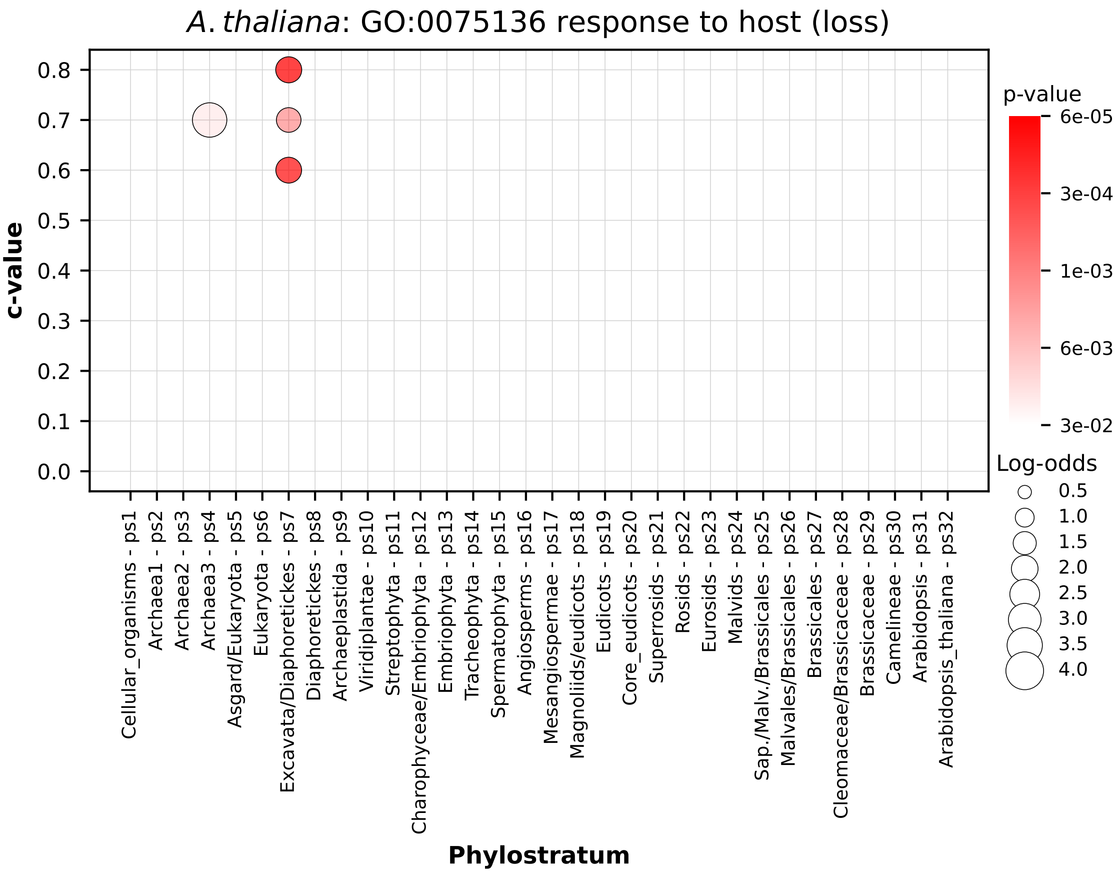

A. thaliana: GO:0075136 response to host (loss)
c-value
0.8
0.7
0.6
0.5
0.4
0.3
0.2
0.1
0.0
p-value
6e-05
3e-04
1e-03
6e-03
3e-02
Log-odds
0.5
1.0
1.5
2.0
2.5
3.0
3.5
4.0
Cellular_organisms - ps1
Archaea1 - ps2
Archaea2 - ps3
Archaea3 - ps4
Asgard/Eukaryota - ps5
Eukaryota - ps6
Excavata/Diaphoretickes - ps7
Diaphoretickes - ps8
Archaeplastida - ps9
Viridiplantae - ps10
Streptophyta - ps11
Charophyceae/Embriophyta - ps12
Embriophyta - ps13
Tracheophyta - ps14
Spermatophyta - ps15
Angiosperms - ps16
Mesangiospermae - ps17
Magnoliids/eudicots - ps18
Eudicots - ps19
Core_eudicots - ps20
Superrosids - ps21
Rosids - ps22
Eurosids - ps23
Malvids - ps24
Sap./Malv./Brassicales - ps25
Malvales/Brassicales - ps26
Brassicales - ps27
Cleomaceae/Brassicaceae - ps28
Brassicaceae - ps29
Camelineae - ps30
Arabidopsis - ps31
Arabidopsis_thaliana - ps32
Phylostratum

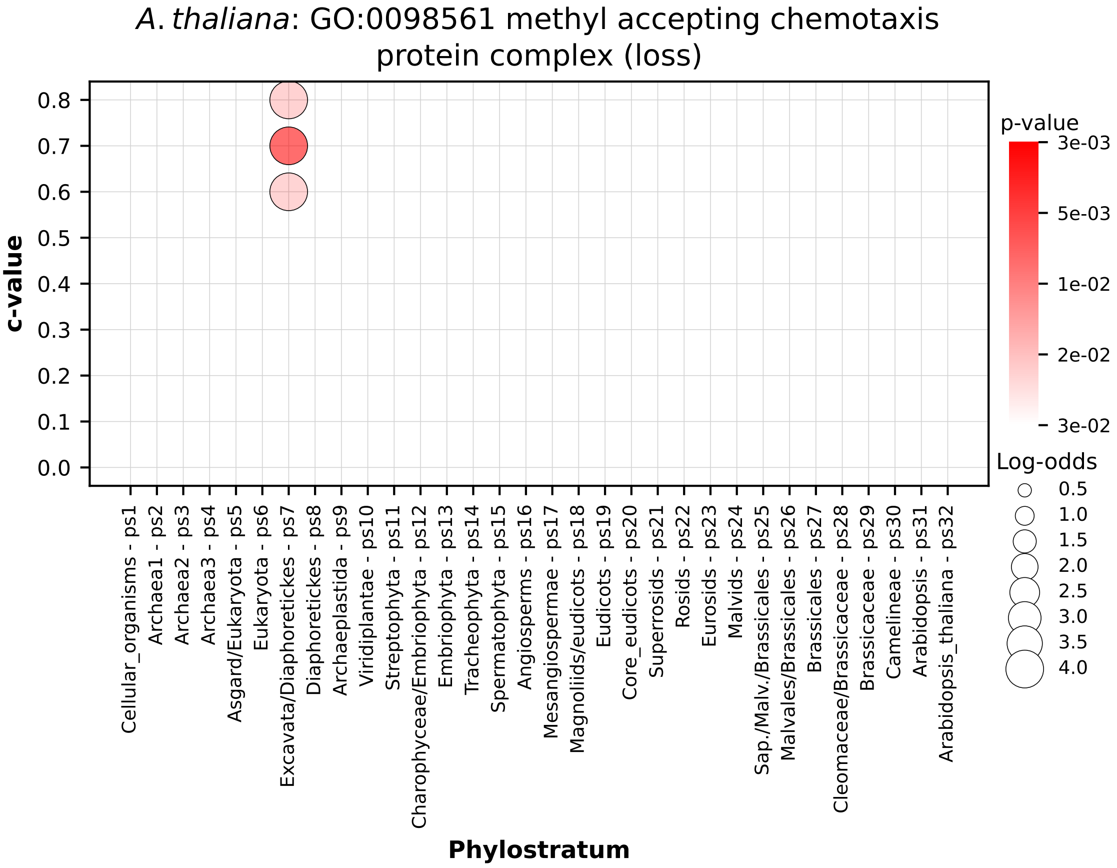

A. thaliana: GO:0098561 methyl accepting chemotaxis protein complex (loss)
c-value
0.8
0.7
0.6
0.5
0.4
0.3
0.2
0.1
0.0
Phylostratum
Cellular_organisms - ps1
Archaea1 - ps2
Archaea2 - ps3
Archaea3 - ps4
Asgard/Eukaryota - ps5
Eukaryota - ps6
Excavata/Diaphoretickes - ps7
Diaphoretickes - ps8
Archaeplastida - ps9
Viridiplantae - ps10
Streptophyta - ps11
Charophyceae/Embriophyta - ps12
Embriophyta - ps13
Tracheophyta - ps14
Spermatophyta - ps15
Angiosperms - ps16
Mesangiospermae - ps17
Magnoliids/eudicots - ps18
Eudicots - ps19
Core_eudicots - ps20
Superrosids - ps21
Rosids - ps22
Eurosids - ps23
Malvids - ps24
Sap./Malv./Brassicales - ps25
Malvales/Brassicales - ps26
Brassicales - ps27
Cleomaceae/Brassicaceae - ps28
Brassicaceae - ps29
Camelineae - ps30
Arabidopsis - ps31
Arabidopsis_thaliana - ps32
p-value
3e-03
5e-03
1e-02
2e-02
3e-02
Log-odds
0.5
1.0
1.5
2.0
2.5
3.0
3.5
4.0

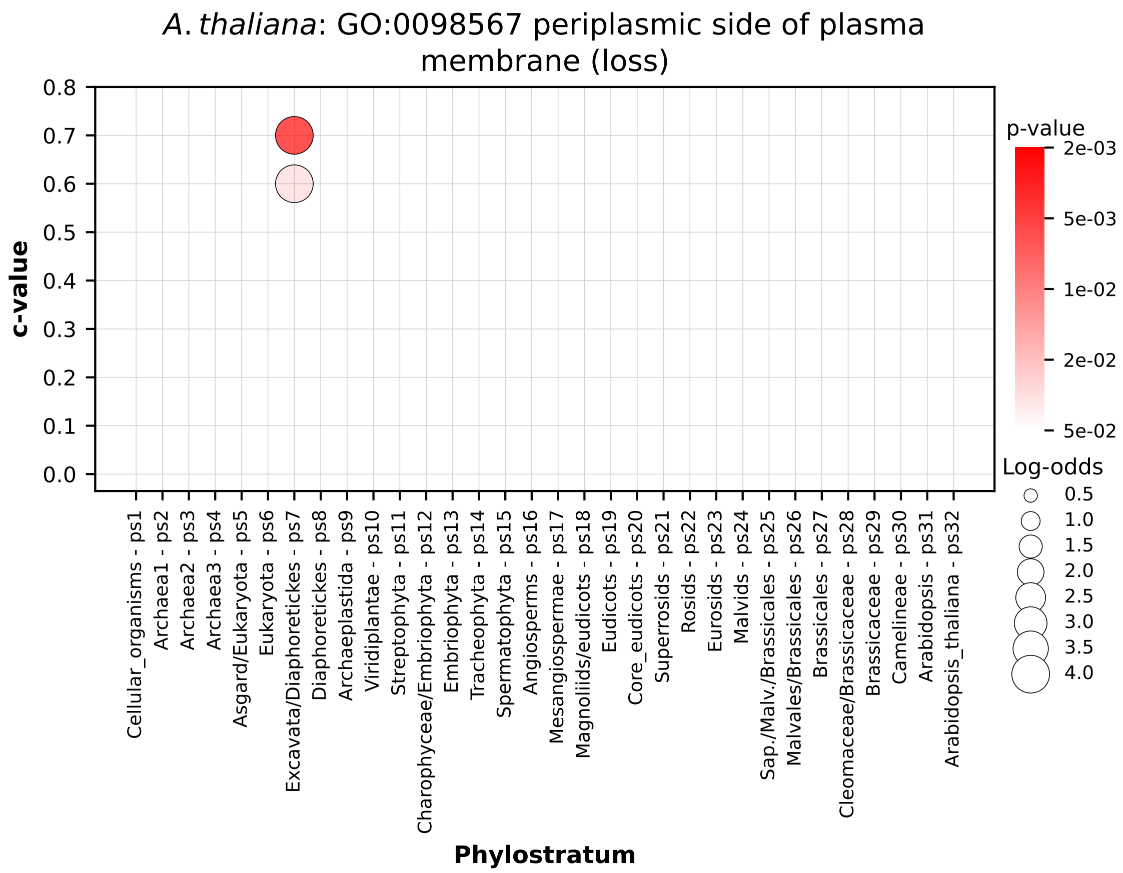

A. thaliana: GO:0098567 periplasmic side of plasma membrane (loss)
c-value
0.8
0.7
0.6
0.5
0.4
0.3
0.2
0.1
0.0
p-value
2e-03
5e-03
1e-02
2e-02
5e-02
Log-odds
0.5
1.0
1.5
2.0
2.5
3.0
3.5
4.0
Cellular_organisms - ps1
Archaea1 - ps2
Archaea2 - ps3
Archaea3 - ps4
Asgard/Eukaryota - ps5
Eukaryota - ps6
Excavata/Diaphoretickes - ps7
Diaphoretickes - ps8
Archaeplastida - ps9
Viridiplantae - ps10
Streptophyta - ps11
Charophyceae/Embriophyta - ps12
Embriophyta - ps13
Tracheophyta - ps14
Spermatophyta - ps15
Angiosperms - ps16
Mesangiospermae - ps17
Magnoliids/eudicots - ps18
Eudicots - ps19
Core_eudicots - ps20
Superrosids - ps21
Rosids - ps22
Eurosids - ps23
Malvids - ps24
Sap./Malv./Brassicales - ps25
Malvales/Brassicales - ps26
Brassicales - ps27
Cleomaceae/Brassicaceae - ps28
Brassicaceae - ps29
Camelineae - ps30
Arabidopsis - ps31
Arabidopsis_thaliana - ps32
Phylostratum

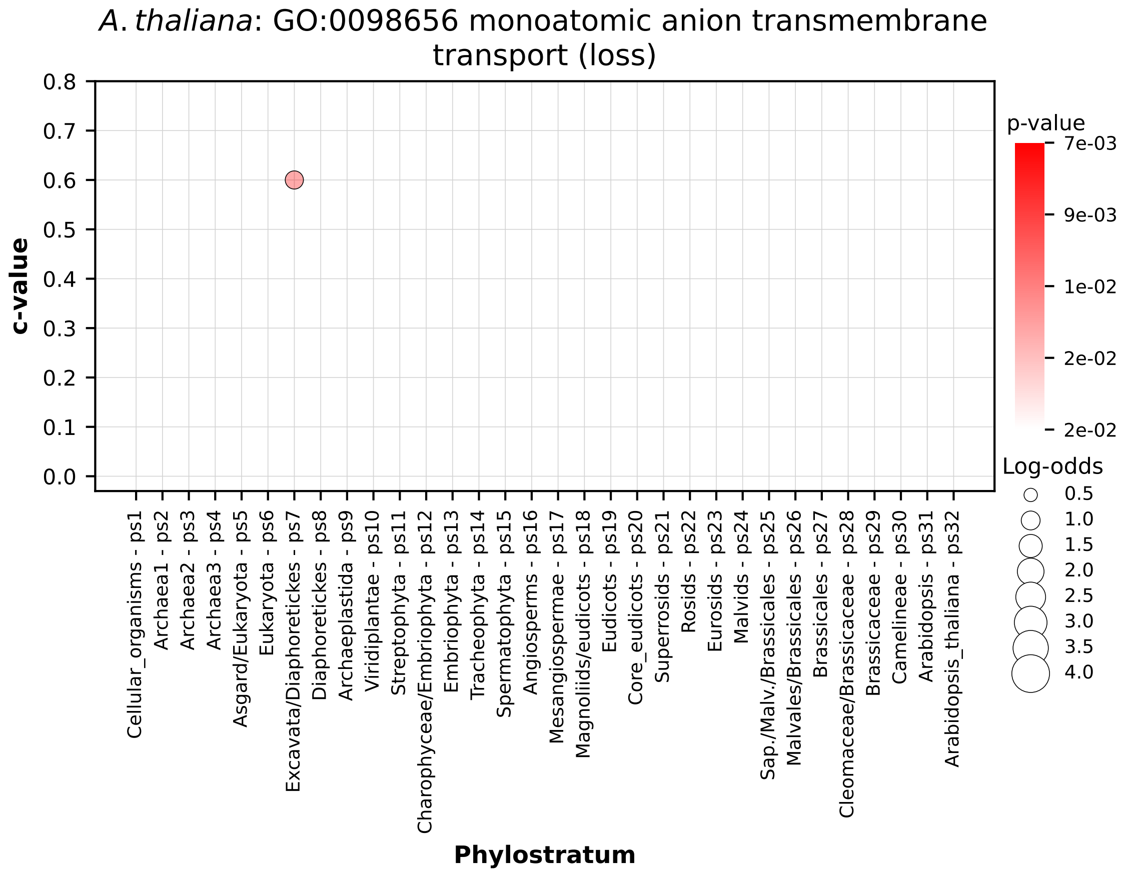

A. thaliana: GO:0098656 monoatomic anion transmembrane transport (loss)
c-value
0.8
0.7
0.6
0.5
0.4
0.3
0.2
0.1
0.0
Phylostratum
Cellular_organisms - ps1
Archaea1 - ps2
Archaea2 - ps3
Archaea3 - ps4
Asgard/Eukaryota - ps5
Eukaryota - ps6
Excavata/Diaphoretickes - ps7
Diaphoretickes - ps8
Archaeplastida - ps9
Viridiplantae - ps10
Streptophyta - ps11
Charophyceae/Embriophyta - ps12
Embriophyta - ps13
Tracheophyta - ps14
Spermatophyta - ps15
Angiosperms - ps16
Mesangiospermae - ps17
Magnoliids/eudicots - ps18
Eudicots - ps19
Core_eudicots - ps20
Superrosids - ps21
Rosids - ps22
Eurosids - ps23
Malvids - ps24
Sap./Malv./Brassicales - ps25
Malvales/Brassicales - ps26
Brassicales - ps27
Cleomaceae/Brassicaceae - ps28
Brassicaceae - ps29
Camelineae - ps30
Arabidopsis - ps31
Arabidopsis_thaliana - ps32
p-value
7e-03
9e-03
1e-02
2e-02
2e-02
Log-odds
0.5
1.0
1.5
2.0
2.5
3.0
3.5
4.0

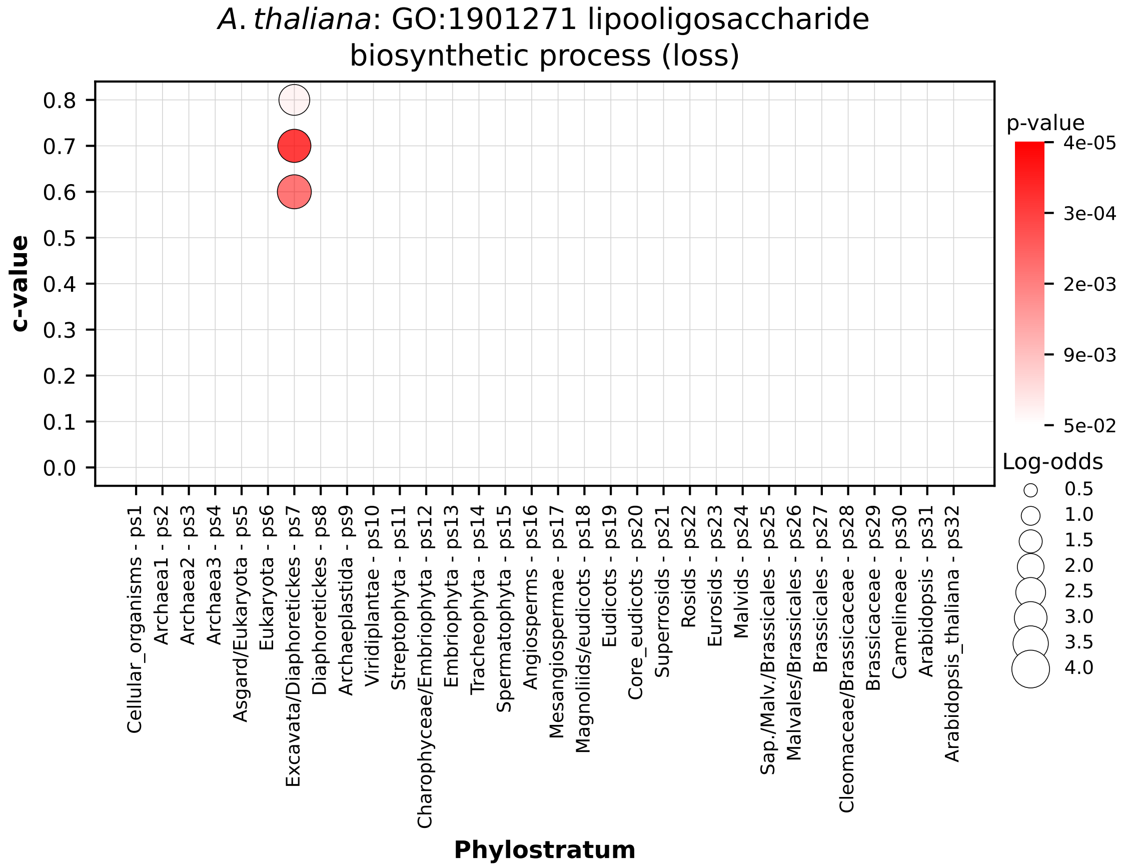

A. thaliana: GO:1901271 lipooligosaccharide biosynthetic process (loss)
c-value
0.8
0.7
0.6
0.5
0.4
0.3
0.2
0.1
0.0
p-value
4e-05
3e-04
2e-03
9e-03
5e-02
Log-odds
0.5
1.0
1.5
2.0
2.5
3.0
3.5
4.0
Cellular_organisms - ps1
Archaea1 - ps2
Archaea2 - ps3
Archaea3 - ps4
Asgard/Eukaryota - ps5
Eukaryota - ps6
Excavata/Diaphoretickes - ps7
Diaphoretickes - ps8
Archaeplastida - ps9
Viridiplantae - ps10
Streptophyta - ps11
Charophyceae/Embriophyta - ps12
Embriophyta - ps13
Tracheophyta - ps14
Spermatophyta - ps15
Angiosperms - ps16
Mesangiospermae - ps17
Magnoliids/eudicots - ps18
Eudicots - ps19
Core_eudicots - ps20
Superrosids - ps21
Rosids - ps22
Eurosids - ps23
Malvids - ps24
Sap./Malv./Brassicales - ps25
Malvales/Brassicales - ps26
Brassicales - ps27
Cleomaceae/Brassicaceae - ps28
Brassicaceae - ps29
Camelineae - ps30
Arabidopsis - ps31
Arabidopsis_thaliana - ps32
Phylostratum

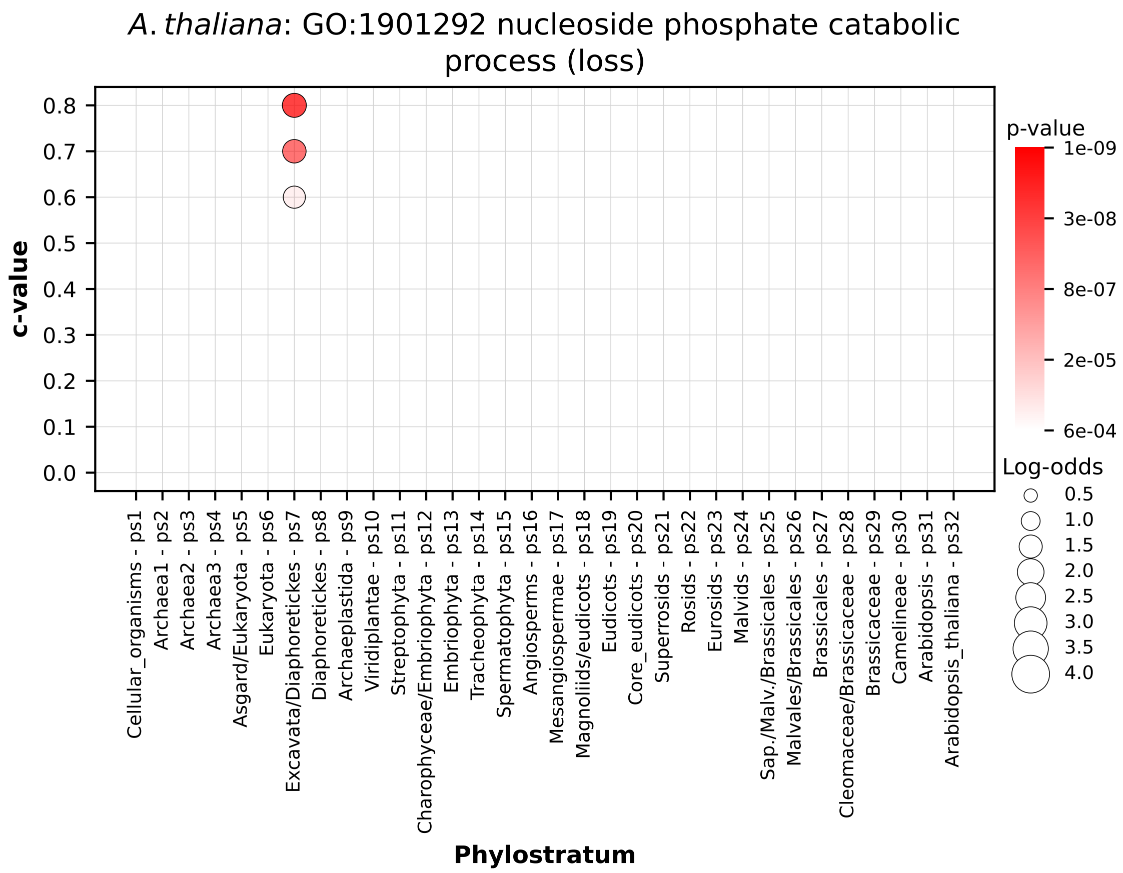

A. thaliana: GO:1901292 nucleoside phosphate catabolic process (loss)
c-value
0.8
0.7
0.6
0.5
0.4
0.3
0.2
0.1
0.0
p-value
1e-09
3e-08
8e-07
2e-05
6e-04
Log-odds
0.5
1.0
1.5
2.0
2.5
3.0
3.5
4.0
Cellular_organisms - ps1
Archaea1 - ps2
Archaea2 - ps3
Archaea3 - ps4
Asgard/Eukaryota - ps5
Eukaryota - ps6
Excavata/Diaphoretickes - ps7
Diaphoretickes - ps8
Archaeplastida - ps9
Viridiplantae - ps10
Streptophyta - ps11
Charophyceae/Embriophyta - ps12
Embriophyta - ps13
Tracheophyta - ps14
Spermatophyta - ps15
Angiosperms - ps16
Mesangiospermae - ps17
Magnoliids/eudicots - ps18
Eudicots - ps19
Core_eudicots - ps20
Superrosids - ps21
Rosids - ps22
Eurosids - ps23
Malvids - ps24
Sap./Malv./Brassicales - ps25
Malvales/Brassicales - ps26
Brassicales - ps27
Cleomaceae/Brassicaceae - ps28
Brassicaceae - ps29
Camelineae - ps30
Arabidopsis - ps31
Arabidopsis_thaliana - ps32
Phylostratum

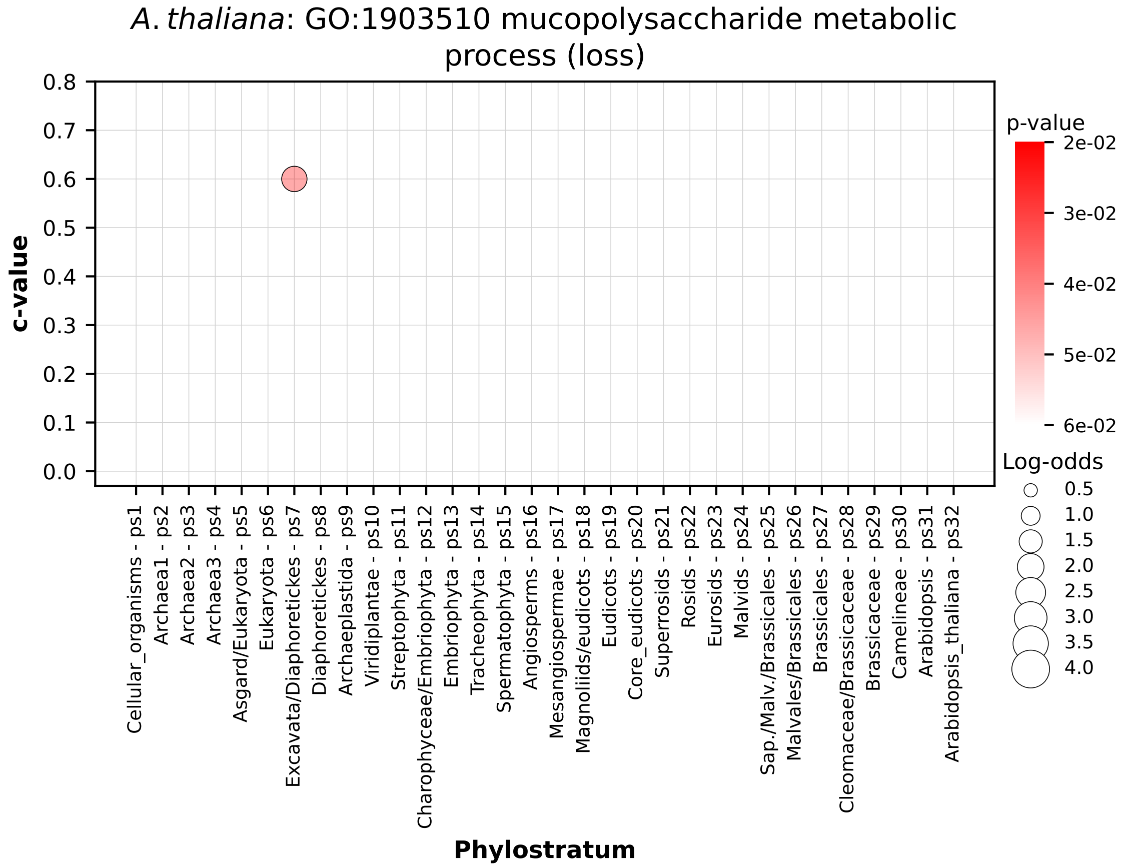

A. thaliana: GO:1903510 mucopolysaccharide metabolic process (loss)
c-value
0.8
0.7
0.6
0.5
0.4
0.3
0.2
0.1
0.0
Phylostratum
Cellular_organisms - ps1
Archaea1 - ps2
Archaea2 - ps3
Archaea3 - ps4
Asgard/Eukaryota - ps5
Eukaryota - ps6
Excavata/Diaphoretickes - ps7
Diaphoretickes - ps8
Archaeplastida - ps9
Viridiplantae - ps10
Streptophyta - ps11
Charophyceae/Embriophyta - ps12
Embriophyta - ps13
Tracheophyta - ps14
Spermatophyta - ps15
Angiosperms - ps16
Mesangiospermae - ps17
Magnoliids/eudicots - ps18
Eudicots - ps19
Core_eudicots - ps20
Superrosids - ps21
Rosids - ps22
Eurosids - ps23
Malvids - ps24
Sap./Malv./Brassicales - ps25
Malvales/Brassicales - ps26
Brassicales - ps27
Cleomaceae/Brassicaceae - ps28
Brassicaceae - ps29
Camelineae - ps30
Arabidopsis - ps31
Arabidopsis_thaliana - ps32
p-value
2e-02
3e-02
4e-02
5e-02
6e-02
Log-odds
0.5
1.0
1.5
2.0
2.5
3.0
3.5
4.0

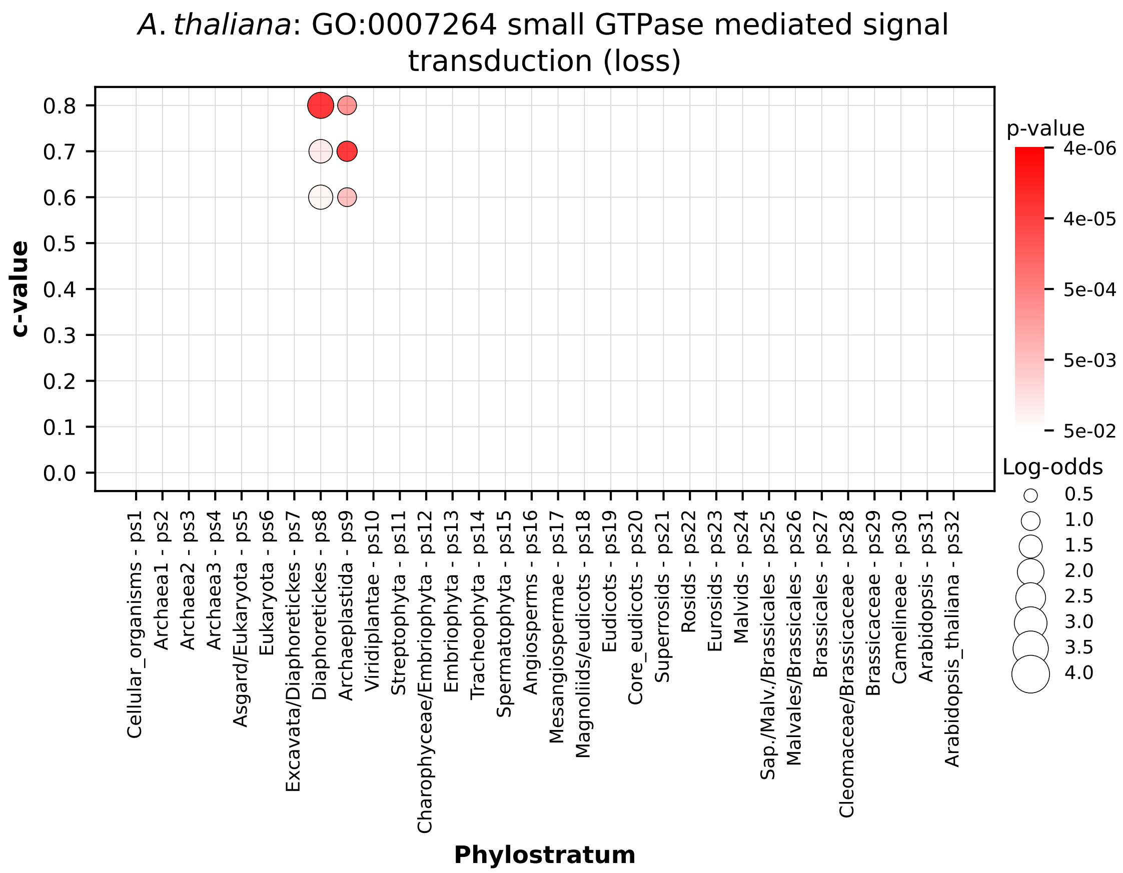

A. thaliana: GO:0007264 small GTPase mediated signal transduction (loss)
c-value
0.8
0.7
0.6
0.5
0.4
0.3
0.2
0.1
0.0
p-value
4e-06
4e-05
5e-04
5e-03
5e-02
Log-odds
0.5
1.0
1.5
2.0
2.5
3.0
3.5
4.0
Cellular_organisms - ps1
Archaea1 - ps2
Archaea2 - ps3
Archaea3 - ps4
Asgard/Eukaryota - ps5
Eukaryota - ps6
Excavata/Diaphoretickes - ps7
Diaphoretickes - ps8
Archaeplastida - ps9
Viridiplantae - ps10
Streptophyta - ps11
Charophyceae/Embriophyta - ps12
Embriophyta - ps13
Tracheophyta - ps14
Spermatophyta - ps15
Angiosperms - ps16
Mesangiospermae - ps17
Magnoliids/eudicots - ps18
Eudicots - ps19
Core_eudicots - ps20
Superrosids - ps21
Rosids - ps22
Eurosids - ps23
Malvids - ps24
Sap./Malv./Brassicales - ps25
Malvales/Brassicales - ps26
Brassicales - ps27
Cleomaceae/Brassicaceae - ps28
Brassicaceae - ps29
Camelineae - ps30
Arabidopsis - ps31
Arabidopsis_thaliana - ps32
Phylostratum

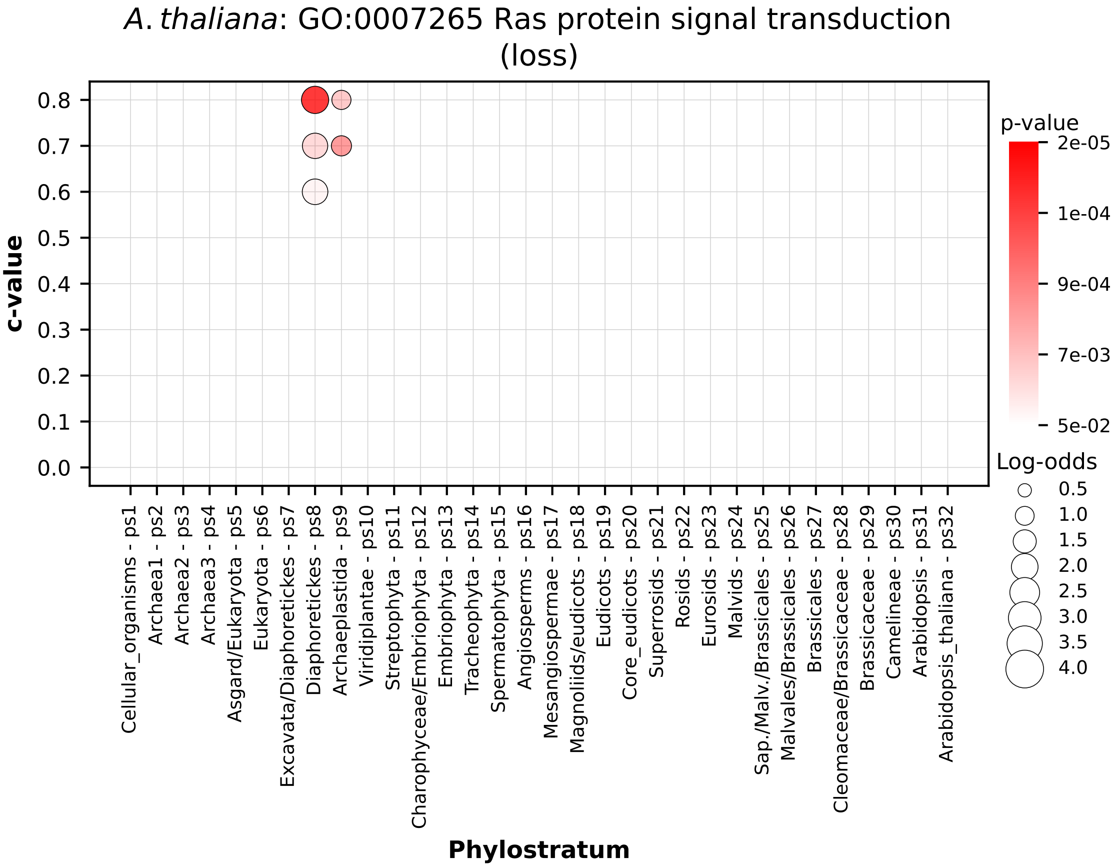

A. thaliana: GO:0007265 Ras protein signal transduction (loss)
c-value
0.0
0.1
0.2
0.3
0.4
0.5
0.6
0.7
0.8
Phylostratum
Cellular_organisms - ps1
Archaea1 - ps2
Archaea2 - ps3
Archaea3 - ps4
Asgard/Eukaryota - ps5
Eukaryota - ps6
Excavata/Diaphoretickes - ps7
Diaphoretickes - ps8
Archaeplastida - ps9
Viridiplantae - ps10
Streptophyta - ps11
Charophyceae/Embriophyta - ps12
Embriophyta - ps13
Tracheophyta - ps14
Spermatophyta - ps15
Angiosperms - ps16
Mesangiospermae - ps17
Magnoliids/eudicots - ps18
Eudicots - ps19
Core_eudicots - ps20
Superrosids - ps21
Rosids - ps22
Eurosids - ps23
Malvids - ps24
Sap./Malv./Brassicales - ps25
Malvales/Brassicales - ps26
Brassicales - ps27
Cleomaceae/Brassicaceae - ps28
Brassicaceae - ps29
Camelineae - ps30
Arabidopsis - ps31
Arabidopsis_thaliana - ps32
p-value
2e-05
1e-04
9e-04
7e-03
5e-02
Log-odds
0.5
1.0
1.5
2.0
2.5
3.0
3.5
4.0

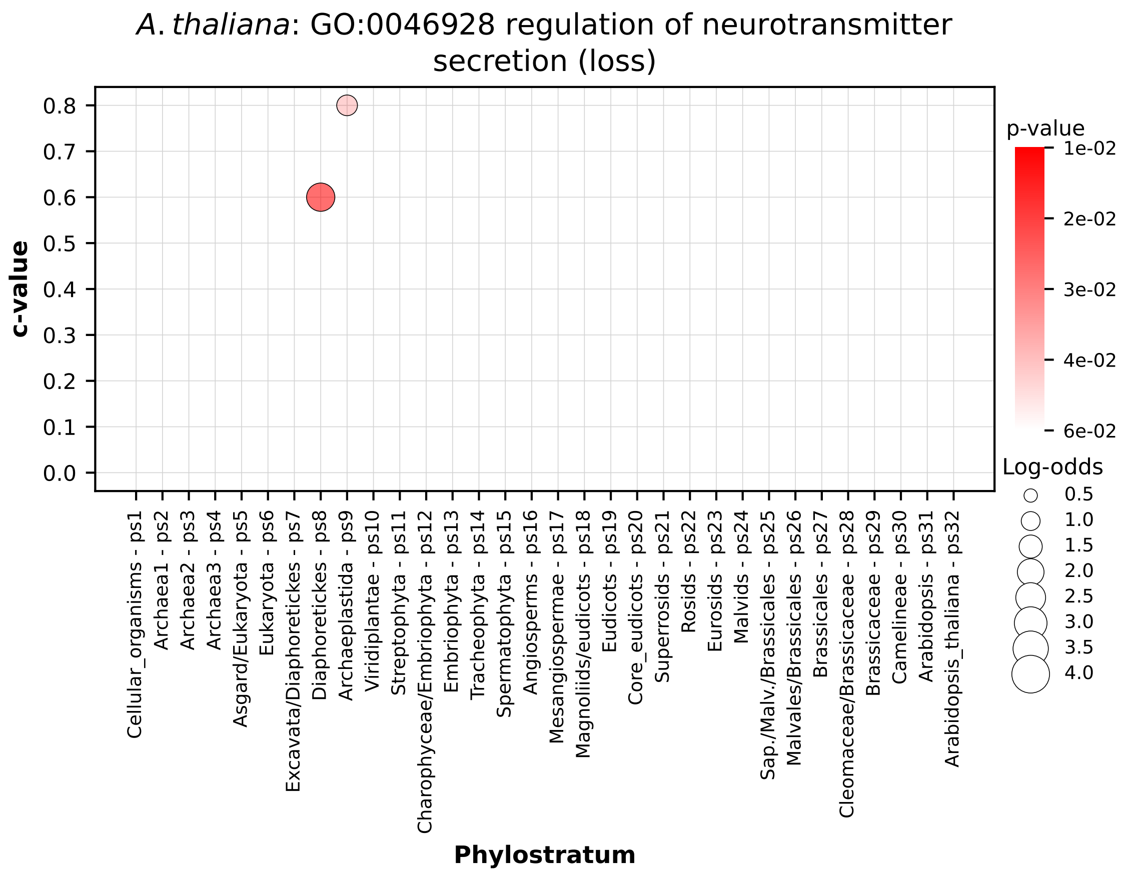
A. thaliana: GO:0046928 regulation of neurotransmitter secretion (loss)
c-value
0.0
0.1
0.2
0.3
0.4
0.5
0.6
0.7
0.8
Phylostratum
Cellular_organisms - ps1
Archaea1 - ps2
Archaea2 - ps3
Archaea3 - ps4
Asgard/Eukaryota - ps5
Eukaryota - ps6
Excavata/Diaphoretickes - ps7
Diaphoretickes - ps8
Archaeplastida - ps9
Viridiplantae - ps10
Streptophyta - ps11
Charophyceae/Embriophyta - ps12
Embriophyta - ps13
Tracheophyta - ps14
Spermatophyta - ps15
Angiosperms - ps16
Mesangiospermae - ps17
Magnoliids/eudicots - ps18
Eudicots - ps19
Core_eudicots - ps20
Superrosids - ps21
Rosids - ps22
Eurosids - ps23
Malvids - ps24
Sap./Malv./Brassicales - ps25
Malvales/Brassicales - ps26
Brassicales - ps27
Cleomaceae/Brassicaceae - ps28
Brassicaceae - ps29
Camelineae - ps30
Arabidopsis - ps31
Arabidopsis_thaliana - ps32
p-value
1e-02
2e-02
3e-02
4e-02
6e-02
Log-odds
0.5
1.0
1.5
2.0
2.5
3.0
3.5
4.0

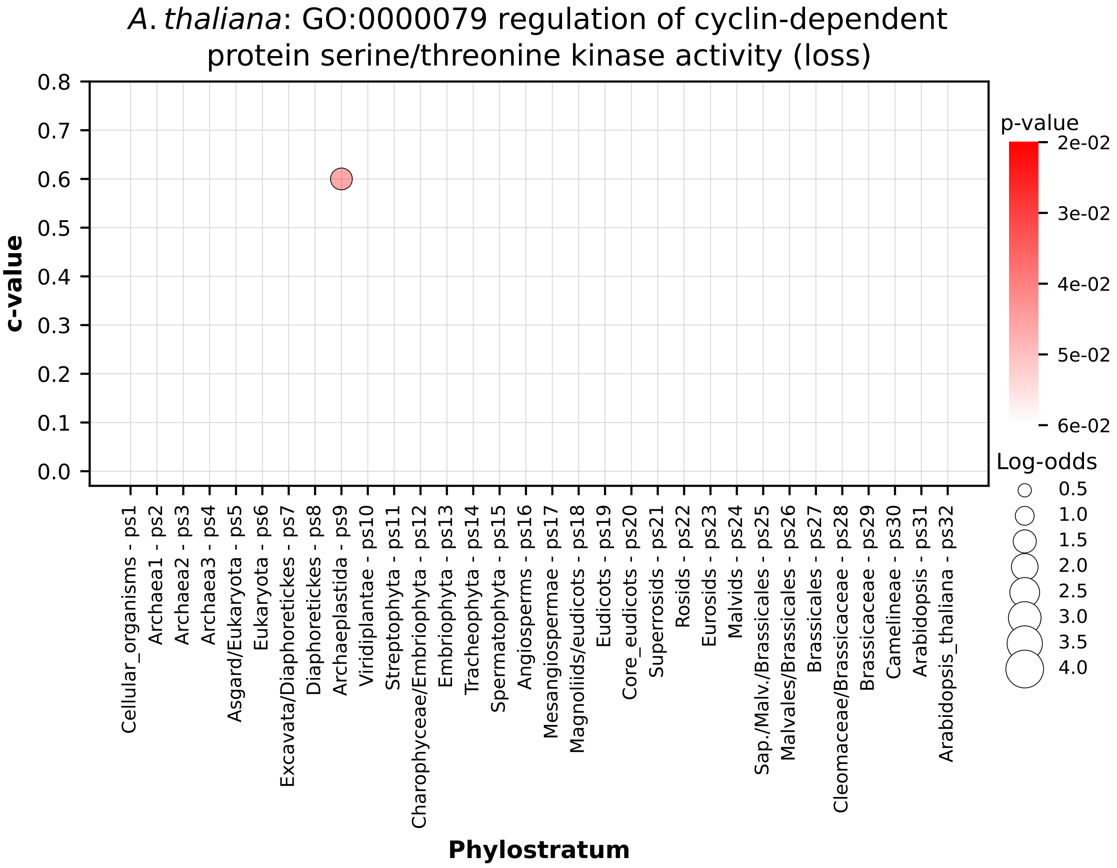

A. thaliana: GO:0000079 regulation of cyclin-dependent protein serine/threonine kinase activity (loss)
c-value
0.8
0.7
0.6
0.5
0.4
0.3
0.2
0.1
0.0
Phylostratum
Cellular_organisms - ps1
Archaea1 - ps2
Archaea2 - ps3
Archaea3 - ps4
Asgard/Eukaryota - ps5
Eukaryota - ps6
Excavata/Diaphoretickes - ps7
Diaphoretickes - ps8
Archaeplastida - ps9
Viridiplantae - ps10
Streptophyta - ps11
Charophyceae/Embriophyta - ps12
Embriophyta - ps13
Tracheophyta - ps14
Spermatophyta - ps15
Angiosperms - ps16
Mesangiospermae - ps17
Magnoliids/eudicots - ps18
Eudicots - ps19
Core_eudicots - ps20
Superrosids - ps21
Rosids - ps22
Eurosids - ps23
Malvids - ps24
Sap./Malv./Brassicales - ps25
Malvales/Brassicales - ps26
Brassicales - ps27
Cleomaceae/Brassicaceae - ps28
Brassicaceae - ps29
Camelineae - ps30
Arabidopsis - ps31
Arabidopsis_thaliana - ps32
p-value
2e-02
3e-02
4e-02
5e-02
6e-02
Log-odds
0.5
1.0
1.5
2.0
2.5
3.0
3.5
4.0

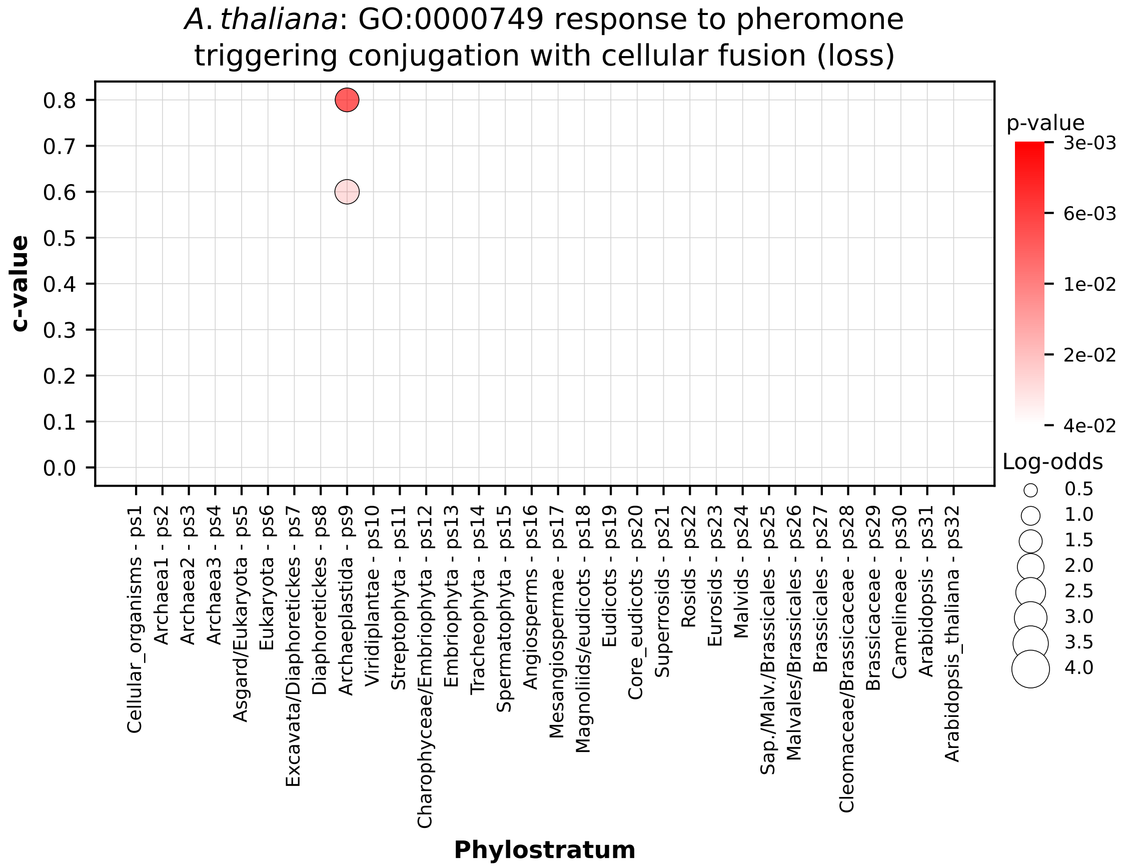

A. thaliana: GO:0000749 response to pheromone triggering conjugation with cellular fusion (loss)
c-value
0.8
0.7
0.6
0.5
0.4
0.3
0.2
0.1
0.0
Phylostratum
Cellular_organisms - ps1
Archaea1 - ps2
Archaea2 - ps3
Archaea3 - ps4
Asgard/Eukaryota - ps5
Eukaryota - ps6
Excavata/Diaphoretickes - ps7
Diaphoretickes - ps8
Archaeplastida - ps9
Viridiplantae - ps10
Streptophyta - ps11
Charophyceae/Embriophyta - ps12
Embriophyta - ps13
Tracheophyta - ps14
Spermatophyta - ps15
Angiosperms - ps16
Mesangiospermae - ps17
Magnoliids/eudicots - ps18
Eudicots - ps19
Core_eudicots - ps20
Superrosids - ps21
Rosids - ps22
Eurosids - ps23
Malvids - ps24
Sap./Malv./Brassicales - ps25
Malvales/Brassicales - ps26
Brassicales - ps27
Cleomaceae/Brassicaceae - ps28
Brassicaceae - ps29
Camelineae - ps30
Arabidopsis - ps31
Arabidopsis_thaliana - ps32
p-value
3e-03
6e-03
1e-02
2e-02
4e-02
Log-odds
0.5
1.0
1.5
2.0
2.5
3.0
3.5
4.0

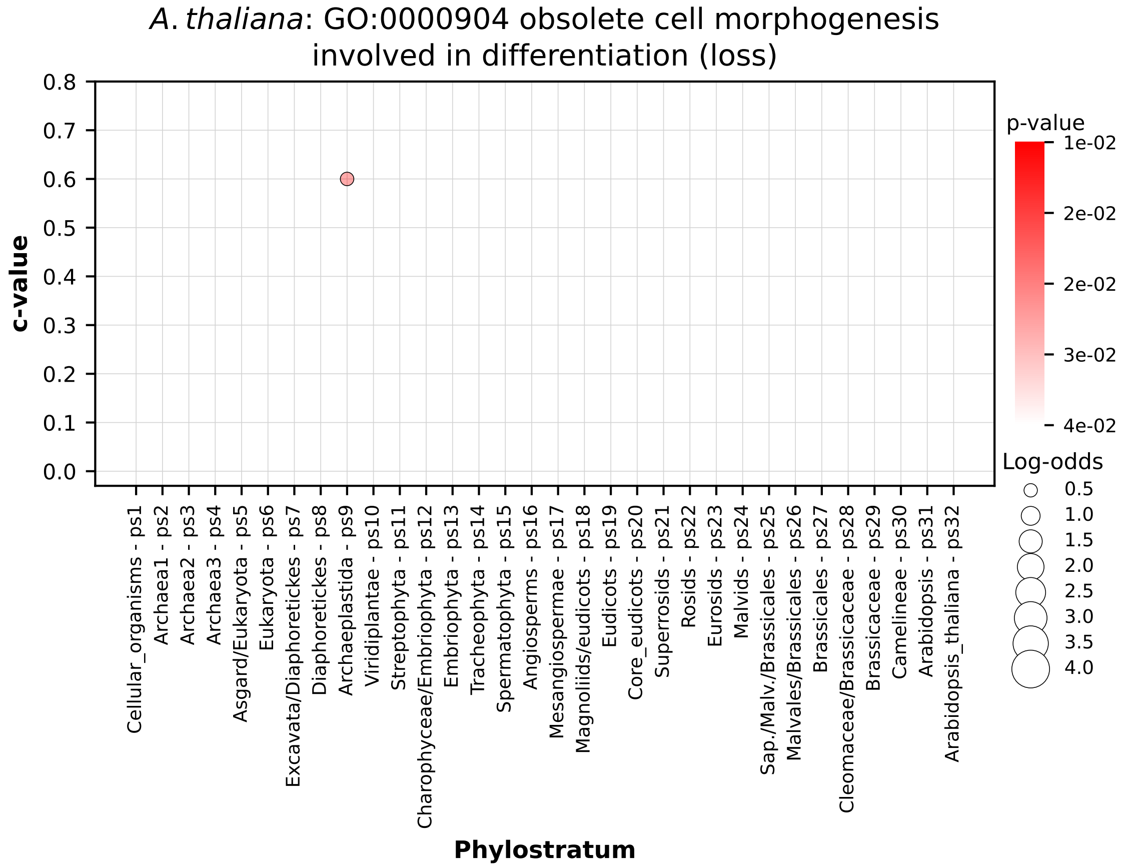

A. thaliana: GO:0000904 obsolete cell morphogenesis involved in differentiation (loss)
c-value
0.8
0.7
0.6
0.5
0.4
0.3
0.2
0.1
0.0
Phylostratum
Cellular_organisms - ps1
Archaea1 - ps2
Archaea2 - ps3
Archaea3 - ps4
Asgard/Eukaryota - ps5
Eukaryota - ps6
Excavata/Diaphoretickes - ps7
Diaphoretickes - ps8
Archaeplastida - ps9
Viridiplantae - ps10
Streptophyta - ps11
Charophyceae/Embriophyta - ps12
Embriophyta - ps13
Tracheophyta - ps14
Spermatophyta - ps15
Angiosperms - ps16
Mesangiospermae - ps17
Magnoliids/eudicots - ps18
Eudicots - ps19
Core_eudicots - ps20
Superrosids - ps21
Rosids - ps22
Eurosids - ps23
Malvids - ps24
Sap./Malv./Brassicales - ps25
Malvales/Brassicales - ps26
Brassicales - ps27
Cleomaceae/Brassicaceae - ps28
Brassicaceae - ps29
Camelineae - ps30
Arabidopsis - ps31
Arabidopsis_thaliana - ps32
p-value
1e-02
2e-02
2e-02
3e-02
4e-02
Log-odds
0.5
1.0
1.5
2.0
2.5
3.0
3.5
4.0

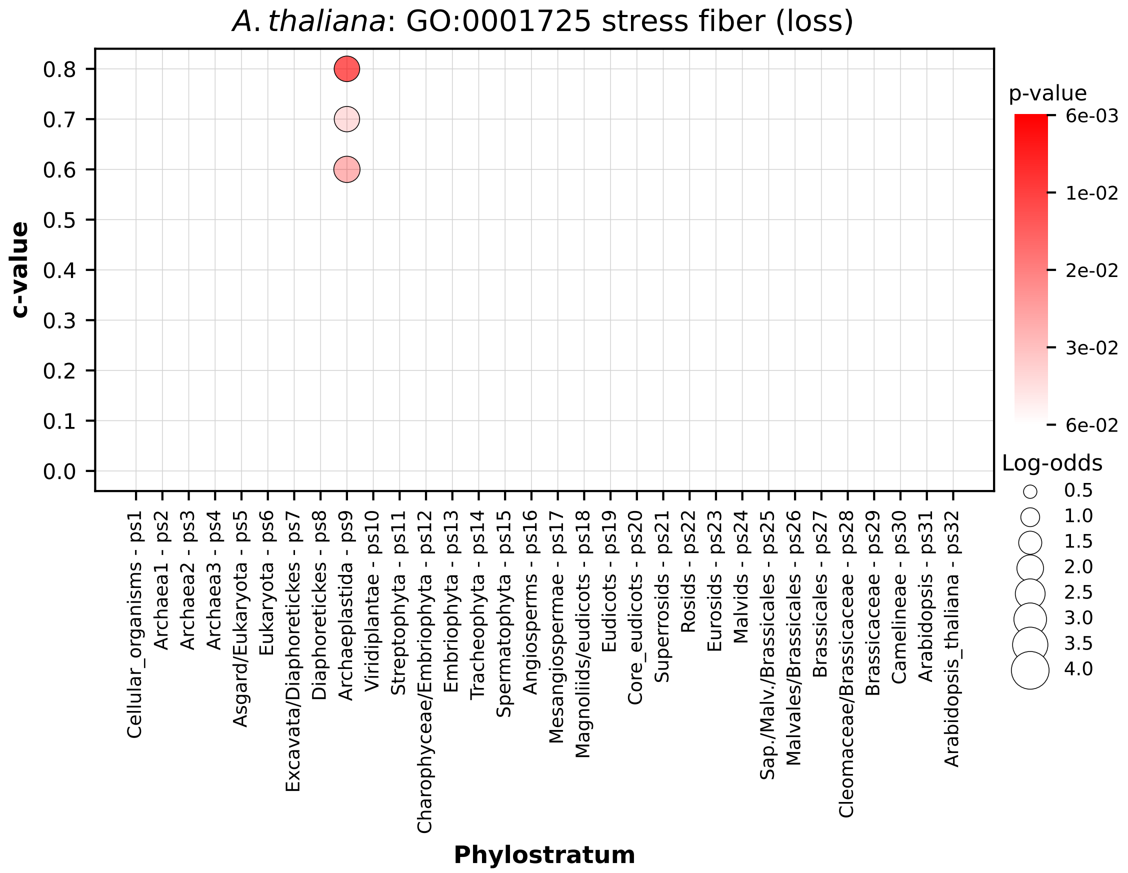

A. thaliana: GO:0001725 stress fiber (loss)
c-value
0.8
0.7
0.6
0.5
0.4
0.3
0.2
0.1
0.0
p-value
6e-03
1e-02
2e-02
3e-02
6e-02
Log-odds
0.5
1.0
1.5
2.0
2.5
3.0
3.5
4.0
Cellular_organisms - ps1
Archaea1 - ps2
Archaea2 - ps3
Archaea3 - ps4
Asgard/Eukaryota - ps5
Eukaryota - ps6
Excavata/Diaphoretickes - ps7
Diaphoretickes - ps8
Archaeplastida - ps9
Viridiplantae - ps10
Streptophyta - ps11
Charophyceae/Embriophyta - ps12
Embriophyta - ps13
Tracheophyta - ps14
Spermatophyta - ps15
Angiosperms - ps16
Mesangiospermae - ps17
Magnoliids/eudicots - ps18
Eudicots - ps19
Core_eudicots - ps20
Superrosids - ps21
Rosids - ps22
Eurosids - ps23
Malvids - ps24
Sap./Malv./Brassicales - ps25
Malvales/Brassicales - ps26
Brassicales - ps27
Cleomaceae/Brassicaceae - ps28
Brassicaceae - ps29
Camelineae - ps30
Arabidopsis - ps31
Arabidopsis_thaliana - ps32
Phylostratum

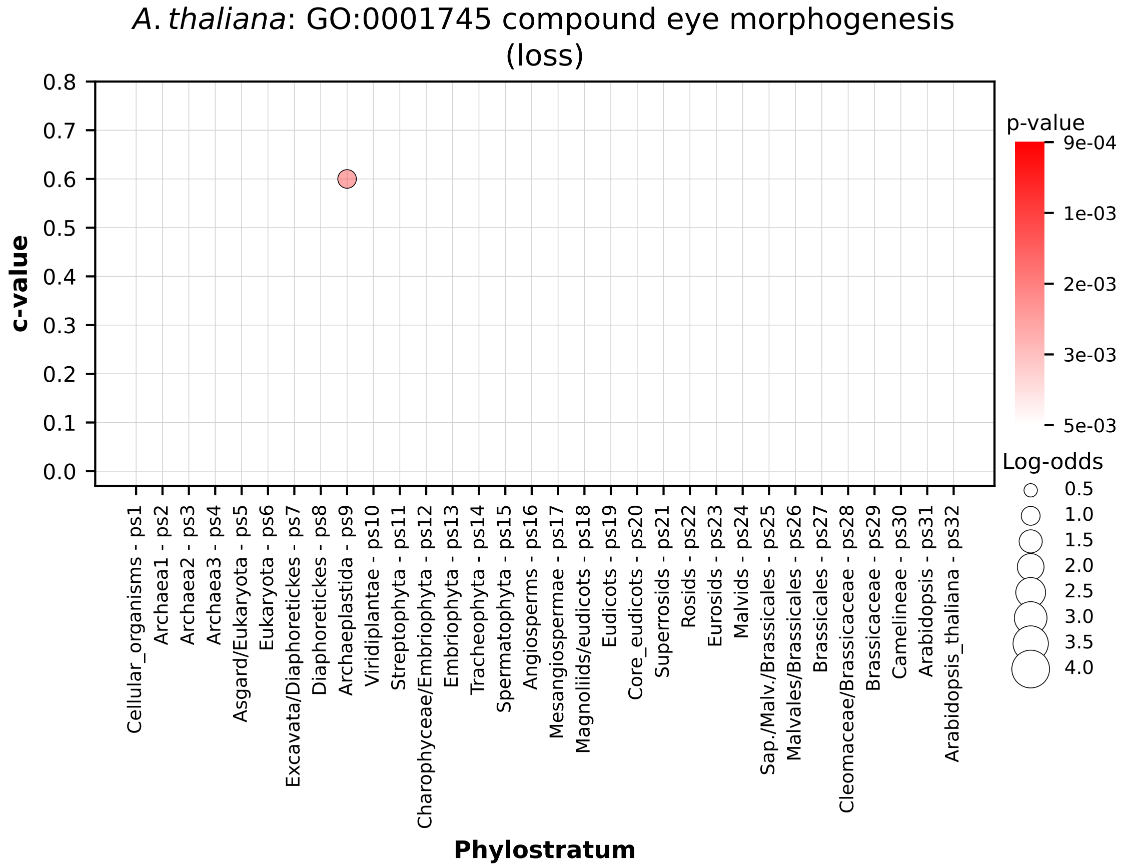

A. thaliana: GO:0001745 compound eye morphogenesis
(loss)
c-value
0.0
0.1
0.2
0.3
0.4
0.5
0.6
0.7
0.8
p-value
9e-04
1e-03
2e-03
3e-03
5e-03
Log-odds
0.5
1.0
1.5
2.0
2.5
3.0
3.5
4.0
Cellular_organisms - ps1
Archaea1 - ps2
Archaea2 - ps3
Archaea3 - ps4
Asgard/Eukaryota - ps5
Eukaryota - ps6
Excavata/Diaphoretickes - ps7
Diaphoretickes - ps8
Archaeplastida - ps9
Viridiplantae - ps10
Streptophyta - ps11
Charophyceae/Embriophyta - ps12
Embriophyta - ps13
Tracheophyta - ps14
Spermatophyta - ps15
Angiosperms - ps16
Mesangiospermae - ps17
Magnoliids/eudicots - ps18
Eudicots - ps19
Core_eudicots - ps20
Superrosids - ps21
Rosids - ps22
Eurosids - ps23
Malvids - ps24
Sap./Malv./Brassicales - ps25
Malvales/Brassicales - ps26
Brassicales - ps27
Cleomaceae/Brassicaceae - ps28
Brassicaceae - ps29
Camelineae - ps30
Arabidopsis - ps31
Arabidopsis_thaliana - ps32
Phylostratum

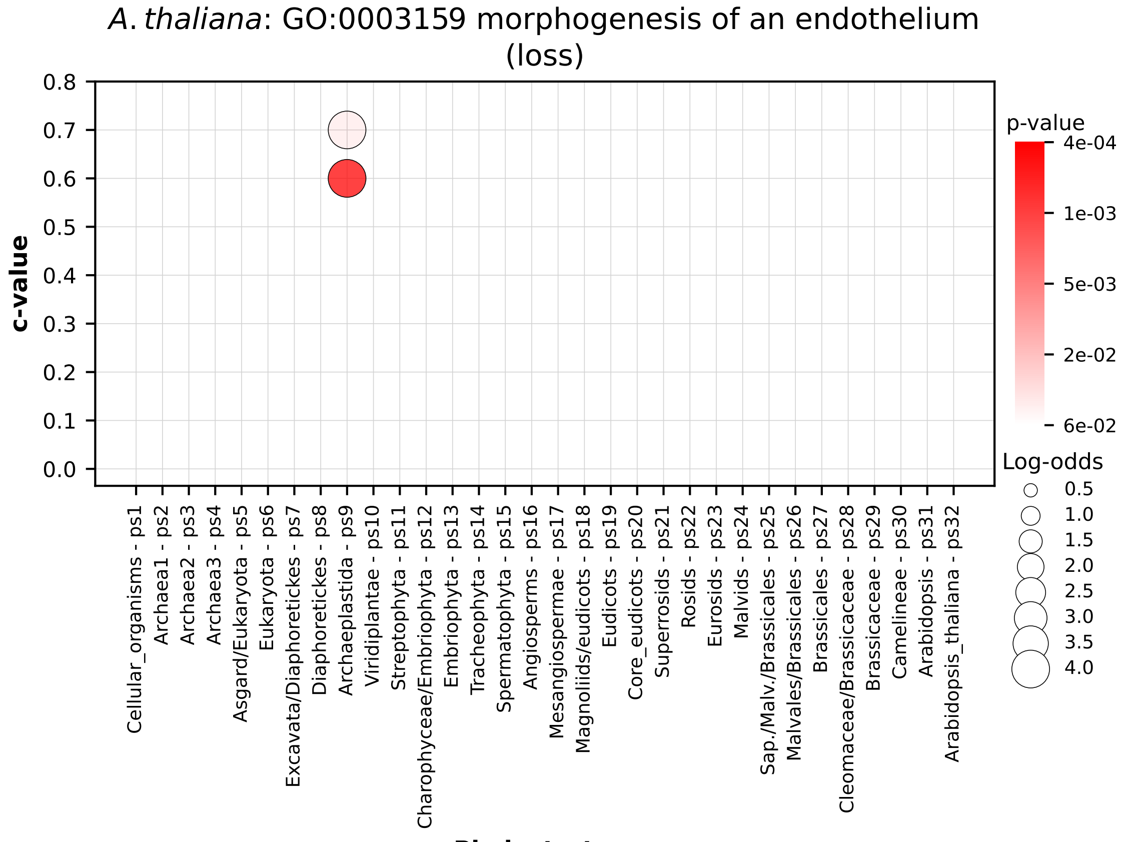
A. thaliana: GO:0003159 morphogenesis of an endothelium
(loss)
c-value
0.8
0.7
0.6
0.5
0.4
0.3
0.2
0.1
0.0
p-value
4e-04
1e-03
5e-03
2e-02
6e-02
Log-odds
0.5
1.0
1.5
2.0
2.5
3.0
3.5
4.0
Cellular_organisms - ps1
Archaea1 - ps2
Archaea2 - ps3
Archaea3 - ps4
Asgard/Eukaryota - ps5
Eukaryota - ps6
Excavata/Diaphoretickes - ps7
Diaphoretickes - ps8
Archaeplastida - ps9
Viridiplantae - ps10
Streptophyta - ps11
Charophyceae/Embriophyta - ps12
Embriophyta - ps13
Tracheophyta - ps14
Spermatophyta - ps15
Angiosperms - ps16
Mesangiospermae - ps17
Magnoliids/eudicots - ps18
Eudicots - ps19
Core_eudicots - ps20
Superrosids - ps21
Rosids - ps22
Eurosids - ps23
Malvids - ps24
Sap./Malv./Brassicales - ps25
Malvales/Brassicales - ps26
Brassicales - ps27
Cleomaceae/Brassicaceae - ps28
Brassicaceae - ps29
Camelineae - ps30
Arabidopsis - ps31
Arabidopsis_thaliana - ps32
Phylostratum

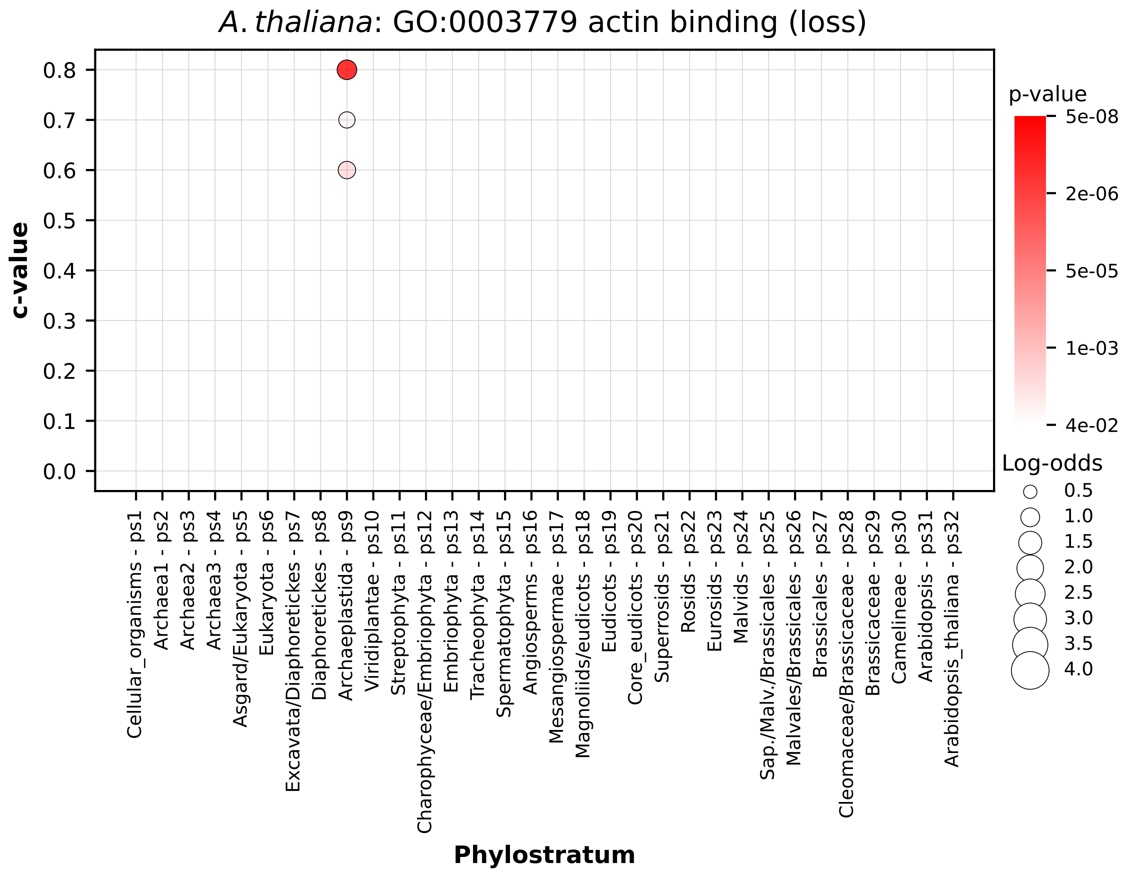

A. thaliana: GO:0003779 actin binding (loss)
c-value
0.8
0.7
0.6
0.5
0.4
0.3
0.2
0.1
0.0
p-value
5e-08
2e-06
5e-05
1e-03
4e-02
Log-odds
0.5
1.0
1.5
2.0
2.5
3.0
3.5
4.0
Cellular_organisms - ps1
Archaea1 - ps2
Archaea2 - ps3
Archaea3 - ps4
Asgard/Eukaryota - ps5
Eukaryota - ps6
Excavata/Diaphoretickes - ps7
Diaphoretickes - ps8
Archaeplastida - ps9
Viridiplantae - ps10
Streptophyta - ps11
Charophyceae/Embriophyta - ps12
Embriophyta - ps13
Tracheophyta - ps14
Spermatophyta - ps15
Angiosperms - ps16
Mesangiospermae - ps17
Magnoliids/eudicots - ps18
Eudicots - ps19
Core_eudicots - ps20
Superrosids - ps21
Rosids - ps22
Eurosids - ps23
Malvids - ps24
Sap./Malv./Brassicales - ps25
Malvales/Brassicales - ps26
Brassicales - ps27
Cleomaceae/Brassicaceae - ps28
Brassicaceae - ps29
Camelineae - ps30
Arabidopsis - ps31
Arabidopsis_thaliana - ps32
Phylostratum

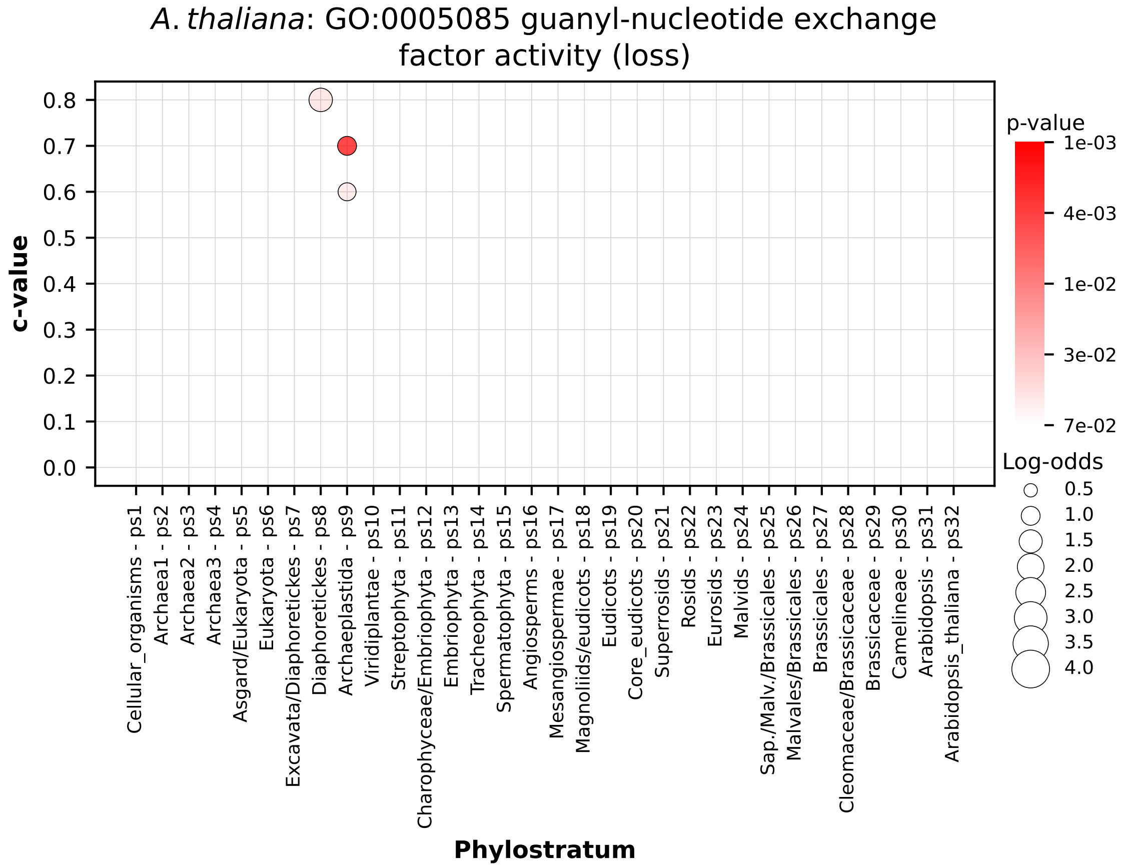

A. thaliana: GO:0005085 guanyl-nucleotide exchange factor activity (loss)
c-value
0.8
0.7
0.6
0.5
0.4
0.3
0.2
0.1
0.0
p-value
1e-03
4e-03
1e-02
3e-02
7e-02
Log-odds
0.5
1.0
1.5
2.0
2.5
3.0
3.5
4.0
Cellular_organisms - ps1
Archaea1 - ps2
Archaea2 - ps3
Archaea3 - ps4
Asgard/Eukaryota - ps5
Eukaryota - ps6
Excavata/Diaphoretickes - ps7
Diaphoretickes - ps8
Archaeplastida - ps9
Viridiplantae - ps10
Streptophyta - ps11
Charophyceae/Embriophyta - ps12
Embriophyta - ps13
Tracheophyta - ps14
Spermatophyta - ps15
Angiosperms - ps16
Mesangiospermae - ps17
Magnoliids/eudicots - ps18
Eudicots - ps19
Core_eudicots - ps20
Superrosids - ps21
Rosids - ps22
Eurosids - ps23
Malvids - ps24
Sap./Malv./Brassicales - ps25
Malvales/Brassicales - ps26
Brassicales - ps27
Cleomaceae/Brassicaceae - ps28
Brassicaceae - ps29
Camelineae - ps30
Arabidopsis - ps31
Arabidopsis_thaliana - ps32
Phylostratum

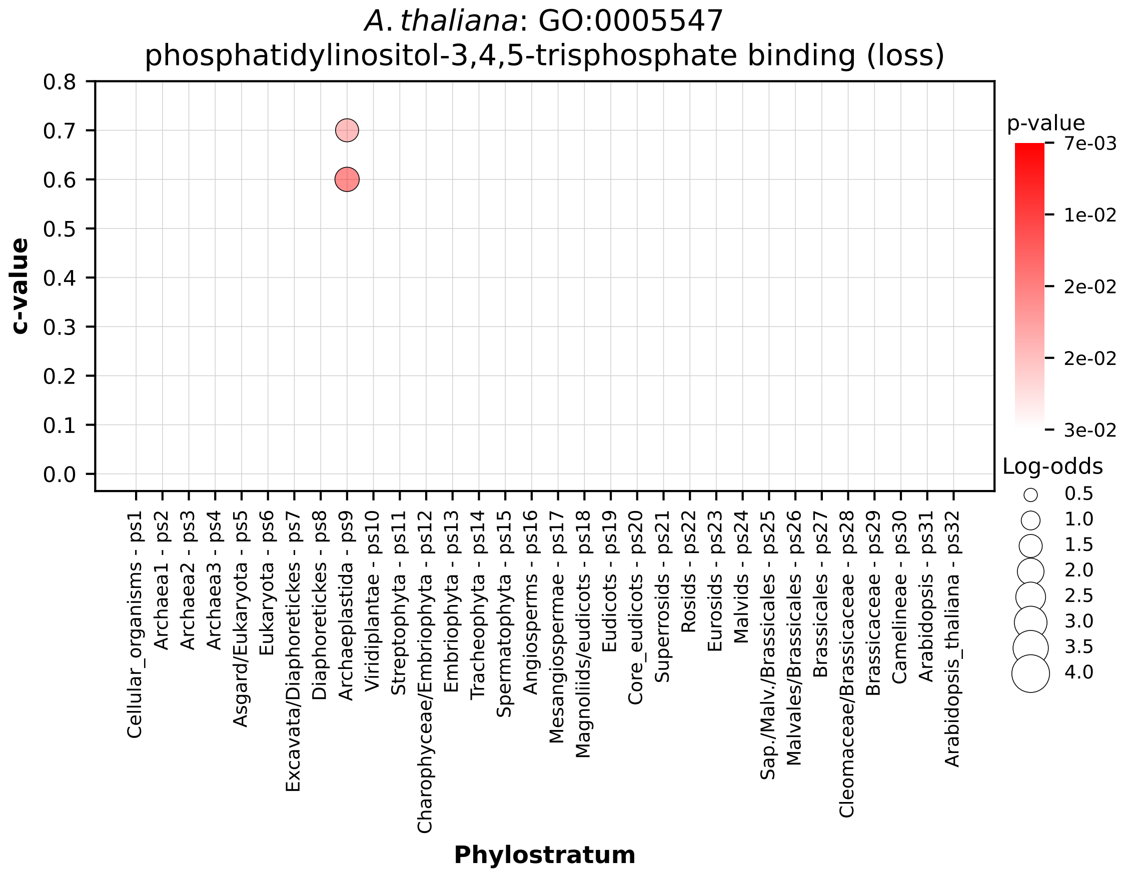
A. thaliana: GO:0005547
phosphatidylinositol-3,4,5-trisphosphate binding (loss)
c-value
0.8
0.7
0.6
0.5
0.4
0.3
0.2
0.1
0.0
p-value
7e-03
1e-02
2e-02
2e-02
3e-02
Log-odds
0.5
1.0
1.5
2.0
2.5
3.0
3.5
4.0
Cellular_organisms - ps1
Archaea1 - ps2
Archaea2 - ps3
Archaea3 - ps4
Asgard/Eukaryota - ps5
Eukaryota - ps6
Excavata/Diaphoretickes - ps7
Diaphoretickes - ps8
Archaeplastida - ps9
Viridiplantae - ps10
Streptophyta - ps11
Charophyceae/Embriophyta - ps12
Embriophyta - ps13
Tracheophyta - ps14
Spermatophyta - ps15
Angiosperms - ps16
Mesangiospermae - ps17
Magnoliids/eudicots - ps18
Eudicots - ps19
Core_eudicots - ps20
Superrosids - ps21
Rosids - ps22
Eurosids - ps23
Malvids - ps24
Sap./Malv./Brassicales - ps25
Malvales/Brassicales - ps26
Brassicales - ps27
Cleomaceae/Brassicaceae - ps28
Brassicaceae - ps29
Camelineae - ps30
Arabidopsis - ps31
Arabidopsis_thaliana - ps32
Phylostratum

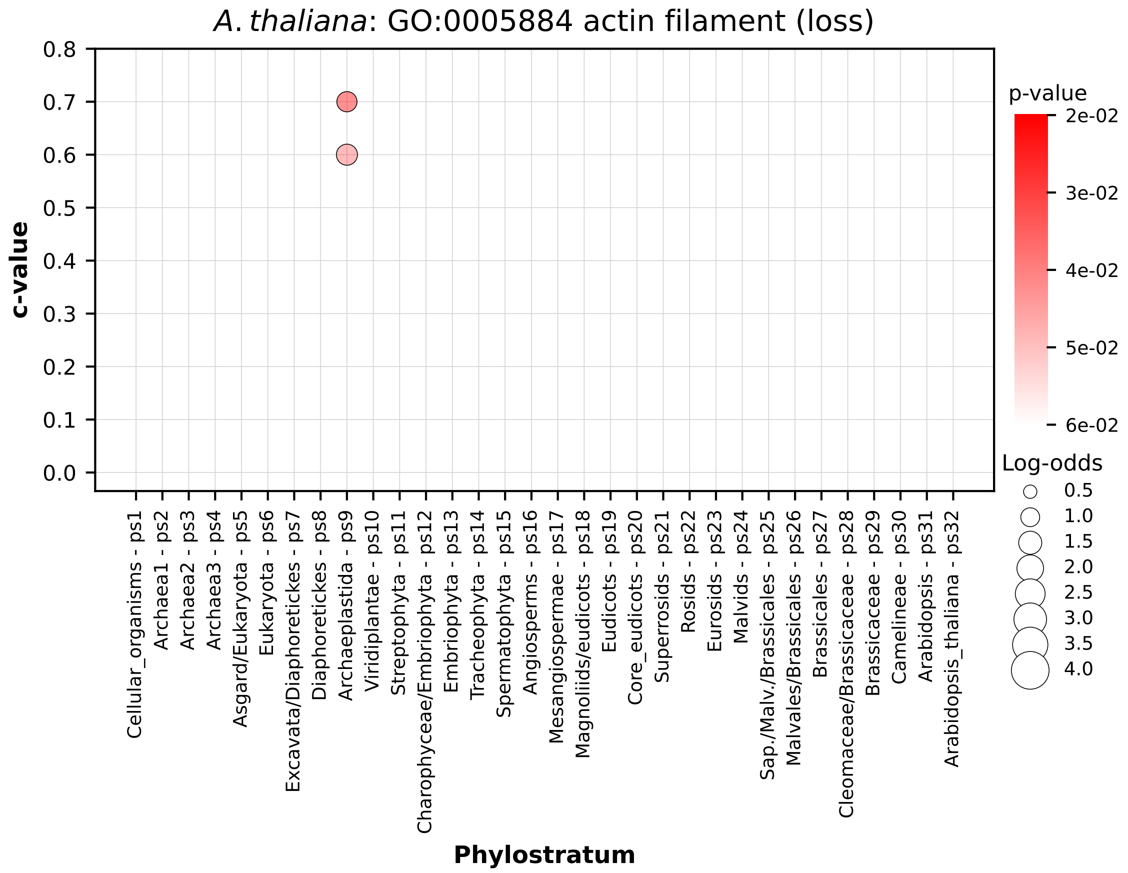

A. thaliana: GO:0005884 actin filament (loss)
c-value
0.8
0.7
0.6
0.5
0.4
0.3
0.2
0.1
0.0
Phylostratum
Cellular_organisms - ps1
Archaea1 - ps2
Archaea2 - ps3
Archaea3 - ps4
Asgard/Eukaryota - ps5
Eukaryota - ps6
Excavata/Diaphoretickes - ps7
Diaphoretickes - ps8
Archaeplastida - ps9
Viridiplantae - ps10
Streptophyta - ps11
Charophyceae/Embriophyta - ps12
Embriophyta - ps13
Tracheophyta - ps14
Spermatophyta - ps15
Angiosperms - ps16
Mesangiospermae - ps17
Magnoliids/eudicots - ps18
Eudicots - ps19
Core_eudicots - ps20
Superrosids - ps21
Rosids - ps22
Eurosids - ps23
Malvids - ps24
Sap./Malv./Brassicales - ps25
Malvales/Brassicales - ps26
Brassicales - ps27
Cleomaceae/Brassicaceae - ps28
Brassicaceae - ps29
Camelineae - ps30
Arabidopsis - ps31
Arabidopsis_thaliana - ps32
p-value
2e-02
3e-02
4e-02
5e-02
6e-02
Log-odds
0.5
1.0
1.5
2.0
2.5
3.0
3.5
4.0

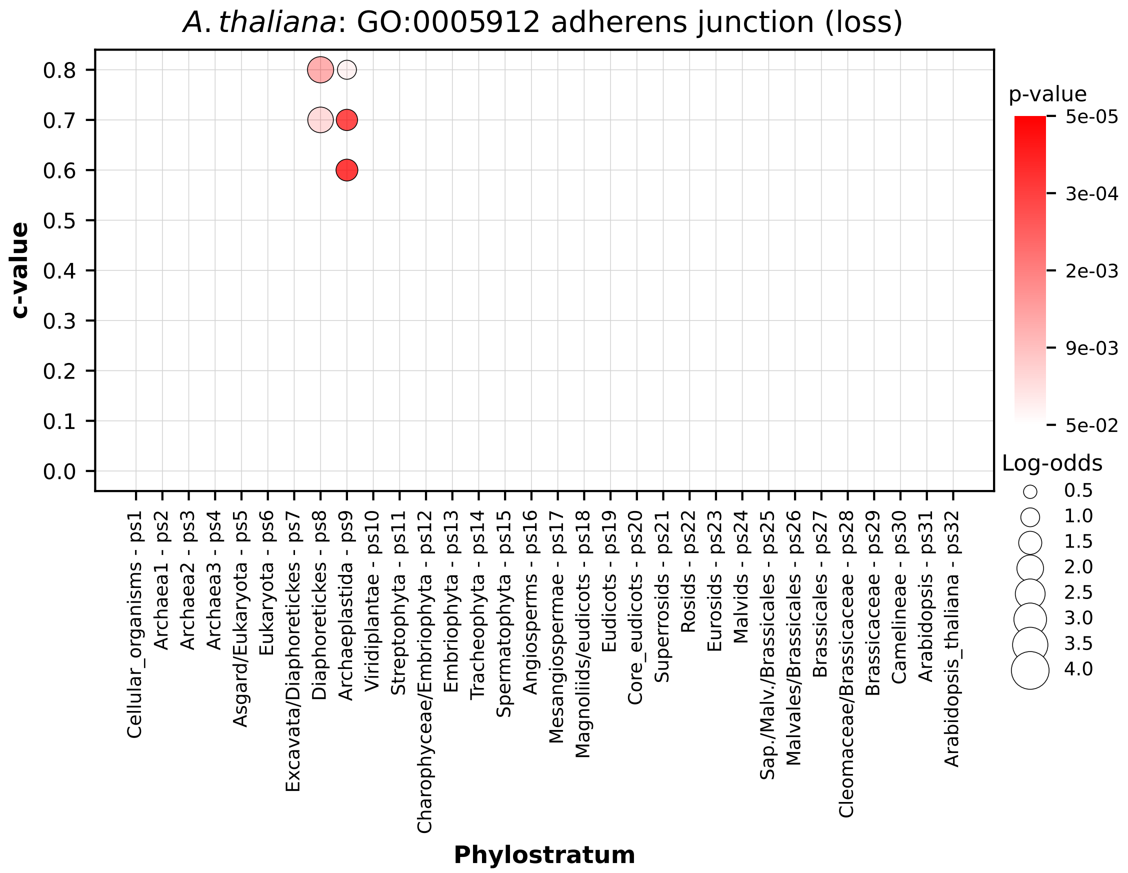

A. thaliana: GO:0005912 adherens junction (loss)
c-value
0.0
0.1
0.2
0.3
0.4
0.5
0.6
0.7
0.8
Phylostratum
Cellular_organisms - ps1
Archaea1 - ps2
Archaea2 - ps3
Archaea3 - ps4
Asgard/Eukaryota - ps5
Eukaryota - ps6
Excavata/Diaphoretickes - ps7
Diaphoretickes - ps8
Archaeplastida - ps9
Viridiplantae - ps10
Streptophyta - ps11
Charophyceae/Embriophyta - ps12
Embriophyta - ps13
Tracheophyta - ps14
Spermatophyta - ps15
Angiosperms - ps16
Mesangiospermae - ps17
Magnoliids/eudicots - ps18
Eudicots - ps19
Core_eudicots - ps20
Superrosids - ps21
Rosids - ps22
Eurosids - ps23
Malvids - ps24
Sap./Malv./Brassicales - ps25
Malvales/Brassicales - ps26
Brassicales - ps27
Cleomaceae/Brassicaceae - ps28
Brassicaceae - ps29
Camelineae - ps30
Arabidopsis - ps31
Arabidopsis_thaliana - ps32
p-value
5e-05
3e-04
2e-03
9e-03
5e-02
Log-odds
0.5
1.0
1.5
2.0
2.5
3.0
3.5
4.0

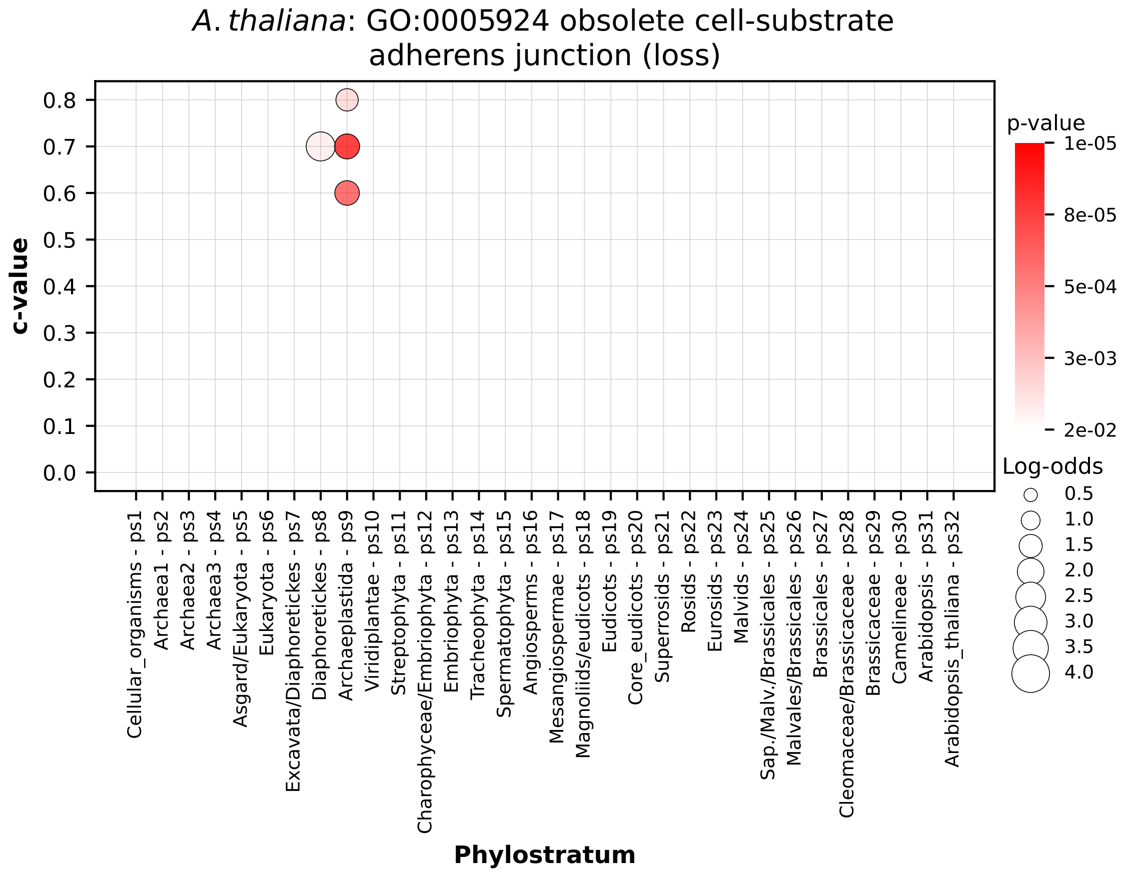

A. thaliana: GO:0005924 obsolete cell-substrate adherens junction (loss)
c-value
0.0
0.1
0.2
0.3
0.4
0.5
0.6
0.7
0.8
Phylostratum
Cellular_organisms - ps1
Archaea1 - ps2
Archaea2 - ps3
Archaea3 - ps4
Asgard/Eukaryota - ps5
Eukaryota - ps6
Excavata/Diaphoretickes - ps7
Diaphoretickes - ps8
Archaeplastida - ps9
Viridiplantae - ps10
Streptophyta - ps11
Charophyceae/Embriophyta - ps12
Embriophyta - ps13
Tracheophyta - ps14
Spermatophyta - ps15
Angiosperms - ps16
Mesangiospermae - ps17
Magnoliids/eudicots - ps18
Eudicots - ps19
Core_eudicots - ps20
Superrosids - ps21
Rosids - ps22
Eurosids - ps23
Malvids - ps24
Sap./Malv./Brassicales - ps25
Malvales/Brassicales - ps26
Brassicales - ps27
Cleomaceae/Brassicaceae - ps28
Brassicaceae - ps29
Camelineae - ps30
Arabidopsis - ps31
Arabidopsis_thaliana - ps32
p-value
1e-05
8e-05
5e-04
3e-03
2e-02
Log-odds
0.5
1.0
1.5
2.0
2.5
3.0
3.5
4.0

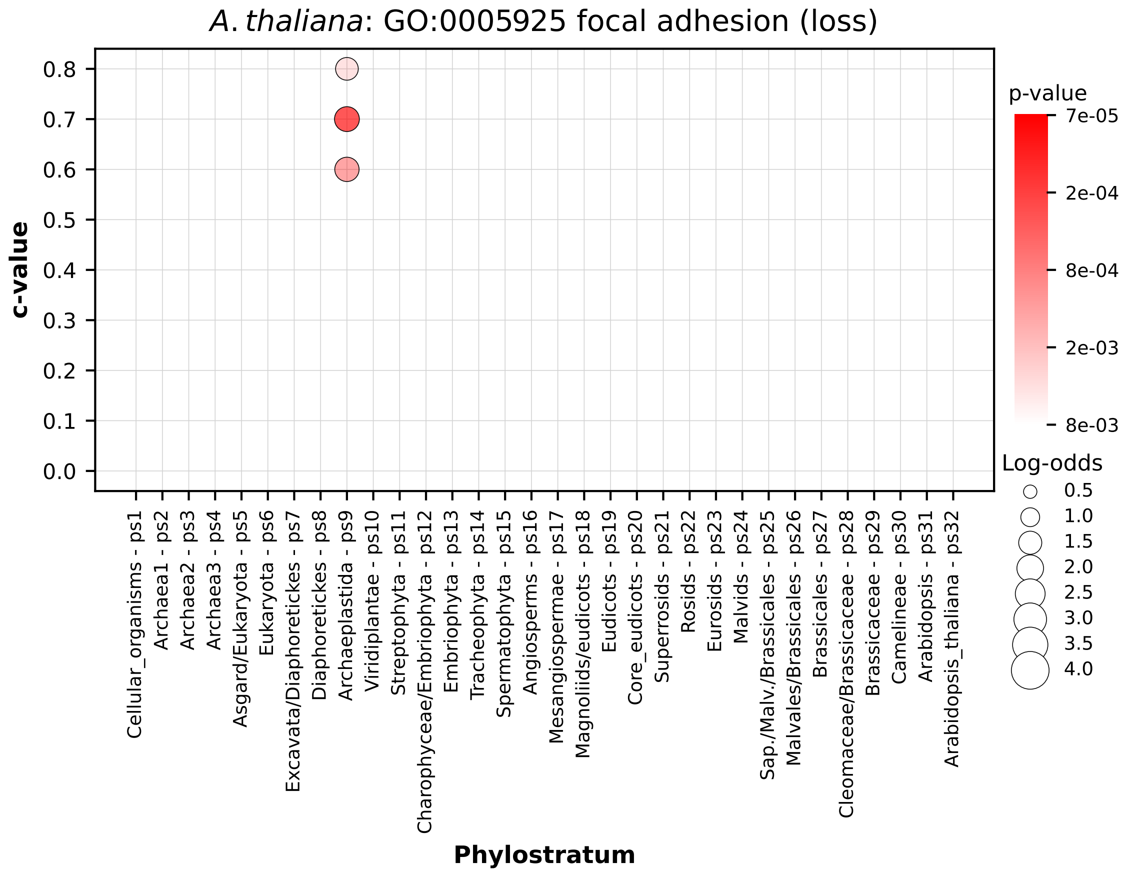

A. thaliana: GO:0005925 focal adhesion (loss)
c-value
0.0
0.1
0.2
0.3
0.4
0.5
0.6
0.7
0.8
p-value
7e-05
2e-04
8e-04
2e-03
8e-03
Log-odds
0.5
1.0
1.5
2.0
2.5
3.0
3.5
4.0
Cellular_organisms - ps1
Archaea1 - ps2
Archaea2 - ps3
Archaea3 - ps4
Asgard/Eukaryota - ps5
Eukaryota - ps6
Excavata/Diaphoretickes - ps7
Diaphoretickes - ps8
Archaeplastida - ps9
Viridiplantae - ps10
Streptophyta - ps11
Charophyceae/Embriophyta - ps12
Embriophyta - ps13
Tracheophyta - ps14
Spermatophyta - ps15
Angiosperms - ps16
Mesangiospermae - ps17
Magnoliids/eudicots - ps18
Eudicots - ps19
Core_eudicots - ps20
Superrosids - ps21
Rosids - ps22
Eurosids - ps23
Malvids - ps24
Sap./Malv./Brassicales - ps25
Malvales/Brassicales - ps26
Brassicales - ps27
Cleomaceae/Brassicaceae - ps28
Brassicaceae - ps29
Camelineae - ps30
Arabidopsis - ps31
Arabidopsis_thaliana - ps32
Phylostratum

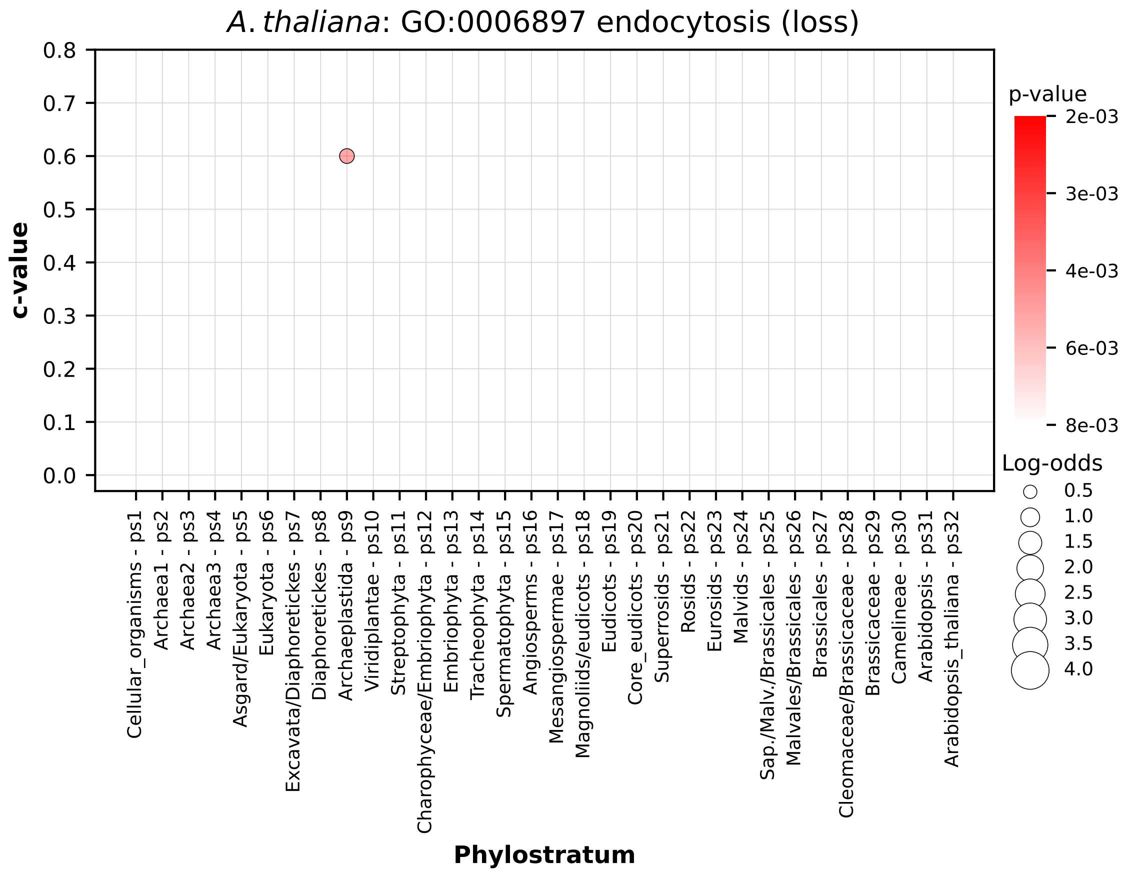

A. thaliana: GO:0006897 endocytosis (loss)
c-value
0.0
0.1
0.2
0.3
0.4
0.5
0.6
0.7
0.8
Phylostratum
Cellular_organisms - ps1
Archaea1 - ps2
Archaea2 - ps3
Archaea3 - ps4
Asgard/Eukaryota - ps5
Eukaryota - ps6
Excavata/Diaphoretickes - ps7
Diaphoretickes - ps8
Archaeplastida - ps9
Viridiplantae - ps10
Streptophyta - ps11
Charophyceae/Embriophyta - ps12
Embriophyta - ps13
Tracheophyta - ps14
Spermatophyta - ps15
Angiosperms - ps16
Mesangiospermae - ps17
Magnoliids/eudicots - ps18
Eudicots - ps19
Core_eudicots - ps20
Superrosids - ps21
Rosids - ps22
Eurosids - ps23
Malvids - ps24
Sap./Malv./Brassicales - ps25
Malvales/Brassicales - ps26
Brassicales - ps27
Cleomaceae/Brassicaceae - ps28
Brassicaceae - ps29
Camelineae - ps30
Arabidopsis - ps31
Arabidopsis_thaliana - ps32
p-value
2e-03
3e-03
4e-03
6e-03
8e-03
Log-odds
0.5
1.0
1.5
2.0
2.5
3.0
3.5
4.0

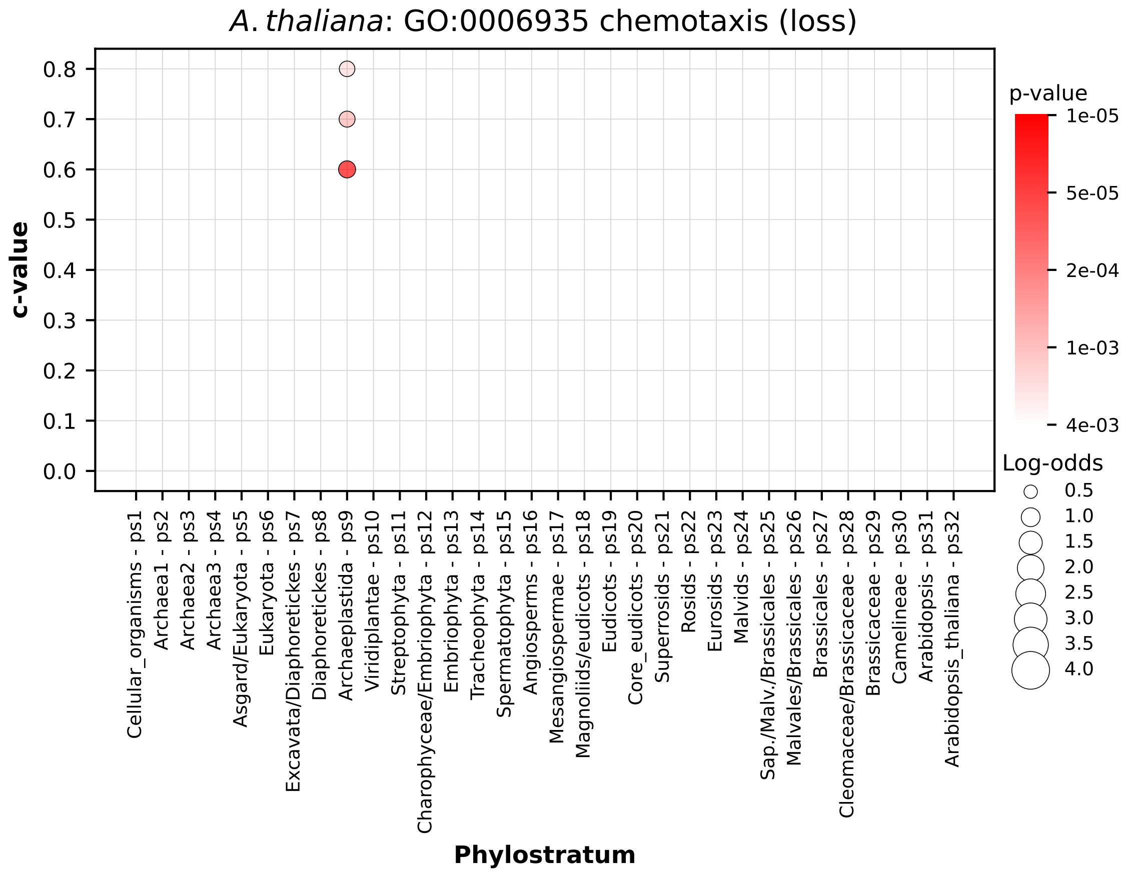

A. thaliana: GO:0006935 chemotaxis (loss)
c-value
0.0
0.1
0.2
0.3
0.4
0.5
0.6
0.7
0.8
Phylostratum
Cellular_organisms - ps1
Archaea1 - ps2
Archaea2 - ps3
Archaea3 - ps4
Asgard/Eukaryota - ps5
Eukaryota - ps6
Excavata/Diaphoretickes - ps7
Diaphoretickes - ps8
Archaeplastida - ps9
Viridiplantae - ps10
Streptophyta - ps11
Charophyceae/Embriophyta - ps12
Embriophyta - ps13
Tracheophyta - ps14
Spermatophyta - ps15
Angiosperms - ps16
Mesangiospermae - ps17
Magnoliids/eudicots - ps18
Eudicots - ps19
Core_eudicots - ps20
Superrosids - ps21
Rosids - ps22
Eurosids - ps23
Malvids - ps24
Sap./Malv./Brassicales - ps25
Malvales/Brassicales - ps26
Brassicales - ps27
Cleomaceae/Brassicaceae - ps28
Brassicaceae - ps29
Camelineae - ps30
Arabidopsis - ps31
Arabidopsis_thaliana - ps32
p-value
1e-05
5e-05
2e-04
1e-03
4e-03
Log-odds
0.5
1.0
1.5
2.0
2.5
3.0
3.5
4.0

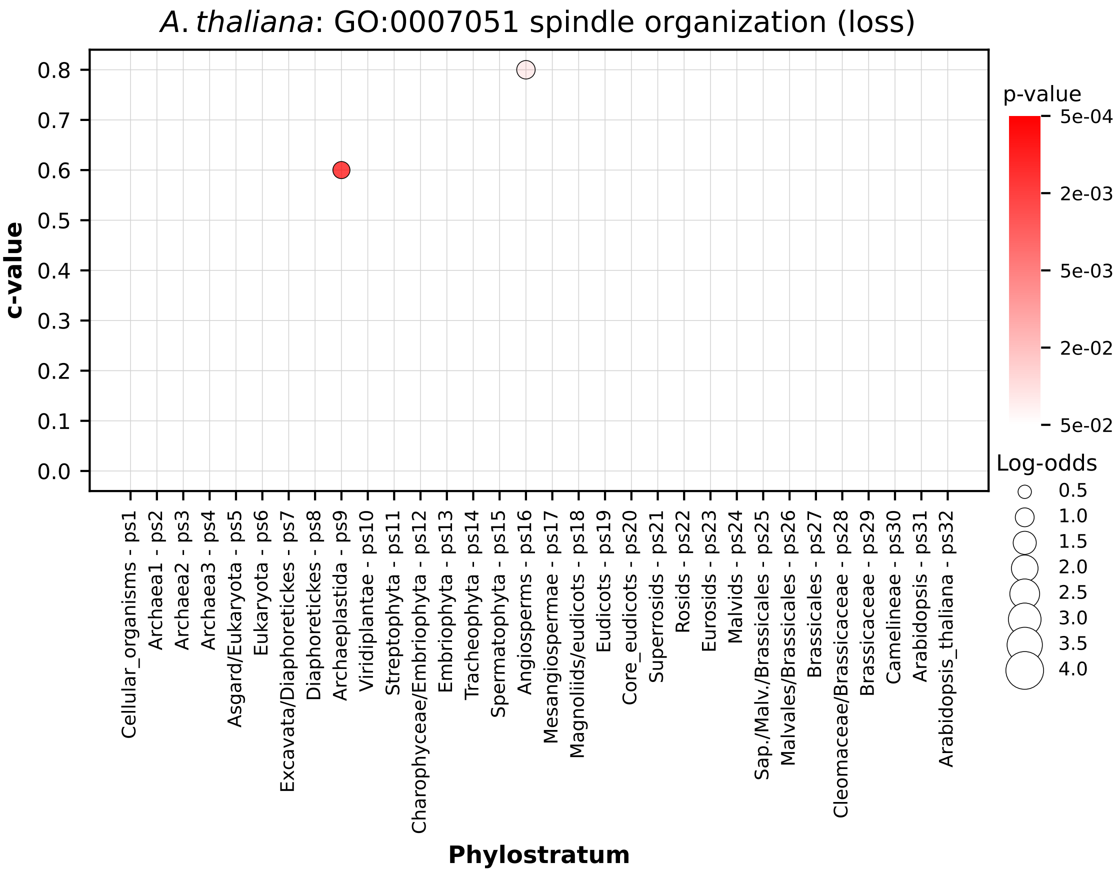
A. thaliana: GO:0007051 spindle organization (loss)
c-value
0.0
0.1
0.2
0.3
0.4
0.5
0.6
0.7
0.8
Phylostratum
Cellular_organisms - ps1
Archaea1 - ps2
Archaea2 - ps3
Archaea3 - ps4
Asgard/Eukaryota - ps5
Eukaryota - ps6
Excavata/Diaphoretickes - ps7
Diaphoretickes - ps8
Archaeplastida - ps9
Viridiplantae - ps10
Streptophyta - ps11
Charophyceae/Embriophyta - ps12
Embriophyta - ps13
Tracheophyta - ps14
Spermatophyta - ps15
Angiosperms - ps16
Mesangiospermae - ps17
Magnoliids/eudicots - ps18
Eudicots - ps19
Core_eudicots - ps20
Superrosids - ps21
Rosids - ps22
Eurosids - ps23
Malvids - ps24
Sap./Malv./Brassicales - ps25
Malvales/Brassicales - ps26
Brassicales - ps27
Cleomaceae/Brassicaceae - ps28
Brassicaceae - ps29
Camelineae - ps30
Arabidopsis - ps31
Arabidopsis_thaliana - ps32
p-value
5e-04
2e-03
5e-03
2e-02
5e-02
Log-odds
0.5
1.0
1.5
2.0
2.5
3.0
3.5
4.0

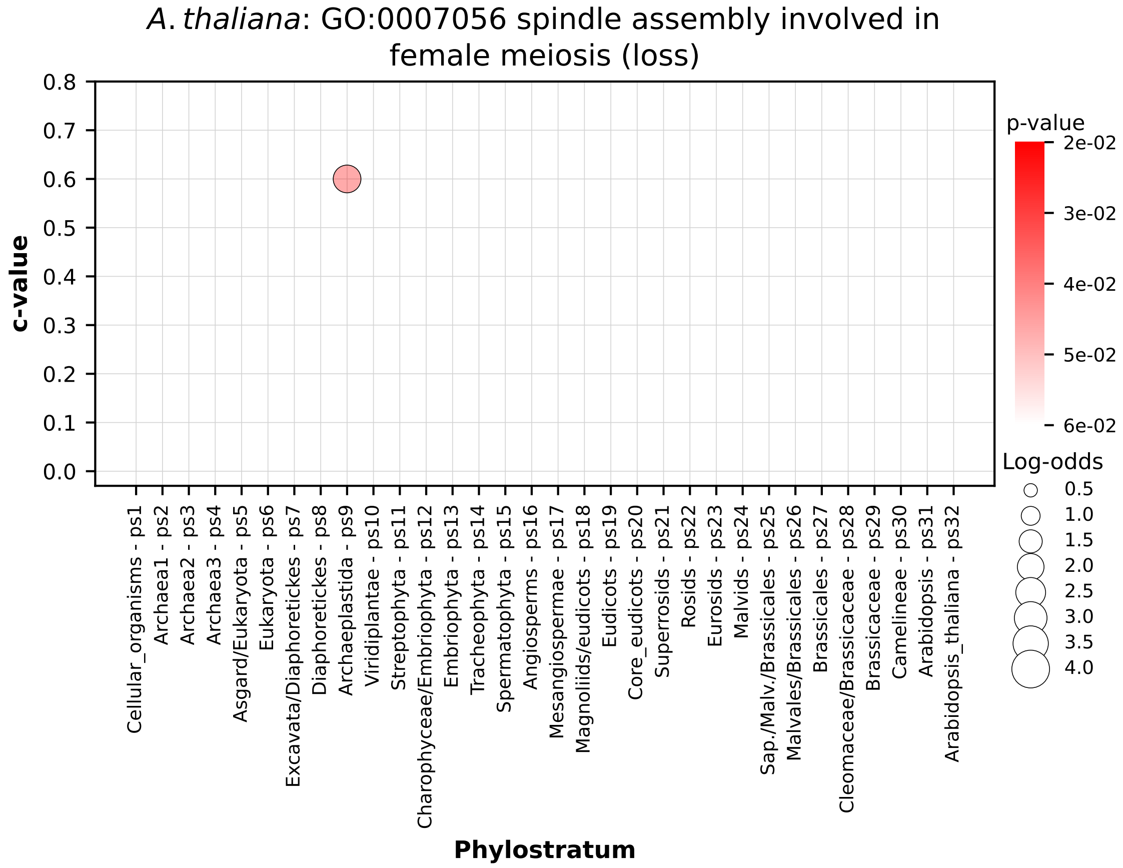

A. thaliana: GO:0007056 spindle assembly involved in female meiosis (loss)
c-value
0.0
0.1
0.2
0.3
0.4
0.5
0.6
0.7
0.8
Phylostratum
Cellular_organisms - ps1
Archaea1 - ps2
Archaea2 - ps3
Archaea3 - ps4
Asgard/Eukaryota - ps5
Eukaryota - ps6
Excavata/Diaphoretickes - ps7
Diaphoretickes - ps8
Archaeplastida - ps9
Viridiplantae - ps10
Streptophyta - ps11
Charophyceae/Embriophyta - ps12
Embriophyta - ps13
Tracheophyta - ps14
Spermatophyta - ps15
Angiosperms - ps16
Mesangiospermae - ps17
Magnoliids/eudicots - ps18
Eudicots - ps19
Core_eudicots - ps20
Superrosids - ps21
Rosids - ps22
Eurosids - ps23
Malvids - ps24
Sap./Malv./Brassicales - ps25
Malvales/Brassicales - ps26
Brassicales - ps27
Cleomaceae/Brassicaceae - ps28
Brassicaceae - ps29
Camelineae - ps30
Arabidopsis - ps31
Arabidopsis_thaliana - ps32
p-value
2e-02
3e-02
4e-02
5e-02
6e-02
Log-odds
0.5
1.0
1.5
2.0
2.5
3.0
3.5
4.0

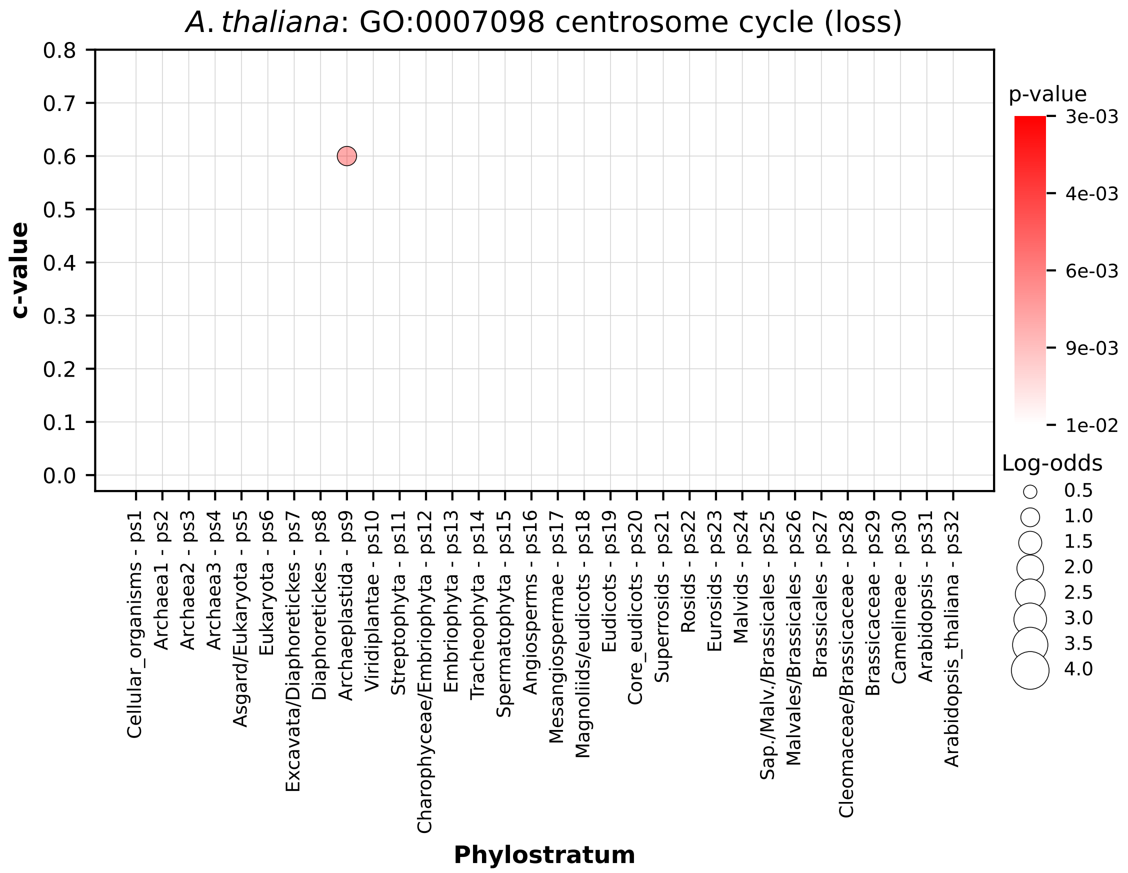

A. thaliana: GO:0007098 centrosome cycle (loss)
c-value
0.8
0.7
0.6
0.5
0.4
0.3
0.2
0.1
0.0
Phylostratum
Cellular_organisms - ps1
Archaea1 - ps2
Archaea2 - ps3
Archaea3 - ps4
Asgard/Eukaryota - ps5
Eukaryota - ps6
Excavata/Diaphoretickes - ps7
Diaphoretickes - ps8
Archaeplastida - ps9
Viridiplantae - ps10
Streptophyta - ps11
Charophyceae/Embriophyta - ps12
Embriophyta - ps13
Tracheophyta - ps14
Spermatophyta - ps15
Angiosperms - ps16
Mesangiospermae - ps17
Magnoliids/eudicots - ps18
Eudicots - ps19
Core_eudicots - ps20
Superrosids - ps21
Rosids - ps22
Eurosids - ps23
Malvids - ps24
Sap./Malv./Brassicales - ps25
Malvales/Brassicales - ps26
Brassicales - ps27
Cleomaceae/Brassicaceae - ps28
Brassicaceae - ps29
Camelineae - ps30
Arabidopsis - ps31
Arabidopsis_thaliana - ps32
p-value
3e-03
4e-03
6e-03
9e-03
1e-02
Log-odds
0.5
1.0
1.5
2.0
2.5
3.0
3.5
4.0

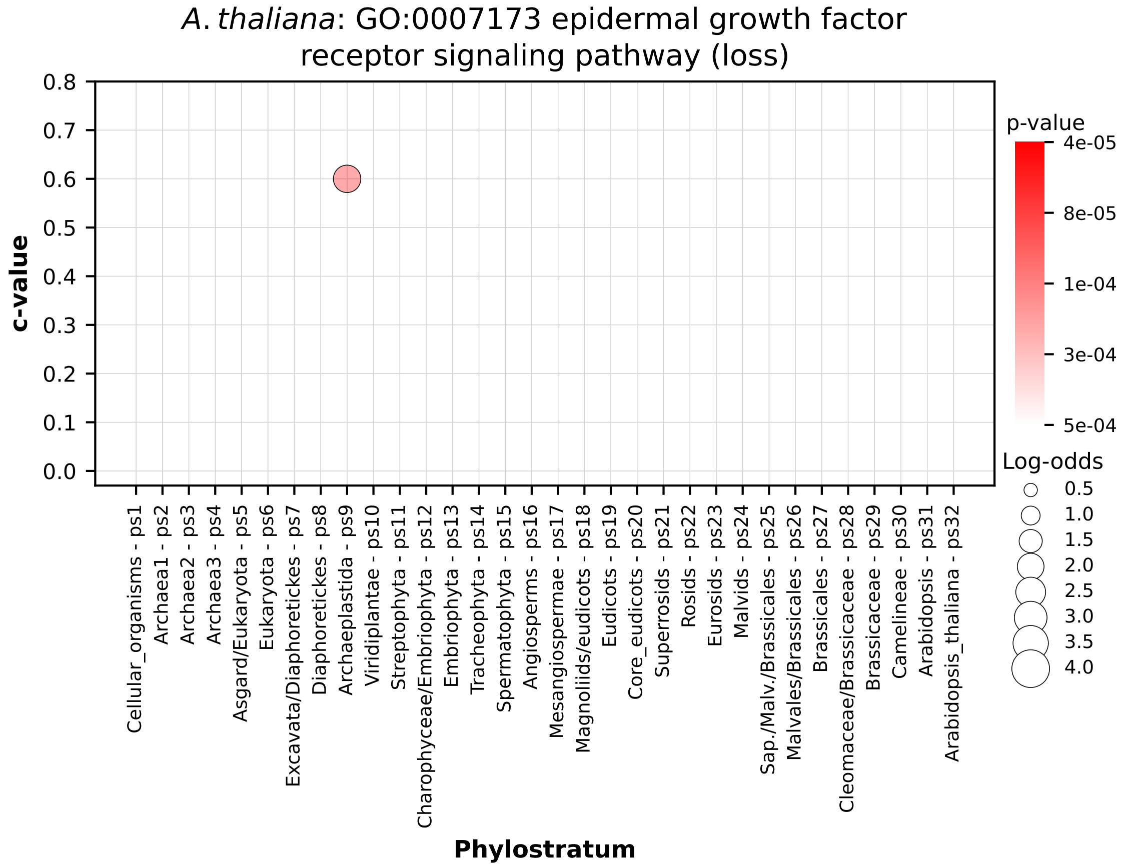

A. thaliana: GO:0007173 epidermal growth factor receptor signaling pathway (loss)
c-value
0.8
0.7
0.6
0.5
0.4
0.3
0.2
0.1
0.0
Phylostratum
Cellular_organisms - ps1
Archaea1 - ps2
Archaea2 - ps3
Archaea3 - ps4
Asgard/Eukaryota - ps5
Eukaryota - ps6
Excavata/Diaphoretickes - ps7
Diaphoretickes - ps8
Archaeplastida - ps9
Viridiplantae - ps10
Streptophyta - ps11
Charophyceae/Embriophyta - ps12
Embriophyta - ps13
Tracheophyta - ps14
Spermatophyta - ps15
Angiosperms - ps16
Mesangiospermae - ps17
Magnoliids/eudicots - ps18
Eudicots - ps19
Core_eudicots - ps20
Superrosids - ps21
Rosids - ps22
Eurosids - ps23
Malvids - ps24
Sap./Malv./Brassicales - ps25
Malvales/Brassicales - ps26
Brassicales - ps27
Cleomaceae/Brassicaceae - ps28
Brassicaceae - ps29
Camelineae - ps30
Arabidopsis - ps31
Arabidopsis_thaliana - ps32
p-value
4e-05
8e-05
1e-04
3e-04
5e-04
Log-odds
0.5
1.0
1.5
2.0
2.5
3.0
3.5
4.0

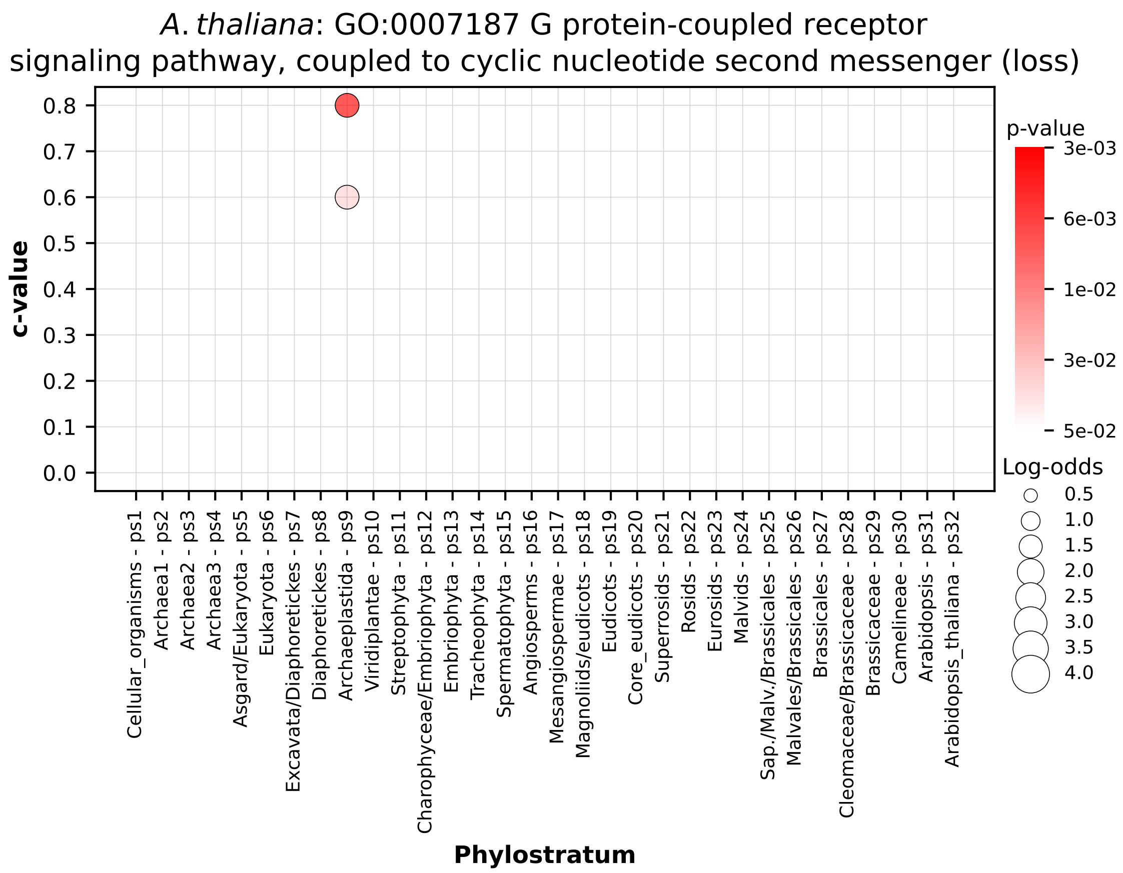

A. thaliana: GO:0007187 G protein-coupled receptor signaling pathway, coupled to cyclic nucleotide second messenger (loss)
c-value
0.8
0.7
0.6
0.5
0.4
0.3
0.2
0.1
0.0
p-value
3e-03
6e-03
1e-02
3e-02
5e-02
Log-odds
0.5
1.0
1.5
2.0
2.5
3.0
3.5
4.0
Cellular_organisms - ps1
Archaea1 - ps2
Archaea2 - ps3
Archaea3 - ps4
Asgard/Eukaryota - ps5
Eukaryota - ps6
Excavata/Diaphoretickes - ps7
Diaphoretickes - ps8
Archaeplastida - ps9
Viridiplantae - ps10
Streptophyta - ps11
Charophyceae/Embriophyta - ps12
Embriophyta - ps13
Tracheophyta - ps14
Spermatophyta - ps15
Angiosperms - ps16
Mesangiospermae - ps17
Magnoliids/eudicots - ps18
Eudicots - ps19
Core_eudicots - ps20
Superrosids - ps21
Rosids - ps22
Eurosids - ps23
Malvids - ps24
Sap./Malv./Brassicales - ps25
Malvales/Brassicales - ps26
Brassicales - ps27
Cleomaceae/Brassicaceae - ps28
Brassicaceae - ps29
Camelineae - ps30
Arabidopsis - ps31
Arabidopsis_thaliana - ps32
Phylostratum

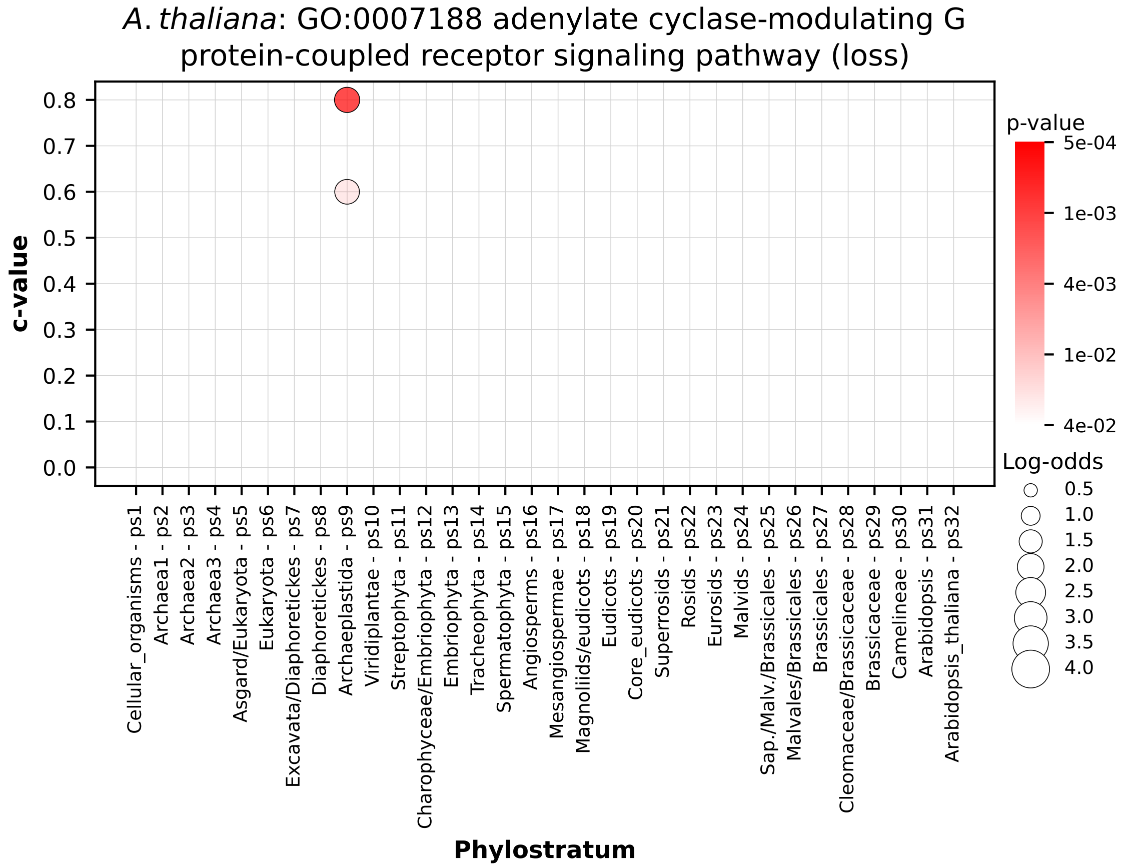

A. thaliana: GO:0007188 adenylate cyclase-modulating G protein-coupled receptor signaling pathway (loss)
c-value
0.8
0.7
0.6
0.5
0.4
0.3
0.2
0.1
0.0
p-value
5e-04
1e-03
4e-03
1e-02
4e-02
Log-odds
0.5
1.0
1.5
2.0
2.5
3.0
3.5
4.0
Cellular_organisms - ps1
Archaea1 - ps2
Archaea2 - ps3
Archaea3 - ps4
Asgard/Eukaryota - ps5
Eukaryota - ps6
Excavata/Diaphoretickes - ps7
Diaphoretickes - ps8
Archaeplastida - ps9
Viridiplantae - ps10
Streptophyta - ps11
Charophyceae/Embriophyta - ps12
Embriophyta - ps13
Tracheophyta - ps14
Spermatophyta - ps15
Angiosperms - ps16
Mesangiospermae - ps17
Magnoliids/eudicots - ps18
Eudicots - ps19
Core_eudicots - ps20
Superrosids - ps21
Rosids - ps22
Eurosids - ps23
Malvids - ps24
Sap./Malv./Brassicales - ps25
Malvales/Brassicales - ps26
Brassicales - ps27
Cleomaceae/Brassicaceae - ps28
Brassicaceae - ps29
Camelineae - ps30
Arabidopsis - ps31
Arabidopsis_thaliana - ps32
Phylostratum

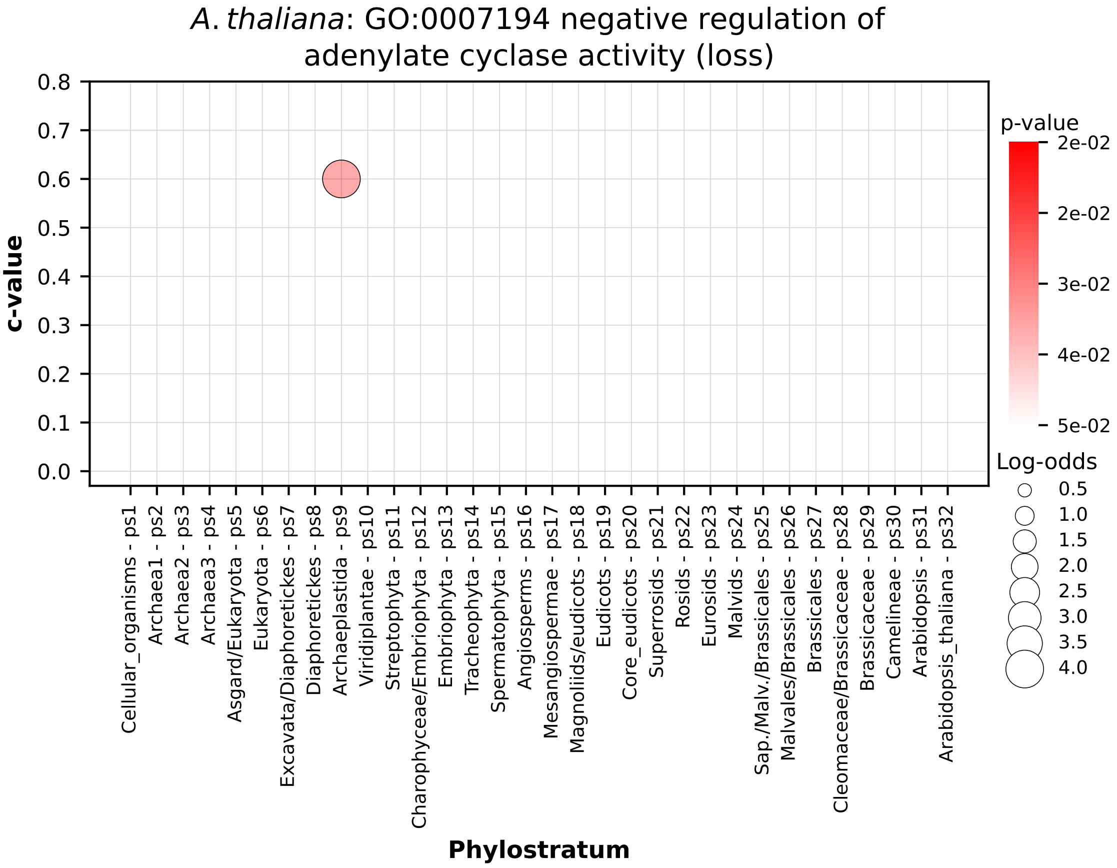

A. thaliana: GO:0007194 negative regulation of adenylate cyclase activity (loss)
c-value
0.8
0.7
0.6
0.5
0.4
0.3
0.2
0.1
0.0
p-value
2e-02
2e-02
3e-02
4e-02
5e-02
Log-odds
0.5
1.0
1.5
2.0
2.5
3.0
3.5
4.0
Cellular_organisms - ps1
Archaea1 - ps2
Archaea2 - ps3
Archaea3 - ps4
Asgard/Eukaryota - ps5
Eukaryota - ps6
Excavata/Diaphoretickes - ps7
Diaphoretickes - ps8
Archaeplastida - ps9
Viridiplantae - ps10
Streptophyta - ps11
Charophyceae/Embriophyta - ps12
Embriophyta - ps13
Tracheophyta - ps14
Spermatophyta - ps15
Angiosperms - ps16
Mesangiospermae - ps17
Magnoliids/eudicots - ps18
Eudicots - ps19
Core_eudicots - ps20
Superrosids - ps21
Rosids - ps22
Eurosids - ps23
Malvids - ps24
Sap./Malv./Brassicales - ps25
Malvales/Brassicales - ps26
Brassicales - ps27
Cleomaceae/Brassicaceae - ps28
Brassicaceae - ps29
Camelineae - ps30
Arabidopsis - ps31
Arabidopsis_thaliana - ps32
Phylostratum

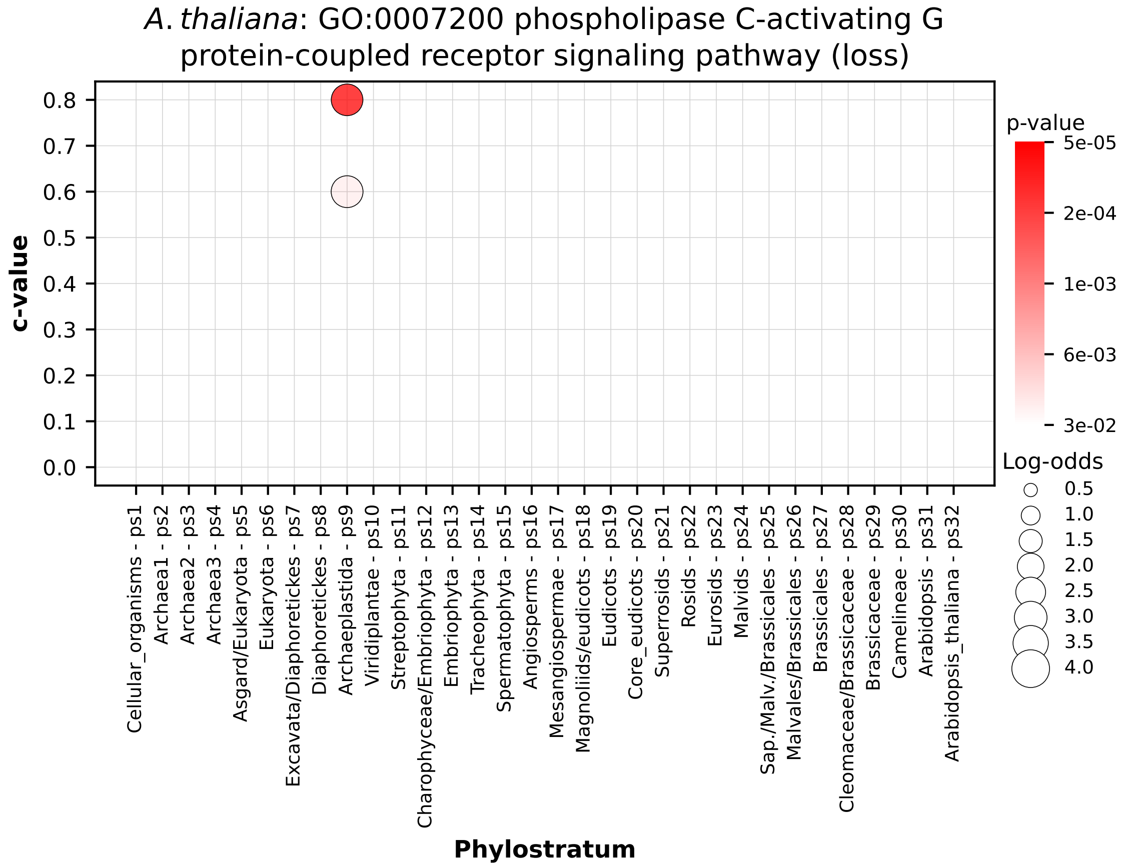

A. thaliana: GO:0007200 phospholipase C-activating G protein-coupled receptor signaling pathway (loss)
c-value
0.0
0.1
0.2
0.3
0.4
0.5
0.6
0.7
0.8
p-value
5e-05
2e-04
1e-03
6e-03
3e-02
Log-odds
0.5
1.0
1.5
2.0
2.5
3.0
3.5
4.0
Cellular_organisms - ps1
Archaea1 - ps2
Archaea2 - ps3
Archaea3 - ps4
Asgard/Eukaryota - ps5
Eukaryota - ps6
Excavata/Diaphoretickes - ps7
Diaphoretickes - ps8
Archaeplastida - ps9
Viridiplantae - ps10
Streptophyta - ps11
Charophyceae/Embriophyta - ps12
Embriophyta - ps13
Tracheophyta - ps14
Spermatophyta - ps15
Angiosperms - ps16
Mesangiospermae - ps17
Magnoliids/eudicots - ps18
Eudicots - ps19
Core_eudicots - ps20
Superrosids - ps21
Rosids - ps22
Eurosids - ps23
Malvids - ps24
Sap./Malv./Brassicales - ps25
Malvales/Brassicales - ps26
Brassicales - ps27
Cleomaceae/Brassicaceae - ps28
Brassicaceae - ps29
Camelineae - ps30
Arabidopsis - ps31
Arabidopsis_thaliana - ps32
Phylostratum

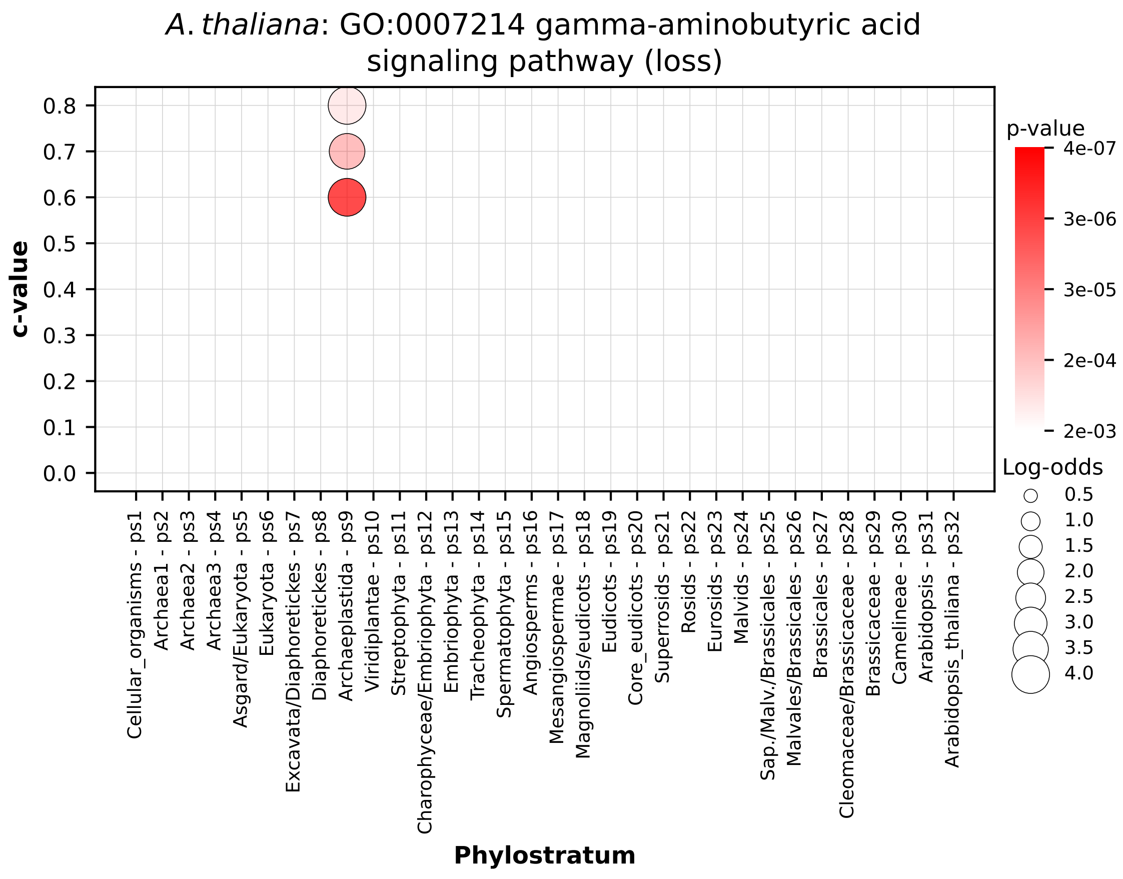
A. thaliana: GO:0007214 gamma-aminobutyric acid signaling pathway (loss)
c-value
0.0
0.1
0.2
0.3
0.4
0.5
0.6
0.7
0.8
p-value
4e-07
3e-06
3e-05
2e-04
2e-03
Log-odds
0.5
1.0
1.5
2.0
2.5
3.0
3.5
4.0
Cellular_organisms - ps1
Archaea1 - ps2
Archaea2 - ps3
Archaea3 - ps4
Asgard/Eukaryota - ps5
Eukaryota - ps6
Excavata/Diaphoretickes - ps7
Diaphoretickes - ps8
Archaeplastida - ps9
Viridiplantae - ps10
Streptophyta - ps11
Charophyceae/Embriophyta - ps12
Embriophyta - ps13
Tracheophyta - ps14
Spermatophyta - ps15
Angiosperms - ps16
Mesangiospermae - ps17
Magnoliids/eudicots - ps18
Eudicots - ps19
Core_eudicots - ps20
Superrosids - ps21
Rosids - ps22
Eurosids - ps23
Malvids - ps24
Sap./Malv./Brassicales - ps25
Malvales/Brassicales - ps26
Brassicales - ps27
Cleomaceae/Brassicaceae - ps28
Brassicaceae - ps29
Camelineae - ps30
Arabidopsis - ps31
Arabidopsis_thaliana - ps32
Phylostratum

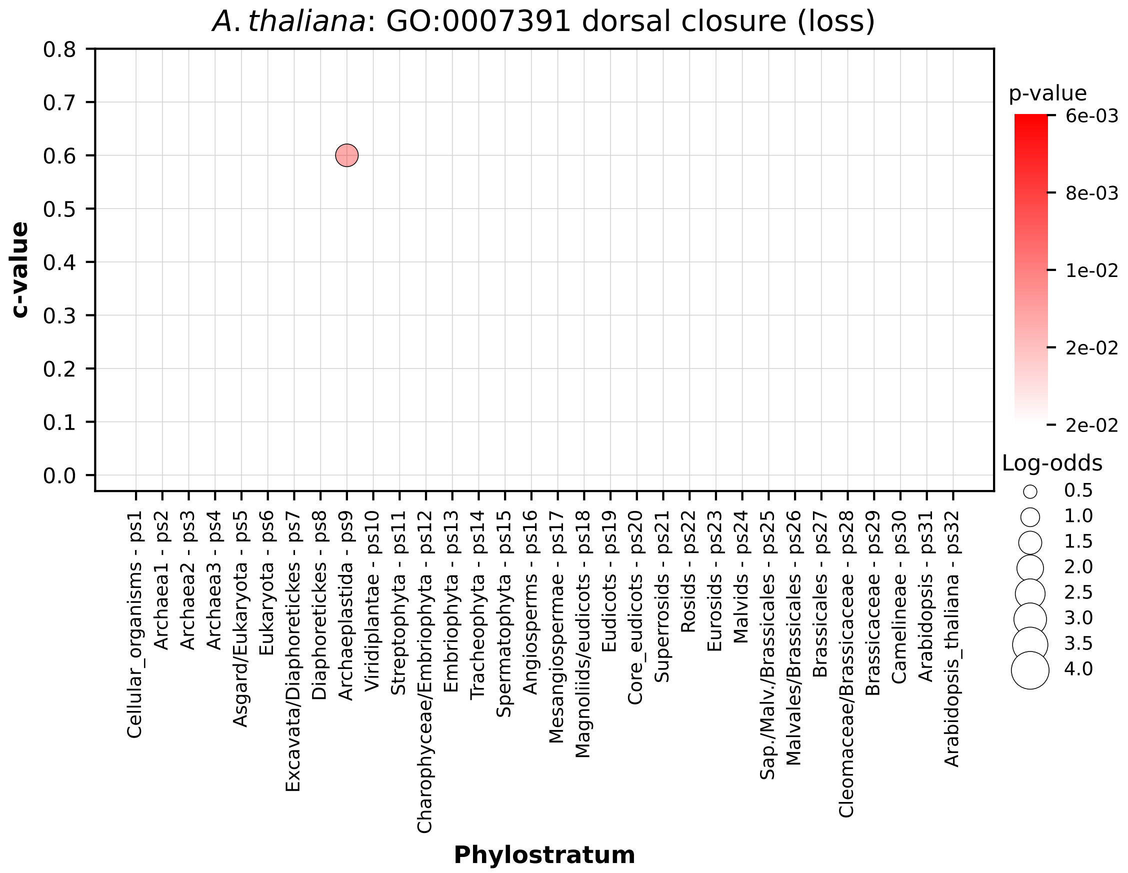
A. thaliana: GO:0007391 dorsal closure (loss)
c-value
0.8
0.7
0.6
0.5
0.4
0.3
0.2
0.1
0.0
p-value
6e-03
8e-03
1e-02
2e-02
2e-02
Log-odds
0.5
1.0
1.5
2.0
2.5
3.0
3.5
4.0
Cellular_organisms - ps1
Archaea1 - ps2
Archaea2 - ps3
Archaea3 - ps4
Asgard/Eukaryota - ps5
Eukaryota - ps6
Excavata/Diaphoretickes - ps7
Diaphoretickes - ps8
Archaeplastida - ps9
Viridiplantae - ps10
Streptophyta - ps11
Charophyceae/Embriophyta - ps12
Embriophyta - ps13
Tracheophyta - ps14
Spermatophyta - ps15
Angiosperms - ps16
Mesangiospermae - ps17
Magnoliids/eudicots - ps18
Eudicots - ps19
Core_eudicots - ps20
Superrosids - ps21
Rosids - ps22
Eurosids - ps23
Malvids - ps24
Sap./Malv./Brassicales - ps25
Malvales/Brassicales - ps26
Brassicales - ps27
Cleomaceae/Brassicaceae - ps28
Brassicaceae - ps29
Camelineae - ps30
Arabidopsis - ps31
Arabidopsis_thaliana - ps32
Phylostratum

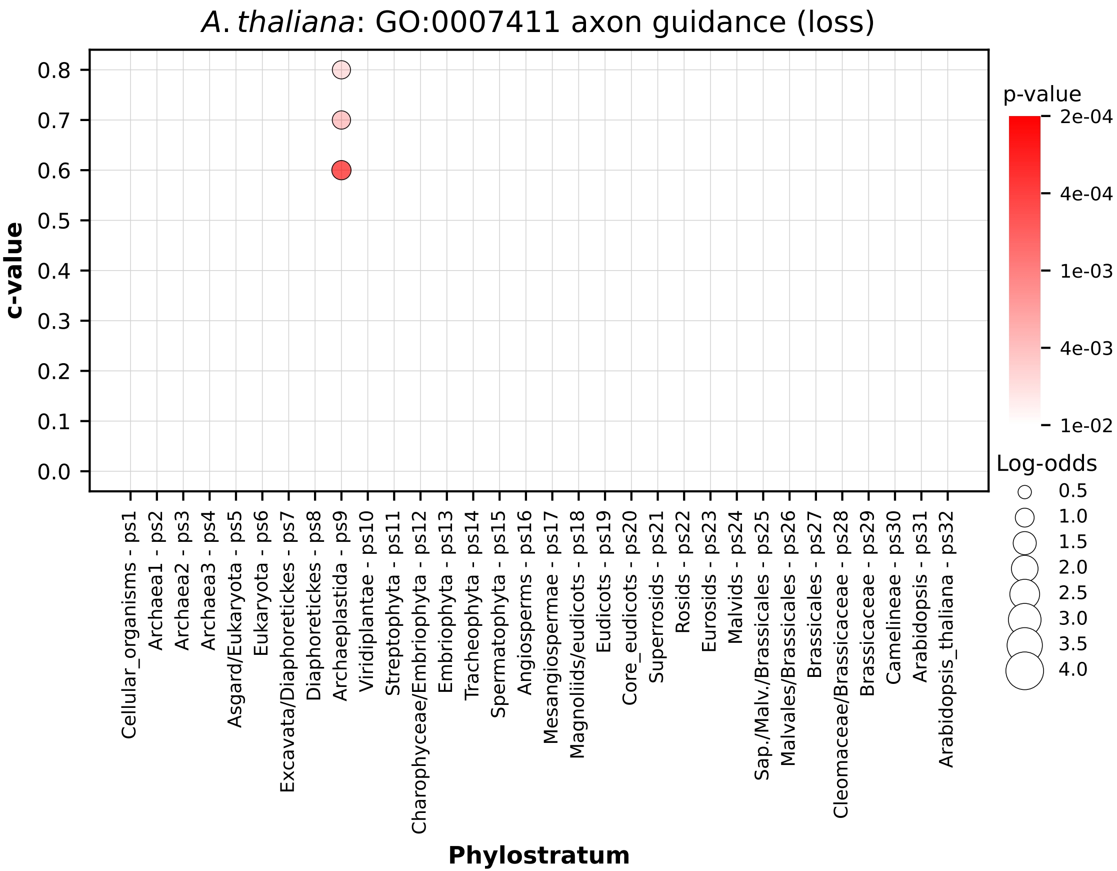

A. thaliana: GO:0007411 axon guidance (loss)
c-value
0.0
0.1
0.2
0.3
0.4
0.5
0.6
0.7
0.8
p-value
2e-04
4e-04
1e-03
4e-03
1e-02
Log-odds
0.5
1.0
1.5
2.0
2.5
3.0
3.5
4.0
Cellular_organisms - ps1
Archaea1 - ps2
Archaea2 - ps3
Archaea3 - ps4
Asgard/Eukaryota - ps5
Eukaryota - ps6
Excavata/Diaphoretickes - ps7
Diaphoretickes - ps8
Archaeplastida - ps9
Viridiplantae - ps10
Streptophyta - ps11
Charophyceae/Embriophyta - ps12
Embriophyta - ps13
Tracheophyta - ps14
Spermatophyta - ps15
Angiosperms - ps16
Mesangiospermae - ps17
Magnoliids/eudicots - ps18
Eudicots - ps19
Core_eudicots - ps20
Superrosids - ps21
Rosids - ps22
Eurosids - ps23
Malvids - ps24
Sap./Malv./Brassicales - ps25
Malvales/Brassicales - ps26
Brassicales - ps27
Cleomaceae/Brassicaceae - ps28
Brassicaceae - ps29
Camelineae - ps30
Arabidopsis - ps31
Arabidopsis_thaliana - ps32
Phylostratum

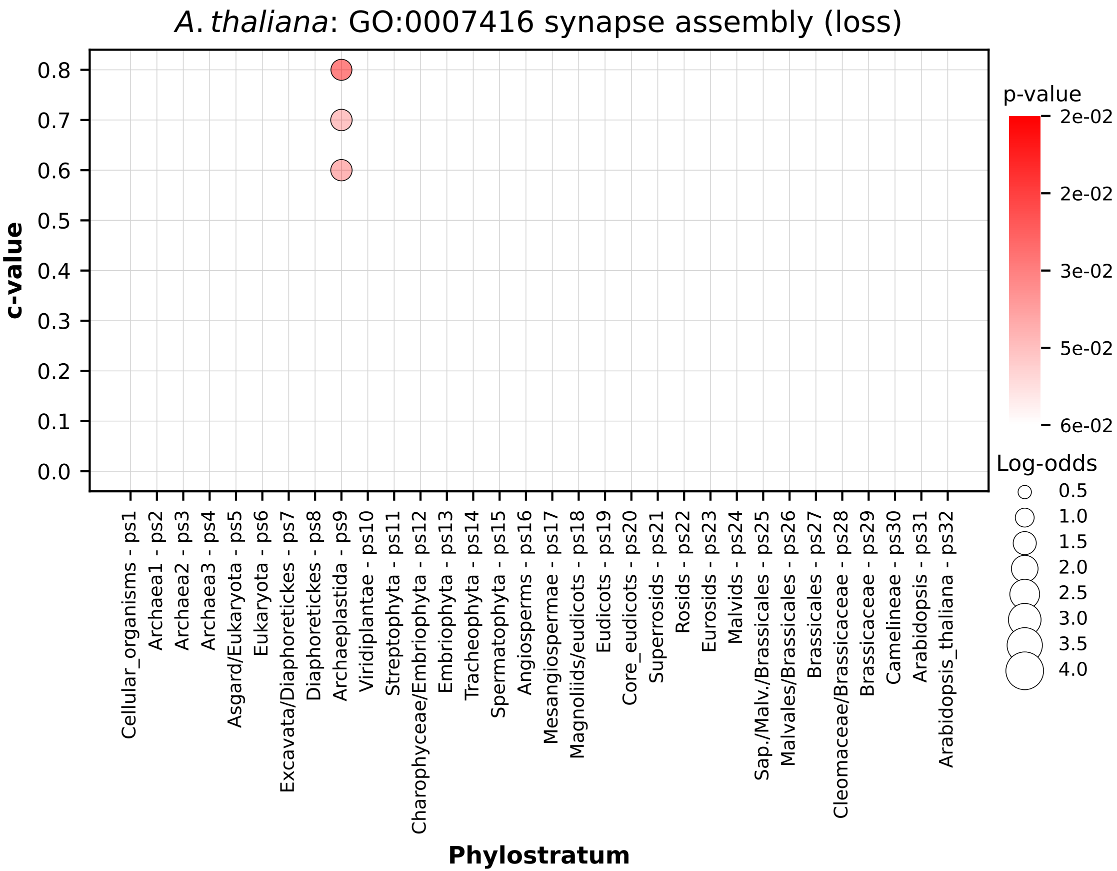

A. thaliana: GO:0007416 synapse assembly (loss)
c-value
0.8
0.7
0.6
0.5
0.4
0.3
0.2
0.1
0.0
p-value
2e-02
2e-02
3e-02
5e-02
6e-02
Log-odds
0.5
1.0
1.5
2.0
2.5
3.0
3.5
4.0
Cellular_organisms - ps1
Archaea1 - ps2
Archaea2 - ps3
Archaea3 - ps4
Asgard/Eukaryota - ps5
Eukaryota - ps6
Excavata/Diaphoretickes - ps7
Diaphoretickes - ps8
Archaeplastida - ps9
Viridiplantae - ps10
Streptophyta - ps11
Charophyceae/Embriophyta - ps12
Embriophyta - ps13
Tracheophyta - ps14
Spermatophyta - ps15
Angiosperms - ps16
Mesangiospermae - ps17
Magnoliids/eudicots - ps18
Eudicots - ps19
Core_eudicots - ps20
Superrosids - ps21
Rosids - ps22
Eurosids - ps23
Malvids - ps24
Sap./Malv./Brassicales - ps25
Malvales/Brassicales - ps26
Brassicales - ps27
Cleomaceae/Brassicaceae - ps28
Brassicaceae - ps29
Camelineae - ps30
Arabidopsis - ps31
Arabidopsis_thaliana - ps32
Phylostratum

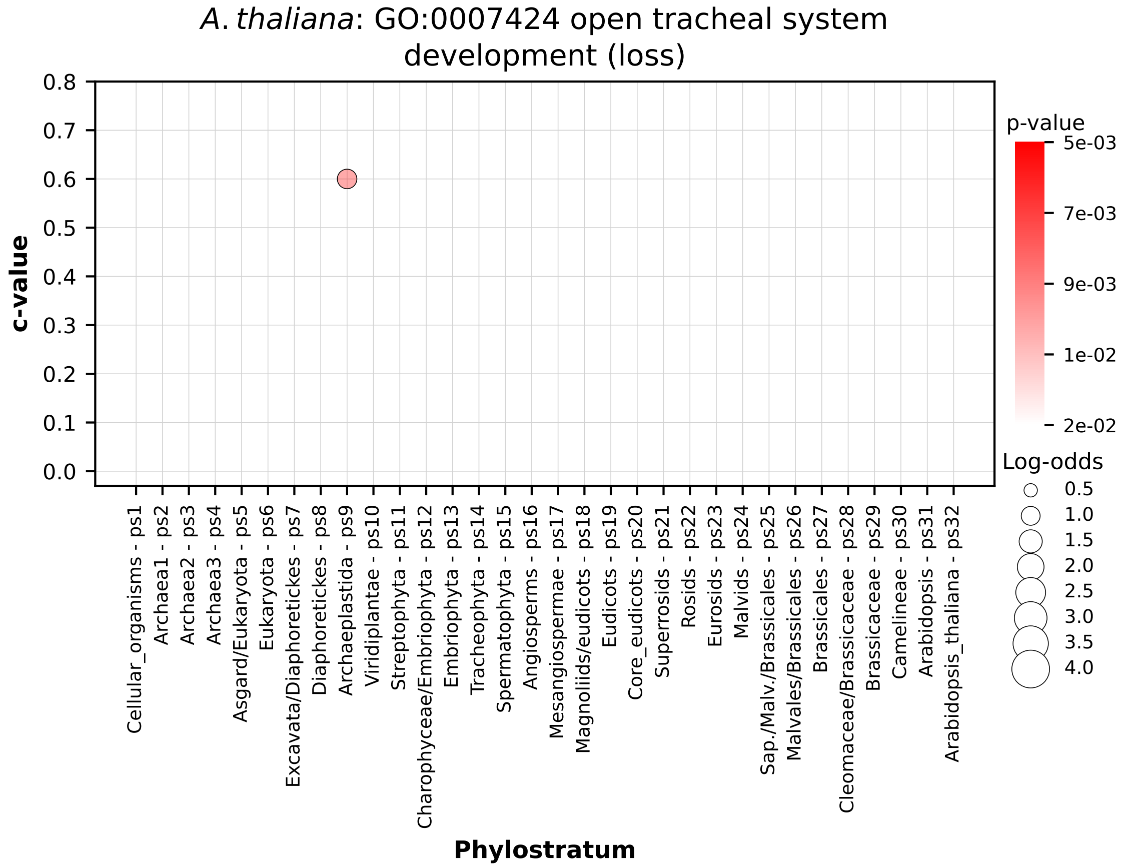

A. thaliana: GO:0007424 open tracheal system development (loss)
c-value
0.8
0.7
0.6
0.5
0.4
0.3
0.2
0.1
0.0
p-value
5e-03
7e-03
9e-03
1e-02
2e-02
Log-odds
0.5
1.0
1.5
2.0
2.5
3.0
3.5
4.0
Cellular_organisms - ps1
Archaea1 - ps2
Archaea2 - ps3
Archaea3 - ps4
Asgard/Eukaryota - ps5
Eukaryota - ps6
Excavata/Diaphoretickes - ps7
Diaphoretickes - ps8
Archaeplastida - ps9
Viridiplantae - ps10
Streptophyta - ps11
Charophyceae/Embriophyta - ps12
Embriophyta - ps13
Tracheophyta - ps14
Spermatophyta - ps15
Angiosperms - ps16
Mesangiospermae - ps17
Magnoliids/eudicots - ps18
Eudicots - ps19
Core_eudicots - ps20
Superrosids - ps21
Rosids - ps22
Eurosids - ps23
Malvids - ps24
Sap./Malv./Brassicales - ps25
Malvales/Brassicales - ps26
Brassicales - ps27
Cleomaceae/Brassicaceae - ps28
Brassicaceae - ps29
Camelineae - ps30
Arabidopsis - ps31
Arabidopsis_thaliana - ps32
Phylostratum

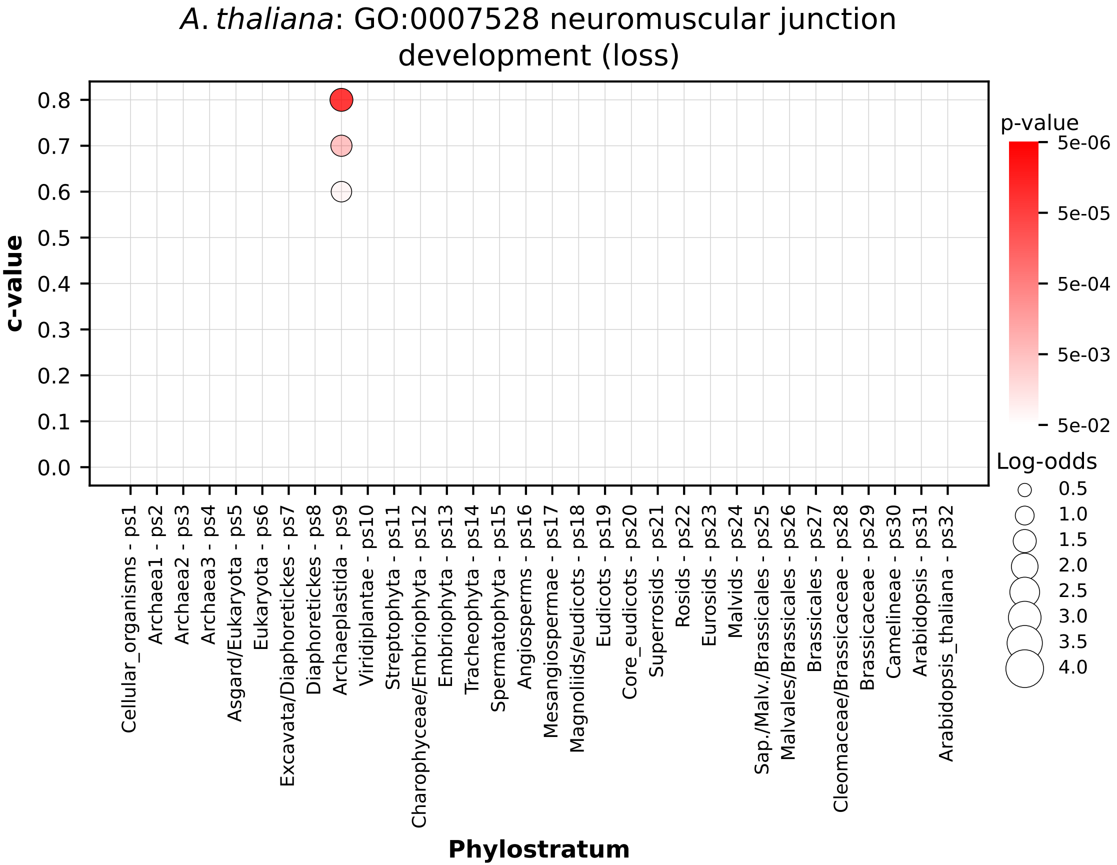

A. thaliana: GO:0007528 neuromuscular junction development (loss)
c-value
0.8
0.7
0.6
0.5
0.4
0.3
0.2
0.1
0.0
p-value
5e-06
5e-05
5e-04
5e-03
5e-02
Log-odds
0.5
1.0
1.5
2.0
2.5
3.0
3.5
4.0
Cellular_organisms - ps1
Archaea1 - ps2
Archaea2 - ps3
Archaea3 - ps4
Asgard/Eukaryota - ps5
Eukaryota - ps6
Excavata/Diaphoretickes - ps7
Diaphoretickes - ps8
Archaeplastida - ps9
Viridiplantae - ps10
Streptophyta - ps11
Charophyceae/Embriophyta - ps12
Embriophyta - ps13
Tracheophyta - ps14
Spermatophyta - ps15
Angiosperms - ps16
Mesangiospermae - ps17
Magnoliids/eudicots - ps18
Eudicots - ps19
Core_eudicots - ps20
Superrosids - ps21
Rosids - ps22
Eurosids - ps23
Malvids - ps24
Sap./Malv./Brassicales - ps25
Malvales/Brassicales - ps26
Brassicales - ps27
Cleomaceae/Brassicaceae - ps28
Brassicaceae - ps29
Camelineae - ps30
Arabidopsis - ps31
Arabidopsis_thaliana - ps32
Phylostratum

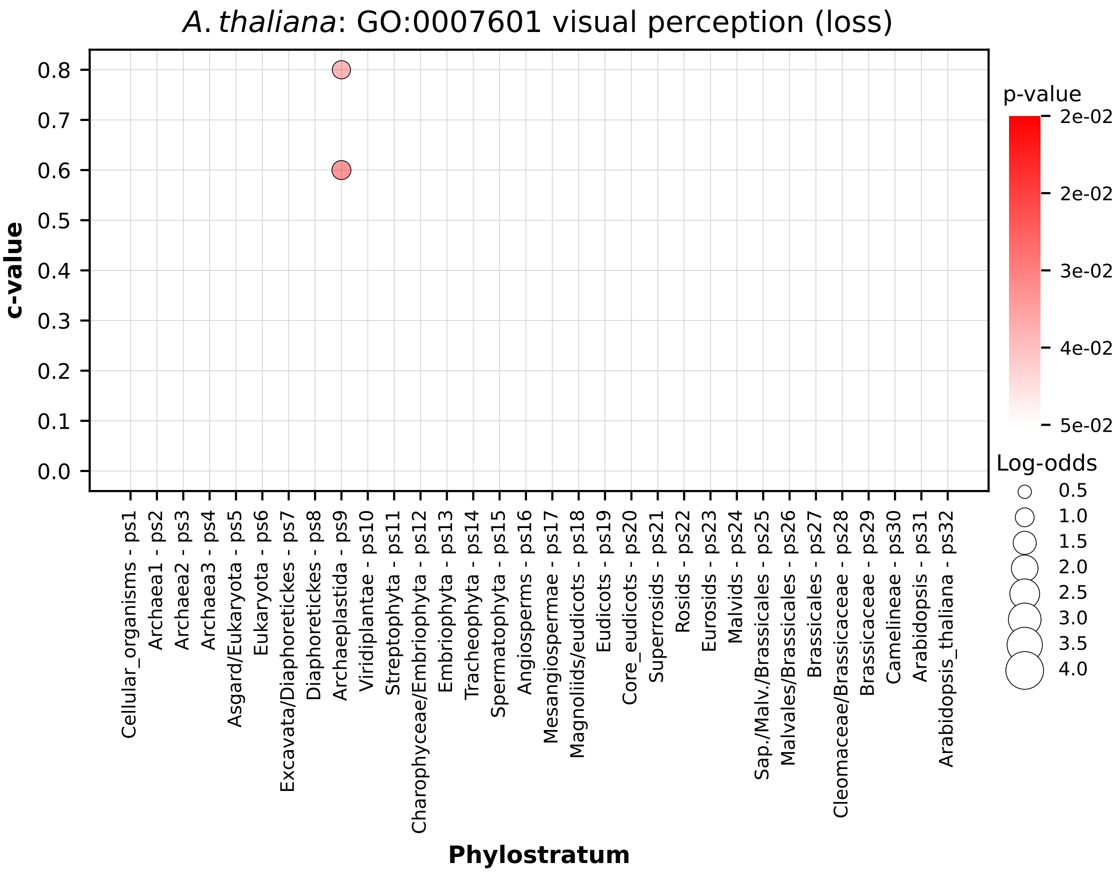
A. thaliana: GO:0007601 visual perception (loss)
c-value
0.0
0.1
0.2
0.3
0.4
0.5
0.6
0.7
0.8
Phylostratum
Cellular_organisms - ps1
Archaea1 - ps2
Archaea2 - ps3
Archaea3 - ps4
Asgard/Eukaryota - ps5
Eukaryota - ps6
Excavata/Diaphoretickes - ps7
Diaphoretickes - ps8
Archaeplastida - ps9
Viridiplantae - ps10
Streptophyta - ps11
Charophyceae/Embriophyta - ps12
Embriophyta - ps13
Tracheophyta - ps14
Spermatophyta - ps15
Angiosperms - ps16
Mesangiospermae - ps17
Magnoliids/eudicots - ps18
Eudicots - ps19
Core_eudicots - ps20
Superrosids - ps21
Rosids - ps22
Eurosids - ps23
Malvids - ps24
Sap./Malv./Brassicales - ps25
Malvales/Brassicales - ps26
Brassicales - ps27
Cleomaceae/Brassicaceae - ps28
Brassicaceae - ps29
Camelineae - ps30
Arabidopsis - ps31
Arabidopsis_thaliana - ps32
p-value
2e-02
2e-02
3e-02
4e-02
5e-02
Log-odds
0.5
1.0
1.5
2.0
2.5
3.0
3.5
4.0

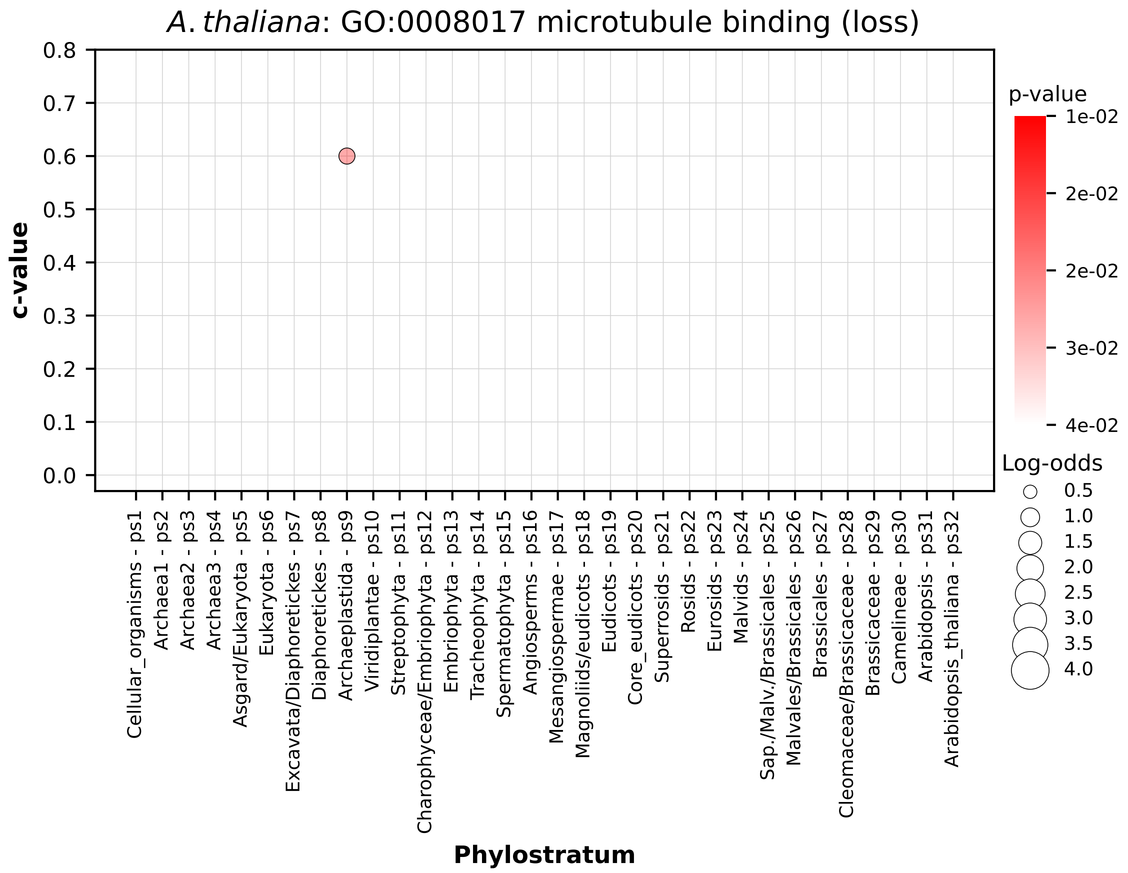

A. thaliana: GO:0008017 microtubule binding (loss)
c-value
0.8
0.7
0.6
0.5
0.4
0.3
0.2
0.1
0.0
Phylostratum
Cellular_organisms - ps1
Archaea1 - ps2
Archaea2 - ps3
Archaea3 - ps4
Asgard/Eukaryota - ps5
Eukaryota - ps6
Excavata/Diaphoretickes - ps7
Diaphoretickes - ps8
Archaeplastida - ps9
Viridiplantae - ps10
Streptophyta - ps11
Charophyceae/Embriophyta - ps12
Embriophyta - ps13
Tracheophyta - ps14
Spermatophyta - ps15
Angiosperms - ps16
Mesangiospermae - ps17
Magnoliids/eudicots - ps18
Eudicots - ps19
Core_eudicots - ps20
Superrosids - ps21
Rosids - ps22
Eurosids - ps23
Malvids - ps24
Sap./Malv./Brassicales - ps25
Malvales/Brassicales - ps26
Brassicales - ps27
Cleomaceae/Brassicaceae - ps28
Brassicaceae - ps29
Camelineae - ps30
Arabidopsis - ps31
Arabidopsis_thaliana - ps32
p-value
1e-02
2e-02
2e-02
3e-02
4e-02
Log-odds
0.5
1.0
1.5
2.0
2.5
3.0
3.5
4.0

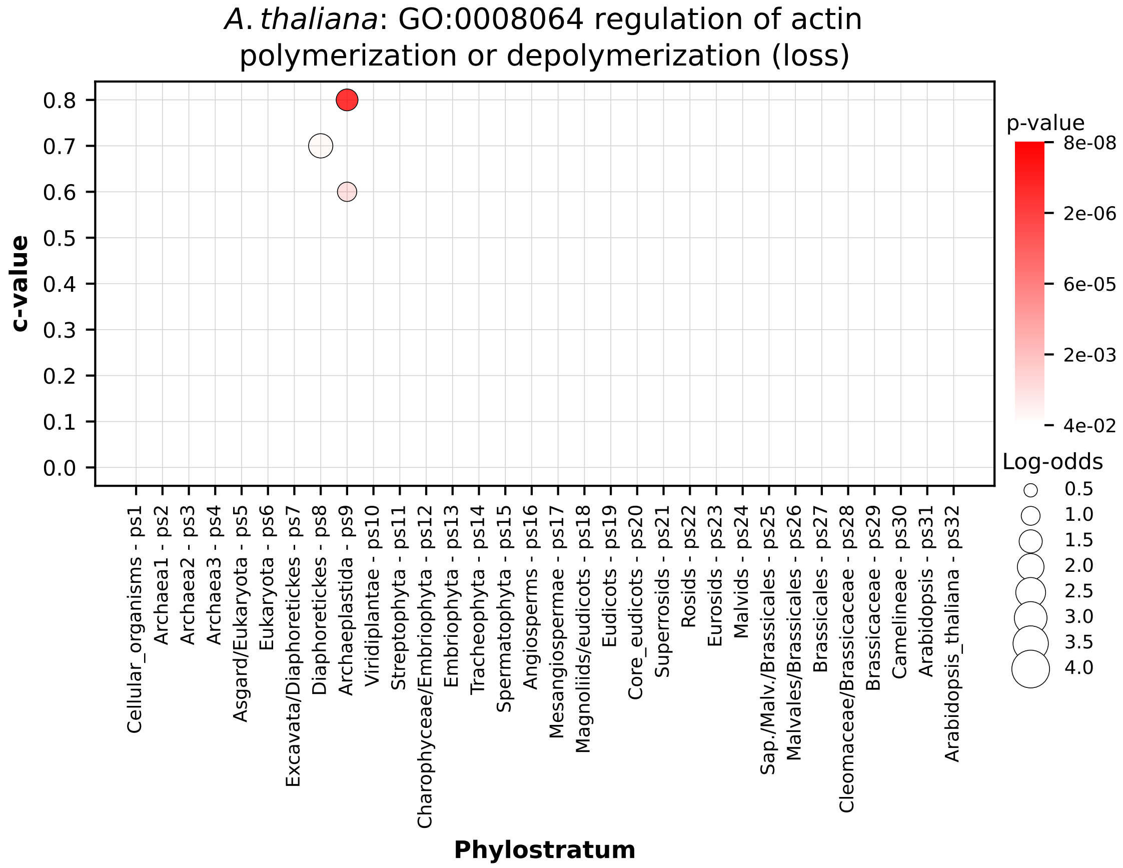

A. thaliana: GO:0008064 regulation of actin polymerization or depolymerization (loss)
c-value
0.8
0.7
0.6
0.5
0.4
0.3
0.2
0.1
0.0
Phylostratum
Cellular_organisms - ps1
Archaea1 - ps2
Archaea2 - ps3
Archaea3 - ps4
Asgard/Eukaryota - ps5
Eukaryota - ps6
Excavata/Diaphoretickes - ps7
Diaphoretickes - ps8
Archaeplastida - ps9
Viridiplantae - ps10
Streptophyta - ps11
Charophyceae/Embriophyta - ps12
Embriophyta - ps13
Tracheophyta - ps14
Spermatophyta - ps15
Angiosperms - ps16
Mesangiospermae - ps17
Magnoliids/eudicots - ps18
Eudicots - ps19
Core_eudicots - ps20
Superrosids - ps21
Rosids - ps22
Eurosids - ps23
Malvids - ps24
Sap./Malv./Brassicales - ps25
Malvales/Brassicales - ps26
Brassicales - ps27
Cleomaceae/Brassicaceae - ps28
Brassicaceae - ps29
Camelineae - ps30
Arabidopsis - ps31
Arabidopsis_thaliana - ps32
p-value
8e-08
2e-06
6e-05
2e-03
4e-02
Log-odds
0.5
1.0
1.5
2.0
2.5
3.0
3.5
4.0

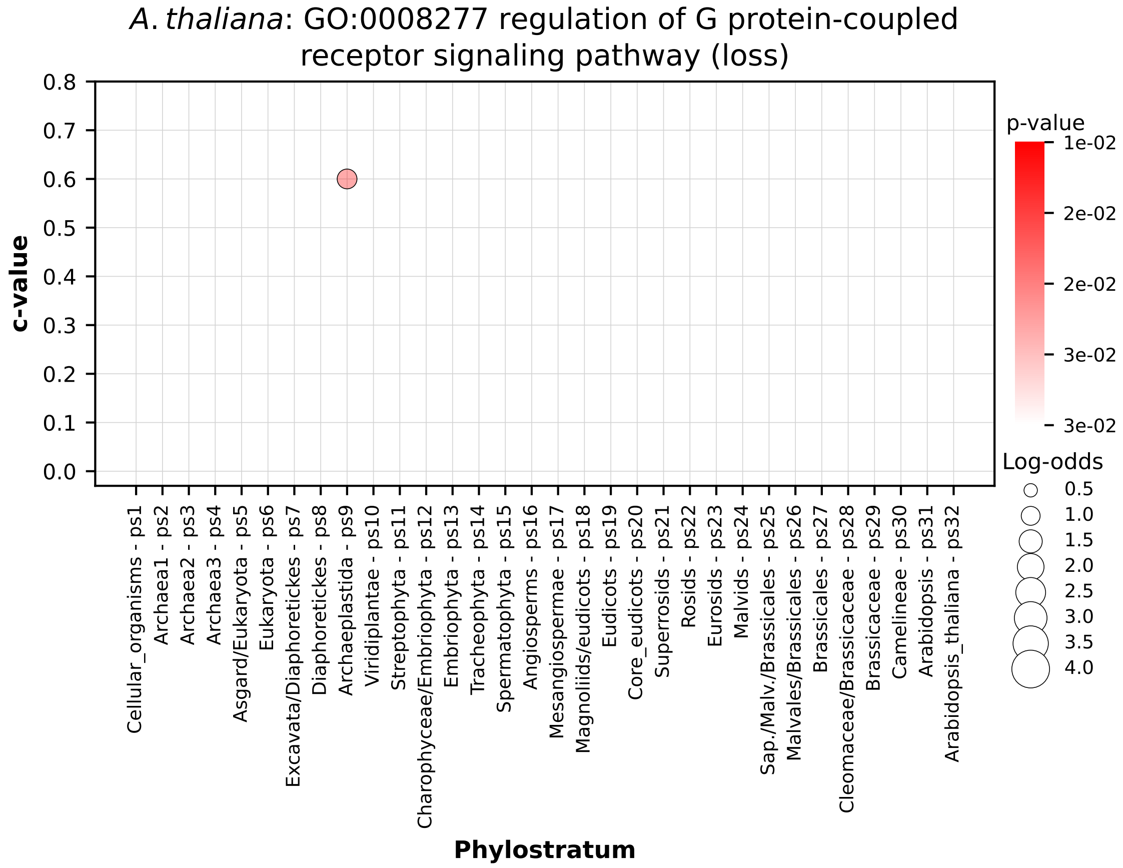
A. thaliana: GO:0008277 regulation of G protein-coupled receptor signaling pathway (loss)
c-value
0.8
0.7
0.6
0.5
0.4
0.3
0.2
0.1
0.0
Phylostratum
Cellular_organisms - ps1
Archaea1 - ps2
Archaea2 - ps3
Archaea3 - ps4
Asgard/Eukaryota - ps5
Eukaryota - ps6
Excavata/Diaphoretickes - ps7
Diaphoretickes - ps8
Archaeplastida - ps9
Viridiplantae - ps10
Streptophyta - ps11
Charophyceae/Embriophyta - ps12
Embriophyta - ps13
Tracheophyta - ps14
Spermatophyta - ps15
Angiosperms - ps16
Mesangiospermae - ps17
Magnoliids/eudicots - ps18
Eudicots - ps19
Core_eudicots - ps20
Superrosids - ps21
Rosids - ps22
Eurosids - ps23
Malvids - ps24
Sap./Malv./Brassicales - ps25
Malvales/Brassicales - ps26
Brassicales - ps27
Cleomaceae/Brassicaceae - ps28
Brassicaceae - ps29
Camelineae - ps30
Arabidopsis - ps31
Arabidopsis_thaliana - ps32
p-value
1e-02
2e-02
2e-02
3e-02
3e-02
Log-odds
0.5
1.0
1.5
2.0
2.5
3.0
3.5
4.0

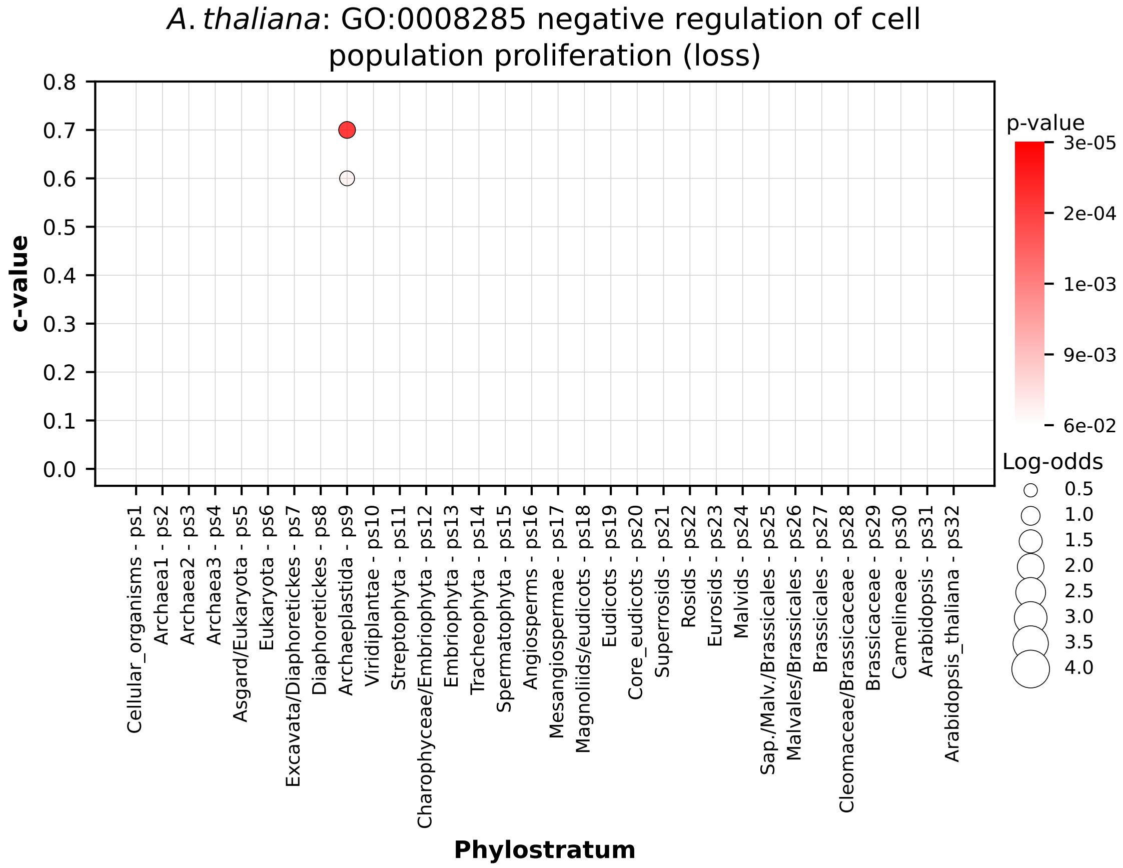

A. thaliana: GO:0008285 negative regulation of cell population proliferation (loss)
c-value
0.8
0.7
0.6
0.5
0.4
0.3
0.2
0.1
0.0
Phylostratum
Cellular_organisms - ps1
Archaea1 - ps2
Archaea2 - ps3
Archaea3 - ps4
Asgard/Eukaryota - ps5
Eukaryota - ps6
Excavata/Diaphoretickes - ps7
Diaphoretickes - ps8
Archaeplastida - ps9
Viridiplantae - ps10
Streptophyta - ps11
Charophyceae/Embriophyta - ps12
Embriophyta - ps13
Tracheophyta - ps14
Spermatophyta - ps15
Angiosperms - ps16
Mesangiospermae - ps17
Magnoliids/eudicots - ps18
Eudicots - ps19
Core_eudicots - ps20
Superrosids - ps21
Rosids - ps22
Eurosids - ps23
Malvids - ps24
Sap./Malv./Brassicales - ps25
Malvales/Brassicales - ps26
Brassicales - ps27
Cleomaceae/Brassicaceae - ps28
Brassicaceae - ps29
Camelineae - ps30
Arabidopsis - ps31
Arabidopsis_thaliana - ps32
p-value
3e-05
2e-04
1e-03
9e-03
6e-02
Log-odds
0.5
1.0
1.5
2.0
2.5
3.0
3.5
4.0

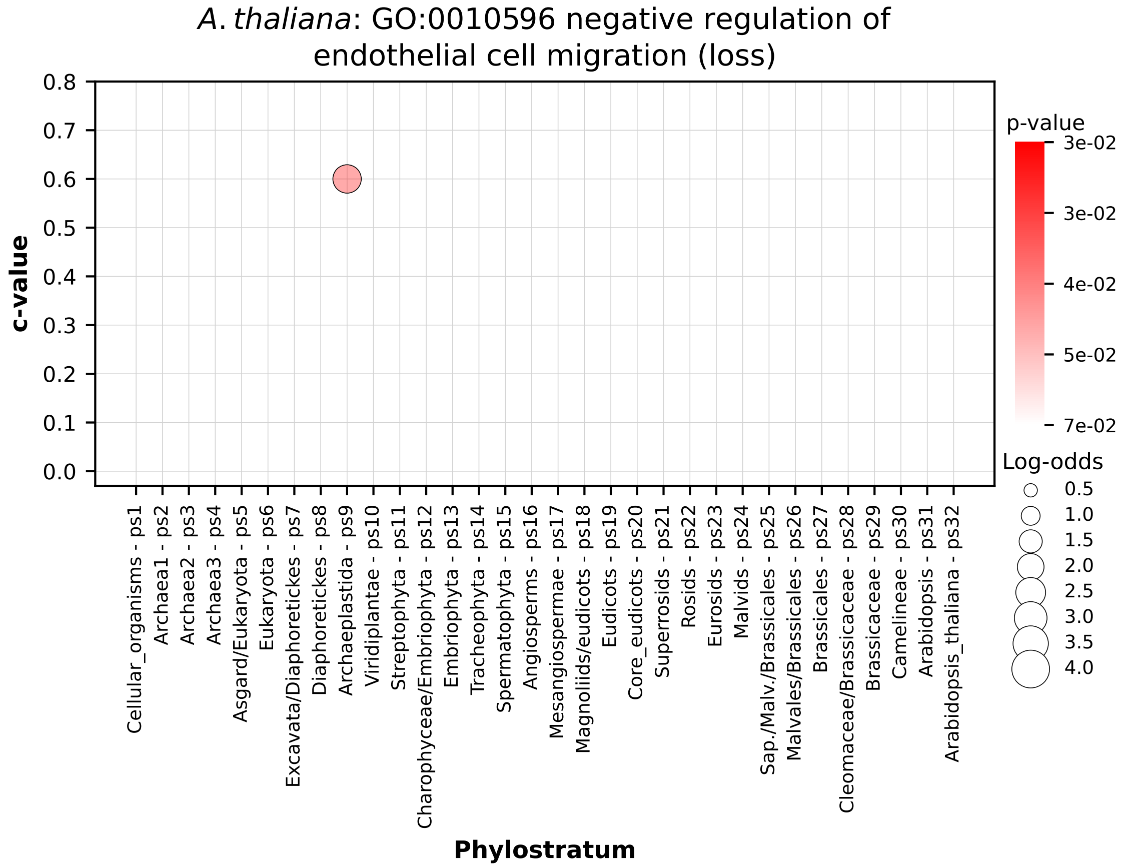

$A. thaliana$: GO:0010596 negative regulation of endothelial cell migration (loss)
c-value
0.8
0.7
0.6
0.5
0.4
0.3
0.2
0.1
0.0
p-value
3e-02
3e-02
4e-02
5e-02
7e-02
Log-odds
0.5
1.0
1.5
2.0
2.5
3.0
3.5
4.0
Cellular_organisms - ps1
Archaea1 - ps2
Archaea2 - ps3
Archaea3 - ps4
Asgard/Eukaryota - ps5
Eukaryota - ps6
Excavata/Diaphoretickes - ps7
Diaphoretickes - ps8
Archaeplastida - ps9
Viridiplantae - ps10
Streptophyta - ps11
Charophyceae/Embriophyta - ps12
Embriophyta - ps13
Tracheophyta - ps14
Spermatophyta - ps15
Angiosperms - ps16
Mesangiospermae - ps17
Magnoliids/eudicots - ps18
Eudicots - ps19
Core_eudicots - ps20
Superrosids - ps21
Rosids - ps22
Eurosids - ps23
Malvids - ps24
Sap./Malv./Brassicales - ps25
Malvales/Brassicales - ps26
Brassicales - ps27
Cleomaceae/Brassicaceae - ps28
Brassicaceae - ps29
Camelineae - ps30
Arabidopsis - ps31
Arabidopsis_thaliana - ps32
Phylostratum

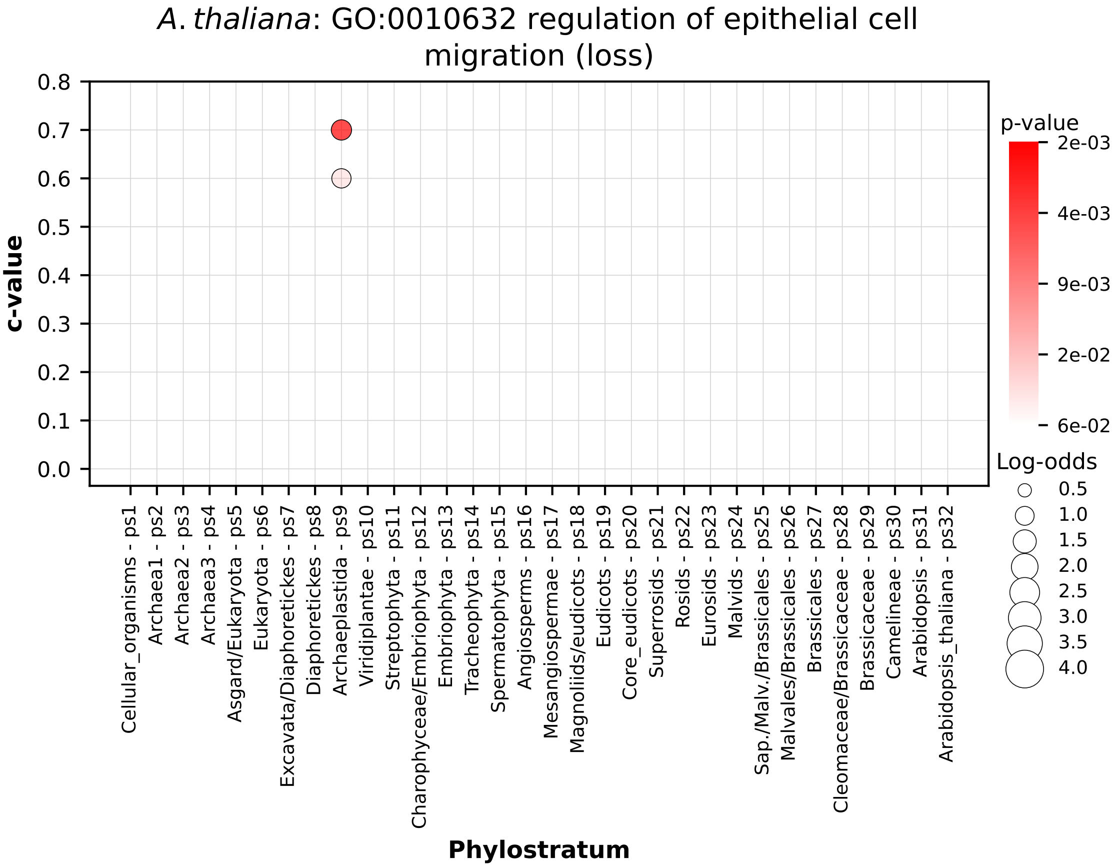

A. thaliana: GO:0010632 regulation of epithelial cell migration (loss)
c-value
0.8
0.7
0.6
0.5
0.4
0.3
0.2
0.1
0.0
p-value
2e-03
4e-03
9e-03
2e-02
6e-02
Log-odds
0.5
1.0
1.5
2.0
2.5
3.0
3.5
4.0
Cellular_organisms - ps1
Archaea1 - ps2
Archaea2 - ps3
Archaea3 - ps4
Asgard/Eukaryota - ps5
Eukaryota - ps6
Excavata/Diaphoretickes - ps7
Diaphoretickes - ps8
Archaeplastida - ps9
Viridiplantae - ps10
Streptophyta - ps11
Charophyceae/Embriophyta - ps12
Embriophyta - ps13
Tracheophyta - ps14
Spermatophyta - ps15
Angiosperms - ps16
Mesangiospermae - ps17
Magnoliids/eudicots - ps18
Eudicots - ps19
Core_eudicots - ps20
Superrosids - ps21
Rosids - ps22
Eurosids - ps23
Malvids - ps24
Sap./Malv./Brassicales - ps25
Malvales/Brassicales - ps26
Brassicales - ps27
Cleomaceae/Brassicaceae - ps28
Brassicaceae - ps29
Camelineae - ps30
Arabidopsis - ps31
Arabidopsis_thaliana - ps32
Phylostratum

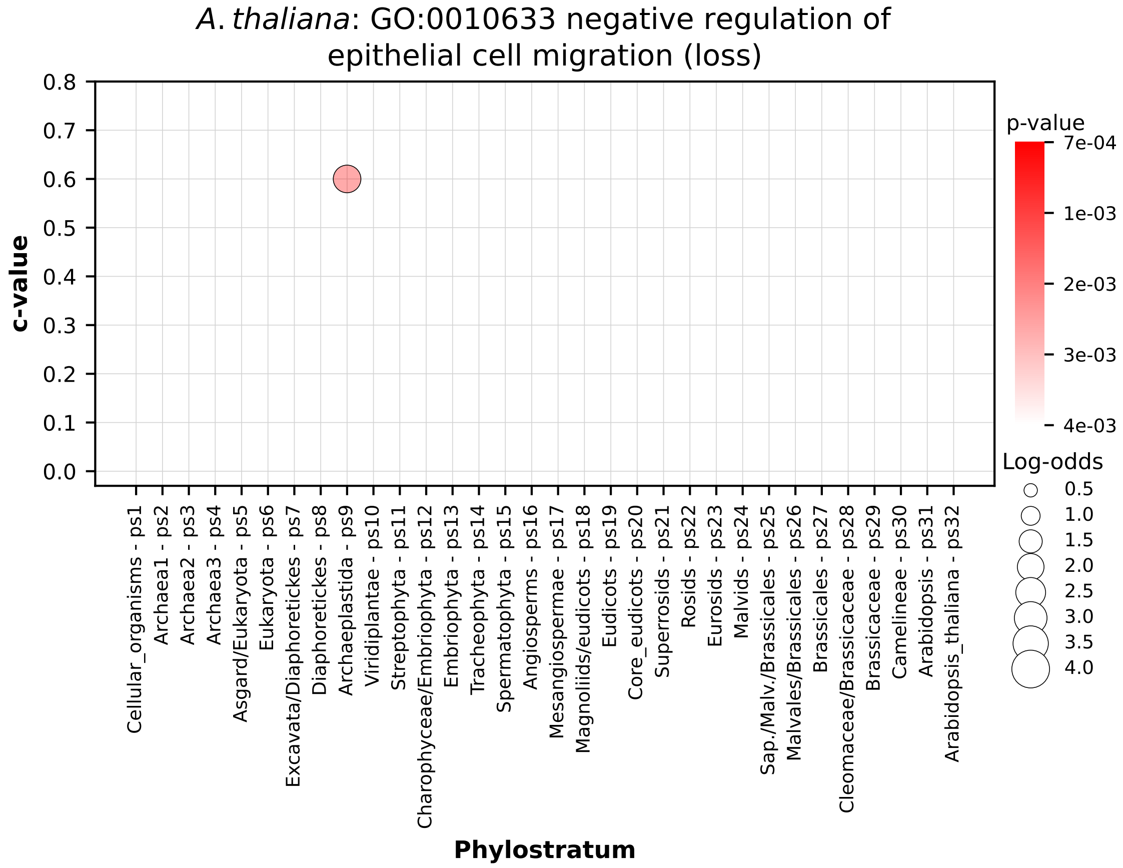

A. thaliana: GO:0010633 negative regulation of epithelial cell migration (loss)
c-value
0.8
0.7
0.6
0.5
0.4
0.3
0.2
0.1
0.0
p-value
7e-04
1e-03
2e-03
3e-03
4e-03
Log-odds
0.5
1.0
1.5
2.0
2.5
3.0
3.5
4.0
Cellular_organisms - ps1
Archaea1 - ps2
Archaea2 - ps3
Archaea3 - ps4
Asgard/Eukaryota - ps5
Eukaryota - ps6
Excavata/Diaphoretickes - ps7
Diaphoretickes - ps8
Archaeplastida - ps9
Viridiplantae - ps10
Streptophyta - ps11
Charophyceae/Embriophyta - ps12
Embriophyta - ps13
Tracheophyta - ps14
Spermatophyta - ps15
Angiosperms - ps16
Mesangiospermae - ps17
Magnoliids/eudicots - ps18
Eudicots - ps19
Core_eudicots - ps20
Superrosids - ps21
Rosids - ps22
Eurosids - ps23
Malvids - ps24
Sap./Malv./Brassicales - ps25
Malvales/Brassicales - ps26
Brassicales - ps27
Cleomaceae/Brassicaceae - ps28
Brassicaceae - ps29
Camelineae - ps30
Arabidopsis - ps31
Arabidopsis_thaliana - ps32
Phylostratum

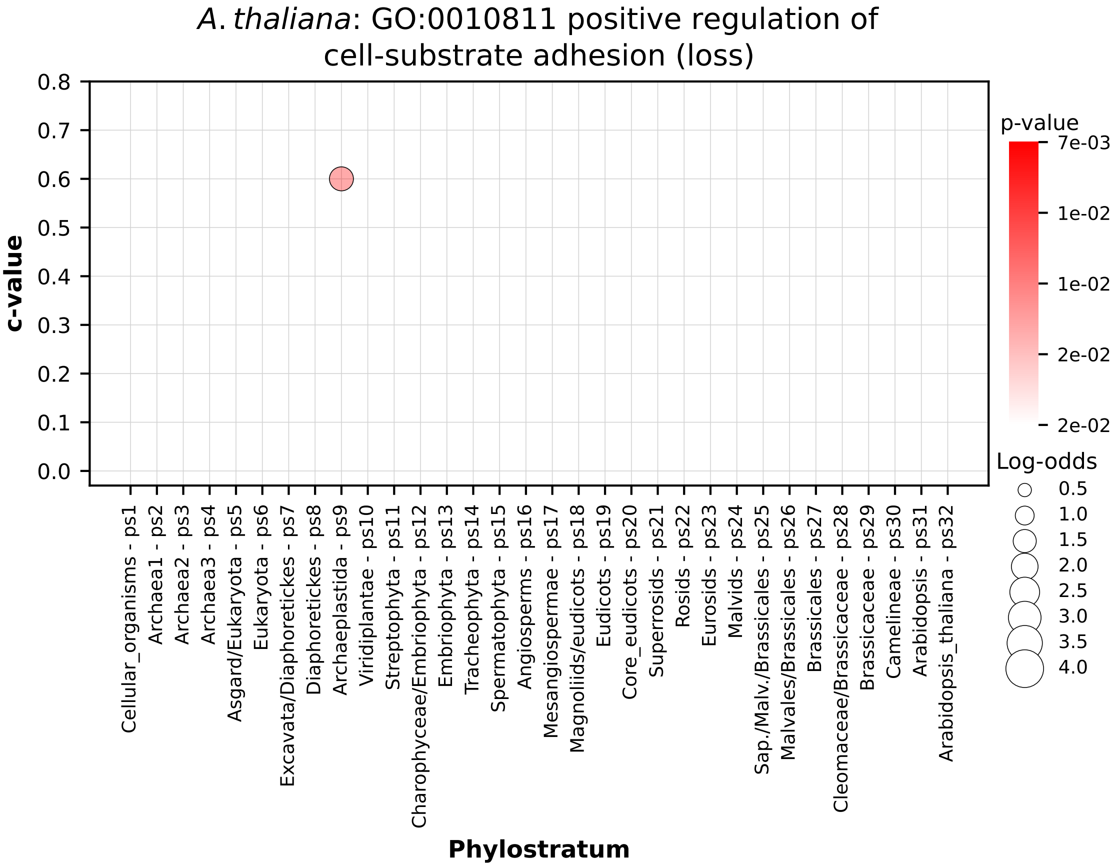

A. thaliana: GO:0010811 positive regulation of cell-substrate adhesion (loss)
c-value
0.8
0.7
0.6
0.5
0.4
0.3
0.2
0.1
0.0
p-value
7e-03
1e-02
1e-02
2e-02
2e-02
Log-odds
0.5
1.0
1.5
2.0
2.5
3.0
3.5
4.0
Cellular_organisms - ps1
Archaea1 - ps2
Archaea2 - ps3
Archaea3 - ps4
Asgard/Eukaryota - ps5
Eukaryota - ps6
Excavata/Diaphoretickes - ps7
Diaphoretickes - ps8
Archaeplastida - ps9
Viridiplantae - ps10
Streptophyta - ps11
Charophyceae/Embriophyta - ps12
Embriophyta - ps13
Tracheophyta - ps14
Spermatophyta - ps15
Angiosperms - ps16
Mesangiospermae - ps17
Magnoliids/eudicots - ps18
Eudicots - ps19
Core_eudicots - ps20
Superrosids - ps21
Rosids - ps22
Eurosids - ps23
Malvids - ps24
Sap./Malv./Brassicales - ps25
Malvales/Brassicales - ps26
Brassicales - ps27
Cleomaceae/Brassicaceae - ps28
Brassicaceae - ps29
Camelineae - ps30
Arabidopsis - ps31
Arabidopsis_thaliana - ps32
Phylostratum

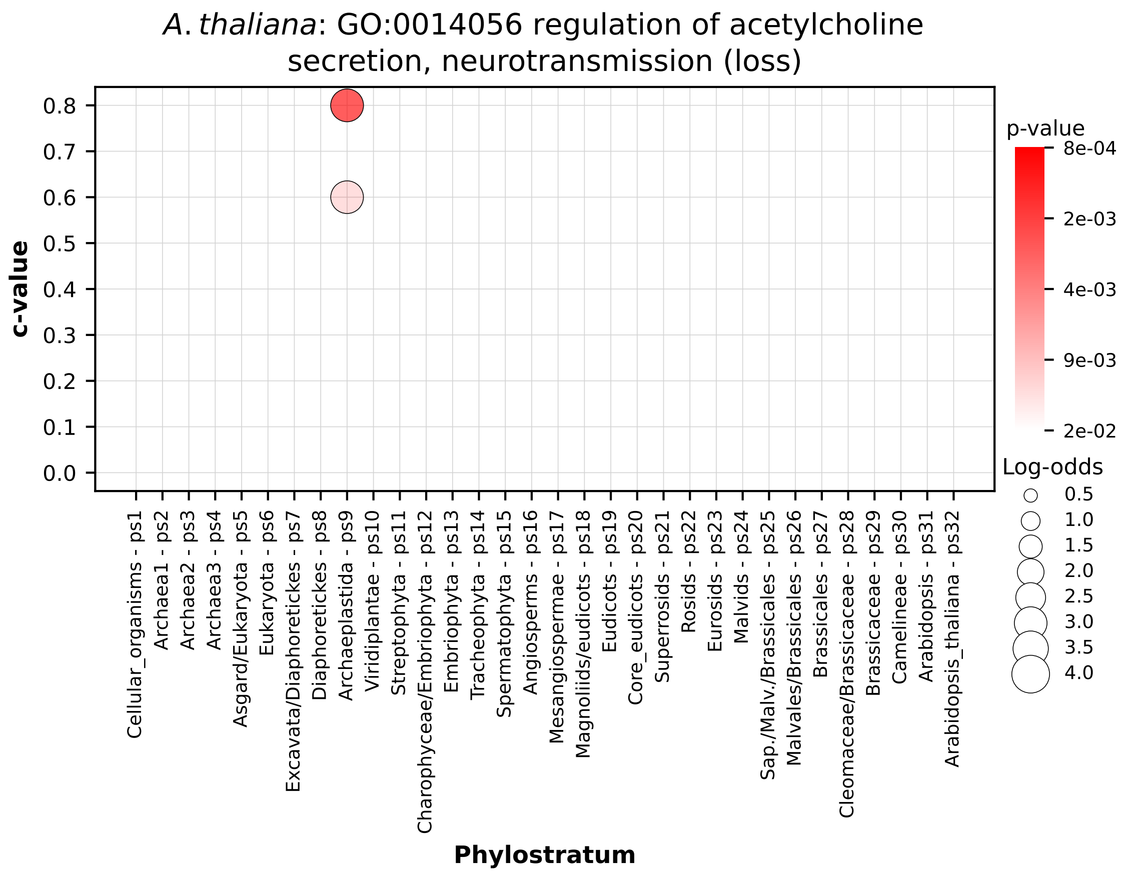

A. thaliana: GO:0014056 regulation of acetylcholine secretion, neurotransmission (loss)
c-value
0.0
0.1
0.2
0.3
0.4
0.5
0.6
0.7
0.8
p-value
8e-04
2e-03
4e-03
9e-03
2e-02
Log-odds
0.5
1.0
1.5
2.0
2.5
3.0
3.5
4.0
Cellular_organisms - ps1
Archaea1 - ps2
Archaea2 - ps3
Archaea3 - ps4
Asgard/Eukaryota - ps5
Eukaryota - ps6
Excavata/Diaphoretickes - ps7
Diaphoretickes - ps8
Archaeplastida - ps9
Viridiplantae - ps10
Streptophyta - ps11
Charophyceae/Embriophyta - ps12
Embriophyta - ps13
Tracheophyta - ps14
Spermatophyta - ps15
Angiosperms - ps16
Mesangiospermae - ps17
Magnoliids/eudicots - ps18
Eudicots - ps19
Core_eudicots - ps20
Superrosids - ps21
Rosids - ps22
Eurosids - ps23
Malvids - ps24
Sap./Malv./Brassicales - ps25
Malvales/Brassicales - ps26
Brassicales - ps27
Cleomaceae/Brassicaceae - ps28
Brassicaceae - ps29
Camelineae - ps30
Arabidopsis - ps31
Arabidopsis_thaliana - ps32
Phylostratum

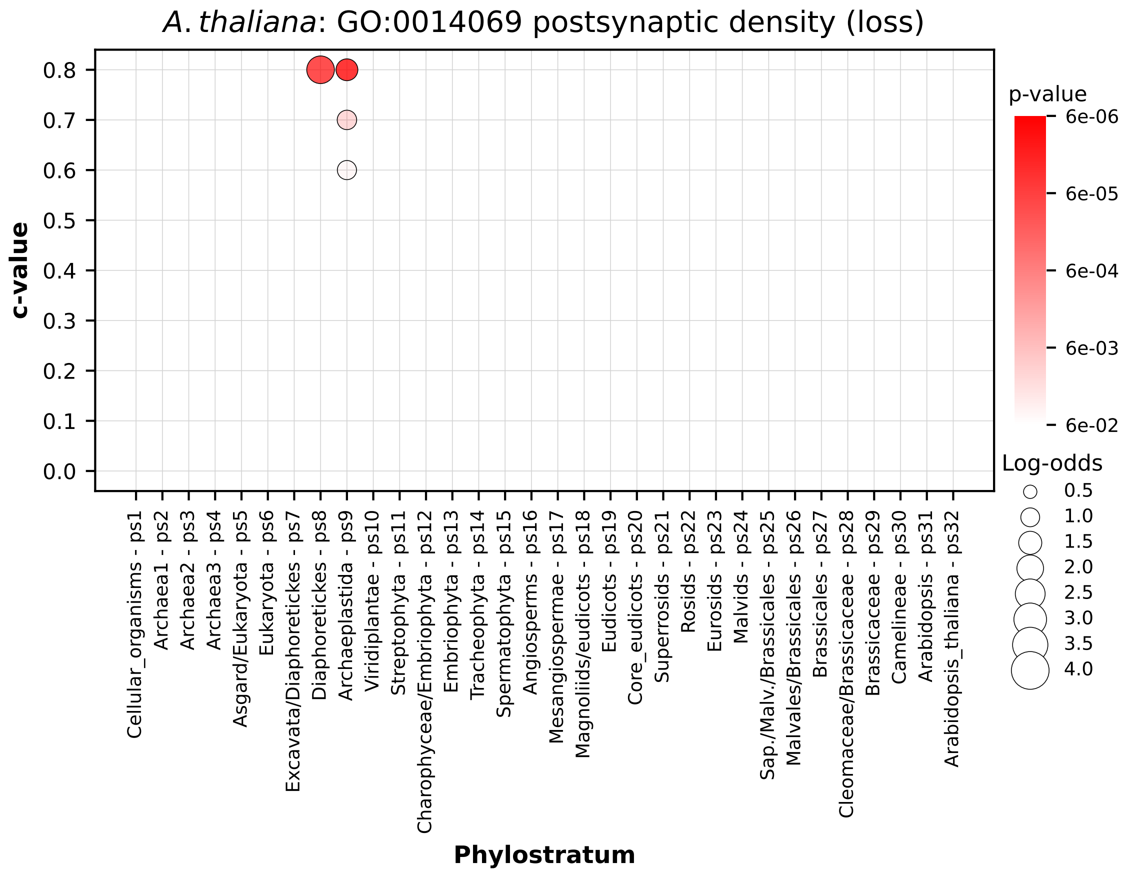
A. thaliana: GO:0014069 postsynaptic density (loss)
c-value
0.0
0.1
0.2
0.3
0.4
0.5
0.6
0.7
0.8
Phylostratum
Cellular_organisms - ps1
Archaea1 - ps2
Archaea2 - ps3
Archaea3 - ps4
Asgard/Eukaryota - ps5
Eukaryota - ps6
Excavata/Diaphoretickes - ps7
Diaphoretickes - ps8
Archaeplastida - ps9
Viridiplantae - ps10
Streptophyta - ps11
Charophyceae/Embriophyta - ps12
Embriophyta - ps13
Tracheophyta - ps14
Spermatophyta - ps15
Angiosperms - ps16
Mesangiospermae - ps17
Magnoliids/eudicots - ps18
Eudicots - ps19
Core_eudicots - ps20
Superrosids - ps21
Rosids - ps22
Eurosids - ps23
Malvids - ps24
Sap./Malv./Brassicales - ps25
Malvales/Brassicales - ps26
Brassicales - ps27
Cleomaceae/Brassicaceae - ps28
Brassicaceae - ps29
Camelineae - ps30
Arabidopsis - ps31
Arabidopsis_thaliana - ps32
p-value
6e-06
6e-05
6e-04
6e-03
6e-02
Log-odds
0.5
1.0
1.5
2.0
2.5
3.0
3.5
4.0

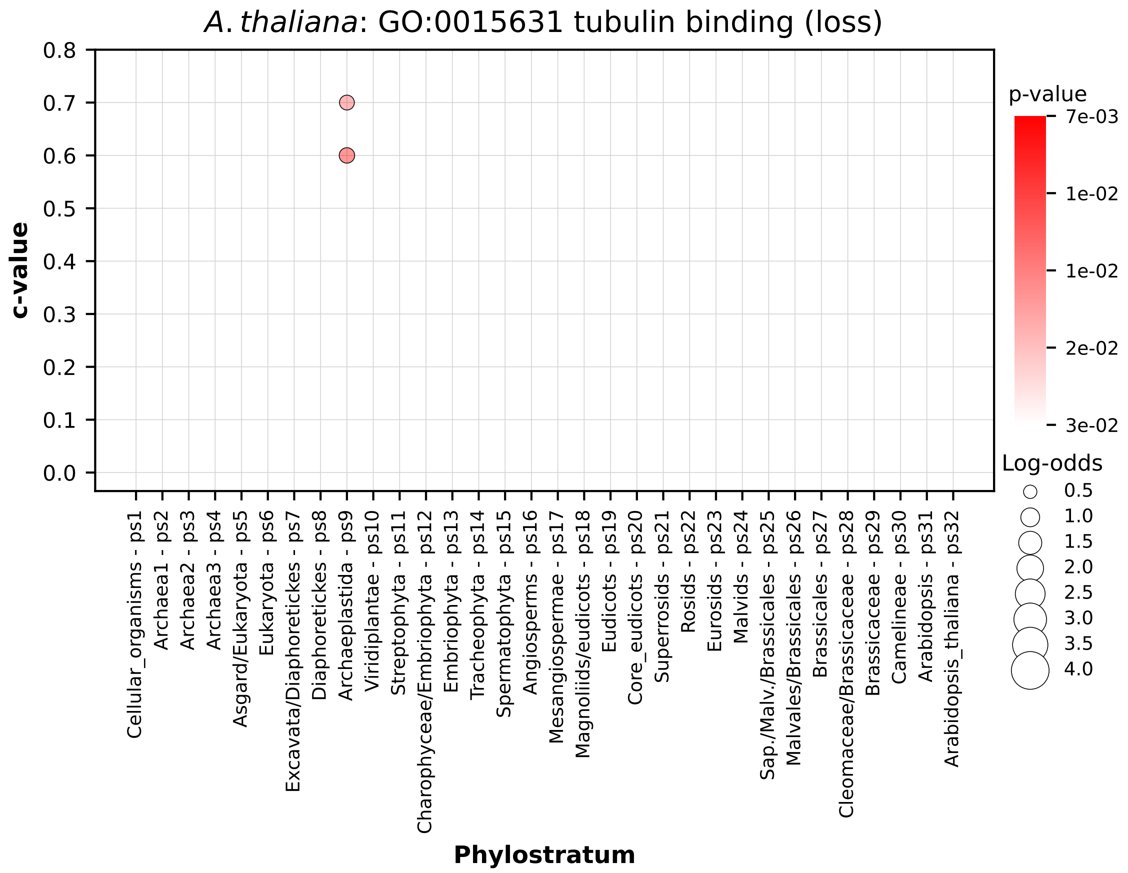

A. thaliana: GO:0015631 tubulin binding (loss)
c-value
0.8
0.7
0.6
0.5
0.4
0.3
0.2
0.1
0.0
Phylostratum
Cellular_organisms - ps1
Archaea1 - ps2
Archaea2 - ps3
Archaea3 - ps4
Asgard/Eukaryota - ps5
Eukaryota - ps6
Excavata/Diaphoretickes - ps7
Diaphoretickes - ps8
Archaeplastida - ps9
Viridiplantae - ps10
Streptophyta - ps11
Charophyceae/Embriophyta - ps12
Embriophyta - ps13
Tracheophyta - ps14
Spermatophyta - ps15
Angiosperms - ps16
Mesangiospermae - ps17
Magnoliids/eudicots - ps18
Eudicots - ps19
Core_eudicots - ps20
Superrosids - ps21
Rosids - ps22
Eurosids - ps23
Malvids - ps24
Sap./Malv./Brassicales - ps25
Malvales/Brassicales - ps26
Brassicales - ps27
Cleomaceae/Brassicaceae - ps28
Brassicaceae - ps29
Camelineae - ps30
Arabidopsis - ps31
Arabidopsis_thaliana - ps32
p-value
7e-03
1e-02
1e-02
2e-02
3e-02
Log-odds
0.5
1.0
1.5
2.0
2.5
3.0
3.5
4.0

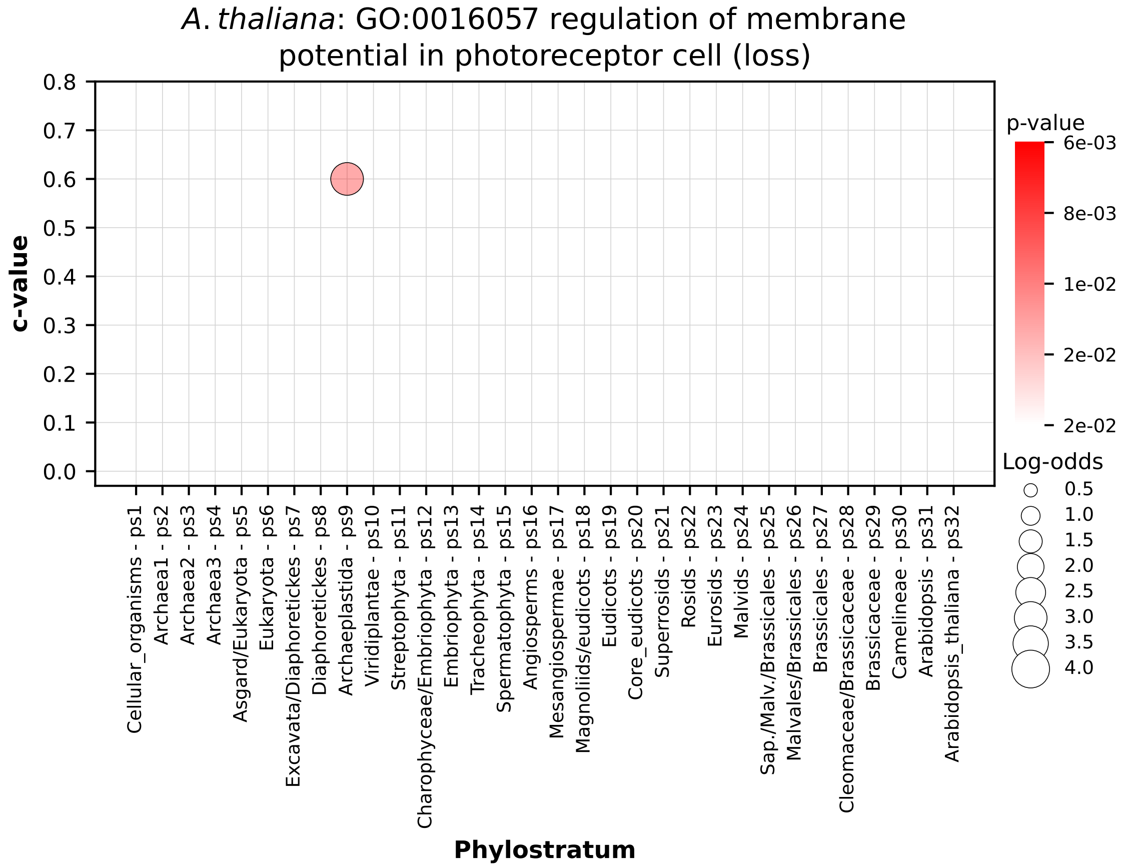
A. thaliana: GO:0016057 regulation of membrane potential in photoreceptor cell (loss)
c-value
0.8
0.7
0.6
0.5
0.4
0.3
0.2
0.1
0.0
Phylostratum
Cellular_organisms - ps1
Archaea1 - ps2
Archaea2 - ps3
Archaea3 - ps4
Asgard/Eukaryota - ps5
Eukaryota - ps6
Excavata/Diaphoretickes - ps7
Diaphoretickes - ps8
Archaeplastida - ps9
Viridiplantae - ps10
Streptophyta - ps11
Charophyceae/Embriophyta - ps12
Embriophyta - ps13
Tracheophyta - ps14
Spermatophyta - ps15
Angiosperms - ps16
Mesangiospermae - ps17
Magnoliids/eudicots - ps18
Eudicots - ps19
Core_eudicots - ps20
Superrosids - ps21
Rosids - ps22
Eurosids - ps23
Malvids - ps24
Sap./Malv./Brassicales - ps25
Malvales/Brassicales - ps26
Brassicales - ps27
Cleomaceae/Brassicaceae - ps28
Brassicaceae - ps29
Camelineae - ps30
Arabidopsis - ps31
Arabidopsis_thaliana - ps32
p-value
6e-03
8e-03
1e-02
2e-02
2e-02
Log-odds
0.5
1.0
1.5
2.0
2.5
3.0
3.5
4.0

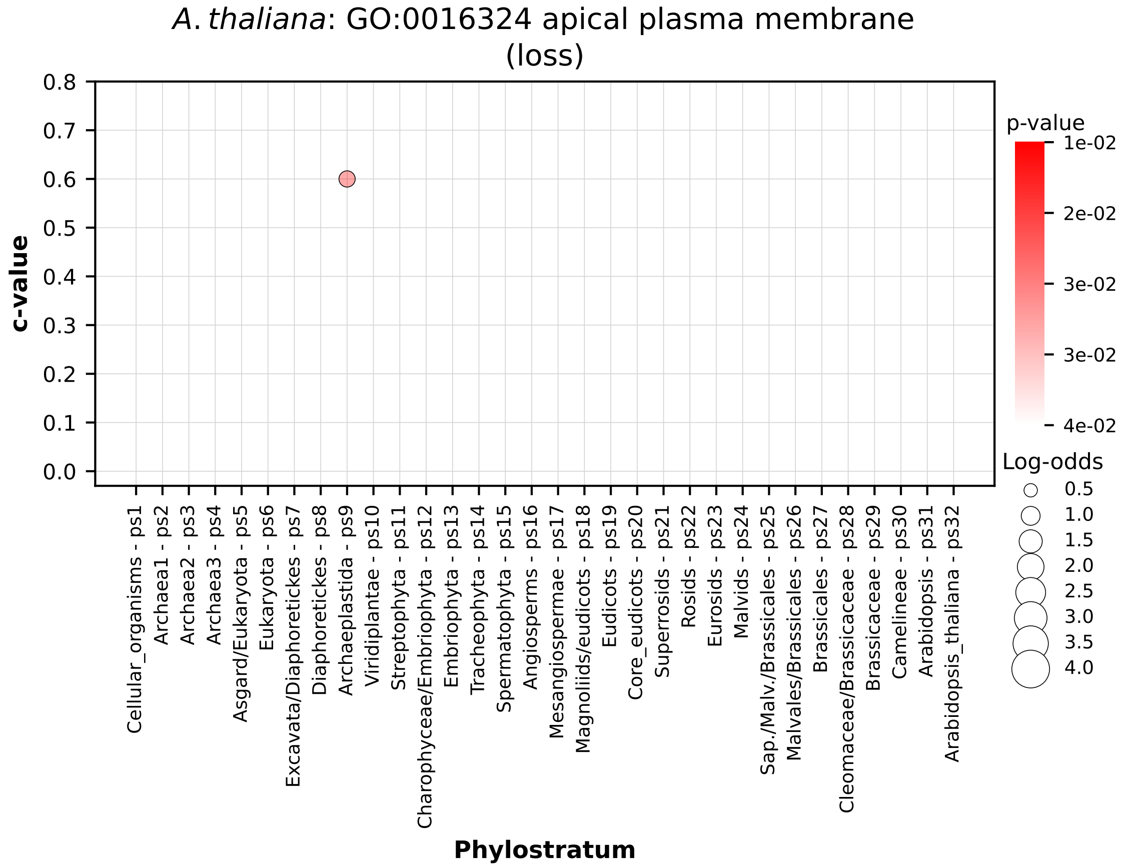

A. thaliana: GO:0016324 apical plasma membrane
(loss)
c-value
0.0
0.1
0.2
0.3
0.4
0.5
0.6
0.7
0.8
Phylostratum
Cellular_organisms - ps1
Archaea1 - ps2
Archaea2 - ps3
Archaea3 - ps4
Asgard/Eukaryota - ps5
Eukaryota - ps6
Excavata/Diaphoretickes - ps7
Diaphoretickes - ps8
Archaeplastida - ps9
Viridiplantae - ps10
Streptophyta - ps11
Charophyceae/Embriophyta - ps12
Embriophyta - ps13
Tracheophyta - ps14
Spermatophyta - ps15
Angiosperms - ps16
Mesangiospermae - ps17
Magnoliids/eudicots - ps18
Eudicots - ps19
Core_eudicots - ps20
Superrosids - ps21
Rosids - ps22
Eurosids - ps23
Malvids - ps24
Sap./Malv./Brassicales - ps25
Malvales/Brassicales - ps26
Brassicales - ps27
Cleomaceae/Brassicaceae - ps28
Brassicaceae - ps29
Camelineae - ps30
Arabidopsis - ps31
Arabidopsis_thaliana - ps32
p-value
1e-02
2e-02
3e-02
3e-02
4e-02
Log-odds
0.5
1.0
1.5
2.0
2.5
3.0
3.5
4.0

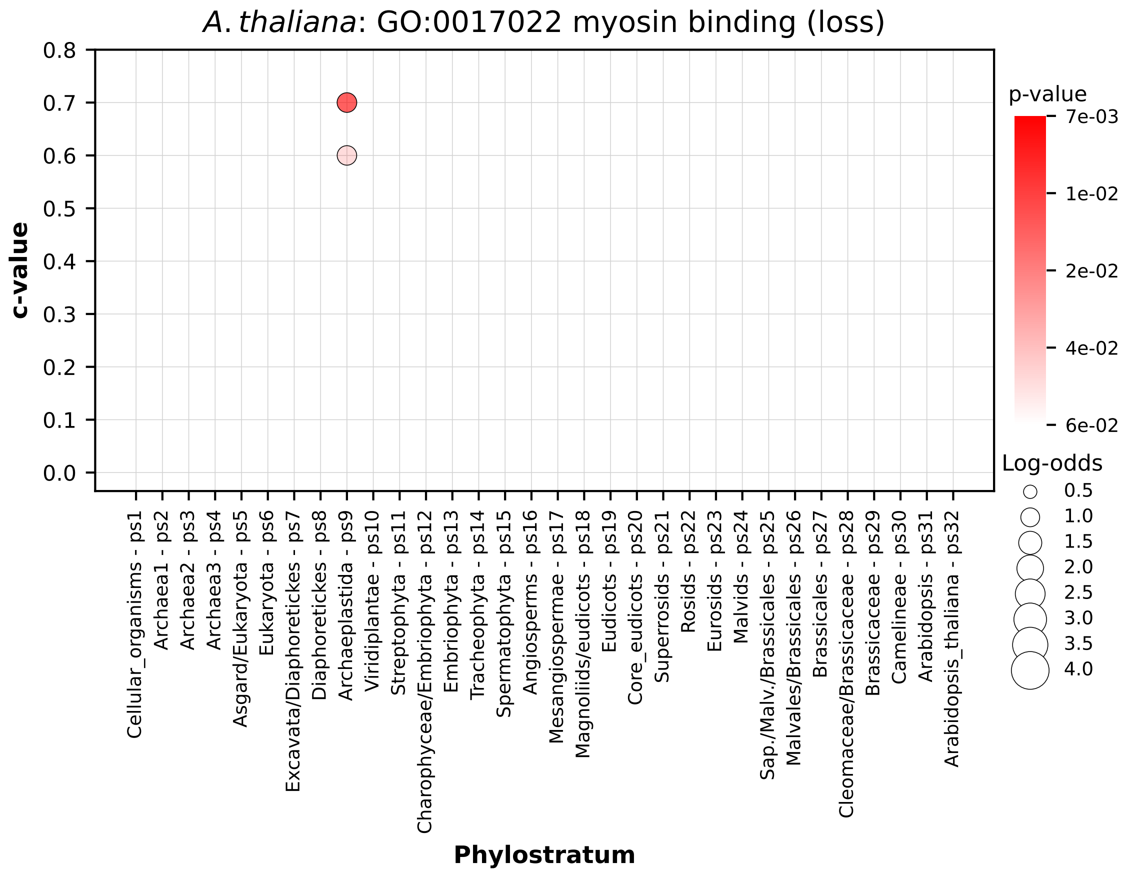

A. thaliana: GO:0017022 myosin binding (loss)
c-value
0.8
0.7
0.6
0.5
0.4
0.3
0.2
0.1
0.0
p-value
7e-03
1e-02
2e-02
4e-02
6e-02
Log-odds
0.5
1.0
1.5
2.0
2.5
3.0
3.5
4.0
Cellular_organisms - ps1
Archaea1 - ps2
Archaea2 - ps3
Archaea3 - ps4
Asgard/Eukaryota - ps5
Eukaryota - ps6
Excavata/Diaphoretickes - ps7
Diaphoretickes - ps8
Archaeplastida - ps9
Viridiplantae - ps10
Streptophyta - ps11
Charophyceae/Embriophyta - ps12
Embriophyta - ps13
Tracheophyta - ps14
Spermatophyta - ps15
Angiosperms - ps16
Mesangiospermae - ps17
Magnoliids/eudicots - ps18
Eudicots - ps19
Core_eudicots - ps20
Superrosids - ps21
Rosids - ps22
Eurosids - ps23
Malvids - ps24
Sap./Malv./Brassicales - ps25
Malvales/Brassicales - ps26
Brassicales - ps27
Cleomaceae/Brassicaceae - ps28
Brassicaceae - ps29
Camelineae - ps30
Arabidopsis - ps31
Arabidopsis_thaliana - ps32
Phylostratum

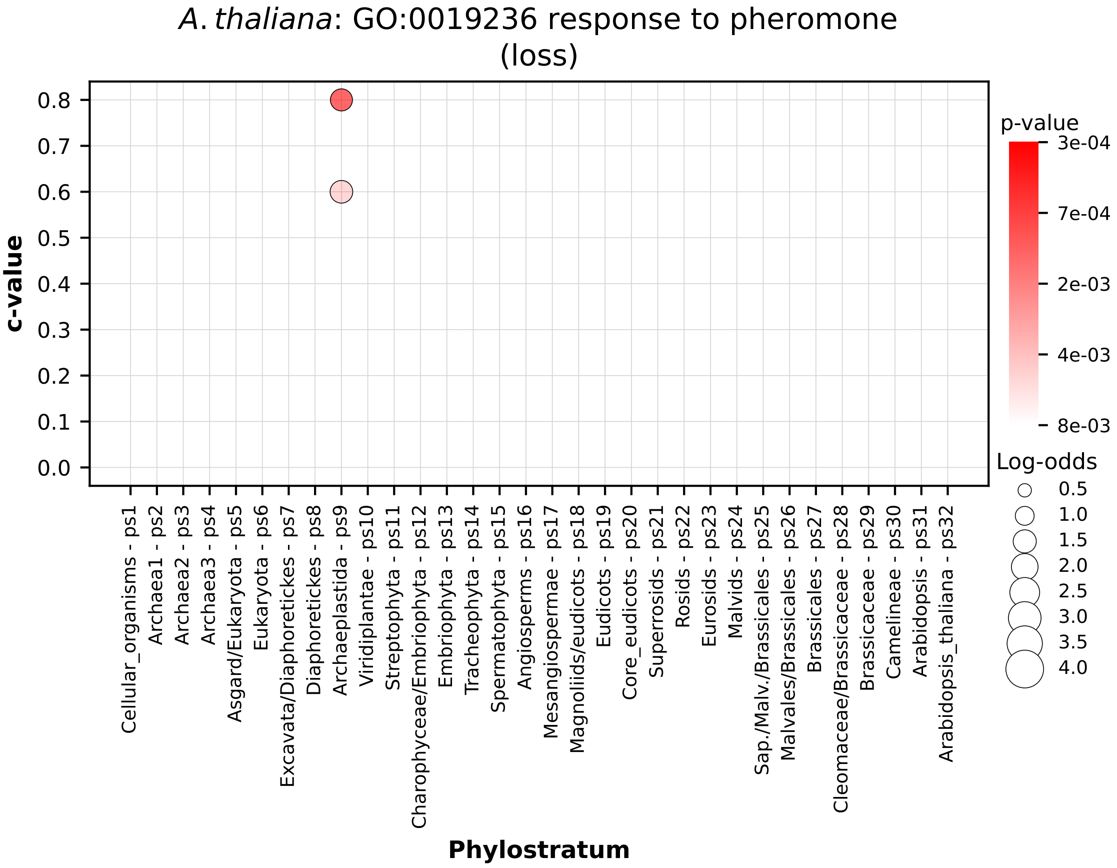

A. thaliana: GO:0019236 response to pheromone
(loss)
c-value
0.0
0.1
0.2
0.3
0.4
0.5
0.6
0.7
0.8
p-value
3e-04
7e-04
2e-03
4e-03
8e-03
Log-odds
0.5
1.0
1.5
2.0
2.5
3.0
3.5
4.0
Cellular_organisms - ps1
Archaea1 - ps2
Archaea2 - ps3
Archaea3 - ps4
Asgard/Eukaryota - ps5
Eukaryota - ps6
Excavata/Diaphoretickes - ps7
Diaphoretickes - ps8
Archaeplastida - ps9
Viridiplantae - ps10
Streptophyta - ps11
Charophyceae/Embriophyta - ps12
Embriophyta - ps13
Tracheophyta - ps14
Spermatophyta - ps15
Angiosperms - ps16
Mesangiospermae - ps17
Magnoliids/eudicots - ps18
Eudicots - ps19
Core_eudicots - ps20
Superrosids - ps21
Rosids - ps22
Eurosids - ps23
Malvids - ps24
Sap./Malv./Brassicales - ps25
Malvales/Brassicales - ps26
Brassicales - ps27
Cleomaceae/Brassicaceae - ps28
Brassicaceae - ps29
Camelineae - ps30
Arabidopsis - ps31
Arabidopsis_thaliana - ps32
Phylostratum

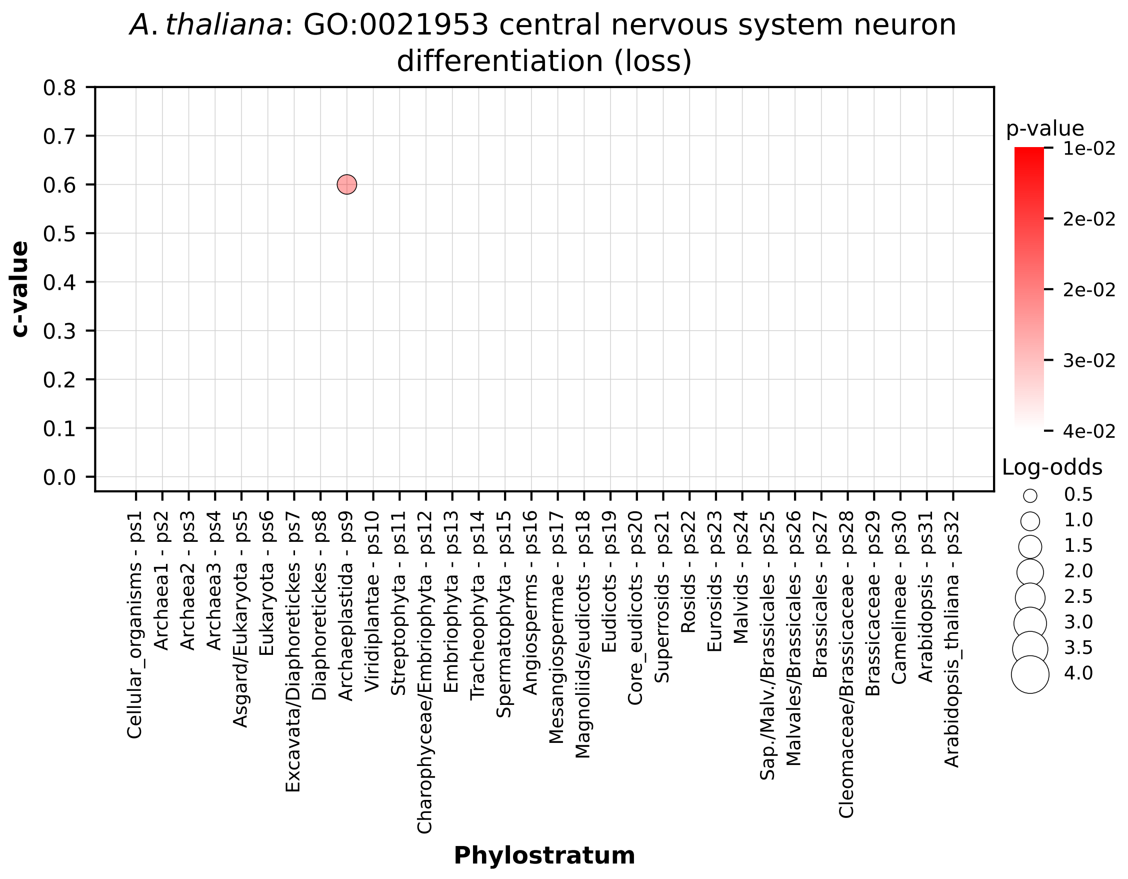

A. thaliana: GO:0021953 central nervous system neuron differentiation (loss)
c-value
0.8
0.7
0.6
0.5
0.4
0.3
0.2
0.1
0.0
Cellular_organisms - ps1
Archaea1 - ps2
Archaea2 - ps3
Archaea3 - ps4
Asgard/Eukaryota - ps5
Eukaryota - ps6
Excavata/Diaphoretickes - ps7
Diaphoretickes - ps8
Archaeplastida - ps9
Viridiplantae - ps10
Streptophyta - ps11
Charophyceae/Embriophyta - ps12
Embriophyta - ps13
Tracheophyta - ps14
Spermatophyta - ps15
Angiosperms - ps16
Mesangiospermae - ps17
Magnoliids/eudicots - ps18
Eudicots - ps19
Core_eudicots - ps20
Superrosids - ps21
Rosids - ps22
Eurosids - ps23
Malvids - ps24
Sap./Malv./Brassicales - ps25
Malvales/Brassicales - ps26
Brassicales - ps27
Cleomaceae/Brassicaceae - ps28
Brassicaceae - ps29
Camelineae - ps30
Arabidopsis - ps31
Arabidopsis_thaliana - ps32
Phylostratum
p-value
1e-02
2e-02
2e-02
3e-02
4e-02
Log-odds
0.5
1.0
1.5
2.0
2.5
3.0
3.5
4.0

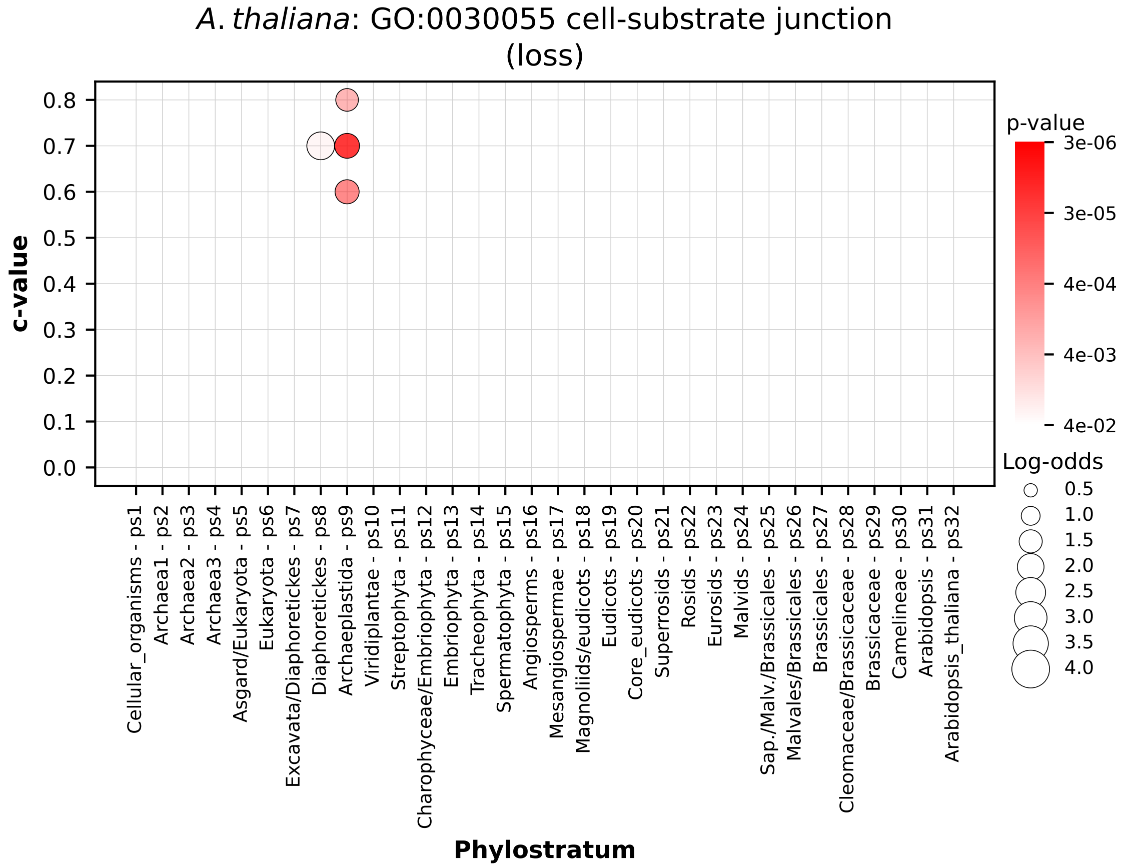

A. thaliana: GO:0030055 cell-substrate junction
(loss)
c-value
0.0
0.1
0.2
0.3
0.4
0.5
0.6
0.7
0.8
Phylostratum
Cellular_organisms - ps1
Archaea1 - ps2
Archaea2 - ps3
Archaea3 - ps4
Asgard/Eukaryota - ps5
Eukaryota - ps6
Excavata/Diaphoretickes - ps7
Diaphoretickes - ps8
Archaeplastida - ps9
Viridiplantae - ps10
Streptophyta - ps11
Charophyceae/Embriophyta - ps12
Embriophyta - ps13
Tracheophyta - ps14
Spermatophyta - ps15
Angiosperms - ps16
Mesangiospermae - ps17
Magnoliids/eudicots - ps18
Eudicots - ps19
Core_eudicots - ps20
Superrosids - ps21
Rosids - ps22
Eurosids - ps23
Malvids - ps24
Sap./Malv./Brassicales - ps25
Malvales/Brassicales - ps26
Brassicales - ps27
Cleomaceae/Brassicaceae - ps28
Brassicaceae - ps29
Camelineae - ps30
Arabidopsis - ps31
Arabidopsis_thaliana - ps32
p-value
3e-06
3e-05
4e-04
4e-03
4e-02
Log-odds
0.5
1.0
1.5
2.0
2.5
3.0
3.5
4.0

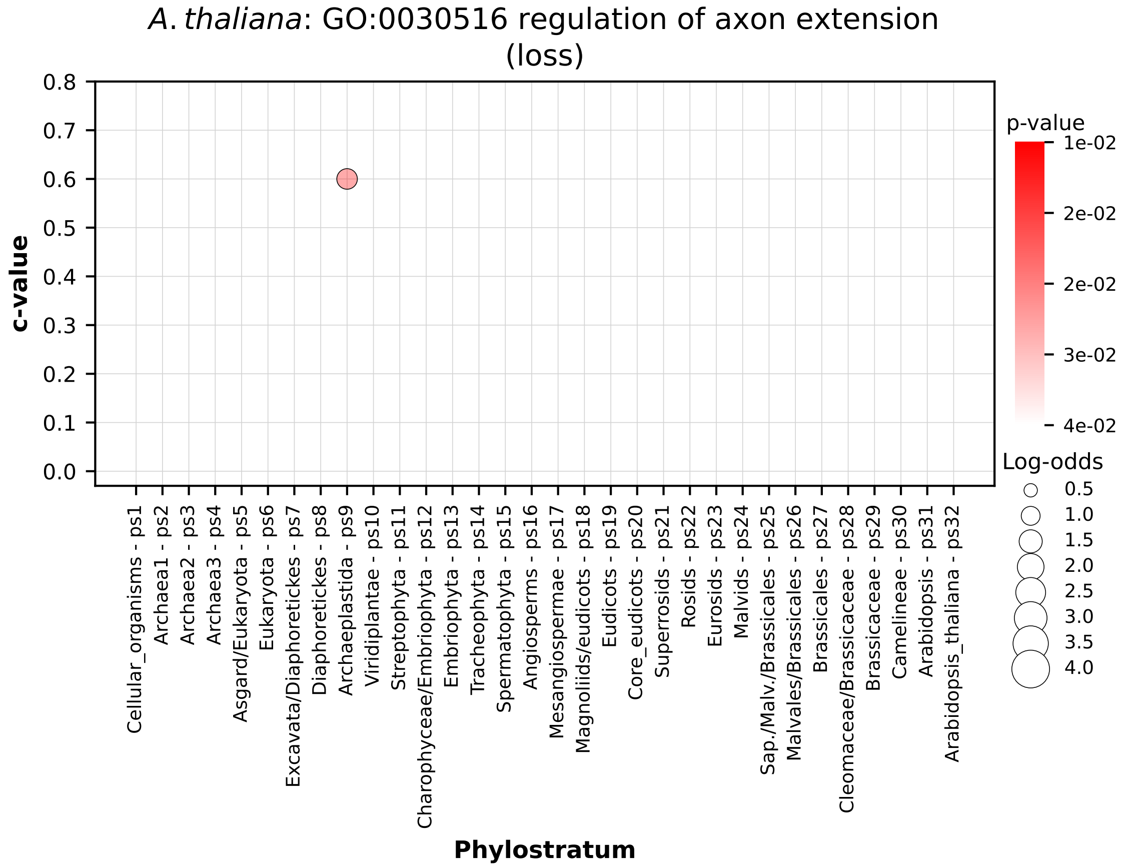

A. thaliana: GO:0030516 regulation of axon extension
(loss)
c-value
0.8
0.7
0.6
0.5
0.4
0.3
0.2
0.1
0.0
p-value
1e-02
2e-02
2e-02
3e-02
4e-02
Log-odds
0.5
1.0
1.5
2.0
2.5
3.0
3.5
4.0
Cellular_organisms - ps1
Archaea1 - ps2
Archaea2 - ps3
Archaea3 - ps4
Asgard/Eukaryota - ps5
Eukaryota - ps6
Excavata/Diaphoretickes - ps7
Diaphoretickes - ps8
Archaeplastida - ps9
Viridiplantae - ps10
Streptophyta - ps11
Charophyceae/Embriophyta - ps12
Embriophyta - ps13
Tracheophyta - ps14
Spermatophyta - ps15
Angiosperms - ps16
Mesangiospermae - ps17
Magnoliids/eudicots - ps18
Eudicots - ps19
Core_eudicots - ps20
Superrosids - ps21
Rosids - ps22
Eurosids - ps23
Malvids - ps24
Sap./Malv./Brassicales - ps25
Malvales/Brassicales - ps26
Brassicales - ps27
Cleomaceae/Brassicaceae - ps28
Brassicaceae - ps29
Camelineae - ps30
Arabidopsis - ps31
Arabidopsis_thaliana - ps32
Phylostratum

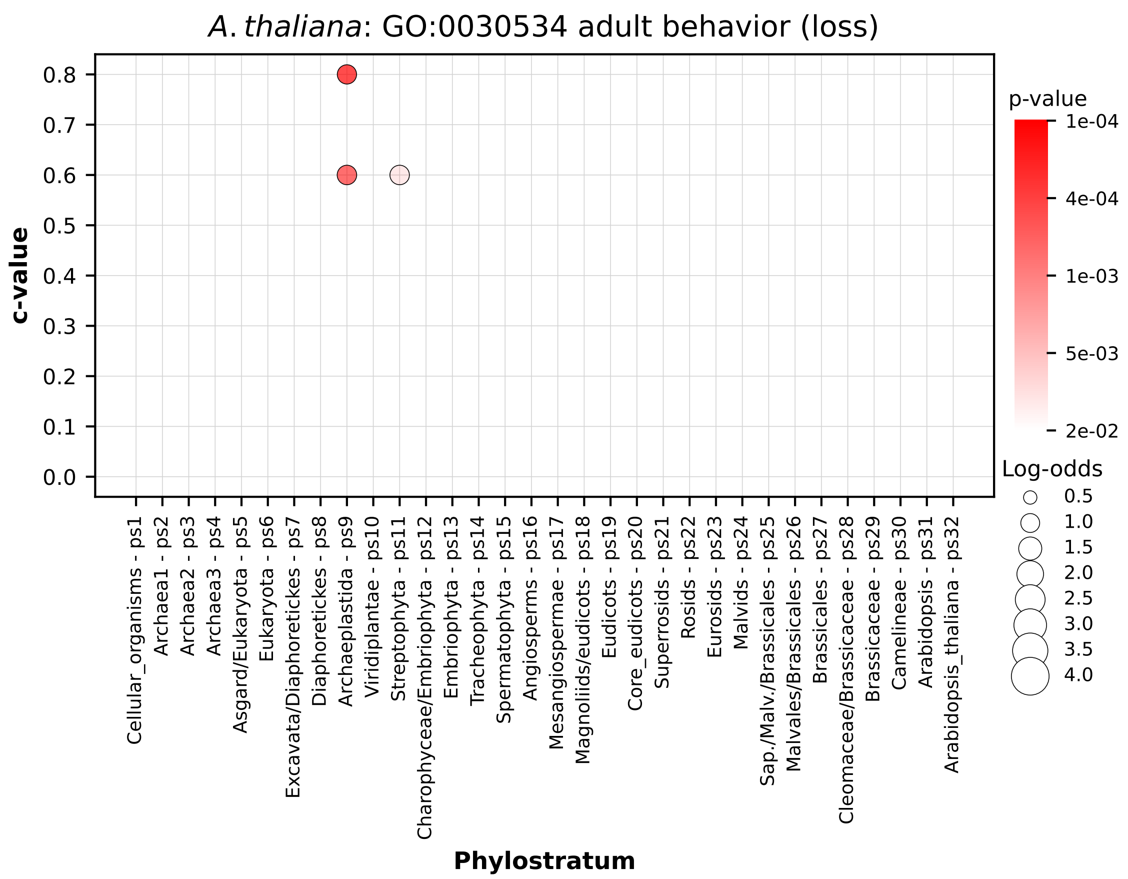

A. thaliana: GO:0030534 adult behavior (loss)
c-value
0.0
0.1
0.2
0.3
0.4
0.5
0.6
0.7
0.8
p-value
1e-04
4e-04
1e-03
5e-03
2e-02
Log-odds
0.5
1.0
1.5
2.0
2.5
3.0
3.5
4.0
Cellular_organisms - ps1
Archaea1 - ps2
Archaea2 - ps3
Archaea3 - ps4
Asgard/Eukaryota - ps5
Eukaryota - ps6
Excavata/Diaphoretickes - ps7
Diaphoretickes - ps8
Archaeplastida - ps9
Viridiplantae - ps10
Streptophyta - ps11
Charophyceae/Embriophyta - ps12
Embriophyta - ps13
Tracheophyta - ps14
Spermatophyta - ps15
Angiosperms - ps16
Mesangiospermae - ps17
Magnoliids/eudicots - ps18
Eudicots - ps19
Core_eudicots - ps20
Superrosids - ps21
Rosids - ps22
Eurosids - ps23
Malvids - ps24
Sap./Malv./Brassicales - ps25
Malvales/Brassicales - ps26
Brassicales - ps27
Cleomaceae/Brassicaceae - ps28
Brassicaceae - ps29
Camelineae - ps30
Arabidopsis - ps31
Arabidopsis_thaliana - ps32
Phylostratum

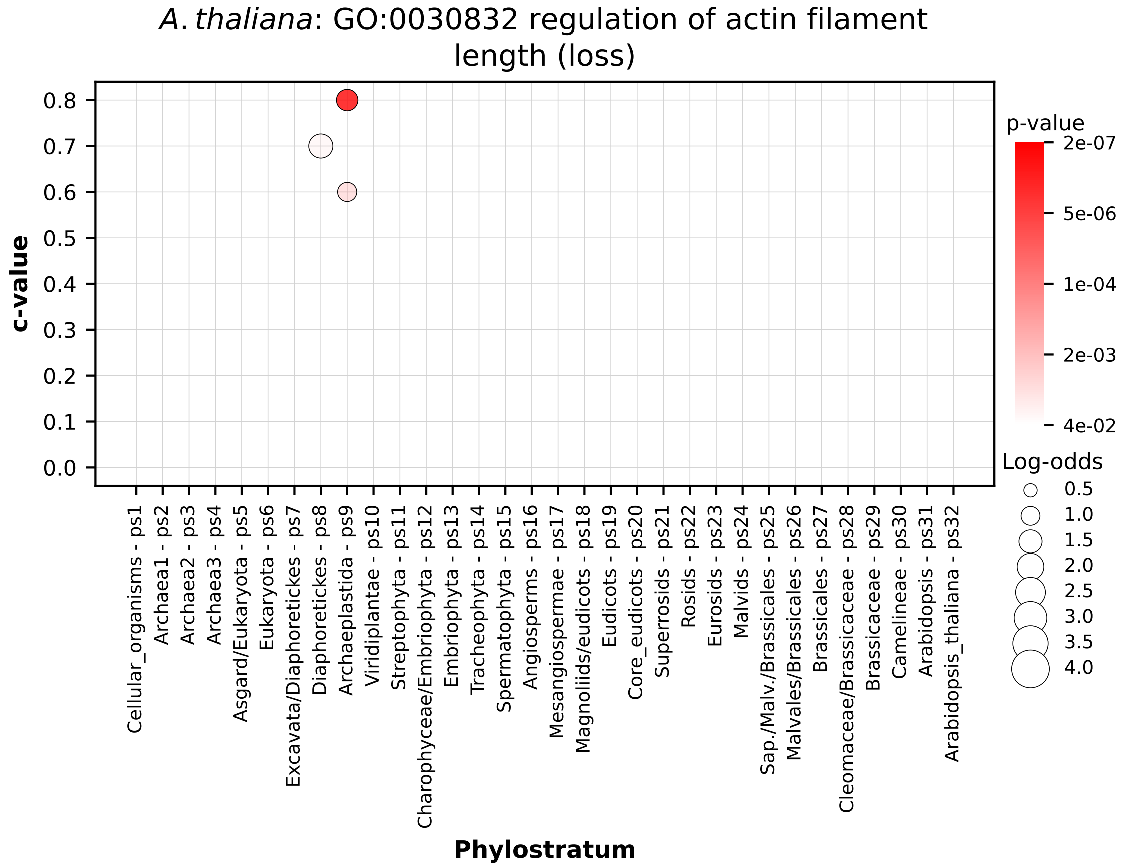

A. thaliana: GO:0030832 regulation of actin filament length (loss)
c-value
0.0
0.1
0.2
0.3
0.4
0.5
0.6
0.7
0.8
Phylostratum
Cellular_organisms - ps1
Archaea1 - ps2
Archaea2 - ps3
Archaea3 - ps4
Asgard/Eukaryota - ps5
Eukaryota - ps6
Excavata/Diaphoretickes - ps7
Diaphoretickes - ps8
Archaeplastida - ps9
Viridiplantae - ps10
Streptophyta - ps11
Charophyceae/Embriophyta - ps12
Embriophyta - ps13
Tracheophyta - ps14
Spermatophyta - ps15
Angiosperms - ps16
Mesangiospermae - ps17
Magnoliids/eudicots - ps18
Eudicots - ps19
Core_eudicots - ps20
Superrosids - ps21
Rosids - ps22
Eurosids - ps23
Malvids - ps24
Sap./Malv./Brassicales - ps25
Malvales/Brassicales - ps26
Brassicales - ps27
Cleomaceae/Brassicaceae - ps28
Brassicaceae - ps29
Camelineae - ps30
Arabidopsis - ps31
Arabidopsis_thaliana - ps32
p-value
2e-07
5e-06
1e-04
2e-03
4e-02
Log-odds
0.5
1.0
1.5
2.0
2.5
3.0
3.5
4.0

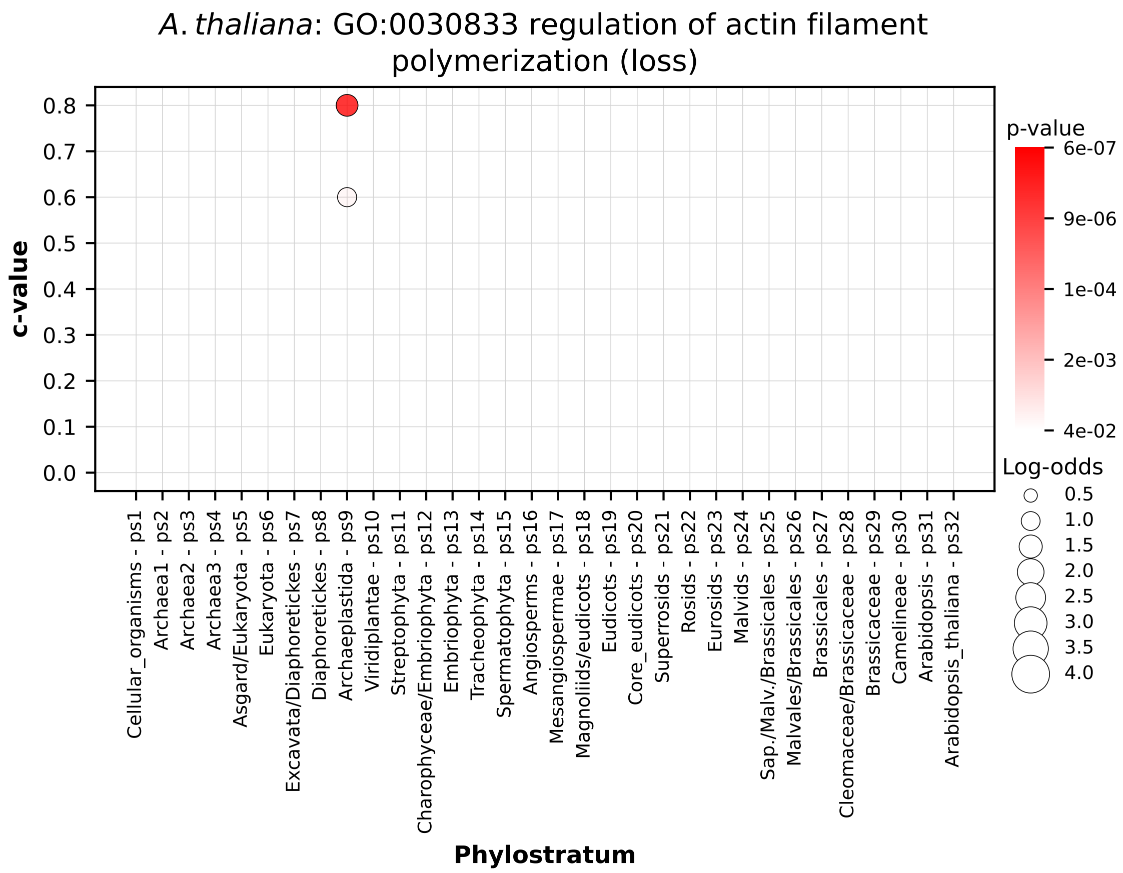
A. thaliana: GO:0030833 regulation of actin filament polymerization (loss)
c-value
0.0
0.1
0.2
0.3
0.4
0.5
0.6
0.7
0.8
p-value
6e-07
9e-06
1e-04
2e-03
4e-02
Log-odds
0.5
1.0
1.5
2.0
2.5
3.0
3.5
4.0
Cellular_organisms - ps1
Archaea1 - ps2
Archaea2 - ps3
Archaea3 - ps4
Asgard/Eukaryota - ps5
Eukaryota - ps6
Excavata/Diaphoretickes - ps7
Diaphoretickes - ps8
Archaeplastida - ps9
Viridiplantae - ps10
Streptophyta - ps11
Charophyceae/Embriophyta - ps12
Embriophyta - ps13
Tracheophyta - ps14
Spermatophyta - ps15
Angiosperms - ps16
Mesangiospermae - ps17
Magnoliids/eudicots - ps18
Eudicots - ps19
Core_eudicots - ps20
Superrosids - ps21
Rosids - ps22
Eurosids - ps23
Malvids - ps24
Sap./Malv./Brassicales - ps25
Malvales/Brassicales - ps26
Brassicales - ps27
Cleomaceae/Brassicaceae - ps28
Brassicaceae - ps29
Camelineae - ps30
Arabidopsis - ps31
Arabidopsis_thaliana - ps32
Phylostratum

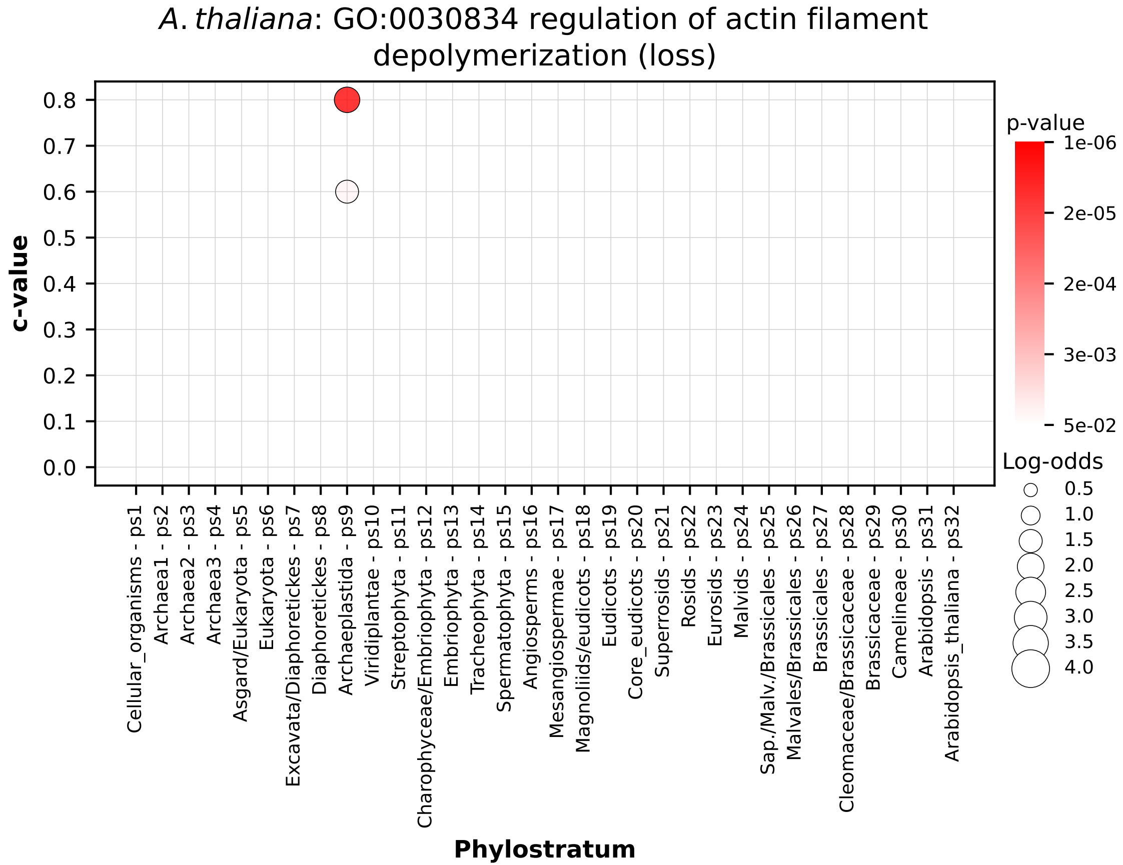

A. thaliana: GO:0030834 regulation of actin filament depolymerization (loss)
c-value
0.8
0.7
0.6
0.5
0.4
0.3
0.2
0.1
0.0
Phylostratum
Cellular_organisms - ps1
Archaea1 - ps2
Archaea2 - ps3
Archaea3 - ps4
Asgard/Eukaryota - ps5
Eukaryota - ps6
Excavata/Diaphoretickes - ps7
Diaphoretickes - ps8
Archaeplastida - ps9
Viridiplantae - ps10
Streptophyta - ps11
Charophyceae/Embriophyta - ps12
Embriophyta - ps13
Tracheophyta - ps14
Spermatophyta - ps15
Angiosperms - ps16
Mesangiospermae - ps17
Magnoliids/eudicots - ps18
Eudicots - ps19
Core_eudicots - ps20
Superrosids - ps21
Rosids - ps22
Eurosids - ps23
Malvids - ps24
Sap./Malv./Brassicales - ps25
Malvales/Brassicales - ps26
Brassicales - ps27
Cleomaceae/Brassicaceae - ps28
Brassicaceae - ps29
Camelineae - ps30
Arabidopsis - ps31
Arabidopsis_thaliana - ps32
p-value
1e-06
2e-05
2e-04
3e-03
5e-02
Log-odds
0.5
1.0
1.5
2.0
2.5
3.0
3.5
4.0

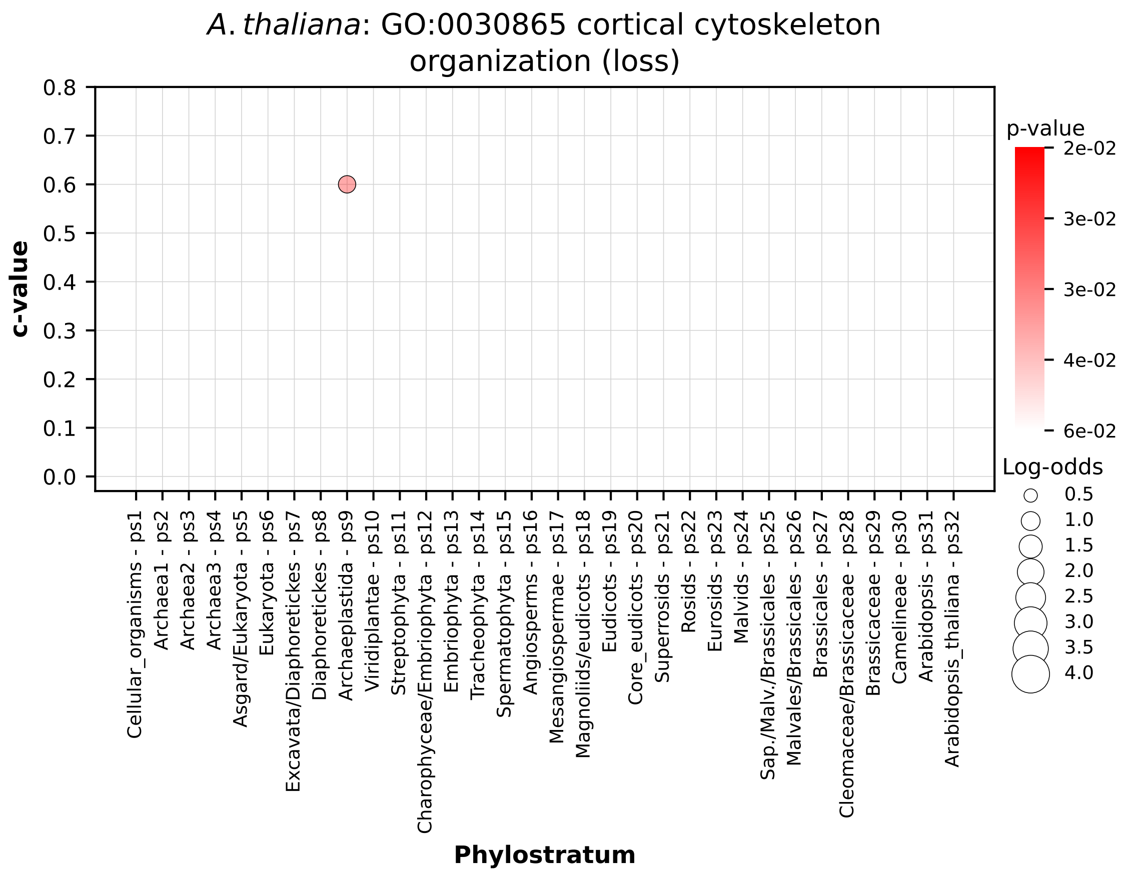

A. thaliana: GO:0030865 cortical cytoskeleton organization (loss)
c-value
0.8
0.7
0.6
0.5
0.4
0.3
0.2
0.1
0.0
p-value
2e-02
3e-02
3e-02
4e-02
6e-02
Log-odds
0.5
1.0
1.5
2.0
2.5
3.0
3.5
4.0
Cellular_organisms - ps1
Archaea1 - ps2
Archaea2 - ps3
Archaea3 - ps4
Asgard/Eukaryota - ps5
Eukaryota - ps6
Excavata/Diaphoretickes - ps7
Diaphoretickes - ps8
Archaeplastida - ps9
Viridiplantae - ps10
Streptophyta - ps11
Charophyceae/Embriophyta - ps12
Embriophyta - ps13
Tracheophyta - ps14
Spermatophyta - ps15
Angiosperms - ps16
Mesangiospermae - ps17
Magnoliids/eudicots - ps18
Eudicots - ps19
Core_eudicots - ps20
Superrosids - ps21
Rosids - ps22
Eurosids - ps23
Malvids - ps24
Sap./Malv./Brassicales - ps25
Malvales/Brassicales - ps26
Brassicales - ps27
Cleomaceae/Brassicaceae - ps28
Brassicaceae - ps29
Camelineae - ps30
Arabidopsis - ps31
Arabidopsis_thaliana - ps32
Phylostratum

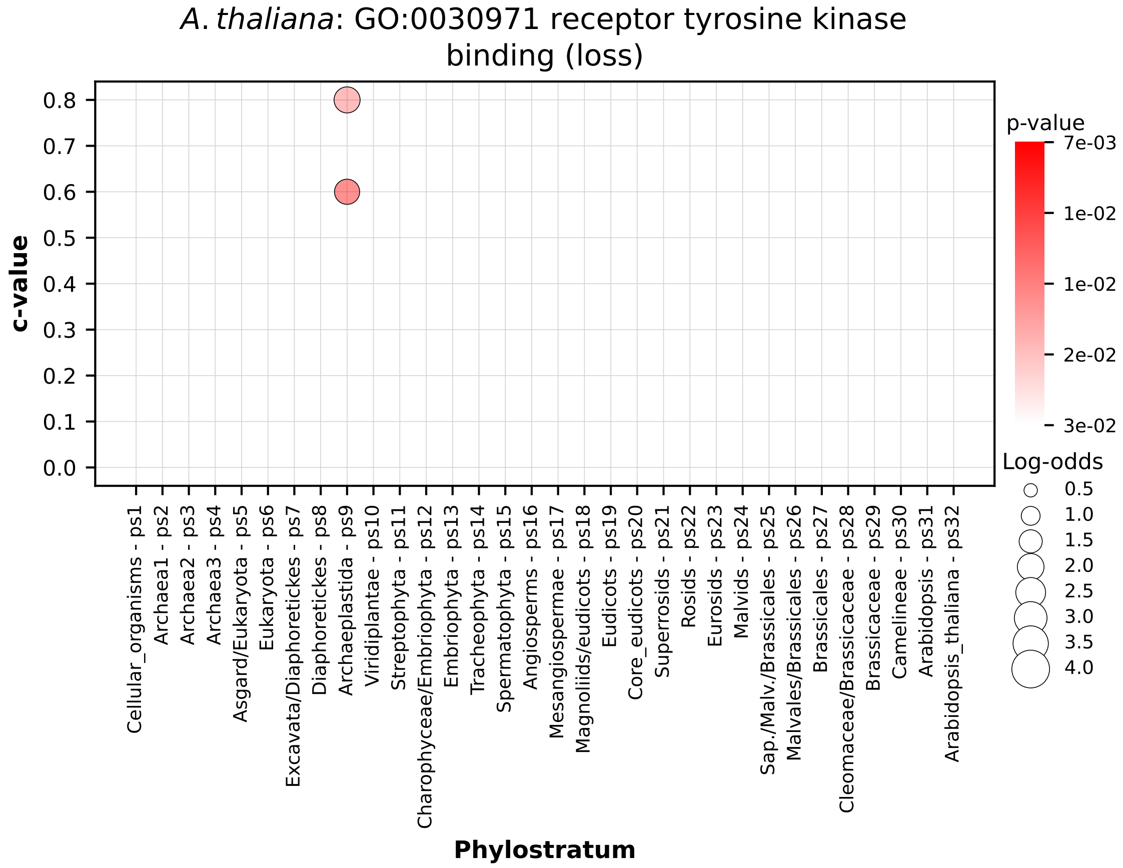
A. thaliana: GO:0030971 receptor tyrosine kinase binding (loss)
c-value
0.0
0.1
0.2
0.3
0.4
0.5
0.6
0.7
0.8
p-value
7e-03
1e-02
1e-02
2e-02
3e-02
Log-odds
0.5
1.0
1.5
2.0
2.5
3.0
3.5
4.0
Cellular_organisms - ps1
Archaea1 - ps2
Archaea2 - ps3
Archaea3 - ps4
Asgard/Eukaryota - ps5
Eukaryota - ps6
Excavata/Diaphoretickes - ps7
Diaphoretickes - ps8
Archaeplastida - ps9
Viridiplantae - ps10
Streptophyta - ps11
Charophyceae/Embriophyta - ps12
Embriophyta - ps13
Tracheophyta - ps14
Spermatophyta - ps15
Angiosperms - ps16
Mesangiospermae - ps17
Magnoliids/eudicots - ps18
Eudicots - ps19
Core_eudicots - ps20
Superrosids - ps21
Rosids - ps22
Eurosids - ps23
Malvids - ps24
Sap./Malv./Brassicales - ps25
Malvales/Brassicales - ps26
Brassicales - ps27
Cleomaceae/Brassicaceae - ps28
Brassicaceae - ps29
Camelineae - ps30
Arabidopsis - ps31
Arabidopsis_thaliana - ps32
Phylostratum

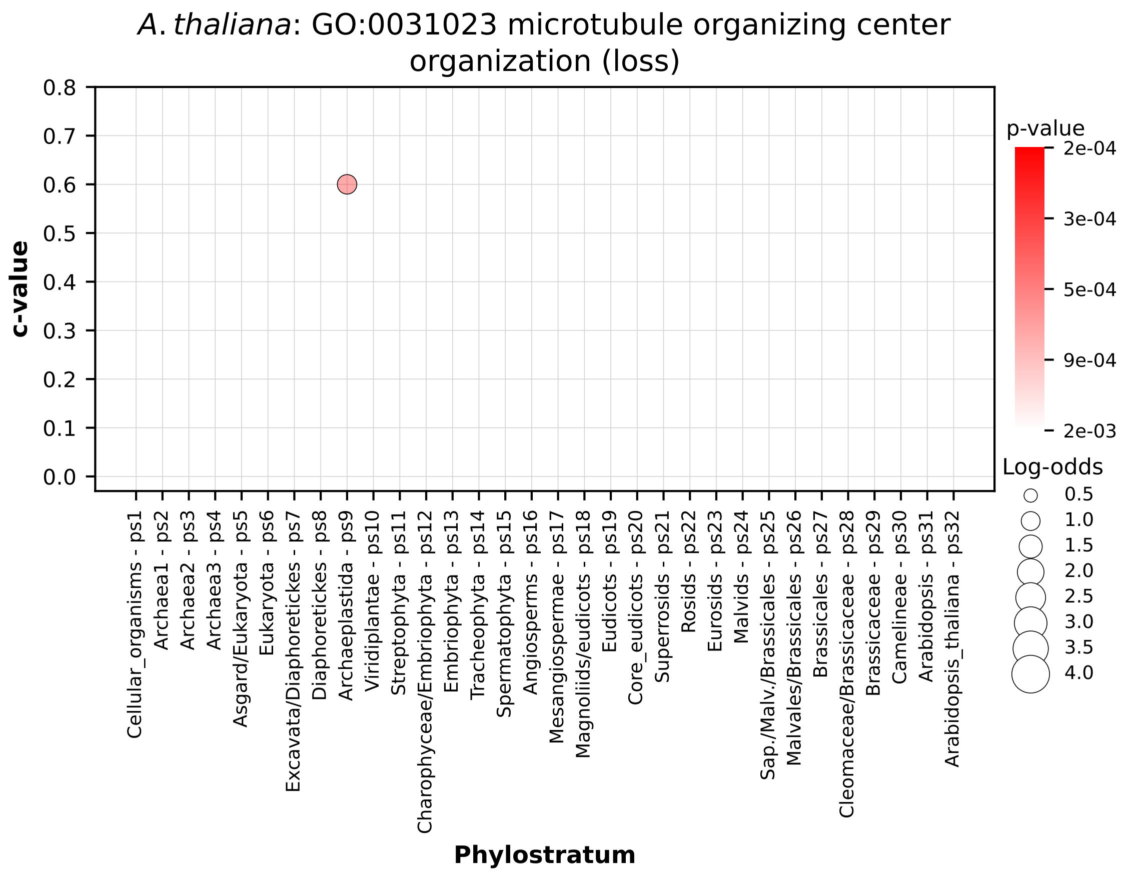
A. thaliana: GO:0031023 microtubule organizing center organization (loss)
c-value
0.0
0.1
0.2
0.3
0.4
0.5
0.6
0.7
0.8
Phylostratum
Cellular_organisms - ps1
Archaea1 - ps2
Archaea2 - ps3
Archaea3 - ps4
Asgard/Eukaryota - ps5
Eukaryota - ps6
Excavata/Diaphoretickes - ps7
Diaphoretickes - ps8
Archaeplastida - ps9
Viridiplantae - ps10
Streptophyta - ps11
Charophyceae/Embriophyta - ps12
Embriophyta - ps13
Tracheophyta - ps14
Spermatophyta - ps15
Angiosperms - ps16
Mesangiospermae - ps17
Magnoliids/eudicots - ps18
Eudicots - ps19
Core_eudicots - ps20
Superrosids - ps21
Rosids - ps22
Eurosids - ps23
Malvids - ps24
Sap./Malv./Brassicales - ps25
Malvales/Brassicales - ps26
Brassicales - ps27
Cleomaceae/Brassicaceae - ps28
Brassicaceae - ps29
Camelineae - ps30
Arabidopsis - ps31
Arabidopsis_thaliana - ps32
p-value
2e-04
3e-04
5e-04
9e-04
2e-03
Log-odds
0.5
1.0
1.5
2.0
2.5
3.0
3.5
4.0

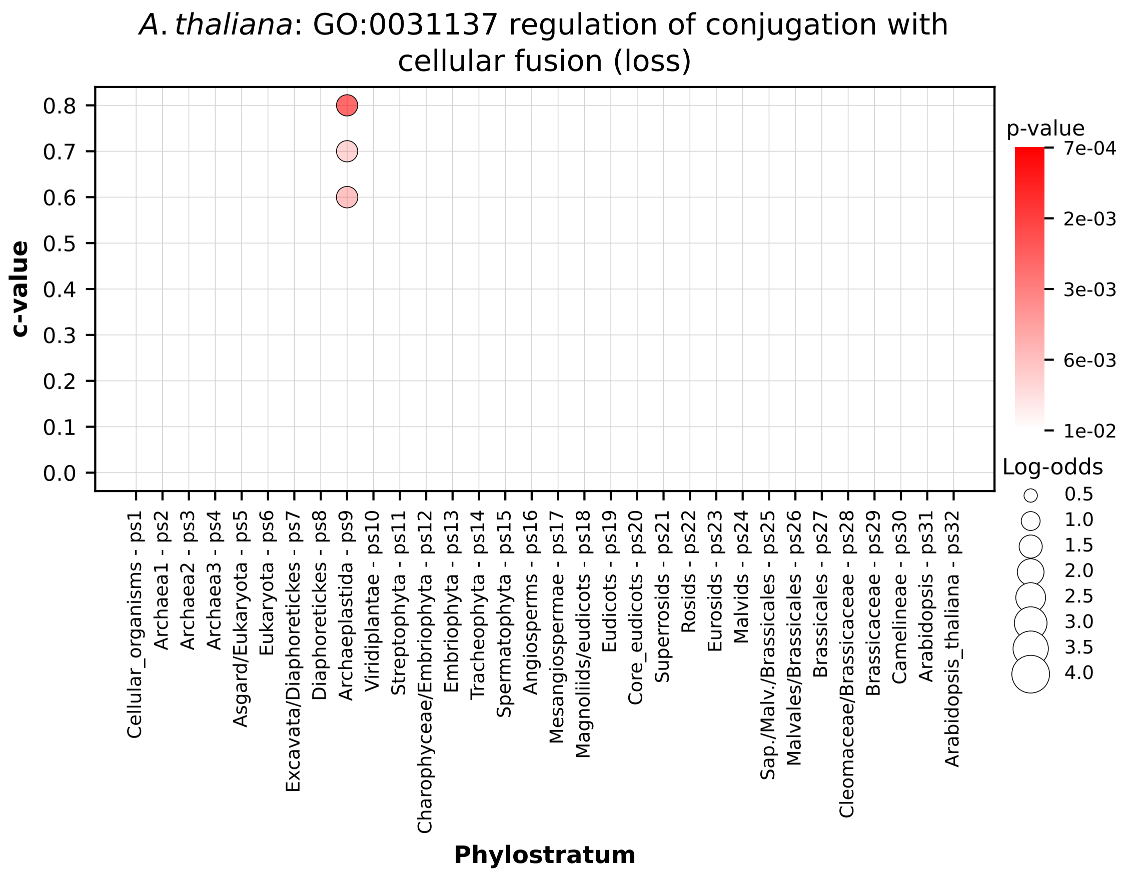

A. thaliana: GO:0031137 regulation of conjugation with cellular fusion (loss)
c-value
0.8
0.7
0.6
0.5
0.4
0.3
0.2
0.1
0.0
Phylostratum
Cellular_organisms - ps1
Archaea1 - ps2
Archaea2 - ps3
Archaea3 - ps4
Asgard/Eukaryota - ps5
Eukaryota - ps6
Excavata/Diaphoretickes - ps7
Diaphoretickes - ps8
Archaeplastida - ps9
Viridiplantae - ps10
Streptophyta - ps11
Charophyceae/Embriophyta - ps12
Embriophyta - ps13
Tracheophyta - ps14
Spermatophyta - ps15
Angiosperms - ps16
Mesangiospermae - ps17
Magnoliids/eudicots - ps18
Eudicots - ps19
Core_eudicots - ps20
Superrosids - ps21
Rosids - ps22
Eurosids - ps23
Malvids - ps24
Sap./Malv./Brassicales - ps25
Malvales/Brassicales - ps26
Brassicales - ps27
Cleomaceae/Brassicaceae - ps28
Brassicaceae - ps29
Camelineae - ps30
Arabidopsis - ps31
Arabidopsis_thaliana - ps32
p-value
7e-04
2e-03
3e-03
6e-03
1e-02
Log-odds
0.5
1.0
1.5
2.0
2.5
3.0
3.5
4.0

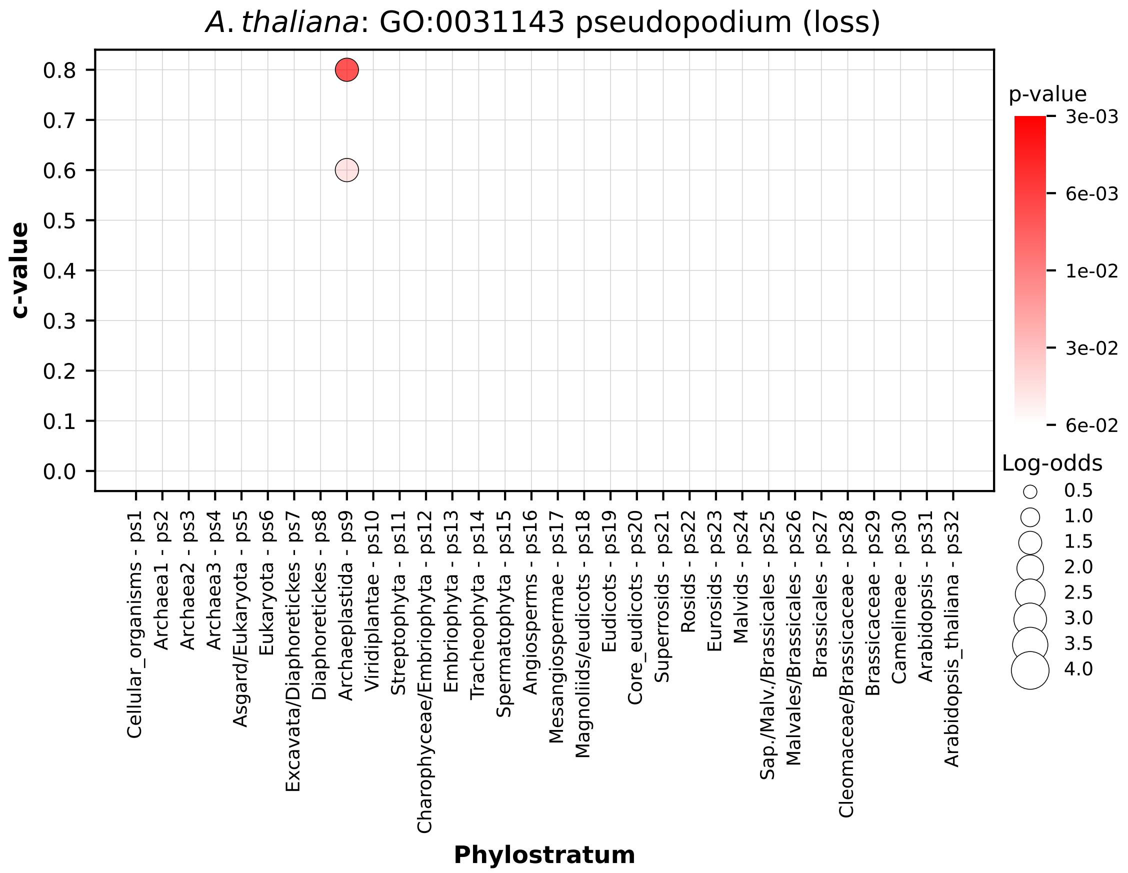

A. thaliana: GO:0031143 pseudopodium (loss)
c-value
0.8
0.7
0.6
0.5
0.4
0.3
0.2
0.1
0.0
Phylostratum
Cellular_organisms - ps1
Archaea1 - ps2
Archaea2 - ps3
Archaea3 - ps4
Asgard/Eukaryota - ps5
Eukaryota - ps6
Excavata/Diaphoretickes - ps7
Diaphoretickes - ps8
Archaeplastida - ps9
Viridiplantae - ps10
Streptophyta - ps11
Charophyceae/Embriophyta - ps12
Embriophyta - ps13
Tracheophyta - ps14
Spermatophyta - ps15
Angiosperms - ps16
Mesangiospermae - ps17
Magnoliids/eudicots - ps18
Eudicots - ps19
Core_eudicots - ps20
Superrosids - ps21
Rosids - ps22
Eurosids - ps23
Malvids - ps24
Sap./Malv./Brassicales - ps25
Malvales/Brassicales - ps26
Brassicales - ps27
Cleomaceae/Brassicaceae - ps28
Brassicaceae - ps29
Camelineae - ps30
Arabidopsis - ps31
Arabidopsis_thaliana - ps32
p-value
3e-03
6e-03
1e-02
3e-02
6e-02
Log-odds
0.5
1.0
1.5
2.0
2.5
3.0
3.5
4.0

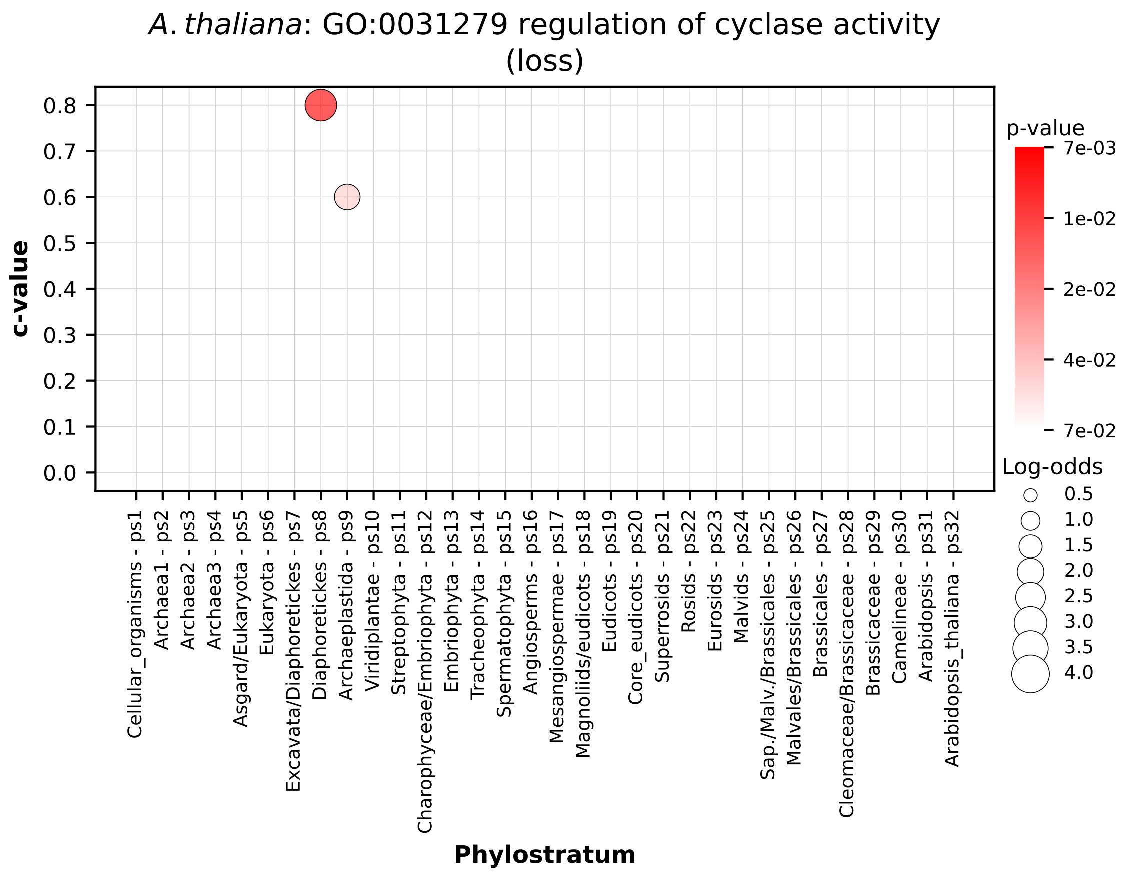

A. thaliana: GO:0031279 regulation of cyclase activity
(loss)
c-value
0.0
0.1
0.2
0.3
0.4
0.5
0.6
0.7
0.8
p-value
7e-03
1e-02
2e-02
4e-02
7e-02
Log-odds
0.5
1.0
1.5
2.0
2.5
3.0
3.5
4.0
Cellular_organisms - ps1
Archaea1 - ps2
Archaea2 - ps3
Archaea3 - ps4
Asgard/Eukaryota - ps5
Eukaryota - ps6
Excavata/Diaphoretickes - ps7
Diaphoretickes - ps8
Archaeplastida - ps9
Viridiplantae - ps10
Streptophyta - ps11
Charophyceae/Embriophyta - ps12
Embriophyta - ps13
Tracheophyta - ps14
Spermatophyta - ps15
Angiosperms - ps16
Mesangiospermae - ps17
Magnoliids/eudicots - ps18
Eudicots - ps19
Core_eudicots - ps20
Superrosids - ps21
Rosids - ps22
Eurosids - ps23
Malvids - ps24
Sap./Malv./Brassicales - ps25
Malvales/Brassicales - ps26
Brassicales - ps27
Cleomaceae/Brassicaceae - ps28
Brassicaceae - ps29
Camelineae - ps30
Arabidopsis - ps31
Arabidopsis_thaliana - ps32
Phylostratum

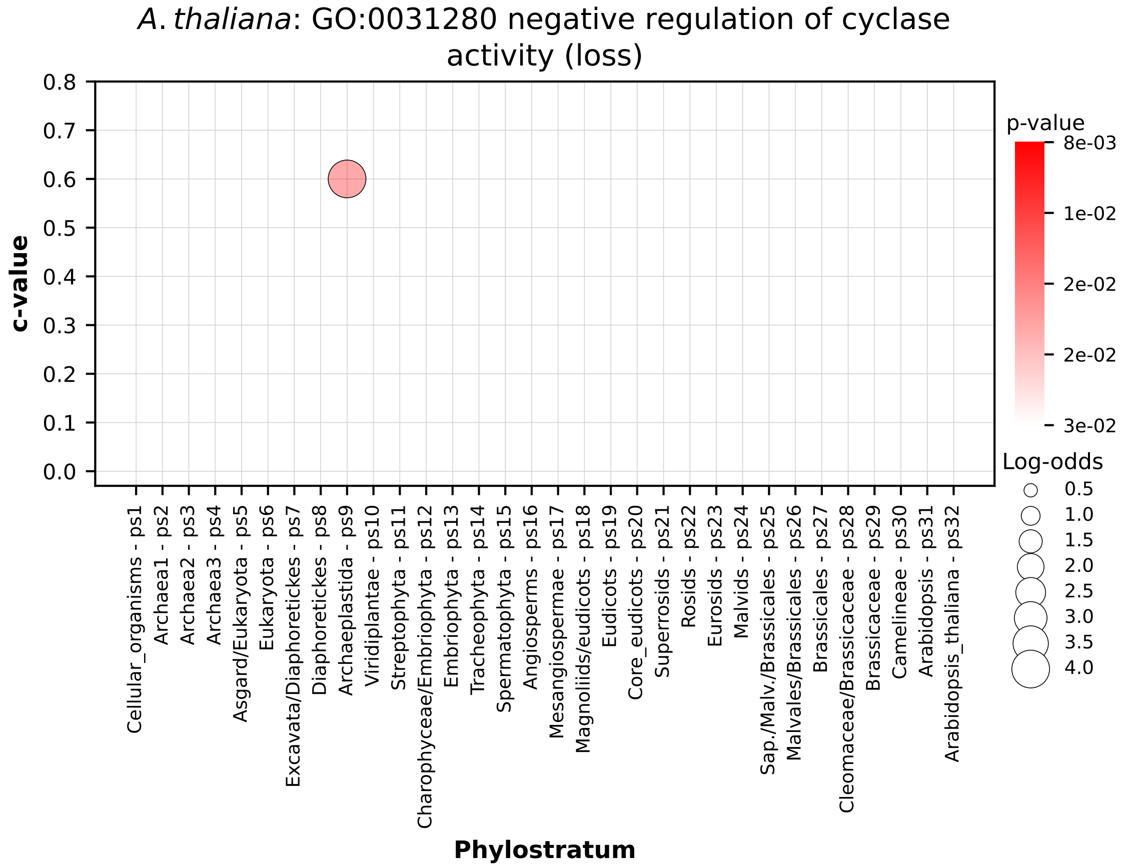

A. thaliana: GO:0031280 negative regulation of cyclase activity (loss)
c-value
0.8
0.7
0.6
0.5
0.4
0.3
0.2
0.1
0.0
p-value
8e-03
1e-02
2e-02
2e-02
3e-02
Log-odds
0.5
1.0
1.5
2.0
2.5
3.0
3.5
4.0
Cellular_organisms - ps1
Archaea1 - ps2
Archaea2 - ps3
Archaea3 - ps4
Asgard/Eukaryota - ps5
Eukaryota - ps6
Excavata/Diaphoretickes - ps7
Diaphoretickes - ps8
Archaeplastida - ps9
Viridiplantae - ps10
Streptophyta - ps11
Charophyceae/Embriophyta - ps12
Embriophyta - ps13
Tracheophyta - ps14
Spermatophyta - ps15
Angiosperms - ps16
Mesangiospermae - ps17
Magnoliids/eudicots - ps18
Eudicots - ps19
Core_eudicots - ps20
Superrosids - ps21
Rosids - ps22
Eurosids - ps23
Malvids - ps24
Sap./Malv./Brassicales - ps25
Malvales/Brassicales - ps26
Brassicales - ps27
Cleomaceae/Brassicaceae - ps28
Brassicaceae - ps29
Camelineae - ps30
Arabidopsis - ps31
Arabidopsis_thaliana - ps32
Phylostratum

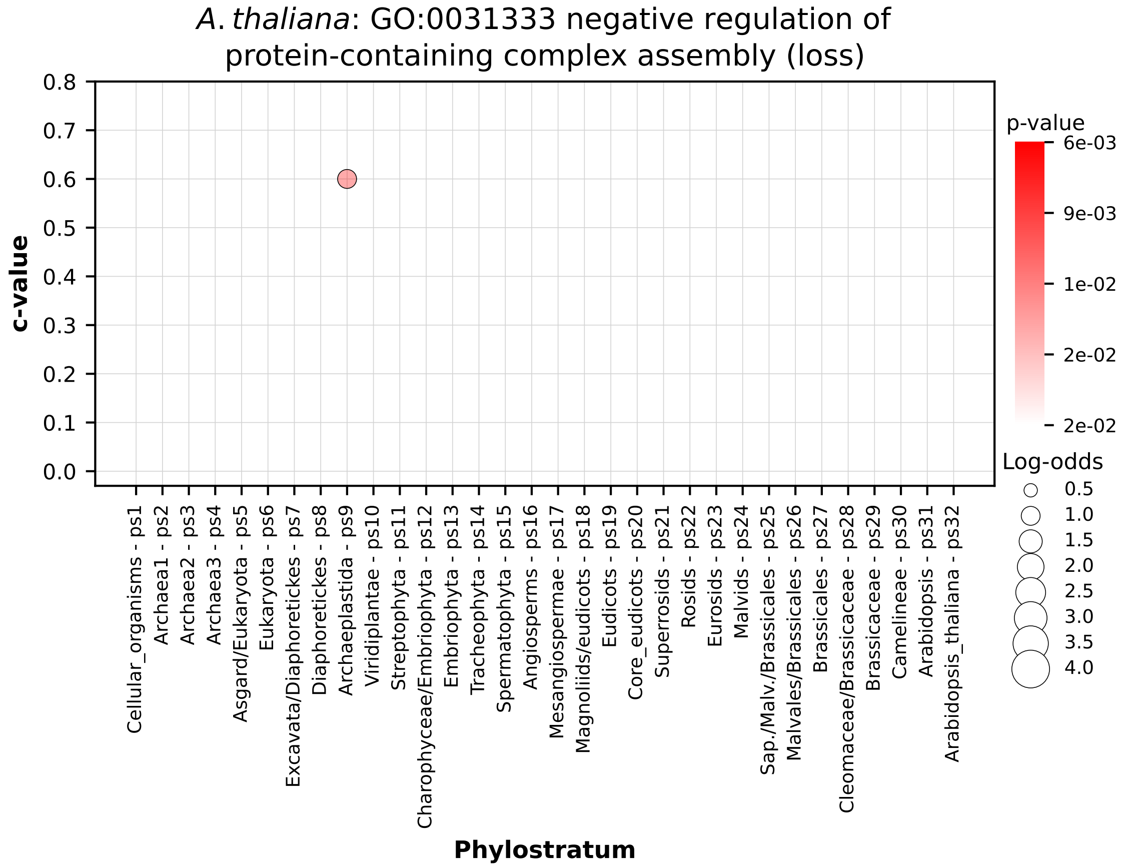

A. thaliana: GO:0031333 negative regulation of protein-containing complex assembly (loss)
c-value
0.8
0.7
0.6
0.5
0.4
0.3
0.2
0.1
0.0
p-value
6e-03
9e-03
1e-02
2e-02
2e-02
Log-odds
0.5
1.0
1.5
2.0
2.5
3.0
3.5
4.0
Cellular_organisms - ps1
Archaea1 - ps2
Archaea2 - ps3
Archaea3 - ps4
Asgard/Eukaryota - ps5
Eukaryota - ps6
Excavata/Diaphoretickes - ps7
Diaphoretickes - ps8
Archaeplastida - ps9
Viridiplantae - ps10
Streptophyta - ps11
Charophyceae/Embriophyta - ps12
Embriophyta - ps13
Tracheophyta - ps14
Spermatophyta - ps15
Angiosperms - ps16
Mesangiospermae - ps17
Magnoliids/eudicots - ps18
Eudicots - ps19
Core_eudicots - ps20
Superrosids - ps21
Rosids - ps22
Eurosids - ps23
Malvids - ps24
Sap./Malv./Brassicales - ps25
Malvales/Brassicales - ps26
Brassicales - ps27
Cleomaceae/Brassicaceae - ps28
Brassicaceae - ps29
Camelineae - ps30
Arabidopsis - ps31
Arabidopsis_thaliana - ps32
Phylostratum

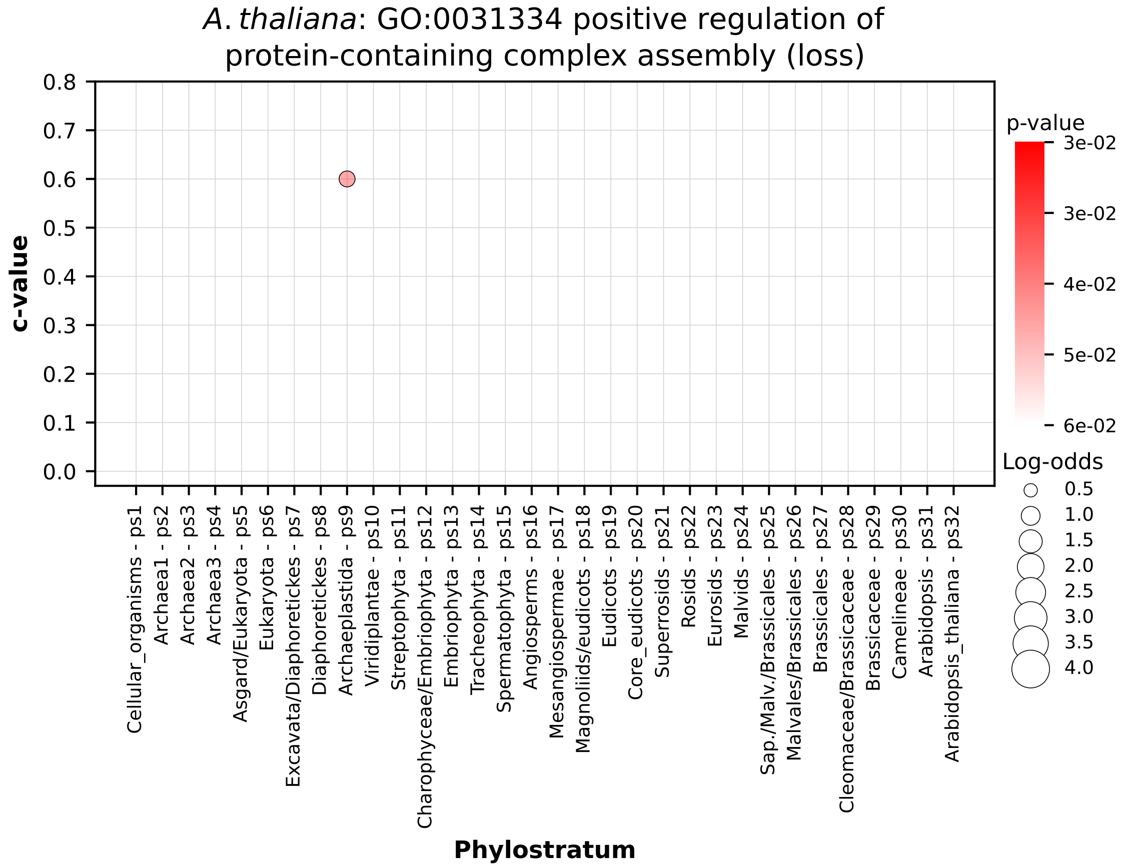

A. thaliana: GO:0031334 positive regulation of protein-containing complex assembly (loss)
c-value
0.8
0.7
0.6
0.5
0.4
0.3
0.2
0.1
0.0
Phylostratum
Cellular_organisms - ps1
Archaea1 - ps2
Archaea2 - ps3
Archaea3 - ps4
Asgard/Eukaryota - ps5
Eukaryota - ps6
Excavata/Diaphoretickes - ps7
Diaphoretickes - ps8
Archaeplastida - ps9
Viridiplantae - ps10
Streptophyta - ps11
Charophyceae/Embriophyta - ps12
Embriophyta - ps13
Tracheophyta - ps14
Spermatophyta - ps15
Angiosperms - ps16
Mesangiospermae - ps17
Magnoliids/eudicots - ps18
Eudicots - ps19
Core_eudicots - ps20
Superrosids - ps21
Rosids - ps22
Eurosids - ps23
Malvids - ps24
Sap./Malv./Brassicales - ps25
Malvales/Brassicales - ps26
Brassicales - ps27
Cleomaceae/Brassicaceae - ps28
Brassicaceae - ps29
Camelineae - ps30
Arabidopsis - ps31
Arabidopsis_thaliana - ps32
p-value
3e-02
3e-02
4e-02
5e-02
6e-02
Log-odds
0.5
1.0
1.5
2.0
2.5
3.0
3.5
4.0

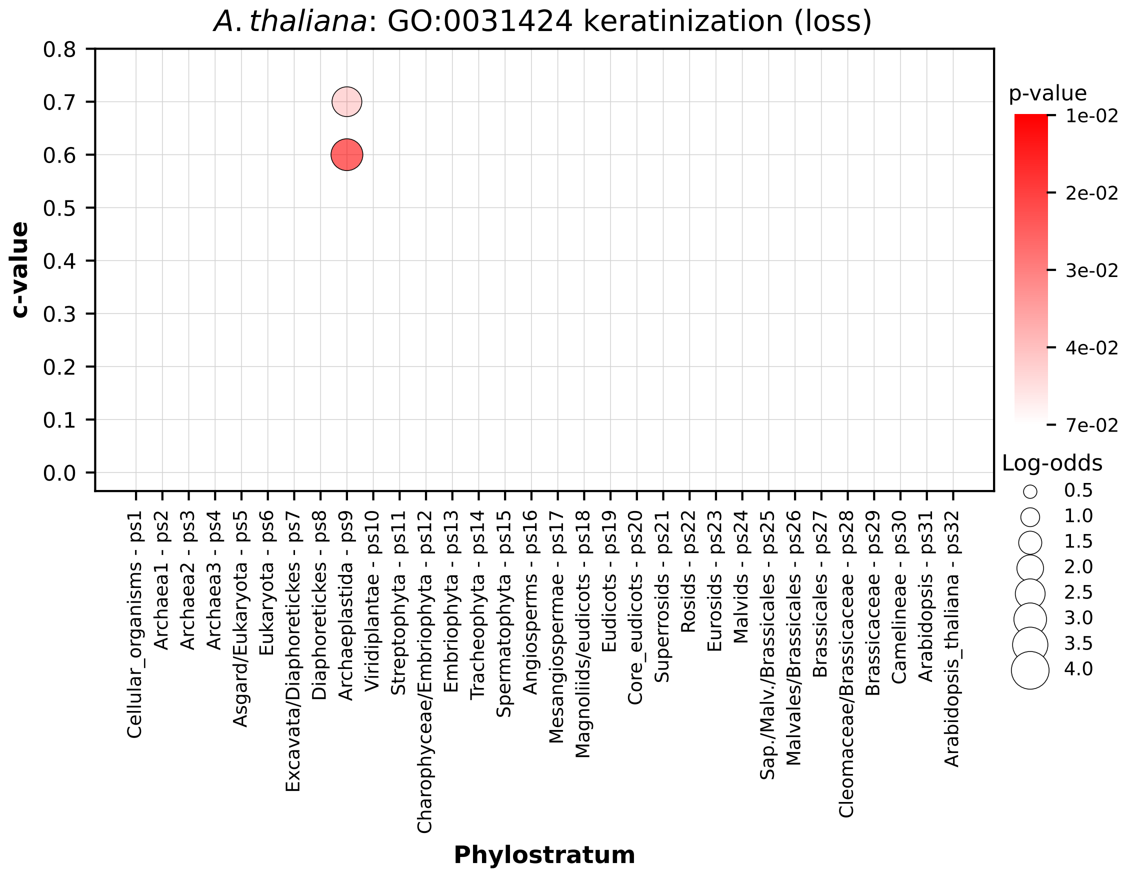
A. thaliana: GO:0031424 keratinization (loss)
c-value
0.8
0.7
0.6
0.5
0.4
0.3
0.2
0.1
0.0
p-value
1e-02
2e-02
3e-02
4e-02
7e-02
Log-odds
0.5
1.0
1.5
2.0
2.5
3.0
3.5
4.0
Cellular_organisms - ps1
Archaea1 - ps2
Archaea2 - ps3
Archaea3 - ps4
Asgard/Eukaryota - ps5
Eukaryota - ps6
Excavata/Diaphoretickes - ps7
Diaphoretickes - ps8
Archaeplastida - ps9
Viridiplantae - ps10
Streptophyta - ps11
Charophyceae/Embriophyta - ps12
Embriophyta - ps13
Tracheophyta - ps14
Spermatophyta - ps15
Angiosperms - ps16
Mesangiospermae - ps17
Magnoliids/eudicots - ps18
Eudicots - ps19
Core_eudicots - ps20
Superrosids - ps21
Rosids - ps22
Eurosids - ps23
Malvids - ps24
Sap./Malv./Brassicales - ps25
Malvales/Brassicales - ps26
Brassicales - ps27
Cleomaceae/Brassicaceae - ps28
Brassicaceae - ps29
Camelineae - ps30
Arabidopsis - ps31
Arabidopsis_thaliana - ps32
Phylostratum

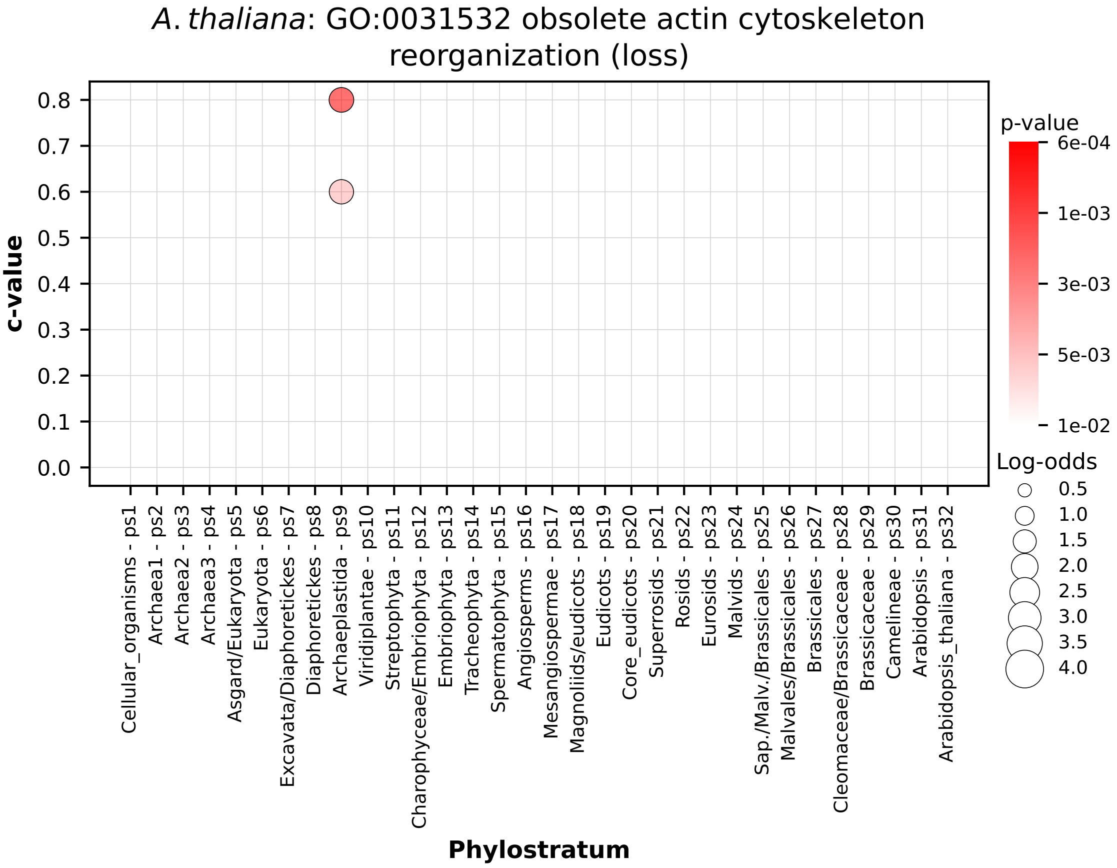

A. thaliana: GO:0031532 obsolete actin cytoskeleton reorganization (loss)
c-value
0.0
0.1
0.2
0.3
0.4
0.5
0.6
0.7
0.8
Phylostratum
Cellular_organisms - ps1
Archaea1 - ps2
Archaea2 - ps3
Archaea3 - ps4
Asgard/Eukaryota - ps5
Eukaryota - ps6
Excavata/Diaphoretickes - ps7
Diaphoretickes - ps8
Archaeplastida - ps9
Viridiplantae - ps10
Streptophyta - ps11
Charophyceae/Embriophyta - ps12
Embriophyta - ps13
Tracheophyta - ps14
Spermatophyta - ps15
Angiosperms - ps16
Mesangiospermae - ps17
Magnoliids/eudicots - ps18
Eudicots - ps19
Core_eudicots - ps20
Superrosids - ps21
Rosids - ps22
Eurosids - ps23
Malvids - ps24
Sap./Malv./Brassicales - ps25
Malvales/Brassicales - ps26
Brassicales - ps27
Cleomaceae/Brassicaceae - ps28
Brassicaceae - ps29
Camelineae - ps30
Arabidopsis - ps31
Arabidopsis_thaliana - ps32
p-value
6e-04
1e-03
3e-03
5e-03
1e-02
Log-odds
0.5
1.0
1.5
2.0
2.5
3.0
3.5
4.0

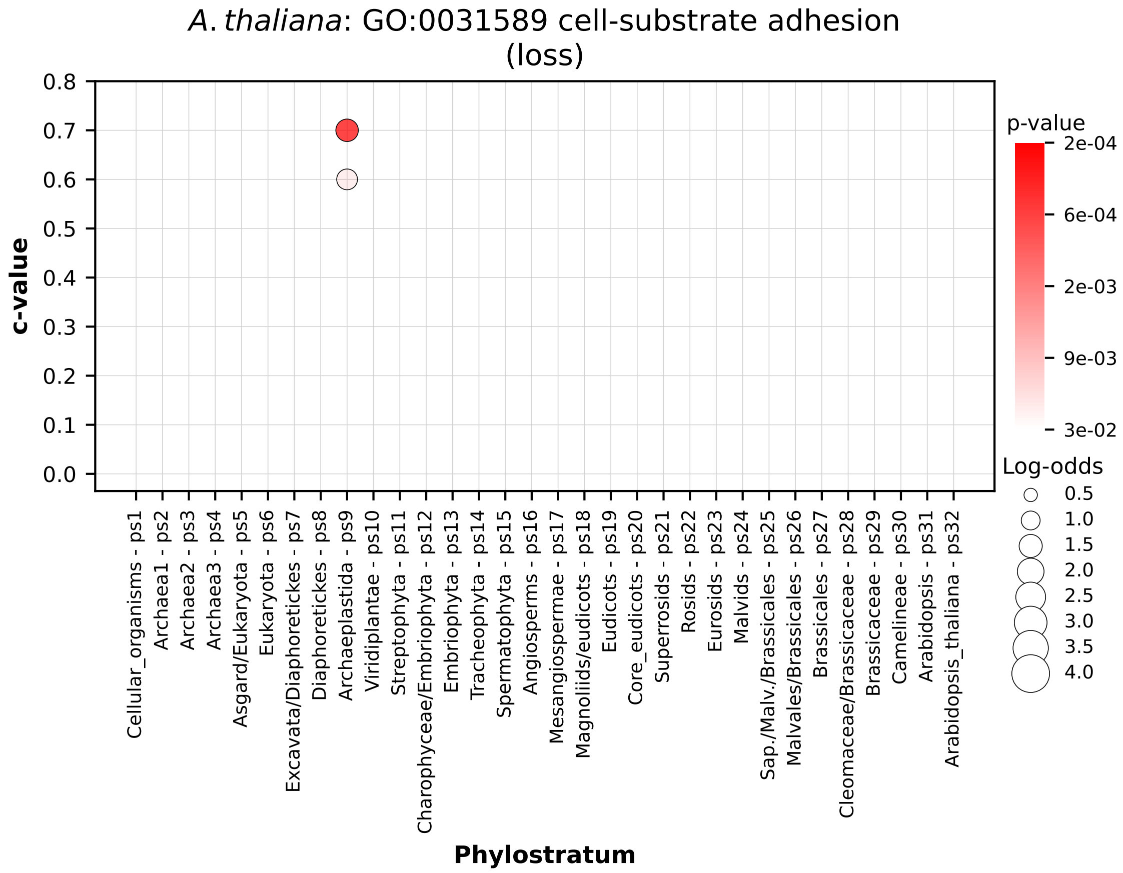

A. thaliana: GO:0031589 cell-substrate adhesion
(loss)
c-value
0.8
0.7
0.6
0.5
0.4
0.3
0.2
0.1
0.0
p-value
2e-04
6e-04
2e-03
9e-03
3e-02
Log-odds
0.5
1.0
1.5
2.0
2.5
3.0
3.5
4.0
Cellular_organisms - ps1
Archaea1 - ps2
Archaea2 - ps3
Archaea3 - ps4
Asgard/Eukaryota - ps5
Eukaryota - ps6
Excavata/Diaphoretickes - ps7
Diaphoretickes - ps8
Archaeplastida - ps9
Viridiplantae - ps10
Streptophyta - ps11
Charophyceae/Embriophyta - ps12
Embriophyta - ps13
Tracheophyta - ps14
Spermatophyta - ps15
Angiosperms - ps16
Mesangiospermae - ps17
Magnoliids/eudicots - ps18
Eudicots - ps19
Core_eudicots - ps20
Superrosids - ps21
Rosids - ps22
Eurosids - ps23
Malvids - ps24
Sap./Malv./Brassicales - ps25
Malvales/Brassicales - ps26
Brassicales - ps27
Cleomaceae/Brassicaceae - ps28
Brassicaceae - ps29
Camelineae - ps30
Arabidopsis - ps31
Arabidopsis_thaliana - ps32
Phylostratum

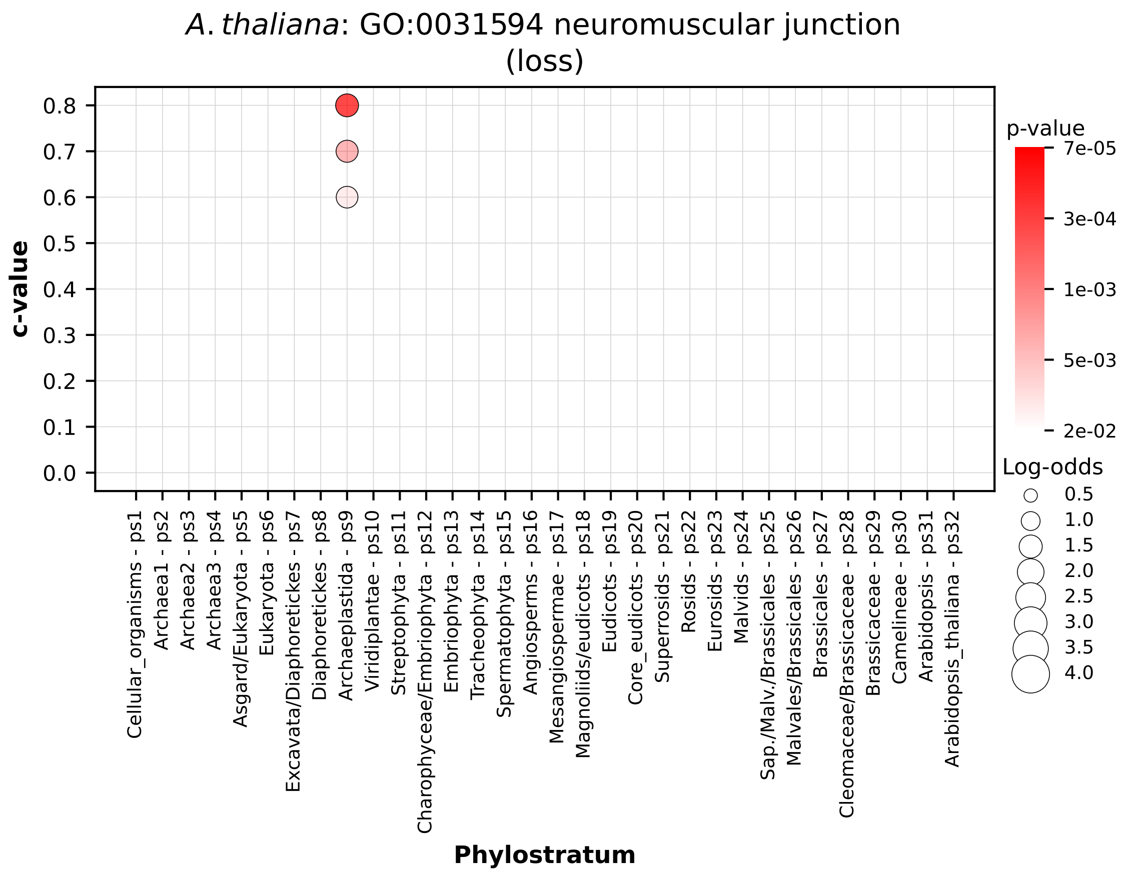

A. thaliana: GO:0031594 neuromuscular junction
(loss)
c-value
0.0
0.1
0.2
0.3
0.4
0.5
0.6
0.7
0.8
Phylostratum
Cellular_organisms - ps1
Archaea1 - ps2
Archaea2 - ps3
Archaea3 - ps4
Asgard/Eukaryota - ps5
Eukaryota - ps6
Excavata/Diaphoretickes - ps7
Diaphoretickes - ps8
Archaeplastida - ps9
Viridiplantae - ps10
Streptophyta - ps11
Charophyceae/Embriophyta - ps12
Embriophyta - ps13
Tracheophyta - ps14
Spermatophyta - ps15
Angiosperms - ps16
Mesangiospermae - ps17
Magnoliids/eudicots - ps18
Eudicots - ps19
Core_eudicots - ps20
Superrosids - ps21
Rosids - ps22
Eurosids - ps23
Malvids - ps24
Sap./Malv./Brassicales - ps25
Malvales/Brassicales - ps26
Brassicales - ps27
Cleomaceae/Brassicaceae - ps28
Brassicaceae - ps29
Camelineae - ps30
Arabidopsis - ps31
Arabidopsis_thaliana - ps32
p-value
7e-05
3e-04
1e-03
5e-03
2e-02
Log-odds
0.5
1.0
1.5
2.0
2.5
3.0
3.5
4.0

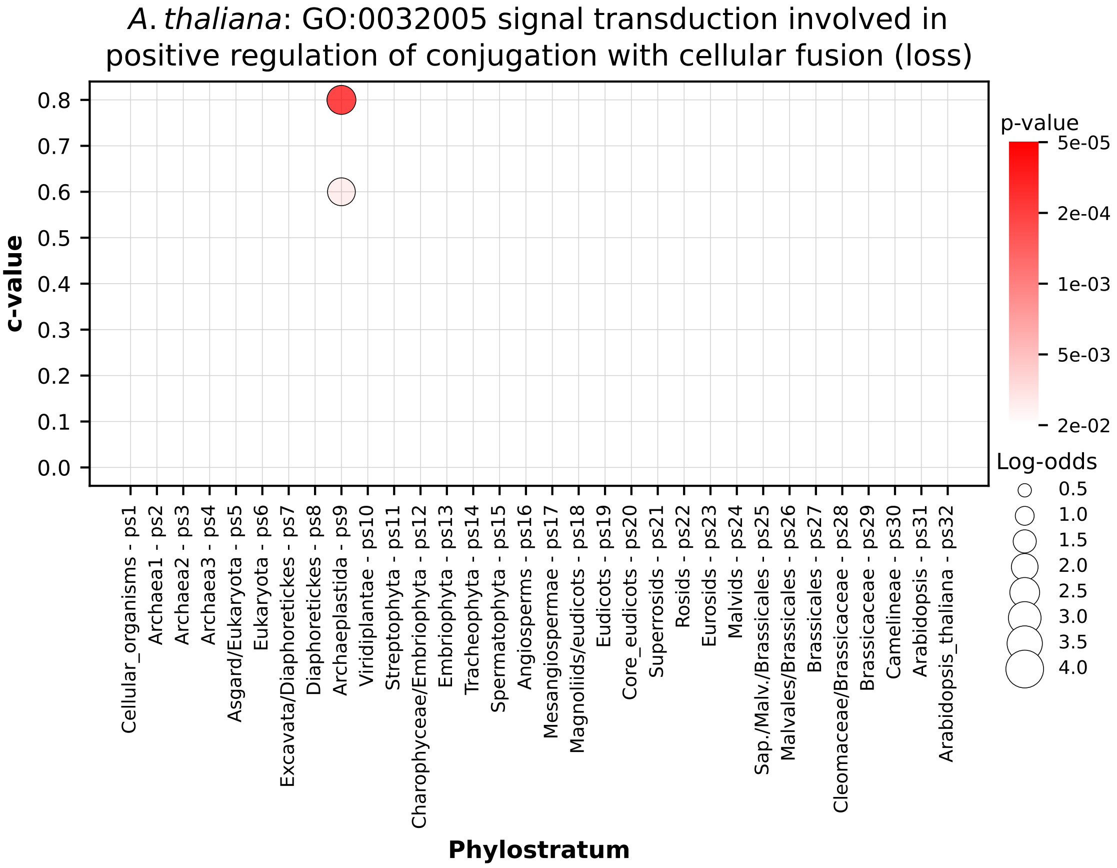

A. thaliana: GO:0032005 signal transduction involved in positive regulation of conjugation with cellular fusion (loss)
c-value
0.0
0.1
0.2
0.3
0.4
0.5
0.6
0.7
0.8
p-value
5e-05
2e-04
1e-03
5e-03
2e-02
Log-odds
0.5
1.0
1.5
2.0
2.5
3.0
3.5
4.0
Cellular_organisms - ps1
Archaea1 - ps2
Archaea2 - ps3
Archaea3 - ps4
Asgard/Eukaryota - ps5
Eukaryota - ps6
Excavata/Diaphoretickes - ps7
Diaphoretickes - ps8
Archaeplastida - ps9
Viridiplantae - ps10
Streptophyta - ps11
Charophyceae/Embriophyta - ps12
Embriophyta - ps13
Tracheophyta - ps14
Spermatophyta - ps15
Angiosperms - ps16
Mesangiospermae - ps17
Magnoliids/eudicots - ps18
Eudicots - ps19
Core_eudicots - ps20
Superrosids - ps21
Rosids - ps22
Eurosids - ps23
Malvids - ps24
Sap./Malv./Brassicales - ps25
Malvales/Brassicales - ps26
Brassicales - ps27
Cleomaceae/Brassicaceae - ps28
Brassicaceae - ps29
Camelineae - ps30
Arabidopsis - ps31
Arabidopsis_thaliana - ps32
Phylostratum

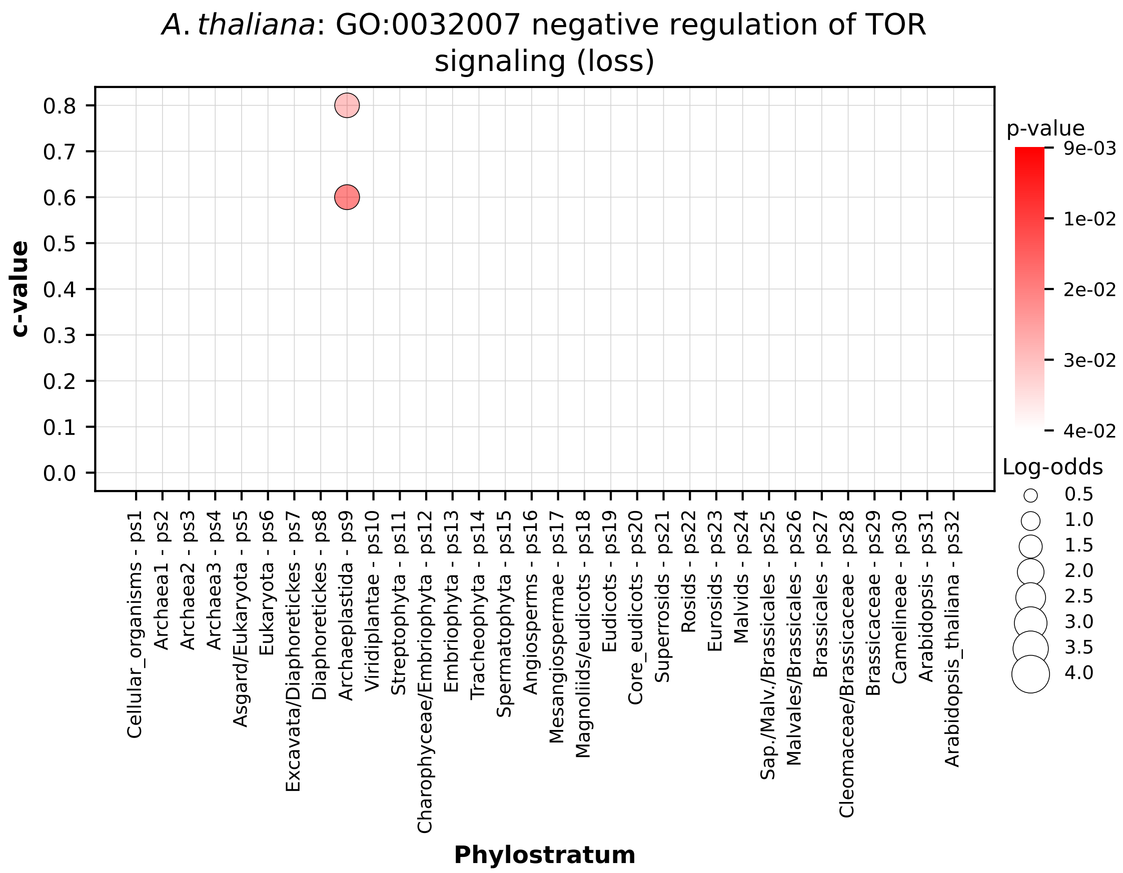

A. thaliana: GO:0032007 negative regulation of TOR signaling (loss)
c-value
0.8
0.7
0.6
0.5
0.4
0.3
0.2
0.1
0.0
p-value
9e-03
1e-02
2e-02
3e-02
4e-02
Log-odds
0.5
1.0
1.5
2.0
2.5
3.0
3.5
4.0
Cellular_organisms - ps1
Archaea1 - ps2
Archaea2 - ps3
Archaea3 - ps4
Asgard/Eukaryota - ps5
Eukaryota - ps6
Excavata/Diaphoretickes - ps7
Diaphoretickes - ps8
Archaeplastida - ps9
Viridiplantae - ps10
Streptophyta - ps11
Charophyceae/Embriophyta - ps12
Embriophyta - ps13
Tracheophyta - ps14
Spermatophyta - ps15
Angiosperms - ps16
Mesangiospermae - ps17
Magnoliids/eudicots - ps18
Eudicots - ps19
Core_eudicots - ps20
Superrosids - ps21
Rosids - ps22
Eurosids - ps23
Malvids - ps24
Sap./Malv./Brassicales - ps25
Malvales/Brassicales - ps26
Brassicales - ps27
Cleomaceae/Brassicaceae - ps28
Brassicaceae - ps29
Camelineae - ps30
Arabidopsis - ps31
Arabidopsis_thaliana - ps32
Phylostratum

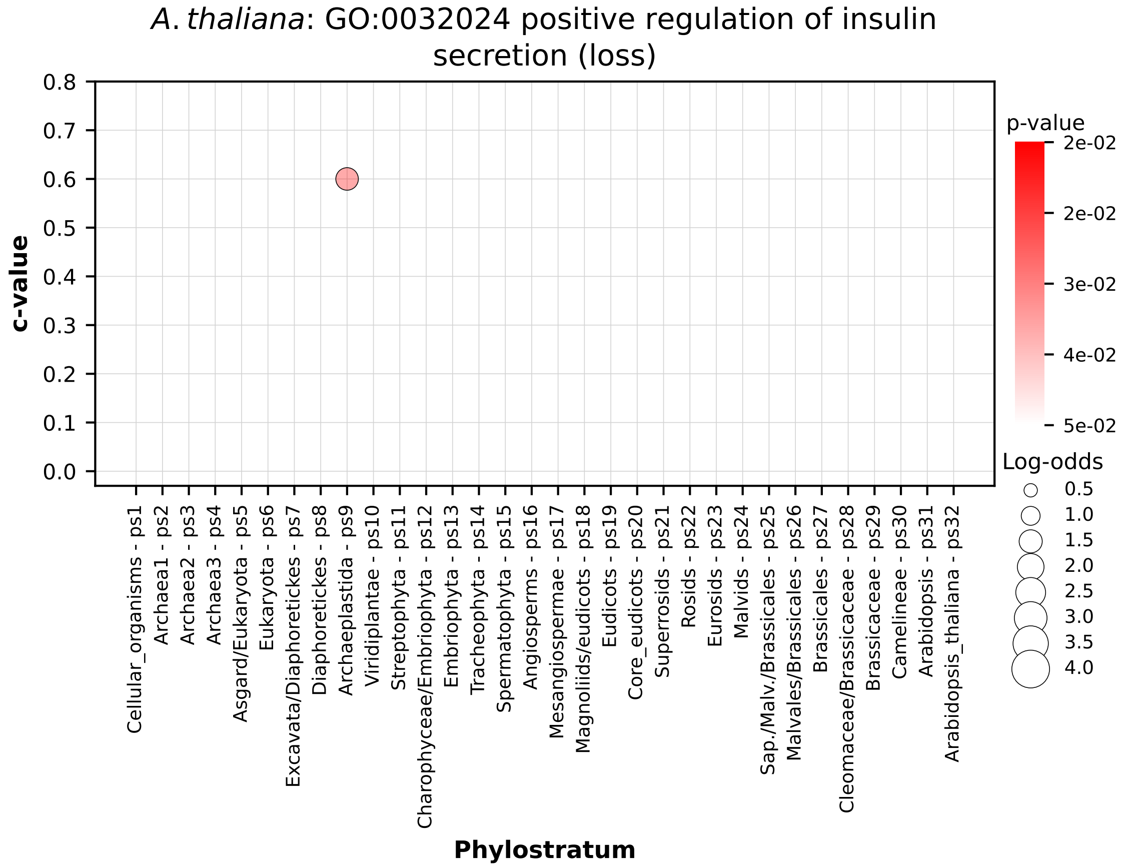

A. thaliana: GO:0032024 positive regulation of insulin secretion (loss)
c-value
0.8
0.7
0.6
0.5
0.4
0.3
0.2
0.1
0.0
p-value
2e-02
2e-02
3e-02
4e-02
5e-02
Log-odds
0.5
1.0
1.5
2.0
2.5
3.0
3.5
4.0
Cellular_organisms - ps1
Archaea1 - ps2
Archaea2 - ps3
Archaea3 - ps4
Asgard/Eukaryota - ps5
Eukaryota - ps6
Excavata/Diaphoretickes - ps7
Diaphoretickes - ps8
Archaeplastida - ps9
Viridiplantae - ps10
Streptophyta - ps11
Charophyceae/Embriophyta - ps12
Embriophyta - ps13
Tracheophyta - ps14
Spermatophyta - ps15
Angiosperms - ps16
Mesangiospermae - ps17
Magnoliids/eudicots - ps18
Eudicots - ps19
Core_eudicots - ps20
Superrosids - ps21
Rosids - ps22
Eurosids - ps23
Malvids - ps24
Sap./Malv./Brassicales - ps25
Malvales/Brassicales - ps26
Brassicales - ps27
Cleomaceae/Brassicaceae - ps28
Brassicaceae - ps29
Camelineae - ps30
Arabidopsis - ps31
Arabidopsis_thaliana - ps32
Phylostratum

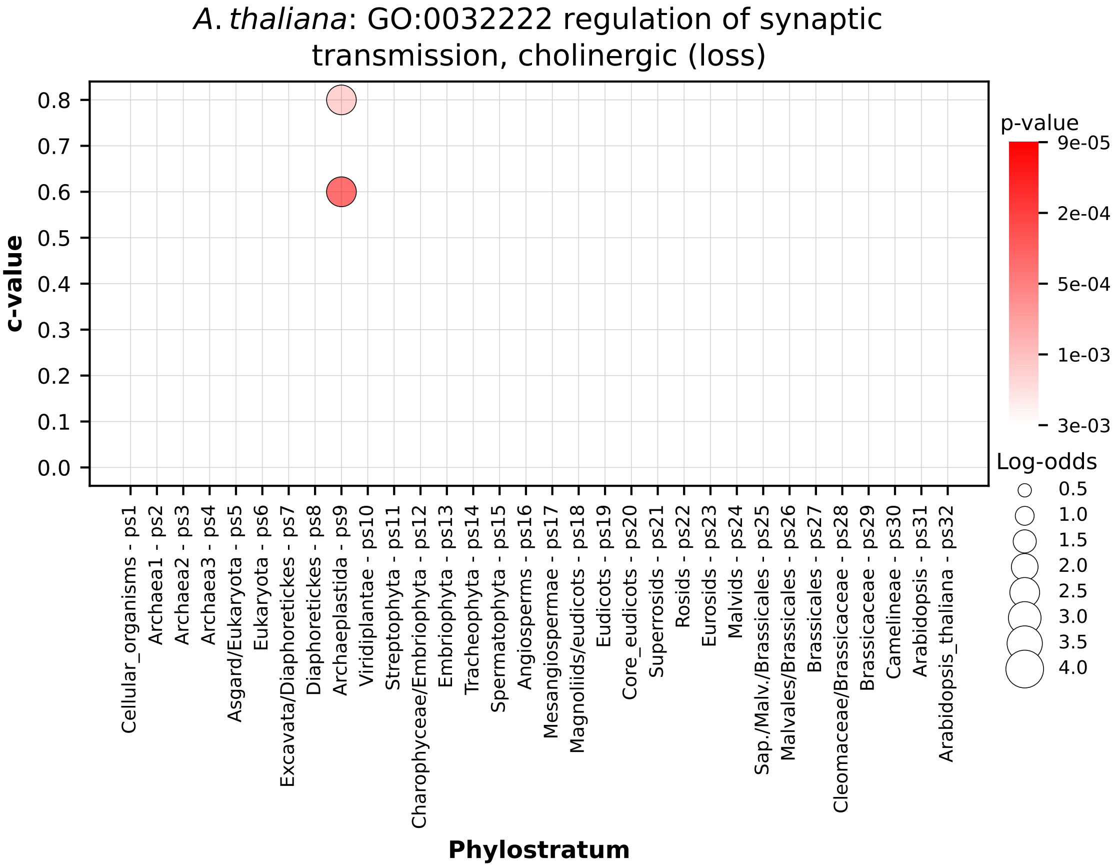

A. thaliana: GO:0032222 regulation of synaptic transmission, cholinergic (loss)
c-value
0.0
0.1
0.2
0.3
0.4
0.5
0.6
0.7
0.8
Phylostratum
Cellular_organisms - ps1
Archaea1 - ps2
Archaea2 - ps3
Archaea3 - ps4
Asgard/Eukaryota - ps5
Eukaryota - ps6
Excavata/Diaphoretickes - ps7
Diaphoretickes - ps8
Archaeplastida - ps9
Viridiplantae - ps10
Streptophyta - ps11
Charophyceae/Embriophyta - ps12
Embriophyta - ps13
Tracheophyta - ps14
Spermatophyta - ps15
Angiosperms - ps16
Mesangiospermae - ps17
Magnoliids/eudicots - ps18
Eudicots - ps19
Core_eudicots - ps20
Superrosids - ps21
Rosids - ps22
Eurosids - ps23
Malvids - ps24
Sap./Malv./Brassicales - ps25
Malvales/Brassicales - ps26
Brassicales - ps27
Cleomaceae/Brassicaceae - ps28
Brassicaceae - ps29
Camelineae - ps30
Arabidopsis - ps31
Arabidopsis_thaliana - ps32
p-value
9e-05
2e-04
5e-04
1e-03
3e-03
Log-odds
0.5
1.0
1.5
2.0
2.5
3.0
3.5
4.0

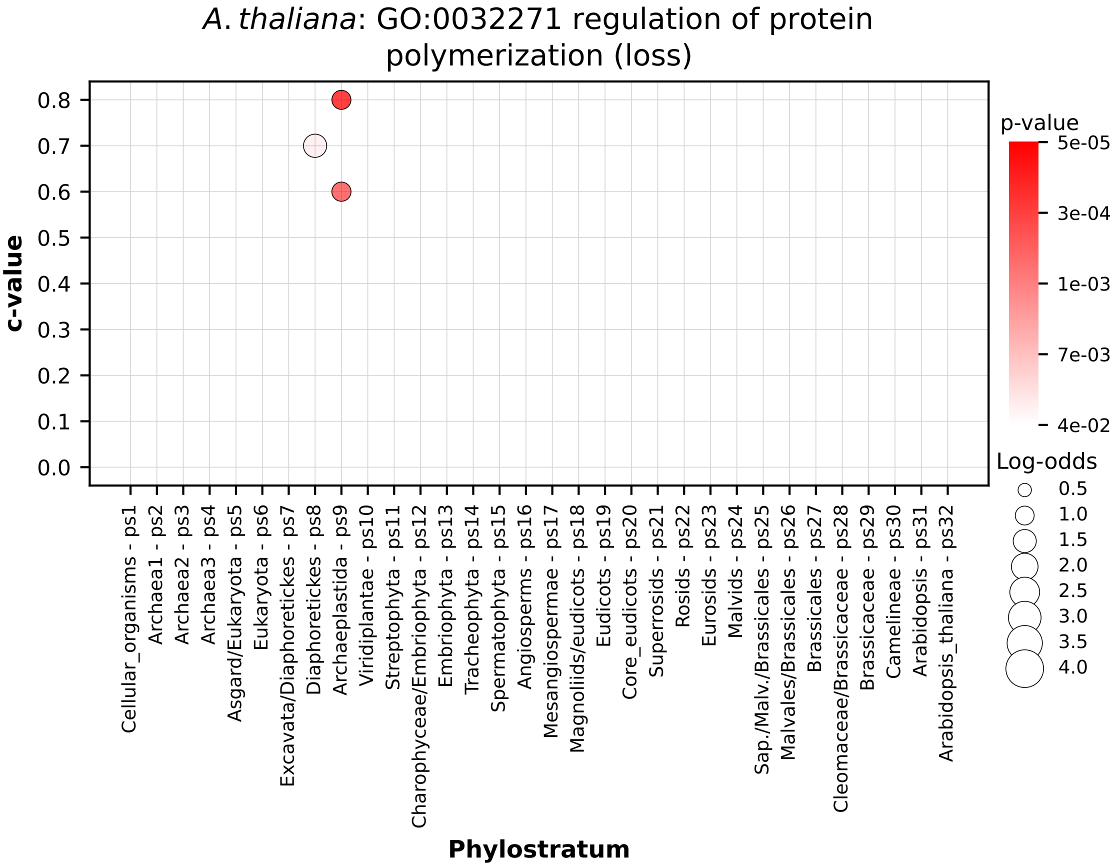

A. thaliana: GO:0032271 regulation of protein polymerization (loss)
c-value
0.8
0.7
0.6
0.5
0.4
0.3
0.2
0.1
0.0
p-value
5e-05
3e-04
1e-03
7e-03
4e-02
Log-odds
0.5
1.0
1.5
2.0
2.5
3.0
3.5
4.0
Cellular_organisms - ps1
Archaea1 - ps2
Archaea2 - ps3
Archaea3 - ps4
Asgard/Eukaryota - ps5
Eukaryota - ps6
Excavata/Diaphoretickes - ps7
Diaphoretickes - ps8
Archaeplastida - ps9
Viridiplantae - ps10
Streptophyta - ps11
Charophyceae/Embriophyta - ps12
Embriophyta - ps13
Tracheophyta - ps14
Spermatophyta - ps15
Angiosperms - ps16
Mesangiospermae - ps17
Magnoliids/eudicots - ps18
Eudicots - ps19
Core_eudicots - ps20
Superrosids - ps21
Rosids - ps22
Eurosids - ps23
Malvids - ps24
Sap./Malv./Brassicales - ps25
Malvales/Brassicales - ps26
Brassicales - ps27
Cleomaceae/Brassicaceae - ps28
Brassicaceae - ps29
Camelineae - ps30
Arabidopsis - ps31
Arabidopsis_thaliana - ps32
Phylostratum

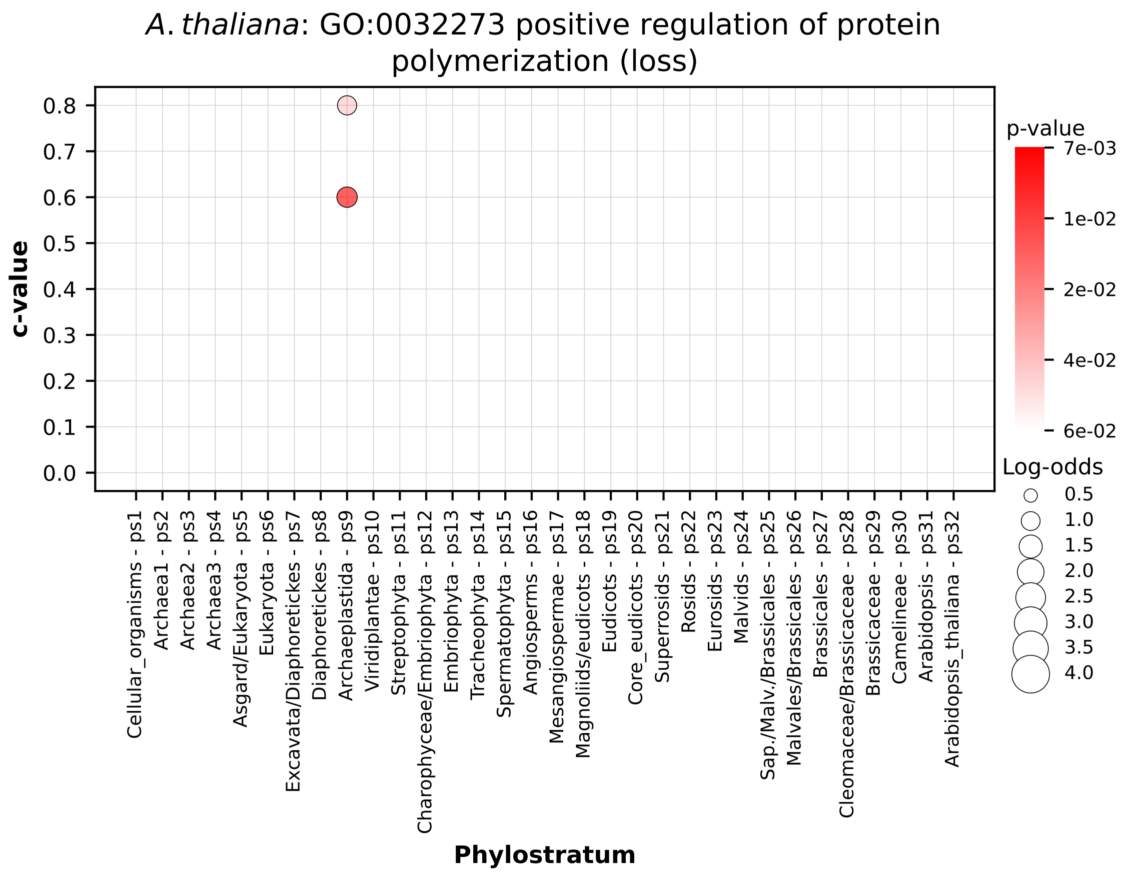
A. thaliana: GO:0032273 positive regulation of protein polymerization (loss)
c-value
0.0
0.1
0.2
0.3
0.4
0.5
0.6
0.7
0.8
p-value
7e-03
1e-02
2e-02
4e-02
6e-02
Log-odds
0.5
1.0
1.5
2.0
2.5
3.0
3.5
4.0
Cellular_organisms - ps1
Archaea1 - ps2
Archaea2 - ps3
Archaea3 - ps4
Asgard/Eukaryota - ps5
Eukaryota - ps6
Excavata/Diaphoretickes - ps7
Diaphoretickes - ps8
Archaeplastida - ps9
Viridiplantae - ps10
Streptophyta - ps11
Charophyceae/Embriophyta - ps12
Embriophyta - ps13
Tracheophyta - ps14
Spermatophyta - ps15
Angiosperms - ps16
Mesangiospermae - ps17
Magnoliids/eudicots - ps18
Eudicots - ps19
Core_eudicots - ps20
Superrosids - ps21
Rosids - ps22
Eurosids - ps23
Malvids - ps24
Sap./Malv./Brassicales - ps25
Malvales/Brassicales - ps26
Brassicales - ps27
Cleomaceae/Brassicaceae - ps28
Brassicaceae - ps29
Camelineae - ps30
Arabidopsis - ps31
Arabidopsis_thaliana - ps32
Phylostratum

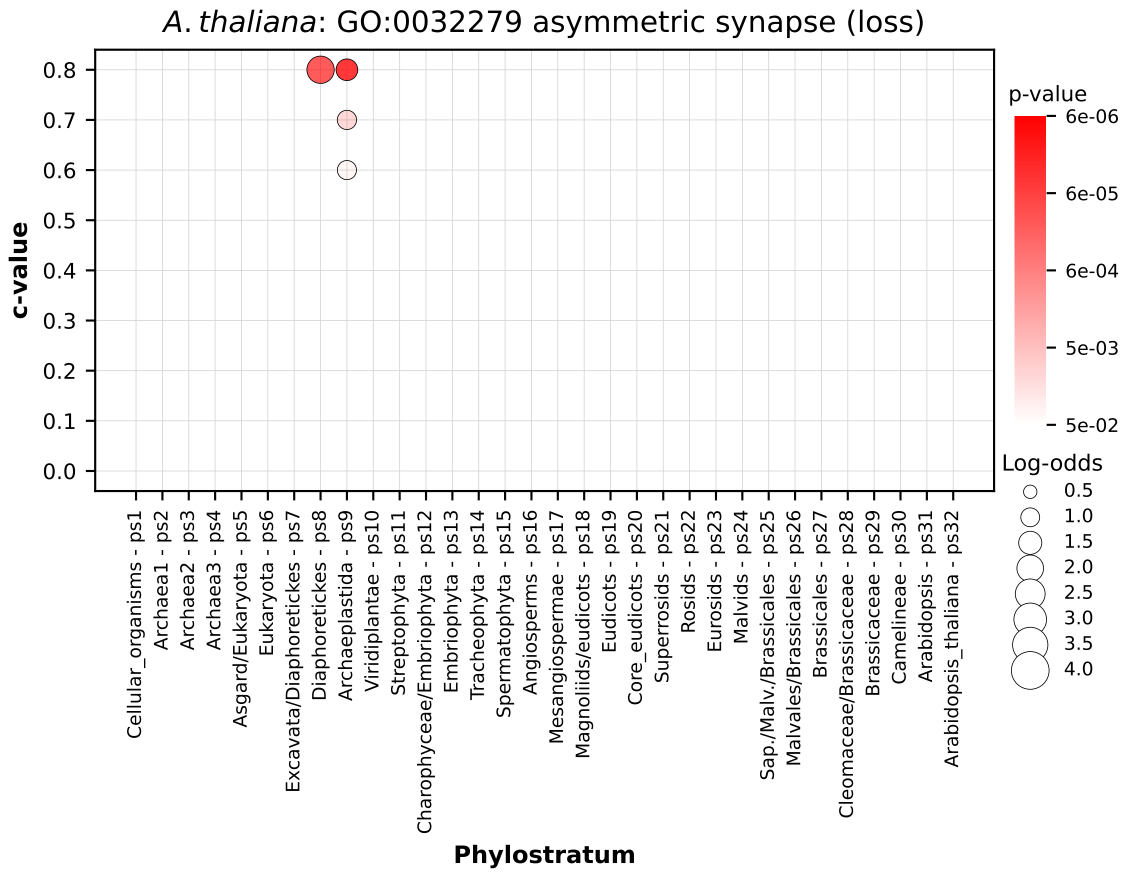

A. thaliana: GO:0032279 asymmetric synapse (loss)
c-value
0.0
0.1
0.2
0.3
0.4
0.5
0.6
0.7
0.8
Phylostratum
Cellular_organisms - ps1
Archaea1 - ps2
Archaea2 - ps3
Archaea3 - ps4
Asgard/Eukaryota - ps5
Eukaryota - ps6
Excavata/Diaphoretickes - ps7
Diaphoretickes - ps8
Archaeplastida - ps9
Viridiplantae - ps10
Streptophyta - ps11
Charophyceae/Embriophyta - ps12
Embriophyta - ps13
Tracheophyta - ps14
Spermatophyta - ps15
Angiosperms - ps16
Mesangiospermae - ps17
Magnoliids/eudicots - ps18
Eudicots - ps19
Core_eudicots - ps20
Superrosids - ps21
Rosids - ps22
Eurosids - ps23
Malvids - ps24
Sap./Malv./Brassicales - ps25
Malvales/Brassicales - ps26
Brassicales - ps27
Cleomaceae/Brassicaceae - ps28
Brassicaceae - ps29
Camelineae - ps30
Arabidopsis - ps31
Arabidopsis_thaliana - ps32
p-value
6e-06
6e-05
6e-04
5e-03
5e-02
Log-odds
0.5
1.0
1.5
2.0
2.5
3.0
3.5
4.0

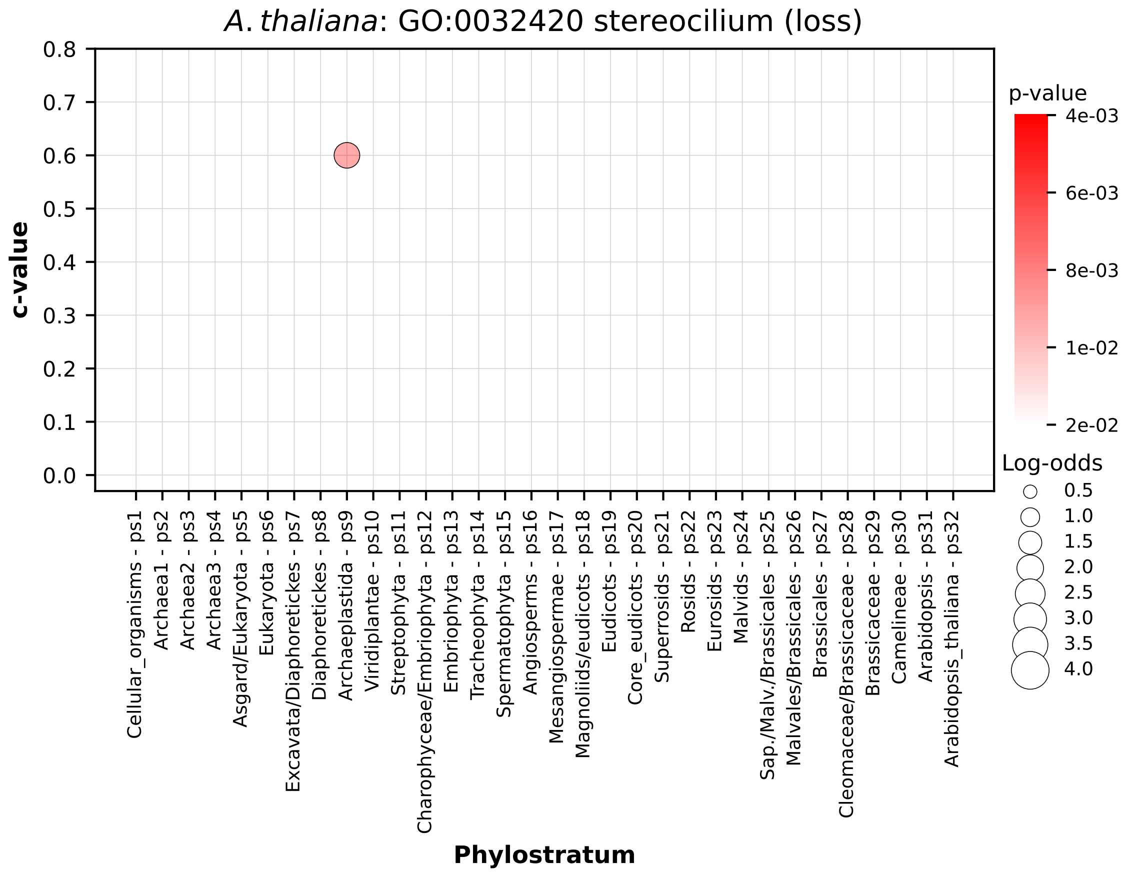

A. thaliana: GO:0032420 stereocilium (loss)
c-value
0.8
0.7
0.6
0.5
0.4
0.3
0.2
0.1
0.0
Phylostratum
Cellular_organisms - ps1
Archaea1 - ps2
Archaea2 - ps3
Archaea3 - ps4
Asgard/Eukaryota - ps5
Eukaryota - ps6
Excavata/Diaphoretickes - ps7
Diaphoretickes - ps8
Archaeplastida - ps9
Viridiplantae - ps10
Streptophyta - ps11
Charophyceae/Embriophyta - ps12
Embriophyta - ps13
Tracheophyta - ps14
Spermatophyta - ps15
Angiosperms - ps16
Mesangiospermae - ps17
Magnoliids/eudicots - ps18
Eudicots - ps19
Core_eudicots - ps20
Superrosids - ps21
Rosids - ps22
Eurosids - ps23
Malvids - ps24
Sap./Malv./Brassicales - ps25
Malvales/Brassicales - ps26
Brassicales - ps27
Cleomaceae/Brassicaceae - ps28
Brassicaceae - ps29
Camelineae - ps30
Arabidopsis - ps31
Arabidopsis_thaliana - ps32
p-value
4e-03
6e-03
8e-03
1e-02
2e-02
Log-odds
0.5
1.0
1.5
2.0
2.5
3.0
3.5
4.0

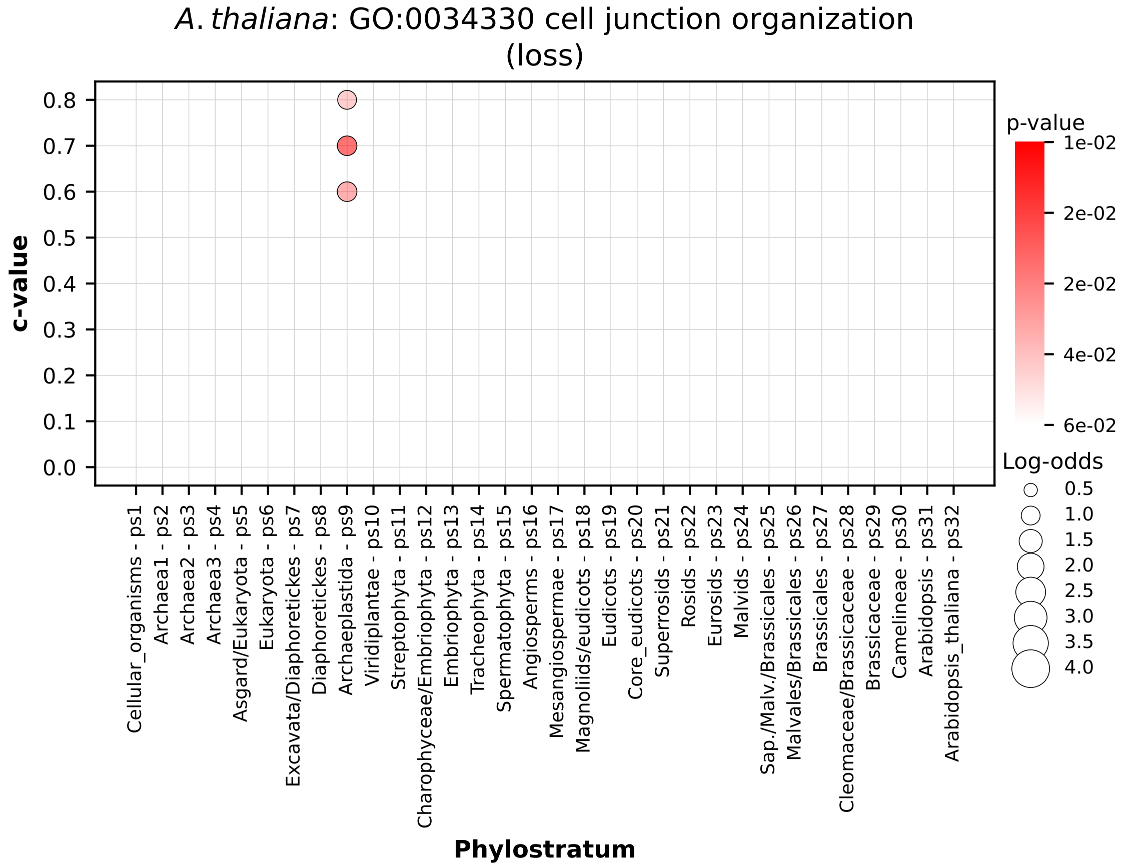

A. thaliana: GO:0034330 cell junction organization
(loss)
c-value
0.8
0.7
0.6
0.5
0.4
0.3
0.2
0.1
0.0
Phylostratum
Cellular_organisms - ps1
Archaea1 - ps2
Archaea2 - ps3
Archaea3 - ps4
Asgard/Eukaryota - ps5
Eukaryota - ps6
Excavata/Diaphoretickes - ps7
Diaphoretickes - ps8
Archaeplastida - ps9
Viridiplantae - ps10
Streptophyta - ps11
Charophyceae/Embriophyta - ps12
Embriophyta - ps13
Tracheophyta - ps14
Spermatophyta - ps15
Angiosperms - ps16
Mesangiospermae - ps17
Magnoliids/eudicots - ps18
Eudicots - ps19
Core_eudicots - ps20
Superrosids - ps21
Rosids - ps22
Eurosids - ps23
Malvids - ps24
Sap./Malv./Brassicales - ps25
Malvales/Brassicales - ps26
Brassicales - ps27
Cleomaceae/Brassicaceae - ps28
Brassicaceae - ps29
Camelineae - ps30
Arabidopsis - ps31
Arabidopsis_thaliana - ps32
p-value
1e-02
2e-02
2e-02
4e-02
6e-02
Log-odds
0.5
1.0
1.5
2.0
2.5
3.0
3.5
4.0

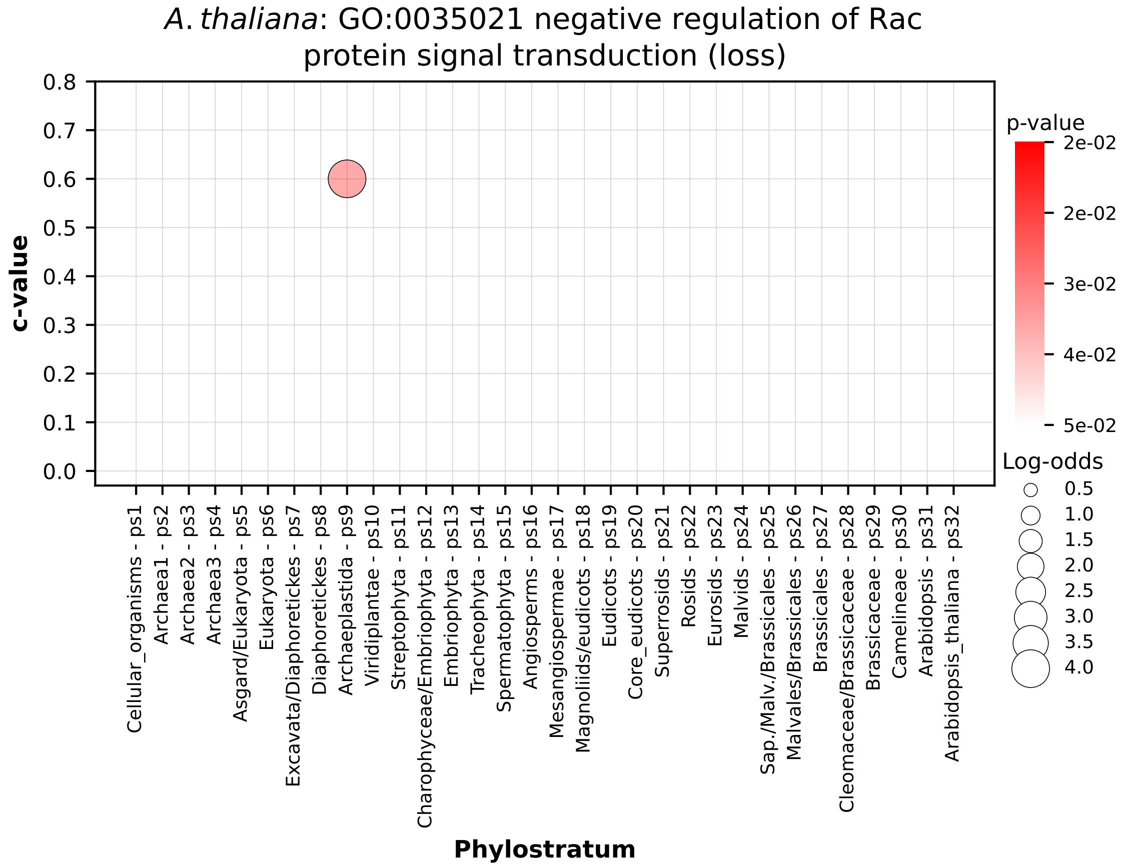

A. thaliana: GO:0035021 negative regulation of Rac protein signal transduction (loss)
c-value
0.8
0.7
0.6
0.5
0.4
0.3
0.2
0.1
0.0
Cellular_organisms - ps1
Archaea1 - ps2
Archaea2 - ps3
Archaea3 - ps4
Asgard/Eukaryota - ps5
Eukaryota - ps6
Excavata/Diaphoretickes - ps7
Diaphoretickes - ps8
Archaeplastida - ps9
Viridiplantae - ps10
Streptophyta - ps11
Charophyceae/Embriophyta - ps12
Embriophyta - ps13
Tracheophyta - ps14
Spermatophyta - ps15
Angiosperms - ps16
Mesangiospermae - ps17
Magnoliids/eudicots - ps18
Eudicots - ps19
Core_eudicots - ps20
Superrosids - ps21
Rosids - ps22
Eurosids - ps23
Malvids - ps24
Sap./Malv./Brassicales - ps25
Malvales/Brassicales - ps26
Brassicales - ps27
Cleomaceae/Brassicaceae - ps28
Brassicaceae - ps29
Camelineae - ps30
Arabidopsis - ps31
Arabidopsis_thaliana - ps32
Phylostratum
p-value
2e-02
2e-02
3e-02
4e-02
5e-02
Log-odds
0.5
1.0
1.5
2.0
2.5
3.0
3.5
4.0

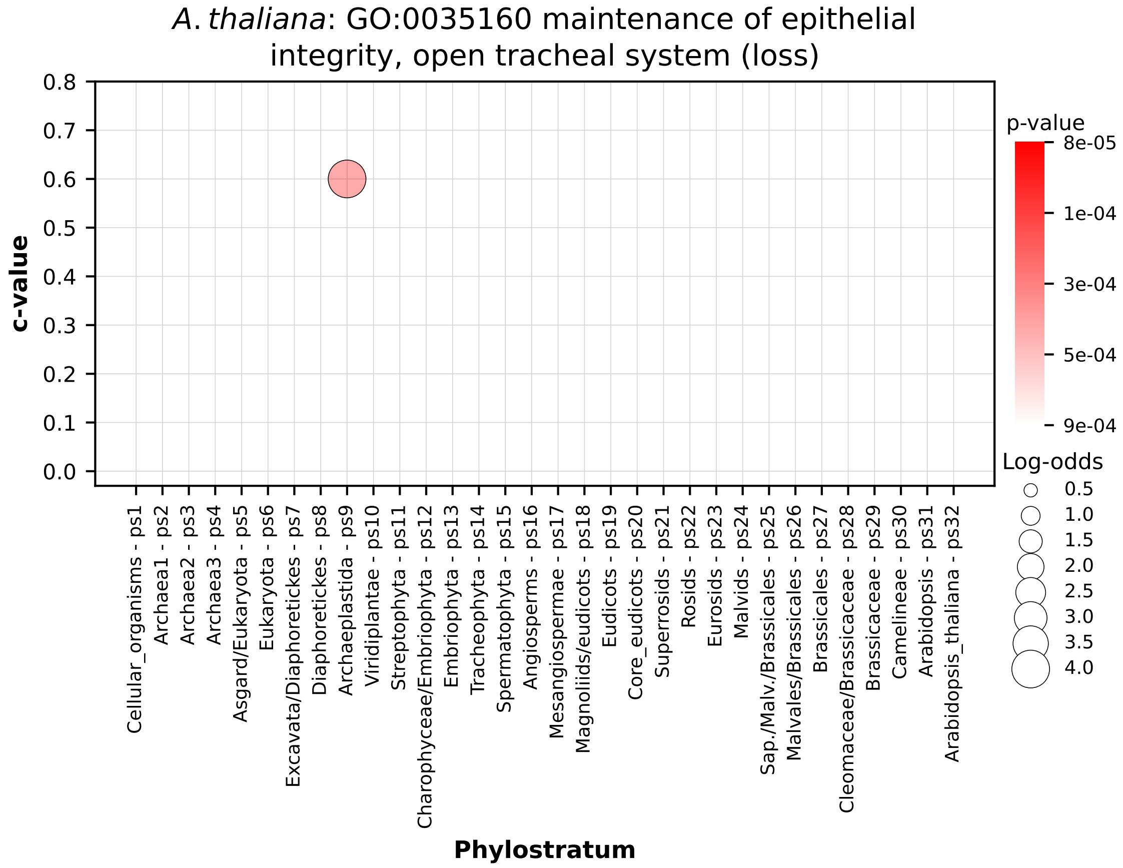
A. thaliana: GO:0035160 maintenance of epithelial integrity, open tracheal system (loss)
c-value
0.8
0.7
0.6
0.5
0.4
0.3
0.2
0.1
0.0
Phylostratum
Cellular_organisms - ps1
Archaea1 - ps2
Archaea2 - ps3
Archaea3 - ps4
Asgard/Eukaryota - ps5
Eukaryota - ps6
Excavata/Diaphoretickes - ps7
Diaphoretickes - ps8
Archaeplastida - ps9
Viridiplantae - ps10
Streptophyta - ps11
Charophyceae/Embriophyta - ps12
Embriophyta - ps13
Tracheophyta - ps14
Spermatophyta - ps15
Angiosperms - ps16
Mesangiospermae - ps17
Magnoliids/eudicots - ps18
Eudicots - ps19
Core_eudicots - ps20
Superrosids - ps21
Rosids - ps22
Eurosids - ps23
Malvids - ps24
Sap./Malv./Brassicales - ps25
Malvales/Brassicales - ps26
Brassicales - ps27
Cleomaceae/Brassicaceae - ps28
Brassicaceae - ps29
Camelineae - ps30
Arabidopsis - ps31
Arabidopsis_thaliana - ps32
p-value
8e-05
1e-04
3e-04
5e-04
9e-04
Log-odds
0.5
1.0
1.5
2.0
2.5
3.0
3.5
4.0

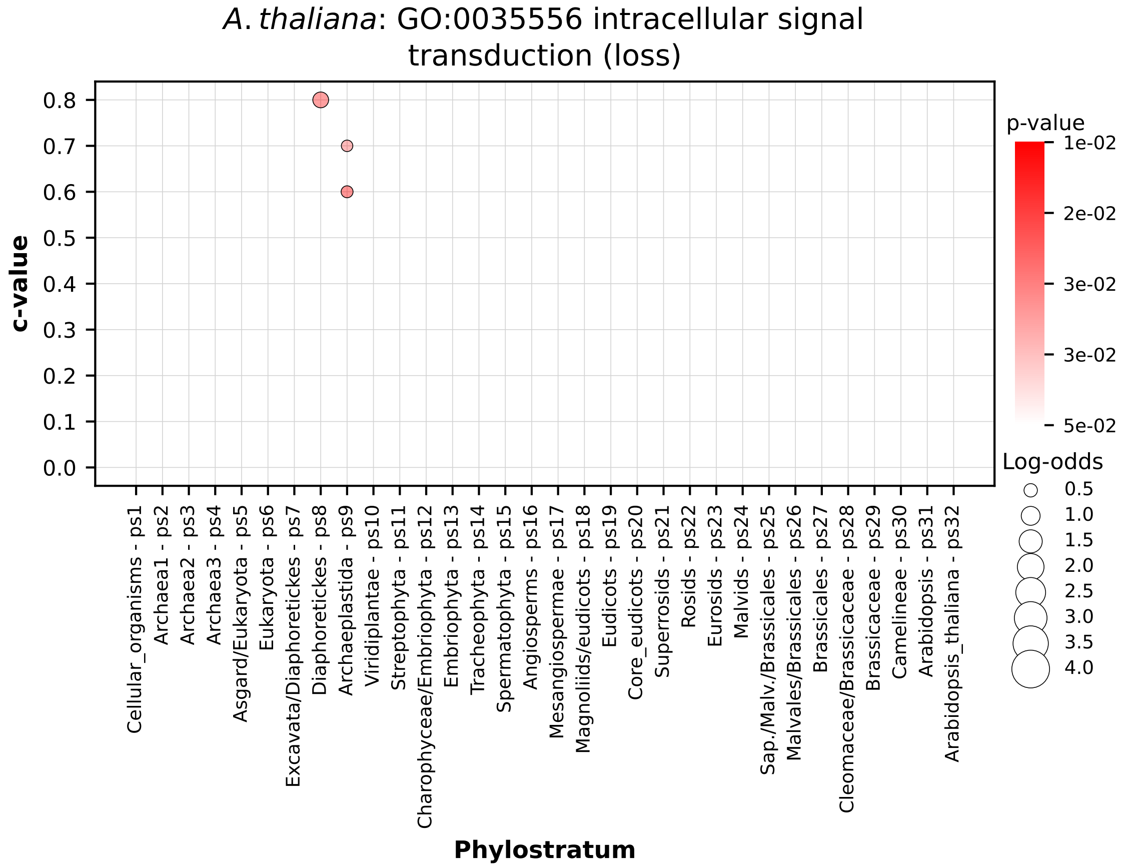
A. thaliana: GO:0035556 intracellular signal transduction (loss)
c-value
0.8
0.7
0.6
0.5
0.4
0.3
0.2
0.1
0.0
p-value
1e-02
2e-02
3e-02
3e-02
5e-02
Log-odds
0.5
1.0
1.5
2.0
2.5
3.0
3.5
4.0
Cellular_organisms - ps1
Archaea1 - ps2
Archaea2 - ps3
Archaea3 - ps4
Asgard/Eukaryota - ps5
Eukaryota - ps6
Excavata/Diaphoretickes - ps7
Diaphoretickes - ps8
Archaeplastida - ps9
Viridiplantae - ps10
Streptophyta - ps11
Charophyceae/Embriophyta - ps12
Embriophyta - ps13
Tracheophyta - ps14
Spermatophyta - ps15
Angiosperms - ps16
Mesangiospermae - ps17
Magnoliids/eudicots - ps18
Eudicots - ps19
Core_eudicots - ps20
Superrosids - ps21
Rosids - ps22
Eurosids - ps23
Malvids - ps24
Sap./Malv./Brassicales - ps25
Malvales/Brassicales - ps26
Brassicales - ps27
Cleomaceae/Brassicaceae - ps28
Brassicaceae - ps29
Camelineae - ps30
Arabidopsis - ps31
Arabidopsis_thaliana - ps32
Phylostratum

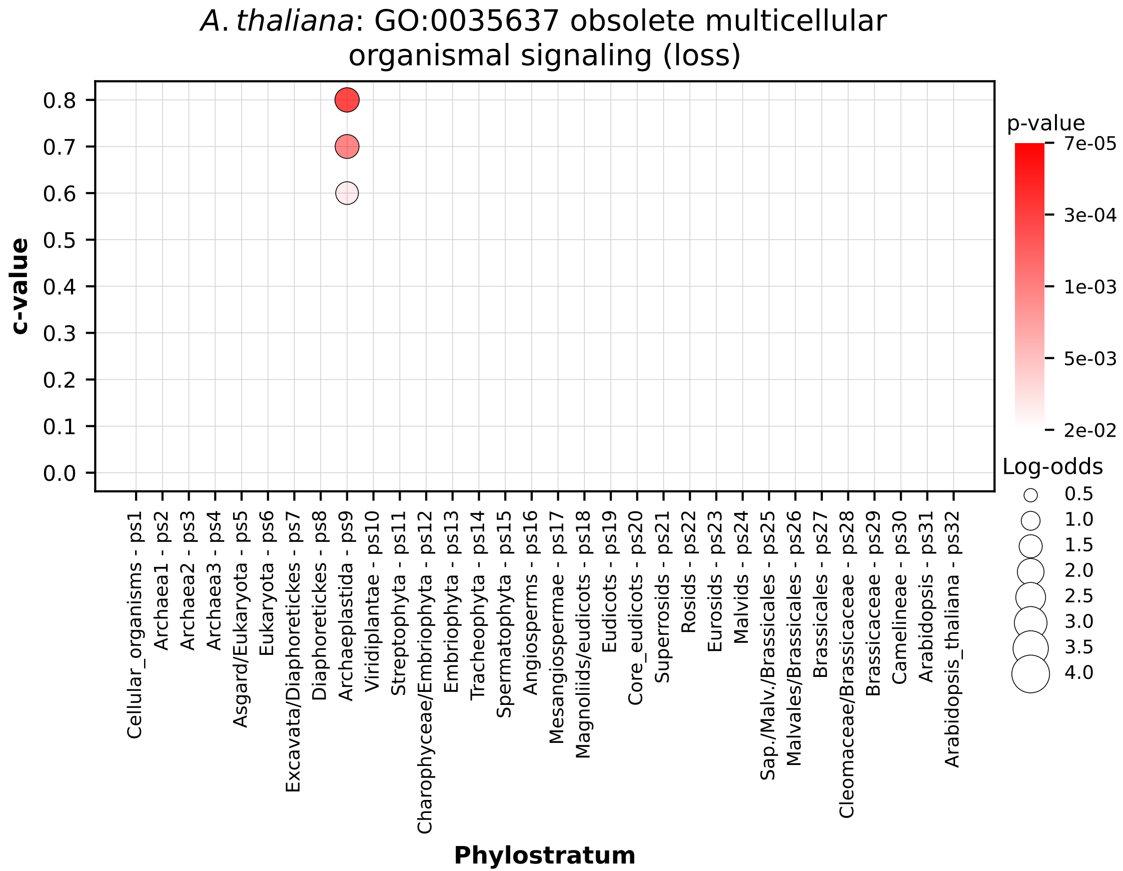

A. thaliana: GO:0035637 obsolete multicellular organismal signaling (loss)
c-value
0.8
0.7
0.6
0.5
0.4
0.3
0.2
0.1
0.0
Phylostratum
Cellular_organisms - ps1
Archaea1 - ps2
Archaea2 - ps3
Archaea3 - ps4
Asgard/Eukaryota - ps5
Eukaryota - ps6
Excavata/Diaphoretickes - ps7
Diaphoretickes - ps8
Archaeplastida - ps9
Viridiplantae - ps10
Streptophyta - ps11
Charophyceae/Embriophyta - ps12
Embriophyta - ps13
Tracheophyta - ps14
Spermatophyta - ps15
Angiosperms - ps16
Mesangiospermae - ps17
Magnoliids/eudicots - ps18
Eudicots - ps19
Core_eudicots - ps20
Superrosids - ps21
Rosids - ps22
Eurosids - ps23
Malvids - ps24
Sap./Malv./Brassicales - ps25
Malvales/Brassicales - ps26
Brassicales - ps27
Cleomaceae/Brassicaceae - ps28
Brassicaceae - ps29
Camelineae - ps30
Arabidopsis - ps31
Arabidopsis_thaliana - ps32
p-value
7e-05
3e-04
1e-03
5e-03
2e-02
Log-odds
0.5
1.0
1.5
2.0
2.5
3.0
3.5
4.0

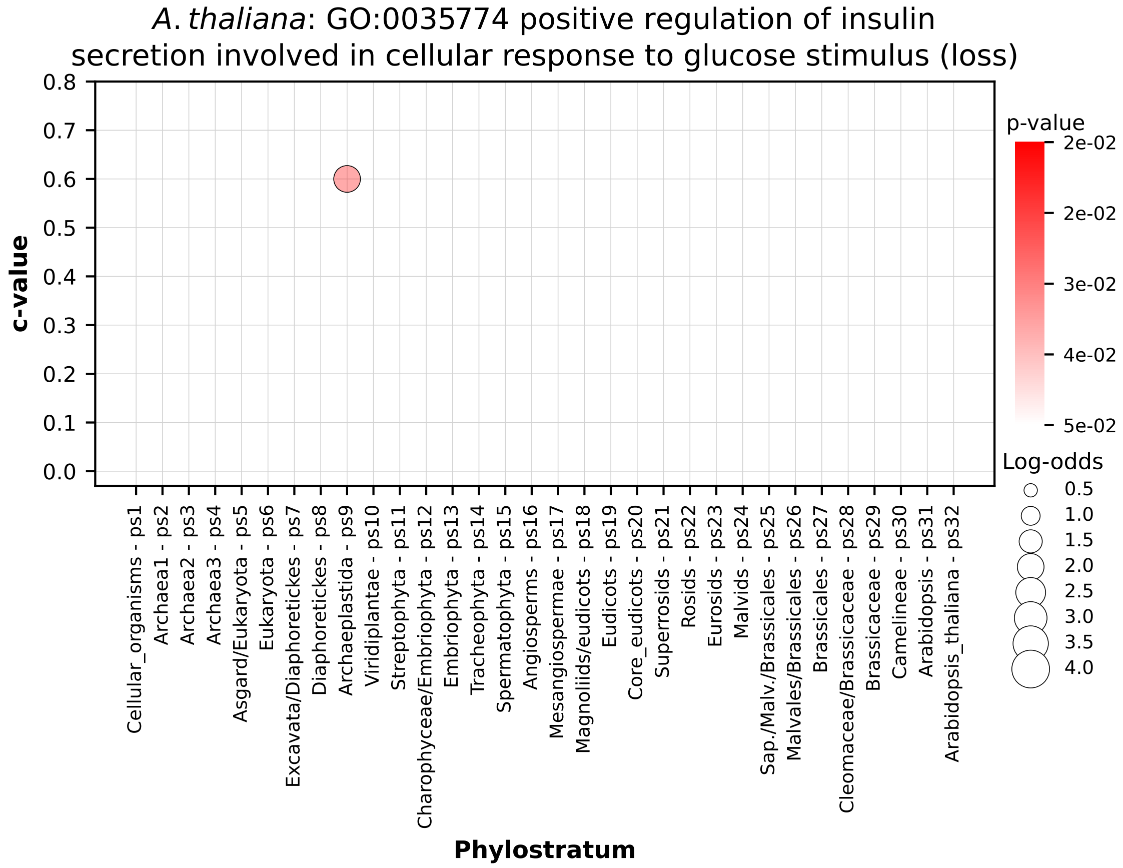

A. thaliana: GO:0035774 positive regulation of insulin secretion involved in cellular response to glucose stimulus (loss)
c-value
0.0
0.1
0.2
0.3
0.4
0.5
0.6
0.7
0.8
Phylostratum
Cellular_organisms - ps1
Archaea1 - ps2
Archaea2 - ps3
Archaea3 - ps4
Asgard/Eukaryota - ps5
Eukaryota - ps6
Excavata/Diaphoretickes - ps7
Diaphoretickes - ps8
Archaeplastida - ps9
Viridiplantae - ps10
Streptophyta - ps11
Charophyceae/Embriophyta - ps12
Embriophyta - ps13
Tracheophyta - ps14
Spermatophyta - ps15
Angiosperms - ps16
Mesangiospermae - ps17
Magnoliids/eudicots - ps18
Eudicots - ps19
Core_eudicots - ps20
Superrosids - ps21
Rosids - ps22
Eurosids - ps23
Malvids - ps24
Sap./Malv./Brassicales - ps25
Malvales/Brassicales - ps26
Brassicales - ps27
Cleomaceae/Brassicaceae - ps28
Brassicaceae - ps29
Camelineae - ps30
Arabidopsis - ps31
Arabidopsis_thaliana - ps32
p-value
2e-02
2e-02
3e-02
4e-02
5e-02
Log-odds
0.5
1.0
1.5
2.0
2.5
3.0
3.5
4.0

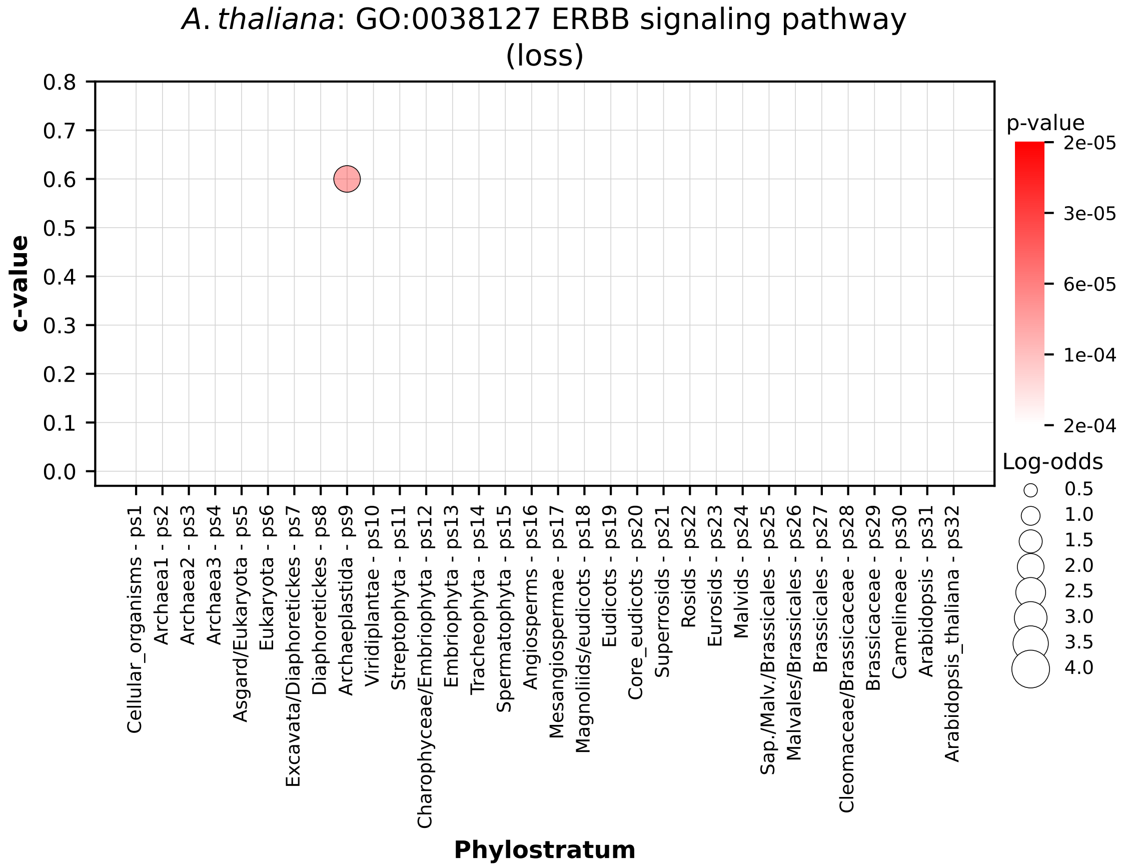

A. thaliana: GO:0038127 ERBB signaling pathway (loss)
c-value
0.8
0.7
0.6
0.5
0.4
0.3
0.2
0.1
0.0
Cellular_organisms - ps1
Archaea1 - ps2
Archaea2 - ps3
Archaea3 - ps4
Asgard/Eukaryota - ps5
Eukaryota - ps6
Excavata/Diaphoretickes - ps7
Diaphoretickes - ps8
Archaeplastida - ps9
Viridiplantae - ps10
Streptophyta - ps11
Charophyceae/Embriophyta - ps12
Embriophyta - ps13
Tracheophyta - ps14
Spermatophyta - ps15
Angiosperms - ps16
Mesangiospermae - ps17
Magnoliids/eudicots - ps18
Eudicots - ps19
Core_eudicots - ps20
Superrosids - ps21
Rosids - ps22
Eurosids - ps23
Malvids - ps24
Sap./Malv./Brassicales - ps25
Malvales/Brassicales - ps26
Brassicales - ps27
Cleomaceae/Brassicaceae - ps28
Brassicaceae - ps29
Camelineae - ps30
Arabidopsis - ps31
Arabidopsis_thaliana - ps32
Phylostratum
p-value
2e-05
3e-05
6e-05
1e-04
2e-04
Log-odds
0.5
1.0
1.5
2.0
2.5
3.0
3.5
4.0

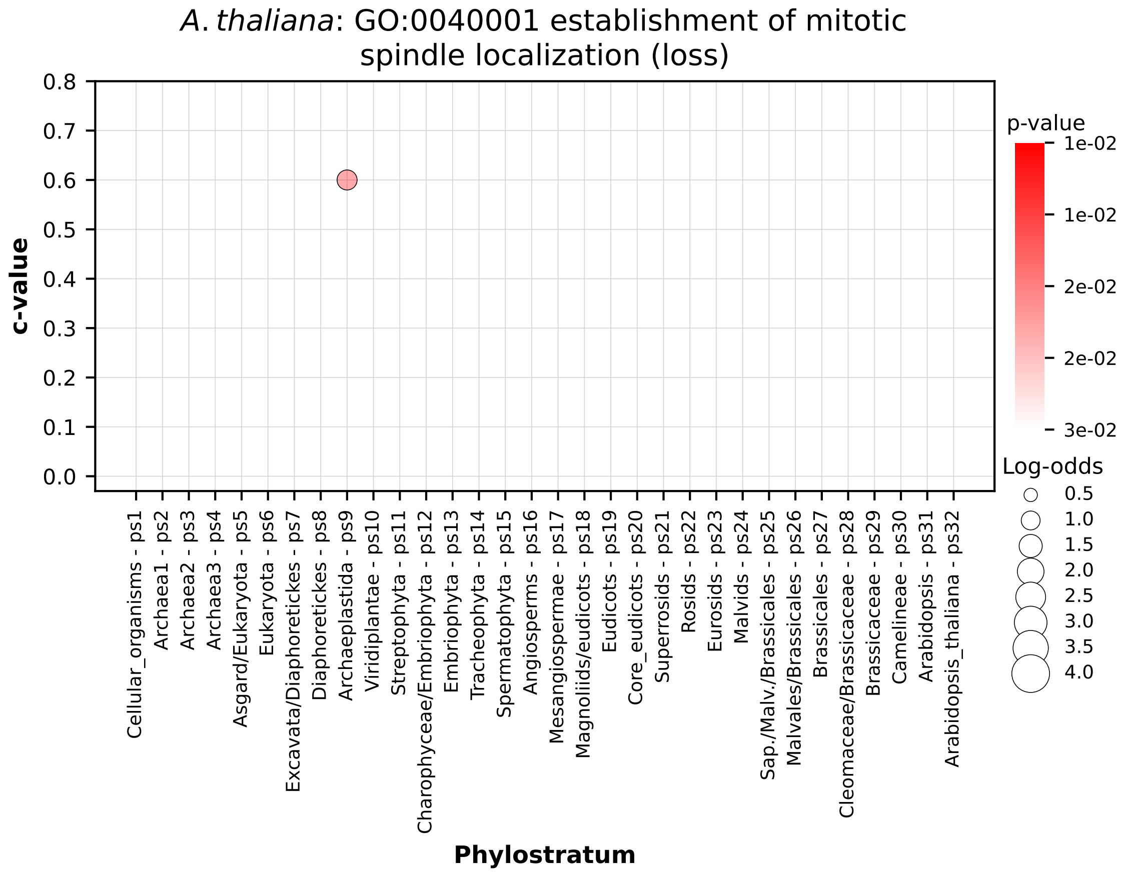

A. thaliana: GO:0040001 establishment of mitotic spindle localization (loss)
c-value
0.8
0.7
0.6
0.5
0.4
0.3
0.2
0.1
0.0
Phylostratum
Cellular_organisms - ps1
Archaea1 - ps2
Archaea2 - ps3
Archaea3 - ps4
Asgard/Eukaryota - ps5
Eukaryota - ps6
Excavata/Diaphoretickes - ps7
Diaphoretickes - ps8
Archaeplastida - ps9
Viridiplantae - ps10
Streptophyta - ps11
Charophyceae/Embriophyta - ps12
Embriophyta - ps13
Tracheophyta - ps14
Spermatophyta - ps15
Angiosperms - ps16
Mesangiospermae - ps17
Magnoliids/eudicots - ps18
Eudicots - ps19
Core_eudicots - ps20
Superrosids - ps21
Rosids - ps22
Eurosids - ps23
Malvids - ps24
Sap./Malv./Brassicales - ps25
Malvales/Brassicales - ps26
Brassicales - ps27
Cleomaceae/Brassicaceae - ps28
Brassicaceae - ps29
Camelineae - ps30
Arabidopsis - ps31
Arabidopsis_thaliana - ps32
p-value
1e-02
1e-02
2e-02
2e-02
3e-02
Log-odds
0.5
1.0
1.5
2.0
2.5
3.0
3.5
4.0

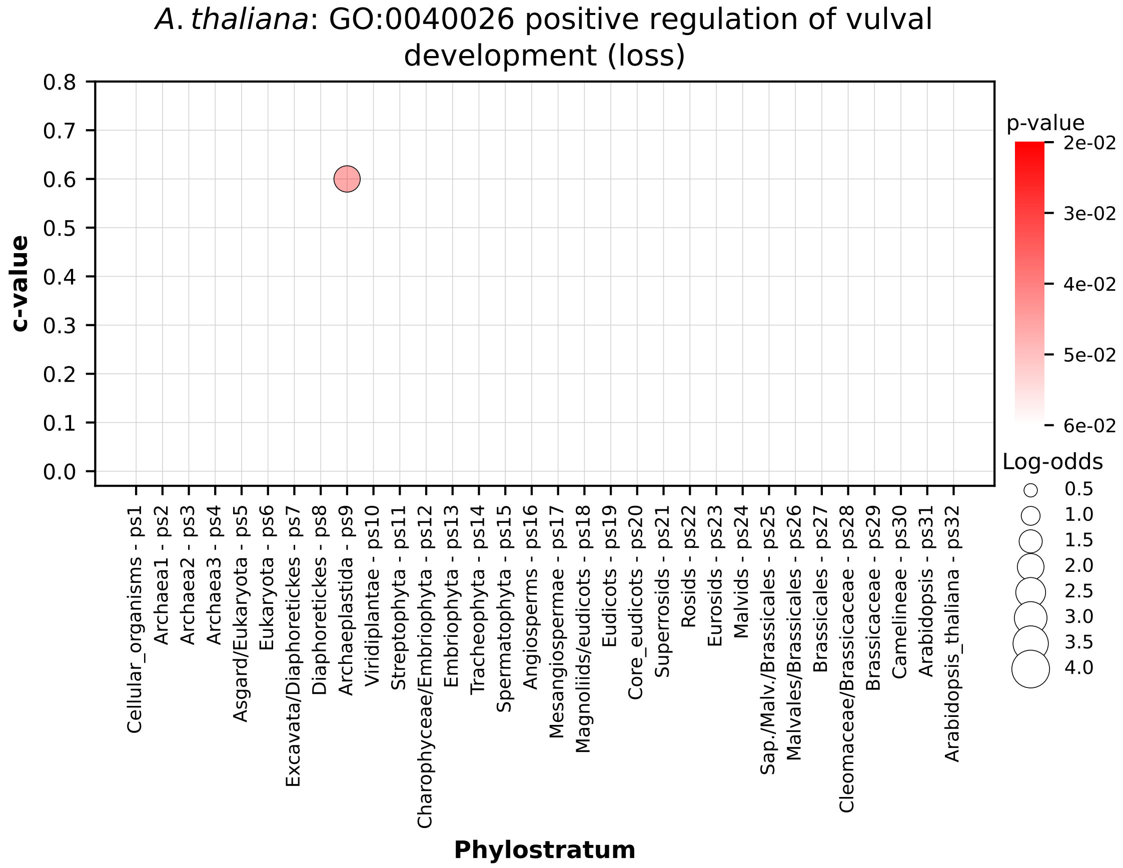

A. thaliana: GO:0040026 positive regulation of vulval development (loss)
c-value
0.8
0.7
0.6
0.5
0.4
0.3
0.2
0.1
0.0
Cellular_organisms - ps1
Archaea1 - ps2
Archaea2 - ps3
Archaea3 - ps4
Asgard/Eukaryota - ps5
Eukaryota - ps6
Excavata/Diaphoretickes - ps7
Diaphoretickes - ps8
Archaeplastida - ps9
Viridiplantae - ps10
Streptophyta - ps11
Charophyceae/Embriophyta - ps12
Embriophyta - ps13
Tracheophyta - ps14
Spermatophyta - ps15
Angiosperms - ps16
Mesangiospermae - ps17
Magnoliids/eudicots - ps18
Eudicots - ps19
Core_eudicots - ps20
Superrosids - ps21
Rosids - ps22
Eurosids - ps23
Malvids - ps24
Sap./Malv./Brassicales - ps25
Malvales/Brassicales - ps26
Brassicales - ps27
Cleomaceae/Brassicaceae - ps28
Brassicaceae - ps29
Camelineae - ps30
Arabidopsis - ps31
Arabidopsis_thaliana - ps32
Phylostratum
p-value
2e-02
3e-02
4e-02
5e-02
6e-02
Log-odds
0.5
1.0
1.5
2.0
2.5
3.0
3.5
4.0

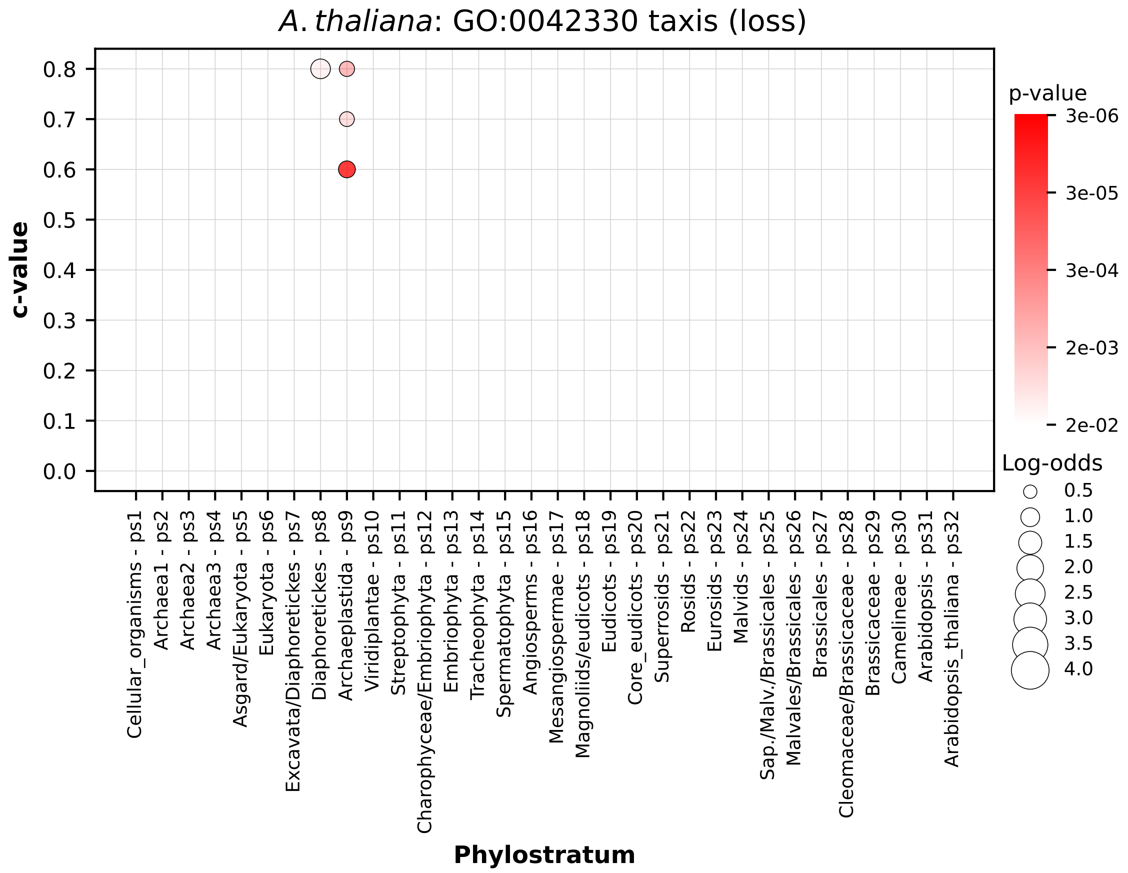

A. thaliana: GO:0042330 taxis (loss)
c-value
0.8
0.7
0.6
0.5
0.4
0.3
0.2
0.1
0.0
p-value
3e-06
3e-05
3e-04
2e-03
2e-02
Log-odds
0.5
1.0
1.5
2.0
2.5
3.0
3.5
4.0
Cellular_organisms - ps1
Archaea1 - ps2
Archaea2 - ps3
Archaea3 - ps4
Asgard/Eukaryota - ps5
Eukaryota - ps6
Excavata/Diaphoretickes - ps7
Diaphoretickes - ps8
Archaeplastida - ps9
Viridiplantae - ps10
Streptophyta - ps11
Charophyceae/Embriophyta - ps12
Embriophyta - ps13
Tracheophyta - ps14
Spermatophyta - ps15
Angiosperms - ps16
Mesangiospermae - ps17
Magnoliids/eudicots - ps18
Eudicots - ps19
Core_eudicots - ps20
Superrosids - ps21
Rosids - ps22
Eurosids - ps23
Malvids - ps24
Sap./Malv./Brassicales - ps25
Malvales/Brassicales - ps26
Brassicales - ps27
Cleomaceae/Brassicaceae - ps28
Brassicaceae - ps29
Camelineae - ps30
Arabidopsis - ps31
Arabidopsis_thaliana - ps32
Phylostratum

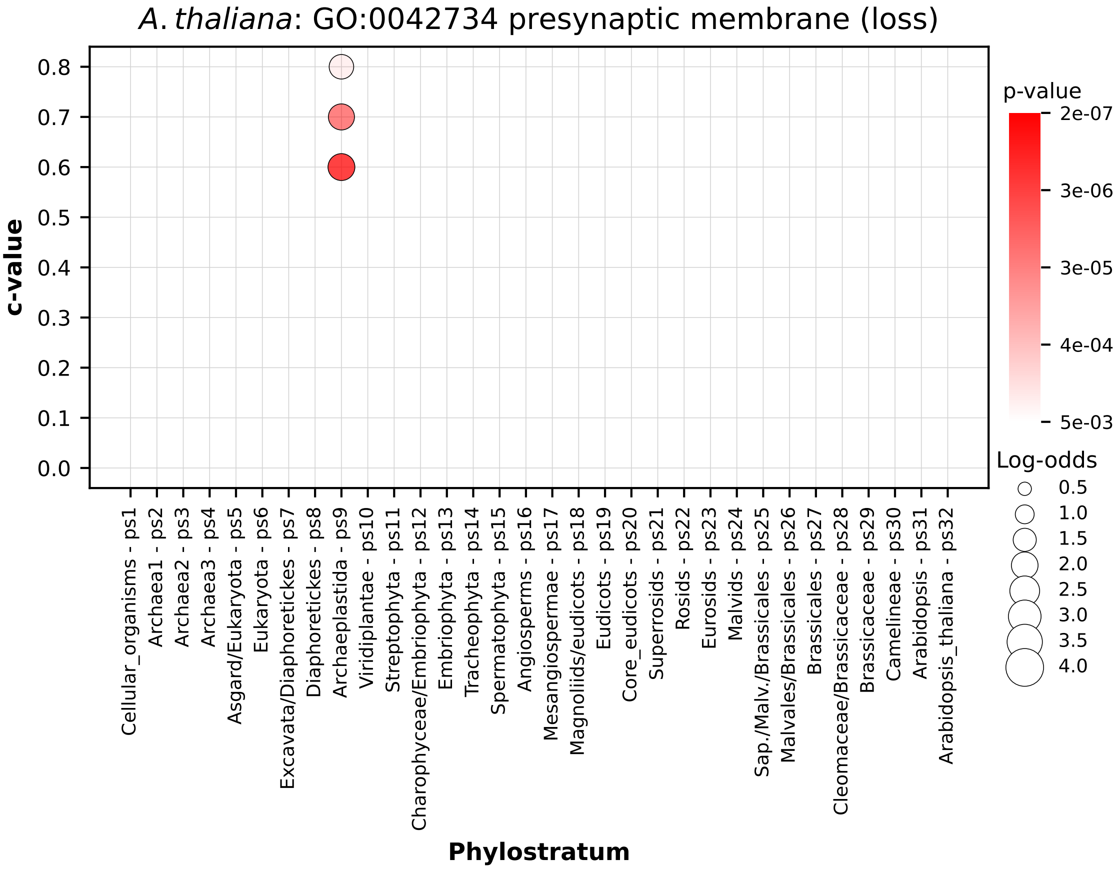

A. thaliana: GO:0042734 presynaptic membrane (loss)
c-value
0.0
0.1
0.2
0.3
0.4
0.5
0.6
0.7
0.8
p-value
2e-07
3e-06
3e-05
4e-04
5e-03
Log-odds
0.5
1.0
1.5
2.0
2.5
3.0
3.5
4.0
Cellular_organisms - ps1
Archaea1 - ps2
Archaea2 - ps3
Archaea3 - ps4
Asgard/Eukaryota - ps5
Eukaryota - ps6
Excavata/Diaphoretickes - ps7
Diaphoretickes - ps8
Archaeplastida - ps9
Viridiplantae - ps10
Streptophyta - ps11
Charophyceae/Embriophyta - ps12
Embriophyta - ps13
Tracheophyta - ps14
Spermatophyta - ps15
Angiosperms - ps16
Mesangiospermae - ps17
Magnoliids/eudicots - ps18
Eudicots - ps19
Core_eudicots - ps20
Superrosids - ps21
Rosids - ps22
Eurosids - ps23
Malvids - ps24
Sap./Malv./Brassicales - ps25
Malvales/Brassicales - ps26
Brassicales - ps27
Cleomaceae/Brassicaceae - ps28
Brassicaceae - ps29
Camelineae - ps30
Arabidopsis - ps31
Arabidopsis_thaliana - ps32
Phylostratum

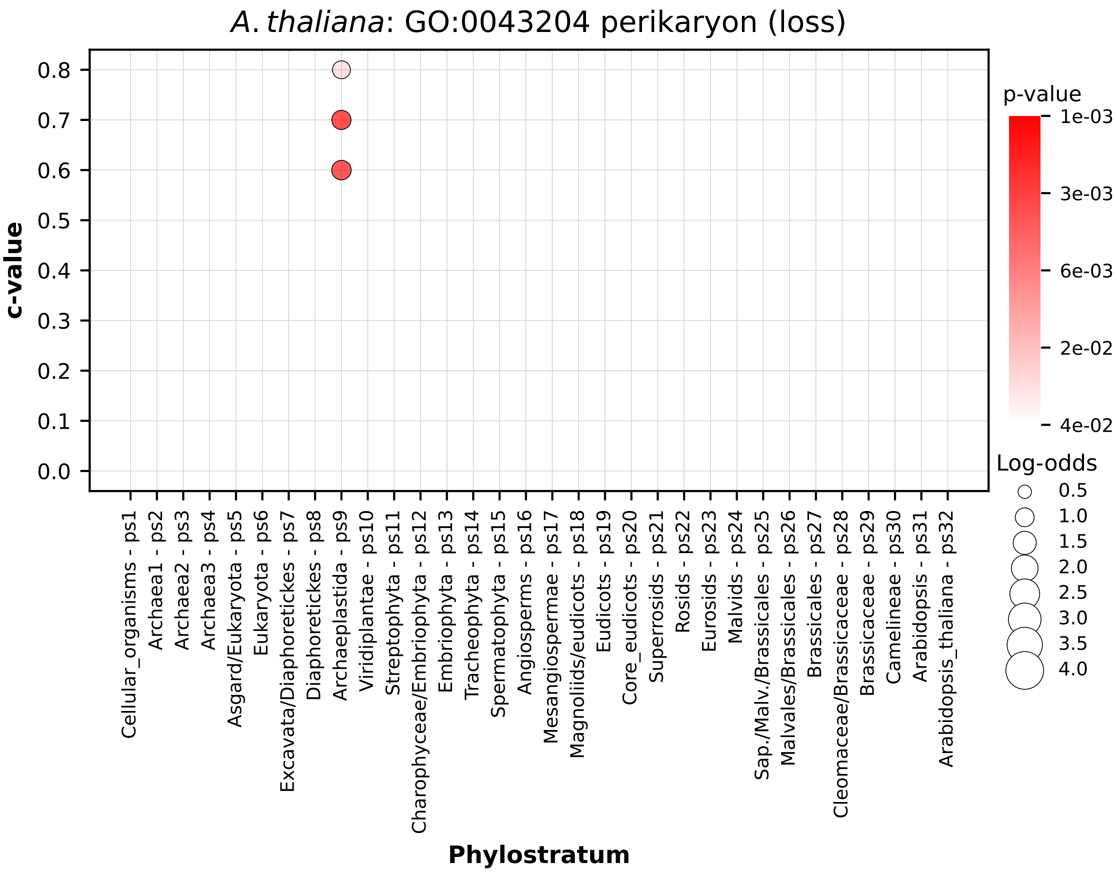

A. thaliana: GO:0043204 perikaryon (loss)
c-value
0.8
0.7
0.6
0.5
0.4
0.3
0.2
0.1
0.0
p-value
1e-03
3e-03
6e-03
2e-02
4e-02
Log-odds
0.5
1.0
1.5
2.0
2.5
3.0
3.5
4.0
Cellular_organisms - ps1
Archaea1 - ps2
Archaea2 - ps3
Archaea3 - ps4
Asgard/Eukaryota - ps5
Eukaryota - ps6
Excavata/Diaphoretickes - ps7
Diaphoretickes - ps8
Archaeplastida - ps9
Viridiplantae - ps10
Streptophyta - ps11
Charophyceae/Embriophyta - ps12
Embriophyta - ps13
Tracheophyta - ps14
Spermatophyta - ps15
Angiosperms - ps16
Mesangiospermae - ps17
Magnoliids/eudicots - ps18
Eudicots - ps19
Core_eudicots - ps20
Superrosids - ps21
Rosids - ps22
Eurosids - ps23
Malvids - ps24
Sap./Malv./Brassicales - ps25
Malvales/Brassicales - ps26
Brassicales - ps27
Cleomaceae/Brassicaceae - ps28
Brassicaceae - ps29
Camelineae - ps30
Arabidopsis - ps31
Arabidopsis_thaliana - ps32
Phylostratum

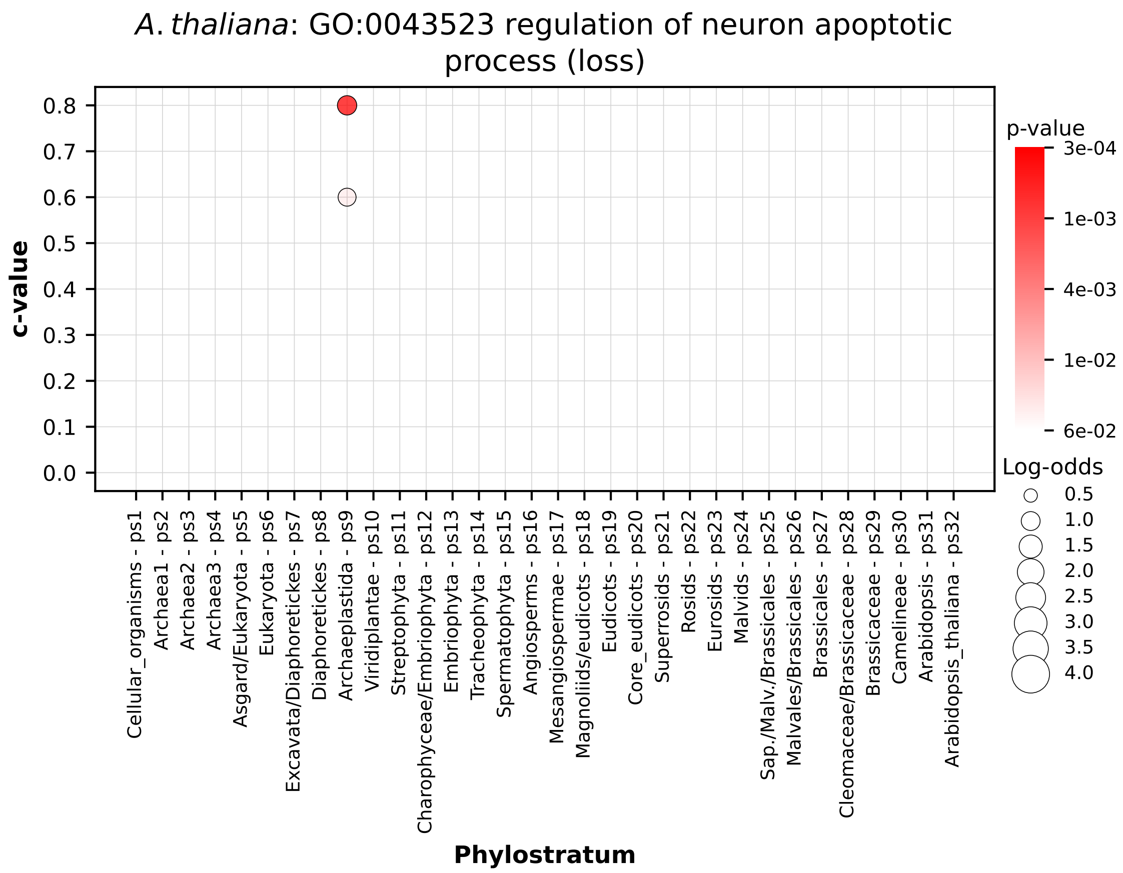

A. thaliana: GO:0043523 regulation of neuron apoptotic process (loss)
c-value
0.8
0.7
0.6
0.5
0.4
0.3
0.2
0.1
0.0
Phylostratum
Cellular_organisms - ps1
Archaea1 - ps2
Archaea2 - ps3
Archaea3 - ps4
Asgard/Eukaryota - ps5
Eukaryota - ps6
Excavata/Diaphoretickes - ps7
Diaphoretickes - ps8
Archaeplastida - ps9
Viridiplantae - ps10
Streptophyta - ps11
Charophyceae/Embriophyta - ps12
Embriophyta - ps13
Tracheophyta - ps14
Spermatophyta - ps15
Angiosperms - ps16
Mesangiospermae - ps17
Magnoliids/eudicots - ps18
Eudicots - ps19
Core_eudicots - ps20
Superrosids - ps21
Rosids - ps22
Eurosids - ps23
Malvids - ps24
Sap./Malv./Brassicales - ps25
Malvales/Brassicales - ps26
Brassicales - ps27
Cleomaceae/Brassicaceae - ps28
Brassicaceae - ps29
Camelineae - ps30
Arabidopsis - ps31
Arabidopsis_thaliana - ps32
p-value
3e-04
1e-03
4e-03
1e-02
6e-02
Log-odds
0.5
1.0
1.5
2.0
2.5
3.0
3.5
4.0

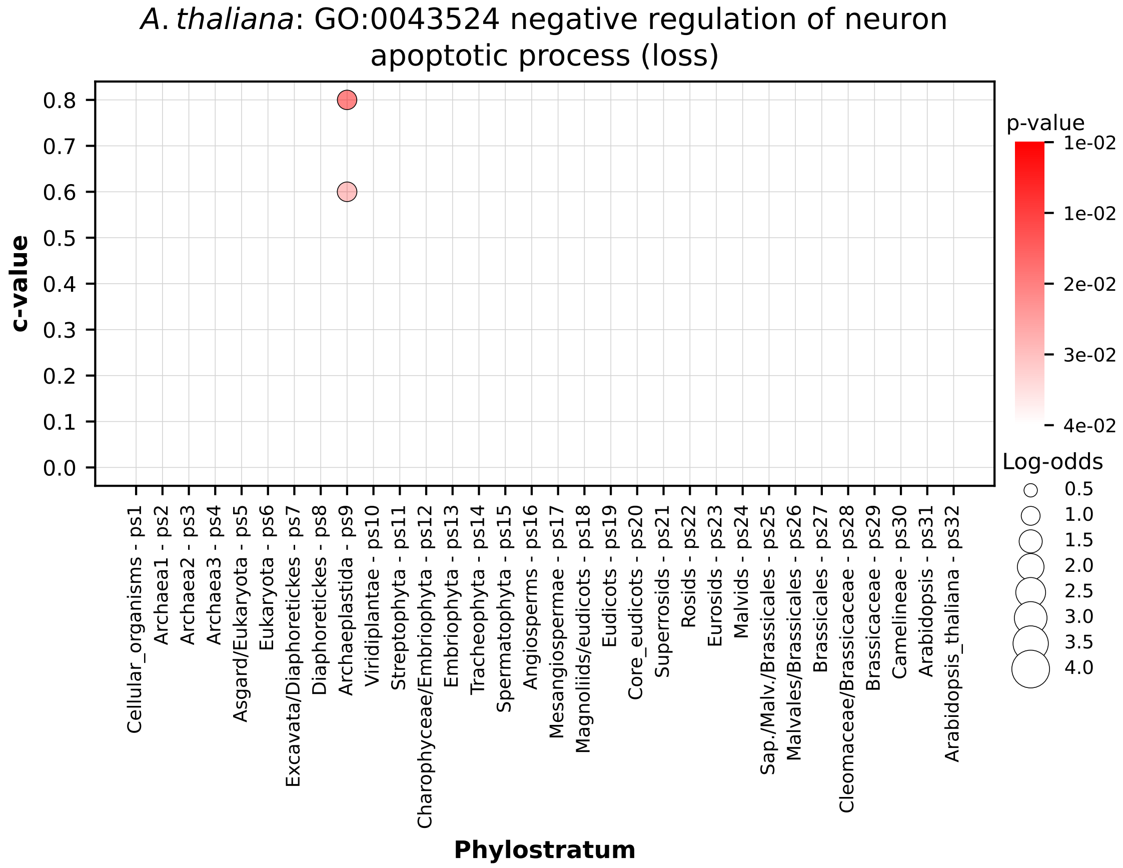
A. thaliana: GO:0043524 negative regulation of neuron apoptotic process (loss)
c-value
0.0
0.1
0.2
0.3
0.4
0.5
0.6
0.7
0.8
p-value
1e-02
1e-02
2e-02
3e-02
4e-02
Log-odds
0.5
1.0
1.5
2.0
2.5
3.0
3.5
4.0
Cellular_organisms - ps1
Archaea1 - ps2
Archaea2 - ps3
Archaea3 - ps4
Asgard/Eukaryota - ps5
Eukaryota - ps6
Excavata/Diaphoretickes - ps7
Diaphoretickes - ps8
Archaeplastida - ps9
Viridiplantae - ps10
Streptophyta - ps11
Charophyceae/Embriophyta - ps12
Embriophyta - ps13
Tracheophyta - ps14
Spermatophyta - ps15
Angiosperms - ps16
Mesangiospermae - ps17
Magnoliids/eudicots - ps18
Eudicots - ps19
Core_eudicots - ps20
Superrosids - ps21
Rosids - ps22
Eurosids - ps23
Malvids - ps24
Sap./Malv./Brassicales - ps25
Malvales/Brassicales - ps26
Brassicales - ps27
Cleomaceae/Brassicaceae - ps28
Brassicaceae - ps29
Camelineae - ps30
Arabidopsis - ps31
Arabidopsis_thaliana - ps32
Phylostratum

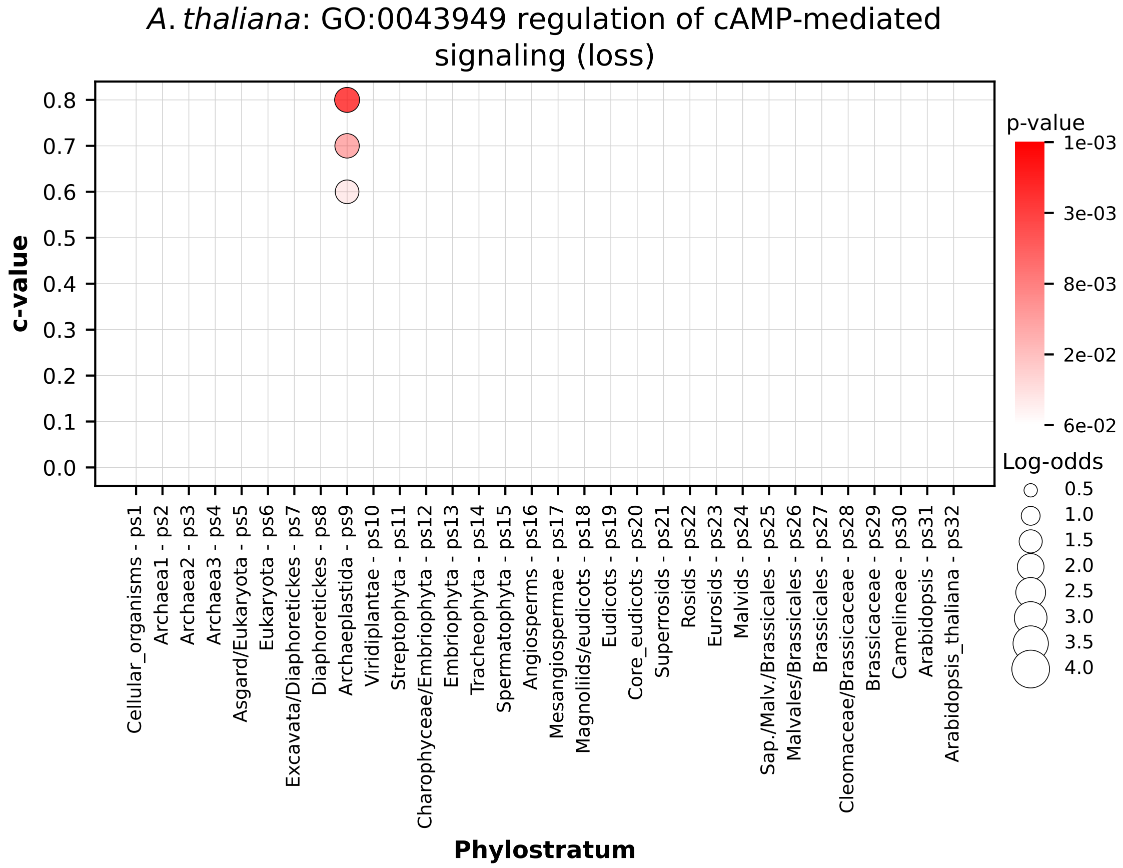

A. thaliana: GO:0043949 regulation of cAMP-mediated signaling (loss)
c-value
0.8
0.7
0.6
0.5
0.4
0.3
0.2
0.1
0.0
p-value
1e-03
3e-03
8e-03
2e-02
6e-02
Log-odds
0.5
1.0
1.5
2.0
2.5
3.0
3.5
4.0
Cellular_organisms - ps1
Archaea1 - ps2
Archaea2 - ps3
Archaea3 - ps4
Asgard/Eukaryota - ps5
Eukaryota - ps6
Excavata/Diaphoretickes - ps7
Diaphoretickes - ps8
Archaeplastida - ps9
Viridiplantae - ps10
Streptophyta - ps11
Charophyceae/Embriophyta - ps12
Embriophyta - ps13
Tracheophyta - ps14
Spermatophyta - ps15
Angiosperms - ps16
Mesangiospermae - ps17
Magnoliids/eudicots - ps18
Eudicots - ps19
Core_eudicots - ps20
Superrosids - ps21
Rosids - ps22
Eurosids - ps23
Malvids - ps24
Sap./Malv./Brassicales - ps25
Malvales/Brassicales - ps26
Brassicales - ps27
Cleomaceae/Brassicaceae - ps28
Brassicaceae - ps29
Camelineae - ps30
Arabidopsis - ps31
Arabidopsis_thaliana - ps32
Phylostratum

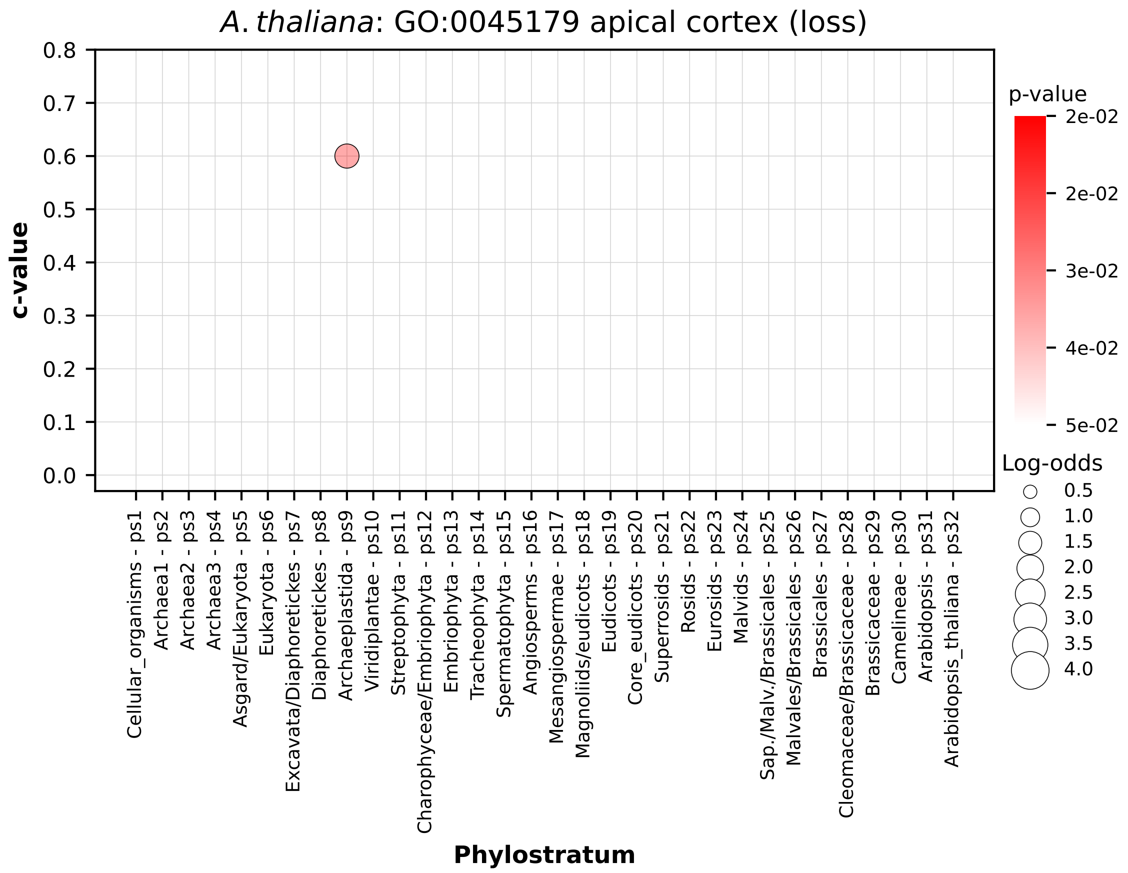

A. thaliana: GO:0045179 apical cortex (loss)
c-value
0.8
0.7
0.6
0.5
0.4
0.3
0.2
0.1
0.0
Phylostratum
Cellular_organisms - ps1
Archaea1 - ps2
Archaea2 - ps3
Archaea3 - ps4
Asgard/Eukaryota - ps5
Eukaryota - ps6
Excavata/Diaphoretickes - ps7
Diaphoretickes - ps8
Archaeplastida - ps9
Viridiplantae - ps10
Streptophyta - ps11
Charophyceae/Embriophyta - ps12
Embriophyta - ps13
Tracheophyta - ps14
Spermatophyta - ps15
Angiosperms - ps16
Mesangiospermae - ps17
Magnoliids/eudicots - ps18
Eudicots - ps19
Core_eudicots - ps20
Superrosids - ps21
Rosids - ps22
Eurosids - ps23
Malvids - ps24
Sap./Malv./Brassicales - ps25
Malvales/Brassicales - ps26
Brassicales - ps27
Cleomaceae/Brassicaceae - ps28
Brassicaceae - ps29
Camelineae - ps30
Arabidopsis - ps31
Arabidopsis_thaliana - ps32
p-value
2e-02
2e-02
3e-02
4e-02
5e-02
Log-odds
0.5
1.0
1.5
2.0
2.5
3.0
3.5
4.0

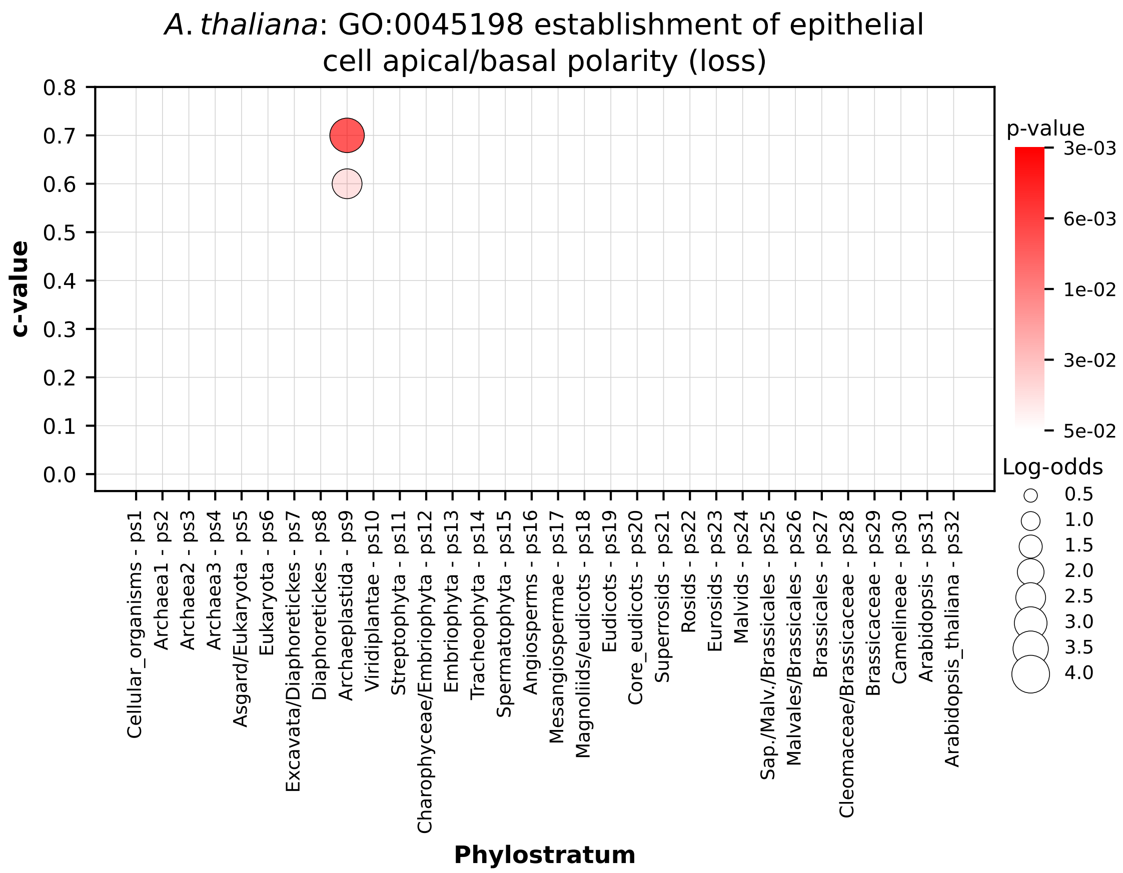

A. thaliana: GO:0045198 establishment of epithelial cell apical/basal polarity (loss)
c-value
0.8
0.7
0.6
0.5
0.4
0.3
0.2
0.1
0.0
Phylostratum
Cellular_organisms - ps1
Archaea1 - ps2
Archaea2 - ps3
Archaea3 - ps4
Asgard/Eukaryota - ps5
Eukaryota - ps6
Excavata/Diaphoretickes - ps7
Diaphoretickes - ps8
Archaeplastida - ps9
Viridiplantae - ps10
Streptophyta - ps11
Charophyceae/Embriophyta - ps12
Embriophyta - ps13
Tracheophyta - ps14
Spermatophyta - ps15
Angiosperms - ps16
Mesangiospermae - ps17
Magnoliids/eudicots - ps18
Eudicots - ps19
Core_eudicots - ps20
Superrosids - ps21
Rosids - ps22
Eurosids - ps23
Malvids - ps24
Sap./Malv./Brassicales - ps25
Malvales/Brassicales - ps26
Brassicales - ps27
Cleomaceae/Brassicaceae - ps28
Brassicaceae - ps29
Camelineae - ps30
Arabidopsis - ps31
Arabidopsis_thaliana - ps32
p-value
3e-03
6e-03
1e-02
3e-02
5e-02
Log-odds
0.5
1.0
1.5
2.0
2.5
3.0
3.5
4.0

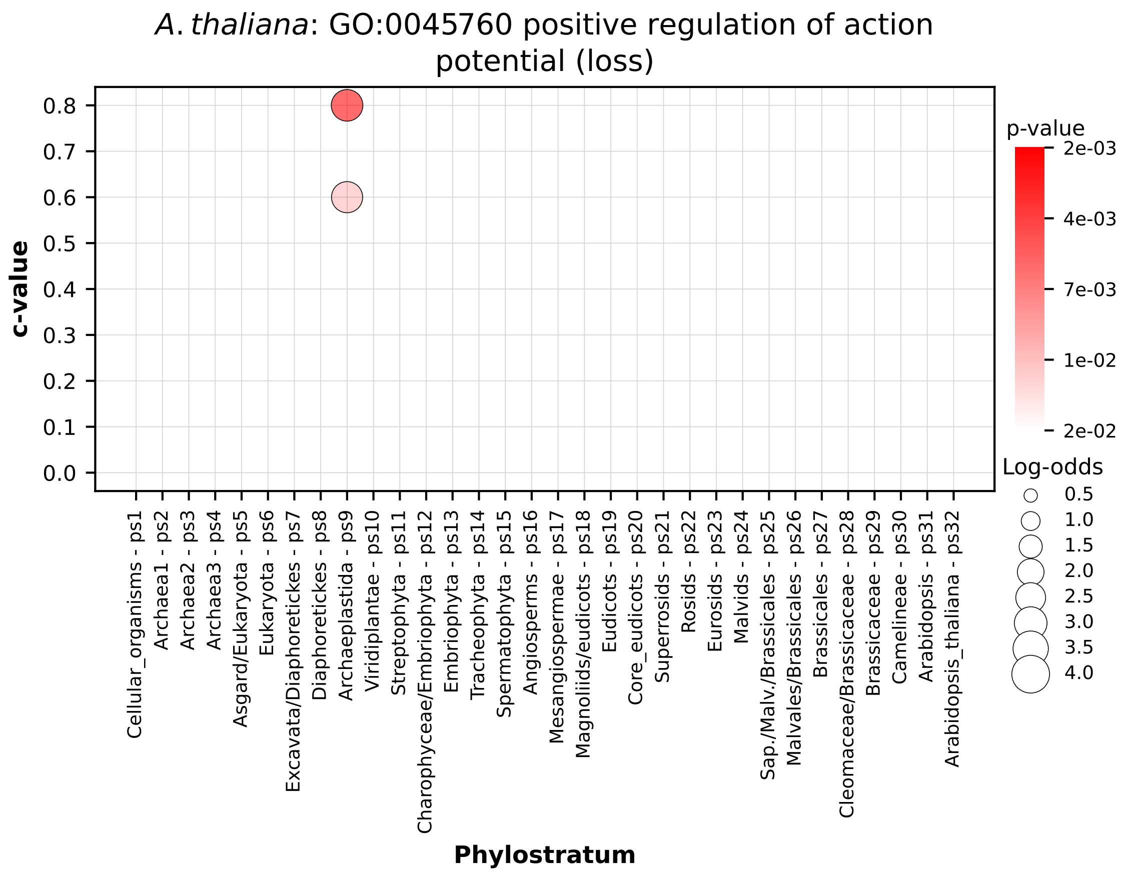

A. thaliana: GO:0045760 positive regulation of action potential (loss)
c-value
0.8
0.7
0.6
0.5
0.4
0.3
0.2
0.1
0.0
p-value
2e-03
4e-03
7e-03
1e-02
2e-02
Log-odds
0.5
1.0
1.5
2.0
2.5
3.0
3.5
4.0
Cellular_organisms - ps1
Archaea1 - ps2
Archaea2 - ps3
Archaea3 - ps4
Asgard/Eukaryota - ps5
Eukaryota - ps6
Excavata/Diaphoretickes - ps7
Diaphoretickes - ps8
Archaeplastida - ps9
Viridiplantae - ps10
Streptophyta - ps11
Charophyceae/Embriophyta - ps12
Embriophyta - ps13
Tracheophyta - ps14
Spermatophyta - ps15
Angiosperms - ps16
Mesangiospermae - ps17
Magnoliids/eudicots - ps18
Eudicots - ps19
Core_eudicots - ps20
Superrosids - ps21
Rosids - ps22
Eurosids - ps23
Malvids - ps24
Sap./Malv./Brassicales - ps25
Malvales/Brassicales - ps26
Brassicales - ps27
Cleomaceae/Brassicaceae - ps28
Brassicaceae - ps29
Camelineae - ps30
Arabidopsis - ps31
Arabidopsis_thaliana - ps32
Phylostratum

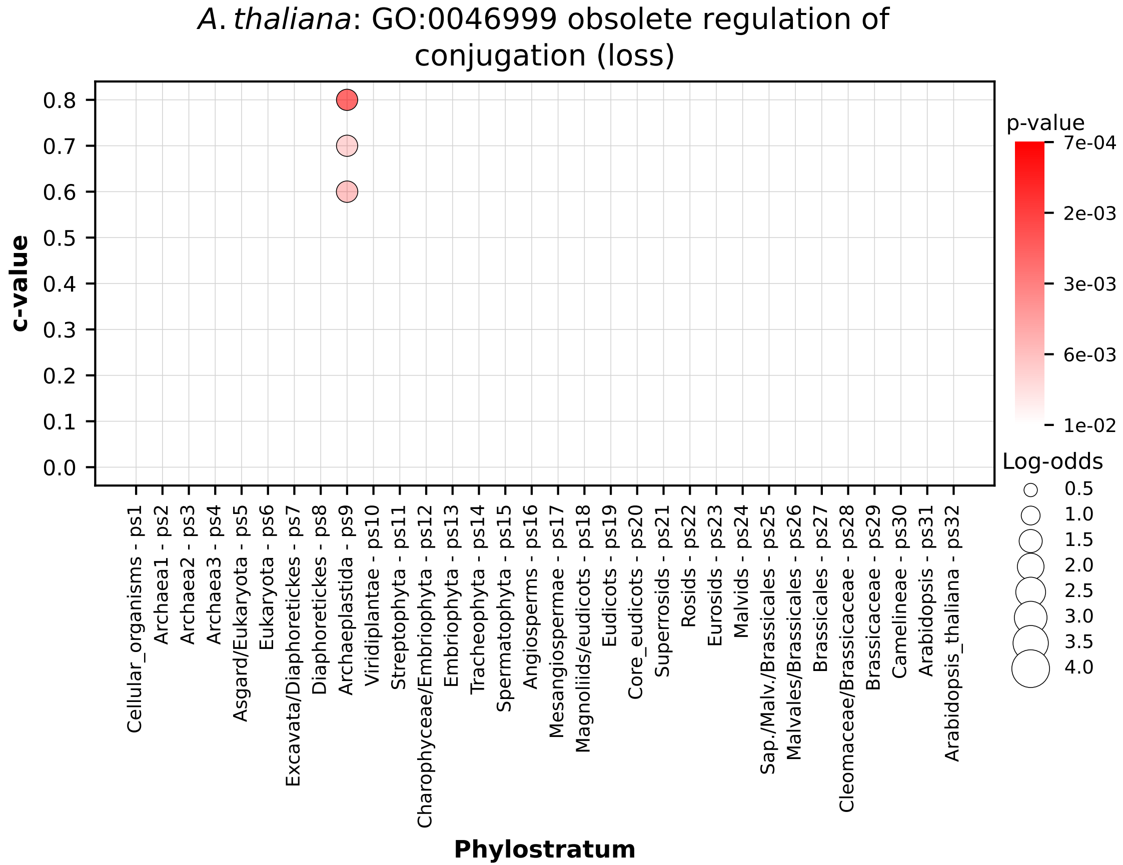

A. thaliana: GO:0046999 obsolete regulation of conjugation (loss)
c-value
0.8
0.7
0.6
0.5
0.4
0.3
0.2
0.1
0.0
p-value
7e-04
2e-03
3e-03
6e-03
1e-02
Log-odds
0.5
1.0
1.5
2.0
2.5
3.0
3.5
4.0
Cellular_organisms - ps1
Archaea1 - ps2
Archaea2 - ps3
Archaea3 - ps4
Asgard/Eukaryota - ps5
Eukaryota - ps6
Excavata/Diaphoretickes - ps7
Diaphoretickes - ps8
Archaeplastida - ps9
Viridiplantae - ps10
Streptophyta - ps11
Charophyceae/Embriophyta - ps12
Embriophyta - ps13
Tracheophyta - ps14
Spermatophyta - ps15
Angiosperms - ps16
Mesangiospermae - ps17
Magnoliids/eudicots - ps18
Eudicots - ps19
Core_eudicots - ps20
Superrosids - ps21
Rosids - ps22
Eurosids - ps23
Malvids - ps24
Sap./Malv./Brassicales - ps25
Malvales/Brassicales - ps26
Brassicales - ps27
Cleomaceae/Brassicaceae - ps28
Brassicaceae - ps29
Camelineae - ps30
Arabidopsis - ps31
Arabidopsis_thaliana - ps32
Phylostratum

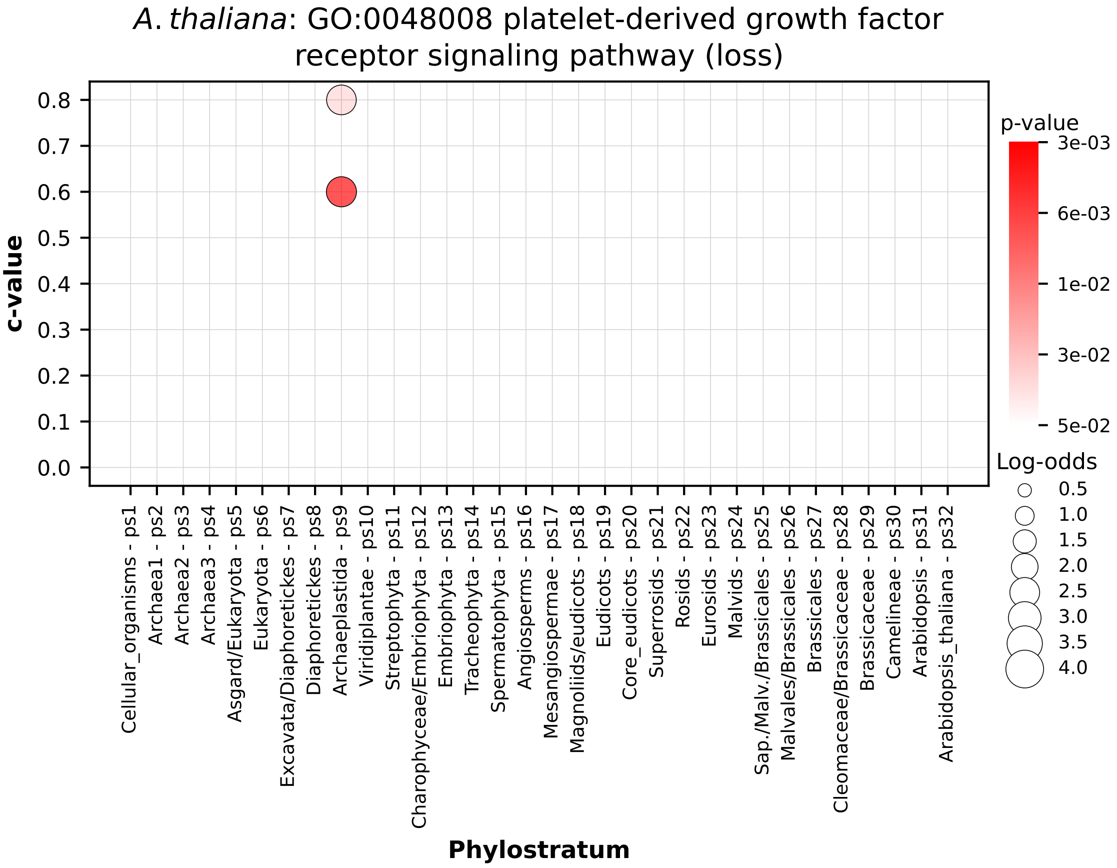

$A. thaliana$: GO:0048008 platelet-derived growth factor receptor signaling pathway (loss)
c-value
0.0
0.1
0.2
0.3
0.4
0.5
0.6
0.7
0.8
Phylostratum
Cellular_organisms - ps1
Archaea1 - ps2
Archaea2 - ps3
Archaea3 - ps4
Asgard/Eukaryota - ps5
Eukaryota - ps6
Excavata/Diaphoretickes - ps7
Diaphoretickes - ps8
Archaeplastida - ps9
Viridiplantae - ps10
Streptophyta - ps11
Charophyceae/Embriophyta - ps12
Embriophyta - ps13
Tracheophyta - ps14
Spermatophyta - ps15
Angiosperms - ps16
Mesangiospermae - ps17
Magnoliids/eudicots - ps18
Eudicots - ps19
Core_eudicots - ps20
Superrosids - ps21
Rosids - ps22
Eurosids - ps23
Malvids - ps24
Sap./Malv./Brassicales - ps25
Malvales/Brassicales - ps26
Brassicales - ps27
Cleomaceae/Brassicaceae - ps28
Brassicaceae - ps29
Camelineae - ps30
Arabidopsis - ps31
Arabidopsis_thaliana - ps32
p-value
3e-03
6e-03
1e-02
3e-02
5e-02
Log-odds
0.5
1.0
1.5
2.0
2.5
3.0
3.5
4.0

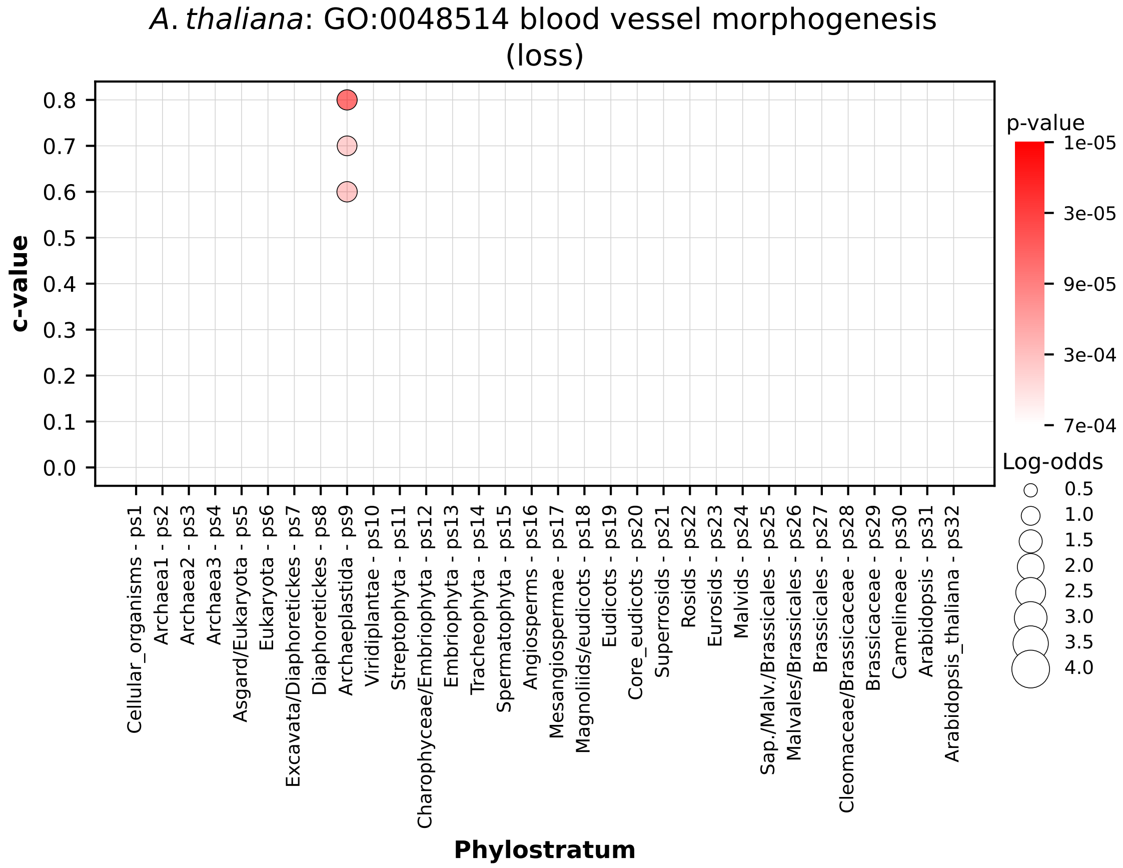

A. thaliana: GO:0048514 blood vessel morphogenesis
(loss)
c-value
0.8
0.7
0.6
0.5
0.4
0.3
0.2
0.1
0.0
Phylostratum
Cellular_organisms - ps1
Archaea1 - ps2
Archaea2 - ps3
Archaea3 - ps4
Asgard/Eukaryota - ps5
Eukaryota - ps6
Excavata/Diaphoretickes - ps7
Diaphoretickes - ps8
Archaeplastida - ps9
Viridiplantae - ps10
Streptophyta - ps11
Charophyceae/Embriophyta - ps12
Embriophyta - ps13
Tracheophyta - ps14
Spermatophyta - ps15
Angiosperms - ps16
Mesangiospermae - ps17
Magnoliids/eudicots - ps18
Eudicots - ps19
Core_eudicots - ps20
Superrosids - ps21
Rosids - ps22
Eurosids - ps23
Malvids - ps24
Sap./Malv./Brassicales - ps25
Malvales/Brassicales - ps26
Brassicales - ps27
Cleomaceae/Brassicaceae - ps28
Brassicaceae - ps29
Camelineae - ps30
Arabidopsis - ps31
Arabidopsis_thaliana - ps32
p-value
1e-05
3e-05
9e-05
3e-04
7e-04
Log-odds
0.5
1.0
1.5
2.0
2.5
3.0
3.5
4.0

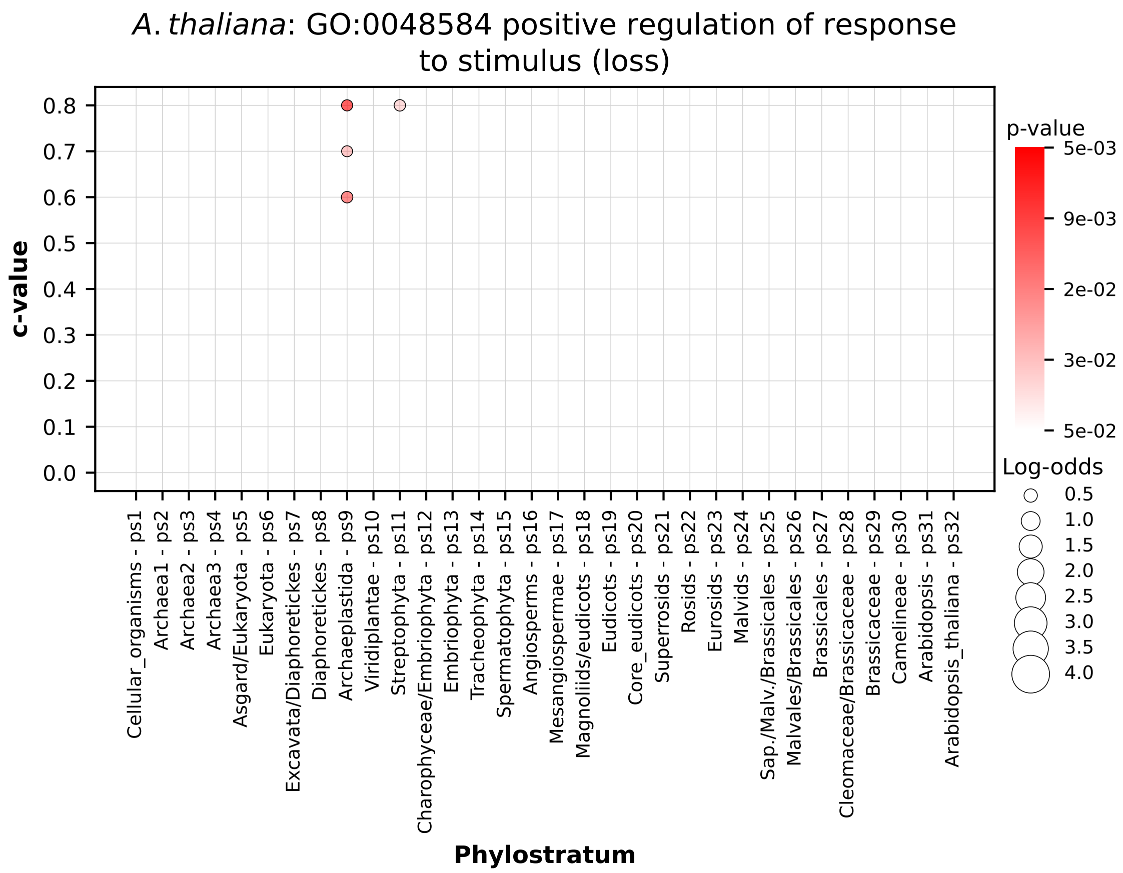
A. thaliana: GO:0048584 positive regulation of response to stimulus (loss)
c-value
0.8
0.7
0.6
0.5
0.4
0.3
0.2
0.1
0.0
p-value
5e-03
9e-03
2e-02
3e-02
5e-02
Log-odds
0.5
1.0
1.5
2.0
2.5
3.0
3.5
4.0
Cellular_organisms - ps1
Archaea1 - ps2
Archaea2 - ps3
Archaea3 - ps4
Asgard/Eukaryota - ps5
Eukaryota - ps6
Excavata/Diaphoretickes - ps7
Diaphoretickes - ps8
Archaeplastida - ps9
Viridiplantae - ps10
Streptophyta - ps11
Charophyceae/Embriophyta - ps12
Embriophyta - ps13
Tracheophyta - ps14
Spermatophyta - ps15
Angiosperms - ps16
Mesangiospermae - ps17
Magnoliids/eudicots - ps18
Eudicots - ps19
Core_eudicots - ps20
Superrosids - ps21
Rosids - ps22
Eurosids - ps23
Malvids - ps24
Sap./Malv./Brassicales - ps25
Malvales/Brassicales - ps26
Brassicales - ps27
Cleomaceae/Brassicaceae - ps28
Brassicaceae - ps29
Camelineae - ps30
Arabidopsis - ps31
Arabidopsis_thaliana - ps32
Phylostratum

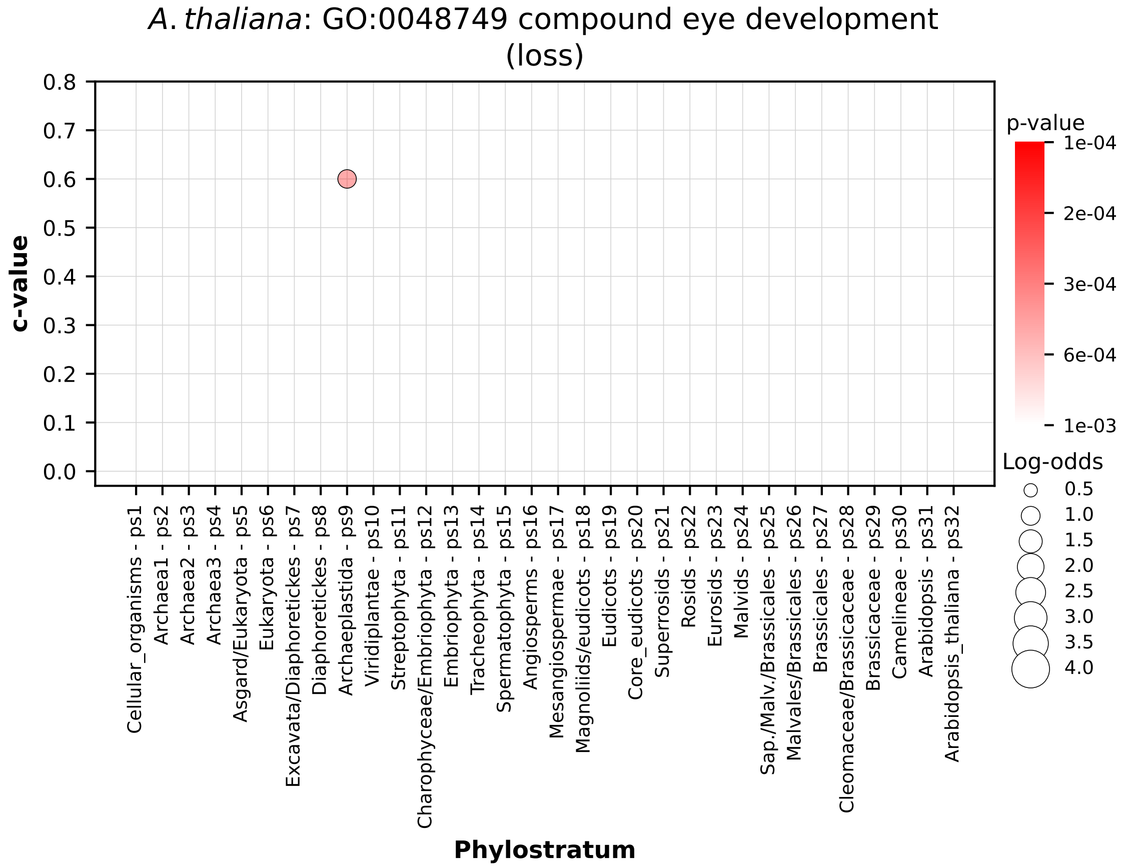
A. thaliana: GO:0048749 compound eye development
(loss)
c-value
0.8
0.7
0.6
0.5
0.4
0.3
0.2
0.1
0.0
p-value
1e-04
2e-04
3e-04
6e-04
1e-03
Log-odds
0.5
1.0
1.5
2.0
2.5
3.0
3.5
4.0
Cellular_organisms - ps1
Archaea1 - ps2
Archaea2 - ps3
Archaea3 - ps4
Asgard/Eukaryota - ps5
Eukaryota - ps6
Excavata/Diaphoretickes - ps7
Diaphoretickes - ps8
Archaeplastida - ps9
Viridiplantae - ps10
Streptophyta - ps11
Charophyceae/Embriophyta - ps12
Embriophyta - ps13
Tracheophyta - ps14
Spermatophyta - ps15
Angiosperms - ps16
Mesangiospermae - ps17
Magnoliids/eudicots - ps18
Eudicots - ps19
Core_eudicots - ps20
Superrosids - ps21
Rosids - ps22
Eurosids - ps23
Malvids - ps24
Sap./Malv./Brassicales - ps25
Malvales/Brassicales - ps26
Brassicales - ps27
Cleomaceae/Brassicaceae - ps28
Brassicaceae - ps29
Camelineae - ps30
Arabidopsis - ps31
Arabidopsis_thaliana - ps32
Phylostratum

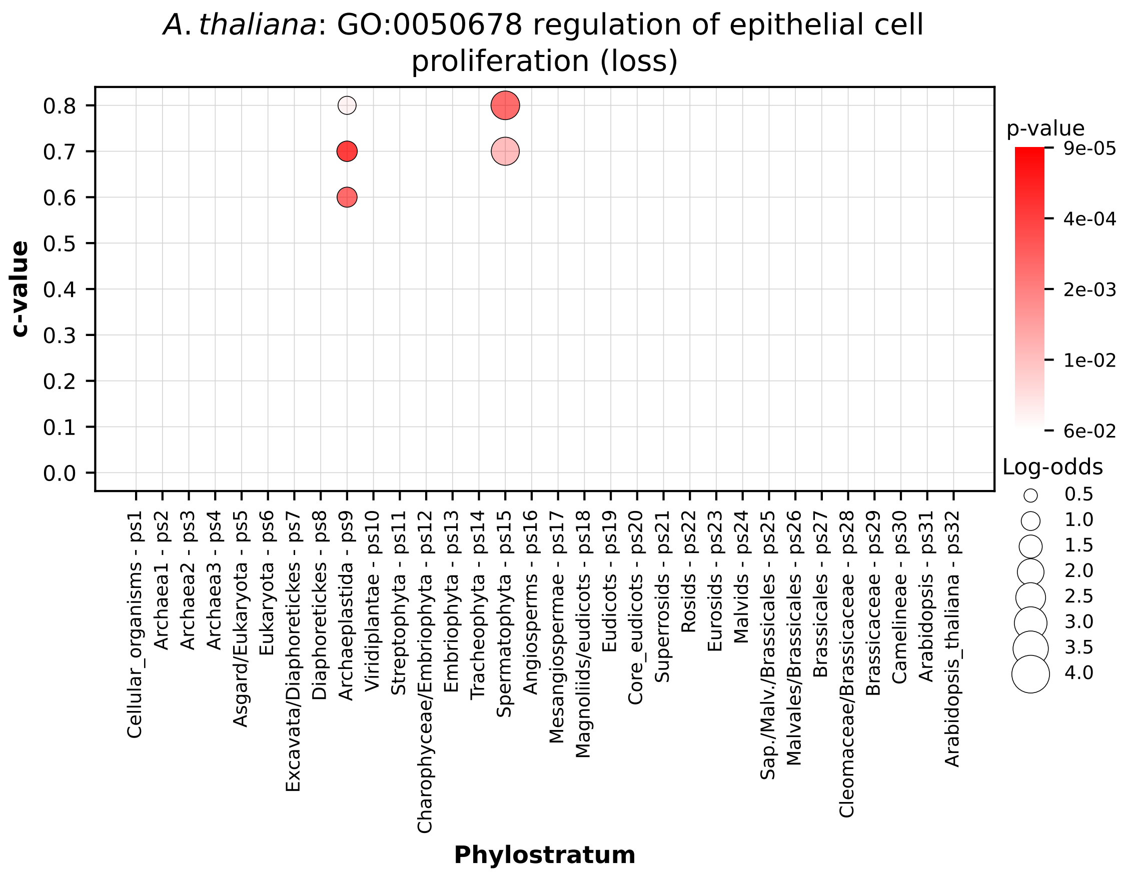

A. thaliana: GO:0050678 regulation of epithelial cell proliferation (loss)
c-value
0.0
0.1
0.2
0.3
0.4
0.5
0.6
0.7
0.8
p-value
9e-05
4e-04
2e-03
1e-02
6e-02
Log-odds
0.5
1.0
1.5
2.0
2.5
3.0
3.5
4.0
Cellular_organisms - ps1
Archaea1 - ps2
Archaea2 - ps3
Archaea3 - ps4
Asgard/Eukaryota - ps5
Eukaryota - ps6
Excavata/Diaphoretickes - ps7
Diaphoretickes - ps8
Archaeplastida - ps9
Viridiplantae - ps10
Streptophyta - ps11
Charophyceae/Embriophyta - ps12
Embriophyta - ps13
Tracheophyta - ps14
Spermatophyta - ps15
Angiosperms - ps16
Mesangiospermae - ps17
Magnoliids/eudicots - ps18
Eudicots - ps19
Core_eudicots - ps20
Superrosids - ps21
Rosids - ps22
Eurosids - ps23
Malvids - ps24
Sap./Malv./Brassicales - ps25
Malvales/Brassicales - ps26
Brassicales - ps27
Cleomaceae/Brassicaceae - ps28
Brassicaceae - ps29
Camelineae - ps30
Arabidopsis - ps31
Arabidopsis_thaliana - ps32
Phylostratum

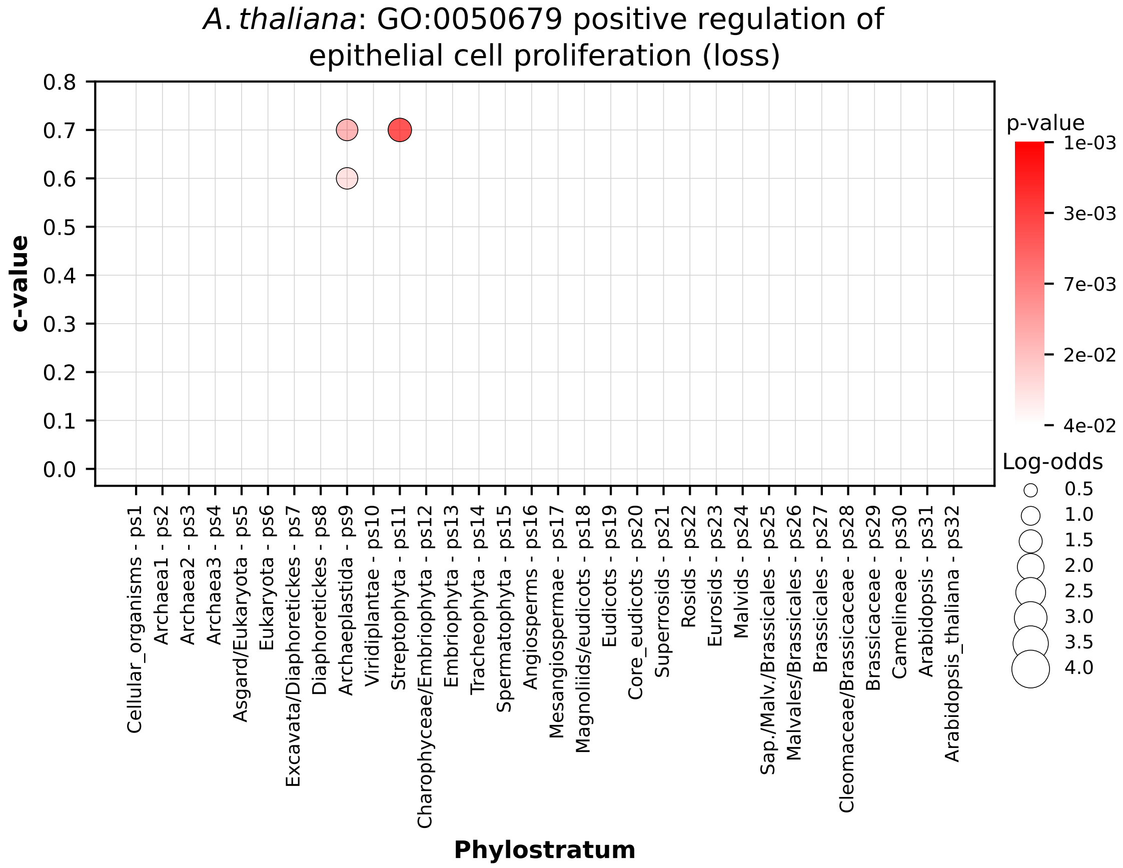

A. thaliana: GO:0050679 positive regulation of epithelial cell proliferation (loss)
c-value
0.8
0.7
0.6
0.5
0.4
0.3
0.2
0.1
0.0
p-value
1e-03
3e-03
7e-03
2e-02
4e-02
Log-odds
0.5
1.0
1.5
2.0
2.5
3.0
3.5
4.0
Cellular_organisms - ps1
Archaea1 - ps2
Archaea2 - ps3
Archaea3 - ps4
Asgard/Eukaryota - ps5
Eukaryota - ps6
Excavata/Diaphoretickes - ps7
Diaphoretickes - ps8
Archaeplastida - ps9
Viridiplantae - ps10
Streptophyta - ps11
Charophyceae/Embriophyta - ps12
Embriophyta - ps13
Tracheophyta - ps14
Spermatophyta - ps15
Angiosperms - ps16
Mesangiospermae - ps17
Magnoliids/eudicots - ps18
Eudicots - ps19
Core_eudicots - ps20
Superrosids - ps21
Rosids - ps22
Eurosids - ps23
Malvids - ps24
Sap./Malv./Brassicales - ps25
Malvales/Brassicales - ps26
Brassicales - ps27
Cleomaceae/Brassicaceae - ps28
Brassicaceae - ps29
Camelineae - ps30
Arabidopsis - ps31
Arabidopsis_thaliana - ps32
Phylostratum

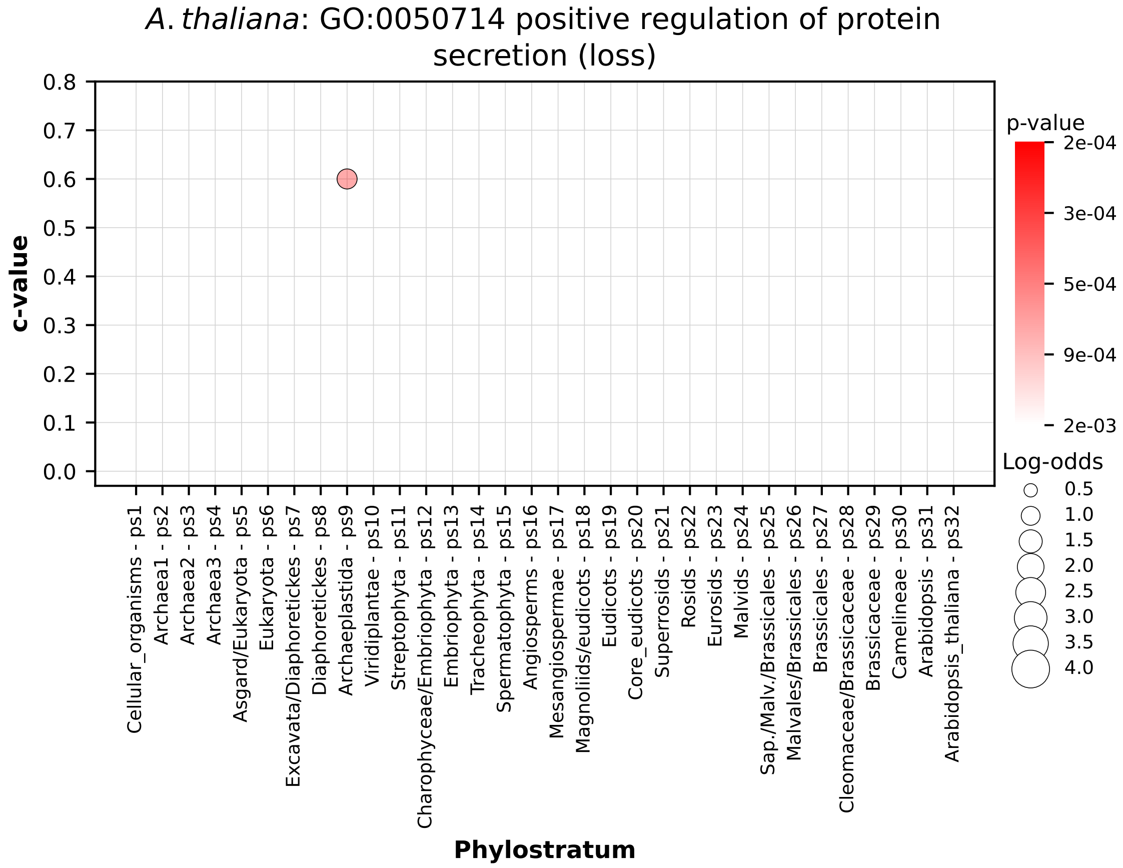

A. thaliana: GO:0050714 positive regulation of protein secretion (loss)
c-value
0.8
0.7
0.6
0.5
0.4
0.3
0.2
0.1
0.0
Phylostratum
Cellular_organisms - ps1
Archaea1 - ps2
Archaea2 - ps3
Archaea3 - ps4
Asgard/Eukaryota - ps5
Eukaryota - ps6
Excavata/Diaphoretickes - ps7
Diaphoretickes - ps8
Archaeplastida - ps9
Viridiplantae - ps10
Streptophyta - ps11
Charophyceae/Embriophyta - ps12
Embriophyta - ps13
Tracheophyta - ps14
Spermatophyta - ps15
Angiosperms - ps16
Mesangiospermae - ps17
Magnoliids/eudicots - ps18
Eudicots - ps19
Core_eudicots - ps20
Superrosids - ps21
Rosids - ps22
Eurosids - ps23
Malvids - ps24
Sap./Malv./Brassicales - ps25
Malvales/Brassicales - ps26
Brassicales - ps27
Cleomaceae/Brassicaceae - ps28
Brassicaceae - ps29
Camelineae - ps30
Arabidopsis - ps31
Arabidopsis_thaliana - ps32
p-value
2e-04
3e-04
5e-04
9e-04
2e-03
Log-odds
0.5
1.0
1.5
2.0
2.5
3.0
3.5
4.0

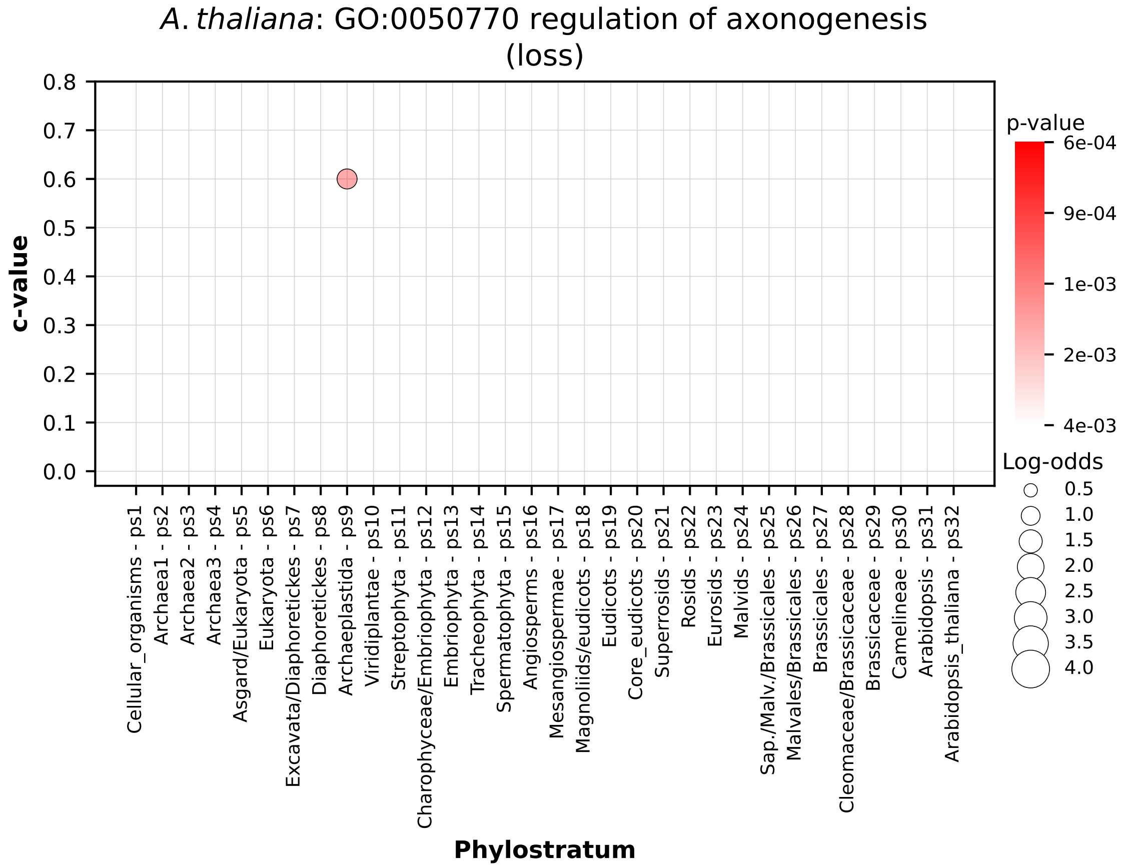

A. thaliana: GO:0050770 regulation of axonogenesis (loss)
c-value
0.8
0.7
0.6
0.5
0.4
0.3
0.2
0.1
0.0
p-value
6e-04
9e-04
1e-03
2e-03
4e-03
Log-odds
0.5
1.0
1.5
2.0
2.5
3.0
3.5
4.0
Cellular_organisms - ps1
Archaea1 - ps2
Archaea2 - ps3
Archaea3 - ps4
Asgard/Eukaryota - ps5
Eukaryota - ps6
Excavata/Diaphoretickes - ps7
Diaphoretickes - ps8
Archaeplastida - ps9
Viridiplantae - ps10
Streptophyta - ps11
Charophyceae/Embriophyta - ps12
Embriophyta - ps13
Tracheophyta - ps14
Spermatophyta - ps15
Angiosperms - ps16
Mesangiospermae - ps17
Magnoliids/eudicots - ps18
Eudicots - ps19
Core_eudicots - ps20
Superrosids - ps21
Rosids - ps22
Eurosids - ps23
Malvids - ps24
Sap./Malv./Brassicales - ps25
Malvales/Brassicales - ps26
Brassicales - ps27
Cleomaceae/Brassicaceae - ps28
Brassicaceae - ps29
Camelineae - ps30
Arabidopsis - ps31
Arabidopsis_thaliana - ps32
Phylostratum

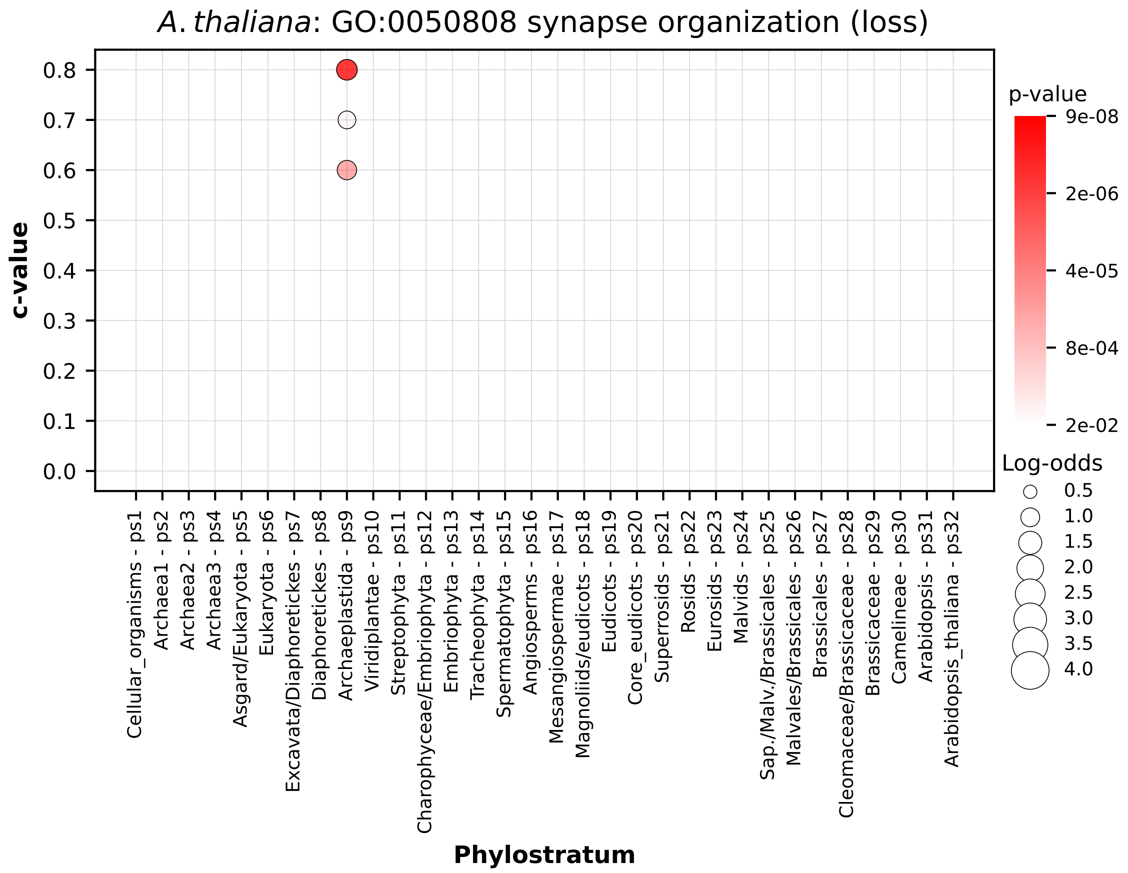
A. thaliana: GO:0050808 synapse organization (loss)
c-value
0.8
0.7
0.6
0.5
0.4
0.3
0.2
0.1
0.0
p-value
9e-08
2e-06
4e-05
8e-04
2e-02
Log-odds
0.5
1.0
1.5
2.0
2.5
3.0
3.5
4.0
Cellular_organisms - ps1
Archaea1 - ps2
Archaea2 - ps3
Archaea3 - ps4
Asgard/Eukaryota - ps5
Eukaryota - ps6
Excavata/Diaphoretickes - ps7
Diaphoretickes - ps8
Archaeplastida - ps9
Viridiplantae - ps10
Streptophyta - ps11
Charophyceae/Embriophyta - ps12
Embriophyta - ps13
Tracheophyta - ps14
Spermatophyta - ps15
Angiosperms - ps16
Mesangiospermae - ps17
Magnoliids/eudicots - ps18
Eudicots - ps19
Core_eudicots - ps20
Superrosids - ps21
Rosids - ps22
Eurosids - ps23
Malvids - ps24
Sap./Malv./Brassicales - ps25
Malvales/Brassicales - ps26
Brassicales - ps27
Cleomaceae/Brassicaceae - ps28
Brassicaceae - ps29
Camelineae - ps30
Arabidopsis - ps31
Arabidopsis_thaliana - ps32
Phylostratum

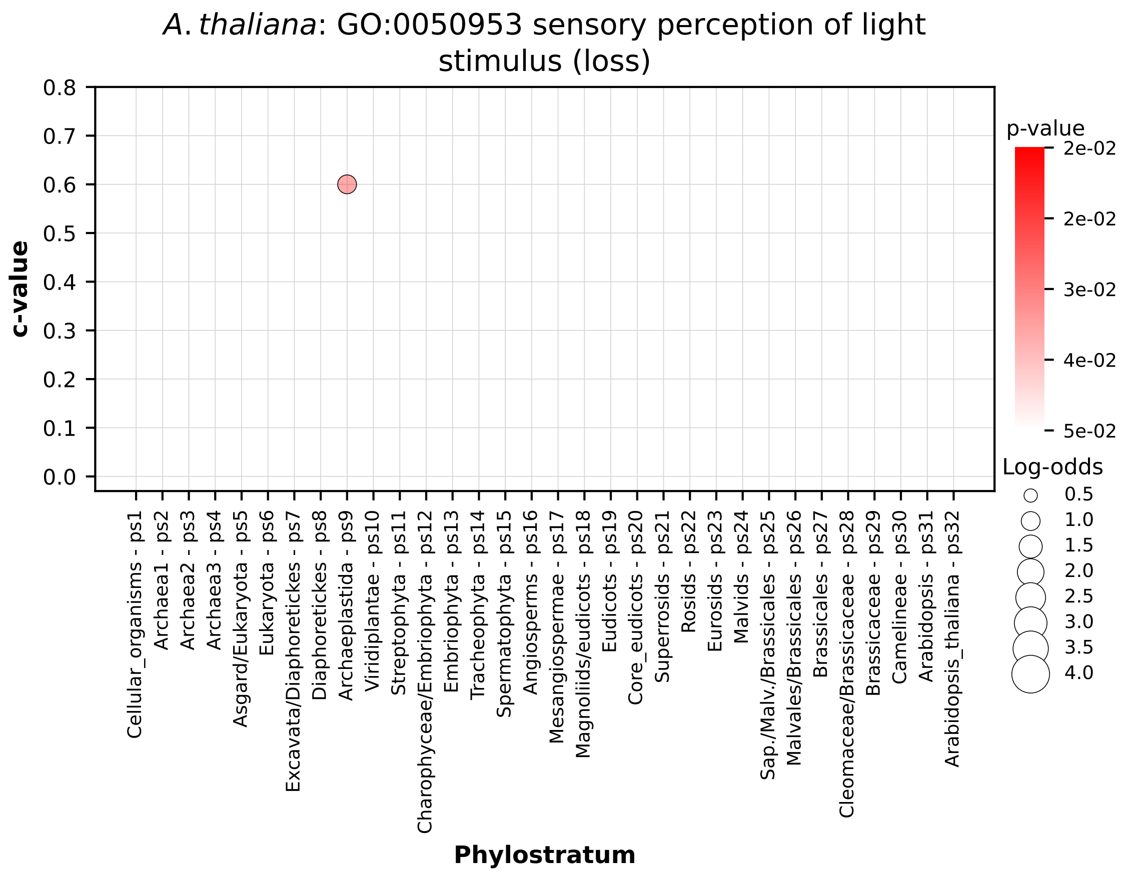

A. thaliana: GO:0050953 sensory perception of light stimulus (loss)
c-value
0.8
0.7
0.6
0.5
0.4
0.3
0.2
0.1
0.0
Phylostratum
Cellular_organisms - ps1
Archaea1 - ps2
Archaea2 - ps3
Archaea3 - ps4
Asgard/Eukaryota - ps5
Eukaryota - ps6
Excavata/Diaphoretickes - ps7
Diaphoretickes - ps8
Archaeplastida - ps9
Viridiplantae - ps10
Streptophyta - ps11
Charophyceae/Embriophyta - ps12
Embriophyta - ps13
Tracheophyta - ps14
Spermatophyta - ps15
Angiosperms - ps16
Mesangiospermae - ps17
Magnoliids/eudicots - ps18
Eudicots - ps19
Core_eudicots - ps20
Superrosids - ps21
Rosids - ps22
Eurosids - ps23
Malvids - ps24
Sap./Malv./Brassicales - ps25
Malvales/Brassicales - ps26
Brassicales - ps27
Cleomaceae/Brassicaceae - ps28
Brassicaceae - ps29
Camelineae - ps30
Arabidopsis - ps31
Arabidopsis_thaliana - ps32
p-value
2e-02
2e-02
3e-02
4e-02
5e-02
Log-odds
0.5
1.0
1.5
2.0
2.5
3.0
3.5
4.0

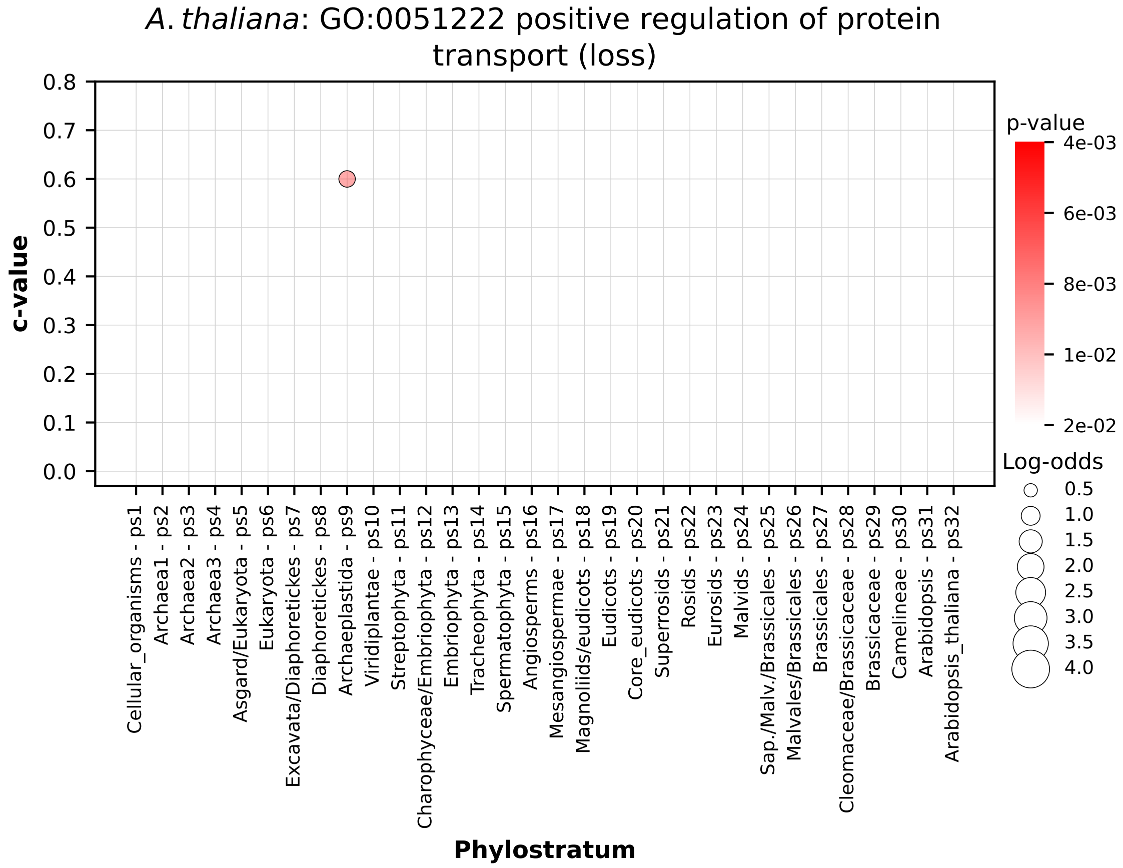
A. thaliana: GO:0051222 positive regulation of protein transport (loss)
c-value
0.8
0.7
0.6
0.5
0.4
0.3
0.2
0.1
0.0
p-value
4e-03
6e-03
8e-03
1e-02
2e-02
Log-odds
0.5
1.0
1.5
2.0
2.5
3.0
3.5
4.0
Cellular_organisms - ps1
Archaea1 - ps2
Archaea2 - ps3
Archaea3 - ps4
Asgard/Eukaryota - ps5
Eukaryota - ps6
Excavata/Diaphoretickes - ps7
Diaphoretickes - ps8
Archaeplastida - ps9
Viridiplantae - ps10
Streptophyta - ps11
Charophyceae/Embriophyta - ps12
Embriophyta - ps13
Tracheophyta - ps14
Spermatophyta - ps15
Angiosperms - ps16
Mesangiospermae - ps17
Magnoliids/eudicots - ps18
Eudicots - ps19
Core_eudicots - ps20
Superrosids - ps21
Rosids - ps22
Eurosids - ps23
Malvids - ps24
Sap./Malv./Brassicales - ps25
Malvales/Brassicales - ps26
Brassicales - ps27
Cleomaceae/Brassicaceae - ps28
Brassicaceae - ps29
Camelineae - ps30
Arabidopsis - ps31
Arabidopsis_thaliana - ps32
Phylostratum

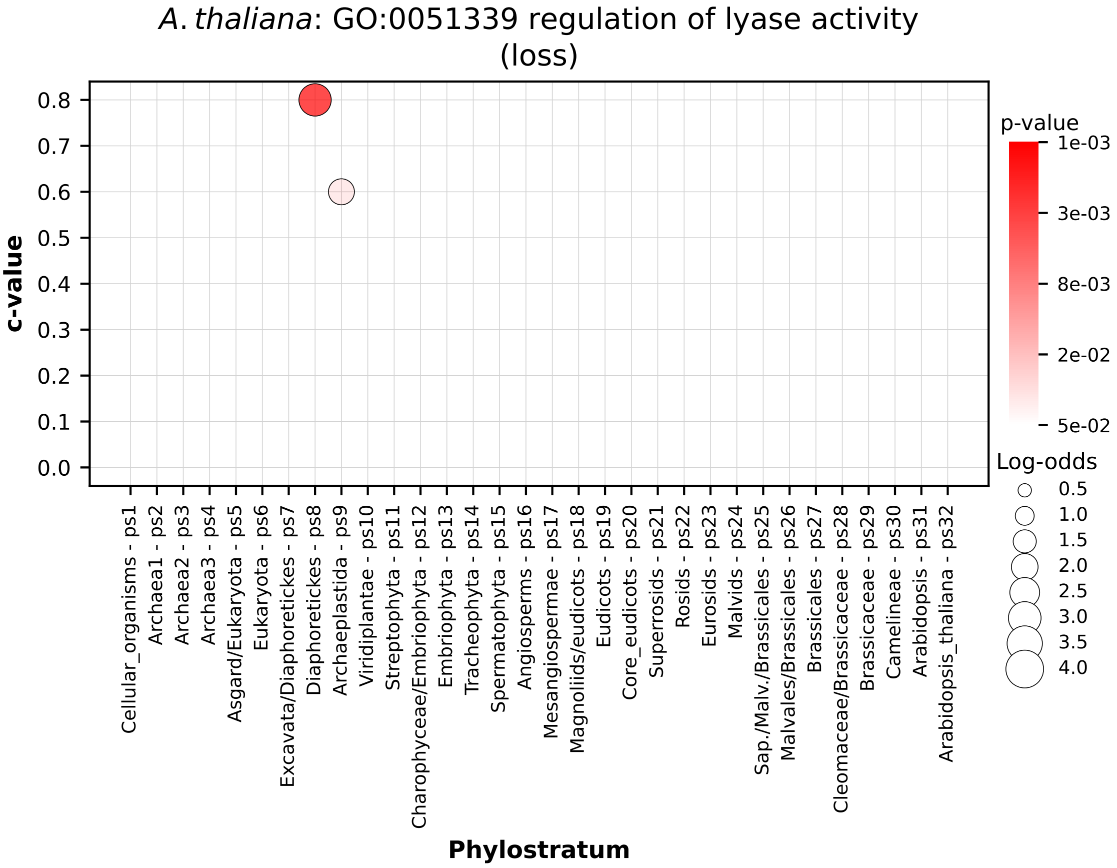
A. thaliana: GO:0051339 regulation of lyase activity
(loss)
c-value
0.8
0.7
0.6
0.5
0.4
0.3
0.2
0.1
0.0
Phylostratum
Cellular_organisms - ps1
Archaea1 - ps2
Archaea2 - ps3
Archaea3 - ps4
Asgard/Eukaryota - ps5
Eukaryota - ps6
Excavata/Diaphoretickes - ps7
Diaphoretickes - ps8
Archaeplastida - ps9
Viridiplantae - ps10
Streptophyta - ps11
Charophyceae/Embriophyta - ps12
Embriophyta - ps13
Tracheophyta - ps14
Spermatophyta - ps15
Angiosperms - ps16
Mesangiospermae - ps17
Magnoliids/eudicots - ps18
Eudicots - ps19
Core_eudicots - ps20
Superrosids - ps21
Rosids - ps22
Eurosids - ps23
Malvids - ps24
Sap./Malv./Brassicales - ps25
Malvales/Brassicales - ps26
Brassicales - ps27
Cleomaceae/Brassicaceae - ps28
Brassicaceae - ps29
Camelineae - ps30
Arabidopsis - ps31
Arabidopsis_thaliana - ps32
p-value
1e-03
3e-03
8e-03
2e-02
5e-02
Log-odds
0.5
1.0
1.5
2.0
2.5
3.0
3.5
4.0

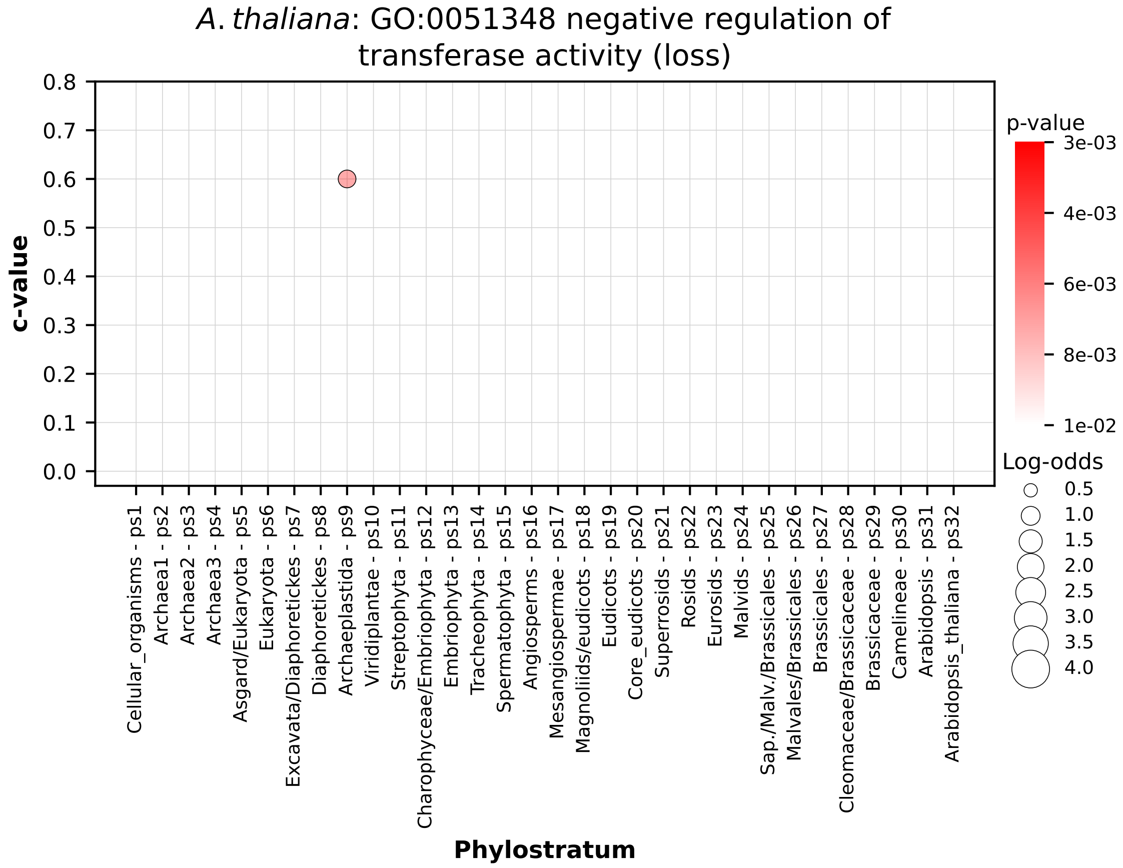
A. thaliana: GO:0051348 negative regulation of transferase activity (loss)
c-value
0.8
0.7
0.6
0.5
0.4
0.3
0.2
0.1
0.0
Phylostratum
Cellular_organisms - ps1
Archaea1 - ps2
Archaea2 - ps3
Archaea3 - ps4
Asgard/Eukaryota - ps5
Eukaryota - ps6
Excavata/Diaphoretickes - ps7
Diaphoretickes - ps8
Archaeplastida - ps9
Viridiplantae - ps10
Streptophyta - ps11
Charophyceae/Embriophyta - ps12
Embriophyta - ps13
Tracheophyta - ps14
Spermatophyta - ps15
Angiosperms - ps16
Mesangiospermae - ps17
Magnoliids/eudicots - ps18
Eudicots - ps19
Core_eudicots - ps20
Superrosids - ps21
Rosids - ps22
Eurosids - ps23
Malvids - ps24
Sap./Malv./Brassicales - ps25
Malvales/Brassicales - ps26
Brassicales - ps27
Cleomaceae/Brassicaceae - ps28
Brassicaceae - ps29
Camelineae - ps30
Arabidopsis - ps31
Arabidopsis_thaliana - ps32
p-value
3e-03
4e-03
6e-03
8e-03
1e-02
Log-odds
0.5
1.0
1.5
2.0
2.5
3.0
3.5
4.0

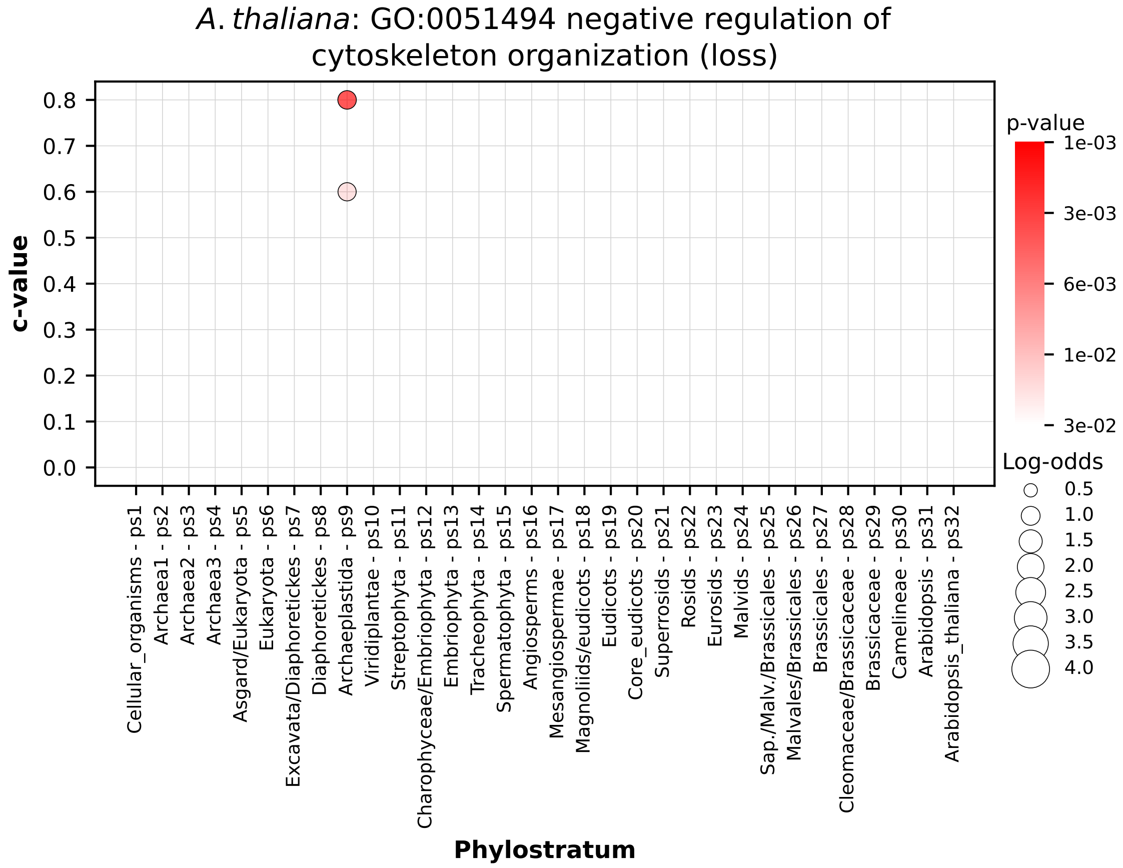
A. thaliana: GO:0051494 negative regulation of cytoskeleton organization (loss)
c-value
0.0
0.1
0.2
0.3
0.4
0.5
0.6
0.7
0.8
Phylostratum
Cellular_organisms - ps1
Archaea1 - ps2
Archaea2 - ps3
Archaea3 - ps4
Asgard/Eukaryota - ps5
Eukaryota - ps6
Excavata/Diaphoretickes - ps7
Diaphoretickes - ps8
Archaeplastida - ps9
Viridiplantae - ps10
Streptophyta - ps11
Charophyceae/Embriophyta - ps12
Embriophyta - ps13
Tracheophyta - ps14
Spermatophyta - ps15
Angiosperms - ps16
Mesangiospermae - ps17
Magnoliids/eudicots - ps18
Eudicots - ps19
Core_eudicots - ps20
Superrosids - ps21
Rosids - ps22
Eurosids - ps23
Malvids - ps24
Sap./Malv./Brassicales - ps25
Malvales/Brassicales - ps26
Brassicales - ps27
Cleomaceae/Brassicaceae - ps28
Brassicaceae - ps29
Camelineae - ps30
Arabidopsis - ps31
Arabidopsis_thaliana - ps32
p-value
1e-03
3e-03
6e-03
1e-02
3e-02
Log-odds
0.5
1.0
1.5
2.0
2.5
3.0
3.5
4.0

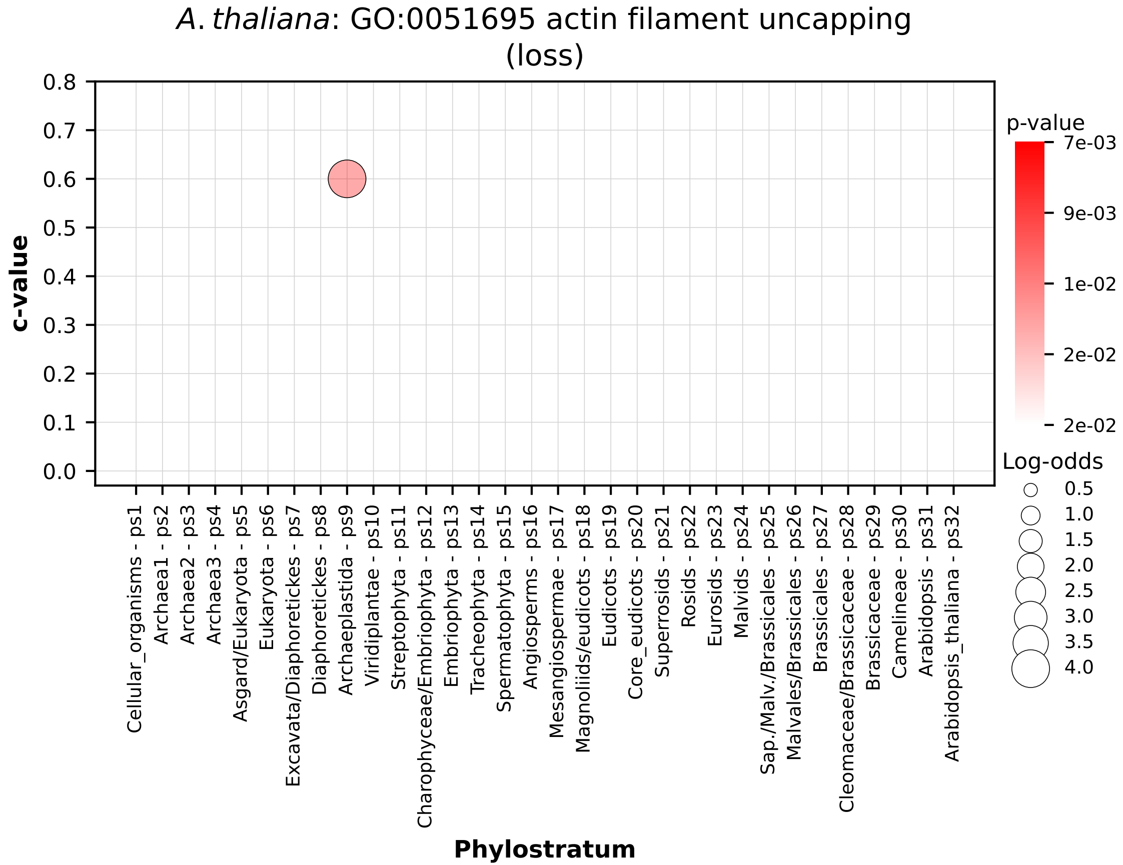

A. thaliana: GO:0051695 actin filament uncapping (loss)
c-value
0.8
0.7
0.6
0.5
0.4
0.3
0.2
0.1
0.0
Phylostratum
Cellular_organisms - ps1
Archaea1 - ps2
Archaea2 - ps3
Archaea3 - ps4
Asgard/Eukaryota - ps5
Eukaryota - ps6
Excavata/Diaphoretickes - ps7
Diaphoretickes - ps8
Archaeplastida - ps9
Viridiplantae - ps10
Streptophyta - ps11
Charophyceae/Embriophyta - ps12
Embriophyta - ps13
Tracheophyta - ps14
Spermatophyta - ps15
Angiosperms - ps16
Mesangiospermae - ps17
Magnoliids/eudicots - ps18
Eudicots - ps19
Core_eudicots - ps20
Superrosids - ps21
Rosids - ps22
Eurosids - ps23
Malvids - ps24
Sap./Malv./Brassicales - ps25
Malvales/Brassicales - ps26
Brassicales - ps27
Cleomaceae/Brassicaceae - ps28
Brassicaceae - ps29
Camelineae - ps30
Arabidopsis - ps31
Arabidopsis_thaliana - ps32
p-value
7e-03
9e-03
1e-02
2e-02
2e-02
Log-odds
0.5
1.0
1.5
2.0
2.5
3.0
3.5
4.0

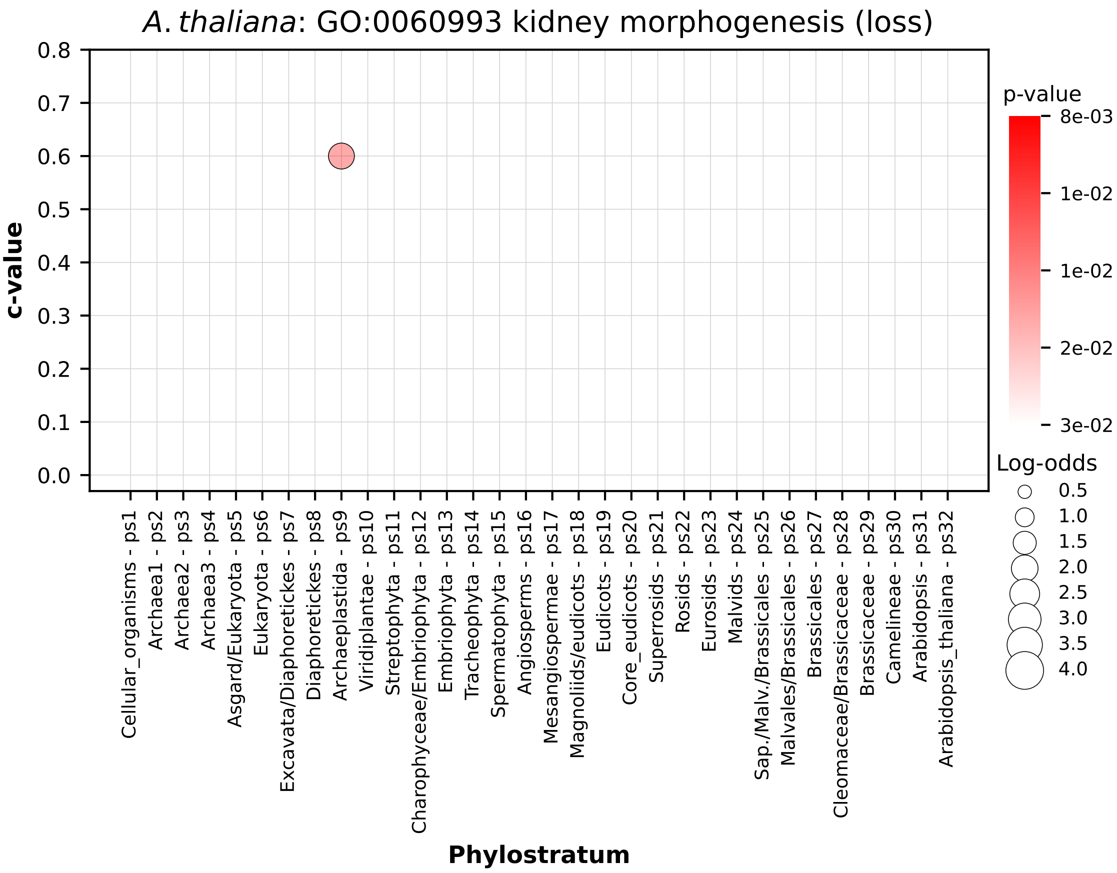

A. thaliana: GO:0060993 kidney morphogenesis (loss)
c-value
0.0
0.1
0.2
0.3
0.4
0.5
0.6
0.7
0.8
p-value
8e-03
1e-02
1e-02
2e-02
3e-02
Log-odds
0.5
1.0
1.5
2.0
2.5
3.0
3.5
4.0
Cellular_organisms - ps1
Archaea1 - ps2
Archaea2 - ps3
Archaea3 - ps4
Asgard/Eukaryota - ps5
Eukaryota - ps6
Excavata/Diaphoretickes - ps7
Diaphoretickes - ps8
Archaeplastida - ps9
Viridiplantae - ps10
Streptophyta - ps11
Charophyceae/Embriophyta - ps12
Embriophyta - ps13
Tracheophyta - ps14
Spermatophyta - ps15
Angiosperms - ps16
Mesangiospermae - ps17
Magnoliids/eudicots - ps18
Eudicots - ps19
Core_eudicots - ps20
Superrosids - ps21
Rosids - ps22
Eurosids - ps23
Malvids - ps24
Sap./Malv./Brassicales - ps25
Malvales/Brassicales - ps26
Brassicales - ps27
Cleomaceae/Brassicaceae - ps28
Brassicaceae - ps29
Camelineae - ps30
Arabidopsis - ps31
Arabidopsis_thaliana - ps32
Phylostratum

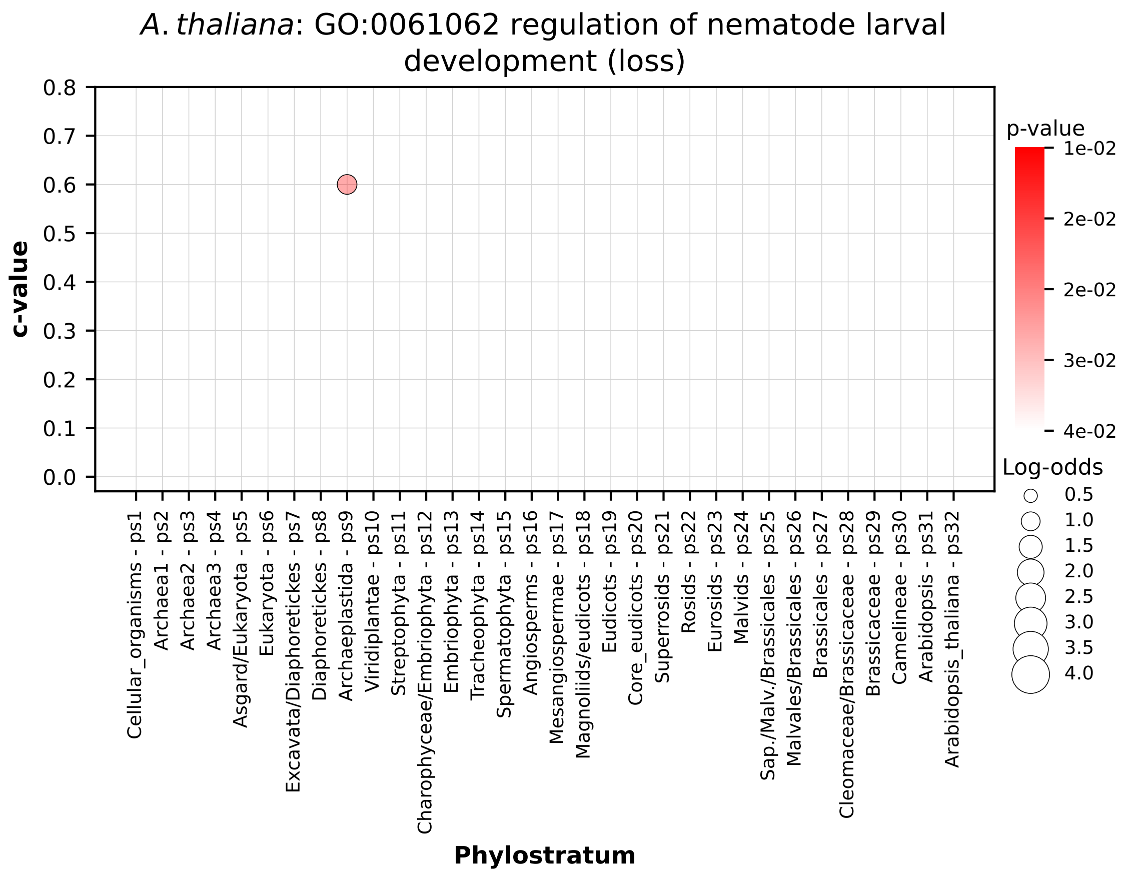

A. thaliana: GO:0061062 regulation of nematode larval development (loss)
c-value
0.8
0.7
0.6
0.5
0.4
0.3
0.2
0.1
0.0
Phylostratum
Cellular_organisms - ps1
Archaea1 - ps2
Archaea2 - ps3
Archaea3 - ps4
Asgard/Eukaryota - ps5
Eukaryota - ps6
Excavata/Diaphoretickes - ps7
Diaphoretickes - ps8
Archaeplastida - ps9
Viridiplantae - ps10
Streptophyta - ps11
Charophyceae/Embriophyta - ps12
Embriophyta - ps13
Tracheophyta - ps14
Spermatophyta - ps15
Angiosperms - ps16
Mesangiospermae - ps17
Magnoliids/eudicots - ps18
Eudicots - ps19
Core_eudicots - ps20
Superrosids - ps21
Rosids - ps22
Eurosids - ps23
Malvids - ps24
Sap./Malv./Brassicales - ps25
Malvales/Brassicales - ps26
Brassicales - ps27
Cleomaceae/Brassicaceae - ps28
Brassicaceae - ps29
Camelineae - ps30
Arabidopsis - ps31
Arabidopsis_thaliana - ps32
p-value
1e-02
2e-02
2e-02
3e-02
4e-02
Log-odds
0.5
1.0
1.5
2.0
2.5
3.0
3.5
4.0

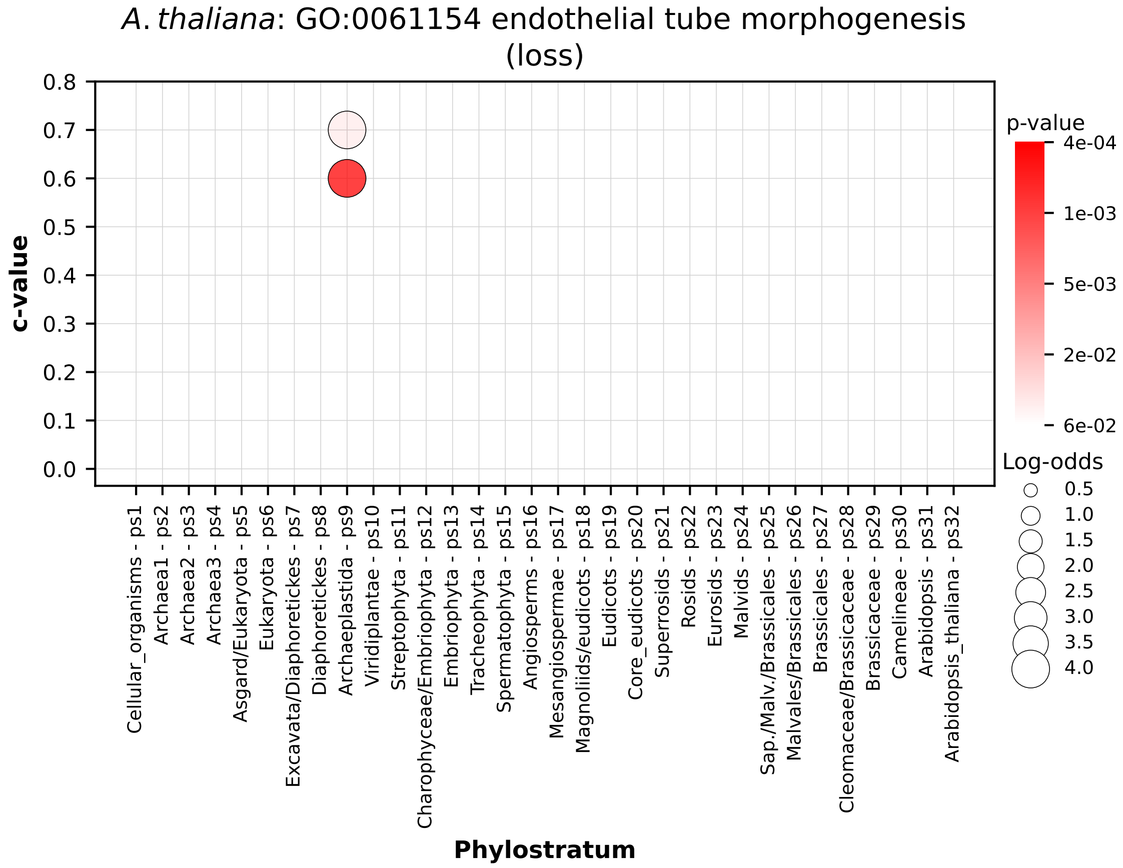

A. thaliana: GO:0061154 endothelial tube morphogenesis
(loss)
c-value
0.8
0.7
0.6
0.5
0.4
0.3
0.2
0.1
0.0
p-value
4e-04
1e-03
5e-03
2e-02
6e-02
Log-odds
0.5
1.0
1.5
2.0
2.5
3.0
3.5
4.0
Cellular_organisms - ps1
Archaea1 - ps2
Archaea2 - ps3
Archaea3 - ps4
Asgard/Eukaryota - ps5
Eukaryota - ps6
Excavata/Diaphoretickes - ps7
Diaphoretickes - ps8
Archaeplastida - ps9
Viridiplantae - ps10
Streptophyta - ps11
Charophyceae/Embriophyta - ps12
Embriophyta - ps13
Tracheophyta - ps14
Spermatophyta - ps15
Angiosperms - ps16
Mesangiospermae - ps17
Magnoliids/eudicots - ps18
Eudicots - ps19
Core_eudicots - ps20
Superrosids - ps21
Rosids - ps22
Eurosids - ps23
Malvids - ps24
Sap./Malv./Brassicales - ps25
Malvales/Brassicales - ps26
Brassicales - ps27
Cleomaceae/Brassicaceae - ps28
Brassicaceae - ps29
Camelineae - ps30
Arabidopsis - ps31
Arabidopsis_thaliana - ps32
Phylostratum

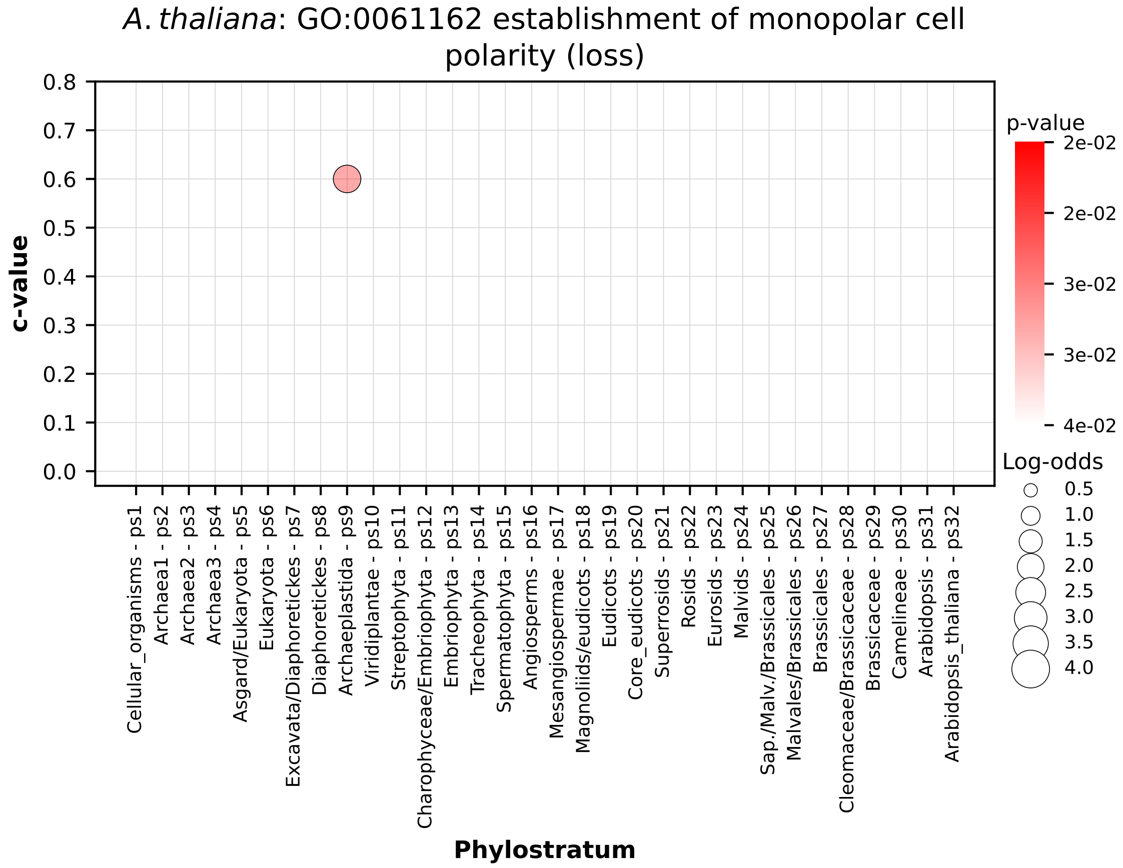

A. thaliana: GO:0061162 establishment of monopolar cell polarity (loss)
c-value
0.8
0.7
0.6
0.5
0.4
0.3
0.2
0.1
0.0
p-value
2e-02
2e-02
3e-02
3e-02
4e-02
Log-odds
0.5
1.0
1.5
2.0
2.5
3.0
3.5
4.0
Cellular_organisms - ps1
Archaea1 - ps2
Archaea2 - ps3
Archaea3 - ps4
Asgard/Eukaryota - ps5
Eukaryota - ps6
Excavata/Diaphoretickes - ps7
Diaphoretickes - ps8
Archaeplastida - ps9
Viridiplantae - ps10
Streptophyta - ps11
Charophyceae/Embriophyta - ps12
Embriophyta - ps13
Tracheophyta - ps14
Spermatophyta - ps15
Angiosperms - ps16
Mesangiospermae - ps17
Magnoliids/eudicots - ps18
Eudicots - ps19
Core_eudicots - ps20
Superrosids - ps21
Rosids - ps22
Eurosids - ps23
Malvids - ps24
Sap./Malv./Brassicales - ps25
Malvales/Brassicales - ps26
Brassicales - ps27
Cleomaceae/Brassicaceae - ps28
Brassicaceae - ps29
Camelineae - ps30
Arabidopsis - ps31
Arabidopsis_thaliana - ps32
Phylostratum

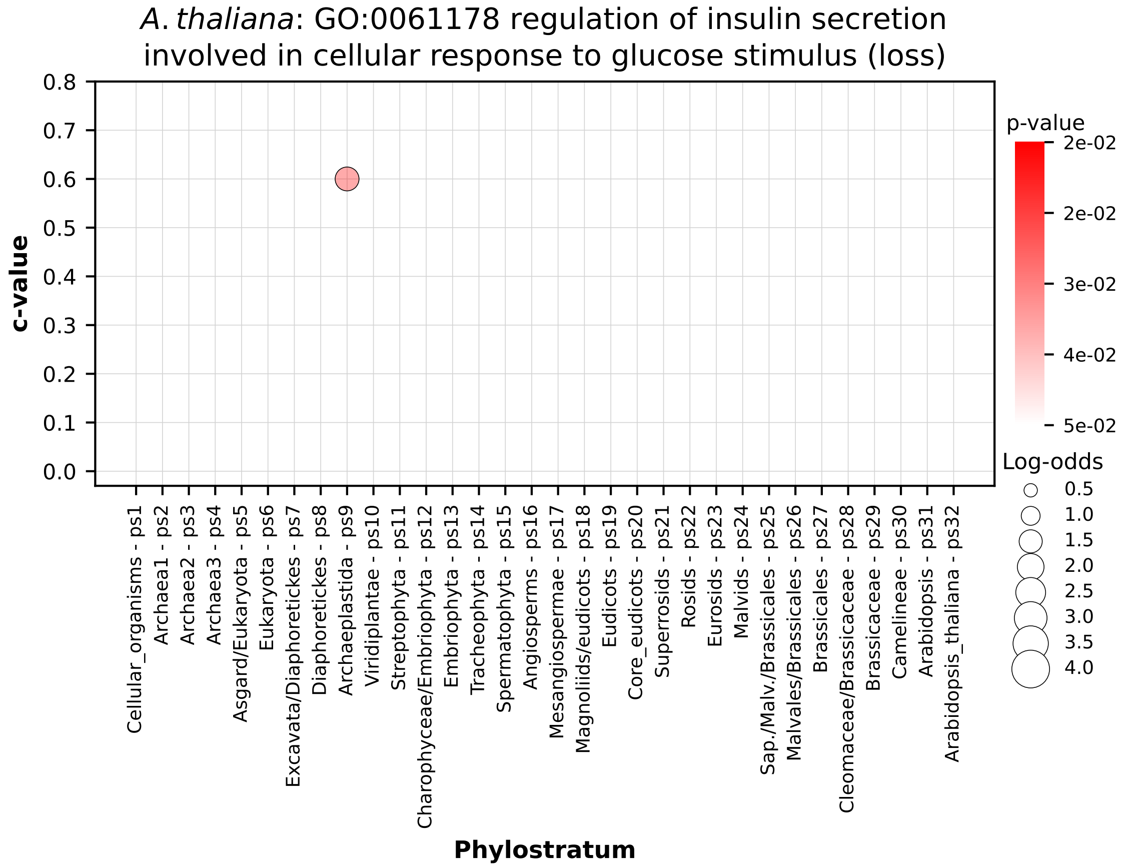
A. thaliana: GO:0061178 regulation of insulin secretion involved in cellular response to glucose stimulus (loss)
c-value
0.8
0.7
0.6
0.5
0.4
0.3
0.2
0.1
0.0
Phylostratum
Cellular_organisms - ps1
Archaea1 - ps2
Archaea2 - ps3
Archaea3 - ps4
Asgard/Eukaryota - ps5
Eukaryota - ps6
Excavata/Diaphoretickes - ps7
Diaphoretickes - ps8
Archaeplastida - ps9
Viridiplantae - ps10
Streptophyta - ps11
Charophyceae/Embriophyta - ps12
Embriophyta - ps13
Tracheophyta - ps14
Spermatophyta - ps15
Angiosperms - ps16
Mesangiospermae - ps17
Magnoliids/eudicots - ps18
Eudicots - ps19
Core_eudicots - ps20
Superrosids - ps21
Rosids - ps22
Eurosids - ps23
Malvids - ps24
Sap./Malv./Brassicales - ps25
Malvales/Brassicales - ps26
Brassicales - ps27
Cleomaceae/Brassicaceae - ps28
Brassicaceae - ps29
Camelineae - ps30
Arabidopsis - ps31
Arabidopsis_thaliana - ps32
p-value
2e-02
2e-02
3e-02
4e-02
5e-02
Log-odds
0.5
1.0
1.5
2.0
2.5
3.0
3.5
4.0

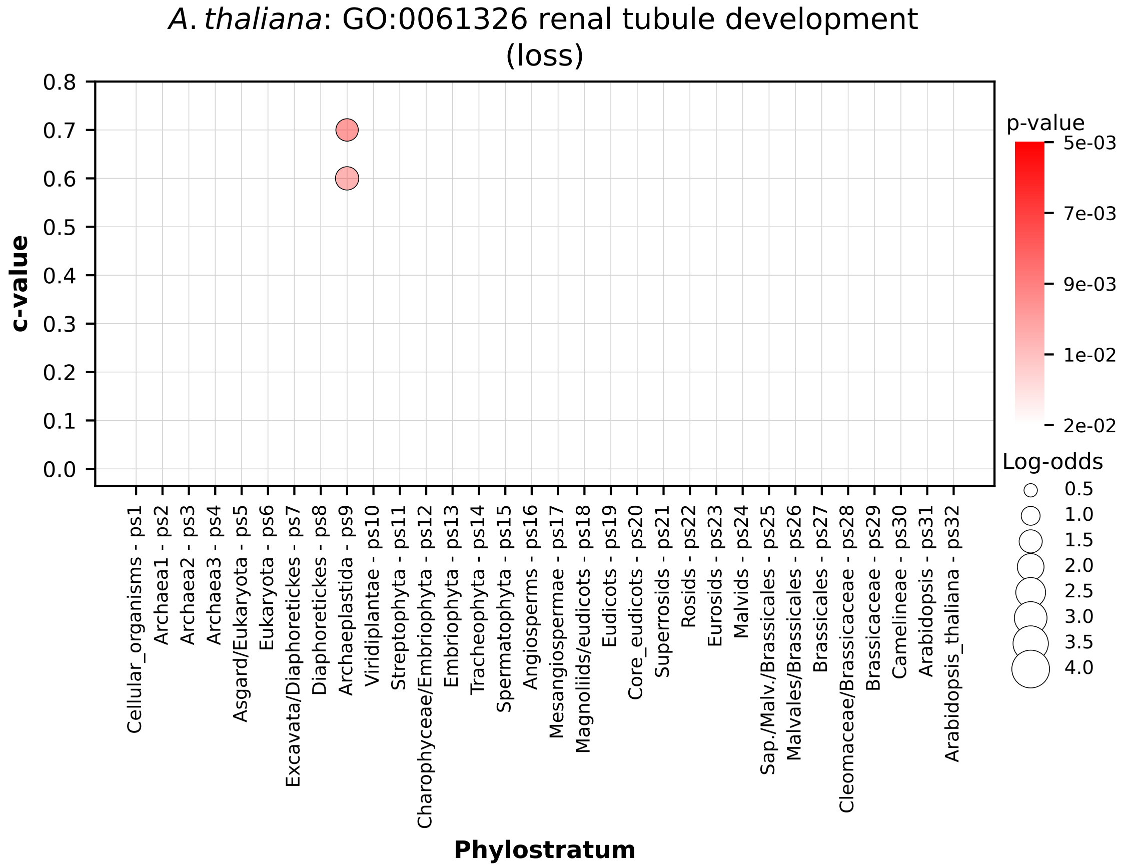

A. thaliana: GO:0061326 renal tubule development
(loss)
c-value
0.8
0.7
0.6
0.5
0.4
0.3
0.2
0.1
0.0
p-value
5e-03
7e-03
9e-03
1e-02
2e-02
Log-odds
0.5
1.0
1.5
2.0
2.5
3.0
3.5
4.0
Cellular_organisms - ps1
Archaea1 - ps2
Archaea2 - ps3
Archaea3 - ps4
Asgard/Eukaryota - ps5
Eukaryota - ps6
Excavata/Diaphoretickes - ps7
Diaphoretickes - ps8
Archaeplastida - ps9
Viridiplantae - ps10
Streptophyta - ps11
Charophyceae/Embriophyta - ps12
Embriophyta - ps13
Tracheophyta - ps14
Spermatophyta - ps15
Angiosperms - ps16
Mesangiospermae - ps17
Magnoliids/eudicots - ps18
Eudicots - ps19
Core_eudicots - ps20
Superrosids - ps21
Rosids - ps22
Eurosids - ps23
Malvids - ps24
Sap./Malv./Brassicales - ps25
Malvales/Brassicales - ps26
Brassicales - ps27
Cleomaceae/Brassicaceae - ps28
Brassicaceae - ps29
Camelineae - ps30
Arabidopsis - ps31
Arabidopsis_thaliana - ps32
Phylostratum

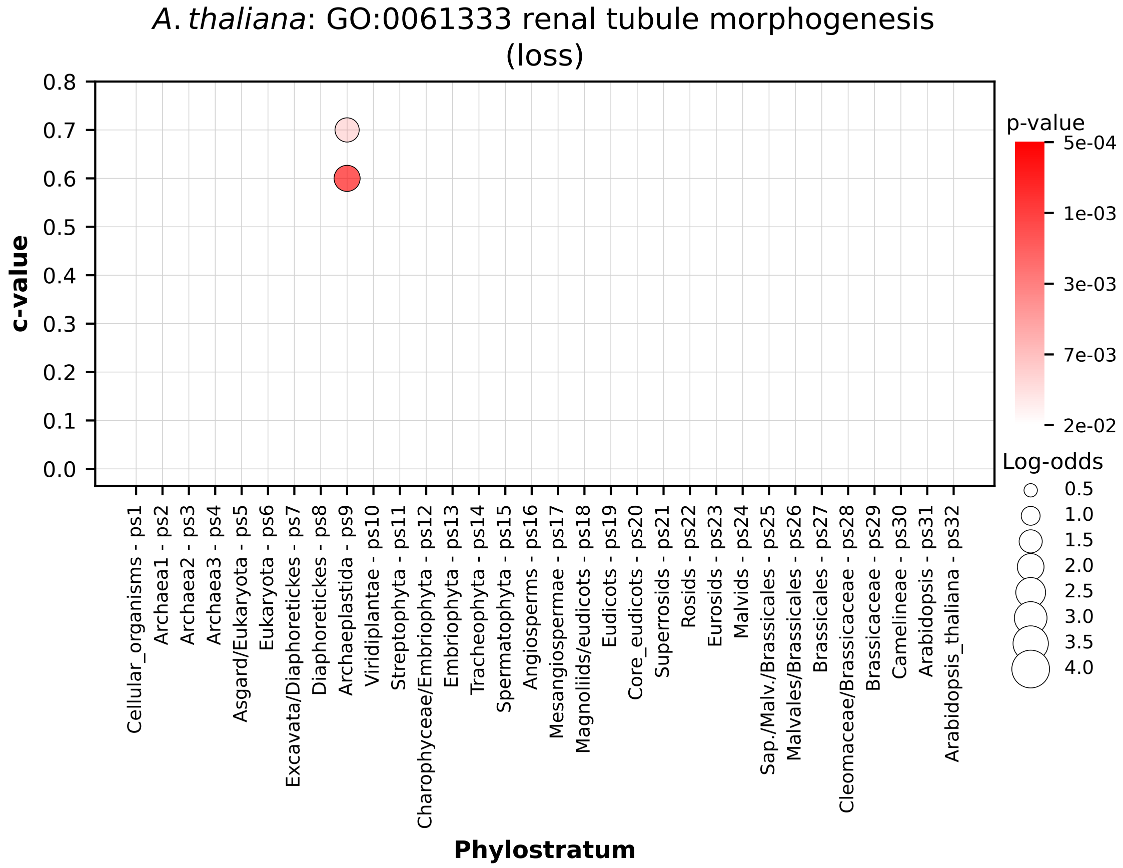
A. thaliana: GO:0061333 renal tubule morphogenesis
(loss)
c-value
0.0
0.1
0.2
0.3
0.4
0.5
0.6
0.7
0.8
p-value
5e-04
1e-03
3e-03
7e-03
2e-02
Log-odds
0.5
1.0
1.5
2.0
2.5
3.0
3.5
4.0
Cellular_organisms - ps1
Archaea1 - ps2
Archaea2 - ps3
Archaea3 - ps4
Asgard/Eukaryota - ps5
Eukaryota - ps6
Excavata/Diaphoretickes - ps7
Diaphoretickes - ps8
Archaeplastida - ps9
Viridiplantae - ps10
Streptophyta - ps11
Charophyceae/Embriophyta - ps12
Embriophyta - ps13
Tracheophyta - ps14
Spermatophyta - ps15
Angiosperms - ps16
Mesangiospermae - ps17
Magnoliids/eudicots - ps18
Eudicots - ps19
Core_eudicots - ps20
Superrosids - ps21
Rosids - ps22
Eurosids - ps23
Malvids - ps24
Sap./Malv./Brassicales - ps25
Malvales/Brassicales - ps26
Brassicales - ps27
Cleomaceae/Brassicaceae - ps28
Brassicaceae - ps29
Camelineae - ps30
Arabidopsis - ps31
Arabidopsis_thaliana - ps32
Phylostratum

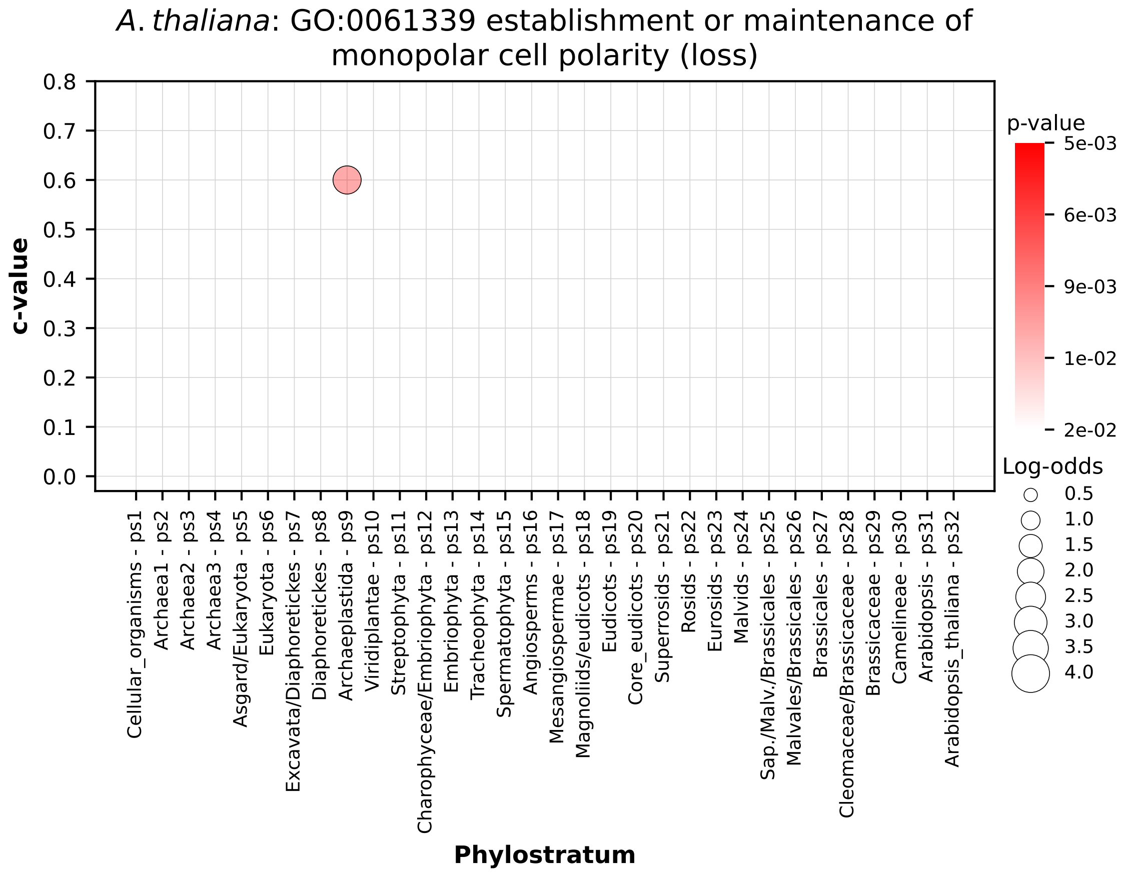

A. thaliana: GO:0061339 establishment or maintenance of monopolar cell polarity (loss)
c-value
0.8
0.7
0.6
0.5
0.4
0.3
0.2
0.1
0.0
Phylostratum
Cellular_organisms - ps1
Archaea1 - ps2
Archaea2 - ps3
Archaea3 - ps4
Asgard/Eukaryota - ps5
Eukaryota - ps6
Excavata/Diaphoretickes - ps7
Diaphoretickes - ps8
Archaeplastida - ps9
Viridiplantae - ps10
Streptophyta - ps11
Charophyceae/Embriophyta - ps12
Embriophyta - ps13
Tracheophyta - ps14
Spermatophyta - ps15
Angiosperms - ps16
Mesangiospermae - ps17
Magnoliids/eudicots - ps18
Eudicots - ps19
Core_eudicots - ps20
Superrosids - ps21
Rosids - ps22
Eurosids - ps23
Malvids - ps24
Sap./Malv./Brassicales - ps25
Malvales/Brassicales - ps26
Brassicales - ps27
Cleomaceae/Brassicaceae - ps28
Brassicaceae - ps29
Camelineae - ps30
Arabidopsis - ps31
Arabidopsis_thaliana - ps32
p-value
5e-03
6e-03
9e-03
1e-02
2e-02
Log-odds
0.5
1.0
1.5
2.0
2.5
3.0
3.5
4.0

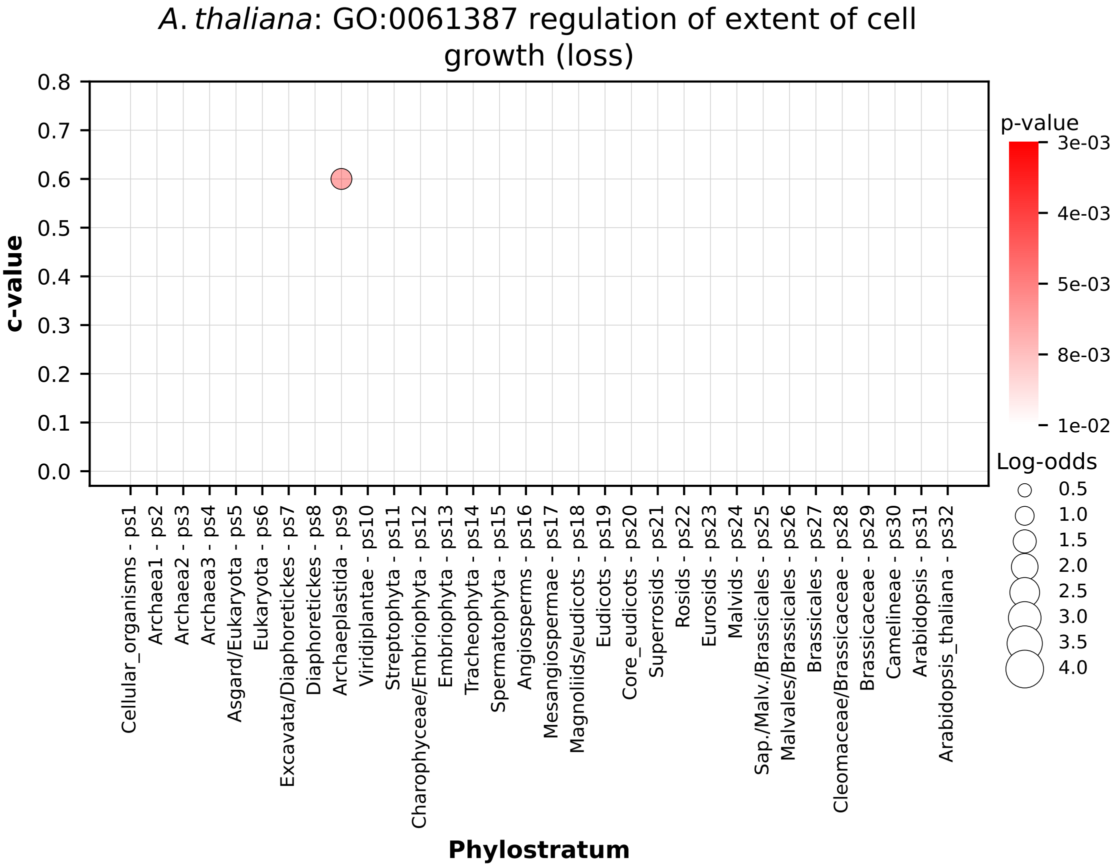

A. thaliana: GO:0061387 regulation of extent of cell growth (loss)
c-value
0.8
0.7
0.6
0.5
0.4
0.3
0.2
0.1
0.0
p-value
3e-03
4e-03
5e-03
8e-03
1e-02
Log-odds
0.5
1.0
1.5
2.0
2.5
3.0
3.5
4.0
Cellular_organisms - ps1
Archaea1 - ps2
Archaea2 - ps3
Archaea3 - ps4
Asgard/Eukaryota - ps5
Eukaryota - ps6
Excavata/Diaphoretickes - ps7
Diaphoretickes - ps8
Archaeplastida - ps9
Viridiplantae - ps10
Streptophyta - ps11
Charophyceae/Embriophyta - ps12
Embriophyta - ps13
Tracheophyta - ps14
Spermatophyta - ps15
Angiosperms - ps16
Mesangiospermae - ps17
Magnoliids/eudicots - ps18
Eudicots - ps19
Core_eudicots - ps20
Superrosids - ps21
Rosids - ps22
Eurosids - ps23
Malvids - ps24
Sap./Malv./Brassicales - ps25
Malvales/Brassicales - ps26
Brassicales - ps27
Cleomaceae/Brassicaceae - ps28
Brassicaceae - ps29
Camelineae - ps30
Arabidopsis - ps31
Arabidopsis_thaliana - ps32
Phylostratum

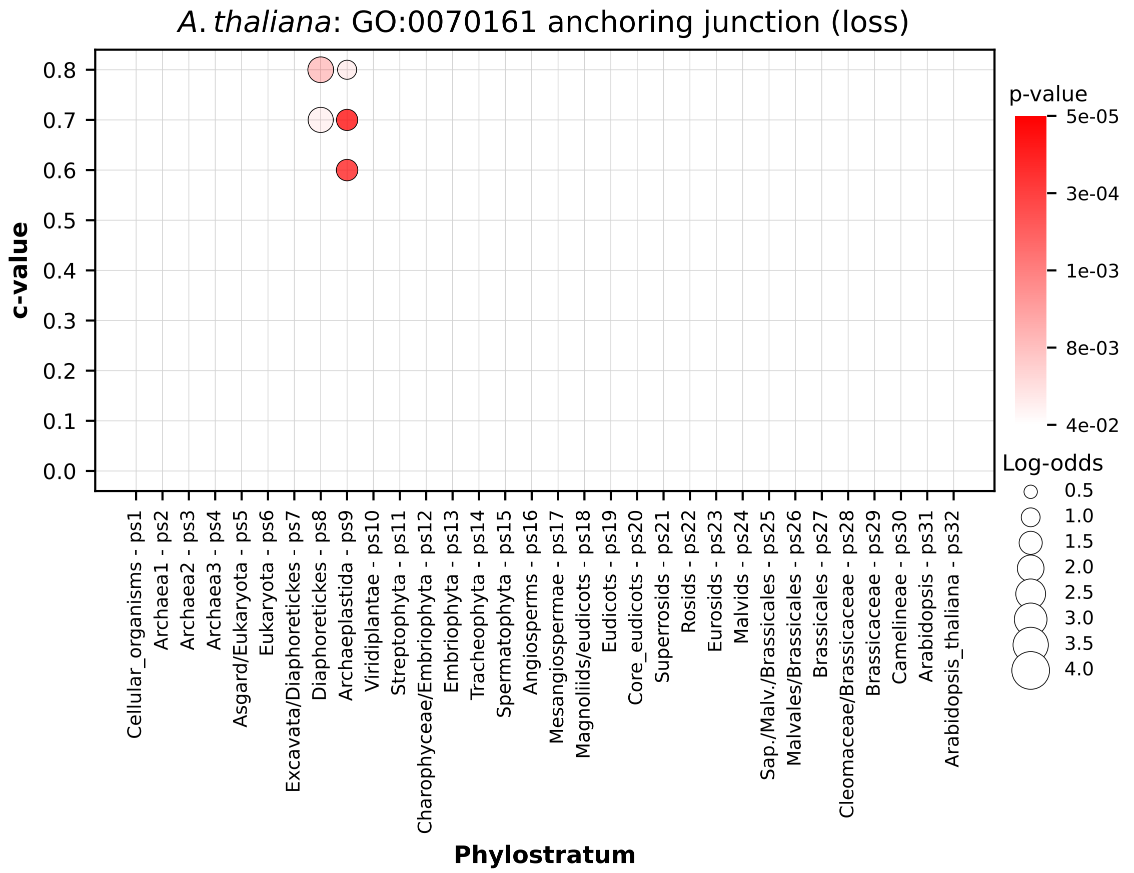
A. thaliana: GO:0070161 anchoring junction (loss)
c-value
0.8
0.7
0.6
0.5
0.4
0.3
0.2
0.1
0.0
Phylostratum
Cellular_organisms - ps1
Archaea1 - ps2
Archaea2 - ps3
Archaea3 - ps4
Asgard/Eukaryota - ps5
Eukaryota - ps6
Excavata/Diaphoretickes - ps7
Diaphoretickes - ps8
Archaeplastida - ps9
Viridiplantae - ps10
Streptophyta - ps11
Charophyceae/Embriophyta - ps12
Embriophyta - ps13
Tracheophyta - ps14
Spermatophyta - ps15
Angiosperms - ps16
Mesangiospermae - ps17
Magnoliids/eudicots - ps18
Eudicots - ps19
Core_eudicots - ps20
Superrosids - ps21
Rosids - ps22
Eurosids - ps23
Malvids - ps24
Sap./Malv./Brassicales - ps25
Malvales/Brassicales - ps26
Brassicales - ps27
Cleomaceae/Brassicaceae - ps28
Brassicaceae - ps29
Camelineae - ps30
Arabidopsis - ps31
Arabidopsis_thaliana - ps32
p-value
5e-05
3e-04
1e-03
8e-03
4e-02
Log-odds
0.5
1.0
1.5
2.0
2.5
3.0
3.5
4.0

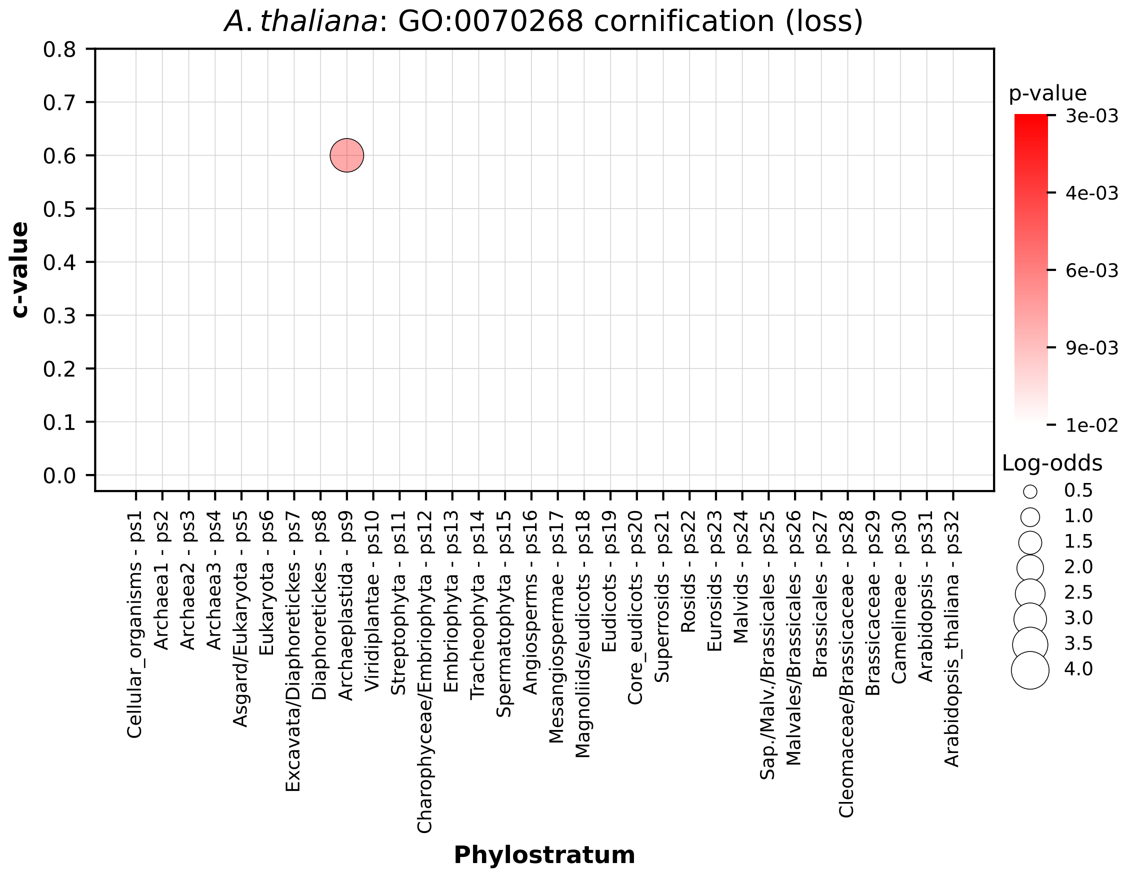

A. thaliana: GO:0070268 cornification (loss)
c-value
0.8
0.7
0.6
0.5
0.4
0.3
0.2
0.1
0.0
p-value
3e-03
4e-03
6e-03
9e-03
1e-02
Log-odds
0.5
1.0
1.5
2.0
2.5
3.0
3.5
4.0
Cellular_organisms - ps1
Archaea1 - ps2
Archaea2 - ps3
Archaea3 - ps4
Asgard/Eukaryota - ps5
Eukaryota - ps6
Excavata/Diaphoretickes - ps7
Diaphoretickes - ps8
Archaeplastida - ps9
Viridiplantae - ps10
Streptophyta - ps11
Charophyceae/Embriophyta - ps12
Embriophyta - ps13
Tracheophyta - ps14
Spermatophyta - ps15
Angiosperms - ps16
Mesangiospermae - ps17
Magnoliids/eudicots - ps18
Eudicots - ps19
Core_eudicots - ps20
Superrosids - ps21
Rosids - ps22
Eurosids - ps23
Malvids - ps24
Sap./Malv./Brassicales - ps25
Malvales/Brassicales - ps26
Brassicales - ps27
Cleomaceae/Brassicaceae - ps28
Brassicaceae - ps29
Camelineae - ps30
Arabidopsis - ps31
Arabidopsis_thaliana - ps32
Phylostratum

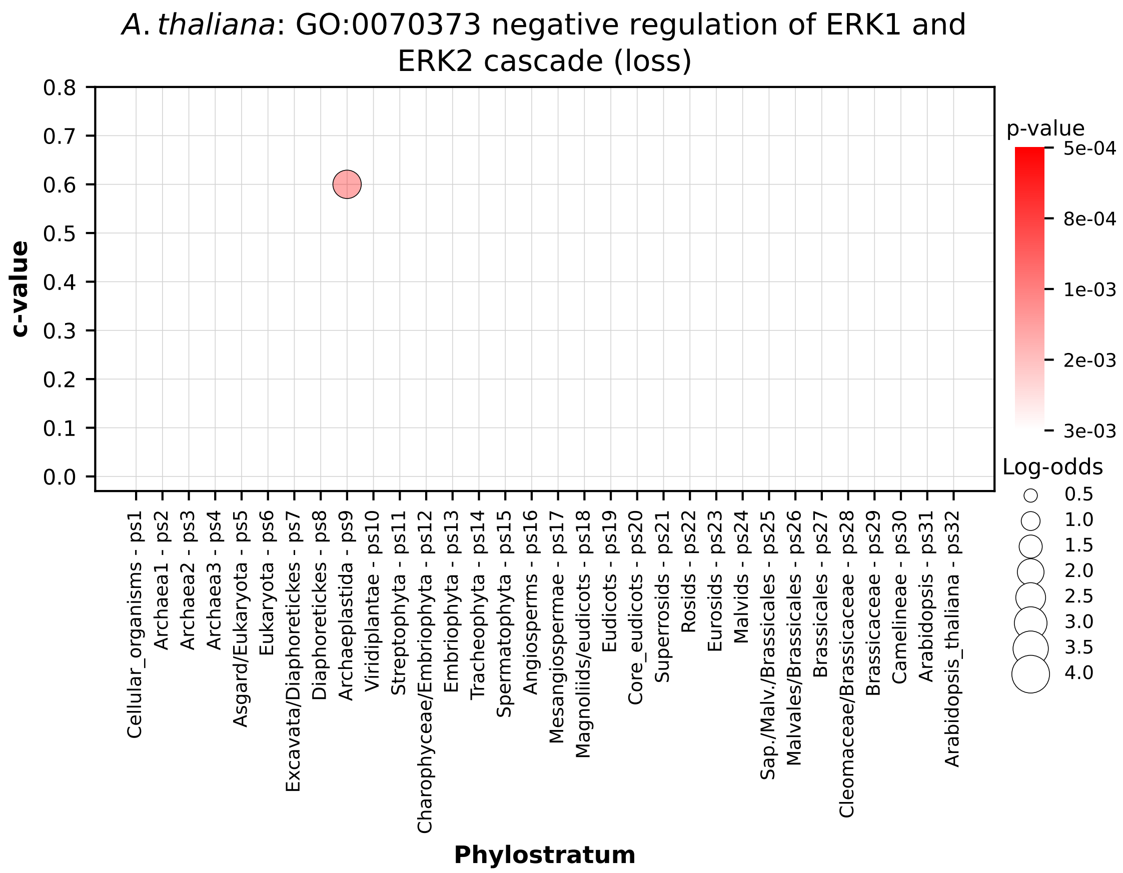

A. thaliana: GO:0070373 negative regulation of ERK1 and ERK2 cascade (loss)
c-value
0.8
0.7
0.6
0.5
0.4
0.3
0.2
0.1
0.0
p-value
5e-04
8e-04
1e-03
2e-03
3e-03
Log-odds
0.5
1.0
1.5
2.0
2.5
3.0
3.5
4.0
Cellular_organisms - ps1
Archaea1 - ps2
Archaea2 - ps3
Archaea3 - ps4
Asgard/Eukaryota - ps5
Eukaryota - ps6
Excavata/Diaphoretickes - ps7
Diaphoretickes - ps8
Archaeplastida - ps9
Viridiplantae - ps10
Streptophyta - ps11
Charophyceae/Embriophyta - ps12
Embriophyta - ps13
Tracheophyta - ps14
Spermatophyta - ps15
Angiosperms - ps16
Mesangiospermae - ps17
Magnoliids/eudicots - ps18
Eudicots - ps19
Core_eudicots - ps20
Superrosids - ps21
Rosids - ps22
Eurosids - ps23
Malvids - ps24
Sap./Malv./Brassicales - ps25
Malvales/Brassicales - ps26
Brassicales - ps27
Cleomaceae/Brassicaceae - ps28
Brassicaceae - ps29
Camelineae - ps30
Arabidopsis - ps31
Arabidopsis_thaliana - ps32
Phylostratum

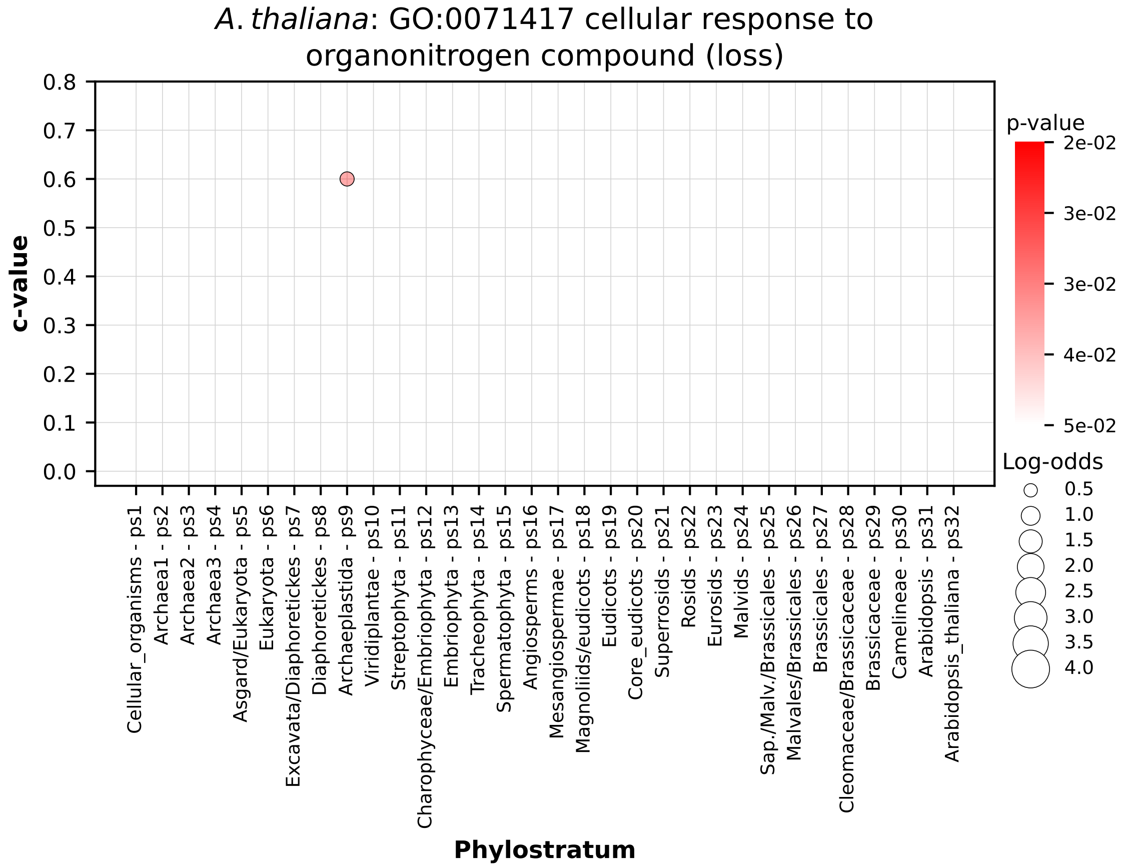

A. thaliana: GO:0071417 cellular response to organonitrogen compound (loss)
c-value
0.8
0.7
0.6
0.5
0.4
0.3
0.2
0.1
0.0
p-value
2e-02
3e-02
3e-02
4e-02
5e-02
Log-odds
0.5
1.0
1.5
2.0
2.5
3.0
3.5
4.0
Cellular_organisms - ps1
Archaea1 - ps2
Archaea2 - ps3
Archaea3 - ps4
Asgard/Eukaryota - ps5
Eukaryota - ps6
Excavata/Diaphoretickes - ps7
Diaphoretickes - ps8
Archaeplastida - ps9
Viridiplantae - ps10
Streptophyta - ps11
Charophyceae/Embriophyta - ps12
Embriophyta - ps13
Tracheophyta - ps14
Spermatophyta - ps15
Angiosperms - ps16
Mesangiospermae - ps17
Magnoliids/eudicots - ps18
Eudicots - ps19
Core_eudicots - ps20
Superrosids - ps21
Rosids - ps22
Eurosids - ps23
Malvids - ps24
Sap./Malv./Brassicales - ps25
Malvales/Brassicales - ps26
Brassicales - ps27
Cleomaceae/Brassicaceae - ps28
Brassicaceae - ps29
Camelineae - ps30
Arabidopsis - ps31
Arabidopsis_thaliana - ps32
Phylostratum

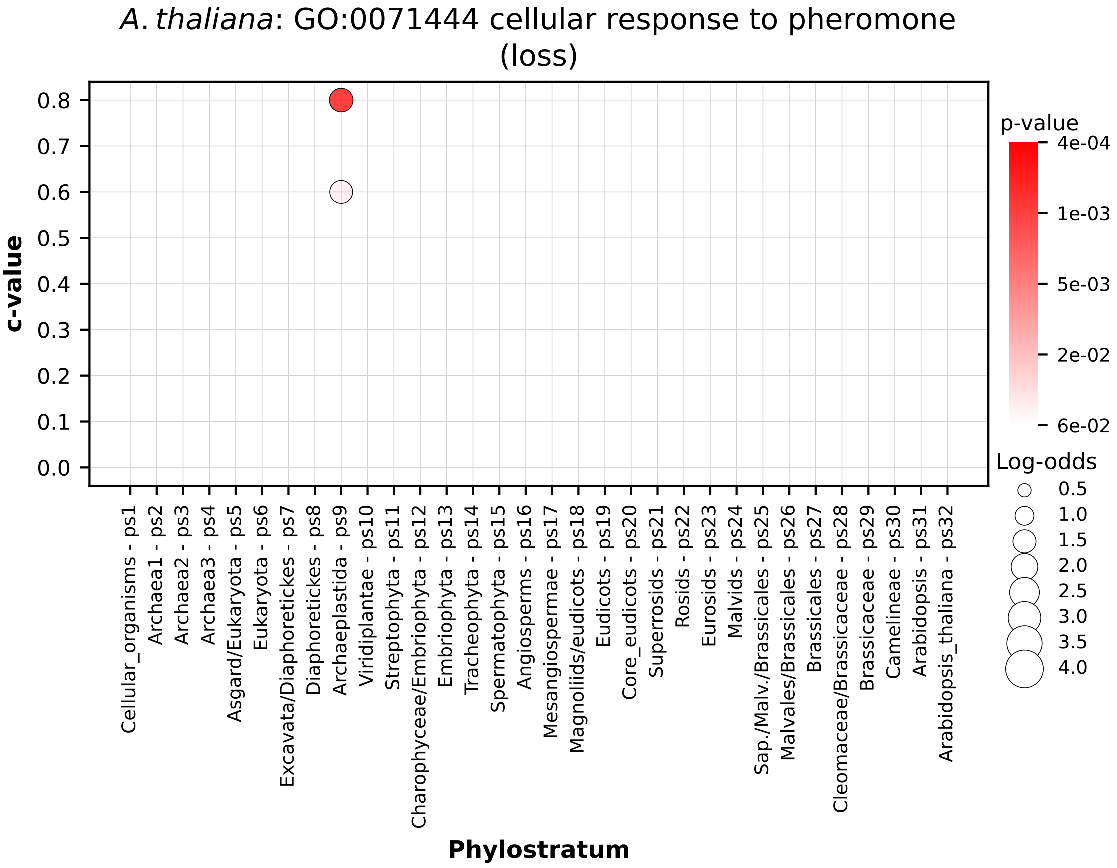
A. thaliana: GO:0071444 cellular response to pheromone (loss)
c-value
0.8
0.7
0.6
0.5
0.4
0.3
0.2
0.1
0.0
p-value
4e-04
1e-03
5e-03
2e-02
6e-02
Log-odds
0.5
1.0
1.5
2.0
2.5
3.0
3.5
4.0
Cellular_organisms - ps1
Archaea1 - ps2
Archaea2 - ps3
Archaea3 - ps4
Asgard/Eukaryota - ps5
Eukaryota - ps6
Excavata/Diaphoretickes - ps7
Diaphoretickes - ps8
Archaeplastida - ps9
Viridiplantae - ps10
Streptophyta - ps11
Charophyceae/Embriophyta - ps12
Embriophyta - ps13
Tracheophyta - ps14
Spermatophyta - ps15
Angiosperms - ps16
Mesangiospermae - ps17
Magnoliids/eudicots - ps18
Eudicots - ps19
Core_eudicots - ps20
Superrosids - ps21
Rosids - ps22
Eurosids - ps23
Malvids - ps24
Sap./Malv./Brassicales - ps25
Malvales/Brassicales - ps26
Brassicales - ps27
Cleomaceae/Brassicaceae - ps28
Brassicaceae - ps29
Camelineae - ps30
Arabidopsis - ps31
Arabidopsis_thaliana - ps32
Phylostratum

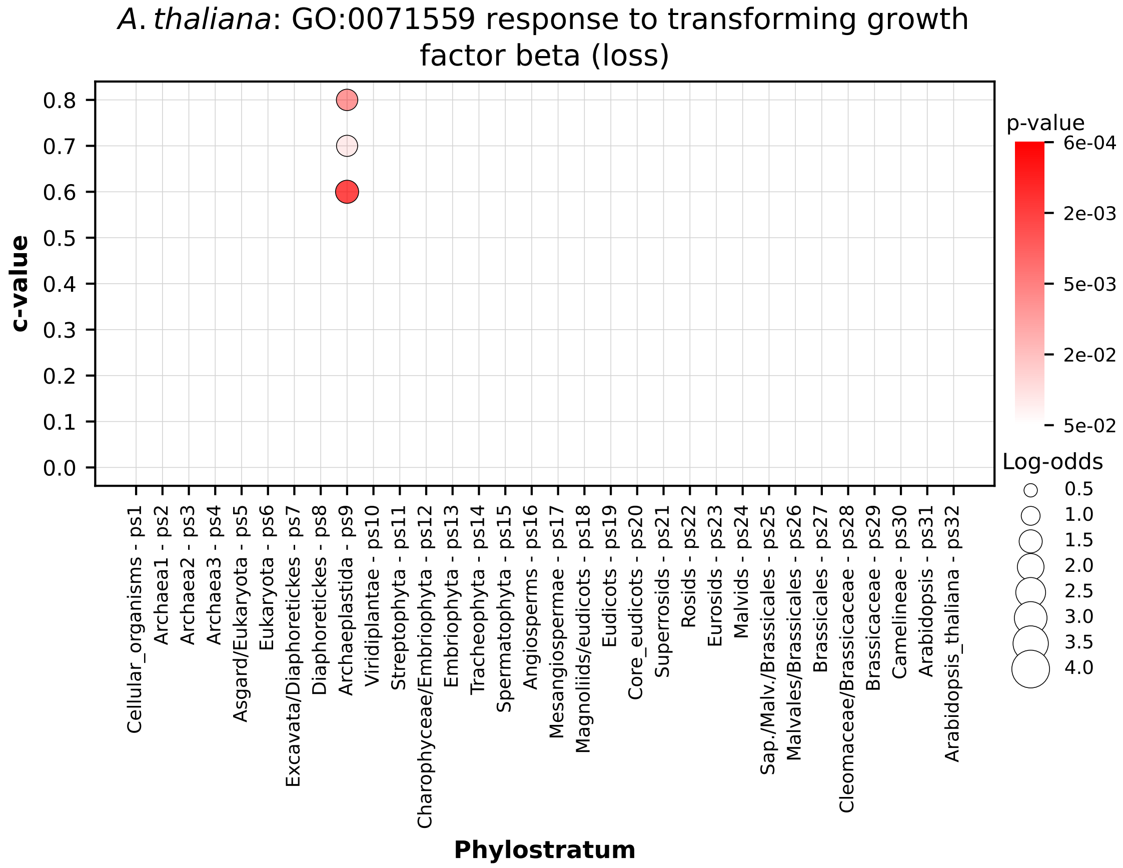

A. thaliana: GO:0071559 response to transforming growth factor beta (loss)
c-value
0.8
0.7
0.6
0.5
0.4
0.3
0.2
0.1
0.0
p-value
6e-04
2e-03
5e-03
2e-02
5e-02
Log-odds
0.5
1.0
1.5
2.0
2.5
3.0
3.5
4.0
Cellular_organisms - ps1
Archaea1 - ps2
Archaea2 - ps3
Archaea3 - ps4
Asgard/Eukaryota - ps5
Eukaryota - ps6
Excavata/Diaphoretickes - ps7
Diaphoretickes - ps8
Archaeplastida - ps9
Viridiplantae - ps10
Streptophyta - ps11
Charophyceae/Embriophyta - ps12
Embriophyta - ps13
Tracheophyta - ps14
Spermatophyta - ps15
Angiosperms - ps16
Mesangiospermae - ps17
Magnoliids/eudicots - ps18
Eudicots - ps19
Core_eudicots - ps20
Superrosids - ps21
Rosids - ps22
Eurosids - ps23
Malvids - ps24
Sap./Malv./Brassicales - ps25
Malvales/Brassicales - ps26
Brassicales - ps27
Cleomaceae/Brassicaceae - ps28
Brassicaceae - ps29
Camelineae - ps30
Arabidopsis - ps31
Arabidopsis_thaliana - ps32
Phylostratum

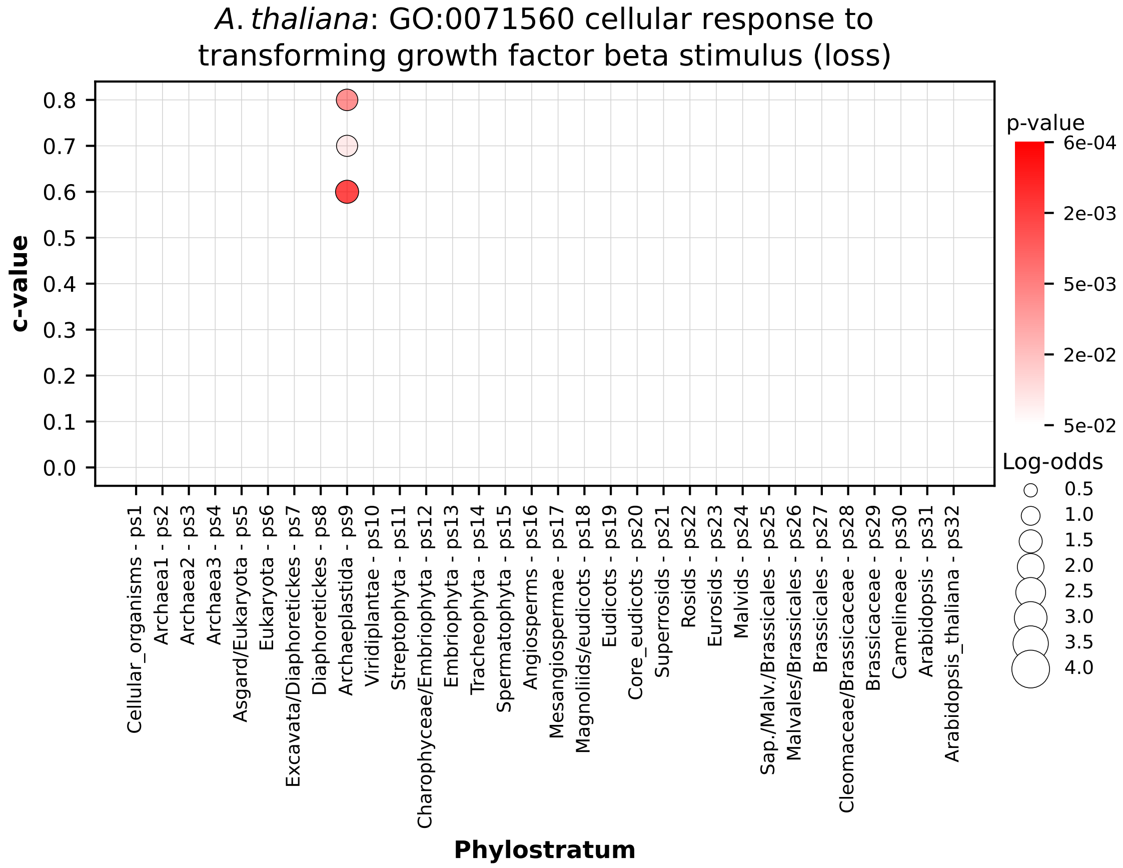

A. thaliana: GO:0071560 cellular response to transforming growth factor beta stimulus (loss)
c-value
0.8
0.7
0.6
0.5
0.4
0.3
0.2
0.1
0.0
p-value
6e-04
2e-03
5e-03
2e-02
5e-02
Log-odds
0.5
1.0
1.5
2.0
2.5
3.0
3.5
4.0
Cellular_organisms - ps1
Archaea1 - ps2
Archaea2 - ps3
Archaea3 - ps4
Asgard/Eukaryota - ps5
Eukaryota - ps6
Excavata/Diaphoretickes - ps7
Diaphoretickes - ps8
Archaeplastida - ps9
Viridiplantae - ps10
Streptophyta - ps11
Charophyceae/Embriophyta - ps12
Embriophyta - ps13
Tracheophyta - ps14
Spermatophyta - ps15
Angiosperms - ps16
Mesangiospermae - ps17
Magnoliids/eudicots - ps18
Eudicots - ps19
Core_eudicots - ps20
Superrosids - ps21
Rosids - ps22
Eurosids - ps23
Malvids - ps24
Sap./Malv./Brassicales - ps25
Malvales/Brassicales - ps26
Brassicales - ps27
Cleomaceae/Brassicaceae - ps28
Brassicaceae - ps29
Camelineae - ps30
Arabidopsis - ps31
Arabidopsis_thaliana - ps32
Phylostratum

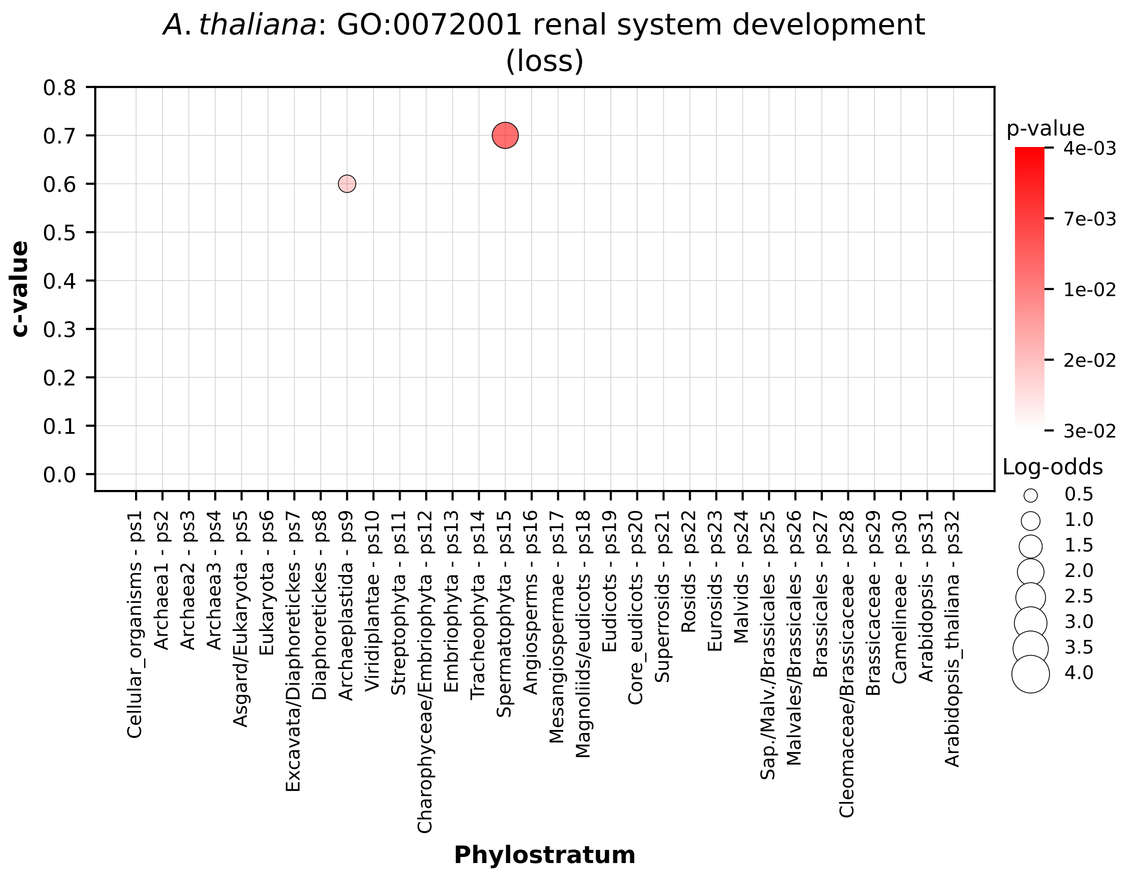

A. thaliana: GO:0072001 renal system development
(loss)
c-value
0.8
0.7
0.6
0.5
0.4
0.3
0.2
0.1
0.0
p-value
4e-03
7e-03
1e-02
2e-02
3e-02
Log-odds
0.5
1.0
1.5
2.0
2.5
3.0
3.5
4.0
Cellular_organisms - ps1
Archaea1 - ps2
Archaea2 - ps3
Archaea3 - ps4
Asgard/Eukaryota - ps5
Eukaryota - ps6
Excavata/Diaphoretickes - ps7
Diaphoretickes - ps8
Archaeplastida - ps9
Viridiplantae - ps10
Streptophyta - ps11
Charophyceae/Embriophyta - ps12
Embriophyta - ps13
Tracheophyta - ps14
Spermatophyta - ps15
Angiosperms - ps16
Mesangiospermae - ps17
Magnoliids/eudicots - ps18
Eudicots - ps19
Core_eudicots - ps20
Superrosids - ps21
Rosids - ps22
Eurosids - ps23
Malvids - ps24
Sap./Malv./Brassicales - ps25
Malvales/Brassicales - ps26
Brassicales - ps27
Cleomaceae/Brassicaceae - ps28
Brassicaceae - ps29
Camelineae - ps30
Arabidopsis - ps31
Arabidopsis_thaliana - ps32
Phylostratum

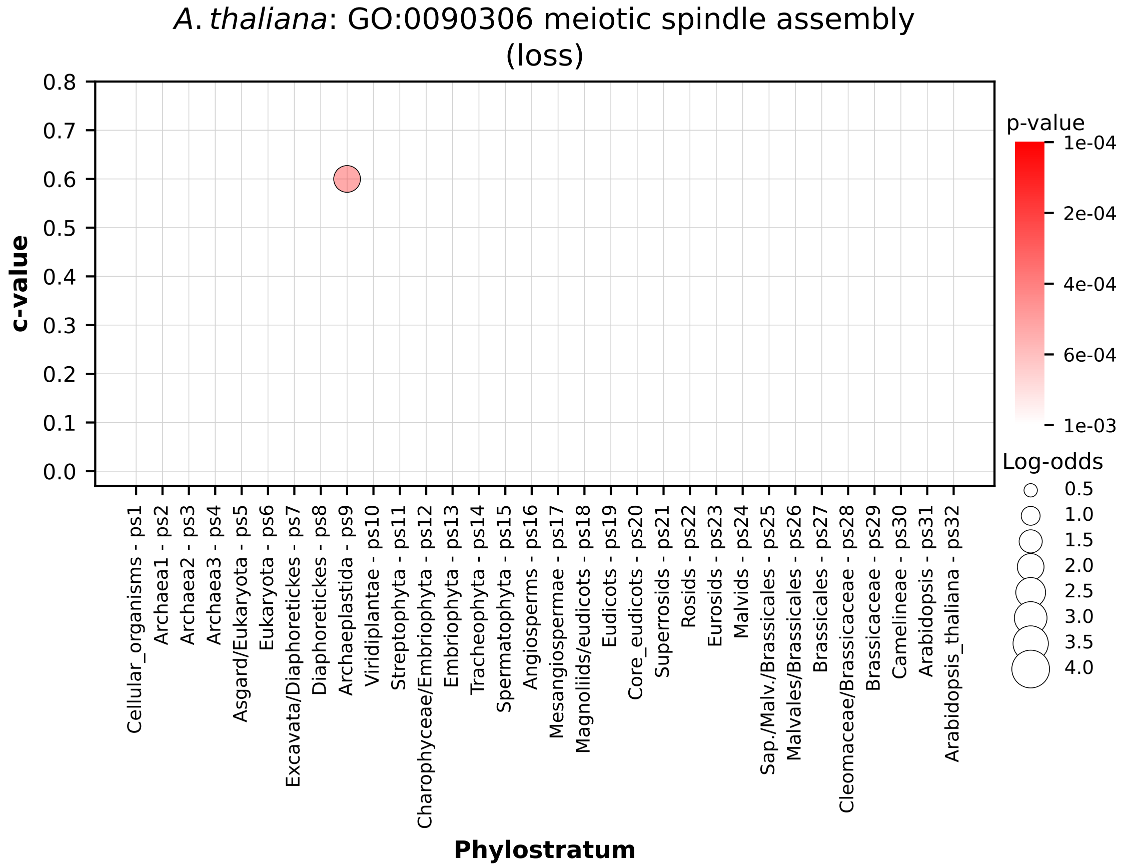
A. thaliana: GO:0090306 meiotic spindle assembly (loss)
c-value
0.8
0.7
0.6
0.5
0.4
0.3
0.2
0.1
0.0
Phylostratum
Cellular_organisms - ps1
Archaea1 - ps2
Archaea2 - ps3
Archaea3 - ps4
Asgard/Eukaryota - ps5
Eukaryota - ps6
Excavata/Diaphoretickes - ps7
Diaphoretickes - ps8
Archaeplastida - ps9
Viridiplantae - ps10
Streptophyta - ps11
Charophyceae/Embriophyta - ps12
Embriophyta - ps13
Tracheophyta - ps14
Spermatophyta - ps15
Angiosperms - ps16
Mesangiospermae - ps17
Magnoliids/eudicots - ps18
Eudicots - ps19
Core_eudicots - ps20
Superrosids - ps21
Rosids - ps22
Eurosids - ps23
Malvids - ps24
Sap./Malv./Brassicales - ps25
Malvales/Brassicales - ps26
Brassicales - ps27
Cleomaceae/Brassicaceae - ps28
Brassicaceae - ps29
Camelineae - ps30
Arabidopsis - ps31
Arabidopsis_thaliana - ps32
p-value
1e-04
2e-04
4e-04
6e-04
1e-03
Log-odds
0.5
1.0
1.5
2.0
2.5
3.0
3.5
4.0

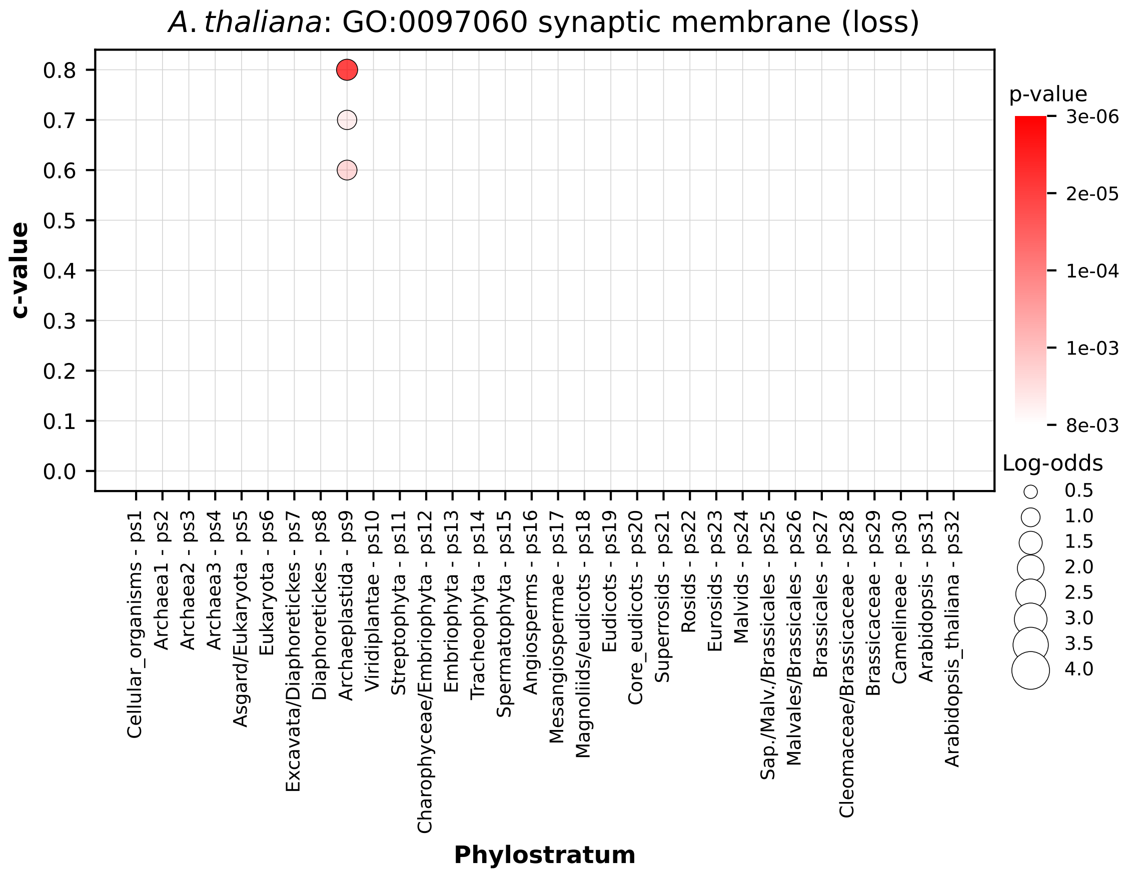

A. thaliana: GO:0097060 synaptic membrane (loss)
c-value
0.8
0.7
0.6
0.5
0.4
0.3
0.2
0.1
0.0
p-value
3e-06
2e-05
1e-04
1e-03
8e-03
Log-odds
0.5
1.0
1.5
2.0
2.5
3.0
3.5
4.0
Cellular_organisms - ps1
Archaea1 - ps2
Archaea2 - ps3
Archaea3 - ps4
Asgard/Eukaryota - ps5
Eukaryota - ps6
Excavata/Diaphoretickes - ps7
Diaphoretickes - ps8
Archaeplastida - ps9
Viridiplantae - ps10
Streptophyta - ps11
Charophyceae/Embriophyta - ps12
Embriophyta - ps13
Tracheophyta - ps14
Spermatophyta - ps15
Angiosperms - ps16
Mesangiospermae - ps17
Magnoliids/eudicots - ps18
Eudicots - ps19
Core_eudicots - ps20
Superrosids - ps21
Rosids - ps22
Eurosids - ps23
Malvids - ps24
Sap./Malv./Brassicales - ps25
Malvales/Brassicales - ps26
Brassicales - ps27
Cleomaceae/Brassicaceae - ps28
Brassicaceae - ps29
Camelineae - ps30
Arabidopsis - ps31
Arabidopsis_thaliana - ps32
Phylostratum

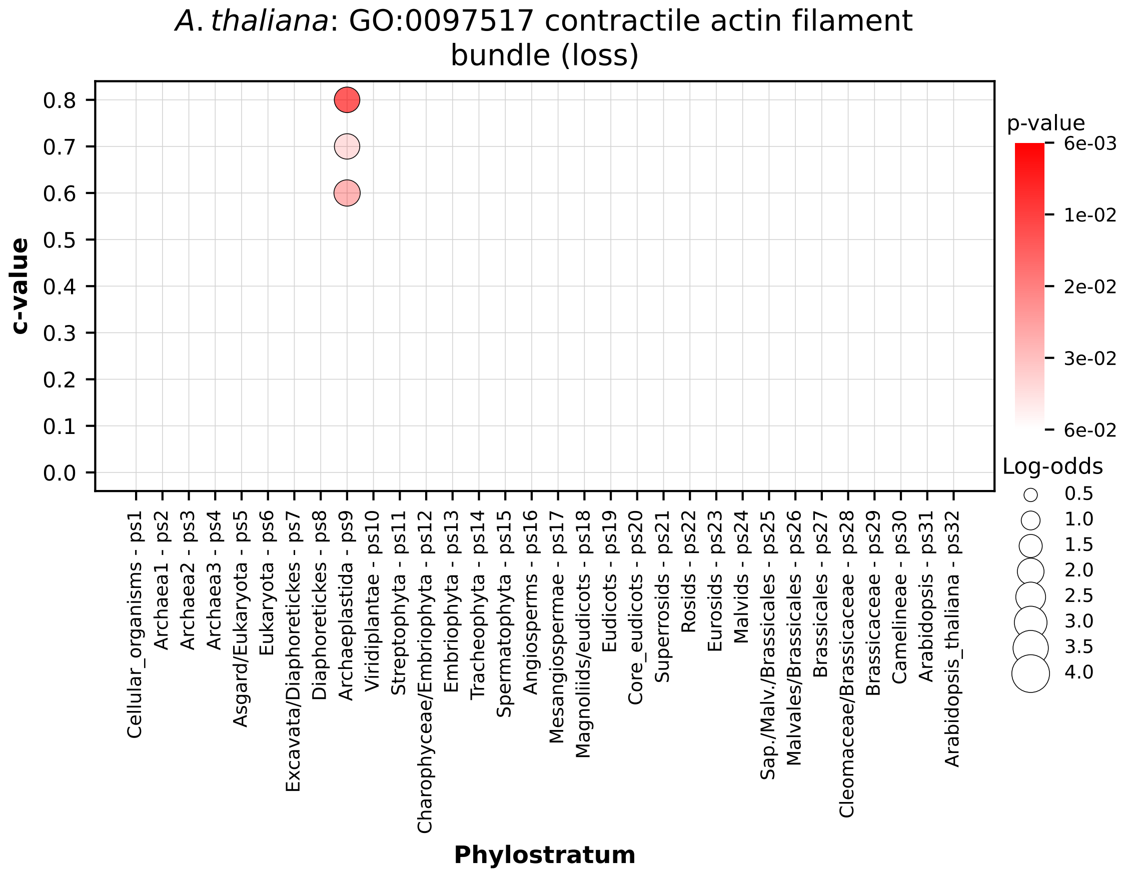
A. thaliana: GO:0097517 contractile actin filament bundle (loss)
c-value
0.8
0.7
0.6
0.5
0.4
0.3
0.2
0.1
0.0
Phylostratum
Cellular_organisms - ps1
Archaea1 - ps2
Archaea2 - ps3
Archaea3 - ps4
Asgard/Eukaryota - ps5
Eukaryota - ps6
Excavata/Diaphoretickes - ps7
Diaphoretickes - ps8
Archaeplastida - ps9
Viridiplantae - ps10
Streptophyta - ps11
Charophyceae/Embriophyta - ps12
Embriophyta - ps13
Tracheophyta - ps14
Spermatophyta - ps15
Angiosperms - ps16
Mesangiospermae - ps17
Magnoliids/eudicots - ps18
Eudicots - ps19
Core_eudicots - ps20
Superrosids - ps21
Rosids - ps22
Eurosids - ps23
Malvids - ps24
Sap./Malv./Brassicales - ps25
Malvales/Brassicales - ps26
Brassicales - ps27
Cleomaceae/Brassicaceae - ps28
Brassicaceae - ps29
Camelineae - ps30
Arabidopsis - ps31
Arabidopsis_thaliana - ps32
p-value
6e-03
1e-02
2e-02
3e-02
6e-02
Log-odds
0.5
1.0
1.5
2.0
2.5
3.0
3.5
4.0

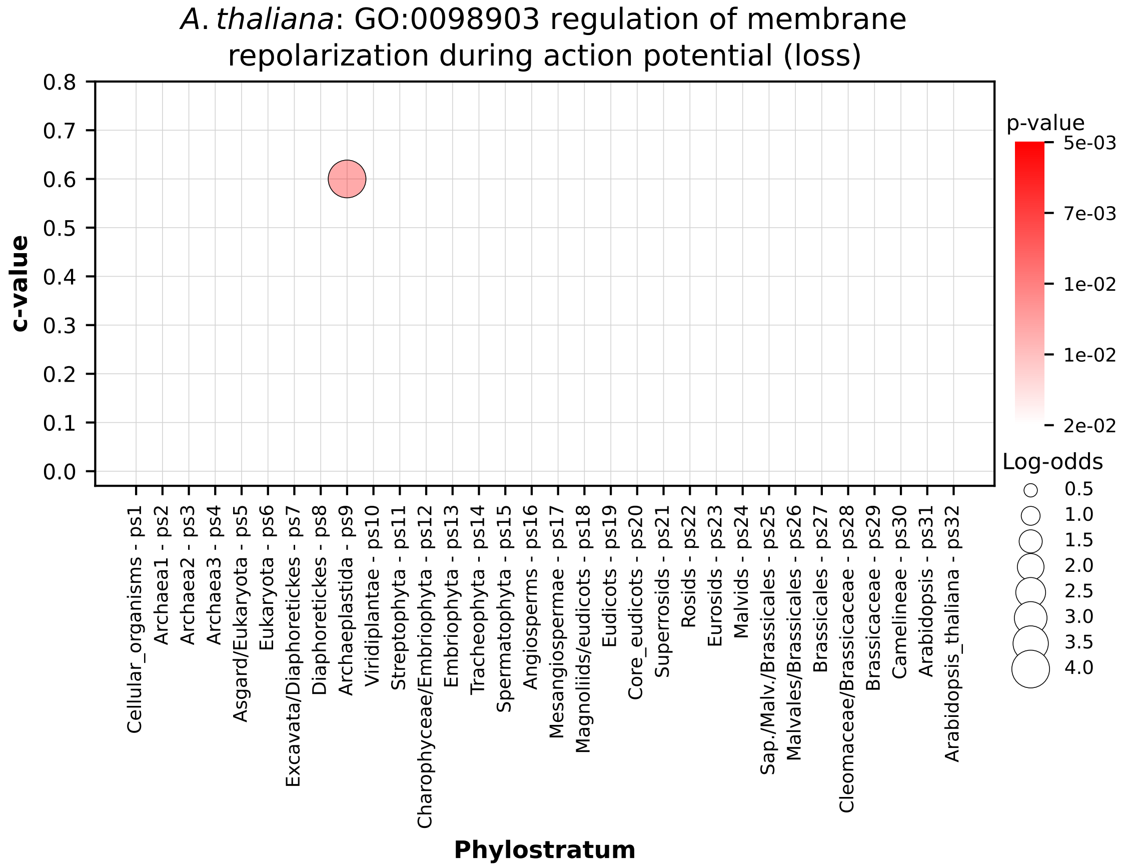

A. thaliana: GO:0098903 regulation of membrane repolarization during action potential (loss)
c-value
0.8
0.7
0.6
0.5
0.4
0.3
0.2
0.1
0.0
p-value
5e-03
7e-03
1e-02
1e-02
2e-02
Log-odds
0.5
1.0
1.5
2.0
2.5
3.0
3.5
4.0
Cellular_organisms - ps1
Archaea1 - ps2
Archaea2 - ps3
Archaea3 - ps4
Asgard/Eukaryota - ps5
Eukaryota - ps6
Excavata/Diaphoretickes - ps7
Diaphoretickes - ps8
Archaeplastida - ps9
Viridiplantae - ps10
Streptophyta - ps11
Charophyceae/Embriophyta - ps12
Embriophyta - ps13
Tracheophyta - ps14
Spermatophyta - ps15
Angiosperms - ps16
Mesangiospermae - ps17
Magnoliids/eudicots - ps18
Eudicots - ps19
Core_eudicots - ps20
Superrosids - ps21
Rosids - ps22
Eurosids - ps23
Malvids - ps24
Sap./Malv./Brassicales - ps25
Malvales/Brassicales - ps26
Brassicales - ps27
Cleomaceae/Brassicaceae - ps28
Brassicaceae - ps29
Camelineae - ps30
Arabidopsis - ps31
Arabidopsis_thaliana - ps32
Phylostratum

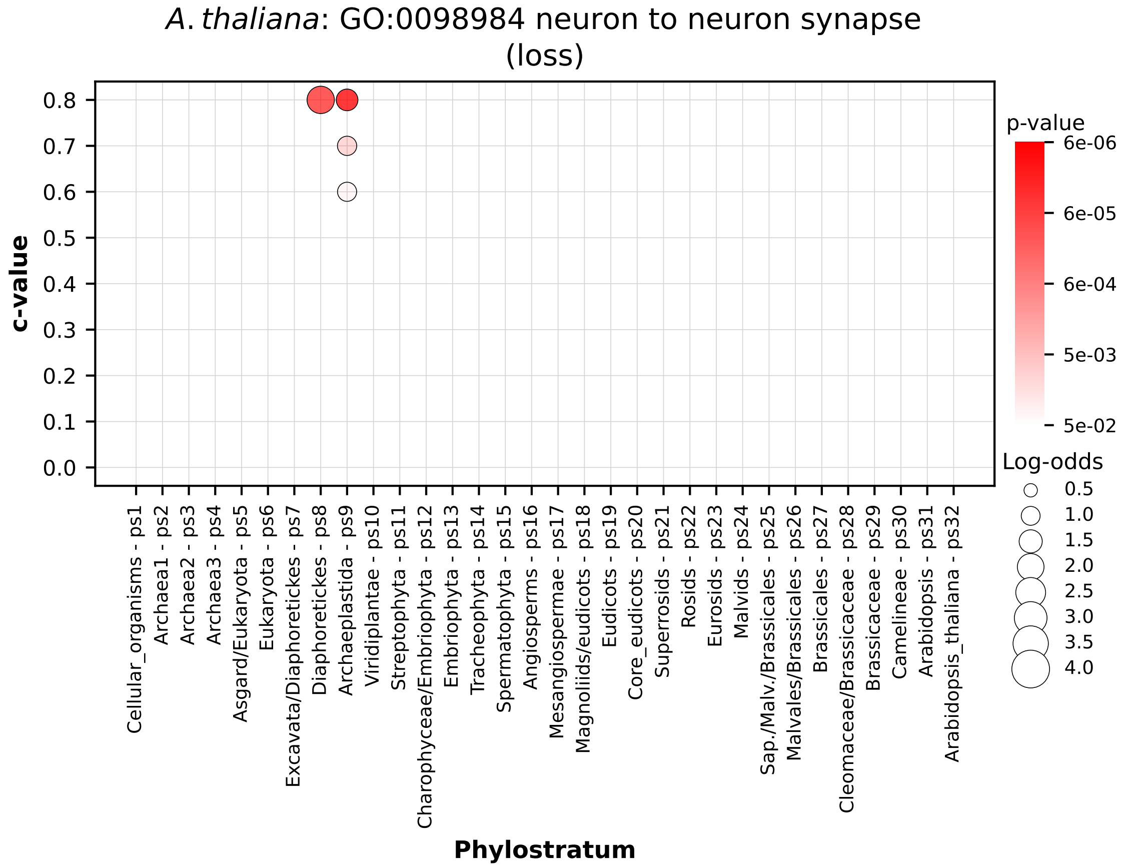

A. thaliana: GO:0098984 neuron to neuron synapse
(loss)
c-value
0.8
0.7
0.6
0.5
0.4
0.3
0.2
0.1
0.0
p-value
6e-06
6e-05
6e-04
5e-03
5e-02
Log-odds
0.5
1.0
1.5
2.0
2.5
3.0
3.5
4.0
Cellular_organisms - ps1
Archaea1 - ps2
Archaea2 - ps3
Archaea3 - ps4
Asgard/Eukaryota - ps5
Eukaryota - ps6
Excavata/Diaphoretickes - ps7
Diaphoretickes - ps8
Archaeplastida - ps9
Viridiplantae - ps10
Streptophyta - ps11
Charophyceae/Embriophyta - ps12
Embriophyta - ps13
Tracheophyta - ps14
Spermatophyta - ps15
Angiosperms - ps16
Mesangiospermae - ps17
Magnoliids/eudicots - ps18
Eudicots - ps19
Core_eudicots - ps20
Superrosids - ps21
Rosids - ps22
Eurosids - ps23
Malvids - ps24
Sap./Malv./Brassicales - ps25
Malvales/Brassicales - ps26
Brassicales - ps27
Cleomaceae/Brassicaceae - ps28
Brassicaceae - ps29
Camelineae - ps30
Arabidopsis - ps31
Arabidopsis_thaliana - ps32
Phylostratum

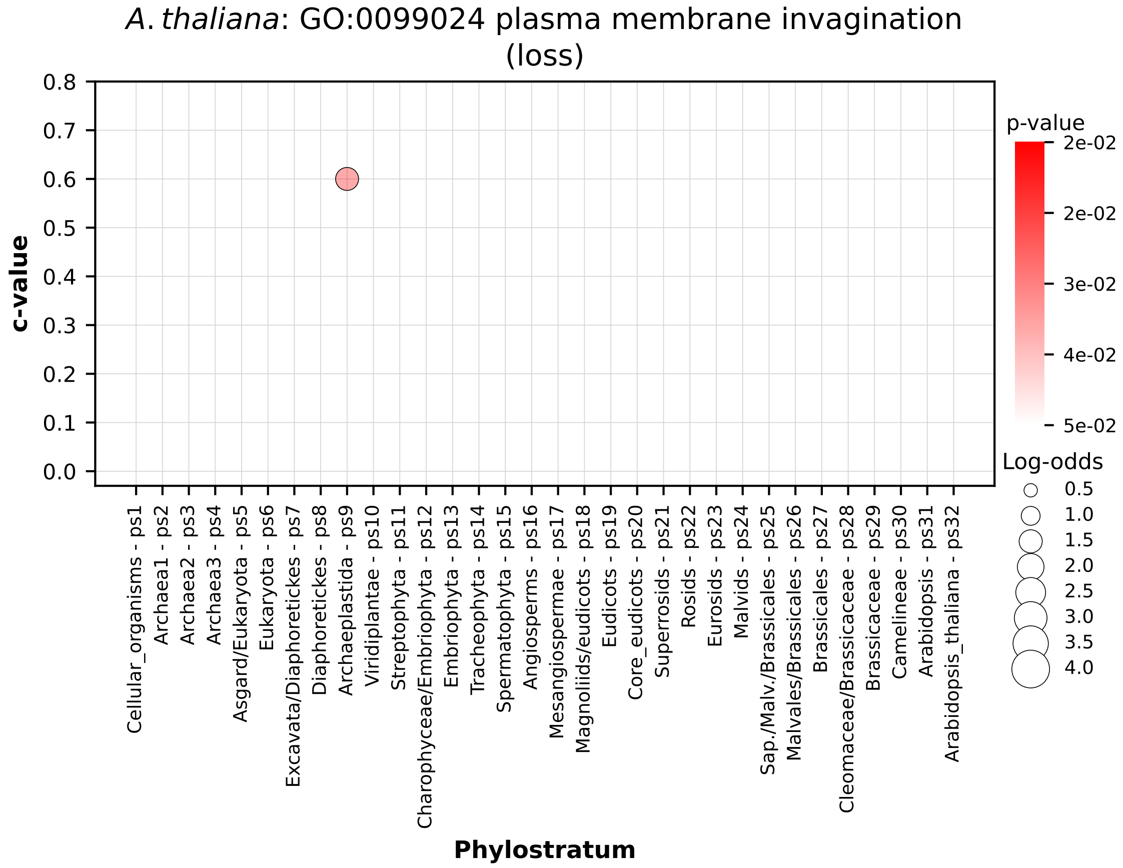

A. thaliana: GO:0099024 plasma membrane invagination
(loss)
c-value
0.8
0.7
0.6
0.5
0.4
0.3
0.2
0.1
0.0
p-value
2e-02
2e-02
3e-02
4e-02
5e-02
Log-odds
0.5
1.0
1.5
2.0
2.5
3.0
3.5
4.0
Cellular_organisms - ps1
Archaea1 - ps2
Archaea2 - ps3
Archaea3 - ps4
Asgard/Eukaryota - ps5
Eukaryota - ps6
Excavata/Diaphoretickes - ps7
Diaphoretickes - ps8
Archaeplastida - ps9
Viridiplantae - ps10
Streptophyta - ps11
Charophyceae/Embriophyta - ps12
Embriophyta - ps13
Tracheophyta - ps14
Spermatophyta - ps15
Angiosperms - ps16
Mesangiospermae - ps17
Magnoliids/eudicots - ps18
Eudicots - ps19
Core_eudicots - ps20
Superrosids - ps21
Rosids - ps22
Eurosids - ps23
Malvids - ps24
Sap./Malv./Brassicales - ps25
Malvales/Brassicales - ps26
Brassicales - ps27
Cleomaceae/Brassicaceae - ps28
Brassicaceae - ps29
Camelineae - ps30
Arabidopsis - ps31
Arabidopsis_thaliana - ps32
Phylostratum

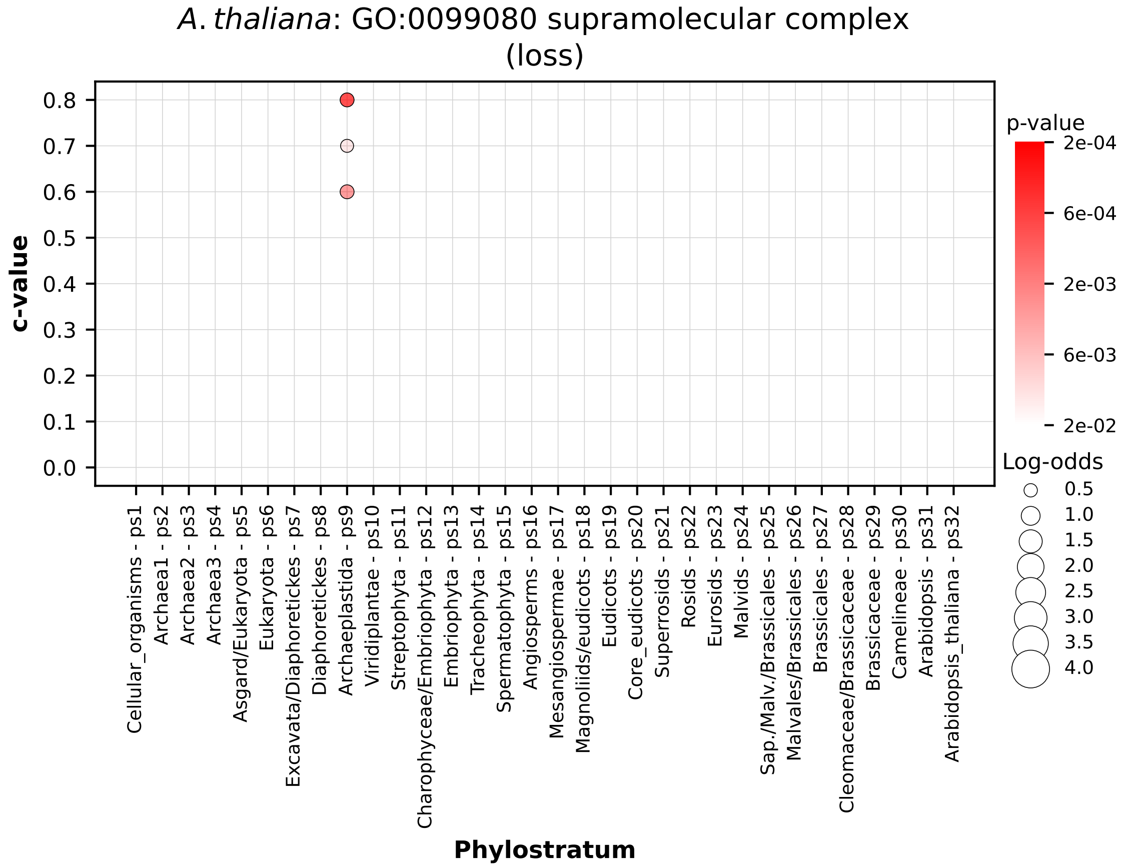

A. thaliana: GO:0099080 supramolecular complex
(loss)
c-value
0.8
0.7
0.6
0.5
0.4
0.3
0.2
0.1
0.0
p-value
2e-04
6e-04
2e-03
6e-03
2e-02
Log-odds
0.5
1.0
1.5
2.0
2.5
3.0
3.5
4.0
Cellular_organisms - ps1
Archaea1 - ps2
Archaea2 - ps3
Archaea3 - ps4
Asgard/Eukaryota - ps5
Eukaryota - ps6
Excavata/Diaphoretickes - ps7
Diaphoretickes - ps8
Archaeplastida - ps9
Viridiplantae - ps10
Streptophyta - ps11
Charophyceae/Embriophyta - ps12
Embriophyta - ps13
Tracheophyta - ps14
Spermatophyta - ps15
Angiosperms - ps16
Mesangiospermae - ps17
Magnoliids/eudicots - ps18
Eudicots - ps19
Core_eudicots - ps20
Superrosids - ps21
Rosids - ps22
Eurosids - ps23
Malvids - ps24
Sap./Malv./Brassicales - ps25
Malvales/Brassicales - ps26
Brassicales - ps27
Cleomaceae/Brassicaceae - ps28
Brassicaceae - ps29
Camelineae - ps30
Arabidopsis - ps31
Arabidopsis_thaliana - ps32
Phylostratum

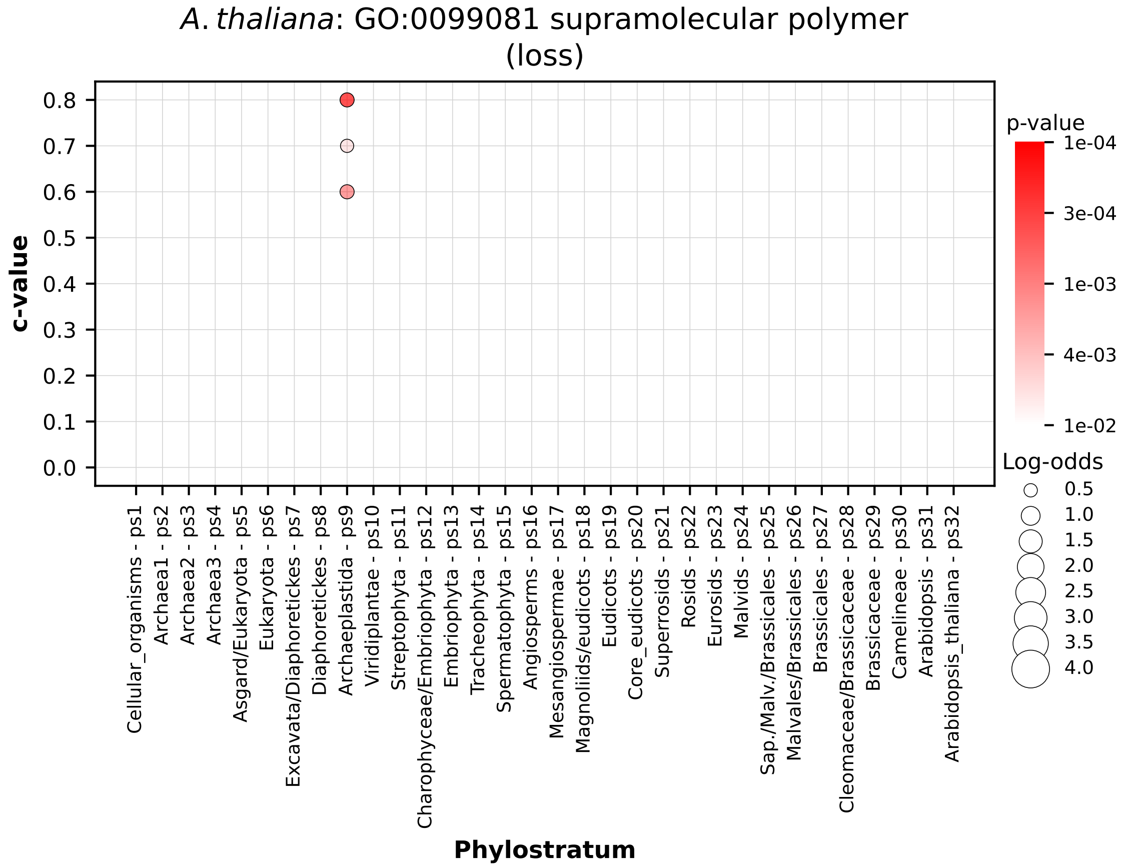

A. thaliana: GO:0099081 supramolecular polymer
(loss)
c-value
0.8
0.7
0.6
0.5
0.4
0.3
0.2
0.1
0.0
p-value
1e-04
3e-04
1e-03
4e-03
1e-02
Log-odds
0.5
1.0
1.5
2.0
2.5
3.0
3.5
4.0
Cellular_organisms - ps1
Archaea1 - ps2
Archaea2 - ps3
Archaea3 - ps4
Asgard/Eukaryota - ps5
Eukaryota - ps6
Excavata/Diaphoretickes - ps7
Diaphoretickes - ps8
Archaeplastida - ps9
Viridiplantae - ps10
Streptophyta - ps11
Charophyceae/Embriophyta - ps12
Embriophyta - ps13
Tracheophyta - ps14
Spermatophyta - ps15
Angiosperms - ps16
Mesangiospermae - ps17
Magnoliids/eudicots - ps18
Eudicots - ps19
Core_eudicots - ps20
Superrosids - ps21
Rosids - ps22
Eurosids - ps23
Malvids - ps24
Sap./Malv./Brassicales - ps25
Malvales/Brassicales - ps26
Brassicales - ps27
Cleomaceae/Brassicaceae - ps28
Brassicaceae - ps29
Camelineae - ps30
Arabidopsis - ps31
Arabidopsis_thaliana - ps32
Phylostratum

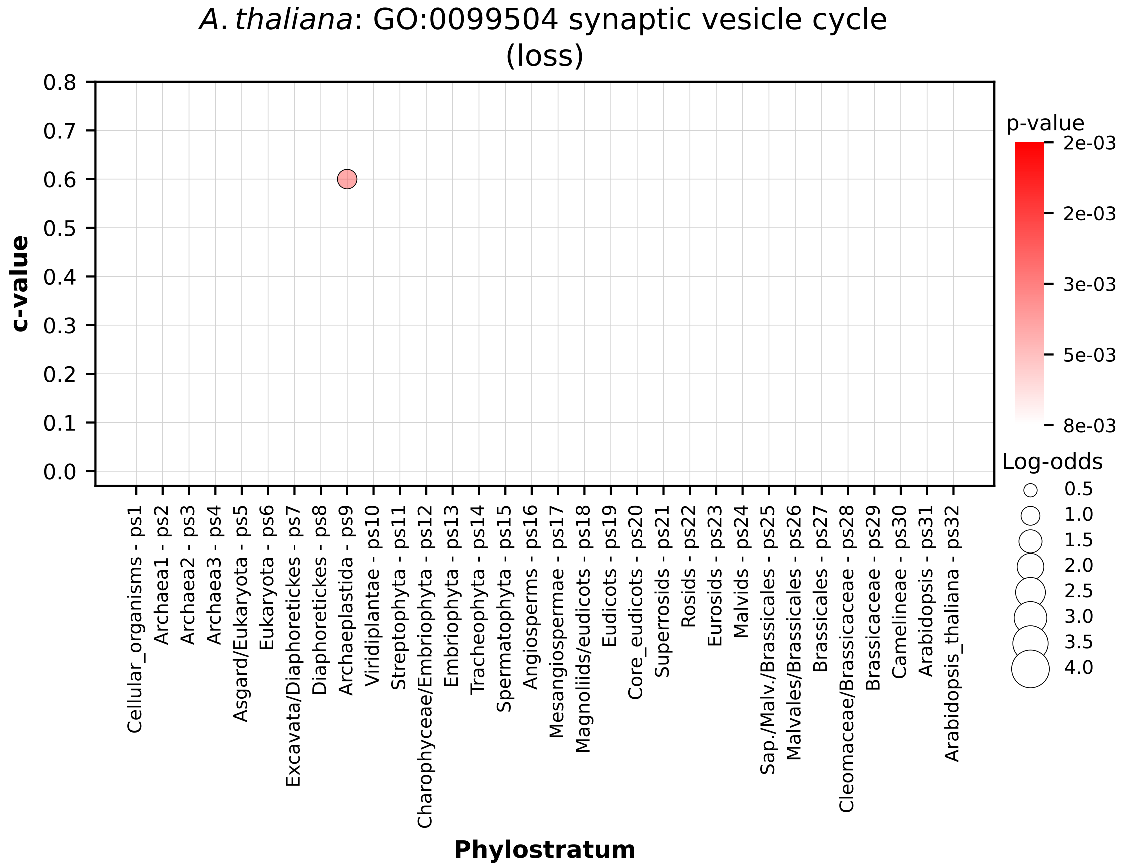
A. thaliana: GO:0099504 synaptic vesicle cycle
(loss)
c-value
0.8
0.7
0.6
0.5
0.4
0.3
0.2
0.1
0.0
p-value
2e-03
2e-03
3e-03
5e-03
8e-03
Log-odds
0.5
1.0
1.5
2.0
2.5
3.0
3.5
4.0
Cellular_organisms - ps1
Archaea1 - ps2
Archaea2 - ps3
Archaea3 - ps4
Asgard/Eukaryota - ps5
Eukaryota - ps6
Excavata/Diaphoretickes - ps7
Diaphoretickes - ps8
Archaeplastida - ps9
Viridiplantae - ps10
Streptophyta - ps11
Charophyceae/Embriophyta - ps12
Embriophyta - ps13
Tracheophyta - ps14
Spermatophyta - ps15
Angiosperms - ps16
Mesangiospermae - ps17
Magnoliids/eudicots - ps18
Eudicots - ps19
Core_eudicots - ps20
Superrosids - ps21
Rosids - ps22
Eurosids - ps23
Malvids - ps24
Sap./Malv./Brassicales - ps25
Malvales/Brassicales - ps26
Brassicales - ps27
Cleomaceae/Brassicaceae - ps28
Brassicaceae - ps29
Camelineae - ps30
Arabidopsis - ps31
Arabidopsis_thaliana - ps32
Phylostratum

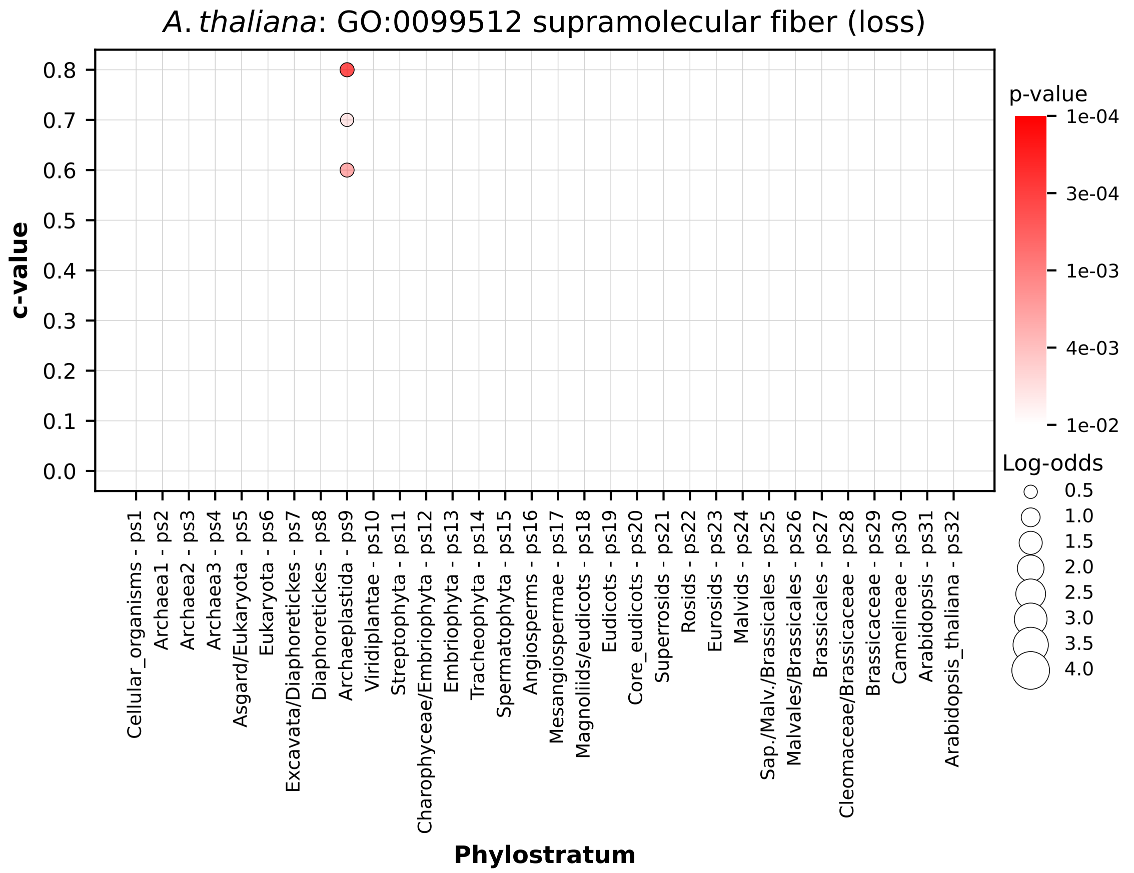

A. thaliana: GO:0099512 supramolecular fiber (loss)
c-value
0.0
0.1
0.2
0.3
0.4
0.5
0.6
0.7
0.8
Phylostratum
Cellular_organisms - ps1
Archaea1 - ps2
Archaea2 - ps3
Archaea3 - ps4
Asgard/Eukaryota - ps5
Eukaryota - ps6
Excavata/Diaphoretickes - ps7
Diaphoretickes - ps8
Archaeplastida - ps9
Viridiplantae - ps10
Streptophyta - ps11
Charophyceae/Embriophyta - ps12
Embriophyta - ps13
Tracheophyta - ps14
Spermatophyta - ps15
Angiosperms - ps16
Mesangiospermae - ps17
Magnoliids/eudicots - ps18
Eudicots - ps19
Core_eudicots - ps20
Superrosids - ps21
Rosids - ps22
Eurosids - ps23
Malvids - ps24
Sap./Malv./Brassicales - ps25
Malvales/Brassicales - ps26
Brassicales - ps27
Cleomaceae/Brassicaceae - ps28
Brassicaceae - ps29
Camelineae - ps30
Arabidopsis - ps31
Arabidopsis_thaliana - ps32
p-value
1e-04
3e-04
1e-03
4e-03
1e-02
Log-odds
0.5
1.0
1.5
2.0
2.5
3.0
3.5
4.0

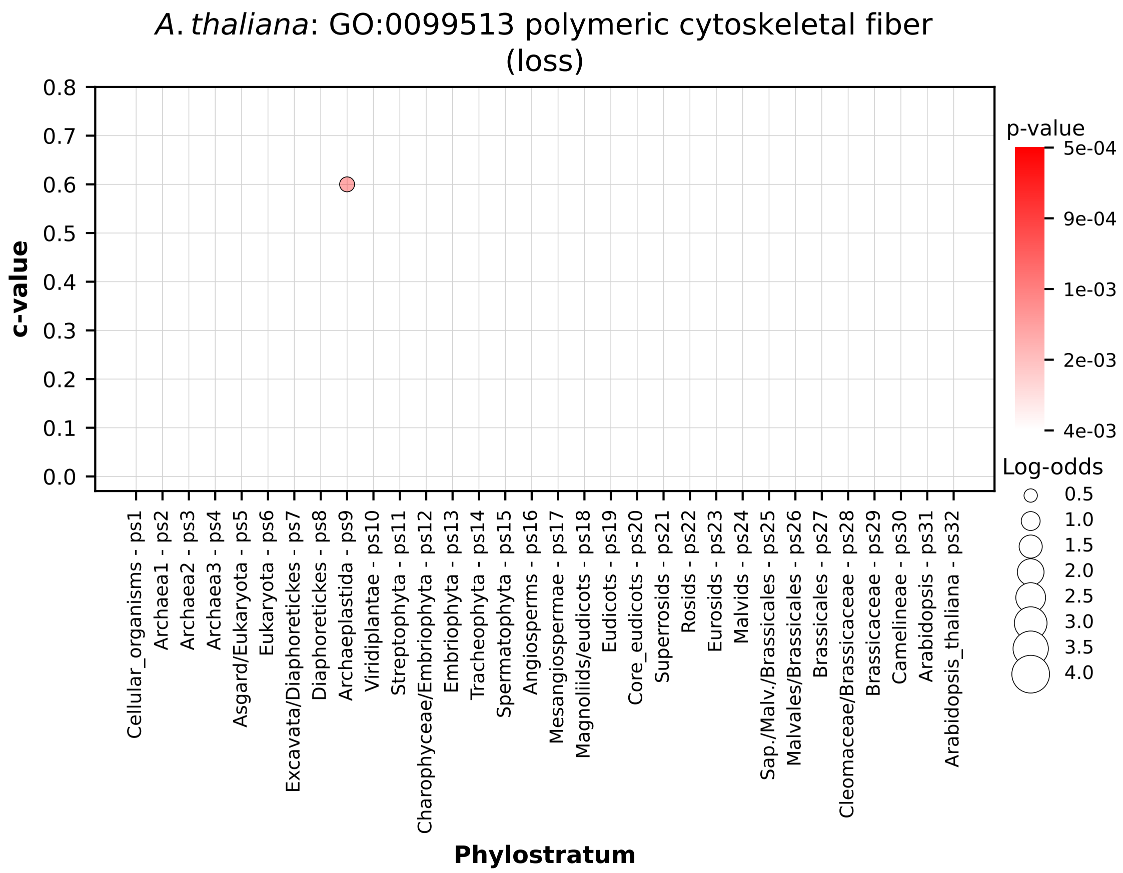

A. thaliana: GO:0099513 polymeric cytoskeletal fiber
(loss)
c-value
0.8
0.7
0.6
0.5
0.4
0.3
0.2
0.1
0.0
Phylostratum
Cellular_organisms - ps1
Archaea1 - ps2
Archaea2 - ps3
Archaea3 - ps4
Asgard/Eukaryota - ps5
Eukaryota - ps6
Excavata/Diaphoretickes - ps7
Diaphoretickes - ps8
Archaeplastida - ps9
Viridiplantae - ps10
Streptophyta - ps11
Charophyceae/Embriophyta - ps12
Embriophyta - ps13
Tracheophyta - ps14
Spermatophyta - ps15
Angiosperms - ps16
Mesangiospermae - ps17
Magnoliids/eudicots - ps18
Eudicots - ps19
Core_eudicots - ps20
Superrosids - ps21
Rosids - ps22
Eurosids - ps23
Malvids - ps24
Sap./Malv./Brassicales - ps25
Malvales/Brassicales - ps26
Brassicales - ps27
Cleomaceae/Brassicaceae - ps28
Brassicaceae - ps29
Camelineae - ps30
Arabidopsis - ps31
Arabidopsis_thaliana - ps32
p-value
5e-04
9e-04
1e-03
2e-03
4e-03
Log-odds
0.5
1.0
1.5
2.0
2.5
3.0
3.5
4.0

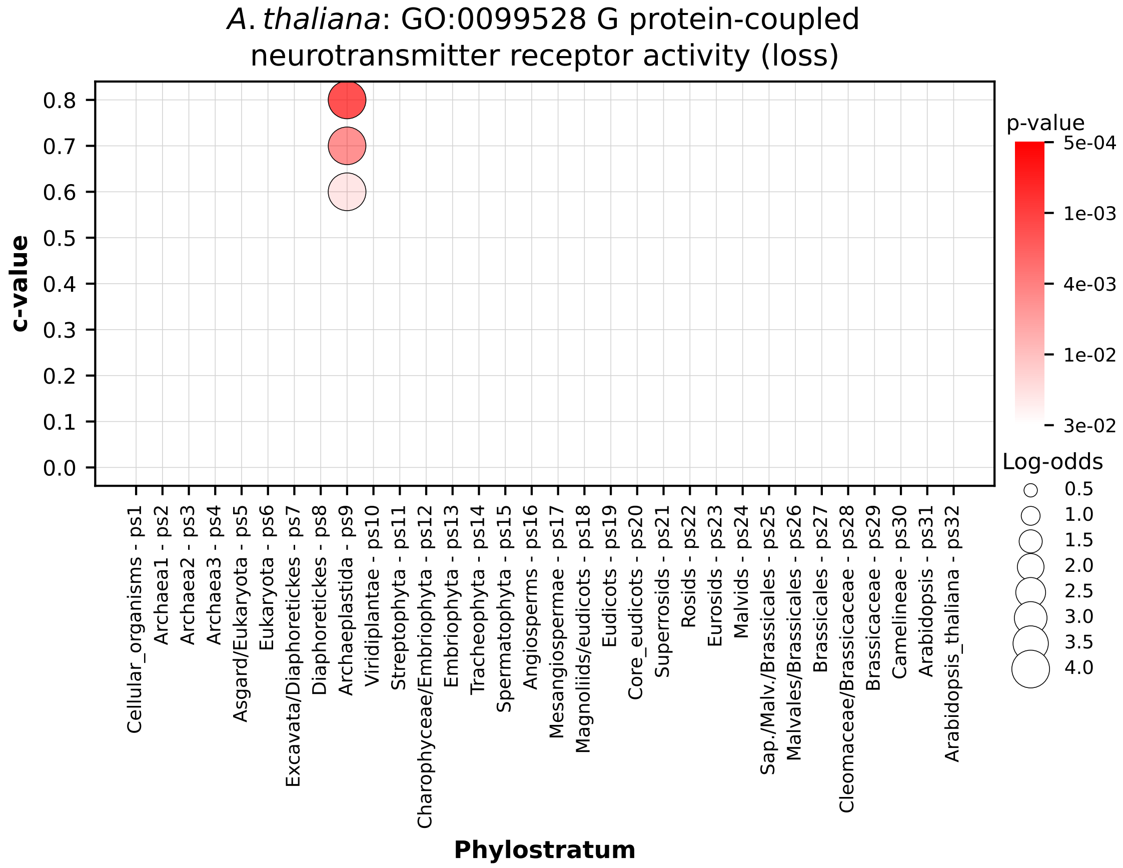

A. thaliana: GO:0099528 G protein-coupled neurotransmitter receptor activity (loss)
c-value
0.0
0.1
0.2
0.3
0.4
0.5
0.6
0.7
0.8
Phylostratum
Cellular_organisms - ps1
Archaea1 - ps2
Archaea2 - ps3
Archaea3 - ps4
Asgard/Eukaryota - ps5
Eukaryota - ps6
Excavata/Diaphoretickes - ps7
Diaphoretickes - ps8
Archaeplastida - ps9
Viridiplantae - ps10
Streptophyta - ps11
Charophyceae/Embriophyta - ps12
Embriophyta - ps13
Tracheophyta - ps14
Spermatophyta - ps15
Angiosperms - ps16
Mesangiospermae - ps17
Magnoliids/eudicots - ps18
Eudicots - ps19
Core_eudicots - ps20
Superrosids - ps21
Rosids - ps22
Eurosids - ps23
Malvids - ps24
Sap./Malv./Brassicales - ps25
Malvales/Brassicales - ps26
Brassicales - ps27
Cleomaceae/Brassicaceae - ps28
Brassicaceae - ps29
Camelineae - ps30
Arabidopsis - ps31
Arabidopsis_thaliana - ps32
p-value
5e-04
1e-03
4e-03
1e-02
3e-02
Log-odds
0.5
1.0
1.5
2.0
2.5
3.0
3.5
4.0

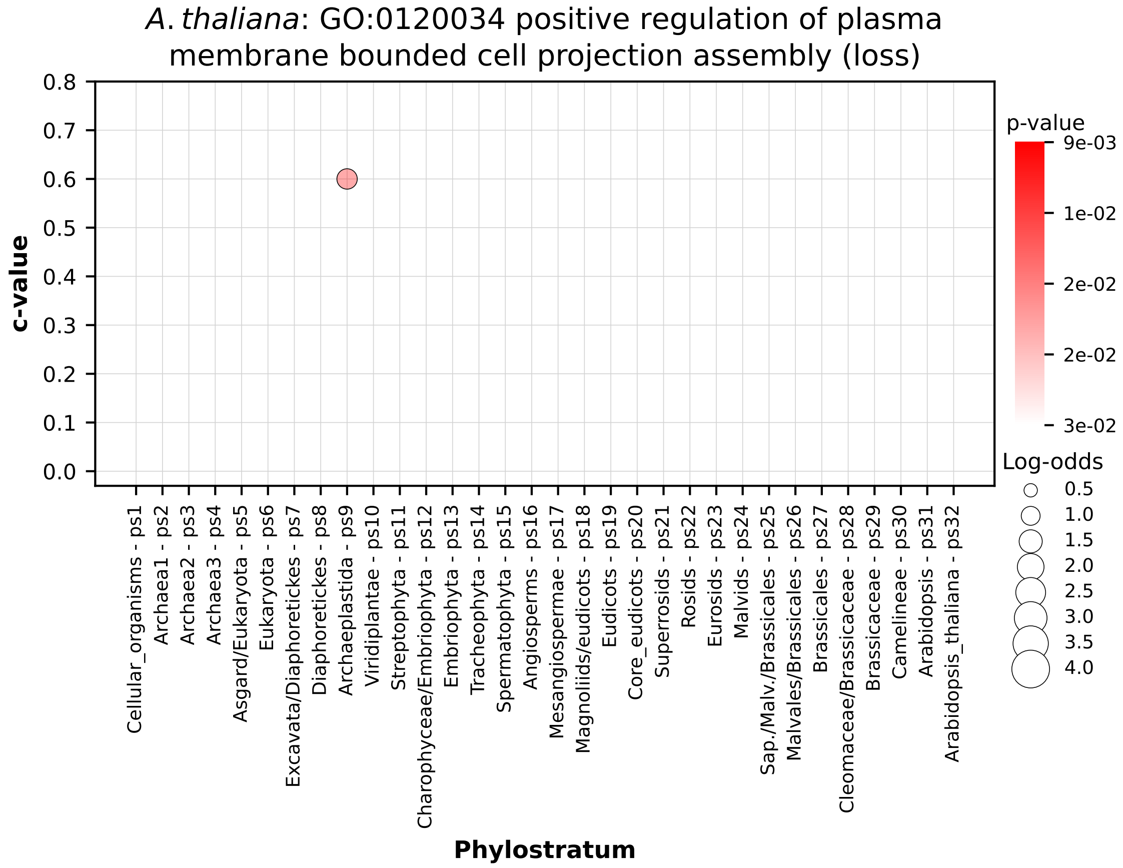

A. thaliana: GO:0120034 positive regulation of plasma membrane bounded cell projection assembly (loss)
c-value
0.8
0.7
0.6
0.5
0.4
0.3
0.2
0.1
0.0
Phylostratum
Cellular_organisms - ps1
Archaea1 - ps2
Archaea2 - ps3
Archaea3 - ps4
Asgard/Eukaryota - ps5
Eukaryota - ps6
Excavata/Diaphoretickes - ps7
Diaphoretickes - ps8
Archaeplastida - ps9
Viridiplantae - ps10
Streptophyta - ps11
Charophyceae/Embriophyta - ps12
Embriophyta - ps13
Tracheophyta - ps14
Spermatophyta - ps15
Angiosperms - ps16
Mesangiospermae - ps17
Magnoliids/eudicots - ps18
Eudicots - ps19
Core_eudicots - ps20
Superrosids - ps21
Rosids - ps22
Eurosids - ps23
Malvids - ps24
Sap./Malv./Brassicales - ps25
Malvales/Brassicales - ps26
Brassicales - ps27
Cleomaceae/Brassicaceae - ps28
Brassicaceae - ps29
Camelineae - ps30
Arabidopsis - ps31
Arabidopsis_thaliana - ps32
p-value
9e-03
1e-02
2e-02
2e-02
3e-02
Log-odds
0.5
1.0
1.5
2.0
2.5
3.0
3.5
4.0

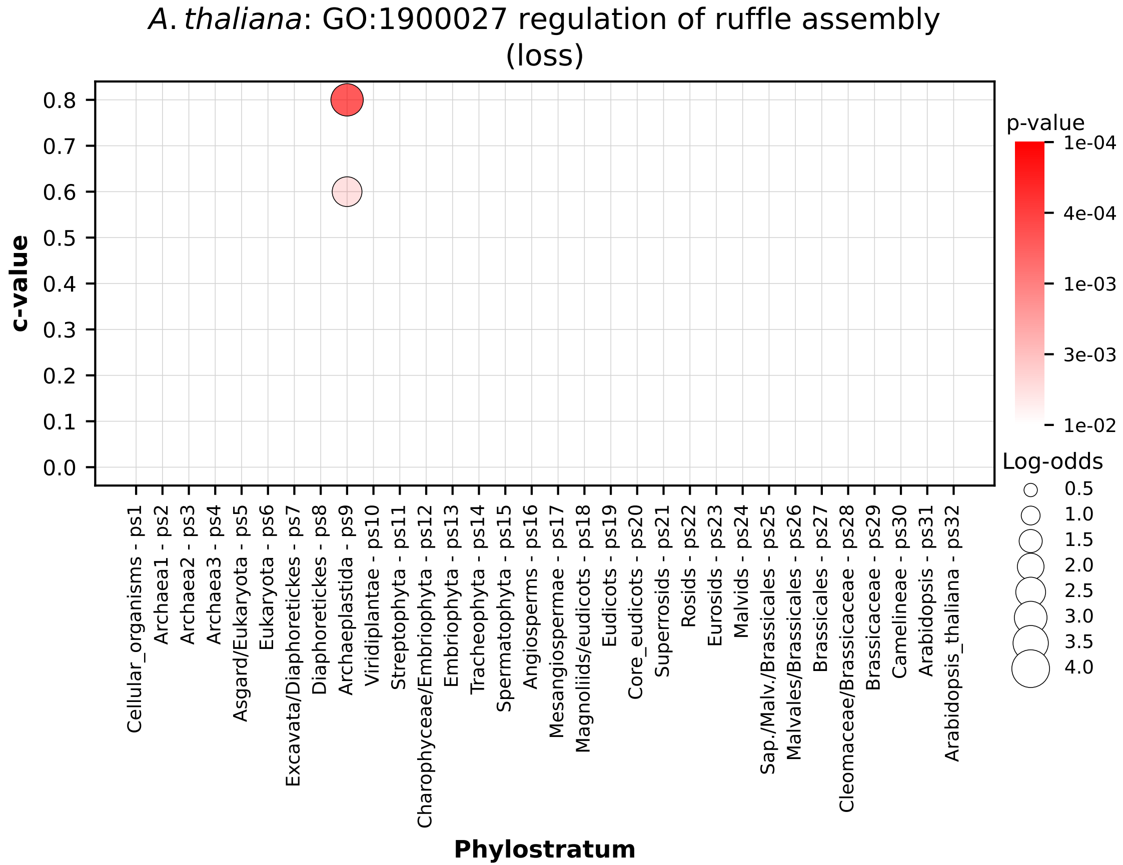

A. thaliana: GO:1900027 regulation of ruffle assembly (loss)
c-value
0.8
0.7
0.6
0.5
0.4
0.3
0.2
0.1
0.0
p-value
1e-04
4e-04
1e-03
3e-03
1e-02
Log-odds
0.5
1.0
1.5
2.0
2.5
3.0
3.5
4.0
Cellular_organisms - ps1
Archaea1 - ps2
Archaea2 - ps3
Archaea3 - ps4
Asgard/Eukaryota - ps5
Eukaryota - ps6
Excavata/Diaphoretickes - ps7
Diaphoretickes - ps8
Archaeplastida - ps9
Viridiplantae - ps10
Streptophyta - ps11
Charophyceae/Embriophyta - ps12
Embriophyta - ps13
Tracheophyta - ps14
Spermatophyta - ps15
Angiosperms - ps16
Mesangiospermae - ps17
Magnoliids/eudicots - ps18
Eudicots - ps19
Core_eudicots - ps20
Superrosids - ps21
Rosids - ps22
Eurosids - ps23
Malvids - ps24
Sap./Malv./Brassicales - ps25
Malvales/Brassicales - ps26
Brassicales - ps27
Cleomaceae/Brassicaceae - ps28
Brassicaceae - ps29
Camelineae - ps30
Arabidopsis - ps31
Arabidopsis_thaliana - ps32
Phylostratum

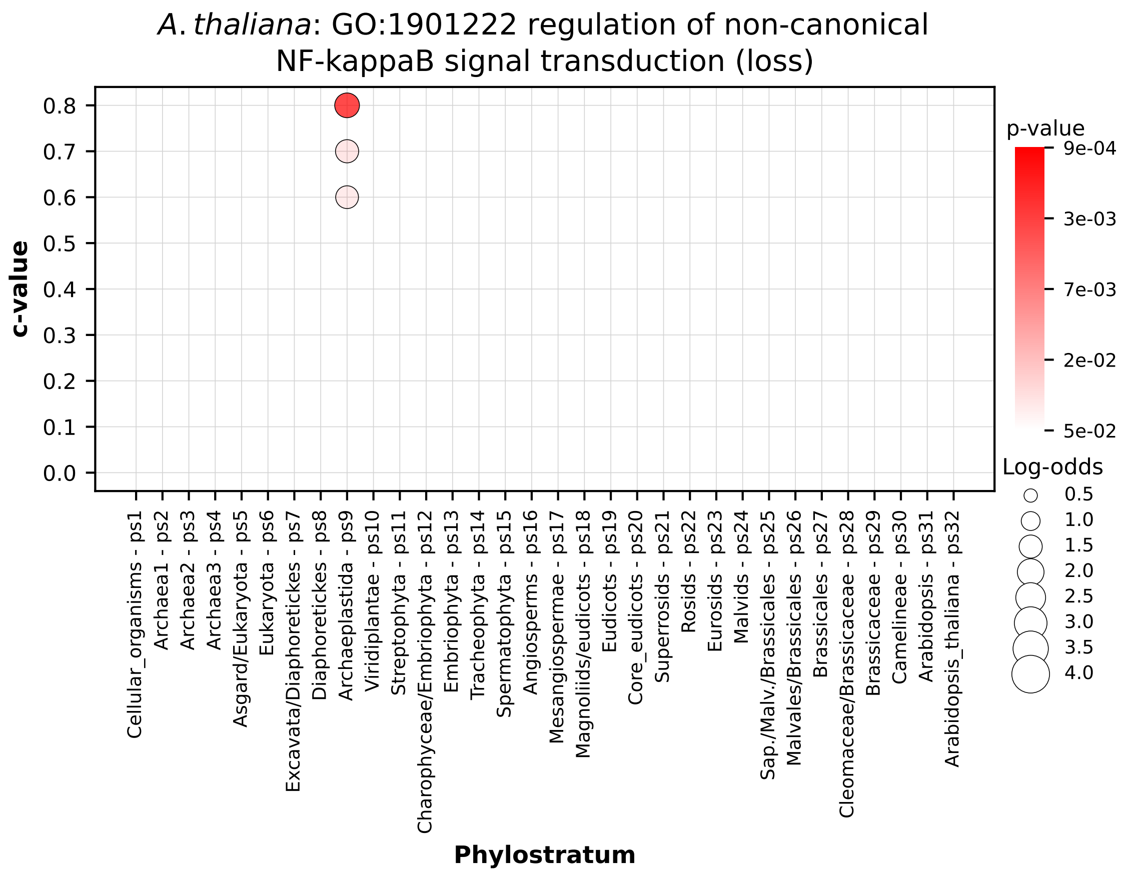
A. thaliana: GO:1901222 regulation of non-canonical NF-kappaB signal transduction (loss)
c-value
0.0
0.1
0.2
0.3
0.4
0.5
0.6
0.7
0.8
p-value
9e-04
3e-03
7e-03
2e-02
5e-02
Log-odds
0.5
1.0
1.5
2.0
2.5
3.0
3.5
4.0
Cellular_organisms - ps1
Archaea1 - ps2
Archaea2 - ps3
Archaea3 - ps4
Asgard/Eukaryota - ps5
Eukaryota - ps6
Excavata/Diaphoretickes - ps7
Diaphoretickes - ps8
Archaeplastida - ps9
Viridiplantae - ps10
Streptophyta - ps11
Charophyceae/Embriophyta - ps12
Embriophyta - ps13
Tracheophyta - ps14
Spermatophyta - ps15
Angiosperms - ps16
Mesangiospermae - ps17
Magnoliids/eudicots - ps18
Eudicots - ps19
Core_eudicots - ps20
Superrosids - ps21
Rosids - ps22
Eurosids - ps23
Malvids - ps24
Sap./Malv./Brassicales - ps25
Malvales/Brassicales - ps26
Brassicales - ps27
Cleomaceae/Brassicaceae - ps28
Brassicaceae - ps29
Camelineae - ps30
Arabidopsis - ps31
Arabidopsis_thaliana - ps32
Phylostratum

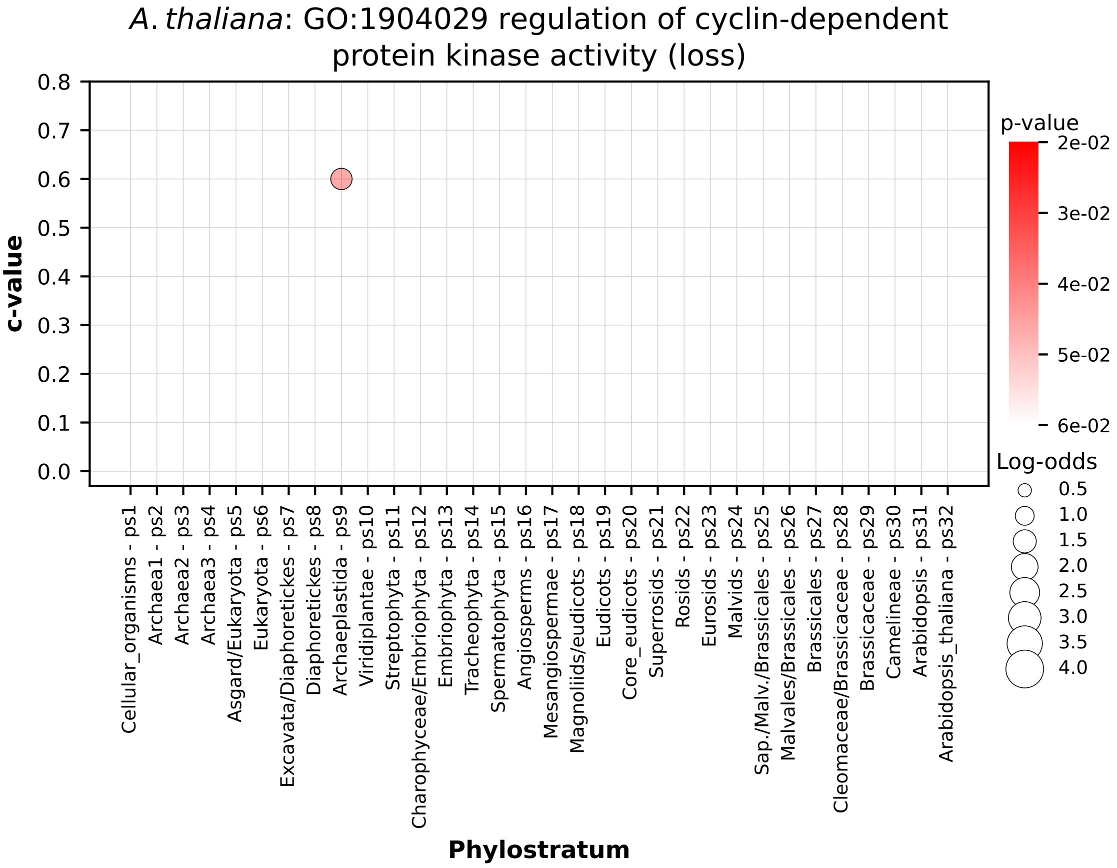

A. thaliana: GO:1904029 regulation of cyclin-dependent protein kinase activity (loss)
c-value
0.8
0.7
0.6
0.5
0.4
0.3
0.2
0.1
0.0
Phylostratum
Cellular_organisms - ps1
Archaea1 - ps2
Archaea2 - ps3
Archaea3 - ps4
Asgard/Eukaryota - ps5
Eukaryota - ps6
Excavata/Diaphoretickes - ps7
Diaphoretickes - ps8
Archaeplastida - ps9
Viridiplantae - ps10
Streptophyta - ps11
Charophyceae/Embriophyta - ps12
Embriophyta - ps13
Tracheophyta - ps14
Spermatophyta - ps15
Angiosperms - ps16
Mesangiospermae - ps17
Magnoliids/eudicots - ps18
Eudicots - ps19
Core_eudicots - ps20
Superrosids - ps21
Rosids - ps22
Eurosids - ps23
Malvids - ps24
Sap./Malv./Brassicales - ps25
Malvales/Brassicales - ps26
Brassicales - ps27
Cleomaceae/Brassicaceae - ps28
Brassicaceae - ps29
Camelineae - ps30
Arabidopsis - ps31
Arabidopsis_thaliana - ps32
p-value
2e-02
3e-02
4e-02
5e-02
6e-02
Log-odds
0.5
1.0
1.5
2.0
2.5
3.0
3.5
4.0

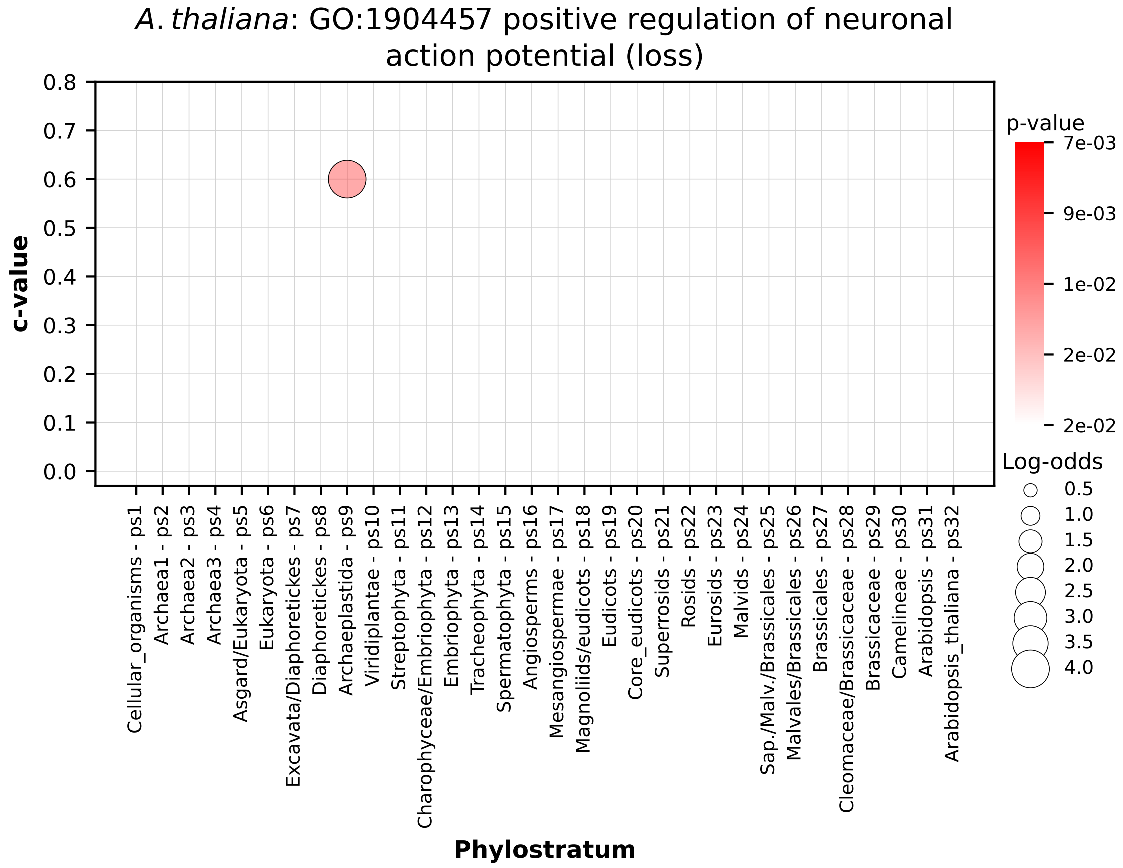

A. thaliana: GO:1904457 positive regulation of neuronal action potential (loss)
c-value
0.8
0.7
0.6
0.5
0.4
0.3
0.2
0.1
0.0
Phylostratum
Cellular_organisms - ps1
Archaea1 - ps2
Archaea2 - ps3
Archaea3 - ps4
Asgard/Eukaryota - ps5
Eukaryota - ps6
Excavata/Diaphoretickes - ps7
Diaphoretickes - ps8
Archaeplastida - ps9
Viridiplantae - ps10
Streptophyta - ps11
Charophyceae/Embriophyta - ps12
Embriophyta - ps13
[truncated: 376,283 more chars]
