# Supplementary material for: Macroevolutionary dynamics of gene family gain and loss along multicellular eukaryotic lineages
Source: Nat Commun. 2024 Mar 26;15:2663. doi: 10.1038/s41467-024-47017-w (PMC10966110; doi:10.1038/s41467-024-47017-w)
Supplement: Supplementary file 22 — Supplementary Dataset 17 [file 41467_2024_47017_MOESM22_ESM.pdf]

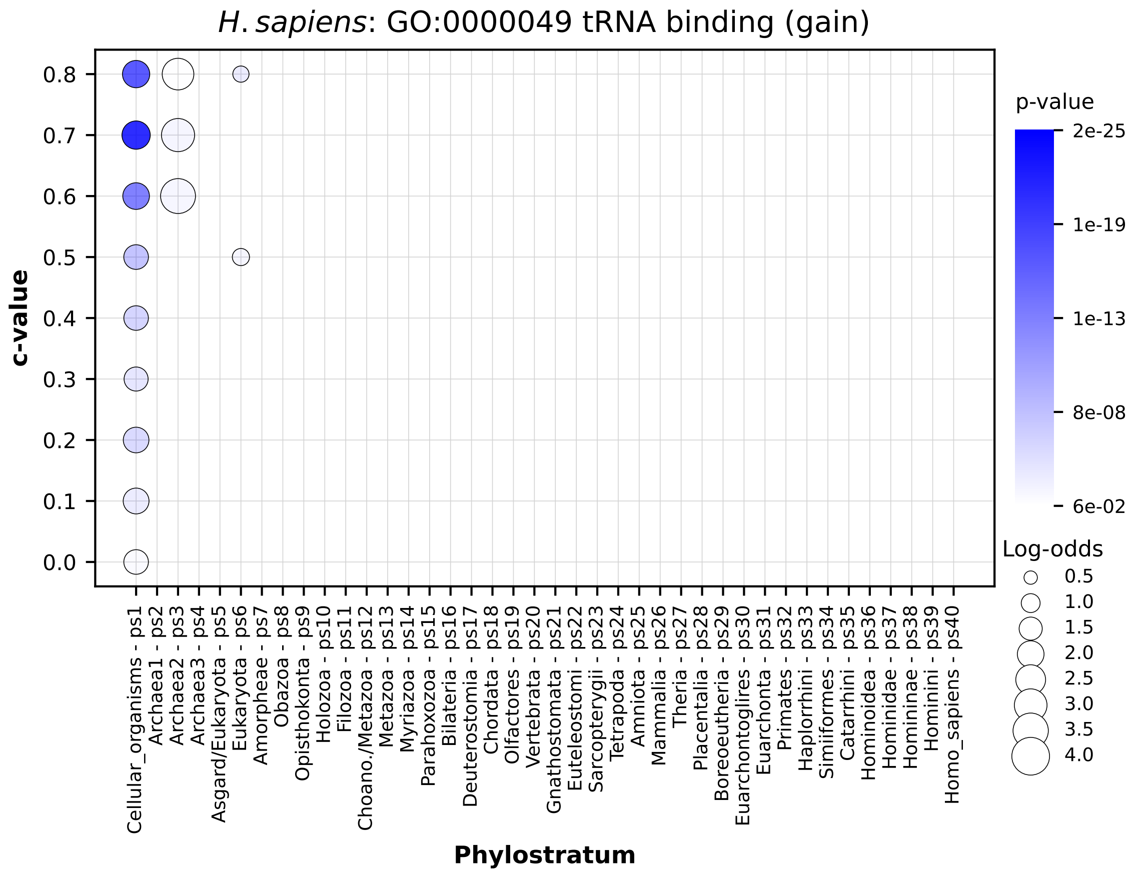
*H. sapiens*: GO:0000049 tRNA binding (gain)
c-value
0.8
0.7
0.6
0.5
0.4
0.3
0.2
0.1
0.0
Phylostratum
Cellular_organisms - ps1
Archaea1 - ps2
Archaea2 - ps3
Archaea3 - ps4
Asgard/Eukaryota - ps5
Eukaryota - ps6
Amorpheae - ps7
Obazoa - ps8
Opisthokonta - ps9
Holozoa - ps10
Filozoa - ps11
Choano./Metazoa - ps12
Metazoa - ps13
Myriazoa - ps14
Parahoxozoa - ps15
Bilateria - ps16
Deuterostomia - ps17
Chordata - ps18
Olfactores - ps19
Vertebrata - ps20
Gnathostomata - ps21
Euteleostomi - ps22
Sarcopterygii - ps23
Tetrapoda - ps24
Amniota - ps25
Mammalia - ps26
Theria - ps27
Placentalia - ps28
Boreoeutheria - ps29
Euarchontoglires - ps30
Euarchonta - ps31
Primates - ps32
Haplorrhini - ps33
Simiiformes - ps34
Catarrhini - ps35
Hominoidea - ps36
Hominidae - ps37
Homininae - ps38
Hominini - ps39
Homo_sapiens - ps40
p-value
2e-25
1e-19
1e-13
8e-08
6e-02
Log-odds
0.5
1.0
1.5
2.0
2.5
3.0
3.5
4.0

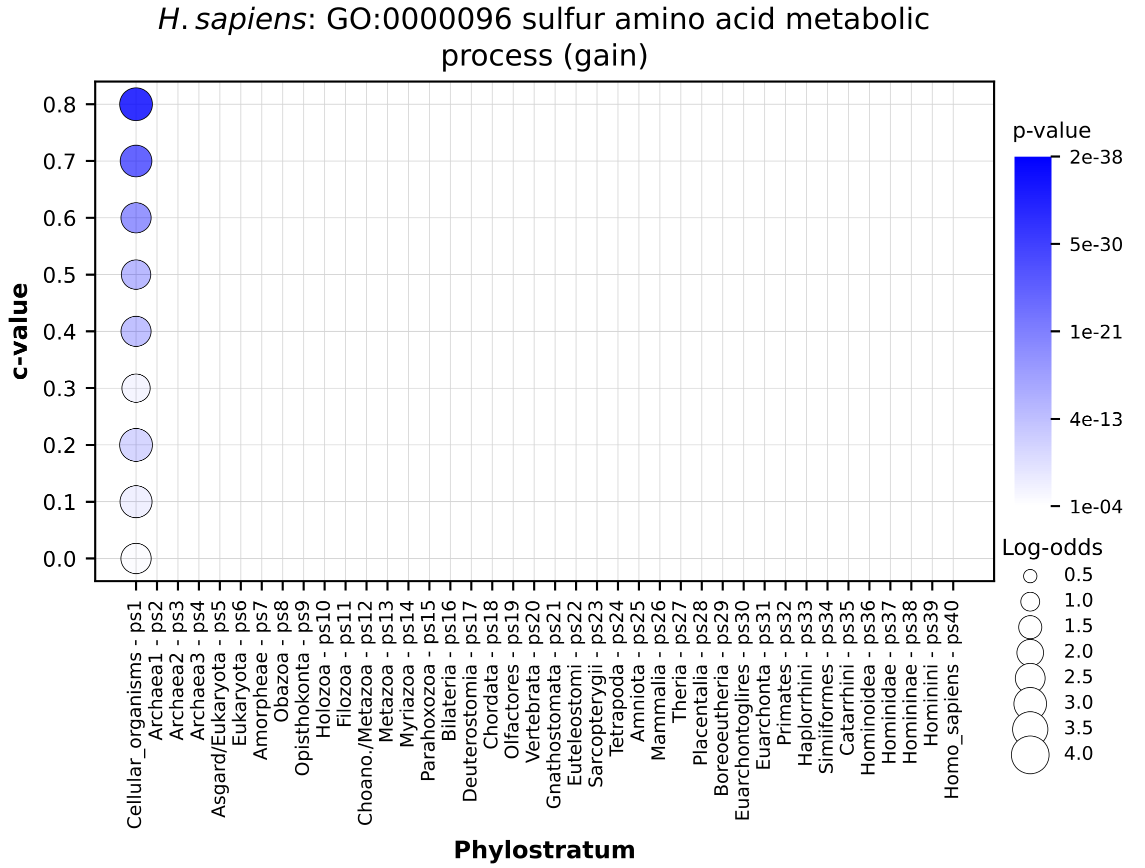

*H. sapiens*: GO:0000096 sulfur amino acid metabolic process (gain)
c-value
0.8
0.7
0.6
0.5
0.4
0.3
0.2
0.1
0.0
p-value
2e-38
5e-30
1e-21
4e-13
1e-04
Log-odds
0.5
1.0
1.5
2.0
2.5
3.0
3.5
4.0
Cellular_organisms - ps1
Archaea1 - ps2
Archaea2 - ps3
Archaea3 - ps4
Asgard/Eukaryota - ps5
Eukaryota - ps6
Amorpheae - ps7
Obazoa - ps8
Opisthokonta - ps9
Holozoa - ps10
Filozoa - ps11
Choano./Metazoa - ps12
Metazoa - ps13
Myriazoa - ps14
Parahoxozoa - ps15
Bilateria - ps16
Deuterostomia - ps17
Chordata - ps18
Olfactores - ps19
Vertebrata - ps20
Gnathostomata - ps21
Euteleostomi - ps22
Sarcopterygii - ps23
Tetrapoda - ps24
Amniota - ps25
Mammalia - ps26
Theria - ps27
Placentalia - ps28
Boreoeutheria - ps29
Euarchontoglires - ps30
Euarchonta - ps31
Primates - ps32
Haplorrhini - ps33
Simiiformes - ps34
Catarrhini - ps35
Hominoidea - ps36
Hominidae - ps37
Homininae - ps38
Hominini - ps39
Homo_sapiens - ps40
Phylostratum

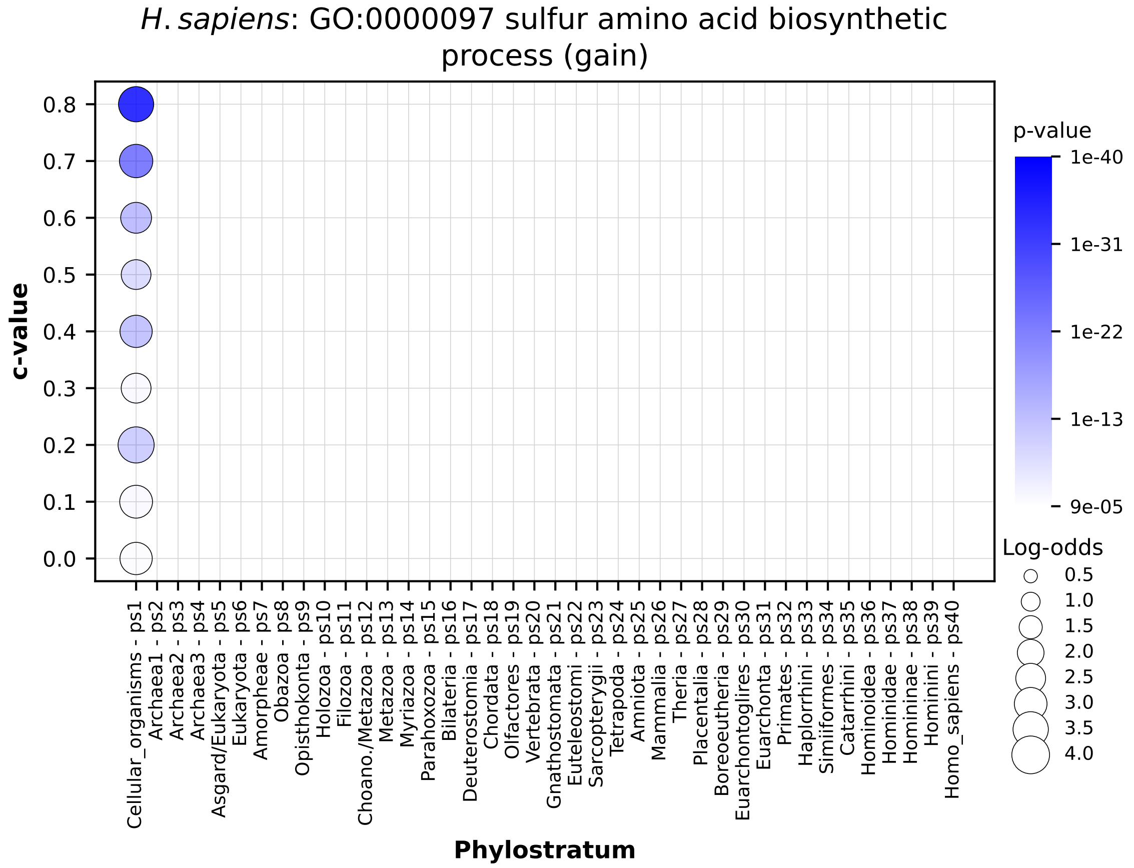

H. sapiens: GO:0000097 sulfur amino acid biosynthetic process (gain)
c-value
0.8
0.7
0.6
0.5
0.4
0.3
0.2
0.1
0.0
p-value
1e-40
1e-31
1e-22
1e-13
9e-05
Log-odds
0.5
1.0
1.5
2.0
2.5
3.0
3.5
4.0
Cellular_organisms - ps1
Archaea1 - ps2
Archaea2 - ps3
Archaea3 - ps4
Asgard/Eukaryota - ps5
Eukaryota - ps6
Amorpheae - ps7
Obazoa - ps8
Opisthokonta - ps9
Holozoa - ps10
Filozoa - ps11
Choano./Metazoa - ps12
Metazoa - ps13
Myriazoa - ps14
Parahoxozoa - ps15
Bilateria - ps16
Deuterostomia - ps17
Chordata - ps18
Olfactores - ps19
Vertebrata - ps20
Gnathostomata - ps21
Euteleostomi - ps22
Sarcopterygii - ps23
Tetrapoda - ps24
Amniota - ps25
Mammalia - ps26
Theria - ps27
Placentalia - ps28
Boreoeutheria - ps29
Euarchontoglires - ps30
Euarchonta - ps31
Primates - ps32
Haplorrhini - ps33
Simiiformes - ps34
Catarrhini - ps35
Hominoidea - ps36
Hominidae - ps37
Homininae - ps38
Hominini - ps39
Homo_sapiens - ps40
Phylostratum

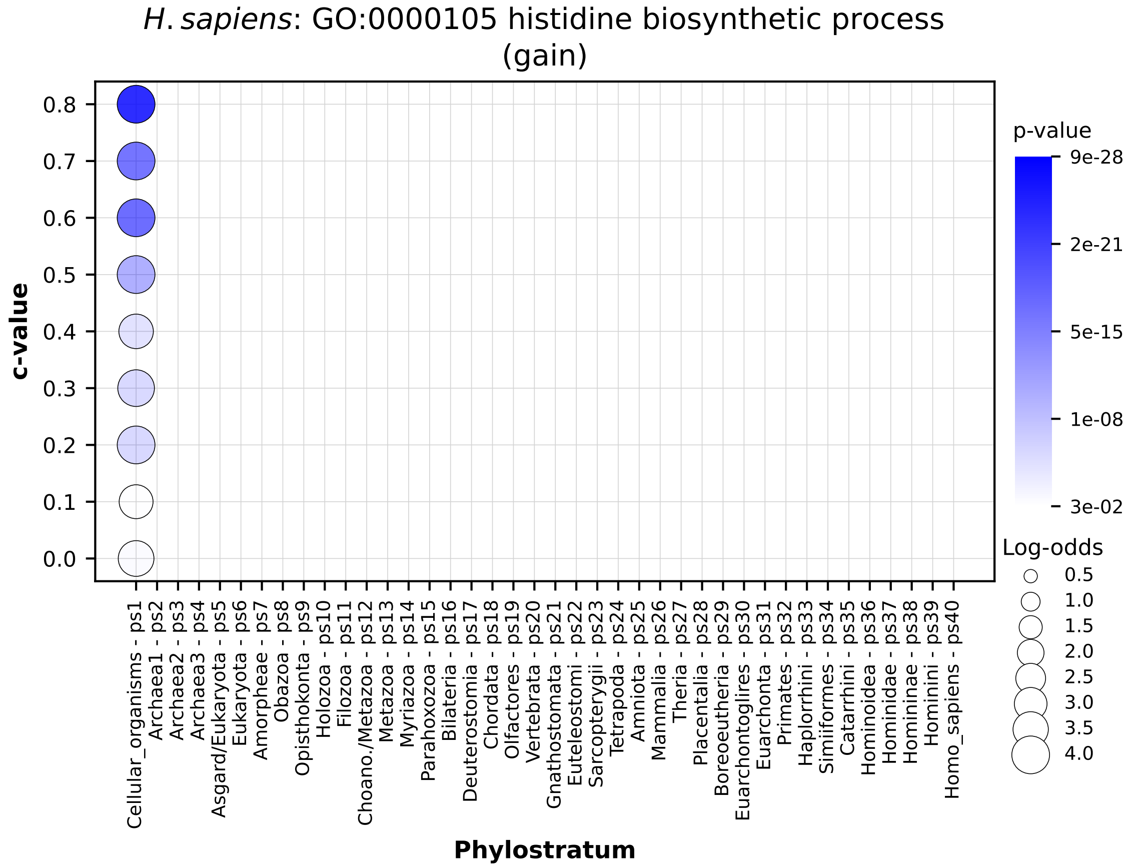

H. sapiens: GO:0000105 histidine biosynthetic process
(gain)
c-value
0.8
0.7
0.6
0.5
0.4
0.3
0.2
0.1
0.0
p-value
9e-28
2e-21
5e-15
1e-08
3e-02
Log-odds
0.5
1.0
1.5
2.0
2.5
3.0
3.5
4.0
Cellular_organisms - ps1
Archaea1 - ps2
Archaea2 - ps3
Archaea3 - ps4
Asgard/Eukaryota - ps5
Eukaryota - ps6
Amorpheae - ps7
Obazoa - ps8
Opisthokonta - ps9
Holozoa - ps10
Filozoa - ps11
Choano./Metazoa - ps12
Metazoa - ps13
Myriazoa - ps14
Parahoxozoa - ps15
Bilateria - ps16
Deuterostomia - ps17
Chordata - ps18
Olfactores - ps19
Vertebrata - ps20
Gnathostomata - ps21
Euteleostomi - ps22
Sarcopterygii - ps23
Tetrapoda - ps24
Amniota - ps25
Mammalia - ps26
Theria - ps27
Placentalia - ps28
Boreoeutheria - ps29
Euarchontoglires - ps30
Euarchonta - ps31
Primates - ps32
Haplorrhini - ps33
Simiiformes - ps34
Catarrhini - ps35
Hominoidea - ps36
Hominidae - ps37
Homininae - ps38
Hominini - ps39
Homo_sapiens - ps40
Phylostratum

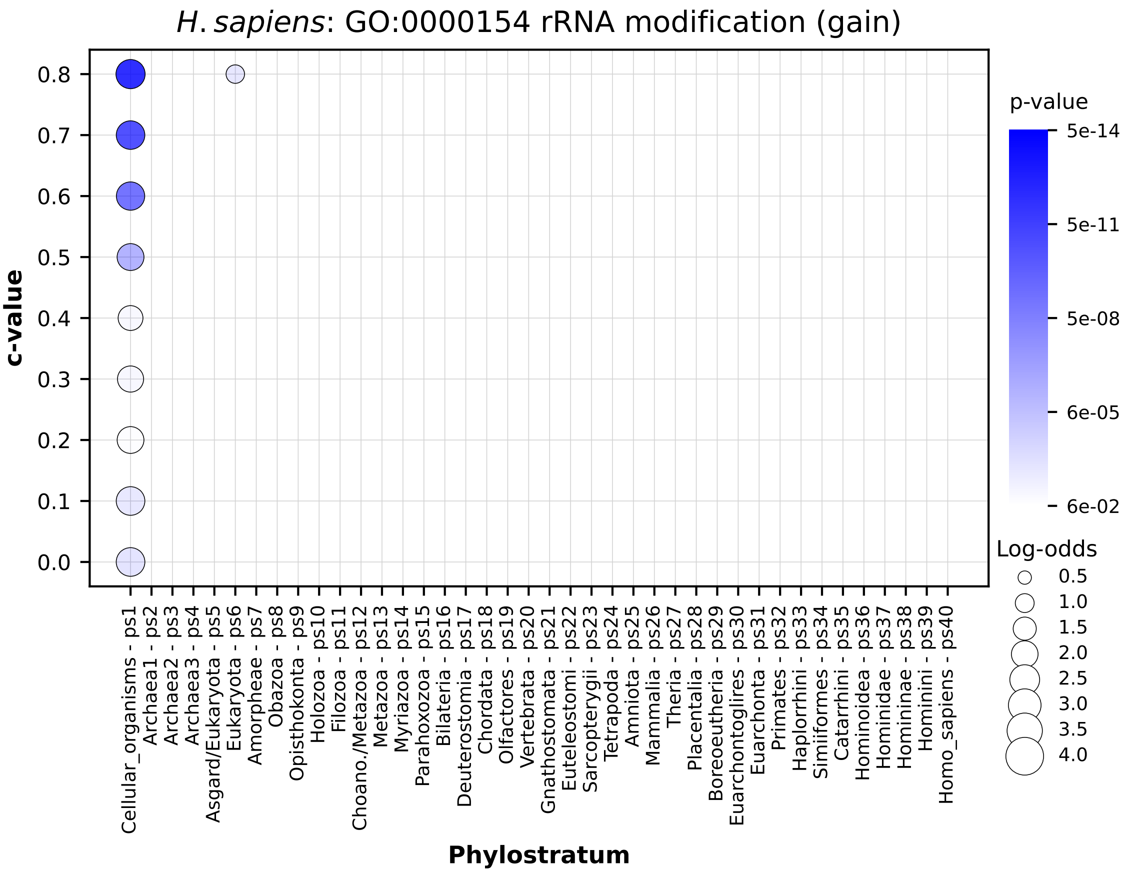

H. sapiens: GO:0000154 rRNA modification (gain)
c-value
0.0
0.1
0.2
0.3
0.4
0.5
0.6
0.7
0.8
Phylostratum
Cellular_organisms - ps1
Archaea1 - ps2
Archaea2 - ps3
Archaea3 - ps4
Asgard/Eukaryota - ps5
Eukaryota - ps6
Amorpheae - ps7
Obazoa - ps8
Opisthokonta - ps9
Holozoa - ps10
Filozoa - ps11
Choano./Metazoa - ps12
Metazoa - ps13
Myriazoa - ps14
Parahoxozoa - ps15
Bilateria - ps16
Deuterostomia - ps17
Chordata - ps18
Olfactores - ps19
Vertebrata - ps20
Gnathostomata - ps21
Euteleostomi - ps22
Sarcopterygii - ps23
Tetrapoda - ps24
Amniota - ps25
Mammalia - ps26
Theria - ps27
Placentalia - ps28
Boreoeutheria - ps29
Euarchontoglires - ps30
Euarchonta - ps31
Primates - ps32
Haplorrhini - ps33
Simiiformes - ps34
Catarrhini - ps35
Hominoidea - ps36
Hominidae - ps37
Homininae - ps38
Hominini - ps39
Homo_sapiens - ps40
p-value
5e-14
5e-11
5e-08
6e-05
6e-02
Log-odds
0.5
1.0
1.5
2.0
2.5
3.0
3.5
4.0

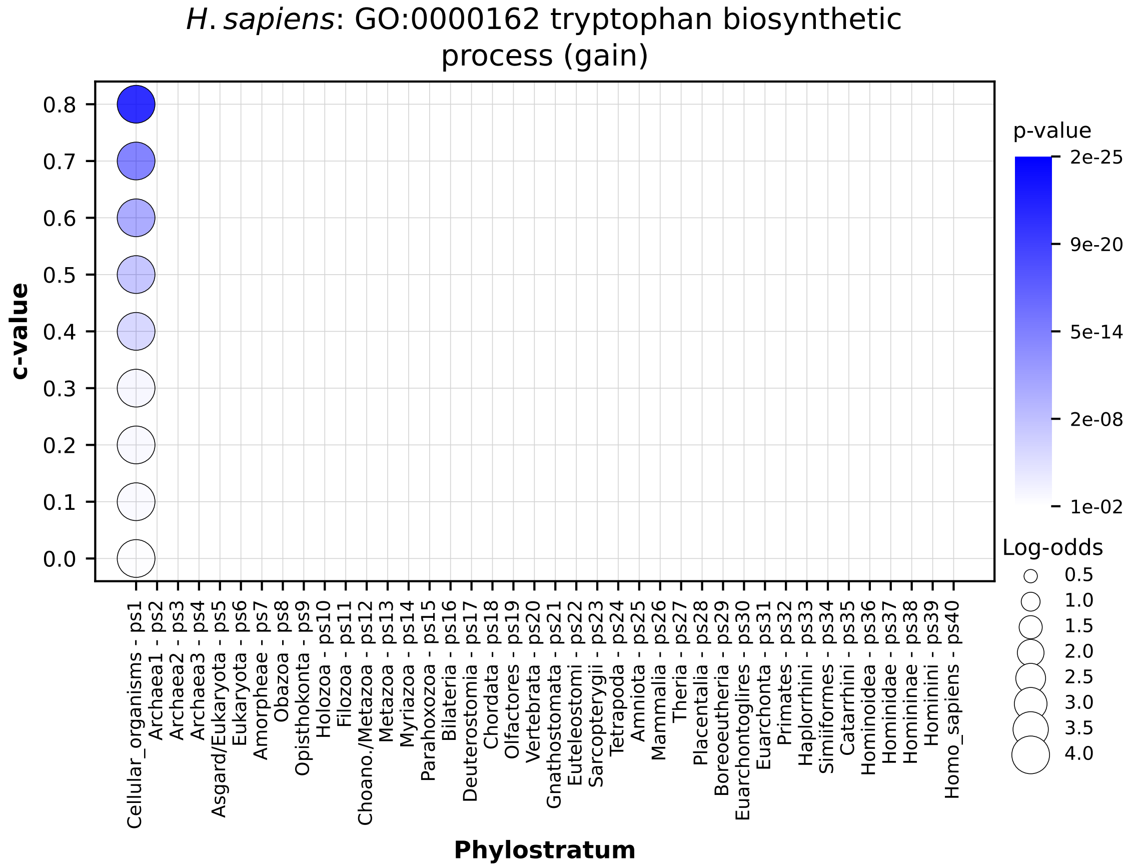

H. sapiens: GO:0000162 tryptophan biosynthetic process (gain)
c-value
0.0
0.1
0.2
0.3
0.4
0.5
0.6
0.7
0.8
Phylostratum
Cellular_organisms - ps1
Archaea1 - ps2
Archaea2 - ps3
Archaea3 - ps4
Asgard/Eukaryota - ps5
Eukaryota - ps6
Amorpheae - ps7
Obazoa - ps8
Opisthokonta - ps9
Holozoa - ps10
Filozoa - ps11
Choano./Metazoa - ps12
Metazoa - ps13
Myriazoa - ps14
Parahoxozoa - ps15
Bilateria - ps16
Deuterostomia - ps17
Chordata - ps18
Olfactores - ps19
Vertebrata - ps20
Gnathostomata - ps21
Euteleostomi - ps22
Sarcopterygii - ps23
Tetrapoda - ps24
Amniota - ps25
Mammalia - ps26
Theria - ps27
Placentalia - ps28
Boreoeutheria - ps29
Euarchontoglires - ps30
Euarchonta - ps31
Primates - ps32
Haplorrhini - ps33
Simiiformes - ps34
Catarrhini - ps35
Hominoidea - ps36
Hominidae - ps37
Homininae - ps38
Hominini - ps39
Homo_sapiens - ps40
p-value
2e-25
9e-20
5e-14
2e-08
1e-02
Log-odds
0.5
1.0
1.5
2.0
2.5
3.0
3.5
4.0

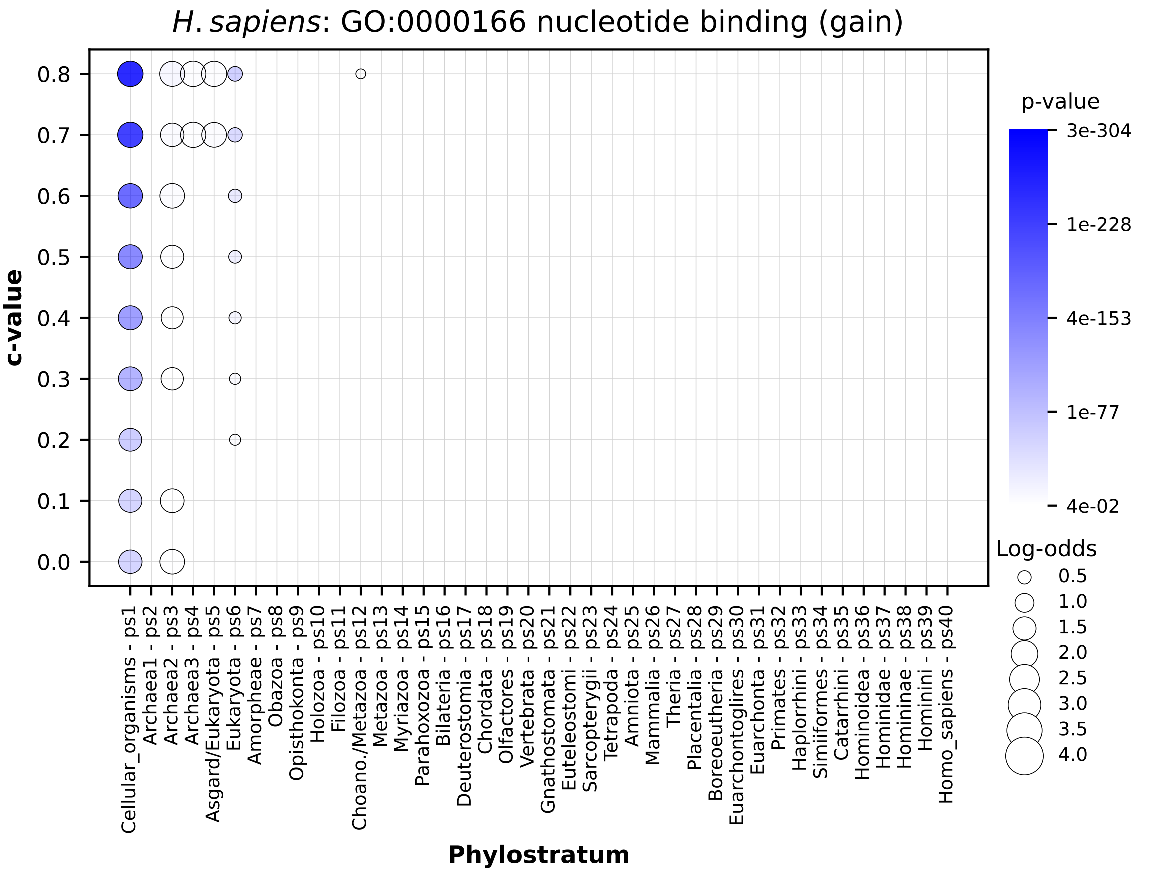

H. sapiens: GO:0000166 nucleotide binding (gain)
c-value
0.8
0.7
0.6
0.5
0.4
0.3
0.2
0.1
0.0
p-value
3e-304
1e-228
4e-153
1e-77
4e-02
Log-odds
0.5
1.0
1.5
2.0
2.5
3.0
3.5
4.0
Cellular_organisms - ps1
Archaea1 - ps2
Archaea2 - ps3
Archaea3 - ps4
Asgard/Eukaryota - ps5
Eukaryota - ps6
Amorpheae - ps7
Obazoa - ps8
Opisthokonta - ps9
Holozoa - ps10
Filozoa - ps11
Choano./Metazoa - ps12
Metazoa - ps13
Myriazoa - ps14
Parahoxozoa - ps15
Bilateria - ps16
Deuterostomia - ps17
Chordata - ps18
Olfactores - ps19
Vertebrata - ps20
Gnathostomata - ps21
Euteleostomi - ps22
Sarcopterygii - ps23
Tetrapoda - ps24
Amniota - ps25
Mammalia - ps26
Theria - ps27
Placentalia - ps28
Boreoeutheria - ps29
Euarchontoglires - ps30
Euarchonta - ps31
Primates - ps32
Haplorrhini - ps33
Simiiformes - ps34
Catarrhini - ps35
Hominoidea - ps36
Hominidae - ps37
Homininae - ps38
Hominini - ps39
Homo_sapiens - ps40
Phylostratum

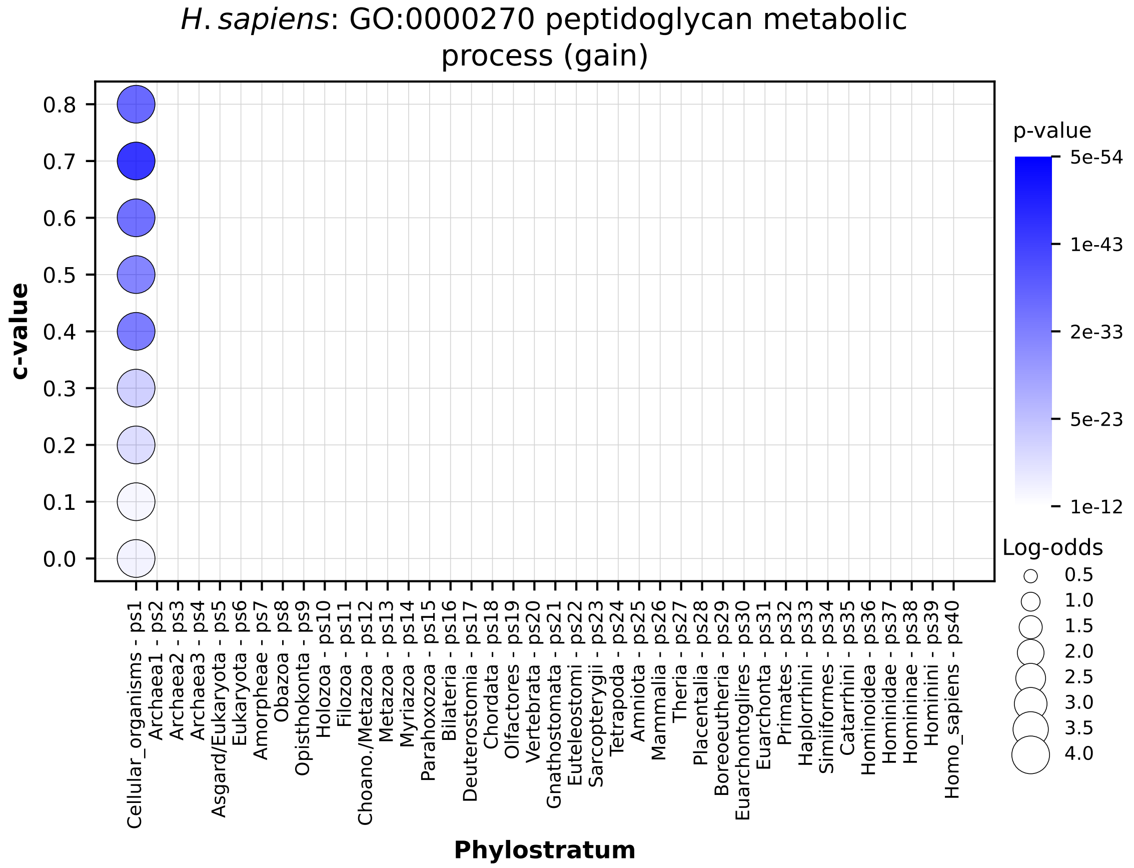

H. sapiens: GO:0000270 peptidoglycan metabolic process (gain)
c-value
0.8
0.7
0.6
0.5
0.4
0.3
0.2
0.1
0.0
p-value
5e-54
1e-43
2e-33
5e-23
1e-12
Log-odds
0.5
1.0
1.5
2.0
2.5
3.0
3.5
4.0
Cellular_organisms - ps1
Archaea1 - ps2
Archaea2 - ps3
Archaea3 - ps4
Asgard/Eukaryota - ps5
Eukaryota - ps6
Amorpheae - ps7
Obazoa - ps8
Opisthokonta - ps9
Holozoa - ps10
Filozoa - ps11
Choano./Metazoa - ps12
Metazoa - ps13
Myriazoa - ps14
Parahoxozoa - ps15
Bilateria - ps16
Deuterostomia - ps17
Chordata - ps18
Olfactores - ps19
Vertebrata - ps20
Gnathostomata - ps21
Euteleostomi - ps22
Sarcopterygii - ps23
Tetrapoda - ps24
Amniota - ps25
Mammalia - ps26
Theria - ps27
Placentalia - ps28
Boreoeutheria - ps29
Euarchontoglires - ps30
Euarchonta - ps31
Primates - ps32
Haplorrhini - ps33
Simiiformes - ps34
Catarrhini - ps35
Hominoidea - ps36
Hominidae - ps37
Homininae - ps38
Hominini - ps39
Homo_sapiens - ps40
Phylostratum

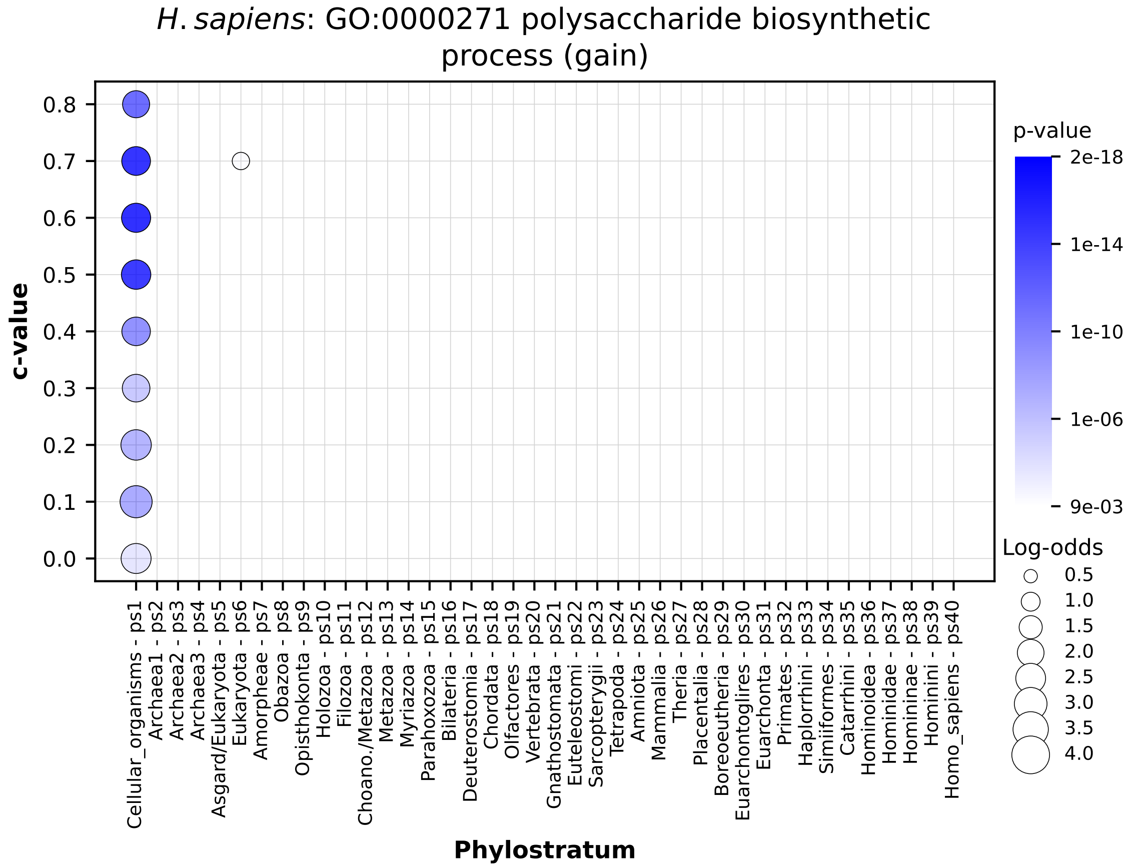

H. sapiens: GO:0000271 polysaccharide biosynthetic process (gain)
c-value
0.0
0.1
0.2
0.3
0.4
0.5
0.6
0.7
0.8
Phylostratum
Cellular_organisms - ps1
Archaea1 - ps2
Archaea2 - ps3
Archaea3 - ps4
Asgard/Eukaryota - ps5
Eukaryota - ps6
Amorpheae - ps7
Obazoa - ps8
Opisthokonta - ps9
Holozoa - ps10
Filozoa - ps11
Choano./Metazoa - ps12
Metazoa - ps13
Myriazoa - ps14
Parahoxozoa - ps15
Bilateria - ps16
Deuterostomia - ps17
Chordata - ps18
Olfactores - ps19
Vertebrata - ps20
Gnathostomata - ps21
Euteleostomi - ps22
Sarcopterygii - ps23
Tetrapoda - ps24
Amniota - ps25
Mammalia - ps26
Theria - ps27
Placentalia - ps28
Boreoeutheria - ps29
Euarchontoglires - ps30
Euarchonta - ps31
Primates - ps32
Haplorrhini - ps33
Simiiformes - ps34
Catarrhini - ps35
Hominoidea - ps36
Hominidae - ps37
Homininae - ps38
Hominini - ps39
Homo_sapiens - ps40
p-value
2e-18
1e-14
1e-10
1e-06
9e-03
Log-odds
0.5
1.0
1.5
2.0
2.5
3.0
3.5
4.0

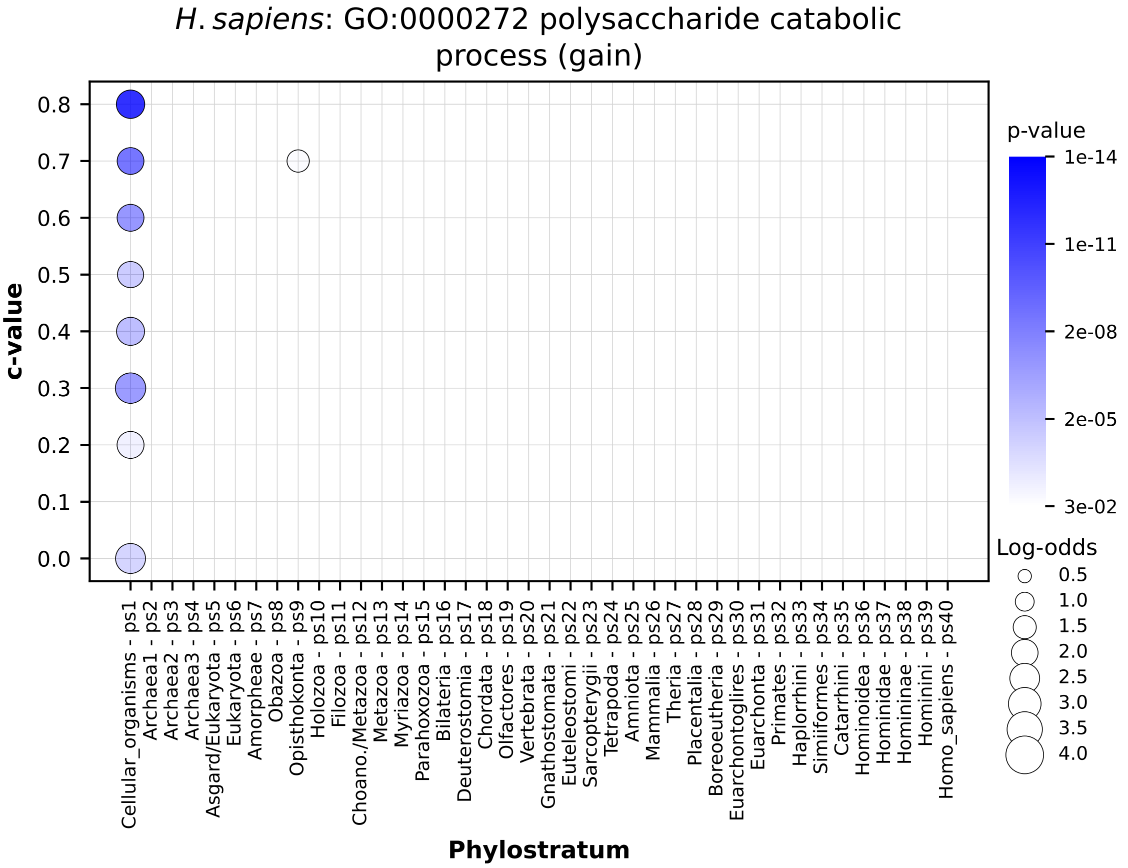
H. sapiens: GO:0000272 polysaccharide catabolic process (gain)
c-value
0.8
0.7
0.6
0.5
0.4
0.3
0.2
0.1
0.0
Phylostratum
Cellular_organisms - ps1
Archaea1 - ps2
Archaea2 - ps3
Archaea3 - ps4
Asgard/Eukaryota - ps5
Eukaryota - ps6
Amorpheae - ps7
Obazoa - ps8
Opisthokonta - ps9
Holozoa - ps10
Filozoa - ps11
Choano./Metazoa - ps12
Metazoa - ps13
Myriazoa - ps14
Parahoxozoa - ps15
Bilateria - ps16
Deuterostomia - ps17
Chordata - ps18
Olfactores - ps19
Vertebrata - ps20
Gnathostomata - ps21
Euteleostomi - ps22
Sarcopterygii - ps23
Tetrapoda - ps24
Amniota - ps25
Mammalia - ps26
Theria - ps27
Placentalia - ps28
Boreoeutheria - ps29
Euarchontoglires - ps30
Euarchonta - ps31
Primates - ps32
Haplorrhini - ps33
Simiiformes - ps34
Catarrhini - ps35
Hominoidea - ps36
Hominidae - ps37
Homininae - ps38
Hominini - ps39
Homo_sapiens - ps40
p-value
1e-14
1e-11
2e-08
2e-05
3e-02
Log-odds
0.5
1.0
1.5
2.0
2.5
3.0
3.5
4.0

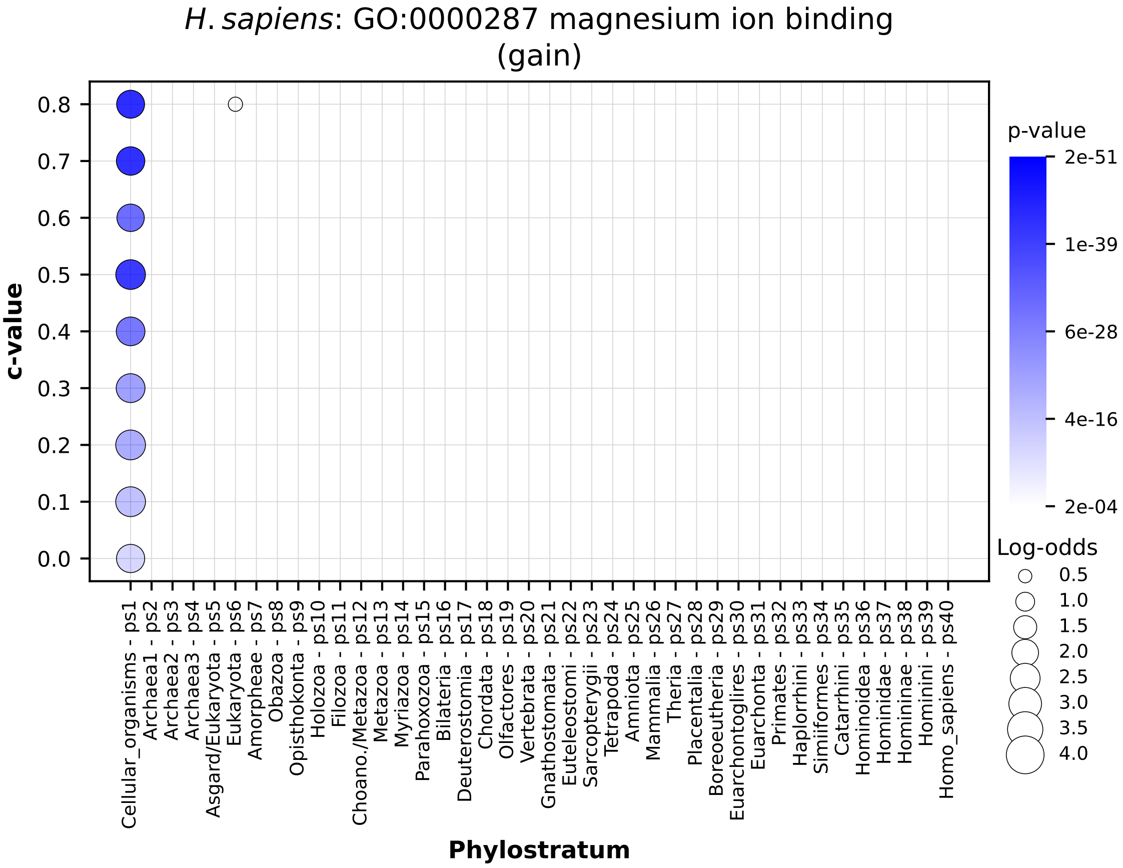

H. sapiens: GO:0000287 magnesium ion binding
(gain)
c-value
0.8
0.7
0.6
0.5
0.4
0.3
0.2
0.1
0.0
p-value
2e-51
1e-39
6e-28
4e-16
2e-04
Log-odds
0.5
1.0
1.5
2.0
2.5
3.0
3.5
4.0
Cellular_organisms - ps1
Archaea1 - ps2
Archaea2 - ps3
Archaea3 - ps4
Asgard/Eukaryota - ps5
Eukaryota - ps6
Amorpheae - ps7
Obazoa - ps8
Opisthokonta - ps9
Holozoa - ps10
Filozoa - ps11
Choano./Metazoa - ps12
Metazoa - ps13
Myriazoa - ps14
Parahoxozoa - ps15
Bilateria - ps16
Deuterostomia - ps17
Chordata - ps18
Olfactores - ps19
Vertebrata - ps20
Gnathostomata - ps21
Euteleostomi - ps22
Sarcopterygii - ps23
Tetrapoda - ps24
Amniota - ps25
Mammalia - ps26
Theria - ps27
Placentalia - ps28
Boreoeutheria - ps29
Euarchontoglires - ps30
Euarchonta - ps31
Primates - ps32
Haplorrhini - ps33
Simiiformes - ps34
Catarrhini - ps35
Hominoidea - ps36
Hominidae - ps37
Homininae - ps38
Hominini - ps39
Homo_sapiens - ps40
Phylostratum

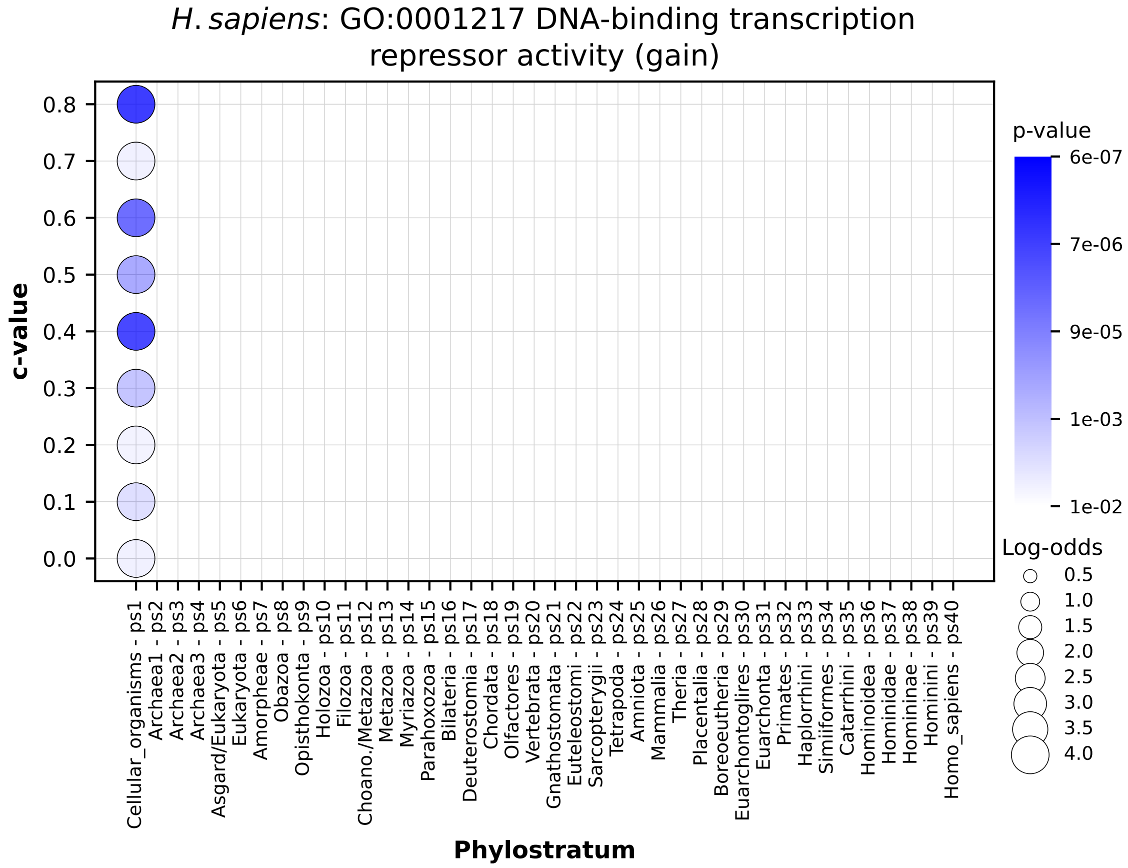

H. sapiens: GO:0001217 DNA-binding transcription repressor activity (gain)
c-value
0.8
0.7
0.6
0.5
0.4
0.3
0.2
0.1
0.0
p-value
6e-07
7e-06
9e-05
1e-03
1e-02
Log-odds
0.5
1.0
1.5
2.0
2.5
3.0
3.5
4.0
Cellular_organisms - ps1
Archaea1 - ps2
Archaea2 - ps3
Archaea3 - ps4
Asgard/Eukaryota - ps5
Eukaryota - ps6
Amorpheae - ps7
Obazoa - ps8
Opisthokonta - ps9
Holozoa - ps10
Filozoa - ps11
Choano./Metazoa - ps12
Metazoa - ps13
Myriazoa - ps14
Parahoxozoa - ps15
Bilateria - ps16
Deuterostomia - ps17
Chordata - ps18
Olfactores - ps19
Vertebrata - ps20
Gnathostomata - ps21
Euteleostomi - ps22
Sarcopterygii - ps23
Tetrapoda - ps24
Amniota - ps25
Mammalia - ps26
Theria - ps27
Placentalia - ps28
Boreoeutheria - ps29
Euarchontoglires - ps30
Euarchonta - ps31
Primates - ps32
Haplorrhini - ps33
Simiiformes - ps34
Catarrhini - ps35
Hominoidea - ps36
Hominidae - ps37
Homininae - ps38
Hominini - ps39
Homo_sapiens - ps40
Phylostratum

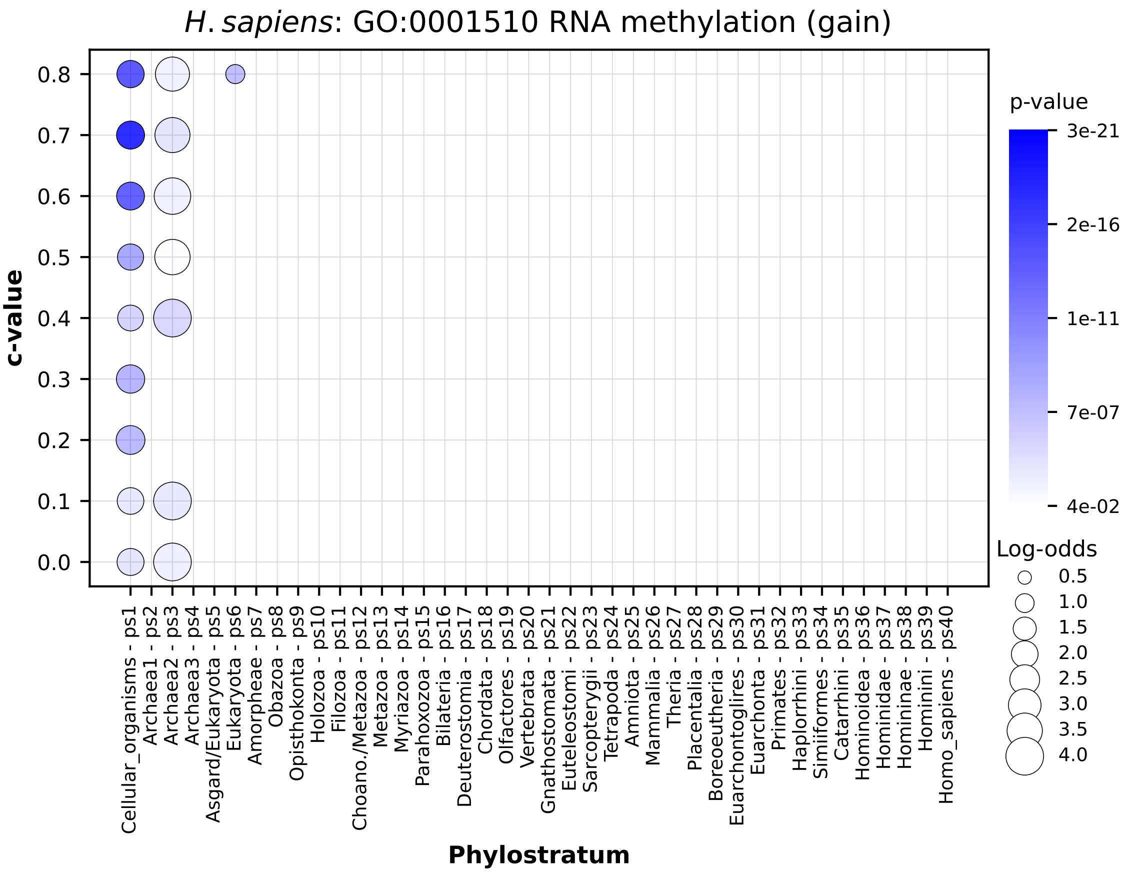

H. sapiens: GO:0001510 RNA methylation (gain)
c-value
0.0
0.1
0.2
0.3
0.4
0.5
0.6
0.7
0.8
Phylostratum
Cellular_organisms - ps1
Archaea1 - ps2
Archaea2 - ps3
Archaea3 - ps4
Asgard/Eukaryota - ps5
Eukaryota - ps6
Amorpheae - ps7
Obazoa - ps8
Opisthokonta - ps9
Holozoa - ps10
Filozoa - ps11
Choano./Metazoa - ps12
Metazoa - ps13
Myriazoa - ps14
Parahoxozoa - ps15
Bilateria - ps16
Deuterostomia - ps17
Chordata - ps18
Olfactores - ps19
Vertebrata - ps20
Gnathostomata - ps21
Euteleostomi - ps22
Sarcopterygii - ps23
Tetrapoda - ps24
Amniota - ps25
Mammalia - ps26
Theria - ps27
Placentalia - ps28
Boreoeutheria - ps29
Euarchontoglires - ps30
Euarchonta - ps31
Primates - ps32
Haplorrhini - ps33
Simiiformes - ps34
Catarrhini - ps35
Hominoidea - ps36
Hominidae - ps37
Homininae - ps38
Hominini - ps39
Homo_sapiens - ps40
p-value
3e-21
2e-16
1e-11
7e-07
4e-02
Log-odds
0.5
1.0
1.5
2.0
2.5
3.0
3.5
4.0

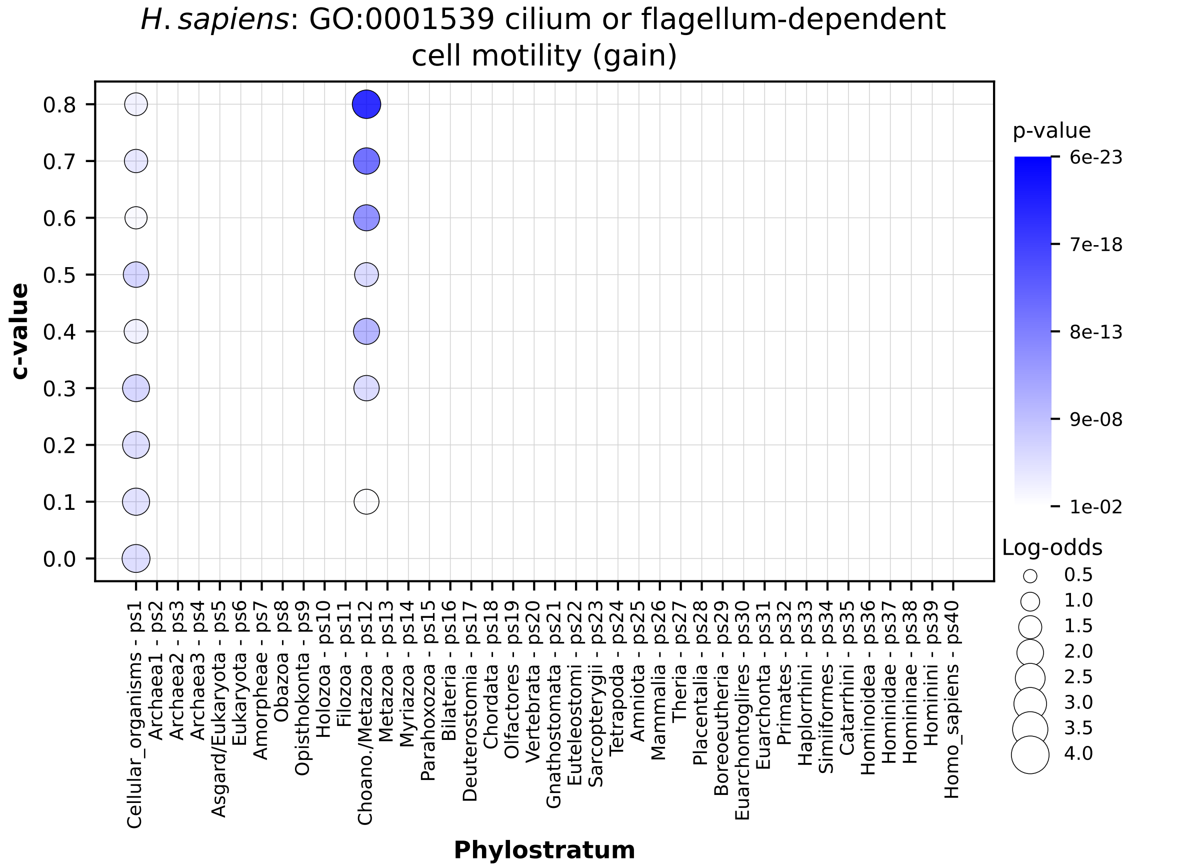

H. sapiens: GO:0001539 cilium or flagellum-dependent cell motility (gain)
c-value
0.8
0.7
0.6
0.5
0.4
0.3
0.2
0.1
0.0
p-value
6e-23
7e-18
8e-13
9e-08
1e-02
Log-odds
0.5
1.0
1.5
2.0
2.5
3.0
3.5
4.0
Cellular_organisms - ps1
Archaea1 - ps2
Archaea2 - ps3
Archaea3 - ps4
Asgard/Eukaryota - ps5
Eukaryota - ps6
Amorpheae - ps7
Obazoa - ps8
Opisthokonta - ps9
Holozoa - ps10
Filozoa - ps11
Choano./Metazoa - ps12
Metazoa - ps13
Myriazoa - ps14
Parahoxozoa - ps15
Bilateria - ps16
Deuterostomia - ps17
Chordata - ps18
Olfactores - ps19
Vertebrata - ps20
Gnathostomata - ps21
Euteleostomi - ps22
Sarcopterygii - ps23
Tetrapoda - ps24
Amniota - ps25
Mammalia - ps26
Theria - ps27
Placentalia - ps28
Boreoeutheria - ps29
Euarchontoglires - ps30
Euarchonta - ps31
Primates - ps32
Haplorrhini - ps33
Simiiformes - ps34
Catarrhini - ps35
Hominoidea - ps36
Hominidae - ps37
Homininae - ps38
Hominini - ps39
Homo_sapiens - ps40
Phylostratum

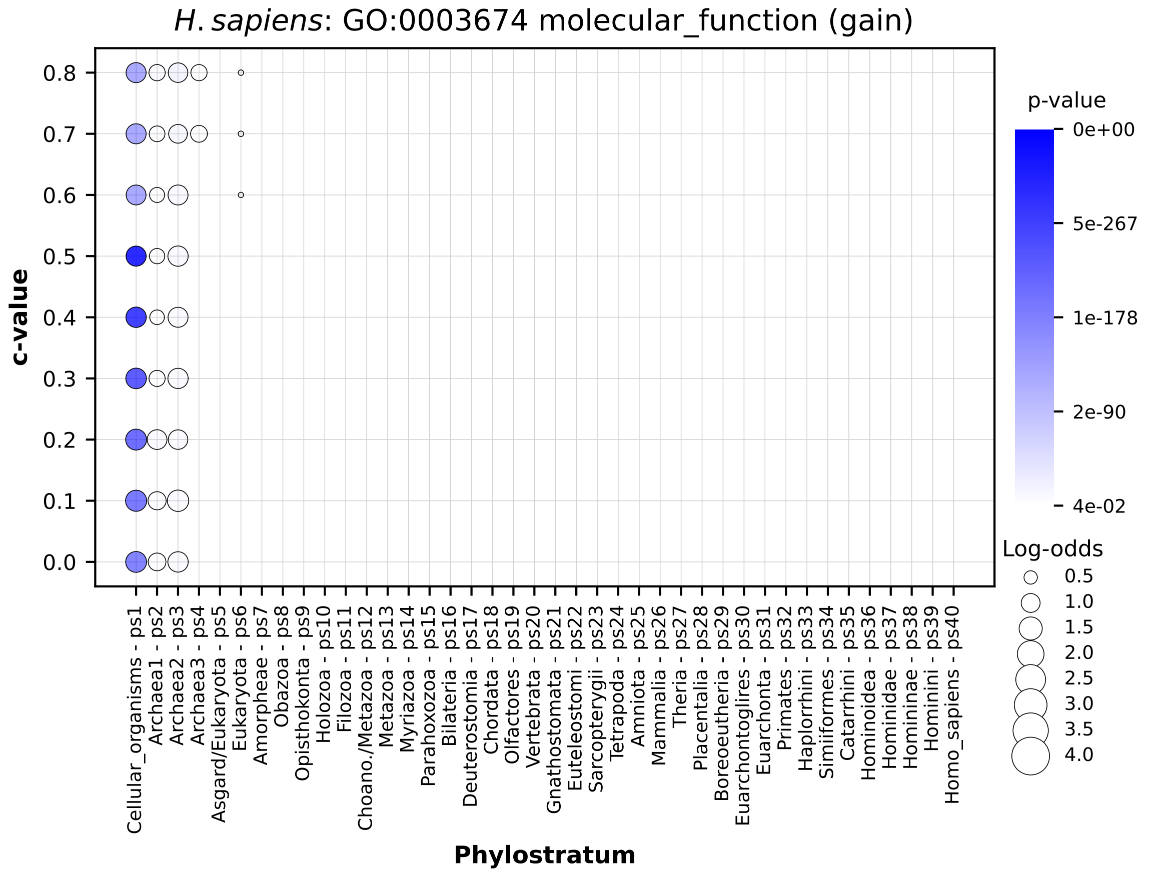

*H. sapiens*: GO:0003674 molecular_function (gain)
c-value
0.8
0.7
0.6
0.5
0.4
0.3
0.2
0.1
0.0
p-value
0e+00
5e-267
1e-178
2e-90
4e-02
Log-odds
0.5
1.0
1.5
2.0
2.5
3.0
3.5
4.0
Cellular_organisms - ps1
Archaea1 - ps2
Archaea2 - ps3
Archaea3 - ps4
Asgard/Eukaryota - ps5
Eukaryota - ps6
Amorpheae - ps7
Obazoa - ps8
Opisthokonta - ps9
Holozoa - ps10
Filozoa - ps11
Choano./Metazoa - ps12
Metazoa - ps13
Myriazoa - ps14
Parahoxozoa - ps15
Bilateria - ps16
Deuterostomia - ps17
Chordata - ps18
Olfactores - ps19
Vertebrata - ps20
Gnathostomata - ps21
Euteleostomi - ps22
Sarcopterygii - ps23
Tetrapoda - ps24
Amniota - ps25
Mammalia - ps26
Theria - ps27
Placentalia - ps28
Boreoeutheria - ps29
Euarchontoglires - ps30
Euarchonta - ps31
Primates - ps32
Haplorrhini - ps33
Simiiformes - ps34
Catarrhini - ps35
Hominoidea - ps36
Hominidae - ps37
Homininae - ps38
Hominini - ps39
Homo_sapiens - ps40
Phylostratum

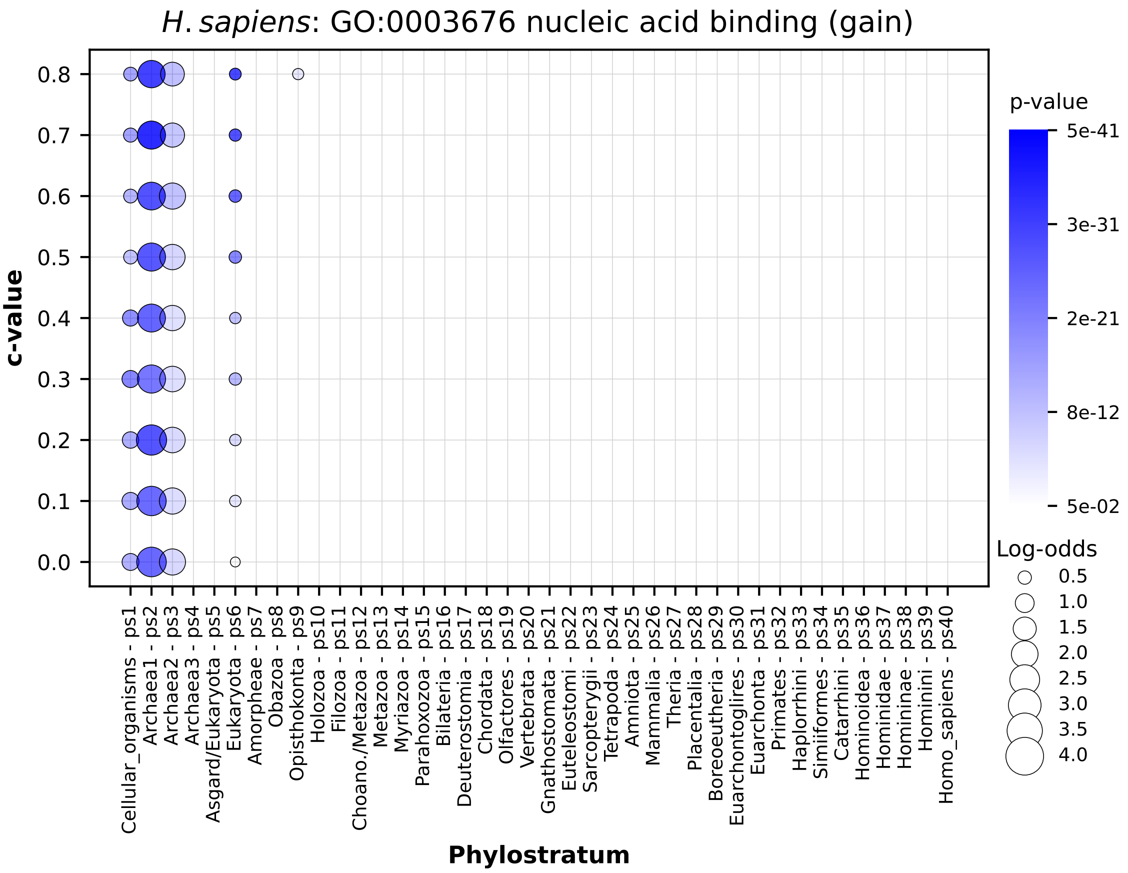
H. sapiens: GO:0003676 nucleic acid binding (gain)
c-value
0.8
0.7
0.6
0.5
0.4
0.3
0.2
0.1
0.0
Phylostratum
Cellular_organisms - ps1
Archaea1 - ps2
Archaea2 - ps3
Archaea3 - ps4
Asgard/Eukaryota - ps5
Eukaryota - ps6
Amorpheae - ps7
Obazoa - ps8
Opisthokonta - ps9
Holozoa - ps10
Filozoa - ps11
Choano./Metazoa - ps12
Metazoa - ps13
Myriazoa - ps14
Parahoxozoa - ps15
Bilateria - ps16
Deuterostomia - ps17
Chordata - ps18
Olfactores - ps19
Vertebrata - ps20
Gnathostomata - ps21
Euteleostomi - ps22
Sarcopterygii - ps23
Tetrapoda - ps24
Amniota - ps25
Mammalia - ps26
Theria - ps27
Placentalia - ps28
Boreoeutheria - ps29
Euarchontoglires - ps30
Euarchonta - ps31
Primates - ps32
Haplorrhini - ps33
Simiiformes - ps34
Catarrhini - ps35
Hominoidea - ps36
Hominidae - ps37
Homininae - ps38
Hominini - ps39
Homo_sapiens - ps40
p-value
5e-41
3e-31
2e-21
8e-12
5e-02
Log-odds
0.5
1.0
1.5
2.0
2.5
3.0
3.5
4.0

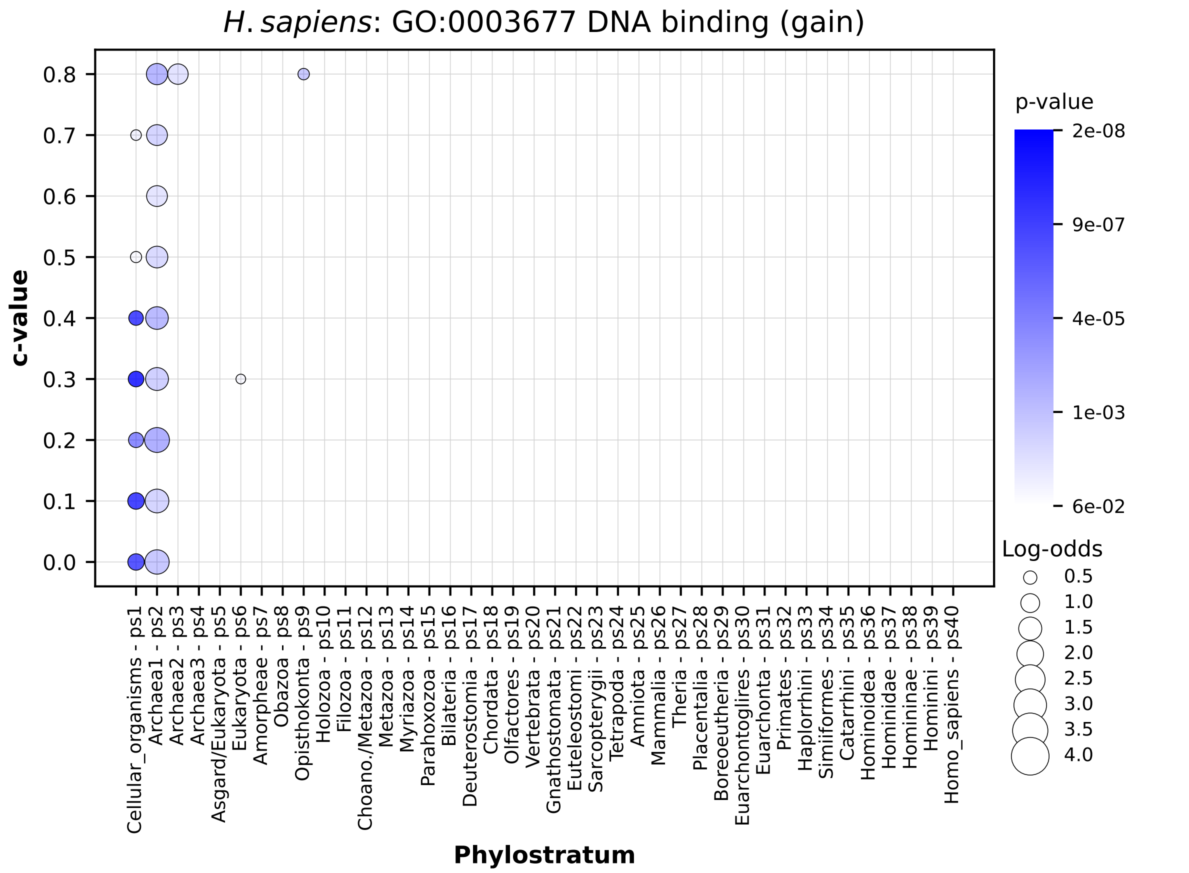
*H. sapiens*: GO:0003677 DNA binding (gain)
c-value
0.0
0.1
0.2
0.3
0.4
0.5
0.6
0.7
0.8
Phylostratum
Cellular_organisms - ps1
Archaea1 - ps2
Archaea2 - ps3
Archaea3 - ps4
Asgard/Eukaryota - ps5
Eukaryota - ps6
Amorpheae - ps7
Obazoa - ps8
Opisthokonta - ps9
Holozoa - ps10
Filozoa - ps11
Choano./Metazoa - ps12
Metazoa - ps13
Myriazoa - ps14
Parahoxozoa - ps15
Bilateria - ps16
Deuterostomia - ps17
Chordata - ps18
Olfactores - ps19
Vertebrata - ps20
Gnathostomata - ps21
Euteleostomi - ps22
Sarcopterygii - ps23
Tetrapoda - ps24
Amniota - ps25
Mammalia - ps26
Theria - ps27
Placentalia - ps28
Boreoeutheria - ps29
Euarchontoglires - ps30
Euarchonta - ps31
Primates - ps32
Haplorrhini - ps33
Simiiformes - ps34
Catarrhini - ps35
Hominoidea - ps36
Hominidae - ps37
Homininae - ps38
Hominini - ps39
Homo_sapiens - ps40
p-value
2e-08
9e-07
4e-05
1e-03
6e-02
Log-odds
0.5
1.0
1.5
2.0
2.5
3.0
3.5
4.0

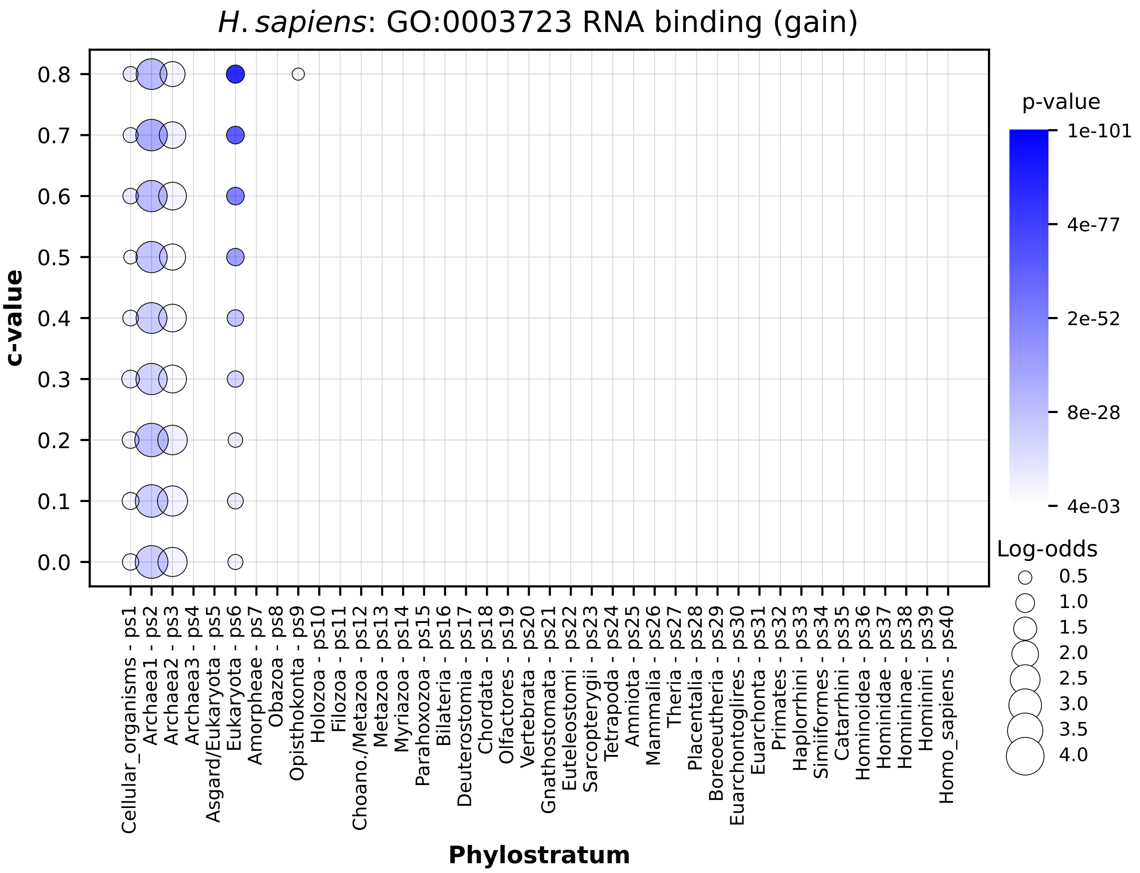

H. sapiens: GO:0003723 RNA binding (gain)
c-value
0.8
0.7
0.6
0.5
0.4
0.3
0.2
0.1
0.0
p-value
1e-101
4e-77
2e-52
8e-28
4e-03
Log-odds
0.5
1.0
1.5
2.0
2.5
3.0
3.5
4.0
Cellular_organisms - ps1
Archaea1 - ps2
Archaea2 - ps3
Archaea3 - ps4
Asgard/Eukaryota - ps5
Eukaryota - ps6
Amorpheae - ps7
Obazoa - ps8
Opisthokonta - ps9
Holozoa - ps10
Filozoa - ps11
Choano./Metazoa - ps12
Metazoa - ps13
Myriazoa - ps14
Parahoxozoa - ps15
Bilateria - ps16
Deuterostomia - ps17
Chordata - ps18
Olfactores - ps19
Vertebrata - ps20
Gnathostomata - ps21
Euteleostomi - ps22
Sarcopterygii - ps23
Tetrapoda - ps24
Amniota - ps25
Mammalia - ps26
Theria - ps27
Placentalia - ps28
Boreoeutheria - ps29
Euarchontoglires - ps30
Euarchonta - ps31
Primates - ps32
Haplorrhini - ps33
Simiiformes - ps34
Catarrhini - ps35
Hominoidea - ps36
Hominidae - ps37
Homininae - ps38
Hominini - ps39
Homo_sapiens - ps40
Phylostratum

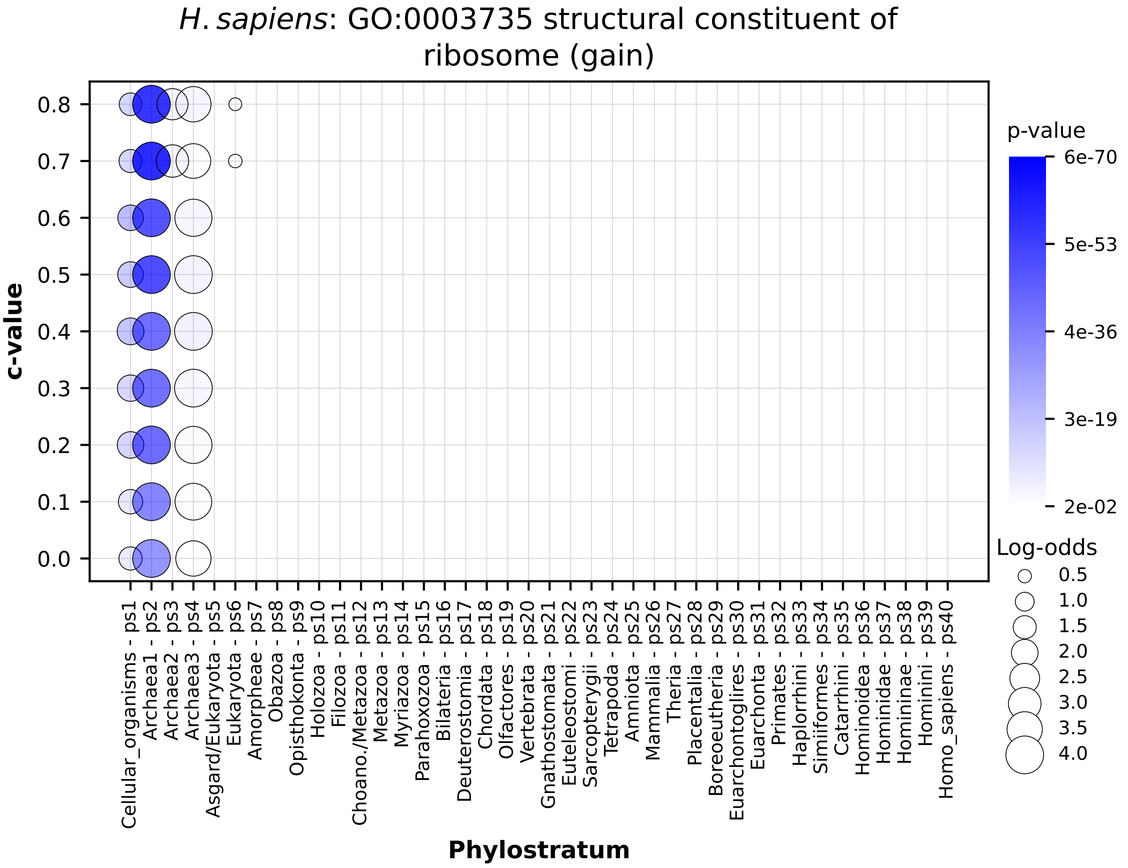

H. sapiens: GO:0003735 structural constituent of ribosome (gain)
c-value
0.8
0.7
0.6
0.5
0.4
0.3
0.2
0.1
0.0
Phylostratum
Cellular_organisms - ps1
Archaea1 - ps2
Archaea2 - ps3
Archaea3 - ps4
Asgard/Eukaryota - ps5
Eukaryota - ps6
Amorpheae - ps7
Obazoa - ps8
Opisthokonta - ps9
Holozoa - ps10
Filozoa - ps11
Choano./Metazoa - ps12
Metazoa - ps13
Myriazoa - ps14
Parahoxozoa - ps15
Bilateria - ps16
Deuterostomia - ps17
Chordata - ps18
Olfactores - ps19
Vertebrata - ps20
Gnathostomata - ps21
Euteleostomi - ps22
Sarcopterygii - ps23
Tetrapoda - ps24
Amniota - ps25
Mammalia - ps26
Theria - ps27
Placentalia - ps28
Boreoeutheria - ps29
Euarchontoglires - ps30
Euarchonta - ps31
Primates - ps32
Haplorrhini - ps33
Simiiformes - ps34
Catarrhini - ps35
Hominoidea - ps36
Hominidae - ps37
Homininae - ps38
Hominini - ps39
Homo_sapiens - ps40
p-value
6e-70
5e-53
4e-36
3e-19
2e-02
Log-odds
0.5
1.0
1.5
2.0
2.5
3.0
3.5
4.0

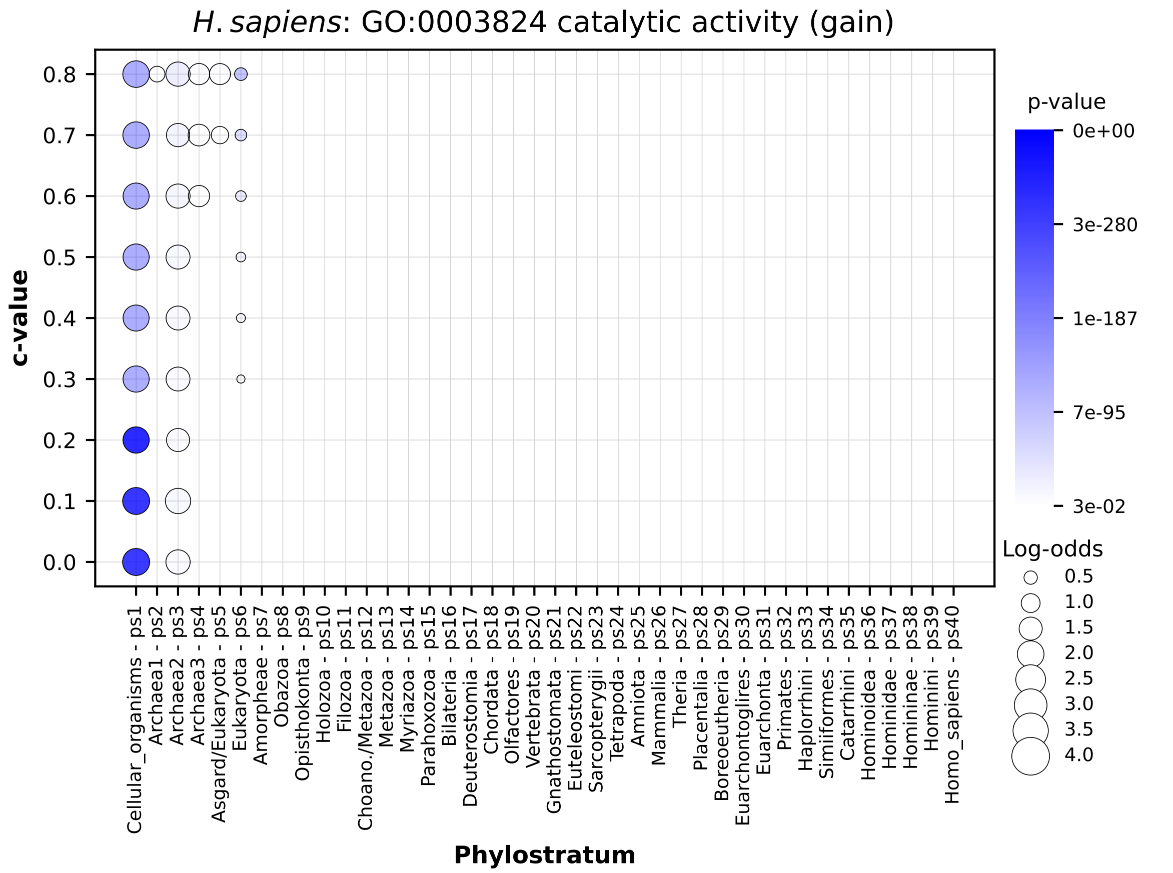
H. sapiens: GO:0003824 catalytic activity (gain)
c-value
0.8
0.7
0.6
0.5
0.4
0.3
0.2
0.1
0.0
Phylostratum
Cellular_organisms - ps1
Archaea1 - ps2
Archaea2 - ps3
Archaea3 - ps4
Asgard/Eukaryota - ps5
Eukaryota - ps6
Amorpheae - ps7
Obazoa - ps8
Opisthokonta - ps9
Holozoa - ps10
Filozoa - ps11
Choano./Metazoa - ps12
Metazoa - ps13
Myriazoa - ps14
Parahoxozoa - ps15
Bilateria - ps16
Deuterostomia - ps17
Chordata - ps18
Olfactores - ps19
Vertebrata - ps20
Gnathostomata - ps21
Euteleostomi - ps22
Sarcopterygii - ps23
Tetrapoda - ps24
Amniota - ps25
Mammalia - ps26
Theria - ps27
Placentalia - ps28
Boreoeutheria - ps29
Euarchontoglires - ps30
Euarchonta - ps31
Primates - ps32
Haplorrhini - ps33
Simiiformes - ps34
Catarrhini - ps35
Hominoidea - ps36
Hominidae - ps37
Homininae - ps38
Hominini - ps39
Homo_sapiens - ps40
p-value
0e+00
3e-280
1e-187
7e-95
3e-02
Log-odds
0.5
1.0
1.5
2.0
2.5
3.0
3.5
4.0

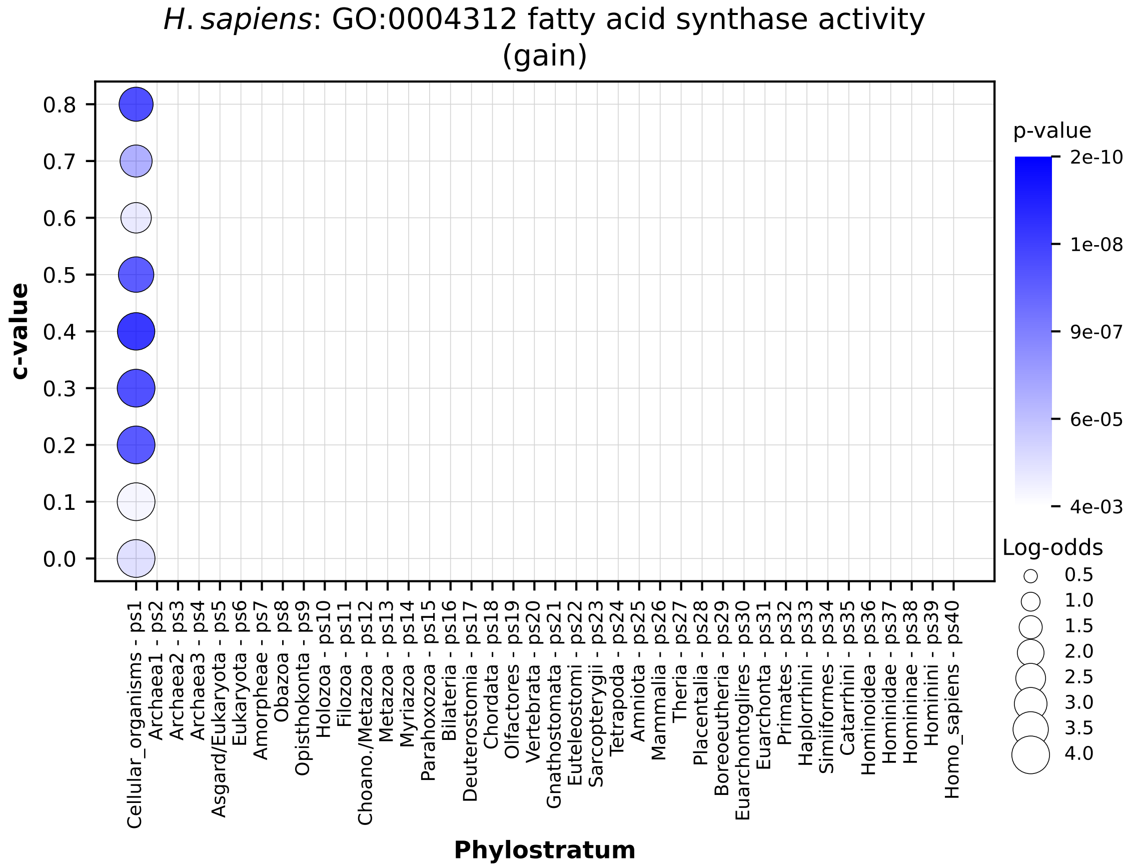

H. sapiens: GO:0004312 fatty acid synthase activity
(gain)
c-value
0.8
0.7
0.6
0.5
0.4
0.3
0.2
0.1
0.0
Phylostratum
Cellular_organisms - ps1
Archaea1 - ps2
Archaea2 - ps3
Archaea3 - ps4
Asgard/Eukaryota - ps5
Eukaryota - ps6
Amorpheae - ps7
Obazoa - ps8
Opisthokonta - ps9
Holozoa - ps10
Filozoa - ps11
Choano./Metazoa - ps12
Metazoa - ps13
Myriazoa - ps14
Parahoxozoa - ps15
Bilateria - ps16
Deuterostomia - ps17
Chordata - ps18
Olfactores - ps19
Vertebrata - ps20
Gnathostomata - ps21
Euteleostomi - ps22
Sarcopterygii - ps23
Tetrapoda - ps24
Amniota - ps25
Mammalia - ps26
Theria - ps27
Placentalia - ps28
Boreoeutheria - ps29
Euarchontoglires - ps30
Euarchonta - ps31
Primates - ps32
Haplorrhini - ps33
Simiiformes - ps34
Catarrhini - ps35
Hominoidea - ps36
Hominidae - ps37
Homininae - ps38
Hominini - ps39
Homo_sapiens - ps40
p-value
2e-10
1e-08
9e-07
6e-05
4e-03
Log-odds
0.5
1.0
1.5
2.0
2.5
3.0
3.5
4.0

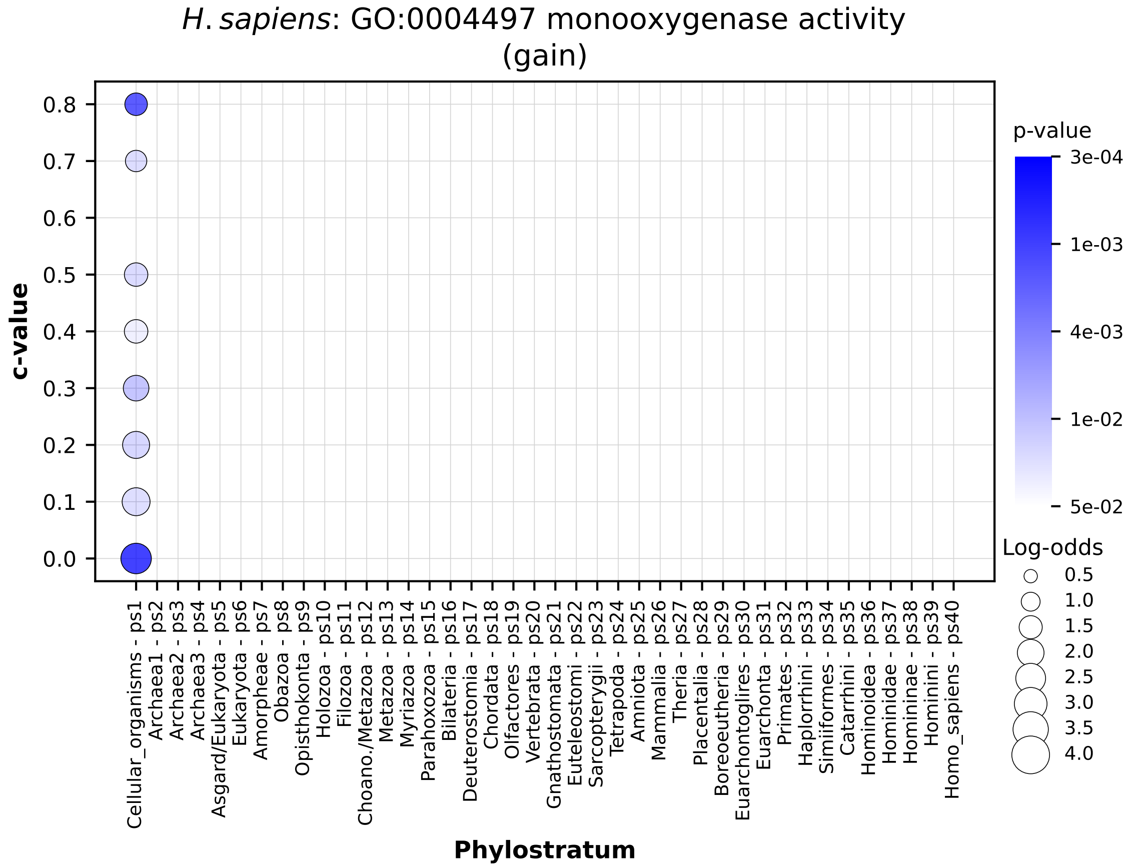

H. sapiens: GO:0004497 monooxygenase activity
(gain)
c-value
0.0
0.1
0.2
0.3
0.4
0.5
0.6
0.7
0.8
Phylostratum
Cellular_organisms - ps1
Archaea1 - ps2
Archaea2 - ps3
Archaea3 - ps4
Asgard/Eukaryota - ps5
Eukaryota - ps6
Amorpheae - ps7
Obazoa - ps8
Opisthokonta - ps9
Holozoa - ps10
Filozoa - ps11
Choano./Metazoa - ps12
Metazoa - ps13
Myriazoa - ps14
Parahoxozoa - ps15
Bilateria - ps16
Deuterostomia - ps17
Chordata - ps18
Olfactores - ps19
Vertebrata - ps20
Gnathostomata - ps21
Euteleostomi - ps22
Sarcopterygii - ps23
Tetrapoda - ps24
Amniota - ps25
Mammalia - ps26
Theria - ps27
Placentalia - ps28
Boreoeutheria - ps29
Euarchontoglires - ps30
Euarchonta - ps31
Primates - ps32
Haplorrhini - ps33
Simiiformes - ps34
Catarrhini - ps35
Hominoidea - ps36
Hominidae - ps37
Homininae - ps38
Hominini - ps39
Homo_sapiens - ps40
p-value
3e-04
1e-03
4e-03
1e-02
5e-02
Log-odds
0.5
1.0
1.5
2.0
2.5
3.0
3.5
4.0

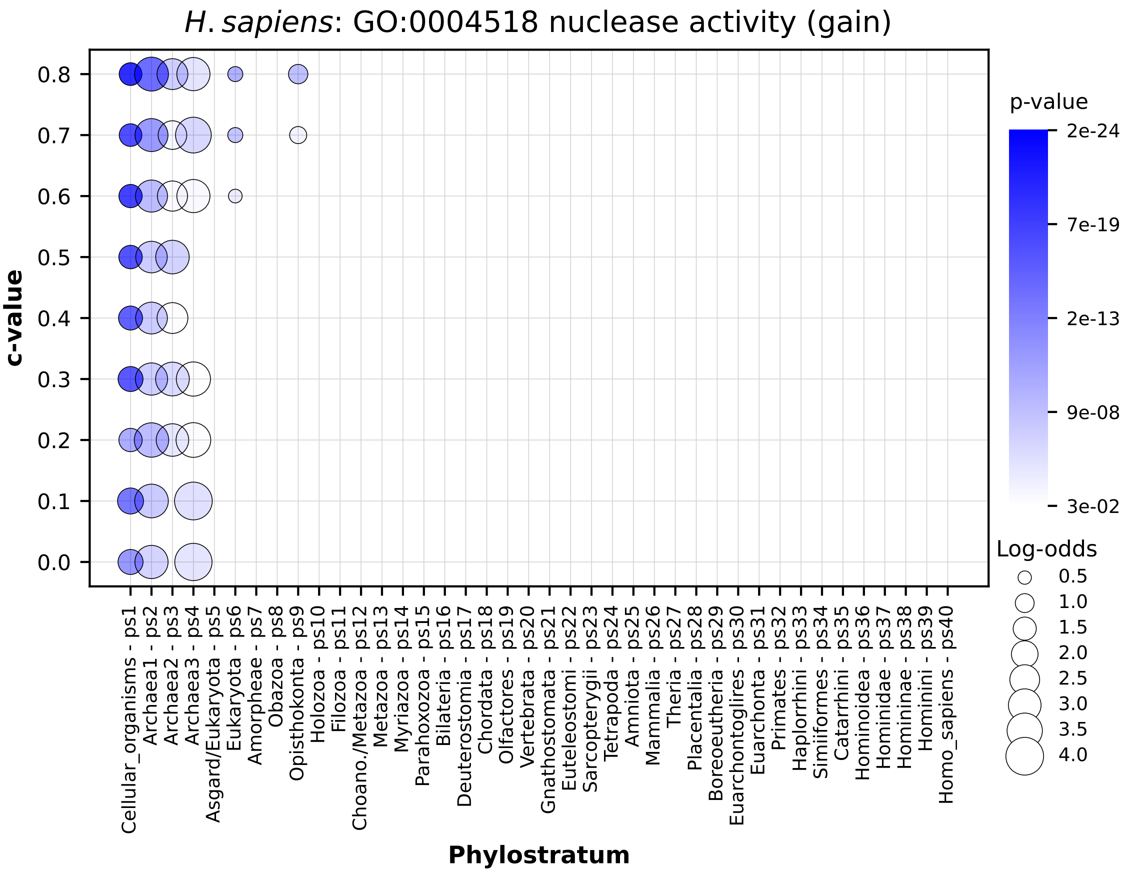

H. sapiens: GO:0004518 nuclease activity (gain)
c-value
0.0
0.1
0.2
0.3
0.4
0.5
0.6
0.7
0.8
Phylostratum
Cellular_organisms - ps1
Archaea1 - ps2
Archaea2 - ps3
Archaea3 - ps4
Asgard/Eukaryota - ps5
Eukaryota - ps6
Amorpheae - ps7
Obazoa - ps8
Opisthokonta - ps9
Holozoa - ps10
Filozoa - ps11
Choano./Metazoa - ps12
Metazoa - ps13
Myriazoa - ps14
Parahoxozoa - ps15
Bilateria - ps16
Deuterostomia - ps17
Chordata - ps18
Olfactores - ps19
Vertebrata - ps20
Gnathostomata - ps21
Euteleostomi - ps22
Sarcopterygii - ps23
Tetrapoda - ps24
Amniota - ps25
Mammalia - ps26
Theria - ps27
Placentalia - ps28
Boreoeutheria - ps29
Euarchontoglires - ps30
Euarchonta - ps31
Primates - ps32
Haplorrhini - ps33
Simiiformes - ps34
Catarrhini - ps35
Hominoidea - ps36
Hominidae - ps37
Homininae - ps38
Hominini - ps39
Homo_sapiens - ps40
p-value
2e-24
7e-19
2e-13
9e-08
3e-02
Log-odds
0.5
1.0
1.5
2.0
2.5
3.0
3.5
4.0

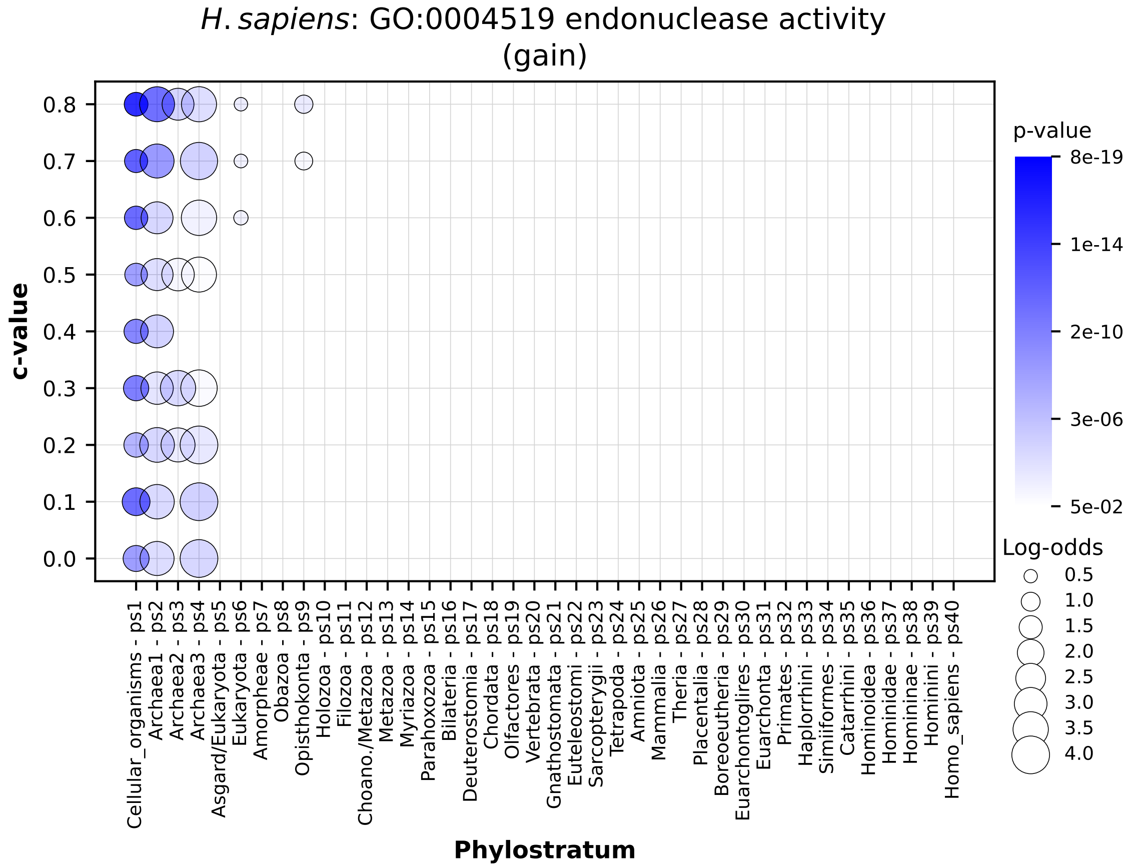
H. sapiens: GO:0004519 endonuclease activity
(gain)
c-value
0.8
0.7
0.6
0.5
0.4
0.3
0.2
0.1
0.0
Phylostratum
Cellular_organisms - ps1
Archaea1 - ps2
Archaea2 - ps3
Archaea3 - ps4
Asgard/Eukaryota - ps5
Eukaryota - ps6
Amorpheae - ps7
Obazoa - ps8
Opisthokonta - ps9
Holozoa - ps10
Filozoa - ps11
Choano./Metazoa - ps12
Metazoa - ps13
Myriazoa - ps14
Parahoxozoa - ps15
Bilateria - ps16
Deuterostomia - ps17
Chordata - ps18
Olfactores - ps19
Vertebrata - ps20
Gnathostomata - ps21
Euteleostomi - ps22
Sarcopterygii - ps23
Tetrapoda - ps24
Amniota - ps25
Mammalia - ps26
Theria - ps27
Placentalia - ps28
Boreoeutheria - ps29
Euarchontoglires - ps30
Euarchonta - ps31
Primates - ps32
Haplorrhini - ps33
Simiiformes - ps34
Catarrhini - ps35
Hominoidea - ps36
Hominidae - ps37
Homininae - ps38
Hominini - ps39
Homo_sapiens - ps40
p-value
8e-19
1e-14
2e-10
3e-06
5e-02
Log-odds
0.5
1.0
1.5
2.0
2.5
3.0
3.5
4.0

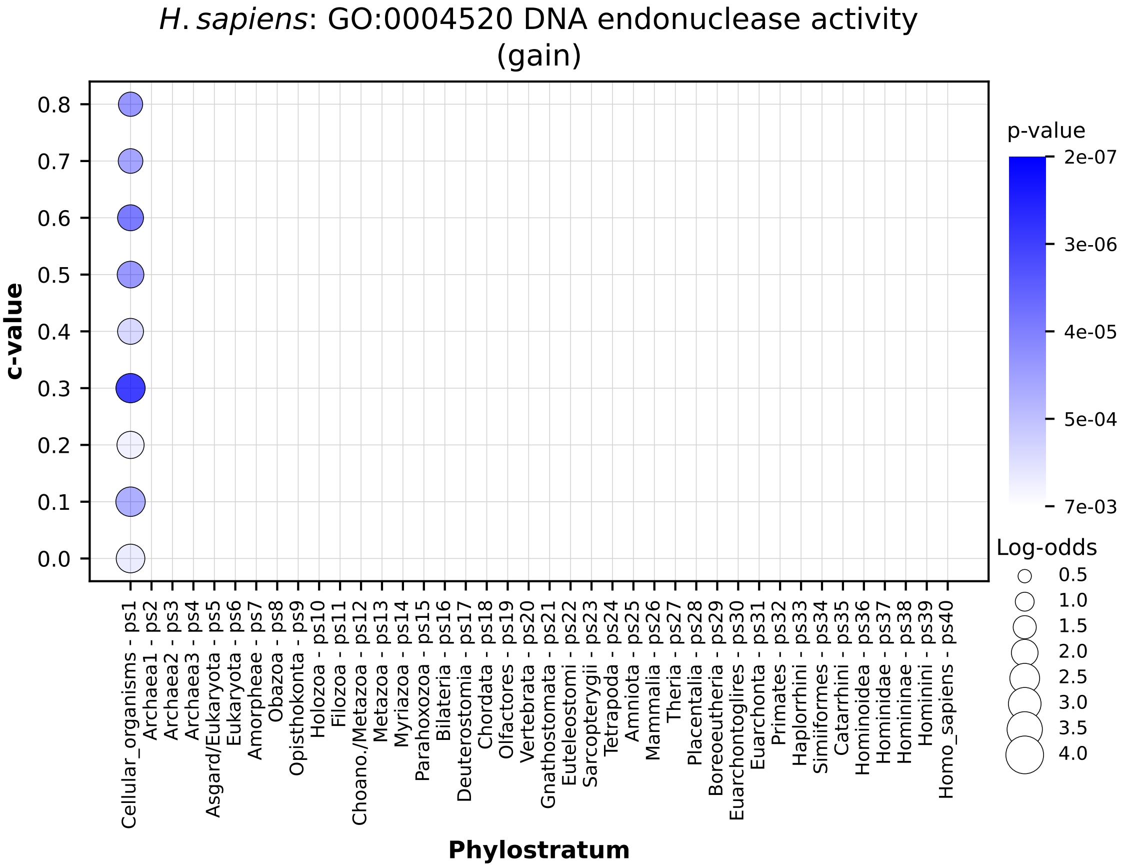

*H. sapiens*: GO:0004520 DNA endonuclease activity (gain)
c-value
0.8
0.7
0.6
0.5
0.4
0.3
0.2
0.1
0.0
p-value
2e-07
3e-06
4e-05
5e-04
7e-03
Log-odds
0.5
1.0
1.5
2.0
2.5
3.0
3.5
4.0
Cellular_organisms - ps1
Archaea1 - ps2
Archaea2 - ps3
Archaea3 - ps4
Asgard/Eukaryota - ps5
Eukaryota - ps6
Amorpheae - ps7
Obazoa - ps8
Opisthokonta - ps9
Holozoa - ps10
Filozoa - ps11
Choano./Metazoa - ps12
Metazoa - ps13
Myriazoa - ps14
Parahoxozoa - ps15
Bilateria - ps16
Deuterostomia - ps17
Chordata - ps18
Olfactores - ps19
Vertebrata - ps20
Gnathostomata - ps21
Euteleostomi - ps22
Sarcopterygii - ps23
Tetrapoda - ps24
Amniota - ps25
Mammalia - ps26
Theria - ps27
Placentalia - ps28
Boreoeutheria - ps29
Euarchontoglires - ps30
Euarchonta - ps31
Primates - ps32
Haplorrhini - ps33
Simiiformes - ps34
Catarrhini - ps35
Hominoidea - ps36
Hominidae - ps37
Homininae - ps38
Hominini - ps39
Homo_sapiens - ps40
Phylostratum

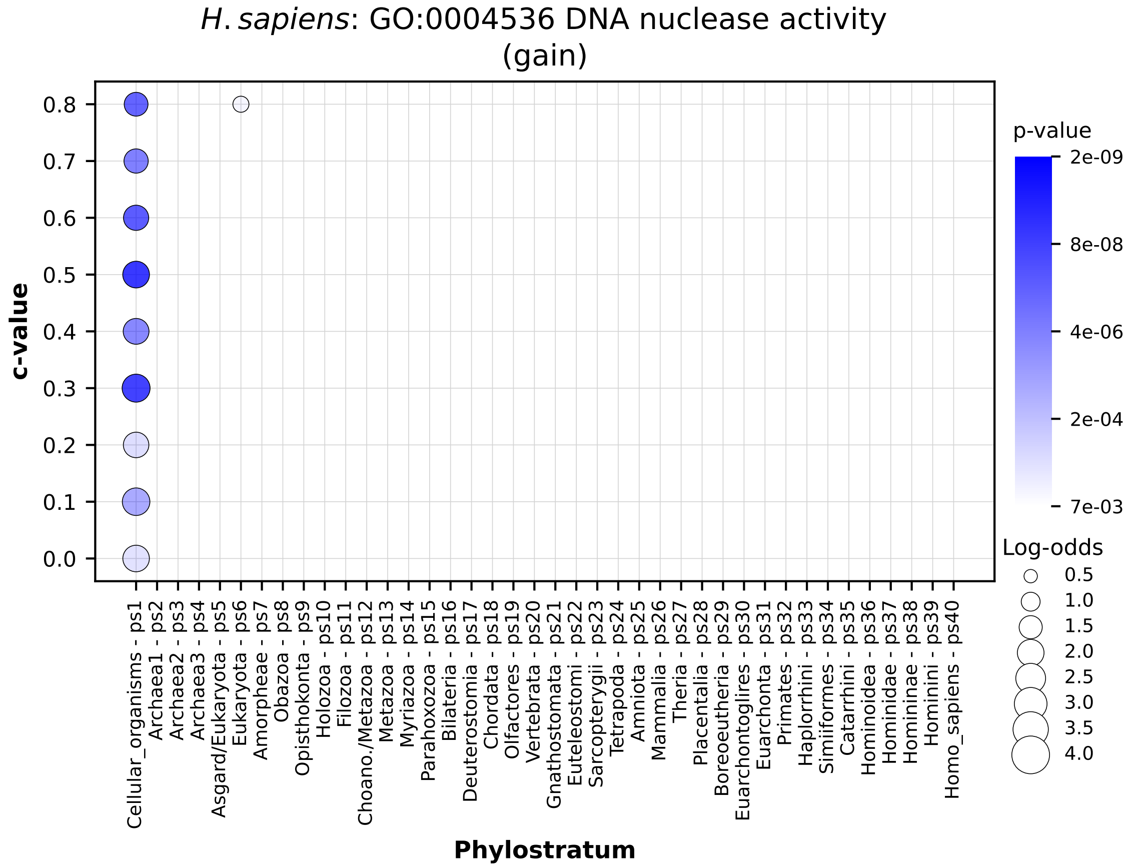

H. sapiens: GO:0004536 DNA nuclease activity
(gain)
c-value
0.8
0.7
0.6
0.5
0.4
0.3
0.2
0.1
0.0
p-value
2e-09
8e-08
4e-06
2e-04
7e-03
Log-odds
0.5
1.0
1.5
2.0
2.5
3.0
3.5
4.0
Cellular_organisms - ps1
Archaea1 - ps2
Archaea2 - ps3
Archaea3 - ps4
Asgard/Eukaryota - ps5
Eukaryota - ps6
Amorpheae - ps7
Obazoa - ps8
Opisthokonta - ps9
Holozoa - ps10
Filozoa - ps11
Choano./Metazoa - ps12
Metazoa - ps13
Myriazoa - ps14
Parahoxozoa - ps15
Bilateria - ps16
Deuterostomia - ps17
Chordata - ps18
Olfactores - ps19
Vertebrata - ps20
Gnathostomata - ps21
Euteleostomi - ps22
Sarcopterygii - ps23
Tetrapoda - ps24
Amniota - ps25
Mammalia - ps26
Theria - ps27
Placentalia - ps28
Boreoeutheria - ps29
Euarchontoglires - ps30
Euarchonta - ps31
Primates - ps32
Haplorrhini - ps33
Simiiformes - ps34
Catarrhini - ps35
Hominoidea - ps36
Hominidae - ps37
Homininae - ps38
Hominini - ps39
Homo_sapiens - ps40
Phylostratum

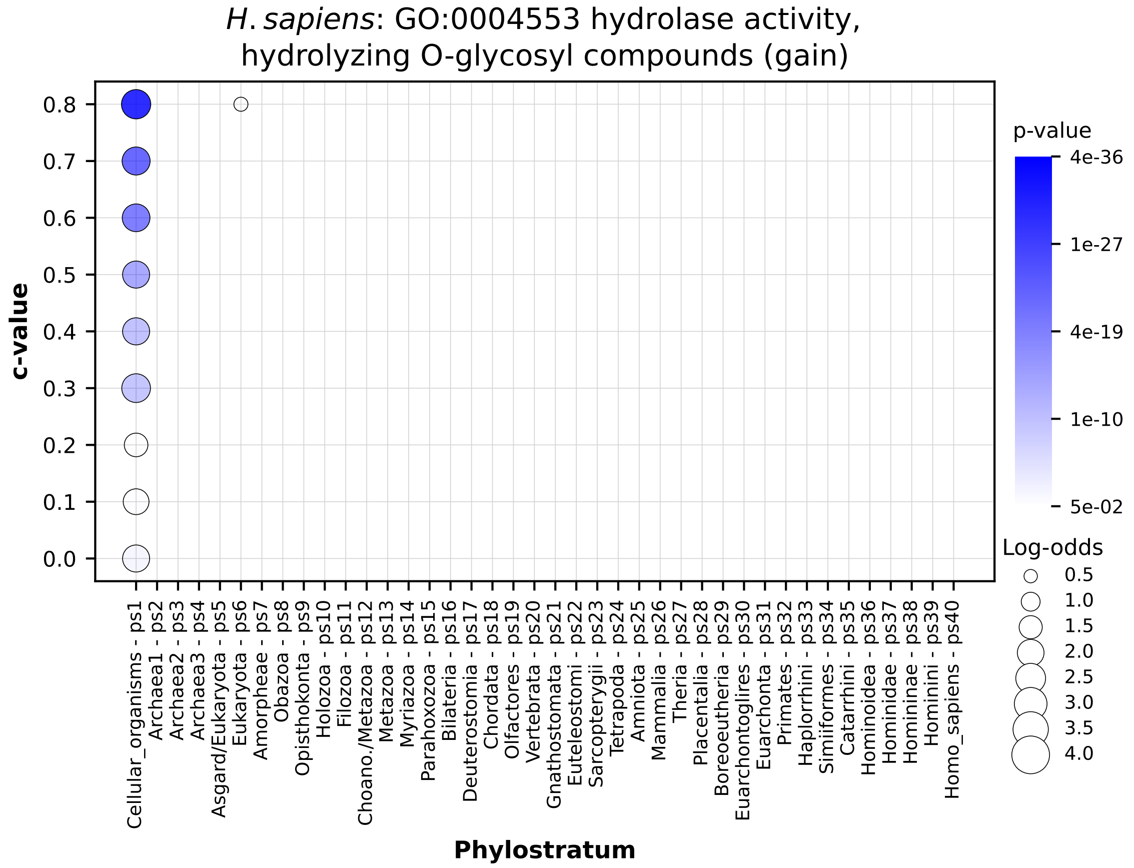

H. sapiens: GO:0004553 hydrolase activity,
hydrolyzing O-glycosyl compounds (gain)
c-value
0.8
0.7
0.6
0.5
0.4
0.3
0.2
0.1
0.0
p-value
4e-36
1e-27
4e-19
1e-10
5e-02
Log-odds
0.5
1.0
1.5
2.0
2.5
3.0
3.5
4.0
Cellular_organisms - ps1
Archaea1 - ps2
Archaea2 - ps3
Archaea3 - ps4
Asgard/Eukaryota - ps5
Eukaryota - ps6
Amorpheae - ps7
Obazoa - ps8
Opisthokonta - ps9
Holozoa - ps10
Filozoa - ps11
Choano./Metazoa - ps12
Metazoa - ps13
Myriazoa - ps14
Parahoxozoa - ps15
Bilateria - ps16
Deuterostomia - ps17
Chordata - ps18
Olfactores - ps19
Vertebrata - ps20
Gnathostomata - ps21
Euteleostomi - ps22
Sarcopterygii - ps23
Tetrapoda - ps24
Amniota - ps25
Mammalia - ps26
Theria - ps27
Placentalia - ps28
Boreoeutheria - ps29
Euarchontoglires - ps30
Euarchonta - ps31
Primates - ps32
Haplorrhini - ps33
Simiiformes - ps34
Catarrhini - ps35
Hominoidea - ps36
Hominidae - ps37
Homininae - ps38
Hominini - ps39
Homo_sapiens - ps40
Phylostratum

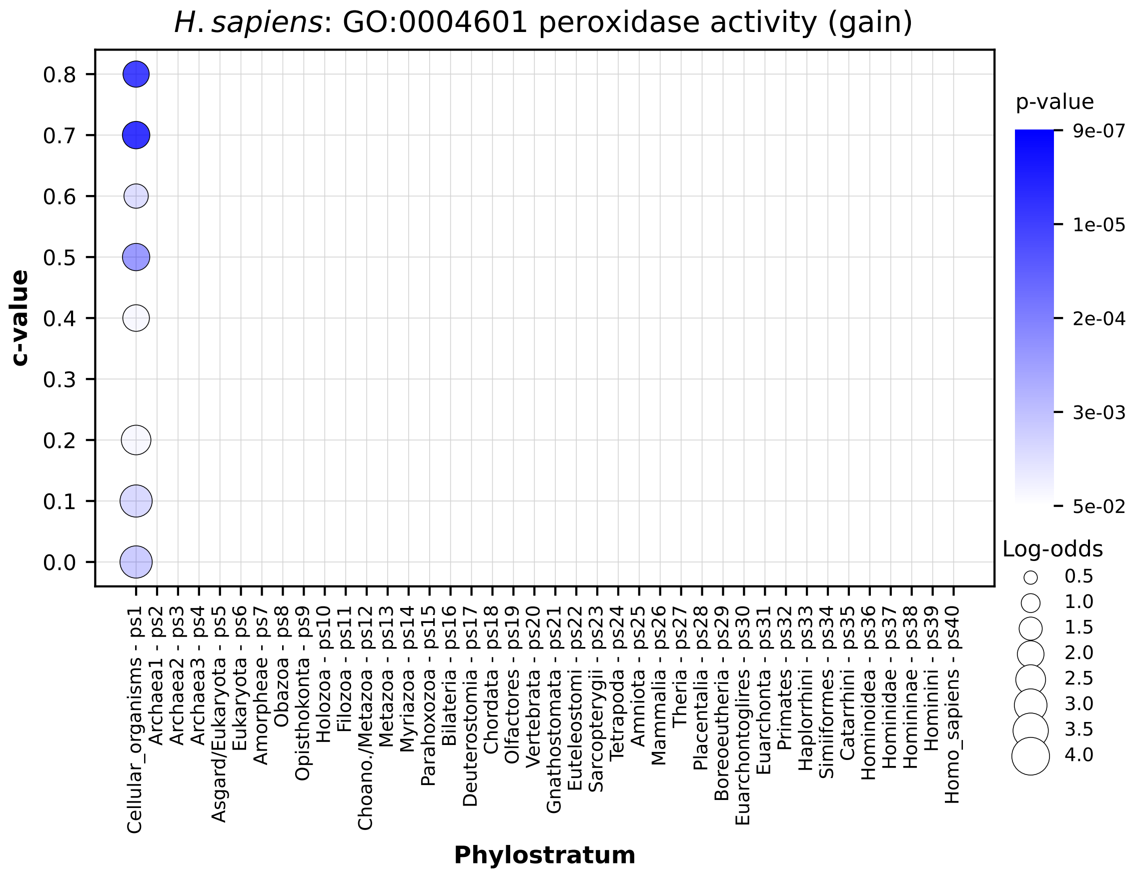
H. sapiens: GO:0004601 peroxidase activity (gain)
c-value
0.8
0.7
0.6
0.5
0.4
0.3
0.2
0.1
0.0
p-value
9e-07
1e-05
2e-04
3e-03
5e-02
Log-odds
0.5
1.0
1.5
2.0
2.5
3.0
3.5
4.0
Cellular_organisms - ps1
Archaea1 - ps2
Archaea2 - ps3
Archaea3 - ps4
Asgard/Eukaryota - ps5
Eukaryota - ps6
Amorpheae - ps7
Obazoa - ps8
Opisthokonta - ps9
Holozoa - ps10
Filozoa - ps11
Choano./Metazoa - ps12
Metazoa - ps13
Myriazoa - ps14
Parahoxozoa - ps15
Bilateria - ps16
Deuterostomia - ps17
Chordata - ps18
Olfactores - ps19
Vertebrata - ps20
Gnathostomata - ps21
Euteleostomi - ps22
Sarcopterygii - ps23
Tetrapoda - ps24
Amniota - ps25
Mammalia - ps26
Theria - ps27
Placentalia - ps28
Boreoeutheria - ps29
Euarchontoglires - ps30
Euarchonta - ps31
Primates - ps32
Haplorrhini - ps33
Simiiformes - ps34
Catarrhini - ps35
Hominoidea - ps36
Hominidae - ps37
Homininae - ps38
Hominini - ps39
Homo_sapiens - ps40
Phylostratum

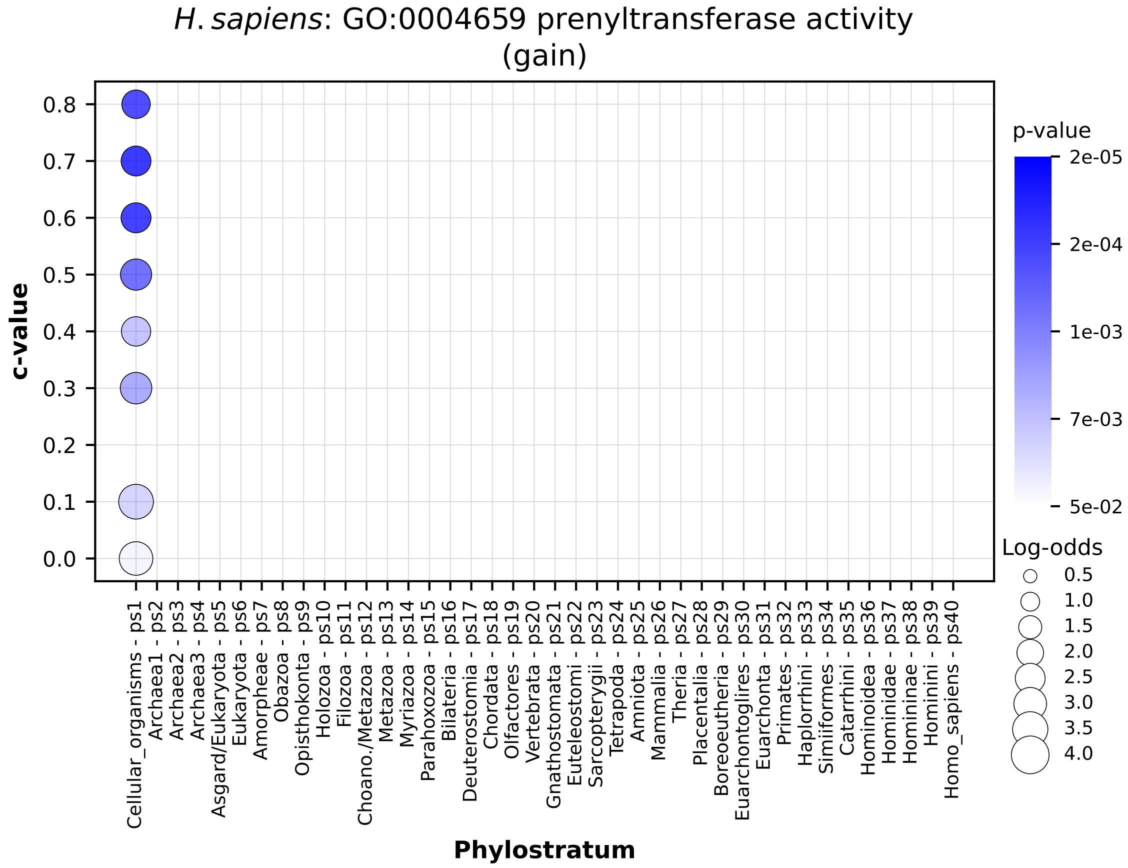

H. sapiens: GO:0004659 prenyltransferase activity (gain)
c-value
0.8
0.7
0.6
0.5
0.4
0.3
0.2
0.1
0.0
Phylostratum
Cellular_organisms - ps1
Archaea1 - ps2
Archaea2 - ps3
Archaea3 - ps4
Asgard/Eukaryota - ps5
Eukaryota - ps6
Amorpheae - ps7
Obazoa - ps8
Opisthokonta - ps9
Holozoa - ps10
Filozoa - ps11
Choano./Metazoa - ps12
Metazoa - ps13
Myriazoa - ps14
Parahoxozoa - ps15
Bilateria - ps16
Deuterostomia - ps17
Chordata - ps18
Olfactores - ps19
Vertebrata - ps20
Gnathostomata - ps21
Euteleostomi - ps22
Sarcopterygii - ps23
Tetrapoda - ps24
Amniota - ps25
Mammalia - ps26
Theria - ps27
Placentalia - ps28
Boreoeutheria - ps29
Euarchontoglires - ps30
Euarchonta - ps31
Primates - ps32
Haplorrhini - ps33
Simiiformes - ps34
Catarrhini - ps35
Hominoidea - ps36
Hominidae - ps37
Homininae - ps38
Hominini - ps39
Homo_sapiens - ps40
p-value
2e-05
2e-04
1e-03
7e-03
5e-02
Log-odds
0.5
1.0
1.5
2.0
2.5
3.0
3.5
4.0

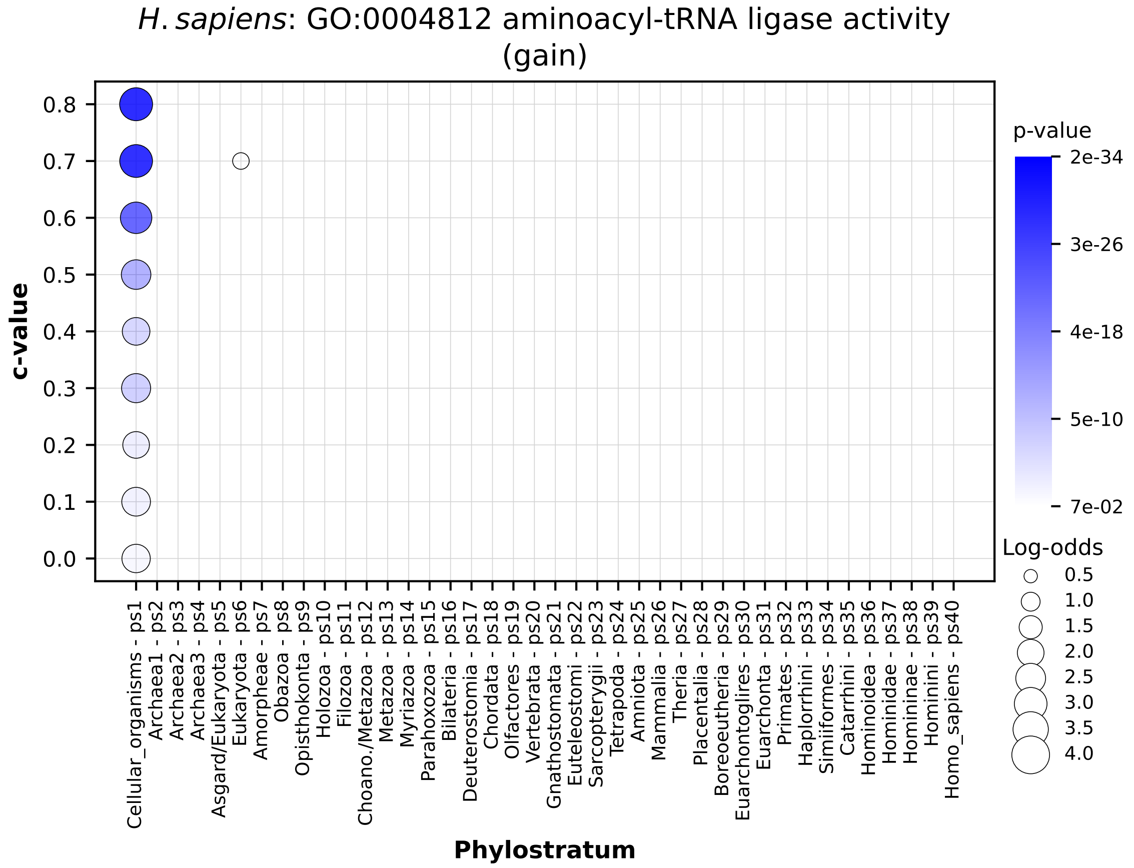

H. sapiens: GO:0004812 aminoacyl-tRNA ligase activity
(gain)
c-value
0.8
0.7
0.6
0.5
0.4
0.3
0.2
0.1
0.0
p-value
2e-34
3e-26
4e-18
5e-10
7e-02
Log-odds
0.5
1.0
1.5
2.0
2.5
3.0
3.5
4.0
Cellular_organisms - ps1
Archaea1 - ps2
Archaea2 - ps3
Archaea3 - ps4
Asgard/Eukaryota - ps5
Eukaryota - ps6
Amorpheae - ps7
Obazoa - ps8
Opisthokonta - ps9
Holozoa - ps10
Filozoa - ps11
Choano./Metazoa - ps12
Metazoa - ps13
Myriazoa - ps14
Parahoxozoa - ps15
Bilateria - ps16
Deuterostomia - ps17
Chordata - ps18
Olfactores - ps19
Vertebrata - ps20
Gnathostomata - ps21
Euteleostomi - ps22
Sarcopterygii - ps23
Tetrapoda - ps24
Amniota - ps25
Mammalia - ps26
Theria - ps27
Placentalia - ps28
Boreoeutheria - ps29
Euarchontoglires - ps30
Euarchonta - ps31
Primates - ps32
Haplorrhini - ps33
Simiiformes - ps34
Catarrhini - ps35
Hominoidea - ps36
Hominidae - ps37
Homininae - ps38
Hominini - ps39
Homo_sapiens - ps40
Phylostratum

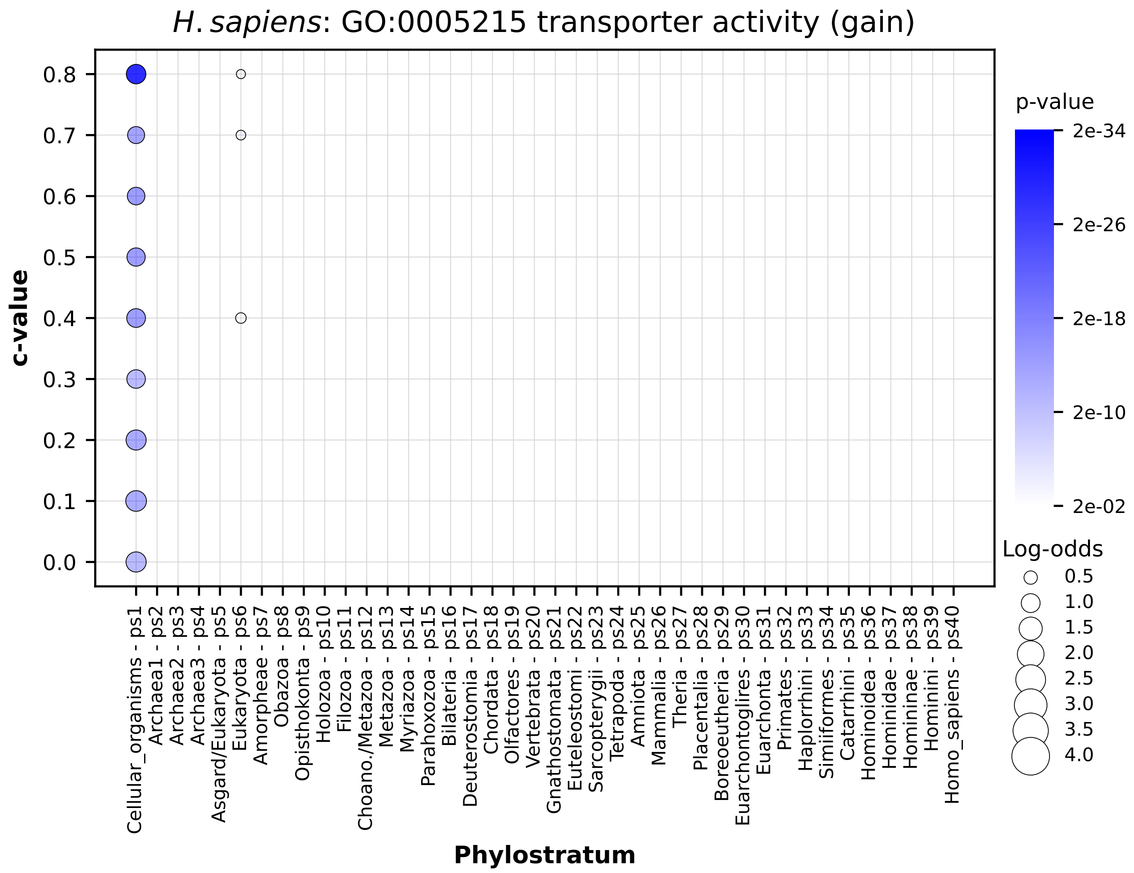

H. sapiens: GO:0005215 transporter activity (gain)
c-value
0.8
0.7
0.6
0.5
0.4
0.3
0.2
0.1
0.0
p-value
2e-34
2e-26
2e-18
2e-10
2e-02
Log-odds
0.5
1.0
1.5
2.0
2.5
3.0
3.5
4.0
Cellular_organisms - ps1
Archaea1 - ps2
Archaea2 - ps3
Archaea3 - ps4
Asgard/Eukaryota - ps5
Eukaryota - ps6
Amorpheae - ps7
Obazoa - ps8
Opisthokonta - ps9
Holozoa - ps10
Filozoa - ps11
Choano./Metazoa - ps12
Metazoa - ps13
Myriazoa - ps14
Parahoxozoa - ps15
Bilateria - ps16
Deuterostomia - ps17
Chordata - ps18
Olfactores - ps19
Vertebrata - ps20
Gnathostomata - ps21
Euteleostomi - ps22
Sarcopterygii - ps23
Tetrapoda - ps24
Amniota - ps25
Mammalia - ps26
Theria - ps27
Placentalia - ps28
Boreoeutheria - ps29
Euarchontoglires - ps30
Euarchonta - ps31
Primates - ps32
Haplorrhini - ps33
Simiiformes - ps34
Catarrhini - ps35
Hominoidea - ps36
Hominidae - ps37
Homininae - ps38
Hominini - ps39
Homo_sapiens - ps40
Phylostratum

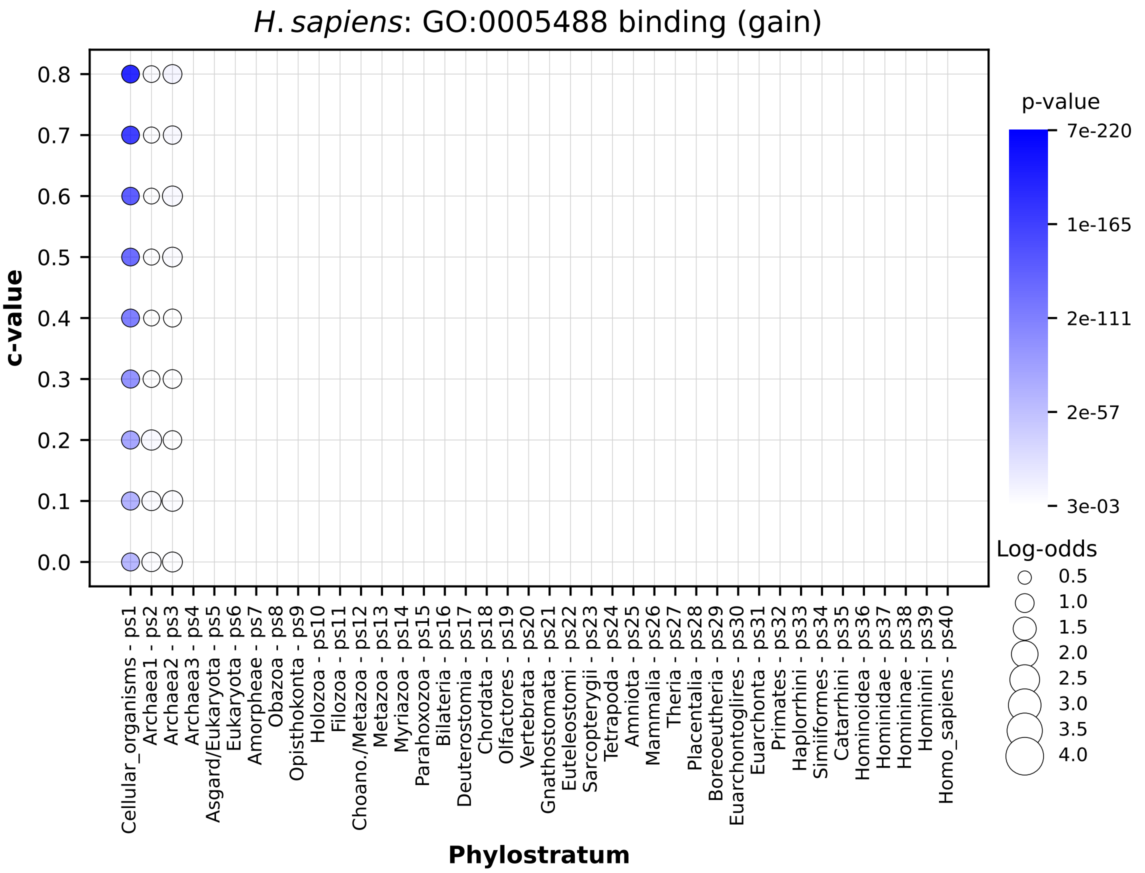
H. sapiens: GO:0005488 binding (gain)
c-value
0.8
0.7
0.6
0.5
0.4
0.3
0.2
0.1
0.0
p-value
7e-220
1e-165
2e-111
2e-57
3e-03
Log-odds
0.5
1.0
1.5
2.0
2.5
3.0
3.5
4.0
Cellular_organisms - ps1
Archaea1 - ps2
Archaea2 - ps3
Archaea3 - ps4
Asgard/Eukaryota - ps5
Eukaryota - ps6
Amorpheae - ps7
Obazoa - ps8
Opisthokonta - ps9
Holozoa - ps10
Filozoa - ps11
Choano./Metazoa - ps12
Metazoa - ps13
Myriazoa - ps14
Parahoxozoa - ps15
Bilateria - ps16
Deuterostomia - ps17
Chordata - ps18
Olfactores - ps19
Vertebrata - ps20
Gnathostomata - ps21
Euteleostomi - ps22
Sarcopterygii - ps23
Tetrapoda - ps24
Amniota - ps25
Mammalia - ps26
Theria - ps27
Placentalia - ps28
Boreoeutheria - ps29
Euarchontoglires - ps30
Euarchonta - ps31
Primates - ps32
Haplorrhini - ps33
Simiiformes - ps34
Catarrhini - ps35
Hominoidea - ps36
Hominidae - ps37
Homininae - ps38
Hominini - ps39
Homo_sapiens - ps40
Phylostratum

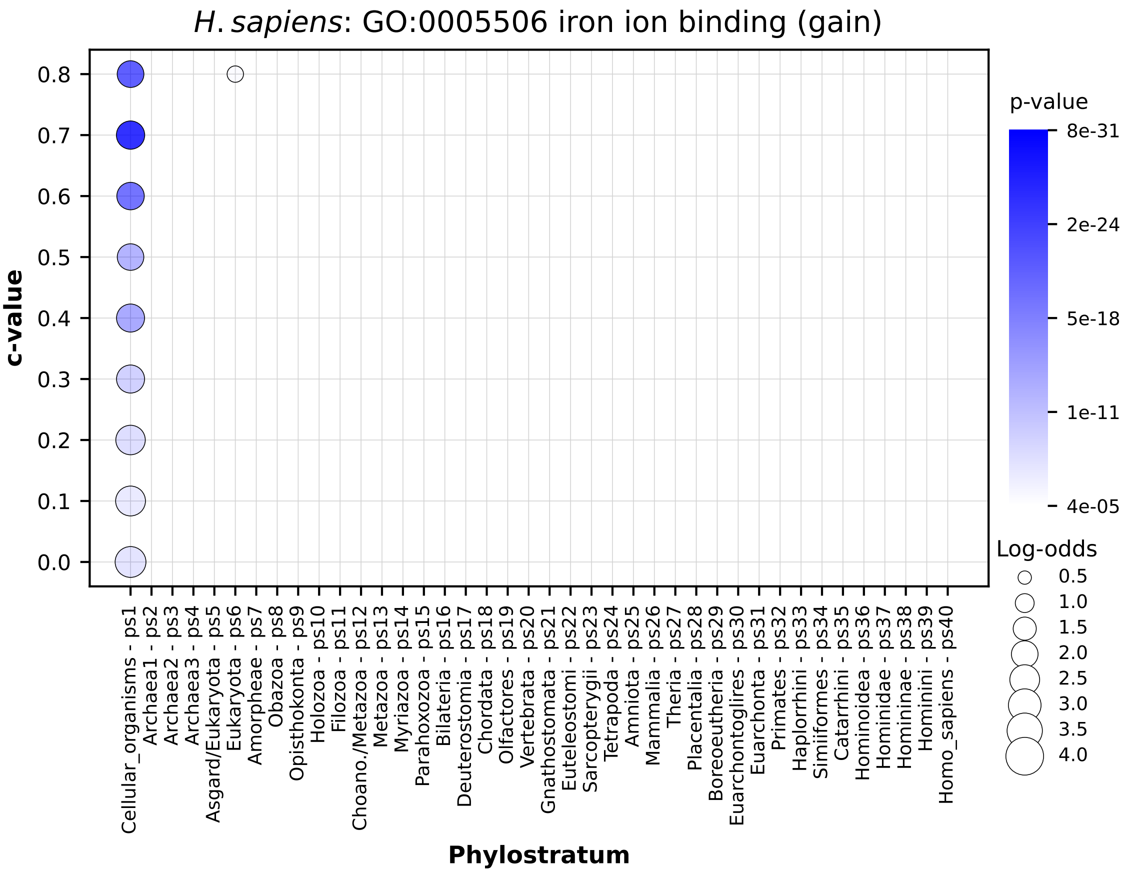
H. sapiens: GO:0005506 iron ion binding (gain)
c-value
0.8
0.7
0.6
0.5
0.4
0.3
0.2
0.1
0.0
p-value
8e-31
2e-24
5e-18
1e-11
4e-05
Log-odds
0.5
1.0
1.5
2.0
2.5
3.0
3.5
4.0
Cellular_organisms - ps1
Archaea1 - ps2
Archaea2 - ps3
Archaea3 - ps4
Asgard/Eukaryota - ps5
Eukaryota - ps6
Amorpheae - ps7
Obazoa - ps8
Opisthokonta - ps9
Holozoa - ps10
Filozoa - ps11
Choano./Metazoa - ps12
Metazoa - ps13
Myriazoa - ps14
Parahoxozoa - ps15
Bilateria - ps16
Deuterostomia - ps17
Chordata - ps18
Olfactores - ps19
Vertebrata - ps20
Gnathostomata - ps21
Euteleostomi - ps22
Sarcopterygii - ps23
Tetrapoda - ps24
Amniota - ps25
Mammalia - ps26
Theria - ps27
Placentalia - ps28
Boreoeutheria - ps29
Euarchontoglires - ps30
Euarchonta - ps31
Primates - ps32
Haplorrhini - ps33
Simiiformes - ps34
Catarrhini - ps35
Hominoidea - ps36
Hominidae - ps37
Homininae - ps38
Hominini - ps39
Homo_sapiens - ps40
Phylostratum

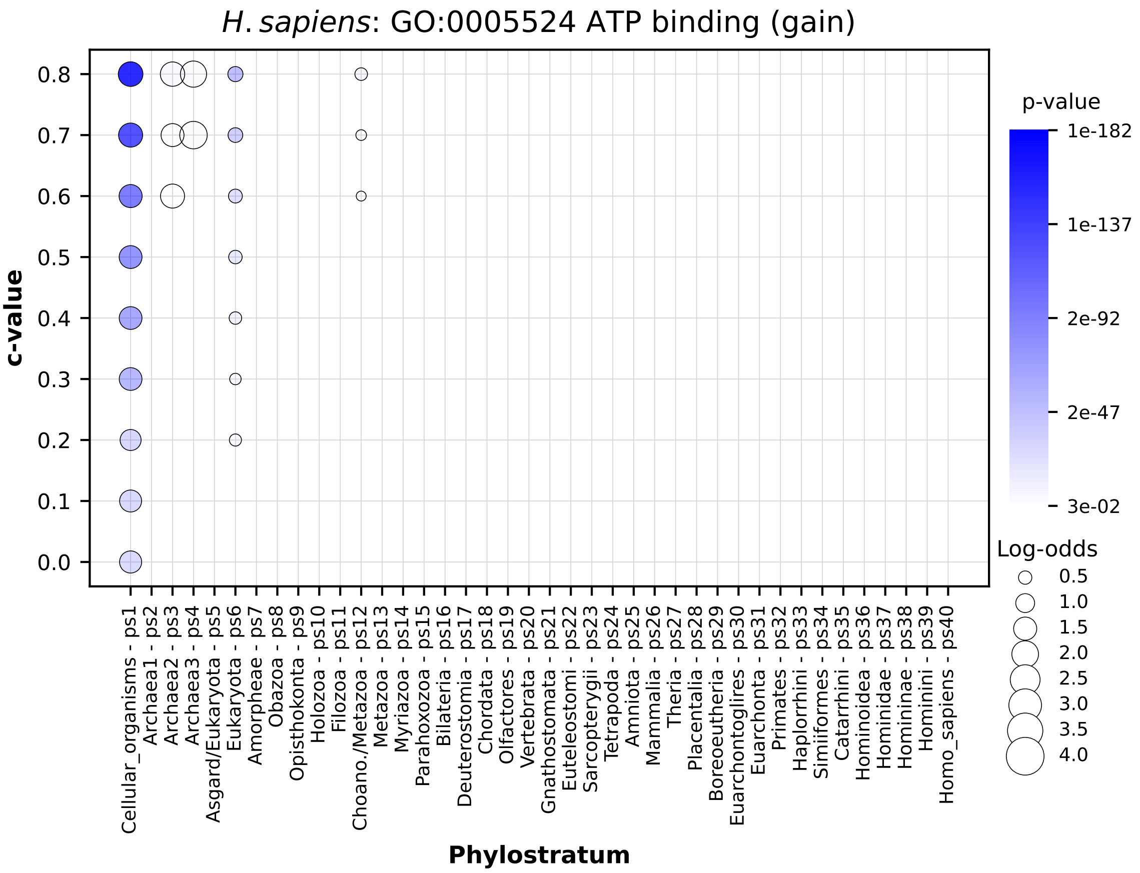
H. sapiens: GO:0005524 ATP binding (gain)
c-value
0.0
0.1
0.2
0.3
0.4
0.5
0.6
0.7
0.8
Phylostratum
Cellular_organisms - ps1
Archaea1 - ps2
Archaea2 - ps3
Archaea3 - ps4
Asgard/Eukaryota - ps5
Eukaryota - ps6
Amorpheae - ps7
Obazoa - ps8
Opisthokonta - ps9
Holozoa - ps10
Filozoa - ps11
Choano./Metazoa - ps12
Metazoa - ps13
Myriazoa - ps14
Parahoxozoa - ps15
Bilateria - ps16
Deuterostomia - ps17
Chordata - ps18
Olfactores - ps19
Vertebrata - ps20
Gnathostomata - ps21
Euteleostomi - ps22
Sarcopterygii - ps23
Tetrapoda - ps24
Amniota - ps25
Mammalia - ps26
Theria - ps27
Placentalia - ps28
Boreoeutheria - ps29
Euarchontoglires - ps30
Euarchonta - ps31
Primates - ps32
Haplorrhini - ps33
Simiiformes - ps34
Catarrhini - ps35
Hominoidea - ps36
Hominidae - ps37
Homininae - ps38
Hominini - ps39
Homo_sapiens - ps40
p-value
1e-182
1e-137
2e-92
2e-47
3e-02
Log-odds
0.5
1.0
1.5
2.0
2.5
3.0
3.5
4.0

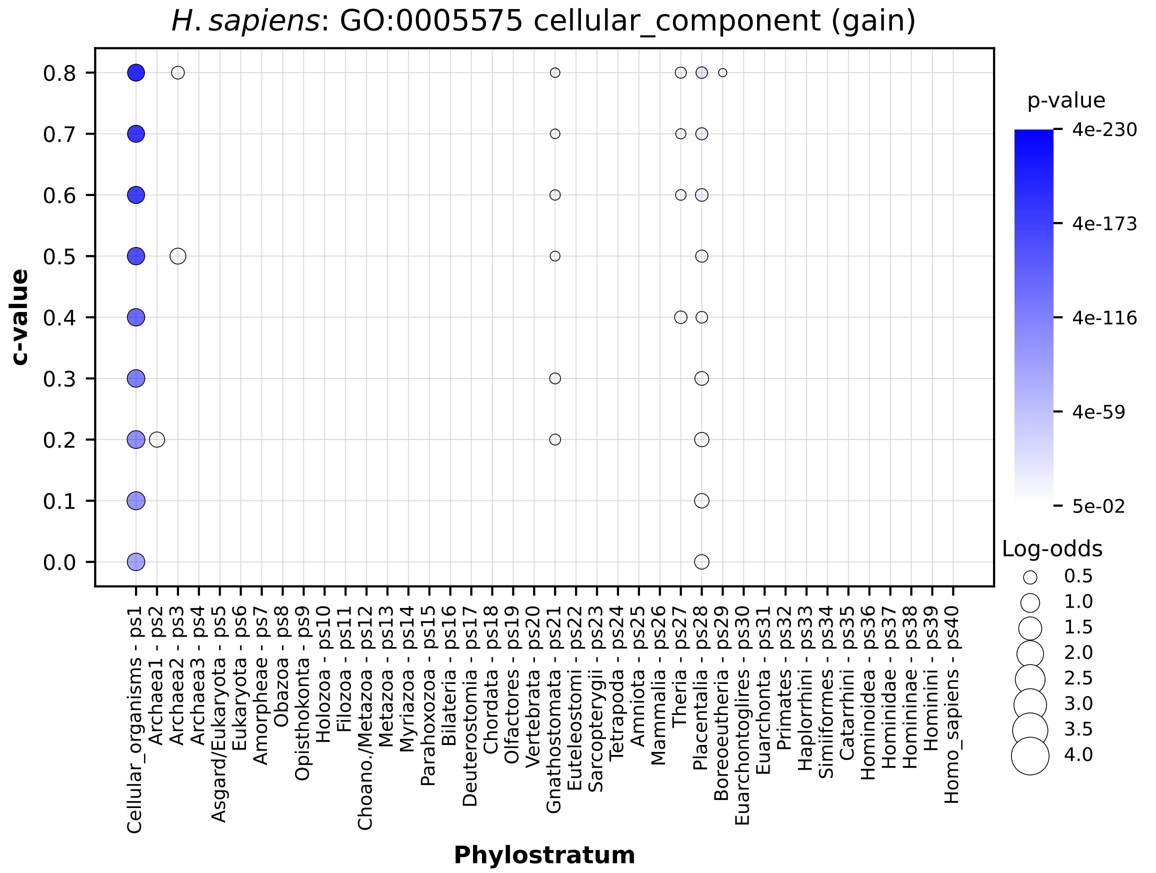
H. sapiens: GO:0005575 cellular_component (gain)
c-value
0.8
0.7
0.6
0.5
0.4
0.3
0.2
0.1
0.0
Phylostratum
Cellular_organisms - ps1
Archaea1 - ps2
Archaea2 - ps3
Archaea3 - ps4
Asgard/Eukaryota - ps5
Eukaryota - ps6
Amorpheae - ps7
Obazoa - ps8
Opisthokonta - ps9
Holozoa - ps10
Filozoa - ps11
Choano./Metazoa - ps12
Metazoa - ps13
Myriazoa - ps14
Parahoxozoa - ps15
Bilateria - ps16
Deuterostomia - ps17
Chordata - ps18
Olfactores - ps19
Vertebrata - ps20
Gnathostomata - ps21
Euteleostomi - ps22
Sarcopterygii - ps23
Tetrapoda - ps24
Amniota - ps25
Mammalia - ps26
Theria - ps27
Placentalia - ps28
Boreoeutheria - ps29
Euarchontoglires - ps30
Euarchonta - ps31
Primates - ps32
Haplorrhini - ps33
Simiiformes - ps34
Catarrhini - ps35
Hominoidea - ps36
Hominidae - ps37
Homininae - ps38
Hominini - ps39
Homo_sapiens - ps40
p-value
4e-230
4e-173
4e-116
4e-59
5e-02
Log-odds
0.5
1.0
1.5
2.0
2.5
3.0
3.5
4.0

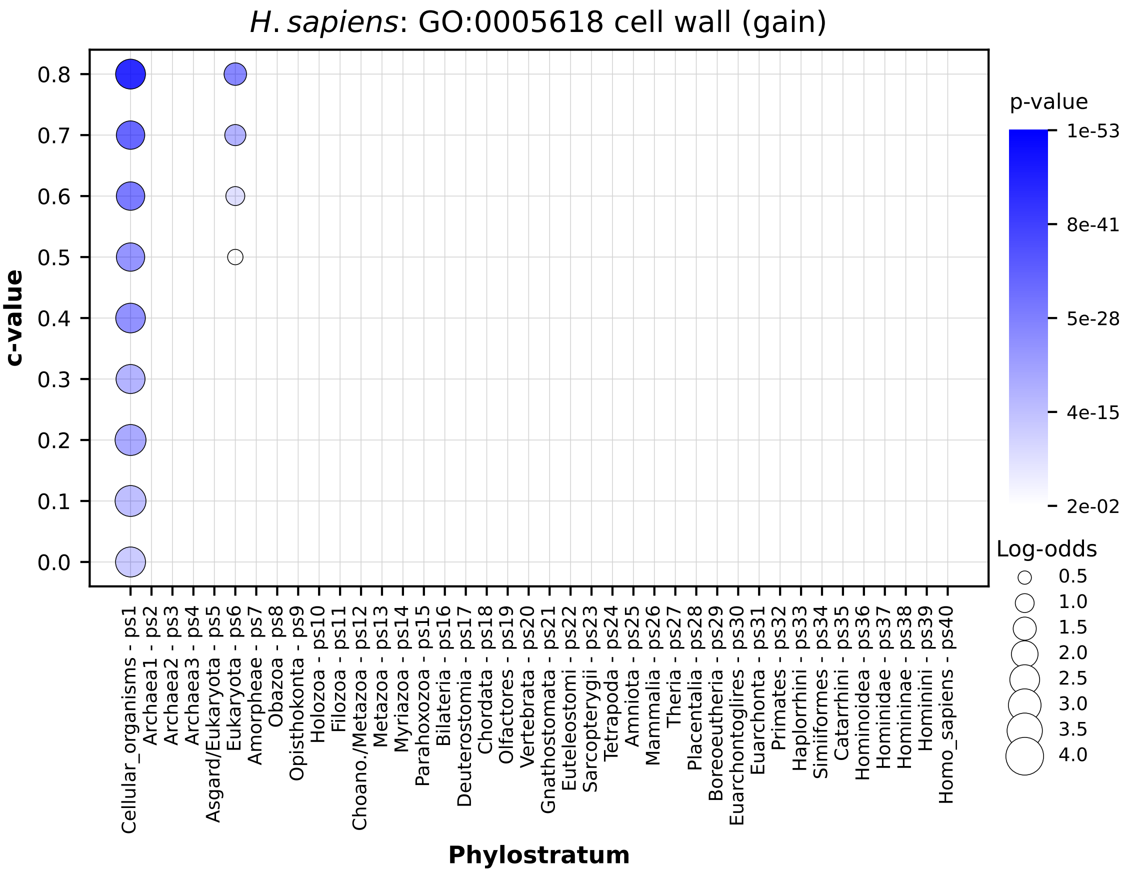

H. sapiens: GO:0005618 cell wall (gain)
c-value
0.8
0.7
0.6
0.5
0.4
0.3
0.2
0.1
0.0
p-value
1e-53
8e-41
5e-28
4e-15
2e-02
Log-odds
0.5
1.0
1.5
2.0
2.5
3.0
3.5
4.0
Cellular_organisms - ps1
Archaea1 - ps2
Archaea2 - ps3
Archaea3 - ps4
Asgard/Eukaryota - ps5
Eukaryota - ps6
Amorpheae - ps7
Obazoa - ps8
Opisthokonta - ps9
Holozoa - ps10
Filozoa - ps11
Choano./Metazoa - ps12
Metazoa - ps13
Myriazoa - ps14
Parahoxozoa - ps15
Bilateria - ps16
Deuterostomia - ps17
Chordata - ps18
Olfactores - ps19
Vertebrata - ps20
Gnathostomata - ps21
Euteleostomi - ps22
Sarcopterygii - ps23
Tetrapoda - ps24
Amniota - ps25
Mammalia - ps26
Theria - ps27
Placentalia - ps28
Boreoeutheria - ps29
Euarchontoglires - ps30
Euarchonta - ps31
Primates - ps32
Haplorrhini - ps33
Simiiformes - ps34
Catarrhini - ps35
Hominoidea - ps36
Hominidae - ps37
Homininae - ps38
Hominini - ps39
Homo_sapiens - ps40
Phylostratum

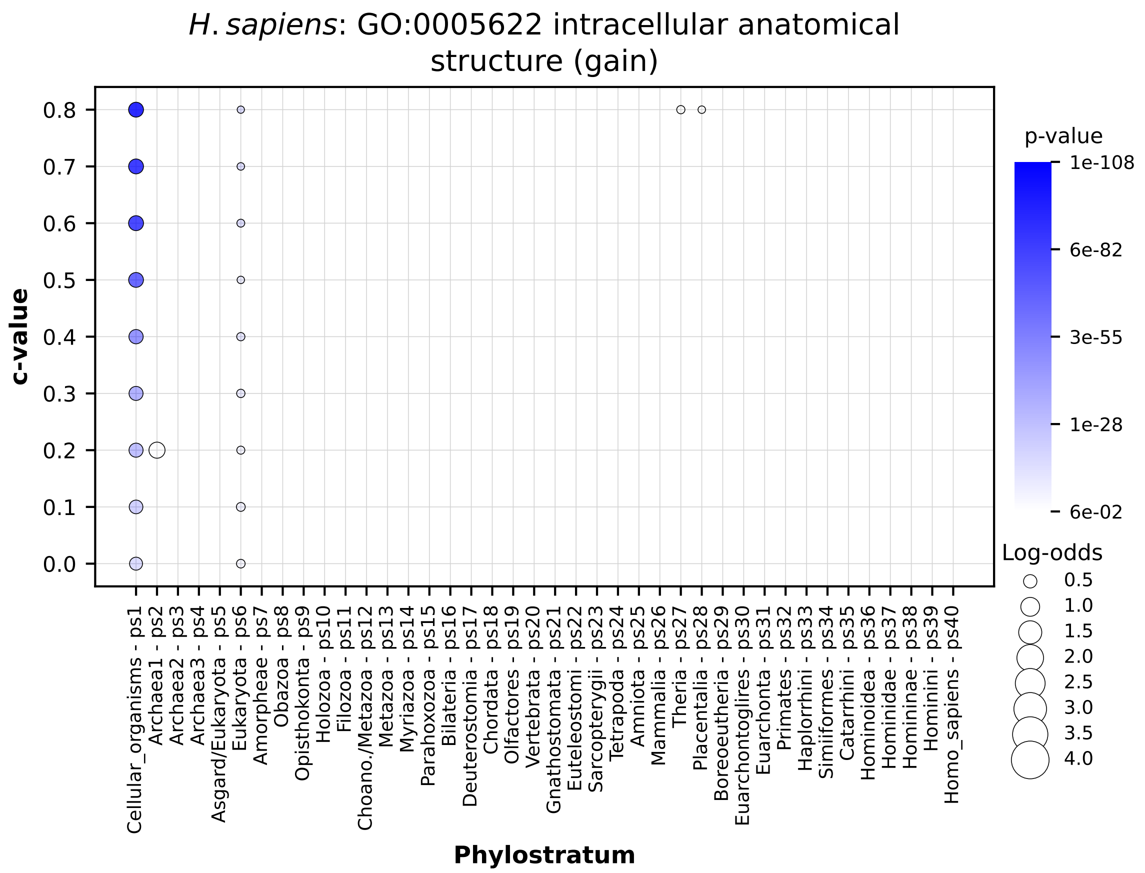
H. sapiens: GO:0005622 intracellular anatomical structure (gain)
c-value
0.8
0.7
0.6
0.5
0.4
0.3
0.2
0.1
0.0
p-value
1e-108
6e-82
3e-55
1e-28
6e-02
Log-odds
0.5
1.0
1.5
2.0
2.5
3.0
3.5
4.0
Cellular_organisms - ps1
Archaea1 - ps2
Archaea2 - ps3
Archaea3 - ps4
Asgard/Eukaryota - ps5
Eukaryota - ps6
Amorpheae - ps7
Obazoa - ps8
Opisthokonta - ps9
Holozoa - ps10
Filozoa - ps11
Choano./Metazoa - ps12
Metazoa - ps13
Myriazoa - ps14
Parahoxozoa - ps15
Bilateria - ps16
Deuterostomia - ps17
Chordata - ps18
Olfactores - ps19
Vertebrata - ps20
Gnathostomata - ps21
Euteleostomi - ps22
Sarcopterygii - ps23
Tetrapoda - ps24
Amniota - ps25
Mammalia - ps26
Theria - ps27
Placentalia - ps28
Boreoeutheria - ps29
Euarchontoglires - ps30
Euarchonta - ps31
Primates - ps32
Haplorrhini - ps33
Simiiformes - ps34
Catarrhini - ps35
Hominoidea - ps36
Hominidae - ps37
Homininae - ps38
Hominini - ps39
Homo_sapiens - ps40
Phylostratum

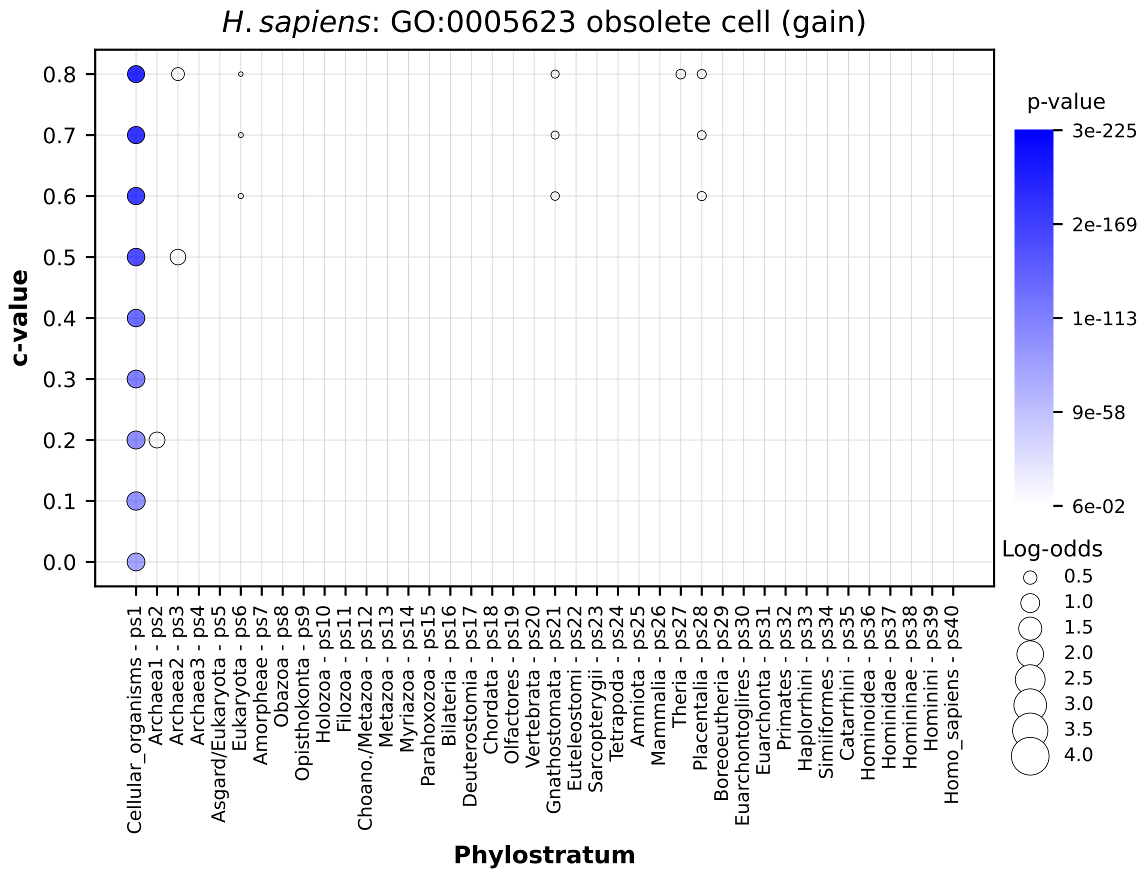
H. sapiens: GO:0005623 obsolete cell (gain)
c-value
0.8
0.7
0.6
0.5
0.4
0.3
0.2
0.1
0.0
p-value
3e-225
2e-169
1e-113
9e-58
6e-02
Log-odds
0.5
1.0
1.5
2.0
2.5
3.0
3.5
4.0
Cellular_organisms - ps1
Archaea1 - ps2
Archaea2 - ps3
Archaea3 - ps4
Asgard/Eukaryota - ps5
Eukaryota - ps6
Amorpheae - ps7
Obazoa - ps8
Opisthokonta - ps9
Holozoa - ps10
Filozoa - ps11
Choano./Metazoa - ps12
Metazoa - ps13
Myriazoa - ps14
Parahoxozoa - ps15
Bilateria - ps16
Deuterostomia - ps17
Chordata - ps18
Olfactores - ps19
Vertebrata - ps20
Gnathostomata - ps21
Euteleostomi - ps22
Sarcopterygii - ps23
Tetrapoda - ps24
Amniota - ps25
Mammalia - ps26
Theria - ps27
Placentalia - ps28
Boreoeutheria - ps29
Euarchontoglires - ps30
Euarchonta - ps31
Primates - ps32
Haplorrhini - ps33
Simiiformes - ps34
Catarrhini - ps35
Hominoidea - ps36
Hominidae - ps37
Homininae - ps38
Hominini - ps39
Homo_sapiens - ps40
Phylostratum

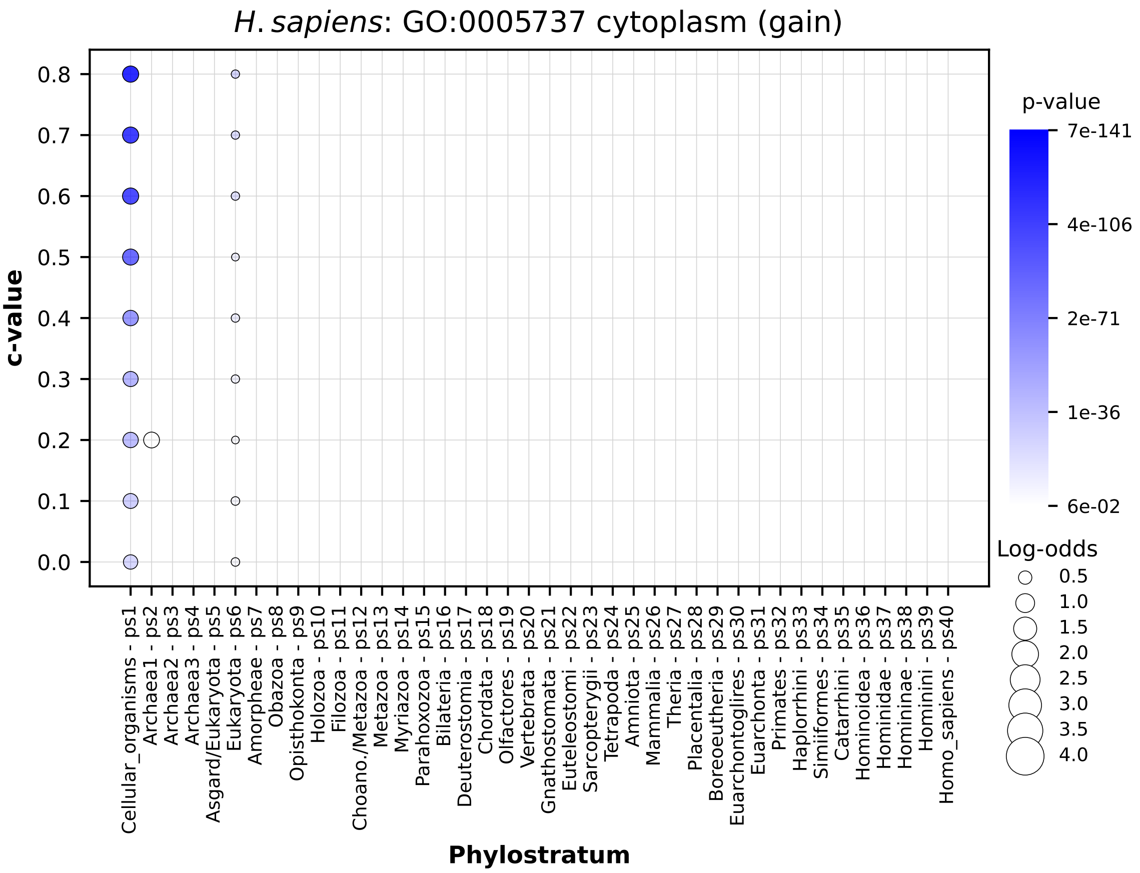

H. sapiens: GO:0005737 cytoplasm (gain)
c-value
0.8
0.7
0.6
0.5
0.4
0.3
0.2
0.1
0.0
p-value
7e-141
4e-106
2e-71
1e-36
6e-02
Log-odds
0.5
1.0
1.5
2.0
2.5
3.0
3.5
4.0
Cellular_organisms - ps1
Archaea1 - ps2
Archaea2 - ps3
Archaea3 - ps4
Asgard/Eukaryota - ps5
Eukaryota - ps6
Amorpheae - ps7
Obazoa - ps8
Opisthokonta - ps9
Holozoa - ps10
Filozoa - ps11
Choano./Metazoa - ps12
Metazoa - ps13
Myriazoa - ps14
Parahoxozoa - ps15
Bilateria - ps16
Deuterostomia - ps17
Chordata - ps18
Olfactores - ps19
Vertebrata - ps20
Gnathostomata - ps21
Euteleostomi - ps22
Sarcopterygii - ps23
Tetrapoda - ps24
Amniota - ps25
Mammalia - ps26
Theria - ps27
Placentalia - ps28
Boreoeutheria - ps29
Euarchontoglires - ps30
Euarchonta - ps31
Primates - ps32
Haplorrhini - ps33
Simiiformes - ps34
Catarrhini - ps35
Hominoidea - ps36
Hominidae - ps37
Homininae - ps38
Hominini - ps39
Homo_sapiens - ps40
Phylostratum

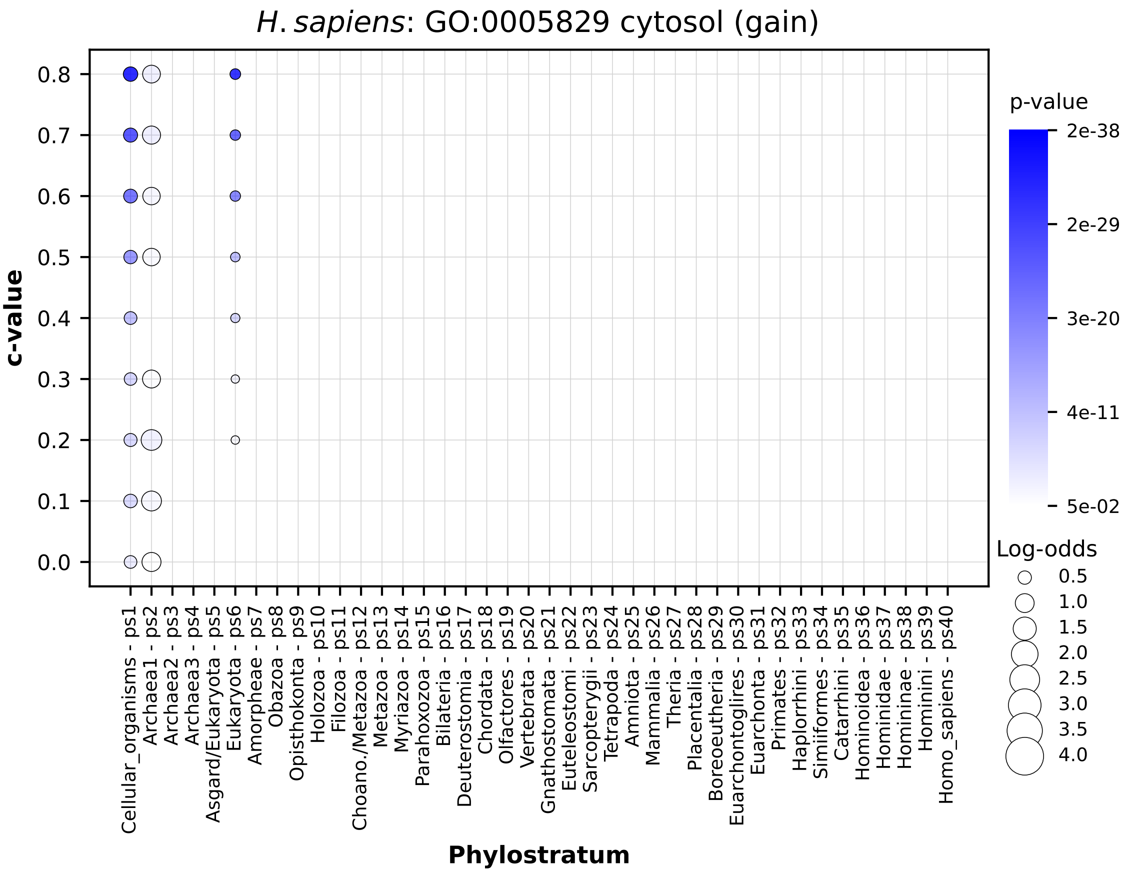

H. sapiens: GO:0005829 cytosol (gain)
c-value
0.0
0.1
0.2
0.3
0.4
0.5
0.6
0.7
0.8
Phylostratum
Cellular_organisms - ps1
Archaea1 - ps2
Archaea2 - ps3
Archaea3 - ps4
Asgard/Eukaryota - ps5
Eukaryota - ps6
Amorpheae - ps7
Obazoa - ps8
Opisthokonta - ps9
Holozoa - ps10
Filozoa - ps11
Choano./Metazoa - ps12
Metazoa - ps13
Myriazoa - ps14
Parahoxozoa - ps15
Bilateria - ps16
Deuterostomia - ps17
Chordata - ps18
Olfactores - ps19
Vertebrata - ps20
Gnathostomata - ps21
Euteleostomi - ps22
Sarcopterygii - ps23
Tetrapoda - ps24
Amniota - ps25
Mammalia - ps26
Theria - ps27
Placentalia - ps28
Boreoeutheria - ps29
Euarchontoglires - ps30
Euarchonta - ps31
Primates - ps32
Haplorrhini - ps33
Simiiformes - ps34
Catarrhini - ps35
Hominoidea - ps36
Hominidae - ps37
Homininae - ps38
Hominini - ps39
Homo_sapiens - ps40
p-value
2e-38
2e-29
3e-20
4e-11
5e-02
Log-odds
0.5
1.0
1.5
2.0
2.5
3.0
3.5
4.0

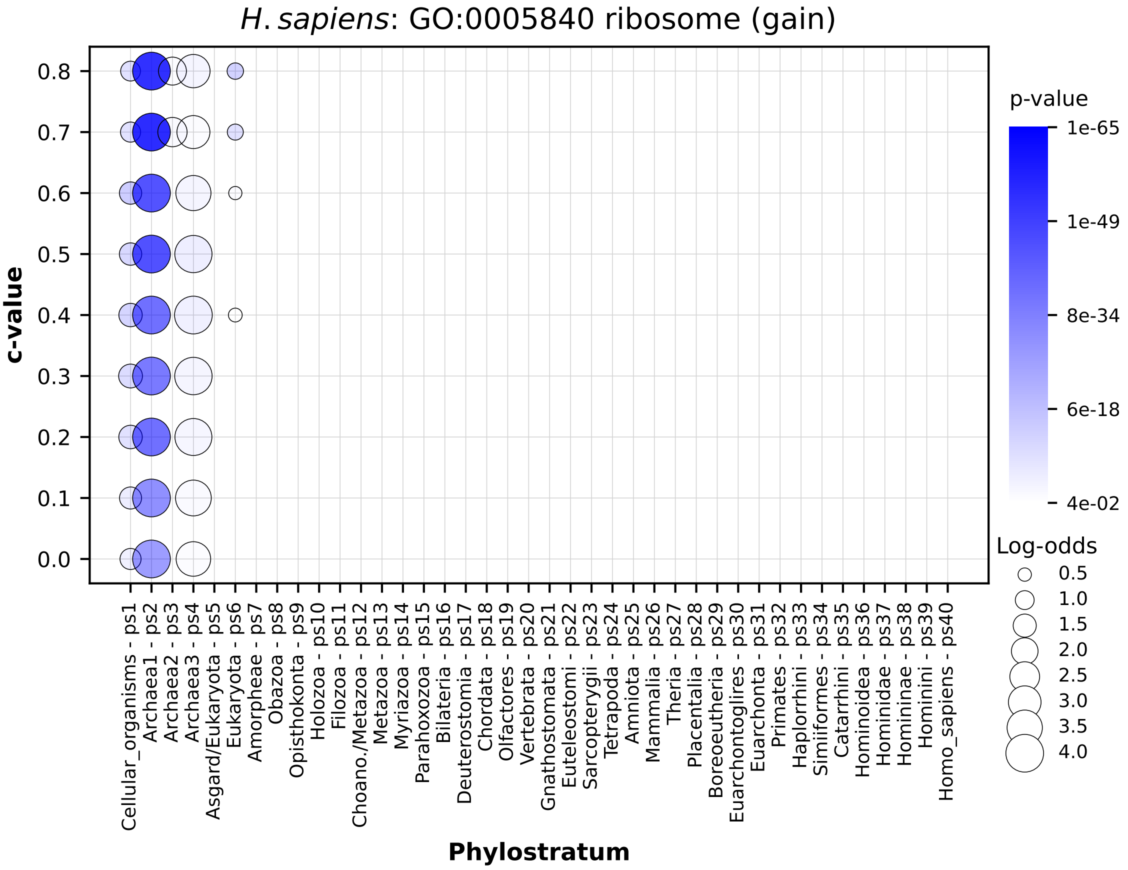

H. sapiens: GO:0005840 ribosome (gain)
c-value
0.8
0.7
0.6
0.5
0.4
0.3
0.2
0.1
0.0
Phylostratum
Cellular_organisms - ps1
Archaea1 - ps2
Archaea2 - ps3
Archaea3 - ps4
Asgard/Eukaryota - ps5
Eukaryota - ps6
Amorpheae - ps7
Obazoa - ps8
Opisthokonta - ps9
Holozoa - ps10
Filozoa - ps11
Choano./Metazoa - ps12
Metazoa - ps13
Myriazoa - ps14
Parahoxozoa - ps15
Bilateria - ps16
Deuterostomia - ps17
Chordata - ps18
Olfactores - ps19
Vertebrata - ps20
Gnathostomata - ps21
Euteleostomi - ps22
Sarcopterygii - ps23
Tetrapoda - ps24
Amniota - ps25
Mammalia - ps26
Theria - ps27
Placentalia - ps28
Boreoeutheria - ps29
Euarchontoglires - ps30
Euarchonta - ps31
Primates - ps32
Haplorrhini - ps33
Simiiformes - ps34
Catarrhini - ps35
Hominoidea - ps36
Hominidae - ps37
Homininae - ps38
Hominini - ps39
Homo_sapiens - ps40
p-value
1e-65
1e-49
8e-34
6e-18
4e-02
Log-odds
0.5
1.0
1.5
2.0
2.5
3.0
3.5
4.0

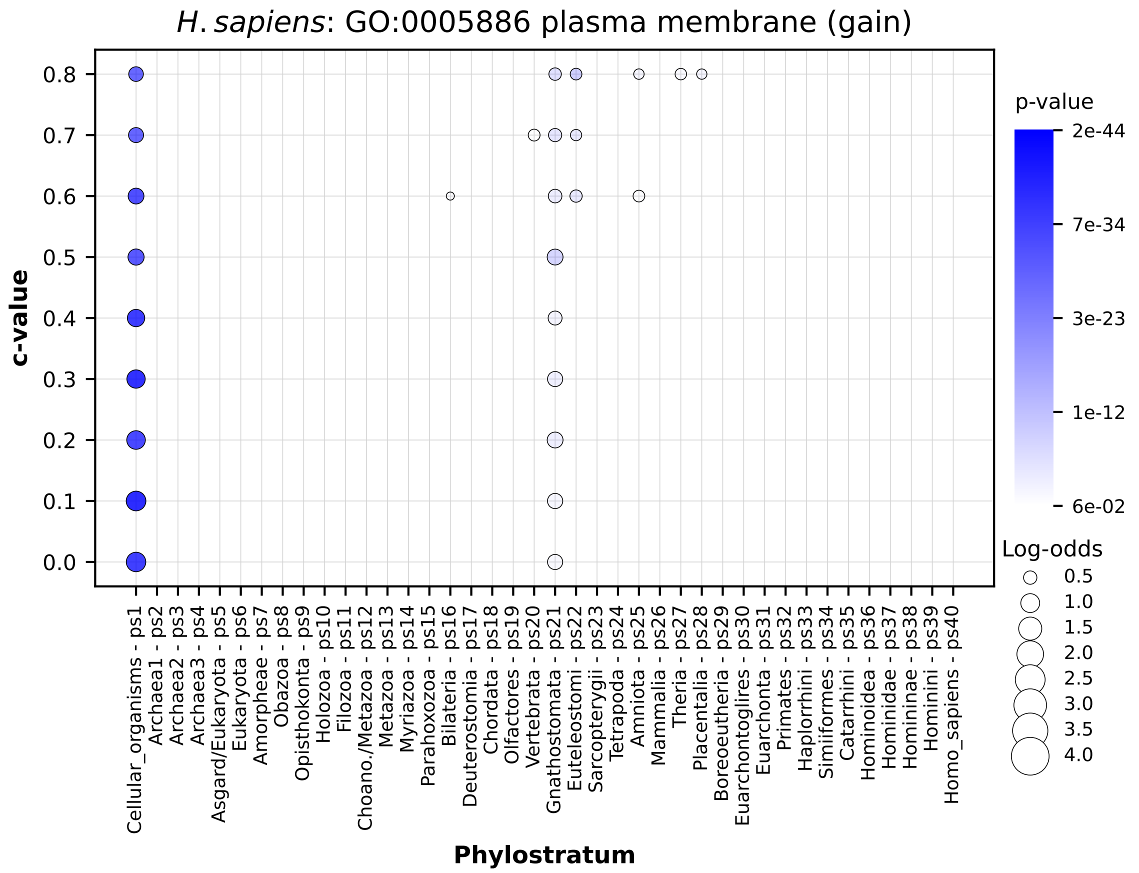
H. sapiens: GO:0005886 plasma membrane (gain)
c-value
0.8
0.7
0.6
0.5
0.4
0.3
0.2
0.1
0.0
Phylostratum
Cellular_organisms - ps1
Archaea1 - ps2
Archaea2 - ps3
Archaea3 - ps4
Asgard/Eukaryota - ps5
Eukaryota - ps6
Amorpheae - ps7
Obazoa - ps8
Opisthokonta - ps9
Holozoa - ps10
Filozoa - ps11
Choano./Metazoa - ps12
Metazoa - ps13
Myriazoa - ps14
Parahoxozoa - ps15
Bilateria - ps16
Deuterostomia - ps17
Chordata - ps18
Olfactores - ps19
Vertebrata - ps20
Gnathostomata - ps21
Euteleostomi - ps22
Sarcopterygii - ps23
Tetrapoda - ps24
Amniota - ps25
Mammalia - ps26
Theria - ps27
Placentalia - ps28
Boreoeutheria - ps29
Euarchontoglires - ps30
Euarchonta - ps31
Primates - ps32
Haplorrhini - ps33
Simiiformes - ps34
Catarrhini - ps35
Hominoidea - ps36
Hominidae - ps37
Homininae - ps38
Hominini - ps39
Homo_sapiens - ps40
p-value
2e-44
7e-34
3e-23
1e-12
6e-02
Log-odds
0.5
1.0
1.5
2.0
2.5
3.0
3.5
4.0

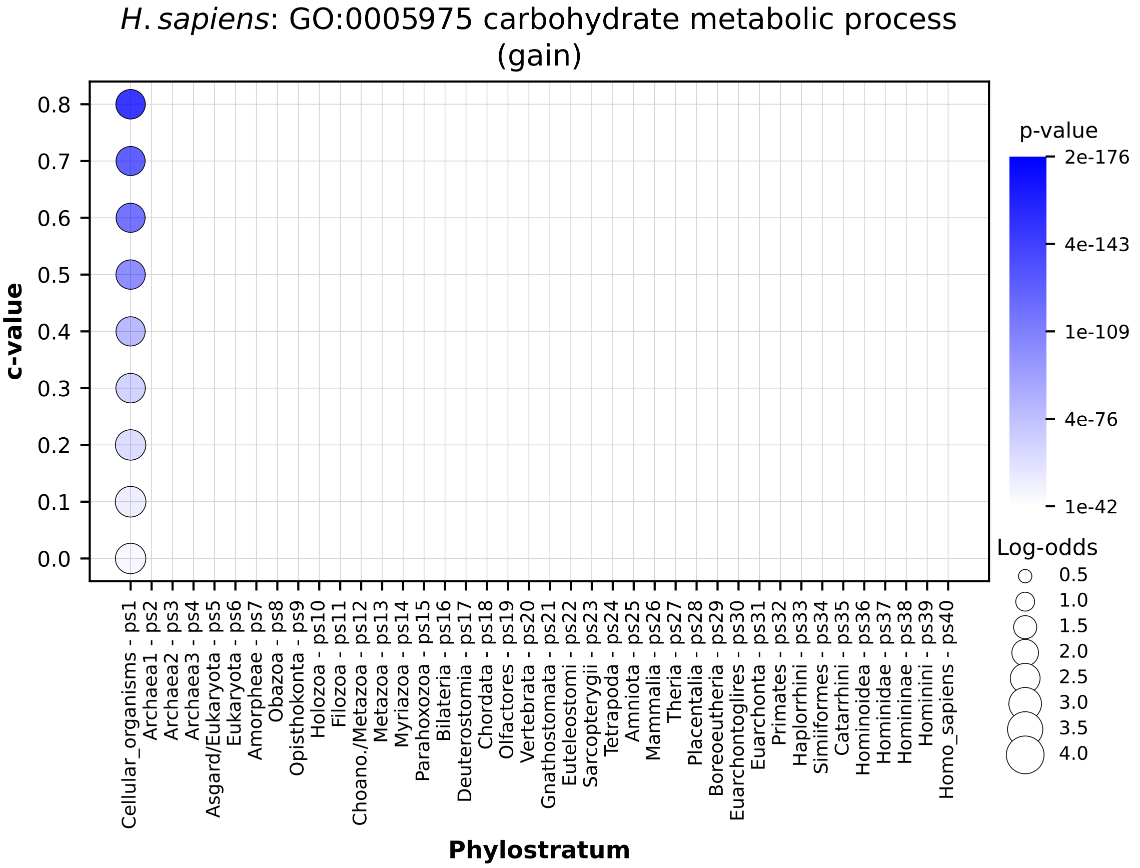

H. sapiens: GO:0005975 carbohydrate metabolic process (gain)
c-value
0.8
0.7
0.6
0.5
0.4
0.3
0.2
0.1
0.0
p-value
2e-176
4e-143
1e-109
4e-76
1e-42
Log-odds
0.5
1.0
1.5
2.0
2.5
3.0
3.5
4.0
Cellular_organisms - ps1
Archaea1 - ps2
Archaea2 - ps3
Archaea3 - ps4
Asgard/Eukaryota - ps5
Eukaryota - ps6
Amorpheae - ps7
Obazoa - ps8
Opisthokonta - ps9
Holozoa - ps10
Filozoa - ps11
Choano./Metazoa - ps12
Metazoa - ps13
Myriazoa - ps14
Parahoxozoa - ps15
Bilateria - ps16
Deuterostomia - ps17
Chordata - ps18
Olfactores - ps19
Vertebrata - ps20
Gnathostomata - ps21
Euteleostomi - ps22
Sarcopterygii - ps23
Tetrapoda - ps24
Amniota - ps25
Mammalia - ps26
Theria - ps27
Placentalia - ps28
Boreoeutheria - ps29
Euarchontoglires - ps30
Euarchonta - ps31
Primates - ps32
Haplorrhini - ps33
Simiiformes - ps34
Catarrhini - ps35
Hominoidea - ps36
Hominidae - ps37
Homininae - ps38
Hominini - ps39
Homo_sapiens - ps40
Phylostratum

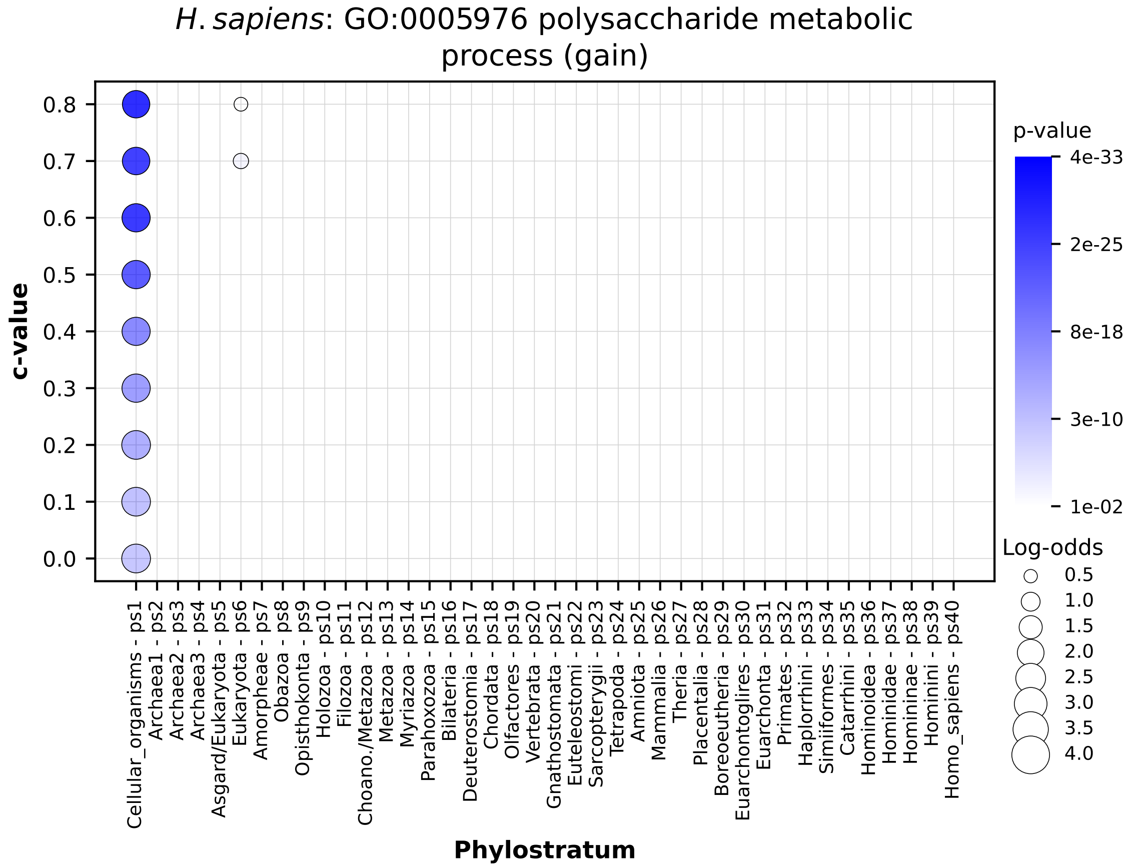

*H. sapiens*: GO:0005976 polysaccharide metabolic process (gain)
c-value
0.8
0.7
0.6
0.5
0.4
0.3
0.2
0.1
0.0
Phylostratum
Cellular_organisms - ps1
Archaea1 - ps2
Archaea2 - ps3
Archaea3 - ps4
Asgard/Eukaryota - ps5
Eukaryota - ps6
Amorpheae - ps7
Obazoa - ps8
Opisthokonta - ps9
Holozoa - ps10
Filozoa - ps11
Choano./Metazoa - ps12
Metazoa - ps13
Myriazoa - ps14
Parahoxozoa - ps15
Bilateria - ps16
Deuterostomia - ps17
Chordata - ps18
Olfactores - ps19
Vertebrata - ps20
Gnathostomata - ps21
Euteleostomi - ps22
Sarcopterygii - ps23
Tetrapoda - ps24
Amniota - ps25
Mammalia - ps26
Theria - ps27
Placentalia - ps28
Boreoeutheria - ps29
Euarchontoglires - ps30
Euarchonta - ps31
Primates - ps32
Haplorrhini - ps33
Simiiformes - ps34
Catarrhini - ps35
Hominoidea - ps36
Hominidae - ps37
Homininae - ps38
Hominini - ps39
Homo_sapiens - ps40
p-value
4e-33
2e-25
8e-18
3e-10
1e-02
Log-odds
0.5
1.0
1.5
2.0
2.5
3.0
3.5
4.0

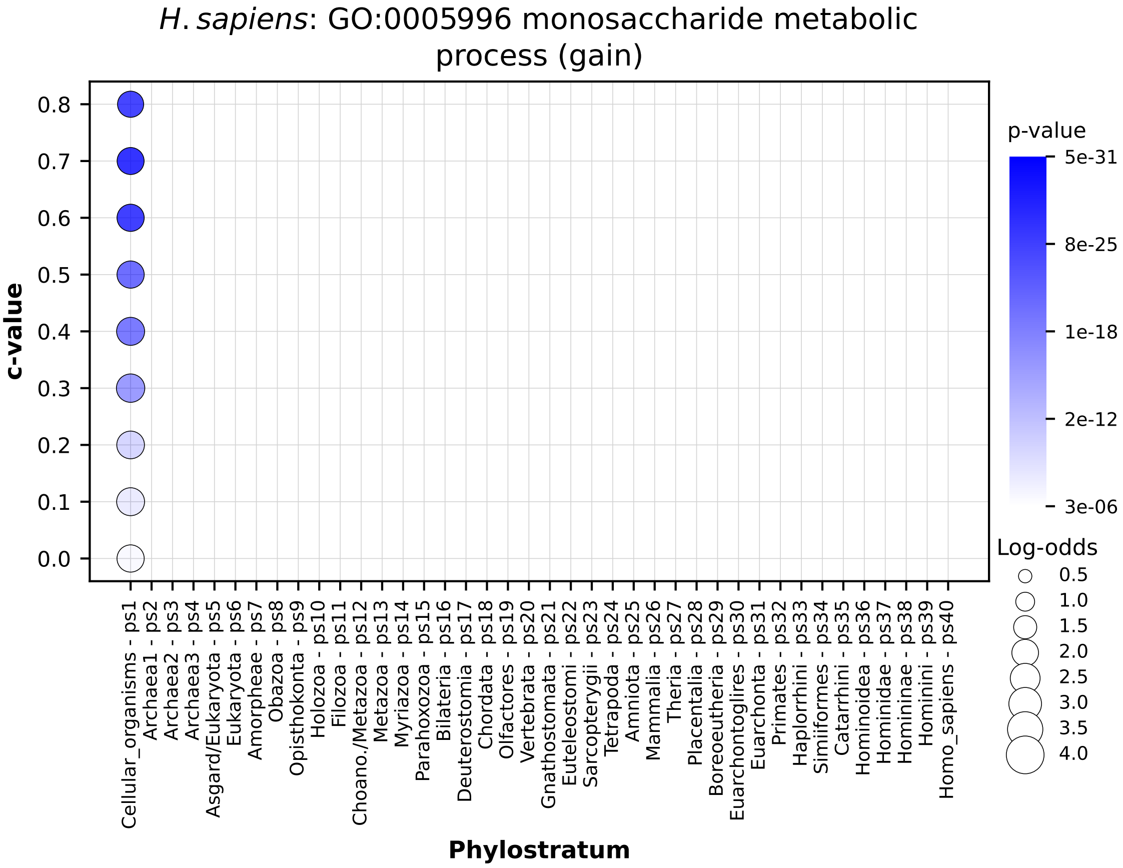

H. sapiens: GO:0005996 monosaccharide metabolic process (gain)
c-value
0.8
0.7
0.6
0.5
0.4
0.3
0.2
0.1
0.0
p-value
5e-31
8e-25
1e-18
2e-12
3e-06
Log-odds
0.5
1.0
1.5
2.0
2.5
3.0
3.5
4.0
Cellular_organisms - ps1
Archaea1 - ps2
Archaea2 - ps3
Archaea3 - ps4
Asgard/Eukaryota - ps5
Eukaryota - ps6
Amorpheae - ps7
Obazoa - ps8
Opisthokonta - ps9
Holozoa - ps10
Filozoa - ps11
Choano./Metazoa - ps12
Metazoa - ps13
Myriazoa - ps14
Parahoxozoa - ps15
Bilateria - ps16
Deuterostomia - ps17
Chordata - ps18
Olfactores - ps19
Vertebrata - ps20
Gnathostomata - ps21
Euteleostomi - ps22
Sarcopterygii - ps23
Tetrapoda - ps24
Amniota - ps25
Mammalia - ps26
Theria - ps27
Placentalia - ps28
Boreoeutheria - ps29
Euarchontoglires - ps30
Euarchonta - ps31
Primates - ps32
Haplorrhini - ps33
Simiiformes - ps34
Catarrhini - ps35
Hominoidea - ps36
Hominidae - ps37
Homininae - ps38
Hominini - ps39
Homo_sapiens - ps40
Phylostratum

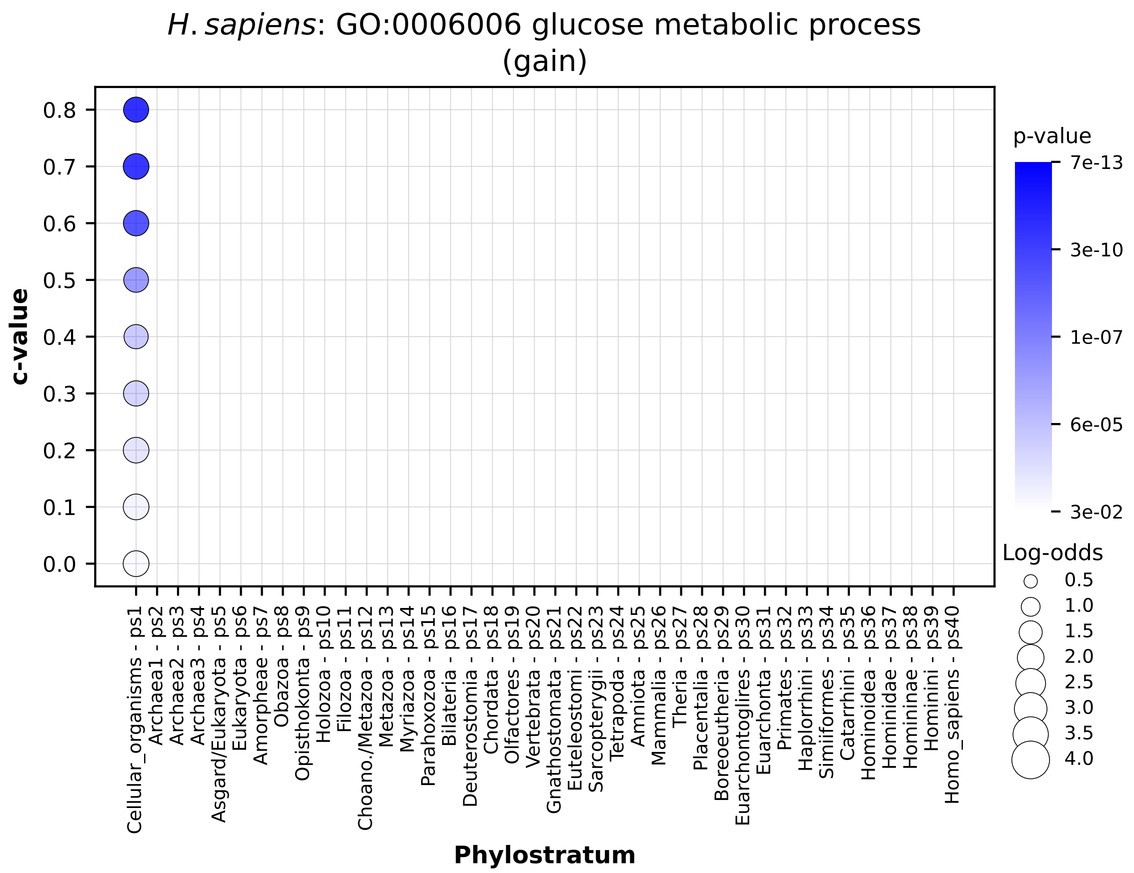

H. sapiens: GO:0006006 glucose metabolic process
(gain)
c-value
0.8
0.7
0.6
0.5
0.4
0.3
0.2
0.1
0.0
p-value
7e-13
3e-10
1e-07
6e-05
3e-02
Log-odds
0.5
1.0
1.5
2.0
2.5
3.0
3.5
4.0
Cellular_organisms - ps1
Archaea1 - ps2
Archaea2 - ps3
Archaea3 - ps4
Asgard/Eukaryota - ps5
Eukaryota - ps6
Amorpheae - ps7
Obazoa - ps8
Opisthokonta - ps9
Holozoa - ps10
Filozoa - ps11
Choano./Metazoa - ps12
Metazoa - ps13
Myriazoa - ps14
Parahoxozoa - ps15
Bilateria - ps16
Deuterostomia - ps17
Chordata - ps18
Olfactores - ps19
Vertebrata - ps20
Gnathostomata - ps21
Euteleostomi - ps22
Sarcopterygii - ps23
Tetrapoda - ps24
Amniota - ps25
Mammalia - ps26
Theria - ps27
Placentalia - ps28
Boreoeutheria - ps29
Euarchontoglires - ps30
Euarchonta - ps31
Primates - ps32
Haplorrhini - ps33
Simiiformes - ps34
Catarrhini - ps35
Hominoidea - ps36
Hominidae - ps37
Homininae - ps38
Hominini - ps39
Homo_sapiens - ps40
Phylostratum

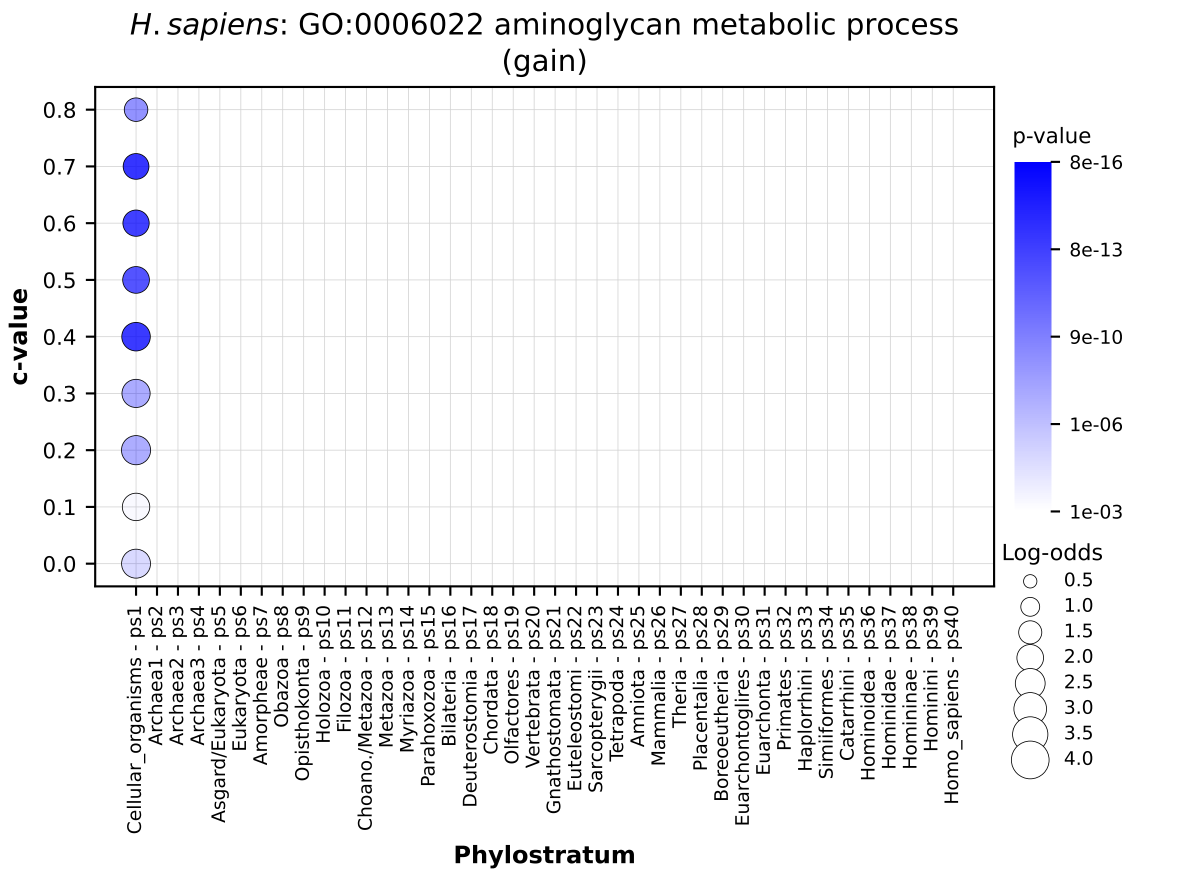

H. sapiens: GO:0006022 aminoglycan metabolic process
(gain)
c-value
0.8
0.7
0.6
0.5
0.4
0.3
0.2
0.1
0.0
p-value
8e-16
8e-13
9e-10
1e-06
1e-03
Log-odds
0.5
1.0
1.5
2.0
2.5
3.0
3.5
4.0
Cellular_organisms - ps1
Archaea1 - ps2
Archaea2 - ps3
Archaea3 - ps4
Asgard/Eukaryota - ps5
Eukaryota - ps6
Amorpheae - ps7
Obazoa - ps8
Opisthokonta - ps9
Holozoa - ps10
Filozoa - ps11
Choano./Metazoa - ps12
Metazoa - ps13
Myriazoa - ps14
Parahoxozoa - ps15
Bilateria - ps16
Deuterostomia - ps17
Chordata - ps18
Olfactores - ps19
Vertebrata - ps20
Gnathostomata - ps21
Euteleostomi - ps22
Sarcopterygii - ps23
Tetrapoda - ps24
Amniota - ps25
Mammalia - ps26
Theria - ps27
Placentalia - ps28
Boreoeutheria - ps29
Euarchontoglires - ps30
Euarchonta - ps31
Primates - ps32
Haplorrhini - ps33
Simiiformes - ps34
Catarrhini - ps35
Hominoidea - ps36
Hominidae - ps37
Homininae - ps38
Hominini - ps39
Homo_sapiens - ps40
Phylostratum

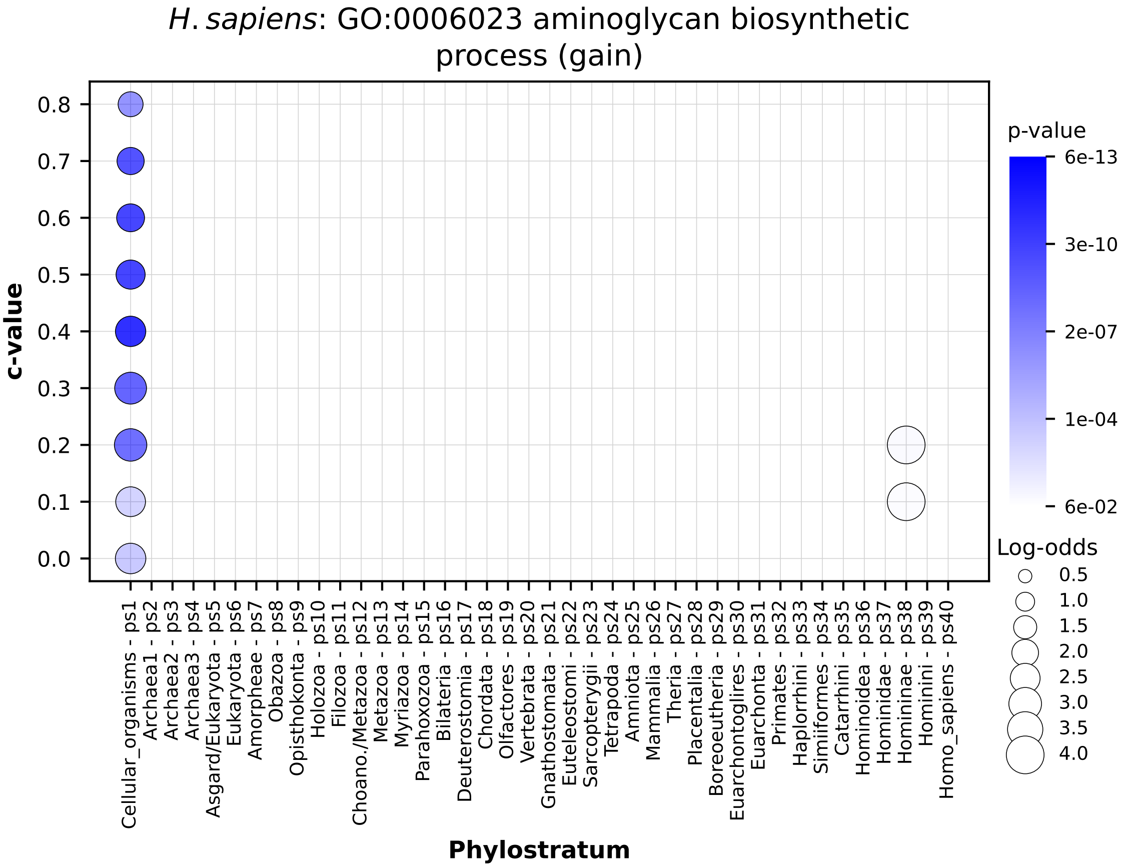

H. sapiens: GO:0006023 aminoglycan biosynthetic process (gain)
c-value
0.8
0.7
0.6
0.5
0.4
0.3
0.2
0.1
0.0
p-value
6e-13
3e-10
2e-07
1e-04
6e-02
Log-odds
0.5
1.0
1.5
2.0
2.5
3.0
3.5
4.0
Cellular_organisms - ps1
Archaea1 - ps2
Archaea2 - ps3
Archaea3 - ps4
Asgard/Eukaryota - ps5
Eukaryota - ps6
Amorpheae - ps7
Obazoa - ps8
Opisthokonta - ps9
Holozoa - ps10
Filozoa - ps11
Choano./Metazoa - ps12
Metazoa - ps13
Myriazoa - ps14
Parahoxozoa - ps15
Bilateria - ps16
Deuterostomia - ps17
Chordata - ps18
Olfactores - ps19
Vertebrata - ps20
Gnathostomata - ps21
Euteleostomi - ps22
Sarcopterygii - ps23
Tetrapoda - ps24
Amniota - ps25
Mammalia - ps26
Theria - ps27
Placentalia - ps28
Boreoeutheria - ps29
Euarchontoglires - ps30
Euarchonta - ps31
Primates - ps32
Haplorrhini - ps33
Simiiformes - ps34
Catarrhini - ps35
Hominoidea - ps36
Hominidae - ps37
Homininae - ps38
Hominini - ps39
Homo_sapiens - ps40
Phylostratum

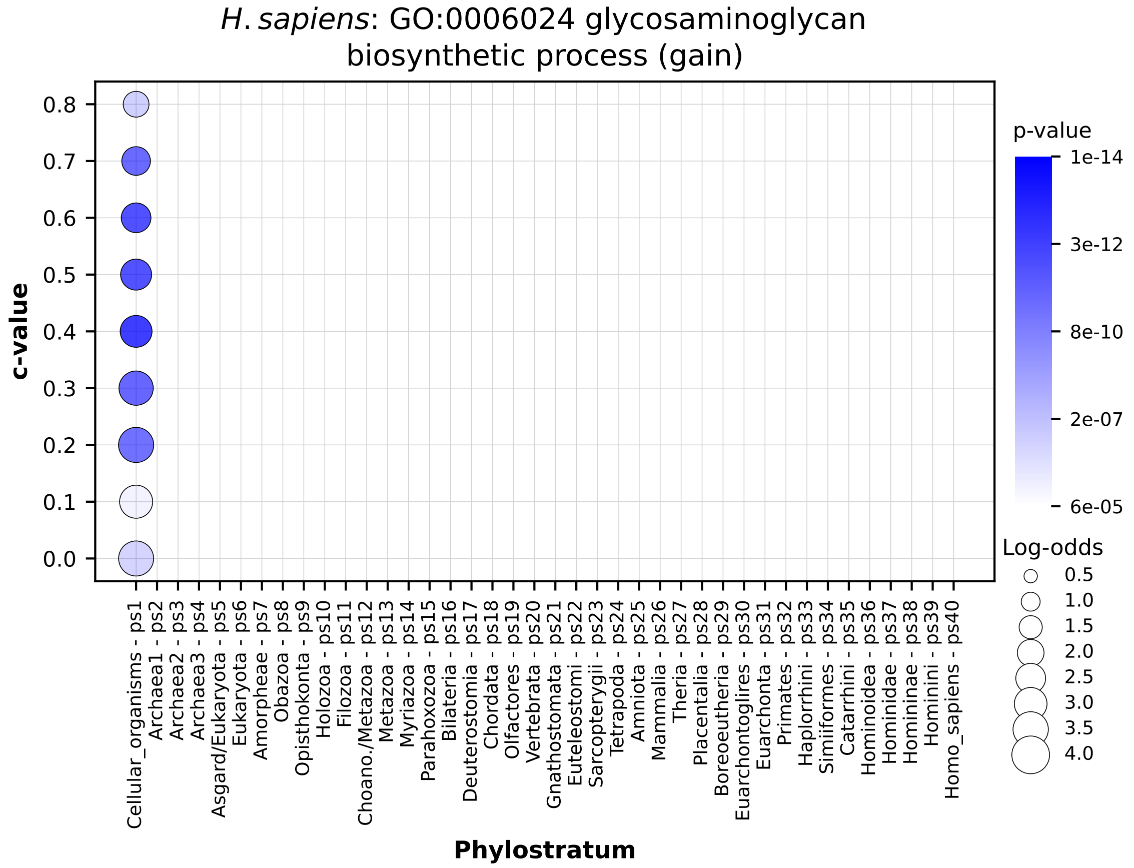
H. sapiens: GO:0006024 glycosaminoglycan biosynthetic process (gain)
c-value
0.0
0.1
0.2
0.3
0.4
0.5
0.6
0.7
0.8
Phylostratum
Cellular_organisms - ps1
Archaea1 - ps2
Archaea2 - ps3
Archaea3 - ps4
Asgard/Eukaryota - ps5
Eukaryota - ps6
Amorpheae - ps7
Obazoa - ps8
Opisthokonta - ps9
Holozoa - ps10
Filozoa - ps11
Choano./Metazoa - ps12
Metazoa - ps13
Myriazoa - ps14
Parahoxozoa - ps15
Bilateria - ps16
Deuterostomia - ps17
Chordata - ps18
Olfactores - ps19
Vertebrata - ps20
Gnathostomata - ps21
Euteleostomi - ps22
Sarcopterygii - ps23
Tetrapoda - ps24
Amniota - ps25
Mammalia - ps26
Theria - ps27
Placentalia - ps28
Boreoeutheria - ps29
Euarchontoglires - ps30
Euarchonta - ps31
Primates - ps32
Haplorrhini - ps33
Simiiformes - ps34
Catarrhini - ps35
Hominoidea - ps36
Hominidae - ps37
Homininae - ps38
Hominini - ps39
Homo_sapiens - ps40
p-value
1e-14
3e-12
8e-10
2e-07
6e-05
Log-odds
0.5
1.0
1.5
2.0
2.5
3.0
3.5
4.0

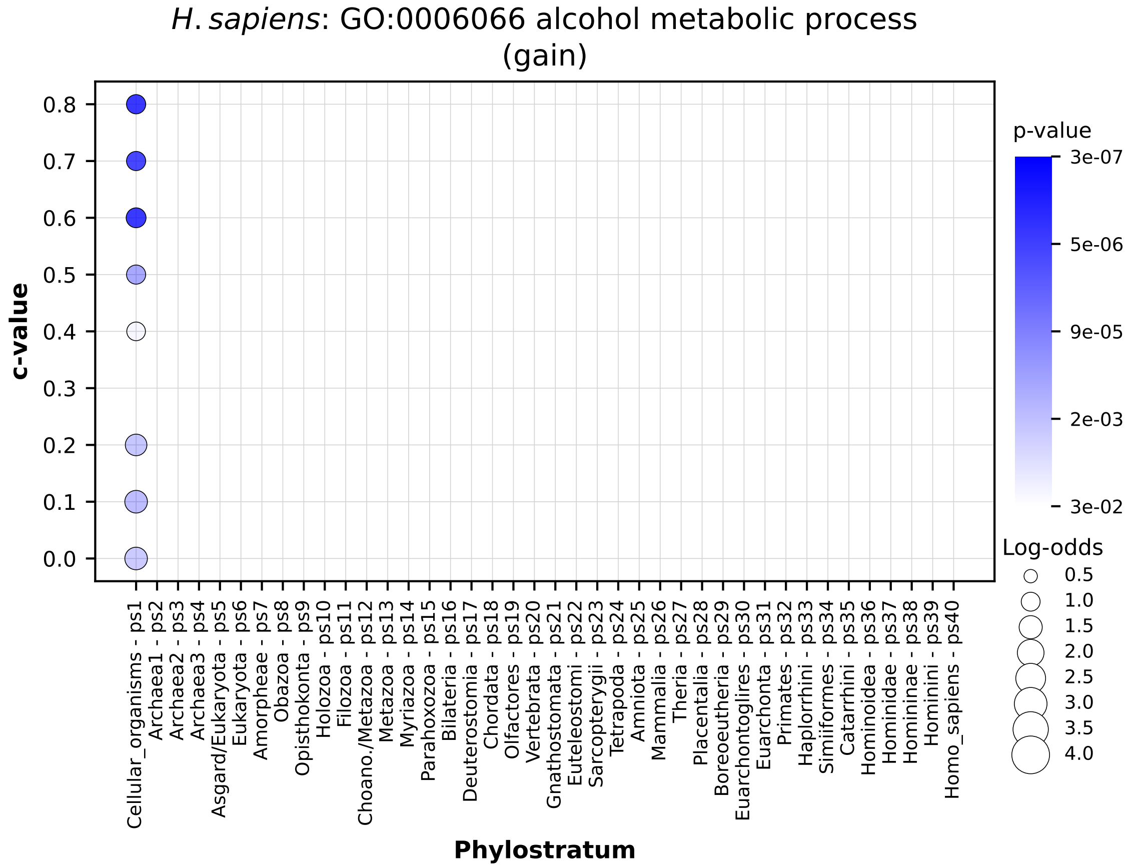

*H. sapiens*: GO:0006066 alcohol metabolic process
(gain)
c-value
0.8
0.7
0.6
0.5
0.4
0.3
0.2
0.1
0.0
p-value
3e-07
5e-06
9e-05
2e-03
3e-02
Log-odds
0.5
1.0
1.5
2.0
2.5
3.0
3.5
4.0
Cellular_organisms - ps1
Archaea1 - ps2
Archaea2 - ps3
Archaea3 - ps4
Asgard/Eukaryota - ps5
Eukaryota - ps6
Amorpheae - ps7
Obazoa - ps8
Opisthokonta - ps9
Holozoa - ps10
Filozoa - ps11
Choano./Metazoa - ps12
Metazoa - ps13
Myriazoa - ps14
Parahoxozoa - ps15
Bilateria - ps16
Deuterostomia - ps17
Chordata - ps18
Olfactores - ps19
Vertebrata - ps20
Gnathostomata - ps21
Euteleostomi - ps22
Sarcopterygii - ps23
Tetrapoda - ps24
Amniota - ps25
Mammalia - ps26
Theria - ps27
Placentalia - ps28
Boreoeutheria - ps29
Euarchontoglires - ps30
Euarchonta - ps31
Primates - ps32
Haplorrhini - ps33
Simiiformes - ps34
Catarrhini - ps35
Hominoidea - ps36
Hominidae - ps37
Homininae - ps38
Hominini - ps39
Homo_sapiens - ps40
Phylostratum

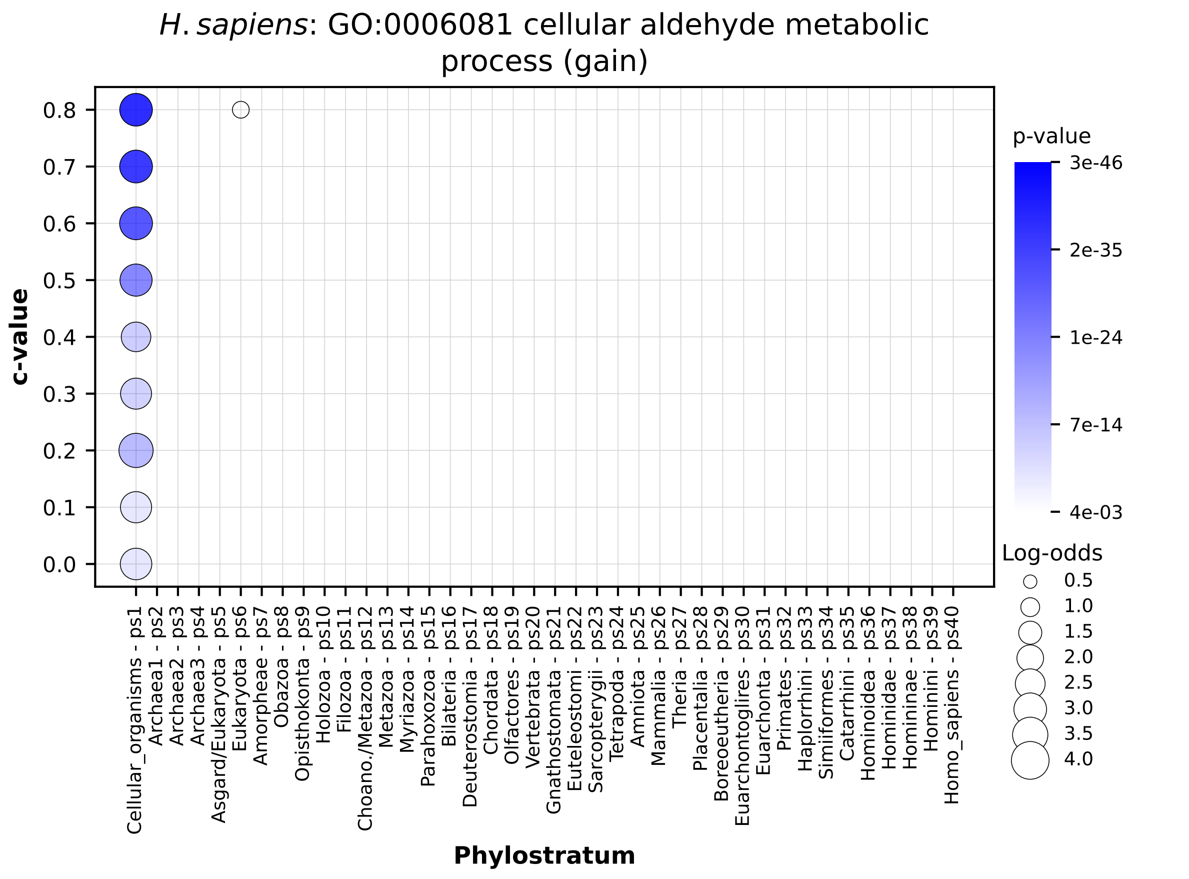

H. sapiens: GO:0006081 cellular aldehyde metabolic process (gain)
c-value
0.8
0.7
0.6
0.5
0.4
0.3
0.2
0.1
0.0
p-value
3e-46
2e-35
1e-24
7e-14
4e-03
Log-odds
0.5
1.0
1.5
2.0
2.5
3.0
3.5
4.0
Cellular_organisms - ps1
Archaea1 - ps2
Archaea2 - ps3
Archaea3 - ps4
Asgard/Eukaryota - ps5
Eukaryota - ps6
Amorpheae - ps7
Obazoa - ps8
Opisthokonta - ps9
Holozoa - ps10
Filozoa - ps11
Choano./Metazoa - ps12
Metazoa - ps13
Myriazoa - ps14
Parahoxozoa - ps15
Bilateria - ps16
Deuterostomia - ps17
Chordata - ps18
Olfactores - ps19
Vertebrata - ps20
Gnathostomata - ps21
Euteleostomi - ps22
Sarcopterygii - ps23
Tetrapoda - ps24
Amniota - ps25
Mammalia - ps26
Theria - ps27
Placentalia - ps28
Boreoeutheria - ps29
Euarchontoglires - ps30
Euarchonta - ps31
Primates - ps32
Haplorrhini - ps33
Simiiformes - ps34
Catarrhini - ps35
Hominoidea - ps36
Hominidae - ps37
Homininae - ps38
Hominini - ps39
Homo_sapiens - ps40
Phylostratum

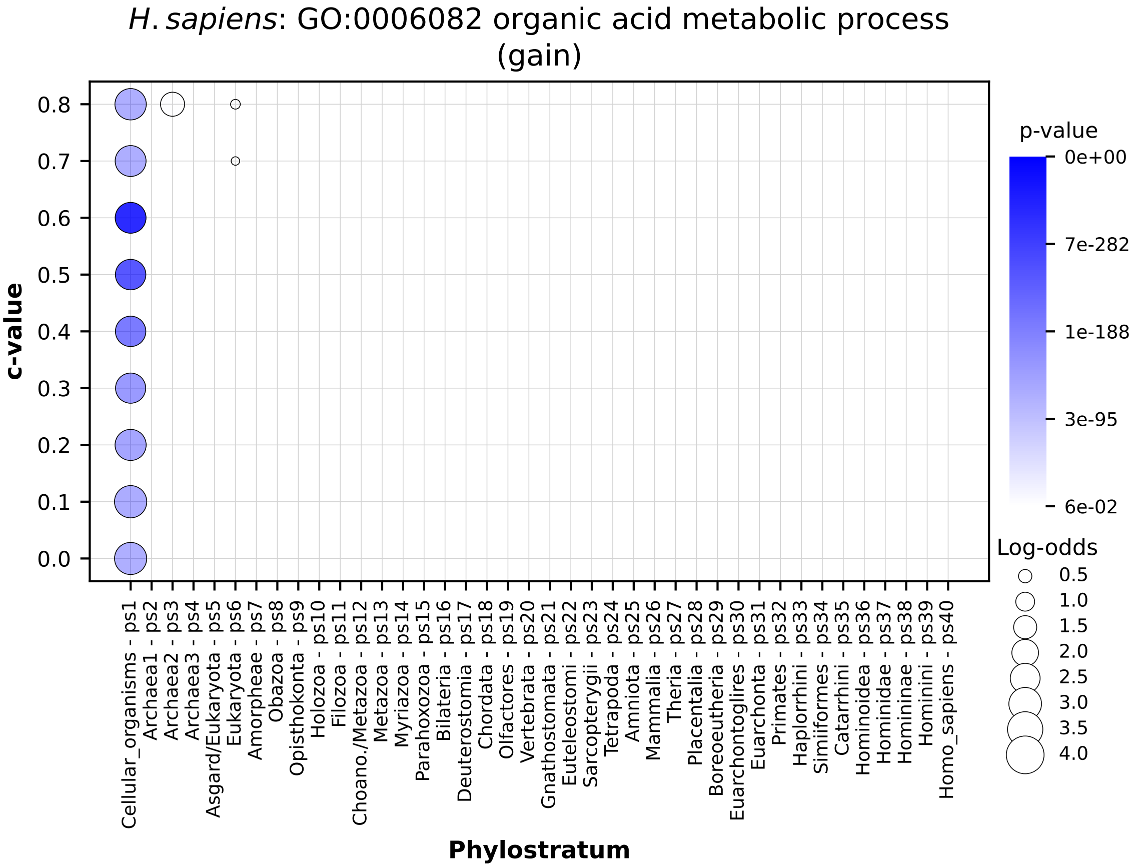
H. sapiens: GO:0006082 organic acid metabolic process
(gain)
c-value
0.8
0.7
0.6
0.5
0.4
0.3
0.2
0.1
0.0
Phylostratum
Cellular_organisms - ps1
Archaea1 - ps2
Archaea2 - ps3
Archaea3 - ps4
Asgard/Eukaryota - ps5
Eukaryota - ps6
Amorpheae - ps7
Obazoa - ps8
Opisthokonta - ps9
Holozoa - ps10
Filozoa - ps11
Choano./Metazoa - ps12
Metazoa - ps13
Myriazoa - ps14
Parahoxozoa - ps15
Bilateria - ps16
Deuterostomia - ps17
Chordata - ps18
Olfactores - ps19
Vertebrata - ps20
Gnathostomata - ps21
Euteleostomi - ps22
Sarcopterygii - ps23
Tetrapoda - ps24
Amniota - ps25
Mammalia - ps26
Theria - ps27
Placentalia - ps28
Boreoeutheria - ps29
Euarchontoglires - ps30
Euarchonta - ps31
Primates - ps32
Haplorrhini - ps33
Simiiformes - ps34
Catarrhini - ps35
Hominoidea - ps36
Hominidae - ps37
Homininae - ps38
Hominini - ps39
Homo_sapiens - ps40
p-value
0e+00
7e-282
1e-188
3e-95
6e-02
Log-odds
0.5
1.0
1.5
2.0
2.5
3.0
3.5
4.0

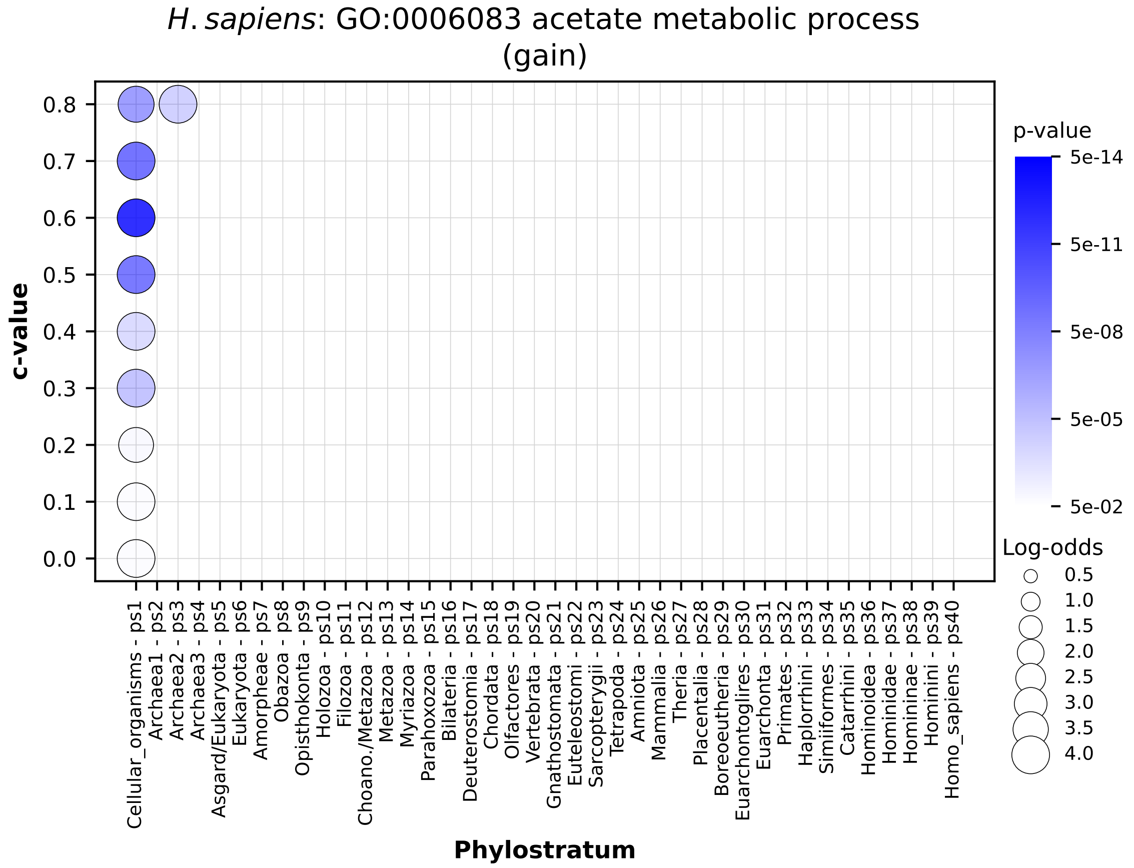
H. sapiens: GO:0006083 acetate metabolic process
(gain)
c-value
0.8
0.7
0.6
0.5
0.4
0.3
0.2
0.1
0.0
p-value
5e-14
5e-11
5e-08
5e-05
5e-02
Log-odds
0.5
1.0
1.5
2.0
2.5
3.0
3.5
4.0
Cellular_organisms - ps1
Archaea1 - ps2
Archaea2 - ps3
Archaea3 - ps4
Asgard/Eukaryota - ps5
Eukaryota - ps6
Amorpheae - ps7
Obazoa - ps8
Opisthokonta - ps9
Holozoa - ps10
Filozoa - ps11
Choano./Metazoa - ps12
Metazoa - ps13
Myriazoa - ps14
Parahoxozoa - ps15
Bilateria - ps16
Deuterostomia - ps17
Chordata - ps18
Olfactores - ps19
Vertebrata - ps20
Gnathostomata - ps21
Euteleostomi - ps22
Sarcopterygii - ps23
Tetrapoda - ps24
Amniota - ps25
Mammalia - ps26
Theria - ps27
Placentalia - ps28
Boreoeutheria - ps29
Euarchontoglires - ps30
Euarchonta - ps31
Primates - ps32
Haplorrhini - ps33
Simiiformes - ps34
Catarrhini - ps35
Hominoidea - ps36
Hominidae - ps37
Homininae - ps38
Hominini - ps39
Homo_sapiens - ps40
Phylostratum

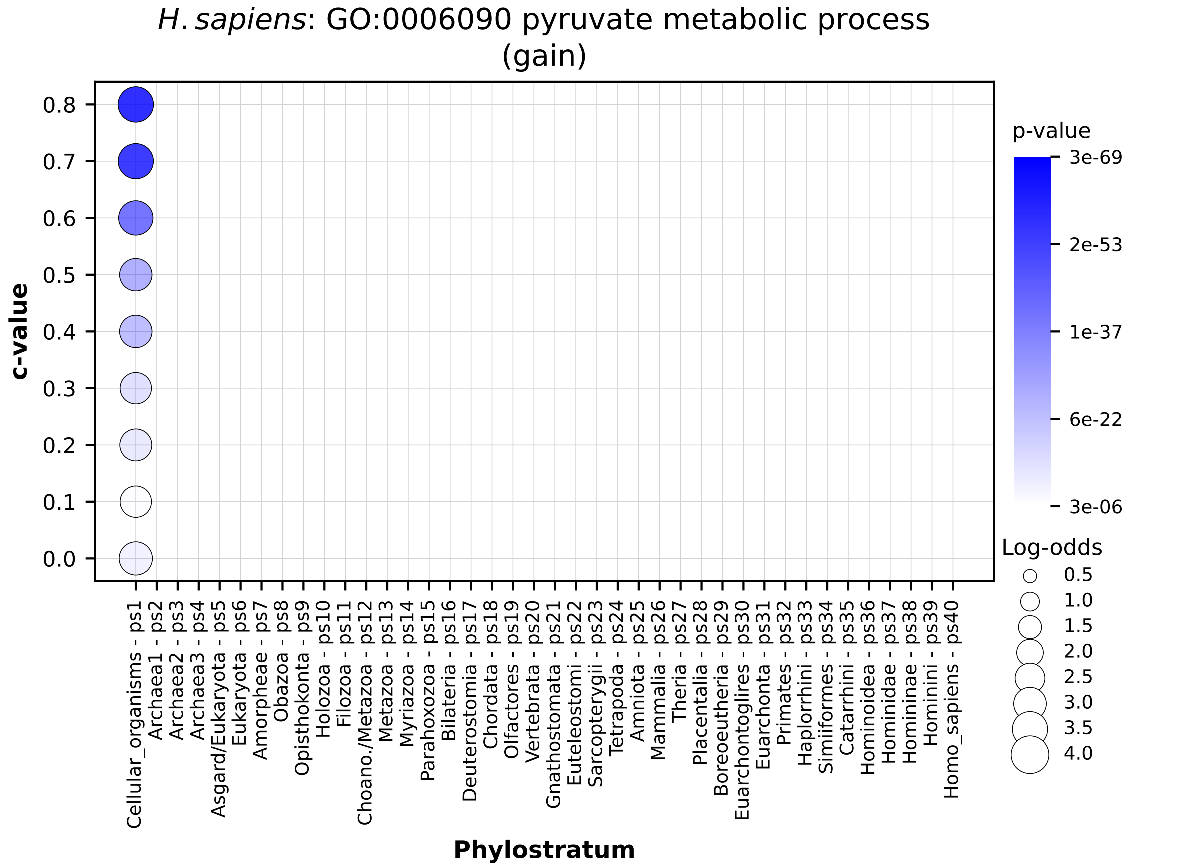

H. sapiens: GO:0006090 pyruvate metabolic process
(gain)
c-value
0.8
0.7
0.6
0.5
0.4
0.3
0.2
0.1
0.0
p-value
3e-69
2e-53
1e-37
6e-22
3e-06
Log-odds
0.5
1.0
1.5
2.0
2.5
3.0
3.5
4.0
Cellular_organisms - ps1
Archaea1 - ps2
Archaea2 - ps3
Archaea3 - ps4
Asgard/Eukaryota - ps5
Eukaryota - ps6
Amorpheae - ps7
Obazoa - ps8
Opisthokonta - ps9
Holozoa - ps10
Filozoa - ps11
Choano./Metazoa - ps12
Metazoa - ps13
Myriazoa - ps14
Parahoxozoa - ps15
Bilateria - ps16
Deuterostomia - ps17
Chordata - ps18
Olfactores - ps19
Vertebrata - ps20
Gnathostomata - ps21
Euteleostomi - ps22
Sarcopterygii - ps23
Tetrapoda - ps24
Amniota - ps25
Mammalia - ps26
Theria - ps27
Placentalia - ps28
Boreoeutheria - ps29
Euarchontoglires - ps30
Euarchonta - ps31
Primates - ps32
Haplorrhini - ps33
Simiiformes - ps34
Catarrhini - ps35
Hominoidea - ps36
Hominidae - ps37
Homininae - ps38
Hominini - ps39
Homo_sapiens - ps40
Phylostratum

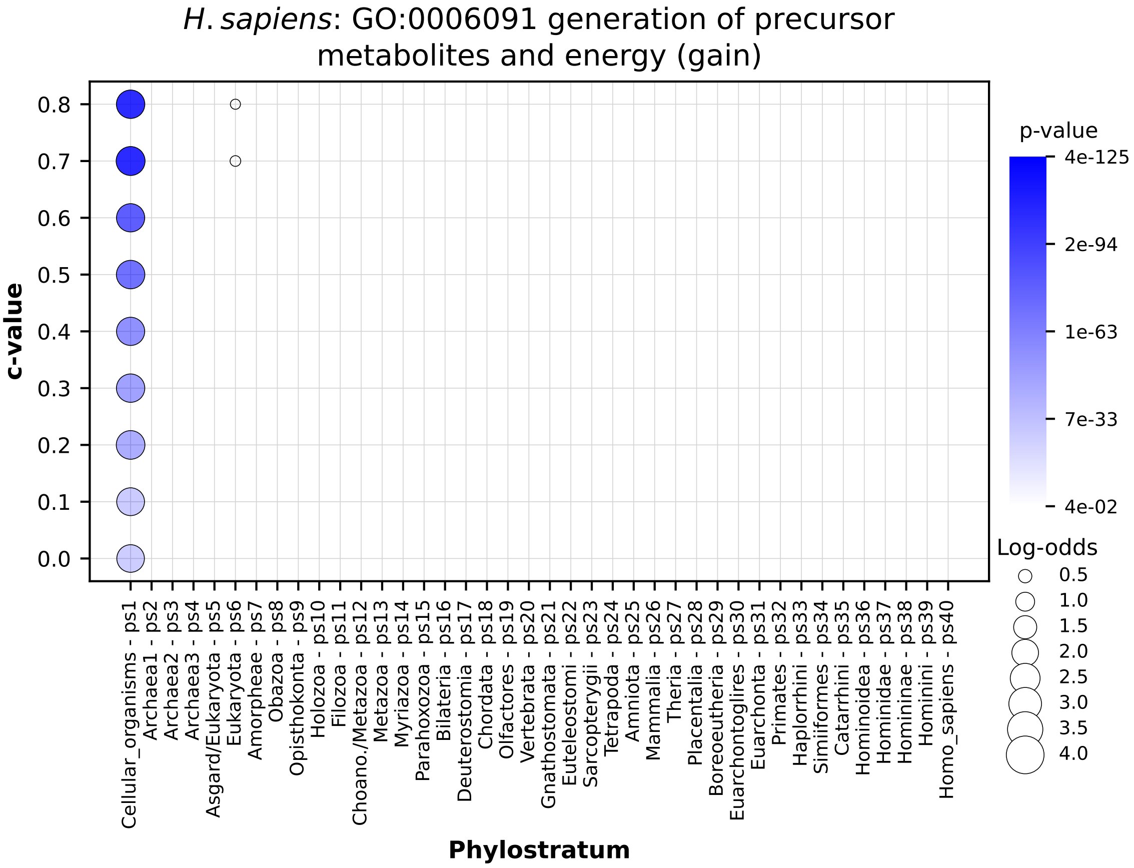

H. sapiens: GO:0006091 generation of precursor metabolites and energy (gain)
c-value
0.8
0.7
0.6
0.5
0.4
0.3
0.2
0.1
0.0
p-value
4e-125
2e-94
1e-63
7e-33
4e-02
Log-odds
0.5
1.0
1.5
2.0
2.5
3.0
3.5
4.0
Cellular_organisms - ps1
Archaea1 - ps2
Archaea2 - ps3
Archaea3 - ps4
Asgard/Eukaryota - ps5
Eukaryota - ps6
Amorpheae - ps7
Obazoa - ps8
Opisthokonta - ps9
Holozoa - ps10
Filozoa - ps11
Choano./Metazoa - ps12
Metazoa - ps13
Myriazoa - ps14
Parahoxozoa - ps15
Bilateria - ps16
Deuterostomia - ps17
Chordata - ps18
Olfactores - ps19
Vertebrata - ps20
Gnathostomata - ps21
Euteleostomi - ps22
Sarcopterygii - ps23
Tetrapoda - ps24
Amniota - ps25
Mammalia - ps26
Theria - ps27
Placentalia - ps28
Boreoeutheria - ps29
Euarchontoglires - ps30
Euarchonta - ps31
Primates - ps32
Haplorrhini - ps33
Simiiformes - ps34
Catarrhini - ps35
Hominoidea - ps36
Hominidae - ps37
Homininae - ps38
Hominini - ps39
Homo_sapiens - ps40
Phylostratum

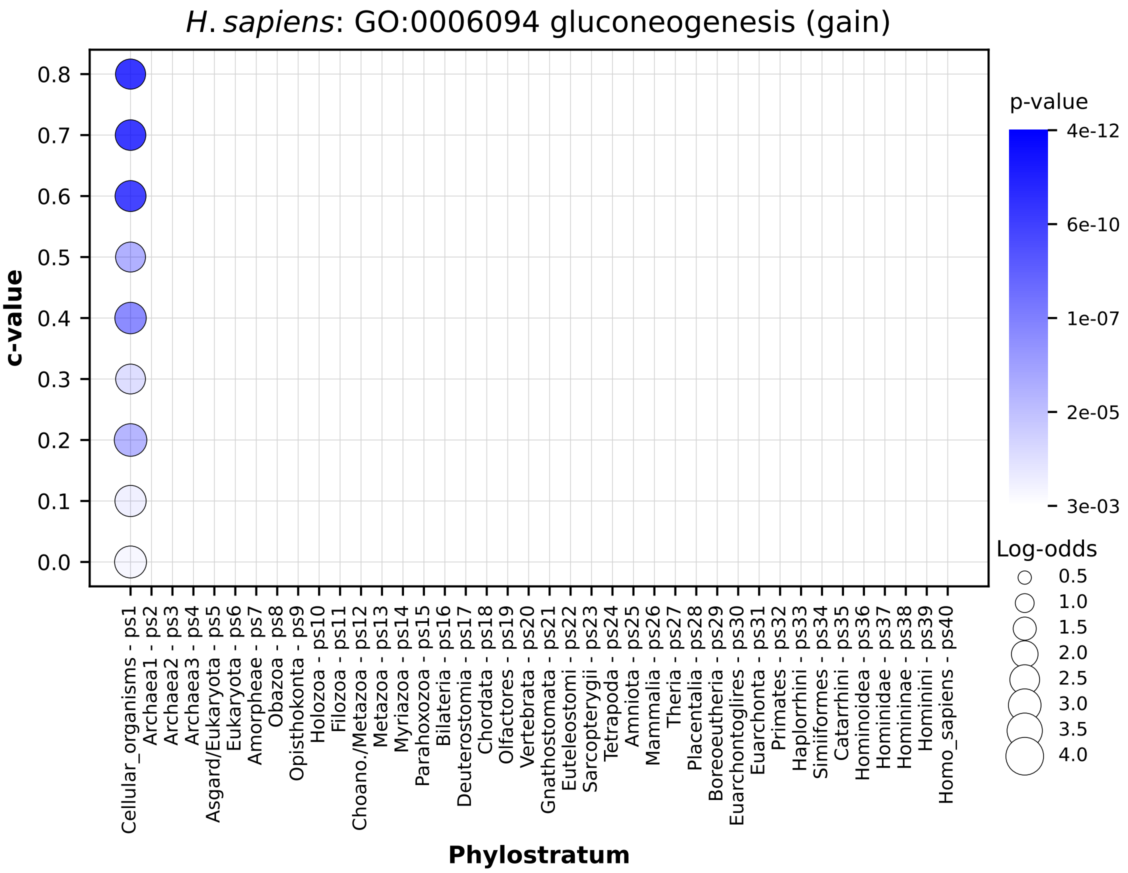

H. sapiens: GO:0006094 gluconeogenesis (gain)
c-value
0.8
0.7
0.6
0.5
0.4
0.3
0.2
0.1
0.0
p-value
4e-12
6e-10
1e-07
2e-05
3e-03
Log-odds
0.5
1.0
1.5
2.0
2.5
3.0
3.5
4.0
Cellular_organisms - ps1
Archaea1 - ps2
Archaea2 - ps3
Archaea3 - ps4
Asgard/Eukaryota - ps5
Eukaryota - ps6
Amorpheae - ps7
Obazoa - ps8
Opisthokonta - ps9
Holozoa - ps10
Filozoa - ps11
Choano./Metazoa - ps12
Metazoa - ps13
Myriazoa - ps14
Parahoxozoa - ps15
Bilateria - ps16
Deuterostomia - ps17
Chordata - ps18
Olfactores - ps19
Vertebrata - ps20
Gnathostomata - ps21
Euteleostomi - ps22
Sarcopterygii - ps23
Tetrapoda - ps24
Amniota - ps25
Mammalia - ps26
Theria - ps27
Placentalia - ps28
Boreoeutheria - ps29
Euarchontoglires - ps30
Euarchonta - ps31
Primates - ps32
Haplorrhini - ps33
Simiiformes - ps34
Catarrhini - ps35
Hominoidea - ps36
Hominidae - ps37
Homininae - ps38
Hominini - ps39
Homo_sapiens - ps40
Phylostratum

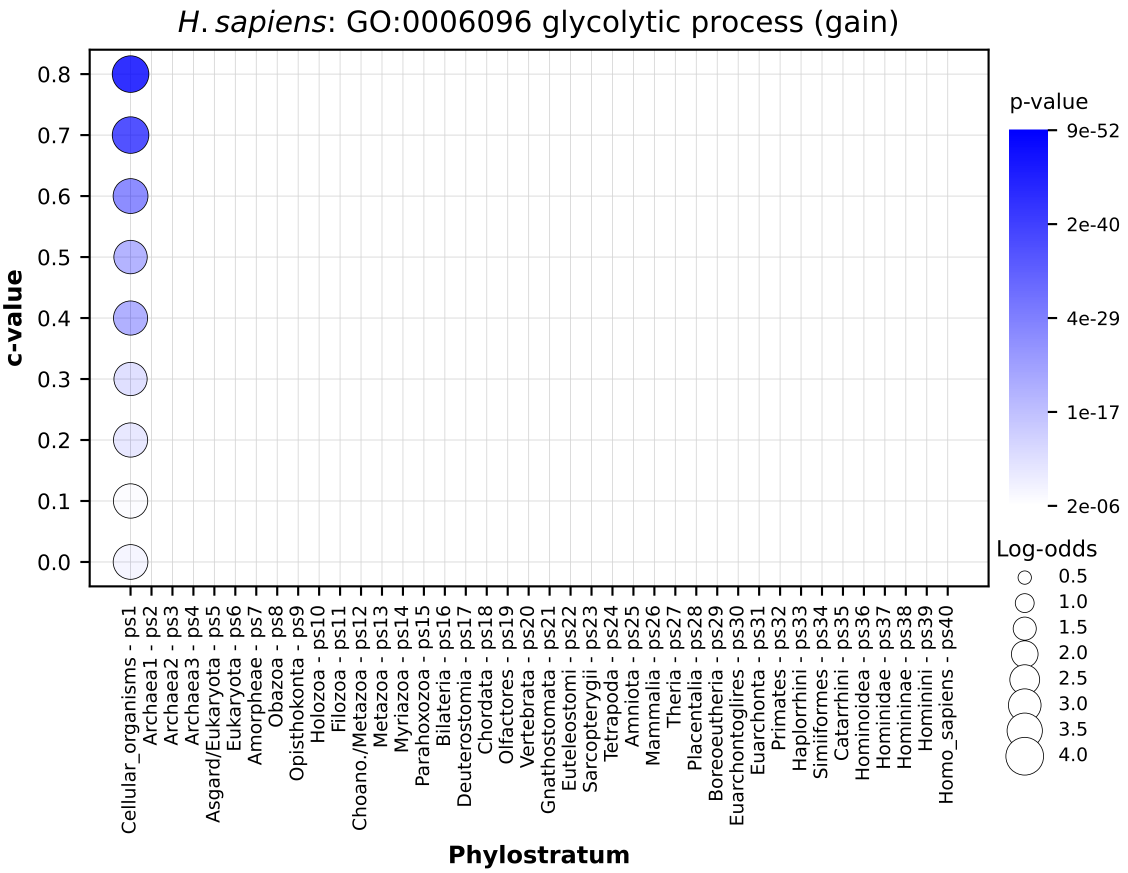

*H. sapiens*: GO:0006096 glycolytic process (gain)
c-value
0.0
0.1
0.2
0.3
0.4
0.5
0.6
0.7
0.8
p-value
9e-52
2e-40
4e-29
1e-17
2e-06
Log-odds
0.5
1.0
1.5
2.0
2.5
3.0
3.5
4.0
Cellular_organisms - ps1
Archaea1 - ps2
Archaea2 - ps3
Archaea3 - ps4
Asgard/Eukaryota - ps5
Eukaryota - ps6
Amorpheae - ps7
Obazoa - ps8
Opisthokonta - ps9
Holozoa - ps10
Filozoa - ps11
Choano./Metazoa - ps12
Metazoa - ps13
Myriazoa - ps14
Parahoxozoa - ps15
Bilateria - ps16
Deuterostomia - ps17
Chordata - ps18
Olfactores - ps19
Vertebrata - ps20
Gnathostomata - ps21
Euteleostomi - ps22
Sarcopterygii - ps23
Tetrapoda - ps24
Amniota - ps25
Mammalia - ps26
Theria - ps27
Placentalia - ps28
Boreoeutheria - ps29
Euarchontoglires - ps30
Euarchonta - ps31
Primates - ps32
Haplorrhini - ps33
Simiiformes - ps34
Catarrhini - ps35
Hominoidea - ps36
Hominidae - ps37
Homininae - ps38
Hominini - ps39
Homo_sapiens - ps40
Phylostratum

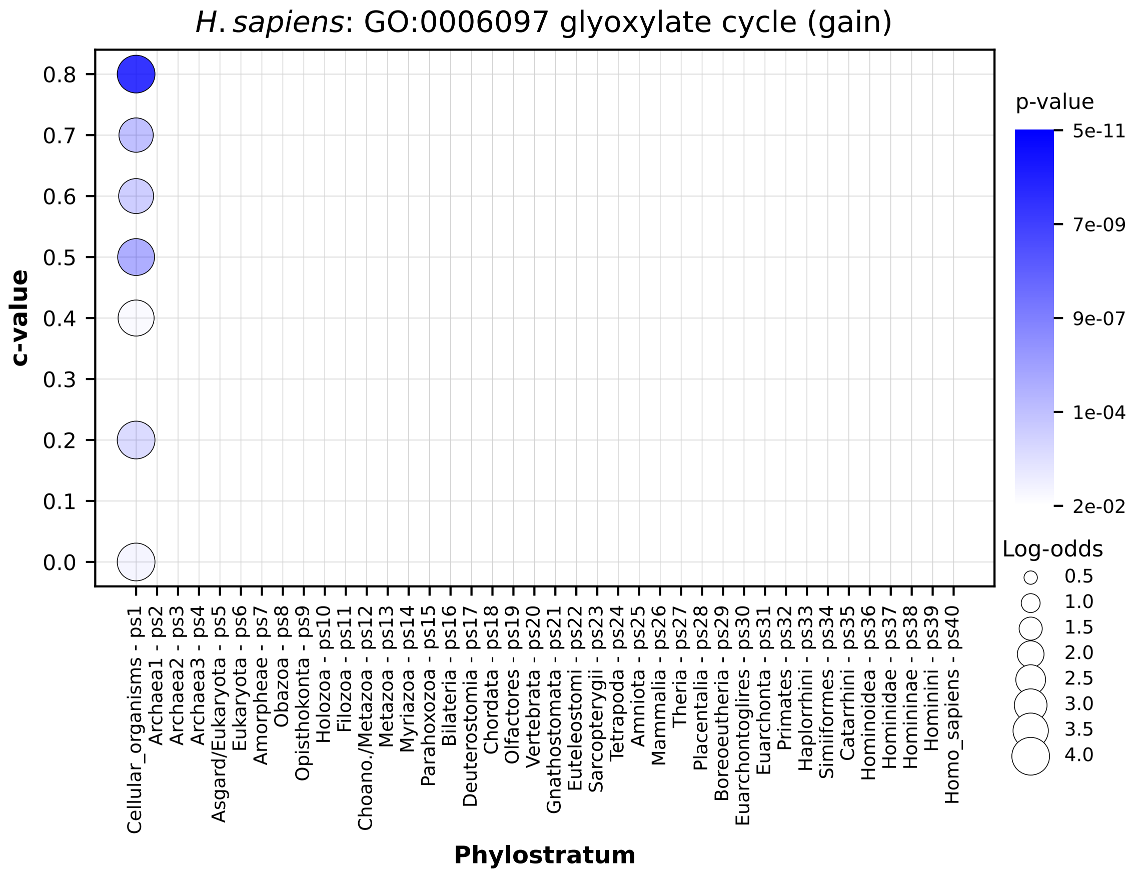

*H. sapiens*: GO:0006097 glyoxylate cycle (gain)
c-value
0.8
0.7
0.6
0.5
0.4
0.3
0.2
0.1
0.0
p-value
5e-11
7e-09
9e-07
1e-04
2e-02
Log-odds
0.5
1.0
1.5
2.0
2.5
3.0
3.5
4.0
Cellular_organisms - ps1
Archaea1 - ps2
Archaea2 - ps3
Archaea3 - ps4
Asgard/Eukaryota - ps5
Eukaryota - ps6
Amorpheae - ps7
Obazoa - ps8
Opisthokonta - ps9
Holozoa - ps10
Filozoa - ps11
Choano./Metazoa - ps12
Metazoa - ps13
Myriazoa - ps14
Parahoxozoa - ps15
Bilateria - ps16
Deuterostomia - ps17
Chordata - ps18
Olfactores - ps19
Vertebrata - ps20
Gnathostomata - ps21
Euteleostomi - ps22
Sarcopterygii - ps23
Tetrapoda - ps24
Amniota - ps25
Mammalia - ps26
Theria - ps27
Placentalia - ps28
Boreoeutheria - ps29
Euarchontoglires - ps30
Euarchonta - ps31
Primates - ps32
Haplorrhini - ps33
Simiiformes - ps34
Catarrhini - ps35
Hominoidea - ps36
Hominidae - ps37
Homininae - ps38
Hominini - ps39
Homo_sapiens - ps40
Phylostratum

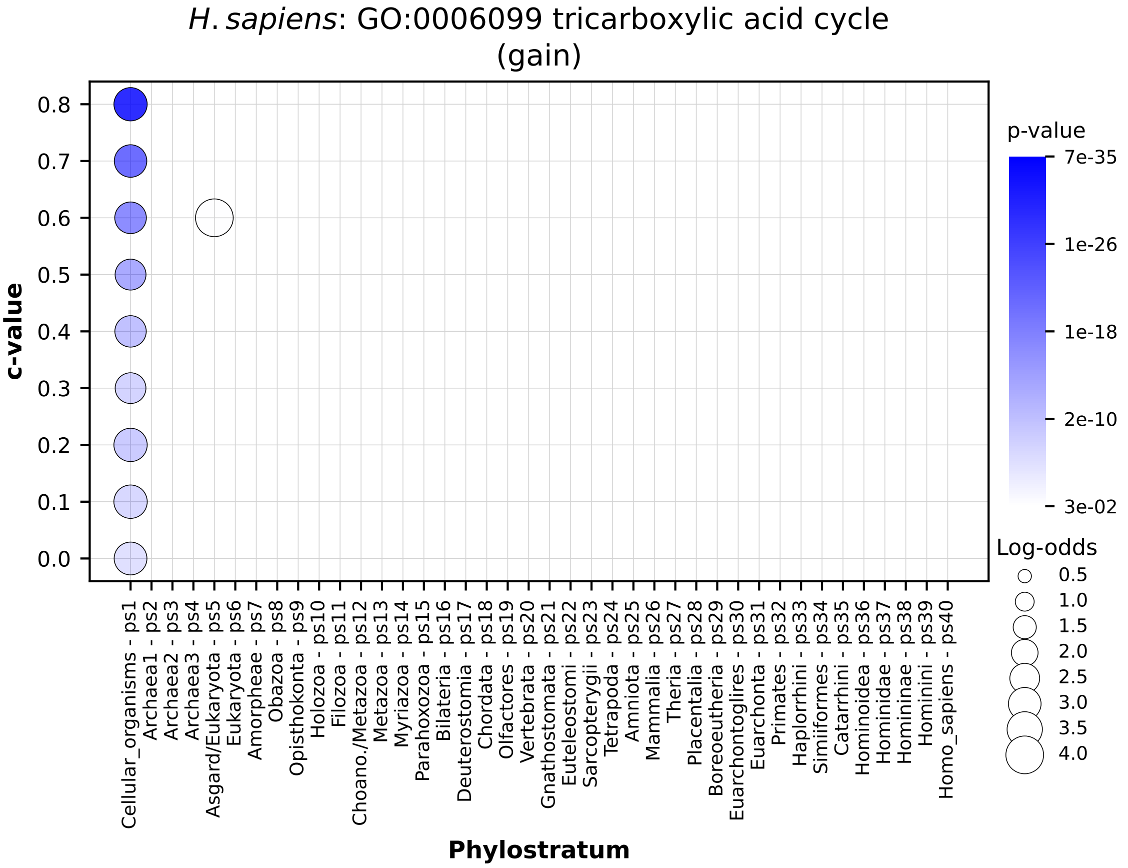

*H. sapiens*: GO:0006099 tricarboxylic acid cycle
(gain)
c-value
0.8
0.7
0.6
0.5
0.4
0.3
0.2
0.1
0.0
p-value
7e-35
1e-26
1e-18
2e-10
3e-02
Log-odds
0.5
1.0
1.5
2.0
2.5
3.0
3.5
4.0
Cellular_organisms - ps1
Archaea1 - ps2
Archaea2 - ps3
Archaea3 - ps4
Asgard/Eukaryota - ps5
Eukaryota - ps6
Amorpheae - ps7
Obazoa - ps8
Opisthokonta - ps9
Holozoa - ps10
Filozoa - ps11
Choano./Metazoa - ps12
Metazoa - ps13
Myriazoa - ps14
Parahoxozoa - ps15
Bilateria - ps16
Deuterostomia - ps17
Chordata - ps18
Olfactores - ps19
Vertebrata - ps20
Gnathostomata - ps21
Euteleostomi - ps22
Sarcopterygii - ps23
Tetrapoda - ps24
Amniota - ps25
Mammalia - ps26
Theria - ps27
Placentalia - ps28
Boreoeutheria - ps29
Euarchontoglires - ps30
Euarchonta - ps31
Primates - ps32
Haplorrhini - ps33
Simiiformes - ps34
Catarrhini - ps35
Hominoidea - ps36
Hominidae - ps37
Homininae - ps38
Hominini - ps39
Homo_sapiens - ps40
Phylostratum

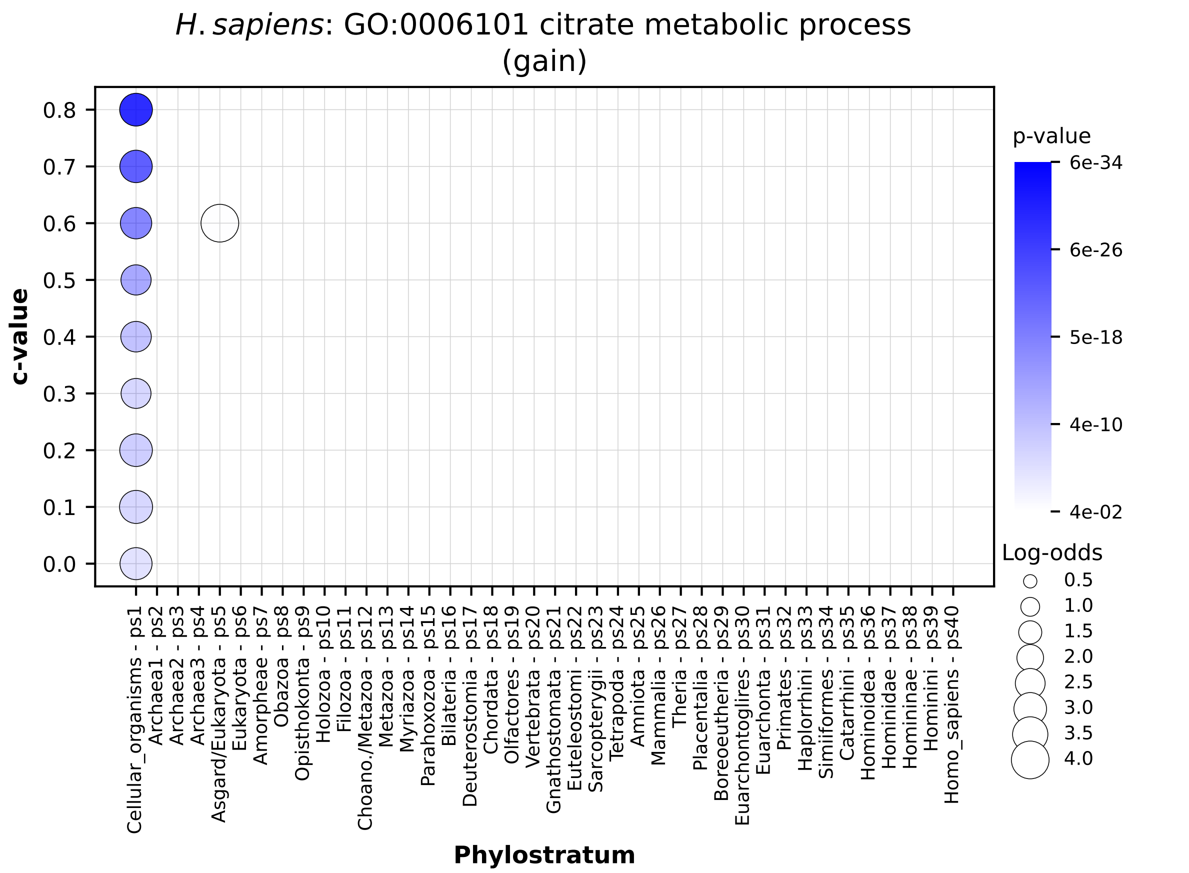
H. sapiens: GO:0006101 citrate metabolic process
(gain)
c-value
0.8
0.7
0.6
0.5
0.4
0.3
0.2
0.1
0.0
p-value
6e-34
6e-26
5e-18
4e-10
4e-02
Log-odds
0.5
1.0
1.5
2.0
2.5
3.0
3.5
4.0
Cellular_organisms - ps1
Archaea1 - ps2
Archaea2 - ps3
Archaea3 - ps4
Asgard/Eukaryota - ps5
Eukaryota - ps6
Amorpheae - ps7
Obazoa - ps8
Opisthokonta - ps9
Holozoa - ps10
Filozoa - ps11
Choano./Metazoa - ps12
Metazoa - ps13
Myriazoa - ps14
Parahoxozoa - ps15
Bilateria - ps16
Deuterostomia - ps17
Chordata - ps18
Olfactores - ps19
Vertebrata - ps20
Gnathostomata - ps21
Euteleostomi - ps22
Sarcopterygii - ps23
Tetrapoda - ps24
Amniota - ps25
Mammalia - ps26
Theria - ps27
Placentalia - ps28
Boreoeutheria - ps29
Euarchontoglires - ps30
Euarchonta - ps31
Primates - ps32
Haplorrhini - ps33
Simiiformes - ps34
Catarrhini - ps35
Hominoidea - ps36
Hominidae - ps37
Homininae - ps38
Hominini - ps39
Homo_sapiens - ps40
Phylostratum

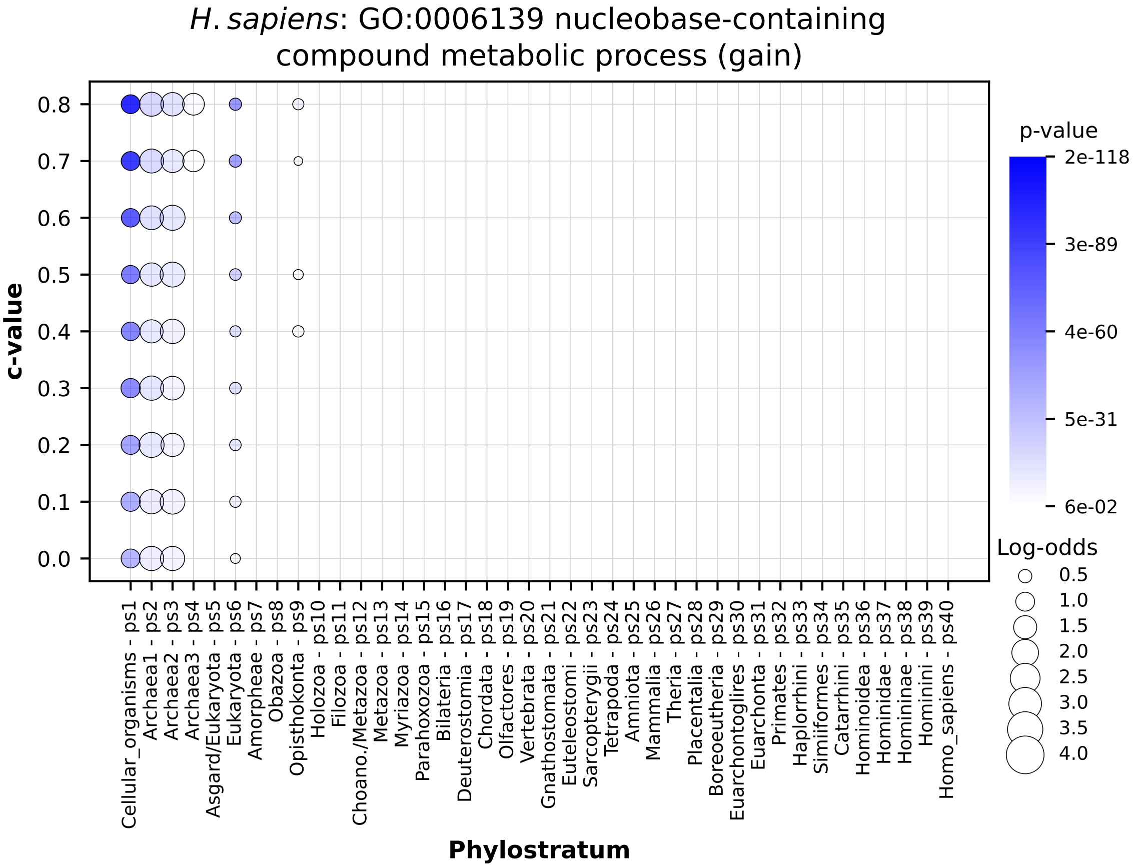
H. sapiens: GO:0006139 nucleobase-containing compound metabolic process (gain)
c-value
0.8
0.7
0.6
0.5
0.4
0.3
0.2
0.1
0.0
Phylostratum
Cellular_organisms - ps1
Archaea1 - ps2
Archaea2 - ps3
Archaea3 - ps4
Asgard/Eukaryota - ps5
Eukaryota - ps6
Amorpheae - ps7
Obazoa - ps8
Opisthokonta - ps9
Holozoa - ps10
Filozoa - ps11
Choano./Metazoa - ps12
Metazoa - ps13
Myriazoa - ps14
Parahoxozoa - ps15
Bilateria - ps16
Deuterostomia - ps17
Chordata - ps18
Olfactores - ps19
Vertebrata - ps20
Gnathostomata - ps21
Euteleostomi - ps22
Sarcopterygii - ps23
Tetrapoda - ps24
Amniota - ps25
Mammalia - ps26
Theria - ps27
Placentalia - ps28
Boreoeutheria - ps29
Euarchontoglires - ps30
Euarchonta - ps31
Primates - ps32
Haplorrhini - ps33
Simiiformes - ps34
Catarrhini - ps35
Hominoidea - ps36
Hominidae - ps37
Homininae - ps38
Hominini - ps39
Homo_sapiens - ps40
p-value
2e-118
3e-89
4e-60
5e-31
6e-02
Log-odds
0.5
1.0
1.5
2.0
2.5
3.0
3.5
4.0

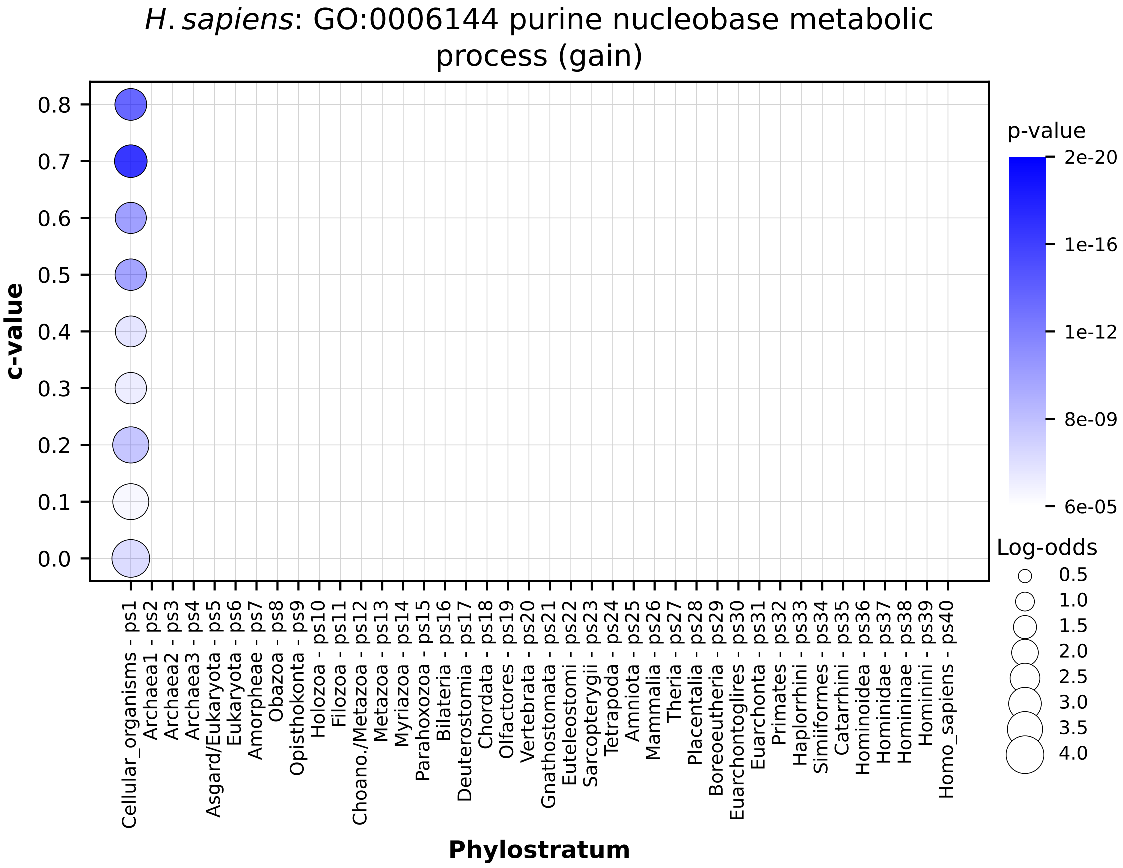
H. sapiens: GO:0006144 purine nucleobase metabolic process (gain)
c-value
0.0
0.1
0.2
0.3
0.4
0.5
0.6
0.7
0.8
Phylostratum
Cellular_organisms - ps1
Archaea1 - ps2
Archaea2 - ps3
Archaea3 - ps4
Asgard/Eukaryota - ps5
Eukaryota - ps6
Amorpheae - ps7
Obazoa - ps8
Opisthokonta - ps9
Holozoa - ps10
Filozoa - ps11
Choano./Metazoa - ps12
Metazoa - ps13
Myriazoa - ps14
Parahoxozoa - ps15
Bilateria - ps16
Deuterostomia - ps17
Chordata - ps18
Olfactores - ps19
Vertebrata - ps20
Gnathostomata - ps21
Euteleostomi - ps22
Sarcopterygii - ps23
Tetrapoda - ps24
Amniota - ps25
Mammalia - ps26
Theria - ps27
Placentalia - ps28
Boreoeutheria - ps29
Euarchontoglires - ps30
Euarchonta - ps31
Primates - ps32
Haplorrhini - ps33
Simiiformes - ps34
Catarrhini - ps35
Hominoidea - ps36
Hominidae - ps37
Homininae - ps38
Hominini - ps39
Homo_sapiens - ps40
p-value
2e-20
1e-16
1e-12
8e-09
6e-05
Log-odds
0.5
1.0
1.5
2.0
2.5
3.0
3.5
4.0

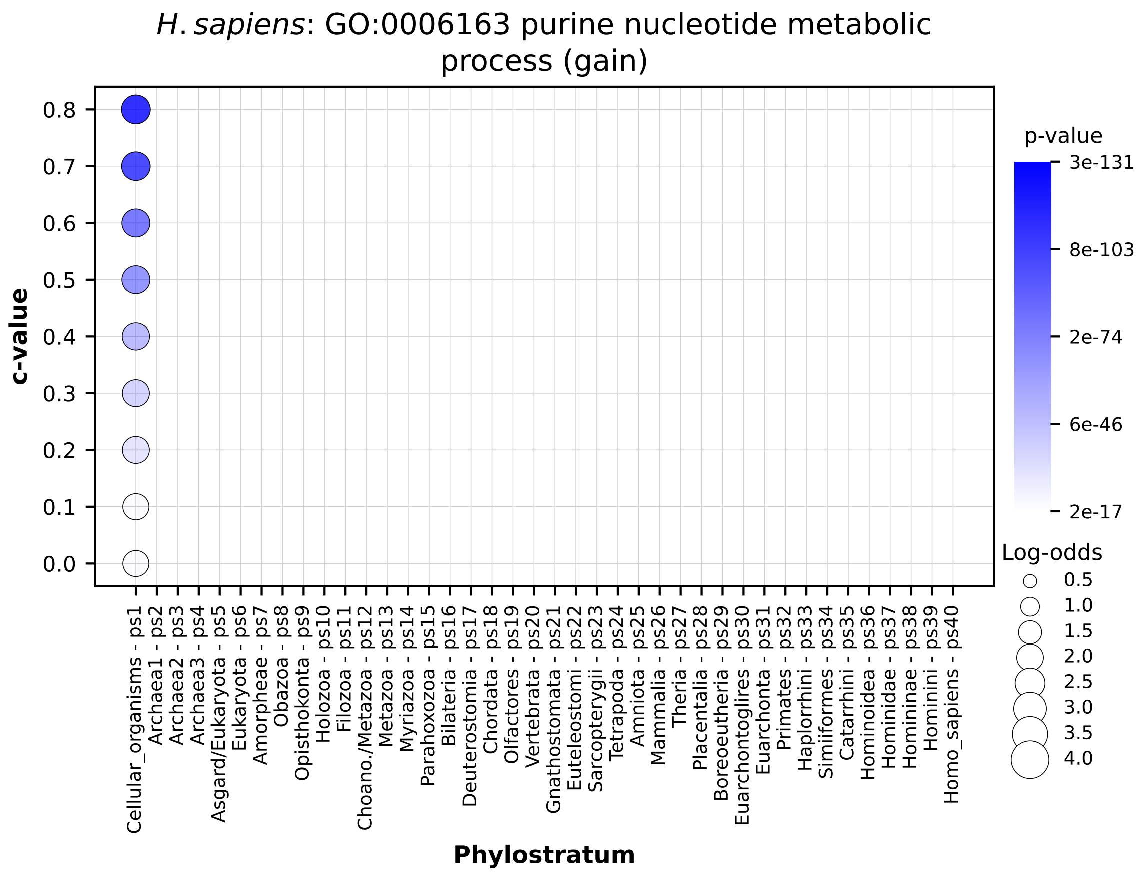
H. sapiens: GO:0006163 purine nucleotide metabolic process (gain)
c-value
0.8
0.7
0.6
0.5
0.4
0.3
0.2
0.1
0.0
Phylostratum
Cellular_organisms - ps1
Archaea1 - ps2
Archaea2 - ps3
Archaea3 - ps4
Asgard/Eukaryota - ps5
Eukaryota - ps6
Amorpheae - ps7
Obazoa - ps8
Opisthokonta - ps9
Holozoa - ps10
Filozoa - ps11
Choano./Metazoa - ps12
Metazoa - ps13
Myriazoa - ps14
Parahoxozoa - ps15
Bilateria - ps16
Deuterostomia - ps17
Chordata - ps18
Olfactores - ps19
Vertebrata - ps20
Gnathostomata - ps21
Euteleostomi - ps22
Sarcopterygii - ps23
Tetrapoda - ps24
Amniota - ps25
Mammalia - ps26
Theria - ps27
Placentalia - ps28
Boreoeutheria - ps29
Euarchontoglires - ps30
Euarchonta - ps31
Primates - ps32
Haplorrhini - ps33
Simiiformes - ps34
Catarrhini - ps35
Hominoidea - ps36
Hominidae - ps37
Homininae - ps38
Hominini - ps39
Homo_sapiens - ps40
p-value
3e-131
8e-103
2e-74
6e-46
2e-17
Log-odds
0.5
1.0
1.5
2.0
2.5
3.0
3.5
4.0

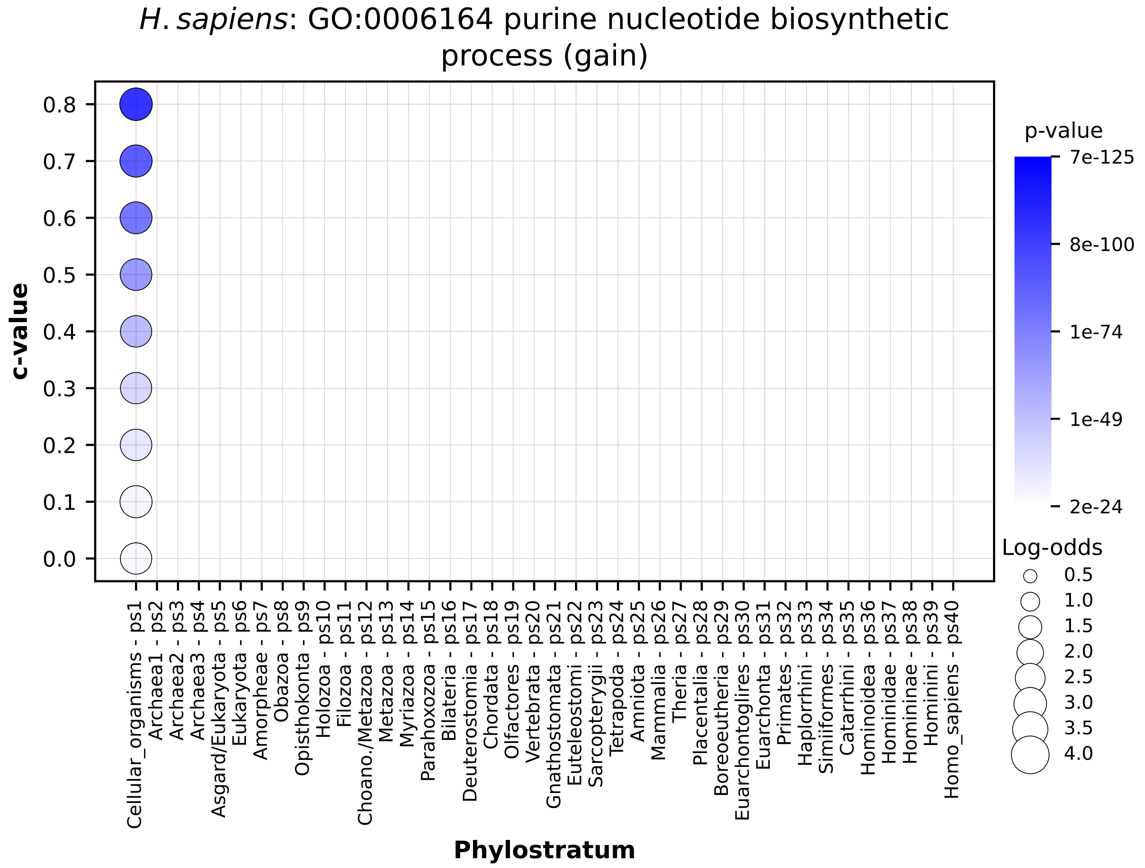

H. sapiens: GO:0006164 purine nucleotide biosynthetic process (gain)
c-value
0.8
0.7
0.6
0.5
0.4
0.3
0.2
0.1
0.0
Phylostratum
Cellular_organisms - ps1
Archaea1 - ps2
Archaea2 - ps3
Archaea3 - ps4
Asgard/Eukaryota - ps5
Eukaryota - ps6
Amorpheae - ps7
Obazoa - ps8
Opisthokonta - ps9
Holozoa - ps10
Filozoa - ps11
Choano./Metazoa - ps12
Metazoa - ps13
Myriazoa - ps14
Parahoxozoa - ps15
Bilateria - ps16
Deuterostomia - ps17
Chordata - ps18
Olfactores - ps19
Vertebrata - ps20
Gnathostomata - ps21
Euteleostomi - ps22
Sarcopterygii - ps23
Tetrapoda - ps24
Amniota - ps25
Mammalia - ps26
Theria - ps27
Placentalia - ps28
Boreoeutheria - ps29
Euarchontoglires - ps30
Euarchonta - ps31
Primates - ps32
Haplorrhini - ps33
Simiiformes - ps34
Catarrhini - ps35
Hominoidea - ps36
Hominidae - ps37
Homininae - ps38
Hominini - ps39
Homo_sapiens - ps40
p-value
7e-125
8e-100
1e-74
1e-49
2e-24
Log-odds
0.5
1.0
1.5
2.0
2.5
3.0
3.5
4.0

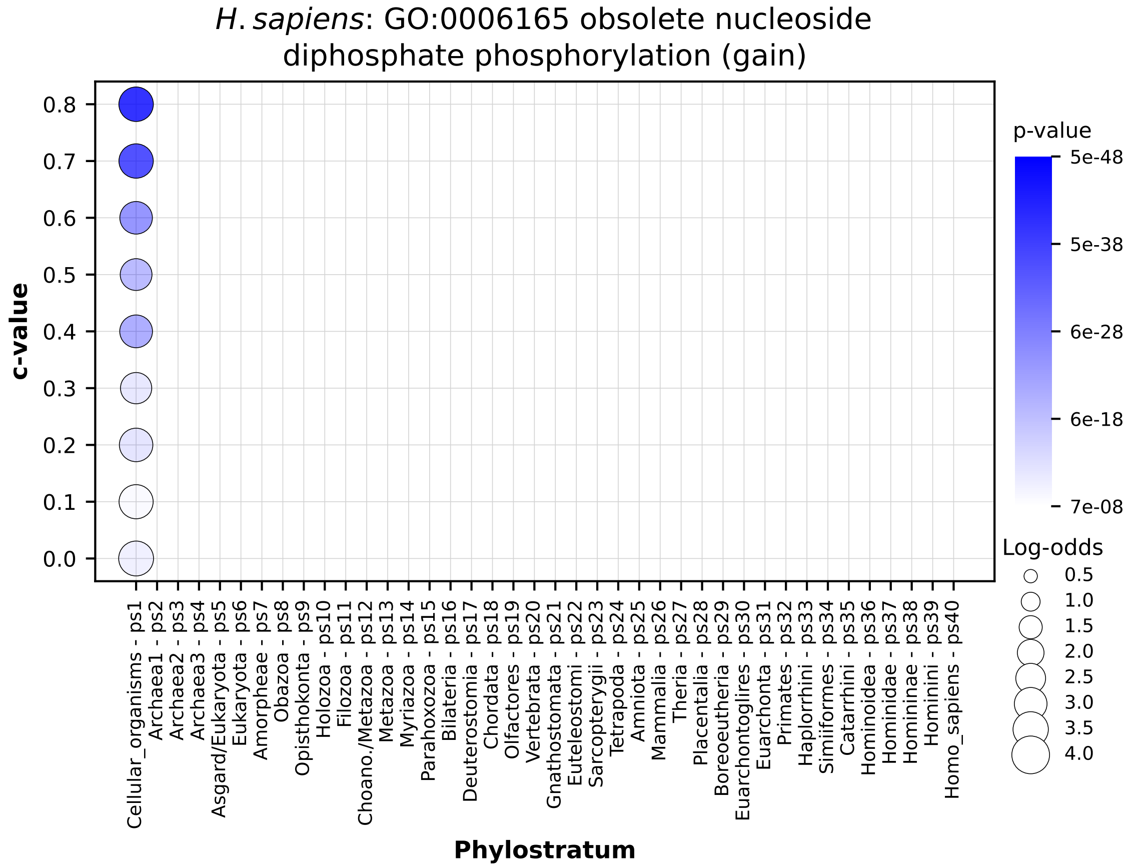
H. sapiens: GO:0006165 obsolete nucleoside diphosphate phosphorylation (gain)
c-value
0.8
0.7
0.6
0.5
0.4
0.3
0.2
0.1
0.0
p-value
5e-48
5e-38
6e-28
6e-18
7e-08
Log-odds
0.5
1.0
1.5
2.0
2.5
3.0
3.5
4.0
Cellular_organisms - ps1
Archaea1 - ps2
Archaea2 - ps3
Archaea3 - ps4
Asgard/Eukaryota - ps5
Eukaryota - ps6
Amorpheae - ps7
Obazoa - ps8
Opisthokonta - ps9
Holozoa - ps10
Filozoa - ps11
Choano./Metazoa - ps12
Metazoa - ps13
Myriazoa - ps14
Parahoxozoa - ps15
Bilateria - ps16
Deuterostomia - ps17
Chordata - ps18
Olfactores - ps19
Vertebrata - ps20
Gnathostomata - ps21
Euteleostomi - ps22
Sarcopterygii - ps23
Tetrapoda - ps24
Amniota - ps25
Mammalia - ps26
Theria - ps27
Placentalia - ps28
Boreoeutheria - ps29
Euarchontoglires - ps30
Euarchonta - ps31
Primates - ps32
Haplorrhini - ps33
Simiiformes - ps34
Catarrhini - ps35
Hominoidea - ps36
Hominidae - ps37
Homininae - ps38
Hominini - ps39
Homo_sapiens - ps40
Phylostratum

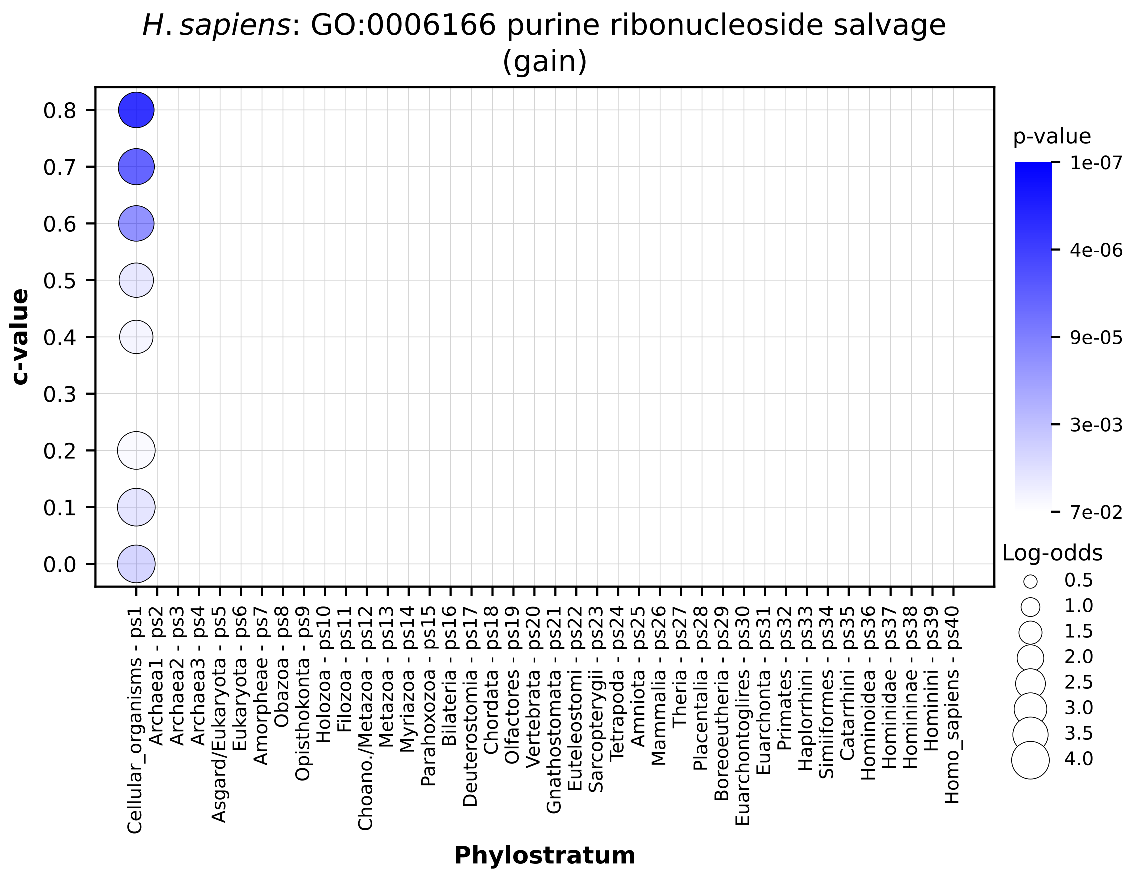

H. sapiens: GO:0006166 purine ribonucleoside salvage
(gain)
c-value
0.8
0.7
0.6
0.5
0.4
0.3
0.2
0.1
0.0
p-value
1e-07
4e-06
9e-05
3e-03
7e-02
Log-odds
0.5
1.0
1.5
2.0
2.5
3.0
3.5
4.0
Cellular_organisms - ps1
Archaea1 - ps2
Archaea2 - ps3
Archaea3 - ps4
Asgard/Eukaryota - ps5
Eukaryota - ps6
Amorpheae - ps7
Obazoa - ps8
Opisthokonta - ps9
Holozoa - ps10
Filozoa - ps11
Choano./Metazoa - ps12
Metazoa - ps13
Myriazoa - ps14
Parahoxozoa - ps15
Bilateria - ps16
Deuterostomia - ps17
Chordata - ps18
Olfactores - ps19
Vertebrata - ps20
Gnathostomata - ps21
Euteleostomi - ps22
Sarcopterygii - ps23
Tetrapoda - ps24
Amniota - ps25
Mammalia - ps26
Theria - ps27
Placentalia - ps28
Boreoeutheria - ps29
Euarchontoglires - ps30
Euarchonta - ps31
Primates - ps32
Haplorrhini - ps33
Simiiformes - ps34
Catarrhini - ps35
Hominoidea - ps36
Hominidae - ps37
Homininae - ps38
Hominini - ps39
Homo_sapiens - ps40
Phylostratum

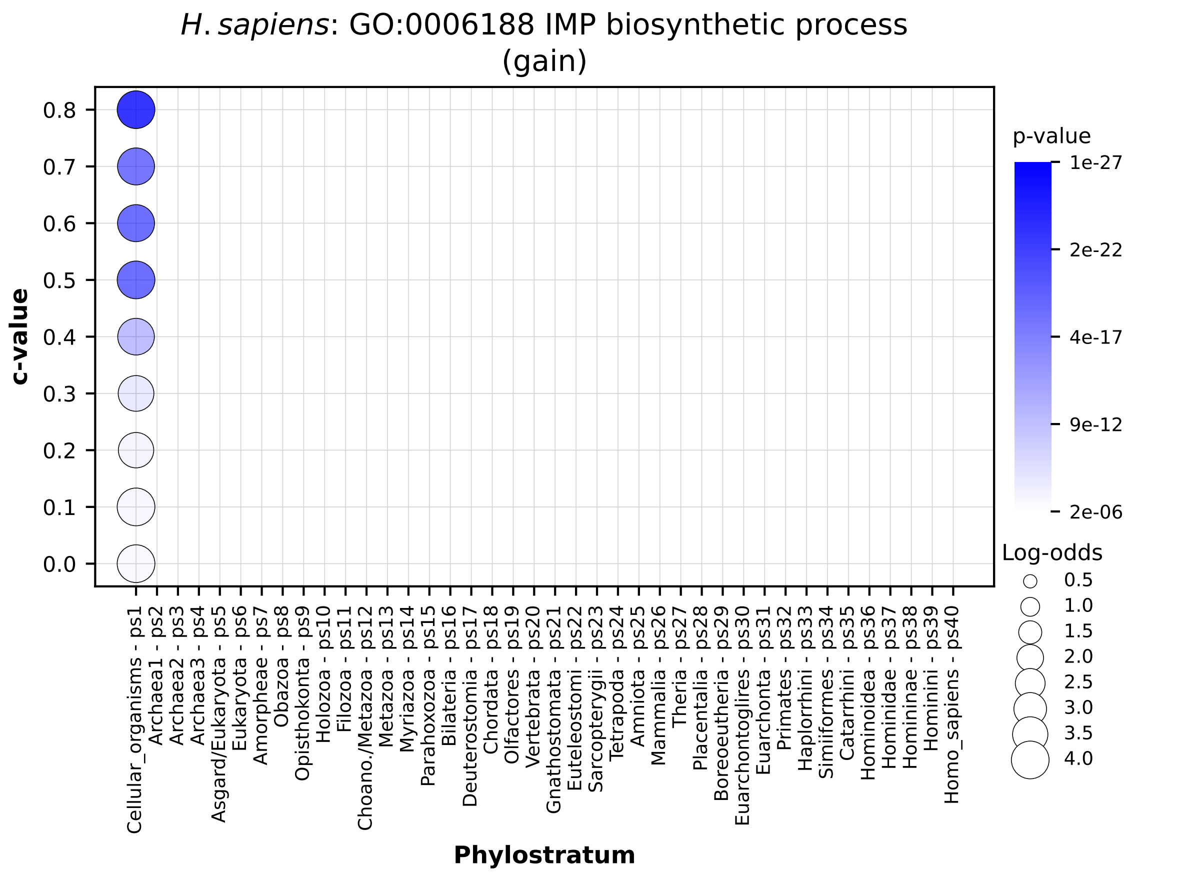

H. sapiens: GO:0006188 IMP biosynthetic process
(gain)
c-value
0.0
0.1
0.2
0.3
0.4
0.5
0.6
0.7
0.8
Phylostratum
Cellular_organisms - ps1
Archaea1 - ps2
Archaea2 - ps3
Archaea3 - ps4
Asgard/Eukaryota - ps5
Eukaryota - ps6
Amorpheae - ps7
Obazoa - ps8
Opisthokonta - ps9
Holozoa - ps10
Filozoa - ps11
Choano./Metazoa - ps12
Metazoa - ps13
Myriazoa - ps14
Parahoxozoa - ps15
Bilateria - ps16
Deuterostomia - ps17
Chordata - ps18
Olfactores - ps19
Vertebrata - ps20
Gnathostomata - ps21
Euteleostomi - ps22
Sarcopterygii - ps23
Tetrapoda - ps24
Amniota - ps25
Mammalia - ps26
Theria - ps27
Placentalia - ps28
Boreoeutheria - ps29
Euarchontoglires - ps30
Euarchonta - ps31
Primates - ps32
Haplorrhini - ps33
Simiiformes - ps34
Catarrhini - ps35
Hominoidea - ps36
Hominidae - ps37
Homininae - ps38
Hominini - ps39
Homo_sapiens - ps40
p-value
1e-27
2e-22
4e-17
9e-12
2e-06
Log-odds
0.5
1.0
1.5
2.0
2.5
3.0
3.5
4.0

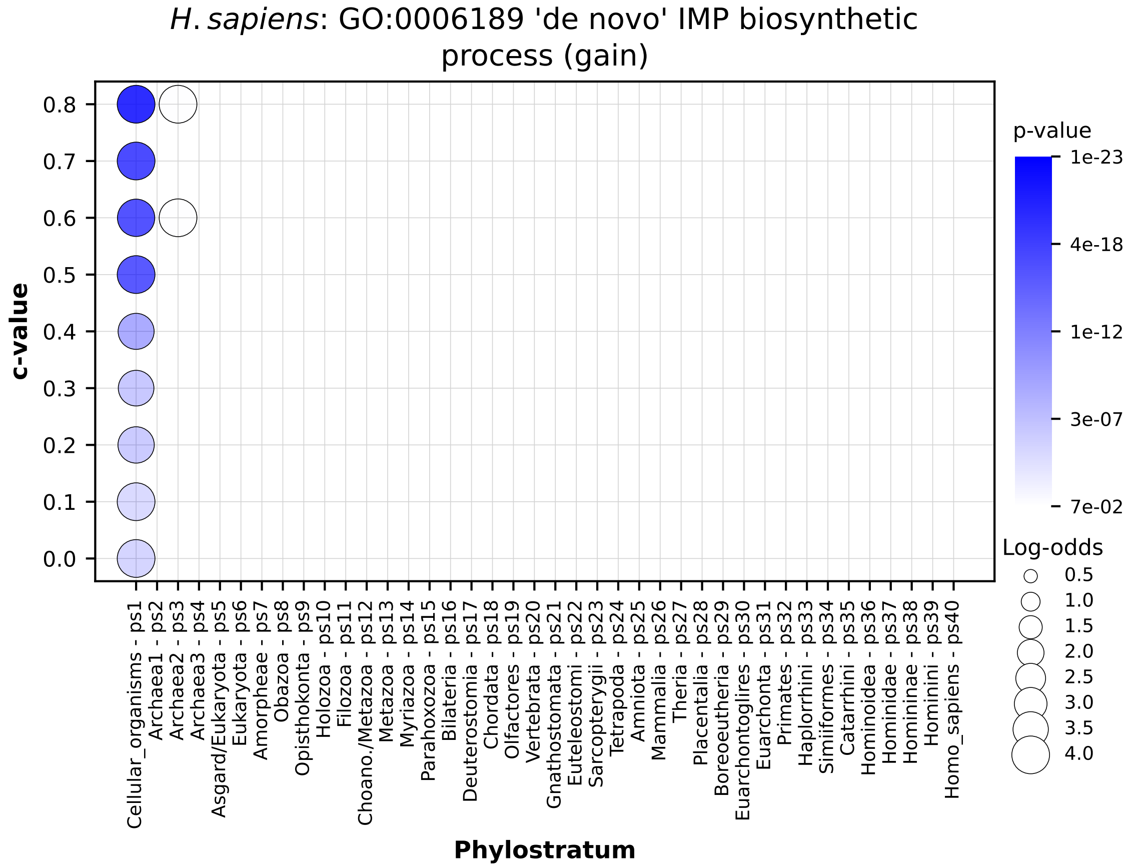
H. sapiens: GO:0006189 'de novo' IMP biosynthetic process (gain)
c-value
0.8
0.7
0.6
0.5
0.4
0.3
0.2
0.1
0.0
p-value
1e-23
4e-18
1e-12
3e-07
7e-02
Log-odds
0.5
1.0
1.5
2.0
2.5
3.0
3.5
4.0
Cellular_organisms - ps1
Archaea1 - ps2
Archaea2 - ps3
Archaea3 - ps4
Asgard/Eukaryota - ps5
Eukaryota - ps6
Amorpheae - ps7
Obazoa - ps8
Opisthokonta - ps9
Holozoa - ps10
Filozoa - ps11
Choano./Metazoa - ps12
Metazoa - ps13
Myriazoa - ps14
Parahoxozoa - ps15
Bilateria - ps16
Deuterostomia - ps17
Chordata - ps18
Olfactores - ps19
Vertebrata - ps20
Gnathostomata - ps21
Euteleostomi - ps22
Sarcopterygii - ps23
Tetrapoda - ps24
Amniota - ps25
Mammalia - ps26
Theria - ps27
Placentalia - ps28
Boreoeutheria - ps29
Euarchontoglires - ps30
Euarchonta - ps31
Primates - ps32
Haplorrhini - ps33
Simiiformes - ps34
Catarrhini - ps35
Hominoidea - ps36
Hominidae - ps37
Homininae - ps38
Hominini - ps39
Homo_sapiens - ps40
Phylostratum

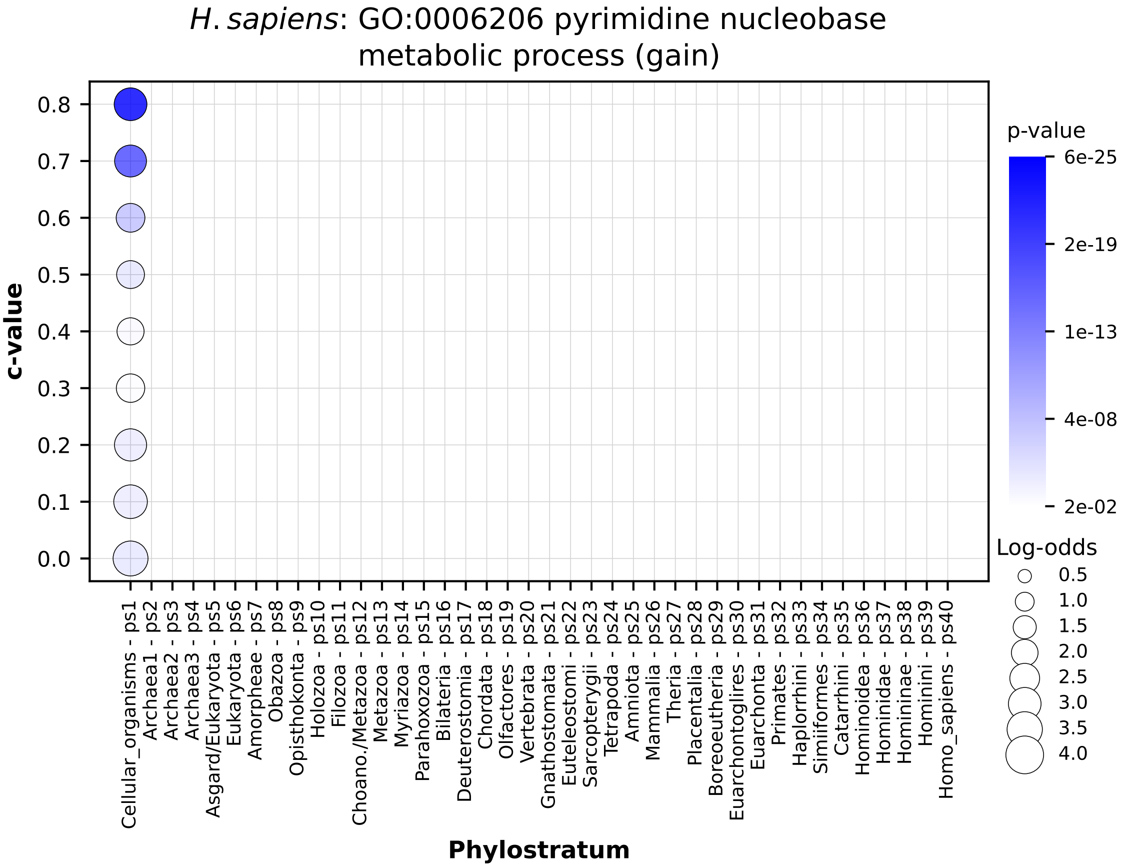
H. sapiens: GO:0006206 pyrimidine nucleobase metabolic process (gain)
c-value
0.8
0.7
0.6
0.5
0.4
0.3
0.2
0.1
0.0
p-value
6e-25
2e-19
1e-13
4e-08
2e-02
Log-odds
0.5
1.0
1.5
2.0
2.5
3.0
3.5
4.0
Cellular_organisms - ps1
Archaea1 - ps2
Archaea2 - ps3
Archaea3 - ps4
Asgard/Eukaryota - ps5
Eukaryota - ps6
Amorpheae - ps7
Obazoa - ps8
Opisthokonta - ps9
Holozoa - ps10
Filozoa - ps11
Choano./Metazoa - ps12
Metazoa - ps13
Myriazoa - ps14
Parahoxozoa - ps15
Bilateria - ps16
Deuterostomia - ps17
Chordata - ps18
Olfactores - ps19
Vertebrata - ps20
Gnathostomata - ps21
Euteleostomi - ps22
Sarcopterygii - ps23
Tetrapoda - ps24
Amniota - ps25
Mammalia - ps26
Theria - ps27
Placentalia - ps28
Boreoeutheria - ps29
Euarchontoglires - ps30
Euarchonta - ps31
Primates - ps32
Haplorrhini - ps33
Simiiformes - ps34
Catarrhini - ps35
Hominoidea - ps36
Hominidae - ps37
Homininae - ps38
Hominini - ps39
Homo_sapiens - ps40
Phylostratum

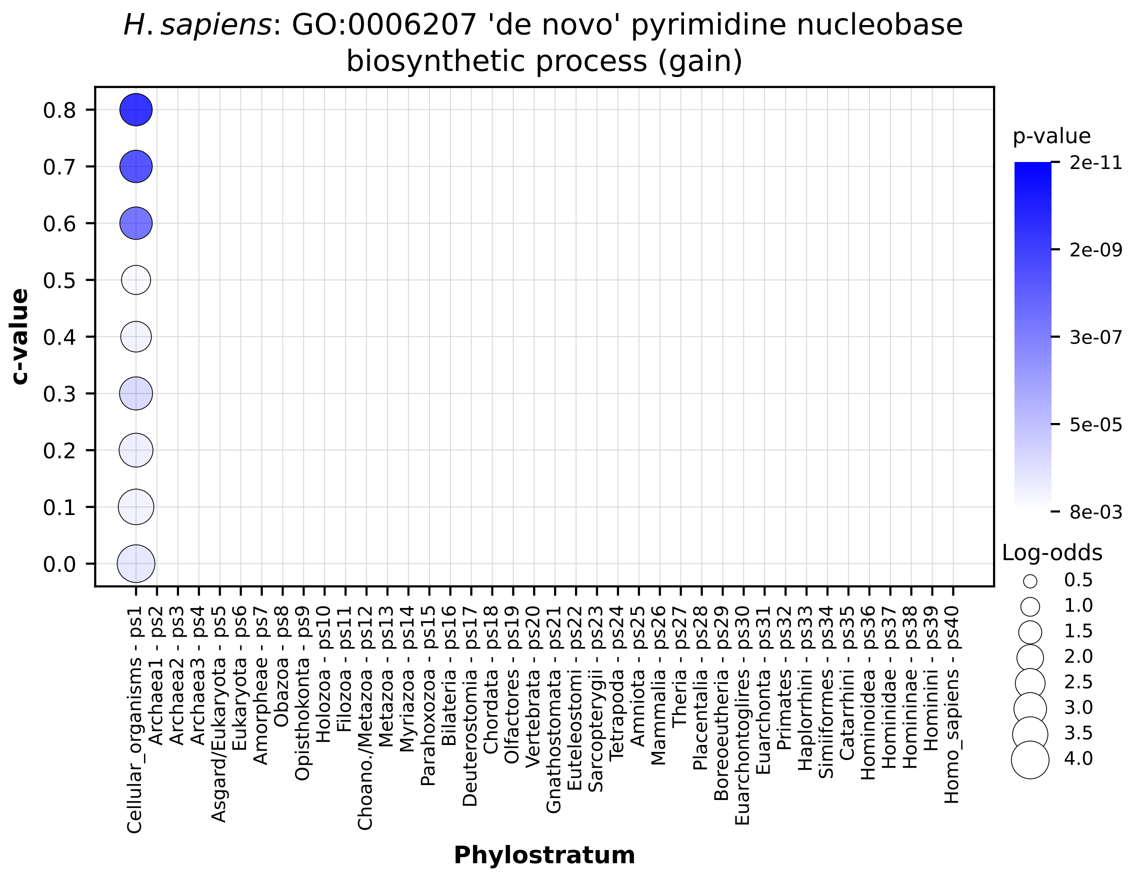

H. sapiens: GO:0006207 'de novo' pyrimidine nucleobase biosynthetic process (gain)
c-value
0.8
0.7
0.6
0.5
0.4
0.3
0.2
0.1
0.0
Phylostratum
Cellular_organisms - ps1
Archaea1 - ps2
Archaea2 - ps3
Archaea3 - ps4
Asgard/Eukaryota - ps5
Eukaryota - ps6
Amorpheae - ps7
Obazoa - ps8
Opisthokonta - ps9
Holozoa - ps10
Filozoa - ps11
Choano./Metazoa - ps12
Metazoa - ps13
Myriazoa - ps14
Parahoxozoa - ps15
Bilateria - ps16
Deuterostomia - ps17
Chordata - ps18
Olfactores - ps19
Vertebrata - ps20
Gnathostomata - ps21
Euteleostomi - ps22
Sarcopterygii - ps23
Tetrapoda - ps24
Amniota - ps25
Mammalia - ps26
Theria - ps27
Placentalia - ps28
Boreoeutheria - ps29
Euarchontoglires - ps30
Euarchonta - ps31
Primates - ps32
Haplorrhini - ps33
Simiiformes - ps34
Catarrhini - ps35
Hominoidea - ps36
Hominidae - ps37
Homininae - ps38
Hominini - ps39
Homo_sapiens - ps40
p-value
2e-11
2e-09
3e-07
5e-05
8e-03
Log-odds
0.5
1.0
1.5
2.0
2.5
3.0
3.5
4.0

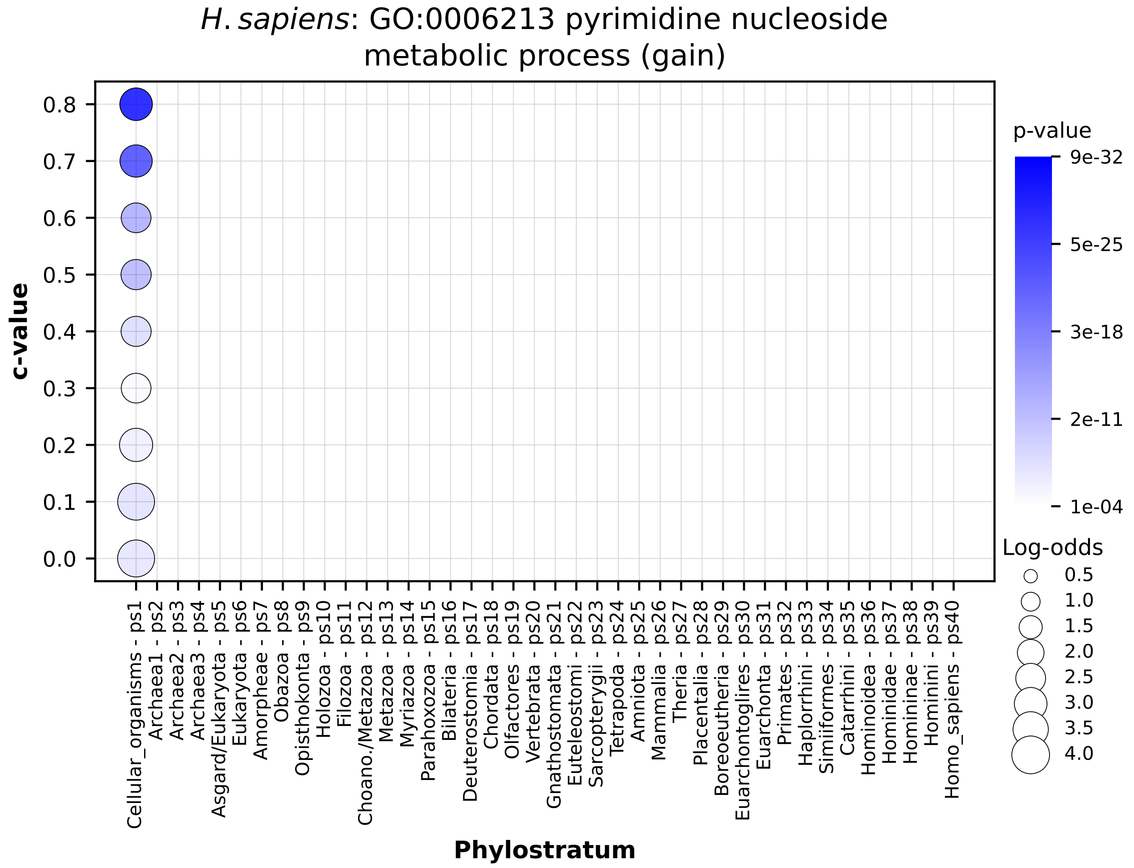
H. sapiens: GO:0006213 pyrimidine nucleoside metabolic process (gain)
c-value
0.0
0.1
0.2
0.3
0.4
0.5
0.6
0.7
0.8
p-value
9e-32
5e-25
3e-18
2e-11
1e-04
Log-odds
0.5
1.0
1.5
2.0
2.5
3.0
3.5
4.0
Cellular_organisms - ps1
Archaea1 - ps2
Archaea2 - ps3
Archaea3 - ps4
Asgard/Eukaryota - ps5
Eukaryota - ps6
Amorpheae - ps7
Obazoa - ps8
Opisthokonta - ps9
Holozoa - ps10
Filozoa - ps11
Choano./Metazoa - ps12
Metazoa - ps13
Myriazoa - ps14
Parahoxozoa - ps15
Bilateria - ps16
Deuterostomia - ps17
Chordata - ps18
Olfactores - ps19
Vertebrata - ps20
Gnathostomata - ps21
Euteleostomi - ps22
Sarcopterygii - ps23
Tetrapoda - ps24
Amniota - ps25
Mammalia - ps26
Theria - ps27
Placentalia - ps28
Boreoeutheria - ps29
Euarchontoglires - ps30
Euarchonta - ps31
Primates - ps32
Haplorrhini - ps33
Simiiformes - ps34
Catarrhini - ps35
Hominoidea - ps36
Hominidae - ps37
Homininae - ps38
Hominini - ps39
Homo_sapiens - ps40
Phylostratum

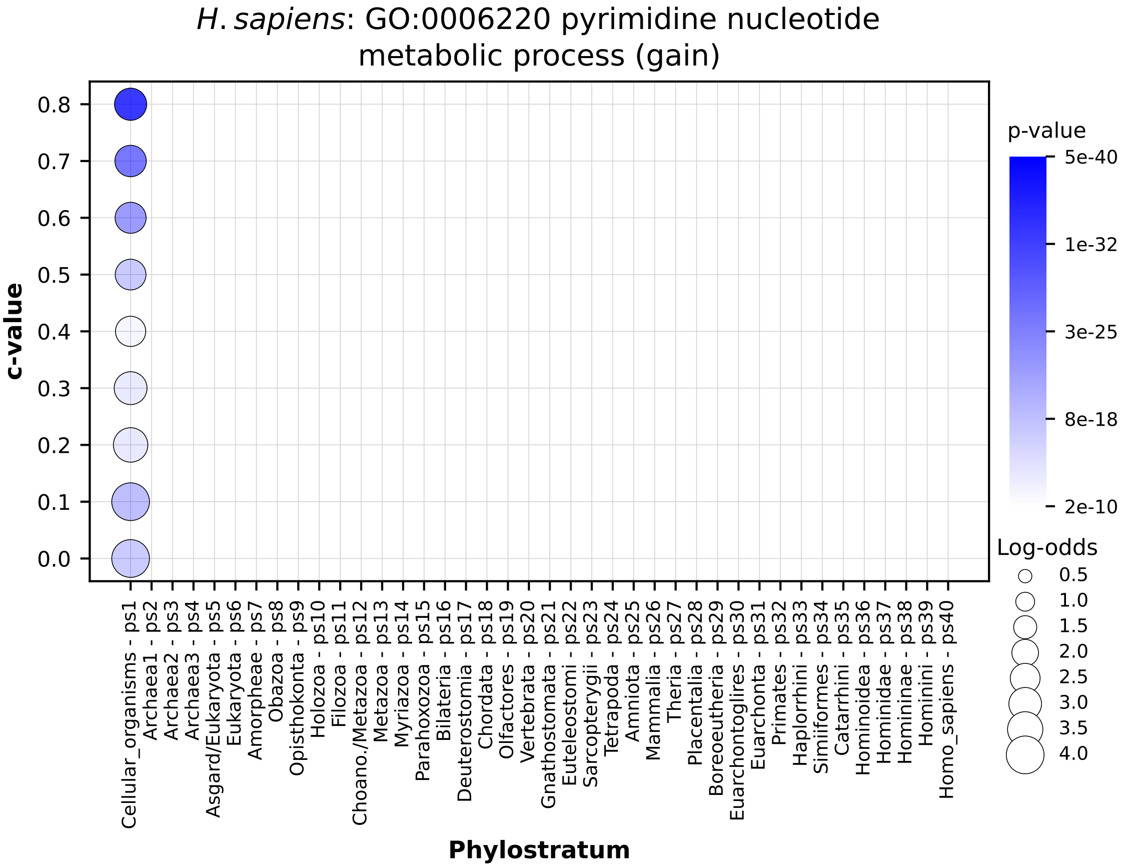

H. sapiens: GO:0006220 pyrimidine nucleotide metabolic process (gain)
c-value
0.8
0.7
0.6
0.5
0.4
0.3
0.2
0.1
0.0
Phylostratum
Cellular_organisms - ps1
Archaea1 - ps2
Archaea2 - ps3
Archaea3 - ps4
Asgard/Eukaryota - ps5
Eukaryota - ps6
Amorpheae - ps7
Obazoa - ps8
Opisthokonta - ps9
Holozoa - ps10
Filozoa - ps11
Choano./Metazoa - ps12
Metazoa - ps13
Myriazoa - ps14
Parahoxozoa - ps15
Bilateria - ps16
Deuterostomia - ps17
Chordata - ps18
Olfactores - ps19
Vertebrata - ps20
Gnathostomata - ps21
Euteleostomi - ps22
Sarcopterygii - ps23
Tetrapoda - ps24
Amniota - ps25
Mammalia - ps26
Theria - ps27
Placentalia - ps28
Boreoeutheria - ps29
Euarchontoglires - ps30
Euarchonta - ps31
Primates - ps32
Haplorrhini - ps33
Simiiformes - ps34
Catarrhini - ps35
Hominoidea - ps36
Hominidae - ps37
Homininae - ps38
Hominini - ps39
Homo_sapiens - ps40
p-value
5e-40
1e-32
3e-25
8e-18
2e-10
Log-odds
0.5
1.0
1.5
2.0
2.5
3.0
3.5
4.0

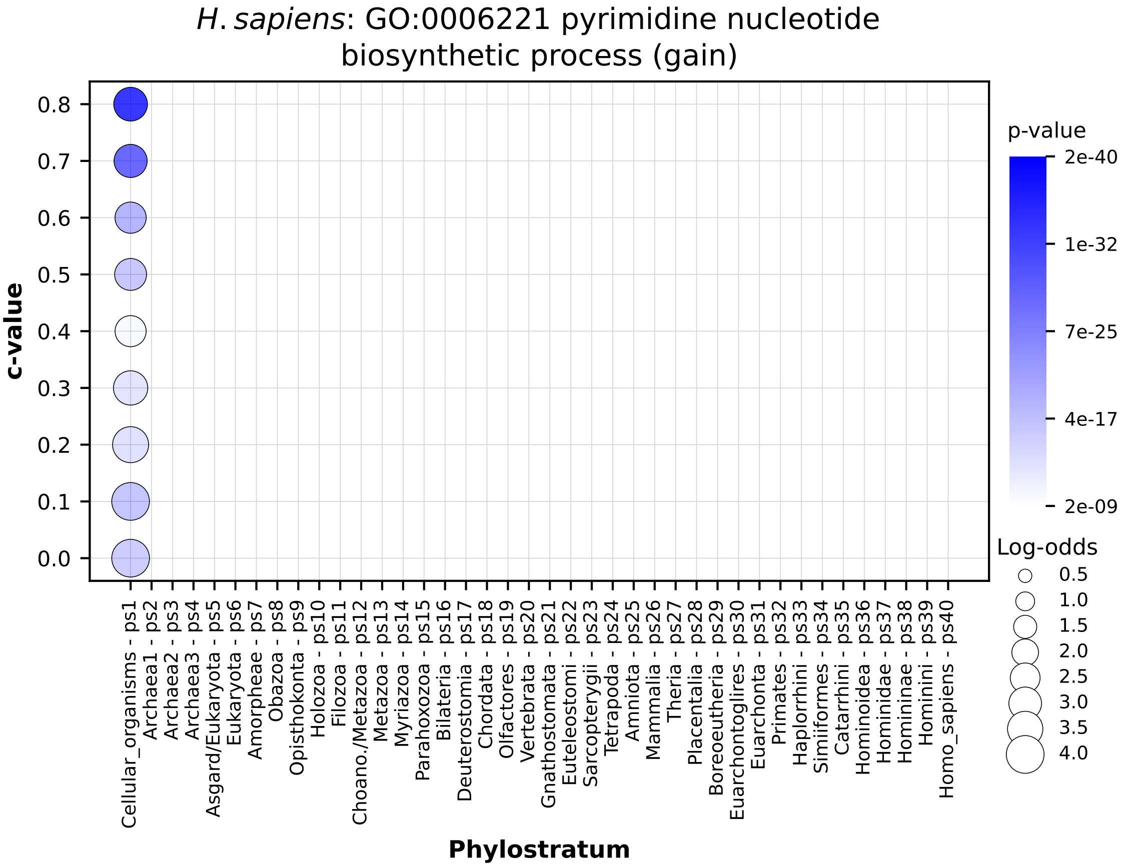
H. sapiens: GO:0006221 pyrimidine nucleotide biosynthetic process (gain)
c-value
0.8
0.7
0.6
0.5
0.4
0.3
0.2
0.1
0.0
p-value
2e-40
1e-32
7e-25
4e-17
2e-09
Log-odds
0.5
1.0
1.5
2.0
2.5
3.0
3.5
4.0
Cellular_organisms - ps1
Archaea1 - ps2
Archaea2 - ps3
Archaea3 - ps4
Asgard/Eukaryota - ps5
Eukaryota - ps6
Amorpheae - ps7
Obazoa - ps8
Opisthokonta - ps9
Holozoa - ps10
Filozoa - ps11
Choano./Metazoa - ps12
Metazoa - ps13
Myriazoa - ps14
Parahoxozoa - ps15
Bilateria - ps16
Deuterostomia - ps17
Chordata - ps18
Olfactores - ps19
Vertebrata - ps20
Gnathostomata - ps21
Euteleostomi - ps22
Sarcopterygii - ps23
Tetrapoda - ps24
Amniota - ps25
Mammalia - ps26
Theria - ps27
Placentalia - ps28
Boreoeutheria - ps29
Euarchontoglires - ps30
Euarchonta - ps31
Primates - ps32
Haplorrhini - ps33
Simiiformes - ps34
Catarrhini - ps35
Hominoidea - ps36
Hominidae - ps37
Homininae - ps38
Hominini - ps39
Homo_sapiens - ps40
Phylostratum

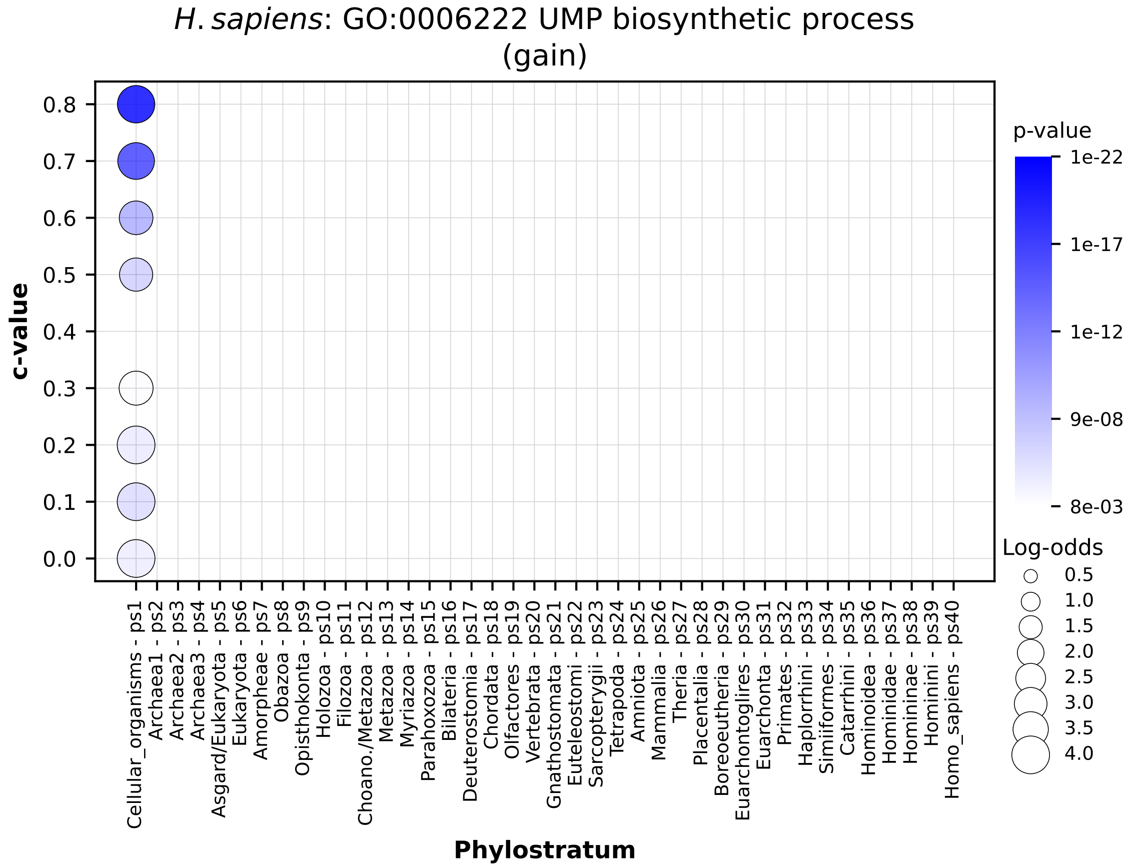
H. sapiens: GO:0006222 UMP biosynthetic process
(gain)
c-value
0.8
0.7
0.6
0.5
0.4
0.3
0.2
0.1
0.0
p-value
1e-22
1e-17
1e-12
9e-08
8e-03
Log-odds
0.5
1.0
1.5
2.0
2.5
3.0
3.5
4.0
Cellular_organisms - ps1
Archaea1 - ps2
Archaea2 - ps3
Archaea3 - ps4
Asgard/Eukaryota - ps5
Eukaryota - ps6
Amorpheae - ps7
Obazoa - ps8
Opisthokonta - ps9
Holozoa - ps10
Filozoa - ps11
Choano./Metazoa - ps12
Metazoa - ps13
Myriazoa - ps14
Parahoxozoa - ps15
Bilateria - ps16
Deuterostomia - ps17
Chordata - ps18
Olfactores - ps19
Vertebrata - ps20
Gnathostomata - ps21
Euteleostomi - ps22
Sarcopterygii - ps23
Tetrapoda - ps24
Amniota - ps25
Mammalia - ps26
Theria - ps27
Placentalia - ps28
Boreoeutheria - ps29
Euarchontoglires - ps30
Euarchonta - ps31
Primates - ps32
Haplorrhini - ps33
Simiiformes - ps34
Catarrhini - ps35
Hominoidea - ps36
Hominidae - ps37
Homininae - ps38
Hominini - ps39
Homo_sapiens - ps40
Phylostratum

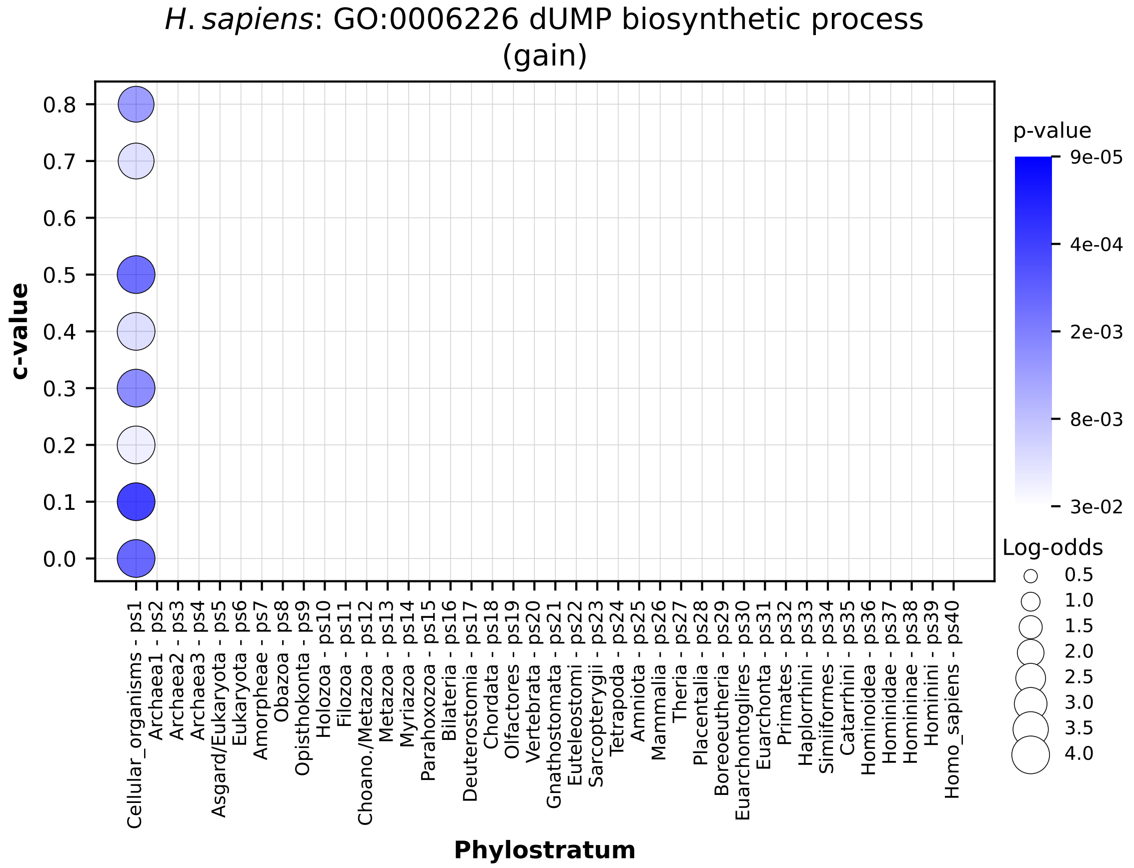

H. sapiens: GO:0006226 dUMP biosynthetic process
(gain)
c-value
0.8
0.7
0.6
0.5
0.4
0.3
0.2
0.1
0.0
p-value
9e-05
4e-04
2e-03
8e-03
3e-02
Log-odds
0.5
1.0
1.5
2.0
2.5
3.0
3.5
4.0
Cellular_organisms - ps1
Archaea1 - ps2
Archaea2 - ps3
Archaea3 - ps4
Asgard/Eukaryota - ps5
Eukaryota - ps6
Amorpheae - ps7
Obazoa - ps8
Opisthokonta - ps9
Holozoa - ps10
Filozoa - ps11
Choano./Metazoa - ps12
Metazoa - ps13
Myriazoa - ps14
Parahoxozoa - ps15
Bilateria - ps16
Deuterostomia - ps17
Chordata - ps18
Olfactores - ps19
Vertebrata - ps20
Gnathostomata - ps21
Euteleostomi - ps22
Sarcopterygii - ps23
Tetrapoda - ps24
Amniota - ps25
Mammalia - ps26
Theria - ps27
Placentalia - ps28
Boreoeutheria - ps29
Euarchontoglires - ps30
Euarchonta - ps31
Primates - ps32
Haplorrhini - ps33
Simiiformes - ps34
Catarrhini - ps35
Hominoidea - ps36
Hominidae - ps37
Homininae - ps38
Hominini - ps39
Homo_sapiens - ps40
Phylostratum

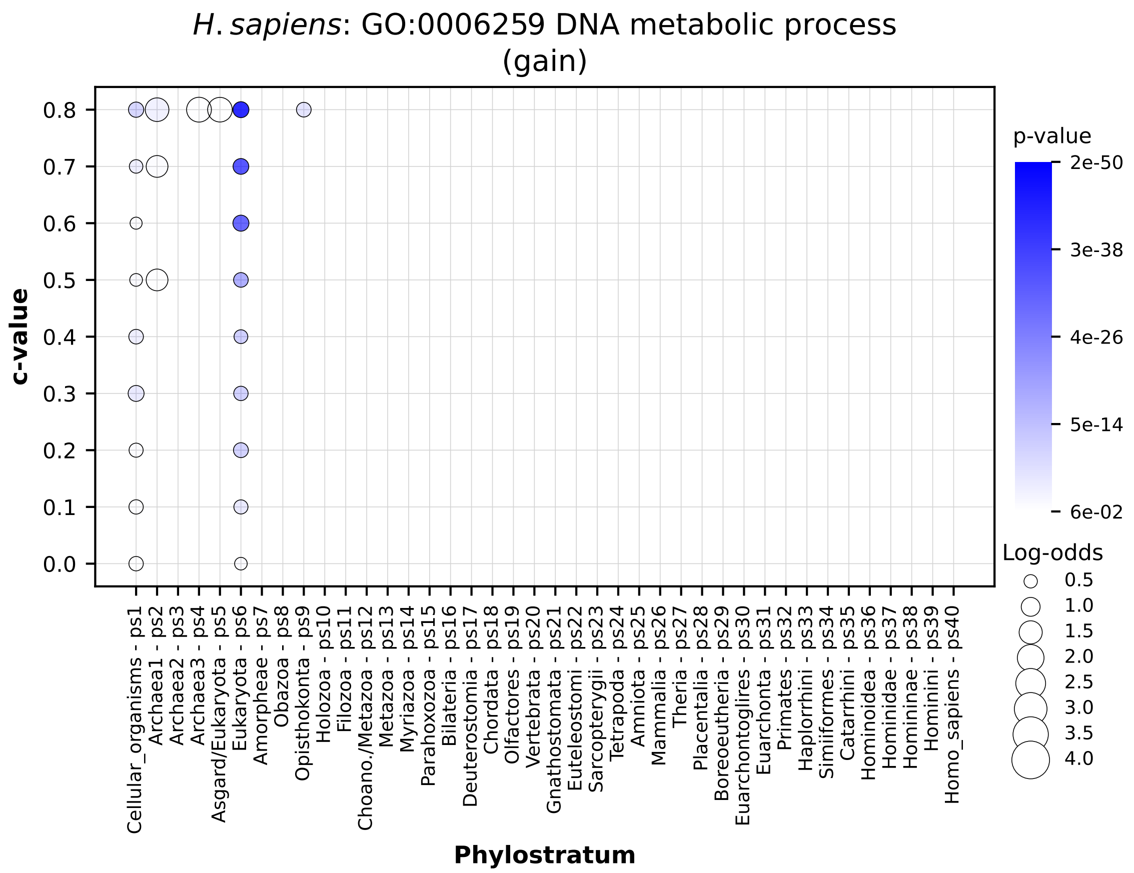

H. sapiens: GO:0006259 DNA metabolic process
(gain)
c-value
0.8
0.7
0.6
0.5
0.4
0.3
0.2
0.1
0.0
p-value
2e-50
3e-38
4e-26
5e-14
6e-02
Log-odds
0.5
1.0
1.5
2.0
2.5
3.0
3.5
4.0
Cellular_organisms - ps1
Archaea1 - ps2
Archaea2 - ps3
Archaea3 - ps4
Asgard/Eukaryota - ps5
Eukaryota - ps6
Amorpheae - ps7
Obazoa - ps8
Opisthokonta - ps9
Holozoa - ps10
Filozoa - ps11
Choano./Metazoa - ps12
Metazoa - ps13
Myriazoa - ps14
Parahoxozoa - ps15
Bilateria - ps16
Deuterostomia - ps17
Chordata - ps18
Olfactores - ps19
Vertebrata - ps20
Gnathostomata - ps21
Euteleostomi - ps22
Sarcopterygii - ps23
Tetrapoda - ps24
Amniota - ps25
Mammalia - ps26
Theria - ps27
Placentalia - ps28
Boreoeutheria - ps29
Euarchontoglires - ps30
Euarchonta - ps31
Primates - ps32
Haplorrhini - ps33
Simiiformes - ps34
Catarrhini - ps35
Hominoidea - ps36
Hominidae - ps37
Homininae - ps38
Hominini - ps39
Homo_sapiens - ps40
Phylostratum

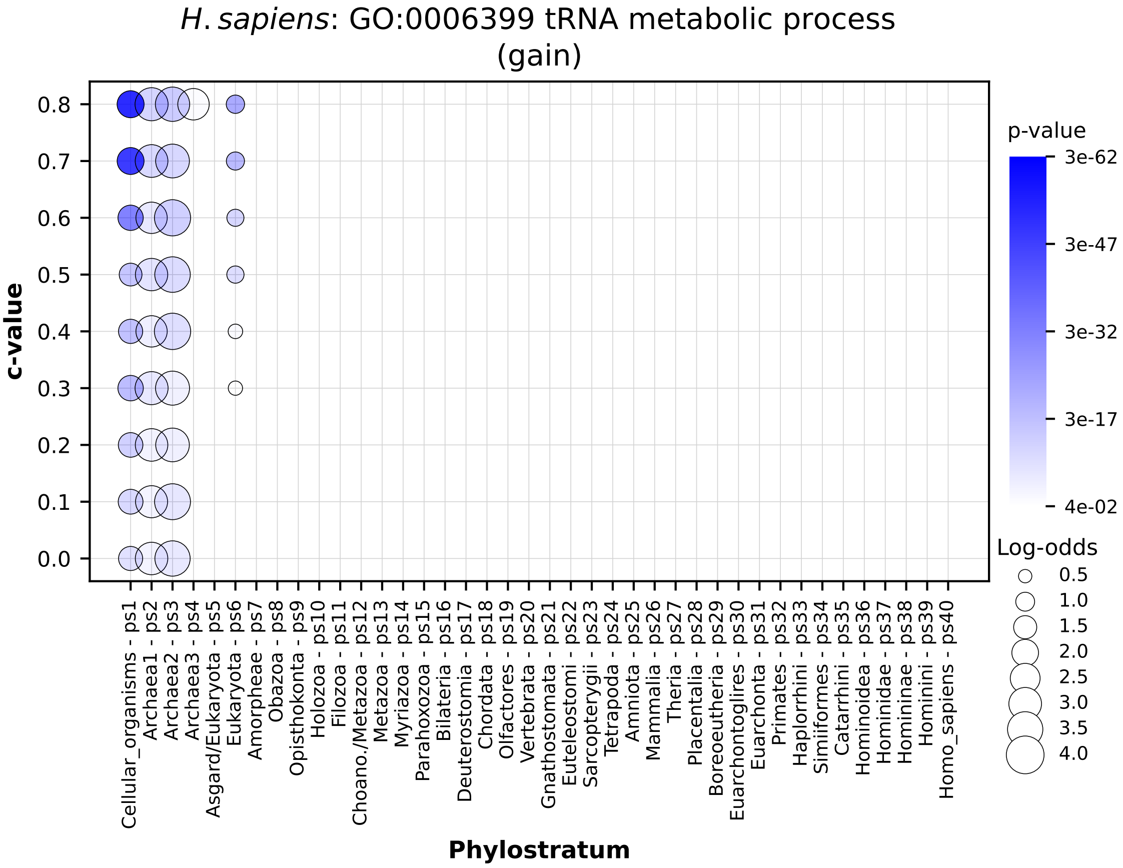

H. sapiens: GO:0006399 tRNA metabolic process
(gain)
c-value
0.8
0.7
0.6
0.5
0.4
0.3
0.2
0.1
0.0
Phylostratum
Cellular_organisms - ps1
Archaea1 - ps2
Archaea2 - ps3
Archaea3 - ps4
Asgard/Eukaryota - ps5
Eukaryota - ps6
Amorpheae - ps7
Obazoa - ps8
Opisthokonta - ps9
Holozoa - ps10
Filozoa - ps11
Choano./Metazoa - ps12
Metazoa - ps13
Myriazoa - ps14
Parahoxozoa - ps15
Bilateria - ps16
Deuterostomia - ps17
Chordata - ps18
Olfactores - ps19
Vertebrata - ps20
Gnathostomata - ps21
Euteleostomi - ps22
Sarcopterygii - ps23
Tetrapoda - ps24
Amniota - ps25
Mammalia - ps26
Theria - ps27
Placentalia - ps28
Boreoeutheria - ps29
Euarchontoglires - ps30
Euarchonta - ps31
Primates - ps32
Haplorrhini - ps33
Simiiformes - ps34
Catarrhini - ps35
Hominoidea - ps36
Hominidae - ps37
Homininae - ps38
Hominini - ps39
Homo_sapiens - ps40
p-value
3e-62
3e-47
3e-32
3e-17
4e-02
Log-odds
0.5
1.0
1.5
2.0
2.5
3.0
3.5
4.0

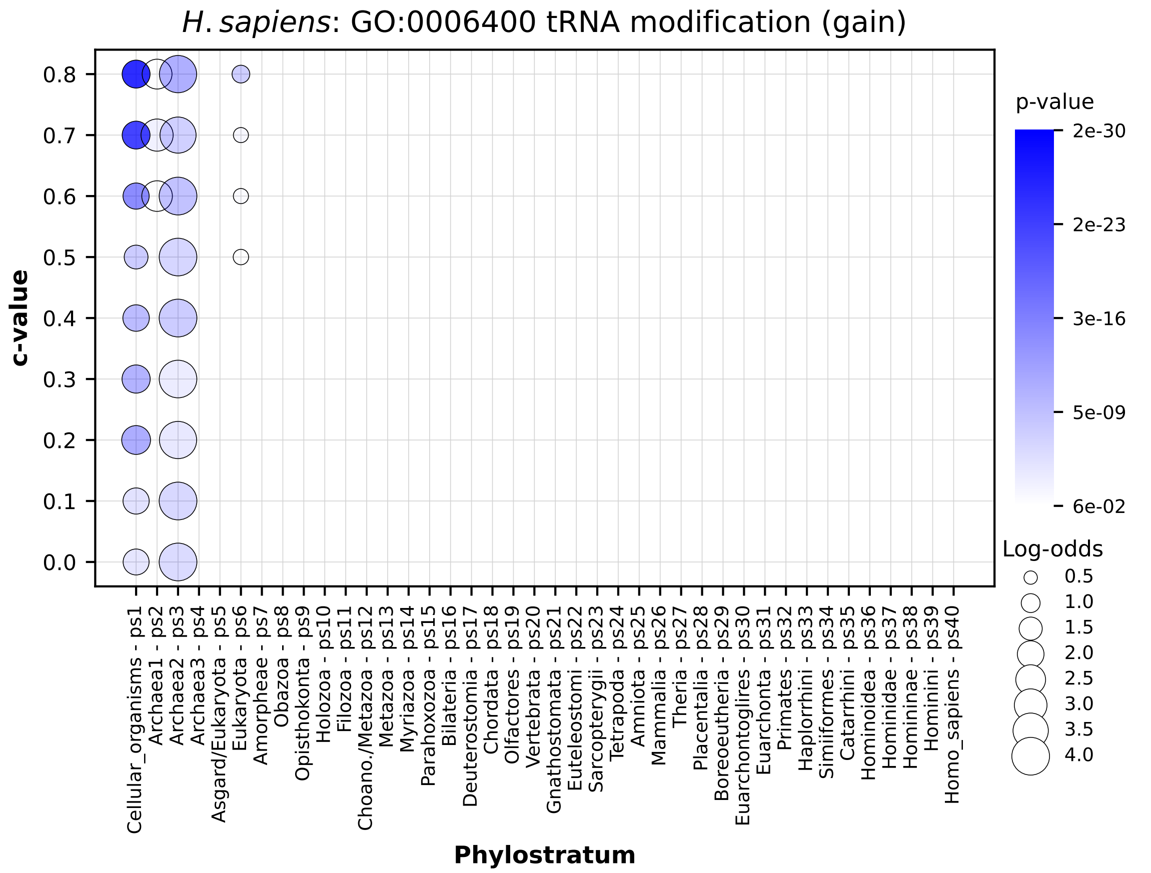

H. sapiens: GO:0006400 tRNA modification (gain)
c-value
0.8
0.7
0.6
0.5
0.4
0.3
0.2
0.1
0.0
Phylostratum
Cellular_organisms - ps1
Archaea1 - ps2
Archaea2 - ps3
Archaea3 - ps4
Asgard/Eukaryota - ps5
Eukaryota - ps6
Amorpheae - ps7
Obazoa - ps8
Opisthokonta - ps9
Holozoa - ps10
Filozoa - ps11
Choano./Metazoa - ps12
Metazoa - ps13
Myriazoa - ps14
Parahoxozoa - ps15
Bilateria - ps16
Deuterostomia - ps17
Chordata - ps18
Olfactores - ps19
Vertebrata - ps20
Gnathostomata - ps21
Euteleostomi - ps22
Sarcopterygii - ps23
Tetrapoda - ps24
Amniota - ps25
Mammalia - ps26
Theria - ps27
Placentalia - ps28
Boreoeutheria - ps29
Euarchontoglires - ps30
Euarchonta - ps31
Primates - ps32
Haplorrhini - ps33
Simiiformes - ps34
Catarrhini - ps35
Hominoidea - ps36
Hominidae - ps37
Homininae - ps38
Hominini - ps39
Homo_sapiens - ps40
p-value
2e-30
2e-23
3e-16
5e-09
6e-02
Log-odds
0.5
1.0
1.5
2.0
2.5
3.0
3.5
4.0

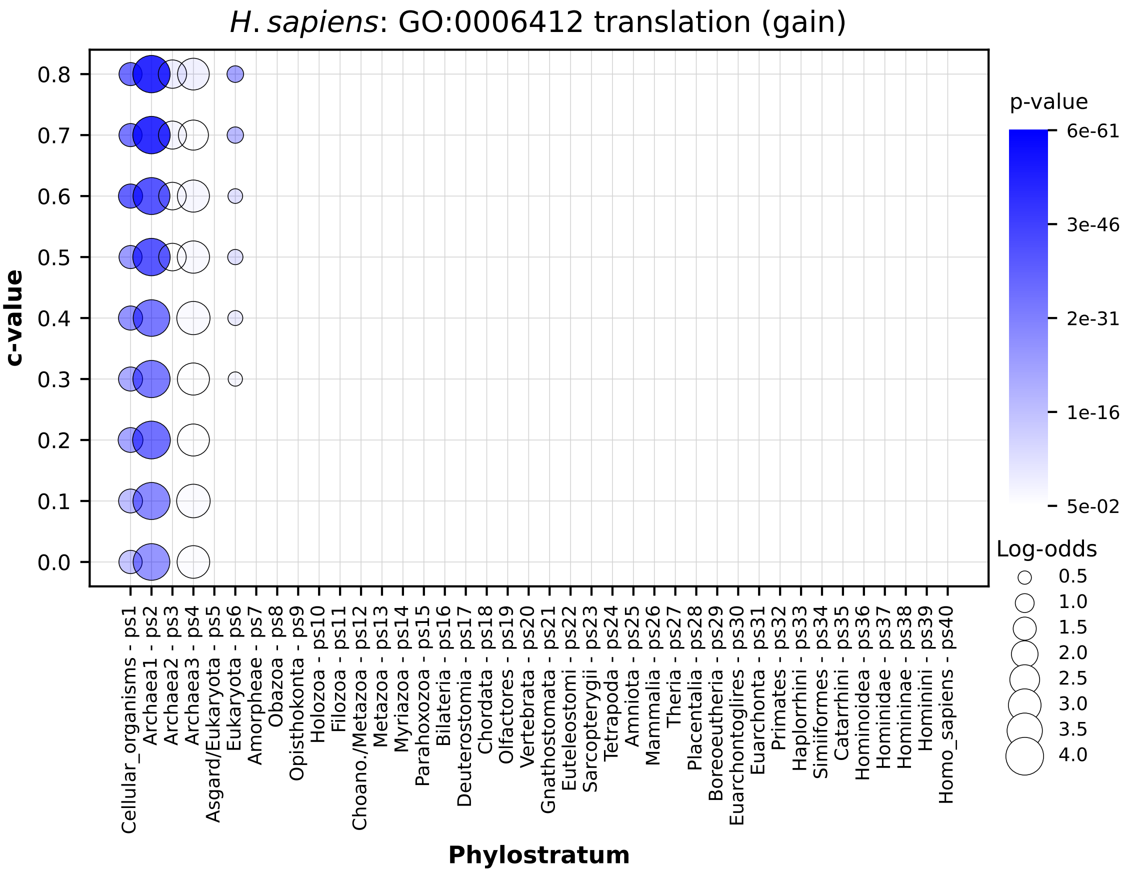
H. sapiens: GO:0006412 translation (gain)
c-value
0.0
0.1
0.2
0.3
0.4
0.5
0.6
0.7
0.8
Phylostratum
Cellular_organisms - ps1
Archaea1 - ps2
Archaea2 - ps3
Archaea3 - ps4
Asgard/Eukaryota - ps5
Eukaryota - ps6
Amorpheae - ps7
Obazoa - ps8
Opisthokonta - ps9
Holozoa - ps10
Filozoa - ps11
Choano./Metazoa - ps12
Metazoa - ps13
Myriazoa - ps14
Parahoxozoa - ps15
Bilateria - ps16
Deuterostomia - ps17
Chordata - ps18
Olfactores - ps19
Vertebrata - ps20
Gnathostomata - ps21
Euteleostomi - ps22
Sarcopterygii - ps23
Tetrapoda - ps24
Amniota - ps25
Mammalia - ps26
Theria - ps27
Placentalia - ps28
Boreoeutheria - ps29
Euarchontoglires - ps30
Euarchonta - ps31
Primates - ps32
Haplorrhini - ps33
Simiiformes - ps34
Catarrhini - ps35
Hominoidea - ps36
Hominidae - ps37
Homininae - ps38
Hominini - ps39
Homo_sapiens - ps40
p-value
6e-61
3e-46
2e-31
1e-16
5e-02
Log-odds
0.5
1.0
1.5
2.0
2.5
3.0
3.5
4.0

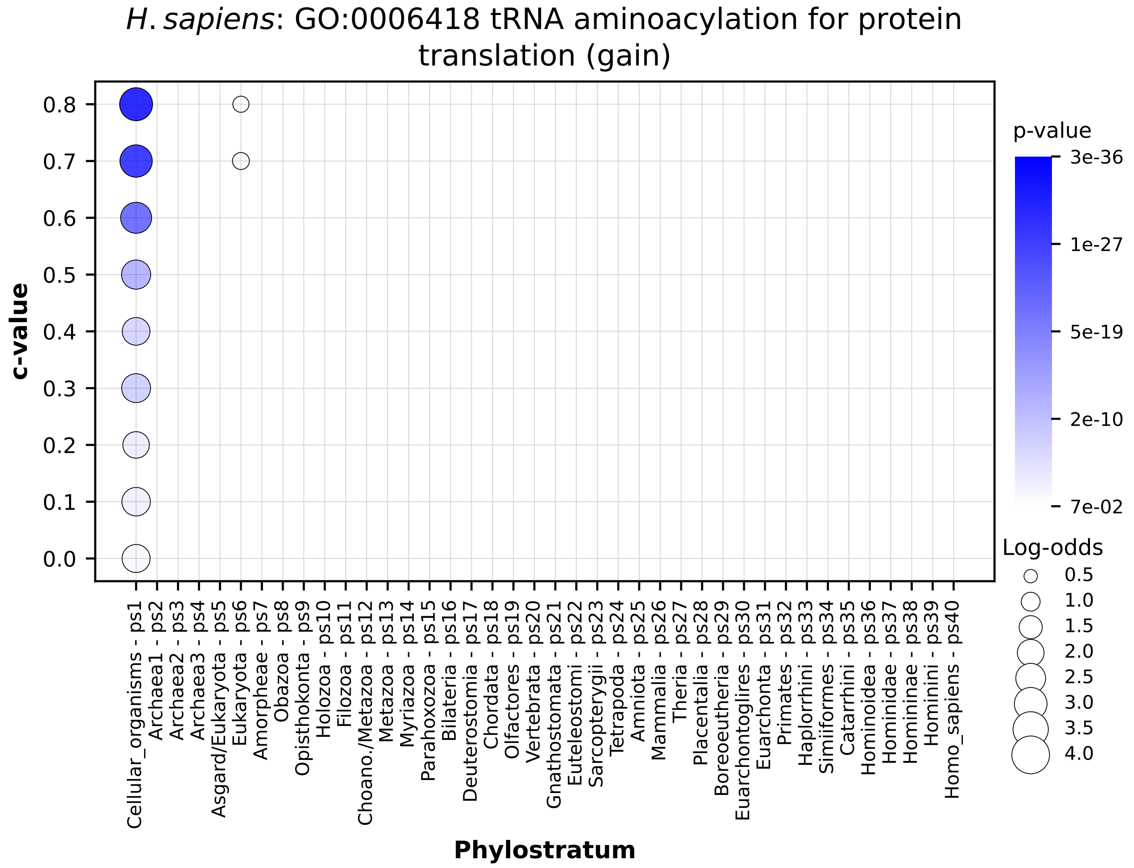

H. sapiens: GO:0006418 tRNA aminoacylation for protein translation (gain)
c-value
0.8
0.7
0.6
0.5
0.4
0.3
0.2
0.1
0.0
Phylostratum
Cellular_organisms - ps1
Archaea1 - ps2
Archaea2 - ps3
Archaea3 - ps4
Asgard/Eukaryota - ps5
Eukaryota - ps6
Amorpheae - ps7
Obazoa - ps8
Opisthokonta - ps9
Holozoa - ps10
Filozoa - ps11
Choano./Metazoa - ps12
Metazoa - ps13
Myriazoa - ps14
Parahoxozoa - ps15
Bilateria - ps16
Deuterostomia - ps17
Chordata - ps18
Olfactores - ps19
Vertebrata - ps20
Gnathostomata - ps21
Euteleostomi - ps22
Sarcopterygii - ps23
Tetrapoda - ps24
Amniota - ps25
Mammalia - ps26
Theria - ps27
Placentalia - ps28
Boreoeutheria - ps29
Euarchontoglires - ps30
Euarchonta - ps31
Primates - ps32
Haplorrhini - ps33
Simiiformes - ps34
Catarrhini - ps35
Hominoidea - ps36
Hominidae - ps37
Homininae - ps38
Hominini - ps39
Homo_sapiens - ps40
p-value
3e-36
1e-27
5e-19
2e-10
7e-02
Log-odds
0.5
1.0
1.5
2.0
2.5
3.0
3.5
4.0

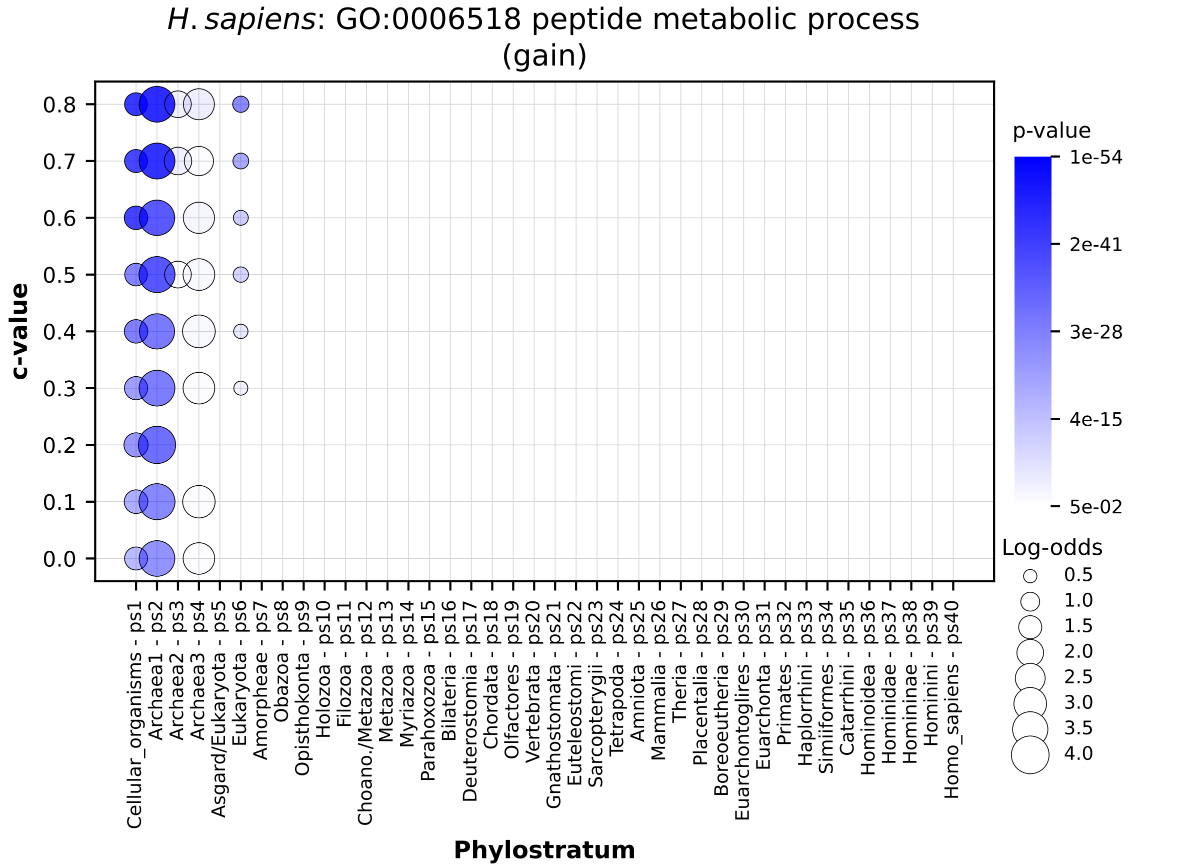
H. sapiens: GO:0006518 peptide metabolic process
(gain)
c-value
0.8
0.7
0.6
0.5
0.4
0.3
0.2
0.1
0.0
p-value
1e-54
2e-41
3e-28
4e-15
5e-02
Log-odds
0.5
1.0
1.5
2.0
2.5
3.0
3.5
4.0
Cellular_organisms - ps1
Archaea1 - ps2
Archaea2 - ps3
Archaea3 - ps4
Asgard/Eukaryota - ps5
Eukaryota - ps6
Amorpheae - ps7
Obazoa - ps8
Opisthokonta - ps9
Holozoa - ps10
Filozoa - ps11
Choano./Metazoa - ps12
Metazoa - ps13
Myriazoa - ps14
Parahoxozoa - ps15
Bilateria - ps16
Deuterostomia - ps17
Chordata - ps18
Olfactores - ps19
Vertebrata - ps20
Gnathostomata - ps21
Euteleostomi - ps22
Sarcopterygii - ps23
Tetrapoda - ps24
Amniota - ps25
Mammalia - ps26
Theria - ps27
Placentalia - ps28
Boreoeutheria - ps29
Euarchontoglires - ps30
Euarchonta - ps31
Primates - ps32
Haplorrhini - ps33
Simiiformes - ps34
Catarrhini - ps35
Hominoidea - ps36
Hominidae - ps37
Homininae - ps38
Hominini - ps39
Homo_sapiens - ps40
Phylostratum

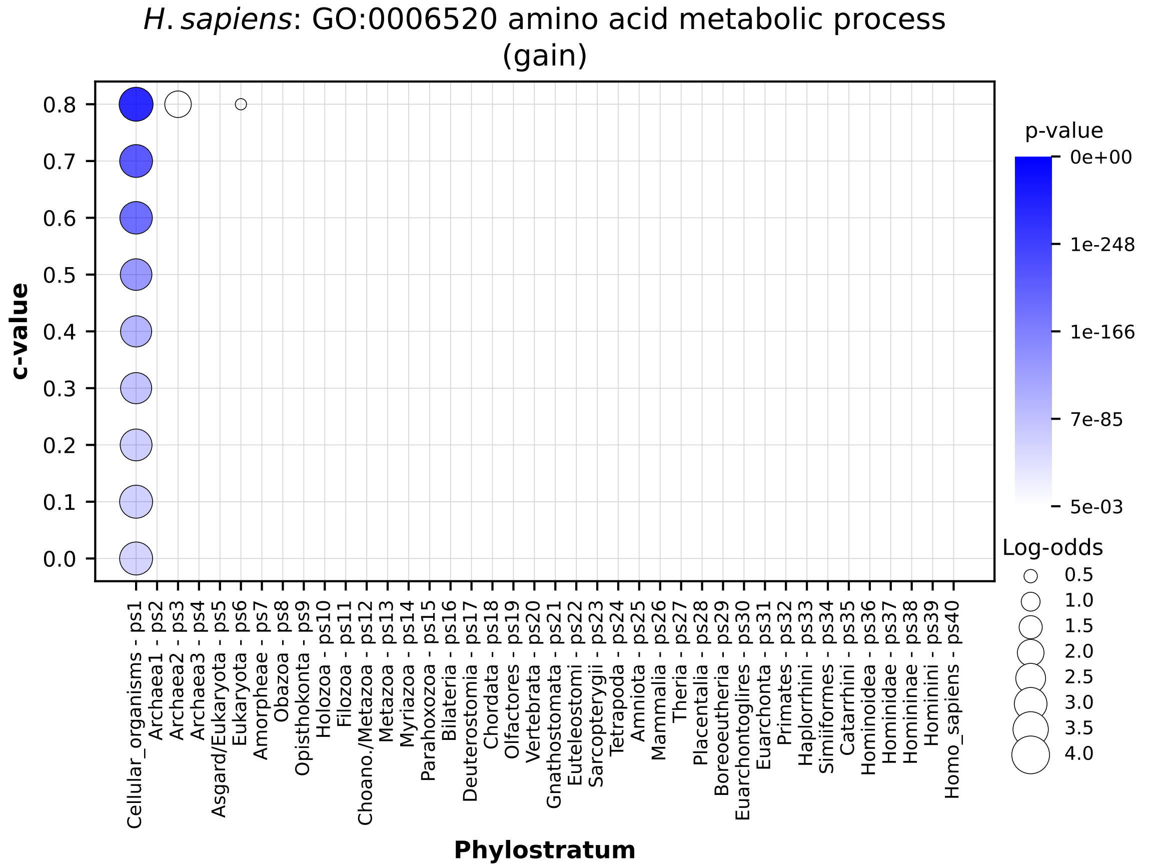

H. sapiens: GO:0006520 amino acid metabolic process
(gain)
c-value
0.8
0.7
0.6
0.5
0.4
0.3
0.2
0.1
0.0
Phylostratum
Cellular_organisms - ps1
Archaea1 - ps2
Archaea2 - ps3
Archaea3 - ps4
Asgard/Eukaryota - ps5
Eukaryota - ps6
Amorpheae - ps7
Obazoa - ps8
Opisthokonta - ps9
Holozoa - ps10
Filozoa - ps11
Choano./Metazoa - ps12
Metazoa - ps13
Myriazoa - ps14
Parahoxozoa - ps15
Bilateria - ps16
Deuterostomia - ps17
Chordata - ps18
Olfactores - ps19
Vertebrata - ps20
Gnathostomata - ps21
Euteleostomi - ps22
Sarcopterygii - ps23
Tetrapoda - ps24
Amniota - ps25
Mammalia - ps26
Theria - ps27
Placentalia - ps28
Boreoeutheria - ps29
Euarchontoglires - ps30
Euarchonta - ps31
Primates - ps32
Haplorrhini - ps33
Simiiformes - ps34
Catarrhini - ps35
Hominoidea - ps36
Hominidae - ps37
Homininae - ps38
Hominini - ps39
Homo_sapiens - ps40
p-value
0e+00
1e-248
1e-166
7e-85
5e-03
Log-odds
0.5
1.0
1.5
2.0
2.5
3.0
3.5
4.0

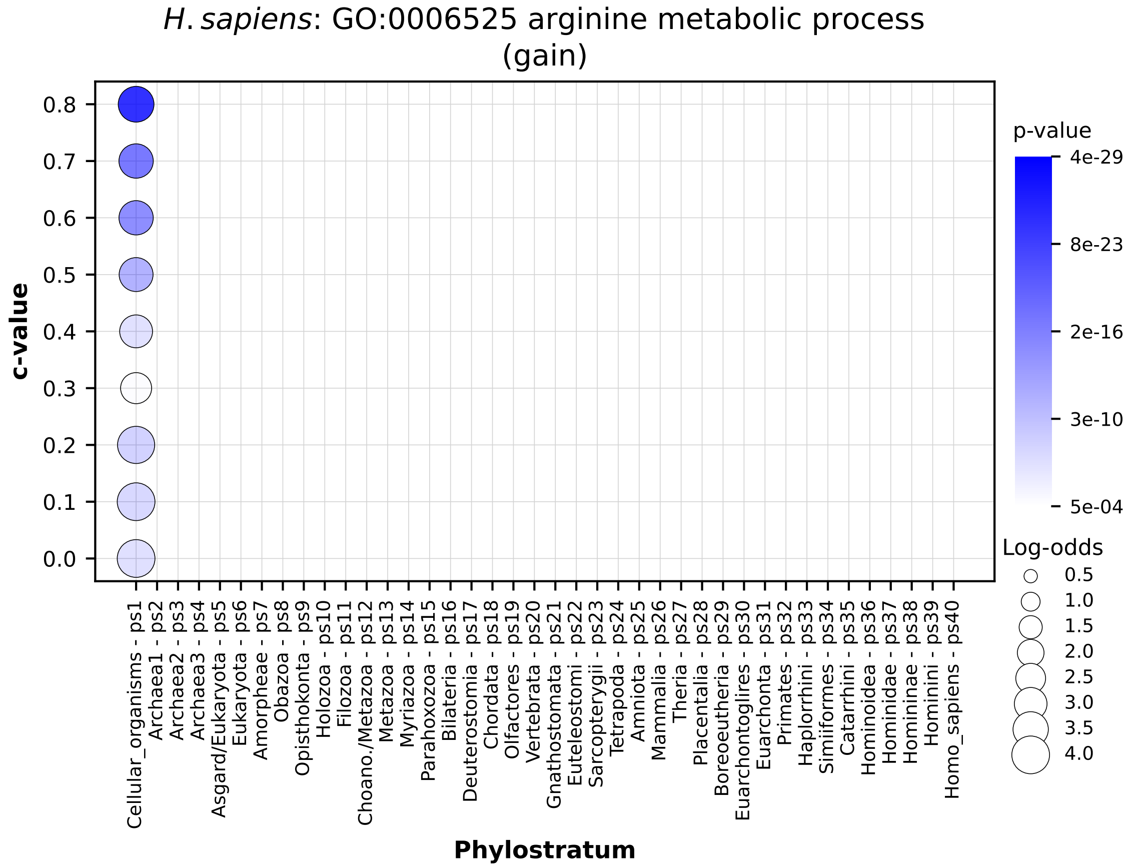

*H. sapiens*: GO:0006525 arginine metabolic process
(gain)
c-value
0.8
0.7
0.6
0.5
0.4
0.3
0.2
0.1
0.0
Phylostratum
Cellular_organisms - ps1
Archaea1 - ps2
Archaea2 - ps3
Archaea3 - ps4
Asgard/Eukaryota - ps5
Eukaryota - ps6
Amorpheae - ps7
Obazoa - ps8
Opisthokonta - ps9
Holozoa - ps10
Filozoa - ps11
Choano./Metazoa - ps12
Metazoa - ps13
Myriazoa - ps14
Parahoxozoa - ps15
Bilateria - ps16
Deuterostomia - ps17
Chordata - ps18
Olfactores - ps19
Vertebrata - ps20
Gnathostomata - ps21
Euteleostomi - ps22
Sarcopterygii - ps23
Tetrapoda - ps24
Amniota - ps25
Mammalia - ps26
Theria - ps27
Placentalia - ps28
Boreoeutheria - ps29
Euarchontoglires - ps30
Euarchonta - ps31
Primates - ps32
Haplorrhini - ps33
Simiiformes - ps34
Catarrhini - ps35
Hominoidea - ps36
Hominidae - ps37
Homininae - ps38
Hominini - ps39
Homo_sapiens - ps40
p-value
4e-29
8e-23
2e-16
3e-10
5e-04
Log-odds
0.5
1.0
1.5
2.0
2.5
3.0
3.5
4.0

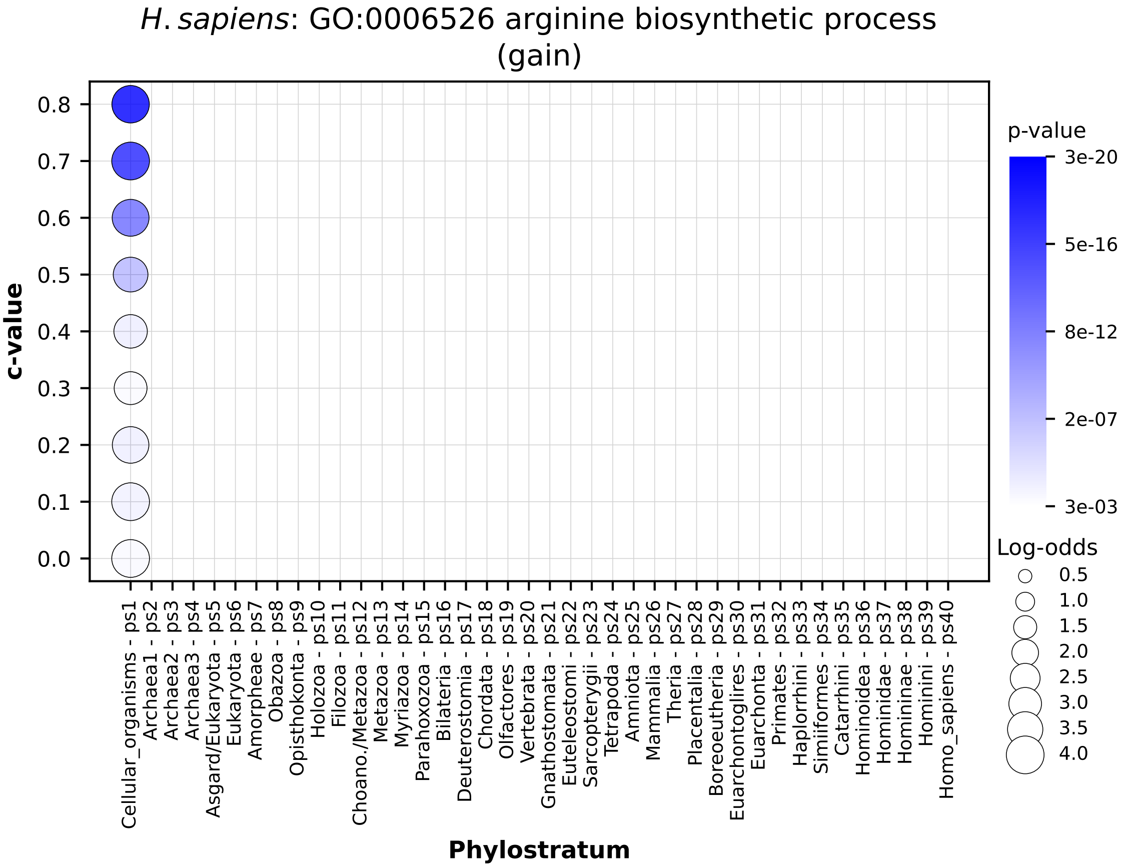

H. sapiens: GO:0006526 arginine biosynthetic process (gain)
c-value
0.8
0.7
0.6
0.5
0.4
0.3
0.2
0.1
0.0
p-value
3e-20
5e-16
8e-12
2e-07
3e-03
Log-odds
0.5
1.0
1.5
2.0
2.5
3.0
3.5
4.0
Cellular_organisms - ps1
Archaea1 - ps2
Archaea2 - ps3
Archaea3 - ps4
Asgard/Eukaryota - ps5
Eukaryota - ps6
Amorpheae - ps7
Obazoa - ps8
Opisthokonta - ps9
Holozoa - ps10
Filozoa - ps11
Choano./Metazoa - ps12
Metazoa - ps13
Myriazoa - ps14
Parahoxozoa - ps15
Bilateria - ps16
Deuterostomia - ps17
Chordata - ps18
Olfactores - ps19
Vertebrata - ps20
Gnathostomata - ps21
Euteleostomi - ps22
Sarcopterygii - ps23
Tetrapoda - ps24
Amniota - ps25
Mammalia - ps26
Theria - ps27
Placentalia - ps28
Boreoeutheria - ps29
Euarchontoglires - ps30
Euarchonta - ps31
Primates - ps32
Haplorrhini - ps33
Simiiformes - ps34
Catarrhini - ps35
Hominoidea - ps36
Hominidae - ps37
Homininae - ps38
Hominini - ps39
Homo_sapiens - ps40
Phylostratum

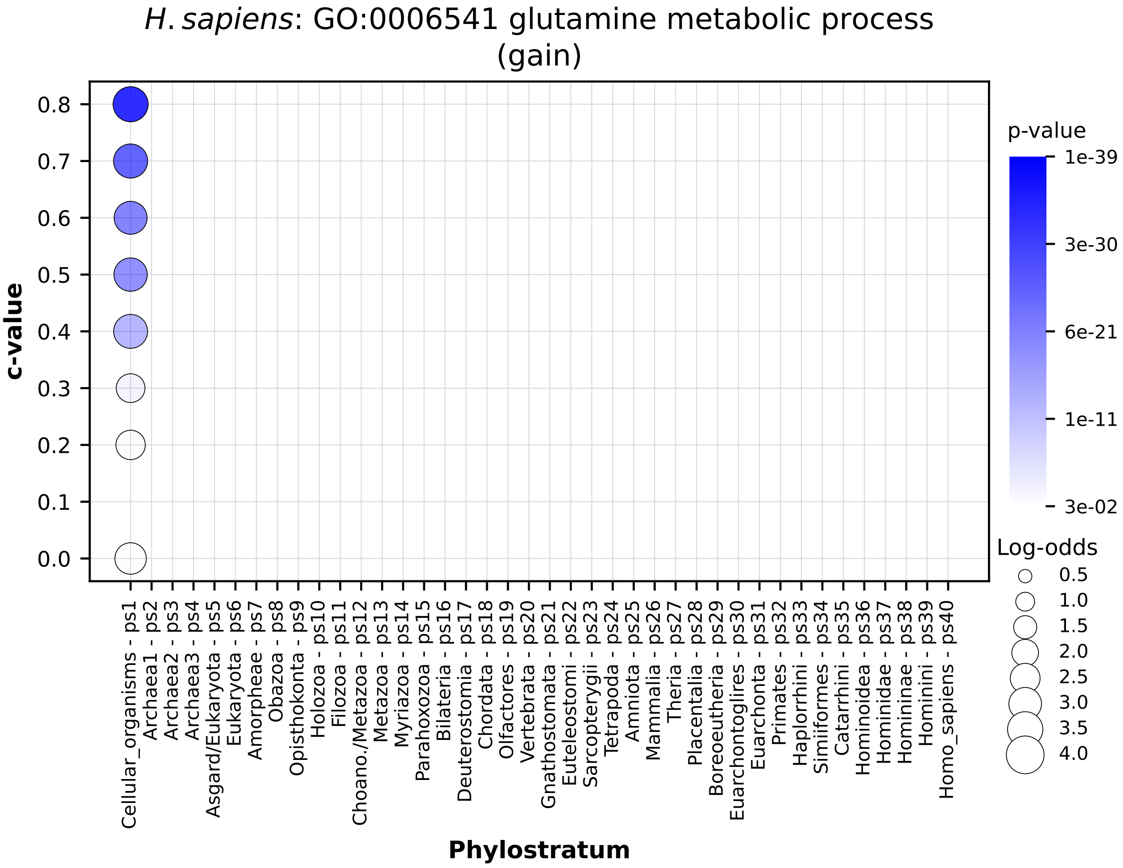
H. sapiens: GO:0006541 glutamine metabolic process
(gain)
c-value
0.8
0.7
0.6
0.5
0.4
0.3
0.2
0.1
0.0
Phylostratum
Cellular_organisms - ps1
Archaea1 - ps2
Archaea2 - ps3
Archaea3 - ps4
Asgard/Eukaryota - ps5
Eukaryota - ps6
Amorpheae - ps7
Obazoa - ps8
Opisthokonta - ps9
Holozoa - ps10
Filozoa - ps11
Choano./Metazoa - ps12
Metazoa - ps13
Myriazoa - ps14
Parahoxozoa - ps15
Bilateria - ps16
Deuterostomia - ps17
Chordata - ps18
Olfactores - ps19
Vertebrata - ps20
Gnathostomata - ps21
Euteleostomi - ps22
Sarcopterygii - ps23
Tetrapoda - ps24
Amniota - ps25
Mammalia - ps26
Theria - ps27
Placentalia - ps28
Boreoeutheria - ps29
Euarchontoglires - ps30
Euarchonta - ps31
Primates - ps32
Haplorrhini - ps33
Simiiformes - ps34
Catarrhini - ps35
Hominoidea - ps36
Hominidae - ps37
Homininae - ps38
Hominini - ps39
Homo_sapiens - ps40
p-value
1e-39
3e-30
6e-21
1e-11
3e-02
Log-odds
0.5
1.0
1.5
2.0
2.5
3.0
3.5
4.0

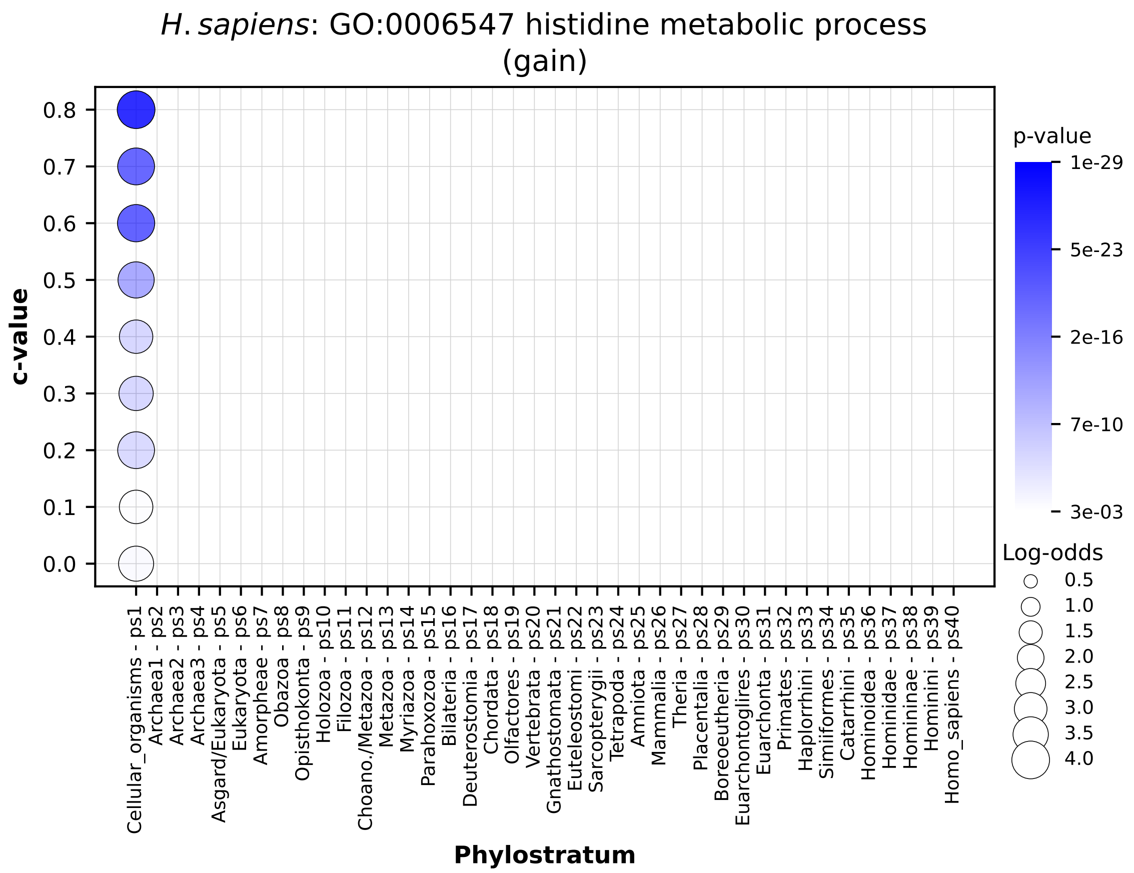
H. sapiens: GO:0006547 histidine metabolic process
(gain)
c-value
0.0
0.1
0.2
0.3
0.4
0.5
0.6
0.7
0.8
Phylostratum
Cellular_organisms - ps1
Archaea1 - ps2
Archaea2 - ps3
Archaea3 - ps4
Asgard/Eukaryota - ps5
Eukaryota - ps6
Amorpheae - ps7
Obazoa - ps8
Opisthokonta - ps9
Holozoa - ps10
Filozoa - ps11
Choano./Metazoa - ps12
Metazoa - ps13
Myriazoa - ps14
Parahoxozoa - ps15
Bilateria - ps16
Deuterostomia - ps17
Chordata - ps18
Olfactores - ps19
Vertebrata - ps20
Gnathostomata - ps21
Euteleostomi - ps22
Sarcopterygii - ps23
Tetrapoda - ps24
Amniota - ps25
Mammalia - ps26
Theria - ps27
Placentalia - ps28
Boreoeutheria - ps29
Euarchontoglires - ps30
Euarchonta - ps31
Primates - ps32
Haplorrhini - ps33
Simiiformes - ps34
Catarrhini - ps35
Hominoidea - ps36
Hominidae - ps37
Homininae - ps38
Hominini - ps39
Homo_sapiens - ps40
p-value
1e-29
5e-23
2e-16
7e-10
3e-03
Log-odds
0.5
1.0
1.5
2.0
2.5
3.0
3.5
4.0

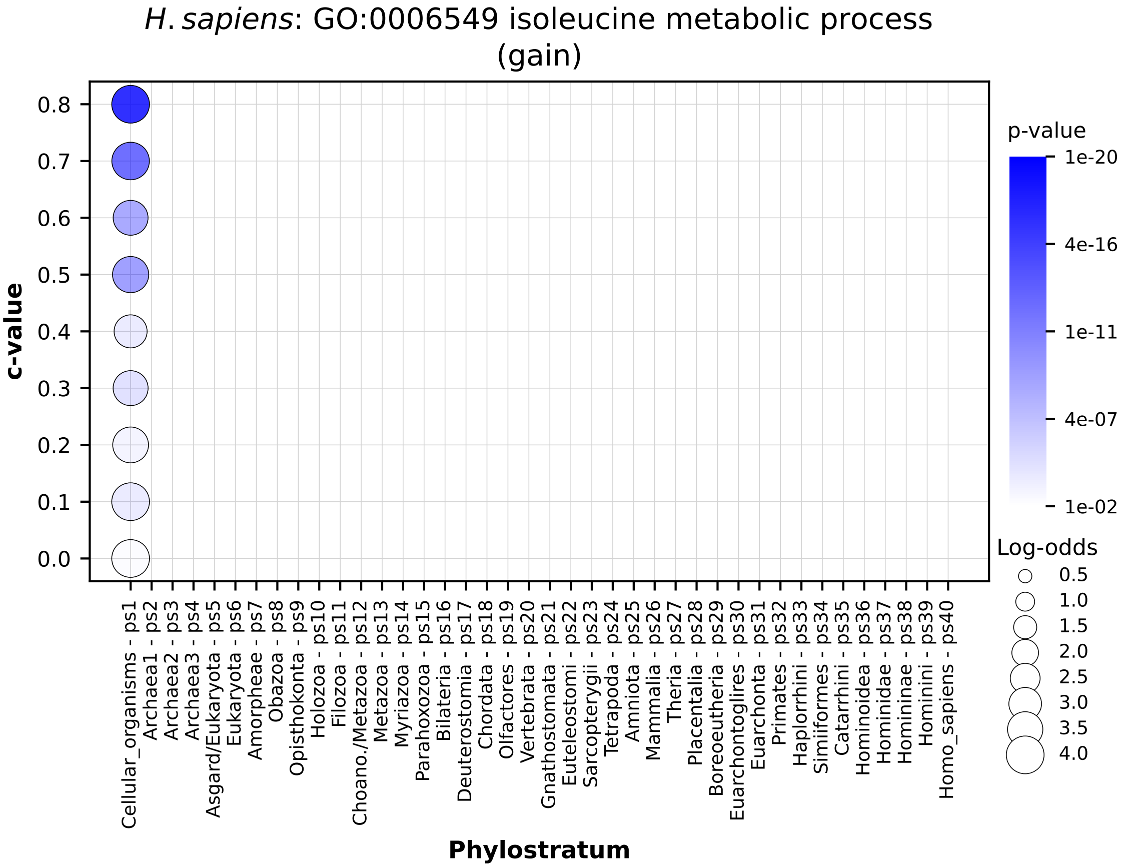

H. sapiens: GO:0006549 isoleucine metabolic process (gain)
c-value
0.8
0.7
0.6
0.5
0.4
0.3
0.2
0.1
0.0
p-value
1e-20
4e-16
1e-11
4e-07
1e-02
Log-odds
0.5
1.0
1.5
2.0
2.5
3.0
3.5
4.0
Cellular_organisms - ps1
Archaea1 - ps2
Archaea2 - ps3
Archaea3 - ps4
Asgard/Eukaryota - ps5
Eukaryota - ps6
Amorpheae - ps7
Obazoa - ps8
Opisthokonta - ps9
Holozoa - ps10
Filozoa - ps11
Choano./Metazoa - ps12
Metazoa - ps13
Myriazoa - ps14
Parahoxozoa - ps15
Bilateria - ps16
Deuterostomia - ps17
Chordata - ps18
Olfactores - ps19
Vertebrata - ps20
Gnathostomata - ps21
Euteleostomi - ps22
Sarcopterygii - ps23
Tetrapoda - ps24
Amniota - ps25
Mammalia - ps26
Theria - ps27
Placentalia - ps28
Boreoeutheria - ps29
Euarchontoglires - ps30
Euarchonta - ps31
Primates - ps32
Haplorrhini - ps33
Simiiformes - ps34
Catarrhini - ps35
Hominoidea - ps36
Hominidae - ps37
Homininae - ps38
Hominini - ps39
Homo_sapiens - ps40
Phylostratum

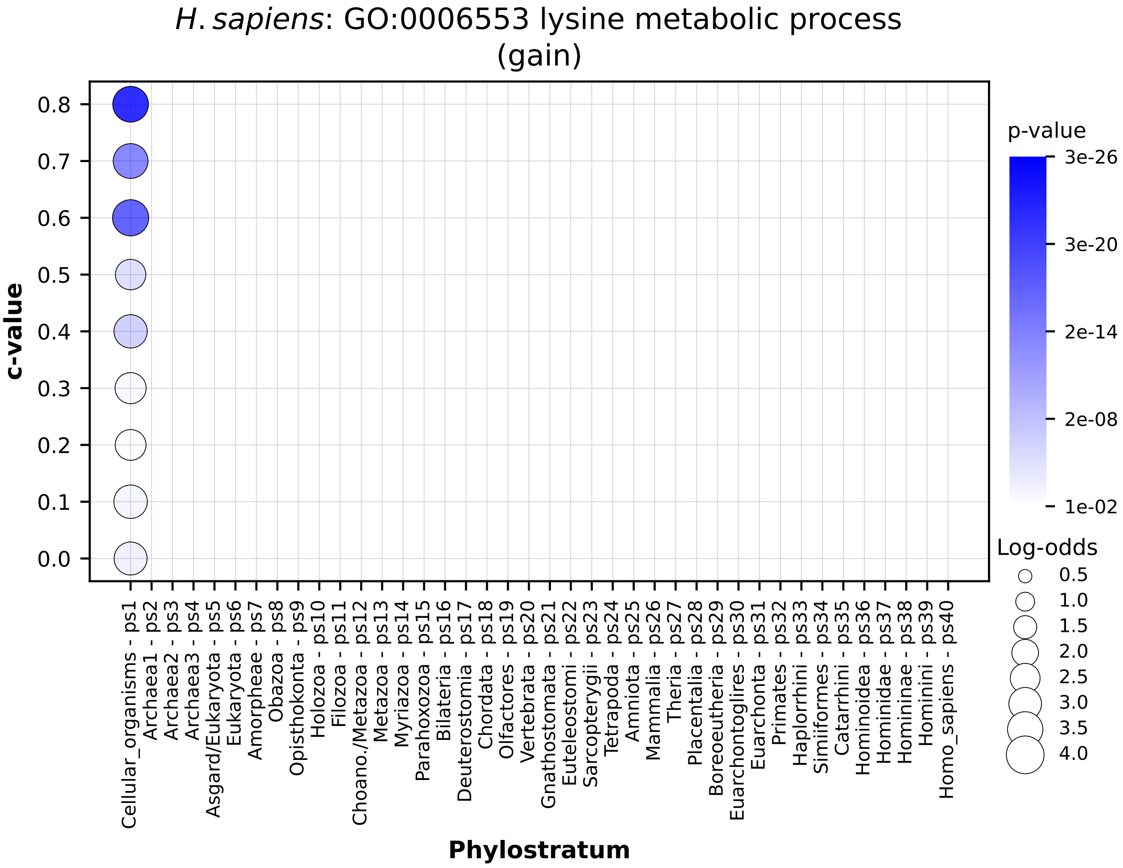

H. sapiens: GO:0006553 lysine metabolic process
(gain)
c-value
0.8
0.7
0.6
0.5
0.4
0.3
0.2
0.1
0.0
p-value
3e-26
3e-20
2e-14
2e-08
1e-02
Log-odds
0.5
1.0
1.5
2.0
2.5
3.0
3.5
4.0
Cellular_organisms - ps1
Archaea1 - ps2
Archaea2 - ps3
Archaea3 - ps4
Asgard/Eukaryota - ps5
Eukaryota - ps6
Amorpheae - ps7
Obazoa - ps8
Opisthokonta - ps9
Holozoa - ps10
Filozoa - ps11
Choano./Metazoa - ps12
Metazoa - ps13
Myriazoa - ps14
Parahoxozoa - ps15
Bilateria - ps16
Deuterostomia - ps17
Chordata - ps18
Olfactores - ps19
Vertebrata - ps20
Gnathostomata - ps21
Euteleostomi - ps22
Sarcopterygii - ps23
Tetrapoda - ps24
Amniota - ps25
Mammalia - ps26
Theria - ps27
Placentalia - ps28
Boreoeutheria - ps29
Euarchontoglires - ps30
Euarchonta - ps31
Primates - ps32
Haplorrhini - ps33
Simiiformes - ps34
Catarrhini - ps35
Hominoidea - ps36
Hominidae - ps37
Homininae - ps38
Hominini - ps39
Homo_sapiens - ps40
Phylostratum

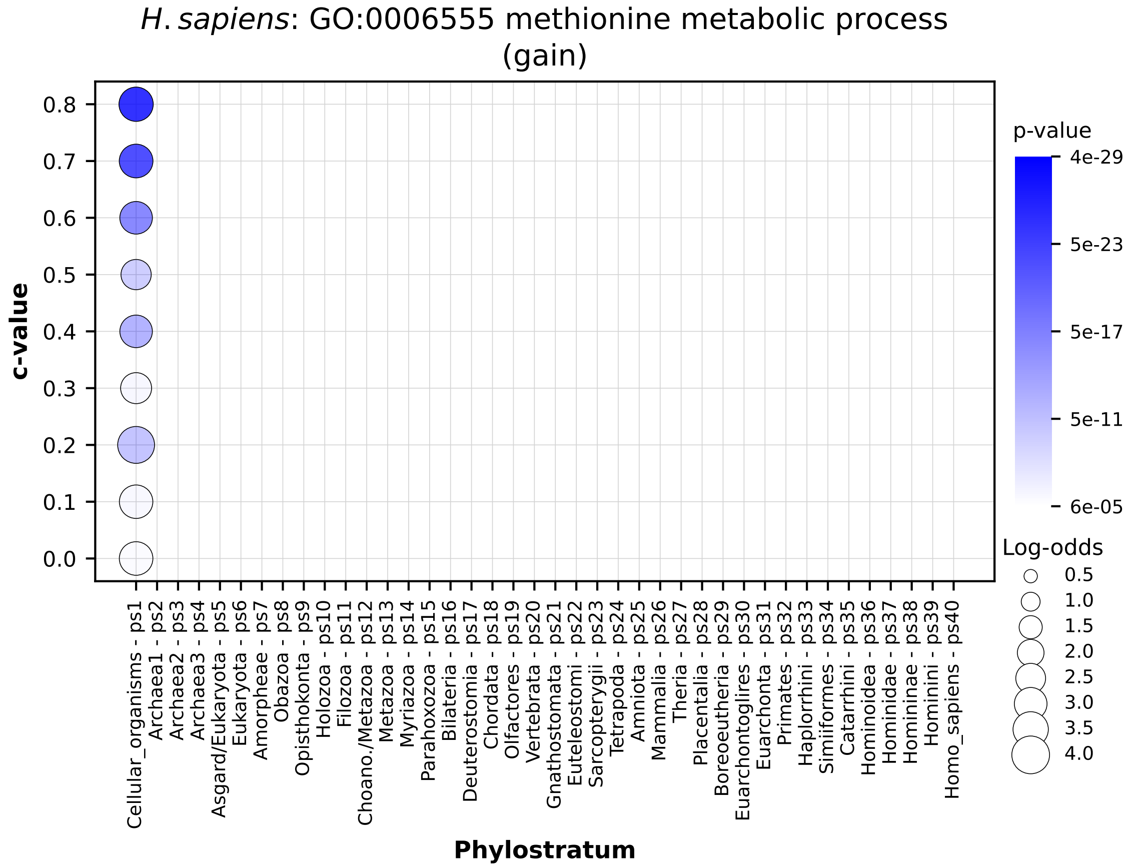

H. sapiens: GO:0006555 methionine metabolic process
(gain)
c-value
0.8
0.7
0.6
0.5
0.4
0.3
0.2
0.1
0.0
p-value
4e-29
5e-23
5e-17
5e-11
6e-05
Log-odds
0.5
1.0
1.5
2.0
2.5
3.0
3.5
4.0
Cellular_organisms - ps1
Archaea1 - ps2
Archaea2 - ps3
Archaea3 - ps4
Asgard/Eukaryota - ps5
Eukaryota - ps6
Amorpheae - ps7
Obazoa - ps8
Opisthokonta - ps9
Holozoa - ps10
Filozoa - ps11
Choano./Metazoa - ps12
Metazoa - ps13
Myriazoa - ps14
Parahoxozoa - ps15
Bilateria - ps16
Deuterostomia - ps17
Chordata - ps18
Olfactores - ps19
Vertebrata - ps20
Gnathostomata - ps21
Euteleostomi - ps22
Sarcopterygii - ps23
Tetrapoda - ps24
Amniota - ps25
Mammalia - ps26
Theria - ps27
Placentalia - ps28
Boreoeutheria - ps29
Euarchontoglires - ps30
Euarchonta - ps31
Primates - ps32
Haplorrhini - ps33
Simiiformes - ps34
Catarrhini - ps35
Hominoidea - ps36
Hominidae - ps37
Homininae - ps38
Hominini - ps39
Homo_sapiens - ps40
Phylostratum

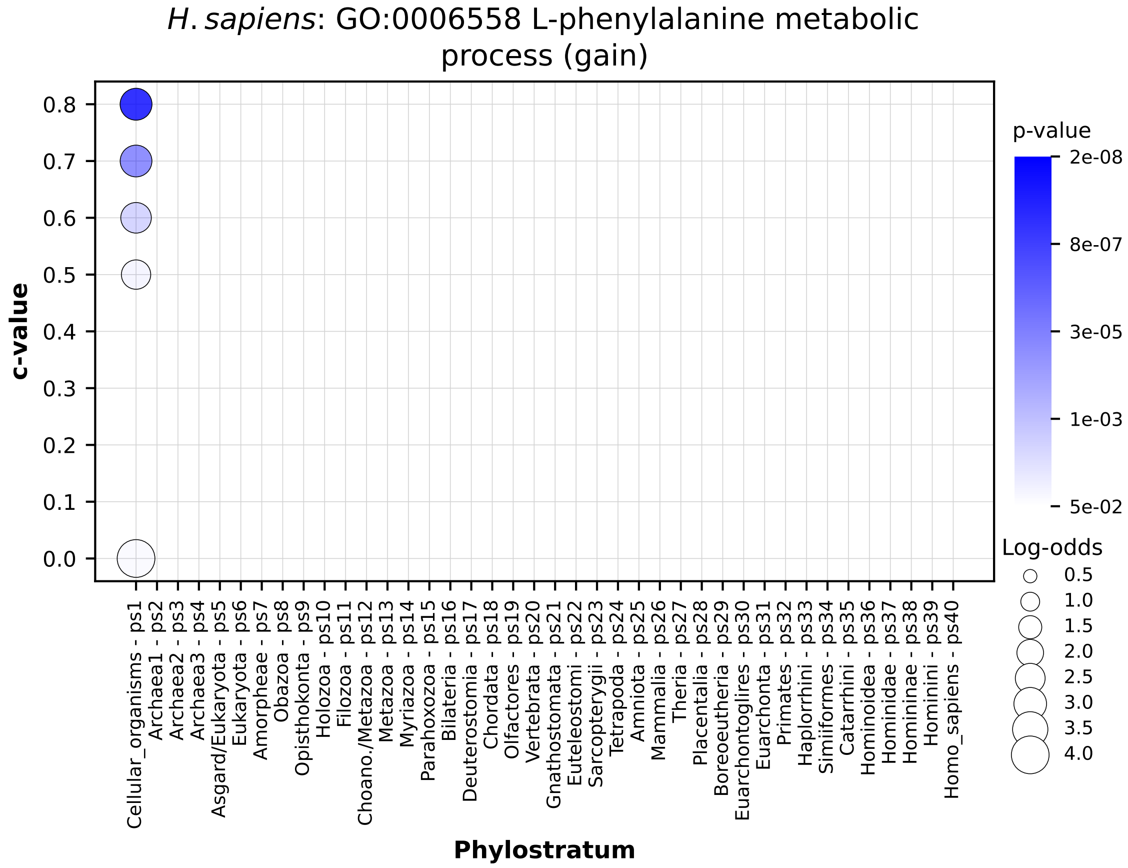

H. sapiens: GO:0006558 L-phenylalanine metabolic process (gain)
c-value
0.8
0.7
0.6
0.5
0.4
0.3
0.2
0.1
0.0
p-value
2e-08
8e-07
3e-05
1e-03
5e-02
Log-odds
0.5
1.0
1.5
2.0
2.5
3.0
3.5
4.0
Cellular_organisms - ps1
Archaea1 - ps2
Archaea2 - ps3
Archaea3 - ps4
Asgard/Eukaryota - ps5
Eukaryota - ps6
Amorpheae - ps7
Obazoa - ps8
Opisthokonta - ps9
Holozoa - ps10
Filozoa - ps11
Choano./Metazoa - ps12
Metazoa - ps13
Myriazoa - ps14
Parahoxozoa - ps15
Bilateria - ps16
Deuterostomia - ps17
Chordata - ps18
Olfactores - ps19
Vertebrata - ps20
Gnathostomata - ps21
Euteleostomi - ps22
Sarcopterygii - ps23
Tetrapoda - ps24
Amniota - ps25
Mammalia - ps26
Theria - ps27
Placentalia - ps28
Boreoeutheria - ps29
Euarchontoglires - ps30
Euarchonta - ps31
Primates - ps32
Haplorrhini - ps33
Simiiformes - ps34
Catarrhini - ps35
Hominoidea - ps36
Hominidae - ps37
Homininae - ps38
Hominini - ps39
Homo_sapiens - ps40
Phylostratum

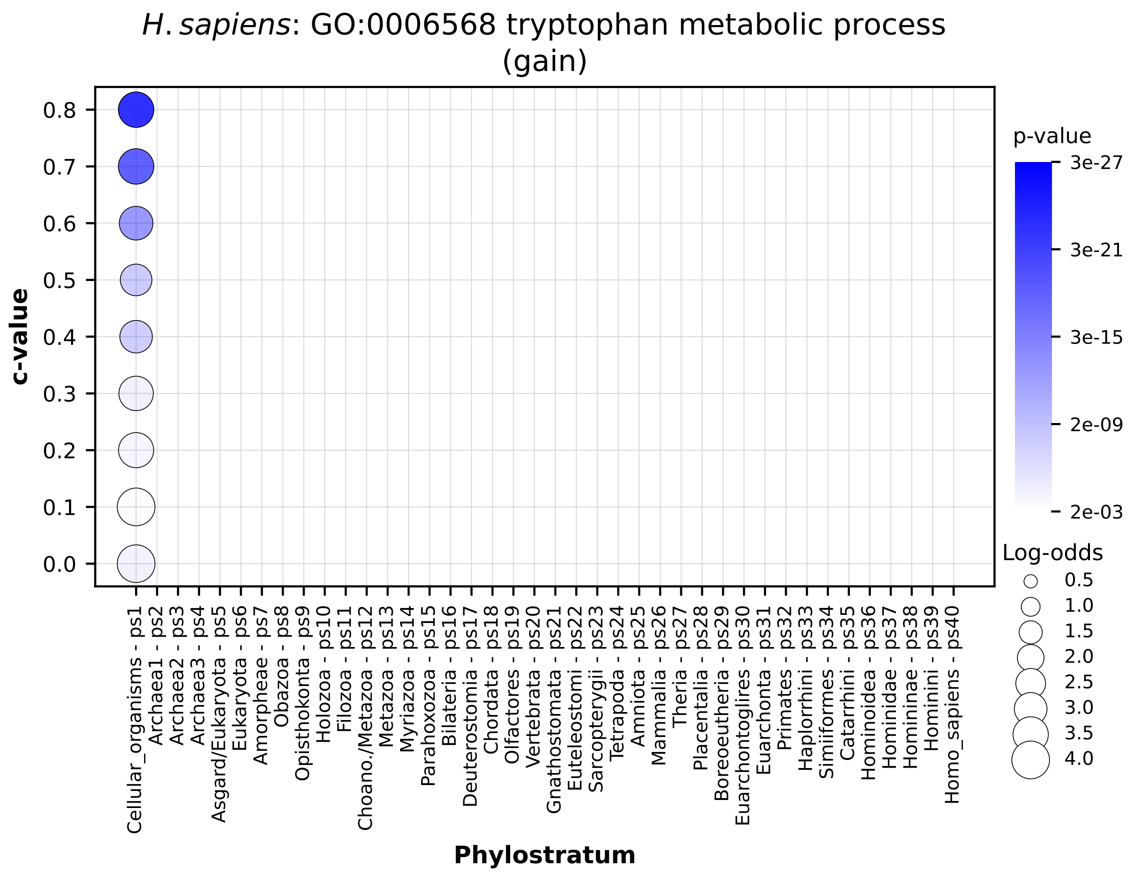

H. sapiens: GO:0006568 tryptophan metabolic process
(gain)
c-value
0.8
0.7
0.6
0.5
0.4
0.3
0.2
0.1
0.0
p-value
3e-27
3e-21
3e-15
2e-09
2e-03
Log-odds
0.5
1.0
1.5
2.0
2.5
3.0
3.5
4.0
Cellular_organisms - ps1
Archaea1 - ps2
Archaea2 - ps3
Archaea3 - ps4
Asgard/Eukaryota - ps5
Eukaryota - ps6
Amorpheae - ps7
Obazoa - ps8
Opisthokonta - ps9
Holozoa - ps10
Filozoa - ps11
Choano./Metazoa - ps12
Metazoa - ps13
Myriazoa - ps14
Parahoxozoa - ps15
Bilateria - ps16
Deuterostomia - ps17
Chordata - ps18
Olfactores - ps19
Vertebrata - ps20
Gnathostomata - ps21
Euteleostomi - ps22
Sarcopterygii - ps23
Tetrapoda - ps24
Amniota - ps25
Mammalia - ps26
Theria - ps27
Placentalia - ps28
Boreoeutheria - ps29
Euarchontoglires - ps30
Euarchonta - ps31
Primates - ps32
Haplorrhini - ps33
Simiiformes - ps34
Catarrhini - ps35
Hominoidea - ps36
Hominidae - ps37
Homininae - ps38
Hominini - ps39
Homo_sapiens - ps40
Phylostratum

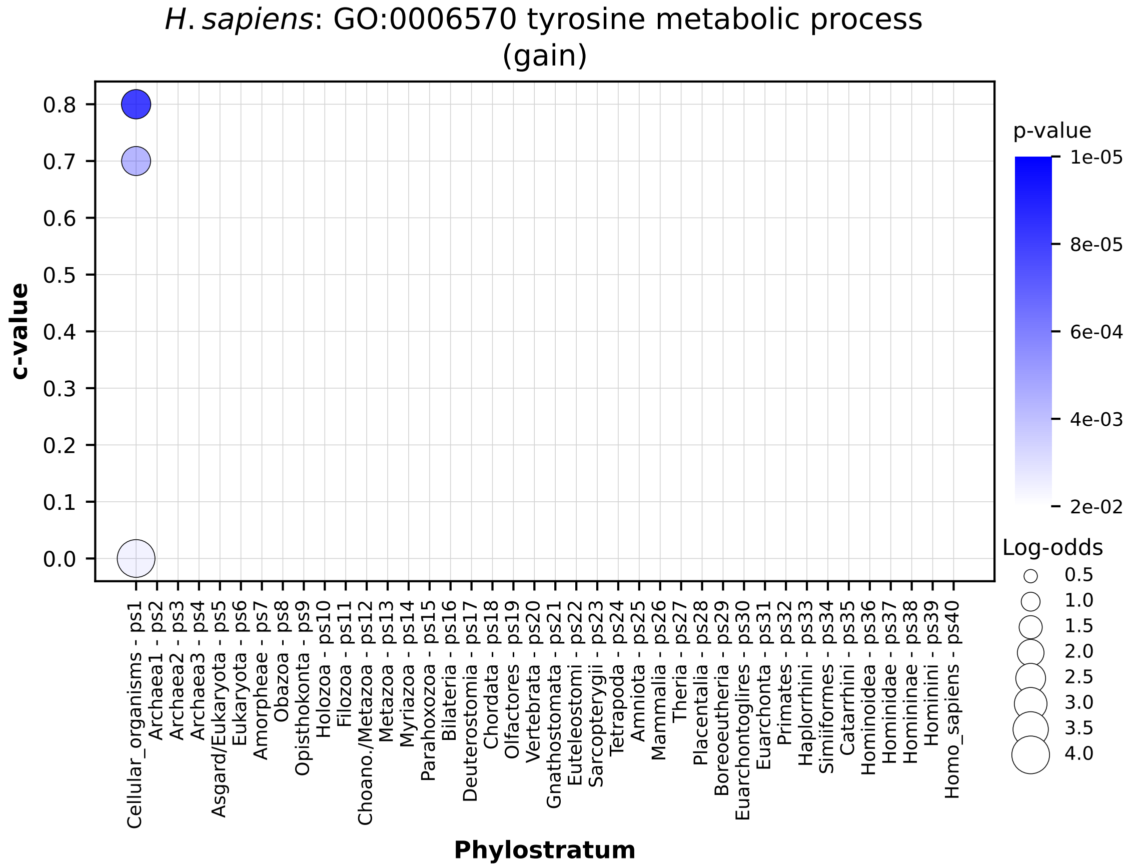

H. sapiens: GO:0006570 tyrosine metabolic process
(gain)
c-value
0.8
0.7
0.6
0.5
0.4
0.3
0.2
0.1
0.0
p-value
1e-05
8e-05
6e-04
4e-03
2e-02
Log-odds
0.5
1.0
1.5
2.0
2.5
3.0
3.5
4.0
Cellular_organisms - ps1
Archaea1 - ps2
Archaea2 - ps3
Archaea3 - ps4
Asgard/Eukaryota - ps5
Eukaryota - ps6
Amorpheae - ps7
Obazoa - ps8
Opisthokonta - ps9
Holozoa - ps10
Filozoa - ps11
Choano./Metazoa - ps12
Metazoa - ps13
Myriazoa - ps14
Parahoxozoa - ps15
Bilateria - ps16
Deuterostomia - ps17
Chordata - ps18
Olfactores - ps19
Vertebrata - ps20
Gnathostomata - ps21
Euteleostomi - ps22
Sarcopterygii - ps23
Tetrapoda - ps24
Amniota - ps25
Mammalia - ps26
Theria - ps27
Placentalia - ps28
Boreoeutheria - ps29
Euarchontoglires - ps30
Euarchonta - ps31
Primates - ps32
Haplorrhini - ps33
Simiiformes - ps34
Catarrhini - ps35
Hominoidea - ps36
Hominidae - ps37
Homininae - ps38
Hominini - ps39
Homo_sapiens - ps40
Phylostratum

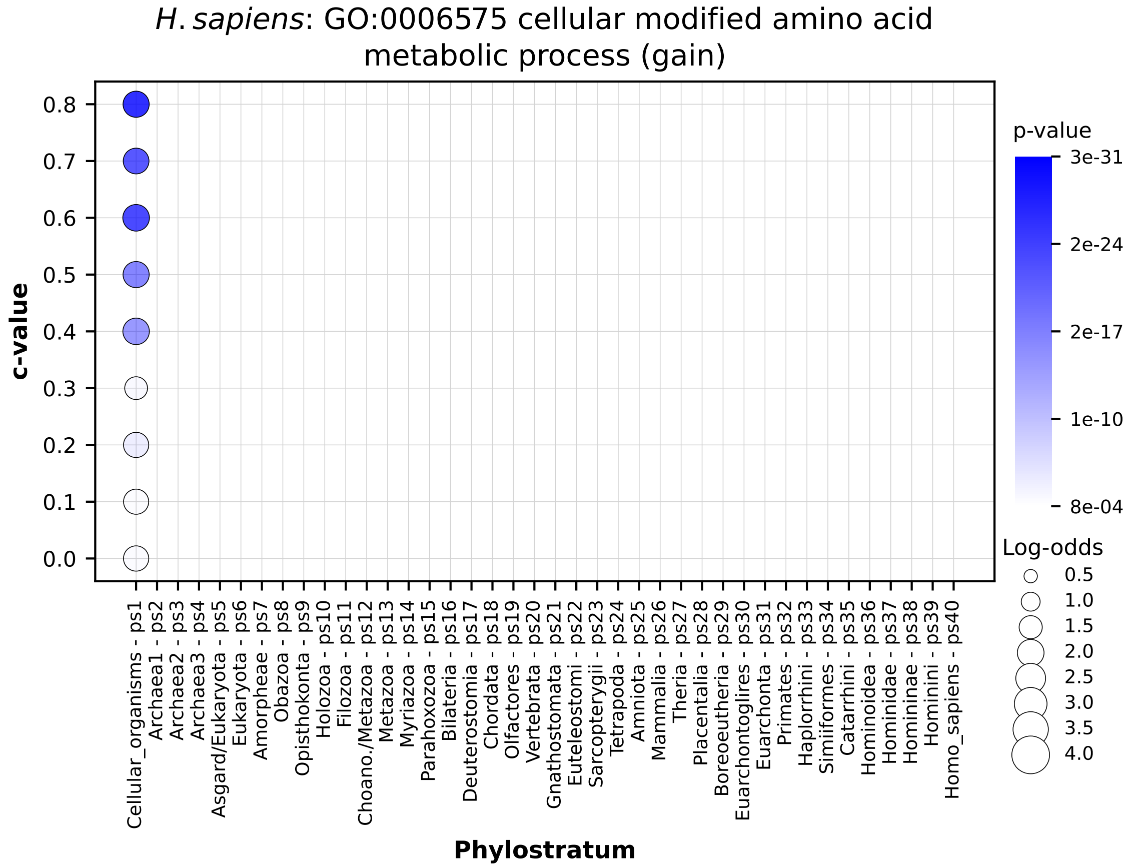
H. sapiens: GO:0006575 cellular modified amino acid metabolic process (gain)
c-value
0.8
0.7
0.6
0.5
0.4
0.3
0.2
0.1
0.0
p-value
3e-31
2e-24
2e-17
1e-10
8e-04
Log-odds
0.5
1.0
1.5
2.0
2.5
3.0
3.5
4.0
Cellular_organisms - ps1
Archaea1 - ps2
Archaea2 - ps3
Archaea3 - ps4
Asgard/Eukaryota - ps5
Eukaryota - ps6
Amorpheae - ps7
Obazoa - ps8
Opisthokonta - ps9
Holozoa - ps10
Filozoa - ps11
Choano./Metazoa - ps12
Metazoa - ps13
Myriazoa - ps14
Parahoxozoa - ps15
Bilateria - ps16
Deuterostomia - ps17
Chordata - ps18
Olfactores - ps19
Vertebrata - ps20
Gnathostomata - ps21
Euteleostomi - ps22
Sarcopterygii - ps23
Tetrapoda - ps24
Amniota - ps25
Mammalia - ps26
Theria - ps27
Placentalia - ps28
Boreoeutheria - ps29
Euarchontoglires - ps30
Euarchonta - ps31
Primates - ps32
Haplorrhini - ps33
Simiiformes - ps34
Catarrhini - ps35
Hominoidea - ps36
Hominidae - ps37
Homininae - ps38
Hominini - ps39
Homo_sapiens - ps40
Phylostratum

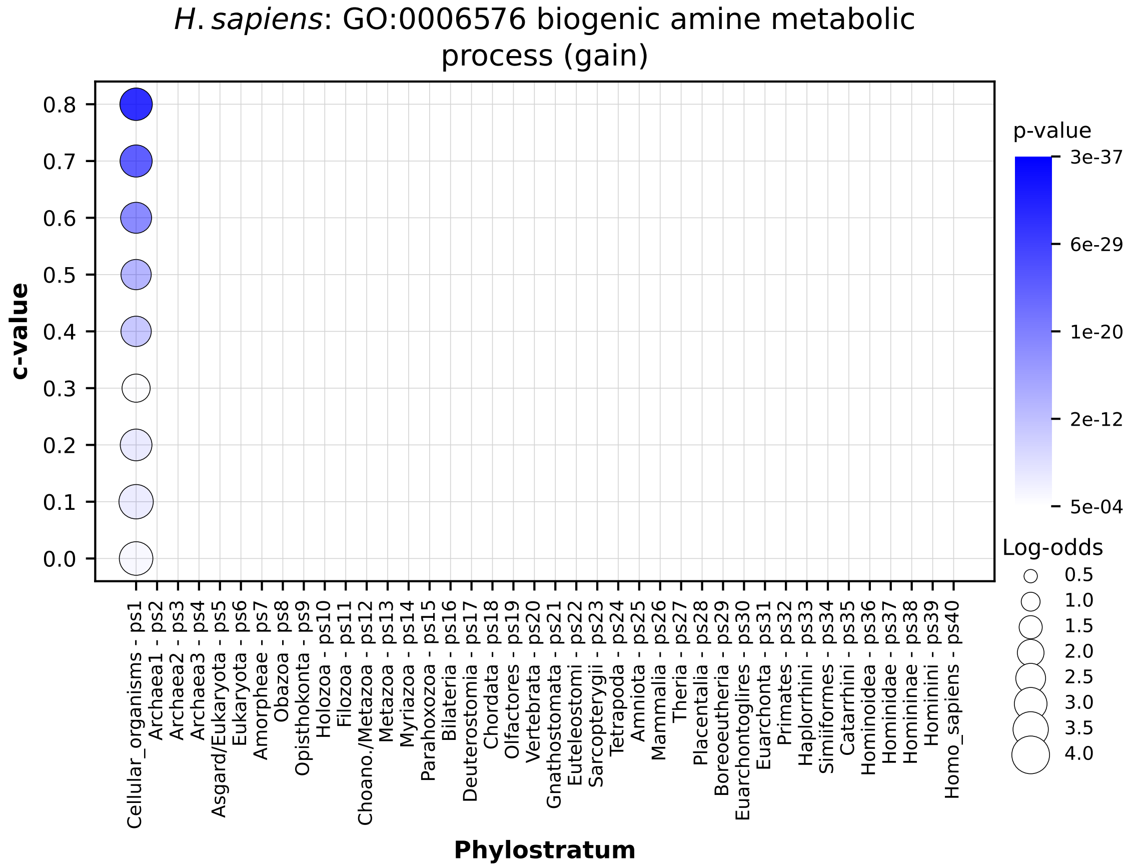

H. sapiens: GO:0006576 biogenic amine metabolic process (gain)
c-value
0.0
0.1
0.2
0.3
0.4
0.5
0.6
0.7
0.8
Phylostratum
Cellular_organisms - ps1
Archaea1 - ps2
Archaea2 - ps3
Archaea3 - ps4
Asgard/Eukaryota - ps5
Eukaryota - ps6
Amorpheae - ps7
Obazoa - ps8
Opisthokonta - ps9
Holozoa - ps10
Filozoa - ps11
Choano./Metazoa - ps12
Metazoa - ps13
Myriazoa - ps14
Parahoxozoa - ps15
Bilateria - ps16
Deuterostomia - ps17
Chordata - ps18
Olfactores - ps19
Vertebrata - ps20
Gnathostomata - ps21
Euteleostomi - ps22
Sarcopterygii - ps23
Tetrapoda - ps24
Amniota - ps25
Mammalia - ps26
Theria - ps27
Placentalia - ps28
Boreoeutheria - ps29
Euarchontoglires - ps30
Euarchonta - ps31
Primates - ps32
Haplorrhini - ps33
Simiiformes - ps34
Catarrhini - ps35
Hominoidea - ps36
Hominidae - ps37
Homininae - ps38
Hominini - ps39
Homo_sapiens - ps40
p-value
3e-37
6e-29
1e-20
2e-12
5e-04
Log-odds
0.5
1.0
1.5
2.0
2.5
3.0
3.5
4.0

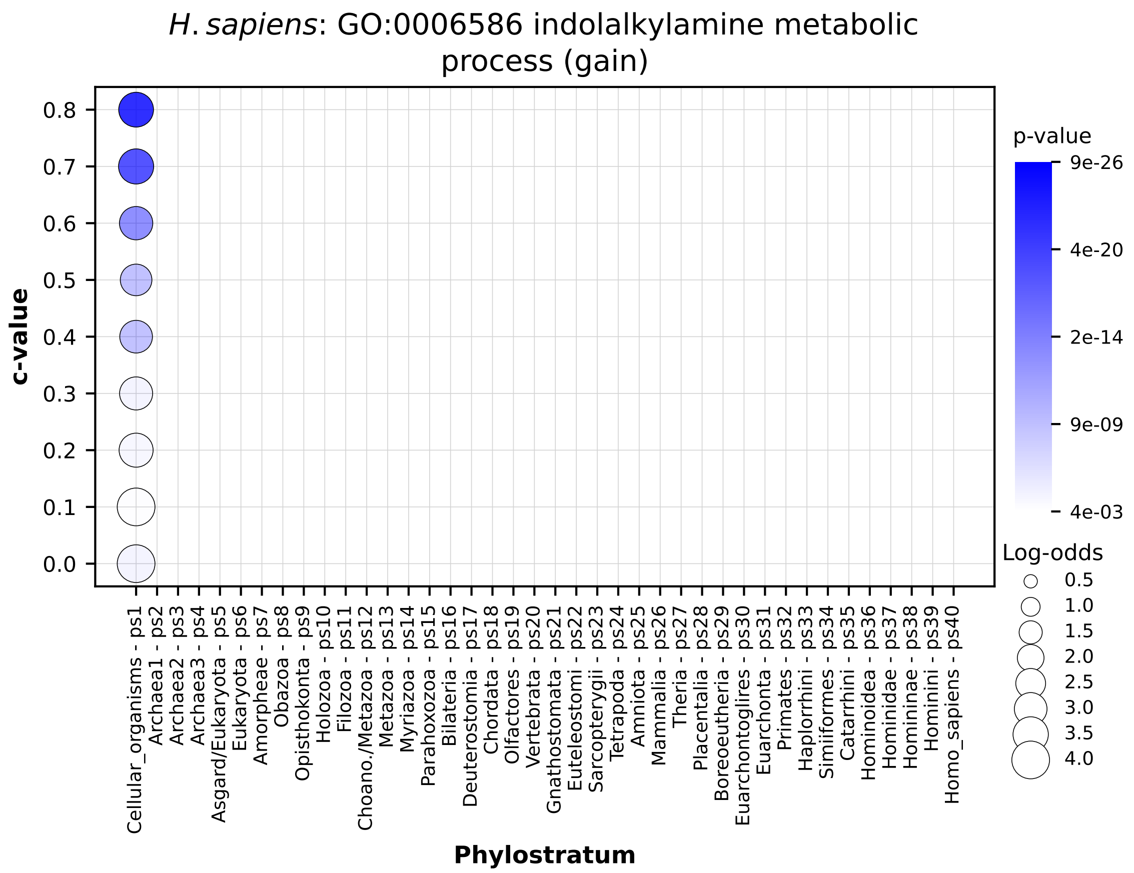

H. sapiens: GO:0006586 indolalkylamine metabolic process (gain)
c-value
0.8
0.7
0.6
0.5
0.4
0.3
0.2
0.1
0.0
Phylostratum
Cellular_organisms - ps1
Archaea1 - ps2
Archaea2 - ps3
Archaea3 - ps4
Asgard/Eukaryota - ps5
Eukaryota - ps6
Amorpheae - ps7
Obazoa - ps8
Opisthokonta - ps9
Holozoa - ps10
Filozoa - ps11
Choano./Metazoa - ps12
Metazoa - ps13
Myriazoa - ps14
Parahoxozoa - ps15
Bilateria - ps16
Deuterostomia - ps17
Chordata - ps18
Olfactores - ps19
Vertebrata - ps20
Gnathostomata - ps21
Euteleostomi - ps22
Sarcopterygii - ps23
Tetrapoda - ps24
Amniota - ps25
Mammalia - ps26
Theria - ps27
Placentalia - ps28
Boreoeutheria - ps29
Euarchontoglires - ps30
Euarchonta - ps31
Primates - ps32
Haplorrhini - ps33
Simiiformes - ps34
Catarrhini - ps35
Hominoidea - ps36
Hominidae - ps37
Homininae - ps38
Hominini - ps39
Homo_sapiens - ps40
p-value
9e-26
4e-20
2e-14
9e-09
4e-03
Log-odds
0.5
1.0
1.5
2.0
2.5
3.0
3.5
4.0

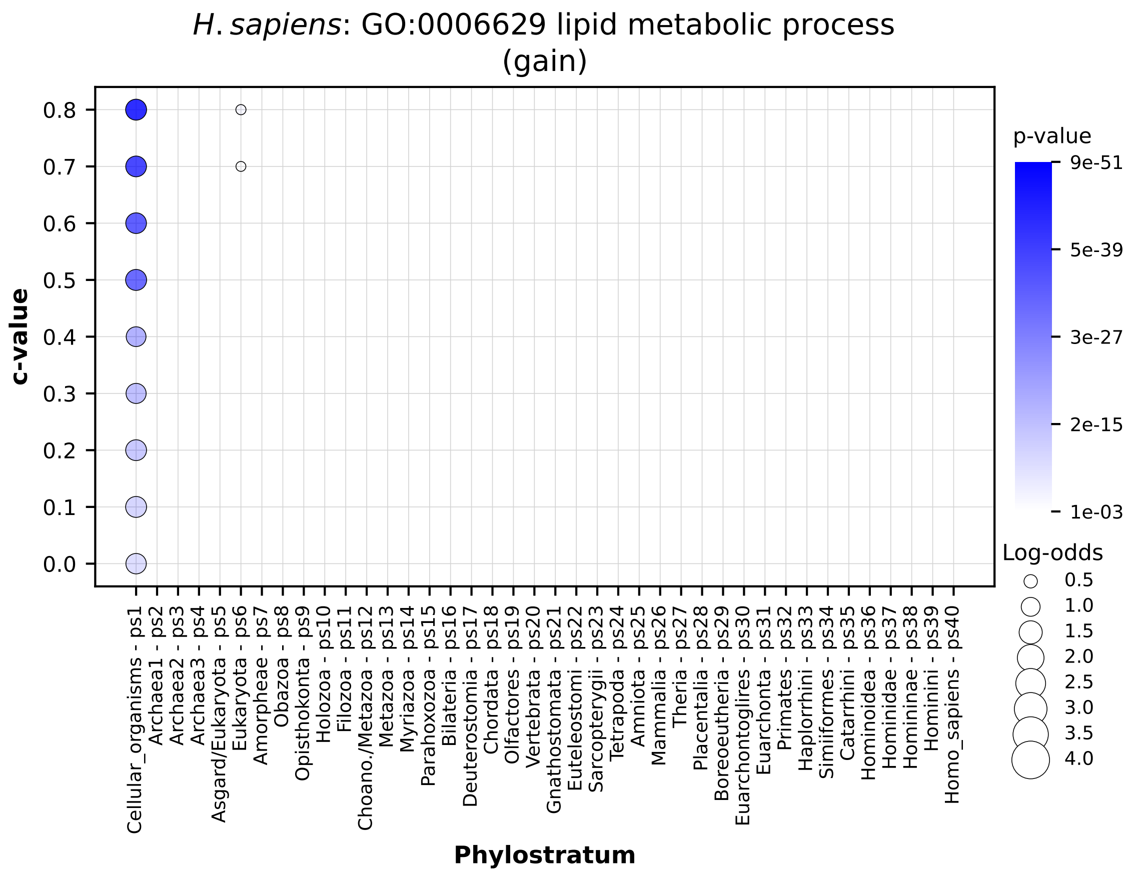

H. sapiens: GO:0006629 lipid metabolic process
(gain)
c-value
0.8
0.7
0.6
0.5
0.4
0.3
0.2
0.1
0.0
Phylostratum
Cellular_organisms - ps1
Archaea1 - ps2
Archaea2 - ps3
Archaea3 - ps4
Asgard/Eukaryota - ps5
Eukaryota - ps6
Amorpheae - ps7
Obazoa - ps8
Opisthokonta - ps9
Holozoa - ps10
Filozoa - ps11
Choano./Metazoa - ps12
Metazoa - ps13
Myriazoa - ps14
Parahoxozoa - ps15
Bilateria - ps16
Deuterostomia - ps17
Chordata - ps18
Olfactores - ps19
Vertebrata - ps20
Gnathostomata - ps21
Euteleostomi - ps22
Sarcopterygii - ps23
Tetrapoda - ps24
Amniota - ps25
Mammalia - ps26
Theria - ps27
Placentalia - ps28
Boreoeutheria - ps29
Euarchontoglires - ps30
Euarchonta - ps31
Primates - ps32
Haplorrhini - ps33
Simiiformes - ps34
Catarrhini - ps35
Hominoidea - ps36
Hominidae - ps37
Homininae - ps38
Hominini - ps39
Homo_sapiens - ps40
p-value
9e-51
5e-39
3e-27
2e-15
1e-03
Log-odds
0.5
1.0
1.5
2.0
2.5
3.0
3.5
4.0

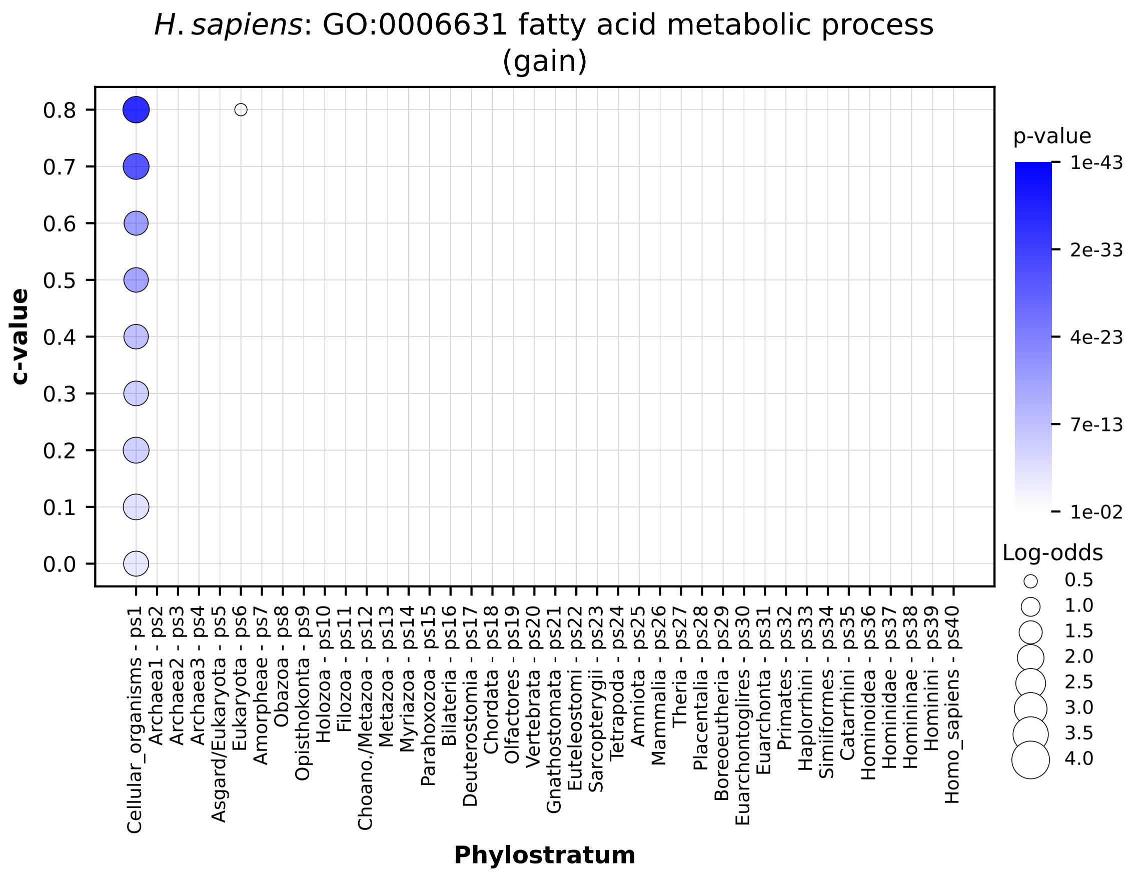
H. sapiens: GO:0006631 fatty acid metabolic process
(gain)
c-value
0.8
0.7
0.6
0.5
0.4
0.3
0.2
0.1
0.0
p-value
1e-43
2e-33
4e-23
7e-13
1e-02
Log-odds
0.5
1.0
1.5
2.0
2.5
3.0
3.5
4.0
Cellular_organisms - ps1
Archaea1 - ps2
Archaea2 - ps3
Archaea3 - ps4
Asgard/Eukaryota - ps5
Eukaryota - ps6
Amorpheae - ps7
Obazoa - ps8
Opisthokonta - ps9
Holozoa - ps10
Filozoa - ps11
Choano./Metazoa - ps12
Metazoa - ps13
Myriazoa - ps14
Parahoxozoa - ps15
Bilateria - ps16
Deuterostomia - ps17
Chordata - ps18
Olfactores - ps19
Vertebrata - ps20
Gnathostomata - ps21
Euteleostomi - ps22
Sarcopterygii - ps23
Tetrapoda - ps24
Amniota - ps25
Mammalia - ps26
Theria - ps27
Placentalia - ps28
Boreoeutheria - ps29
Euarchontoglires - ps30
Euarchonta - ps31
Primates - ps32
Haplorrhini - ps33
Simiiformes - ps34
Catarrhini - ps35
Hominoidea - ps36
Hominidae - ps37
Homininae - ps38
Hominini - ps39
Homo_sapiens - ps40
Phylostratum

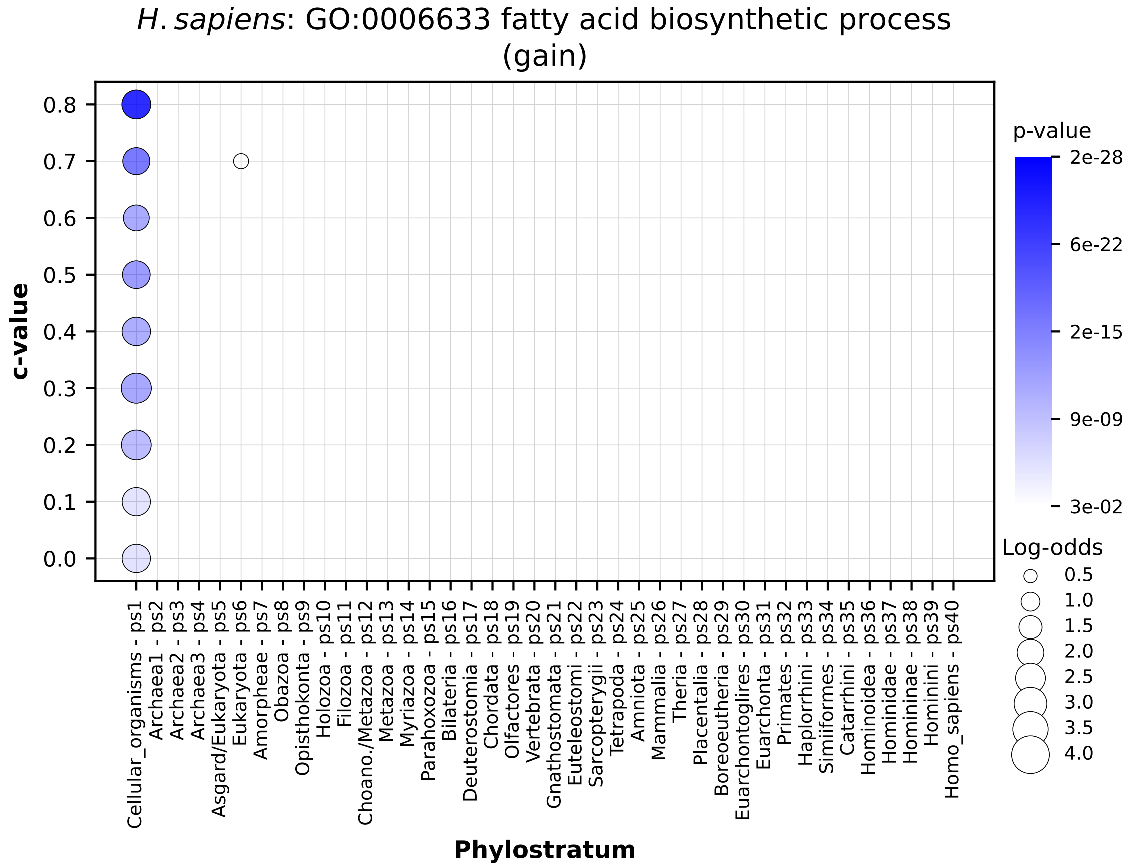

H. sapiens: GO:0006633 fatty acid biosynthetic process
(gain)
c-value
0.8
0.7
0.6
0.5
0.4
0.3
0.2
0.1
0.0
p-value
2e-28
6e-22
2e-15
9e-09
3e-02
Log-odds
0.5
1.0
1.5
2.0
2.5
3.0
3.5
4.0
Cellular_organisms - ps1
Archaea1 - ps2
Archaea2 - ps3
Archaea3 - ps4
Asgard/Eukaryota - ps5
Eukaryota - ps6
Amorpheae - ps7
Obazoa - ps8
Opisthokonta - ps9
Holozoa - ps10
Filozoa - ps11
Choano./Metazoa - ps12
Metazoa - ps13
Myriazoa - ps14
Parahoxozoa - ps15
Bilateria - ps16
Deuterostomia - ps17
Chordata - ps18
Olfactores - ps19
Vertebrata - ps20
Gnathostomata - ps21
Euteleostomi - ps22
Sarcopterygii - ps23
Tetrapoda - ps24
Amniota - ps25
Mammalia - ps26
Theria - ps27
Placentalia - ps28
Boreoeutheria - ps29
Euarchontoglires - ps30
Euarchonta - ps31
Primates - ps32
Haplorrhini - ps33
Simiiformes - ps34
Catarrhini - ps35
Hominoidea - ps36
Hominidae - ps37
Homininae - ps38
Hominini - ps39
Homo_sapiens - ps40
Phylostratum

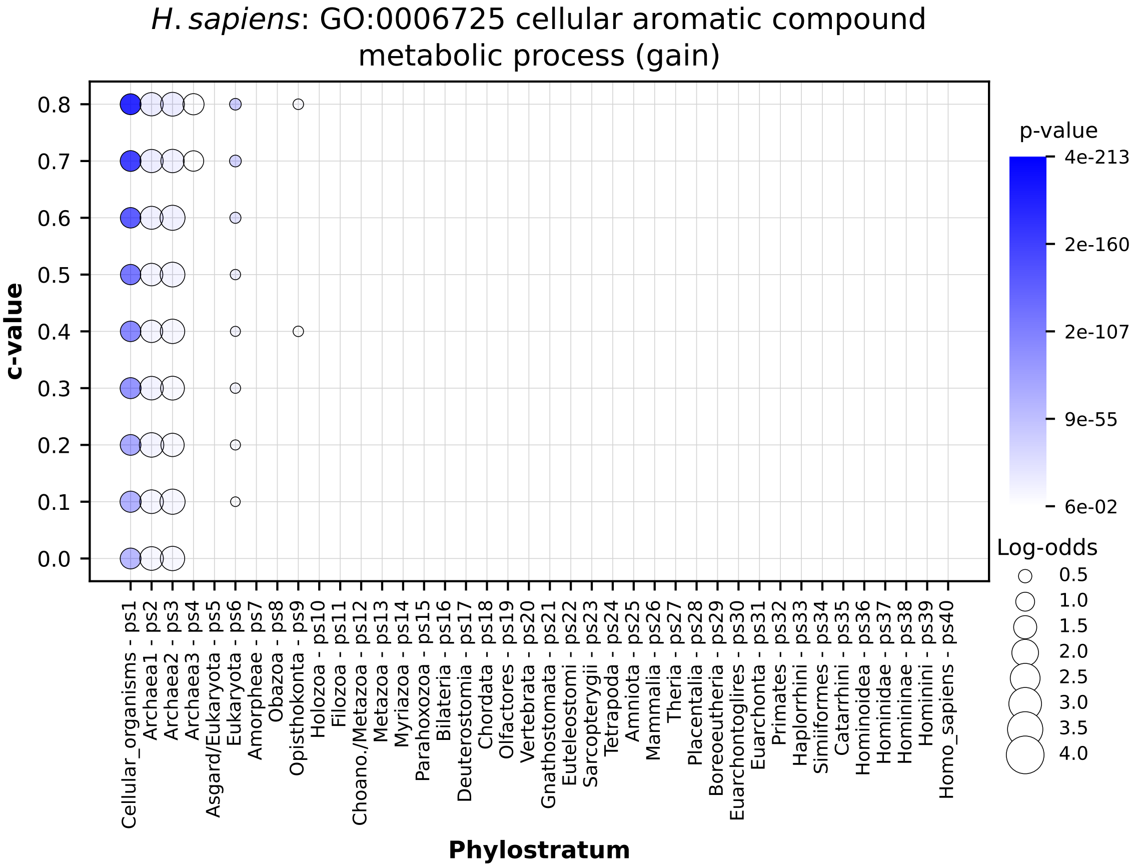
H. sapiens: GO:0006725 cellular aromatic compound metabolic process (gain)
c-value
0.8
0.7
0.6
0.5
0.4
0.3
0.2
0.1
0.0
p-value
4e-213
2e-160
2e-107
9e-55
6e-02
Log-odds
0.5
1.0
1.5
2.0
2.5
3.0
3.5
4.0
Cellular_organisms - ps1
Archaea1 - ps2
Archaea2 - ps3
Archaea3 - ps4
Asgard/Eukaryota - ps5
Eukaryota - ps6
Amorpheae - ps7
Obazoa - ps8
Opisthokonta - ps9
Holozoa - ps10
Filozoa - ps11
Choano./Metazoa - ps12
Metazoa - ps13
Myriazoa - ps14
Parahoxozoa - ps15
Bilateria - ps16
Deuterostomia - ps17
Chordata - ps18
Olfactores - ps19
Vertebrata - ps20
Gnathostomata - ps21
Euteleostomi - ps22
Sarcopterygii - ps23
Tetrapoda - ps24
Amniota - ps25
Mammalia - ps26
Theria - ps27
Placentalia - ps28
Boreoeutheria - ps29
Euarchontoglires - ps30
Euarchonta - ps31
Primates - ps32
Haplorrhini - ps33
Simiiformes - ps34
Catarrhini - ps35
Hominoidea - ps36
Hominidae - ps37
Homininae - ps38
Hominini - ps39
Homo_sapiens - ps40
Phylostratum

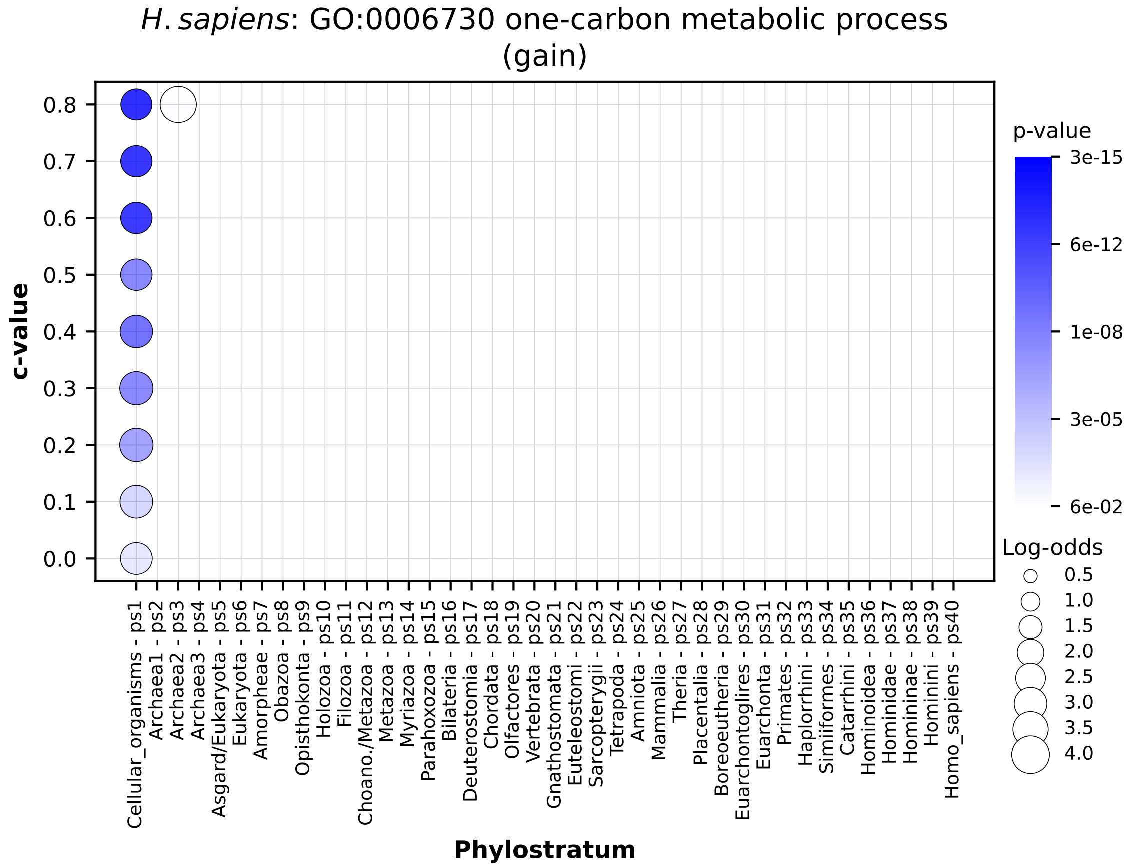

H. sapiens: GO:0006730 one-carbon metabolic process
(gain)
c-value
0.0
0.1
0.2
0.3
0.4
0.5
0.6
0.7
0.8
Phylostratum
Cellular_organisms - ps1
Archaea1 - ps2
Archaea2 - ps3
Archaea3 - ps4
Asgard/Eukaryota - ps5
Eukaryota - ps6
Amorpheae - ps7
Obazoa - ps8
Opisthokonta - ps9
Holozoa - ps10
Filozoa - ps11
Choano./Metazoa - ps12
Metazoa - ps13
Myriazoa - ps14
Parahoxozoa - ps15
Bilateria - ps16
Deuterostomia - ps17
Chordata - ps18
Olfactores - ps19
Vertebrata - ps20
Gnathostomata - ps21
Euteleostomi - ps22
Sarcopterygii - ps23
Tetrapoda - ps24
Amniota - ps25
Mammalia - ps26
Theria - ps27
Placentalia - ps28
Boreoeutheria - ps29
Euarchontoglires - ps30
Euarchonta - ps31
Primates - ps32
Haplorrhini - ps33
Simiiformes - ps34
Catarrhini - ps35
Hominoidea - ps36
Hominidae - ps37
Homininae - ps38
Hominini - ps39
Homo_sapiens - ps40
p-value
3e-15
6e-12
1e-08
3e-05
6e-02
Log-odds
0.5
1.0
1.5
2.0
2.5
3.0
3.5
4.0

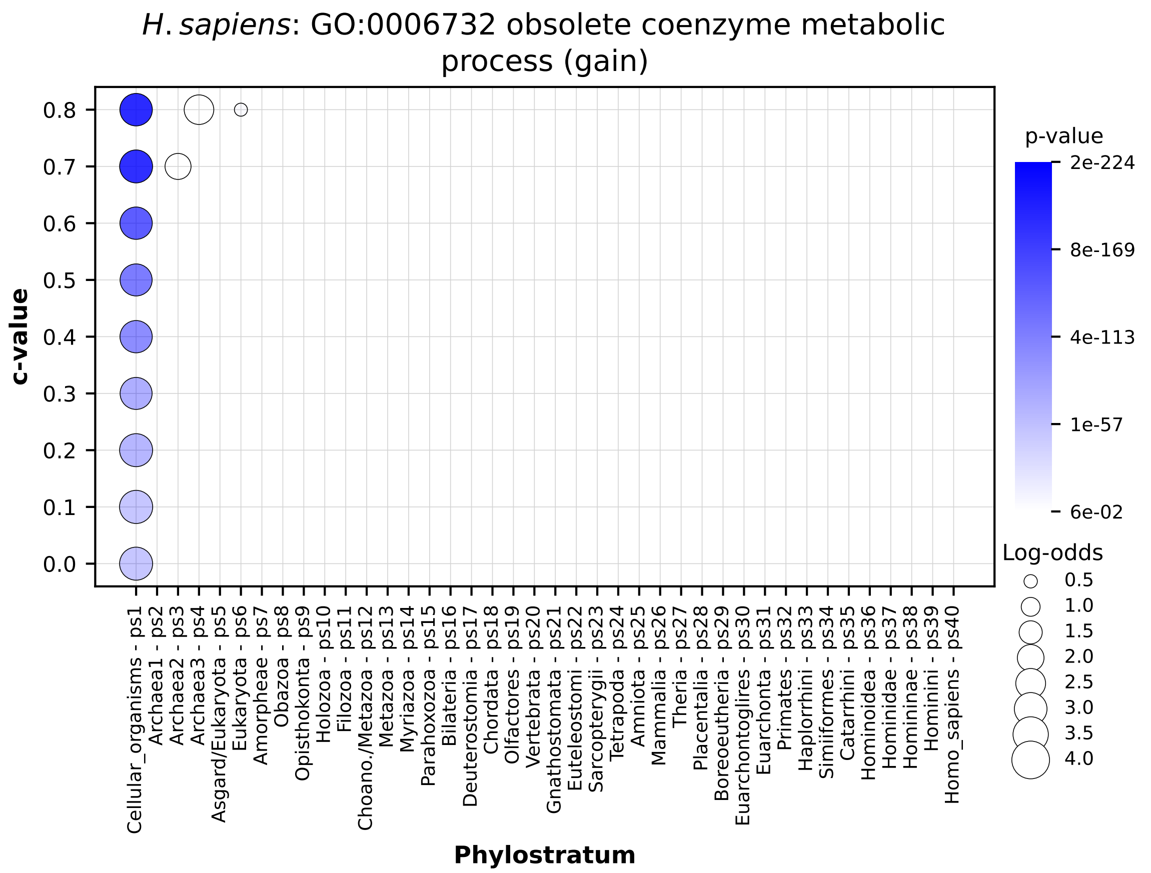

H. sapiens: GO:0006732 obsolete coenzyme metabolic process (gain)
c-value
0.8
0.7
0.6
0.5
0.4
0.3
0.2
0.1
0.0
p-value
2e-224
8e-169
4e-113
1e-57
6e-02
Log-odds
0.5
1.0
1.5
2.0
2.5
3.0
3.5
4.0
Cellular_organisms - ps1
Archaea1 - ps2
Archaea2 - ps3
Archaea3 - ps4
Asgard/Eukaryota - ps5
Eukaryota - ps6
Amorpheae - ps7
Obazoa - ps8
Opisthokonta - ps9
Holozoa - ps10
Filozoa - ps11
Choano./Metazoa - ps12
Metazoa - ps13
Myriazoa - ps14
Parahoxozoa - ps15
Bilateria - ps16
Deuterostomia - ps17
Chordata - ps18
Olfactores - ps19
Vertebrata - ps20
Gnathostomata - ps21
Euteleostomi - ps22
Sarcopterygii - ps23
Tetrapoda - ps24
Amniota - ps25
Mammalia - ps26
Theria - ps27
Placentalia - ps28
Boreoeutheria - ps29
Euarchontoglires - ps30
Euarchonta - ps31
Primates - ps32
Haplorrhini - ps33
Simiiformes - ps34
Catarrhini - ps35
Hominoidea - ps36
Hominidae - ps37
Homininae - ps38
Hominini - ps39
Homo_sapiens - ps40
Phylostratum

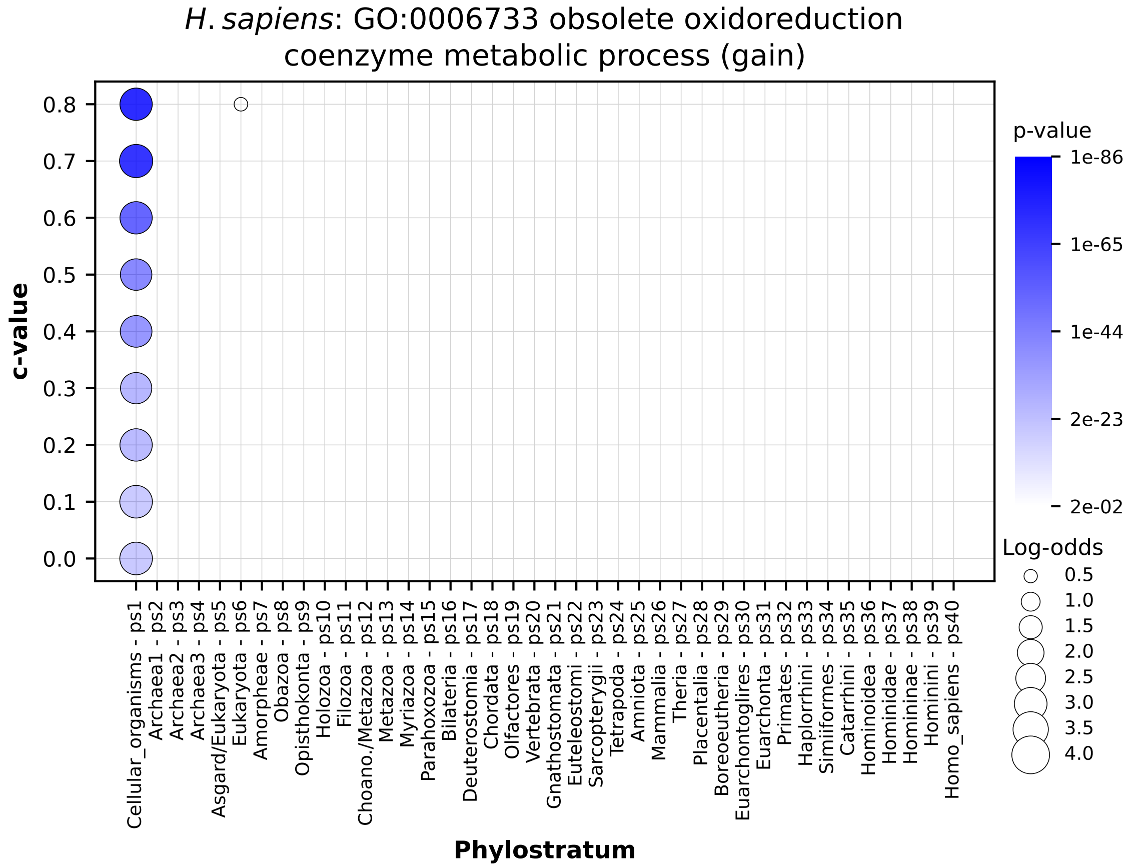

H. sapiens: GO:0006733 obsolete oxidoreduction coenzyme metabolic process (gain)
c-value
0.8
0.7
0.6
0.5
0.4
0.3
0.2
0.1
0.0
Phylostratum
Cellular_organisms - ps1
Archaea1 - ps2
Archaea2 - ps3
Archaea3 - ps4
Asgard/Eukaryota - ps5
Eukaryota - ps6
Amorpheae - ps7
Obazoa - ps8
Opisthokonta - ps9
Holozoa - ps10
Filozoa - ps11
Choano./Metazoa - ps12
Metazoa - ps13
Myriazoa - ps14
Parahoxozoa - ps15
Bilateria - ps16
Deuterostomia - ps17
Chordata - ps18
Olfactores - ps19
Vertebrata - ps20
Gnathostomata - ps21
Euteleostomi - ps22
Sarcopterygii - ps23
Tetrapoda - ps24
Amniota - ps25
Mammalia - ps26
Theria - ps27
Placentalia - ps28
Boreoeutheria - ps29
Euarchontoglires - ps30
Euarchonta - ps31
Primates - ps32
Haplorrhini - ps33
Simiiformes - ps34
Catarrhini - ps35
Hominoidea - ps36
Hominidae - ps37
Homininae - ps38
Hominini - ps39
Homo_sapiens - ps40
p-value
1e-86
1e-65
1e-44
2e-23
2e-02
Log-odds
0.5
1.0
1.5
2.0
2.5
3.0
3.5
4.0

*H. sapiens*: GO:0006743 ubiquinone metabolic process (gain)

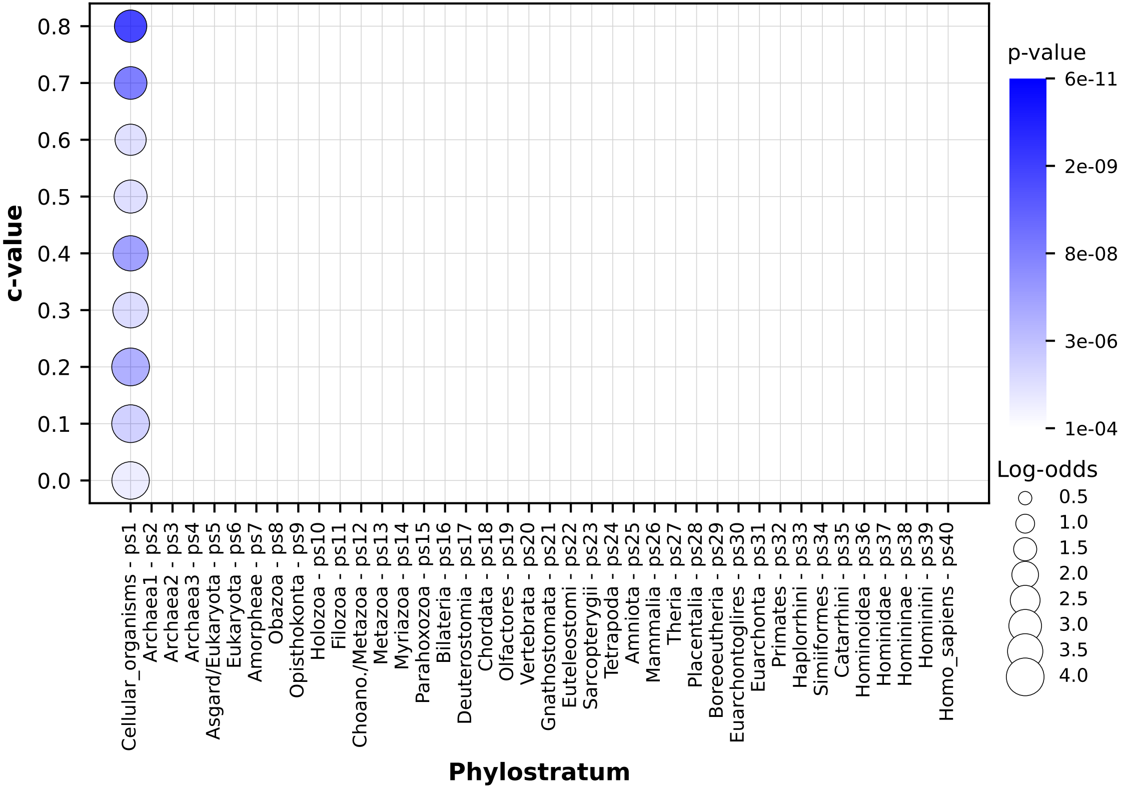

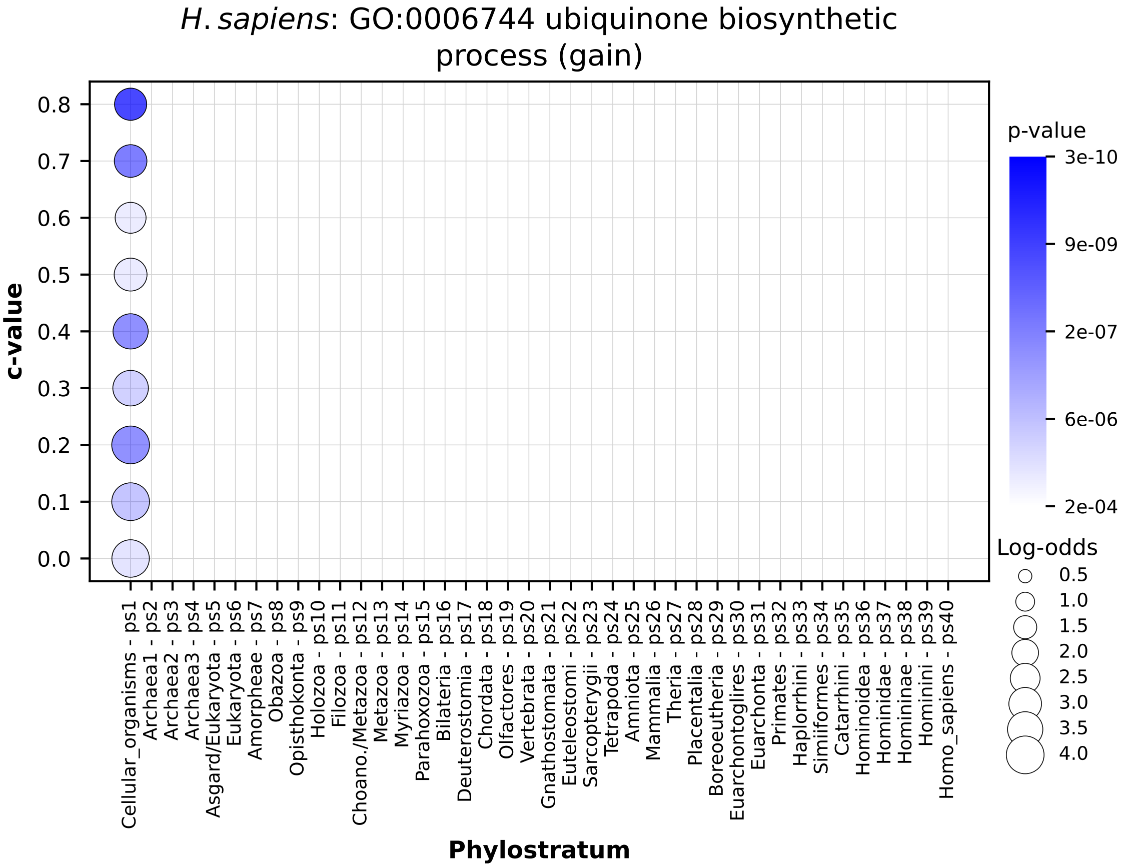

H. sapiens: GO:0006744 ubiquinone biosynthetic process (gain)
c-value
0.8
0.7
0.6
0.5
0.4
0.3
0.2
0.1
0.0
Phylostratum
Cellular_organisms - ps1
Archaea1 - ps2
Archaea2 - ps3
Archaea3 - ps4
Asgard/Eukaryota - ps5
Eukaryota - ps6
Amorpheae - ps7
Obazoa - ps8
Opisthokonta - ps9
Holozoa - ps10
Filozoa - ps11
Choano./Metazoa - ps12
Metazoa - ps13
Myriazoa - ps14
Parahoxozoa - ps15
Bilateria - ps16
Deuterostomia - ps17
Chordata - ps18
Olfactores - ps19
Vertebrata - ps20
Gnathostomata - ps21
Euteleostomi - ps22
Sarcopterygii - ps23
Tetrapoda - ps24
Amniota - ps25
Mammalia - ps26
Theria - ps27
Placentalia - ps28
Boreoeutheria - ps29
Euarchontoglires - ps30
Euarchonta - ps31
Primates - ps32
Haplorrhini - ps33
Simiiformes - ps34
Catarrhini - ps35
Hominoidea - ps36
Hominidae - ps37
Homininae - ps38
Hominini - ps39
Homo_sapiens - ps40
p-value
3e-10
9e-09
2e-07
6e-06
2e-04
Log-odds
0.5
1.0
1.5
2.0
2.5
3.0
3.5
4.0

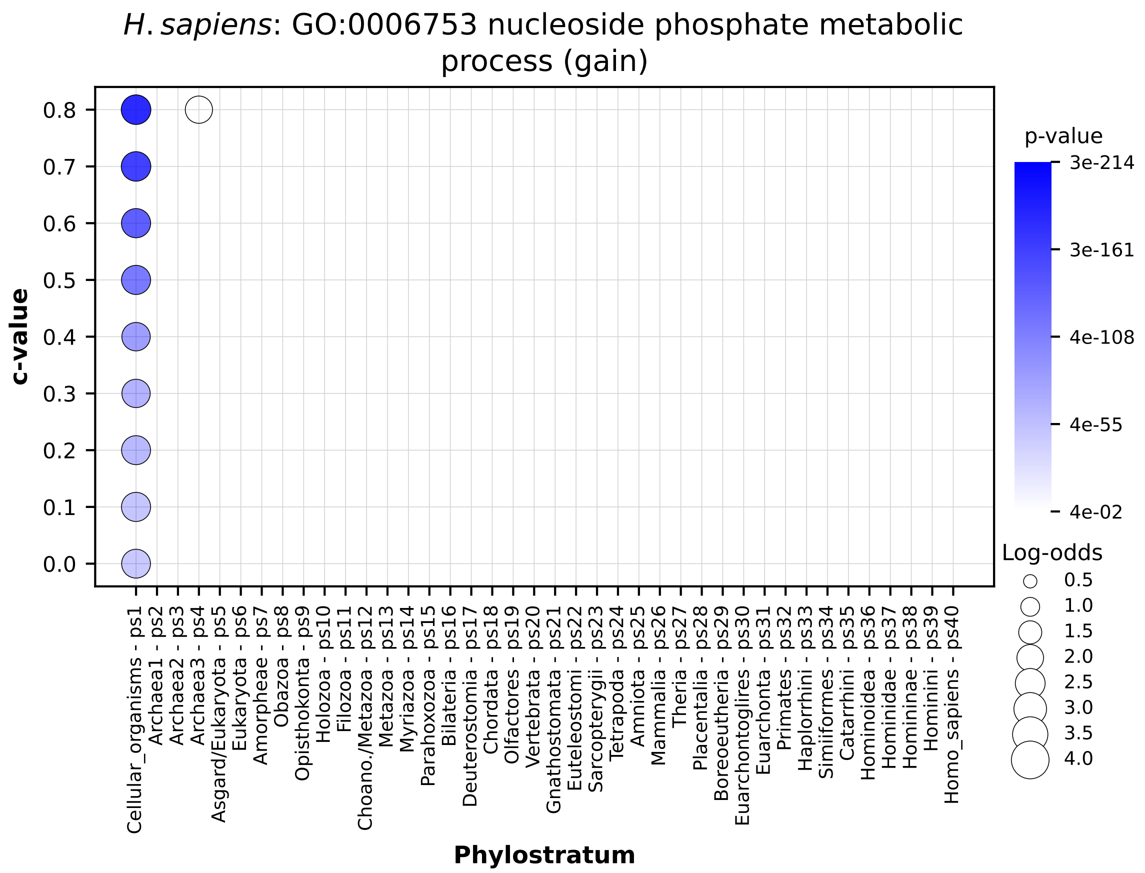
H. sapiens: GO:0006753 nucleoside phosphate metabolic process (gain)
c-value
0.8
0.7
0.6
0.5
0.4
0.3
0.2
0.1
0.0
Phylostratum
Cellular_organisms - ps1
Archaea1 - ps2
Archaea2 - ps3
Archaea3 - ps4
Asgard/Eukaryota - ps5
Eukaryota - ps6
Amorpheae - ps7
Obazoa - ps8
Opisthokonta - ps9
Holozoa - ps10
Filozoa - ps11
Choano./Metazoa - ps12
Metazoa - ps13
Myriazoa - ps14
Parahoxozoa - ps15
Bilateria - ps16
Deuterostomia - ps17
Chordata - ps18
Olfactores - ps19
Vertebrata - ps20
Gnathostomata - ps21
Euteleostomi - ps22
Sarcopterygii - ps23
Tetrapoda - ps24
Amniota - ps25
Mammalia - ps26
Theria - ps27
Placentalia - ps28
Boreoeutheria - ps29
Euarchontoglires - ps30
Euarchonta - ps31
Primates - ps32
Haplorrhini - ps33
Simiiformes - ps34
Catarrhini - ps35
Hominoidea - ps36
Hominidae - ps37
Homininae - ps38
Hominini - ps39
Homo_sapiens - ps40
p-value
3e-214
3e-161
4e-108
4e-55
4e-02
Log-odds
0.5
1.0
1.5
2.0
2.5
3.0
3.5
4.0

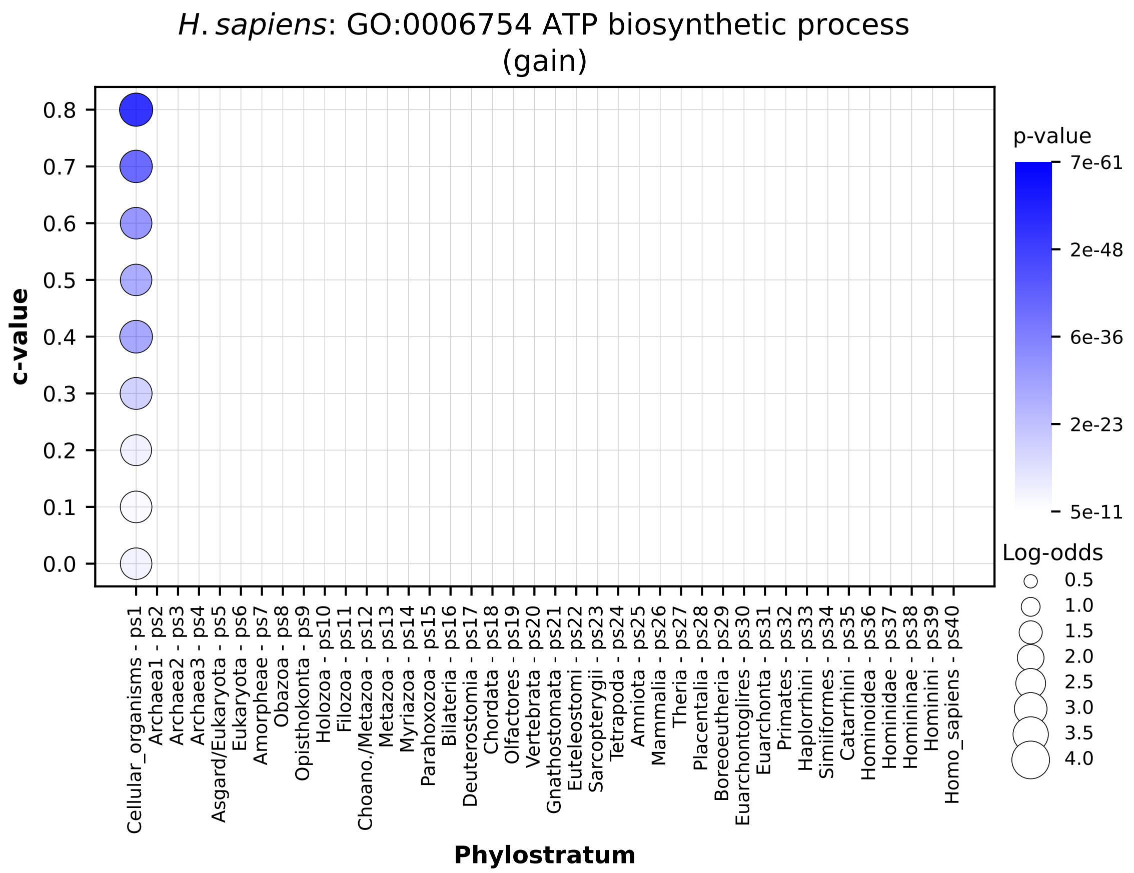
H. sapiens: GO:0006754 ATP biosynthetic process
(gain)
c-value
Phylostratum
p-value
7e-61
2e-48
6e-36
2e-23
5e-11
Log-odds
0.5
1.0
1.5
2.0
2.5
3.0
3.5
4.0
Cellular_organisms - ps1
Archaea1 - ps2
Archaea2 - ps3
Archaea3 - ps4
Asgard/Eukaryota - ps5
Eukaryota - ps6
Amorpheae - ps7
Obazoa - ps8
Opisthokonta - ps9
Holozoa - ps10
Filozoa - ps11
Choano./Metazoa - ps12
Metazoa - ps13
Myriazoa - ps14
Parahoxozoa - ps15
Bilateria - ps16
Deuterostomia - ps17
Chordata - ps18
Olfactores - ps19
Vertebrata - ps20
Gnathostomata - ps21
Euteleostomi - ps22
Sarcopterygii - ps23
Tetrapoda - ps24
Amniota - ps25
Mammalia - ps26
Theria - ps27
Placentalia - ps28
Boreoeutheria - ps29
Euarchontoglires - ps30
Euarchonta - ps31
Primates - ps32
Haplorrhini - ps33
Simiiformes - ps34
Catarrhini - ps35
Hominoidea - ps36
Hominidae - ps37
Homininae - ps38
Hominini - ps39
Homo_sapiens - ps40

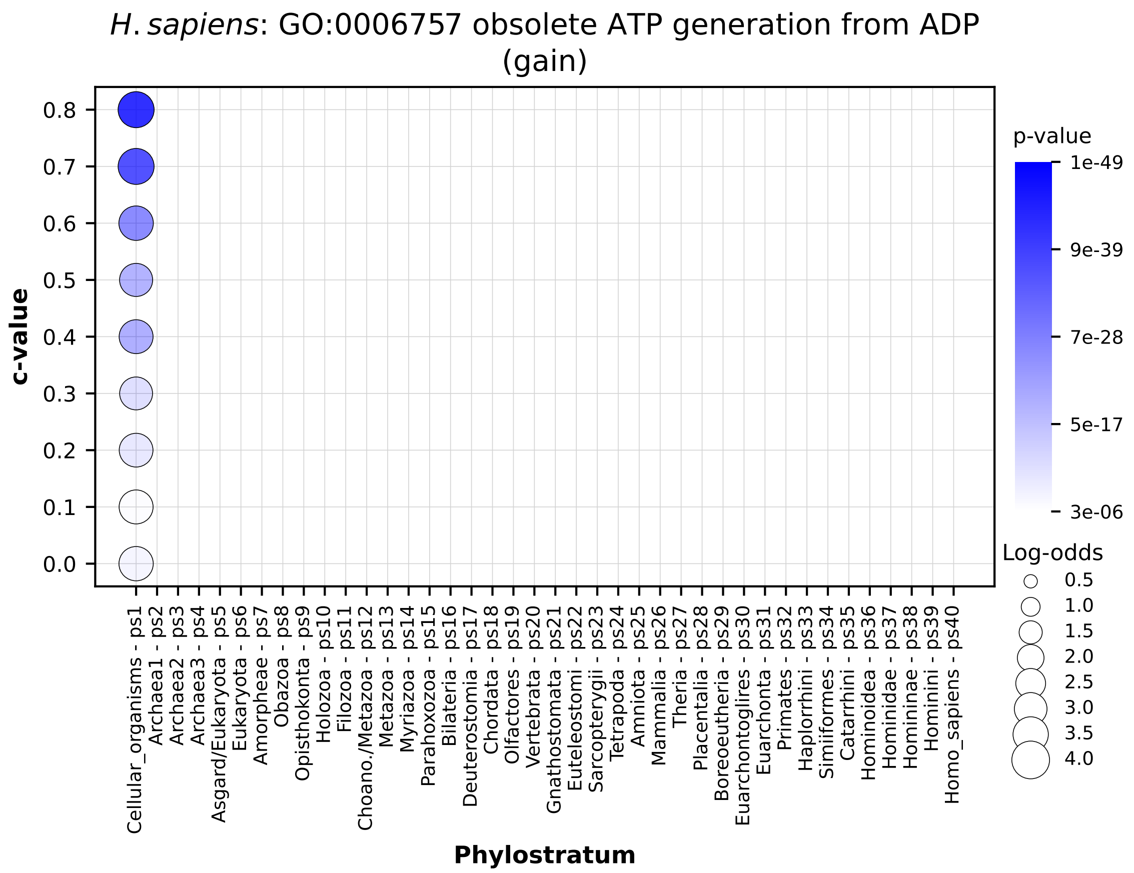

H. sapiens: GO:0006757 obsolete ATP generation from ADP (gain)
c-value
0.8
0.7
0.6
0.5
0.4
0.3
0.2
0.1
0.0
p-value
1e-49
9e-39
7e-28
5e-17
3e-06
Log-odds
0.5
1.0
1.5
2.0
2.5
3.0
3.5
4.0
Cellular_organisms - ps1
Archaea1 - ps2
Archaea2 - ps3
Archaea3 - ps4
Asgard/Eukaryota - ps5
Eukaryota - ps6
Amorpheae - ps7
Obazoa - ps8
Opisthokonta - ps9
Holozoa - ps10
Filozoa - ps11
Choano./Metazoa - ps12
Metazoa - ps13
Myriazoa - ps14
Parahoxozoa - ps15
Bilateria - ps16
Deuterostomia - ps17
Chordata - ps18
Olfactores - ps19
Vertebrata - ps20
Gnathostomata - ps21
Euteleostomi - ps22
Sarcopterygii - ps23
Tetrapoda - ps24
Amniota - ps25
Mammalia - ps26
Theria - ps27
Placentalia - ps28
Boreoeutheria - ps29
Euarchontoglires - ps30
Euarchonta - ps31
Primates - ps32
Haplorrhini - ps33
Simiiformes - ps34
Catarrhini - ps35
Hominoidea - ps36
Hominidae - ps37
Homininae - ps38
Hominini - ps39
Homo_sapiens - ps40
Phylostratum

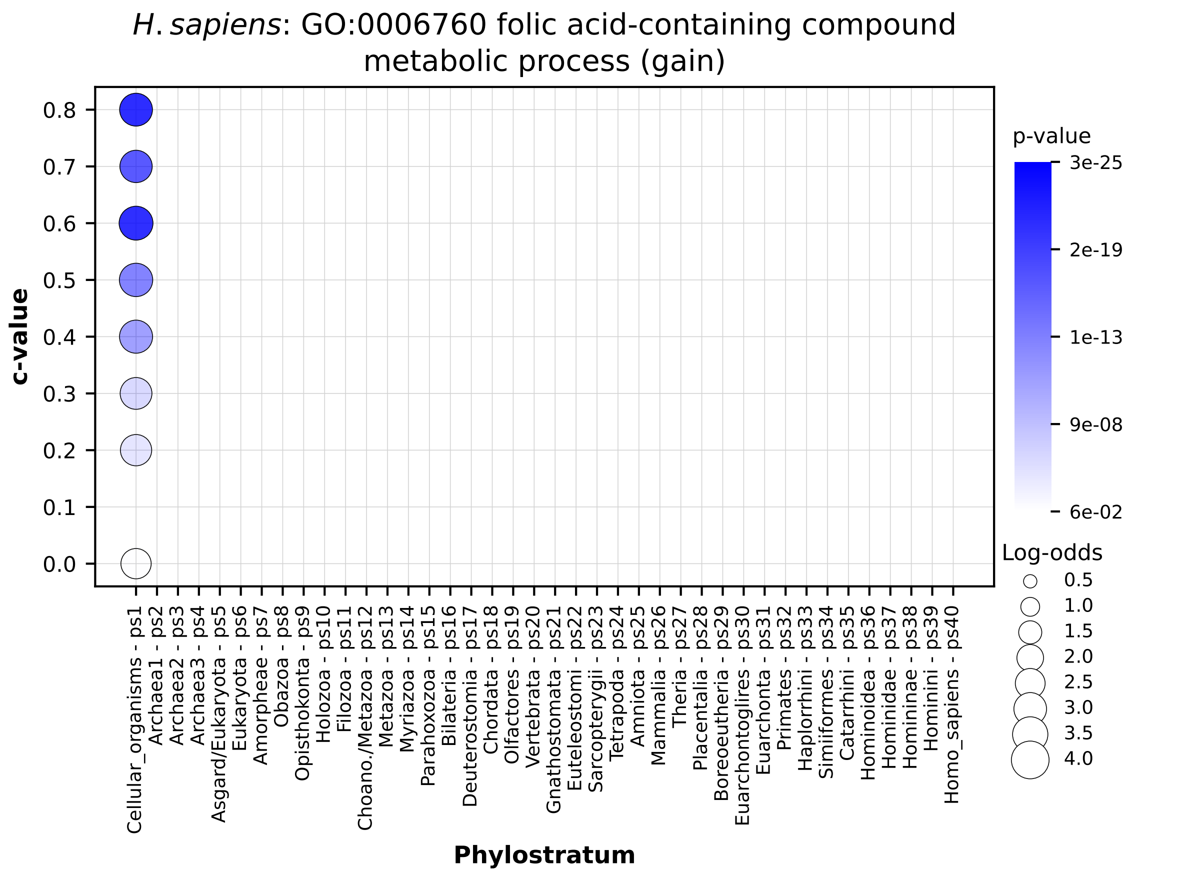

H. sapiens: GO:0006760 folic acid-containing compound metabolic process (gain)
c-value
0.8
0.7
0.6
0.5
0.4
0.3
0.2
0.1
0.0
p-value
3e-25
2e-19
1e-13
9e-08
6e-02
Log-odds
0.5
1.0
1.5
2.0
2.5
3.0
3.5
4.0
Cellular_organisms - ps1
Archaea1 - ps2
Archaea2 - ps3
Archaea3 - ps4
Asgard/Eukaryota - ps5
Eukaryota - ps6
Amorpheae - ps7
Obazoa - ps8
Opisthokonta - ps9
Holozoa - ps10
Filozoa - ps11
Choano./Metazoa - ps12
Metazoa - ps13
Myriazoa - ps14
Parahoxozoa - ps15
Bilateria - ps16
Deuterostomia - ps17
Chordata - ps18
Olfactores - ps19
Vertebrata - ps20
Gnathostomata - ps21
Euteleostomi - ps22
Sarcopterygii - ps23
Tetrapoda - ps24
Amniota - ps25
Mammalia - ps26
Theria - ps27
Placentalia - ps28
Boreoeutheria - ps29
Euarchontoglires - ps30
Euarchonta - ps31
Primates - ps32
Haplorrhini - ps33
Simiiformes - ps34
Catarrhini - ps35
Hominoidea - ps36
Hominidae - ps37
Homininae - ps38
Hominini - ps39
Homo_sapiens - ps40
Phylostratum

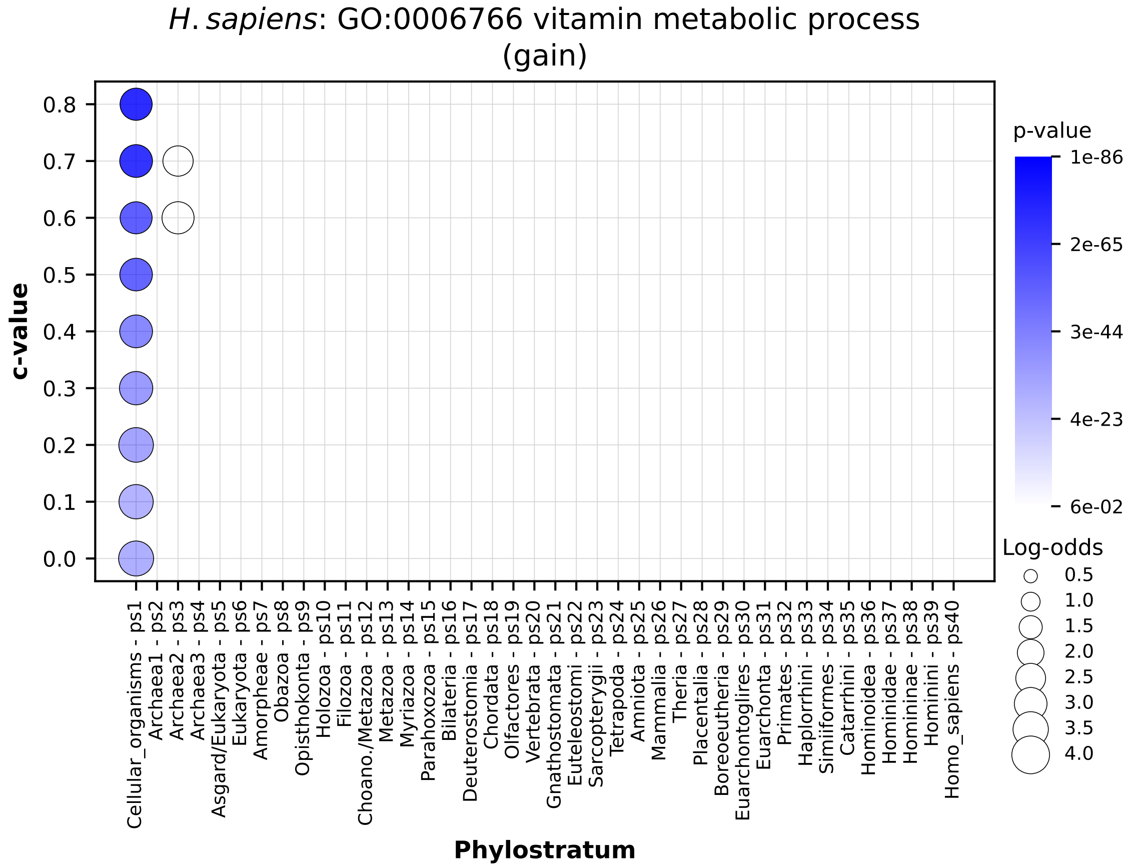

H. sapiens: GO:0006766 vitamin metabolic process
(gain)
c-value
0.8
0.7
0.6
0.5
0.4
0.3
0.2
0.1
0.0
p-value
1e-86
2e-65
3e-44
4e-23
6e-02
Log-odds
0.5
1.0
1.5
2.0
2.5
3.0
3.5
4.0
Cellular_organisms - ps1
Archaea1 - ps2
Archaea2 - ps3
Archaea3 - ps4
Asgard/Eukaryota - ps5
Eukaryota - ps6
Amorpheae - ps7
Obazoa - ps8
Opisthokonta - ps9
Holozoa - ps10
Filozoa - ps11
Choano./Metazoa - ps12
Metazoa - ps13
Myriazoa - ps14
Parahoxozoa - ps15
Bilateria - ps16
Deuterostomia - ps17
Chordata - ps18
Olfactores - ps19
Vertebrata - ps20
Gnathostomata - ps21
Euteleostomi - ps22
Sarcopterygii - ps23
Tetrapoda - ps24
Amniota - ps25
Mammalia - ps26
Theria - ps27
Placentalia - ps28
Boreoeutheria - ps29
Euarchontoglires - ps30
Euarchonta - ps31
Primates - ps32
Haplorrhini - ps33
Simiiformes - ps34
Catarrhini - ps35
Hominoidea - ps36
Hominidae - ps37
Homininae - ps38
Hominini - ps39
Homo_sapiens - ps40
Phylostratum

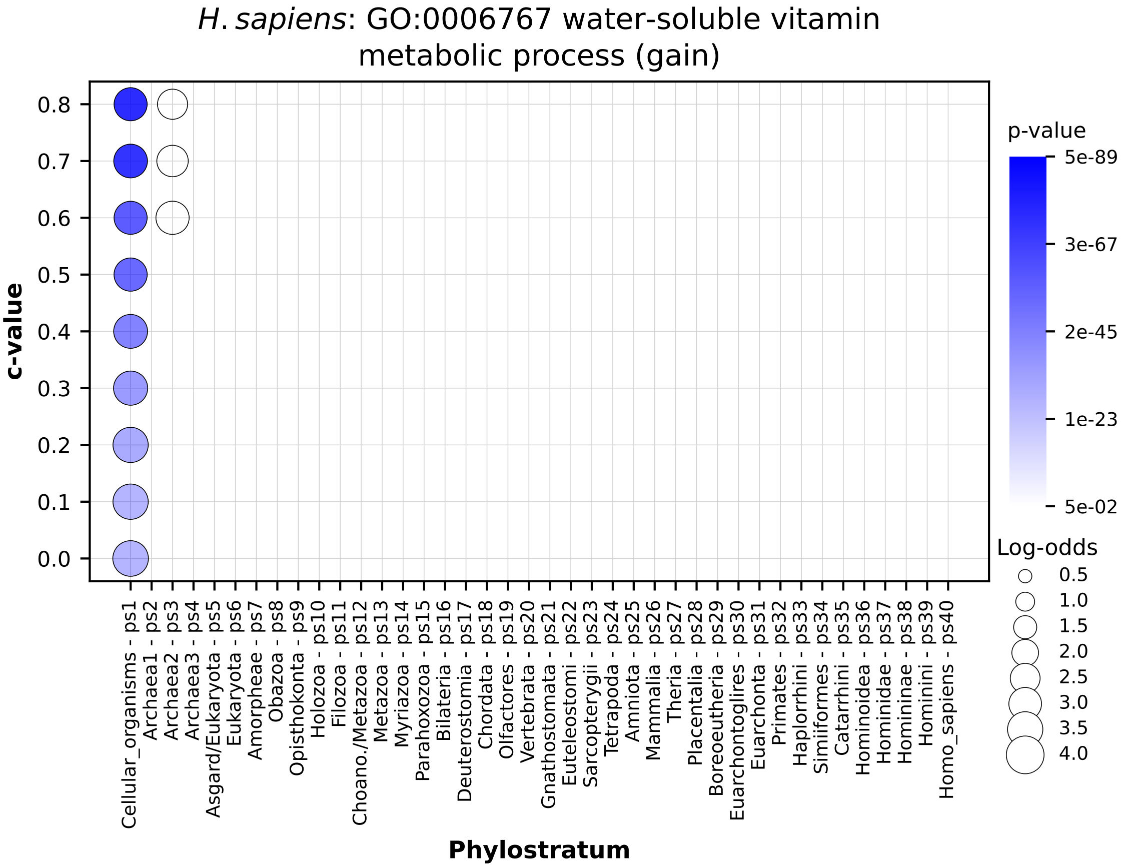
H. sapiens: GO:0006767 water-soluble vitamin metabolic process (gain)
c-value
0.8
0.7
0.6
0.5
0.4
0.3
0.2
0.1
0.0
p-value
5e-89
3e-67
2e-45
1e-23
5e-02
Log-odds
0.5
1.0
1.5
2.0
2.5
3.0
3.5
4.0
Cellular_organisms - ps1
Archaea1 - ps2
Archaea2 - ps3
Archaea3 - ps4
Asgard/Eukaryota - ps5
Eukaryota - ps6
Amorpheae - ps7
Obazoa - ps8
Opisthokonta - ps9
Holozoa - ps10
Filozoa - ps11
Choano./Metazoa - ps12
Metazoa - ps13
Myriazoa - ps14
Parahoxozoa - ps15
Bilateria - ps16
Deuterostomia - ps17
Chordata - ps18
Olfactores - ps19
Vertebrata - ps20
Gnathostomata - ps21
Euteleostomi - ps22
Sarcopterygii - ps23
Tetrapoda - ps24
Amniota - ps25
Mammalia - ps26
Theria - ps27
Placentalia - ps28
Boreoeutheria - ps29
Euarchontoglires - ps30
Euarchonta - ps31
Primates - ps32
Haplorrhini - ps33
Simiiformes - ps34
Catarrhini - ps35
Hominoidea - ps36
Hominidae - ps37
Homininae - ps38
Hominini - ps39
Homo_sapiens - ps40
Phylostratum

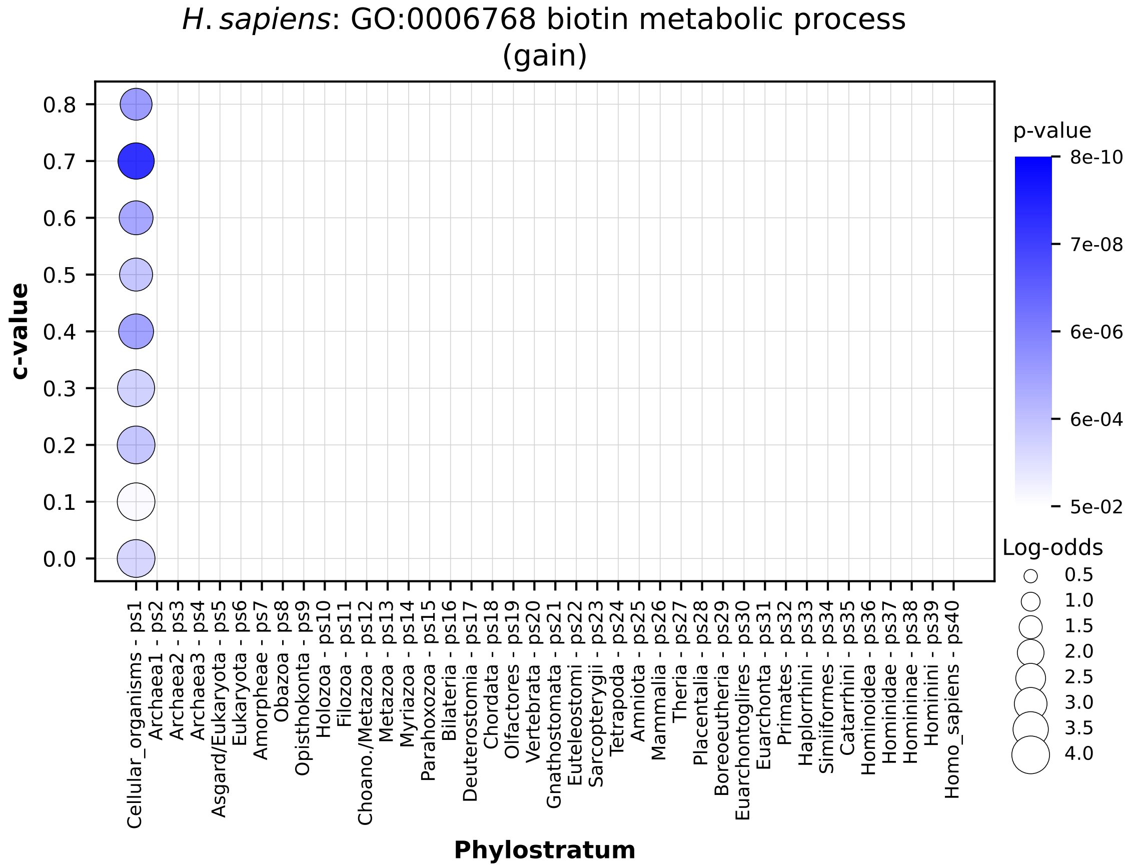

H. sapiens: GO:0006768 biotin metabolic process
(gain)
c-value
0.8
0.7
0.6
0.5
0.4
0.3
0.2
0.1
0.0
p-value
8e-10
7e-08
6e-06
6e-04
5e-02
Log-odds
0.5
1.0
1.5
2.0
2.5
3.0
3.5
4.0
Cellular_organisms - ps1
Archaea1 - ps2
Archaea2 - ps3
Archaea3 - ps4
Asgard/Eukaryota - ps5
Eukaryota - ps6
Amorpheae - ps7
Obazoa - ps8
Opisthokonta - ps9
Holozoa - ps10
Filozoa - ps11
Choano./Metazoa - ps12
Metazoa - ps13
Myriazoa - ps14
Parahoxozoa - ps15
Bilateria - ps16
Deuterostomia - ps17
Chordata - ps18
Olfactores - ps19
Vertebrata - ps20
Gnathostomata - ps21
Euteleostomi - ps22
Sarcopterygii - ps23
Tetrapoda - ps24
Amniota - ps25
Mammalia - ps26
Theria - ps27
Placentalia - ps28
Boreoeutheria - ps29
Euarchontoglires - ps30
Euarchonta - ps31
Primates - ps32
Haplorrhini - ps33
Simiiformes - ps34
Catarrhini - ps35
Hominoidea - ps36
Hominidae - ps37
Homininae - ps38
Hominini - ps39
Homo_sapiens - ps40
Phylostratum

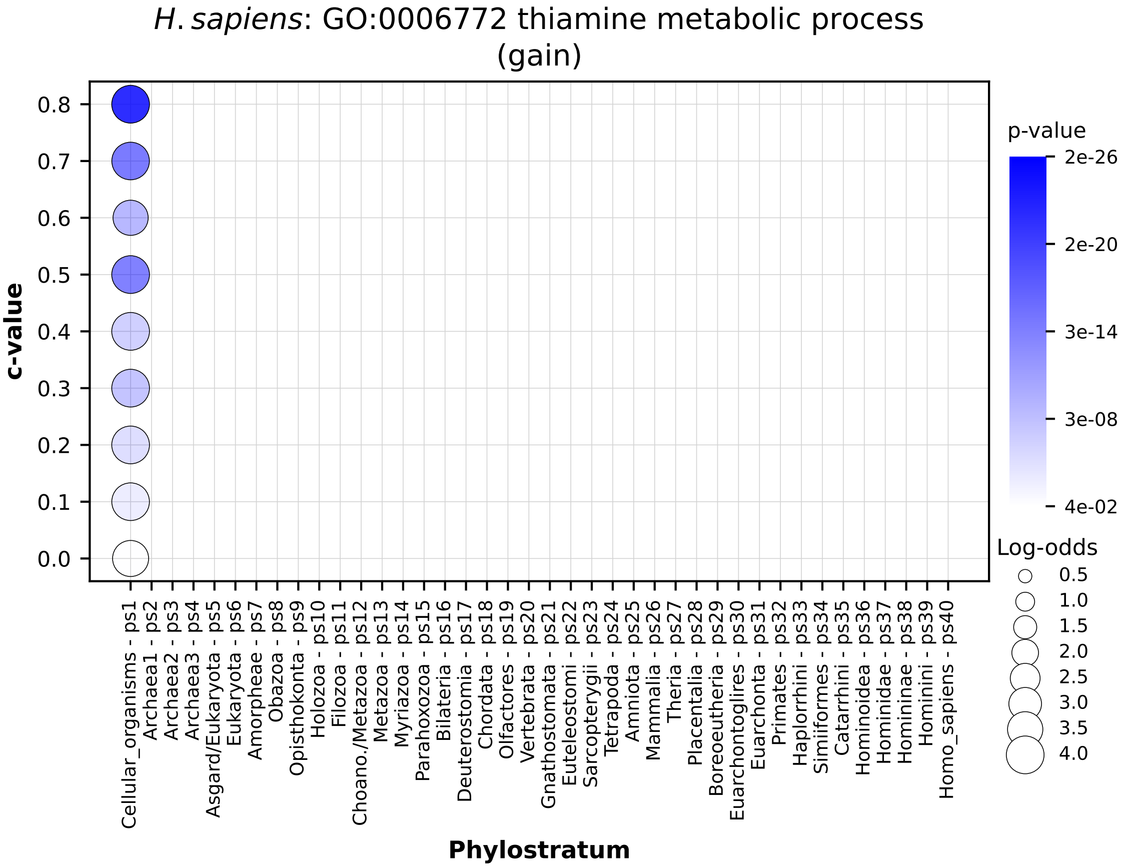

*H. sapiens*: GO:0006772 thiamine metabolic process
(gain)
c-value
0.8
0.7
0.6
0.5
0.4
0.3
0.2
0.1
0.0
p-value
2e-26
2e-20
3e-14
3e-08
4e-02
Log-odds
0.5
1.0
1.5
2.0
2.5
3.0
3.5
4.0
Cellular_organisms - ps1
Archaea1 - ps2
Archaea2 - ps3
Archaea3 - ps4
Asgard/Eukaryota - ps5
Eukaryota - ps6
Amorpheae - ps7
Obazoa - ps8
Opisthokonta - ps9
Holozoa - ps10
Filozoa - ps11
Choano./Metazoa - ps12
Metazoa - ps13
Myriazoa - ps14
Parahoxozoa - ps15
Bilateria - ps16
Deuterostomia - ps17
Chordata - ps18
Olfactores - ps19
Vertebrata - ps20
Gnathostomata - ps21
Euteleostomi - ps22
Sarcopterygii - ps23
Tetrapoda - ps24
Amniota - ps25
Mammalia - ps26
Theria - ps27
Placentalia - ps28
Boreoeutheria - ps29
Euarchontoglires - ps30
Euarchonta - ps31
Primates - ps32
Haplorrhini - ps33
Simiiformes - ps34
Catarrhini - ps35
Hominoidea - ps36
Hominidae - ps37
Homininae - ps38
Hominini - ps39
Homo_sapiens - ps40
Phylostratum

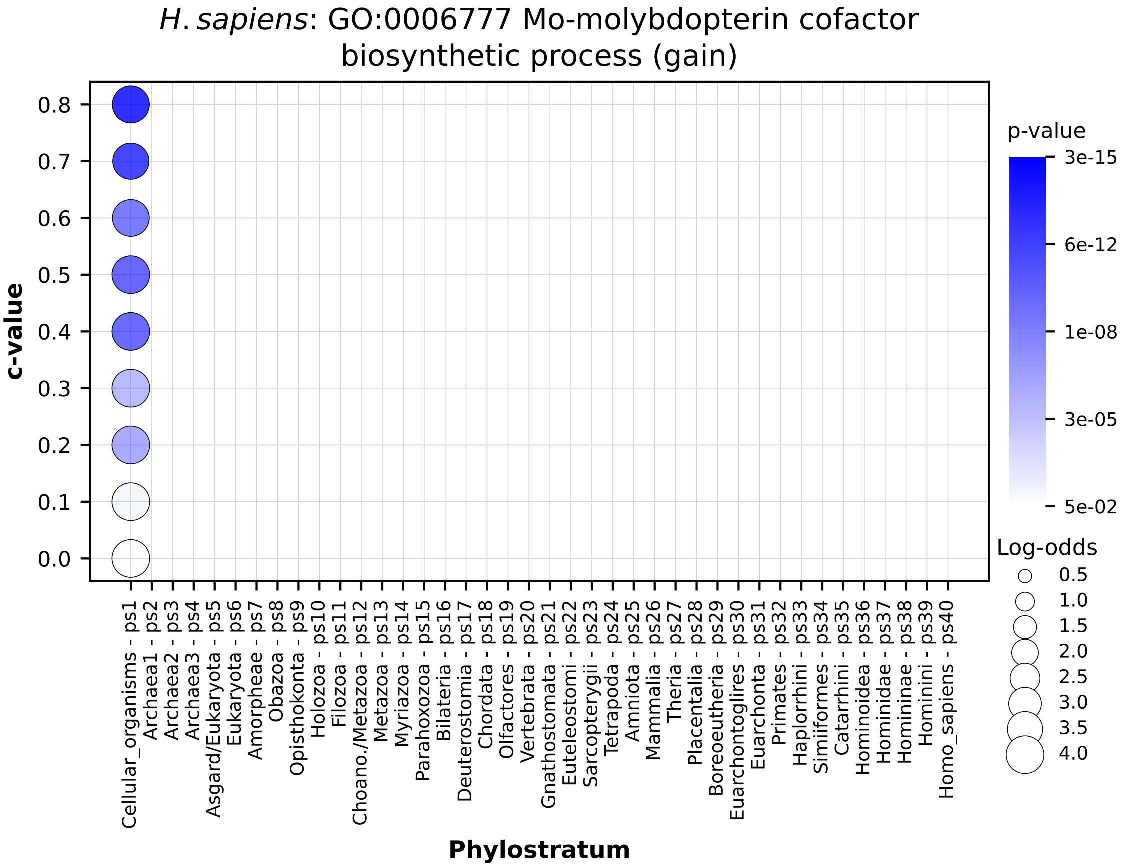

H. sapiens: GO:0006777 Mo-molybdopterin cofactor biosynthetic process (gain)
c-value
0.8
0.7
0.6
0.5
0.4
0.3
0.2
0.1
0.0
p-value
3e-15
6e-12
1e-08
3e-05
5e-02
Log-odds
0.5
1.0
1.5
2.0
2.5
3.0
3.5
4.0
Cellular_organisms - ps1
Archaea1 - ps2
Archaea2 - ps3
Archaea3 - ps4
Asgard/Eukaryota - ps5
Eukaryota - ps6
Amorpheae - ps7
Obazoa - ps8
Opisthokonta - ps9
Holozoa - ps10
Filozoa - ps11
Choano./Metazoa - ps12
Metazoa - ps13
Myriazoa - ps14
Parahoxozoa - ps15
Bilateria - ps16
Deuterostomia - ps17
Chordata - ps18
Olfactores - ps19
Vertebrata - ps20
Gnathostomata - ps21
Euteleostomi - ps22
Sarcopterygii - ps23
Tetrapoda - ps24
Amniota - ps25
Mammalia - ps26
Theria - ps27
Placentalia - ps28
Boreoeutheria - ps29
Euarchontoglires - ps30
Euarchonta - ps31
Primates - ps32
Haplorrhini - ps33
Simiiformes - ps34
Catarrhini - ps35
Hominoidea - ps36
Hominidae - ps37
Homininae - ps38
Hominini - ps39
Homo_sapiens - ps40
Phylostratum

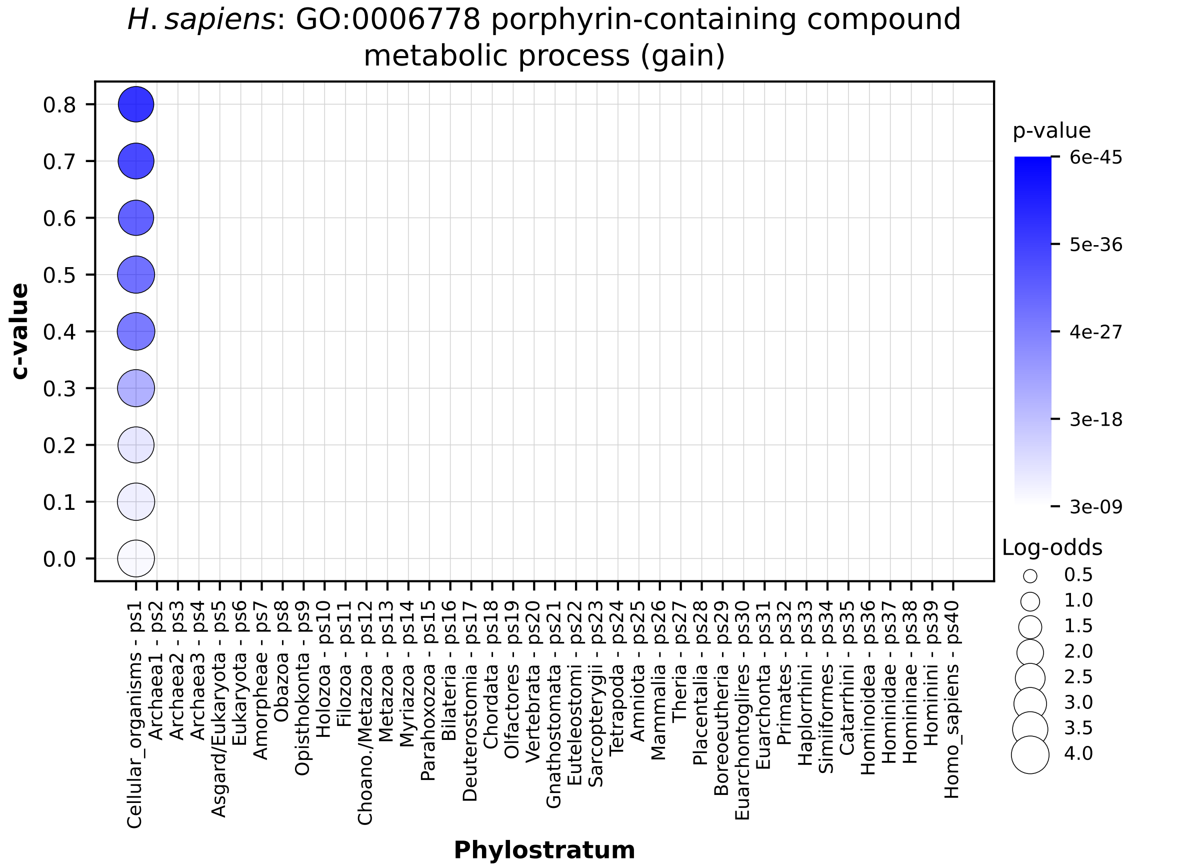
H. sapiens: GO:0006778 porphyrin-containing compound metabolic process (gain)
c-value
0.0
0.1
0.2
0.3
0.4
0.5
0.6
0.7
0.8
p-value
6e-45
5e-36
4e-27
3e-18
3e-09
Log-odds
0.5
1.0
1.5
2.0
2.5
3.0
3.5
4.0
Cellular_organisms - ps1
Archaea1 - ps2
Archaea2 - ps3
Archaea3 - ps4
Asgard/Eukaryota - ps5
Eukaryota - ps6
Amorpheae - ps7
Obazoa - ps8
Opisthokonta - ps9
Holozoa - ps10
Filozoa - ps11
Choano./Metazoa - ps12
Metazoa - ps13
Myriazoa - ps14
Parahoxozoa - ps15
Bilateria - ps16
Deuterostomia - ps17
Chordata - ps18
Olfactores - ps19
Vertebrata - ps20
Gnathostomata - ps21
Euteleostomi - ps22
Sarcopterygii - ps23
Tetrapoda - ps24
Amniota - ps25
Mammalia - ps26
Theria - ps27
Placentalia - ps28
Boreoeutheria - ps29
Euarchontoglires - ps30
Euarchonta - ps31
Primates - ps32
Haplorrhini - ps33
Similformes - ps34
Catarrhini - ps35
Hominoidea - ps36
Hominidae - ps37
Homininae - ps38
Hominini - ps39
Homo_sapiens - ps40
Phylostratum

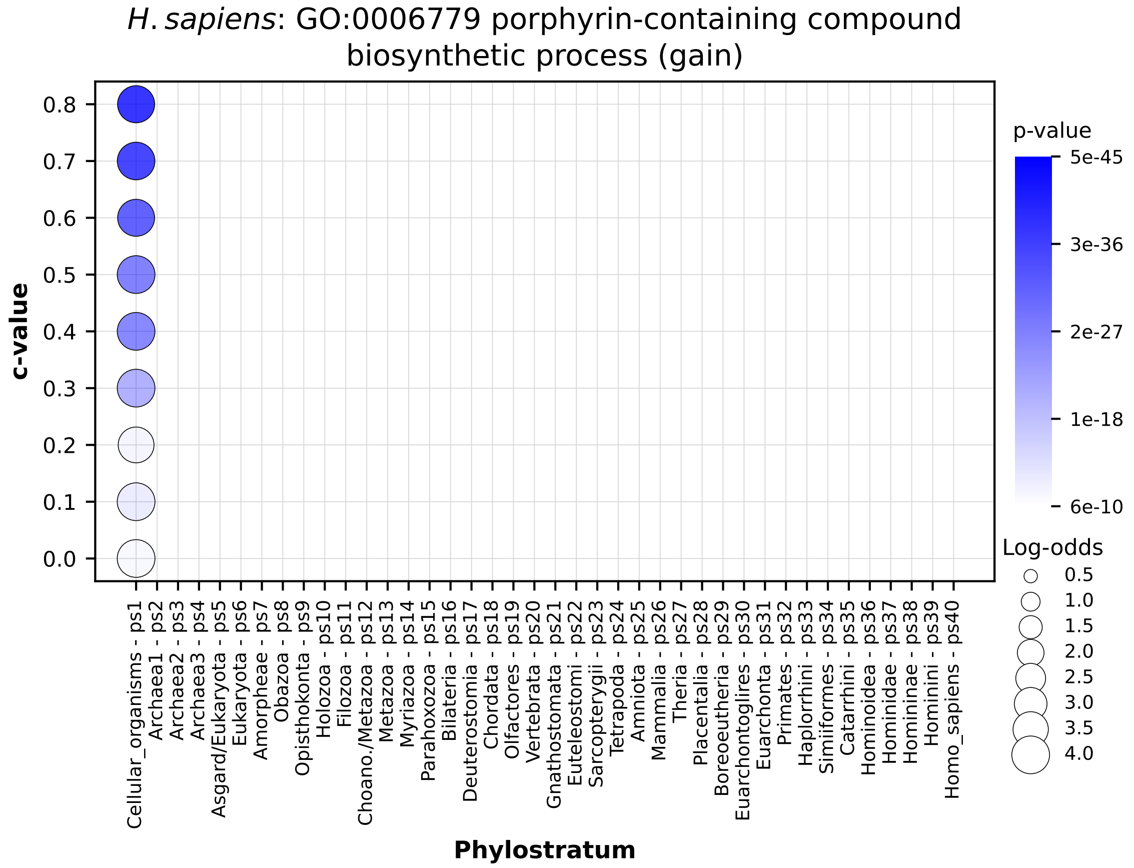
H. sapiens: GO:0006779 porphyrin-containing compound biosynthetic process (gain)
c-value
0.8
0.7
0.6
0.5
0.4
0.3
0.2
0.1
0.0
p-value
5e-45
3e-36
2e-27
1e-18
6e-10
Log-odds
0.5
1.0
1.5
2.0
2.5
3.0
3.5
4.0
Cellular_organisms - ps1
Archaea1 - ps2
Archaea2 - ps3
Archaea3 - ps4
Asgard/Eukaryota - ps5
Eukaryota - ps6
Amorpheae - ps7
Obazoa - ps8
Opisthokonta - ps9
Holozoa - ps10
Filozoa - ps11
Choano./Metazoa - ps12
Metazoa - ps13
Myriazoa - ps14
Parahoxozoa - ps15
Bilateria - ps16
Deuterostomia - ps17
Chordata - ps18
Olfactores - ps19
Vertebrata - ps20
Gnathostomata - ps21
Euteleostomi - ps22
Sarcopterygii - ps23
Tetrapoda - ps24
Amniota - ps25
Mammalia - ps26
Theria - ps27
Placentalia - ps28
Boreoeutheria - ps29
Euarchontoglires - ps30
Euarchonta - ps31
Primates - ps32
Haplorrhini - ps33
Simiiformes - ps34
Catarrhini - ps35
Hominoidea - ps36
Hominidae - ps37
Homininae - ps38
Hominini - ps39
Homo_sapiens - ps40
Phylostratum

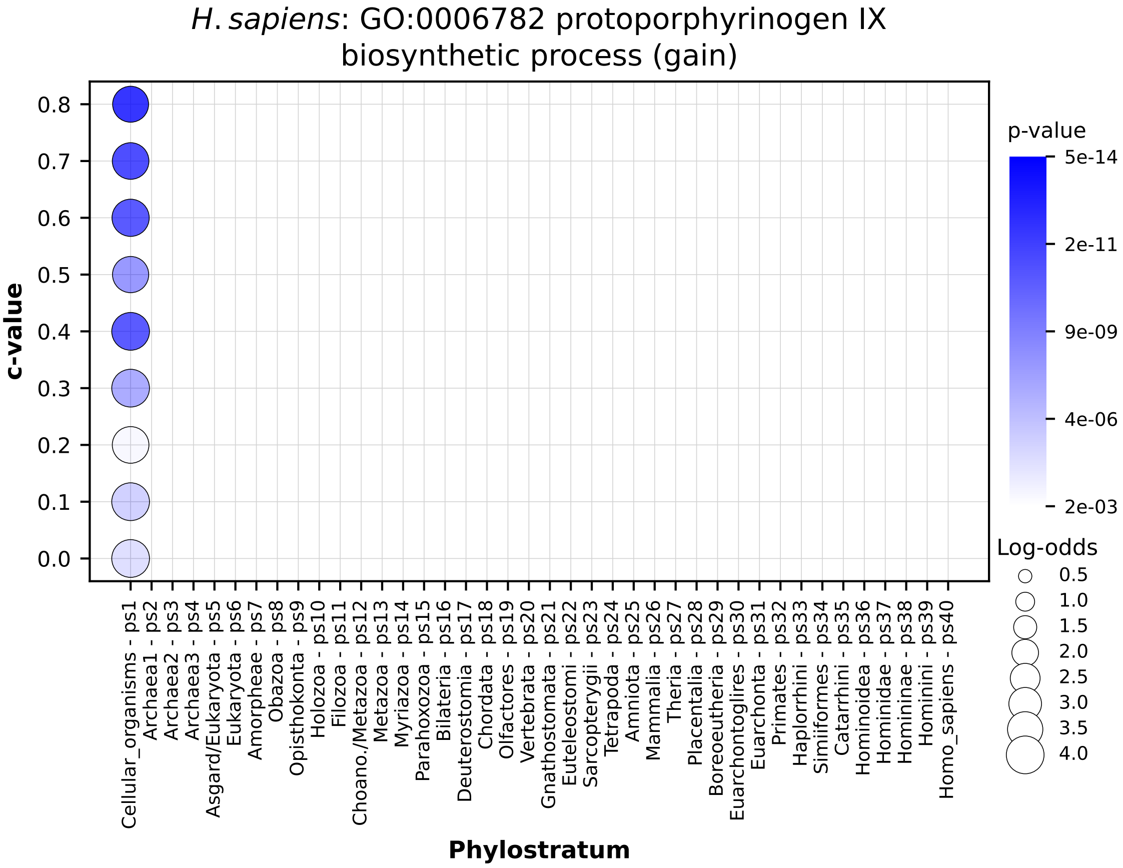

*H. sapiens*: GO:0006782 protoporphyrinogen IX biosynthetic process (gain)
c-value
0.8
0.7
0.6
0.5
0.4
0.3
0.2
0.1
0.0
p-value
5e-14
2e-11
9e-09
4e-06
2e-03
Log-odds
0.5
1.0
1.5
2.0
2.5
3.0
3.5
4.0
Cellular_organisms - ps1
Archaea1 - ps2
Archaea2 - ps3
Archaea3 - ps4
Asgard/Eukaryota - ps5
Eukaryota - ps6
Amorpheae - ps7
Obazoa - ps8
Opisthokonta - ps9
Holozoa - ps10
Filozoa - ps11
Choano./Metazoa - ps12
Metazoa - ps13
Myriazoa - ps14
Parahoxozoa - ps15
Bilateria - ps16
Deuterostomia - ps17
Chordata - ps18
Olfactores - ps19
Vertebrata - ps20
Gnathostomata - ps21
Euteleostomi - ps22
Sarcopterygii - ps23
Tetrapoda - ps24
Amniota - ps25
Mammalia - ps26
Theria - ps27
Placentalia - ps28
Boreoeutheria - ps29
Euarchontoglires - ps30
Euarchonta - ps31
Primates - ps32
Haplorrhini - ps33
Simiiformes - ps34
Catarrhini - ps35
Hominoidea - ps36
Hominidae - ps37
Homininae - ps38
Hominini - ps39
Homo_sapiens - ps40
Phylostratum

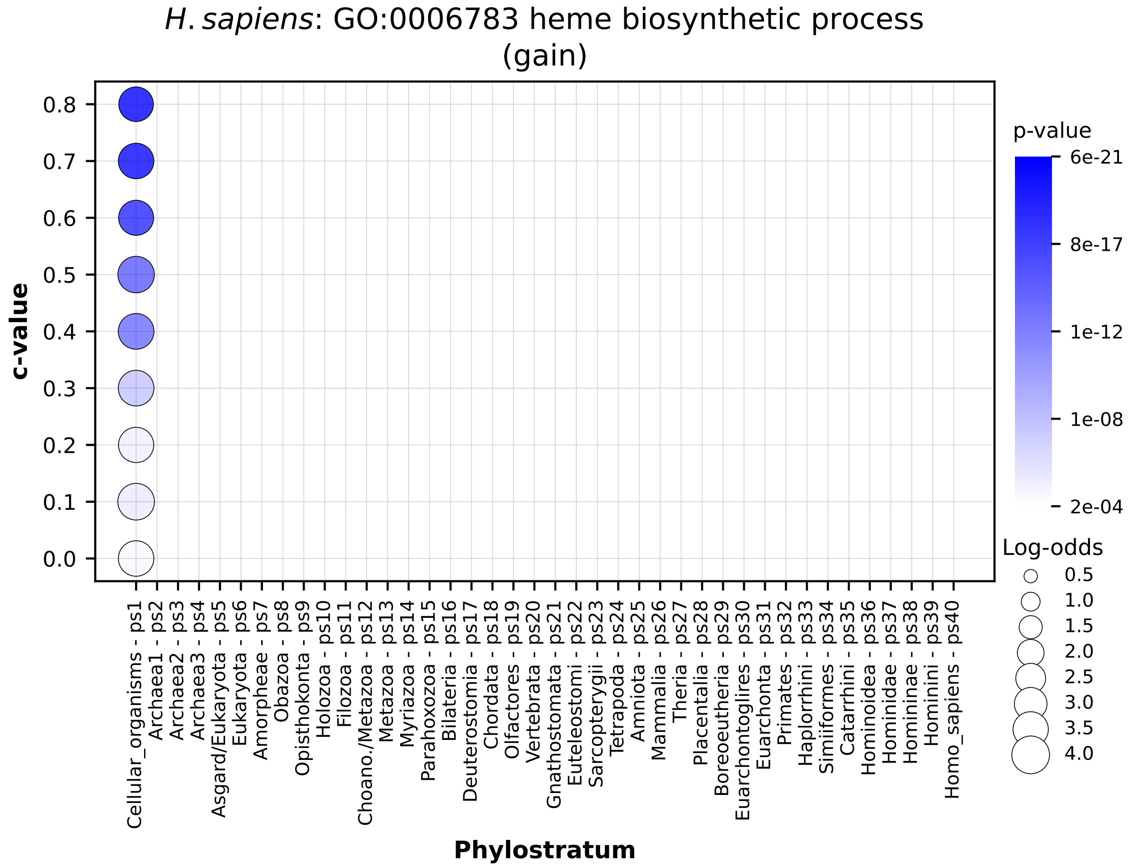
H. sapiens: GO:0006783 heme biosynthetic process
(gain)
c-value
0.8
0.7
0.6
0.5
0.4
0.3
0.2
0.1
0.0
p-value
6e-21
8e-17
1e-12
1e-08
2e-04
Log-odds
0.5
1.0
1.5
2.0
2.5
3.0
3.5
4.0
Cellular_organisms - ps1
Archaea1 - ps2
Archaea2 - ps3
Archaea3 - ps4
Asgard/Eukaryota - ps5
Eukaryota - ps6
Amorpheae - ps7
Obazoa - ps8
Opisthokonta - ps9
Holozoa - ps10
Filozoa - ps11
Choano./Metazoa - ps12
Metazoa - ps13
Myriazoa - ps14
Parahoxozoa - ps15
Bilateria - ps16
Deuterostomia - ps17
Chordata - ps18
Olfactores - ps19
Vertebrata - ps20
Gnathostomata - ps21
Euteleostomi - ps22
Sarcopterygii - ps23
Tetrapoda - ps24
Amniota - ps25
Mammalia - ps26
Theria - ps27
Placentalia - ps28
Boreoeutheria - ps29
Euarchontoglires - ps30
Euarchonta - ps31
Primates - ps32
Haplorrhini - ps33
Simiiformes - ps34
Catarrhini - ps35
Hominoidea - ps36
Hominidae - ps37
Homininae - ps38
Hominini - ps39
Homo_sapiens - ps40
Phylostratum

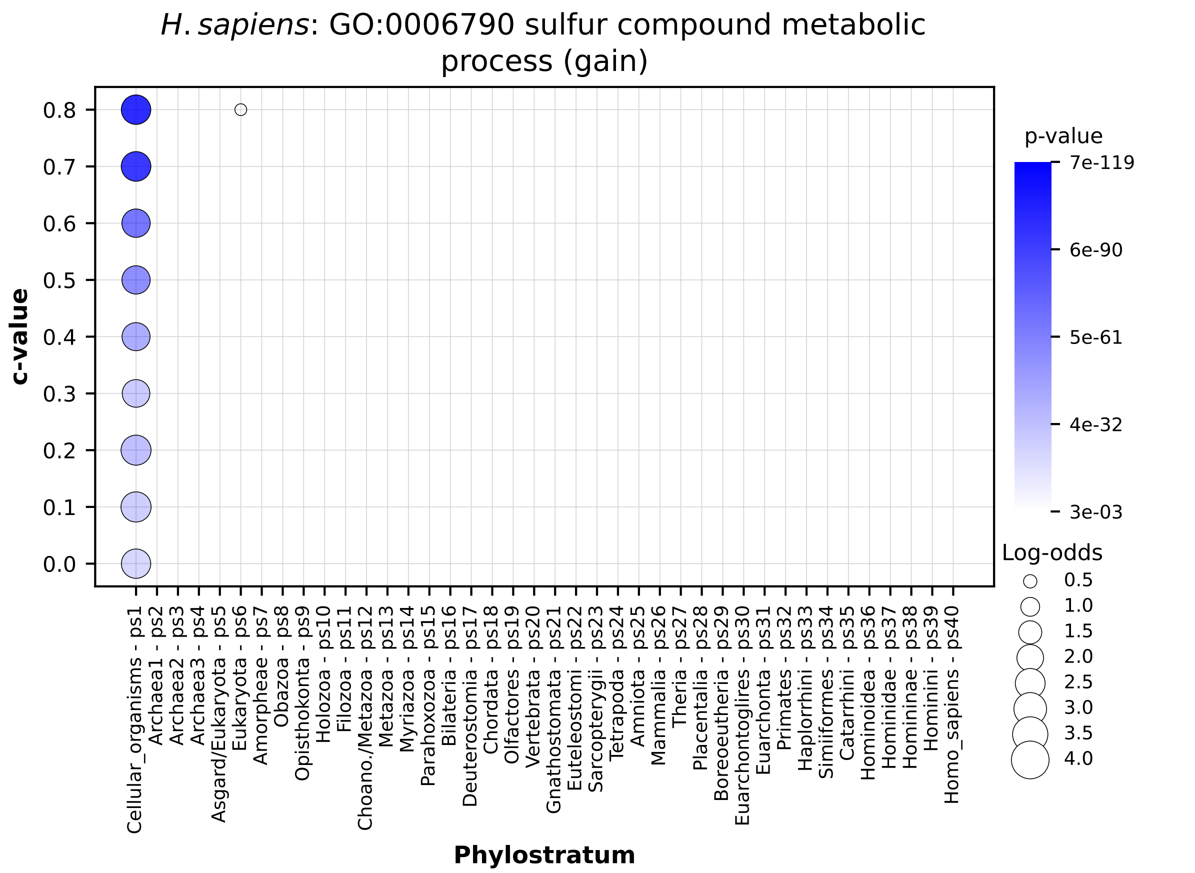

H. sapiens: GO:0006790 sulfur compound metabolic process (gain)
c-value
0.8
0.7
0.6
0.5
0.4
0.3
0.2
0.1
0.0
Phylostratum
Cellular_organisms - ps1
Archaea1 - ps2
Archaea2 - ps3
Archaea3 - ps4
Asgard/Eukaryota - ps5
Eukaryota - ps6
Amorpheae - ps7
Obazoa - ps8
Opisthokonta - ps9
Holozoa - ps10
Filozoa - ps11
Choano./Metazoa - ps12
Metazoa - ps13
Myriazoa - ps14
Parahoxozoa - ps15
Bilateria - ps16
Deuterostomia - ps17
Chordata - ps18
Olfactores - ps19
Vertebrata - ps20
Gnathostomata - ps21
Euteleostomi - ps22
Sarcopterygii - ps23
Tetrapoda - ps24
Amniota - ps25
Mammalia - ps26
Theria - ps27
Placentalia - ps28
Boreoeutheria - ps29
Euarchontoglires - ps30
Euarchonta - ps31
Primates - ps32
Haplorrhini - ps33
Simiiformes - ps34
Catarrhini - ps35
Hominoidea - ps36
Hominidae - ps37
Homininae - ps38
Hominini - ps39
Homo_sapiens - ps40
p-value
7e-119
6e-90
5e-61
4e-32
3e-03
Log-odds
0.5
1.0
1.5
2.0
2.5
3.0
3.5
4.0

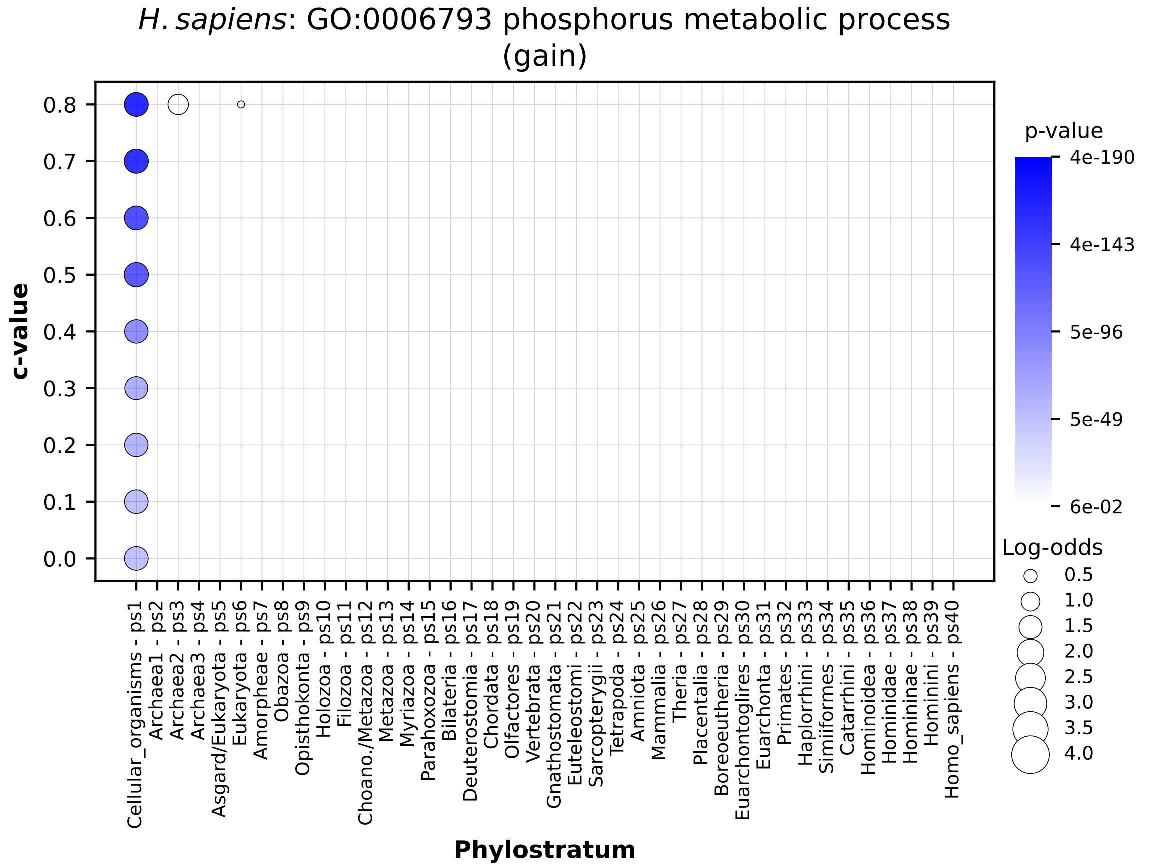

H. sapiens: GO:0006793 phosphorus metabolic process
(gain)
c-value
0.8
0.7
0.6
0.5
0.4
0.3
0.2
0.1
0.0
p-value
4e-190
4e-143
5e-96
5e-49
6e-02
Log-odds
0.5
1.0
1.5
2.0
2.5
3.0
3.5
4.0
Cellular_organisms - ps1
Archaea1 - ps2
Archaea2 - ps3
Archaea3 - ps4
Asgard/Eukaryota - ps5
Eukaryota - ps6
Amorpheae - ps7
Obazoa - ps8
Opisthokonta - ps9
Holozoa - ps10
Filozoa - ps11
Choano./Metazoa - ps12
Metazoa - ps13
Myriazoa - ps14
Parahoxozoa - ps15
Bilateria - ps16
Deuterostomia - ps17
Chordata - ps18
Olfactores - ps19
Vertebrata - ps20
Gnathostomata - ps21
Euteleostomi - ps22
Sarcopterygii - ps23
Tetrapoda - ps24
Amniota - ps25
Mammalia - ps26
Theria - ps27
Placentalia - ps28
Boreoeutheria - ps29
Euarchontoglires - ps30
Euarchonta - ps31
Primates - ps32
Haplorrhini - ps33
Simiiformes - ps34
Catarrhini - ps35
Hominoidea - ps36
Hominidae - ps37
Homininae - ps38
Hominini - ps39
Homo_sapiens - ps40
Phylostratum

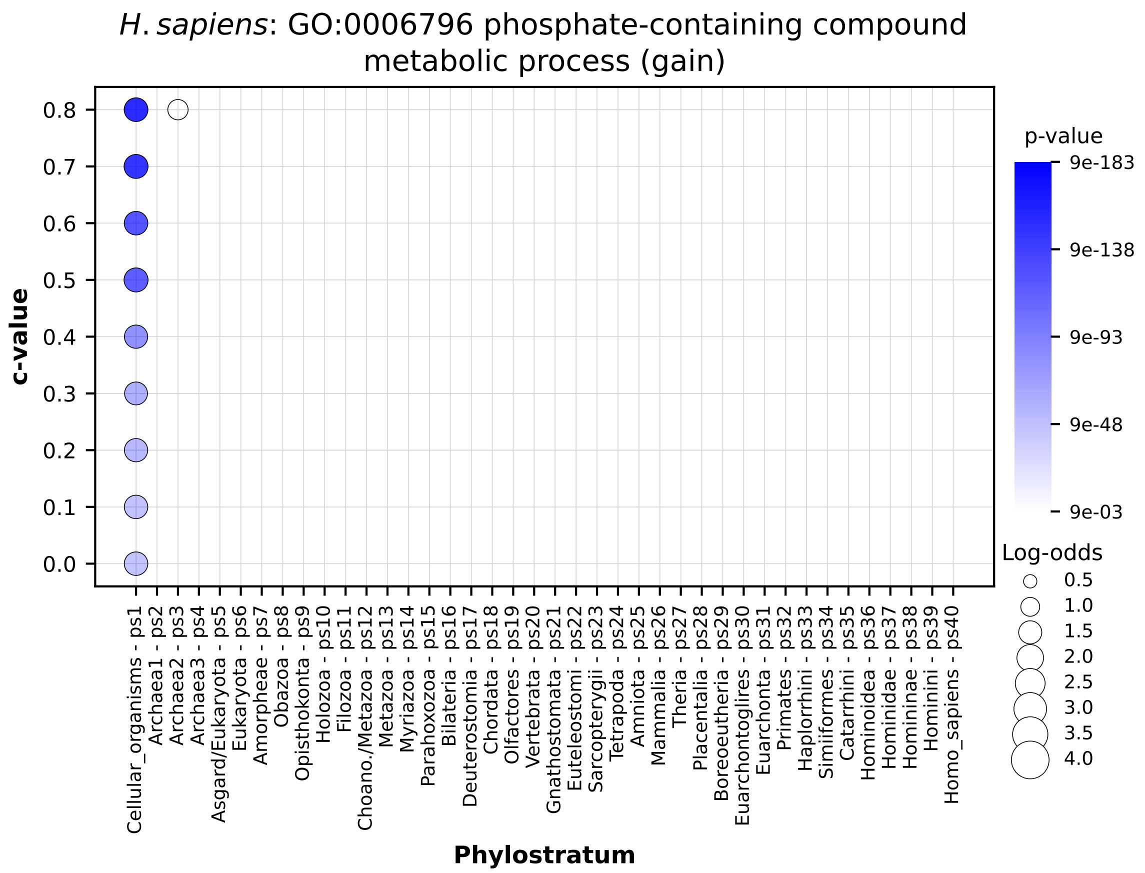
H. sapiens: GO:0006796 phosphate-containing compound metabolic process (gain)
c-value
0.8
0.7
0.6
0.5
0.4
0.3
0.2
0.1
0.0
p-value
9e-183
9e-138
9e-93
9e-48
9e-03
Log-odds
0.5
1.0
1.5
2.0
2.5
3.0
3.5
4.0
Cellular_organisms - ps1
Archaea1 - ps2
Archaea2 - ps3
Archaea3 - ps4
Asgard/Eukaryota - ps5
Eukaryota - ps6
Amorpheae - ps7
Obazoa - ps8
Opisthokonta - ps9
Holozoa - ps10
Filozoa - ps11
Choano./Metazoa - ps12
Metazoa - ps13
Myriazoa - ps14
Parahoxozoa - ps15
Bilateria - ps16
Deuterostomia - ps17
Chordata - ps18
Olfactores - ps19
Vertebrata - ps20
Gnathostomata - ps21
Euteleostomi - ps22
Sarcopterygii - ps23
Tetrapoda - ps24
Amniota - ps25
Mammalia - ps26
Theria - ps27
Placentalia - ps28
Boreoeutheria - ps29
Euarchontoglires - ps30
Euarchonta - ps31
Primates - ps32
Haplorrhini - ps33
Simiiformes - ps34
Catarrhini - ps35
Hominoidea - ps36
Hominidae - ps37
Homininae - ps38
Hominini - ps39
Homo_sapiens - ps40
Phylostratum

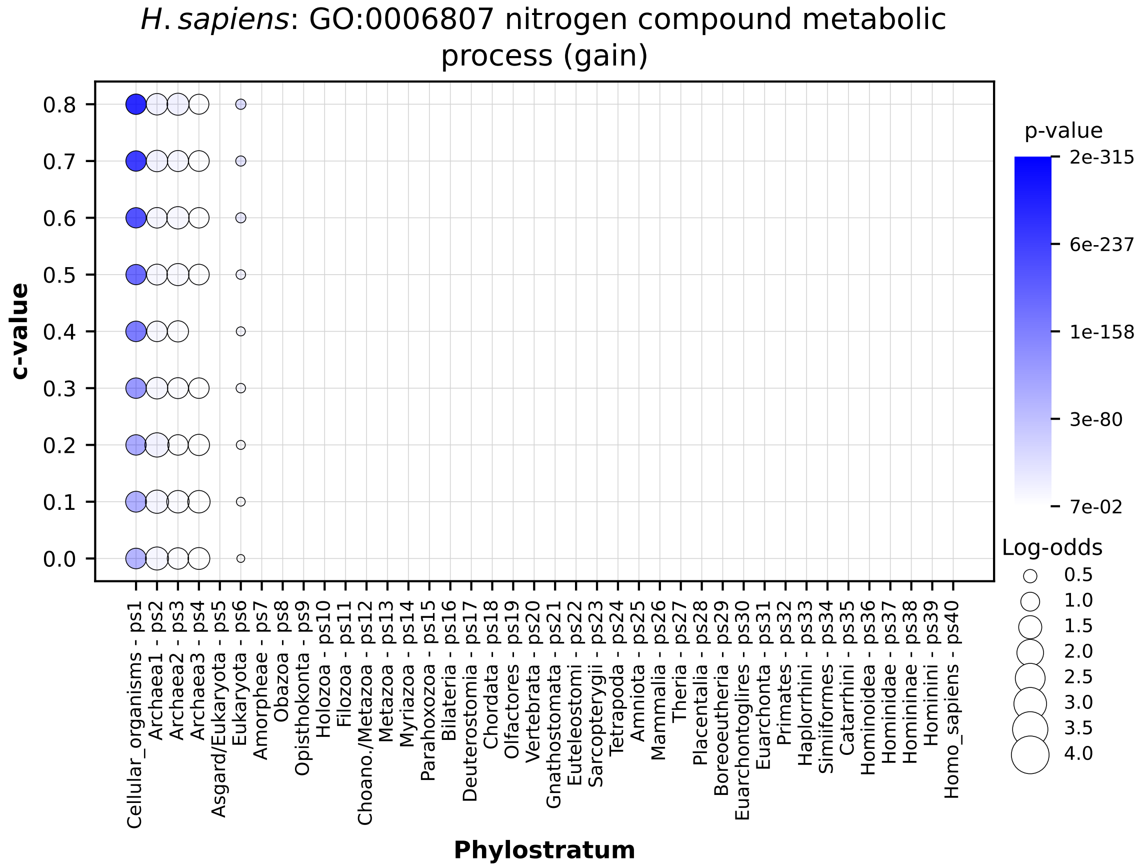

H. sapiens: GO:0006807 nitrogen compound metabolic process (gain)
c-value
0.8
0.7
0.6
0.5
0.4
0.3
0.2
0.1
0.0
p-value
2e-315
6e-237
1e-158
3e-80
7e-02
Log-odds
0.5
1.0
1.5
2.0
2.5
3.0
3.5
4.0
Cellular_organisms - ps1
Archaea1 - ps2
Archaea2 - ps3
Archaea3 - ps4
Asgard/Eukaryota - ps5
Eukaryota - ps6
Amorpheae - ps7
Obazoa - ps8
Opisthokonta - ps9
Holozoa - ps10
Filozoa - ps11
Choano./Metazoa - ps12
Metazoa - ps13
Myriazoa - ps14
Parahoxozoa - ps15
Bilateria - ps16
Deuterostomia - ps17
Chordata - ps18
Olfactores - ps19
Vertebrata - ps20
Gnathostomata - ps21
Euteleostomi - ps22
Sarcopterygii - ps23
Tetrapoda - ps24
Amniota - ps25
Mammalia - ps26
Theria - ps27
Placentalia - ps28
Boreoeutheria - ps29
Euarchontoglires - ps30
Euarchonta - ps31
Primates - ps32
Haplorrhini - ps33
Simiiformes - ps34
Catarrhini - ps35
Hominoidea - ps36
Hominidae - ps37
Homininae - ps38
Hominini - ps39
Homo_sapiens - ps40
Phylostratum

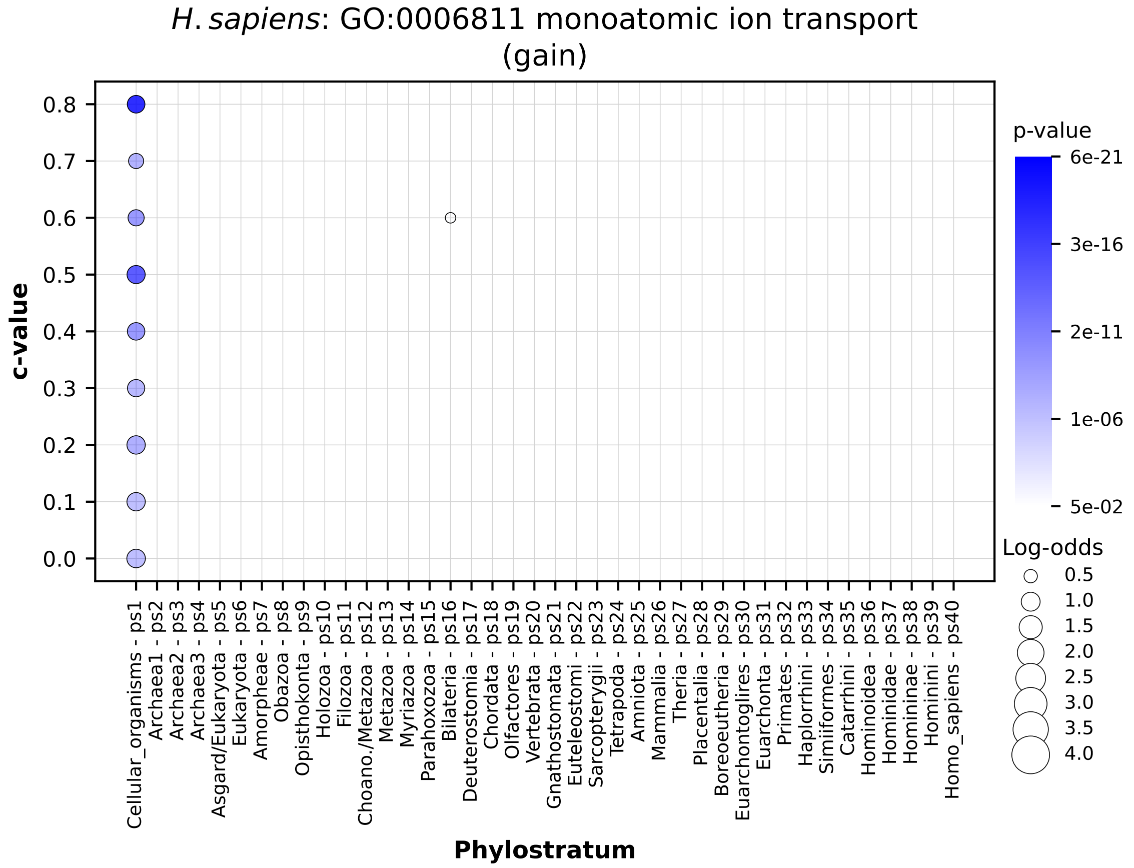

H. sapiens: GO:0006811 monoatomic ion transport
(gain)
c-value
0.8
0.7
0.6
0.5
0.4
0.3
0.2
0.1
0.0
p-value
6e-21
3e-16
2e-11
1e-06
5e-02
Log-odds
0.5
1.0
1.5
2.0
2.5
3.0
3.5
4.0
Cellular_organisms - ps1
Archaea1 - ps2
Archaea2 - ps3
Archaea3 - ps4
Asgard/Eukaryota - ps5
Eukaryota - ps6
Amorpheae - ps7
Obazoa - ps8
Opisthokonta - ps9
Holozoa - ps10
Filozoa - ps11
Choano./Metazoa - ps12
Metazoa - ps13
Myriazoa - ps14
Parahoxozoa - ps15
Bilateria - ps16
Deuterostomia - ps17
Chordata - ps18
Olfactores - ps19
Vertebrata - ps20
Gnathostomata - ps21
Euteleostomi - ps22
Sarcopterygii - ps23
Tetrapoda - ps24
Amniota - ps25
Mammalia - ps26
Theria - ps27
Placentalia - ps28
Boreoeutheria - ps29
Euarchontoglires - ps30
Euarchonta - ps31
Primates - ps32
Haplorrhini - ps33
Simiiformes - ps34
Catarrhini - ps35
Hominoidea - ps36
Hominidae - ps37
Homininae - ps38
Hominini - ps39
Homo_sapiens - ps40
Phylostratum

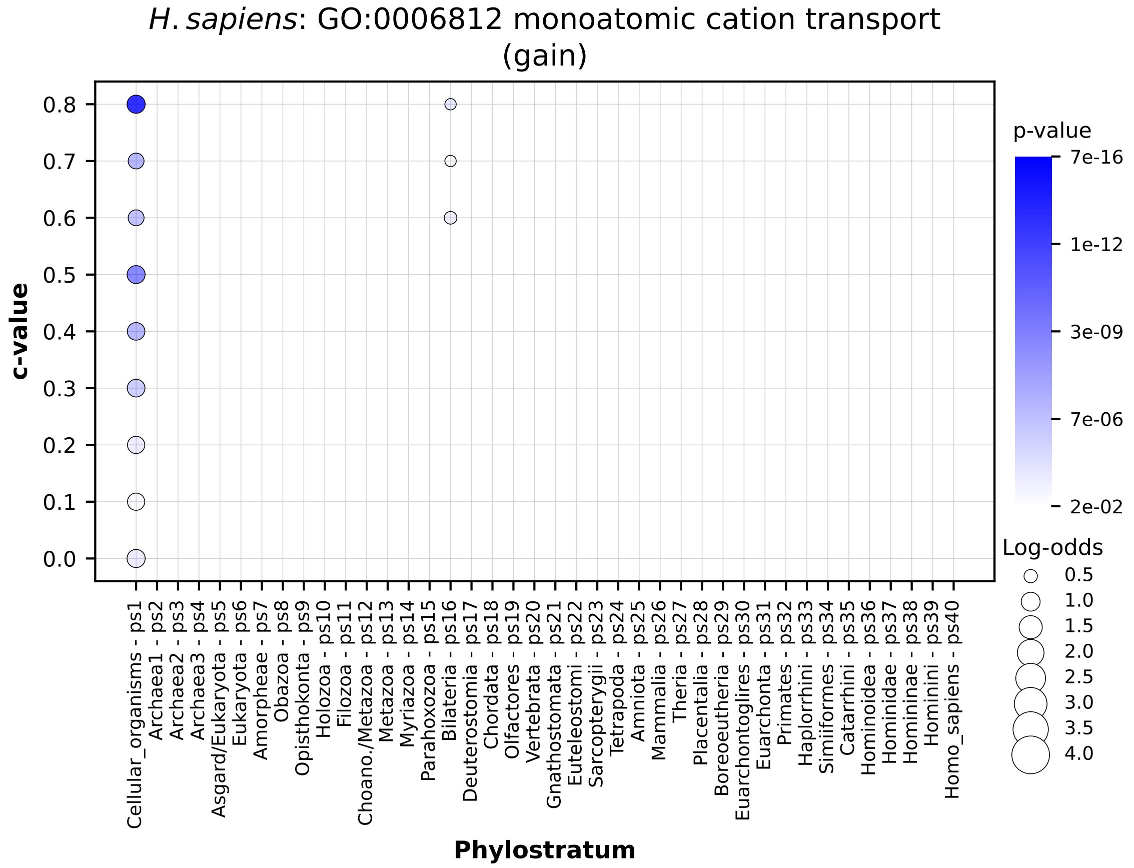
H. sapiens: GO:0006812 monoatomic cation transport
(gain)
c-value
0.8
0.7
0.6
0.5
0.4
0.3
0.2
0.1
0.0
Phylostratum
Cellular_organisms - ps1
Archaea1 - ps2
Archaea2 - ps3
Archaea3 - ps4
Asgard/Eukaryota - ps5
Eukaryota - ps6
Amorpheae - ps7
Obazoa - ps8
Opisthokonta - ps9
Holozoa - ps10
Filozoa - ps11
Choano./Metazoa - ps12
Metazoa - ps13
Myriazoa - ps14
Parahoxozoa - ps15
Bilateria - ps16
Deuterostomia - ps17
Chordata - ps18
Olfactores - ps19
Vertebrata - ps20
Gnathostomata - ps21
Euteleostomi - ps22
Sarcopterygii - ps23
Tetrapoda - ps24
Amniota - ps25
Mammalia - ps26
Theria - ps27
Placentalia - ps28
Boreoeutheria - ps29
Euarchontoglires - ps30
Euarchonta - ps31
Primates - ps32
Haplorrhini - ps33
Simiiformes - ps34
Catarrhini - ps35
Hominoidea - ps36
Hominidae - ps37
Homininae - ps38
Hominini - ps39
Homo_sapiens - ps40
p-value
7e-16
1e-12
3e-09
7e-06
2e-02
Log-odds
0.5
1.0
1.5
2.0
2.5
3.0
3.5
4.0

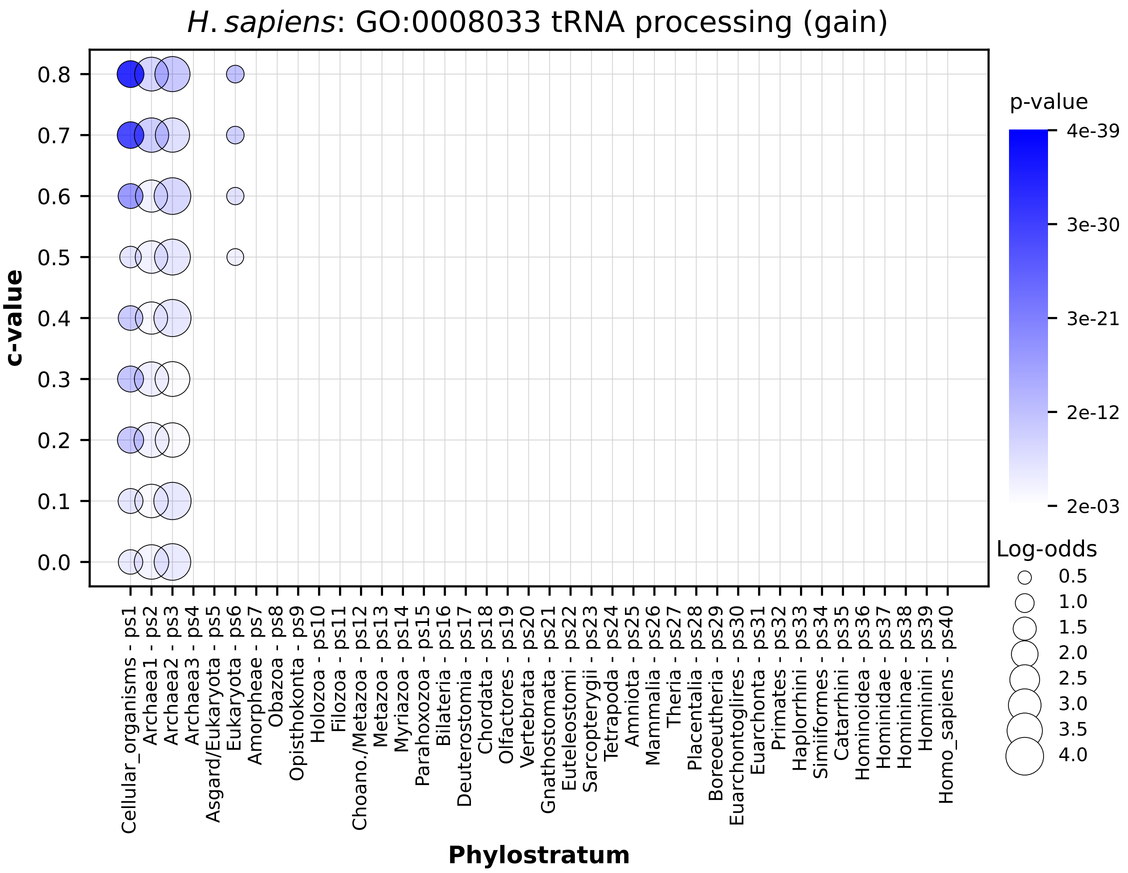
H. sapiens: GO:0008033 tRNA processing (gain)
c-value
0.8
0.7
0.6
0.5
0.4
0.3
0.2
0.1
0.0
Phylostratum
Cellular_organisms - ps1
Archaea1 - ps2
Archaea2 - ps3
Archaea3 - ps4
Asgard/Eukaryota - ps5
Eukaryota - ps6
Amorpheae - ps7
Obazoa - ps8
Opisthokonta - ps9
Holozoa - ps10
Filozoa - ps11
Choano./Metazoa - ps12
Metazoa - ps13
Myriazoa - ps14
Parahoxozoa - ps15
Bilateria - ps16
Deuterostomia - ps17
Chordata - ps18
Olfactores - ps19
Vertebrata - ps20
Gnathostomata - ps21
Euteleostomi - ps22
Sarcopterygii - ps23
Tetrapoda - ps24
Amniota - ps25
Mammalia - ps26
Theria - ps27
Placentalia - ps28
Boreoeutheria - ps29
Euarchontoglires - ps30
Euarchonta - ps31
Primates - ps32
Haplorrhini - ps33
Simiiformes - ps34
Catarrhini - ps35
Hominoidea - ps36
Hominidae - ps37
Homininae - ps38
Hominini - ps39
Homo_sapiens - ps40
p-value
4e-39
3e-30
3e-21
2e-12
2e-03
Log-odds
0.5
1.0
1.5
2.0
2.5
3.0
3.5
4.0

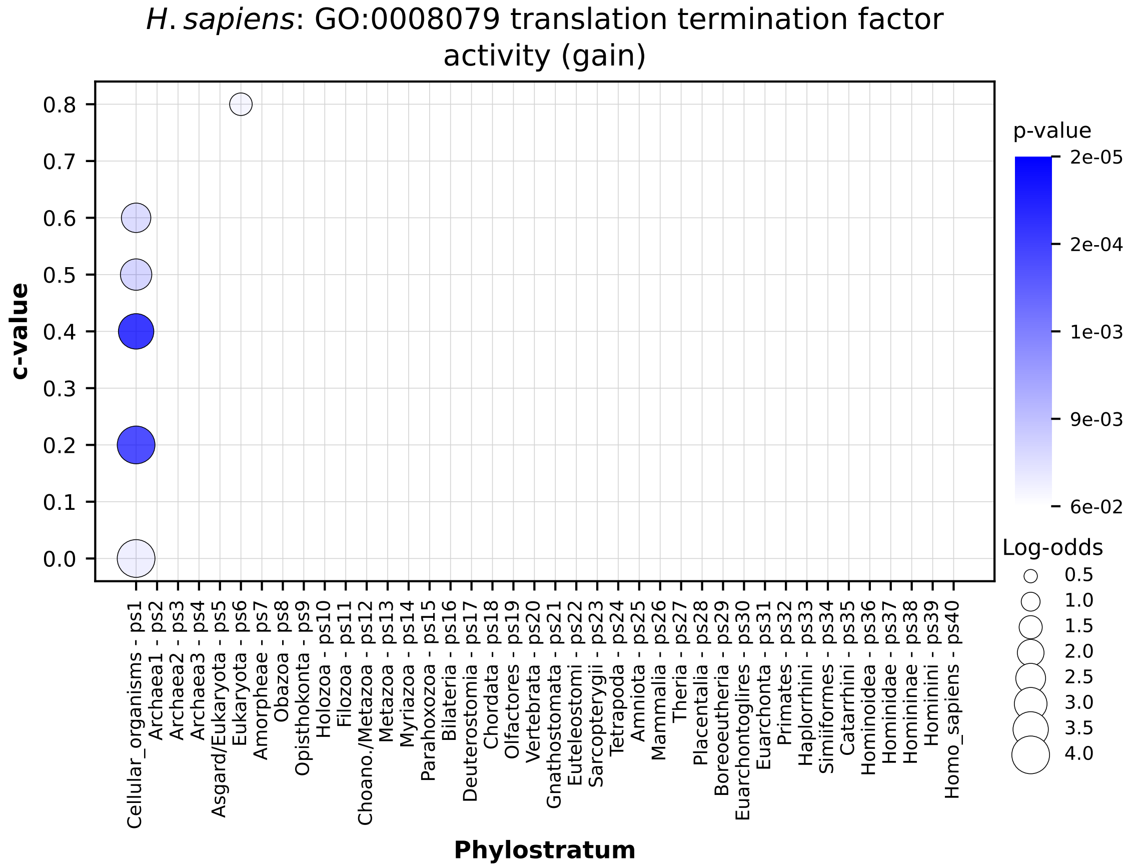
H. sapiens: GO:0008079 translation termination factor activity (gain)
c-value
0.8
0.7
0.6
0.5
0.4
0.3
0.2
0.1
0.0
Phylostratum
Cellular_organisms - ps1
Archaea1 - ps2
Archaea2 - ps3
Archaea3 - ps4
Asgard/Eukaryota - ps5
Eukaryota - ps6
Amorpheae - ps7
Obazoa - ps8
Opisthokonta - ps9
Holozoa - ps10
Filozoa - ps11
Choano./Metazoa - ps12
Metazoa - ps13
Myriazoa - ps14
Parahoxozoa - ps15
Bilateria - ps16
Deuterostomia - ps17
Chordata - ps18
Olfactores - ps19
Vertebrata - ps20
Gnathostomata - ps21
Euteleostomi - ps22
Sarcopterygii - ps23
Tetrapoda - ps24
Amniota - ps25
Mammalia - ps26
Theria - ps27
Placentalia - ps28
Boreoeutheria - ps29
Euarchontoglires - ps30
Euarchonta - ps31
Primates - ps32
Haplorrhini - ps33
Simiiformes - ps34
Catarrhini - ps35
Hominoidea - ps36
Hominidae - ps37
Homininae - ps38
Hominini - ps39
Homo_sapiens - ps40
p-value
2e-05
2e-04
1e-03
9e-03
6e-02
Log-odds
0.5
1.0
1.5
2.0
2.5
3.0
3.5
4.0

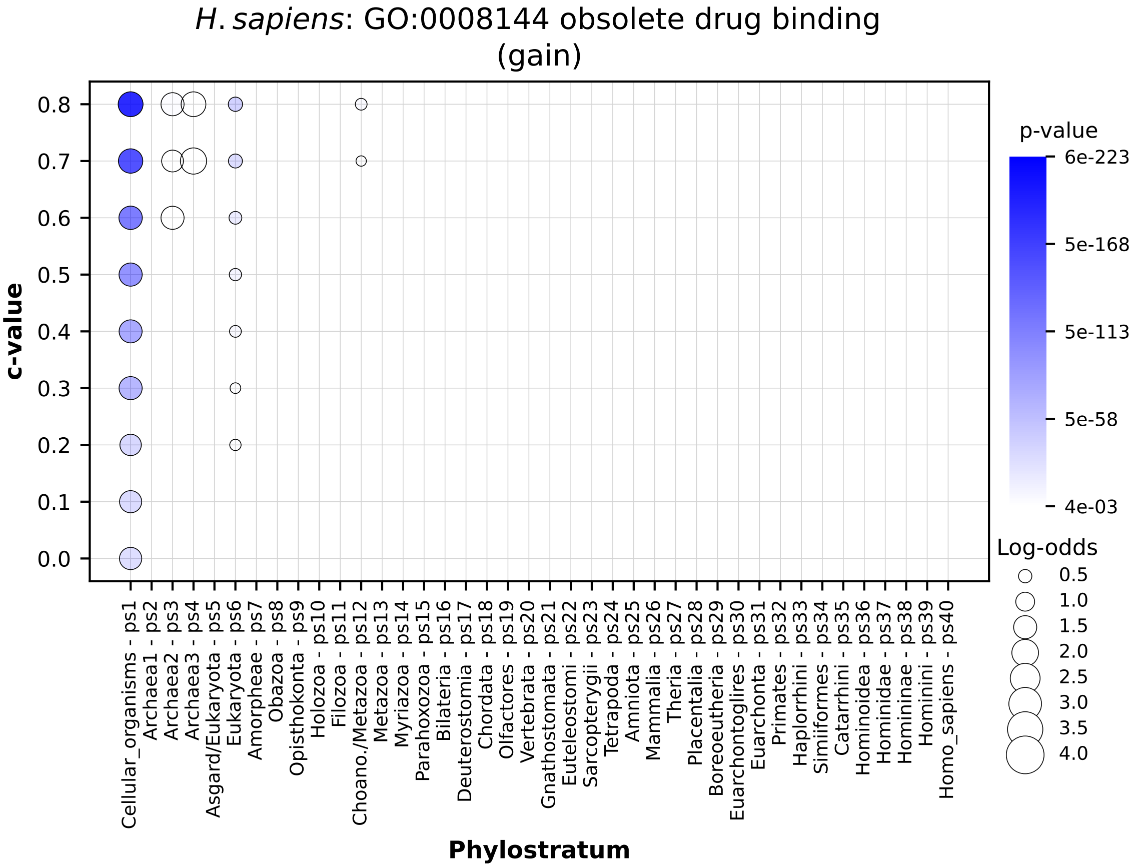

H. sapiens: GO:0008144 obsolete drug binding
(gain)
c-value
0.8
0.7
0.6
0.5
0.4
0.3
0.2
0.1
0.0
Phylostratum
Cellular_organisms - ps1
Archaea1 - ps2
Archaea2 - ps3
Archaea3 - ps4
Asgard/Eukaryota - ps5
Eukaryota - ps6
Amorpheae - ps7
Obazoa - ps8
Opisthokonta - ps9
Holozoa - ps10
Filozoa - ps11
Choano./Metazoa - ps12
Metazoa - ps13
Myriazoa - ps14
Parahoxozoa - ps15
Bilateria - ps16
Deuterostomia - ps17
Chordata - ps18
Olfactores - ps19
Vertebrata - ps20
Gnathostomata - ps21
Euteleostomi - ps22
Sarcopterygii - ps23
Tetrapoda - ps24
Amniota - ps25
Mammalia - ps26
Theria - ps27
Placentalia - ps28
Boreoeutheria - ps29
Euarchontoglires - ps30
Euarchonta - ps31
Primates - ps32
Haplorrhini - ps33
Simiiformes - ps34
Catarrhini - ps35
Hominoidea - ps36
Hominidae - ps37
Homininae - ps38
Hominini - ps39
Homo_sapiens - ps40
p-value
6e-223
5e-168
5e-113
5e-58
4e-03
Log-odds
0.5
1.0
1.5
2.0
2.5
3.0
3.5
4.0

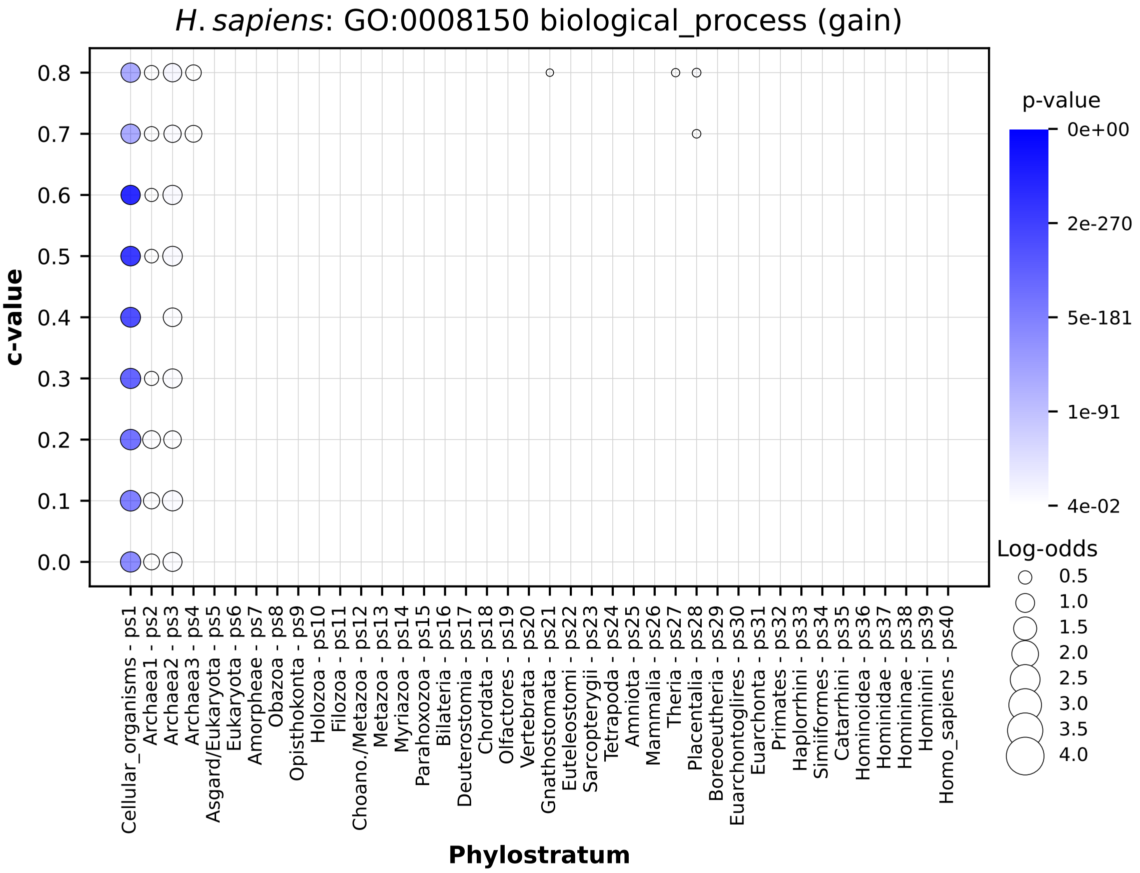

H. sapiens: GO:0008150 biological_process (gain)
c-value
0.0
0.1
0.2
0.3
0.4
0.5
0.6
0.7
0.8
p-value
0e+00
2e-270
5e-181
1e-91
4e-02
Log-odds
0.5
1.0
1.5
2.0
2.5
3.0
3.5
4.0
Cellular_organisms - ps1
Archaea1 - ps2
Archaea2 - ps3
Archaea3 - ps4
Asgard/Eukaryota - ps5
Eukaryota - ps6
Amorpheae - ps7
Obazoa - ps8
Opisthokonta - ps9
Holozoa - ps10
Filozoa - ps11
Choano./Metazoa - ps12
Metazoa - ps13
Myriazoa - ps14
Parahoxozoa - ps15
Bilateria - ps16
Deuterostomia - ps17
Chordata - ps18
Olfactores - ps19
Vertebrata - ps20
Gnathostomata - ps21
Euteleostomi - ps22
Sarcopterygii - ps23
Tetrapoda - ps24
Amniota - ps25
Mammalia - ps26
Theria - ps27
Placentalia - ps28
Boreoeutheria - ps29
Euarchontoglires - ps30
Euarchonta - ps31
Primates - ps32
Haplorrhini - ps33
Simiiformes - ps34
Catarrhini - ps35
Hominoidea - ps36
Hominidae - ps37
Homininae - ps38
Hominini - ps39
Homo_sapiens - ps40
Phylostratum

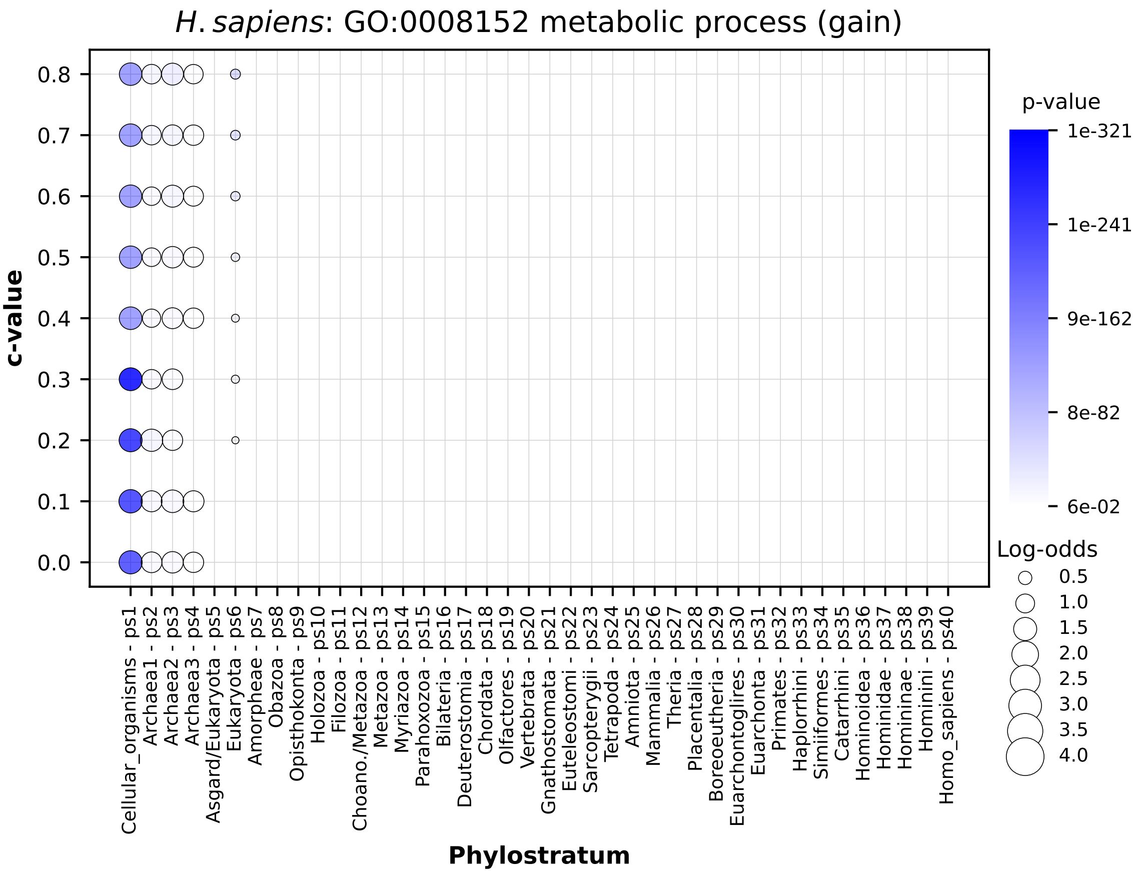

H. sapiens: GO:0008152 metabolic process (gain)
c-value
0.0
0.1
0.2
0.3
0.4
0.5
0.6
0.7
0.8
Phylostratum
Cellular_organisms - ps1
Archaea1 - ps2
Archaea2 - ps3
Archaea3 - ps4
Asgard/Eukaryota - ps5
Eukaryota - ps6
Amorpheae - ps7
Obazoa - ps8
Opisthokonta - ps9
Holozoa - ps10
Filozoa - ps11
Choano./Metazoa - ps12
Metazoa - ps13
Myriazoa - ps14
Parahoxozoa - ps15
Bilateria - ps16
Deuterostomia - ps17
Chordata - ps18
Olfactores - ps19
Vertebrata - ps20
Gnathostomata - ps21
Euteleostomi - ps22
Sarcopterygii - ps23
Tetrapoda - ps24
Amniota - ps25
Mammalia - ps26
Theria - ps27
Placentalia - ps28
Boreoeutheria - ps29
Euarchontoglires - ps30
Euarchonta - ps31
Primates - ps32
Haplorrhini - ps33
Simiiformes - ps34
Catarrhini - ps35
Hominoidea - ps36
Hominidae - ps37
Homininae - ps38
Hominini - ps39
Homo_sapiens - ps40
p-value
1e-321
1e-241
9e-162
8e-82
6e-02
Log-odds
0.5
1.0
1.5
2.0
2.5
3.0
3.5
4.0

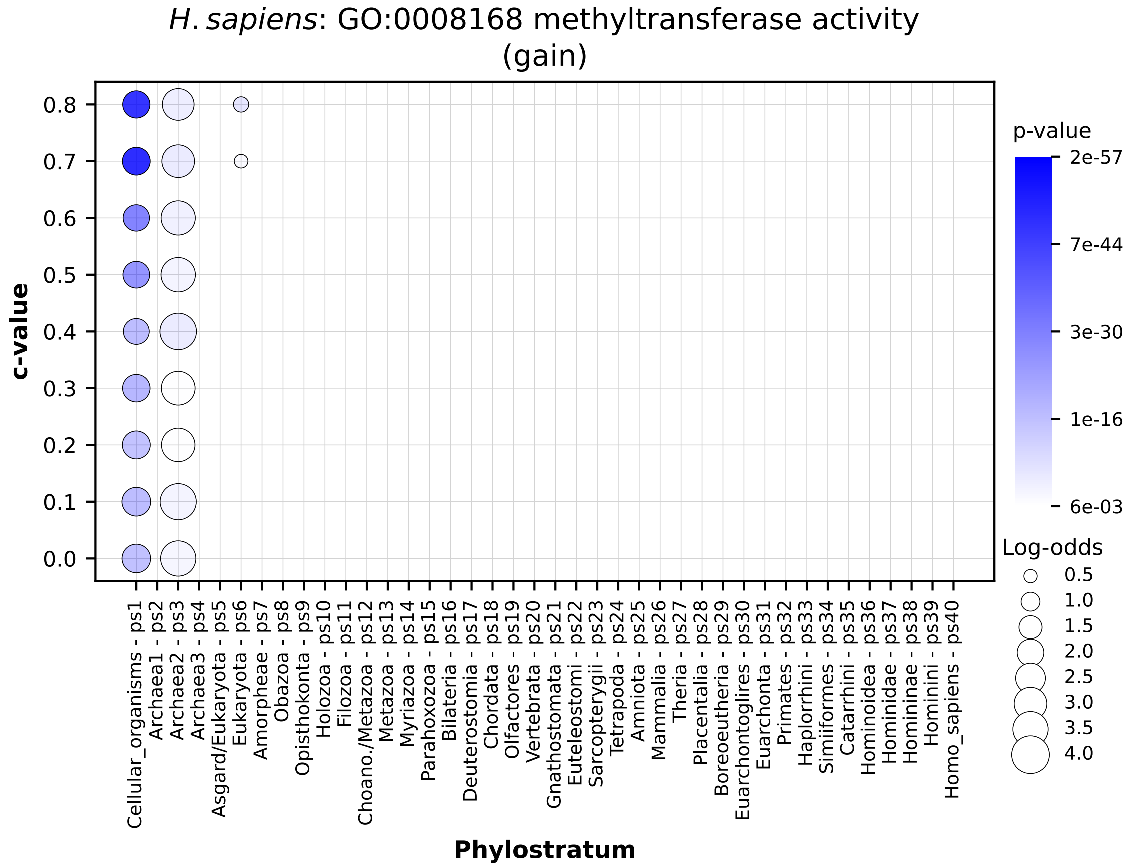
H. sapiens: GO:0008168 methyltransferase activity
(gain)
c-value
0.8
0.7
0.6
0.5
0.4
0.3
0.2
0.1
0.0
p-value
2e-57
7e-44
3e-30
1e-16
6e-03
Log-odds
0.5
1.0
1.5
2.0
2.5
3.0
3.5
4.0
Cellular_organisms - ps1
Archaea1 - ps2
Archaea2 - ps3
Archaea3 - ps4
Asgard/Eukaryota - ps5
Eukaryota - ps6
Amorpheae - ps7
Obazoa - ps8
Opisthokonta - ps9
Holozoa - ps10
Filozoa - ps11
Choano./Metazoa - ps12
Metazoa - ps13
Myriazoa - ps14
Parahoxozoa - ps15
Bilateria - ps16
Deuterostomia - ps17
Chordata - ps18
Olfactores - ps19
Vertebrata - ps20
Gnathostomata - ps21
Euteleostomi - ps22
Sarcopterygii - ps23
Tetrapoda - ps24
Amniota - ps25
Mammalia - ps26
Theria - ps27
Placentalia - ps28
Boreoeutheria - ps29
Euarchontoglires - ps30
Euarchonta - ps31
Primates - ps32
Haplorrhini - ps33
Simiiformes - ps34
Catarrhini - ps35
Hominoidea - ps36
Hominidae - ps37
Homininae - ps38
Hominini - ps39
Homo_sapiens - ps40
Phylostratum

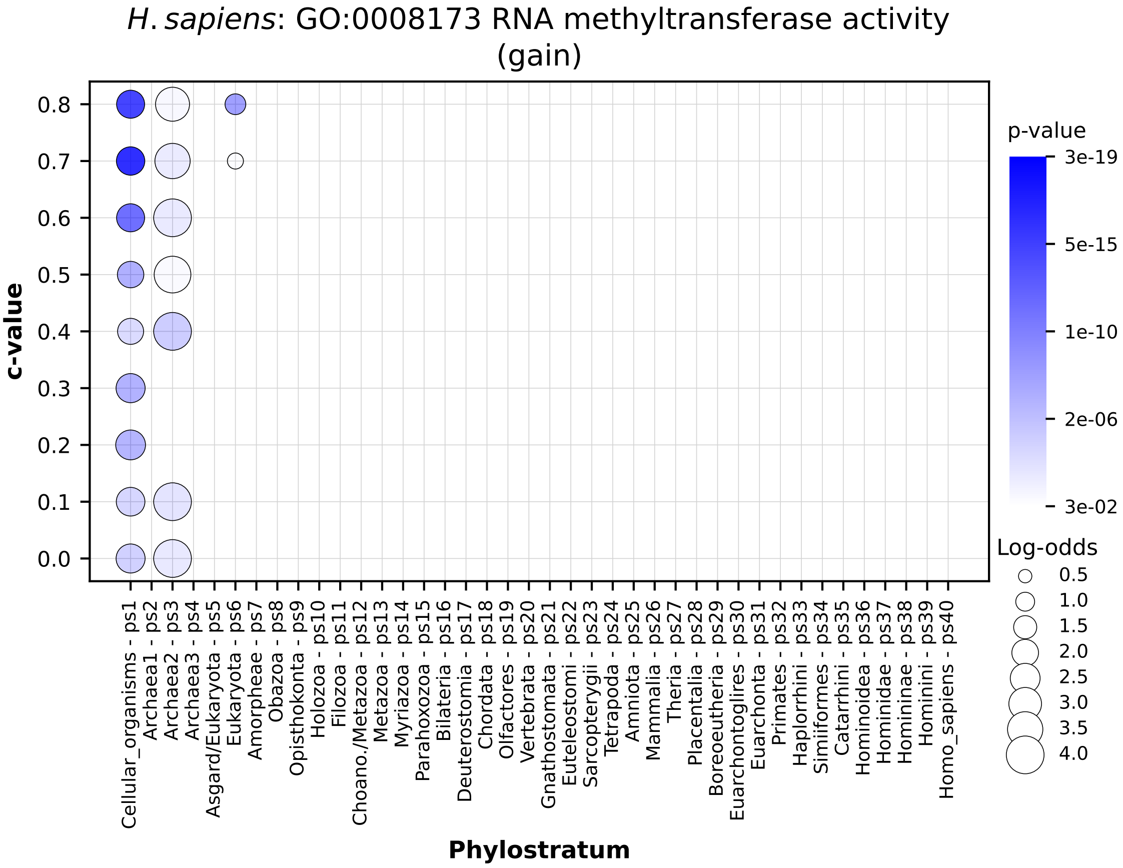

*H. sapiens*: GO:0008173 RNA methyltransferase activity (gain)
c-value
0.8
0.7
0.6
0.5
0.4
0.3
0.2
0.1
0.0
Phylostratum
Cellular_organisms - ps1
Archaea1 - ps2
Archaea2 - ps3
Archaea3 - ps4
Asgard/Eukaryota - ps5
Eukaryota - ps6
Amorpheae - ps7
Obazoa - ps8
Opisthokonta - ps9
Holozoa - ps10
Filozoa - ps11
Choano./Metazoa - ps12
Metazoa - ps13
Myriazoa - ps14
Parahoxozoa - ps15
Bilateria - ps16
Deuterostomia - ps17
Chordata - ps18
Olfactores - ps19
Vertebrata - ps20
Gnathostomata - ps21
Euteleostomi - ps22
Sarcopterygii - ps23
Tetrapoda - ps24
Amniota - ps25
Mammalia - ps26
Theria - ps27
Placentalia - ps28
Boreoeutheria - ps29
Euarchontoglires - ps30
Euarchonta - ps31
Primates - ps32
Haplorrhini - ps33
Simiiformes - ps34
Catarrhini - ps35
Hominoidea - ps36
Hominidae - ps37
Homininae - ps38
Hominini - ps39
Homo_sapiens - ps40
p-value
3e-19
5e-15
1e-10
2e-06
3e-02
Log-odds
0.5
1.0
1.5
2.0
2.5
3.0
3.5
4.0

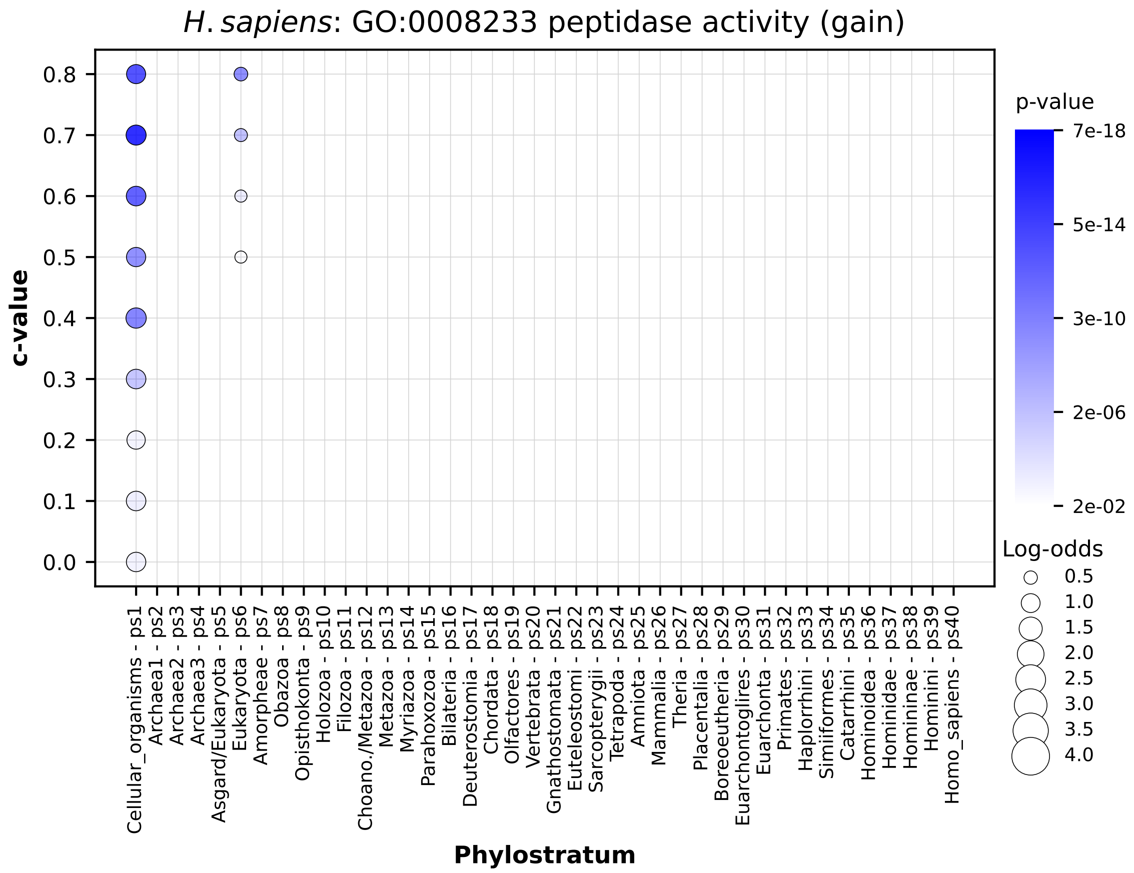

H. sapiens: GO:0008233 peptidase activity (gain)
c-value
0.8
0.7
0.6
0.5
0.4
0.3
0.2
0.1
0.0
Phylostratum
Cellular_organisms - ps1
Archaea1 - ps2
Archaea2 - ps3
Archaea3 - ps4
Asgard/Eukaryota - ps5
Eukaryota - ps6
Amorpheae - ps7
Obazoa - ps8
Opisthokonta - ps9
Holozoa - ps10
Filozoa - ps11
Choano./Metazoa - ps12
Metazoa - ps13
Myriazoa - ps14
Parahoxozoa - ps15
Bilateria - ps16
Deuterostomia - ps17
Chordata - ps18
Olfactores - ps19
Vertebrata - ps20
Gnathostomata - ps21
Euteleostomi - ps22
Sarcopterygii - ps23
Tetrapoda - ps24
Amniota - ps25
Mammalia - ps26
Theria - ps27
Placentalia - ps28
Boreoeutheria - ps29
Euarchontoglires - ps30
Euarchonta - ps31
Primates - ps32
Haplorrhini - ps33
Simiiformes - ps34
Catarrhini - ps35
Hominoidea - ps36
Hominidae - ps37
Homininae - ps38
Hominini - ps39
Homo_sapiens - ps40
p-value
7e-18
5e-14
3e-10
2e-06
2e-02
Log-odds
0.5
1.0
1.5
2.0
2.5
3.0
3.5
4.0

*H. sapiens*: GO:0008236 serine-type peptidase activity (gain)

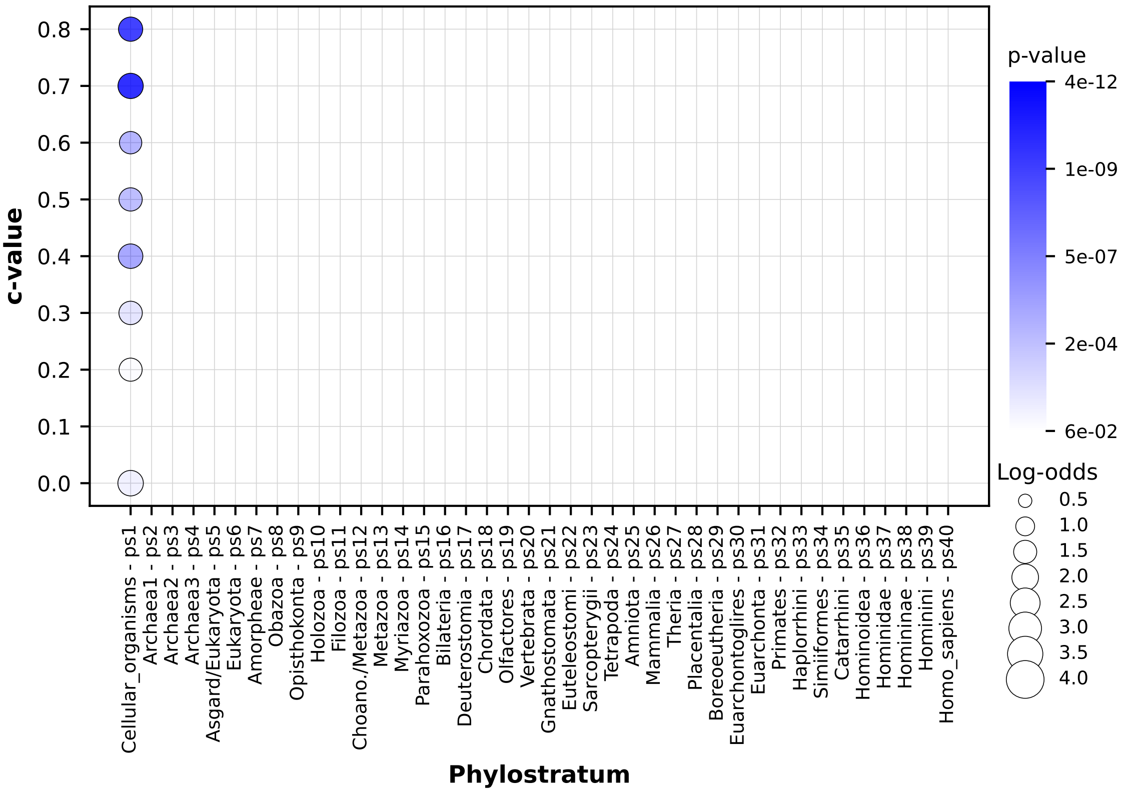

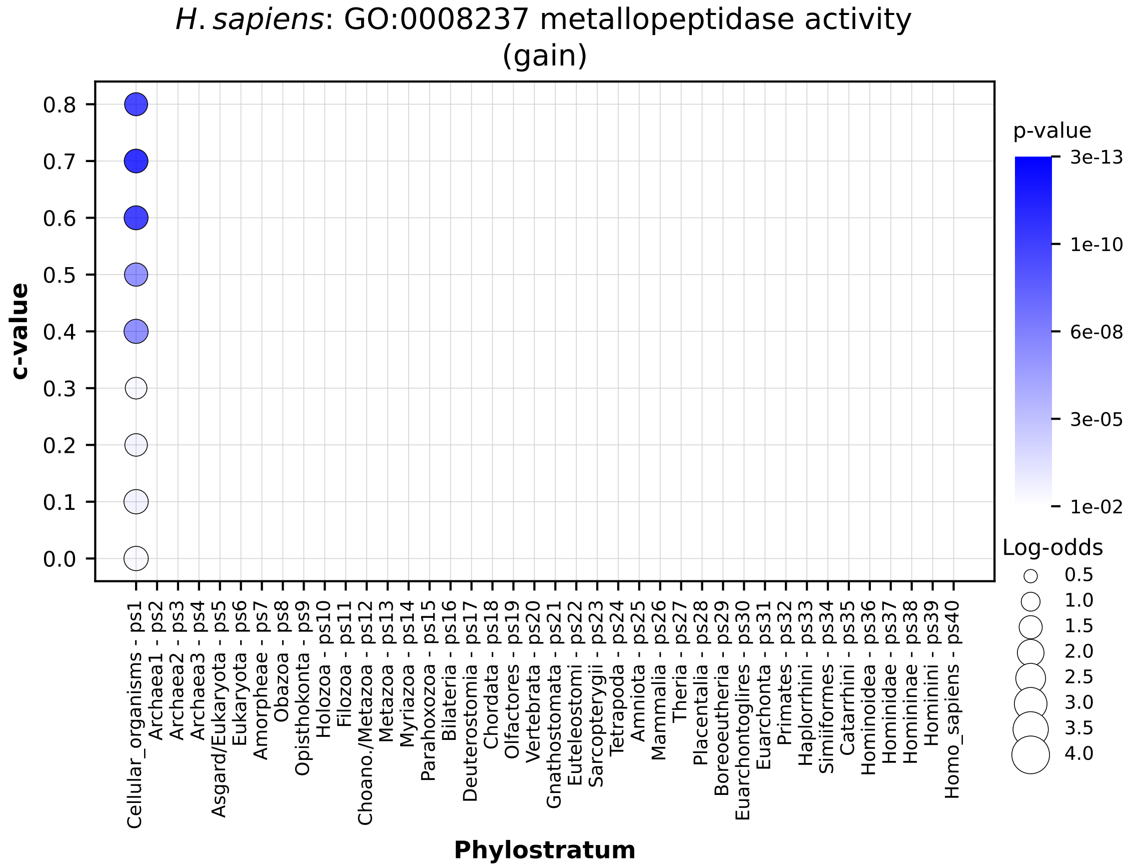
H. sapiens: GO:0008237 metallopeptidase activity
(gain)
c-value
0.8
0.7
0.6
0.5
0.4
0.3
0.2
0.1
0.0
Phylostratum
Cellular_organisms - ps1
Archaea1 - ps2
Archaea2 - ps3
Archaea3 - ps4
Asgard/Eukaryota - ps5
Eukaryota - ps6
Amorpheae - ps7
Obazoa - ps8
Opisthokonta - ps9
Holozoa - ps10
Filozoa - ps11
Choano./Metazoa - ps12
Metazoa - ps13
Myriazoa - ps14
Parahoxozoa - ps15
Bilateria - ps16
Deuterostomia - ps17
Chordata - ps18
Olfactores - ps19
Vertebrata - ps20
Gnathostomata - ps21
Euteleostomi - ps22
Sarcopterygii - ps23
Tetrapoda - ps24
Amniota - ps25
Mammalia - ps26
Theria - ps27
Placentalia - ps28
Boreoeutheria - ps29
Euarchontoglires - ps30
Euarchonta - ps31
Primates - ps32
Haplorrhini - ps33
Simiiformes - ps34
Catarrhini - ps35
Hominoidea - ps36
Hominidae - ps37
Homininae - ps38
Hominini - ps39
Homo_sapiens - ps40
p-value
3e-13
1e-10
6e-08
3e-05
1e-02
Log-odds
0.5
1.0
1.5
2.0
2.5
3.0
3.5
4.0

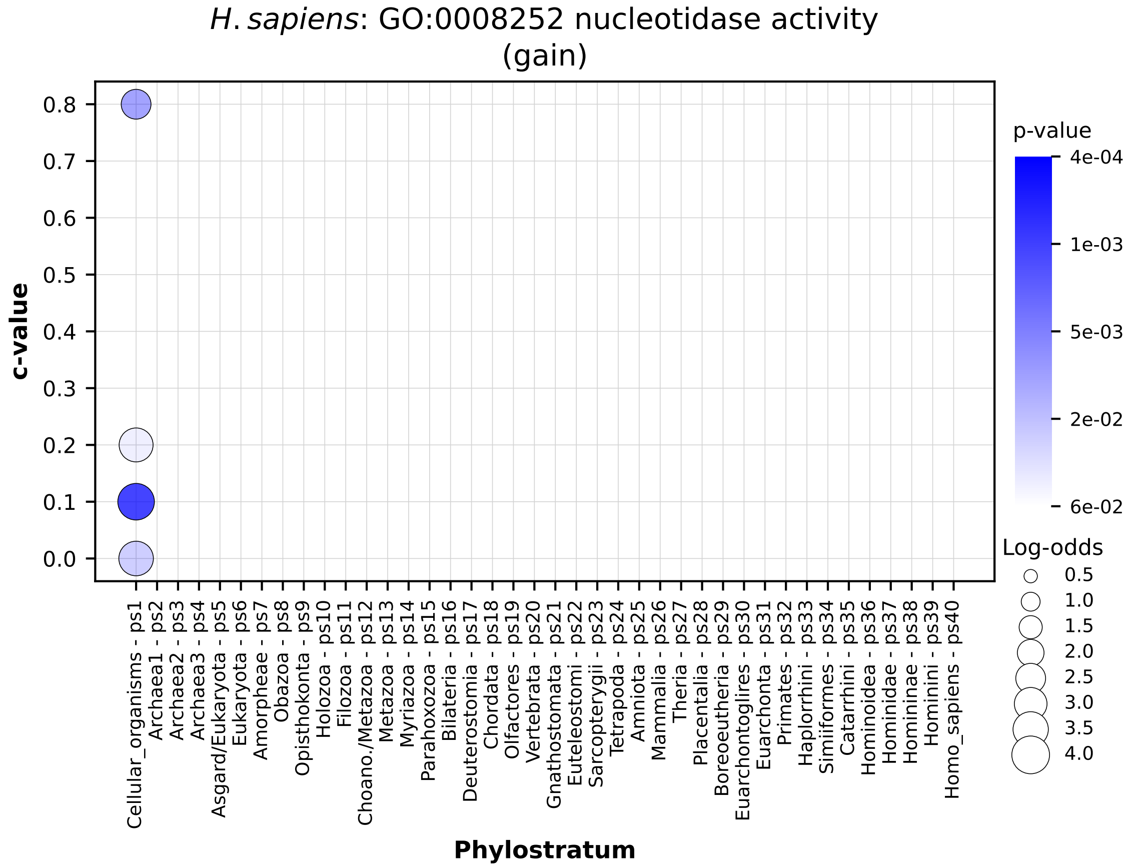
H. sapiens: GO:0008252 nucleotidase activity
(gain)
c-value
0.8
0.7
0.6
0.5
0.4
0.3
0.2
0.1
0.0
p-value
4e-04
1e-03
5e-03
2e-02
6e-02
Log-odds
0.5
1.0
1.5
2.0
2.5
3.0
3.5
4.0
Cellular_organisms - ps1
Archaea1 - ps2
Archaea2 - ps3
Archaea3 - ps4
Asgard/Eukaryota - ps5
Eukaryota - ps6
Amorpheae - ps7
Obazoa - ps8
Opisthokonta - ps9
Holozoa - ps10
Filozoa - ps11
Choano./Metazoa - ps12
Metazoa - ps13
Myriazoa - ps14
Parahoxozoa - ps15
Bilateria - ps16
Deuterostomia - ps17
Chordata - ps18
Olfactores - ps19
Vertebrata - ps20
Gnathostomata - ps21
Euteleostomi - ps22
Sarcopterygii - ps23
Tetrapoda - ps24
Amniota - ps25
Mammalia - ps26
Theria - ps27
Placentalia - ps28
Boreoeutheria - ps29
Euarchontoglires - ps30
Euarchonta - ps31
Primates - ps32
Haplorrhini - ps33
Simiiformes - ps34
Catarrhini - ps35
Hominoidea - ps36
Hominidae - ps37
Homininae - ps38
Hominini - ps39
Homo_sapiens - ps40
Phylostratum

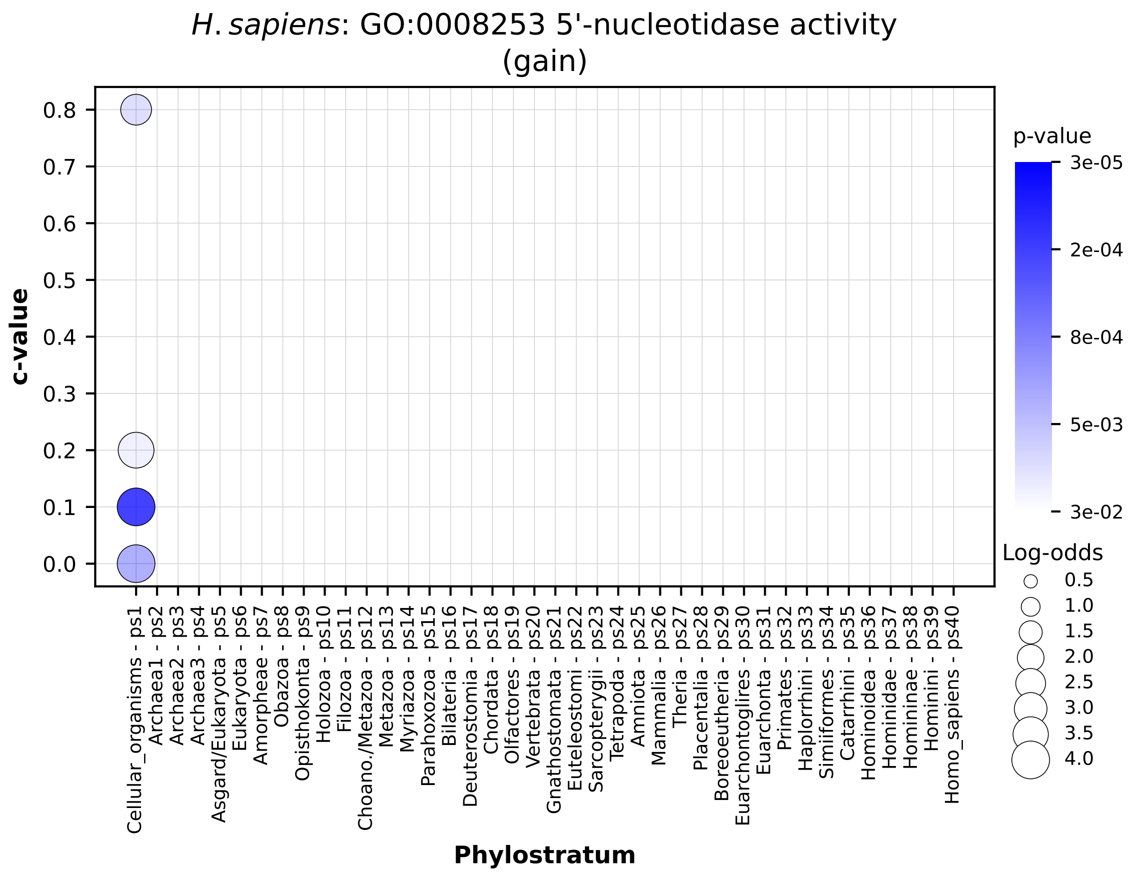

H. sapiens: GO:0008253 5'-nucleotidase activity
(gain)
c-value
0.8
0.7
0.6
0.5
0.4
0.3
0.2
0.1
0.0
p-value
3e-05
2e-04
8e-04
5e-03
3e-02
Log-odds
0.5
1.0
1.5
2.0
2.5
3.0
3.5
4.0
Cellular_organisms - ps1
Archaea1 - ps2
Archaea2 - ps3
Archaea3 - ps4
Asgard/Eukaryota - ps5
Eukaryota - ps6
Amorpheae - ps7
Obazoa - ps8
Opisthokonta - ps9
Holozoa - ps10
Filozoa - ps11
Choano./Metazoa - ps12
Metazoa - ps13
Myriazoa - ps14
Parahoxozoa - ps15
Bilateria - ps16
Deuterostomia - ps17
Chordata - ps18
Olfactores - ps19
Vertebrata - ps20
Gnathostomata - ps21
Euteleostomi - ps22
Sarcopterygii - ps23
Tetrapoda - ps24
Amniota - ps25
Mammalia - ps26
Theria - ps27
Placentalia - ps28
Boreoeutheria - ps29
Euarchontoglires - ps30
Euarchonta - ps31
Primates - ps32
Haplorrhini - ps33
Simiiformes - ps34
Catarrhini - ps35
Hominoidea - ps36
Hominidae - ps37
Homininae - ps38
Hominini - ps39
Homo_sapiens - ps40
Phylostratum

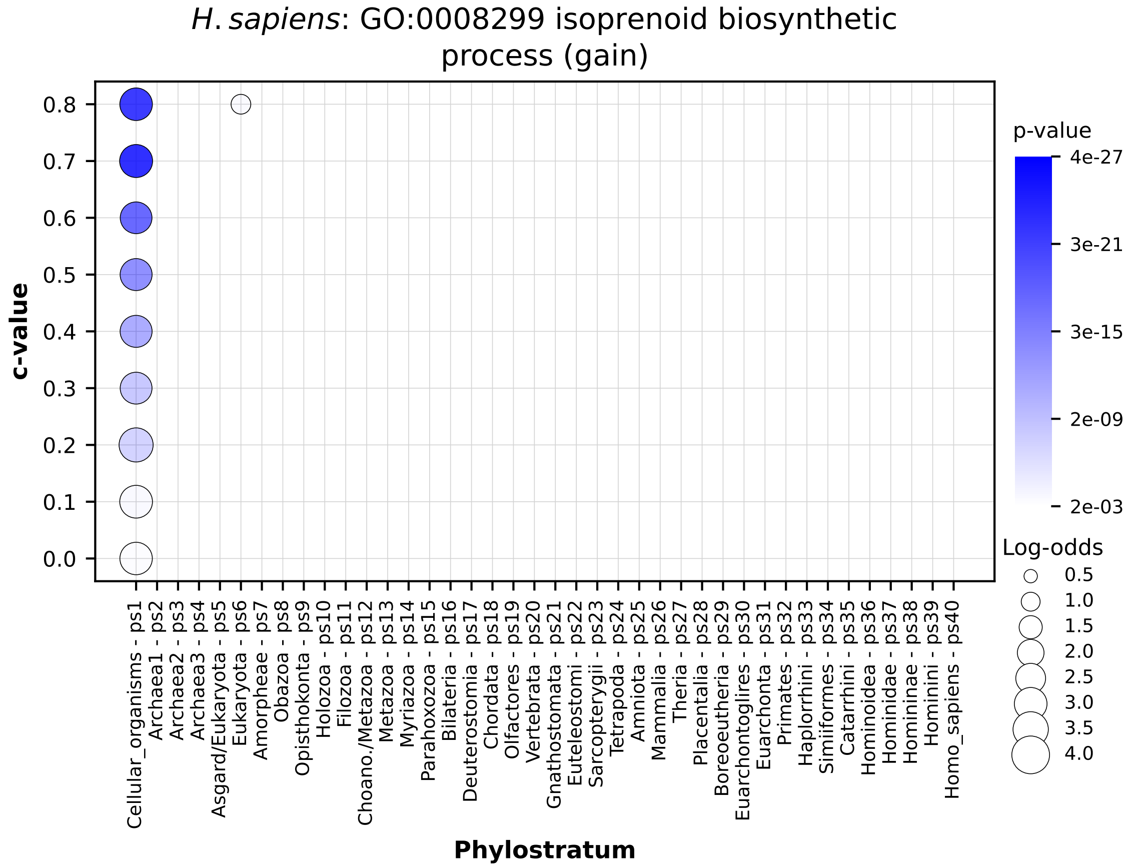

H. sapiens: GO:0008299 isoprenoid biosynthetic process (gain)
c-value
0.8
0.7
0.6
0.5
0.4
0.3
0.2
0.1
0.0
p-value
4e-27
3e-21
3e-15
2e-09
2e-03
Log-odds
0.5
1.0
1.5
2.0
2.5
3.0
3.5
4.0
Cellular_organisms - ps1
Archaea1 - ps2
Archaea2 - ps3
Archaea3 - ps4
Asgard/Eukaryota - ps5
Eukaryota - ps6
Amorpheae - ps7
Obazoa - ps8
Opisthokonta - ps9
Holozoa - ps10
Filozoa - ps11
Choano./Metazoa - ps12
Metazoa - ps13
Myriazoa - ps14
Parahoxozoa - ps15
Bilateria - ps16
Deuterostomia - ps17
Chordata - ps18
Olfactores - ps19
Vertebrata - ps20
Gnathostomata - ps21
Euteleostomi - ps22
Sarcopterygii - ps23
Tetrapoda - ps24
Amniota - ps25
Mammalia - ps26
Theria - ps27
Placentalia - ps28
Boreoeutheria - ps29
Euarchontoglires - ps30
Euarchonta - ps31
Primates - ps32
Haplorrhini - ps33
Simiiformes - ps34
Catarrhini - ps35
Hominoidea - ps36
Hominidae - ps37
Homininae - ps38
Hominini - ps39
Homo_sapiens - ps40
Phylostratum

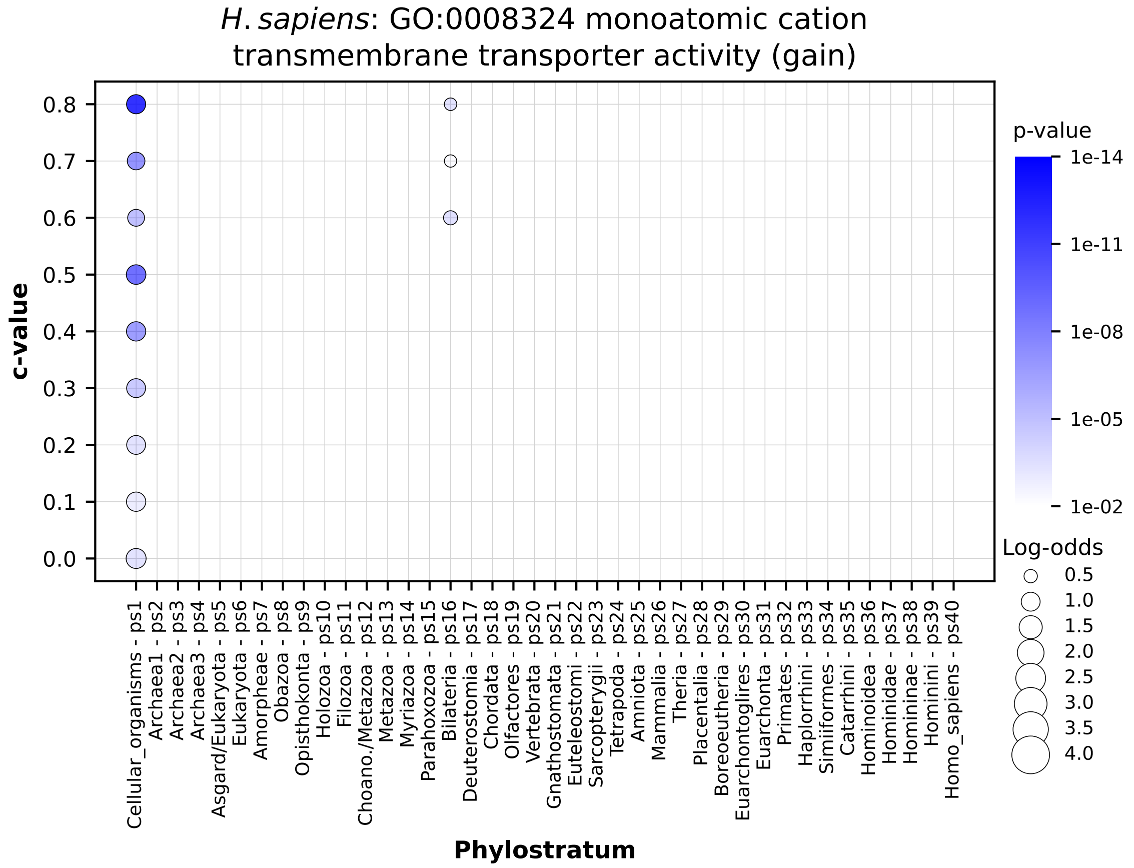
H. sapiens: GO:0008324 monoatomic cation transmembrane transporter activity (gain)
c-value
0.8
0.7
0.6
0.5
0.4
0.3
0.2
0.1
0.0
Phylostratum
Cellular_organisms - ps1
Archaea1 - ps2
Archaea2 - ps3
Archaea3 - ps4
Asgard/Eukaryota - ps5
Eukaryota - ps6
Amorpheae - ps7
Obazoa - ps8
Opisthokonta - ps9
Holozoa - ps10
Filozoa - ps11
Choano./Metazoa - ps12
Metazoa - ps13
Myriazoa - ps14
Parahoxozoa - ps15
Bilateria - ps16
Deuterostomia - ps17
Chordata - ps18
Olfactores - ps19
Vertebrata - ps20
Gnathostomata - ps21
Euteleostomi - ps22
Sarcopterygii - ps23
Tetrapoda - ps24
Amniota - ps25
Mammalia - ps26
Theria - ps27
Placentalia - ps28
Boreoeutheria - ps29
Euarchontoglires - ps30
Euarchonta - ps31
Primates - ps32
Haplorrhini - ps33
Simiiformes - ps34
Catarrhini - ps35
Hominoidea - ps36
Hominidae - ps37
Homininae - ps38
Hominini - ps39
Homo_sapiens - ps40
p-value
1e-14
1e-11
1e-08
1e-05
1e-02
Log-odds
0.5
1.0
1.5
2.0
2.5
3.0
3.5
4.0

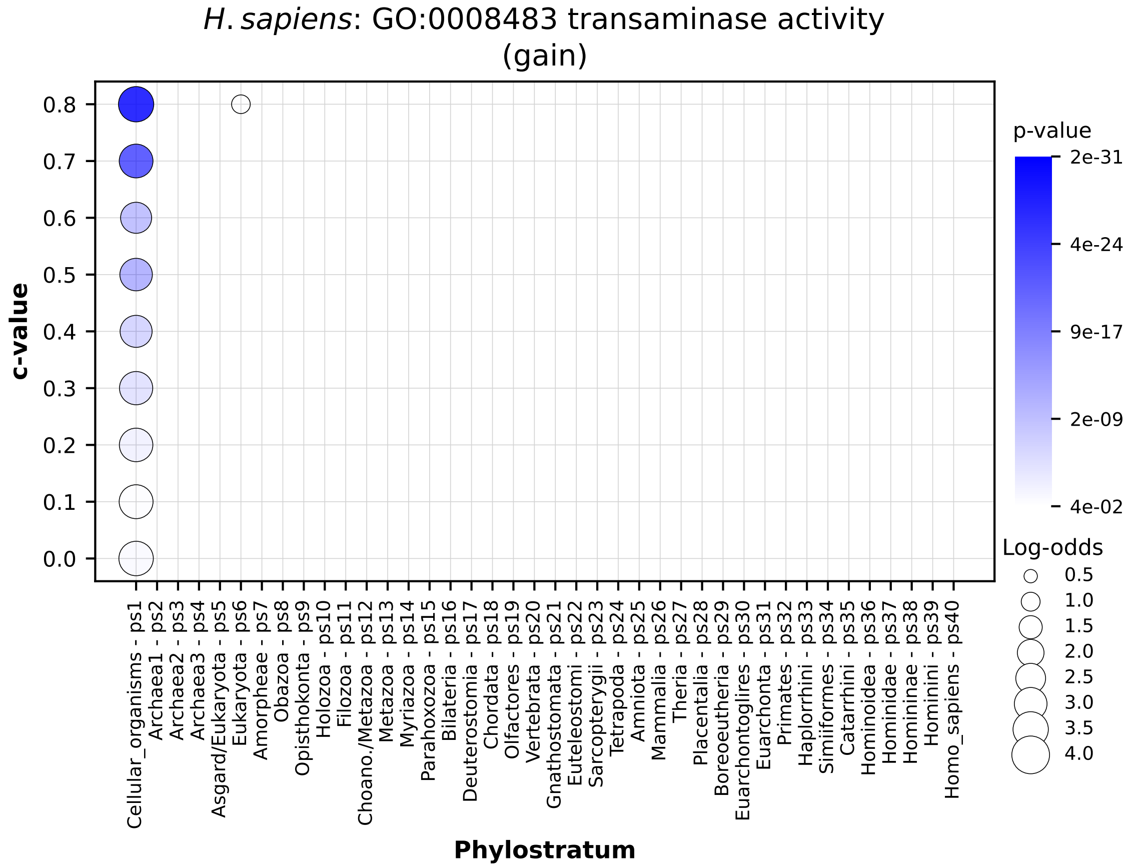

H. sapiens: GO:0008483 transaminase activity
(gain)
c-value
0.8
0.7
0.6
0.5
0.4
0.3
0.2
0.1
0.0
p-value
2e-31
4e-24
9e-17
2e-09
4e-02
Log-odds
0.5
1.0
1.5
2.0
2.5
3.0
3.5
4.0
Cellular_organisms - ps1
Archaea1 - ps2
Archaea2 - ps3
Archaea3 - ps4
Asgard/Eukaryota - ps5
Eukaryota - ps6
Amorpheae - ps7
Obazoa - ps8
Opisthokonta - ps9
Holozoa - ps10
Filozoa - ps11
Choano./Metazoa - ps12
Metazoa - ps13
Myriazoa - ps14
Parahoxozoa - ps15
Bilateria - ps16
Deuterostomia - ps17
Chordata - ps18
Olfactores - ps19
Vertebrata - ps20
Gnathostomata - ps21
Euteleostomi - ps22
Sarcopterygii - ps23
Tetrapoda - ps24
Amniota - ps25
Mammalia - ps26
Theria - ps27
Placentalia - ps28
Boreoeutheria - ps29
Euarchontoglires - ps30
Euarchonta - ps31
Primates - ps32
Haplorrhini - ps33
Simiiformes - ps34
Catarrhini - ps35
Hominoidea - ps36
Hominidae - ps37
Homininae - ps38
Hominini - ps39
Homo_sapiens - ps40
Phylostratum

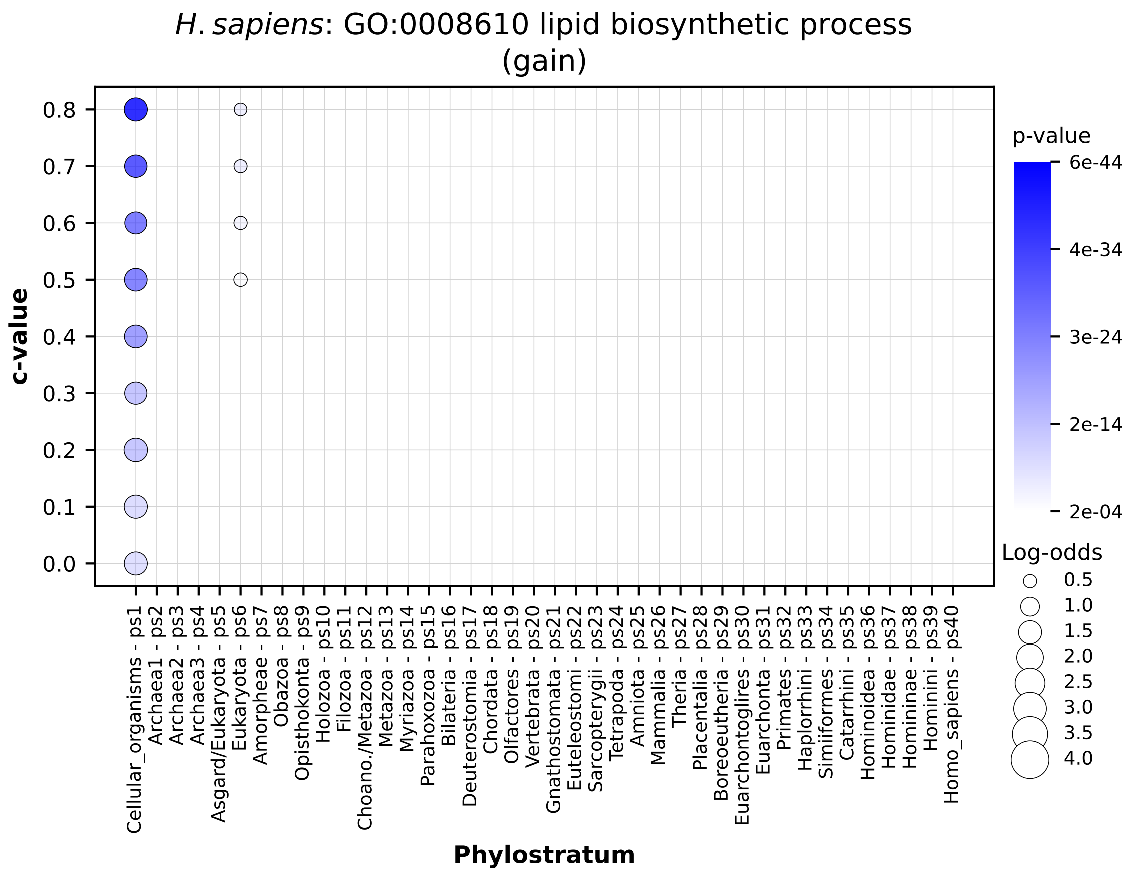

H. sapiens: GO:0008610 lipid biosynthetic process
(gain)
c-value
0.8
0.7
0.6
0.5
0.4
0.3
0.2
0.1
0.0
p-value
6e-44
4e-34
3e-24
2e-14
2e-04
Log-odds
0.5
1.0
1.5
2.0
2.5
3.0
3.5
4.0
Cellular_organisms - ps1
Archaea1 - ps2
Archaea2 - ps3
Archaea3 - ps4
Asgard/Eukaryota - ps5
Eukaryota - ps6
Amorpheae - ps7
Obazoa - ps8
Opisthokonta - ps9
Holozoa - ps10
Filozoa - ps11
Choano./Metazoa - ps12
Metazoa - ps13
Myriazoa - ps14
Parahoxozoa - ps15
Bilateria - ps16
Deuterostomia - ps17
Chordata - ps18
Olfactores - ps19
Vertebrata - ps20
Gnathostomata - ps21
Euteleostomi - ps22
Sarcopterygii - ps23
Tetrapoda - ps24
Amniota - ps25
Mammalia - ps26
Theria - ps27
Placentalia - ps28
Boreoeutheria - ps29
Euarchontoglires - ps30
Euarchonta - ps31
Primates - ps32
Haplorrhini - ps33
Simiiformes - ps34
Catarrhini - ps35
Hominoidea - ps36
Hominidae - ps37
Homininae - ps38
Hominini - ps39
Homo_sapiens - ps40
Phylostratum

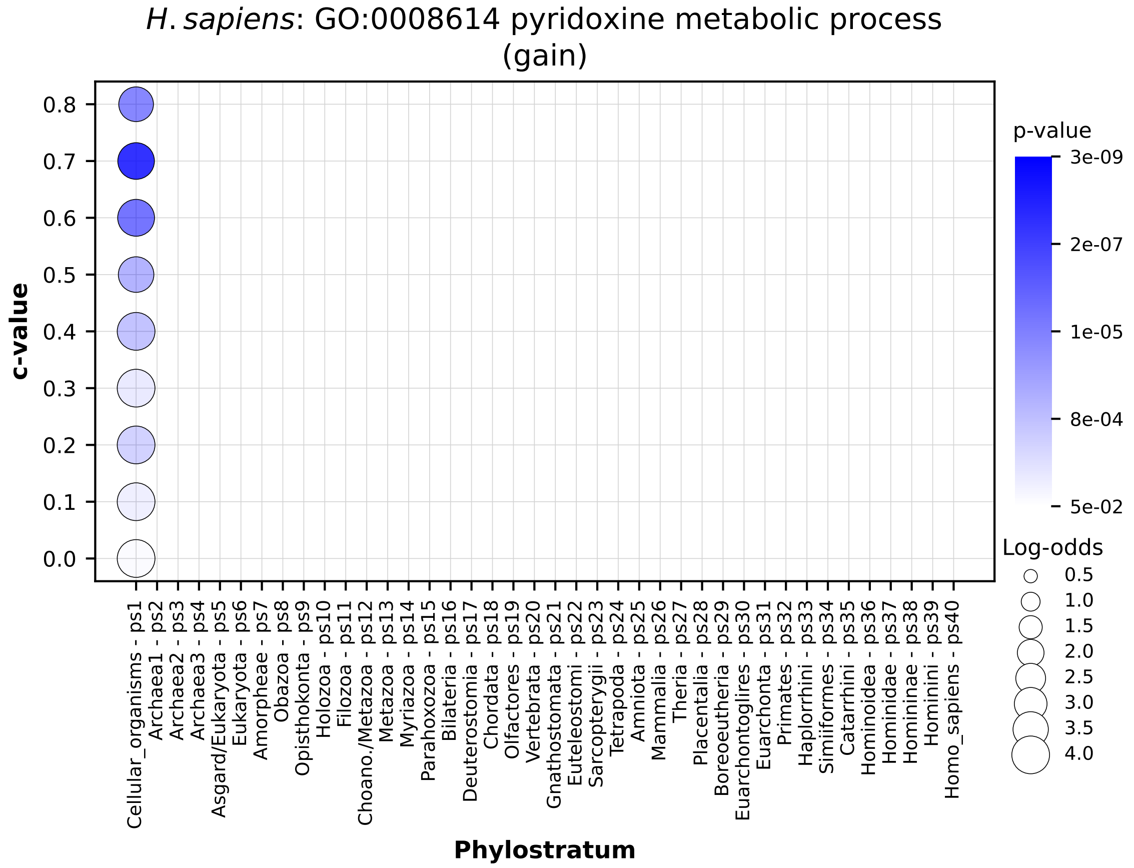
H. sapiens: GO:0008614 pyridoxine metabolic process
(gain)
c-value
0.8
0.7
0.6
0.5
0.4
0.3
0.2
0.1
0.0
p-value
3e-09
2e-07
1e-05
8e-04
5e-02
Log-odds
0.5
1.0
1.5
2.0
2.5
3.0
3.5
4.0
Cellular_organisms - ps1
Archaea1 - ps2
Archaea2 - ps3
Archaea3 - ps4
Asgard/Eukaryota - ps5
Eukaryota - ps6
Amorpheae - ps7
Obazoa - ps8
Opisthokonta - ps9
Holozoa - ps10
Filozoa - ps11
Choano./Metazoa - ps12
Metazoa - ps13
Myriazoa - ps14
Parahoxozoa - ps15
Bilateria - ps16
Deuterostomia - ps17
Chordata - ps18
Olfactores - ps19
Vertebrata - ps20
Gnathostomata - ps21
Euteleostomi - ps22
Sarcopterygii - ps23
Tetrapoda - ps24
Amniota - ps25
Mammalia - ps26
Theria - ps27
Placentalia - ps28
Boreoeutheria - ps29
Euarchontoglires - ps30
Euarchonta - ps31
Primates - ps32
Haplorrhini - ps33
Simiiformes - ps34
Catarrhini - ps35
Hominoidea - ps36
Hominidae - ps37
Homininae - ps38
Hominini - ps39
Homo_sapiens - ps40
Phylostratum

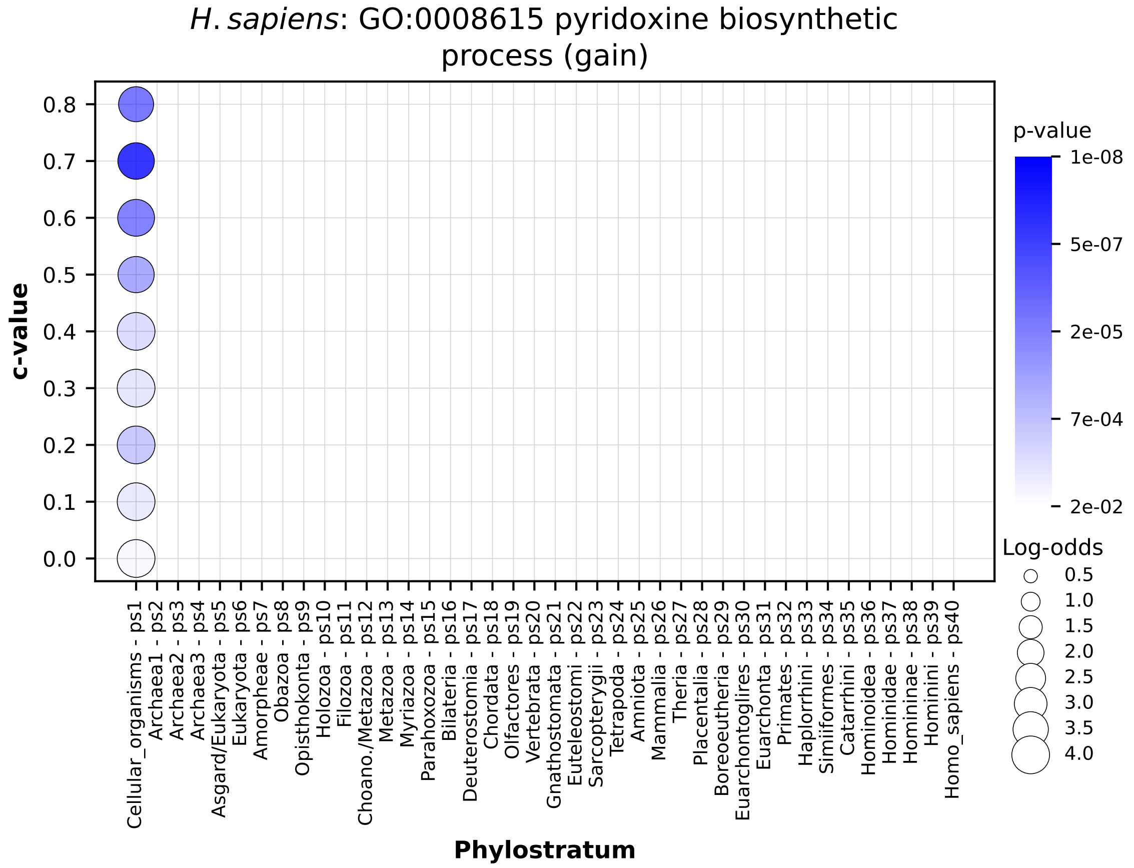
H. sapiens: GO:0008615 pyridoxine biosynthetic process (gain)
c-value
0.0
0.1
0.2
0.3
0.4
0.5
0.6
0.7
0.8
Phylostratum
Cellular_organisms - ps1
Archaea1 - ps2
Archaea2 - ps3
Archaea3 - ps4
Asgard/Eukaryota - ps5
Eukaryota - ps6
Amorpheae - ps7
Obazoa - ps8
Opisthokonta - ps9
Holozoa - ps10
Filozoa - ps11
Choano./Metazoa - ps12
Metazoa - ps13
Myriazoa - ps14
Parahoxozoa - ps15
Bilateria - ps16
Deuterostomia - ps17
Chordata - ps18
Olfactores - ps19
Vertebrata - ps20
Gnathostomata - ps21
Euteleostomi - ps22
Sarcopterygii - ps23
Tetrapoda - ps24
Amniota - ps25
Mammalia - ps26
Theria - ps27
Placentalia - ps28
Boreoeutheria - ps29
Euarchontoglires - ps30
Euarchonta - ps31
Primates - ps32
Haplorrhini - ps33
Simiiformes - ps34
Catarrhini - ps35
Hominoidea - ps36
Hominidae - ps37
Homininae - ps38
Hominini - ps39
Homo_sapiens - ps40
p-value
1e-08
5e-07
2e-05
7e-04
2e-02
Log-odds
0.5
1.0
1.5
2.0
2.5
3.0
3.5
4.0

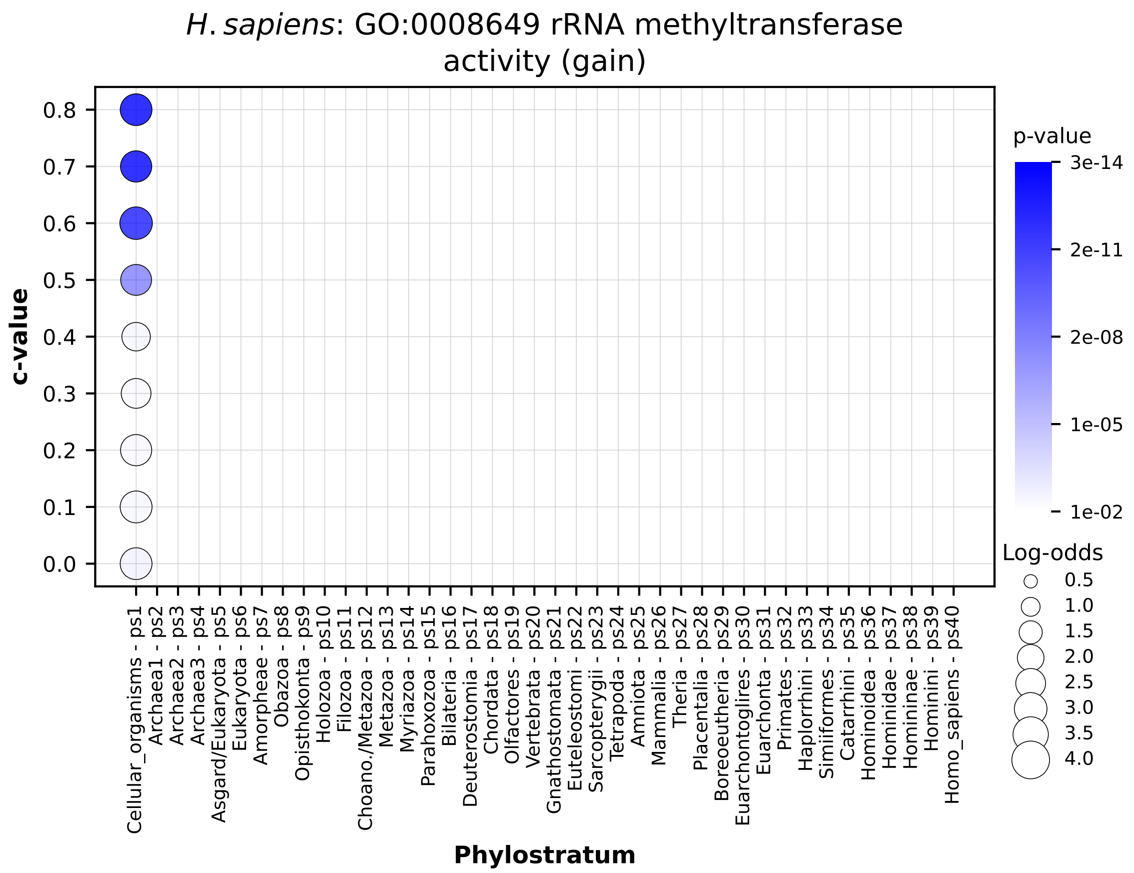
H. sapiens: GO:0008649 rRNA methyltransferase activity (gain)
c-value
0.8
0.7
0.6
0.5
0.4
0.3
0.2
0.1
0.0
Phylostratum
Cellular_organisms - ps1
Archaea1 - ps2
Archaea2 - ps3
Archaea3 - ps4
Asgard/Eukaryota - ps5
Eukaryota - ps6
Amorpheae - ps7
Obazoa - ps8
Opisthokonta - ps9
Holozoa - ps10
Filozoa - ps11
Choano./Metazoa - ps12
Metazoa - ps13
Myriazoa - ps14
Parahoxozoa - ps15
Bilateria - ps16
Deuterostomia - ps17
Chordata - ps18
Olfactores - ps19
Vertebrata - ps20
Gnathostomata - ps21
Euteleostomi - ps22
Sarcopterygii - ps23
Tetrapoda - ps24
Amniota - ps25
Mammalia - ps26
Theria - ps27
Placentalia - ps28
Boreoeutheria - ps29
Euarchontoglires - ps30
Euarchonta - ps31
Primates - ps32
Haplorrhini - ps33
Simiiformes - ps34
Catarrhini - ps35
Hominoidea - ps36
Hominidae - ps37
Homininae - ps38
Hominini - ps39
Homo_sapiens - ps40
p-value
3e-14
2e-11
2e-08
1e-05
1e-02
Log-odds
0.5
1.0
1.5
2.0
2.5
3.0
3.5
4.0

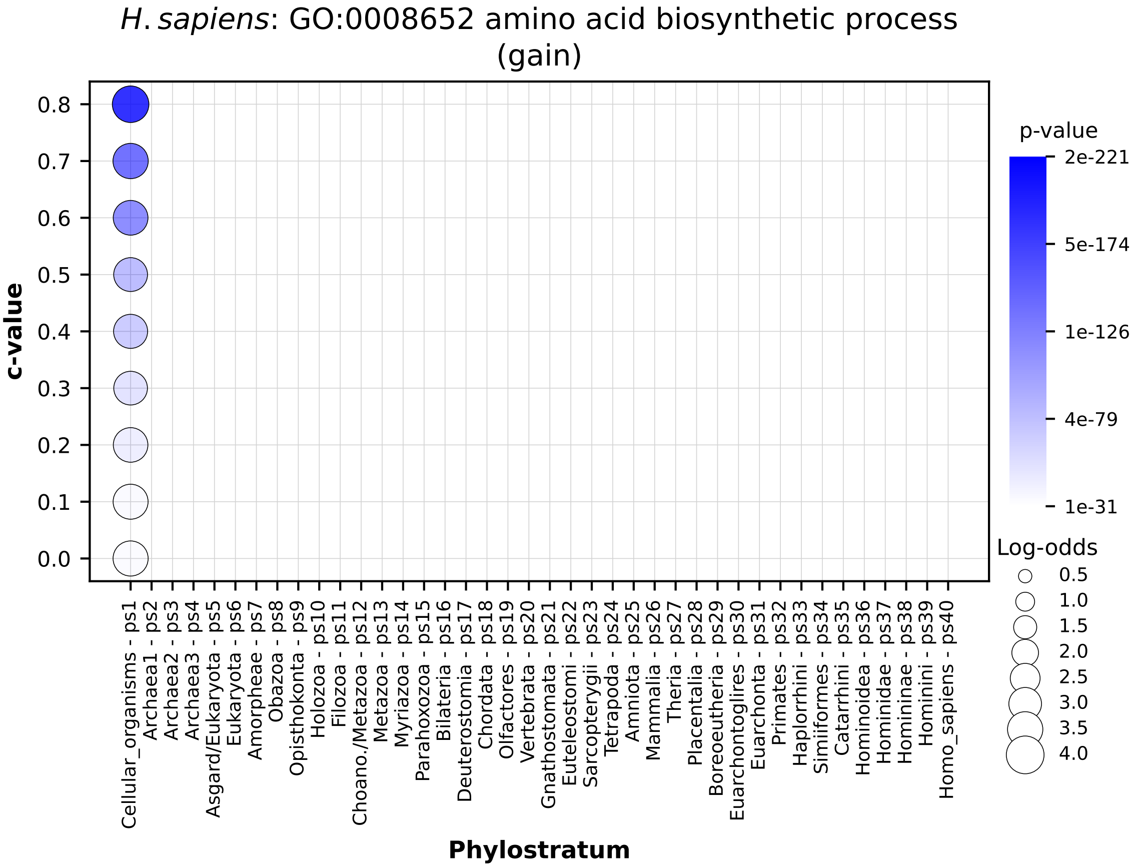

*H. sapiens*: GO:0008652 amino acid biosynthetic process (gain)
c-value
0.8
0.7
0.6
0.5
0.4
0.3
0.2
0.1
0.0
Phylostratum
Cellular_organisms - ps1
Archaea1 - ps2
Archaea2 - ps3
Archaea3 - ps4
Asgard/Eukaryota - ps5
Eukaryota - ps6
Amorpheae - ps7
Obazoa - ps8
Opisthokonta - ps9
Holozoa - ps10
Filozoa - ps11
Choano./Metazoa - ps12
Metazoa - ps13
Myriazoa - ps14
Parahoxozoa - ps15
Bilateria - ps16
Deuterostomia - ps17
Chordata - ps18
Olfactores - ps19
Vertebrata - ps20
Gnathostomata - ps21
Euteleostomi - ps22
Sarcopterygii - ps23
Tetrapoda - ps24
Amniota - ps25
Mammalia - ps26
Theria - ps27
Placentalia - ps28
Boreoeutheria - ps29
Euarchontoglires - ps30
Euarchonta - ps31
Primates - ps32
Haplorrhini - ps33
Simiiformes - ps34
Catarrhini - ps35
Hominoidea - ps36
Hominidae - ps37
Homininae - ps38
Hominini - ps39
Homo_sapiens - ps40
p-value
2e-221
5e-174
1e-126
4e-79
1e-31
Log-odds
0.5
1.0
1.5
2.0
2.5
3.0
3.5
4.0

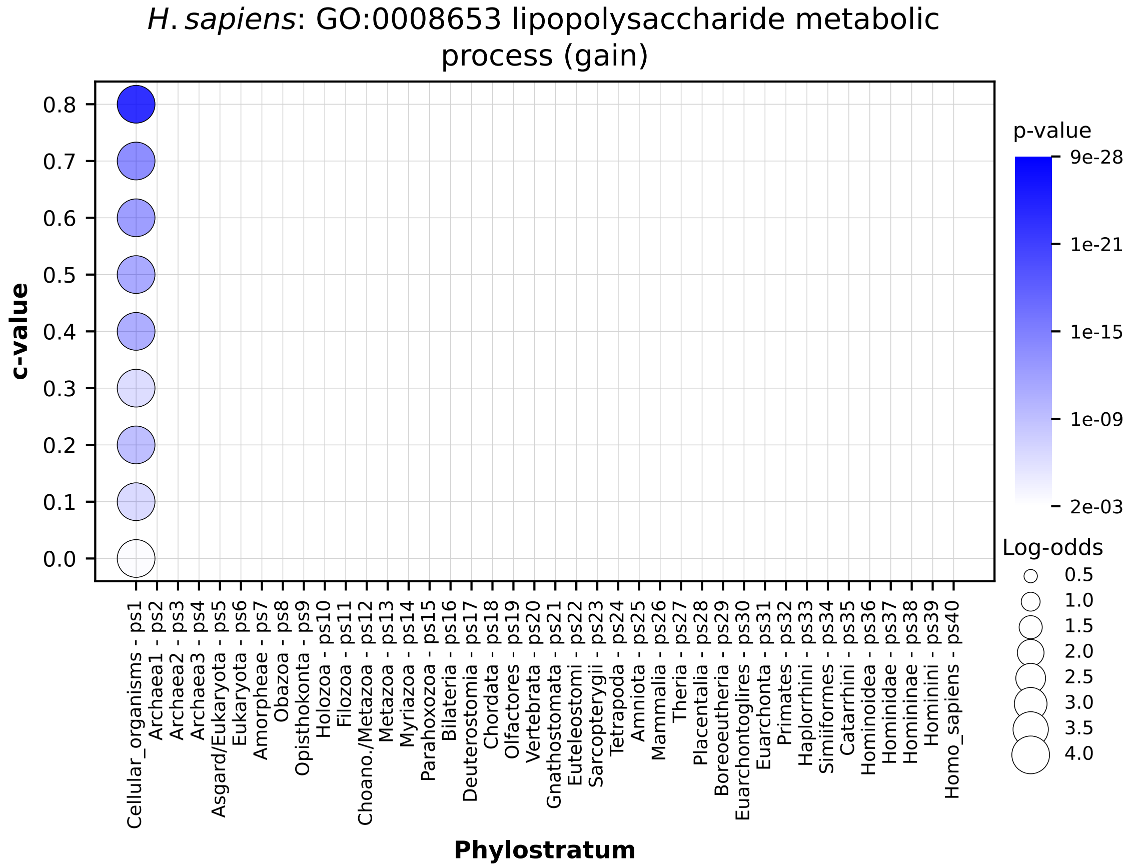

*H. sapiens*: GO:0008653 lipopolysaccharide metabolic process (gain)
c-value
0.8
0.7
0.6
0.5
0.4
0.3
0.2
0.1
0.0
p-value
9e-28
1e-21
1e-15
1e-09
2e-03
Log-odds
0.5
1.0
1.5
2.0
2.5
3.0
3.5
4.0
Cellular_organisms - ps1
Archaea1 - ps2
Archaea2 - ps3
Archaea3 - ps4
Asgard/Eukaryota - ps5
Eukaryota - ps6
Amorpheae - ps7
Obazoa - ps8
Opisthokonta - ps9
Holozoa - ps10
Filozoa - ps11
Choano./Metazoa - ps12
Metazoa - ps13
Myriazoa - ps14
Parahoxozoa - ps15
Bilateria - ps16
Deuterostomia - ps17
Chordata - ps18
Olfactores - ps19
Vertebrata - ps20
Gnathostomata - ps21
Euteleostomi - ps22
Sarcopterygii - ps23
Tetrapoda - ps24
Amniota - ps25
Mammalia - ps26
Theria - ps27
Placentalia - ps28
Boreoeutheria - ps29
Euarchontoglires - ps30
Euarchonta - ps31
Primates - ps32
Haplorrhini - ps33
Simiiformes - ps34
Catarrhini - ps35
Hominoidea - ps36
Hominidae - ps37
Homininae - ps38
Hominini - ps39
Homo_sapiens - ps40
Phylostratum

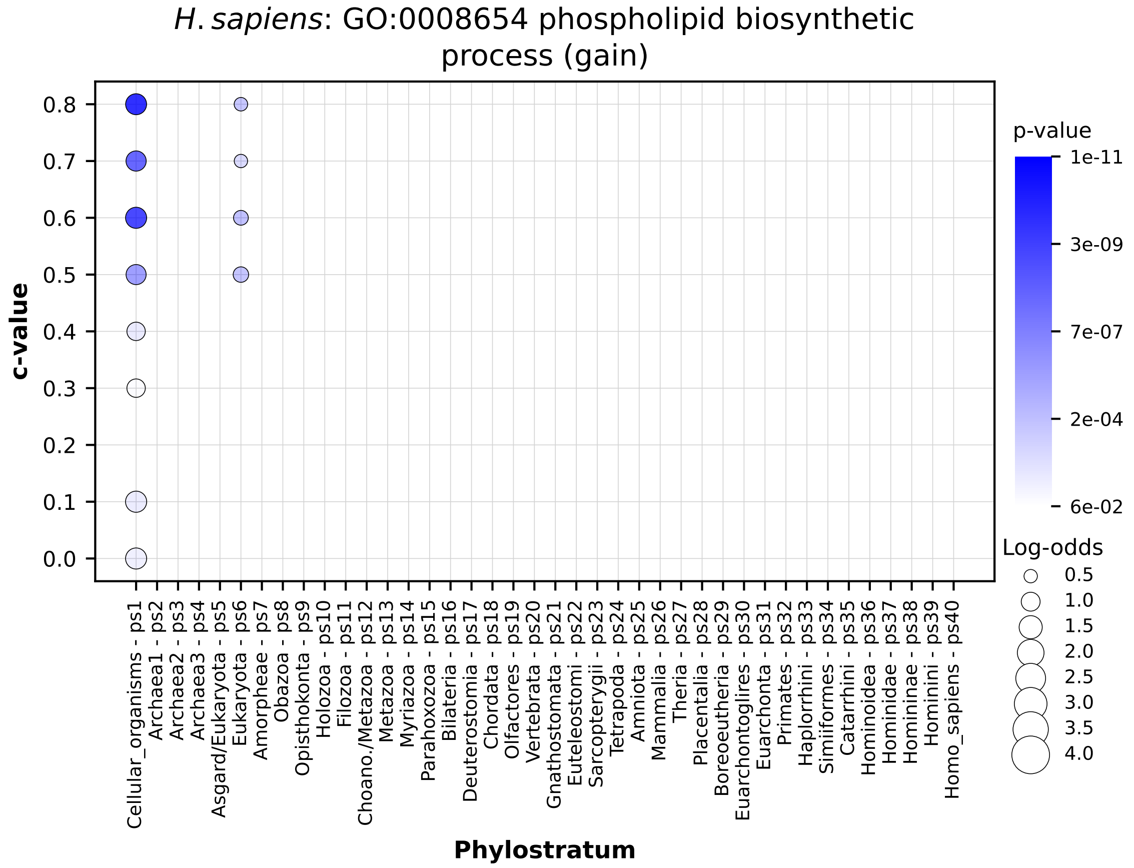

*H. sapiens*: GO:0008654 phospholipid biosynthetic process (gain)
c-value
0.8
0.7
0.6
0.5
0.4
0.3
0.2
0.1
0.0
Phylostratum
Cellular_organisms - ps1
Archaea1 - ps2
Archaea2 - ps3
Archaea3 - ps4
Asgard/Eukaryota - ps5
Eukaryota - ps6
Amorpheae - ps7
Obazoa - ps8
Opisthokonta - ps9
Holozoa - ps10
Filozoa - ps11
Choano./Metazoa - ps12
Metazoa - ps13
Myriazoa - ps14
Parahoxozoa - ps15
Bilateria - ps16
Deuterostomia - ps17
Chordata - ps18
Olfactores - ps19
Vertebrata - ps20
Gnathostomata - ps21
Euteleostomi - ps22
Sarcopterygii - ps23
Tetrapoda - ps24
Amniota - ps25
Mammalia - ps26
Theria - ps27
Placentalia - ps28
Boreoeutheria - ps29
Euarchontoglires - ps30
Euarchonta - ps31
Primates - ps32
Haplorrhini - ps33
Simiiformes - ps34
Catarrhini - ps35
Hominoidea - ps36
Hominidae - ps37
Homininae - ps38
Hominini - ps39
Homo_sapiens - ps40
p-value
1e-11
3e-09
7e-07
2e-04
6e-02
Log-odds
0.5
1.0
1.5
2.0
2.5
3.0
3.5
4.0

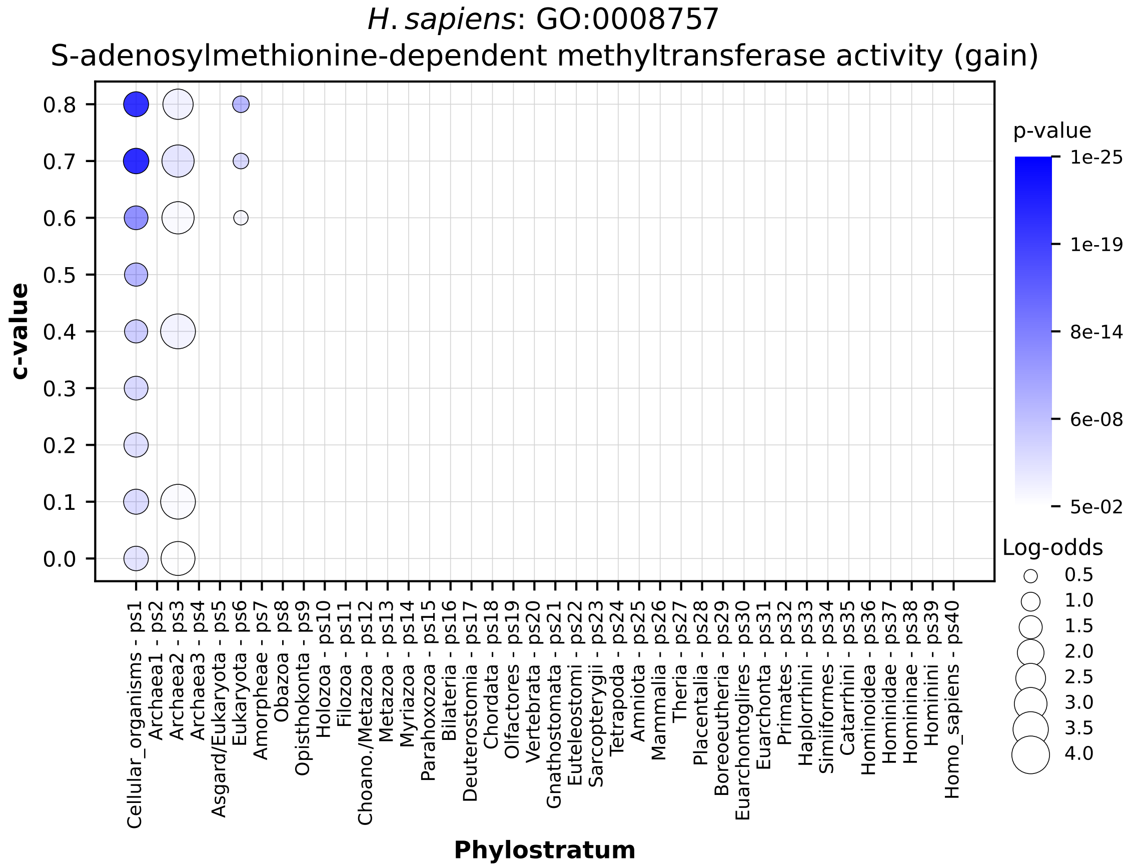

H. sapiens: GO:0008757
S-adenosylmethionine-dependent methyltransferase activity (gain)
c-value
0.8
0.7
0.6
0.5
0.4
0.3
0.2
0.1
0.0
p-value
1e-25
1e-19
8e-14
6e-08
5e-02
Log-odds
0.5
1.0
1.5
2.0
2.5
3.0
3.5
4.0
Cellular_organisms - ps1
Archaea1 - ps2
Archaea2 - ps3
Archaea3 - ps4
Asgard/Eukaryota - ps5
Eukaryota - ps6
Amorpheae - ps7
Obazoa - ps8
Opisthokonta - ps9
Holozoa - ps10
Filozoa - ps11
Choano./Metazoa - ps12
Metazoa - ps13
Myriazoa - ps14
Parahoxozoa - ps15
Bilateria - ps16
Deuterostomia - ps17
Chordata - ps18
Olfactores - ps19
Vertebrata - ps20
Gnathostomata - ps21
Euteleostomi - ps22
Sarcopterygii - ps23
Tetrapoda - ps24
Amniota - ps25
Mammalia - ps26
Theria - ps27
Placentalia - ps28
Boreoeutheria - ps29
Euarchontoglires - ps30
Euarchonta - ps31
Primates - ps32
Haplorrhini - ps33
Simiiformes - ps34
Catarrhini - ps35
Hominoidea - ps36
Hominidae - ps37
Homininae - ps38
Hominini - ps39
Homo_sapiens - ps40
Phylostratum

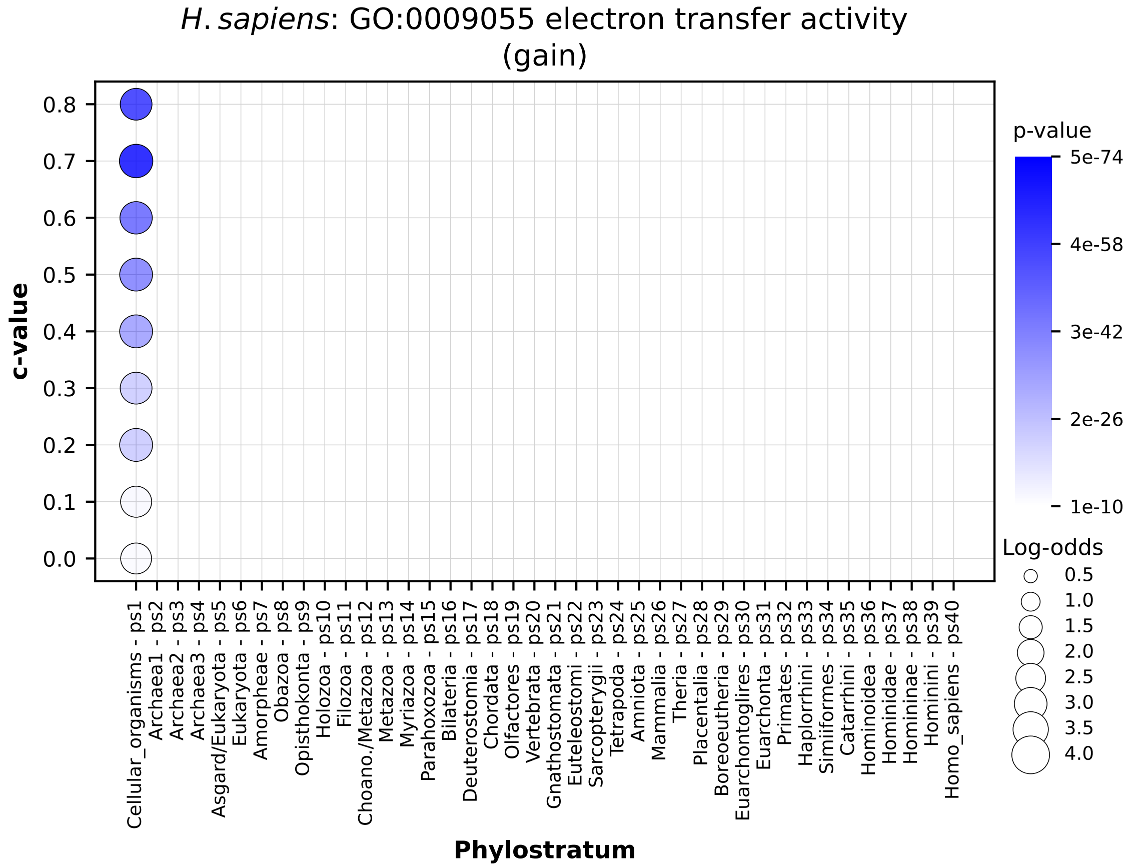

H. sapiens: GO:0009055 electron transfer activity
(gain)
c-value
0.0
0.1
0.2
0.3
0.4
0.5
0.6
0.7
0.8
p-value
5e-74
4e-58
3e-42
2e-26
1e-10
Log-odds
0.5
1.0
1.5
2.0
2.5
3.0
3.5
4.0
Cellular_organisms - ps1
Archaea1 - ps2
Archaea2 - ps3
Archaea3 - ps4
Asgard/Eukaryota - ps5
Eukaryota - ps6
Amorpheae - ps7
Obazoa - ps8
Opisthokonta - ps9
Holozoa - ps10
Filozoa - ps11
Choano./Metazoa - ps12
Metazoa - ps13
Myriazoa - ps14
Parahoxozoa - ps15
Bilateria - ps16
Deuterostomia - ps17
Chordata - ps18
Olfactores - ps19
Vertebrata - ps20
Gnathostomata - ps21
Euteleostomi - ps22
Sarcopterygii - ps23
Tetrapoda - ps24
Amniota - ps25
Mammalia - ps26
Theria - ps27
Placentalia - ps28
Boreoeutheria - ps29
Euarchontoglires - ps30
Euarchonta - ps31
Primates - ps32
Haplorrhini - ps33
Simiiformes - ps34
Catarrhini - ps35
Hominoidea - ps36
Hominidae - ps37
Homininae - ps38
Hominini - ps39
Homo_sapiens - ps40
Phylostratum

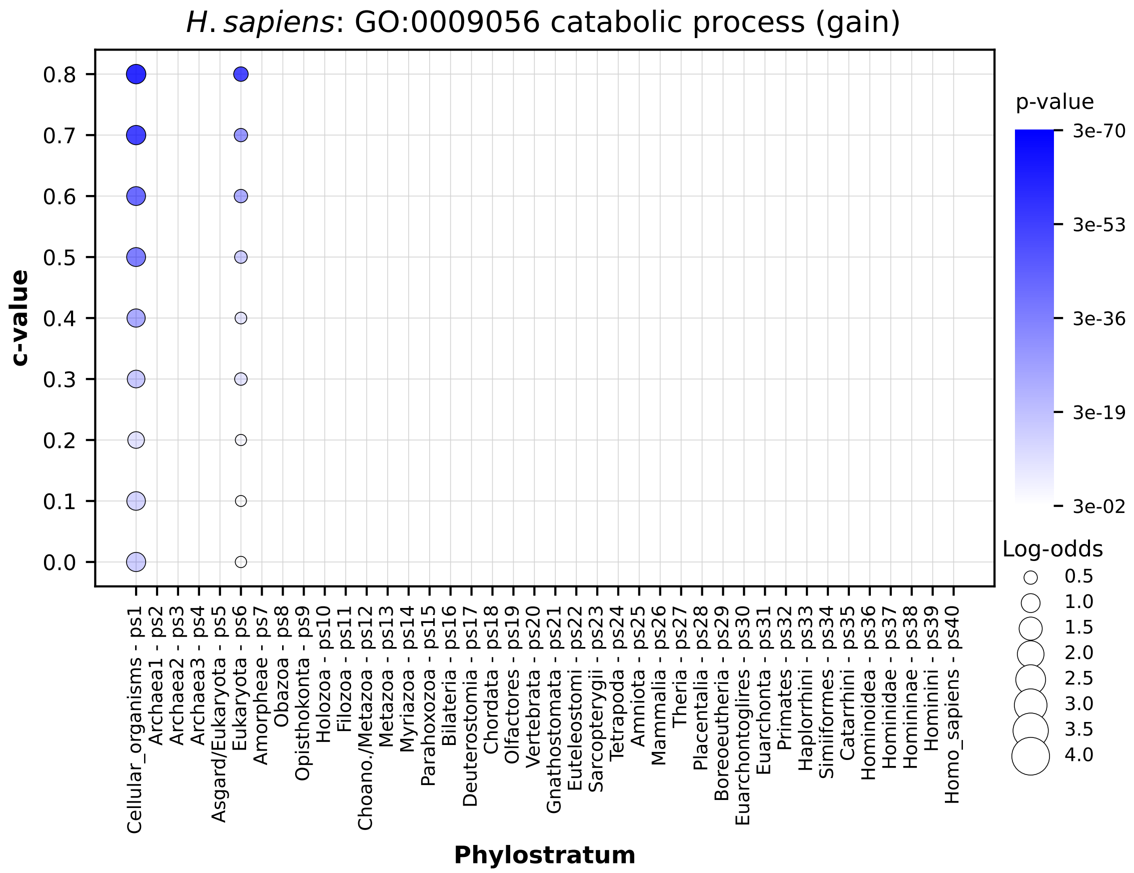

H. sapiens: GO:0009056 catabolic process (gain)
c-value
0.8
0.7
0.6
0.5
0.4
0.3
0.2
0.1
0.0
Phylostratum
Cellular_organisms - ps1
Archaea1 - ps2
Archaea2 - ps3
Archaea3 - ps4
Asgard/Eukaryota - ps5
Eukaryota - ps6
Amorpheae - ps7
Obazoa - ps8
Opisthokonta - ps9
Holozoa - ps10
Filozoa - ps11
Choano./Metazoa - ps12
Metazoa - ps13
Myriazoa - ps14
Parahoxozoa - ps15
Bilateria - ps16
Deuterostomia - ps17
Chordata - ps18
Olfactores - ps19
Vertebrata - ps20
Gnathostomata - ps21
Euteleostomi - ps22
Sarcopterygii - ps23
Tetrapoda - ps24
Amniota - ps25
Mammalia - ps26
Theria - ps27
Placentalia - ps28
Boreoeutheria - ps29
Euarchontoglires - ps30
Euarchonta - ps31
Primates - ps32
Haplorrhini - ps33
Simiiformes - ps34
Catarrhini - ps35
Hominoidea - ps36
Hominidae - ps37
Homininae - ps38
Hominini - ps39
Homo_sapiens - ps40
p-value
3e-70
3e-53
3e-36
3e-19
3e-02
Log-odds
0.5
1.0
1.5
2.0
2.5
3.0
3.5
4.0

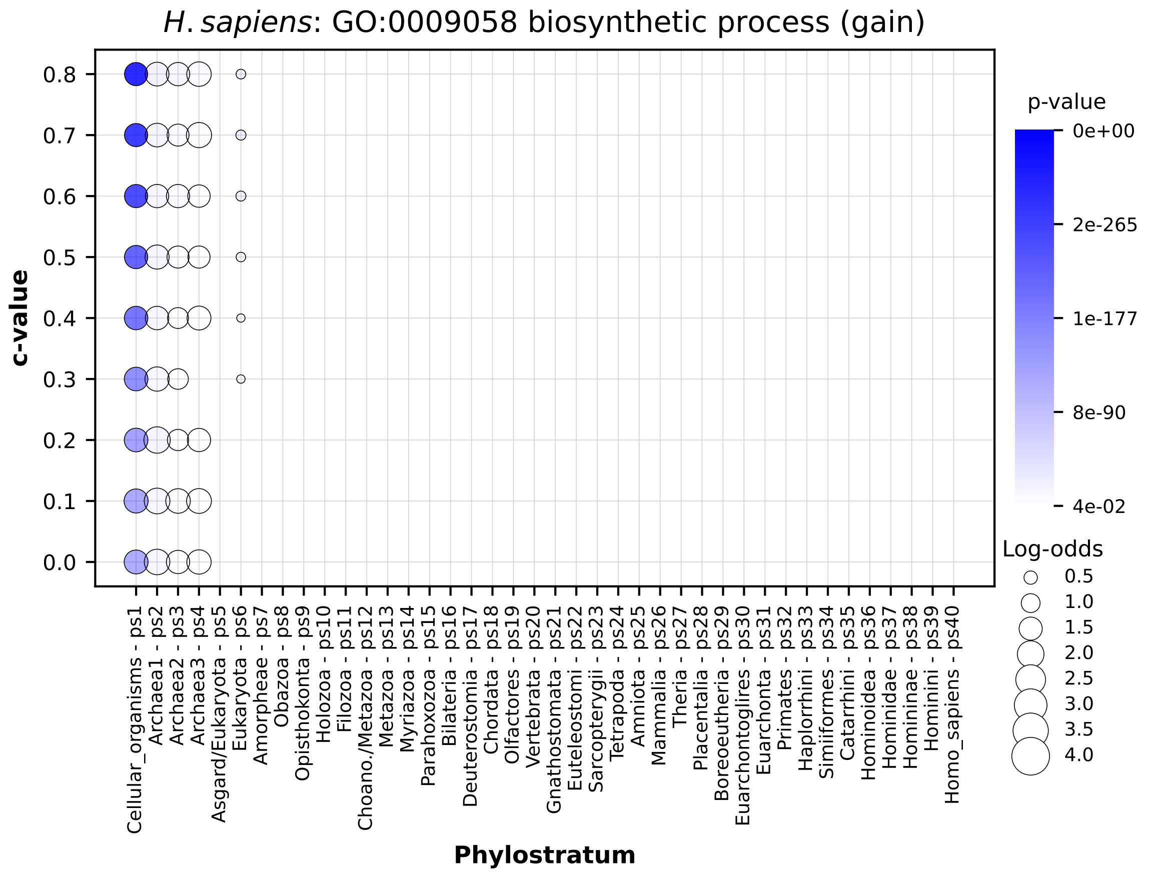

H. sapiens: GO:0009058 biosynthetic process (gain)
c-value
0.8
0.7
0.6
0.5
0.4
0.3
0.2
0.1
0.0
p-value
0e+00
2e-265
1e-177
8e-90
4e-02
Log-odds
0.5
1.0
1.5
2.0
2.5
3.0
3.5
4.0
Cellular_organisms - ps1
Archaea1 - ps2
Archaea2 - ps3
Archaea3 - ps4
Asgard/Eukaryota - ps5
Eukaryota - ps6
Amorpheae - ps7
Obazoa - ps8
Opisthokonta - ps9
Holozoa - ps10
Filozoa - ps11
Choano./Metazoa - ps12
Metazoa - ps13
Myriazoa - ps14
Parahoxozoa - ps15
Bilateria - ps16
Deuterostomia - ps17
Chordata - ps18
Olfactores - ps19
Vertebrata - ps20
Gnathostomata - ps21
Euteleostomi - ps22
Sarcopterygii - ps23
Tetrapoda - ps24
Amniota - ps25
Mammalia - ps26
Theria - ps27
Placentalia - ps28
Boreoeutheria - ps29
Euarchontoglires - ps30
Euarchonta - ps31
Primates - ps32
Haplorrhini - ps33
Simiiformes - ps34
Catarrhini - ps35
Hominoidea - ps36
Hominidae - ps37
Homininae - ps38
Hominini - ps39
Homo_sapiens - ps40
Phylostratum

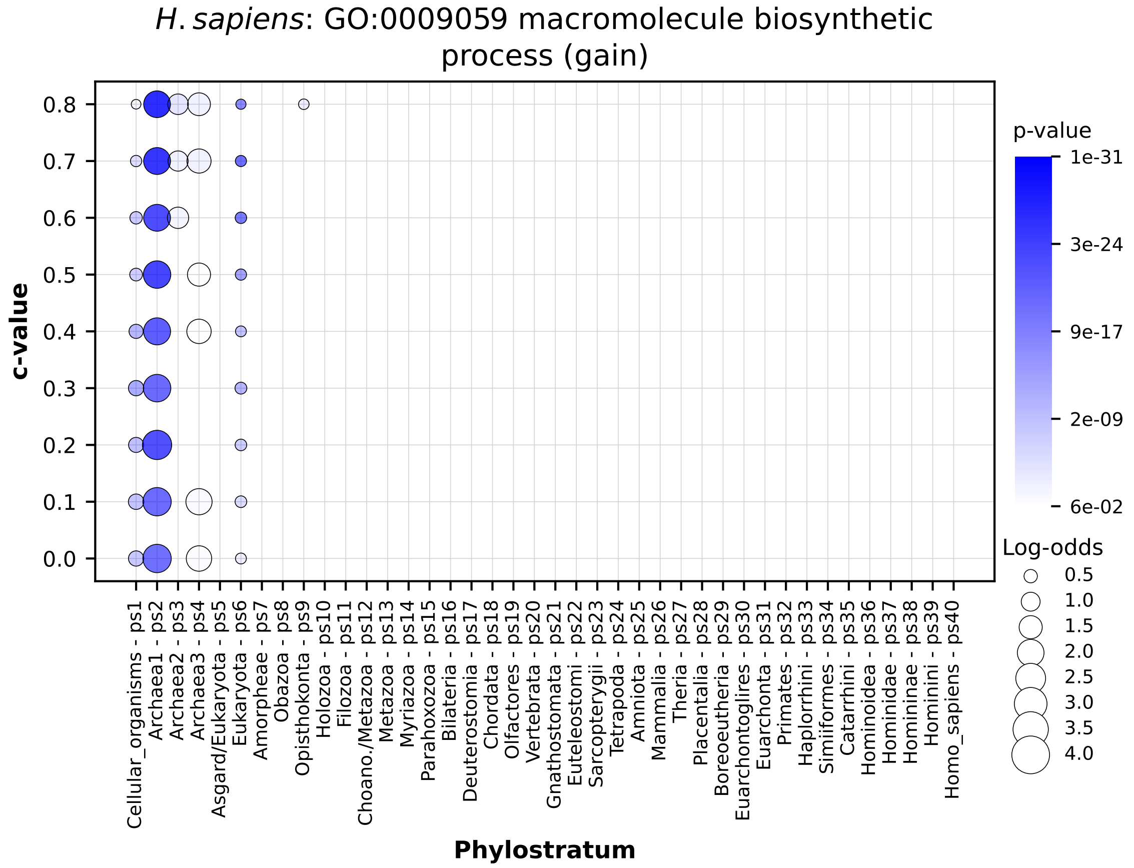

H. sapiens: GO:0009059 macromolecule biosynthetic process (gain)
c-value
0.8
0.7
0.6
0.5
0.4
0.3
0.2
0.1
0.0
Phylostratum
Cellular_organisms - ps1
Archaea1 - ps2
Archaea2 - ps3
Archaea3 - ps4
Asgard/Eukaryota - ps5
Eukaryota - ps6
Amorpheae - ps7
Obazoa - ps8
Opisthokonta - ps9
Holozoa - ps10
Filozoa - ps11
Choano./Metazoa - ps12
Metazoa - ps13
Myriazoa - ps14
Parahoxozoa - ps15
Bilateria - ps16
Deuterostomia - ps17
Chordata - ps18
Olfactores - ps19
Vertebrata - ps20
Gnathostomata - ps21
Euteleostomi - ps22
Sarcopterygii - ps23
Tetrapoda - ps24
Amniota - ps25
Mammalia - ps26
Theria - ps27
Placentalia - ps28
Boreoeutheria - ps29
Euarchontoglires - ps30
Euarchonta - ps31
Primates - ps32
Haplorrhini - ps33
Simiiformes - ps34
Catarrhini - ps35
Hominoidea - ps36
Hominidae - ps37
Homininae - ps38
Hominini - ps39
Homo_sapiens - ps40
p-value
1e-31
3e-24
9e-17
2e-09
6e-02
Log-odds
0.5
1.0
1.5
2.0
2.5
3.0
3.5
4.0

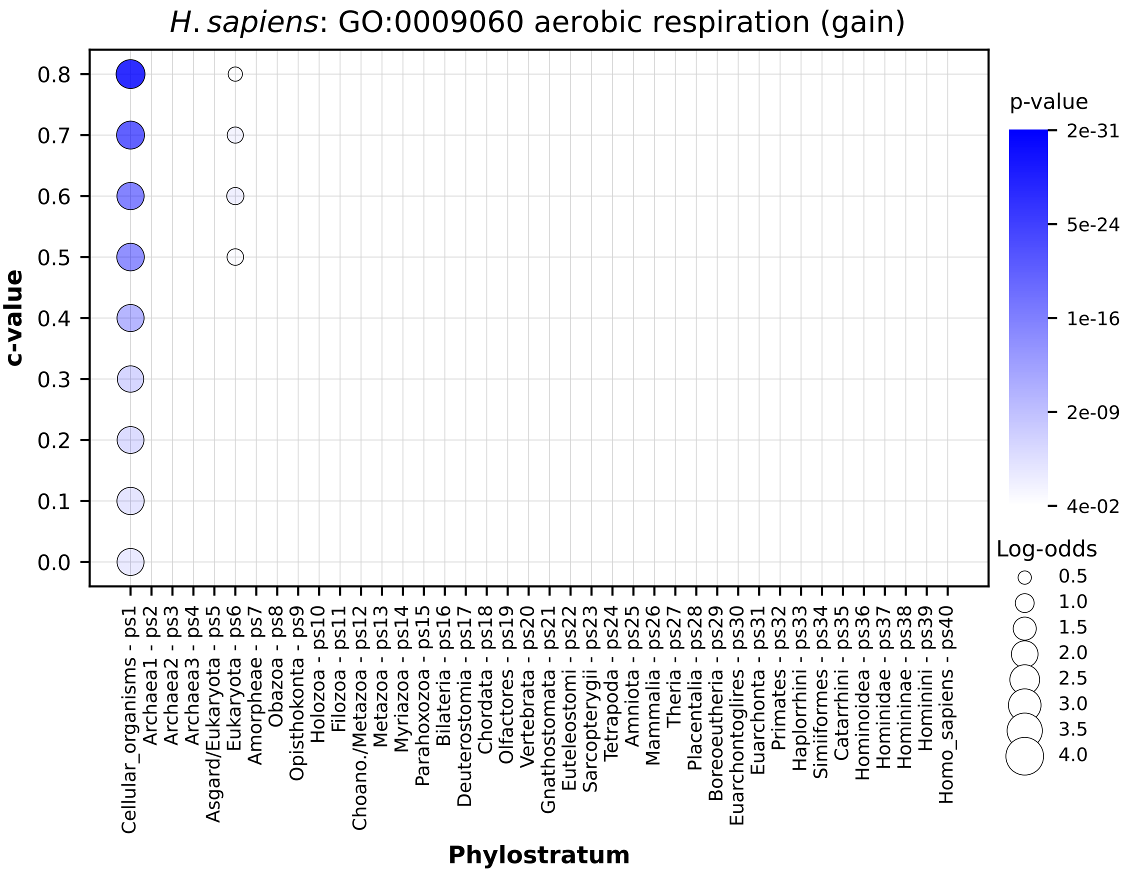

H. sapiens: GO:0009060 aerobic respiration (gain)
c-value
0.8
0.7
0.6
0.5
0.4
0.3
0.2
0.1
0.0
p-value
2e-31
5e-24
1e-16
2e-09
4e-02
Log-odds
0.5
1.0
1.5
2.0
2.5
3.0
3.5
4.0
Cellular_organisms - ps1
Archaea1 - ps2
Archaea2 - ps3
Archaea3 - ps4
Asgard/Eukaryota - ps5
Eukaryota - ps6
Amorpheae - ps7
Obazoa - ps8
Opisthokonta - ps9
Holozoa - ps10
Filozoa - ps11
Choano./Metazoa - ps12
Metazoa - ps13
Myriazoa - ps14
Parahoxozoa - ps15
Bilateria - ps16
Deuterostomia - ps17
Chordata - ps18
Olfactores - ps19
Vertebrata - ps20
Gnathostomata - ps21
Euteleostomi - ps22
Sarcopterygii - ps23
Tetrapoda - ps24
Amniota - ps25
Mammalia - ps26
Theria - ps27
Placentalia - ps28
Boreoeutheria - ps29
Euarchontoglires - ps30
Euarchonta - ps31
Primates - ps32
Haplorrhini - ps33
Simiiformes - ps34
Catarrhini - ps35
Hominoidea - ps36
Hominidae - ps37
Homininae - ps38
Hominini - ps39
Homo_sapiens - ps40
Phylostratum

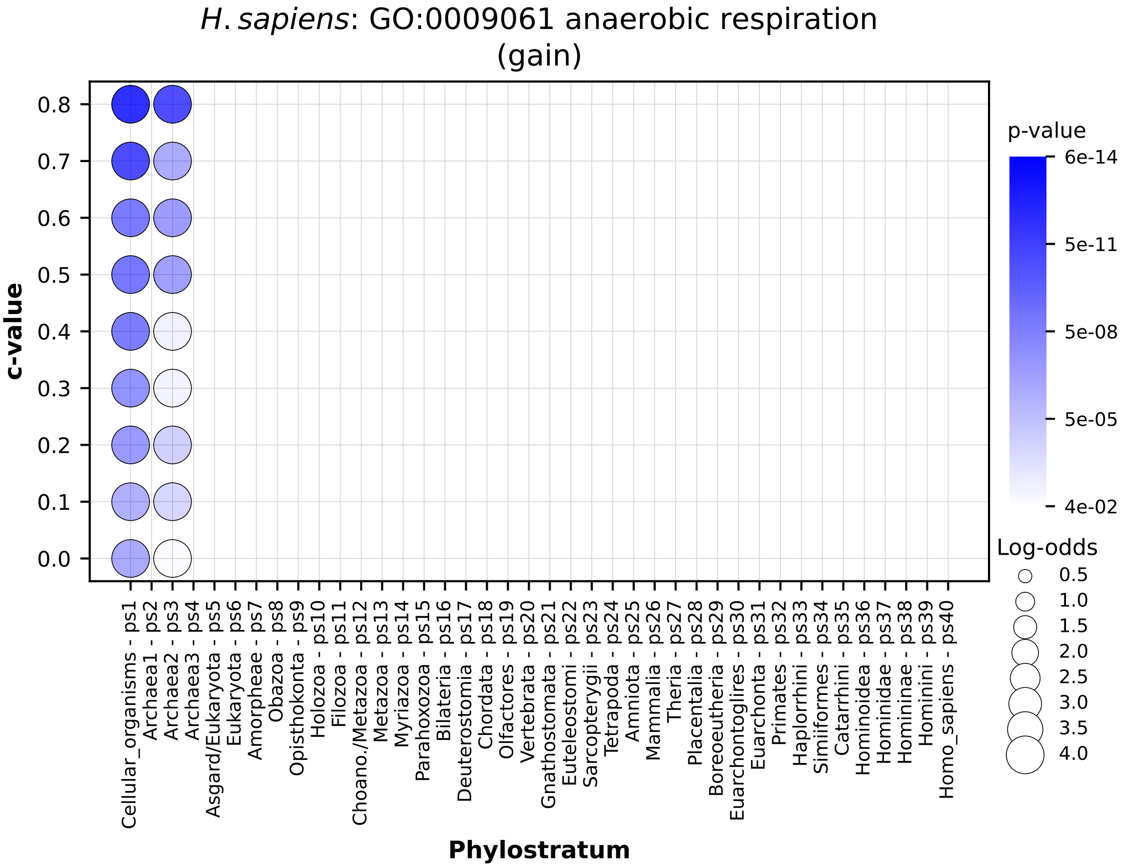

H. sapiens: GO:0009061 anaerobic respiration
(gain)
c-value
0.8
0.7
0.6
0.5
0.4
0.3
0.2
0.1
0.0
p-value
6e-14
5e-11
5e-08
5e-05
4e-02
Log-odds
0.5
1.0
1.5
2.0
2.5
3.0
3.5
4.0
Cellular_organisms - ps1
Archaea1 - ps2
Archaea2 - ps3
Archaea3 - ps4
Asgard/Eukaryota - ps5
Eukaryota - ps6
Amorpheae - ps7
Obazoa - ps8
Opisthokonta - ps9
Holozoa - ps10
Filozoa - ps11
Choano./Metazoa - ps12
Metazoa - ps13
Myriazoa - ps14
Parahoxozoa - ps15
Bilateria - ps16
Deuterostomia - ps17
Chordata - ps18
Olfactores - ps19
Vertebrata - ps20
Gnathostomata - ps21
Euteleostomi - ps22
Sarcopterygii - ps23
Tetrapoda - ps24
Amniota - ps25
Mammalia - ps26
Theria - ps27
Placentalia - ps28
Boreoeutheria - ps29
Euarchontoglires - ps30
Euarchonta - ps31
Primates - ps32
Haplorrhini - ps33
Simiiformes - ps34
Catarrhini - ps35
Hominoidea - ps36
Hominidae - ps37
Homininae - ps38
Hominini - ps39
Homo_sapiens - ps40
Phylostratum

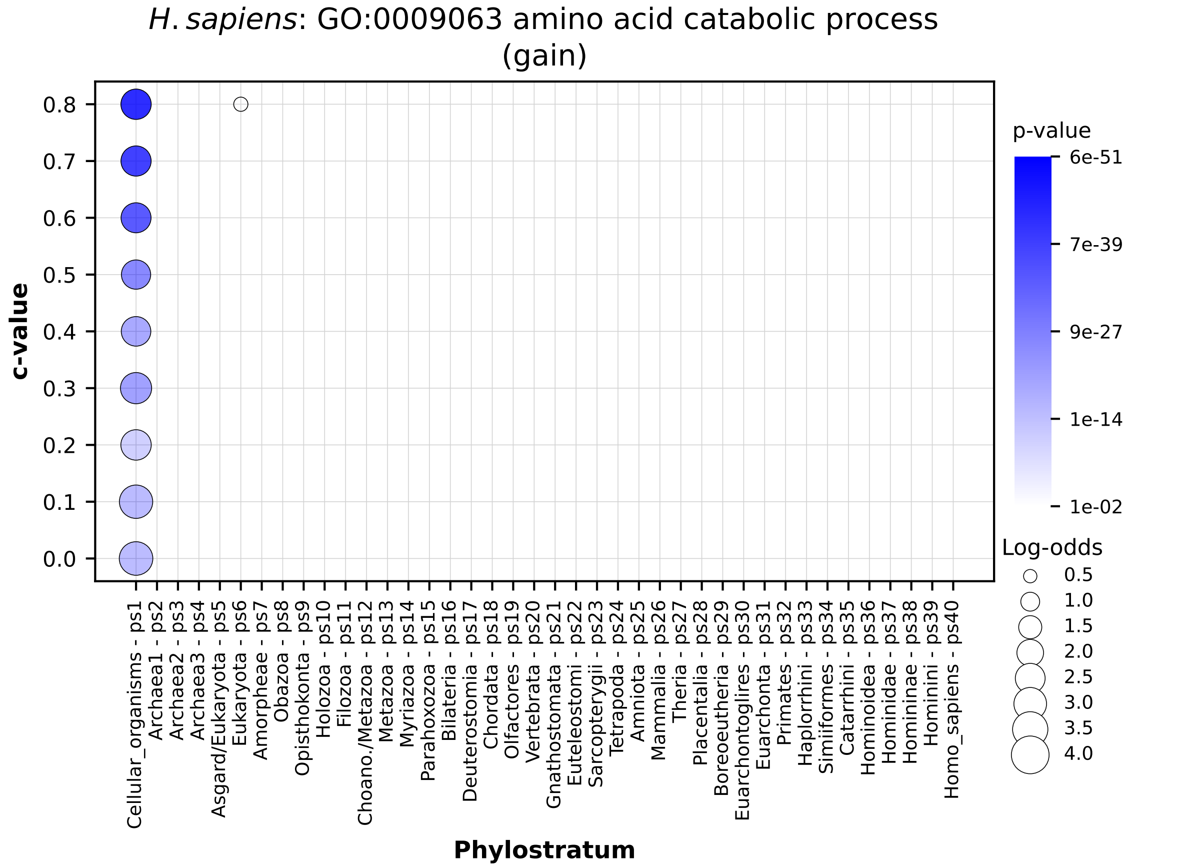

H. sapiens: GO:0009063 amino acid catabolic process
(gain)
c-value
0.8
0.7
0.6
0.5
0.4
0.3
0.2
0.1
0.0
p-value
6e-51
7e-39
9e-27
1e-14
1e-02
Log-odds
0.5
1.0
1.5
2.0
2.5
3.0
3.5
4.0
Cellular_organisms - ps1
Archaea1 - ps2
Archaea2 - ps3
Archaea3 - ps4
Asgard/Eukaryota - ps5
Eukaryota - ps6
Amorpheae - ps7
Obazoa - ps8
Opisthokonta - ps9
Holozoa - ps10
Filozoa - ps11
Choano./Metazoa - ps12
Metazoa - ps13
Myriazoa - ps14
Parahoxozoa - ps15
Bilateria - ps16
Deuterostomia - ps17
Chordata - ps18
Olfactores - ps19
Vertebrata - ps20
Gnathostomata - ps21
Euteleostomi - ps22
Sarcopterygii - ps23
Tetrapoda - ps24
Amniota - ps25
Mammalia - ps26
Theria - ps27
Placentalia - ps28
Boreoeutheria - ps29
Euarchontoglires - ps30
Euarchonta - ps31
Primates - ps32
Haplorrhini - ps33
Simiiformes - ps34
Catarrhini - ps35
Hominoidea - ps36
Hominidae - ps37
Homininae - ps38
Hominini - ps39
Homo_sapiens - ps40
Phylostratum

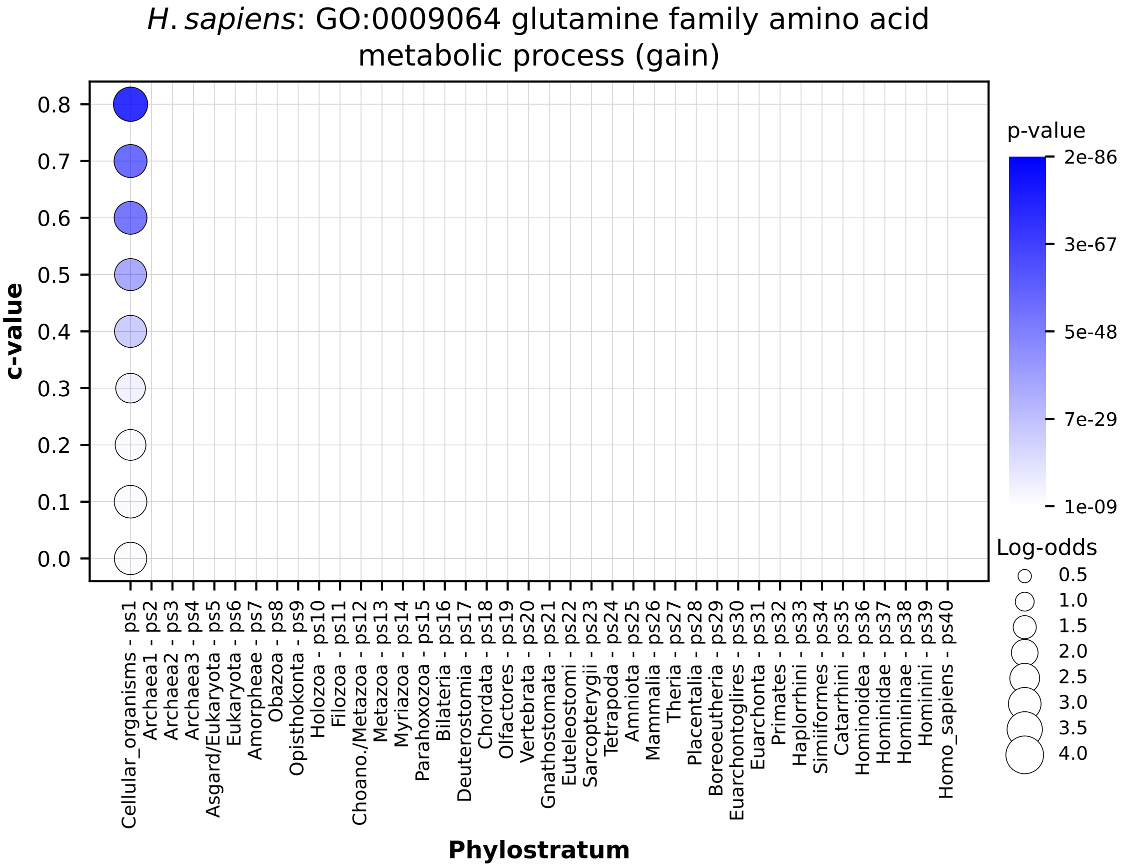
H. sapiens: GO:0009064 glutamine family amino acid metabolic process (gain)
c-value
0.0
0.1
0.2
0.3
0.4
0.5
0.6
0.7
0.8
p-value
2e-86
3e-67
5e-48
7e-29
1e-09
Log-odds
0.5
1.0
1.5
2.0
2.5
3.0
3.5
4.0
Cellular_organisms - ps1
Archaea1 - ps2
Archaea2 - ps3
Archaea3 - ps4
Asgard/Eukaryota - ps5
Eukaryota - ps6
Amorpheae - ps7
Obazoa - ps8
Opisthokonta - ps9
Holozoa - ps10
Filozoa - ps11
Choano./Metazoa - ps12
Metazoa - ps13
Myriazoa - ps14
Parahoxozoa - ps15
Bilateria - ps16
Deuterostomia - ps17
Chordata - ps18
Olfactores - ps19
Vertebrata - ps20
Gnathostomata - ps21
Euteleostomi - ps22
Sarcopterygii - ps23
Tetrapoda - ps24
Amniota - ps25
Mammalia - ps26
Theria - ps27
Placentalia - ps28
Boreoeutheria - ps29
Euarchontoglires - ps30
Euarchonta - ps31
Primates - ps32
Haplorrhini - ps33
Simiiformes - ps34
Catarrhini - ps35
Hominoidea - ps36
Hominidae - ps37
Homininae - ps38
Hominini - ps39
Homo_sapiens - ps40
Phylostratum

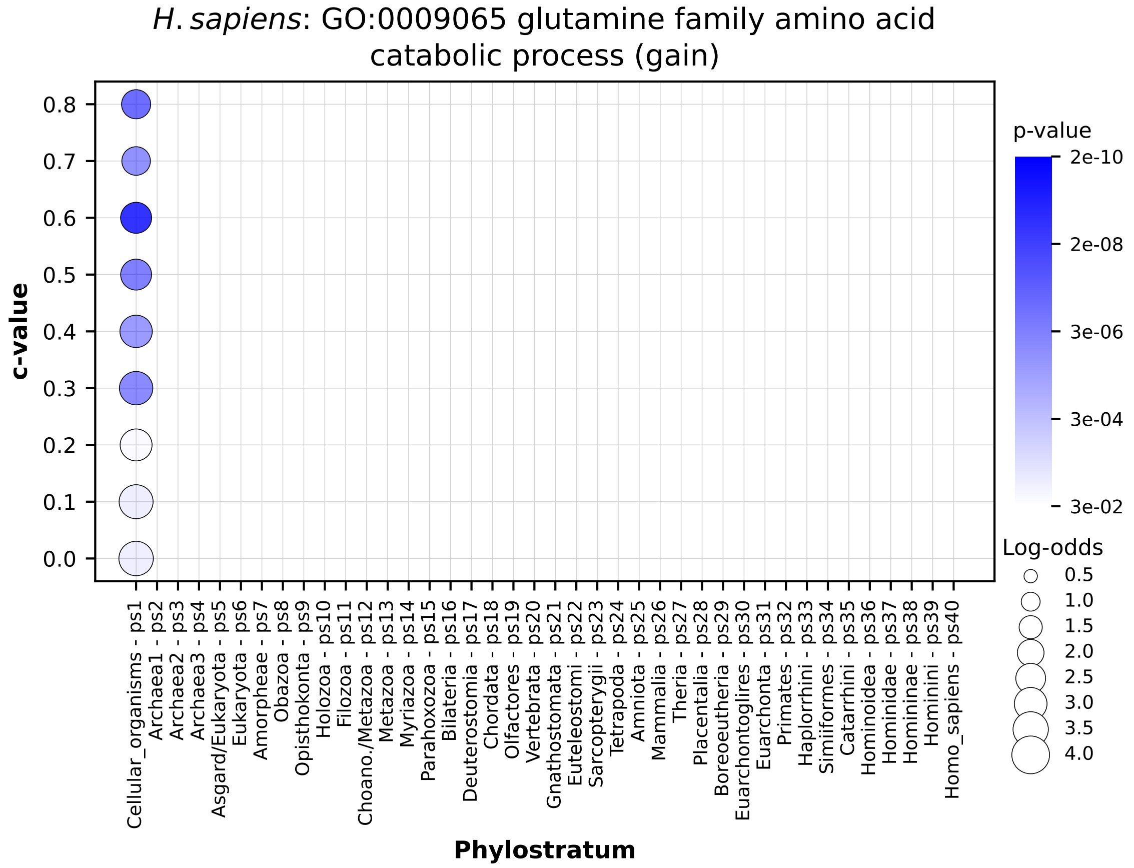
H. sapiens: GO:0009065 glutamine family amino acid catabolic process (gain)
c-value
0.0
0.1
0.2
0.3
0.4
0.5
0.6
0.7
0.8
p-value
2e-10
2e-08
3e-06
3e-04
3e-02
Log-odds
0.5
1.0
1.5
2.0
2.5
3.0
3.5
4.0
Cellular_organisms - ps1
Archaea1 - ps2
Archaea2 - ps3
Archaea3 - ps4
Asgard/Eukaryota - ps5
Eukaryota - ps6
Amorpheae - ps7
Obazoa - ps8
Opisthokonta - ps9
Holozoa - ps10
Filozoa - ps11
Choano./Metazoa - ps12
Metazoa - ps13
Myriazoa - ps14
Parahoxozoa - ps15
Bilateria - ps16
Deuterostomia - ps17
Chordata - ps18
Olfactores - ps19
Vertebrata - ps20
Gnathostomata - ps21
Euteleostomi - ps22
Sarcopterygii - ps23
Tetrapoda - ps24
Amniota - ps25
Mammalia - ps26
Theria - ps27
Placentalia - ps28
Boreoeutheria - ps29
Euarchontoglires - ps30
Euarchonta - ps31
Primates - ps32
Haplorrhini - ps33
Simiiformes - ps34
Catarrhini - ps35
Hominoidea - ps36
Hominidae - ps37
Homininae - ps38
Hominini - ps39
Homo_sapiens - ps40
Phylostratum

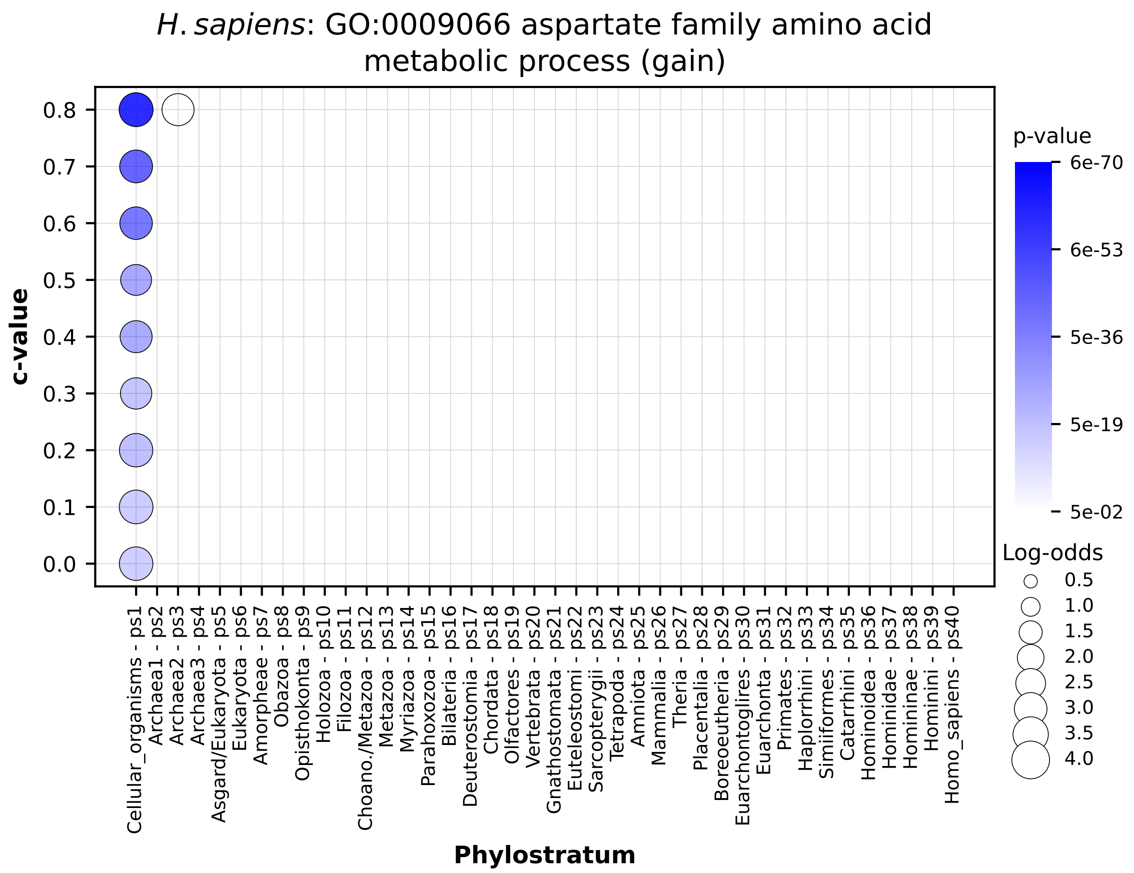

*H. sapiens*: GO:0009066 aspartate family amino acid metabolic process (gain)
c-value
0.8
0.7
0.6
0.5
0.4
0.3
0.2
0.1
0.0
p-value
6e-70
6e-53
5e-36
5e-19
5e-02
Log-odds
0.5
1.0
1.5
2.0
2.5
3.0
3.5
4.0
Cellular_organisms - ps1
Archaea1 - ps2
Archaea2 - ps3
Archaea3 - ps4
Asgard/Eukaryota - ps5
Eukaryota - ps6
Amorpheae - ps7
Obazoa - ps8
Opisthokonta - ps9
Holozoa - ps10
Filozoa - ps11
Choano./Metazoa - ps12
Metazoa - ps13
Myriazoa - ps14
Parahoxozoa - ps15
Bilateria - ps16
Deuterostomia - ps17
Chordata - ps18
Olfactores - ps19
Vertebrata - ps20
Gnathostomata - ps21
Euteleostomi - ps22
Sarcopterygii - ps23
Tetrapoda - ps24
Amniota - ps25
Mammalia - ps26
Theria - ps27
Placentalia - ps28
Boreoeutheria - ps29
Euarchontoglires - ps30
Euarchonta - ps31
Primates - ps32
Haplorrhini - ps33
Simiiformes - ps34
Catarrhini - ps35
Hominoidea - ps36
Hominidae - ps37
Homininae - ps38
Hominini - ps39
Homo_sapiens - ps40
Phylostratum

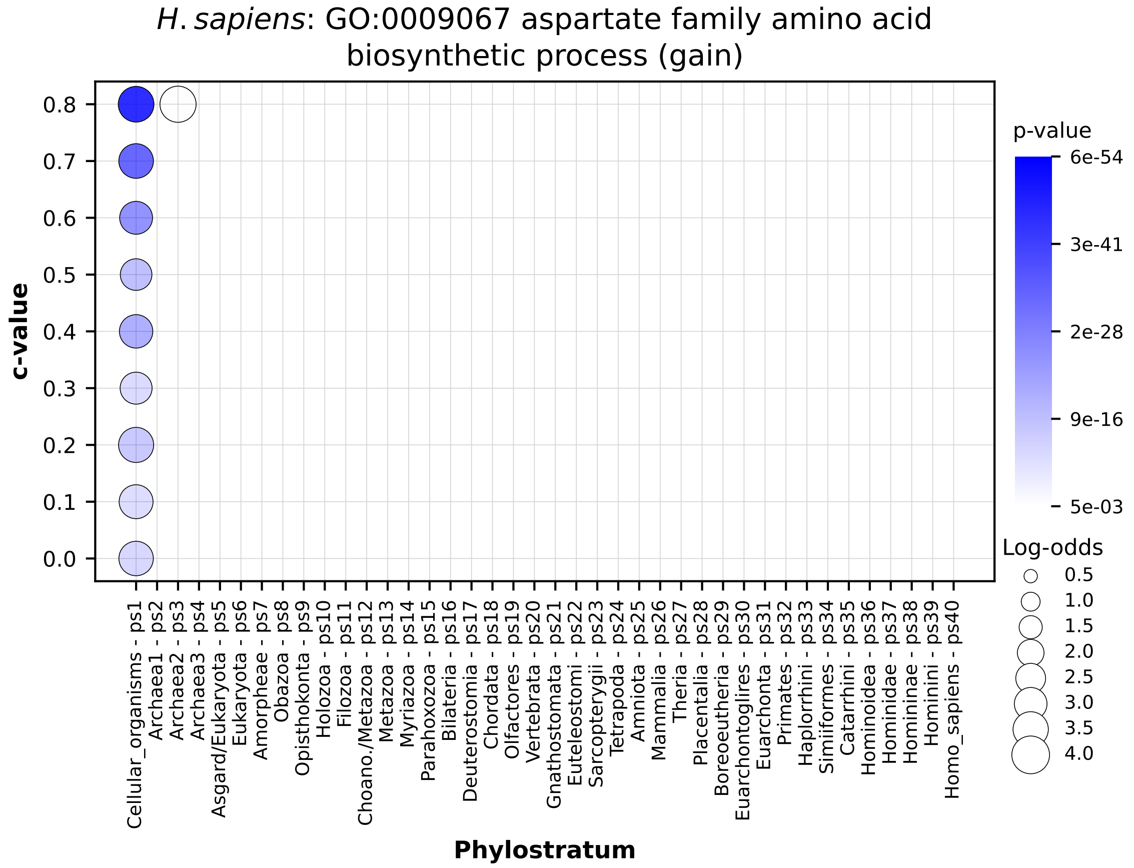
H. sapiens: GO:0009067 aspartate family amino acid biosynthetic process (gain)
c-value
0.8
0.7
0.6
0.5
0.4
0.3
0.2
0.1
0.0
p-value
6e-54
3e-41
2e-28
9e-16
5e-03
Log-odds
0.5
1.0
1.5
2.0
2.5
3.0
3.5
4.0
Cellular_organisms - ps1
Archaea1 - ps2
Archaea2 - ps3
Archaea3 - ps4
Asgard/Eukaryota - ps5
Eukaryota - ps6
Amorpheae - ps7
Obazoa - ps8
Opisthokonta - ps9
Holozoa - ps10
Filozoa - ps11
Choano./Metazoa - ps12
Metazoa - ps13
Myriazoa - ps14
Parahoxozoa - ps15
Bilateria - ps16
Deuterostomia - ps17
Chordata - ps18
Olfactores - ps19
Vertebrata - ps20
Gnathostomata - ps21
Euteleostomi - ps22
Sarcopterygii - ps23
Tetrapoda - ps24
Amniota - ps25
Mammalia - ps26
Theria - ps27
Placentalia - ps28
Boreoeutheria - ps29
Euarchontoglires - ps30
Euarchonta - ps31
Primates - ps32
Haplorrhini - ps33
Simiiformes - ps34
Catarrhini - ps35
Hominoidea - ps36
Hominidae - ps37
Homininae - ps38
Hominini - ps39
Homo_sapiens - ps40
Phylostratum

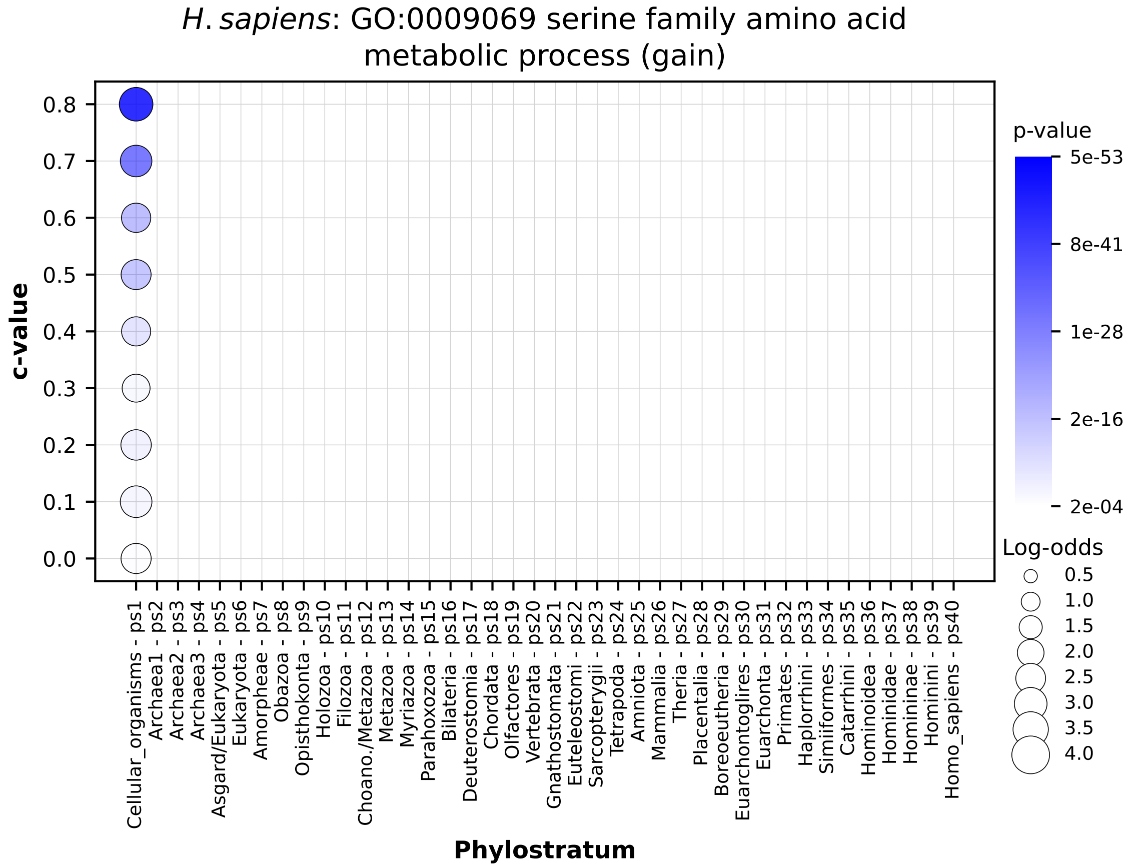

H. sapiens: GO:0009069 serine family amino acid metabolic process (gain)
c-value
0.8
0.7
0.6
0.5
0.4
0.3
0.2
0.1
0.0
Phylostratum
Cellular_organisms - ps1
Archaea1 - ps2
Archaea2 - ps3
Archaea3 - ps4
Asgard/Eukaryota - ps5
Eukaryota - ps6
Amorpheae - ps7
Obazoa - ps8
Opisthokonta - ps9
Holozoa - ps10
Filozoa - ps11
Choano./Metazoa - ps12
Metazoa - ps13
Myriazoa - ps14
Parahoxozoa - ps15
Bilateria - ps16
Deuterostomia - ps17
Chordata - ps18
Olfactores - ps19
Vertebrata - ps20
Gnathostomata - ps21
Euteleostomi - ps22
Sarcopterygii - ps23
Tetrapoda - ps24
Amniota - ps25
Mammalia - ps26
Theria - ps27
Placentalia - ps28
Boreoeutheria - ps29
Euarchontoglires - ps30
Euarchonta - ps31
Primates - ps32
Haplorrhini - ps33
Simiiformes - ps34
Catarrhini - ps35
Hominoidea - ps36
Hominidae - ps37
Homininae - ps38
Hominini - ps39
Homo_sapiens - ps40
p-value
5e-53
8e-41
1e-28
2e-16
2e-04
Log-odds
0.5
1.0
1.5
2.0
2.5
3.0
3.5
4.0

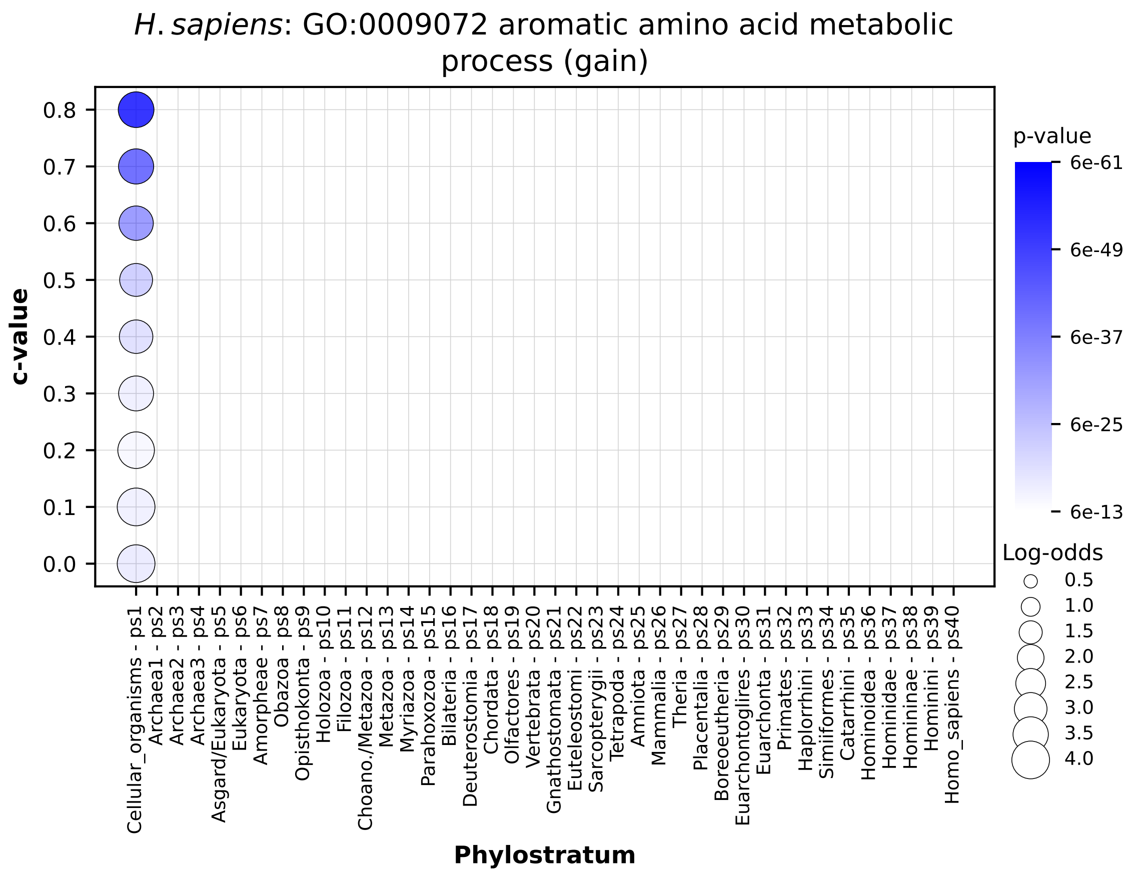
H. sapiens: GO:0009072 aromatic amino acid metabolic process (gain)
c-value
0.8
0.7
0.6
0.5
0.4
0.3
0.2
0.1
0.0
p-value
6e-61
6e-49
6e-37
6e-25
6e-13
Log-odds
0.5
1.0
1.5
2.0
2.5
3.0
3.5
4.0
Cellular_organisms - ps1
Archaea1 - ps2
Archaea2 - ps3
Archaea3 - ps4
Asgard/Eukaryota - ps5
Eukaryota - ps6
Amorpheae - ps7
Obazoa - ps8
Opisthokonta - ps9
Holozoa - ps10
Filozoa - ps11
Choano./Metazoa - ps12
Metazoa - ps13
Myriazoa - ps14
Parahoxozoa - ps15
Bilateria - ps16
Deuterostomia - ps17
Chordata - ps18
Olfactores - ps19
Vertebrata - ps20
Gnathostomata - ps21
Euteleostomi - ps22
Sarcopterygii - ps23
Tetrapoda - ps24
Amniota - ps25
Mammalia - ps26
Theria - ps27
Placentalia - ps28
Boreoeutheria - ps29
Euarchontoglires - ps30
Euarchonta - ps31
Primates - ps32
Haplorrhini - ps33
Simiiformes - ps34
Catarrhini - ps35
Hominoidea - ps36
Hominidae - ps37
Homininae - ps38
Hominini - ps39
Homo_sapiens - ps40
Phylostratum

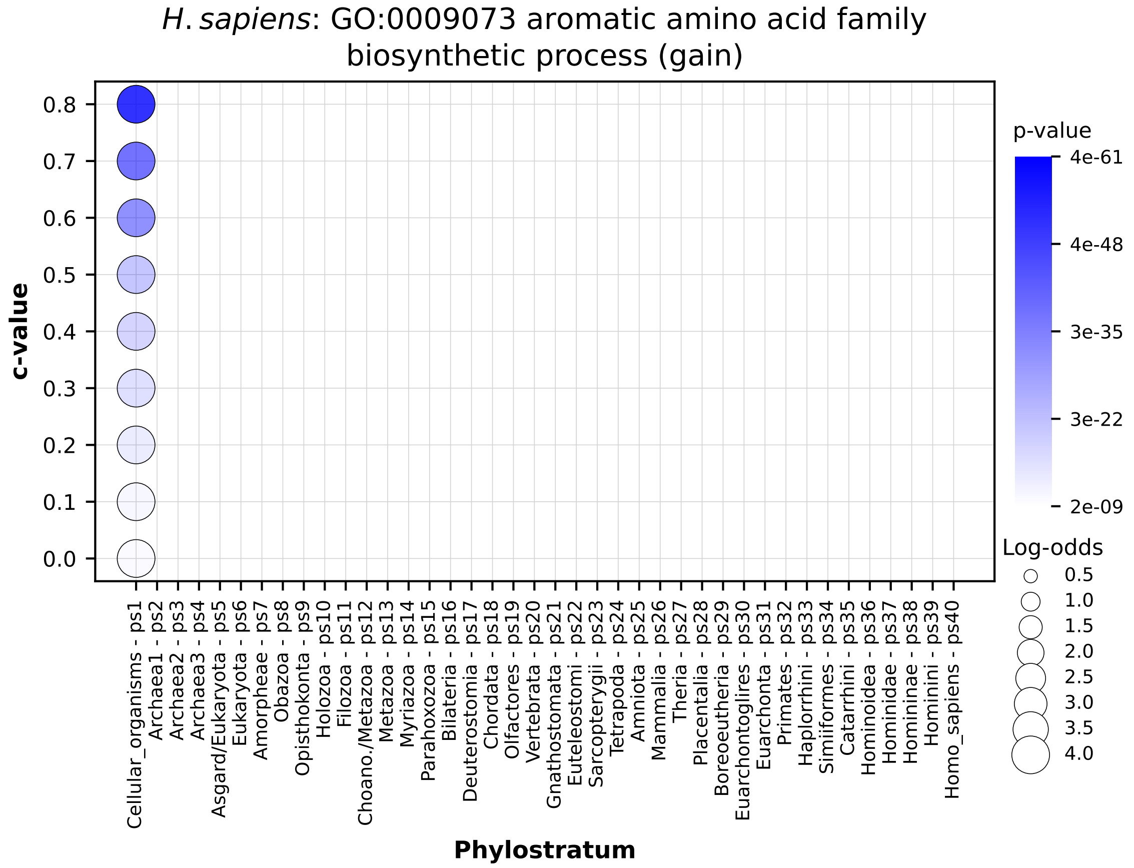
H. sapiens: GO:0009073 aromatic amino acid family biosynthetic process (gain)
c-value
0.8
0.7
0.6
0.5
0.4
0.3
0.2
0.1
0.0
p-value
4e-61
4e-48
3e-35
3e-22
2e-09
Log-odds
0.5
1.0
1.5
2.0
2.5
3.0
3.5
4.0
Cellular_organisms - ps1
Archaea1 - ps2
Archaea2 - ps3
Archaea3 - ps4
Asgard/Eukaryota - ps5
Eukaryota - ps6
Amorpheae - ps7
Obazoa - ps8
Opisthokonta - ps9
Holozoa - ps10
Filozoa - ps11
Choano./Metazoa - ps12
Metazoa - ps13
Myriazoa - ps14
Parahoxozoa - ps15
Bilateria - ps16
Deuterostomia - ps17
Chordata - ps18
Olfactores - ps19
Vertebrata - ps20
Gnathostomata - ps21
Euteleostomi - ps22
Sarcopterygii - ps23
Tetrapoda - ps24
Amniota - ps25
Mammalia - ps26
Theria - ps27
Placentalia - ps28
Boreoeutheria - ps29
Euarchontoglires - ps30
Euarchonta - ps31
Primates - ps32
Haplorrhini - ps33
Simiiformes - ps34
Catarrhini - ps35
Hominoidea - ps36
Hominidae - ps37
Homininae - ps38
Hominini - ps39
Homo_sapiens - ps40
Phylostratum

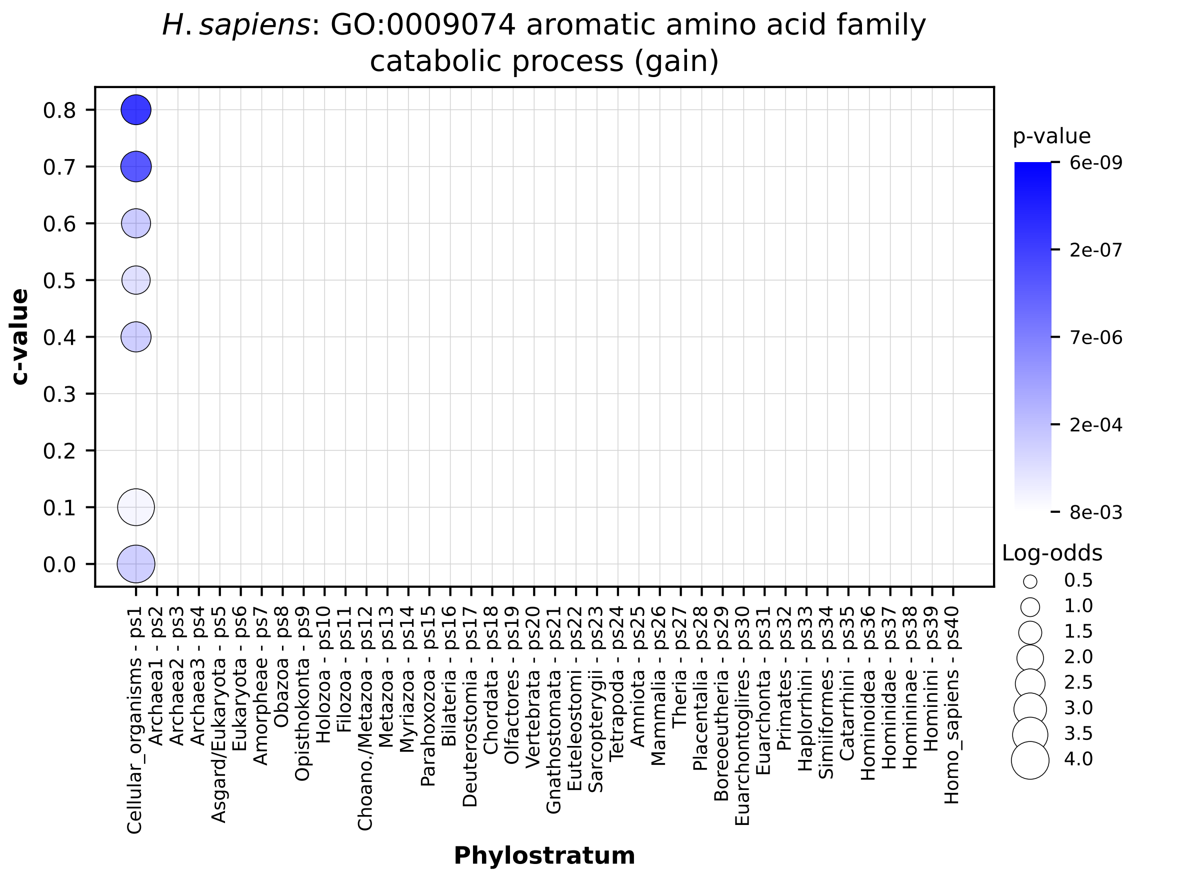

H. sapiens: GO:0009074 aromatic amino acid family catabolic process (gain)
c-value
0.8
0.7
0.6
0.5
0.4
0.3
0.2
0.1
0.0
p-value
6e-09
2e-07
7e-06
2e-04
8e-03
Log-odds
0.5
1.0
1.5
2.0
2.5
3.0
3.5
4.0
Cellular_organisms - ps1
Archaea1 - ps2
Archaea2 - ps3
Archaea3 - ps4
Asgard/Eukaryota - ps5
Eukaryota - ps6
Amorpheae - ps7
Obazoa - ps8
Opisthokonta - ps9
Holozoa - ps10
Filozoa - ps11
Choano./Metazoa - ps12
Metazoa - ps13
Myriazoa - ps14
Parahoxozoa - ps15
Bilateria - ps16
Deuterostomia - ps17
Chordata - ps18
Olfactores - ps19
Vertebrata - ps20
Gnathostomata - ps21
Euteleostomi - ps22
Sarcopterygii - ps23
Tetrapoda - ps24
Amniota - ps25
Mammalia - ps26
Theria - ps27
Placentalia - ps28
Boreoeutheria - ps29
Euarchontoglires - ps30
Euarchonta - ps31
Primates - ps32
Haplorrhini - ps33
Simiiformes - ps34
Catarrhini - ps35
Hominoidea - ps36
Hominidae - ps37
Homininae - ps38
Hominini - ps39
Homo_sapiens - ps40
Phylostratum

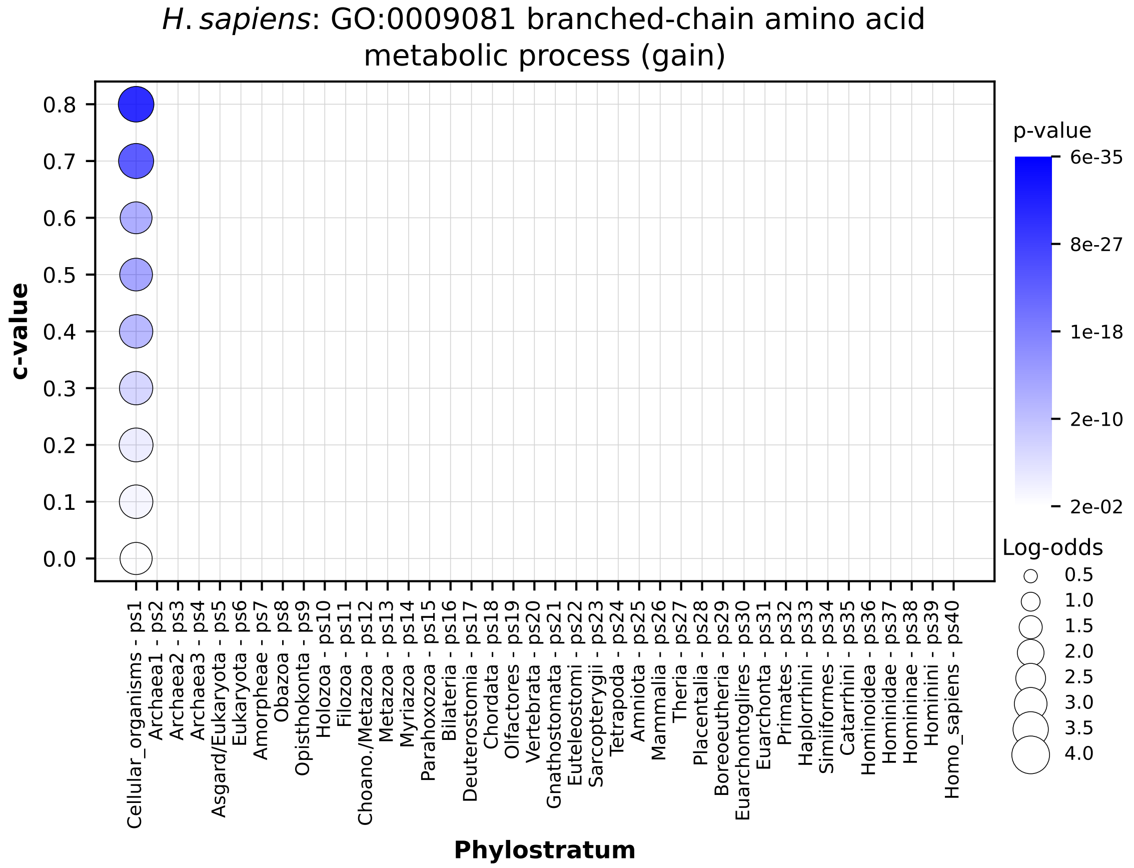

H. sapiens: GO:0009081 branched-chain amino acid metabolic process (gain)
c-value
0.8
0.7
0.6
0.5
0.4
0.3
0.2
0.1
0.0
p-value
6e-35
8e-27
1e-18
2e-10
2e-02
Log-odds
0.5
1.0
1.5
2.0
2.5
3.0
3.5
4.0
Cellular_organisms - ps1
Archaea1 - ps2
Archaea2 - ps3
Archaea3 - ps4
Asgard/Eukaryota - ps5
Eukaryota - ps6
Amorpheae - ps7
Obazoa - ps8
Opisthokonta - ps9
Holozoa - ps10
Filozoa - ps11
Choano./Metazoa - ps12
Metazoa - ps13
Myriazoa - ps14
Parahoxozoa - ps15
Bilateria - ps16
Deuterostomia - ps17
Chordata - ps18
Olfactores - ps19
Vertebrata - ps20
Gnathostomata - ps21
Euteleostomi - ps22
Sarcopterygii - ps23
Tetrapoda - ps24
Amniota - ps25
Mammalia - ps26
Theria - ps27
Placentalia - ps28
Boreoeutheria - ps29
Euarchontoglires - ps30
Euarchonta - ps31
Primates - ps32
Haplorrhini - ps33
Simiiformes - ps34
Catarrhini - ps35
Hominoidea - ps36
Hominidae - ps37
Homininae - ps38
Hominini - ps39
Homo_sapiens - ps40
Phylostratum

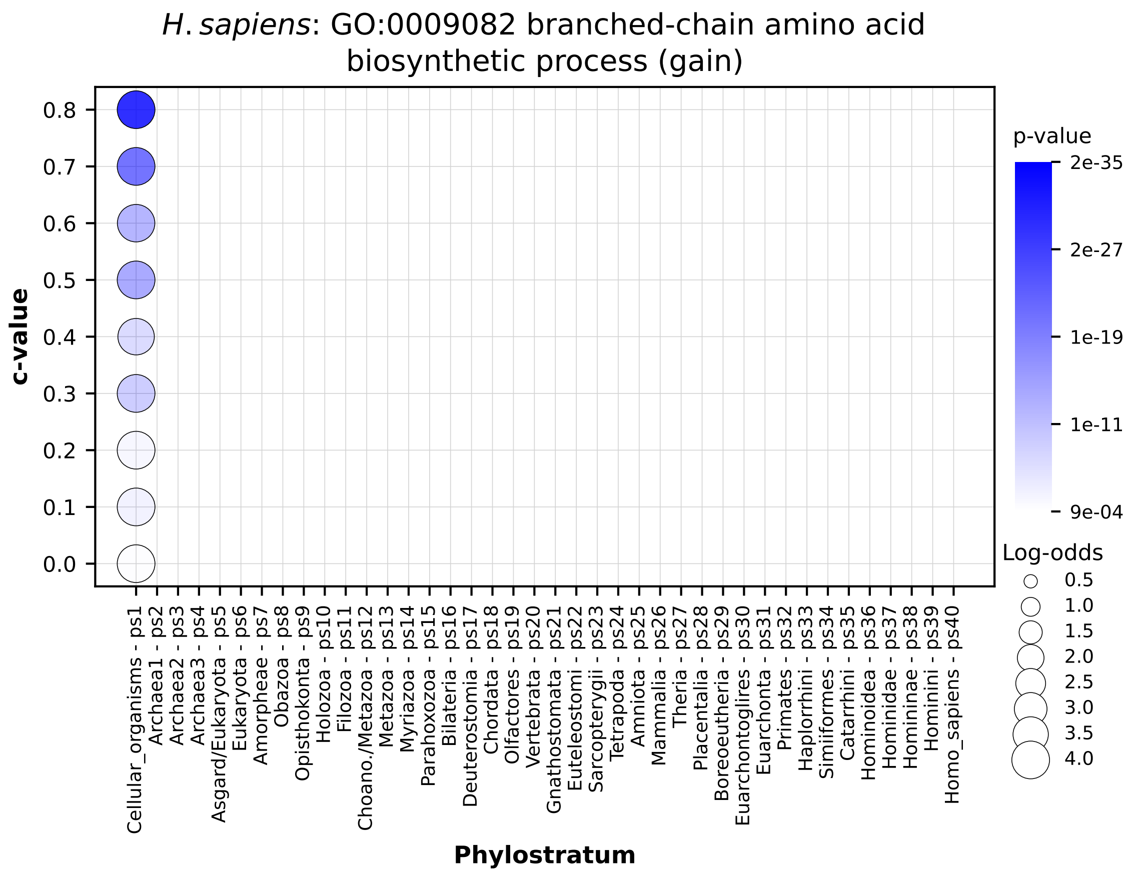

H. sapiens: GO:0009082 branched-chain amino acid biosynthetic process (gain)
c-value
0.0
0.1
0.2
0.3
0.4
0.5
0.6
0.7
0.8
Phylostratum
Cellular_organisms - ps1
Archaea1 - ps2
Archaea2 - ps3
Archaea3 - ps4
Asgard/Eukaryota - ps5
Eukaryota - ps6
Amorpheae - ps7
Obazoa - ps8
Opisthokonta - ps9
Holozoa - ps10
Filozoa - ps11
Choano./Metazoa - ps12
Metazoa - ps13
Myriazoa - ps14
Parahoxozoa - ps15
Bilateria - ps16
Deuterostomia - ps17
Chordata - ps18
Olfactores - ps19
Vertebrata - ps20
Gnathostomata - ps21
Euteleostomi - ps22
Sarcopterygii - ps23
Tetrapoda - ps24
Amniota - ps25
Mammalia - ps26
Theria - ps27
Placentalia - ps28
Boreoeutheria - ps29
Euarchontoglires - ps30
Euarchonta - ps31
Primates - ps32
Haplorrhini - ps33
Simiiformes - ps34
Catarrhini - ps35
Hominoidea - ps36
Hominidae - ps37
Homininae - ps38
Hominini - ps39
Homo_sapiens - ps40
p-value
2e-35
2e-27
1e-19
1e-11
9e-04
Log-odds
0.5
1.0
1.5
2.0
2.5
3.0
3.5
4.0

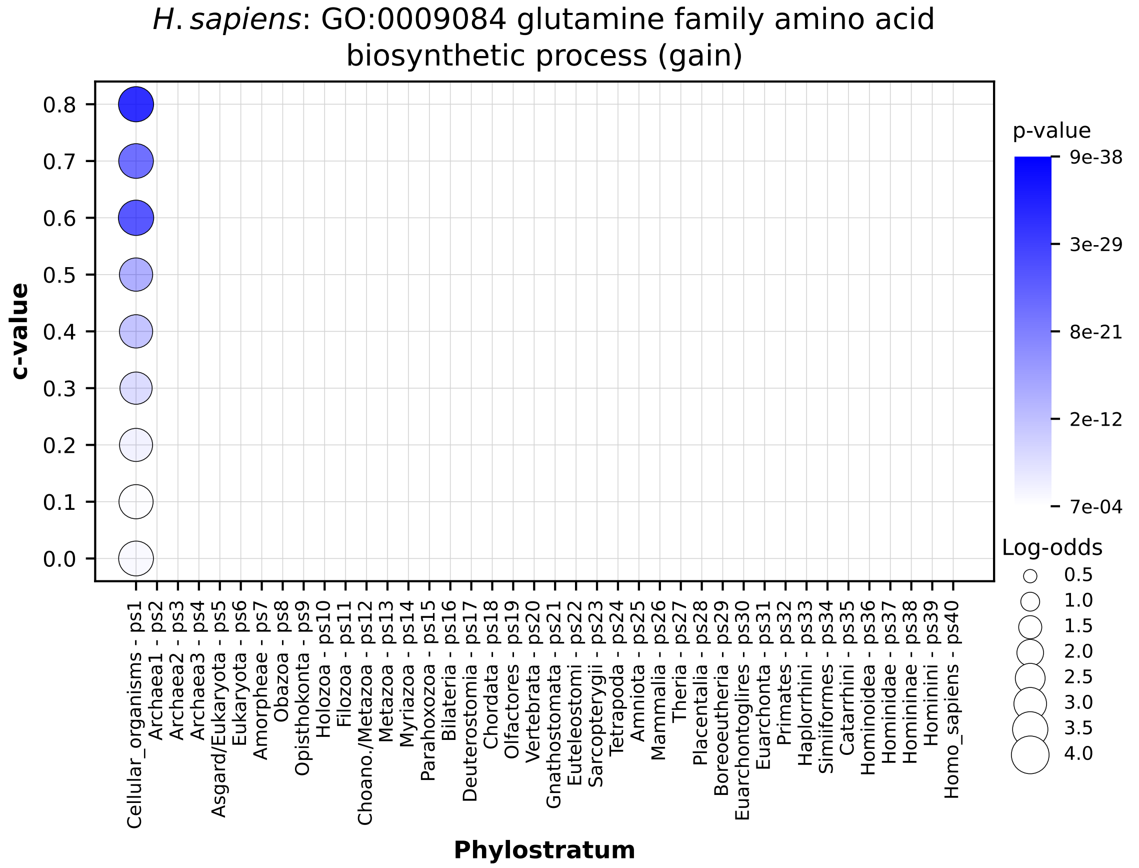

*H. sapiens*: GO:0009084 glutamine family amino acid biosynthetic process (gain)
c-value
0.8
0.7
0.6
0.5
0.4
0.3
0.2
0.1
0.0
p-value
9e-38
3e-29
8e-21
2e-12
7e-04
Log-odds
0.5
1.0
1.5
2.0
2.5
3.0
3.5
4.0
Cellular_organisms - ps1
Archaea1 - ps2
Archaea2 - ps3
Archaea3 - ps4
Asgard/Eukaryota - ps5
Eukaryota - ps6
Amorpheae - ps7
Obazoa - ps8
Opisthokonta - ps9
Holozoa - ps10
Filozoa - ps11
Choano./Metazoa - ps12
Metazoa - ps13
Myriazoa - ps14
Parahoxozoa - ps15
Bilateria - ps16
Deuterostomia - ps17
Chordata - ps18
Olfactores - ps19
Vertebrata - ps20
Gnathostomata - ps21
Euteleostomi - ps22
Sarcopterygii - ps23
Tetrapoda - ps24
Amniota - ps25
Mammalia - ps26
Theria - ps27
Placentalia - ps28
Boreoeutheria - ps29
Euarchontoglires - ps30
Euarchonta - ps31
Primates - ps32
Haplorrhini - ps33
Simiiformes - ps34
Catarrhini - ps35
Hominoidea - ps36
Hominidae - ps37
Homininae - ps38
Hominini - ps39
Homo_sapiens - ps40
Phylostratum

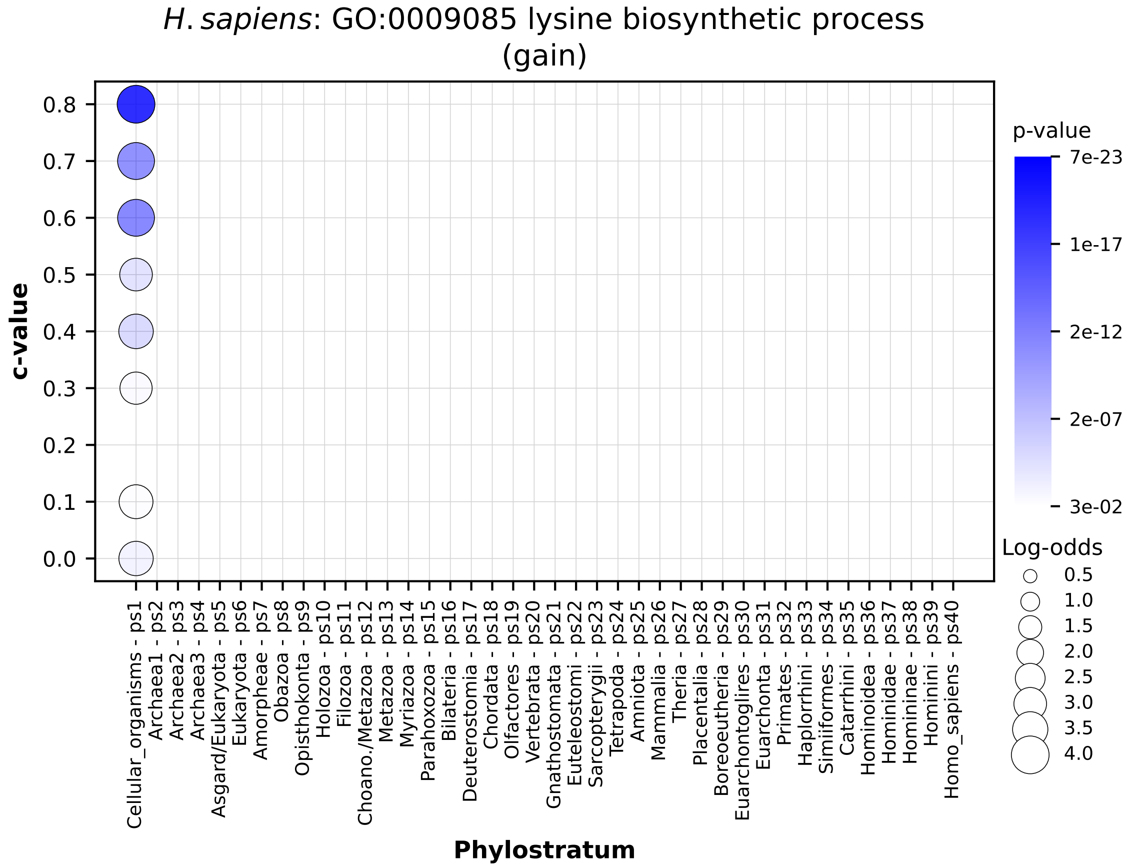

H. sapiens: GO:0009085 lysine biosynthetic process
(gain)
c-value
0.8
0.7
0.6
0.5
0.4
0.3
0.2
0.1
0.0
p-value
7e-23
1e-17
2e-12
2e-07
3e-02
Log-odds
0.5
1.0
1.5
2.0
2.5
3.0
3.5
4.0
Cellular_organisms - ps1
Archaea1 - ps2
Archaea2 - ps3
Archaea3 - ps4
Asgard/Eukaryota - ps5
Eukaryota - ps6
Amorpheae - ps7
Obazoa - ps8
Opisthokonta - ps9
Holozoa - ps10
Filozoa - ps11
Choano./Metazoa - ps12
Metazoa - ps13
Myriazoa - ps14
Parahoxozoa - ps15
Bilateria - ps16
Deuterostomia - ps17
Chordata - ps18
Olfactores - ps19
Vertebrata - ps20
Gnathostomata - ps21
Euteleostomi - ps22
Sarcopterygii - ps23
Tetrapoda - ps24
Amniota - ps25
Mammalia - ps26
Theria - ps27
Placentalia - ps28
Boreoeutheria - ps29
Euarchontoglires - ps30
Euarchonta - ps31
Primates - ps32
Haplorrhini - ps33
Simiiformes - ps34
Catarrhini - ps35
Hominoidea - ps36
Hominidae - ps37
Homininae - ps38
Hominini - ps39
Homo_sapiens - ps40
Phylostratum

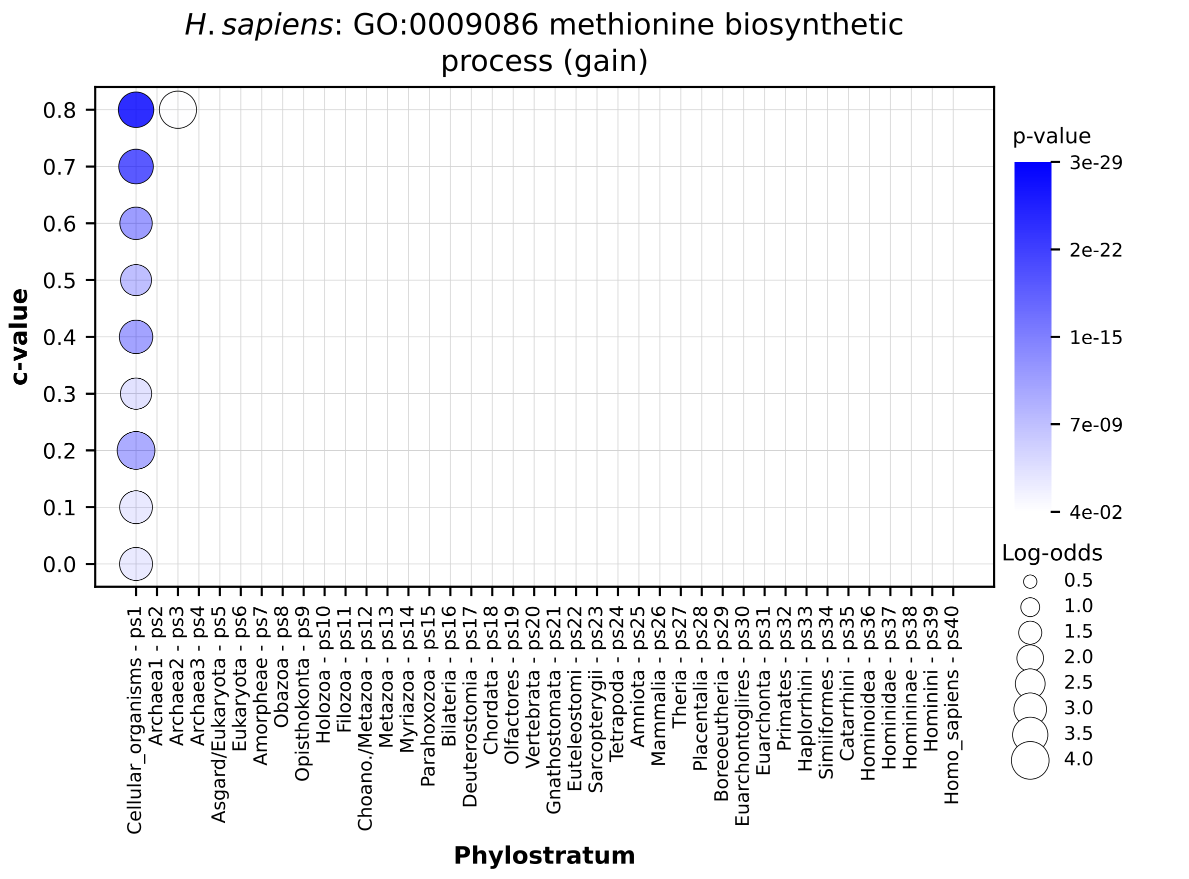

H. sapiens: GO:0009086 methionine biosynthetic process (gain)
c-value
0.8
0.7
0.6
0.5
0.4
0.3
0.2
0.1
0.0
p-value
3e-29
2e-22
1e-15
7e-09
4e-02
Log-odds
0.5
1.0
1.5
2.0
2.5
3.0
3.5
4.0
Cellular_organisms - ps1
Archaea1 - ps2
Archaea2 - ps3
Archaea3 - ps4
Asgard/Eukaryota - ps5
Eukaryota - ps6
Amorpheae - ps7
Obazoa - ps8
Opisthokonta - ps9
Holozoa - ps10
Filozoa - ps11
Choano./Metazoa - ps12
Metazoa - ps13
Myriazoa - ps14
Parahoxozoa - ps15
Bilateria - ps16
Deuterostomia - ps17
Chordata - ps18
Olfactores - ps19
Vertebrata - ps20
Gnathostomata - ps21
Euteleostomi - ps22
Sarcopterygii - ps23
Tetrapoda - ps24
Amniota - ps25
Mammalia - ps26
Theria - ps27
Placentalia - ps28
Boreoeutheria - ps29
Euarchontoglires - ps30
Euarchonta - ps31
Primates - ps32
Haplorrhini - ps33
Simiiformes - ps34
Catarrhini - ps35
Hominoidea - ps36
Hominidae - ps37
Homininae - ps38
Hominini - ps39
Homo_sapiens - ps40
Phylostratum

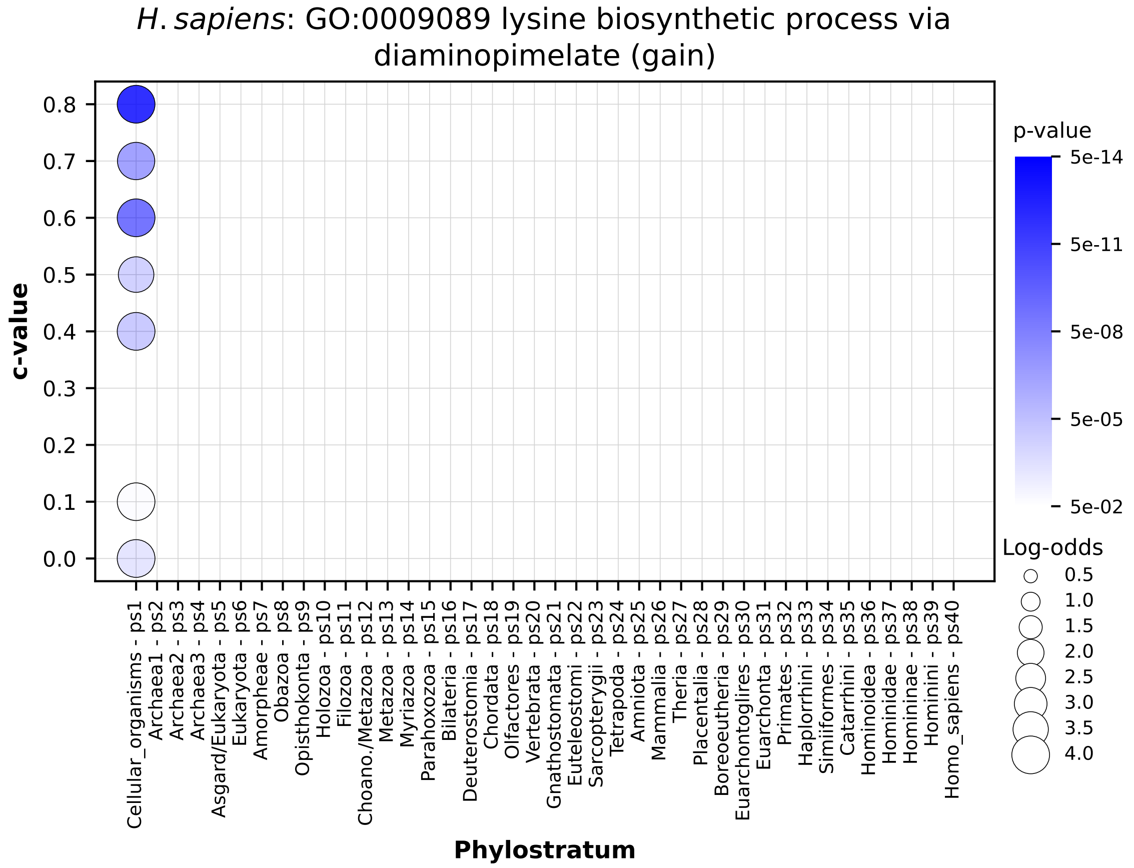

H. sapiens: GO:0009089 lysine biosynthetic process via diaminopimelate (gain)
c-value
0.0
0.1
0.2
0.3
0.4
0.5
0.6
0.7
0.8
Phylostratum
Cellular_organisms - ps1
Archaea1 - ps2
Archaea2 - ps3
Archaea3 - ps4
Asgard/Eukaryota - ps5
Eukaryota - ps6
Amorpheae - ps7
Obazoa - ps8
Opisthokonta - ps9
Holozoa - ps10
Filozoa - ps11
Choano./Metazoa - ps12
Metazoa - ps13
Myriazoa - ps14
Parahoxozoa - ps15
Bilateria - ps16
Deuterostomia - ps17
Chordata - ps18
Olfactores - ps19
Vertebrata - ps20
Gnathostomata - ps21
Euteleostomi - ps22
Sarcopterygii - ps23
Tetrapoda - ps24
Amniota - ps25
Mammalia - ps26
Theria - ps27
Placentalia - ps28
Boreoeutheria - ps29
Euarchontoglires - ps30
Euarchonta - ps31
Primates - ps32
Haplorrhini - ps33
Simiiformes - ps34
Catarrhini - ps35
Hominoidea - ps36
Hominidae - ps37
Homininae - ps38
Hominini - ps39
Homo_sapiens - ps40
p-value
5e-14
5e-11
5e-08
5e-05
5e-02
Log-odds
0.5
1.0
1.5
2.0
2.5
3.0
3.5
4.0

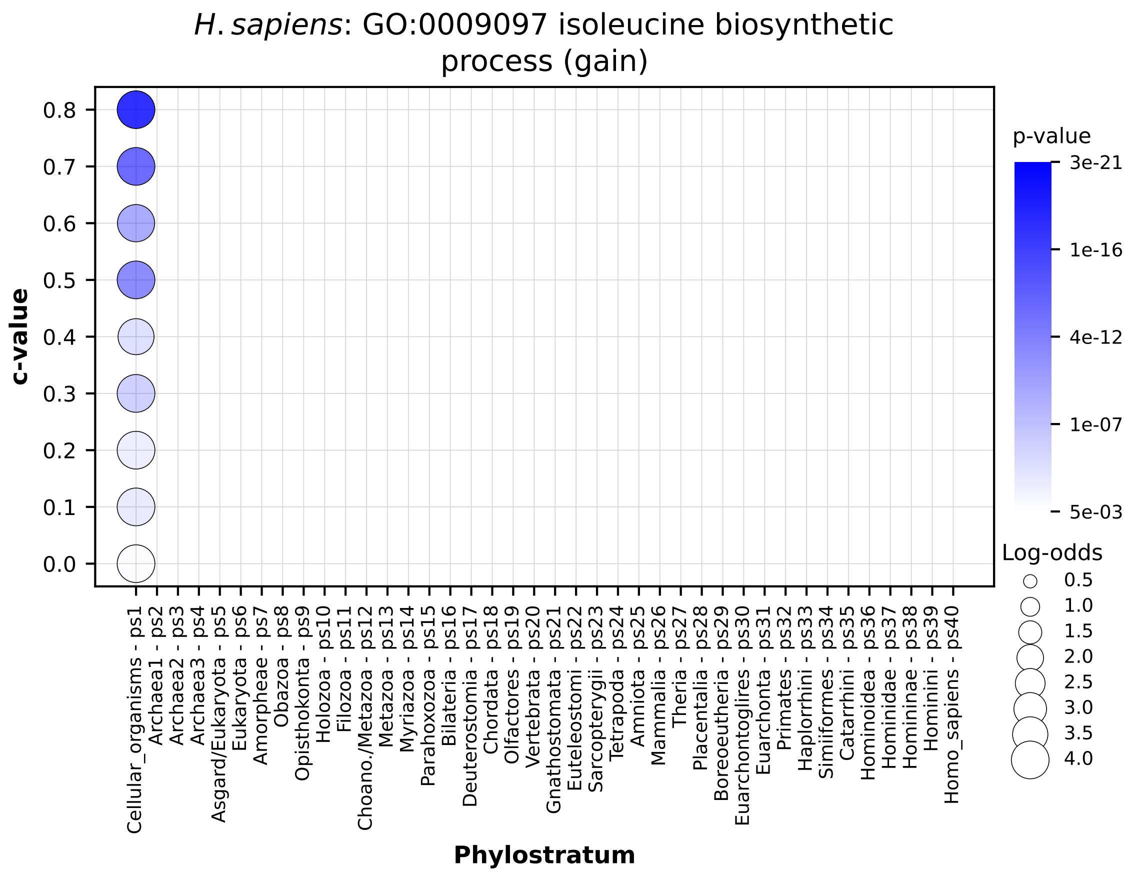
*H. sapiens*: GO:0009097 isoleucine biosynthetic process (gain)
c-value
0.8
0.7
0.6
0.5
0.4
0.3
0.2
0.1
0.0
Phylostratum
Cellular_organisms - ps1
Archaea1 - ps2
Archaea2 - ps3
Archaea3 - ps4
Asgard/Eukaryota - ps5
Eukaryota - ps6
Amorpheae - ps7
Obazoa - ps8
Opisthokonta - ps9
Holozoa - ps10
Filozoa - ps11
Choano./Metazoa - ps12
Metazoa - ps13
Myriazoa - ps14
Parahoxozoa - ps15
Bilateria - ps16
Deuterostomia - ps17
Chordata - ps18
Olfactores - ps19
Vertebrata - ps20
Gnathostomata - ps21
Euteleostomi - ps22
Sarcopterygii - ps23
Tetrapoda - ps24
Amniota - ps25
Mammalia - ps26
Theria - ps27
Placentalia - ps28
Boreoeutheria - ps29
Euarchontoglires - ps30
Euarchonta - ps31
Primates - ps32
Haplorrhini - ps33
Simiiformes - ps34
Catarrhini - ps35
Hominoidea - ps36
Hominidae - ps37
Homininae - ps38
Hominini - ps39
Homo_sapiens - ps40
p-value
3e-21
1e-16
4e-12
1e-07
5e-03
Log-odds
0.5
1.0
1.5
2.0
2.5
3.0
3.5
4.0

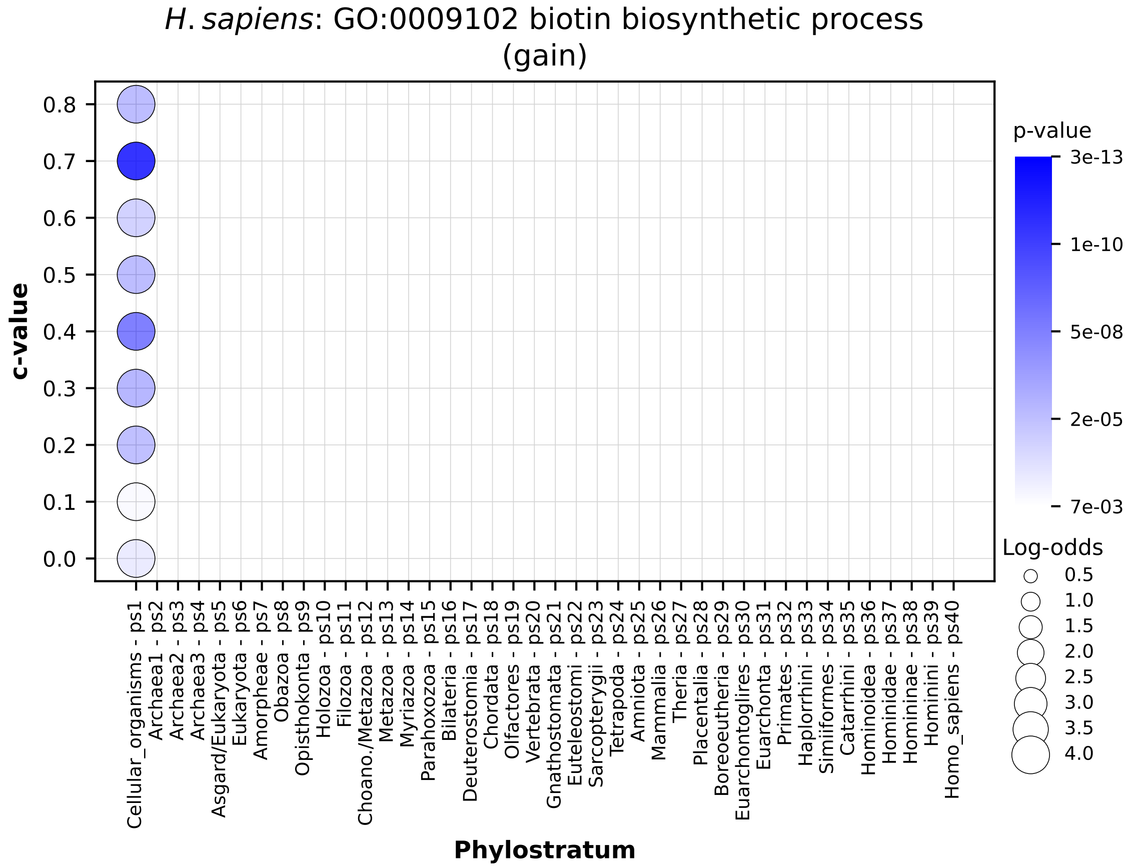
H. sapiens: GO:0009102 biotin biosynthetic process
(gain)
c-value
0.8
0.7
0.6
0.5
0.4
0.3
0.2
0.1
0.0
p-value
3e-13
1e-10
5e-08
2e-05
7e-03
Log-odds
0.5
1.0
1.5
2.0
2.5
3.0
3.5
4.0
Cellular_organisms - ps1
Archaea1 - ps2
Archaea2 - ps3
Archaea3 - ps4
Asgard/Eukaryota - ps5
Eukaryota - ps6
Amorpheae - ps7
Obazoa - ps8
Opisthokonta - ps9
Holozoa - ps10
Filozoa - ps11
Choano./Metazoa - ps12
Metazoa - ps13
Myriazoa - ps14
Parahoxozoa - ps15
Bilateria - ps16
Deuterostomia - ps17
Chordata - ps18
Olfactores - ps19
Vertebrata - ps20
Gnathostomata - ps21
Euteleostomi - ps22
Sarcopterygii - ps23
Tetrapoda - ps24
Amniota - ps25
Mammalia - ps26
Theria - ps27
Placentalia - ps28
Boreoeutheria - ps29
Euarchontoglires - ps30
Euarchonta - ps31
Primates - ps32
Haplorrhini - ps33
Simiiformes - ps34
Catarrhini - ps35
Hominoidea - ps36
Hominidae - ps37
Homininae - ps38
Hominini - ps39
Homo_sapiens - ps40
Phylostratum

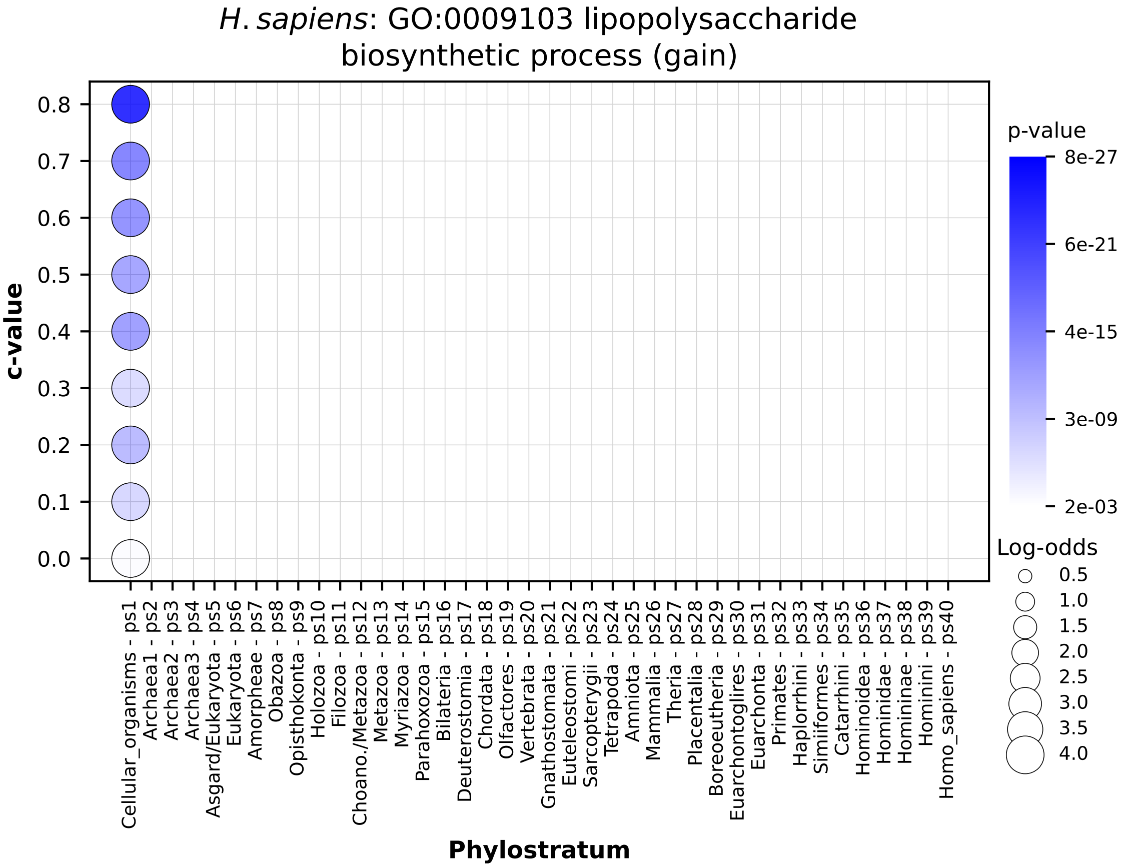

*H. sapiens*: GO:0009103 lipopolysaccharide biosynthetic process (gain)
c-value
0.8
0.7
0.6
0.5
0.4
0.3
0.2
0.1
0.0
p-value
8e-27
6e-21
4e-15
3e-09
2e-03
Log-odds
0.5
1.0
1.5
2.0
2.5
3.0
3.5
4.0
Cellular_organisms - ps1
Archaea1 - ps2
Archaea2 - ps3
Archaea3 - ps4
Asgard/Eukaryota - ps5
Eukaryota - ps6
Amorpheae - ps7
Obazoa - ps8
Opisthokonta - ps9
Holozoa - ps10
Filozoa - ps11
Choano./Metazoa - ps12
Metazoa - ps13
Myriazoa - ps14
Parahoxozoa - ps15
Bilateria - ps16
Deuterostomia - ps17
Chordata - ps18
Olfactores - ps19
Vertebrata - ps20
Gnathostomata - ps21
Euteleostomi - ps22
Sarcopterygii - ps23
Tetrapoda - ps24
Amniota - ps25
Mammalia - ps26
Theria - ps27
Placentalia - ps28
Boreoeutheria - ps29
Euarchontoglires - ps30
Euarchonta - ps31
Primates - ps32
Haplorrhini - ps33
Simiiformes - ps34
Catarrhini - ps35
Hominoidea - ps36
Hominidae - ps37
Homininae - ps38
Hominini - ps39
Homo_sapiens - ps40
Phylostratum

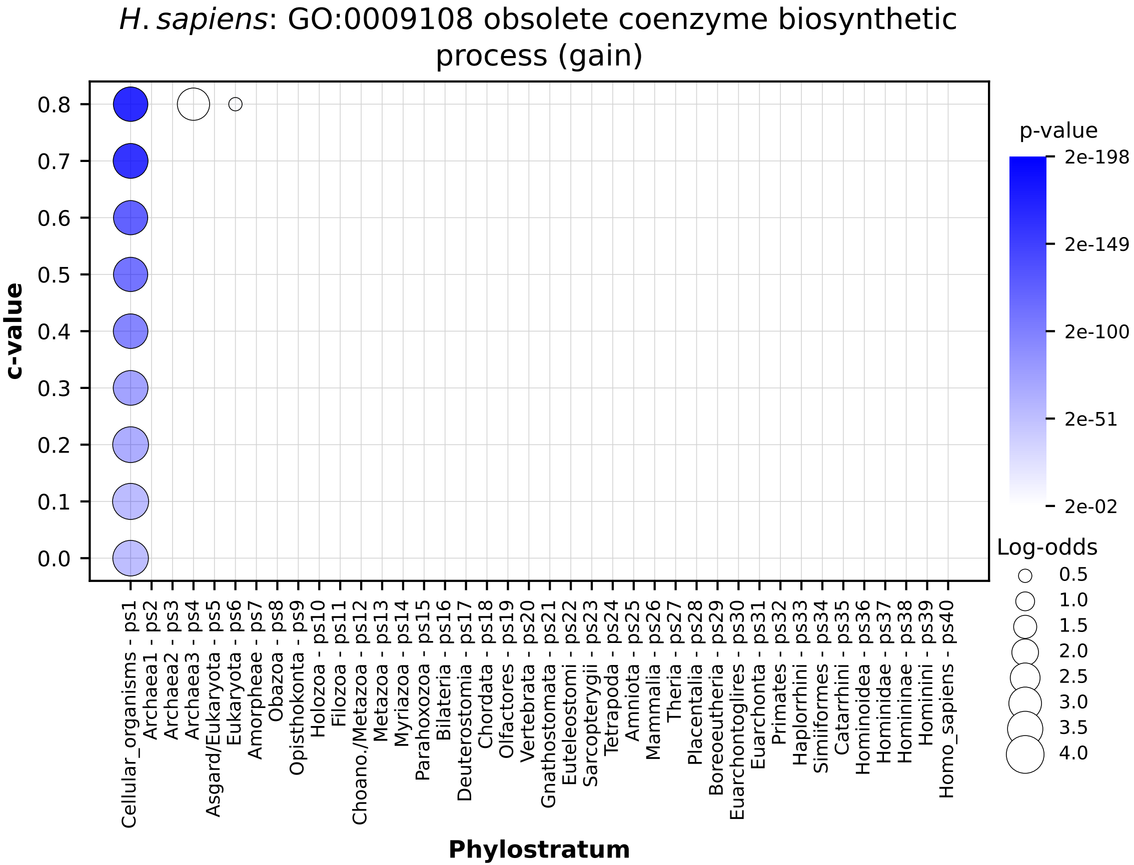

H. sapiens: GO:0009108 obsolete coenzyme biosynthetic process (gain)
c-value
0.8
0.7
0.6
0.5
0.4
0.3
0.2
0.1
0.0
p-value
2e-198
2e-149
2e-100
2e-51
2e-02
Log-odds
0.5
1.0
1.5
2.0
2.5
3.0
3.5
4.0
Cellular_organisms - ps1
Archaea1 - ps2
Archaea2 - ps3
Archaea3 - ps4
Asgard/Eukaryota - ps5
Eukaryota - ps6
Amorpheae - ps7
Obazoa - ps8
Opisthokonta - ps9
Holozoa - ps10
Filozoa - ps11
Choano./Metazoa - ps12
Metazoa - ps13
Myriazoa - ps14
Parahoxozoa - ps15
Bilateria - ps16
Deuterostomia - ps17
Chordata - ps18
Olfactores - ps19
Vertebrata - ps20
Gnathostomata - ps21
Euteleostomi - ps22
Sarcopterygii - ps23
Tetrapoda - ps24
Amniota - ps25
Mammalia - ps26
Theria - ps27
Placentalia - ps28
Boreoeutheria - ps29
Euarchontoglires - ps30
Euarchonta - ps31
Primates - ps32
Haplorrhini - ps33
Simiiformes - ps34
Catarrhini - ps35
Hominoidea - ps36
Hominidae - ps37
Homininae - ps38
Hominini - ps39
Homo_sapiens - ps40
Phylostratum

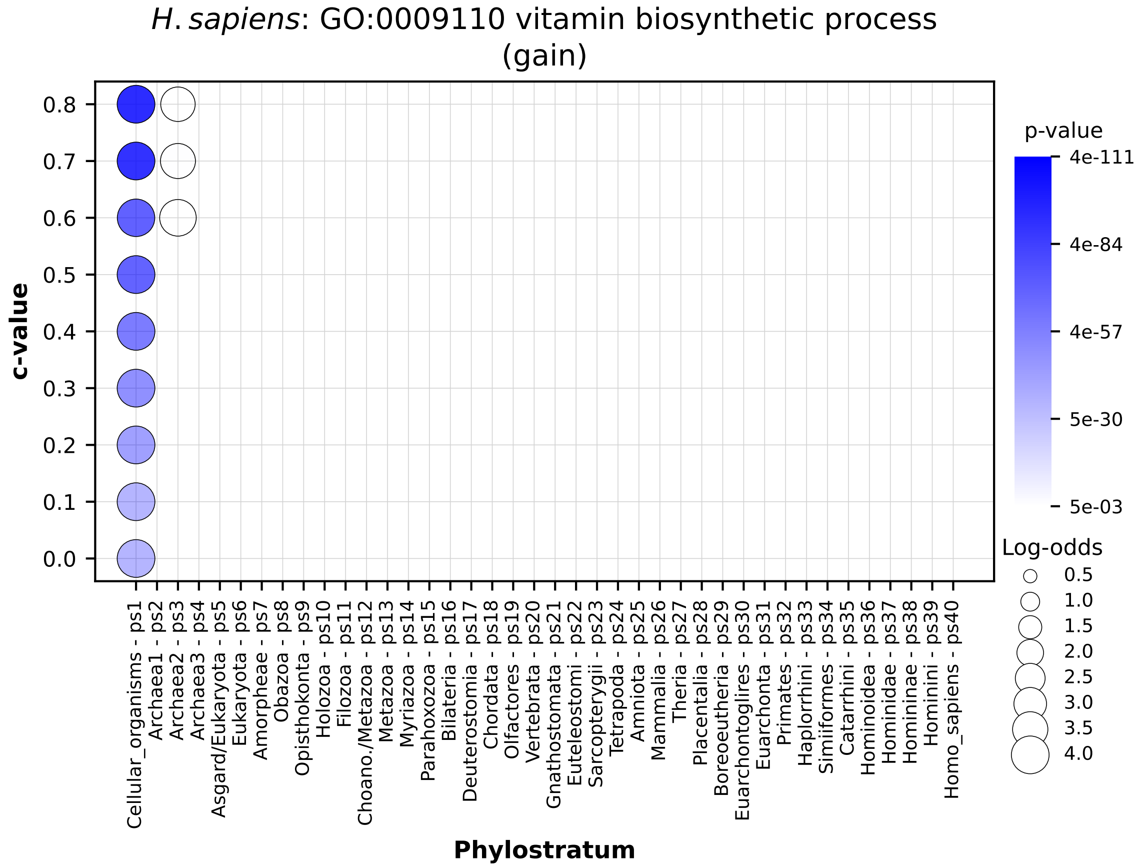

*H. sapiens*: GO:0009110 vitamin biosynthetic process
(gain)
c-value
0.8
0.7
0.6
0.5
0.4
0.3
0.2
0.1
0.0
p-value
4e-111
4e-84
4e-57
5e-30
5e-03
Log-odds
0.5
1.0
1.5
2.0
2.5
3.0
3.5
4.0
Cellular_organisms - ps1
Archaea1 - ps2
Archaea2 - ps3
Archaea3 - ps4
Asgard/Eukaryota - ps5
Eukaryota - ps6
Amorpheae - ps7
Obazoa - ps8
Opisthokonta - ps9
Holozoa - ps10
Filozoa - ps11
Choano./Metazoa - ps12
Metazoa - ps13
Myriazoa - ps14
Parahoxozoa - ps15
Bilateria - ps16
Deuterostomia - ps17
Chordata - ps18
Olfactores - ps19
Vertebrata - ps20
Gnathostomata - ps21
Euteleostomi - ps22
Sarcopterygii - ps23
Tetrapoda - ps24
Amniota - ps25
Mammalia - ps26
Theria - ps27
Placentalia - ps28
Boreoeutheria - ps29
Euarchontoglires - ps30
Euarchonta - ps31
Primates - ps32
Haplorrhini - ps33
Simiiformes - ps34
Catarrhini - ps35
Hominoidea - ps36
Hominidae - ps37
Homininae - ps38
Hominini - ps39
Homo_sapiens - ps40
Phylostratum

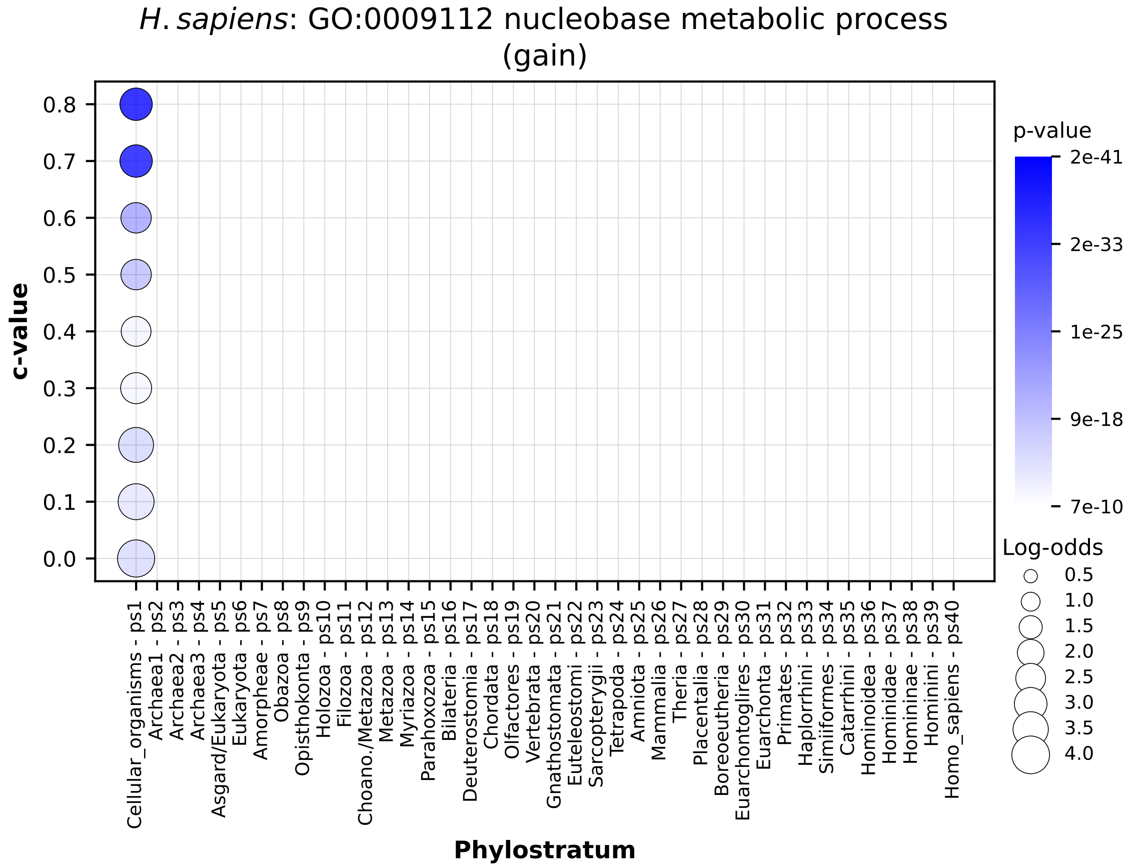

*H. sapiens*: GO:0009112 nucleobase metabolic process (gain)
c-value
0.8
0.7
0.6
0.5
0.4
0.3
0.2
0.1
0.0
p-value
2e-41
2e-33
1e-25
9e-18
7e-10
Log-odds
0.5
1.0
1.5
2.0
2.5
3.0
3.5
4.0
Cellular_organisms - ps1
Archaea1 - ps2
Archaea2 - ps3
Archaea3 - ps4
Asgard/Eukaryota - ps5
Eukaryota - ps6
Amorpheae - ps7
Obazoa - ps8
Opisthokonta - ps9
Holozoa - ps10
Filozoa - ps11
Choano./Metazoa - ps12
Metazoa - ps13
Myriazoa - ps14
Parahoxozoa - ps15
Bilateria - ps16
Deuterostomia - ps17
Chordata - ps18
Olfactores - ps19
Vertebrata - ps20
Gnathostomata - ps21
Euteleostomi - ps22
Sarcopterygii - ps23
Tetrapoda - ps24
Amniota - ps25
Mammalia - ps26
Theria - ps27
Placentalia - ps28
Boreoeutheria - ps29
Euarchontoglires - ps30
Euarchonta - ps31
Primates - ps32
Haplorrhini - ps33
Simiiformes - ps34
Catarrhini - ps35
Hominoidea - ps36
Hominidae - ps37
Homininae - ps38
Hominini - ps39
Homo_sapiens - ps40
Phylostratum

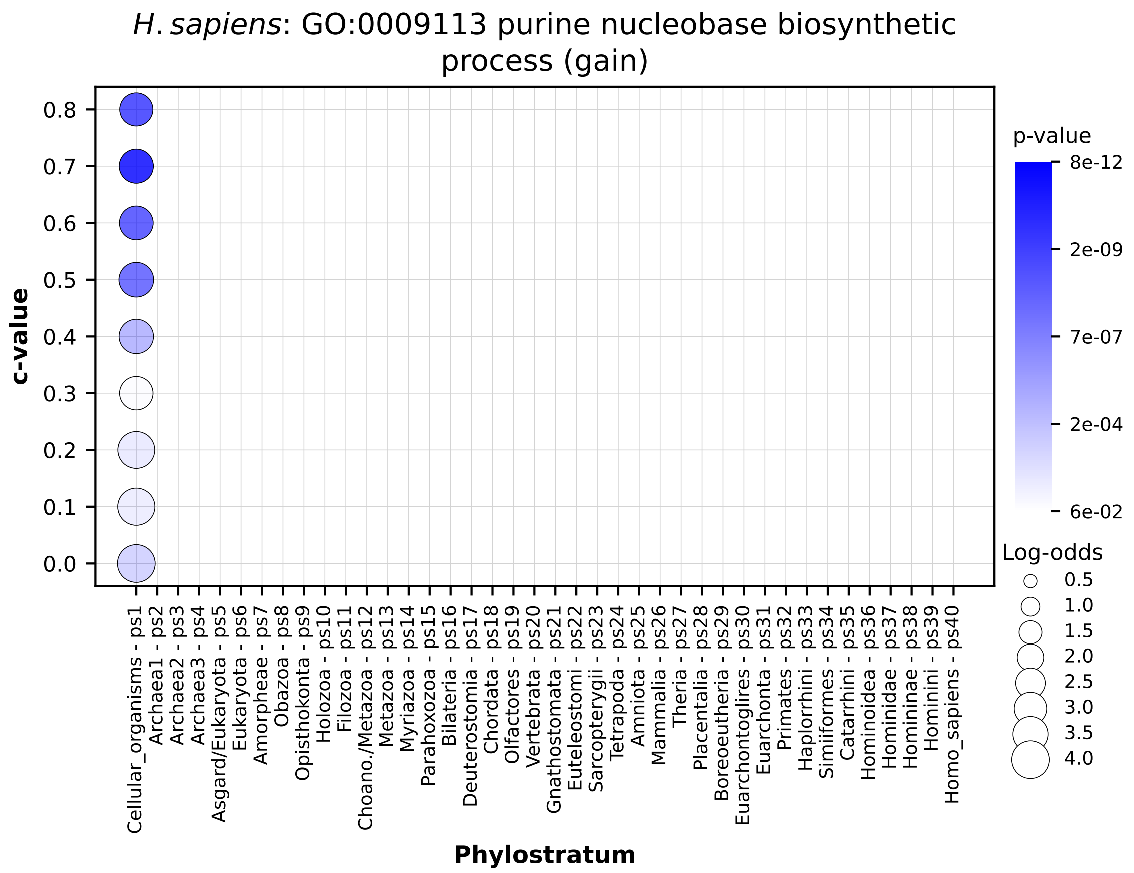

H. sapiens: GO:0009113 purine nucleobase biosynthetic process (gain)
c-value
0.8
0.7
0.6
0.5
0.4
0.3
0.2
0.1
0.0
Phylostratum
Cellular_organisms - ps1
Archaea1 - ps2
Archaea2 - ps3
Archaea3 - ps4
Asgard/Eukaryota - ps5
Eukaryota - ps6
Amorpheae - ps7
Obazoa - ps8
Opisthokonta - ps9
Holozoa - ps10
Filozoa - ps11
Choano./Metazoa - ps12
Metazoa - ps13
Myriazoa - ps14
Parahoxozoa - ps15
Bilateria - ps16
Deuterostomia - ps17
Chordata - ps18
Olfactores - ps19
Vertebrata - ps20
Gnathostomata - ps21
Euteleostomi - ps22
Sarcopterygii - ps23
Tetrapoda - ps24
Amniota - ps25
Mammalia - ps26
Theria - ps27
Placentalia - ps28
Boreoeutheria - ps29
Euarchontoglires - ps30
Euarchonta - ps31
Primates - ps32
Haplorrhini - ps33
Simiiformes - ps34
Catarrhini - ps35
Hominoidea - ps36
Hominidae - ps37
Homininae - ps38
Hominini - ps39
Homo_sapiens - ps40
p-value
8e-12
2e-09
7e-07
2e-04
6e-02
Log-odds
0.5
1.0
1.5
2.0
2.5
3.0
3.5
4.0

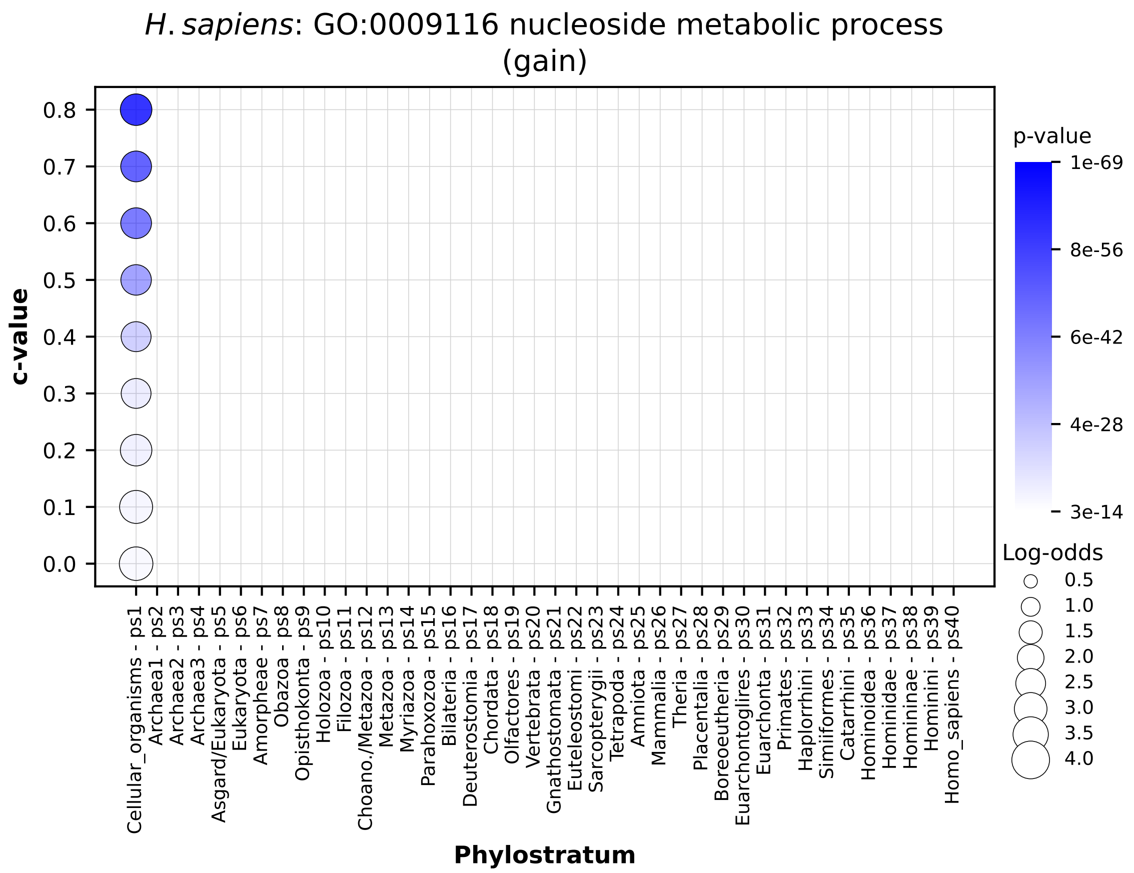

*H. sapiens*: GO:0009116 nucleoside metabolic process
(gain)
c-value
0.8
0.7
0.6
0.5
0.4
0.3
0.2
0.1
0.0
p-value
1e-69
8e-56
6e-42
4e-28
3e-14
Log-odds
0.5
1.0
1.5
2.0
2.5
3.0
3.5
4.0
Cellular_organisms - ps1
Archaea1 - ps2
Archaea2 - ps3
Archaea3 - ps4
Asgard/Eukaryota - ps5
Eukaryota - ps6
Amorpheae - ps7
Obazoa - ps8
Opisthokonta - ps9
Holozoa - ps10
Filozoa - ps11
Choano./Metazoa - ps12
Metazoa - ps13
Myriazoa - ps14
Parahoxozoa - ps15
Bilateria - ps16
Deuterostomia - ps17
Chordata - ps18
Olfactores - ps19
Vertebrata - ps20
Gnathostomata - ps21
Euteleostomi - ps22
Sarcopterygii - ps23
Tetrapoda - ps24
Amniota - ps25
Mammalia - ps26
Theria - ps27
Placentalia - ps28
Boreoeutheria - ps29
Euarchontoglires - ps30
Euarchonta - ps31
Primates - ps32
Haplorrhini - ps33
Simiiformes - ps34
Catarrhini - ps35
Hominoidea - ps36
Hominidae - ps37
Homininae - ps38
Hominini - ps39
Homo_sapiens - ps40
Phylostratum

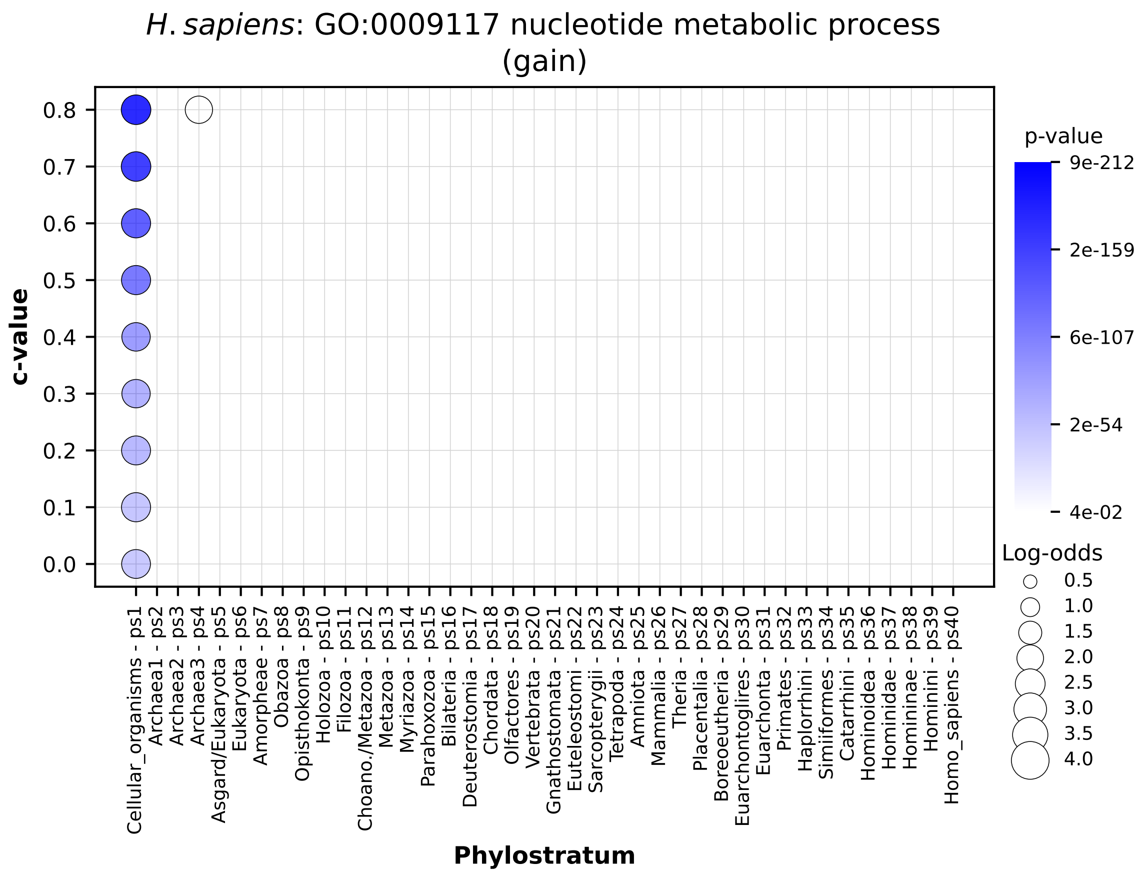

H. sapiens: GO:0009117 nucleotide metabolic process
(gain)
c-value
0.8
0.7
0.6
0.5
0.4
0.3
0.2
0.1
0.0
p-value
9e-212
2e-159
6e-107
2e-54
4e-02
Log-odds
0.5
1.0
1.5
2.0
2.5
3.0
3.5
4.0
Cellular_organisms - ps1
Archaea1 - ps2
Archaea2 - ps3
Archaea3 - ps4
Asgard/Eukaryota - ps5
Eukaryota - ps6
Amorpheae - ps7
Obazoa - ps8
Opisthokonta - ps9
Holozoa - ps10
Filozoa - ps11
Choano./Metazoa - ps12
Metazoa - ps13
Myriazoa - ps14
Parahoxozoa - ps15
Bilateria - ps16
Deuterostomia - ps17
Chordata - ps18
Olfactores - ps19
Vertebrata - ps20
Gnathostomata - ps21
Euteleostomi - ps22
Sarcopterygii - ps23
Tetrapoda - ps24
Amniota - ps25
Mammalia - ps26
Theria - ps27
Placentalia - ps28
Boreoeutheria - ps29
Euarchontoglires - ps30
Euarchonta - ps31
Primates - ps32
Haplorrhini - ps33
Simiiformes - ps34
Catarrhini - ps35
Hominoidea - ps36
Hominidae - ps37
Homininae - ps38
Hominini - ps39
Homo_sapiens - ps40
Phylostratum

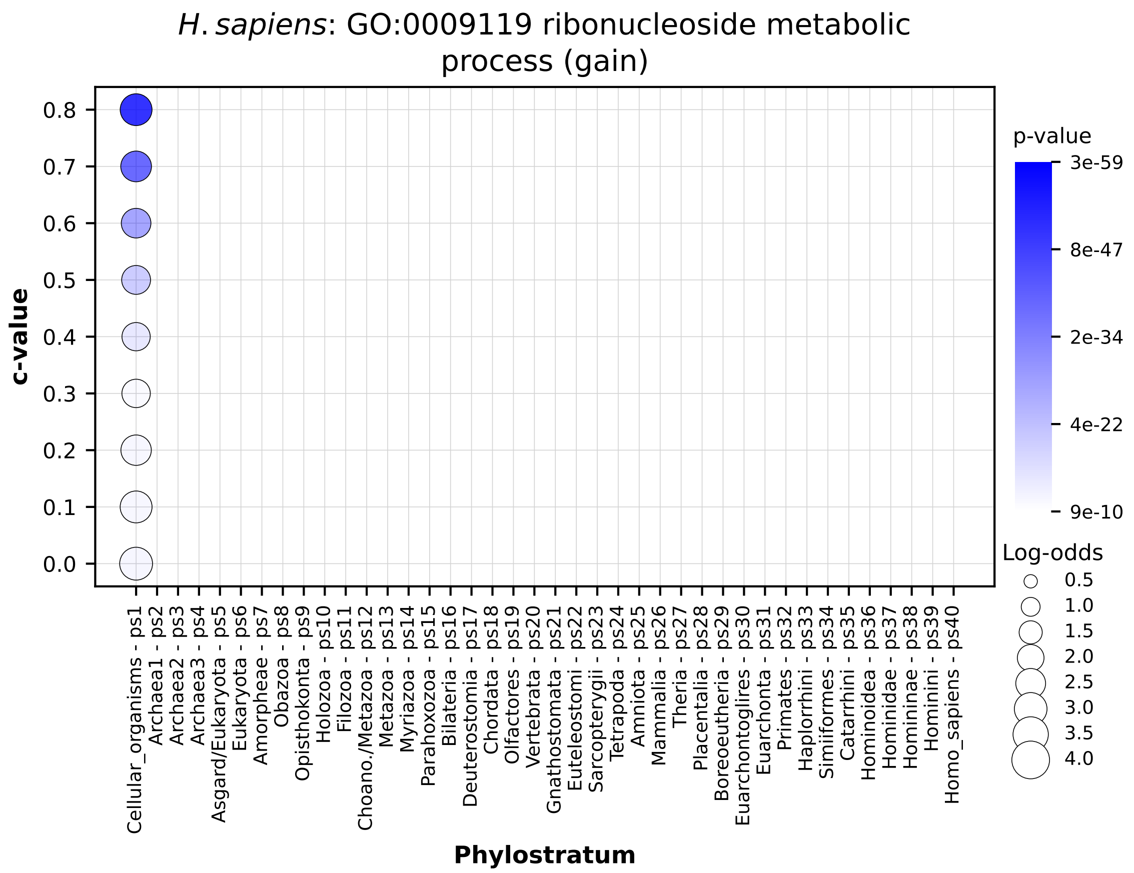

H. sapiens: GO:0009119 ribonucleoside metabolic process (gain)
c-value
0.0
0.1
0.2
0.3
0.4
0.5
0.6
0.7
0.8
Phylostratum
Cellular_organisms - ps1
Archaea1 - ps2
Archaea2 - ps3
Archaea3 - ps4
Asgard/Eukaryota - ps5
Eukaryota - ps6
Amorpheae - ps7
Obazoa - ps8
Opisthokonta - ps9
Holozoa - ps10
Filozoa - ps11
Choano./Metazoa - ps12
Metazoa - ps13
Myriazoa - ps14
Parahoxozoa - ps15
Bilateria - ps16
Deuterostomia - ps17
Chordata - ps18
Olfactores - ps19
Vertebrata - ps20
Gnathostomata - ps21
Euteleostomi - ps22
Sarcopterygii - ps23
Tetrapoda - ps24
Amniota - ps25
Mammalia - ps26
Theria - ps27
Placentalia - ps28
Boreoeutheria - ps29
Euarchontoglires - ps30
Euarchonta - ps31
Primates - ps32
Haplorrhini - ps33
Simiiformes - ps34
Catarrhini - ps35
Hominoidea - ps36
Hominidae - ps37
Homininae - ps38
Hominini - ps39
Homo_sapiens - ps40
p-value
3e-59
8e-47
2e-34
4e-22
9e-10
Log-odds
0.5
1.0
1.5
2.0
2.5
3.0
3.5
4.0

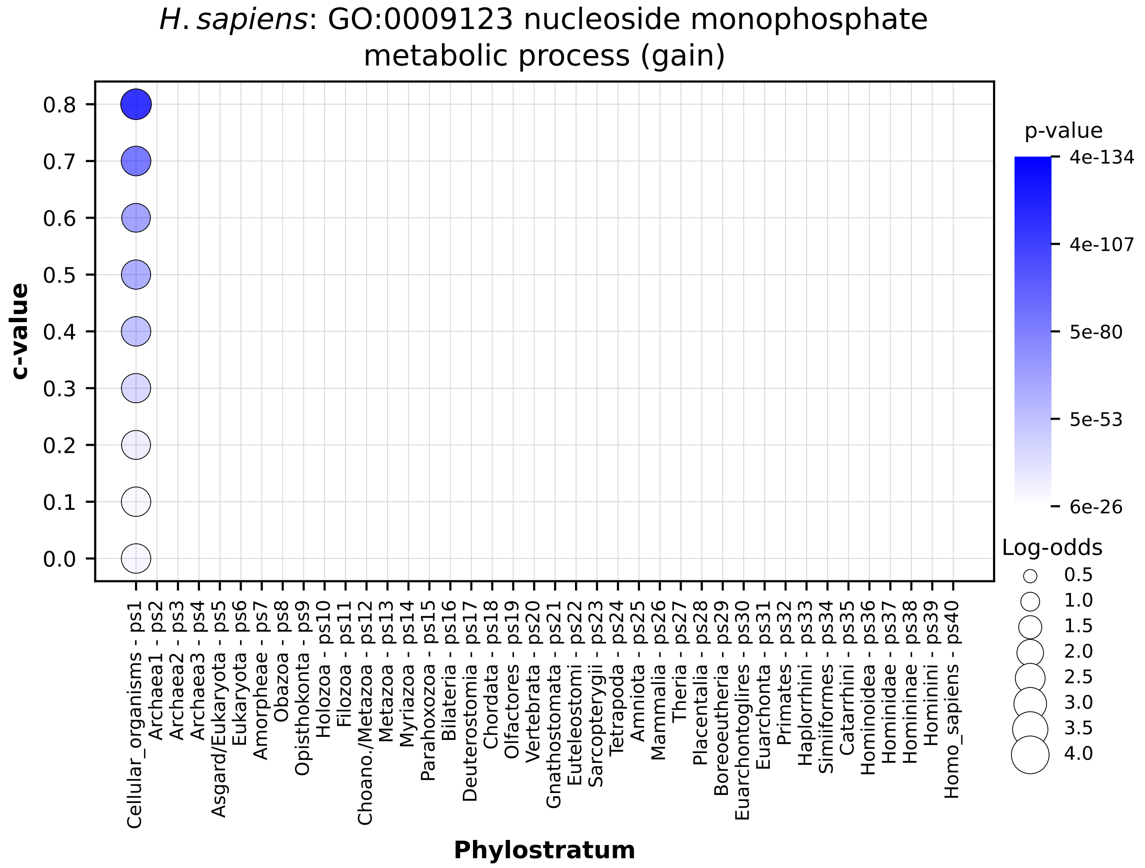

H. sapiens: GO:0009123 nucleoside monophosphate metabolic process (gain)
c-value
0.8
0.7
0.6
0.5
0.4
0.3
0.2
0.1
0.0
Phylostratum
Cellular_organisms - ps1
Archaea1 - ps2
Archaea2 - ps3
Archaea3 - ps4
Asgard/Eukaryota - ps5
Eukaryota - ps6
Amorpheae - ps7
Obazoa - ps8
Opisthokonta - ps9
Holozoa - ps10
Filozoa - ps11
Choano./Metazoa - ps12
Metazoa - ps13
Myriazoa - ps14
Parahoxozoa - ps15
Bilateria - ps16
Deuterostomia - ps17
Chordata - ps18
Olfactores - ps19
Vertebrata - ps20
Gnathostomata - ps21
Euteleostomi - ps22
Sarcopterygii - ps23
Tetrapoda - ps24
Amniota - ps25
Mammalia - ps26
Theria - ps27
Placentalia - ps28
Boreoeutheria - ps29
Euarchontoglires - ps30
Euarchonta - ps31
Primates - ps32
Haplorrhini - ps33
Simiiformes - ps34
Catarrhini - ps35
Hominoidea - ps36
Hominidae - ps37
Homininae - ps38
Hominini - ps39
Homo_sapiens - ps40
p-value
4e-134
4e-107
5e-80
5e-53
6e-26
Log-odds
0.5
1.0
1.5
2.0
2.5
3.0
3.5
4.0

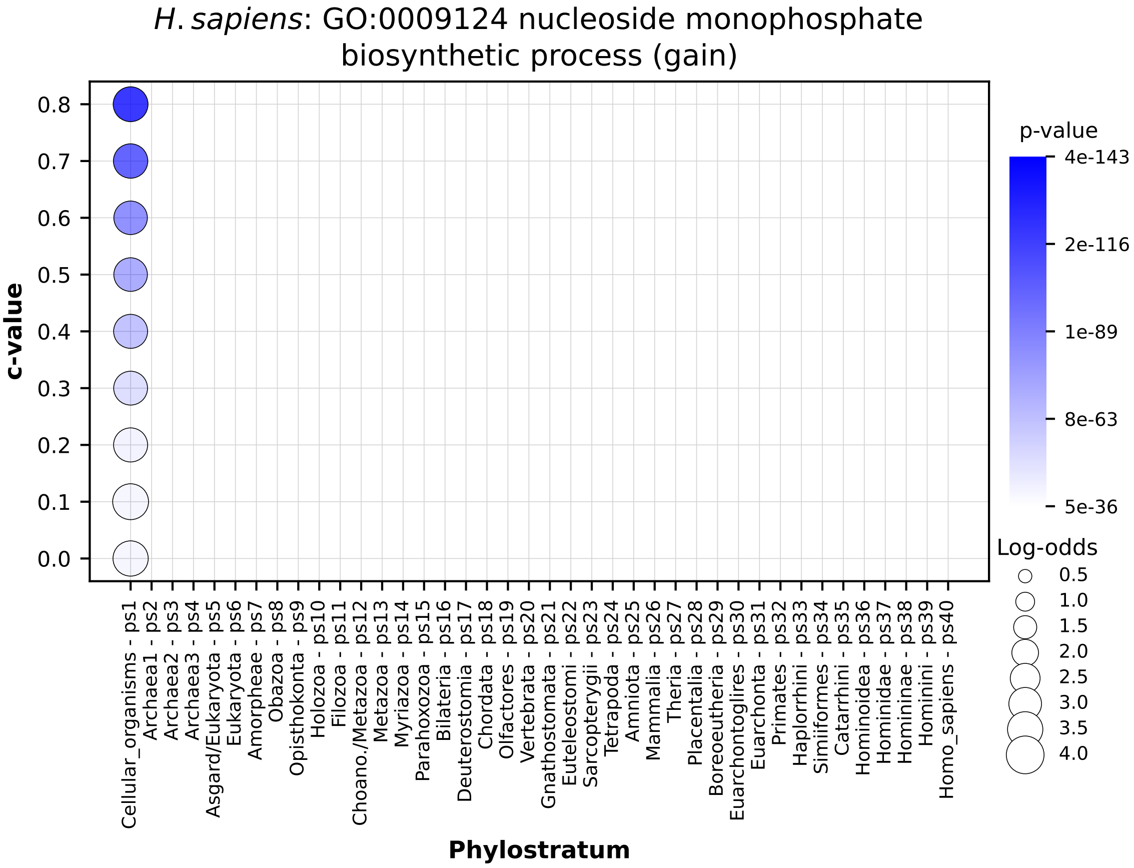

H. sapiens: GO:0009124 nucleoside monophosphate biosynthetic process (gain)
c-value
0.8
0.7
0.6
0.5
0.4
0.3
0.2
0.1
0.0
Phylostratum
Cellular_organisms - ps1
Archaea1 - ps2
Archaea2 - ps3
Archaea3 - ps4
Asgard/Eukaryota - ps5
Eukaryota - ps6
Amorpheae - ps7
Obazoa - ps8
Opisthokonta - ps9
Holozoa - ps10
Filozoa - ps11
Choano./Metazoa - ps12
Metazoa - ps13
Myriazoa - ps14
Parahoxozoa - ps15
Bilateria - ps16
Deuterostomia - ps17
Chordata - ps18
Olfactores - ps19
Vertebrata - ps20
Gnathostomata - ps21
Euteleostomi - ps22
Sarcopterygii - ps23
Tetrapoda - ps24
Amniota - ps25
Mammalia - ps26
Theria - ps27
Placentalia - ps28
Boreoeutheria - ps29
Euarchontoglires - ps30
Euarchonta - ps31
Primates - ps32
Haplorrhini - ps33
Simiiformes - ps34
Catarrhini - ps35
Hominoidea - ps36
Hominidae - ps37
Homininae - ps38
Hominini - ps39
Homo_sapiens - ps40
p-value
4e-143
2e-116
1e-89
8e-63
5e-36
Log-odds
0.5
1.0
1.5
2.0
2.5
3.0
3.5
4.0

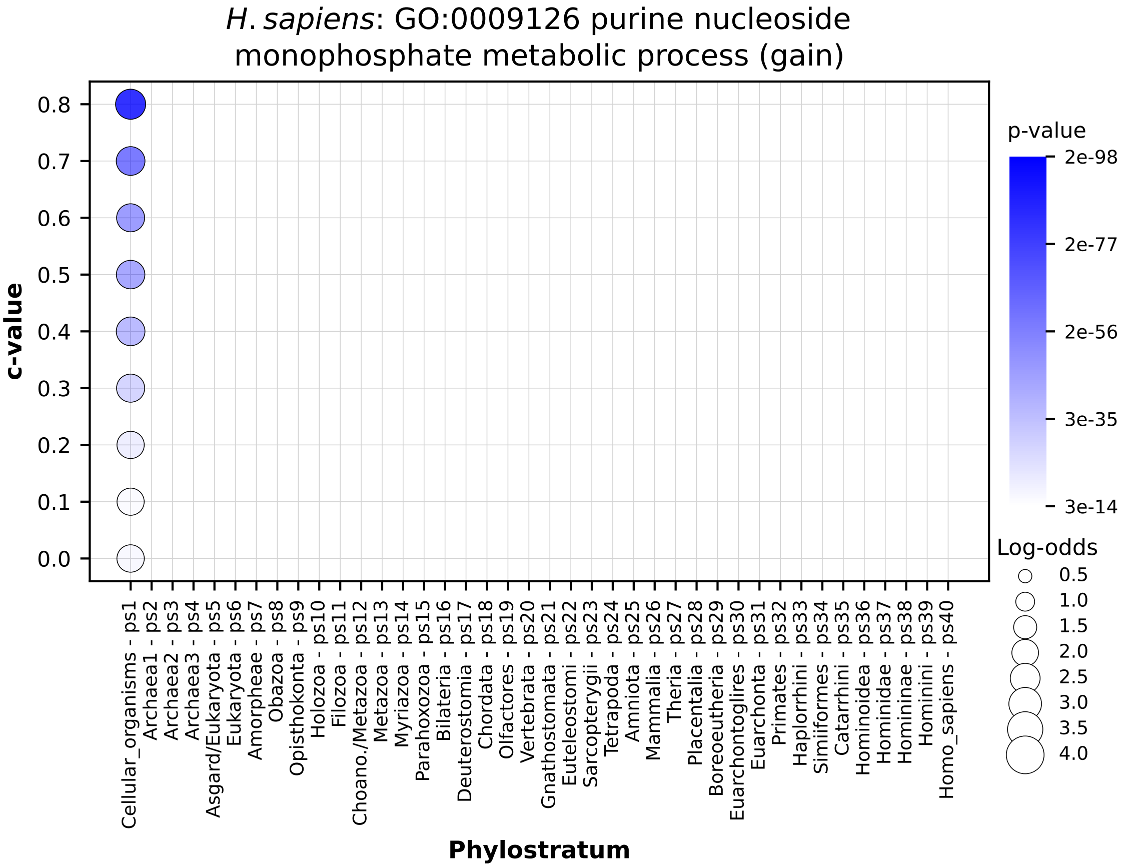

H. sapiens: GO:0009126 purine nucleoside monophosphate metabolic process (gain)
c-value
0.8
0.7
0.6
0.5
0.4
0.3
0.2
0.1
0.0
p-value
2e-98
2e-77
2e-56
3e-35
3e-14
Log-odds
0.5
1.0
1.5
2.0
2.5
3.0
3.5
4.0
Cellular_organisms - ps1
Archaea1 - ps2
Archaea2 - ps3
Archaea3 - ps4
Asgard/Eukaryota - ps5
Eukaryota - ps6
Amorpheae - ps7
Obazoa - ps8
Opisthokonta - ps9
Holozoa - ps10
Filozoa - ps11
Choano./Metazoa - ps12
Metazoa - ps13
Myriazoa - ps14
Parahoxozoa - ps15
Bilateria - ps16
Deuterostomia - ps17
Chordata - ps18
Olfactores - ps19
Vertebrata - ps20
Gnathostomata - ps21
Euteleostomi - ps22
Sarcopterygii - ps23
Tetrapoda - ps24
Amniota - ps25
Mammalia - ps26
Theria - ps27
Placentalia - ps28
Boreoeutheria - ps29
Euarchontoglires - ps30
Euarchonta - ps31
Primates - ps32
Haplorrhini - ps33
Simiiformes - ps34
Catarrhini - ps35
Hominoidea - ps36
Hominidae - ps37
Homininae - ps38
Hominini - ps39
Homo_sapiens - ps40
Phylostratum

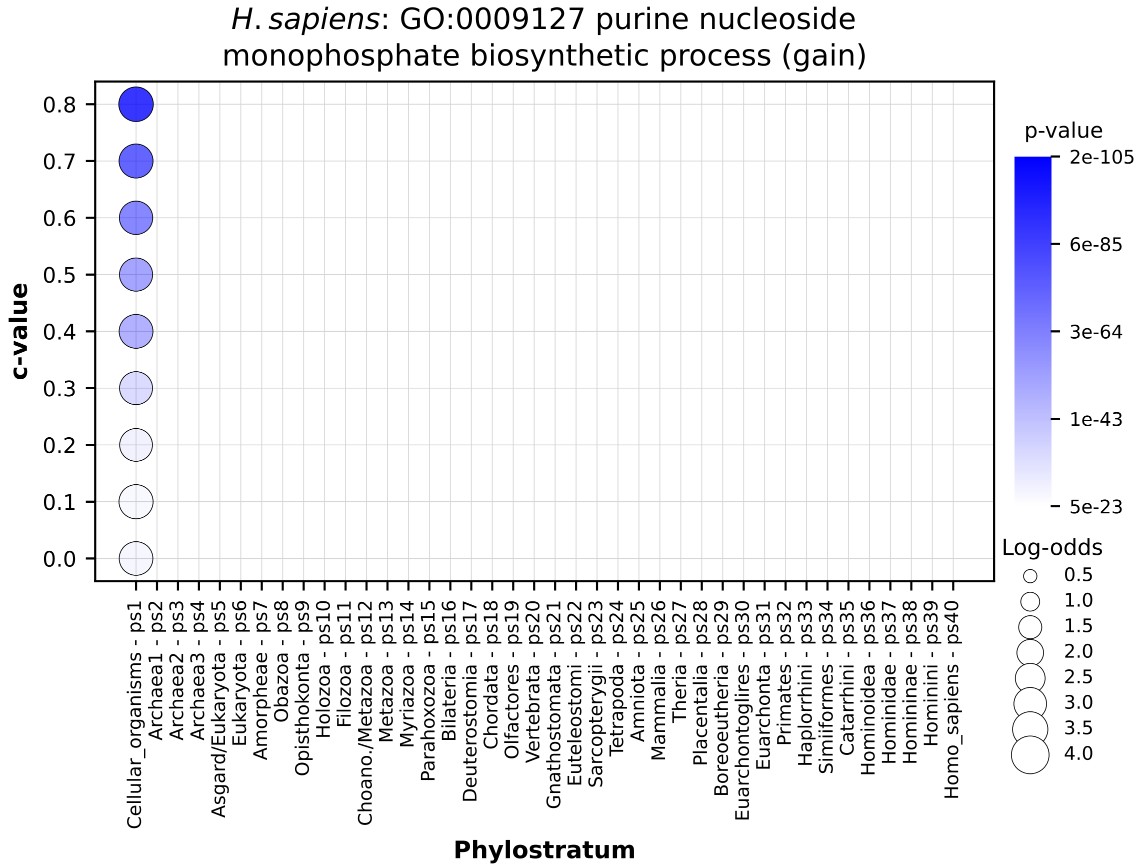

*H. sapiens*: GO:0009127 purine nucleoside monophosphate biosynthetic process (gain)
c-value
0.8
0.7
0.6
0.5
0.4
0.3
0.2
0.1
0.0
p-value
2e-105
6e-85
3e-64
1e-43
5e-23
Log-odds
0.5
1.0
1.5
2.0
2.5
3.0
3.5
4.0
Cellular_organisms - ps1
Archaea1 - ps2
Archaea2 - ps3
Archaea3 - ps4
Asgard/Eukaryota - ps5
Eukaryota - ps6
Amorpheae - ps7
Obazoa - ps8
Opisthokonta - ps9
Holozoa - ps10
Filozoa - ps11
Choano./Metazoa - ps12
Metazoa - ps13
Myriazoa - ps14
Parahoxozoa - ps15
Bilateria - ps16
Deuterostomia - ps17
Chordata - ps18
Olfactores - ps19
Vertebrata - ps20
Gnathostomata - ps21
Euteleostomi - ps22
Sarcopterygii - ps23
Tetrapoda - ps24
Amniota - ps25
Mammalia - ps26
Theria - ps27
Placentalia - ps28
Boreoeutheria - ps29
Euarchontoglires - ps30
Euarchonta - ps31
Primates - ps32
Haplorrhini - ps33
Simiiformes - ps34
Catarrhini - ps35
Hominoidea - ps36
Hominidae - ps37
Homininae - ps38
Hominini - ps39
Homo_sapiens - ps40
Phylostratum

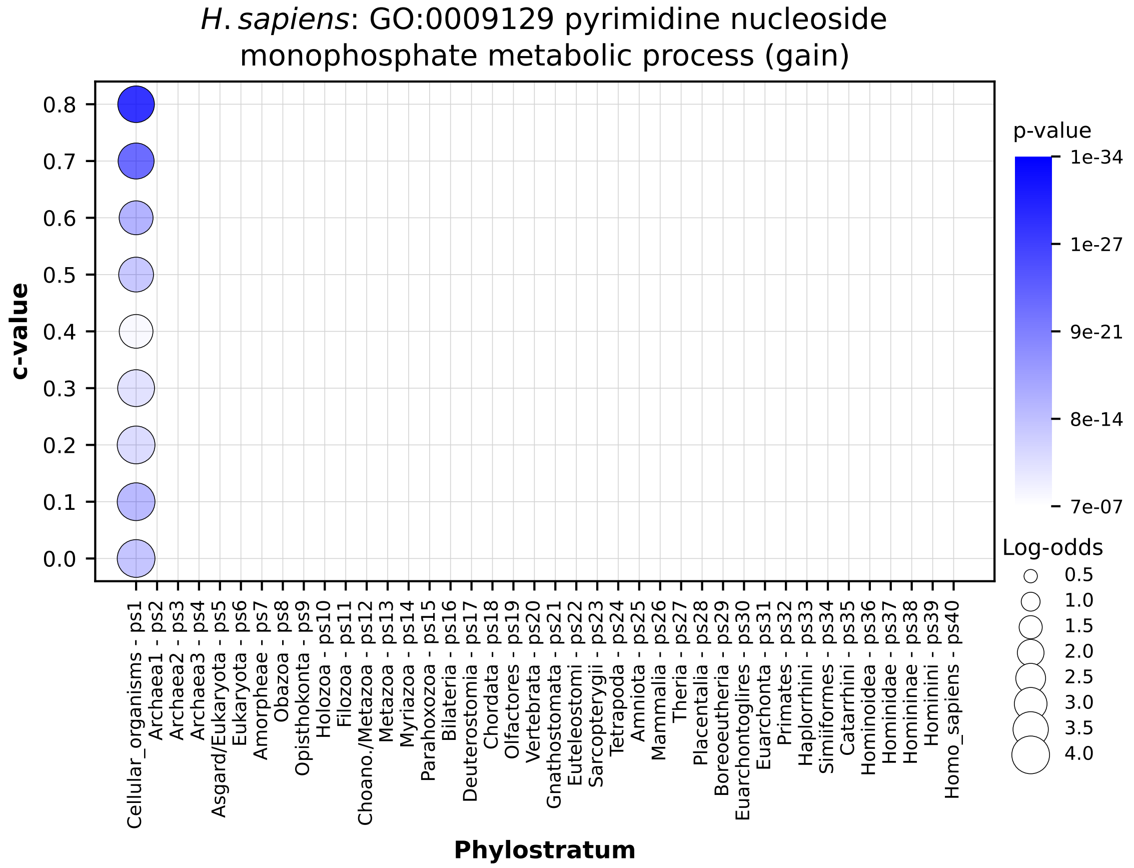

H. sapiens: GO:0009129 pyrimidine nucleoside monophosphate metabolic process (gain)
c-value
0.8
0.7
0.6
0.5
0.4
0.3
0.2
0.1
0.0
Phylostratum
Cellular_organisms - ps1
Archaea1 - ps2
Archaea2 - ps3
Archaea3 - ps4
Asgard/Eukaryota - ps5
Eukaryota - ps6
Amorpheae - ps7
Obazoa - ps8
Opisthokonta - ps9
Holozoa - ps10
Filozoa - ps11
Choano./Metazoa - ps12
Metazoa - ps13
Myriazoa - ps14
Parahoxozoa - ps15
Bilateria - ps16
Deuterostomia - ps17
Chordata - ps18
Olfactores - ps19
Vertebrata - ps20
Gnathostomata - ps21
Euteleostomi - ps22
Sarcopterygii - ps23
Tetrapoda - ps24
Amniota - ps25
Mammalia - ps26
Theria - ps27
Placentalia - ps28
Boreoeutheria - ps29
Euarchontoglires - ps30
Euarchonta - ps31
Primates - ps32
Haplorrhini - ps33
Simiiformes - ps34
Catarrhini - ps35
Hominoidea - ps36
Hominidae - ps37
Homininae - ps38
Hominini - ps39
Homo_sapiens - ps40
p-value
1e-34
1e-27
9e-21
8e-14
7e-07
Log-odds
0.5
1.0
1.5
2.0
2.5
3.0
3.5
4.0

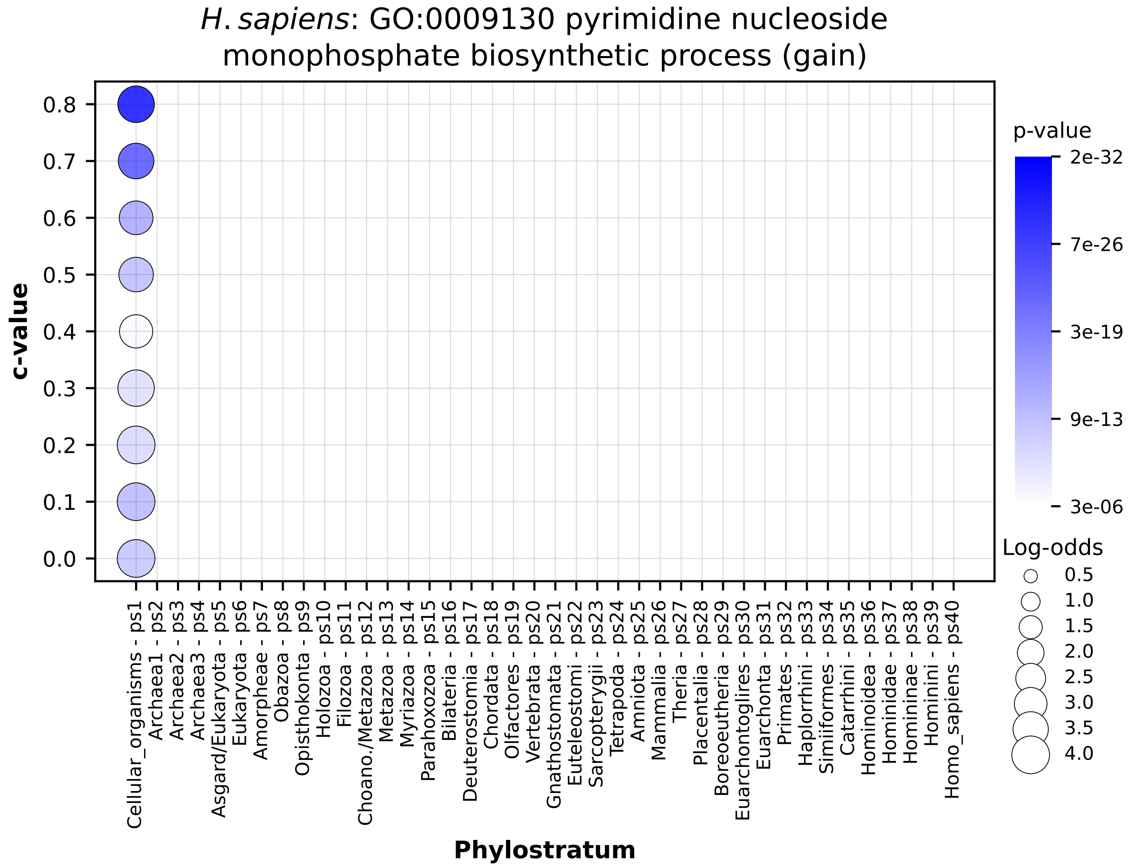
H. sapiens: GO:0009130 pyrimidine nucleoside monophosphate biosynthetic process (gain)
c-value
0.8
0.7
0.6
0.5
0.4
0.3
0.2
0.1
0.0
p-value
2e-32
7e-26
3e-19
9e-13
3e-06
Log-odds
0.5
1.0
1.5
2.0
2.5
3.0
3.5
4.0
Cellular_organisms - ps1
Archaea1 - ps2
Archaea2 - ps3
Archaea3 - ps4
Asgard/Eukaryota - ps5
Eukaryota - ps6
Amorpheae - ps7
Obazoa - ps8
Opisthokonta - ps9
Holozoa - ps10
Filozoa - ps11
Choano./Metazoa - ps12
Metazoa - ps13
Myriazoa - ps14
Parahoxozoa - ps15
Bilateria - ps16
Deuterostomia - ps17
Chordata - ps18
Olfactores - ps19
Vertebrata - ps20
Gnathostomata - ps21
Euteleostomi - ps22
Sarcopterygii - ps23
Tetrapoda - ps24
Amniota - ps25
Mammalia - ps26
Theria - ps27
Placentalia - ps28
Boreoeutheria - ps29
Euarchontoglires - ps30
Euarchonta - ps31
Primates - ps32
Haplorrhini - ps33
Simiiformes - ps34
Catarrhini - ps35
Hominoidea - ps36
Hominidae - ps37
Homininae - ps38
Hominini - ps39
Homo_sapiens - ps40
Phylostratum

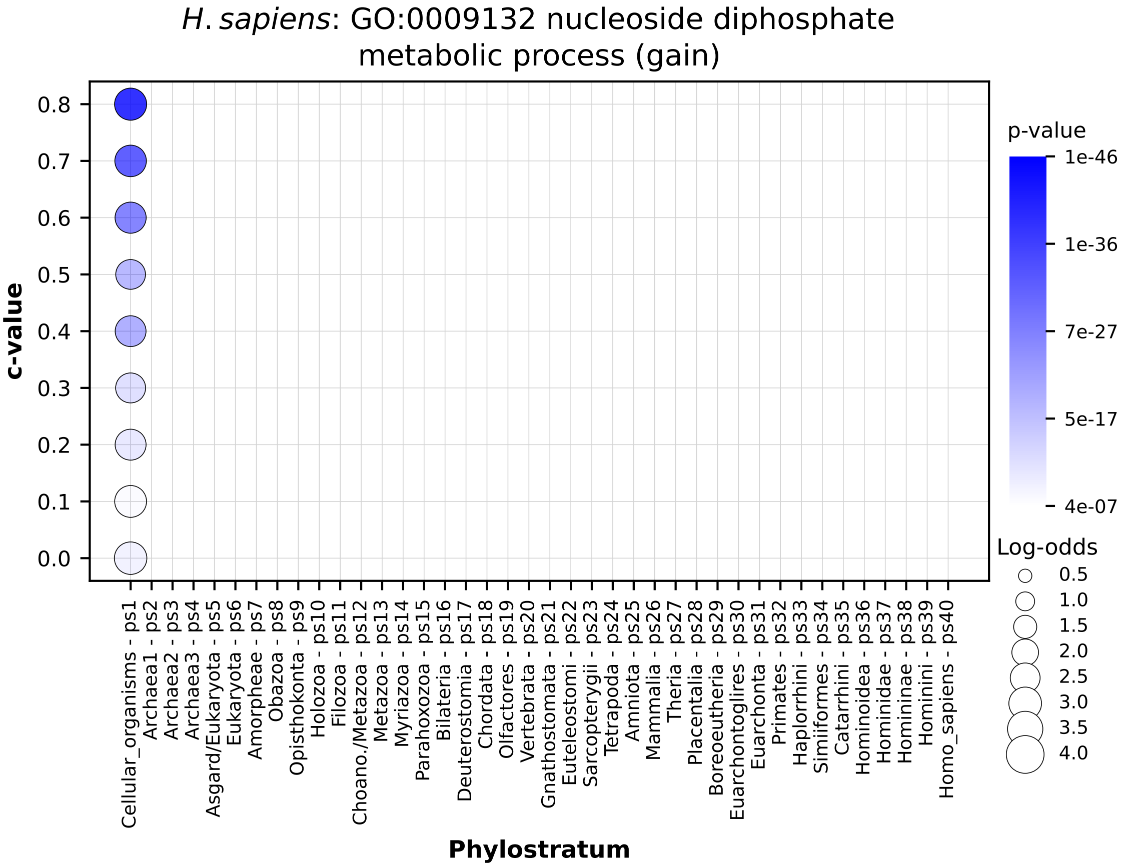
H. sapiens: GO:0009132 nucleoside diphosphate metabolic process (gain)
c-value
0.8
0.7
0.6
0.5
0.4
0.3
0.2
0.1
0.0
p-value
1e-46
1e-36
7e-27
5e-17
4e-07
Log-odds
0.5
1.0
1.5
2.0
2.5
3.0
3.5
4.0
Cellular_organisms - ps1
Archaea1 - ps2
Archaea2 - ps3
Archaea3 - ps4
Asgard/Eukaryota - ps5
Eukaryota - ps6
Amorpheae - ps7
Obazoa - ps8
Opisthokonta - ps9
Holozoa - ps10
Filozoa - ps11
Choano./Metazoa - ps12
Metazoa - ps13
Myriazoa - ps14
Parahoxozoa - ps15
Bilateria - ps16
Deuterostomia - ps17
Chordata - ps18
Olfactores - ps19
Vertebrata - ps20
Gnathostomata - ps21
Euteleostomi - ps22
Sarcopterygii - ps23
Tetrapoda - ps24
Amniota - ps25
Mammalia - ps26
Theria - ps27
Placentalia - ps28
Boreoeutheria - ps29
Euarchontoglires - ps30
Euarchonta - ps31
Primates - ps32
Haplorrhini - ps33
Simiiformes - ps34
Catarrhini - ps35
Hominoidea - ps36
Hominidae - ps37
Homininae - ps38
Hominini - ps39
Homo_sapiens - ps40
Phylostratum

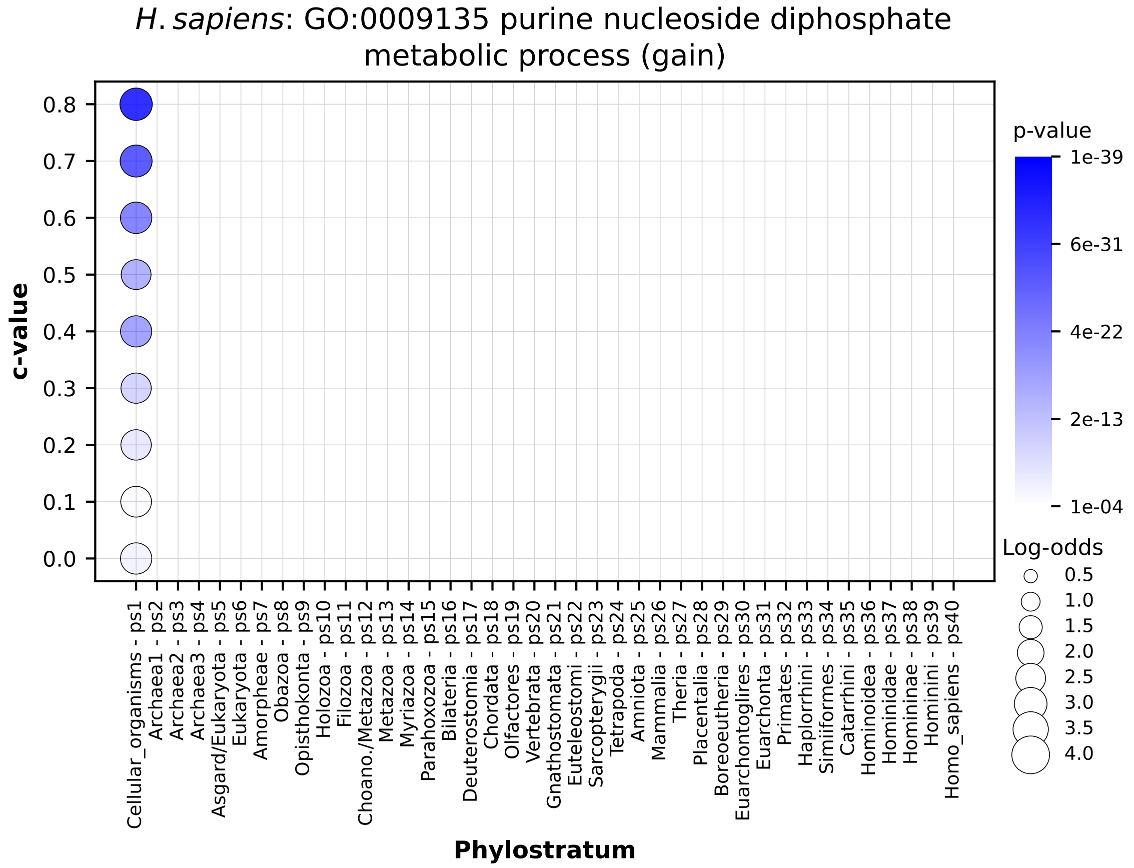

H. sapiens: GO:0009135 purine nucleoside diphosphate metabolic process (gain)
c-value
0.8
0.7
0.6
0.5
0.4
0.3
0.2
0.1
0.0
Phylostratum
Cellular_organisms - ps1
Archaea1 - ps2
Archaea2 - ps3
Archaea3 - ps4
Asgard/Eukaryota - ps5
Eukaryota - ps6
Amorpheae - ps7
Obazoa - ps8
Opisthokonta - ps9
Holozoa - ps10
Filozoa - ps11
Choano./Metazoa - ps12
Metazoa - ps13
Myriazoa - ps14
Parahoxozoa - ps15
Bilateria - ps16
Deuterostomia - ps17
Chordata - ps18
Olfactores - ps19
Vertebrata - ps20
Gnathostomata - ps21
Euteleostomi - ps22
Sarcopterygii - ps23
Tetrapoda - ps24
Amniota - ps25
Mammalia - ps26
Theria - ps27
Placentalia - ps28
Boreoeutheria - ps29
Euarchontoglires - ps30
Euarchonta - ps31
Primates - ps32
Haplorrhini - ps33
Simiiformes - ps34
Catarrhini - ps35
Hominoidea - ps36
Hominidae - ps37
Homininae - ps38
Hominini - ps39
Homo_sapiens - ps40
p-value
1e-39
6e-31
4e-22
2e-13
1e-04
Log-odds
0.5
1.0
1.5
2.0
2.5
3.0
3.5
4.0

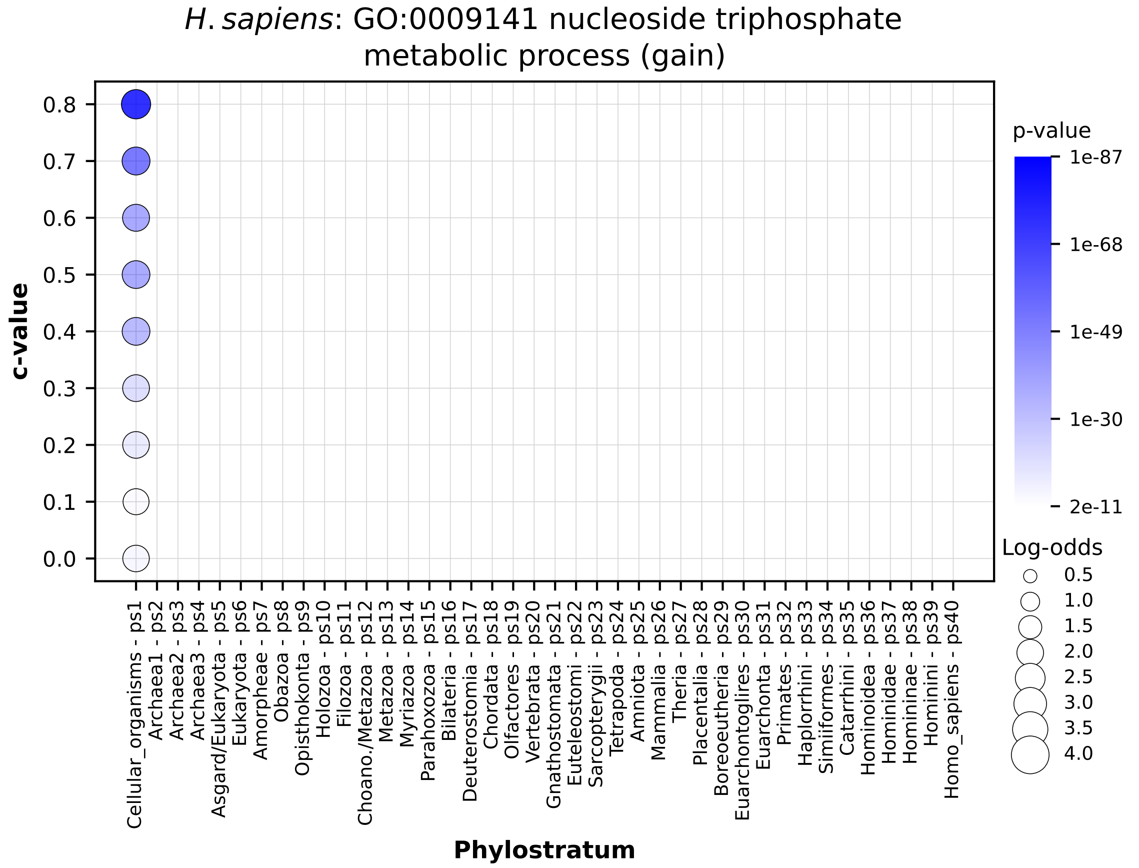

H. sapiens: GO:0009141 nucleoside triphosphate metabolic process (gain)
c-value
0.0
0.1
0.2
0.3
0.4
0.5
0.6
0.7
0.8
Phylostratum
Cellular_organisms - ps1
Archaea1 - ps2
Archaea2 - ps3
Archaea3 - ps4
Asgard/Eukaryota - ps5
Eukaryota - ps6
Amorpheae - ps7
Obazoa - ps8
Opisthokonta - ps9
Holozoa - ps10
Filozoa - ps11
Choano./Metazoa - ps12
Metazoa - ps13
Myriazoa - ps14
Parahoxozoa - ps15
Bilateria - ps16
Deuterostomia - ps17
Chordata - ps18
Olfactores - ps19
Vertebrata - ps20
Gnathostomata - ps21
Euteleostomi - ps22
Sarcopterygii - ps23
Tetrapoda - ps24
Amniota - ps25
Mammalia - ps26
Theria - ps27
Placentalia - ps28
Boreoeutheria - ps29
Euarchontoglires - ps30
Euarchonta - ps31
Primates - ps32
Haplorrhini - ps33
Simiiformes - ps34
Catarrhini - ps35
Hominoidea - ps36
Hominidae - ps37
Homininae - ps38
Hominini - ps39
Homo_sapiens - ps40
p-value
1e-87
1e-68
1e-49
1e-30
2e-11
Log-odds
0.5
1.0
1.5
2.0
2.5
3.0
3.5
4.0

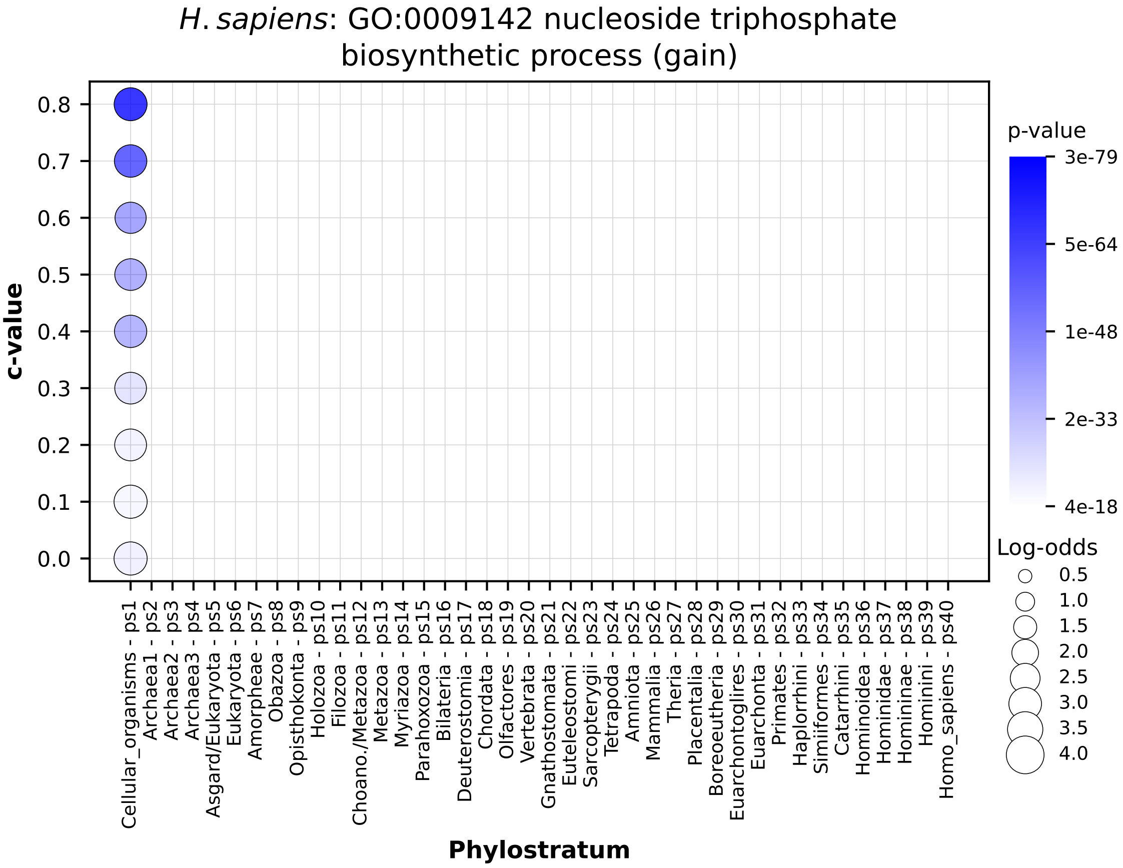

H. sapiens: GO:0009142 nucleoside triphosphate biosynthetic process (gain)
c-value
0.8
0.7
0.6
0.5
0.4
0.3
0.2
0.1
0.0
p-value
3e-79
5e-64
1e-48
2e-33
4e-18
Log-odds
0.5
1.0
1.5
2.0
2.5
3.0
3.5
4.0
Cellular_organisms - ps1
Archaea1 - ps2
Archaea2 - ps3
Archaea3 - ps4
Asgard/Eukaryota - ps5
Eukaryota - ps6
Amorpheae - ps7
Obazoa - ps8
Opisthokonta - ps9
Holozoa - ps10
Filozoa - ps11
Choano./Metazoa - ps12
Metazoa - ps13
Myriazoa - ps14
Parahoxozoa - ps15
Bilateria - ps16
Deuterostomia - ps17
Chordata - ps18
Olfactores - ps19
Vertebrata - ps20
Gnathostomata - ps21
Euteleostomi - ps22
Sarcopterygii - ps23
Tetrapoda - ps24
Amniota - ps25
Mammalia - ps26
Theria - ps27
Placentalia - ps28
Boreoeutheria - ps29
Euarchontoglires - ps30
Euarchonta - ps31
Primates - ps32
Haplorrhini - ps33
Simiiformes - ps34
Catarrhini - ps35
Hominoidea - ps36
Hominidae - ps37
Homininae - ps38
Hominini - ps39
Homo_sapiens - ps40
Phylostratum

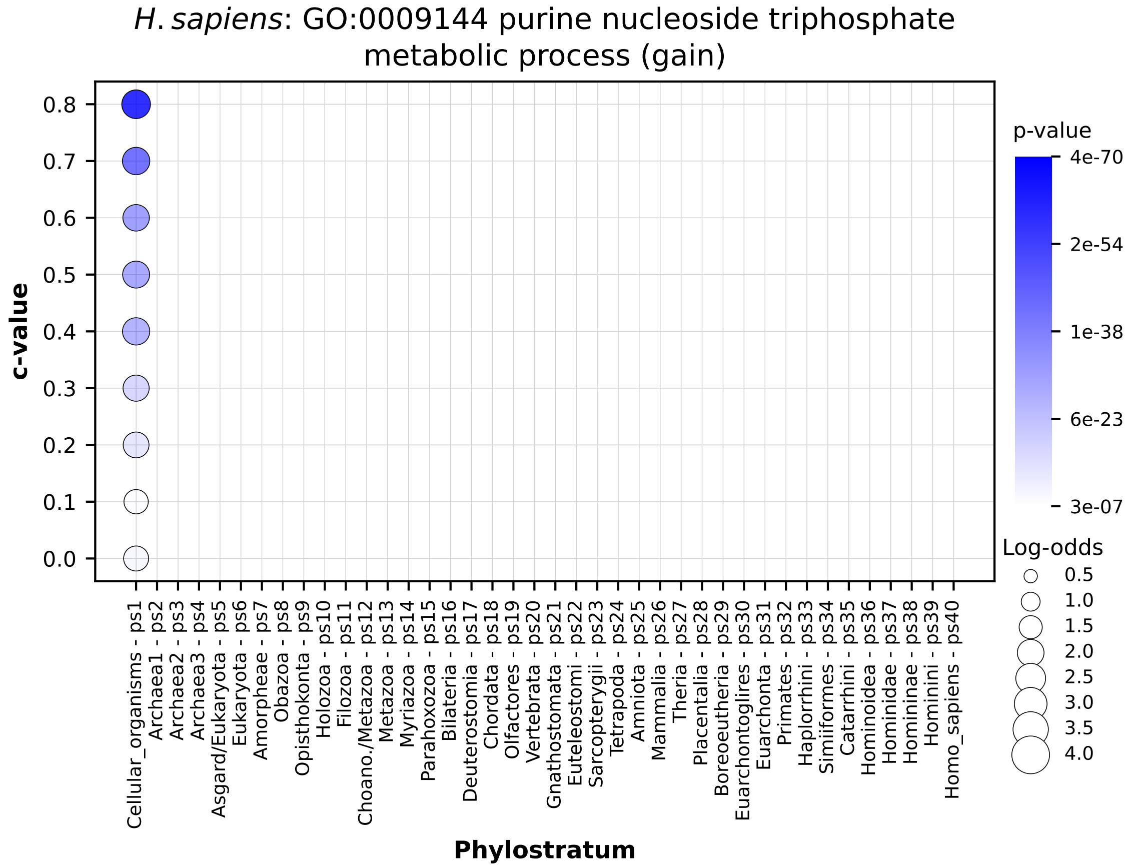
H. sapiens: GO:0009144 purine nucleoside triphosphate metabolic process (gain)
c-value
0.8
0.7
0.6
0.5
0.4
0.3
0.2
0.1
0.0
p-value
4e-70
2e-54
1e-38
6e-23
3e-07
Log-odds
0.5
1.0
1.5
2.0
2.5
3.0
3.5
4.0
Cellular_organisms - ps1
Archaea1 - ps2
Archaea2 - ps3
Archaea3 - ps4
Asgard/Eukaryota - ps5
Eukaryota - ps6
Amorpheae - ps7
Obazoa - ps8
Opisthokonta - ps9
Holozoa - ps10
Filozoa - ps11
Choano./Metazoa - ps12
Metazoa - ps13
Myriazoa - ps14
Parahoxozoa - ps15
Bilateria - ps16
Deuterostomia - ps17
Chordata - ps18
Olfactores - ps19
Vertebrata - ps20
Gnathostomata - ps21
Euteleostomi - ps22
Sarcopterygii - ps23
Tetrapoda - ps24
Amniota - ps25
Mammalia - ps26
Theria - ps27
Placentalia - ps28
Boreoeutheria - ps29
Euarchontoglires - ps30
Euarchonta - ps31
Primates - ps32
Haplorrhini - ps33
Simiiformes - ps34
Catarrhini - ps35
Hominoidea - ps36
Hominidae - ps37
Homininae - ps38
Hominini - ps39
Homo_sapiens - ps40
Phylostratum

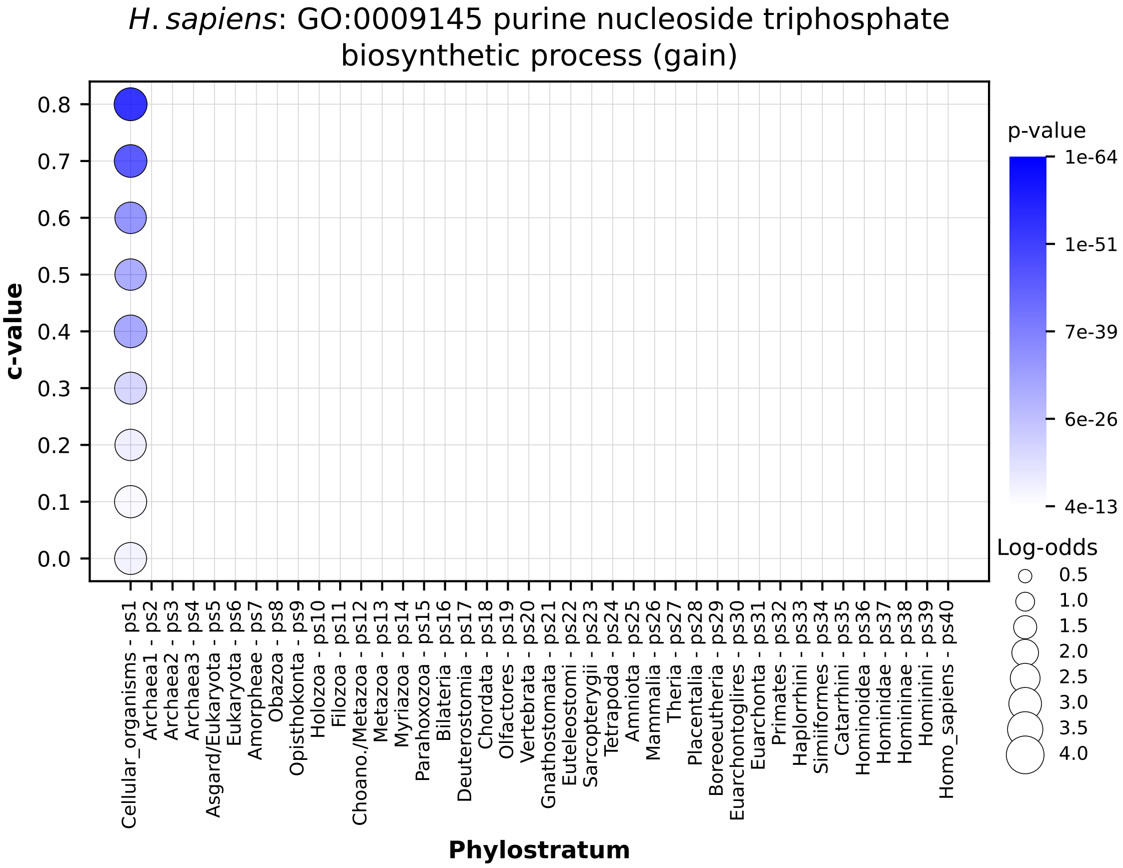

H. sapiens: GO:0009145 purine nucleoside triphosphate biosynthetic process (gain)
c-value
0.8
0.7
0.6
0.5
0.4
0.3
0.2
0.1
0.0
p-value
1e-64
1e-51
7e-39
6e-26
4e-13
Log-odds
0.5
1.0
1.5
2.0
2.5
3.0
3.5
4.0
Cellular_organisms - ps1
Archaea1 - ps2
Archaea2 - ps3
Archaea3 - ps4
Asgard/Eukaryota - ps5
Eukaryota - ps6
Amorpheae - ps7
Obazoa - ps8
Opisthokonta - ps9
Holozoa - ps10
Filozoa - ps11
Choano./Metazoa - ps12
Metazoa - ps13
Myriazoa - ps14
Parahoxozoa - ps15
Bilateria - ps16
Deuterostomia - ps17
Chordata - ps18
Olfactores - ps19
Vertebrata - ps20
Gnathostomata - ps21
Euteleostomi - ps22
Sarcopterygii - ps23
Tetrapoda - ps24
Amniota - ps25
Mammalia - ps26
Theria - ps27
Placentalia - ps28
Boreoeutheria - ps29
Euarchontoglires - ps30
Euarchonta - ps31
Primates - ps32
Haplorrhini - ps33
Simiiformes - ps34
Catarrhini - ps35
Hominoidea - ps36
Hominidae - ps37
Homininae - ps38
Hominini - ps39
Homo_sapiens - ps40
Phylostratum

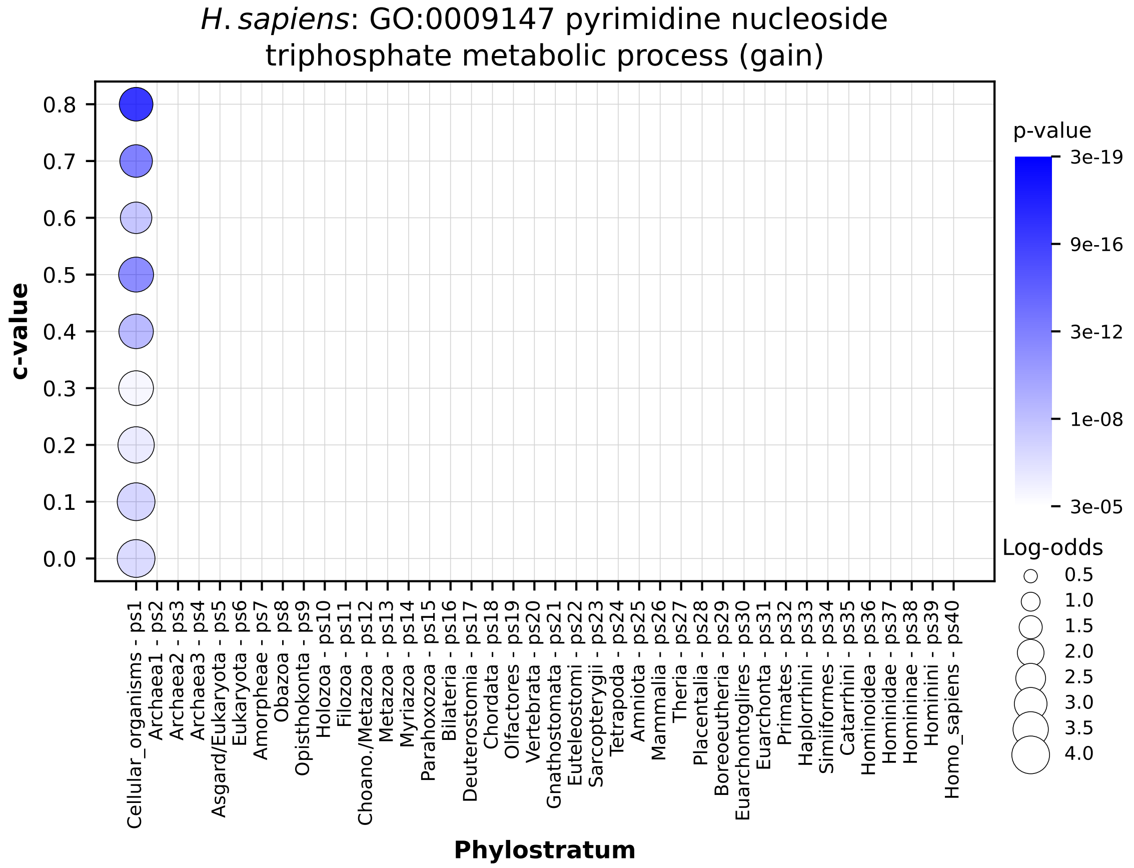
H. sapiens: GO:0009147 pyrimidine nucleoside triphosphate metabolic process (gain)
c-value
0.8
0.7
0.6
0.5
0.4
0.3
0.2
0.1
0.0
p-value
3e-19
9e-16
3e-12
1e-08
3e-05
Log-odds
0.5
1.0
1.5
2.0
2.5
3.0
3.5
4.0
Cellular_organisms - ps1
Archaea1 - ps2
Archaea2 - ps3
Archaea3 - ps4
Asgard/Eukaryota - ps5
Eukaryota - ps6
Amorpheae - ps7
Obazoa - ps8
Opisthokonta - ps9
Holozoa - ps10
Filozoa - ps11
Choano./Metazoa - ps12
Metazoa - ps13
Myriazoa - ps14
Parahoxozoa - ps15
Bilateria - ps16
Deuterostomia - ps17
Chordata - ps18
Olfactores - ps19
Vertebrata - ps20
Gnathostomata - ps21
Euteleostomi - ps22
Sarcopterygii - ps23
Tetrapoda - ps24
Amniota - ps25
Mammalia - ps26
Theria - ps27
Placentalia - ps28
Boreoeutheria - ps29
Euarchontoglires - ps30
Euarchonta - ps31
Primates - ps32
Haplorrhini - ps33
Simiiformes - ps34
Catarrhini - ps35
Hominoidea - ps36
Hominidae - ps37
Homininae - ps38
Hominini - ps39
Homo_sapiens - ps40
Phylostratum

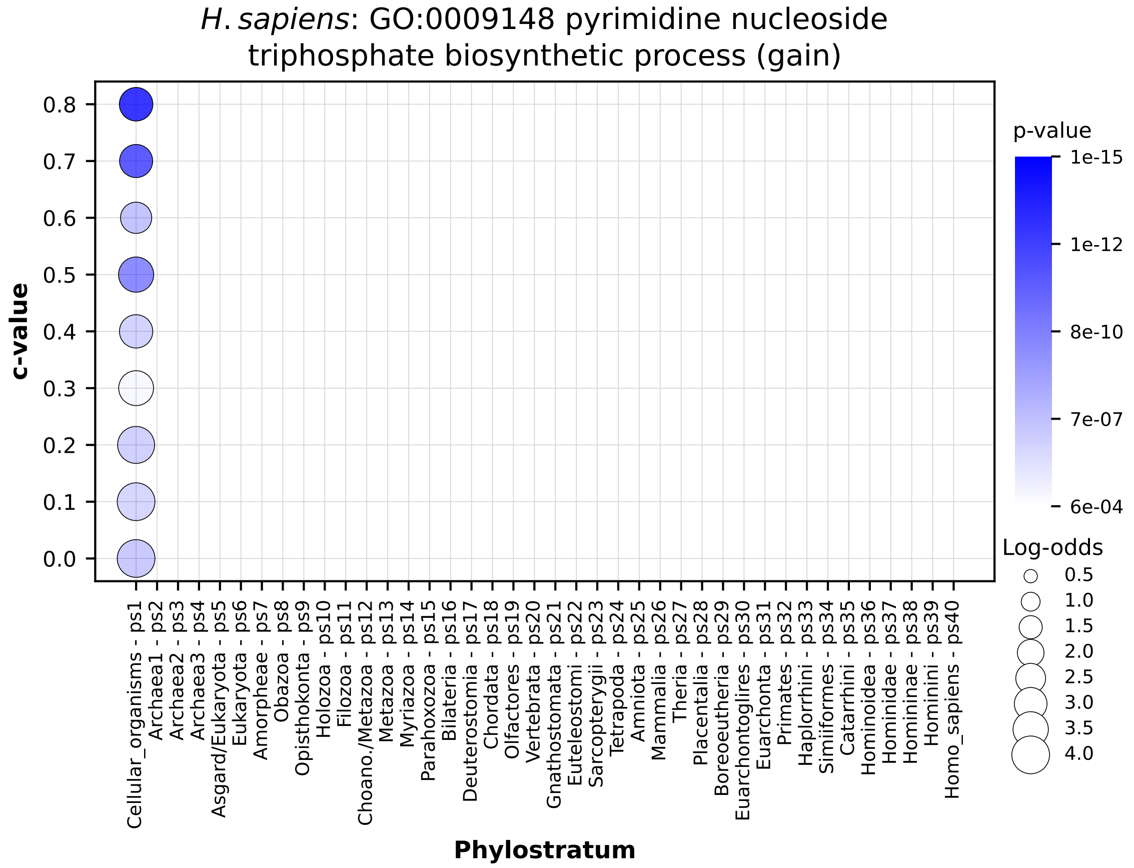

H. sapiens: GO:0009148 pyrimidine nucleoside triphosphate biosynthetic process (gain)
c-value
0.8
0.7
0.6
0.5
0.4
0.3
0.2
0.1
0.0
p-value
1e-15
1e-12
8e-10
7e-07
6e-04
Log-odds
0.5
1.0
1.5
2.0
2.5
3.0
3.5
4.0
Cellular_organisms - ps1
Archaea1 - ps2
Archaea2 - ps3
Archaea3 - ps4
Asgard/Eukaryota - ps5
Eukaryota - ps6
Amorpheae - ps7
Obazoa - ps8
Opisthokonta - ps9
Holozoa - ps10
Filozoa - ps11
Choano./Metazoa - ps12
Metazoa - ps13
Myriazoa - ps14
Parahoxozoa - ps15
Bilateria - ps16
Deuterostomia - ps17
Chordata - ps18
Olfactores - ps19
Vertebrata - ps20
Gnathostomata - ps21
Euteleostomi - ps22
Sarcopterygii - ps23
Tetrapoda - ps24
Amniota - ps25
Mammalia - ps26
Theria - ps27
Placentalia - ps28
Boreoeutheria - ps29
Euarchontoglires - ps30
Euarchonta - ps31
Primates - ps32
Haplorrhini - ps33
Simiiformes - ps34
Catarrhini - ps35
Hominoidea - ps36
Hominidae - ps37
Homininae - ps38
Hominini - ps39
Homo_sapiens - ps40
Phylostratum

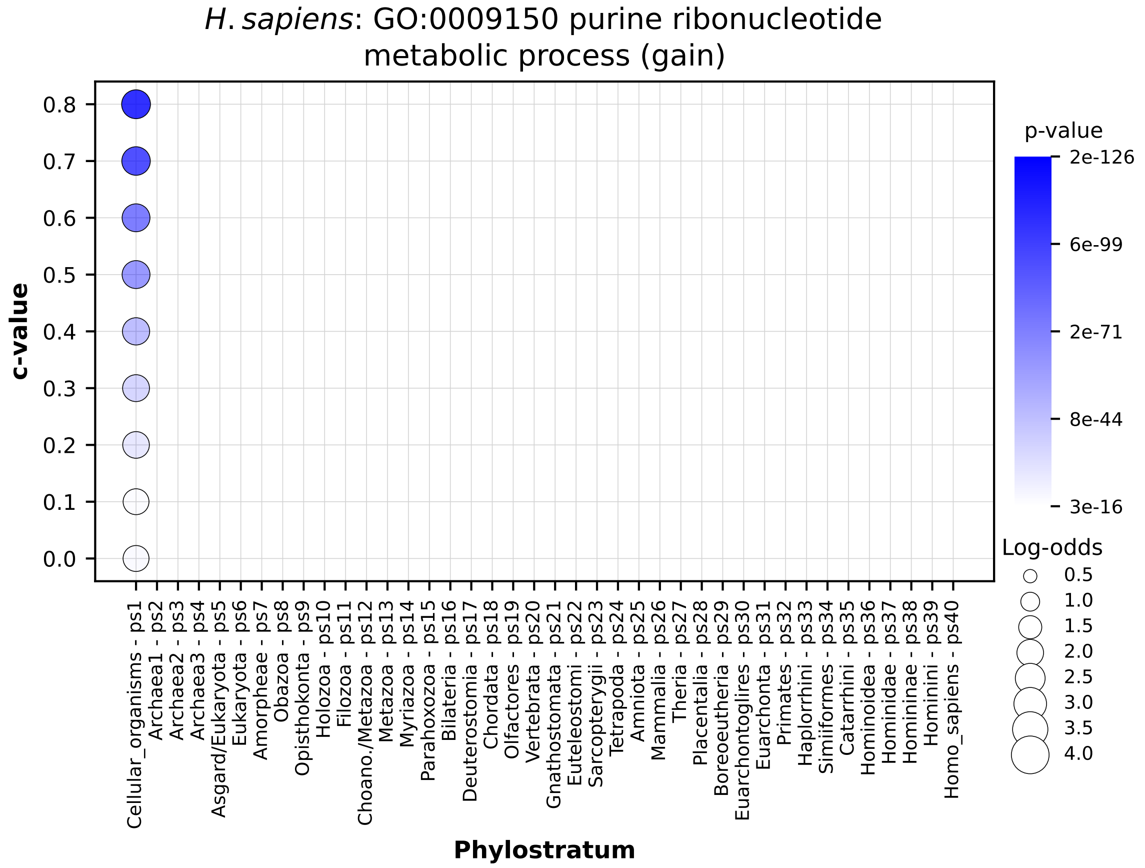

H. sapiens: GO:0009150 purine ribonucleotide metabolic process (gain)
c-value
0.0
0.1
0.2
0.3
0.4
0.5
0.6
0.7
0.8
Phylostratum
Cellular_organisms - ps1
Archaea1 - ps2
Archaea2 - ps3
Archaea3 - ps4
Asgard/Eukaryota - ps5
Eukaryota - ps6
Amorpheae - ps7
Obazoa - ps8
Opisthokonta - ps9
Holozoa - ps10
Filozoa - ps11
Choano./Metazoa - ps12
Metazoa - ps13
Myriazoa - ps14
Parahoxozoa - ps15
Bilateria - ps16
Deuterostomia - ps17
Chordata - ps18
Olfactores - ps19
Vertebrata - ps20
Gnathostomata - ps21
Euteleostomi - ps22
Sarcopterygii - ps23
Tetrapoda - ps24
Amniota - ps25
Mammalia - ps26
Theria - ps27
Placentalia - ps28
Boreoeutheria - ps29
Euarchontoglires - ps30
Euarchonta - ps31
Primates - ps32
Haplorrhini - ps33
Simiiformes - ps34
Catarrhini - ps35
Hominoidea - ps36
Hominidae - ps37
Homininae - ps38
Hominini - ps39
Homo_sapiens - ps40
p-value
2e-126
6e-99
2e-71
8e-44
3e-16
Log-odds
0.5
1.0
1.5
2.0
2.5
3.0
3.5
4.0

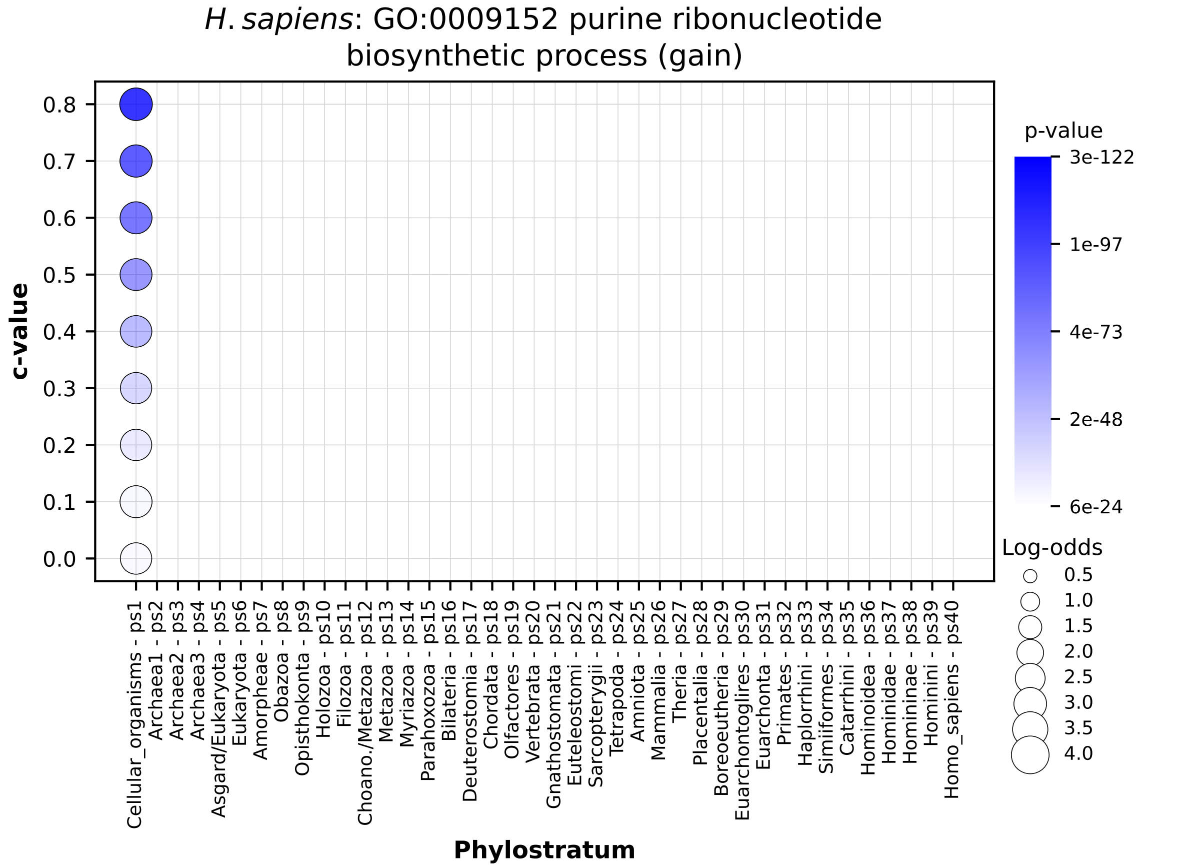

H. sapiens: GO:0009152 purine ribonucleotide biosynthetic process (gain)
c-value
0.8
0.7
0.6
0.5
0.4
0.3
0.2
0.1
0.0
p-value
3e-122
1e-97
4e-73
2e-48
6e-24
Log-odds
0.5
1.0
1.5
2.0
2.5
3.0
3.5
4.0
Cellular_organisms - ps1
Archaea1 - ps2
Archaea2 - ps3
Archaea3 - ps4
Asgard/Eukaryota - ps5
Eukaryota - ps6
Amorpheae - ps7
Obazoa - ps8
Opisthokonta - ps9
Holozoa - ps10
Filozoa - ps11
Choano./Metazoa - ps12
Metazoa - ps13
Myriazoa - ps14
Parahoxozoa - ps15
Bilateria - ps16
Deuterostomia - ps17
Chordata - ps18
Olfactores - ps19
Vertebrata - ps20
Gnathostomata - ps21
Euteleostomi - ps22
Sarcopterygii - ps23
Tetrapoda - ps24
Amniota - ps25
Mammalia - ps26
Theria - ps27
Placentalia - ps28
Boreoeutheria - ps29
Euarchontoglires - ps30
Euarchonta - ps31
Primates - ps32
Haplorrhini - ps33
Simiiformes - ps34
Catarrhini - ps35
Hominoidea - ps36
Hominidae - ps37
Homininae - ps38
Hominini - ps39
Homo_sapiens - ps40
Phylostratum

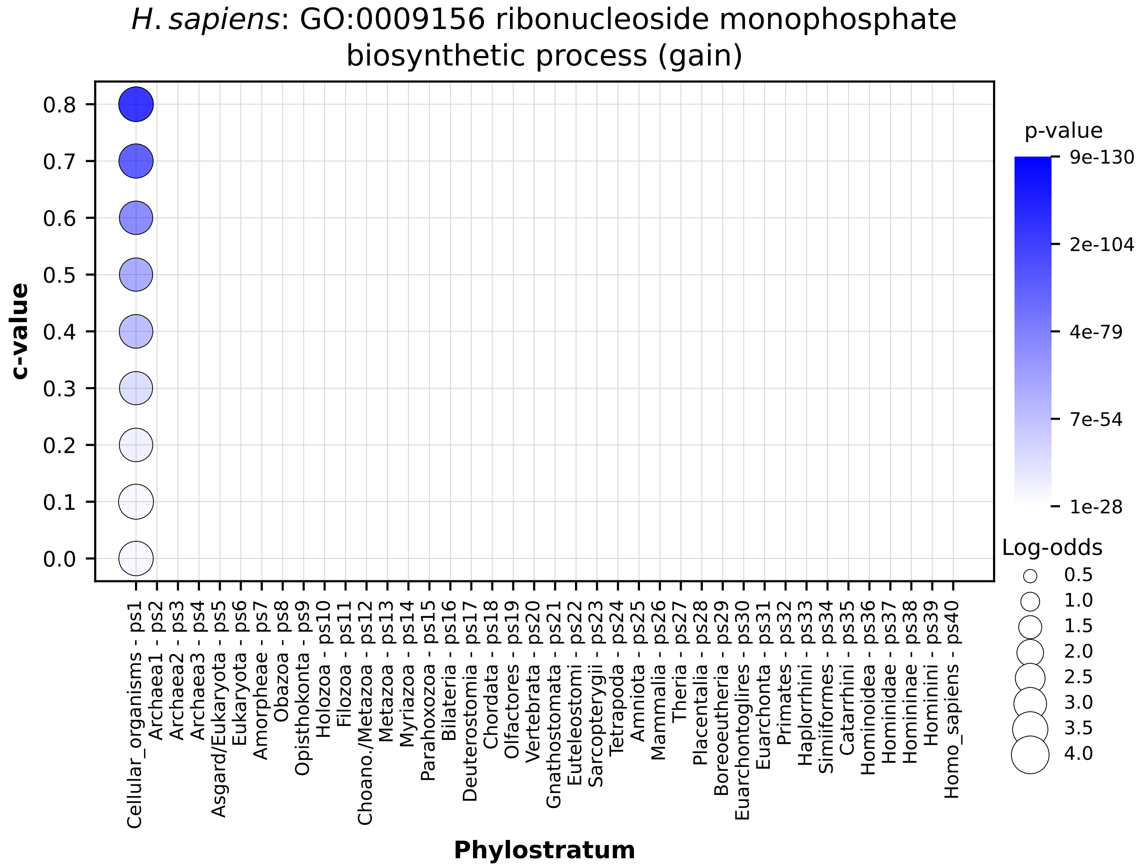

H. sapiens: GO:0009156 ribonucleoside monophosphate biosynthetic process (gain)
c-value
0.8
0.7
0.6
0.5
0.4
0.3
0.2
0.1
0.0
p-value
9e-130
2e-104
4e-79
7e-54
1e-28
Log-odds
0.5
1.0
1.5
2.0
2.5
3.0
3.5
4.0
Cellular_organisms - ps1
Archaea1 - ps2
Archaea2 - ps3
Archaea3 - ps4
Asgard/Eukaryota - ps5
Eukaryota - ps6
Amorpheae - ps7
Obazoa - ps8
Opisthokonta - ps9
Holozoa - ps10
Filozoa - ps11
Choano./Metazoa - ps12
Metazoa - ps13
Myriazoa - ps14
Parahoxozoa - ps15
Bilateria - ps16
Deuterostomia - ps17
Chordata - ps18
Olfactores - ps19
Vertebrata - ps20
Gnathostomata - ps21
Euteleostomi - ps22
Sarcopterygii - ps23
Tetrapoda - ps24
Amniota - ps25
Mammalia - ps26
Theria - ps27
Placentalia - ps28
Boreoeutheria - ps29
Euarchontoglires - ps30
Euarchonta - ps31
Primates - ps32
Haplorrhini - ps33
Simiiformes - ps34
Catarrhini - ps35
Hominoidea - ps36
Hominidae - ps37
Homininae - ps38
Hominini - ps39
Homo_sapiens - ps40
Phylostratum

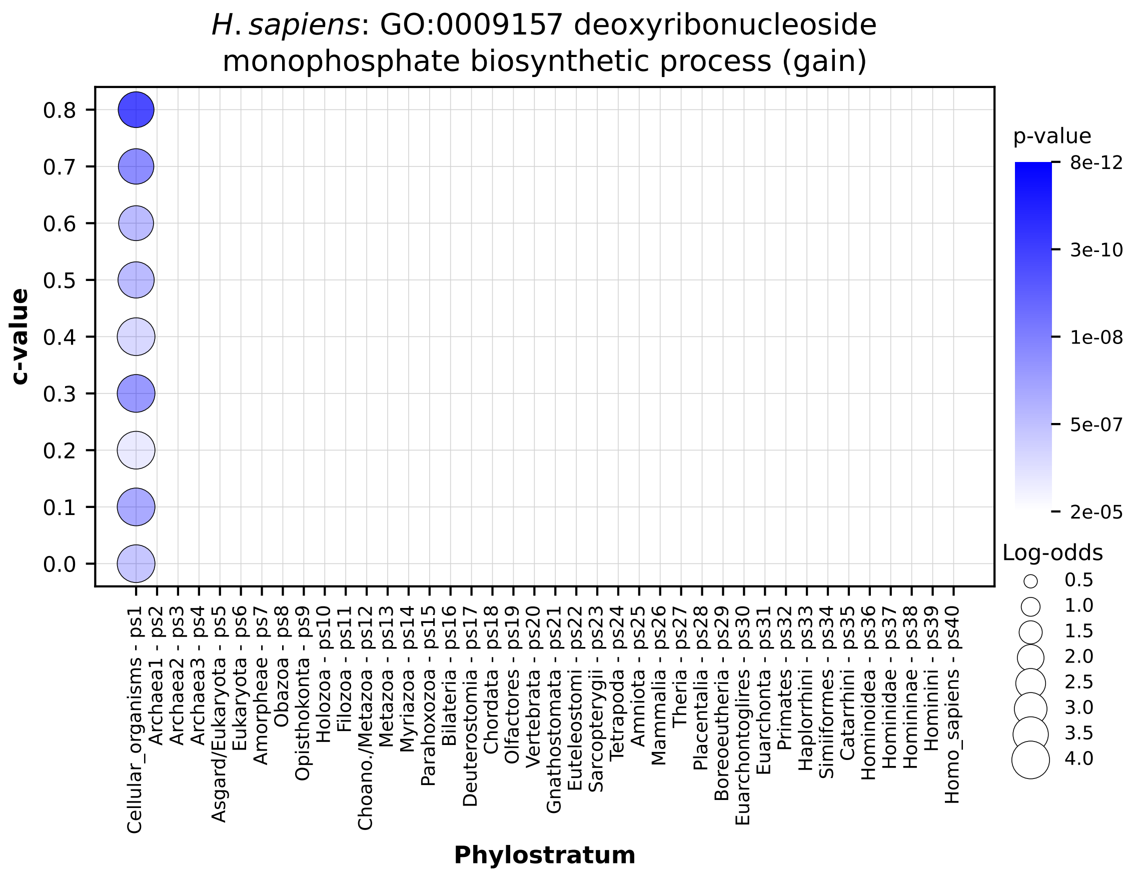
H. sapiens: GO:0009157 deoxyribonucleoside monophosphate biosynthetic process (gain)
c-value
0.8
0.7
0.6
0.5
0.4
0.3
0.2
0.1
0.0
p-value
8e-12
3e-10
1e-08
5e-07
2e-05
Log-odds
0.5
1.0
1.5
2.0
2.5
3.0
3.5
4.0
Cellular_organisms - ps1
Archaea1 - ps2
Archaea2 - ps3
Archaea3 - ps4
Asgard/Eukaryota - ps5
Eukaryota - ps6
Amorpheae - ps7
Obazoa - ps8
Opisthokonta - ps9
Holozoa - ps10
Filozoa - ps11
Choano./Metazoa - ps12
Metazoa - ps13
Myriazoa - ps14
Parahoxozoa - ps15
Bilateria - ps16
Deuterostomia - ps17
Chordata - ps18
Olfactores - ps19
Vertebrata - ps20
Gnathostomata - ps21
Euteleostomi - ps22
Sarcopterygii - ps23
Tetrapoda - ps24
Amniota - ps25
Mammalia - ps26
Theria - ps27
Placentalia - ps28
Boreoeutheria - ps29
Euarchontoglires - ps30
Euarchonta - ps31
Primates - ps32
Haplorrhini - ps33
Simiiformes - ps34
Catarrhini - ps35
Hominoidea - ps36
Hominidae - ps37
Homininae - ps38
Hominini - ps39
Homo_sapiens - ps40
Phylostratum

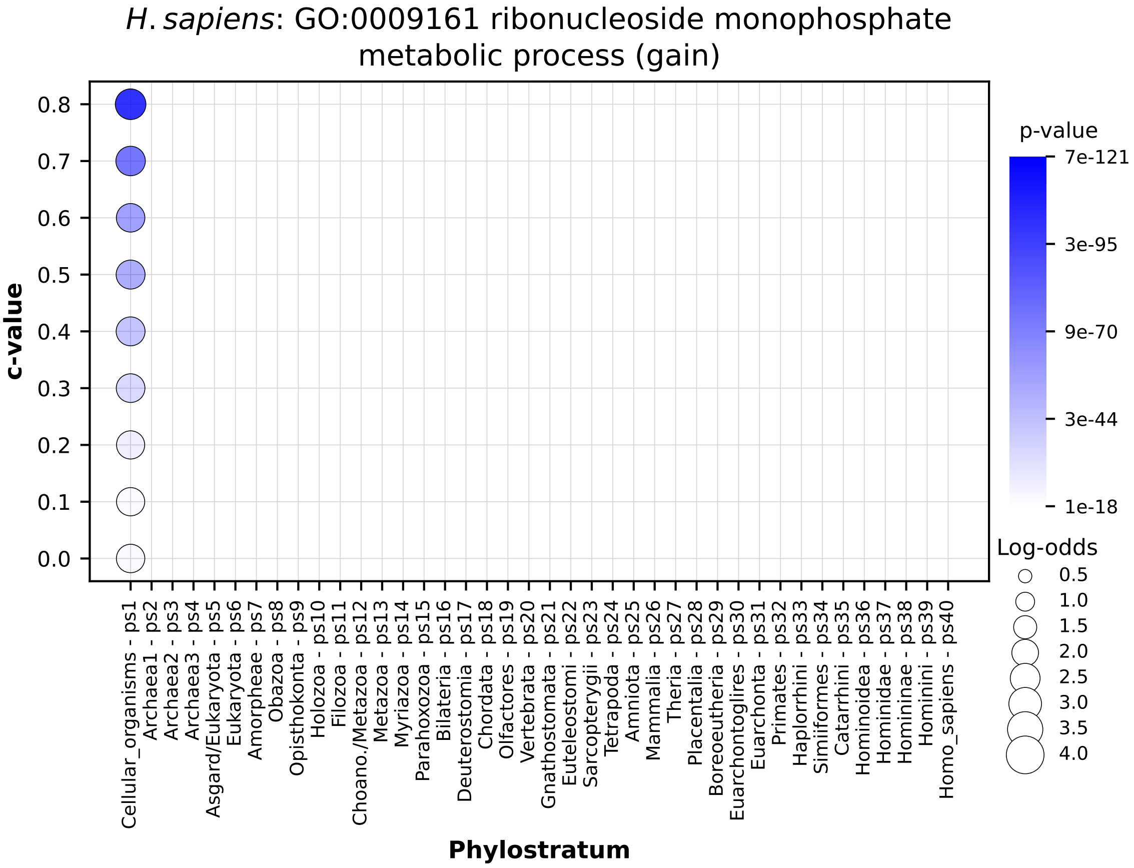
H. sapiens: GO:0009161 ribonucleoside monophosphate metabolic process (gain)
c-value
0.0
0.1
0.2
0.3
0.4
0.5
0.6
0.7
0.8
p-value
7e-121
3e-95
9e-70
3e-44
1e-18
Log-odds
0.5
1.0
1.5
2.0
2.5
3.0
3.5
4.0
Phylostratum
Cellular_organisms - ps1
Archaea1 - ps2
Archaea2 - ps3
Archaea3 - ps4
Asgard/Eukaryota - ps5
Eukaryota - ps6
Amorpheae - ps7
Obazoa - ps8
Opisthokonta - ps9
Holozoa - ps10
Filozoa - ps11
Choano./Metazoa - ps12
Metazoa - ps13
Myriazoa - ps14
Parahoxozoa - ps15
Bilateria - ps16
Deuterostomia - ps17
Chordata - ps18
Olfactores - ps19
Vertebrata - ps20
Gnathostomata - ps21
Euteleostomi - ps22
Sarcopterygii - ps23
Tetrapoda - ps24
Amniota - ps25
Mammalia - ps26
Theria - ps27
Placentalia - ps28
Boreoeutheria - ps29
Euarchontoglires - ps30
Euarchonta - ps31
Primates - ps32
Haplorrhini - ps33
Simiiformes - ps34
Catarrhini - ps35
Hominoidea - ps36
Hominidae - ps37
Homininae - ps38
Hominini - ps39
Homo_sapiens - ps40

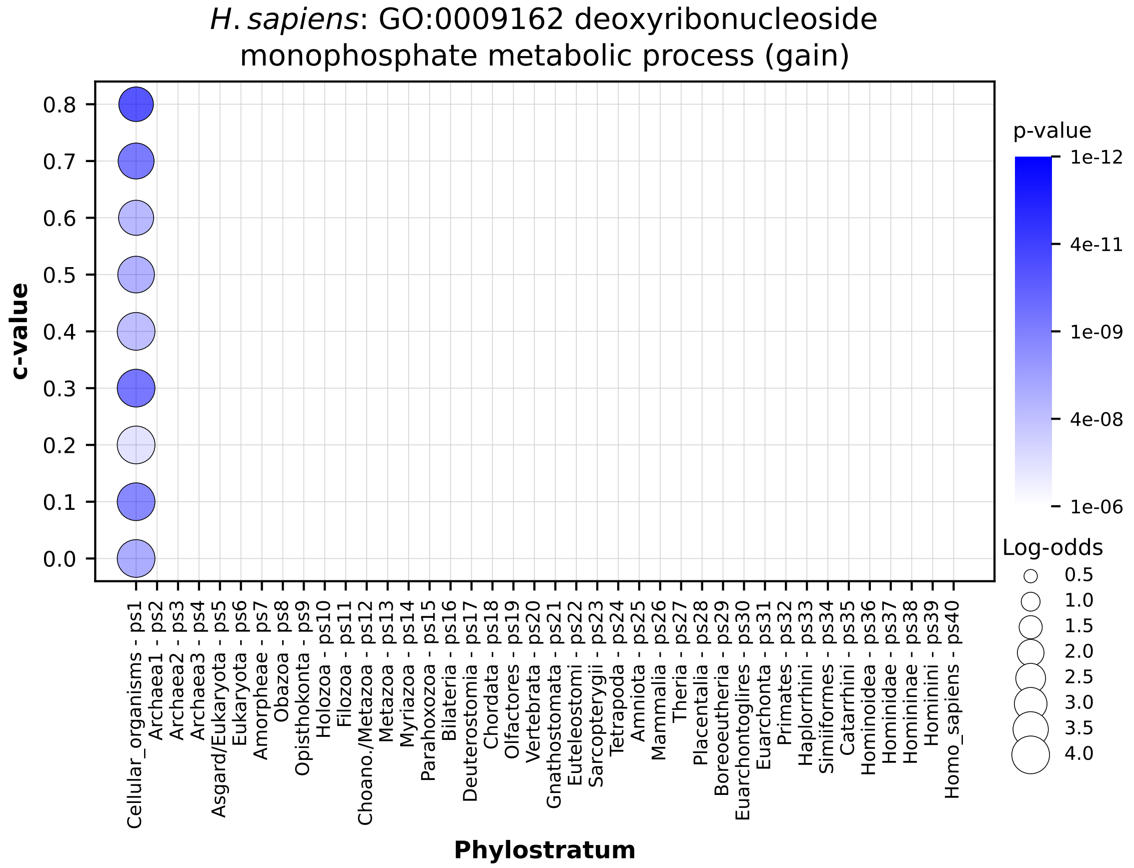

*H. sapiens*: GO:0009162 deoxyribonucleoside monophosphate metabolic process (gain)
c-value
0.8
0.7
0.6
0.5
0.4
0.3
0.2
0.1
0.0
p-value
1e-12
4e-11
1e-09
4e-08
1e-06
Log-odds
0.5
1.0
1.5
2.0
2.5
3.0
3.5
4.0
Cellular_organisms - ps1
Archaea1 - ps2
Archaea2 - ps3
Archaea3 - ps4
Asgard/Eukaryota - ps5
Eukaryota - ps6
Amorpheae - ps7
Obazoa - ps8
Opisthokonta - ps9
Holozoa - ps10
Filozoa - ps11
Choano./Metazoa - ps12
Metazoa - ps13
Myriazoa - ps14
Parahoxozoa - ps15
Bilateria - ps16
Deuterostomia - ps17
Chordata - ps18
Olfactores - ps19
Vertebrata - ps20
Gnathostomata - ps21
Euteleostomi - ps22
Sarcopterygii - ps23
Tetrapoda - ps24
Amniota - ps25
Mammalia - ps26
Theria - ps27
Placentalia - ps28
Boreoeutheria - ps29
Euarchontoglires - ps30
Euarchonta - ps31
Primates - ps32
Haplorrhini - ps33
Simiiformes - ps34
Catarrhini - ps35
Hominoidea - ps36
Hominidae - ps37
Homininae - ps38
Hominini - ps39
Homo_sapiens - ps40
Phylostratum

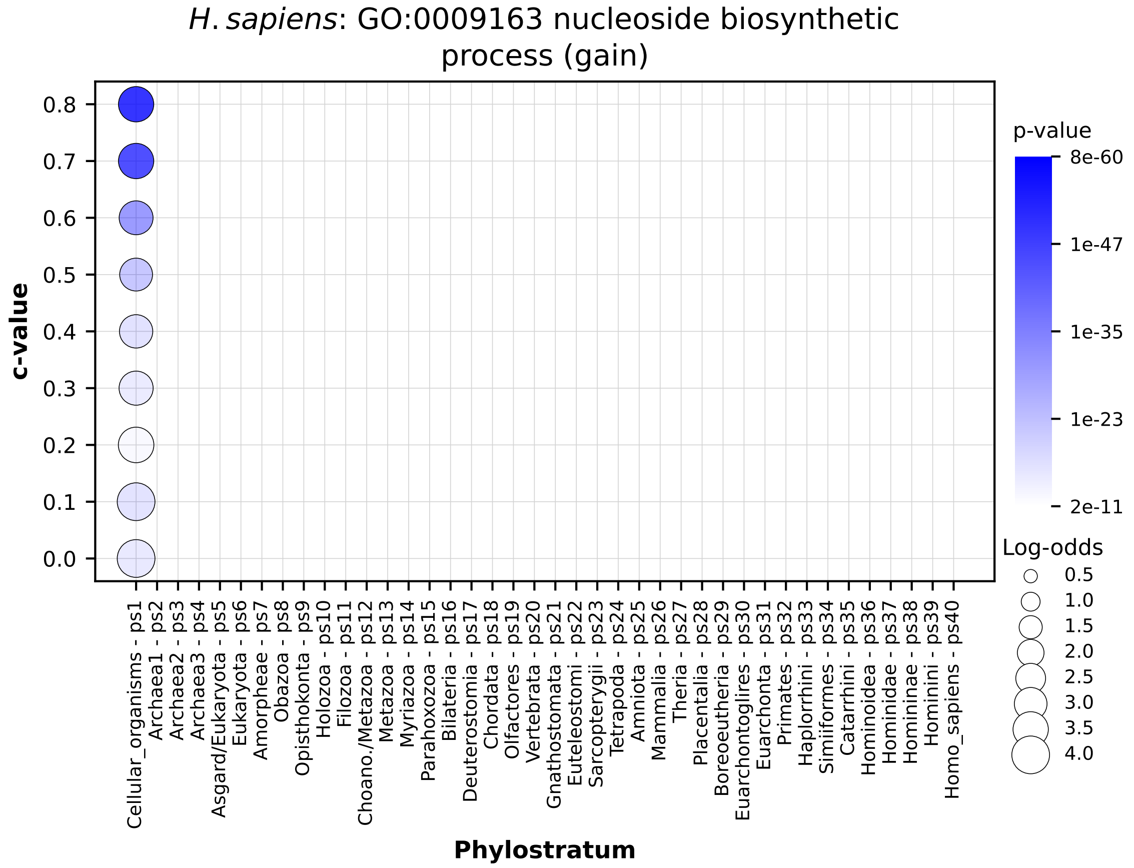
H. sapiens: GO:0009163 nucleoside biosynthetic process (gain)
c-value
0.0
0.1
0.2
0.3
0.4
0.5
0.6
0.7
0.8
p-value
8e-60
1e-47
1e-35
1e-23
2e-11
Log-odds
0.5
1.0
1.5
2.0
2.5
3.0
3.5
4.0
Cellular_organisms - ps1
Archaea1 - ps2
Archaea2 - ps3
Archaea3 - ps4
Asgard/Eukaryota - ps5
Eukaryota - ps6
Amorpheae - ps7
Obazoa - ps8
Opisthokonta - ps9
Holozoa - ps10
Filozoa - ps11
Choano./Metazoa - ps12
Metazoa - ps13
Myriazoa - ps14
Parahoxozoa - ps15
Bilateria - ps16
Deuterostomia - ps17
Chordata - ps18
Olfactores - ps19
Vertebrata - ps20
Gnathostomata - ps21
Euteleostomi - ps22
Sarcopterygii - ps23
Tetrapoda - ps24
Amniota - ps25
Mammalia - ps26
Theria - ps27
Placentalia - ps28
Boreoeutheria - ps29
Euarchontoglires - ps30
Euarchonta - ps31
Primates - ps32
Haplorrhini - ps33
Simiiformes - ps34
Catarrhini - ps35
Hominoidea - ps36
Hominidae - ps37
Homininae - ps38
Hominini - ps39
Homo_sapiens - ps40
Phylostratum

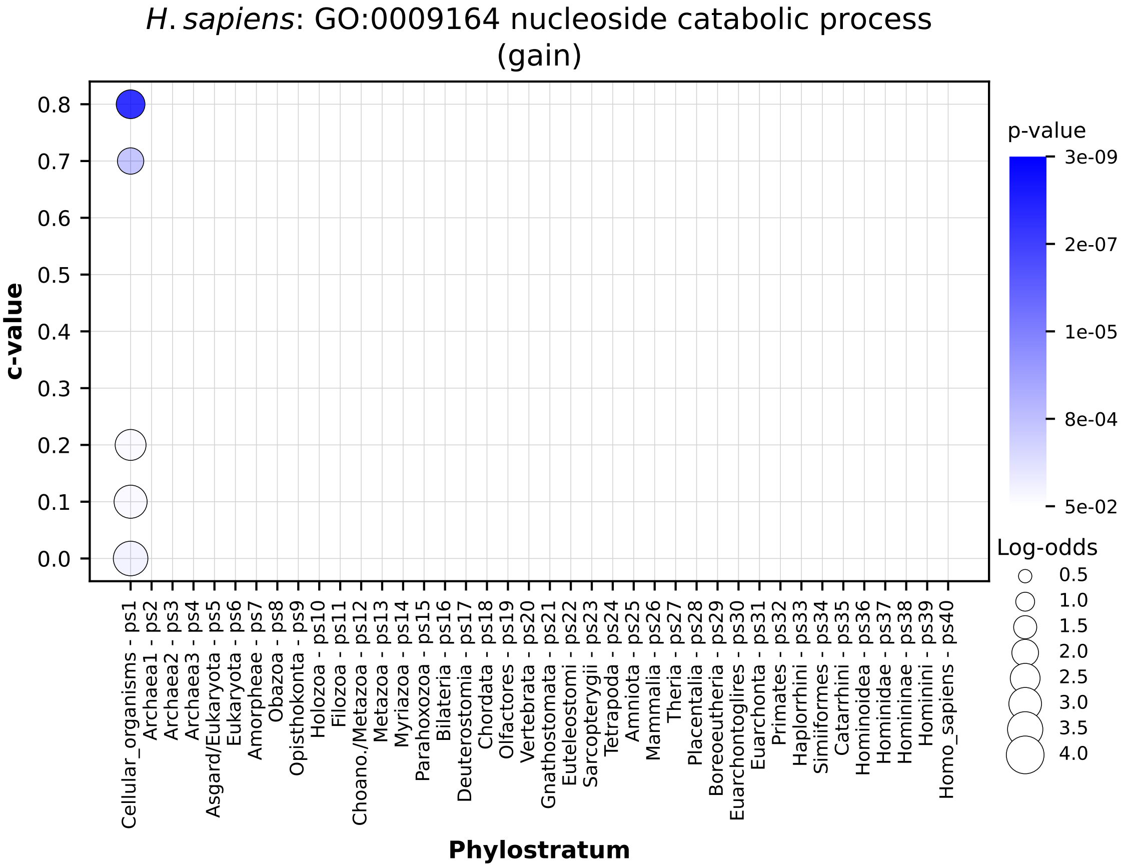

H. sapiens: GO:0009164 nucleoside catabolic process
(gain)
c-value
0.8
0.7
0.6
0.5
0.4
0.3
0.2
0.1
0.0
p-value
3e-09
2e-07
1e-05
8e-04
5e-02
Log-odds
0.5
1.0
1.5
2.0
2.5
3.0
3.5
4.0
Cellular_organisms - ps1
Archaea1 - ps2
Archaea2 - ps3
Archaea3 - ps4
Asgard/Eukaryota - ps5
Eukaryota - ps6
Amorpheae - ps7
Obazoa - ps8
Opisthokonta - ps9
Holozoa - ps10
Filozoa - ps11
Choano./Metazoa - ps12
Metazoa - ps13
Myriazoa - ps14
Parahoxozoa - ps15
Bilateria - ps16
Deuterostomia - ps17
Chordata - ps18
Olfactores - ps19
Vertebrata - ps20
Gnathostomata - ps21
Euteleostomi - ps22
Sarcopterygii - ps23
Tetrapoda - ps24
Amniota - ps25
Mammalia - ps26
Theria - ps27
Placentalia - ps28
Boreoeutheria - ps29
Euarchontoglires - ps30
Euarchonta - ps31
Primates - ps32
Haplorrhini - ps33
Simiiformes - ps34
Catarrhini - ps35
Hominoidea - ps36
Hominidae - ps37
Homininae - ps38
Hominini - ps39
Homo_sapiens - ps40
Phylostratum

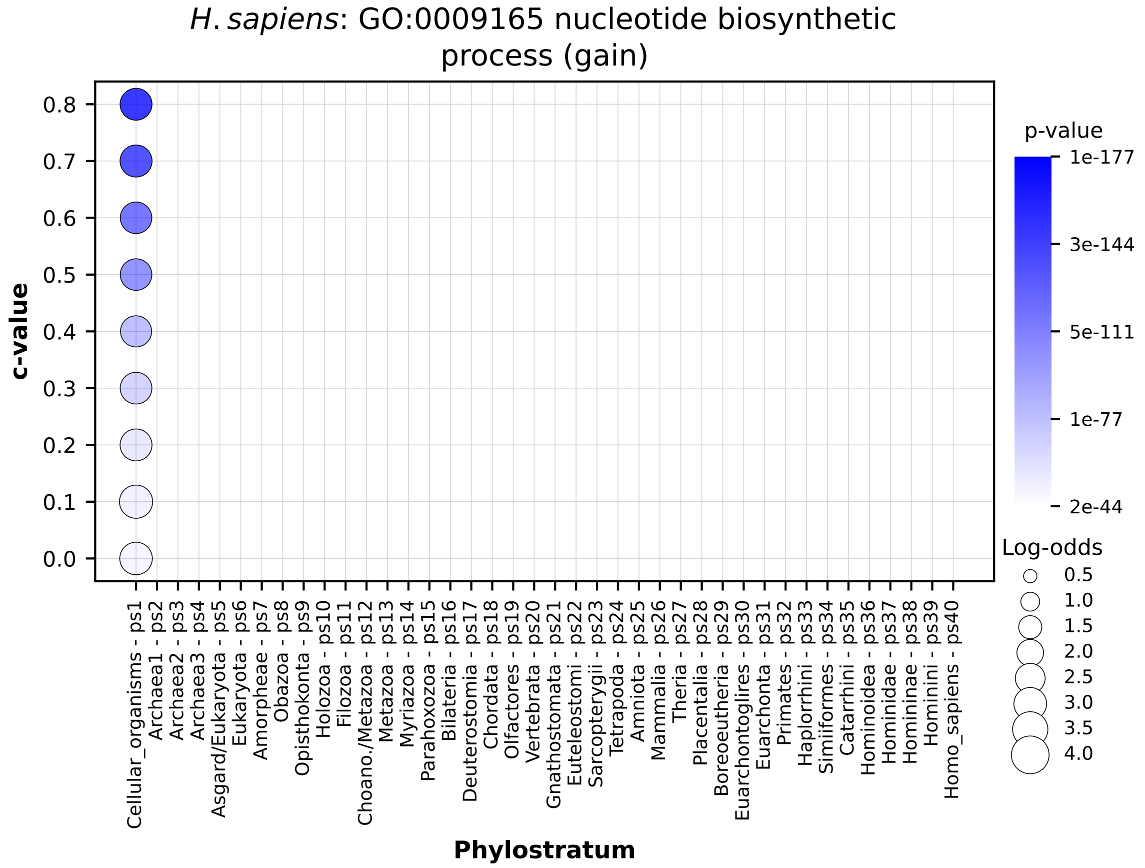
H. sapiens: GO:0009165 nucleotide biosynthetic process (gain)
c-value
0.8
0.7
0.6
0.5
0.4
0.3
0.2
0.1
0.0
p-value
1e-177
3e-144
5e-111
1e-77
2e-44
Log-odds
0.5
1.0
1.5
2.0
2.5
3.0
3.5
4.0
Cellular_organisms - ps1
Archaea1 - ps2
Archaea2 - ps3
Archaea3 - ps4
Asgard/Eukaryota - ps5
Eukaryota - ps6
Amorpheae - ps7
Obazoa - ps8
Opisthokonta - ps9
Holozoa - ps10
Filozoa - ps11
Choano./Metazoa - ps12
Metazoa - ps13
Myriazoa - ps14
Parahoxozoa - ps15
Bilateria - ps16
Deuterostomia - ps17
Chordata - ps18
Olfactores - ps19
Vertebrata - ps20
Gnathostomata - ps21
Euteleostomi - ps22
Sarcopterygii - ps23
Tetrapoda - ps24
Amniota - ps25
Mammalia - ps26
Theria - ps27
Placentalia - ps28
Boreoeutheria - ps29
Euarchontoglires - ps30
Euarchonta - ps31
Primates - ps32
Haplorrhini - ps33
Simiiformes - ps34
Catarrhini - ps35
Hominoidea - ps36
Hominidae - ps37
Homininae - ps38
Hominini - ps39
Homo_sapiens - ps40
Phylostratum

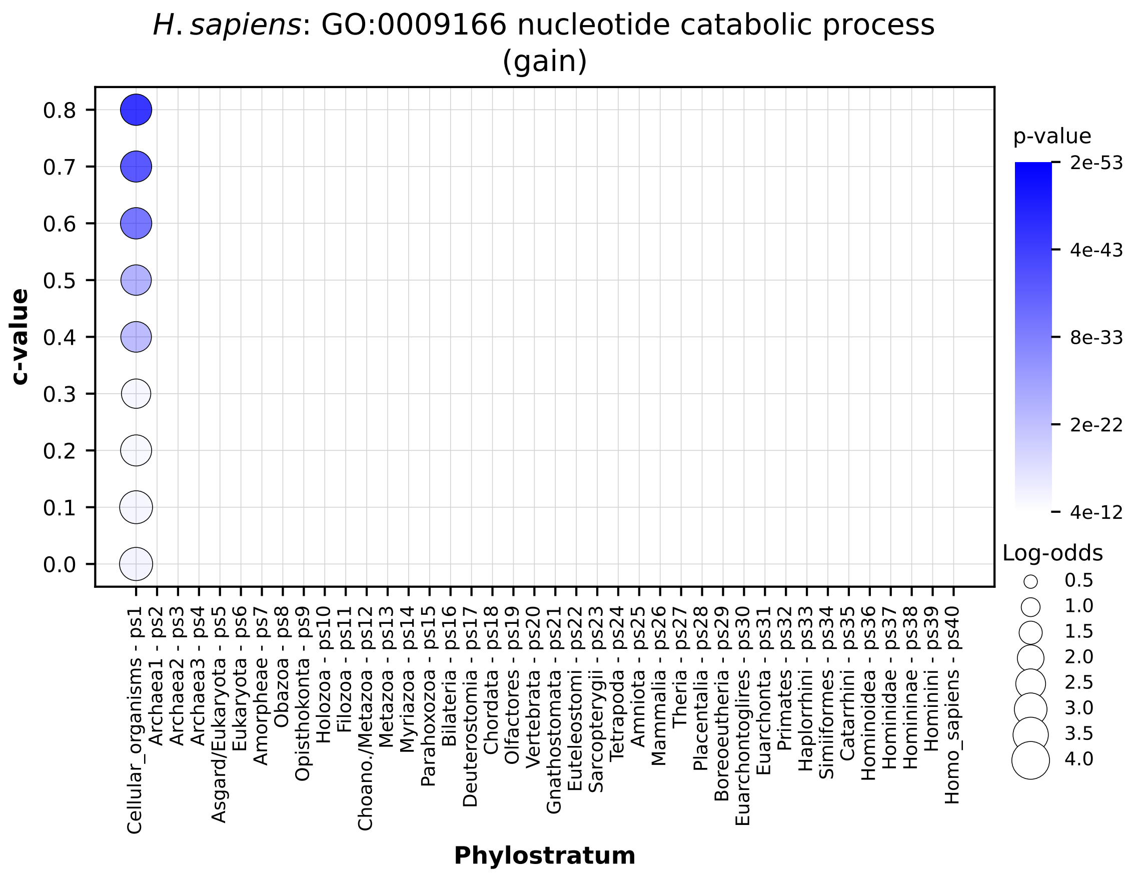

H. sapiens: GO:0009166 nucleotide catabolic process
(gain)
c-value
0.8
0.7
0.6
0.5
0.4
0.3
0.2
0.1
0.0
p-value
2e-53
4e-43
8e-33
2e-22
4e-12
Log-odds
0.5
1.0
1.5
2.0
2.5
3.0
3.5
4.0
Cellular_organisms - ps1
Archaea1 - ps2
Archaea2 - ps3
Archaea3 - ps4
Asgard/Eukaryota - ps5
Eukaryota - ps6
Amorpheae - ps7
Obazoa - ps8
Opisthokonta - ps9
Holozoa - ps10
Filozoa - ps11
Choano./Metazoa - ps12
Metazoa - ps13
Myriazoa - ps14
Parahoxozoa - ps15
Bilateria - ps16
Deuterostomia - ps17
Chordata - ps18
Olfactores - ps19
Vertebrata - ps20
Gnathostomata - ps21
Euteleostomi - ps22
Sarcopterygii - ps23
Tetrapoda - ps24
Amniota - ps25
Mammalia - ps26
Theria - ps27
Placentalia - ps28
Boreoeutheria - ps29
Euarchontoglires - ps30
Euarchonta - ps31
Primates - ps32
Haplorrhini - ps33
Simiiformes - ps34
Catarrhini - ps35
Hominoidea - ps36
Hominidae - ps37
Homininae - ps38
Hominini - ps39
Homo_sapiens - ps40
Phylostratum

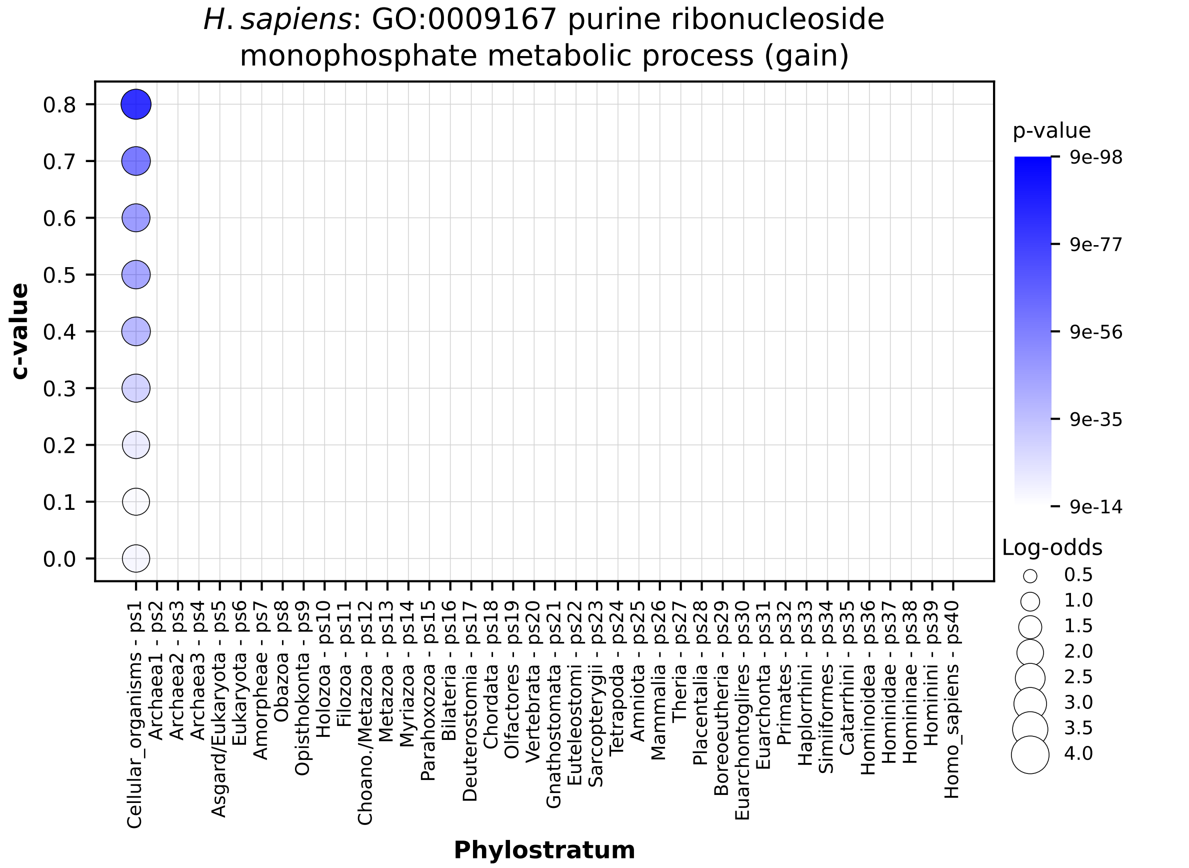

H. sapiens: GO:0009167 purine ribonucleoside monophosphate metabolic process (gain)
c-value
0.8
0.7
0.6
0.5
0.4
0.3
0.2
0.1
0.0
Phylostratum
Cellular_organisms - ps1
Archaea1 - ps2
Archaea2 - ps3
Archaea3 - ps4
Asgard/Eukaryota - ps5
Eukaryota - ps6
Amorpheae - ps7
Obazoa - ps8
Opisthokonta - ps9
Holozoa - ps10
Filozoa - ps11
Choano./Metazoa - ps12
Metazoa - ps13
Myriazoa - ps14
Parahoxozoa - ps15
Bilateria - ps16
Deuterostomia - ps17
Chordata - ps18
Olfactores - ps19
Vertebrata - ps20
Gnathostomata - ps21
Euteleostomi - ps22
Sarcopterygii - ps23
Tetrapoda - ps24
Amniota - ps25
Mammalia - ps26
Theria - ps27
Placentalia - ps28
Boreoeutheria - ps29
Euarchontoglires - ps30
Euarchonta - ps31
Primates - ps32
Haplorrhini - ps33
Simiiformes - ps34
Catarrhini - ps35
Hominoidea - ps36
Hominidae - ps37
Homininae - ps38
Hominini - ps39
Homo_sapiens - ps40
p-value
9e-98
9e-77
9e-56
9e-35
9e-14
Log-odds
0.5
1.0
1.5
2.0
2.5
3.0
3.5
4.0

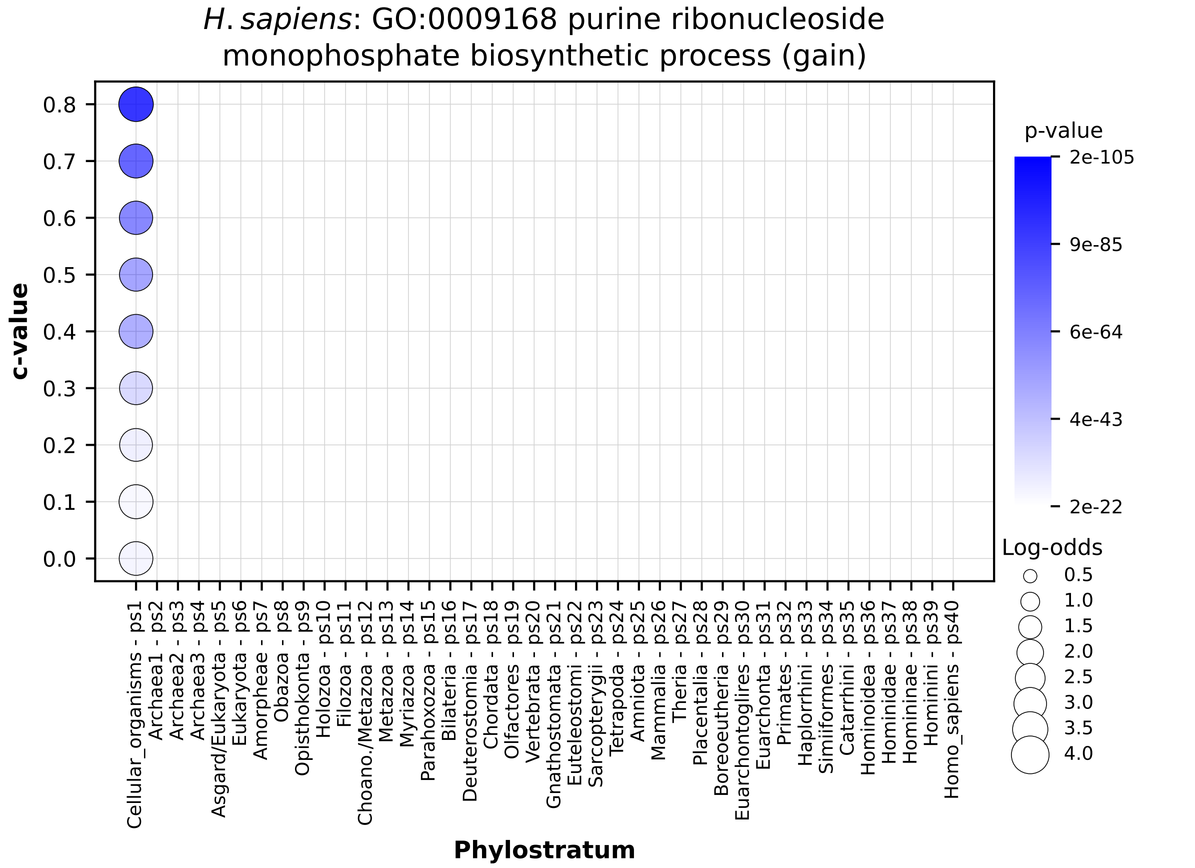

H. sapiens: GO:0009168 purine ribonucleoside monophosphate biosynthetic process (gain)
c-value
0.0
0.1
0.2
0.3
0.4
0.5
0.6
0.7
0.8
p-value
2e-105
9e-85
6e-64
4e-43
2e-22
Log-odds
0.5
1.0
1.5
2.0
2.5
3.0
3.5
4.0
Cellular_organisms - ps1
Archaea1 - ps2
Archaea2 - ps3
Archaea3 - ps4
Asgard/Eukaryota - ps5
Eukaryota - ps6
Amorpheae - ps7
Obazoa - ps8
Opisthokonta - ps9
Holozoa - ps10
Filozoa - ps11
Choano./Metazoa - ps12
Metazoa - ps13
Myriazoa - ps14
Parahoxozoa - ps15
Bilateria - ps16
Deuterostomia - ps17
Chordata - ps18
Olfactores - ps19
Vertebrata - ps20
Gnathostomata - ps21
Euteleostomi - ps22
Sarcopterygii - ps23
Tetrapoda - ps24
Amniota - ps25
Mammalia - ps26
Theria - ps27
Placentalia - ps28
Boreoeutheria - ps29
Euarchontoglires - ps30
Euarchonta - ps31
Primates - ps32
Haplorrhini - ps33
Simiiformes - ps34
Catarrhini - ps35
Hominoidea - ps36
Hominidae - ps37
Homininae - ps38
Hominini - ps39
Homo_sapiens - ps40
Phylostratum

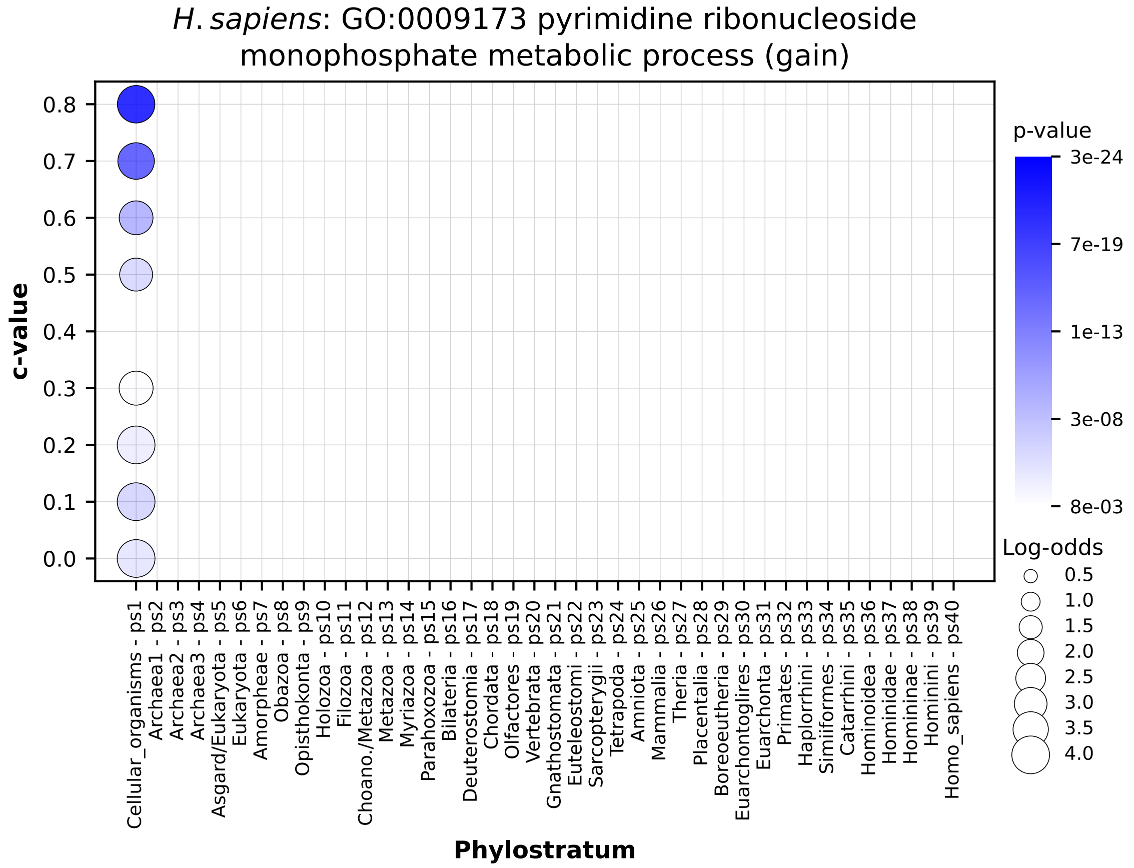

*H. sapiens*: GO:0009173 pyrimidine ribonucleoside monophosphate metabolic process (gain)
c-value
0.8
0.7
0.6
0.5
0.4
0.3
0.2
0.1
0.0
Phylostratum
Cellular_organisms - ps1
Archaea1 - ps2
Archaea2 - ps3
Archaea3 - ps4
Asgard/Eukaryota - ps5
Eukaryota - ps6
Amorpheae - ps7
Obazoa - ps8
Opisthokonta - ps9
Holozoa - ps10
Filozoa - ps11
Choano./Metazoa - ps12
Metazoa - ps13
Myriazoa - ps14
Parahoxozoa - ps15
Bilateria - ps16
Deuterostomia - ps17
Chordata - ps18
Olfactores - ps19
Vertebrata - ps20
Gnathostomata - ps21
Euteleostomi - ps22
Sarcopterygii - ps23
Tetrapoda - ps24
Amniota - ps25
Mammalia - ps26
Theria - ps27
Placentalia - ps28
Boreoeutheria - ps29
Euarchontoglires - ps30
Euarchonta - ps31
Primates - ps32
Haplorrhini - ps33
Simiiformes - ps34
Catarrhini - ps35
Hominoidea - ps36
Hominidae - ps37
Homininae - ps38
Hominini - ps39
Homo_sapiens - ps40
p-value
3e-24
7e-19
1e-13
3e-08
8e-03
Log-odds
0.5
1.0
1.5
2.0
2.5
3.0
3.5
4.0

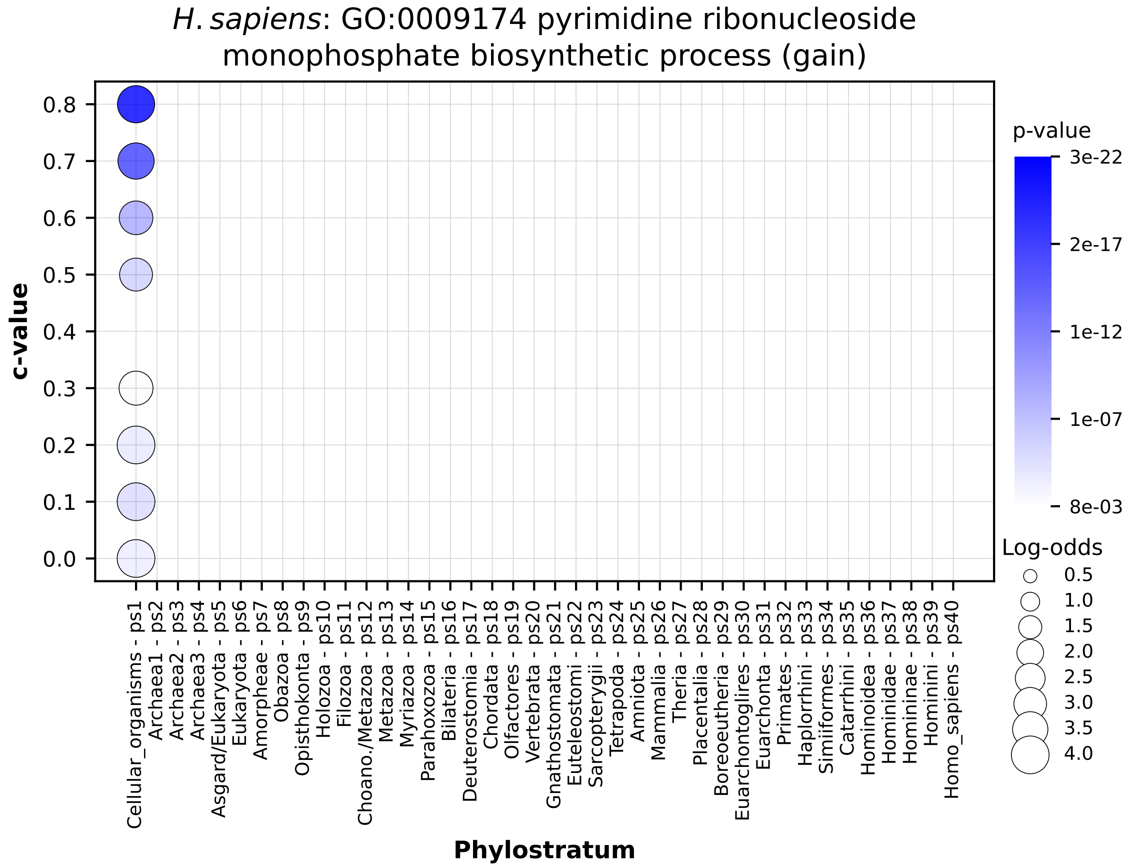
H. sapiens: GO:0009174 pyrimidine ribonucleoside monophosphate biosynthetic process (gain)
c-value
Phylostratum
p-value
3e-22
2e-17
1e-12
1e-07
8e-03
Log-odds
0.5
1.0
1.5
2.0
2.5
3.0
3.5
4.0
Cellular_organisms - ps1
Archaea1 - ps2
Archaea2 - ps3
Archaea3 - ps4
Asgard/Eukaryota - ps5
Eukaryota - ps6
Amorpheae - ps7
Obazoa - ps8
Opisthokonta - ps9
Holozoa - ps10
Filozoa - ps11
Choano./Metazoa - ps12
Metazoa - ps13
Myriazoa - ps14
Parahoxozoa - ps15
Bilateria - ps16
Deuterostomia - ps17
Chordata - ps18
Olfactores - ps19
Vertebrata - ps20
Gnathostomata - ps21
Euteleostomi - ps22
Sarcopterygii - ps23
Tetrapoda - ps24
Amniota - ps25
Mammalia - ps26
Theria - ps27
Placentalia - ps28
Boreoeutheria - ps29
Euarchontoglires - ps30
Euarchonta - ps31
Primates - ps32
Haplorrhini - ps33
Simiiformes - ps34
Catarrhini - ps35
Hominoidea - ps36
Hominidae - ps37
Homininae - ps38
Hominini - ps39
Homo_sapiens - ps40

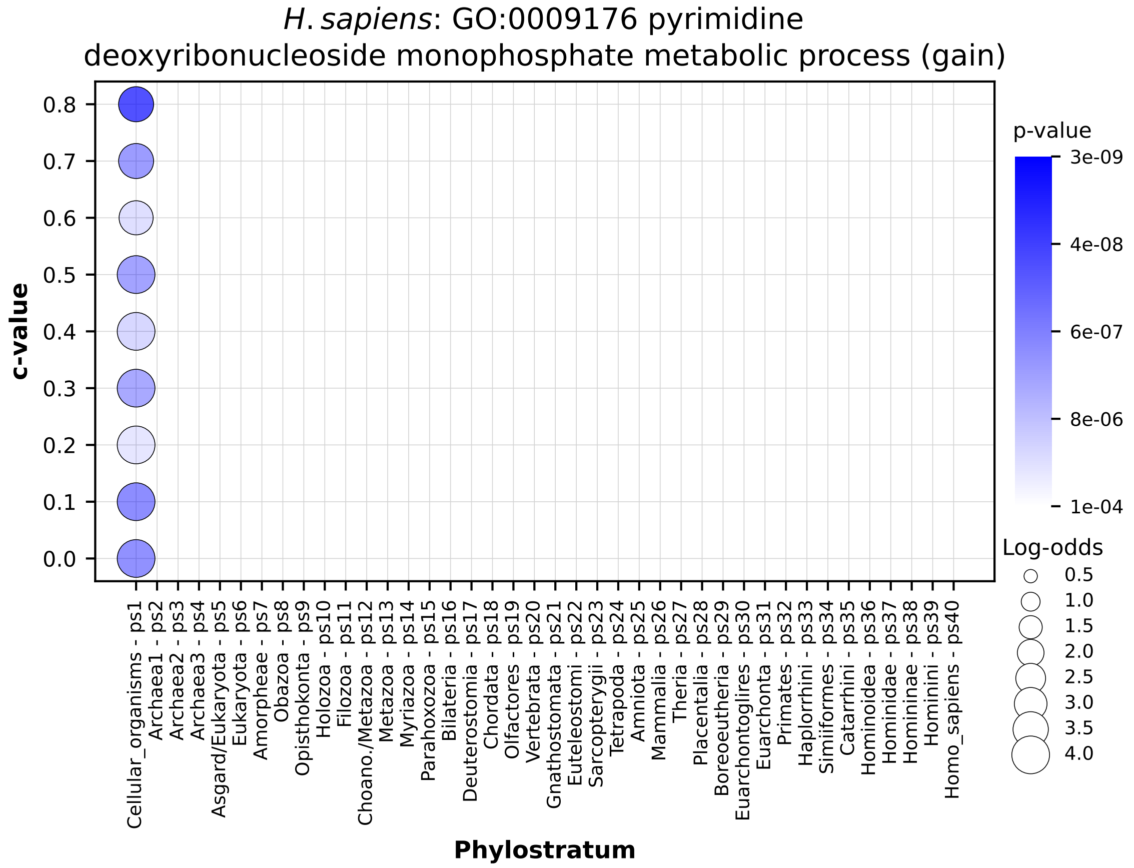

*H. sapiens*: GO:0009176 pyrimidine deoxyribonucleoside monophosphate metabolic process (gain)
c-value
0.8
0.7
0.6
0.5
0.4
0.3
0.2
0.1
0.0
Phylostratum
Cellular_organisms - ps1
Archaea1 - ps2
Archaea2 - ps3
Archaea3 - ps4
Asgard/Eukaryota - ps5
Eukaryota - ps6
Amorpheae - ps7
Obazoa - ps8
Opisthokonta - ps9
Holozoa - ps10
Filozoa - ps11
Choano./Metazoa - ps12
Metazoa - ps13
Myriazoa - ps14
Parahoxozoa - ps15
Bilateria - ps16
Deuterostomia - ps17
Chordata - ps18
Olfactores - ps19
Vertebrata - ps20
Gnathostomata - ps21
Euteleostomi - ps22
Sarcopterygii - ps23
Tetrapoda - ps24
Amniota - ps25
Mammalia - ps26
Theria - ps27
Placentalia - ps28
Boreoeutheria - ps29
Euarchontoglires - ps30
Euarchonta - ps31
Primates - ps32
Haplorrhini - ps33
Simiiformes - ps34
Catarrhini - ps35
Hominoidea - ps36
Hominidae - ps37
Homininae - ps38
Hominini - ps39
Homo_sapiens - ps40
p-value
3e-09
4e-08
6e-07
8e-06
1e-04
Log-odds
0.5
1.0
1.5
2.0
2.5
3.0
3.5
4.0

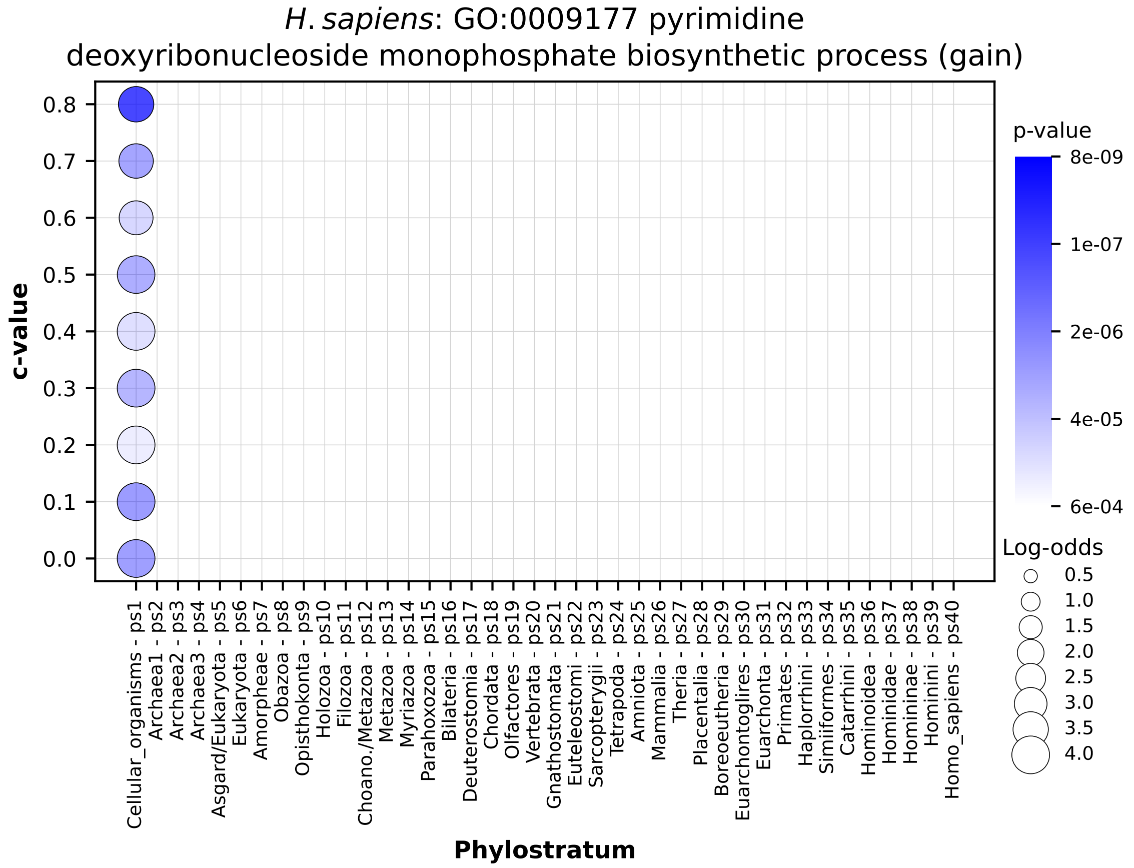

H. sapiens: GO:0009177 pyrimidine deoxyribonucleoside monophosphate biosynthetic process (gain)
c-value
0.0
0.1
0.2
0.3
0.4
0.5
0.6
0.7
0.8
Phylostratum
Cellular_organisms - ps1
Archaea1 - ps2
Archaea2 - ps3
Archaea3 - ps4
Asgard/Eukaryota - ps5
Eukaryota - ps6
Amorpheae - ps7
Obazoa - ps8
Opisthokonta - ps9
Holozoa - ps10
Filozoa - ps11
Choano./Metazoa - ps12
Metazoa - ps13
Myriazoa - ps14
Parahoxozoa - ps15
Bilateria - ps16
Deuterostomia - ps17
Chordata - ps18
Olfactores - ps19
Vertebrata - ps20
Gnathostomata - ps21
Euteleostomi - ps22
Sarcopterygii - ps23
Tetrapoda - ps24
Amniota - ps25
Mammalia - ps26
Theria - ps27
Placentalia - ps28
Boreoeutheria - ps29
Euarchontoglires - ps30
Euarchonta - ps31
Primates - ps32
Haplorrhini - ps33
Simiiformes - ps34
Catarrhini - ps35
Hominoidea - ps36
Hominidae - ps37
Homininae - ps38
Hominini - ps39
Homo_sapiens - ps40
p-value
8e-09
1e-07
2e-06
4e-05
6e-04
Log-odds
0.5
1.0
1.5
2.0
2.5
3.0
3.5
4.0

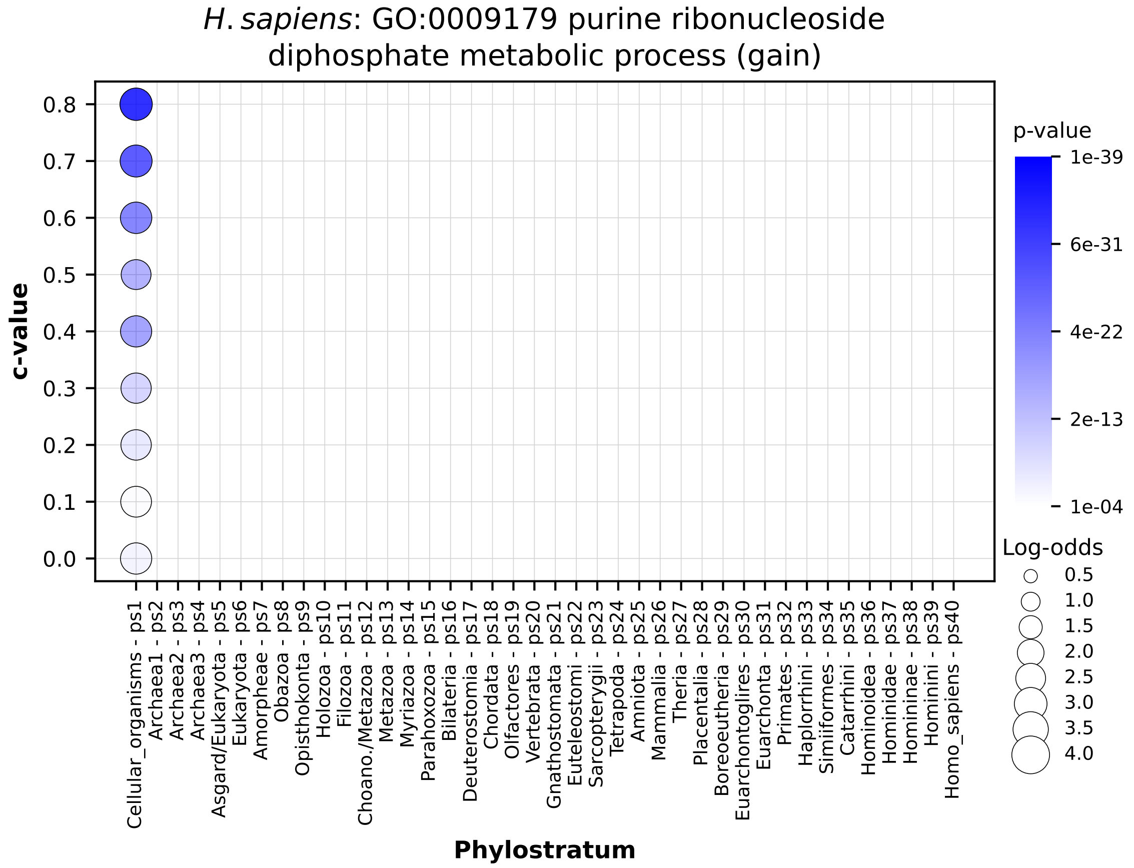

*H. sapiens*: GO:0009179 purine ribonucleoside diphosphate metabolic process (gain)
c-value
0.8
0.7
0.6
0.5
0.4
0.3
0.2
0.1
0.0
p-value
1e-39
6e-31
4e-22
2e-13
1e-04
Log-odds
0.5
1.0
1.5
2.0
2.5
3.0
3.5
4.0
Cellular_organisms - ps1
Archaea1 - ps2
Archaea2 - ps3
Archaea3 - ps4
Asgard/Eukaryota - ps5
Eukaryota - ps6
Amorpheae - ps7
Obazoa - ps8
Opisthokonta - ps9
Holozoa - ps10
Filozoa - ps11
Choano./Metazoa - ps12
Metazoa - ps13
Myriazoa - ps14
Parahoxozoa - ps15
Bilateria - ps16
Deuterostomia - ps17
Chordata - ps18
Olfactores - ps19
Vertebrata - ps20
Gnathostomata - ps21
Euteleostomi - ps22
Sarcopterygii - ps23
Tetrapoda - ps24
Amniota - ps25
Mammalia - ps26
Theria - ps27
Placentalia - ps28
Boreoeutheria - ps29
Euarchontoglires - ps30
Euarchonta - ps31
Primates - ps32
Haplorrhini - ps33
Simiiformes - ps34
Catarrhini - ps35
Hominoidea - ps36
Hominidae - ps37
Homininae - ps38
Hominini - ps39
Homo_sapiens - ps40
Phylostratum

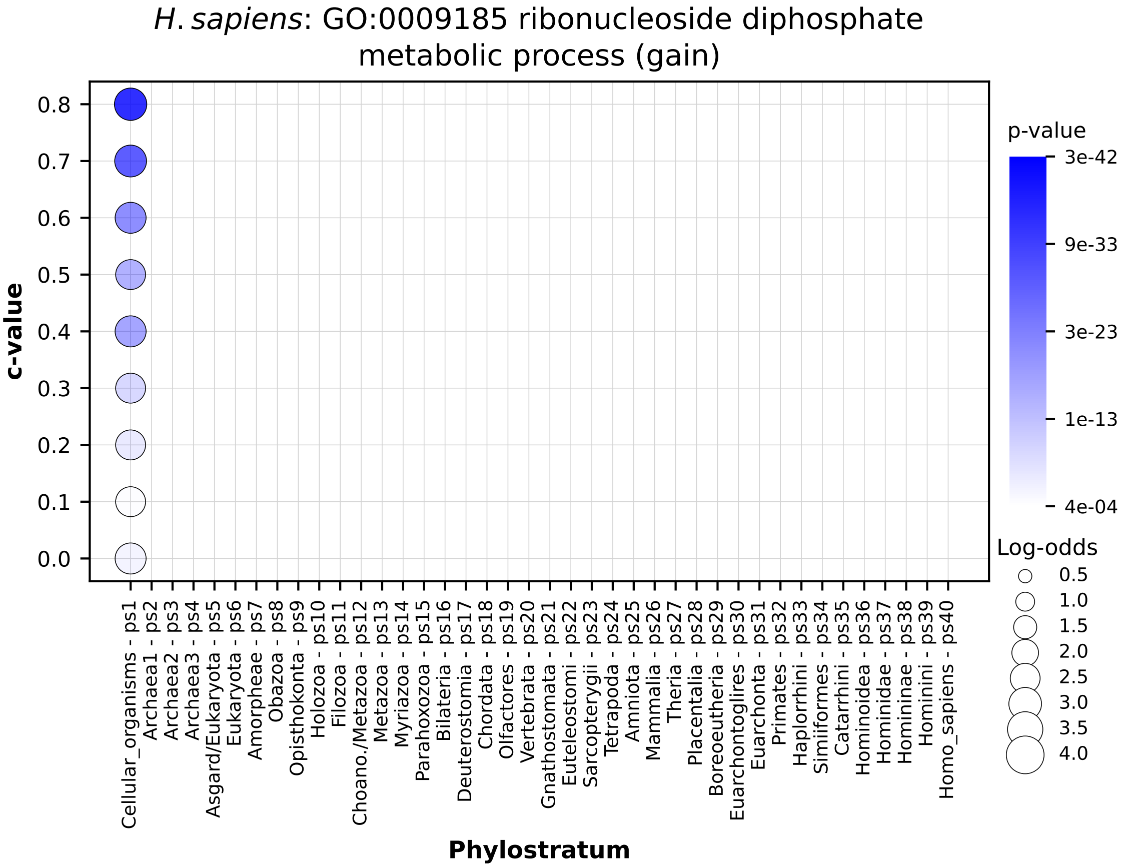
H. sapiens: GO:0009185 ribonucleoside diphosphate metabolic process (gain)
c-value
0.8
0.7
0.6
0.5
0.4
0.3
0.2
0.1
0.0
p-value
3e-42
9e-33
3e-23
1e-13
4e-04
Log-odds
0.5
1.0
1.5
2.0
2.5
3.0
3.5
4.0
Cellular_organisms - ps1
Archaea1 - ps2
Archaea2 - ps3
Archaea3 - ps4
Asgard/Eukaryota - ps5
Eukaryota - ps6
Amorpheae - ps7
Obazoa - ps8
Opisthokonta - ps9
Holozoa - ps10
Filozoa - ps11
Choano./Metazoa - ps12
Metazoa - ps13
Myriazoa - ps14
Parahoxozoa - ps15
Bilateria - ps16
Deuterostomia - ps17
Chordata - ps18
Olfactores - ps19
Vertebrata - ps20
Gnathostomata - ps21
Euteleostomi - ps22
Sarcopterygii - ps23
Tetrapoda - ps24
Amniota - ps25
Mammalia - ps26
Theria - ps27
Placentalia - ps28
Boreoeutheria - ps29
Euarchontoglires - ps30
Euarchonta - ps31
Primates - ps32
Haplorrhini - ps33
Simiiformes - ps34
Catarrhini - ps35
Hominoidea - ps36
Hominidae - ps37
Homininae - ps38
Hominini - ps39
Homo_sapiens - ps40
Phylostratum

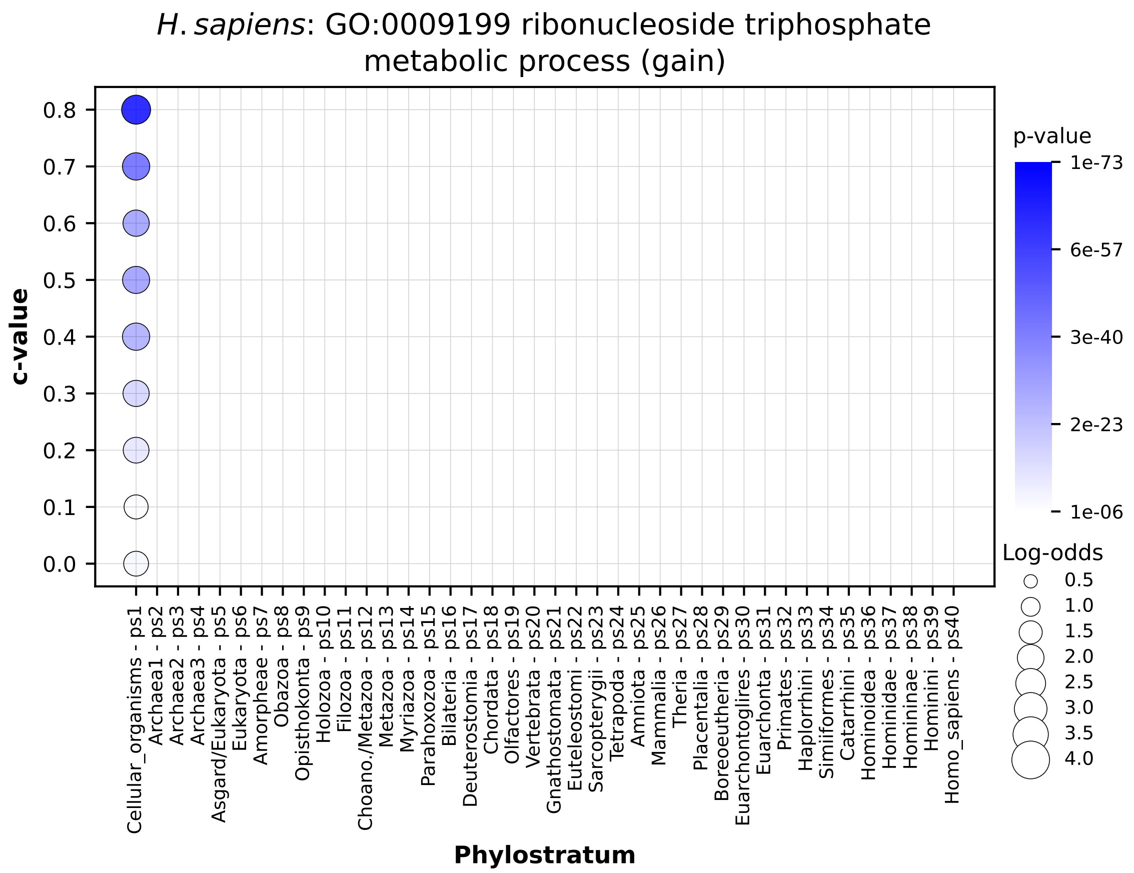

*H. sapiens*: GO:0009199 ribonucleoside triphosphate metabolic process (gain)
c-value
0.0
0.1
0.2
0.3
0.4
0.5
0.6
0.7
0.8
p-value
1e-73
6e-57
3e-40
2e-23
1e-06
Log-odds
0.5
1.0
1.5
2.0
2.5
3.0
3.5
4.0
Cellular_organisms - ps1
Archaea1 - ps2
Archaea2 - ps3
Archaea3 - ps4
Asgard/Eukaryota - ps5
Eukaryota - ps6
Amorpheae - ps7
Obazoa - ps8
Opisthokonta - ps9
Holozoa - ps10
Filozoa - ps11
Choano./Metazoa - ps12
Metazoa - ps13
Myriazoa - ps14
Parahoxozoa - ps15
Bilateria - ps16
Deuterostomia - ps17
Chordata - ps18
Olfactores - ps19
Vertebrata - ps20
Gnathostomata - ps21
Euteleostomi - ps22
Sarcopterygii - ps23
Tetrapoda - ps24
Amniota - ps25
Mammalia - ps26
Theria - ps27
Placentalia - ps28
Boreoeutheria - ps29
Euarchontoglires - ps30
Euarchonta - ps31
Primates - ps32
Haplorrhini - ps33
Simiiformes - ps34
Catarrhini - ps35
Hominoidea - ps36
Hominidae - ps37
Homininae - ps38
Hominini - ps39
Homo_sapiens - ps40
Phylostratum

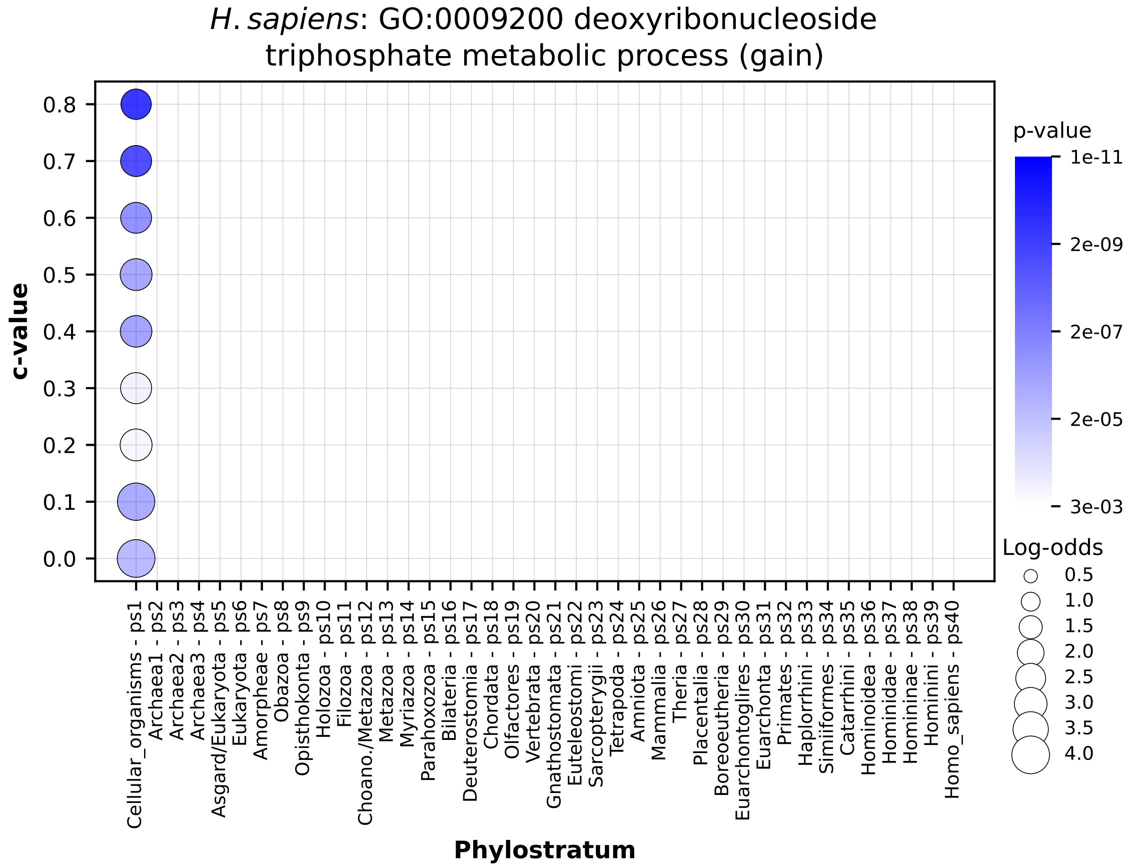

*H. sapiens*: GO:0009200 deoxyribonucleoside triphosphate metabolic process (gain)
c-value
0.0
0.1
0.2
0.3
0.4
0.5
0.6
0.7
0.8
p-value
1e-11
2e-09
2e-07
2e-05
3e-03
Log-odds
0.5
1.0
1.5
2.0
2.5
3.0
3.5
4.0
Cellular_organisms - ps1
Archaea1 - ps2
Archaea2 - ps3
Archaea3 - ps4
Asgard/Eukaryota - ps5
Eukaryota - ps6
Amorpheae - ps7
Obazoa - ps8
Opisthokonta - ps9
Holozoa - ps10
Filozoa - ps11
Choano./Metazoa - ps12
Metazoa - ps13
Myriazoa - ps14
Parahoxozoa - ps15
Bilateria - ps16
Deuterostomia - ps17
Chordata - ps18
Olfactores - ps19
Vertebrata - ps20
Gnathostomata - ps21
Euteleostomi - ps22
Sarcopterygii - ps23
Tetrapoda - ps24
Amniota - ps25
Mammalia - ps26
Theria - ps27
Placentalia - ps28
Boreoeutheria - ps29
Euarchontoglires - ps30
Euarchonta - ps31
Primates - ps32
Haplorrhini - ps33
Simiiformes - ps34
Catarrhini - ps35
Hominoidea - ps36
Hominidae - ps37
Homininae - ps38
Hominini - ps39
Homo_sapiens - ps40
Phylostratum

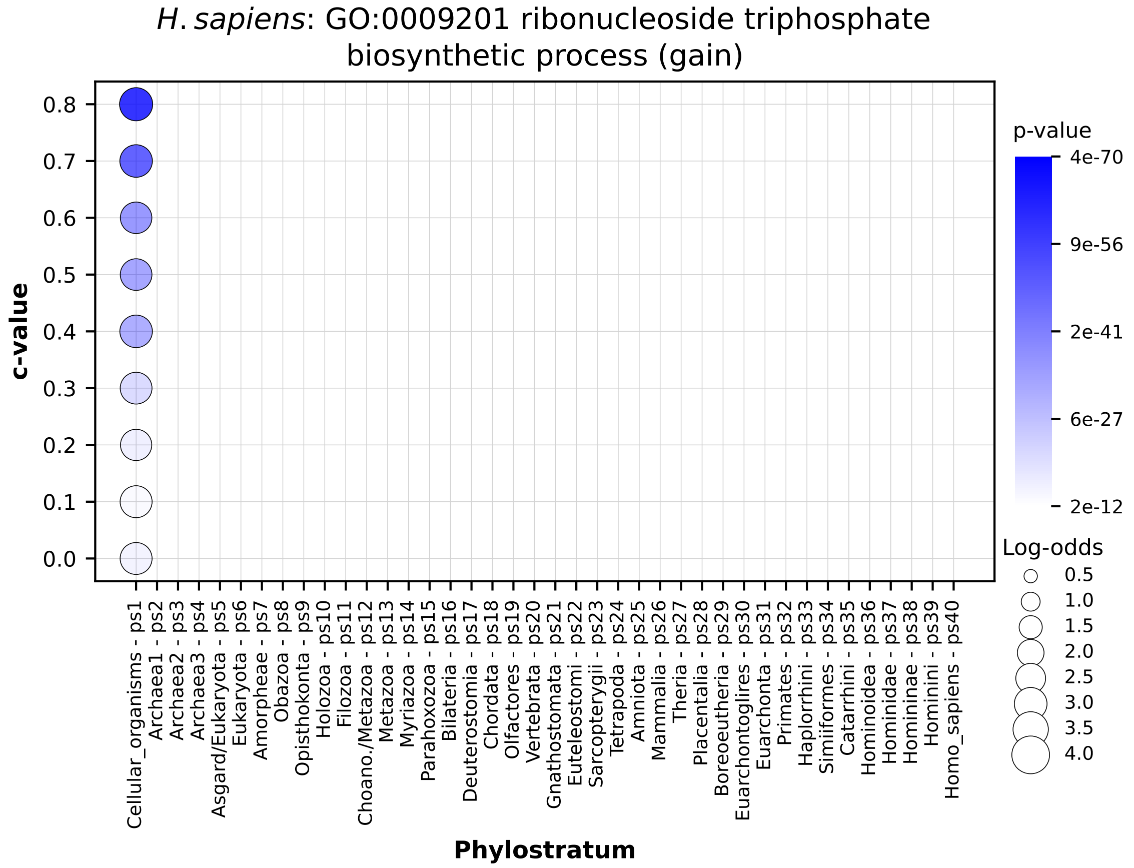

H. sapiens: GO:0009201 ribonucleoside triphosphate biosynthetic process (gain)
c-value
0.8
0.7
0.6
0.5
0.4
0.3
0.2
0.1
0.0
p-value
4e-70
9e-56
2e-41
6e-27
2e-12
Log-odds
0.5
1.0
1.5
2.0
2.5
3.0
3.5
4.0
Cellular_organisms - ps1
Archaea1 - ps2
Archaea2 - ps3
Archaea3 - ps4
Asgard/Eukaryota - ps5
Eukaryota - ps6
Amorpheae - ps7
Obazoa - ps8
Opisthokonta - ps9
Holozoa - ps10
Filozoa - ps11
Choano./Metazoa - ps12
Metazoa - ps13
Myriazoa - ps14
Parahoxozoa - ps15
Bilateria - ps16
Deuterostomia - ps17
Chordata - ps18
Olfactores - ps19
Vertebrata - ps20
Gnathostomata - ps21
Euteleostomi - ps22
Sarcopterygii - ps23
Tetrapoda - ps24
Amniota - ps25
Mammalia - ps26
Theria - ps27
Placentalia - ps28
Boreoeutheria - ps29
Euarchontoglires - ps30
Euarchonta - ps31
Primates - ps32
Haplorrhini - ps33
Simiiformes - ps34
Catarrhini - ps35
Hominoidea - ps36
Hominidae - ps37
Homininae - ps38
Hominini - ps39
Homo_sapiens - ps40
Phylostratum

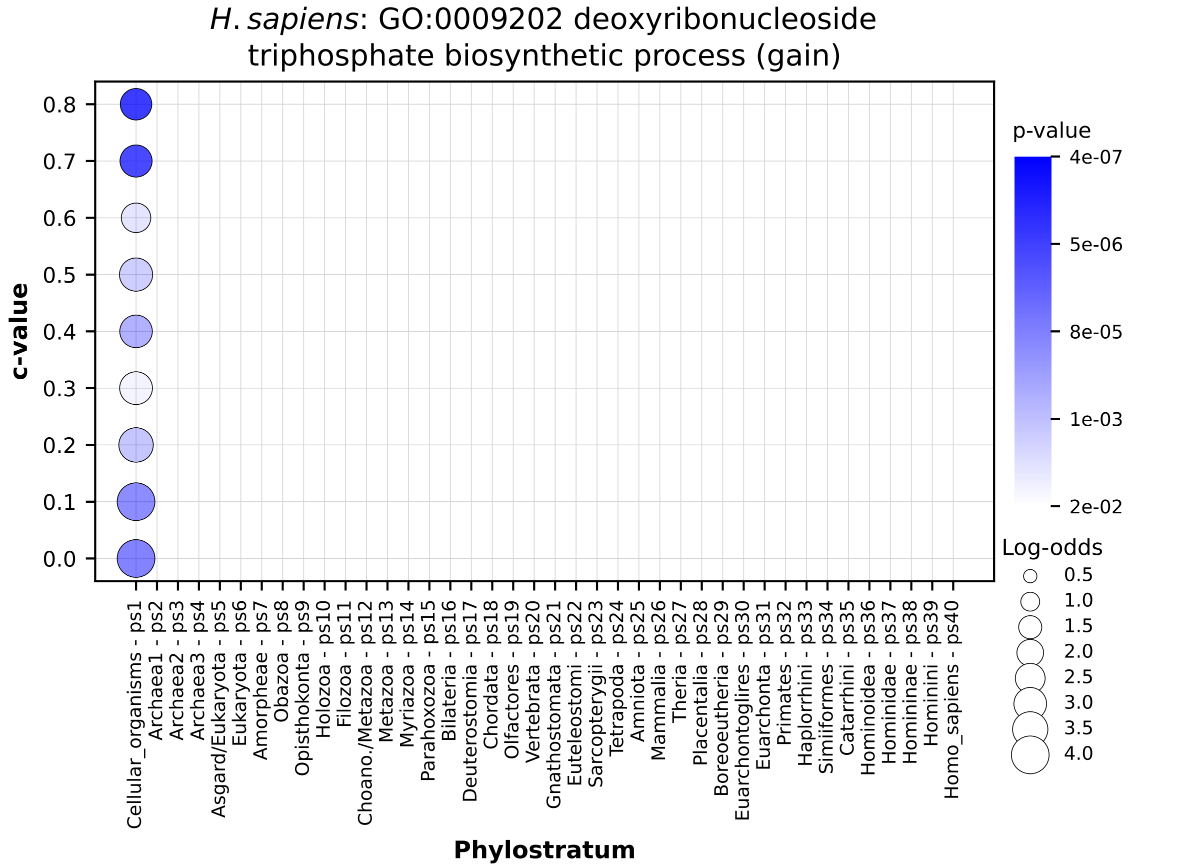

*H. sapiens*: GO:0009202 deoxyribonucleoside triphosphate biosynthetic process (gain)
c-value
0.8
0.7
0.6
0.5
0.4
0.3
0.2
0.1
0.0
p-value
4e-07
5e-06
8e-05
1e-03
2e-02
Log-odds
0.5
1.0
1.5
2.0
2.5
3.0
3.5
4.0
Cellular_organisms - ps1
Archaea1 - ps2
Archaea2 - ps3
Archaea3 - ps4
Asgard/Eukaryota - ps5
Eukaryota - ps6
Amorpheae - ps7
Obazoa - ps8
Opisthokonta - ps9
Holozoa - ps10
Filozoa - ps11
Choano./Metazoa - ps12
Metazoa - ps13
Myriazoa - ps14
Parahoxozoa - ps15
Bilateria - ps16
Deuterostomia - ps17
Chordata - ps18
Olfactores - ps19
Vertebrata - ps20
Gnathostomata - ps21
Euteleostomi - ps22
Sarcopterygii - ps23
Tetrapoda - ps24
Amniota - ps25
Mammalia - ps26
Theria - ps27
Placentalia - ps28
Boreoeutheria - ps29
Euarchontoglires - ps30
Euarchonta - ps31
Primates - ps32
Haplorrhini - ps33
Simiiformes - ps34
Catarrhini - ps35
Hominoidea - ps36
Hominidae - ps37
Homininae - ps38
Hominini - ps39
Homo_sapiens - ps40
Phylostratum

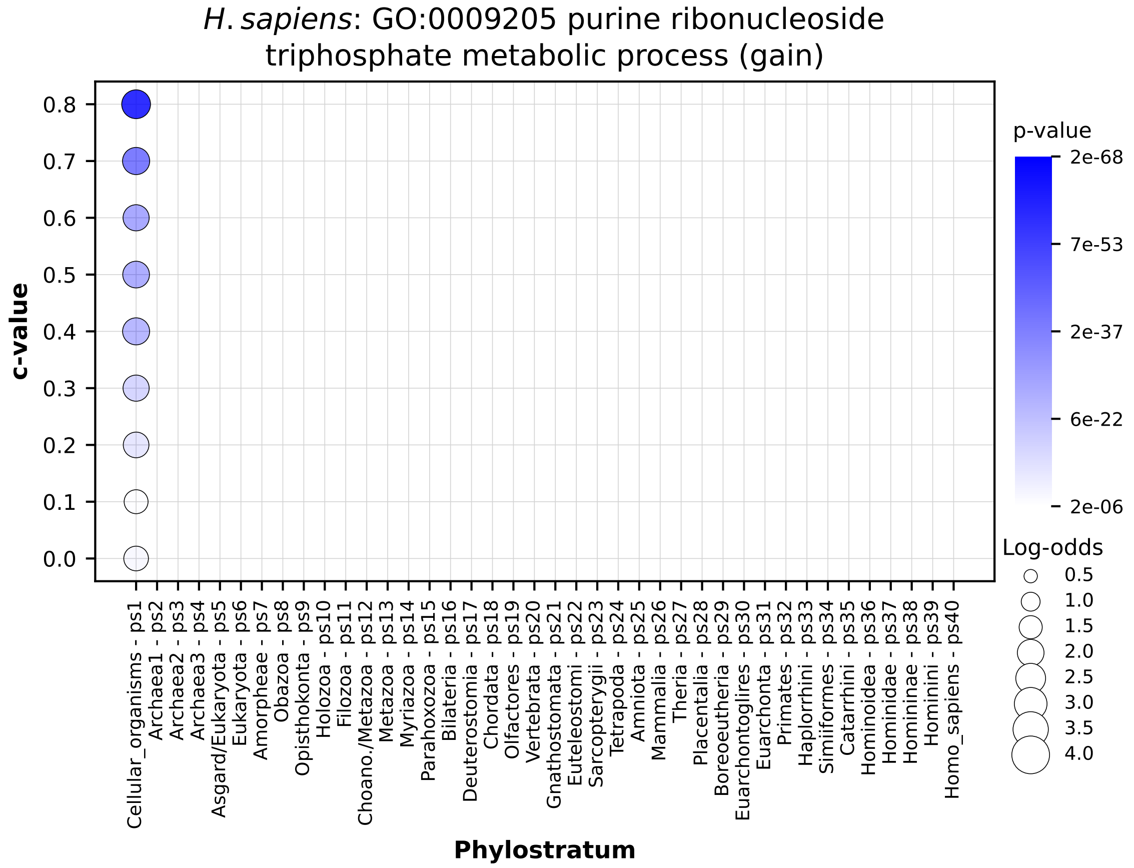

H. sapiens: GO:0009205 purine ribonucleoside triphosphate metabolic process (gain)
c-value
0.8
0.7
0.6
0.5
0.4
0.3
0.2
0.1
0.0
p-value
2e-68
7e-53
2e-37
6e-22
2e-06
Log-odds
0.5
1.0
1.5
2.0
2.5
3.0
3.5
4.0
Cellular_organisms - ps1
Archaea1 - ps2
Archaea2 - ps3
Archaea3 - ps4
Asgard/Eukaryota - ps5
Eukaryota - ps6
Amorpheae - ps7
Obazoa - ps8
Opisthokonta - ps9
Holozoa - ps10
Filozoa - ps11
Choano./Metazoa - ps12
Metazoa - ps13
Myriazoa - ps14
Parahoxozoa - ps15
Bilateria - ps16
Deuterostomia - ps17
Chordata - ps18
Olfactores - ps19
Vertebrata - ps20
Gnathostomata - ps21
Euteleostomi - ps22
Sarcopterygii - ps23
Tetrapoda - ps24
Amniota - ps25
Mammalia - ps26
Theria - ps27
Placentalia - ps28
Boreoeutheria - ps29
Euarchontoglires - ps30
Euarchonta - ps31
Primates - ps32
Haplorrhini - ps33
Simiiformes - ps34
Catarrhini - ps35
Hominoidea - ps36
Hominidae - ps37
Homininae - ps38
Hominini - ps39
Homo_sapiens - ps40
Phylostratum

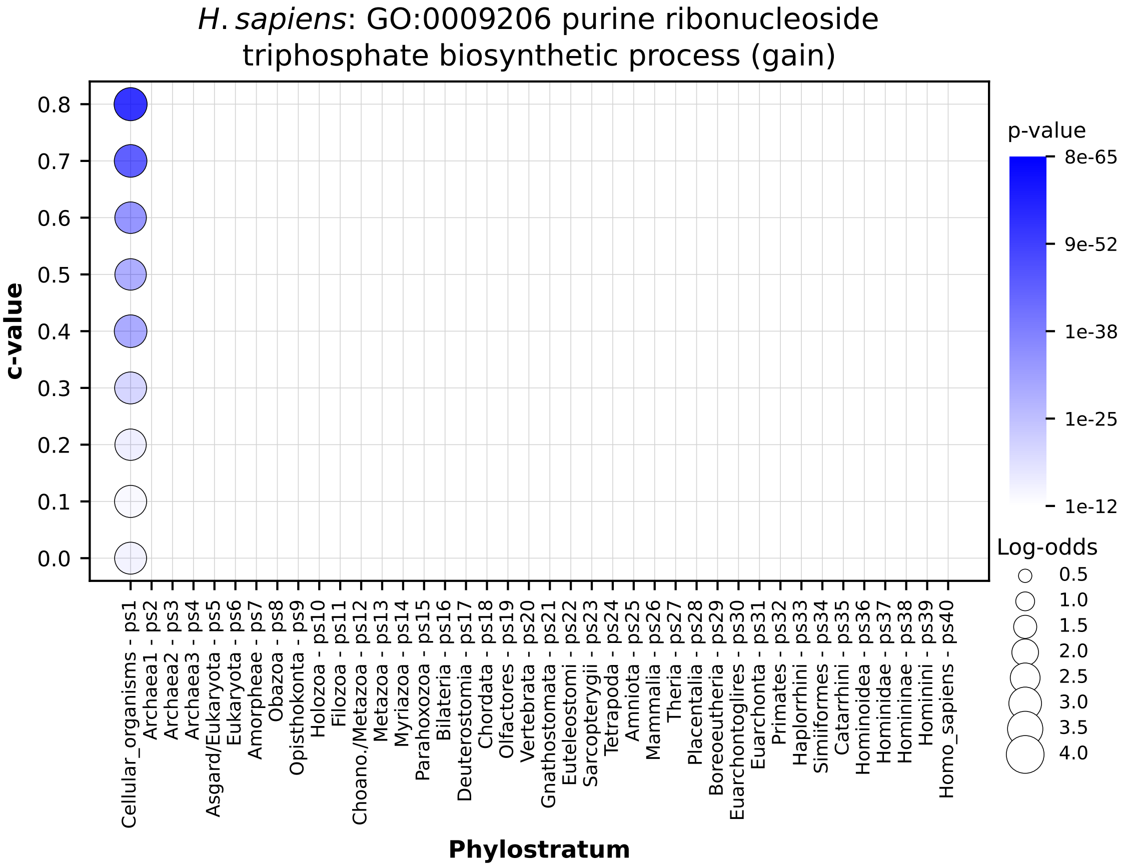

H. sapiens: GO:0009206 purine ribonucleoside triphosphate biosynthetic process (gain)
c-value
0.8
0.7
0.6
0.5
0.4
0.3
0.2
0.1
0.0
Phylostratum
Cellular_organisms - ps1
Archaea1 - ps2
Archaea2 - ps3
Archaea3 - ps4
Asgard/Eukaryota - ps5
Eukaryota - ps6
Amorpheae - ps7
Obazoa - ps8
Opisthokonta - ps9
Holozoa - ps10
Filozoa - ps11
Choano./Metazoa - ps12
Metazoa - ps13
Myriazoa - ps14
Parahoxozoa - ps15
Bilateria - ps16
Deuterostomia - ps17
Chordata - ps18
Olfactores - ps19
Vertebrata - ps20
Gnathostomata - ps21
Euteleostomi - ps22
Sarcopterygii - ps23
Tetrapoda - ps24
Amniota - ps25
Mammalia - ps26
Theria - ps27
Placentalia - ps28
Boreoeutheria - ps29
Euarchontoglires - ps30
Euarchonta - ps31
Primates - ps32
Haplorrhini - ps33
Simiiformes - ps34
Catarrhini - ps35
Hominoidea - ps36
Hominidae - ps37
Homininae - ps38
Hominini - ps39
Homo_sapiens - ps40
p-value
8e-65
9e-52
1e-38
1e-25
1e-12
Log-odds
0.5
1.0
1.5
2.0
2.5
3.0
3.5
4.0

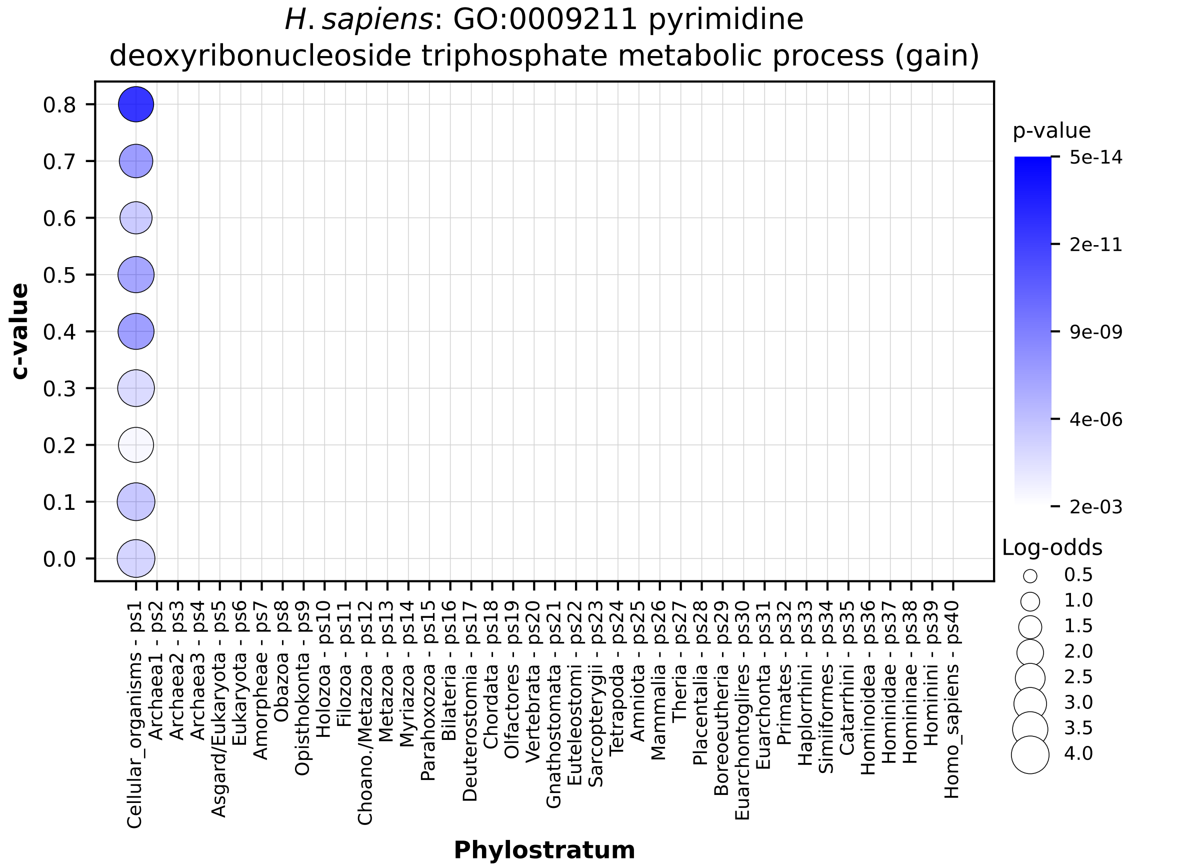
H. sapiens: GO:0009211 pyrimidine deoxyribonucleoside triphosphate metabolic process (gain)
c-value
0.8
0.7
0.6
0.5
0.4
0.3
0.2
0.1
0.0
Phylostratum
Cellular_organisms - ps1
Archaea1 - ps2
Archaea2 - ps3
Archaea3 - ps4
Asgard/Eukaryota - ps5
Eukaryota - ps6
Amorpheae - ps7
Obazoa - ps8
Opisthokonta - ps9
Holozoa - ps10
Filozoa - ps11
Choano./Metazoa - ps12
Metazoa - ps13
Myriazoa - ps14
Parahoxozoa - ps15
Bilateria - ps16
Deuterostomia - ps17
Chordata - ps18
Olfactores - ps19
Vertebrata - ps20
Gnathostomata - ps21
Euteleostomi - ps22
Sarcopterygii - ps23
Tetrapoda - ps24
Amniota - ps25
Mammalia - ps26
Theria - ps27
Placentalia - ps28
Boreoeutheria - ps29
Euarchontoglires - ps30
Euarchonta - ps31
Primates - ps32
Haplorrhini - ps33
Simiiformes - ps34
Catarrhini - ps35
Hominoidea - ps36
Hominidae - ps37
Homininae - ps38
Hominini - ps39
Homo_sapiens - ps40
p-value
5e-14
2e-11
9e-09
4e-06
2e-03
Log-odds
0.5
1.0
1.5
2.0
2.5
3.0
3.5
4.0

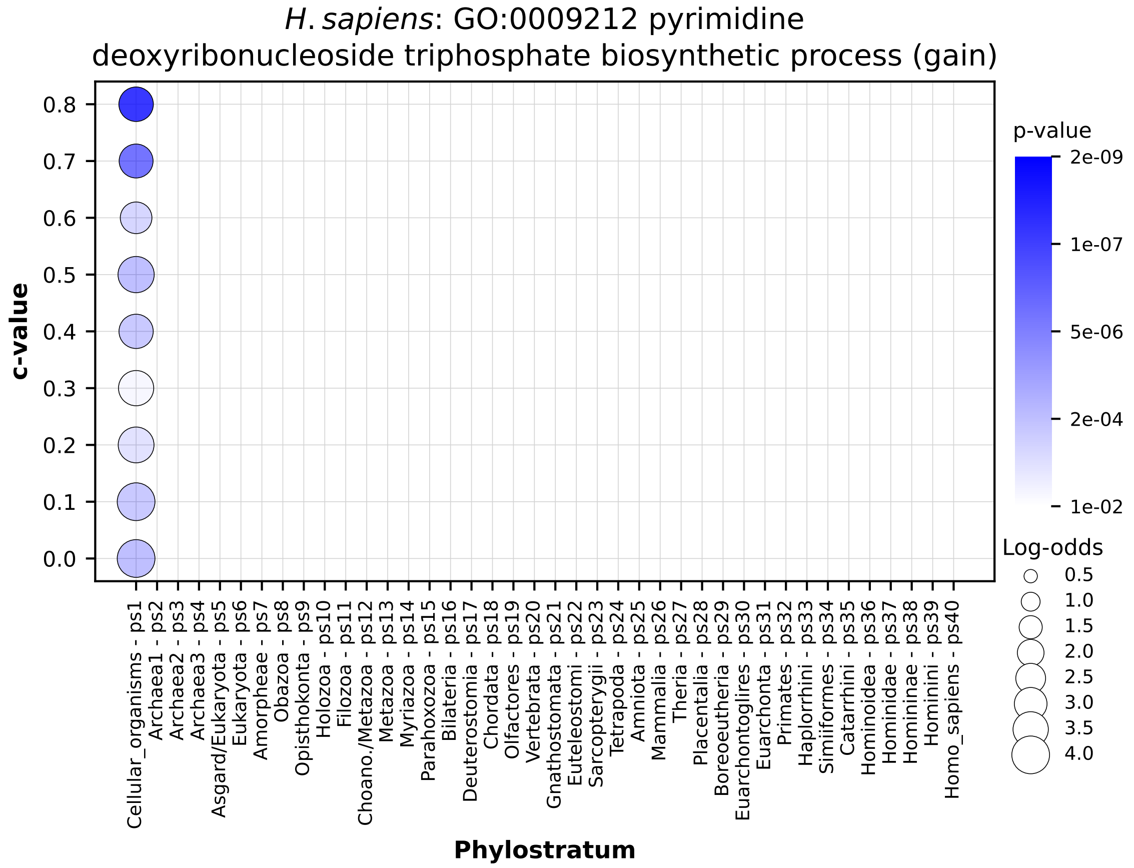
H. sapiens: GO:0009212 pyrimidine deoxyribonucleoside triphosphate biosynthetic process (gain)
c-value
0.0
0.1
0.2
0.3
0.4
0.5
0.6
0.7
0.8
Phylostratum
Cellular_organisms - ps1
Archaea1 - ps2
Archaea2 - ps3
Archaea3 - ps4
Asgard/Eukaryota - ps5
Eukaryota - ps6
Amorpheae - ps7
Obazoa - ps8
Opisthokonta - ps9
Holozoa - ps10
Filozoa - ps11
Choano./Metazoa - ps12
Metazoa - ps13
Myriazoa - ps14
Parahoxozoa - ps15
Bilateria - ps16
Deuterostomia - ps17
Chordata - ps18
Olfactores - ps19
Vertebrata - ps20
Gnathostomata - ps21
Euteleostomi - ps22
Sarcopterygii - ps23
Tetrapoda - ps24
Amniota - ps25
Mammalia - ps26
Theria - ps27
Placentalia - ps28
Boreoeutheria - ps29
Euarchontoglires - ps30
Euarchonta - ps31
Primates - ps32
Haplorrhini - ps33
Simiiformes - ps34
Catarrhini - ps35
Hominoidea - ps36
Hominidae - ps37
Homininae - ps38
Hominini - ps39
Homo_sapiens - ps40
p-value
2e-09
1e-07
5e-06
2e-04
1e-02
Log-odds
0.5
1.0
1.5
2.0
2.5
3.0
3.5
4.0

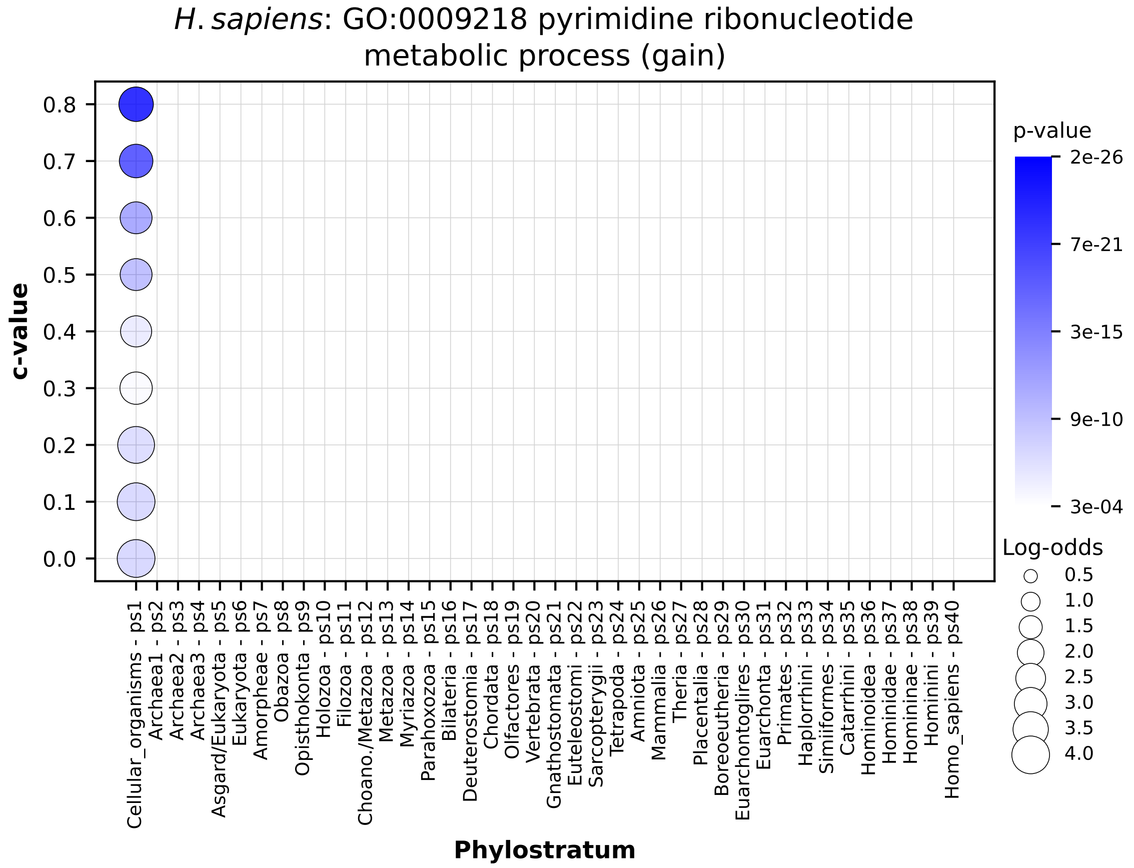
H. sapiens: GO:0009218 pyrimidine ribonucleotide metabolic process (gain)
c-value
0.0
0.1
0.2
0.3
0.4
0.5
0.6
0.7
0.8
Phylostratum
Cellular_organisms - ps1
Archaea1 - ps2
Archaea2 - ps3
Archaea3 - ps4
Asgard/Eukaryota - ps5
Eukaryota - ps6
Amorpheae - ps7
Obazoa - ps8
Opisthokonta - ps9
Holozoa - ps10
Filozoa - ps11
Choano./Metazoa - ps12
Metazoa - ps13
Myriazoa - ps14
Parahoxozoa - ps15
Bilateria - ps16
Deuterostomia - ps17
Chordata - ps18
Olfactores - ps19
Vertebrata - ps20
Gnathostomata - ps21
Euteleostomi - ps22
Sarcopterygii - ps23
Tetrapoda - ps24
Amniota - ps25
Mammalia - ps26
Theria - ps27
Placentalia - ps28
Boreoeutheria - ps29
Euarchontoglires - ps30
Euarchonta - ps31
Primates - ps32
Haplorrhini - ps33
Simiiformes - ps34
Catarrhini - ps35
Hominoidea - ps36
Hominidae - ps37
Homininae - ps38
Hominini - ps39
Homo_sapiens - ps40
p-value
2e-26
7e-21
3e-15
9e-10
3e-04
Log-odds
0.5
1.0
1.5
2.0
2.5
3.0
3.5
4.0

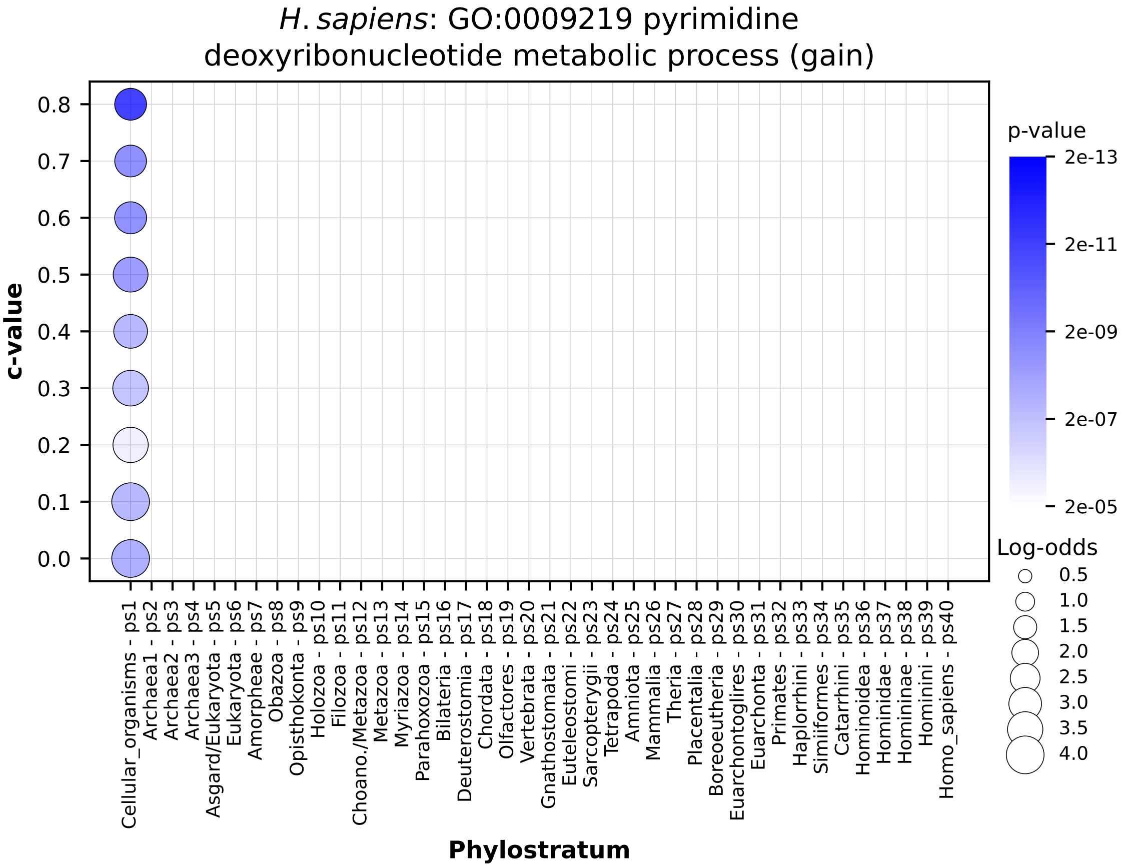

H. sapiens: GO:0009219 pyrimidine deoxyribonucleotide metabolic process (gain)
c-value
0.8
0.7
0.6
0.5
0.4
0.3
0.2
0.1
0.0
Phylostratum
Cellular_organisms - ps1
Archaea1 - ps2
Archaea2 - ps3
Archaea3 - ps4
Asgard/Eukaryota - ps5
Eukaryota - ps6
Amorpheae - ps7
Obazoa - ps8
Opisthokonta - ps9
Holozoa - ps10
Filozoa - ps11
Choano./Metazoa - ps12
Metazoa - ps13
Myriazoa - ps14
Parahoxozoa - ps15
Bilateria - ps16
Deuterostomia - ps17
Chordata - ps18
Olfactores - ps19
Vertebrata - ps20
Gnathostomata - ps21
Euteleostomi - ps22
Sarcopterygii - ps23
Tetrapoda - ps24
Amniota - ps25
Mammalia - ps26
Theria - ps27
Placentalia - ps28
Boreoeutheria - ps29
Euarchontoglires - ps30
Euarchonta - ps31
Primates - ps32
Haplorrhini - ps33
Simiiformes - ps34
Catarrhini - ps35
Hominoidea - ps36
Hominidae - ps37
Homininae - ps38
Hominini - ps39
Homo_sapiens - ps40
p-value
2e-13
2e-11
2e-09
2e-07
2e-05
Log-odds
0.5
1.0
1.5
2.0
2.5
3.0
3.5
4.0

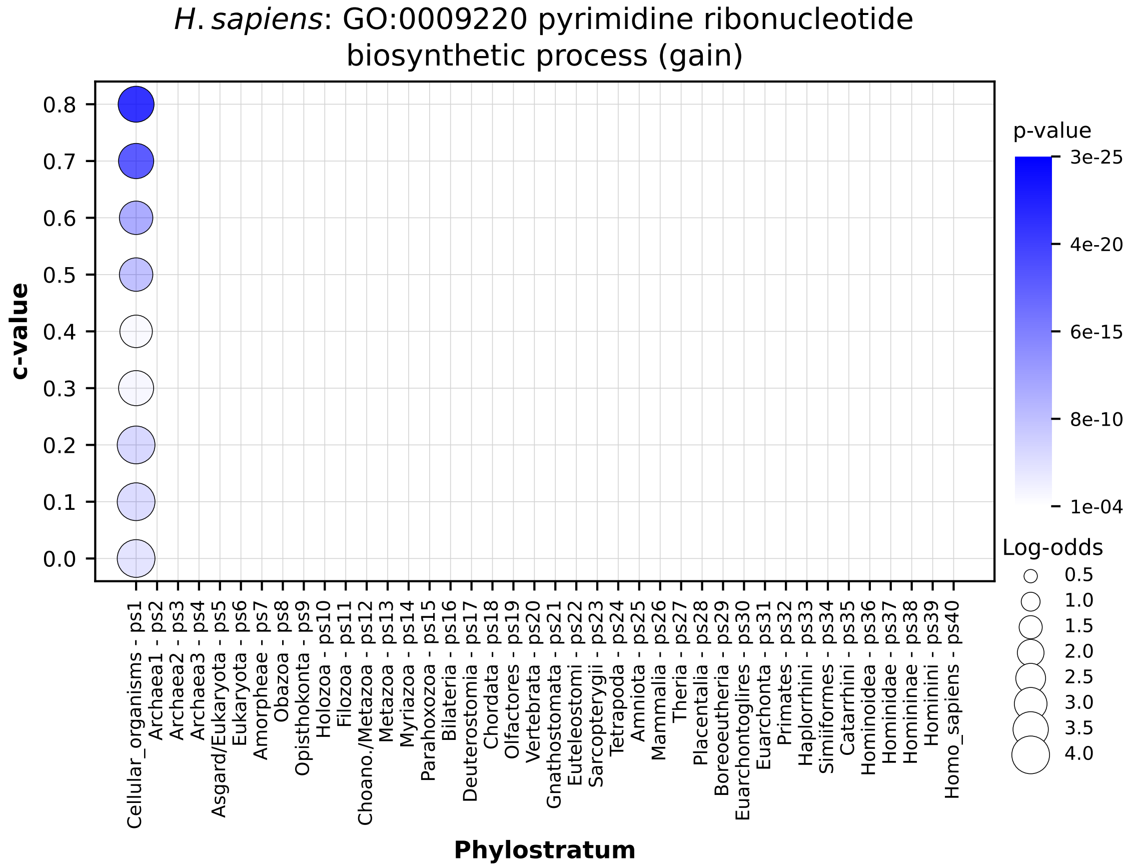
H. sapiens: GO:0009220 pyrimidine ribonucleotide biosynthetic process (gain)
c-value
0.8
0.7
0.6
0.5
0.4
0.3
0.2
0.1
0.0
p-value
3e-25
4e-20
6e-15
8e-10
1e-04
Log-odds
0.5
1.0
1.5
2.0
2.5
3.0
3.5
4.0
Cellular_organisms - ps1
Archaea1 - ps2
Archaea2 - ps3
Archaea3 - ps4
Asgard/Eukaryota - ps5
Eukaryota - ps6
Amorpheae - ps7
Obazoa - ps8
Opisthokonta - ps9
Holozoa - ps10
Filozoa - ps11
Choano./Metazoa - ps12
Metazoa - ps13
Myriazoa - ps14
Parahoxozoa - ps15
Bilateria - ps16
Deuterostomia - ps17
Chordata - ps18
Olfactores - ps19
Vertebrata - ps20
Gnathostomata - ps21
Euteleostomi - ps22
Sarcopterygii - ps23
Tetrapoda - ps24
Amniota - ps25
Mammalia - ps26
Theria - ps27
Placentalia - ps28
Boreoeutheria - ps29
Euarchontoglires - ps30
Euarchonta - ps31
Primates - ps32
Haplorrhini - ps33
Simiiformes - ps34
Catarrhini - ps35
Hominoidea - ps36
Hominidae - ps37
Homininae - ps38
Hominini - ps39
Homo_sapiens - ps40
Phylostratum

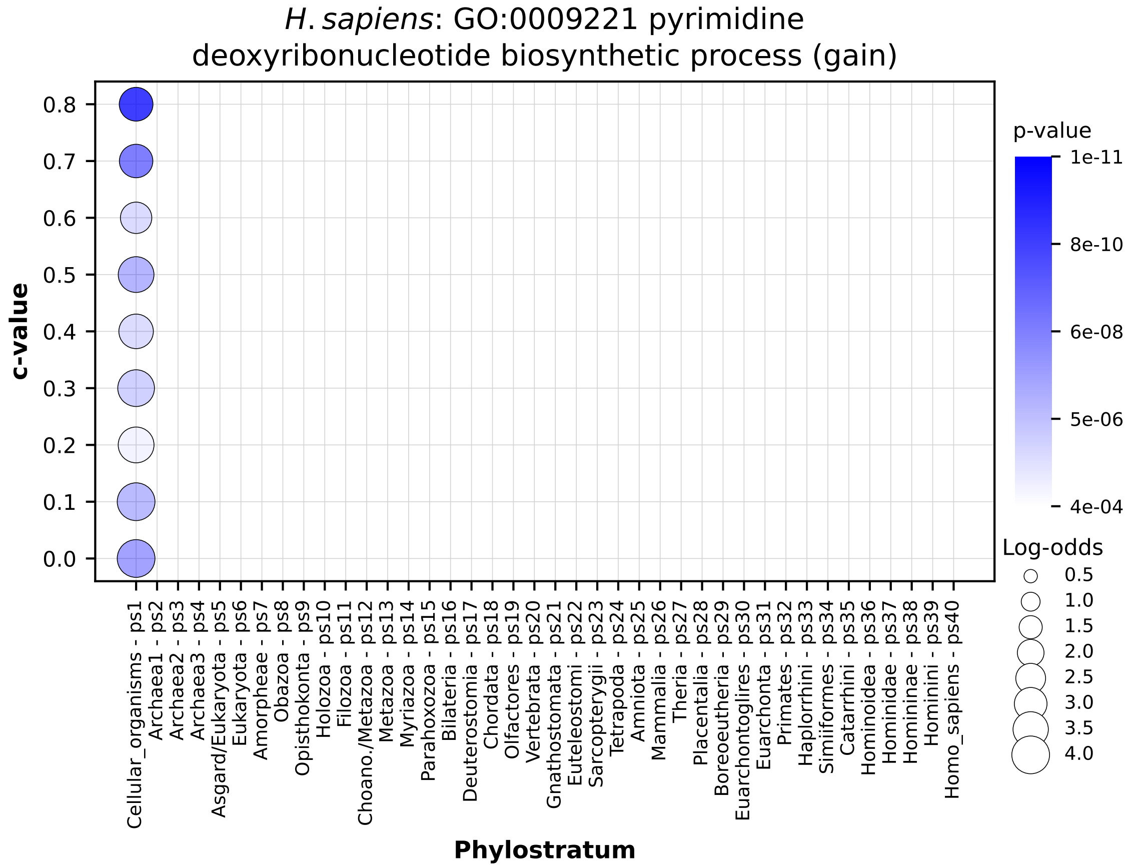

H. sapiens: GO:0009221 pyrimidine deoxyribonucleotide biosynthetic process (gain)
c-value
0.8
0.7
0.6
0.5
0.4
0.3
0.2
0.1
0.0
p-value
1e-11
8e-10
6e-08
5e-06
4e-04
Log-odds
0.5
1.0
1.5
2.0
2.5
3.0
3.5
4.0
Cellular_organisms - ps1
Archaea1 - ps2
Archaea2 - ps3
Archaea3 - ps4
Asgard/Eukaryota - ps5
Eukaryota - ps6
Amorpheae - ps7
Obazoa - ps8
Opisthokonta - ps9
Holozoa - ps10
Filozoa - ps11
Choano./Metazoa - ps12
Metazoa - ps13
Myriazoa - ps14
Parahoxozoa - ps15
Bilateria - ps16
Deuterostomia - ps17
Chordata - ps18
Olfactores - ps19
Vertebrata - ps20
Gnathostomata - ps21
Euteleostomi - ps22
Sarcopterygii - ps23
Tetrapoda - ps24
Amniota - ps25
Mammalia - ps26
Theria - ps27
Placentalia - ps28
Boreoeutheria - ps29
Euarchontoglires - ps30
Euarchonta - ps31
Primates - ps32
Haplorrhini - ps33
Simiiformes - ps34
Catarrhini - ps35
Hominoidea - ps36
Hominidae - ps37
Homininae - ps38
Hominini - ps39
Homo_sapiens - ps40
Phylostratum

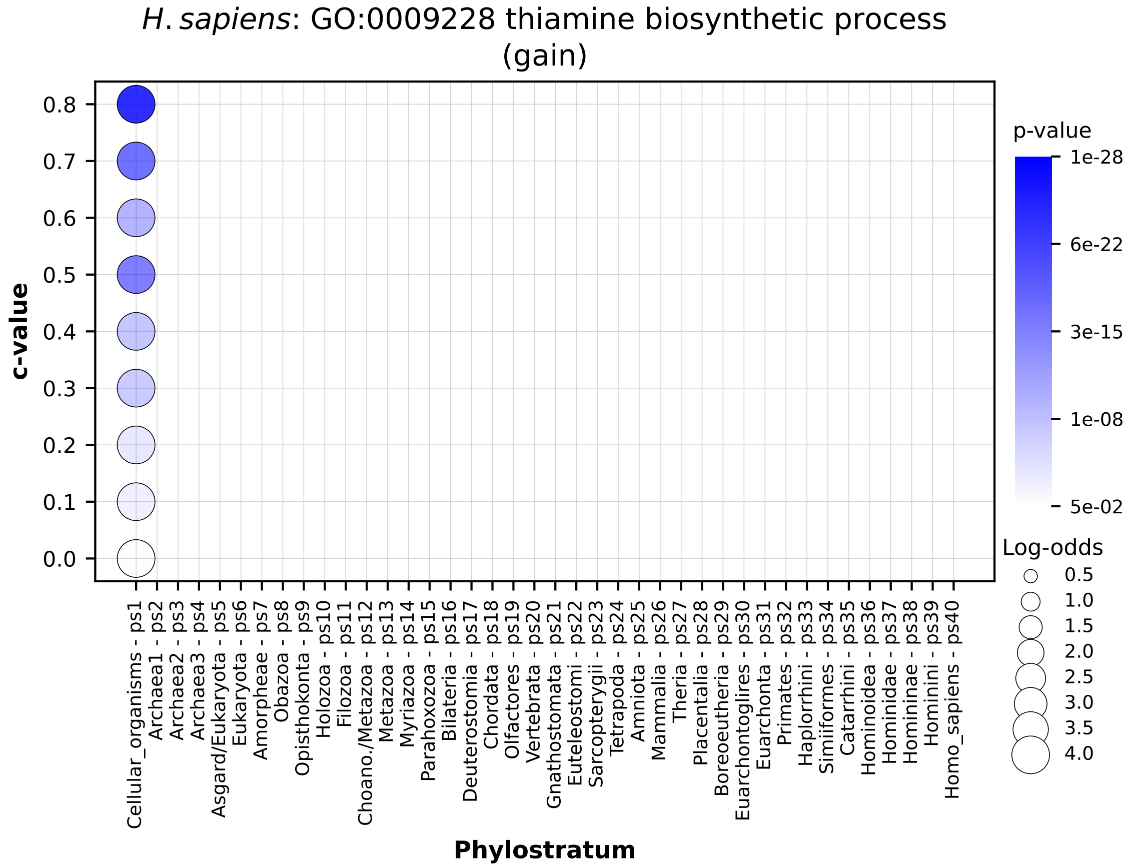

H. sapiens: GO:0009228 thiamine biosynthetic process
(gain)
c-value
0.0
0.1
0.2
0.3
0.4
0.5
0.6
0.7
0.8
p-value
1e-28
6e-22
3e-15
1e-08
5e-02
Log-odds
0.5
1.0
1.5
2.0
2.5
3.0
3.5
4.0
Cellular_organisms - ps1
Archaea1 - ps2
Archaea2 - ps3
Archaea3 - ps4
Asgard/Eukaryota - ps5
Eukaryota - ps6
Amorpheae - ps7
Obazoa - ps8
Opisthokonta - ps9
Holozoa - ps10
Filozoa - ps11
Choano./Metazoa - ps12
Metazoa - ps13
Myriazoa - ps14
Parahoxozoa - ps15
Bilateria - ps16
Deuterostomia - ps17
Chordata - ps18
Olfactores - ps19
Vertebrata - ps20
Gnathostomata - ps21
Euteleostomi - ps22
Sarcopterygii - ps23
Tetrapoda - ps24
Amniota - ps25
Mammalia - ps26
Theria - ps27
Placentalia - ps28
Boreoeutheria - ps29
Euarchontoglires - ps30
Euarchonta - ps31
Primates - ps32
Haplorrhini - ps33
Simiiformes - ps34
Catarrhini - ps35
Hominoidea - ps36
Hominidae - ps37
Homininae - ps38
Hominini - ps39
Homo_sapiens - ps40
Phylostratum

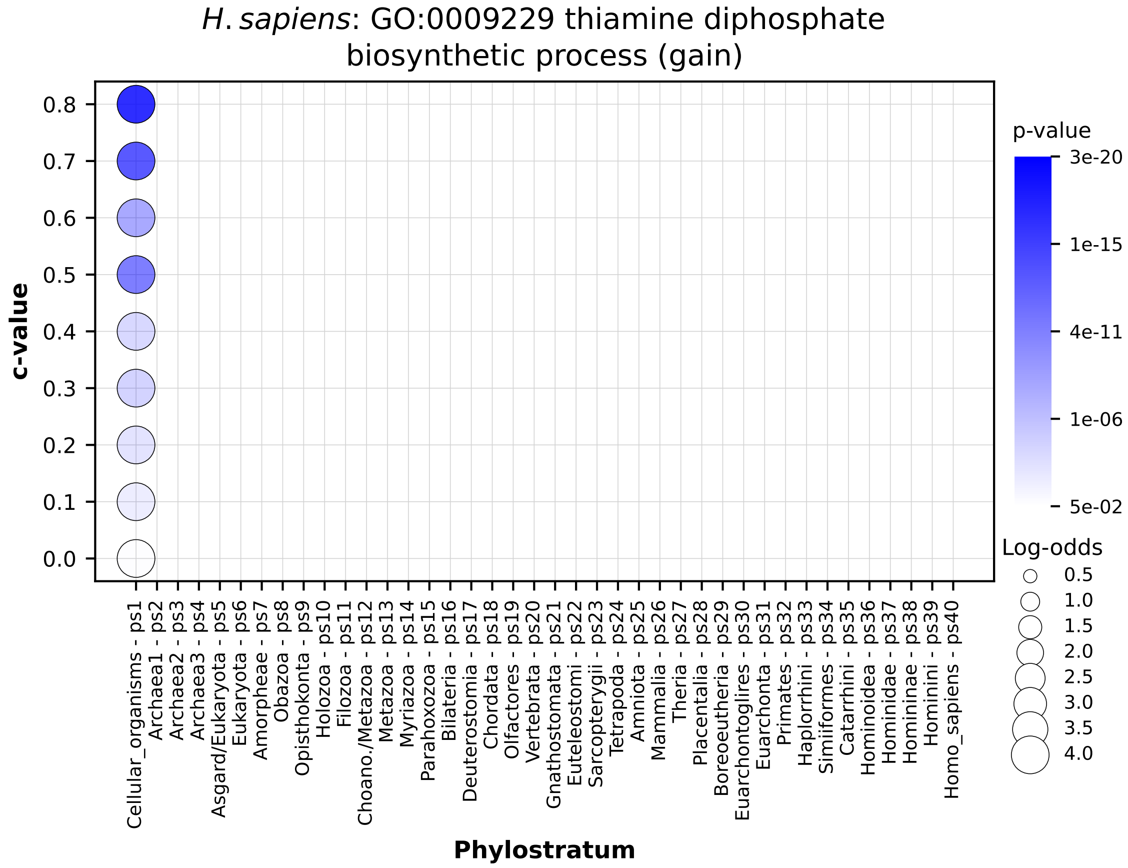
H. sapiens: GO:0009229 thiamine diphosphate biosynthetic process (gain)
c-value
0.8
0.7
0.6
0.5
0.4
0.3
0.2
0.1
0.0
p-value
3e-20
1e-15
4e-11
1e-06
5e-02
Log-odds
0.5
1.0
1.5
2.0
2.5
3.0
3.5
4.0
Cellular_organisms - ps1
Archaea1 - ps2
Archaea2 - ps3
Archaea3 - ps4
Asgard/Eukaryota - ps5
Eukaryota - ps6
Amorpheae - ps7
Obazoa - ps8
Opisthokonta - ps9
Holozoa - ps10
Filozoa - ps11
Choano./Metazoa - ps12
Metazoa - ps13
Myriazoa - ps14
Parahoxozoa - ps15
Bilateria - ps16
Deuterostomia - ps17
Chordata - ps18
Olfactores - ps19
Vertebrata - ps20
Gnathostomata - ps21
Euteleostomi - ps22
Sarcopterygii - ps23
Tetrapoda - ps24
Amniota - ps25
Mammalia - ps26
Theria - ps27
Placentalia - ps28
Boreoeutheria - ps29
Euarchontoglires - ps30
Euarchonta - ps31
Primates - ps32
Haplorrhini - ps33
Simiiformes - ps34
Catarrhini - ps35
Hominoidea - ps36
Hominidae - ps37
Homininae - ps38
Hominini - ps39
Homo_sapiens - ps40
Phylostratum

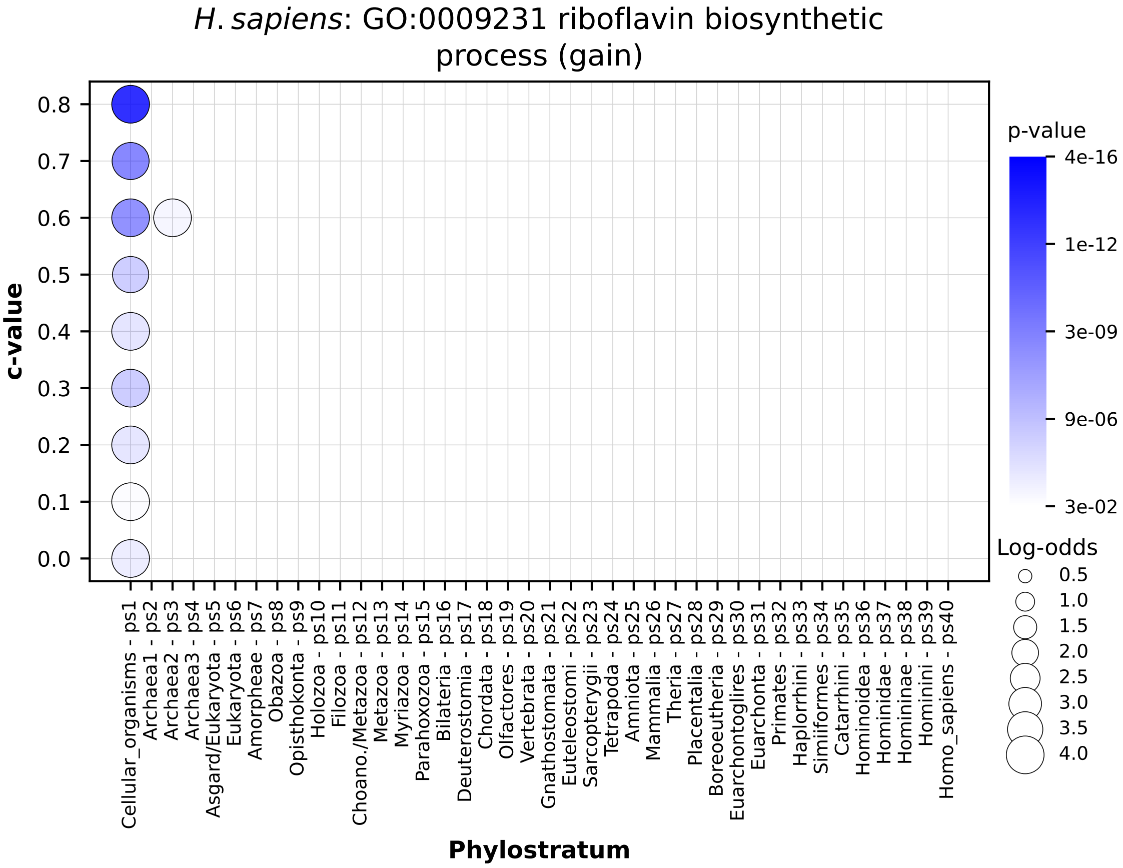
H. sapiens: GO:0009231 riboflavin biosynthetic process (gain)
c-value
0.8
0.7
0.6
0.5
0.4
0.3
0.2
0.1
0.0
Phylostratum
Cellular_organisms - ps1
Archaea1 - ps2
Archaea2 - ps3
Archaea3 - ps4
Asgard/Eukaryota - ps5
Eukaryota - ps6
Amorpheae - ps7
Obazoa - ps8
Opisthokonta - ps9
Holozoa - ps10
Filozoa - ps11
Choano./Metazoa - ps12
Metazoa - ps13
Myriazoa - ps14
Parahoxozoa - ps15
Bilateria - ps16
Deuterostomia - ps17
Chordata - ps18
Olfactores - ps19
Vertebrata - ps20
Gnathostomata - ps21
Euteleostomi - ps22
Sarcopterygii - ps23
Tetrapoda - ps24
Amniota - ps25
Mammalia - ps26
Theria - ps27
Placentalia - ps28
Boreoeutheria - ps29
Euarchontoglires - ps30
Euarchonta - ps31
Primates - ps32
Haplorrhini - ps33
Simiiformes - ps34
Catarrhini - ps35
Hominoidea - ps36
Hominidae - ps37
Homininae - ps38
Hominini - ps39
Homo_sapiens - ps40
p-value
4e-16
1e-12
3e-09
9e-06
3e-02
Log-odds
0.5
1.0
1.5
2.0
2.5
3.0
3.5
4.0

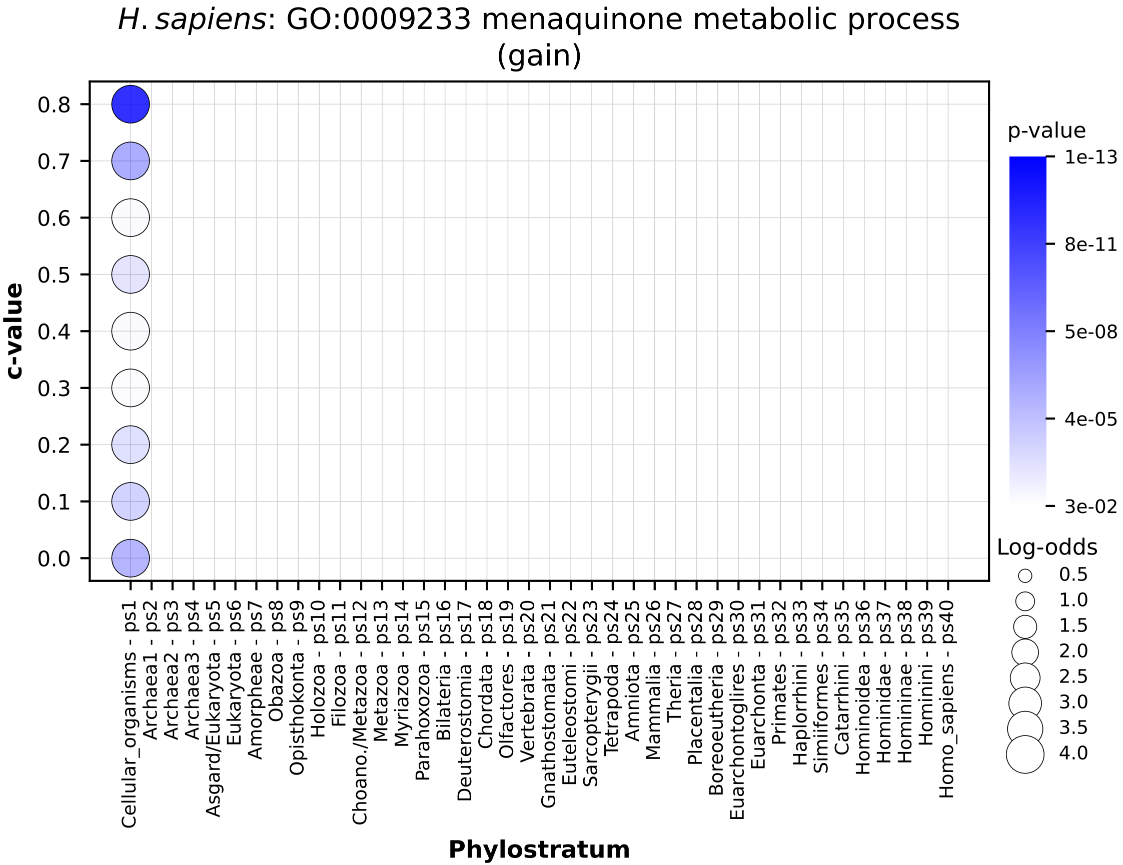

H. sapiens: GO:0009233 menaquinone metabolic process (gain)
c-value
0.8
0.7
0.6
0.5
0.4
0.3
0.2
0.1
0.0
p-value
1e-13
8e-11
5e-08
4e-05
3e-02
Log-odds
0.5
1.0
1.5
2.0
2.5
3.0
3.5
4.0
Cellular_organisms - ps1
Archaea1 - ps2
Archaea2 - ps3
Archaea3 - ps4
Asgard/Eukaryota - ps5
Eukaryota - ps6
Amorpheae - ps7
Obazoa - ps8
Opisthokonta - ps9
Holozoa - ps10
Filozoa - ps11
Choano./Metazoa - ps12
Metazoa - ps13
Myriazoa - ps14
Parahoxozoa - ps15
Bilateria - ps16
Deuterostomia - ps17
Chordata - ps18
Olfactores - ps19
Vertebrata - ps20
Gnathostomata - ps21
Euteleostomi - ps22
Sarcopterygii - ps23
Tetrapoda - ps24
Amniota - ps25
Mammalia - ps26
Theria - ps27
Placentalia - ps28
Boreoeutheria - ps29
Euarchontoglires - ps30
Euarchonta - ps31
Primates - ps32
Haplorrhini - ps33
Simiiformes - ps34
Catarrhini - ps35
Hominoidea - ps36
Hominidae - ps37
Homininae - ps38
Hominini - ps39
Homo_sapiens - ps40
Phylostratum

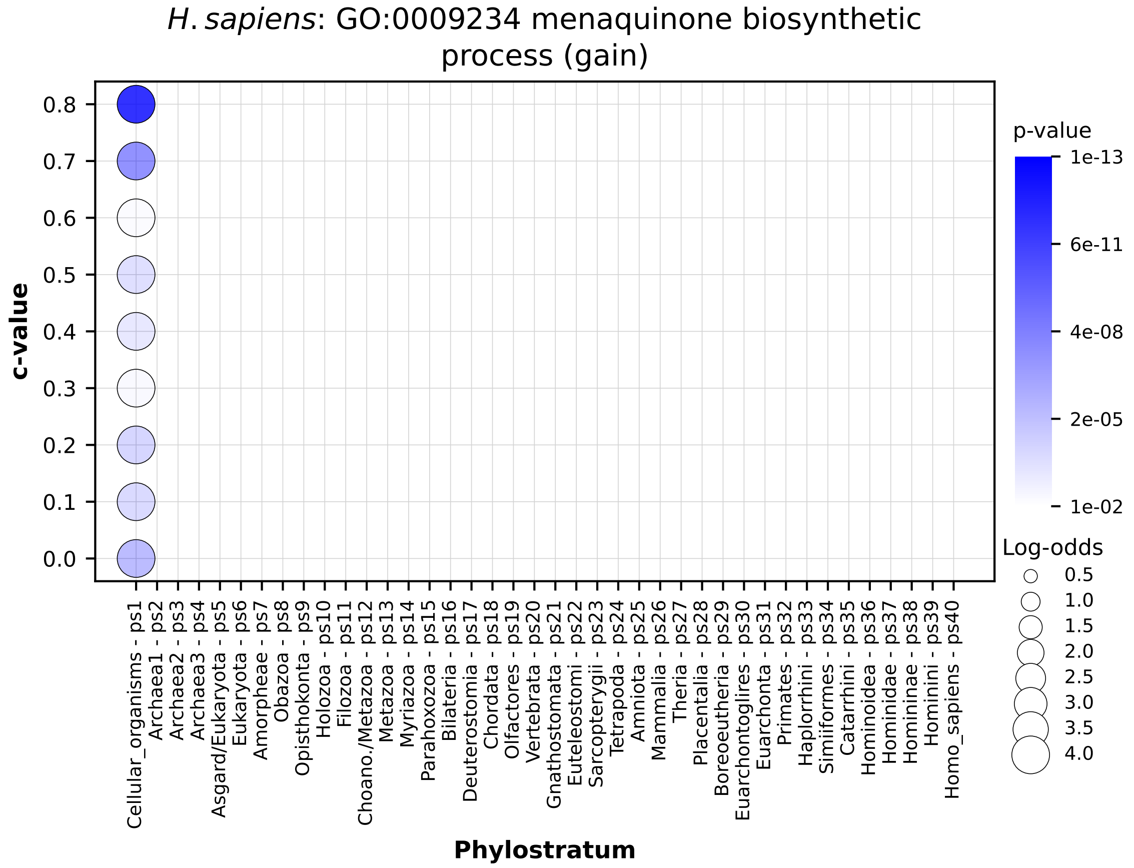

H. sapiens: GO:0009234 menaquinone biosynthetic process (gain)
c-value
0.0
0.1
0.2
0.3
0.4
0.5
0.6
0.7
0.8
Phylostratum
Cellular_organisms - ps1
Archaea1 - ps2
Archaea2 - ps3
Archaea3 - ps4
Asgard/Eukaryota - ps5
Eukaryota - ps6
Amorpheae - ps7
Obazoa - ps8
Opisthokonta - ps9
Holozoa - ps10
Filozoa - ps11
Choano./Metazoa - ps12
Metazoa - ps13
Myriazoa - ps14
Parahoxozoa - ps15
Bilateria - ps16
Deuterostomia - ps17
Chordata - ps18
Olfactores - ps19
Vertebrata - ps20
Gnathostomata - ps21
Euteleostomi - ps22
Sarcopterygii - ps23
Tetrapoda - ps24
Amniota - ps25
Mammalia - ps26
Theria - ps27
Placentalia - ps28
Boreoeutheria - ps29
Euarchontoglires - ps30
Euarchonta - ps31
Primates - ps32
Haplorrhini - ps33
Simiiformes - ps34
Catarrhini - ps35
Hominoidea - ps36
Hominidae - ps37
Homininae - ps38
Hominini - ps39
Homo_sapiens - ps40
p-value
1e-13
6e-11
4e-08
2e-05
1e-02
Log-odds
0.5
1.0
1.5
2.0
2.5
3.0
3.5
4.0

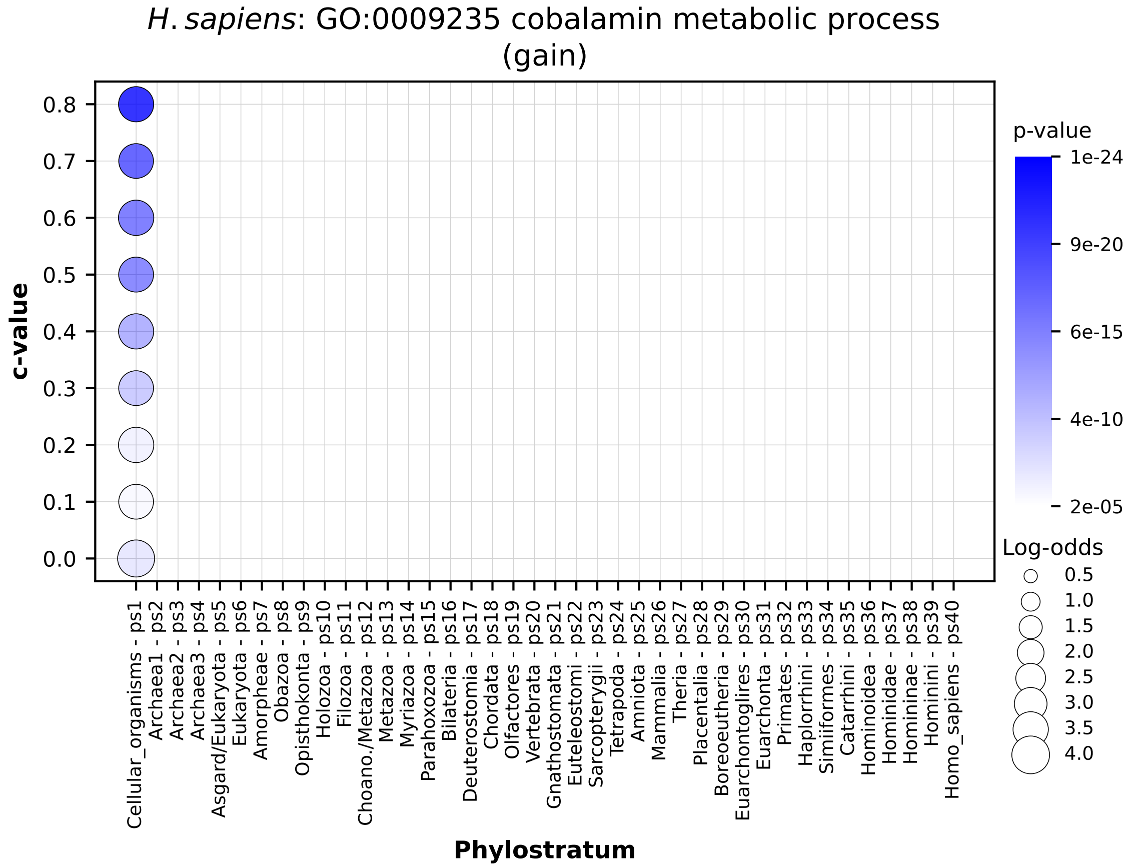

*H. sapiens*: GO:0009235 cobalamin metabolic process (gain)
c-value
0.8
0.7
0.6
0.5
0.4
0.3
0.2
0.1
0.0
p-value
1e-24
9e-20
6e-15
4e-10
2e-05
Log-odds
0.5
1.0
1.5
2.0
2.5
3.0
3.5
4.0
Cellular_organisms - ps1
Archaea1 - ps2
Archaea2 - ps3
Archaea3 - ps4
Asgard/Eukaryota - ps5
Eukaryota - ps6
Amorpheae - ps7
Obazoa - ps8
Opisthokonta - ps9
Holozoa - ps10
Filozoa - ps11
Choano./Metazoa - ps12
Metazoa - ps13
Myriazoa - ps14
Parahoxozoa - ps15
Bilateria - ps16
Deuterostomia - ps17
Chordata - ps18
Olfactores - ps19
Vertebrata - ps20
Gnathostomata - ps21
Euteleostomi - ps22
Sarcopterygii - ps23
Tetrapoda - ps24
Amniota - ps25
Mammalia - ps26
Theria - ps27
Placentalia - ps28
Boreoeutheria - ps29
Euarchontoglires - ps30
Euarchonta - ps31
Primates - ps32
Haplorrhini - ps33
Simiiformes - ps34
Catarrhini - ps35
Hominoidea - ps36
Hominidae - ps37
Homininae - ps38
Hominini - ps39
Homo_sapiens - ps40
Phylostratum

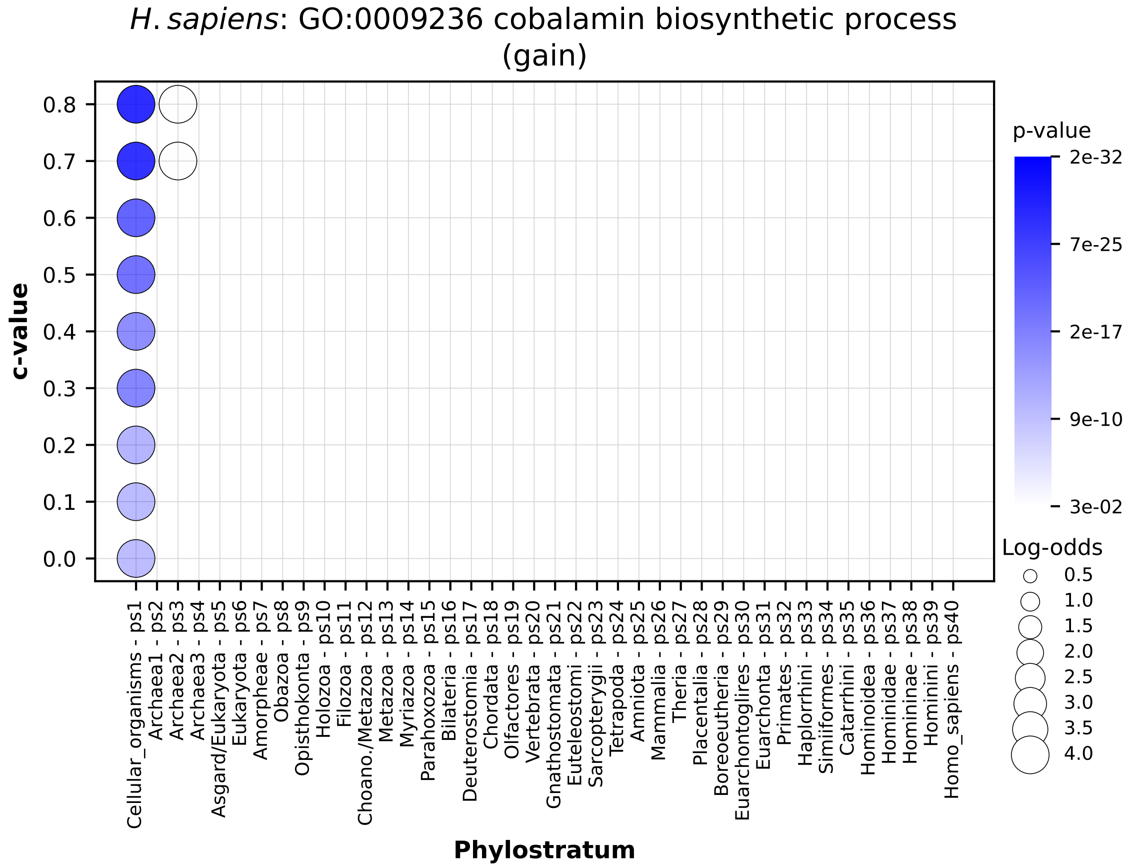

H. sapiens: GO:0009236 cobalamin biosynthetic process
(gain)
c-value
0.8
0.7
0.6
0.5
0.4
0.3
0.2
0.1
0.0
p-value
2e-32
7e-25
2e-17
9e-10
3e-02
Log-odds
0.5
1.0
1.5
2.0
2.5
3.0
3.5
4.0
Cellular_organisms - ps1
Archaea1 - ps2
Archaea2 - ps3
Archaea3 - ps4
Asgard/Eukaryota - ps5
Eukaryota - ps6
Amorpheae - ps7
Obazoa - ps8
Opisthokonta - ps9
Holozoa - ps10
Filozoa - ps11
Choano./Metazoa - ps12
Metazoa - ps13
Myriazoa - ps14
Parahoxozoa - ps15
Bilateria - ps16
Deuterostomia - ps17
Chordata - ps18
Olfactores - ps19
Vertebrata - ps20
Gnathostomata - ps21
Euteleostomi - ps22
Sarcopterygii - ps23
Tetrapoda - ps24
Amniota - ps25
Mammalia - ps26
Theria - ps27
Placentalia - ps28
Boreoeutheria - ps29
Euarchontoglires - ps30
Euarchonta - ps31
Primates - ps32
Haplorrhini - ps33
Simiiformes - ps34
Catarrhini - ps35
Hominoidea - ps36
Hominidae - ps37
Homininae - ps38
Hominini - ps39
Homo_sapiens - ps40
Phylostratum

# *H. sapiens*: GO:0009245 lipid A biosynthetic process (gain)

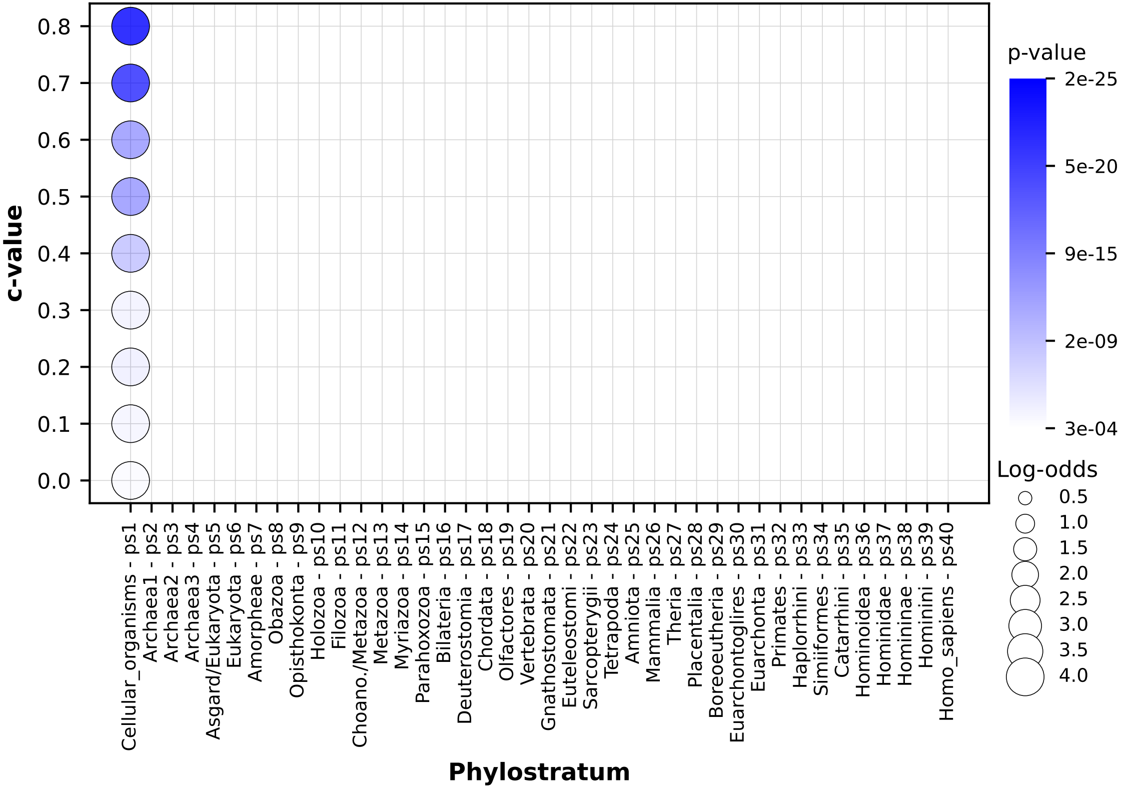

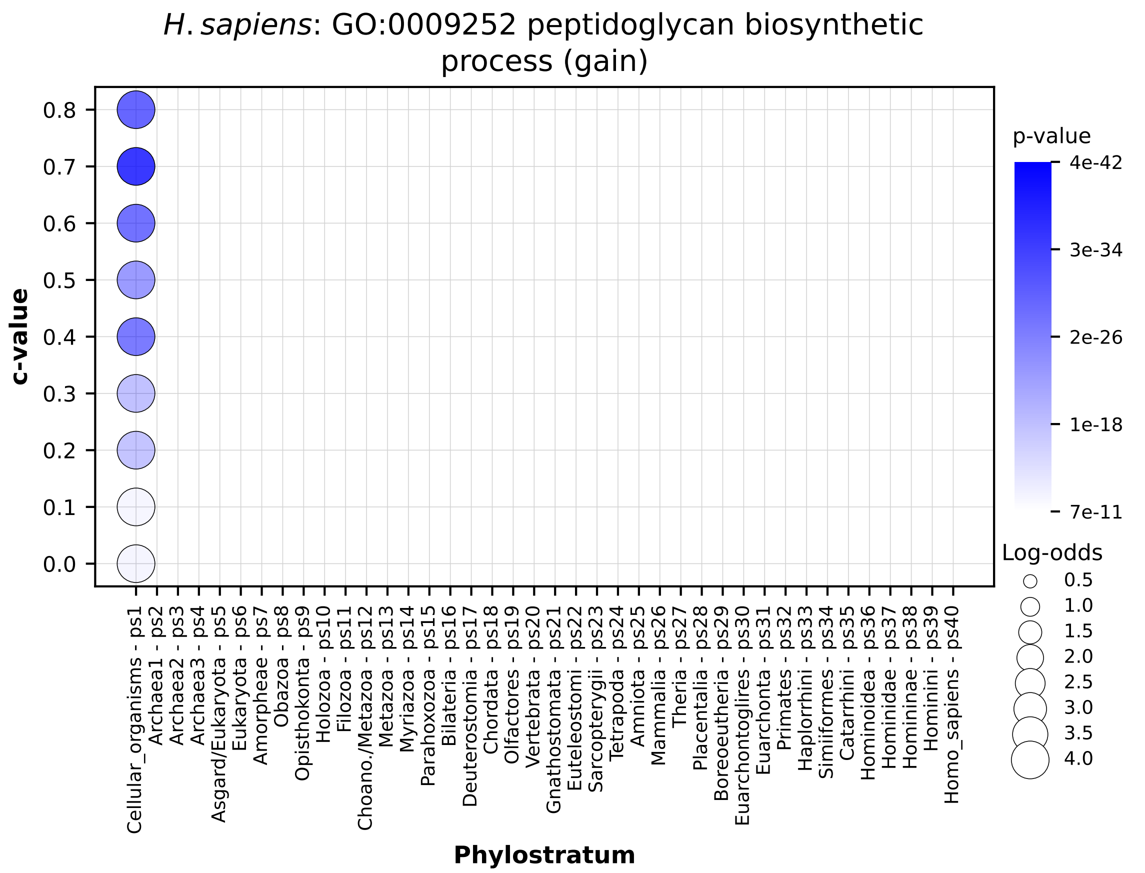

H. sapiens: GO:0009252 peptidoglycan biosynthetic process (gain)
c-value
0.0
0.1
0.2
0.3
0.4
0.5
0.6
0.7
0.8
Phylostratum
Cellular_organisms - ps1
Archaea1 - ps2
Archaea2 - ps3
Archaea3 - ps4
Asgard/Eukaryota - ps5
Eukaryota - ps6
Amorpheae - ps7
Obazoa - ps8
Opisthokonta - ps9
Holozoa - ps10
Filozoa - ps11
Choano./Metazoa - ps12
Metazoa - ps13
Myriazoa - ps14
Parahoxozoa - ps15
Bilateria - ps16
Deuterostomia - ps17
Chordata - ps18
Olfactores - ps19
Vertebrata - ps20
Gnathostomata - ps21
Euteleostomi - ps22
Sarcopterygii - ps23
Tetrapoda - ps24
Amniota - ps25
Mammalia - ps26
Theria - ps27
Placentalia - ps28
Boreoeutheria - ps29
Euarchontoglires - ps30
Euarchonta - ps31
Primates - ps32
Haplorrhini - ps33
Simiiformes - ps34
Catarrhini - ps35
Hominoidea - ps36
Hominidae - ps37
Homininae - ps38
Hominini - ps39
Homo_sapiens - ps40
p-value
4e-42
3e-34
2e-26
1e-18
7e-11
Log-odds
0.5
1.0
1.5
2.0
2.5
3.0
3.5
4.0

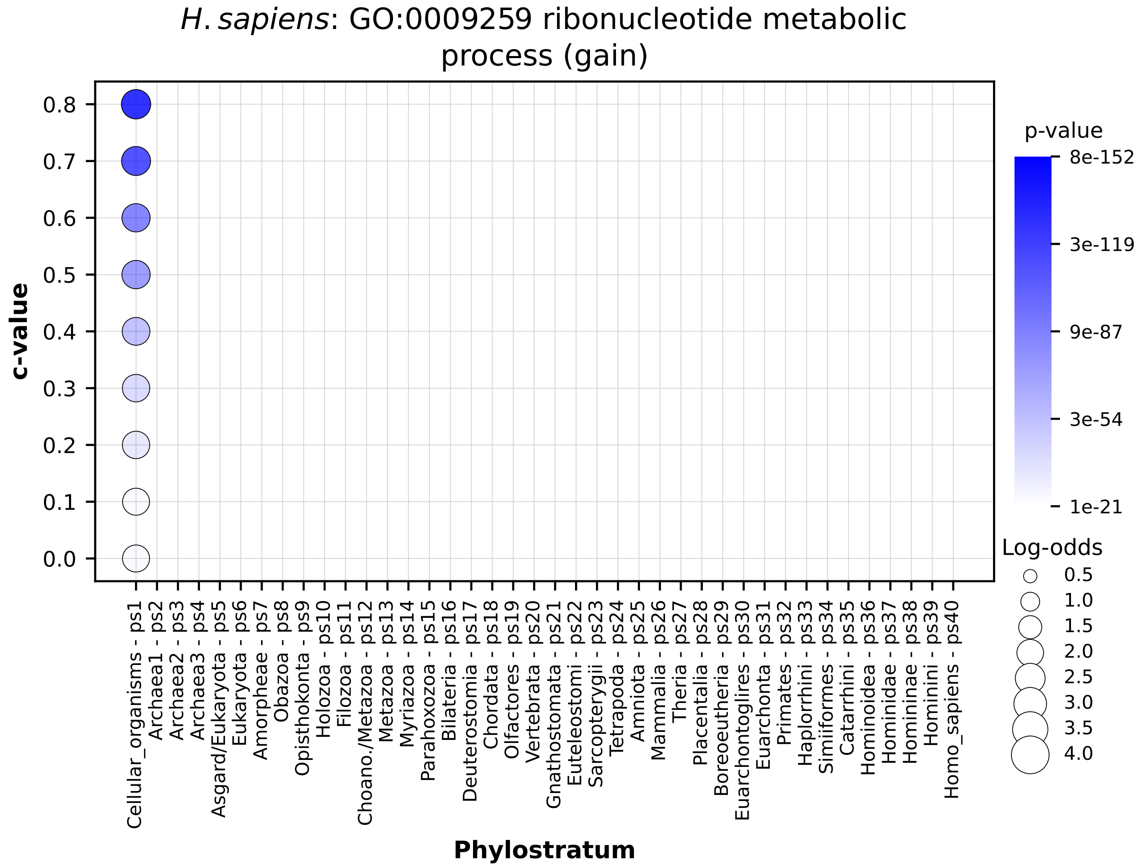
H. sapiens: GO:0009259 ribonucleotide metabolic process (gain)
c-value
0.8
0.7
0.6
0.5
0.4
0.3
0.2
0.1
0.0
Phylostratum
Cellular_organisms - ps1
Archaea1 - ps2
Archaea2 - ps3
Archaea3 - ps4
Asgard/Eukaryota - ps5
Eukaryota - ps6
Amorpheae - ps7
Obazoa - ps8
Opisthokonta - ps9
Holozoa - ps10
Filozoa - ps11
Choano./Metazoa - ps12
Metazoa - ps13
Myriazoa - ps14
Parahoxozoa - ps15
Bilateria - ps16
Deuterostomia - ps17
Chordata - ps18
Olfactores - ps19
Vertebrata - ps20
Gnathostomata - ps21
Euteleostomi - ps22
Sarcopterygii - ps23
Tetrapoda - ps24
Amniota - ps25
Mammalia - ps26
Theria - ps27
Placentalia - ps28
Boreoeutheria - ps29
Euarchontoglires - ps30
Euarchonta - ps31
Primates - ps32
Haplorrhini - ps33
Simiiformes - ps34
Catarrhini - ps35
Hominoidea - ps36
Hominidae - ps37
Homininae - ps38
Hominini - ps39
Homo_sapiens - ps40
p-value
8e-152
3e-119
9e-87
3e-54
1e-21
Log-odds
0.5
1.0
1.5
2.0
2.5
3.0
3.5
4.0

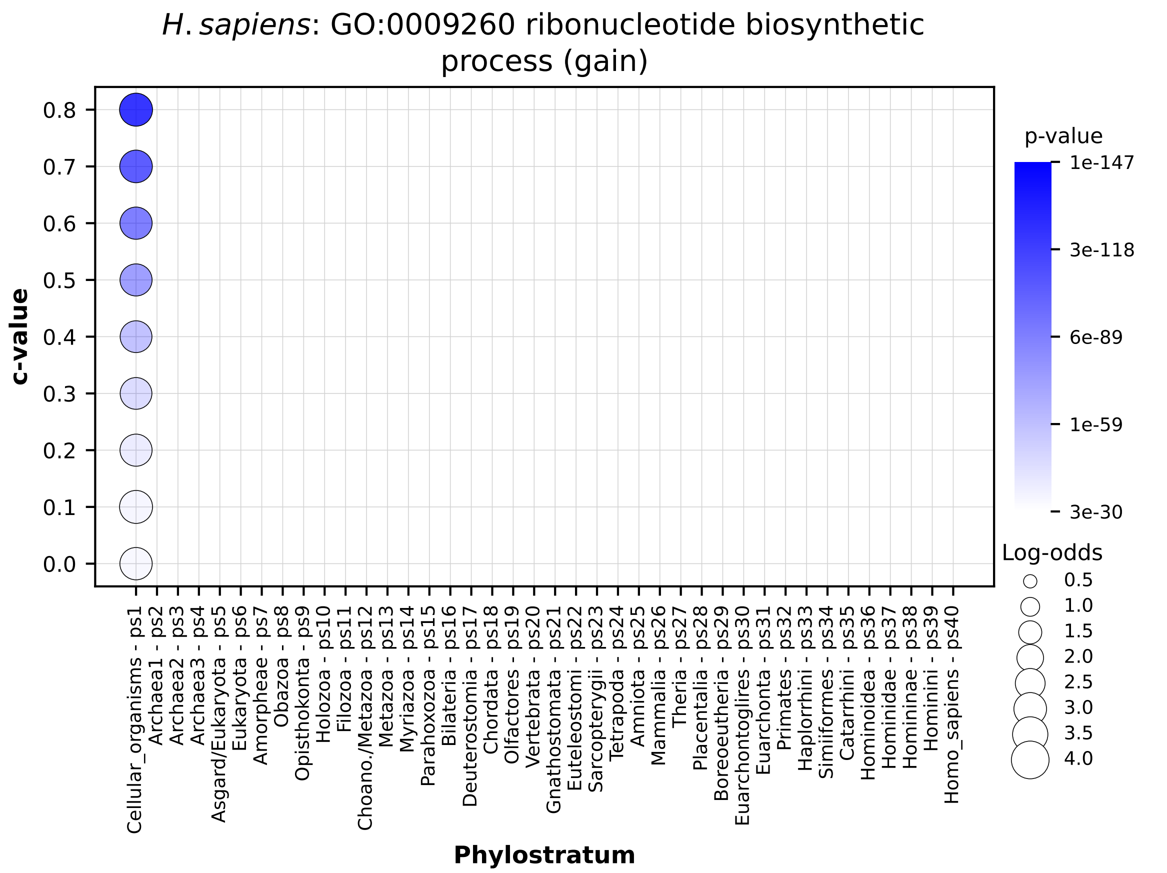

H. sapiens: GO:0009260 ribonucleotide biosynthetic process (gain)
c-value
0.8
0.7
0.6
0.5
0.4
0.3
0.2
0.1
0.0
p-value
1e-147
3e-118
6e-89
1e-59
3e-30
Log-odds
0.5
1.0
1.5
2.0
2.5
3.0
3.5
4.0
Cellular_organisms - ps1
Archaea1 - ps2
Archaea2 - ps3
Archaea3 - ps4
Asgard/Eukaryota - ps5
Eukaryota - ps6
Amorpheae - ps7
Obazoa - ps8
Opisthokonta - ps9
Holozoa - ps10
Filozoa - ps11
Choano./Metazoa - ps12
Metazoa - ps13
Myriazoa - ps14
Parahoxozoa - ps15
Bilateria - ps16
Deuterostomia - ps17
Chordata - ps18
Olfactores - ps19
Vertebrata - ps20
Gnathostomata - ps21
Euteleostomi - ps22
Sarcopterygii - ps23
Tetrapoda - ps24
Amniota - ps25
Mammalia - ps26
Theria - ps27
Placentalia - ps28
Boreoeutheria - ps29
Euarchontoglires - ps30
Euarchonta - ps31
Primates - ps32
Haplorrhini - ps33
Simiiformes - ps34
Catarrhini - ps35
Hominoidea - ps36
Hominidae - ps37
Homininae - ps38
Hominini - ps39
Homo_sapiens - ps40
Phylostratum

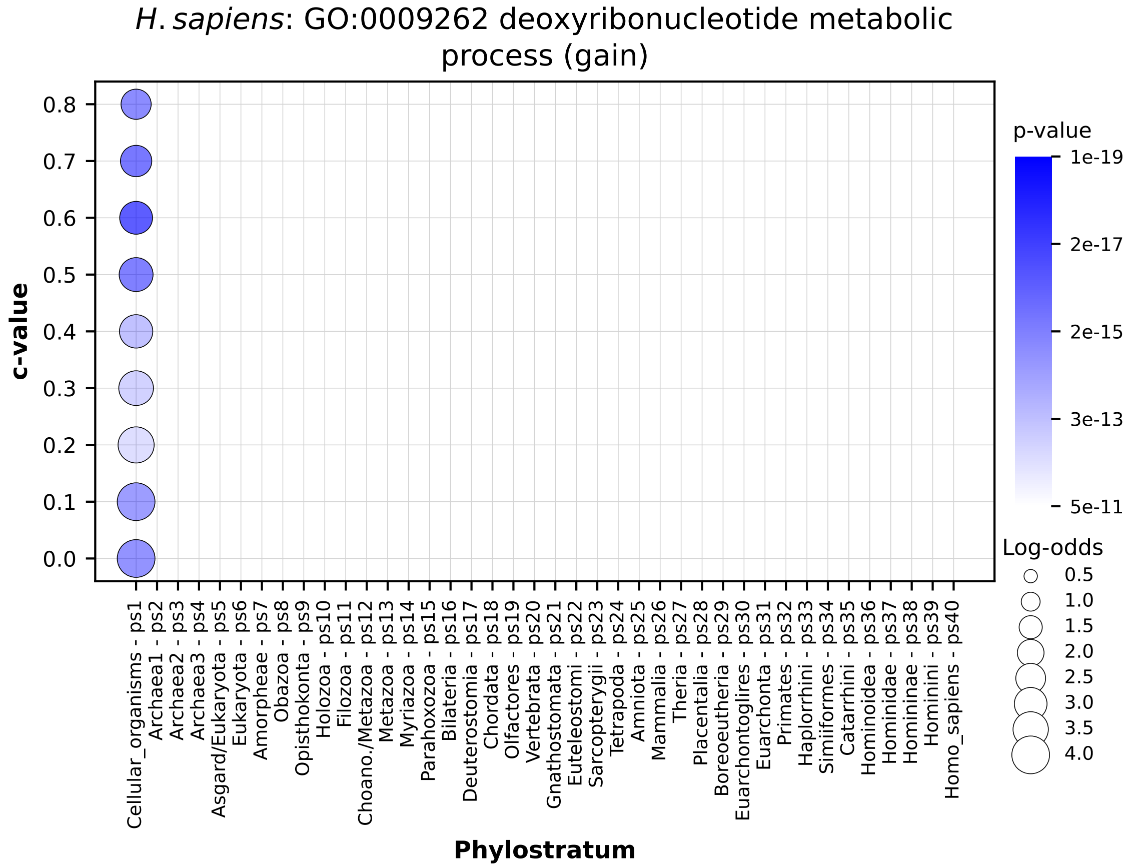

H. sapiens: GO:0009262 deoxyribonucleotide metabolic process (gain)
c-value
0.8
0.7
0.6
0.5
0.4
0.3
0.2
0.1
0.0
Phylostratum
Cellular_organisms - ps1
Archaea1 - ps2
Archaea2 - ps3
Archaea3 - ps4
Asgard/Eukaryota - ps5
Eukaryota - ps6
Amorpheae - ps7
Obazoa - ps8
Opisthokonta - ps9
Holozoa - ps10
Filozoa - ps11
Choano./Metazoa - ps12
Metazoa - ps13
Myriazoa - ps14
Parahoxozoa - ps15
Bilateria - ps16
Deuterostomia - ps17
Chordata - ps18
Olfactores - ps19
Vertebrata - ps20
Gnathostomata - ps21
Euteleostomi - ps22
Sarcopterygii - ps23
Tetrapoda - ps24
Amniota - ps25
Mammalia - ps26
Theria - ps27
Placentalia - ps28
Boreoeutheria - ps29
Euarchontoglires - ps30
Euarchonta - ps31
Primates - ps32
Haplorrhini - ps33
Simiiformes - ps34
Catarrhini - ps35
Hominoidea - ps36
Hominidae - ps37
Homininae - ps38
Hominini - ps39
Homo_sapiens - ps40
p-value
1e-19
2e-17
2e-15
3e-13
5e-11
Log-odds
0.5
1.0
1.5
2.0
2.5
3.0
3.5
4.0

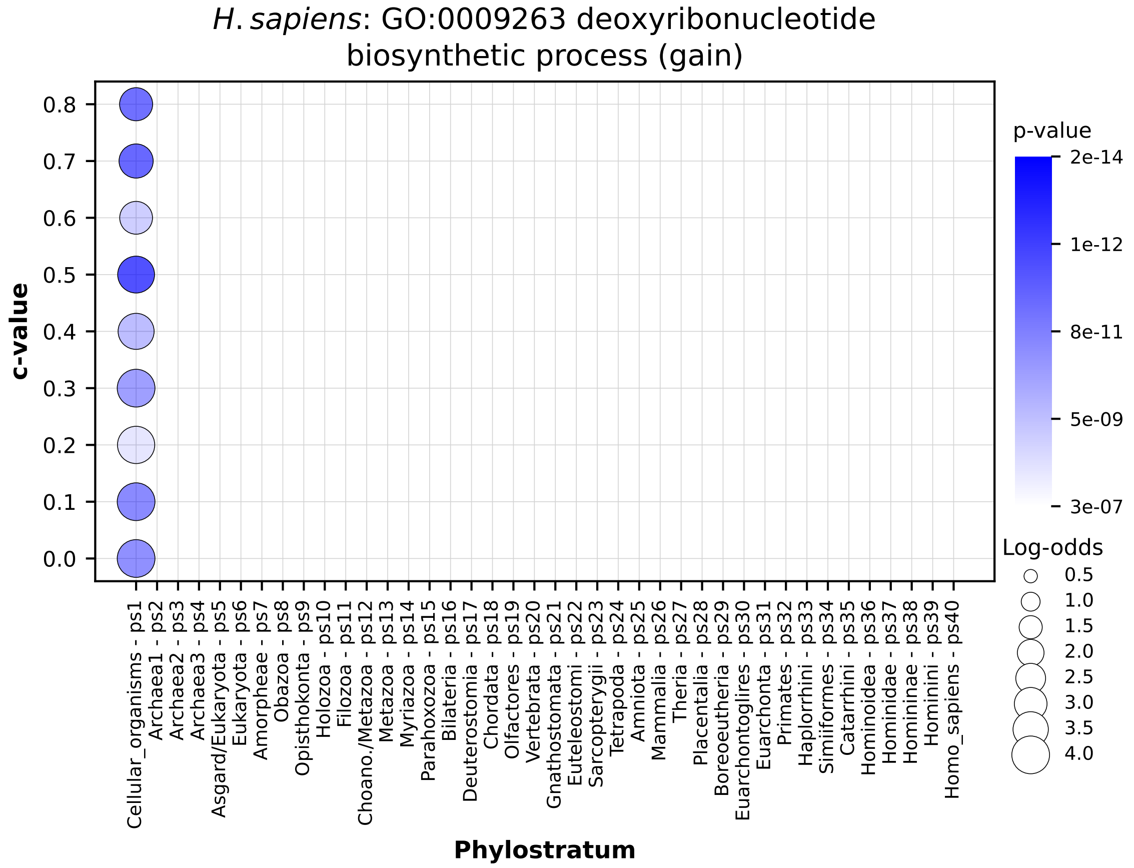

H. sapiens: GO:0009263 deoxyribonucleotide biosynthetic process (gain)
c-value
0.8
0.7
0.6
0.5
0.4
0.3
0.2
0.1
0.0
p-value
2e-14
1e-12
8e-11
5e-09
3e-07
Log-odds
0.5
1.0
1.5
2.0
2.5
3.0
3.5
4.0
Cellular_organisms - ps1
Archaea1 - ps2
Archaea2 - ps3
Archaea3 - ps4
Asgard/Eukaryota - ps5
Eukaryota - ps6
Amorpheae - ps7
Obazoa - ps8
Opisthokonta - ps9
Holozoa - ps10
Filozoa - ps11
Choano./Metazoa - ps12
Metazoa - ps13
Myriazoa - ps14
Parahoxozoa - ps15
Bilateria - ps16
Deuterostomia - ps17
Chordata - ps18
Olfactores - ps19
Vertebrata - ps20
Gnathostomata - ps21
Euteleostomi - ps22
Sarcopterygii - ps23
Tetrapoda - ps24
Amniota - ps25
Mammalia - ps26
Theria - ps27
Placentalia - ps28
Boreoeutheria - ps29
Euarchontoglires - ps30
Euarchonta - ps31
Primates - ps32
Haplorrhini - ps33
Simiiformes - ps34
Catarrhini - ps35
Hominoidea - ps36
Hominidae - ps37
Homininae - ps38
Hominini - ps39
Homo_sapiens - ps40
Phylostratum

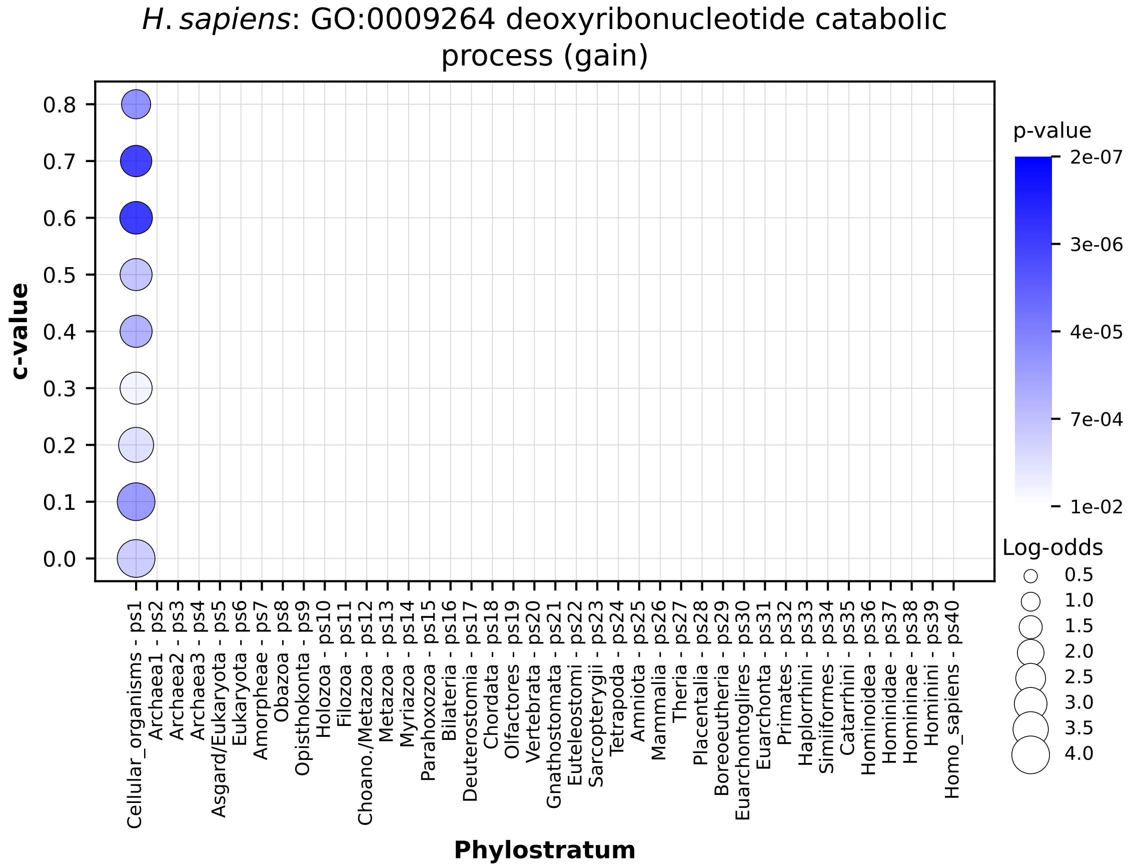
H. sapiens: GO:0009264 deoxyribonucleotide catabolic process (gain)
c-value
0.8
0.7
0.6
0.5
0.4
0.3
0.2
0.1
0.0
p-value
2e-07
3e-06
4e-05
7e-04
1e-02
Log-odds
0.5
1.0
1.5
2.0
2.5
3.0
3.5
4.0
Cellular_organisms - ps1
Archaea1 - ps2
Archaea2 - ps3
Archaea3 - ps4
Asgard/Eukaryota - ps5
Eukaryota - ps6
Amorpheae - ps7
Obazoa - ps8
Opisthokonta - ps9
Holozoa - ps10
Filozoa - ps11
Choano./Metazoa - ps12
Metazoa - ps13
Myriazoa - ps14
Parahoxozoa - ps15
Bilateria - ps16
Deuterostomia - ps17
Chordata - ps18
Olfactores - ps19
Vertebrata - ps20
Gnathostomata - ps21
Euteleostomi - ps22
Sarcopterygii - ps23
Tetrapoda - ps24
Amniota - ps25
Mammalia - ps26
Theria - ps27
Placentalia - ps28
Boreoeutheria - ps29
Euarchontoglires - ps30
Euarchonta - ps31
Primates - ps32
Haplorrhini - ps33
Simiiformes - ps34
Catarrhini - ps35
Hominoidea - ps36
Hominidae - ps37
Homininae - ps38
Hominini - ps39
Homo_sapiens - ps40
Phylostratum

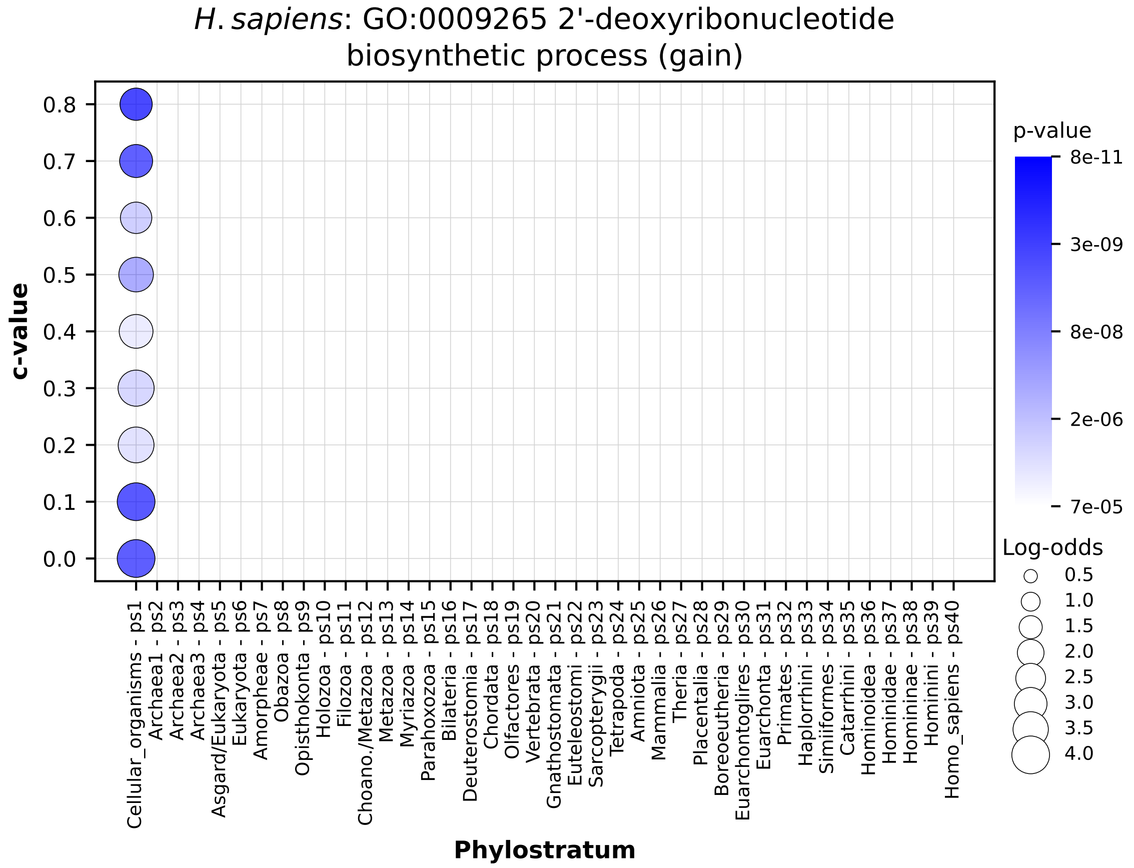

H. sapiens: GO:0009265 2'-deoxyribonucleotide biosynthetic process (gain)
c-value
0.0
0.1
0.2
0.3
0.4
0.5
0.6
0.7
0.8
Phylostratum
Cellular_organisms - ps1
Archaea1 - ps2
Archaea2 - ps3
Archaea3 - ps4
Asgard/Eukaryota - ps5
Eukaryota - ps6
Amorpheae - ps7
Obazoa - ps8
Opisthokonta - ps9
Holozoa - ps10
Filozoa - ps11
Choano./Metazoa - ps12
Metazoa - ps13
Myriazoa - ps14
Parahoxozoa - ps15
Bilateria - ps16
Deuterostomia - ps17
Chordata - ps18
Olfactores - ps19
Vertebrata - ps20
Gnathostomata - ps21
Euteleostomi - ps22
Sarcopterygii - ps23
Tetrapoda - ps24
Amniota - ps25
Mammalia - ps26
Theria - ps27
Placentalia - ps28
Boreoeutheria - ps29
Euarchontoglires - ps30
Euarchonta - ps31
Primates - ps32
Haplorrhini - ps33
Simiiformes - ps34
Catarrhini - ps35
Hominoidea - ps36
Hominidae - ps37
Homininae - ps38
Hominini - ps39
Homo_sapiens - ps40
p-value
8e-11
3e-09
8e-08
2e-06
7e-05
Log-odds
0.5
1.0
1.5
2.0
2.5
3.0
3.5
4.0

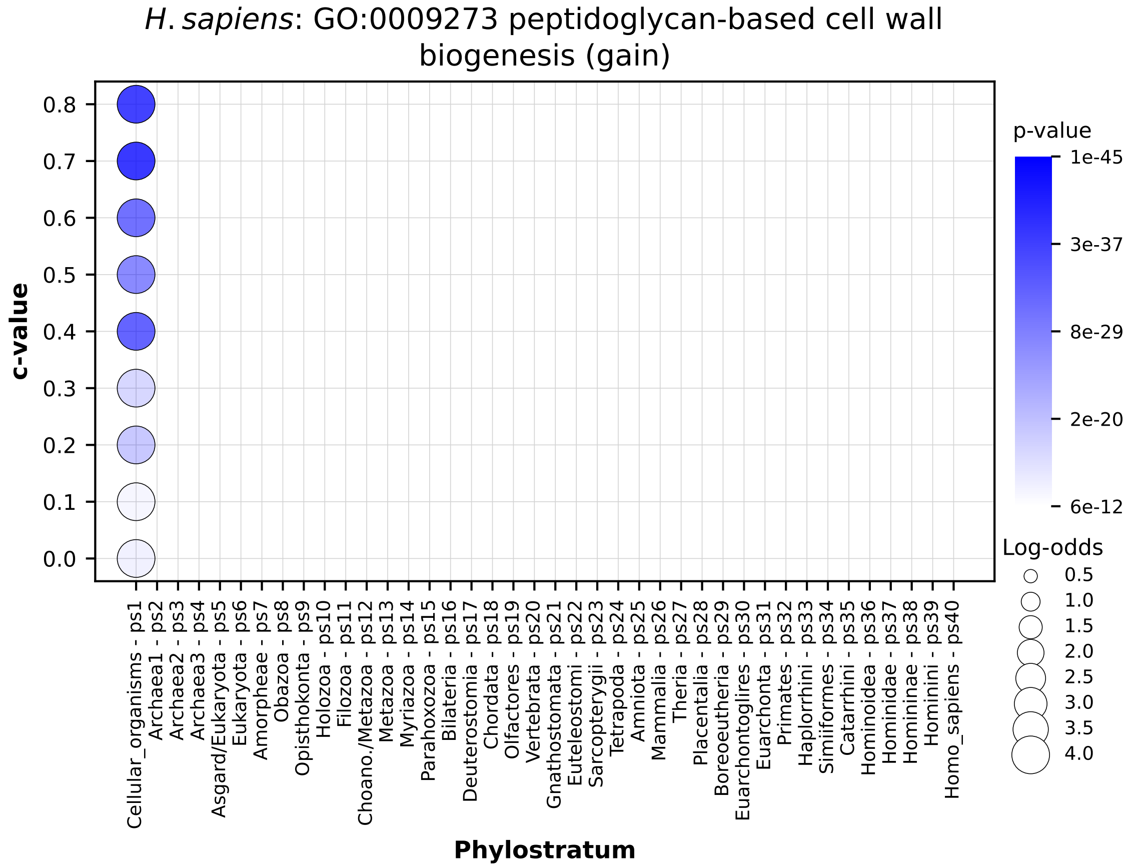

*H. sapiens*: GO:0009273 peptidoglycan-based cell wall biogenesis (gain)
c-value
0.8
0.7
0.6
0.5
0.4
0.3
0.2
0.1
0.0
p-value
1e-45
3e-37
8e-29
2e-20
6e-12
Log-odds
0.5
1.0
1.5
2.0
2.5
3.0
3.5
4.0
Cellular_organisms - ps1
Archaea1 - ps2
Archaea2 - ps3
Archaea3 - ps4
Asgard/Eukaryota - ps5
Eukaryota - ps6
Amorpheae - ps7
Obazoa - ps8
Opisthokonta - ps9
Holozoa - ps10
Filozoa - ps11
Choano./Metazoa - ps12
Metazoa - ps13
Myriazoa - ps14
Parahoxozoa - ps15
Bilateria - ps16
Deuterostomia - ps17
Chordata - ps18
Olfactores - ps19
Vertebrata - ps20
Gnathostomata - ps21
Euteleostomi - ps22
Sarcopterygii - ps23
Tetrapoda - ps24
Amniota - ps25
Mammalia - ps26
Theria - ps27
Placentalia - ps28
Boreoeutheria - ps29
Euarchontoglires - ps30
Euarchonta - ps31
Primates - ps32
Haplorrhini - ps33
Simiiformes - ps34
Catarrhini - ps35
Hominoidea - ps36
Hominidae - ps37
Homininae - ps38
Hominini - ps39
Homo_sapiens - ps40
Phylostratum

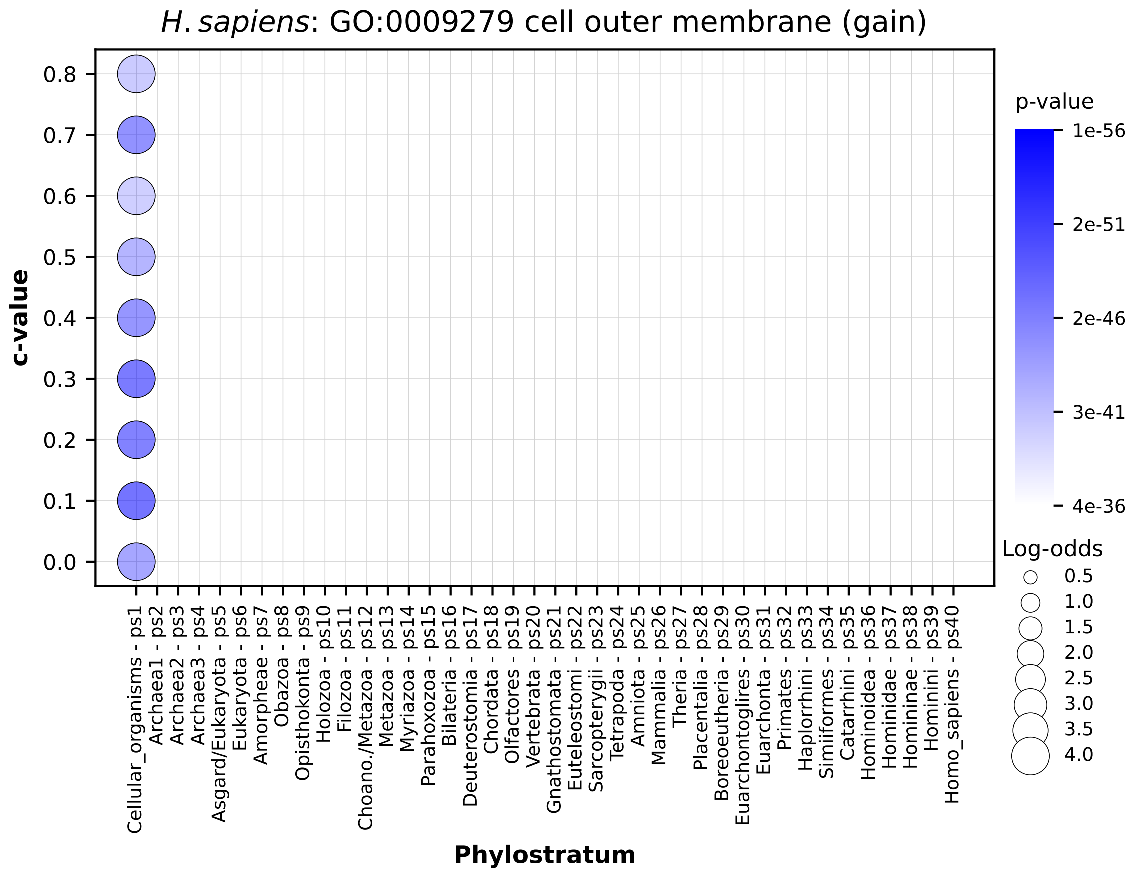

*H. sapiens*: GO:0009279 cell outer membrane (gain)
c-value
0.8
0.7
0.6
0.5
0.4
0.3
0.2
0.1
0.0
p-value
1e-56
2e-51
2e-46
3e-41
4e-36
Log-odds
0.5
1.0
1.5
2.0
2.5
3.0
3.5
4.0
Cellular_organisms - ps1
Archaea1 - ps2
Archaea2 - ps3
Archaea3 - ps4
Asgard/Eukaryota - ps5
Eukaryota - ps6
Amorpheae - ps7
Obazoa - ps8
Opisthokonta - ps9
Holozoa - ps10
Filozoa - ps11
Choano./Metazoa - ps12
Metazoa - ps13
Myriazoa - ps14
Parahoxozoa - ps15
Bilateria - ps16
Deuterostomia - ps17
Chordata - ps18
Olfactores - ps19
Vertebrata - ps20
Gnathostomata - ps21
Euteleostomi - ps22
Sarcopterygii - ps23
Tetrapoda - ps24
Amniota - ps25
Mammalia - ps26
Theria - ps27
Placentalia - ps28
Boreoeutheria - ps29
Euarchontoglires - ps30
Euarchonta - ps31
Primates - ps32
Haplorrhini - ps33
Simiiformes - ps34
Catarrhini - ps35
Hominoidea - ps36
Hominidae - ps37
Homininae - ps38
Hominini - ps39
Homo_sapiens - ps40
Phylostratum

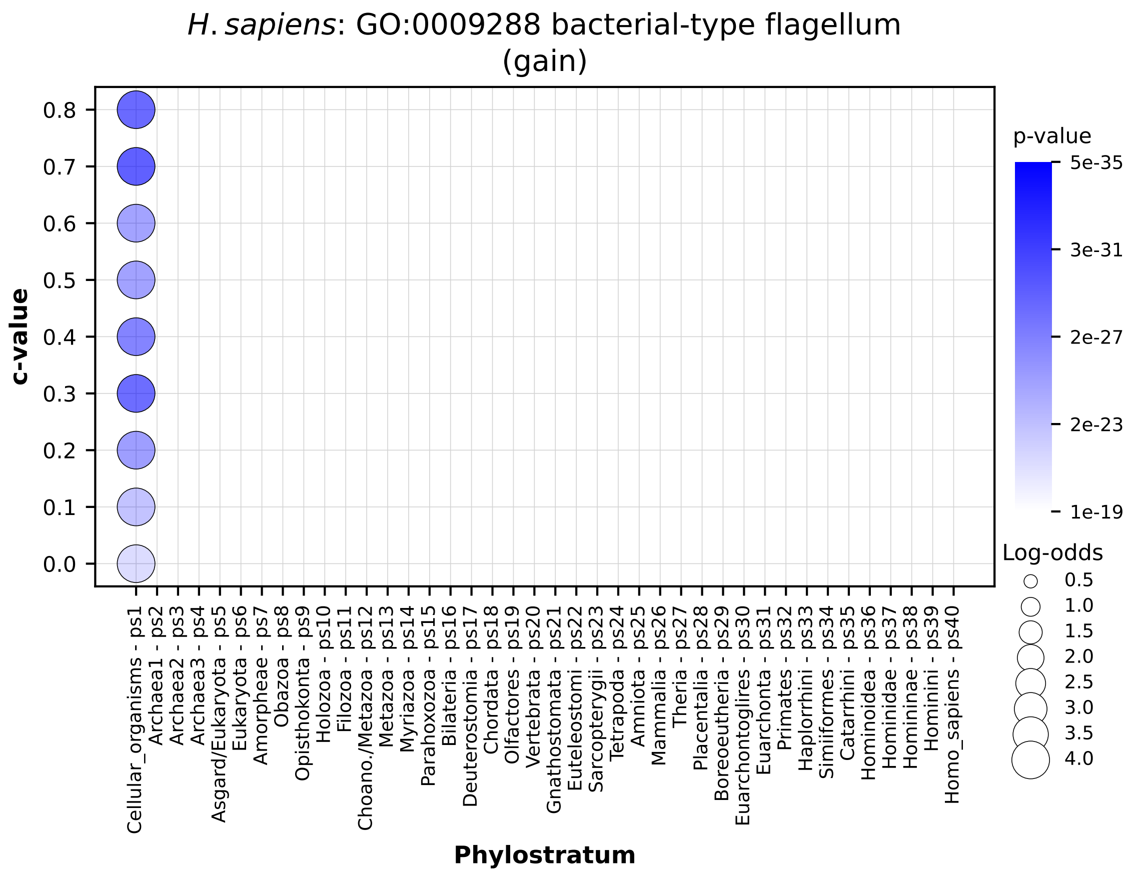
H. sapiens: GO:0009288 bacterial-type flagellum
(gain)
c-value
0.8
0.7
0.6
0.5
0.4
0.3
0.2
0.1
0.0
p-value
5e-35
3e-31
2e-27
2e-23
1e-19
Log-odds
0.5
1.0
1.5
2.0
2.5
3.0
3.5
4.0
Cellular_organisms - ps1
Archaea1 - ps2
Archaea2 - ps3
Archaea3 - ps4
Asgard/Eukaryota - ps5
Eukaryota - ps6
Amorpheae - ps7
Obazoa - ps8
Opisthokonta - ps9
Holozoa - ps10
Filozoa - ps11
Choano./Metazoa - ps12
Metazoa - ps13
Myriazoa - ps14
Parahoxozoa - ps15
Bilateria - ps16
Deuterostomia - ps17
Chordata - ps18
Olfactores - ps19
Vertebrata - ps20
Gnathostomata - ps21
Euteleostomi - ps22
Sarcopterygii - ps23
Tetrapoda - ps24
Amniota - ps25
Mammalia - ps26
Theria - ps27
Placentalia - ps28
Boreoeutheria - ps29
Euarchontoglires - ps30
Euarchonta - ps31
Primates - ps32
Haplorrhini - ps33
Simiiformes - ps34
Catarrhini - ps35
Hominoidea - ps36
Hominidae - ps37
Homininae - ps38
Hominini - ps39
Homo_sapiens - ps40
Phylostratum

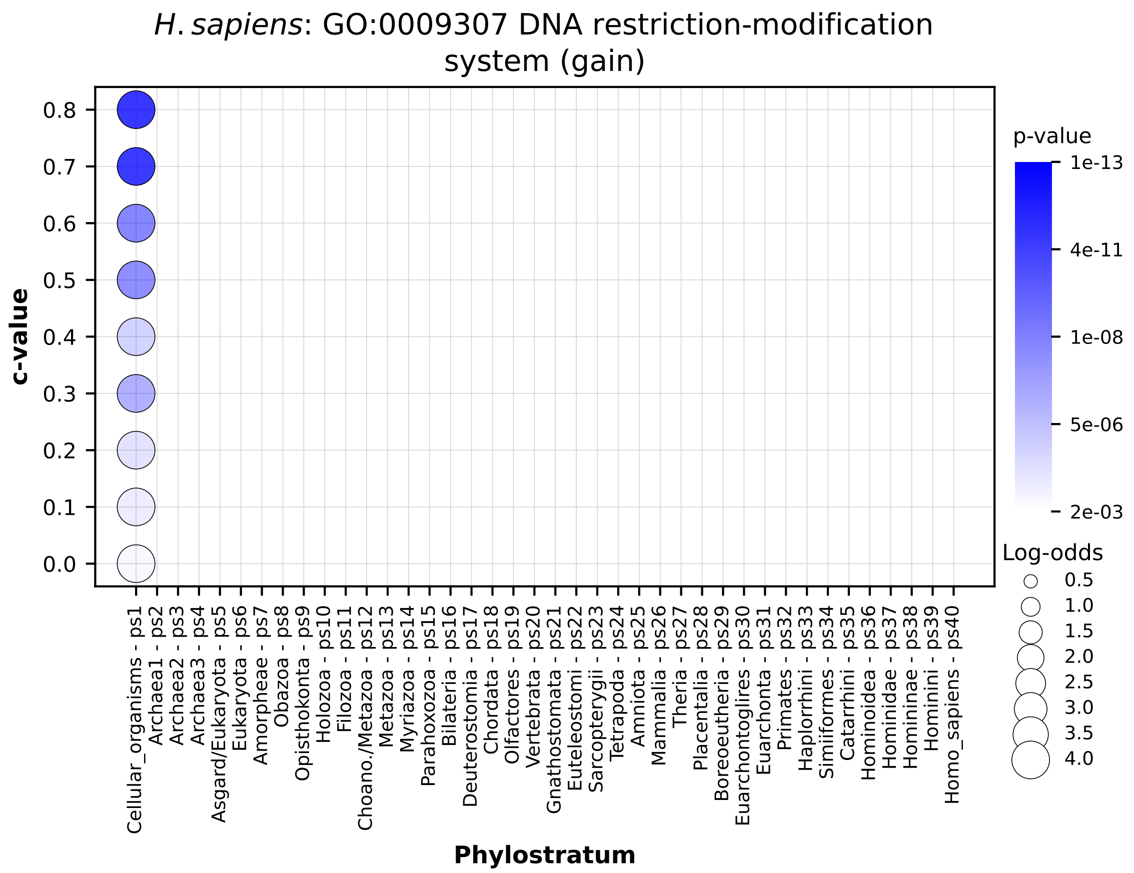

*H. sapiens*: GO:0009307 DNA restriction-modification system (gain)
c-value
0.8
0.7
0.6
0.5
0.4
0.3
0.2
0.1
0.0
Phylostratum
Cellular_organisms - ps1
Archaea1 - ps2
Archaea2 - ps3
Archaea3 - ps4
Asgard/Eukaryota - ps5
Eukaryota - ps6
Amorpheae - ps7
Obazoa - ps8
Opisthokonta - ps9
Holozoa - ps10
Filozoa - ps11
Choano./Metazoa - ps12
Metazoa - ps13
Myriazoa - ps14
Parahoxozoa - ps15
Bilateria - ps16
Deuterostomia - ps17
Chordata - ps18
Olfactores - ps19
Vertebrata - ps20
Gnathostomata - ps21
Euteleostomi - ps22
Sarcopterygii - ps23
Tetrapoda - ps24
Amniota - ps25
Mammalia - ps26
Theria - ps27
Placentalia - ps28
Boreoeutheria - ps29
Euarchontoglires - ps30
Euarchonta - ps31
Primates - ps32
Haplorrhini - ps33
Simiiformes - ps34
Catarrhini - ps35
Hominoidea - ps36
Hominidae - ps37
Homininae - ps38
Hominini - ps39
Homo_sapiens - ps40
p-value
1e-13
4e-11
1e-08
5e-06
2e-03
Log-odds
0.5
1.0
1.5
2.0
2.5
3.0
3.5
4.0

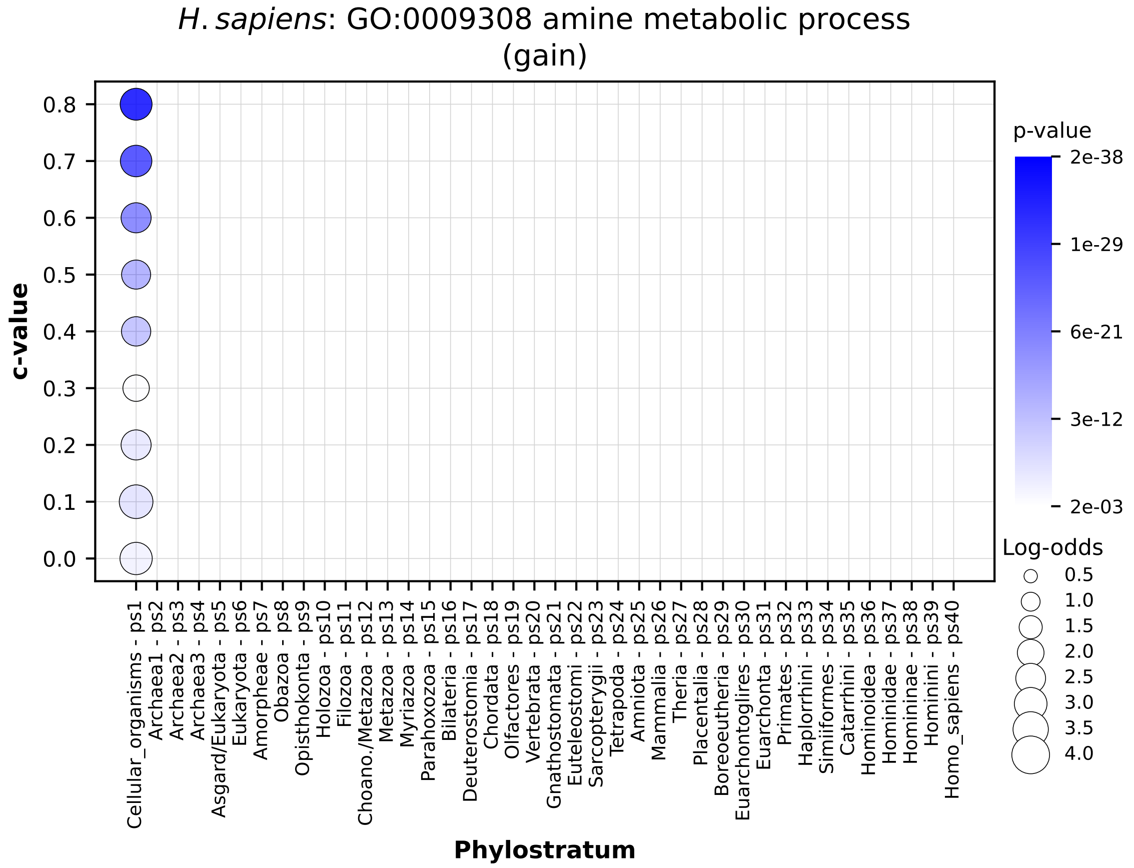
H. sapiens: GO:0009308 amine metabolic process
(gain)
c-value
0.8
0.7
0.6
0.5
0.4
0.3
0.2
0.1
0.0
p-value
2e-38
1e-29
6e-21
3e-12
2e-03
Log-odds
0.5
1.0
1.5
2.0
2.5
3.0
3.5
4.0
Cellular_organisms - ps1
Archaea1 - ps2
Archaea2 - ps3
Archaea3 - ps4
Asgard/Eukaryota - ps5
Eukaryota - ps6
Amorpheae - ps7
Obazoa - ps8
Opisthokonta - ps9
Holozoa - ps10
Filozoa - ps11
Choano./Metazoa - ps12
Metazoa - ps13
Myriazoa - ps14
Parahoxozoa - ps15
Bilateria - ps16
Deuterostomia - ps17
Chordata - ps18
Olfactores - ps19
Vertebrata - ps20
Gnathostomata - ps21
Euteleostomi - ps22
Sarcopterygii - ps23
Tetrapoda - ps24
Amniota - ps25
Mammalia - ps26
Theria - ps27
Placentalia - ps28
Boreoeutheria - ps29
Euarchontoglires - ps30
Euarchonta - ps31
Primates - ps32
Haplorrhini - ps33
Simiiformes - ps34
Catarrhini - ps35
Hominoidea - ps36
Hominidae - ps37
Homininae - ps38
Hominini - ps39
Homo_sapiens - ps40
Phylostratum

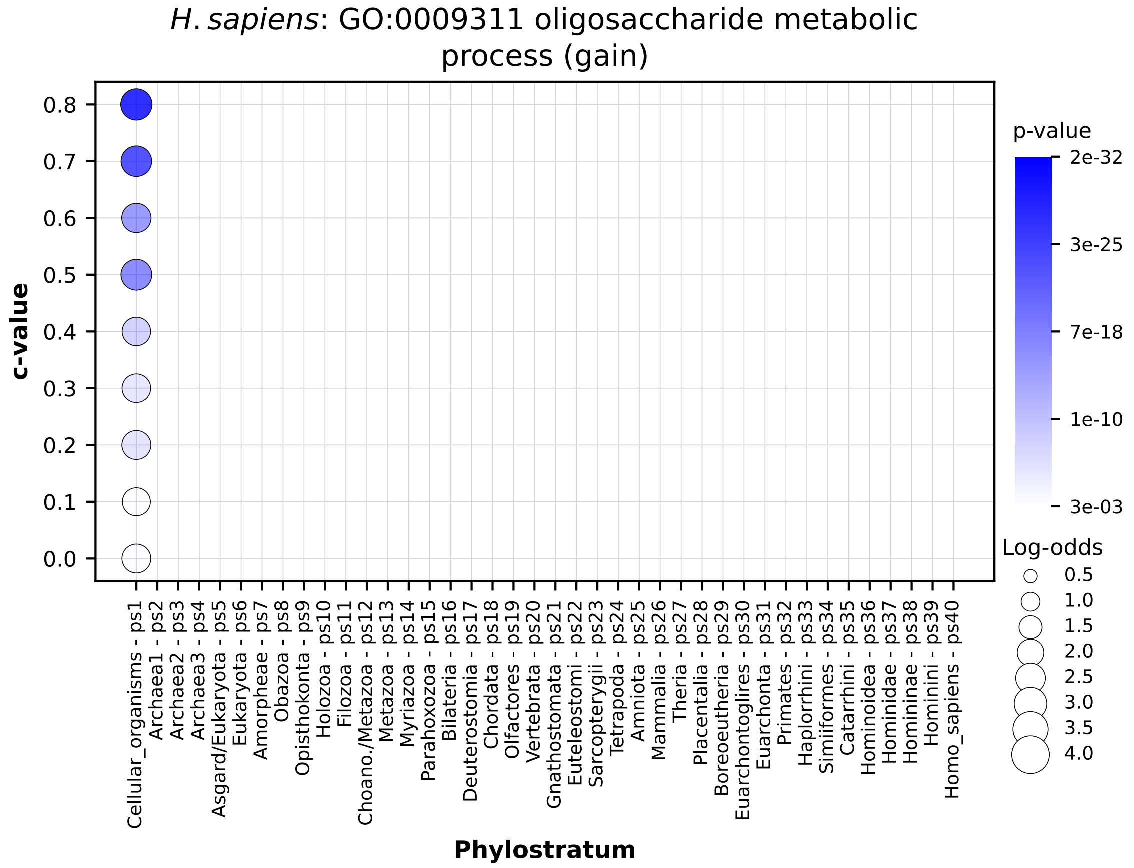

H. sapiens: GO:0009311 oligosaccharide metabolic process (gain)
c-value
0.8
0.7
0.6
0.5
0.4
0.3
0.2
0.1
0.0
p-value
2e-32
3e-25
7e-18
1e-10
3e-03
Log-odds
0.5
1.0
1.5
2.0
2.5
3.0
3.5
4.0
Cellular_organisms - ps1
Archaea1 - ps2
Archaea2 - ps3
Archaea3 - ps4
Asgard/Eukaryota - ps5
Eukaryota - ps6
Amorpheae - ps7
Obazoa - ps8
Opisthokonta - ps9
Holozoa - ps10
Filozoa - ps11
Choano./Metazoa - ps12
Metazoa - ps13
Myriazoa - ps14
Parahoxozoa - ps15
Bilateria - ps16
Deuterostomia - ps17
Chordata - ps18
Olfactores - ps19
Vertebrata - ps20
Gnathostomata - ps21
Euteleostomi - ps22
Sarcopterygii - ps23
Tetrapoda - ps24
Amniota - ps25
Mammalia - ps26
Theria - ps27
Placentalia - ps28
Boreoeutheria - ps29
Euarchontoglires - ps30
Euarchonta - ps31
Primates - ps32
Haplorrhini - ps33
Simiiformes - ps34
Catarrhini - ps35
Hominoidea - ps36
Hominidae - ps37
Homininae - ps38
Hominini - ps39
Homo_sapiens - ps40
Phylostratum

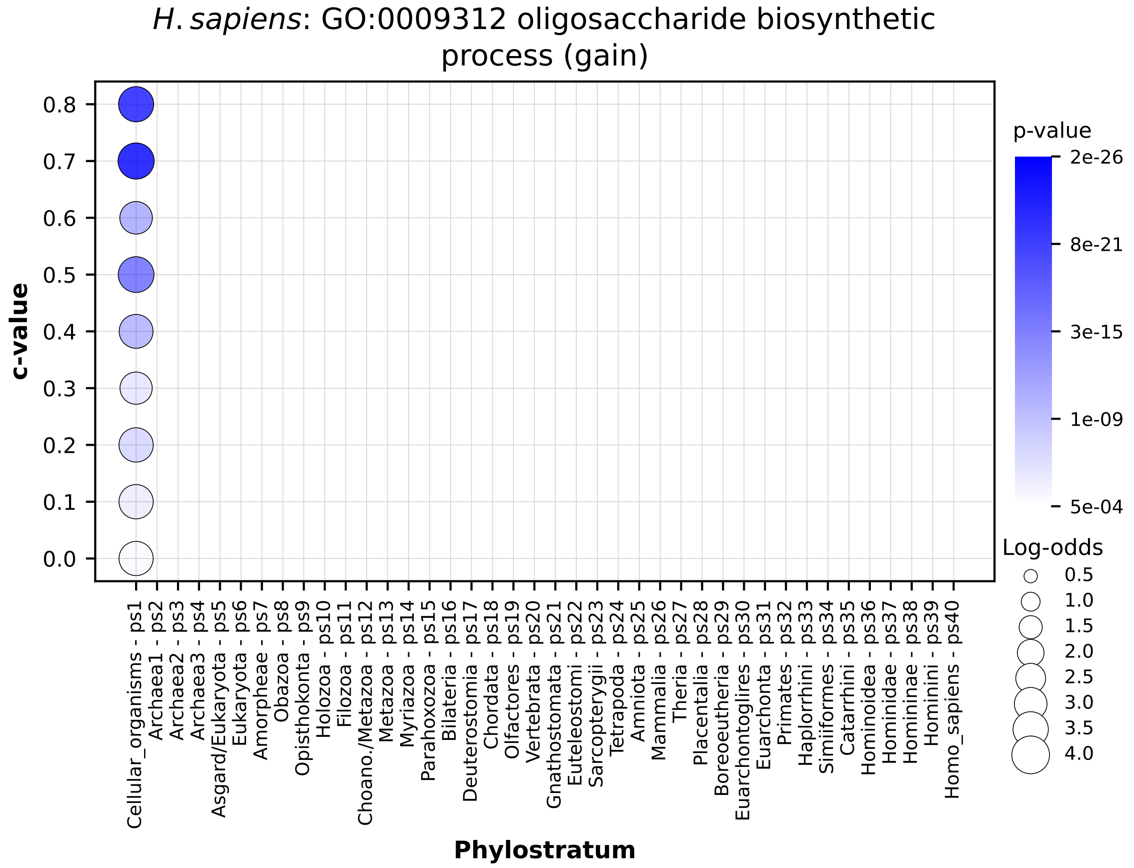
H. sapiens: GO:0009312 oligosaccharide biosynthetic process (gain)
c-value
0.8
0.7
0.6
0.5
0.4
0.3
0.2
0.1
0.0
Phylostratum
Cellular_organisms - ps1
Archaea1 - ps2
Archaea2 - ps3
Archaea3 - ps4
Asgard/Eukaryota - ps5
Eukaryota - ps6
Amorpheae - ps7
Obazoa - ps8
Opisthokonta - ps9
Holozoa - ps10
Filozoa - ps11
Choano./Metazoa - ps12
Metazoa - ps13
Myriazoa - ps14
Parahoxozoa - ps15
Bilateria - ps16
Deuterostomia - ps17
Chordata - ps18
Olfactores - ps19
Vertebrata - ps20
Gnathostomata - ps21
Euteleostomi - ps22
Sarcopterygii - ps23
Tetrapoda - ps24
Amniota - ps25
Mammalia - ps26
Theria - ps27
Placentalia - ps28
Boreoeutheria - ps29
Euarchontoglires - ps30
Euarchonta - ps31
Primates - ps32
Haplorrhini - ps33
Simiiformes - ps34
Catarrhini - ps35
Hominoidea - ps36
Hominidae - ps37
Homininae - ps38
Hominini - ps39
Homo_sapiens - ps40
p-value
2e-26
8e-21
3e-15
1e-09
5e-04
Log-odds
0.5
1.0
1.5
2.0
2.5
3.0
3.5
4.0

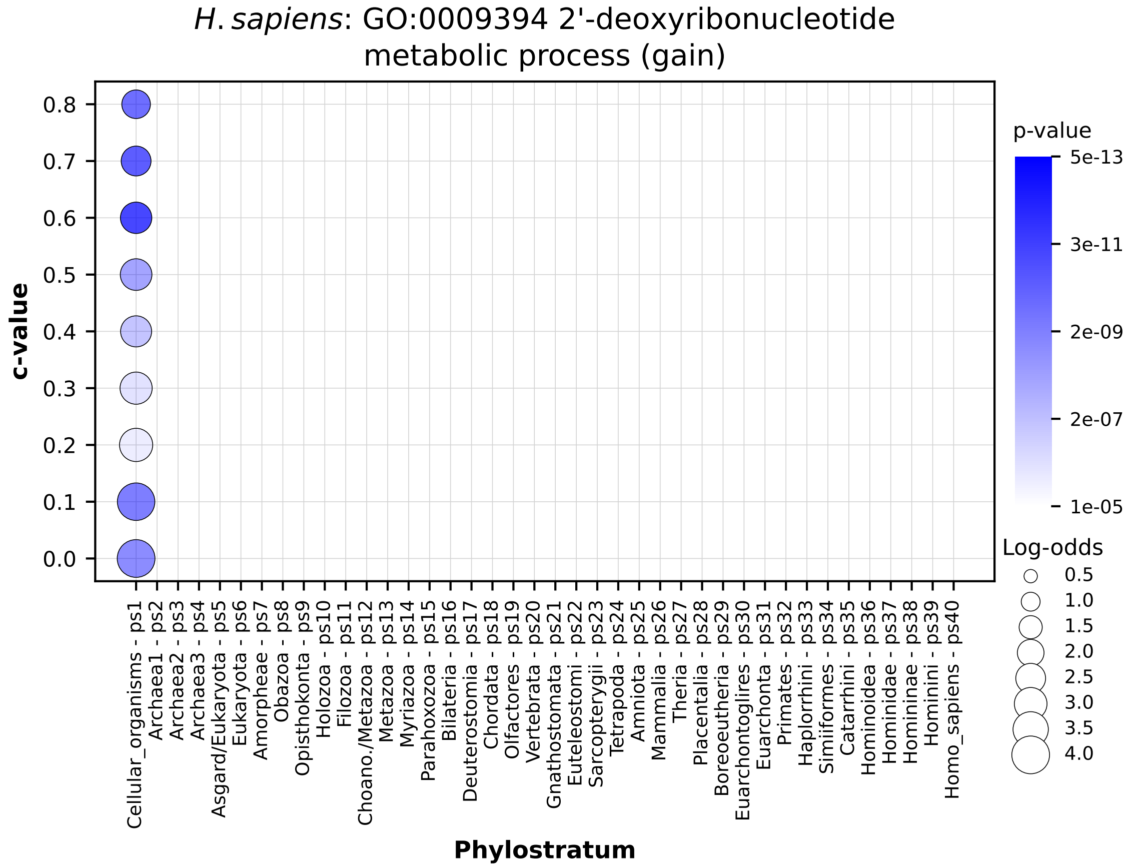
H. sapiens: GO:0009394 2'-deoxyribonucleotide metabolic process (gain)
c-value
0.0
0.1
0.2
0.3
0.4
0.5
0.6
0.7
0.8
Phylostratum
Cellular_organisms - ps1
Archaea1 - ps2
Archaea2 - ps3
Archaea3 - ps4
Asgard/Eukaryota - ps5
Eukaryota - ps6
Amorpheae - ps7
Obazoa - ps8
Opisthokonta - ps9
Holozoa - ps10
Filozoa - ps11
Choano./Metazoa - ps12
Metazoa - ps13
Myriazoa - ps14
Parahoxozoa - ps15
Bilateria - ps16
Deuterostomia - ps17
Chordata - ps18
Olfactores - ps19
Vertebrata - ps20
Gnathostomata - ps21
Euteleostomi - ps22
Sarcopterygii - ps23
Tetrapoda - ps24
Amniota - ps25
Mammalia - ps26
Theria - ps27
Placentalia - ps28
Boreoeutheria - ps29
Euarchontoglires - ps30
Euarchonta - ps31
Primates - ps32
Haplorrhini - ps33
Simiiformes - ps34
Catarrhini - ps35
Hominoidea - ps36
Hominidae - ps37
Homininae - ps38
Hominini - ps39
Homo_sapiens - ps40
p-value
5e-13
3e-11
2e-09
2e-07
1e-05
Log-odds
0.5
1.0
1.5
2.0
2.5
3.0
3.5
4.0

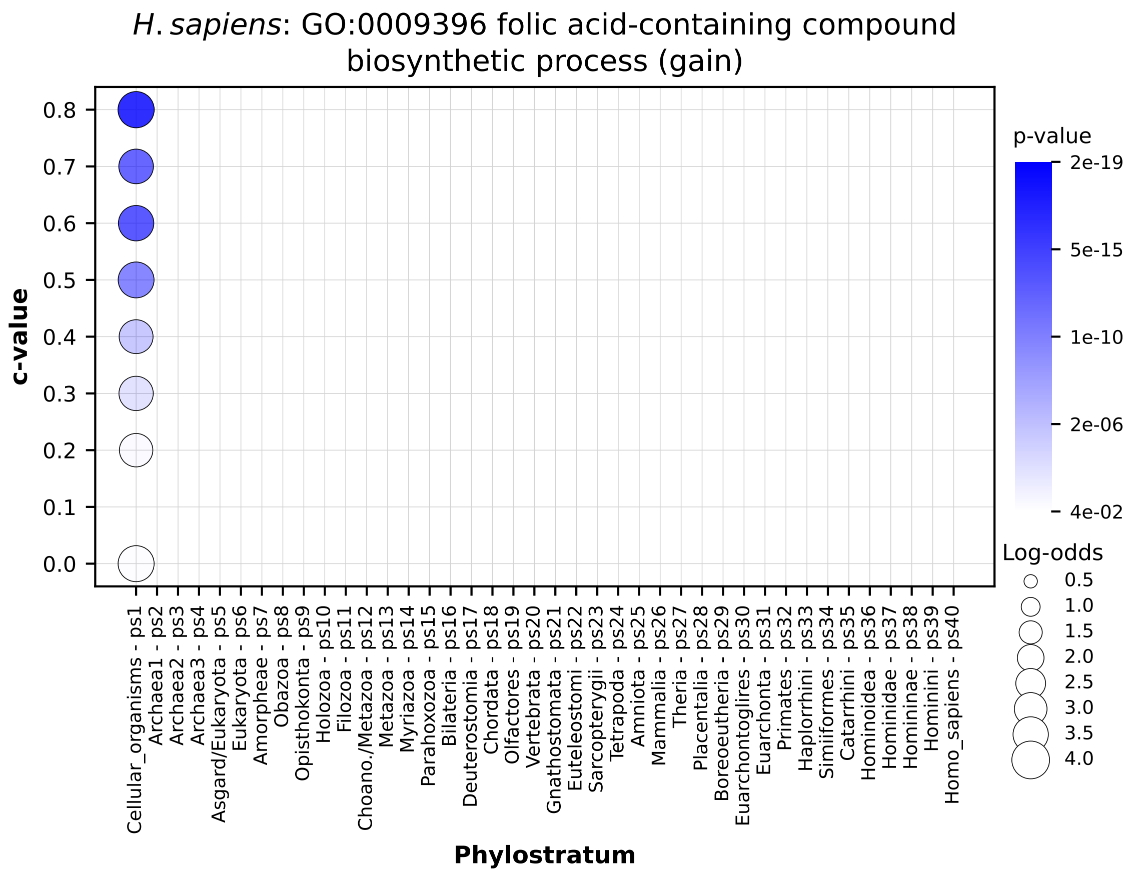
H. sapiens: GO:0009396 folic acid-containing compound biosynthetic process (gain)
c-value
0.8
0.7
0.6
0.5
0.4
0.3
0.2
0.1
0.0
Phylostratum
Cellular_organisms - ps1
Archaea1 - ps2
Archaea2 - ps3
Archaea3 - ps4
Asgard/Eukaryota - ps5
Eukaryota - ps6
Amorpheae - ps7
Obazoa - ps8
Opisthokonta - ps9
Holozoa - ps10
Filozoa - ps11
Choano./Metazoa - ps12
Metazoa - ps13
Myriazoa - ps14
Parahoxozoa - ps15
Bilateria - ps16
Deuterostomia - ps17
Chordata - ps18
Olfactores - ps19
Vertebrata - ps20
Gnathostomata - ps21
Euteleostomi - ps22
Sarcopterygii - ps23
Tetrapoda - ps24
Amniota - ps25
Mammalia - ps26
Theria - ps27
Placentalia - ps28
Boreoeutheria - ps29
Euarchontoglires - ps30
Euarchonta - ps31
Primates - ps32
Haplorrhini - ps33
Simiiformes - ps34
Catarrhini - ps35
Hominoidea - ps36
Hominidae - ps37
Homininae - ps38
Hominini - ps39
Homo_sapiens - ps40
p-value
2e-19
5e-15
1e-10
2e-06
4e-02
Log-odds
0.5
1.0
1.5
2.0
2.5
3.0
3.5
4.0

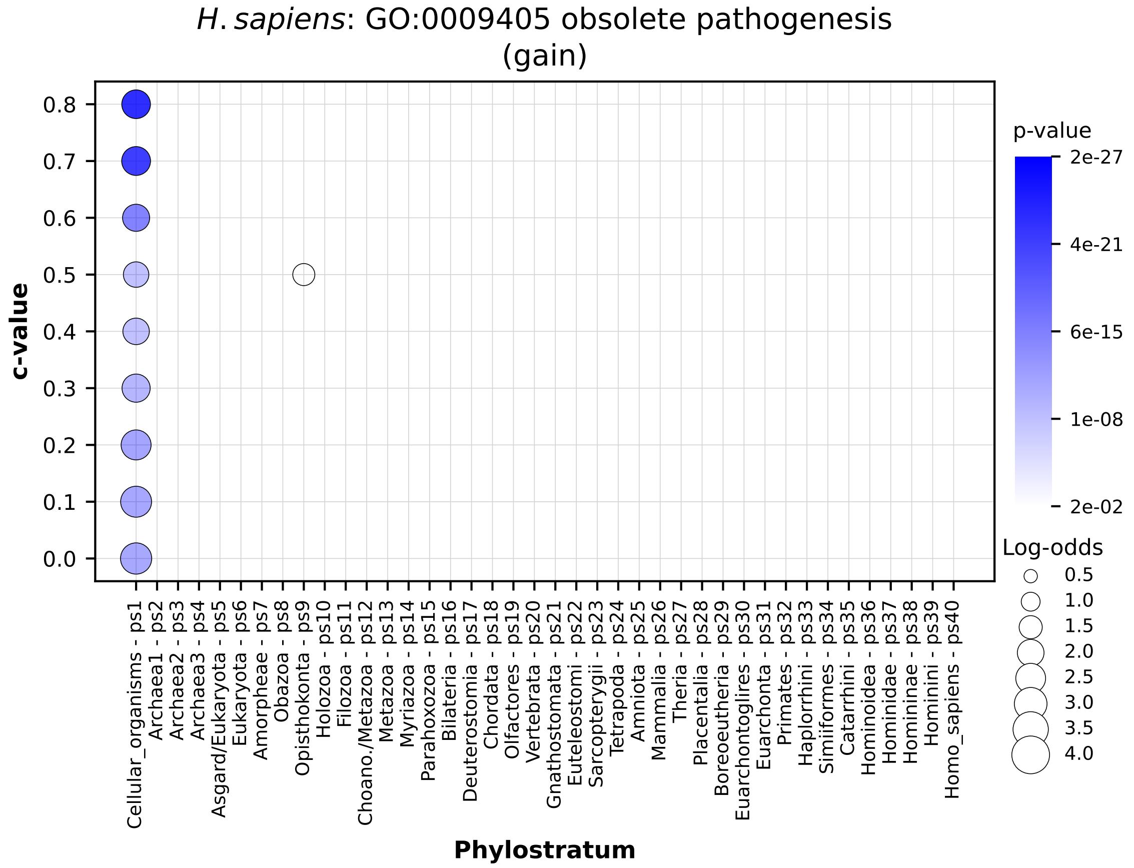

H. sapiens: GO:0009405 obsolete pathogenesis (gain)
c-value
0.8
0.7
0.6
0.5
0.4
0.3
0.2
0.1
0.0
p-value
2e-27
4e-21
6e-15
1e-08
2e-02
Log-odds
0.5
1.0
1.5
2.0
2.5
3.0
3.5
4.0
Cellular_organisms - ps1
Archaea1 - ps2
Archaea2 - ps3
Archaea3 - ps4
Asgard/Eukaryota - ps5
Eukaryota - ps6
Amorpheae - ps7
Obazoa - ps8
Opisthokonta - ps9
Holozoa - ps10
Filozoa - ps11
Choano./Metazoa - ps12
Metazoa - ps13
Myriazoa - ps14
Parahoxozoa - ps15
Bilateria - ps16
Deuterostomia - ps17
Chordata - ps18
Olfactores - ps19
Vertebrata - ps20
Gnathostomata - ps21
Euteleostomi - ps22
Sarcopterygii - ps23
Tetrapoda - ps24
Amniota - ps25
Mammalia - ps26
Theria - ps27
Placentalia - ps28
Boreoeutheria - ps29
Euarchontoglires - ps30
Euarchonta - ps31
Primates - ps32
Haplorrhini - ps33
Simiiformes - ps34
Catarrhini - ps35
Hominoidea - ps36
Hominidae - ps37
Homininae - ps38
Hominini - ps39
Homo_sapiens - ps40
Phylostratum

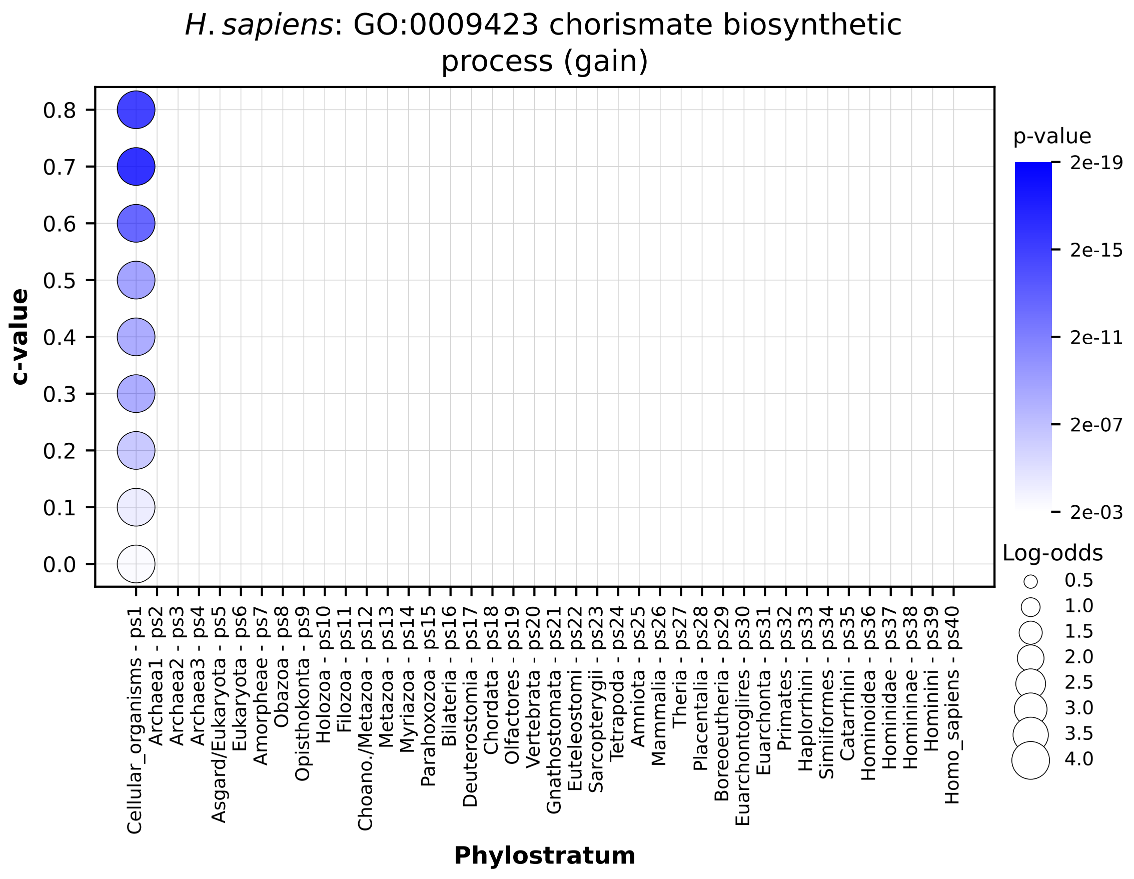

H. sapiens: GO:0009423 chorismate biosynthetic process (gain)
c-value
0.8
0.7
0.6
0.5
0.4
0.3
0.2
0.1
0.0
p-value
2e-19
2e-15
2e-11
2e-07
2e-03
Log-odds
0.5
1.0
1.5
2.0
2.5
3.0
3.5
4.0
Cellular_organisms - ps1
Archaea1 - ps2
Archaea2 - ps3
Archaea3 - ps4
Asgard/Eukaryota - ps5
Eukaryota - ps6
Amorpheae - ps7
Obazoa - ps8
Opisthokonta - ps9
Holozoa - ps10
Filozoa - ps11
Choano./Metazoa - ps12
Metazoa - ps13
Myriazoa - ps14
Parahoxozoa - ps15
Bilateria - ps16
Deuterostomia - ps17
Chordata - ps18
Olfactores - ps19
Vertebrata - ps20
Gnathostomata - ps21
Euteleostomi - ps22
Sarcopterygii - ps23
Tetrapoda - ps24
Amniota - ps25
Mammalia - ps26
Theria - ps27
Placentalia - ps28
Boreoeutheria - ps29
Euarchontoglires - ps30
Euarchonta - ps31
Primates - ps32
Haplorrhini - ps33
Simiiformes - ps34
Catarrhini - ps35
Hominoidea - ps36
Hominidae - ps37
Homininae - ps38
Hominini - ps39
Homo_sapiens - ps40
Phylostratum

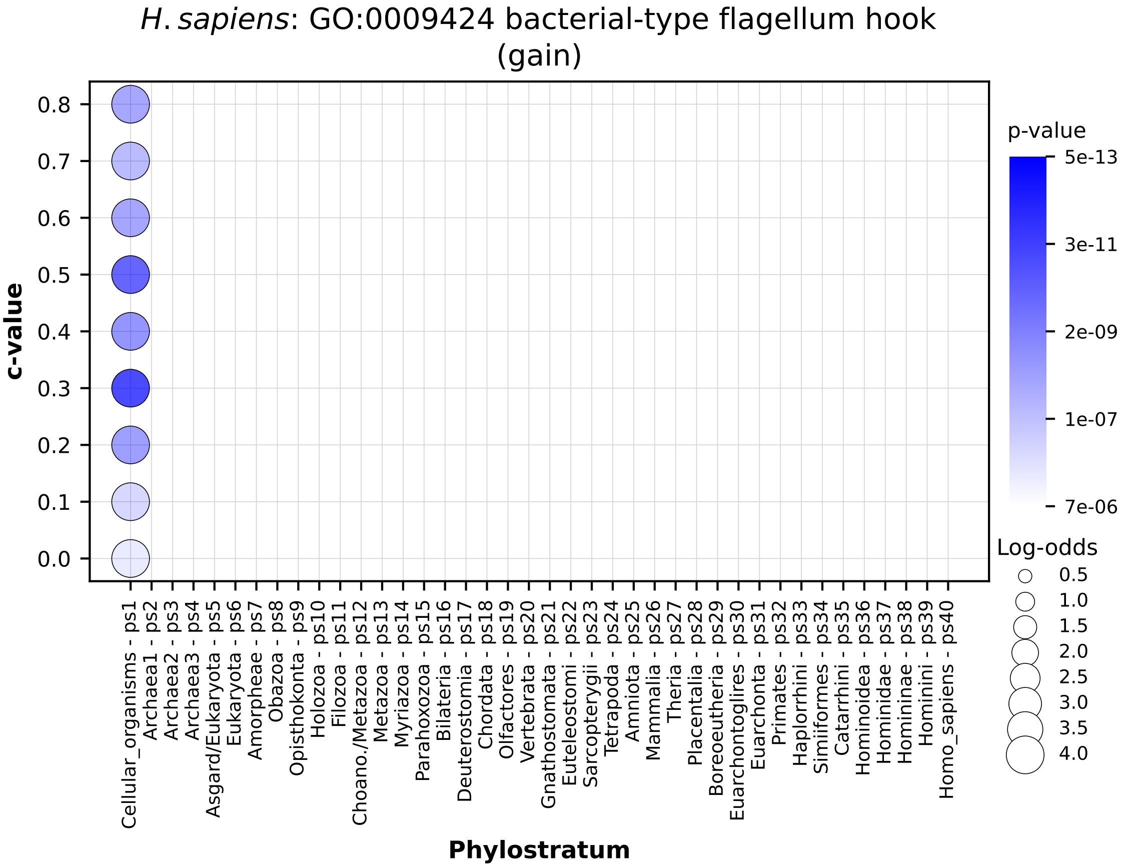

*H. sapiens*: GO:0009424 bacterial-type flagellum hook
(gain)
c-value
0.0
0.1
0.2
0.3
0.4
0.5
0.6
0.7
0.8
Phylostratum
Cellular_organisms - ps1
Archaea1 - ps2
Archaea2 - ps3
Archaea3 - ps4
Asgard/Eukaryota - ps5
Eukaryota - ps6
Amorpheae - ps7
Obazoa - ps8
Opisthokonta - ps9
Holozoa - ps10
Filozoa - ps11
Choano./Metazoa - ps12
Metazoa - ps13
Myriazoa - ps14
Parahoxozoa - ps15
Bilateria - ps16
Deuterostomia - ps17
Chordata - ps18
Olfactores - ps19
Vertebrata - ps20
Gnathostomata - ps21
Euteleostomi - ps22
Sarcopterygii - ps23
Tetrapoda - ps24
Amniota - ps25
Mammalia - ps26
Theria - ps27
Placentalia - ps28
Boreoeutheria - ps29
Euarchontoglires - ps30
Euarchonta - ps31
Primates - ps32
Haplorrhini - ps33
Simiiformes - ps34
Catarrhini - ps35
Hominoidea - ps36
Hominidae - ps37
Homininae - ps38
Hominini - ps39
Homo_sapiens - ps40
p-value
5e-13
3e-11
2e-09
1e-07
7e-06
Log-odds
0.5
1.0
1.5
2.0
2.5
3.0
3.5
4.0

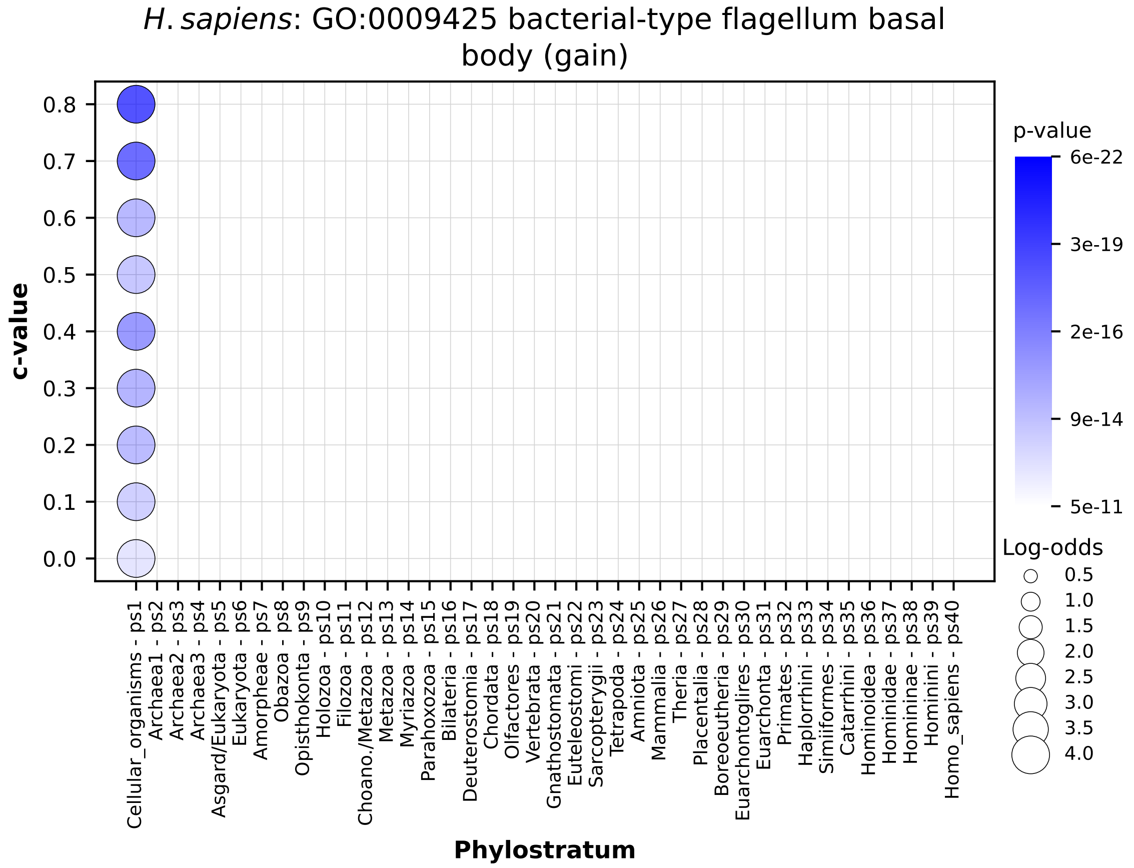

H. sapiens: GO:0009425 bacterial-type flagellum basal body (gain)
c-value
0.0
0.1
0.2
0.3
0.4
0.5
0.6
0.7
0.8
Phylostratum
Cellular_organisms - ps1
Archaea1 - ps2
Archaea2 - ps3
Archaea3 - ps4
Asgard/Eukaryota - ps5
Eukaryota - ps6
Amorpheae - ps7
Obazoa - ps8
Opisthokonta - ps9
Holozoa - ps10
Filozoa - ps11
Choano./Metazoa - ps12
Metazoa - ps13
Myriazoa - ps14
Parahoxozoa - ps15
Bilateria - ps16
Deuterostomia - ps17
Chordata - ps18
Olfactores - ps19
Vertebrata - ps20
Gnathostomata - ps21
Euteleostomi - ps22
Sarcopterygii - ps23
Tetrapoda - ps24
Amniota - ps25
Mammalia - ps26
Theria - ps27
Placentalia - ps28
Boreoeutheria - ps29
Euarchontoglires - ps30
Euarchonta - ps31
Primates - ps32
Haplorrhini - ps33
Simiiformes - ps34
Catarrhini - ps35
Hominoidea - ps36
Hominidae - ps37
Homininae - ps38
Hominini - ps39
Homo_sapiens - ps40
p-value
6e-22
3e-19
2e-16
9e-14
5e-11
Log-odds
0.5
1.0
1.5
2.0
2.5
3.0
3.5
4.0

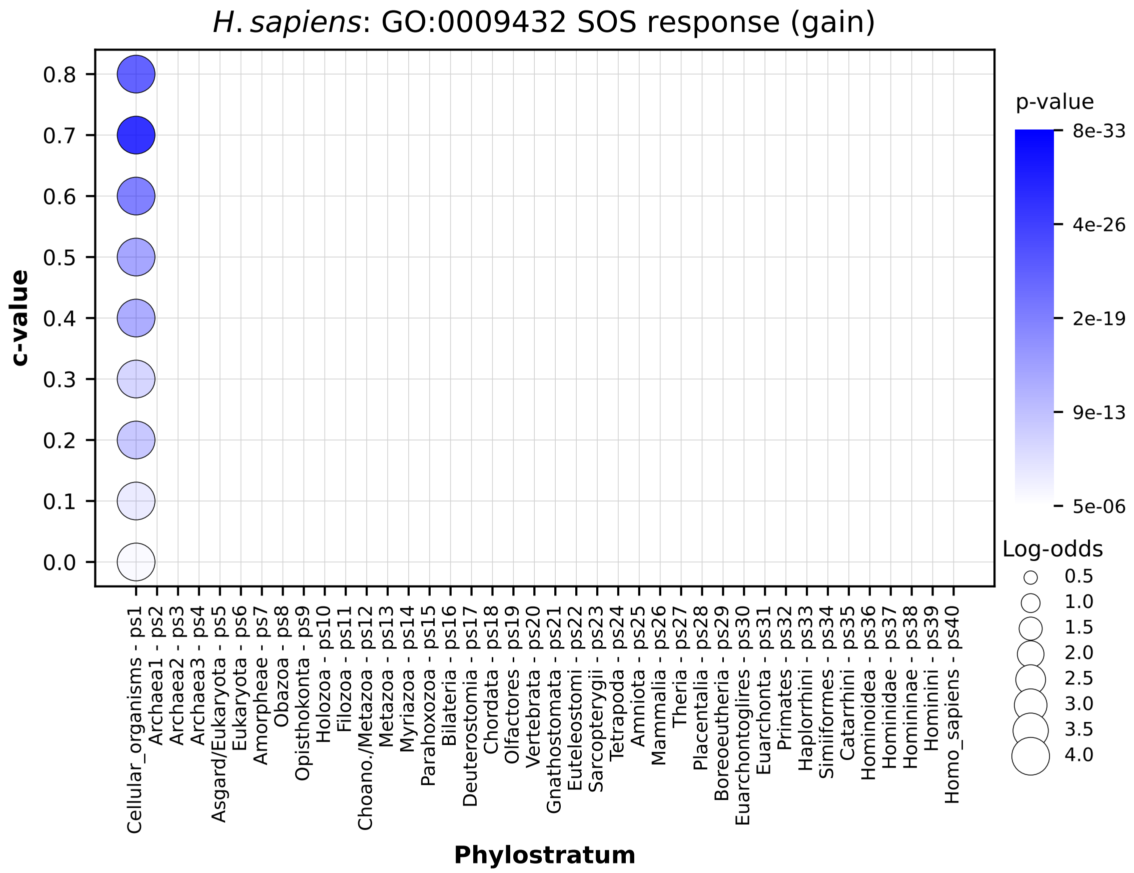

H. sapiens: GO:0009432 SOS response (gain)
c-value
0.8
0.7
0.6
0.5
0.4
0.3
0.2
0.1
0.0
Phylostratum
Cellular_organisms - ps1
Archaea1 - ps2
Archaea2 - ps3
Archaea3 - ps4
Asgard/Eukaryota - ps5
Eukaryota - ps6
Amorpheae - ps7
Obazoa - ps8
Opisthokonta - ps9
Holozoa - ps10
Filozoa - ps11
Choano./Metazoa - ps12
Metazoa - ps13
Myriazoa - ps14
Parahoxozoa - ps15
Bilateria - ps16
Deuterostomia - ps17
Chordata - ps18
Olfactores - ps19
Vertebrata - ps20
Gnathostomata - ps21
Euteleostomi - ps22
Sarcopterygii - ps23
Tetrapoda - ps24
Amniota - ps25
Mammalia - ps26
Theria - ps27
Placentalia - ps28
Boreoeutheria - ps29
Euarchontoglires - ps30
Euarchonta - ps31
Primates - ps32
Haplorrhini - ps33
Simiiformes - ps34
Catarrhini - ps35
Hominoidea - ps36
Hominidae - ps37
Homininae - ps38
Hominini - ps39
Homo_sapiens - ps40
p-value
8e-33
4e-26
2e-19
9e-13
5e-06
Log-odds
0.5
1.0
1.5
2.0
2.5
3.0
3.5
4.0

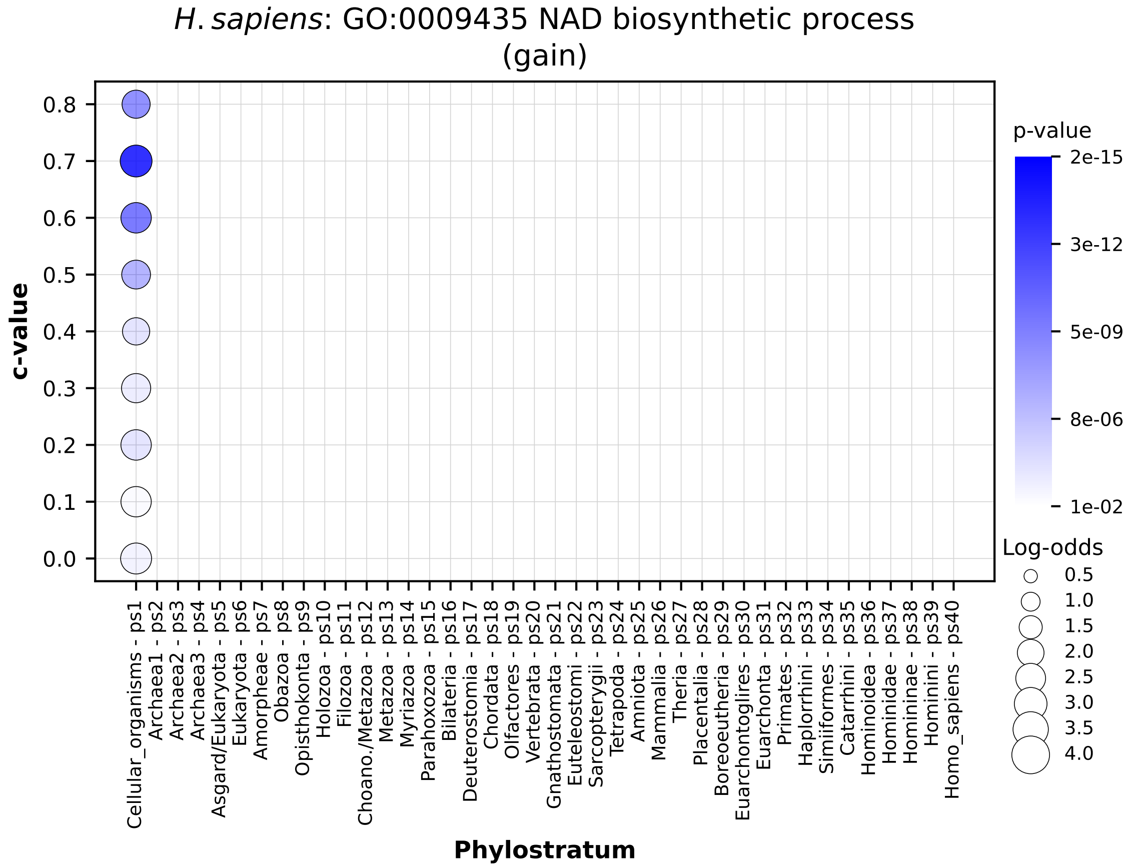

H. sapiens: GO:0009435 NAD biosynthetic process
(gain)
c-value
0.8
0.7
0.6
0.5
0.4
0.3
0.2
0.1
0.0
p-value
2e-15
3e-12
5e-09
8e-06
1e-02
Log-odds
0.5
1.0
1.5
2.0
2.5
3.0
3.5
4.0
Cellular_organisms - ps1
Archaea1 - ps2
Archaea2 - ps3
Archaea3 - ps4
Asgard/Eukaryota - ps5
Eukaryota - ps6
Amorpheae - ps7
Obazoa - ps8
Opisthokonta - ps9
Holozoa - ps10
Filozoa - ps11
Choano./Metazoa - ps12
Metazoa - ps13
Myriazoa - ps14
Parahoxozoa - ps15
Bilateria - ps16
Deuterostomia - ps17
Chordata - ps18
Olfactores - ps19
Vertebrata - ps20
Gnathostomata - ps21
Euteleostomi - ps22
Sarcopterygii - ps23
Tetrapoda - ps24
Amniota - ps25
Mammalia - ps26
Theria - ps27
Placentalia - ps28
Boreoeutheria - ps29
Euarchontoglires - ps30
Euarchonta - ps31
Primates - ps32
Haplorrhini - ps33
Simiiformes - ps34
Catarrhini - ps35
Hominoidea - ps36
Hominidae - ps37
Homininae - ps38
Hominini - ps39
Homo_sapiens - ps40
Phylostratum

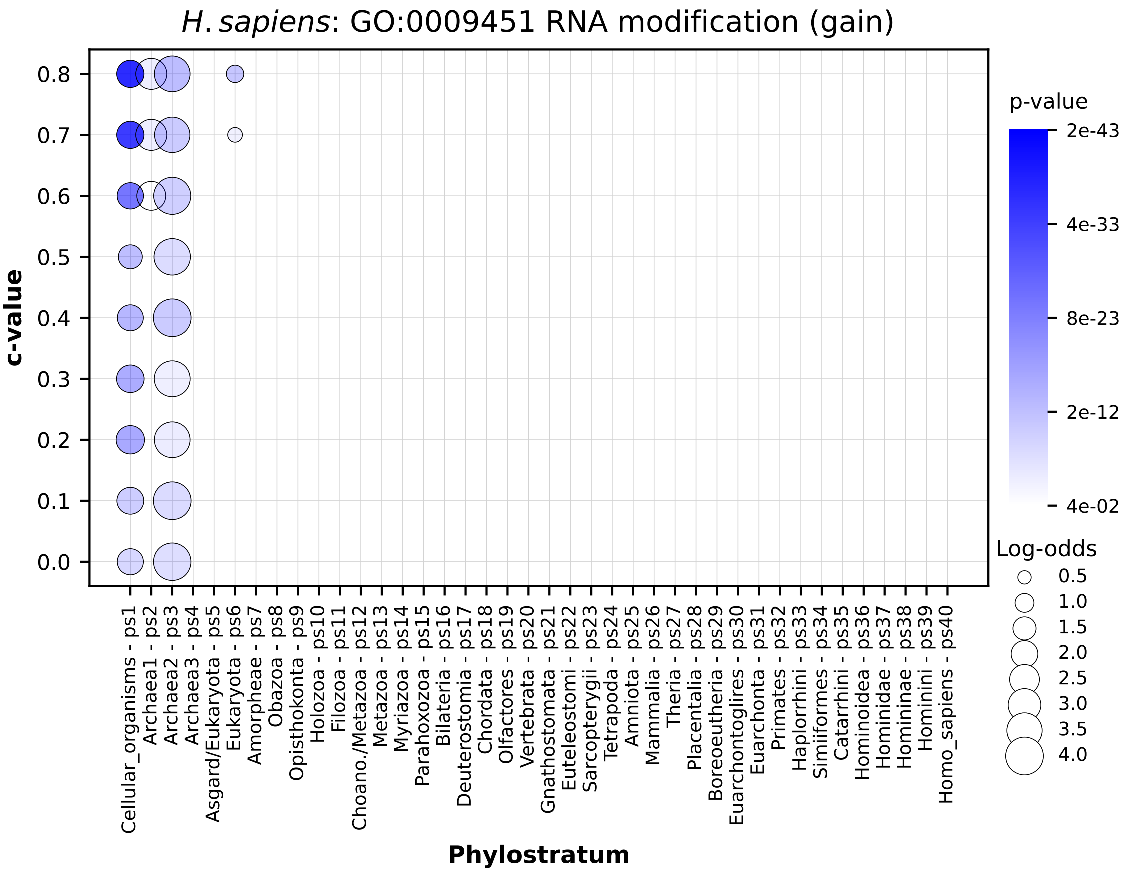

H. sapiens: GO:0009451 RNA modification (gain)
c-value
0.0
0.1
0.2
0.3
0.4
0.5
0.6
0.7
0.8
Phylostratum
Cellular_organisms - ps1
Archaea1 - ps2
Archaea2 - ps3
Archaea3 - ps4
Asgard/Eukaryota - ps5
Eukaryota - ps6
Amorpheae - ps7
Obazoa - ps8
Opisthokonta - ps9
Holozoa - ps10
Filozoa - ps11
Choano./Metazoa - ps12
Metazoa - ps13
Myriazoa - ps14
Parahoxozoa - ps15
Bilateria - ps16
Deuterostomia - ps17
Chordata - ps18
Olfactores - ps19
Vertebrata - ps20
Gnathostomata - ps21
Euteleostomi - ps22
Sarcopterygii - ps23
Tetrapoda - ps24
Amniota - ps25
Mammalia - ps26
Theria - ps27
Placentalia - ps28
Boreoeutheria - ps29
Euarchontoglires - ps30
Euarchonta - ps31
Primates - ps32
Haplorrhini - ps33
Simiiformes - ps34
Catarrhini - ps35
Hominoidea - ps36
Hominidae - ps37
Homininae - ps38
Hominini - ps39
Homo_sapiens - ps40
p-value
2e-43
4e-33
8e-23
2e-12
4e-02
Log-odds
0.5
1.0
1.5
2.0
2.5
3.0
3.5
4.0

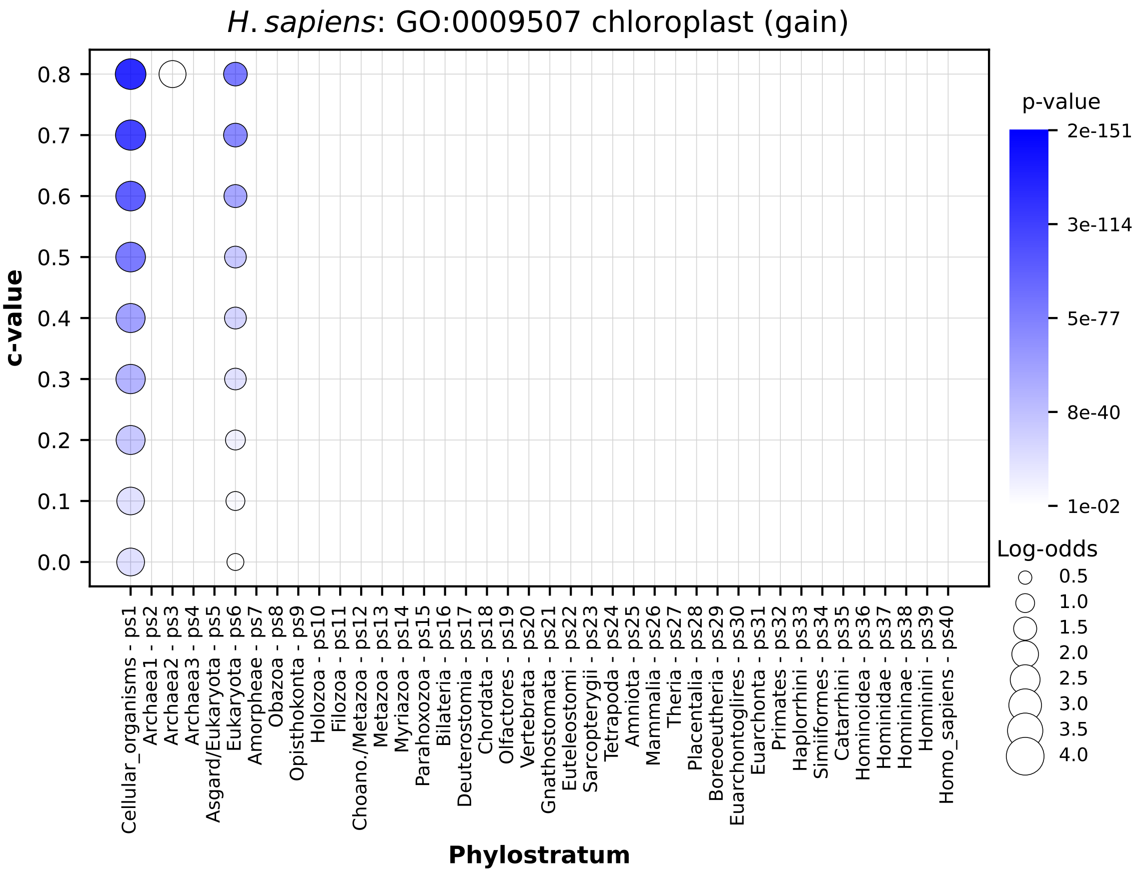
H. sapiens: GO:0009507 chloroplast (gain)
c-value
0.8
0.7
0.6
0.5
0.4
0.3
0.2
0.1
0.0
p-value
2e-151
3e-114
5e-77
8e-40
1e-02
Log-odds
0.5
1.0
1.5
2.0
2.5
3.0
3.5
4.0
Cellular_organisms - ps1
Archaea1 - ps2
Archaea2 - ps3
Archaea3 - ps4
Asgard/Eukaryota - ps5
Eukaryota - ps6
Amorpheae - ps7
Obazoa - ps8
Opisthokonta - ps9
Holozoa - ps10
Filozoa - ps11
Choano./Metazoa - ps12
Metazoa - ps13
Myriazoa - ps14
Parahoxozoa - ps15
Bilateria - ps16
Deuterostomia - ps17
Chordata - ps18
Olfactores - ps19
Vertebrata - ps20
Gnathostomata - ps21
Euteleostomi - ps22
Sarcopterygii - ps23
Tetrapoda - ps24
Amniota - ps25
Mammalia - ps26
Theria - ps27
Placentalia - ps28
Boreoeutheria - ps29
Euarchontoglires - ps30
Euarchonta - ps31
Primates - ps32
Haplorrhini - ps33
Simiiformes - ps34
Catarrhini - ps35
Hominoidea - ps36
Hominidae - ps37
Homininae - ps38
Hominini - ps39
Homo_sapiens - ps40
Phylostratum

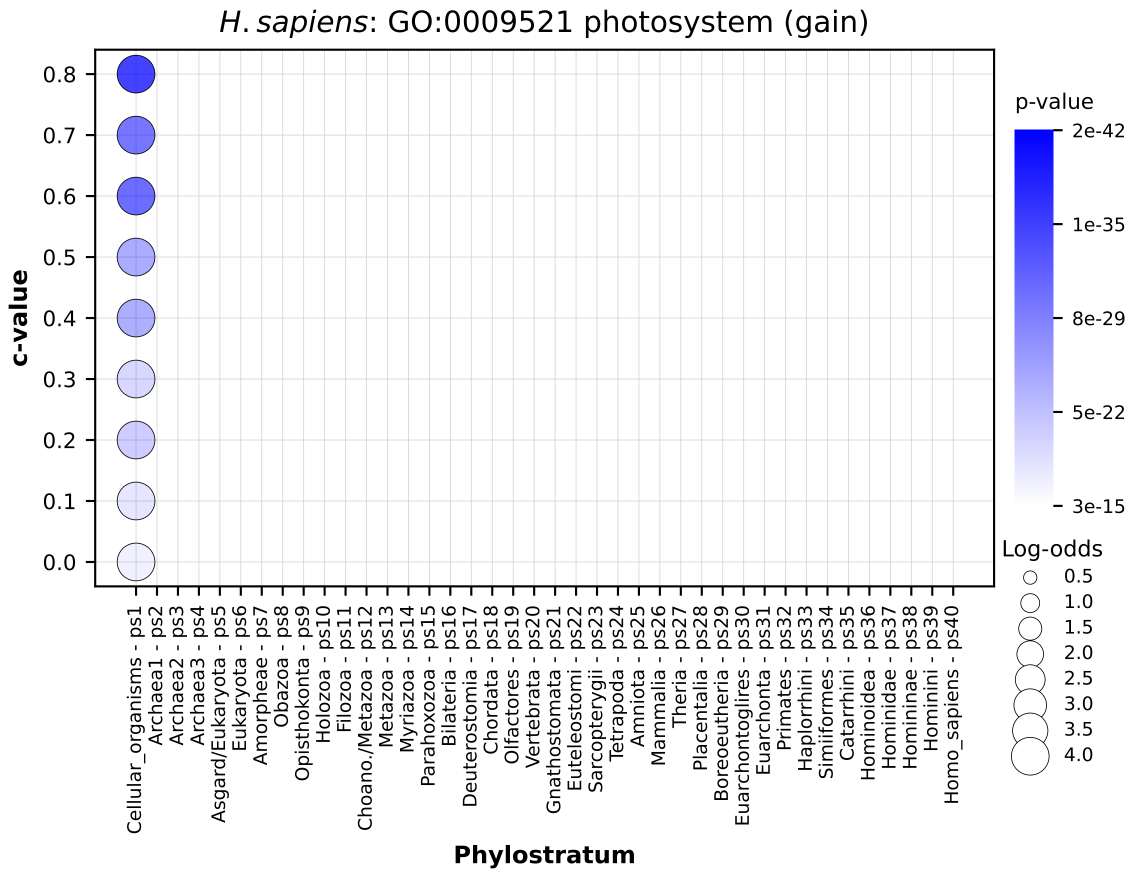

H. sapiens: GO:0009521 photosystem (gain)
c-value
0.8
0.7
0.6
0.5
0.4
0.3
0.2
0.1
0.0
p-value
2e-42
1e-35
8e-29
5e-22
3e-15
Log-odds
0.5
1.0
1.5
2.0
2.5
3.0
3.5
4.0
Cellular_organisms - ps1
Archaea1 - ps2
Archaea2 - ps3
Archaea3 - ps4
Asgard/Eukaryota - ps5
Eukaryota - ps6
Amorpheae - ps7
Obazoa - ps8
Opisthokonta - ps9
Holozoa - ps10
Filozoa - ps11
Choano./Metazoa - ps12
Metazoa - ps13
Myriazoa - ps14
Parahoxozoa - ps15
Bilateria - ps16
Deuterostomia - ps17
Chordata - ps18
Olfactores - ps19
Vertebrata - ps20
Gnathostomata - ps21
Euteleostomi - ps22
Sarcopterygii - ps23
Tetrapoda - ps24
Amniota - ps25
Mammalia - ps26
Theria - ps27
Placentalia - ps28
Boreoeutheria - ps29
Euarchontoglires - ps30
Euarchonta - ps31
Primates - ps32
Haplorrhini - ps33
Simiiformes - ps34
Catarrhini - ps35
Hominoidea - ps36
Hominidae - ps37
Homininae - ps38
Hominini - ps39
Homo_sapiens - ps40
Phylostratum

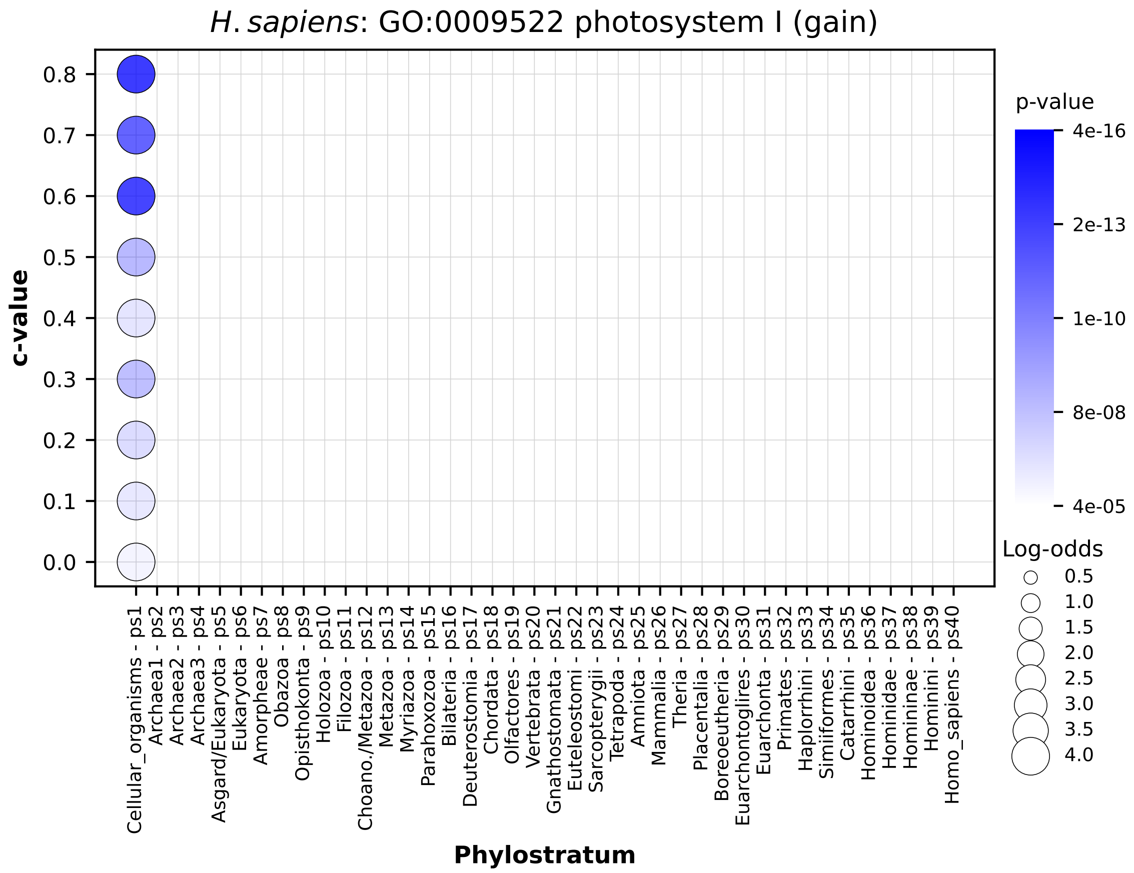

H. sapiens: GO:0009522 photosystem I (gain)
c-value
0.8
0.7
0.6
0.5
0.4
0.3
0.2
0.1
0.0
p-value
4e-16
2e-13
1e-10
8e-08
4e-05
Log-odds
0.5
1.0
1.5
2.0
2.5
3.0
3.5
4.0
Cellular_organisms - ps1
Archaea1 - ps2
Archaea2 - ps3
Archaea3 - ps4
Asgard/Eukaryota - ps5
Eukaryota - ps6
Amorpheae - ps7
Obazoa - ps8
Opisthokonta - ps9
Holozoa - ps10
Filozoa - ps11
Choano./Metazoa - ps12
Metazoa - ps13
Myriazoa - ps14
Parahoxozoa - ps15
Bilateria - ps16
Deuterostomia - ps17
Chordata - ps18
Olfactores - ps19
Vertebrata - ps20
Gnathostomata - ps21
Euteleostomi - ps22
Sarcopterygii - ps23
Tetrapoda - ps24
Amniota - ps25
Mammalia - ps26
Theria - ps27
Placentalia - ps28
Boreoeutheria - ps29
Euarchontoglires - ps30
Euarchonta - ps31
Primates - ps32
Haplorrhini - ps33
Simiiformes - ps34
Catarrhini - ps35
Hominoidea - ps36
Hominidae - ps37
Homininae - ps38
Hominini - ps39
Homo_sapiens - ps40
Phylostratum

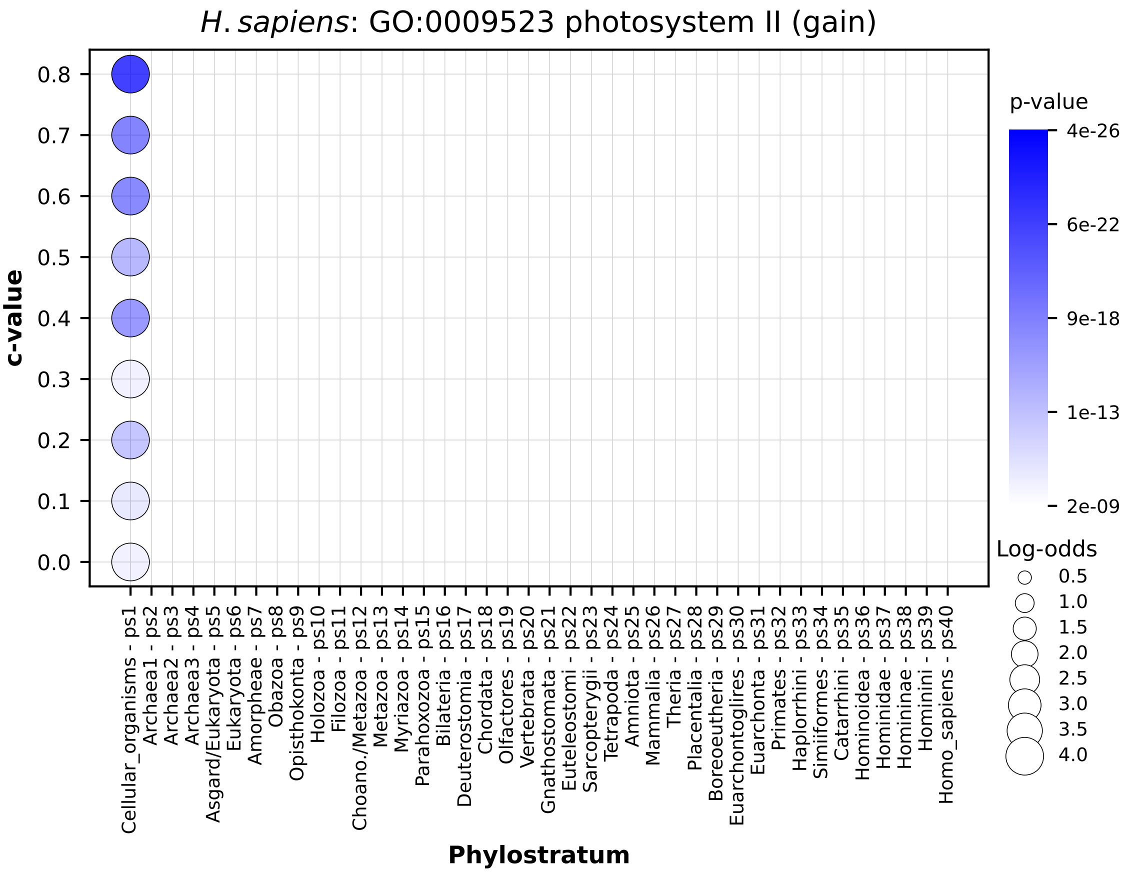
H. sapiens: GO:0009523 photosystem II (gain)
c-value
0.8
0.7
0.6
0.5
0.4
0.3
0.2
0.1
0.0
Phylostratum
Cellular_organisms - ps1
Archaea1 - ps2
Archaea2 - ps3
Archaea3 - ps4
Asgard/Eukaryota - ps5
Eukaryota - ps6
Amorpheae - ps7
Obazoa - ps8
Opisthokonta - ps9
Holozoa - ps10
Filozoa - ps11
Choano./Metazoa - ps12
Metazoa - ps13
Myriazoa - ps14
Parahoxozoa - ps15
Bilateria - ps16
Deuterostomia - ps17
Chordata - ps18
Olfactores - ps19
Vertebrata - ps20
Gnathostomata - ps21
Euteleostomi - ps22
Sarcopterygii - ps23
Tetrapoda - ps24
Amniota - ps25
Mammalia - ps26
Theria - ps27
Placentalia - ps28
Boreoeutheria - ps29
Euarchontoglires - ps30
Euarchonta - ps31
Primates - ps32
Haplorrhini - ps33
Simiiformes - ps34
Catarrhini - ps35
Hominoidea - ps36
Hominidae - ps37
Homininae - ps38
Hominini - ps39
Homo_sapiens - ps40
p-value
4e-26
6e-22
9e-18
1e-13
2e-09
Log-odds
0.5
1.0
1.5
2.0
2.5
3.0
3.5
4.0

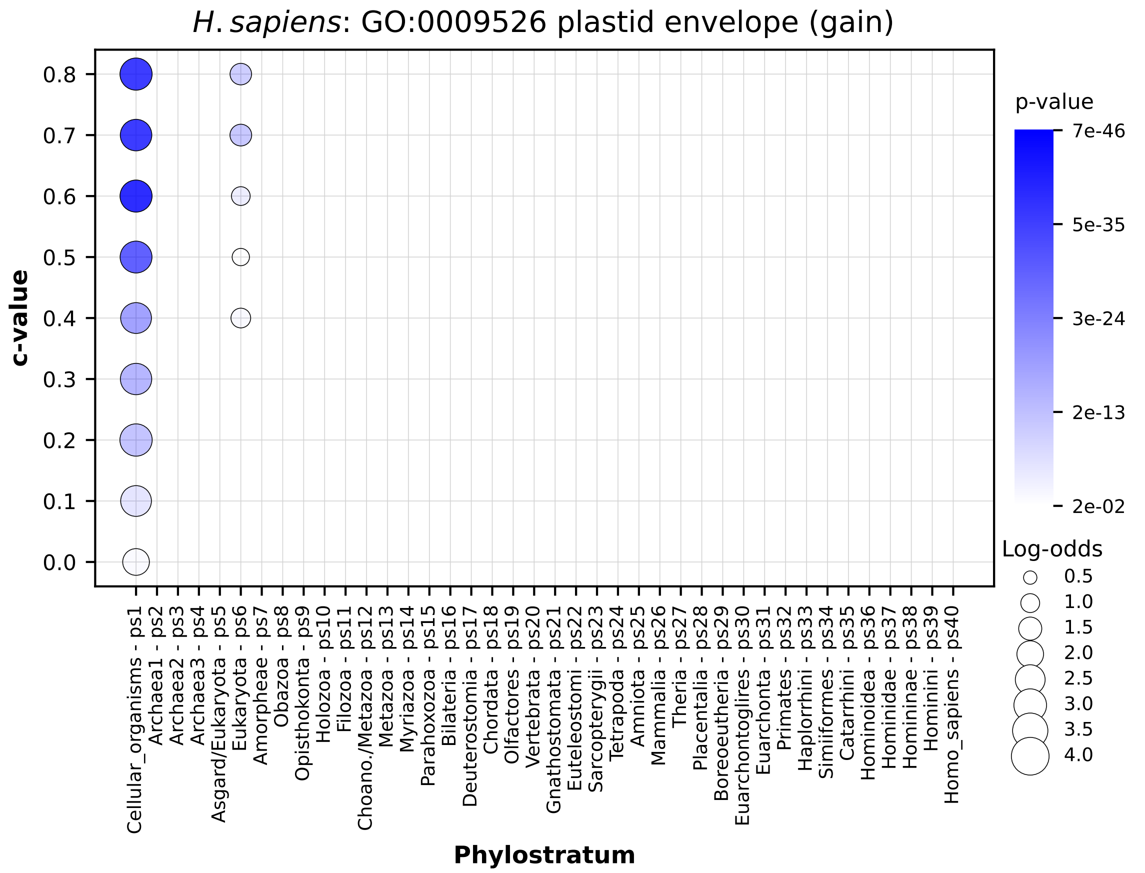
H. sapiens: GO:0009526 plastid envelope (gain)
c-value
0.8
0.7
0.6
0.5
0.4
0.3
0.2
0.1
0.0
p-value
7e-46
5e-35
3e-24
2e-13
2e-02
Log-odds
0.5
1.0
1.5
2.0
2.5
3.0
3.5
4.0
Cellular_organisms - ps1
Archaea1 - ps2
Archaea2 - ps3
Archaea3 - ps4
Asgard/Eukaryota - ps5
Eukaryota - ps6
Amorpheae - ps7
Obazoa - ps8
Opisthokonta - ps9
Holozoa - ps10
Filozoa - ps11
Choano./Metazoa - ps12
Metazoa - ps13
Myriazoa - ps14
Parahoxozoa - ps15
Bilateria - ps16
Deuterostomia - ps17
Chordata - ps18
Olfactores - ps19
Vertebrata - ps20
Gnathostomata - ps21
Euteleostomi - ps22
Sarcopterygii - ps23
Tetrapoda - ps24
Amniota - ps25
Mammalia - ps26
Theria - ps27
Placentalia - ps28
Boreoeutheria - ps29
Euarchontoglires - ps30
Euarchonta - ps31
Primates - ps32
Haplorrhini - ps33
Simiiformes - ps34
Catarrhini - ps35
Hominoidea - ps36
Hominidae - ps37
Homininae - ps38
Hominini - ps39
Homo_sapiens - ps40
Phylostratum

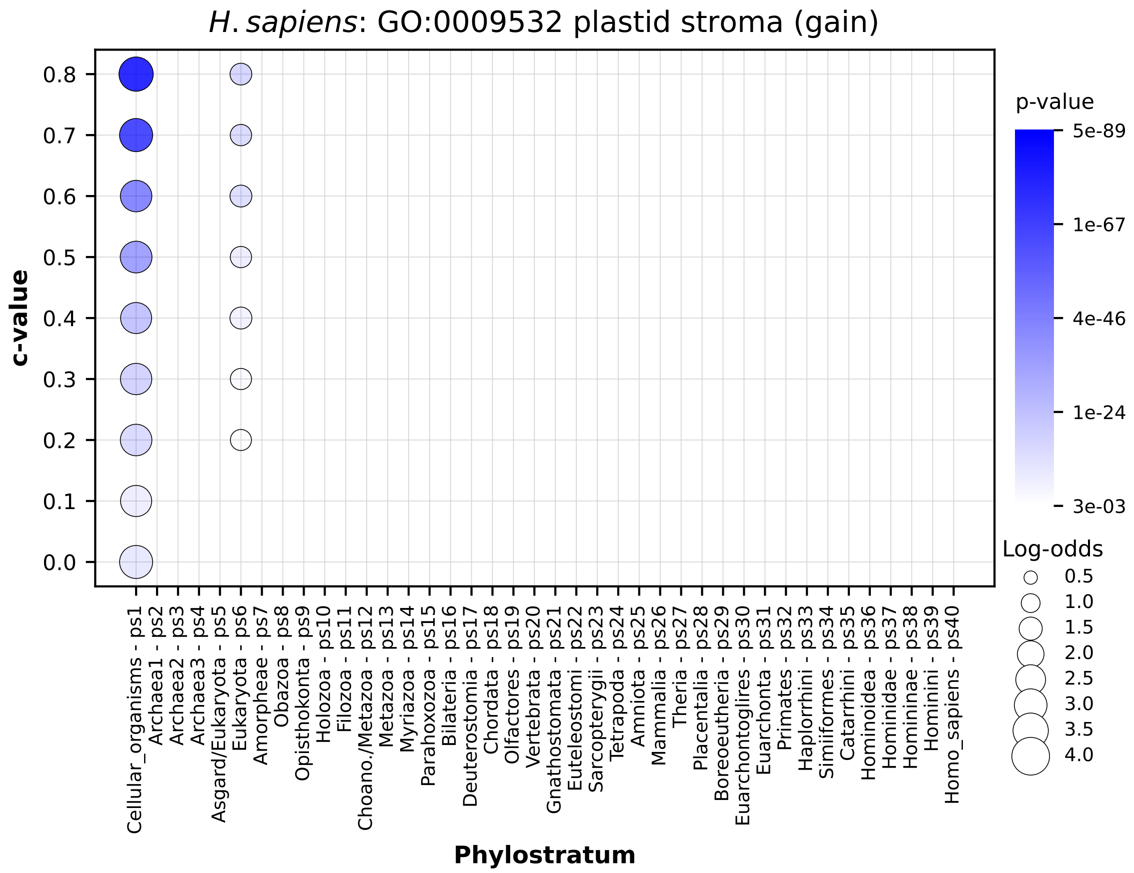

*H. sapiens*: GO:0009532 plastid stroma (gain)
c-value
0.8
0.7
0.6
0.5
0.4
0.3
0.2
0.1
0.0
Phylostratum
Cellular_organisms - ps1
Archaea1 - ps2
Archaea2 - ps3
Archaea3 - ps4
Asgard/Eukaryota - ps5
Eukaryota - ps6
Amorpheae - ps7
Obazoa - ps8
Opisthokonta - ps9
Holozoa - ps10
Filozoa - ps11
Choano./Metazoa - ps12
Metazoa - ps13
Myriazoa - ps14
Parahoxozoa - ps15
Bilateria - ps16
Deuterostomia - ps17
Chordata - ps18
Olfactores - ps19
Vertebrata - ps20
Gnathostomata - ps21
Euteleostomi - ps22
Sarcopterygii - ps23
Tetrapoda - ps24
Amniota - ps25
Mammalia - ps26
Theria - ps27
Placentalia - ps28
Boreoeutheria - ps29
Euarchontoglires - ps30
Euarchonta - ps31
Primates - ps32
Haplorrhini - ps33
Simiiformes - ps34
Catarrhini - ps35
Hominoidea - ps36
Hominidae - ps37
Homininae - ps38
Hominini - ps39
Homo_sapiens - ps40
p-value
5e-89
1e-67
4e-46
1e-24
3e-03
Log-odds
0.5
1.0
1.5
2.0
2.5
3.0
3.5
4.0

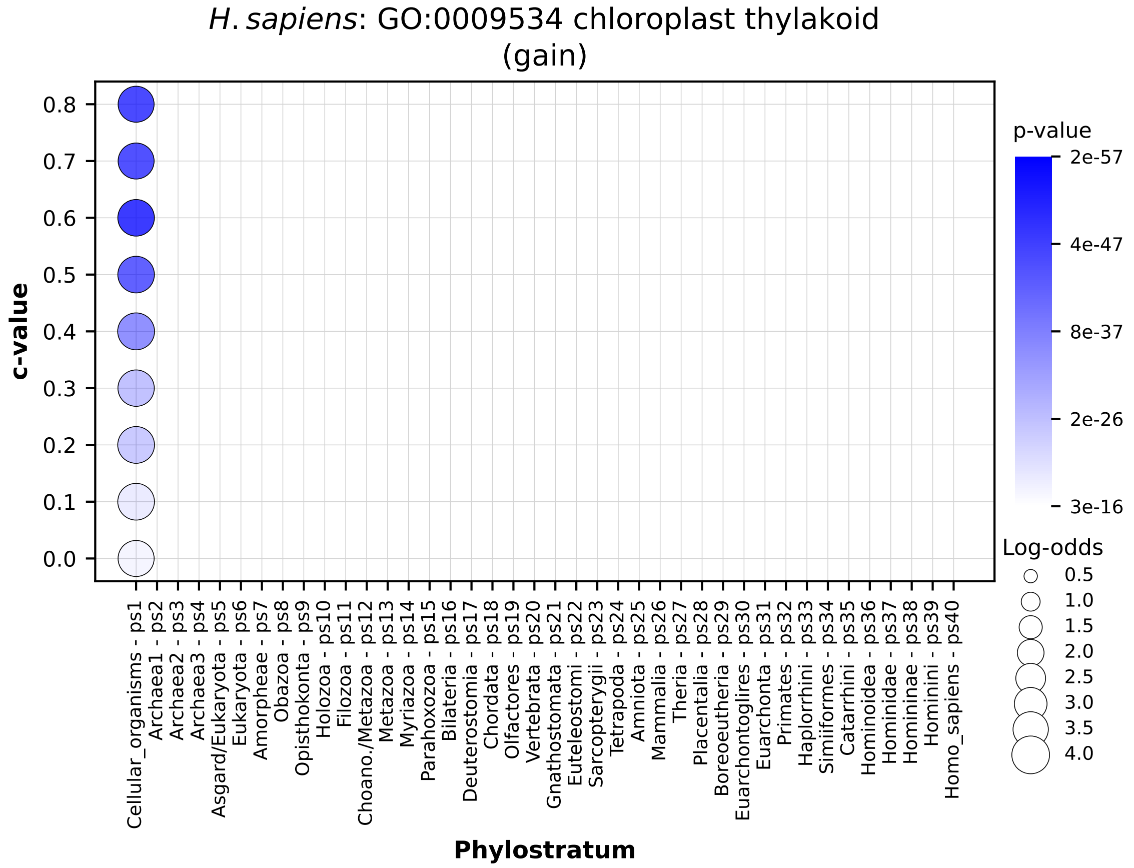

H. sapiens: GO:0009534 chloroplast thylakoid
(gain)
c-value
0.8
0.7
0.6
0.5
0.4
0.3
0.2
0.1
0.0
p-value
2e-57
4e-47
8e-37
2e-26
3e-16
Log-odds
0.5
1.0
1.5
2.0
2.5
3.0
3.5
4.0
Cellular_organisms - ps1
Archaea1 - ps2
Archaea2 - ps3
Archaea3 - ps4
Asgard/Eukaryota - ps5
Eukaryota - ps6
Amorpheae - ps7
Obazoa - ps8
Opisthokonta - ps9
Holozoa - ps10
Filozoa - ps11
Choano./Metazoa - ps12
Metazoa - ps13
Myriazoa - ps14
Parahoxozoa - ps15
Bilateria - ps16
Deuterostomia - ps17
Chordata - ps18
Olfactores - ps19
Vertebrata - ps20
Gnathostomata - ps21
Euteleostomi - ps22
Sarcopterygii - ps23
Tetrapoda - ps24
Amniota - ps25
Mammalia - ps26
Theria - ps27
Placentalia - ps28
Boreoeutheria - ps29
Euarchontoglires - ps30
Euarchonta - ps31
Primates - ps32
Haplorrhini - ps33
Simiiformes - ps34
Catarrhini - ps35
Hominoidea - ps36
Hominidae - ps37
Homininae - ps38
Hominini - ps39
Homo_sapiens - ps40
Phylostratum

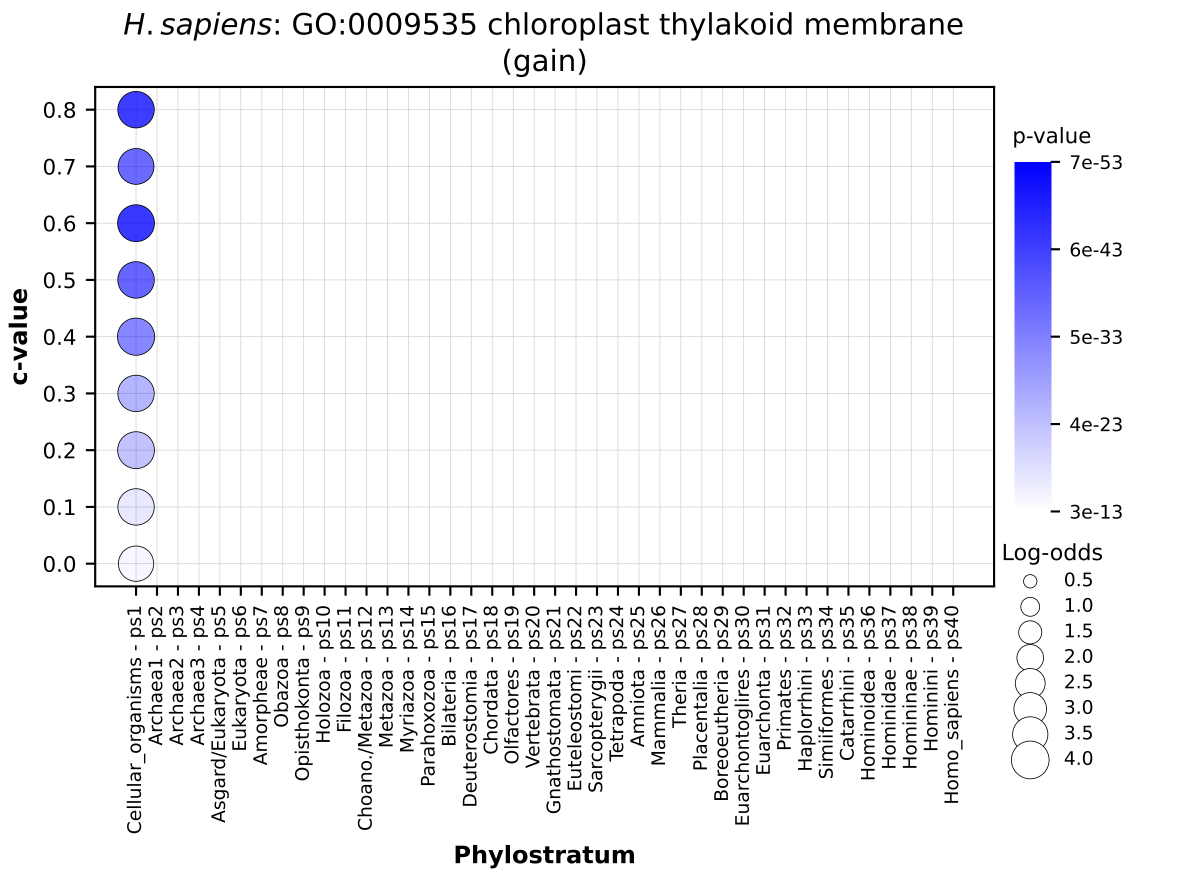

H. sapiens: GO:0009535 chloroplast thylakoid membrane (gain)
c-value
0.0
0.1
0.2
0.3
0.4
0.5
0.6
0.7
0.8
Phylostratum
Cellular_organisms - ps1
Archaea1 - ps2
Archaea2 - ps3
Archaea3 - ps4
Asgard/Eukaryota - ps5
Eukaryota - ps6
Amorpheae - ps7
Obazoa - ps8
Opisthokonta - ps9
Holozoa - ps10
Filozoa - ps11
Choano./Metazoa - ps12
Metazoa - ps13
Myriazoa - ps14
Parahoxozoa - ps15
Bilateria - ps16
Deuterostomia - ps17
Chordata - ps18
Olfactores - ps19
Vertebrata - ps20
Gnathostomata - ps21
Euteleostomi - ps22
Sarcopterygii - ps23
Tetrapoda - ps24
Amniota - ps25
Mammalia - ps26
Theria - ps27
Placentalia - ps28
Boreoeutheria - ps29
Euarchontoglires - ps30
Euarchonta - ps31
Primates - ps32
Haplorrhini - ps33
Simiiformes - ps34
Catarrhini - ps35
Hominoidea - ps36
Hominidae - ps37
Homininae - ps38
Hominini - ps39
Homo_sapiens - ps40
p-value
7e-53
6e-43
5e-33
4e-23
3e-13
Log-odds
0.5
1.0
1.5
2.0
2.5
3.0
3.5
4.0

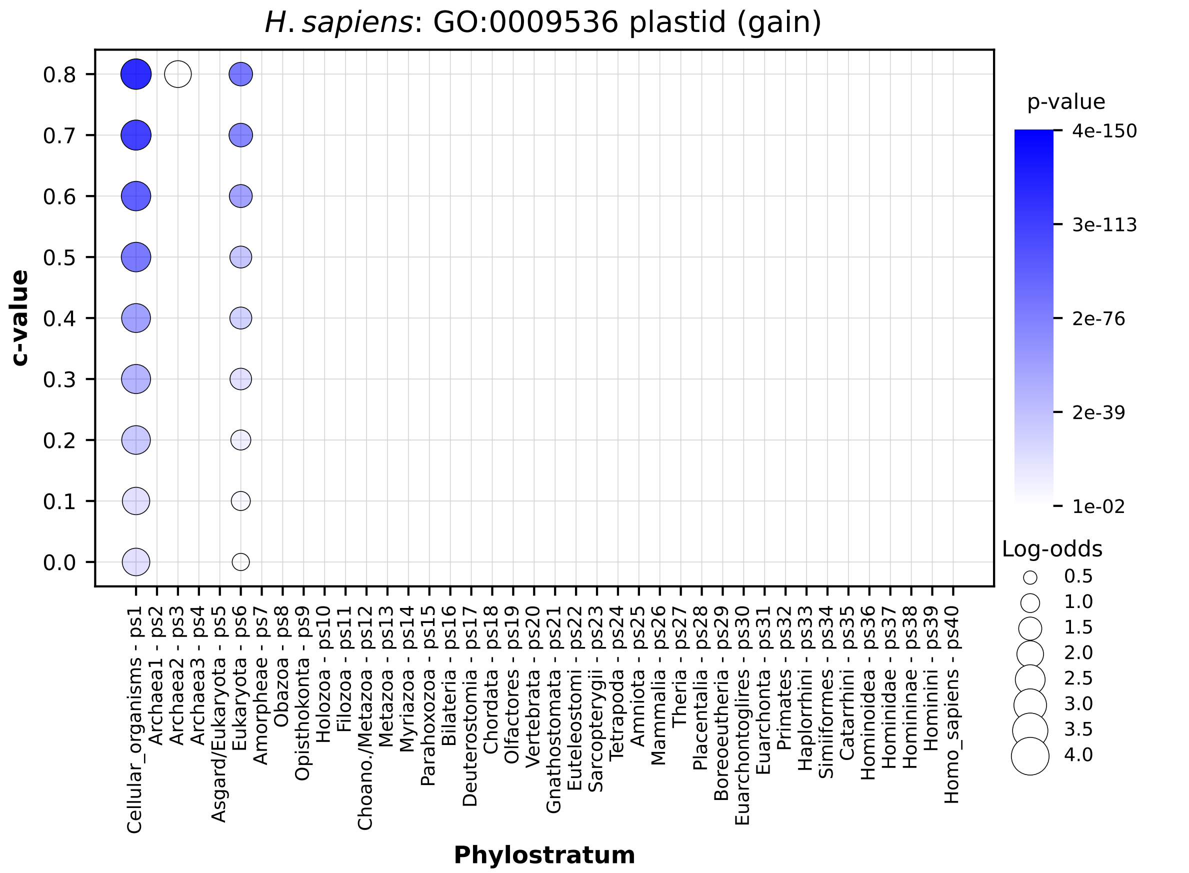

H. sapiens: GO:0009536 plastid (gain)
c-value
0.8
0.7
0.6
0.5
0.4
0.3
0.2
0.1
0.0
p-value
4e-150
3e-113
2e-76
2e-39
1e-02
Log-odds
0.5
1.0
1.5
2.0
2.5
3.0
3.5
4.0
Cellular_organisms - ps1
Archaea1 - ps2
Archaea2 - ps3
Archaea3 - ps4
Asgard/Eukaryota - ps5
Eukaryota - ps6
Amorpheae - ps7
Obazoa - ps8
Opisthokonta - ps9
Holozoa - ps10
Filozoa - ps11
Choano./Metazoa - ps12
Metazoa - ps13
Myriazoa - ps14
Parahoxozoa - ps15
Bilateria - ps16
Deuterostomia - ps17
Chordata - ps18
Olfactores - ps19
Vertebrata - ps20
Gnathostomata - ps21
Euteleostomi - ps22
Sarcopterygii - ps23
Tetrapoda - ps24
Amniota - ps25
Mammalia - ps26
Theria - ps27
Placentalia - ps28
Boreoeutheria - ps29
Euarchontoglires - ps30
Euarchonta - ps31
Primates - ps32
Haplorrhini - ps33
Simiiformes - ps34
Catarrhini - ps35
Hominoidea - ps36
Hominidae - ps37
Homininae - ps38
Hominini - ps39
Homo_sapiens - ps40
Phylostratum

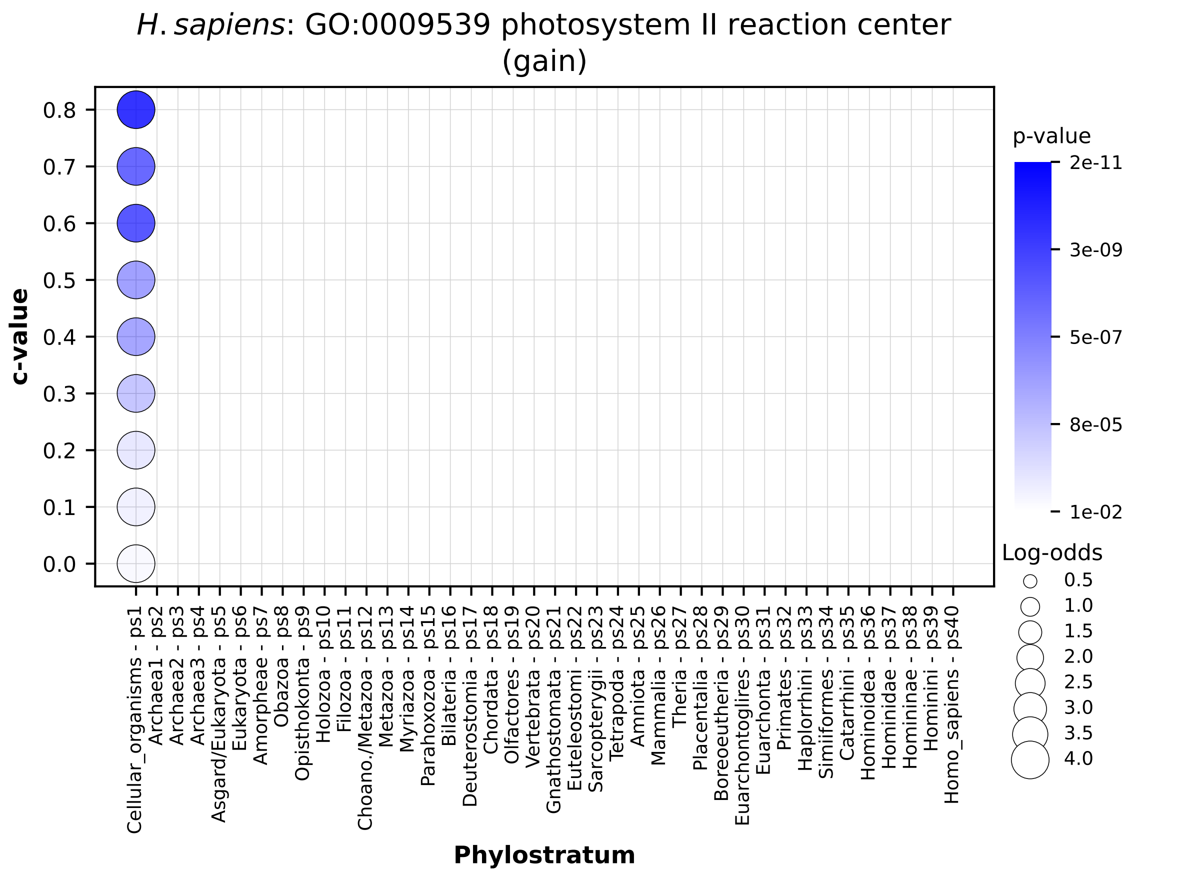
H. sapiens: GO:0009539 photosystem II reaction center (gain)
c-value
0.8
0.7
0.6
0.5
0.4
0.3
0.2
0.1
0.0
p-value
2e-11
3e-09
5e-07
8e-05
1e-02
Log-odds
0.5
1.0
1.5
2.0
2.5
3.0
3.5
4.0
Cellular_organisms - ps1
Archaea1 - ps2
Archaea2 - ps3
Archaea3 - ps4
Asgard/Eukaryota - ps5
Eukaryota - ps6
Amorpheae - ps7
Obazoa - ps8
Opisthokonta - ps9
Holozoa - ps10
Filozoa - ps11
Choano./Metazoa - ps12
Metazoa - ps13
Myriazoa - ps14
Parahoxozoa - ps15
Bilateria - ps16
Deuterostomia - ps17
Chordata - ps18
Olfactores - ps19
Vertebrata - ps20
Gnathostomata - ps21
Euteleostomi - ps22
Sarcopterygii - ps23
Tetrapoda - ps24
Amniota - ps25
Mammalia - ps26
Theria - ps27
Placentalia - ps28
Boreoeutheria - ps29
Euarchontoglires - ps30
Euarchonta - ps31
Primates - ps32
Haplorrhini - ps33
Simiiformes - ps34
Catarrhini - ps35
Hominoidea - ps36
Hominidae - ps37
Homininae - ps38
Hominini - ps39
Homo_sapiens - ps40
Phylostratum

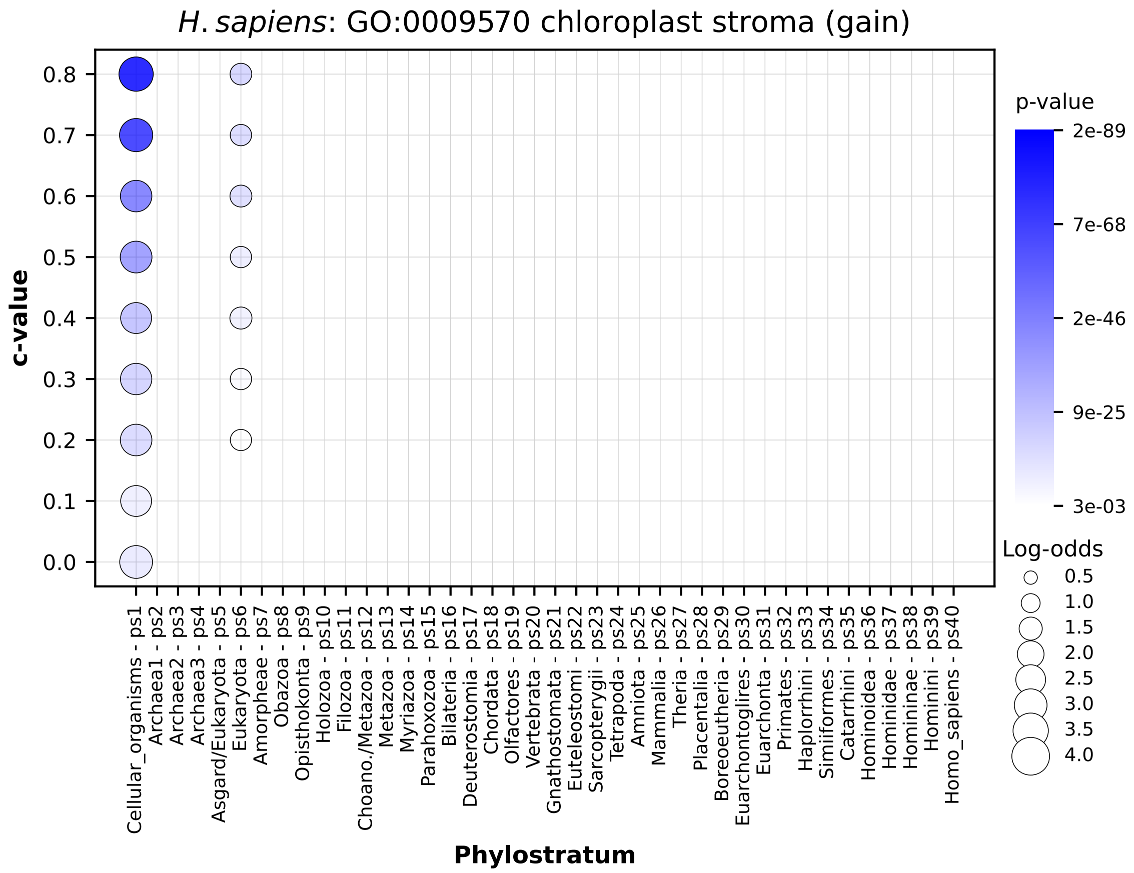

H. sapiens: GO:0009570 chloroplast stroma (gain)
c-value
0.0
0.1
0.2
0.3
0.4
0.5
0.6
0.7
0.8
Phylostratum
Cellular_organisms - ps1
Archaea1 - ps2
Archaea2 - ps3
Archaea3 - ps4
Asgard/Eukaryota - ps5
Eukaryota - ps6
Amorpheae - ps7
Obazoa - ps8
Opisthokonta - ps9
Holozoa - ps10
Filozoa - ps11
Choano./Metazoa - ps12
Metazoa - ps13
Myriazoa - ps14
Parahoxozoa - ps15
Bilateria - ps16
Deuterostomia - ps17
Chordata - ps18
Olfactores - ps19
Vertebrata - ps20
Gnathostomata - ps21
Euteleostomi - ps22
Sarcopterygii - ps23
Tetrapoda - ps24
Amniota - ps25
Mammalia - ps26
Theria - ps27
Placentalia - ps28
Boreoeutheria - ps29
Euarchontoglires - ps30
Euarchonta - ps31
Primates - ps32
Haplorrhini - ps33
Simiiformes - ps34
Catarrhini - ps35
Hominoidea - ps36
Hominidae - ps37
Homininae - ps38
Hominini - ps39
Homo_sapiens - ps40
p-value
2e-89
7e-68
2e-46
9e-25
3e-03
Log-odds
0.5
1.0
1.5
2.0
2.5
3.0
3.5
4.0

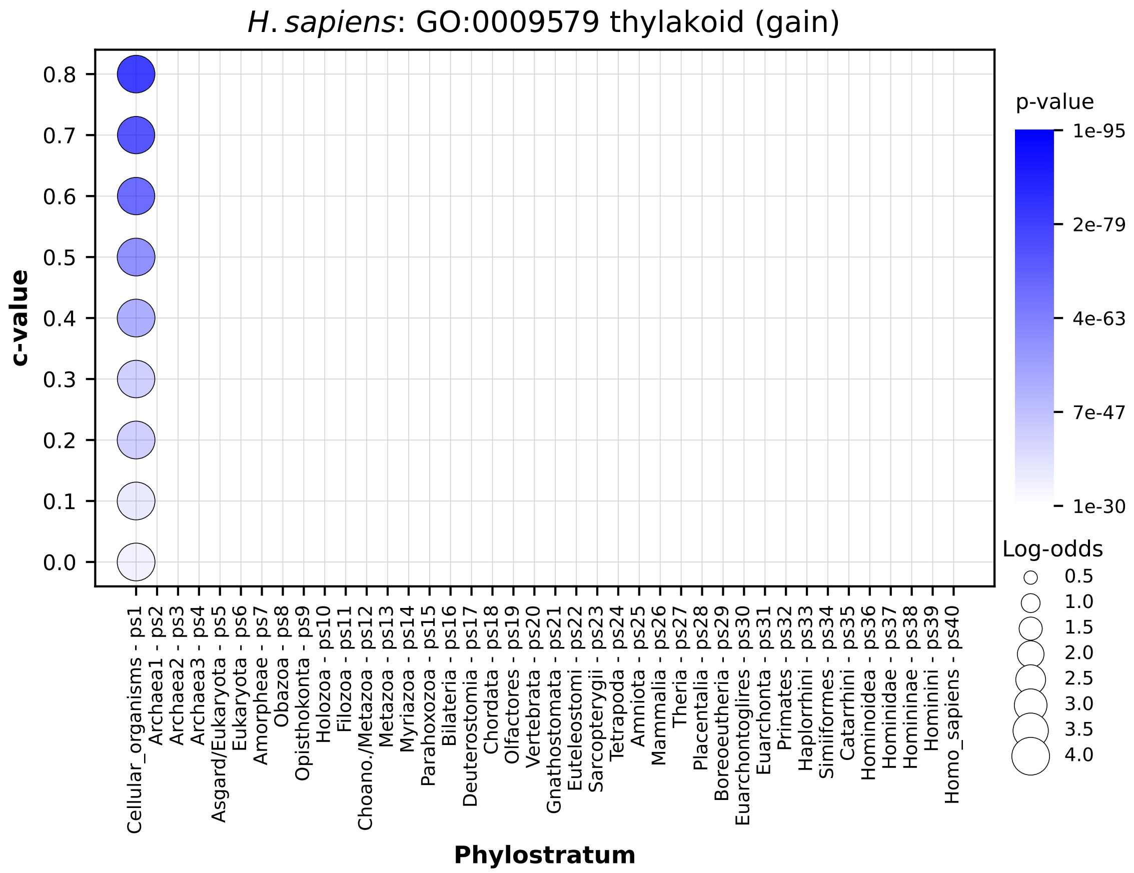

H. sapiens: GO:0009579 thylakoid (gain)
c-value
0.8
0.7
0.6
0.5
0.4
0.3
0.2
0.1
0.0
p-value
1e-95
2e-79
4e-63
7e-47
1e-30
Log-odds
0.5
1.0
1.5
2.0
2.5
3.0
3.5
4.0
Cellular_organisms - ps1
Archaea1 - ps2
Archaea2 - ps3
Archaea3 - ps4
Asgard/Eukaryota - ps5
Eukaryota - ps6
Amorpheae - ps7
Obazoa - ps8
Opisthokonta - ps9
Holozoa - ps10
Filozoa - ps11
Choano./Metazoa - ps12
Metazoa - ps13
Myriazoa - ps14
Parahoxozoa - ps15
Bilateria - ps16
Deuterostomia - ps17
Chordata - ps18
Olfactores - ps19
Vertebrata - ps20
Gnathostomata - ps21
Euteleostomi - ps22
Sarcopterygii - ps23
Tetrapoda - ps24
Amniota - ps25
Mammalia - ps26
Theria - ps27
Placentalia - ps28
Boreoeutheria - ps29
Euarchontoglires - ps30
Euarchonta - ps31
Primates - ps32
Haplorrhini - ps33
Simiiformes - ps34
Catarrhini - ps35
Hominoidea - ps36
Hominidae - ps37
Homininae - ps38
Hominini - ps39
Homo_sapiens - ps40
Phylostratum

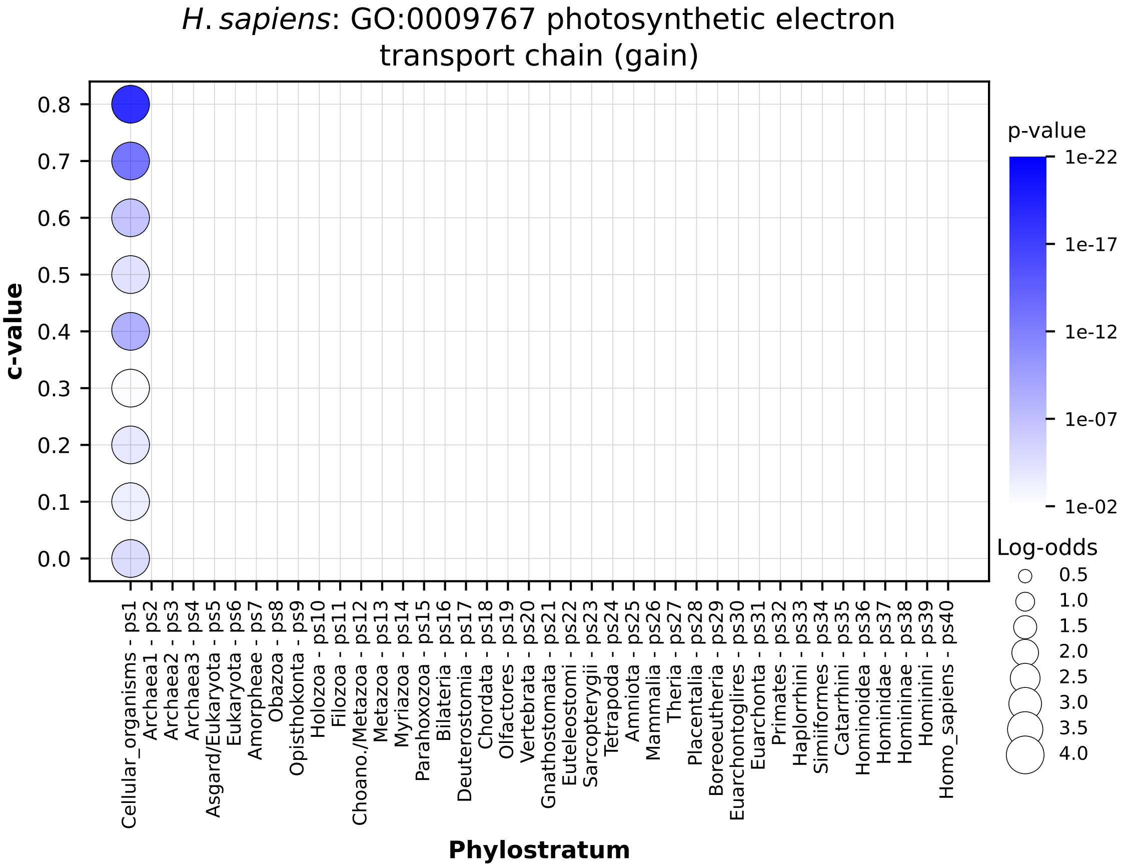
H. sapiens: GO:0009767 photosynthetic electron transport chain (gain)
c-value
0.8
0.7
0.6
0.5
0.4
0.3
0.2
0.1
0.0
p-value
1e-22
1e-17
1e-12
1e-07
1e-02
Log-odds
0.5
1.0
1.5
2.0
2.5
3.0
3.5
4.0
Cellular_organisms - ps1
Archaea1 - ps2
Archaea2 - ps3
Archaea3 - ps4
Asgard/Eukaryota - ps5
Eukaryota - ps6
Amorpheae - ps7
Obazoa - ps8
Opisthokonta - ps9
Holozoa - ps10
Filozoa - ps11
Choano./Metazoa - ps12
Metazoa - ps13
Myriazoa - ps14
Parahoxozoa - ps15
Bilateria - ps16
Deuterostomia - ps17
Chordata - ps18
Olfactores - ps19
Vertebrata - ps20
Gnathostomata - ps21
Euteleostomi - ps22
Sarcopterygii - ps23
Tetrapoda - ps24
Amniota - ps25
Mammalia - ps26
Theria - ps27
Placentalia - ps28
Boreoeutheria - ps29
Euarchontoglires - ps30
Euarchonta - ps31
Primates - ps32
Haplorrhini - ps33
Simiiformes - ps34
Catarrhini - ps35
Hominoidea - ps36
Hominidae - ps37
Homininae - ps38
Hominini - ps39
Homo_sapiens - ps40
Phylostratum

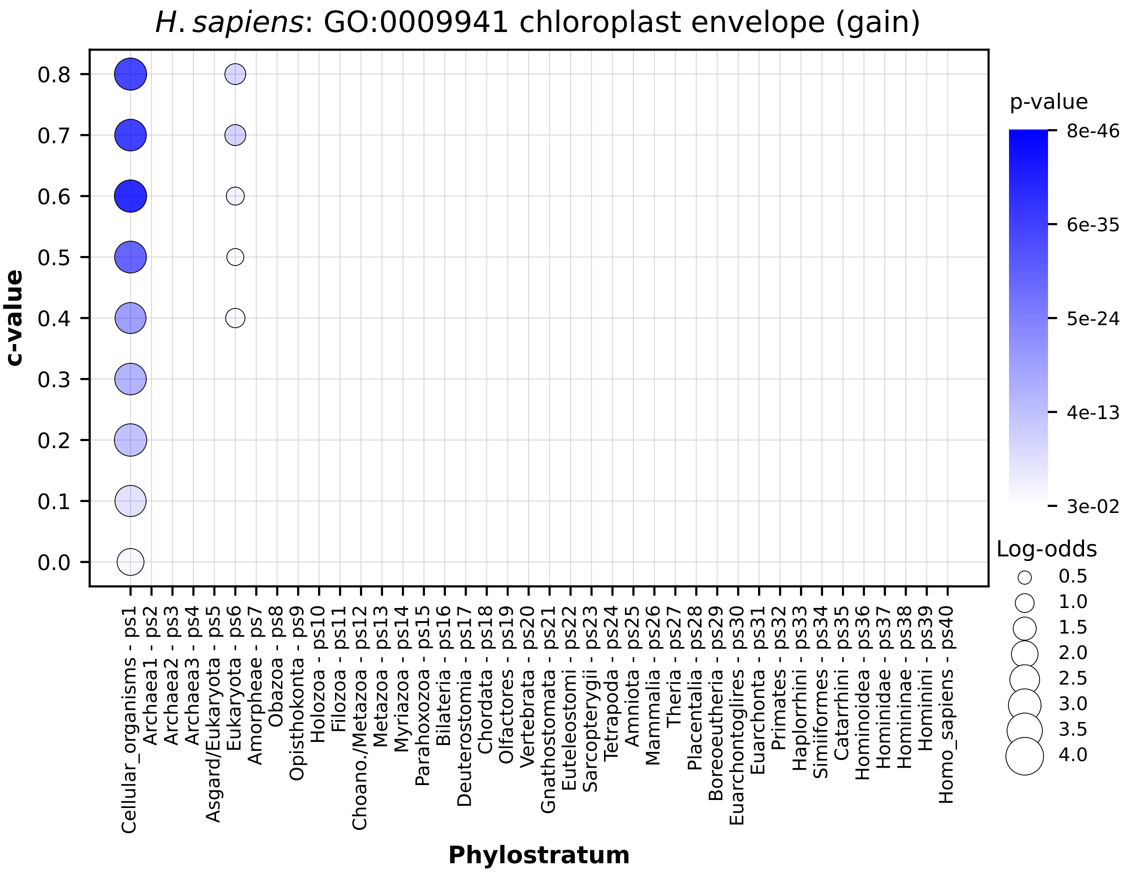
H. sapiens: GO:0009941 chloroplast envelope (gain)
c-value
0.0
0.1
0.2
0.3
0.4
0.5
0.6
0.7
0.8
Phylostratum
Cellular_organisms - ps1
Archaea1 - ps2
Archaea2 - ps3
Archaea3 - ps4
Asgard/Eukaryota - ps5
Eukaryota - ps6
Amorpheae - ps7
Obazoa - ps8
Opisthokonta - ps9
Holozoa - ps10
Filozoa - ps11
Choano./Metazoa - ps12
Metazoa - ps13
Myriazoa - ps14
Parahoxozoa - ps15
Bilateria - ps16
Deuterostomia - ps17
Chordata - ps18
Olfactores - ps19
Vertebrata - ps20
Gnathostomata - ps21
Euteleostomi - ps22
Sarcopterygii - ps23
Tetrapoda - ps24
Amniota - ps25
Mammalia - ps26
Theria - ps27
Placentalia - ps28
Boreoeutheria - ps29
Euarchontoglires - ps30
Euarchonta - ps31
Primates - ps32
Haplorrhini - ps33
Simiiformes - ps34
Catarrhini - ps35
Hominoidea - ps36
Hominidae - ps37
Homininae - ps38
Hominini - ps39
Homo_sapiens - ps40
p-value
8e-46
6e-35
5e-24
4e-13
3e-02
Log-odds
0.5
1.0
1.5
2.0
2.5
3.0
3.5
4.0

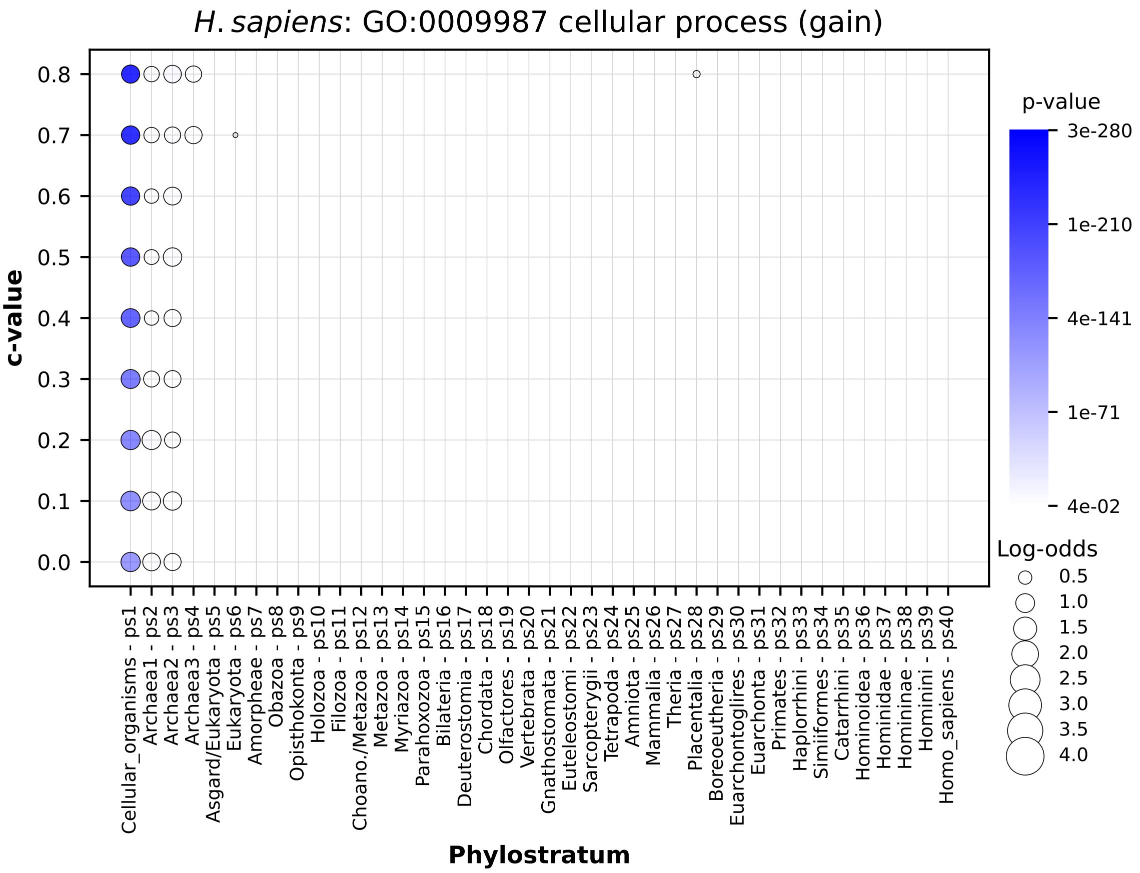
H. sapiens: GO:0009987 cellular process (gain)
c-value
0.8
0.7
0.6
0.5
0.4
0.3
0.2
0.1
0.0
Phylostratum
Cellular_organisms - ps1
Archaea1 - ps2
Archaea2 - ps3
Archaea3 - ps4
Asgard/Eukaryota - ps5
Eukaryota - ps6
Amorpheae - ps7
Obazoa - ps8
Opisthokonta - ps9
Holozoa - ps10
Filozoa - ps11
Choano./Metazoa - ps12
Metazoa - ps13
Myriazoa - ps14
Parahoxozoa - ps15
Bilateria - ps16
Deuterostomia - ps17
Chordata - ps18
Olfactores - ps19
Vertebrata - ps20
Gnathostomata - ps21
Euteleostomi - ps22
Sarcopterygii - ps23
Tetrapoda - ps24
Amniota - ps25
Mammalia - ps26
Theria - ps27
Placentalia - ps28
Boreoeutheria - ps29
Euarchontoglires - ps30
Euarchonta - ps31
Primates - ps32
Haplorrhini - ps33
Simiiformes - ps34
Catarrhini - ps35
Hominoidea - ps36
Hominidae - ps37
Homininae - ps38
Hominini - ps39
Homo_sapiens - ps40
p-value
3e-280
1e-210
4e-141
1e-71
4e-02
Log-odds
0.5
1.0
1.5
2.0
2.5
3.0
3.5
4.0

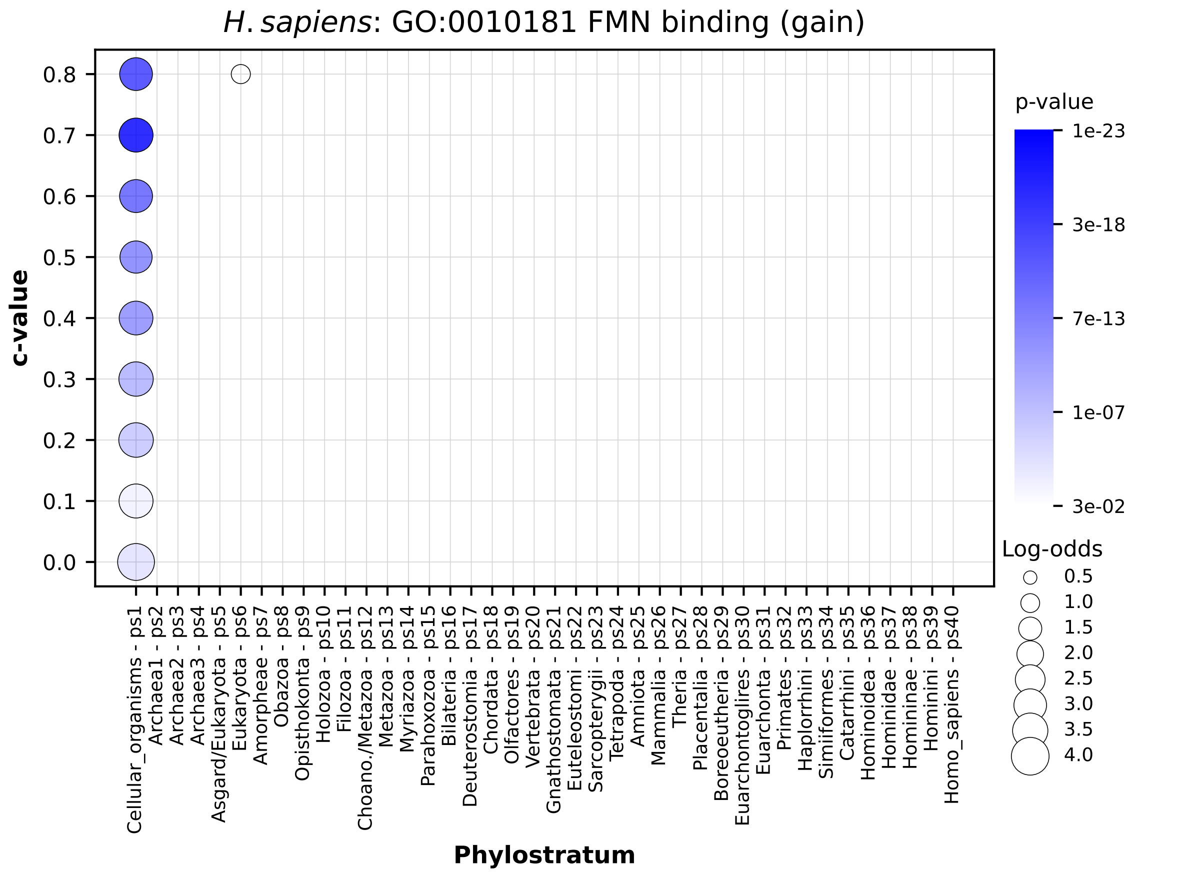
H. sapiens: GO:0010181 FMN binding (gain)
c-value
0.8
0.7
0.6
0.5
0.4
0.3
0.2
0.1
0.0
p-value
1e-23
3e-18
7e-13
1e-07
3e-02
Log-odds
0.5
1.0
1.5
2.0
2.5
3.0
3.5
4.0
Cellular_organisms - ps1
Archaea1 - ps2
Archaea2 - ps3
Archaea3 - ps4
Asgard/Eukaryota - ps5
Eukaryota - ps6
Amorpheae - ps7
Obazoa - ps8
Opisthokonta - ps9
Holozoa - ps10
Filozoa - ps11
Choano./Metazoa - ps12
Metazoa - ps13
Myriazoa - ps14
Parahoxozoa - ps15
Bilateria - ps16
Deuterostomia - ps17
Chordata - ps18
Olfactores - ps19
Vertebrata - ps20
Gnathostomata - ps21
Euteleostomi - ps22
Sarcopterygii - ps23
Tetrapoda - ps24
Amniota - ps25
Mammalia - ps26
Theria - ps27
Placentalia - ps28
Boreoeutheria - ps29
Euarchontoglires - ps30
Euarchonta - ps31
Primates - ps32
Haplorrhini - ps33
Simiiformes - ps34
Catarrhini - ps35
Hominoidea - ps36
Hominidae - ps37
Homininae - ps38
Hominini - ps39
Homo_sapiens - ps40
Phylostratum

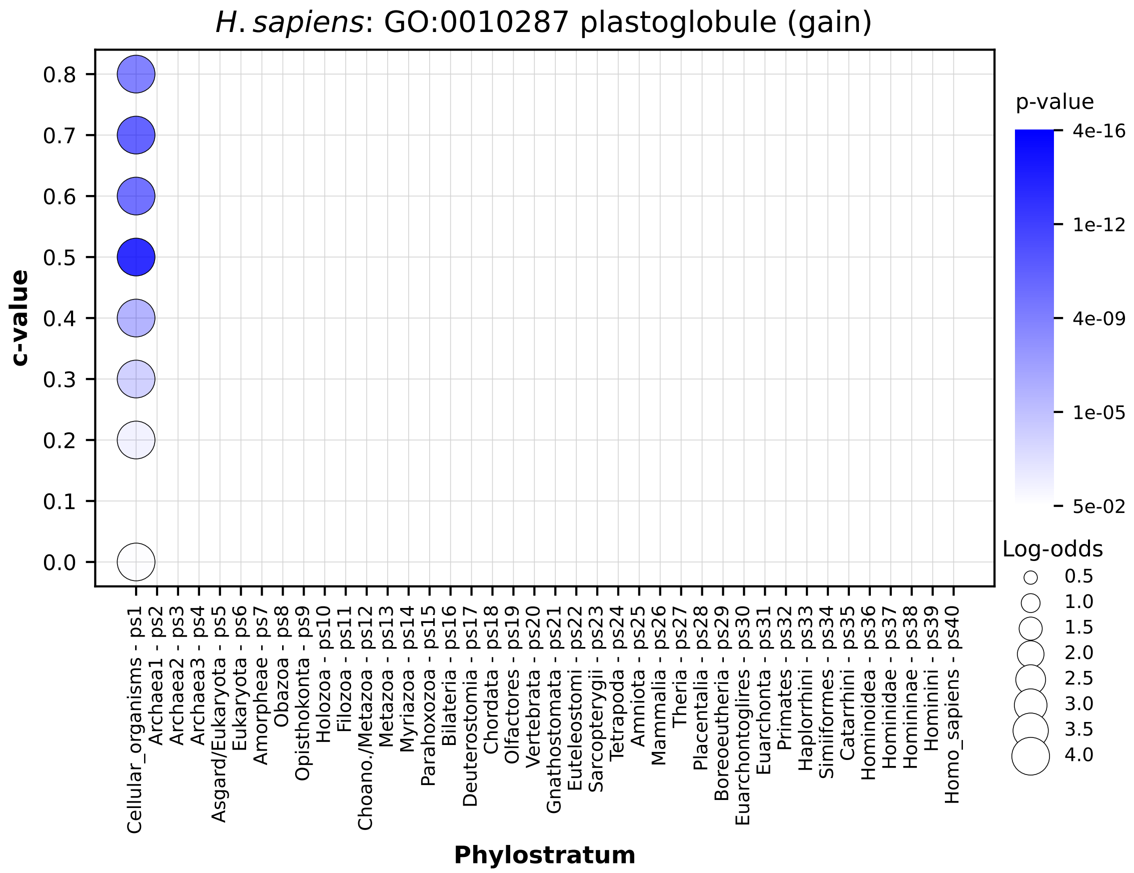

H. sapiens: GO:0010287 plastoglobule (gain)
c-value
0.0
0.1
0.2
0.3
0.4
0.5
0.6
0.7
0.8
p-value
4e-16
1e-12
4e-09
1e-05
5e-02
Log-odds
0.5
1.0
1.5
2.0
2.5
3.0
3.5
4.0
Cellular_organisms - ps1
Archaea1 - ps2
Archaea2 - ps3
Archaea3 - ps4
Asgard/Eukaryota - ps5
Eukaryota - ps6
Amorpheae - ps7
Obazoa - ps8
Opisthokonta - ps9
Holozoa - ps10
Filozoa - ps11
Choano./Metazoa - ps12
Metazoa - ps13
Myriazoa - ps14
Parahoxozoa - ps15
Bilateria - ps16
Deuterostomia - ps17
Chordata - ps18
Olfactores - ps19
Vertebrata - ps20
Gnathostomata - ps21
Euteleostomi - ps22
Sarcopterygii - ps23
Tetrapoda - ps24
Amniota - ps25
Mammalia - ps26
Theria - ps27
Placentalia - ps28
Boreoeutheria - ps29
Euarchontoglires - ps30
Euarchonta - ps31
Primates - ps32
Haplorrhini - ps33
Simiiformes - ps34
Catarrhini - ps35
Hominoidea - ps36
Hominidae - ps37
Homininae - ps38
Hominini - ps39
Homo_sapiens - ps40
Phylostratum

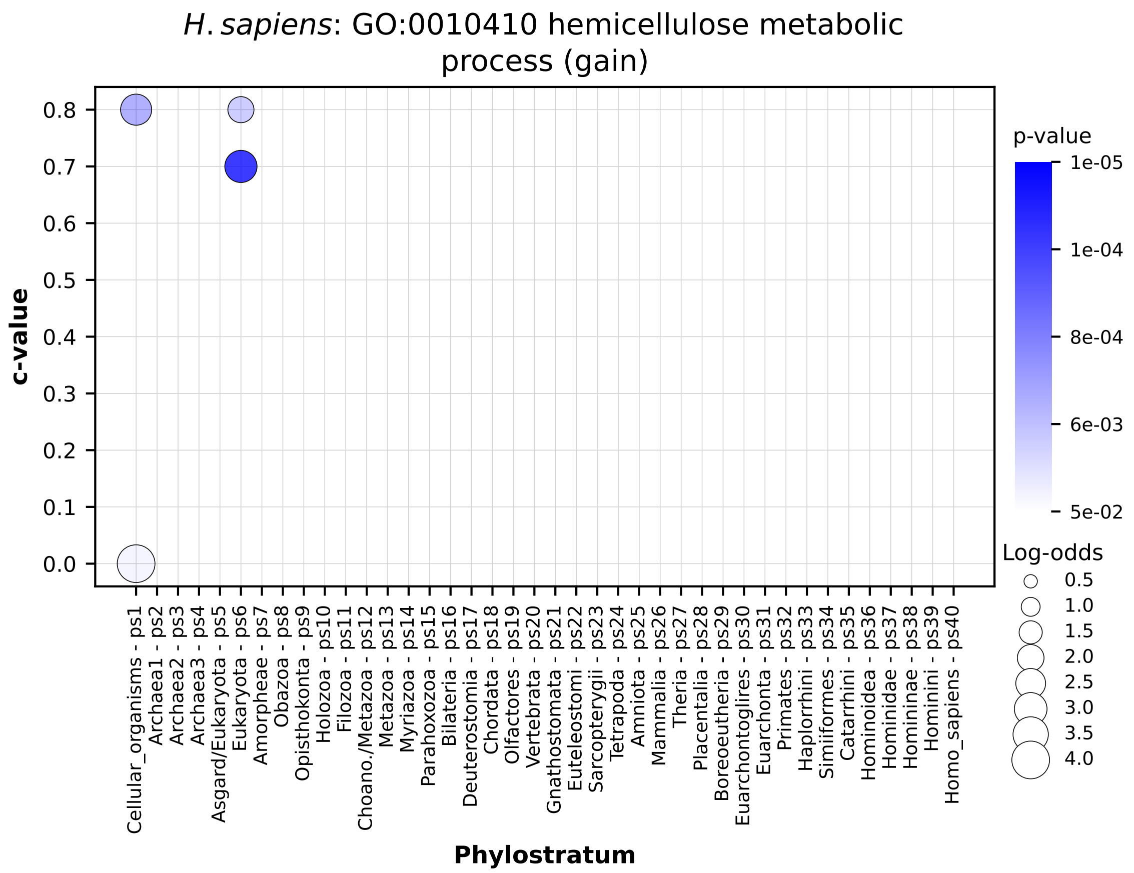
H. sapiens: GO:0010410 hemicellulose metabolic process (gain)
c-value
0.0
0.1
0.2
0.3
0.4
0.5
0.6
0.7
0.8
Phylostratum
Cellular_organisms - ps1
Archaea1 - ps2
Archaea2 - ps3
Archaea3 - ps4
Asgard/Eukaryota - ps5
Eukaryota - ps6
Amorpheae - ps7
Obazoa - ps8
Opisthokonta - ps9
Holozoa - ps10
Filozoa - ps11
Choano./Metazoa - ps12
Metazoa - ps13
Myriazoa - ps14
Parahoxozoa - ps15
Bilateria - ps16
Deuterostomia - ps17
Chordata - ps18
Olfactores - ps19
Vertebrata - ps20
Gnathostomata - ps21
Euteleostomi - ps22
Sarcopterygii - ps23
Tetrapoda - ps24
Amniota - ps25
Mammalia - ps26
Theria - ps27
Placentalia - ps28
Boreoeutheria - ps29
Euarchontoglires - ps30
Euarchonta - ps31
Primates - ps32
Haplorrhini - ps33
Simiiformes - ps34
Catarrhini - ps35
Hominoidea - ps36
Hominidae - ps37
Homininae - ps38
Hominini - ps39
Homo_sapiens - ps40
p-value
1e-05
1e-04
8e-04
6e-03
5e-02
Log-odds
0.5
1.0
1.5
2.0
2.5
3.0
3.5
4.0

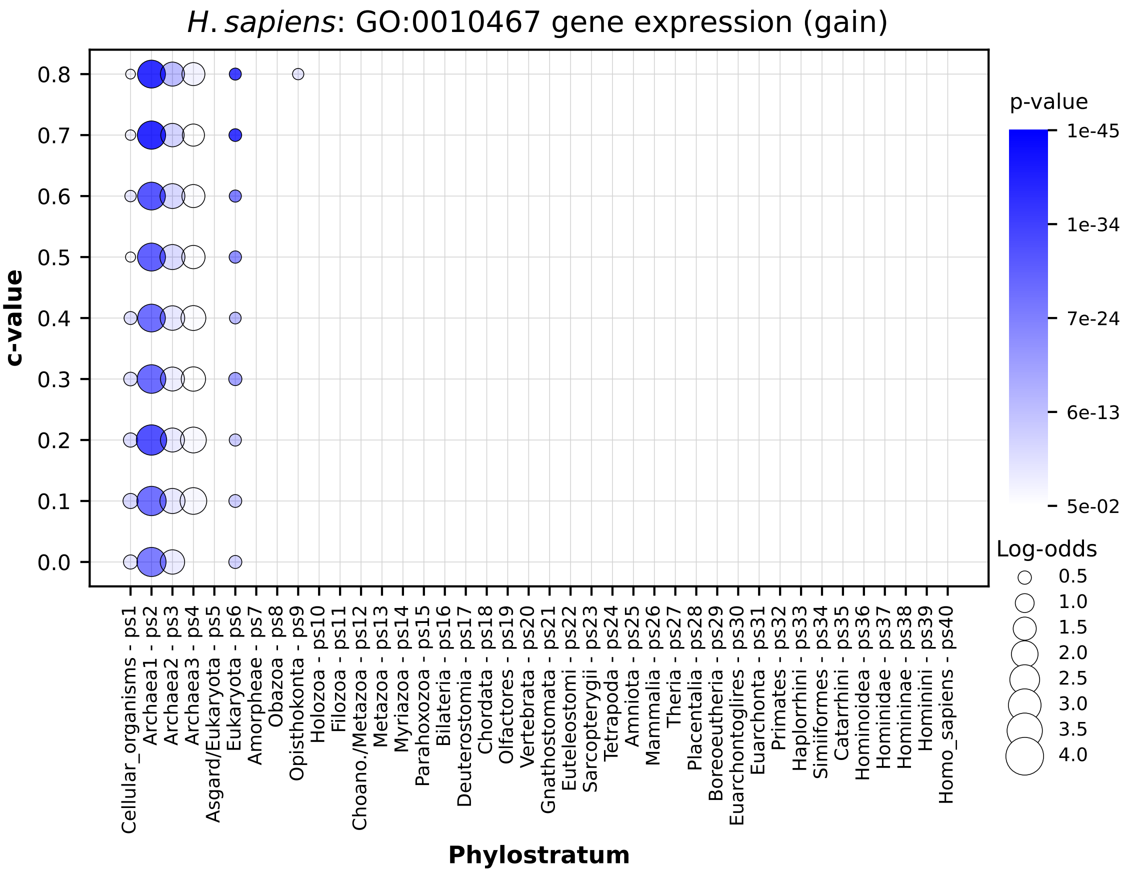

H. sapiens: GO:0010467 gene expression (gain)
c-value
0.8
0.7
0.6
0.5
0.4
0.3
0.2
0.1
0.0
Phylostratum
Cellular_organisms - ps1
Archaea1 - ps2
Archaea2 - ps3
Archaea3 - ps4
Asgard/Eukaryota - ps5
Eukaryota - ps6
Amorpheae - ps7
Obazoa - ps8
Opisthokonta - ps9
Holozoa - ps10
Filozoa - ps11
Choano./Metazoa - ps12
Metazoa - ps13
Myriazoa - ps14
Parahoxozoa - ps15
Bilateria - ps16
Deuterostomia - ps17
Chordata - ps18
Olfactores - ps19
Vertebrata - ps20
Gnathostomata - ps21
Euteleostomi - ps22
Sarcopterygii - ps23
Tetrapoda - ps24
Amniota - ps25
Mammalia - ps26
Theria - ps27
Placentalia - ps28
Boreoeutheria - ps29
Euarchontoglires - ps30
Euarchonta - ps31
Primates - ps32
Haplorrhini - ps33
Simiiformes - ps34
Catarrhini - ps35
Hominoidea - ps36
Hominidae - ps37
Homininae - ps38
Hominini - ps39
Homo_sapiens - ps40
p-value
1e-45
1e-34
7e-24
6e-13
5e-02
Log-odds
0.5
1.0
1.5
2.0
2.5
3.0
3.5
4.0

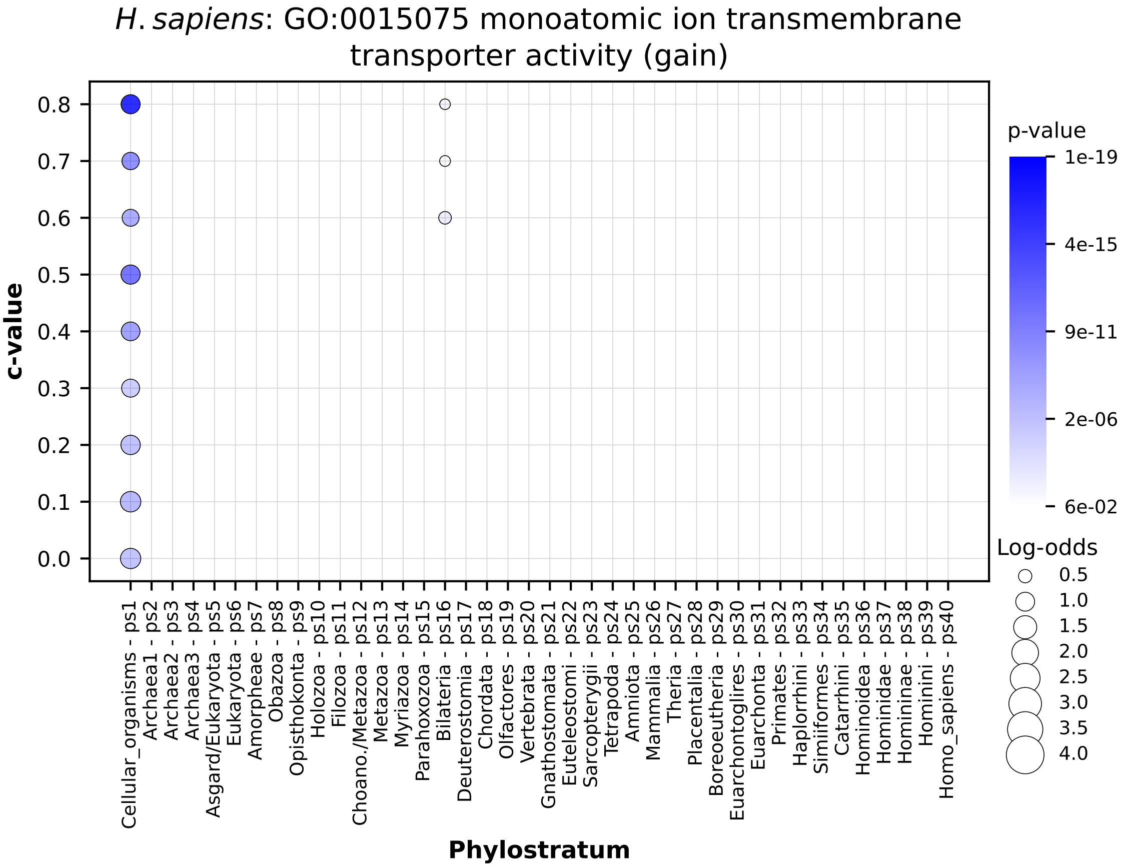

*H. sapiens*: GO:0015075 monoatomic ion transmembrane transporter activity (gain)
c-value
0.8
0.7
0.6
0.5
0.4
0.3
0.2
0.1
0.0
p-value
1e-19
4e-15
9e-11
2e-06
6e-02
Log-odds
0.5
1.0
1.5
2.0
2.5
3.0
3.5
4.0
Cellular_organisms - ps1
Archaea1 - ps2
Archaea2 - ps3
Archaea3 - ps4
Asgard/Eukaryota - ps5
Eukaryota - ps6
Amorpheae - ps7
Obazoa - ps8
Opisthokonta - ps9
Holozoa - ps10
Filozoa - ps11
Choano./Metazoa - ps12
Metazoa - ps13
Myriazoa - ps14
Parahoxozoa - ps15
Bilateria - ps16
Deuterostomia - ps17
Chordata - ps18
Olfactores - ps19
Vertebrata - ps20
Gnathostomata - ps21
Euteleostomi - ps22
Sarcopterygii - ps23
Tetrapoda - ps24
Amniota - ps25
Mammalia - ps26
Theria - ps27
Placentalia - ps28
Boreoeutheria - ps29
Euarchontoglires - ps30
Euarchonta - ps31
Primates - ps32
Haplorrhini - ps33
Simiiformes - ps34
Catarrhini - ps35
Hominoidea - ps36
Hominidae - ps37
Homininae - ps38
Hominini - ps39
Homo_sapiens - ps40
Phylostratum

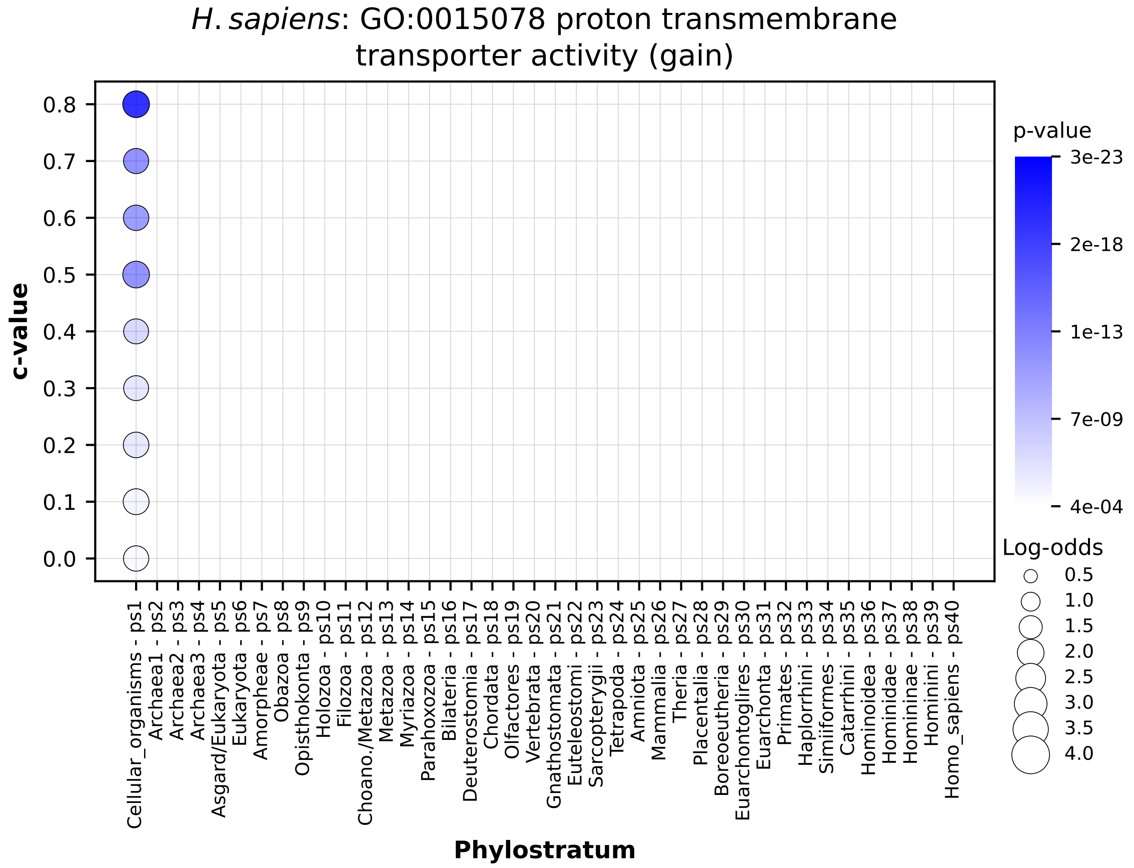

*H. sapiens*: GO:0015078 proton transmembrane transporter activity (gain)
c-value
0.8
0.7
0.6
0.5
0.4
0.3
0.2
0.1
0.0
p-value
3e-23
2e-18
1e-13
7e-09
4e-04
Log-odds
0.5
1.0
1.5
2.0
2.5
3.0
3.5
4.0
Cellular_organisms - ps1
Archaea1 - ps2
Archaea2 - ps3
Archaea3 - ps4
Asgard/Eukaryota - ps5
Eukaryota - ps6
Amorpheae - ps7
Obazoa - ps8
Opisthokonta - ps9
Holozoa - ps10
Filozoa - ps11
Choano./Metazoa - ps12
Metazoa - ps13
Myriazoa - ps14
Parahoxozoa - ps15
Bilateria - ps16
Deuterostomia - ps17
Chordata - ps18
Olfactores - ps19
Vertebrata - ps20
Gnathostomata - ps21
Euteleostomi - ps22
Sarcopterygii - ps23
Tetrapoda - ps24
Amniota - ps25
Mammalia - ps26
Theria - ps27
Placentalia - ps28
Boreoeutheria - ps29
Euarchontoglires - ps30
Euarchonta - ps31
Primates - ps32
Haplorrhini - ps33
Simiiformes - ps34
Catarrhini - ps35
Hominoidea - ps36
Hominidae - ps37
Homininae - ps38
Hominini - ps39
Homo_sapiens - ps40
Phylostratum

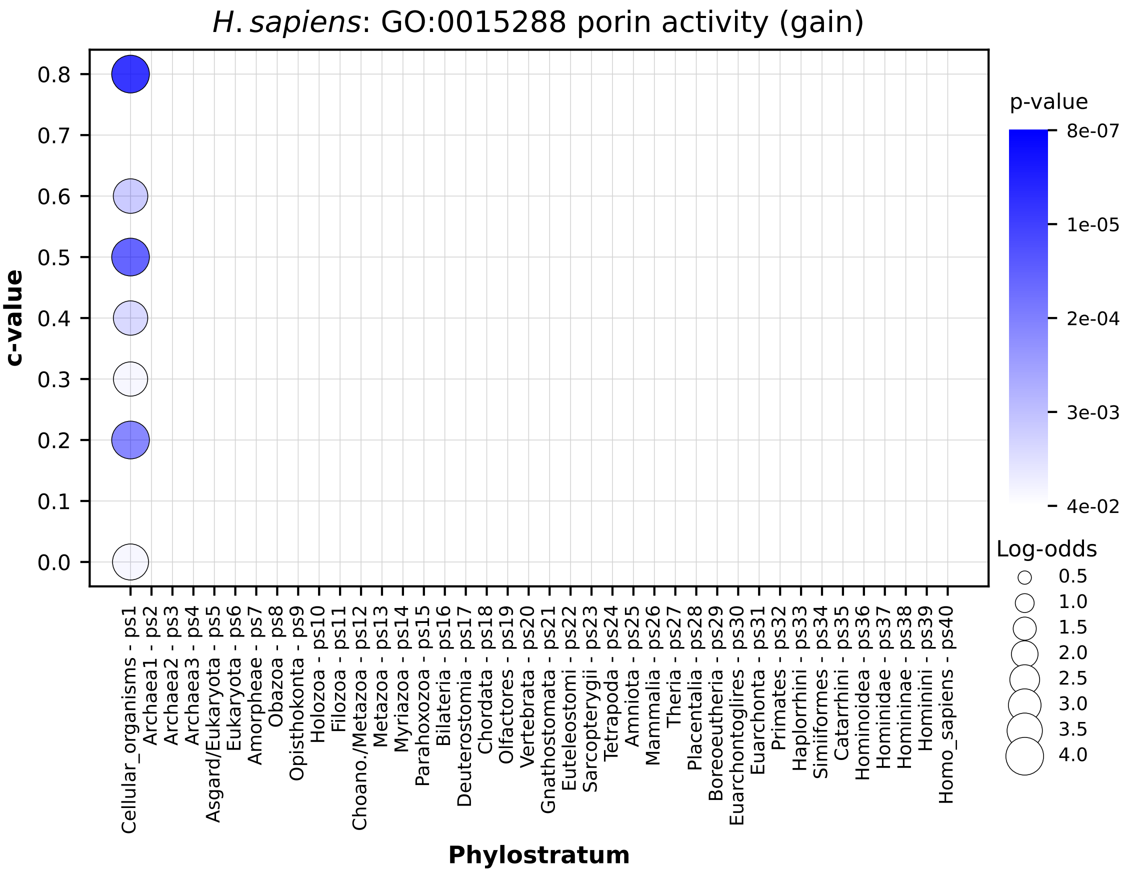

H. sapiens: GO:0015288 porin activity (gain)
c-value
0.8
0.7
0.6
0.5
0.4
0.3
0.2
0.1
0.0
p-value
8e-07
1e-05
2e-04
3e-03
4e-02
Log-odds
0.5
1.0
1.5
2.0
2.5
3.0
3.5
4.0
Cellular_organisms - ps1
Archaea1 - ps2
Archaea2 - ps3
Archaea3 - ps4
Asgard/Eukaryota - ps5
Eukaryota - ps6
Amorpheae - ps7
Obazoa - ps8
Opisthokonta - ps9
Holozoa - ps10
Filozoa - ps11
Choano./Metazoa - ps12
Metazoa - ps13
Myriazoa - ps14
Parahoxozoa - ps15
Bilateria - ps16
Deuterostomia - ps17
Chordata - ps18
Olfactores - ps19
Vertebrata - ps20
Gnathostomata - ps21
Euteleostomi - ps22
Sarcopterygii - ps23
Tetrapoda - ps24
Amniota - ps25
Mammalia - ps26
Theria - ps27
Placentalia - ps28
Boreoeutheria - ps29
Euarchontoglires - ps30
Euarchonta - ps31
Primates - ps32
Haplorrhini - ps33
Simiiformes - ps34
Catarrhini - ps35
Hominoidea - ps36
Hominidae - ps37
Homininae - ps38
Hominini - ps39
Homo_sapiens - ps40
Phylostratum

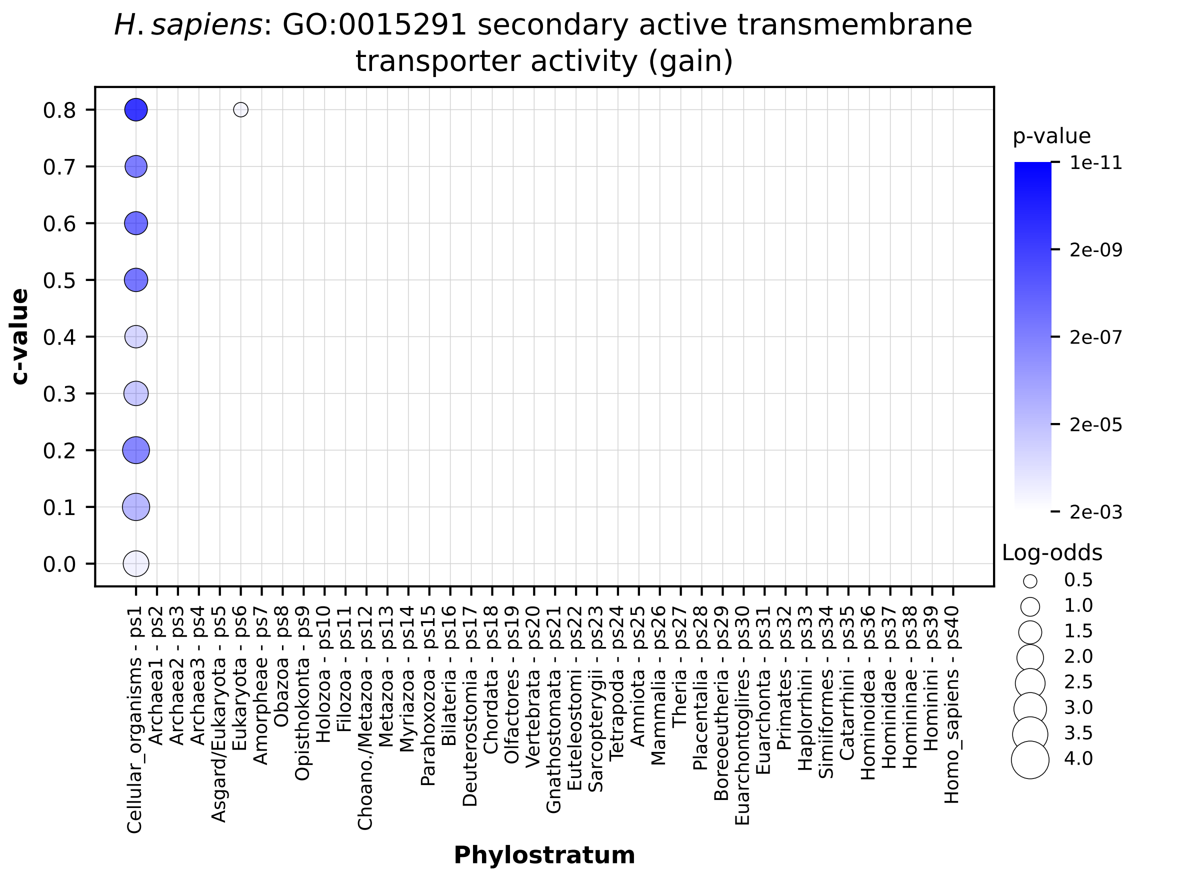

H. sapiens: GO:0015291 secondary active transmembrane transporter activity (gain)
c-value
0.0
0.1
0.2
0.3
0.4
0.5
0.6
0.7
0.8
Phylostratum
Cellular_organisms - ps1
Archaea1 - ps2
Archaea2 - ps3
Archaea3 - ps4
Asgard/Eukaryota - ps5
Eukaryota - ps6
Amorpheae - ps7
Obazoa - ps8
Opisthokonta - ps9
Holozoa - ps10
Filozoa - ps11
Choano./Metazoa - ps12
Metazoa - ps13
Myriazoa - ps14
Parahoxozoa - ps15
Bilateria - ps16
Deuterostomia - ps17
Chordata - ps18
Olfactores - ps19
Vertebrata - ps20
Gnathostomata - ps21
Euteleostomi - ps22
Sarcopterygii - ps23
Tetrapoda - ps24
Amniota - ps25
Mammalia - ps26
Theria - ps27
Placentalia - ps28
Boreoeutheria - ps29
Euarchontoglires - ps30
Euarchonta - ps31
Primates - ps32
Haplorrhini - ps33
Simiiformes - ps34
Catarrhini - ps35
Hominoidea - ps36
Hominidae - ps37
Homininae - ps38
Hominini - ps39
Homo_sapiens - ps40
p-value
1e-11
2e-09
2e-07
2e-05
2e-03
Log-odds
0.5
1.0
1.5
2.0
2.5
3.0
3.5
4.0

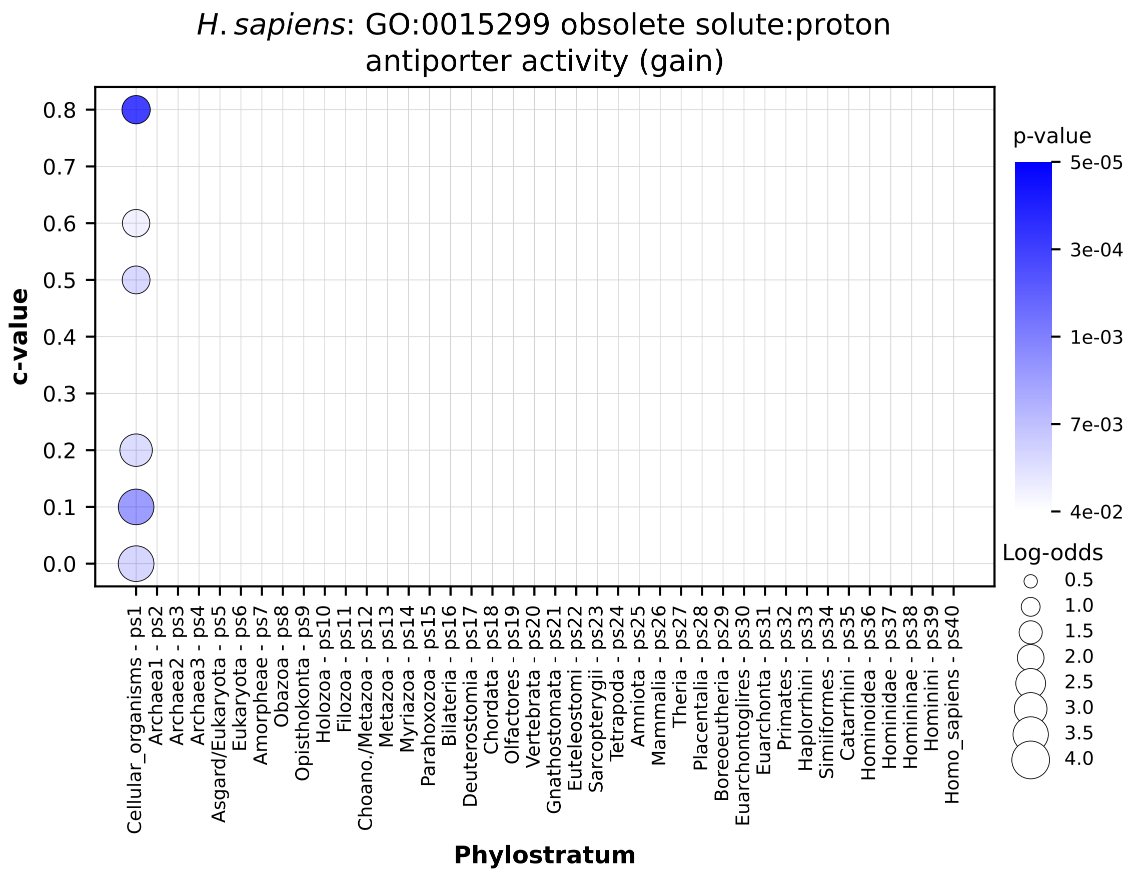

H. sapiens: GO:0015299 obsolete solute:proton antiporter activity (gain)
c-value
0.8
0.7
0.6
0.5
0.4
0.3
0.2
0.1
0.0
p-value
5e-05
3e-04
1e-03
7e-03
4e-02
Log-odds
0.5
1.0
1.5
2.0
2.5
3.0
3.5
4.0
Cellular_organisms - ps1
Archaea1 - ps2
Archaea2 - ps3
Archaea3 - ps4
Asgard/Eukaryota - ps5
Eukaryota - ps6
Amorpheae - ps7
Obazoa - ps8
Opisthokonta - ps9
Holozoa - ps10
Filozoa - ps11
Choano./Metazoa - ps12
Metazoa - ps13
Myriazoa - ps14
Parahoxozoa - ps15
Bilateria - ps16
Deuterostomia - ps17
Chordata - ps18
Olfactores - ps19
Vertebrata - ps20
Gnathostomata - ps21
Euteleostomi - ps22
Sarcopterygii - ps23
Tetrapoda - ps24
Amniota - ps25
Mammalia - ps26
Theria - ps27
Placentalia - ps28
Boreoeutheria - ps29
Euarchontoglires - ps30
Euarchonta - ps31
Primates - ps32
Haplorrhini - ps33
Simiiformes - ps34
Catarrhini - ps35
Hominoidea - ps36
Hominidae - ps37
Homininae - ps38
Hominini - ps39
Homo_sapiens - ps40
Phylostratum

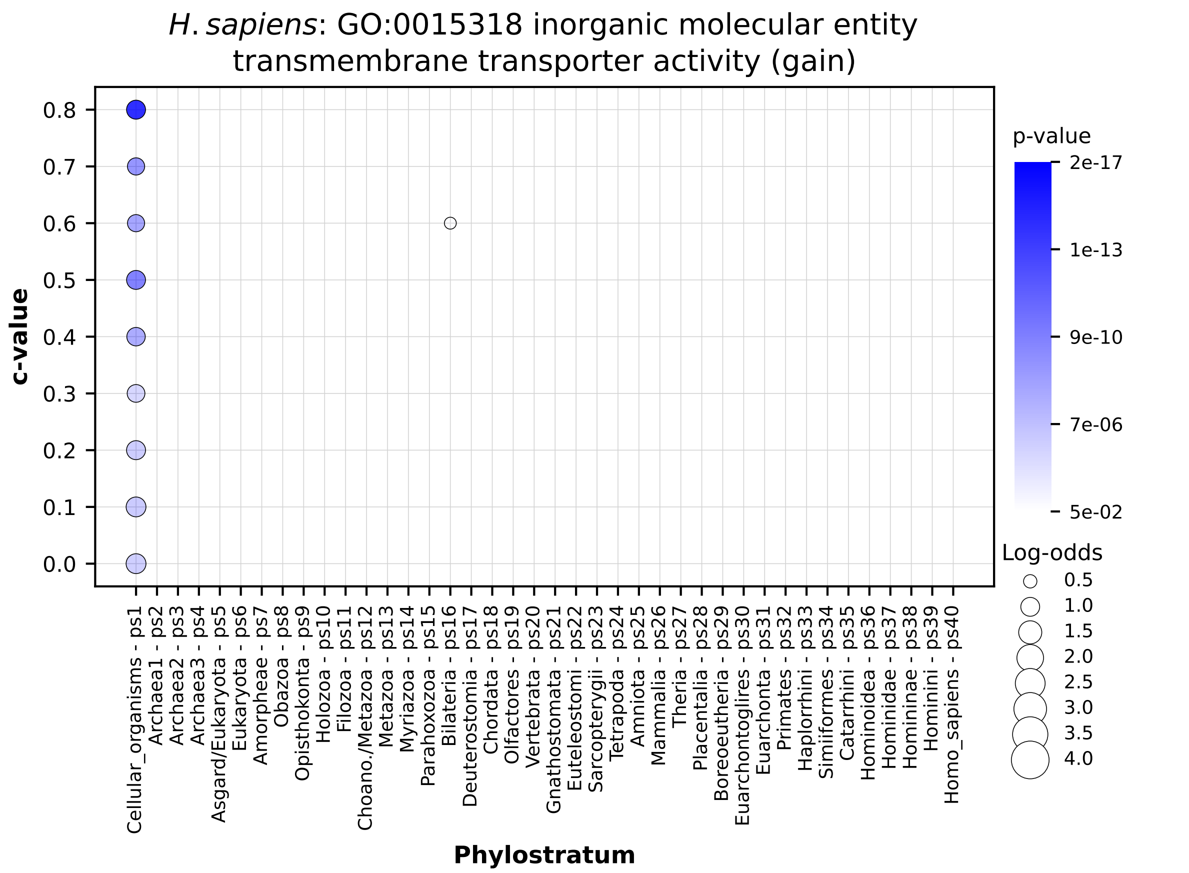

H. sapiens: GO:0015318 inorganic molecular entity transmembrane transporter activity (gain)
c-value
0.0
0.1
0.2
0.3
0.4
0.5
0.6
0.7
0.8
Phylostratum
Cellular_organisms - ps1
Archaea1 - ps2
Archaea2 - ps3
Archaea3 - ps4
Asgard/Eukaryota - ps5
Eukaryota - ps6
Amorpheae - ps7
Obazoa - ps8
Opisthokonta - ps9
Holozoa - ps10
Filozoa - ps11
Choano./Metazoa - ps12
Metazoa - ps13
Myriazoa - ps14
Parahoxozoa - ps15
Bilateria - ps16
Deuterostomia - ps17
Chordata - ps18
Olfactores - ps19
Vertebrata - ps20
Gnathostomata - ps21
Euteleostomi - ps22
Sarcopterygii - ps23
Tetrapoda - ps24
Amniota - ps25
Mammalia - ps26
Theria - ps27
Placentalia - ps28
Boreoeutheria - ps29
Euarchontoglires - ps30
Euarchonta - ps31
Primates - ps32
Haplorrhini - ps33
Simiiformes - ps34
Catarrhini - ps35
Hominoidea - ps36
Hominidae - ps37
Homininae - ps38
Hominini - ps39
Homo_sapiens - ps40
p-value
2e-17
1e-13
9e-10
7e-06
5e-02
Log-odds
0.5
1.0
1.5
2.0
2.5
3.0
3.5
4.0

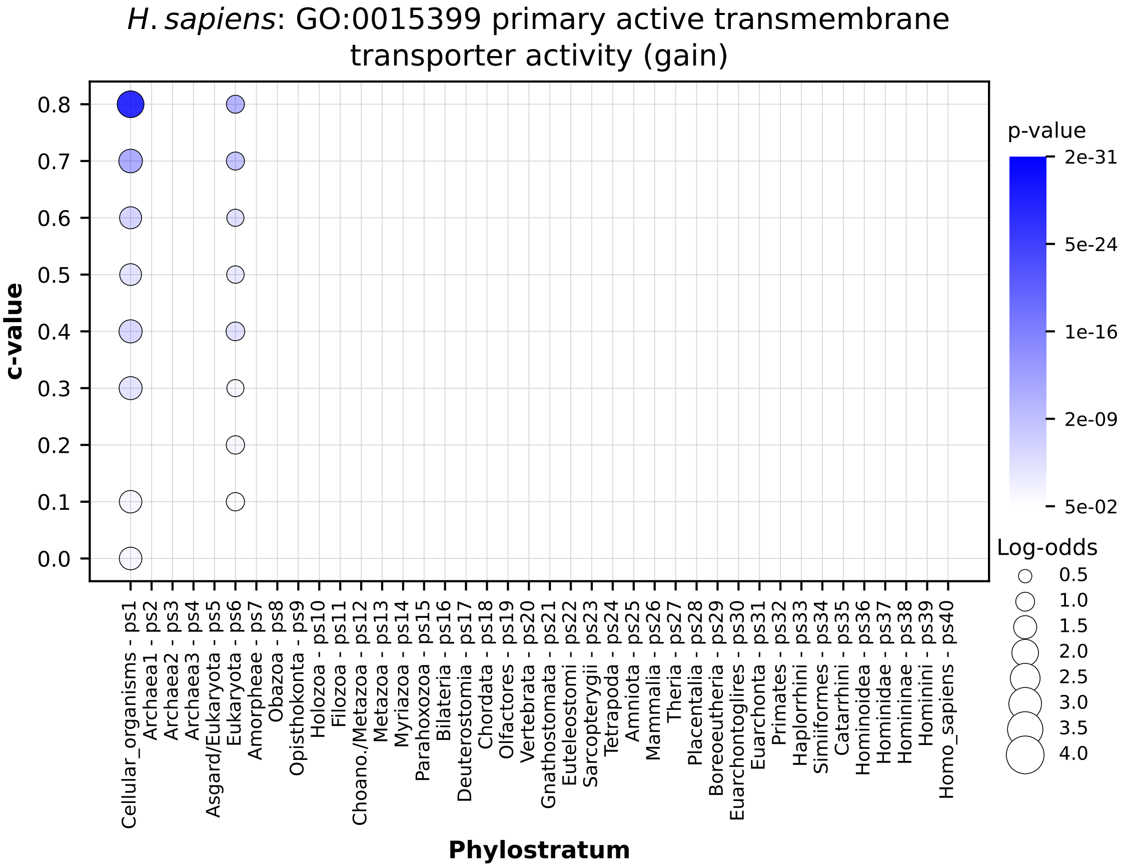

H. sapiens: GO:0015399 primary active transmembrane transporter activity (gain)
c-value
0.8
0.7
0.6
0.5
0.4
0.3
0.2
0.1
0.0
p-value
2e-31
5e-24
1e-16
2e-09
5e-02
Log-odds
0.5
1.0
1.5
2.0
2.5
3.0
3.5
4.0
Cellular_organisms - ps1
Archaea1 - ps2
Archaea2 - ps3
Archaea3 - ps4
Asgard/Eukaryota - ps5
Eukaryota - ps6
Amorpheae - ps7
Obazoa - ps8
Opisthokonta - ps9
Holozoa - ps10
Filozoa - ps11
Choano./Metazoa - ps12
Metazoa - ps13
Myriazoa - ps14
Parahoxozoa - ps15
Bilateria - ps16
Deuterostomia - ps17
Chordata - ps18
Olfactores - ps19
Vertebrata - ps20
Gnathostomata - ps21
Euteleostomi - ps22
Sarcopterygii - ps23
Tetrapoda - ps24
Amniota - ps25
Mammalia - ps26
Theria - ps27
Placentalia - ps28
Boreoeutheria - ps29
Euarchontoglires - ps30
Euarchonta - ps31
Primates - ps32
Haplorrhini - ps33
Simiiformes - ps34
Catarrhini - ps35
Hominoidea - ps36
Hominidae - ps37
Homininae - ps38
Hominini - ps39
Homo_sapiens - ps40
Phylostratum

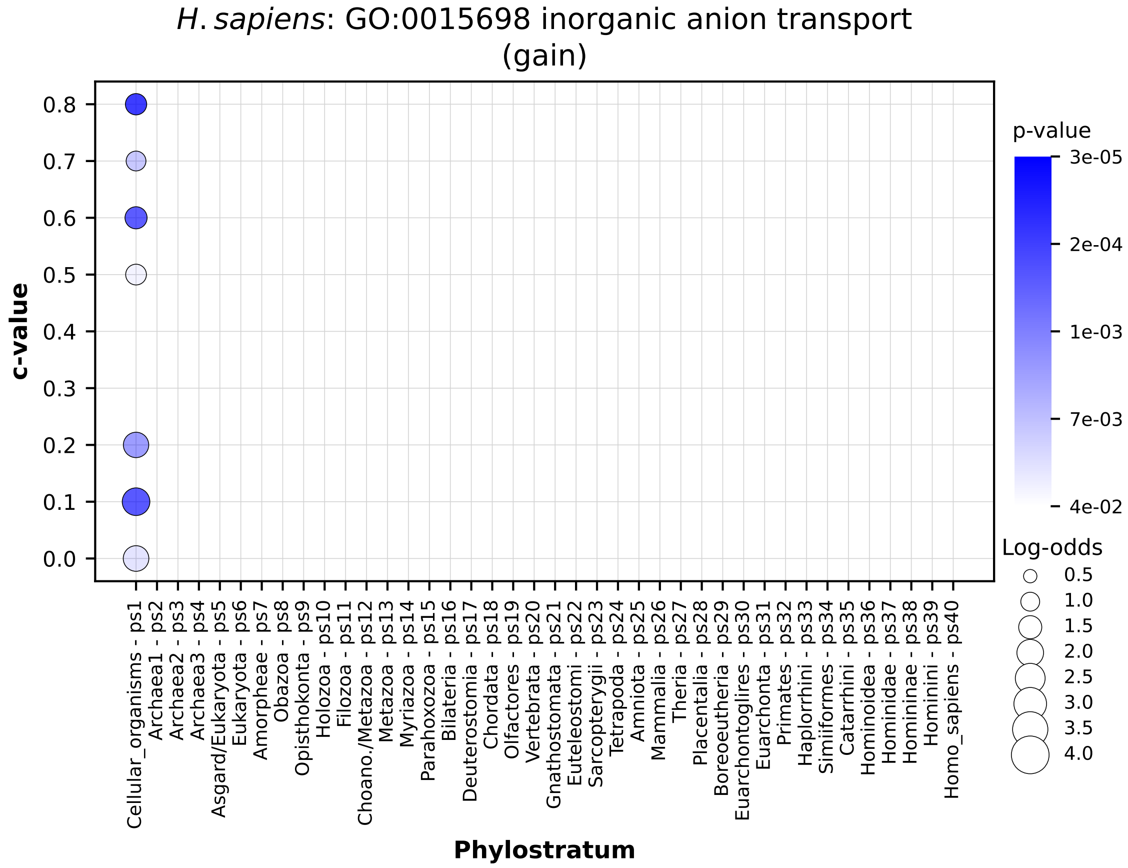

H. sapiens: GO:0015698 inorganic anion transport
(gain)
c-value
0.8
0.7
0.6
0.5
0.4
0.3
0.2
0.1
0.0
p-value
3e-05
2e-04
1e-03
7e-03
4e-02
Log-odds
0.5
1.0
1.5
2.0
2.5
3.0
3.5
4.0
Cellular_organisms - ps1
Archaea1 - ps2
Archaea2 - ps3
Archaea3 - ps4
Asgard/Eukaryota - ps5
Eukaryota - ps6
Amorpheae - ps7
Obazoa - ps8
Opisthokonta - ps9
Holozoa - ps10
Filozoa - ps11
Choano./Metazoa - ps12
Metazoa - ps13
Myriazoa - ps14
Parahoxozoa - ps15
Bilateria - ps16
Deuterostomia - ps17
Chordata - ps18
Olfactores - ps19
Vertebrata - ps20
Gnathostomata - ps21
Euteleostomi - ps22
Sarcopterygii - ps23
Tetrapoda - ps24
Amniota - ps25
Mammalia - ps26
Theria - ps27
Placentalia - ps28
Boreoeutheria - ps29
Euarchontoglires - ps30
Euarchonta - ps31
Primates - ps32
Haplorrhini - ps33
Simiiformes - ps34
Catarrhini - ps35
Hominoidea - ps36
Hominidae - ps37
Homininae - ps38
Hominini - ps39
Homo_sapiens - ps40
Phylostratum

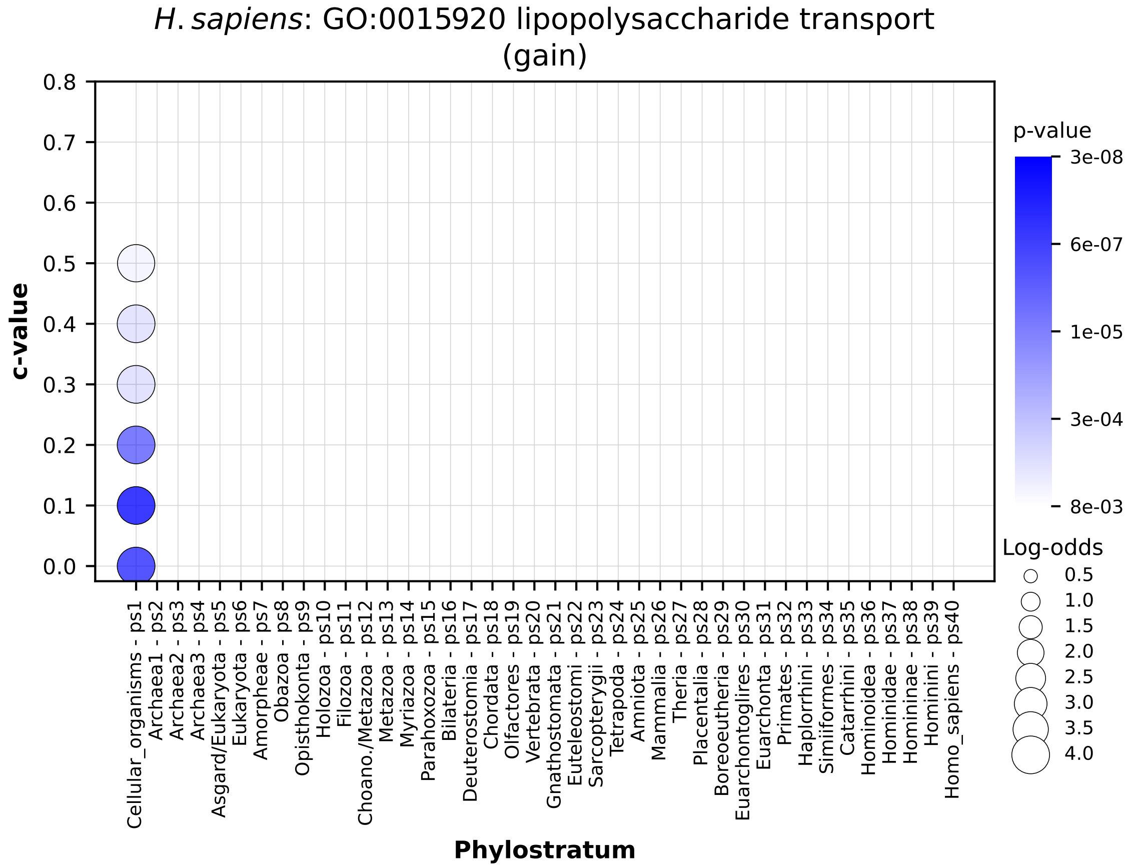

*H. sapiens*: GO:0015920 lipopolysaccharide transport
(gain)
c-value
0.8
0.7
0.6
0.5
0.4
0.3
0.2
0.1
0.0
Phylostratum
Cellular_organisms - ps1
Archaea1 - ps2
Archaea2 - ps3
Archaea3 - ps4
Asgard/Eukaryota - ps5
Eukaryota - ps6
Amorpheae - ps7
Obazoa - ps8
Opisthokonta - ps9
Holozoa - ps10
Filozoa - ps11
Choano./Metazoa - ps12
Metazoa - ps13
Myriazoa - ps14
Parahoxozoa - ps15
Bilateria - ps16
Deuterostomia - ps17
Chordata - ps18
Olfactores - ps19
Vertebrata - ps20
Gnathostomata - ps21
Euteleostomi - ps22
Sarcopterygii - ps23
Tetrapoda - ps24
Amniota - ps25
Mammalia - ps26
Theria - ps27
Placentalia - ps28
Boreoeutheria - ps29
Euarchontoglires - ps30
Euarchonta - ps31
Primates - ps32
Haplorrhini - ps33
Simiiformes - ps34
Catarrhini - ps35
Hominoidea - ps36
Hominidae - ps37
Homininae - ps38
Hominini - ps39
Homo_sapiens - ps40
p-value
3e-08
6e-07
1e-05
3e-04
8e-03
Log-odds
0.5
1.0
1.5
2.0
2.5
3.0
3.5
4.0

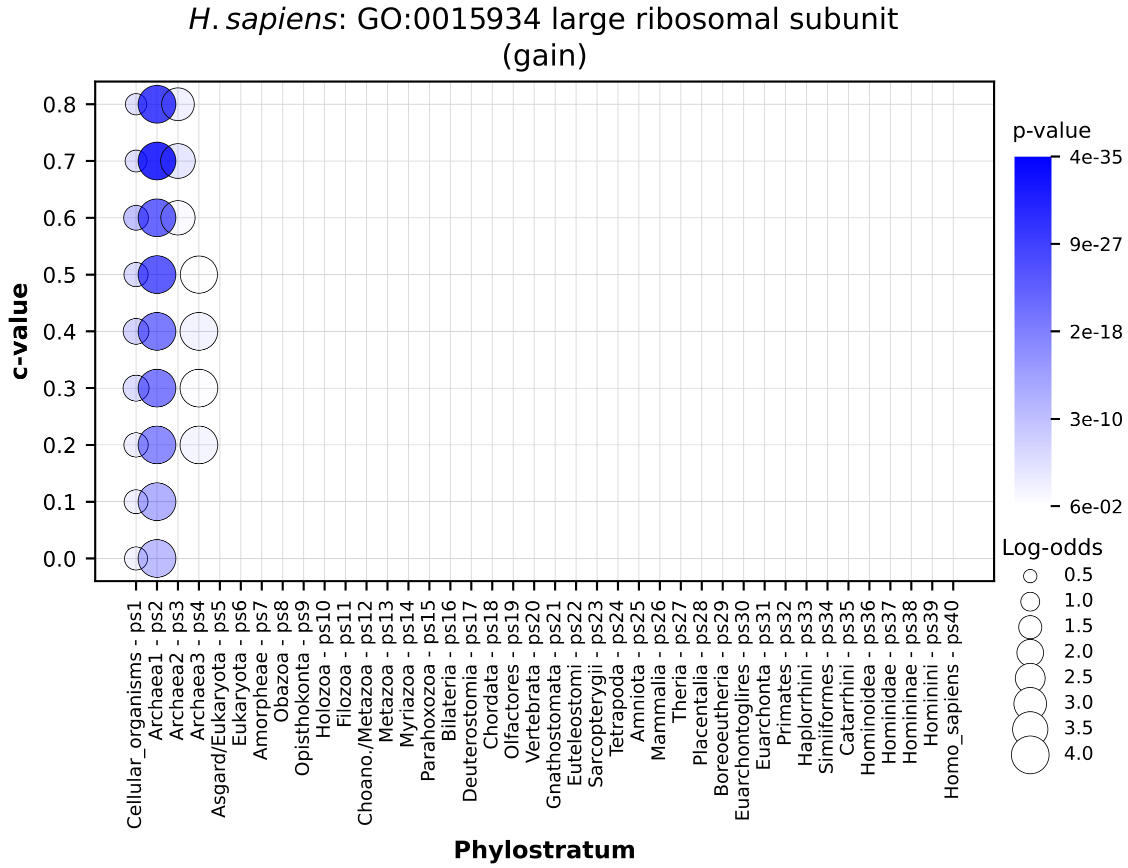

H. sapiens: GO:0015934 large ribosomal subunit
(gain)
c-value
0.8
0.7
0.6
0.5
0.4
0.3
0.2
0.1
0.0
p-value
4e-35
9e-27
2e-18
3e-10
6e-02
Log-odds
0.5
1.0
1.5
2.0
2.5
3.0
3.5
4.0
Cellular_organisms - ps1
Archaea1 - ps2
Archaea2 - ps3
Archaea3 - ps4
Asgard/Eukaryota - ps5
Eukaryota - ps6
Amorpheae - ps7
Obazoa - ps8
Opisthokonta - ps9
Holozoa - ps10
Filozoa - ps11
Choano./Metazoa - ps12
Metazoa - ps13
Myriazoa - ps14
Parahoxozoa - ps15
Bilateria - ps16
Deuterostomia - ps17
Chordata - ps18
Olfactores - ps19
Vertebrata - ps20
Gnathostomata - ps21
Euteleostomi - ps22
Sarcopterygii - ps23
Tetrapoda - ps24
Amniota - ps25
Mammalia - ps26
Theria - ps27
Placentalia - ps28
Boreoeutheria - ps29
Euarchontoglires - ps30
Euarchonta - ps31
Primates - ps32
Haplorrhini - ps33
Simiiformes - ps34
Catarrhini - ps35
Hominoidea - ps36
Hominidae - ps37
Homininae - ps38
Hominini - ps39
Homo_sapiens - ps40
Phylostratum

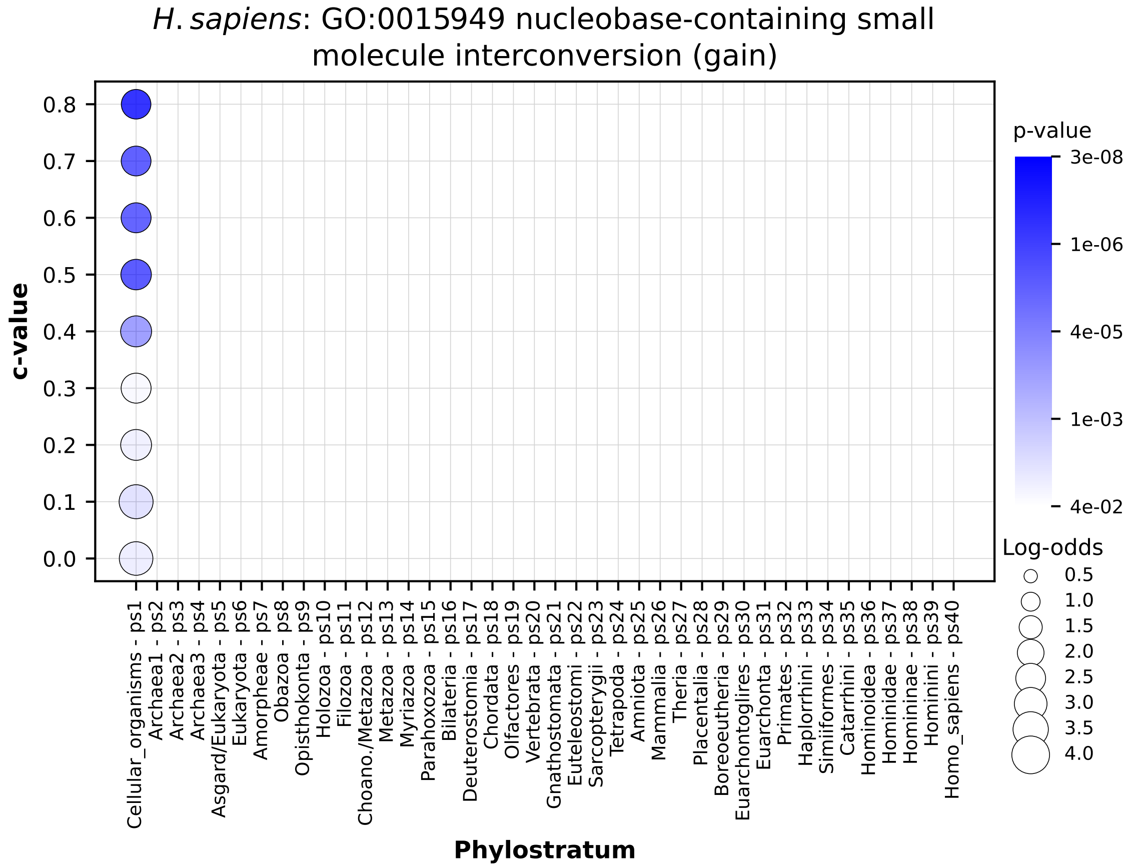

*H. sapiens*: GO:0015949 nucleobase-containing small molecule interconversion (gain)
c-value
0.8
0.7
0.6
0.5
0.4
0.3
0.2
0.1
0.0
p-value
3e-08
1e-06
4e-05
1e-03
4e-02
Log-odds
0.5
1.0
1.5
2.0
2.5
3.0
3.5
4.0
Cellular_organisms - ps1
Archaea1 - ps2
Archaea2 - ps3
Archaea3 - ps4
Asgard/Eukaryota - ps5
Eukaryota - ps6
Amorpheae - ps7
Obazoa - ps8
Opisthokonta - ps9
Holozoa - ps10
Filozoa - ps11
Choano./Metazoa - ps12
Metazoa - ps13
Myriazoa - ps14
Parahoxozoa - ps15
Bilateria - ps16
Deuterostomia - ps17
Chordata - ps18
Olfactores - ps19
Vertebrata - ps20
Gnathostomata - ps21
Euteleostomi - ps22
Sarcopterygii - ps23
Tetrapoda - ps24
Amniota - ps25
Mammalia - ps26
Theria - ps27
Placentalia - ps28
Boreoeutheria - ps29
Euarchontoglires - ps30
Euarchonta - ps31
Primates - ps32
Haplorrhini - ps33
Simiiformes - ps34
Catarrhini - ps35
Hominoidea - ps36
Hominidae - ps37
Homininae - ps38
Hominini - ps39
Homo_sapiens - ps40
Phylostratum

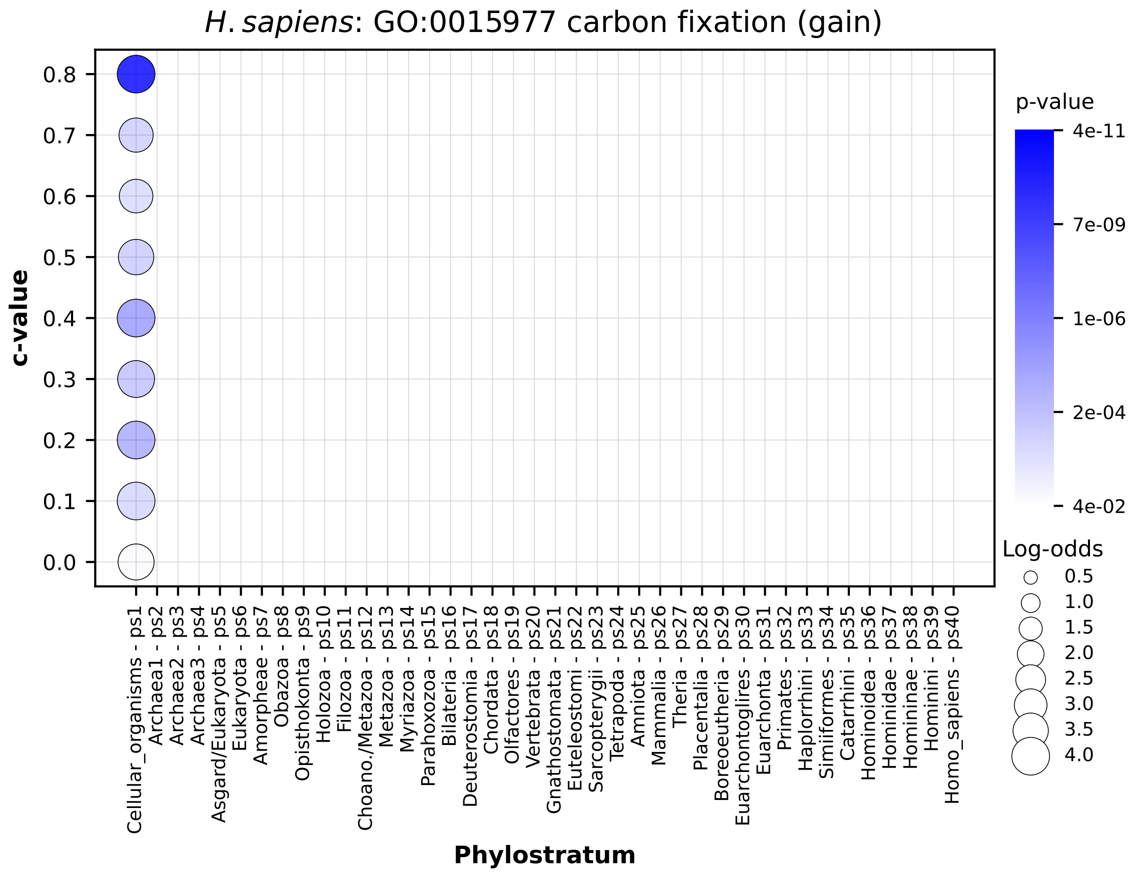

H. sapiens: GO:0015977 carbon fixation (gain)
c-value
0.8
0.7
0.6
0.5
0.4
0.3
0.2
0.1
0.0
p-value
4e-11
7e-09
1e-06
2e-04
4e-02
Log-odds
0.5
1.0
1.5
2.0
2.5
3.0
3.5
4.0
Cellular_organisms - ps1
Archaea1 - ps2
Archaea2 - ps3
Archaea3 - ps4
Asgard/Eukaryota - ps5
Eukaryota - ps6
Amorpheae - ps7
Obazoa - ps8
Opisthokonta - ps9
Holozoa - ps10
Filozoa - ps11
Choano./Metazoa - ps12
Metazoa - ps13
Myriazoa - ps14
Parahoxozoa - ps15
Bilateria - ps16
Deuterostomia - ps17
Chordata - ps18
Olfactores - ps19
Vertebrata - ps20
Gnathostomata - ps21
Euteleostomi - ps22
Sarcopterygii - ps23
Tetrapoda - ps24
Amniota - ps25
Mammalia - ps26
Theria - ps27
Placentalia - ps28
Boreoeutheria - ps29
Euarchontoglires - ps30
Euarchonta - ps31
Primates - ps32
Haplorrhini - ps33
Simiiformes - ps34
Catarrhini - ps35
Hominoidea - ps36
Hominidae - ps37
Homininae - ps38
Hominini - ps39
Homo_sapiens - ps40
Phylostratum

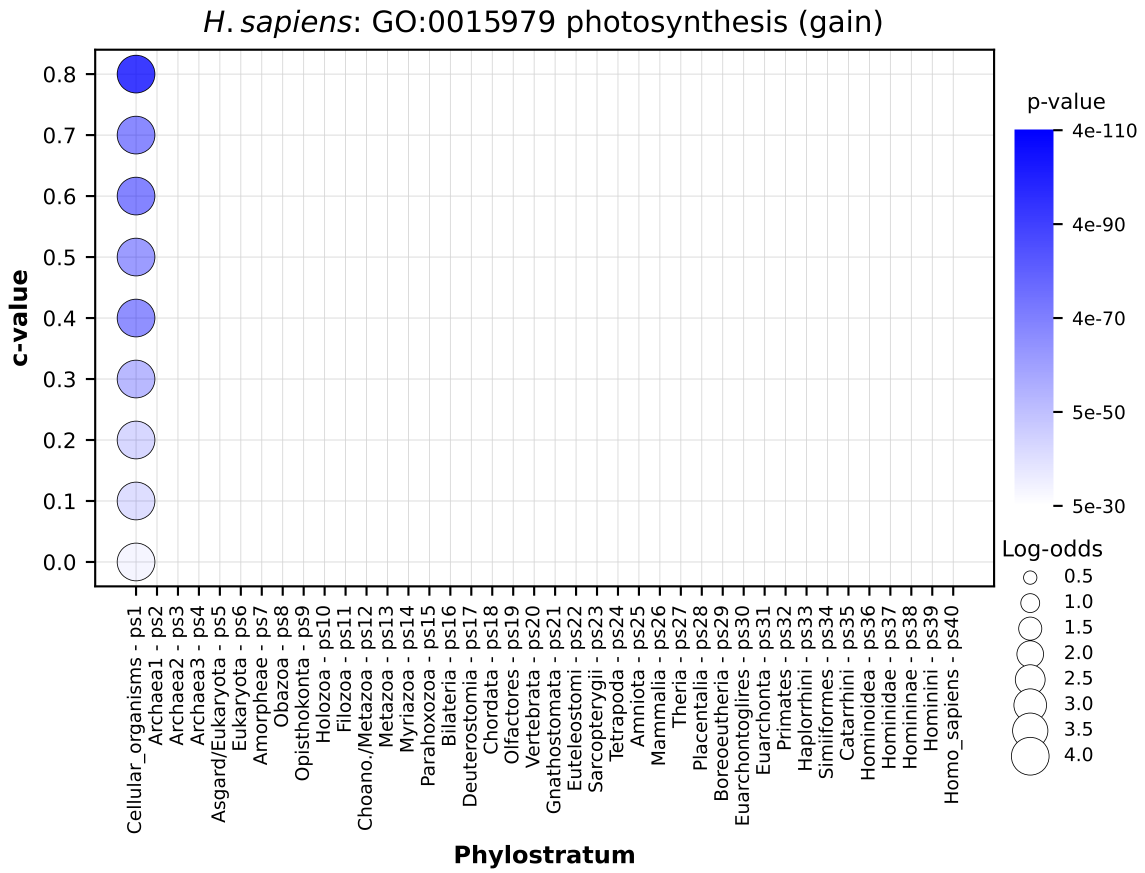
H. sapiens: GO:0015979 photosynthesis (gain)
c-value
0.8
0.7
0.6
0.5
0.4
0.3
0.2
0.1
0.0
Phylostratum
Cellular_organisms - ps1
Archaea1 - ps2
Archaea2 - ps3
Archaea3 - ps4
Asgard/Eukaryota - ps5
Eukaryota - ps6
Amorpheae - ps7
Obazoa - ps8
Opisthokonta - ps9
Holozoa - ps10
Filozoa - ps11
Choano./Metazoa - ps12
Metazoa - ps13
Myriazoa - ps14
Parahoxozoa - ps15
Bilateria - ps16
Deuterostomia - ps17
Chordata - ps18
Olfactores - ps19
Vertebrata - ps20
Gnathostomata - ps21
Euteleostomi - ps22
Sarcopterygii - ps23
Tetrapoda - ps24
Amniota - ps25
Mammalia - ps26
Theria - ps27
Placentalia - ps28
Boreoeutheria - ps29
Euarchontoglires - ps30
Euarchonta - ps31
Primates - ps32
Haplorrhini - ps33
Simiiformes - ps34
Catarrhini - ps35
Hominoidea - ps36
Hominidae - ps37
Homininae - ps38
Hominini - ps39
Homo_sapiens - ps40
p-value
4e-110
4e-90
4e-70
5e-50
5e-30
Log-odds
0.5
1.0
1.5
2.0
2.5
3.0
3.5
4.0

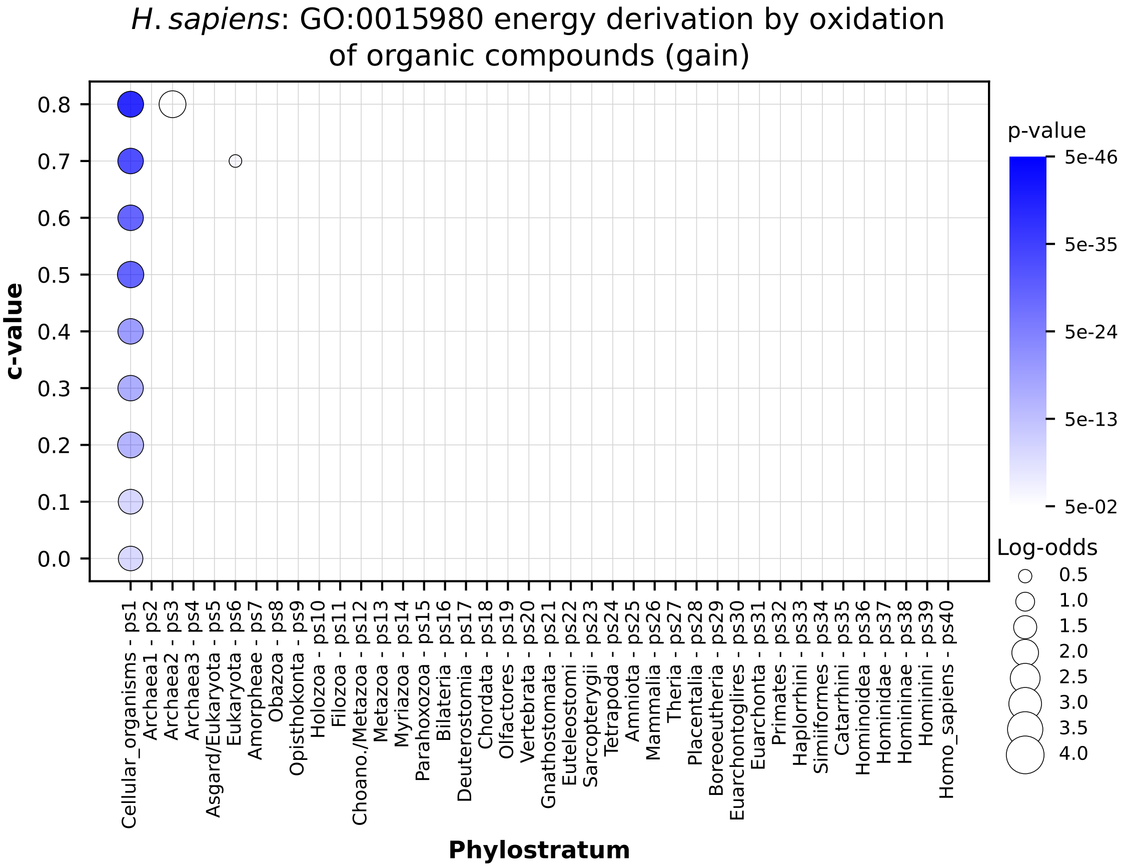

H. sapiens: GO:0015980 energy derivation by oxidation of organic compounds (gain)
c-value
0.8
0.7
0.6
0.5
0.4
0.3
0.2
0.1
0.0
Phylostratum
Cellular_organisms - ps1
Archaea1 - ps2
Archaea2 - ps3
Archaea3 - ps4
Asgard/Eukaryota - ps5
Eukaryota - ps6
Amorpheae - ps7
Obazoa - ps8
Opisthokonta - ps9
Holozoa - ps10
Filozoa - ps11
Choano./Metazoa - ps12
Metazoa - ps13
Myriazoa - ps14
Parahoxozoa - ps15
Bilateria - ps16
Deuterostomia - ps17
Chordata - ps18
Olfactores - ps19
Vertebrata - ps20
Gnathostomata - ps21
Euteleostomi - ps22
Sarcopterygii - ps23
Tetrapoda - ps24
Amniota - ps25
Mammalia - ps26
Theria - ps27
Placentalia - ps28
Boreoeutheria - ps29
Euarchontoglires - ps30
Euarchonta - ps31
Primates - ps32
Haplorrhini - ps33
Simiiformes - ps34
Catarrhini - ps35
Hominoidea - ps36
Hominidae - ps37
Homininae - ps38
Hominini - ps39
Homo_sapiens - ps40
p-value
5e-46
5e-35
5e-24
5e-13
5e-02
Log-odds
0.5
1.0
1.5
2.0
2.5
3.0
3.5
4.0

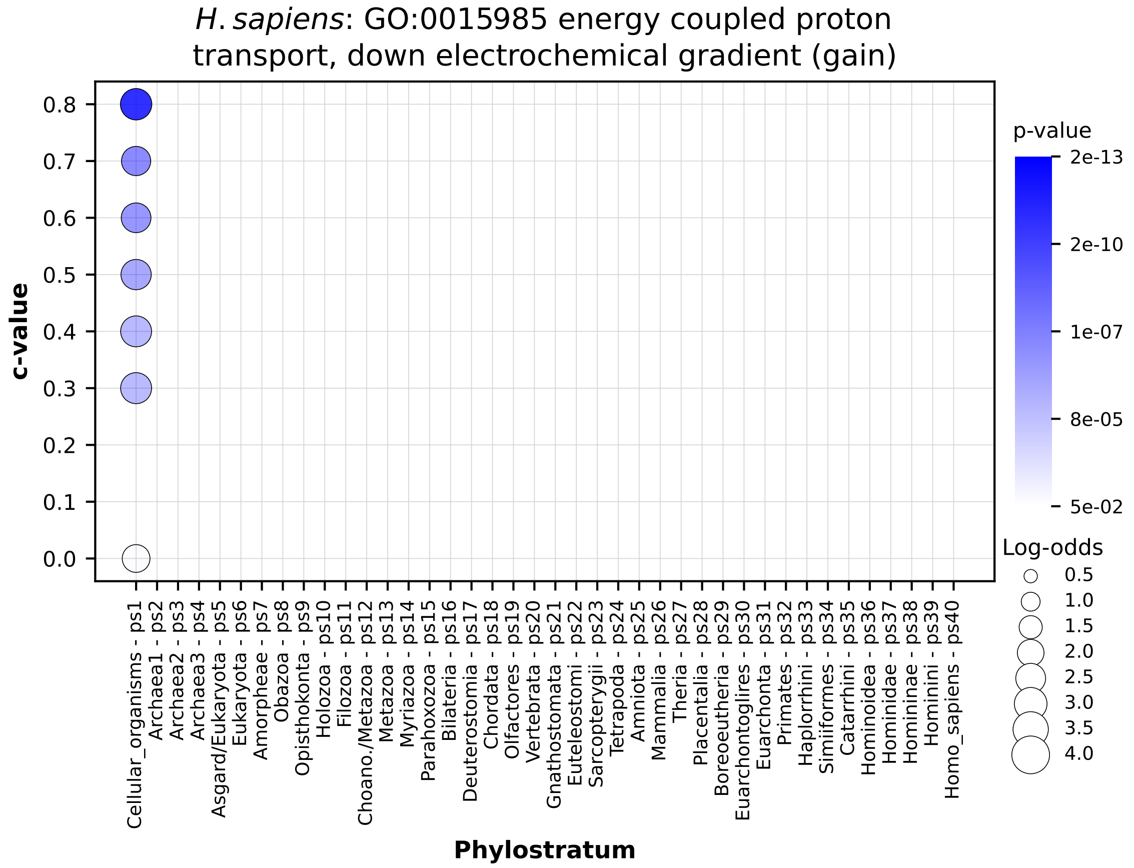

H. sapiens: GO:0015985 energy coupled proton transport, down electrochemical gradient (gain)
c-value
0.8
0.7
0.6
0.5
0.4
0.3
0.2
0.1
0.0
p-value
2e-13
2e-10
1e-07
8e-05
5e-02
Log-odds
0.5
1.0
1.5
2.0
2.5
3.0
3.5
4.0
Cellular_organisms - ps1
Archaea1 - ps2
Archaea2 - ps3
Archaea3 - ps4
Asgard/Eukaryota - ps5
Eukaryota - ps6
Amorpheae - ps7
Obazoa - ps8
Opisthokonta - ps9
Holozoa - ps10
Filozoa - ps11
Choano./Metazoa - ps12
Metazoa - ps13
Myriazoa - ps14
Parahoxozoa - ps15
Bilateria - ps16
Deuterostomia - ps17
Chordata - ps18
Olfactores - ps19
Vertebrata - ps20
Gnathostomata - ps21
Euteleostomi - ps22
Sarcopterygii - ps23
Tetrapoda - ps24
Amniota - ps25
Mammalia - ps26
Theria - ps27
Placentalia - ps28
Boreoeutheria - ps29
Euarchontoglires - ps30
Euarchonta - ps31
Primates - ps32
Haplorrhini - ps33
Simiiformes - ps34
Catarrhini - ps35
Hominoidea - ps36
Hominidae - ps37
Homininae - ps38
Hominini - ps39
Homo_sapiens - ps40
Phylostratum

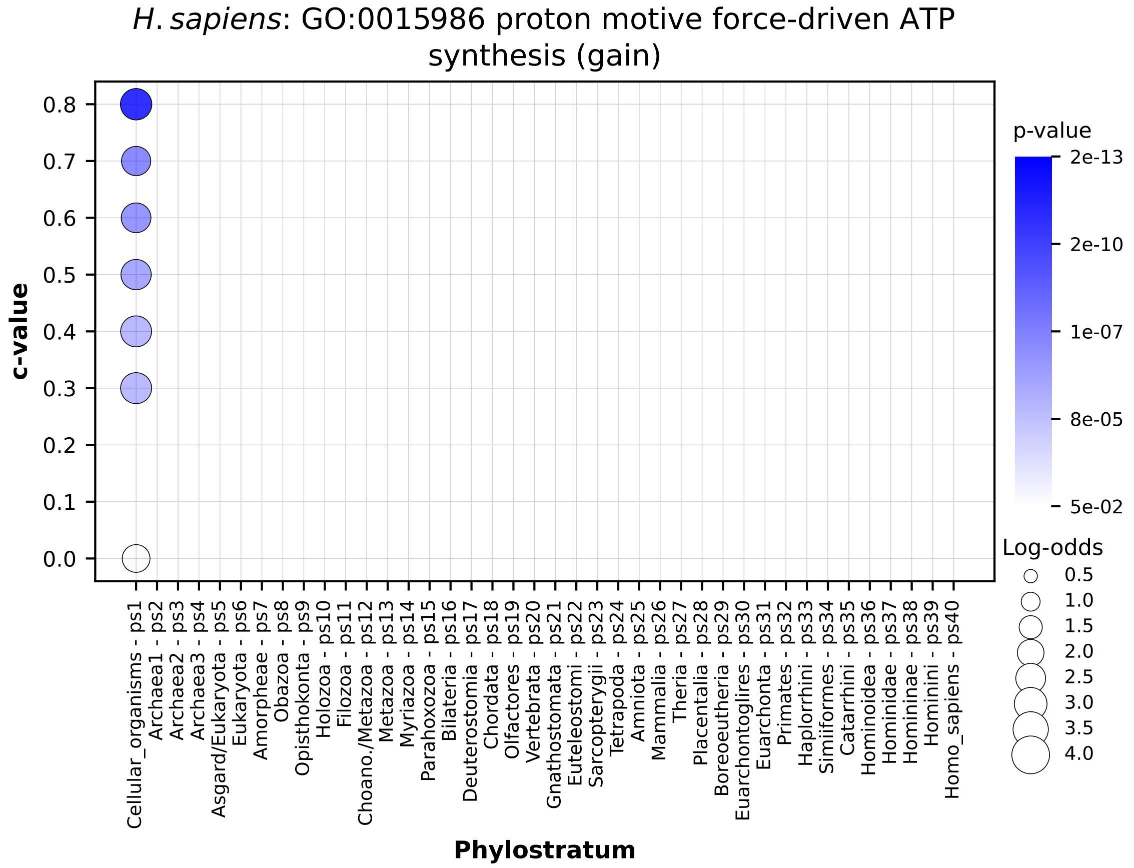
H. sapiens: GO:0015986 proton motive force-driven ATP synthesis (gain)
c-value
0.8
0.7
0.6
0.5
0.4
0.3
0.2
0.1
0.0
p-value
2e-13
2e-10
1e-07
8e-05
5e-02
Log-odds
0.5
1.0
1.5
2.0
2.5
3.0
3.5
4.0
Cellular_organisms - ps1
Archaea1 - ps2
Archaea2 - ps3
Archaea3 - ps4
Asgard/Eukaryota - ps5
Eukaryota - ps6
Amorpheae - ps7
Obazoa - ps8
Opisthokonta - ps9
Holozoa - ps10
Filozoa - ps11
Choano./Metazoa - ps12
Metazoa - ps13
Myriazoa - ps14
Parahoxozoa - ps15
Bilateria - ps16
Deuterostomia - ps17
Chordata - ps18
Olfactores - ps19
Vertebrata - ps20
Gnathostomata - ps21
Euteleostomi - ps22
Sarcopterygii - ps23
Tetrapoda - ps24
Amniota - ps25
Mammalia - ps26
Theria - ps27
Placentalia - ps28
Boreoeutheria - ps29
Euarchontoglires - ps30
Euarchonta - ps31
Primates - ps32
Haplorrhini - ps33
Simiiformes - ps34
Catarrhini - ps35
Hominoidea - ps36
Hominidae - ps37
Homininae - ps38
Hominini - ps39
Homo_sapiens - ps40
Phylostratum

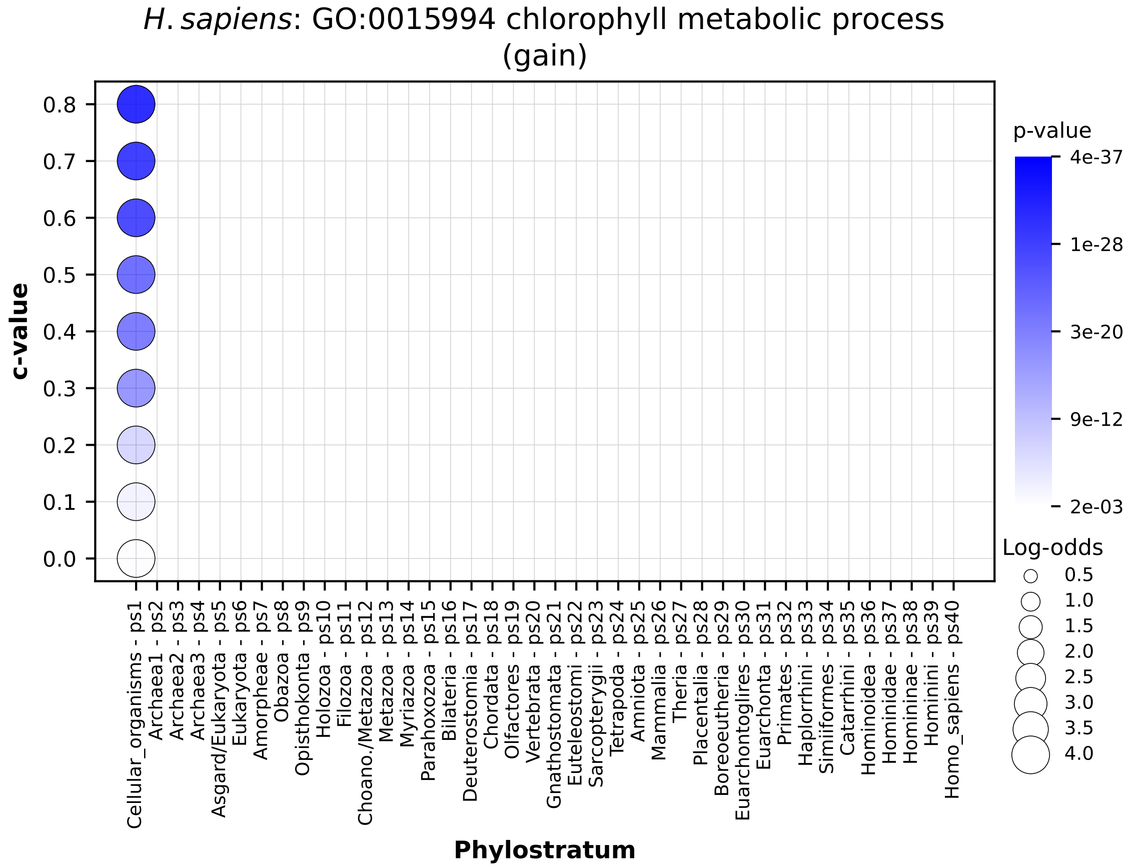

H. sapiens: GO:0015994 chlorophyll metabolic process
(gain)
c-value
0.8
0.7
0.6
0.5
0.4
0.3
0.2
0.1
0.0
p-value
4e-37
1e-28
3e-20
9e-12
2e-03
Log-odds
0.5
1.0
1.5
2.0
2.5
3.0
3.5
4.0
Cellular_organisms - ps1
Archaea1 - ps2
Archaea2 - ps3
Archaea3 - ps4
Asgard/Eukaryota - ps5
Eukaryota - ps6
Amorpheae - ps7
Obazoa - ps8
Opisthokonta - ps9
Holozoa - ps10
Filozoa - ps11
Choano./Metazoa - ps12
Metazoa - ps13
Myriazoa - ps14
Parahoxozoa - ps15
Bilateria - ps16
Deuterostomia - ps17
Chordata - ps18
Olfactores - ps19
Vertebrata - ps20
Gnathostomata - ps21
Euteleostomi - ps22
Sarcopterygii - ps23
Tetrapoda - ps24
Amniota - ps25
Mammalia - ps26
Theria - ps27
Placentalia - ps28
Boreoeutheria - ps29
Euarchontoglires - ps30
Euarchonta - ps31
Primates - ps32
Haplorrhini - ps33
Simiiformes - ps34
Catarrhini - ps35
Hominoidea - ps36
Hominidae - ps37
Homininae - ps38
Hominini - ps39
Homo_sapiens - ps40
Phylostratum

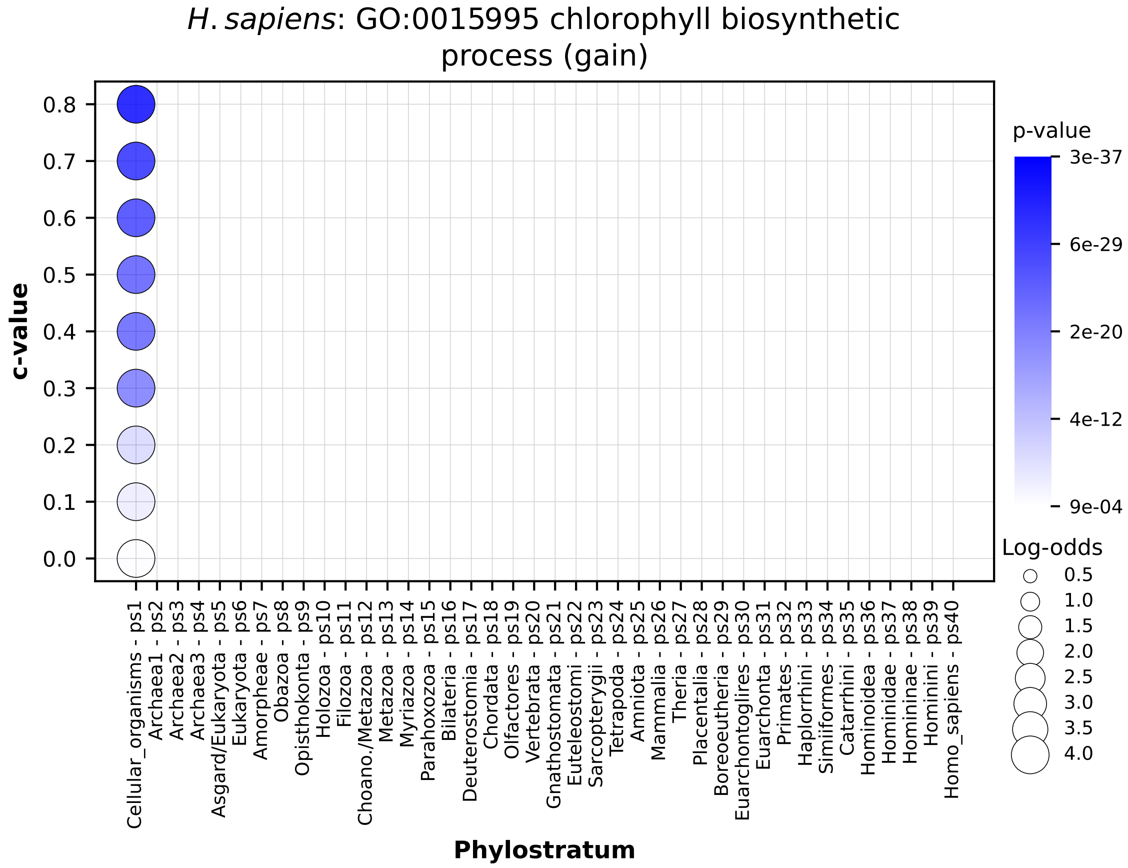

H. sapiens: GO:0015995 chlorophyll biosynthetic process (gain)
c-value
0.0
0.1
0.2
0.3
0.4
0.5
0.6
0.7
0.8
Phylostratum
Cellular_organisms - ps1
Archaea1 - ps2
Archaea2 - ps3
Archaea3 - ps4
Asgard/Eukaryota - ps5
Eukaryota - ps6
Amorpheae - ps7
Obazoa - ps8
Opisthokonta - ps9
Holozoa - ps10
Filozoa - ps11
Choano./Metazoa - ps12
Metazoa - ps13
Myriazoa - ps14
Parahoxozoa - ps15
Bilateria - ps16
Deuterostomia - ps17
Chordata - ps18
Olfactores - ps19
Vertebrata - ps20
Gnathostomata - ps21
Euteleostomi - ps22
Sarcopterygii - ps23
Tetrapoda - ps24
Amniota - ps25
Mammalia - ps26
Theria - ps27
Placentalia - ps28
Boreoeutheria - ps29
Euarchontoglires - ps30
Euarchonta - ps31
Primates - ps32
Haplorrhini - ps33
Simiiformes - ps34
Catarrhini - ps35
Hominoidea - ps36
Hominidae - ps37
Homininae - ps38
Hominini - ps39
Homo_sapiens - ps40
p-value
3e-37
6e-29
2e-20
4e-12
9e-04
Log-odds
0.5
1.0
1.5
2.0
2.5
3.0
3.5
4.0

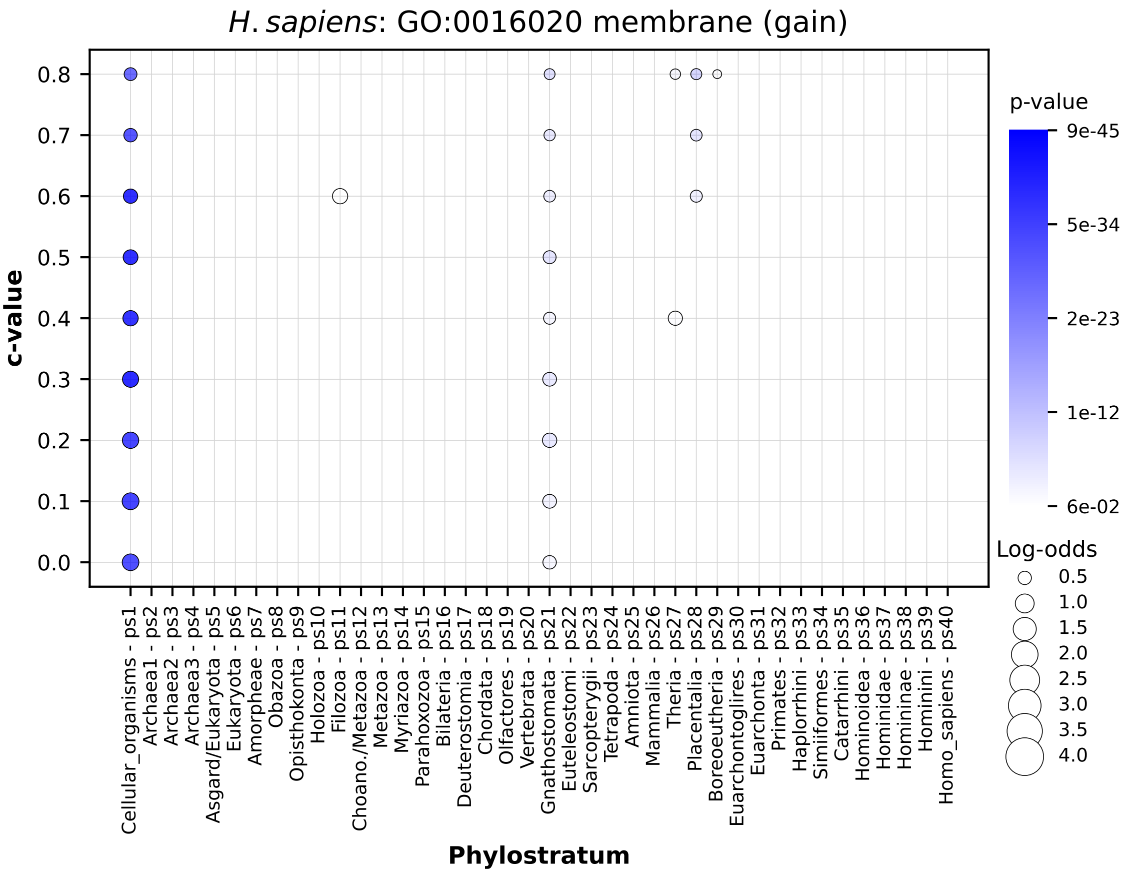

*H. sapiens*: GO:0016020 membrane (gain)
c-value
0.8
0.7
0.6
0.5
0.4
0.3
0.2
0.1
0.0
p-value
9e-45
5e-34
2e-23
1e-12
6e-02
Log-odds
0.5
1.0
1.5
2.0
2.5
3.0
3.5
4.0
Cellular_organisms - ps1
Archaea1 - ps2
Archaea2 - ps3
Archaea3 - ps4
Asgard/Eukaryota - ps5
Eukaryota - ps6
Amorpheae - ps7
Obazoa - ps8
Opisthokonta - ps9
Holozoa - ps10
Filozoa - ps11
Choano./Metazoa - ps12
Metazoa - ps13
Myriazoa - ps14
Parahoxozoa - ps15
Bilateria - ps16
Deuterostomia - ps17
Chordata - ps18
Olfactores - ps19
Vertebrata - ps20
Gnathostomata - ps21
Euteleostomi - ps22
Sarcopterygii - ps23
Tetrapoda - ps24
Amniota - ps25
Mammalia - ps26
Theria - ps27
Placentalia - ps28
Boreoeutheria - ps29
Euarchontoglires - ps30
Euarchonta - ps31
Primates - ps32
Haplorrhini - ps33
Simiiformes - ps34
Catarrhini - ps35
Hominoidea - ps36
Hominidae - ps37
Homininae - ps38
Hominini - ps39
Homo_sapiens - ps40
Phylostratum

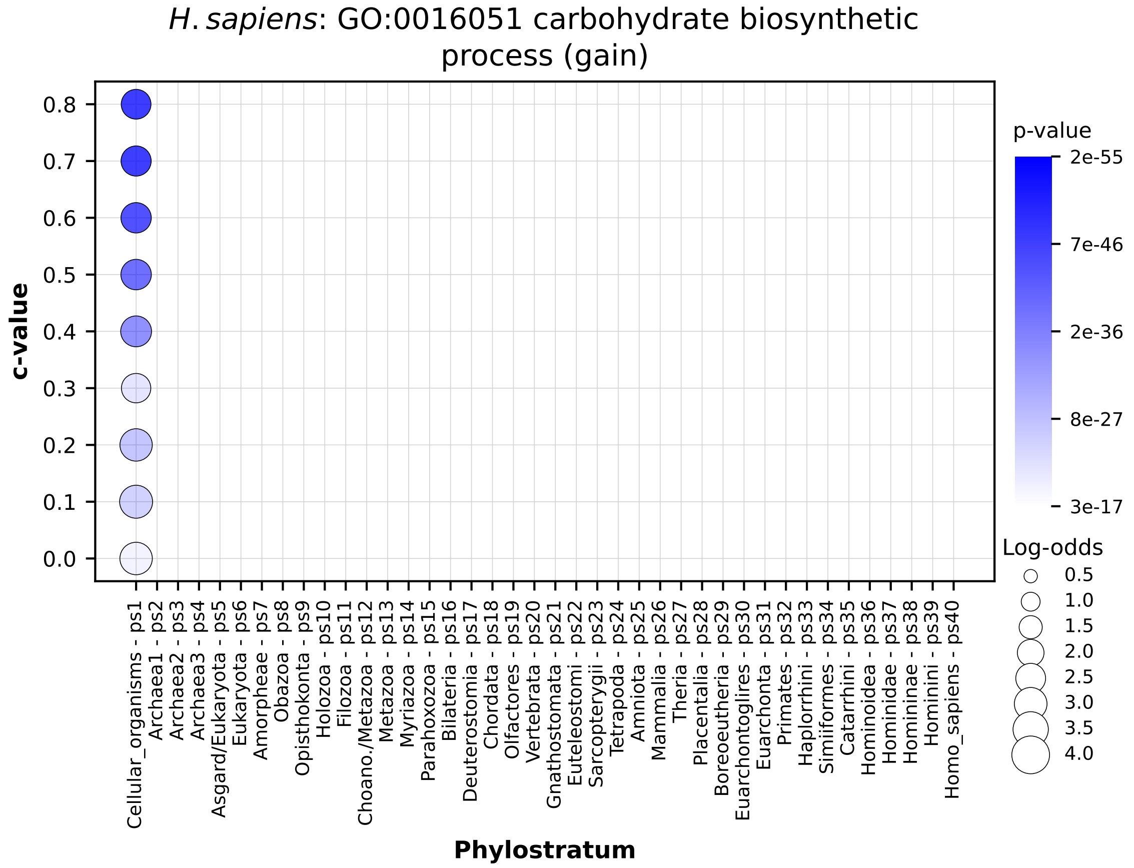

H. sapiens: GO:0016051 carbohydrate biosynthetic process (gain)
c-value
0.8
0.7
0.6
0.5
0.4
0.3
0.2
0.1
0.0
Phylostratum
Cellular_organisms - ps1
Archaea1 - ps2
Archaea2 - ps3
Archaea3 - ps4
Asgard/Eukaryota - ps5
Eukaryota - ps6
Amorpheae - ps7
Obazoa - ps8
Opisthokonta - ps9
Holozoa - ps10
Filozoa - ps11
Choano./Metazoa - ps12
Metazoa - ps13
Myriazoa - ps14
Parahoxozoa - ps15
Bilateria - ps16
Deuterostomia - ps17
Chordata - ps18
Olfactores - ps19
Vertebrata - ps20
Gnathostomata - ps21
Euteleostomi - ps22
Sarcopterygii - ps23
Tetrapoda - ps24
Amniota - ps25
Mammalia - ps26
Theria - ps27
Placentalia - ps28
Boreoeutheria - ps29
Euarchontoglires - ps30
Euarchonta - ps31
Primates - ps32
Haplorrhini - ps33
Simiiformes - ps34
Catarrhini - ps35
Hominoidea - ps36
Hominidae - ps37
Homininae - ps38
Hominini - ps39
Homo_sapiens - ps40
p-value
2e-55
7e-46
2e-36
8e-27
3e-17
Log-odds
0.5
1.0
1.5
2.0
2.5
3.0
3.5
4.0

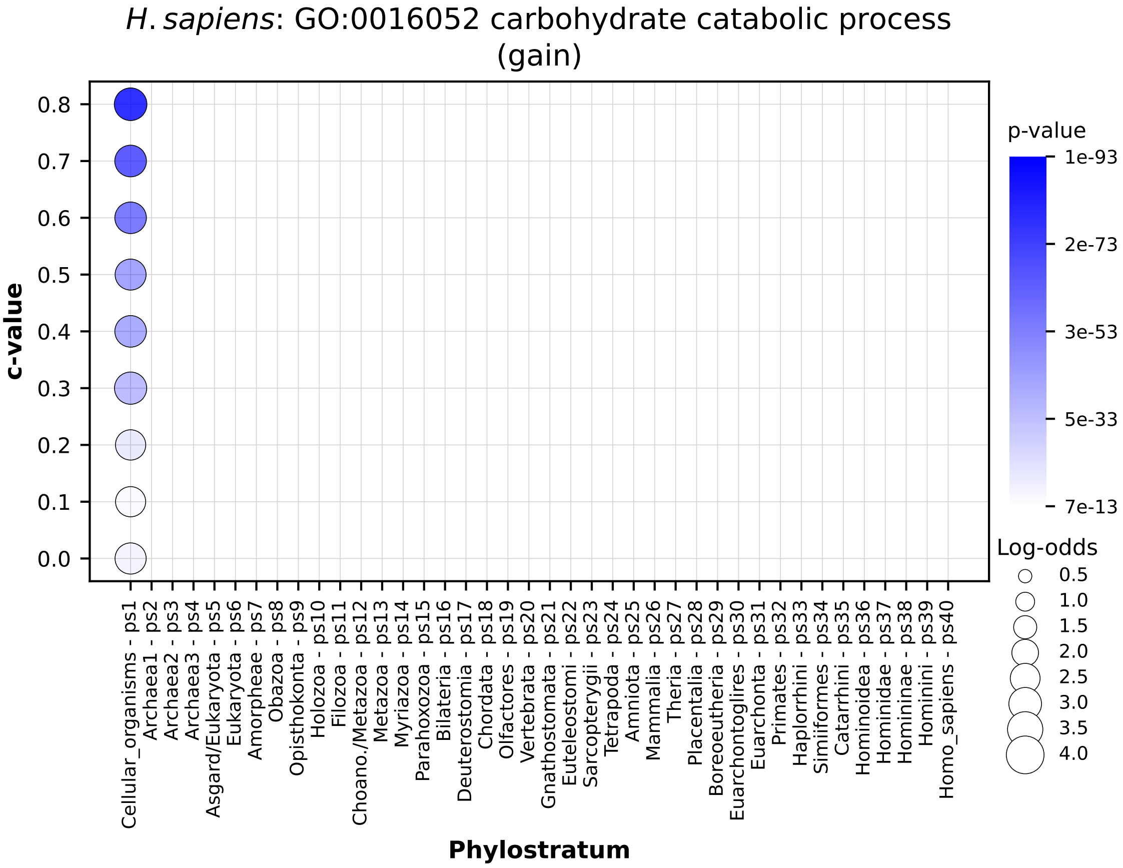

H. sapiens: GO:0016052 carbohydrate catabolic process (gain)
c-value
0.8
0.7
0.6
0.5
0.4
0.3
0.2
0.1
0.0
p-value
1e-93
2e-73
3e-53
5e-33
7e-13
Log-odds
0.5
1.0
1.5
2.0
2.5
3.0
3.5
4.0
Cellular_organisms - ps1
Archaea1 - ps2
Archaea2 - ps3
Archaea3 - ps4
Asgard/Eukaryota - ps5
Eukaryota - ps6
Amorpheae - ps7
Obazoa - ps8
Opisthokonta - ps9
Holozoa - ps10
Filozoa - ps11
Choano./Metazoa - ps12
Metazoa - ps13
Myriazoa - ps14
Parahoxozoa - ps15
Bilateria - ps16
Deuterostomia - ps17
Chordata - ps18
Olfactores - ps19
Vertebrata - ps20
Gnathostomata - ps21
Euteleostomi - ps22
Sarcopterygii - ps23
Tetrapoda - ps24
Amniota - ps25
Mammalia - ps26
Theria - ps27
Placentalia - ps28
Boreoeutheria - ps29
Euarchontoglires - ps30
Euarchonta - ps31
Primates - ps32
Haplorrhini - ps33
Simiiformes - ps34
Catarrhini - ps35
Hominoidea - ps36
Hominidae - ps37
Homininae - ps38
Hominini - ps39
Homo_sapiens - ps40
Phylostratum

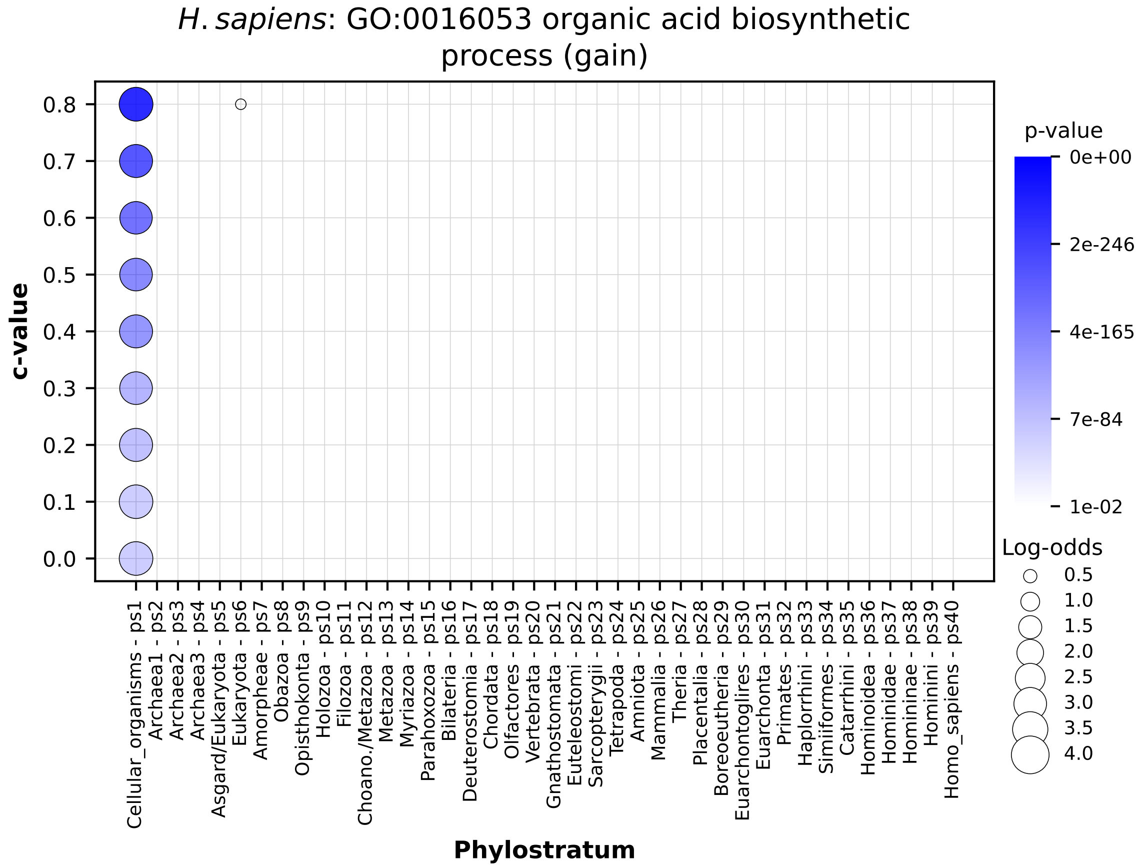
H. sapiens: GO:0016053 organic acid biosynthetic process (gain)
c-value
0.8
0.7
0.6
0.5
0.4
0.3
0.2
0.1
0.0
Phylostratum
Cellular_organisms - ps1
Archaea1 - ps2
Archaea2 - ps3
Archaea3 - ps4
Asgard/Eukaryota - ps5
Eukaryota - ps6
Amorpheae - ps7
Obazoa - ps8
Opisthokonta - ps9
Holozoa - ps10
Filozoa - ps11
Choano./Metazoa - ps12
Metazoa - ps13
Myriazoa - ps14
Parahoxozoa - ps15
Bilateria - ps16
Deuterostomia - ps17
Chordata - ps18
Olfactores - ps19
Vertebrata - ps20
Gnathostomata - ps21
Euteleostomi - ps22
Sarcopterygii - ps23
Tetrapoda - ps24
Amniota - ps25
Mammalia - ps26
Theria - ps27
Placentalia - ps28
Boreoeutheria - ps29
Euarchontoglires - ps30
Euarchonta - ps31
Primates - ps32
Haplorrhini - ps33
Simiiformes - ps34
Catarrhini - ps35
Hominoidea - ps36
Hominidae - ps37
Homininae - ps38
Hominini - ps39
Homo_sapiens - ps40
p-value
0e+00
2e-246
4e-165
7e-84
1e-02
Log-odds
0.5
1.0
1.5
2.0
2.5
3.0
3.5
4.0

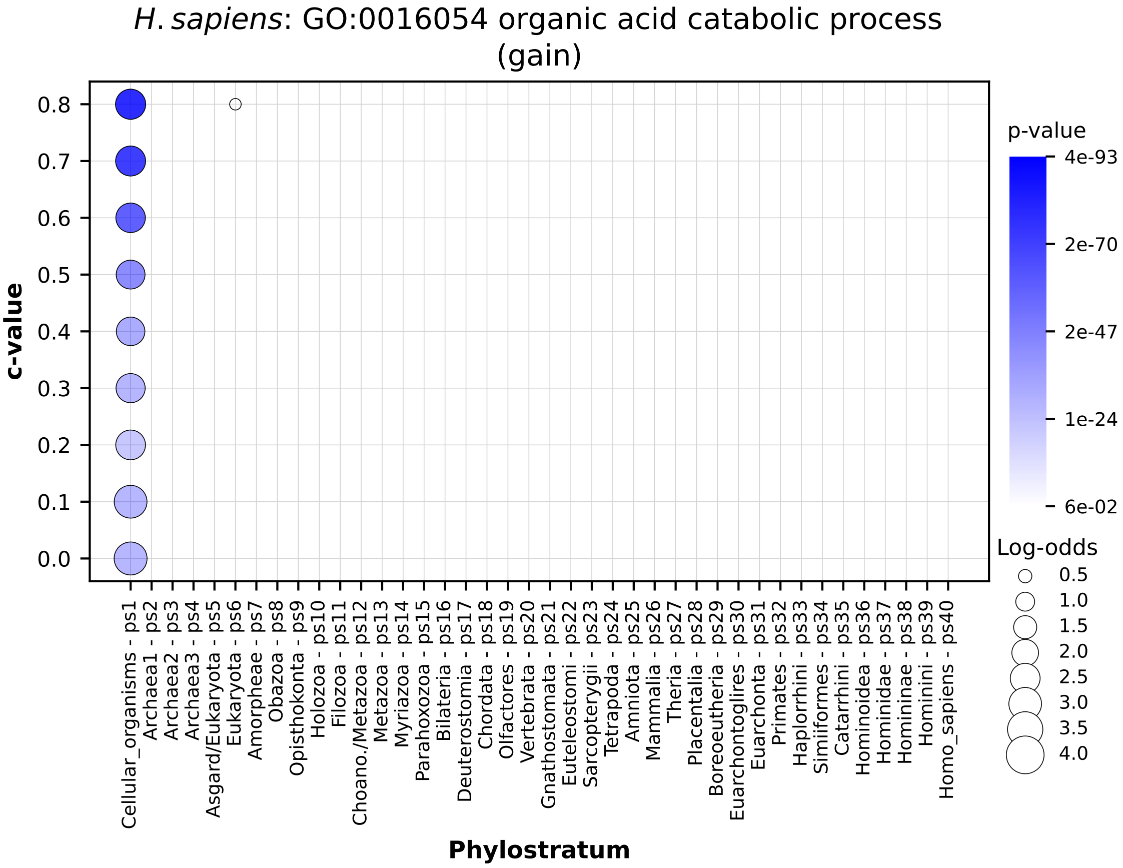
H. sapiens: GO:0016054 organic acid catabolic process
(gain)
c-value
0.8
0.7
0.6
0.5
0.4
0.3
0.2
0.1
0.0
p-value
4e-93
2e-70
2e-47
1e-24
6e-02
Log-odds
0.5
1.0
1.5
2.0
2.5
3.0
3.5
4.0
Cellular_organisms - ps1
Archaea1 - ps2
Archaea2 - ps3
Archaea3 - ps4
Asgard/Eukaryota - ps5
Eukaryota - ps6
Amorpheae - ps7
Obazoa - ps8
Opisthokonta - ps9
Holozoa - ps10
Filozoa - ps11
Choano./Metazoa - ps12
Metazoa - ps13
Myriazoa - ps14
Parahoxozoa - ps15
Bilateria - ps16
Deuterostomia - ps17
Chordata - ps18
Olfactores - ps19
Vertebrata - ps20
Gnathostomata - ps21
Euteleostomi - ps22
Sarcopterygii - ps23
Tetrapoda - ps24
Amniota - ps25
Mammalia - ps26
Theria - ps27
Placentalia - ps28
Boreoeutheria - ps29
Euarchontoglires - ps30
Euarchonta - ps31
Primates - ps32
Haplorrhini - ps33
Simiiformes - ps34
Catarrhini - ps35
Hominoidea - ps36
Hominidae - ps37
Homininae - ps38
Hominini - ps39
Homo_sapiens - ps40
Phylostratum

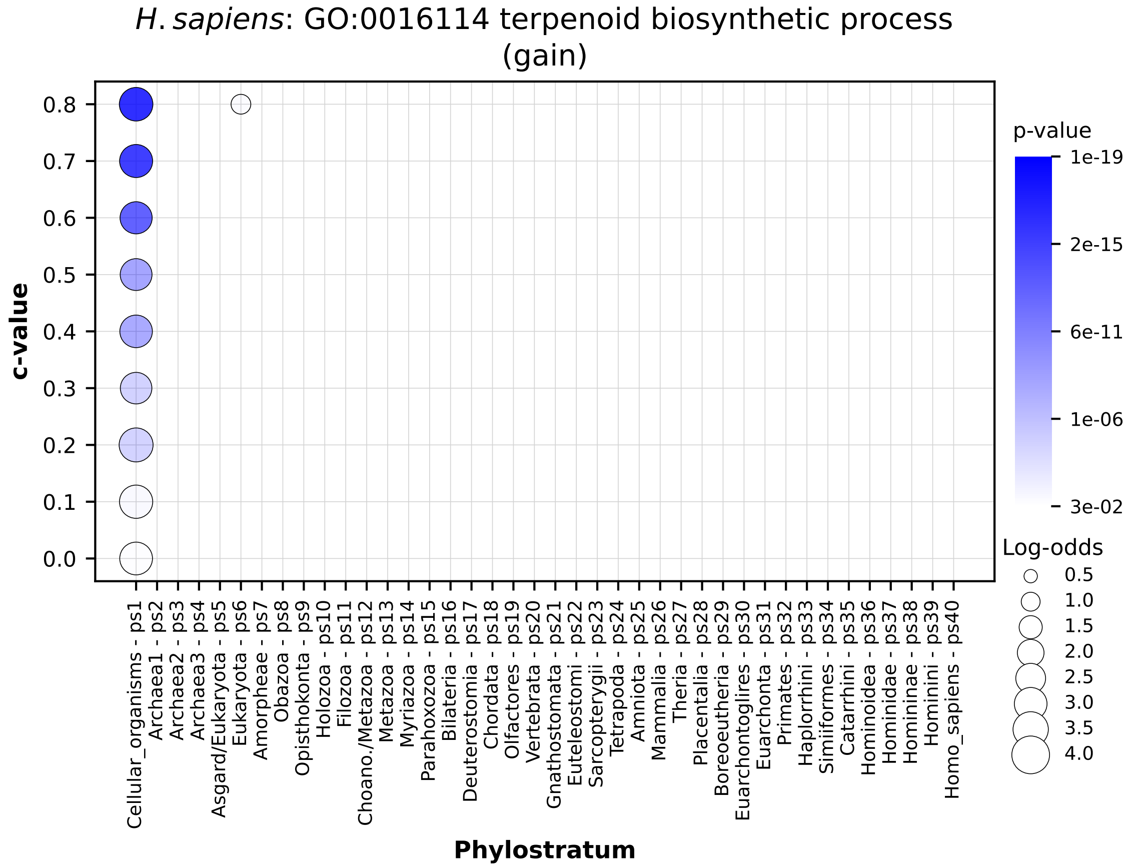
H. sapiens: GO:0016114 terpenoid biosynthetic process
(gain)
c-value
0.8
0.7
0.6
0.5
0.4
0.3
0.2
0.1
0.0
p-value
1e-19
2e-15
6e-11
1e-06
3e-02
Log-odds
0.5
1.0
1.5
2.0
2.5
3.0
3.5
4.0
Cellular_organisms - ps1
Archaea1 - ps2
Archaea2 - ps3
Archaea3 - ps4
Asgard/Eukaryota - ps5
Eukaryota - ps6
Amorpheae - ps7
Obazoa - ps8
Opisthokonta - ps9
Holozoa - ps10
Filozoa - ps11
Choano./Metazoa - ps12
Metazoa - ps13
Myriazoa - ps14
Parahoxozoa - ps15
Bilateria - ps16
Deuterostomia - ps17
Chordata - ps18
Olfactores - ps19
Vertebrata - ps20
Gnathostomata - ps21
Euteleostomi - ps22
Sarcopterygii - ps23
Tetrapoda - ps24
Amniota - ps25
Mammalia - ps26
Theria - ps27
Placentalia - ps28
Boreoeutheria - ps29
Euarchontoglires - ps30
Euarchonta - ps31
Primates - ps32
Haplorrhini - ps33
Simiiformes - ps34
Catarrhini - ps35
Hominoidea - ps36
Hominidae - ps37
Homininae - ps38
Hominini - ps39
Homo_sapiens - ps40
Phylostratum

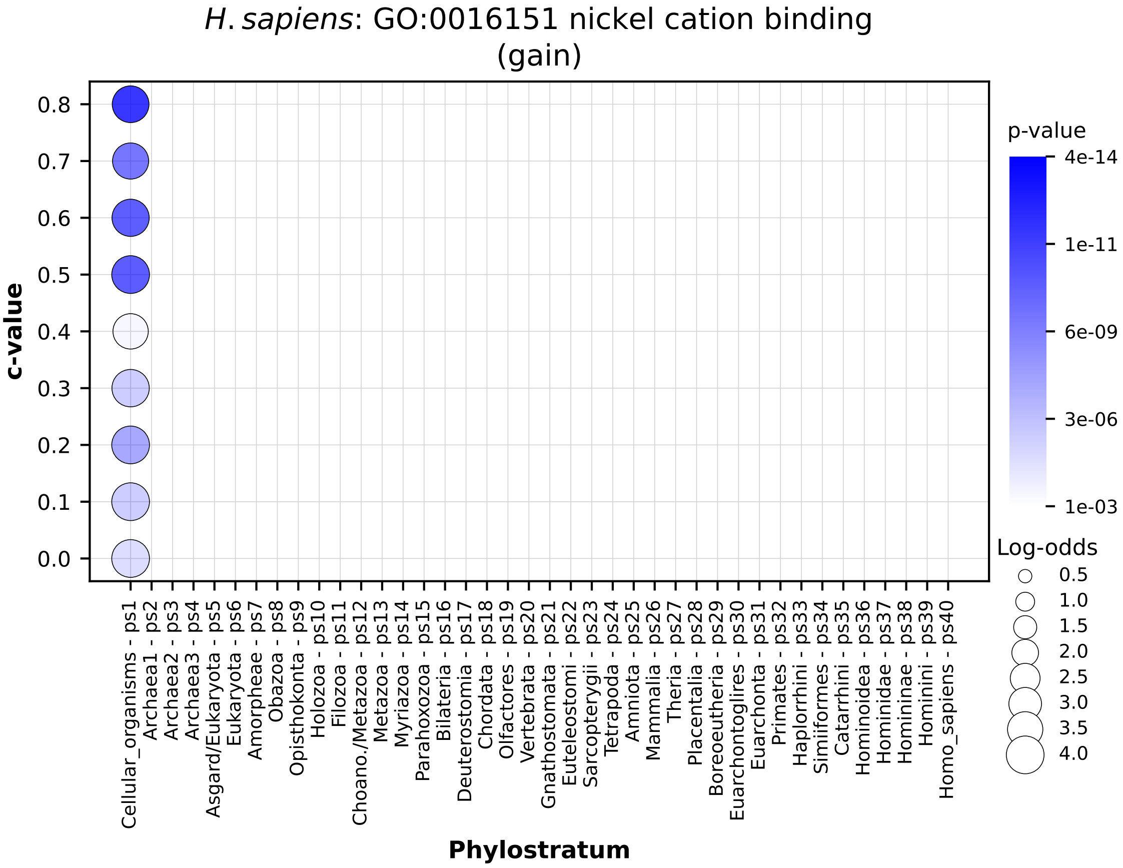

H. sapiens: GO:0016151 nickel cation binding
(gain)
c-value
0.8
0.7
0.6
0.5
0.4
0.3
0.2
0.1
0.0
Phylostratum
Cellular_organisms - ps1
Archaea1 - ps2
Archaea2 - ps3
Archaea3 - ps4
Asgard/Eukaryota - ps5
Eukaryota - ps6
Amorpheae - ps7
Obazoa - ps8
Opisthokonta - ps9
Holozoa - ps10
Filozoa - ps11
Choano./Metazoa - ps12
Metazoa - ps13
Myriazoa - ps14
Parahoxozoa - ps15
Bilateria - ps16
Deuterostomia - ps17
Chordata - ps18
Olfactores - ps19
Vertebrata - ps20
Gnathostomata - ps21
Euteleostomi - ps22
Sarcopterygii - ps23
Tetrapoda - ps24
Amniota - ps25
Mammalia - ps26
Theria - ps27
Placentalia - ps28
Boreoeutheria - ps29
Euarchontoglires - ps30
Euarchonta - ps31
Primates - ps32
Haplorrhini - ps33
Simiiformes - ps34
Catarrhini - ps35
Hominoidea - ps36
Hominidae - ps37
Homininae - ps38
Hominini - ps39
Homo_sapiens - ps40
p-value
4e-14
1e-11
6e-09
3e-06
1e-03
Log-odds
0.5
1.0
1.5
2.0
2.5
3.0
3.5
4.0

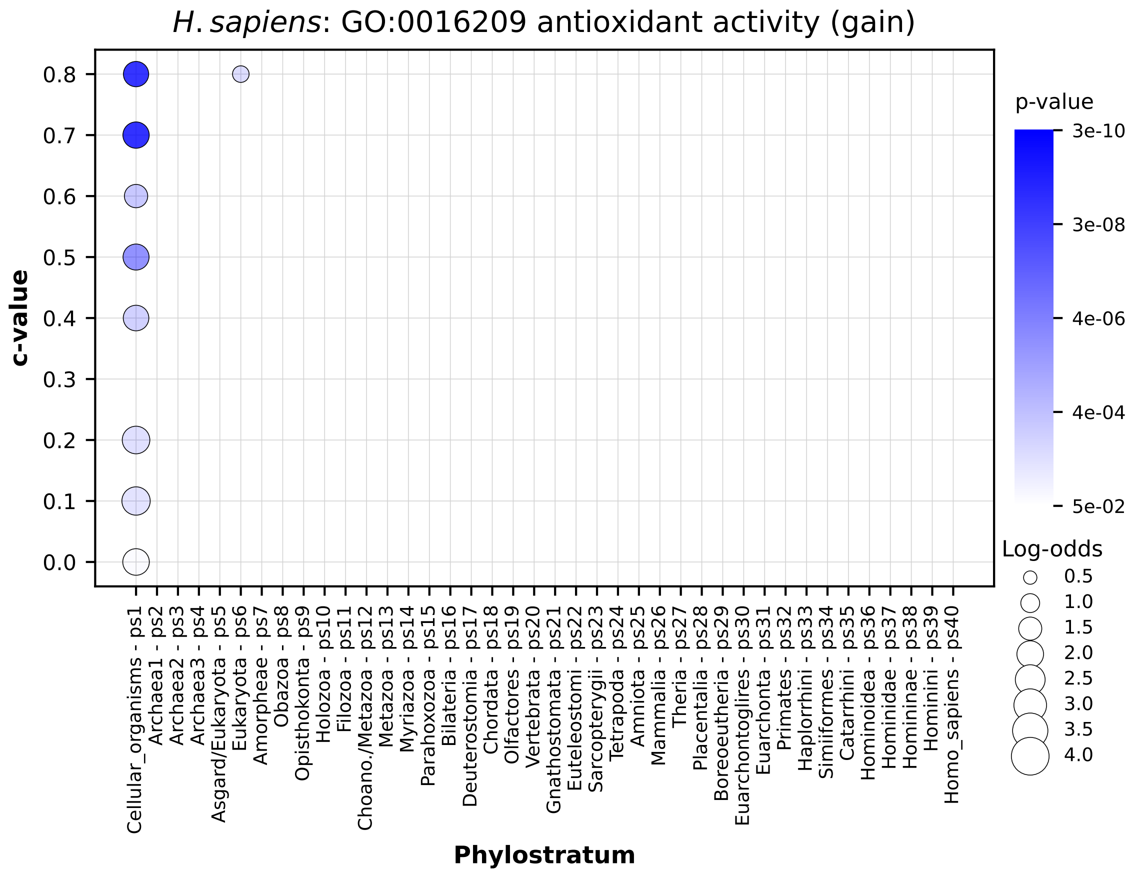

H. sapiens: GO:0016209 antioxidant activity (gain)
c-value
0.8
0.7
0.6
0.5
0.4
0.3
0.2
0.1
0.0
p-value
3e-10
3e-08
4e-06
4e-04
5e-02
Log-odds
0.5
1.0
1.5
2.0
2.5
3.0
3.5
4.0
Cellular_organisms - ps1
Archaea1 - ps2
Archaea2 - ps3
Archaea3 - ps4
Asgard/Eukaryota - ps5
Eukaryota - ps6
Amorpheae - ps7
Obazoa - ps8
Opisthokonta - ps9
Holozoa - ps10
Filozoa - ps11
Choano./Metazoa - ps12
Metazoa - ps13
Myriazoa - ps14
Parahoxozoa - ps15
Bilateria - ps16
Deuterostomia - ps17
Chordata - ps18
Olfactores - ps19
Vertebrata - ps20
Gnathostomata - ps21
Euteleostomi - ps22
Sarcopterygii - ps23
Tetrapoda - ps24
Amniota - ps25
Mammalia - ps26
Theria - ps27
Placentalia - ps28
Boreoeutheria - ps29
Euarchontoglires - ps30
Euarchonta - ps31
Primates - ps32
Haplorrhini - ps33
Simiiformes - ps34
Catarrhini - ps35
Hominoidea - ps36
Hominidae - ps37
Homininae - ps38
Hominini - ps39
Homo_sapiens - ps40
Phylostratum

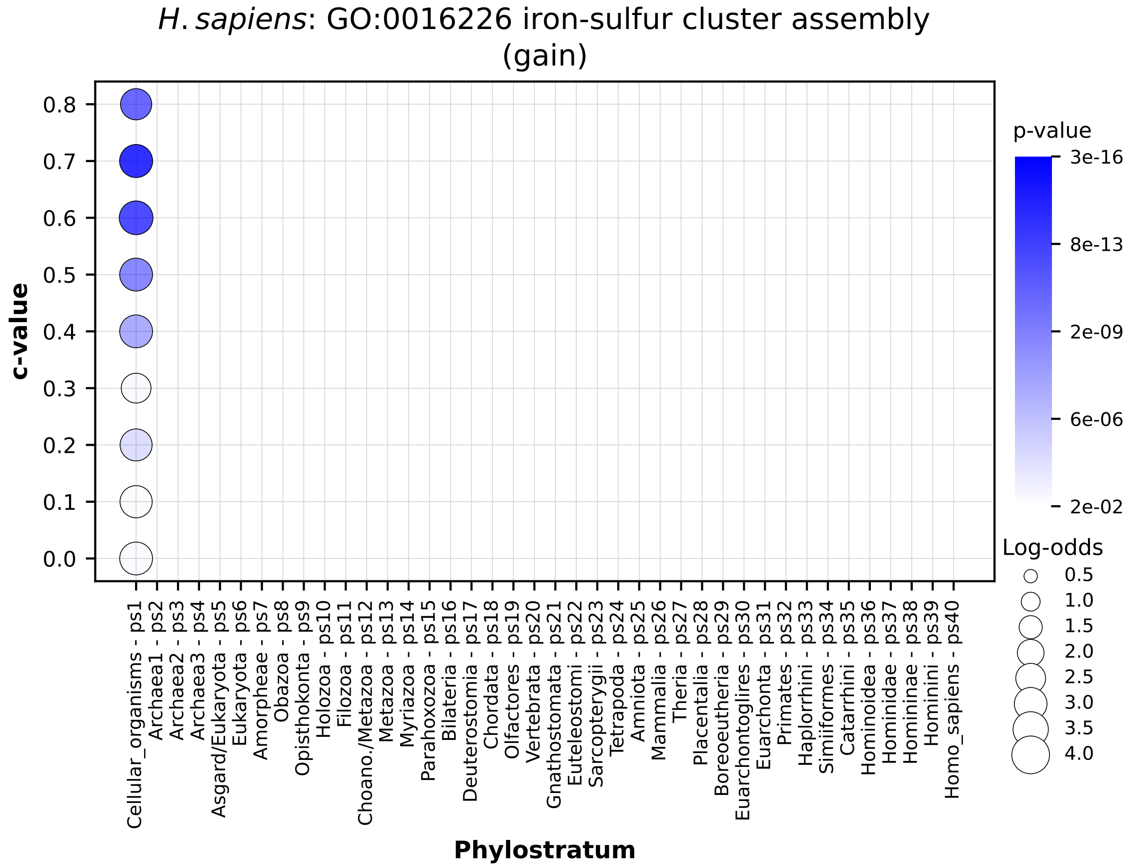

*H. sapiens*: GO:0016226 iron-sulfur cluster assembly
(gain)
c-value
0.8
0.7
0.6
0.5
0.4
0.3
0.2
0.1
0.0
p-value
3e-16
8e-13
2e-09
6e-06
2e-02
Log-odds
0.5
1.0
1.5
2.0
2.5
3.0
3.5
4.0
Cellular_organisms - ps1
Archaea1 - ps2
Archaea2 - ps3
Archaea3 - ps4
Asgard/Eukaryota - ps5
Eukaryota - ps6
Amorpheae - ps7
Obazoa - ps8
Opisthokonta - ps9
Holozoa - ps10
Filozoa - ps11
Choano./Metazoa - ps12
Metazoa - ps13
Myriazoa - ps14
Parahoxozoa - ps15
Bilateria - ps16
Deuterostomia - ps17
Chordata - ps18
Olfactores - ps19
Vertebrata - ps20
Gnathostomata - ps21
Euteleostomi - ps22
Sarcopterygii - ps23
Tetrapoda - ps24
Amniota - ps25
Mammalia - ps26
Theria - ps27
Placentalia - ps28
Boreoeutheria - ps29
Euarchontoglires - ps30
Euarchonta - ps31
Primates - ps32
Haplorrhini - ps33
Simiiformes - ps34
Catarrhini - ps35
Hominoidea - ps36
Hominidae - ps37
Homininae - ps38
Hominini - ps39
Homo_sapiens - ps40
Phylostratum

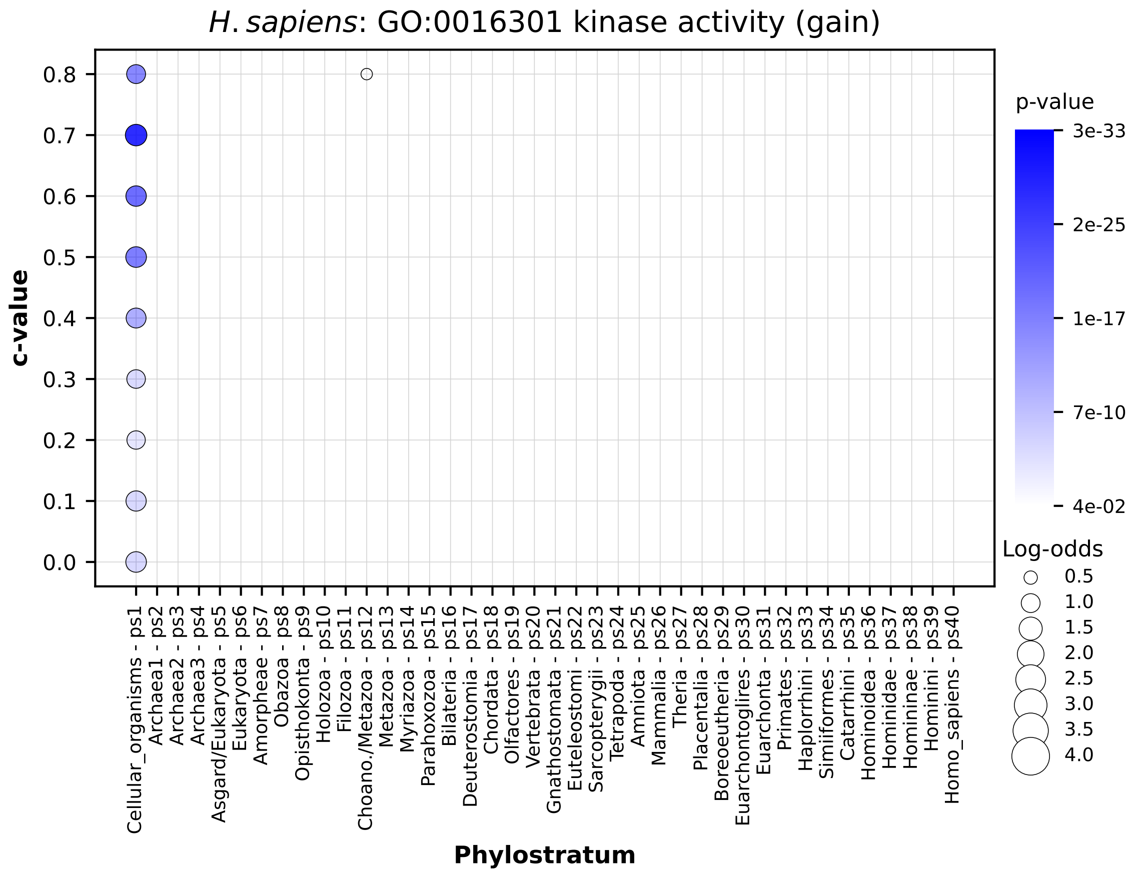
H. sapiens: GO:0016301 kinase activity (gain)
c-value
0.8
0.7
0.6
0.5
0.4
0.3
0.2
0.1
0.0
p-value
3e-33
2e-25
1e-17
7e-10
4e-02
Log-odds
0.5
1.0
1.5
2.0
2.5
3.0
3.5
4.0
Cellular_organisms - ps1
Archaea1 - ps2
Archaea2 - ps3
Archaea3 - ps4
Asgard/Eukaryota - ps5
Eukaryota - ps6
Amorpheae - ps7
Obazoa - ps8
Opisthokonta - ps9
Holozoa - ps10
Filozoa - ps11
Choano./Metazoa - ps12
Metazoa - ps13
Myriazoa - ps14
Parahoxozoa - ps15
Bilateria - ps16
Deuterostomia - ps17
Chordata - ps18
Olfactores - ps19
Vertebrata - ps20
Gnathostomata - ps21
Euteleostomi - ps22
Sarcopterygii - ps23
Tetrapoda - ps24
Amniota - ps25
Mammalia - ps26
Theria - ps27
Placentalia - ps28
Boreoeutheria - ps29
Euarchontoglires - ps30
Euarchonta - ps31
Primates - ps32
Haplorrhini - ps33
Simiiformes - ps34
Catarrhini - ps35
Hominoidea - ps36
Hominidae - ps37
Homininae - ps38
Hominini - ps39
Homo_sapiens - ps40
Phylostratum

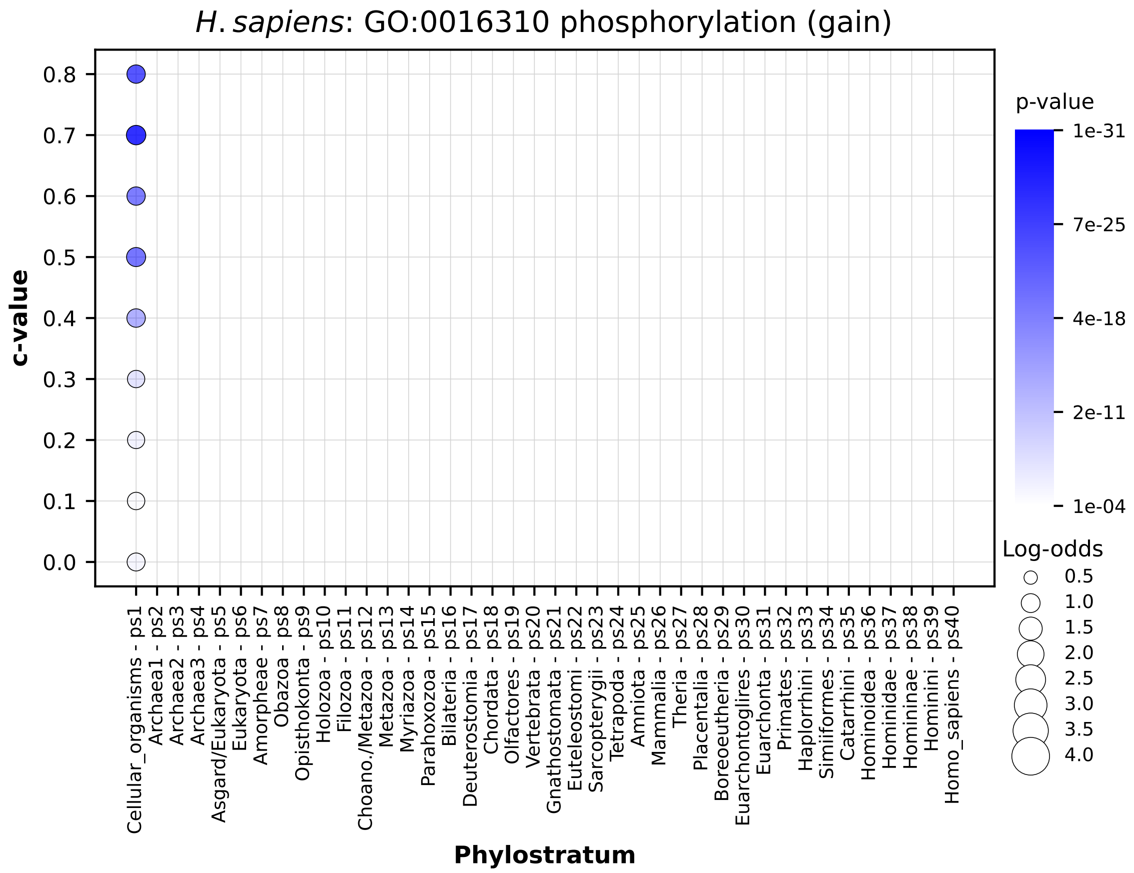

H. sapiens: GO:0016310 phosphorylation (gain)
c-value
0.8
0.7
0.6
0.5
0.4
0.3
0.2
0.1
0.0
p-value
1e-31
7e-25
4e-18
2e-11
1e-04
Log-odds
0.5
1.0
1.5
2.0
2.5
3.0
3.5
4.0
Cellular_organisms - ps1
Archaea1 - ps2
Archaea2 - ps3
Archaea3 - ps4
Asgard/Eukaryota - ps5
Eukaryota - ps6
Amorpheae - ps7
Obazoa - ps8
Opisthokonta - ps9
Holozoa - ps10
Filozoa - ps11
Choano./Metazoa - ps12
Metazoa - ps13
Myriazoa - ps14
Parahoxozoa - ps15
Bilateria - ps16
Deuterostomia - ps17
Chordata - ps18
Olfactores - ps19
Vertebrata - ps20
Gnathostomata - ps21
Euteleostomi - ps22
Sarcopterygii - ps23
Tetrapoda - ps24
Amniota - ps25
Mammalia - ps26
Theria - ps27
Placentalia - ps28
Boreoeutheria - ps29
Euarchontoglires - ps30
Euarchonta - ps31
Primates - ps32
Haplorrhini - ps33
Simiiformes - ps34
Catarrhini - ps35
Hominoidea - ps36
Hominidae - ps37
Homininae - ps38
Hominini - ps39
Homo_sapiens - ps40
Phylostratum

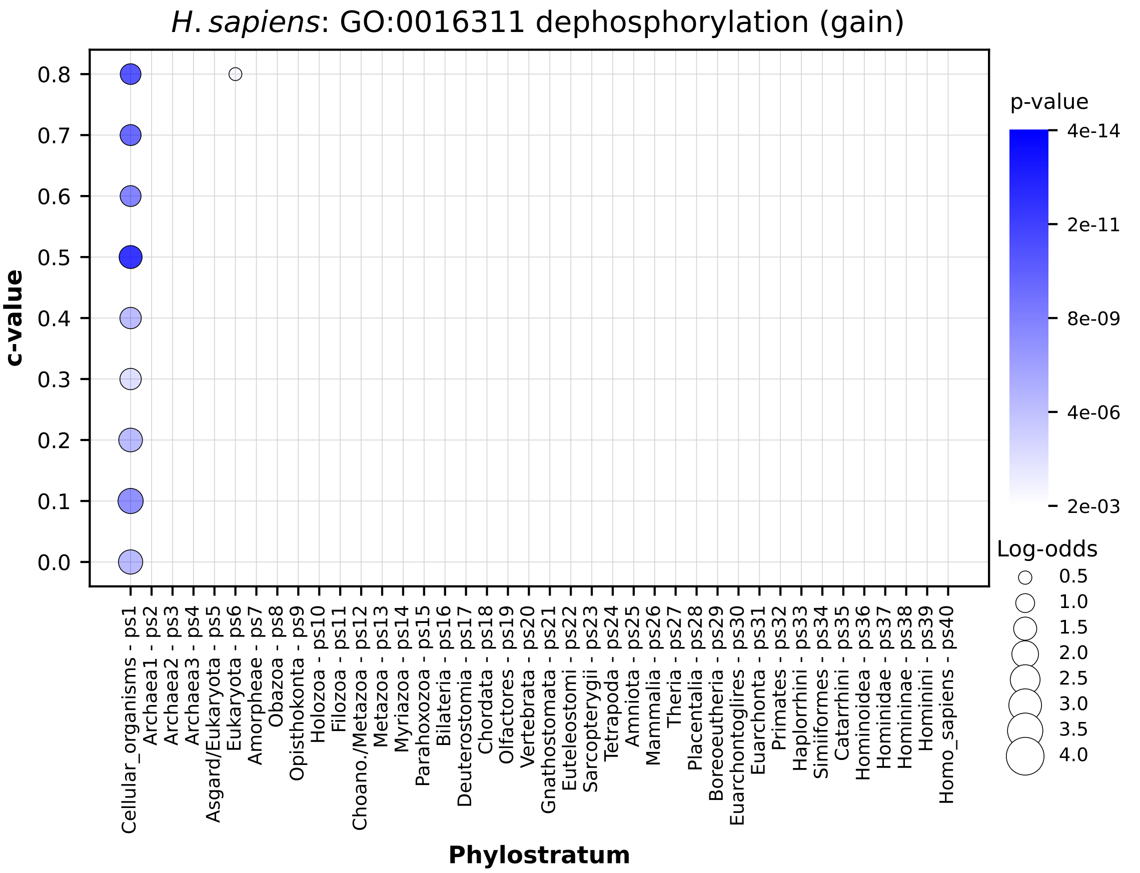
H. sapiens: GO:0016311 dephosphorylation (gain)
c-value
0.8
0.7
0.6
0.5
0.4
0.3
0.2
0.1
0.0
p-value
4e-14
2e-11
8e-09
4e-06
2e-03
Log-odds
0.5
1.0
1.5
2.0
2.5
3.0
3.5
4.0
Cellular_organisms - ps1
Archaea1 - ps2
Archaea2 - ps3
Archaea3 - ps4
Asgard/Eukaryota - ps5
Eukaryota - ps6
Amorpheae - ps7
Obazoa - ps8
Opisthokonta - ps9
Holozoa - ps10
Filozoa - ps11
Choano./Metazoa - ps12
Metazoa - ps13
Myriazoa - ps14
Parahoxozoa - ps15
Bilateria - ps16
Deuterostomia - ps17
Chordata - ps18
Olfactores - ps19
Vertebrata - ps20
Gnathostomata - ps21
Euteleostomi - ps22
Sarcopterygii - ps23
Tetrapoda - ps24
Amniota - ps25
Mammalia - ps26
Theria - ps27
Placentalia - ps28
Boreoeutheria - ps29
Euarchontoglires - ps30
Euarchonta - ps31
Primates - ps32
Haplorrhini - ps33
Simiiformes - ps34
Catarrhini - ps35
Hominoidea - ps36
Hominidae - ps37
Homininae - ps38
Hominini - ps39
Homo_sapiens - ps40
Phylostratum

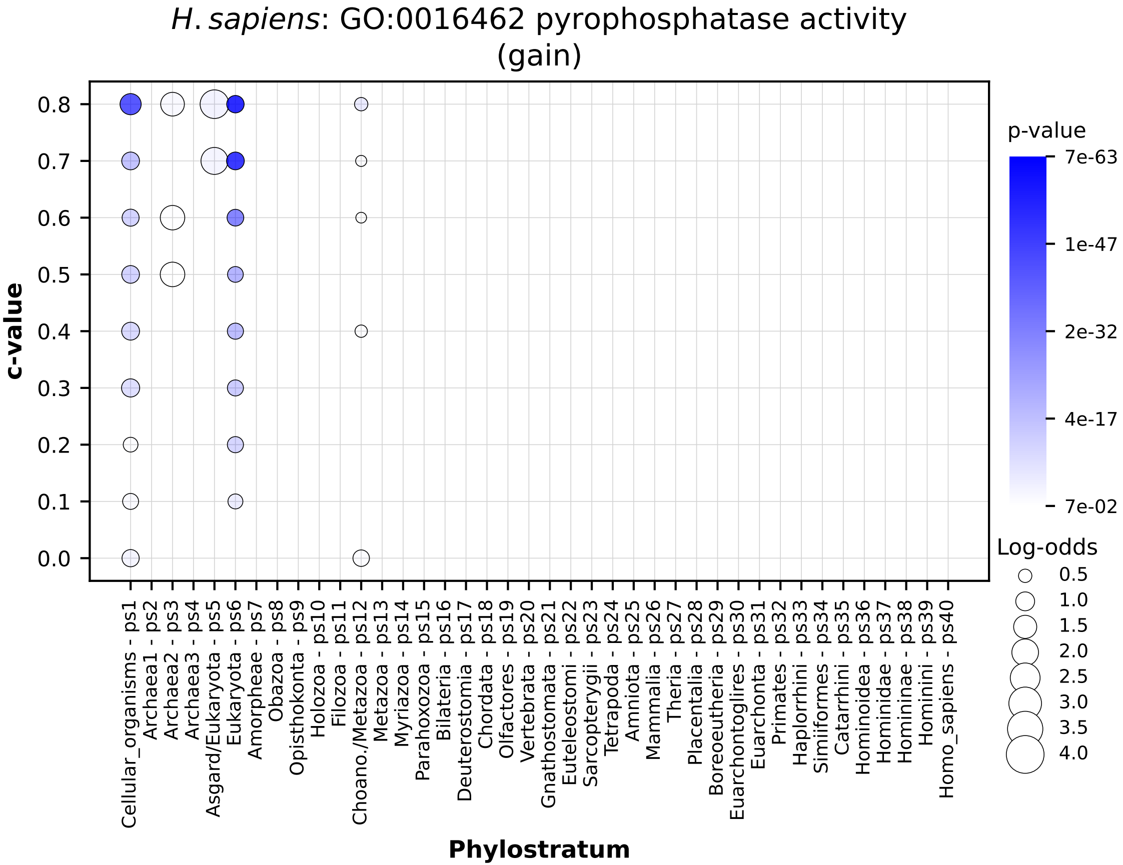
H. sapiens: GO:0016462 pyrophosphatase activity
(gain)
c-value
0.8
0.7
0.6
0.5
0.4
0.3
0.2
0.1
0.0
p-value
7e-63
1e-47
2e-32
4e-17
7e-02
Log-odds
0.5
1.0
1.5
2.0
2.5
3.0
3.5
4.0
Cellular_organisms - ps1
Archaea1 - ps2
Archaea2 - ps3
Archaea3 - ps4
Asgard/Eukaryota - ps5
Eukaryota - ps6
Amorpheae - ps7
Obazoa - ps8
Opisthokonta - ps9
Holozoa - ps10
Filozoa - ps11
Choano./Metazoa - ps12
Metazoa - ps13
Myriazoa - ps14
Parahoxozoa - ps15
Bilateria - ps16
Deuterostomia - ps17
Chordata - ps18
Olfactores - ps19
Vertebrata - ps20
Gnathostomata - ps21
Euteleostomi - ps22
Sarcopterygii - ps23
Tetrapoda - ps24
Amniota - ps25
Mammalia - ps26
Theria - ps27
Placentalia - ps28
Boreoeutheria - ps29
Euarchontoglires - ps30
Euarchonta - ps31
Primates - ps32
Haplorrhini - ps33
Simiiformes - ps34
Catarrhini - ps35
Hominoidea - ps36
Hominidae - ps37
Homininae - ps38
Hominini - ps39
Homo_sapiens - ps40
Phylostratum

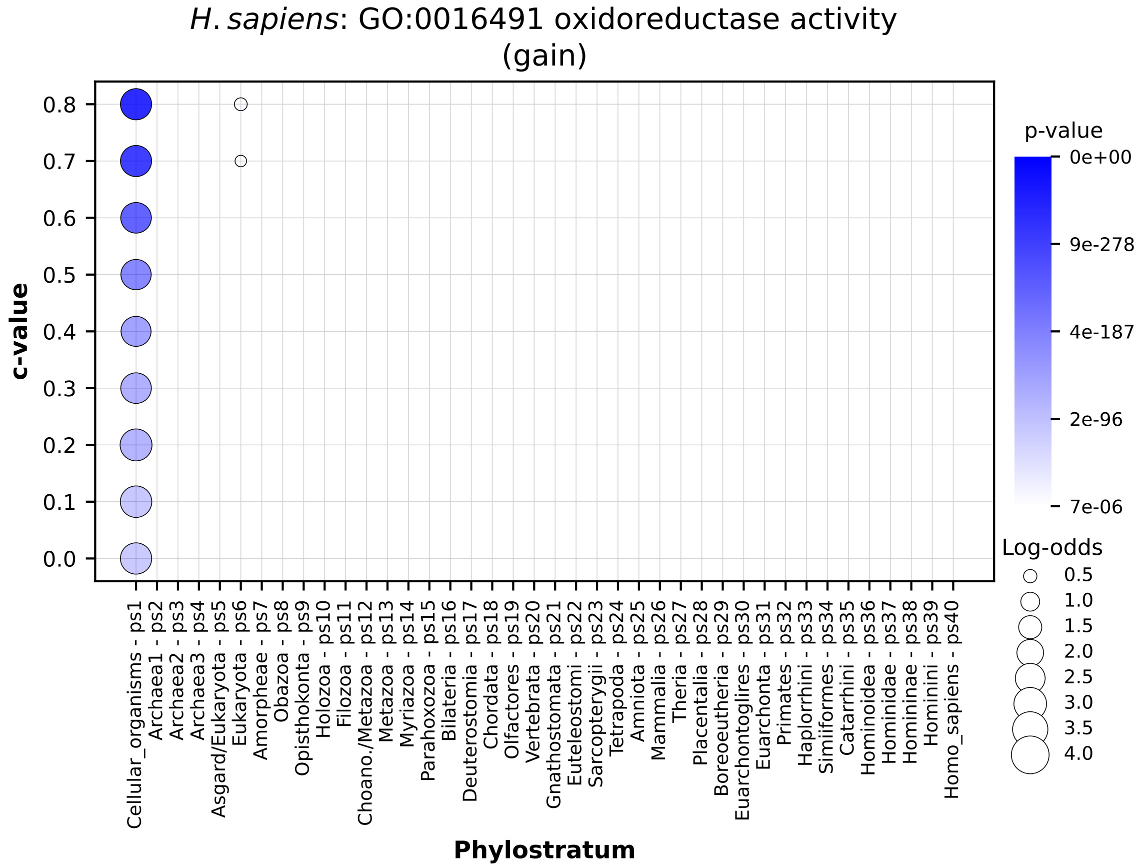

H. sapiens: GO:0016491 oxidoreductase activity
(gain)
c-value
0.8
0.7
0.6
0.5
0.4
0.3
0.2
0.1
0.0
Phylostratum
Cellular_organisms - ps1
Archaea1 - ps2
Archaea2 - ps3
Archaea3 - ps4
Asgard/Eukaryota - ps5
Eukaryota - ps6
Amorpheae - ps7
Obazoa - ps8
Opisthokonta - ps9
Holozoa - ps10
Filozoa - ps11
Choano./Metazoa - ps12
Metazoa - ps13
Myriazoa - ps14
Parahoxozoa - ps15
Bilateria - ps16
Deuterostomia - ps17
Chordata - ps18
Olfactores - ps19
Vertebrata - ps20
Gnathostomata - ps21
Euteleostomi - ps22
Sarcopterygii - ps23
Tetrapoda - ps24
Amniota - ps25
Mammalia - ps26
Theria - ps27
Placentalia - ps28
Boreoeutheria - ps29
Euarchontoglires - ps30
Euarchonta - ps31
Primates - ps32
Haplorrhini - ps33
Simiiformes - ps34
Catarrhini - ps35
Hominoidea - ps36
Hominidae - ps37
Homininae - ps38
Hominini - ps39
Homo_sapiens - ps40
p-value
0e+00
9e-278
4e-187
2e-96
7e-06
Log-odds
0.5
1.0
1.5
2.0
2.5
3.0
3.5
4.0

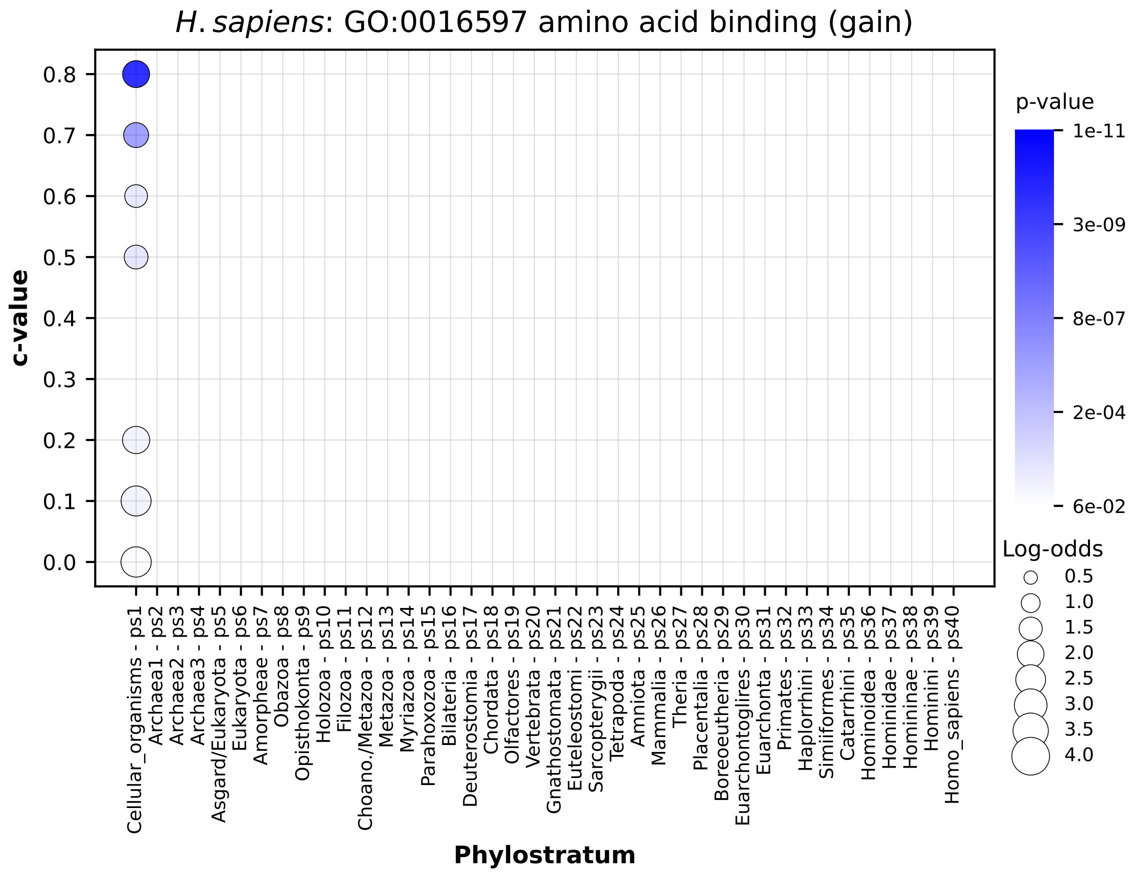

H. sapiens: GO:0016597 amino acid binding (gain)
c-value
0.8
0.7
0.6
0.5
0.4
0.3
0.2
0.1
0.0
Phylostratum
Cellular_organisms - ps1
Archaea1 - ps2
Archaea2 - ps3
Archaea3 - ps4
Asgard/Eukaryota - ps5
Eukaryota - ps6
Amorpheae - ps7
Obazoa - ps8
Opisthokonta - ps9
Holozoa - ps10
Filozoa - ps11
Choano./Metazoa - ps12
Metazoa - ps13
Myriazoa - ps14
Parahoxozoa - ps15
Bilateria - ps16
Deuterostomia - ps17
Chordata - ps18
Olfactores - ps19
Vertebrata - ps20
Gnathostomata - ps21
Euteleostomi - ps22
Sarcopterygii - ps23
Tetrapoda - ps24
Amniota - ps25
Mammalia - ps26
Theria - ps27
Placentalia - ps28
Boreoeutheria - ps29
Euarchontoglires - ps30
Euarchonta - ps31
Primates - ps32
Haplorrhini - ps33
Simiiformes - ps34
Catarrhini - ps35
Hominoidea - ps36
Hominidae - ps37
Homininae - ps38
Hominini - ps39
Homo_sapiens - ps40
p-value
1e-11
3e-09
8e-07
2e-04
6e-02
Log-odds
0.5
1.0
1.5
2.0
2.5
3.0
3.5
4.0

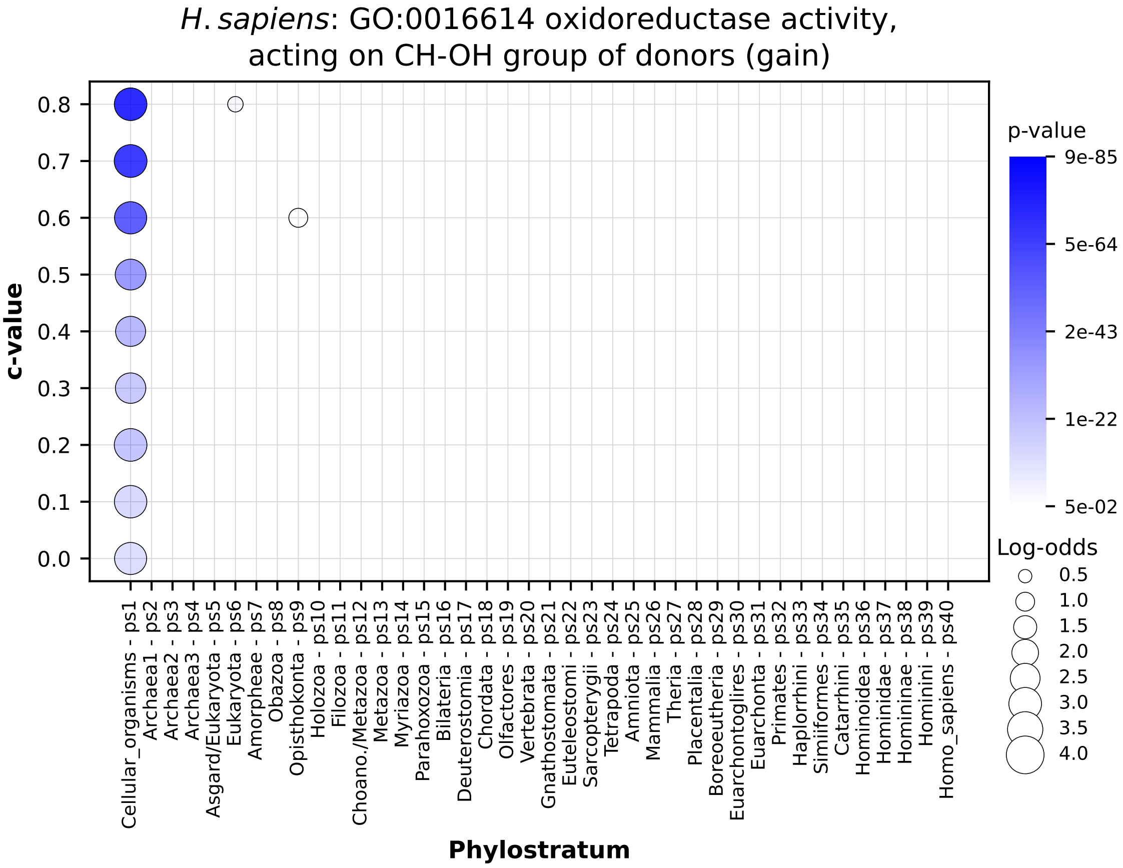

*H. sapiens*: GO:0016614 oxidoreductase activity, acting on CH-OH group of donors (gain)
c-value
0.8
0.7
0.6
0.5
0.4
0.3
0.2
0.1
0.0
Phylostratum
Cellular_organisms - ps1
Archaea1 - ps2
Archaea2 - ps3
Archaea3 - ps4
Asgard/Eukaryota - ps5
Eukaryota - ps6
Amorpheae - ps7
Obazoa - ps8
Opisthokonta - ps9
Holozoa - ps10
Filozoa - ps11
Choano./Metazoa - ps12
Metazoa - ps13
Myriazoa - ps14
Parahoxozoa - ps15
Bilateria - ps16
Deuterostomia - ps17
Chordata - ps18
Olfactores - ps19
Vertebrata - ps20
Gnathostomata - ps21
Euteleostomi - ps22
Sarcopterygii - ps23
Tetrapoda - ps24
Amniota - ps25
Mammalia - ps26
Theria - ps27
Placentalia - ps28
Boreoeutheria - ps29
Euarchontoglires - ps30
Euarchonta - ps31
Primates - ps32
Haplorrhini - ps33
Simiiformes - ps34
Catarrhini - ps35
Hominoidea - ps36
Hominidae - ps37
Homininae - ps38
Hominini - ps39
Homo_sapiens - ps40
p-value
9e-85
5e-64
2e-43
1e-22
5e-02
Log-odds
0.5
1.0
1.5
2.0
2.5
3.0
3.5
4.0

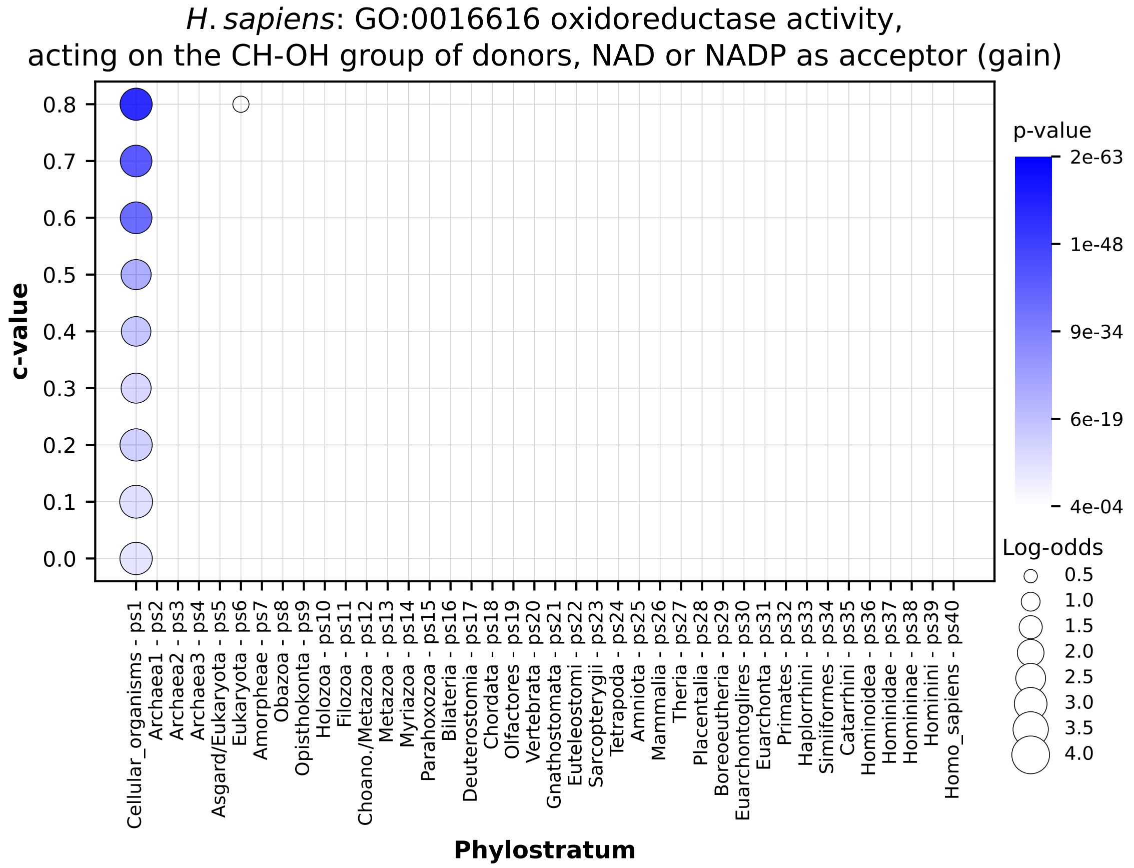

H. sapiens: GO:0016616 oxidoreductase activity,
acting on the CH-OH group of donors, NAD or NADP as acceptor (gain)
c-value
0.0
0.1
0.2
0.3
0.4
0.5
0.6
0.7
0.8
Phylostratum
Cellular_organisms - ps1
Archaea1 - ps2
Archaea2 - ps3
Archaea3 - ps4
Asgard/Eukaryota - ps5
Eukaryota - ps6
Amorpheae - ps7
Obazoa - ps8
Opisthokonta - ps9
Holozoa - ps10
Filozoa - ps11
Choano./Metazoa - ps12
Metazoa - ps13
Myriazoa - ps14
Parahoxozoa - ps15
Bilateria - ps16
Deuterostomia - ps17
Chordata - ps18
Olfactores - ps19
Vertebrata - ps20
Gnathostomata - ps21
Euteleostomi - ps22
Sarcopterygii - ps23
Tetrapoda - ps24
Amniota - ps25
Mammalia - ps26
Theria - ps27
Placentalia - ps28
Boreoeutheria - ps29
Euarchontoglires - ps30
Euarchonta - ps31
Primates - ps32
Haplorrhini - ps33
Simiiformes - ps34
Catarrhini - ps35
Hominoidea - ps36
Hominidae - ps37
Homininae - ps38
Hominini - ps39
Homo_sapiens - ps40
p-value
2e-63
1e-48
9e-34
6e-19
4e-04
Log-odds
0.5
1.0
1.5
2.0
2.5
3.0
3.5
4.0

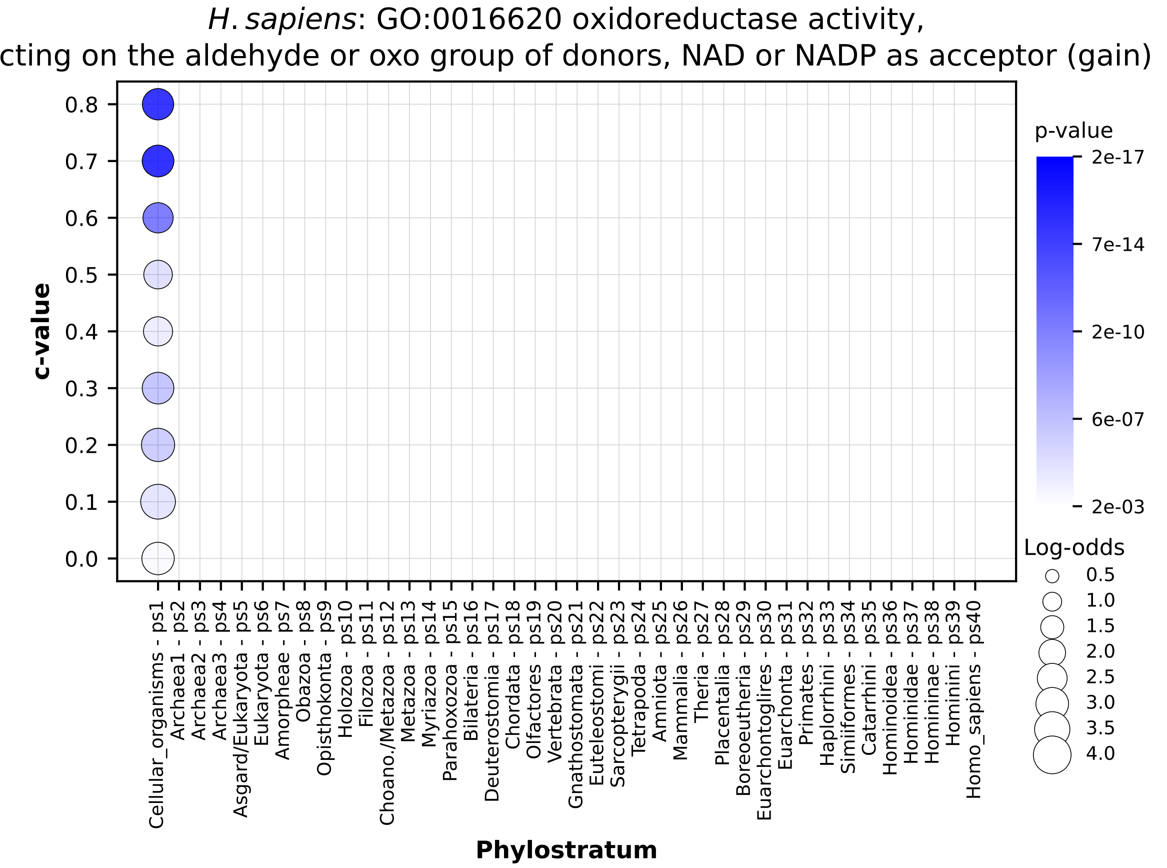

*H. sapiens*: GO:0016620 oxidoreductase activity,
cting on the aldehyde or oxo group of donors, NAD or NADP as acceptor (gain)
c-value
0.8
0.7
0.6
0.5
0.4
0.3
0.2
0.1
0.0
p-value
2e-17
7e-14
2e-10
6e-07
2e-03
Log-odds
0.5
1.0
1.5
2.0
2.5
3.0
3.5
4.0
Cellular_organisms - ps1
Archaea1 - ps2
Archaea2 - ps3
Archaea3 - ps4
Asgard/Eukaryota - ps5
Eukaryota - ps6
Amorpheae - ps7
Obazoa - ps8
Opisthokonta - ps9
Holozoa - ps10
Filozoa - ps11
Choano./Metazoa - ps12
Metazoa - ps13
Myriazoa - ps14
Parahoxozoa - ps15
Bilateria - ps16
Deuterostomia - ps17
Chordata - ps18
Olfactores - ps19
Vertebrata - ps20
Gnathostomata - ps21
Euteleostomi - ps22
Sarcopterygii - ps23
Tetrapoda - ps24
Amniota - ps25
Mammalia - ps26
Theria - ps27
Placentalia - ps28
Boreoeutheria - ps29
Euarchontoglires - ps30
Euarchonta - ps31
Primates - ps32
Haplorrhini - ps33
Simiiformes - ps34
Catarrhini - ps35
Hominoidea - ps36
Hominidae - ps37
Homininae - ps38
Hominini - ps39
Homo_sapiens - ps40
Phylostratum

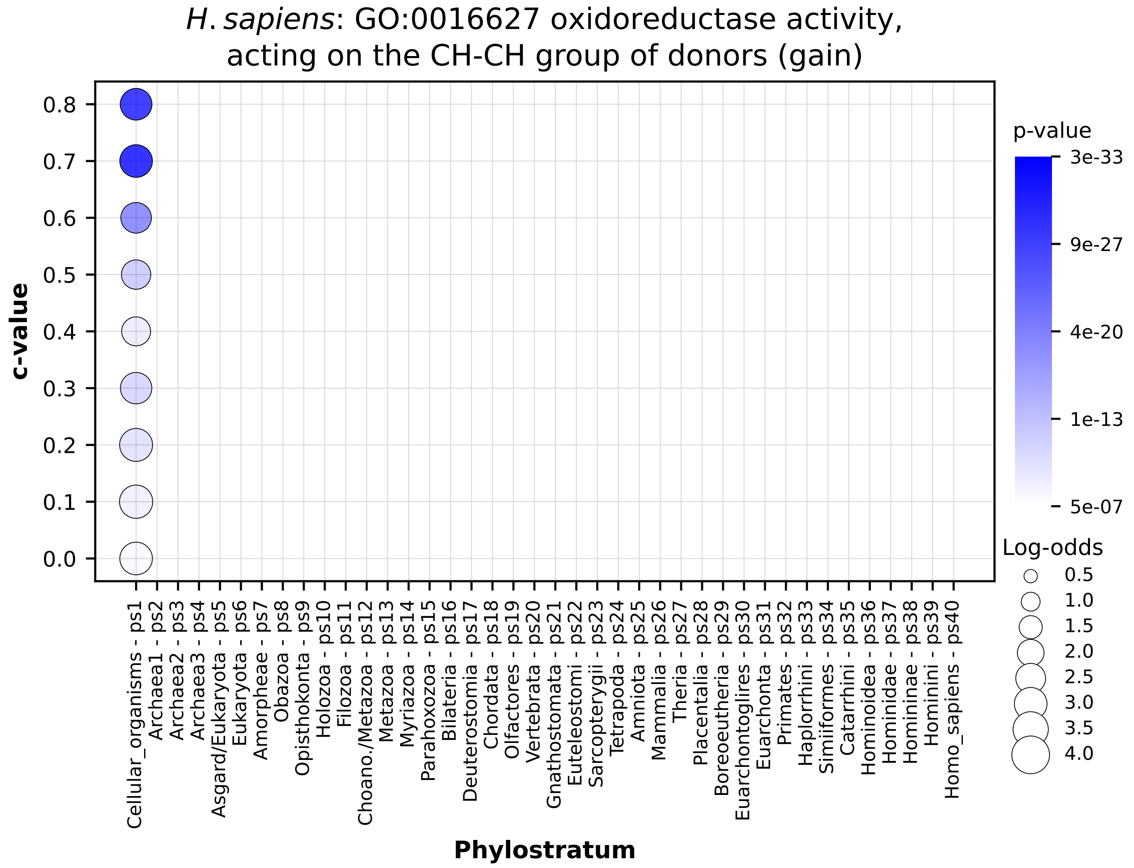

H. sapiens: GO:0016627 oxidoreductase activity, acting on the CH-CH group of donors (gain)
c-value
0.0
0.1
0.2
0.3
0.4
0.5
0.6
0.7
0.8
Phylostratum
Cellular_organisms - ps1
Archaea1 - ps2
Archaea2 - ps3
Archaea3 - ps4
Asgard/Eukaryota - ps5
Eukaryota - ps6
Amorpheae - ps7
Obazoa - ps8
Opisthokonta - ps9
Holozoa - ps10
Filozoa - ps11
Choano./Metazoa - ps12
Metazoa - ps13
Myriazoa - ps14
Parahoxozoa - ps15
Bilateria - ps16
Deuterostomia - ps17
Chordata - ps18
Olfactores - ps19
Vertebrata - ps20
Gnathostomata - ps21
Euteleostomi - ps22
Sarcopterygii - ps23
Tetrapoda - ps24
Amniota - ps25
Mammalia - ps26
Theria - ps27
Placentalia - ps28
Boreoeutheria - ps29
Euarchontoglires - ps30
Euarchonta - ps31
Primates - ps32
Haplorrhini - ps33
Simiiformes - ps34
Catarrhini - ps35
Hominoidea - ps36
Hominidae - ps37
Homininae - ps38
Hominini - ps39
Homo_sapiens - ps40
p-value
3e-33
9e-27
4e-20
1e-13
5e-07
Log-odds
0.5
1.0
1.5
2.0
2.5
3.0
3.5
4.0

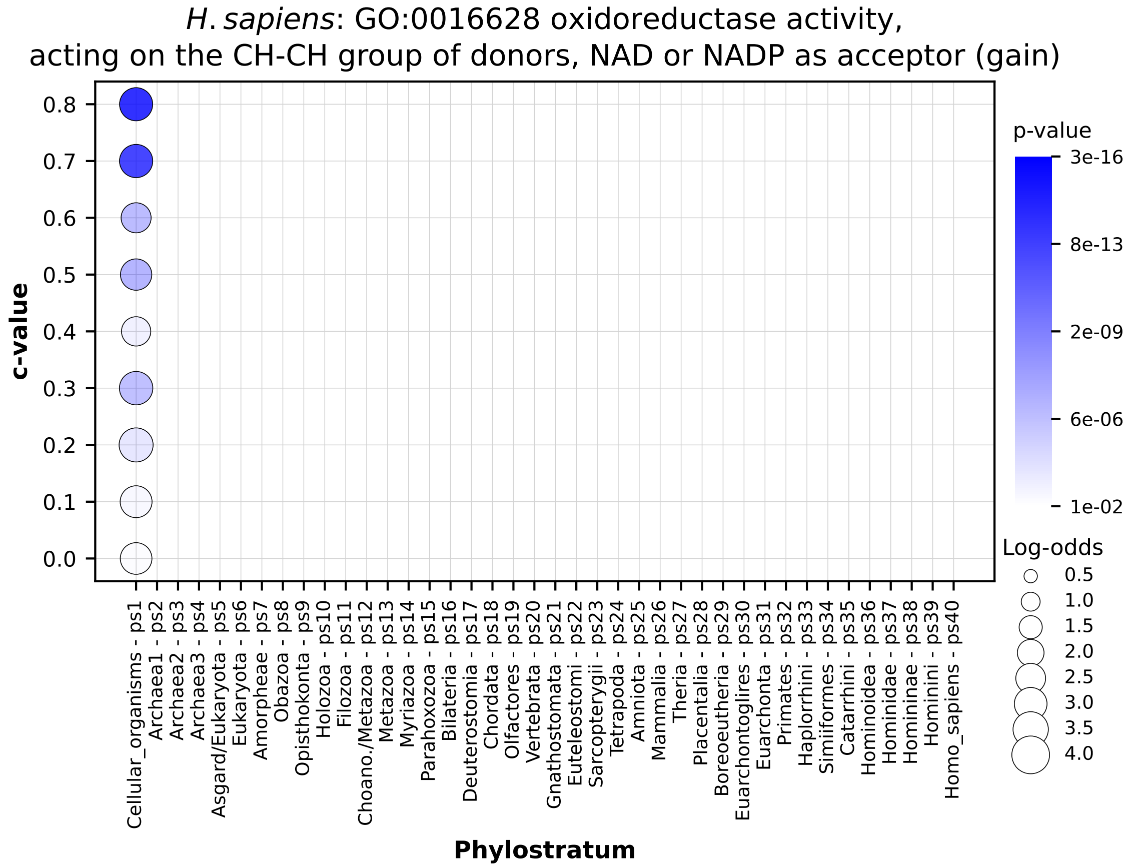
H. sapiens: GO:0016628 oxidoreductase activity,
acting on the CH-CH group of donors, NAD or NADP as acceptor (gain)
c-value
0.8
0.7
0.6
0.5
0.4
0.3
0.2
0.1
0.0
p-value
3e-16
8e-13
2e-09
6e-06
1e-02
Log-odds
0.5
1.0
1.5
2.0
2.5
3.0
3.5
4.0
Cellular_organisms - ps1
Archaea1 - ps2
Archaea2 - ps3
Archaea3 - ps4
Asgard/Eukaryota - ps5
Eukaryota - ps6
Amorpheae - ps7
Obazoa - ps8
Opisthokonta - ps9
Holozoa - ps10
Filozoa - ps11
Choano./Metazoa - ps12
Metazoa - ps13
Myriazoa - ps14
Parahoxozoa - ps15
Bilateria - ps16
Deuterostomia - ps17
Chordata - ps18
Olfactores - ps19
Vertebrata - ps20
Gnathostomata - ps21
Euteleostomi - ps22
Sarcopterygii - ps23
Tetrapoda - ps24
Amniota - ps25
Mammalia - ps26
Theria - ps27
Placentalia - ps28
Boreoeutheria - ps29
Euarchontoglires - ps30
Euarchonta - ps31
Primates - ps32
Haplorrhini - ps33
Simiiformes - ps34
Catarrhini - ps35
Hominoidea - ps36
Hominidae - ps37
Homininae - ps38
Hominini - ps39
Homo_sapiens - ps40
Phylostratum

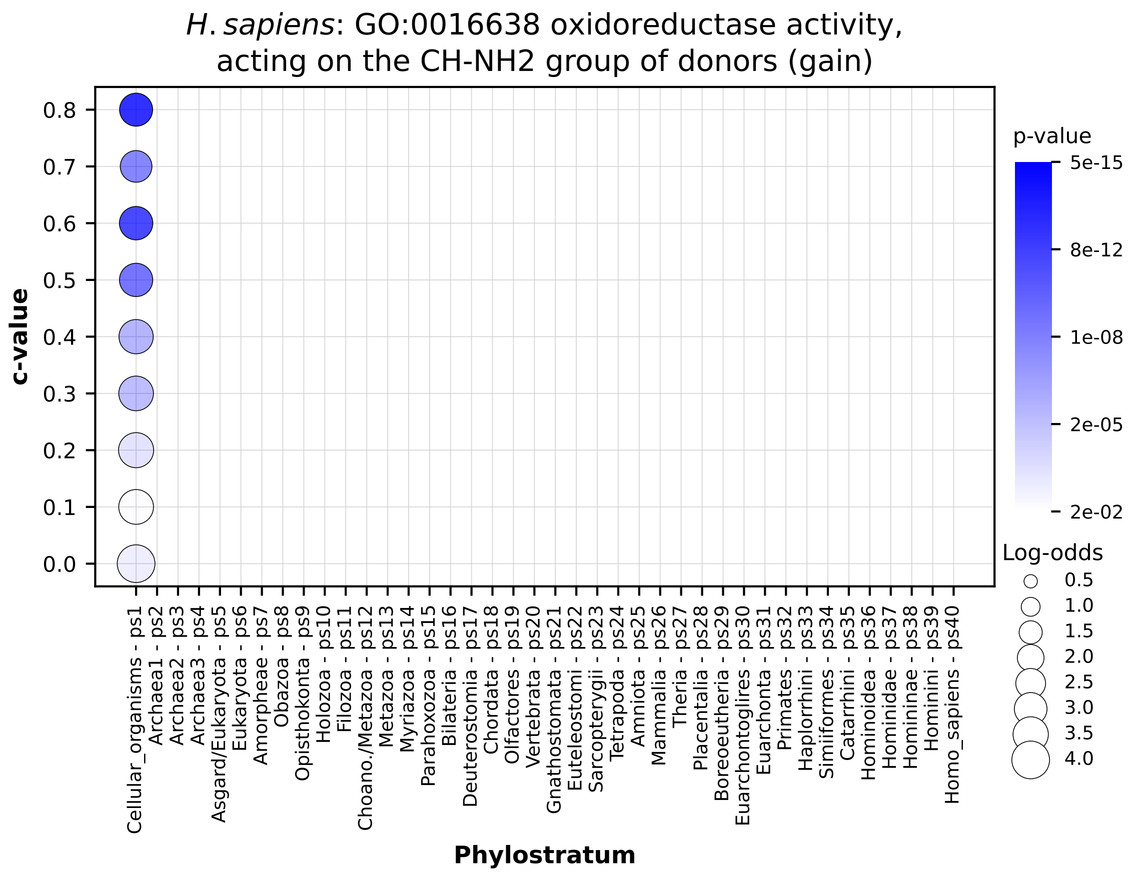

H. sapiens: GO:0016638 oxidoreductase activity, acting on the CH-NH2 group of donors (gain)
c-value
0.8
0.7
0.6
0.5
0.4
0.3
0.2
0.1
0.0
Phylostratum
Cellular_organisms - ps1
Archaea1 - ps2
Archaea2 - ps3
Archaea3 - ps4
Asgard/Eukaryota - ps5
Eukaryota - ps6
Amorpheae - ps7
Obazoa - ps8
Opisthokonta - ps9
Holozoa - ps10
Filozoa - ps11
Choano./Metazoa - ps12
Metazoa - ps13
Myriazoa - ps14
Parahoxozoa - ps15
Bilateria - ps16
Deuterostomia - ps17
Chordata - ps18
Olfactores - ps19
Vertebrata - ps20
Gnathostomata - ps21
Euteleostomi - ps22
Sarcopterygii - ps23
Tetrapoda - ps24
Amniota - ps25
Mammalia - ps26
Theria - ps27
Placentalia - ps28
Boreoeutheria - ps29
Euarchontoglires - ps30
Euarchonta - ps31
Primates - ps32
Haplorrhini - ps33
Simiiformes - ps34
Catarrhini - ps35
Hominoidea - ps36
Hominidae - ps37
Homininae - ps38
Hominini - ps39
Homo_sapiens - ps40
p-value
5e-15
8e-12
1e-08
2e-05
2e-02
Log-odds
0.5
1.0
1.5
2.0
2.5
3.0
3.5
4.0

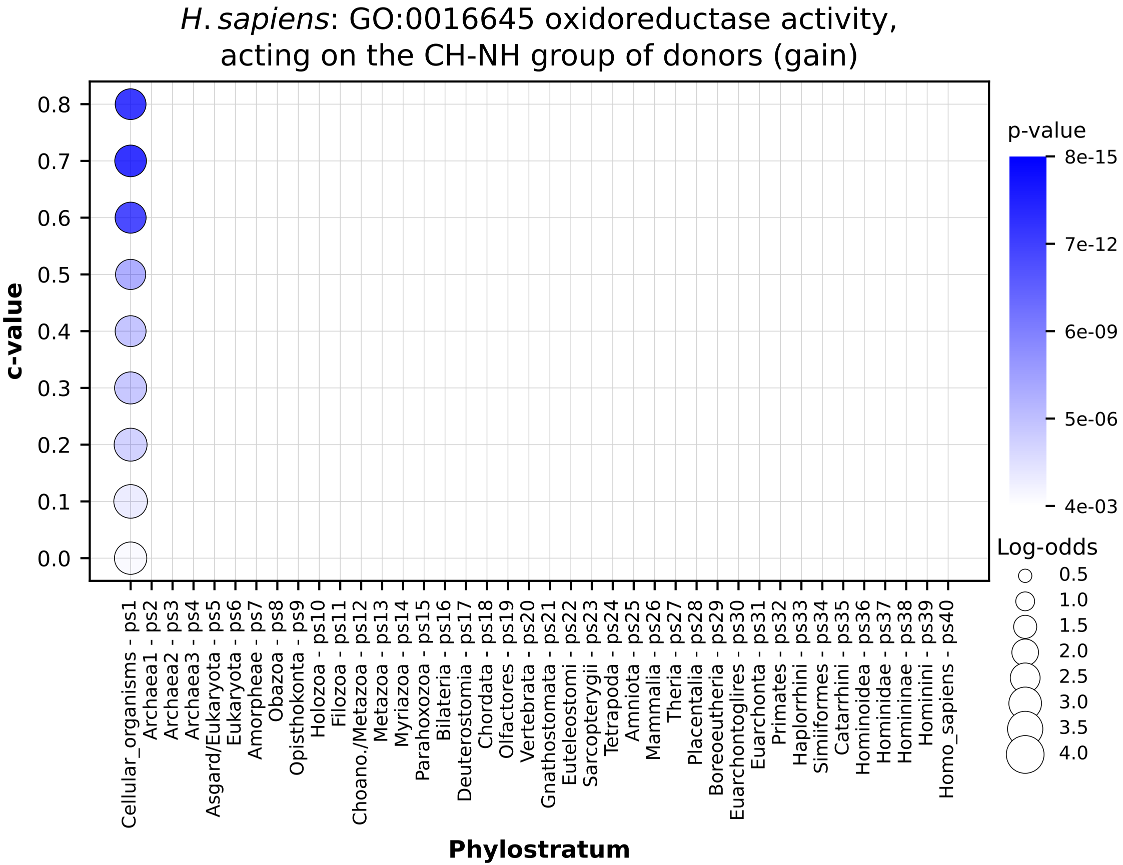

*H. sapiens*: GO:0016645 oxidoreductase activity, acting on the CH-NH group of donors (gain)
c-value
0.8
0.7
0.6
0.5
0.4
0.3
0.2
0.1
0.0
Phylostratum
Cellular_organisms - ps1
Archaea1 - ps2
Archaea2 - ps3
Archaea3 - ps4
Asgard/Eukaryota - ps5
Eukaryota - ps6
Amorpheae - ps7
Obazoa - ps8
Opisthokonta - ps9
Holozoa - ps10
Filozoa - ps11
Choano./Metazoa - ps12
Metazoa - ps13
Myriazoa - ps14
Parahoxozoa - ps15
Bilateria - ps16
Deuterostomia - ps17
Chordata - ps18
Olfactores - ps19
Vertebrata - ps20
Gnathostomata - ps21
Euteleostomi - ps22
Sarcopterygii - ps23
Tetrapoda - ps24
Amniota - ps25
Mammalia - ps26
Theria - ps27
Placentalia - ps28
Boreoeutheria - ps29
Euarchontoglires - ps30
Euarchonta - ps31
Primates - ps32
Haplorrhini - ps33
Simiiformes - ps34
Catarrhini - ps35
Hominoidea - ps36
Hominidae - ps37
Homininae - ps38
Hominini - ps39
Homo_sapiens - ps40
p-value
8e-15
7e-12
6e-09
5e-06
4e-03
Log-odds
0.5
1.0
1.5
2.0
2.5
3.0
3.5
4.0

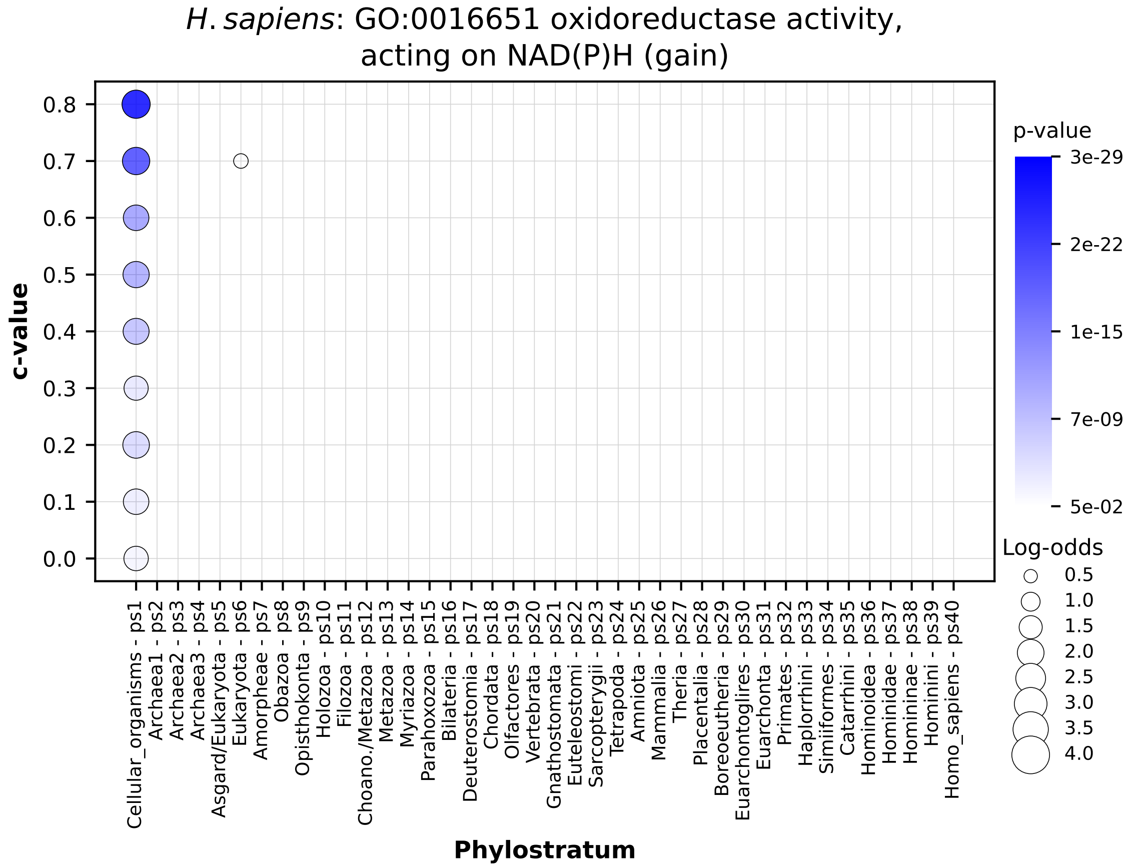
H. sapiens: GO:0016651 oxidoreductase activity, acting on NAD(P)H (gain)
c-value
0.8
0.7
0.6
0.5
0.4
0.3
0.2
0.1
0.0
Phylostratum
Cellular_organisms - ps1
Archaea1 - ps2
Archaea2 - ps3
Archaea3 - ps4
Asgard/Eukaryota - ps5
Eukaryota - ps6
Amorpheae - ps7
Obazoa - ps8
Opisthokonta - ps9
Holozoa - ps10
Filozoa - ps11
Choano./Metazoa - ps12
Metazoa - ps13
Myriazoa - ps14
Parahoxozoa - ps15
Bilateria - ps16
Deuterostomia - ps17
Chordata - ps18
Olfactores - ps19
Vertebrata - ps20
Gnathostomata - ps21
Euteleostomi - ps22
Sarcopterygii - ps23
Tetrapoda - ps24
Amniota - ps25
Mammalia - ps26
Theria - ps27
Placentalia - ps28
Boreoeutheria - ps29
Euarchontoglires - ps30
Euarchonta - ps31
Primates - ps32
Haplorrhini - ps33
Simiiformes - ps34
Catarrhini - ps35
Hominoidea - ps36
Hominidae - ps37
Homininae - ps38
Hominini - ps39
Homo_sapiens - ps40
p-value
3e-29
2e-22
1e-15
7e-09
5e-02
Log-odds
0.5
1.0
1.5
2.0
2.5
3.0
3.5
4.0

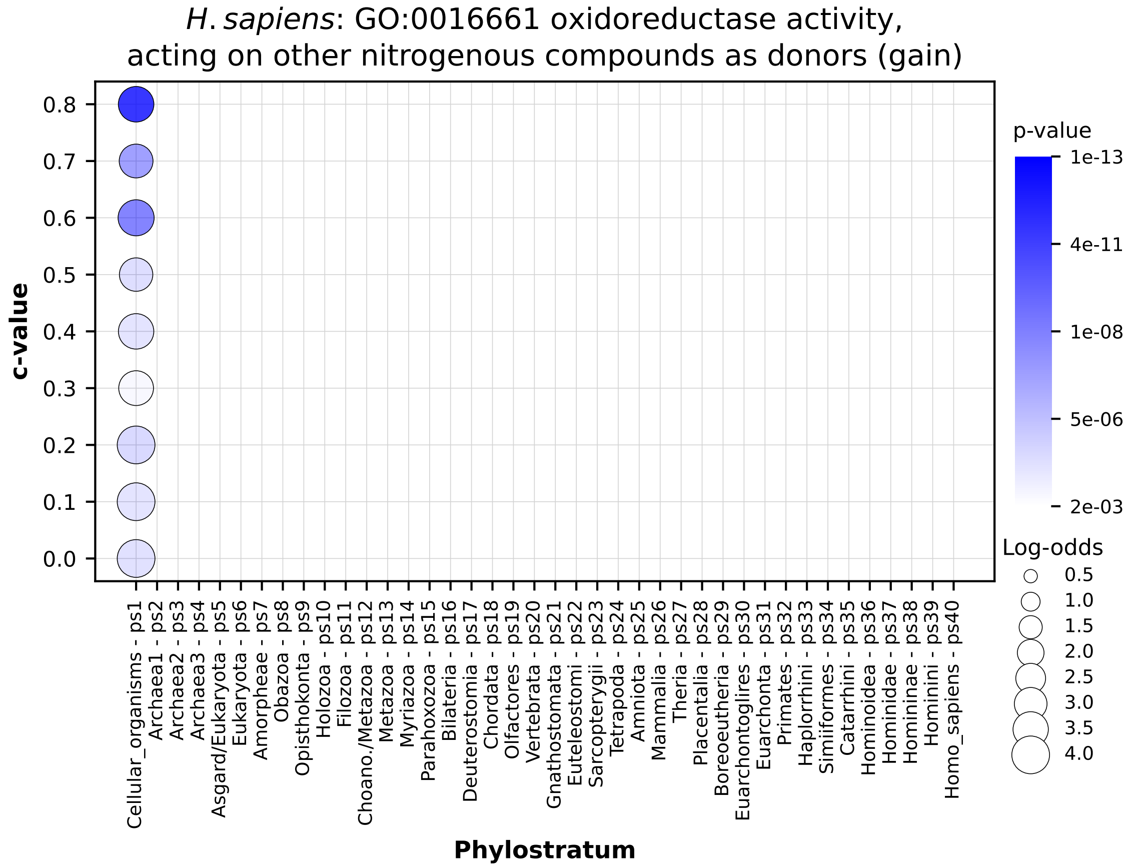

H. sapiens: GO:0016661 oxidoreductase activity,
acting on other nitrogenous compounds as donors (gain)
c-value
0.0
0.1
0.2
0.3
0.4
0.5
0.6
0.7
0.8
Phylostratum
Cellular_organisms - ps1
Archaea1 - ps2
Archaea2 - ps3
Archaea3 - ps4
Asgard/Eukaryota - ps5
Eukaryota - ps6
Amorpheae - ps7
Obazoa - ps8
Opisthokonta - ps9
Holozoa - ps10
Filozoa - ps11
Choano./Metazoa - ps12
Metazoa - ps13
Myriazoa - ps14
Parahoxozoa - ps15
Bilateria - ps16
Deuterostomia - ps17
Chordata - ps18
Olfactores - ps19
Vertebrata - ps20
Gnathostomata - ps21
Euteleostomi - ps22
Sarcopterygii - ps23
Tetrapoda - ps24
Amniota - ps25
Mammalia - ps26
Theria - ps27
Placentalia - ps28
Boreoeutheria - ps29
Euarchontoglires - ps30
Euarchonta - ps31
Primates - ps32
Haplorrhini - ps33
Simiiformes - ps34
Catarrhini - ps35
Hominoidea - ps36
Hominidae - ps37
Homininae - ps38
Hominini - ps39
Homo_sapiens - ps40
p-value
1e-13
4e-11
1e-08
5e-06
2e-03
Log-odds
0.5
1.0
1.5
2.0
2.5
3.0
3.5
4.0

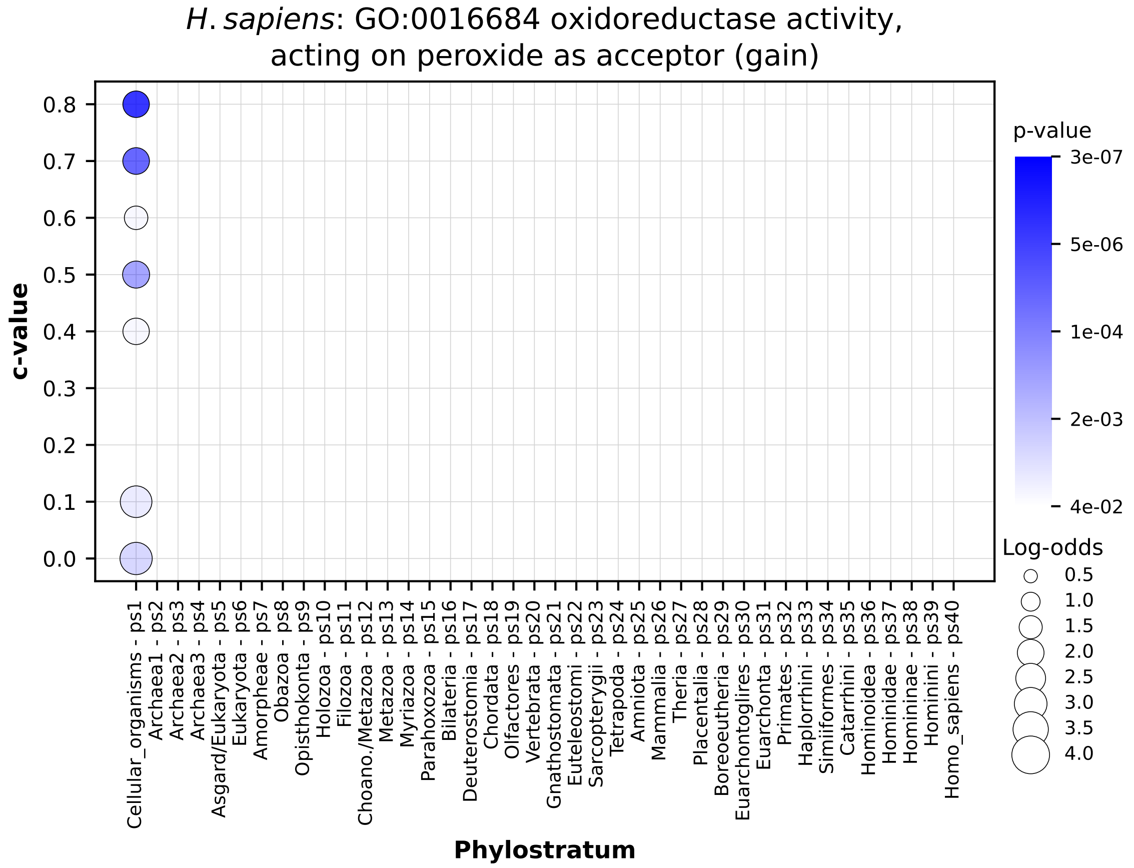
H. sapiens: GO:0016684 oxidoreductase activity, acting on peroxide as acceptor (gain)
c-value
0.8
0.7
0.6
0.5
0.4
0.3
0.2
0.1
0.0
Phylostratum
Cellular_organisms - ps1
Archaea1 - ps2
Archaea2 - ps3
Archaea3 - ps4
Asgard/Eukaryota - ps5
Eukaryota - ps6
Amorpheae - ps7
Obazoa - ps8
Opisthokonta - ps9
Holozoa - ps10
Filozoa - ps11
Choano./Metazoa - ps12
Metazoa - ps13
Myriazoa - ps14
Parahoxozoa - ps15
Bilateria - ps16
Deuterostomia - ps17
Chordata - ps18
Olfactores - ps19
Vertebrata - ps20
Gnathostomata - ps21
Euteleostomi - ps22
Sarcopterygii - ps23
Tetrapoda - ps24
Amniota - ps25
Mammalia - ps26
Theria - ps27
Placentalia - ps28
Boreoeutheria - ps29
Euarchontoglires - ps30
Euarchonta - ps31
Primates - ps32
Haplorrhini - ps33
Simiiformes - ps34
Catarrhini - ps35
Hominoidea - ps36
Hominidae - ps37
Homininae - ps38
Hominini - ps39
Homo_sapiens - ps40
p-value
3e-07
5e-06
1e-04
2e-03
4e-02
Log-odds
0.5
1.0
1.5
2.0
2.5
3.0
3.5
4.0

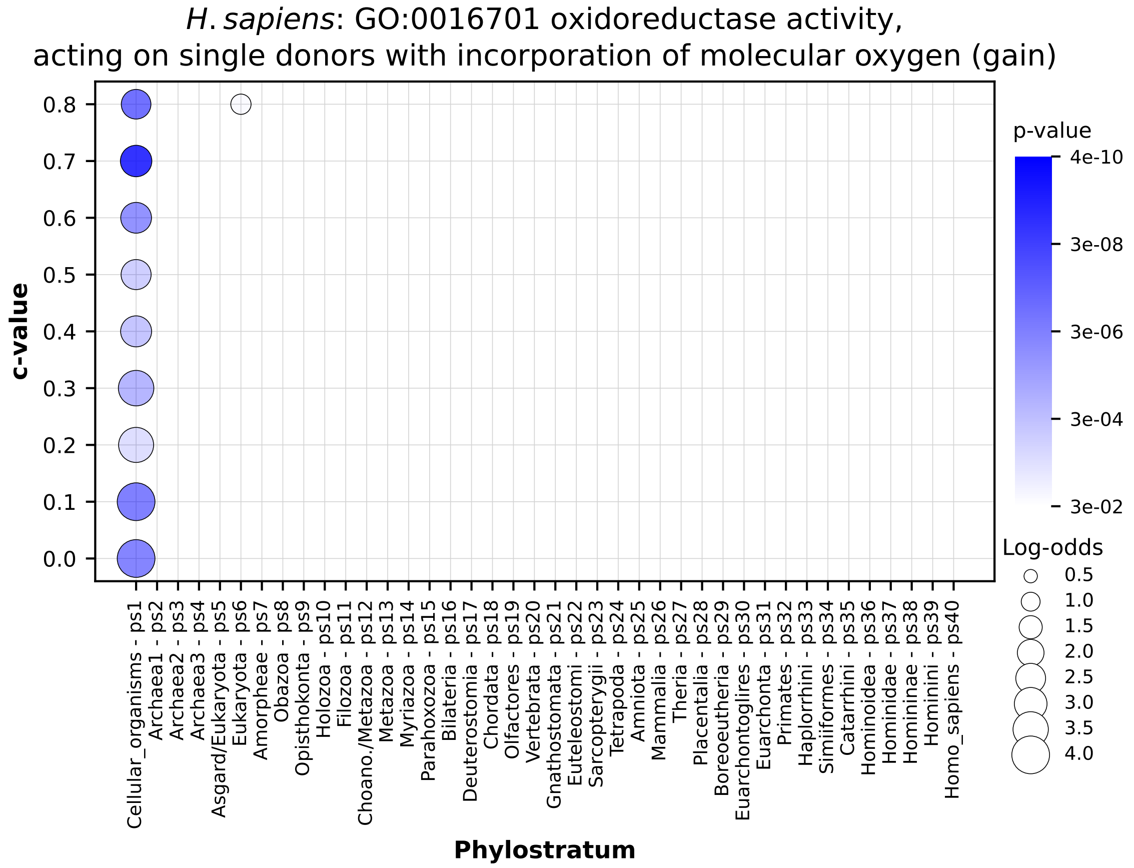

*H. sapiens*: GO:0016701 oxidoreductase activity,
acting on single donors with incorporation of molecular oxygen (gain)
c-value
0.0
0.1
0.2
0.3
0.4
0.5
0.6
0.7
0.8
p-value
4e-10
3e-08
3e-06
3e-04
3e-02
Log-odds
0.5
1.0
1.5
2.0
2.5
3.0
3.5
4.0
Cellular_organisms - ps1
Archaea1 - ps2
Archaea2 - ps3
Archaea3 - ps4
Asgard/Eukaryota - ps5
Eukaryota - ps6
Amorpheae - ps7
Obazoa - ps8
Opisthokonta - ps9
Holozoa - ps10
Filozoa - ps11
Choano./Metazoa - ps12
Metazoa - ps13
Myriazoa - ps14
Parahoxozoa - ps15
Bilateria - ps16
Deuterostomia - ps17
Chordata - ps18
Olfactores - ps19
Vertebrata - ps20
Gnathostomata - ps21
Euteleostomi - ps22
Sarcopterygii - ps23
Tetrapoda - ps24
Amniota - ps25
Mammalia - ps26
Theria - ps27
Placentalia - ps28
Boreoeutheria - ps29
Euarchontoglires - ps30
Euarchonta - ps31
Primates - ps32
Haplorrhini - ps33
Simiiformes - ps34
Catarrhini - ps35
Hominoidea - ps36
Hominidae - ps37
Homininae - ps38
Hominini - ps39
Homo_sapiens - ps40
Phylostratum

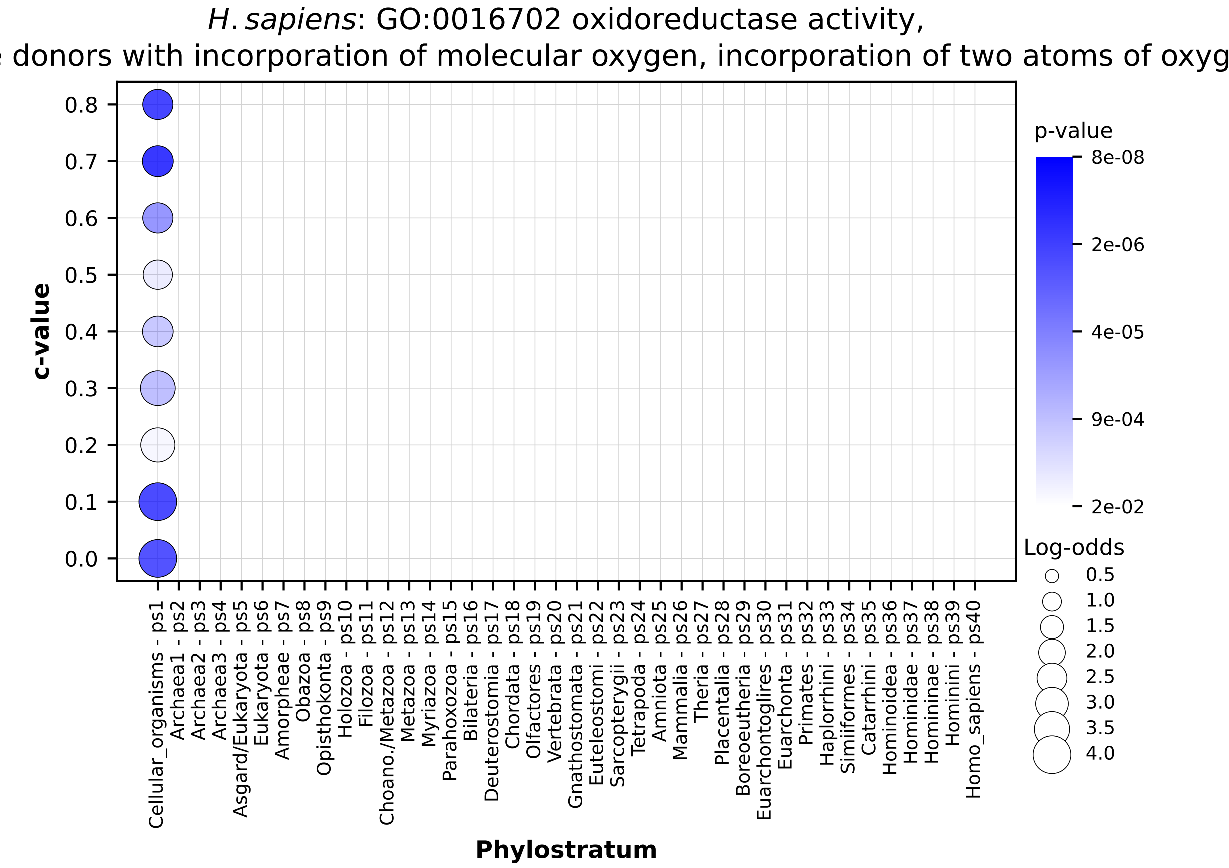

*H. sapiens*: GO:0016702 oxidoreductase activity,
e donors with incorporation of molecular oxygen, incorporation of two atoms of oxyg
c-value
0.8
0.7
0.6
0.5
0.4
0.3
0.2
0.1
0.0
p-value
8e-08
2e-06
4e-05
9e-04
2e-02
Log-odds
0.5
1.0
1.5
2.0
2.5
3.0
3.5
4.0
Cellular_organisms - ps1
Archaea1 - ps2
Archaea2 - ps3
Archaea3 - ps4
Asgard/Eukaryota - ps5
Eukaryota - ps6
Amorpheae - ps7
Obazoa - ps8
Opisthokonta - ps9
Holozoa - ps10
Filozoa - ps11
Choano./Metazoa - ps12
Metazoa - ps13
Myriazoa - ps14
Parahoxozoa - ps15
Bilateria - ps16
Deuterostomia - ps17
Chordata - ps18
Olfactores - ps19
Vertebrata - ps20
Gnathostomata - ps21
Euteleostomi - ps22
Sarcopterygii - ps23
Tetrapoda - ps24
Amniota - ps25
Mammalia - ps26
Theria - ps27
Placentalia - ps28
Boreoeutheria - ps29
Euarchontoglires - ps30
Euarchonta - ps31
Primates - ps32
Haplorrhini - ps33
Simiiformes - ps34
Catarrhini - ps35
Hominoidea - ps36
Hominidae - ps37
Homininae - ps38
Hominini - ps39
Homo_sapiens - ps40
Phylostratum

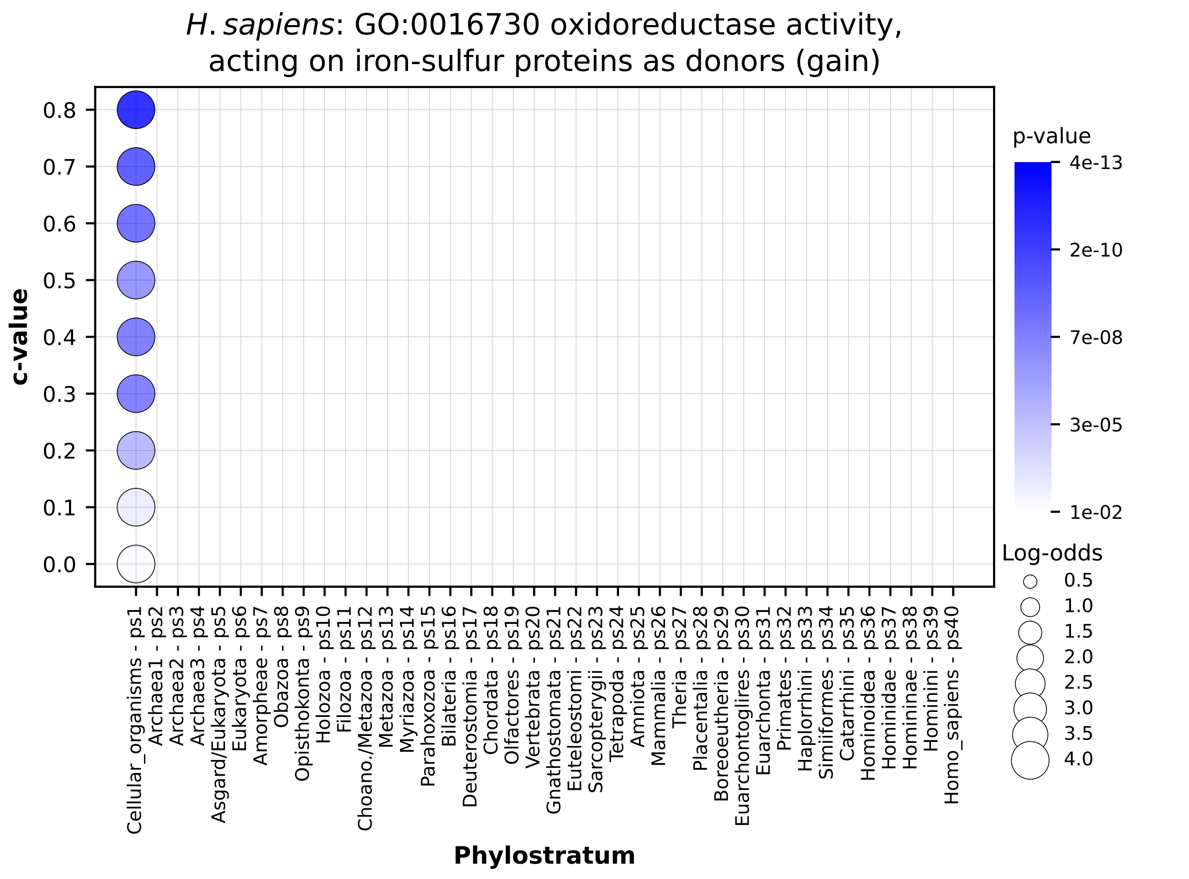
H. sapiens: GO:0016730 oxidoreductase activity, acting on iron-sulfur proteins as donors (gain)
c-value
0.8
0.7
0.6
0.5
0.4
0.3
0.2
0.1
0.0
p-value
4e-13
2e-10
7e-08
3e-05
1e-02
Log-odds
0.5
1.0
1.5
2.0
2.5
3.0
3.5
4.0
Cellular_organisms - ps1
Archaea1 - ps2
Archaea2 - ps3
Archaea3 - ps4
Asgard/Eukaryota - ps5
Eukaryota - ps6
Amorpheae - ps7
Obazoa - ps8
Opisthokonta - ps9
Holozoa - ps10
Filozoa - ps11
Choano./Metazoa - ps12
Metazoa - ps13
Myriazoa - ps14
Parahoxozoa - ps15
Bilateria - ps16
Deuterostomia - ps17
Chordata - ps18
Olfactores - ps19
Vertebrata - ps20
Gnathostomata - ps21
Euteleostomi - ps22
Sarcopterygii - ps23
Tetrapoda - ps24
Amniota - ps25
Mammalia - ps26
Theria - ps27
Placentalia - ps28
Boreoeutheria - ps29
Euarchontoglires - ps30
Euarchonta - ps31
Primates - ps32
Haplorrhini - ps33
Simiiformes - ps34
Catarrhini - ps35
Hominoidea - ps36
Hominidae - ps37
Homininae - ps38
Hominini - ps39
Homo_sapiens - ps40
Phylostratum

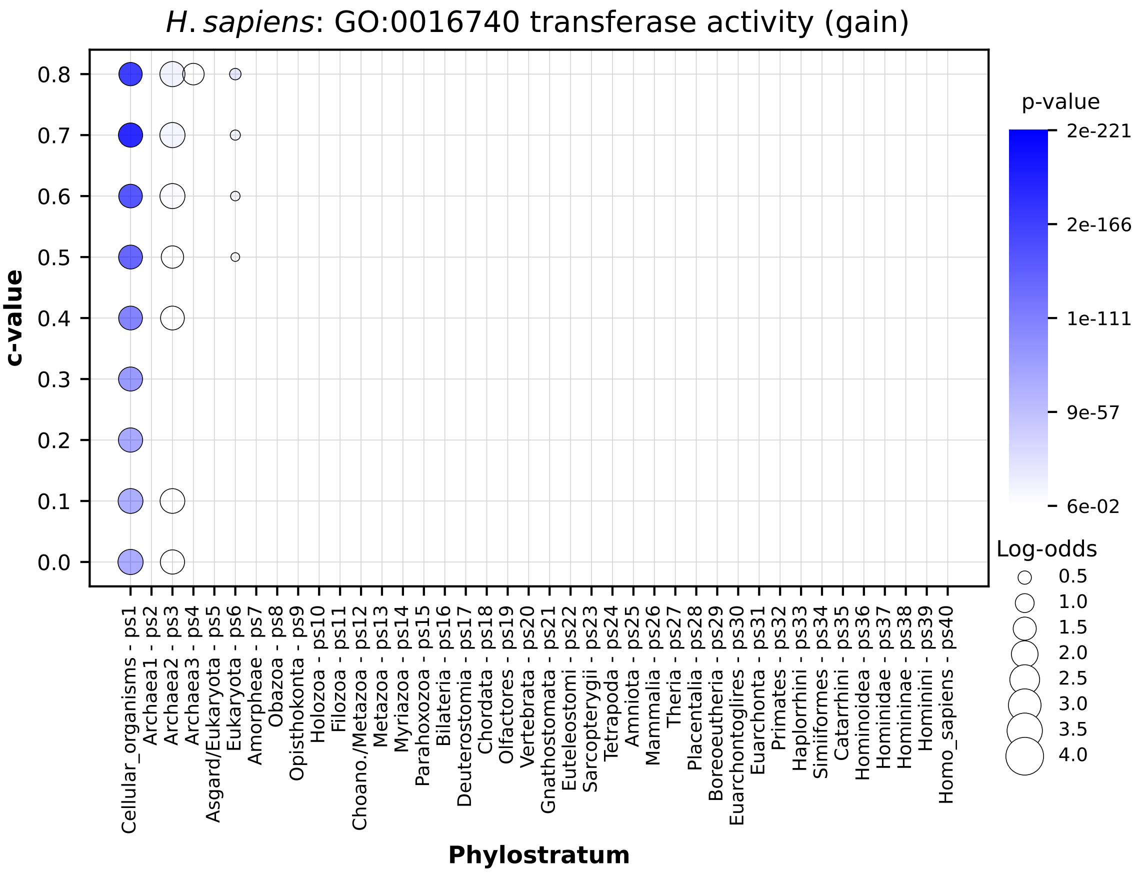

H. sapiens: GO:0016740 transferase activity (gain)
c-value
0.0
0.1
0.2
0.3
0.4
0.5
0.6
0.7
0.8
Phylostratum
Cellular_organisms - ps1
Archaea1 - ps2
Archaea2 - ps3
Archaea3 - ps4
Asgard/Eukaryota - ps5
Eukaryota - ps6
Amorpheae - ps7
Obazoa - ps8
Opisthokonta - ps9
Holozoa - ps10
Filozoa - ps11
Choano./Metazoa - ps12
Metazoa - ps13
Myriazoa - ps14
Parahoxozoa - ps15
Bilateria - ps16
Deuterostomia - ps17
Chordata - ps18
Olfactores - ps19
Vertebrata - ps20
Gnathostomata - ps21
Euteleostomi - ps22
Sarcopterygii - ps23
Tetrapoda - ps24
Amniota - ps25
Mammalia - ps26
Theria - ps27
Placentalia - ps28
Boreoeutheria - ps29
Euarchontoglires - ps30
Euarchonta - ps31
Primates - ps32
Haplorrhini - ps33
Simiiformes - ps34
Catarrhini - ps35
Hominoidea - ps36
Hominidae - ps37
Homininae - ps38
Hominini - ps39
Homo_sapiens - ps40
p-value
2e-221
2e-166
1e-111
9e-57
6e-02
Log-odds
0.5
1.0
1.5
2.0
2.5
3.0
3.5
4.0

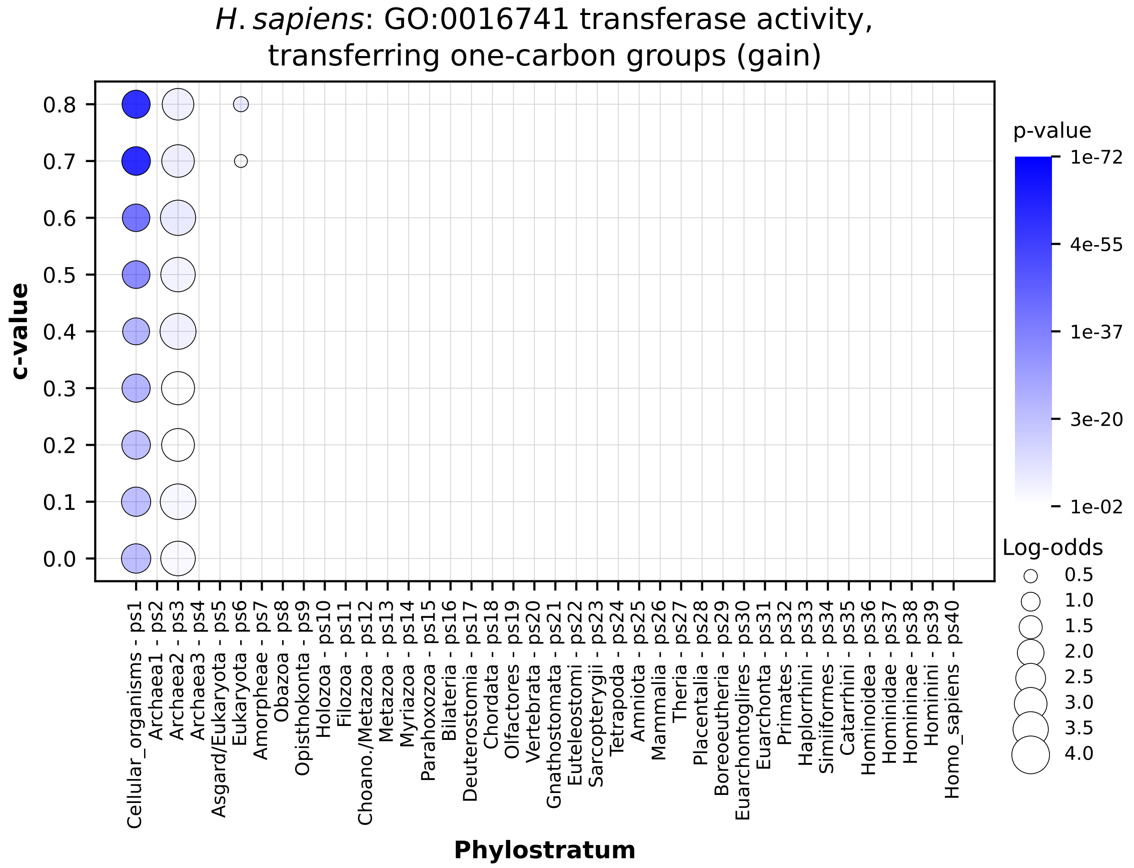

*H. sapiens*: GO:0016741 transferase activity, transferring one-carbon groups (gain)
c-value
0.8
0.7
0.6
0.5
0.4
0.3
0.2
0.1
0.0
p-value
1e-72
4e-55
1e-37
3e-20
1e-02
Log-odds
0.5
1.0
1.5
2.0
2.5
3.0
3.5
4.0
Cellular_organisms - ps1
Archaea1 - ps2
Archaea2 - ps3
Archaea3 - ps4
Asgard/Eukaryota - ps5
Eukaryota - ps6
Amorpheae - ps7
Obazoa - ps8
Opisthokonta - ps9
Holozoa - ps10
Filozoa - ps11
Choano./Metazoa - ps12
Metazoa - ps13
Myriazoa - ps14
Parahoxozoa - ps15
Bilateria - ps16
Deuterostomia - ps17
Chordata - ps18
Olfactores - ps19
Vertebrata - ps20
Gnathostomata - ps21
Euteleostomi - ps22
Sarcopterygii - ps23
Tetrapoda - ps24
Amniota - ps25
Mammalia - ps26
Theria - ps27
Placentalia - ps28
Boreoeutheria - ps29
Euarchontoglires - ps30
Euarchonta - ps31
Primates - ps32
Haplorrhini - ps33
Simiiformes - ps34
Catarrhini - ps35
Hominoidea - ps36
Hominidae - ps37
Homininae - ps38
Hominini - ps39
Homo_sapiens - ps40
Phylostratum

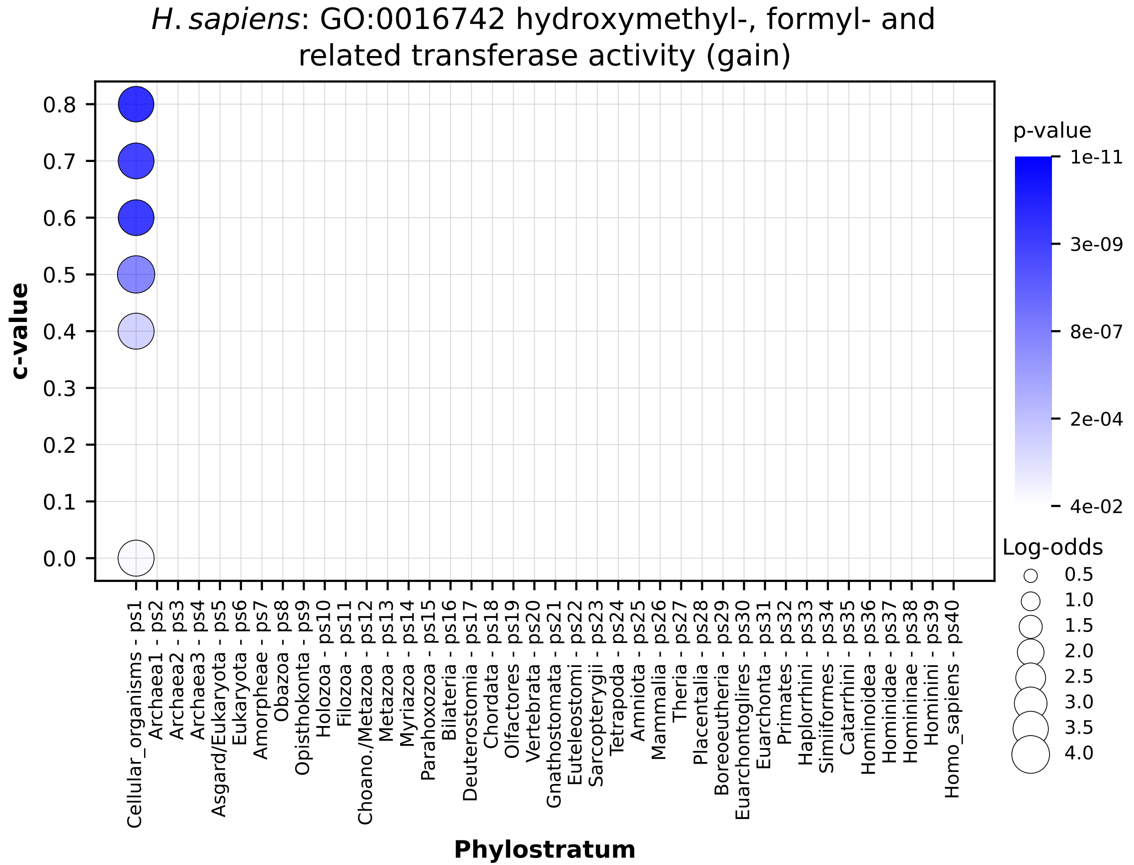

H. sapiens: GO:0016742 hydroxymethyl-, formyl- and related transferase activity (gain)
c-value
0.8
0.7
0.6
0.5
0.4
0.3
0.2
0.1
0.0
Phylostratum
Cellular_organisms - ps1
Archaea1 - ps2
Archaea2 - ps3
Archaea3 - ps4
Asgard/Eukaryota - ps5
Eukaryota - ps6
Amorpheae - ps7
Obazoa - ps8
Opisthokonta - ps9
Holozoa - ps10
Filozoa - ps11
Choano./Metazoa - ps12
Metazoa - ps13
Myriazoa - ps14
Parahoxozoa - ps15
Bilateria - ps16
Deuterostomia - ps17
Chordata - ps18
Olfactores - ps19
Vertebrata - ps20
Gnathostomata - ps21
Euteleostomi - ps22
Sarcopterygii - ps23
Tetrapoda - ps24
Amniota - ps25
Mammalia - ps26
Theria - ps27
Placentalia - ps28
Boreoeutheria - ps29
Euarchontoglires - ps30
Euarchonta - ps31
Primates - ps32
Haplorrhini - ps33
Simiiformes - ps34
Catarrhini - ps35
Hominoidea - ps36
Hominidae - ps37
Homininae - ps38
Hominini - ps39
Homo_sapiens - ps40
p-value
1e-11
3e-09
8e-07
2e-04
4e-02
Log-odds
0.5
1.0
1.5
2.0
2.5
3.0
3.5
4.0

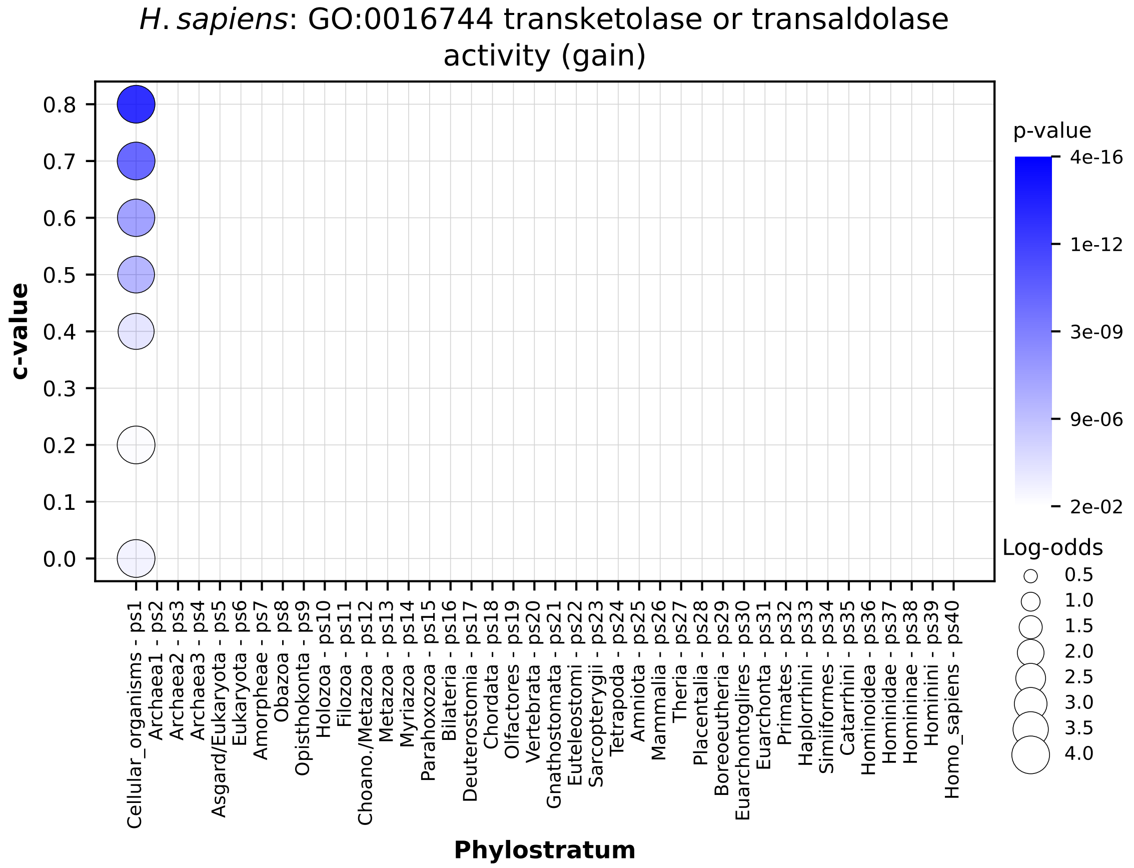

H. sapiens: GO:0016744 transketolase or transaldolase activity (gain)
c-value
0.0
0.1
0.2
0.3
0.4
0.5
0.6
0.7
0.8
p-value
4e-16
1e-12
3e-09
9e-06
2e-02
Log-odds
0.5
1.0
1.5
2.0
2.5
3.0
3.5
4.0
Cellular_organisms - ps1
Archaea1 - ps2
Archaea2 - ps3
Archaea3 - ps4
Asgard/Eukaryota - ps5
Eukaryota - ps6
Amorpheae - ps7
Obazoa - ps8
Opisthokonta - ps9
Holozoa - ps10
Filozoa - ps11
Choano./Metazoa - ps12
Metazoa - ps13
Myriazoa - ps14
Parahoxozoa - ps15
Bilateria - ps16
Deuterostomia - ps17
Chordata - ps18
Olfactores - ps19
Vertebrata - ps20
Gnathostomata - ps21
Euteleostomi - ps22
Sarcopterygii - ps23
Tetrapoda - ps24
Amniota - ps25
Mammalia - ps26
Theria - ps27
Placentalia - ps28
Boreoeutheria - ps29
Euarchontoglires - ps30
Euarchonta - ps31
Primates - ps32
Haplorrhini - ps33
Simiiformes - ps34
Catarrhini - ps35
Hominoidea - ps36
Hominidae - ps37
Homininae - ps38
Hominini - ps39
Homo_sapiens - ps40
Phylostratum

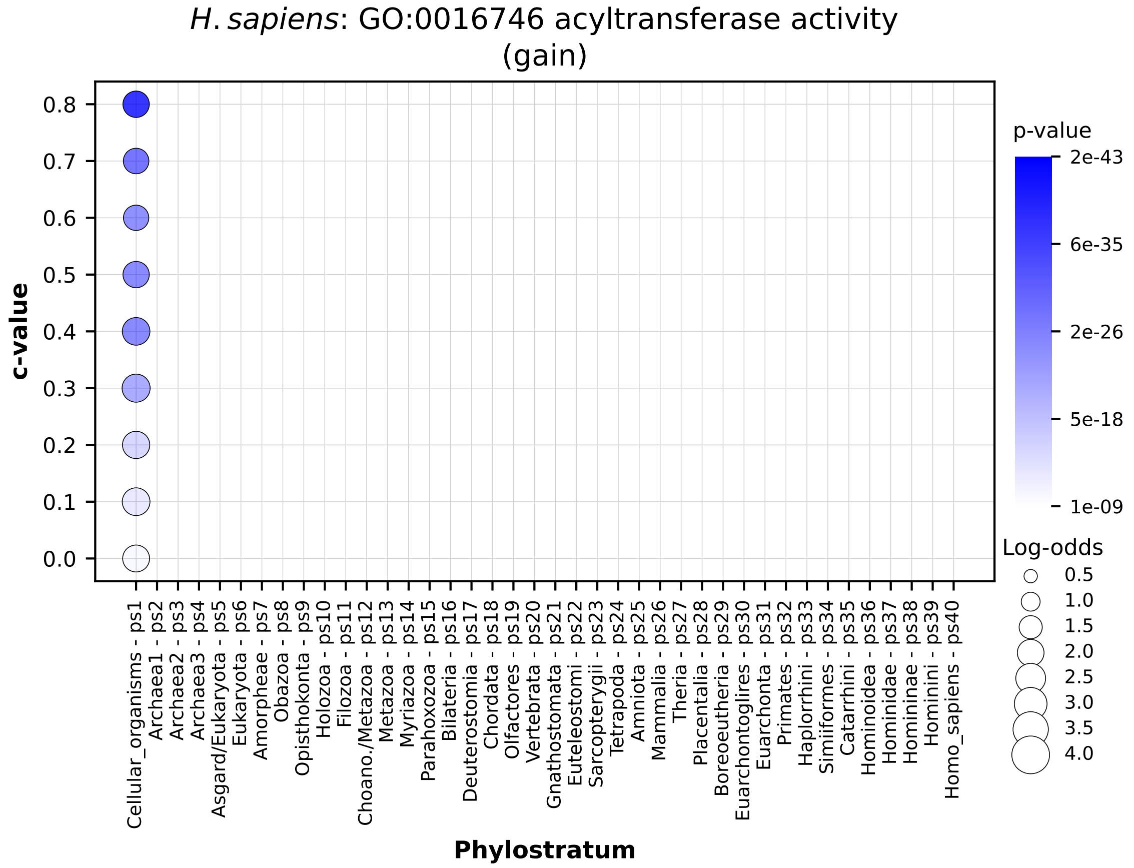

H. sapiens: GO:0016746 acyltransferase activity
(gain)
c-value
0.0
0.1
0.2
0.3
0.4
0.5
0.6
0.7
0.8
Phylostratum
Cellular_organisms - ps1
Archaea1 - ps2
Archaea2 - ps3
Archaea3 - ps4
Asgard/Eukaryota - ps5
Eukaryota - ps6
Amorpheae - ps7
Obazoa - ps8
Opisthokonta - ps9
Holozoa - ps10
Filozoa - ps11
Choano./Metazoa - ps12
Metazoa - ps13
Myriazoa - ps14
Parahoxozoa - ps15
Bilateria - ps16
Deuterostomia - ps17
Chordata - ps18
Olfactores - ps19
Vertebrata - ps20
Gnathostomata - ps21
Euteleostomi - ps22
Sarcopterygii - ps23
Tetrapoda - ps24
Amniota - ps25
Mammalia - ps26
Theria - ps27
Placentalia - ps28
Boreoeutheria - ps29
Euarchontoglires - ps30
Euarchonta - ps31
Primates - ps32
Haplorrhini - ps33
Simiiformes - ps34
Catarrhini - ps35
Hominoidea - ps36
Hominidae - ps37
Homininae - ps38
Hominini - ps39
Homo_sapiens - ps40
p-value
2e-43
6e-35
2e-26
5e-18
1e-09
Log-odds
0.5
1.0
1.5
2.0
2.5
3.0
3.5
4.0

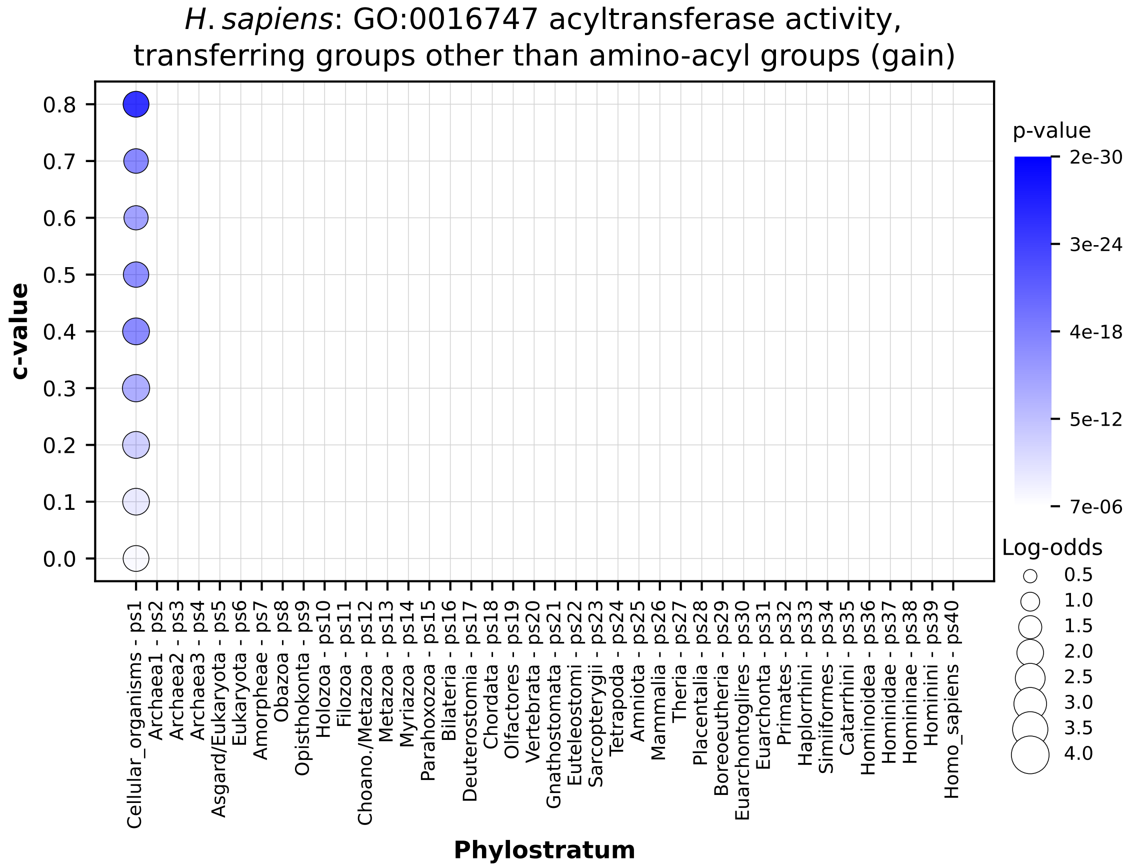
H. sapiens: GO:0016747 acyltransferase activity, transferring groups other than amino-acyl groups (gain)
c-value
0.8
0.7
0.6
0.5
0.4
0.3
0.2
0.1
0.0
p-value
2e-30
3e-24
4e-18
5e-12
7e-06
Log-odds
0.5
1.0
1.5
2.0
2.5
3.0
3.5
4.0
Cellular_organisms - ps1
Archaea1 - ps2
Archaea2 - ps3
Archaea3 - ps4
Asgard/Eukaryota - ps5
Eukaryota - ps6
Amorpheae - ps7
Obazoa - ps8
Opisthokonta - ps9
Holozoa - ps10
Filozoa - ps11
Choano./Metazoa - ps12
Metazoa - ps13
Myriazoa - ps14
Parahoxozoa - ps15
Bilateria - ps16
Deuterostomia - ps17
Chordata - ps18
Olfactores - ps19
Vertebrata - ps20
Gnathostomata - ps21
Euteleostomi - ps22
Sarcopterygii - ps23
Tetrapoda - ps24
Amniota - ps25
Mammalia - ps26
Theria - ps27
Placentalia - ps28
Boreoeutheria - ps29
Euarchontoglires - ps30
Euarchonta - ps31
Primates - ps32
Haplorrhini - ps33
Simiiformes - ps34
Catarrhini - ps35
Hominoidea - ps36
Hominidae - ps37
Homininae - ps38
Hominini - ps39
Homo_sapiens - ps40
Phylostratum

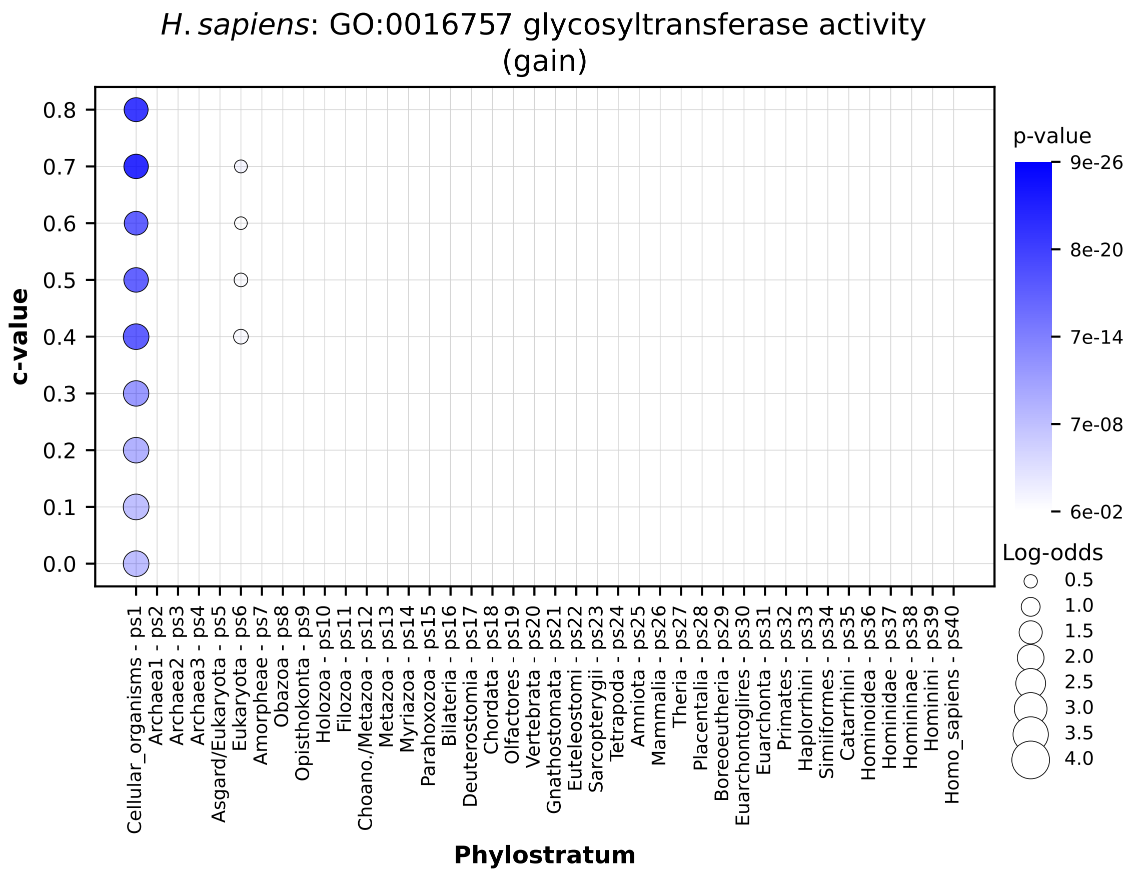

*H. sapiens*: GO:0016757 glycosyltransferase activity (gain)
c-value
0.8
0.7
0.6
0.5
0.4
0.3
0.2
0.1
0.0
p-value
9e-26
8e-20
7e-14
7e-08
6e-02
Log-odds
0.5
1.0
1.5
2.0
2.5
3.0
3.5
4.0
Cellular_organisms - ps1
Archaea1 - ps2
Archaea2 - ps3
Archaea3 - ps4
Asgard/Eukaryota - ps5
Eukaryota - ps6
Amorpheae - ps7
Obazoa - ps8
Opisthokonta - ps9
Holozoa - ps10
Filozoa - ps11
Choano./Metazoa - ps12
Metazoa - ps13
Myriazoa - ps14
Parahoxozoa - ps15
Bilateria - ps16
Deuterostomia - ps17
Chordata - ps18
Olfactores - ps19
Vertebrata - ps20
Gnathostomata - ps21
Euteleostomi - ps22
Sarcopterygii - ps23
Tetrapoda - ps24
Amniota - ps25
Mammalia - ps26
Theria - ps27
Placentalia - ps28
Boreoeutheria - ps29
Euarchontoglires - ps30
Euarchonta - ps31
Primates - ps32
Haplorrhini - ps33
Simiiformes - ps34
Catarrhini - ps35
Hominoidea - ps36
Hominidae - ps37
Homininae - ps38
Hominini - ps39
Homo_sapiens - ps40
Phylostratum

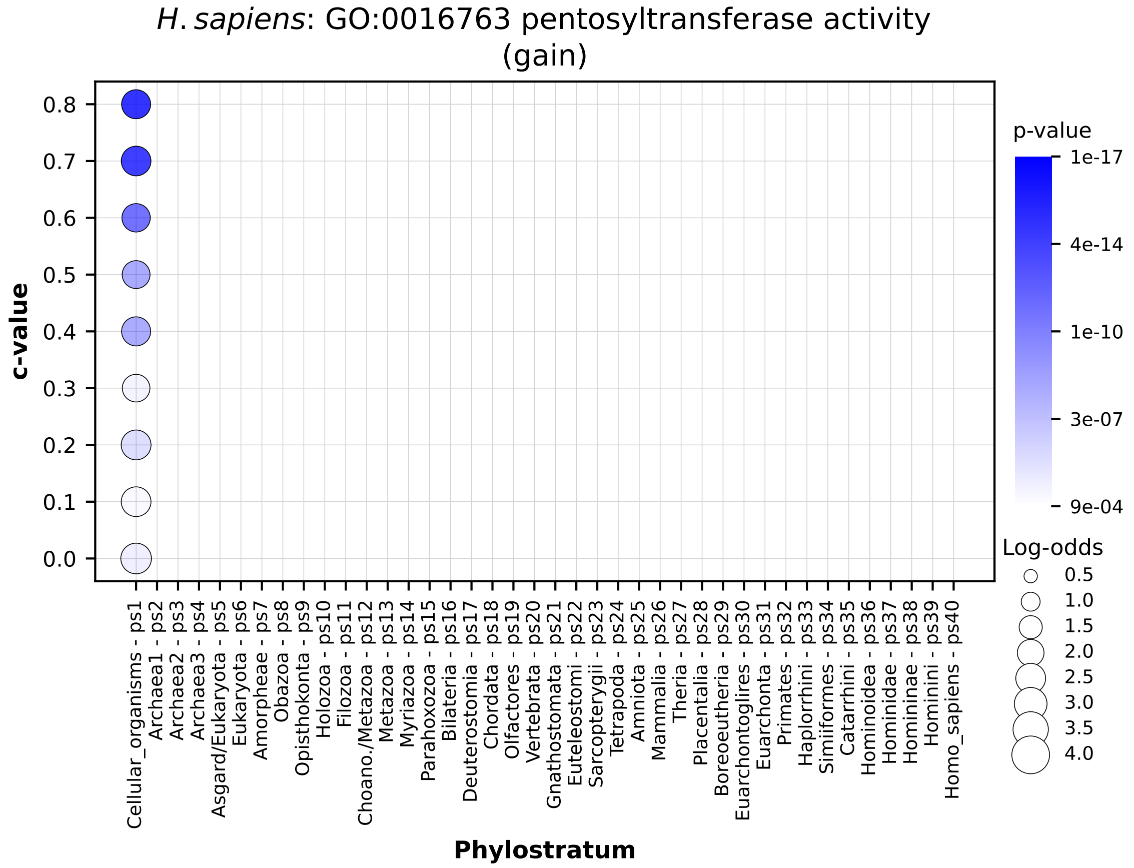

H. sapiens: GO:0016763 pentosyltransferase activity
(gain)
c-value
0.8
0.7
0.6
0.5
0.4
0.3
0.2
0.1
0.0
p-value
1e-17
4e-14
1e-10
3e-07
9e-04
Log-odds
0.5
1.0
1.5
2.0
2.5
3.0
3.5
4.0
Cellular_organisms - ps1
Archaea1 - ps2
Archaea2 - ps3
Archaea3 - ps4
Asgard/Eukaryota - ps5
Eukaryota - ps6
Amorpheae - ps7
Obazoa - ps8
Opisthokonta - ps9
Holozoa - ps10
Filozoa - ps11
Choano./Metazoa - ps12
Metazoa - ps13
Myriazoa - ps14
Parahoxozoa - ps15
Bilateria - ps16
Deuterostomia - ps17
Chordata - ps18
Olfactores - ps19
Vertebrata - ps20
Gnathostomata - ps21
Euteleostomi - ps22
Sarcopterygii - ps23
Tetrapoda - ps24
Amniota - ps25
Mammalia - ps26
Theria - ps27
Placentalia - ps28
Boreoeutheria - ps29
Euarchontoglires - ps30
Euarchonta - ps31
Primates - ps32
Haplorrhini - ps33
Simiiformes - ps34
Catarrhini - ps35
Hominoidea - ps36
Hominidae - ps37
Homininae - ps38
Hominini - ps39
Homo_sapiens - ps40
Phylostratum

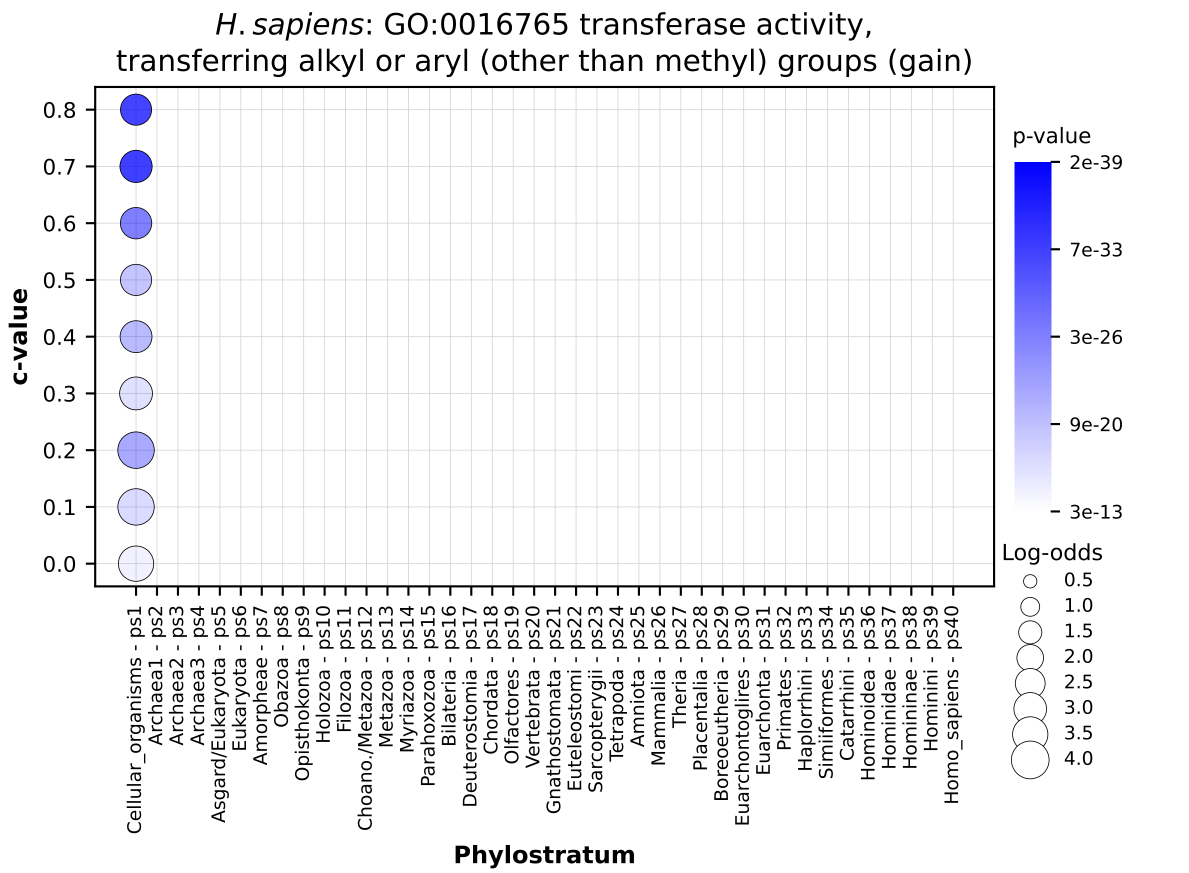
H. sapiens: GO:0016765 transferase activity, transferring alkyl or aryl (other than methyl) groups (gain)
c-value
0.0
0.1
0.2
0.3
0.4
0.5
0.6
0.7
0.8
Phylostratum
Cellular_organisms - ps1
Archaea1 - ps2
Archaea2 - ps3
Archaea3 - ps4
Asgard/Eukaryota - ps5
Eukaryota - ps6
Amorpheae - ps7
Obazoa - ps8
Opisthokonta - ps9
Holozoa - ps10
Filozoa - ps11
Choano./Metazoa - ps12
Metazoa - ps13
Myriazoa - ps14
Parahoxozoa - ps15
Bilateria - ps16
Deuterostomia - ps17
Chordata - ps18
Olfactores - ps19
Vertebrata - ps20
Gnathostomata - ps21
Euteleostomi - ps22
Sarcopterygii - ps23
Tetrapoda - ps24
Amniota - ps25
Mammalia - ps26
Theria - ps27
Placentalia - ps28
Boreoeutheria - ps29
Euarchontoglires - ps30
Euarchonta - ps31
Primates - ps32
Haplorrhini - ps33
Simiiformes - ps34
Catarrhini - ps35
Hominoidea - ps36
Hominidae - ps37
Homininae - ps38
Hominini - ps39
Homo_sapiens - ps40
p-value
2e-39
7e-33
3e-26
9e-20
3e-13
Log-odds
0.5
1.0
1.5
2.0
2.5
3.0
3.5
4.0

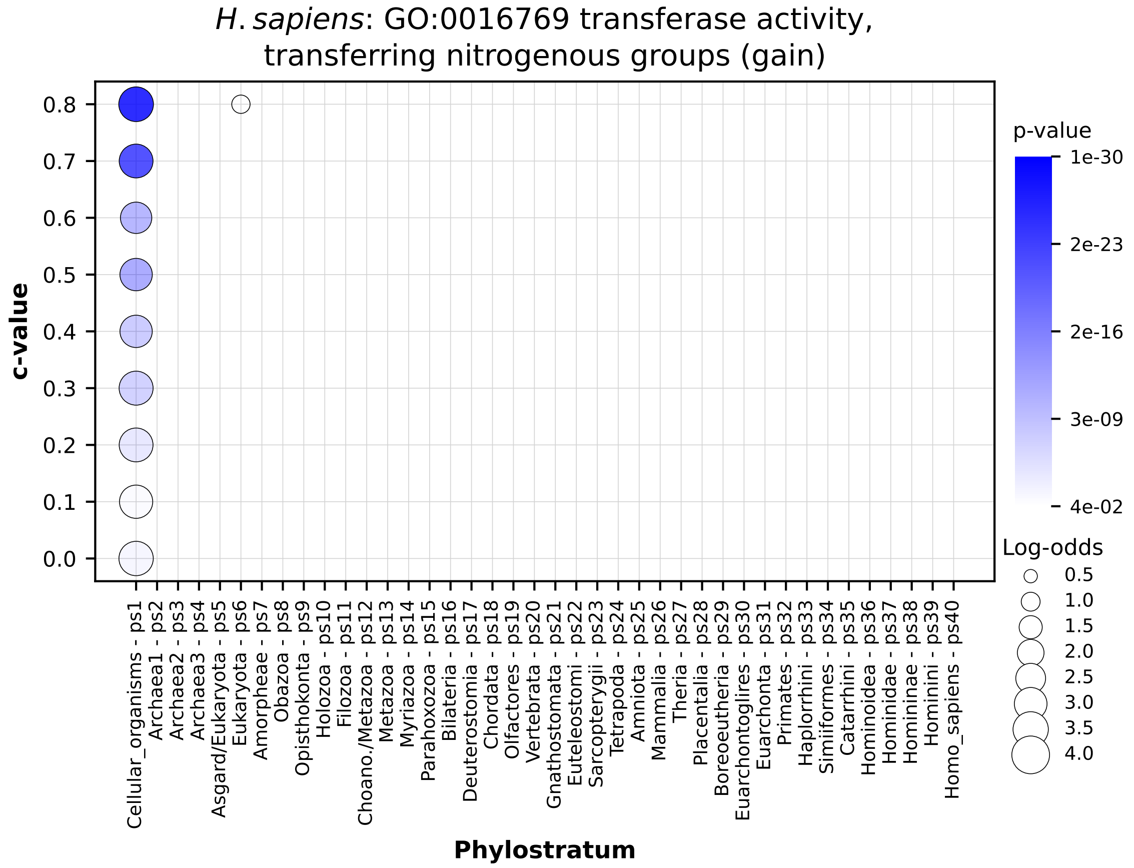

H. sapiens: GO:0016769 transferase activity, transferring nitrogenous groups (gain)
c-value
0.8
0.7
0.6
0.5
0.4
0.3
0.2
0.1
0.0
Phylostratum
Cellular_organisms - ps1
Archaea1 - ps2
Archaea2 - ps3
Archaea3 - ps4
Asgard/Eukaryota - ps5
Eukaryota - ps6
Amorpheae - ps7
Obazoa - ps8
Opisthokonta - ps9
Holozoa - ps10
Filozoa - ps11
Choano./Metazoa - ps12
Metazoa - ps13
Myriazoa - ps14
Parahoxozoa - ps15
Bilateria - ps16
Deuterostomia - ps17
Chordata - ps18
Olfactores - ps19
Vertebrata - ps20
Gnathostomata - ps21
Euteleostomi - ps22
Sarcopterygii - ps23
Tetrapoda - ps24
Amniota - ps25
Mammalia - ps26
Theria - ps27
Placentalia - ps28
Boreoeutheria - ps29
Euarchontoglires - ps30
Euarchonta - ps31
Primates - ps32
Haplorrhini - ps33
Simiiformes - ps34
Catarrhini - ps35
Hominoidea - ps36
Hominidae - ps37
Homininae - ps38
Hominini - ps39
Homo_sapiens - ps40
p-value
1e-30
2e-23
2e-16
3e-09
4e-02
Log-odds
0.5
1.0
1.5
2.0
2.5
3.0
3.5
4.0

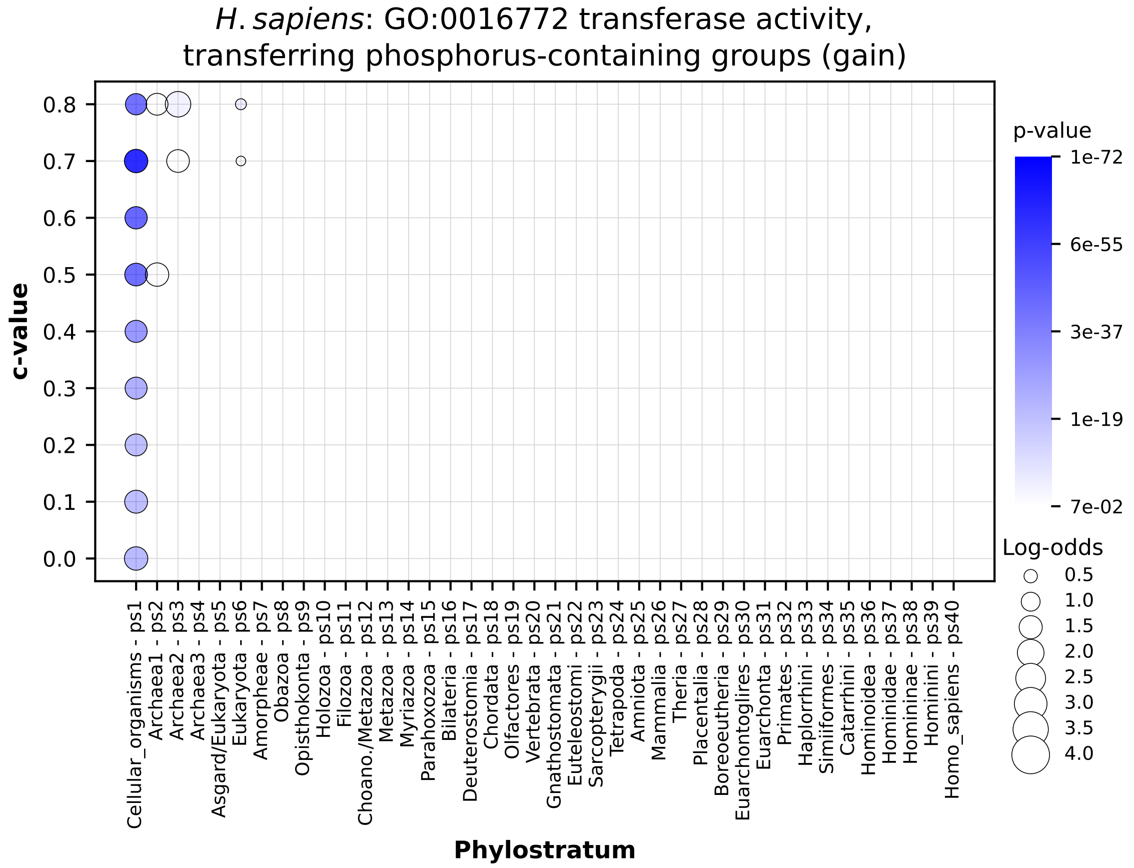

H. sapiens: GO:0016772 transferase activity, transferring phosphorus-containing groups (gain)
c-value
0.8
0.7
0.6
0.5
0.4
0.3
0.2
0.1
0.0
p-value
1e-72
6e-55
3e-37
1e-19
7e-02
Log-odds
0.5
1.0
1.5
2.0
2.5
3.0
3.5
4.0
Cellular_organisms - ps1
Archaea1 - ps2
Archaea2 - ps3
Archaea3 - ps4
Asgard/Eukaryota - ps5
Eukaryota - ps6
Amorpheae - ps7
Obazoa - ps8
Opisthokonta - ps9
Holozoa - ps10
Filozoa - ps11
Choano./Metazoa - ps12
Metazoa - ps13
Myriazoa - ps14
Parahoxozoa - ps15
Bilateria - ps16
Deuterostomia - ps17
Chordata - ps18
Olfactores - ps19
Vertebrata - ps20
Gnathostomata - ps21
Euteleostomi - ps22
Sarcopterygii - ps23
Tetrapoda - ps24
Amniota - ps25
Mammalia - ps26
Theria - ps27
Placentalia - ps28
Boreoeutheria - ps29
Euarchontoglires - ps30
Euarchonta - ps31
Primates - ps32
Haplorrhini - ps33
Simiiformes - ps34
Catarrhini - ps35
Hominoidea - ps36
Hominidae - ps37
Homininae - ps38
Hominini - ps39
Homo_sapiens - ps40
Phylostratum

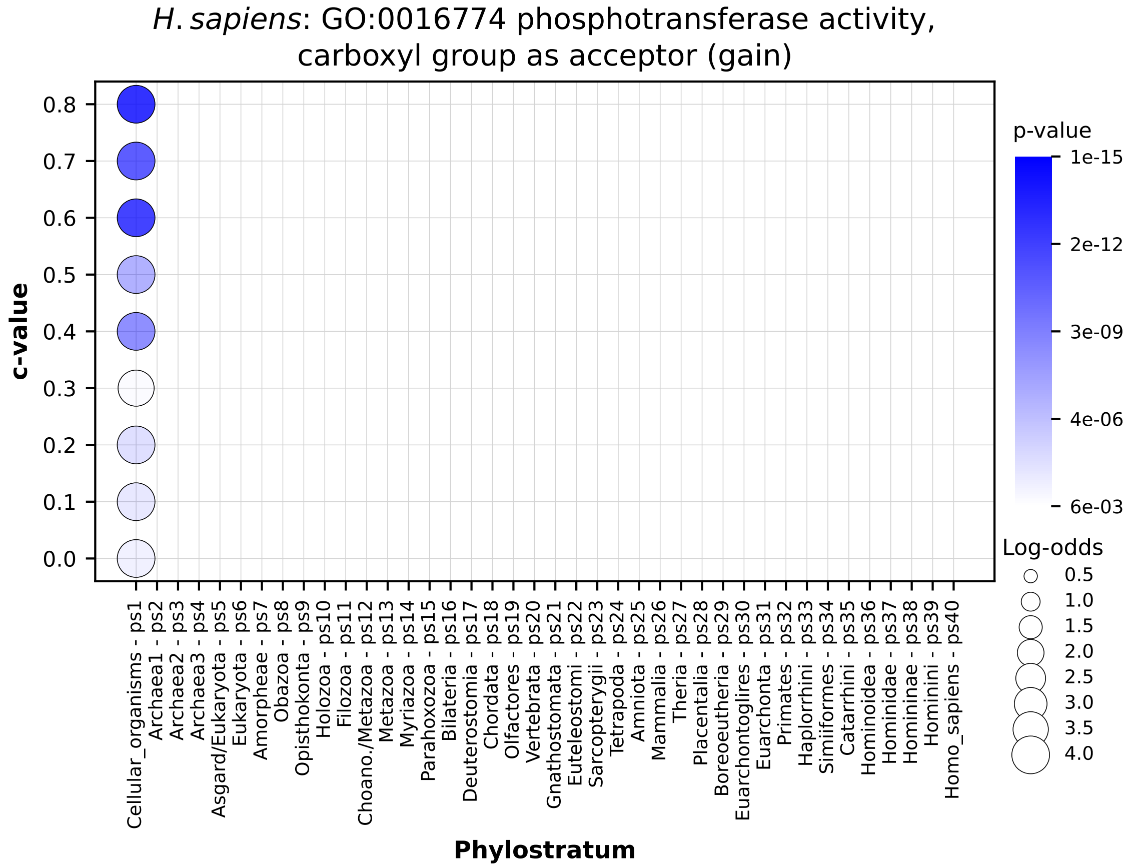
H. sapiens: GO:0016774 phosphotransferase activity, carboxyl group as acceptor (gain)
c-value
0.8
0.7
0.6
0.5
0.4
0.3
0.2
0.1
0.0
p-value
1e-15
2e-12
3e-09
4e-06
6e-03
Log-odds
0.5
1.0
1.5
2.0
2.5
3.0
3.5
4.0
Cellular_organisms - ps1
Archaea1 - ps2
Archaea2 - ps3
Archaea3 - ps4
Asgard/Eukaryota - ps5
Eukaryota - ps6
Amorpheae - ps7
Obazoa - ps8
Opisthokonta - ps9
Holozoa - ps10
Filozoa - ps11
Choano./Metazoa - ps12
Metazoa - ps13
Myriazoa - ps14
Parahoxozoa - ps15
Bilateria - ps16
Deuterostomia - ps17
Chordata - ps18
Olfactores - ps19
Vertebrata - ps20
Gnathostomata - ps21
Euteleostomi - ps22
Sarcopterygii - ps23
Tetrapoda - ps24
Amniota - ps25
Mammalia - ps26
Theria - ps27
Placentalia - ps28
Boreoeutheria - ps29
Euarchontoglires - ps30
Euarchonta - ps31
Primates - ps32
Haplorrhini - ps33
Simiiformes - ps34
Catarrhini - ps35
Hominoidea - ps36
Hominidae - ps37
Homininae - ps38
Hominini - ps39
Homo_sapiens - ps40
Phylostratum

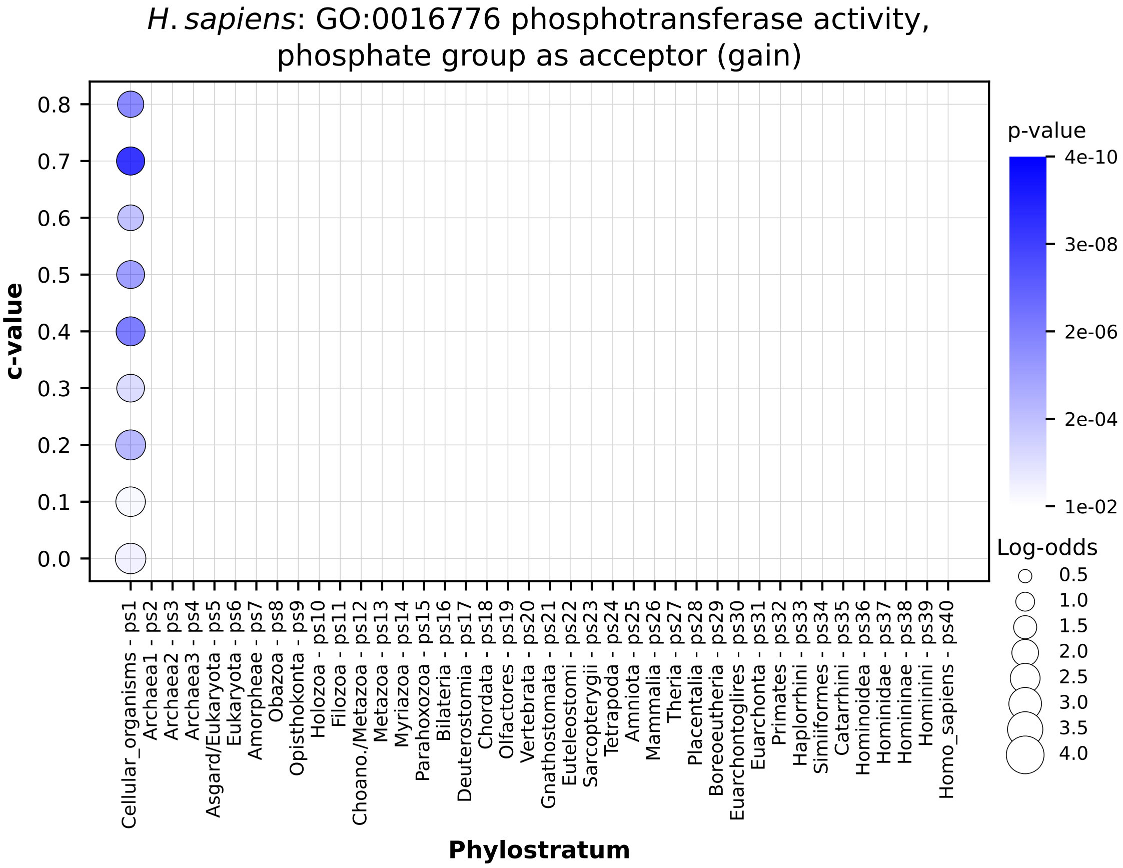
H. sapiens: GO:0016776 phosphotransferase activity, phosphate group as acceptor (gain)
c-value
0.8
0.7
0.6
0.5
0.4
0.3
0.2
0.1
0.0
p-value
4e-10
3e-08
2e-06
2e-04
1e-02
Log-odds
0.5
1.0
1.5
2.0
2.5
3.0
3.5
4.0
Cellular_organisms - ps1
Archaea1 - ps2
Archaea2 - ps3
Archaea3 - ps4
Asgard/Eukaryota - ps5
Eukaryota - ps6
Amorpheae - ps7
Obazoa - ps8
Opisthokonta - ps9
Holozoa - ps10
Filozoa - ps11
Choano./Metazoa - ps12
Metazoa - ps13
Myriazoa - ps14
Parahoxozoa - ps15
Bilateria - ps16
Deuterostomia - ps17
Chordata - ps18
Olfactores - ps19
Vertebrata - ps20
Gnathostomata - ps21
Euteleostomi - ps22
Sarcopterygii - ps23
Tetrapoda - ps24
Amniota - ps25
Mammalia - ps26
Theria - ps27
Placentalia - ps28
Boreoeutheria - ps29
Euarchontoglires - ps30
Euarchonta - ps31
Primates - ps32
Haplorrhini - ps33
Simiiformes - ps34
Catarrhini - ps35
Hominoidea - ps36
Hominidae - ps37
Homininae - ps38
Hominini - ps39
Homo_sapiens - ps40
Phylostratum

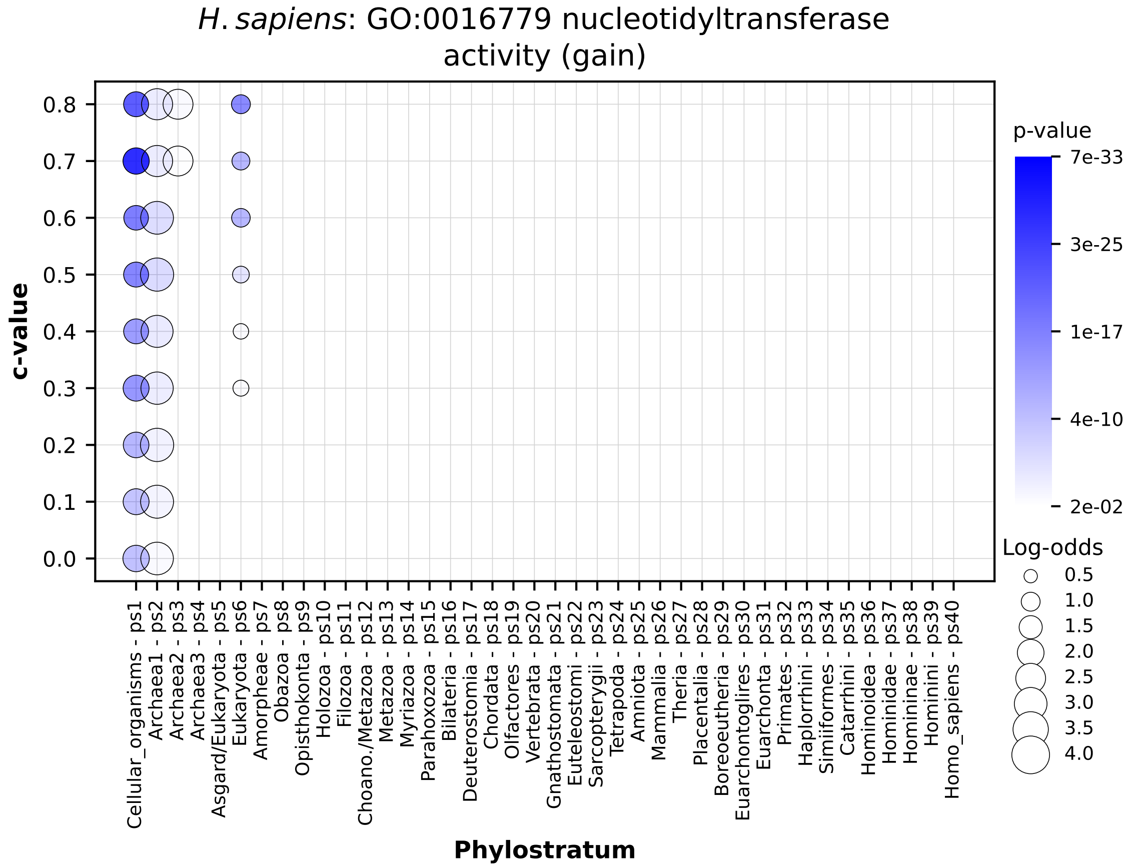

H. sapiens: GO:0016779 nucleotidyltransferase activity (gain)
c-value
0.8
0.7
0.6
0.5
0.4
0.3
0.2
0.1
0.0
p-value
7e-33
3e-25
1e-17
4e-10
2e-02
Log-odds
0.5
1.0
1.5
2.0
2.5
3.0
3.5
4.0
Cellular_organisms - ps1
Archaea1 - ps2
Archaea2 - ps3
Archaea3 - ps4
Asgard/Eukaryota - ps5
Eukaryota - ps6
Amorpheae - ps7
Obazoa - ps8
Opisthokonta - ps9
Holozoa - ps10
Filozoa - ps11
Choano./Metazoa - ps12
Metazoa - ps13
Myriazoa - ps14
Parahoxozoa - ps15
Bilateria - ps16
Deuterostomia - ps17
Chordata - ps18
Olfactores - ps19
Vertebrata - ps20
Gnathostomata - ps21
Euteleostomi - ps22
Sarcopterygii - ps23
Tetrapoda - ps24
Amniota - ps25
Mammalia - ps26
Theria - ps27
Placentalia - ps28
Boreoeutheria - ps29
Euarchontoglires - ps30
Euarchonta - ps31
Primates - ps32
Haplorrhini - ps33
Simiiformes - ps34
Catarrhini - ps35
Hominoidea - ps36
Hominidae - ps37
Homininae - ps38
Hominini - ps39
Homo_sapiens - ps40
Phylostratum

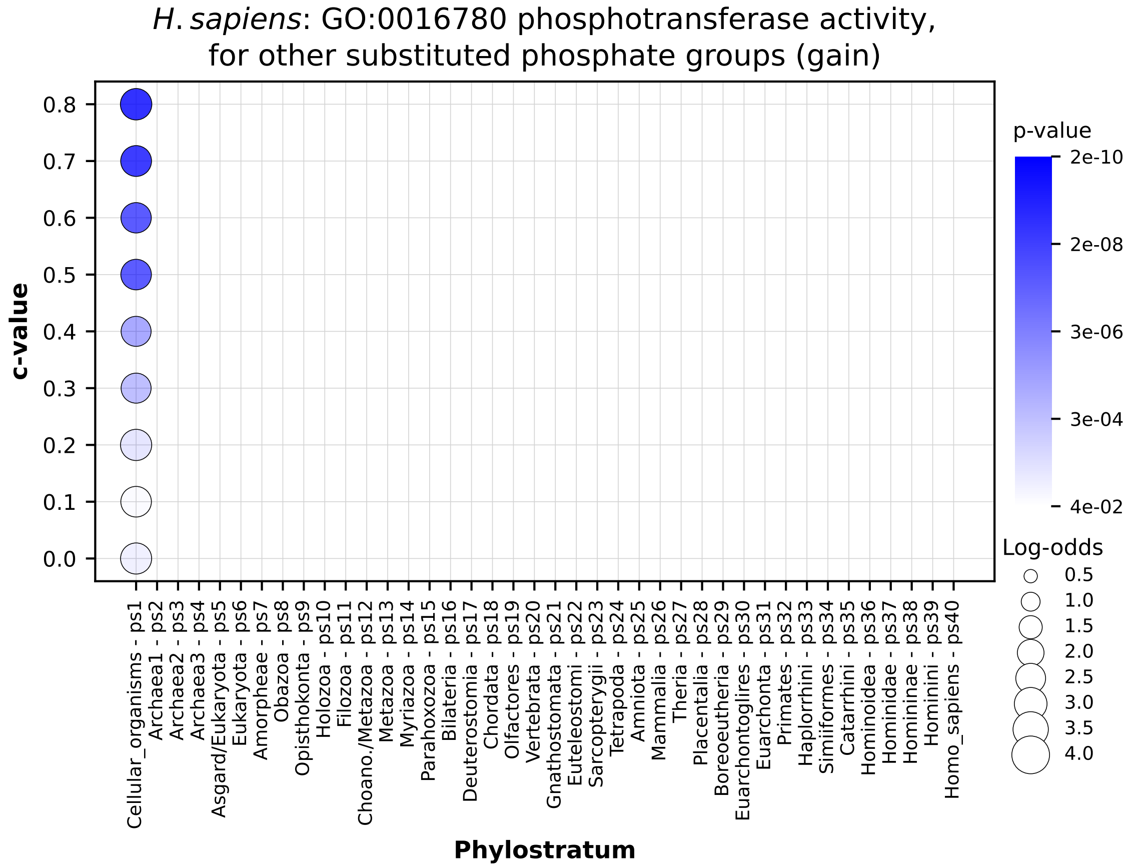

*H. sapiens*: GO:0016780 phosphotransferase activity,
for other substituted phosphate groups (gain)
c-value
0.8
0.7
0.6
0.5
0.4
0.3
0.2
0.1
0.0
p-value
2e-10
2e-08
3e-06
3e-04
4e-02
Log-odds
0.5
1.0
1.5
2.0
2.5
3.0
3.5
4.0
Cellular_organisms - ps1
Archaea1 - ps2
Archaea2 - ps3
Archaea3 - ps4
Asgard/Eukaryota - ps5
Eukaryota - ps6
Amorpheae - ps7
Obazoa - ps8
Opisthokonta - ps9
Holozoa - ps10
Filozoa - ps11
Choano./Metazoa - ps12
Metazoa - ps13
Myriazoa - ps14
Parahoxozoa - ps15
Bilateria - ps16
Deuterostomia - ps17
Chordata - ps18
Olfactores - ps19
Vertebrata - ps20
Gnathostomata - ps21
Euteleostomi - ps22
Sarcopterygii - ps23
Tetrapoda - ps24
Amniota - ps25
Mammalia - ps26
Theria - ps27
Placentalia - ps28
Boreoeutheria - ps29
Euarchontoglires - ps30
Euarchonta - ps31
Primates - ps32
Haplorrhini - ps33
Simiiformes - ps34
Catarrhini - ps35
Hominoidea - ps36
Hominidae - ps37
Homininae - ps38
Hominini - ps39
Homo_sapiens - ps40
Phylostratum

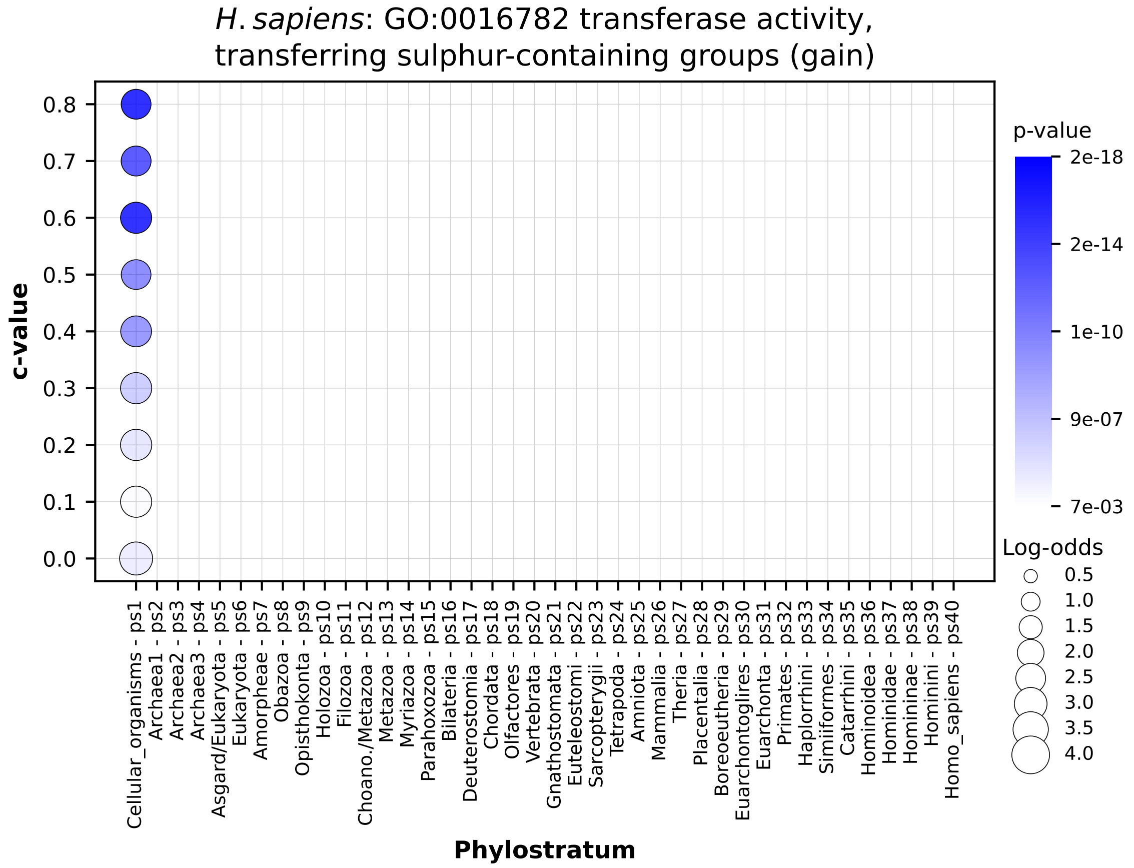
H. sapiens: GO:0016782 transferase activity, transferring sulphur-containing groups (gain)
c-value
0.0
0.1
0.2
0.3
0.4
0.5
0.6
0.7
0.8
Phylostratum
Cellular_organisms - ps1
Archaea1 - ps2
Archaea2 - ps3
Archaea3 - ps4
Asgard/Eukaryota - ps5
Eukaryota - ps6
Amorpheae - ps7
Obazoa - ps8
Opisthokonta - ps9
Holozoa - ps10
Filozoa - ps11
Choano./Metazoa - ps12
Metazoa - ps13
Myriazoa - ps14
Parahoxozoa - ps15
Bilateria - ps16
Deuterostomia - ps17
Chordata - ps18
Olfactores - ps19
Vertebrata - ps20
Gnathostomata - ps21
Euteleostomi - ps22
Sarcopterygii - ps23
Tetrapoda - ps24
Amniota - ps25
Mammalia - ps26
Theria - ps27
Placentalia - ps28
Boreoeutheria - ps29
Euarchontoglires - ps30
Euarchonta - ps31
Primates - ps32
Haplorrhini - ps33
Simiiformes - ps34
Catarrhini - ps35
Hominoidea - ps36
Hominidae - ps37
Homininae - ps38
Hominini - ps39
Homo_sapiens - ps40
p-value
2e-18
2e-14
1e-10
9e-07
7e-03
Log-odds
0.5
1.0
1.5
2.0
2.5
3.0
3.5
4.0

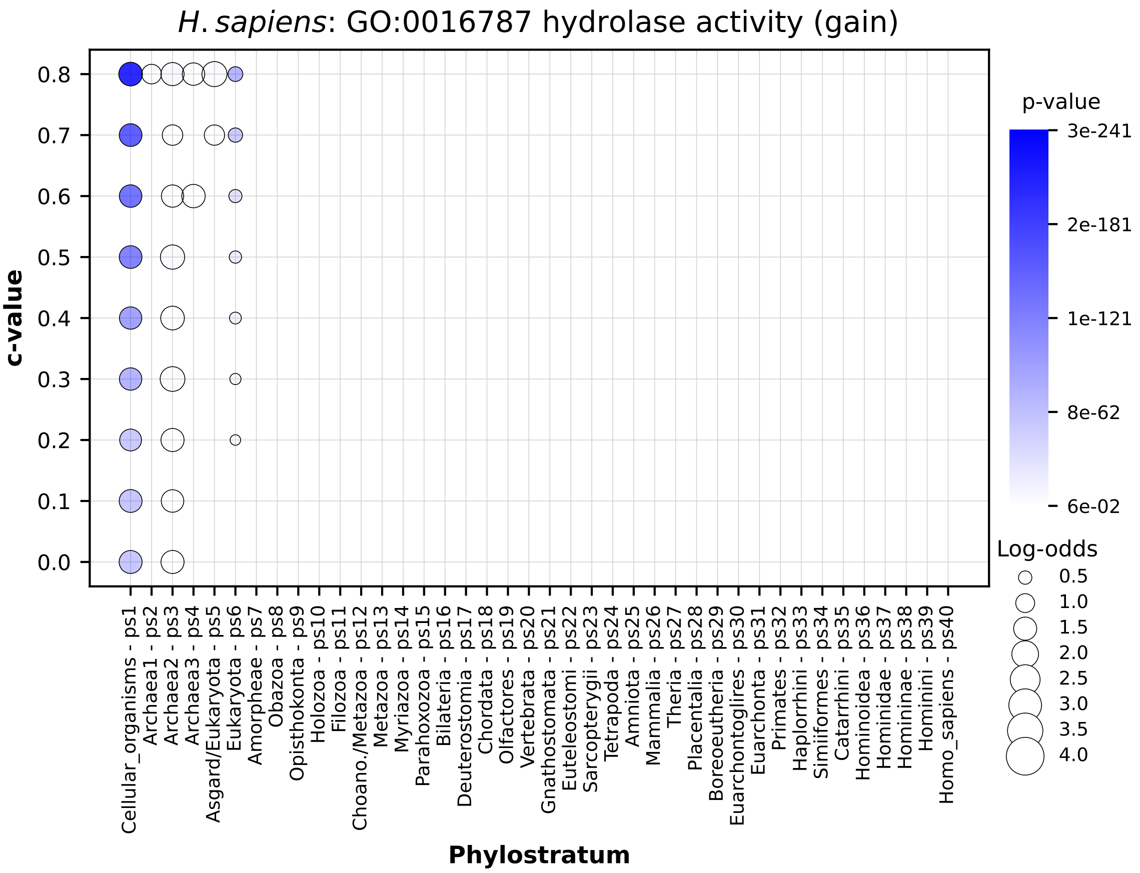
H. sapiens: GO:0016787 hydrolase activity (gain)
c-value
0.8
0.7
0.6
0.5
0.4
0.3
0.2
0.1
0.0
p-value
3e-241
2e-181
1e-121
8e-62
6e-02
Log-odds
0.5
1.0
1.5
2.0
2.5
3.0
3.5
4.0
Cellular_organisms - ps1
Archaea1 - ps2
Archaea2 - ps3
Archaea3 - ps4
Asgard/Eukaryota - ps5
Eukaryota - ps6
Amorpheae - ps7
Obazoa - ps8
Opisthokonta - ps9
Holozoa - ps10
Filozoa - ps11
Choano./Metazoa - ps12
Metazoa - ps13
Myriazoa - ps14
Parahoxozoa - ps15
Bilateria - ps16
Deuterostomia - ps17
Chordata - ps18
Olfactores - ps19
Vertebrata - ps20
Gnathostomata - ps21
Euteleostomi - ps22
Sarcopterygii - ps23
Tetrapoda - ps24
Amniota - ps25
Mammalia - ps26
Theria - ps27
Placentalia - ps28
Boreoeutheria - ps29
Euarchontoglires - ps30
Euarchonta - ps31
Primates - ps32
Haplorrhini - ps33
Simiiformes - ps34
Catarrhini - ps35
Hominoidea - ps36
Hominidae - ps37
Homininae - ps38
Hominini - ps39
Homo_sapiens - ps40
Phylostratum

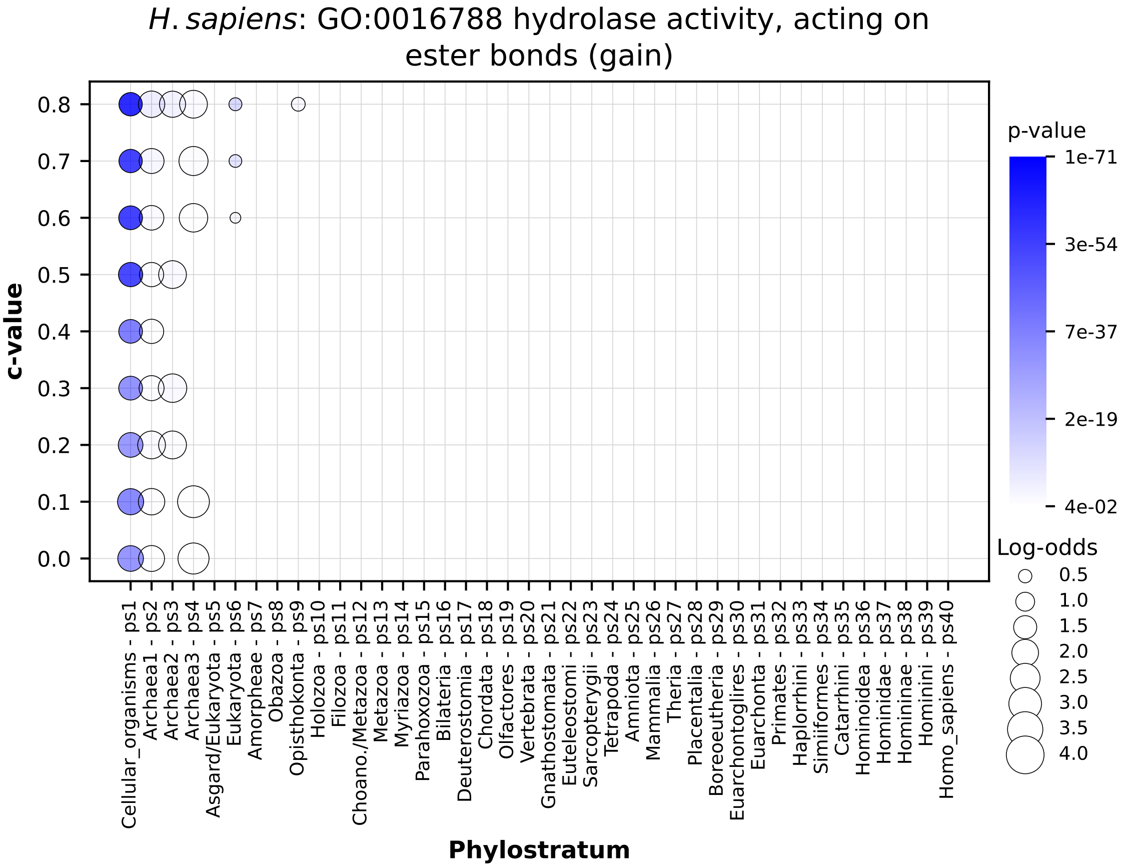

H. sapiens: GO:0016788 hydrolase activity, acting on ester bonds (gain)
c-value
0.8
0.7
0.6
0.5
0.4
0.3
0.2
0.1
0.0
p-value
1e-71
3e-54
7e-37
2e-19
4e-02
Log-odds
0.5
1.0
1.5
2.0
2.5
3.0
3.5
4.0
Cellular_organisms - ps1
Archaea1 - ps2
Archaea2 - ps3
Archaea3 - ps4
Asgard/Eukaryota - ps5
Eukaryota - ps6
Amorpheae - ps7
Obazoa - ps8
Opisthokonta - ps9
Holozoa - ps10
Filozoa - ps11
Choano./Metazoa - ps12
Metazoa - ps13
Myriazoa - ps14
Parahoxozoa - ps15
Bilateria - ps16
Deuterostomia - ps17
Chordata - ps18
Olfactores - ps19
Vertebrata - ps20
Gnathostomata - ps21
Euteleostomi - ps22
Sarcopterygii - ps23
Tetrapoda - ps24
Amniota - ps25
Mammalia - ps26
Theria - ps27
Placentalia - ps28
Boreoeutheria - ps29
Euarchontoglires - ps30
Euarchonta - ps31
Primates - ps32
Haplorrhini - ps33
Simiiformes - ps34
Catarrhini - ps35
Hominoidea - ps36
Hominidae - ps37
Homininae - ps38
Hominini - ps39
Homo_sapiens - ps40
Phylostratum

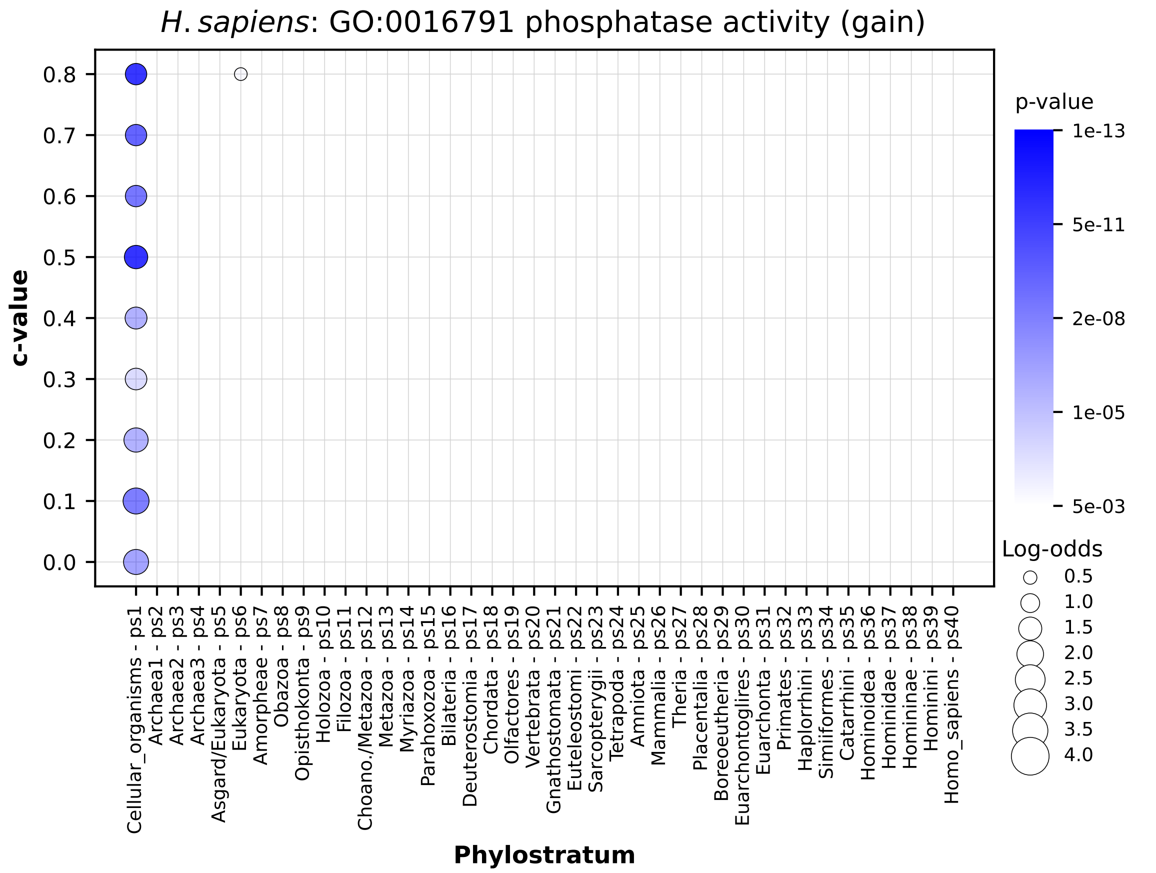
H. sapiens: GO:0016791 phosphatase activity (gain)
c-value
Phylostratum
p-value
1e-13
5e-11
2e-08
1e-05
5e-03
Log-odds
0.5
1.0
1.5
2.0
2.5
3.0
3.5
4.0
Cellular_organisms - ps1
Archaea1 - ps2
Archaea2 - ps3
Archaea3 - ps4
Asgard/Eukaryota - ps5
Eukaryota - ps6
Amorpheae - ps7
Obazoa - ps8
Opisthokonta - ps9
Holozoa - ps10
Filozoa - ps11
Choano./Metazoa - ps12
Metazoa - ps13
Myriazoa - ps14
Parahoxozoa - ps15
Bilateria - ps16
Deuterostomia - ps17
Chordata - ps18
Olfactores - ps19
Vertebrata - ps20
Gnathostomata - ps21
Euteleostomi - ps22
Sarcopterygii - ps23
Tetrapoda - ps24
Amniota - ps25
Mammalia - ps26
Theria - ps27
Placentalia - ps28
Boreoeutheria - ps29
Euarchontoglires - ps30
Euarchonta - ps31
Primates - ps32
Haplorrhini - ps33
Simiiformes - ps34
Catarrhini - ps35
Hominoidea - ps36
Hominidae - ps37
Homininae - ps38
Hominini - ps39
Homo_sapiens - ps40

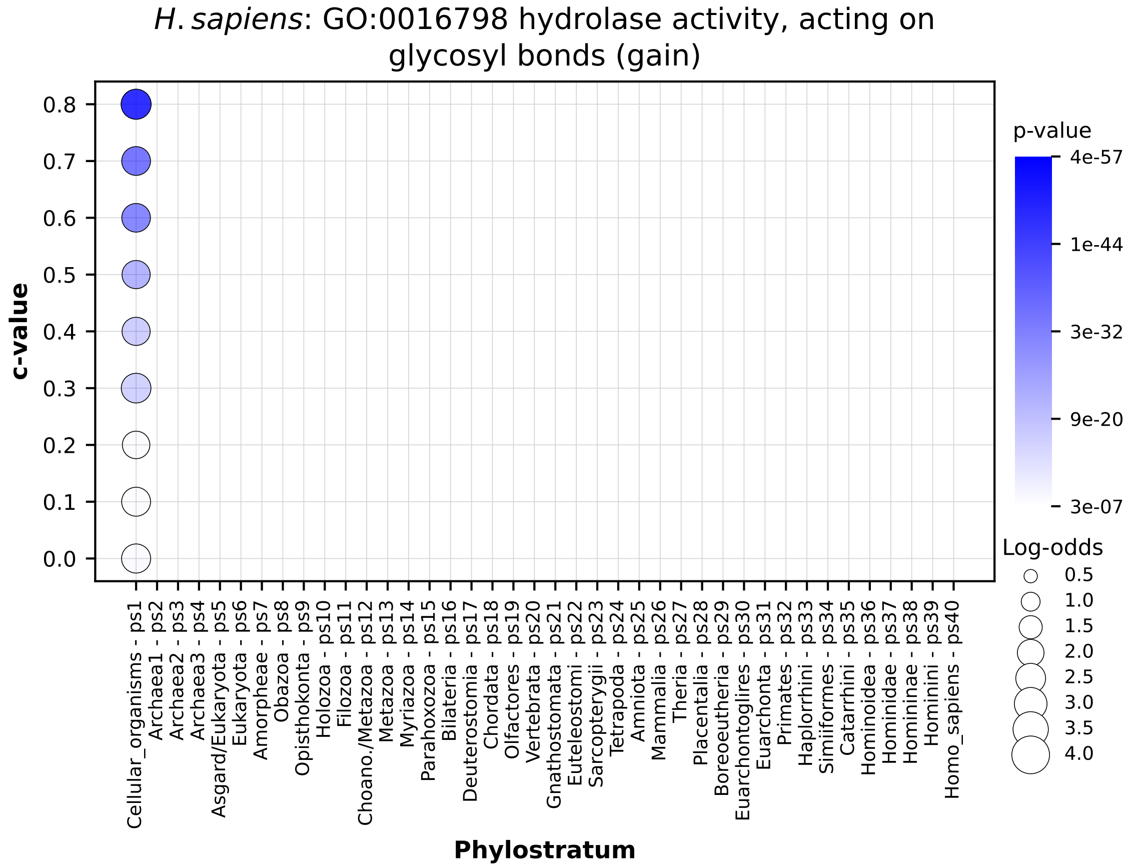

H. sapiens: GO:0016798 hydrolase activity, acting on glycosyl bonds (gain)
c-value
0.8
0.7
0.6
0.5
0.4
0.3
0.2
0.1
0.0
p-value
4e-57
1e-44
3e-32
9e-20
3e-07
Log-odds
0.5
1.0
1.5
2.0
2.5
3.0
3.5
4.0
Cellular_organisms - ps1
Archaea1 - ps2
Archaea2 - ps3
Archaea3 - ps4
Asgard/Eukaryota - ps5
Eukaryota - ps6
Amorpheae - ps7
Obazoa - ps8
Opisthokonta - ps9
Holozoa - ps10
Filozoa - ps11
Choano./Metazoa - ps12
Metazoa - ps13
Myriazoa - ps14
Parahoxozoa - ps15
Bilateria - ps16
Deuterostomia - ps17
Chordata - ps18
Olfactores - ps19
Vertebrata - ps20
Gnathostomata - ps21
Euteleostomi - ps22
Sarcopterygii - ps23
Tetrapoda - ps24
Amniota - ps25
Mammalia - ps26
Theria - ps27
Placentalia - ps28
Boreoeutheria - ps29
Euarchontoglires - ps30
Euarchonta - ps31
Primates - ps32
Haplorrhini - ps33
Simiiformes - ps34
Catarrhini - ps35
Hominoidea - ps36
Hominidae - ps37
Homininae - ps38
Hominini - ps39
Homo_sapiens - ps40
Phylostratum

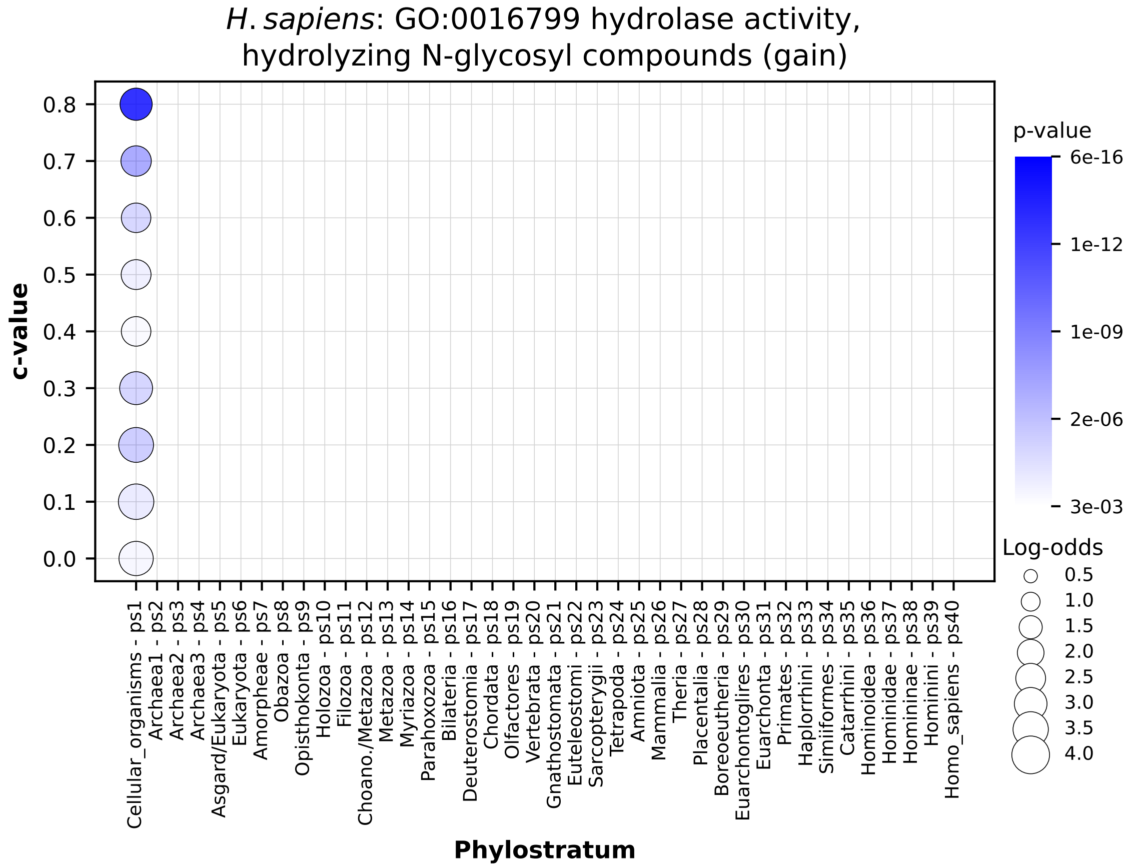
H. sapiens: GO:0016799 hydrolase activity, hydrolyzing N-glycosyl compounds (gain)
c-value
0.8
0.7
0.6
0.5
0.4
0.3
0.2
0.1
0.0
p-value
6e-16
1e-12
1e-09
2e-06
3e-03
Log-odds
0.5
1.0
1.5
2.0
2.5
3.0
3.5
4.0
Cellular_organisms - ps1
Archaea1 - ps2
Archaea2 - ps3
Archaea3 - ps4
Asgard/Eukaryota - ps5
Eukaryota - ps6
Amorpheae - ps7
Obazoa - ps8
Opisthokonta - ps9
Holozoa - ps10
Filozoa - ps11
Choano./Metazoa - ps12
Metazoa - ps13
Myriazoa - ps14
Parahoxozoa - ps15
Bilateria - ps16
Deuterostomia - ps17
Chordata - ps18
Olfactores - ps19
Vertebrata - ps20
Gnathostomata - ps21
Euteleostomi - ps22
Sarcopterygii - ps23
Tetrapoda - ps24
Amniota - ps25
Mammalia - ps26
Theria - ps27
Placentalia - ps28
Boreoeutheria - ps29
Euarchontoglires - ps30
Euarchonta - ps31
Primates - ps32
Haplorrhini - ps33
Simiiformes - ps34
Catarrhini - ps35
Hominoidea - ps36
Hominidae - ps37
Homininae - ps38
Hominini - ps39
Homo_sapiens - ps40
Phylostratum

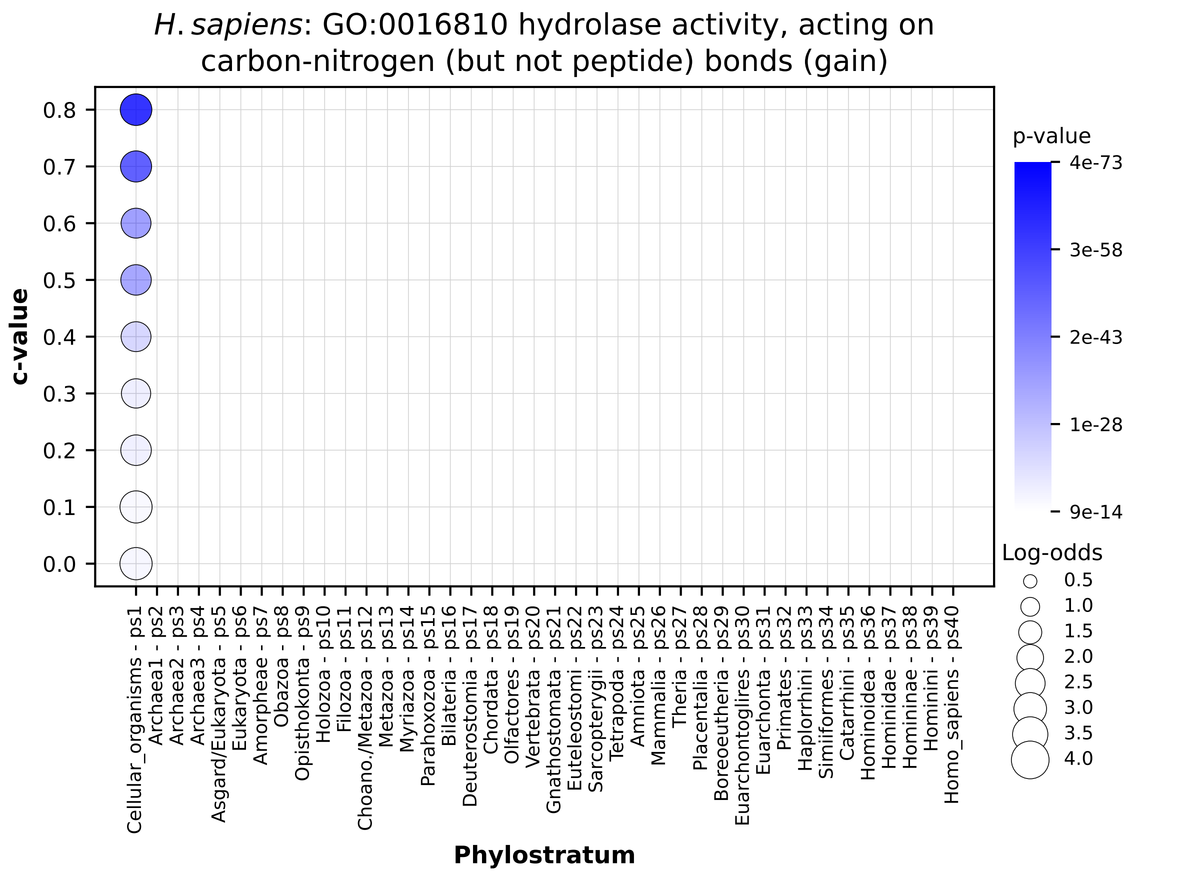

*H. sapiens*: GO:0016810 hydrolase activity, acting on carbon-nitrogen (but not peptide) bonds (gain)
c-value
0.0
0.1
0.2
0.3
0.4
0.5
0.6
0.7
0.8
p-value
4e-73
3e-58
2e-43
1e-28
9e-14
Log-odds
0.5
1.0
1.5
2.0
2.5
3.0
3.5
4.0
Cellular_organisms - ps1
Archaea1 - ps2
Archaea2 - ps3
Archaea3 - ps4
Asgard/Eukaryota - ps5
Eukaryota - ps6
Amorpheae - ps7
Obazoa - ps8
Opisthokonta - ps9
Holozoa - ps10
Filozoa - ps11
Choano./Metazoa - ps12
Metazoa - ps13
Myriazoa - ps14
Parahoxozoa - ps15
Bilateria - ps16
Deuterostomia - ps17
Chordata - ps18
Olfactores - ps19
Vertebrata - ps20
Gnathostomata - ps21
Euteleostomi - ps22
Sarcopterygii - ps23
Tetrapoda - ps24
Amniota - ps25
Mammalia - ps26
Theria - ps27
Placentalia - ps28
Boreoeutheria - ps29
Euarchontoglires - ps30
Euarchonta - ps31
Primates - ps32
Haplorrhini - ps33
Simiiformes - ps34
Catarrhini - ps35
Hominoidea - ps36
Hominidae - ps37
Homininae - ps38
Hominini - ps39
Homo_sapiens - ps40
Phylostratum

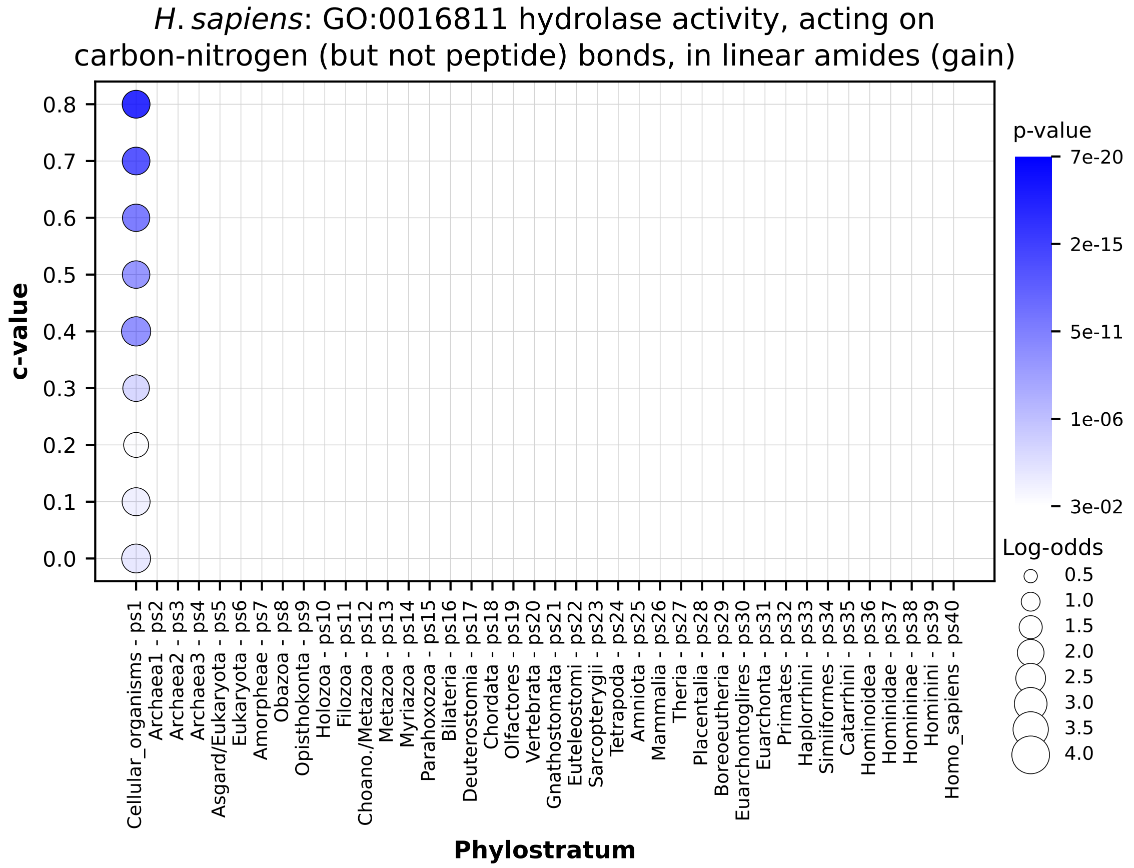
H. sapiens: GO:0016811 hydrolase activity, acting on carbon-nitrogen (but not peptide) bonds, in linear amides (gain)
c-value
Phylostratum
p-value
7e-20
2e-15
5e-11
1e-06
3e-02
Log-odds
0.5
1.0
1.5
2.0
2.5
3.0
3.5
4.0
Cellular_organisms - ps1
Archaea1 - ps2
Archaea2 - ps3
Archaea3 - ps4
Asgard/Eukaryota - ps5
Eukaryota - ps6
Amorpheae - ps7
Obazoa - ps8
Opisthokonta - ps9
Holozoa - ps10
Filozoa - ps11
Choano./Metazoa - ps12
Metazoa - ps13
Myriazoa - ps14
Parahoxozoa - ps15
Bilateria - ps16
Deuterostomia - ps17
Chordata - ps18
Olfactores - ps19
Vertebrata - ps20
Gnathostomata - ps21
Euteleostomi - ps22
Sarcopterygii - ps23
Tetrapoda - ps24
Amniota - ps25
Mammalia - ps26
Theria - ps27
Placentalia - ps28
Boreoeutheria - ps29
Euarchontoglires - ps30
Euarchonta - ps31
Primates - ps32
Haplorrhini - ps33
Simiiformes - ps34
Catarrhini - ps35
Hominoidea - ps36
Hominidae - ps37
Homininae - ps38
Hominini - ps39
Homo_sapiens - ps40

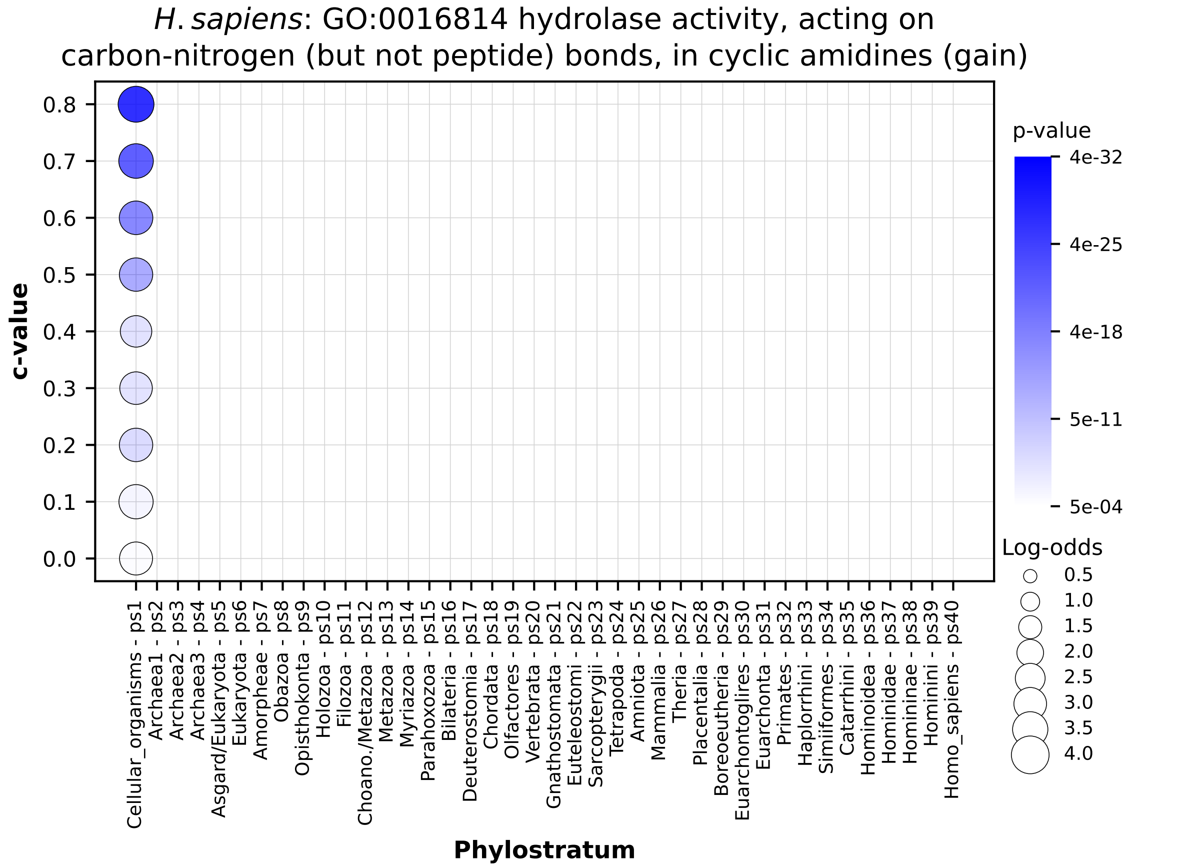

H. sapiens: GO:0016814 hydrolase activity, acting on carbon-nitrogen (but not peptide) bonds, in cyclic amidines (gain)
c-value
0.8
0.7
0.6
0.5
0.4
0.3
0.2
0.1
0.0
p-value
4e-32
4e-25
4e-18
5e-11
5e-04
Log-odds
0.5
1.0
1.5
2.0
2.5
3.0
3.5
4.0
Cellular_organisms - ps1
Archaea1 - ps2
Archaea2 - ps3
Archaea3 - ps4
Asgard/Eukaryota - ps5
Eukaryota - ps6
Amorpheae - ps7
Obazoa - ps8
Opisthokonta - ps9
Holozoa - ps10
Filozoa - ps11
Choano./Metazoa - ps12
Metazoa - ps13
Myriazoa - ps14
Parahoxozoa - ps15
Bilateria - ps16
Deuterostomia - ps17
Chordata - ps18
Olfactores - ps19
Vertebrata - ps20
Gnathostomata - ps21
Euteleostomi - ps22
Sarcopterygii - ps23
Tetrapoda - ps24
Amniota - ps25
Mammalia - ps26
Theria - ps27
Placentalia - ps28
Boreoeutheria - ps29
Euarchontoglires - ps30
Euarchonta - ps31
Primates - ps32
Haplorrhini - ps33
Simiiformes - ps34
Catarrhini - ps35
Hominoidea - ps36
Hominidae - ps37
Homininae - ps38
Hominini - ps39
Homo_sapiens - ps40
Phylostratum

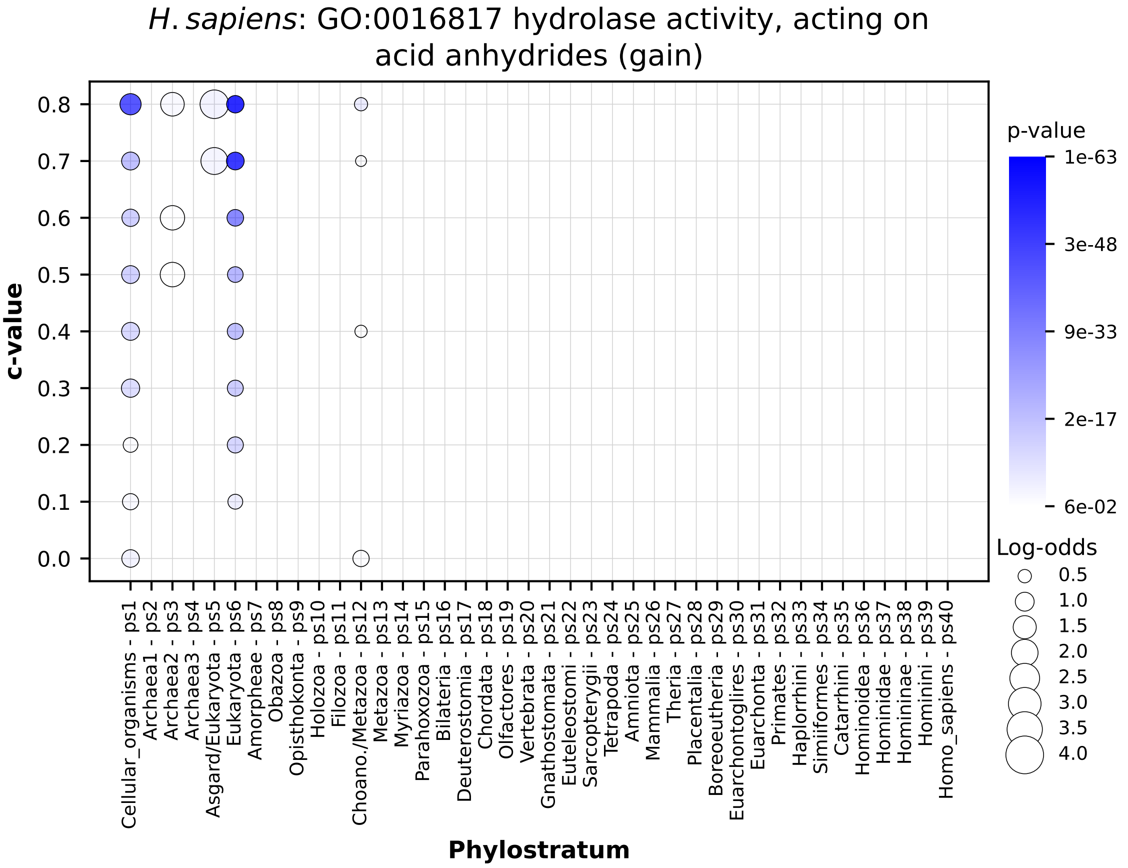

H. sapiens: GO:0016817 hydrolase activity, acting on acid anhydrides (gain)
c-value
0.0
0.1
0.2
0.3
0.4
0.5
0.6
0.7
0.8
Phylostratum
Cellular_organisms - ps1
Archaea1 - ps2
Archaea2 - ps3
Archaea3 - ps4
Asgard/Eukaryota - ps5
Eukaryota - ps6
Amorpheae - ps7
Obazoa - ps8
Opisthokonta - ps9
Holozoa - ps10
Filozoa - ps11
Choano./Metazoa - ps12
Metazoa - ps13
Myriazoa - ps14
Parahoxozoa - ps15
Bilateria - ps16
Deuterostomia - ps17
Chordata - ps18
Olfactores - ps19
Vertebrata - ps20
Gnathostomata - ps21
Euteleostomi - ps22
Sarcopterygii - ps23
Tetrapoda - ps24
Amniota - ps25
Mammalia - ps26
Theria - ps27
Placentalia - ps28
Boreoeutheria - ps29
Euarchontoglires - ps30
Euarchonta - ps31
Primates - ps32
Haplorrhini - ps33
Simiiformes - ps34
Catarrhini - ps35
Hominoidea - ps36
Hominidae - ps37
Homininae - ps38
Hominini - ps39
Homo_sapiens - ps40
p-value
1e-63
3e-48
9e-33
2e-17
6e-02
Log-odds
0.5
1.0
1.5
2.0
2.5
3.0
3.5
4.0

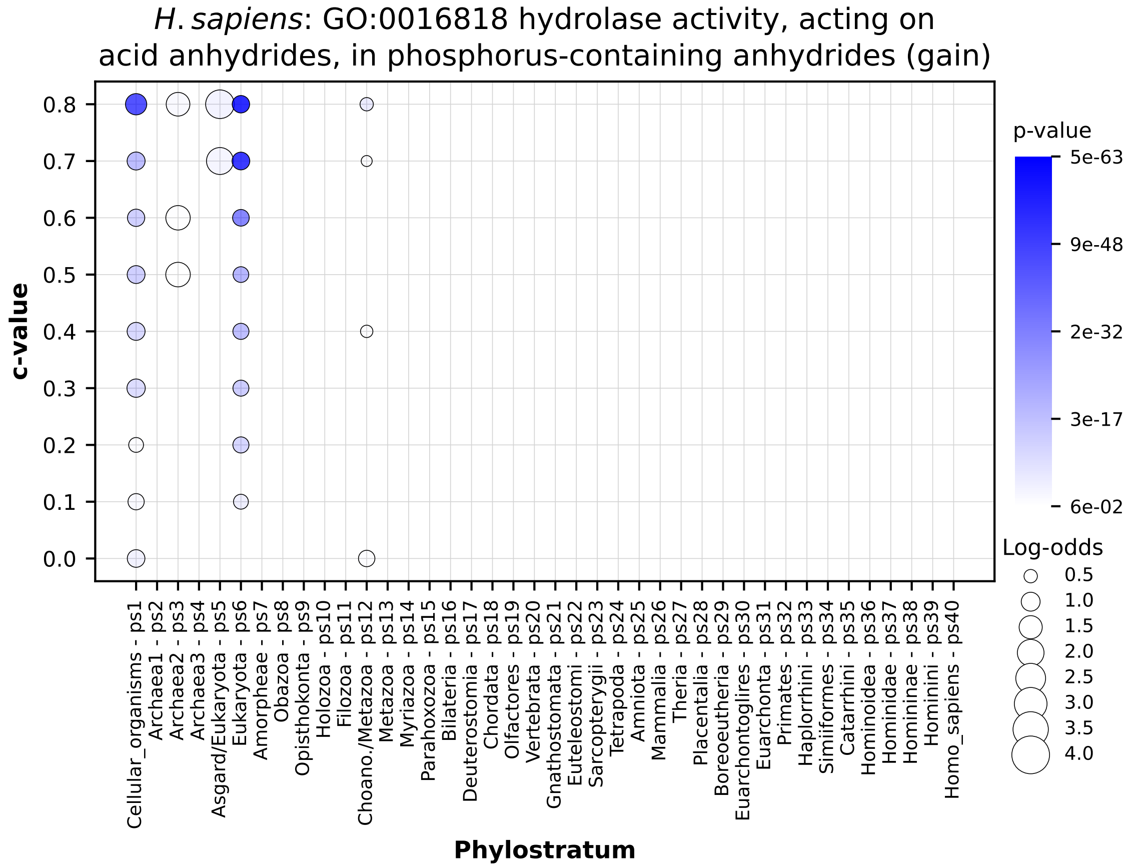

H. sapiens: GO:0016818 hydrolase activity, acting on acid anhydrides, in phosphorus-containing anhydrides (gain)
c-value
Phylostratum
p-value
5e-63
9e-48
2e-32
3e-17
6e-02
Log-odds
0.5
1.0
1.5
2.0
2.5
3.0
3.5
4.0
Cellular_organisms - ps1
Archaea1 - ps2
Archaea2 - ps3
Archaea3 - ps4
Asgard/Eukaryota - ps5
Eukaryota - ps6
Amorpheae - ps7
Obazoa - ps8
Opisthokonta - ps9
Holozoa - ps10
Filozoa - ps11
Choano./Metazoa - ps12
Metazoa - ps13
Myriazoa - ps14
Parahoxozoa - ps15
Bilateria - ps16
Deuterostomia - ps17
Chordata - ps18
Olfactores - ps19
Vertebrata - ps20
Gnathostomata - ps21
Euteleostomi - ps22
Sarcopterygii - ps23
Tetrapoda - ps24
Amniota - ps25
Mammalia - ps26
Theria - ps27
Placentalia - ps28
Boreoeutheria - ps29
Euarchontoglires - ps30
Euarchonta - ps31
Primates - ps32
Haplorrhini - ps33
Simiiformes - ps34
Catarrhini - ps35
Hominoidea - ps36
Hominidae - ps37
Homininae - ps38
Hominini - ps39
Homo_sapiens - ps40

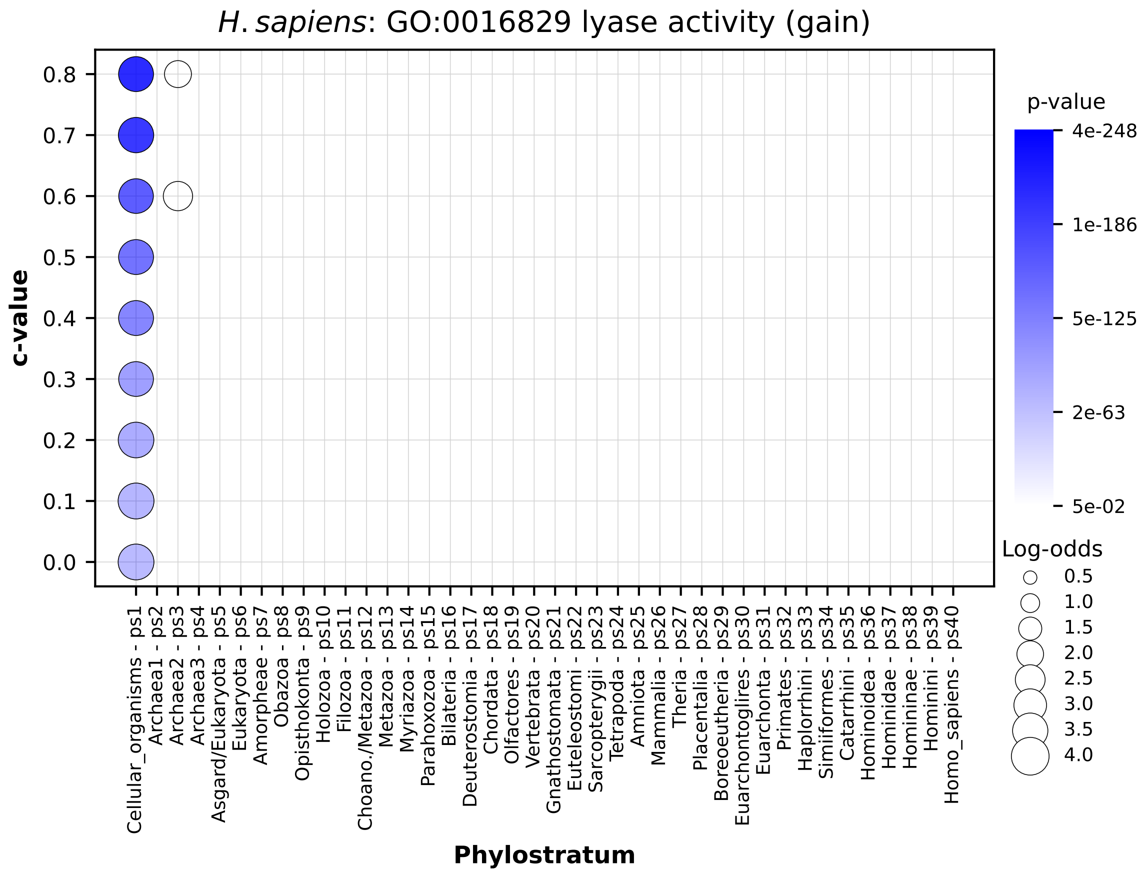
H. sapiens: GO:0016829 lyase activity (gain)
c-value
0.8
0.7
0.6
0.5
0.4
0.3
0.2
0.1
0.0
p-value
4e-248
1e-186
5e-125
2e-63
5e-02
Log-odds
0.5
1.0
1.5
2.0
2.5
3.0
3.5
4.0
Cellular_organisms - ps1
Archaea1 - ps2
Archaea2 - ps3
Archaea3 - ps4
Asgard/Eukaryota - ps5
Eukaryota - ps6
Amorpheae - ps7
Obazoa - ps8
Opisthokonta - ps9
Holozoa - ps10
Filozoa - ps11
Choano./Metazoa - ps12
Metazoa - ps13
Myriazoa - ps14
Parahoxozoa - ps15
Bilateria - ps16
Deuterostomia - ps17
Chordata - ps18
Olfactores - ps19
Vertebrata - ps20
Gnathostomata - ps21
Euteleostomi - ps22
Sarcopterygii - ps23
Tetrapoda - ps24
Amniota - ps25
Mammalia - ps26
Theria - ps27
Placentalia - ps28
Boreoeutheria - ps29
Euarchontoglires - ps30
Euarchonta - ps31
Primates - ps32
Haplorrhini - ps33
Simiiformes - ps34
Catarrhini - ps35
Hominoidea - ps36
Hominidae - ps37
Homininae - ps38
Hominini - ps39
Homo_sapiens - ps40
Phylostratum

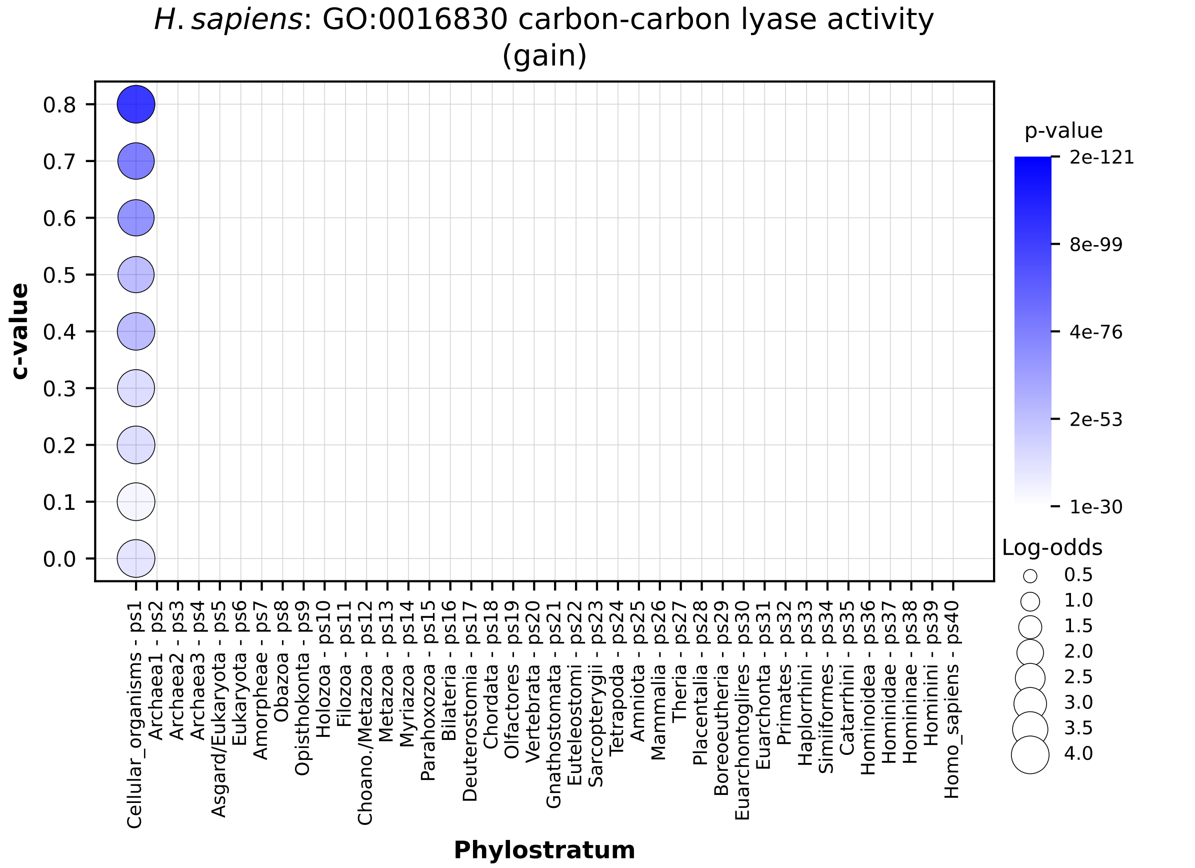
H. sapiens: GO:0016830 carbon-carbon lyase activity (gain)
c-value
0.0
0.1
0.2
0.3
0.4
0.5
0.6
0.7
0.8
p-value
2e-121
8e-99
4e-76
2e-53
1e-30
Log-odds
0.5
1.0
1.5
2.0
2.5
3.0
3.5
4.0
Cellular_organisms - ps1
Archaea1 - ps2
Archaea2 - ps3
Archaea3 - ps4
Asgard/Eukaryota - ps5
Eukaryota - ps6
Amorpheae - ps7
Obazoa - ps8
Opisthokonta - ps9
Holozoa - ps10
Filozoa - ps11
Choano./Metazoa - ps12
Metazoa - ps13
Myriazoa - ps14
Parahoxozoa - ps15
Bilateria - ps16
Deuterostomia - ps17
Chordata - ps18
Olfactores - ps19
Vertebrata - ps20
Gnathostomata - ps21
Euteleostomi - ps22
Sarcopterygii - ps23
Tetrapoda - ps24
Amniota - ps25
Mammalia - ps26
Theria - ps27
Placentalia - ps28
Boreoeutheria - ps29
Euarchontoglires - ps30
Euarchonta - ps31
Primates - ps32
Haplorrhini - ps33
Simiiformes - ps34
Catarrhini - ps35
Hominoidea - ps36
Hominidae - ps37
Homininae - ps38
Hominini - ps39
Homo_sapiens - ps40
Phylostratum

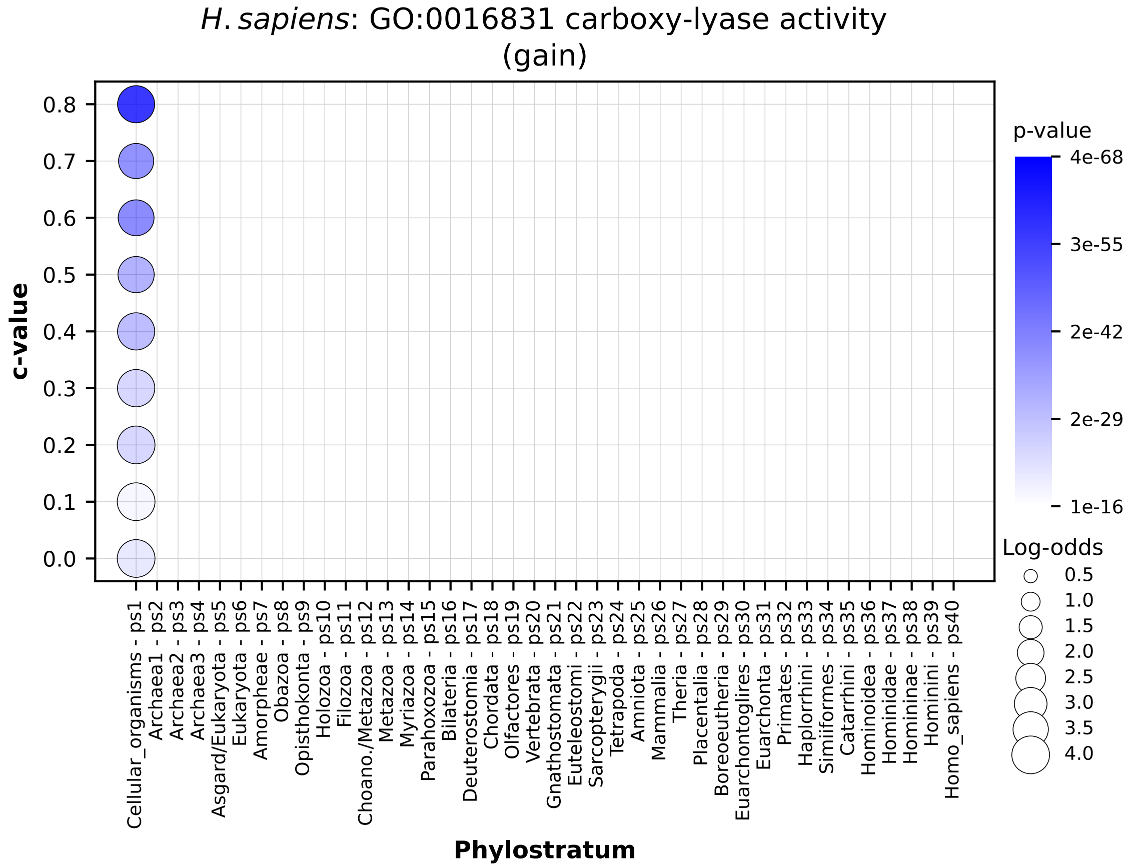

H. sapiens: GO:0016831 carboxy-lyase activity
(gain)
c-value
0.8
0.7
0.6
0.5
0.4
0.3
0.2
0.1
0.0
p-value
4e-68
3e-55
2e-42
2e-29
1e-16
Log-odds
0.5
1.0
1.5
2.0
2.5
3.0
3.5
4.0
Cellular_organisms - ps1
Archaea1 - ps2
Archaea2 - ps3
Archaea3 - ps4
Asgard/Eukaryota - ps5
Eukaryota - ps6
Amorpheae - ps7
Obazoa - ps8
Opisthokonta - ps9
Holozoa - ps10
Filozoa - ps11
Choano./Metazoa - ps12
Metazoa - ps13
Myriazoa - ps14
Parahoxozoa - ps15
Bilateria - ps16
Deuterostomia - ps17
Chordata - ps18
Olfactores - ps19
Vertebrata - ps20
Gnathostomata - ps21
Euteleostomi - ps22
Sarcopterygii - ps23
Tetrapoda - ps24
Amniota - ps25
Mammalia - ps26
Theria - ps27
Placentalia - ps28
Boreoeutheria - ps29
Euarchontoglires - ps30
Euarchonta - ps31
Primates - ps32
Haplorrhini - ps33
Simiiformes - ps34
Catarrhini - ps35
Hominoidea - ps36
Hominidae - ps37
Homininae - ps38
Hominini - ps39
Homo_sapiens - ps40
Phylostratum

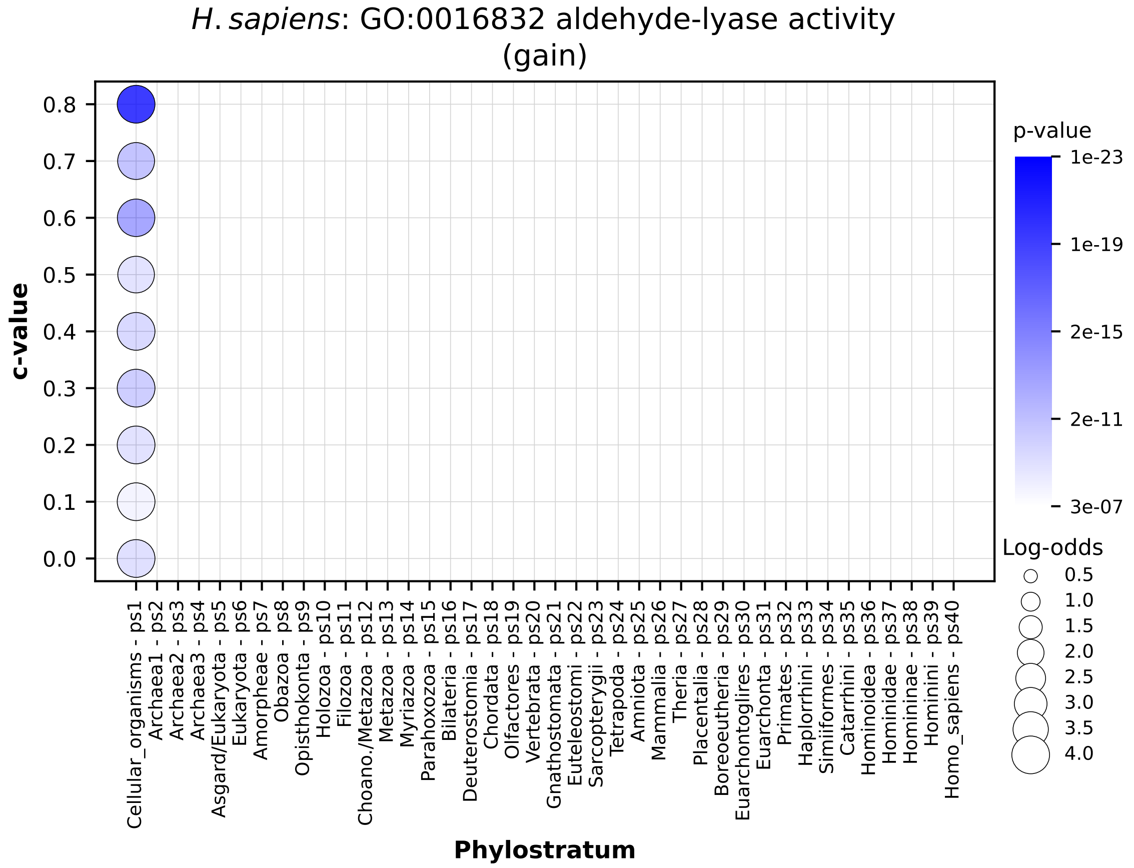
H. sapiens: GO:0016832 aldehyde-lyase activity
(gain)
c-value
0.8
0.7
0.6
0.5
0.4
0.3
0.2
0.1
0.0
p-value
1e-23
1e-19
2e-15
2e-11
3e-07
Log-odds
0.5
1.0
1.5
2.0
2.5
3.0
3.5
4.0
Cellular_organisms - ps1
Archaea1 - ps2
Archaea2 - ps3
Archaea3 - ps4
Asgard/Eukaryota - ps5
Eukaryota - ps6
Amorpheae - ps7
Obazoa - ps8
Opisthokonta - ps9
Holozoa - ps10
Filozoa - ps11
Choano./Metazoa - ps12
Metazoa - ps13
Myriazoa - ps14
Parahoxozoa - ps15
Bilateria - ps16
Deuterostomia - ps17
Chordata - ps18
Olfactores - ps19
Vertebrata - ps20
Gnathostomata - ps21
Euteleostomi - ps22
Sarcopterygii - ps23
Tetrapoda - ps24
Amniota - ps25
Mammalia - ps26
Theria - ps27
Placentalia - ps28
Boreoeutheria - ps29
Euarchontoglires - ps30
Euarchonta - ps31
Primates - ps32
Haplorrhini - ps33
Simiiformes - ps34
Catarrhini - ps35
Hominoidea - ps36
Hominidae - ps37
Homininae - ps38
Hominini - ps39
Homo_sapiens - ps40
Phylostratum

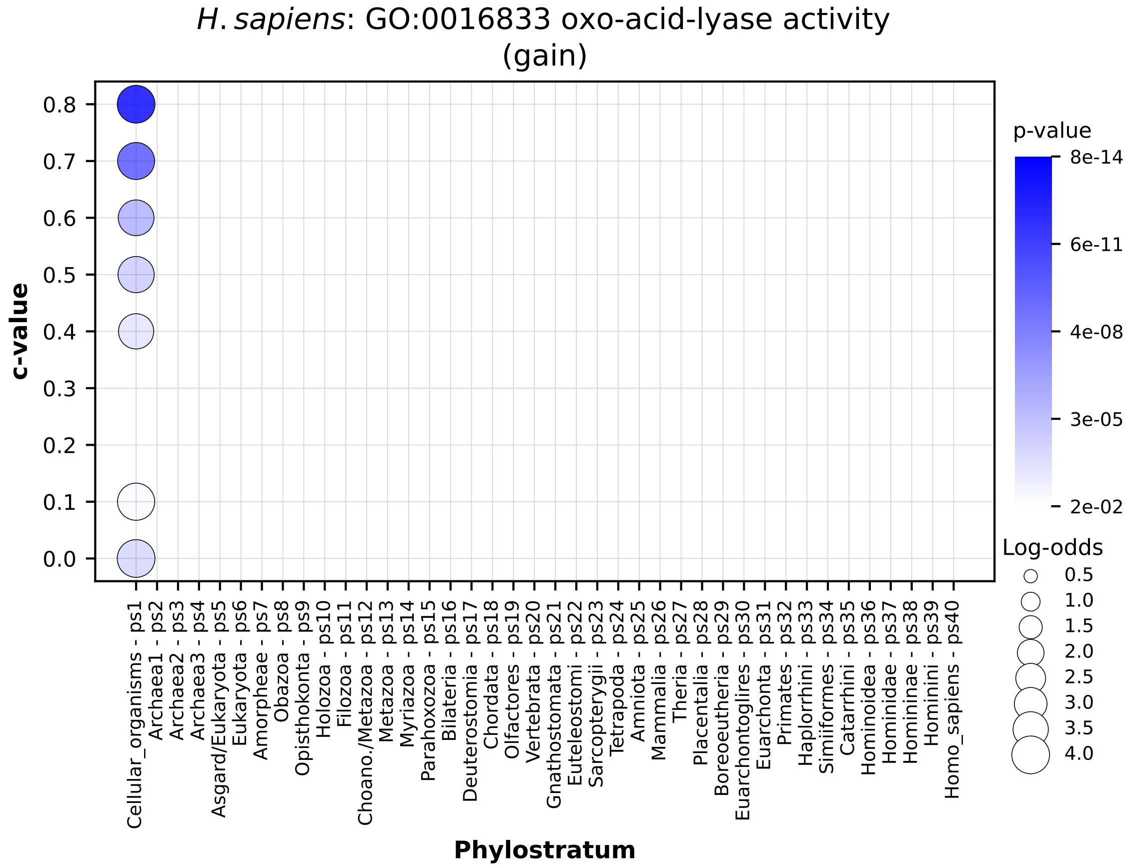

H. sapiens: GO:0016833 oxo-acid-lyase activity
(gain)
c-value
0.8
0.7
0.6
0.5
0.4
0.3
0.2
0.1
0.0
p-value
8e-14
6e-11
4e-08
3e-05
2e-02
Log-odds
0.5
1.0
1.5
2.0
2.5
3.0
3.5
4.0
Cellular_organisms - ps1
Archaea1 - ps2
Archaea2 - ps3
Archaea3 - ps4
Asgard/Eukaryota - ps5
Eukaryota - ps6
Amorpheae - ps7
Obazoa - ps8
Opisthokonta - ps9
Holozoa - ps10
Filozoa - ps11
Choano./Metazoa - ps12
Metazoa - ps13
Myriazoa - ps14
Parahoxozoa - ps15
Bilateria - ps16
Deuterostomia - ps17
Chordata - ps18
Olfactores - ps19
Vertebrata - ps20
Gnathostomata - ps21
Euteleostomi - ps22
Sarcopterygii - ps23
Tetrapoda - ps24
Amniota - ps25
Mammalia - ps26
Theria - ps27
Placentalia - ps28
Boreoeutheria - ps29
Euarchontoglires - ps30
Euarchonta - ps31
Primates - ps32
Haplorrhini - ps33
Simiiformes - ps34
Catarrhini - ps35
Hominoidea - ps36
Hominidae - ps37
Homininae - ps38
Hominini - ps39
Homo_sapiens - ps40
Phylostratum

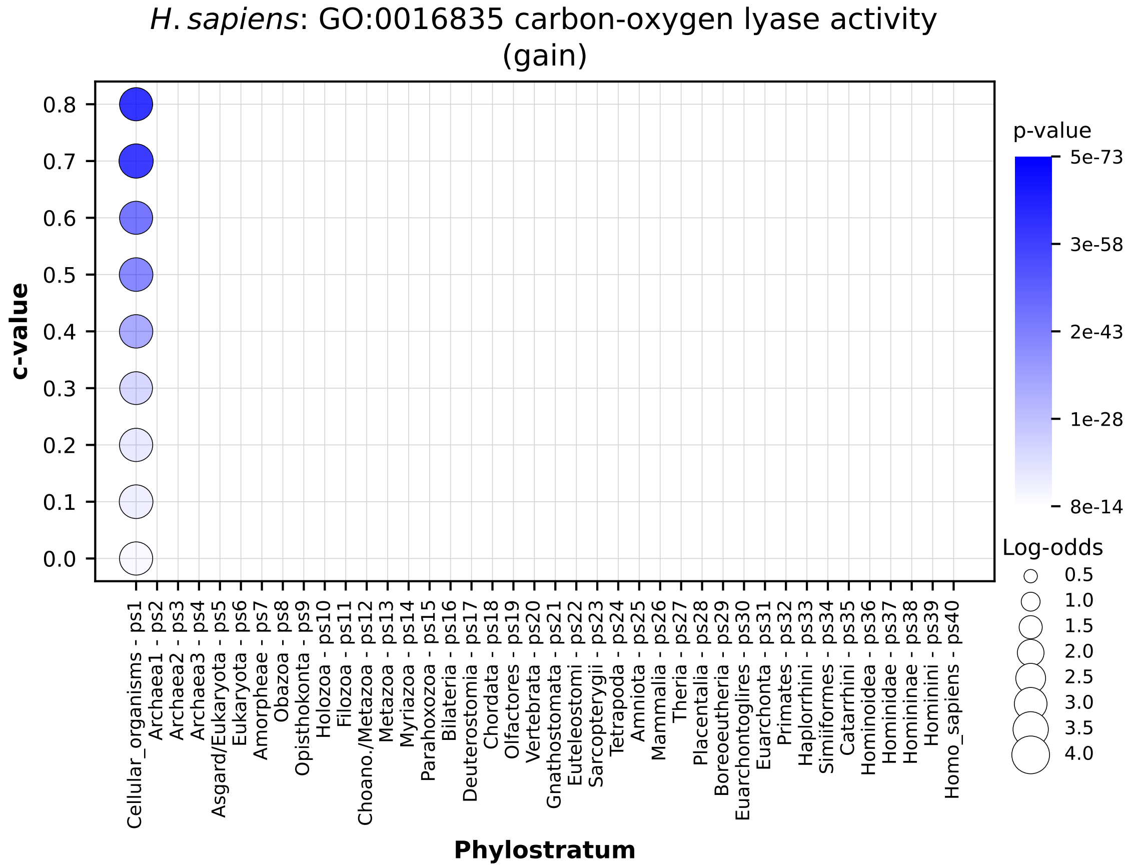

H. sapiens: GO:0016835 carbon-oxygen lyase activity
(gain)
c-value
0.8
0.7
0.6
0.5
0.4
0.3
0.2
0.1
0.0
p-value
5e-73
3e-58
2e-43
1e-28
8e-14
Log-odds
0.5
1.0
1.5
2.0
2.5
3.0
3.5
4.0
Cellular_organisms - ps1
Archaea1 - ps2
Archaea2 - ps3
Archaea3 - ps4
Asgard/Eukaryota - ps5
Eukaryota - ps6
Amorpheae - ps7
Obazoa - ps8
Opisthokonta - ps9
Holozoa - ps10
Filozoa - ps11
Choano./Metazoa - ps12
Metazoa - ps13
Myriazoa - ps14
Parahoxozoa - ps15
Bilateria - ps16
Deuterostomia - ps17
Chordata - ps18
Olfactores - ps19
Vertebrata - ps20
Gnathostomata - ps21
Euteleostomi - ps22
Sarcopterygii - ps23
Tetrapoda - ps24
Amniota - ps25
Mammalia - ps26
Theria - ps27
Placentalia - ps28
Boreoeutheria - ps29
Euarchontoglires - ps30
Euarchonta - ps31
Primates - ps32
Haplorrhini - ps33
Simiiformes - ps34
Catarrhini - ps35
Hominoidea - ps36
Hominidae - ps37
Homininae - ps38
Hominini - ps39
Homo_sapiens - ps40
Phylostratum

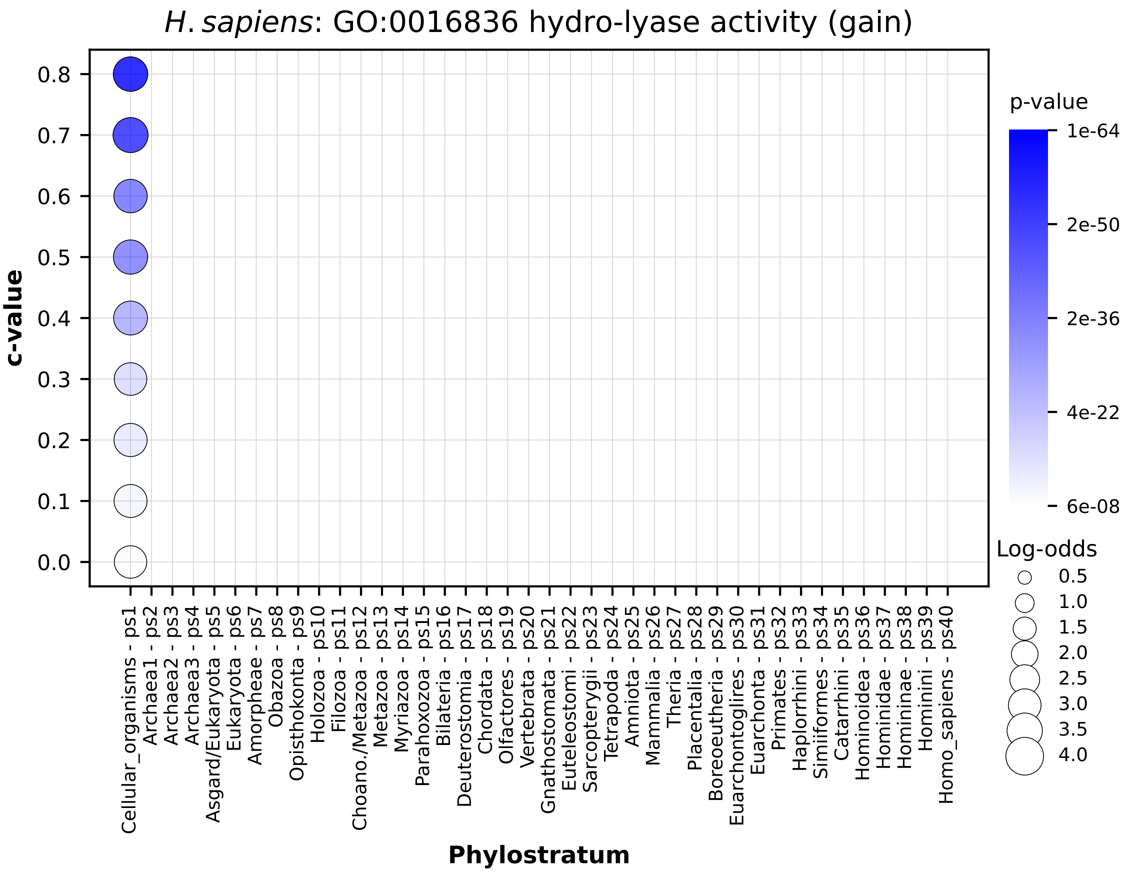

H. sapiens: GO:0016836 hydro-lyase activity (gain)
c-value
0.8
0.7
0.6
0.5
0.4
0.3
0.2
0.1
0.0
Phylostratum
Cellular_organisms - ps1
Archaea1 - ps2
Archaea2 - ps3
Archaea3 - ps4
Asgard/Eukaryota - ps5
Eukaryota - ps6
Amorpheae - ps7
Obazoa - ps8
Opisthokonta - ps9
Holozoa - ps10
Filozoa - ps11
Choano./Metazoa - ps12
Metazoa - ps13
Myriazoa - ps14
Parahoxozoa - ps15
Bilateria - ps16
Deuterostomia - ps17
Chordata - ps18
Olfactores - ps19
Vertebrata - ps20
Gnathostomata - ps21
Euteleostomi - ps22
Sarcopterygii - ps23
Tetrapoda - ps24
Amniota - ps25
Mammalia - ps26
Theria - ps27
Placentalia - ps28
Boreoeutheria - ps29
Euarchontoglires - ps30
Euarchonta - ps31
Primates - ps32
Haplorrhini - ps33
Simiiformes - ps34
Catarrhini - ps35
Hominoidea - ps36
Hominidae - ps37
Homininae - ps38
Hominini - ps39
Homo_sapiens - ps40
p-value
1e-64
2e-50
2e-36
4e-22
6e-08
Log-odds
0.5
1.0
1.5
2.0
2.5
3.0
3.5
4.0

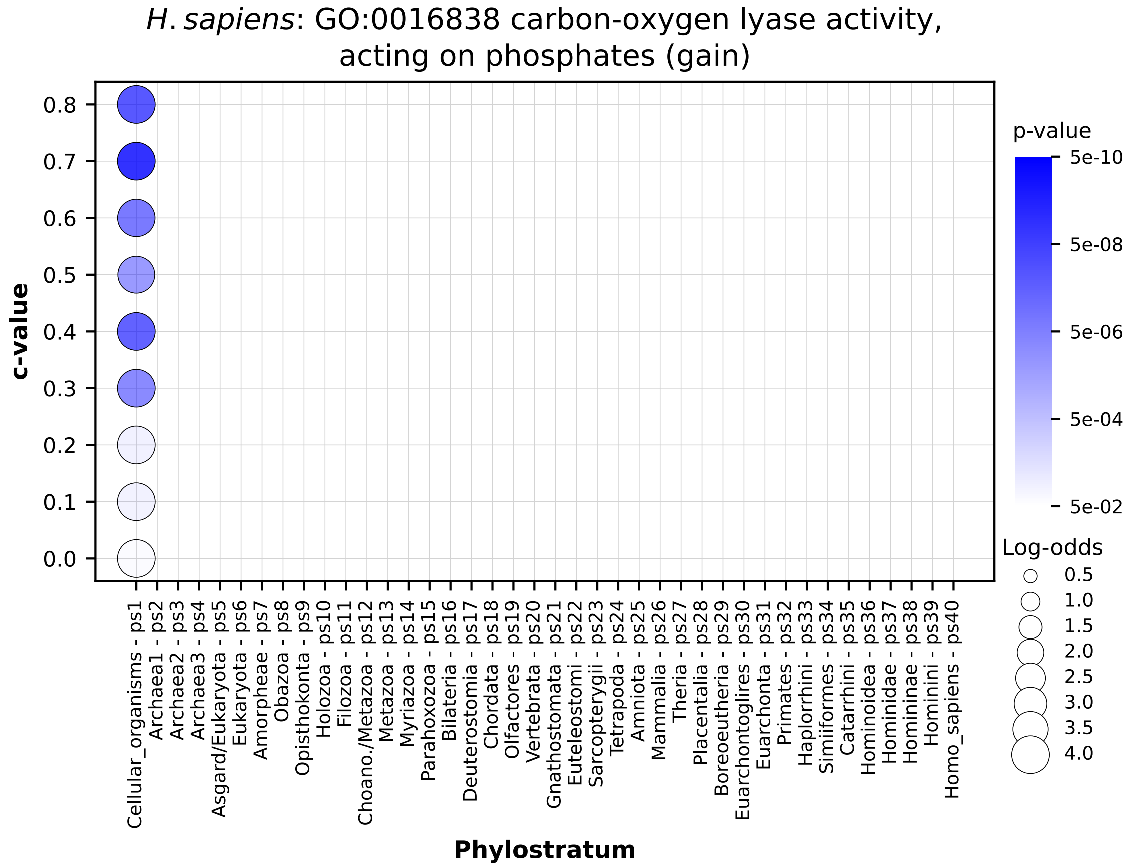

*H. sapiens*: GO:0016838 carbon-oxygen lyase activity, acting on phosphates (gain)
c-value
0.8
0.7
0.6
0.5
0.4
0.3
0.2
0.1
0.0
p-value
5e-10
5e-08
5e-06
5e-04
5e-02
Log-odds
0.5
1.0
1.5
2.0
2.5
3.0
3.5
4.0
Cellular_organisms - ps1
Archaea1 - ps2
Archaea2 - ps3
Archaea3 - ps4
Asgard/Eukaryota - ps5
Eukaryota - ps6
Amorpheae - ps7
Obazoa - ps8
Opisthokonta - ps9
Holozoa - ps10
Filozoa - ps11
Choano./Metazoa - ps12
Metazoa - ps13
Myriazoa - ps14
Parahoxozoa - ps15
Bilateria - ps16
Deuterostomia - ps17
Chordata - ps18
Olfactores - ps19
Vertebrata - ps20
Gnathostomata - ps21
Euteleostomi - ps22
Sarcopterygii - ps23
Tetrapoda - ps24
Amniota - ps25
Mammalia - ps26
Theria - ps27
Placentalia - ps28
Boreoeutheria - ps29
Euarchontoglires - ps30
Euarchonta - ps31
Primates - ps32
Haplorrhini - ps33
Simiiformes - ps34
Catarrhini - ps35
Hominoidea - ps36
Hominidae - ps37
Homininae - ps38
Hominini - ps39
Homo_sapiens - ps40
Phylostratum

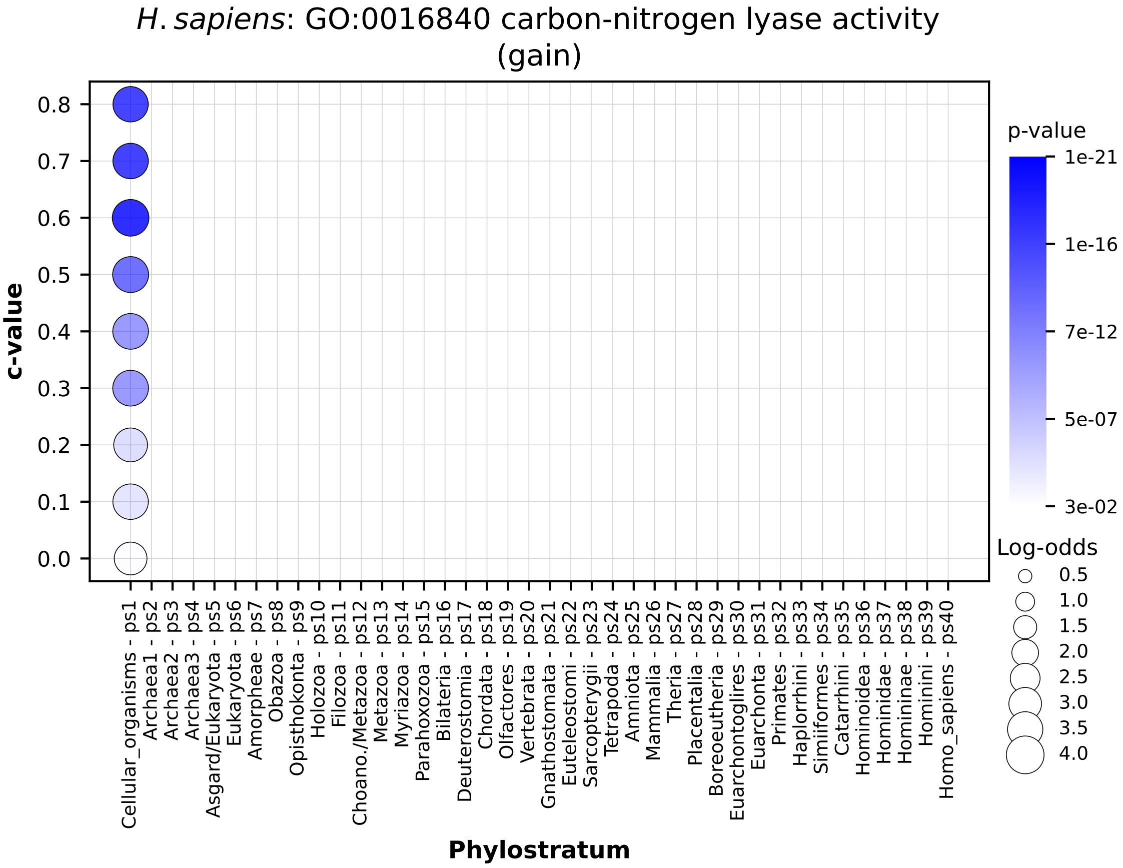

*H. sapiens*: GO:0016840 carbon-nitrogen lyase activity (gain)
c-value
0.8
0.7
0.6
0.5
0.4
0.3
0.2
0.1
0.0
p-value
1e-21
1e-16
7e-12
5e-07
3e-02
Log-odds
0.5
1.0
1.5
2.0
2.5
3.0
3.5
4.0
Cellular_organisms - ps1
Archaea1 - ps2
Archaea2 - ps3
Archaea3 - ps4
Asgard/Eukaryota - ps5
Eukaryota - ps6
Amorpheae - ps7
Obazoa - ps8
Opisthokonta - ps9
Holozoa - ps10
Filozoa - ps11
Choano./Metazoa - ps12
Metazoa - ps13
Myriazoa - ps14
Parahoxozoa - ps15
Bilateria - ps16
Deuterostomia - ps17
Chordata - ps18
Olfactores - ps19
Vertebrata - ps20
Gnathostomata - ps21
Euteleostomi - ps22
Sarcopterygii - ps23
Tetrapoda - ps24
Amniota - ps25
Mammalia - ps26
Theria - ps27
Placentalia - ps28
Boreoeutheria - ps29
Euarchontoglires - ps30
Euarchonta - ps31
Primates - ps32
Haplorrhini - ps33
Simiiformes - ps34
Catarrhini - ps35
Hominoidea - ps36
Hominidae - ps37
Homininae - ps38
Hominini - ps39
Homo_sapiens - ps40
Phylostratum

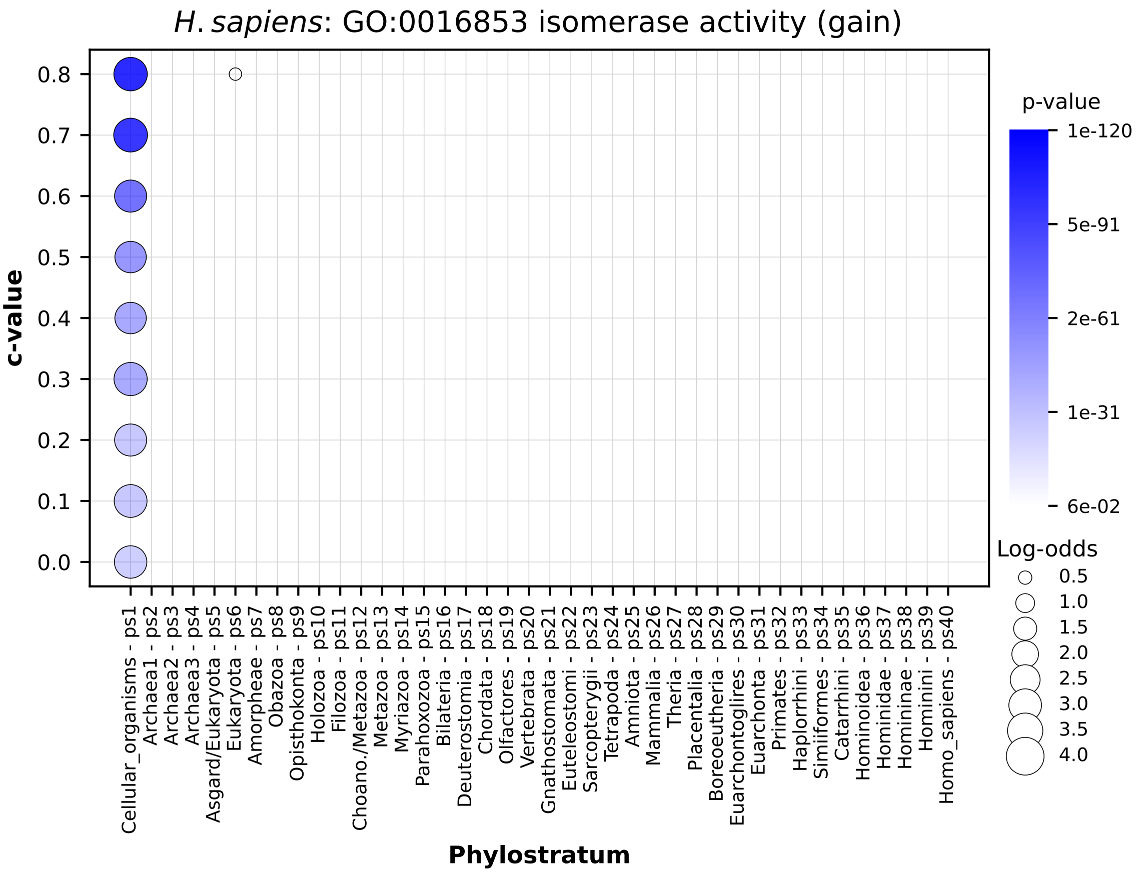

H. sapiens: GO:0016853 isomerase activity (gain)
c-value
0.8
0.7
0.6
0.5
0.4
0.3
0.2
0.1
0.0
p-value
1e-120
5e-91
2e-61
1e-31
6e-02
Log-odds
0.5
1.0
1.5
2.0
2.5
3.0
3.5
4.0
Cellular_organisms - ps1
Archaea1 - ps2
Archaea2 - ps3
Archaea3 - ps4
Asgard/Eukaryota - ps5
Eukaryota - ps6
Amorpheae - ps7
Obazoa - ps8
Opisthokonta - ps9
Holozoa - ps10
Filozoa - ps11
Choano./Metazoa - ps12
Metazoa - ps13
Myriazoa - ps14
Parahoxozoa - ps15
Bilateria - ps16
Deuterostomia - ps17
Chordata - ps18
Olfactores - ps19
Vertebrata - ps20
Gnathostomata - ps21
Euteleostomi - ps22
Sarcopterygii - ps23
Tetrapoda - ps24
Amniota - ps25
Mammalia - ps26
Theria - ps27
Placentalia - ps28
Boreoeutheria - ps29
Euarchontoglires - ps30
Euarchonta - ps31
Primates - ps32
Haplorrhini - ps33
Simiiformes - ps34
Catarrhini - ps35
Hominoidea - ps36
Hominidae - ps37
Homininae - ps38
Hominini - ps39
Homo_sapiens - ps40
Phylostratum

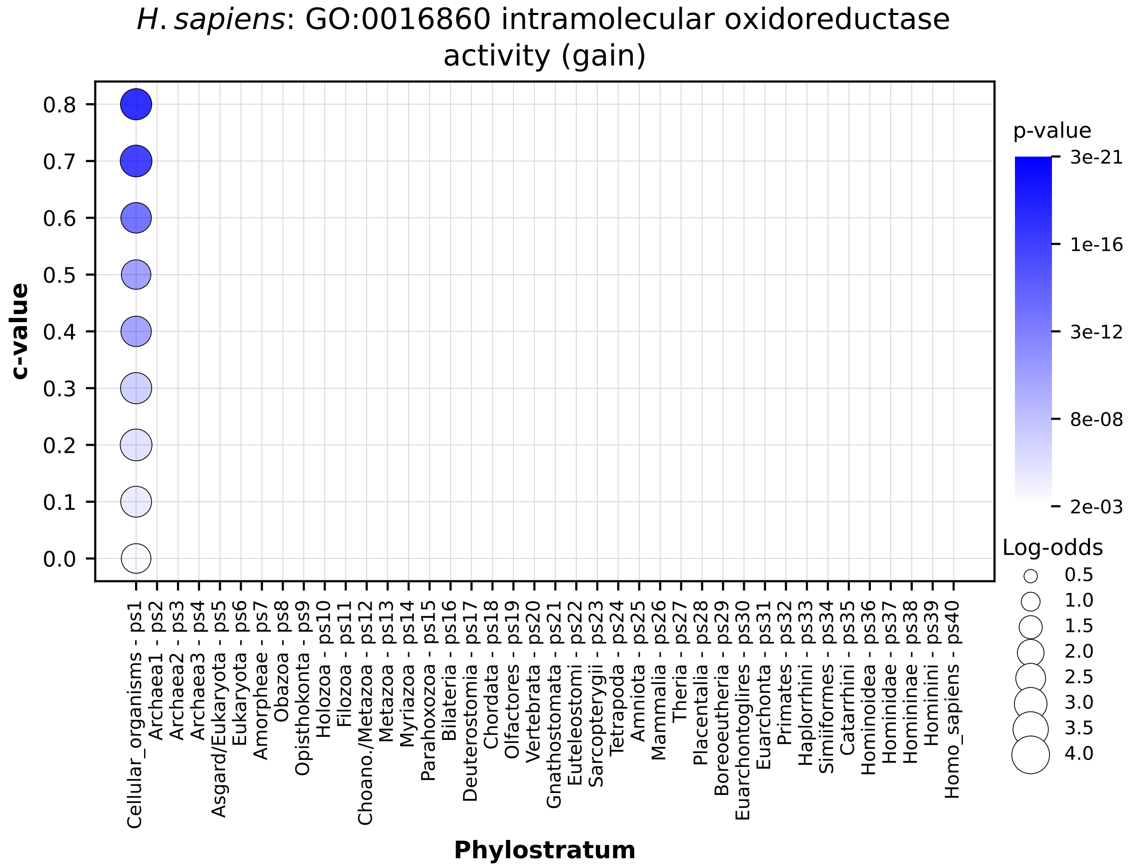

H. sapiens: GO:0016860 intramolecular oxidoreductase activity (gain)
c-value
0.8
0.7
0.6
0.5
0.4
0.3
0.2
0.1
0.0
p-value
3e-21
1e-16
3e-12
8e-08
2e-03
Log-odds
0.5
1.0
1.5
2.0
2.5
3.0
3.5
4.0
Cellular_organisms - ps1
Archaea1 - ps2
Archaea2 - ps3
Archaea3 - ps4
Asgard/Eukaryota - ps5
Eukaryota - ps6
Amorpheae - ps7
Obazoa - ps8
Opisthokonta - ps9
Holozoa - ps10
Filozoa - ps11
Choano./Metazoa - ps12
Metazoa - ps13
Myriazoa - ps14
Parahoxozoa - ps15
Bilateria - ps16
Deuterostomia - ps17
Chordata - ps18
Olfactores - ps19
Vertebrata - ps20
Gnathostomata - ps21
Euteleostomi - ps22
Sarcopterygii - ps23
Tetrapoda - ps24
Amniota - ps25
Mammalia - ps26
Theria - ps27
Placentalia - ps28
Boreoeutheria - ps29
Euarchontoglires - ps30
Euarchonta - ps31
Primates - ps32
Haplorrhini - ps33
Simiiformes - ps34
Catarrhini - ps35
Hominoidea - ps36
Hominidae - ps37
Homininae - ps38
Hominini - ps39
Homo_sapiens - ps40
Phylostratum

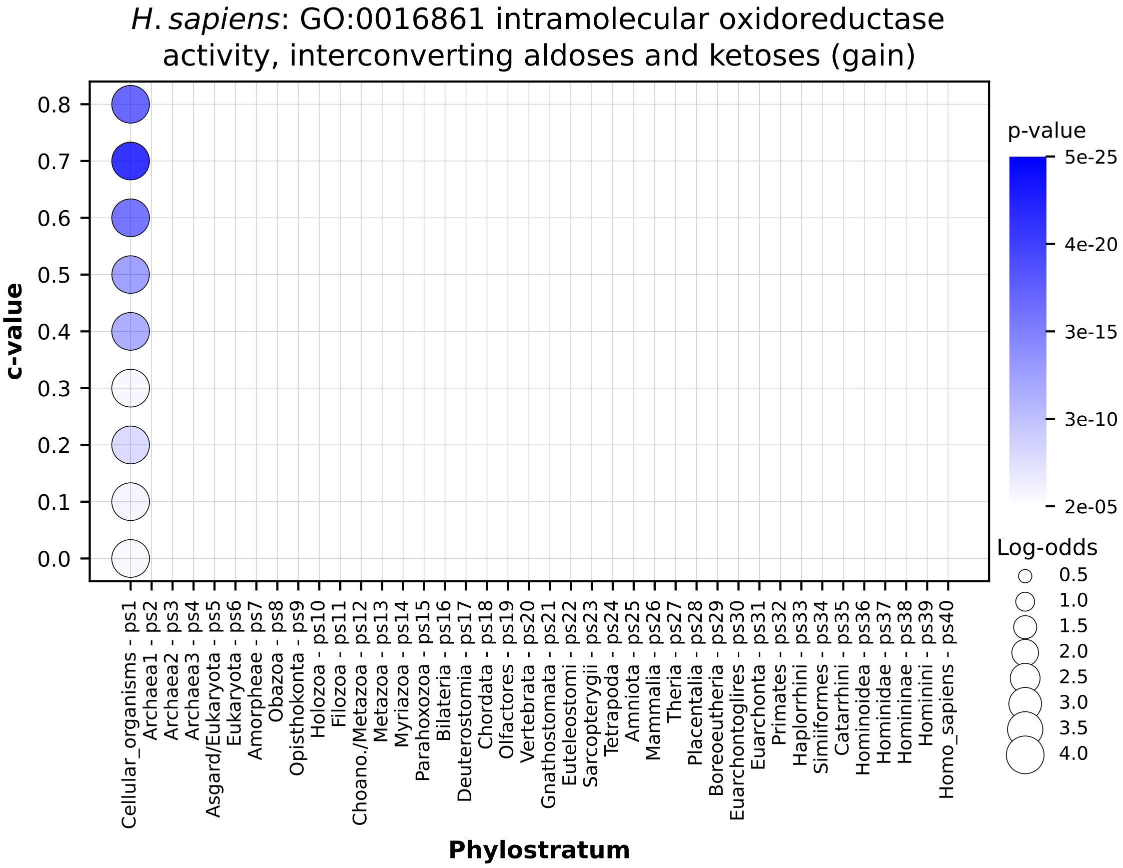

H. sapiens: GO:0016861 intramolecular oxidoreductase activity, interconverting aldoses and ketoses (gain)
c-value
0.8
0.7
0.6
0.5
0.4
0.3
0.2
0.1
0.0
p-value
5e-25
4e-20
3e-15
3e-10
2e-05
Log-odds
0.5
1.0
1.5
2.0
2.5
3.0
3.5
4.0
Cellular_organisms - ps1
Archaea1 - ps2
Archaea2 - ps3
Archaea3 - ps4
Asgard/Eukaryota - ps5
Eukaryota - ps6
Amorpheae - ps7
Obazoa - ps8
Opisthokonta - ps9
Holozoa - ps10
Filozoa - ps11
Choano./Metazoa - ps12
Metazoa - ps13
Myriazoa - ps14
Parahoxozoa - ps15
Bilateria - ps16
Deuterostomia - ps17
Chordata - ps18
Olfactores - ps19
Vertebrata - ps20
Gnathostomata - ps21
Euteleostomi - ps22
Sarcopterygii - ps23
Tetrapoda - ps24
Amniota - ps25
Mammalia - ps26
Theria - ps27
Placentalia - ps28
Boreoeutheria - ps29
Euarchontoglires - ps30
Euarchonta - ps31
Primates - ps32
Haplorrhini - ps33
Simiiformes - ps34
Catarrhini - ps35
Hominoidea - ps36
Hominidae - ps37
Homininae - ps38
Hominini - ps39
Homo_sapiens - ps40
Phylostratum

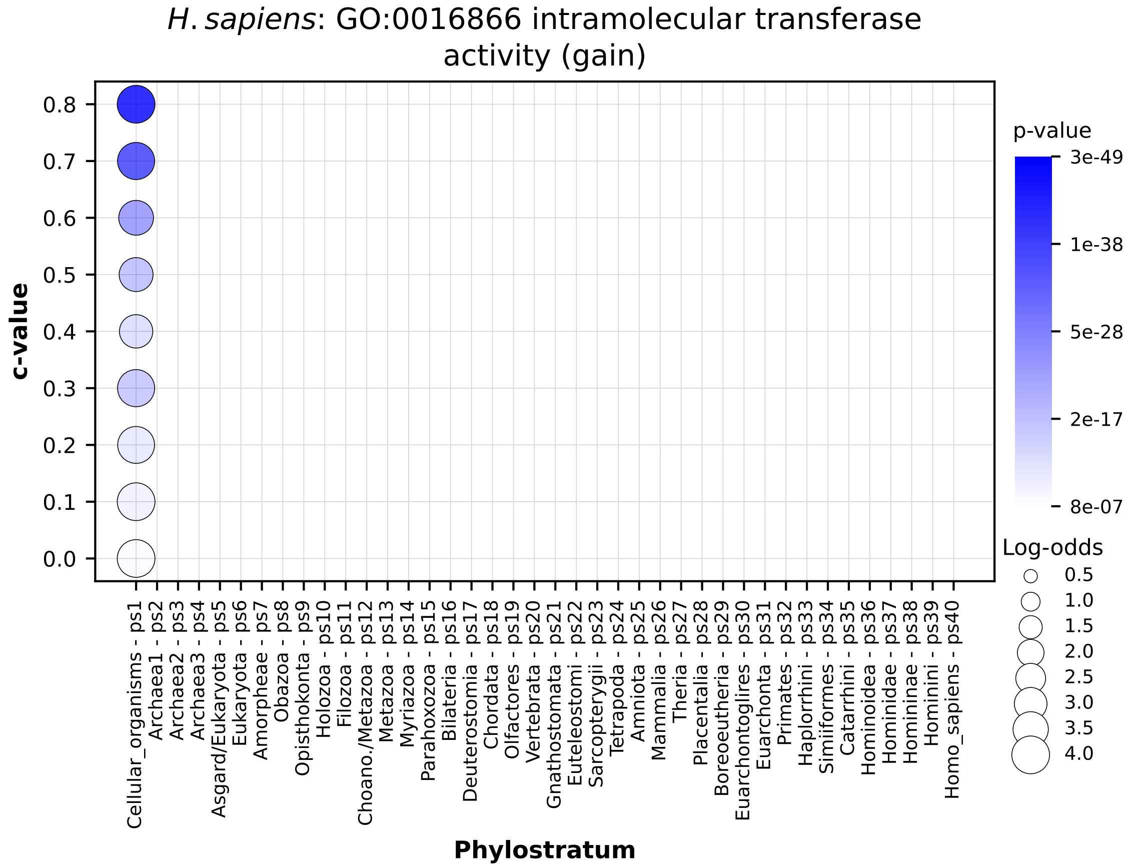
H. sapiens: GO:0016866 intramolecular transferase activity (gain)
c-value
0.8
0.7
0.6
0.5
0.4
0.3
0.2
0.1
0.0
p-value
3e-49
1e-38
5e-28
2e-17
8e-07
Log-odds
0.5
1.0
1.5
2.0
2.5
3.0
3.5
4.0
Cellular_organisms - ps1
Archaea1 - ps2
Archaea2 - ps3
Archaea3 - ps4
Asgard/Eukaryota - ps5
Eukaryota - ps6
Amorpheae - ps7
Obazoa - ps8
Opisthokonta - ps9
Holozoa - ps10
Filozoa - ps11
Choano./Metazoa - ps12
Metazoa - ps13
Myriazoa - ps14
Parahoxozoa - ps15
Bilateria - ps16
Deuterostomia - ps17
Chordata - ps18
Olfactores - ps19
Vertebrata - ps20
Gnathostomata - ps21
Euteleostomi - ps22
Sarcopterygii - ps23
Tetrapoda - ps24
Amniota - ps25
Mammalia - ps26
Theria - ps27
Placentalia - ps28
Boreoeutheria - ps29
Euarchontoglires - ps30
Euarchonta - ps31
Primates - ps32
Haplorrhini - ps33
Simiiformes - ps34
Catarrhini - ps35
Hominoidea - ps36
Hominidae - ps37
Homininae - ps38
Hominini - ps39
Homo_sapiens - ps40
Phylostratum

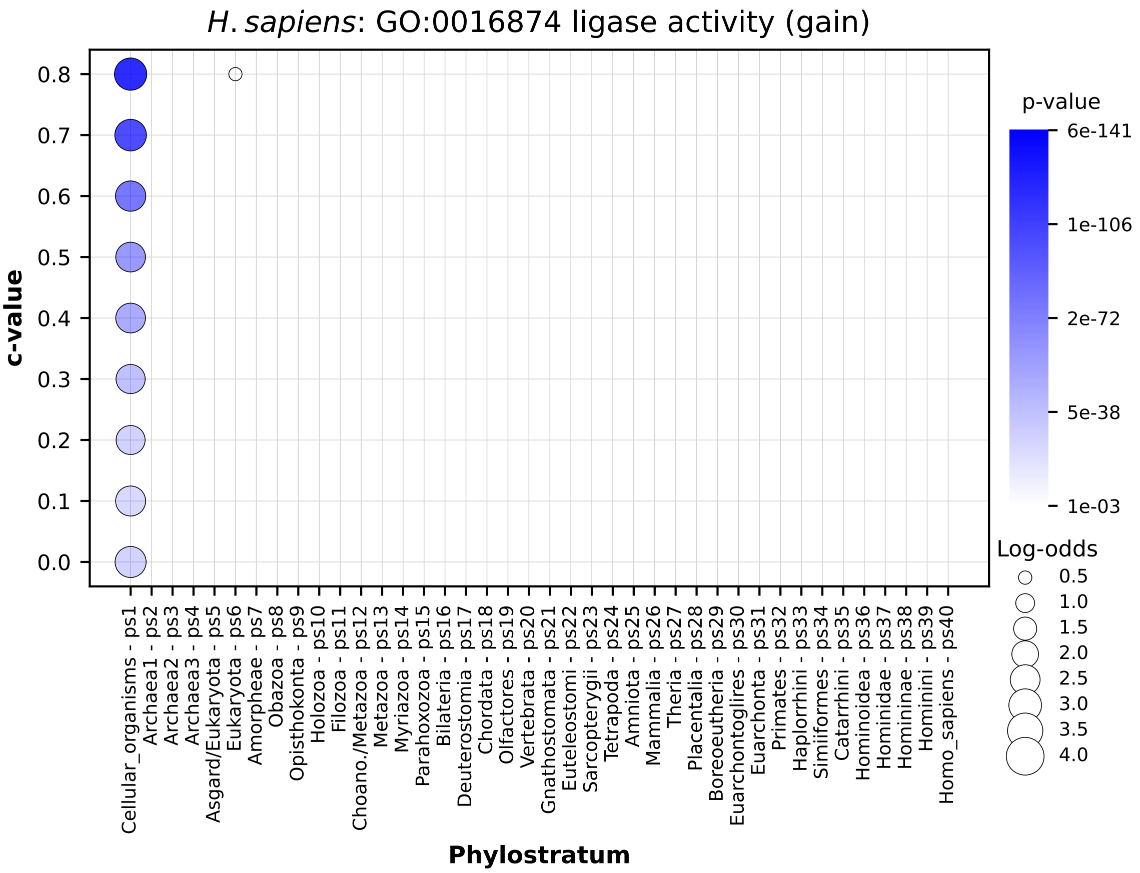
H. sapiens: GO:0016874 ligase activity (gain)
c-value
0.0
0.1
0.2
0.3
0.4
0.5
0.6
0.7
0.8
p-value
6e-141
1e-106
2e-72
5e-38
1e-03
Log-odds
0.5
1.0
1.5
2.0
2.5
3.0
3.5
4.0
Phylostratum
Cellular_organisms - ps1
Archaea1 - ps2
Archaea2 - ps3
Archaea3 - ps4
Asgard/Eukaryota - ps5
Eukaryota - ps6
Amorpheae - ps7
Obazoa - ps8
Opisthokonta - ps9
Holozoa - ps10
Filozoa - ps11
Choano./Metazoa - ps12
Metazoa - ps13
Myriazoa - ps14
Parahoxozoa - ps15
Bilateria - ps16
Deuterostomia - ps17
Chordata - ps18
Olfactores - ps19
Vertebrata - ps20
Gnathostomata - ps21
Euteleostomi - ps22
Sarcopterygii - ps23
Tetrapoda - ps24
Amniota - ps25
Mammalia - ps26
Theria - ps27
Placentalia - ps28
Boreoeutheria - ps29
Euarchontoglires - ps30
Euarchonta - ps31
Primates - ps32
Haplorrhini - ps33
Simiiformes - ps34
Catarrhini - ps35
Hominoidea - ps36
Hominidae - ps37
Homininae - ps38
Hominini - ps39
Homo_sapiens - ps40

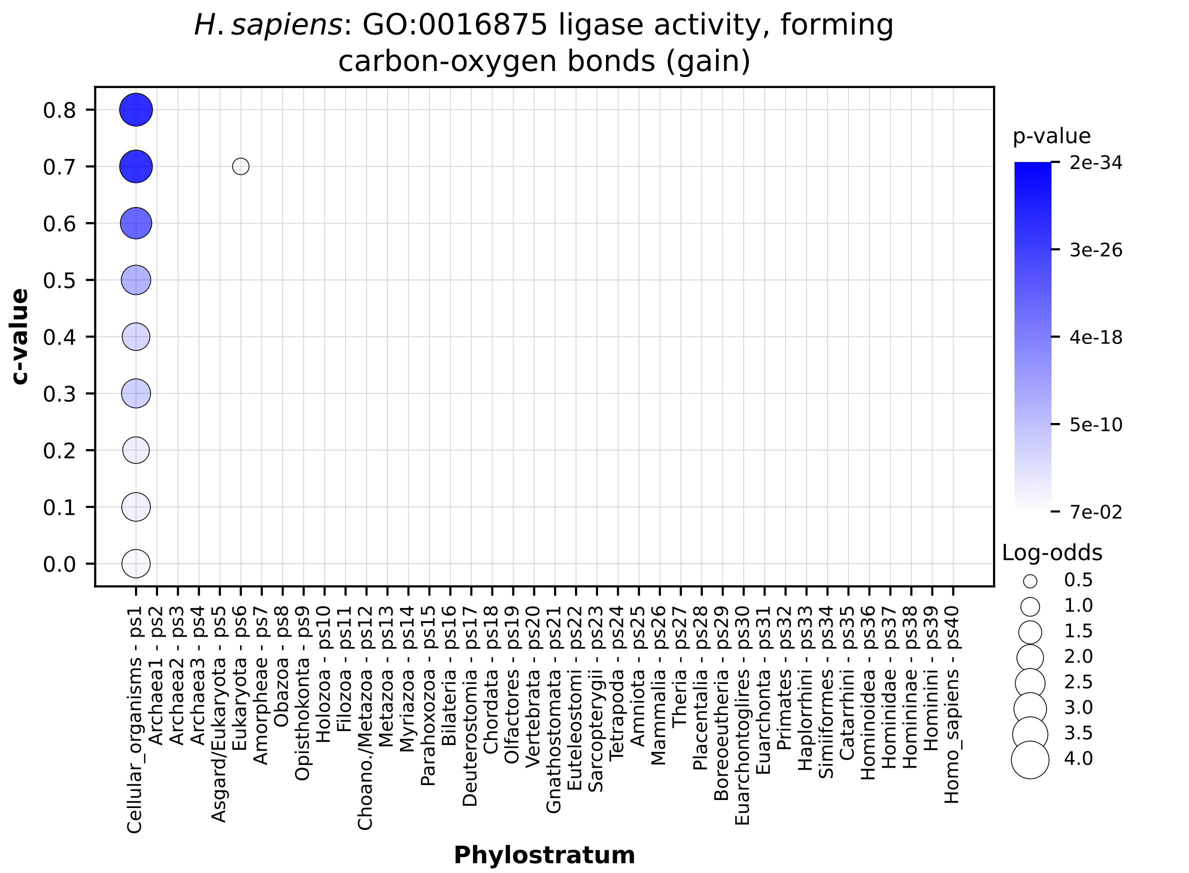

H. sapiens: GO:0016875 ligase activity, forming carbon-oxygen bonds (gain)
c-value
0.8
0.7
0.6
0.5
0.4
0.3
0.2
0.1
0.0
p-value
2e-34
3e-26
4e-18
5e-10
7e-02
Log-odds
0.5
1.0
1.5
2.0
2.5
3.0
3.5
4.0
Cellular_organisms - ps1
Archaea1 - ps2
Archaea2 - ps3
Archaea3 - ps4
Asgard/Eukaryota - ps5
Eukaryota - ps6
Amorpheae - ps7
Obazoa - ps8
Opisthokonta - ps9
Holozoa - ps10
Filozoa - ps11
Choano./Metazoa - ps12
Metazoa - ps13
Myriazoa - ps14
Parahoxozoa - ps15
Bilateria - ps16
Deuterostomia - ps17
Chordata - ps18
Olfactores - ps19
Vertebrata - ps20
Gnathostomata - ps21
Euteleostomi - ps22
Sarcopterygii - ps23
Tetrapoda - ps24
Amniota - ps25
Mammalia - ps26
Theria - ps27
Placentalia - ps28
Boreoeutheria - ps29
Euarchontoglires - ps30
Euarchonta - ps31
Primates - ps32
Haplorrhini - ps33
Simiiformes - ps34
Catarrhini - ps35
Hominoidea - ps36
Hominidae - ps37
Homininae - ps38
Hominini - ps39
Homo_sapiens - ps40
Phylostratum

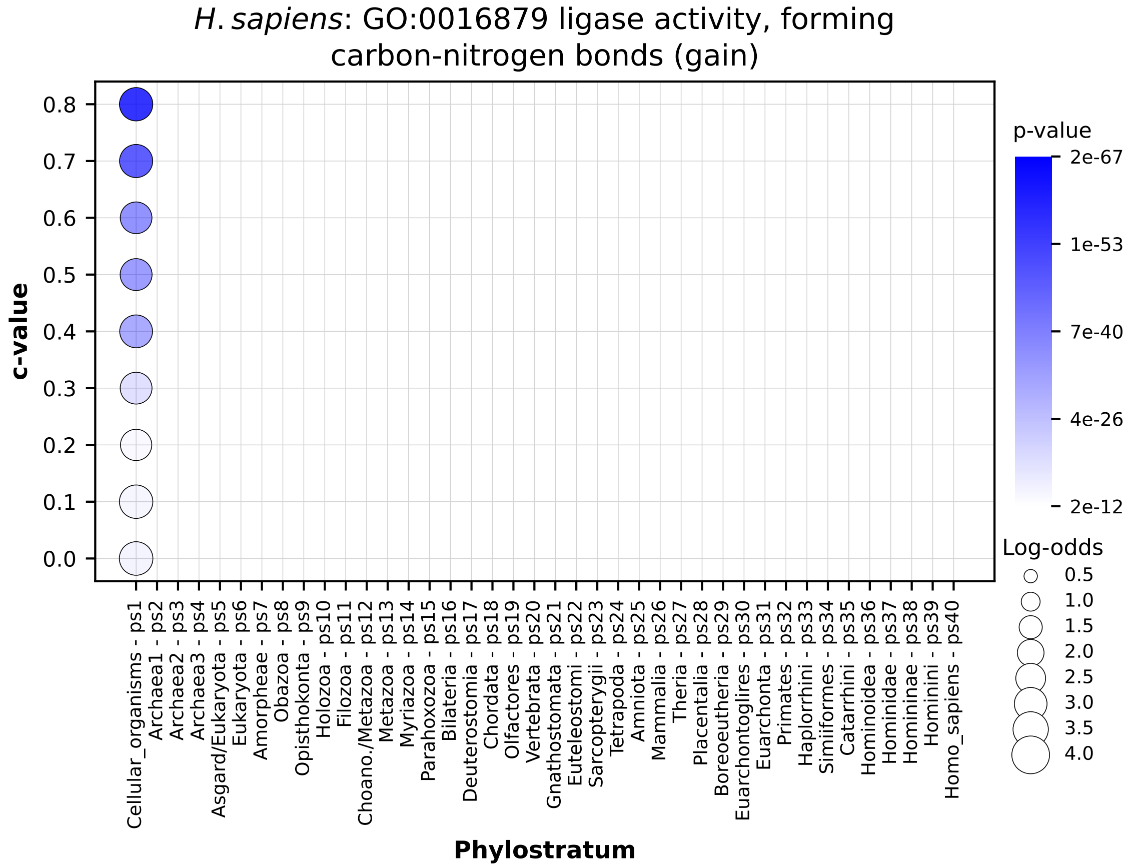
H. sapiens: GO:0016879 ligase activity, forming carbon-nitrogen bonds (gain)
c-value
0.8
0.7
0.6
0.5
0.4
0.3
0.2
0.1
0.0
p-value
2e-67
1e-53
7e-40
4e-26
2e-12
Log-odds
0.5
1.0
1.5
2.0
2.5
3.0
3.5
4.0
Cellular_organisms - ps1
Archaea1 - ps2
Archaea2 - ps3
Archaea3 - ps4
Asgard/Eukaryota - ps5
Eukaryota - ps6
Amorpheae - ps7
Obazoa - ps8
Opisthokonta - ps9
Holozoa - ps10
Filozoa - ps11
Choano./Metazoa - ps12
Metazoa - ps13
Myriazoa - ps14
Parahoxozoa - ps15
Bilateria - ps16
Deuterostomia - ps17
Chordata - ps18
Olfactores - ps19
Vertebrata - ps20
Gnathostomata - ps21
Euteleostomi - ps22
Sarcopterygii - ps23
Tetrapoda - ps24
Amniota - ps25
Mammalia - ps26
Theria - ps27
Placentalia - ps28
Boreoeutheria - ps29
Euarchontoglires - ps30
Euarchonta - ps31
Primates - ps32
Haplorrhini - ps33
Simiiformes - ps34
Catarrhini - ps35
Hominoidea - ps36
Hominidae - ps37
Homininae - ps38
Hominini - ps39
Homo_sapiens - ps40
Phylostratum

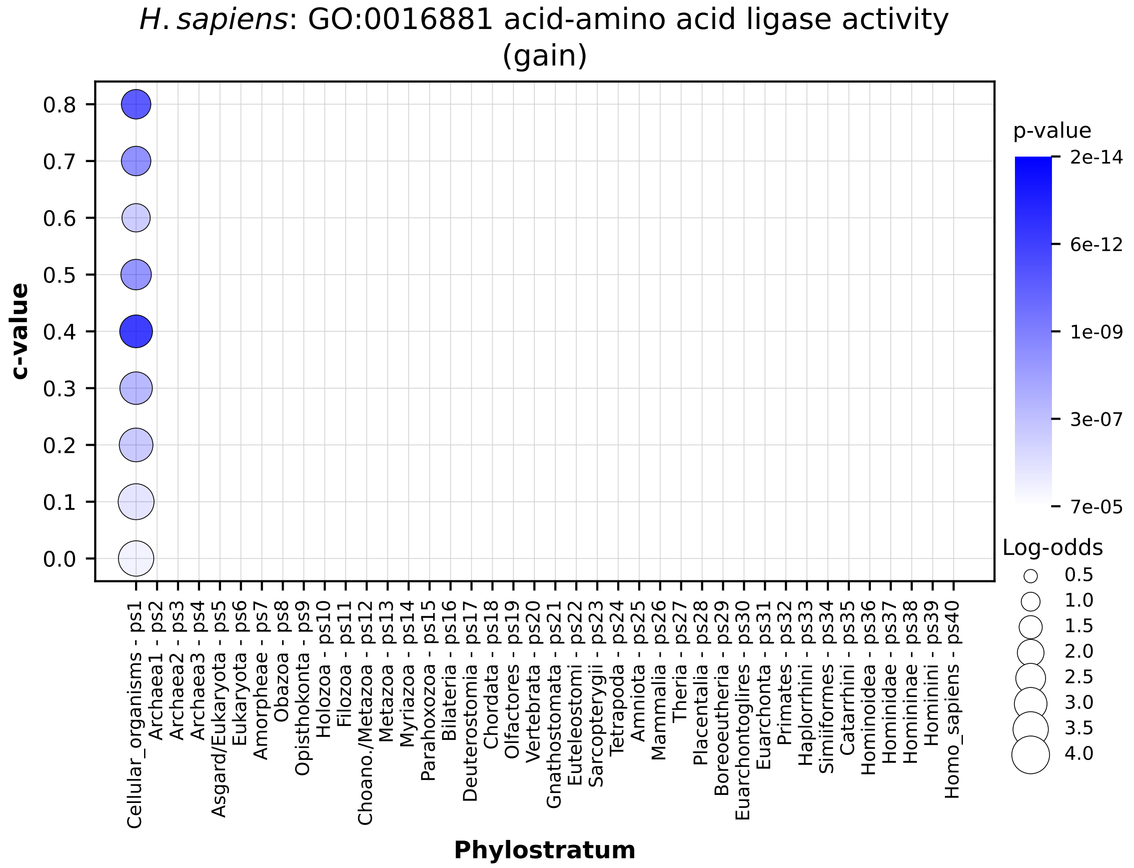
H. sapiens: GO:0016881 acid-amino acid ligase activity (gain)
c-value
0.8
0.7
0.6
0.5
0.4
0.3
0.2
0.1
0.0
p-value
2e-14
6e-12
1e-09
3e-07
7e-05
Log-odds
0.5
1.0
1.5
2.0
2.5
3.0
3.5
4.0
Cellular_organisms - ps1
Archaea1 - ps2
Archaea2 - ps3
Archaea3 - ps4
Asgard/Eukaryota - ps5
Eukaryota - ps6
Amorpheae - ps7
Obazoa - ps8
Opisthokonta - ps9
Holozoa - ps10
Filozoa - ps11
Choano./Metazoa - ps12
Metazoa - ps13
Myriazoa - ps14
Parahoxozoa - ps15
Bilateria - ps16
Deuterostomia - ps17
Chordata - ps18
Olfactores - ps19
Vertebrata - ps20
Gnathostomata - ps21
Euteleostomi - ps22
Sarcopterygii - ps23
Tetrapoda - ps24
Amniota - ps25
Mammalia - ps26
Theria - ps27
Placentalia - ps28
Boreoeutheria - ps29
Euarchontoglires - ps30
Euarchonta - ps31
Primates - ps32
Haplorrhini - ps33
Simiiformes - ps34
Catarrhini - ps35
Hominoidea - ps36
Hominidae - ps37
Homininae - ps38
Hominini - ps39
Homo_sapiens - ps40
Phylostratum

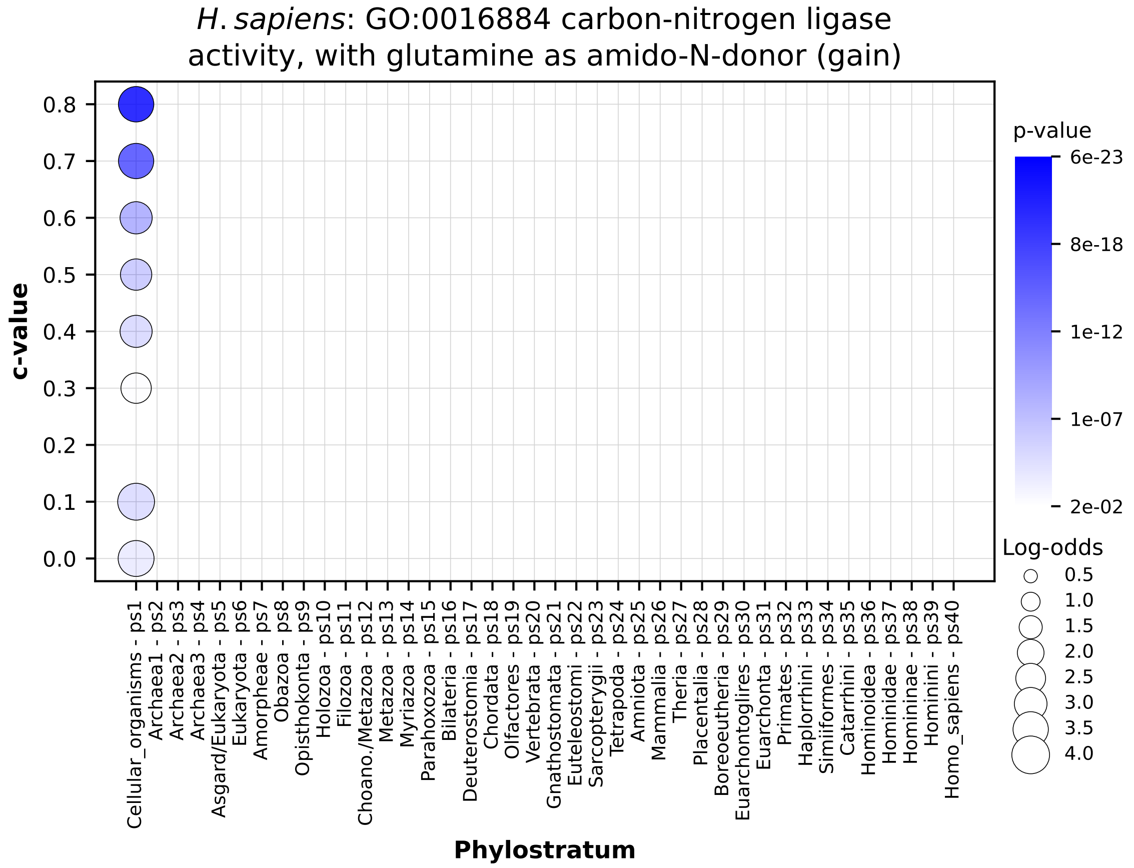

H. sapiens: GO:0016884 carbon-nitrogen ligase activity, with glutamine as amido-N-donor (gain)
c-value
0.8
0.7
0.6
0.5
0.4
0.3
0.2
0.1
0.0
p-value
6e-23
8e-18
1e-12
1e-07
2e-02
Log-odds
0.5
1.0
1.5
2.0
2.5
3.0
3.5
4.0
Cellular_organisms - ps1
Archaea1 - ps2
Archaea2 - ps3
Archaea3 - ps4
Asgard/Eukaryota - ps5
Eukaryota - ps6
Amorpheae - ps7
Obazoa - ps8
Opisthokonta - ps9
Holozoa - ps10
Filozoa - ps11
Choano./Metazoa - ps12
Metazoa - ps13
Myriazoa - ps14
Parahoxozoa - ps15
Bilateria - ps16
Deuterostomia - ps17
Chordata - ps18
Olfactores - ps19
Vertebrata - ps20
Gnathostomata - ps21
Euteleostomi - ps22
Sarcopterygii - ps23
Tetrapoda - ps24
Amniota - ps25
Mammalia - ps26
Theria - ps27
Placentalia - ps28
Boreoeutheria - ps29
Euarchontoglires - ps30
Euarchonta - ps31
Primates - ps32
Haplorrhini - ps33
Simiiformes - ps34
Catarrhini - ps35
Hominoidea - ps36
Hominidae - ps37
Homininae - ps38
Hominini - ps39
Homo_sapiens - ps40
Phylostratum

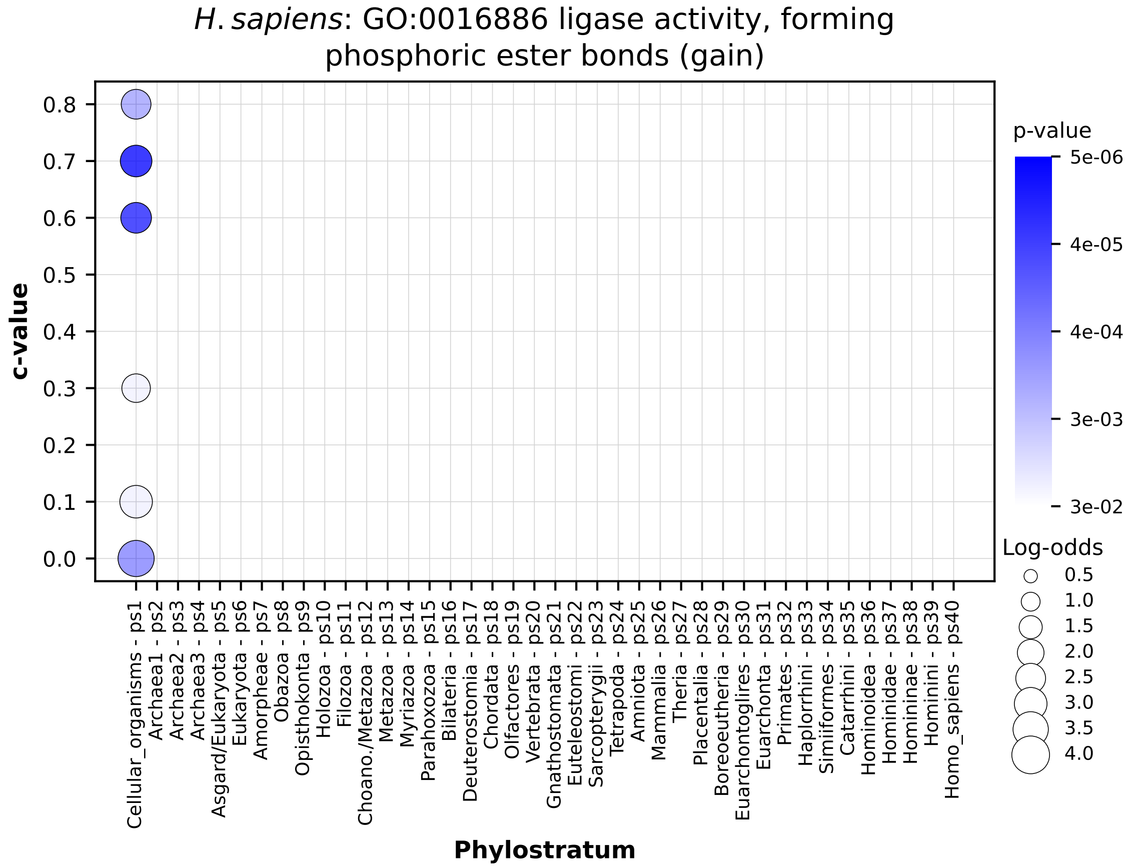

*H. sapiens*: GO:0016886 ligase activity, forming phosphoric ester bonds (gain)
c-value
0.8
0.7
0.6
0.5
0.4
0.3
0.2
0.1
0.0
p-value
5e-06
4e-05
4e-04
3e-03
3e-02
Log-odds
0.5
1.0
1.5
2.0
2.5
3.0
3.5
4.0
Cellular_organisms - ps1
Archaea1 - ps2
Archaea2 - ps3
Archaea3 - ps4
Asgard/Eukaryota - ps5
Eukaryota - ps6
Amorpheae - ps7
Obazoa - ps8
Opisthokonta - ps9
Holozoa - ps10
Filozoa - ps11
Choano./Metazoa - ps12
Metazoa - ps13
Myriazoa - ps14
Parahoxozoa - ps15
Bilateria - ps16
Deuterostomia - ps17
Chordata - ps18
Olfactores - ps19
Vertebrata - ps20
Gnathostomata - ps21
Euteleostomi - ps22
Sarcopterygii - ps23
Tetrapoda - ps24
Amniota - ps25
Mammalia - ps26
Theria - ps27
Placentalia - ps28
Boreoeutheria - ps29
Euarchontoglires - ps30
Euarchonta - ps31
Primates - ps32
Haplorrhini - ps33
Simiiformes - ps34
Catarrhini - ps35
Hominoidea - ps36
Hominidae - ps37
Homininae - ps38
Hominini - ps39
Homo_sapiens - ps40
Phylostratum

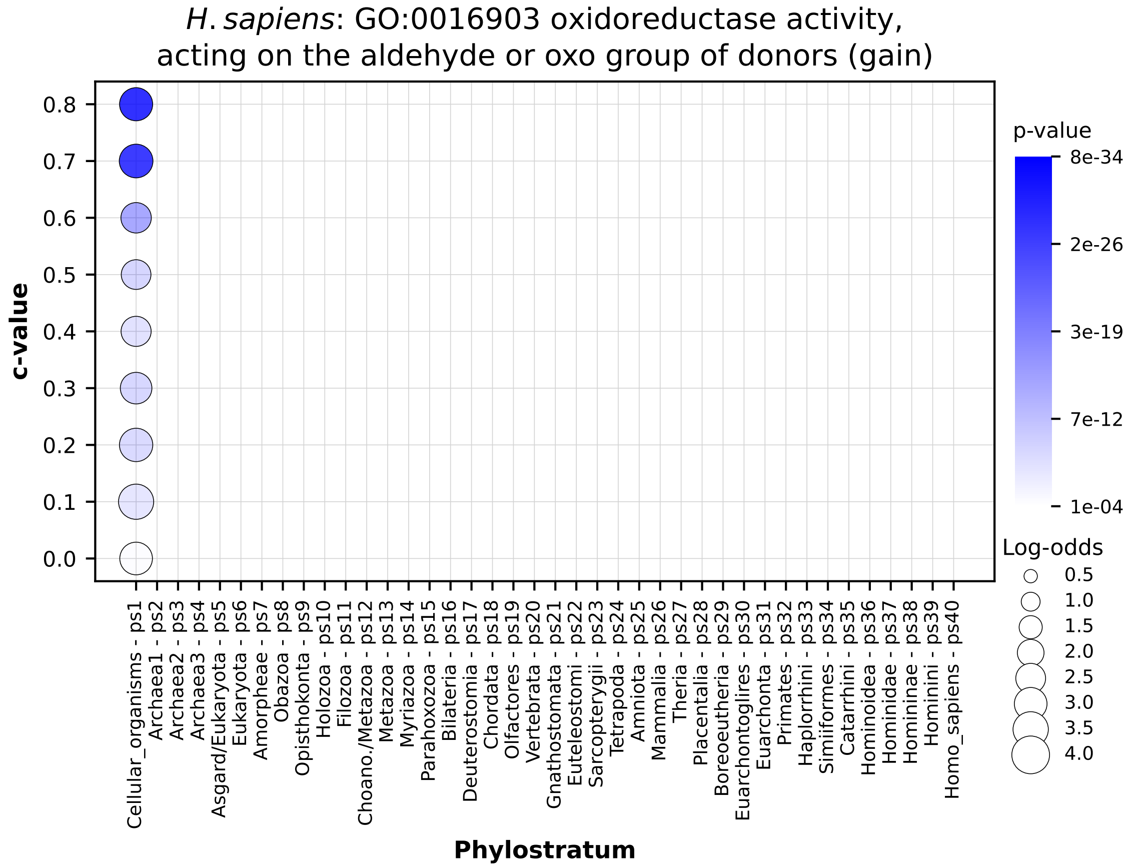
H. sapiens: GO:0016903 oxidoreductase activity,
acting on the aldehyde or oxo group of donors (gain)
c-value
0.0
0.1
0.2
0.3
0.4
0.5
0.6
0.7
0.8
Phylostratum
Cellular_organisms - ps1
Archaea1 - ps2
Archaea2 - ps3
Archaea3 - ps4
Asgard/Eukaryota - ps5
Eukaryota - ps6
Amorpheae - ps7
Obazoa - ps8
Opisthokonta - ps9
Holozoa - ps10
Filozoa - ps11
Choano./Metazoa - ps12
Metazoa - ps13
Myriazoa - ps14
Parahoxozoa - ps15
Bilateria - ps16
Deuterostomia - ps17
Chordata - ps18
Olfactores - ps19
Vertebrata - ps20
Gnathostomata - ps21
Euteleostomi - ps22
Sarcopterygii - ps23
Tetrapoda - ps24
Amniota - ps25
Mammalia - ps26
Theria - ps27
Placentalia - ps28
Boreoeutheria - ps29
Euarchontoglires - ps30
Euarchonta - ps31
Primates - ps32
Haplorrhini - ps33
Simiiformes - ps34
Catarrhini - ps35
Hominoidea - ps36
Hominidae - ps37
Homininae - ps38
Hominini - ps39
Homo_sapiens - ps40
p-value
8e-34
2e-26
3e-19
7e-12
1e-04
Log-odds
0.5
1.0
1.5
2.0
2.5
3.0
3.5
4.0

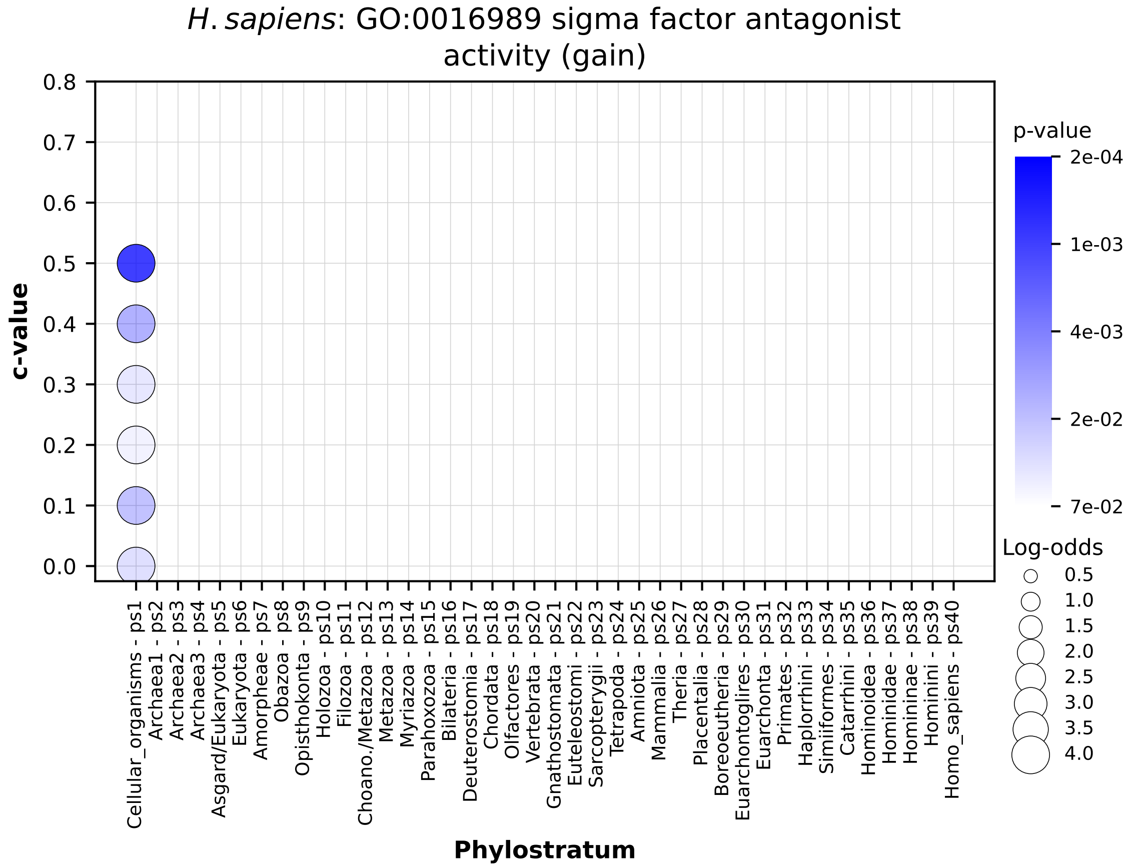

H. sapiens: GO:0016989 sigma factor antagonist activity (gain)
c-value
0.0
0.1
0.2
0.3
0.4
0.5
0.6
0.7
0.8
Phylostratum
Cellular_organisms - ps1
Archaea1 - ps2
Archaea2 - ps3
Archaea3 - ps4
Asgard/Eukaryota - ps5
Eukaryota - ps6
Amorpheae - ps7
Obazoa - ps8
Opisthokonta - ps9
Holozoa - ps10
Filozoa - ps11
Choano./Metazoa - ps12
Metazoa - ps13
Myriazoa - ps14
Parahoxozoa - ps15
Bilateria - ps16
Deuterostomia - ps17
Chordata - ps18
Olfactores - ps19
Vertebrata - ps20
Gnathostomata - ps21
Euteleostomi - ps22
Sarcopterygii - ps23
Tetrapoda - ps24
Amniota - ps25
Mammalia - ps26
Theria - ps27
Placentalia - ps28
Boreoeutheria - ps29
Euarchontoglires - ps30
Euarchonta - ps31
Primates - ps32
Haplorrhini - ps33
Simiiformes - ps34
Catarrhini - ps35
Hominoidea - ps36
Hominidae - ps37
Homininae - ps38
Hominini - ps39
Homo_sapiens - ps40
p-value
2e-04
1e-03
4e-03
2e-02
7e-02
Log-odds
0.5
1.0
1.5
2.0
2.5
3.0
3.5
4.0

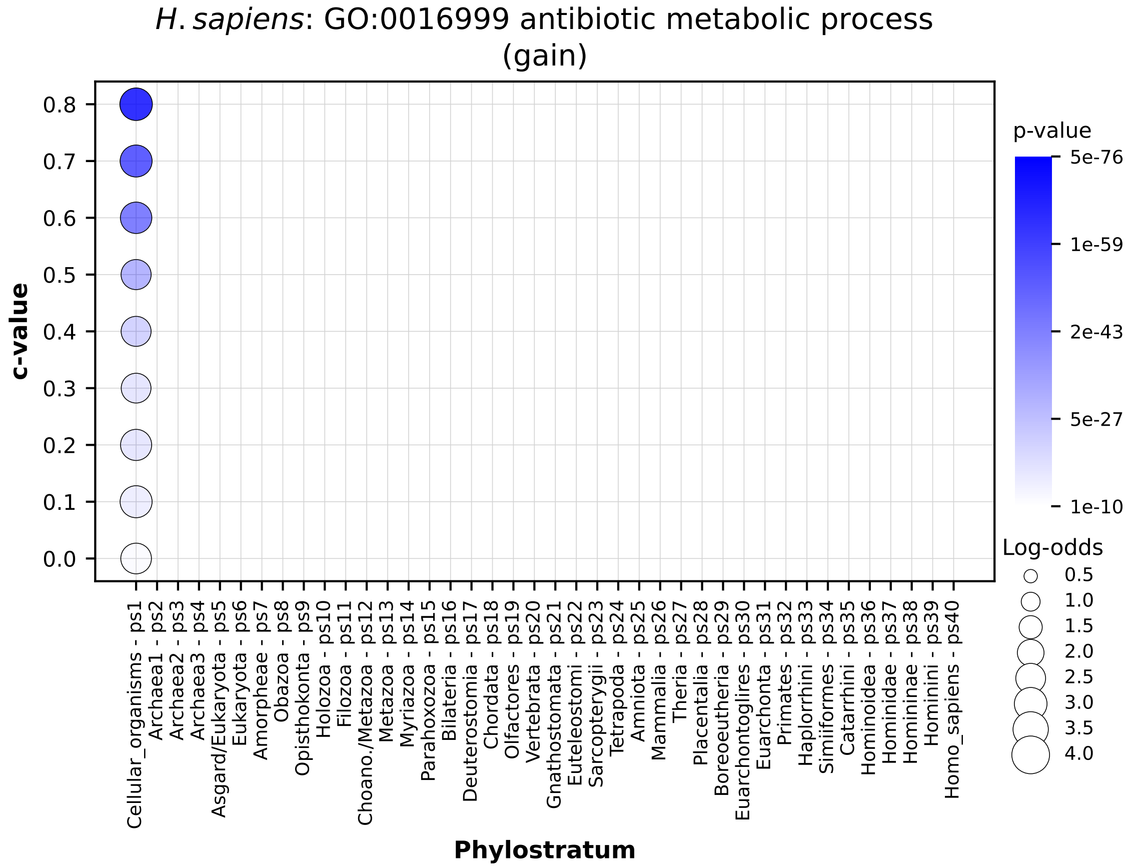

H. sapiens: GO:0016999 antibiotic metabolic process
(gain)
c-value
0.0
0.1
0.2
0.3
0.4
0.5
0.6
0.7
0.8
p-value
5e-76
1e-59
2e-43
5e-27
1e-10
Log-odds
0.5
1.0
1.5
2.0
2.5
3.0
3.5
4.0
Cellular_organisms - ps1
Archaea1 - ps2
Archaea2 - ps3
Archaea3 - ps4
Asgard/Eukaryota - ps5
Eukaryota - ps6
Amorpheae - ps7
Obazoa - ps8
Opisthokonta - ps9
Holozoa - ps10
Filozoa - ps11
Choano./Metazoa - ps12
Metazoa - ps13
Myriazoa - ps14
Parahoxozoa - ps15
Bilateria - ps16
Deuterostomia - ps17
Chordata - ps18
Olfactores - ps19
Vertebrata - ps20
Gnathostomata - ps21
Euteleostomi - ps22
Sarcopterygii - ps23
Tetrapoda - ps24
Amniota - ps25
Mammalia - ps26
Theria - ps27
Placentalia - ps28
Boreoeutheria - ps29
Euarchontoglires - ps30
Euarchonta - ps31
Primates - ps32
Haplorrhini - ps33
Simiiformes - ps34
Catarrhini - ps35
Hominoidea - ps36
Hominidae - ps37
Homininae - ps38
Hominini - ps39
Homo_sapiens - ps40
Phylostratum

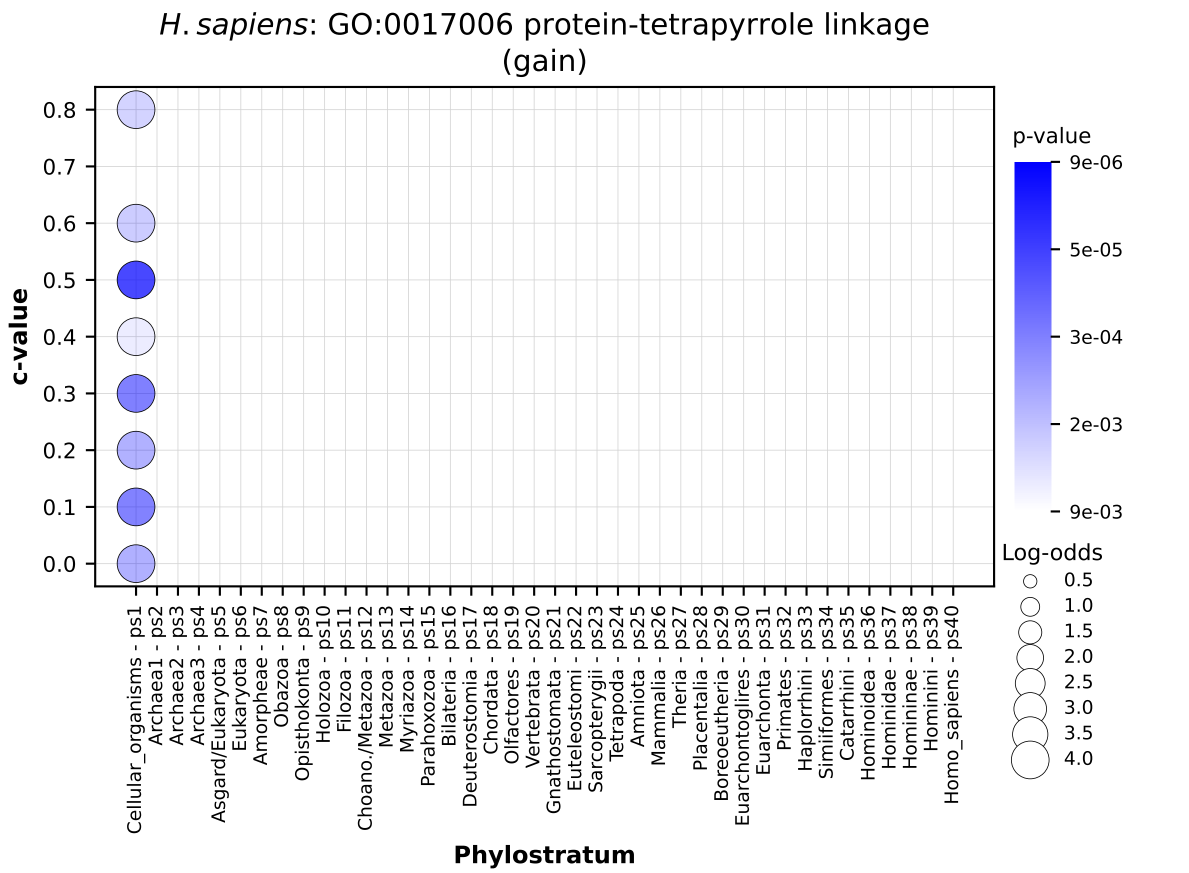

H. sapiens: GO:0017006 protein-tetrapyrrole linkage
(gain)
c-value
0.0
0.1
0.2
0.3
0.4
0.5
0.6
0.7
0.8
p-value
9e-06
5e-05
3e-04
2e-03
9e-03
Log-odds
0.5
1.0
1.5
2.0
2.5
3.0
3.5
4.0
Cellular_organisms - ps1
Archaea1 - ps2
Archaea2 - ps3
Archaea3 - ps4
Asgard/Eukaryota - ps5
Eukaryota - ps6
Amorpheae - ps7
Obazoa - ps8
Opisthokonta - ps9
Holozoa - ps10
Filozoa - ps11
Choano./Metazoa - ps12
Metazoa - ps13
Myriazoa - ps14
Parahoxozoa - ps15
Bilateria - ps16
Deuterostomia - ps17
Chordata - ps18
Olfactores - ps19
Vertebrata - ps20
Gnathostomata - ps21
Euteleostomi - ps22
Sarcopterygii - ps23
Tetrapoda - ps24
Amniota - ps25
Mammalia - ps26
Theria - ps27
Placentalia - ps28
Boreoeutheria - ps29
Euarchontoglires - ps30
Euarchonta - ps31
Primates - ps32
Haplorrhini - ps33
Simiiformes - ps34
Catarrhini - ps35
Hominoidea - ps36
Hominidae - ps37
Homininae - ps38
Hominini - ps39
Homo_sapiens - ps40
Phylostratum

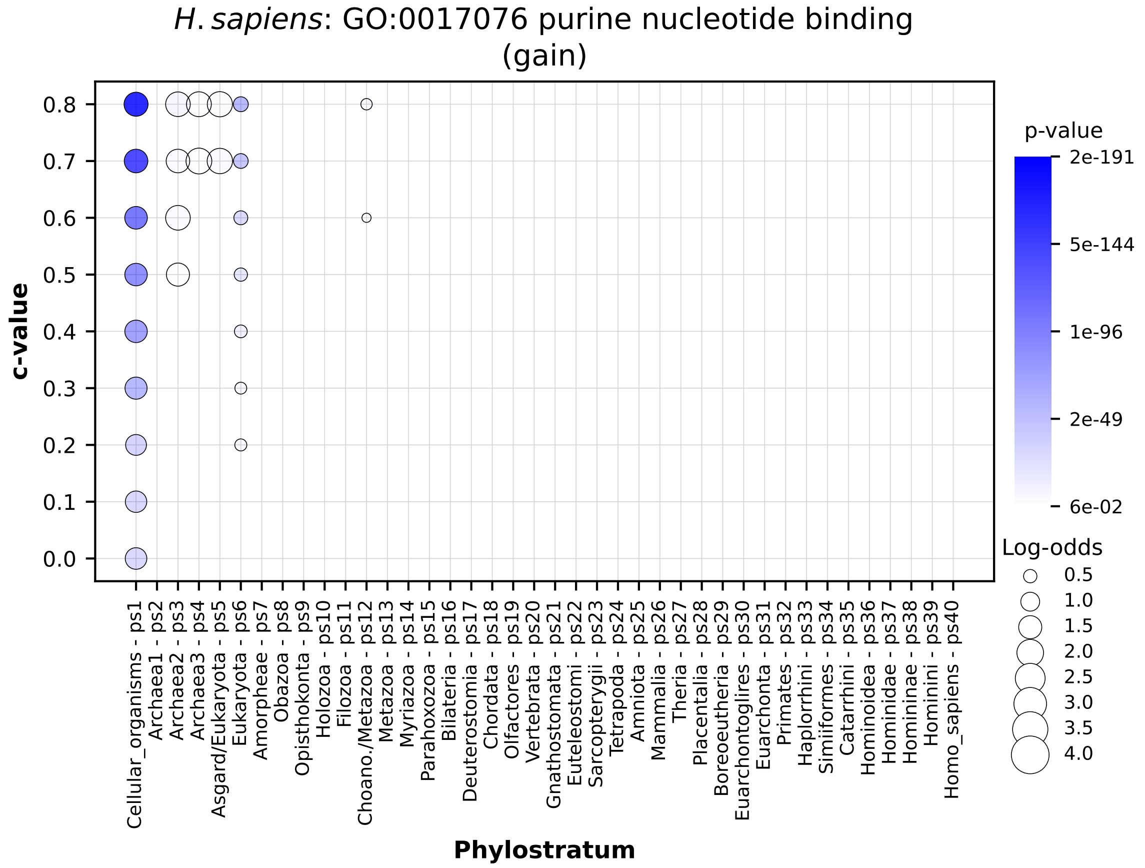
H. sapiens: GO:0017076 purine nucleotide binding
(gain)
c-value
0.8
0.7
0.6
0.5
0.4
0.3
0.2
0.1
0.0
p-value
2e-191
5e-144
1e-96
2e-49
6e-02
Log-odds
0.5
1.0
1.5
2.0
2.5
3.0
3.5
4.0
Cellular_organisms - ps1
Archaea1 - ps2
Archaea2 - ps3
Archaea3 - ps4
Asgard/Eukaryota - ps5
Eukaryota - ps6
Amorpheae - ps7
Obazoa - ps8
Opisthokonta - ps9
Holozoa - ps10
Filozoa - ps11
Choano./Metazoa - ps12
Metazoa - ps13
Myriazoa - ps14
Parahoxozoa - ps15
Bilateria - ps16
Deuterostomia - ps17
Chordata - ps18
Olfactores - ps19
Vertebrata - ps20
Gnathostomata - ps21
Euteleostomi - ps22
Sarcopterygii - ps23
Tetrapoda - ps24
Amniota - ps25
Mammalia - ps26
Theria - ps27
Placentalia - ps28
Boreoeutheria - ps29
Euarchontoglires - ps30
Euarchonta - ps31
Primates - ps32
Haplorrhini - ps33
Simiiformes - ps34
Catarrhini - ps35
Hominoidea - ps36
Hominidae - ps37
Homininae - ps38
Hominini - ps39
Homo_sapiens - ps40
Phylostratum

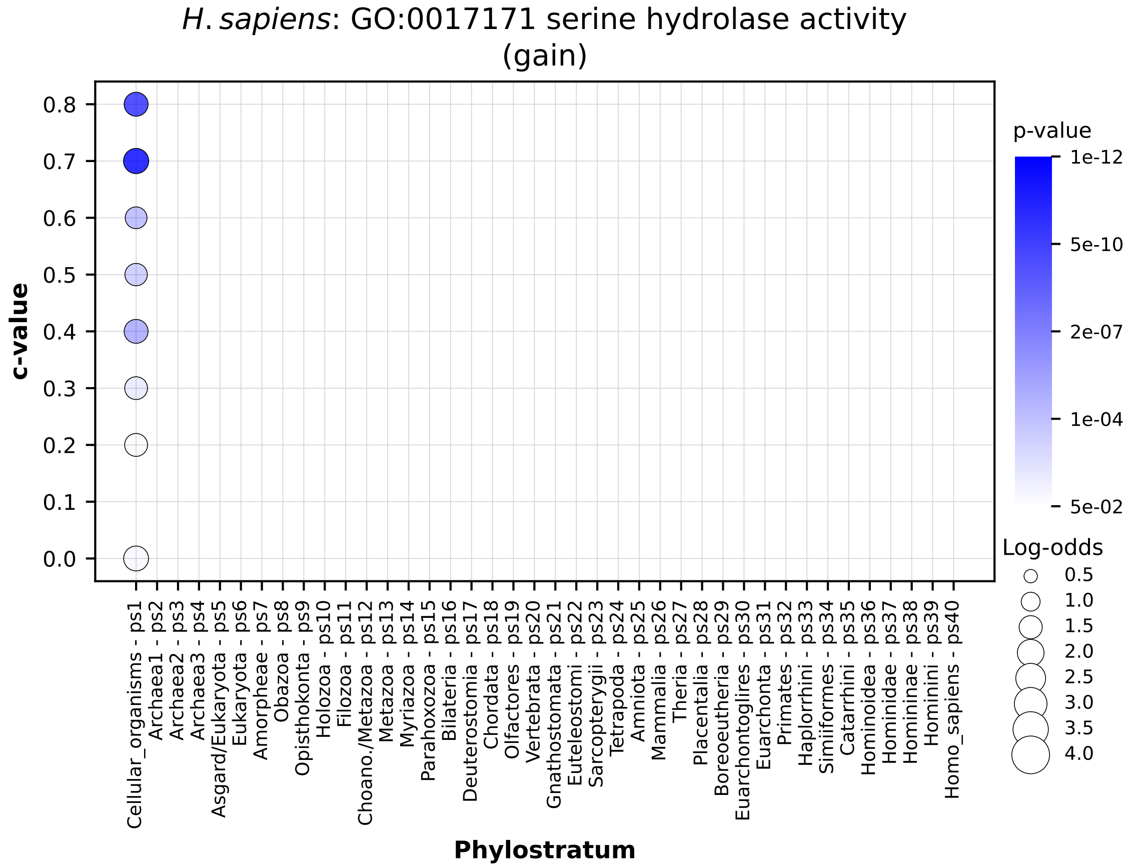

H. sapiens: GO:0017171 serine hydrolase activity
(gain)
c-value
0.8
0.7
0.6
0.5
0.4
0.3
0.2
0.1
0.0
p-value
1e-12
5e-10
2e-07
1e-04
5e-02
Log-odds
0.5
1.0
1.5
2.0
2.5
3.0
3.5
4.0
Cellular_organisms - ps1
Archaea1 - ps2
Archaea2 - ps3
Archaea3 - ps4
Asgard/Eukaryota - ps5
Eukaryota - ps6
Amorpheae - ps7
Obazoa - ps8
Opisthokonta - ps9
Holozoa - ps10
Filozoa - ps11
Choano./Metazoa - ps12
Metazoa - ps13
Myriazoa - ps14
Parahoxozoa - ps15
Bilateria - ps16
Deuterostomia - ps17
Chordata - ps18
Olfactores - ps19
Vertebrata - ps20
Gnathostomata - ps21
Euteleostomi - ps22
Sarcopterygii - ps23
Tetrapoda - ps24
Amniota - ps25
Mammalia - ps26
Theria - ps27
Placentalia - ps28
Boreoeutheria - ps29
Euarchontoglires - ps30
Euarchonta - ps31
Primates - ps32
Haplorrhini - ps33
Simiiformes - ps34
Catarrhini - ps35
Hominoidea - ps36
Hominidae - ps37
Homininae - ps38
Hominini - ps39
Homo_sapiens - ps40
Phylostratum

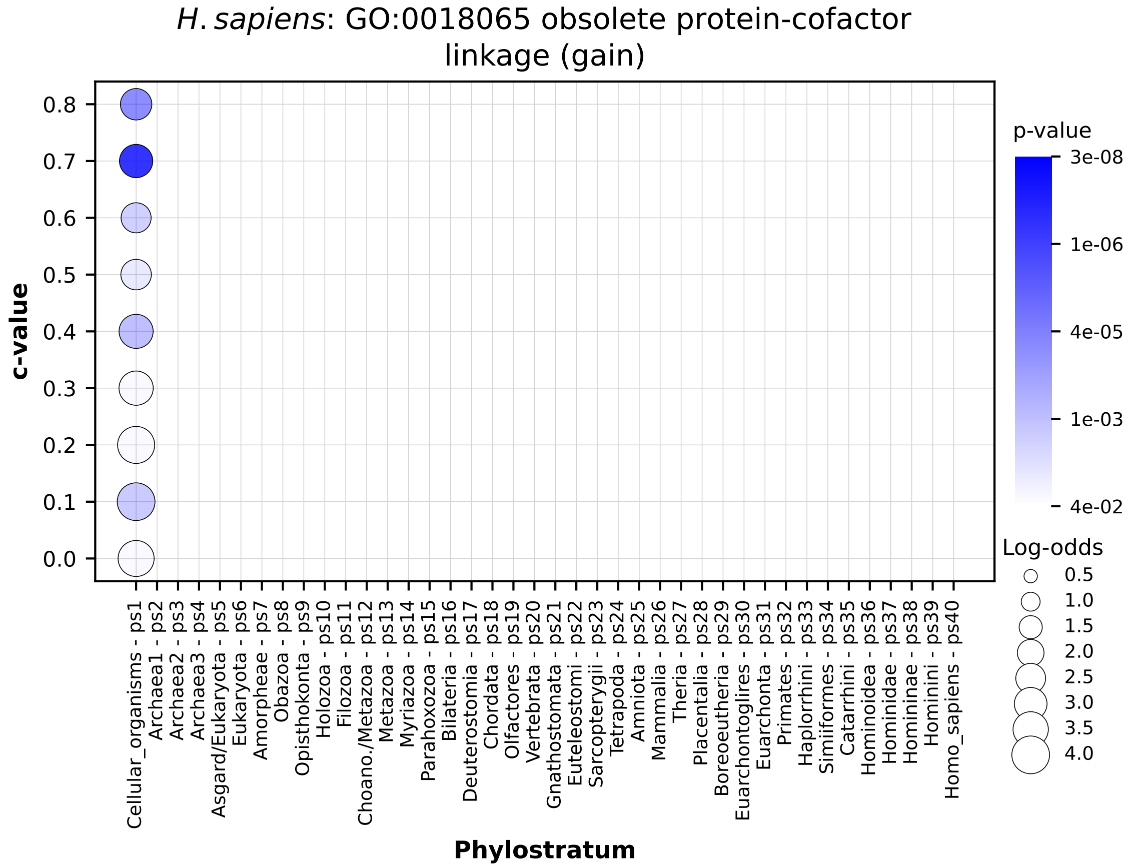

H. sapiens: GO:0018065 obsolete protein-cofactor linkage (gain)
c-value
0.0
0.1
0.2
0.3
0.4
0.5
0.6
0.7
0.8
Phylostratum
Cellular_organisms - ps1
Archaea1 - ps2
Archaea2 - ps3
Archaea3 - ps4
Asgard/Eukaryota - ps5
Eukaryota - ps6
Amorpheae - ps7
Obazoa - ps8
Opisthokonta - ps9
Holozoa - ps10
Filozoa - ps11
Choano./Metazoa - ps12
Metazoa - ps13
Myriazoa - ps14
Parahoxozoa - ps15
Bilateria - ps16
Deuterostomia - ps17
Chordata - ps18
Olfactores - ps19
Vertebrata - ps20
Gnathostomata - ps21
Euteleostomi - ps22
Sarcopterygii - ps23
Tetrapoda - ps24
Amniota - ps25
Mammalia - ps26
Theria - ps27
Placentalia - ps28
Boreoeutheria - ps29
Euarchontoglires - ps30
Euarchonta - ps31
Primates - ps32
Haplorrhini - ps33
Simiiformes - ps34
Catarrhini - ps35
Hominoidea - ps36
Hominidae - ps37
Homininae - ps38
Hominini - ps39
Homo_sapiens - ps40
p-value
3e-08
1e-06
4e-05
1e-03
4e-02
Log-odds
0.5
1.0
1.5
2.0
2.5
3.0
3.5
4.0

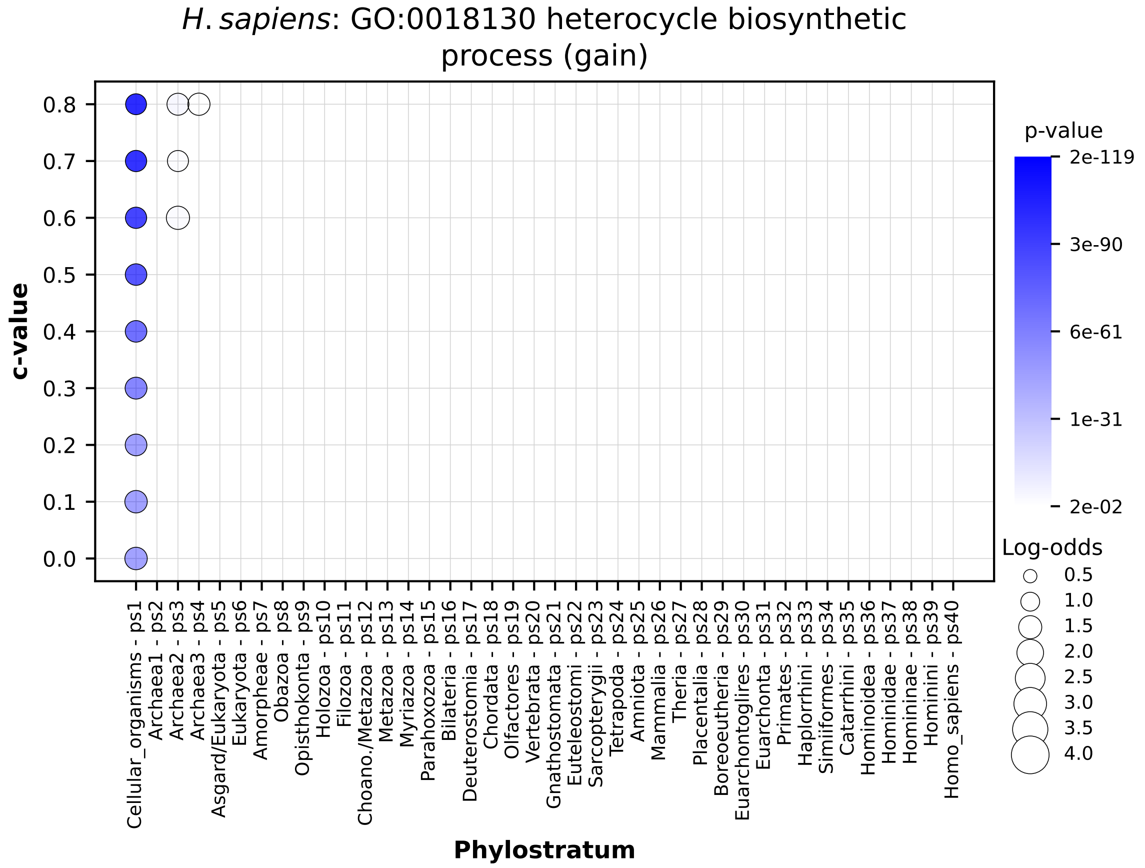

H. sapiens: GO:0018130 heterocycle biosynthetic process (gain)
c-value
0.8
0.7
0.6
0.5
0.4
0.3
0.2
0.1
0.0
p-value
2e-119
3e-90
6e-61
1e-31
2e-02
Log-odds
0.5
1.0
1.5
2.0
2.5
3.0
3.5
4.0
Cellular_organisms - ps1
Archaea1 - ps2
Archaea2 - ps3
Archaea3 - ps4
Asgard/Eukaryota - ps5
Eukaryota - ps6
Amorpheae - ps7
Obazoa - ps8
Opisthokonta - ps9
Holozoa - ps10
Filozoa - ps11
Choano./Metazoa - ps12
Metazoa - ps13
Myriazoa - ps14
Parahoxozoa - ps15
Bilateria - ps16
Deuterostomia - ps17
Chordata - ps18
Olfactores - ps19
Vertebrata - ps20
Gnathostomata - ps21
Euteleostomi - ps22
Sarcopterygii - ps23
Tetrapoda - ps24
Amniota - ps25
Mammalia - ps26
Theria - ps27
Placentalia - ps28
Boreoeutheria - ps29
Euarchontoglires - ps30
Euarchonta - ps31
Primates - ps32
Haplorrhini - ps33
Simiiformes - ps34
Catarrhini - ps35
Hominoidea - ps36
Hominidae - ps37
Homininae - ps38
Hominini - ps39
Homo_sapiens - ps40
Phylostratum

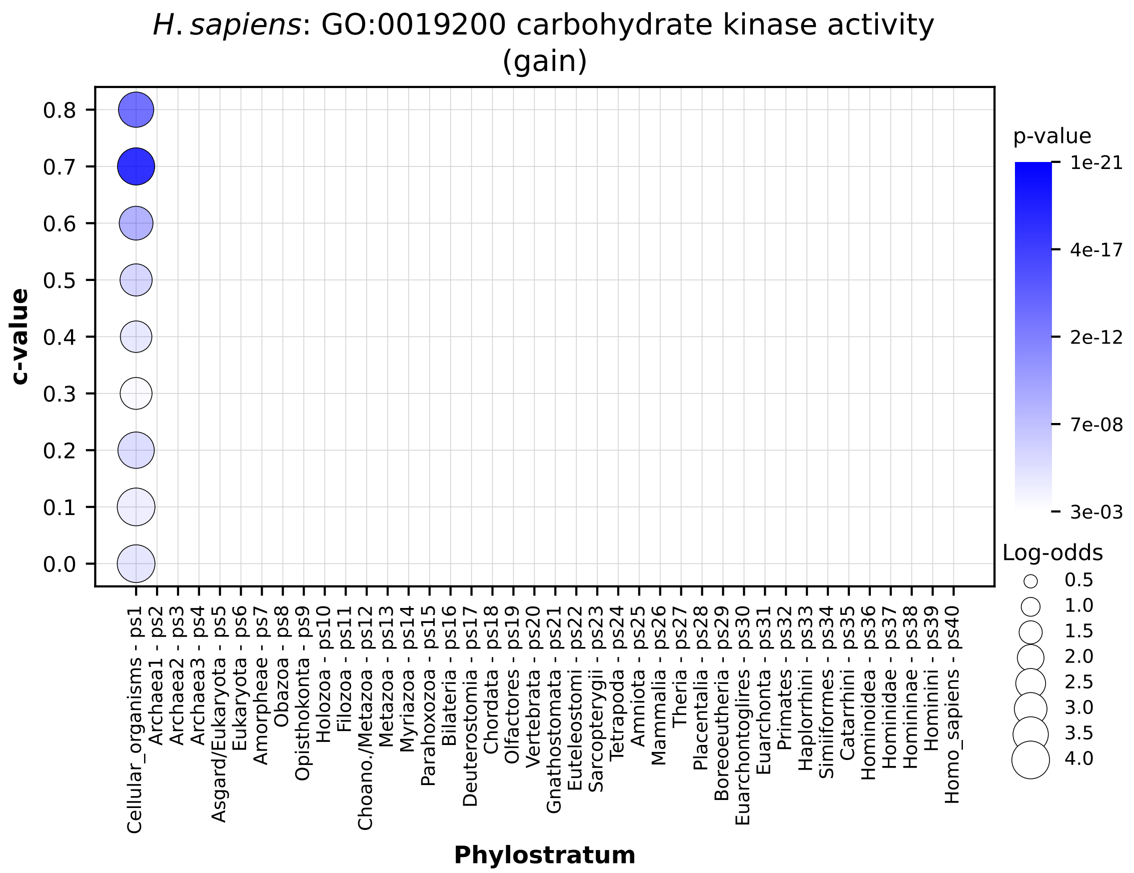

H. sapiens: GO:0019200 carbohydrate kinase activity
(gain)
c-value
0.8
0.7
0.6
0.5
0.4
0.3
0.2
0.1
0.0
p-value
1e-21
4e-17
2e-12
7e-08
3e-03
Log-odds
0.5
1.0
1.5
2.0
2.5
3.0
3.5
4.0
Cellular_organisms - ps1
Archaea1 - ps2
Archaea2 - ps3
Archaea3 - ps4
Asgard/Eukaryota - ps5
Eukaryota - ps6
Amorpheae - ps7
Obazoa - ps8
Opisthokonta - ps9
Holozoa - ps10
Filozoa - ps11
Choano./Metazoa - ps12
Metazoa - ps13
Myriazoa - ps14
Parahoxozoa - ps15
Bilateria - ps16
Deuterostomia - ps17
Chordata - ps18
Olfactores - ps19
Vertebrata - ps20
Gnathostomata - ps21
Euteleostomi - ps22
Sarcopterygii - ps23
Tetrapoda - ps24
Amniota - ps25
Mammalia - ps26
Theria - ps27
Placentalia - ps28
Boreoeutheria - ps29
Euarchontoglires - ps30
Euarchonta - ps31
Primates - ps32
Haplorrhini - ps33
Simiiformes - ps34
Catarrhini - ps35
Hominoidea - ps36
Hominidae - ps37
Homininae - ps38
Hominini - ps39
Homo_sapiens - ps40
Phylostratum

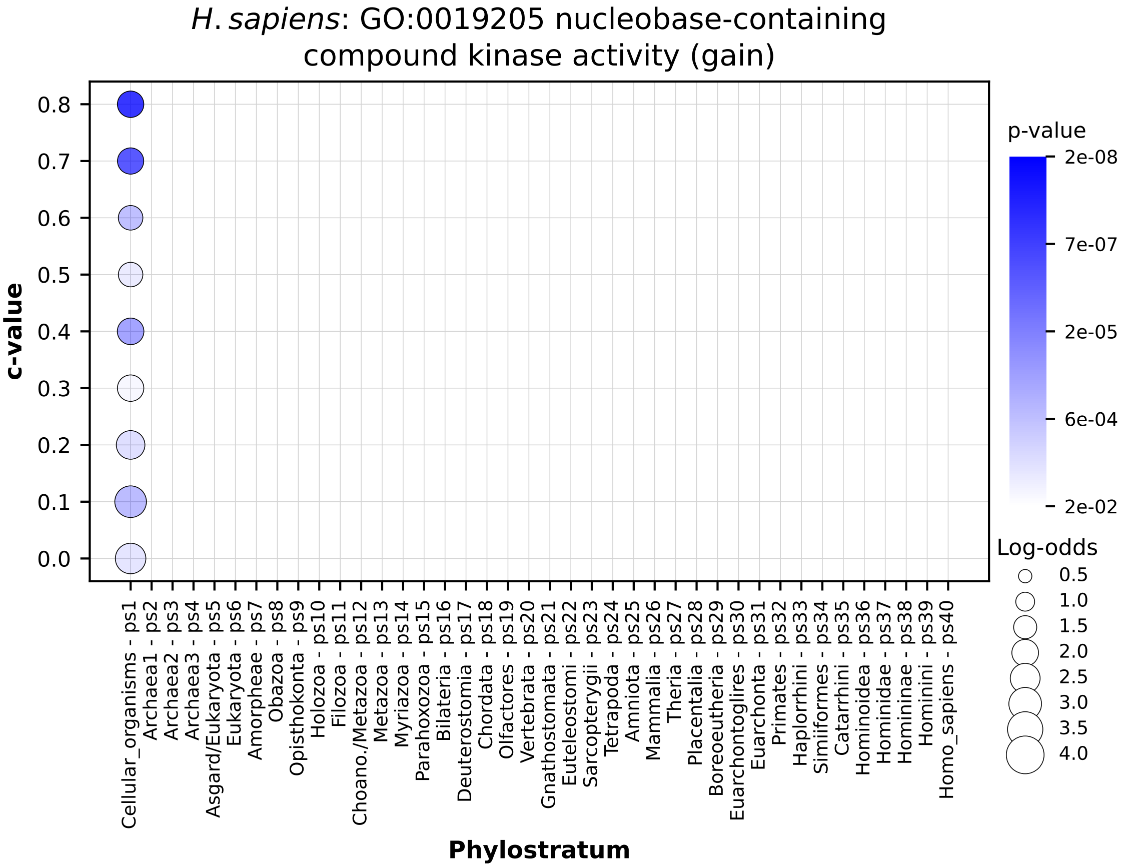
H. sapiens: GO:0019205 nucleobase-containing compound kinase activity (gain)
c-value
0.0
0.1
0.2
0.3
0.4
0.5
0.6
0.7
0.8
p-value
2e-08
7e-07
2e-05
6e-04
2e-02
Log-odds
0.5
1.0
1.5
2.0
2.5
3.0
3.5
4.0
Phylostratum
Cellular_organisms - ps1
Archaea1 - ps2
Archaea2 - ps3
Archaea3 - ps4
Asgard/Eukaryota - ps5
Eukaryota - ps6
Amorpheae - ps7
Obazoa - ps8
Opisthokonta - ps9
Holozoa - ps10
Filozoa - ps11
Choano./Metazoa - ps12
Metazoa - ps13
Myriazoa - ps14
Parahoxozoa - ps15
Bilateria - ps16
Deuterostomia - ps17
Chordata - ps18
Olfactores - ps19
Vertebrata - ps20
Gnathostomata - ps21
Euteleostomi - ps22
Sarcopterygii - ps23
Tetrapoda - ps24
Amniota - ps25
Mammalia - ps26
Theria - ps27
Placentalia - ps28
Boreoeutheria - ps29
Euarchontoglires - ps30
Euarchonta - ps31
Primates - ps32
Haplorrhini - ps33
Simiiformes - ps34
Catarrhini - ps35
Hominoidea - ps36
Hominidae - ps37
Homininae - ps38
Hominini - ps39
Homo_sapiens - ps40

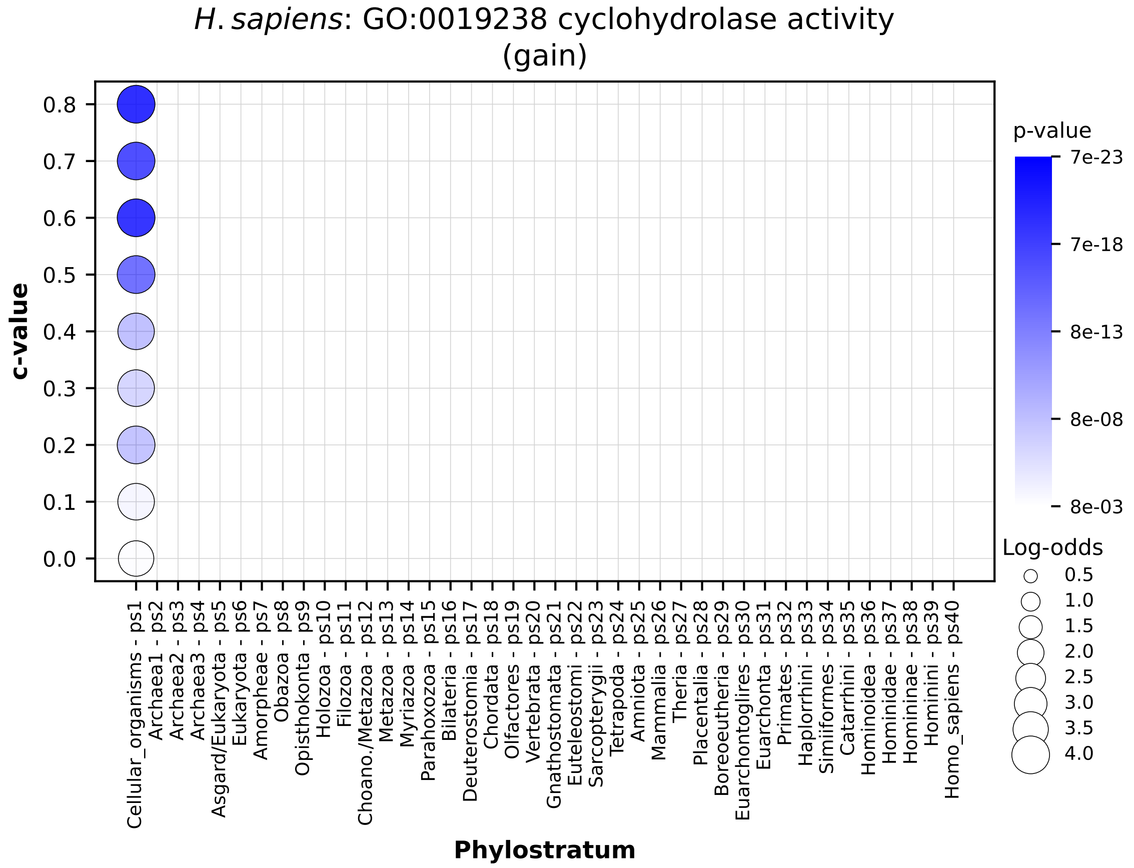

H. sapiens: GO:0019238 cyclohydrolase activity
(gain)
c-value
0.8
0.7
0.6
0.5
0.4
0.3
0.2
0.1
0.0
p-value
7e-23
7e-18
8e-13
8e-08
8e-03
Log-odds
0.5
1.0
1.5
2.0
2.5
3.0
3.5
4.0
Cellular_organisms - ps1
Archaea1 - ps2
Archaea2 - ps3
Archaea3 - ps4
Asgard/Eukaryota - ps5
Eukaryota - ps6
Amorpheae - ps7
Obazoa - ps8
Opisthokonta - ps9
Holozoa - ps10
Filozoa - ps11
Choano./Metazoa - ps12
Metazoa - ps13
Myriazoa - ps14
Parahoxozoa - ps15
Bilateria - ps16
Deuterostomia - ps17
Chordata - ps18
Olfactores - ps19
Vertebrata - ps20
Gnathostomata - ps21
Euteleostomi - ps22
Sarcopterygii - ps23
Tetrapoda - ps24
Amniota - ps25
Mammalia - ps26
Theria - ps27
Placentalia - ps28
Boreoeutheria - ps29
Euarchontoglires - ps30
Euarchonta - ps31
Primates - ps32
Haplorrhini - ps33
Simiiformes - ps34
Catarrhini - ps35
Hominoidea - ps36
Hominidae - ps37
Homininae - ps38
Hominini - ps39
Homo_sapiens - ps40
Phylostratum

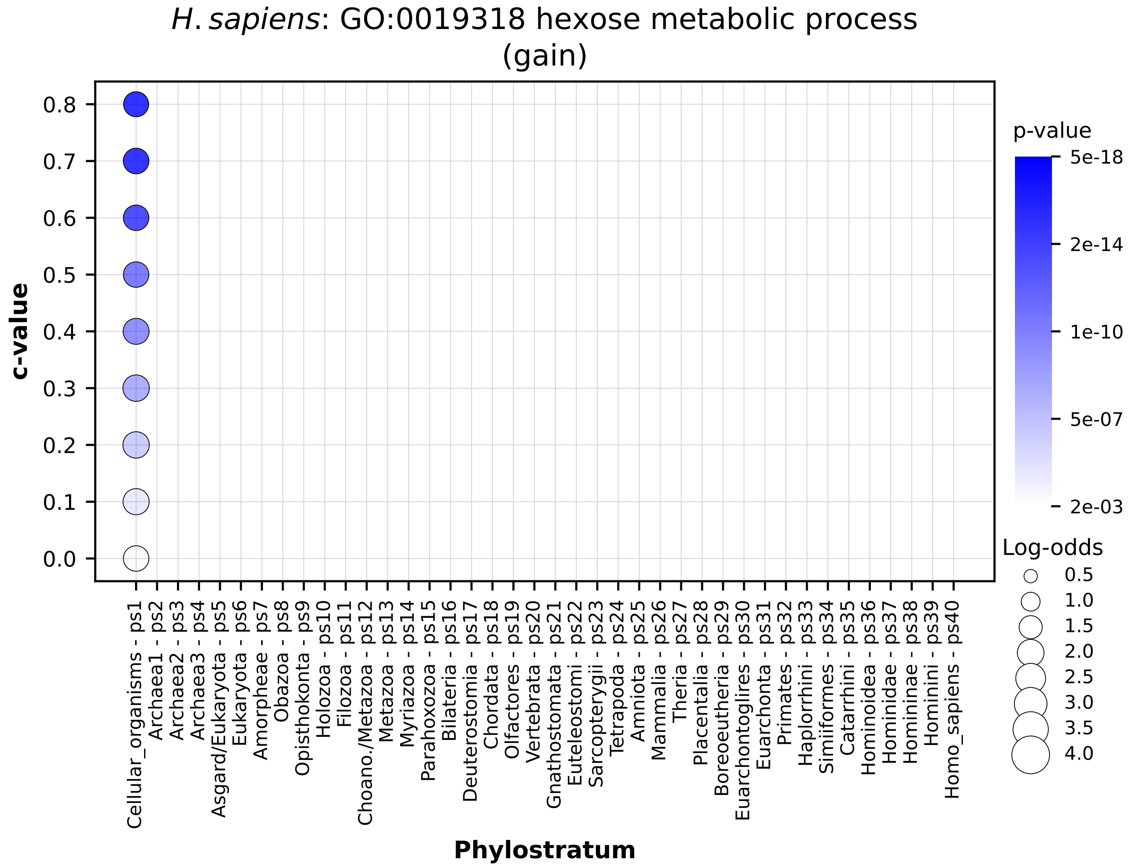

H. sapiens: GO:0019318 hexose metabolic process
(gain)
c-value
0.8
0.7
0.6
0.5
0.4
0.3
0.2
0.1
0.0
p-value
5e-18
2e-14
1e-10
5e-07
2e-03
Log-odds
0.5
1.0
1.5
2.0
2.5
3.0
3.5
4.0
Cellular_organisms - ps1
Archaea1 - ps2
Archaea2 - ps3
Archaea3 - ps4
Asgard/Eukaryota - ps5
Eukaryota - ps6
Amorpheae - ps7
Obazoa - ps8
Opisthokonta - ps9
Holozoa - ps10
Filozoa - ps11
Choano./Metazoa - ps12
Metazoa - ps13
Myriazoa - ps14
Parahoxozoa - ps15
Bilateria - ps16
Deuterostomia - ps17
Chordata - ps18
Olfactores - ps19
Vertebrata - ps20
Gnathostomata - ps21
Euteleostomi - ps22
Sarcopterygii - ps23
Tetrapoda - ps24
Amniota - ps25
Mammalia - ps26
Theria - ps27
Placentalia - ps28
Boreoeutheria - ps29
Euarchontoglires - ps30
Euarchonta - ps31
Primates - ps32
Haplorrhini - ps33
Simiiformes - ps34
Catarrhini - ps35
Hominoidea - ps36
Hominidae - ps37
Homininae - ps38
Hominini - ps39
Homo_sapiens - ps40
Phylostratum

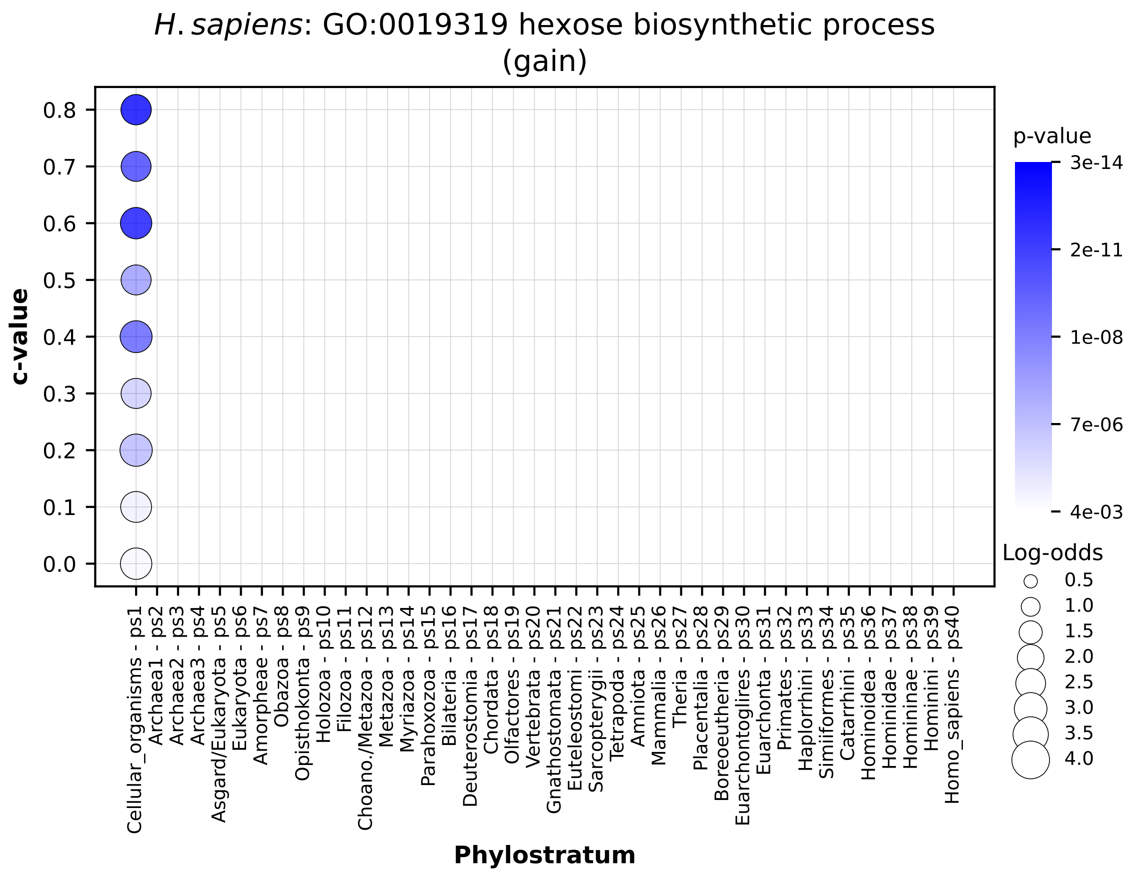

H. sapiens: GO:0019319 hexose biosynthetic process
(gain)
c-value
0.8
0.7
0.6
0.5
0.4
0.3
0.2
0.1
0.0
p-value
3e-14
2e-11
1e-08
7e-06
4e-03
Log-odds
0.5
1.0
1.5
2.0
2.5
3.0
3.5
4.0
Cellular_organisms - ps1
Archaea1 - ps2
Archaea2 - ps3
Archaea3 - ps4
Asgard/Eukaryota - ps5
Eukaryota - ps6
Amorpheae - ps7
Obazoa - ps8
Opisthokonta - ps9
Holozoa - ps10
Filozoa - ps11
Choano./Metazoa - ps12
Metazoa - ps13
Myriazoa - ps14
Parahoxozoa - ps15
Bilateria - ps16
Deuterostomia - ps17
Chordata - ps18
Olfactores - ps19
Vertebrata - ps20
Gnathostomata - ps21
Euteleostomi - ps22
Sarcopterygii - ps23
Tetrapoda - ps24
Amniota - ps25
Mammalia - ps26
Theria - ps27
Placentalia - ps28
Boreoeutheria - ps29
Euarchontoglires - ps30
Euarchonta - ps31
Primates - ps32
Haplorrhini - ps33
Simiiformes - ps34
Catarrhini - ps35
Hominoidea - ps36
Hominidae - ps37
Homininae - ps38
Hominini - ps39
Homo_sapiens - ps40
Phylostratum

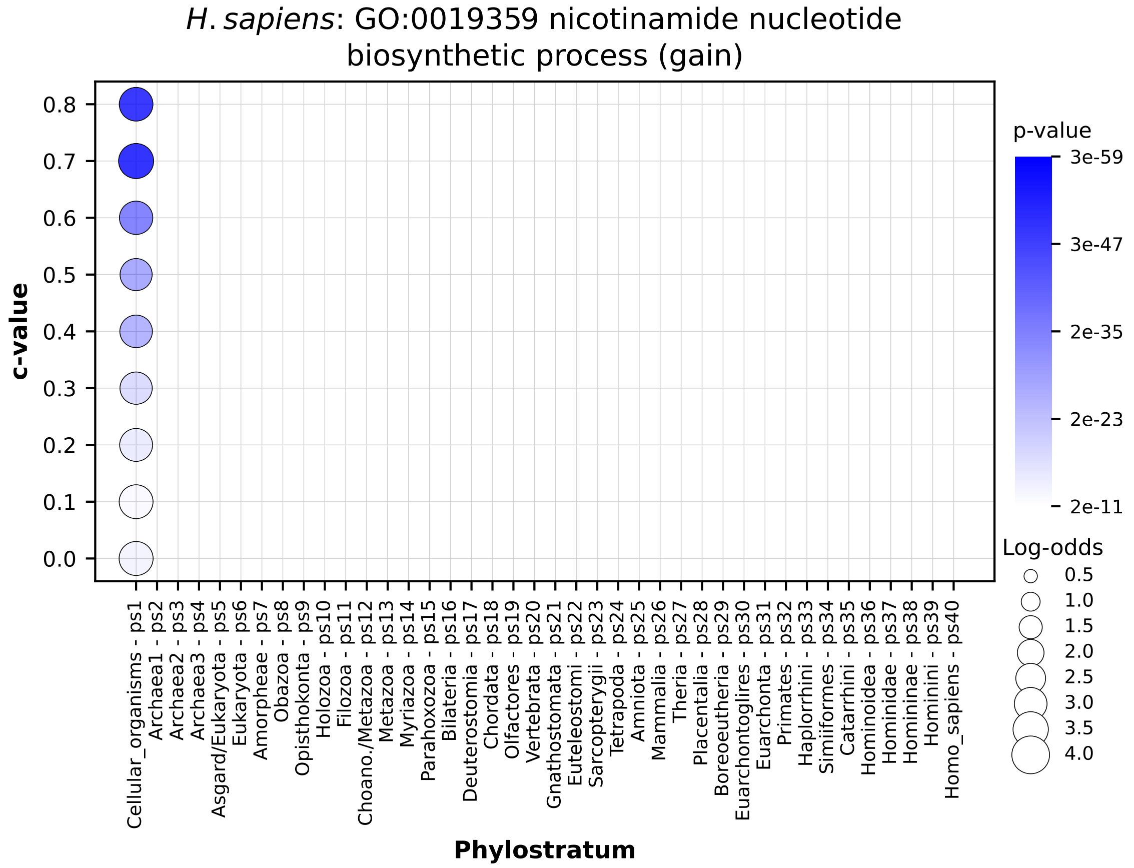

H. sapiens: GO:0019359 nicotinamide nucleotide biosynthetic process (gain)
c-value
0.8
0.7
0.6
0.5
0.4
0.3
0.2
0.1
0.0
p-value
3e-59
3e-47
2e-35
2e-23
2e-11
Log-odds
0.5
1.0
1.5
2.0
2.5
3.0
3.5
4.0
Cellular_organisms - ps1
Archaea1 - ps2
Archaea2 - ps3
Archaea3 - ps4
Asgard/Eukaryota - ps5
Eukaryota - ps6
Amorpheae - ps7
Obazoa - ps8
Opisthokonta - ps9
Holozoa - ps10
Filozoa - ps11
Choano./Metazoa - ps12
Metazoa - ps13
Myriazoa - ps14
Parahoxozoa - ps15
Bilateria - ps16
Deuterostomia - ps17
Chordata - ps18
Olfactores - ps19
Vertebrata - ps20
Gnathostomata - ps21
Euteleostomi - ps22
Sarcopterygii - ps23
Tetrapoda - ps24
Amniota - ps25
Mammalia - ps26
Theria - ps27
Placentalia - ps28
Boreoeutheria - ps29
Euarchontoglires - ps30
Euarchonta - ps31
Primates - ps32
Haplorrhini - ps33
Simiiformes - ps34
Catarrhini - ps35
Hominoidea - ps36
Hominidae - ps37
Homininae - ps38
Hominini - ps39
Homo_sapiens - ps40
Phylostratum

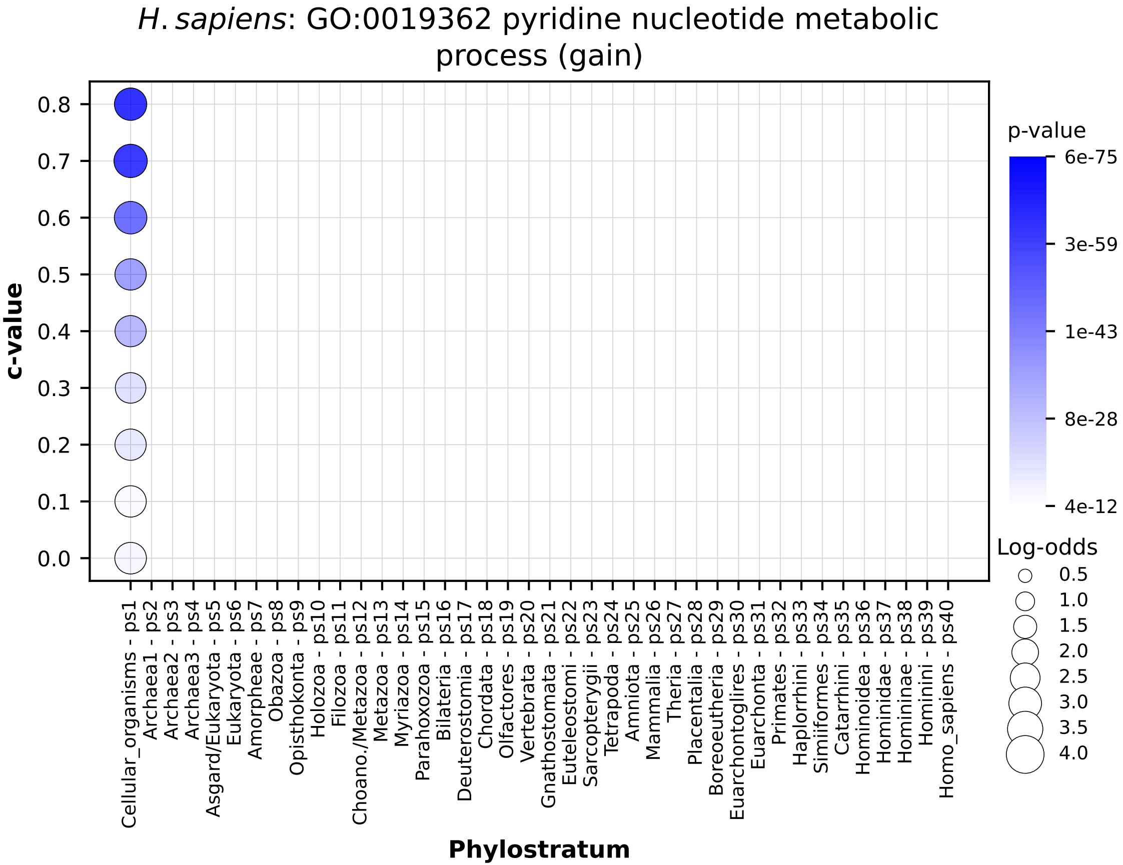

H. sapiens: GO:0019362 pyridine nucleotide metabolic process (gain)
c-value
0.0
0.1
0.2
0.3
0.4
0.5
0.6
0.7
0.8
Phylostratum
Cellular_organisms - ps1
Archaea1 - ps2
Archaea2 - ps3
Archaea3 - ps4
Asgard/Eukaryota - ps5
Eukaryota - ps6
Amorpheae - ps7
Obazoa - ps8
Opisthokonta - ps9
Holozoa - ps10
Filozoa - ps11
Choano./Metazoa - ps12
Metazoa - ps13
Myriazoa - ps14
Parahoxozoa - ps15
Bilateria - ps16
Deuterostomia - ps17
Chordata - ps18
Olfactores - ps19
Vertebrata - ps20
Gnathostomata - ps21
Euteleostomi - ps22
Sarcopterygii - ps23
Tetrapoda - ps24
Amniota - ps25
Mammalia - ps26
Theria - ps27
Placentalia - ps28
Boreoeutheria - ps29
Euarchontoglires - ps30
Euarchonta - ps31
Primates - ps32
Haplorrhini - ps33
Simiiformes - ps34
Catarrhini - ps35
Hominoidea - ps36
Hominidae - ps37
Homininae - ps38
Hominini - ps39
Homo_sapiens - ps40
p-value
6e-75
3e-59
1e-43
8e-28
4e-12
Log-odds
0.5
1.0
1.5
2.0
2.5
3.0
3.5
4.0

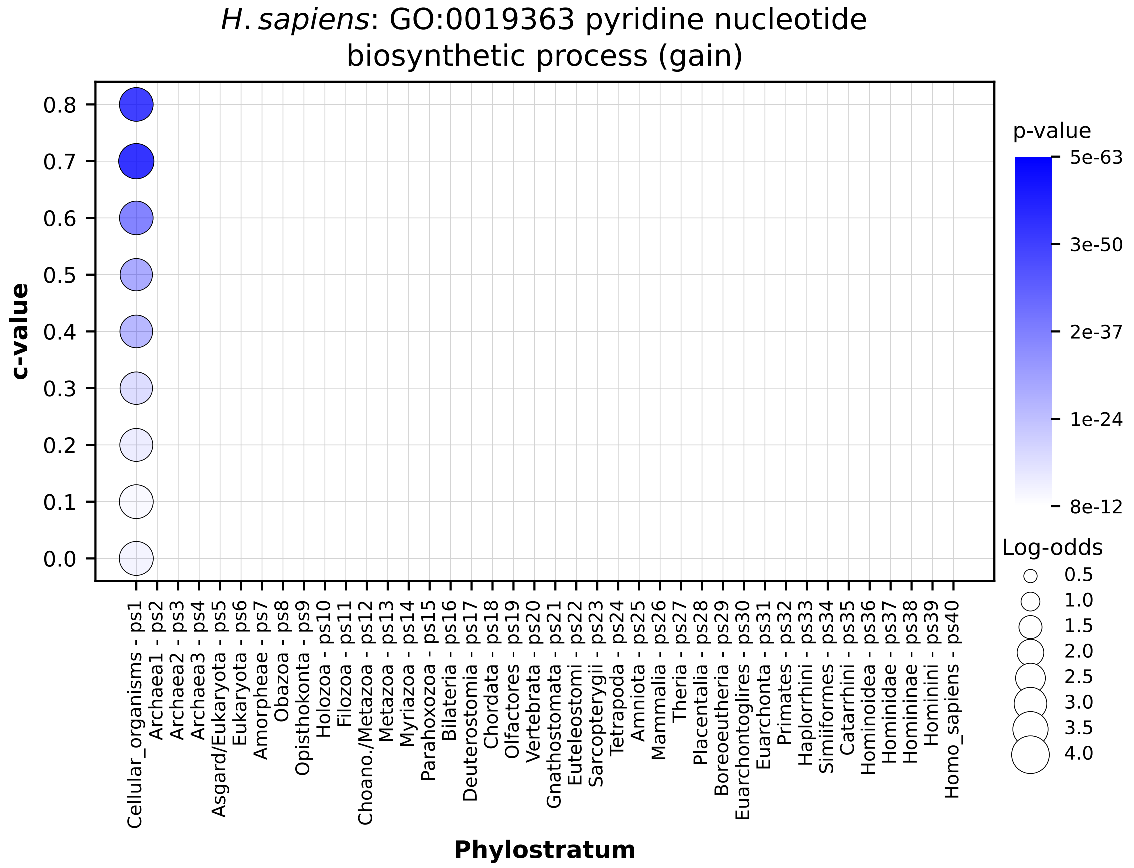
H. sapiens: GO:0019363 pyridine nucleotide biosynthetic process (gain)
c-value
0.8
0.7
0.6
0.5
0.4
0.3
0.2
0.1
0.0
Phylostratum
Cellular_organisms - ps1
Archaea1 - ps2
Archaea2 - ps3
Archaea3 - ps4
Asgard/Eukaryota - ps5
Eukaryota - ps6
Amorpheae - ps7
Obazoa - ps8
Opisthokonta - ps9
Holozoa - ps10
Filozoa - ps11
Choano./Metazoa - ps12
Metazoa - ps13
Myriazoa - ps14
Parahoxozoa - ps15
Bilateria - ps16
Deuterostomia - ps17
Chordata - ps18
Olfactores - ps19
Vertebrata - ps20
Gnathostomata - ps21
Euteleostomi - ps22
Sarcopterygii - ps23
Tetrapoda - ps24
Amniota - ps25
Mammalia - ps26
Theria - ps27
Placentalia - ps28
Boreoeutheria - ps29
Euarchontoglires - ps30
Euarchonta - ps31
Primates - ps32
Haplorrhini - ps33
Simiiformes - ps34
Catarrhini - ps35
Hominoidea - ps36
Hominidae - ps37
Homininae - ps38
Hominini - ps39
Homo_sapiens - ps40
p-value
5e-63
3e-50
2e-37
1e-24
8e-12
Log-odds
0.5
1.0
1.5
2.0
2.5
3.0
3.5
4.0

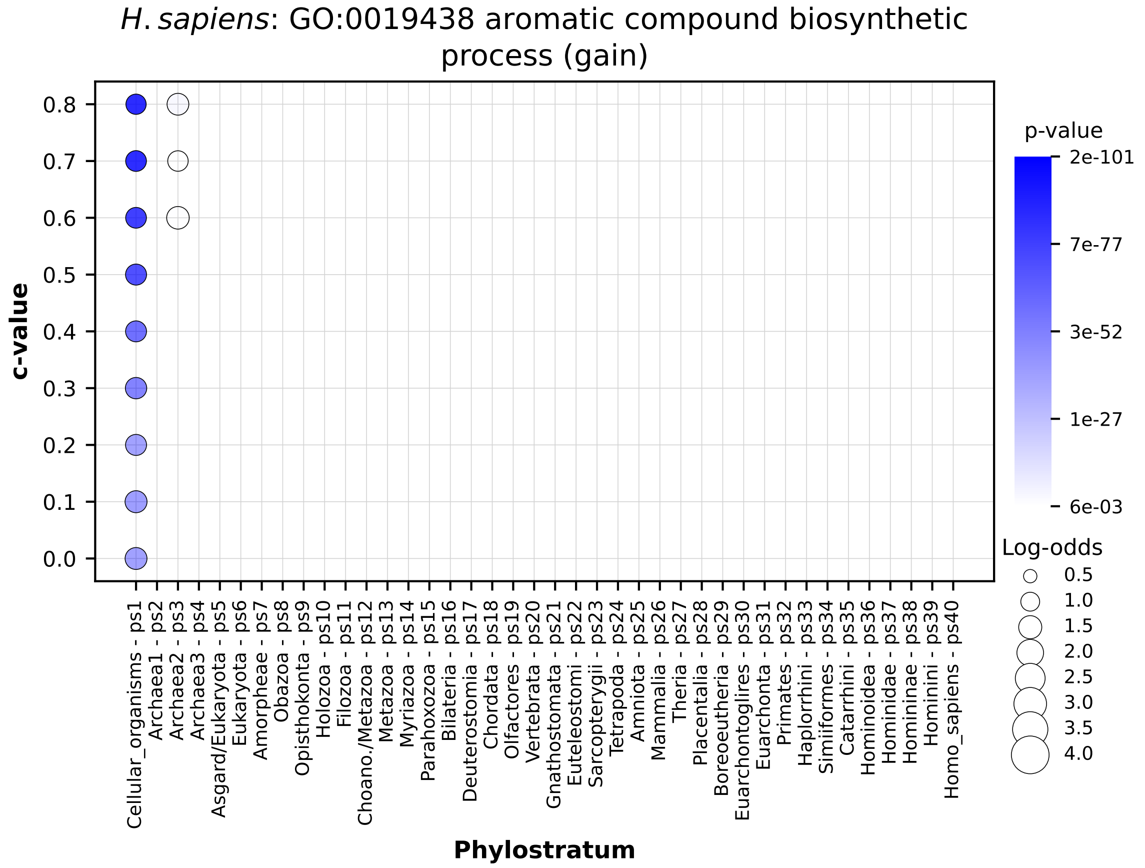

H. sapiens: GO:0019438 aromatic compound biosynthetic process (gain)
c-value
0.0
0.1
0.2
0.3
0.4
0.5
0.6
0.7
0.8
Phylostratum
Cellular_organisms - ps1
Archaea1 - ps2
Archaea2 - ps3
Archaea3 - ps4
Asgard/Eukaryota - ps5
Eukaryota - ps6
Amorpheae - ps7
Obazoa - ps8
Opisthokonta - ps9
Holozoa - ps10
Filozoa - ps11
Choano./Metazoa - ps12
Metazoa - ps13
Myriazoa - ps14
Parahoxozoa - ps15
Bilateria - ps16
Deuterostomia - ps17
Chordata - ps18
Olfactores - ps19
Vertebrata - ps20
Gnathostomata - ps21
Euteleostomi - ps22
Sarcopterygii - ps23
Tetrapoda - ps24
Amniota - ps25
Mammalia - ps26
Theria - ps27
Placentalia - ps28
Boreoeutheria - ps29
Euarchontoglires - ps30
Euarchonta - ps31
Primates - ps32
Haplorrhini - ps33
Simiiformes - ps34
Catarrhini - ps35
Hominoidea - ps36
Hominidae - ps37
Homininae - ps38
Hominini - ps39
Homo_sapiens - ps40
p-value
2e-101
7e-77
3e-52
1e-27
6e-03
Log-odds
0.5
1.0
1.5
2.0
2.5
3.0
3.5
4.0

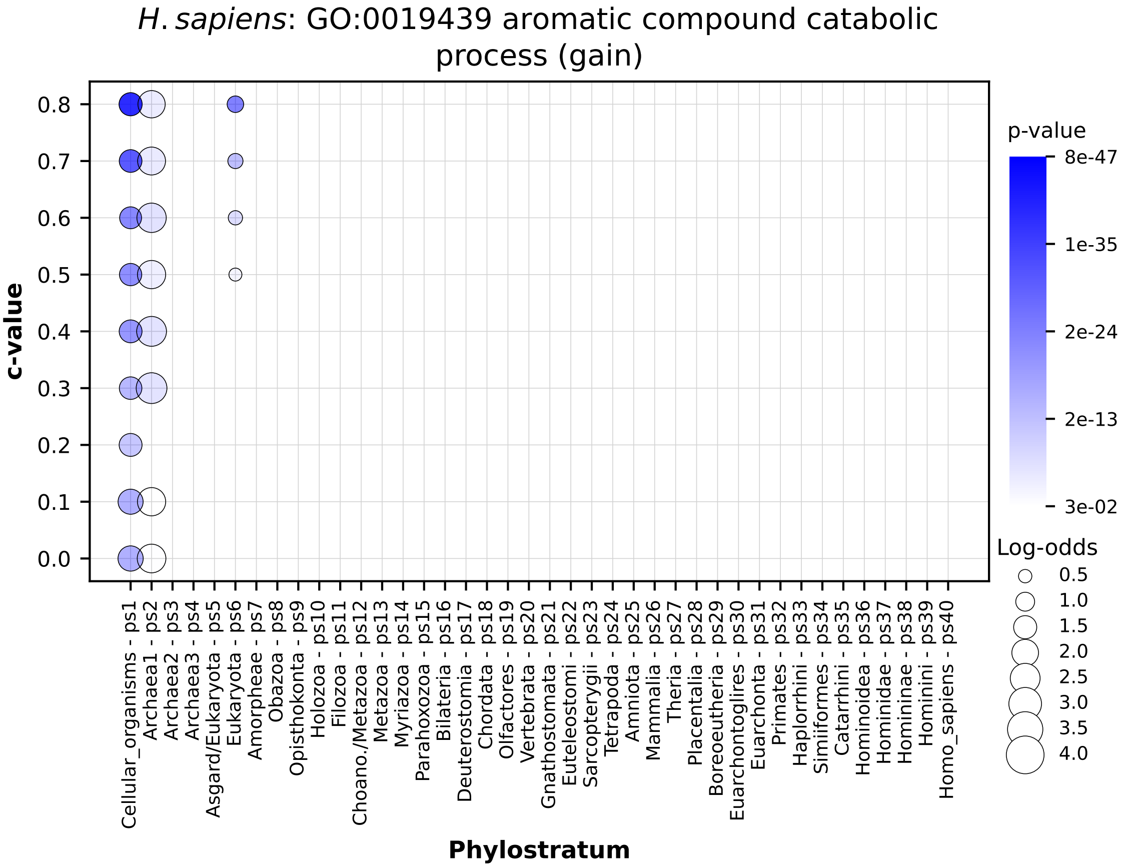
H. sapiens: GO:0019439 aromatic compound catabolic process (gain)
c-value
0.8
0.7
0.6
0.5
0.4
0.3
0.2
0.1
0.0
p-value
8e-47
1e-35
2e-24
2e-13
3e-02
Log-odds
0.5
1.0
1.5
2.0
2.5
3.0
3.5
4.0
Cellular_organisms - ps1
Archaea1 - ps2
Archaea2 - ps3
Archaea3 - ps4
Asgard/Eukaryota - ps5
Eukaryota - ps6
Amorpheae - ps7
Obazoa - ps8
Opisthokonta - ps9
Holozoa - ps10
Filozoa - ps11
Choano./Metazoa - ps12
Metazoa - ps13
Myriazoa - ps14
Parahoxozoa - ps15
Bilateria - ps16
Deuterostomia - ps17
Chordata - ps18
Olfactores - ps19
Vertebrata - ps20
Gnathostomata - ps21
Euteleostomi - ps22
Sarcopterygii - ps23
Tetrapoda - ps24
Amniota - ps25
Mammalia - ps26
Theria - ps27
Placentalia - ps28
Boreoeutheria - ps29
Euarchontoglires - ps30
Euarchonta - ps31
Primates - ps32
Haplorrhini - ps33
Simiiformes - ps34
Catarrhini - ps35
Hominoidea - ps36
Hominidae - ps37
Homininae - ps38
Hominini - ps39
Homo_sapiens - ps40
Phylostratum

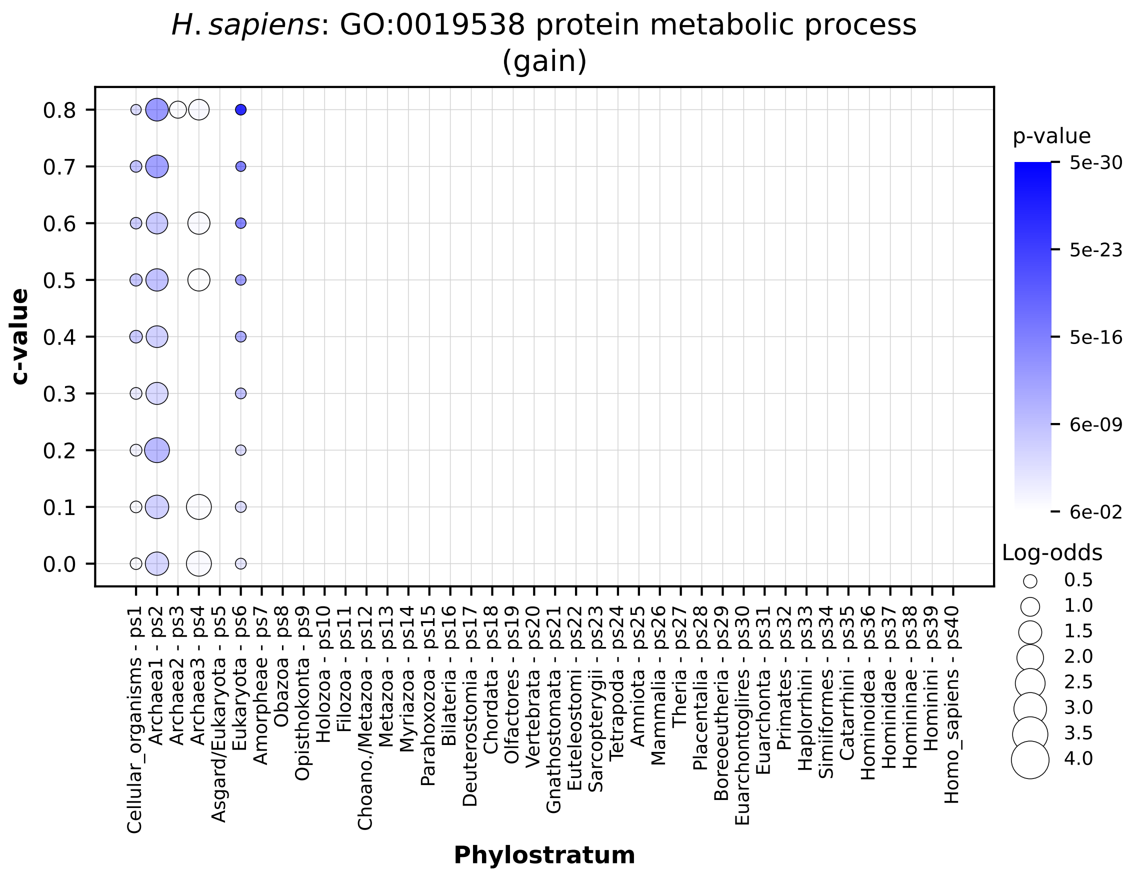
H. sapiens: GO:0019538 protein metabolic process
(gain)
c-value
0.8
0.7
0.6
0.5
0.4
0.3
0.2
0.1
0.0
p-value
5e-30
5e-23
5e-16
6e-09
6e-02
Log-odds
0.5
1.0
1.5
2.0
2.5
3.0
3.5
4.0
Cellular_organisms - ps1
Archaea1 - ps2
Archaea2 - ps3
Archaea3 - ps4
Asgard/Eukaryota - ps5
Eukaryota - ps6
Amorpheae - ps7
Obazoa - ps8
Opisthokonta - ps9
Holozoa - ps10
Filozoa - ps11
Choano./Metazoa - ps12
Metazoa - ps13
Myriazoa - ps14
Parahoxozoa - ps15
Bilateria - ps16
Deuterostomia - ps17
Chordata - ps18
Olfactores - ps19
Vertebrata - ps20
Gnathostomata - ps21
Euteleostomi - ps22
Sarcopterygii - ps23
Tetrapoda - ps24
Amniota - ps25
Mammalia - ps26
Theria - ps27
Placentalia - ps28
Boreoeutheria - ps29
Euarchontoglires - ps30
Euarchonta - ps31
Primates - ps32
Haplorrhini - ps33
Simiiformes - ps34
Catarrhini - ps35
Hominoidea - ps36
Hominidae - ps37
Homininae - ps38
Hominini - ps39
Homo_sapiens - ps40
Phylostratum

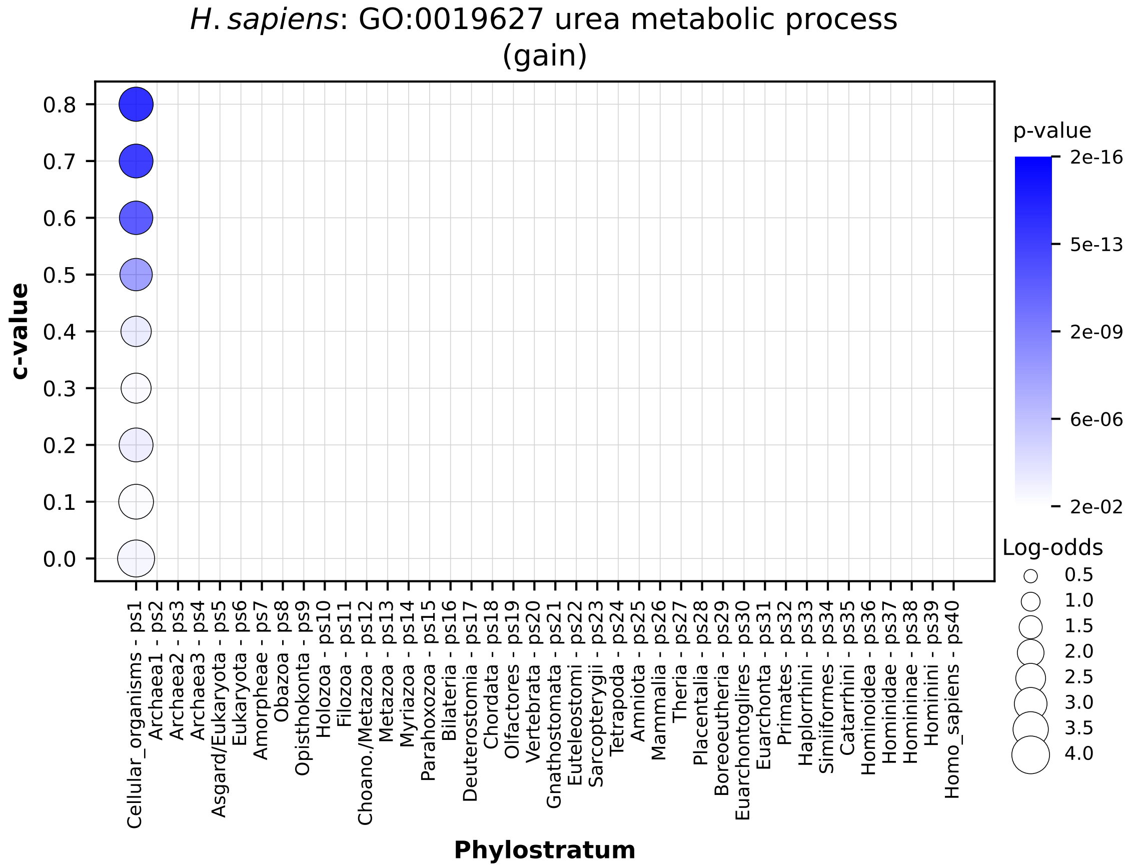
*H. sapiens*: GO:0019627 urea metabolic process
(gain)
c-value
0.8
0.7
0.6
0.5
0.4
0.3
0.2
0.1
0.0
Phylostratum
Cellular_organisms - ps1
Archaea1 - ps2
Archaea2 - ps3
Archaea3 - ps4
Asgard/Eukaryota - ps5
Eukaryota - ps6
Amorpheae - ps7
Obazoa - ps8
Opisthokonta - ps9
Holozoa - ps10
Filozoa - ps11
Choano./Metazoa - ps12
Metazoa - ps13
Myriazoa - ps14
Parahoxozoa - ps15
Bilateria - ps16
Deuterostomia - ps17
Chordata - ps18
Olfactores - ps19
Vertebrata - ps20
Gnathostomata - ps21
Euteleostomi - ps22
Sarcopterygii - ps23
Tetrapoda - ps24
Amniota - ps25
Mammalia - ps26
Theria - ps27
Placentalia - ps28
Boreoeutheria - ps29
Euarchontoglires - ps30
Euarchonta - ps31
Primates - ps32
Haplorrhini - ps33
Simiiformes - ps34
Catarrhini - ps35
Hominoidea - ps36
Hominidae - ps37
Homininae - ps38
Hominini - ps39
Homo_sapiens - ps40
p-value
2e-16
5e-13
2e-09
6e-06
2e-02
Log-odds
0.5
1.0
1.5
2.0
2.5
3.0
3.5
4.0

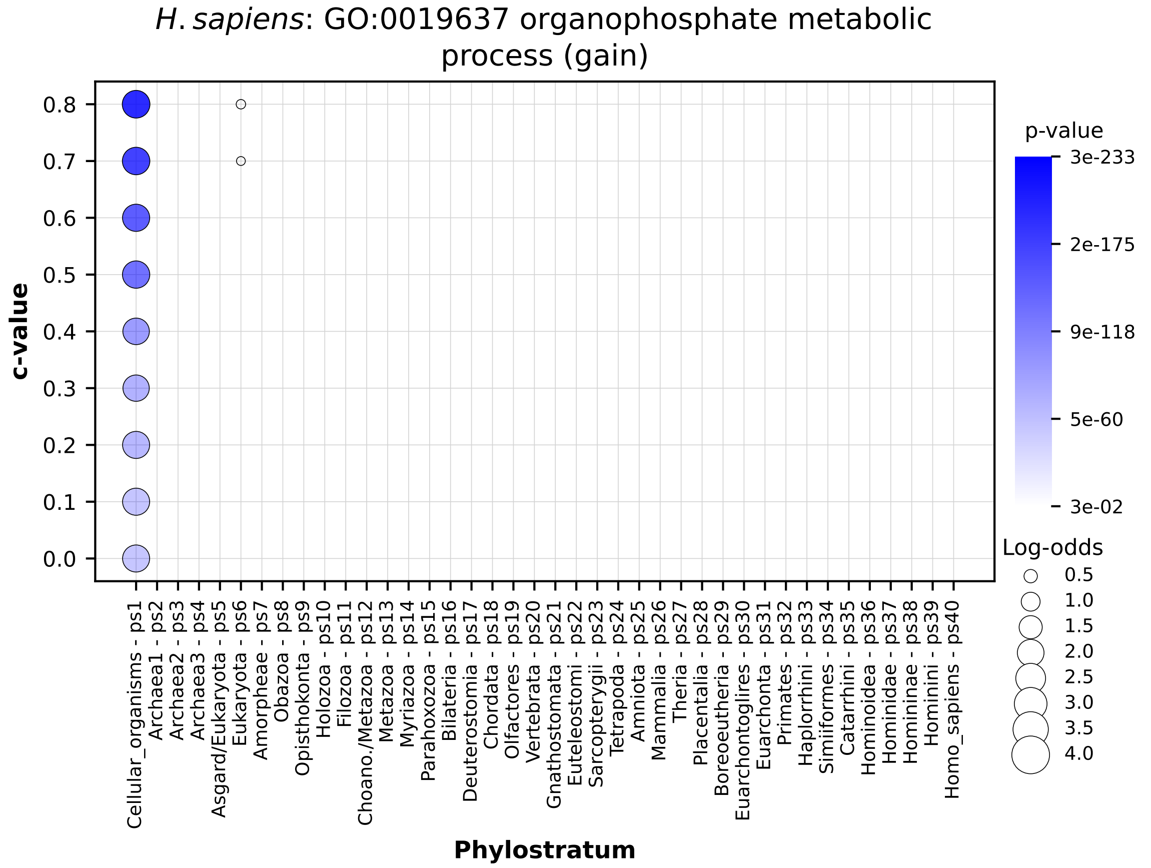

H. sapiens: GO:0019637 organophosphate metabolic process (gain)
c-value
0.8
0.7
0.6
0.5
0.4
0.3
0.2
0.1
0.0
p-value
3e-233
2e-175
9e-118
5e-60
3e-02
Log-odds
0.5
1.0
1.5
2.0
2.5
3.0
3.5
4.0
Cellular_organisms - ps1
Archaea1 - ps2
Archaea2 - ps3
Archaea3 - ps4
Asgard/Eukaryota - ps5
Eukaryota - ps6
Amorpheae - ps7
Obazoa - ps8
Opisthokonta - ps9
Holozoa - ps10
Filozoa - ps11
Choano./Metazoa - ps12
Metazoa - ps13
Myriazoa - ps14
Parahoxozoa - ps15
Bilateria - ps16
Deuterostomia - ps17
Chordata - ps18
Olfactores - ps19
Vertebrata - ps20
Gnathostomata - ps21
Euteleostomi - ps22
Sarcopterygii - ps23
Tetrapoda - ps24
Amniota - ps25
Mammalia - ps26
Theria - ps27
Placentalia - ps28
Boreoeutheria - ps29
Euarchontoglires - ps30
Euarchonta - ps31
Primates - ps32
Haplorrhini - ps33
Simiiformes - ps34
Catarrhini - ps35
Hominoidea - ps36
Hominidae - ps37
Homininae - ps38
Hominini - ps39
Homo_sapiens - ps40
Phylostratum

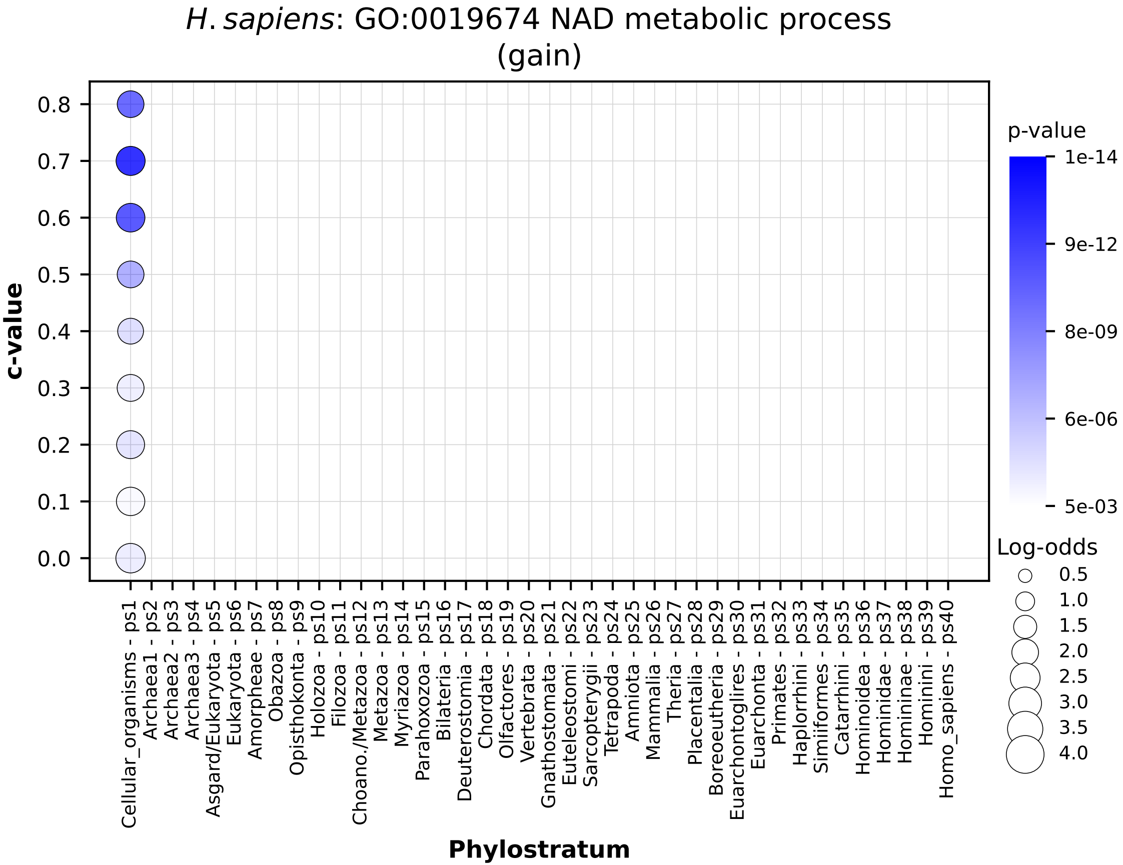

H. sapiens: GO:0019674 NAD metabolic process
(gain)
c-value
0.8
0.7
0.6
0.5
0.4
0.3
0.2
0.1
0.0
Phylostratum
Cellular_organisms - ps1
Archaea1 - ps2
Archaea2 - ps3
Archaea3 - ps4
Asgard/Eukaryota - ps5
Eukaryota - ps6
Amorpheae - ps7
Obazoa - ps8
Opisthokonta - ps9
Holozoa - ps10
Filozoa - ps11
Choano./Metazoa - ps12
Metazoa - ps13
Myriazoa - ps14
Parahoxozoa - ps15
Bilateria - ps16
Deuterostomia - ps17
Chordata - ps18
Olfactores - ps19
Vertebrata - ps20
Gnathostomata - ps21
Euteleostomi - ps22
Sarcopterygii - ps23
Tetrapoda - ps24
Amniota - ps25
Mammalia - ps26
Theria - ps27
Placentalia - ps28
Boreoeutheria - ps29
Euarchontoglires - ps30
Euarchonta - ps31
Primates - ps32
Haplorrhini - ps33
Simiiformes - ps34
Catarrhini - ps35
Hominoidea - ps36
Hominidae - ps37
Homininae - ps38
Hominini - ps39
Homo_sapiens - ps40
p-value
1e-14
9e-12
8e-09
6e-06
5e-03
Log-odds
0.5
1.0
1.5
2.0
2.5
3.0
3.5
4.0

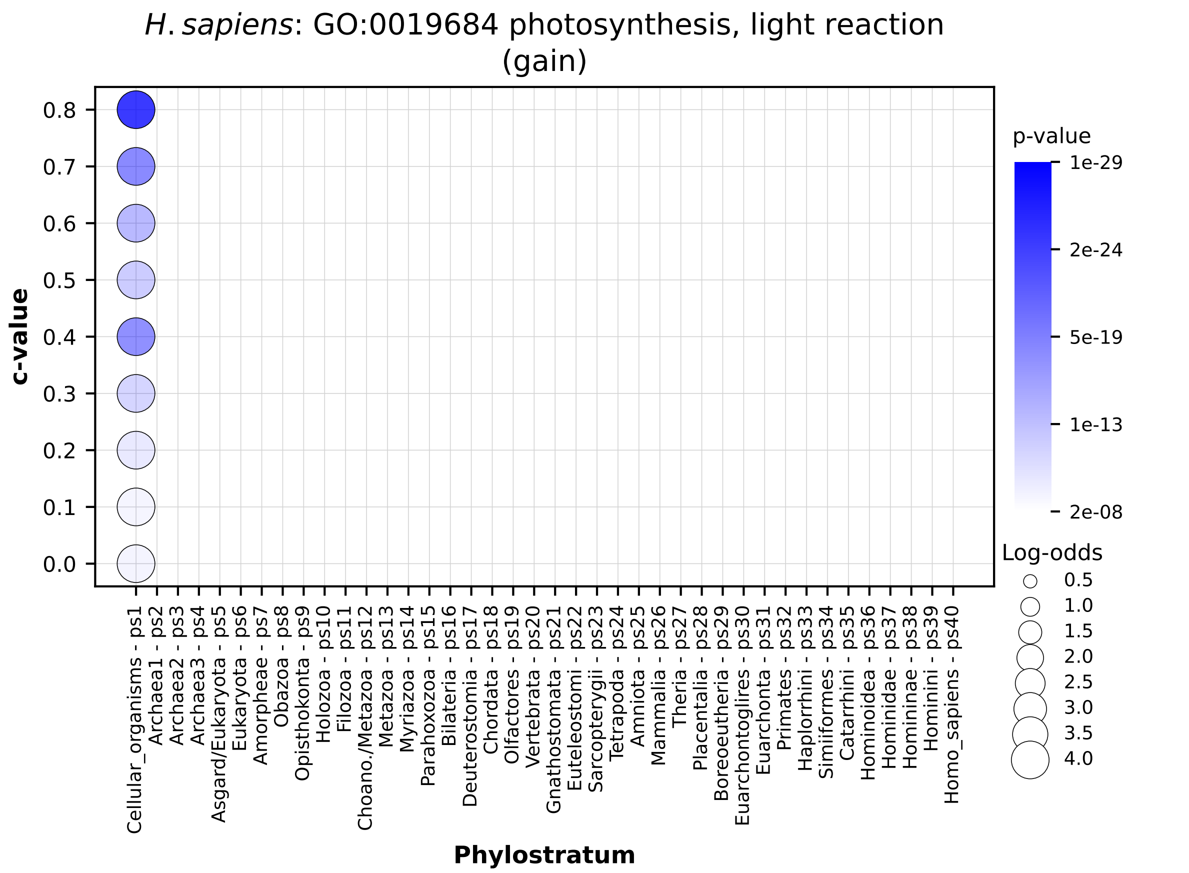

H. sapiens: GO:0019684 photosynthesis, light reaction
(gain)
c-value
0.8
0.7
0.6
0.5
0.4
0.3
0.2
0.1
0.0
Phylostratum
Cellular_organisms - ps1
Archaea1 - ps2
Archaea2 - ps3
Archaea3 - ps4
Asgard/Eukaryota - ps5
Eukaryota - ps6
Amorpheae - ps7
Obazoa - ps8
Opisthokonta - ps9
Holozoa - ps10
Filozoa - ps11
Choano./Metazoa - ps12
Metazoa - ps13
Myriazoa - ps14
Parahoxozoa - ps15
Bilateria - ps16
Deuterostomia - ps17
Chordata - ps18
Olfactores - ps19
Vertebrata - ps20
Gnathostomata - ps21
Euteleostomi - ps22
Sarcopterygii - ps23
Tetrapoda - ps24
Amniota - ps25
Mammalia - ps26
Theria - ps27
Placentalia - ps28
Boreoeutheria - ps29
Euarchontoglires - ps30
Euarchonta - ps31
Primates - ps32
Haplorrhini - ps33
Simiiformes - ps34
Catarrhini - ps35
Hominoidea - ps36
Hominidae - ps37
Homininae - ps38
Hominini - ps39
Homo_sapiens - ps40
p-value
1e-29
2e-24
5e-19
1e-13
2e-08
Log-odds
0.5
1.0
1.5
2.0
2.5
3.0
3.5
4.0

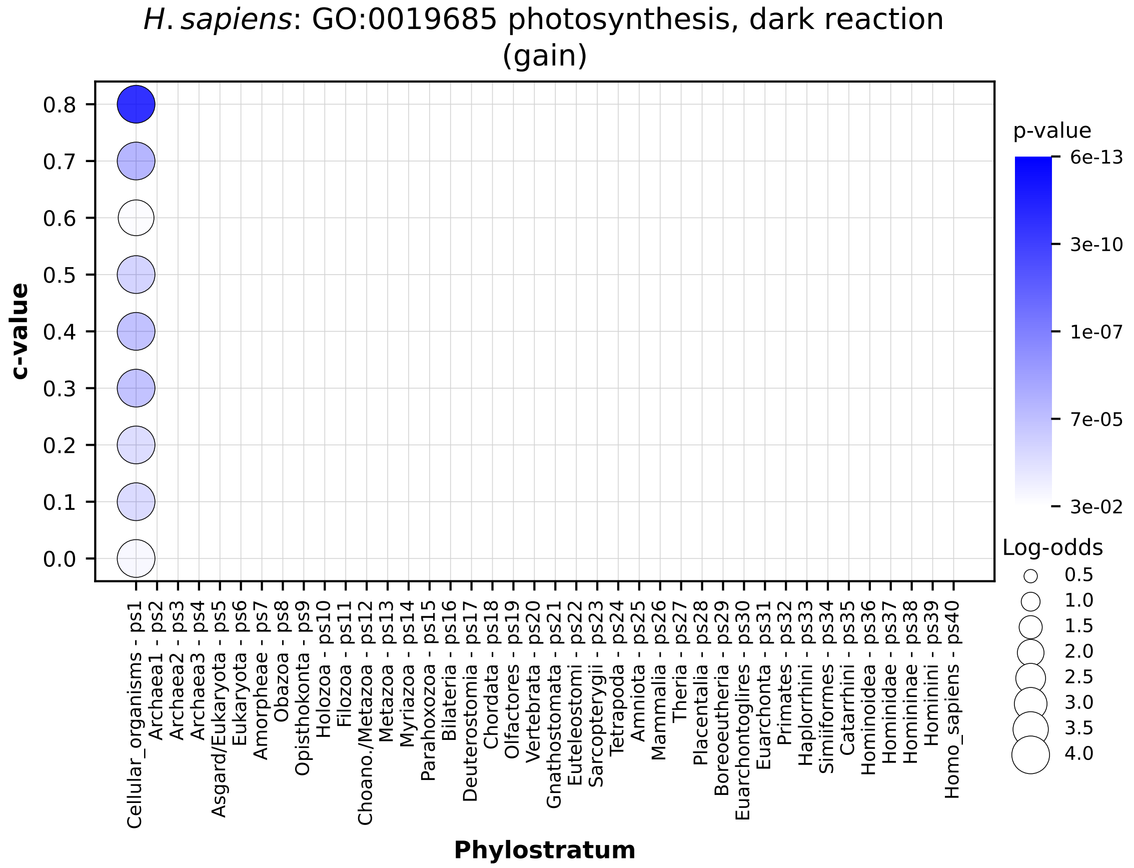

H. sapiens: GO:0019685 photosynthesis, dark reaction (gain)
c-value
0.0
0.1
0.2
0.3
0.4
0.5
0.6
0.7
0.8
p-value
6e-13
3e-10
1e-07
7e-05
3e-02
Log-odds
0.5
1.0
1.5
2.0
2.5
3.0
3.5
4.0
Cellular_organisms - ps1
Archaea1 - ps2
Archaea2 - ps3
Archaea3 - ps4
Asgard/Eukaryota - ps5
Eukaryota - ps6
Amorpheae - ps7
Obazoa - ps8
Opisthokonta - ps9
Holozoa - ps10
Filozoa - ps11
Choano./Metazoa - ps12
Metazoa - ps13
Myriazoa - ps14
Parahoxozoa - ps15
Bilateria - ps16
Deuterostomia - ps17
Chordata - ps18
Olfactores - ps19
Vertebrata - ps20
Gnathostomata - ps21
Euteleostomi - ps22
Sarcopterygii - ps23
Tetrapoda - ps24
Amniota - ps25
Mammalia - ps26
Theria - ps27
Placentalia - ps28
Boreoeutheria - ps29
Euarchontoglires - ps30
Euarchonta - ps31
Primates - ps32
Haplorrhini - ps33
Simiiformes - ps34
Catarrhini - ps35
Hominoidea - ps36
Hominidae - ps37
Homininae - ps38
Hominini - ps39
Homo_sapiens - ps40
Phylostratum

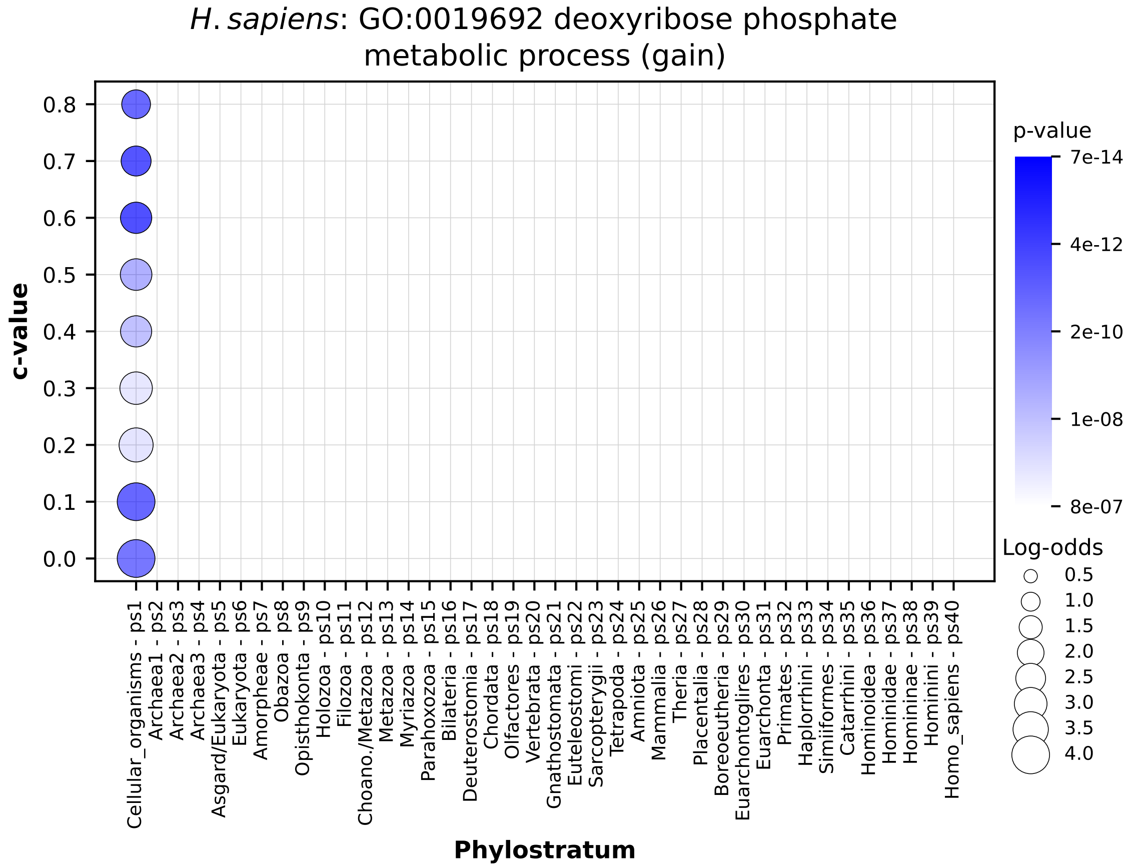

H. sapiens: GO:0019692 deoxyribose phosphate metabolic process (gain)
c-value
0.8
0.7
0.6
0.5
0.4
0.3
0.2
0.1
0.0
p-value
7e-14
4e-12
2e-10
1e-08
8e-07
Log-odds
0.5
1.0
1.5
2.0
2.5
3.0
3.5
4.0
Cellular_organisms - ps1
Archaea1 - ps2
Archaea2 - ps3
Archaea3 - ps4
Asgard/Eukaryota - ps5
Eukaryota - ps6
Amorpheae - ps7
Obazoa - ps8
Opisthokonta - ps9
Holozoa - ps10
Filozoa - ps11
Choano./Metazoa - ps12
Metazoa - ps13
Myriazoa - ps14
Parahoxozoa - ps15
Bilateria - ps16
Deuterostomia - ps17
Chordata - ps18
Olfactores - ps19
Vertebrata - ps20
Gnathostomata - ps21
Euteleostomi - ps22
Sarcopterygii - ps23
Tetrapoda - ps24
Amniota - ps25
Mammalia - ps26
Theria - ps27
Placentalia - ps28
Boreoeutheria - ps29
Euarchontoglires - ps30
Euarchonta - ps31
Primates - ps32
Haplorrhini - ps33
Simiiformes - ps34
Catarrhini - ps35
Hominoidea - ps36
Hominidae - ps37
Homininae - ps38
Hominini - ps39
Homo_sapiens - ps40
Phylostratum

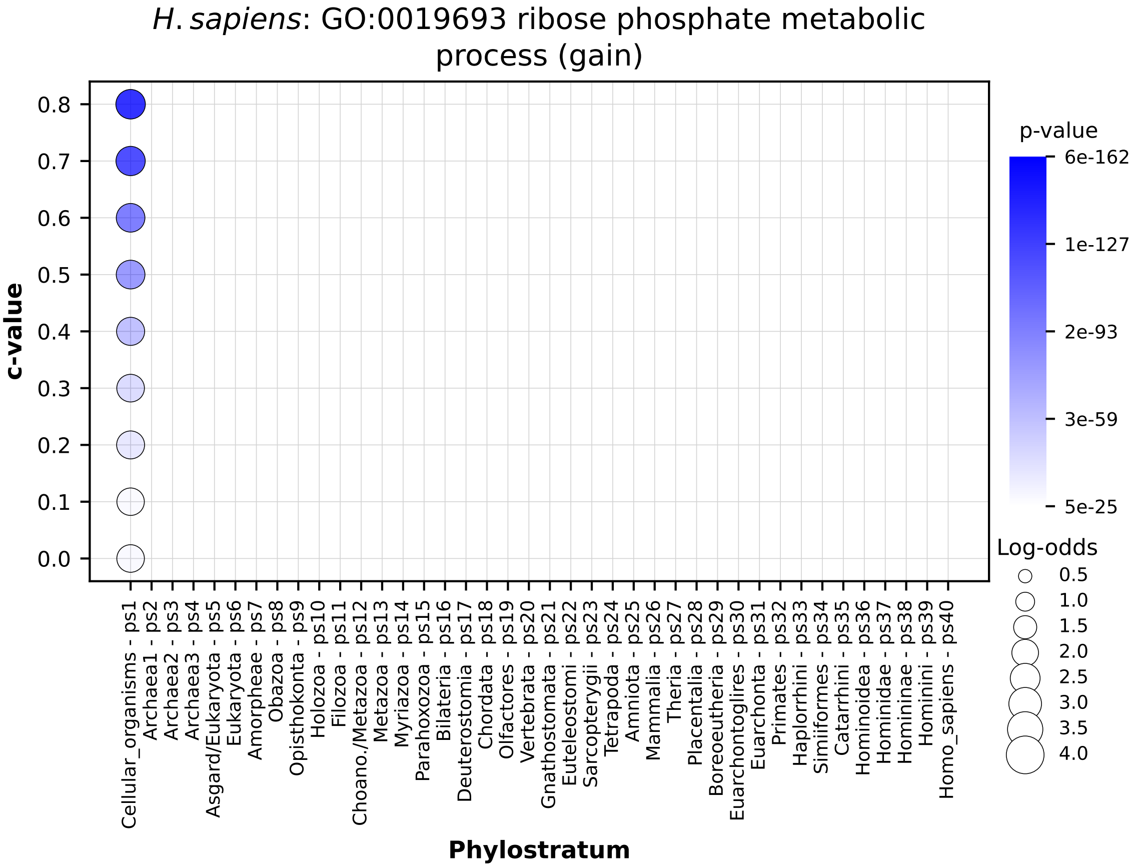

H. sapiens: GO:0019693 ribose phosphate metabolic process (gain)
c-value
0.8
0.7
0.6
0.5
0.4
0.3
0.2
0.1
0.0
p-value
6e-162
1e-127
2e-93
3e-59
5e-25
Log-odds
0.5
1.0
1.5
2.0
2.5
3.0
3.5
4.0
Cellular_organisms - ps1
Archaea1 - ps2
Archaea2 - ps3
Archaea3 - ps4
Asgard/Eukaryota - ps5
Eukaryota - ps6
Amorpheae - ps7
Obazoa - ps8
Opisthokonta - ps9
Holozoa - ps10
Filozoa - ps11
Choano./Metazoa - ps12
Metazoa - ps13
Myriazoa - ps14
Parahoxozoa - ps15
Bilateria - ps16
Deuterostomia - ps17
Chordata - ps18
Olfactores - ps19
Vertebrata - ps20
Gnathostomata - ps21
Euteleostomi - ps22
Sarcopterygii - ps23
Tetrapoda - ps24
Amniota - ps25
Mammalia - ps26
Theria - ps27
Placentalia - ps28
Boreoeutheria - ps29
Euarchontoglires - ps30
Euarchonta - ps31
Primates - ps32
Haplorrhini - ps33
Simiiformes - ps34
Catarrhini - ps35
Hominoidea - ps36
Hominidae - ps37
Homininae - ps38
Hominini - ps39
Homo_sapiens - ps40
Phylostratum

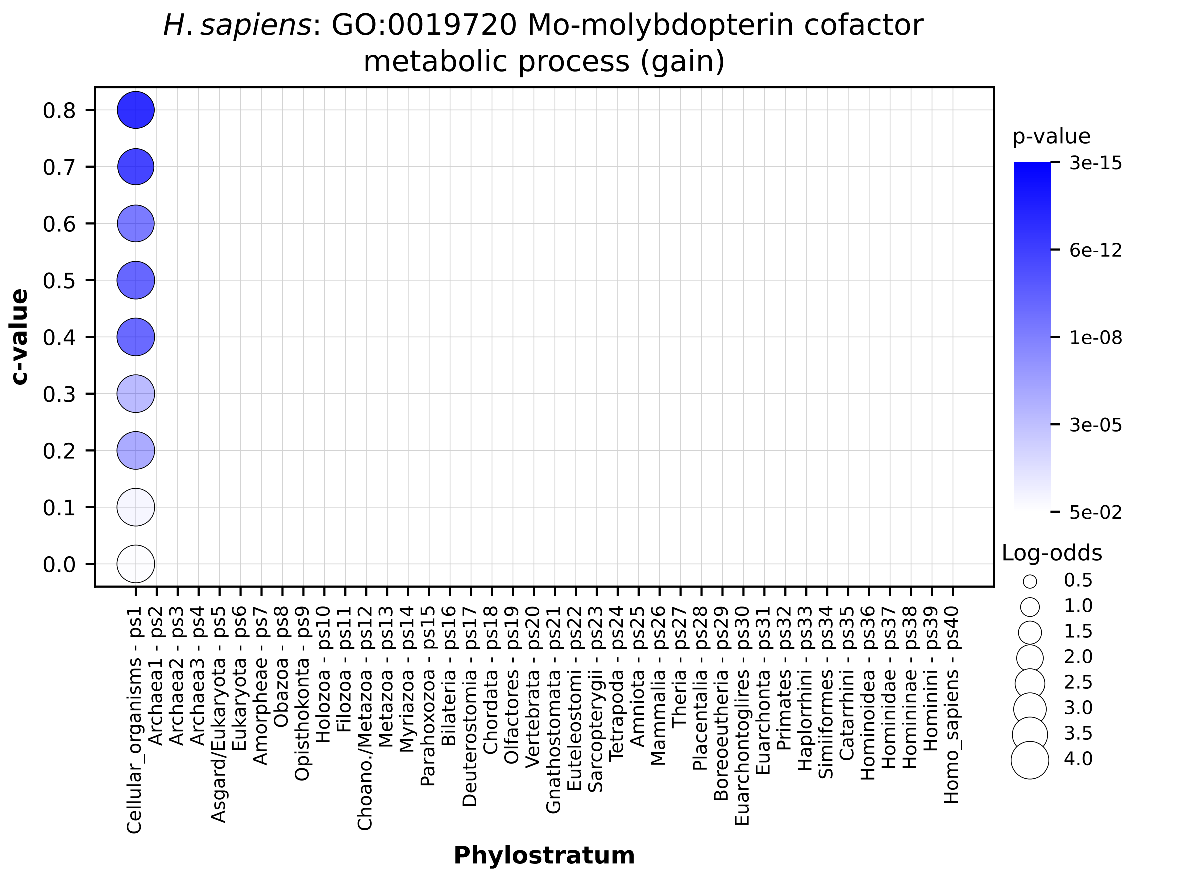

H. sapiens: GO:0019720 Mo-molybdopterin cofactor metabolic process (gain)
c-value
0.8
0.7
0.6
0.5
0.4
0.3
0.2
0.1
0.0
p-value
3e-15
6e-12
1e-08
3e-05
5e-02
Log-odds
0.5
1.0
1.5
2.0
2.5
3.0
3.5
4.0
Cellular_organisms - ps1
Archaea1 - ps2
Archaea2 - ps3
Archaea3 - ps4
Asgard/Eukaryota - ps5
Eukaryota - ps6
Amorpheae - ps7
Obazoa - ps8
Opisthokonta - ps9
Holozoa - ps10
Filozoa - ps11
Choano./Metazoa - ps12
Metazoa - ps13
Myriazoa - ps14
Parahoxozoa - ps15
Bilateria - ps16
Deuterostomia - ps17
Chordata - ps18
Olfactores - ps19
Vertebrata - ps20
Gnathostomata - ps21
Euteleostomi - ps22
Sarcopterygii - ps23
Tetrapoda - ps24
Amniota - ps25
Mammalia - ps26
Theria - ps27
Placentalia - ps28
Boreoeutheria - ps29
Euarchontoglires - ps30
Euarchonta - ps31
Primates - ps32
Haplorrhini - ps33
Simiiformes - ps34
Catarrhini - ps35
Hominoidea - ps36
Hominidae - ps37
Homininae - ps38
Hominini - ps39
Homo_sapiens - ps40
Phylostratum

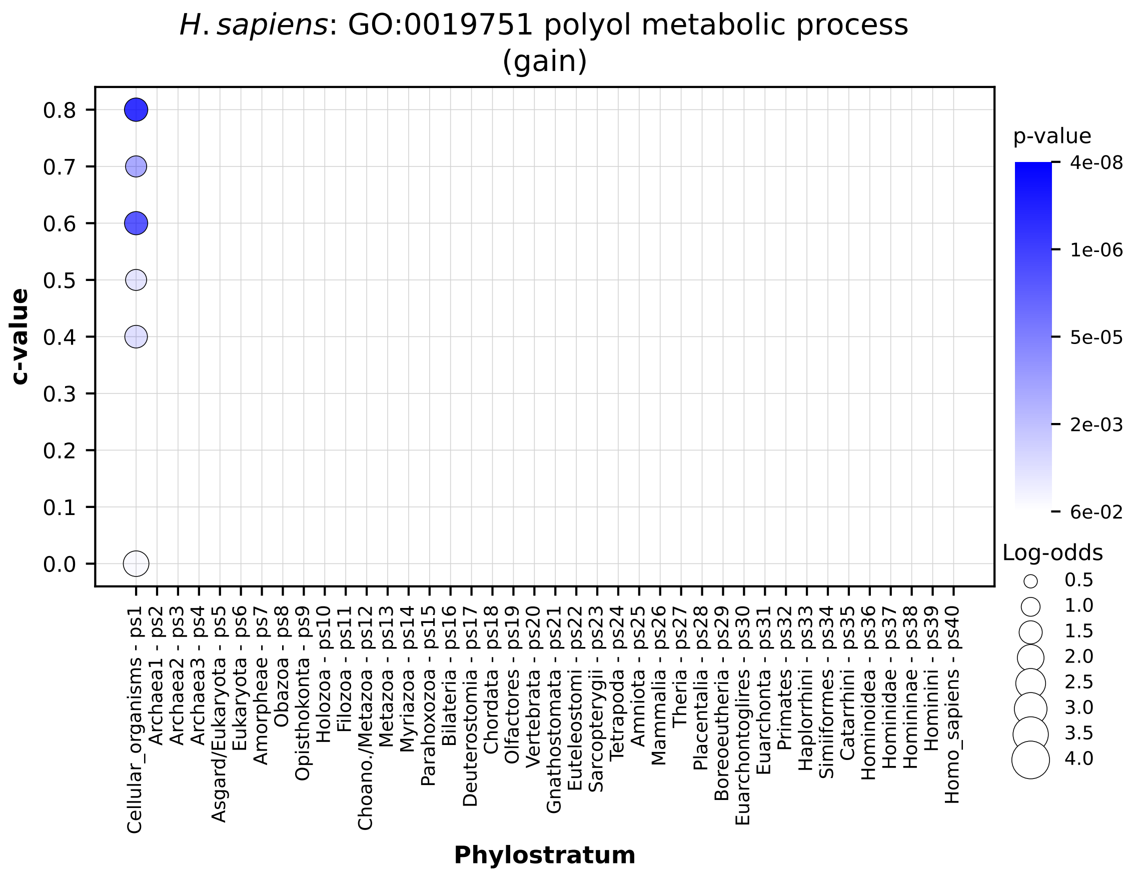
H. sapiens: GO:0019751 polyol metabolic process
(gain)
c-value
0.8
0.7
0.6
0.5
0.4
0.3
0.2
0.1
0.0
p-value
4e-08
1e-06
5e-05
2e-03
6e-02
Log-odds
0.5
1.0
1.5
2.0
2.5
3.0
3.5
4.0
Cellular_organisms - ps1
Archaea1 - ps2
Archaea2 - ps3
Archaea3 - ps4
Asgard/Eukaryota - ps5
Eukaryota - ps6
Amorpheae - ps7
Obazoa - ps8
Opisthokonta - ps9
Holozoa - ps10
Filozoa - ps11
Choano./Metazoa - ps12
Metazoa - ps13
Myriazoa - ps14
Parahoxozoa - ps15
Bilateria - ps16
Deuterostomia - ps17
Chordata - ps18
Olfactores - ps19
Vertebrata - ps20
Gnathostomata - ps21
Euteleostomi - ps22
Sarcopterygii - ps23
Tetrapoda - ps24
Amniota - ps25
Mammalia - ps26
Theria - ps27
Placentalia - ps28
Boreoeutheria - ps29
Euarchontoglires - ps30
Euarchonta - ps31
Primates - ps32
Haplorrhini - ps33
Simiiformes - ps34
Catarrhini - ps35
Hominoidea - ps36
Hominidae - ps37
Homininae - ps38
Hominini - ps39
Homo_sapiens - ps40
Phylostratum

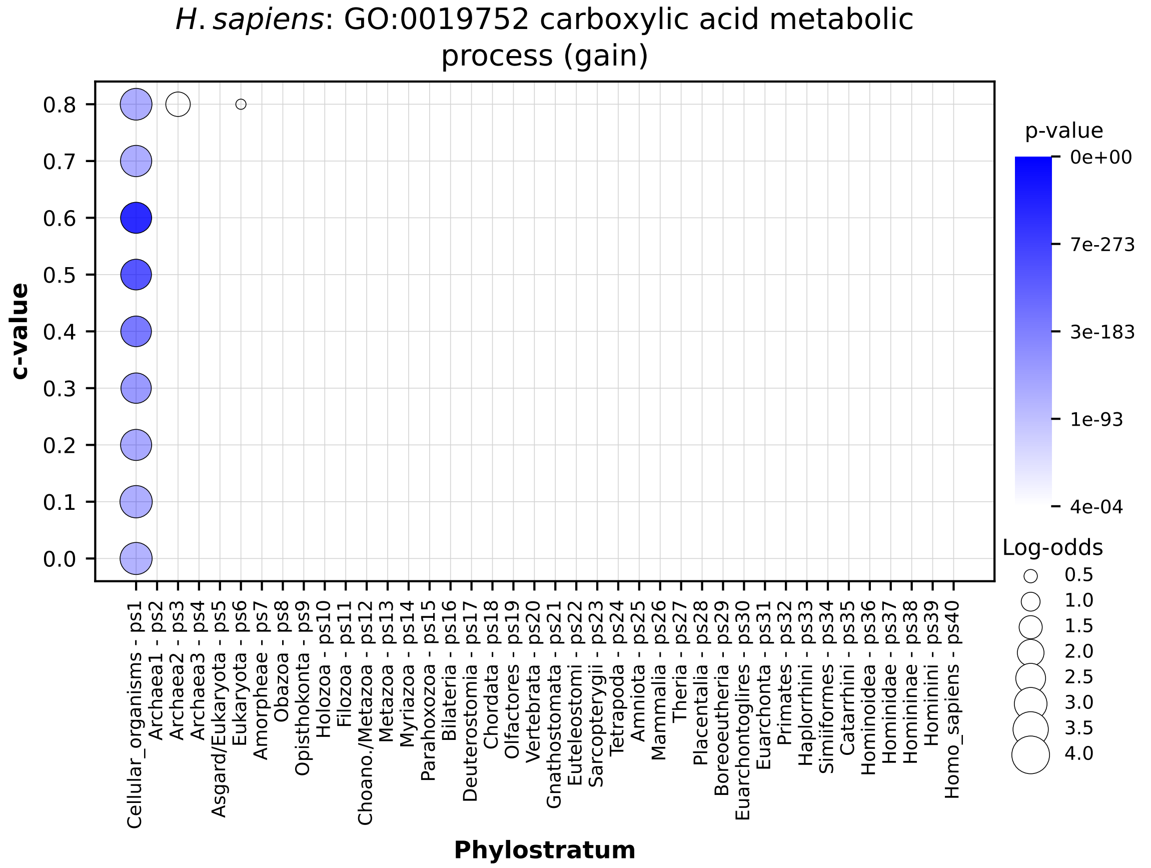
H. sapiens: GO:0019752 carboxylic acid metabolic process (gain)
c-value
0.8
0.7
0.6
0.5
0.4
0.3
0.2
0.1
0.0
Phylostratum
Cellular_organisms - ps1
Archaea1 - ps2
Archaea2 - ps3
Archaea3 - ps4
Asgard/Eukaryota - ps5
Eukaryota - ps6
Amorpheae - ps7
Obazoa - ps8
Opisthokonta - ps9
Holozoa - ps10
Filozoa - ps11
Choano./Metazoa - ps12
Metazoa - ps13
Myriazoa - ps14
Parahoxozoa - ps15
Bilateria - ps16
Deuterostomia - ps17
Chordata - ps18
Olfactores - ps19
Vertebrata - ps20
Gnathostomata - ps21
Euteleostomi - ps22
Sarcopterygii - ps23
Tetrapoda - ps24
Amniota - ps25
Mammalia - ps26
Theria - ps27
Placentalia - ps28
Boreoeutheria - ps29
Euarchontoglires - ps30
Euarchonta - ps31
Primates - ps32
Haplorrhini - ps33
Simiiformes - ps34
Catarrhini - ps35
Hominoidea - ps36
Hominidae - ps37
Homininae - ps38
Hominini - ps39
Homo_sapiens - ps40
p-value
0e+00
7e-273
3e-183
1e-93
4e-04
Log-odds
0.5
1.0
1.5
2.0
2.5
3.0
3.5
4.0

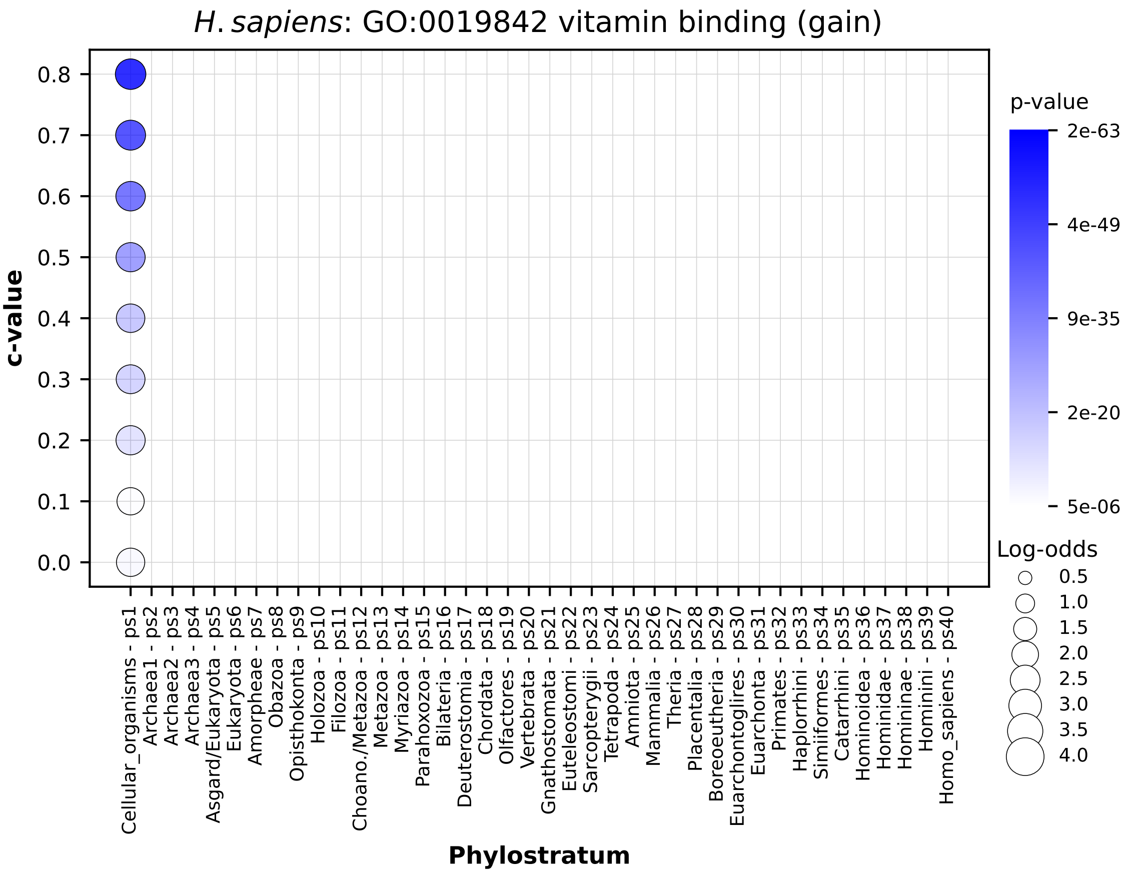

H. sapiens: GO:0019842 vitamin binding (gain)
c-value
0.8
0.7
0.6
0.5
0.4
0.3
0.2
0.1
0.0
Phylostratum
Cellular_organisms - ps1
Archaea1 - ps2
Archaea2 - ps3
Archaea3 - ps4
Asgard/Eukaryota - ps5
Eukaryota - ps6
Amorpheae - ps7
Obazoa - ps8
Opisthokonta - ps9
Holozoa - ps10
Filozoa - ps11
Choano./Metazoa - ps12
Metazoa - ps13
Myriazoa - ps14
Parahoxozoa - ps15
Bilateria - ps16
Deuterostomia - ps17
Chordata - ps18
Olfactores - ps19
Vertebrata - ps20
Gnathostomata - ps21
Euteleostomi - ps22
Sarcopterygii - ps23
Tetrapoda - ps24
Amniota - ps25
Mammalia - ps26
Theria - ps27
Placentalia - ps28
Boreoeutheria - ps29
Euarchontoglires - ps30
Euarchonta - ps31
Primates - ps32
Haplorrhini - ps33
Simiiformes - ps34
Catarrhini - ps35
Hominoidea - ps36
Hominidae - ps37
Homininae - ps38
Hominini - ps39
Homo_sapiens - ps40
p-value
2e-63
4e-49
9e-35
2e-20
5e-06
Log-odds
0.5
1.0
1.5
2.0
2.5
3.0
3.5
4.0

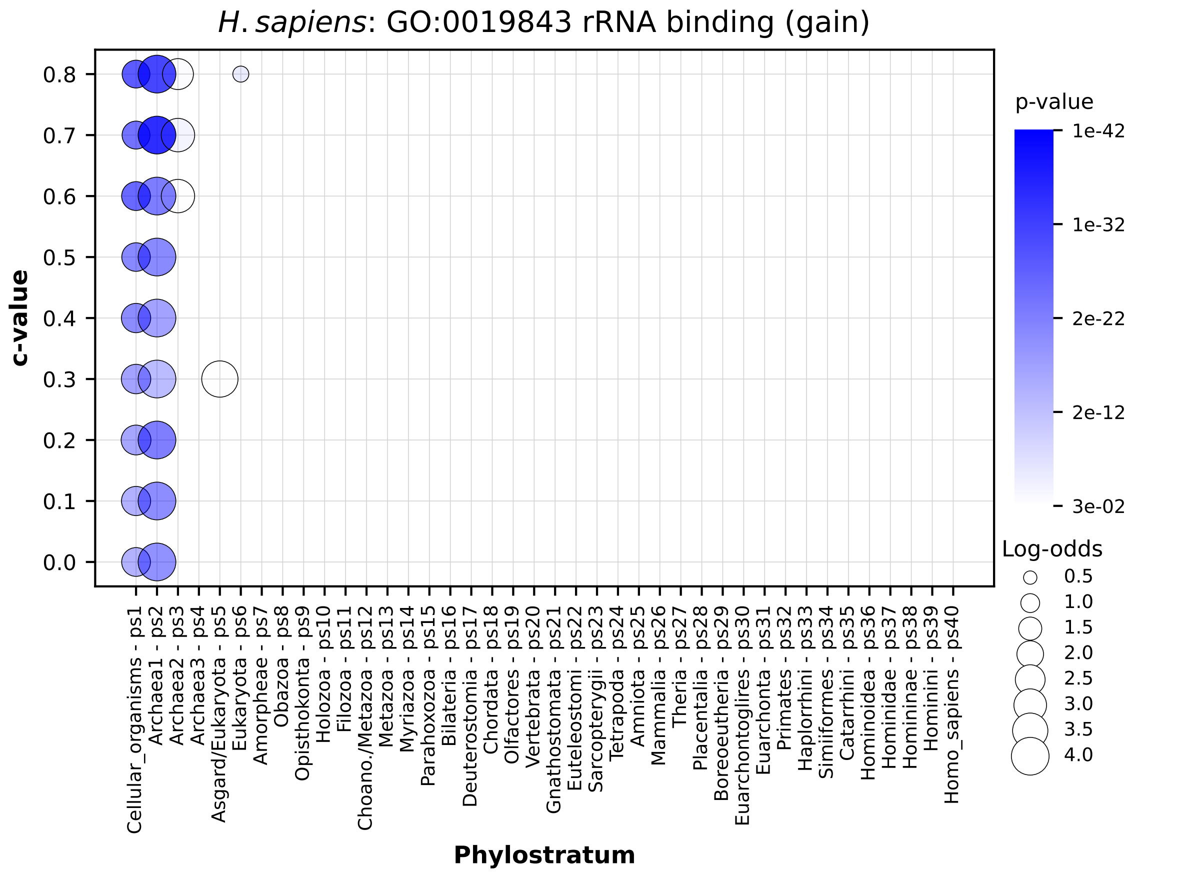

H. sapiens: GO:0019843 rRNA binding (gain)
c-value
0.8
0.7
0.6
0.5
0.4
0.3
0.2
0.1
0.0
p-value
1e-42
1e-32
2e-22
2e-12
3e-02
Log-odds
0.5
1.0
1.5
2.0
2.5
3.0
3.5
4.0
Cellular_organisms - ps1
Archaea1 - ps2
Archaea2 - ps3
Archaea3 - ps4
Asgard/Eukaryota - ps5
Eukaryota - ps6
Amorpheae - ps7
Obazoa - ps8
Opisthokonta - ps9
Holozoa - ps10
Filozoa - ps11
Choano./Metazoa - ps12
Metazoa - ps13
Myriazoa - ps14
Parahoxozoa - ps15
Bilateria - ps16
Deuterostomia - ps17
Chordata - ps18
Olfactores - ps19
Vertebrata - ps20
Gnathostomata - ps21
Euteleostomi - ps22
Sarcopterygii - ps23
Tetrapoda - ps24
Amniota - ps25
Mammalia - ps26
Theria - ps27
Placentalia - ps28
Boreoeutheria - ps29
Euarchontoglires - ps30
Euarchonta - ps31
Primates - ps32
Haplorrhini - ps33
Simiiformes - ps34
Catarrhini - ps35
Hominoidea - ps36
Hominidae - ps37
Homininae - ps38
Hominini - ps39
Homo_sapiens - ps40
Phylostratum

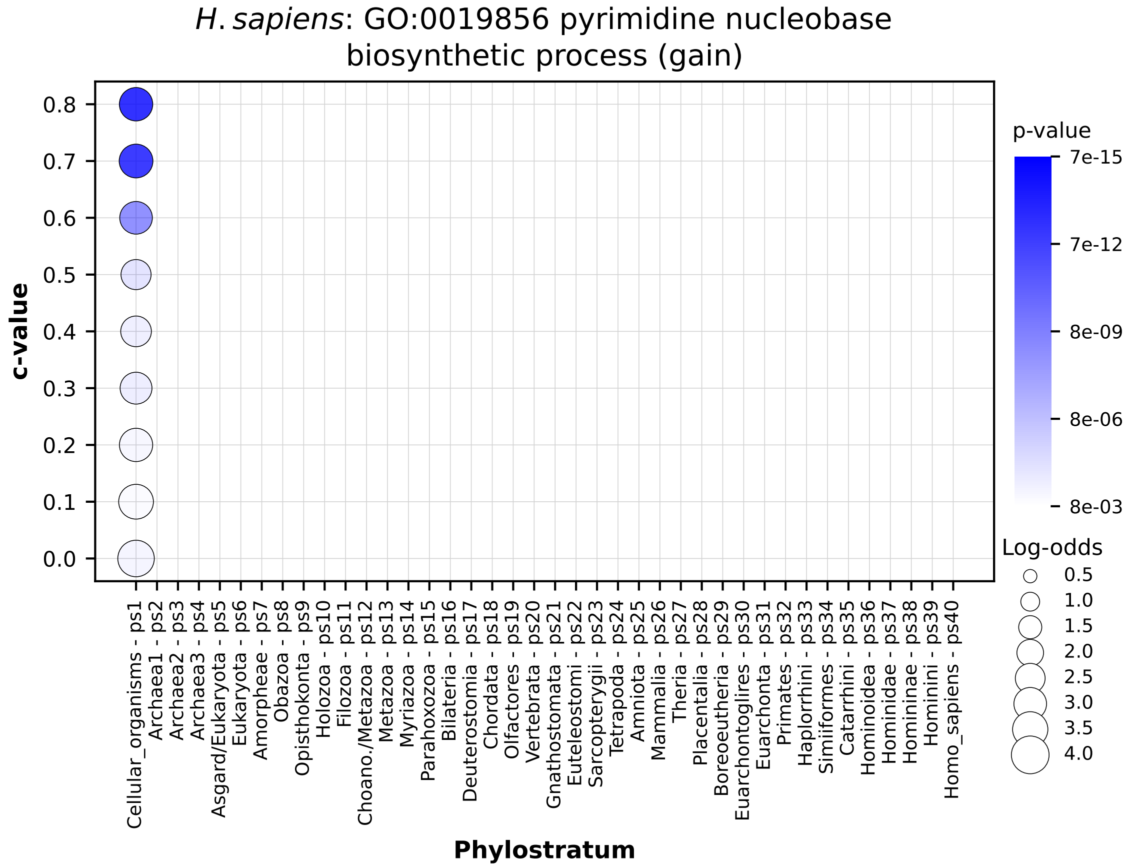

H. sapiens: GO:0019856 pyrimidine nucleobase biosynthetic process (gain)
c-value
0.0
0.1
0.2
0.3
0.4
0.5
0.6
0.7
0.8
p-value
7e-15
7e-12
8e-09
8e-06
8e-03
Log-odds
0.5
1.0
1.5
2.0
2.5
3.0
3.5
4.0
Cellular_organisms - ps1
Archaea1 - ps2
Archaea2 - ps3
Archaea3 - ps4
Asgard/Eukaryota - ps5
Eukaryota - ps6
Amorpheae - ps7
Obazoa - ps8
Opisthokonta - ps9
Holozoa - ps10
Filozoa - ps11
Choano./Metazoa - ps12
Metazoa - ps13
Myriazoa - ps14
Parahoxozoa - ps15
Bilateria - ps16
Deuterostomia - ps17
Chordata - ps18
Olfactores - ps19
Vertebrata - ps20
Gnathostomata - ps21
Euteleostomi - ps22
Sarcopterygii - ps23
Tetrapoda - ps24
Amniota - ps25
Mammalia - ps26
Theria - ps27
Placentalia - ps28
Boreoeutheria - ps29
Euarchontoglires - ps30
Euarchonta - ps31
Primates - ps32
Haplorrhini - ps33
Simiiformes - ps34
Catarrhini - ps35
Hominoidea - ps36
Hominidae - ps37
Homininae - ps38
Hominini - ps39
Homo_sapiens - ps40
Phylostratum

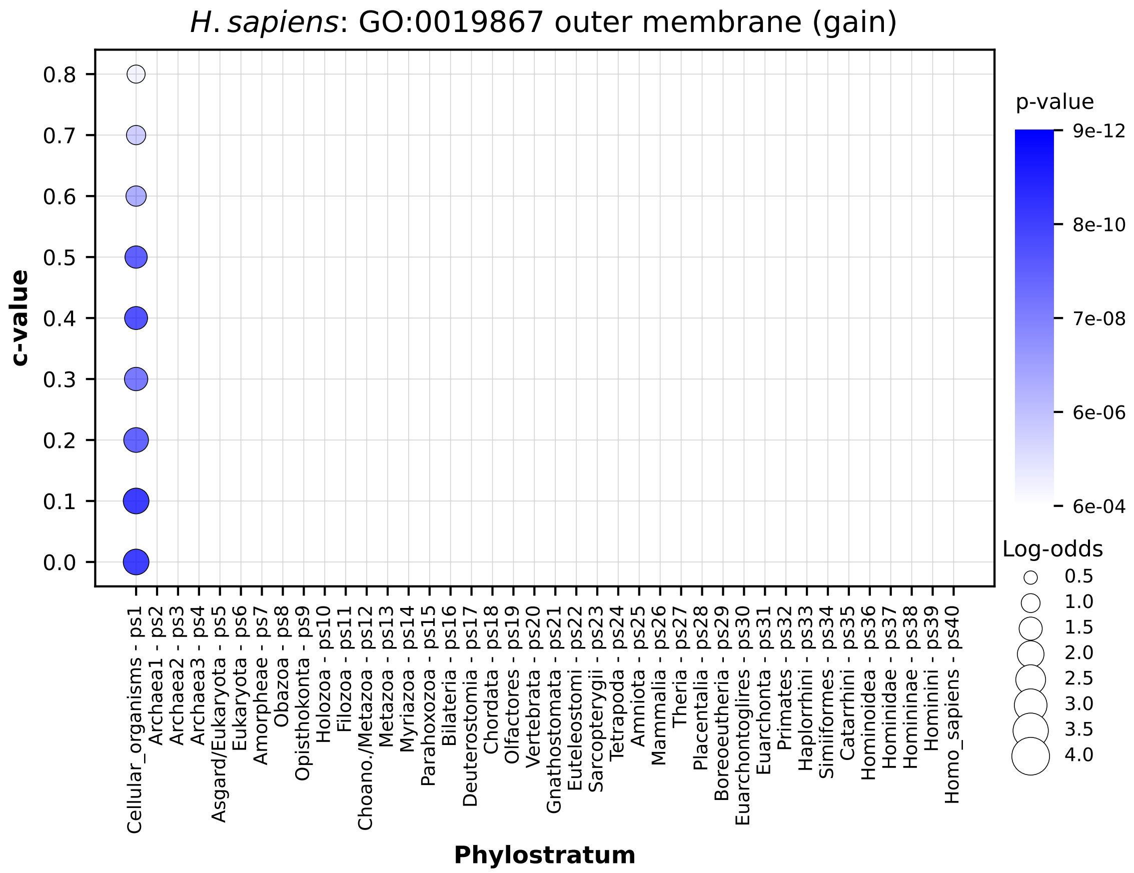

H. sapiens: GO:0019867 outer membrane (gain)
c-value
0.0
0.1
0.2
0.3
0.4
0.5
0.6
0.7
0.8
Phylostratum
Cellular_organisms - ps1
Archaea1 - ps2
Archaea2 - ps3
Archaea3 - ps4
Asgard/Eukaryota - ps5
Eukaryota - ps6
Amorpheae - ps7
Obazoa - ps8
Opisthokonta - ps9
Holozoa - ps10
Filozoa - ps11
Choano./Metazoa - ps12
Metazoa - ps13
Myriazoa - ps14
Parahoxozoa - ps15
Bilateria - ps16
Deuterostomia - ps17
Chordata - ps18
Olfactores - ps19
Vertebrata - ps20
Gnathostomata - ps21
Euteleostomi - ps22
Sarcopterygii - ps23
Tetrapoda - ps24
Amniota - ps25
Mammalia - ps26
Theria - ps27
Placentalia - ps28
Boreoeutheria - ps29
Euarchontoglires - ps30
Euarchonta - ps31
Primates - ps32
Haplorrhini - ps33
Simiiformes - ps34
Catarrhini - ps35
Hominoidea - ps36
Hominidae - ps37
Homininae - ps38
Hominini - ps39
Homo_sapiens - ps40
p-value
9e-12
8e-10
7e-08
6e-06
6e-04
Log-odds
0.5
1.0
1.5
2.0
2.5
3.0
3.5
4.0

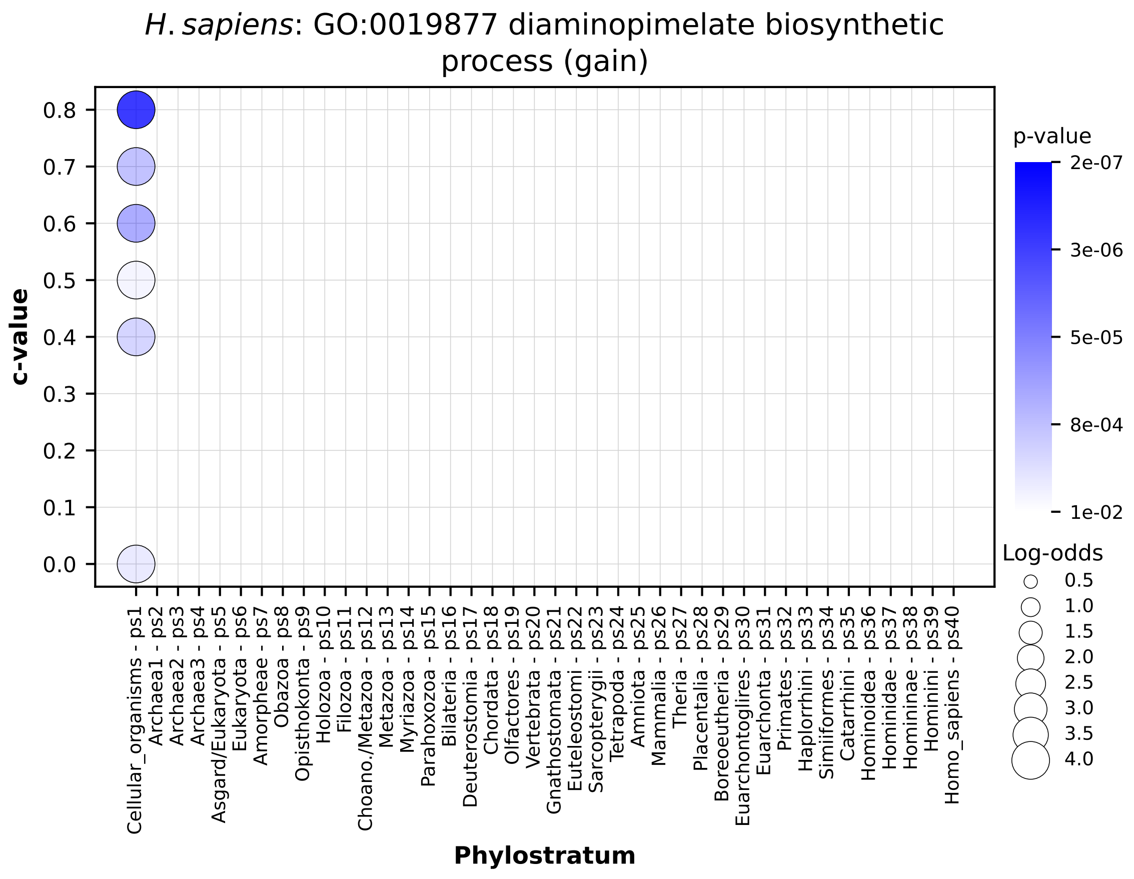

H. sapiens: GO:0019877 diaminopimelate biosynthetic process (gain)
c-value
0.0
0.1
0.2
0.3
0.4
0.5
0.6
0.7
0.8
p-value
2e-07
3e-06
5e-05
8e-04
1e-02
Log-odds
0.5
1.0
1.5
2.0
2.5
3.0
3.5
4.0
Cellular_organisms - ps1
Archaea1 - ps2
Archaea2 - ps3
Archaea3 - ps4
Asgard/Eukaryota - ps5
Eukaryota - ps6
Amorpheae - ps7
Obazoa - ps8
Opisthokonta - ps9
Holozoa - ps10
Filozoa - ps11
Choano./Metazoa - ps12
Metazoa - ps13
Myriazoa - ps14
Parahoxozoa - ps15
Bilateria - ps16
Deuterostomia - ps17
Chordata - ps18
Olfactores - ps19
Vertebrata - ps20
Gnathostomata - ps21
Euteleostomi - ps22
Sarcopterygii - ps23
Tetrapoda - ps24
Amniota - ps25
Mammalia - ps26
Theria - ps27
Placentalia - ps28
Boreoeutheria - ps29
Euarchontoglires - ps30
Euarchonta - ps31
Primates - ps32
Haplorrhini - ps33
Simiiformes - ps34
Catarrhini - ps35
Hominoidea - ps36
Hominidae - ps37
Homininae - ps38
Hominini - ps39
Homo_sapiens - ps40
Phylostratum

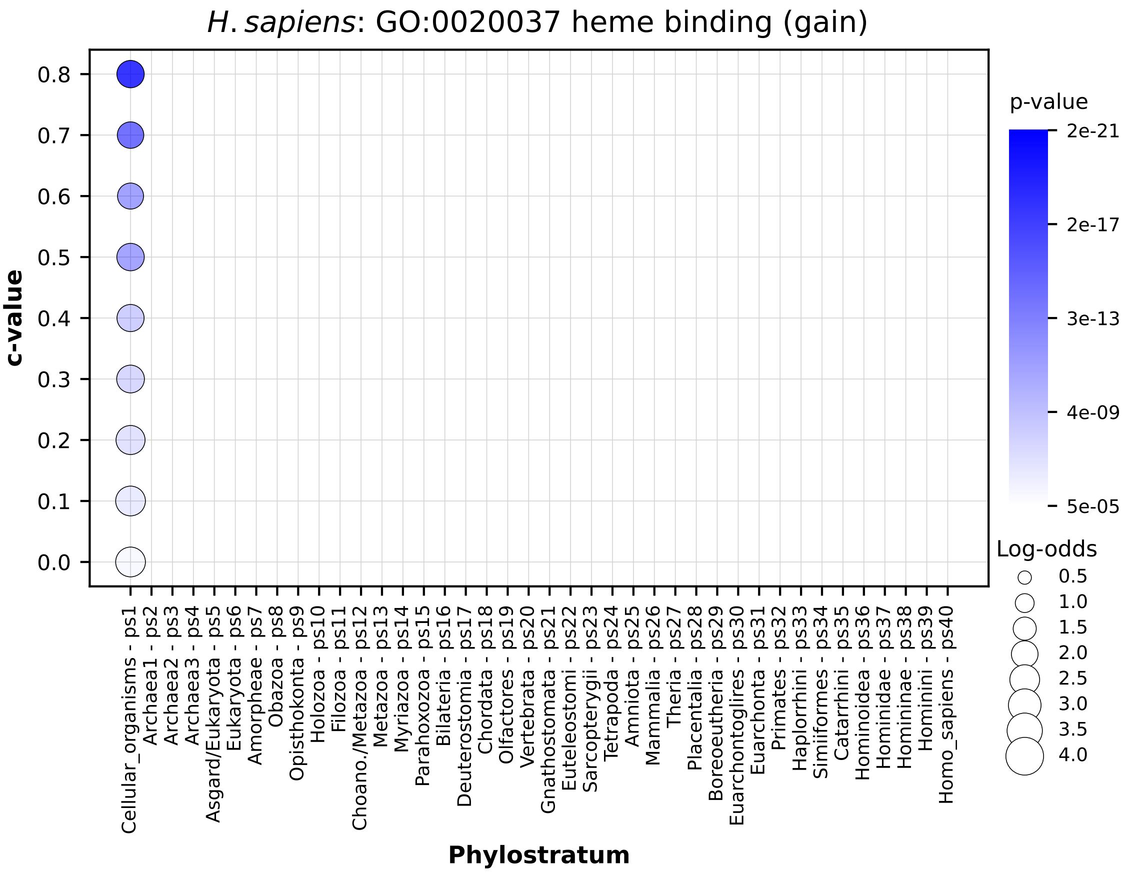
H. sapiens: GO:0020037 heme binding (gain)
c-value
0.8
0.7
0.6
0.5
0.4
0.3
0.2
0.1
0.0
Phylostratum
Cellular_organisms - ps1
Archaea1 - ps2
Archaea2 - ps3
Archaea3 - ps4
Asgard/Eukaryota - ps5
Eukaryota - ps6
Amorpheae - ps7
Obazoa - ps8
Opisthokonta - ps9
Holozoa - ps10
Filozoa - ps11
Choano./Metazoa - ps12
Metazoa - ps13
Myriazoa - ps14
Parahoxozoa - ps15
Bilateria - ps16
Deuterostomia - ps17
Chordata - ps18
Olfactores - ps19
Vertebrata - ps20
Gnathostomata - ps21
Euteleostomi - ps22
Sarcopterygii - ps23
Tetrapoda - ps24
Amniota - ps25
Mammalia - ps26
Theria - ps27
Placentalia - ps28
Boreoeutheria - ps29
Euarchontoglires - ps30
Euarchonta - ps31
Primates - ps32
Haplorrhini - ps33
Simiiformes - ps34
Catarrhini - ps35
Hominoidea - ps36
Hominidae - ps37
Homininae - ps38
Hominini - ps39
Homo_sapiens - ps40
p-value
2e-21
2e-17
3e-13
4e-09
5e-05
Log-odds
0.5
1.0
1.5
2.0
2.5
3.0
3.5
4.0

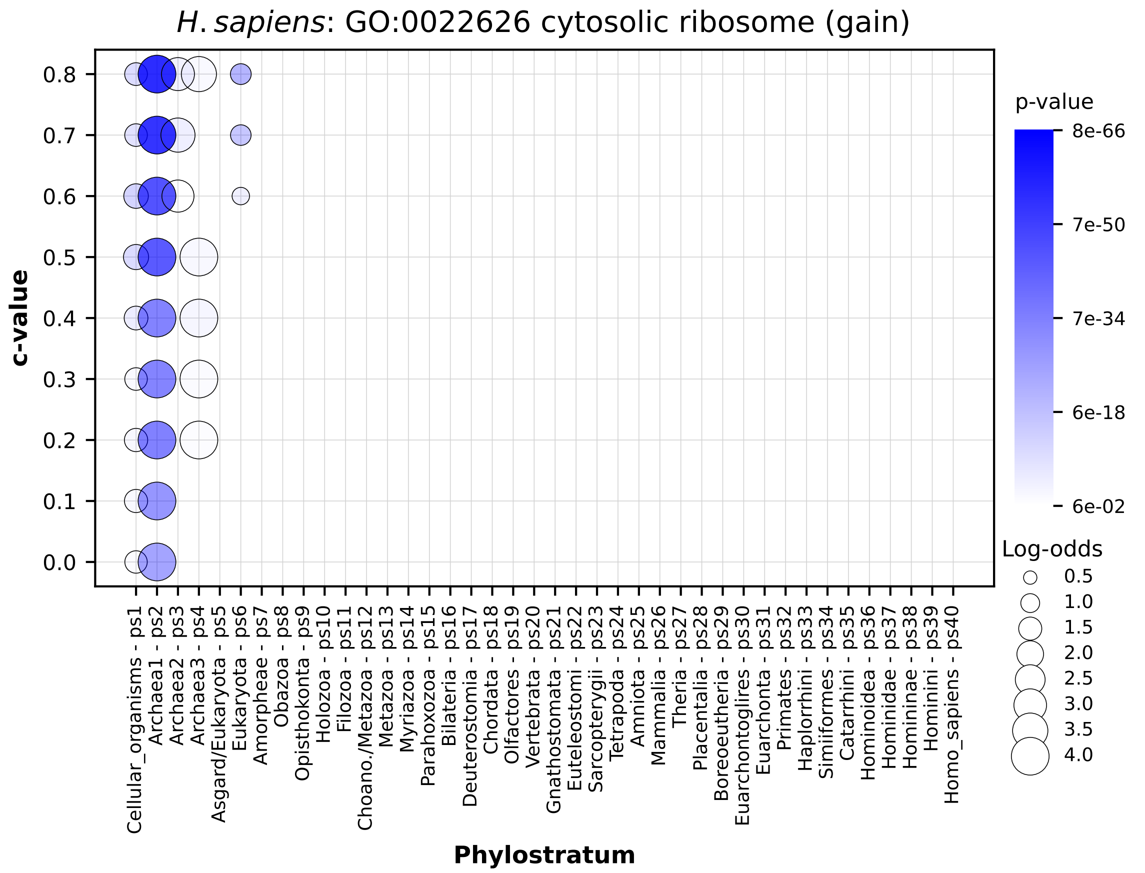
H. sapiens: GO:0022626 cytosolic ribosome (gain)
c-value
0.8
0.7
0.6
0.5
0.4
0.3
0.2
0.1
0.0
Phylostratum
Cellular_organisms - ps1
Archaea1 - ps2
Archaea2 - ps3
Archaea3 - ps4
Asgard/Eukaryota - ps5
Eukaryota - ps6
Amorpheae - ps7
Obazoa - ps8
Opisthokonta - ps9
Holozoa - ps10
Filozoa - ps11
Choano./Metazoa - ps12
Metazoa - ps13
Myriazoa - ps14
Parahoxozoa - ps15
Bilateria - ps16
Deuterostomia - ps17
Chordata - ps18
Olfactores - ps19
Vertebrata - ps20
Gnathostomata - ps21
Euteleostomi - ps22
Sarcopterygii - ps23
Tetrapoda - ps24
Amniota - ps25
Mammalia - ps26
Theria - ps27
Placentalia - ps28
Boreoeutheria - ps29
Euarchontoglires - ps30
Euarchonta - ps31
Primates - ps32
Haplorrhini - ps33
Simiiformes - ps34
Catarrhini - ps35
Hominoidea - ps36
Hominidae - ps37
Homininae - ps38
Hominini - ps39
Homo_sapiens - ps40
p-value
8e-66
7e-50
7e-34
6e-18
6e-02
Log-odds
0.5
1.0
1.5
2.0
2.5
3.0
3.5
4.0

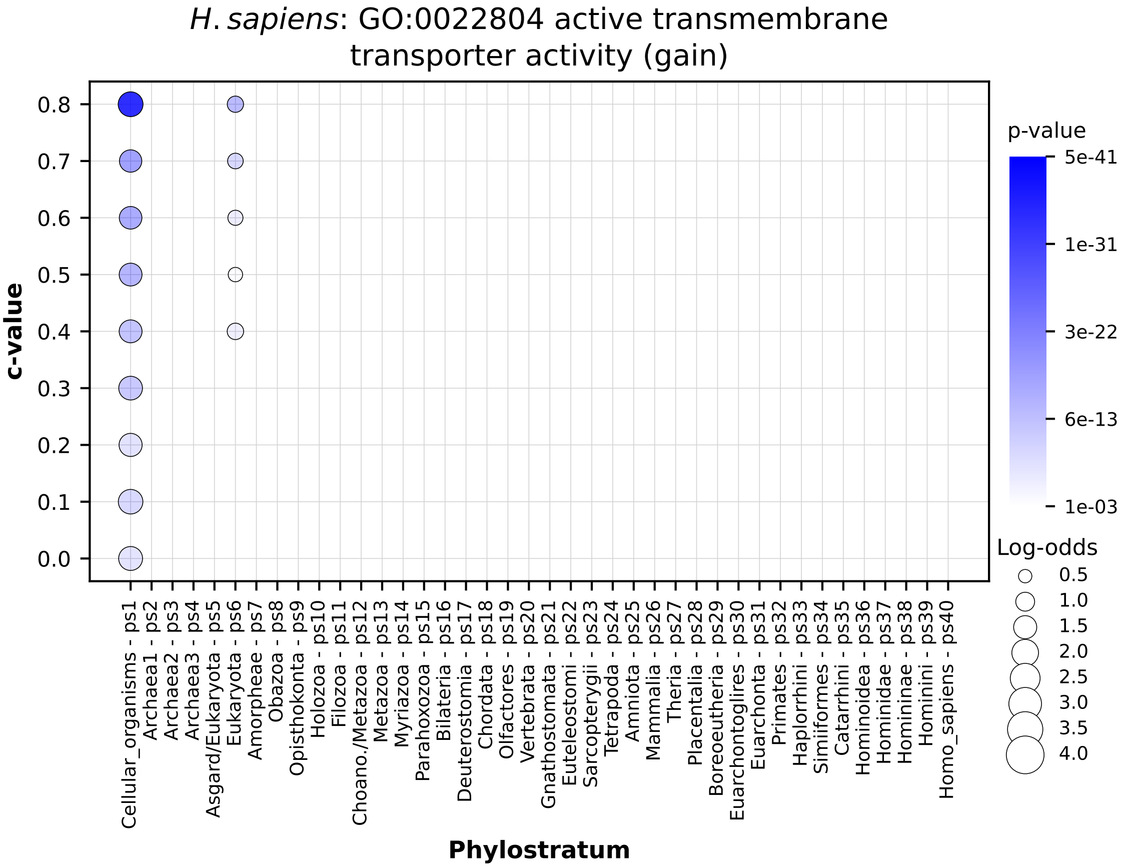

H. sapiens: GO:0022804 active transmembrane transporter activity (gain)
c-value
0.0
0.1
0.2
0.3
0.4
0.5
0.6
0.7
0.8
Phylostratum
Cellular_organisms - ps1
Archaea1 - ps2
Archaea2 - ps3
Archaea3 - ps4
Asgard/Eukaryota - ps5
Eukaryota - ps6
Amorpheae - ps7
Obazoa - ps8
Opisthokonta - ps9
Holozoa - ps10
Filozoa - ps11
Choano./Metazoa - ps12
Metazoa - ps13
Myriazoa - ps14
Parahoxozoa - ps15
Bilateria - ps16
Deuterostomia - ps17
Chordata - ps18
Olfactores - ps19
Vertebrata - ps20
Gnathostomata - ps21
Euteleostomi - ps22
Sarcopterygii - ps23
Tetrapoda - ps24
Amniota - ps25
Mammalia - ps26
Theria - ps27
Placentalia - ps28
Boreoeutheria - ps29
Euarchontoglires - ps30
Euarchonta - ps31
Primates - ps32
Haplorrhini - ps33
Simiiformes - ps34
Catarrhini - ps35
Hominoidea - ps36
Hominidae - ps37
Homininae - ps38
Hominini - ps39
Homo_sapiens - ps40
p-value
5e-41
1e-31
3e-22
6e-13
1e-03
Log-odds
0.5
1.0
1.5
2.0
2.5
3.0
3.5
4.0

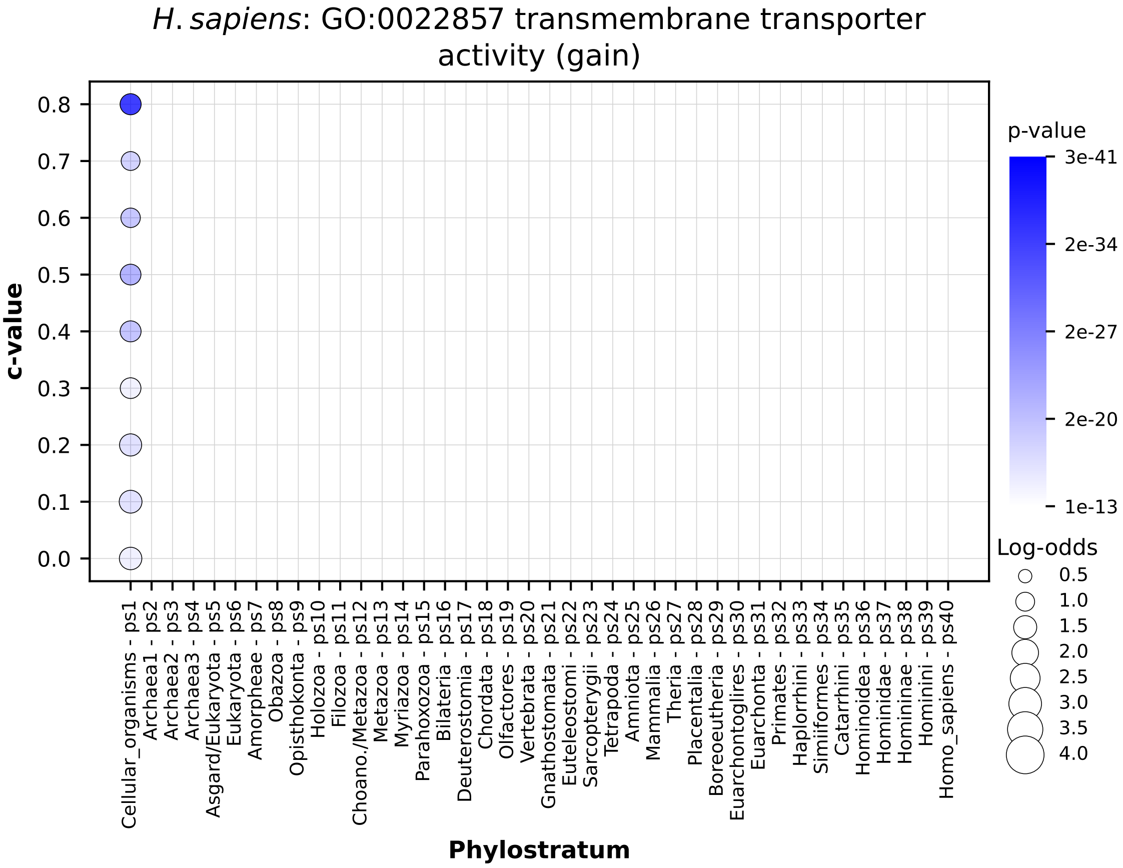

H. sapiens: GO:0022857 transmembrane transporter activity (gain)
c-value
0.8
0.7
0.6
0.5
0.4
0.3
0.2
0.1
0.0
Phylostratum
Cellular_organisms - ps1
Archaea1 - ps2
Archaea2 - ps3
Archaea3 - ps4
Asgard/Eukaryota - ps5
Eukaryota - ps6
Amorpheae - ps7
Obazoa - ps8
Opisthokonta - ps9
Holozoa - ps10
Filozoa - ps11
Choano./Metazoa - ps12
Metazoa - ps13
Myriazoa - ps14
Parahoxozoa - ps15
Bilateria - ps16
Deuterostomia - ps17
Chordata - ps18
Olfactores - ps19
Vertebrata - ps20
Gnathostomata - ps21
Euteleostomi - ps22
Sarcopterygii - ps23
Tetrapoda - ps24
Amniota - ps25
Mammalia - ps26
Theria - ps27
Placentalia - ps28
Boreoeutheria - ps29
Euarchontoglires - ps30
Euarchonta - ps31
Primates - ps32
Haplorrhini - ps33
Simiiformes - ps34
Catarrhini - ps35
Hominoidea - ps36
Hominidae - ps37
Homininae - ps38
Hominini - ps39
Homo_sapiens - ps40
p-value
3e-41
2e-34
2e-27
2e-20
1e-13
Log-odds
0.5
1.0
1.5
2.0
2.5
3.0
3.5
4.0

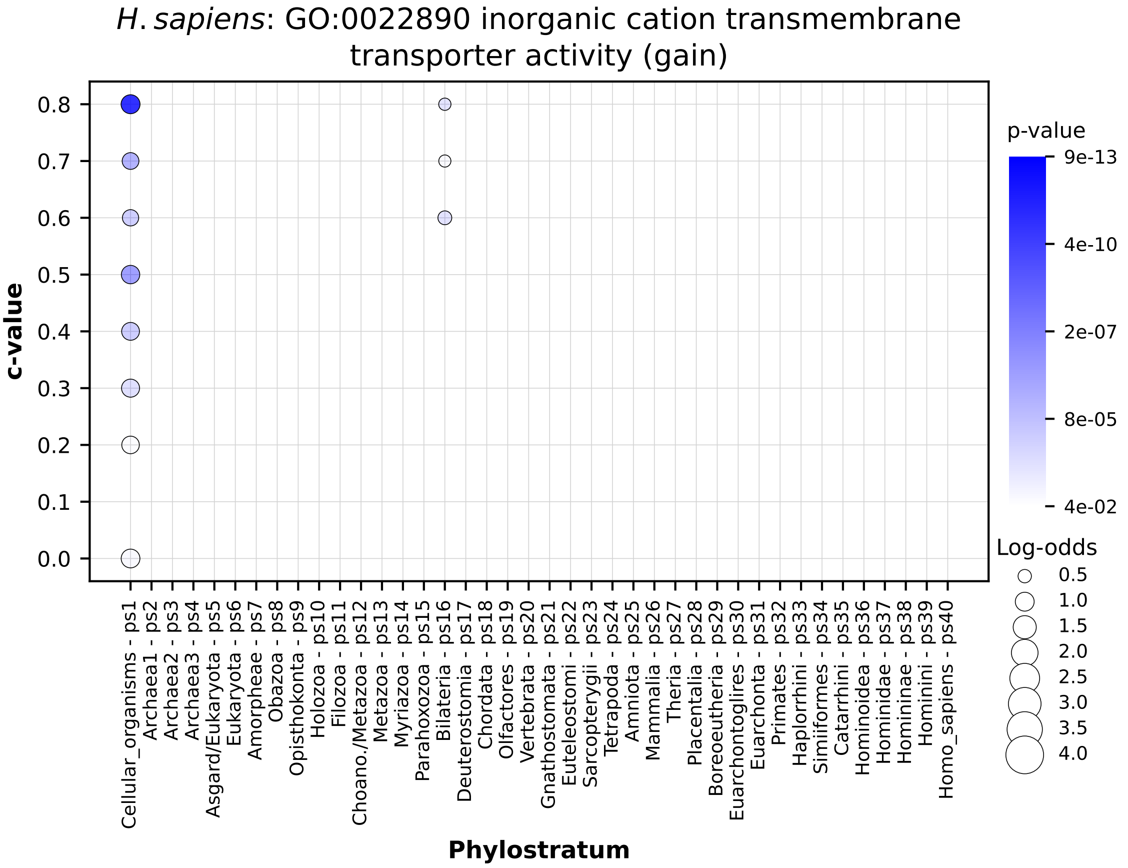

H. sapiens: GO:0022890 inorganic cation transmembrane transporter activity (gain)
c-value
0.0
0.1
0.2
0.3
0.4
0.5
0.6
0.7
0.8
Phylostratum
Cellular_organisms - ps1
Archaea1 - ps2
Archaea2 - ps3
Archaea3 - ps4
Asgard/Eukaryota - ps5
Eukaryota - ps6
Amorpheae - ps7
Obazoa - ps8
Opisthokonta - ps9
Holozoa - ps10
Filozoa - ps11
Choano./Metazoa - ps12
Metazoa - ps13
Myriazoa - ps14
Parahoxozoa - ps15
Bilateria - ps16
Deuterostomia - ps17
Chordata - ps18
Olfactores - ps19
Vertebrata - ps20
Gnathostomata - ps21
Euteleostomi - ps22
Sarcopterygii - ps23
Tetrapoda - ps24
Amniota - ps25
Mammalia - ps26
Theria - ps27
Placentalia - ps28
Boreoeutheria - ps29
Euarchontoglires - ps30
Euarchonta - ps31
Primates - ps32
Haplorrhini - ps33
Simiiformes - ps34
Catarrhini - ps35
Hominoidea - ps36
Hominidae - ps37
Homininae - ps38
Hominini - ps39
Homo_sapiens - ps40
p-value
9e-13
4e-10
2e-07
8e-05
4e-02
Log-odds
0.5
1.0
1.5
2.0
2.5
3.0
3.5
4.0

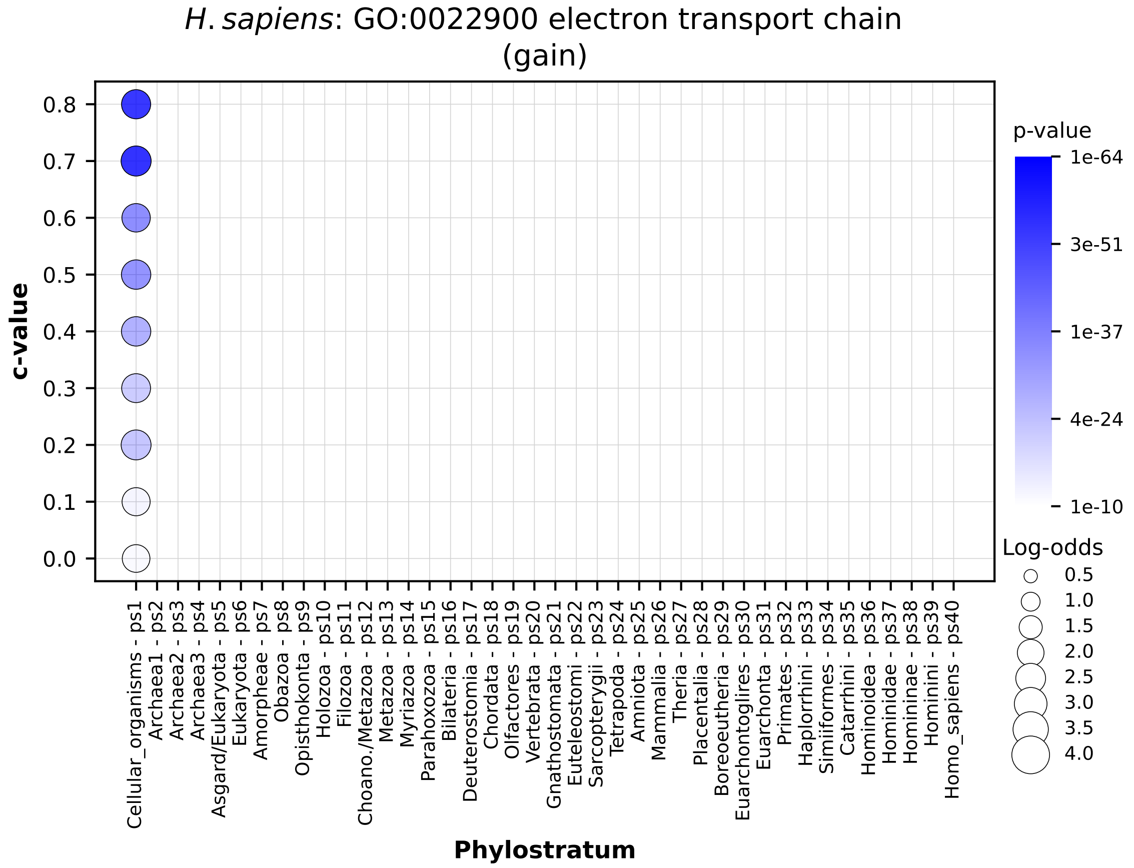

*H. sapiens*: GO:0022900 electron transport chain (gain)
c-value
0.0
0.1
0.2
0.3
0.4
0.5
0.6
0.7
0.8
p-value
1e-64
3e-51
1e-37
4e-24
1e-10
Log-odds
0.5
1.0
1.5
2.0
2.5
3.0
3.5
4.0
Cellular_organisms - ps1
Archaea1 - ps2
Archaea2 - ps3
Archaea3 - ps4
Asgard/Eukaryota - ps5
Eukaryota - ps6
Amorpheae - ps7
Obazoa - ps8
Opisthokonta - ps9
Holozoa - ps10
Filozoa - ps11
Choano./Metazoa - ps12
Metazoa - ps13
Myriazoa - ps14
Parahoxozoa - ps15
Bilateria - ps16
Deuterostomia - ps17
Chordata - ps18
Olfactores - ps19
Vertebrata - ps20
Gnathostomata - ps21
Euteleostomi - ps22
Sarcopterygii - ps23
Tetrapoda - ps24
Amniota - ps25
Mammalia - ps26
Theria - ps27
Placentalia - ps28
Boreoeutheria - ps29
Euarchontoglires - ps30
Euarchonta - ps31
Primates - ps32
Haplorrhini - ps33
Simiiformes - ps34
Catarrhini - ps35
Hominoidea - ps36
Hominidae - ps37
Homininae - ps38
Hominini - ps39
Homo_sapiens - ps40
Phylostratum

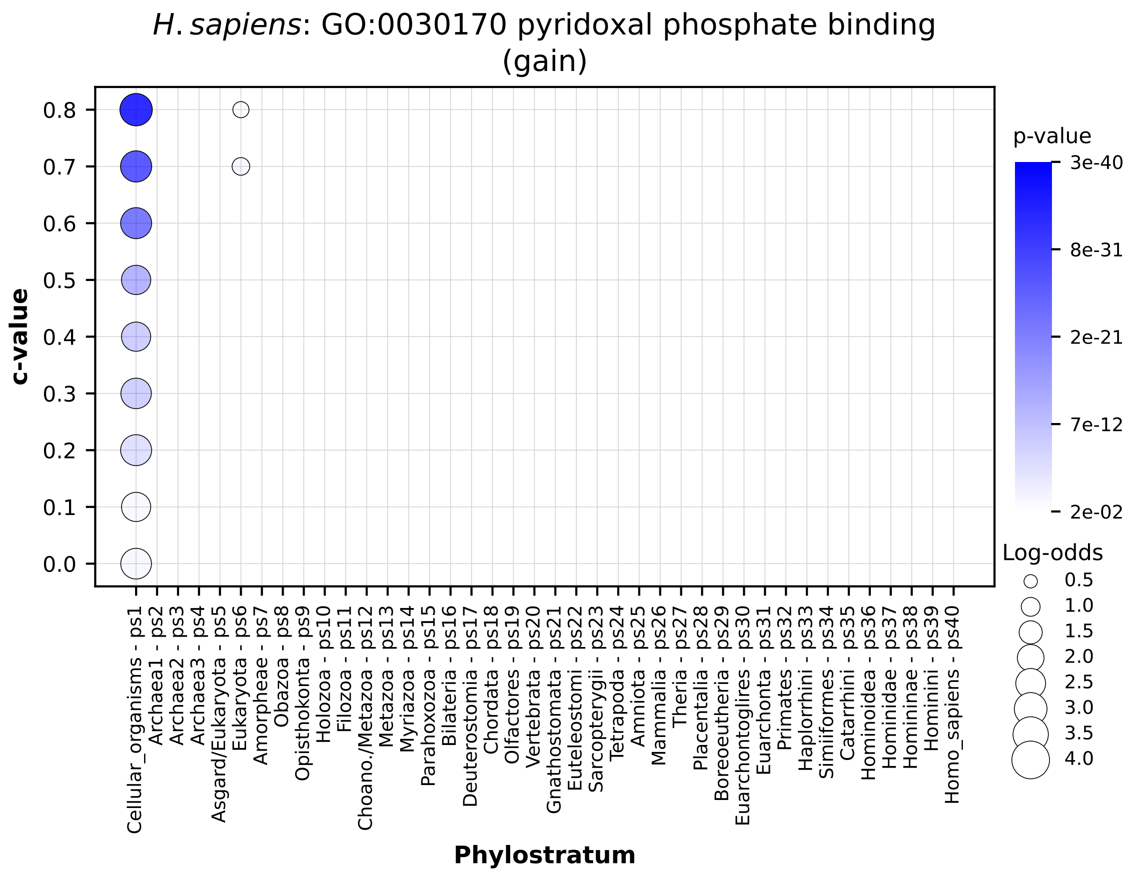

H. sapiens: GO:0030170 pyridoxal phosphate binding (gain)
c-value
0.8
0.7
0.6
0.5
0.4
0.3
0.2
0.1
0.0
p-value
3e-40
8e-31
2e-21
7e-12
2e-02
Log-odds
0.5
1.0
1.5
2.0
2.5
3.0
3.5
4.0
Cellular_organisms - ps1
Archaea1 - ps2
Archaea2 - ps3
Archaea3 - ps4
Asgard/Eukaryota - ps5
Eukaryota - ps6
Amorpheae - ps7
Obazoa - ps8
Opisthokonta - ps9
Holozoa - ps10
Filozoa - ps11
Choano./Metazoa - ps12
Metazoa - ps13
Myriazoa - ps14
Parahoxozoa - ps15
Bilateria - ps16
Deuterostomia - ps17
Chordata - ps18
Olfactores - ps19
Vertebrata - ps20
Gnathostomata - ps21
Euteleostomi - ps22
Sarcopterygii - ps23
Tetrapoda - ps24
Amniota - ps25
Mammalia - ps26
Theria - ps27
Placentalia - ps28
Boreoeutheria - ps29
Euarchontoglires - ps30
Euarchonta - ps31
Primates - ps32
Haplorrhini - ps33
Simiiformes - ps34
Catarrhini - ps35
Hominoidea - ps36
Hominidae - ps37
Homininae - ps38
Hominini - ps39
Homo_sapiens - ps40
Phylostratum

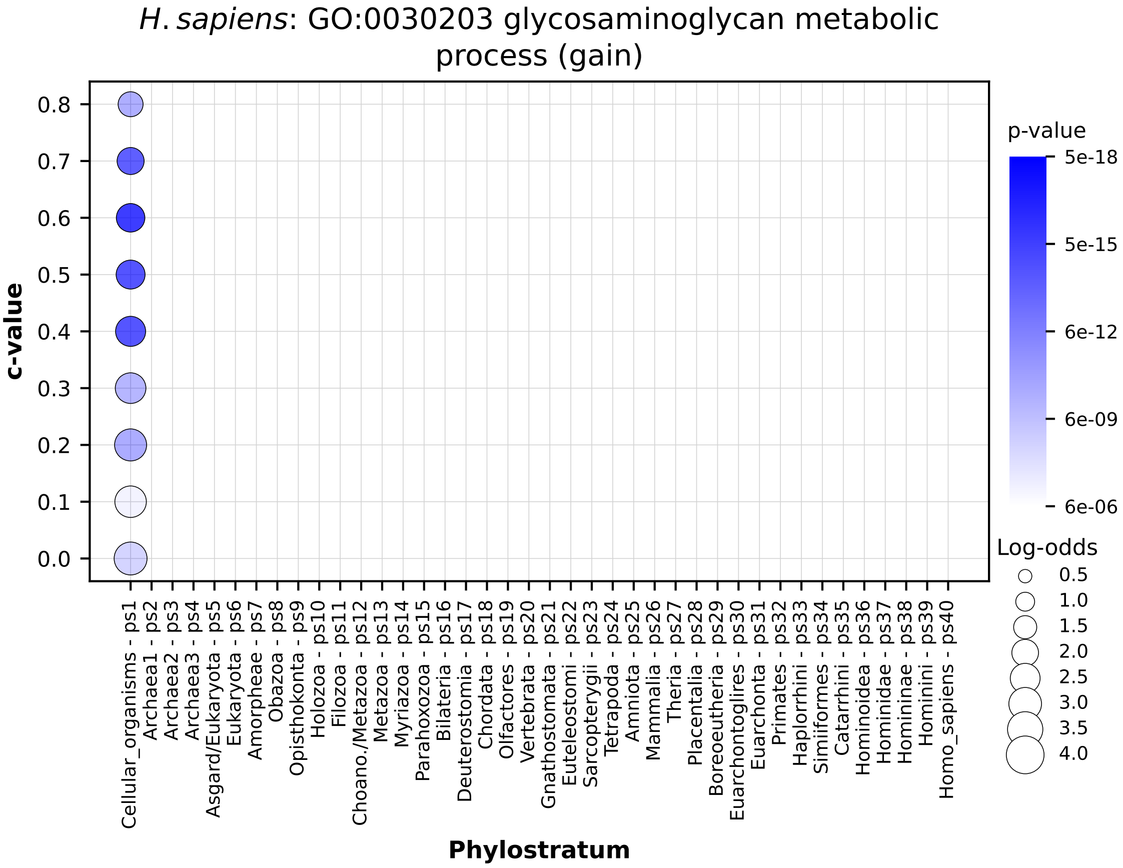

H. sapiens: GO:0030203 glycosaminoglycan metabolic process (gain)
c-value
0.8
0.7
0.6
0.5
0.4
0.3
0.2
0.1
0.0
p-value
5e-18
5e-15
6e-12
6e-09
6e-06
Log-odds
0.5
1.0
1.5
2.0
2.5
3.0
3.5
4.0
Cellular_organisms - ps1
Archaea1 - ps2
Archaea2 - ps3
Archaea3 - ps4
Asgard/Eukaryota - ps5
Eukaryota - ps6
Amorpheae - ps7
Obazoa - ps8
Opisthokonta - ps9
Holozoa - ps10
Filozoa - ps11
Choano./Metazoa - ps12
Metazoa - ps13
Myriazoa - ps14
Parahoxozoa - ps15
Bilateria - ps16
Deuterostomia - ps17
Chordata - ps18
Olfactores - ps19
Vertebrata - ps20
Gnathostomata - ps21
Euteleostomi - ps22
Sarcopterygii - ps23
Tetrapoda - ps24
Amniota - ps25
Mammalia - ps26
Theria - ps27
Placentalia - ps28
Boreoeutheria - ps29
Euarchontoglires - ps30
Euarchonta - ps31
Primates - ps32
Haplorrhini - ps33
Simiiformes - ps34
Catarrhini - ps35
Hominoidea - ps36
Hominidae - ps37
Homininae - ps38
Hominini - ps39
Homo_sapiens - ps40
Phylostratum

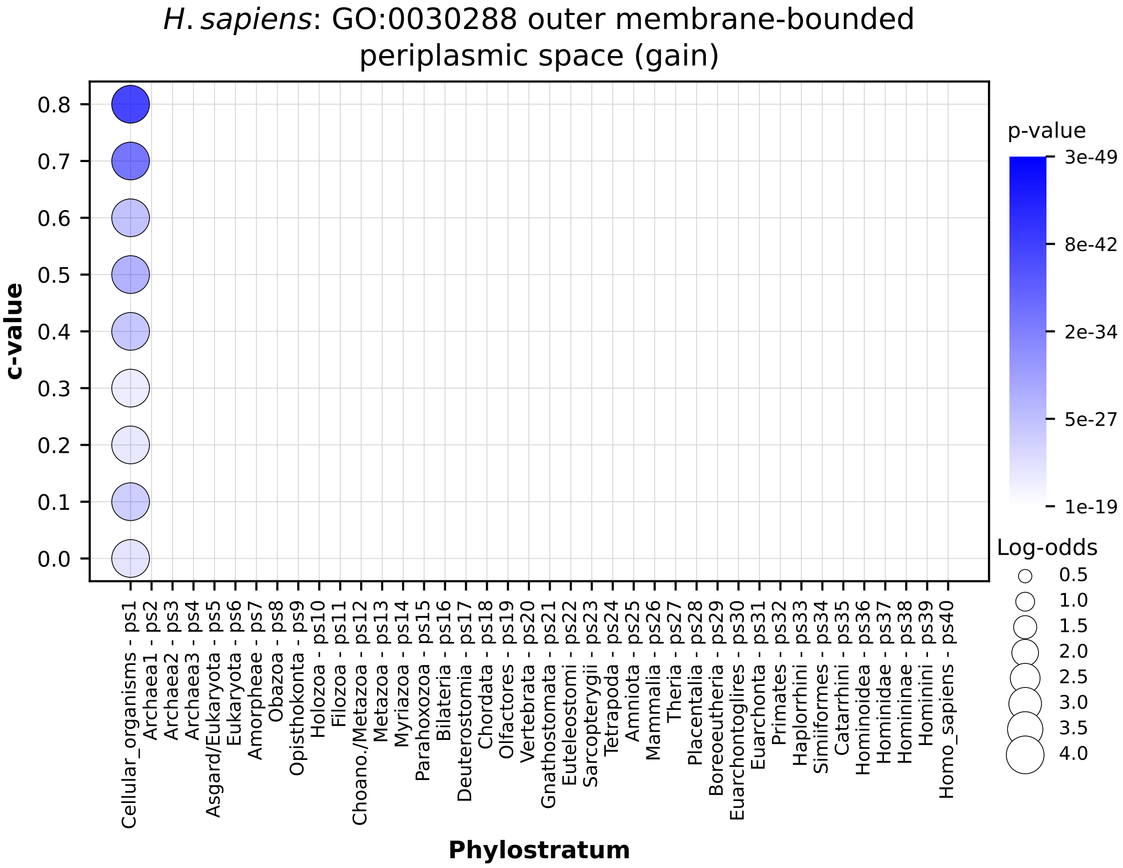

*H. sapiens*: GO:0030288 outer membrane-bounded periplasmic space (gain)
c-value
0.8
0.7
0.6
0.5
0.4
0.3
0.2
0.1
0.0
p-value
3e-49
8e-42
2e-34
5e-27
1e-19
Log-odds
0.5
1.0
1.5
2.0
2.5
3.0
3.5
4.0
Cellular_organisms - ps1
Archaea1 - ps2
Archaea2 - ps3
Archaea3 - ps4
Asgard/Eukaryota - ps5
Eukaryota - ps6
Amorpheae - ps7
Obazoa - ps8
Opisthokonta - ps9
Holozoa - ps10
Filozoa - ps11
Choano./Metazoa - ps12
Metazoa - ps13
Myriazoa - ps14
Parahoxozoa - ps15
Bilateria - ps16
Deuterostomia - ps17
Chordata - ps18
Olfactores - ps19
Vertebrata - ps20
Gnathostomata - ps21
Euteleostomi - ps22
Sarcopterygii - ps23
Tetrapoda - ps24
Amniota - ps25
Mammalia - ps26
Theria - ps27
Placentalia - ps28
Boreoeutheria - ps29
Euarchontoglires - ps30
Euarchonta - ps31
Primates - ps32
Haplorrhini - ps33
Simiiformes - ps34
Catarrhini - ps35
Hominoidea - ps36
Hominidae - ps37
Homininae - ps38
Hominini - ps39
Homo_sapiens - ps40
Phylostratum

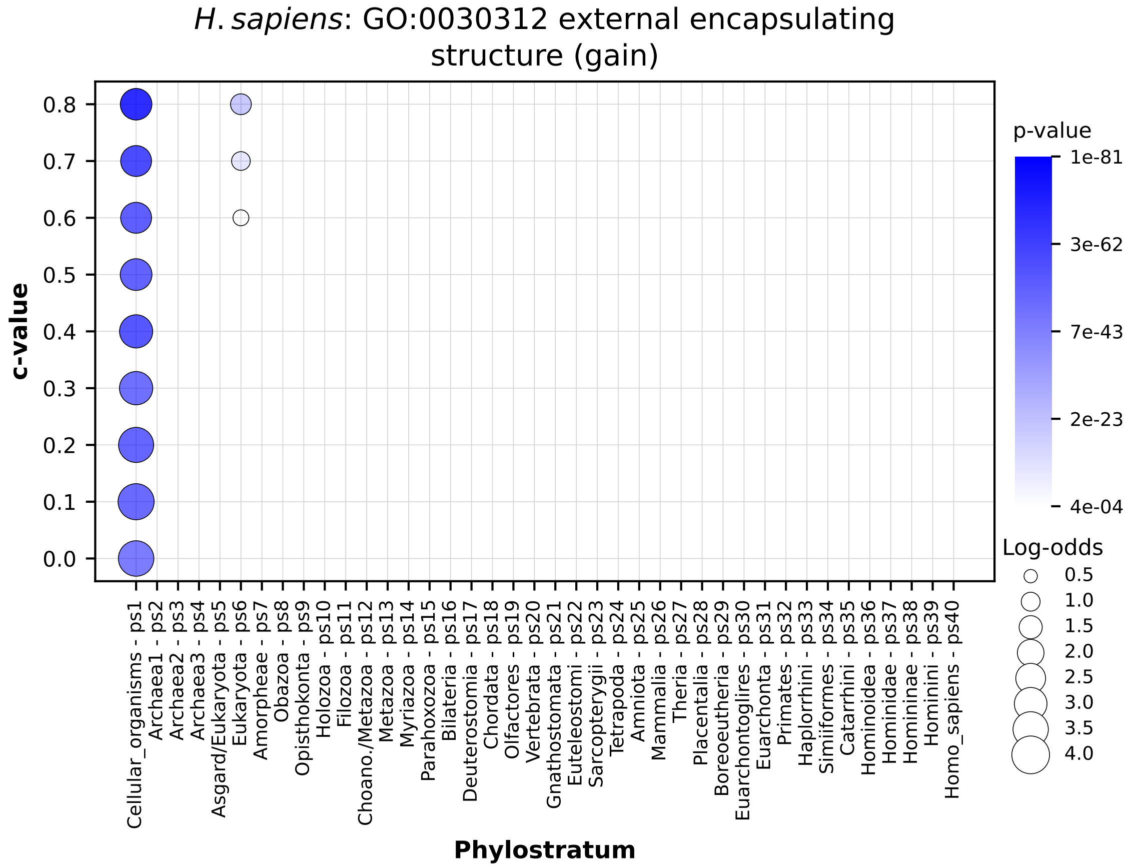

H. sapiens: GO:0030312 external encapsulating structure (gain)
c-value
0.8
0.7
0.6
0.5
0.4
0.3
0.2
0.1
0.0
Phylostratum
Cellular_organisms - ps1
Archaea1 - ps2
Archaea2 - ps3
Archaea3 - ps4
Asgard/Eukaryota - ps5
Eukaryota - ps6
Amorpheae - ps7
Obazoa - ps8
Opisthokonta - ps9
Holozoa - ps10
Filozoa - ps11
Choano./Metazoa - ps12
Metazoa - ps13
Myriazoa - ps14
Parahoxozoa - ps15
Bilateria - ps16
Deuterostomia - ps17
Chordata - ps18
Olfactores - ps19
Vertebrata - ps20
Gnathostomata - ps21
Euteleostomi - ps22
Sarcopterygii - ps23
Tetrapoda - ps24
Amniota - ps25
Mammalia - ps26
Theria - ps27
Placentalia - ps28
Boreoeutheria - ps29
Euarchontoglires - ps30
Euarchonta - ps31
Primates - ps32
Haplorrhini - ps33
Simiiformes - ps34
Catarrhini - ps35
Hominoidea - ps36
Hominidae - ps37
Homininae - ps38
Hominini - ps39
Homo_sapiens - ps40
p-value
1e-81
3e-62
7e-43
2e-23
4e-04
Log-odds
0.5
1.0
1.5
2.0
2.5
3.0
3.5
4.0

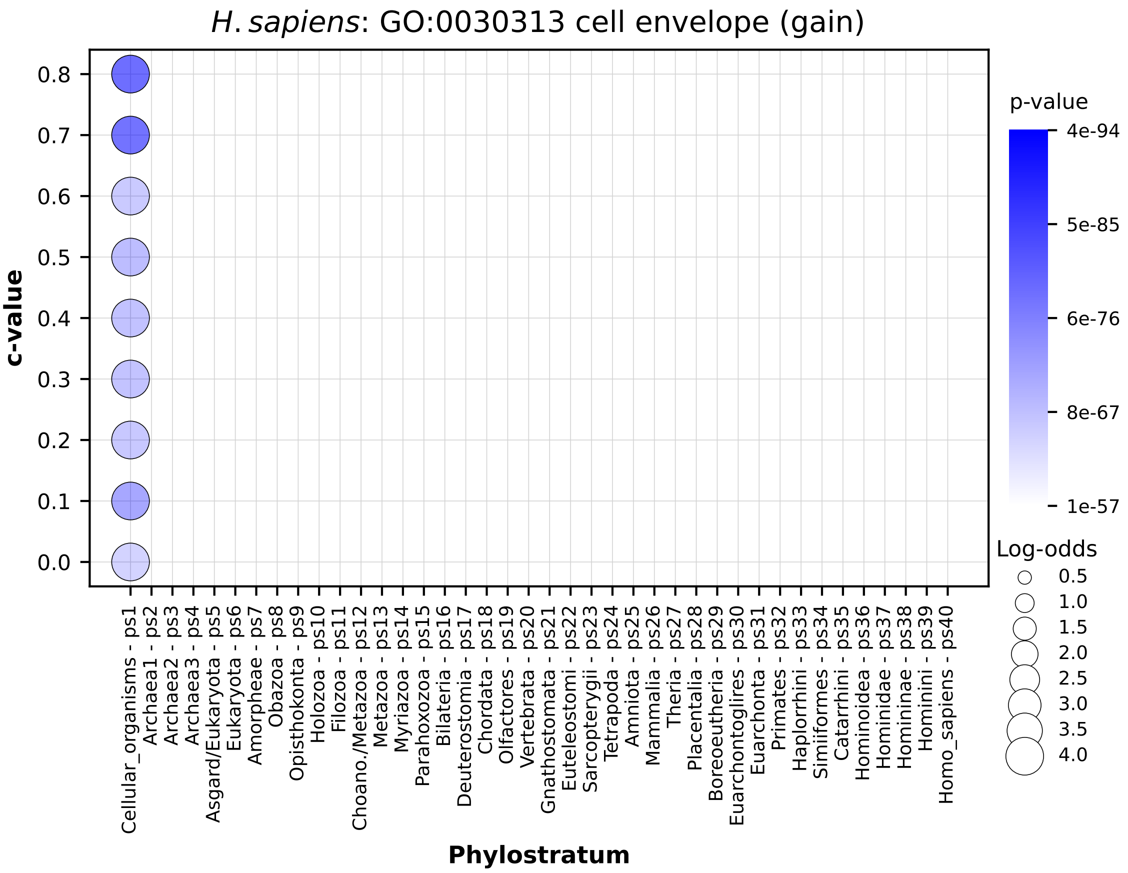

H. sapiens: GO:0030313 cell envelope (gain)
c-value
0.0
0.1
0.2
0.3
0.4
0.5
0.6
0.7
0.8
Phylostratum
Cellular_organisms - ps1
Archaea1 - ps2
Archaea2 - ps3
Archaea3 - ps4
Asgard/Eukaryota - ps5
Eukaryota - ps6
Amorpheae - ps7
Obazoa - ps8
Opisthokonta - ps9
Holozoa - ps10
Filozoa - ps11
Choano./Metazoa - ps12
Metazoa - ps13
Myriazoa - ps14
Parahoxozoa - ps15
Bilateria - ps16
Deuterostomia - ps17
Chordata - ps18
Olfactores - ps19
Vertebrata - ps20
Gnathostomata - ps21
Euteleostomi - ps22
Sarcopterygii - ps23
Tetrapoda - ps24
Amniota - ps25
Mammalia - ps26
Theria - ps27
Placentalia - ps28
Boreoeutheria - ps29
Euarchontoglires - ps30
Euarchonta - ps31
Primates - ps32
Haplorrhini - ps33
Simiiformes - ps34
Catarrhini - ps35
Hominoidea - ps36
Hominidae - ps37
Homininae - ps38
Hominini - ps39
Homo_sapiens - ps40
p-value
4e-94
5e-85
6e-76
8e-67
1e-57
Log-odds
0.5
1.0
1.5
2.0
2.5
3.0
3.5
4.0

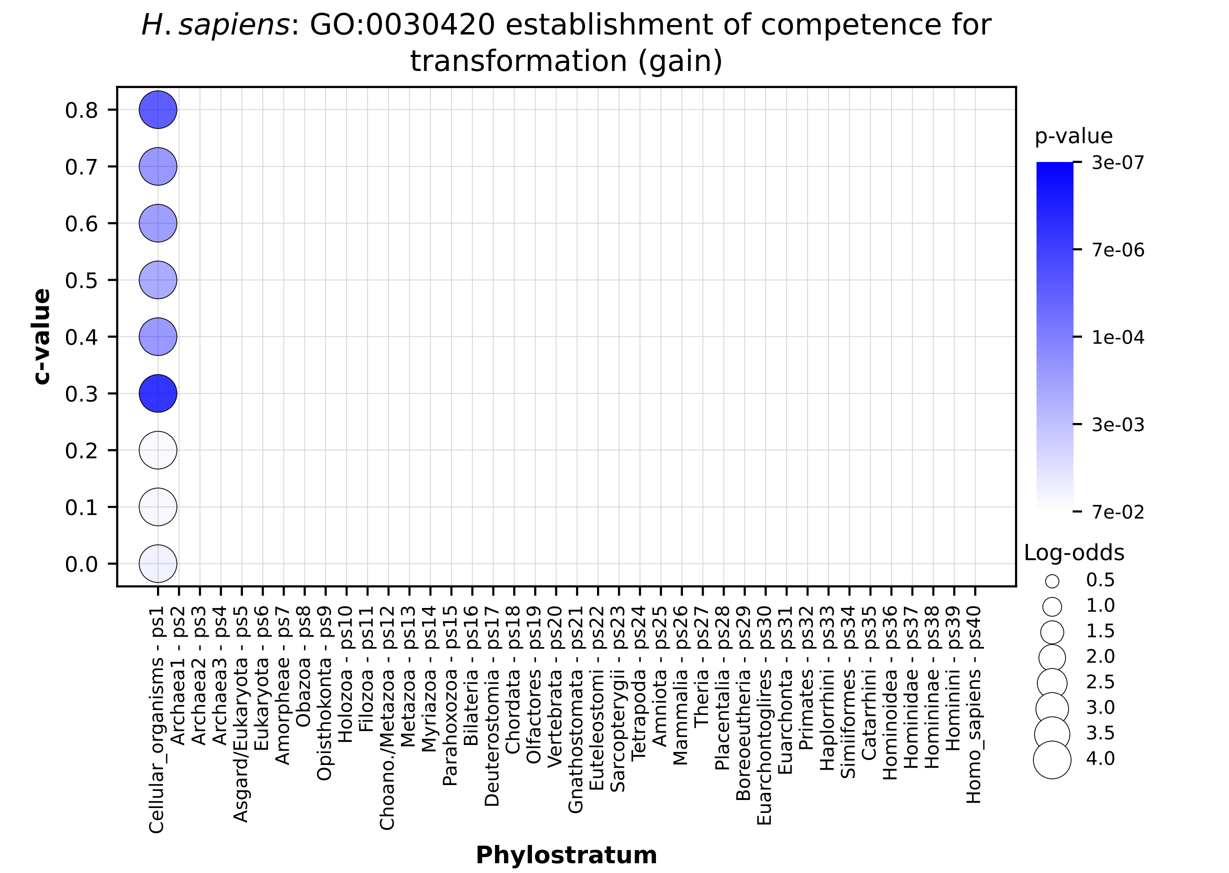
H. sapiens: GO:0030420 establishment of competence for transformation (gain)
c-value
Phylostratum
p-value
3e-07
7e-06
1e-04
3e-03
7e-02
Log-odds
0.5
1.0
1.5
2.0
2.5
3.0
3.5
4.0
0.0
0.1
0.2
0.3
0.4
0.5
0.6
0.7
0.8
Cellular_organisms - ps1
Archaea1 - ps2
Archaea2 - ps3
Archaea3 - ps4
Asgard/Eukaryota - ps5
Eukaryota - ps6
Amorpheae - ps7
Obazoa - ps8
Opisthokonta - ps9
Holozoa - ps10
Filozoa - ps11
Choano./Metazoa - ps12
Metazoa - ps13
Myriazoa - ps14
Parahoxozoa - ps15
Bilateria - ps16
Deuterostomia - ps17
Chordata - ps18
Olfactores - ps19
Vertebrata - ps20
Gnathostomata - ps21
Euteleostomi - ps22
Sarcopterygii - ps23
Tetrapoda - ps24
Amniota - ps25
Mammalia - ps26
Theria - ps27
Placentalia - ps28
Boreoeutheria - ps29
Euarchontoglires - ps30
Euarchonta - ps31
Primates - ps32
Haplorrhini - ps33
Simiiformes - ps34
Catarrhini - ps35
Hominoidea - ps36
Hominidae - ps37
Homininae - ps38
Hominini - ps39
Homo_sapiens - ps40

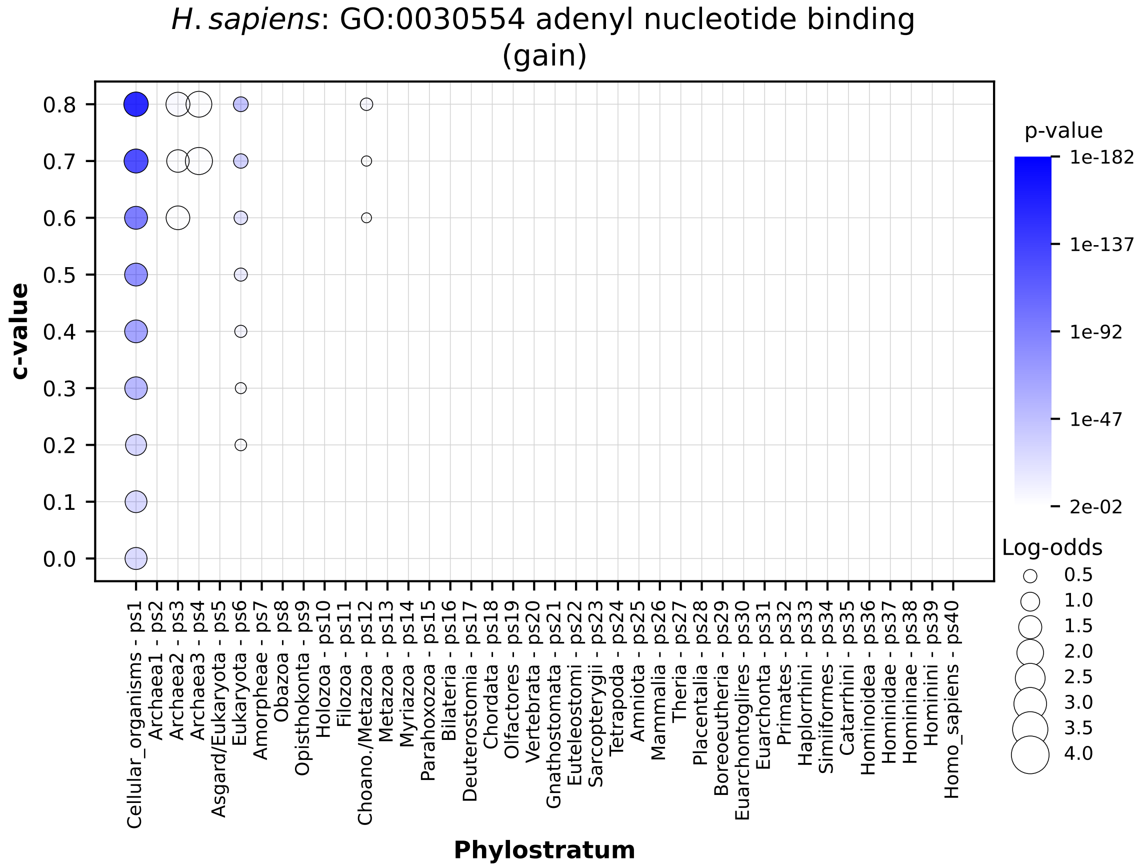

H. sapiens: GO:0030554 adenyl nucleotide binding
(gain)
c-value
0.8
0.7
0.6
0.5
0.4
0.3
0.2
0.1
0.0
Phylostratum
Cellular_organisms - ps1
Archaea1 - ps2
Archaea2 - ps3
Archaea3 - ps4
Asgard/Eukaryota - ps5
Eukaryota - ps6
Amorpheae - ps7
Obazoa - ps8
Opisthokonta - ps9
Holozoa - ps10
Filozoa - ps11
Choano./Metazoa - ps12
Metazoa - ps13
Myriazoa - ps14
Parahoxozoa - ps15
Bilateria - ps16
Deuterostomia - ps17
Chordata - ps18
Olfactores - ps19
Vertebrata - ps20
Gnathostomata - ps21
Euteleostomi - ps22
Sarcopterygii - ps23
Tetrapoda - ps24
Amniota - ps25
Mammalia - ps26
Theria - ps27
Placentalia - ps28
Boreoeutheria - ps29
Euarchontoglires - ps30
Euarchonta - ps31
Primates - ps32
Haplorrhini - ps33
Simiiformes - ps34
Catarrhini - ps35
Hominoidea - ps36
Hominidae - ps37
Homininae - ps38
Hominini - ps39
Homo_sapiens - ps40
p-value
1e-182
1e-137
1e-92
1e-47
2e-02
Log-odds
0.5
1.0
1.5
2.0
2.5
3.0
3.5
4.0

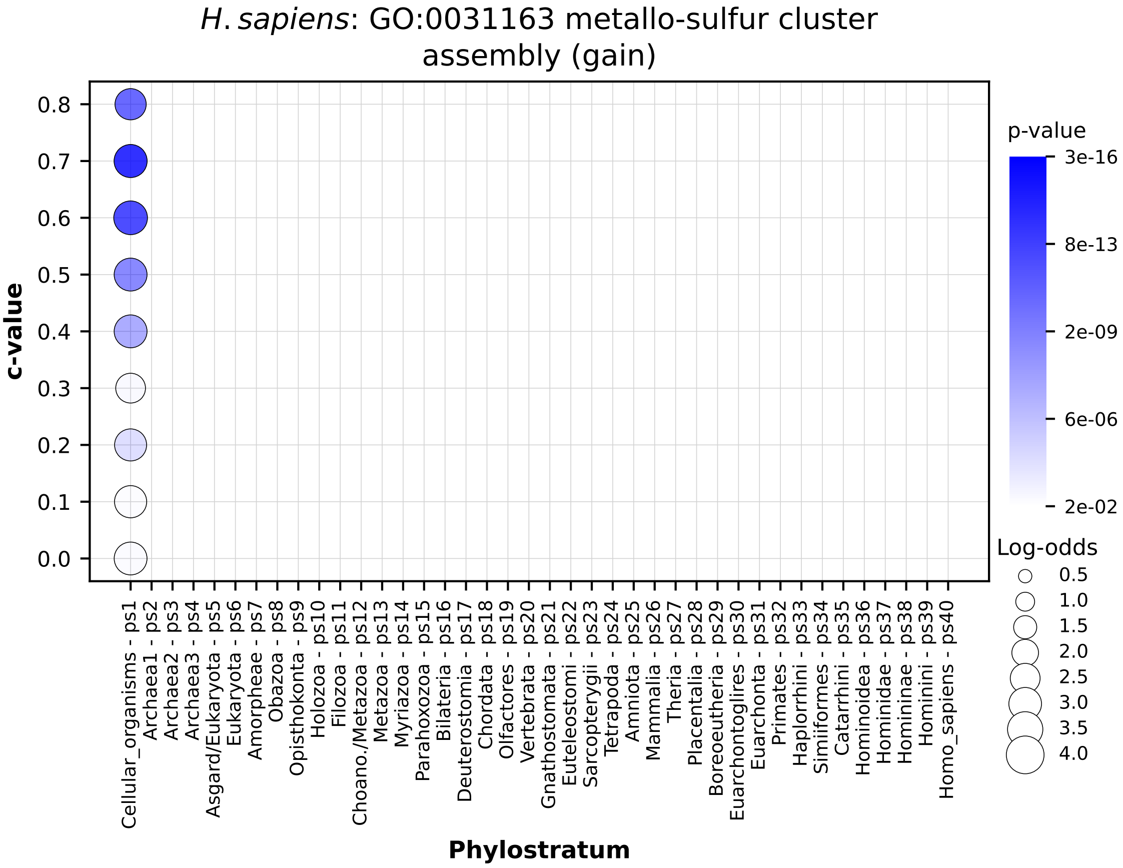

*H. sapiens*: GO:0031163 metallo-sulfur cluster assembly (gain)
c-value
0.0
0.1
0.2
0.3
0.4
0.5
0.6
0.7
0.8
p-value
3e-16
8e-13
2e-09
6e-06
2e-02
Log-odds
0.5
1.0
1.5
2.0
2.5
3.0
3.5
4.0
Cellular_organisms - ps1
Archaea1 - ps2
Archaea2 - ps3
Archaea3 - ps4
Asgard/Eukaryota - ps5
Eukaryota - ps6
Amorpheae - ps7
Obazoa - ps8
Opisthokonta - ps9
Holozoa - ps10
Filozoa - ps11
Choano./Metazoa - ps12
Metazoa - ps13
Myriazoa - ps14
Parahoxozoa - ps15
Bilateria - ps16
Deuterostomia - ps17
Chordata - ps18
Olfactores - ps19
Vertebrata - ps20
Gnathostomata - ps21
Euteleostomi - ps22
Sarcopterygii - ps23
Tetrapoda - ps24
Amniota - ps25
Mammalia - ps26
Theria - ps27
Placentalia - ps28
Boreoeutheria - ps29
Euarchontoglires - ps30
Euarchonta - ps31
Primates - ps32
Haplorrhini - ps33
Simiiformes - ps34
Catarrhini - ps35
Hominoidea - ps36
Hominidae - ps37
Homininae - ps38
Hominini - ps39
Homo_sapiens - ps40
Phylostratum

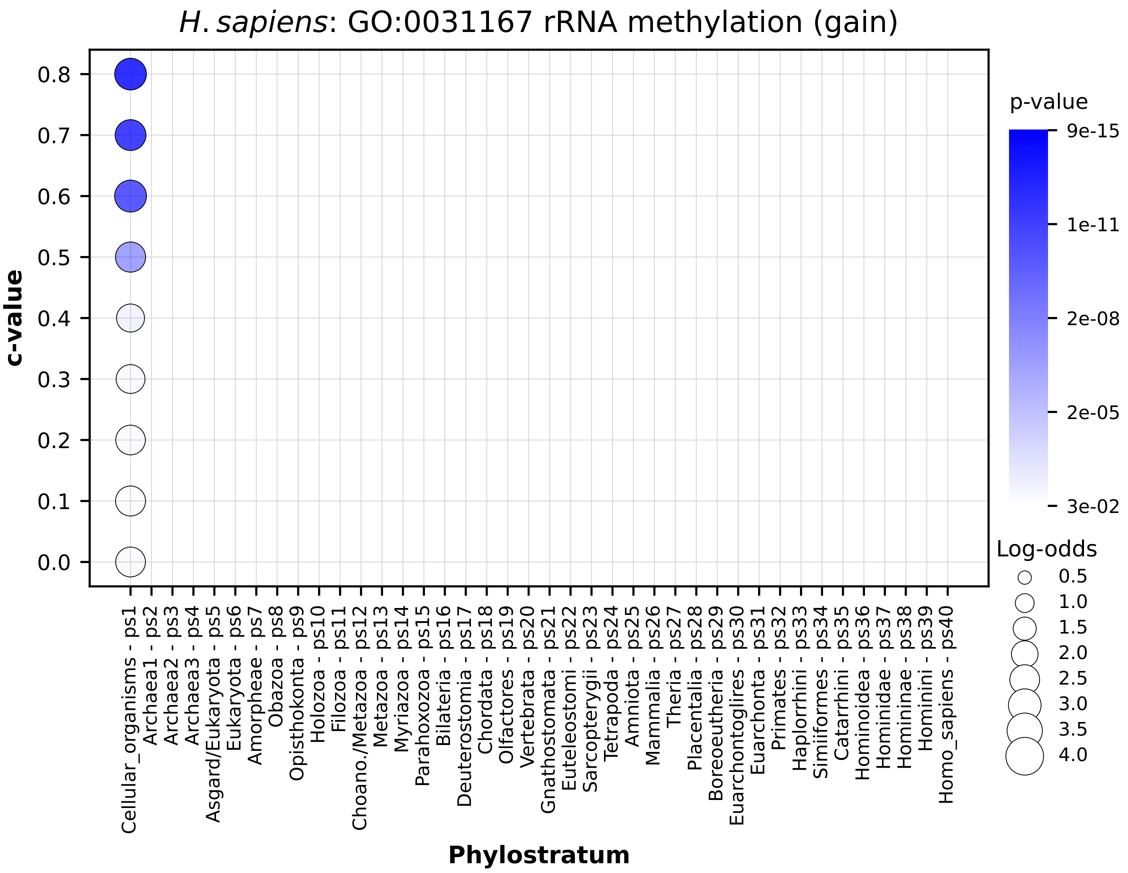
H. sapiens: GO:0031167 rRNA methylation (gain)
c-value
0.8
0.7
0.6
0.5
0.4
0.3
0.2
0.1
0.0
Phylostratum
Cellular_organisms - ps1
Archaea1 - ps2
Archaea2 - ps3
Archaea3 - ps4
Asgard/Eukaryota - ps5
Eukaryota - ps6
Amorpheae - ps7
Obazoa - ps8
Opisthokonta - ps9
Holozoa - ps10
Filozoa - ps11
Choano./Metazoa - ps12
Metazoa - ps13
Myriazoa - ps14
Parahoxozoa - ps15
Bilateria - ps16
Deuterostomia - ps17
Chordata - ps18
Olfactores - ps19
Vertebrata - ps20
Gnathostomata - ps21
Euteleostomi - ps22
Sarcopterygii - ps23
Tetrapoda - ps24
Amniota - ps25
Mammalia - ps26
Theria - ps27
Placentalia - ps28
Boreoeutheria - ps29
Euarchontoglires - ps30
Euarchonta - ps31
Primates - ps32
Haplorrhini - ps33
Simiiformes - ps34
Catarrhini - ps35
Hominoidea - ps36
Hominidae - ps37
Homininae - ps38
Hominini - ps39
Homo_sapiens - ps40
p-value
9e-15
1e-11
2e-08
2e-05
3e-02
Log-odds
0.5
1.0
1.5
2.0
2.5
3.0
3.5
4.0

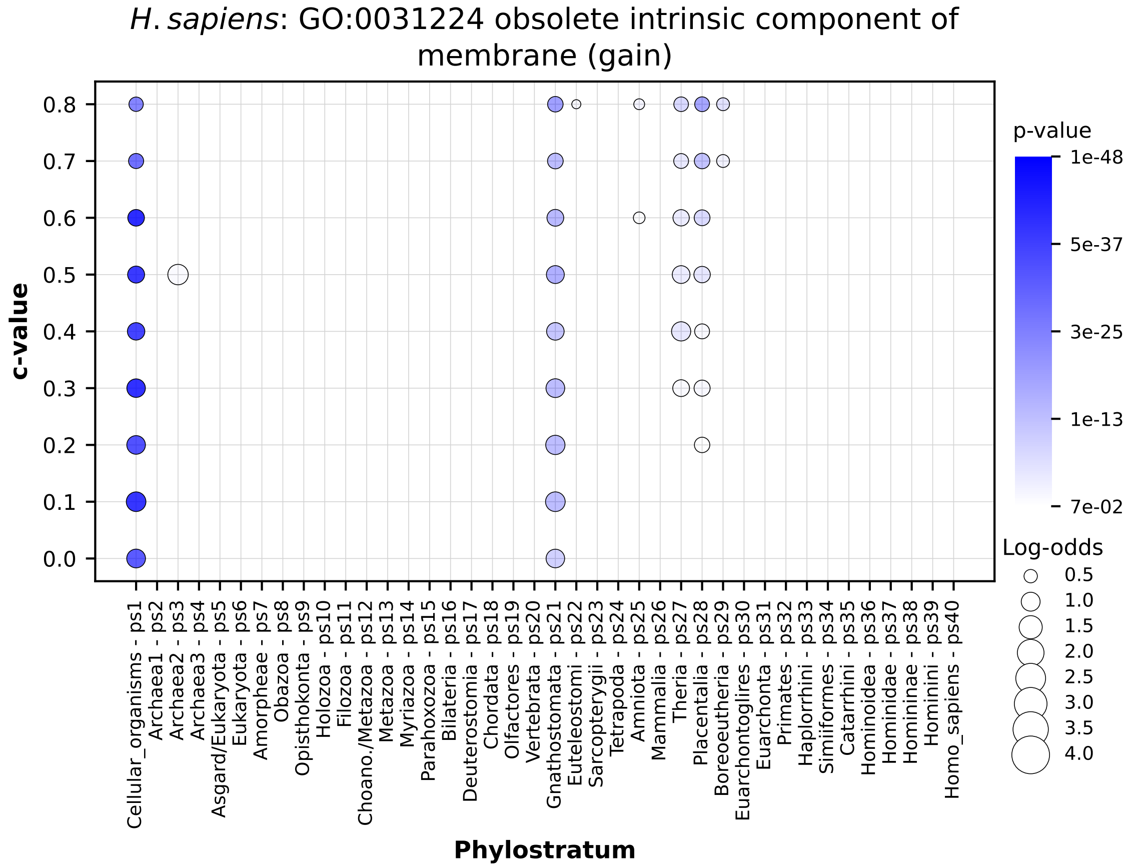

H. sapiens: GO:0031224 obsolete intrinsic component of membrane (gain)
c-value
0.0
0.1
0.2
0.3
0.4
0.5
0.6
0.7
0.8
Phylostratum
Cellular_organisms - ps1
Archaea1 - ps2
Archaea2 - ps3
Archaea3 - ps4
Asgard/Eukaryota - ps5
Eukaryota - ps6
Amorpheae - ps7
Obazoa - ps8
Opisthokonta - ps9
Holozoa - ps10
Filozoa - ps11
Choano./Metazoa - ps12
Metazoa - ps13
Myriazoa - ps14
Parahoxozoa - ps15
Bilateria - ps16
Deuterostomia - ps17
Chordata - ps18
Olfactores - ps19
Vertebrata - ps20
Gnathostomata - ps21
Euteleostomi - ps22
Sarcopterygii - ps23
Tetrapoda - ps24
Amniota - ps25
Mammalia - ps26
Theria - ps27
Placentalia - ps28
Boreoeutheria - ps29
Euarchontoglires - ps30
Euarchonta - ps31
Primates - ps32
Haplorrhini - ps33
Simiiformes - ps34
Catarrhini - ps35
Hominoidea - ps36
Hominidae - ps37
Homininae - ps38
Hominini - ps39
Homo_sapiens - ps40
p-value
1e-48
5e-37
3e-25
1e-13
7e-02
Log-odds
0.5
1.0
1.5
2.0
2.5
3.0
3.5
4.0

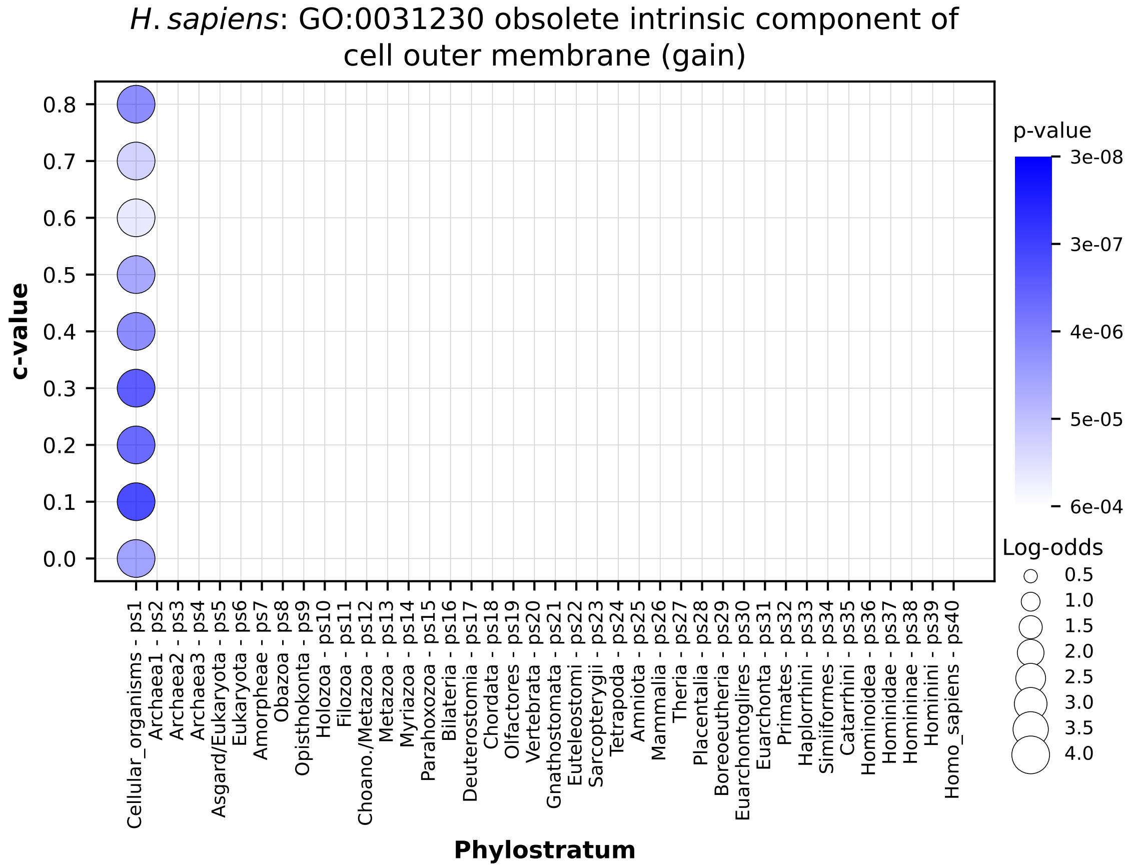

H. sapiens: GO:0031230 obsolete intrinsic component of cell outer membrane (gain)
c-value
0.0
0.1
0.2
0.3
0.4
0.5
0.6
0.7
0.8
Phylostratum
Cellular_organisms - ps1
Archaea1 - ps2
Archaea2 - ps3
Archaea3 - ps4
Asgard/Eukaryota - ps5
Eukaryota - ps6
Amorpheae - ps7
Obazoa - ps8
Opisthokonta - ps9
Holozoa - ps10
Filozoa - ps11
Choano./Metazoa - ps12
Metazoa - ps13
Myriazoa - ps14
Parahoxozoa - ps15
Bilateria - ps16
Deuterostomia - ps17
Chordata - ps18
Olfactores - ps19
Vertebrata - ps20
Gnathostomata - ps21
Euteleostomi - ps22
Sarcopterygii - ps23
Tetrapoda - ps24
Amniota - ps25
Mammalia - ps26
Theria - ps27
Placentalia - ps28
Boreoeutheria - ps29
Euarchontoglires - ps30
Euarchonta - ps31
Primates - ps32
Haplorrhini - ps33
Simiiformes - ps34
Catarrhini - ps35
Hominoidea - ps36
Hominidae - ps37
Homininae - ps38
Hominini - ps39
Homo_sapiens - ps40
p-value
3e-08
3e-07
4e-06
5e-05
6e-04
Log-odds
0.5
1.0
1.5
2.0
2.5
3.0
3.5
4.0

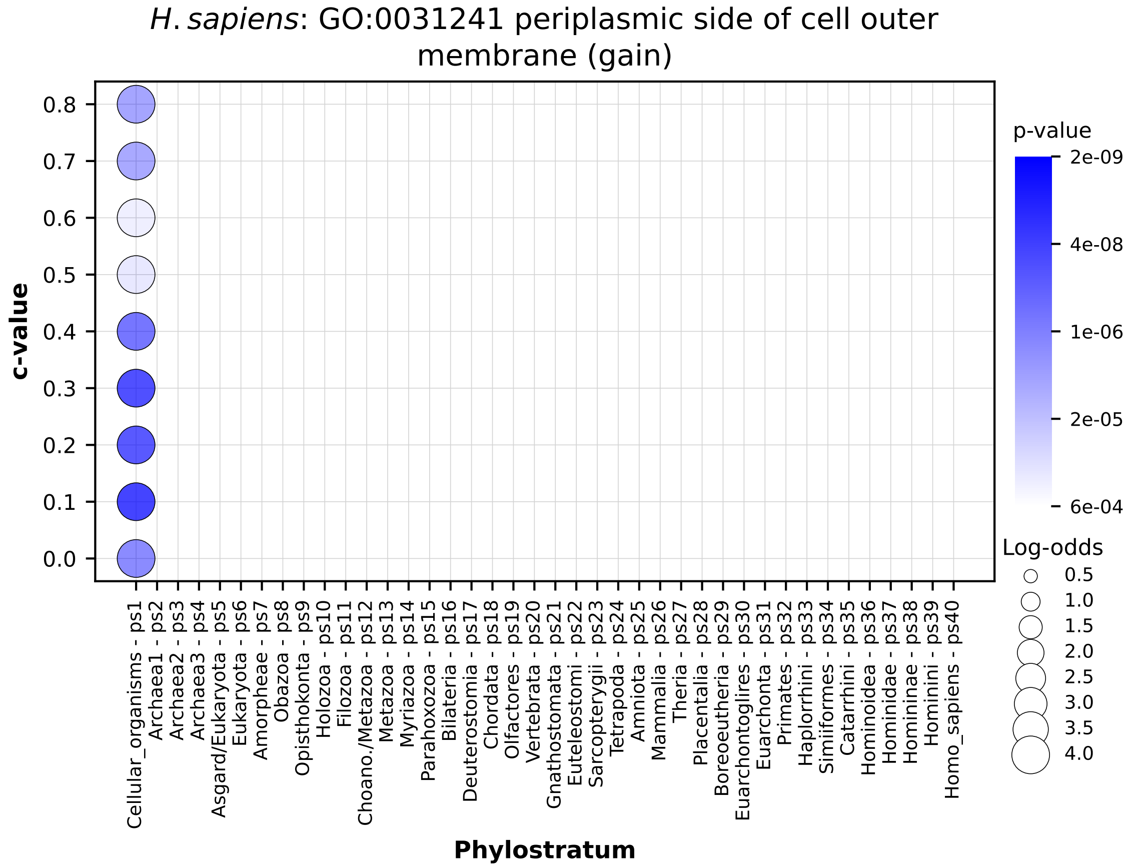

H. sapiens: GO:0031241 periplasmic side of cell outer membrane (gain)
c-value
0.8
0.7
0.6
0.5
0.4
0.3
0.2
0.1
0.0
Phylostratum
Cellular_organisms - ps1
Archaea1 - ps2
Archaea2 - ps3
Archaea3 - ps4
Asgard/Eukaryota - ps5
Eukaryota - ps6
Amorpheae - ps7
Obazoa - ps8
Opisthokonta - ps9
Holozoa - ps10
Filozoa - ps11
Choano./Metazoa - ps12
Metazoa - ps13
Myriazoa - ps14
Parahoxozoa - ps15
Bilateria - ps16
Deuterostomia - ps17
Chordata - ps18
Olfactores - ps19
Vertebrata - ps20
Gnathostomata - ps21
Euteleostomi - ps22
Sarcopterygii - ps23
Tetrapoda - ps24
Amniota - ps25
Mammalia - ps26
Theria - ps27
Placentalia - ps28
Boreoeutheria - ps29
Euarchontoglires - ps30
Euarchonta - ps31
Primates - ps32
Haplorrhini - ps33
Simiiformes - ps34
Catarrhini - ps35
Hominoidea - ps36
Hominidae - ps37
Homininae - ps38
Hominini - ps39
Homo_sapiens - ps40
p-value
2e-09
4e-08
1e-06
2e-05
6e-04
Log-odds
0.5
1.0
1.5
2.0
2.5
3.0
3.5
4.0

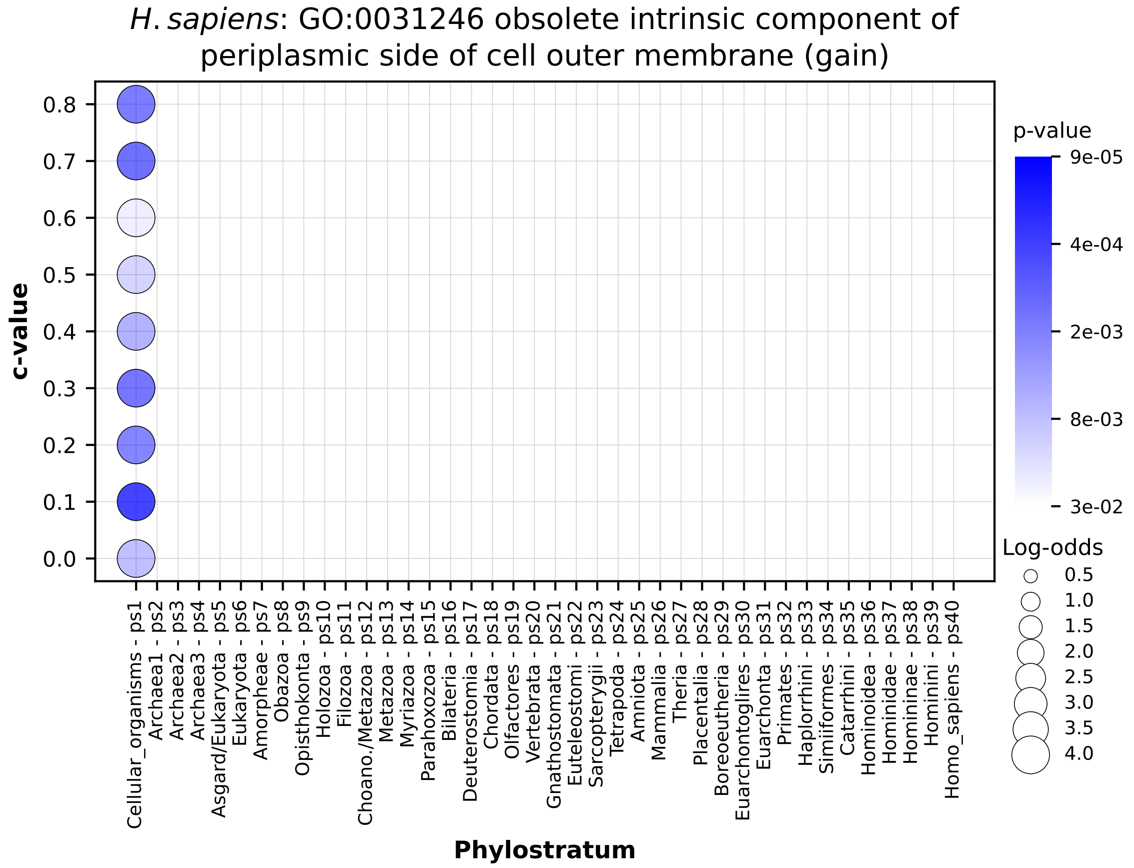

*H. sapiens*: GO:0031246 obsolete intrinsic component of periplasmic side of cell outer membrane (gain)
c-value
0.8
0.7
0.6
0.5
0.4
0.3
0.2
0.1
0.0
p-value
9e-05
4e-04
2e-03
8e-03
3e-02
Log-odds
0.5
1.0
1.5
2.0
2.5
3.0
3.5
4.0
Cellular_organisms - ps1
Archaea1 - ps2
Archaea2 - ps3
Archaea3 - ps4
Asgard/Eukaryota - ps5
Eukaryota - ps6
Amorpheae - ps7
Obazoa - ps8
Opisthokonta - ps9
Holozoa - ps10
Filozoa - ps11
Choano./Metazoa - ps12
Metazoa - ps13
Myriazoa - ps14
Parahoxozoa - ps15
Bilateria - ps16
Deuterostomia - ps17
Chordata - ps18
Olfactores - ps19
Vertebrata - ps20
Gnathostomata - ps21
Euteleostomi - ps22
Sarcopterygii - ps23
Tetrapoda - ps24
Amniota - ps25
Mammalia - ps26
Theria - ps27
Placentalia - ps28
Boreoeutheria - ps29
Euarchontoglires - ps30
Euarchonta - ps31
Primates - ps32
Haplorrhini - ps33
Simiiformes - ps34
Catarrhini - ps35
Hominoidea - ps36
Hominidae - ps37
Homininae - ps38
Hominini - ps39
Homo_sapiens - ps40
Phylostratum

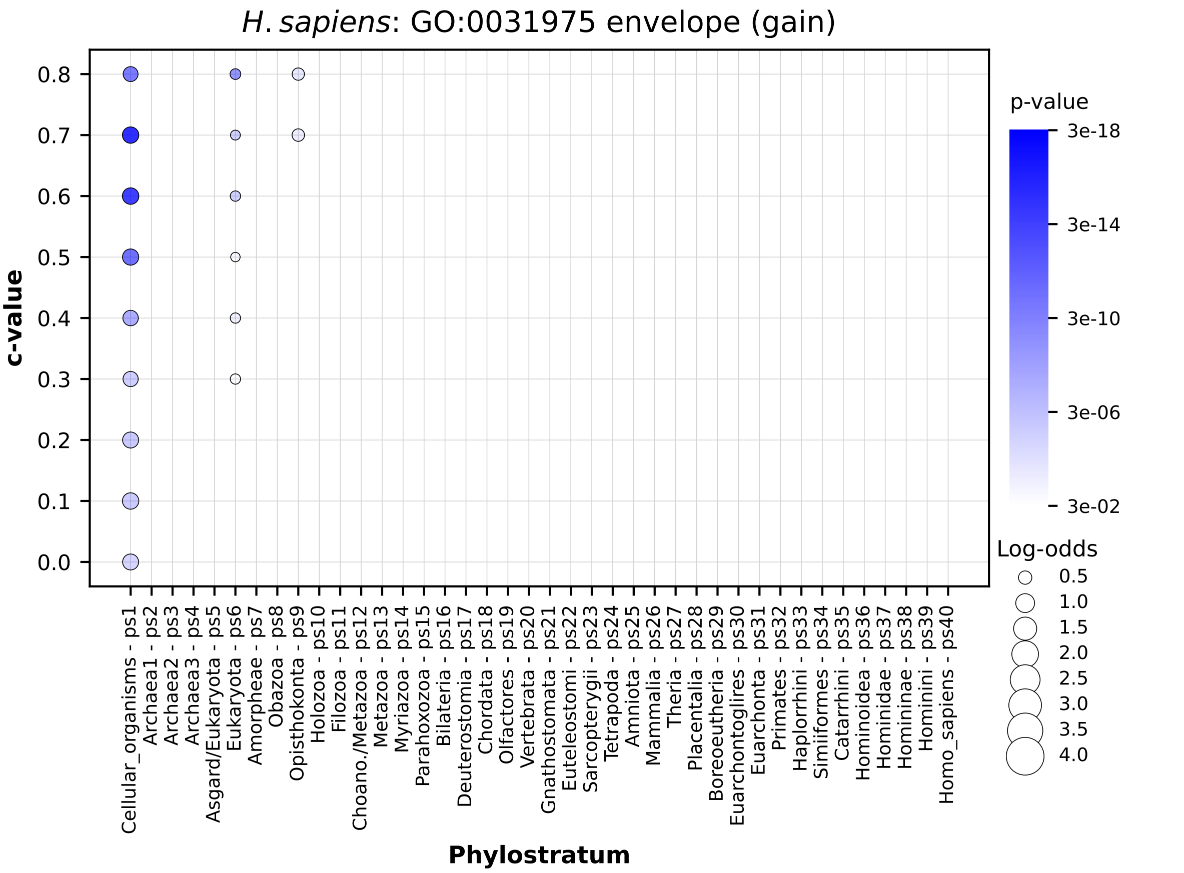

H. sapiens: GO:0031975 envelope (gain)
c-value
0.8
0.7
0.6
0.5
0.4
0.3
0.2
0.1
0.0
Phylostratum
Cellular_organisms - ps1
Archaea1 - ps2
Archaea2 - ps3
Archaea3 - ps4
Asgard/Eukaryota - ps5
Eukaryota - ps6
Amorpheae - ps7
Obazoa - ps8
Opisthokonta - ps9
Holozoa - ps10
Filozoa - ps11
Choano./Metazoa - ps12
Metazoa - ps13
Myriazoa - ps14
Parahoxozoa - ps15
Bilateria - ps16
Deuterostomia - ps17
Chordata - ps18
Olfactores - ps19
Vertebrata - ps20
Gnathostomata - ps21
Euteleostomi - ps22
Sarcopterygii - ps23
Tetrapoda - ps24
Amniota - ps25
Mammalia - ps26
Theria - ps27
Placentalia - ps28
Boreoeutheria - ps29
Euarchontoglires - ps30
Euarchonta - ps31
Primates - ps32
Haplorrhini - ps33
Simiiformes - ps34
Catarrhini - ps35
Hominoidea - ps36
Hominidae - ps37
Homininae - ps38
Hominini - ps39
Homo_sapiens - ps40
p-value
3e-18
3e-14
3e-10
3e-06
3e-02
Log-odds
0.5
1.0
1.5
2.0
2.5
3.0
3.5
4.0

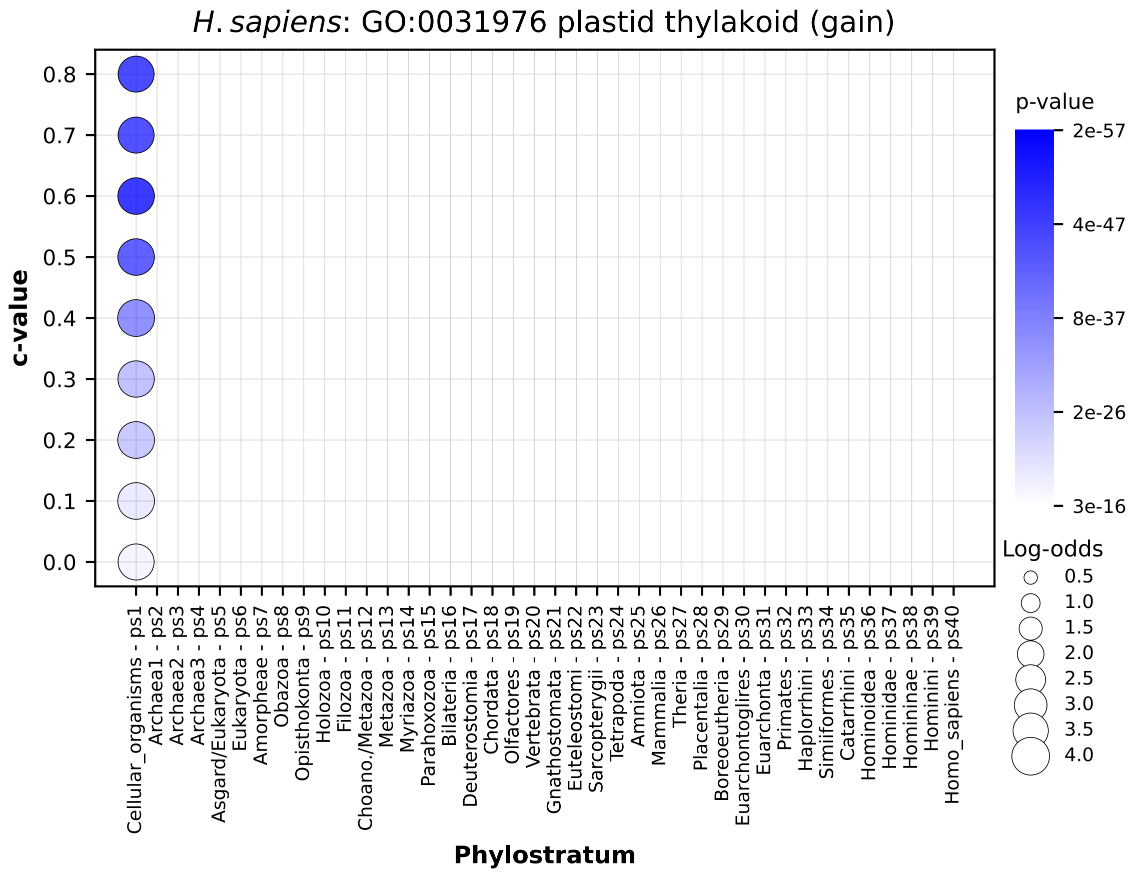

H. sapiens: GO:0031976 plastid thylakoid (gain)
c-value
0.8
0.7
0.6
0.5
0.4
0.3
0.2
0.1
0.0
p-value
2e-57
4e-47
8e-37
2e-26
3e-16
Log-odds
0.5
1.0
1.5
2.0
2.5
3.0
3.5
4.0
Cellular_organisms - ps1
Archaea1 - ps2
Archaea2 - ps3
Archaea3 - ps4
Asgard/Eukaryota - ps5
Eukaryota - ps6
Amorpheae - ps7
Obazoa - ps8
Opisthokonta - ps9
Holozoa - ps10
Filozoa - ps11
Choano./Metazoa - ps12
Metazoa - ps13
Myriazoa - ps14
Parahoxozoa - ps15
Bilateria - ps16
Deuterostomia - ps17
Chordata - ps18
Olfactores - ps19
Vertebrata - ps20
Gnathostomata - ps21
Euteleostomi - ps22
Sarcopterygii - ps23
Tetrapoda - ps24
Amniota - ps25
Mammalia - ps26
Theria - ps27
Placentalia - ps28
Boreoeutheria - ps29
Euarchontoglires - ps30
Euarchonta - ps31
Primates - ps32
Haplorrhini - ps33
Simiiformes - ps34
Catarrhini - ps35
Hominoidea - ps36
Hominidae - ps37
Homininae - ps38
Hominini - ps39
Homo_sapiens - ps40
Phylostratum

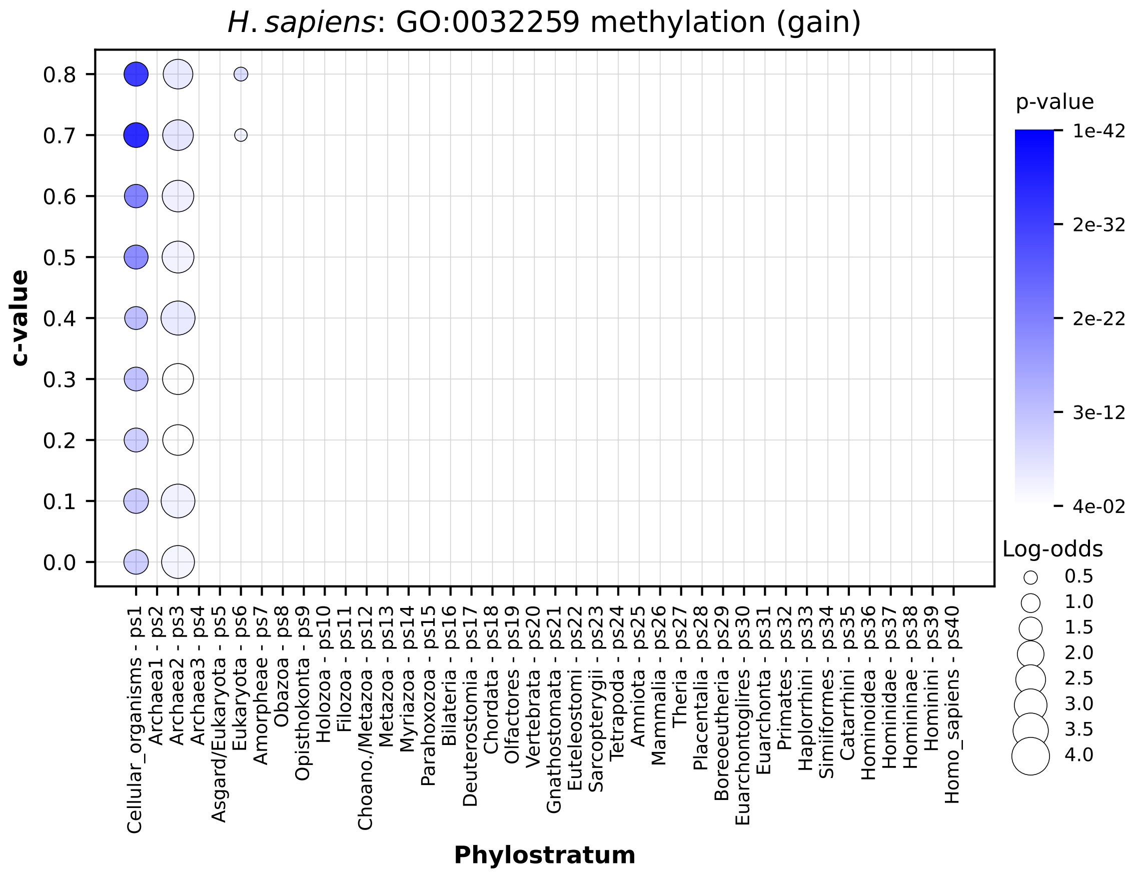

H. sapiens: GO:0032259 methylation (gain)
c-value
0.0
0.1
0.2
0.3
0.4
0.5
0.6
0.7
0.8
Phylostratum
Cellular_organisms - ps1
Archaea1 - ps2
Archaea2 - ps3
Archaea3 - ps4
Asgard/Eukaryota - ps5
Eukaryota - ps6
Amorpheae - ps7
Obazoa - ps8
Opisthokonta - ps9
Holozoa - ps10
Filozoa - ps11
Choano./Metazoa - ps12
Metazoa - ps13
Myriazoa - ps14
Parahoxozoa - ps15
Bilateria - ps16
Deuterostomia - ps17
Chordata - ps18
Olfactores - ps19
Vertebrata - ps20
Gnathostomata - ps21
Euteleostomi - ps22
Sarcopterygii - ps23
Tetrapoda - ps24
Amniota - ps25
Mammalia - ps26
Theria - ps27
Placentalia - ps28
Boreoeutheria - ps29
Euarchontoglires - ps30
Euarchonta - ps31
Primates - ps32
Haplorrhini - ps33
Simiiformes - ps34
Catarrhini - ps35
Hominoidea - ps36
Hominidae - ps37
Homininae - ps38
Hominini - ps39
Homo_sapiens - ps40
p-value
1e-42
2e-32
2e-22
3e-12
4e-02
Log-odds
0.5
1.0
1.5
2.0
2.5
3.0
3.5
4.0

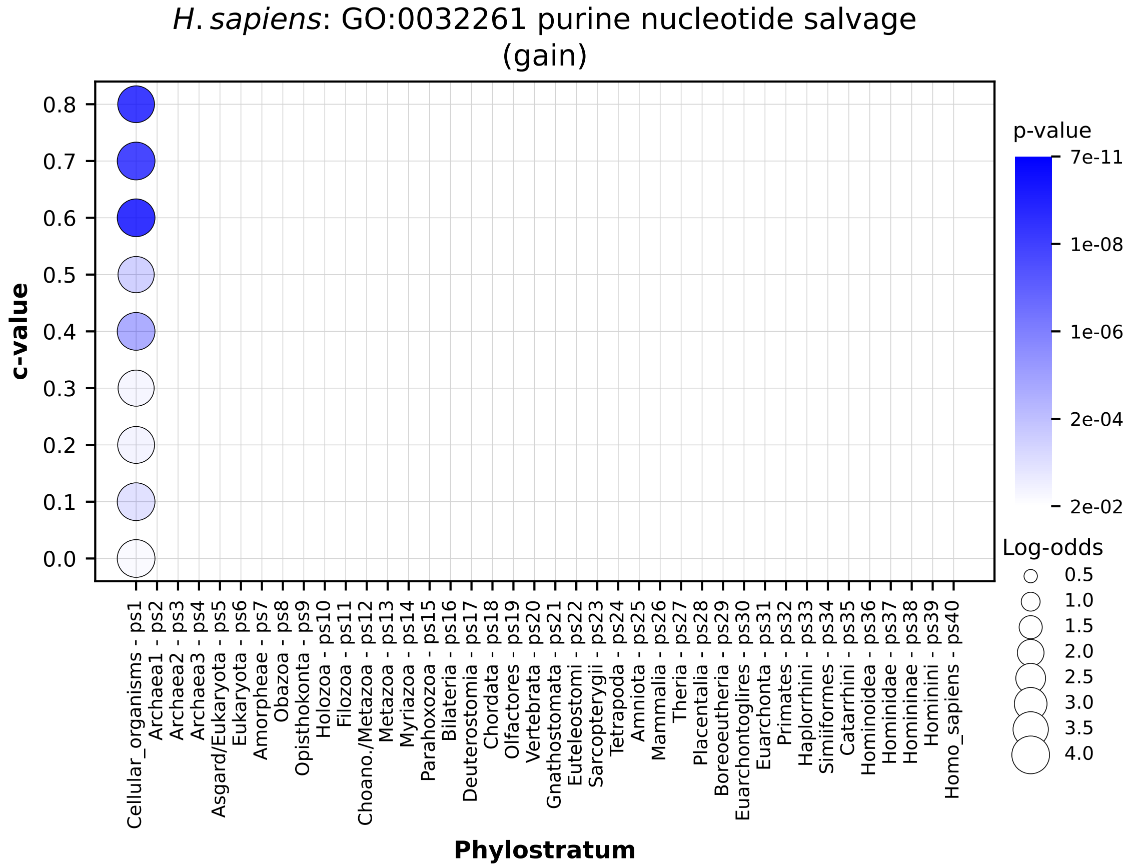
H. sapiens: GO:0032261 purine nucleotide salvage
(gain)
c-value
0.8
0.7
0.6
0.5
0.4
0.3
0.2
0.1
0.0
p-value
7e-11
1e-08
1e-06
2e-04
2e-02
Log-odds
0.5
1.0
1.5
2.0
2.5
3.0
3.5
4.0
Cellular_organisms - ps1
Archaea1 - ps2
Archaea2 - ps3
Archaea3 - ps4
Asgard/Eukaryota - ps5
Eukaryota - ps6
Amorpheae - ps7
Obazoa - ps8
Opisthokonta - ps9
Holozoa - ps10
Filozoa - ps11
Choano./Metazoa - ps12
Metazoa - ps13
Myriazoa - ps14
Parahoxozoa - ps15
Bilateria - ps16
Deuterostomia - ps17
Chordata - ps18
Olfactores - ps19
Vertebrata - ps20
Gnathostomata - ps21
Euteleostomi - ps22
Sarcopterygii - ps23
Tetrapoda - ps24
Amniota - ps25
Mammalia - ps26
Theria - ps27
Placentalia - ps28
Boreoeutheria - ps29
Euarchontoglires - ps30
Euarchonta - ps31
Primates - ps32
Haplorrhini - ps33
Simiiformes - ps34
Catarrhini - ps35
Hominoidea - ps36
Hominidae - ps37
Homininae - ps38
Hominini - ps39
Homo_sapiens - ps40
Phylostratum

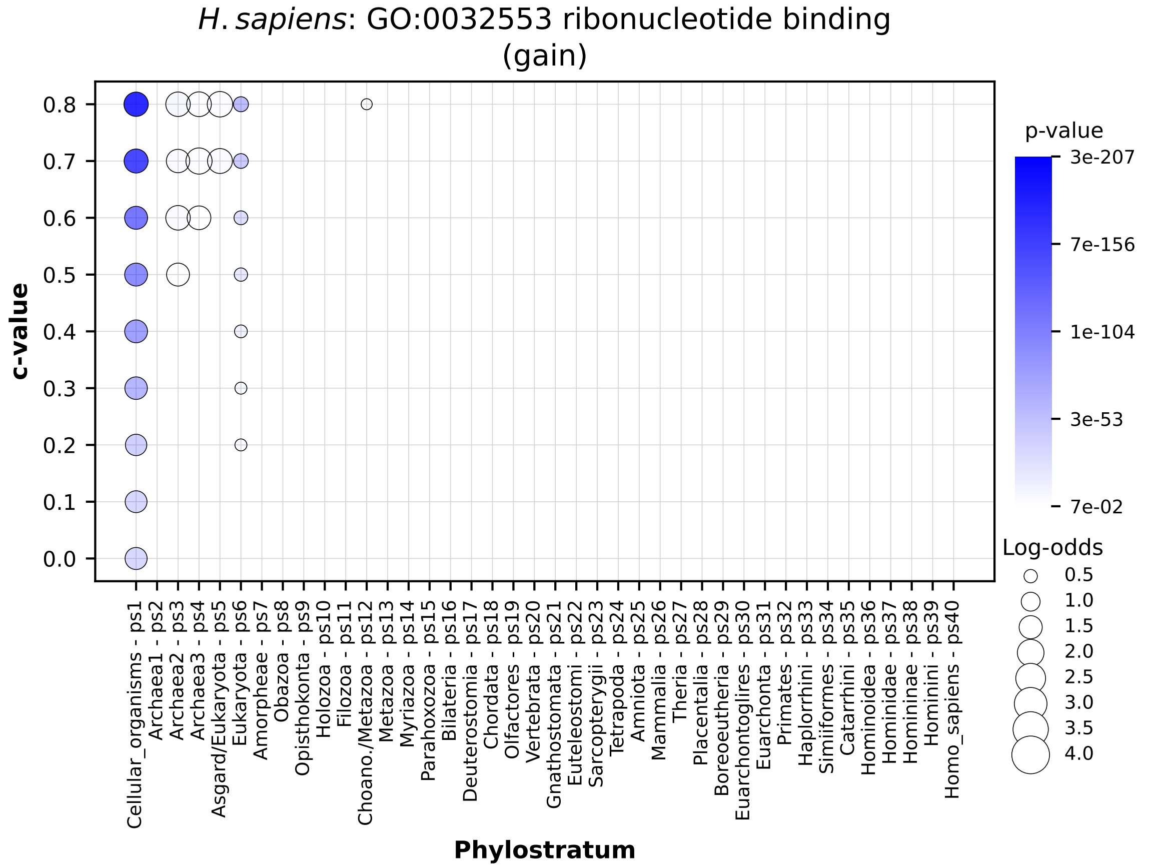

H. sapiens: GO:0032553 ribonucleotide binding
(gain)
c-value
0.8
0.7
0.6
0.5
0.4
0.3
0.2
0.1
0.0
p-value
3e-207
7e-156
1e-104
3e-53
7e-02
Log-odds
0.5
1.0
1.5
2.0
2.5
3.0
3.5
4.0
Cellular_organisms - ps1
Archaea1 - ps2
Archaea2 - ps3
Archaea3 - ps4
Asgard/Eukaryota - ps5
Eukaryota - ps6
Amorpheae - ps7
Obazoa - ps8
Opisthokonta - ps9
Holozoa - ps10
Filozoa - ps11
Choano./Metazoa - ps12
Metazoa - ps13
Myriazoa - ps14
Parahoxozoa - ps15
Bilateria - ps16
Deuterostomia - ps17
Chordata - ps18
Olfactores - ps19
Vertebrata - ps20
Gnathostomata - ps21
Euteleostomi - ps22
Sarcopterygii - ps23
Tetrapoda - ps24
Amniota - ps25
Mammalia - ps26
Theria - ps27
Placentalia - ps28
Boreoeutheria - ps29
Euarchontoglires - ps30
Euarchonta - ps31
Primates - ps32
Haplorrhini - ps33
Simiiformes - ps34
Catarrhini - ps35
Hominoidea - ps36
Hominidae - ps37
Homininae - ps38
Hominini - ps39
Homo_sapiens - ps40
Phylostratum

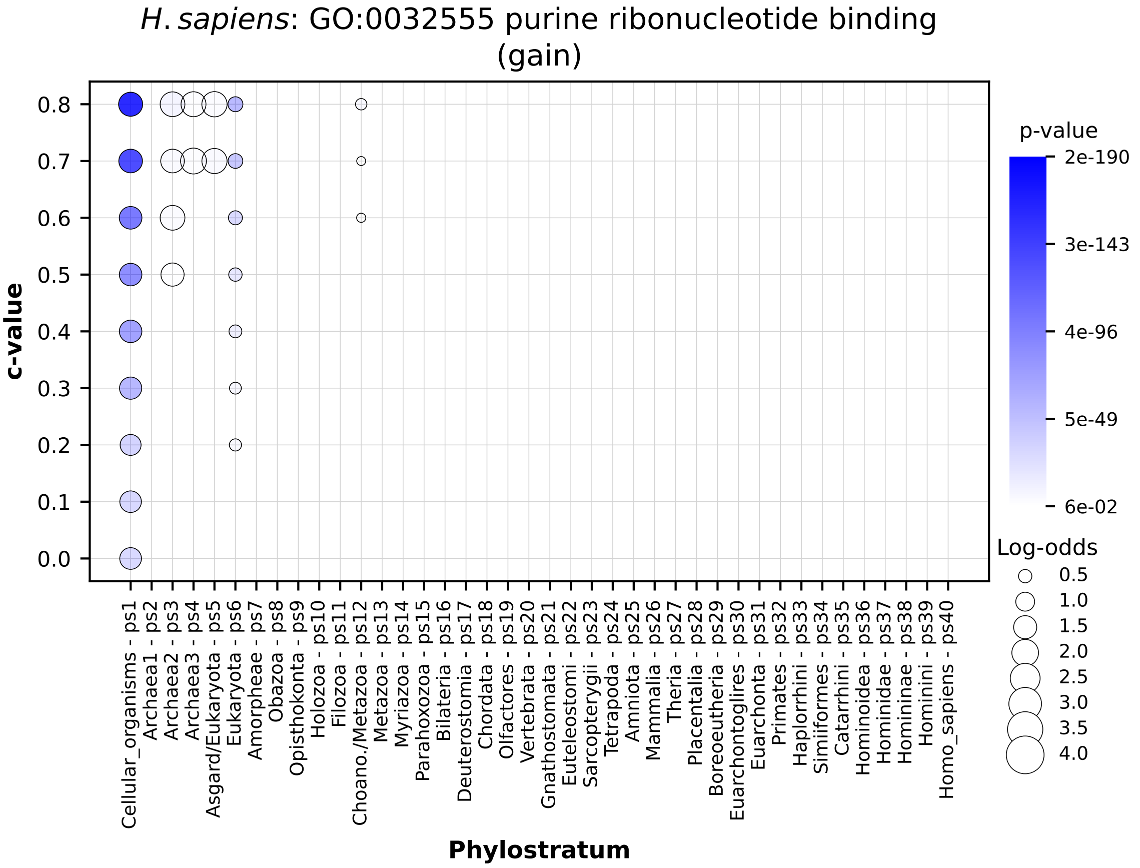

H. sapiens: GO:0032555 purine ribonucleotide binding (gain)
c-value
0.8
0.7
0.6
0.5
0.4
0.3
0.2
0.1
0.0
p-value
2e-190
3e-143
4e-96
5e-49
6e-02
Log-odds
0.5
1.0
1.5
2.0
2.5
3.0
3.5
4.0
Cellular_organisms - ps1
Archaea1 - ps2
Archaea2 - ps3
Archaea3 - ps4
Asgard/Eukaryota - ps5
Eukaryota - ps6
Amorpheae - ps7
Obazoa - ps8
Opisthokonta - ps9
Holozoa - ps10
Filozoa - ps11
Choano./Metazoa - ps12
Metazoa - ps13
Myriazoa - ps14
Parahoxozoa - ps15
Bilateria - ps16
Deuterostomia - ps17
Chordata - ps18
Olfactores - ps19
Vertebrata - ps20
Gnathostomata - ps21
Euteleostomi - ps22
Sarcopterygii - ps23
Tetrapoda - ps24
Amniota - ps25
Mammalia - ps26
Theria - ps27
Placentalia - ps28
Boreoeutheria - ps29
Euarchontoglires - ps30
Euarchonta - ps31
Primates - ps32
Haplorrhini - ps33
Simiiformes - ps34
Catarrhini - ps35
Hominoidea - ps36
Hominidae - ps37
Homininae - ps38
Hominini - ps39
Homo_sapiens - ps40
Phylostratum

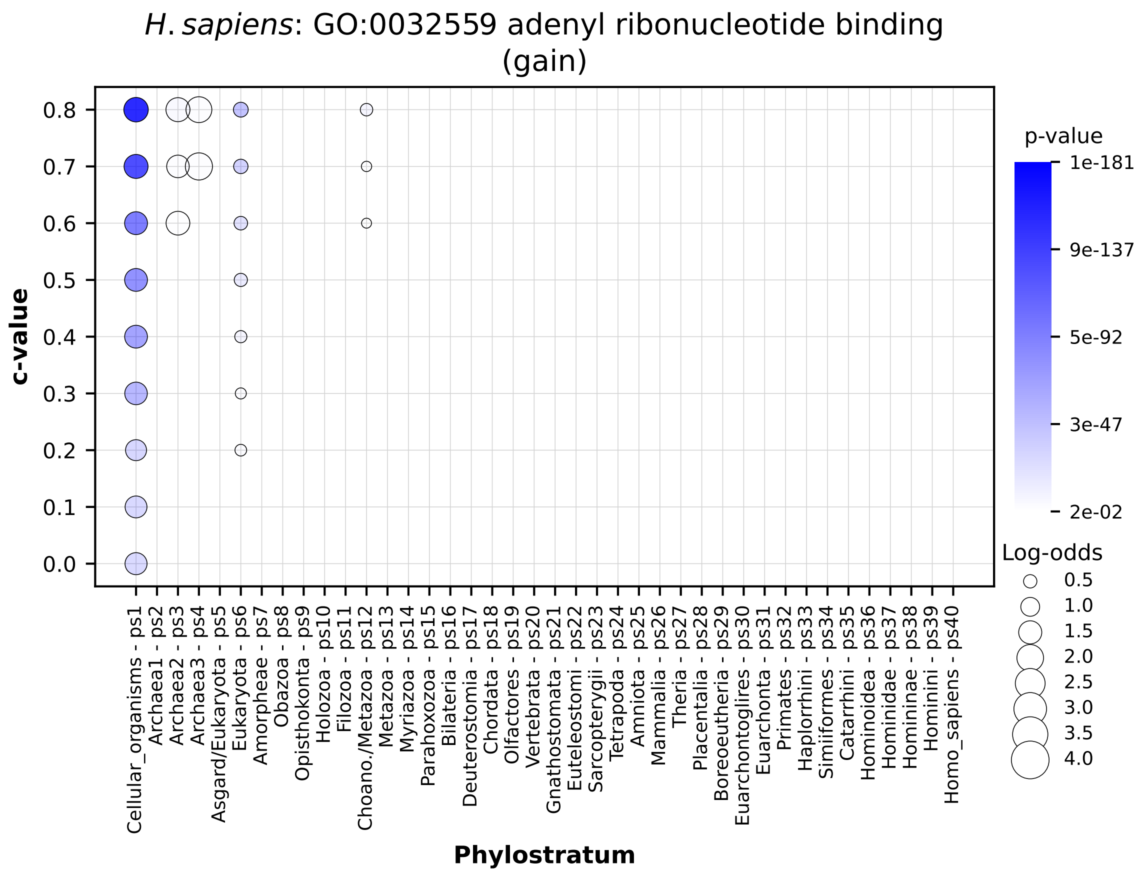

H. sapiens: GO:0032559 adenyl ribonucleotide binding (gain)
c-value
0.0
0.1
0.2
0.3
0.4
0.5
0.6
0.7
0.8
Phylostratum
Cellular_organisms - ps1
Archaea1 - ps2
Archaea2 - ps3
Archaea3 - ps4
Asgard/Eukaryota - ps5
Eukaryota - ps6
Amorpheae - ps7
Obazoa - ps8
Opisthokonta - ps9
Holozoa - ps10
Filozoa - ps11
Choano./Metazoa - ps12
Metazoa - ps13
Myriazoa - ps14
Parahoxozoa - ps15
Bilateria - ps16
Deuterostomia - ps17
Chordata - ps18
Olfactores - ps19
Vertebrata - ps20
Gnathostomata - ps21
Euteleostomi - ps22
Sarcopterygii - ps23
Tetrapoda - ps24
Amniota - ps25
Mammalia - ps26
Theria - ps27
Placentalia - ps28
Boreoeutheria - ps29
Euarchontoglires - ps30
Euarchonta - ps31
Primates - ps32
Haplorrhini - ps33
Simiiformes - ps34
Catarrhini - ps35
Hominoidea - ps36
Hominidae - ps37
Homininae - ps38
Hominini - ps39
Homo_sapiens - ps40
p-value
1e-181
9e-137
5e-92
3e-47
2e-02
Log-odds
0.5
1.0
1.5
2.0
2.5
3.0
3.5
4.0

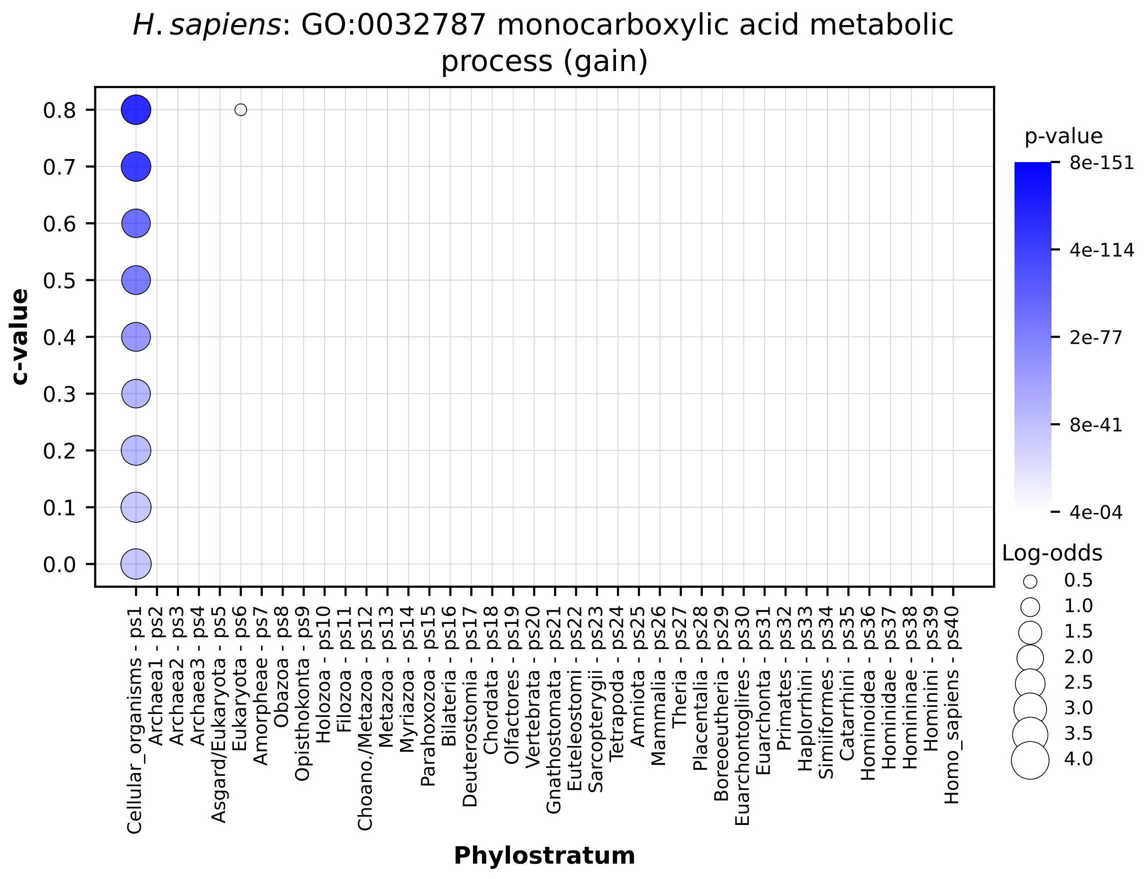

H. sapiens: GO:0032787 monocarboxylic acid metabolic process (gain)
c-value
0.8
0.7
0.6
0.5
0.4
0.3
0.2
0.1
0.0
Phylostratum
Cellular_organisms - ps1
Archaea1 - ps2
Archaea2 - ps3
Archaea3 - ps4
Asgard/Eukaryota - ps5
Eukaryota - ps6
Amorpheae - ps7
Obazoa - ps8
Opisthokonta - ps9
Holozoa - ps10
Filozoa - ps11
Choano./Metazoa - ps12
Metazoa - ps13
Myriazoa - ps14
Parahoxozoa - ps15
Bilateria - ps16
Deuterostomia - ps17
Chordata - ps18
Olfactores - ps19
Vertebrata - ps20
Gnathostomata - ps21
Euteleostomi - ps22
Sarcopterygii - ps23
Tetrapoda - ps24
Amniota - ps25
Mammalia - ps26
Theria - ps27
Placentalia - ps28
Boreoeutheria - ps29
Euarchontoglires - ps30
Euarchonta - ps31
Primates - ps32
Haplorrhini - ps33
Simiiformes - ps34
Catarrhini - ps35
Hominoidea - ps36
Hominidae - ps37
Homininae - ps38
Hominini - ps39
Homo_sapiens - ps40
p-value
8e-151
4e-114
2e-77
8e-41
4e-04
Log-odds
0.5
1.0
1.5
2.0
2.5
3.0
3.5
4.0

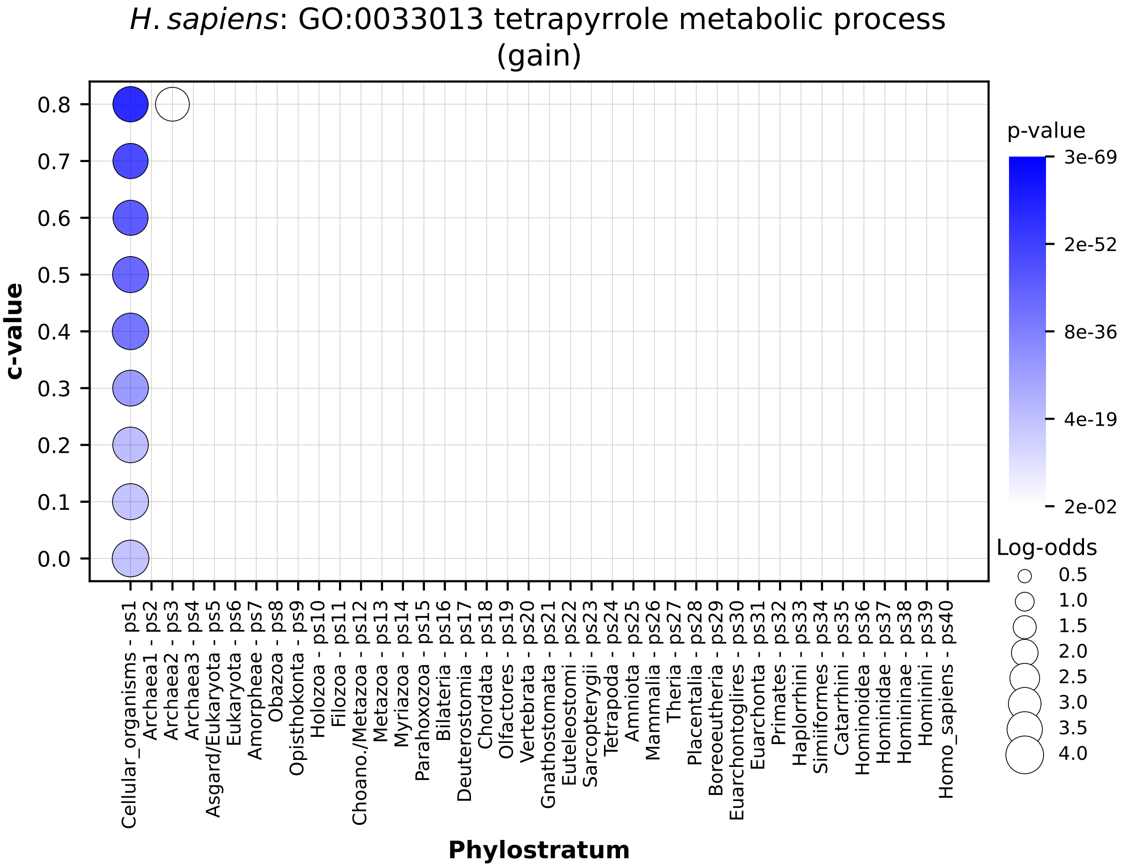
H. sapiens: GO:0033013 tetrapyrrole metabolic process
(gain)
c-value
0.8
0.7
0.6
0.5
0.4
0.3
0.2
0.1
0.0
p-value
3e-69
2e-52
8e-36
4e-19
2e-02
Log-odds
0.5
1.0
1.5
2.0
2.5
3.0
3.5
4.0
Cellular_organisms - ps1
Archaea1 - ps2
Archaea2 - ps3
Archaea3 - ps4
Asgard/Eukaryota - ps5
Eukaryota - ps6
Amorpheae - ps7
Obazoa - ps8
Opisthokonta - ps9
Holozoa - ps10
Filozoa - ps11
Choano./Metazoa - ps12
Metazoa - ps13
Myriazoa - ps14
Parahoxozoa - ps15
Bilateria - ps16
Deuterostomia - ps17
Chordata - ps18
Olfactores - ps19
Vertebrata - ps20
Gnathostomata - ps21
Euteleostomi - ps22
Sarcopterygii - ps23
Tetrapoda - ps24
Amniota - ps25
Mammalia - ps26
Theria - ps27
Placentalia - ps28
Boreoeutheria - ps29
Euarchontoglires - ps30
Euarchonta - ps31
Primates - ps32
Haplorrhini - ps33
Simiiformes - ps34
Catarrhini - ps35
Hominoidea - ps36
Hominidae - ps37
Homininae - ps38
Hominini - ps39
Homo_sapiens - ps40
Phylostratum

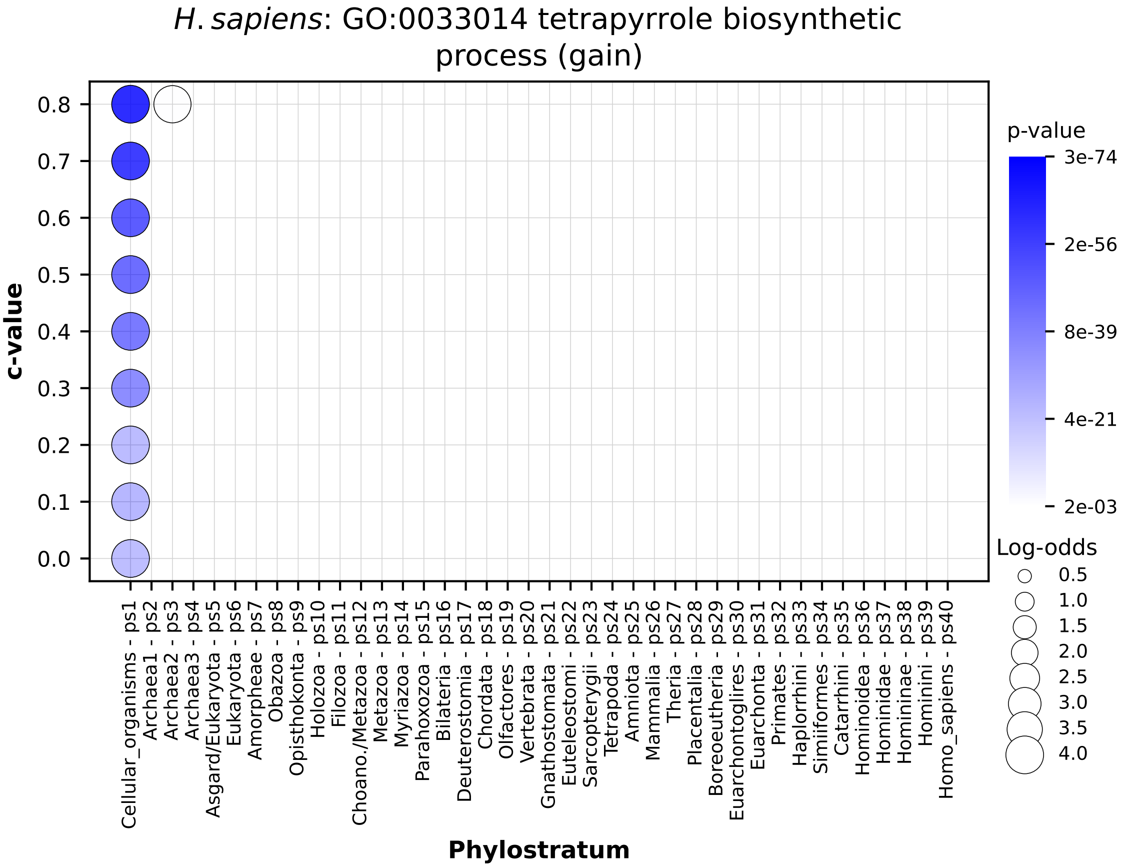

H. sapiens: GO:0033014 tetrapyrrole biosynthetic process (gain)
c-value
0.8
0.7
0.6
0.5
0.4
0.3
0.2
0.1
0.0
p-value
3e-74
2e-56
8e-39
4e-21
2e-03
Log-odds
0.5
1.0
1.5
2.0
2.5
3.0
3.5
4.0
Cellular_organisms - ps1
Archaea1 - ps2
Archaea2 - ps3
Archaea3 - ps4
Asgard/Eukaryota - ps5
Eukaryota - ps6
Amorpheae - ps7
Obazoa - ps8
Opisthokonta - ps9
Holozoa - ps10
Filozoa - ps11
Choano./Metazoa - ps12
Metazoa - ps13
Myriazoa - ps14
Parahoxozoa - ps15
Bilateria - ps16
Deuterostomia - ps17
Chordata - ps18
Olfactores - ps19
Vertebrata - ps20
Gnathostomata - ps21
Euteleostomi - ps22
Sarcopterygii - ps23
Tetrapoda - ps24
Amniota - ps25
Mammalia - ps26
Theria - ps27
Placentalia - ps28
Boreoeutheria - ps29
Euarchontoglires - ps30
Euarchonta - ps31
Primates - ps32
Haplorrhini - ps33
Simiiformes - ps34
Catarrhini - ps35
Hominoidea - ps36
Hominidae - ps37
Homininae - ps38
Hominini - ps39
Homo_sapiens - ps40
Phylostratum

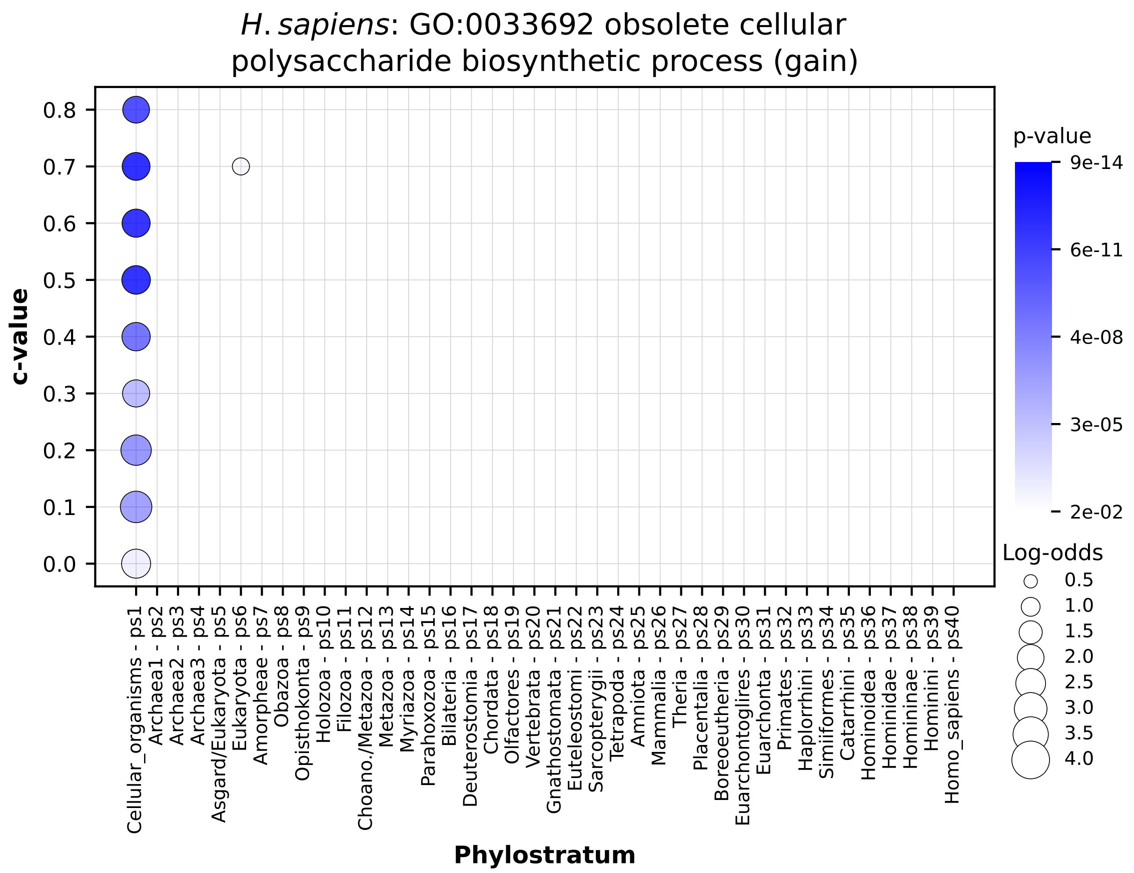

H. sapiens: GO:0033692 obsolete cellular polysaccharide biosynthetic process (gain)
c-value
0.8
0.7
0.6
0.5
0.4
0.3
0.2
0.1
0.0
Phylostratum
Cellular_organisms - ps1
Archaea1 - ps2
Archaea2 - ps3
Archaea3 - ps4
Asgard/Eukaryota - ps5
Eukaryota - ps6
Amorpheae - ps7
Obazoa - ps8
Opisthokonta - ps9
Holozoa - ps10
Filozoa - ps11
Choano./Metazoa - ps12
Metazoa - ps13
Myriazoa - ps14
Parahoxozoa - ps15
Bilateria - ps16
Deuterostomia - ps17
Chordata - ps18
Olfactores - ps19
Vertebrata - ps20
Gnathostomata - ps21
Euteleostomi - ps22
Sarcopterygii - ps23
Tetrapoda - ps24
Amniota - ps25
Mammalia - ps26
Theria - ps27
Placentalia - ps28
Boreoeutheria - ps29
Euarchontoglires - ps30
Euarchonta - ps31
Primates - ps32
Haplorrhini - ps33
Simiiformes - ps34
Catarrhini - ps35
Hominoidea - ps36
Hominidae - ps37
Homininae - ps38
Hominini - ps39
Homo_sapiens - ps40
p-value
9e-14
6e-11
4e-08
3e-05
2e-02
Log-odds
0.5
1.0
1.5
2.0
2.5
3.0
3.5
4.0

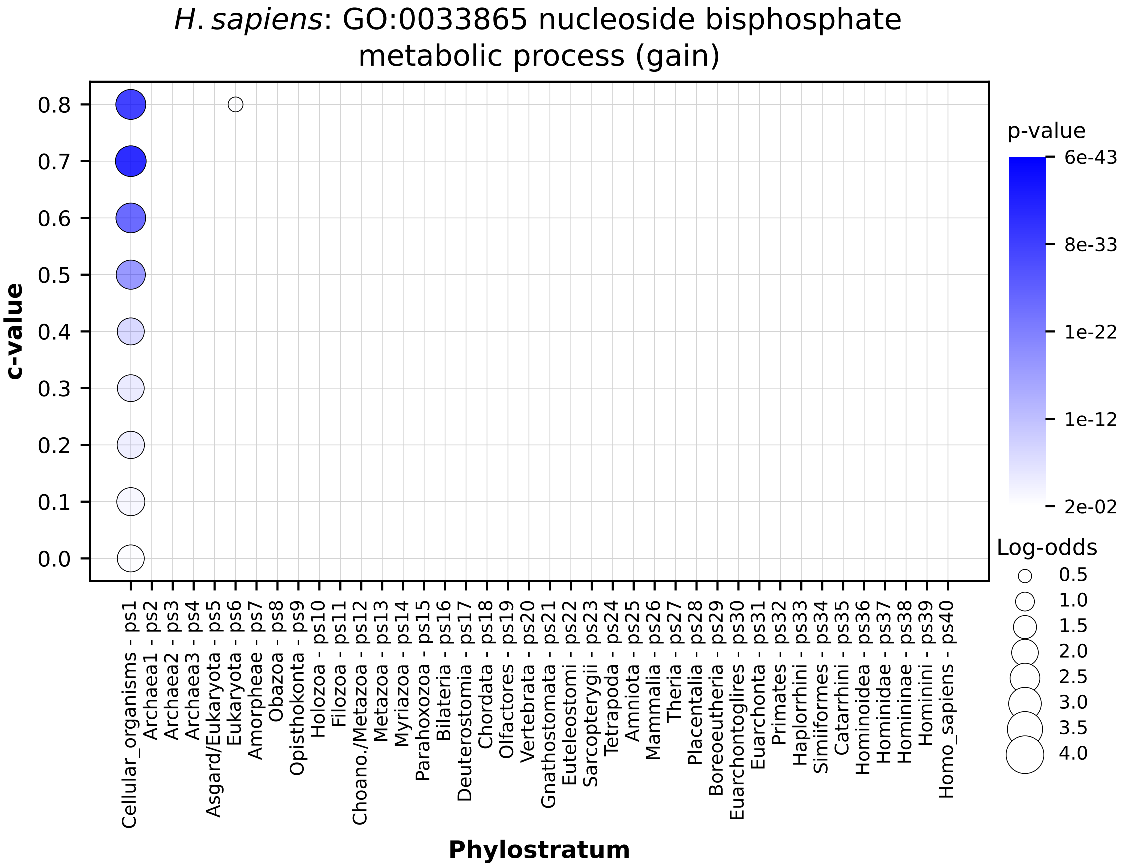

H. sapiens: GO:0033865 nucleoside bisphosphate metabolic process (gain)
c-value
0.8
0.7
0.6
0.5
0.4
0.3
0.2
0.1
0.0
p-value
6e-43
8e-33
1e-22
1e-12
2e-02
Log-odds
0.5
1.0
1.5
2.0
2.5
3.0
3.5
4.0
Cellular_organisms - ps1
Archaea1 - ps2
Archaea2 - ps3
Archaea3 - ps4
Asgard/Eukaryota - ps5
Eukaryota - ps6
Amorpheae - ps7
Obazoa - ps8
Opisthokonta - ps9
Holozoa - ps10
Filozoa - ps11
Choano./Metazoa - ps12
Metazoa - ps13
Myriazoa - ps14
Parahoxozoa - ps15
Bilateria - ps16
Deuterostomia - ps17
Chordata - ps18
Olfactores - ps19
Vertebrata - ps20
Gnathostomata - ps21
Euteleostomi - ps22
Sarcopterygii - ps23
Tetrapoda - ps24
Amniota - ps25
Mammalia - ps26
Theria - ps27
Placentalia - ps28
Boreoeutheria - ps29
Euarchontoglires - ps30
Euarchonta - ps31
Primates - ps32
Haplorrhini - ps33
Simiiformes - ps34
Catarrhini - ps35
Hominoidea - ps36
Hominidae - ps37
Homininae - ps38
Hominini - ps39
Homo_sapiens - ps40
Phylostratum

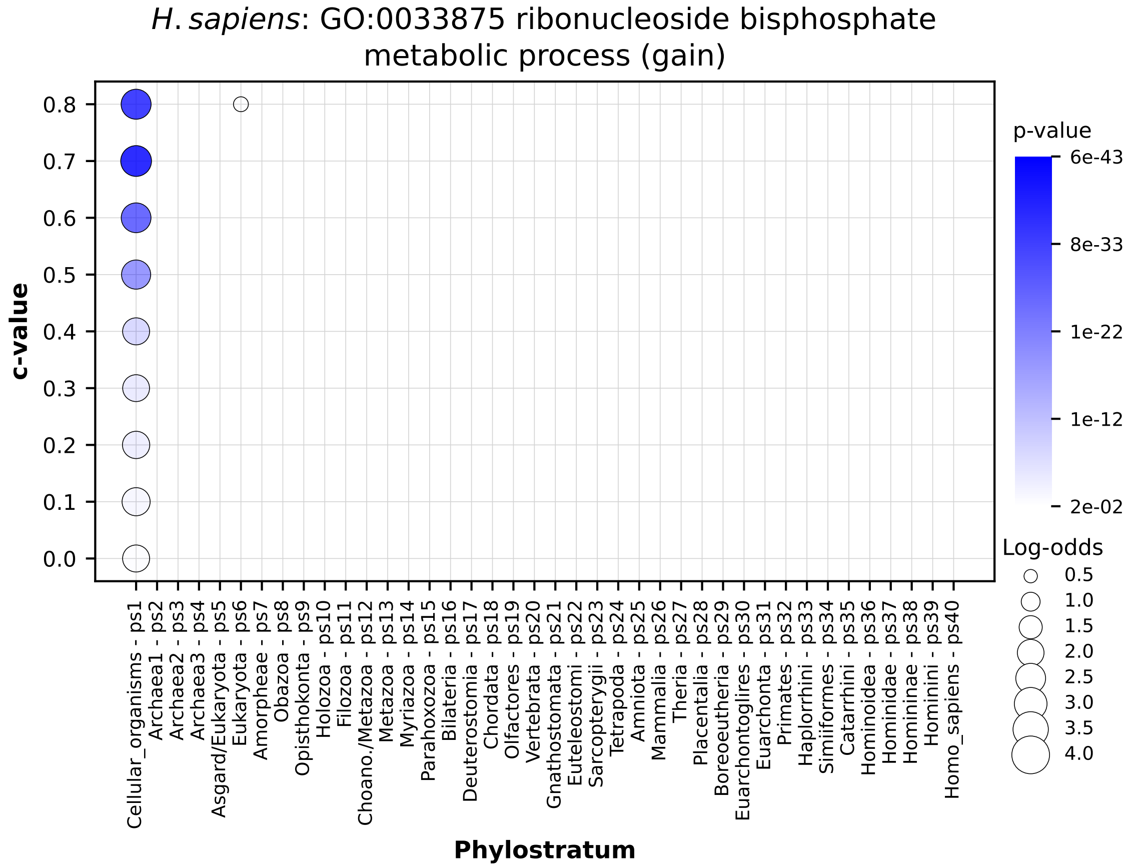
H. sapiens: GO:0033875 ribonucleoside bisphosphate metabolic process (gain)
c-value
0.8
0.7
0.6
0.5
0.4
0.3
0.2
0.1
0.0
p-value
6e-43
8e-33
1e-22
1e-12
2e-02
Log-odds
0.5
1.0
1.5
2.0
2.5
3.0
3.5
4.0
Cellular_organisms - ps1
Archaea1 - ps2
Archaea2 - ps3
Archaea3 - ps4
Asgard/Eukaryota - ps5
Eukaryota - ps6
Amorpheae - ps7
Obazoa - ps8
Opisthokonta - ps9
Holozoa - ps10
Filozoa - ps11
Choano./Metazoa - ps12
Metazoa - ps13
Myriazoa - ps14
Parahoxozoa - ps15
Bilateria - ps16
Deuterostomia - ps17
Chordata - ps18
Olfactores - ps19
Vertebrata - ps20
Gnathostomata - ps21
Euteleostomi - ps22
Sarcopterygii - ps23
Tetrapoda - ps24
Amniota - ps25
Mammalia - ps26
Theria - ps27
Placentalia - ps28
Boreoeutheria - ps29
Euarchontoglires - ps30
Euarchonta - ps31
Primates - ps32
Haplorrhini - ps33
Simiiformes - ps34
Catarrhini - ps35
Hominoidea - ps36
Hominidae - ps37
Homininae - ps38
Hominini - ps39
Homo_sapiens - ps40
Phylostratum

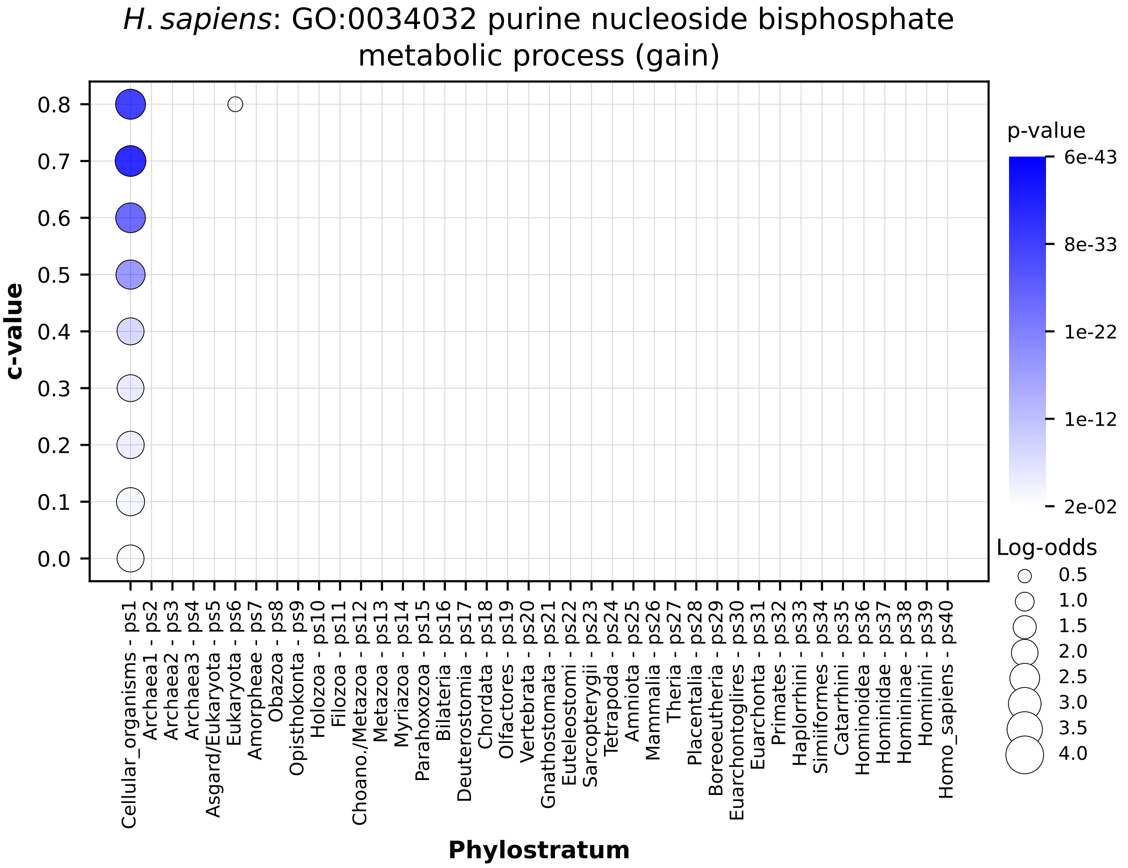

H. sapiens: GO:0034032 purine nucleoside bisphosphate metabolic process (gain)
c-value
0.8
0.7
0.6
0.5
0.4
0.3
0.2
0.1
0.0
Phylostratum
Cellular_organisms - ps1
Archaea1 - ps2
Archaea2 - ps3
Archaea3 - ps4
Asgard/Eukaryota - ps5
Eukaryota - ps6
Amorpheae - ps7
Obazoa - ps8
Opisthokonta - ps9
Holozoa - ps10
Filozoa - ps11
Choano./Metazoa - ps12
Metazoa - ps13
Myriazoa - ps14
Parahoxozoa - ps15
Bilateria - ps16
Deuterostomia - ps17
Chordata - ps18
Olfactores - ps19
Vertebrata - ps20
Gnathostomata - ps21
Euteleostomi - ps22
Sarcopterygii - ps23
Tetrapoda - ps24
Amniota - ps25
Mammalia - ps26
Theria - ps27
Placentalia - ps28
Boreoeutheria - ps29
Euarchontoglires - ps30
Euarchonta - ps31
Primates - ps32
Haplorrhini - ps33
Simiiformes - ps34
Catarrhini - ps35
Hominoidea - ps36
Hominidae - ps37
Homininae - ps38
Hominini - ps39
Homo_sapiens - ps40
p-value
6e-43
8e-33
1e-22
1e-12
2e-02
Log-odds
0.5
1.0
1.5
2.0
2.5
3.0
3.5
4.0

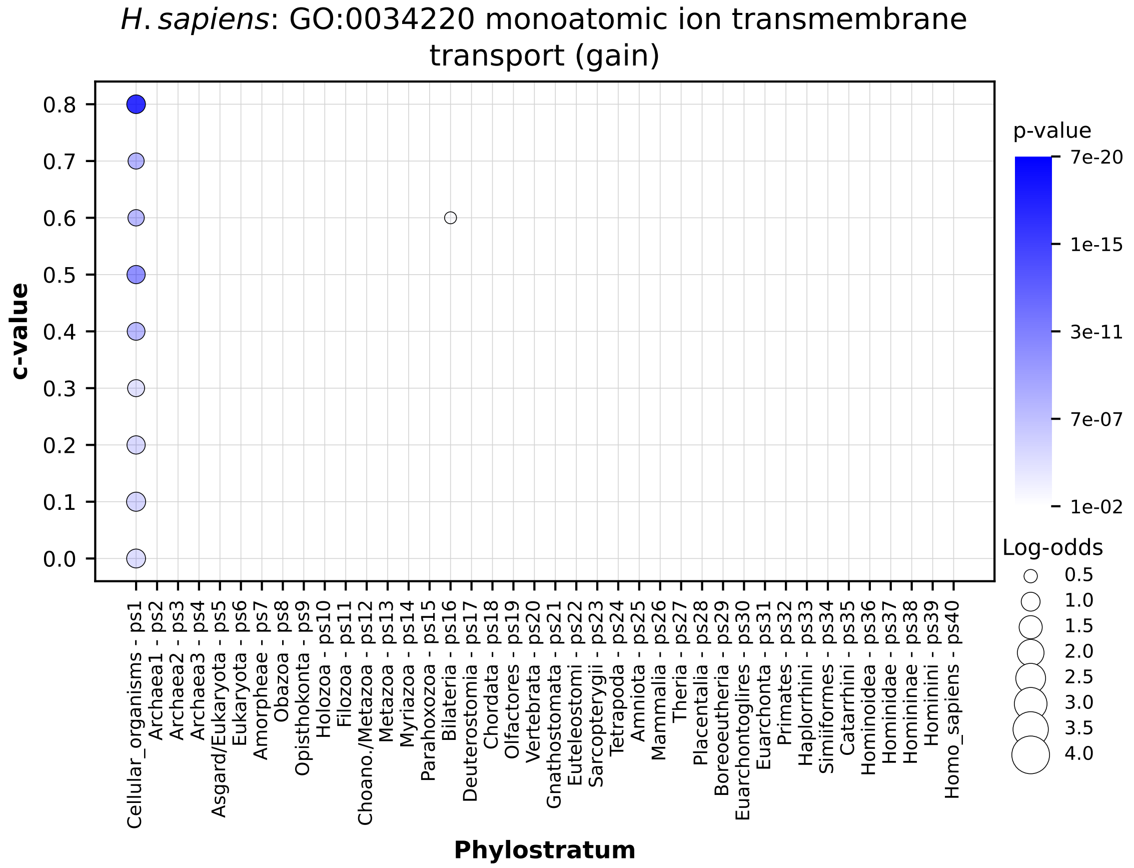

H. sapiens: GO:0034220 monoatomic ion transmembrane transport (gain)
c-value
0.8
0.7
0.6
0.5
0.4
0.3
0.2
0.1
0.0
Phylostratum
Cellular_organisms - ps1
Archaea1 - ps2
Archaea2 - ps3
Archaea3 - ps4
Asgard/Eukaryota - ps5
Eukaryota - ps6
Amorpheae - ps7
Obazoa - ps8
Opisthokonta - ps9
Holozoa - ps10
Filozoa - ps11
Choano./Metazoa - ps12
Metazoa - ps13
Myriazoa - ps14
Parahoxozoa - ps15
Bilateria - ps16
Deuterostomia - ps17
Chordata - ps18
Olfactores - ps19
Vertebrata - ps20
Gnathostomata - ps21
Euteleostomi - ps22
Sarcopterygii - ps23
Tetrapoda - ps24
Amniota - ps25
Mammalia - ps26
Theria - ps27
Placentalia - ps28
Boreoeutheria - ps29
Euarchontoglires - ps30
Euarchonta - ps31
Primates - ps32
Haplorrhini - ps33
Simiiformes - ps34
Catarrhini - ps35
Hominoidea - ps36
Hominidae - ps37
Homininae - ps38
Hominini - ps39
Homo_sapiens - ps40
p-value
7e-20
1e-15
3e-11
7e-07
1e-02
Log-odds
0.5
1.0
1.5
2.0
2.5
3.0
3.5
4.0

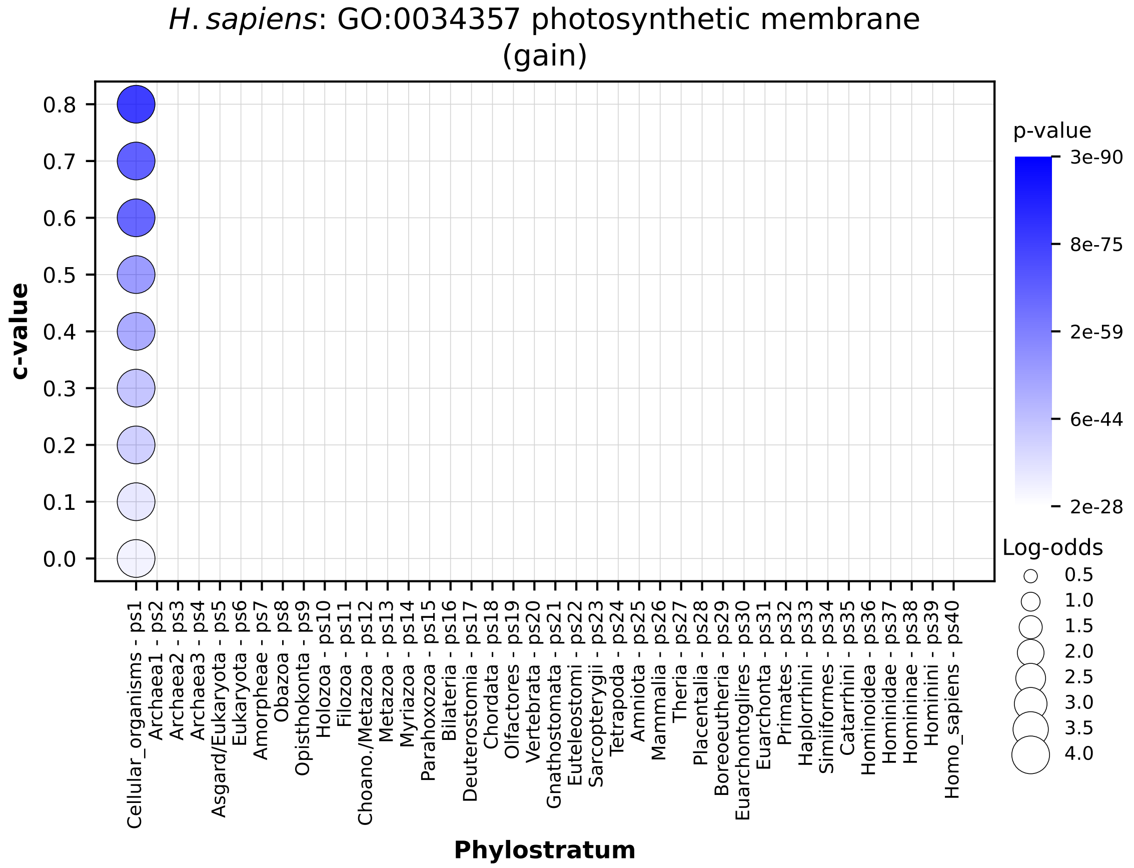

H. sapiens: GO:0034357 photosynthetic membrane
(gain)
c-value
0.0
0.1
0.2
0.3
0.4
0.5
0.6
0.7
0.8
p-value
3e-90
8e-75
2e-59
6e-44
2e-28
Log-odds
0.5
1.0
1.5
2.0
2.5
3.0
3.5
4.0
Cellular_organisms - ps1
Archaea1 - ps2
Archaea2 - ps3
Archaea3 - ps4
Asgard/Eukaryota - ps5
Eukaryota - ps6
Amorpheae - ps7
Obazoa - ps8
Opisthokonta - ps9
Holozoa - ps10
Filozoa - ps11
Choano./Metazoa - ps12
Metazoa - ps13
Myriazoa - ps14
Parahoxozoa - ps15
Bilateria - ps16
Deuterostomia - ps17
Chordata - ps18
Olfactores - ps19
Vertebrata - ps20
Gnathostomata - ps21
Euteleostomi - ps22
Sarcopterygii - ps23
Tetrapoda - ps24
Amniota - ps25
Mammalia - ps26
Theria - ps27
Placentalia - ps28
Boreoeutheria - ps29
Euarchontoglires - ps30
Euarchonta - ps31
Primates - ps32
Haplorrhini - ps33
Simiiformes - ps34
Catarrhini - ps35
Hominoidea - ps36
Hominidae - ps37
Homininae - ps38
Hominini - ps39
Homo_sapiens - ps40
Phylostratum

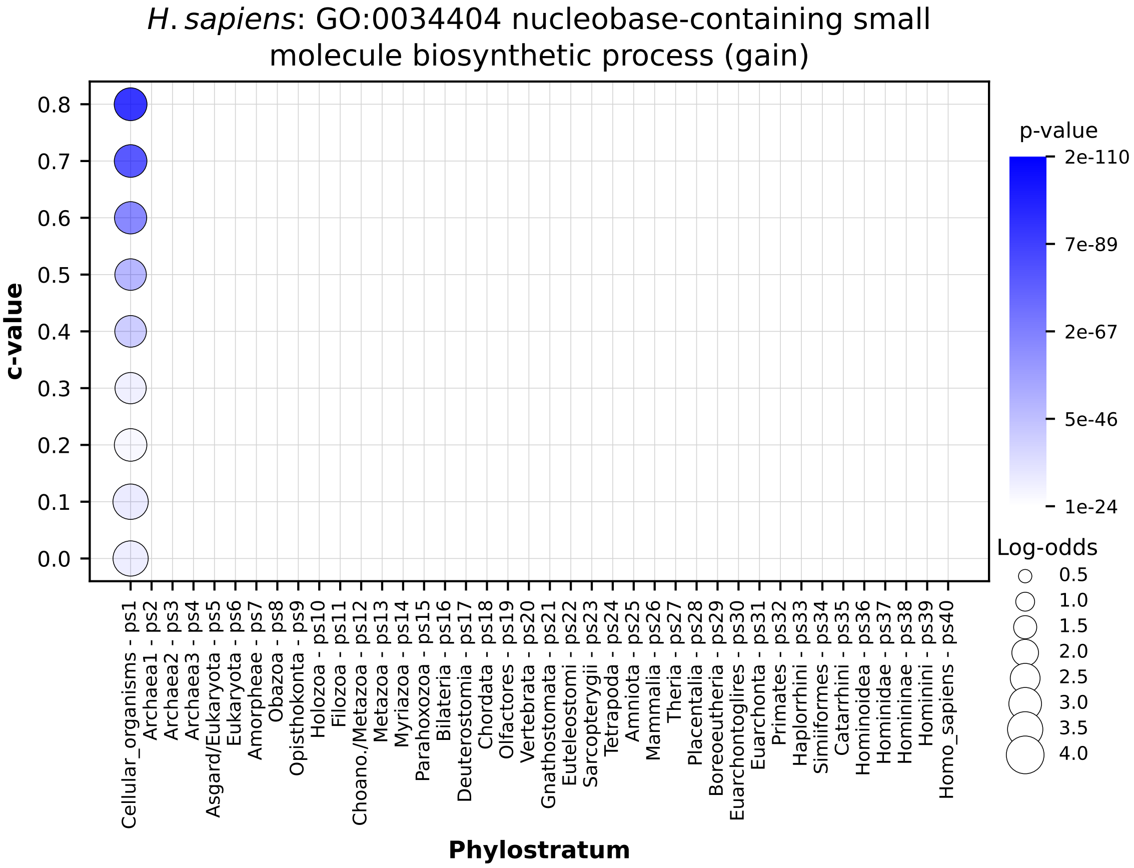

H. sapiens: GO:0034404 nucleobase-containing small molecule biosynthetic process (gain)
c-value
0.8
0.7
0.6
0.5
0.4
0.3
0.2
0.1
0.0
Phylostratum
Cellular_organisms - ps1
Archaea1 - ps2
Archaea2 - ps3
Archaea3 - ps4
Asgard/Eukaryota - ps5
Eukaryota - ps6
Amorpheae - ps7
Obazoa - ps8
Opisthokonta - ps9
Holozoa - ps10
Filozoa - ps11
Choano./Metazoa - ps12
Metazoa - ps13
Myriazoa - ps14
Parahoxozoa - ps15
Bilateria - ps16
Deuterostomia - ps17
Chordata - ps18
Olfactores - ps19
Vertebrata - ps20
Gnathostomata - ps21
Euteleostomi - ps22
Sarcopterygii - ps23
Tetrapoda - ps24
Amniota - ps25
Mammalia - ps26
Theria - ps27
Placentalia - ps28
Boreoeutheria - ps29
Euarchontoglires - ps30
Euarchonta - ps31
Primates - ps32
Haplorrhini - ps33
Simiiformes - ps34
Catarrhini - ps35
Hominoidea - ps36
Hominidae - ps37
Homininae - ps38
Hominini - ps39
Homo_sapiens - ps40
p-value
2e-110
7e-89
2e-67
5e-46
1e-24
Log-odds
0.5
1.0
1.5
2.0
2.5
3.0
3.5
4.0

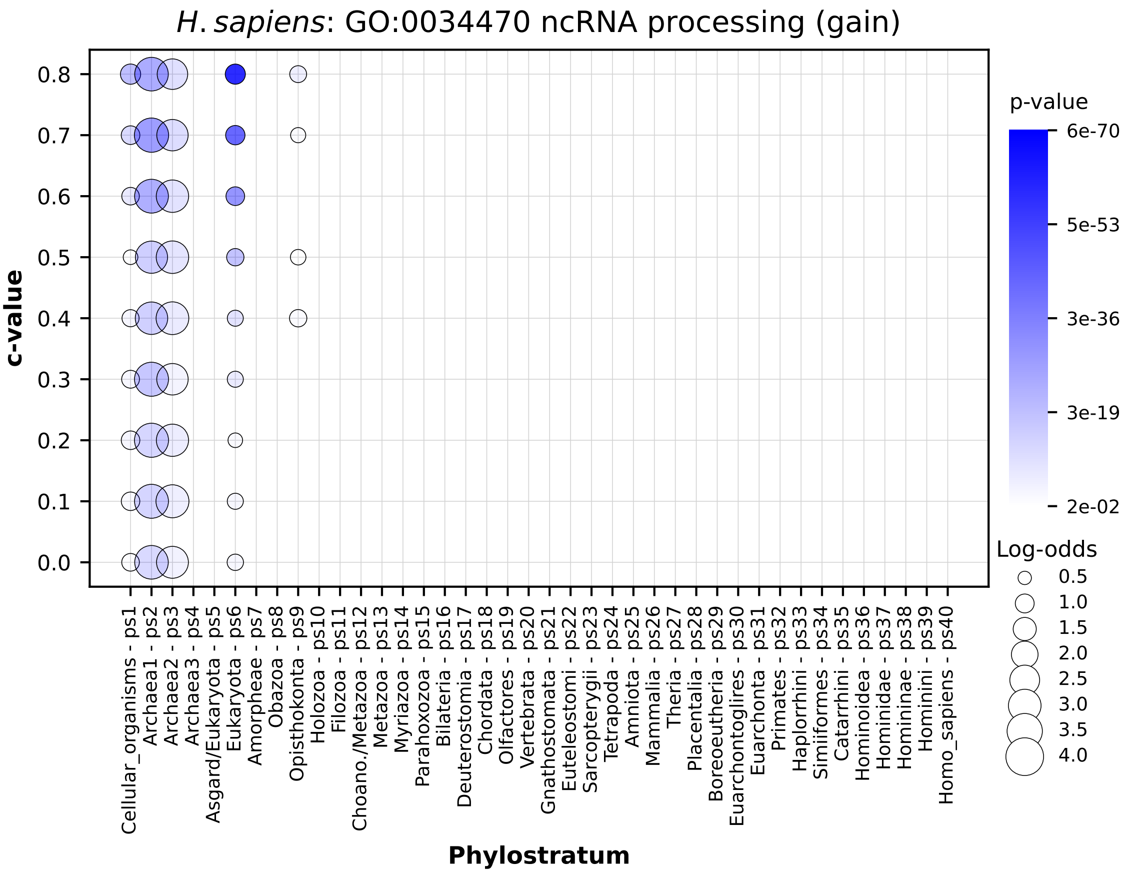

H. sapiens: GO:0034470 ncRNA processing (gain)
c-value
0.0
0.1
0.2
0.3
0.4
0.5
0.6
0.7
0.8
p-value
6e-70
5e-53
3e-36
3e-19
2e-02
Log-odds
0.5
1.0
1.5
2.0
2.5
3.0
3.5
4.0
Cellular_organisms - ps1
Archaea1 - ps2
Archaea2 - ps3
Archaea3 - ps4
Asgard/Eukaryota - ps5
Eukaryota - ps6
Amorpheae - ps7
Obazoa - ps8
Opisthokonta - ps9
Holozoa - ps10
Filozoa - ps11
Choano./Metazoa - ps12
Metazoa - ps13
Myriazoa - ps14
Parahoxozoa - ps15
Bilateria - ps16
Deuterostomia - ps17
Chordata - ps18
Olfactores - ps19
Vertebrata - ps20
Gnathostomata - ps21
Euteleostomi - ps22
Sarcopterygii - ps23
Tetrapoda - ps24
Amniota - ps25
Mammalia - ps26
Theria - ps27
Placentalia - ps28
Boreoeutheria - ps29
Euarchontoglires - ps30
Euarchonta - ps31
Primates - ps32
Haplorrhini - ps33
Simiiformes - ps34
Catarrhini - ps35
Hominoidea - ps36
Hominidae - ps37
Homininae - ps38
Hominini - ps39
Homo_sapiens - ps40
Phylostratum

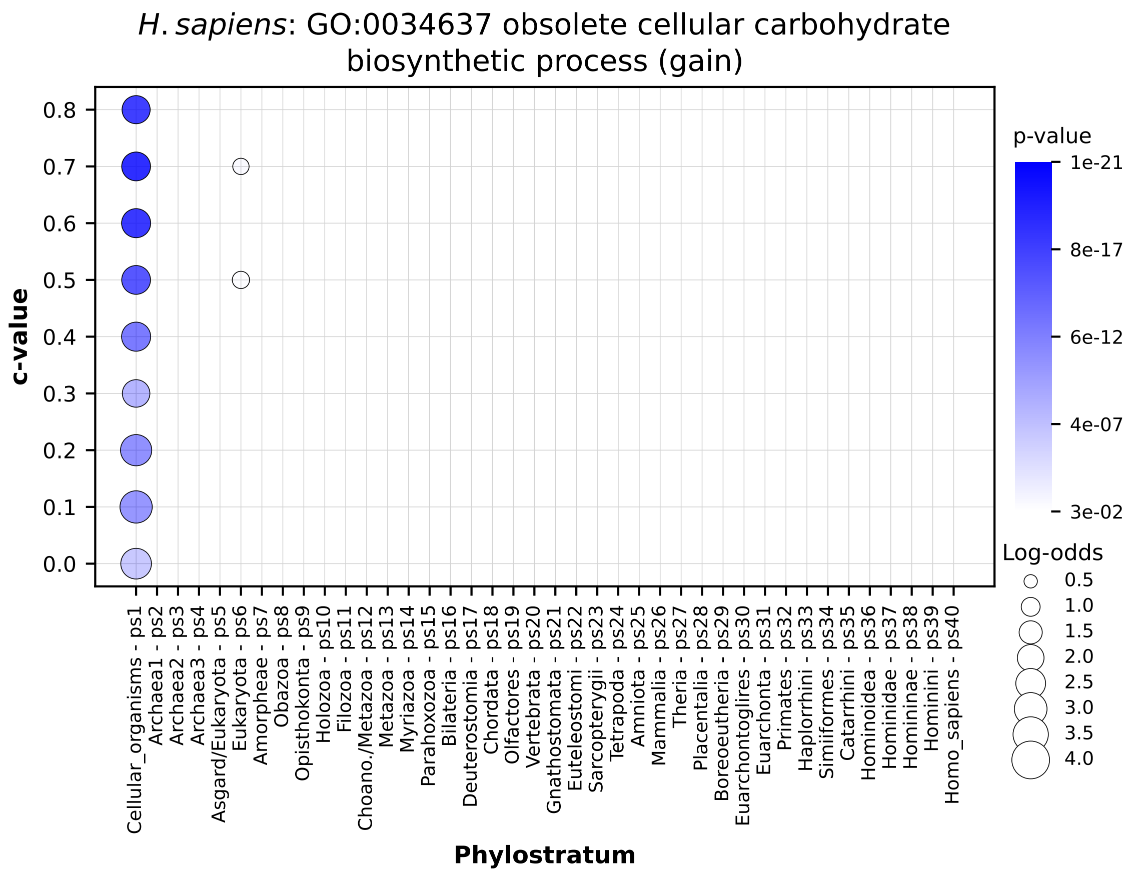
H. sapiens: GO:0034637 obsolete cellular carbohydrate biosynthetic process (gain)
c-value
0.8
0.7
0.6
0.5
0.4
0.3
0.2
0.1
0.0
Phylostratum
Cellular_organisms - ps1
Archaea1 - ps2
Archaea2 - ps3
Archaea3 - ps4
Asgard/Eukaryota - ps5
Eukaryota - ps6
Amorpheae - ps7
Obazoa - ps8
Opisthokonta - ps9
Holozoa - ps10
Filozoa - ps11
Choano./Metazoa - ps12
Metazoa - ps13
Myriazoa - ps14
Parahoxozoa - ps15
Bilateria - ps16
Deuterostomia - ps17
Chordata - ps18
Olfactores - ps19
Vertebrata - ps20
Gnathostomata - ps21
Euteleostomi - ps22
Sarcopterygii - ps23
Tetrapoda - ps24
Amniota - ps25
Mammalia - ps26
Theria - ps27
Placentalia - ps28
Boreoeutheria - ps29
Euarchontoglires - ps30
Euarchonta - ps31
Primates - ps32
Haplorrhini - ps33
Simiiformes - ps34
Catarrhini - ps35
Hominoidea - ps36
Hominidae - ps37
Homininae - ps38
Hominini - ps39
Homo_sapiens - ps40
p-value
1e-21
8e-17
6e-12
4e-07
3e-02
Log-odds
0.5
1.0
1.5
2.0
2.5
3.0
3.5
4.0

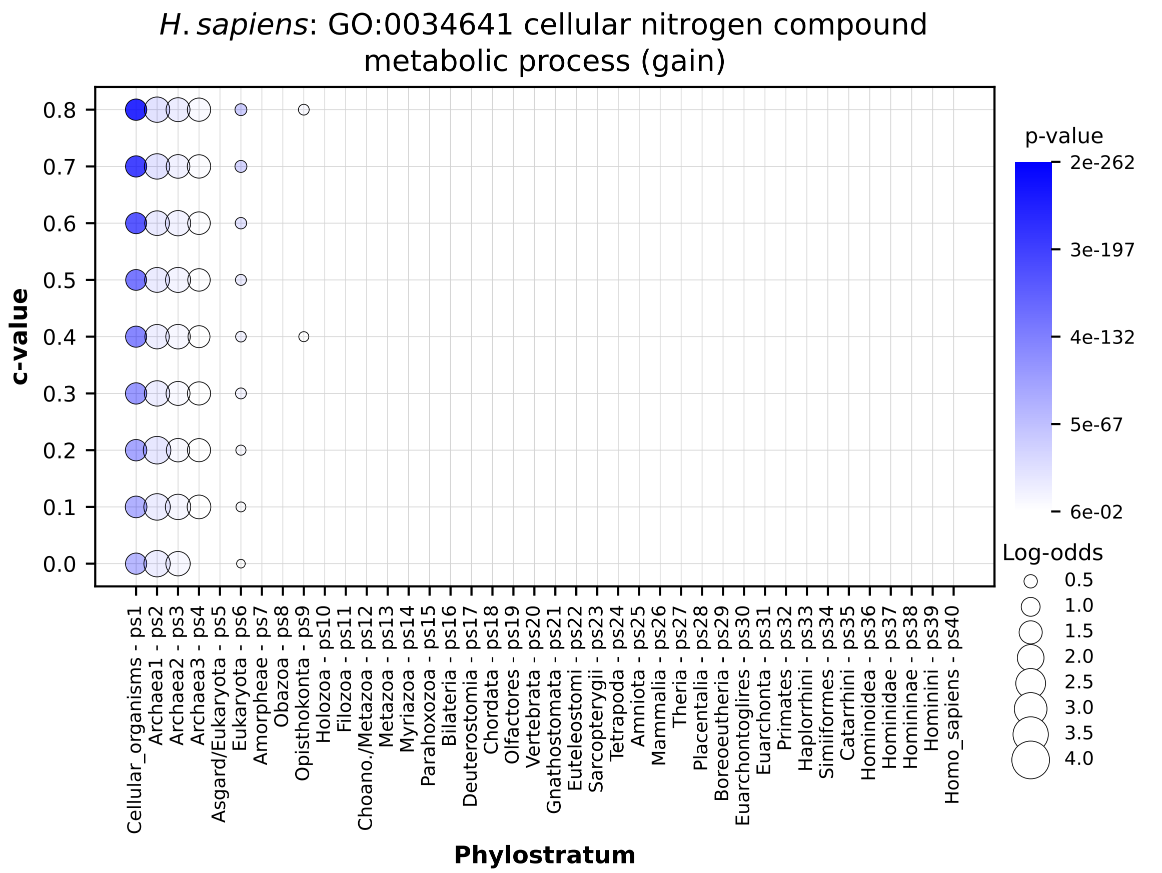

H. sapiens: GO:0034641 cellular nitrogen compound metabolic process (gain)
c-value
0.8
0.7
0.6
0.5
0.4
0.3
0.2
0.1
0.0
p-value
2e-262
3e-197
4e-132
5e-67
6e-02
Log-odds
0.5
1.0
1.5
2.0
2.5
3.0
3.5
4.0
Cellular_organisms - ps1
Archaea1 - ps2
Archaea2 - ps3
Archaea3 - ps4
Asgard/Eukaryota - ps5
Eukaryota - ps6
Amorpheae - ps7
Obazoa - ps8
Opisthokonta - ps9
Holozoa - ps10
Filozoa - ps11
Choano./Metazoa - ps12
Metazoa - ps13
Myriazoa - ps14
Parahoxozoa - ps15
Bilateria - ps16
Deuterostomia - ps17
Chordata - ps18
Olfactores - ps19
Vertebrata - ps20
Gnathostomata - ps21
Euteleostomi - ps22
Sarcopterygii - ps23
Tetrapoda - ps24
Amniota - ps25
Mammalia - ps26
Theria - ps27
Placentalia - ps28
Boreoeutheria - ps29
Euarchontoglires - ps30
Euarchonta - ps31
Primates - ps32
Haplorrhini - ps33
Simiiformes - ps34
Catarrhini - ps35
Hominoidea - ps36
Hominidae - ps37
Homininae - ps38
Hominini - ps39
Homo_sapiens - ps40
Phylostratum

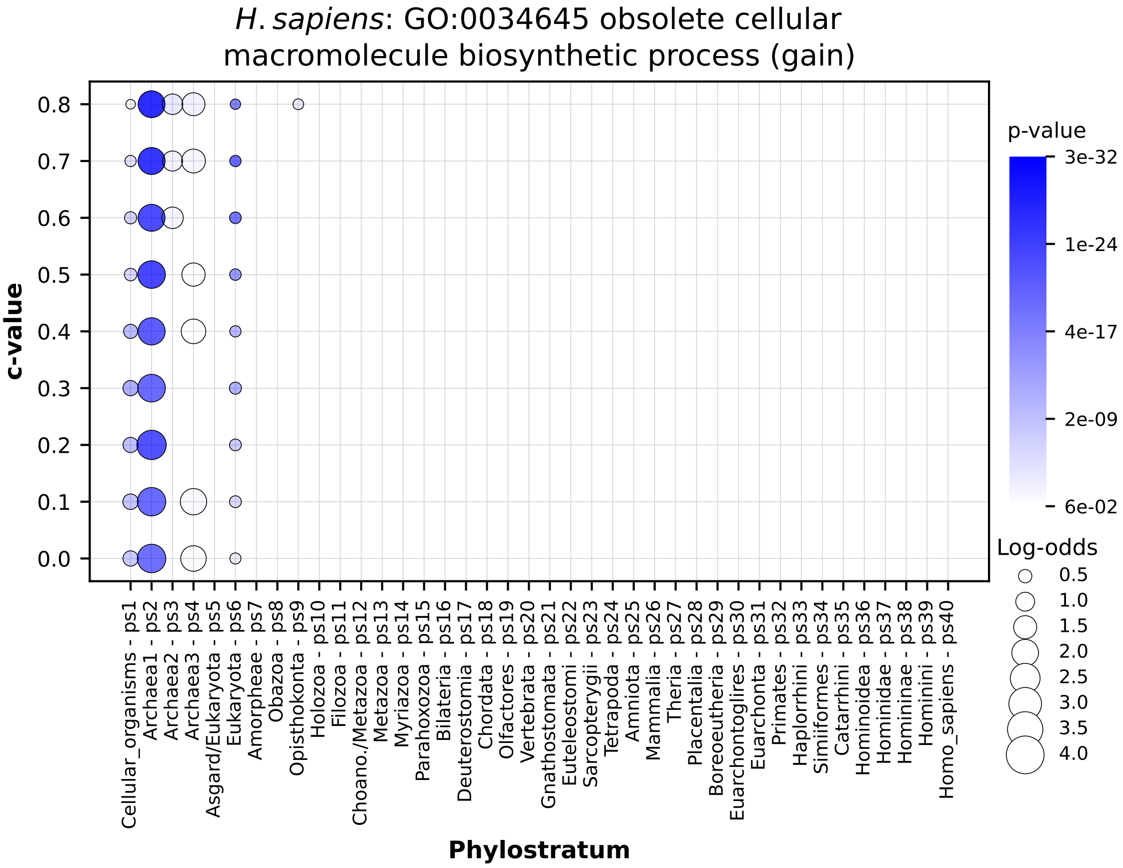
H. sapiens: GO:0034645 obsolete cellular macromolecule biosynthetic process (gain)
c-value
0.8
0.7
0.6
0.5
0.4
0.3
0.2
0.1
0.0
p-value
3e-32
1e-24
4e-17
2e-09
6e-02
Log-odds
0.5
1.0
1.5
2.0
2.5
3.0
3.5
4.0
Cellular_organisms - ps1
Archaea1 - ps2
Archaea2 - ps3
Archaea3 - ps4
Asgard/Eukaryota - ps5
Eukaryota - ps6
Amorpheae - ps7
Obazoa - ps8
Opisthokonta - ps9
Holozoa - ps10
Filozoa - ps11
Choano./Metazoa - ps12
Metazoa - ps13
Myriazoa - ps14
Parahoxozoa - ps15
Bilateria - ps16
Deuterostomia - ps17
Chordata - ps18
Olfactores - ps19
Vertebrata - ps20
Gnathostomata - ps21
Euteleostomi - ps22
Sarcopterygii - ps23
Tetrapoda - ps24
Amniota - ps25
Mammalia - ps26
Theria - ps27
Placentalia - ps28
Boreoeutheria - ps29
Euarchontoglires - ps30
Euarchonta - ps31
Primates - ps32
Haplorrhini - ps33
Simiiformes - ps34
Catarrhini - ps35
Hominoidea - ps36
Hominidae - ps37
Homininae - ps38
Hominini - ps39
Homo_sapiens - ps40
Phylostratum

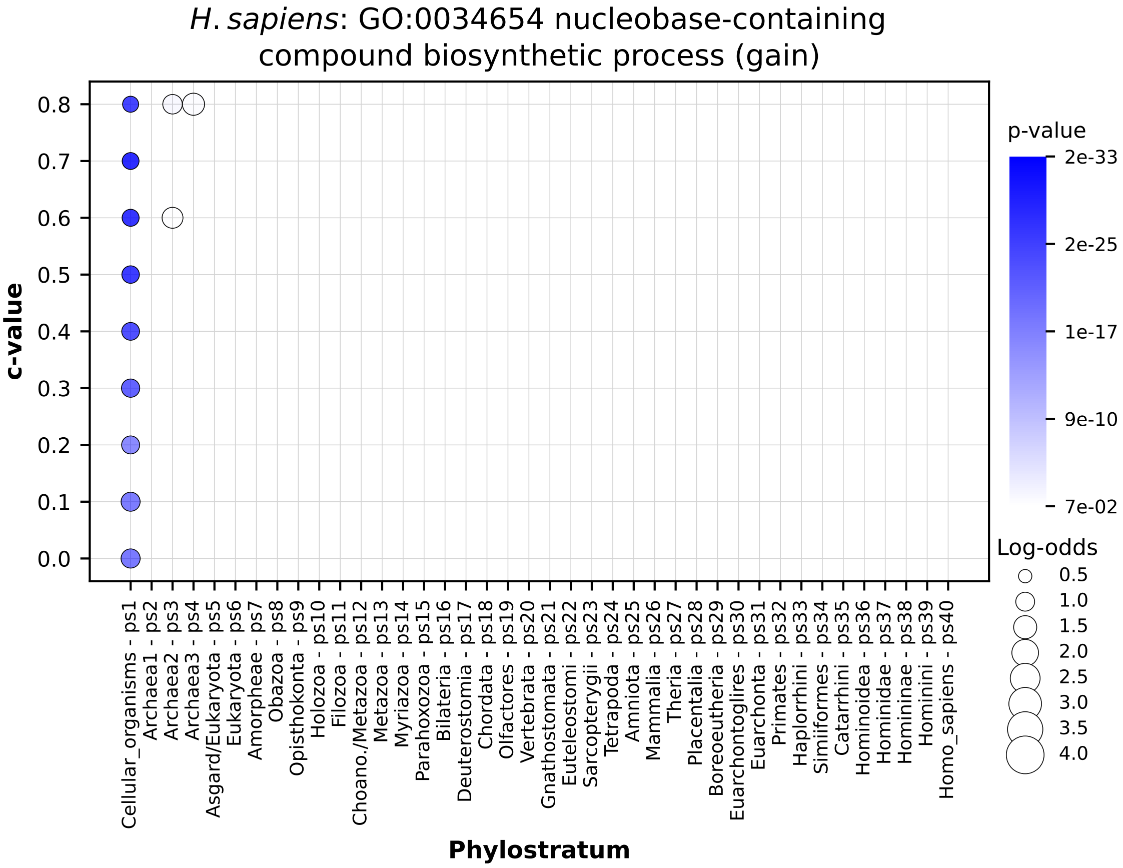

H. sapiens: GO:0034654 nucleobase-containing compound biosynthetic process (gain)
c-value
0.8
0.7
0.6
0.5
0.4
0.3
0.2
0.1
0.0
p-value
2e-33
2e-25
1e-17
9e-10
7e-02
Log-odds
0.5
1.0
1.5
2.0
2.5
3.0
3.5
4.0
Cellular_organisms - ps1
Archaea1 - ps2
Archaea2 - ps3
Archaea3 - ps4
Asgard/Eukaryota - ps5
Eukaryota - ps6
Amorpheae - ps7
Obazoa - ps8
Opisthokonta - ps9
Holozoa - ps10
Filozoa - ps11
Choano./Metazoa - ps12
Metazoa - ps13
Myriazoa - ps14
Parahoxozoa - ps15
Bilateria - ps16
Deuterostomia - ps17
Chordata - ps18
Olfactores - ps19
Vertebrata - ps20
Gnathostomata - ps21
Euteleostomi - ps22
Sarcopterygii - ps23
Tetrapoda - ps24
Amniota - ps25
Mammalia - ps26
Theria - ps27
Placentalia - ps28
Boreoeutheria - ps29
Euarchontoglires - ps30
Euarchonta - ps31
Primates - ps32
Haplorrhini - ps33
Simiiformes - ps34
Catarrhini - ps35
Hominoidea - ps36
Hominidae - ps37
Homininae - ps38
Hominini - ps39
Homo_sapiens - ps40
Phylostratum

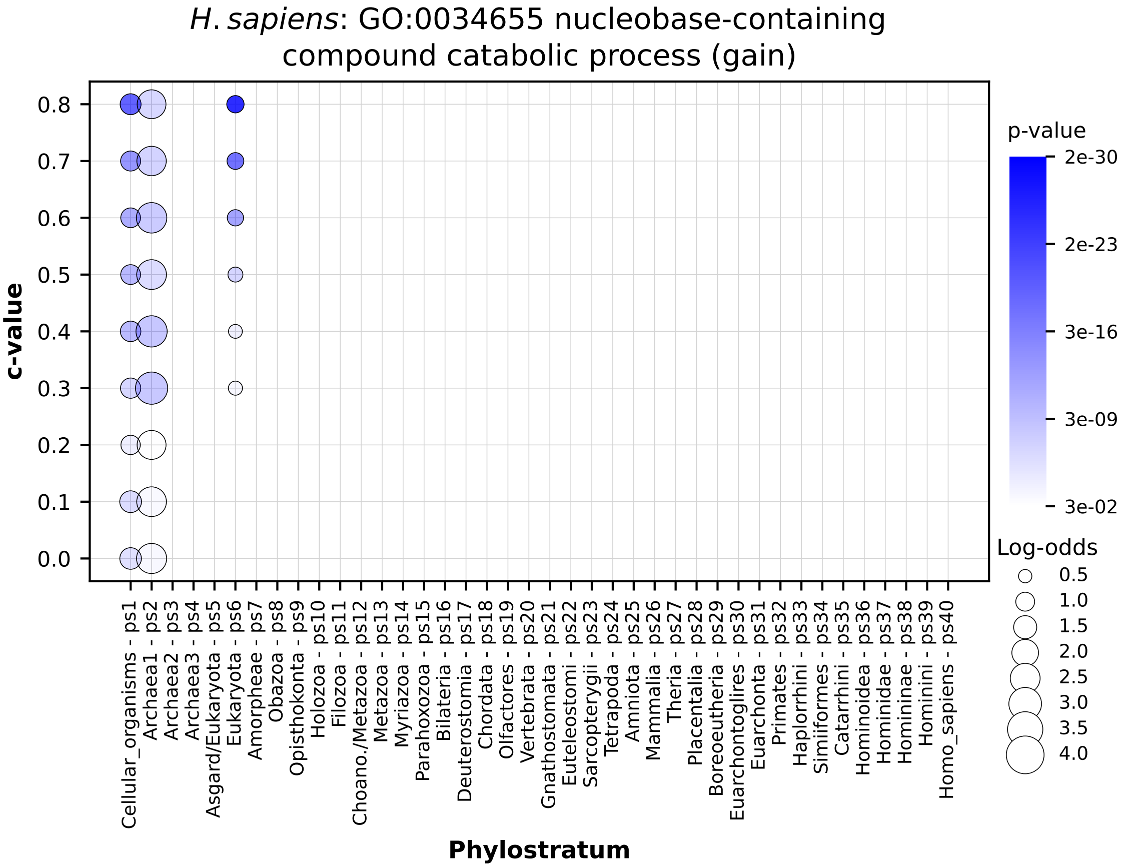
H. sapiens: GO:0034655 nucleobase-containing compound catabolic process (gain)
c-value
0.8
0.7
0.6
0.5
0.4
0.3
0.2
0.1
0.0
p-value
2e-30
2e-23
3e-16
3e-09
3e-02
Log-odds
0.5
1.0
1.5
2.0
2.5
3.0
3.5
4.0
Cellular_organisms - ps1
Archaea1 - ps2
Archaea2 - ps3
Archaea3 - ps4
Asgard/Eukaryota - ps5
Eukaryota - ps6
Amorpheae - ps7
Obazoa - ps8
Opisthokonta - ps9
Holozoa - ps10
Filozoa - ps11
Choano./Metazoa - ps12
Metazoa - ps13
Myriazoa - ps14
Parahoxozoa - ps15
Bilateria - ps16
Deuterostomia - ps17
Chordata - ps18
Olfactores - ps19
Vertebrata - ps20
Gnathostomata - ps21
Euteleostomi - ps22
Sarcopterygii - ps23
Tetrapoda - ps24
Amniota - ps25
Mammalia - ps26
Theria - ps27
Placentalia - ps28
Boreoeutheria - ps29
Euarchontoglires - ps30
Euarchonta - ps31
Primates - ps32
Haplorrhini - ps33
Simiiformes - ps34
Catarrhini - ps35
Hominoidea - ps36
Hominidae - ps37
Homininae - ps38
Hominini - ps39
Homo_sapiens - ps40
Phylostratum

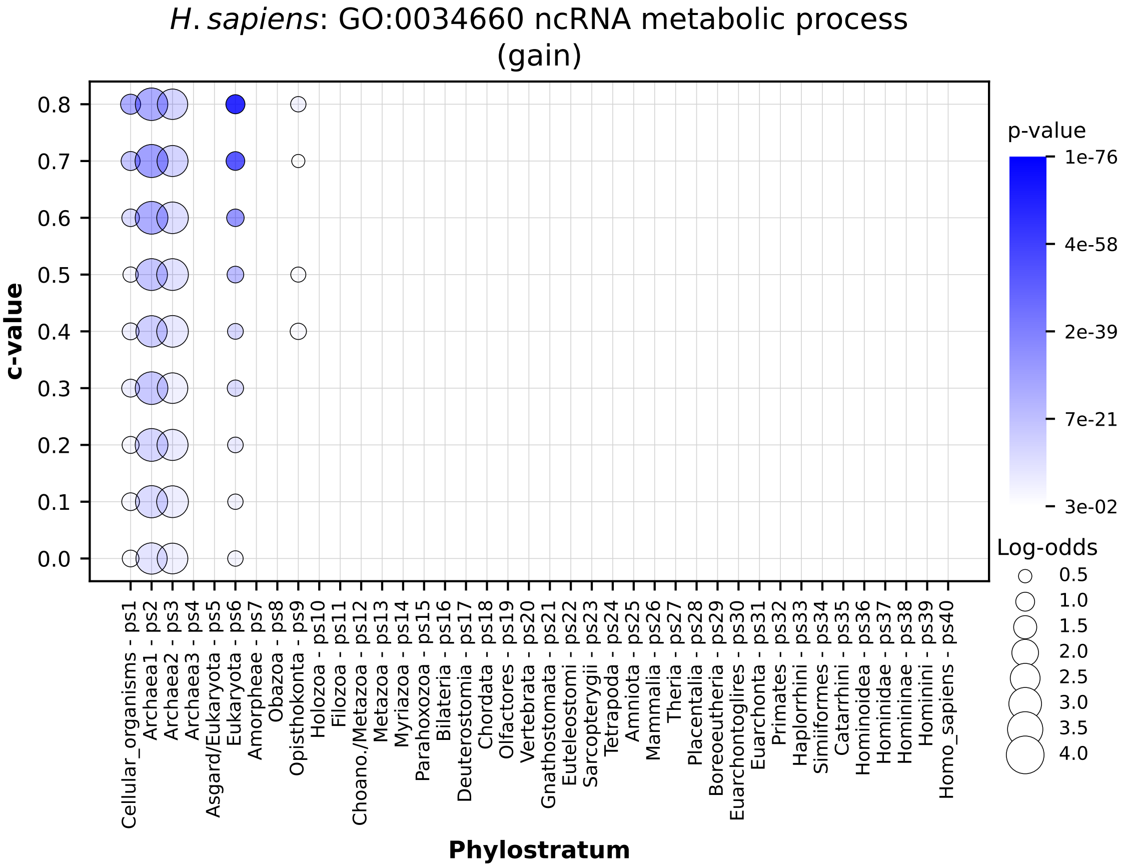

H. sapiens: GO:0034660 ncRNA metabolic process (gain)
c-value
0.0
0.1
0.2
0.3
0.4
0.5
0.6
0.7
0.8
Phylostratum
Cellular_organisms - ps1
Archaea1 - ps2
Archaea2 - ps3
Archaea3 - ps4
Asgard/Eukaryota - ps5
Eukaryota - ps6
Amorpheae - ps7
Obazoa - ps8
Opisthokonta - ps9
Holozoa - ps10
Filozoa - ps11
Choano./Metazoa - ps12
Metazoa - ps13
Myriazoa - ps14
Parahoxozoa - ps15
Bilateria - ps16
Deuterostomia - ps17
Chordata - ps18
Olfactores - ps19
Vertebrata - ps20
Gnathostomata - ps21
Euteleostomi - ps22
Sarcopterygii - ps23
Tetrapoda - ps24
Amniota - ps25
Mammalia - ps26
Theria - ps27
Placentalia - ps28
Boreoeutheria - ps29
Euarchontoglires - ps30
Euarchonta - ps31
Primates - ps32
Haplorrhini - ps33
Simiiformes - ps34
Catarrhini - ps35
Hominoidea - ps36
Hominidae - ps37
Homininae - ps38
Hominini - ps39
Homo_sapiens - ps40
p-value
1e-76
4e-58
2e-39
7e-21
3e-02
Log-odds
0.5
1.0
1.5
2.0
2.5
3.0
3.5
4.0

H. sapiens: GO:0035639 purine ribonucleoside triphosphate binding (gain)

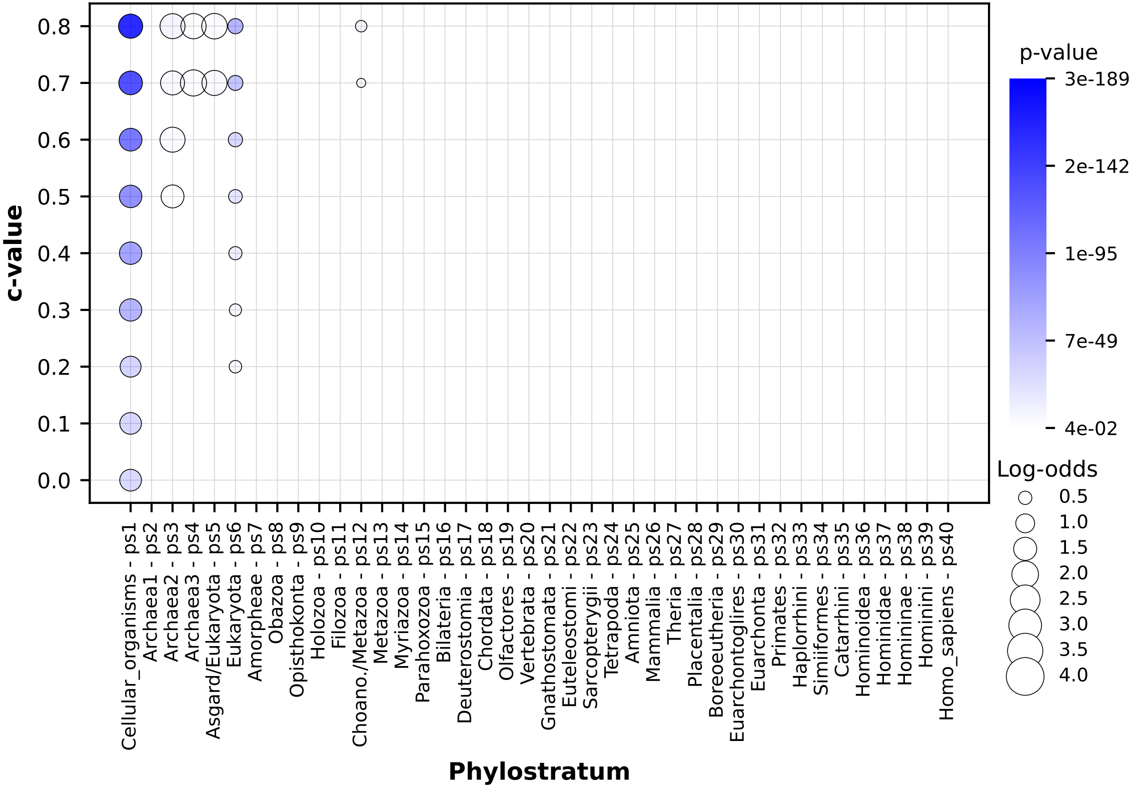

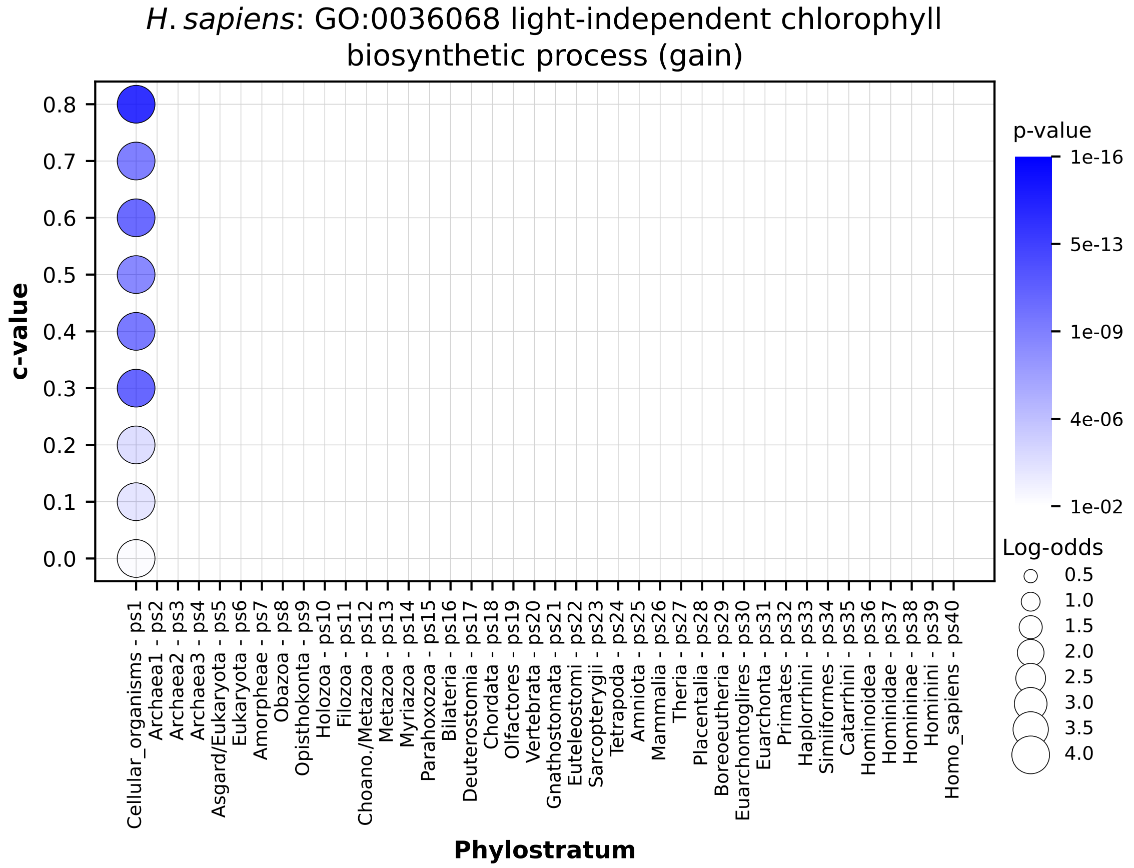
H. sapiens: GO:0036068 light-independent chlorophyll biosynthetic process (gain)
c-value
0.8
0.7
0.6
0.5
0.4
0.3
0.2
0.1
0.0
p-value
1e-16
5e-13
1e-09
4e-06
1e-02
Log-odds
0.5
1.0
1.5
2.0
2.5
3.0
3.5
4.0
Cellular_organisms - ps1
Archaea1 - ps2
Archaea2 - ps3
Archaea3 - ps4
Asgard/Eukaryota - ps5
Eukaryota - ps6
Amorpheae - ps7
Obazoa - ps8
Opisthokonta - ps9
Holozoa - ps10
Filozoa - ps11
Choano./Metazoa - ps12
Metazoa - ps13
Myriazoa - ps14
Parahoxozoa - ps15
Bilateria - ps16
Deuterostomia - ps17
Chordata - ps18
Olfactores - ps19
Vertebrata - ps20
Gnathostomata - ps21
Euteleostomi - ps22
Sarcopterygii - ps23
Tetrapoda - ps24
Amniota - ps25
Mammalia - ps26
Theria - ps27
Placentalia - ps28
Boreoeutheria - ps29
Euarchontoglires - ps30
Euarchonta - ps31
Primates - ps32
Haplorrhini - ps33
Simiiformes - ps34
Catarrhini - ps35
Hominoidea - ps36
Hominidae - ps37
Homininae - ps38
Hominini - ps39
Homo_sapiens - ps40
Phylostratum

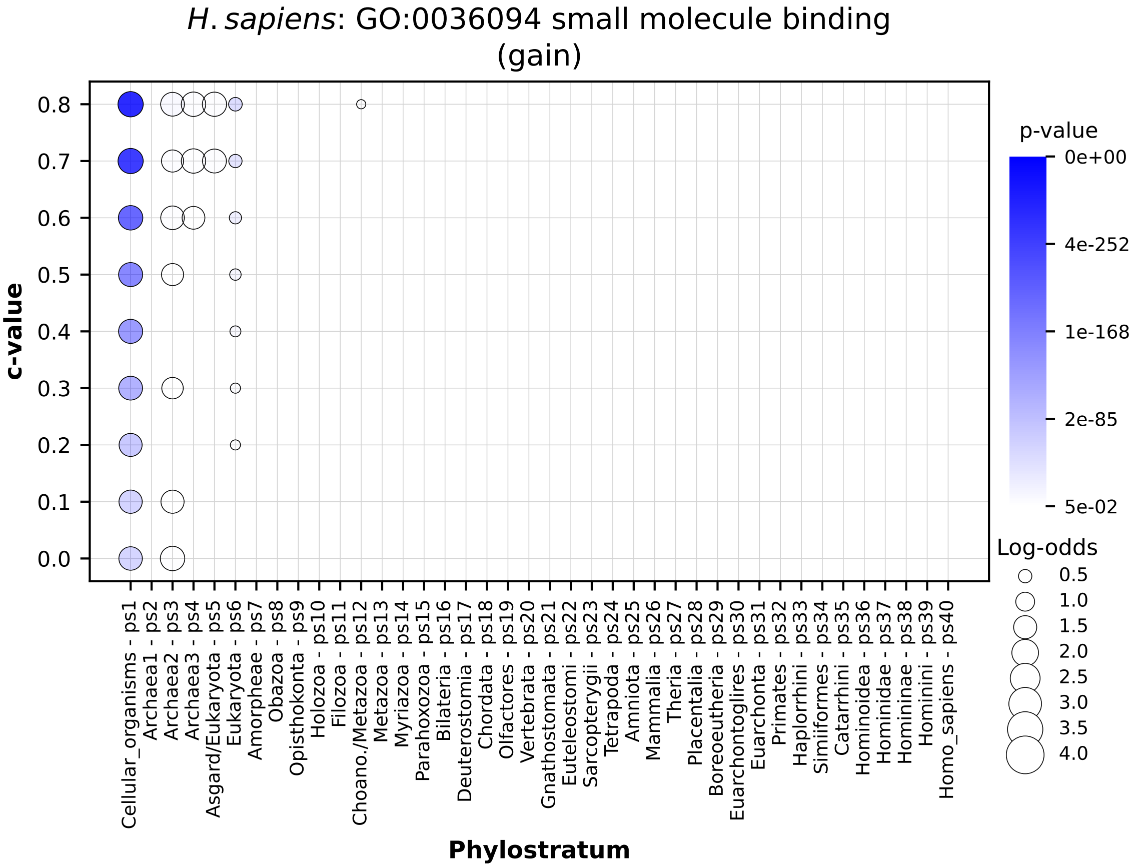
H. sapiens: GO:0036094 small molecule binding
(gain)
c-value
0.8
0.7
0.6
0.5
0.4
0.3
0.2
0.1
0.0
Phylostratum
Cellular_organisms - ps1
Archaea1 - ps2
Archaea2 - ps3
Archaea3 - ps4
Asgard/Eukaryota - ps5
Eukaryota - ps6
Amorpheae - ps7
Obazoa - ps8
Opisthokonta - ps9
Holozoa - ps10
Filozoa - ps11
Choano./Metazoa - ps12
Metazoa - ps13
Myriazoa - ps14
Parahoxozoa - ps15
Bilateria - ps16
Deuterostomia - ps17
Chordata - ps18
Olfactores - ps19
Vertebrata - ps20
Gnathostomata - ps21
Euteleostomi - ps22
Sarcopterygii - ps23
Tetrapoda - ps24
Amniota - ps25
Mammalia - ps26
Theria - ps27
Placentalia - ps28
Boreoeutheria - ps29
Euarchontoglires - ps30
Euarchonta - ps31
Primates - ps32
Haplorrhini - ps33
Simiiformes - ps34
Catarrhini - ps35
Hominoidea - ps36
Hominidae - ps37
Homininae - ps38
Hominini - ps39
Homo_sapiens - ps40
p-value
0e+00
4e-252
1e-168
2e-85
5e-02
Log-odds
0.5
1.0
1.5
2.0
2.5
3.0
3.5
4.0

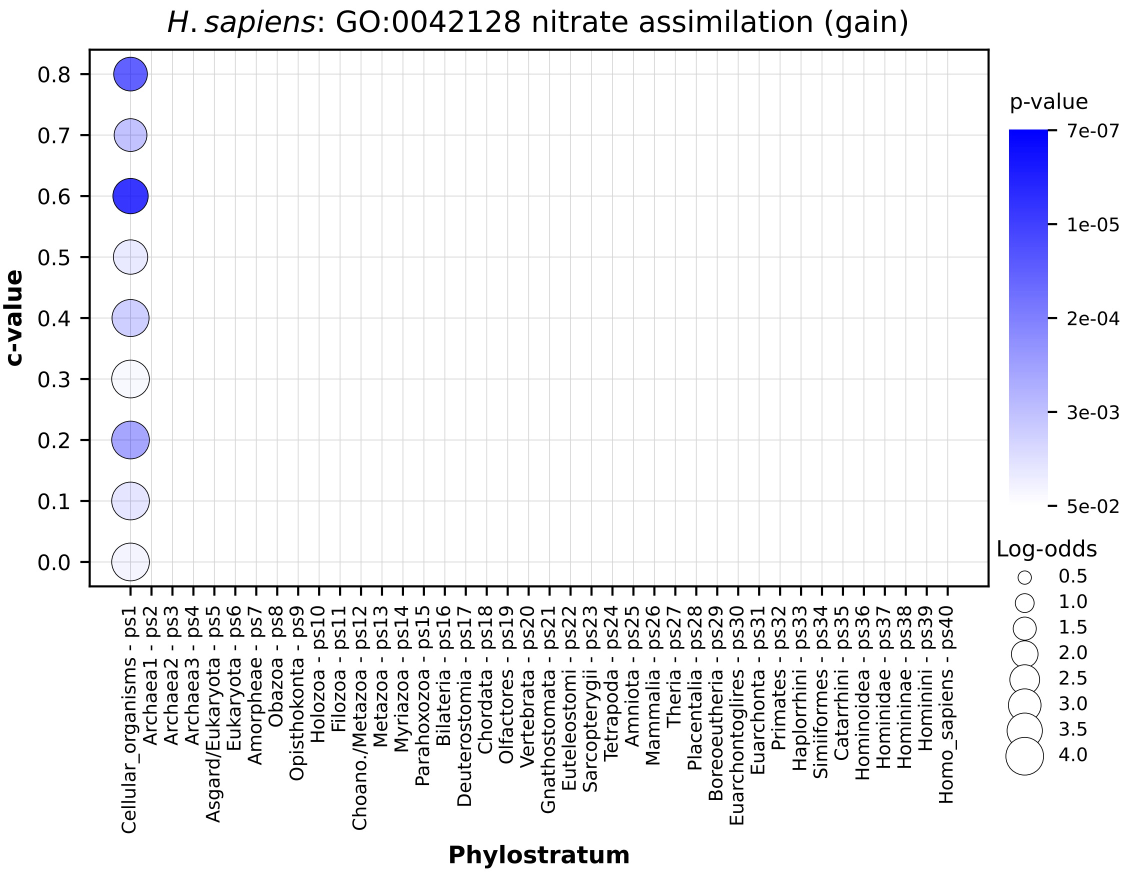
H. sapiens: GO:0042128 nitrate assimilation (gain)
c-value
0.8
0.7
0.6
0.5
0.4
0.3
0.2
0.1
0.0
Phylostratum
Cellular_organisms - ps1
Archaea1 - ps2
Archaea2 - ps3
Archaea3 - ps4
Asgard/Eukaryota - ps5
Eukaryota - ps6
Amorpheae - ps7
Obazoa - ps8
Opisthokonta - ps9
Holozoa - ps10
Filozoa - ps11
Choano./Metazoa - ps12
Metazoa - ps13
Myriazoa - ps14
Parahoxozoa - ps15
Bilateria - ps16
Deuterostomia - ps17
Chordata - ps18
Olfactores - ps19
Vertebrata - ps20
Gnathostomata - ps21
Euteleostomi - ps22
Sarcopterygii - ps23
Tetrapoda - ps24
Amniota - ps25
Mammalia - ps26
Theria - ps27
Placentalia - ps28
Boreoeutheria - ps29
Euarchontoglires - ps30
Euarchonta - ps31
Primates - ps32
Haplorrhini - ps33
Simiiformes - ps34
Catarrhini - ps35
Hominoidea - ps36
Hominidae - ps37
Homininae - ps38
Hominini - ps39
Homo_sapiens - ps40
p-value
7e-07
1e-05
2e-04
3e-03
5e-02
Log-odds
0.5
1.0
1.5
2.0
2.5
3.0
3.5
4.0

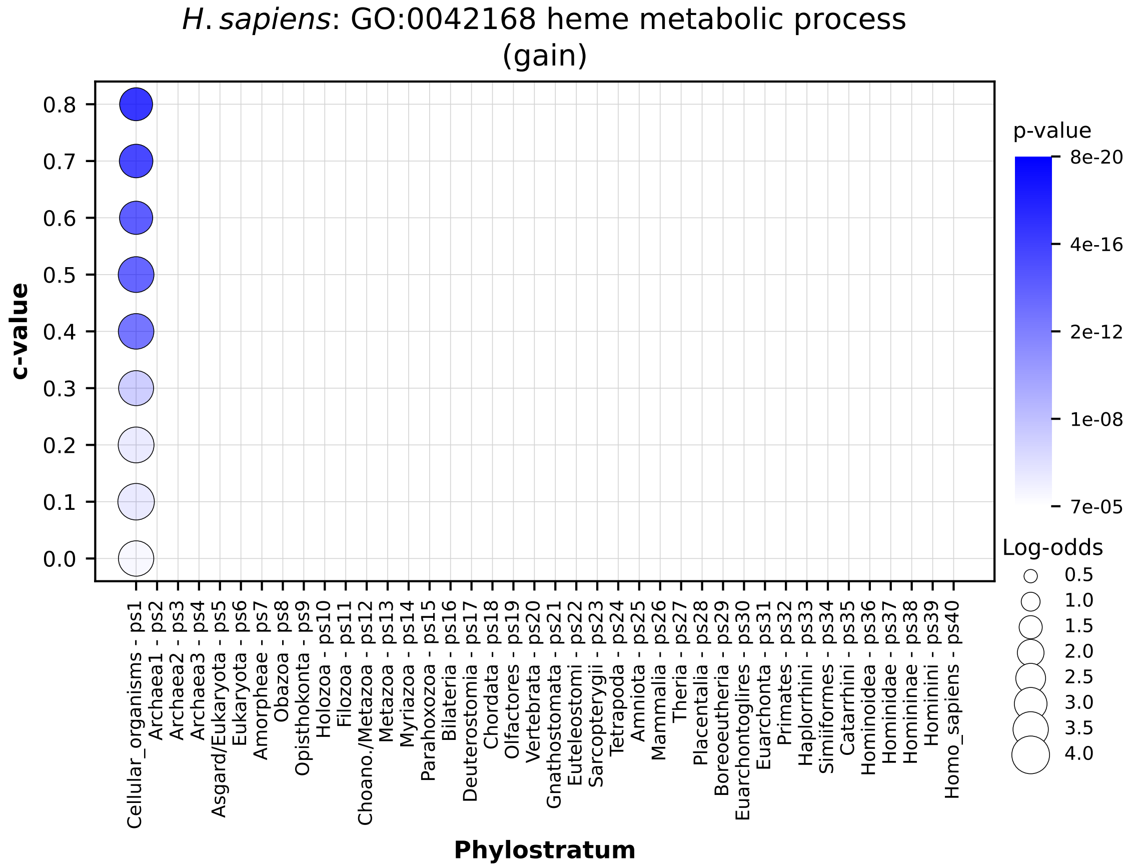

$H. sapiens$: GO:0042168 heme metabolic process
(gain)
c-value
0.8
0.7
0.6
0.5
0.4
0.3
0.2
0.1
0.0
p-value
8e-20
4e-16
2e-12
1e-08
7e-05
Log-odds
0.5
1.0
1.5
2.0
2.5
3.0
3.5
4.0
Cellular_organisms - ps1
Archaea1 - ps2
Archaea2 - ps3
Archaea3 - ps4
Asgard/Eukaryota - ps5
Eukaryota - ps6
Amorpheae - ps7
Obazoa - ps8
Opisthokonta - ps9
Holozoa - ps10
Filozoa - ps11
Choano./Metazoa - ps12
Metazoa - ps13
Myriazoa - ps14
Parahoxozoa - ps15
Bilateria - ps16
Deuterostomia - ps17
Chordata - ps18
Olfactores - ps19
Vertebrata - ps20
Gnathostomata - ps21
Euteleostomi - ps22
Sarcopterygii - ps23
Tetrapoda - ps24
Amniota - ps25
Mammalia - ps26
Theria - ps27
Placentalia - ps28
Boreoeutheria - ps29
Euarchontoglires - ps30
Euarchonta - ps31
Primates - ps32
Haplorrhini - ps33
Simiiformes - ps34
Catarrhini - ps35
Hominoidea - ps36
Hominidae - ps37
Homininae - ps38
Hominini - ps39
Homo_sapiens - ps40
Phylostratum

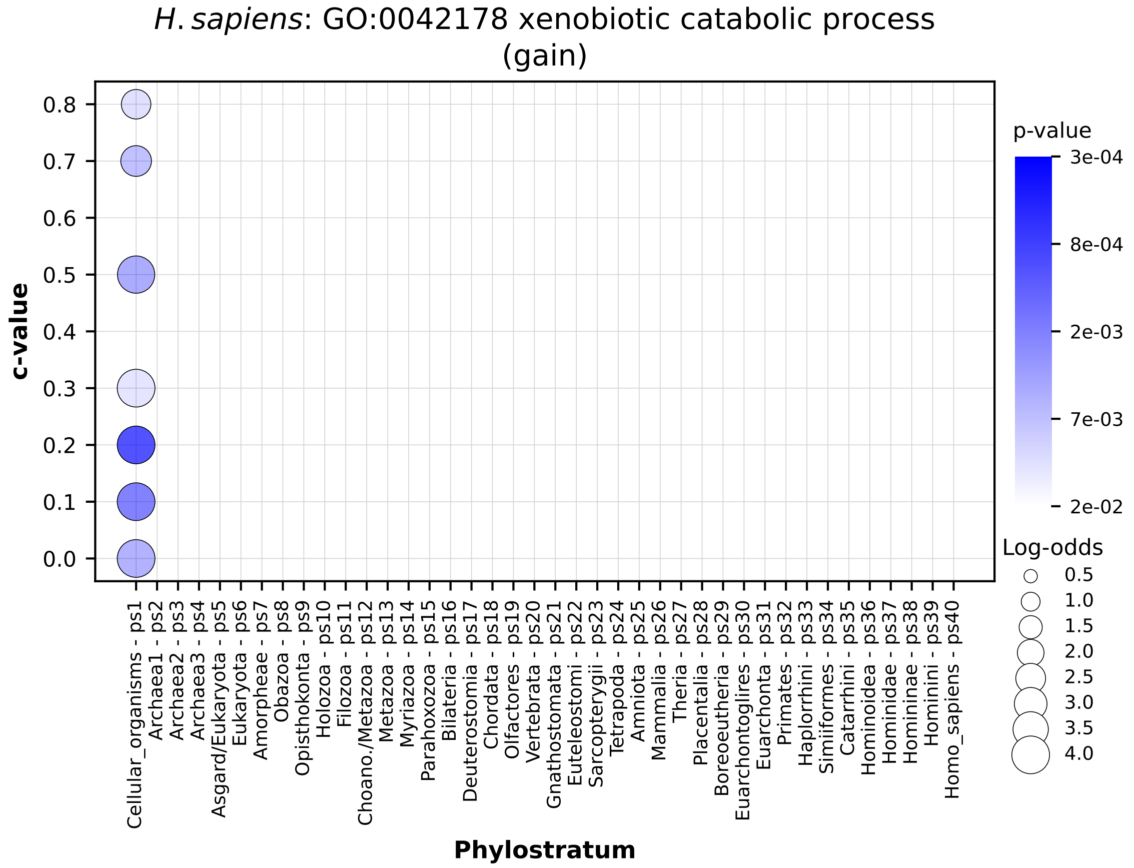

H. sapiens: GO:0042178 xenobiotic catabolic process
(gain)
c-value
0.8
0.7
0.6
0.5
0.4
0.3
0.2
0.1
0.0
p-value
3e-04
8e-04
2e-03
7e-03
2e-02
Log-odds
0.5
1.0
1.5
2.0
2.5
3.0
3.5
4.0
Cellular_organisms - ps1
Archaea1 - ps2
Archaea2 - ps3
Archaea3 - ps4
Asgard/Eukaryota - ps5
Eukaryota - ps6
Amorpheae - ps7
Obazoa - ps8
Opisthokonta - ps9
Holozoa - ps10
Filozoa - ps11
Choano./Metazoa - ps12
Metazoa - ps13
Myriazoa - ps14
Parahoxozoa - ps15
Bilateria - ps16
Deuterostomia - ps17
Chordata - ps18
Olfactores - ps19
Vertebrata - ps20
Gnathostomata - ps21
Euteleostomi - ps22
Sarcopterygii - ps23
Tetrapoda - ps24
Amniota - ps25
Mammalia - ps26
Theria - ps27
Placentalia - ps28
Boreoeutheria - ps29
Euarchontoglires - ps30
Euarchonta - ps31
Primates - ps32
Haplorrhini - ps33
Simiiformes - ps34
Catarrhini - ps35
Hominoidea - ps36
Hominidae - ps37
Homininae - ps38
Hominini - ps39
Homo_sapiens - ps40
Phylostratum

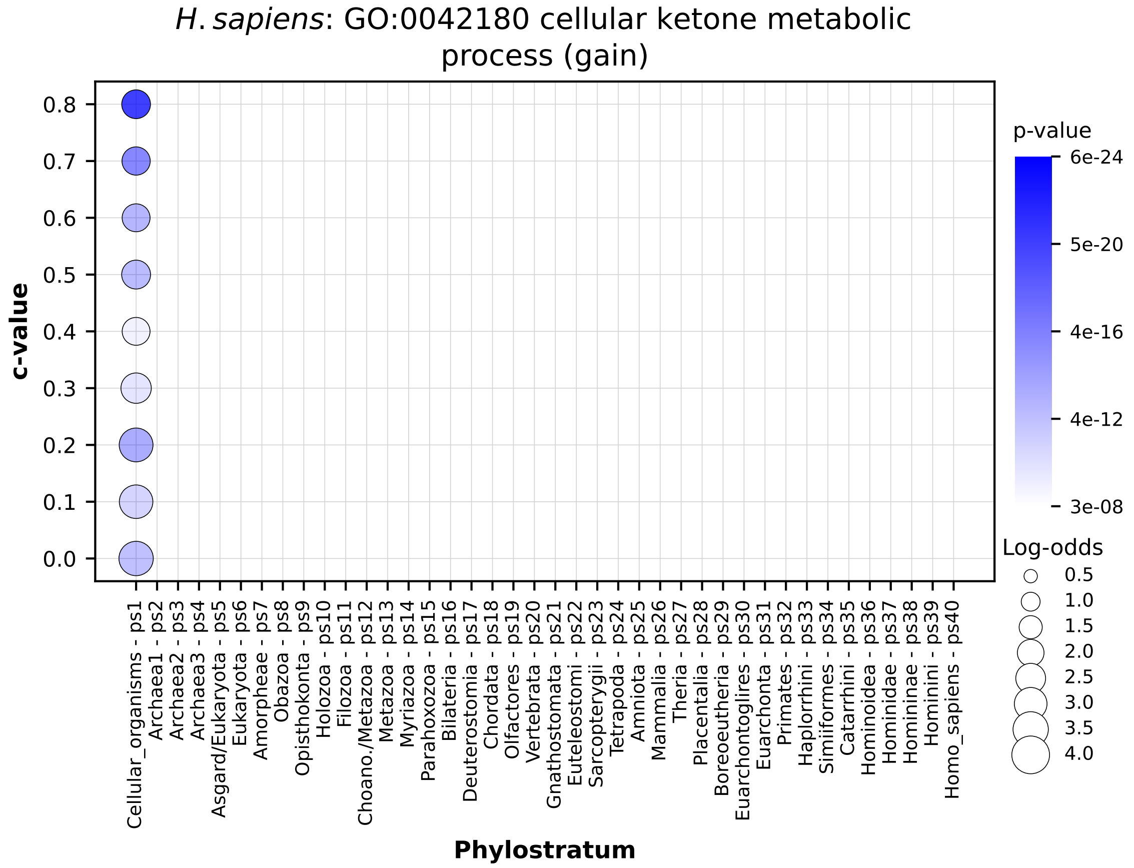

H. sapiens: GO:0042180 cellular ketone metabolic process (gain)
c-value
0.8
0.7
0.6
0.5
0.4
0.3
0.2
0.1
0.0
Phylostratum
Cellular_organisms - ps1
Archaea1 - ps2
Archaea2 - ps3
Archaea3 - ps4
Asgard/Eukaryota - ps5
Eukaryota - ps6
Amorpheae - ps7
Obazoa - ps8
Opisthokonta - ps9
Holozoa - ps10
Filozoa - ps11
Choano./Metazoa - ps12
Metazoa - ps13
Myriazoa - ps14
Parahoxozoa - ps15
Bilateria - ps16
Deuterostomia - ps17
Chordata - ps18
Olfactores - ps19
Vertebrata - ps20
Gnathostomata - ps21
Euteleostomi - ps22
Sarcopterygii - ps23
Tetrapoda - ps24
Amniota - ps25
Mammalia - ps26
Theria - ps27
Placentalia - ps28
Boreoeutheria - ps29
Euarchontoglires - ps30
Euarchonta - ps31
Primates - ps32
Haplorrhini - ps33
Simiiformes - ps34
Catarrhini - ps35
Hominoidea - ps36
Hominidae - ps37
Homininae - ps38
Hominini - ps39
Homo_sapiens - ps40
p-value
6e-24
5e-20
4e-16
4e-12
3e-08
Log-odds
0.5
1.0
1.5
2.0
2.5
3.0
3.5
4.0

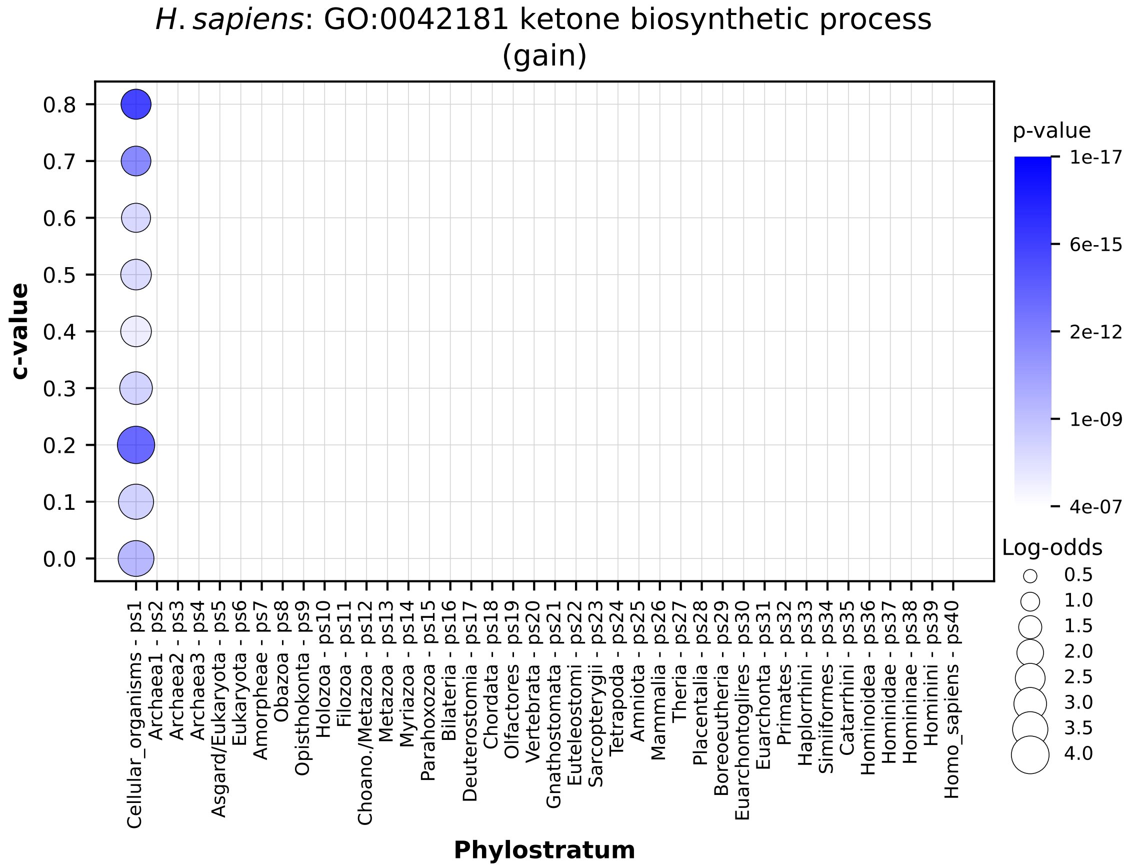
H. sapiens: GO:0042181 ketone biosynthetic process
(gain)
c-value
0.8
0.7
0.6
0.5
0.4
0.3
0.2
0.1
0.0
p-value
1e-17
6e-15
2e-12
1e-09
4e-07
Log-odds
0.5
1.0
1.5
2.0
2.5
3.0
3.5
4.0
Cellular_organisms - ps1
Archaea1 - ps2
Archaea2 - ps3
Archaea3 - ps4
Asgard/Eukaryota - ps5
Eukaryota - ps6
Amorpheae - ps7
Obazoa - ps8
Opisthokonta - ps9
Holozoa - ps10
Filozoa - ps11
Choano./Metazoa - ps12
Metazoa - ps13
Myriazoa - ps14
Parahoxozoa - ps15
Bilateria - ps16
Deuterostomia - ps17
Chordata - ps18
Olfactores - ps19
Vertebrata - ps20
Gnathostomata - ps21
Euteleostomi - ps22
Sarcopterygii - ps23
Tetrapoda - ps24
Amniota - ps25
Mammalia - ps26
Theria - ps27
Placentalia - ps28
Boreoeutheria - ps29
Euarchontoglires - ps30
Euarchonta - ps31
Primates - ps32
Haplorrhini - ps33
Simiiformes - ps34
Catarrhini - ps35
Hominoidea - ps36
Hominidae - ps37
Homininae - ps38
Hominini - ps39
Homo_sapiens - ps40
Phylostratum

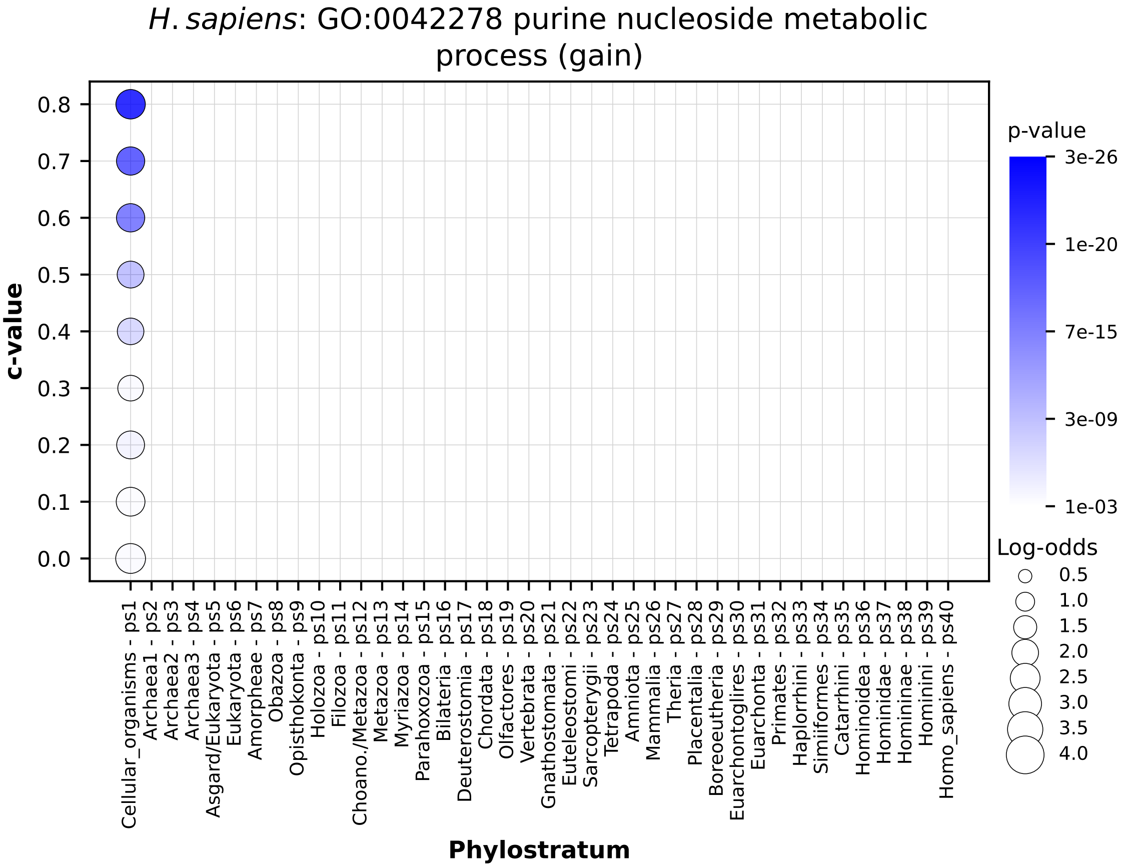

H. sapiens: GO:0042278 purine nucleoside metabolic process (gain)
c-value
0.8
0.7
0.6
0.5
0.4
0.3
0.2
0.1
0.0
Phylostratum
Cellular_organisms - ps1
Archaea1 - ps2
Archaea2 - ps3
Archaea3 - ps4
Asgard/Eukaryota - ps5
Eukaryota - ps6
Amorpheae - ps7
Obazoa - ps8
Opisthokonta - ps9
Holozoa - ps10
Filozoa - ps11
Choano./Metazoa - ps12
Metazoa - ps13
Myriazoa - ps14
Parahoxozoa - ps15
Bilateria - ps16
Deuterostomia - ps17
Chordata - ps18
Olfactores - ps19
Vertebrata - ps20
Gnathostomata - ps21
Euteleostomi - ps22
Sarcopterygii - ps23
Tetrapoda - ps24
Amniota - ps25
Mammalia - ps26
Theria - ps27
Placentalia - ps28
Boreoeutheria - ps29
Euarchontoglires - ps30
Euarchonta - ps31
Primates - ps32
Haplorrhini - ps33
Simiiformes - ps34
Catarrhini - ps35
Hominoidea - ps36
Hominidae - ps37
Homininae - ps38
Hominini - ps39
Homo_sapiens - ps40
p-value
3e-26
1e-20
7e-15
3e-09
1e-03
Log-odds
0.5
1.0
1.5
2.0
2.5
3.0
3.5
4.0

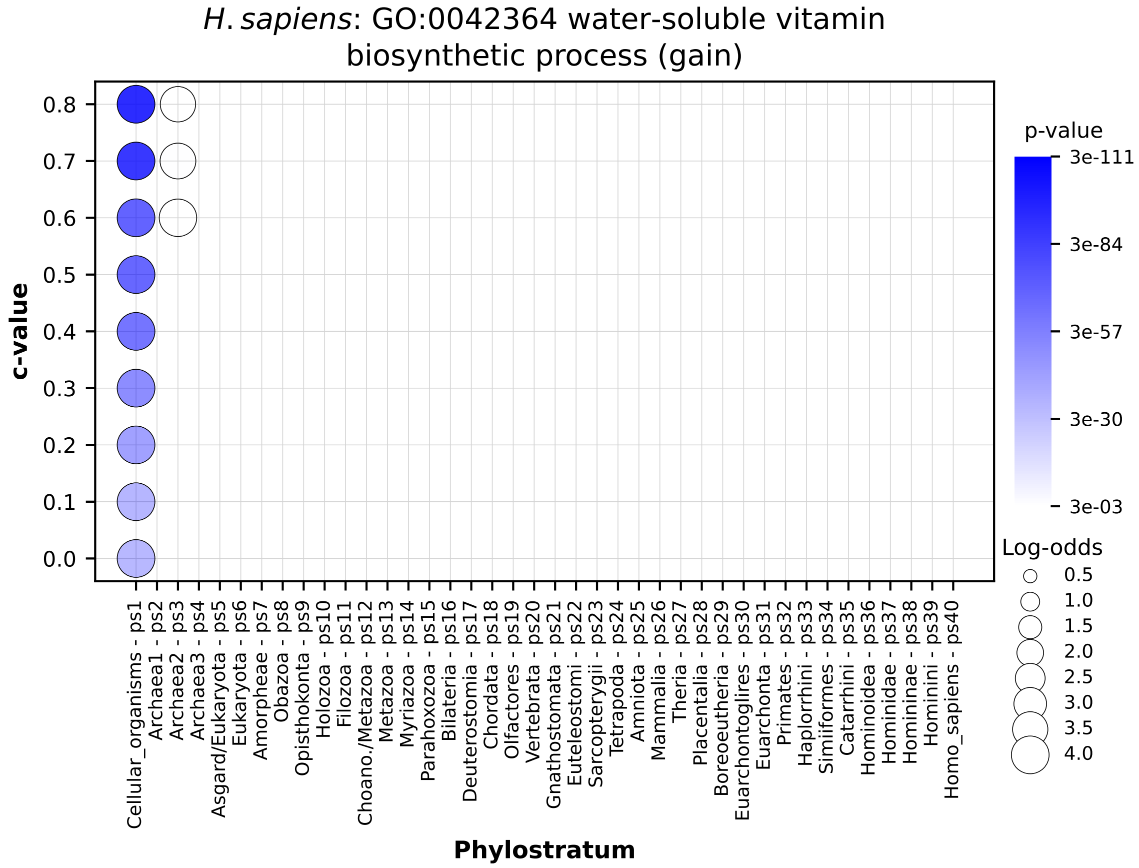
H. sapiens: GO:0042364 water-soluble vitamin biosynthetic process (gain)
c-value
0.8
0.7
0.6
0.5
0.4
0.3
0.2
0.1
0.0
Phylostratum
Cellular_organisms - ps1
Archaea1 - ps2
Archaea2 - ps3
Archaea3 - ps4
Asgard/Eukaryota - ps5
Eukaryota - ps6
Amorpheae - ps7
Obazoa - ps8
Opisthokonta - ps9
Holozoa - ps10
Filozoa - ps11
Choano./Metazoa - ps12
Metazoa - ps13
Myriazoa - ps14
Parahoxozoa - ps15
Bilateria - ps16
Deuterostomia - ps17
Chordata - ps18
Olfactores - ps19
Vertebrata - ps20
Gnathostomata - ps21
Euteleostomi - ps22
Sarcopterygii - ps23
Tetrapoda - ps24
Amniota - ps25
Mammalia - ps26
Theria - ps27
Placentalia - ps28
Boreoeutheria - ps29
Euarchontoglires - ps30
Euarchonta - ps31
Primates - ps32
Haplorrhini - ps33
Simiiformes - ps34
Catarrhini - ps35
Hominoidea - ps36
Hominidae - ps37
Homininae - ps38
Hominini - ps39
Homo_sapiens - ps40
p-value
3e-111
3e-84
3e-57
3e-30
3e-03
Log-odds
0.5
1.0
1.5
2.0
2.5
3.0
3.5
4.0

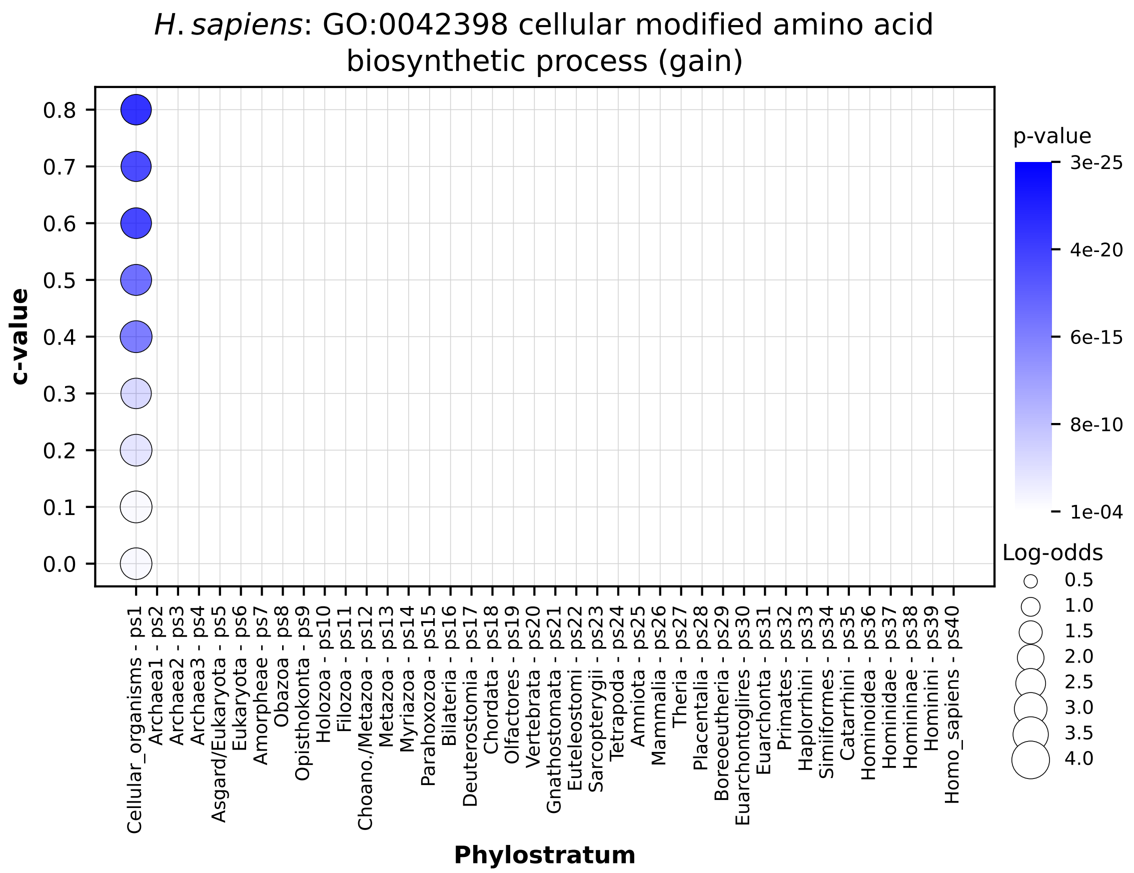

H. sapiens: GO:0042398 cellular modified amino acid biosynthetic process (gain)
c-value
0.8
0.7
0.6
0.5
0.4
0.3
0.2
0.1
0.0
Phylostratum
Cellular_organisms - ps1
Archaea1 - ps2
Archaea2 - ps3
Archaea3 - ps4
Asgard/Eukaryota - ps5
Eukaryota - ps6
Amorpheae - ps7
Obazoa - ps8
Opisthokonta - ps9
Holozoa - ps10
Filozoa - ps11
Choano./Metazoa - ps12
Metazoa - ps13
Myriazoa - ps14
Parahoxozoa - ps15
Bilateria - ps16
Deuterostomia - ps17
Chordata - ps18
Olfactores - ps19
Vertebrata - ps20
Gnathostomata - ps21
Euteleostomi - ps22
Sarcopterygii - ps23
Tetrapoda - ps24
Amniota - ps25
Mammalia - ps26
Theria - ps27
Placentalia - ps28
Boreoeutheria - ps29
Euarchontoglires - ps30
Euarchonta - ps31
Primates - ps32
Haplorrhini - ps33
Simiiformes - ps34
Catarrhini - ps35
Hominoidea - ps36
Hominidae - ps37
Homininae - ps38
Hominini - ps39
Homo_sapiens - ps40
p-value
3e-25
4e-20
6e-15
8e-10
1e-04
Log-odds
0.5
1.0
1.5
2.0
2.5
3.0
3.5
4.0

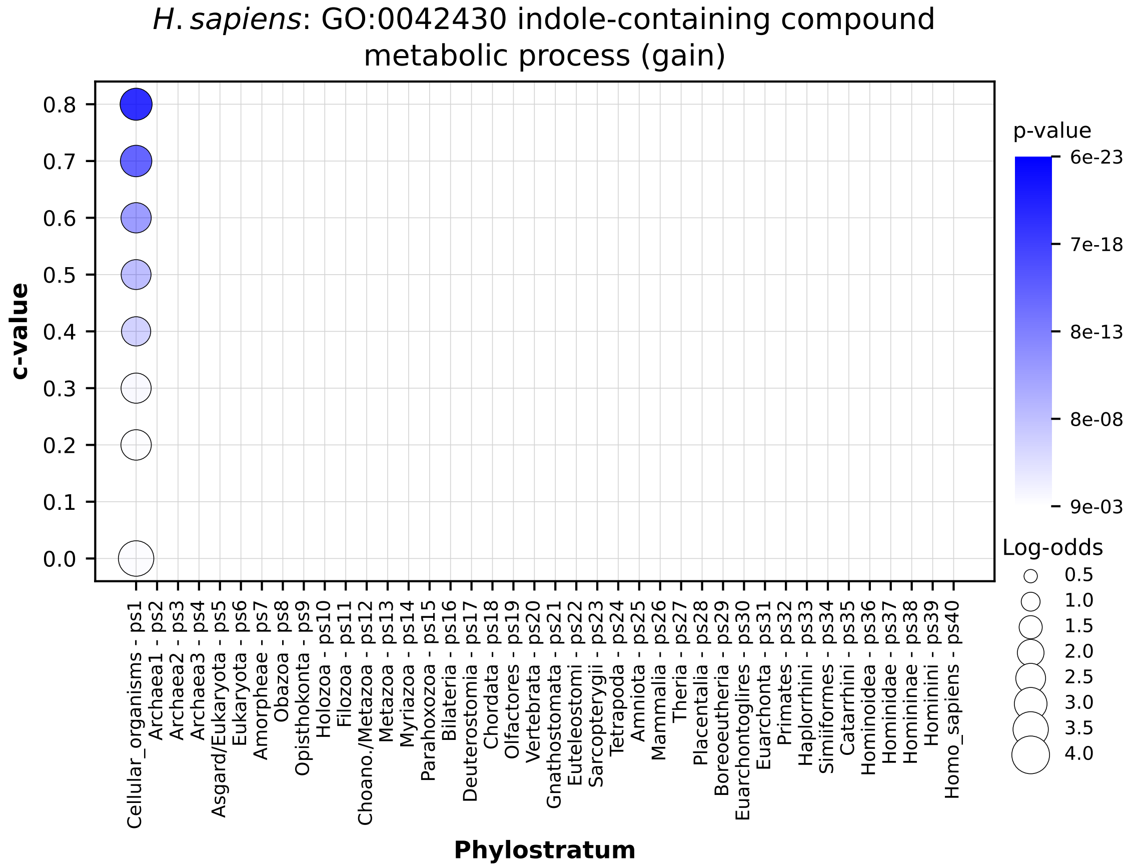

*H. sapiens*: GO:0042430 indole-containing compound metabolic process (gain)
c-value
0.8
0.7
0.6
0.5
0.4
0.3
0.2
0.1
0.0
p-value
6e-23
7e-18
8e-13
8e-08
9e-03
Log-odds
0.5
1.0
1.5
2.0
2.5
3.0
3.5
4.0
Cellular_organisms - ps1
Archaea1 - ps2
Archaea2 - ps3
Archaea3 - ps4
Asgard/Eukaryota - ps5
Eukaryota - ps6
Amorpheae - ps7
Obazoa - ps8
Opisthokonta - ps9
Holozoa - ps10
Filozoa - ps11
Choano./Metazoa - ps12
Metazoa - ps13
Myriazoa - ps14
Parahoxozoa - ps15
Bilateria - ps16
Deuterostomia - ps17
Chordata - ps18
Olfactores - ps19
Vertebrata - ps20
Gnathostomata - ps21
Euteleostomi - ps22
Sarcopterygii - ps23
Tetrapoda - ps24
Amniota - ps25
Mammalia - ps26
Theria - ps27
Placentalia - ps28
Boreoeutheria - ps29
Euarchontoglires - ps30
Euarchonta - ps31
Primates - ps32
Haplorrhini - ps33
Simiiformes - ps34
Catarrhini - ps35
Hominoidea - ps36
Hominidae - ps37
Homininae - ps38
Hominini - ps39
Homo_sapiens - ps40
Phylostratum

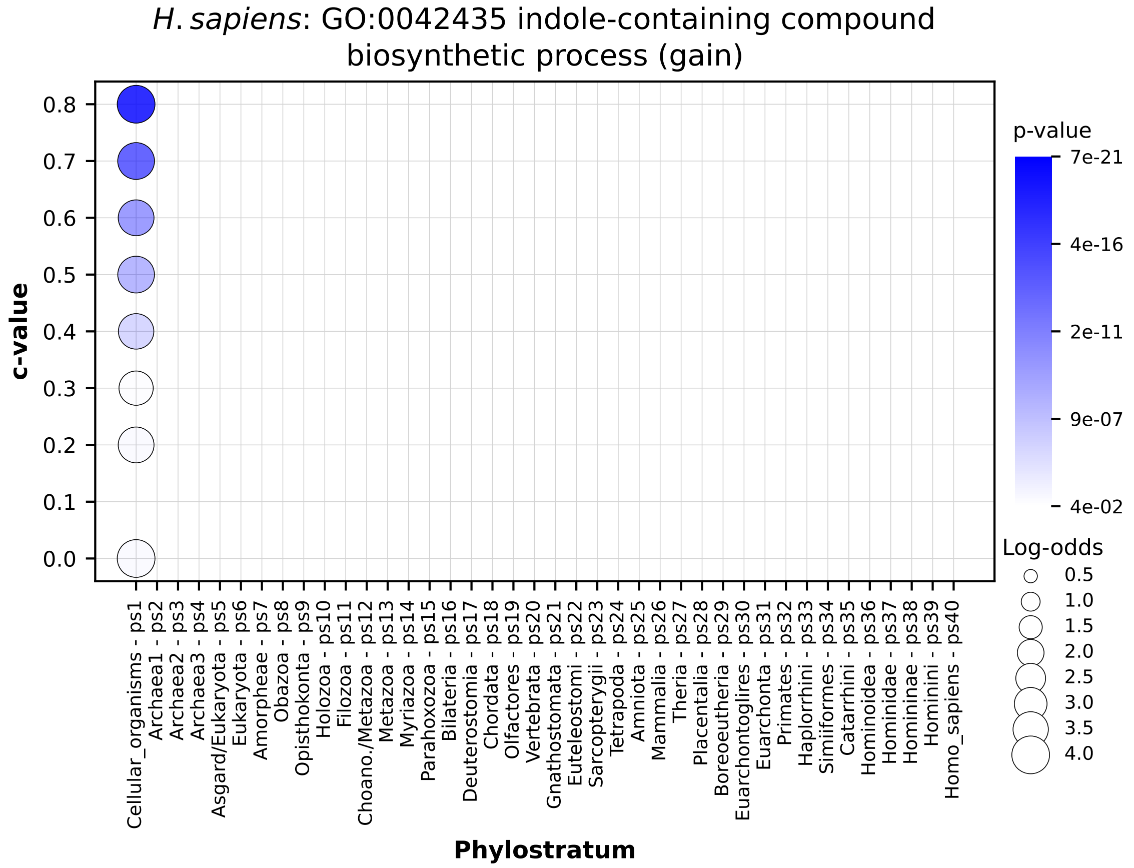

H. sapiens: GO:0042435 indole-containing compound biosynthetic process (gain)
c-value
0.8
0.7
0.6
0.5
0.4
0.3
0.2
0.1
0.0
p-value
7e-21
4e-16
2e-11
9e-07
4e-02
Log-odds
0.5
1.0
1.5
2.0
2.5
3.0
3.5
4.0
Cellular_organisms - ps1
Archaea1 - ps2
Archaea2 - ps3
Archaea3 - ps4
Asgard/Eukaryota - ps5
Eukaryota - ps6
Amorpheae - ps7
Obazoa - ps8
Opisthokonta - ps9
Holozoa - ps10
Filozoa - ps11
Choano./Metazoa - ps12
Metazoa - ps13
Myriazoa - ps14
Parahoxozoa - ps15
Bilateria - ps16
Deuterostomia - ps17
Chordata - ps18
Olfactores - ps19
Vertebrata - ps20
Gnathostomata - ps21
Euteleostomi - ps22
Sarcopterygii - ps23
Tetrapoda - ps24
Amniota - ps25
Mammalia - ps26
Theria - ps27
Placentalia - ps28
Boreoeutheria - ps29
Euarchontoglires - ps30
Euarchonta - ps31
Primates - ps32
Haplorrhini - ps33
Simiiformes - ps34
Catarrhini - ps35
Hominoidea - ps36
Hominidae - ps37
Homininae - ps38
Hominini - ps39
Homo_sapiens - ps40
Phylostratum

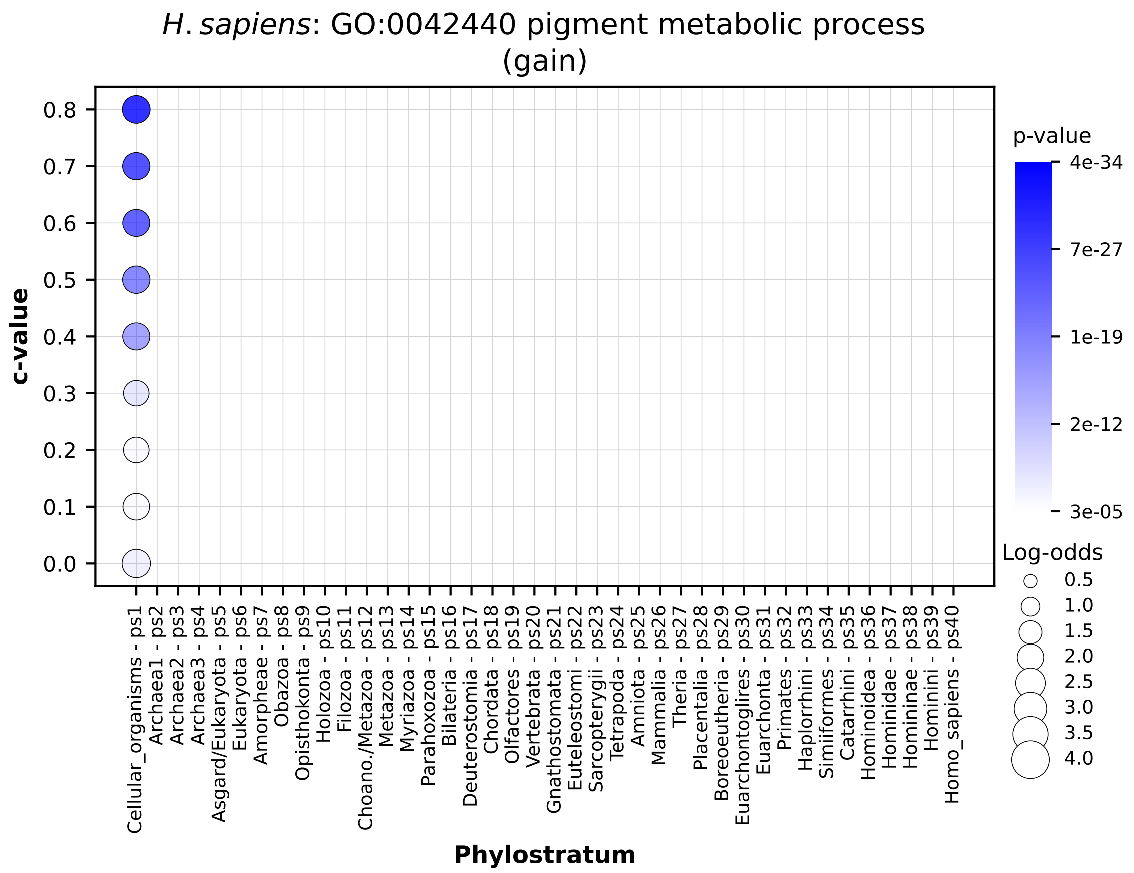

H. sapiens: GO:0042440 pigment metabolic process
(gain)
c-value
0.8
0.7
0.6
0.5
0.4
0.3
0.2
0.1
0.0
p-value
4e-34
7e-27
1e-19
2e-12
3e-05
Log-odds
0.5
1.0
1.5
2.0
2.5
3.0
3.5
4.0
Cellular_organisms - ps1
Archaea1 - ps2
Archaea2 - ps3
Archaea3 - ps4
Asgard/Eukaryota - ps5
Eukaryota - ps6
Amorpheae - ps7
Obazoa - ps8
Opisthokonta - ps9
Holozoa - ps10
Filozoa - ps11
Choano./Metazoa - ps12
Metazoa - ps13
Myriazoa - ps14
Parahoxozoa - ps15
Bilateria - ps16
Deuterostomia - ps17
Chordata - ps18
Olfactores - ps19
Vertebrata - ps20
Gnathostomata - ps21
Euteleostomi - ps22
Sarcopterygii - ps23
Tetrapoda - ps24
Amniota - ps25
Mammalia - ps26
Theria - ps27
Placentalia - ps28
Boreoeutheria - ps29
Euarchontoglires - ps30
Euarchonta - ps31
Primates - ps32
Haplorrhini - ps33
Simiiformes - ps34
Catarrhini - ps35
Hominoidea - ps36
Hominidae - ps37
Homininae - ps38
Hominini - ps39
Homo_sapiens - ps40
Phylostratum

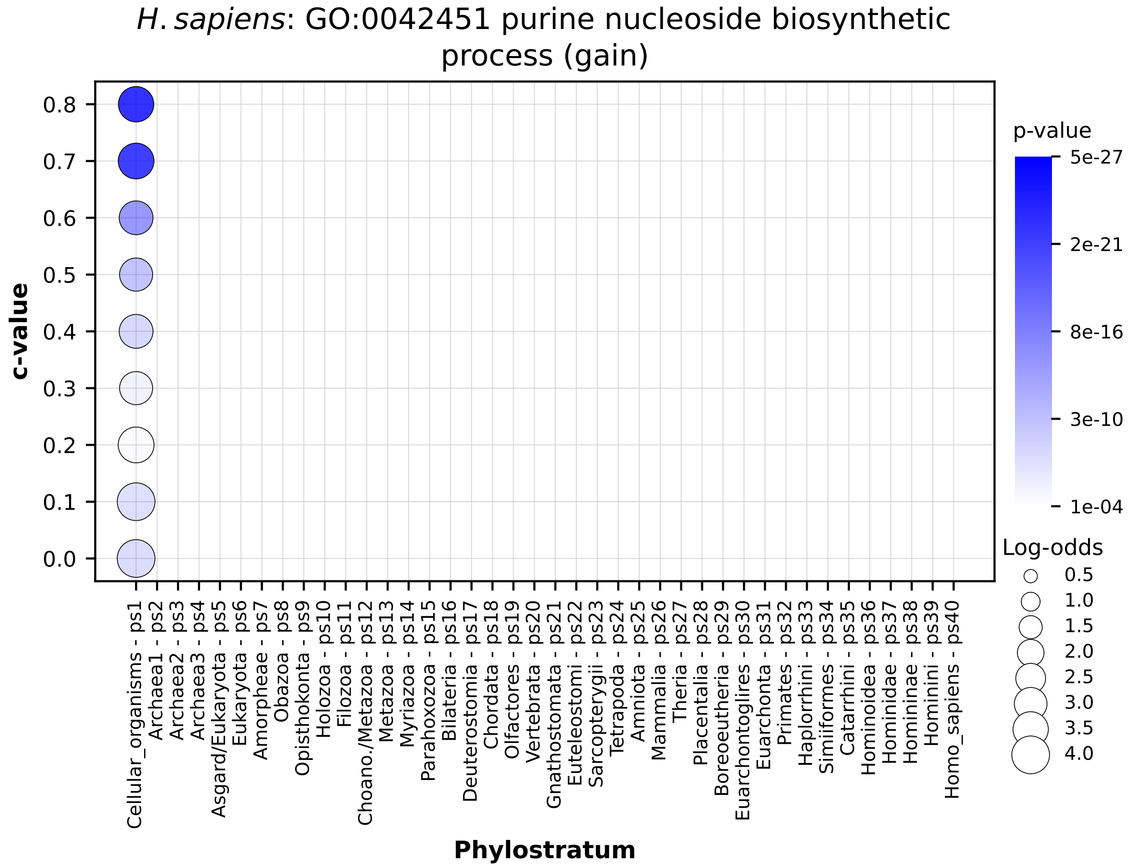
H. sapiens: GO:0042451 purine nucleoside biosynthetic process (gain)
c-value
0.8
0.7
0.6
0.5
0.4
0.3
0.2
0.1
0.0
p-value
5e-27
2e-21
8e-16
3e-10
1e-04
Log-odds
0.5
1.0
1.5
2.0
2.5
3.0
3.5
4.0
Cellular_organisms - ps1
Archaea1 - ps2
Archaea2 - ps3
Archaea3 - ps4
Asgard/Eukaryota - ps5
Eukaryota - ps6
Amorpheae - ps7
Obazoa - ps8
Opisthokonta - ps9
Holozoa - ps10
Filozoa - ps11
Choano./Metazoa - ps12
Metazoa - ps13
Myriazoa - ps14
Parahoxozoa - ps15
Bilateria - ps16
Deuterostomia - ps17
Chordata - ps18
Olfactores - ps19
Vertebrata - ps20
Gnathostomata - ps21
Euteleostomi - ps22
Sarcopterygii - ps23
Tetrapoda - ps24
Amniota - ps25
Mammalia - ps26
Theria - ps27
Placentalia - ps28
Boreoeutheria - ps29
Euarchontoglires - ps30
Euarchonta - ps31
Primates - ps32
Haplorrhini - ps33
Simiiformes - ps34
Catarrhini - ps35
Hominoidea - ps36
Hominidae - ps37
Homininae - ps38
Hominini - ps39
Homo_sapiens - ps40
Phylostratum

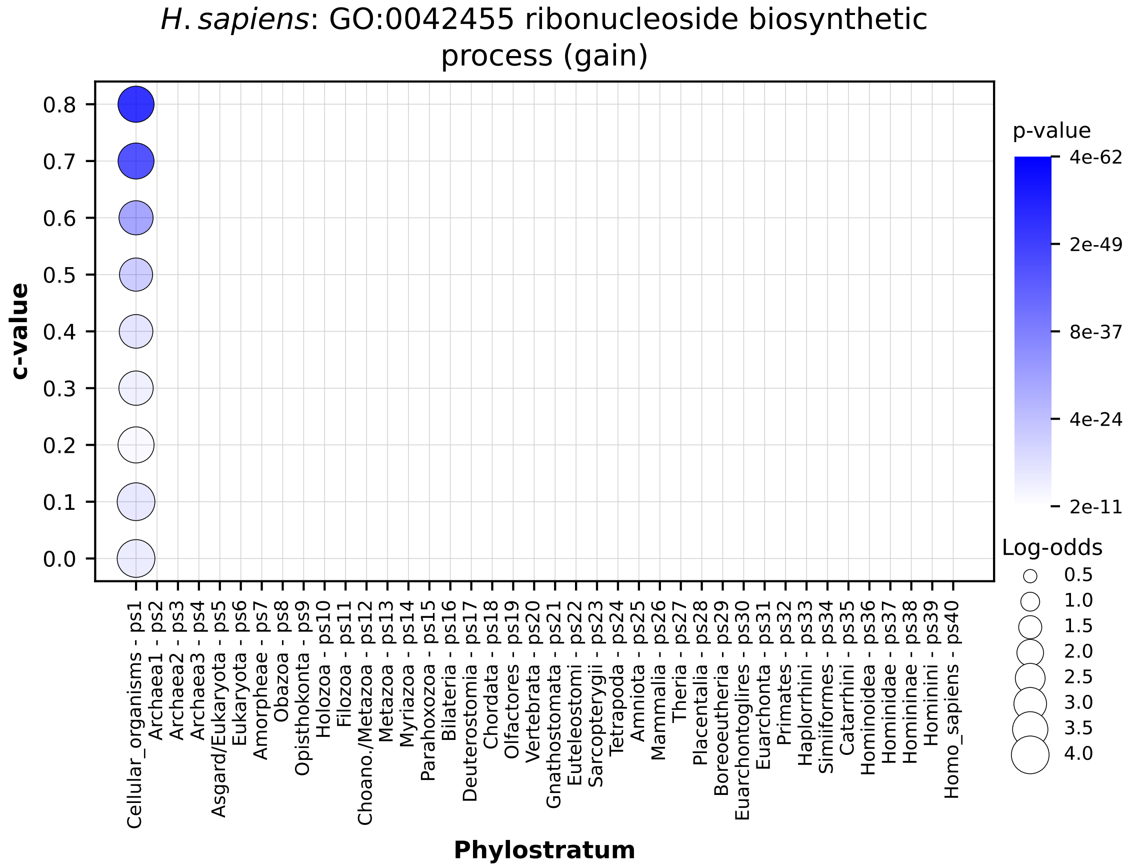

*H. sapiens*: GO:0042455 ribonucleoside biosynthetic process (gain)
c-value
0.8
0.7
0.6
0.5
0.4
0.3
0.2
0.1
0.0
p-value
4e-62
2e-49
8e-37
4e-24
2e-11
Log-odds
0.5
1.0
1.5
2.0
2.5
3.0
3.5
4.0
Cellular_organisms - ps1
Archaea1 - ps2
Archaea2 - ps3
Archaea3 - ps4
Asgard/Eukaryota - ps5
Eukaryota - ps6
Amorpheae - ps7
Obazoa - ps8
Opisthokonta - ps9
Holozoa - ps10
Filozoa - ps11
Choano./Metazoa - ps12
Metazoa - ps13
Myriazoa - ps14
Parahoxozoa - ps15
Bilateria - ps16
Deuterostomia - ps17
Chordata - ps18
Olfactores - ps19
Vertebrata - ps20
Gnathostomata - ps21
Euteleostomi - ps22
Sarcopterygii - ps23
Tetrapoda - ps24
Amniota - ps25
Mammalia - ps26
Theria - ps27
Placentalia - ps28
Boreoeutheria - ps29
Euarchontoglires - ps30
Euarchonta - ps31
Primates - ps32
Haplorrhini - ps33
Simiiformes - ps34
Catarrhini - ps35
Hominoidea - ps36
Hominidae - ps37
Homininae - ps38
Hominini - ps39
Homo_sapiens - ps40
Phylostratum

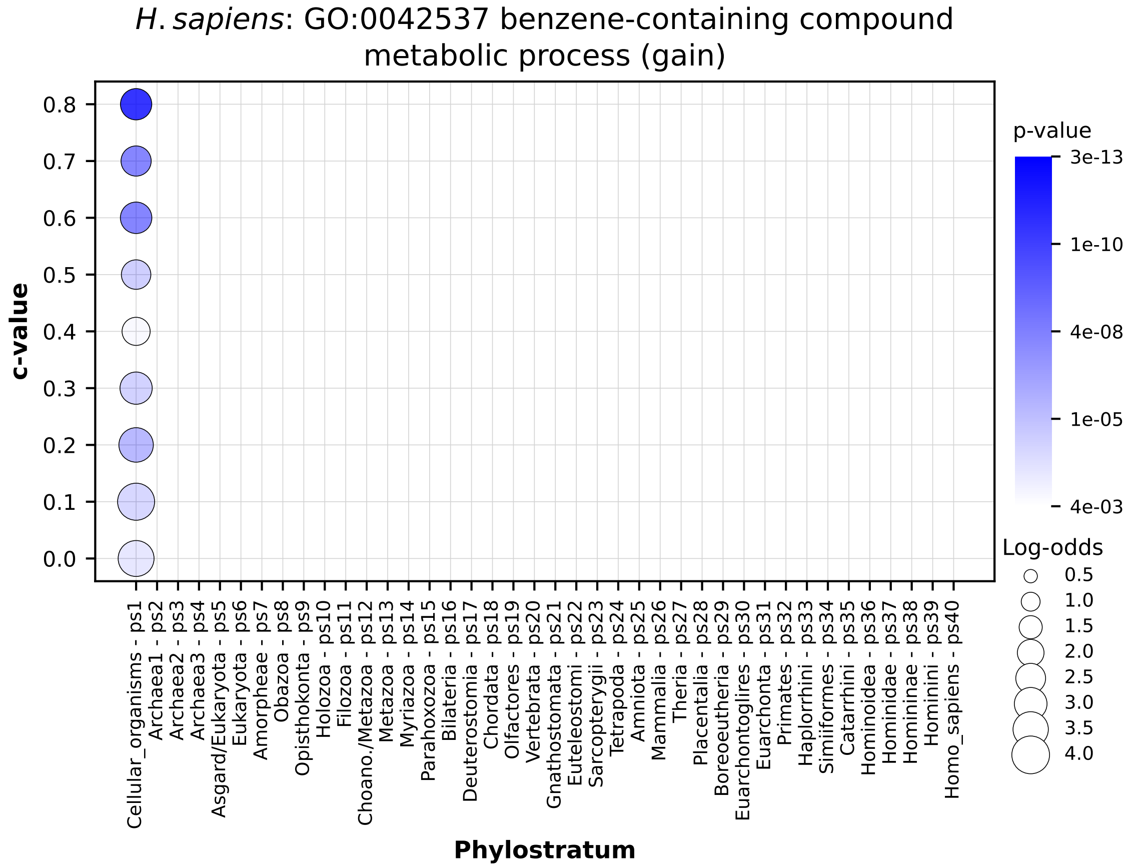

H. sapiens: GO:0042537 benzene-containing compound metabolic process (gain)
c-value
0.8
0.7
0.6
0.5
0.4
0.3
0.2
0.1
0.0
p-value
3e-13
1e-10
4e-08
1e-05
4e-03
Log-odds
0.5
1.0
1.5
2.0
2.5
3.0
3.5
4.0
Cellular_organisms - ps1
Archaea1 - ps2
Archaea2 - ps3
Archaea3 - ps4
Asgard/Eukaryota - ps5
Eukaryota - ps6
Amorpheae - ps7
Obazoa - ps8
Opisthokonta - ps9
Holozoa - ps10
Filozoa - ps11
Choano./Metazoa - ps12
Metazoa - ps13
Myriazoa - ps14
Parahoxozoa - ps15
Bilateria - ps16
Deuterostomia - ps17
Chordata - ps18
Olfactores - ps19
Vertebrata - ps20
Gnathostomata - ps21
Euteleostomi - ps22
Sarcopterygii - ps23
Tetrapoda - ps24
Amniota - ps25
Mammalia - ps26
Theria - ps27
Placentalia - ps28
Boreoeutheria - ps29
Euarchontoglires - ps30
Euarchonta - ps31
Primates - ps32
Haplorrhini - ps33
Simiiformes - ps34
Catarrhini - ps35
Hominoidea - ps36
Hominidae - ps37
Homininae - ps38
Hominini - ps39
Homo_sapiens - ps40
Phylostratum

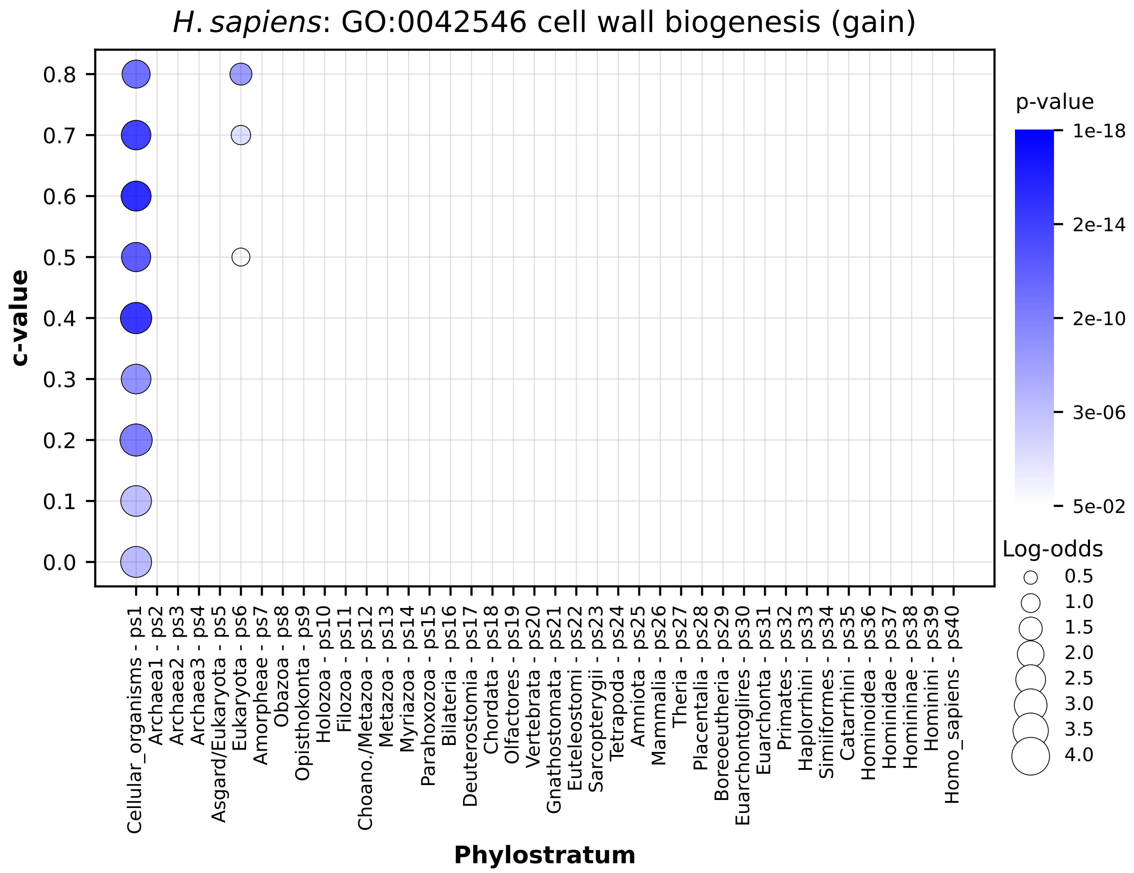

H. sapiens: GO:0042546 cell wall biogenesis (gain)
c-value
0.8
0.7
0.6
0.5
0.4
0.3
0.2
0.1
0.0
p-value
1e-18
2e-14
2e-10
3e-06
5e-02
Log-odds
0.5
1.0
1.5
2.0
2.5
3.0
3.5
4.0
Cellular_organisms - ps1
Archaea1 - ps2
Archaea2 - ps3
Archaea3 - ps4
Asgard/Eukaryota - ps5
Eukaryota - ps6
Amorpheae - ps7
Obazoa - ps8
Opisthokonta - ps9
Holozoa - ps10
Filozoa - ps11
Choano./Metazoa - ps12
Metazoa - ps13
Myriazoa - ps14
Parahoxozoa - ps15
Bilateria - ps16
Deuterostomia - ps17
Chordata - ps18
Olfactores - ps19
Vertebrata - ps20
Gnathostomata - ps21
Euteleostomi - ps22
Sarcopterygii - ps23
Tetrapoda - ps24
Amniota - ps25
Mammalia - ps26
Theria - ps27
Placentalia - ps28
Boreoeutheria - ps29
Euarchontoglires - ps30
Euarchonta - ps31
Primates - ps32
Haplorrhini - ps33
Simiiformes - ps34
Catarrhini - ps35
Hominoidea - ps36
Hominidae - ps37
Homininae - ps38
Hominini - ps39
Homo_sapiens - ps40
Phylostratum

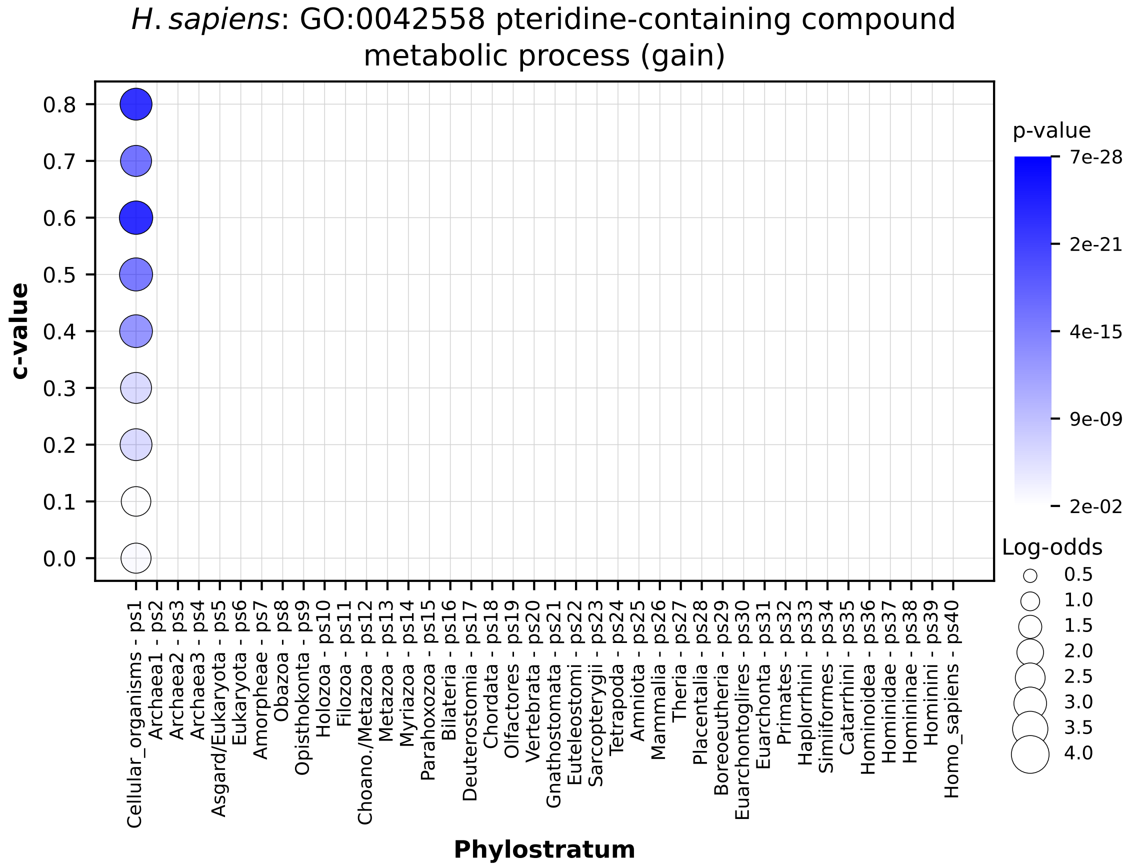

H. sapiens: GO:0042558 pteridine-containing compound metabolic process (gain)
c-value
0.8
0.7
0.6
0.5
0.4
0.3
0.2
0.1
0.0
Phylostratum
Cellular_organisms - ps1
Archaea1 - ps2
Archaea2 - ps3
Archaea3 - ps4
Asgard/Eukaryota - ps5
Eukaryota - ps6
Amorpheae - ps7
Obazoa - ps8
Opisthokonta - ps9
Holozoa - ps10
Filozoa - ps11
Choano./Metazoa - ps12
Metazoa - ps13
Myriazoa - ps14
Parahoxozoa - ps15
Bilateria - ps16
Deuterostomia - ps17
Chordata - ps18
Olfactores - ps19
Vertebrata - ps20
Gnathostomata - ps21
Euteleostomi - ps22
Sarcopterygii - ps23
Tetrapoda - ps24
Amniota - ps25
Mammalia - ps26
Theria - ps27
Placentalia - ps28
Boreoeutheria - ps29
Euarchontoglires - ps30
Euarchonta - ps31
Primates - ps32
Haplorrhini - ps33
Simiiformes - ps34
Catarrhini - ps35
Hominoidea - ps36
Hominidae - ps37
Homininae - ps38
Hominini - ps39
Homo_sapiens - ps40
p-value
7e-28
2e-21
4e-15
9e-09
2e-02
Log-odds
0.5
1.0
1.5
2.0
2.5
3.0
3.5
4.0

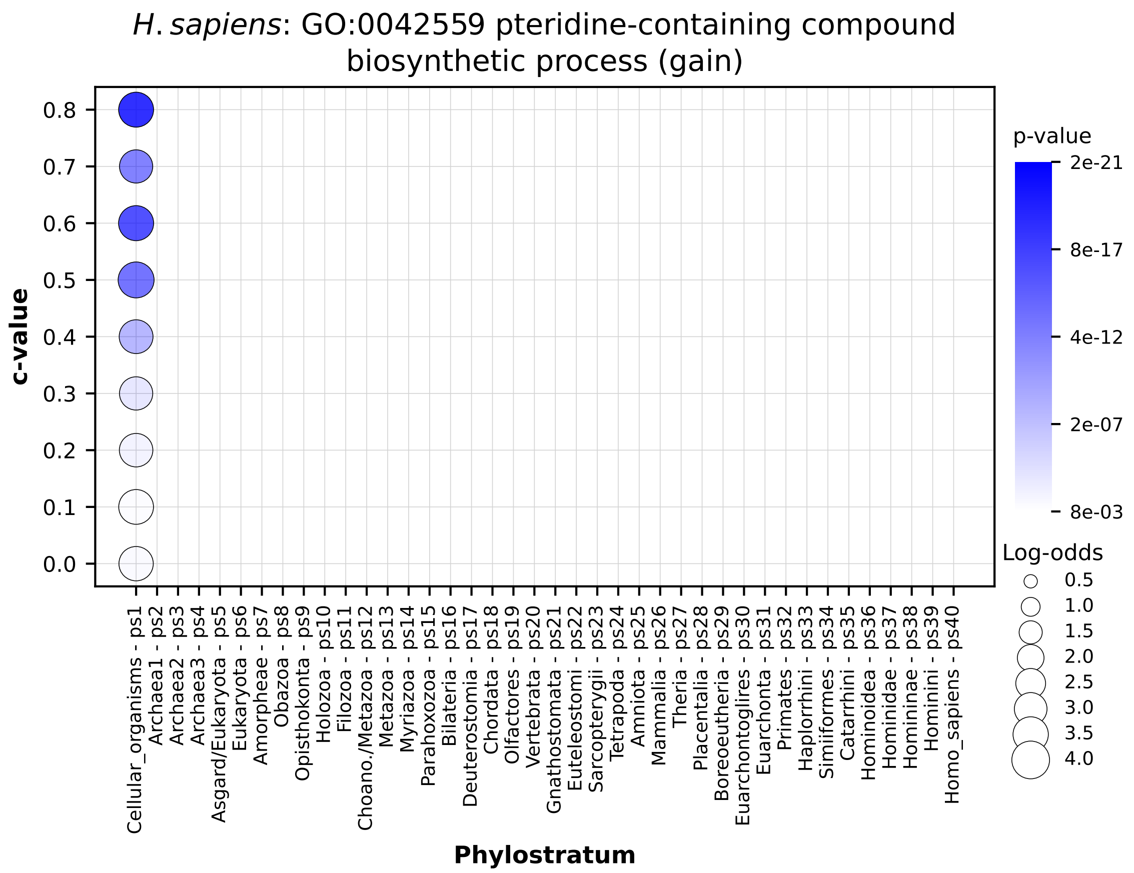

H. sapiens: GO:0042559 pteridine-containing compound biosynthetic process (gain)
c-value
0.8
0.7
0.6
0.5
0.4
0.3
0.2
0.1
0.0
p-value
2e-21
8e-17
4e-12
2e-07
8e-03
Log-odds
0.5
1.0
1.5
2.0
2.5
3.0
3.5
4.0
Cellular_organisms - ps1
Archaea1 - ps2
Archaea2 - ps3
Archaea3 - ps4
Asgard/Eukaryota - ps5
Eukaryota - ps6
Amorpheae - ps7
Obazoa - ps8
Opisthokonta - ps9
Holozoa - ps10
Filozoa - ps11
Choano./Metazoa - ps12
Metazoa - ps13
Myriazoa - ps14
Parahoxozoa - ps15
Bilateria - ps16
Deuterostomia - ps17
Chordata - ps18
Olfactores - ps19
Vertebrata - ps20
Gnathostomata - ps21
Euteleostomi - ps22
Sarcopterygii - ps23
Tetrapoda - ps24
Amniota - ps25
Mammalia - ps26
Theria - ps27
Placentalia - ps28
Boreoeutheria - ps29
Euarchontoglires - ps30
Euarchonta - ps31
Primates - ps32
Haplorrhini - ps33
Simiiformes - ps34
Catarrhini - ps35
Hominoidea - ps36
Hominidae - ps37
Homininae - ps38
Hominini - ps39
Homo_sapiens - ps40
Phylostratum

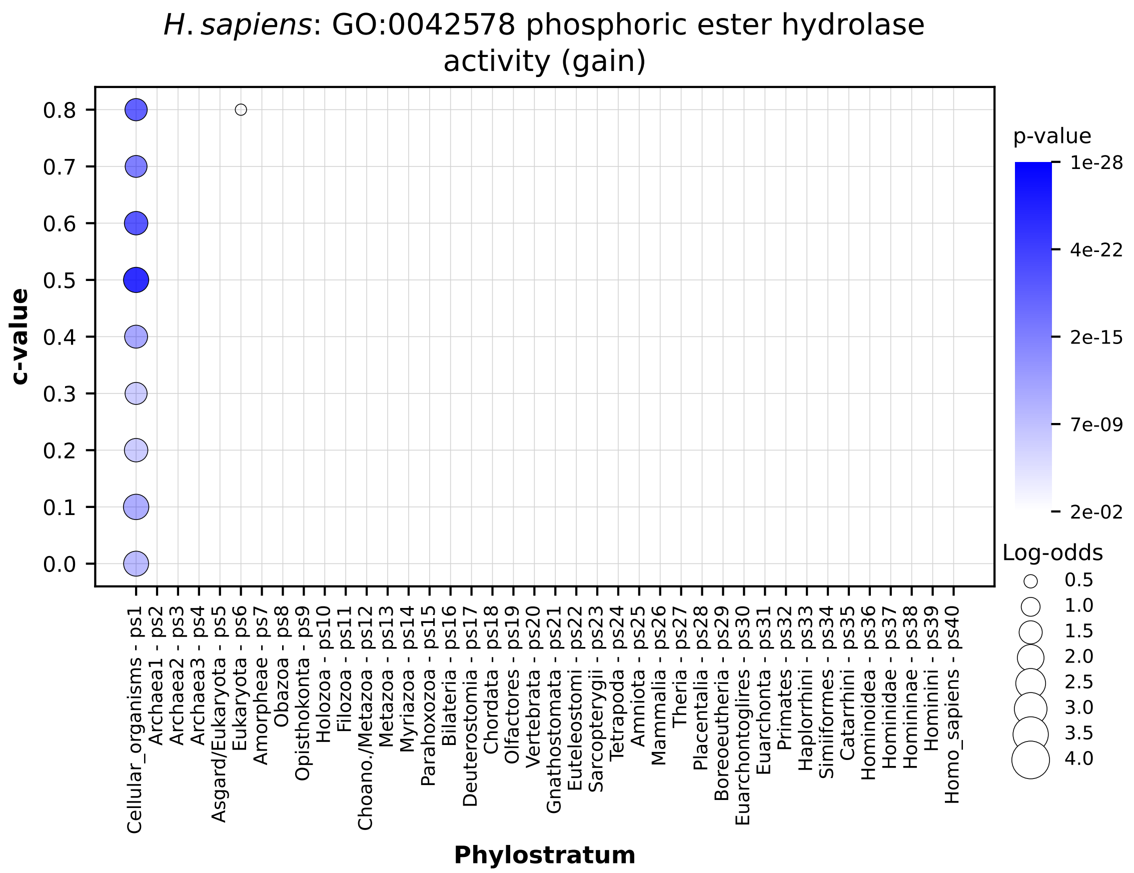

H. sapiens: GO:0042578 phosphoric ester hydrolase activity (gain)
c-value
0.8
0.7
0.6
0.5
0.4
0.3
0.2
0.1
0.0
p-value
1e-28
4e-22
2e-15
7e-09
2e-02
Log-odds
0.5
1.0
1.5
2.0
2.5
3.0
3.5
4.0
Cellular_organisms - ps1
Archaea1 - ps2
Archaea2 - ps3
Archaea3 - ps4
Asgard/Eukaryota - ps5
Eukaryota - ps6
Amorpheae - ps7
Obazoa - ps8
Opisthokonta - ps9
Holozoa - ps10
Filozoa - ps11
Choano./Metazoa - ps12
Metazoa - ps13
Myriazoa - ps14
Parahoxozoa - ps15
Bilateria - ps16
Deuterostomia - ps17
Chordata - ps18
Olfactores - ps19
Vertebrata - ps20
Gnathostomata - ps21
Euteleostomi - ps22
Sarcopterygii - ps23
Tetrapoda - ps24
Amniota - ps25
Mammalia - ps26
Theria - ps27
Placentalia - ps28
Boreoeutheria - ps29
Euarchontoglires - ps30
Euarchonta - ps31
Primates - ps32
Haplorrhini - ps33
Simiiformes - ps34
Catarrhini - ps35
Hominoidea - ps36
Hominidae - ps37
Homininae - ps38
Hominini - ps39
Homo_sapiens - ps40
Phylostratum

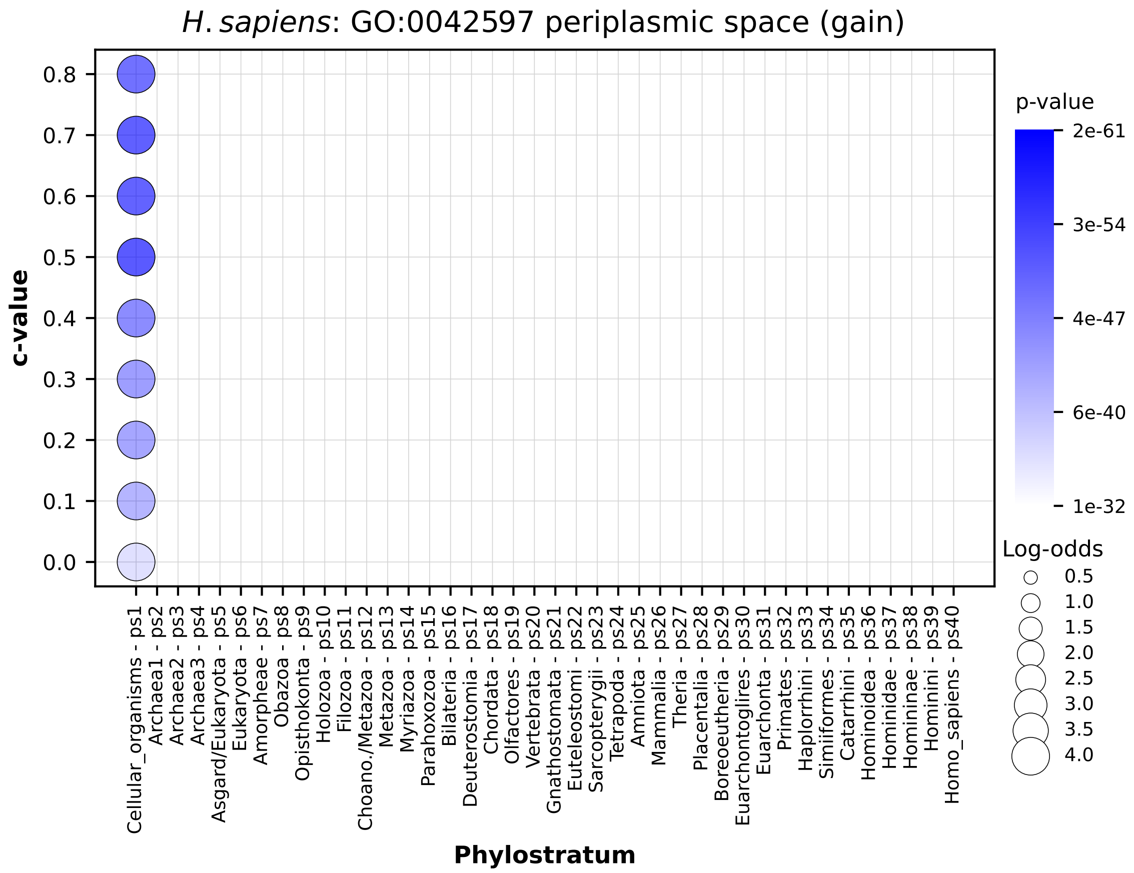

H. sapiens: GO:0042597 periplasmic space (gain)
c-value
0.8
0.7
0.6
0.5
0.4
0.3
0.2
0.1
0.0
p-value
2e-61
3e-54
4e-47
6e-40
1e-32
Log-odds
0.5
1.0
1.5
2.0
2.5
3.0
3.5
4.0
Phylostratum
Cellular_organisms - ps1
Archaea1 - ps2
Archaea2 - ps3
Archaea3 - ps4
Asgard/Eukaryota - ps5
Eukaryota - ps6
Amorpheae - ps7
Obazoa - ps8
Opisthokonta - ps9
Holozoa - ps10
Filozoa - ps11
Choano./Metazoa - ps12
Metazoa - ps13
Myriazoa - ps14
Parahoxozoa - ps15
Bilateria - ps16
Deuterostomia - ps17
Chordata - ps18
Olfactores - ps19
Vertebrata - ps20
Gnathostomata - ps21
Euteleostomi - ps22
Sarcopterygii - ps23
Tetrapoda - ps24
Amniota - ps25
Mammalia - ps26
Theria - ps27
Placentalia - ps28
Boreoeutheria - ps29
Euarchontoglires - ps30
Euarchonta - ps31
Primates - ps32
Haplorrhini - ps33
Simiiformes - ps34
Catarrhini - ps35
Hominoidea - ps36
Hominidae - ps37
Homininae - ps38
Hominini - ps39
Homo_sapiens - ps40

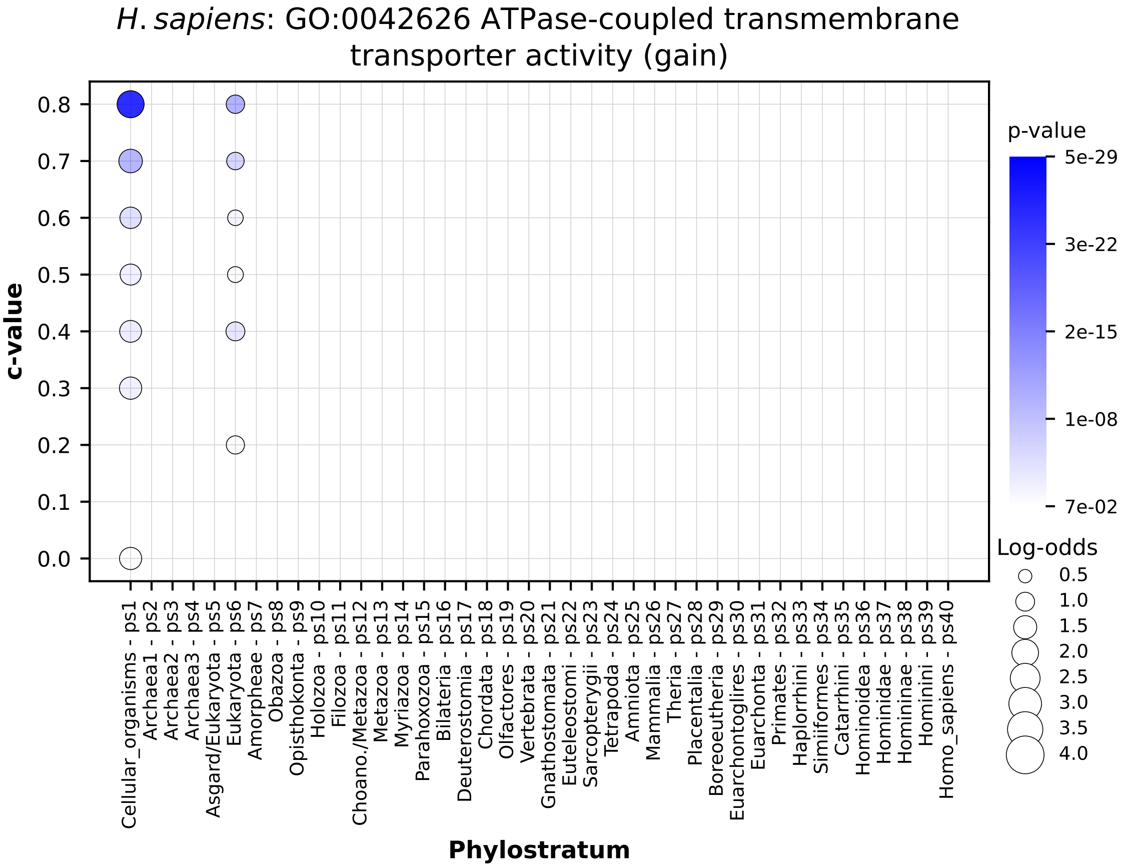

*H. sapiens*: GO:0042626 ATPase-coupled transmembrane transporter activity (gain)
c-value
0.0
0.1
0.2
0.3
0.4
0.5
0.6
0.7
0.8
Phylostratum
Cellular_organisms - ps1
Archaea1 - ps2
Archaea2 - ps3
Archaea3 - ps4
Asgard/Eukaryota - ps5
Eukaryota - ps6
Amorpheae - ps7
Obazoa - ps8
Opisthokonta - ps9
Holozoa - ps10
Filozoa - ps11
Choano./Metazoa - ps12
Metazoa - ps13
Myriazoa - ps14
Parahoxozoa - ps15
Bilateria - ps16
Deuterostomia - ps17
Chordata - ps18
Olfactores - ps19
Vertebrata - ps20
Gnathostomata - ps21
Euteleostomi - ps22
Sarcopterygii - ps23
Tetrapoda - ps24
Amniota - ps25
Mammalia - ps26
Theria - ps27
Placentalia - ps28
Boreoeutheria - ps29
Euarchontoglires - ps30
Euarchonta - ps31
Primates - ps32
Haplorrhini - ps33
Simiiformes - ps34
Catarrhini - ps35
Hominoidea - ps36
Hominidae - ps37
Homininae - ps38
Hominini - ps39
Homo_sapiens - ps40
p-value
5e-29
3e-22
2e-15
1e-08
7e-02
Log-odds
0.5
1.0
1.5
2.0
2.5
3.0
3.5
4.0

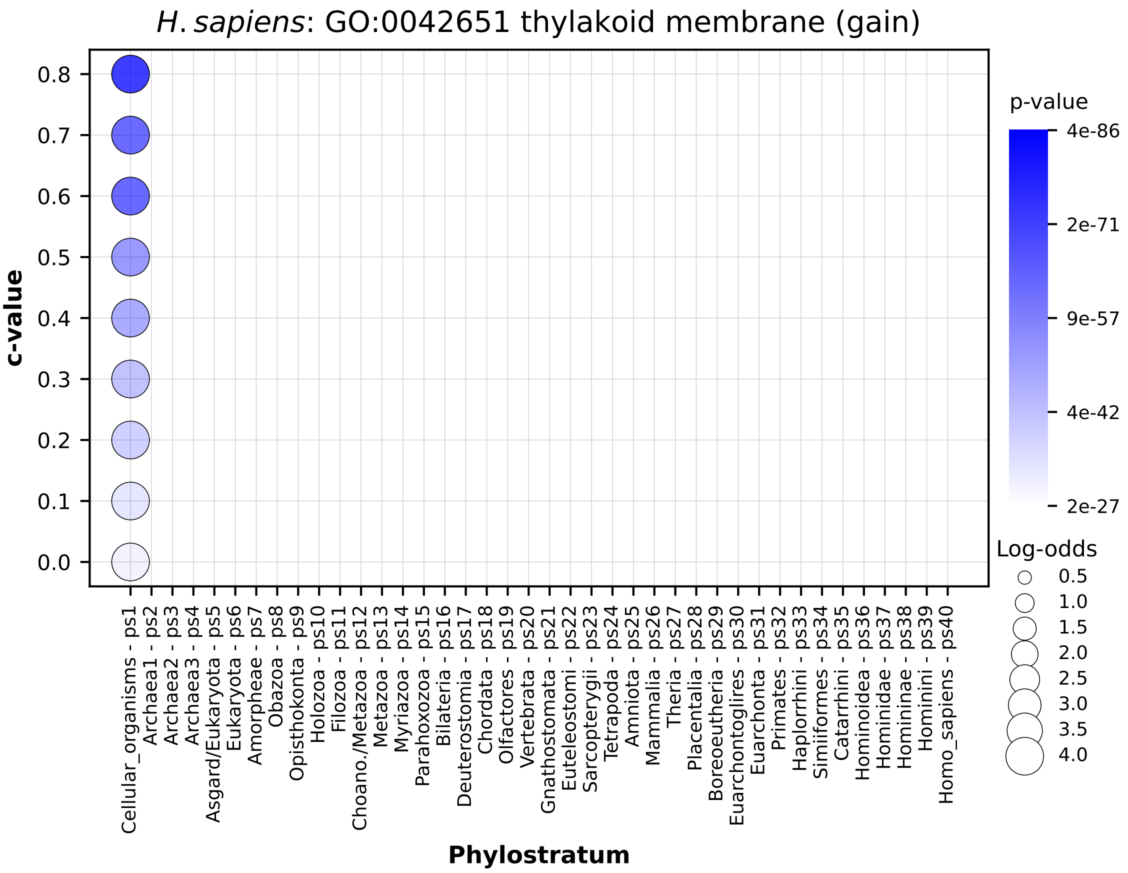

H. sapiens: GO:0042651 thylakoid membrane (gain)
c-value
0.8
0.7
0.6
0.5
0.4
0.3
0.2
0.1
0.0
p-value
4e-86
2e-71
9e-57
4e-42
2e-27
Log-odds
0.5
1.0
1.5
2.0
2.5
3.0
3.5
4.0
Cellular_organisms - ps1
Archaea1 - ps2
Archaea2 - ps3
Archaea3 - ps4
Asgard/Eukaryota - ps5
Eukaryota - ps6
Amorpheae - ps7
Obazoa - ps8
Opisthokonta - ps9
Holozoa - ps10
Filozoa - ps11
Choano./Metazoa - ps12
Metazoa - ps13
Myriazoa - ps14
Parahoxozoa - ps15
Bilateria - ps16
Deuterostomia - ps17
Chordata - ps18
Olfactores - ps19
Vertebrata - ps20
Gnathostomata - ps21
Euteleostomi - ps22
Sarcopterygii - ps23
Tetrapoda - ps24
Amniota - ps25
Mammalia - ps26
Theria - ps27
Placentalia - ps28
Boreoeutheria - ps29
Euarchontoglires - ps30
Euarchonta - ps31
Primates - ps32
Haplorrhini - ps33
Simiiformes - ps34
Catarrhini - ps35
Hominoidea - ps36
Hominidae - ps37
Homininae - ps38
Hominini - ps39
Homo_sapiens - ps40
Phylostratum

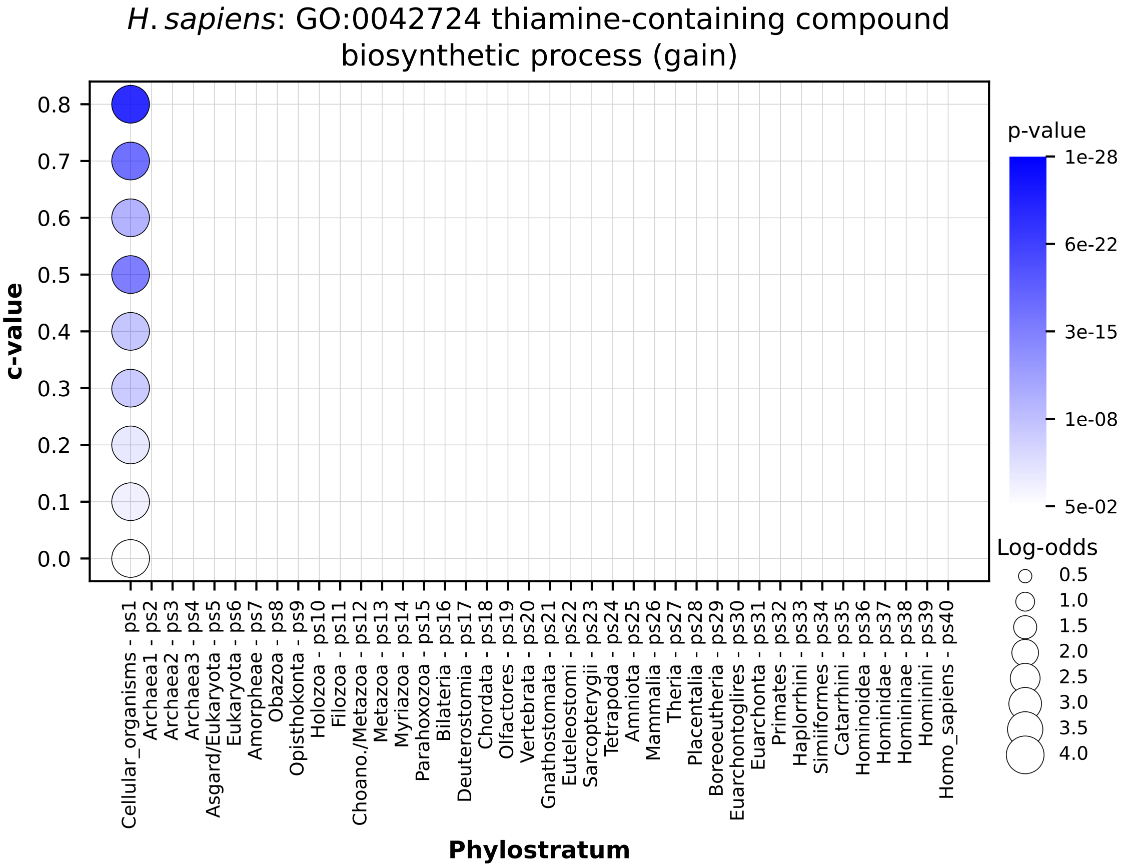

H. sapiens: GO:0042724 thiamine-containing compound biosynthetic process (gain)
c-value
0.8
0.7
0.6
0.5
0.4
0.3
0.2
0.1
0.0
Phylostratum
Cellular_organisms - ps1
Archaea1 - ps2
Archaea2 - ps3
Archaea3 - ps4
Asgard/Eukaryota - ps5
Eukaryota - ps6
Amorpheae - ps7
Obazoa - ps8
Opisthokonta - ps9
Holozoa - ps10
Filozoa - ps11
Choano./Metazoa - ps12
Metazoa - ps13
Myriazoa - ps14
Parahoxozoa - ps15
Bilateria - ps16
Deuterostomia - ps17
Chordata - ps18
Olfactores - ps19
Vertebrata - ps20
Gnathostomata - ps21
Euteleostomi - ps22
Sarcopterygii - ps23
Tetrapoda - ps24
Amniota - ps25
Mammalia - ps26
Theria - ps27
Placentalia - ps28
Boreoeutheria - ps29
Euarchontoglires - ps30
Euarchonta - ps31
Primates - ps32
Haplorrhini - ps33
Simiiformes - ps34
Catarrhini - ps35
Hominoidea - ps36
Hominidae - ps37
Homininae - ps38
Hominini - ps39
Homo_sapiens - ps40
p-value
1e-28
6e-22
3e-15
1e-08
5e-02
Log-odds
0.5
1.0
1.5
2.0
2.5
3.0
3.5
4.0

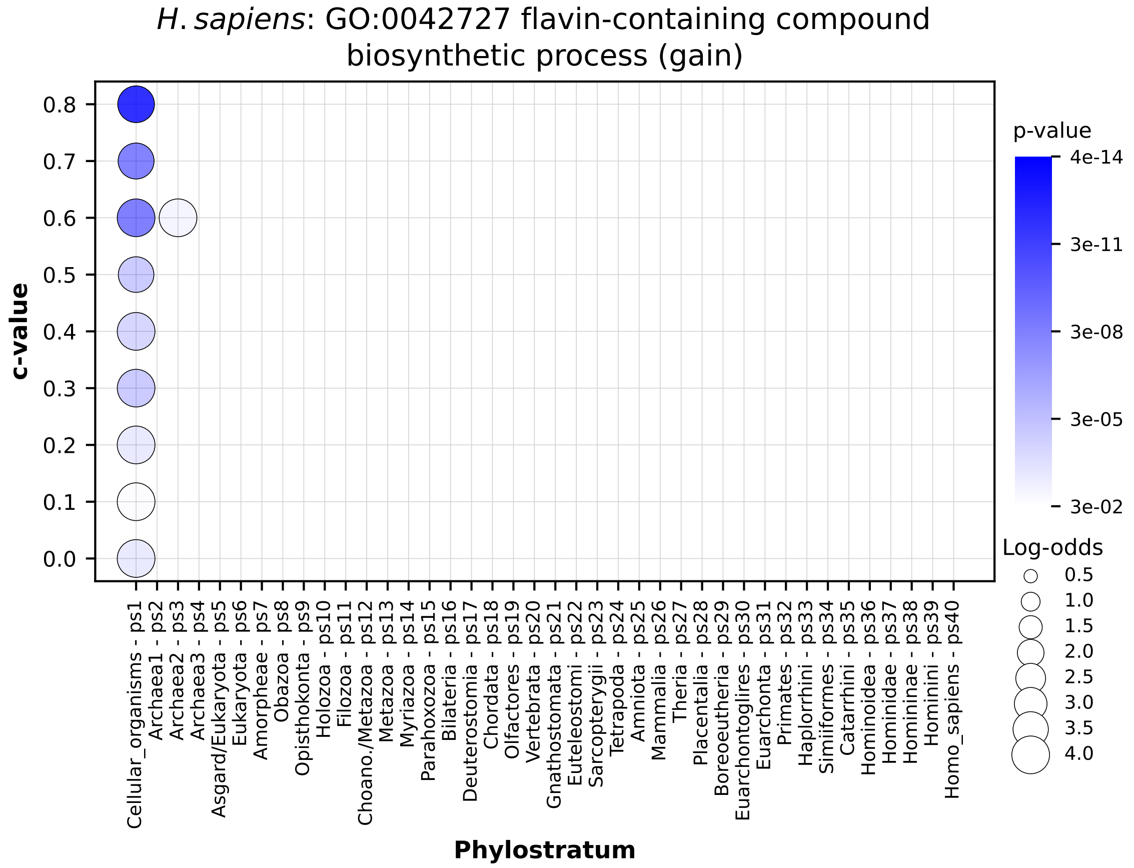

H. sapiens: GO:0042727 flavin-containing compound biosynthetic process (gain)
c-value
0.0
0.1
0.2
0.3
0.4
0.5
0.6
0.7
0.8
Phylostratum
Cellular_organisms - ps1
Archaea1 - ps2
Archaea2 - ps3
Archaea3 - ps4
Asgard/Eukaryota - ps5
Eukaryota - ps6
Amorpheae - ps7
Obazoa - ps8
Opisthokonta - ps9
Holozoa - ps10
Filozoa - ps11
Choano./Metazoa - ps12
Metazoa - ps13
Myriazoa - ps14
Parahoxozoa - ps15
Bilateria - ps16
Deuterostomia - ps17
Chordata - ps18
Olfactores - ps19
Vertebrata - ps20
Gnathostomata - ps21
Euteleostomi - ps22
Sarcopterygii - ps23
Tetrapoda - ps24
Amniota - ps25
Mammalia - ps26
Theria - ps27
Placentalia - ps28
Boreoeutheria - ps29
Euarchontoglires - ps30
Euarchonta - ps31
Primates - ps32
Haplorrhini - ps33
Simiiformes - ps34
Catarrhini - ps35
Hominoidea - ps36
Hominidae - ps37
Homininae - ps38
Hominini - ps39
Homo_sapiens - ps40
p-value
4e-14
3e-11
3e-08
3e-05
3e-02
Log-odds
0.5
1.0
1.5
2.0
2.5
3.0
3.5
4.0

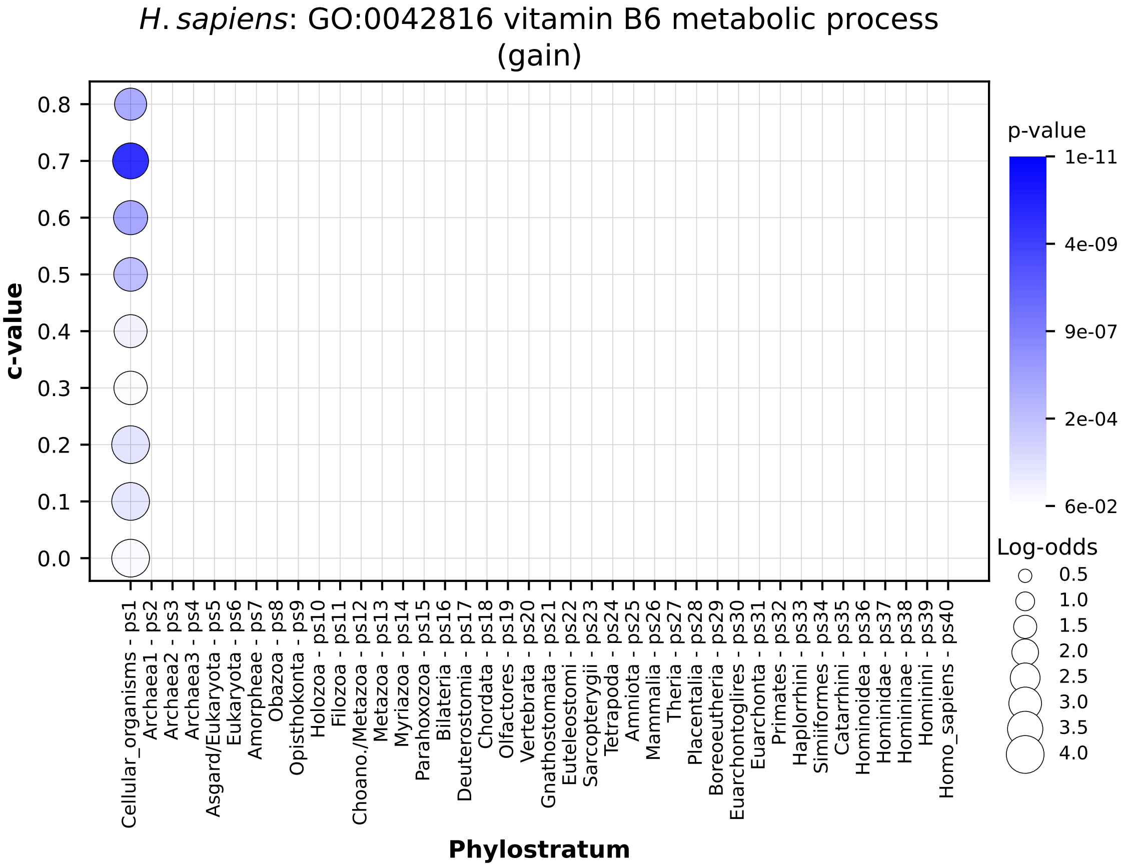
H. sapiens: GO:0042816 vitamin B6 metabolic process
(gain)
c-value
0.8
0.7
0.6
0.5
0.4
0.3
0.2
0.1
0.0
p-value
1e-11
4e-09
9e-07
2e-04
6e-02
Log-odds
0.5
1.0
1.5
2.0
2.5
3.0
3.5
4.0
Cellular_organisms - ps1
Archaea1 - ps2
Archaea2 - ps3
Archaea3 - ps4
Asgard/Eukaryota - ps5
Eukaryota - ps6
Amorpheae - ps7
Obazoa - ps8
Opisthokonta - ps9
Holozoa - ps10
Filozoa - ps11
Choano./Metazoa - ps12
Metazoa - ps13
Myriazoa - ps14
Parahoxozoa - ps15
Bilateria - ps16
Deuterostomia - ps17
Chordata - ps18
Olfactores - ps19
Vertebrata - ps20
Gnathostomata - ps21
Euteleostomi - ps22
Sarcopterygii - ps23
Tetrapoda - ps24
Amniota - ps25
Mammalia - ps26
Theria - ps27
Placentalia - ps28
Boreoeutheria - ps29
Euarchontoglires - ps30
Euarchonta - ps31
Primates - ps32
Haplorrhini - ps33
Simiiformes - ps34
Catarrhini - ps35
Hominoidea - ps36
Hominidae - ps37
Homininae - ps38
Hominini - ps39
Homo_sapiens - ps40
Phylostratum

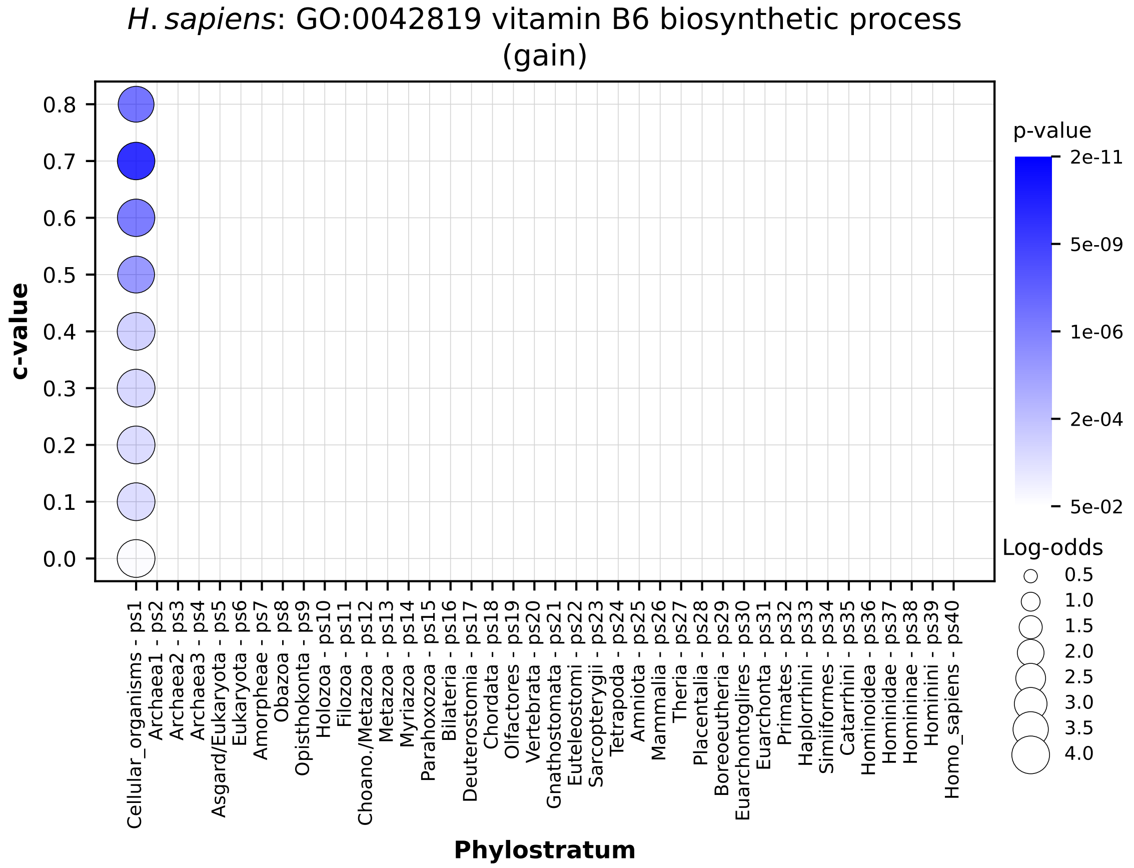

H. sapiens: GO:0042819 vitamin B6 biosynthetic process
(gain)
c-value
0.8
0.7
0.6
0.5
0.4
0.3
0.2
0.1
0.0
Phylostratum
Cellular_organisms - ps1
Archaea1 - ps2
Archaea2 - ps3
Archaea3 - ps4
Asgard/Eukaryota - ps5
Eukaryota - ps6
Amorpheae - ps7
Obazoa - ps8
Opisthokonta - ps9
Holozoa - ps10
Filozoa - ps11
Choano./Metazoa - ps12
Metazoa - ps13
Myriazoa - ps14
Parahoxozoa - ps15
Bilateria - ps16
Deuterostomia - ps17
Chordata - ps18
Olfactores - ps19
Vertebrata - ps20
Gnathostomata - ps21
Euteleostomi - ps22
Sarcopterygii - ps23
Tetrapoda - ps24
Amniota - ps25
Mammalia - ps26
Theria - ps27
Placentalia - ps28
Boreoeutheria - ps29
Euarchontoglires - ps30
Euarchonta - ps31
Primates - ps32
Haplorrhini - ps33
Simiiformes - ps34
Catarrhini - ps35
Hominoidea - ps36
Hominidae - ps37
Homininae - ps38
Hominini - ps39
Homo_sapiens - ps40
p-value
2e-11
5e-09
1e-06
2e-04
5e-02
Log-odds
0.5
1.0
1.5
2.0
2.5
3.0
3.5
4.0

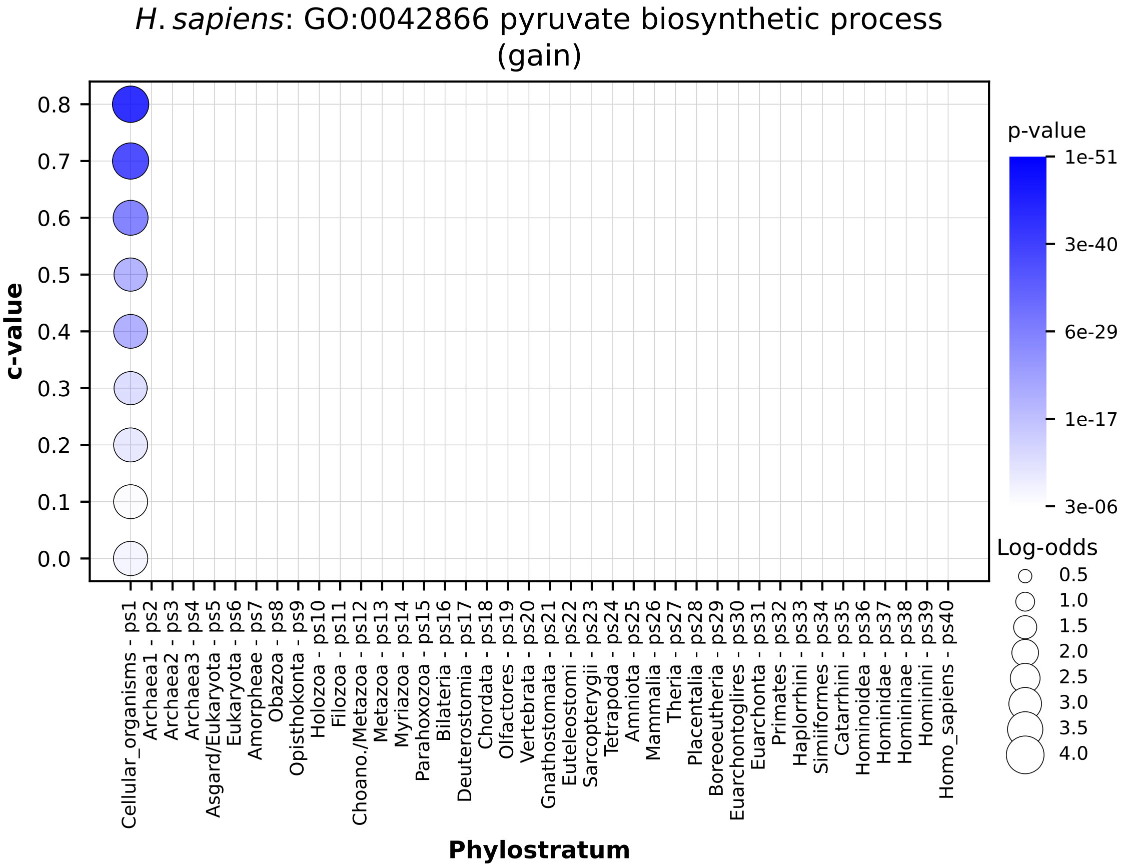
*H. sapiens*: GO:0042866 pyruvate biosynthetic process (gain)
c-value
0.0
0.1
0.2
0.3
0.4
0.5
0.6
0.7
0.8
Phylostratum
Cellular_organisms - ps1
Archaea1 - ps2
Archaea2 - ps3
Archaea3 - ps4
Asgard/Eukaryota - ps5
Eukaryota - ps6
Amorpheae - ps7
Obazoa - ps8
Opisthokonta - ps9
Holozoa - ps10
Filozoa - ps11
Choano./Metazoa - ps12
Metazoa - ps13
Myriazoa - ps14
Parahoxozoa - ps15
Bilateria - ps16
Deuterostomia - ps17
Chordata - ps18
Olfactores - ps19
Vertebrata - ps20
Gnathostomata - ps21
Euteleostomi - ps22
Sarcopterygii - ps23
Tetrapoda - ps24
Amniota - ps25
Mammalia - ps26
Theria - ps27
Placentalia - ps28
Boreoeutheria - ps29
Euarchontoglires - ps30
Euarchonta - ps31
Primates - ps32
Haplorrhini - ps33
Simiiformes - ps34
Catarrhini - ps35
Hominoidea - ps36
Hominidae - ps37
Homininae - ps38
Hominini - ps39
Homo_sapiens - ps40
p-value
1e-51
3e-40
6e-29
1e-17
3e-06
Log-odds
0.5
1.0
1.5
2.0
2.5
3.0
3.5
4.0

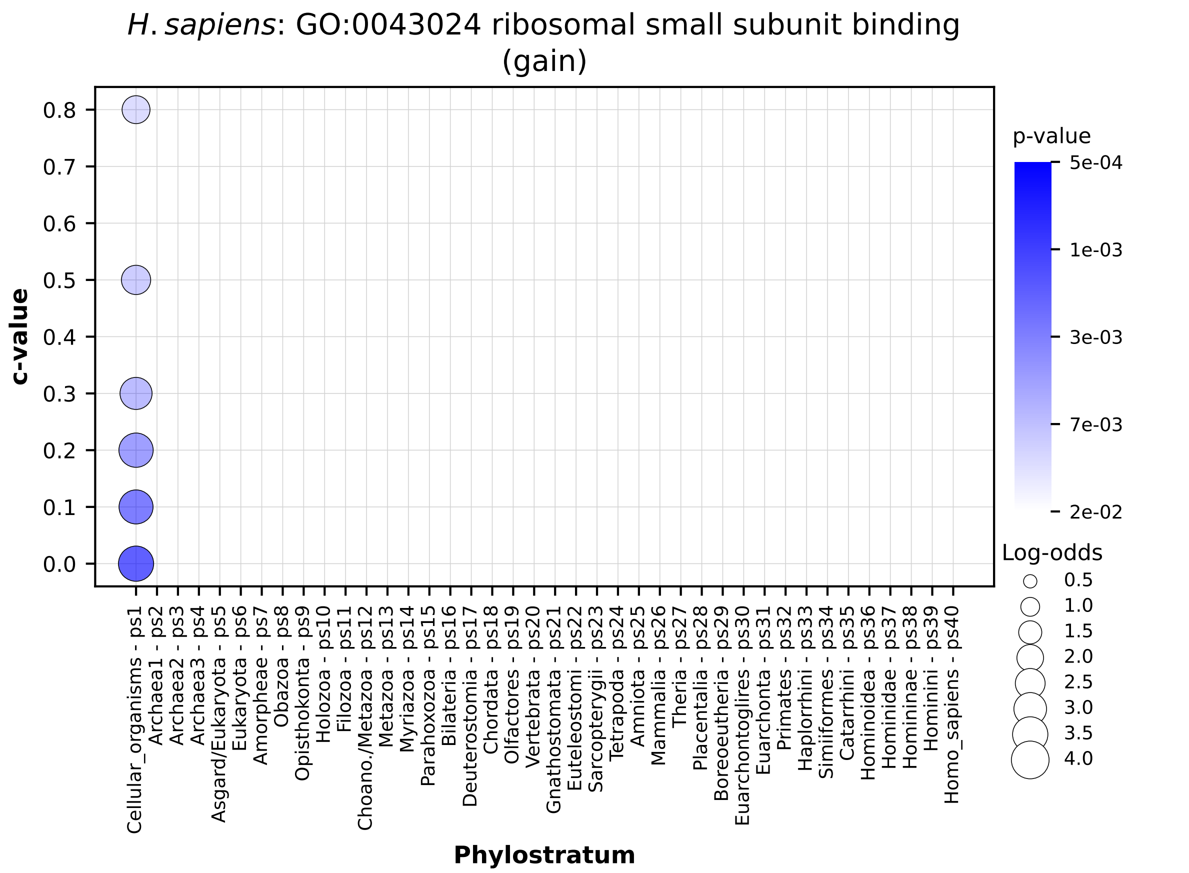

H. sapiens: GO:0043024 ribosomal small subunit binding (gain)
c-value
0.0
0.1
0.2
0.3
0.4
0.5
0.6
0.7
0.8
Phylostratum
Cellular_organisms - ps1
Archaea1 - ps2
Archaea2 - ps3
Archaea3 - ps4
Asgard/Eukaryota - ps5
Eukaryota - ps6
Amorpheae - ps7
Obazoa - ps8
Opisthokonta - ps9
Holozoa - ps10
Filozoa - ps11
Choano./Metazoa - ps12
Metazoa - ps13
Myriazoa - ps14
Parahoxozoa - ps15
Bilateria - ps16
Deuterostomia - ps17
Chordata - ps18
Olfactores - ps19
Vertebrata - ps20
Gnathostomata - ps21
Euteleostomi - ps22
Sarcopterygii - ps23
Tetrapoda - ps24
Amniota - ps25
Mammalia - ps26
Theria - ps27
Placentalia - ps28
Boreoeutheria - ps29
Euarchontoglires - ps30
Euarchonta - ps31
Primates - ps32
Haplorrhini - ps33
Simiiformes - ps34
Catarrhini - ps35
Hominoidea - ps36
Hominidae - ps37
Homininae - ps38
Hominini - ps39
Homo_sapiens - ps40
p-value
5e-04
1e-03
3e-03
7e-03
2e-02
Log-odds
0.5
1.0
1.5
2.0
2.5
3.0
3.5
4.0

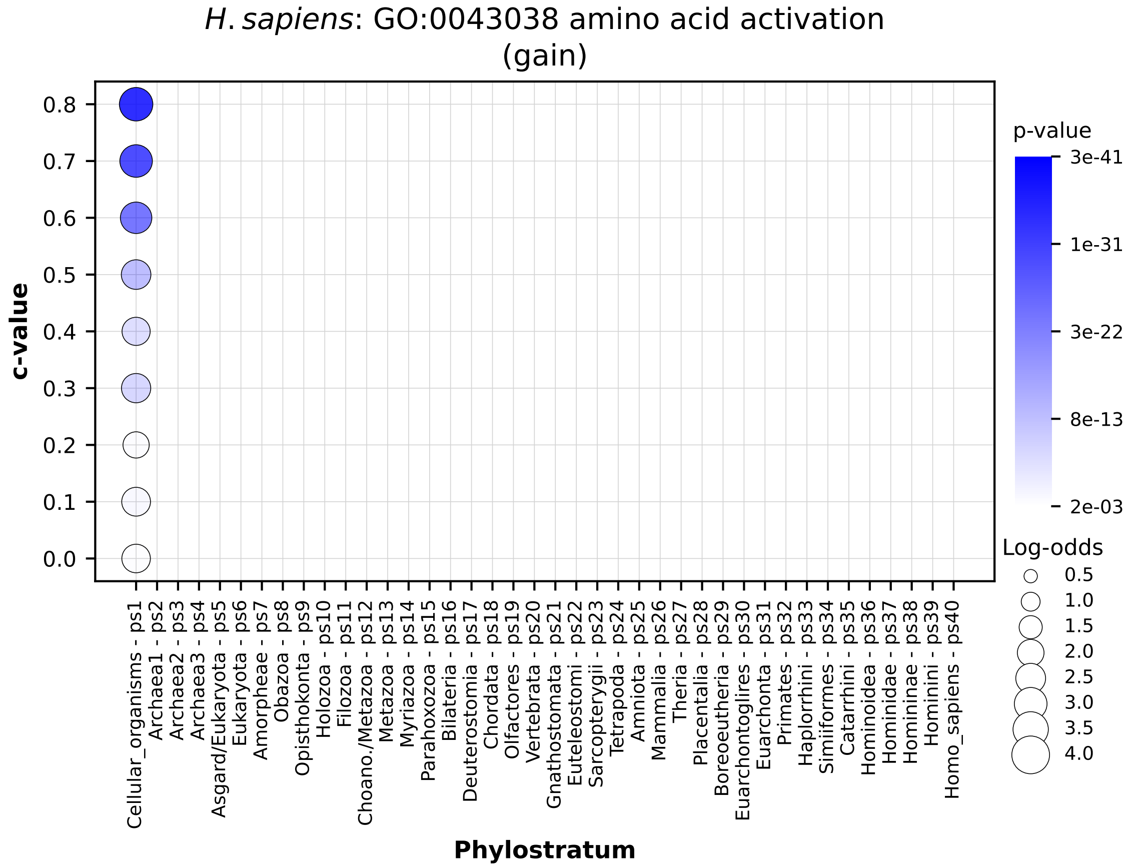
H. sapiens: GO:0043038 amino acid activation
(gain)
c-value
0.0
0.1
0.2
0.3
0.4
0.5
0.6
0.7
0.8
p-value
3e-41
1e-31
3e-22
8e-13
2e-03
Log-odds
0.5
1.0
1.5
2.0
2.5
3.0
3.5
4.0
Cellular_organisms - ps1
Archaea1 - ps2
Archaea2 - ps3
Archaea3 - ps4
Asgard/Eukaryota - ps5
Eukaryota - ps6
Amorpheae - ps7
Obazoa - ps8
Opisthokonta - ps9
Holozoa - ps10
Filozoa - ps11
Choano./Metazoa - ps12
Metazoa - ps13
Myriazoa - ps14
Parahoxozoa - ps15
Bilateria - ps16
Deuterostomia - ps17
Chordata - ps18
Olfactores - ps19
Vertebrata - ps20
Gnathostomata - ps21
Euteleostomi - ps22
Sarcopterygii - ps23
Tetrapoda - ps24
Amniota - ps25
Mammalia - ps26
Theria - ps27
Placentalia - ps28
Boreoeutheria - ps29
Euarchontoglires - ps30
Euarchonta - ps31
Primates - ps32
Haplorrhini - ps33
Simiiformes - ps34
Catarrhini - ps35
Hominoidea - ps36
Hominidae - ps37
Homininae - ps38
Hominini - ps39
Homo_sapiens - ps40
Phylostratum

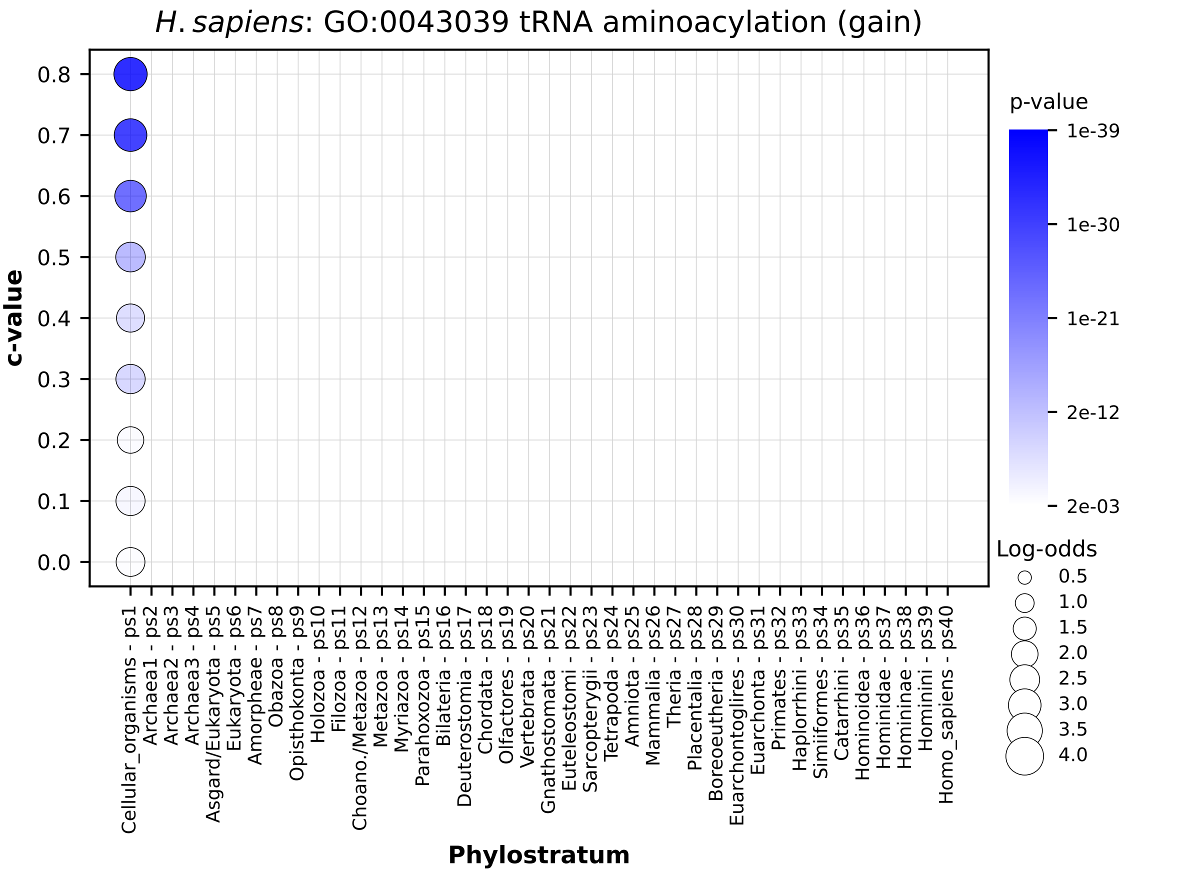
H. sapiens: GO:0043039 tRNA aminoacylation (gain)
c-value
0.8
0.7
0.6
0.5
0.4
0.3
0.2
0.1
0.0
Phylostratum
Cellular_organisms - ps1
Archaea1 - ps2
Archaea2 - ps3
Archaea3 - ps4
Asgard/Eukaryota - ps5
Eukaryota - ps6
Amorpheae - ps7
Obazoa - ps8
Opisthokonta - ps9
Holozoa - ps10
Filozoa - ps11
Choano./Metazoa - ps12
Metazoa - ps13
Myriazoa - ps14
Parahoxozoa - ps15
Bilateria - ps16
Deuterostomia - ps17
Chordata - ps18
Olfactores - ps19
Vertebrata - ps20
Gnathostomata - ps21
Euteleostomi - ps22
Sarcopterygii - ps23
Tetrapoda - ps24
Amniota - ps25
Mammalia - ps26
Theria - ps27
Placentalia - ps28
Boreoeutheria - ps29
Euarchontoglires - ps30
Euarchonta - ps31
Primates - ps32
Haplorrhini - ps33
Simiiformes - ps34
Catarrhini - ps35
Hominoidea - ps36
Hominidae - ps37
Homininae - ps38
Hominini - ps39
Homo_sapiens - ps40
p-value
1e-39
1e-30
1e-21
2e-12
2e-03
Log-odds
0.5
1.0
1.5
2.0
2.5
3.0
3.5
4.0

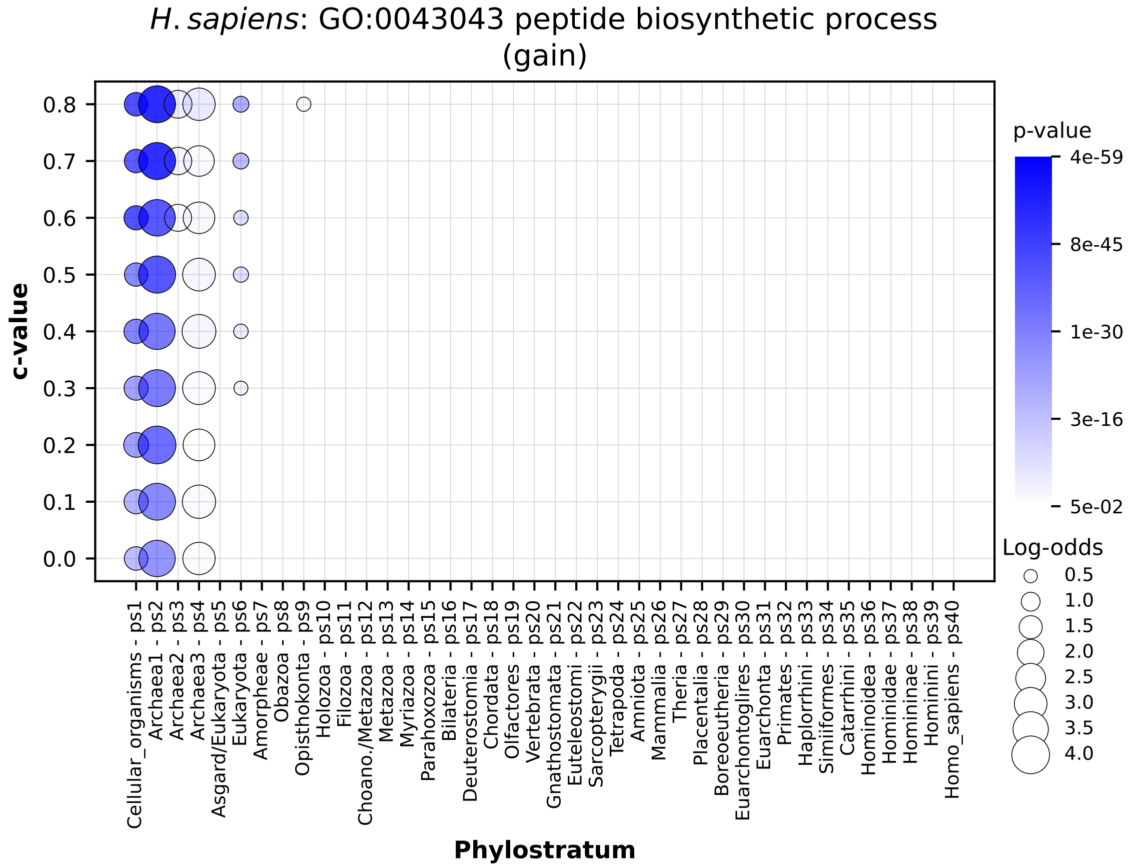

*H. sapiens*: GO:0043043 peptide biosynthetic process (gain)
c-value
0.8
0.7
0.6
0.5
0.4
0.3
0.2
0.1
0.0
Cellular_organisms - ps1
Archaea1 - ps2
Archaea2 - ps3
Archaea3 - ps4
Asgard/Eukaryota - ps5
Eukaryota - ps6
Amorpheae - ps7
Obazoa - ps8
Opisthokonta - ps9
Holozoa - ps10
Filozoa - ps11
Choano./Metazoa - ps12
Metazoa - ps13
Myriazoa - ps14
Parahoxozoa - ps15
Bilateria - ps16
Deuterostomia - ps17
Chordata - ps18
Olfactores - ps19
Vertebrata - ps20
Gnathostomata - ps21
Euteleostomi - ps22
Sarcopterygii - ps23
Tetrapoda - ps24
Amniota - ps25
Mammalia - ps26
Theria - ps27
Placentalia - ps28
Boreoeutheria - ps29
Euarchontoglires - ps30
Euarchonta - ps31
Primates - ps32
Haplorrhini - ps33
Simiiformes - ps34
Catarrhini - ps35
Hominoidea - ps36
Hominidae - ps37
Homininae - ps38
Hominini - ps39
Homo_sapiens - ps40
Phylostratum
p-value
4e-59
8e-45
1e-30
3e-16
5e-02
Log-odds
0.5
1.0
1.5
2.0
2.5
3.0
3.5
4.0

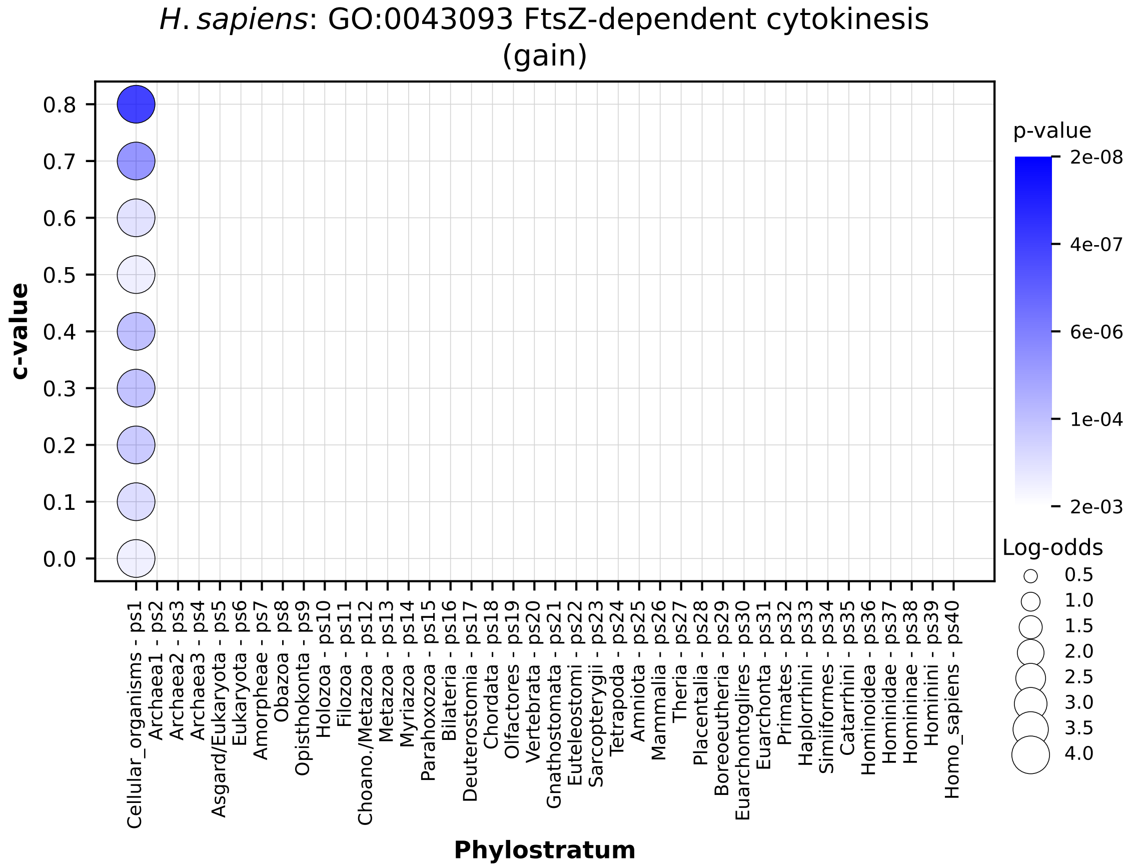

*H. sapiens*: GO:0043093 FtsZ-dependent cytokinesis
(gain)
c-value
0.8
0.7
0.6
0.5
0.4
0.3
0.2
0.1
0.0
p-value
2e-08
4e-07
6e-06
1e-04
2e-03
Log-odds
0.5
1.0
1.5
2.0
2.5
3.0
3.5
4.0
Cellular_organisms - ps1
Archaea1 - ps2
Archaea2 - ps3
Archaea3 - ps4
Asgard/Eukaryota - ps5
Eukaryota - ps6
Amorpheae - ps7
Obazoa - ps8
Opisthokonta - ps9
Holozoa - ps10
Filozoa - ps11
Choano./Metazoa - ps12
Metazoa - ps13
Myriazoa - ps14
Parahoxozoa - ps15
Bilateria - ps16
Deuterostomia - ps17
Chordata - ps18
Olfactores - ps19
Vertebrata - ps20
Gnathostomata - ps21
Euteleostomi - ps22
Sarcopterygii - ps23
Tetrapoda - ps24
Amniota - ps25
Mammalia - ps26
Theria - ps27
Placentalia - ps28
Boreoeutheria - ps29
Euarchontoglires - ps30
Euarchonta - ps31
Primates - ps32
Haplorrhini - ps33
Simiiformes - ps34
Catarrhini - ps35
Hominoidea - ps36
Hominidae - ps37
Homininae - ps38
Hominini - ps39
Homo_sapiens - ps40
Phylostratum

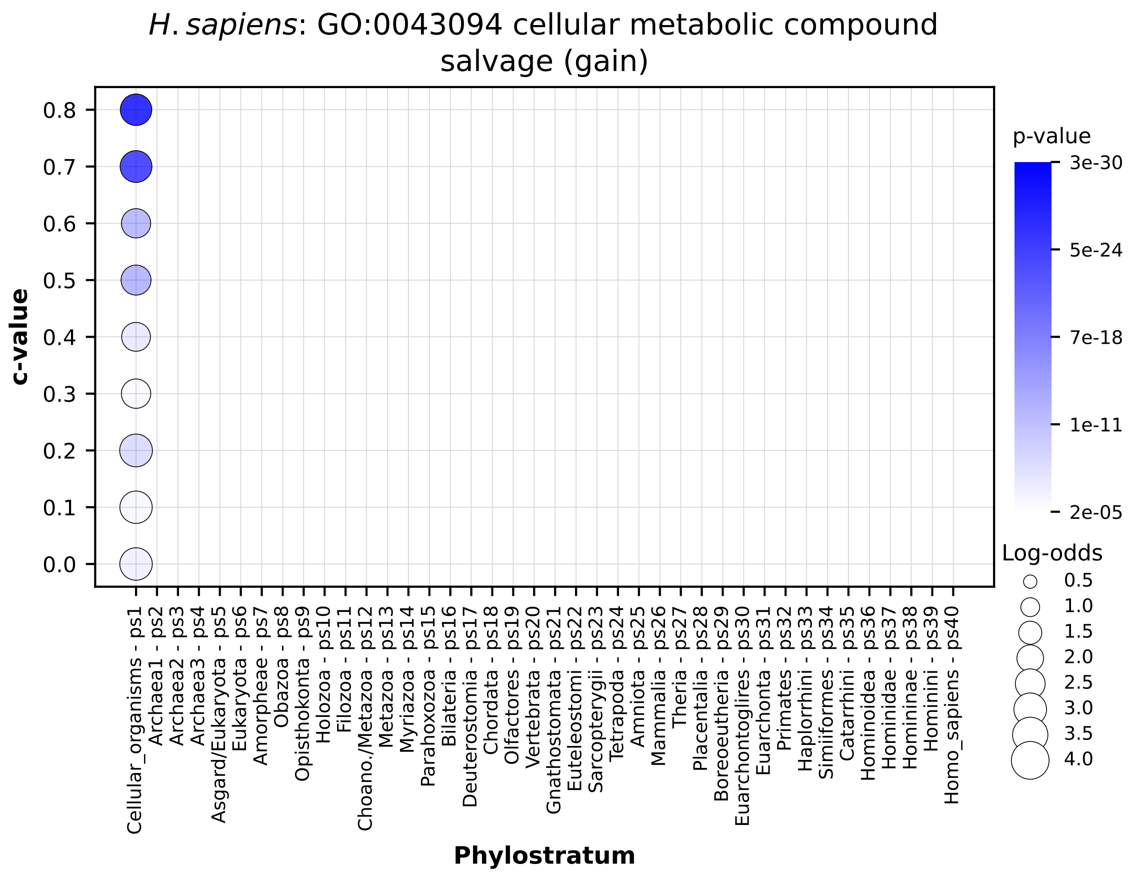
H. sapiens: GO:0043094 cellular metabolic compound salvage (gain)
c-value
0.8
0.7
0.6
0.5
0.4
0.3
0.2
0.1
0.0
p-value
3e-30
5e-24
7e-18
1e-11
2e-05
Log-odds
0.5
1.0
1.5
2.0
2.5
3.0
3.5
4.0
Cellular_organisms - ps1
Archaea1 - ps2
Archaea2 - ps3
Archaea3 - ps4
Asgard/Eukaryota - ps5
Eukaryota - ps6
Amorpheae - ps7
Obazoa - ps8
Opisthokonta - ps9
Holozoa - ps10
Filozoa - ps11
Choano./Metazoa - ps12
Metazoa - ps13
Myriazoa - ps14
Parahoxozoa - ps15
Bilateria - ps16
Deuterostomia - ps17
Chordata - ps18
Olfactores - ps19
Vertebrata - ps20
Gnathostomata - ps21
Euteleostomi - ps22
Sarcopterygii - ps23
Tetrapoda - ps24
Amniota - ps25
Mammalia - ps26
Theria - ps27
Placentalia - ps28
Boreoeutheria - ps29
Euarchontoglires - ps30
Euarchonta - ps31
Primates - ps32
Haplorrhini - ps33
Simiiformes - ps34
Catarrhini - ps35
Hominoidea - ps36
Hominidae - ps37
Homininae - ps38
Hominini - ps39
Homo_sapiens - ps40
Phylostratum

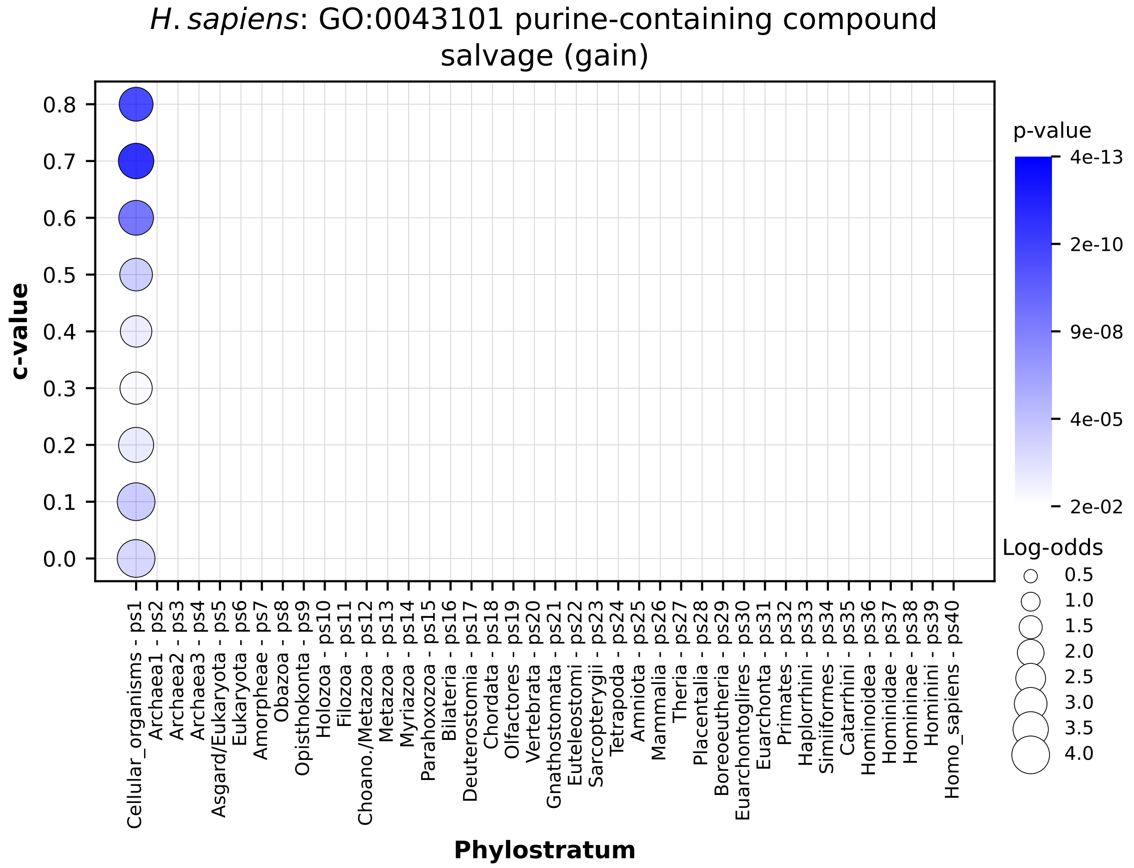

H. sapiens: GO:0043101 purine-containing compound salvage (gain)
c-value
0.0
0.1
0.2
0.3
0.4
0.5
0.6
0.7
0.8
p-value
4e-13
2e-10
9e-08
4e-05
2e-02
Log-odds
0.5
1.0
1.5
2.0
2.5
3.0
3.5
4.0
Cellular_organisms - ps1
Archaea1 - ps2
Archaea2 - ps3
Archaea3 - ps4
Asgard/Eukaryota - ps5
Eukaryota - ps6
Amorpheae - ps7
Obazoa - ps8
Opisthokonta - ps9
Holozoa - ps10
Filozoa - ps11
Choano./Metazoa - ps12
Metazoa - ps13
Myriazoa - ps14
Parahoxozoa - ps15
Bilateria - ps16
Deuterostomia - ps17
Chordata - ps18
Olfactores - ps19
Vertebrata - ps20
Gnathostomata - ps21
Euteleostomi - ps22
Sarcopterygii - ps23
Tetrapoda - ps24
Amniota - ps25
Mammalia - ps26
Theria - ps27
Placentalia - ps28
Boreoeutheria - ps29
Euarchontoglires - ps30
Euarchonta - ps31
Primates - ps32
Haplorrhini - ps33
Simiiformes - ps34
Catarrhini - ps35
Hominoidea - ps36
Hominidae - ps37
Homininae - ps38
Hominini - ps39
Homo_sapiens - ps40
Phylostratum

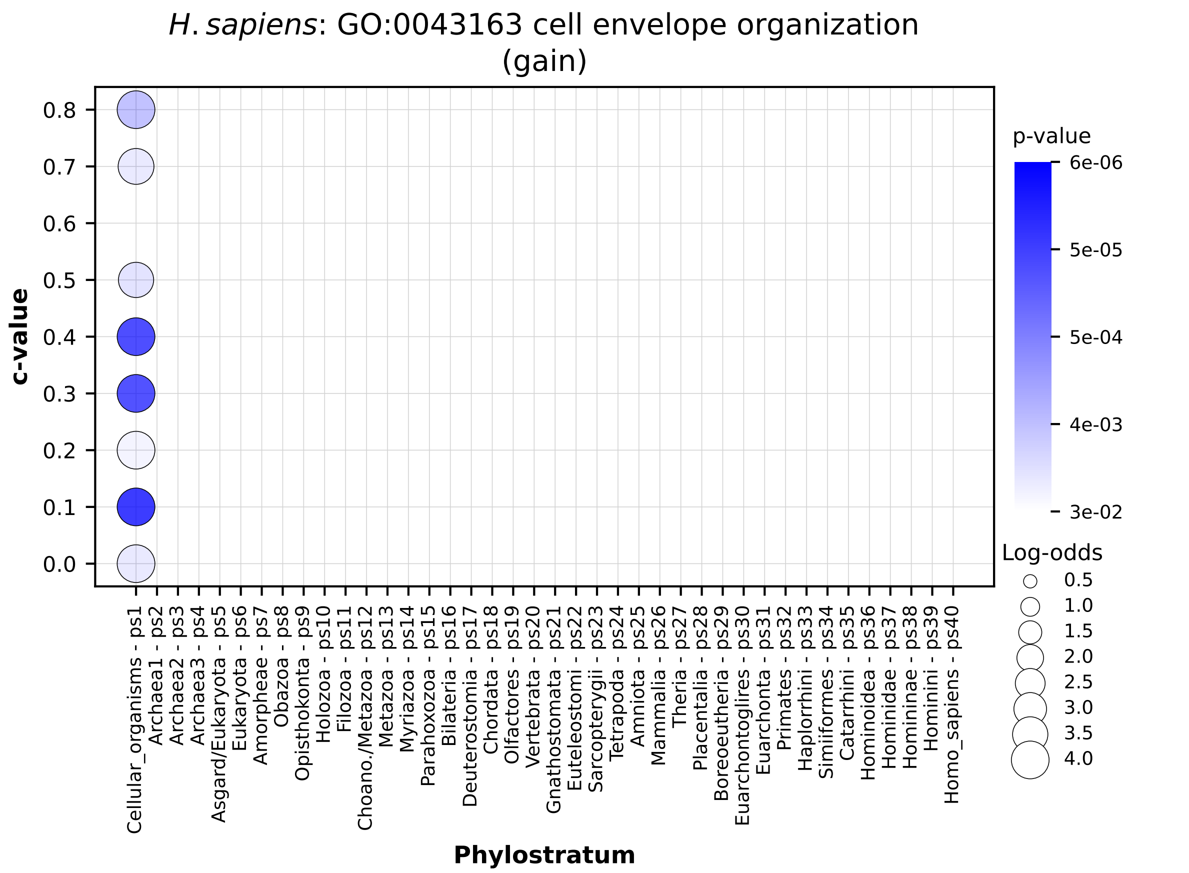
H. sapiens: GO:0043163 cell envelope organization
(gain)
c-value
0.0
0.1
0.2
0.3
0.4
0.5
0.6
0.7
0.8
Phylostratum
Cellular_organisms - ps1
Archaea1 - ps2
Archaea2 - ps3
Archaea3 - ps4
Asgard/Eukaryota - ps5
Eukaryota - ps6
Amorpheae - ps7
Obazoa - ps8
Opisthokonta - ps9
Holozoa - ps10
Filozoa - ps11
Choano./Metazoa - ps12
Metazoa - ps13
Myriazoa - ps14
Parahoxozoa - ps15
Bilateria - ps16
Deuterostomia - ps17
Chordata - ps18
Olfactores - ps19
Vertebrata - ps20
Gnathostomata - ps21
Euteleostomi - ps22
Sarcopterygii - ps23
Tetrapoda - ps24
Amniota - ps25
Mammalia - ps26
Theria - ps27
Placentalia - ps28
Boreoeutheria - ps29
Euarchontoglires - ps30
Euarchonta - ps31
Primates - ps32
Haplorrhini - ps33
Simiiformes - ps34
Catarrhini - ps35
Hominoidea - ps36
Hominidae - ps37
Homininae - ps38
Hominini - ps39
Homo_sapiens - ps40
p-value
6e-06
5e-05
5e-04
4e-03
3e-02
Log-odds
0.5
1.0
1.5
2.0
2.5
3.0
3.5
4.0

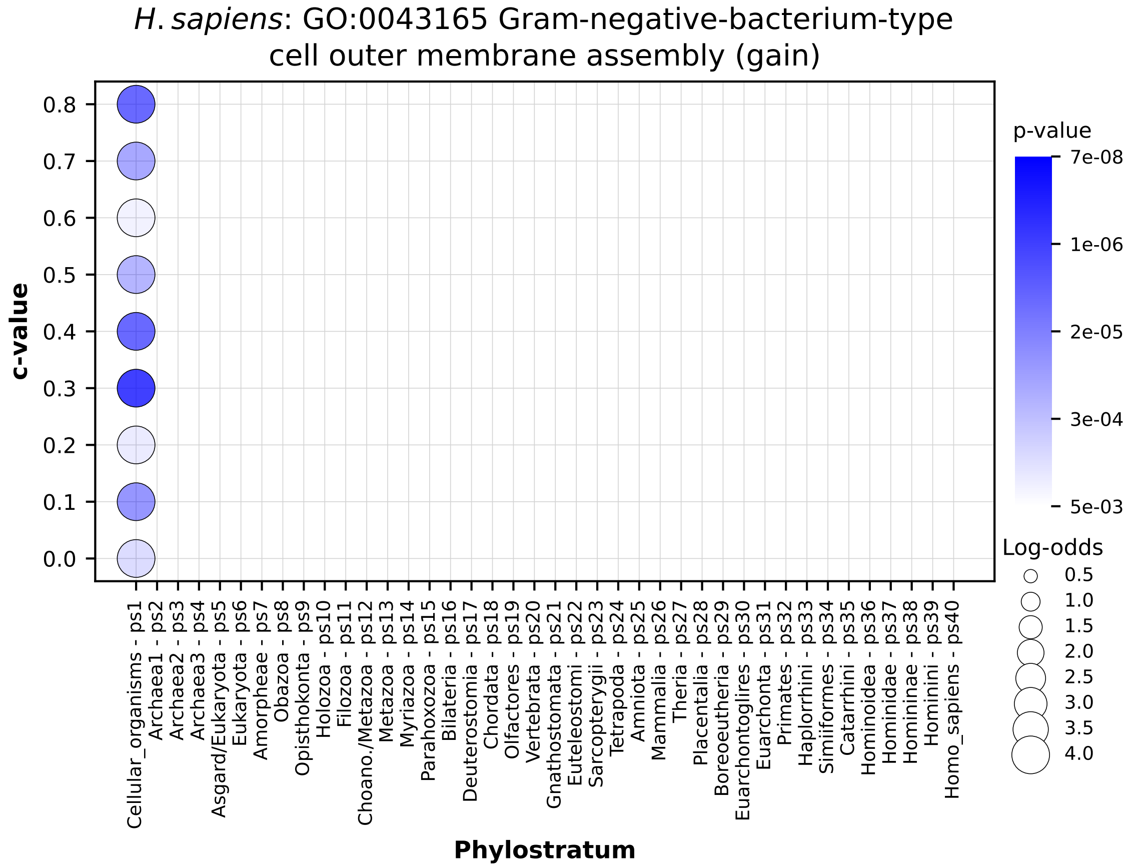
H. sapiens: GO:0043165 Gram-negative-bacterium-type cell outer membrane assembly (gain)
c-value
0.8
0.7
0.6
0.5
0.4
0.3
0.2
0.1
0.0
Phylostratum
Cellular_organisms - ps1
Archaea1 - ps2
Archaea2 - ps3
Archaea3 - ps4
Asgard/Eukaryota - ps5
Eukaryota - ps6
Amorpheae - ps7
Obazoa - ps8
Opisthokonta - ps9
Holozoa - ps10
Filozoa - ps11
Choano./Metazoa - ps12
Metazoa - ps13
Myriazoa - ps14
Parahoxozoa - ps15
Bilateria - ps16
Deuterostomia - ps17
Chordata - ps18
Olfactores - ps19
Vertebrata - ps20
Gnathostomata - ps21
Euteleostomi - ps22
Sarcopterygii - ps23
Tetrapoda - ps24
Amniota - ps25
Mammalia - ps26
Theria - ps27
Placentalia - ps28
Boreoeutheria - ps29
Euarchontoglires - ps30
Euarchonta - ps31
Primates - ps32
Haplorrhini - ps33
Simiiformes - ps34
Catarrhini - ps35
Hominoidea - ps36
Hominidae - ps37
Homininae - ps38
Hominini - ps39
Homo_sapiens - ps40
p-value
7e-08
1e-06
2e-05
3e-04
5e-03
Log-odds
0.5
1.0
1.5
2.0
2.5
3.0
3.5
4.0

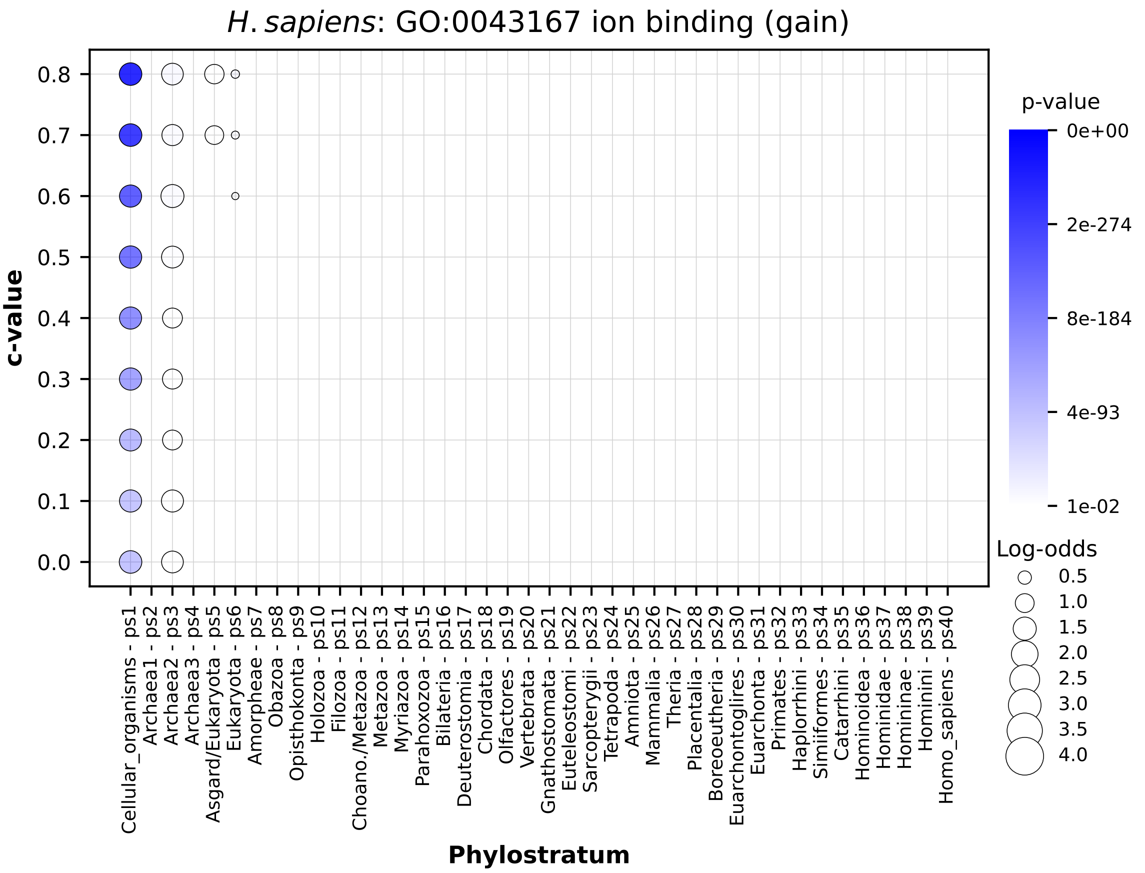

H. sapiens: GO:0043167 ion binding (gain)
c-value
0.0
0.1
0.2
0.3
0.4
0.5
0.6
0.7
0.8
Phylostratum
Cellular_organisms - ps1
Archaea1 - ps2
Archaea2 - ps3
Archaea3 - ps4
Asgard/Eukaryota - ps5
Eukaryota - ps6
Amorpheae - ps7
Obazoa - ps8
Opisthokonta - ps9
Holozoa - ps10
Filozoa - ps11
Choano./Metazoa - ps12
Metazoa - ps13
Myriazoa - ps14
Parahoxozoa - ps15
Bilateria - ps16
Deuterostomia - ps17
Chordata - ps18
Olfactores - ps19
Vertebrata - ps20
Gnathostomata - ps21
Euteleostomi - ps22
Sarcopterygii - ps23
Tetrapoda - ps24
Amniota - ps25
Mammalia - ps26
Theria - ps27
Placentalia - ps28
Boreoeutheria - ps29
Euarchontoglires - ps30
Euarchonta - ps31
Primates - ps32
Haplorrhini - ps33
Simiiformes - ps34
Catarrhini - ps35
Hominoidea - ps36
Hominidae - ps37
Homininae - ps38
Hominini - ps39
Homo_sapiens - ps40
p-value
0e+00
2e-274
8e-184
4e-93
1e-02
Log-odds
0.5
1.0
1.5
2.0
2.5
3.0
3.5
4.0

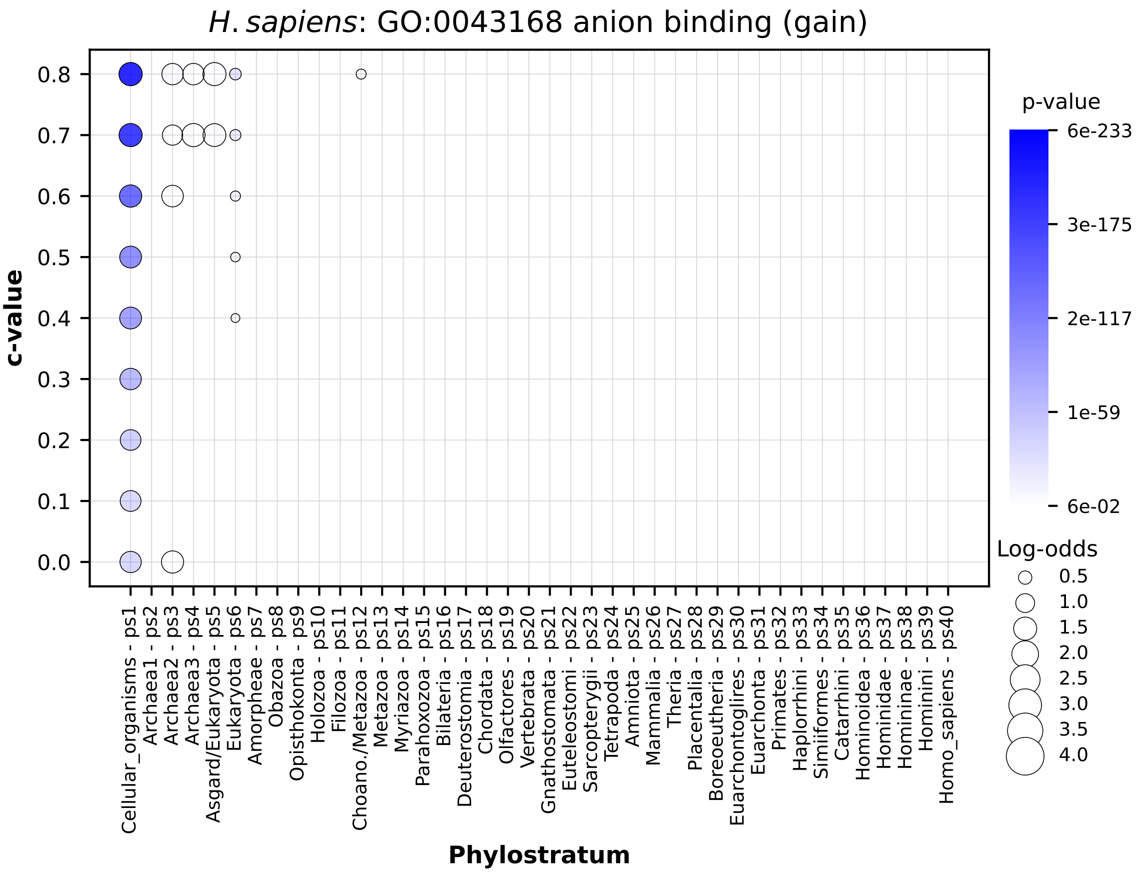

H. sapiens: GO:0043168 anion binding (gain)
c-value
0.8
0.7
0.6
0.5
0.4
0.3
0.2
0.1
0.0
Phylostratum
Cellular_organisms - ps1
Archaea1 - ps2
Archaea2 - ps3
Archaea3 - ps4
Asgard/Eukaryota - ps5
Eukaryota - ps6
Amorpheae - ps7
Obazoa - ps8
Opisthokonta - ps9
Holozoa - ps10
Filozoa - ps11
Choano./Metazoa - ps12
Metazoa - ps13
Myriazoa - ps14
Parahoxozoa - ps15
Bilateria - ps16
Deuterostomia - ps17
Chordata - ps18
Olfactores - ps19
Vertebrata - ps20
Gnathostomata - ps21
Euteleostomi - ps22
Sarcopterygii - ps23
Tetrapoda - ps24
Amniota - ps25
Mammalia - ps26
Theria - ps27
Placentalia - ps28
Boreoeutheria - ps29
Euarchontoglires - ps30
Euarchonta - ps31
Primates - ps32
Haplorrhini - ps33
Simiiformes - ps34
Catarrhini - ps35
Hominoidea - ps36
Hominidae - ps37
Homininae - ps38
Hominini - ps39
Homo_sapiens - ps40
p-value
6e-233
3e-175
2e-117
1e-59
6e-02
Log-odds
0.5
1.0
1.5
2.0
2.5
3.0
3.5
4.0

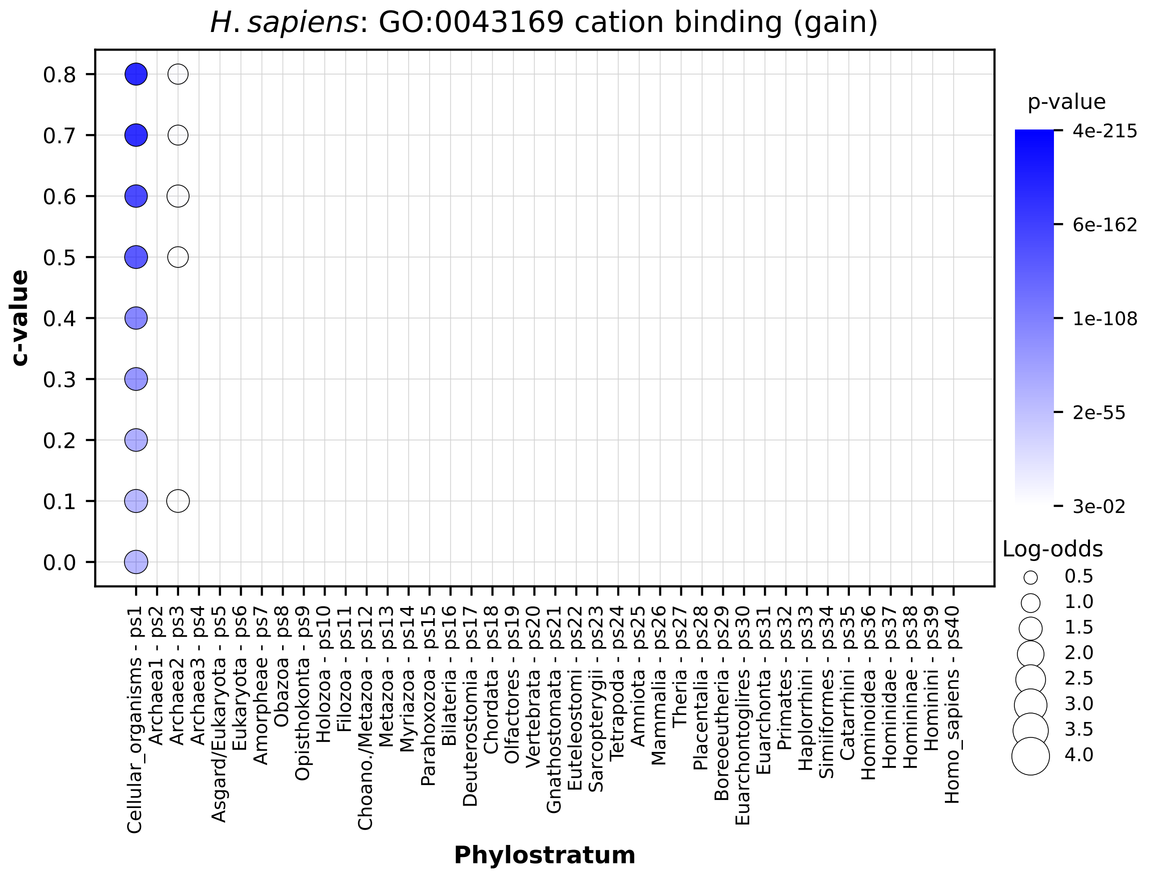
H. sapiens: GO:0043169 cation binding (gain)
c-value
0.8
0.7
0.6
0.5
0.4
0.3
0.2
0.1
0.0
Phylostratum
Cellular_organisms - ps1
Archaea1 - ps2
Archaea2 - ps3
Archaea3 - ps4
Asgard/Eukaryota - ps5
Eukaryota - ps6
Amorpheae - ps7
Obazoa - ps8
Opisthokonta - ps9
Holozoa - ps10
Filozoa - ps11
Choano./Metazoa - ps12
Metazoa - ps13
Myriazoa - ps14
Parahoxozoa - ps15
Bilateria - ps16
Deuterostomia - ps17
Chordata - ps18
Olfactores - ps19
Vertebrata - ps20
Gnathostomata - ps21
Euteleostomi - ps22
Sarcopterygii - ps23
Tetrapoda - ps24
Amniota - ps25
Mammalia - ps26
Theria - ps27
Placentalia - ps28
Boreoeutheria - ps29
Euarchontoglires - ps30
Euarchonta - ps31
Primates - ps32
Haplorrhini - ps33
Simiiformes - ps34
Catarrhini - ps35
Hominoidea - ps36
Hominidae - ps37
Homininae - ps38
Hominini - ps39
Homo_sapiens - ps40
p-value
4e-215
6e-162
1e-108
2e-55
3e-02
Log-odds
0.5
1.0
1.5
2.0
2.5
3.0
3.5
4.0

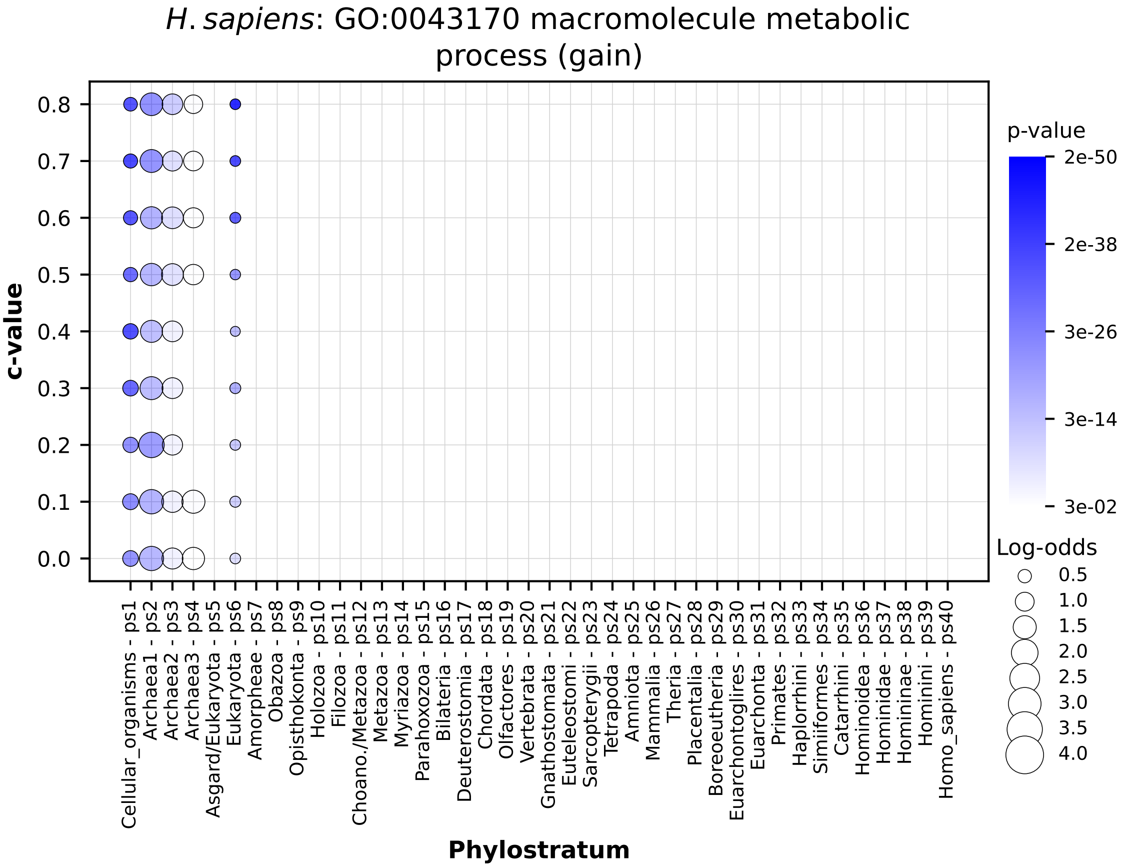

H. sapiens: GO:0043170 macromolecule metabolic process (gain)
c-value
0.8
0.7
0.6
0.5
0.4
0.3
0.2
0.1
0.0
Phylostratum
Cellular_organisms - ps1
Archaea1 - ps2
Archaea2 - ps3
Archaea3 - ps4
Asgard/Eukaryota - ps5
Eukaryota - ps6
Amorpheae - ps7
Obazoa - ps8
Opisthokonta - ps9
Holozoa - ps10
Filozoa - ps11
Choano./Metazoa - ps12
Metazoa - ps13
Myriazoa - ps14
Parahoxozoa - ps15
Bilateria - ps16
Deuterostomia - ps17
Chordata - ps18
Olfactores - ps19
Vertebrata - ps20
Gnathostomata - ps21
Euteleostomi - ps22
Sarcopterygii - ps23
Tetrapoda - ps24
Amniota - ps25
Mammalia - ps26
Theria - ps27
Placentalia - ps28
Boreoeutheria - ps29
Euarchontoglires - ps30
Euarchonta - ps31
Primates - ps32
Haplorrhini - ps33
Simiiformes - ps34
Catarrhini - ps35
Hominoidea - ps36
Hominidae - ps37
Homininae - ps38
Hominini - ps39
Homo_sapiens - ps40
p-value
2e-50
2e-38
3e-26
3e-14
3e-02
Log-odds
0.5
1.0
1.5
2.0
2.5
3.0
3.5
4.0

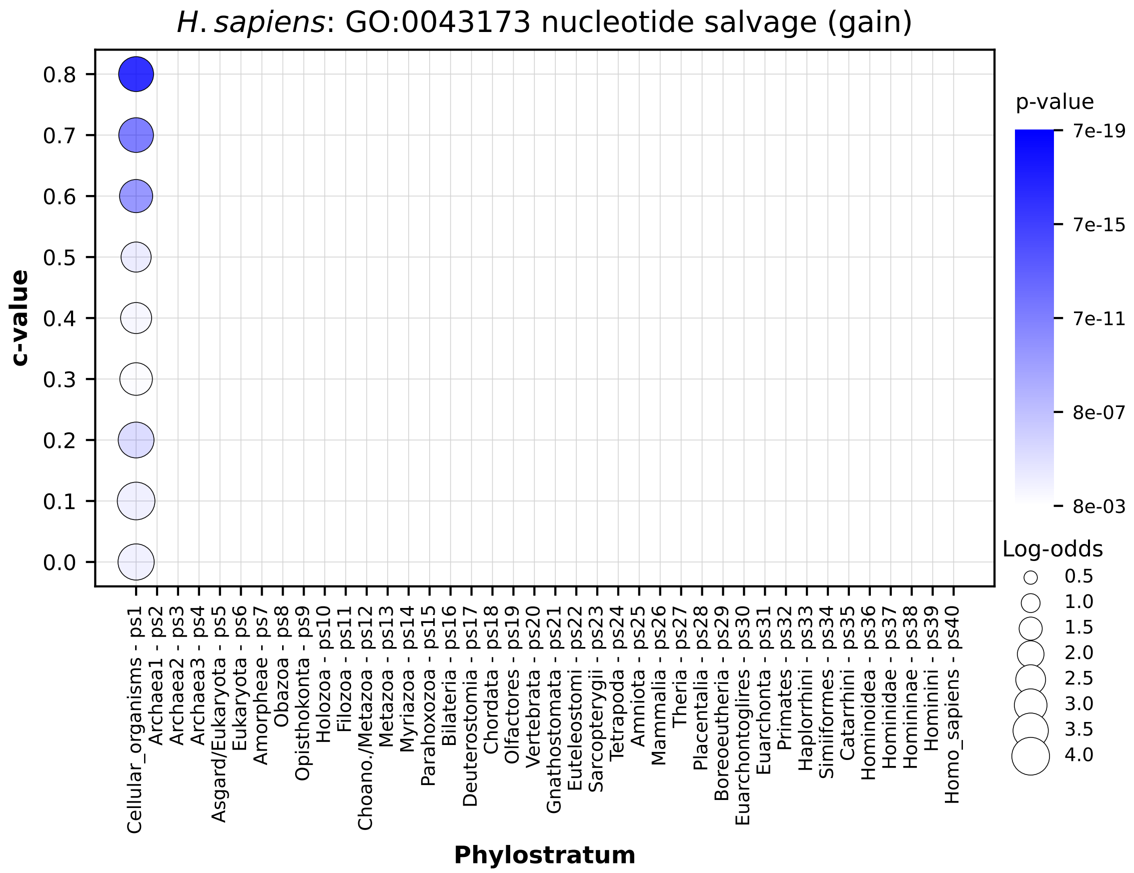

H. sapiens: GO:0043173 nucleotide salvage (gain)
c-value
0.0
0.1
0.2
0.3
0.4
0.5
0.6
0.7
0.8
Phylostratum
Cellular_organisms - ps1
Archaea1 - ps2
Archaea2 - ps3
Archaea3 - ps4
Asgard/Eukaryota - ps5
Eukaryota - ps6
Amorpheae - ps7
Obazoa - ps8
Opisthokonta - ps9
Holozoa - ps10
Filozoa - ps11
Choano./Metazoa - ps12
Metazoa - ps13
Myriazoa - ps14
Parahoxozoa - ps15
Bilateria - ps16
Deuterostomia - ps17
Chordata - ps18
Olfactores - ps19
Vertebrata - ps20
Gnathostomata - ps21
Euteleostomi - ps22
Sarcopterygii - ps23
Tetrapoda - ps24
Amniota - ps25
Mammalia - ps26
Theria - ps27
Placentalia - ps28
Boreoeutheria - ps29
Euarchontoglires - ps30
Euarchonta - ps31
Primates - ps32
Haplorrhini - ps33
Simiiformes - ps34
Catarrhini - ps35
Hominoidea - ps36
Hominidae - ps37
Homininae - ps38
Hominini - ps39
Homo_sapiens - ps40
p-value
7e-19
7e-15
7e-11
8e-07
8e-03
Log-odds
0.5
1.0
1.5
2.0
2.5
3.0
3.5
4.0

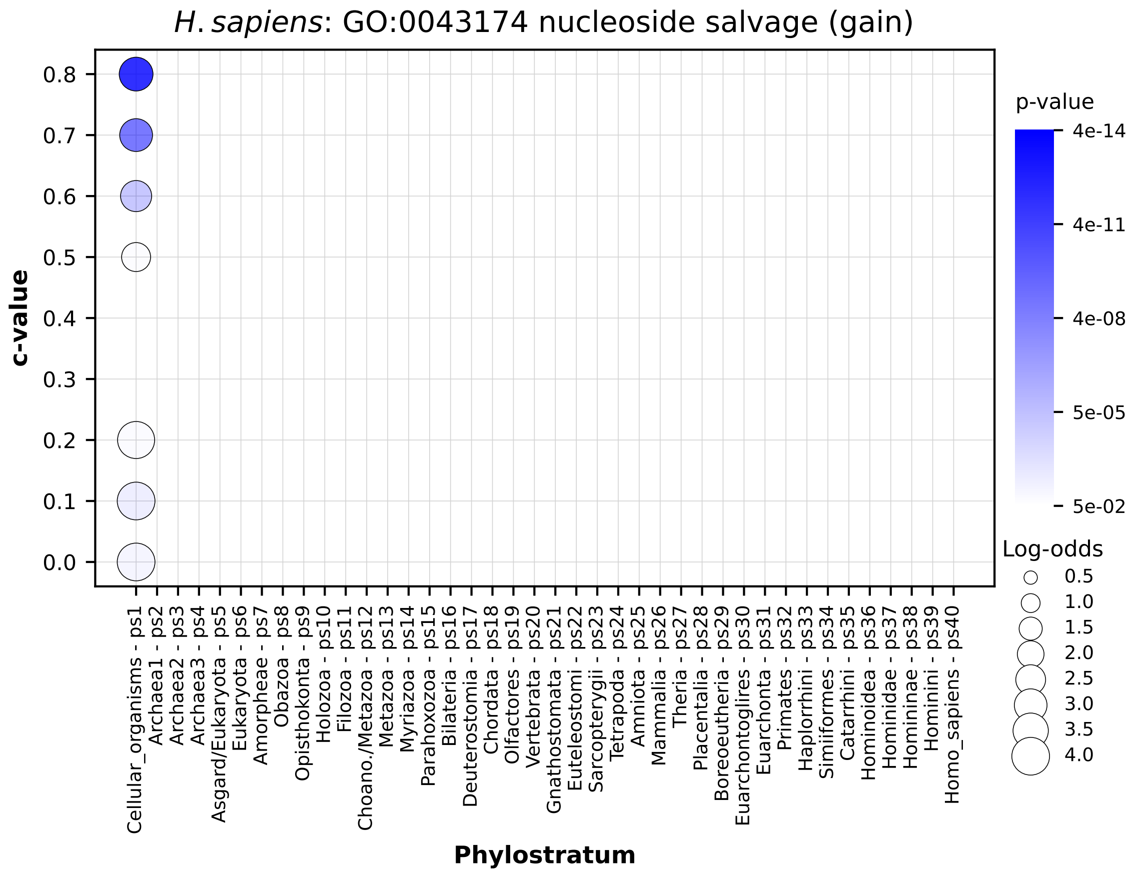

H. sapiens: GO:0043174 nucleoside salvage (gain)
c-value
0.8
0.7
0.6
0.5
0.4
0.3
0.2
0.1
0.0
p-value
4e-14
4e-11
4e-08
5e-05
5e-02
Log-odds
0.5
1.0
1.5
2.0
2.5
3.0
3.5
4.0
Cellular_organisms - ps1
Archaea1 - ps2
Archaea2 - ps3
Archaea3 - ps4
Asgard/Eukaryota - ps5
Eukaryota - ps6
Amorpheae - ps7
Obazoa - ps8
Opisthokonta - ps9
Holozoa - ps10
Filozoa - ps11
Choano./Metazoa - ps12
Metazoa - ps13
Myriazoa - ps14
Parahoxozoa - ps15
Bilateria - ps16
Deuterostomia - ps17
Chordata - ps18
Olfactores - ps19
Vertebrata - ps20
Gnathostomata - ps21
Euteleostomi - ps22
Sarcopterygii - ps23
Tetrapoda - ps24
Amniota - ps25
Mammalia - ps26
Theria - ps27
Placentalia - ps28
Boreoeutheria - ps29
Euarchontoglires - ps30
Euarchonta - ps31
Primates - ps32
Haplorrhini - ps33
Simiiformes - ps34
Catarrhini - ps35
Hominoidea - ps36
Hominidae - ps37
Homininae - ps38
Hominini - ps39
Homo_sapiens - ps40
Phylostratum

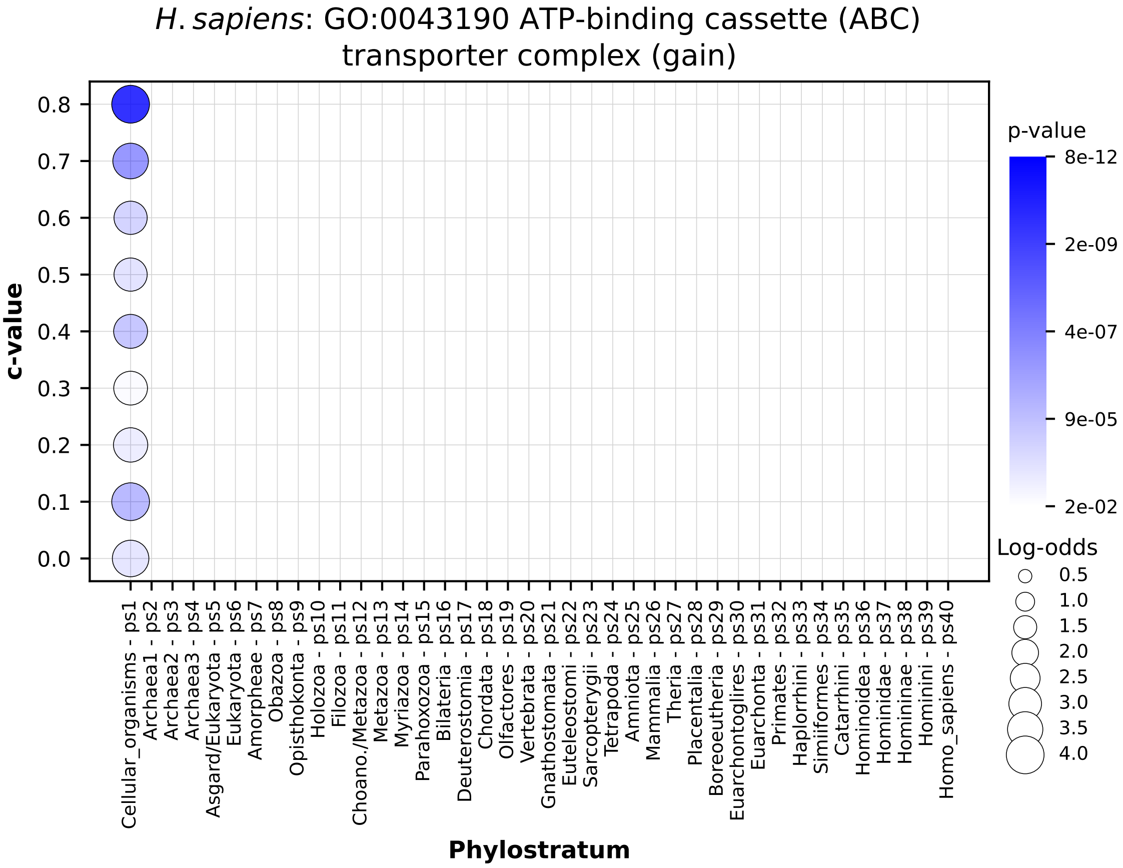

H. sapiens: GO:0043190 ATP-binding cassette (ABC) transporter complex (gain)
c-value
0.8
0.7
0.6
0.5
0.4
0.3
0.2
0.1
0.0
p-value
8e-12
2e-09
4e-07
9e-05
2e-02
Log-odds
0.5
1.0
1.5
2.0
2.5
3.0
3.5
4.0
Cellular_organisms - ps1
Archaea1 - ps2
Archaea2 - ps3
Archaea3 - ps4
Asgard/Eukaryota - ps5
Eukaryota - ps6
Amorpheae - ps7
Obazoa - ps8
Opisthokonta - ps9
Holozoa - ps10
Filozoa - ps11
Choano./Metazoa - ps12
Metazoa - ps13
Myriazoa - ps14
Parahoxozoa - ps15
Bilateria - ps16
Deuterostomia - ps17
Chordata - ps18
Olfactores - ps19
Vertebrata - ps20
Gnathostomata - ps21
Euteleostomi - ps22
Sarcopterygii - ps23
Tetrapoda - ps24
Amniota - ps25
Mammalia - ps26
Theria - ps27
Placentalia - ps28
Boreoeutheria - ps29
Euarchontoglires - ps30
Euarchonta - ps31
Primates - ps32
Haplorrhini - ps33
Simiiformes - ps34
Catarrhini - ps35
Hominoidea - ps36
Hominidae - ps37
Homininae - ps38
Hominini - ps39
Homo_sapiens - ps40
Phylostratum

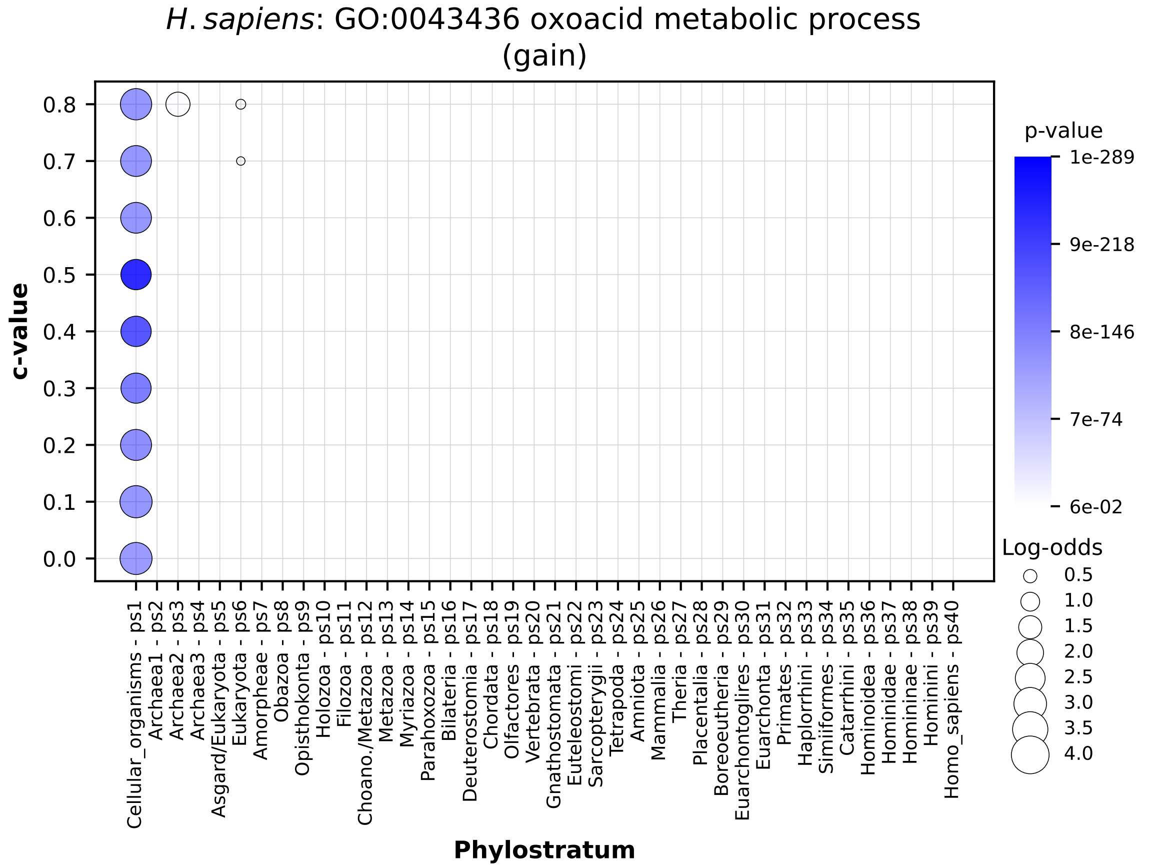

H. sapiens: GO:0043436 oxoacid metabolic process (gain)
c-value
0.0
0.1
0.2
0.3
0.4
0.5
0.6
0.7
0.8
Phylostratum
Cellular_organisms - ps1
Archaea1 - ps2
Archaea2 - ps3
Archaea3 - ps4
Asgard/Eukaryota - ps5
Eukaryota - ps6
Amorpheae - ps7
Obazoa - ps8
Opisthokonta - ps9
Holozoa - ps10
Filozoa - ps11
Choano./Metazoa - ps12
Metazoa - ps13
Myriazoa - ps14
Parahoxozoa - ps15
Bilateria - ps16
Deuterostomia - ps17
Chordata - ps18
Olfactores - ps19
Vertebrata - ps20
Gnathostomata - ps21
Euteleostomi - ps22
Sarcopterygii - ps23
Tetrapoda - ps24
Amniota - ps25
Mammalia - ps26
Theria - ps27
Placentalia - ps28
Boreoeutheria - ps29
Euarchontoglires - ps30
Euarchonta - ps31
Primates - ps32
Haplorrhini - ps33
Simiiformes - ps34
Catarrhini - ps35
Hominoidea - ps36
Hominidae - ps37
Homininae - ps38
Hominini - ps39
Homo_sapiens - ps40
p-value
1e-289
9e-218
8e-146
7e-74
6e-02
Log-odds
0.5
1.0
1.5
2.0
2.5
3.0
3.5
4.0

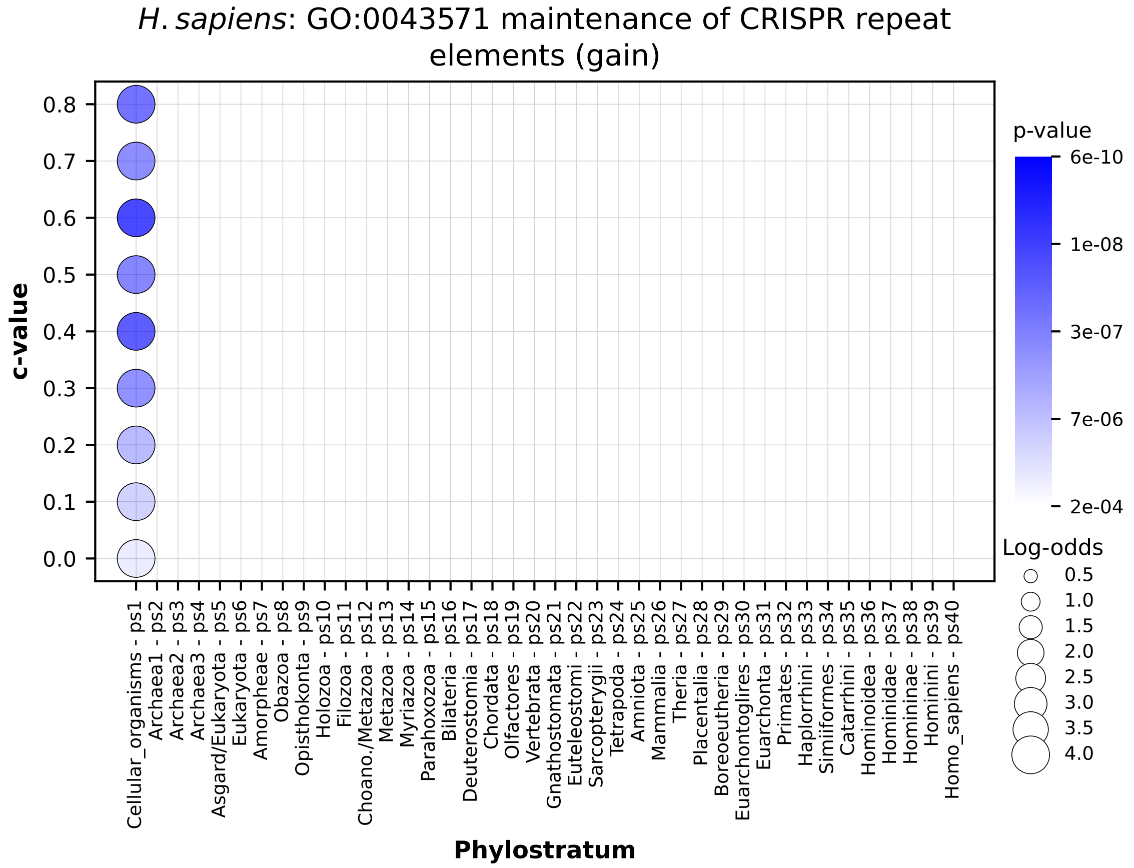

H. sapiens: GO:0043571 maintenance of CRISPR repeat elements (gain)
c-value
0.0
0.1
0.2
0.3
0.4
0.5
0.6
0.7
0.8
Phylostratum
Cellular_organisms - ps1
Archaea1 - ps2
Archaea2 - ps3
Archaea3 - ps4
Asgard/Eukaryota - ps5
Eukaryota - ps6
Amorpheae - ps7
Obazoa - ps8
Opisthokonta - ps9
Holozoa - ps10
Filozoa - ps11
Choano./Metazoa - ps12
Metazoa - ps13
Myriazoa - ps14
Parahoxozoa - ps15
Bilateria - ps16
Deuterostomia - ps17
Chordata - ps18
Olfactores - ps19
Vertebrata - ps20
Gnathostomata - ps21
Euteleostomi - ps22
Sarcopterygii - ps23
Tetrapoda - ps24
Amniota - ps25
Mammalia - ps26
Theria - ps27
Placentalia - ps28
Boreoeutheria - ps29
Euarchontoglires - ps30
Euarchonta - ps31
Primates - ps32
Haplorrhini - ps33
Simiiformes - ps34
Catarrhini - ps35
Hominoidea - ps36
Hominidae - ps37
Homininae - ps38
Hominini - ps39
Homo_sapiens - ps40
p-value
6e-10
1e-08
3e-07
7e-06
2e-04
Log-odds
0.5
1.0
1.5
2.0
2.5
3.0
3.5
4.0

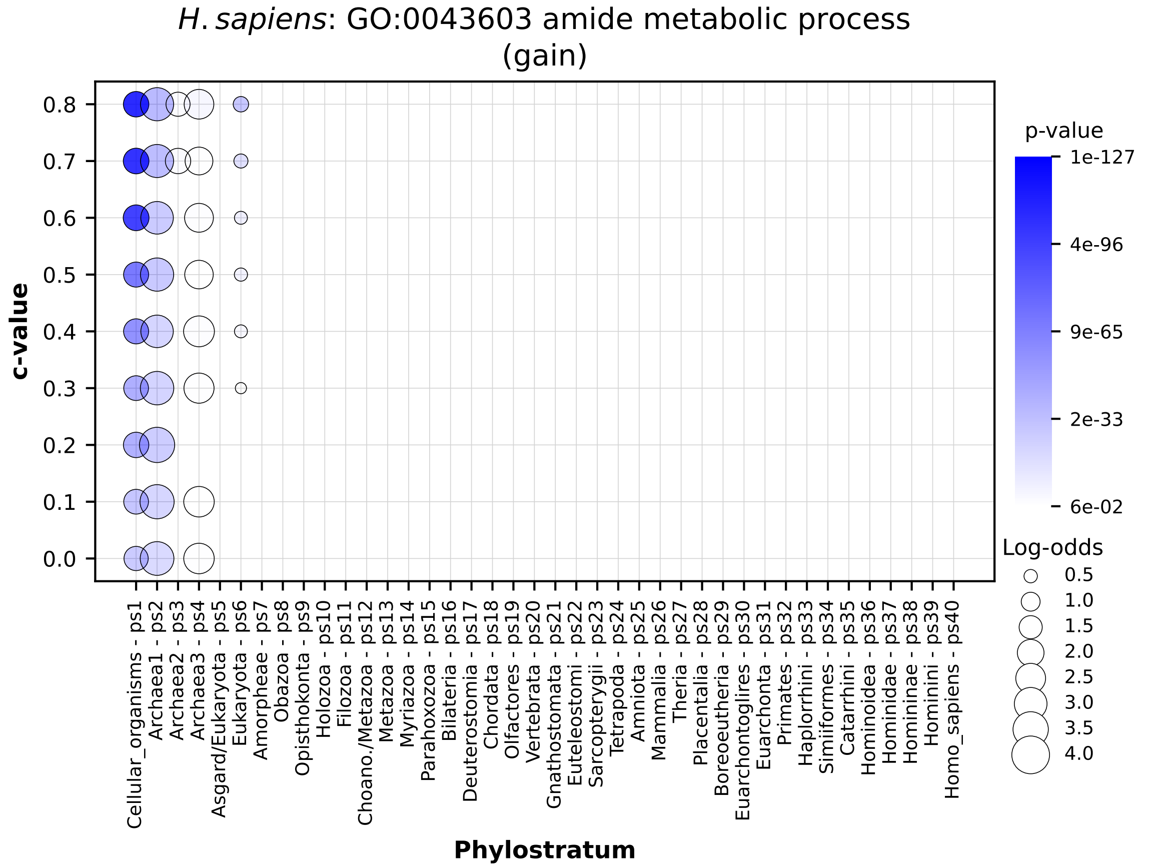

H. sapiens: GO:0043603 amide metabolic process
(gain)
c-value
0.8
0.7
0.6
0.5
0.4
0.3
0.2
0.1
0.0
p-value
1e-127
4e-96
9e-65
2e-33
6e-02
Log-odds
0.5
1.0
1.5
2.0
2.5
3.0
3.5
4.0
Cellular_organisms - ps1
Archaea1 - ps2
Archaea2 - ps3
Archaea3 - ps4
Asgard/Eukaryota - ps5
Eukaryota - ps6
Amorpheae - ps7
Obazoa - ps8
Opisthokonta - ps9
Holozoa - ps10
Filozoa - ps11
Choano./Metazoa - ps12
Metazoa - ps13
Myriazoa - ps14
Parahoxozoa - ps15
Bilateria - ps16
Deuterostomia - ps17
Chordata - ps18
Olfactores - ps19
Vertebrata - ps20
Gnathostomata - ps21
Euteleostomi - ps22
Sarcopterygii - ps23
Tetrapoda - ps24
Amniota - ps25
Mammalia - ps26
Theria - ps27
Placentalia - ps28
Boreoeutheria - ps29
Euarchontoglires - ps30
Euarchonta - ps31
Primates - ps32
Haplorrhini - ps33
Simiiformes - ps34
Catarrhini - ps35
Hominoidea - ps36
Hominidae - ps37
Homininae - ps38
Hominini - ps39
Homo_sapiens - ps40
Phylostratum

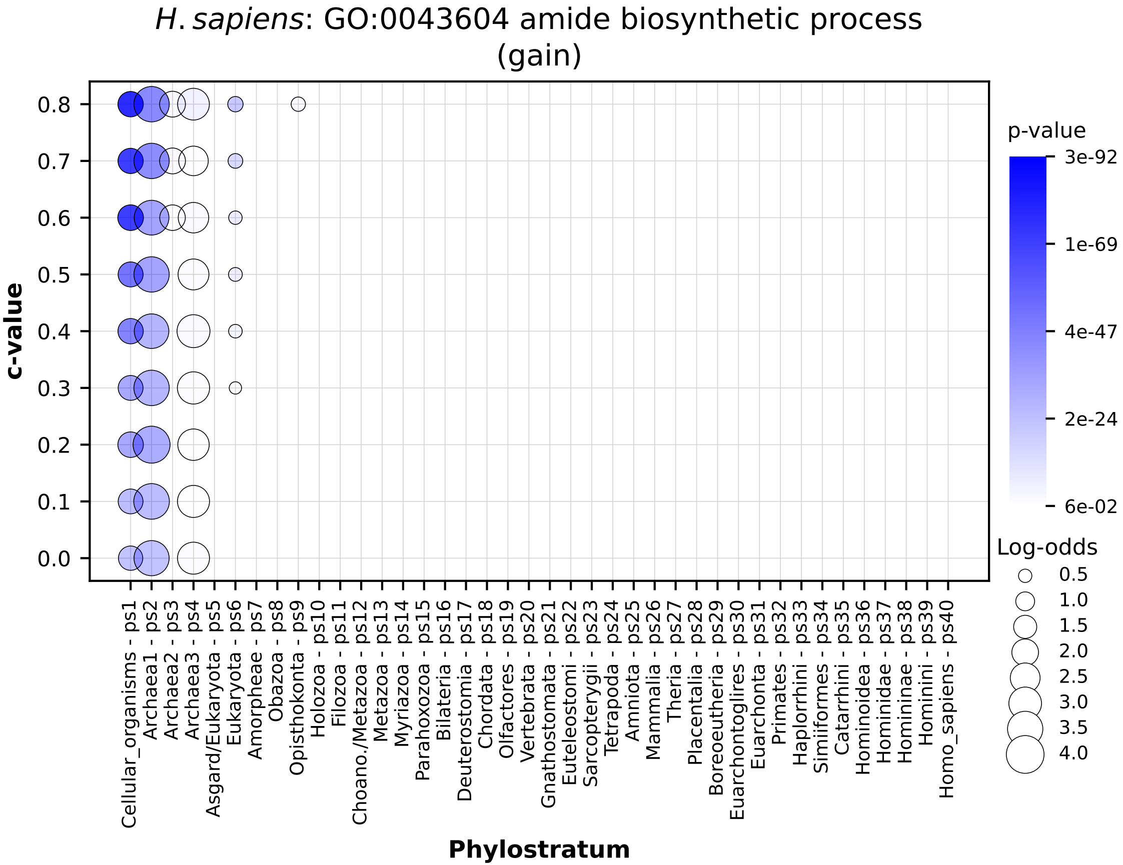

H. sapiens: GO:0043604 amide biosynthetic process
(gain)
c-value
0.8
0.7
0.6
0.5
0.4
0.3
0.2
0.1
0.0
p-value
3e-92
1e-69
4e-47
2e-24
6e-02
Log-odds
0.5
1.0
1.5
2.0
2.5
3.0
3.5
4.0
Cellular_organisms - ps1
Archaea1 - ps2
Archaea2 - ps3
Archaea3 - ps4
Asgard/Eukaryota - ps5
Eukaryota - ps6
Amorpheae - ps7
Obazoa - ps8
Opisthokonta - ps9
Holozoa - ps10
Filozoa - ps11
Choano./Metazoa - ps12
Metazoa - ps13
Myriazoa - ps14
Parahoxozoa - ps15
Bilateria - ps16
Deuterostomia - ps17
Chordata - ps18
Olfactores - ps19
Vertebrata - ps20
Gnathostomata - ps21
Euteleostomi - ps22
Sarcopterygii - ps23
Tetrapoda - ps24
Amniota - ps25
Mammalia - ps26
Theria - ps27
Placentalia - ps28
Boreoeutheria - ps29
Euarchontoglires - ps30
Euarchonta - ps31
Primates - ps32
Haplorrhini - ps33
Simiiformes - ps34
Catarrhini - ps35
Hominoidea - ps36
Hominidae - ps37
Homininae - ps38
Hominini - ps39
Homo_sapiens - ps40
Phylostratum

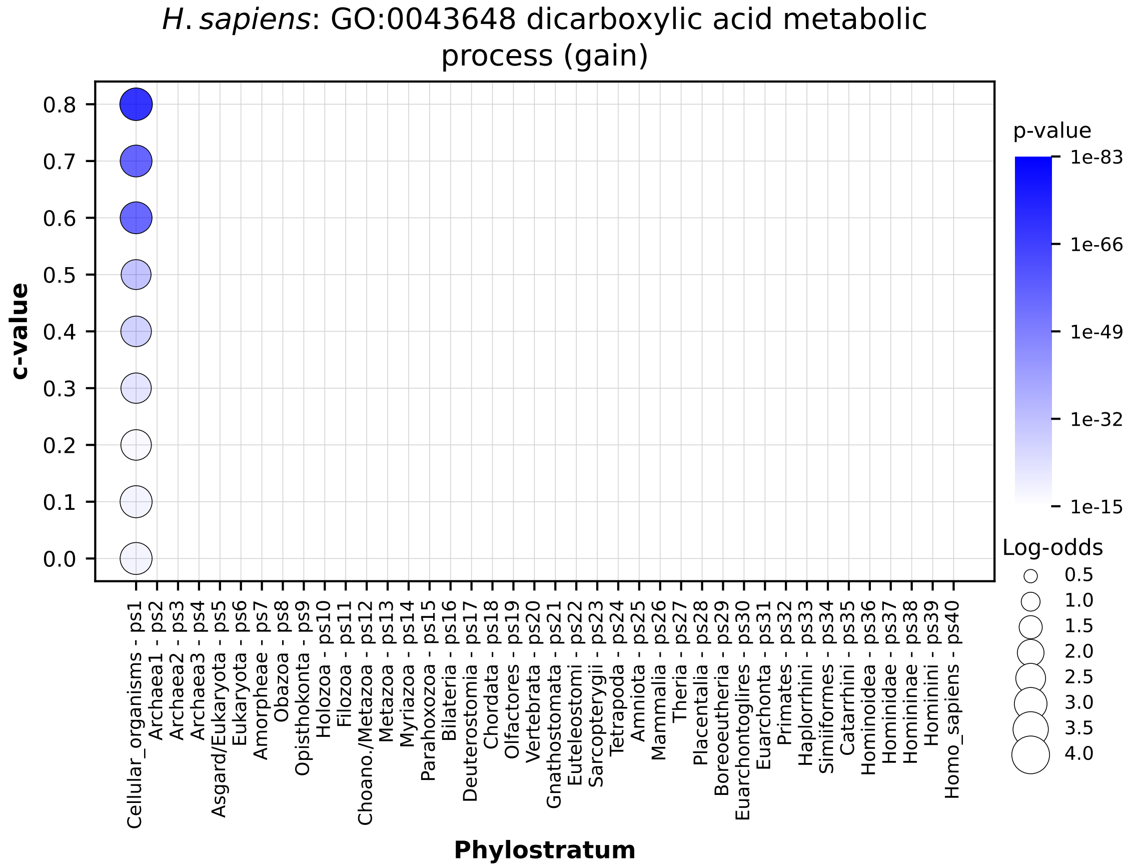

H. sapiens: GO:0043648 dicarboxylic acid metabolic process (gain)
c-value
0.8
0.7
0.6
0.5
0.4
0.3
0.2
0.1
0.0
Phylostratum
Cellular_organisms - ps1
Archaea1 - ps2
Archaea2 - ps3
Archaea3 - ps4
Asgard/Eukaryota - ps5
Eukaryota - ps6
Amorpheae - ps7
Obazoa - ps8
Opisthokonta - ps9
Holozoa - ps10
Filozoa - ps11
Choano./Metazoa - ps12
Metazoa - ps13
Myriazoa - ps14
Parahoxozoa - ps15
Bilateria - ps16
Deuterostomia - ps17
Chordata - ps18
Olfactores - ps19
Vertebrata - ps20
Gnathostomata - ps21
Euteleostomi - ps22
Sarcopterygii - ps23
Tetrapoda - ps24
Amniota - ps25
Mammalia - ps26
Theria - ps27
Placentalia - ps28
Boreoeutheria - ps29
Euarchontoglires - ps30
Euarchonta - ps31
Primates - ps32
Haplorrhini - ps33
Simiiformes - ps34
Catarrhini - ps35
Hominoidea - ps36
Hominidae - ps37
Homininae - ps38
Hominini - ps39
Homo_sapiens - ps40
p-value
1e-83
1e-66
1e-49
1e-32
1e-15
Log-odds
0.5
1.0
1.5
2.0
2.5
3.0
3.5
4.0

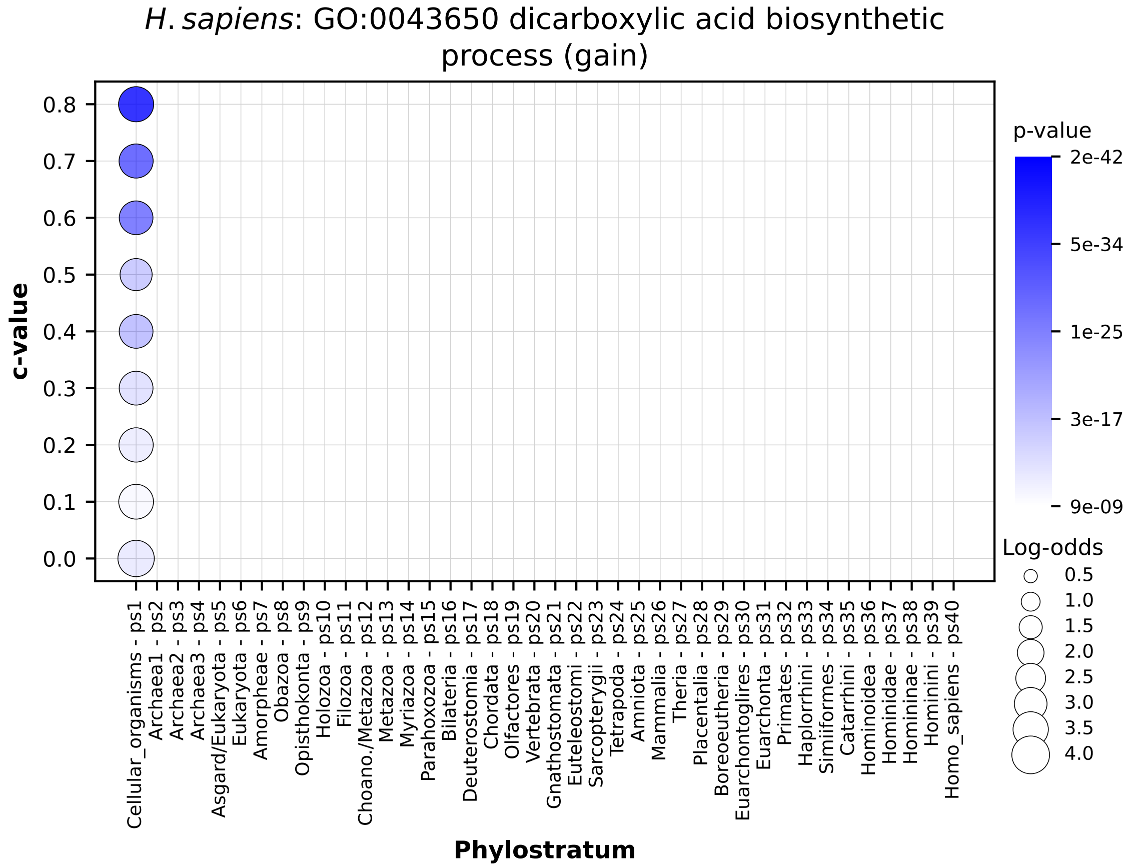

H. sapiens: GO:0043650 dicarboxylic acid biosynthetic process (gain)
c-value
0.8
0.7
0.6
0.5
0.4
0.3
0.2
0.1
0.0
Phylostratum
Cellular_organisms - ps1
Archaea1 - ps2
Archaea2 - ps3
Archaea3 - ps4
Asgard/Eukaryota - ps5
Eukaryota - ps6
Amorpheae - ps7
Obazoa - ps8
Opisthokonta - ps9
Holozoa - ps10
Filozoa - ps11
Choano./Metazoa - ps12
Metazoa - ps13
Myriazoa - ps14
Parahoxozoa - ps15
Bilateria - ps16
Deuterostomia - ps17
Chordata - ps18
Olfactores - ps19
Vertebrata - ps20
Gnathostomata - ps21
Euteleostomi - ps22
Sarcopterygii - ps23
Tetrapoda - ps24
Amniota - ps25
Mammalia - ps26
Theria - ps27
Placentalia - ps28
Boreoeutheria - ps29
Euarchontoglires - ps30
Euarchonta - ps31
Primates - ps32
Haplorrhini - ps33
Simiiformes - ps34
Catarrhini - ps35
Hominoidea - ps36
Hominidae - ps37
Homininae - ps38
Hominini - ps39
Homo_sapiens - ps40
p-value
2e-42
5e-34
1e-25
3e-17
9e-09
Log-odds
0.5
1.0
1.5
2.0
2.5
3.0
3.5
4.0

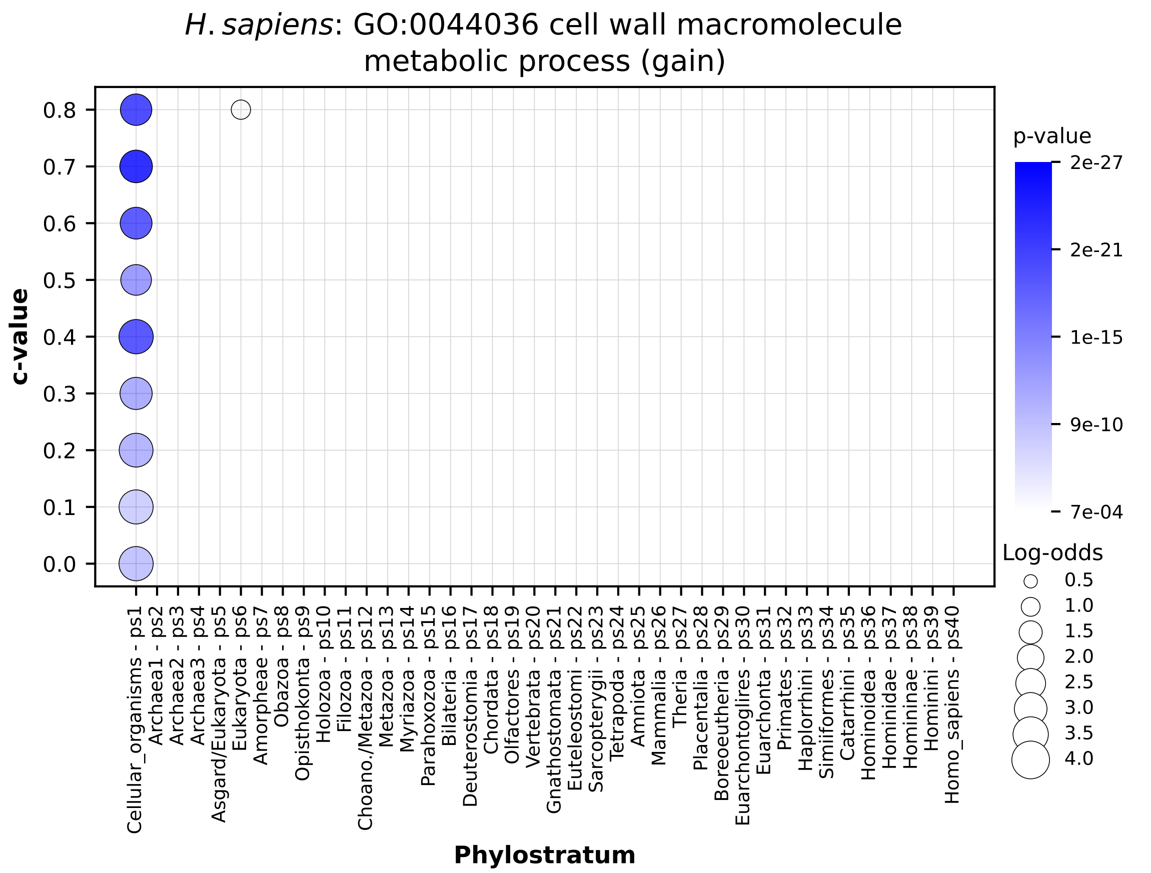

H. sapiens: GO:0044036 cell wall macromolecule metabolic process (gain)
c-value
0.0
0.1
0.2
0.3
0.4
0.5
0.6
0.7
0.8
Phylostratum
Cellular_organisms - ps1
Archaea1 - ps2
Archaea2 - ps3
Archaea3 - ps4
Asgard/Eukaryota - ps5
Eukaryota - ps6
Amorpheae - ps7
Obazoa - ps8
Opisthokonta - ps9
Holozoa - ps10
Filozoa - ps11
Choano./Metazoa - ps12
Metazoa - ps13
Myriazoa - ps14
Parahoxozoa - ps15
Bilateria - ps16
Deuterostomia - ps17
Chordata - ps18
Olfactores - ps19
Vertebrata - ps20
Gnathostomata - ps21
Euteleostomi - ps22
Sarcopterygii - ps23
Tetrapoda - ps24
Amniota - ps25
Mammalia - ps26
Theria - ps27
Placentalia - ps28
Boreoeutheria - ps29
Euarchontoglires - ps30
Euarchonta - ps31
Primates - ps32
Haplorrhini - ps33
Simiiformes - ps34
Catarrhini - ps35
Hominoidea - ps36
Hominidae - ps37
Homininae - ps38
Hominini - ps39
Homo_sapiens - ps40
p-value
2e-27
2e-21
1e-15
9e-10
7e-04
Log-odds
0.5
1.0
1.5
2.0
2.5
3.0
3.5
4.0

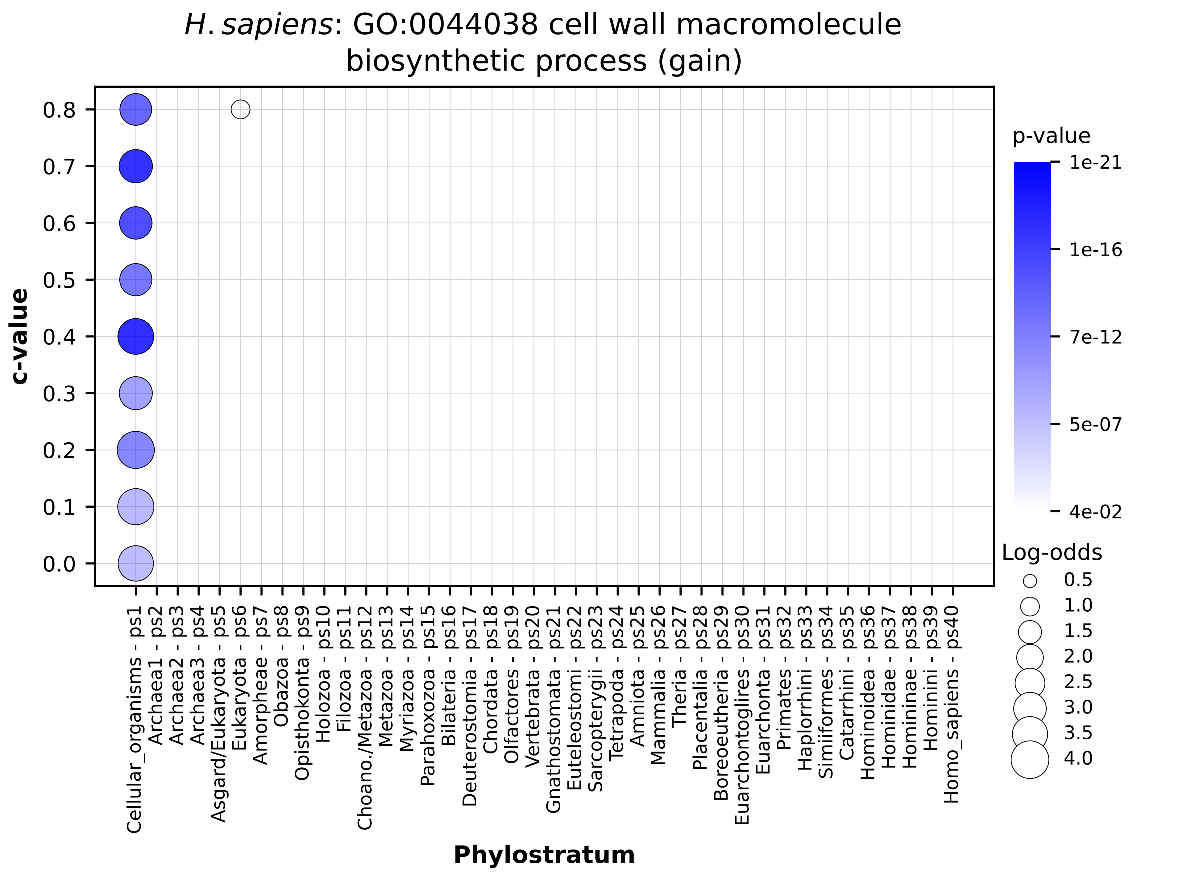
H. sapiens: GO:0044038 cell wall macromolecule biosynthetic process (gain)
c-value
0.8
0.7
0.6
0.5
0.4
0.3
0.2
0.1
0.0
p-value
1e-21
1e-16
7e-12
5e-07
4e-02
Log-odds
0.5
1.0
1.5
2.0
2.5
3.0
3.5
4.0
Cellular_organisms - ps1
Archaea1 - ps2
Archaea2 - ps3
Archaea3 - ps4
Asgard/Eukaryota - ps5
Eukaryota - ps6
Amorpheae - ps7
Obazoa - ps8
Opisthokonta - ps9
Holozoa - ps10
Filozoa - ps11
Choano./Metazoa - ps12
Metazoa - ps13
Myriazoa - ps14
Parahoxozoa - ps15
Bilateria - ps16
Deuterostomia - ps17
Chordata - ps18
Olfactores - ps19
Vertebrata - ps20
Gnathostomata - ps21
Euteleostomi - ps22
Sarcopterygii - ps23
Tetrapoda - ps24
Amniota - ps25
Mammalia - ps26
Theria - ps27
Placentalia - ps28
Boreoeutheria - ps29
Euarchontoglires - ps30
Euarchonta - ps31
Primates - ps32
Haplorrhini - ps33
Simiiformes - ps34
Catarrhini - ps35
Hominoidea - ps36
Hominidae - ps37
Homininae - ps38
Hominini - ps39
Homo_sapiens - ps40
Phylostratum

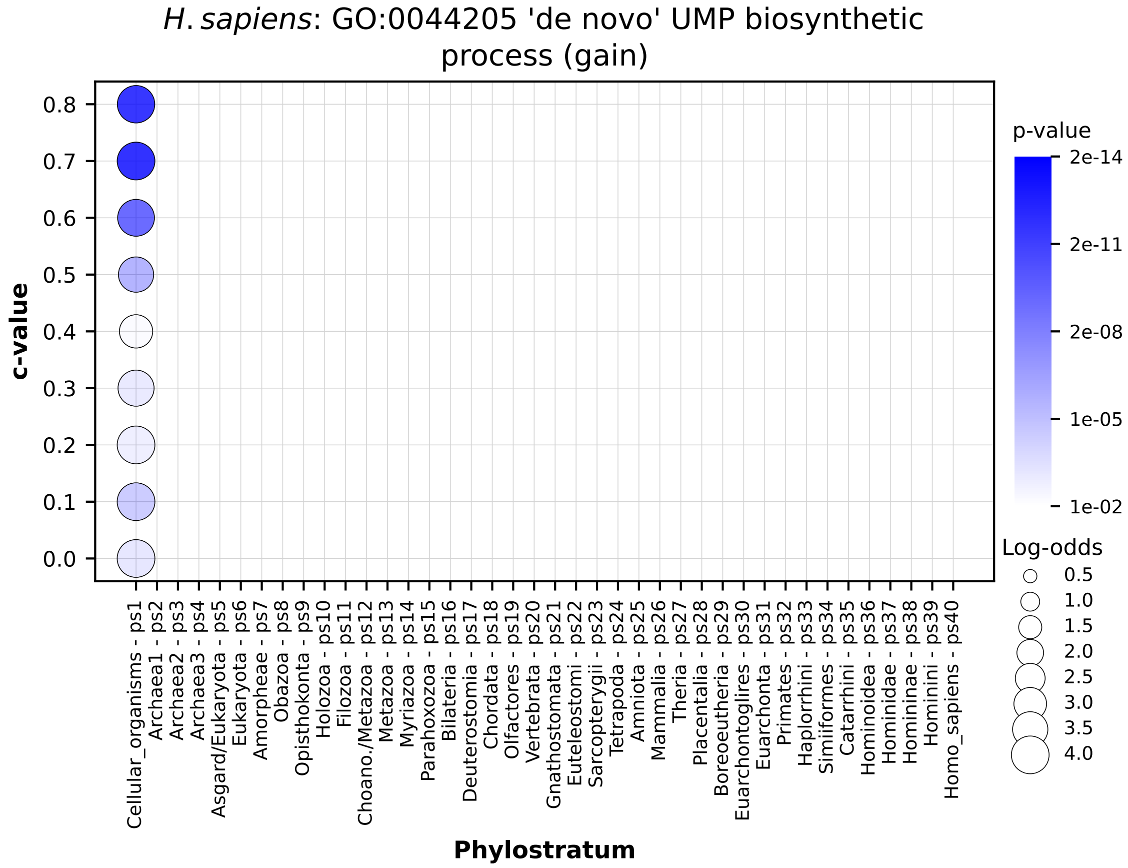
H. sapiens: GO:0044205 'de novo' UMP biosynthetic process (gain)
c-value
0.8
0.7
0.6
0.5
0.4
0.3
0.2
0.1
0.0
p-value
2e-14
2e-11
2e-08
1e-05
1e-02
Log-odds
0.5
1.0
1.5
2.0
2.5
3.0
3.5
4.0
Cellular_organisms - ps1
Archaea1 - ps2
Archaea2 - ps3
Archaea3 - ps4
Asgard/Eukaryota - ps5
Eukaryota - ps6
Amorpheae - ps7
Obazoa - ps8
Opisthokonta - ps9
Holozoa - ps10
Filozoa - ps11
Choano./Metazoa - ps12
Metazoa - ps13
Myriazoa - ps14
Parahoxozoa - ps15
Bilateria - ps16
Deuterostomia - ps17
Chordata - ps18
Olfactores - ps19
Vertebrata - ps20
Gnathostomata - ps21
Euteleostomi - ps22
Sarcopterygii - ps23
Tetrapoda - ps24
Amniota - ps25
Mammalia - ps26
Theria - ps27
Placentalia - ps28
Boreoeutheria - ps29
Euarchontoglires - ps30
Euarchonta - ps31
Primates - ps32
Haplorrhini - ps33
Simiiformes - ps34
Catarrhini - ps35
Hominoidea - ps36
Hominidae - ps37
Homininae - ps38
Hominini - ps39
Homo_sapiens - ps40
Phylostratum

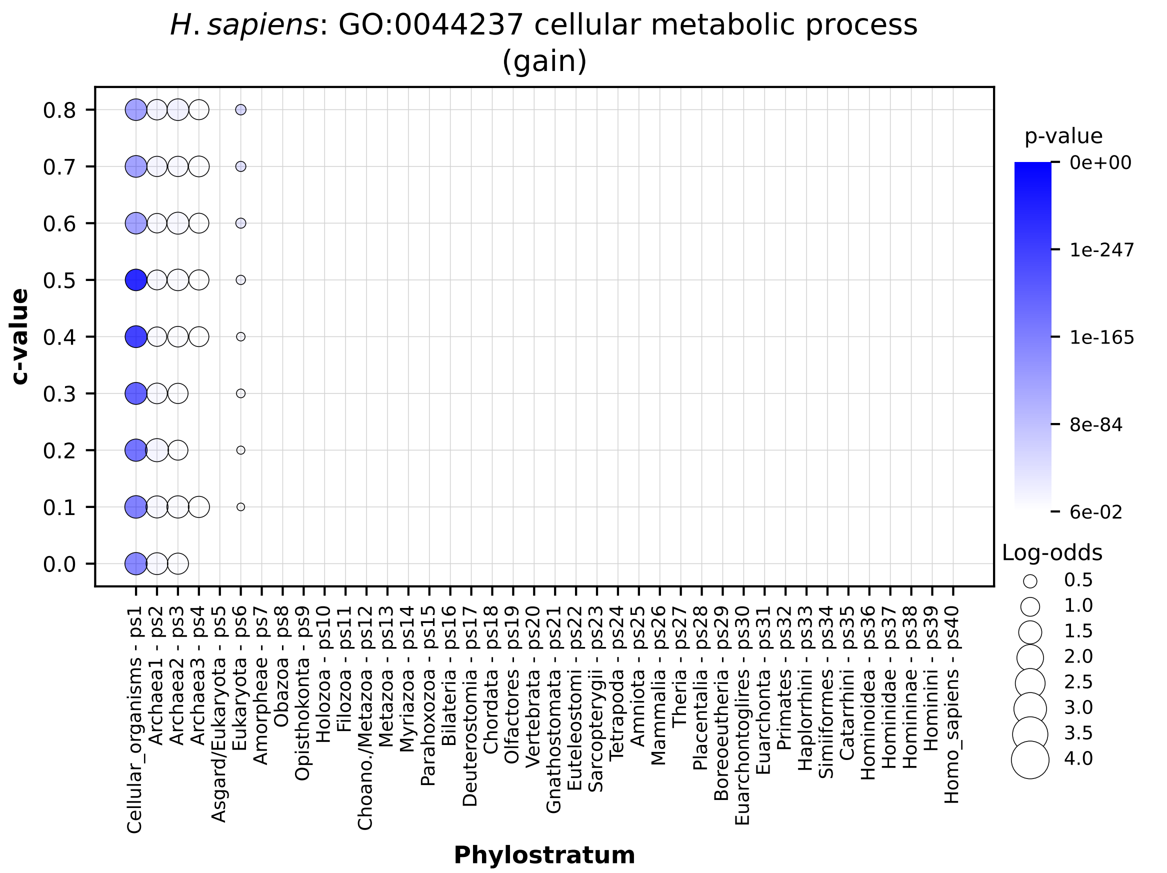

H. sapiens: GO:0044237 cellular metabolic process
(gain)
c-value
0.8
0.7
0.6
0.5
0.4
0.3
0.2
0.1
0.0
p-value
0e+00
1e-247
1e-165
8e-84
6e-02
Log-odds
0.5
1.0
1.5
2.0
2.5
3.0
3.5
4.0
Cellular_organisms - ps1
Archaea1 - ps2
Archaea2 - ps3
Archaea3 - ps4
Asgard/Eukaryota - ps5
Eukaryota - ps6
Amorpheae - ps7
Obazoa - ps8
Opisthokonta - ps9
Holozoa - ps10
Filozoa - ps11
Choano./Metazoa - ps12
Metazoa - ps13
Myriazoa - ps14
Parahoxozoa - ps15
Bilateria - ps16
Deuterostomia - ps17
Chordata - ps18
Olfactores - ps19
Vertebrata - ps20
Gnathostomata - ps21
Euteleostomi - ps22
Sarcopterygii - ps23
Tetrapoda - ps24
Amniota - ps25
Mammalia - ps26
Theria - ps27
Placentalia - ps28
Boreoeutheria - ps29
Euarchontoglires - ps30
Euarchonta - ps31
Primates - ps32
Haplorrhini - ps33
Simiiformes - ps34
Catarrhini - ps35
Hominoidea - ps36
Hominidae - ps37
Homininae - ps38
Hominini - ps39
Homo_sapiens - ps40
Phylostratum

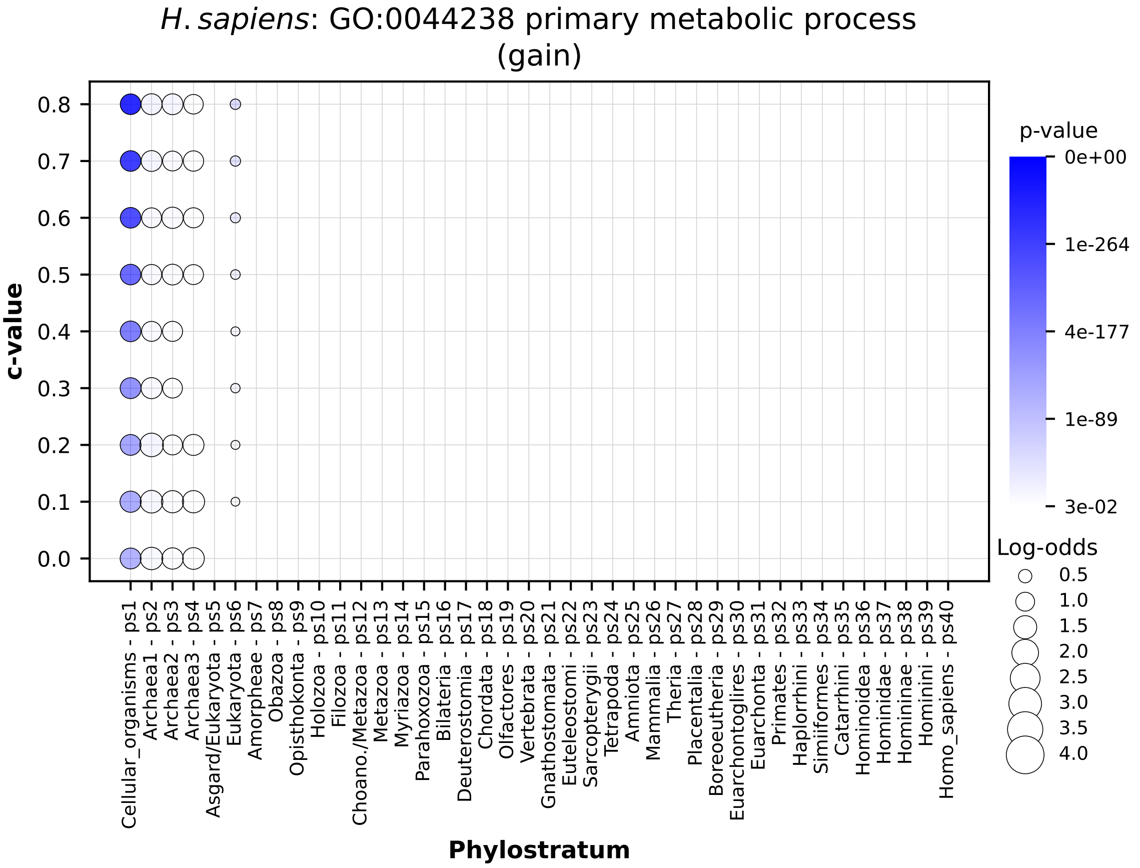

H. sapiens: GO:0044238 primary metabolic process (gain)
c-value
0.8
0.7
0.6
0.5
0.4
0.3
0.2
0.1
0.0
Phylostratum
Cellular_organisms - ps1
Archaea1 - ps2
Archaea2 - ps3
Archaea3 - ps4
Asgard/Eukaryota - ps5
Eukaryota - ps6
Amorpheae - ps7
Obazoa - ps8
Opisthokonta - ps9
Holozoa - ps10
Filozoa - ps11
Choano./Metazoa - ps12
Metazoa - ps13
Myriazoa - ps14
Parahoxozoa - ps15
Bilateria - ps16
Deuterostomia - ps17
Chordata - ps18
Olfactores - ps19
Vertebrata - ps20
Gnathostomata - ps21
Euteleostomi - ps22
Sarcopterygii - ps23
Tetrapoda - ps24
Amniota - ps25
Mammalia - ps26
Theria - ps27
Placentalia - ps28
Boreoeutheria - ps29
Euarchontoglires - ps30
Euarchonta - ps31
Primates - ps32
Haplorrhini - ps33
Simiiformes - ps34
Catarrhini - ps35
Hominoidea - ps36
Hominidae - ps37
Homininae - ps38
Hominini - ps39
Homo_sapiens - ps40
p-value
0e+00
1e-264
4e-177
1e-89
3e-02
Log-odds
0.5
1.0
1.5
2.0
2.5
3.0
3.5
4.0

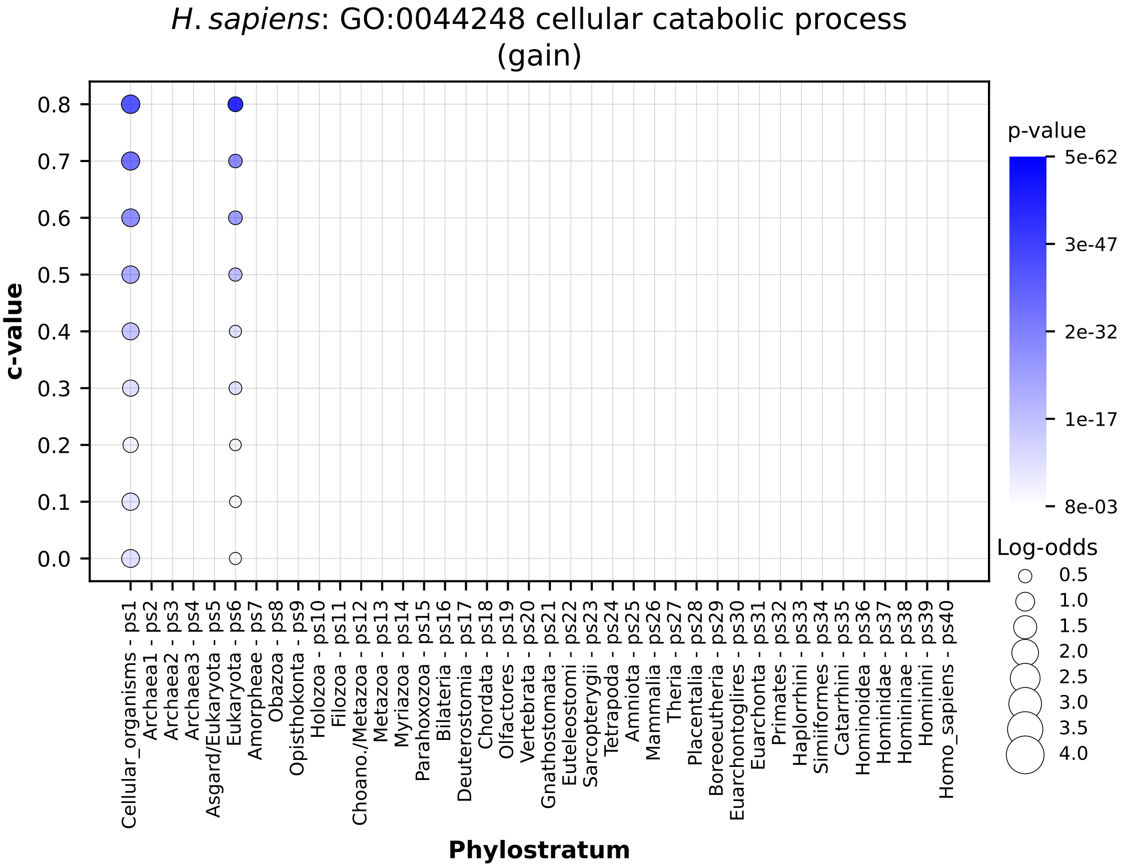

*H. sapiens*: GO:0044248 cellular catabolic process
(gain)
c-value
0.8
0.7
0.6
0.5
0.4
0.3
0.2
0.1
0.0
p-value
5e-62
3e-47
2e-32
1e-17
8e-03
Log-odds
0.5
1.0
1.5
2.0
2.5
3.0
3.5
4.0
Cellular_organisms - ps1
Archaea1 - ps2
Archaea2 - ps3
Archaea3 - ps4
Asgard/Eukaryota - ps5
Eukaryota - ps6
Amorpheae - ps7
Obazoa - ps8
Opisthokonta - ps9
Holozoa - ps10
Filozoa - ps11
Choano./Metazoa - ps12
Metazoa - ps13
Myriazoa - ps14
Parahoxozoa - ps15
Bilateria - ps16
Deuterostomia - ps17
Chordata - ps18
Olfactores - ps19
Vertebrata - ps20
Gnathostomata - ps21
Euteleostomi - ps22
Sarcopterygii - ps23
Tetrapoda - ps24
Amniota - ps25
Mammalia - ps26
Theria - ps27
Placentalia - ps28
Boreoeutheria - ps29
Euarchontoglires - ps30
Euarchonta - ps31
Primates - ps32
Haplorrhini - ps33
Simiiformes - ps34
Catarrhini - ps35
Hominoidea - ps36
Hominidae - ps37
Homininae - ps38
Hominini - ps39
Homo_sapiens - ps40
Phylostratum

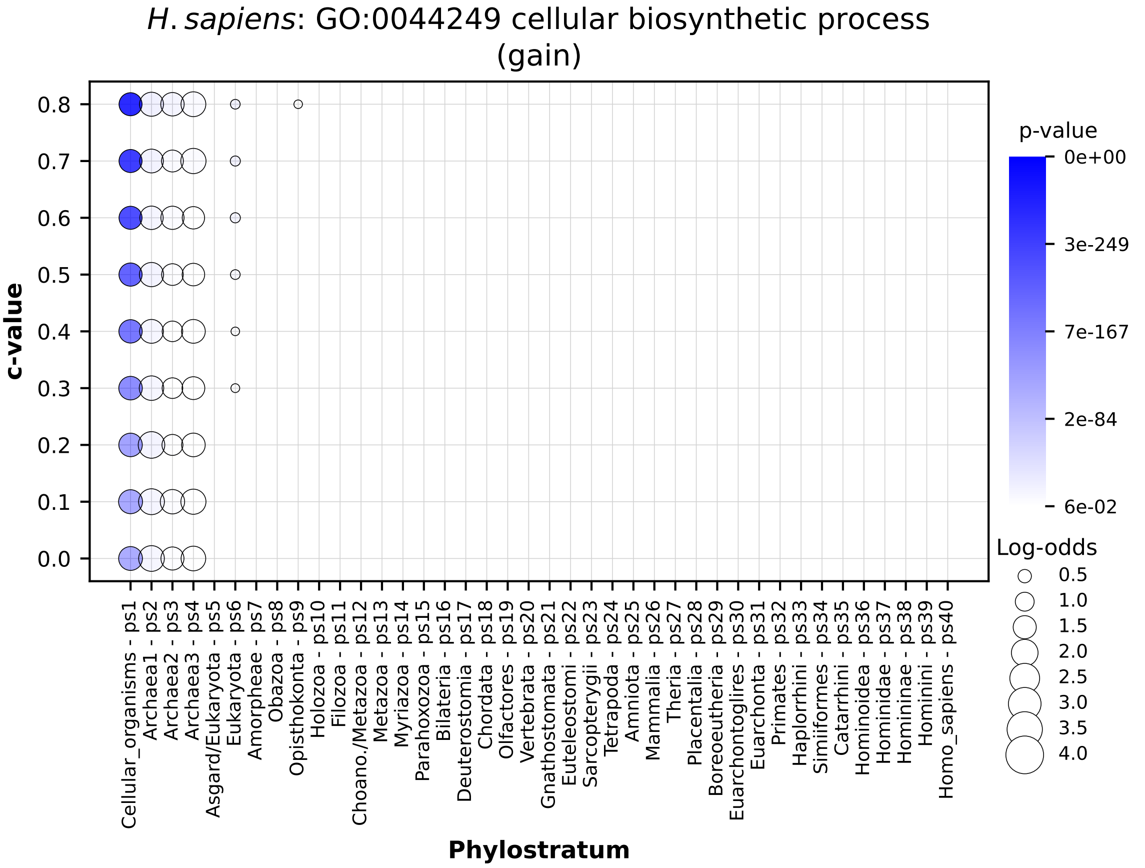

H. sapiens: GO:0044249 cellular biosynthetic process
(gain)
c-value
0.8
0.7
0.6
0.5
0.4
0.3
0.2
0.1
0.0
Phylostratum
Cellular_organisms - ps1
Archaea1 - ps2
Archaea2 - ps3
Archaea3 - ps4
Asgard/Eukaryota - ps5
Eukaryota - ps6
Amorpheae - ps7
Obazoa - ps8
Opisthokonta - ps9
Holozoa - ps10
Filozoa - ps11
Choano./Metazoa - ps12
Metazoa - ps13
Myriazoa - ps14
Parahoxozoa - ps15
Bilateria - ps16
Deuterostomia - ps17
Chordata - ps18
Olfactores - ps19
Vertebrata - ps20
Gnathostomata - ps21
Euteleostomi - ps22
Sarcopterygii - ps23
Tetrapoda - ps24
Amniota - ps25
Mammalia - ps26
Theria - ps27
Placentalia - ps28
Boreoeutheria - ps29
Euarchontoglires - ps30
Euarchonta - ps31
Primates - ps32
Haplorrhini - ps33
Simiiformes - ps34
Catarrhini - ps35
Hominoidea - ps36
Hominidae - ps37
Homininae - ps38
Hominini - ps39
Homo_sapiens - ps40
p-value
0e+00
3e-249
7e-167
2e-84
6e-02
Log-odds
0.5
1.0
1.5
2.0
2.5
3.0
3.5
4.0

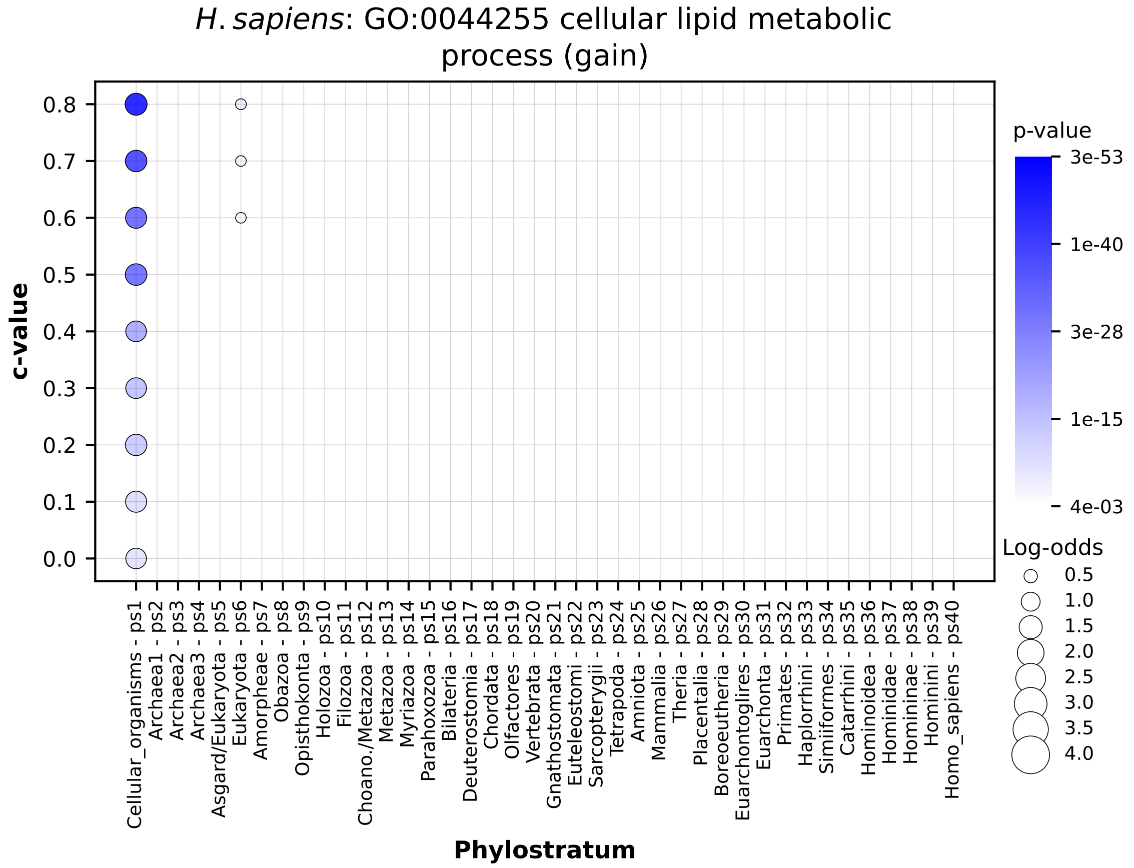
H. sapiens: GO:0044255 cellular lipid metabolic process (gain)
c-value
0.8
0.7
0.6
0.5
0.4
0.3
0.2
0.1
0.0
p-value
3e-53
1e-40
3e-28
1e-15
4e-03
Log-odds
0.5
1.0
1.5
2.0
2.5
3.0
3.5
4.0
Cellular_organisms - ps1
Archaea1 - ps2
Archaea2 - ps3
Archaea3 - ps4
Asgard/Eukaryota - ps5
Eukaryota - ps6
Amorpheae - ps7
Obazoa - ps8
Opisthokonta - ps9
Holozoa - ps10
Filozoa - ps11
Choano./Metazoa - ps12
Metazoa - ps13
Myriazoa - ps14
Parahoxozoa - ps15
Bilateria - ps16
Deuterostomia - ps17
Chordata - ps18
Olfactores - ps19
Vertebrata - ps20
Gnathostomata - ps21
Euteleostomi - ps22
Sarcopterygii - ps23
Tetrapoda - ps24
Amniota - ps25
Mammalia - ps26
Theria - ps27
Placentalia - ps28
Boreoeutheria - ps29
Euarchontoglires - ps30
Euarchonta - ps31
Primates - ps32
Haplorrhini - ps33
Simiiformes - ps34
Catarrhini - ps35
Hominoidea - ps36
Hominidae - ps37
Homininae - ps38
Hominini - ps39
Homo_sapiens - ps40
Phylostratum

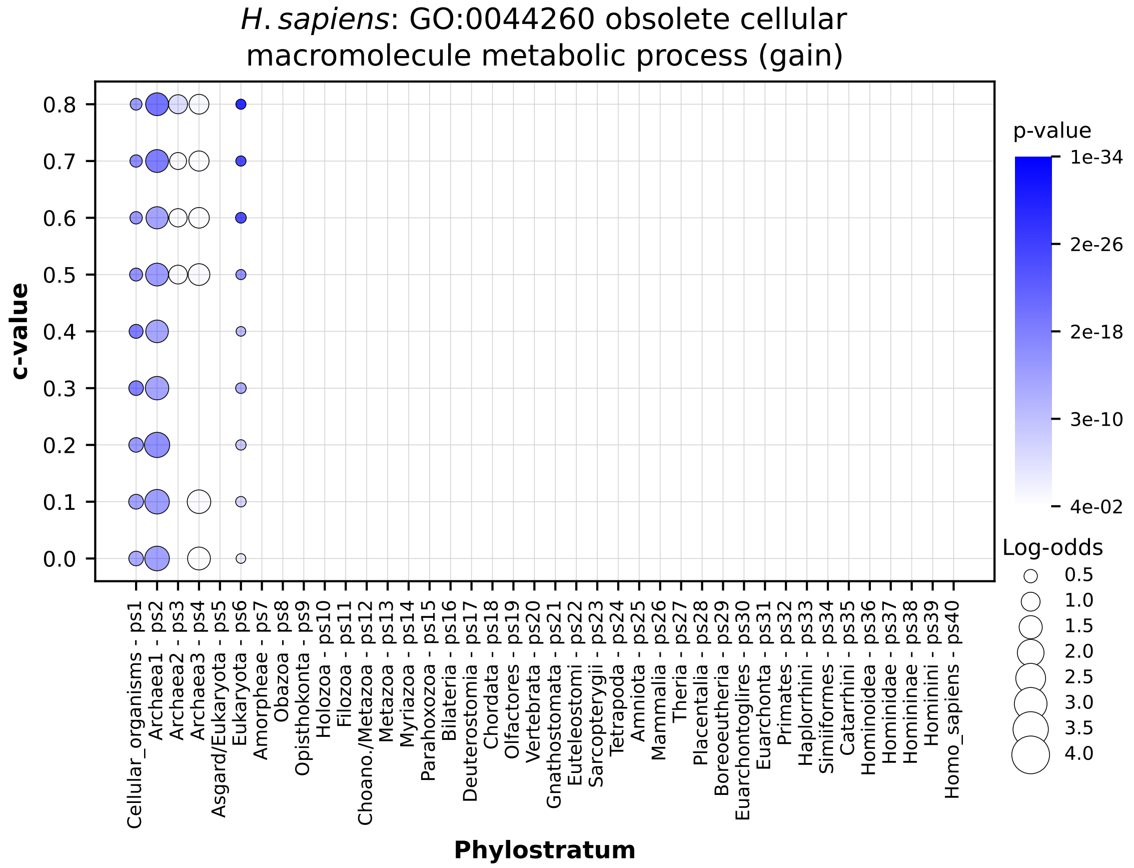

H. sapiens: GO:0044260 obsolete cellular macromolecule metabolic process (gain)
c-value
0.8
0.7
0.6
0.5
0.4
0.3
0.2
0.1
0.0
Phylostratum
Cellular_organisms - ps1
Archaea1 - ps2
Archaea2 - ps3
Archaea3 - ps4
Asgard/Eukaryota - ps5
Eukaryota - ps6
Amorpheae - ps7
Obazoa - ps8
Opisthokonta - ps9
Holozoa - ps10
Filozoa - ps11
Choano./Metazoa - ps12
Metazoa - ps13
Myriazoa - ps14
Parahoxozoa - ps15
Bilateria - ps16
Deuterostomia - ps17
Chordata - ps18
Olfactores - ps19
Vertebrata - ps20
Gnathostomata - ps21
Euteleostomi - ps22
Sarcopterygii - ps23
Tetrapoda - ps24
Amniota - ps25
Mammalia - ps26
Theria - ps27
Placentalia - ps28
Boreoeutheria - ps29
Euarchontoglires - ps30
Euarchonta - ps31
Primates - ps32
Haplorrhini - ps33
Simiiformes - ps34
Catarrhini - ps35
Hominoidea - ps36
Hominidae - ps37
Homininae - ps38
Hominini - ps39
Homo_sapiens - ps40
p-value
1e-34
2e-26
2e-18
3e-10
4e-02
Log-odds
0.5
1.0
1.5
2.0
2.5
3.0
3.5
4.0

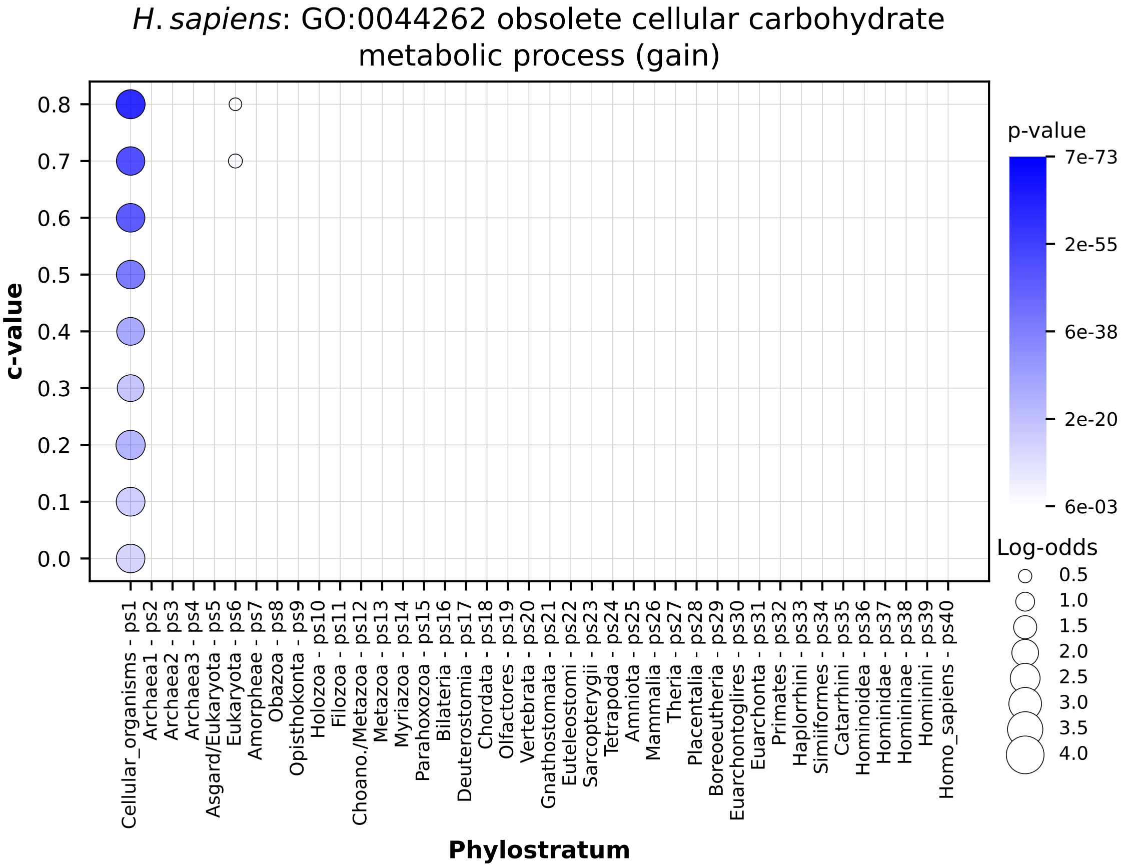
H. sapiens: GO:0044262 obsolete cellular carbohydrate metabolic process (gain)
c-value
0.8
0.7
0.6
0.5
0.4
0.3
0.2
0.1
0.0
Phylostratum
Cellular_organisms - ps1
Archaea1 - ps2
Archaea2 - ps3
Archaea3 - ps4
Asgard/Eukaryota - ps5
Eukaryota - ps6
Amorpheae - ps7
Obazoa - ps8
Opisthokonta - ps9
Holozoa - ps10
Filozoa - ps11
Choano./Metazoa - ps12
Metazoa - ps13
Myriazoa - ps14
Parahoxozoa - ps15
Bilateria - ps16
Deuterostomia - ps17
Chordata - ps18
Olfactores - ps19
Vertebrata - ps20
Gnathostomata - ps21
Euteleostomi - ps22
Sarcopterygii - ps23
Tetrapoda - ps24
Amniota - ps25
Mammalia - ps26
Theria - ps27
Placentalia - ps28
Boreoeutheria - ps29
Euarchontoglires - ps30
Euarchonta - ps31
Primates - ps32
Haplorrhini - ps33
Simiiformes - ps34
Catarrhini - ps35
Hominoidea - ps36
Hominidae - ps37
Homininae - ps38
Hominini - ps39
Homo_sapiens - ps40
p-value
7e-73
2e-55
6e-38
2e-20
6e-03
Log-odds
0.5
1.0
1.5
2.0
2.5
3.0
3.5
4.0

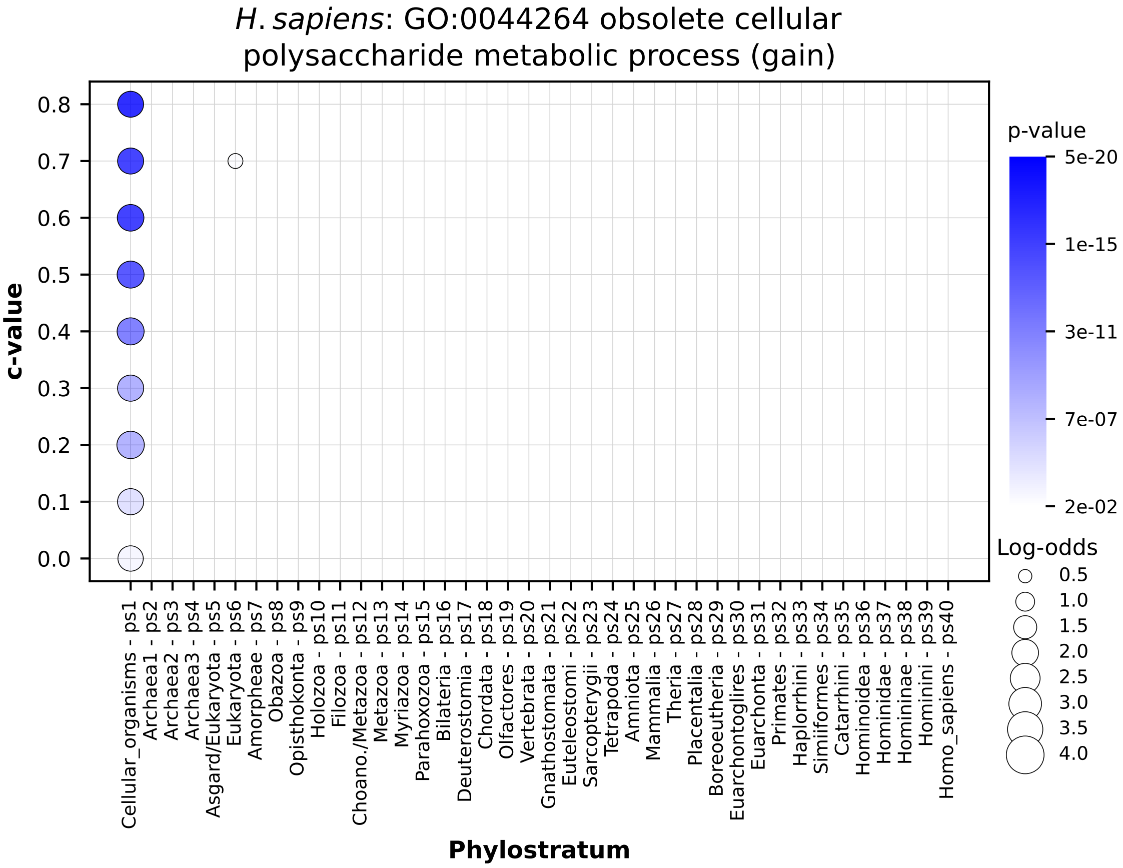
H. sapiens: GO:0044264 obsolete cellular polysaccharide metabolic process (gain)
c-value
0.8
0.7
0.6
0.5
0.4
0.3
0.2
0.1
0.0
Phylostratum
Cellular_organisms - ps1
Archaea1 - ps2
Archaea2 - ps3
Archaea3 - ps4
Asgard/Eukaryota - ps5
Eukaryota - ps6
Amorpheae - ps7
Obazoa - ps8
Opisthokonta - ps9
Holozoa - ps10
Filozoa - ps11
Choano./Metazoa - ps12
Metazoa - ps13
Myriazoa - ps14
Parahoxozoa - ps15
Bilateria - ps16
Deuterostomia - ps17
Chordata - ps18
Olfactores - ps19
Vertebrata - ps20
Gnathostomata - ps21
Euteleostomi - ps22
Sarcopterygii - ps23
Tetrapoda - ps24
Amniota - ps25
Mammalia - ps26
Theria - ps27
Placentalia - ps28
Boreoeutheria - ps29
Euarchontoglires - ps30
Euarchonta - ps31
Primates - ps32
Haplorrhini - ps33
Simiiformes - ps34
Catarrhini - ps35
Hominoidea - ps36
Hominidae - ps37
Homininae - ps38
Hominini - ps39
Homo_sapiens - ps40
p-value
5e-20
1e-15
3e-11
7e-07
2e-02
Log-odds
0.5
1.0
1.5
2.0
2.5
3.0
3.5
4.0

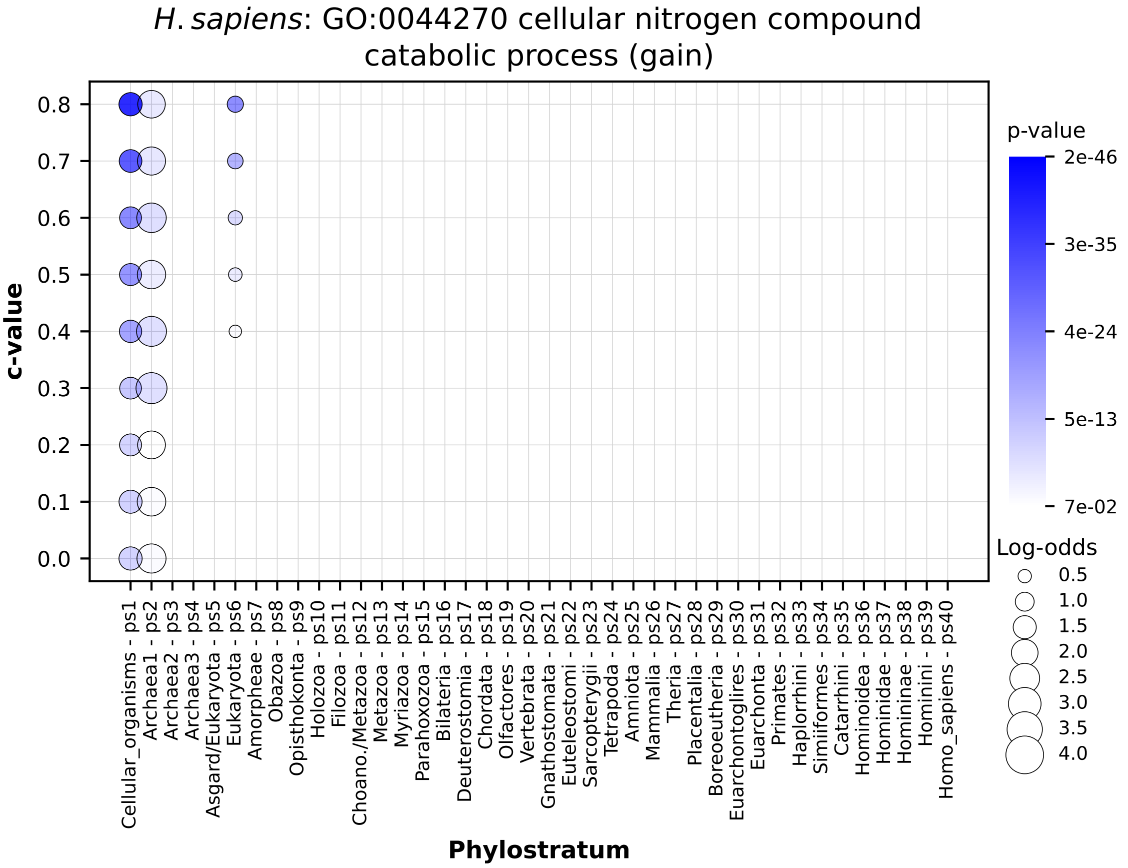

*H. sapiens*: GO:0044270 cellular nitrogen compound catabolic process (gain)
c-value
0.0
0.1
0.2
0.3
0.4
0.5
0.6
0.7
0.8
Phylostratum
Cellular_organisms - ps1
Archaea1 - ps2
Archaea2 - ps3
Archaea3 - ps4
Asgard/Eukaryota - ps5
Eukaryota - ps6
Amorpheae - ps7
Obazoa - ps8
Opisthokonta - ps9
Holozoa - ps10
Filozoa - ps11
Choano./Metazoa - ps12
Metazoa - ps13
Myriazoa - ps14
Parahoxozoa - ps15
Bilateria - ps16
Deuterostomia - ps17
Chordata - ps18
Olfactores - ps19
Vertebrata - ps20
Gnathostomata - ps21
Euteleostomi - ps22
Sarcopterygii - ps23
Tetrapoda - ps24
Amniota - ps25
Mammalia - ps26
Theria - ps27
Placentalia - ps28
Boreoeutheria - ps29
Euarchontoglires - ps30
Euarchonta - ps31
Primates - ps32
Haplorrhini - ps33
Simiiformes - ps34
Catarrhini - ps35
Hominoidea - ps36
Hominidae - ps37
Homininae - ps38
Hominini - ps39
Homo_sapiens - ps40
p-value
2e-46
3e-35
4e-24
5e-13
7e-02
Log-odds
0.5
1.0
1.5
2.0
2.5
3.0
3.5
4.0

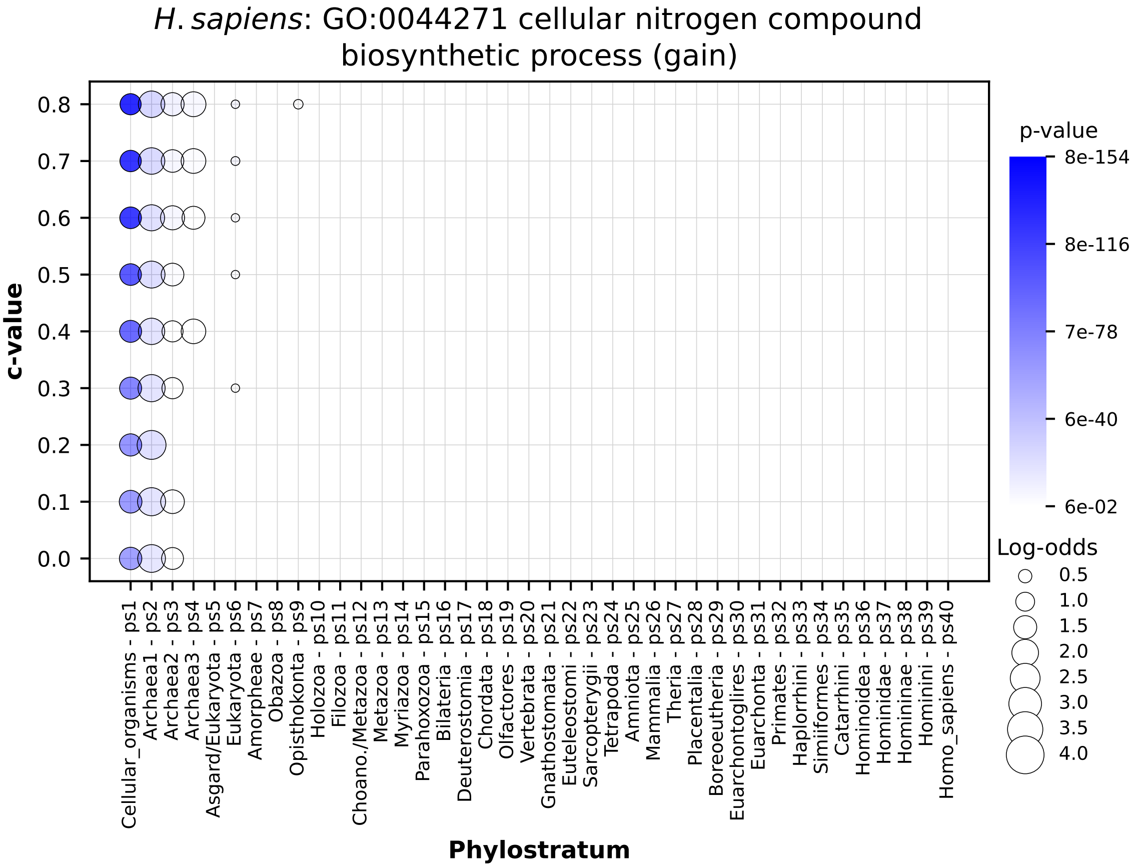

H. sapiens: GO:0044271 cellular nitrogen compound biosynthetic process (gain)
c-value
0.8
0.7
0.6
0.5
0.4
0.3
0.2
0.1
0.0
Phylostratum
Cellular_organisms - ps1
Archaea1 - ps2
Archaea2 - ps3
Archaea3 - ps4
Asgard/Eukaryota - ps5
Eukaryota - ps6
Amorpheae - ps7
Obazoa - ps8
Opisthokonta - ps9
Holozoa - ps10
Filozoa - ps11
Choano./Metazoa - ps12
Metazoa - ps13
Myriazoa - ps14
Parahoxozoa - ps15
Bilateria - ps16
Deuterostomia - ps17
Chordata - ps18
Olfactores - ps19
Vertebrata - ps20
Gnathostomata - ps21
Euteleostomi - ps22
Sarcopterygii - ps23
Tetrapoda - ps24
Amniota - ps25
Mammalia - ps26
Theria - ps27
Placentalia - ps28
Boreoeutheria - ps29
Euarchontoglires - ps30
Euarchonta - ps31
Primates - ps32
Haplorrhini - ps33
Simiiformes - ps34
Catarrhini - ps35
Hominoidea - ps36
Hominidae - ps37
Homininae - ps38
Hominini - ps39
Homo_sapiens - ps40
p-value
8e-154
8e-116
7e-78
6e-40
6e-02
Log-odds
0.5
1.0
1.5
2.0
2.5
3.0
3.5
4.0

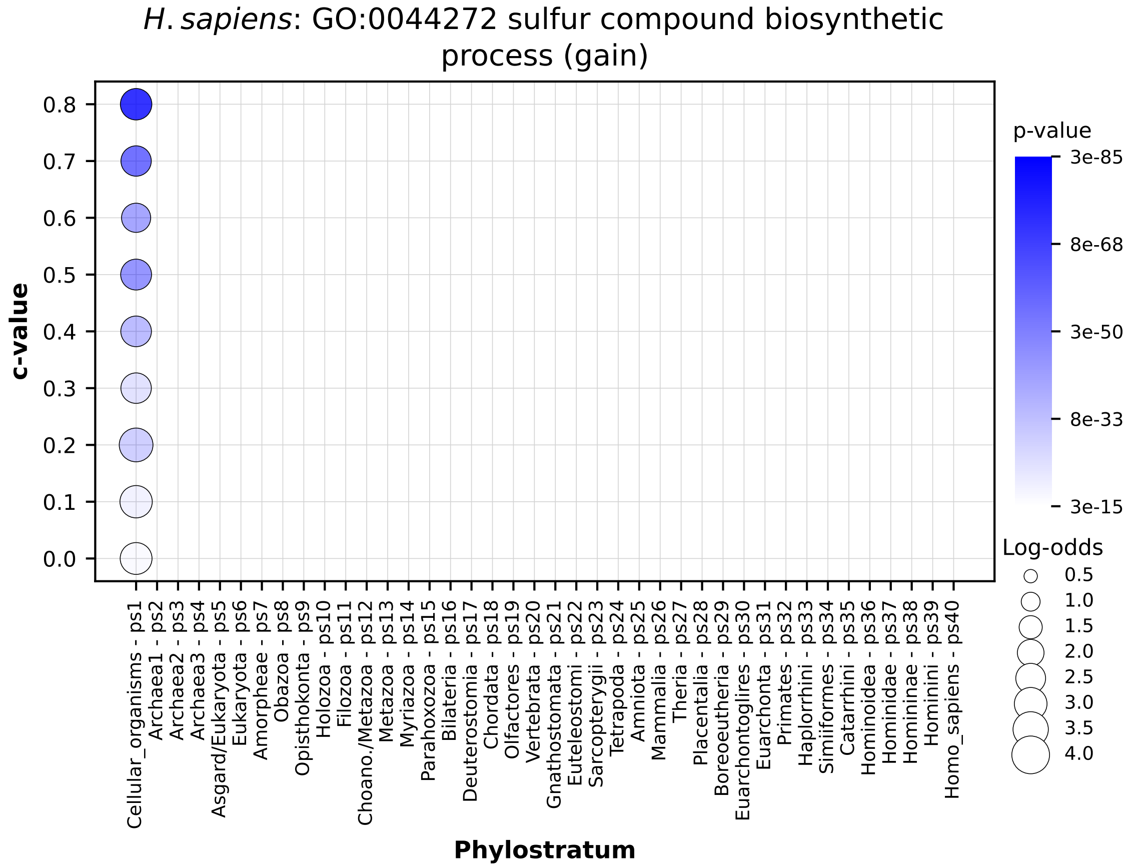

H. sapiens: GO:0044272 sulfur compound biosynthetic process (gain)
c-value
0.8
0.7
0.6
0.5
0.4
0.3
0.2
0.1
0.0
p-value
3e-85
8e-68
3e-50
8e-33
3e-15
Log-odds
0.5
1.0
1.5
2.0
2.5
3.0
3.5
4.0
Cellular_organisms - ps1
Archaea1 - ps2
Archaea2 - ps3
Archaea3 - ps4
Asgard/Eukaryota - ps5
Eukaryota - ps6
Amorpheae - ps7
Obazoa - ps8
Opisthokonta - ps9
Holozoa - ps10
Filozoa - ps11
Choano./Metazoa - ps12
Metazoa - ps13
Myriazoa - ps14
Parahoxozoa - ps15
Bilateria - ps16
Deuterostomia - ps17
Chordata - ps18
Olfactores - ps19
Vertebrata - ps20
Gnathostomata - ps21
Euteleostomi - ps22
Sarcopterygii - ps23
Tetrapoda - ps24
Amniota - ps25
Mammalia - ps26
Theria - ps27
Placentalia - ps28
Boreoeutheria - ps29
Euarchontoglires - ps30
Euarchonta - ps31
Primates - ps32
Haplorrhini - ps33
Simiiformes - ps34
Catarrhini - ps35
Hominoidea - ps36
Hominidae - ps37
Homininae - ps38
Hominini - ps39
Homo_sapiens - ps40
Phylostratum

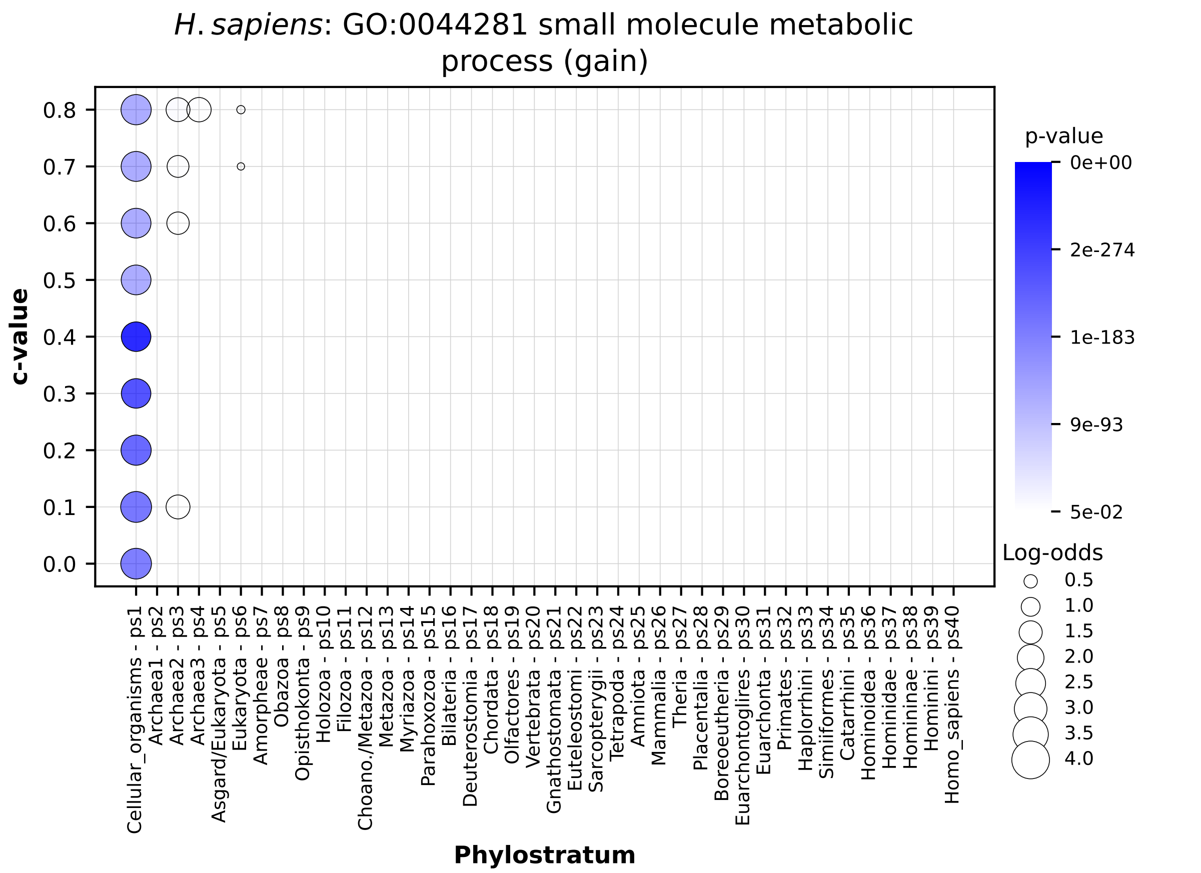

H. sapiens: GO:0044281 small molecule metabolic process (gain)
c-value
0.8
0.7
0.6
0.5
0.4
0.3
0.2
0.1
0.0
Phylostratum
Cellular_organisms - ps1
Archaea1 - ps2
Archaea2 - ps3
Archaea3 - ps4
Asgard/Eukaryota - ps5
Eukaryota - ps6
Amorpheae - ps7
Obazoa - ps8
Opisthokonta - ps9
Holozoa - ps10
Filozoa - ps11
Choano./Metazoa - ps12
Metazoa - ps13
Myriazoa - ps14
Parahoxozoa - ps15
Bilateria - ps16
Deuterostomia - ps17
Chordata - ps18
Olfactores - ps19
Vertebrata - ps20
Gnathostomata - ps21
Euteleostomi - ps22
Sarcopterygii - ps23
Tetrapoda - ps24
Amniota - ps25
Mammalia - ps26
Theria - ps27
Placentalia - ps28
Boreoeutheria - ps29
Euarchontoglires - ps30
Euarchonta - ps31
Primates - ps32
Haplorrhini - ps33
Simiiformes - ps34
Catarrhini - ps35
Hominoidea - ps36
Hominidae - ps37
Homininae - ps38
Hominini - ps39
Homo_sapiens - ps40
p-value
0e+00
2e-274
1e-183
9e-93
5e-02
Log-odds
0.5
1.0
1.5
2.0
2.5
3.0
3.5
4.0

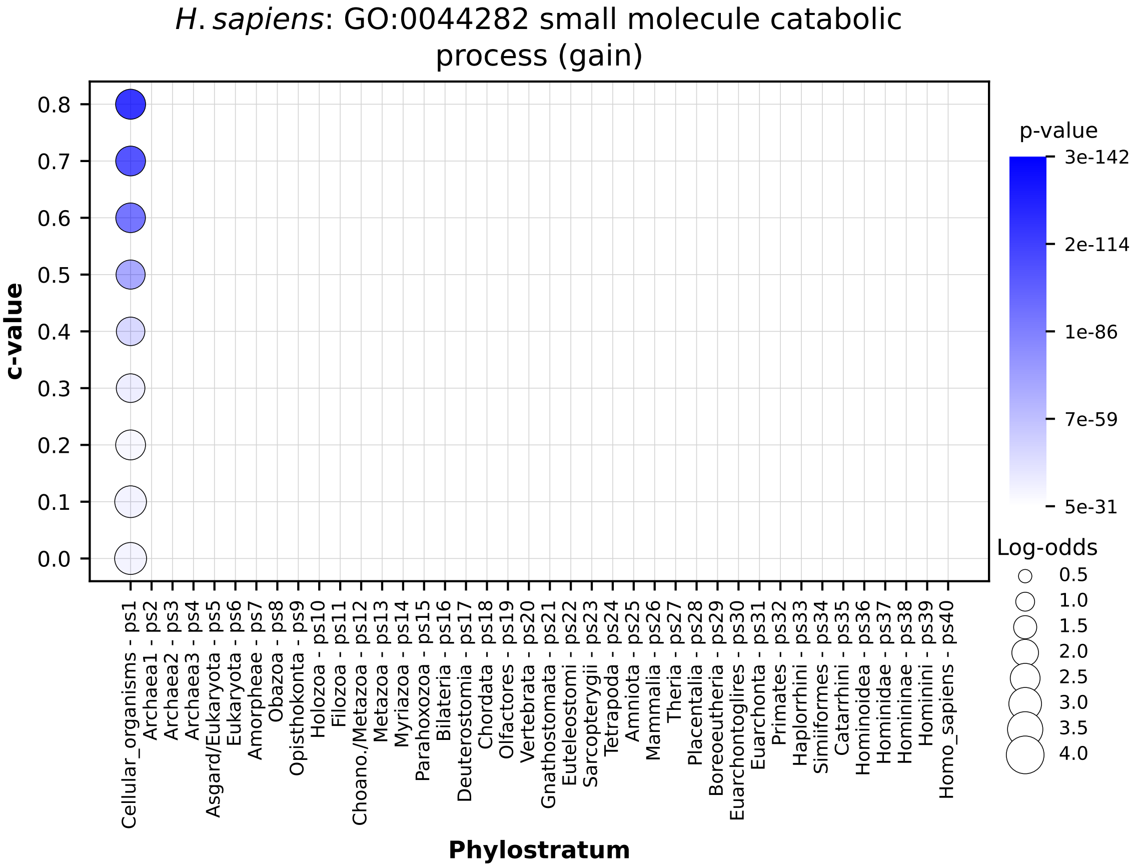
H. sapiens: GO:0044282 small molecule catabolic process (gain)
c-value
0.0
0.1
0.2
0.3
0.4
0.5
0.6
0.7
0.8
p-value
3e-142
2e-114
1e-86
7e-59
5e-31
Log-odds
0.5
1.0
1.5
2.0
2.5
3.0
3.5
4.0
Cellular_organisms - ps1
Archaea1 - ps2
Archaea2 - ps3
Archaea3 - ps4
Asgard/Eukaryota - ps5
Eukaryota - ps6
Amorpheae - ps7
Obazoa - ps8
Opisthokonta - ps9
Holozoa - ps10
Filozoa - ps11
Choano./Metazoa - ps12
Metazoa - ps13
Myriazoa - ps14
Parahoxozoa - ps15
Bilateria - ps16
Deuterostomia - ps17
Chordata - ps18
Olfactores - ps19
Vertebrata - ps20
Gnathostomata - ps21
Euteleostomi - ps22
Sarcopterygii - ps23
Tetrapoda - ps24
Amniota - ps25
Mammalia - ps26
Theria - ps27
Placentalia - ps28
Boreoeutheria - ps29
Euarchontoglires - ps30
Euarchonta - ps31
Primates - ps32
Haplorrhini - ps33
Simiiformes - ps34
Catarrhini - ps35
Hominoidea - ps36
Hominidae - ps37
Homininae - ps38
Hominini - ps39
Homo_sapiens - ps40
Phylostratum

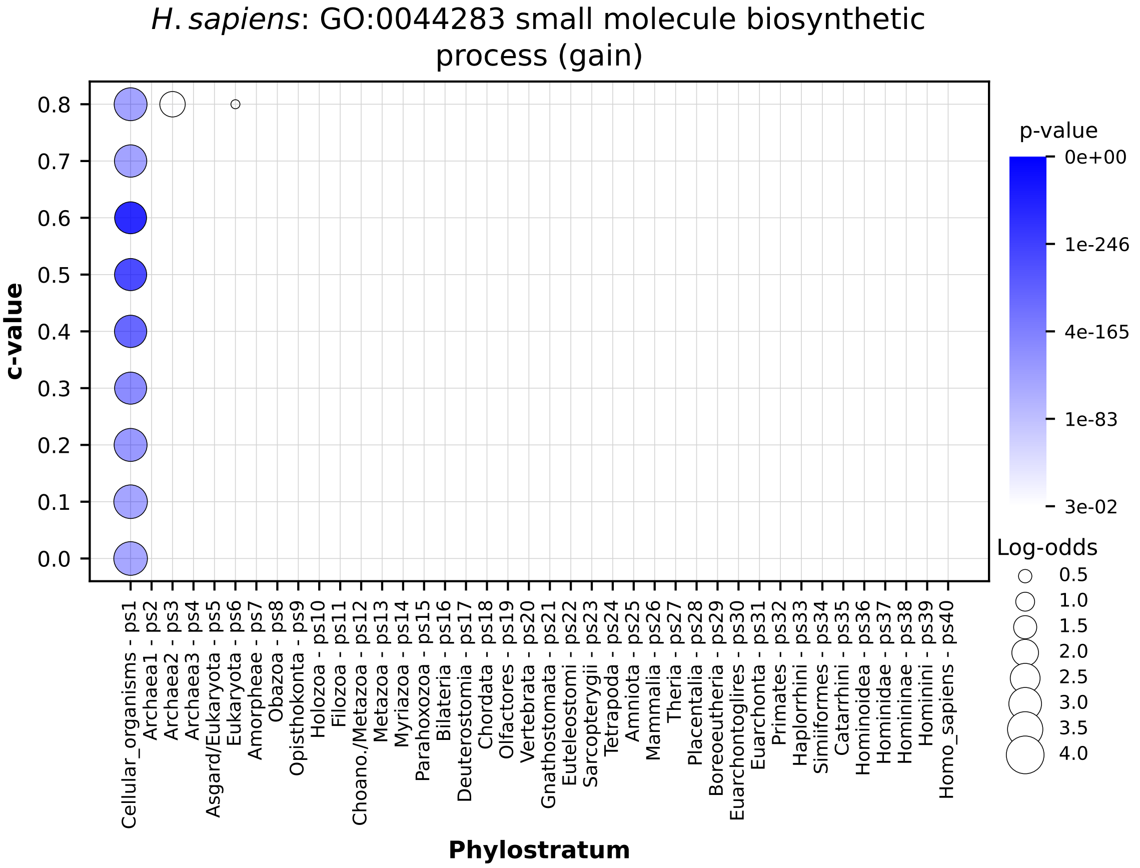

H. sapiens: GO:0044283 small molecule biosynthetic process (gain)
c-value
0.8
0.7
0.6
0.5
0.4
0.3
0.2
0.1
0.0
Phylostratum
Cellular_organisms - ps1
Archaea1 - ps2
Archaea2 - ps3
Archaea3 - ps4
Asgard/Eukaryota - ps5
Eukaryota - ps6
Amorpheae - ps7
Obazoa - ps8
Opisthokonta - ps9
Holozoa - ps10
Filozoa - ps11
Choano./Metazoa - ps12
Metazoa - ps13
Myriazoa - ps14
Parahoxozoa - ps15
Bilateria - ps16
Deuterostomia - ps17
Chordata - ps18
Olfactores - ps19
Vertebrata - ps20
Gnathostomata - ps21
Euteleostomi - ps22
Sarcopterygii - ps23
Tetrapoda - ps24
Amniota - ps25
Mammalia - ps26
Theria - ps27
Placentalia - ps28
Boreoeutheria - ps29
Euarchontoglires - ps30
Euarchonta - ps31
Primates - ps32
Haplorrhini - ps33
Simiiformes - ps34
Catarrhini - ps35
Hominoidea - ps36
Hominidae - ps37
Homininae - ps38
Hominini - ps39
Homo_sapiens - ps40
p-value
0e+00
1e-246
4e-165
1e-83
3e-02
Log-odds
0.5
1.0
1.5
2.0
2.5
3.0
3.5
4.0

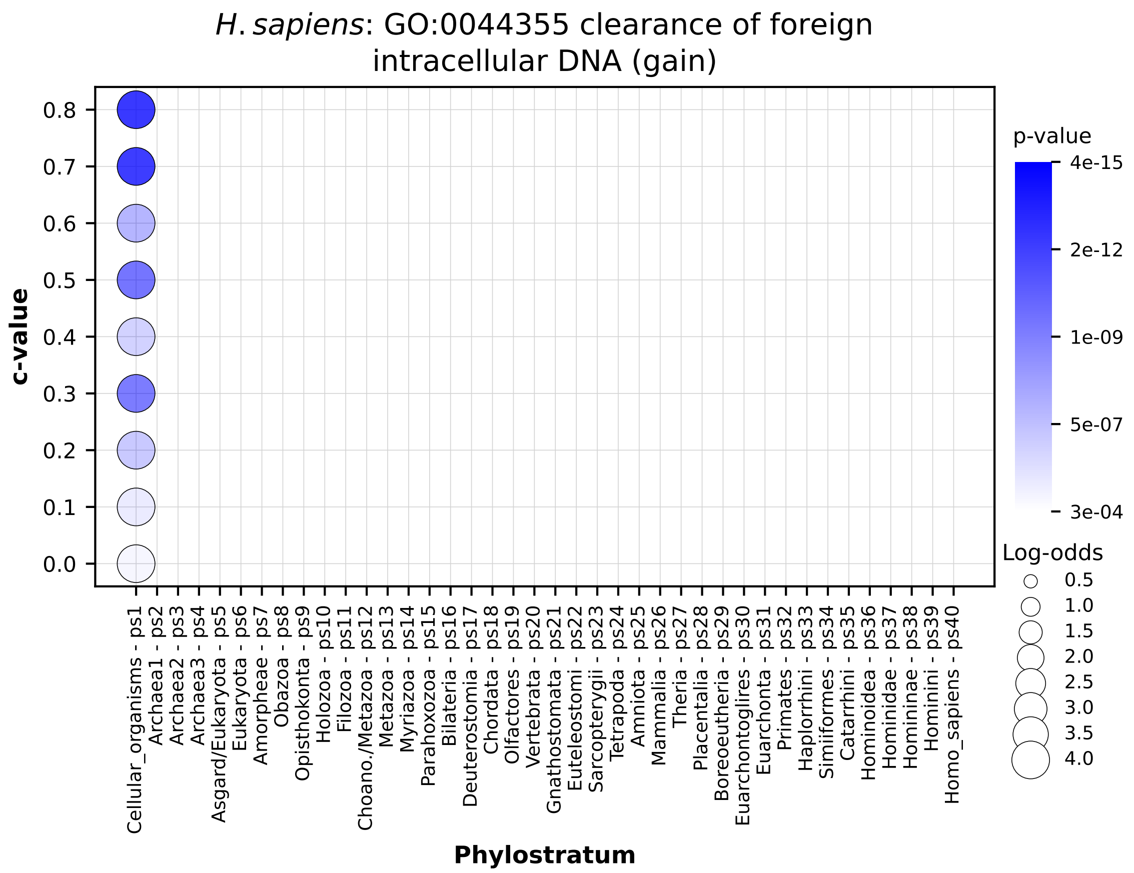

H. sapiens: GO:0044355 clearance of foreign intracellular DNA (gain)
c-value
0.8
0.7
0.6
0.5
0.4
0.3
0.2
0.1
0.0
p-value
4e-15
2e-12
1e-09
5e-07
3e-04
Log-odds
0.5
1.0
1.5
2.0
2.5
3.0
3.5
4.0
Cellular_organisms - ps1
Archaea1 - ps2
Archaea2 - ps3
Archaea3 - ps4
Asgard/Eukaryota - ps5
Eukaryota - ps6
Amorpheae - ps7
Obazoa - ps8
Opisthokonta - ps9
Holozoa - ps10
Filozoa - ps11
Choano./Metazoa - ps12
Metazoa - ps13
Myriazoa - ps14
Parahoxozoa - ps15
Bilateria - ps16
Deuterostomia - ps17
Chordata - ps18
Olfactores - ps19
Vertebrata - ps20
Gnathostomata - ps21
Euteleostomi - ps22
Sarcopterygii - ps23
Tetrapoda - ps24
Amniota - ps25
Mammalia - ps26
Theria - ps27
Placentalia - ps28
Boreoeutheria - ps29
Euarchontoglires - ps30
Euarchonta - ps31
Primates - ps32
Haplorrhini - ps33
Simiiformes - ps34
Catarrhini - ps35
Hominoidea - ps36
Hominidae - ps37
Homininae - ps38
Hominini - ps39
Homo_sapiens - ps40
Phylostratum

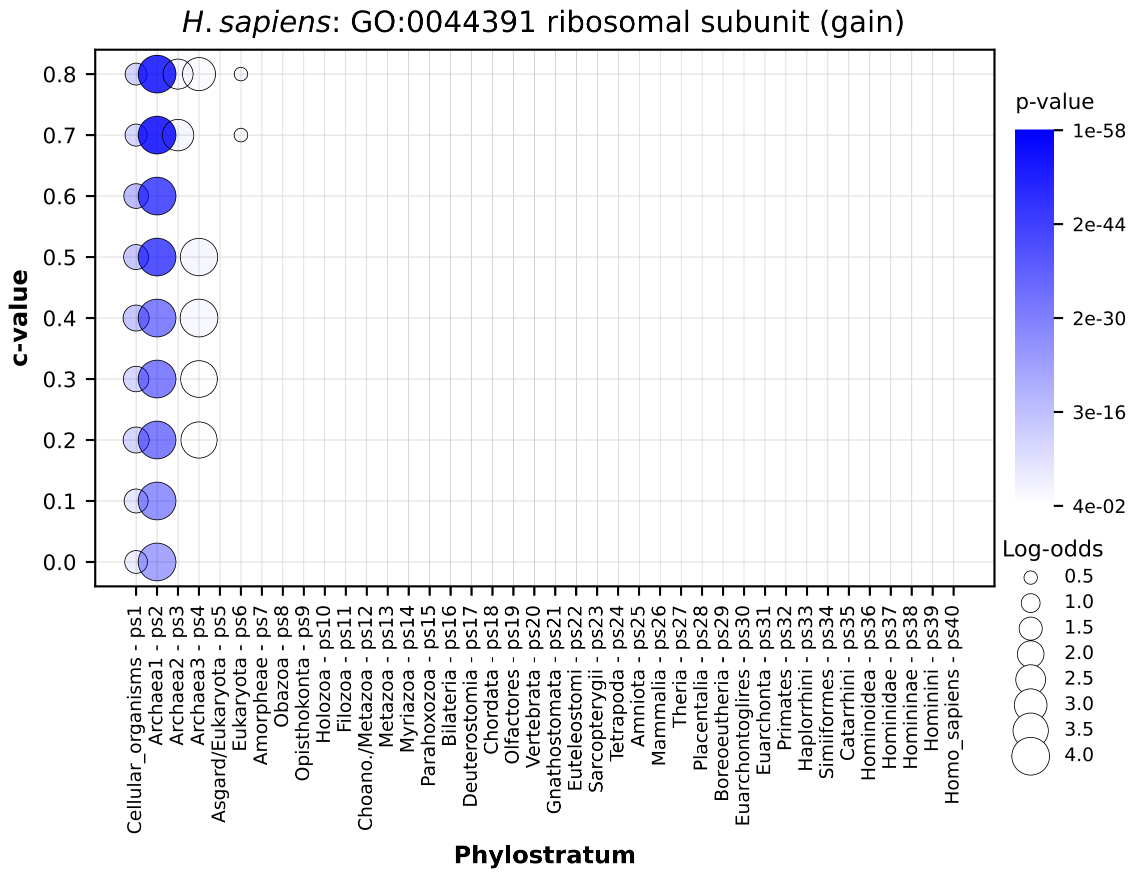

H. sapiens: GO:0044391 ribosomal subunit (gain)
c-value
0.8
0.7
0.6
0.5
0.4
0.3
0.2
0.1
0.0
p-value
1e-58
2e-44
2e-30
3e-16
4e-02
Log-odds
0.5
1.0
1.5
2.0
2.5
3.0
3.5
4.0
Cellular_organisms - ps1
Archaea1 - ps2
Archaea2 - ps3
Archaea3 - ps4
Asgard/Eukaryota - ps5
Eukaryota - ps6
Amorpheae - ps7
Obazoa - ps8
Opisthokonta - ps9
Holozoa - ps10
Filozoa - ps11
Choano./Metazoa - ps12
Metazoa - ps13
Myriazoa - ps14
Parahoxozoa - ps15
Bilateria - ps16
Deuterostomia - ps17
Chordata - ps18
Olfactores - ps19
Vertebrata - ps20
Gnathostomata - ps21
Euteleostomi - ps22
Sarcopterygii - ps23
Tetrapoda - ps24
Amniota - ps25
Mammalia - ps26
Theria - ps27
Placentalia - ps28
Boreoeutheria - ps29
Euarchontoglires - ps30
Euarchonta - ps31
Primates - ps32
Haplorrhini - ps33
Simiiformes - ps34
Catarrhini - ps35
Hominoidea - ps36
Hominidae - ps37
Homininae - ps38
Hominini - ps39
Homo_sapiens - ps40
Phylostratum

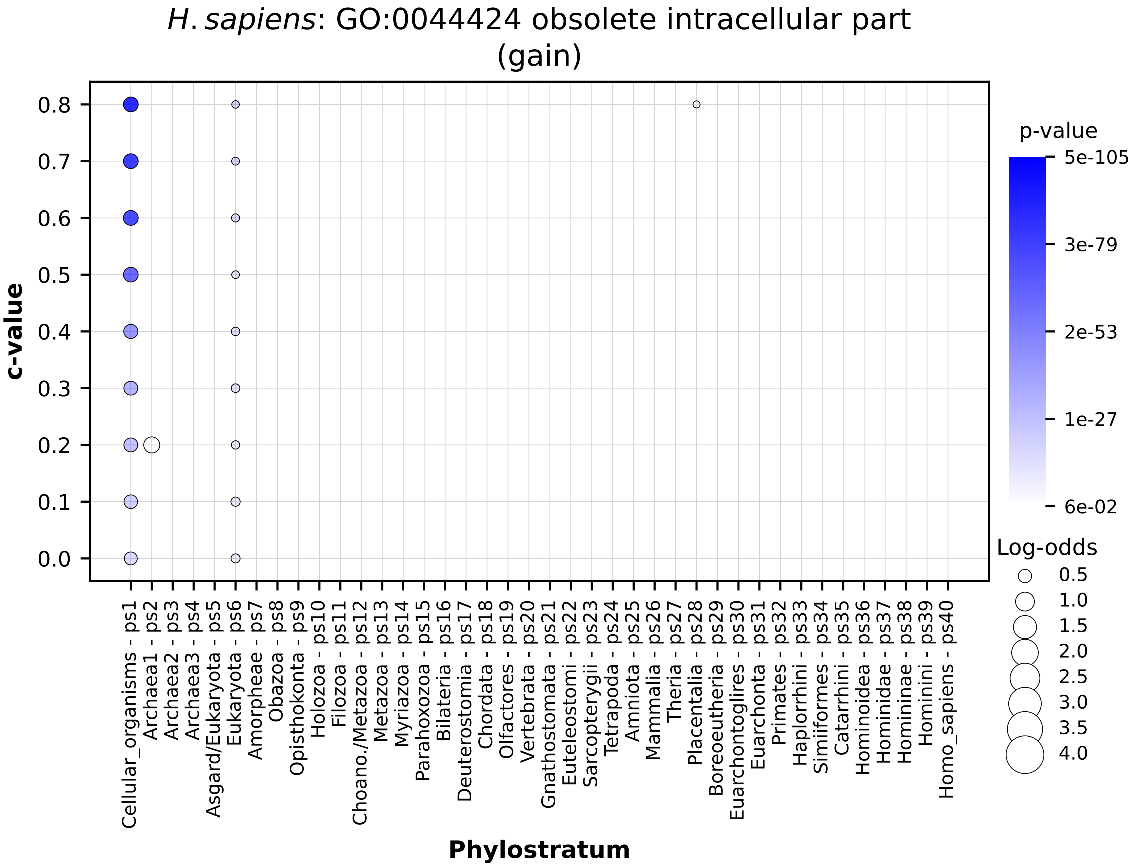

H. sapiens: GO:0044424 obsolete intracellular part
(gain)
c-value
0.8
0.7
0.6
0.5
0.4
0.3
0.2
0.1
0.0
p-value
5e-105
3e-79
2e-53
1e-27
6e-02
Log-odds
0.5
1.0
1.5
2.0
2.5
3.0
3.5
4.0
Cellular_organisms - ps1
Archaea1 - ps2
Archaea2 - ps3
Archaea3 - ps4
Asgard/Eukaryota - ps5
Eukaryota - ps6
Amorpheae - ps7
Obazoa - ps8
Opisthokonta - ps9
Holozoa - ps10
Filozoa - ps11
Choano./Metazoa - ps12
Metazoa - ps13
Myriazoa - ps14
Parahoxozoa - ps15
Bilateria - ps16
Deuterostomia - ps17
Chordata - ps18
Olfactores - ps19
Vertebrata - ps20
Gnathostomata - ps21
Euteleostomi - ps22
Sarcopterygii - ps23
Tetrapoda - ps24
Amniota - ps25
Mammalia - ps26
Theria - ps27
Placentalia - ps28
Boreoeutheria - ps29
Euarchontoglires - ps30
Euarchonta - ps31
Primates - ps32
Haplorrhini - ps33
Simiiformes - ps34
Catarrhini - ps35
Hominoidea - ps36
Hominidae - ps37
Homininae - ps38
Hominini - ps39
Homo_sapiens - ps40
Phylostratum

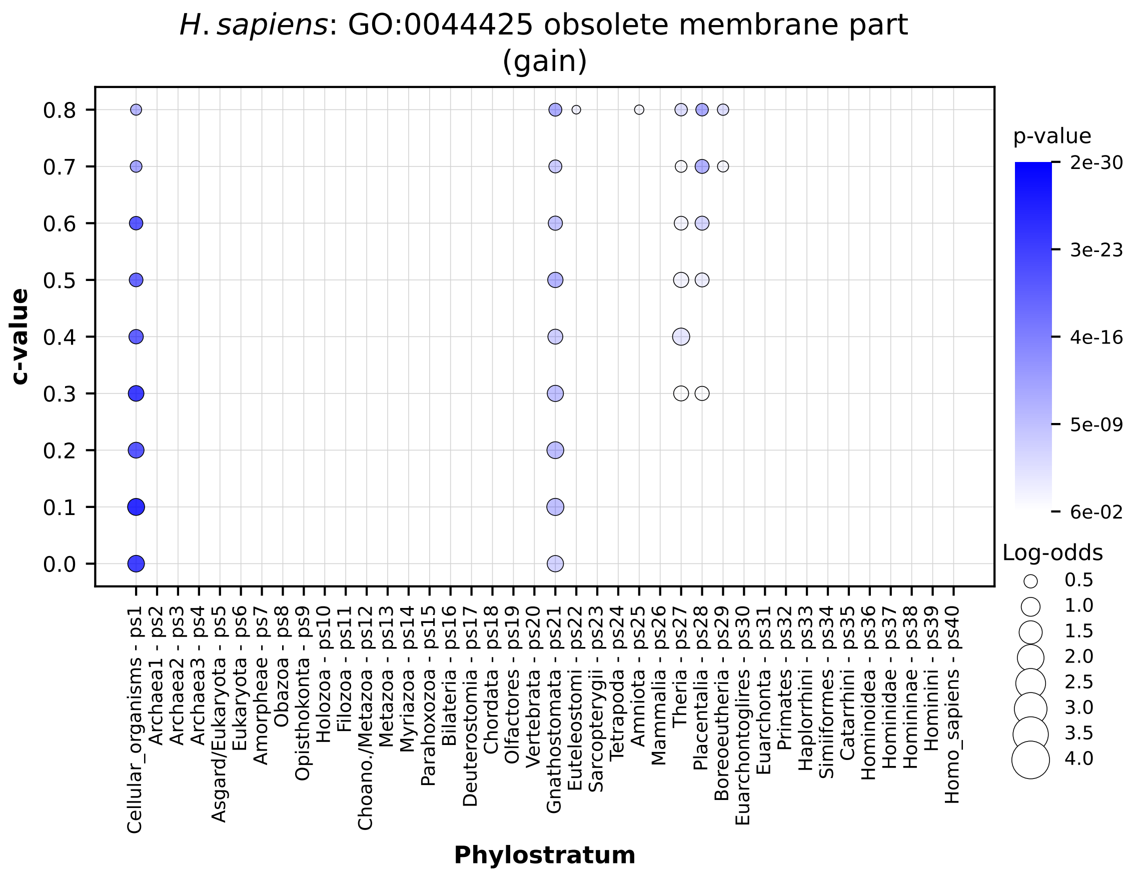
H. sapiens: GO:0044425 obsolete membrane part
(gain)
c-value
0.8
0.7
0.6
0.5
0.4
0.3
0.2
0.1
0.0
p-value
2e-30
3e-23
4e-16
5e-09
6e-02
Log-odds
0.5
1.0
1.5
2.0
2.5
3.0
3.5
4.0
Cellular_organisms - ps1
Archaea1 - ps2
Archaea2 - ps3
Archaea3 - ps4
Asgard/Eukaryota - ps5
Eukaryota - ps6
Amorpheae - ps7
Obazoa - ps8
Opisthokonta - ps9
Holozoa - ps10
Filozoa - ps11
Choano./Metazoa - ps12
Metazoa - ps13
Myriazoa - ps14
Parahoxozoa - ps15
Bilateria - ps16
Deuterostomia - ps17
Chordata - ps18
Olfactores - ps19
Vertebrata - ps20
Gnathostomata - ps21
Euteleostomi - ps22
Sarcopterygii - ps23
Tetrapoda - ps24
Amniota - ps25
Mammalia - ps26
Theria - ps27
Placentalia - ps28
Boreoeutheria - ps29
Euarchontoglires - ps30
Euarchonta - ps31
Primates - ps32
Haplorrhini - ps33
Simiiformes - ps34
Catarrhini - ps35
Hominoidea - ps36
Hominidae - ps37
Homininae - ps38
Hominini - ps39
Homo_sapiens - ps40
Phylostratum

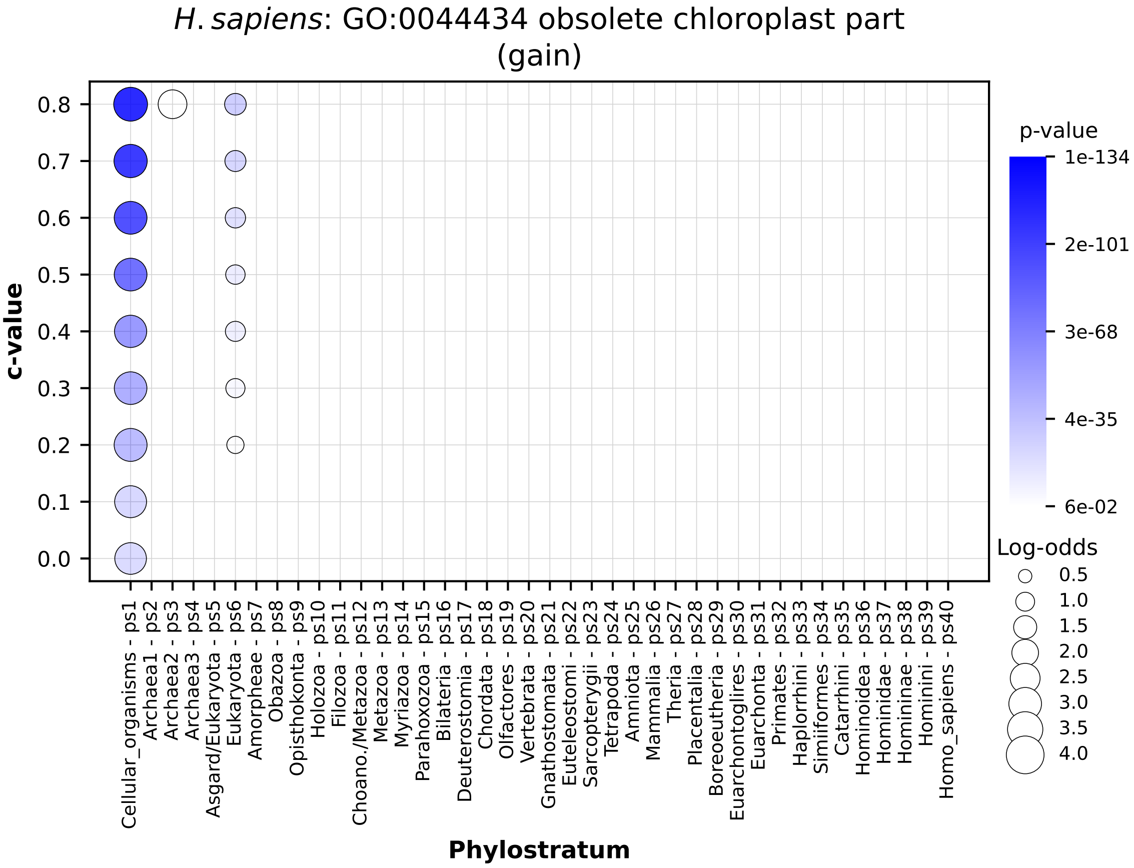

H. sapiens: GO:0044434 obsolete chloroplast part
(gain)
c-value
0.8
0.7
0.6
0.5
0.4
0.3
0.2
0.1
0.0
Phylostratum
Cellular_organisms - ps1
Archaea1 - ps2
Archaea2 - ps3
Archaea3 - ps4
Asgard/Eukaryota - ps5
Eukaryota - ps6
Amorpheae - ps7
Obazoa - ps8
Opisthokonta - ps9
Holozoa - ps10
Filozoa - ps11
Choano./Metazoa - ps12
Metazoa - ps13
Myriazoa - ps14
Parahoxozoa - ps15
Bilateria - ps16
Deuterostomia - ps17
Chordata - ps18
Olfactores - ps19
Vertebrata - ps20
Gnathostomata - ps21
Euteleostomi - ps22
Sarcopterygii - ps23
Tetrapoda - ps24
Amniota - ps25
Mammalia - ps26
Theria - ps27
Placentalia - ps28
Boreoeutheria - ps29
Euarchontoglires - ps30
Euarchonta - ps31
Primates - ps32
Haplorrhini - ps33
Simiiformes - ps34
Catarrhini - ps35
Hominoidea - ps36
Hominidae - ps37
Homininae - ps38
Hominini - ps39
Homo_sapiens - ps40
p-value
1e-134
2e-101
3e-68
4e-35
6e-02
Log-odds
0.5
1.0
1.5
2.0
2.5
3.0
3.5
4.0

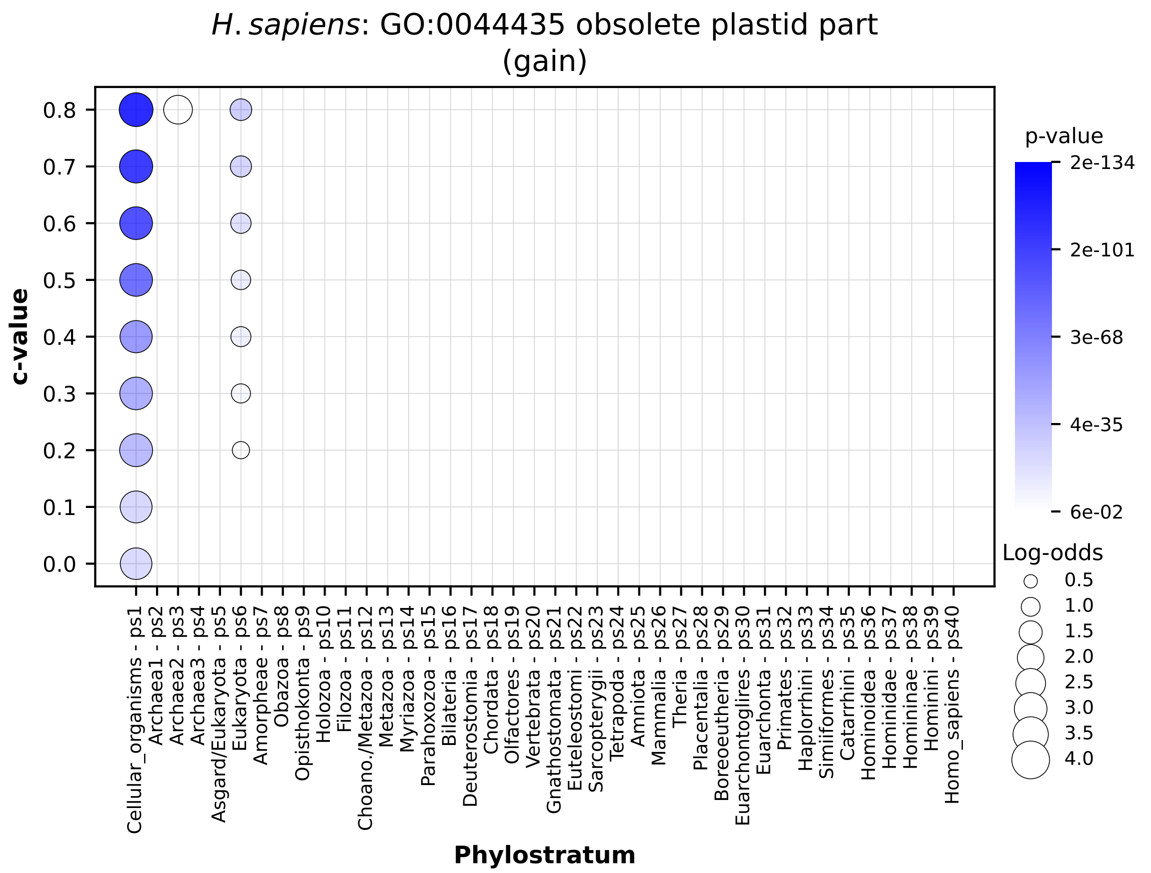

*H. sapiens*: GO:0044435 obsolete plastid part
(gain)
c-value
0.0
0.1
0.2
0.3
0.4
0.5
0.6
0.7
0.8
Phylostratum
Cellular_organisms - ps1
Archaea1 - ps2
Archaea2 - ps3
Archaea3 - ps4
Asgard/Eukaryota - ps5
Eukaryota - ps6
Amorpheae - ps7
Obazoa - ps8
Opisthokonta - ps9
Holozoa - ps10
Filozoa - ps11
Choano./Metazoa - ps12
Metazoa - ps13
Myriazoa - ps14
Parahoxozoa - ps15
Bilateria - ps16
Deuterostomia - ps17
Chordata - ps18
Olfactores - ps19
Vertebrata - ps20
Gnathostomata - ps21
Euteleostomi - ps22
Sarcopterygii - ps23
Tetrapoda - ps24
Amniota - ps25
Mammalia - ps26
Theria - ps27
Placentalia - ps28
Boreoeutheria - ps29
Euarchontoglires - ps30
Euarchonta - ps31
Primates - ps32
Haplorrhini - ps33
Simiiformes - ps34
Catarrhini - ps35
Hominoidea - ps36
Hominidae - ps37
Homininae - ps38
Hominini - ps39
Homo_sapiens - ps40
p-value
2e-134
2e-101
3e-68
4e-35
6e-02
Log-odds
0.5
1.0
1.5
2.0
2.5
3.0
3.5
4.0

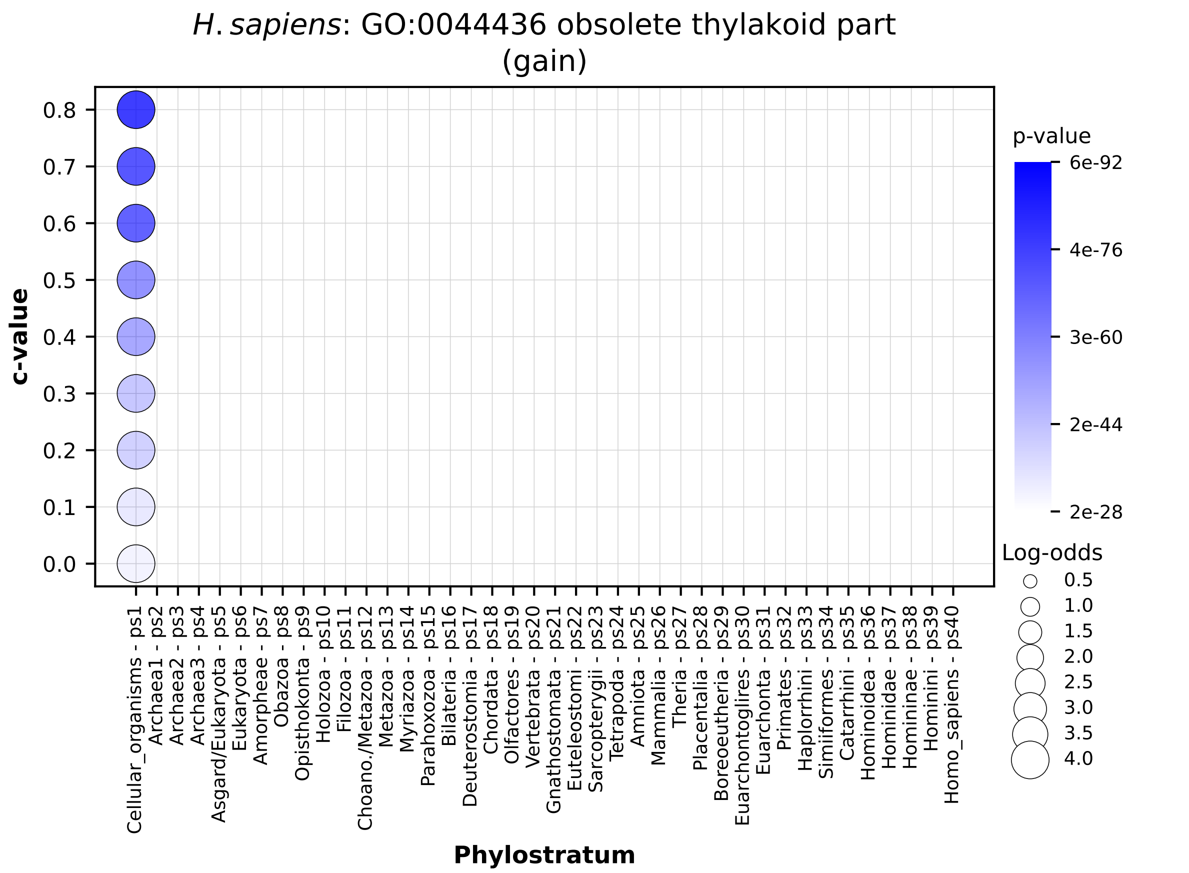

H. sapiens: GO:0044436 obsolete thylakoid part
(gain)
c-value
0.0
0.1
0.2
0.3
0.4
0.5
0.6
0.7
0.8
p-value
6e-92
4e-76
3e-60
2e-44
2e-28
Log-odds
0.5
1.0
1.5
2.0
2.5
3.0
3.5
4.0
Cellular_organisms - ps1
Archaea1 - ps2
Archaea2 - ps3
Archaea3 - ps4
Asgard/Eukaryota - ps5
Eukaryota - ps6
Amorpheae - ps7
Obazoa - ps8
Opisthokonta - ps9
Holozoa - ps10
Filozoa - ps11
Choano./Metazoa - ps12
Metazoa - ps13
Myriazoa - ps14
Parahoxozoa - ps15
Bilateria - ps16
Deuterostomia - ps17
Chordata - ps18
Olfactores - ps19
Vertebrata - ps20
Gnathostomata - ps21
Euteleostomi - ps22
Sarcopterygii - ps23
Tetrapoda - ps24
Amniota - ps25
Mammalia - ps26
Theria - ps27
Placentalia - ps28
Boreoeutheria - ps29
Euarchontoglires - ps30
Euarchonta - ps31
Primates - ps32
Haplorrhini - ps33
Simiiformes - ps34
Catarrhini - ps35
Hominoidea - ps36
Hominidae - ps37
Homininae - ps38
Hominini - ps39
Homo_sapiens - ps40
Phylostratum

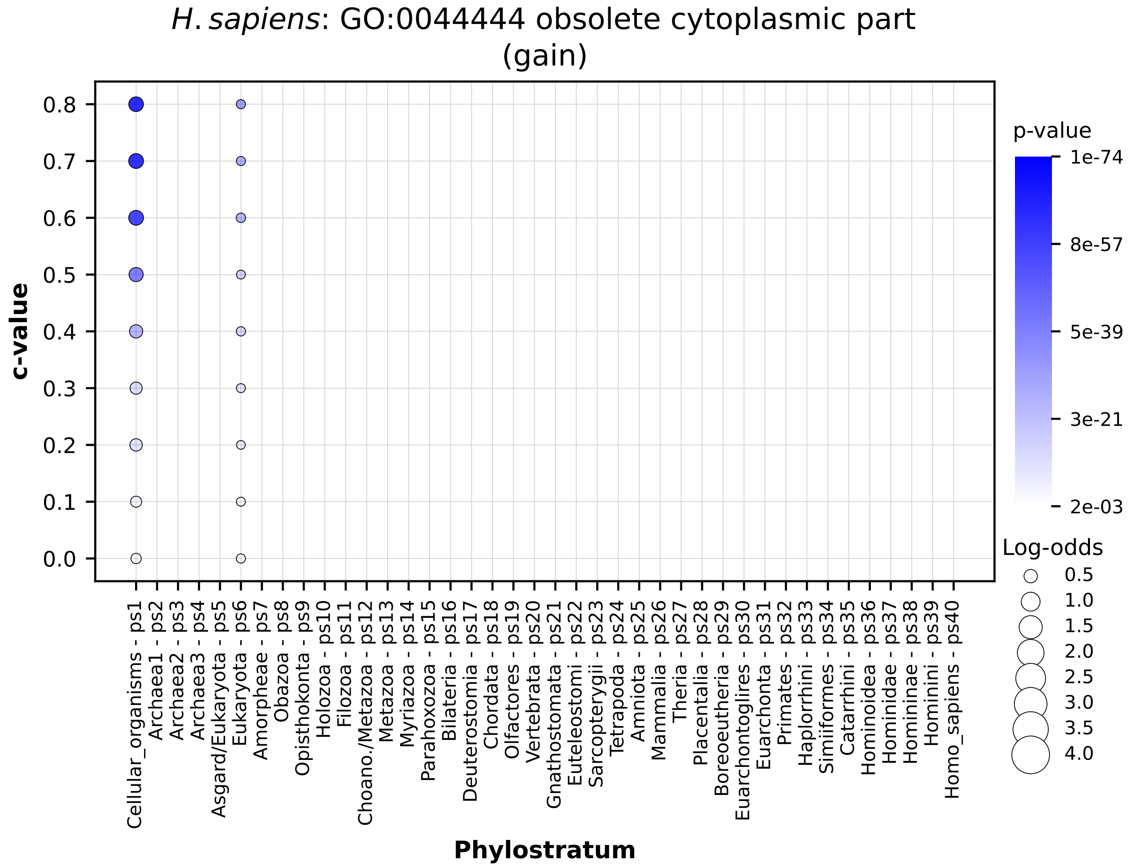

H. sapiens: GO:0044444 obsolete cytoplasmic part
(gain)
c-value
0.8
0.7
0.6
0.5
0.4
0.3
0.2
0.1
0.0
p-value
1e-74
8e-57
5e-39
3e-21
2e-03
Log-odds
0.5
1.0
1.5
2.0
2.5
3.0
3.5
4.0
Cellular_organisms - ps1
Archaea1 - ps2
Archaea2 - ps3
Archaea3 - ps4
Asgard/Eukaryota - ps5
Eukaryota - ps6
Amorpheae - ps7
Obazoa - ps8
Opisthokonta - ps9
Holozoa - ps10
Filozoa - ps11
Choano./Metazoa - ps12
Metazoa - ps13
Myriazoa - ps14
Parahoxozoa - ps15
Bilateria - ps16
Deuterostomia - ps17
Chordata - ps18
Olfactores - ps19
Vertebrata - ps20
Gnathostomata - ps21
Euteleostomi - ps22
Sarcopterygii - ps23
Tetrapoda - ps24
Amniota - ps25
Mammalia - ps26
Theria - ps27
Placentalia - ps28
Boreoeutheria - ps29
Euarchontoglires - ps30
Euarchonta - ps31
Primates - ps32
Haplorrhini - ps33
Simiiformes - ps34
Catarrhini - ps35
Hominoidea - ps36
Hominidae - ps37
Homininae - ps38
Hominini - ps39
Homo_sapiens - ps40
Phylostratum

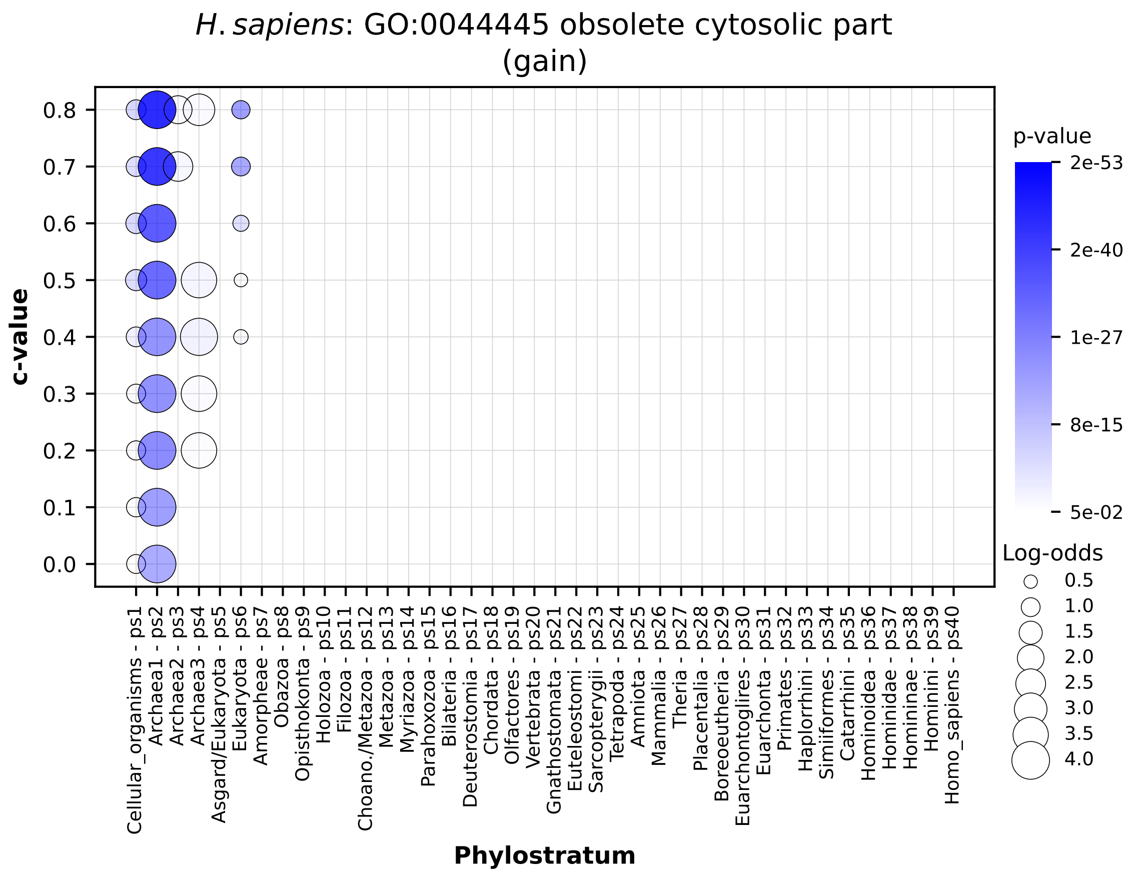

H. sapiens: GO:0044445 obsolete cytosolic part
(gain)
c-value
0.8
0.7
0.6
0.5
0.4
0.3
0.2
0.1
0.0
p-value
2e-53
2e-40
1e-27
8e-15
5e-02
Log-odds
0.5
1.0
1.5
2.0
2.5
3.0
3.5
4.0
Cellular_organisms - ps1
Archaea1 - ps2
Archaea2 - ps3
Archaea3 - ps4
Asgard/Eukaryota - ps5
Eukaryota - ps6
Amorpheae - ps7
Obazoa - ps8
Opisthokonta - ps9
Holozoa - ps10
Filozoa - ps11
Choano./Metazoa - ps12
Metazoa - ps13
Myriazoa - ps14
Parahoxozoa - ps15
Bilateria - ps16
Deuterostomia - ps17
Chordata - ps18
Olfactores - ps19
Vertebrata - ps20
Gnathostomata - ps21
Euteleostomi - ps22
Sarcopterygii - ps23
Tetrapoda - ps24
Amniota - ps25
Mammalia - ps26
Theria - ps27
Placentalia - ps28
Boreoeutheria - ps29
Euarchontoglires - ps30
Euarchonta - ps31
Primates - ps32
Haplorrhini - ps33
Simiiformes - ps34
Catarrhini - ps35
Hominoidea - ps36
Hominidae - ps37
Homininae - ps38
Hominini - ps39
Homo_sapiens - ps40
Phylostratum

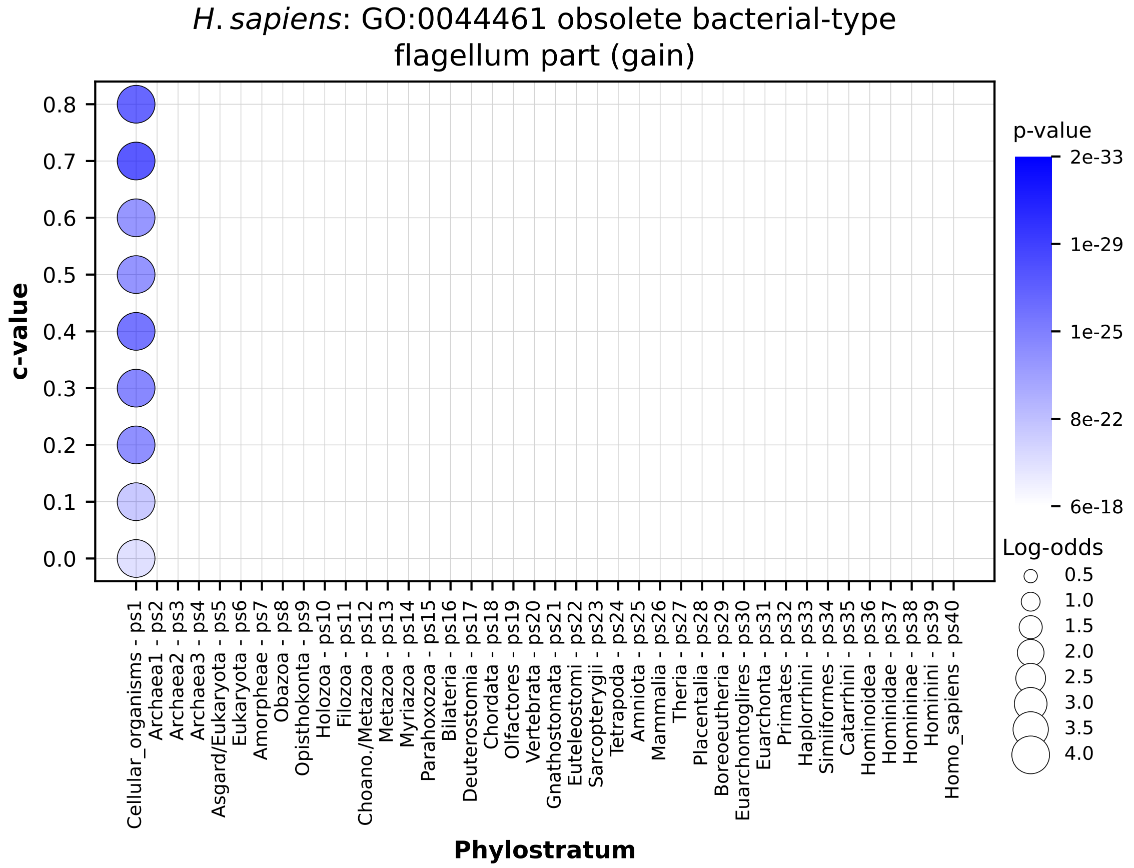

H. sapiens: GO:0044461 obsolete bacterial-type flagellum part (gain)
c-value
0.8
0.7
0.6
0.5
0.4
0.3
0.2
0.1
0.0
p-value
2e-33
1e-29
1e-25
8e-22
6e-18
Log-odds
0.5
1.0
1.5
2.0
2.5
3.0
3.5
4.0
Cellular_organisms - ps1
Archaea1 - ps2
Archaea2 - ps3
Archaea3 - ps4
Asgard/Eukaryota - ps5
Eukaryota - ps6
Amorpheae - ps7
Obazoa - ps8
Opisthokonta - ps9
Holozoa - ps10
Filozoa - ps11
Choano./Metazoa - ps12
Metazoa - ps13
Myriazoa - ps14
Parahoxozoa - ps15
Bilateria - ps16
Deuterostomia - ps17
Chordata - ps18
Olfactores - ps19
Vertebrata - ps20
Gnathostomata - ps21
Euteleostomi - ps22
Sarcopterygii - ps23
Tetrapoda - ps24
Amniota - ps25
Mammalia - ps26
Theria - ps27
Placentalia - ps28
Boreoeutheria - ps29
Euarchontoglires - ps30
Euarchonta - ps31
Primates - ps32
Haplorrhini - ps33
Simiiformes - ps34
Catarrhini - ps35
Hominoidea - ps36
Hominidae - ps37
Homininae - ps38
Hominini - ps39
Homo_sapiens - ps40
Phylostratum

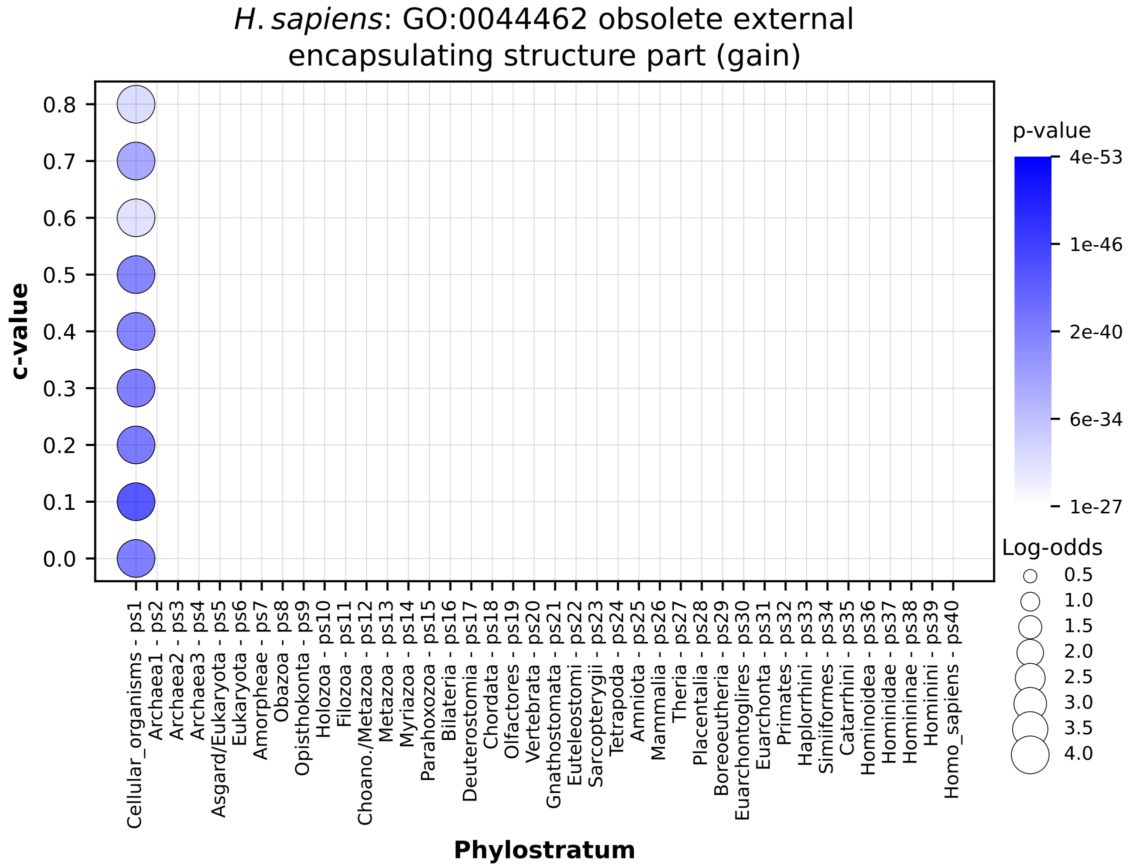

H. sapiens: GO:0044462 obsolete external encapsulating structure part (gain)
c-value
0.8
0.7
0.6
0.5
0.4
0.3
0.2
0.1
0.0
p-value
4e-53
1e-46
2e-40
6e-34
1e-27
Log-odds
0.5
1.0
1.5
2.0
2.5
3.0
3.5
4.0
Cellular_organisms - ps1
Archaea1 - ps2
Archaea2 - ps3
Archaea3 - ps4
Asgard/Eukaryota - ps5
Eukaryota - ps6
Amorpheae - ps7
Obazoa - ps8
Opisthokonta - ps9
Holozoa - ps10
Filozoa - ps11
Choano./Metazoa - ps12
Metazoa - ps13
Myriazoa - ps14
Parahoxozoa - ps15
Bilateria - ps16
Deuterostomia - ps17
Chordata - ps18
Olfactores - ps19
Vertebrata - ps20
Gnathostomata - ps21
Euteleostomi - ps22
Sarcopterygii - ps23
Tetrapoda - ps24
Amniota - ps25
Mammalia - ps26
Theria - ps27
Placentalia - ps28
Boreoeutheria - ps29
Euarchontoglires - ps30
Euarchonta - ps31
Primates - ps32
Haplorrhini - ps33
Simiiformes - ps34
Catarrhini - ps35
Hominoidea - ps36
Hominidae - ps37
Homininae - ps38
Hominini - ps39
Homo_sapiens - ps40
Phylostratum

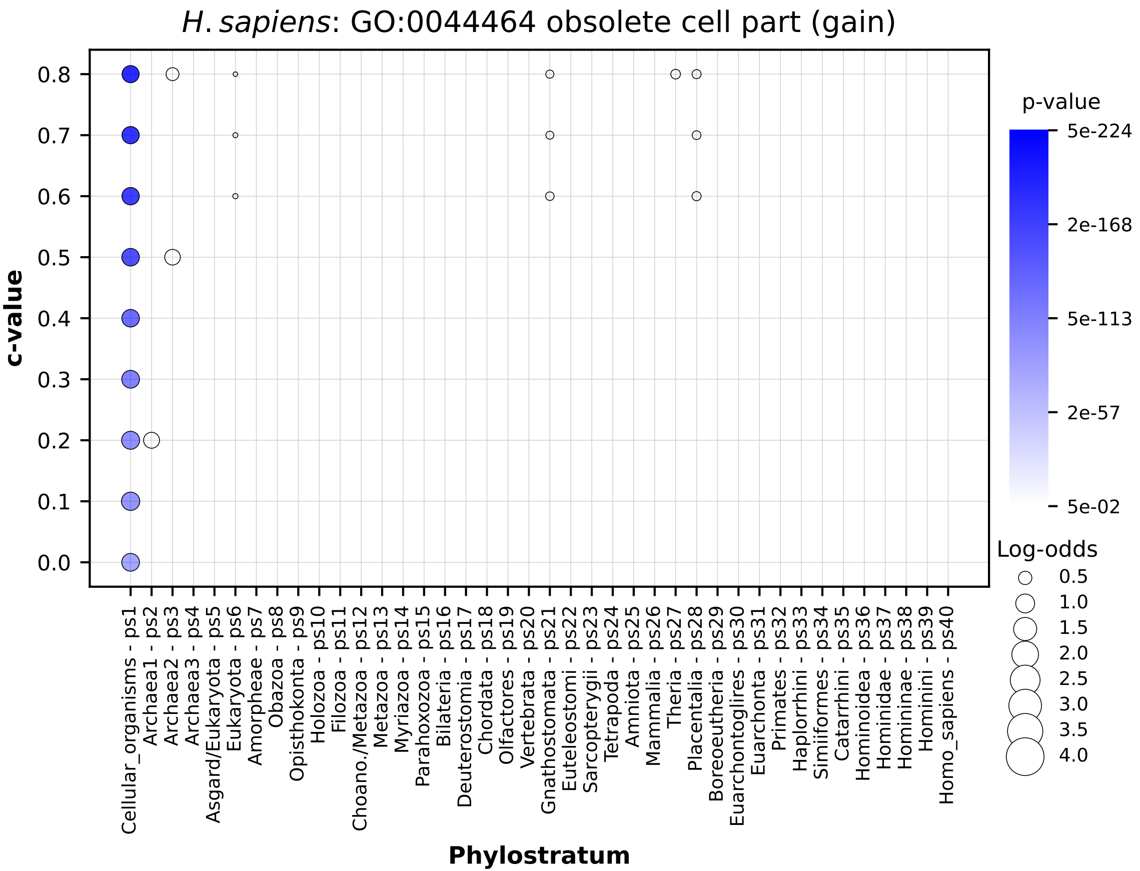

H. sapiens: GO:0044464 obsolete cell part (gain)
c-value
0.8
0.7
0.6
0.5
0.4
0.3
0.2
0.1
0.0
Phylostratum
Cellular_organisms - ps1
Archaea1 - ps2
Archaea2 - ps3
Archaea3 - ps4
Asgard/Eukaryota - ps5
Eukaryota - ps6
Amorpheae - ps7
Obazoa - ps8
Opisthokonta - ps9
Holozoa - ps10
Filozoa - ps11
Choano./Metazoa - ps12
Metazoa - ps13
Myriazoa - ps14
Parahoxozoa - ps15
Bilateria - ps16
Deuterostomia - ps17
Chordata - ps18
Olfactores - ps19
Vertebrata - ps20
Gnathostomata - ps21
Euteleostomi - ps22
Sarcopterygii - ps23
Tetrapoda - ps24
Amniota - ps25
Mammalia - ps26
Theria - ps27
Placentalia - ps28
Boreoeutheria - ps29
Euarchontoglires - ps30
Euarchonta - ps31
Primates - ps32
Haplorrhini - ps33
Simiiformes - ps34
Catarrhini - ps35
Hominoidea - ps36
Hominidae - ps37
Homininae - ps38
Hominini - ps39
Homo_sapiens - ps40
p-value
5e-224
2e-168
5e-113
2e-57
5e-02
Log-odds
0.5
1.0
1.5
2.0
2.5
3.0
3.5
4.0

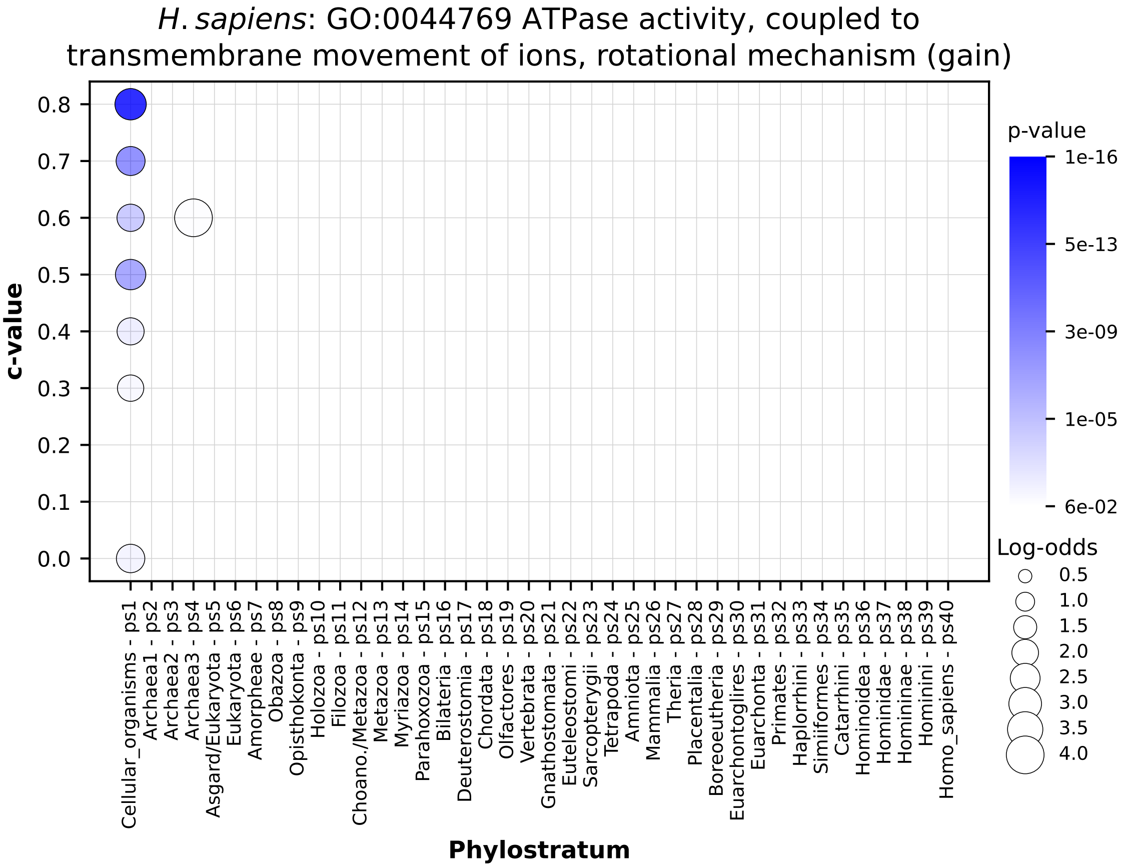

*H. sapiens*: GO:0044769 ATPase activity, coupled to transmembrane movement of ions, rotational mechanism (gain)
c-value
0.8
0.7
0.6
0.5
0.4
0.3
0.2
0.1
0.0
p-value
1e-16
5e-13
3e-09
1e-05
6e-02
Log-odds
0.5
1.0
1.5
2.0
2.5
3.0
3.5
4.0
Cellular_organisms - ps1
Archaea1 - ps2
Archaea2 - ps3
Archaea3 - ps4
Asgard/Eukaryota - ps5
Eukaryota - ps6
Amorpheae - ps7
Obazoa - ps8
Opisthokonta - ps9
Holozoa - ps10
Filozoa - ps11
Choano./Metazoa - ps12
Metazoa - ps13
Myriazoa - ps14
Parahoxozoa - ps15
Bilateria - ps16
Deuterostomia - ps17
Chordata - ps18
Olfactores - ps19
Vertebrata - ps20
Gnathostomata - ps21
Euteleostomi - ps22
Sarcopterygii - ps23
Tetrapoda - ps24
Amniota - ps25
Mammalia - ps26
Theria - ps27
Placentalia - ps28
Boreoeutheria - ps29
Euarchontoglires - ps30
Euarchonta - ps31
Primates - ps32
Haplorrhini - ps33
Simiiformes - ps34
Catarrhini - ps35
Hominoidea - ps36
Hominidae - ps37
Homininae - ps38
Hominini - ps39
Homo_sapiens - ps40
Phylostratum

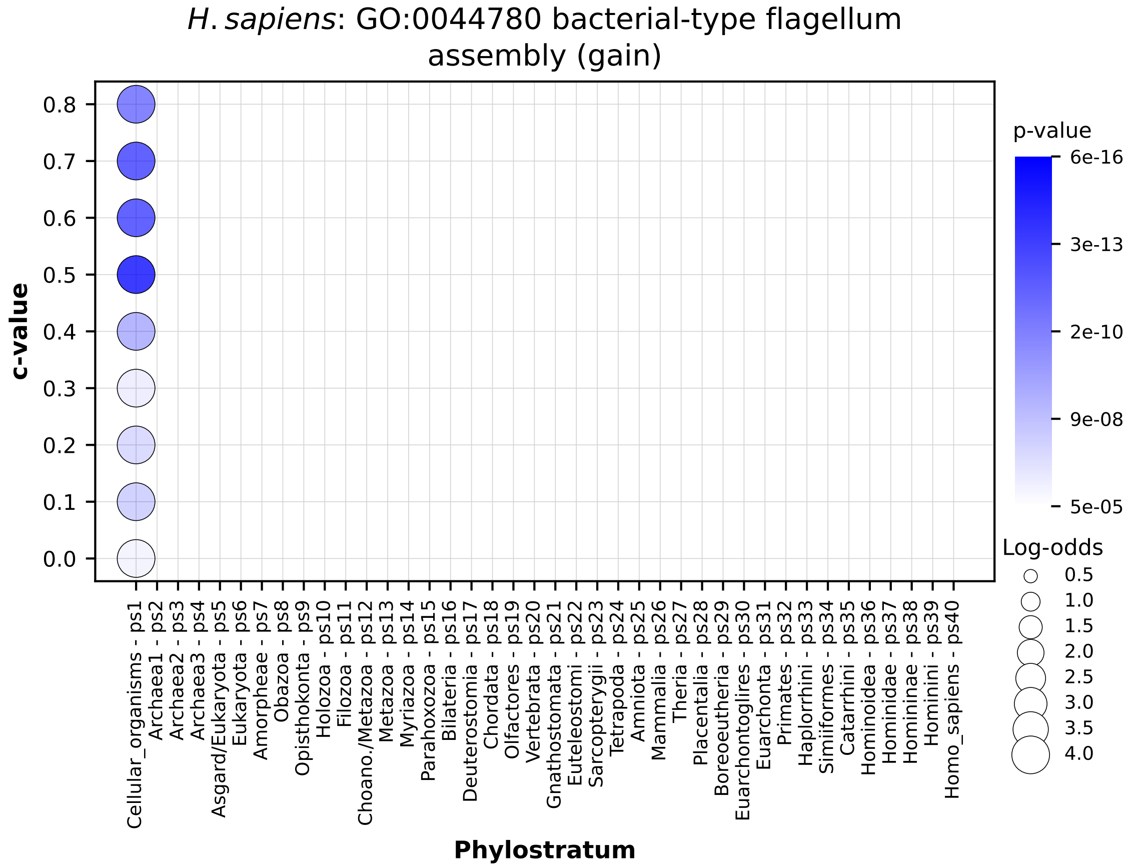
H. sapiens: GO:0044780 bacterial-type flagellum assembly (gain)
c-value
0.8
0.7
0.6
0.5
0.4
0.3
0.2
0.1
0.0
p-value
6e-16
3e-13
2e-10
9e-08
5e-05
Log-odds
0.5
1.0
1.5
2.0
2.5
3.0
3.5
4.0
Cellular_organisms - ps1
Archaea1 - ps2
Archaea2 - ps3
Archaea3 - ps4
Asgard/Eukaryota - ps5
Eukaryota - ps6
Amorpheae - ps7
Obazoa - ps8
Opisthokonta - ps9
Holozoa - ps10
Filozoa - ps11
Choano./Metazoa - ps12
Metazoa - ps13
Myriazoa - ps14
Parahoxozoa - ps15
Bilateria - ps16
Deuterostomia - ps17
Chordata - ps18
Olfactores - ps19
Vertebrata - ps20
Gnathostomata - ps21
Euteleostomi - ps22
Sarcopterygii - ps23
Tetrapoda - ps24
Amniota - ps25
Mammalia - ps26
Theria - ps27
Placentalia - ps28
Boreoeutheria - ps29
Euarchontoglires - ps30
Euarchonta - ps31
Primates - ps32
Haplorrhini - ps33
Simiiformes - ps34
Catarrhini - ps35
Hominoidea - ps36
Hominidae - ps37
Homininae - ps38
Hominini - ps39
Homo_sapiens - ps40
Phylostratum

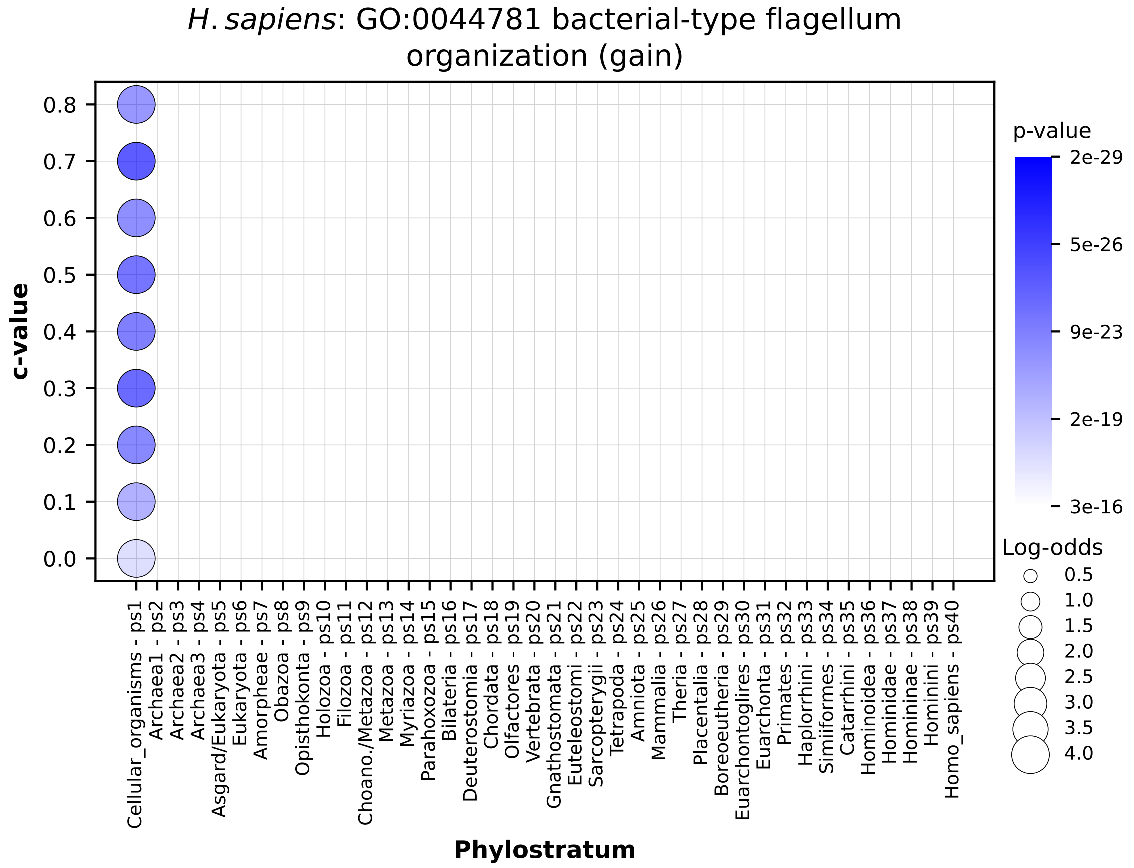
H. sapiens: GO:0044781 bacterial-type flagellum organization (gain)
c-value
0.0
0.1
0.2
0.3
0.4
0.5
0.6
0.7
0.8
p-value
2e-29
5e-26
9e-23
2e-19
3e-16
Log-odds
0.5
1.0
1.5
2.0
2.5
3.0
3.5
4.0
Cellular_organisms - ps1
Archaea1 - ps2
Archaea2 - ps3
Archaea3 - ps4
Asgard/Eukaryota - ps5
Eukaryota - ps6
Amorpheae - ps7
Obazoa - ps8
Opisthokonta - ps9
Holozoa - ps10
Filozoa - ps11
Choano./Metazoa - ps12
Metazoa - ps13
Myriazoa - ps14
Parahoxozoa - ps15
Bilateria - ps16
Deuterostomia - ps17
Chordata - ps18
Olfactores - ps19
Vertebrata - ps20
Gnathostomata - ps21
Euteleostomi - ps22
Sarcopterygii - ps23
Tetrapoda - ps24
Amniota - ps25
Mammalia - ps26
Theria - ps27
Placentalia - ps28
Boreoeutheria - ps29
Euarchontoglires - ps30
Euarchonta - ps31
Primates - ps32
Haplorrhini - ps33
Simiiformes - ps34
Catarrhini - ps35
Hominoidea - ps36
Hominidae - ps37
Homininae - ps38
Hominini - ps39
Homo_sapiens - ps40
Phylostratum

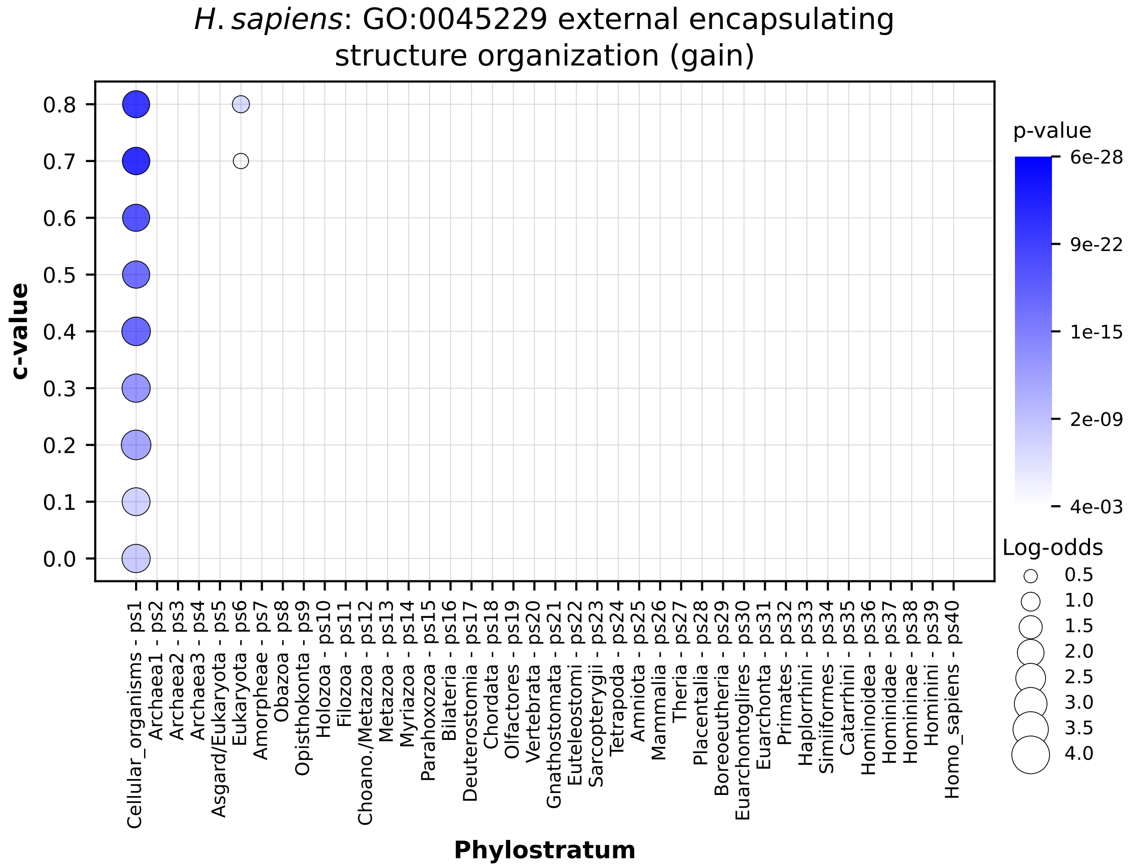

H. sapiens: GO:0045229 external encapsulating structure organization (gain)
c-value
0.8
0.7
0.6
0.5
0.4
0.3
0.2
0.1
0.0
p-value
6e-28
9e-22
1e-15
2e-09
4e-03
Log-odds
0.5
1.0
1.5
2.0
2.5
3.0
3.5
4.0
Cellular_organisms - ps1
Archaea1 - ps2
Archaea2 - ps3
Archaea3 - ps4
Asgard/Eukaryota - ps5
Eukaryota - ps6
Amorpheae - ps7
Obazoa - ps8
Opisthokonta - ps9
Holozoa - ps10
Filozoa - ps11
Choano./Metazoa - ps12
Metazoa - ps13
Myriazoa - ps14
Parahoxozoa - ps15
Bilateria - ps16
Deuterostomia - ps17
Chordata - ps18
Olfactores - ps19
Vertebrata - ps20
Gnathostomata - ps21
Euteleostomi - ps22
Sarcopterygii - ps23
Tetrapoda - ps24
Amniota - ps25
Mammalia - ps26
Theria - ps27
Placentalia - ps28
Boreoeutheria - ps29
Euarchontoglires - ps30
Euarchonta - ps31
Primates - ps32
Haplorrhini - ps33
Simiiformes - ps34
Catarrhini - ps35
Hominoidea - ps36
Hominidae - ps37
Homininae - ps38
Hominini - ps39
Homo_sapiens - ps40
Phylostratum

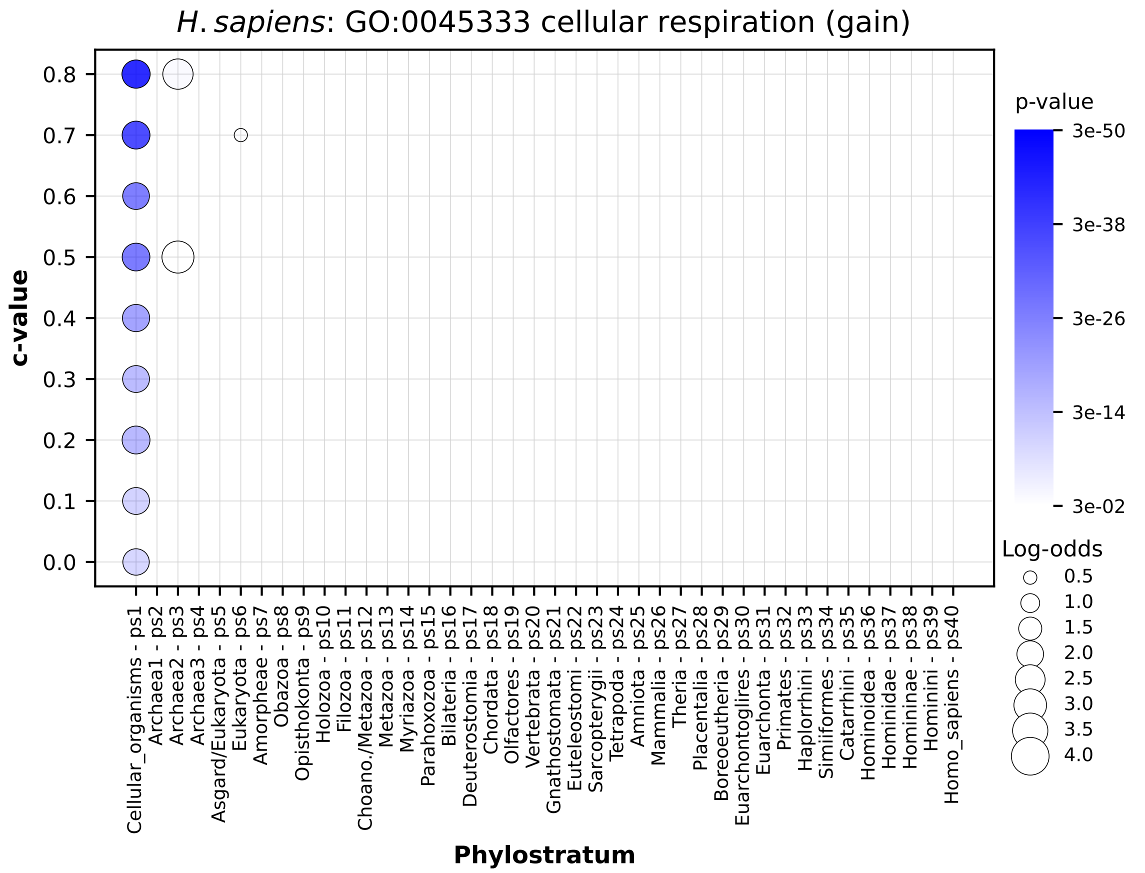

H. sapiens: GO:0045333 cellular respiration (gain)
c-value
0.8
0.7
0.6
0.5
0.4
0.3
0.2
0.1
0.0
Phylostratum
Cellular_organisms - ps1
Archaea1 - ps2
Archaea2 - ps3
Archaea3 - ps4
Asgard/Eukaryota - ps5
Eukaryota - ps6
Amorpheae - ps7
Obazoa - ps8
Opisthokonta - ps9
Holozoa - ps10
Filozoa - ps11
Choano./Metazoa - ps12
Metazoa - ps13
Myriazoa - ps14
Parahoxozoa - ps15
Bilateria - ps16
Deuterostomia - ps17
Chordata - ps18
Olfactores - ps19
Vertebrata - ps20
Gnathostomata - ps21
Euteleostomi - ps22
Sarcopterygii - ps23
Tetrapoda - ps24
Amniota - ps25
Mammalia - ps26
Theria - ps27
Placentalia - ps28
Boreoeutheria - ps29
Euarchontoglires - ps30
Euarchonta - ps31
Primates - ps32
Haplorrhini - ps33
Simiiformes - ps34
Catarrhini - ps35
Hominoidea - ps36
Hominidae - ps37
Homininae - ps38
Hominini - ps39
Homo_sapiens - ps40
p-value
3e-50
3e-38
3e-26
3e-14
3e-02
Log-odds
0.5
1.0
1.5
2.0
2.5
3.0
3.5
4.0

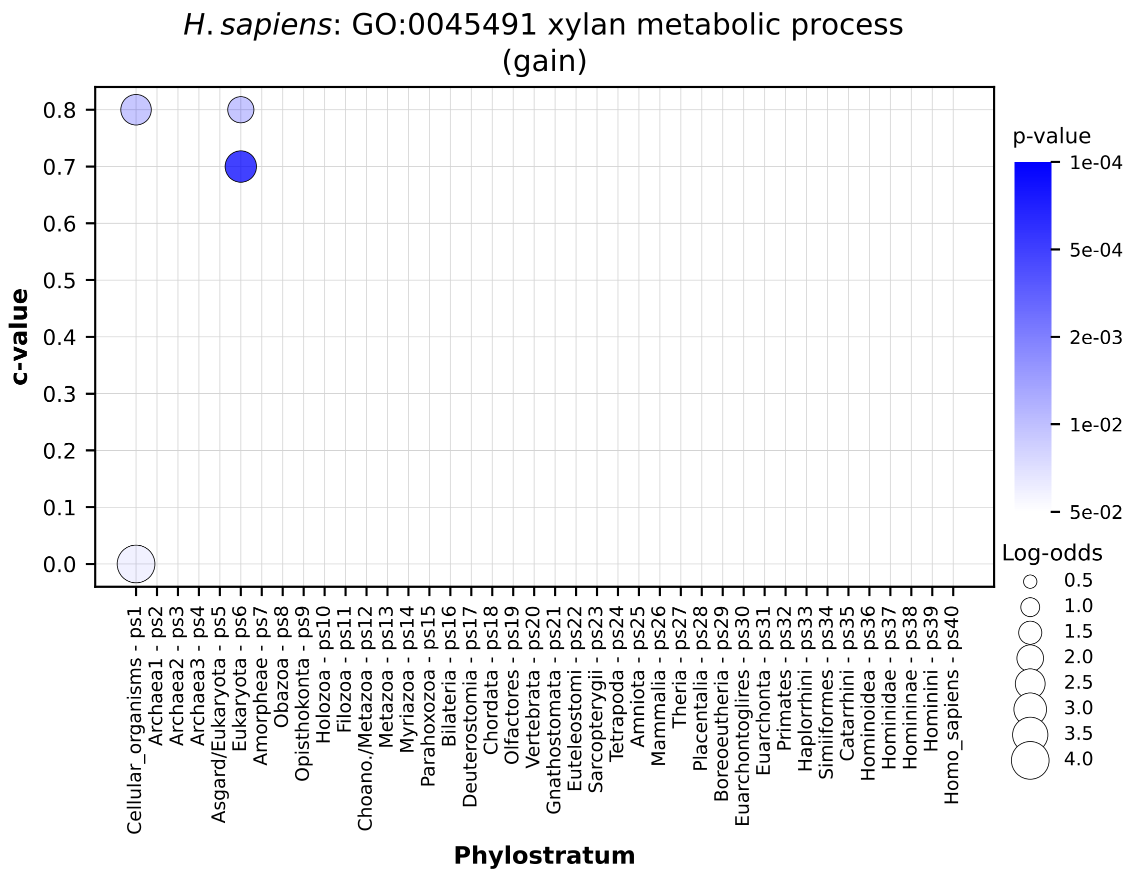

H. sapiens: GO:0045491 xylan metabolic process
(gain)
c-value
0.8
0.7
0.6
0.5
0.4
0.3
0.2
0.1
0.0
p-value
1e-04
5e-04
2e-03
1e-02
5e-02
Log-odds
0.5
1.0
1.5
2.0
2.5
3.0
3.5
4.0
Cellular_organisms - ps1
Archaea1 - ps2
Archaea2 - ps3
Archaea3 - ps4
Asgard/Eukaryota - ps5
Eukaryota - ps6
Amorpheae - ps7
Obazoa - ps8
Opisthokonta - ps9
Holozoa - ps10
Filozoa - ps11
Choano./Metazoa - ps12
Metazoa - ps13
Myriazoa - ps14
Parahoxozoa - ps15
Bilateria - ps16
Deuterostomia - ps17
Chordata - ps18
Olfactores - ps19
Vertebrata - ps20
Gnathostomata - ps21
Euteleostomi - ps22
Sarcopterygii - ps23
Tetrapoda - ps24
Amniota - ps25
Mammalia - ps26
Theria - ps27
Placentalia - ps28
Boreoeutheria - ps29
Euarchontoglires - ps30
Euarchonta - ps31
Primates - ps32
Haplorrhini - ps33
Simiiformes - ps34
Catarrhini - ps35
Hominoidea - ps36
Hominidae - ps37
Homininae - ps38
Hominini - ps39
Homo_sapiens - ps40
Phylostratum

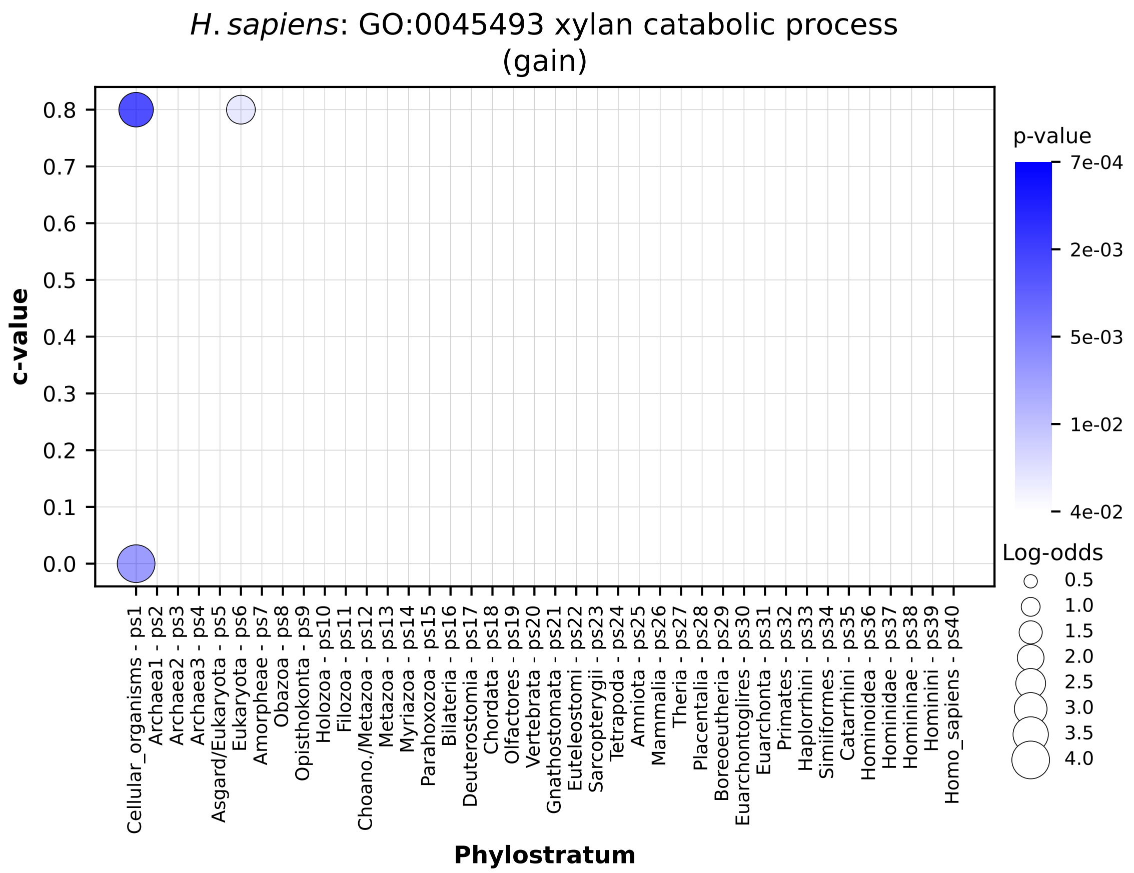

H. sapiens: GO:0045493 xylan catabolic process
(gain)
c-value
0.8
0.7
0.6
0.5
0.4
0.3
0.2
0.1
0.0
Phylostratum
Cellular_organisms - ps1
Archaea1 - ps2
Archaea2 - ps3
Archaea3 - ps4
Asgard/Eukaryota - ps5
Eukaryota - ps6
Amorpheae - ps7
Obazoa - ps8
Opisthokonta - ps9
Holozoa - ps10
Filozoa - ps11
Choano./Metazoa - ps12
Metazoa - ps13
Myriazoa - ps14
Parahoxozoa - ps15
Bilateria - ps16
Deuterostomia - ps17
Chordata - ps18
Olfactores - ps19
Vertebrata - ps20
Gnathostomata - ps21
Euteleostomi - ps22
Sarcopterygii - ps23
Tetrapoda - ps24
Amniota - ps25
Mammalia - ps26
Theria - ps27
Placentalia - ps28
Boreoeutheria - ps29
Euarchontoglires - ps30
Euarchonta - ps31
Primates - ps32
Haplorrhini - ps33
Simiiformes - ps34
Catarrhini - ps35
Hominoidea - ps36
Hominidae - ps37
Homininae - ps38
Hominini - ps39
Homo_sapiens - ps40
p-value
7e-04
2e-03
5e-03
1e-02
4e-02
Log-odds
0.5
1.0
1.5
2.0
2.5
3.0
3.5
4.0

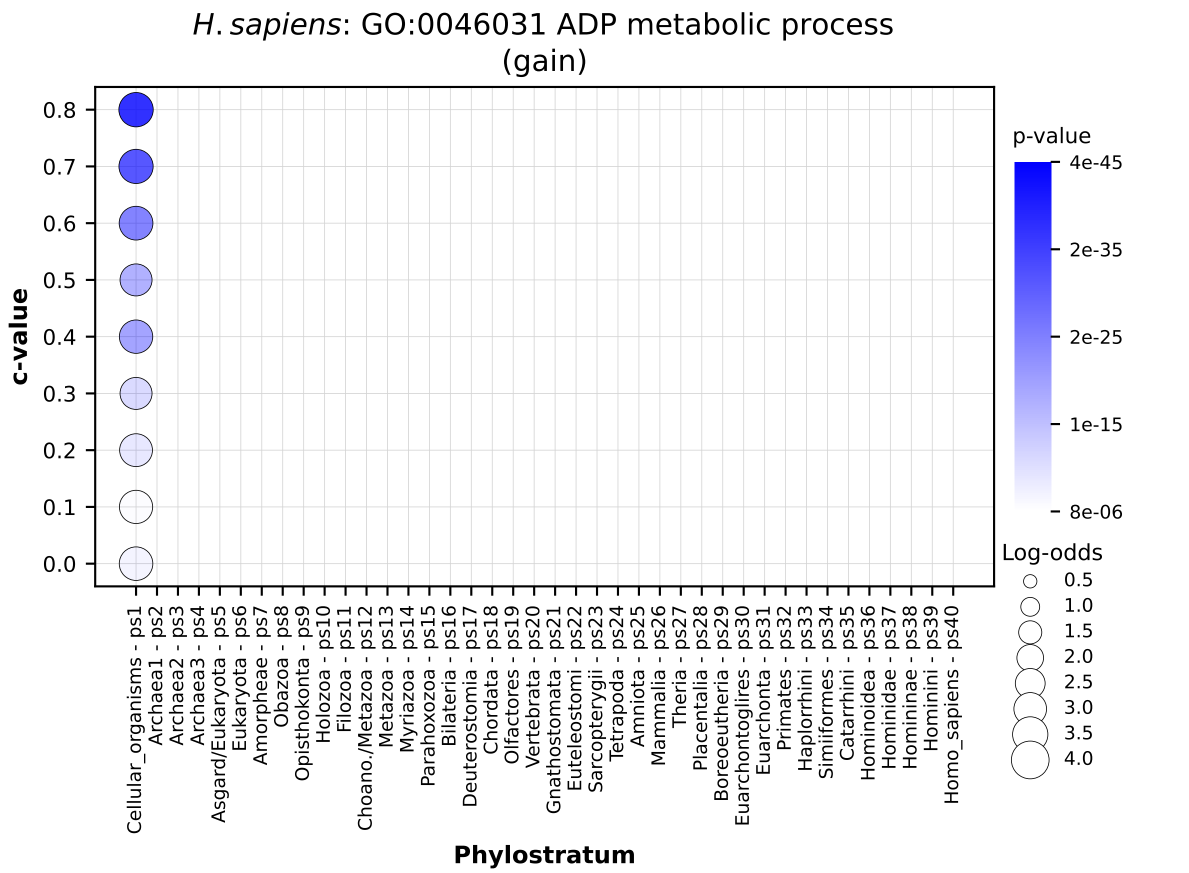

H. sapiens: GO:0046031 ADP metabolic process
(gain)
c-value
0.8
0.7
0.6
0.5
0.4
0.3
0.2
0.1
0.0
p-value
4e-45
2e-35
2e-25
1e-15
8e-06
Log-odds
0.5
1.0
1.5
2.0
2.5
3.0
3.5
4.0
Cellular_organisms - ps1
Archaea1 - ps2
Archaea2 - ps3
Archaea3 - ps4
Asgard/Eukaryota - ps5
Eukaryota - ps6
Amorpheae - ps7
Obazoa - ps8
Opisthokonta - ps9
Holozoa - ps10
Filozoa - ps11
Choano./Metazoa - ps12
Metazoa - ps13
Myriazoa - ps14
Parahoxozoa - ps15
Bilateria - ps16
Deuterostomia - ps17
Chordata - ps18
Olfactores - ps19
Vertebrata - ps20
Gnathostomata - ps21
Euteleostomi - ps22
Sarcopterygii - ps23
Tetrapoda - ps24
Amniota - ps25
Mammalia - ps26
Theria - ps27
Placentalia - ps28
Boreoeutheria - ps29
Euarchontoglires - ps30
Euarchonta - ps31
Primates - ps32
Haplorrhini - ps33
Simiiformes - ps34
Catarrhini - ps35
Hominoidea - ps36
Hominidae - ps37
Homininae - ps38
Hominini - ps39
Homo_sapiens - ps40
Phylostratum

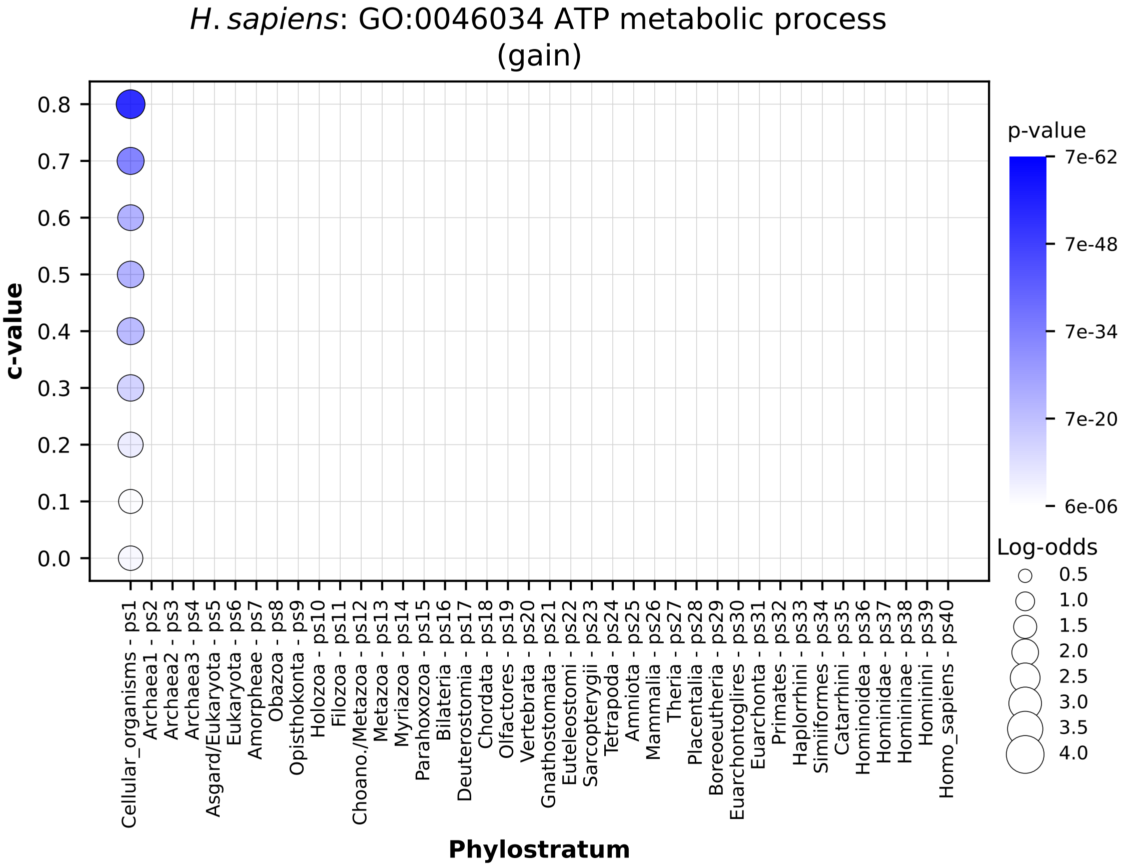

*H. sapiens*: GO:0046034 ATP metabolic process
(gain)
c-value
0.8
0.7
0.6
0.5
0.4
0.3
0.2
0.1
0.0
p-value
7e-62
7e-48
7e-34
7e-20
6e-06
Log-odds
0.5
1.0
1.5
2.0
2.5
3.0
3.5
4.0
Cellular_organisms - ps1
Archaea1 - ps2
Archaea2 - ps3
Archaea3 - ps4
Asgard/Eukaryota - ps5
Eukaryota - ps6
Amorpheae - ps7
Obazoa - ps8
Opisthokonta - ps9
Holozoa - ps10
Filozoa - ps11
Choano./Metazoa - ps12
Metazoa - ps13
Myriazoa - ps14
Parahoxozoa - ps15
Bilateria - ps16
Deuterostomia - ps17
Chordata - ps18
Olfactores - ps19
Vertebrata - ps20
Gnathostomata - ps21
Euteleostomi - ps22
Sarcopterygii - ps23
Tetrapoda - ps24
Amniota - ps25
Mammalia - ps26
Theria - ps27
Placentalia - ps28
Boreoeutheria - ps29
Euarchontoglires - ps30
Euarchonta - ps31
Primates - ps32
Haplorrhini - ps33
Simiiformes - ps34
Catarrhini - ps35
Hominoidea - ps36
Hominidae - ps37
Homininae - ps38
Hominini - ps39
Homo_sapiens - ps40
Phylostratum

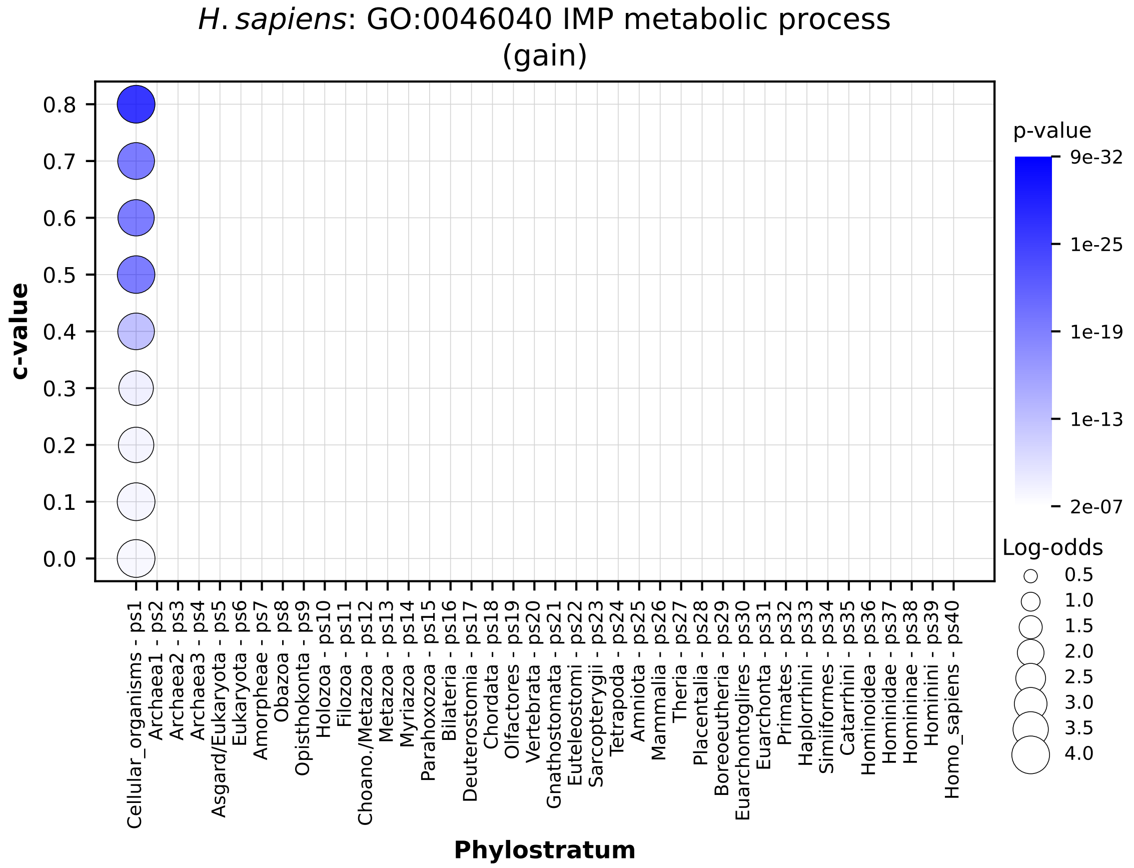
H. sapiens: GO:0046040 IMP metabolic process
(gain)
c-value
0.8
0.7
0.6
0.5
0.4
0.3
0.2
0.1
0.0
p-value
9e-32
1e-25
1e-19
1e-13
2e-07
Log-odds
0.5
1.0
1.5
2.0
2.5
3.0
3.5
4.0
Cellular_organisms - ps1
Archaea1 - ps2
Archaea2 - ps3
Archaea3 - ps4
Asgard/Eukaryota - ps5
Eukaryota - ps6
Amorpheae - ps7
Obazoa - ps8
Opisthokonta - ps9
Holozoa - ps10
Filozoa - ps11
Choano./Metazoa - ps12
Metazoa - ps13
Myriazoa - ps14
Parahoxozoa - ps15
Bilateria - ps16
Deuterostomia - ps17
Chordata - ps18
Olfactores - ps19
Vertebrata - ps20
Gnathostomata - ps21
Euteleostomi - ps22
Sarcopterygii - ps23
Tetrapoda - ps24
Amniota - ps25
Mammalia - ps26
Theria - ps27
Placentalia - ps28
Boreoeutheria - ps29
Euarchontoglires - ps30
Euarchonta - ps31
Primates - ps32
Haplorrhini - ps33
Simiiformes - ps34
Catarrhini - ps35
Hominoidea - ps36
Hominidae - ps37
Homininae - ps38
Hominini - ps39
Homo_sapiens - ps40
Phylostratum

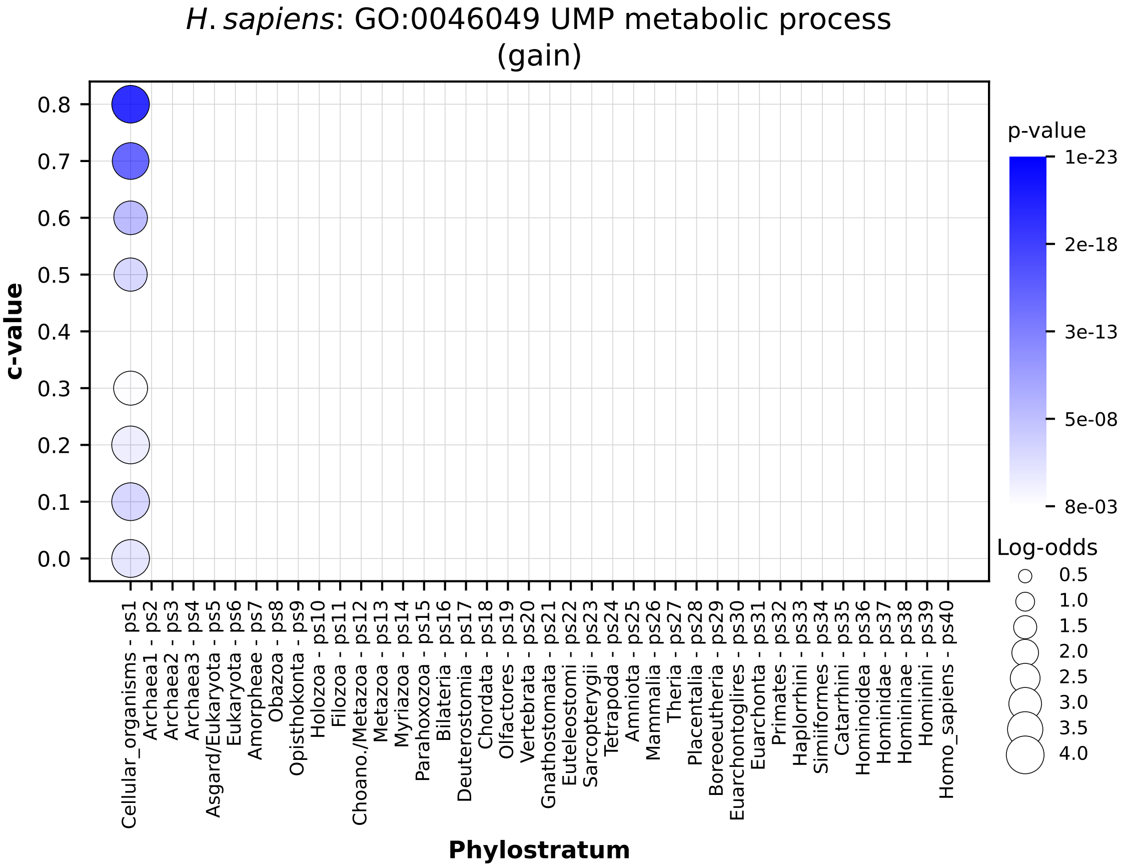

*H. sapiens*: GO:0046049 UMP metabolic process
(gain)
c-value
0.8
0.7
0.6
0.5
0.4
0.3
0.2
0.1
0.0
p-value
1e-23
2e-18
3e-13
5e-08
8e-03
Log-odds
0.5
1.0
1.5
2.0
2.5
3.0
3.5
4.0
Cellular_organisms - ps1
Archaea1 - ps2
Archaea2 - ps3
Archaea3 - ps4
Asgard/Eukaryota - ps5
Eukaryota - ps6
Amorpheae - ps7
Obazoa - ps8
Opisthokonta - ps9
Holozoa - ps10
Filozoa - ps11
Choano./Metazoa - ps12
Metazoa - ps13
Myriazoa - ps14
Parahoxozoa - ps15
Bilateria - ps16
Deuterostomia - ps17
Chordata - ps18
Olfactores - ps19
Vertebrata - ps20
Gnathostomata - ps21
Euteleostomi - ps22
Sarcopterygii - ps23
Tetrapoda - ps24
Amniota - ps25
Mammalia - ps26
Theria - ps27
Placentalia - ps28
Boreoeutheria - ps29
Euarchontoglires - ps30
Euarchonta - ps31
Primates - ps32
Haplorrhini - ps33
Simiiformes - ps34
Catarrhini - ps35
Hominoidea - ps36
Hominidae - ps37
Homininae - ps38
Hominini - ps39
Homo_sapiens - ps40
Phylostratum

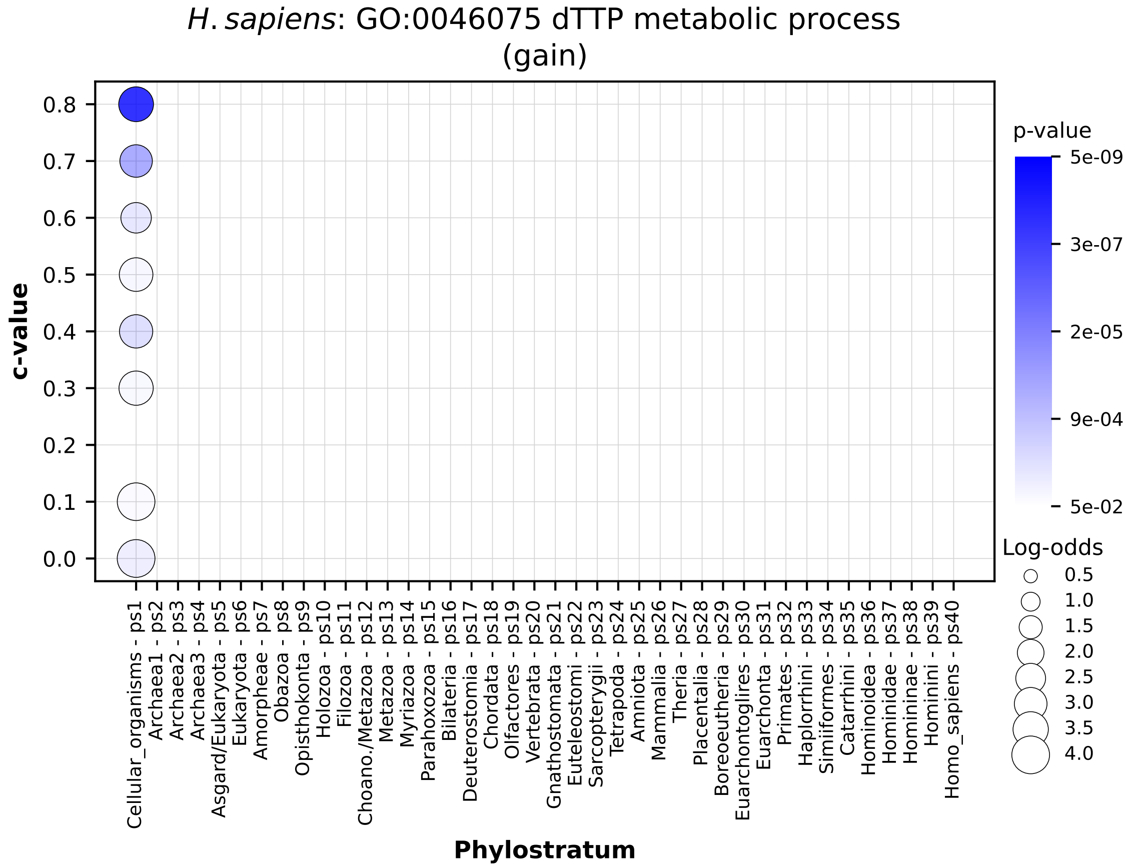

H. sapiens: GO:0046075 dTTP metabolic process
(gain)
c-value
0.8
0.7
0.6
0.5
0.4
0.3
0.2
0.1
0.0
p-value
5e-09
3e-07
2e-05
9e-04
5e-02
Log-odds
0.5
1.0
1.5
2.0
2.5
3.0
3.5
4.0
Cellular_organisms - ps1
Archaea1 - ps2
Archaea2 - ps3
Archaea3 - ps4
Asgard/Eukaryota - ps5
Eukaryota - ps6
Amorpheae - ps7
Obazoa - ps8
Opisthokonta - ps9
Holozoa - ps10
Filozoa - ps11
Choano./Metazoa - ps12
Metazoa - ps13
Myriazoa - ps14
Parahoxozoa - ps15
Bilateria - ps16
Deuterostomia - ps17
Chordata - ps18
Olfactores - ps19
Vertebrata - ps20
Gnathostomata - ps21
Euteleostomi - ps22
Sarcopterygii - ps23
Tetrapoda - ps24
Amniota - ps25
Mammalia - ps26
Theria - ps27
Placentalia - ps28
Boreoeutheria - ps29
Euarchontoglires - ps30
Euarchonta - ps31
Primates - ps32
Haplorrhini - ps33
Simiiformes - ps34
Catarrhini - ps35
Hominoidea - ps36
Hominidae - ps37
Homininae - ps38
Hominini - ps39
Homo_sapiens - ps40
Phylostratum

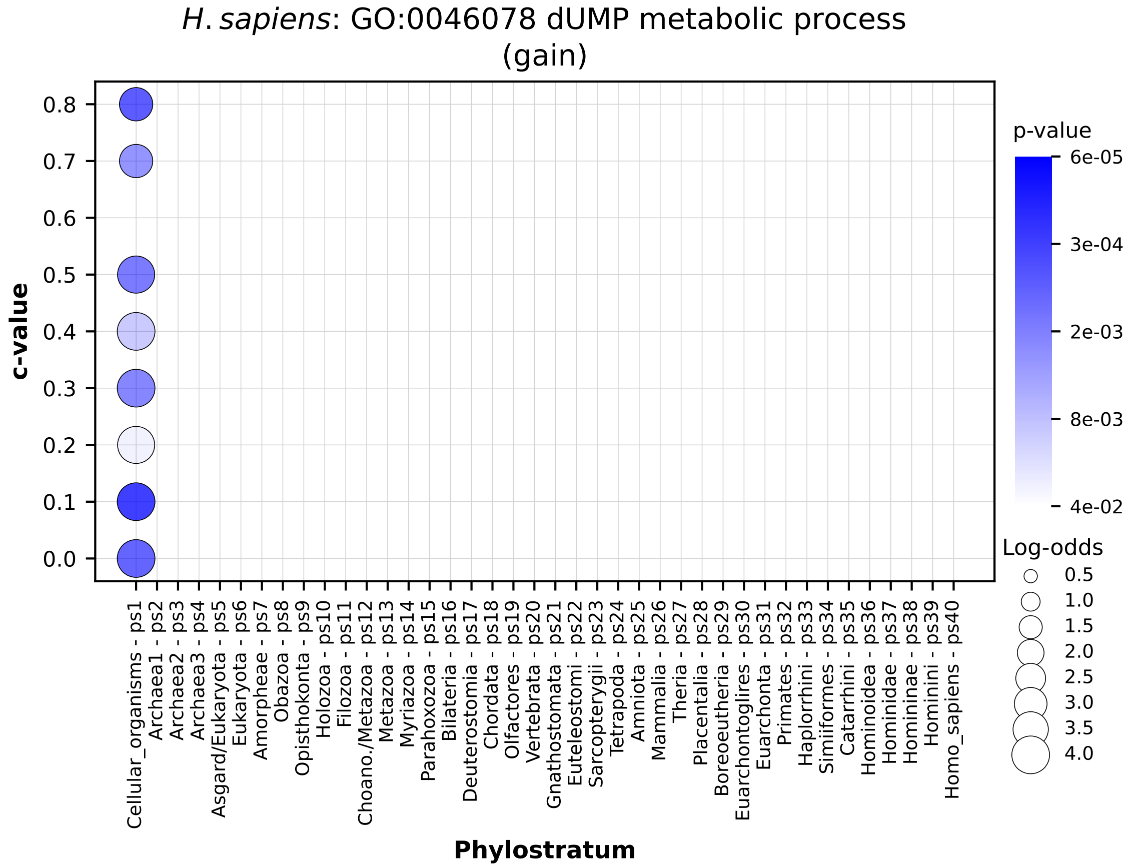

H. sapiens: GO:0046078 dUMP metabolic process
(gain)
c-value
0.8
0.7
0.6
0.5
0.4
0.3
0.2
0.1
0.0
p-value
6e-05
3e-04
2e-03
8e-03
4e-02
Log-odds
0.5
1.0
1.5
2.0
2.5
3.0
3.5
4.0
Cellular_organisms - ps1
Archaea1 - ps2
Archaea2 - ps3
Archaea3 - ps4
Asgard/Eukaryota - ps5
Eukaryota - ps6
Amorpheae - ps7
Obazoa - ps8
Opisthokonta - ps9
Holozoa - ps10
Filozoa - ps11
Choano./Metazoa - ps12
Metazoa - ps13
Myriazoa - ps14
Parahoxozoa - ps15
Bilateria - ps16
Deuterostomia - ps17
Chordata - ps18
Olfactores - ps19
Vertebrata - ps20
Gnathostomata - ps21
Euteleostomi - ps22
Sarcopterygii - ps23
Tetrapoda - ps24
Amniota - ps25
Mammalia - ps26
Theria - ps27
Placentalia - ps28
Boreoeutheria - ps29
Euarchontoglires - ps30
Euarchonta - ps31
Primates - ps32
Haplorrhini - ps33
Simiiformes - ps34
Catarrhini - ps35
Hominoidea - ps36
Hominidae - ps37
Homininae - ps38
Hominini - ps39
Homo_sapiens - ps40
Phylostratum

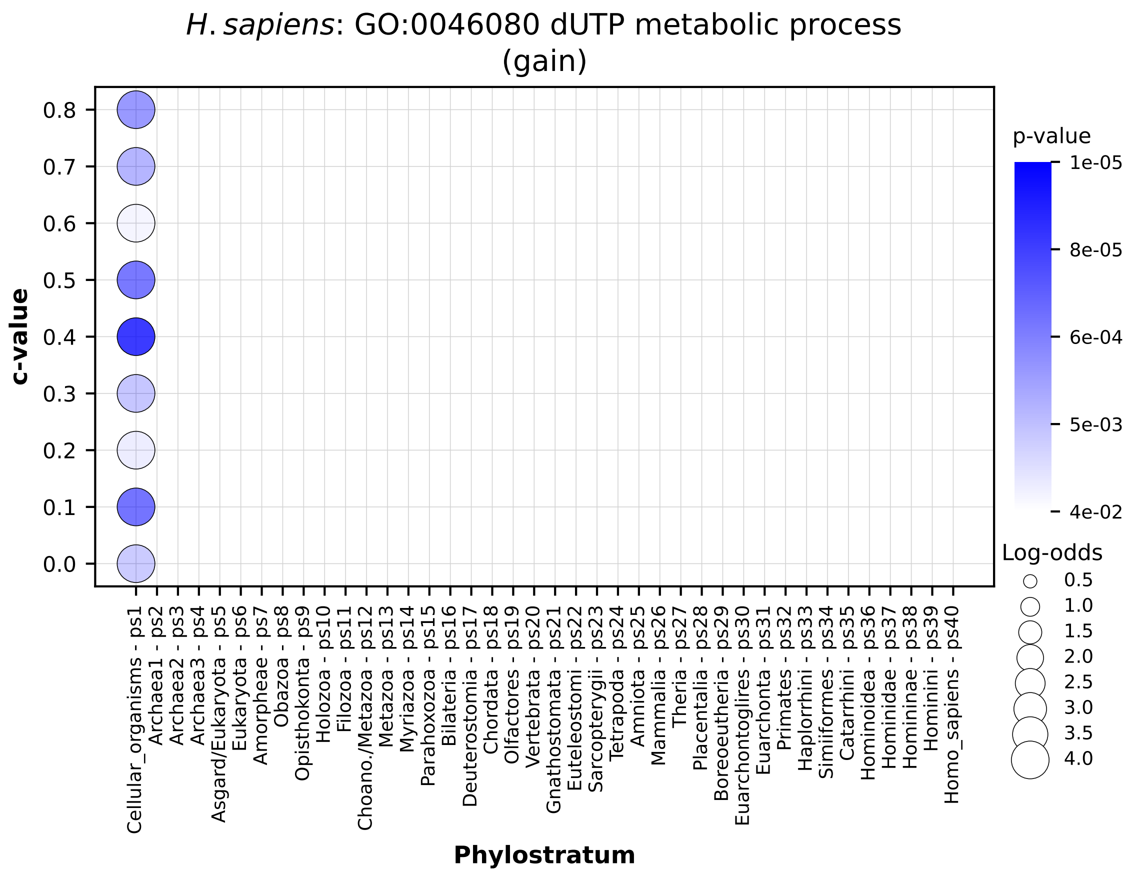

H. sapiens: GO:0046080 dUTP metabolic process
(gain)
c-value
0.8
0.7
0.6
0.5
0.4
0.3
0.2
0.1
0.0
p-value
1e-05
8e-05
6e-04
5e-03
4e-02
Log-odds
0.5
1.0
1.5
2.0
2.5
3.0
3.5
4.0
Cellular_organisms - ps1
Archaea1 - ps2
Archaea2 - ps3
Archaea3 - ps4
Asgard/Eukaryota - ps5
Eukaryota - ps6
Amorpheae - ps7
Obazoa - ps8
Opisthokonta - ps9
Holozoa - ps10
Filozoa - ps11
Choano./Metazoa - ps12
Metazoa - ps13
Myriazoa - ps14
Parahoxozoa - ps15
Bilateria - ps16
Deuterostomia - ps17
Chordata - ps18
Olfactores - ps19
Vertebrata - ps20
Gnathostomata - ps21
Euteleostomi - ps22
Sarcopterygii - ps23
Tetrapoda - ps24
Amniota - ps25
Mammalia - ps26
Theria - ps27
Placentalia - ps28
Boreoeutheria - ps29
Euarchontoglires - ps30
Euarchonta - ps31
Primates - ps32
Haplorrhini - ps33
Simiiformes - ps34
Catarrhini - ps35
Hominoidea - ps36
Hominidae - ps37
Homininae - ps38
Hominini - ps39
Homo_sapiens - ps40
Phylostratum

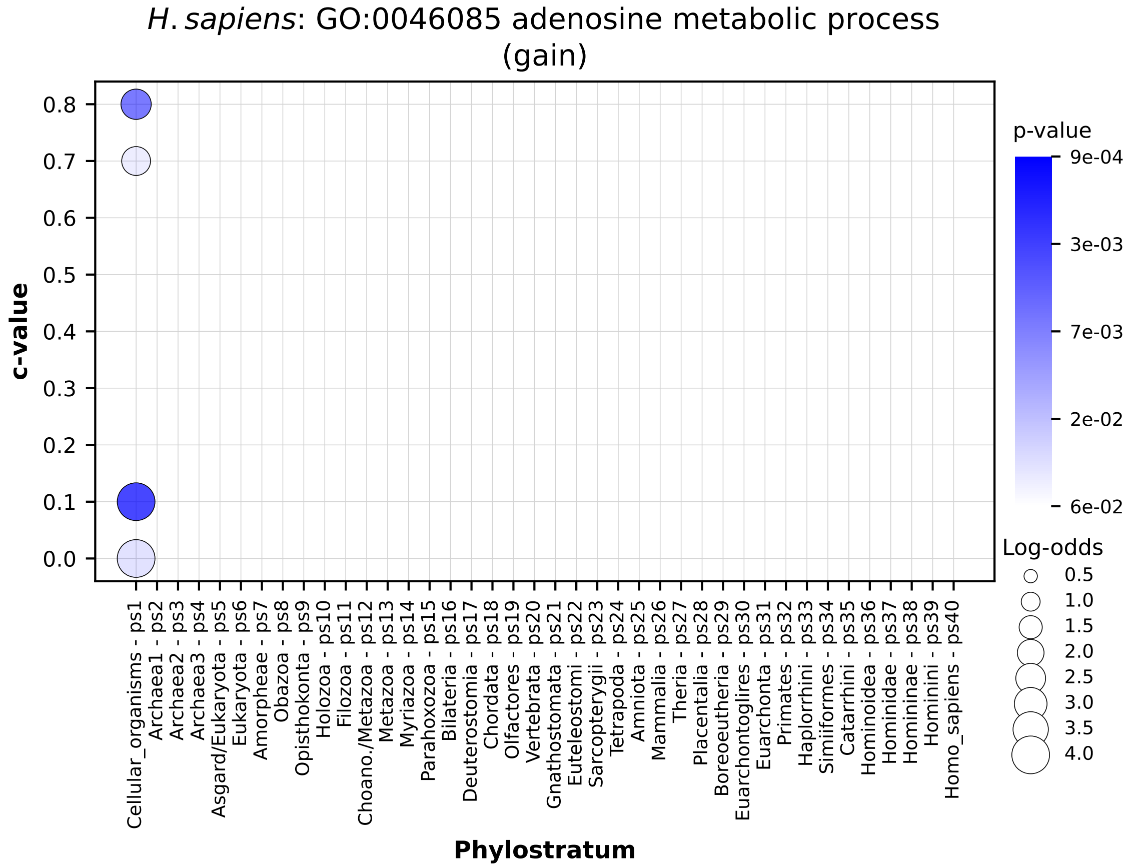
H. sapiens: GO:0046085 adenosine metabolic process (gain)
c-value
0.8
0.7
0.6
0.5
0.4
0.3
0.2
0.1
0.0
Phylostratum
Cellular_organisms - ps1
Archaea1 - ps2
Archaea2 - ps3
Archaea3 - ps4
Asgard/Eukaryota - ps5
Eukaryota - ps6
Amorpheae - ps7
Obazoa - ps8
Opisthokonta - ps9
Holozoa - ps10
Filozoa - ps11
Choano./Metazoa - ps12
Metazoa - ps13
Myriazoa - ps14
Parahoxozoa - ps15
Bilateria - ps16
Deuterostomia - ps17
Chordata - ps18
Olfactores - ps19
Vertebrata - ps20
Gnathostomata - ps21
Euteleostomi - ps22
Sarcopterygii - ps23
Tetrapoda - ps24
Amniota - ps25
Mammalia - ps26
Theria - ps27
Placentalia - ps28
Boreoeutheria - ps29
Euarchontoglires - ps30
Euarchonta - ps31
Primates - ps32
Haplorrhini - ps33
Simiiformes - ps34
Catarrhini - ps35
Hominoidea - ps36
Hominidae - ps37
Homininae - ps38
Hominini - ps39
Homo_sapiens - ps40
p-value
9e-04
3e-03
7e-03
2e-02
6e-02
Log-odds
0.5
1.0
1.5
2.0
2.5
3.0
3.5
4.0

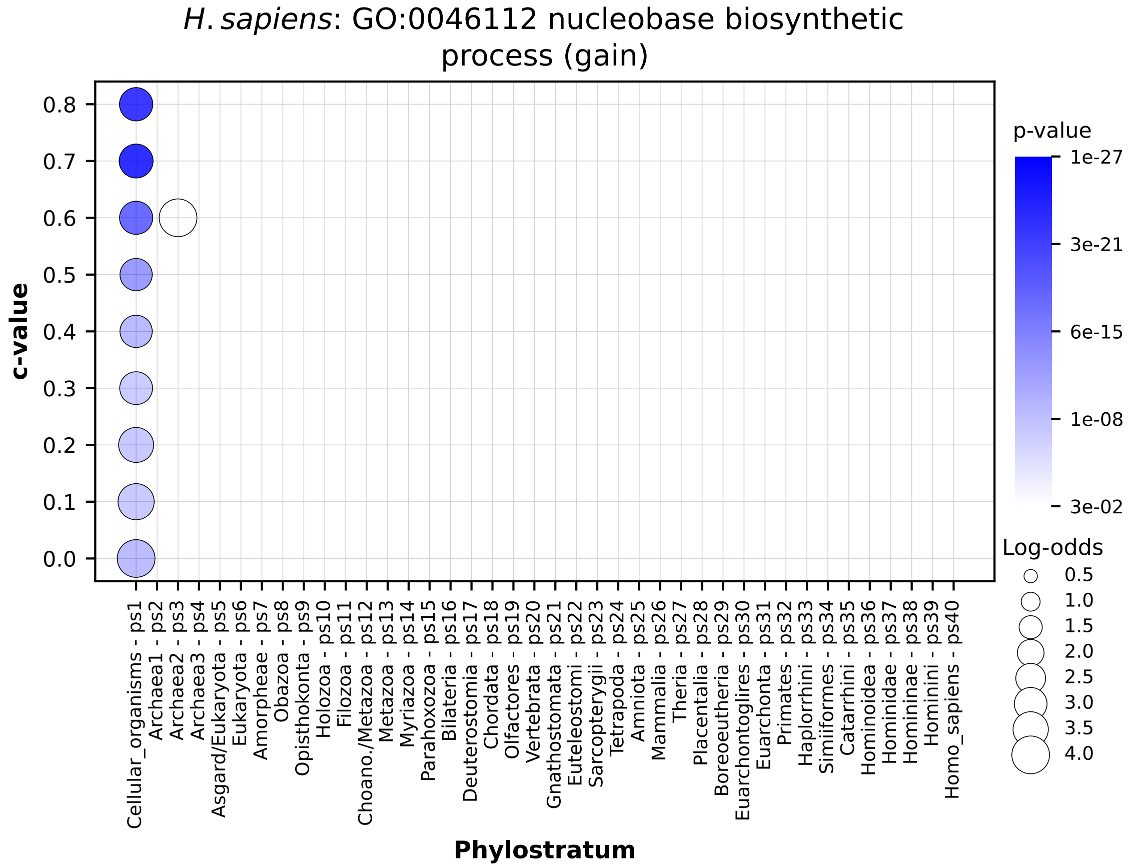

H. sapiens: GO:0046112 nucleobase biosynthetic process (gain)
c-value
0.8
0.7
0.6
0.5
0.4
0.3
0.2
0.1
0.0
Phylostratum
Cellular_organisms - ps1
Archaea1 - ps2
Archaea2 - ps3
Archaea3 - ps4
Asgard/Eukaryota - ps5
Eukaryota - ps6
Amorpheae - ps7
Obazoa - ps8
Opisthokonta - ps9
Holozoa - ps10
Filozoa - ps11
Choano./Metazoa - ps12
Metazoa - ps13
Myriazoa - ps14
Parahoxozoa - ps15
Bilateria - ps16
Deuterostomia - ps17
Chordata - ps18
Olfactores - ps19
Vertebrata - ps20
Gnathostomata - ps21
Euteleostomi - ps22
Sarcopterygii - ps23
Tetrapoda - ps24
Amniota - ps25
Mammalia - ps26
Theria - ps27
Placentalia - ps28
Boreoeutheria - ps29
Euarchontoglires - ps30
Euarchonta - ps31
Primates - ps32
Haplorrhini - ps33
Simiiformes - ps34
Catarrhini - ps35
Hominoidea - ps36
Hominidae - ps37
Homininae - ps38
Hominini - ps39
Homo_sapiens - ps40
p-value
1e-27
3e-21
6e-15
1e-08
3e-02
Log-odds
0.5
1.0
1.5
2.0
2.5
3.0
3.5
4.0

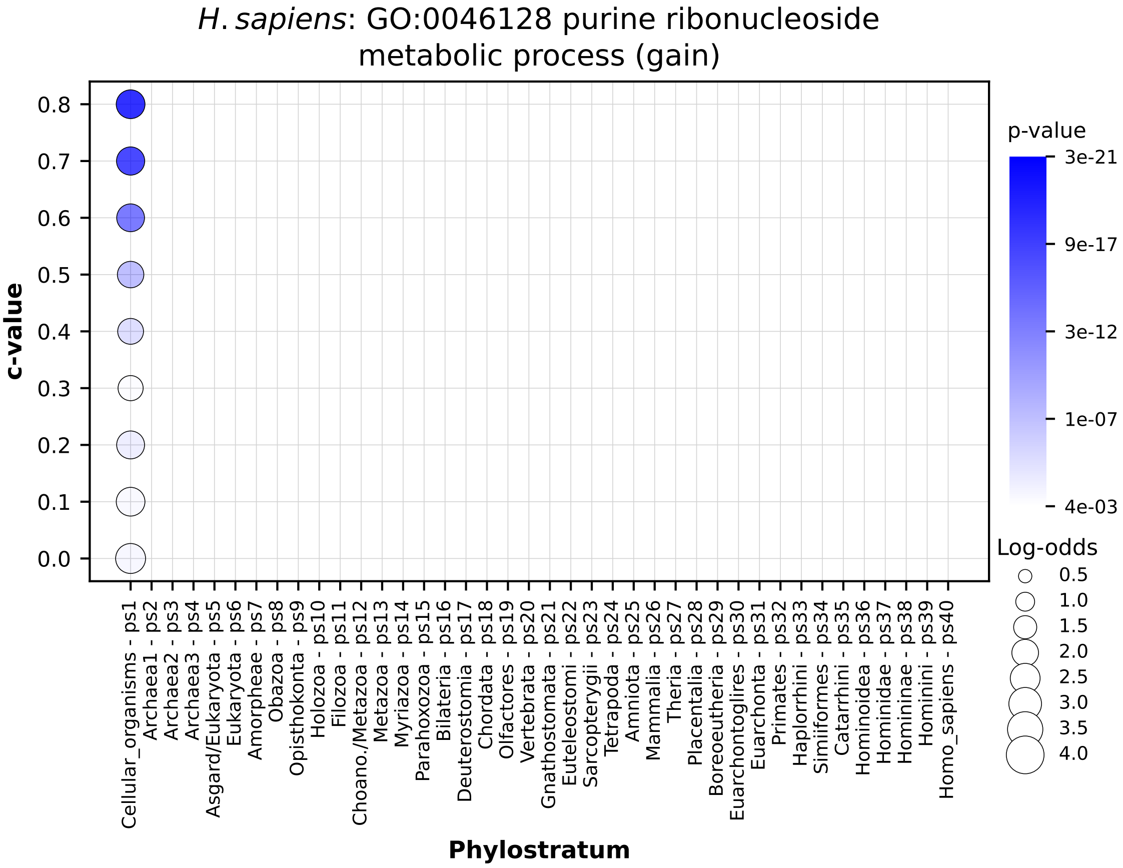

H. sapiens: GO:0046128 purine ribonucleoside metabolic process (gain)
c-value
0.8
0.7
0.6
0.5
0.4
0.3
0.2
0.1
0.0
Phylostratum
Cellular_organisms - ps1
Archaea1 - ps2
Archaea2 - ps3
Archaea3 - ps4
Asgard/Eukaryota - ps5
Eukaryota - ps6
Amorpheae - ps7
Obazoa - ps8
Opisthokonta - ps9
Holozoa - ps10
Filozoa - ps11
Choano./Metazoa - ps12
Metazoa - ps13
Myriazoa - ps14
Parahoxozoa - ps15
Bilateria - ps16
Deuterostomia - ps17
Chordata - ps18
Olfactores - ps19
Vertebrata - ps20
Gnathostomata - ps21
Euteleostomi - ps22
Sarcopterygii - ps23
Tetrapoda - ps24
Amniota - ps25
Mammalia - ps26
Theria - ps27
Placentalia - ps28
Boreoeutheria - ps29
Euarchontoglires - ps30
Euarchonta - ps31
Primates - ps32
Haplorrhini - ps33
Simiiformes - ps34
Catarrhini - ps35
Hominoidea - ps36
Hominidae - ps37
Homininae - ps38
Hominini - ps39
Homo_sapiens - ps40
p-value
3e-21
9e-17
3e-12
1e-07
4e-03
Log-odds
0.5
1.0
1.5
2.0
2.5
3.0
3.5
4.0

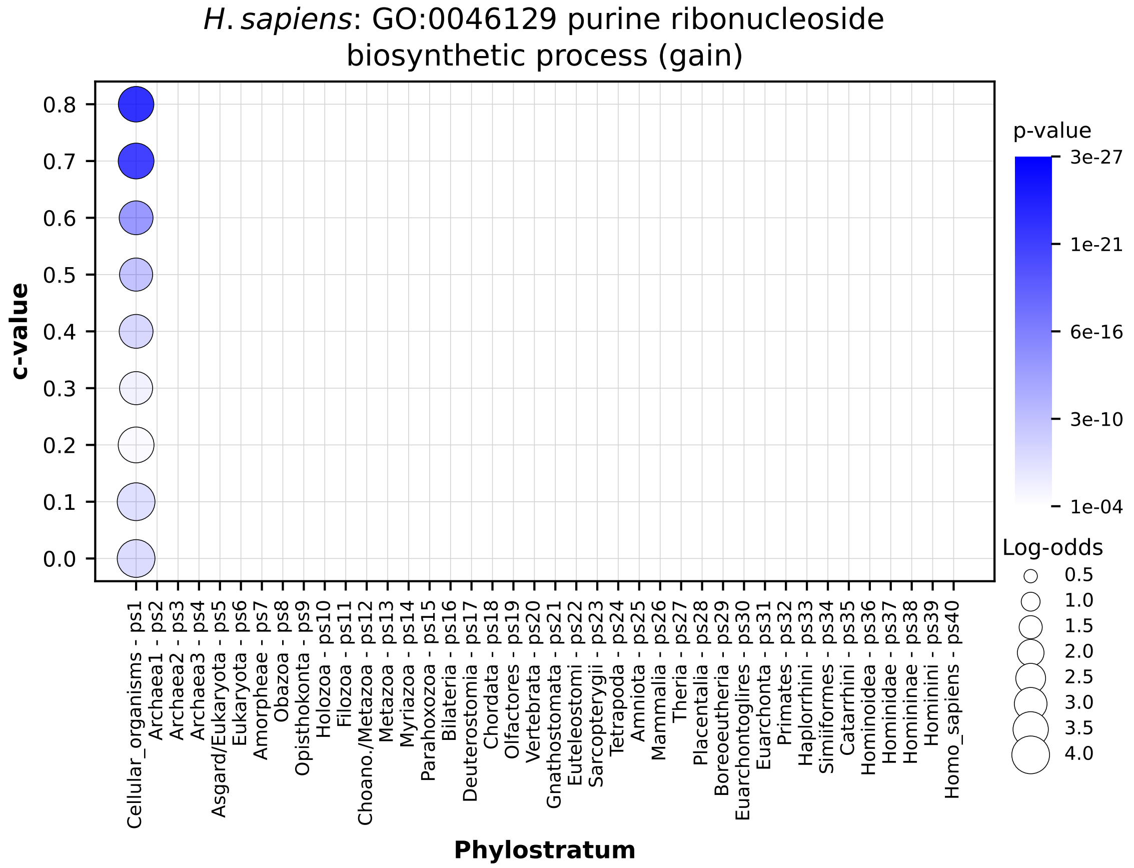
H. sapiens: GO:0046129 purine ribonucleoside biosynthetic process (gain)
c-value
0.8
0.7
0.6
0.5
0.4
0.3
0.2
0.1
0.0
Phylostratum
Cellular_organisms - ps1
Archaea1 - ps2
Archaea2 - ps3
Archaea3 - ps4
Asgard/Eukaryota - ps5
Eukaryota - ps6
Amorpheae - ps7
Obazoa - ps8
Opisthokonta - ps9
Holozoa - ps10
Filozoa - ps11
Choano./Metazoa - ps12
Metazoa - ps13
Myriazoa - ps14
Parahoxozoa - ps15
Bilateria - ps16
Deuterostomia - ps17
Chordata - ps18
Olfactores - ps19
Vertebrata - ps20
Gnathostomata - ps21
Euteleostomi - ps22
Sarcopterygii - ps23
Tetrapoda - ps24
Amniota - ps25
Mammalia - ps26
Theria - ps27
Placentalia - ps28
Boreoeutheria - ps29
Euarchontoglires - ps30
Euarchonta - ps31
Primates - ps32
Haplorrhini - ps33
Simiiformes - ps34
Catarrhini - ps35
Hominoidea - ps36
Hominidae - ps37
Homininae - ps38
Hominini - ps39
Homo_sapiens - ps40
p-value
3e-27
1e-21
6e-16
3e-10
1e-04
Log-odds
0.5
1.0
1.5
2.0
2.5
3.0
3.5
4.0

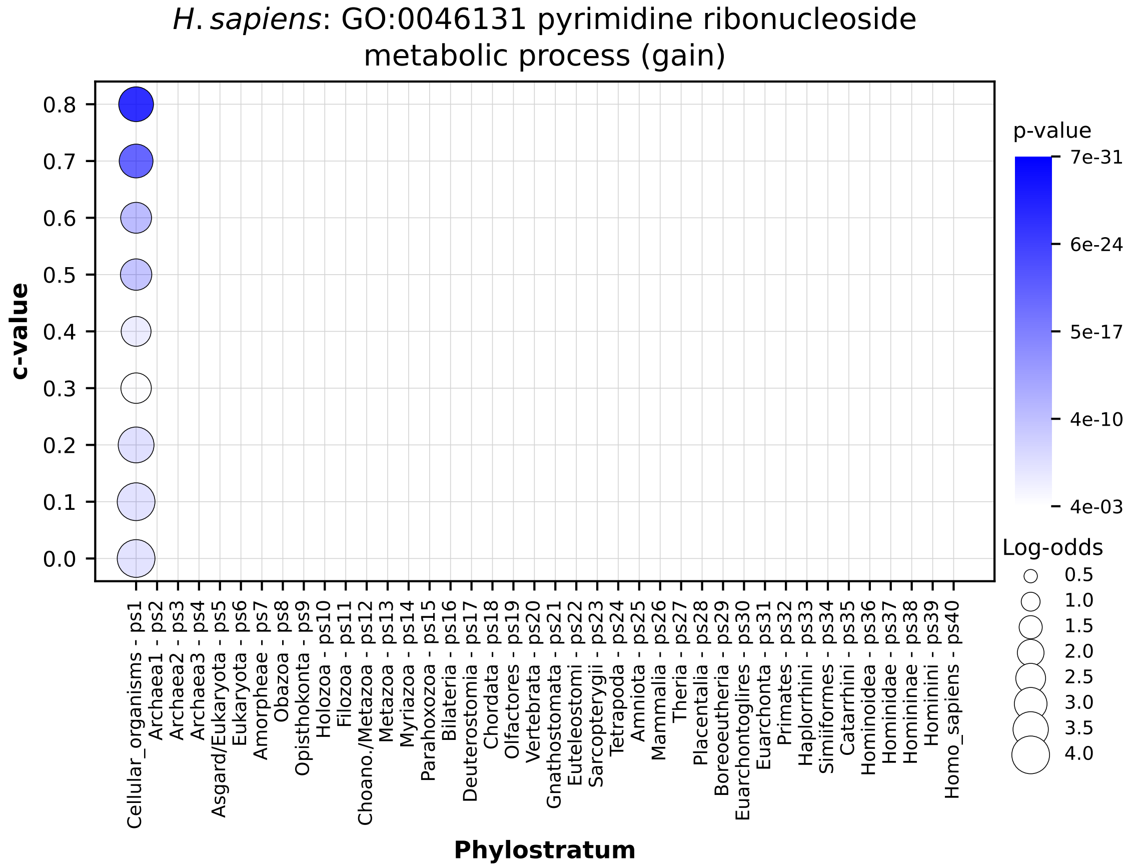

H. sapiens: GO:0046131 pyrimidine ribonucleoside metabolic process (gain)
c-value
0.8
0.7
0.6
0.5
0.4
0.3
0.2
0.1
0.0
p-value
7e-31
6e-24
5e-17
4e-10
4e-03
Log-odds
0.5
1.0
1.5
2.0
2.5
3.0
3.5
4.0
Cellular_organisms - ps1
Archaea1 - ps2
Archaea2 - ps3
Archaea3 - ps4
Asgard/Eukaryota - ps5
Eukaryota - ps6
Amorpheae - ps7
Obazoa - ps8
Opisthokonta - ps9
Holozoa - ps10
Filozoa - ps11
Choano./Metazoa - ps12
Metazoa - ps13
Myriazoa - ps14
Parahoxozoa - ps15
Bilateria - ps16
Deuterostomia - ps17
Chordata - ps18
Olfactores - ps19
Vertebrata - ps20
Gnathostomata - ps21
Euteleostomi - ps22
Sarcopterygii - ps23
Tetrapoda - ps24
Amniota - ps25
Mammalia - ps26
Theria - ps27
Placentalia - ps28
Boreoeutheria - ps29
Euarchontoglires - ps30
Euarchonta - ps31
Primates - ps32
Haplorrhini - ps33
Simiiformes - ps34
Catarrhini - ps35
Hominoidea - ps36
Hominidae - ps37
Homininae - ps38
Hominini - ps39
Homo_sapiens - ps40
Phylostratum

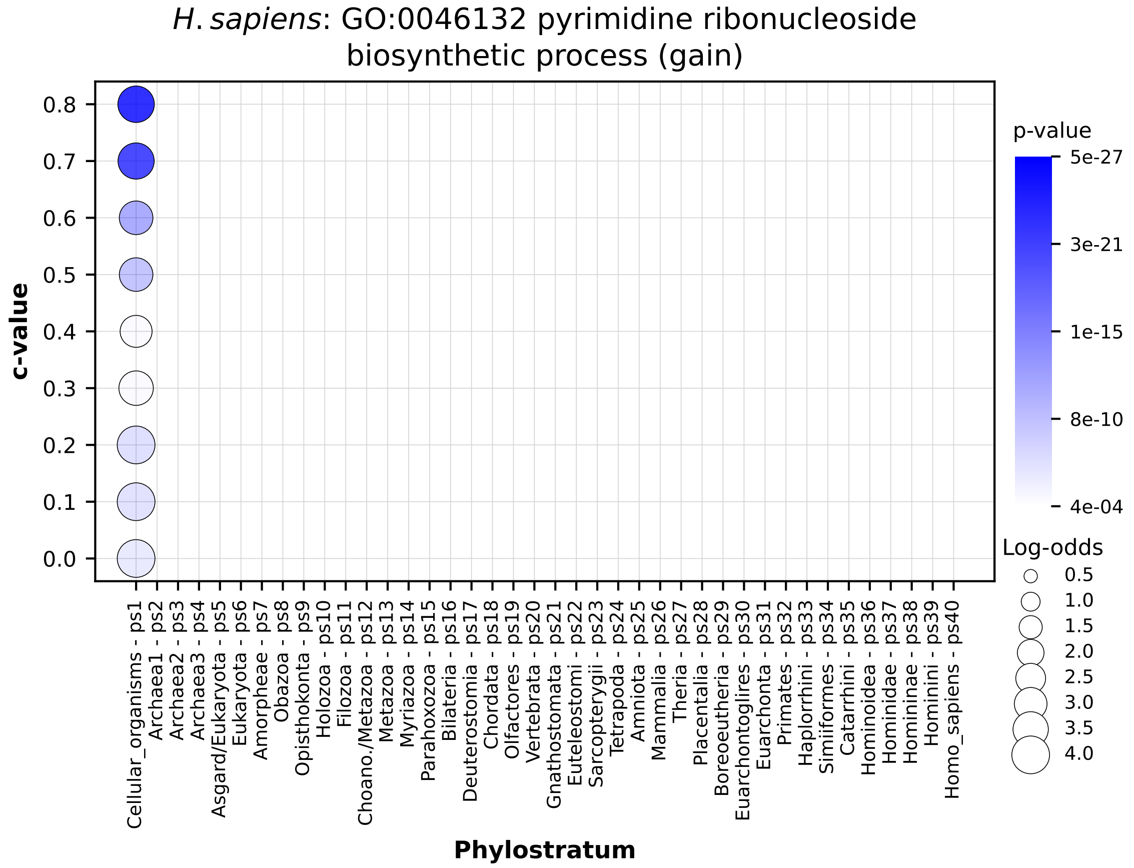

H. sapiens: GO:0046132 pyrimidine ribonucleoside biosynthetic process (gain)
c-value
0.0
0.1
0.2
0.3
0.4
0.5
0.6
0.7
0.8
p-value
5e-27
3e-21
1e-15
8e-10
4e-04
Log-odds
0.5
1.0
1.5
2.0
2.5
3.0
3.5
4.0
Phylostratum
Cellular_organisms - ps1
Archaea1 - ps2
Archaea2 - ps3
Archaea3 - ps4
Asgard/Eukaryota - ps5
Eukaryota - ps6
Amorpheae - ps7
Obazoa - ps8
Opisthokonta - ps9
Holozoa - ps10
Filozoa - ps11
Choano./Metazoa - ps12
Metazoa - ps13
Myriazoa - ps14
Parahoxozoa - ps15
Bilateria - ps16
Deuterostomia - ps17
Chordata - ps18
Olfactores - ps19
Vertebrata - ps20
Gnathostomata - ps21
Euteleostomi - ps22
Sarcopterygii - ps23
Tetrapoda - ps24
Amniota - ps25
Mammalia - ps26
Theria - ps27
Placentalia - ps28
Boreoeutheria - ps29
Euarchontoglires - ps30
Euarchonta - ps31
Primates - ps32
Haplorrhini - ps33
Simiiformes - ps34
Catarrhini - ps35
Hominoidea - ps36
Hominidae - ps37
Homininae - ps38
Hominini - ps39
Homo_sapiens - ps40

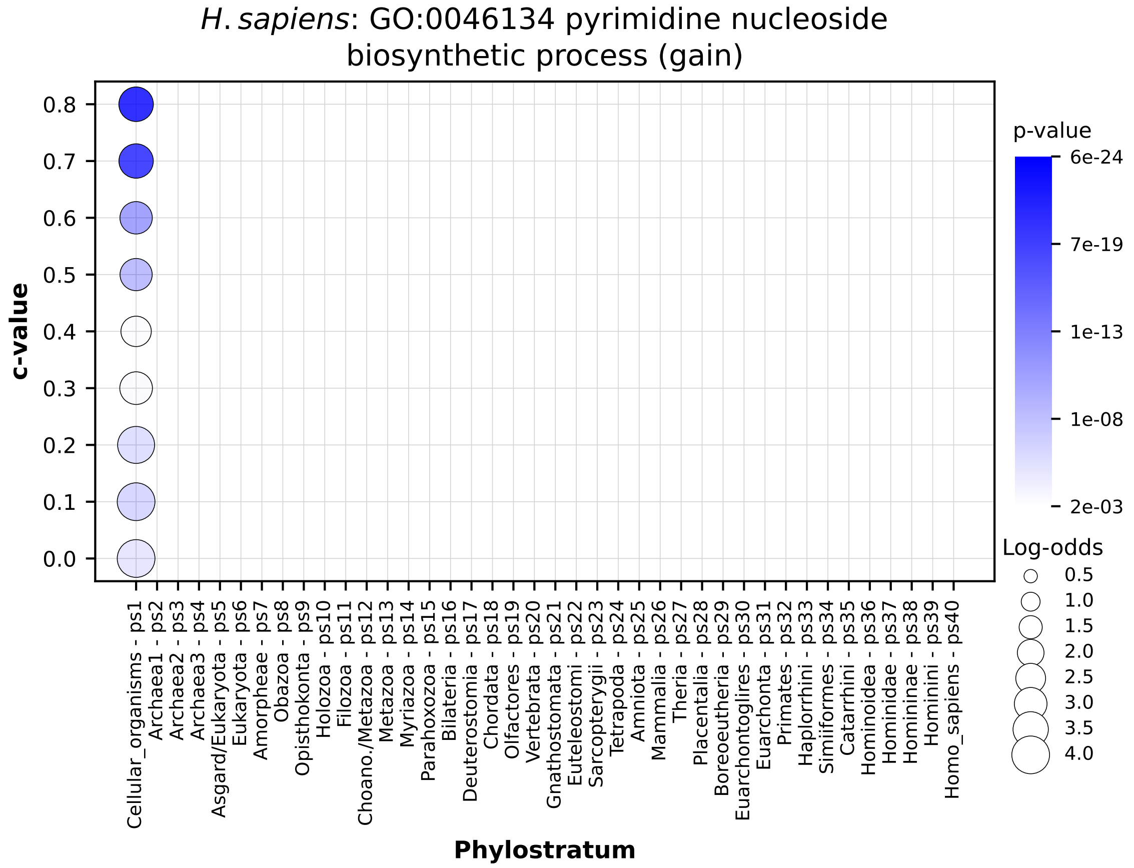

H. sapiens: GO:0046134 pyrimidine nucleoside biosynthetic process (gain)
c-value
0.8
0.7
0.6
0.5
0.4
0.3
0.2
0.1
0.0
p-value
6e-24
7e-19
1e-13
1e-08
2e-03
Log-odds
0.5
1.0
1.5
2.0
2.5
3.0
3.5
4.0
Cellular_organisms - ps1
Archaea1 - ps2
Archaea2 - ps3
Archaea3 - ps4
Asgard/Eukaryota - ps5
Eukaryota - ps6
Amorpheae - ps7
Obazoa - ps8
Opisthokonta - ps9
Holozoa - ps10
Filozoa - ps11
Choano./Metazoa - ps12
Metazoa - ps13
Myriazoa - ps14
Parahoxozoa - ps15
Bilateria - ps16
Deuterostomia - ps17
Chordata - ps18
Olfactores - ps19
Vertebrata - ps20
Gnathostomata - ps21
Euteleostomi - ps22
Sarcopterygii - ps23
Tetrapoda - ps24
Amniota - ps25
Mammalia - ps26
Theria - ps27
Placentalia - ps28
Boreoeutheria - ps29
Euarchontoglires - ps30
Euarchonta - ps31
Primates - ps32
Haplorrhini - ps33
Simiiformes - ps34
Catarrhini - ps35
Hominoidea - ps36
Hominidae - ps37
Homininae - ps38
Hominini - ps39
Homo_sapiens - ps40
Phylostratum

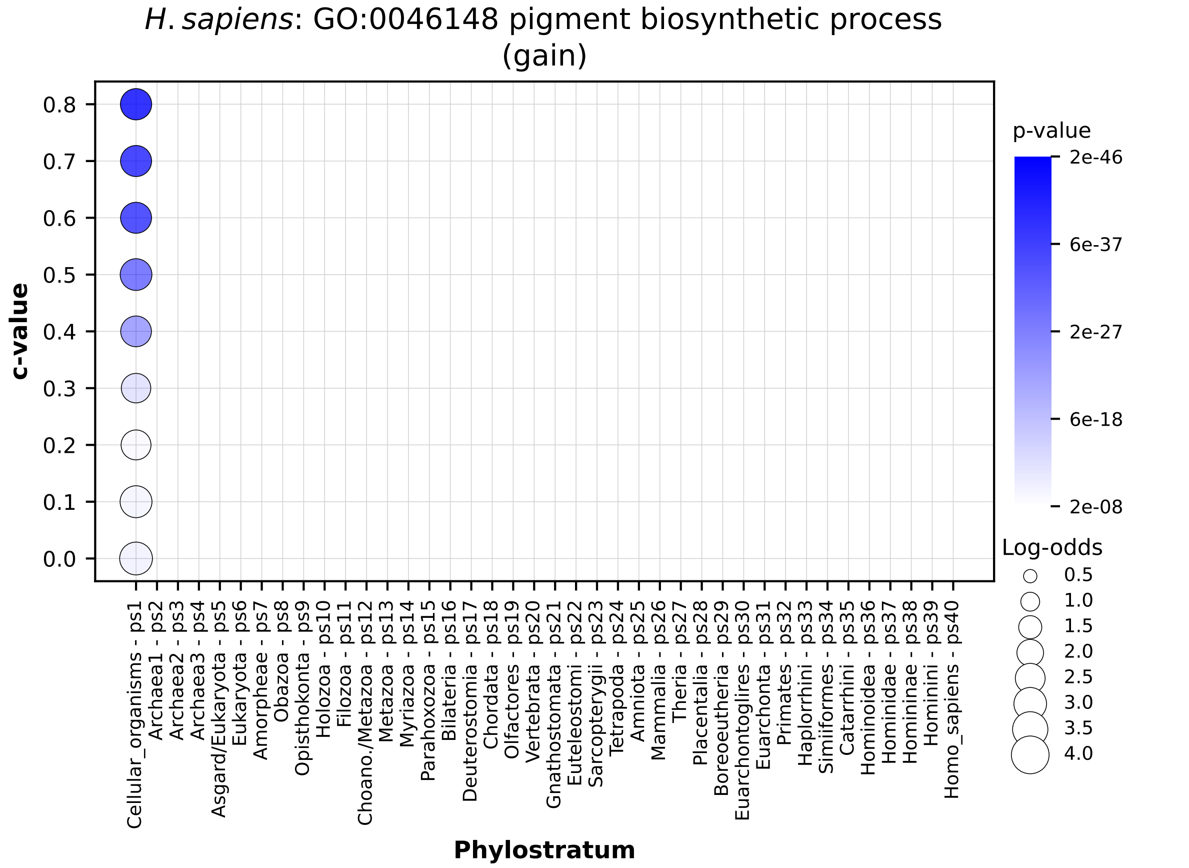
H. sapiens: GO:0046148 pigment biosynthetic process
(gain)
c-value
0.0
0.1
0.2
0.3
0.4
0.5
0.6
0.7
0.8
p-value
2e-46
6e-37
2e-27
6e-18
2e-08
Log-odds
0.5
1.0
1.5
2.0
2.5
3.0
3.5
4.0
Cellular_organisms - ps1
Archaea1 - ps2
Archaea2 - ps3
Archaea3 - ps4
Asgard/Eukaryota - ps5
Eukaryota - ps6
Amorpheae - ps7
Obazoa - ps8
Opisthokonta - ps9
Holozoa - ps10
Filozoa - ps11
Choano./Metazoa - ps12
Metazoa - ps13
Myriazoa - ps14
Parahoxozoa - ps15
Bilateria - ps16
Deuterostomia - ps17
Chordata - ps18
Olfactores - ps19
Vertebrata - ps20
Gnathostomata - ps21
Euteleostomi - ps22
Sarcopterygii - ps23
Tetrapoda - ps24
Amniota - ps25
Mammalia - ps26
Theria - ps27
Placentalia - ps28
Boreoeutheria - ps29
Euarchontoglires - ps30
Euarchonta - ps31
Primates - ps32
Haplorrhini - ps33
Simiiformes - ps34
Catarrhini - ps35
Hominoidea - ps36
Hominidae - ps37
Homininae - ps38
Hominini - ps39
Homo_sapiens - ps40
Phylostratum

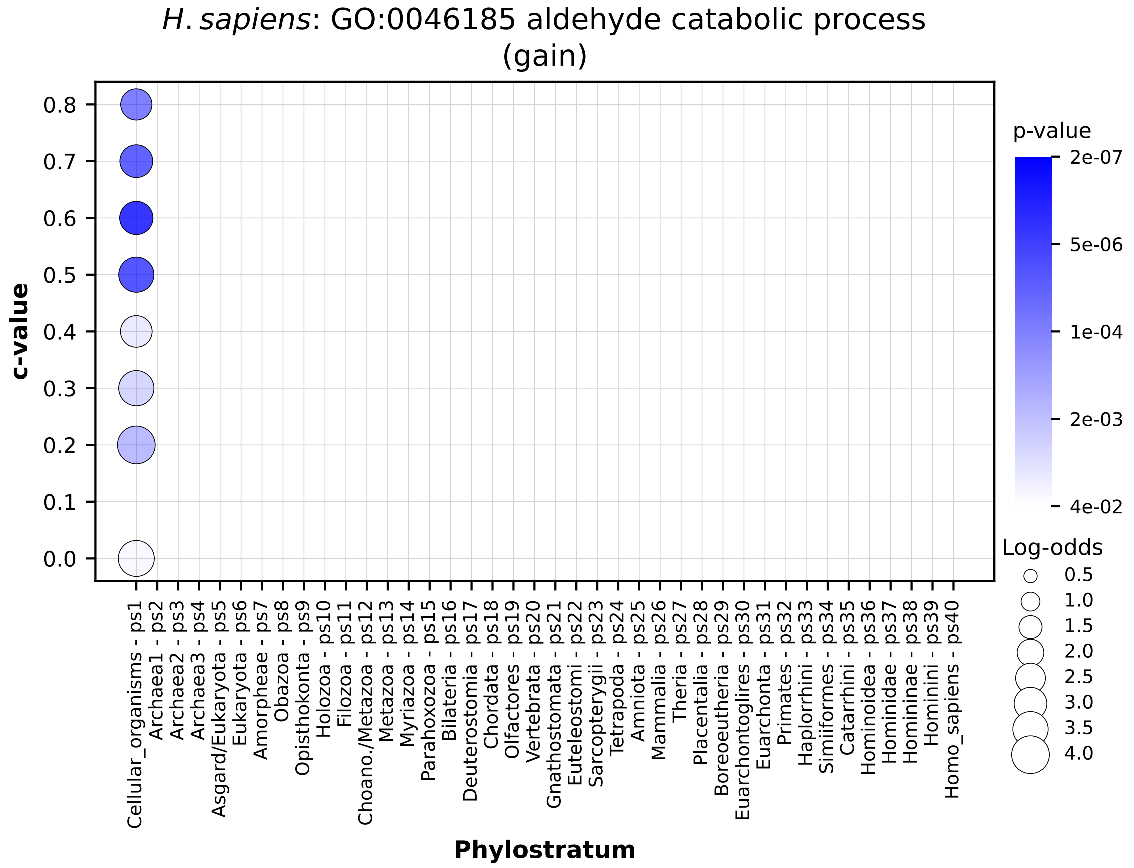

*H. sapiens*: GO:0046185 aldehyde catabolic process
(gain)
c-value
0.8
0.7
0.6
0.5
0.4
0.3
0.2
0.1
0.0
p-value
2e-07
5e-06
1e-04
2e-03
4e-02
Log-odds
0.5
1.0
1.5
2.0
2.5
3.0
3.5
4.0
Cellular_organisms - ps1
Archaea1 - ps2
Archaea2 - ps3
Archaea3 - ps4
Asgard/Eukaryota - ps5
Eukaryota - ps6
Amorpheae - ps7
Obazoa - ps8
Opisthokonta - ps9
Holozoa - ps10
Filozoa - ps11
Choano./Metazoa - ps12
Metazoa - ps13
Myriazoa - ps14
Parahoxozoa - ps15
Bilateria - ps16
Deuterostomia - ps17
Chordata - ps18
Olfactores - ps19
Vertebrata - ps20
Gnathostomata - ps21
Euteleostomi - ps22
Sarcopterygii - ps23
Tetrapoda - ps24
Amniota - ps25
Mammalia - ps26
Theria - ps27
Placentalia - ps28
Boreoeutheria - ps29
Euarchontoglires - ps30
Euarchonta - ps31
Primates - ps32
Haplorrhini - ps33
Simiiformes - ps34
Catarrhini - ps35
Hominoidea - ps36
Hominidae - ps37
Homininae - ps38
Hominini - ps39
Homo_sapiens - ps40
Phylostratum

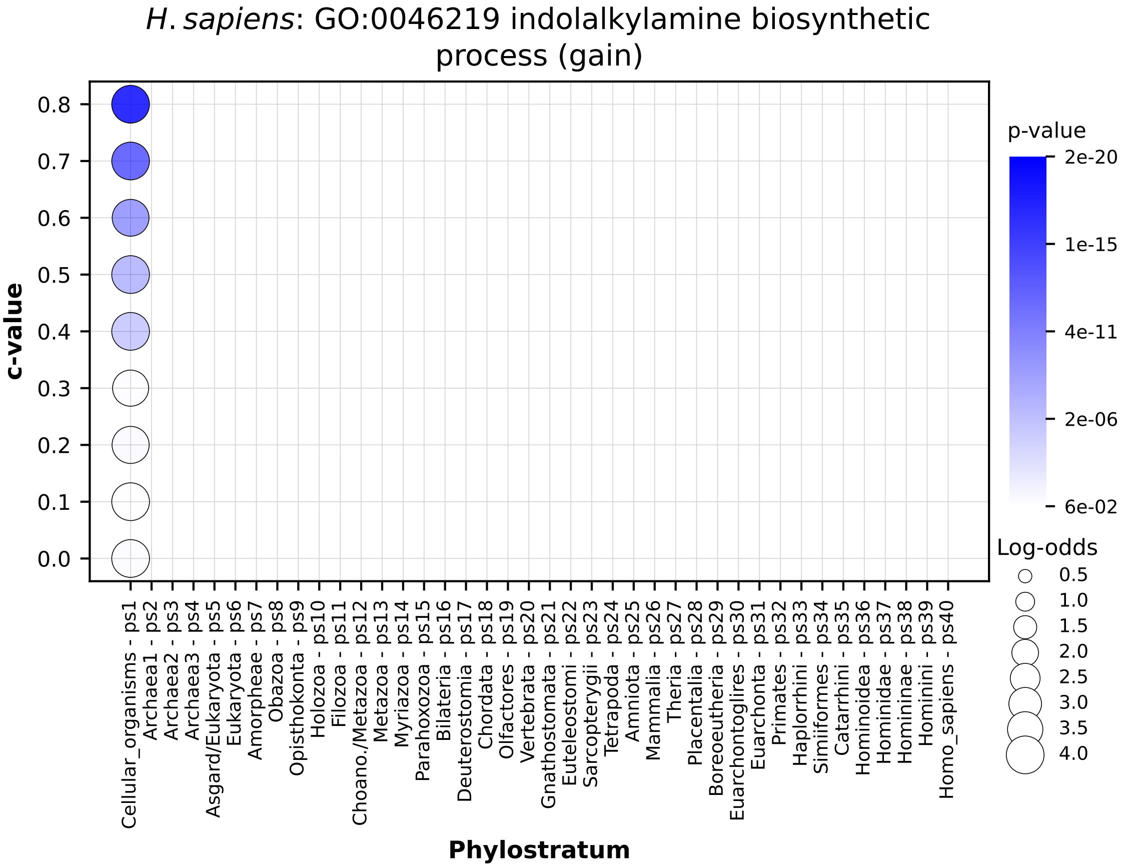

H. sapiens: GO:0046219 indolalkylamine biosynthetic process (gain)
c-value
0.0
0.1
0.2
0.3
0.4
0.5
0.6
0.7
0.8
Phylostratum
Cellular_organisms - ps1
Archaea1 - ps2
Archaea2 - ps3
Archaea3 - ps4
Asgard/Eukaryota - ps5
Eukaryota - ps6
Amorpheae - ps7
Obazoa - ps8
Opisthokonta - ps9
Holozoa - ps10
Filozoa - ps11
Choano./Metazoa - ps12
Metazoa - ps13
Myriazoa - ps14
Parahoxozoa - ps15
Bilateria - ps16
Deuterostomia - ps17
Chordata - ps18
Olfactores - ps19
Vertebrata - ps20
Gnathostomata - ps21
Euteleostomi - ps22
Sarcopterygii - ps23
Tetrapoda - ps24
Amniota - ps25
Mammalia - ps26
Theria - ps27
Placentalia - ps28
Boreoeutheria - ps29
Euarchontoglires - ps30
Euarchonta - ps31
Primates - ps32
Haplorrhini - ps33
Simiiformes - ps34
Catarrhini - ps35
Hominoidea - ps36
Hominidae - ps37
Homininae - ps38
Hominini - ps39
Homo_sapiens - ps40
p-value
2e-20
1e-15
4e-11
2e-06
6e-02
Log-odds
0.5
1.0
1.5
2.0
2.5
3.0
3.5
4.0

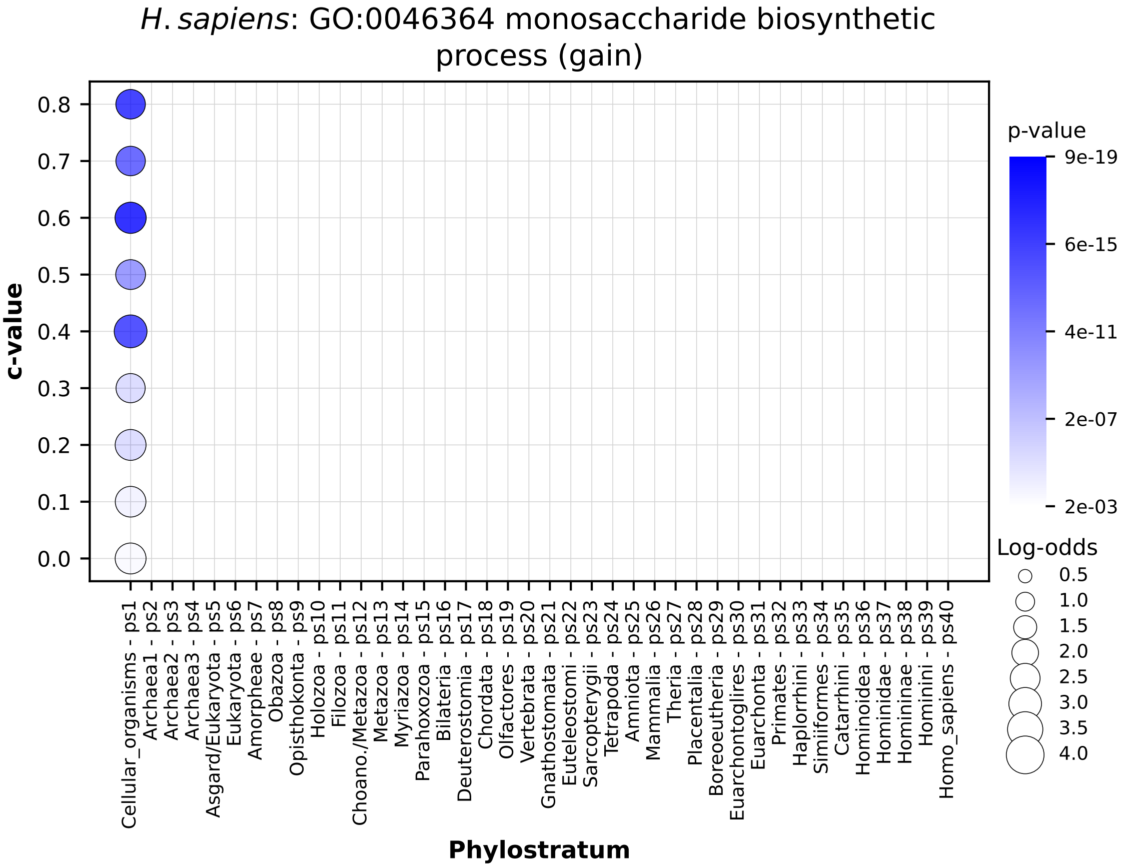

H. sapiens: GO:0046364 monosaccharide biosynthetic process (gain)
c-value
0.0
0.1
0.2
0.3
0.4
0.5
0.6
0.7
0.8
Phylostratum
Cellular_organisms - ps1
Archaea1 - ps2
Archaea2 - ps3
Archaea3 - ps4
Asgard/Eukaryota - ps5
Eukaryota - ps6
Amorpheae - ps7
Obazoa - ps8
Opisthokonta - ps9
Holozoa - ps10
Filozoa - ps11
Choano./Metazoa - ps12
Metazoa - ps13
Myriazoa - ps14
Parahoxozoa - ps15
Bilateria - ps16
Deuterostomia - ps17
Chordata - ps18
Olfactores - ps19
Vertebrata - ps20
Gnathostomata - ps21
Euteleostomi - ps22
Sarcopterygii - ps23
Tetrapoda - ps24
Amniota - ps25
Mammalia - ps26
Theria - ps27
Placentalia - ps28
Boreoeutheria - ps29
Euarchontoglires - ps30
Euarchonta - ps31
Primates - ps32
Haplorrhini - ps33
Simiiformes - ps34
Catarrhini - ps35
Hominoidea - ps36
Hominidae - ps37
Homininae - ps38
Hominini - ps39
Homo_sapiens - ps40
p-value
9e-19
6e-15
4e-11
2e-07
2e-03
Log-odds
0.5
1.0
1.5
2.0
2.5
3.0
3.5
4.0

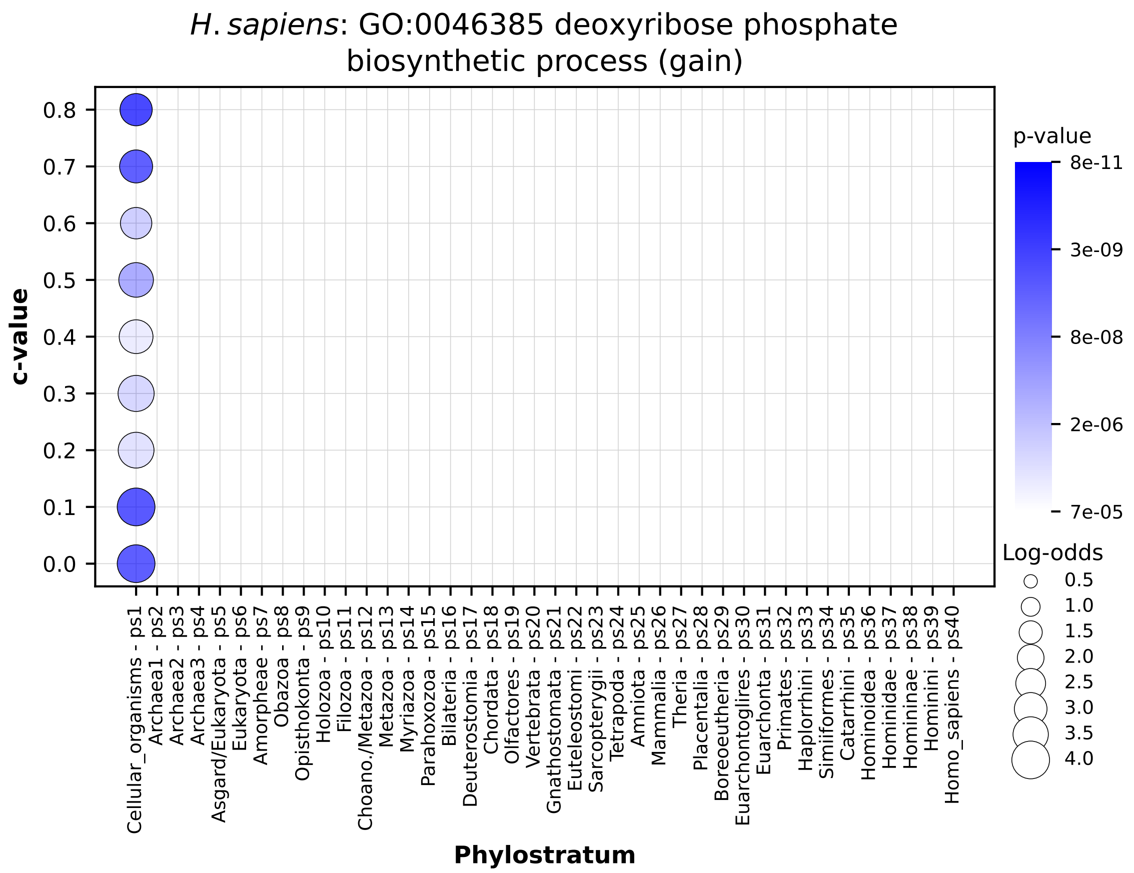
H. sapiens: GO:0046385 deoxyribose phosphate biosynthetic process (gain)
c-value
0.0
0.1
0.2
0.3
0.4
0.5
0.6
0.7
0.8
Phylostratum
Cellular_organisms - ps1
Archaea1 - ps2
Archaea2 - ps3
Archaea3 - ps4
Asgard/Eukaryota - ps5
Eukaryota - ps6
Amorpheae - ps7
Obazoa - ps8
Opisthokonta - ps9
Holozoa - ps10
Filozoa - ps11
Choano./Metazoa - ps12
Metazoa - ps13
Myriazoa - ps14
Parahoxozoa - ps15
Bilateria - ps16
Deuterostomia - ps17
Chordata - ps18
Olfactores - ps19
Vertebrata - ps20
Gnathostomata - ps21
Euteleostomi - ps22
Sarcopterygii - ps23
Tetrapoda - ps24
Amniota - ps25
Mammalia - ps26
Theria - ps27
Placentalia - ps28
Boreoeutheria - ps29
Euarchontoglires - ps30
Euarchonta - ps31
Primates - ps32
Haplorrhini - ps33
Simiiformes - ps34
Catarrhini - ps35
Hominoidea - ps36
Hominidae - ps37
Homininae - ps38
Hominini - ps39
Homo_sapiens - ps40
p-value
8e-11
3e-09
8e-08
2e-06
7e-05
Log-odds
0.5
1.0
1.5
2.0
2.5
3.0
3.5
4.0

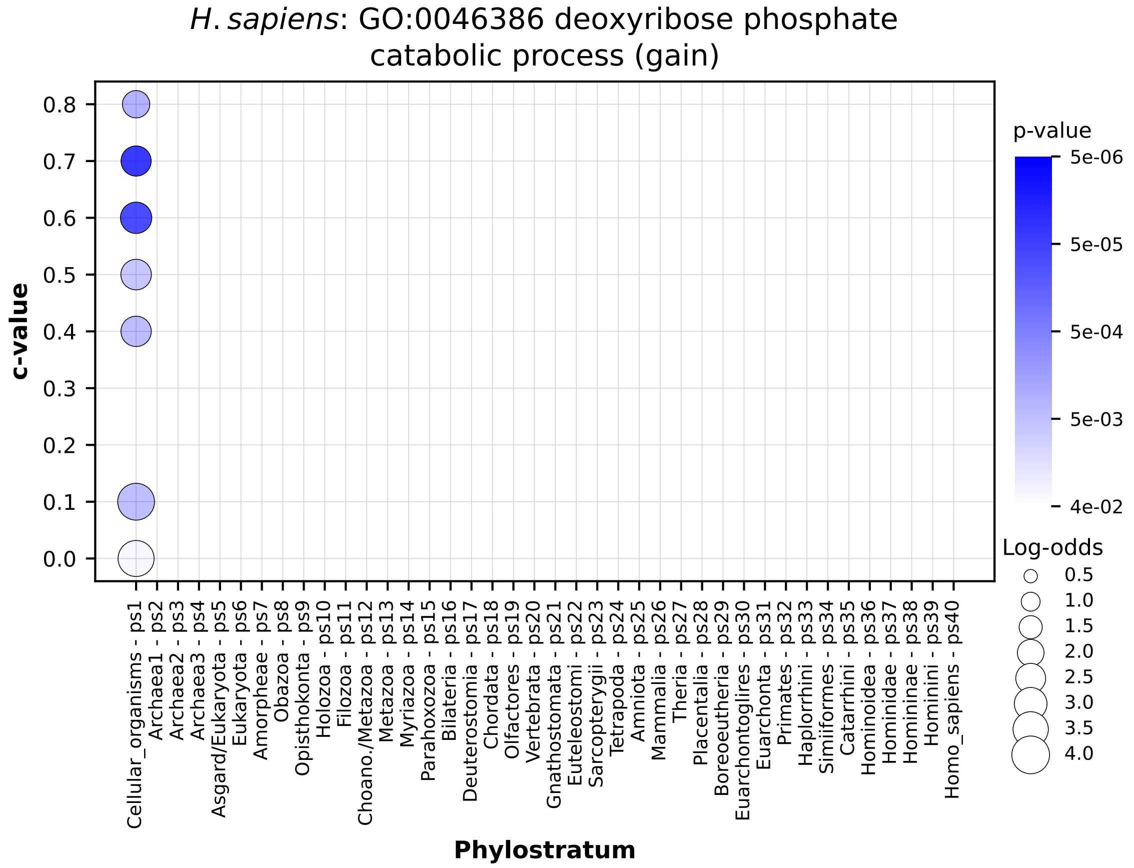

H. sapiens: GO:0046386 deoxyribose phosphate catabolic process (gain)
c-value
0.0
0.1
0.2
0.3
0.4
0.5
0.6
0.7
0.8
Phylostratum
Cellular_organisms - ps1
Archaea1 - ps2
Archaea2 - ps3
Archaea3 - ps4
Asgard/Eukaryota - ps5
Eukaryota - ps6
Amorpheae - ps7
Obazoa - ps8
Opisthokonta - ps9
Holozoa - ps10
Filozoa - ps11
Choano./Metazoa - ps12
Metazoa - ps13
Myriazoa - ps14
Parahoxozoa - ps15
Bilateria - ps16
Deuterostomia - ps17
Chordata - ps18
Olfactores - ps19
Vertebrata - ps20
Gnathostomata - ps21
Euteleostomi - ps22
Sarcopterygii - ps23
Tetrapoda - ps24
Amniota - ps25
Mammalia - ps26
Theria - ps27
Placentalia - ps28
Boreoeutheria - ps29
Euarchontoglires - ps30
Euarchonta - ps31
Primates - ps32
Haplorrhini - ps33
Simiiformes - ps34
Catarrhini - ps35
Hominoidea - ps36
Hominidae - ps37
Homininae - ps38
Hominini - ps39
Homo_sapiens - ps40
p-value
5e-06
5e-05
5e-04
5e-03
4e-02
Log-odds
0.5
1.0
1.5
2.0
2.5
3.0
3.5
4.0

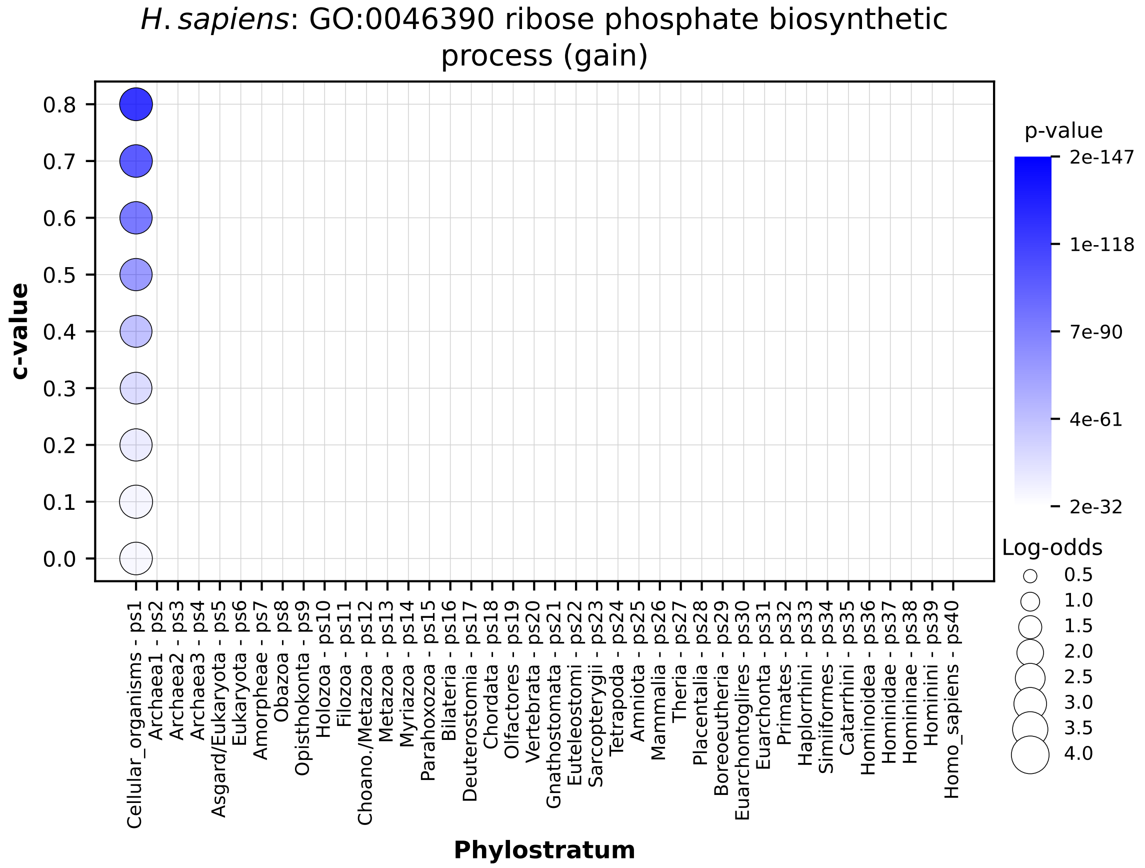
H. sapiens: GO:0046390 ribose phosphate biosynthetic process (gain)
c-value
0.8
0.7
0.6
0.5
0.4
0.3
0.2
0.1
0.0
p-value
2e-147
1e-118
7e-90
4e-61
2e-32
Log-odds
0.5
1.0
1.5
2.0
2.5
3.0
3.5
4.0
Cellular_organisms - ps1
Archaea1 - ps2
Archaea2 - ps3
Archaea3 - ps4
Asgard/Eukaryota - ps5
Eukaryota - ps6
Amorpheae - ps7
Obazoa - ps8
Opisthokonta - ps9
Holozoa - ps10
Filozoa - ps11
Choano./Metazoa - ps12
Metazoa - ps13
Myriazoa - ps14
Parahoxozoa - ps15
Bilateria - ps16
Deuterostomia - ps17
Chordata - ps18
Olfactores - ps19
Vertebrata - ps20
Gnathostomata - ps21
Euteleostomi - ps22
Sarcopterygii - ps23
Tetrapoda - ps24
Amniota - ps25
Mammalia - ps26
Theria - ps27
Placentalia - ps28
Boreoeutheria - ps29
Euarchontoglires - ps30
Euarchonta - ps31
Primates - ps32
Haplorrhini - ps33
Simiiformes - ps34
Catarrhini - ps35
Hominoidea - ps36
Hominidae - ps37
Homininae - ps38
Hominini - ps39
Homo_sapiens - ps40
Phylostratum

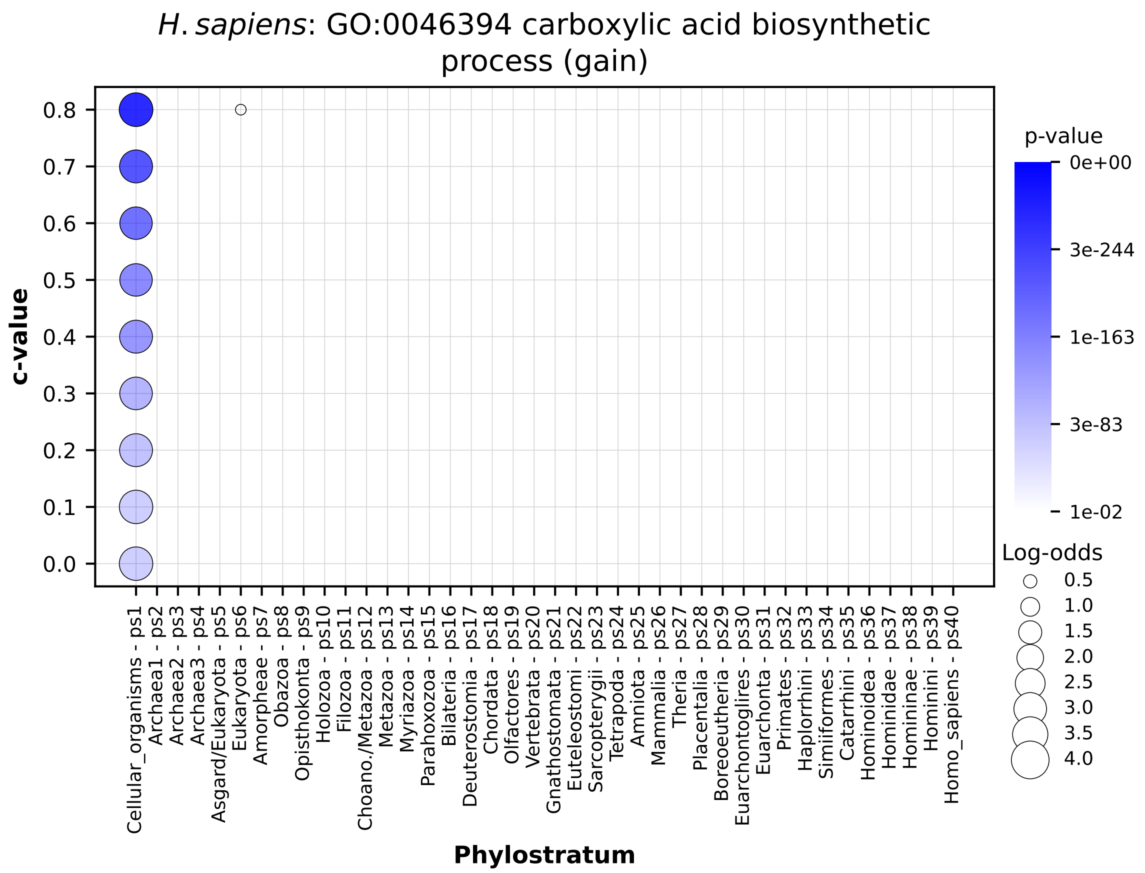

H. sapiens: GO:0046394 carboxylic acid biosynthetic process (gain)
c-value
0.8
0.7
0.6
0.5
0.4
0.3
0.2
0.1
0.0
Phylostratum
Cellular_organisms - ps1
Archaea1 - ps2
Archaea2 - ps3
Archaea3 - ps4
Asgard/Eukaryota - ps5
Eukaryota - ps6
Amorpheae - ps7
Obazoa - ps8
Opisthokonta - ps9
Holozoa - ps10
Filozoa - ps11
Choano./Metazoa - ps12
Metazoa - ps13
Myriazoa - ps14
Parahoxozoa - ps15
Bilateria - ps16
Deuterostomia - ps17
Chordata - ps18
Olfactores - ps19
Vertebrata - ps20
Gnathostomata - ps21
Euteleostomi - ps22
Sarcopterygii - ps23
Tetrapoda - ps24
Amniota - ps25
Mammalia - ps26
Theria - ps27
Placentalia - ps28
Boreoeutheria - ps29
Euarchontoglires - ps30
Euarchonta - ps31
Primates - ps32
Haplorrhini - ps33
Simiiformes - ps34
Catarrhini - ps35
Hominoidea - ps36
Hominidae - ps37
Homininae - ps38
Hominini - ps39
Homo_sapiens - ps40
p-value
0e+00
3e-244
1e-163
3e-83
1e-02
Log-odds
0.5
1.0
1.5
2.0
2.5
3.0
3.5
4.0

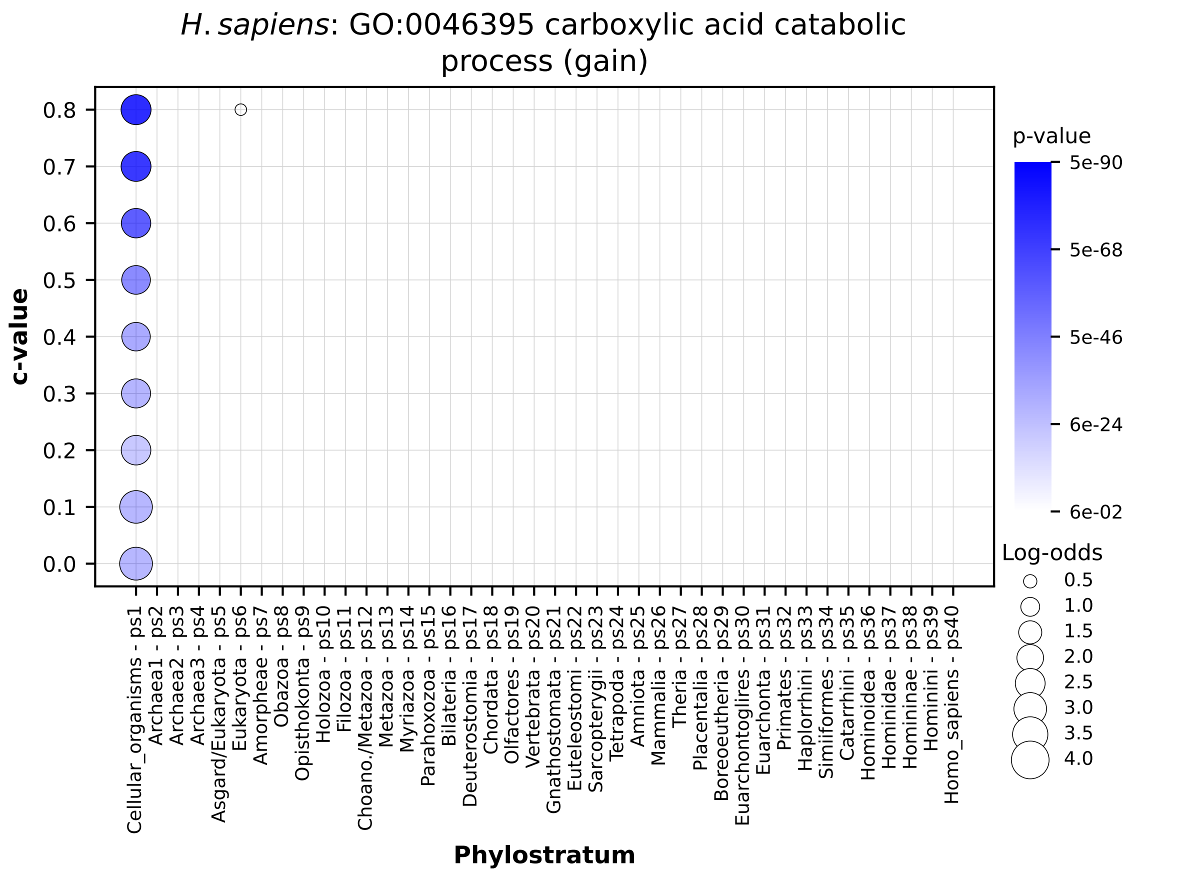
H. sapiens: GO:0046395 carboxylic acid catabolic process (gain)
c-value
0.8
0.7
0.6
0.5
0.4
0.3
0.2
0.1
0.0
p-value
5e-90
5e-68
5e-46
6e-24
6e-02
Log-odds
0.5
1.0
1.5
2.0
2.5
3.0
3.5
4.0
Cellular_organisms - ps1
Archaea1 - ps2
Archaea2 - ps3
Archaea3 - ps4
Asgard/Eukaryota - ps5
Eukaryota - ps6
Amorpheae - ps7
Obazoa - ps8
Opisthokonta - ps9
Holozoa - ps10
Filozoa - ps11
Choano./Metazoa - ps12
Metazoa - ps13
Myriazoa - ps14
Parahoxozoa - ps15
Bilateria - ps16
Deuterostomia - ps17
Chordata - ps18
Olfactores - ps19
Vertebrata - ps20
Gnathostomata - ps21
Euteleostomi - ps22
Sarcopterygii - ps23
Tetrapoda - ps24
Amniota - ps25
Mammalia - ps26
Theria - ps27
Placentalia - ps28
Boreoeutheria - ps29
Euarchontoglires - ps30
Euarchonta - ps31
Primates - ps32
Haplorrhini - ps33
Simiiformes - ps34
Catarrhini - ps35
Hominoidea - ps36
Hominidae - ps37
Homininae - ps38
Hominini - ps39
Homo_sapiens - ps40
Phylostratum

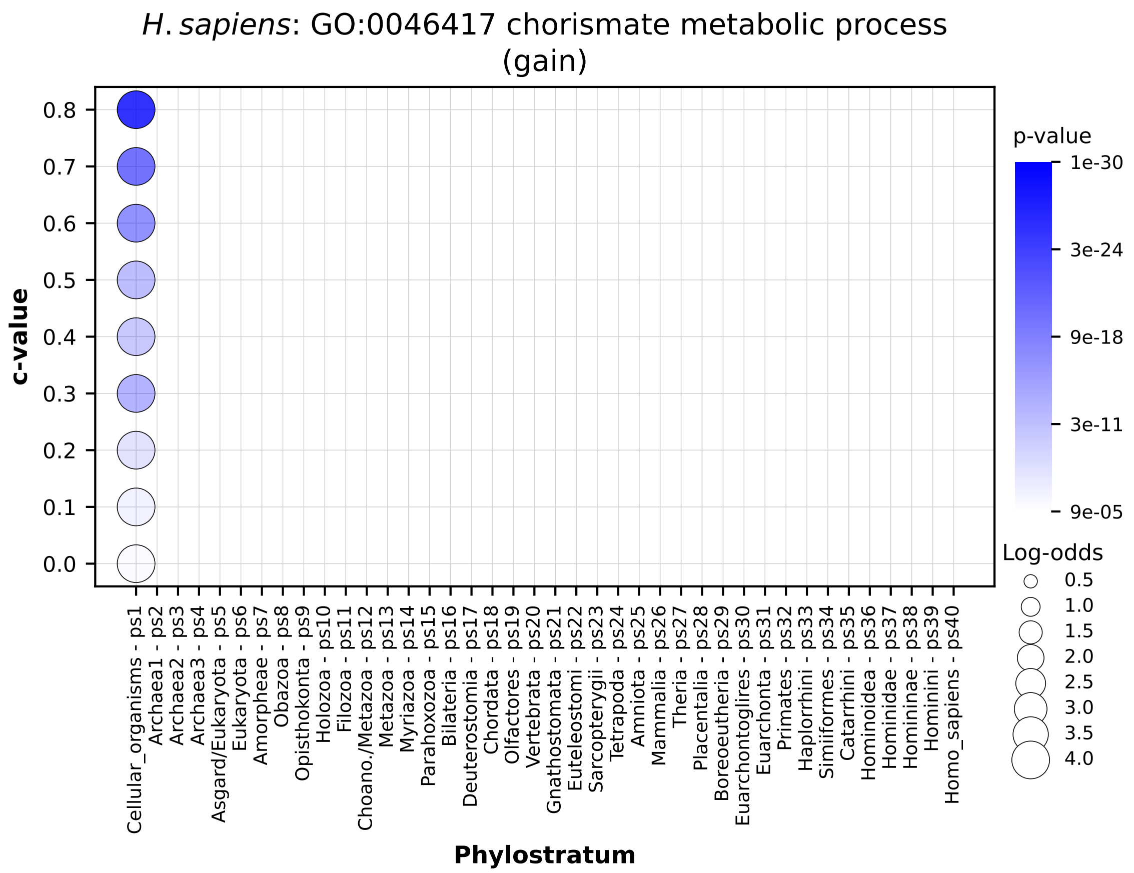
H. sapiens: GO:0046417 chorismate metabolic process
(gain)
c-value
0.8
0.7
0.6
0.5
0.4
0.3
0.2
0.1
0.0
p-value
1e-30
3e-24
9e-18
3e-11
9e-05
Log-odds
0.5
1.0
1.5
2.0
2.5
3.0
3.5
4.0
Cellular_organisms - ps1
Archaea1 - ps2
Archaea2 - ps3
Archaea3 - ps4
Asgard/Eukaryota - ps5
Eukaryota - ps6
Amorpheae - ps7
Obazoa - ps8
Opisthokonta - ps9
Holozoa - ps10
Filozoa - ps11
Choano./Metazoa - ps12
Metazoa - ps13
Myriazoa - ps14
Parahoxozoa - ps15
Bilateria - ps16
Deuterostomia - ps17
Chordata - ps18
Olfactores - ps19
Vertebrata - ps20
Gnathostomata - ps21
Euteleostomi - ps22
Sarcopterygii - ps23
Tetrapoda - ps24
Amniota - ps25
Mammalia - ps26
Theria - ps27
Placentalia - ps28
Boreoeutheria - ps29
Euarchontoglires - ps30
Euarchonta - ps31
Primates - ps32
Haplorrhini - ps33
Simiiformes - ps34
Catarrhini - ps35
Hominoidea - ps36
Hominidae - ps37
Homininae - ps38
Hominini - ps39
Homo_sapiens - ps40
Phylostratum

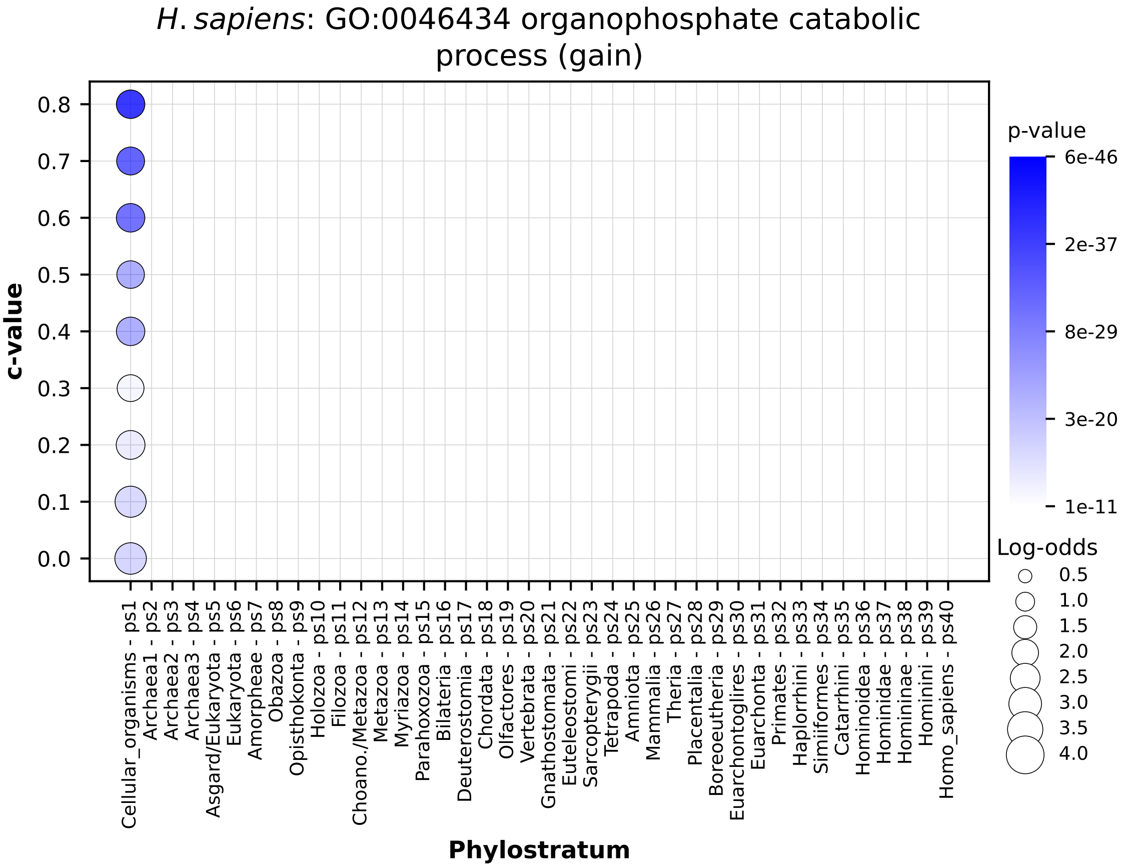
H. sapiens: GO:0046434 organophosphate catabolic process (gain)
c-value
0.8
0.7
0.6
0.5
0.4
0.3
0.2
0.1
0.0
Phylostratum
Cellular_organisms - ps1
Archaea1 - ps2
Archaea2 - ps3
Archaea3 - ps4
Asgard/Eukaryota - ps5
Eukaryota - ps6
Amorpheae - ps7
Obazoa - ps8
Opisthokonta - ps9
Holozoa - ps10
Filozoa - ps11
Choano./Metazoa - ps12
Metazoa - ps13
Myriazoa - ps14
Parahoxozoa - ps15
Bilateria - ps16
Deuterostomia - ps17
Chordata - ps18
Olfactores - ps19
Vertebrata - ps20
Gnathostomata - ps21
Euteleostomi - ps22
Sarcopterygii - ps23
Tetrapoda - ps24
Amniota - ps25
Mammalia - ps26
Theria - ps27
Placentalia - ps28
Boreoeutheria - ps29
Euarchontoglires - ps30
Euarchonta - ps31
Primates - ps32
Haplorrhini - ps33
Simiiformes - ps34
Catarrhini - ps35
Hominoidea - ps36
Hominidae - ps37
Homininae - ps38
Hominini - ps39
Homo_sapiens - ps40
p-value
6e-46
2e-37
8e-29
3e-20
1e-11
Log-odds
0.5
1.0
1.5
2.0
2.5
3.0
3.5
4.0

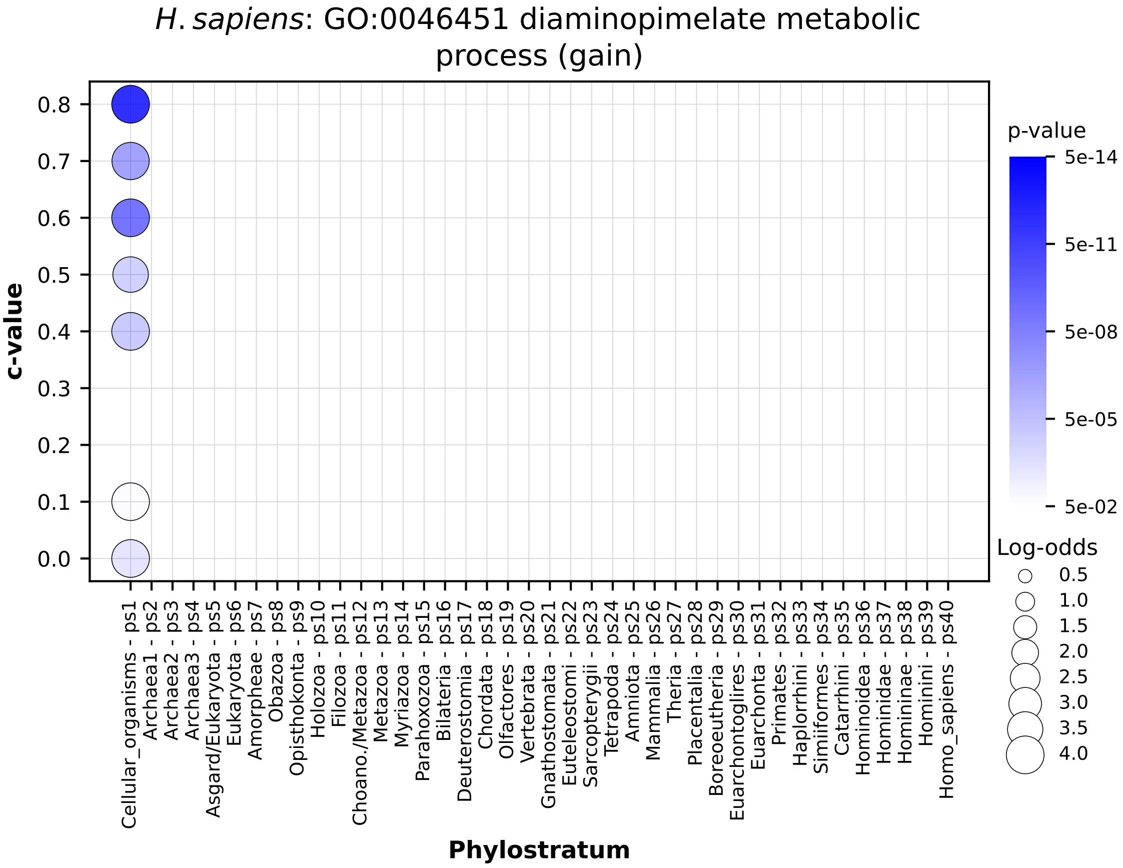

H. sapiens: GO:0046451 diaminopimelate metabolic process (gain)
c-value
0.8
0.7
0.6
0.5
0.4
0.3
0.2
0.1
0.0
p-value
5e-14
5e-11
5e-08
5e-05
5e-02
Log-odds
0.5
1.0
1.5
2.0
2.5
3.0
3.5
4.0
Cellular_organisms - ps1
Archaea1 - ps2
Archaea2 - ps3
Archaea3 - ps4
Asgard/Eukaryota - ps5
Eukaryota - ps6
Amorpheae - ps7
Obazoa - ps8
Opisthokonta - ps9
Holozoa - ps10
Filozoa - ps11
Choano./Metazoa - ps12
Metazoa - ps13
Myriazoa - ps14
Parahoxozoa - ps15
Bilateria - ps16
Deuterostomia - ps17
Chordata - ps18
Olfactores - ps19
Vertebrata - ps20
Gnathostomata - ps21
Euteleostomi - ps22
Sarcopterygii - ps23
Tetrapoda - ps24
Amniota - ps25
Mammalia - ps26
Theria - ps27
Placentalia - ps28
Boreoeutheria - ps29
Euarchontoglires - ps30
Euarchonta - ps31
Primates - ps32
Haplorrhini - ps33
Simiiformes - ps34
Catarrhini - ps35
Hominoidea - ps36
Hominidae - ps37
Homininae - ps38
Hominini - ps39
Homo_sapiens - ps40
Phylostratum

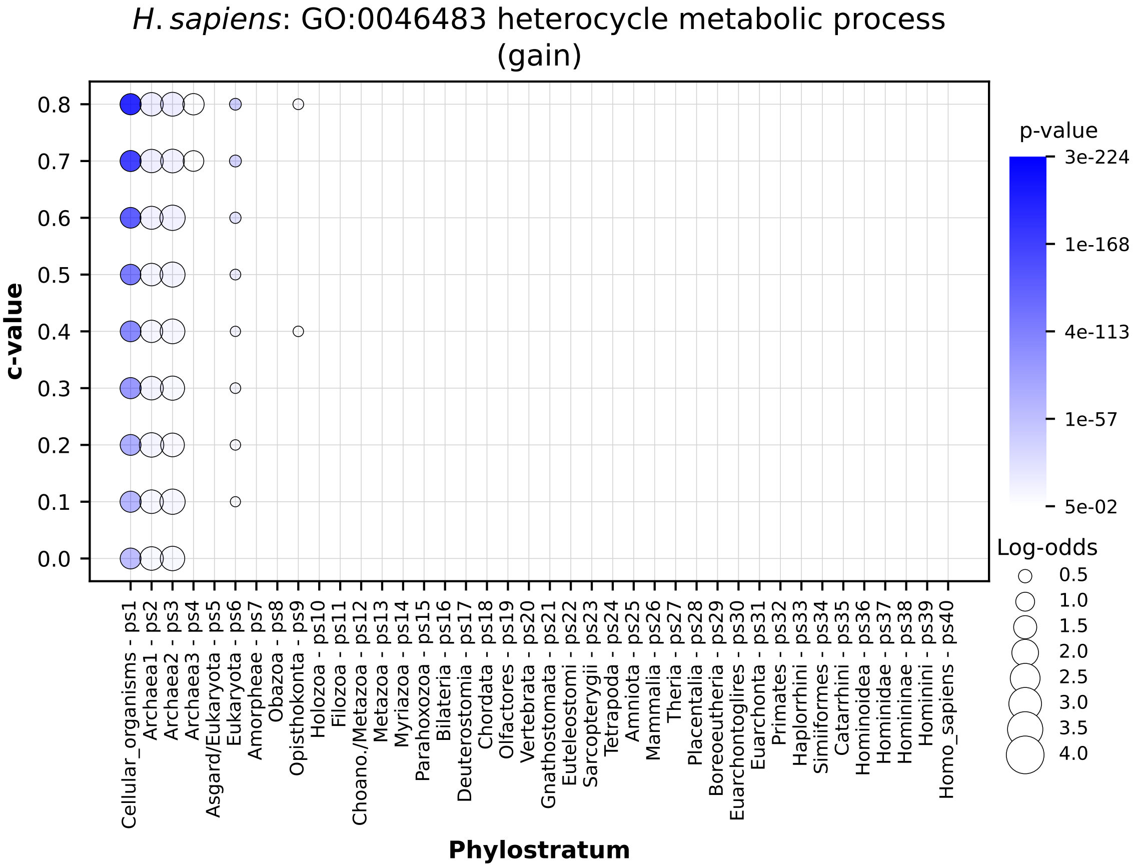
H. sapiens: GO:0046483 heterocycle metabolic process
(gain)
c-value
0.8
0.7
0.6
0.5
0.4
0.3
0.2
0.1
0.0
p-value
3e-224
1e-168
4e-113
1e-57
5e-02
Log-odds
0.5
1.0
1.5
2.0
2.5
3.0
3.5
4.0
Cellular_organisms - ps1
Archaea1 - ps2
Archaea2 - ps3
Archaea3 - ps4
Asgard/Eukaryota - ps5
Eukaryota - ps6
Amorpheae - ps7
Obazoa - ps8
Opisthokonta - ps9
Holozoa - ps10
Filozoa - ps11
Choano./Metazoa - ps12
Metazoa - ps13
Myriazoa - ps14
Parahoxozoa - ps15
Bilateria - ps16
Deuterostomia - ps17
Chordata - ps18
Olfactores - ps19
Vertebrata - ps20
Gnathostomata - ps21
Euteleostomi - ps22
Sarcopterygii - ps23
Tetrapoda - ps24
Amniota - ps25
Mammalia - ps26
Theria - ps27
Placentalia - ps28
Boreoeutheria - ps29
Euarchontoglires - ps30
Euarchonta - ps31
Primates - ps32
Haplorrhini - ps33
Simiiformes - ps34
Catarrhini - ps35
Hominoidea - ps36
Hominidae - ps37
Homininae - ps38
Hominini - ps39
Homo_sapiens - ps40
Phylostratum

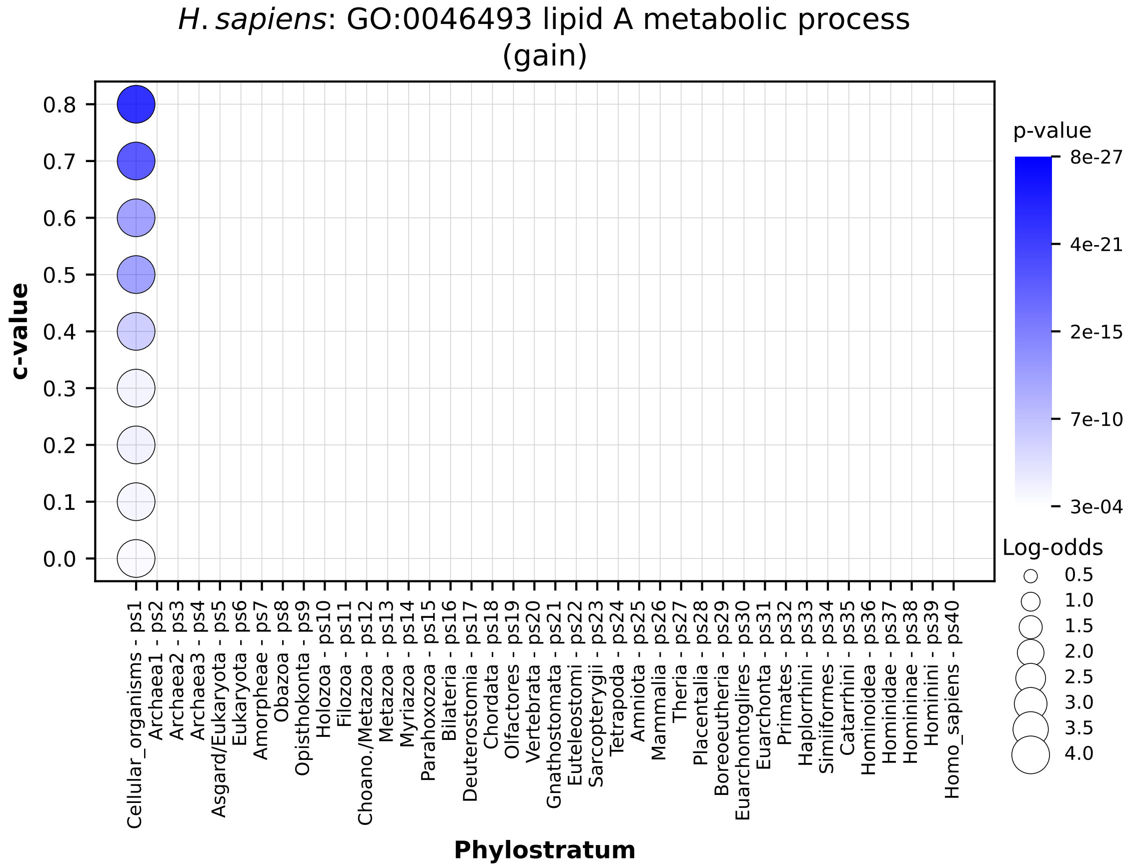

*H. sapiens*: GO:0046493 lipid A metabolic process
(gain)
c-value
0.8
0.7
0.6
0.5
0.4
0.3
0.2
0.1
0.0
p-value
8e-27
4e-21
2e-15
7e-10
3e-04
Log-odds
0.5
1.0
1.5
2.0
2.5
3.0
3.5
4.0
Cellular_organisms - ps1
Archaea1 - ps2
Archaea2 - ps3
Archaea3 - ps4
Asgard/Eukaryota - ps5
Eukaryota - ps6
Amorpheae - ps7
Obazoa - ps8
Opisthokonta - ps9
Holozoa - ps10
Filozoa - ps11
Choano./Metazoa - ps12
Metazoa - ps13
Myriazoa - ps14
Parahoxozoa - ps15
Bilateria - ps16
Deuterostomia - ps17
Chordata - ps18
Olfactores - ps19
Vertebrata - ps20
Gnathostomata - ps21
Euteleostomi - ps22
Sarcopterygii - ps23
Tetrapoda - ps24
Amniota - ps25
Mammalia - ps26
Theria - ps27
Placentalia - ps28
Boreoeutheria - ps29
Euarchontoglires - ps30
Euarchonta - ps31
Primates - ps32
Haplorrhini - ps33
Simiiformes - ps34
Catarrhini - ps35
Hominoidea - ps36
Hominidae - ps37
Homininae - ps38
Hominini - ps39
Homo_sapiens - ps40
Phylostratum

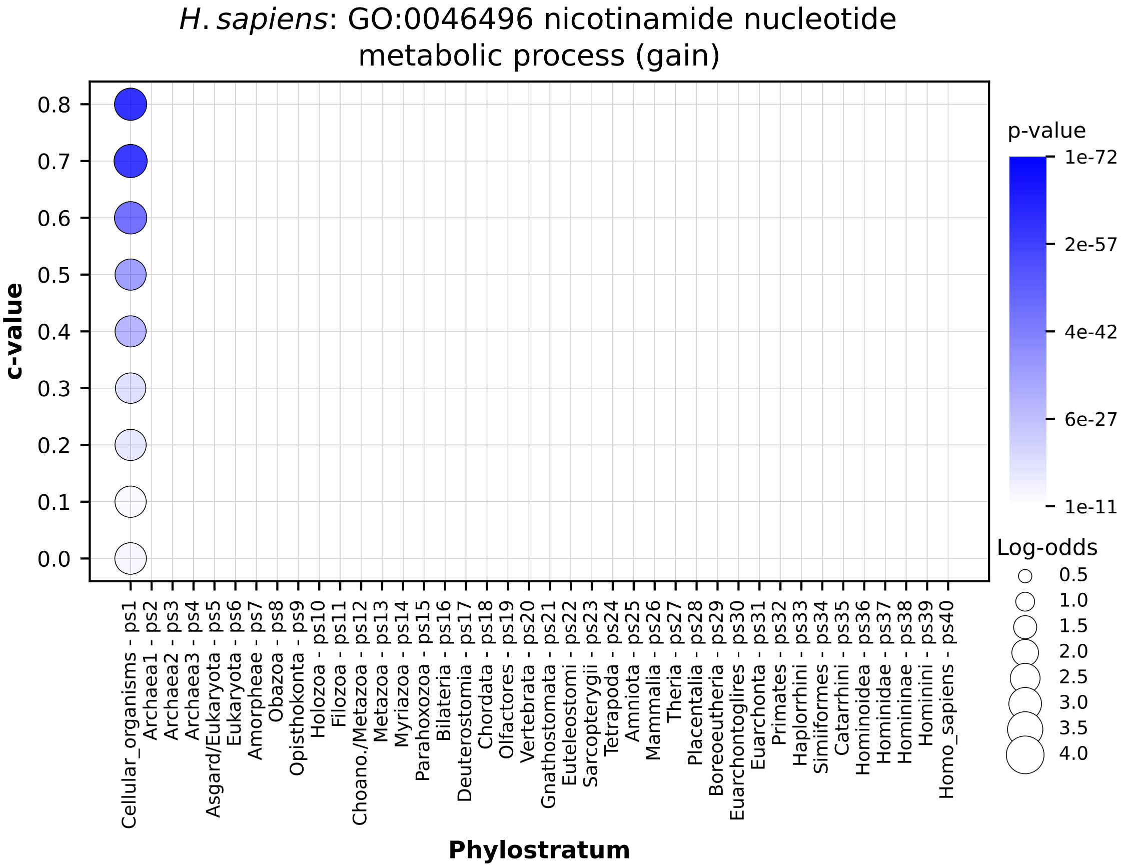

H. sapiens: GO:0046496 nicotinamide nucleotide metabolic process (gain)
c-value
0.8
0.7
0.6
0.5
0.4
0.3
0.2
0.1
0.0
p-value
1e-72
2e-57
4e-42
6e-27
1e-11
Log-odds
0.5
1.0
1.5
2.0
2.5
3.0
3.5
4.0
Cellular_organisms - ps1
Archaea1 - ps2
Archaea2 - ps3
Archaea3 - ps4
Asgard/Eukaryota - ps5
Eukaryota - ps6
Amorpheae - ps7
Obazoa - ps8
Opisthokonta - ps9
Holozoa - ps10
Filozoa - ps11
Choano./Metazoa - ps12
Metazoa - ps13
Myriazoa - ps14
Parahoxozoa - ps15
Bilateria - ps16
Deuterostomia - ps17
Chordata - ps18
Olfactores - ps19
Vertebrata - ps20
Gnathostomata - ps21
Euteleostomi - ps22
Sarcopterygii - ps23
Tetrapoda - ps24
Amniota - ps25
Mammalia - ps26
Theria - ps27
Placentalia - ps28
Boreoeutheria - ps29
Euarchontoglires - ps30
Euarchonta - ps31
Primates - ps32
Haplorrhini - ps33
Simiiformes - ps34
Catarrhini - ps35
Hominoidea - ps36
Hominidae - ps37
Homininae - ps38
Hominini - ps39
Homo_sapiens - ps40
Phylostratum

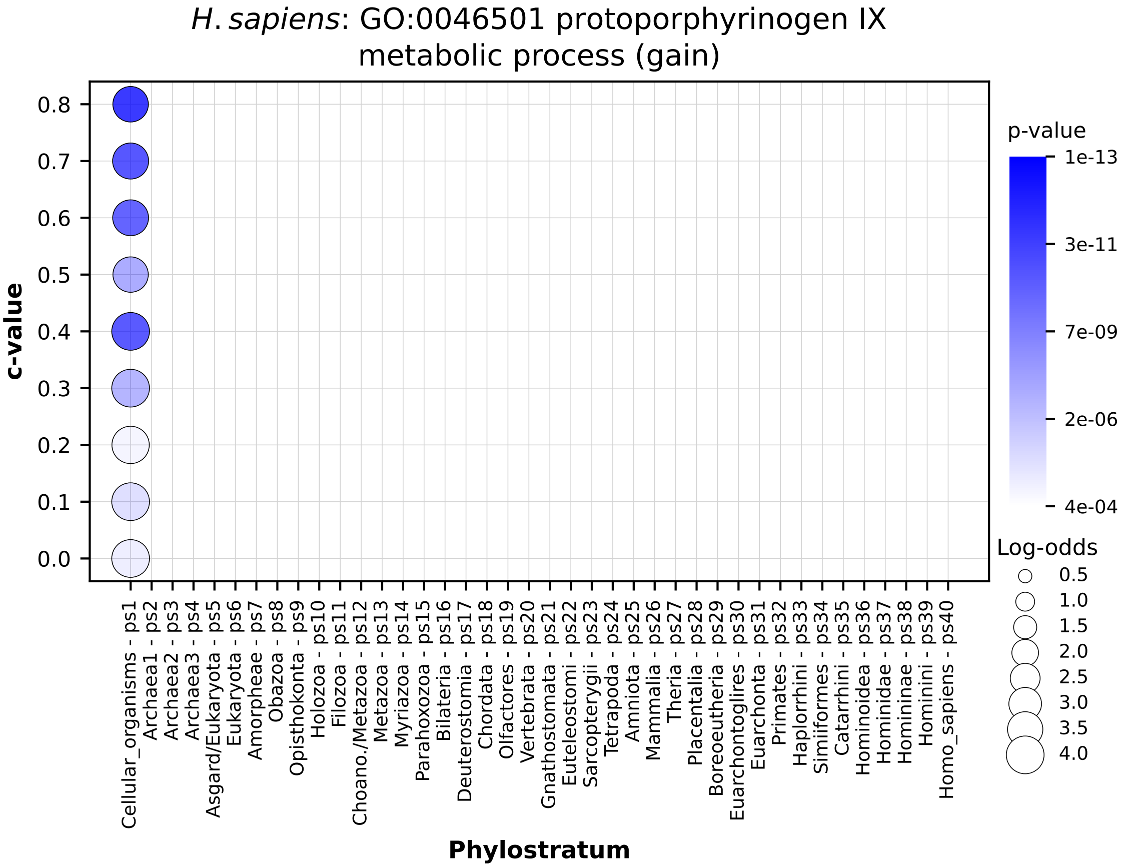

H. sapiens: GO:0046501 protoporphyrinogen IX metabolic process (gain)
c-value
0.8
0.7
0.6
0.5
0.4
0.3
0.2
0.1
0.0
Phylostratum
Cellular_organisms - ps1
Archaea1 - ps2
Archaea2 - ps3
Archaea3 - ps4
Asgard/Eukaryota - ps5
Eukaryota - ps6
Amorpheae - ps7
Obazoa - ps8
Opisthokonta - ps9
Holozoa - ps10
Filozoa - ps11
Choano./Metazoa - ps12
Metazoa - ps13
Myriazoa - ps14
Parahoxozoa - ps15
Bilateria - ps16
Deuterostomia - ps17
Chordata - ps18
Olfactores - ps19
Vertebrata - ps20
Gnathostomata - ps21
Euteleostomi - ps22
Sarcopterygii - ps23
Tetrapoda - ps24
Amniota - ps25
Mammalia - ps26
Theria - ps27
Placentalia - ps28
Boreoeutheria - ps29
Euarchontoglires - ps30
Euarchonta - ps31
Primates - ps32
Haplorrhini - ps33
Simiiformes - ps34
Catarrhini - ps35
Hominoidea - ps36
Hominidae - ps37
Homininae - ps38
Hominini - ps39
Homo_sapiens - ps40
p-value
1e-13
3e-11
7e-09
2e-06
4e-04
Log-odds
0.5
1.0
1.5
2.0
2.5
3.0
3.5
4.0

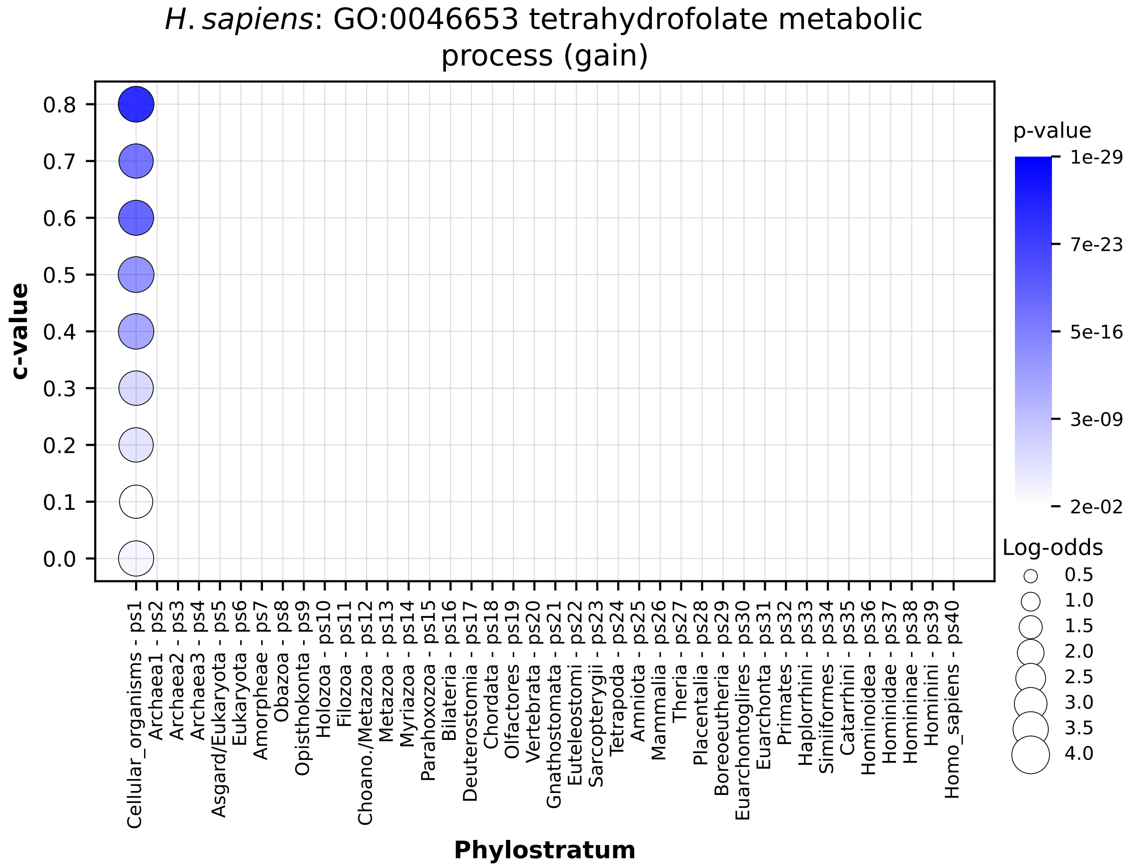

H. sapiens: GO:0046653 tetrahydrofolate metabolic process (gain)
c-value
0.8
0.7
0.6
0.5
0.4
0.3
0.2
0.1
0.0
p-value
1e-29
7e-23
5e-16
3e-09
2e-02
Log-odds
0.5
1.0
1.5
2.0
2.5
3.0
3.5
4.0
Cellular_organisms - ps1
Archaea1 - ps2
Archaea2 - ps3
Archaea3 - ps4
Asgard/Eukaryota - ps5
Eukaryota - ps6
Amorpheae - ps7
Obazoa - ps8
Opisthokonta - ps9
Holozoa - ps10
Filozoa - ps11
Choano./Metazoa - ps12
Metazoa - ps13
Myriazoa - ps14
Parahoxozoa - ps15
Bilateria - ps16
Deuterostomia - ps17
Chordata - ps18
Olfactores - ps19
Vertebrata - ps20
Gnathostomata - ps21
Euteleostomi - ps22
Sarcopterygii - ps23
Tetrapoda - ps24
Amniota - ps25
Mammalia - ps26
Theria - ps27
Placentalia - ps28
Boreoeutheria - ps29
Euarchontoglires - ps30
Euarchonta - ps31
Primates - ps32
Haplorrhini - ps33
Simiiformes - ps34
Catarrhini - ps35
Hominoidea - ps36
Hominidae - ps37
Homininae - ps38
Hominini - ps39
Homo_sapiens - ps40
Phylostratum

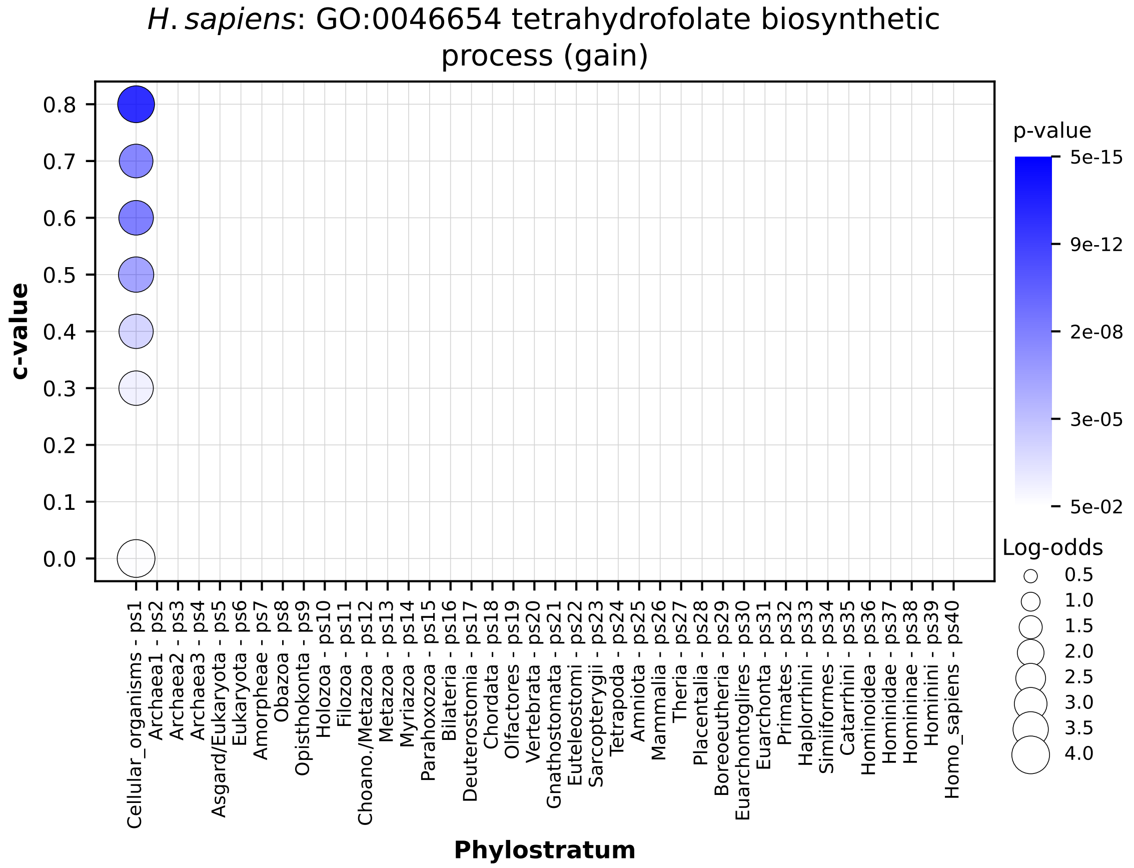

H. sapiens: GO:0046654 tetrahydrofolate biosynthetic process (gain)
c-value
0.8
0.7
0.6
0.5
0.4
0.3
0.2
0.1
0.0
p-value
5e-15
9e-12
2e-08
3e-05
5e-02
Log-odds
0.5
1.0
1.5
2.0
2.5
3.0
3.5
4.0
Cellular_organisms - ps1
Archaea1 - ps2
Archaea2 - ps3
Archaea3 - ps4
Asgard/Eukaryota - ps5
Eukaryota - ps6
Amorpheae - ps7
Obazoa - ps8
Opisthokonta - ps9
Holozoa - ps10
Filozoa - ps11
Choano./Metazoa - ps12
Metazoa - ps13
Myriazoa - ps14
Parahoxozoa - ps15
Bilateria - ps16
Deuterostomia - ps17
Chordata - ps18
Olfactores - ps19
Vertebrata - ps20
Gnathostomata - ps21
Euteleostomi - ps22
Sarcopterygii - ps23
Tetrapoda - ps24
Amniota - ps25
Mammalia - ps26
Theria - ps27
Placentalia - ps28
Boreoeutheria - ps29
Euarchontoglires - ps30
Euarchonta - ps31
Primates - ps32
Haplorrhini - ps33
Simiiformes - ps34
Catarrhini - ps35
Hominoidea - ps36
Hominidae - ps37
Homininae - ps38
Hominini - ps39
Homo_sapiens - ps40
Phylostratum

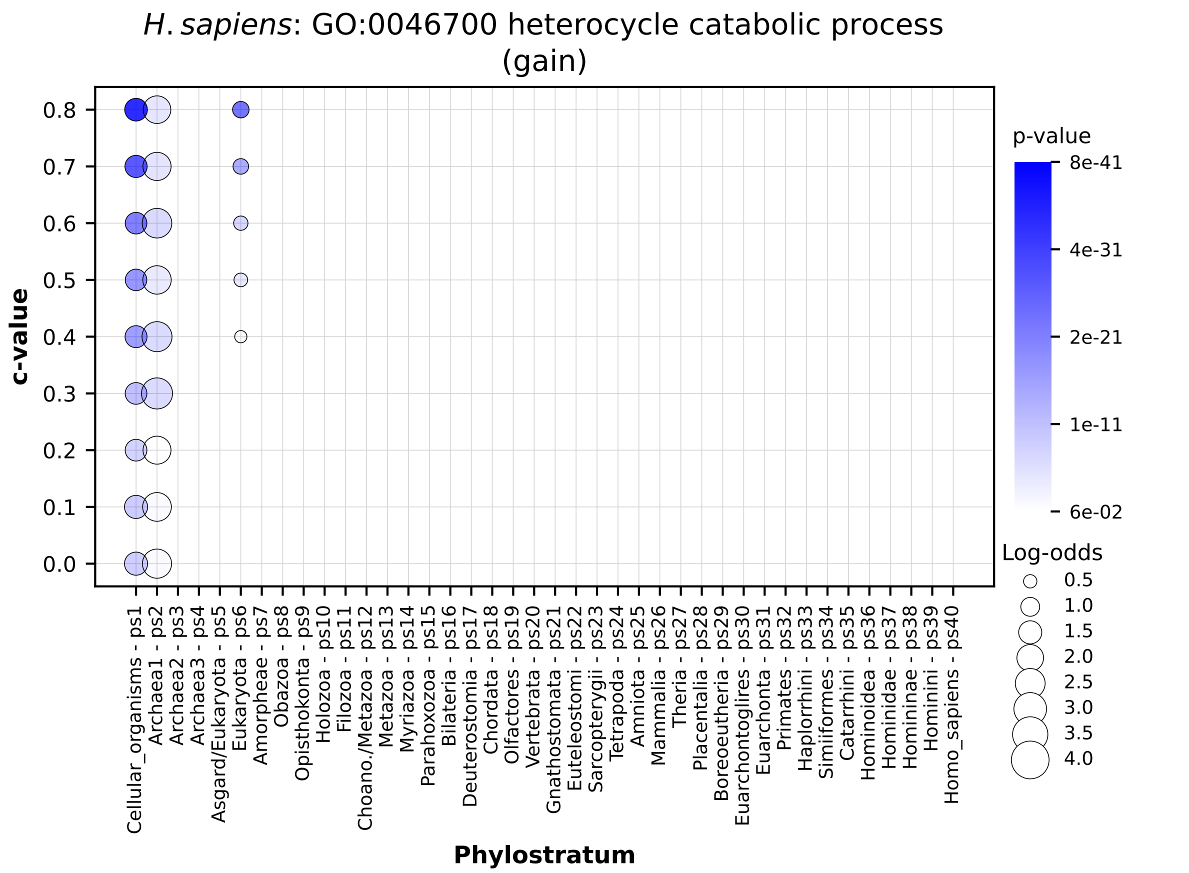

H. sapiens: GO:0046700 heterocycle catabolic process
(gain)
c-value
0.8
0.7
0.6
0.5
0.4
0.3
0.2
0.1
0.0
p-value
8e-41
4e-31
2e-21
1e-11
6e-02
Log-odds
0.5
1.0
1.5
2.0
2.5
3.0
3.5
4.0
Cellular_organisms - ps1
Archaea1 - ps2
Archaea2 - ps3
Archaea3 - ps4
Asgard/Eukaryota - ps5
Eukaryota - ps6
Amorpheae - ps7
Obazoa - ps8
Opisthokonta - ps9
Holozoa - ps10
Filozoa - ps11
Choano./Metazoa - ps12
Metazoa - ps13
Myriazoa - ps14
Parahoxozoa - ps15
Bilateria - ps16
Deuterostomia - ps17
Chordata - ps18
Olfactores - ps19
Vertebrata - ps20
Gnathostomata - ps21
Euteleostomi - ps22
Sarcopterygii - ps23
Tetrapoda - ps24
Amniota - ps25
Mammalia - ps26
Theria - ps27
Placentalia - ps28
Boreoeutheria - ps29
Euarchontoglires - ps30
Euarchonta - ps31
Primates - ps32
Haplorrhini - ps33
Simiiformes - ps34
Catarrhini - ps35
Hominoidea - ps36
Hominidae - ps37
Homininae - ps38
Hominini - ps39
Homo_sapiens - ps40
Phylostratum

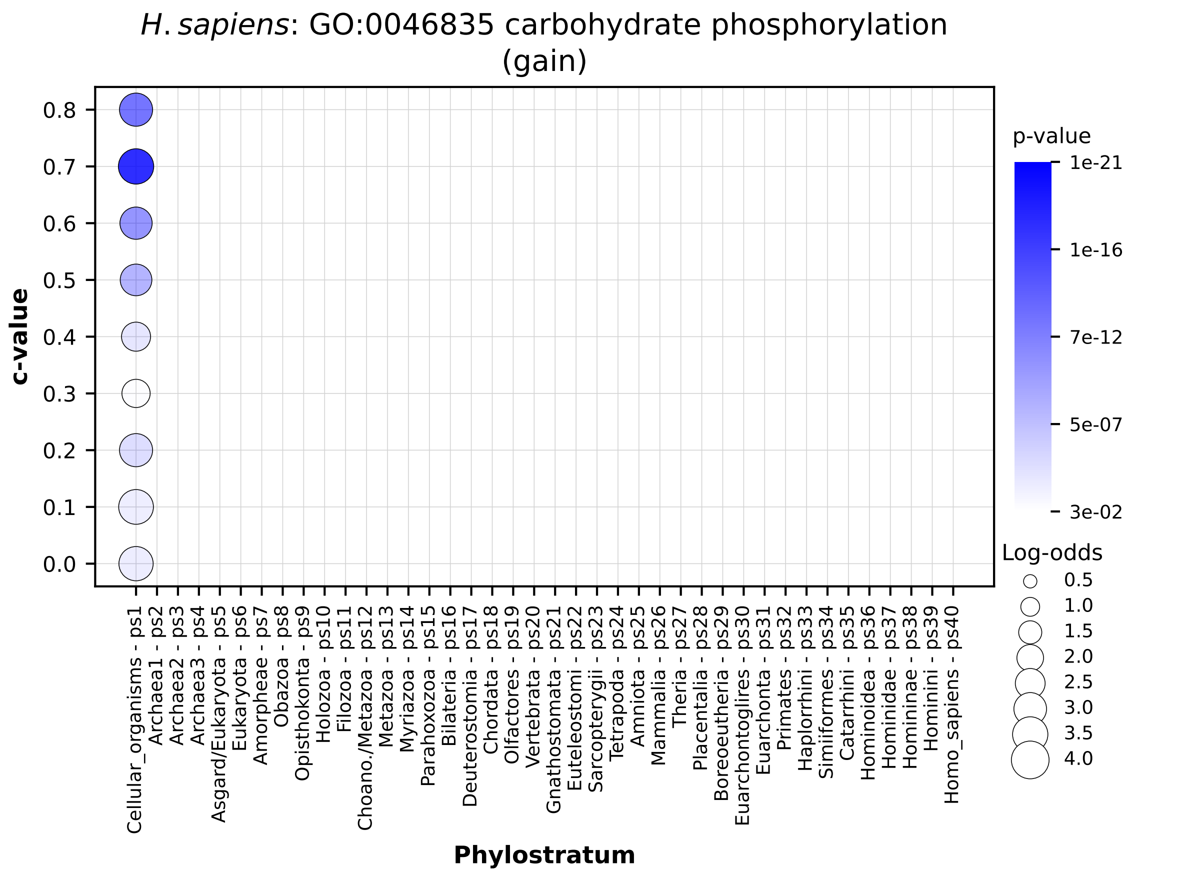

H. sapiens: GO:0046835 carbohydrate phosphorylation
(gain)
c-value
0.8
0.7
0.6
0.5
0.4
0.3
0.2
0.1
0.0
p-value
1e-21
1e-16
7e-12
5e-07
3e-02
Log-odds
0.5
1.0
1.5
2.0
2.5
3.0
3.5
4.0
Cellular_organisms - ps1
Archaea1 - ps2
Archaea2 - ps3
Archaea3 - ps4
Asgard/Eukaryota - ps5
Eukaryota - ps6
Amorpheae - ps7
Obazoa - ps8
Opisthokonta - ps9
Holozoa - ps10
Filozoa - ps11
Choano./Metazoa - ps12
Metazoa - ps13
Myriazoa - ps14
Parahoxozoa - ps15
Bilateria - ps16
Deuterostomia - ps17
Chordata - ps18
Olfactores - ps19
Vertebrata - ps20
Gnathostomata - ps21
Euteleostomi - ps22
Sarcopterygii - ps23
Tetrapoda - ps24
Amniota - ps25
Mammalia - ps26
Theria - ps27
Placentalia - ps28
Boreoeutheria - ps29
Euarchontoglires - ps30
Euarchonta - ps31
Primates - ps32
Haplorrhini - ps33
Simiiformes - ps34
Catarrhini - ps35
Hominoidea - ps36
Hominidae - ps37
Homininae - ps38
Hominini - ps39
Homo_sapiens - ps40
Phylostratum

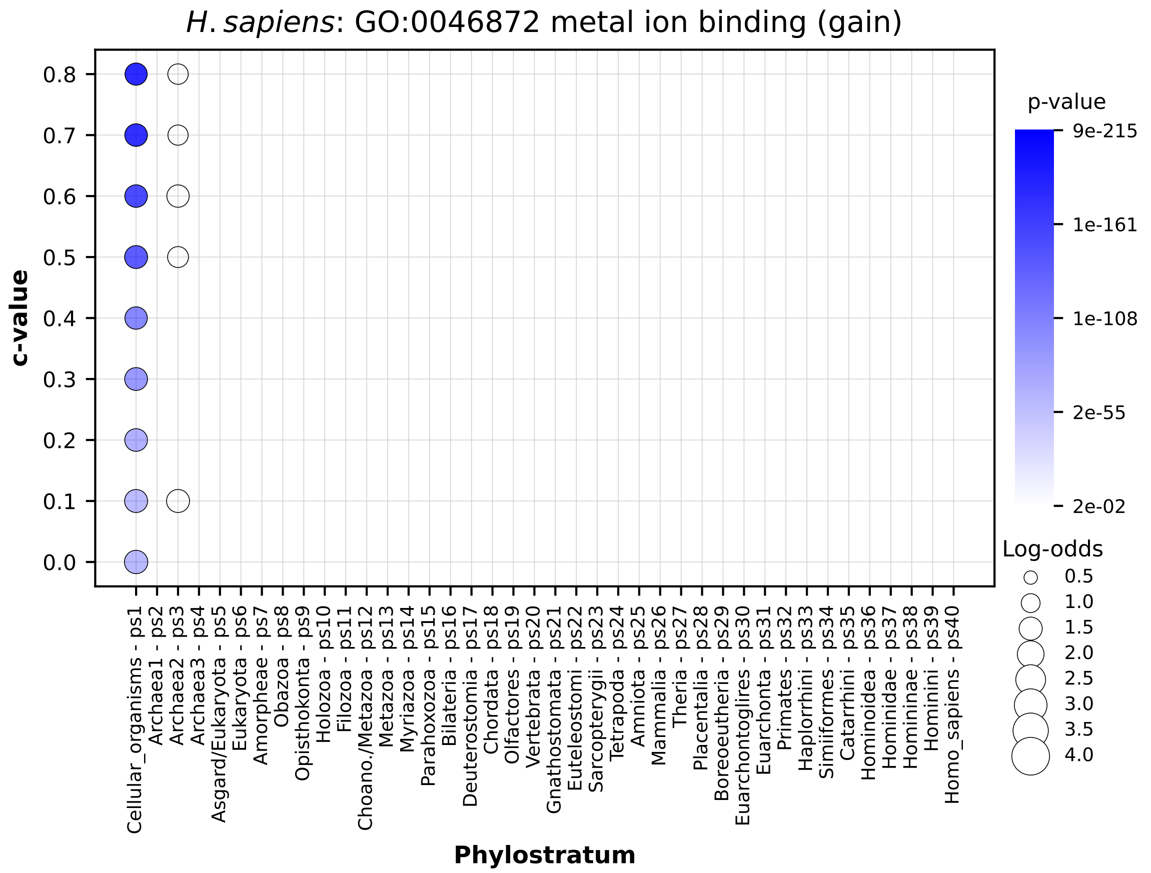

H. sapiens: GO:0046872 metal ion binding (gain)
c-value
0.8
0.7
0.6
0.5
0.4
0.3
0.2
0.1
0.0
p-value
9e-215
1e-161
1e-108
2e-55
2e-02
Log-odds
0.5
1.0
1.5
2.0
2.5
3.0
3.5
4.0
Cellular_organisms - ps1
Archaea1 - ps2
Archaea2 - ps3
Archaea3 - ps4
Asgard/Eukaryota - ps5
Eukaryota - ps6
Amorpheae - ps7
Obazoa - ps8
Opisthokonta - ps9
Holozoa - ps10
Filozoa - ps11
Choano./Metazoa - ps12
Metazoa - ps13
Myriazoa - ps14
Parahoxozoa - ps15
Bilateria - ps16
Deuterostomia - ps17
Chordata - ps18
Olfactores - ps19
Vertebrata - ps20
Gnathostomata - ps21
Euteleostomi - ps22
Sarcopterygii - ps23
Tetrapoda - ps24
Amniota - ps25
Mammalia - ps26
Theria - ps27
Placentalia - ps28
Boreoeutheria - ps29
Euarchontoglires - ps30
Euarchonta - ps31
Primates - ps32
Haplorrhini - ps33
Simiiformes - ps34
Catarrhini - ps35
Hominoidea - ps36
Hominidae - ps37
Homininae - ps38
Hominini - ps39
Homo_sapiens - ps40
Phylostratum

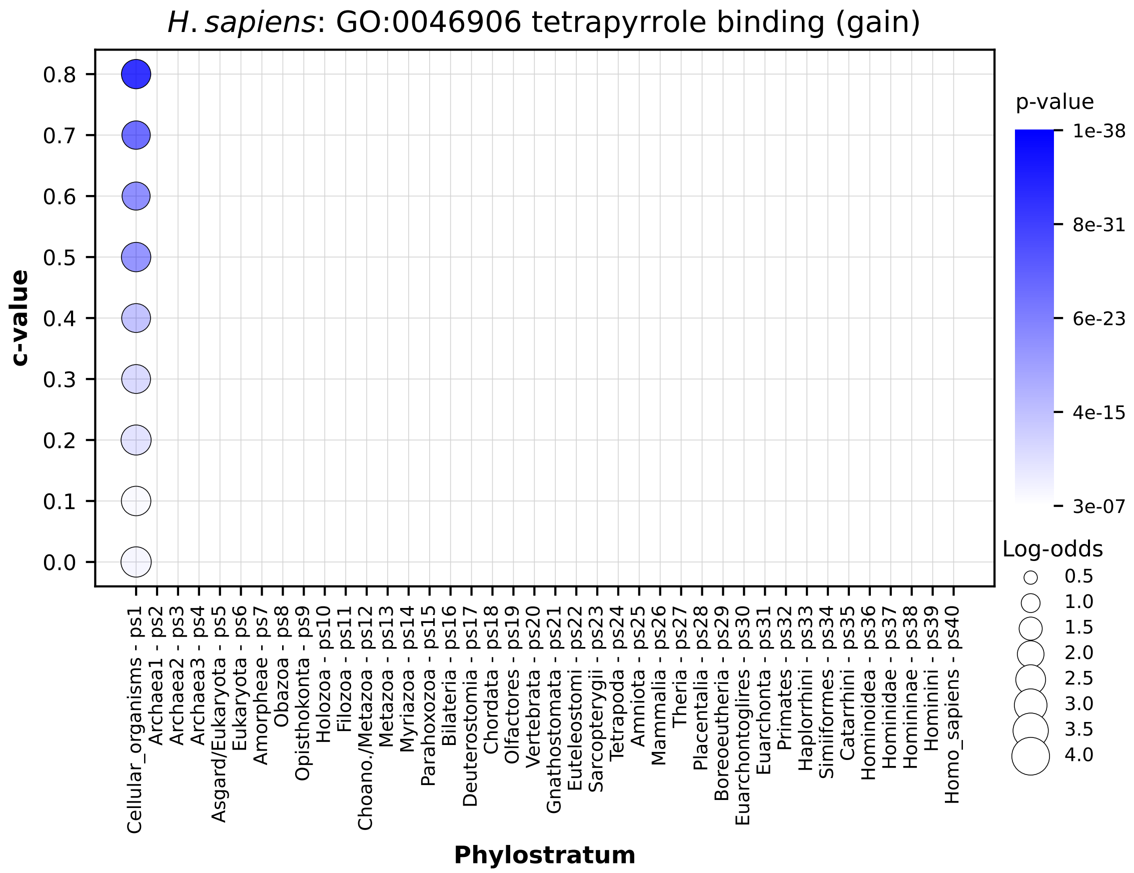

H. sapiens: GO:0046906 tetrapyrrole binding (gain)
c-value
0.8
0.7
0.6
0.5
0.4
0.3
0.2
0.1
0.0
p-value
1e-38
8e-31
6e-23
4e-15
3e-07
Log-odds
0.5
1.0
1.5
2.0
2.5
3.0
3.5
4.0
Cellular_organisms - ps1
Archaea1 - ps2
Archaea2 - ps3
Archaea3 - ps4
Asgard/Eukaryota - ps5
Eukaryota - ps6
Amorpheae - ps7
Obazoa - ps8
Opisthokonta - ps9
Holozoa - ps10
Filozoa - ps11
Choano./Metazoa - ps12
Metazoa - ps13
Myriazoa - ps14
Parahoxozoa - ps15
Bilateria - ps16
Deuterostomia - ps17
Chordata - ps18
Olfactores - ps19
Vertebrata - ps20
Gnathostomata - ps21
Euteleostomi - ps22
Sarcopterygii - ps23
Tetrapoda - ps24
Amniota - ps25
Mammalia - ps26
Theria - ps27
Placentalia - ps28
Boreoeutheria - ps29
Euarchontoglires - ps30
Euarchonta - ps31
Primates - ps32
Haplorrhini - ps33
Simiiformes - ps34
Catarrhini - ps35
Hominoidea - ps36
Hominidae - ps37
Homininae - ps38
Hominini - ps39
Homo_sapiens - ps40
Phylostratum

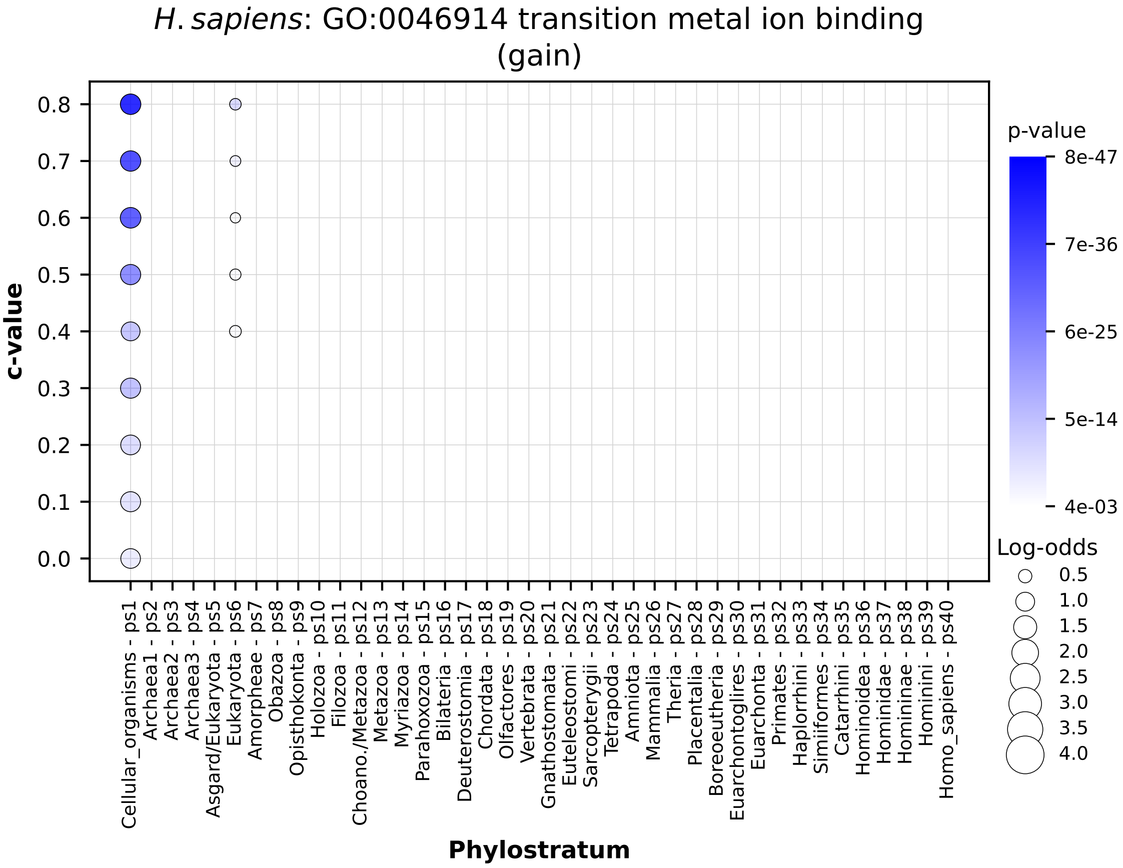

H. sapiens: GO:0046914 transition metal ion binding
(gain)
c-value
0.8
0.7
0.6
0.5
0.4
0.3
0.2
0.1
0.0
Phylostratum
Cellular_organisms - ps1
Archaea1 - ps2
Archaea2 - ps3
Archaea3 - ps4
Asgard/Eukaryota - ps5
Eukaryota - ps6
Amorpheae - ps7
Obazoa - ps8
Opisthokonta - ps9
Holozoa - ps10
Filozoa - ps11
Choano./Metazoa - ps12
Metazoa - ps13
Myriazoa - ps14
Parahoxozoa - ps15
Bilateria - ps16
Deuterostomia - ps17
Chordata - ps18
Olfactores - ps19
Vertebrata - ps20
Gnathostomata - ps21
Euteleostomi - ps22
Sarcopterygii - ps23
Tetrapoda - ps24
Amniota - ps25
Mammalia - ps26
Theria - ps27
Placentalia - ps28
Boreoeutheria - ps29
Euarchontoglires - ps30
Euarchonta - ps31
Primates - ps32
Haplorrhini - ps33
Simiiformes - ps34
Catarrhini - ps35
Hominoidea - ps36
Hominidae - ps37
Homininae - ps38
Hominini - ps39
Homo_sapiens - ps40
p-value
8e-47
7e-36
6e-25
5e-14
4e-03
Log-odds
0.5
1.0
1.5
2.0
2.5
3.0
3.5
4.0

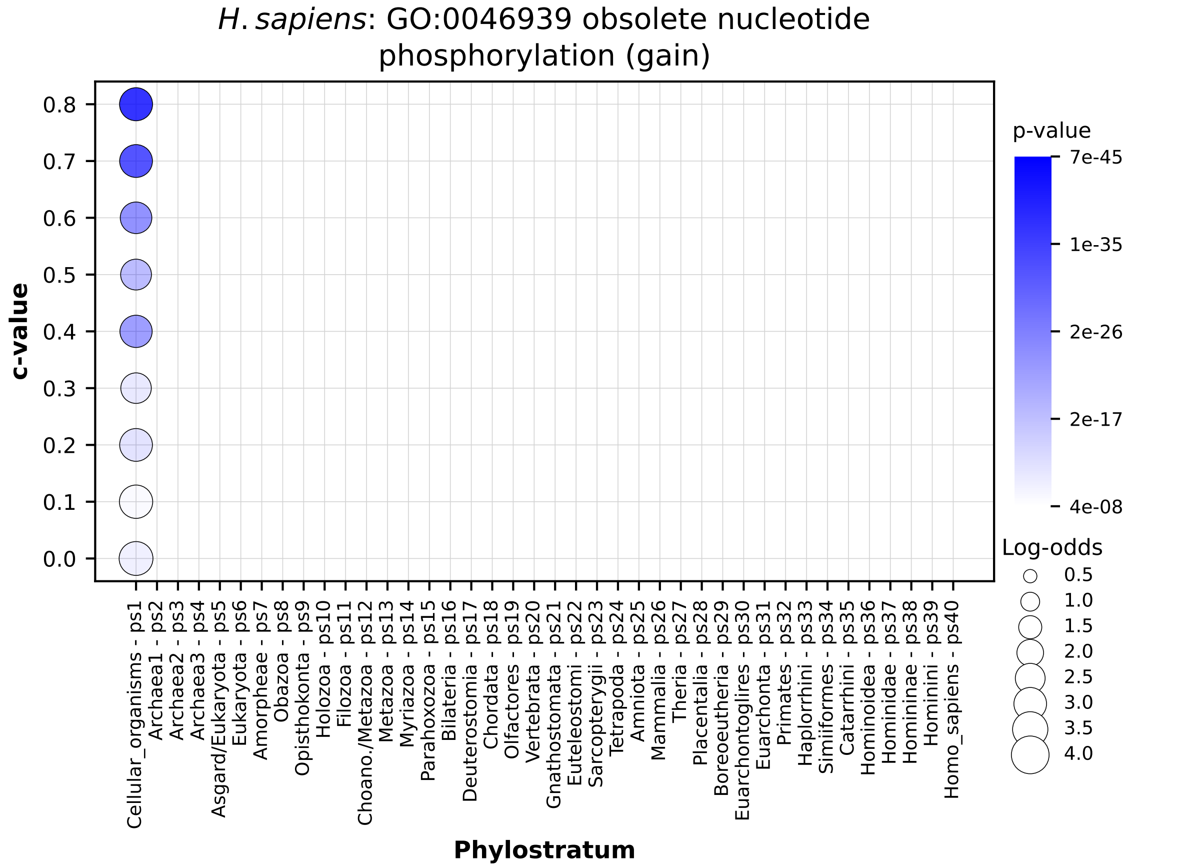

H. sapiens: GO:0046939 obsolete nucleotide phosphorylation (gain)
c-value
0.8
0.7
0.6
0.5
0.4
0.3
0.2
0.1
0.0
p-value
7e-45
1e-35
2e-26
2e-17
4e-08
Log-odds
0.5
1.0
1.5
2.0
2.5
3.0
3.5
4.0
Cellular_organisms - ps1
Archaea1 - ps2
Archaea2 - ps3
Archaea3 - ps4
Asgard/Eukaryota - ps5
Eukaryota - ps6
Amorpheae - ps7
Obazoa - ps8
Opisthokonta - ps9
Holozoa - ps10
Filozoa - ps11
Choano./Metazoa - ps12
Metazoa - ps13
Myriazoa - ps14
Parahoxozoa - ps15
Bilateria - ps16
Deuterostomia - ps17
Chordata - ps18
Olfactores - ps19
Vertebrata - ps20
Gnathostomata - ps21
Euteleostomi - ps22
Sarcopterygii - ps23
Tetrapoda - ps24
Amniota - ps25
Mammalia - ps26
Theria - ps27
Placentalia - ps28
Boreoeutheria - ps29
Euarchontoglires - ps30
Euarchonta - ps31
Primates - ps32
Haplorrhini - ps33
Simiiformes - ps34
Catarrhini - ps35
Hominoidea - ps36
Hominidae - ps37
Homininae - ps38
Hominini - ps39
Homo_sapiens - ps40
Phylostratum

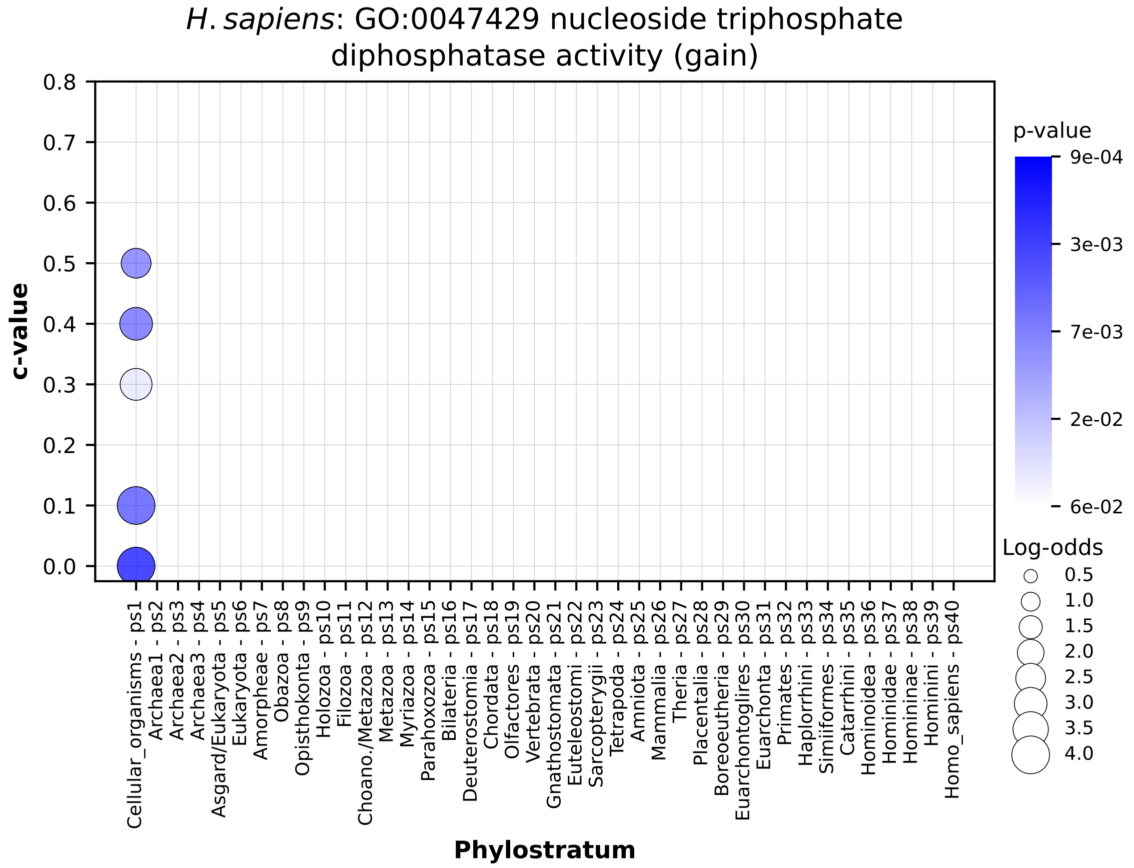
H. sapiens: GO:0047429 nucleoside triphosphate diphosphatase activity (gain)
c-value
0.8
0.7
0.6
0.5
0.4
0.3
0.2
0.1
0.0
p-value
9e-04
3e-03
7e-03
2e-02
6e-02
Log-odds
0.5
1.0
1.5
2.0
2.5
3.0
3.5
4.0
Cellular_organisms - ps1
Archaea1 - ps2
Archaea2 - ps3
Archaea3 - ps4
Asgard/Eukaryota - ps5
Eukaryota - ps6
Amorpheae - ps7
Obazoa - ps8
Opisthokonta - ps9
Holozoa - ps10
Filozoa - ps11
Choano./Metazoa - ps12
Metazoa - ps13
Myriazoa - ps14
Parahoxozoa - ps15
Bilateria - ps16
Deuterostomia - ps17
Chordata - ps18
Olfactores - ps19
Vertebrata - ps20
Gnathostomata - ps21
Euteleostomi - ps22
Sarcopterygii - ps23
Tetrapoda - ps24
Amniota - ps25
Mammalia - ps26
Theria - ps27
Placentalia - ps28
Boreoeutheria - ps29
Euarchontoglires - ps30
Euarchonta - ps31
Primates - ps32
Haplorrhini - ps33
Simiiformes - ps34
Catarrhini - ps35
Hominoidea - ps36
Hominidae - ps37
Homininae - ps38
Hominini - ps39
Homo_sapiens - ps40
Phylostratum

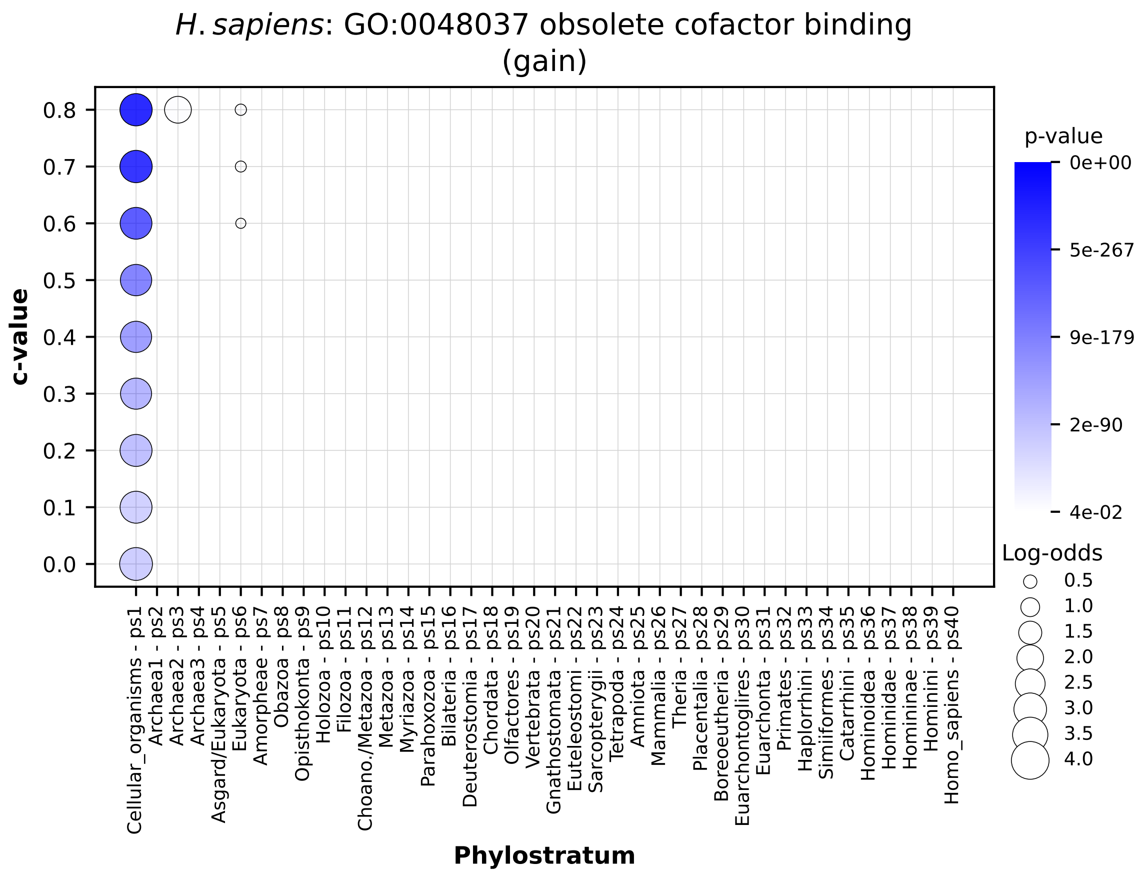

H. sapiens: GO:0048037 obsolete cofactor binding
(gain)
c-value
0.8
0.7
0.6
0.5
0.4
0.3
0.2
0.1
0.0
p-value
0e+00
5e-267
9e-179
2e-90
4e-02
Log-odds
0.5
1.0
1.5
2.0
2.5
3.0
3.5
4.0
Cellular_organisms - ps1
Archaea1 - ps2
Archaea2 - ps3
Archaea3 - ps4
Asgard/Eukaryota - ps5
Eukaryota - ps6
Amorpheae - ps7
Obazoa - ps8
Opisthokonta - ps9
Holozoa - ps10
Filozoa - ps11
Choano./Metazoa - ps12
Metazoa - ps13
Myriazoa - ps14
Parahoxozoa - ps15
Bilateria - ps16
Deuterostomia - ps17
Chordata - ps18
Olfactores - ps19
Vertebrata - ps20
Gnathostomata - ps21
Euteleostomi - ps22
Sarcopterygii - ps23
Tetrapoda - ps24
Amniota - ps25
Mammalia - ps26
Theria - ps27
Placentalia - ps28
Boreoeutheria - ps29
Euarchontoglires - ps30
Euarchonta - ps31
Primates - ps32
Haplorrhini - ps33
Simiiformes - ps34
Catarrhini - ps35
Hominoidea - ps36
Hominidae - ps37
Homininae - ps38
Hominini - ps39
Homo_sapiens - ps40
Phylostratum

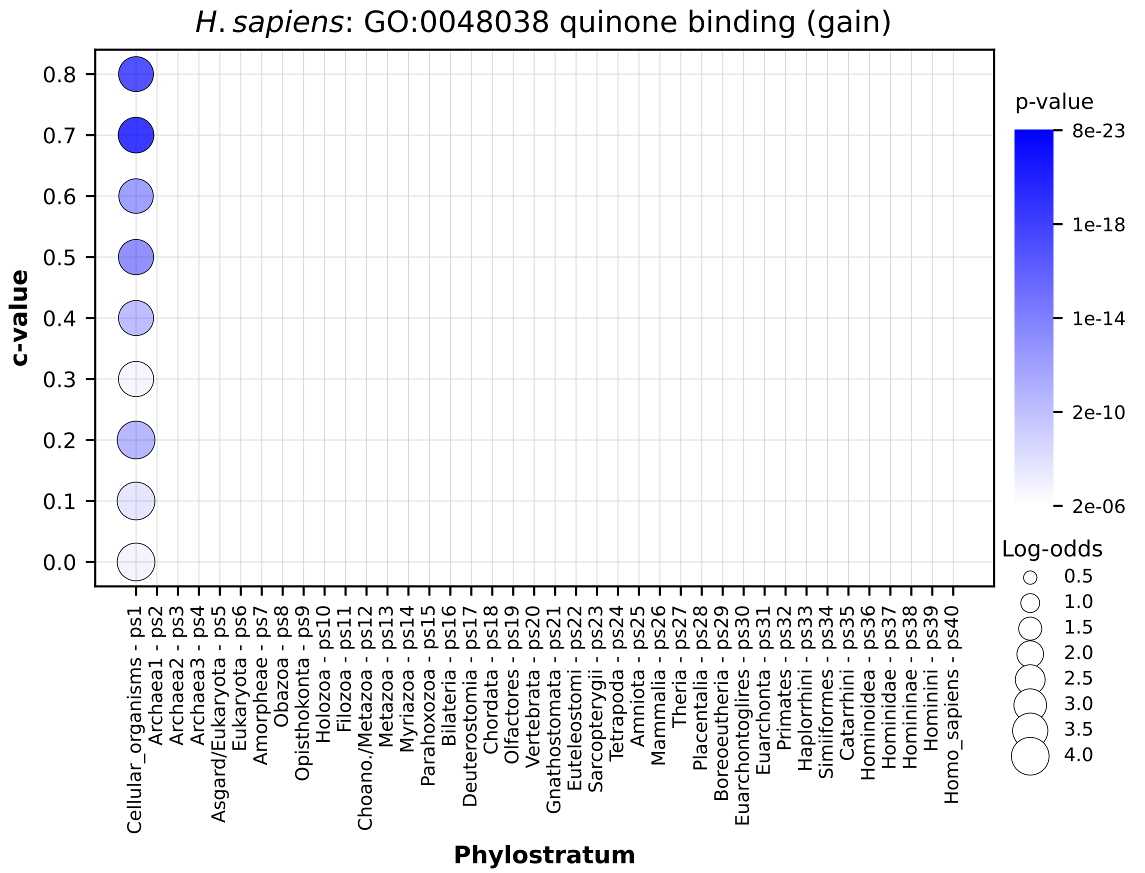

H. sapiens: GO:0048038 quinone binding (gain)
c-value
0.0
0.1
0.2
0.3
0.4
0.5
0.6
0.7
0.8
Phylostratum
Cellular_organisms - ps1
Archaea1 - ps2
Archaea2 - ps3
Archaea3 - ps4
Asgard/Eukaryota - ps5
Eukaryota - ps6
Amorpheae - ps7
Obazoa - ps8
Opisthokonta - ps9
Holozoa - ps10
Filozoa - ps11
Choano./Metazoa - ps12
Metazoa - ps13
Myriazoa - ps14
Parahoxozoa - ps15
Bilateria - ps16
Deuterostomia - ps17
Chordata - ps18
Olfactores - ps19
Vertebrata - ps20
Gnathostomata - ps21
Euteleostomi - ps22
Sarcopterygii - ps23
Tetrapoda - ps24
Amniota - ps25
Mammalia - ps26
Theria - ps27
Placentalia - ps28
Boreoeutheria - ps29
Euarchontoglires - ps30
Euarchonta - ps31
Primates - ps32
Haplorrhini - ps33
Simiiformes - ps34
Catarrhini - ps35
Hominoidea - ps36
Hominidae - ps37
Homininae - ps38
Hominini - ps39
Homo_sapiens - ps40
p-value
8e-23
1e-18
1e-14
2e-10
2e-06
Log-odds
0.5
1.0
1.5
2.0
2.5
3.0
3.5
4.0

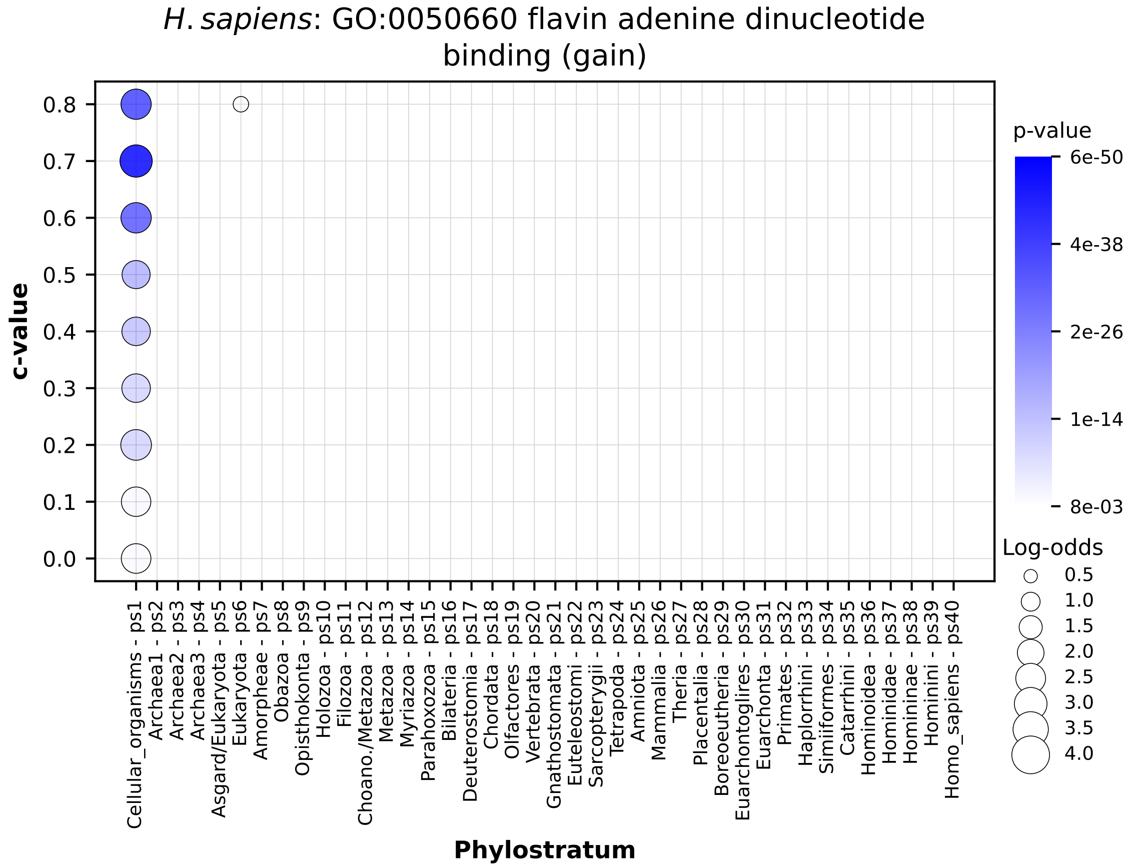

H. sapiens: GO:0050660 flavin adenine dinucleotide binding (gain)
c-value
0.8
0.7
0.6
0.5
0.4
0.3
0.2
0.1
0.0
p-value
6e-50
4e-38
2e-26
1e-14
8e-03
Log-odds
0.5
1.0
1.5
2.0
2.5
3.0
3.5
4.0
Cellular_organisms - ps1
Archaea1 - ps2
Archaea2 - ps3
Archaea3 - ps4
Asgard/Eukaryota - ps5
Eukaryota - ps6
Amorpheae - ps7
Obazoa - ps8
Opisthokonta - ps9
Holozoa - ps10
Filozoa - ps11
Choano./Metazoa - ps12
Metazoa - ps13
Myriazoa - ps14
Parahoxozoa - ps15
Bilateria - ps16
Deuterostomia - ps17
Chordata - ps18
Olfactores - ps19
Vertebrata - ps20
Gnathostomata - ps21
Euteleostomi - ps22
Sarcopterygii - ps23
Tetrapoda - ps24
Amniota - ps25
Mammalia - ps26
Theria - ps27
Placentalia - ps28
Boreoeutheria - ps29
Euarchontoglires - ps30
Euarchonta - ps31
Primates - ps32
Haplorrhini - ps33
Simiiformes - ps34
Catarrhini - ps35
Hominoidea - ps36
Hominidae - ps37
Homininae - ps38
Hominini - ps39
Homo_sapiens - ps40
Phylostratum

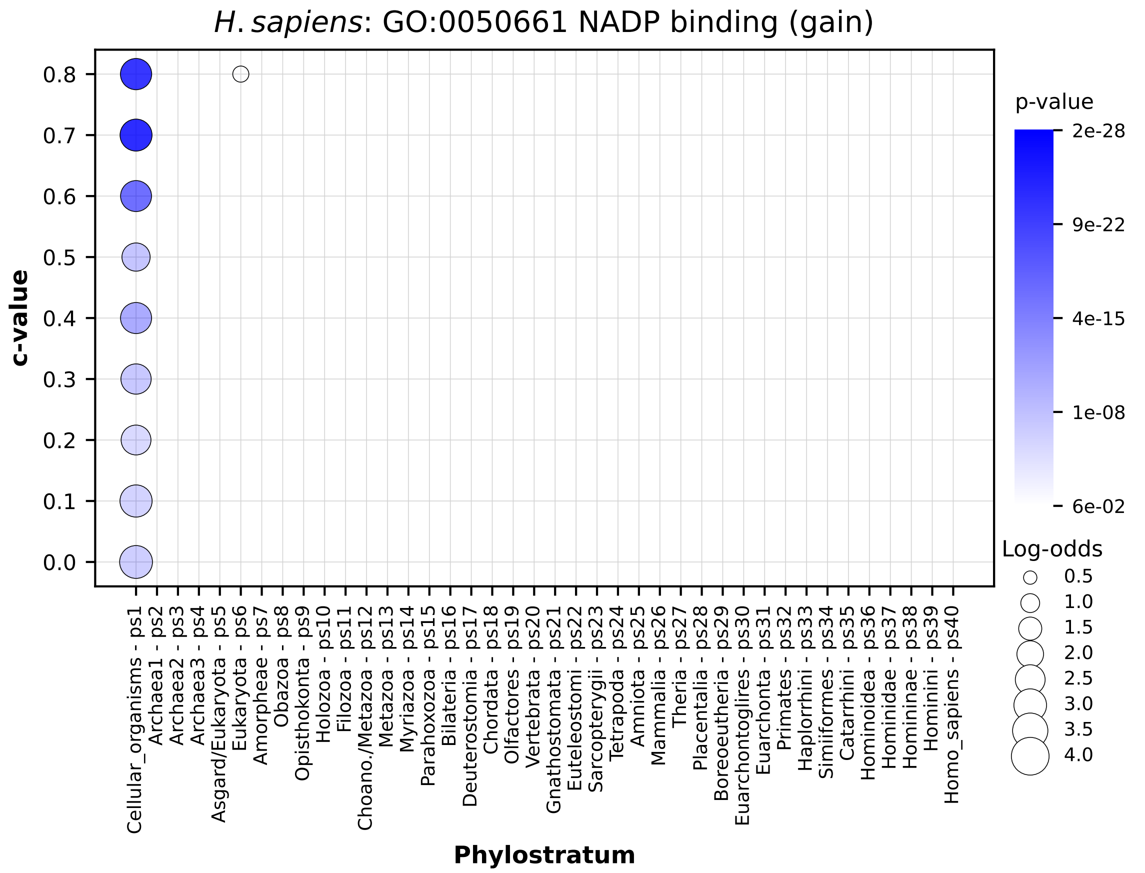

*H. sapiens*: GO:0050661 NADP binding (gain)
c-value
0.8
0.7
0.6
0.5
0.4
0.3
0.2
0.1
0.0
p-value
2e-28
9e-22
4e-15
1e-08
6e-02
Log-odds
0.5
1.0
1.5
2.0
2.5
3.0
3.5
4.0
Cellular_organisms - ps1
Archaea1 - ps2
Archaea2 - ps3
Archaea3 - ps4
Asgard/Eukaryota - ps5
Eukaryota - ps6
Amorpheae - ps7
Obazoa - ps8
Opisthokonta - ps9
Holozoa - ps10
Filozoa - ps11
Choano./Metazoa - ps12
Metazoa - ps13
Myriazoa - ps14
Parahoxozoa - ps15
Bilateria - ps16
Deuterostomia - ps17
Chordata - ps18
Olfactores - ps19
Vertebrata - ps20
Gnathostomata - ps21
Euteleostomi - ps22
Sarcopterygii - ps23
Tetrapoda - ps24
Amniota - ps25
Mammalia - ps26
Theria - ps27
Placentalia - ps28
Boreoeutheria - ps29
Euarchontoglires - ps30
Euarchonta - ps31
Primates - ps32
Haplorrhini - ps33
Simiiformes - ps34
Catarrhini - ps35
Hominoidea - ps36
Hominidae - ps37
Homininae - ps38
Hominini - ps39
Homo_sapiens - ps40
Phylostratum

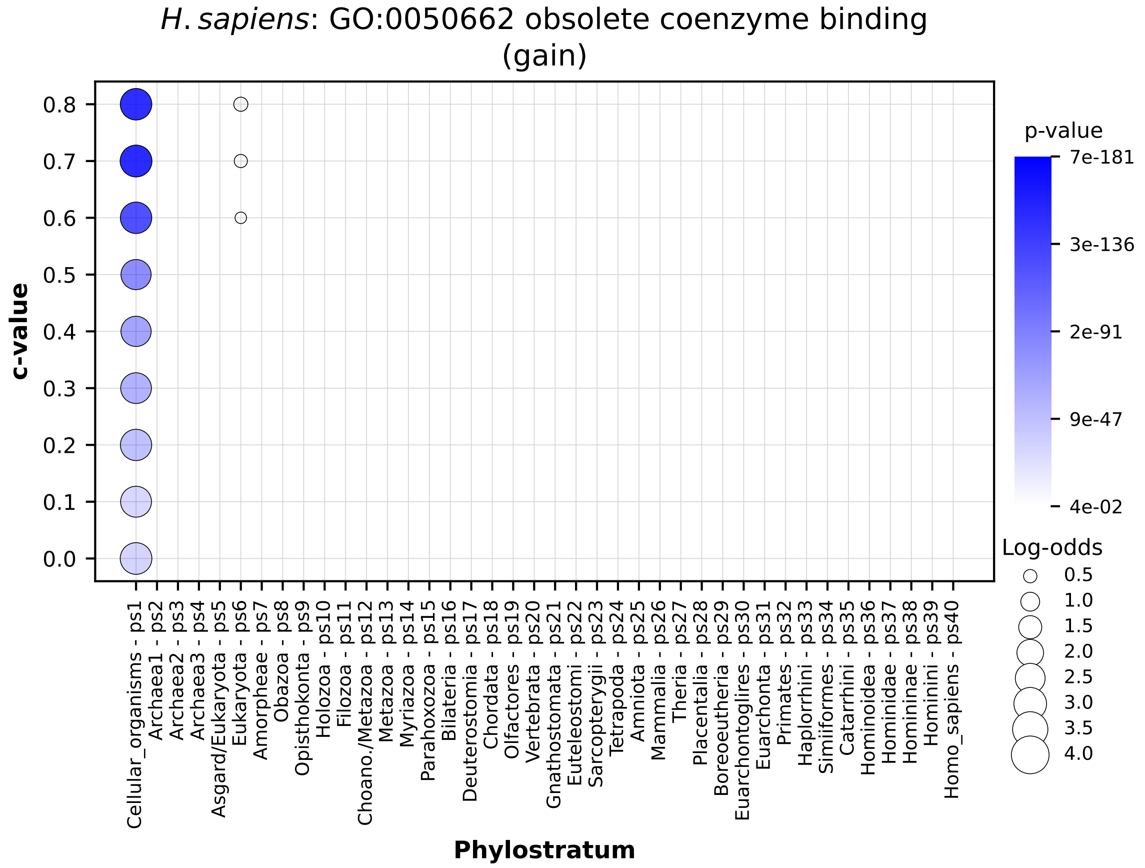

H. sapiens: GO:0050662 obsolete coenzyme binding (gain)
c-value
0.8
0.7
0.6
0.5
0.4
0.3
0.2
0.1
0.0
Phylostratum
Cellular_organisms - ps1
Archaea1 - ps2
Archaea2 - ps3
Archaea3 - ps4
Asgard/Eukaryota - ps5
Eukaryota - ps6
Amorpheae - ps7
Obazoa - ps8
Opisthokonta - ps9
Holozoa - ps10
Filozoa - ps11
Choano./Metazoa - ps12
Metazoa - ps13
Myriazoa - ps14
Parahoxozoa - ps15
Bilateria - ps16
Deuterostomia - ps17
Chordata - ps18
Olfactores - ps19
Vertebrata - ps20
Gnathostomata - ps21
Euteleostomi - ps22
Sarcopterygii - ps23
Tetrapoda - ps24
Amniota - ps25
Mammalia - ps26
Theria - ps27
Placentalia - ps28
Boreoeutheria - ps29
Euarchontoglires - ps30
Euarchonta - ps31
Primates - ps32
Haplorrhini - ps33
Simiiformes - ps34
Catarrhini - ps35
Hominoidea - ps36
Hominidae - ps37
Homininae - ps38
Hominini - ps39
Homo_sapiens - ps40
p-value
7e-181
3e-136
2e-91
9e-47
4e-02
Log-odds
0.5
1.0
1.5
2.0
2.5
3.0
3.5
4.0

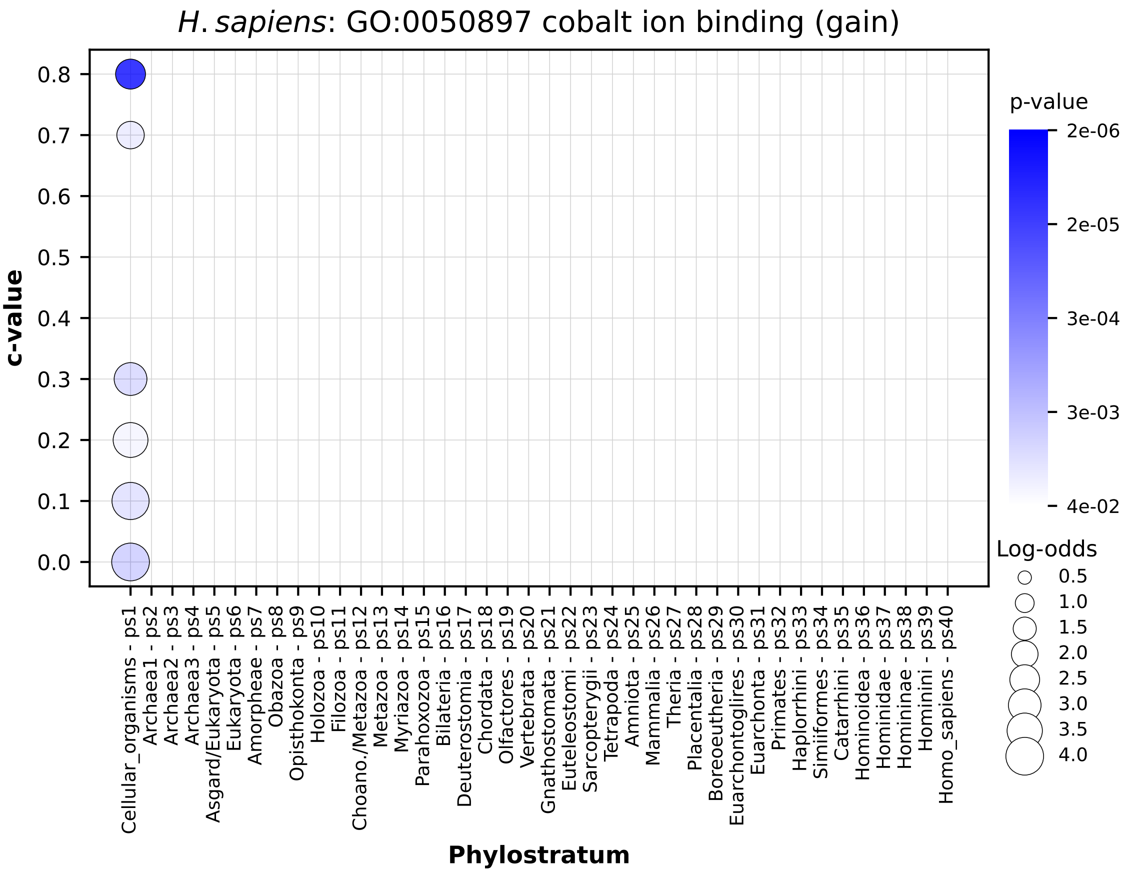
H. sapiens: GO:0050897 cobalt ion binding (gain)
c-value
0.0
0.1
0.2
0.3
0.4
0.5
0.6
0.7
0.8
Phylostratum
Cellular_organisms - ps1
Archaea1 - ps2
Archaea2 - ps3
Archaea3 - ps4
Asgard/Eukaryota - ps5
Eukaryota - ps6
Amorpheae - ps7
Obazoa - ps8
Opisthokonta - ps9
Holozoa - ps10
Filozoa - ps11
Choano./Metazoa - ps12
Metazoa - ps13
Myriazoa - ps14
Parahoxozoa - ps15
Bilateria - ps16
Deuterostomia - ps17
Chordata - ps18
Olfactores - ps19
Vertebrata - ps20
Gnathostomata - ps21
Euteleostomi - ps22
Sarcopterygii - ps23
Tetrapoda - ps24
Amniota - ps25
Mammalia - ps26
Theria - ps27
Placentalia - ps28
Boreoeutheria - ps29
Euarchontoglires - ps30
Euarchonta - ps31
Primates - ps32
Haplorrhini - ps33
Simiiformes - ps34
Catarrhini - ps35
Hominoidea - ps36
Hominidae - ps37
Homininae - ps38
Hominini - ps39
Homo_sapiens - ps40
p-value
2e-06
2e-05
3e-04
3e-03
4e-02
Log-odds
0.5
1.0
1.5
2.0
2.5
3.0
3.5
4.0

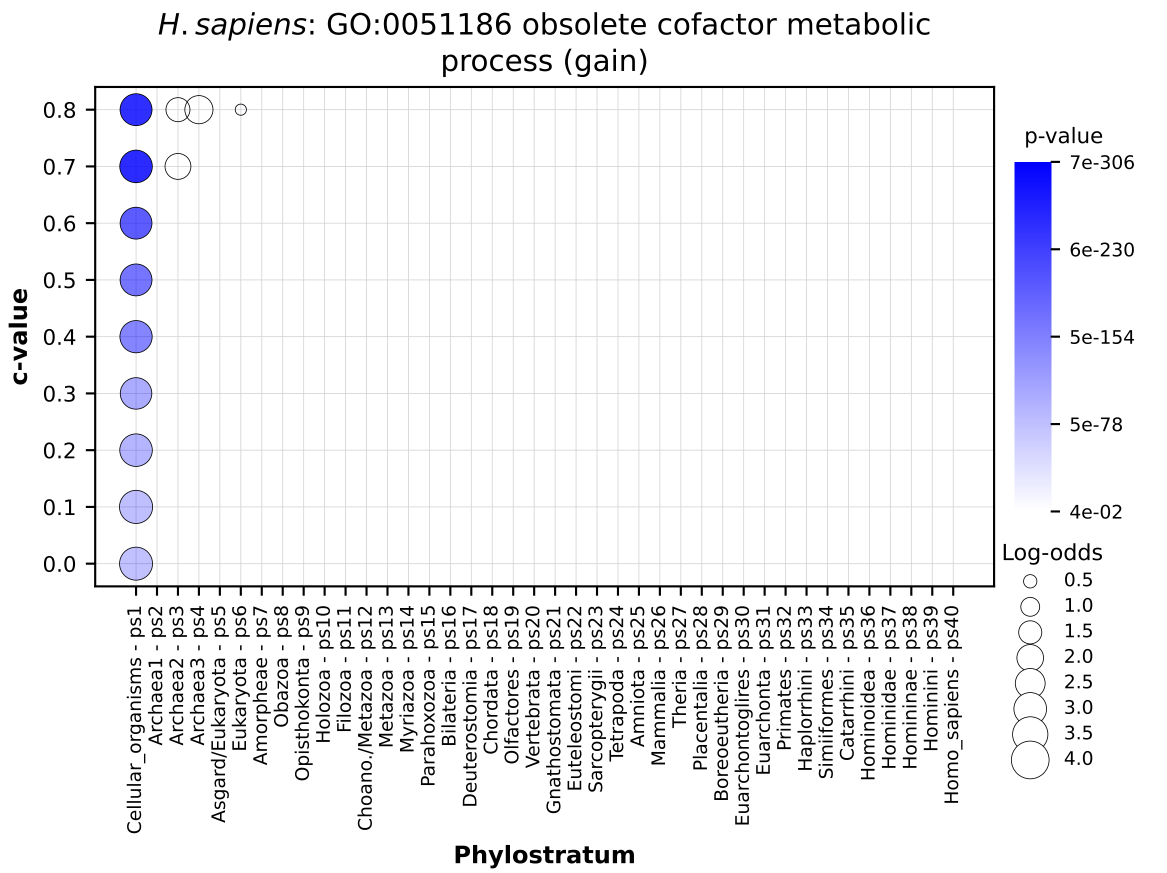

H. sapiens: GO:0051186 obsolete cofactor metabolic process (gain)
c-value
0.8
0.7
0.6
0.5
0.4
0.3
0.2
0.1
0.0
p-value
7e-306
6e-230
5e-154
5e-78
4e-02
Log-odds
0.5
1.0
1.5
2.0
2.5
3.0
3.5
4.0
Cellular_organisms - ps1
Archaea1 - ps2
Archaea2 - ps3
Archaea3 - ps4
Asgard/Eukaryota - ps5
Eukaryota - ps6
Amorpheae - ps7
Obazoa - ps8
Opisthokonta - ps9
Holozoa - ps10
Filozoa - ps11
Choano./Metazoa - ps12
Metazoa - ps13
Myriazoa - ps14
Parahoxozoa - ps15
Bilateria - ps16
Deuterostomia - ps17
Chordata - ps18
Olfactores - ps19
Vertebrata - ps20
Gnathostomata - ps21
Euteleostomi - ps22
Sarcopterygii - ps23
Tetrapoda - ps24
Amniota - ps25
Mammalia - ps26
Theria - ps27
Placentalia - ps28
Boreoeutheria - ps29
Euarchontoglires - ps30
Euarchonta - ps31
Primates - ps32
Haplorrhini - ps33
Simiiformes - ps34
Catarrhini - ps35
Hominoidea - ps36
Hominidae - ps37
Homininae - ps38
Hominini - ps39
Homo_sapiens - ps40
Phylostratum

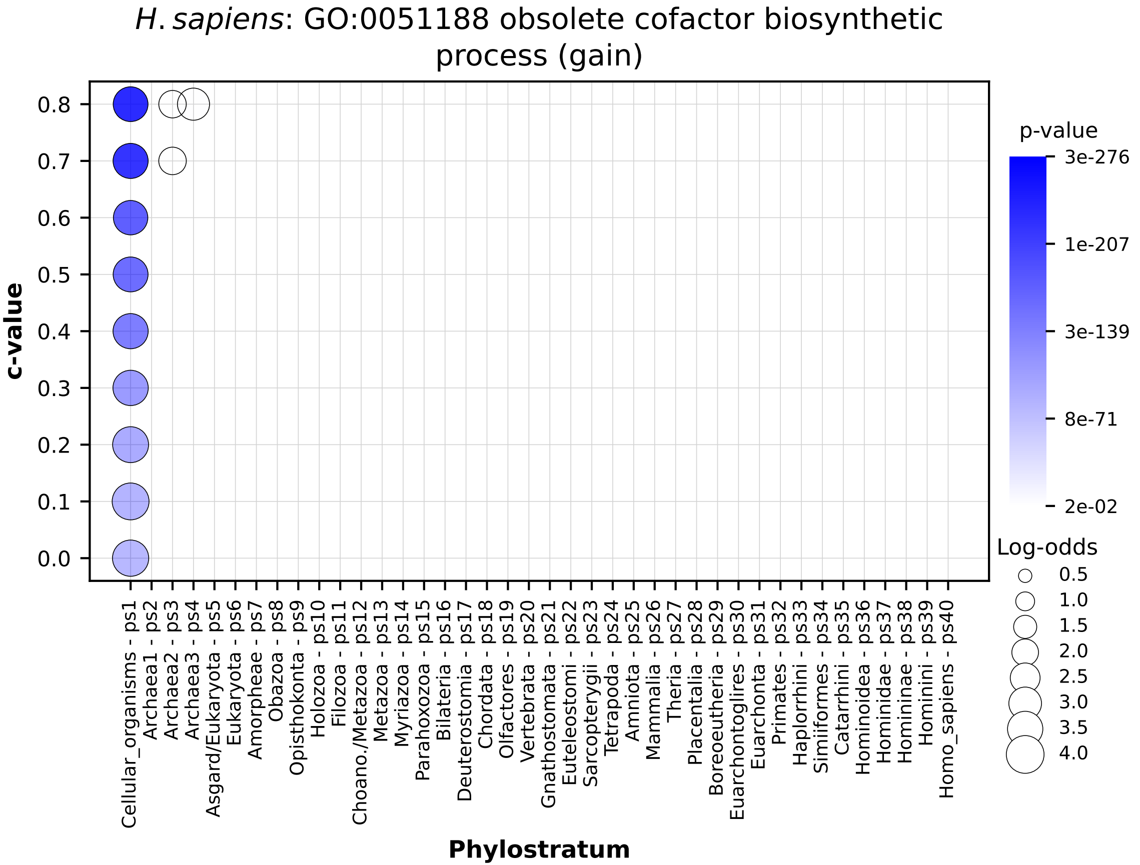

H. sapiens: GO:0051188 obsolete cofactor biosynthetic process (gain)
c-value
0.8
0.7
0.6
0.5
0.4
0.3
0.2
0.1
0.0
p-value
3e-276
1e-207
3e-139
8e-71
2e-02
Log-odds
0.5
1.0
1.5
2.0
2.5
3.0
3.5
4.0
Cellular_organisms - ps1
Archaea1 - ps2
Archaea2 - ps3
Archaea3 - ps4
Asgard/Eukaryota - ps5
Eukaryota - ps6
Amorpheae - ps7
Obazoa - ps8
Opisthokonta - ps9
Holozoa - ps10
Filozoa - ps11
Choano./Metazoa - ps12
Metazoa - ps13
Myriazoa - ps14
Parahoxozoa - ps15
Bilateria - ps16
Deuterostomia - ps17
Chordata - ps18
Olfactores - ps19
Vertebrata - ps20
Gnathostomata - ps21
Euteleostomi - ps22
Sarcopterygii - ps23
Tetrapoda - ps24
Amniota - ps25
Mammalia - ps26
Theria - ps27
Placentalia - ps28
Boreoeutheria - ps29
Euarchontoglires - ps30
Euarchonta - ps31
Primates - ps32
Haplorrhini - ps33
Simiiformes - ps34
Catarrhini - ps35
Hominoidea - ps36
Hominidae - ps37
Homininae - ps38
Hominini - ps39
Homo_sapiens - ps40
Phylostratum

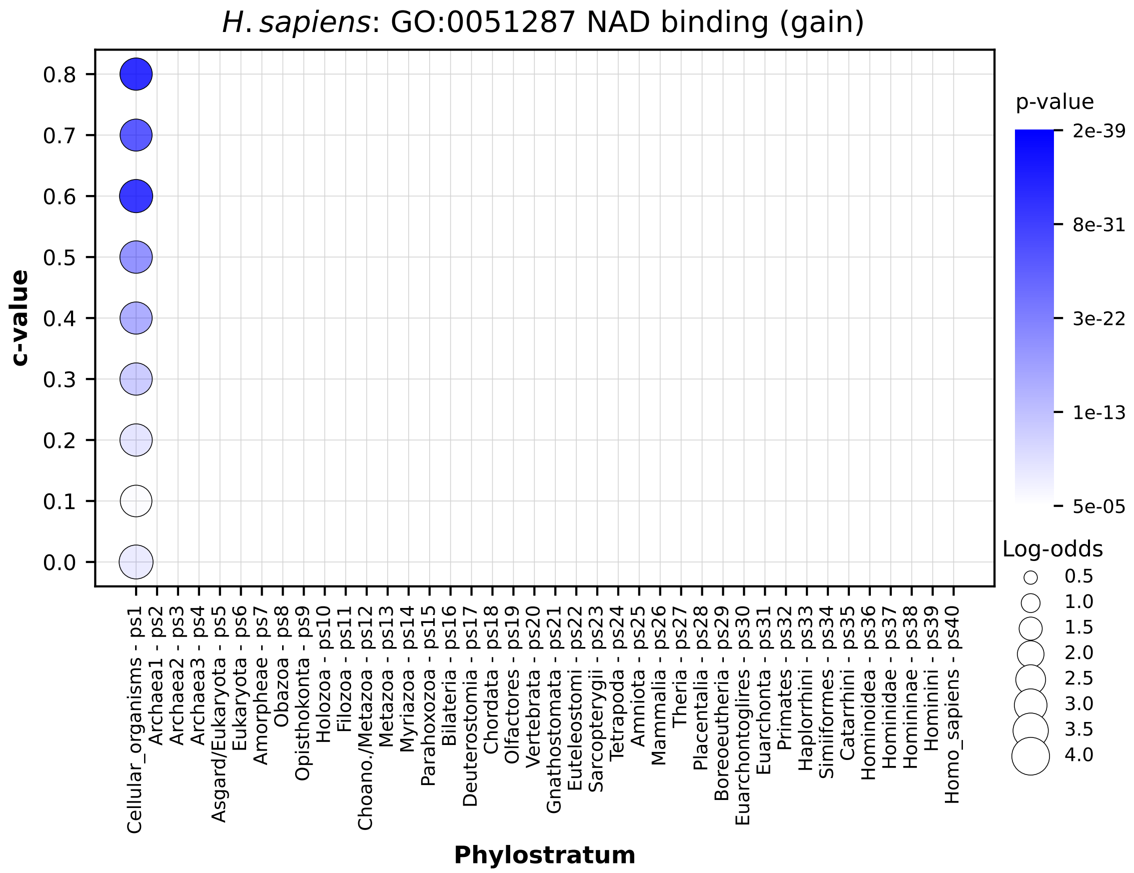

H. sapiens: GO:0051287 NAD binding (gain)
c-value
0.8
0.7
0.6
0.5
0.4
0.3
0.2
0.1
0.0
Cellular_organisms - ps1
Archaea1 - ps2
Archaea2 - ps3
Archaea3 - ps4
Asgard/Eukaryota - ps5
Eukaryota - ps6
Amorpheae - ps7
Obazoa - ps8
Opisthokonta - ps9
Holozoa - ps10
Filozoa - ps11
Choano./Metazoa - ps12
Metazoa - ps13
Myriazoa - ps14
Parahoxozoa - ps15
Bilateria - ps16
Deuterostomia - ps17
Chordata - ps18
Olfactores - ps19
Vertebrata - ps20
Gnathostomata - ps21
Euteleostomi - ps22
Sarcopterygii - ps23
Tetrapoda - ps24
Amniota - ps25
Mammalia - ps26
Theria - ps27
Placentalia - ps28
Boreoeutheria - ps29
Euarchontoglires - ps30
Euarchonta - ps31
Primates - ps32
Haplorrhini - ps33
Simiiformes - ps34
Catarrhini - ps35
Hominoidea - ps36
Hominidae - ps37
Homininae - ps38
Hominini - ps39
Homo_sapiens - ps40
Phylostratum
p-value
2e-39
8e-31
3e-22
1e-13
5e-05
Log-odds
0.5
1.0
1.5
2.0
2.5
3.0
3.5
4.0

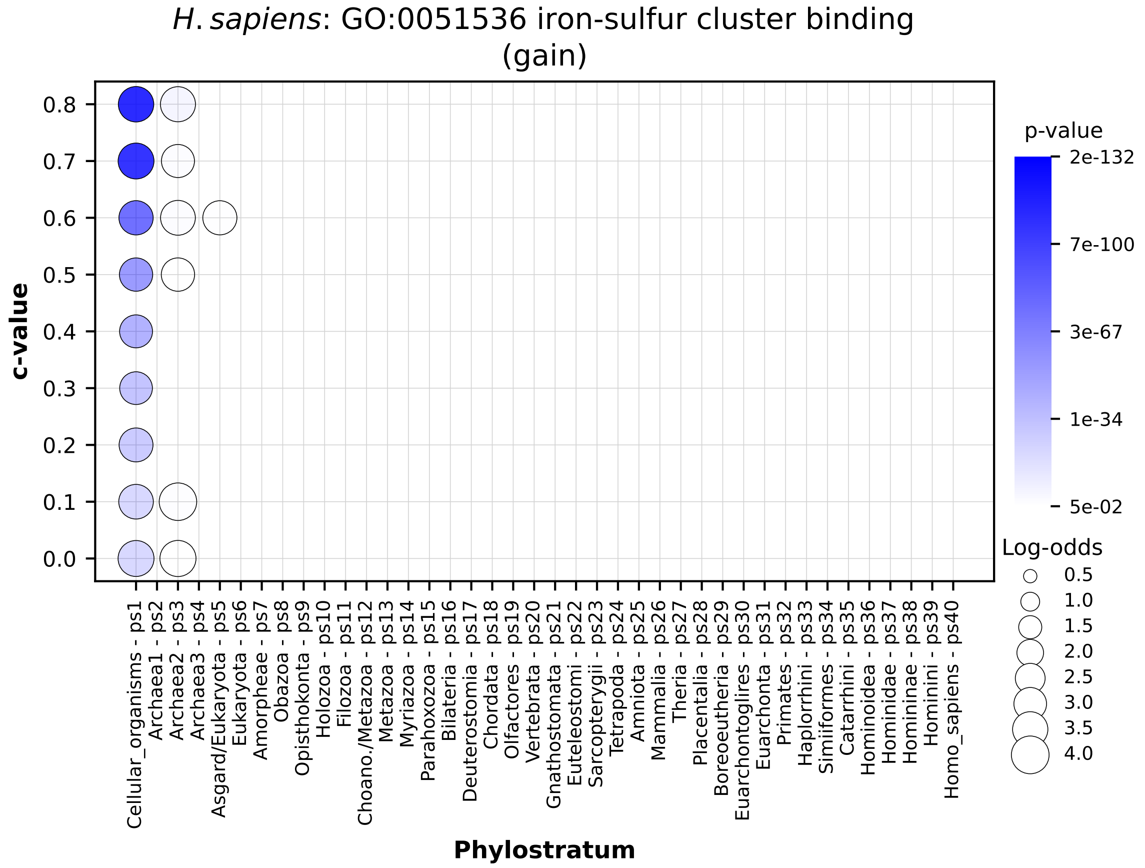

H. sapiens: GO:0051536 iron-sulfur cluster binding
(gain)
c-value
0.8
0.7
0.6
0.5
0.4
0.3
0.2
0.1
0.0
p-value
2e-132
7e-100
3e-67
1e-34
5e-02
Log-odds
0.5
1.0
1.5
2.0
2.5
3.0
3.5
4.0
Cellular_organisms - ps1
Archaea1 - ps2
Archaea2 - ps3
Archaea3 - ps4
Asgard/Eukaryota - ps5
Eukaryota - ps6
Amorpheae - ps7
Obazoa - ps8
Opisthokonta - ps9
Holozoa - ps10
Filozoa - ps11
Choano./Metazoa - ps12
Metazoa - ps13
Myriazoa - ps14
Parahoxozoa - ps15
Bilateria - ps16
Deuterostomia - ps17
Chordata - ps18
Olfactores - ps19
Vertebrata - ps20
Gnathostomata - ps21
Euteleostomi - ps22
Sarcopterygii - ps23
Tetrapoda - ps24
Amniota - ps25
Mammalia - ps26
Theria - ps27
Placentalia - ps28
Boreoeutheria - ps29
Euarchontoglires - ps30
Euarchonta - ps31
Primates - ps32
Haplorrhini - ps33
Simiiformes - ps34
Catarrhini - ps35
Hominoidea - ps36
Hominidae - ps37
Homininae - ps38
Hominini - ps39
Homo_sapiens - ps40
Phylostratum

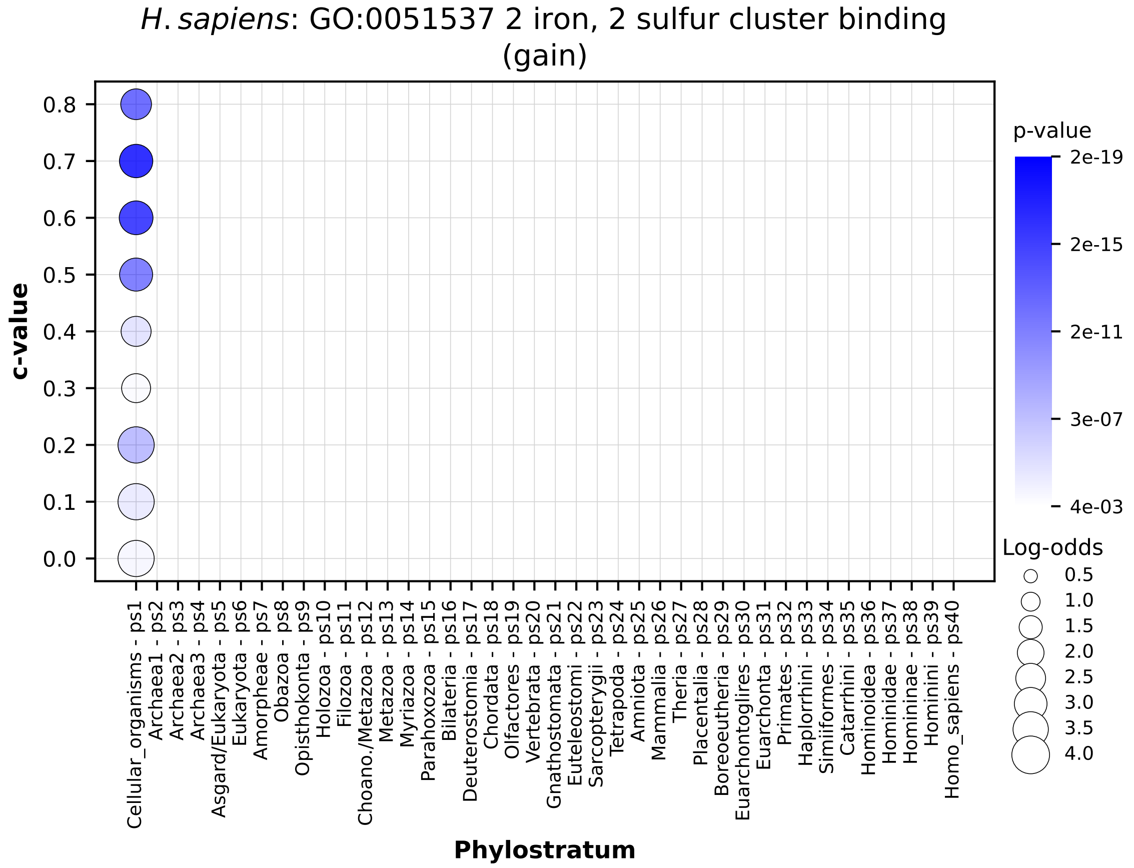
H. sapiens: GO:0051537 2 iron, 2 sulfur cluster binding
(gain)
c-value
0.8
0.7
0.6
0.5
0.4
0.3
0.2
0.1
0.0
p-value
2e-19
2e-15
2e-11
3e-07
4e-03
Log-odds
0.5
1.0
1.5
2.0
2.5
3.0
3.5
4.0
Cellular_organisms - ps1
Archaea1 - ps2
Archaea2 - ps3
Archaea3 - ps4
Asgard/Eukaryota - ps5
Eukaryota - ps6
Amorpheae - ps7
Obazoa - ps8
Opisthokonta - ps9
Holozoa - ps10
Filozoa - ps11
Choano./Metazoa - ps12
Metazoa - ps13
Myriazoa - ps14
Parahoxozoa - ps15
Bilateria - ps16
Deuterostomia - ps17
Chordata - ps18
Olfactores - ps19
Vertebrata - ps20
Gnathostomata - ps21
Euteleostomi - ps22
Sarcopterygii - ps23
Tetrapoda - ps24
Amniota - ps25
Mammalia - ps26
Theria - ps27
Placentalia - ps28
Boreoeutheria - ps29
Euarchontoglires - ps30
Euarchonta - ps31
Primates - ps32
Haplorrhini - ps33
Simiiformes - ps34
Catarrhini - ps35
Hominoidea - ps36
Hominidae - ps37
Homininae - ps38
Hominini - ps39
Homo_sapiens - ps40
Phylostratum

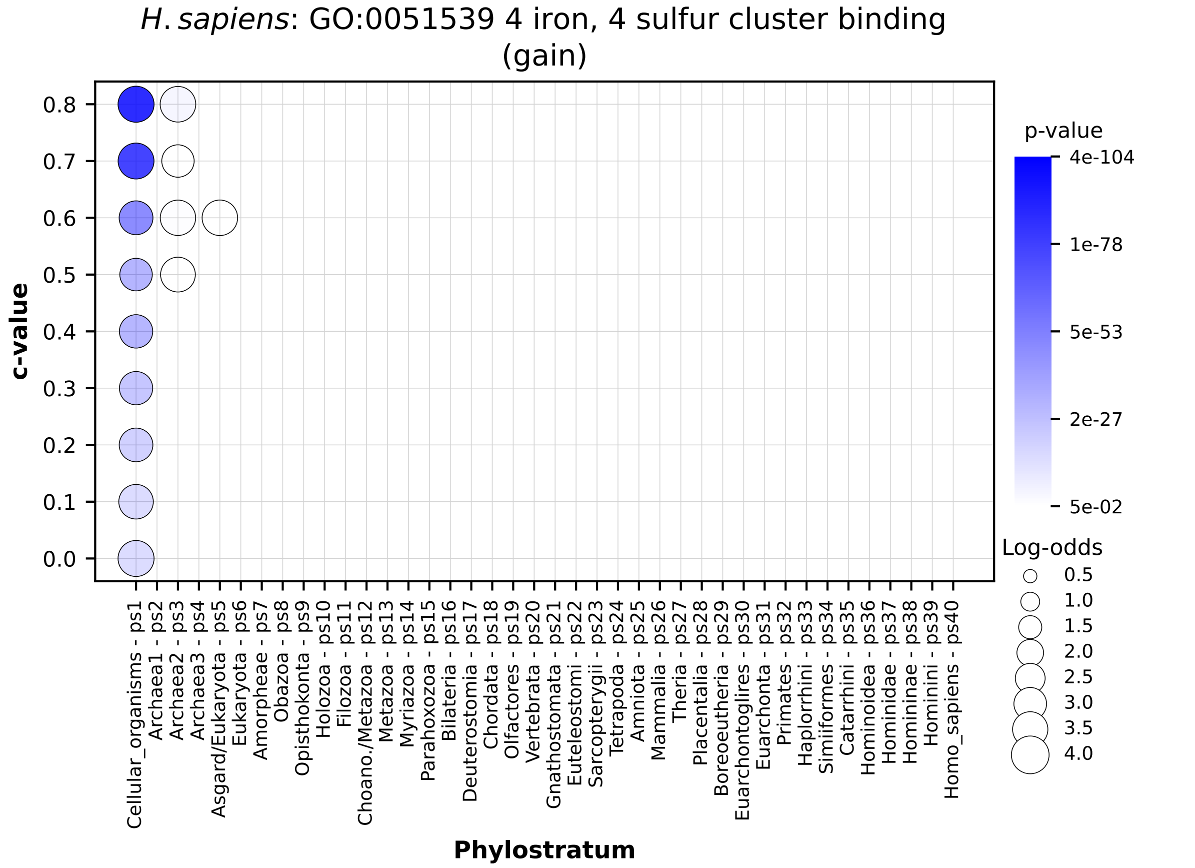
H. sapiens: GO:0051539 4 iron, 4 sulfur cluster binding (gain)
c-value
0.8
0.7
0.6
0.5
0.4
0.3
0.2
0.1
0.0
p-value
4e-104
1e-78
5e-53
2e-27
5e-02
Log-odds
0.5
1.0
1.5
2.0
2.5
3.0
3.5
4.0
Cellular_organisms - ps1
Archaea1 - ps2
Archaea2 - ps3
Archaea3 - ps4
Asgard/Eukaryota - ps5
Eukaryota - ps6
Amorpheae - ps7
Obazoa - ps8
Opisthokonta - ps9
Holozoa - ps10
Filozoa - ps11
Choano./Metazoa - ps12
Metazoa - ps13
Myriazoa - ps14
Parahoxozoa - ps15
Bilateria - ps16
Deuterostomia - ps17
Chordata - ps18
Olfactores - ps19
Vertebrata - ps20
Gnathostomata - ps21
Euteleostomi - ps22
Sarcopterygii - ps23
Tetrapoda - ps24
Amniota - ps25
Mammalia - ps26
Theria - ps27
Placentalia - ps28
Boreoeutheria - ps29
Euarchontoglires - ps30
Euarchonta - ps31
Primates - ps32
Haplorrhini - ps33
Simiiformes - ps34
Catarrhini - ps35
Hominoidea - ps36
Hominidae - ps37
Homininae - ps38
Hominini - ps39
Homo_sapiens - ps40
Phylostratum

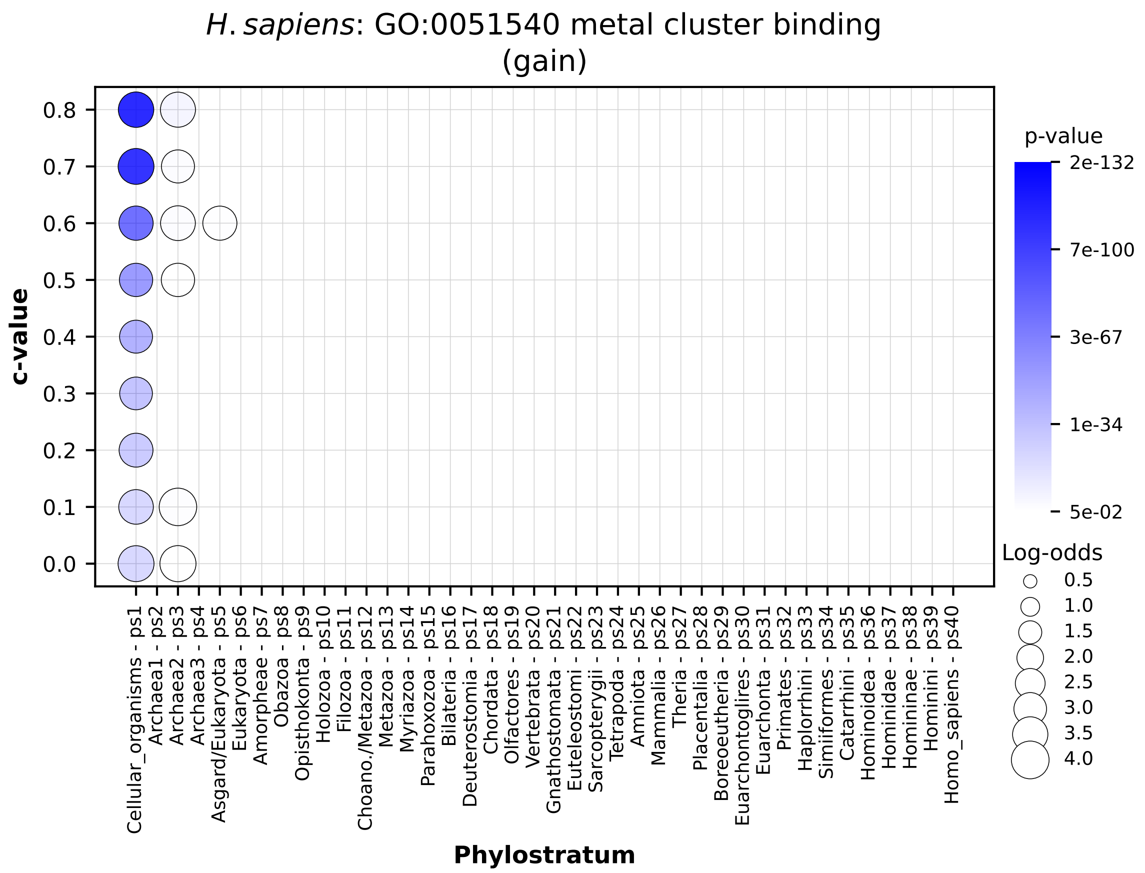

*H. sapiens*: GO:0051540 metal cluster binding
(gain)
c-value
0.8
0.7
0.6
0.5
0.4
0.3
0.2
0.1
0.0
p-value
2e-132
7e-100
3e-67
1e-34
5e-02
Log-odds
0.5
1.0
1.5
2.0
2.5
3.0
3.5
4.0
Cellular_organisms - ps1
Archaea1 - ps2
Archaea2 - ps3
Archaea3 - ps4
Asgard/Eukaryota - ps5
Eukaryota - ps6
Amorpheae - ps7
Obazoa - ps8
Opisthokonta - ps9
Holozoa - ps10
Filozoa - ps11
Choano./Metazoa - ps12
Metazoa - ps13
Myriazoa - ps14
Parahoxozoa - ps15
Bilateria - ps16
Deuterostomia - ps17
Chordata - ps18
Olfactores - ps19
Vertebrata - ps20
Gnathostomata - ps21
Euteleostomi - ps22
Sarcopterygii - ps23
Tetrapoda - ps24
Amniota - ps25
Mammalia - ps26
Theria - ps27
Placentalia - ps28
Boreoeutheria - ps29
Euarchontoglires - ps30
Euarchonta - ps31
Primates - ps32
Haplorrhini - ps33
Simiiformes - ps34
Catarrhini - ps35
Hominoidea - ps36
Hominidae - ps37
Homininae - ps38
Hominini - ps39
Homo_sapiens - ps40
Phylostratum

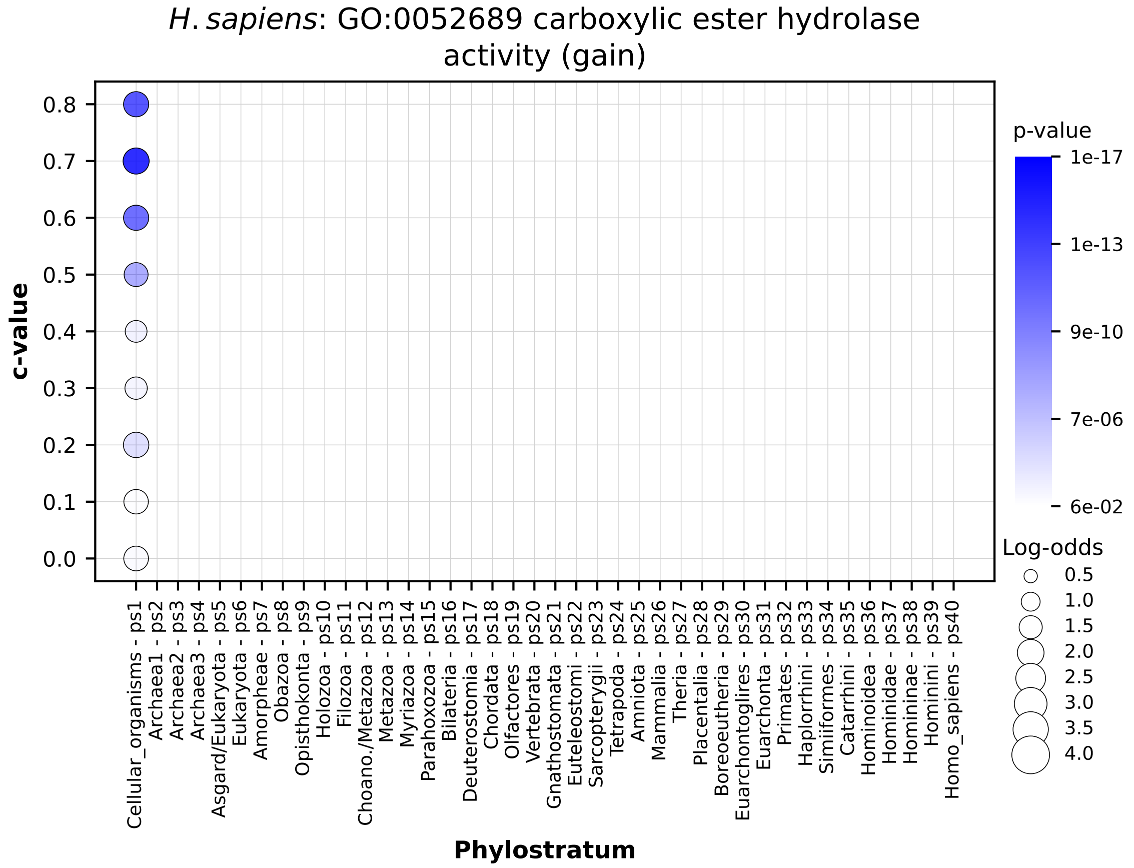

H. sapiens: GO:0052689 carboxylic ester hydrolase activity (gain)
c-value
0.8
0.7
0.6
0.5
0.4
0.3
0.2
0.1
0.0
Phylostratum
Cellular_organisms - ps1
Archaea1 - ps2
Archaea2 - ps3
Archaea3 - ps4
Asgard/Eukaryota - ps5
Eukaryota - ps6
Amorpheae - ps7
Obazoa - ps8
Opisthokonta - ps9
Holozoa - ps10
Filozoa - ps11
Choano./Metazoa - ps12
Metazoa - ps13
Myriazoa - ps14
Parahoxozoa - ps15
Bilateria - ps16
Deuterostomia - ps17
Chordata - ps18
Olfactores - ps19
Vertebrata - ps20
Gnathostomata - ps21
Euteleostomi - ps22
Sarcopterygii - ps23
Tetrapoda - ps24
Amniota - ps25
Mammalia - ps26
Theria - ps27
Placentalia - ps28
Boreoeutheria - ps29
Euarchontoglires - ps30
Euarchonta - ps31
Primates - ps32
Haplorrhini - ps33
Simiiformes - ps34
Catarrhini - ps35
Hominoidea - ps36
Hominidae - ps37
Homininae - ps38
Hominini - ps39
Homo_sapiens - ps40
p-value
1e-17
1e-13
9e-10
7e-06
6e-02
Log-odds
0.5
1.0
1.5
2.0
2.5
3.0
3.5
4.0

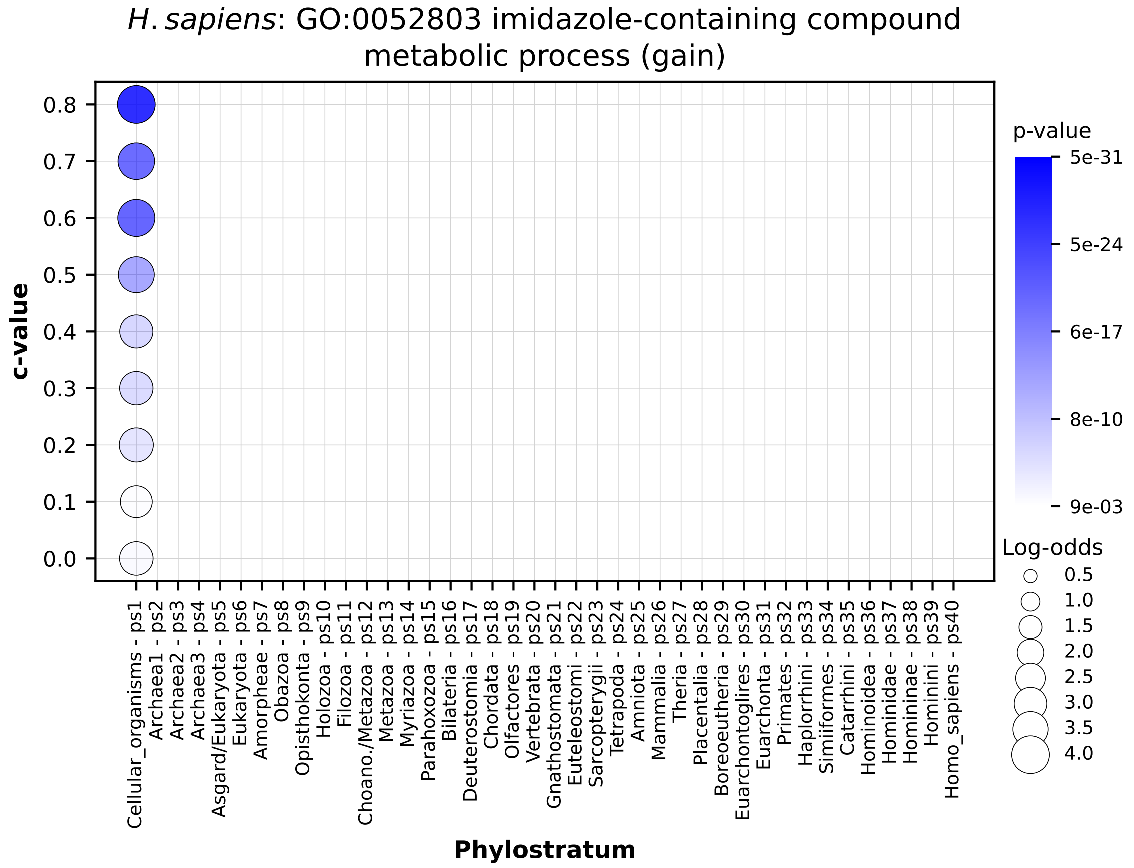
H. sapiens: GO:0052803 imidazole-containing compound metabolic process (gain)
c-value
0.8
0.7
0.6
0.5
0.4
0.3
0.2
0.1
0.0
p-value
5e-31
5e-24
6e-17
8e-10
9e-03
Log-odds
0.5
1.0
1.5
2.0
2.5
3.0
3.5
4.0
Cellular_organisms - ps1
Archaea1 - ps2
Archaea2 - ps3
Archaea3 - ps4
Asgard/Eukaryota - ps5
Eukaryota - ps6
Amorpheae - ps7
Obazoa - ps8
Opisthokonta - ps9
Holozoa - ps10
Filozoa - ps11
Choano./Metazoa - ps12
Metazoa - ps13
Myriazoa - ps14
Parahoxozoa - ps15
Bilateria - ps16
Deuterostomia - ps17
Chordata - ps18
Olfactores - ps19
Vertebrata - ps20
Gnathostomata - ps21
Euteleostomi - ps22
Sarcopterygii - ps23
Tetrapoda - ps24
Amniota - ps25
Mammalia - ps26
Theria - ps27
Placentalia - ps28
Boreoeutheria - ps29
Euarchontoglires - ps30
Euarchonta - ps31
Primates - ps32
Haplorrhini - ps33
Simiiformes - ps34
Catarrhini - ps35
Hominoidea - ps36
Hominidae - ps37
Homininae - ps38
Hominini - ps39
Homo_sapiens - ps40
Phylostratum

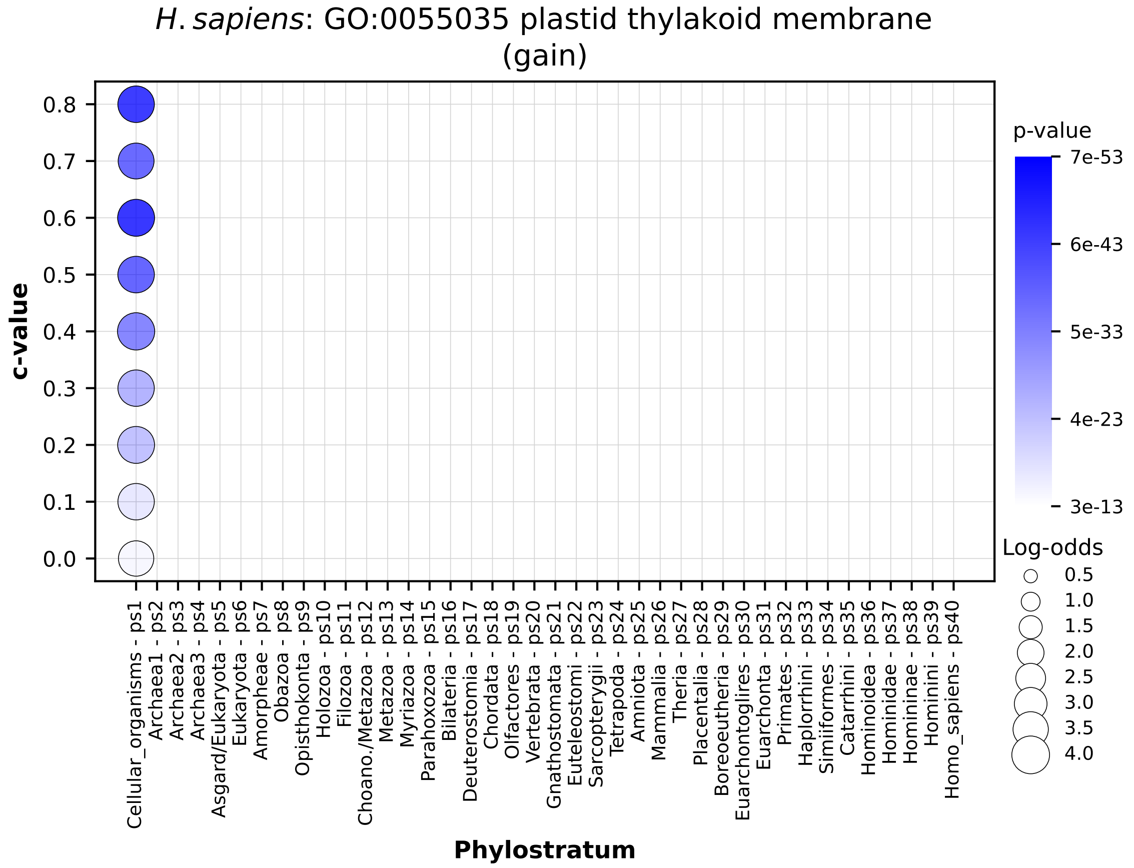

*H. sapiens*: GO:0055035 plastid thylakoid membrane (gain)
c-value
0.8
0.7
0.6
0.5
0.4
0.3
0.2
0.1
0.0
p-value
7e-53
6e-43
5e-33
4e-23
3e-13
Log-odds
0.5
1.0
1.5
2.0
2.5
3.0
3.5
4.0
Cellular_organisms - ps1
Archaea1 - ps2
Archaea2 - ps3
Archaea3 - ps4
Asgard/Eukaryota - ps5
Eukaryota - ps6
Amorpheae - ps7
Obazoa - ps8
Opisthokonta - ps9
Holozoa - ps10
Filozoa - ps11
Choano./Metazoa - ps12
Metazoa - ps13
Myriazoa - ps14
Parahoxozoa - ps15
Bilateria - ps16
Deuterostomia - ps17
Chordata - ps18
Olfactores - ps19
Vertebrata - ps20
Gnathostomata - ps21
Euteleostomi - ps22
Sarcopterygii - ps23
Tetrapoda - ps24
Amniota - ps25
Mammalia - ps26
Theria - ps27
Placentalia - ps28
Boreoeutheria - ps29
Euarchontoglires - ps30
Euarchonta - ps31
Primates - ps32
Haplorrhini - ps33
Simiiformes - ps34
Catarrhini - ps35
Hominoidea - ps36
Hominidae - ps37
Homininae - ps38
Hominini - ps39
Homo_sapiens - ps40
Phylostratum

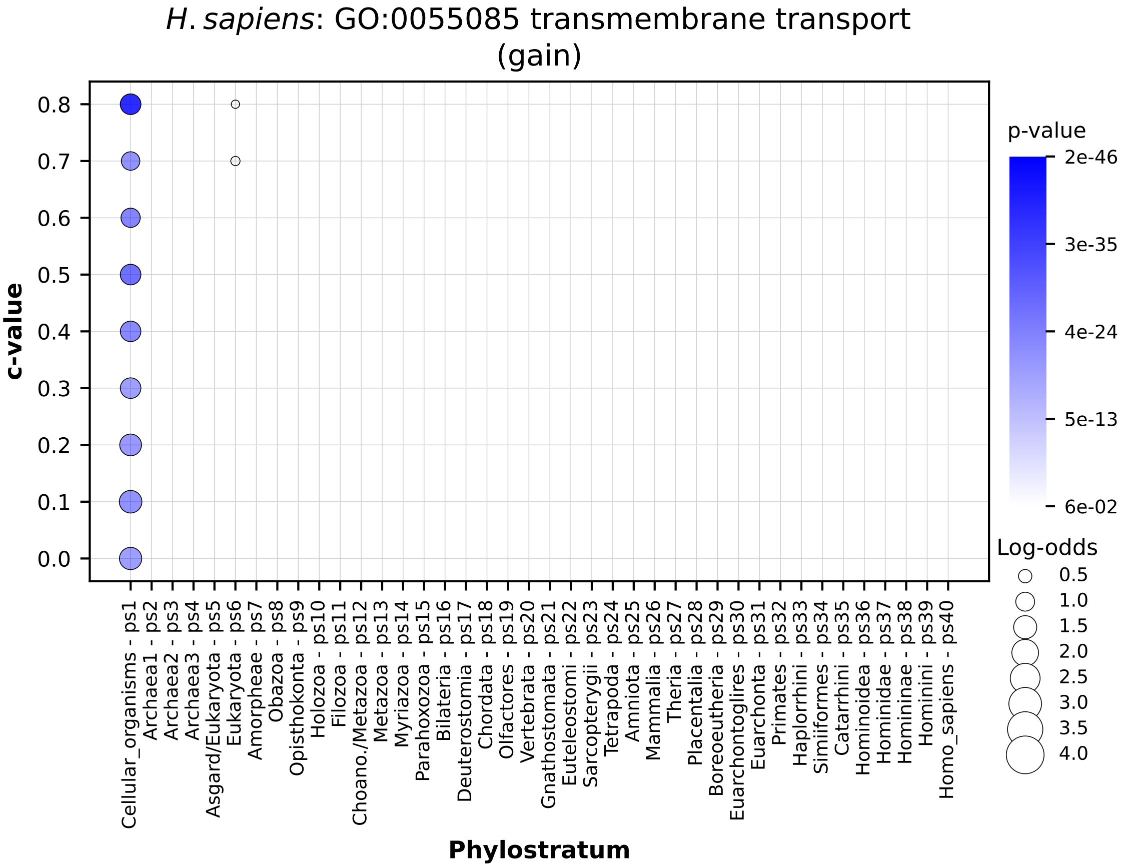

H. sapiens: GO:0055085 transmembrane transport
(gain)
c-value
0.0
0.1
0.2
0.3
0.4
0.5
0.6
0.7
0.8
Phylostratum
Cellular_organisms - ps1
Archaea1 - ps2
Archaea2 - ps3
Archaea3 - ps4
Asgard/Eukaryota - ps5
Eukaryota - ps6
Amorpheae - ps7
Obazoa - ps8
Opisthokonta - ps9
Holozoa - ps10
Filozoa - ps11
Choano./Metazoa - ps12
Metazoa - ps13
Myriazoa - ps14
Parahoxozoa - ps15
Bilateria - ps16
Deuterostomia - ps17
Chordata - ps18
Olfactores - ps19
Vertebrata - ps20
Gnathostomata - ps21
Euteleostomi - ps22
Sarcopterygii - ps23
Tetrapoda - ps24
Amniota - ps25
Mammalia - ps26
Theria - ps27
Placentalia - ps28
Boreoeutheria - ps29
Euarchontoglires - ps30
Euarchonta - ps31
Primates - ps32
Haplorrhini - ps33
Simiiformes - ps34
Catarrhini - ps35
Hominoidea - ps36
Hominidae - ps37
Homininae - ps38
Hominini - ps39
Homo_sapiens - ps40
p-value
2e-46
3e-35
4e-24
5e-13
6e-02
Log-odds
0.5
1.0
1.5
2.0
2.5
3.0
3.5
4.0

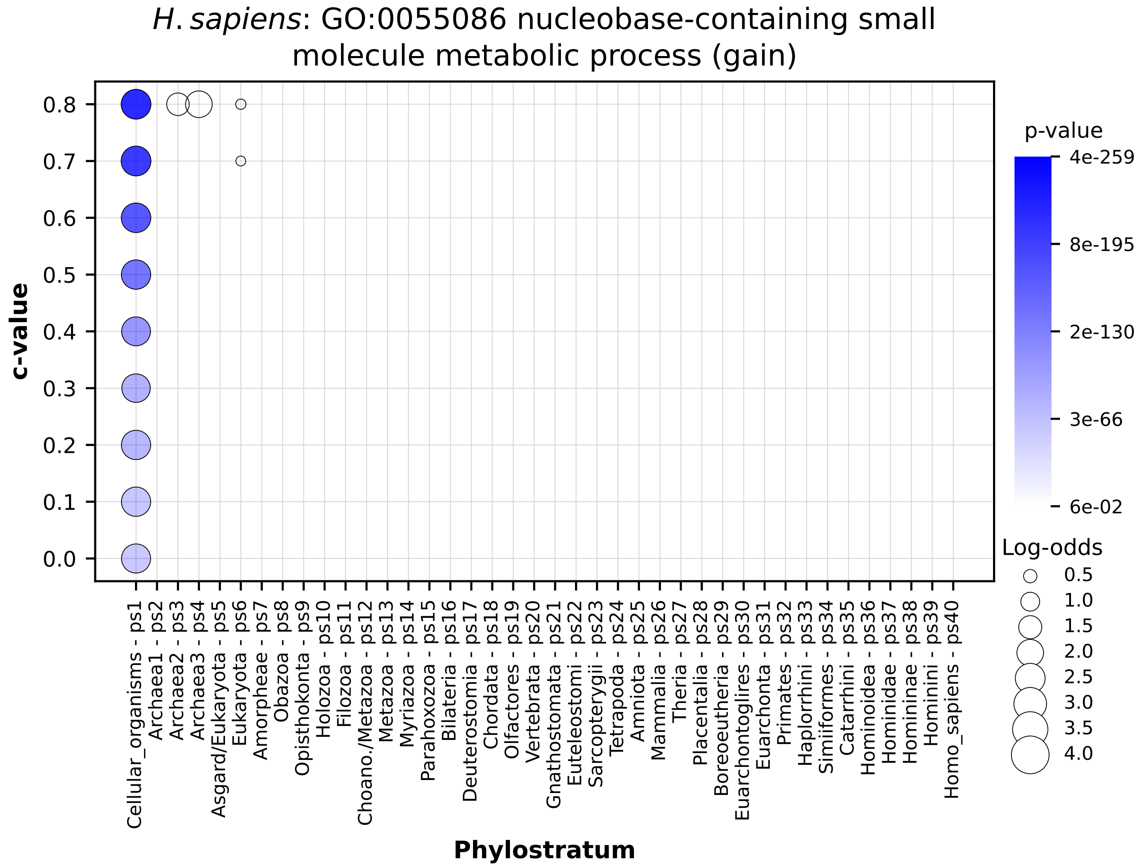
H. sapiens: GO:0055086 nucleobase-containing small molecule metabolic process (gain)
c-value
0.8
0.7
0.6
0.5
0.4
0.3
0.2
0.1
0.0
p-value
4e-259
8e-195
2e-130
3e-66
6e-02
Log-odds
0.5
1.0
1.5
2.0
2.5
3.0
3.5
4.0
Cellular_organisms - ps1
Archaea1 - ps2
Archaea2 - ps3
Archaea3 - ps4
Asgard/Eukaryota - ps5
Eukaryota - ps6
Amorpheae - ps7
Obazoa - ps8
Opisthokonta - ps9
Holozoa - ps10
Filozoa - ps11
Choano./Metazoa - ps12
Metazoa - ps13
Myriazoa - ps14
Parahoxozoa - ps15
Bilateria - ps16
Deuterostomia - ps17
Chordata - ps18
Olfactores - ps19
Vertebrata - ps20
Gnathostomata - ps21
Euteleostomi - ps22
Sarcopterygii - ps23
Tetrapoda - ps24
Amniota - ps25
Mammalia - ps26
Theria - ps27
Placentalia - ps28
Boreoeutheria - ps29
Euarchontoglires - ps30
Euarchonta - ps31
Primates - ps32
Haplorrhini - ps33
Simiiformes - ps34
Catarrhini - ps35
Hominoidea - ps36
Hominidae - ps37
Homininae - ps38
Hominini - ps39
Homo_sapiens - ps40
Phylostratum

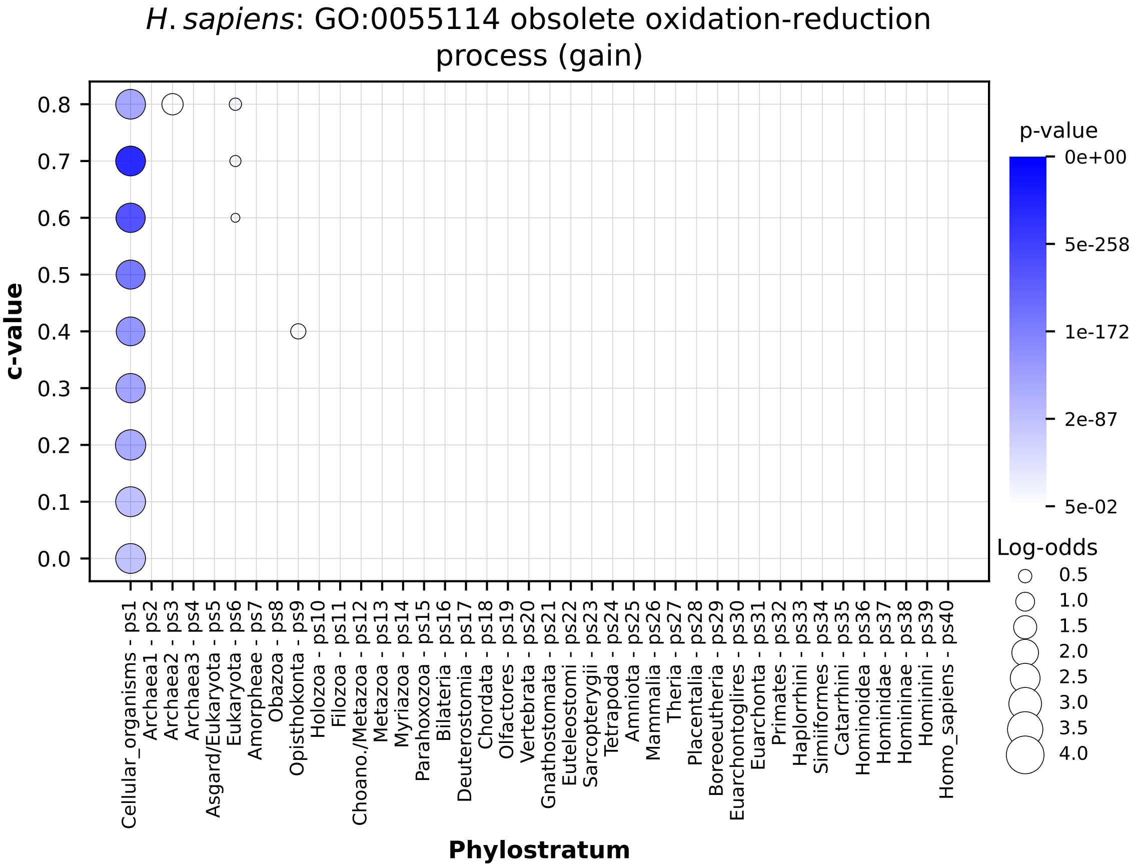

H. sapiens: GO:0055114 obsolete oxidation-reduction process (gain)
c-value
0.8
0.7
0.6
0.5
0.4
0.3
0.2
0.1
0.0
p-value
0e+00
5e-258
1e-172
2e-87
5e-02
Log-odds
0.5
1.0
1.5
2.0
2.5
3.0
3.5
4.0
Cellular_organisms - ps1
Archaea1 - ps2
Archaea2 - ps3
Archaea3 - ps4
Asgard/Eukaryota - ps5
Eukaryota - ps6
Amorpheae - ps7
Obazoa - ps8
Opisthokonta - ps9
Holozoa - ps10
Filozoa - ps11
Choano./Metazoa - ps12
Metazoa - ps13
Myriazoa - ps14
Parahoxozoa - ps15
Bilateria - ps16
Deuterostomia - ps17
Chordata - ps18
Olfactores - ps19
Vertebrata - ps20
Gnathostomata - ps21
Euteleostomi - ps22
Sarcopterygii - ps23
Tetrapoda - ps24
Amniota - ps25
Mammalia - ps26
Theria - ps27
Placentalia - ps28
Boreoeutheria - ps29
Euarchontoglires - ps30
Euarchonta - ps31
Primates - ps32
Haplorrhini - ps33
Simiiformes - ps34
Catarrhini - ps35
Hominoidea - ps36
Hominidae - ps37
Homininae - ps38
Hominini - ps39
Homo_sapiens - ps40
Phylostratum

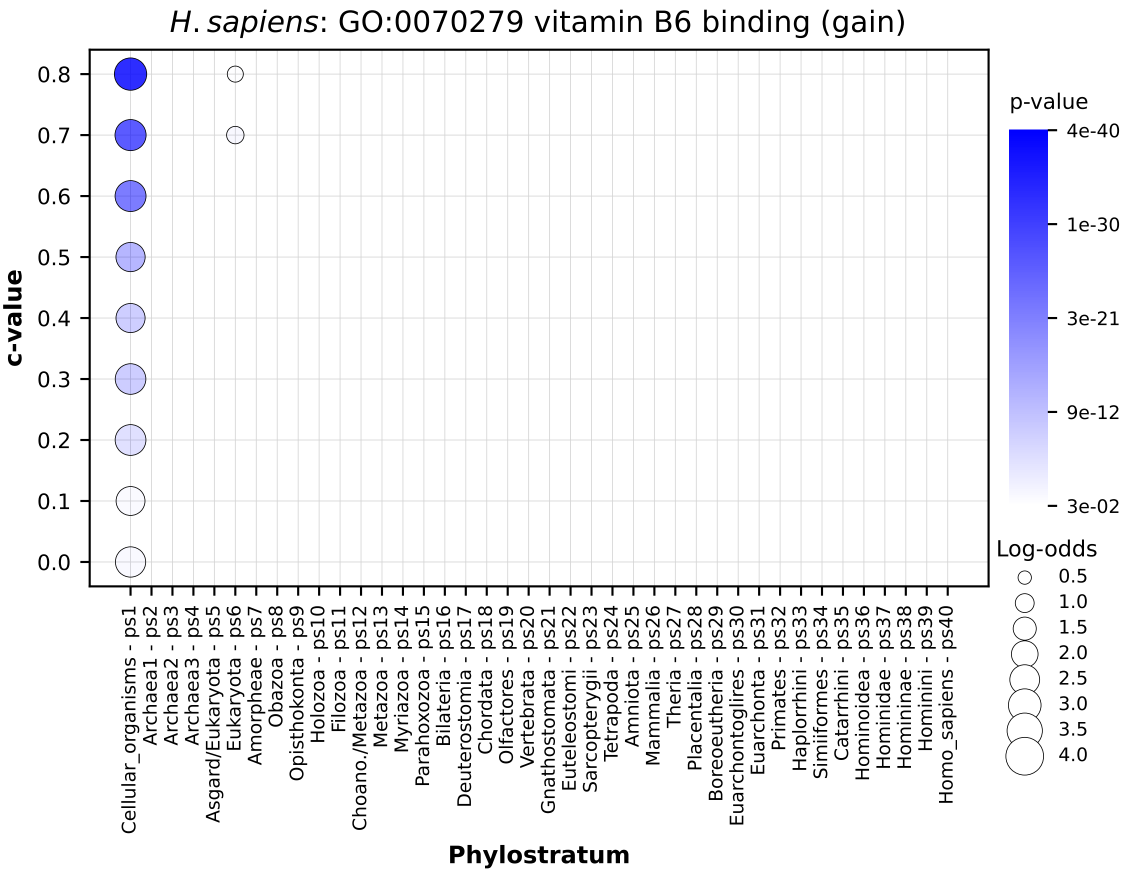

H. sapiens: GO:0070279 vitamin B6 binding (gain)
c-value
0.8
0.7
0.6
0.5
0.4
0.3
0.2
0.1
0.0
p-value
4e-40
1e-30
3e-21
9e-12
3e-02
Log-odds
0.5
1.0
1.5
2.0
2.5
3.0
3.5
4.0
Cellular_organisms - ps1
Archaea1 - ps2
Archaea2 - ps3
Archaea3 - ps4
Asgard/Eukaryota - ps5
Eukaryota - ps6
Amorpheae - ps7
Obazoa - ps8
Opisthokonta - ps9
Holozoa - ps10
Filozoa - ps11
Choano./Metazoa - ps12
Metazoa - ps13
Myriazoa - ps14
Parahoxozoa - ps15
Bilateria - ps16
Deuterostomia - ps17
Chordata - ps18
Olfactores - ps19
Vertebrata - ps20
Gnathostomata - ps21
Euteleostomi - ps22
Sarcopterygii - ps23
Tetrapoda - ps24
Amniota - ps25
Mammalia - ps26
Theria - ps27
Placentalia - ps28
Boreoeutheria - ps29
Euarchontoglires - ps30
Euarchonta - ps31
Primates - ps32
Haplorrhini - ps33
Simiiformes - ps34
Catarrhini - ps35
Hominoidea - ps36
Hominidae - ps37
Homininae - ps38
Hominini - ps39
Homo_sapiens - ps40
Phylostratum

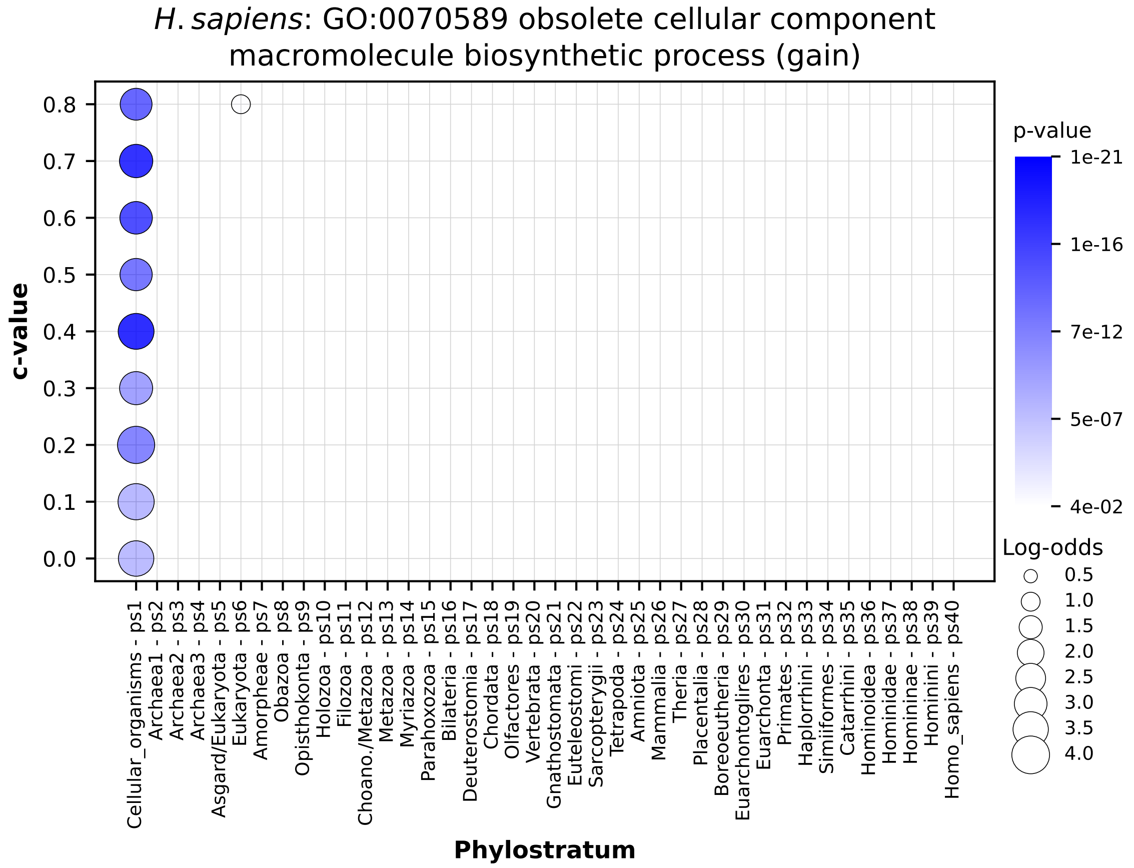

H. sapiens: GO:0070589 obsolete cellular component macromolecule biosynthetic process (gain)
c-value
0.8
0.7
0.6
0.5
0.4
0.3
0.2
0.1
0.0
Phylostratum
Cellular_organisms - ps1
Archaea1 - ps2
Archaea2 - ps3
Archaea3 - ps4
Asgard/Eukaryota - ps5
Eukaryota - ps6
Amorpheae - ps7
Obazoa - ps8
Opisthokonta - ps9
Holozoa - ps10
Filozoa - ps11
Choano./Metazoa - ps12
Metazoa - ps13
Myriazoa - ps14
Parahoxozoa - ps15
Bilateria - ps16
Deuterostomia - ps17
Chordata - ps18
Olfactores - ps19
Vertebrata - ps20
Gnathostomata - ps21
Euteleostomi - ps22
Sarcopterygii - ps23
Tetrapoda - ps24
Amniota - ps25
Mammalia - ps26
Theria - ps27
Placentalia - ps28
Boreoeutheria - ps29
Euarchontoglires - ps30
Euarchonta - ps31
Primates - ps32
Haplorrhini - ps33
Simiiformes - ps34
Catarrhini - ps35
Hominoidea - ps36
Hominidae - ps37
Homininae - ps38
Hominini - ps39
Homo_sapiens - ps40
p-value
1e-21
1e-16
7e-12
5e-07
4e-02
Log-odds
0.5
1.0
1.5
2.0
2.5
3.0
3.5
4.0

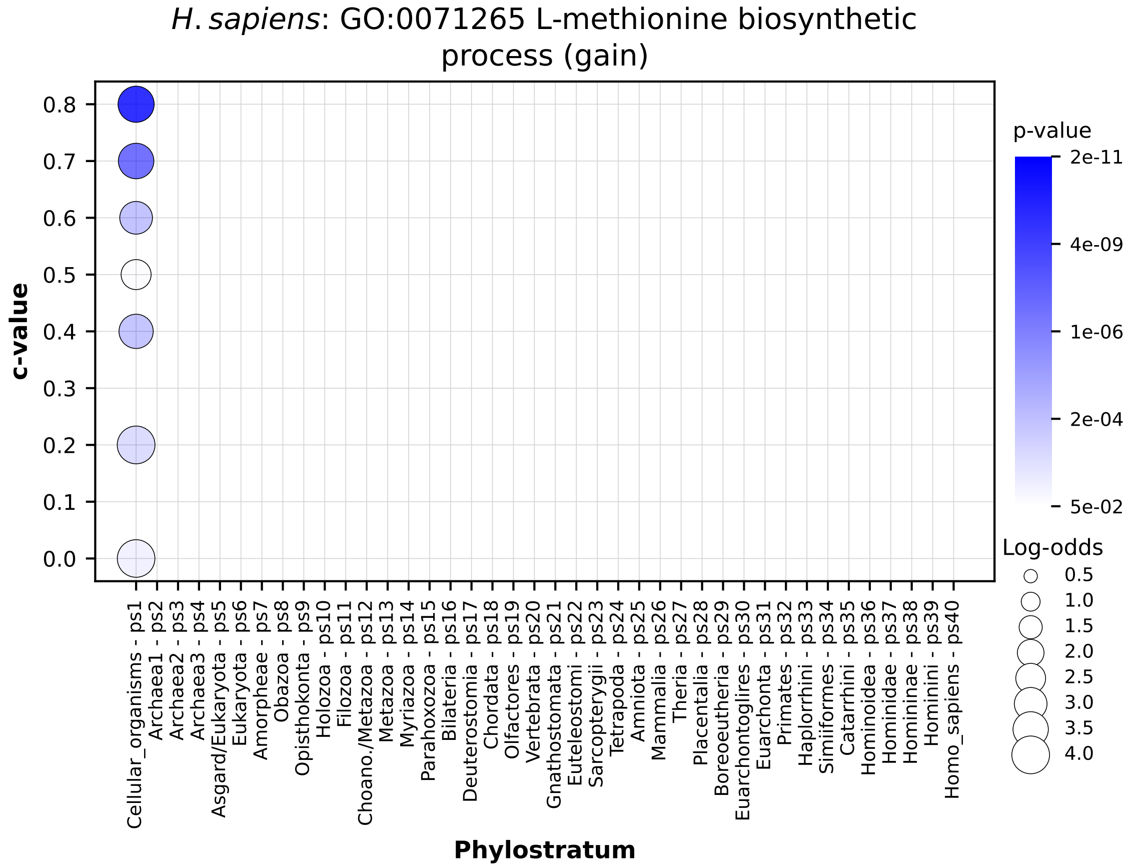

H. sapiens: GO:0071265 L-methionine biosynthetic process (gain)
c-value
0.0
0.1
0.2
0.3
0.4
0.5
0.6
0.7
0.8
p-value
2e-11
4e-09
1e-06
2e-04
5e-02
Log-odds
0.5
1.0
1.5
2.0
2.5
3.0
3.5
4.0
Cellular_organisms - ps1
Archaea1 - ps2
Archaea2 - ps3
Archaea3 - ps4
Asgard/Eukaryota - ps5
Eukaryota - ps6
Amorpheae - ps7
Obazoa - ps8
Opisthokonta - ps9
Holozoa - ps10
Filozoa - ps11
Choano./Metazoa - ps12
Metazoa - ps13
Myriazoa - ps14
Parahoxozoa - ps15
Bilateria - ps16
Deuterostomia - ps17
Chordata - ps18
Olfactores - ps19
Vertebrata - ps20
Gnathostomata - ps21
Euteleostomi - ps22
Sarcopterygii - ps23
Tetrapoda - ps24
Amniota - ps25
Mammalia - ps26
Theria - ps27
Placentalia - ps28
Boreoeutheria - ps29
Euarchontoglires - ps30
Euarchonta - ps31
Primates - ps32
Haplorrhini - ps33
Simiiformes - ps34
Catarrhini - ps35
Hominoidea - ps36
Hominidae - ps37
Homininae - ps38
Hominini - ps39
Homo_sapiens - ps40
Phylostratum

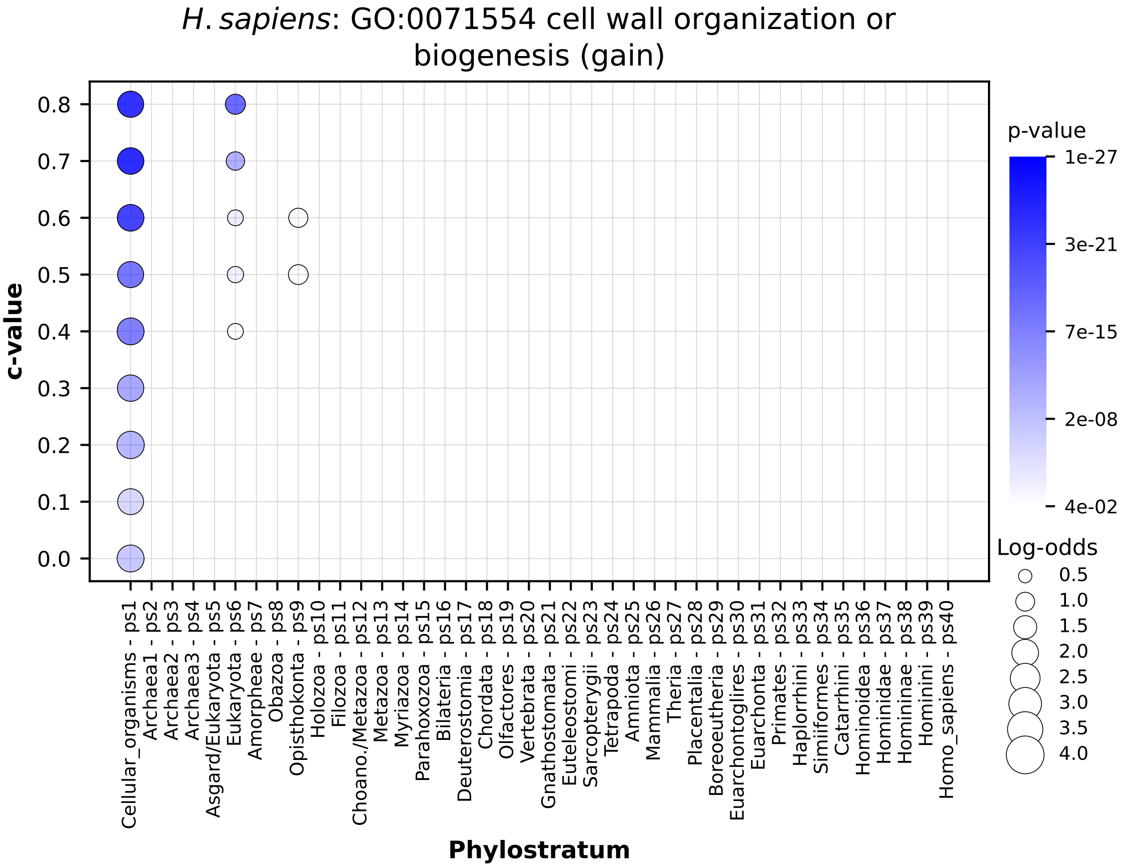

H. sapiens: GO:0071554 cell wall organization or biogenesis (gain)
c-value
0.8
0.7
0.6
0.5
0.4
0.3
0.2
0.1
0.0
Phylostratum
Cellular_organisms - ps1
Archaea1 - ps2
Archaea2 - ps3
Archaea3 - ps4
Asgard/Eukaryota - ps5
Eukaryota - ps6
Amorpheae - ps7
Obazoa - ps8
Opisthokonta - ps9
Holozoa - ps10
Filozoa - ps11
Choano./Metazoa - ps12
Metazoa - ps13
Myriazoa - ps14
Parahoxozoa - ps15
Bilateria - ps16
Deuterostomia - ps17
Chordata - ps18
Olfactores - ps19
Vertebrata - ps20
Gnathostomata - ps21
Euteleostomi - ps22
Sarcopterygii - ps23
Tetrapoda - ps24
Amniota - ps25
Mammalia - ps26
Theria - ps27
Placentalia - ps28
Boreoeutheria - ps29
Euarchontoglires - ps30
Euarchonta - ps31
Primates - ps32
Haplorrhini - ps33
Simiiformes - ps34
Catarrhini - ps35
Hominoidea - ps36
Hominidae - ps37
Homininae - ps38
Hominini - ps39
Homo_sapiens - ps40
p-value
1e-27
3e-21
7e-15
2e-08
4e-02
Log-odds
0.5
1.0
1.5
2.0
2.5
3.0
3.5
4.0

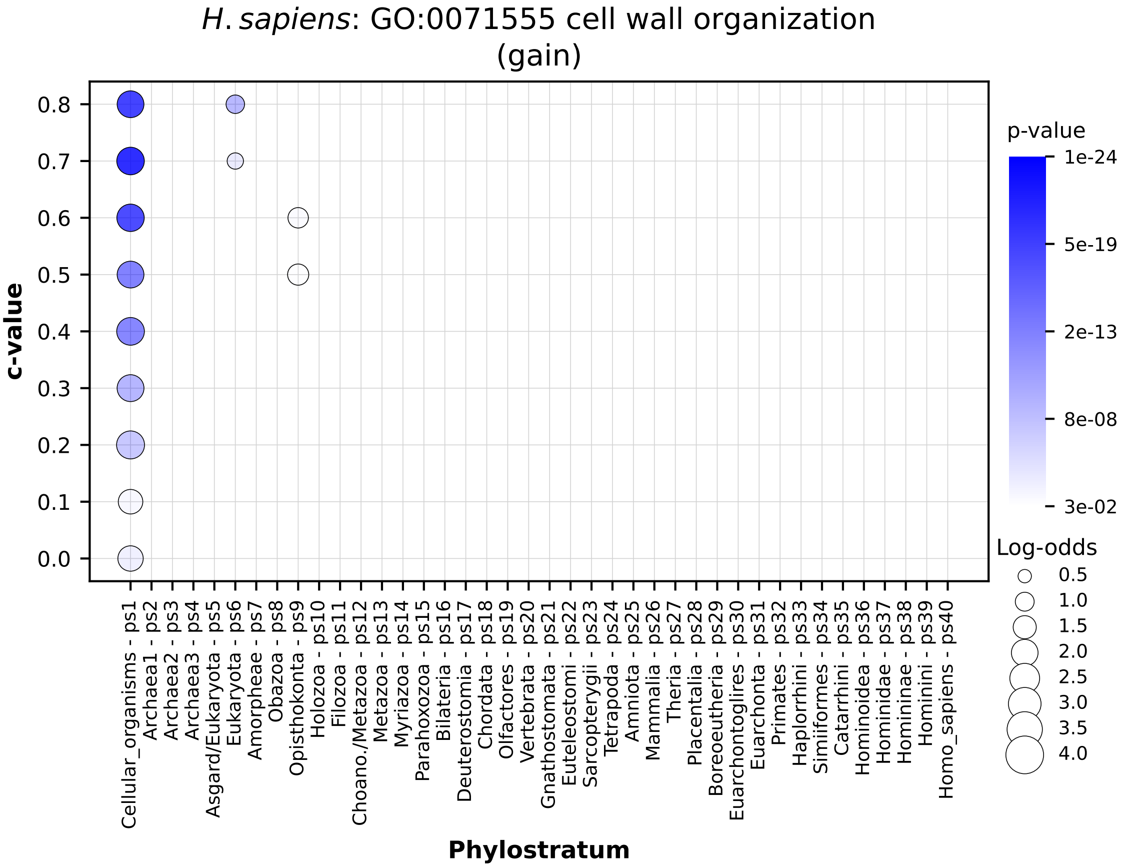

H. sapiens: GO:0071555 cell wall organization
(gain)
c-value
0.8
0.7
0.6
0.5
0.4
0.3
0.2
0.1
0.0
Phylostratum
Cellular_organisms - ps1
Archaea1 - ps2
Archaea2 - ps3
Archaea3 - ps4
Asgard/Eukaryota - ps5
Eukaryota - ps6
Amorpheae - ps7
Obazoa - ps8
Opisthokonta - ps9
Holozoa - ps10
Filozoa - ps11
Choano./Metazoa - ps12
Metazoa - ps13
Myriazoa - ps14
Parahoxozoa - ps15
Bilateria - ps16
Deuterostomia - ps17
Chordata - ps18
Olfactores - ps19
Vertebrata - ps20
Gnathostomata - ps21
Euteleostomi - ps22
Sarcopterygii - ps23
Tetrapoda - ps24
Amniota - ps25
Mammalia - ps26
Theria - ps27
Placentalia - ps28
Boreoeutheria - ps29
Euarchontoglires - ps30
Euarchonta - ps31
Primates - ps32
Haplorrhini - ps33
Simiiformes - ps34
Catarrhini - ps35
Hominoidea - ps36
Hominidae - ps37
Homininae - ps38
Hominini - ps39
Homo_sapiens - ps40
p-value
1e-24
5e-19
2e-13
8e-08
3e-02
Log-odds
0.5
1.0
1.5
2.0
2.5
3.0
3.5
4.0

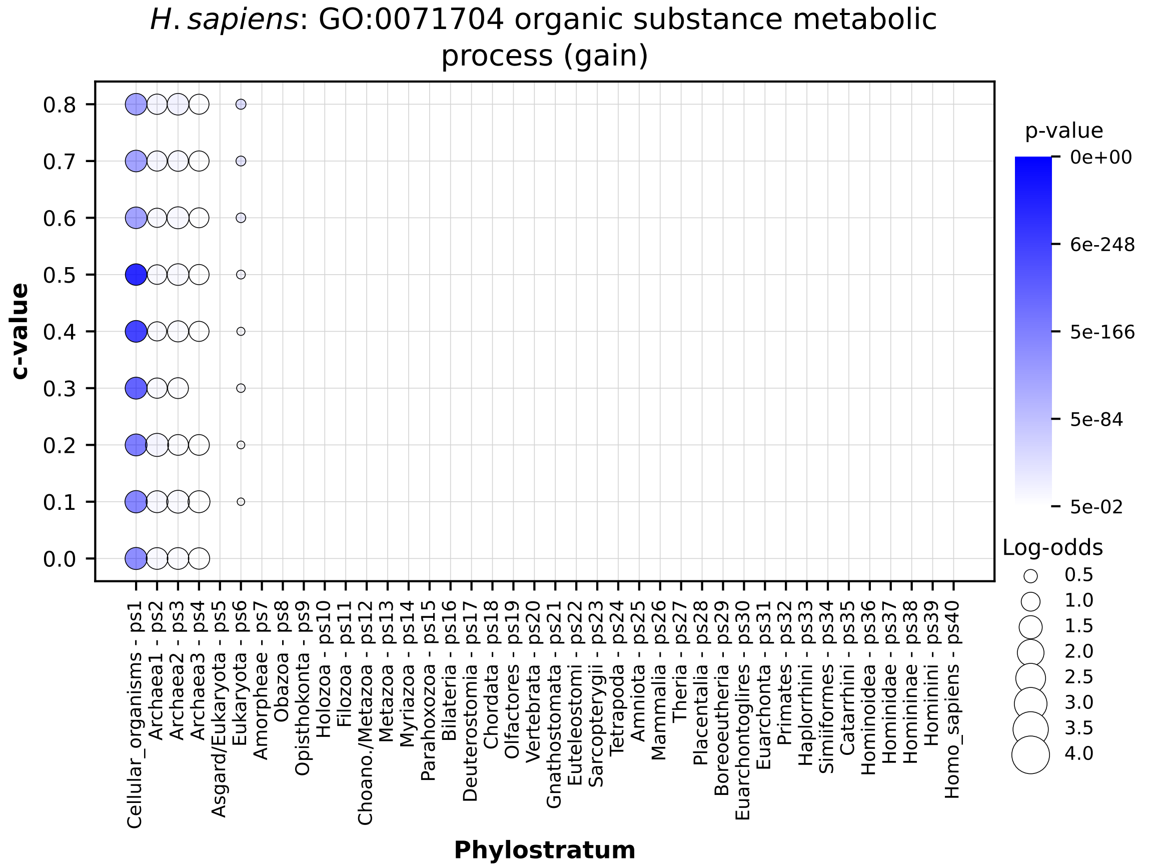

*H. sapiens*: GO:0071704 organic substance metabolic process (gain)
c-value
0.0
0.1
0.2
0.3
0.4
0.5
0.6
0.7
0.8
Phylostratum
Cellular_organisms - ps1
Archaea1 - ps2
Archaea2 - ps3
Archaea3 - ps4
Asgard/Eukaryota - ps5
Eukaryota - ps6
Amorpheae - ps7
Obazoa - ps8
Opisthokonta - ps9
Holozoa - ps10
Filozoa - ps11
Choano./Metazoa - ps12
Metazoa - ps13
Myriazoa - ps14
Parahoxozoa - ps15
Bilateria - ps16
Deuterostomia - ps17
Chordata - ps18
Olfactores - ps19
Vertebrata - ps20
Gnathostomata - ps21
Euteleostomi - ps22
Sarcopterygii - ps23
Tetrapoda - ps24
Amniota - ps25
Mammalia - ps26
Theria - ps27
Placentalia - ps28
Boreoeutheria - ps29
Euarchontoglires - ps30
Euarchonta - ps31
Primates - ps32
Haplorrhini - ps33
Simiiformes - ps34
Catarrhini - ps35
Hominoidea - ps36
Hominidae - ps37
Homininae - ps38
Hominini - ps39
Homo_sapiens - ps40
p-value
0e+00
6e-248
5e-166
5e-84
5e-02
Log-odds
0.5
1.0
1.5
2.0
2.5
3.0
3.5
4.0

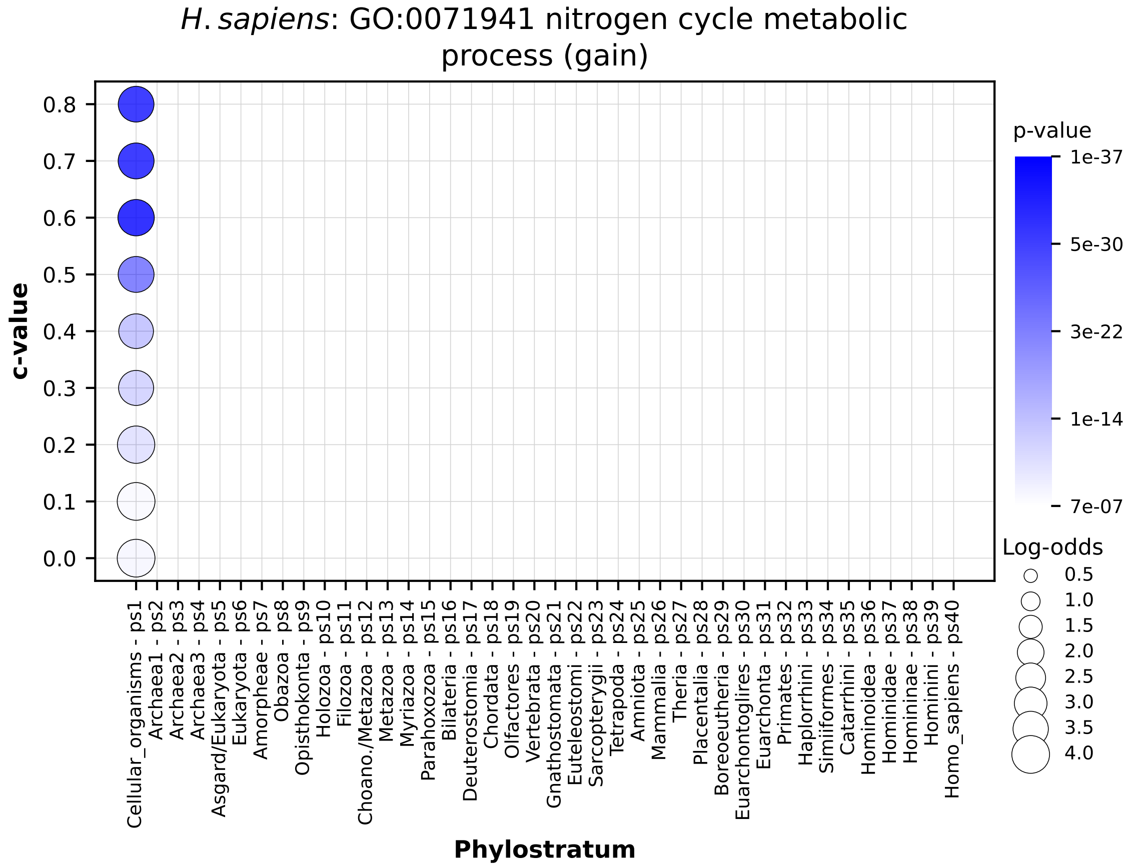

*H. sapiens*: GO:0071941 nitrogen cycle metabolic process (gain)
c-value
0.8
0.7
0.6
0.5
0.4
0.3
0.2
0.1
0.0
Phylostratum
Cellular_organisms - ps1
Archaea1 - ps2
Archaea2 - ps3
Archaea3 - ps4
Asgard/Eukaryota - ps5
Eukaryota - ps6
Amorpheae - ps7
Obazoa - ps8
Opisthokonta - ps9
Holozoa - ps10
Filozoa - ps11
Choano./Metazoa - ps12
Metazoa - ps13
Myriazoa - ps14
Parahoxozoa - ps15
Bilateria - ps16
Deuterostomia - ps17
Chordata - ps18
Olfactores - ps19
Vertebrata - ps20
Gnathostomata - ps21
Euteleostomi - ps22
Sarcopterygii - ps23
Tetrapoda - ps24
Amniota - ps25
Mammalia - ps26
Theria - ps27
Placentalia - ps28
Boreoeutheria - ps29
Euarchontoglires - ps30
Euarchonta - ps31
Primates - ps32
Haplorrhini - ps33
Simiiformes - ps34
Catarrhini - ps35
Hominoidea - ps36
Hominidae - ps37
Homininae - ps38
Hominini - ps39
Homo_sapiens - ps40
p-value
1e-37
5e-30
3e-22
1e-14
7e-07
Log-odds
0.5
1.0
1.5
2.0
2.5
3.0
3.5
4.0

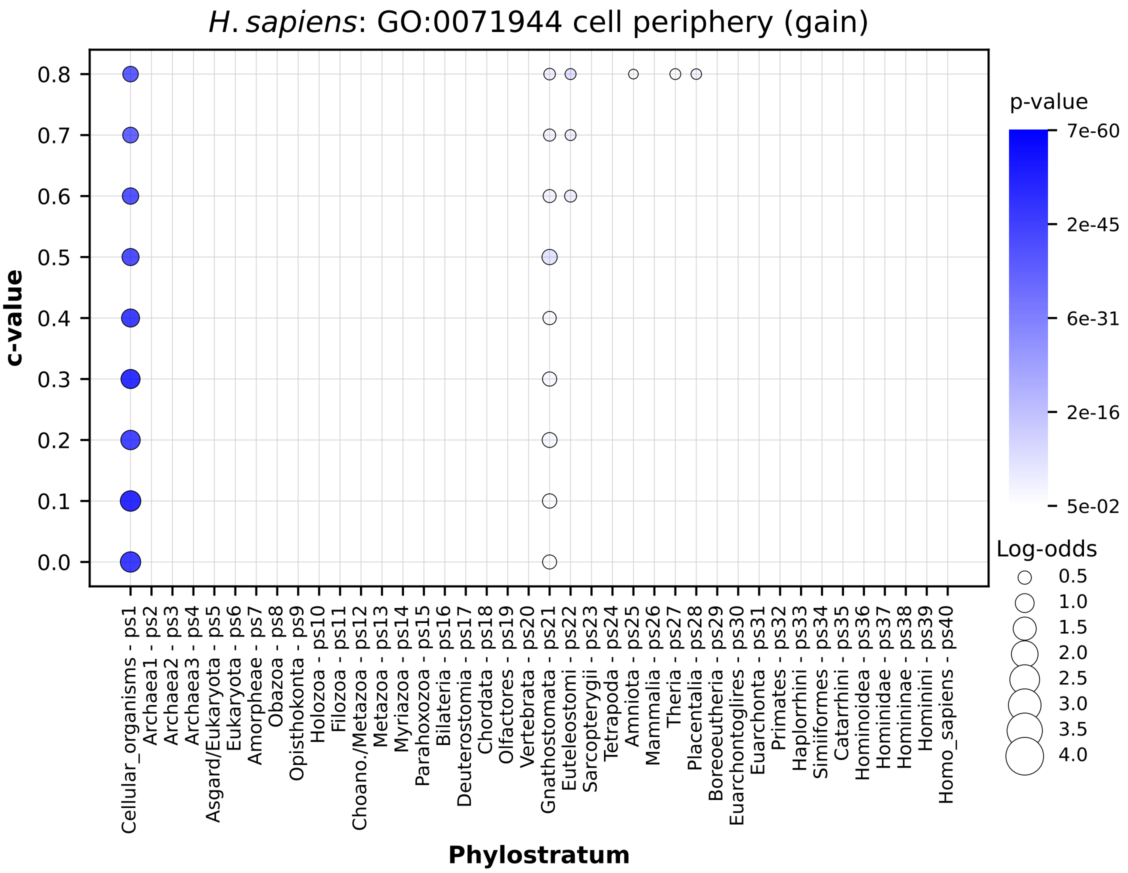
H. sapiens: GO:0071944 cell periphery (gain)
c-value
0.8
0.7
0.6
0.5
0.4
0.3
0.2
0.1
0.0
p-value
7e-60
2e-45
6e-31
2e-16
5e-02
Log-odds
0.5
1.0
1.5
2.0
2.5
3.0
3.5
4.0
Cellular_organisms - ps1
Archaea1 - ps2
Archaea2 - ps3
Archaea3 - ps4
Asgard/Eukaryota - ps5
Eukaryota - ps6
Amorpheae - ps7
Obazoa - ps8
Opisthokonta - ps9
Holozoa - ps10
Filozoa - ps11
Choano./Metazoa - ps12
Metazoa - ps13
Myriazoa - ps14
Parahoxozoa - ps15
Bilateria - ps16
Deuterostomia - ps17
Chordata - ps18
Olfactores - ps19
Vertebrata - ps20
Gnathostomata - ps21
Euteleostomi - ps22
Sarcopterygii - ps23
Tetrapoda - ps24
Amniota - ps25
Mammalia - ps26
Theria - ps27
Placentalia - ps28
Boreoeutheria - ps29
Euarchontoglires - ps30
Euarchonta - ps31
Primates - ps32
Haplorrhini - ps33
Simiiformes - ps34
Catarrhini - ps35
Hominoidea - ps36
Hominidae - ps37
Homininae - ps38
Hominini - ps39
Homo_sapiens - ps40
Phylostratum

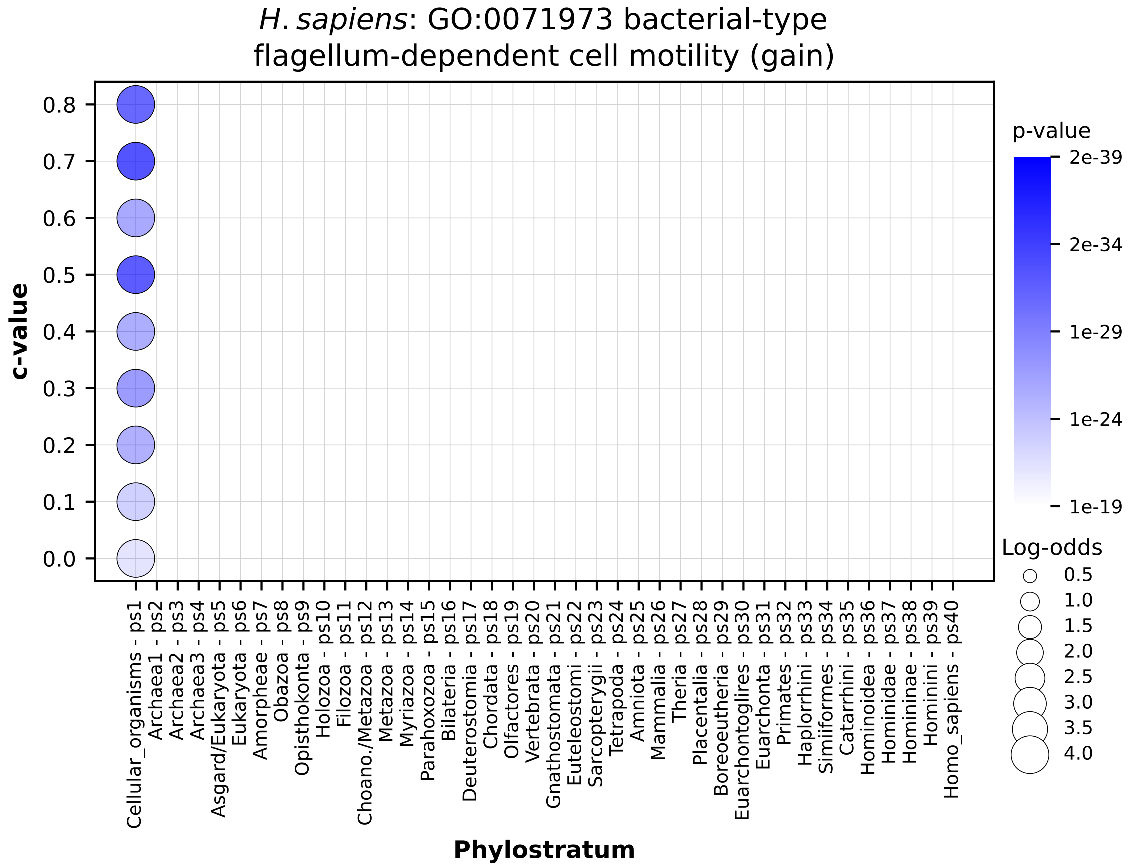

H. sapiens: GO:0071973 bacterial-type flagellum-dependent cell motility (gain)
c-value
0.8
0.7
0.6
0.5
0.4
0.3
0.2
0.1
0.0
Phylostratum
Cellular_organisms - ps1
Archaea1 - ps2
Archaea2 - ps3
Archaea3 - ps4
Asgard/Eukaryota - ps5
Eukaryota - ps6
Amorpheae - ps7
Obazoa - ps8
Opisthokonta - ps9
Holozoa - ps10
Filozoa - ps11
Choano./Metazoa - ps12
Metazoa - ps13
Myriazoa - ps14
Parahoxozoa - ps15
Bilateria - ps16
Deuterostomia - ps17
Chordata - ps18
Olfactores - ps19
Vertebrata - ps20
Gnathostomata - ps21
Euteleostomi - ps22
Sarcopterygii - ps23
Tetrapoda - ps24
Amniota - ps25
Mammalia - ps26
Theria - ps27
Placentalia - ps28
Boreoeutheria - ps29
Euarchontoglires - ps30
Euarchonta - ps31
Primates - ps32
Haplorrhini - ps33
Simiiformes - ps34
Catarrhini - ps35
Hominoidea - ps36
Hominidae - ps37
Homininae - ps38
Hominini - ps39
Homo_sapiens - ps40
p-value
2e-39
2e-34
1e-29
1e-24
1e-19
Log-odds
0.5
1.0
1.5
2.0
2.5
3.0
3.5
4.0

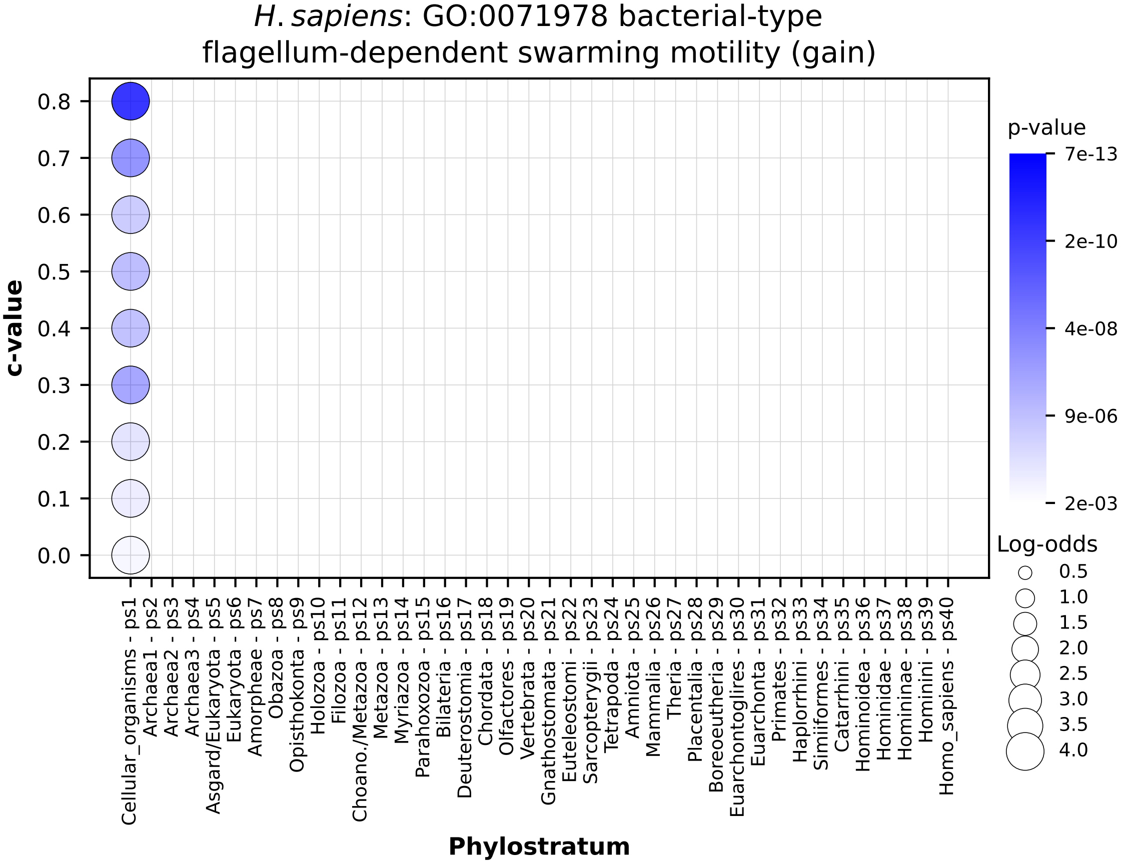

*H. sapiens*: GO:0071978 bacterial-type flagellum-dependent swarming motility (gain)
c-value
0.8
0.7
0.6
0.5
0.4
0.3
0.2
0.1
0.0
p-value
7e-13
2e-10
4e-08
9e-06
2e-03
Log-odds
0.5
1.0
1.5
2.0
2.5
3.0
3.5
4.0
Cellular_organisms - ps1
Archaea1 - ps2
Archaea2 - ps3
Archaea3 - ps4
Asgard/Eukaryota - ps5
Eukaryota - ps6
Amorpheae - ps7
Obazoa - ps8
Opisthokonta - ps9
Holozoa - ps10
Filozoa - ps11
Choano./Metazoa - ps12
Metazoa - ps13
Myriazoa - ps14
Parahoxozoa - ps15
Bilateria - ps16
Deuterostomia - ps17
Chordata - ps18
Olfactores - ps19
Vertebrata - ps20
Gnathostomata - ps21
Euteleostomi - ps22
Sarcopterygii - ps23
Tetrapoda - ps24
Amniota - ps25
Mammalia - ps26
Theria - ps27
Placentalia - ps28
Boreoeutheria - ps29
Euarchontoglires - ps30
Euarchonta - ps31
Primates - ps32
Haplorrhini - ps33
Simiiformes - ps34
Catarrhini - ps35
Hominoidea - ps36
Hominidae - ps37
Homininae - ps38
Hominini - ps39
Homo_sapiens - ps40
Phylostratum

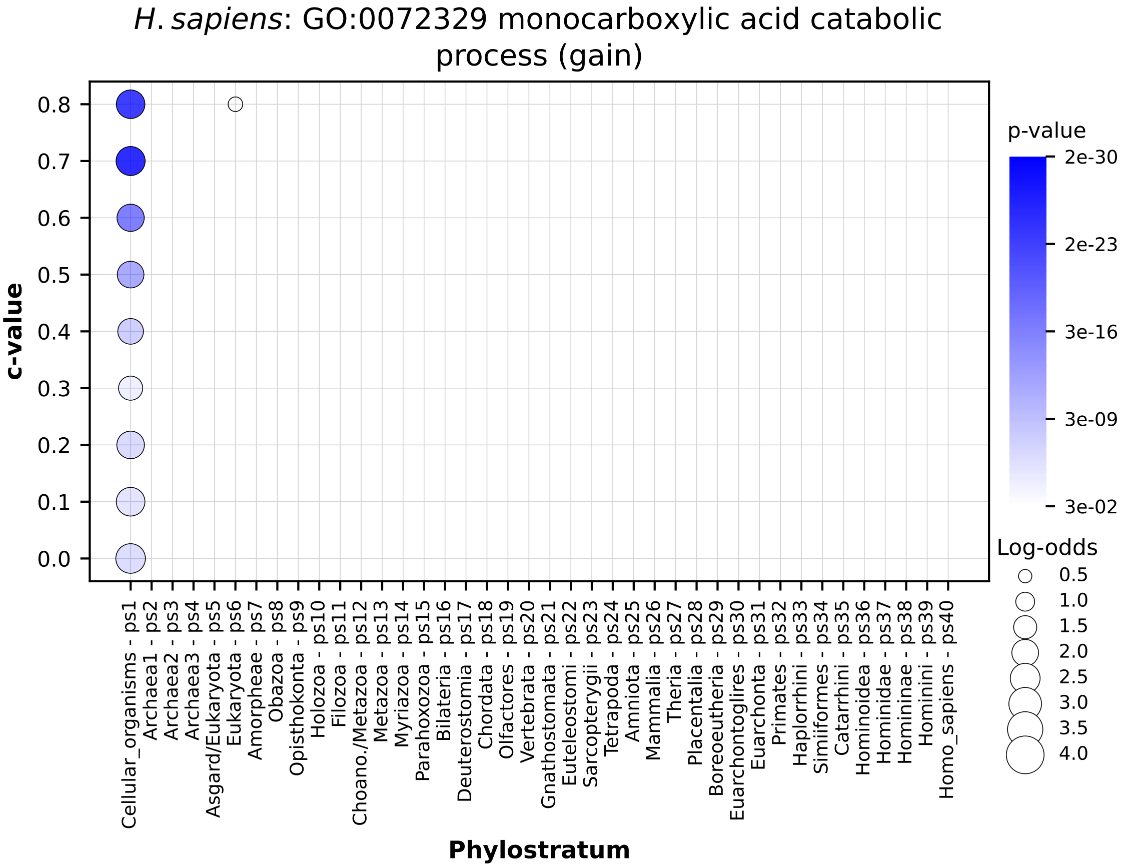

H. sapiens: GO:0072329 monocarboxylic acid catabolic process (gain)
c-value
0.0
0.1
0.2
0.3
0.4
0.5
0.6
0.7
0.8
Phylostratum
Cellular_organisms - ps1
Archaea1 - ps2
Archaea2 - ps3
Archaea3 - ps4
Asgard/Eukaryota - ps5
Eukaryota - ps6
Amorpheae - ps7
Obazoa - ps8
Opisthokonta - ps9
Holozoa - ps10
Filozoa - ps11
Choano./Metazoa - ps12
Metazoa - ps13
Myriazoa - ps14
Parahoxozoa - ps15
Bilateria - ps16
Deuterostomia - ps17
Chordata - ps18
Olfactores - ps19
Vertebrata - ps20
Gnathostomata - ps21
Euteleostomi - ps22
Sarcopterygii - ps23
Tetrapoda - ps24
Amniota - ps25
Mammalia - ps26
Theria - ps27
Placentalia - ps28
Boreoeutheria - ps29
Euarchontoglires - ps30
Euarchonta - ps31
Primates - ps32
Haplorrhini - ps33
Simiiformes - ps34
Catarrhini - ps35
Hominoidea - ps36
Hominidae - ps37
Homininae - ps38
Hominini - ps39
Homo_sapiens - ps40
p-value
2e-30
2e-23
3e-16
3e-09
3e-02
Log-odds
0.5
1.0
1.5
2.0
2.5
3.0
3.5
4.0

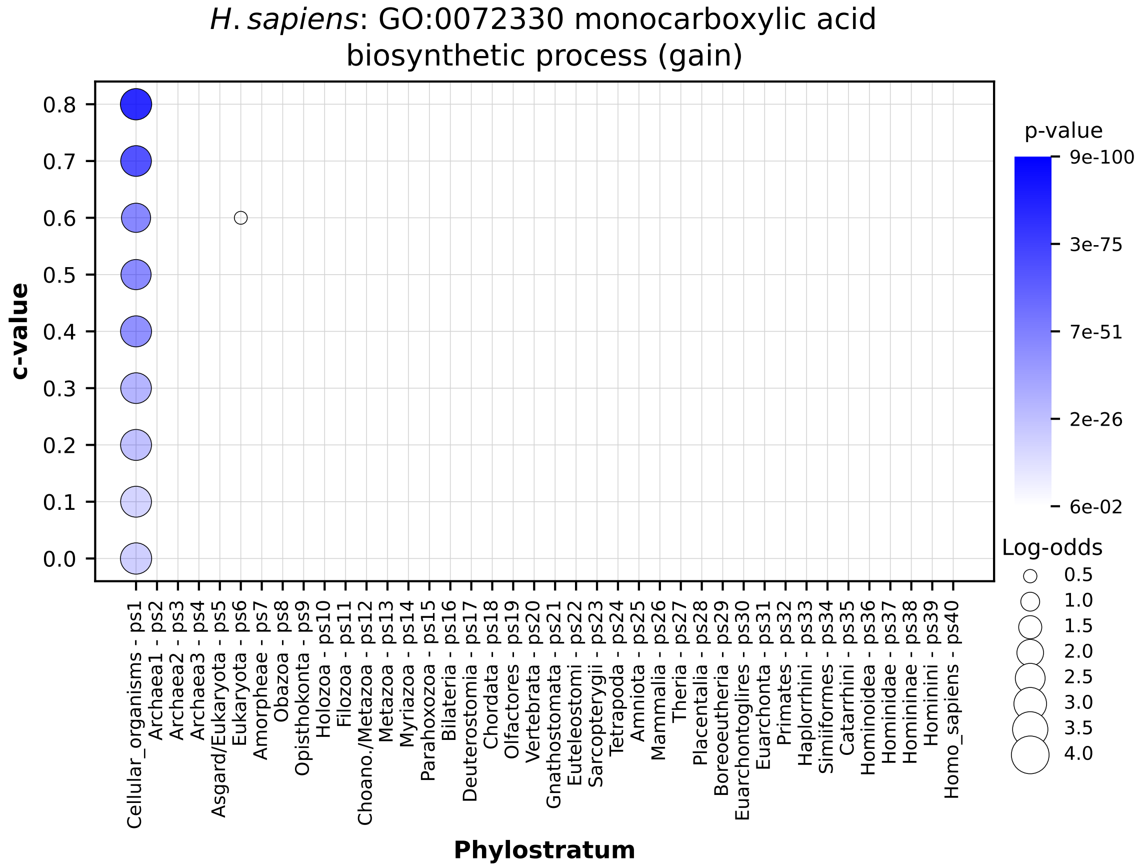

H. sapiens: GO:0072330 monocarboxylic acid biosynthetic process (gain)
c-value
0.8
0.7
0.6
0.5
0.4
0.3
0.2
0.1
0.0
Phylostratum
Cellular_organisms - ps1
Archaea1 - ps2
Archaea2 - ps3
Archaea3 - ps4
Asgard/Eukaryota - ps5
Eukaryota - ps6
Amorpheae - ps7
Obazoa - ps8
Opisthokonta - ps9
Holozoa - ps10
Filozoa - ps11
Choano./Metazoa - ps12
Metazoa - ps13
Myriazoa - ps14
Parahoxozoa - ps15
Bilateria - ps16
Deuterostomia - ps17
Chordata - ps18
Olfactores - ps19
Vertebrata - ps20
Gnathostomata - ps21
Euteleostomi - ps22
Sarcopterygii - ps23
Tetrapoda - ps24
Amniota - ps25
Mammalia - ps26
Theria - ps27
Placentalia - ps28
Boreoeutheria - ps29
Euarchontoglires - ps30
Euarchonta - ps31
Primates - ps32
Haplorrhini - ps33
Simiiformes - ps34
Catarrhini - ps35
Hominoidea - ps36
Hominidae - ps37
Homininae - ps38
Hominini - ps39
Homo_sapiens - ps40
p-value
9e-100
3e-75
7e-51
2e-26
6e-02
Log-odds
0.5
1.0
1.5
2.0
2.5
3.0
3.5
4.0

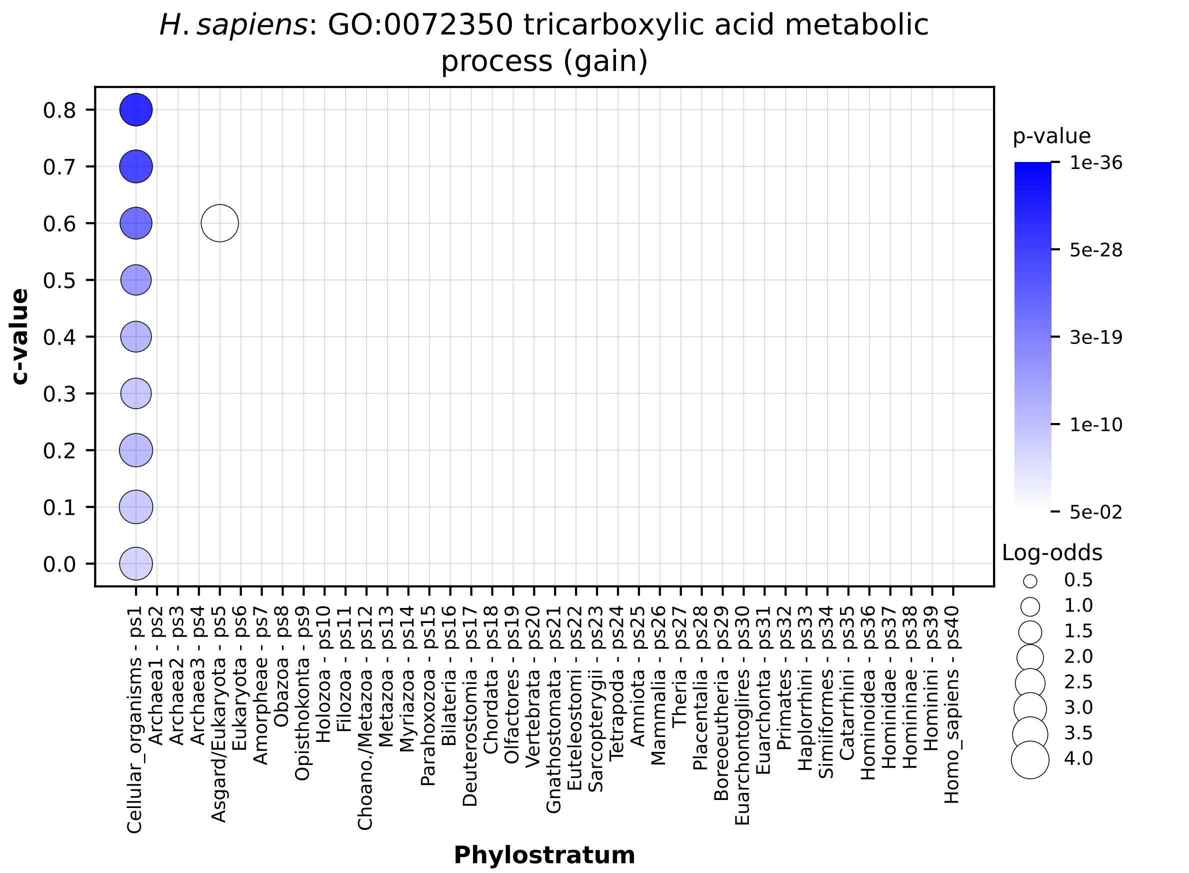
H. sapiens: GO:0072350 tricarboxylic acid metabolic process (gain)
c-value
0.8
0.7
0.6
0.5
0.4
0.3
0.2
0.1
0.0
p-value
1e-36
5e-28
3e-19
1e-10
5e-02
Log-odds
0.5
1.0
1.5
2.0
2.5
3.0
3.5
4.0
Cellular_organisms - ps1
Archaea1 - ps2
Archaea2 - ps3
Archaea3 - ps4
Asgard/Eukaryota - ps5
Eukaryota - ps6
Amorpheae - ps7
Obazoa - ps8
Opisthokonta - ps9
Holozoa - ps10
Filozoa - ps11
Choano./Metazoa - ps12
Metazoa - ps13
Myriazoa - ps14
Parahoxozoa - ps15
Bilateria - ps16
Deuterostomia - ps17
Chordata - ps18
Olfactores - ps19
Vertebrata - ps20
Gnathostomata - ps21
Euteleostomi - ps22
Sarcopterygii - ps23
Tetrapoda - ps24
Amniota - ps25
Mammalia - ps26
Theria - ps27
Placentalia - ps28
Boreoeutheria - ps29
Euarchontoglires - ps30
Euarchonta - ps31
Primates - ps32
Haplorrhini - ps33
Simiiformes - ps34
Catarrhini - ps35
Hominoidea - ps36
Hominidae - ps37
Homininae - ps38
Hominini - ps39
Homo_sapiens - ps40
Phylostratum

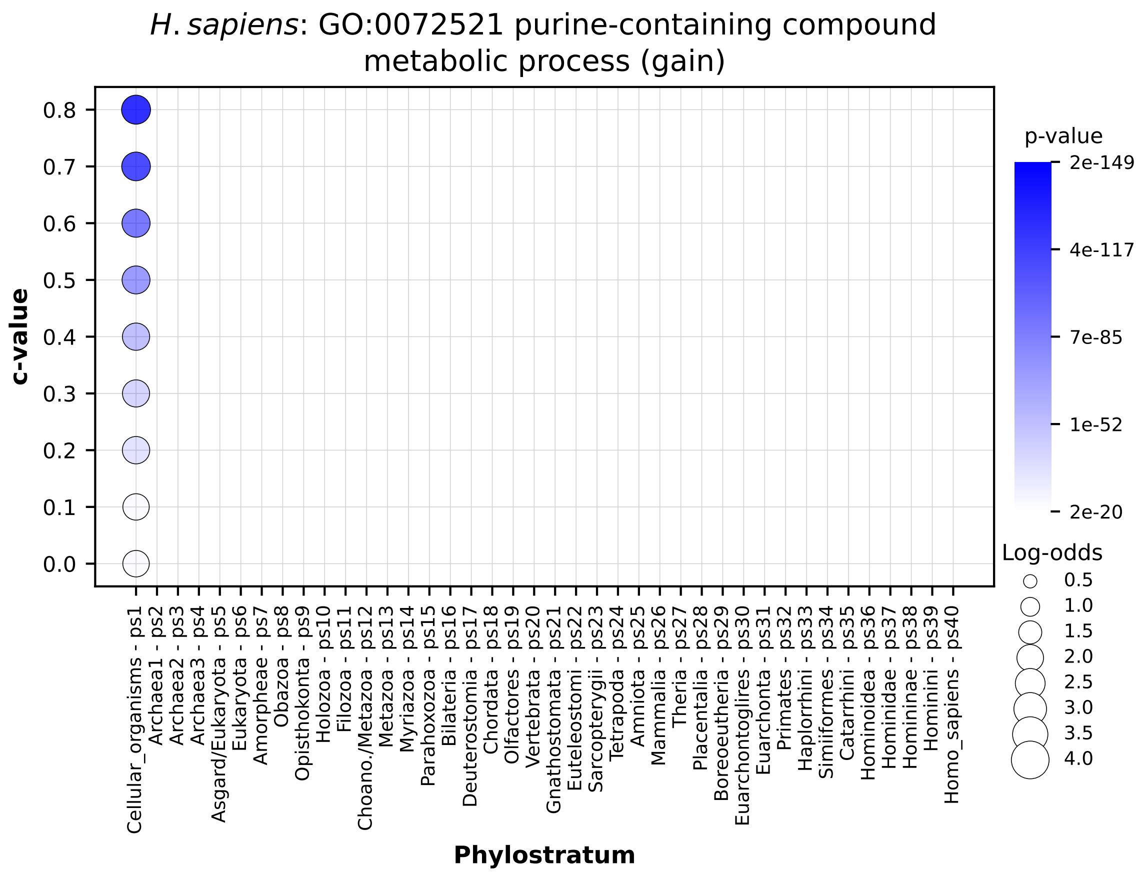

*H. sapiens*: GO:0072521 purine-containing compound metabolic process (gain)
c-value
0.8
0.7
0.6
0.5
0.4
0.3
0.2
0.1
0.0
p-value
2e-149
4e-117
7e-85
1e-52
2e-20
Log-odds
0.5
1.0
1.5
2.0
2.5
3.0
3.5
4.0
Cellular_organisms - ps1
Archaea1 - ps2
Archaea2 - ps3
Archaea3 - ps4
Asgard/Eukaryota - ps5
Eukaryota - ps6
Amorpheae - ps7
Obazoa - ps8
Opisthokonta - ps9
Holozoa - ps10
Filozoa - ps11
Choano./Metazoa - ps12
Metazoa - ps13
Myriazoa - ps14
Parahoxozoa - ps15
Bilateria - ps16
Deuterostomia - ps17
Chordata - ps18
Olfactores - ps19
Vertebrata - ps20
Gnathostomata - ps21
Euteleostomi - ps22
Sarcopterygii - ps23
Tetrapoda - ps24
Amniota - ps25
Mammalia - ps26
Theria - ps27
Placentalia - ps28
Boreoeutheria - ps29
Euarchontoglires - ps30
Euarchonta - ps31
Primates - ps32
Haplorrhini - ps33
Simiiformes - ps34
Catarrhini - ps35
Hominoidea - ps36
Hominidae - ps37
Homininae - ps38
Hominini - ps39
Homo_sapiens - ps40
Phylostratum

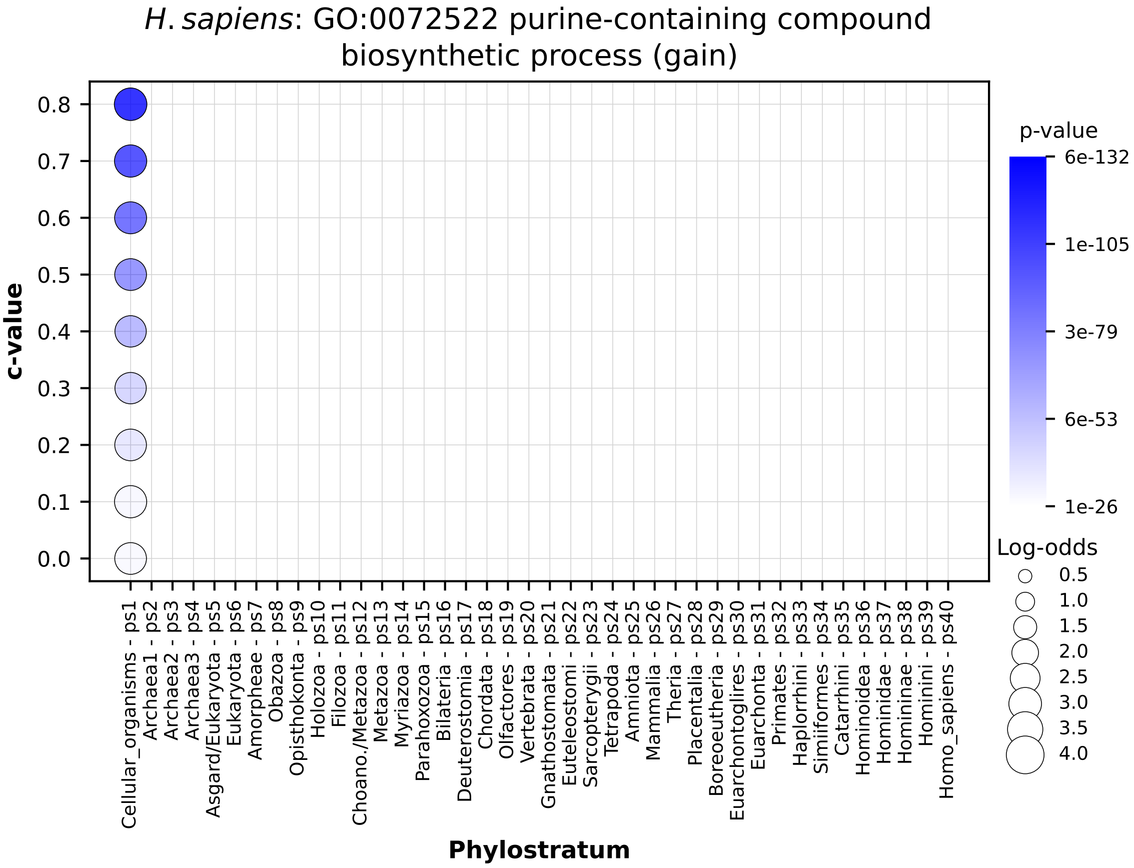

H. sapiens: GO:0072522 purine-containing compound biosynthetic process (gain)
c-value
0.8
0.7
0.6
0.5
0.4
0.3
0.2
0.1
0.0
p-value
6e-132
1e-105
3e-79
6e-53
1e-26
Log-odds
0.5
1.0
1.5
2.0
2.5
3.0
3.5
4.0
Cellular_organisms - ps1
Archaea1 - ps2
Archaea2 - ps3
Archaea3 - ps4
Asgard/Eukaryota - ps5
Eukaryota - ps6
Amorpheae - ps7
Obazoa - ps8
Opisthokonta - ps9
Holozoa - ps10
Filozoa - ps11
Choano./Metazoa - ps12
Metazoa - ps13
Myriazoa - ps14
Parahoxozoa - ps15
Bilateria - ps16
Deuterostomia - ps17
Chordata - ps18
Olfactores - ps19
Vertebrata - ps20
Gnathostomata - ps21
Euteleostomi - ps22
Sarcopterygii - ps23
Tetrapoda - ps24
Amniota - ps25
Mammalia - ps26
Theria - ps27
Placentalia - ps28
Boreoeutheria - ps29
Euarchontoglires - ps30
Euarchonta - ps31
Primates - ps32
Haplorrhini - ps33
Simiiformes - ps34
Catarrhini - ps35
Hominoidea - ps36
Hominidae - ps37
Homininae - ps38
Hominini - ps39
Homo_sapiens - ps40
Phylostratum

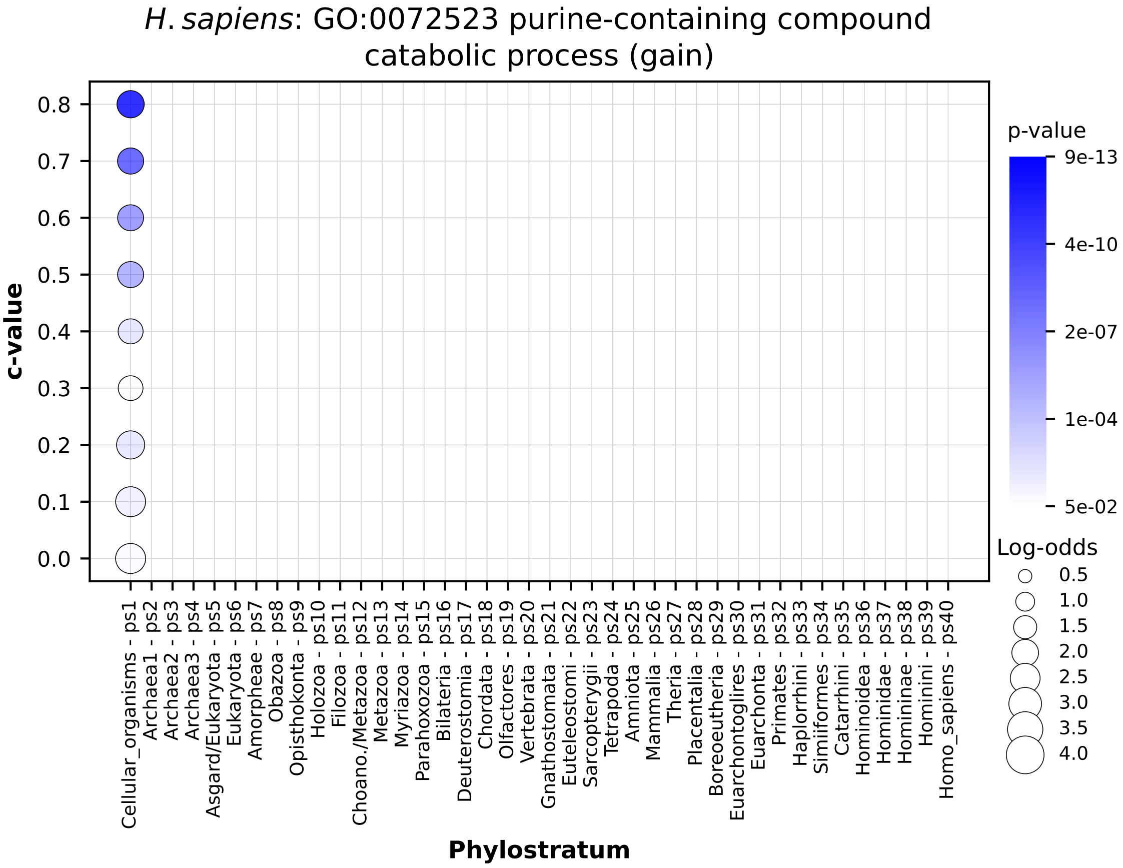

H. sapiens: GO:0072523 purine-containing compound catabolic process (gain)
c-value
0.8
0.7
0.6
0.5
0.4
0.3
0.2
0.1
0.0
p-value
9e-13
4e-10
2e-07
1e-04
5e-02
Log-odds
0.5
1.0
1.5
2.0
2.5
3.0
3.5
4.0
Cellular_organisms - ps1
Archaea1 - ps2
Archaea2 - ps3
Archaea3 - ps4
Asgard/Eukaryota - ps5
Eukaryota - ps6
Amorpheae - ps7
Obazoa - ps8
Opisthokonta - ps9
Holozoa - ps10
Filozoa - ps11
Choano./Metazoa - ps12
Metazoa - ps13
Myriazoa - ps14
Parahoxozoa - ps15
Bilateria - ps16
Deuterostomia - ps17
Chordata - ps18
Olfactores - ps19
Vertebrata - ps20
Gnathostomata - ps21
Euteleostomi - ps22
Sarcopterygii - ps23
Tetrapoda - ps24
Amniota - ps25
Mammalia - ps26
Theria - ps27
Placentalia - ps28
Boreoeutheria - ps29
Euarchontoglires - ps30
Euarchonta - ps31
Primates - ps32
Haplorrhini - ps33
Simiiformes - ps34
Catarrhini - ps35
Hominoidea - ps36
Hominidae - ps37
Homininae - ps38
Hominini - ps39
Homo_sapiens - ps40
Phylostratum

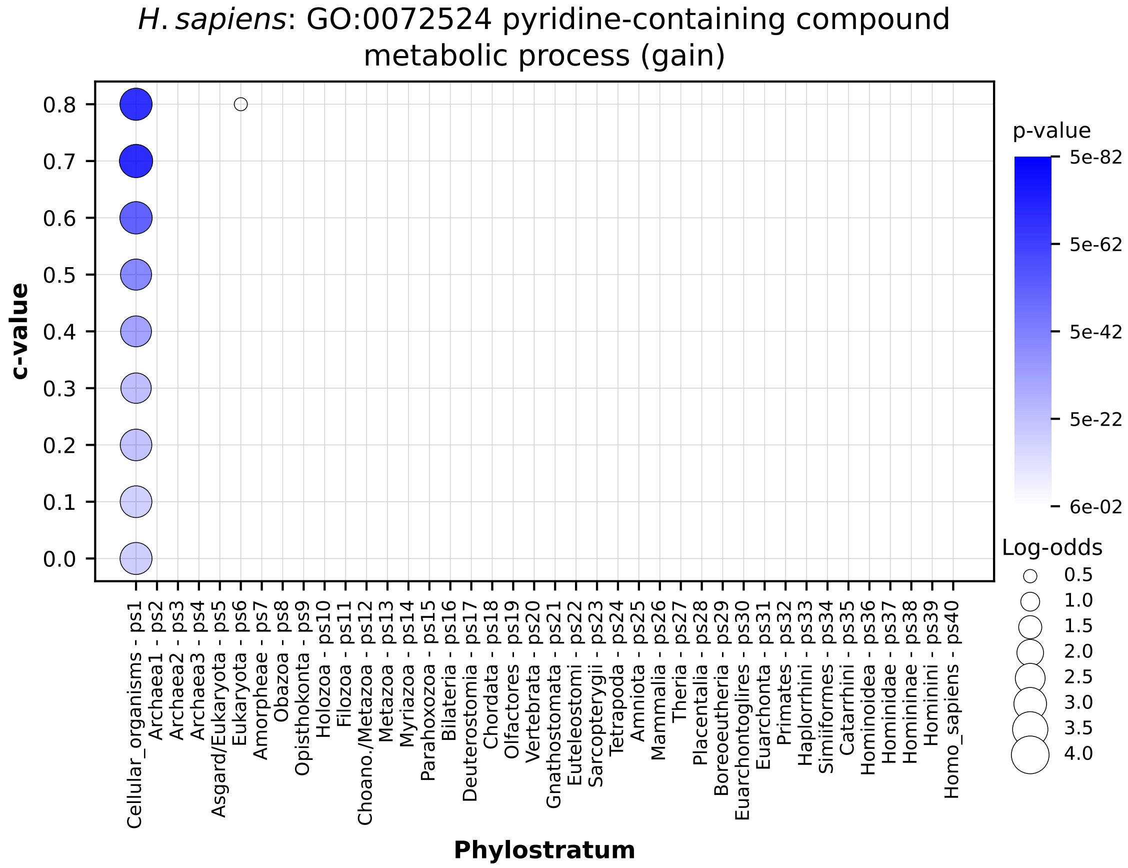

*H. sapiens*: GO:0072524 pyridine-containing compound metabolic process (gain)
c-value
0.0
0.1
0.2
0.3
0.4
0.5
0.6
0.7
0.8
Phylostratum
Cellular_organisms - ps1
Archaea1 - ps2
Archaea2 - ps3
Archaea3 - ps4
Asgard/Eukaryota - ps5
Eukaryota - ps6
Amorpheae - ps7
Obazoa - ps8
Opisthokonta - ps9
Holozoa - ps10
Filozoa - ps11
Choano./Metazoa - ps12
Metazoa - ps13
Myriazoa - ps14
Parahoxozoa - ps15
Bilateria - ps16
Deuterostomia - ps17
Chordata - ps18
Olfactores - ps19
Vertebrata - ps20
Gnathostomata - ps21
Euteleostomi - ps22
Sarcopterygii - ps23
Tetrapoda - ps24
Amniota - ps25
Mammalia - ps26
Theria - ps27
Placentalia - ps28
Boreoeutheria - ps29
Euarchontoglires - ps30
Euarchonta - ps31
Primates - ps32
Haplorrhini - ps33
Simiiformes - ps34
Catarrhini - ps35
Hominoidea - ps36
Hominidae - ps37
Homininae - ps38
Hominini - ps39
Homo_sapiens - ps40
p-value
5e-82
5e-62
5e-42
5e-22
6e-02
Log-odds
0.5
1.0
1.5
2.0
2.5
3.0
3.5
4.0

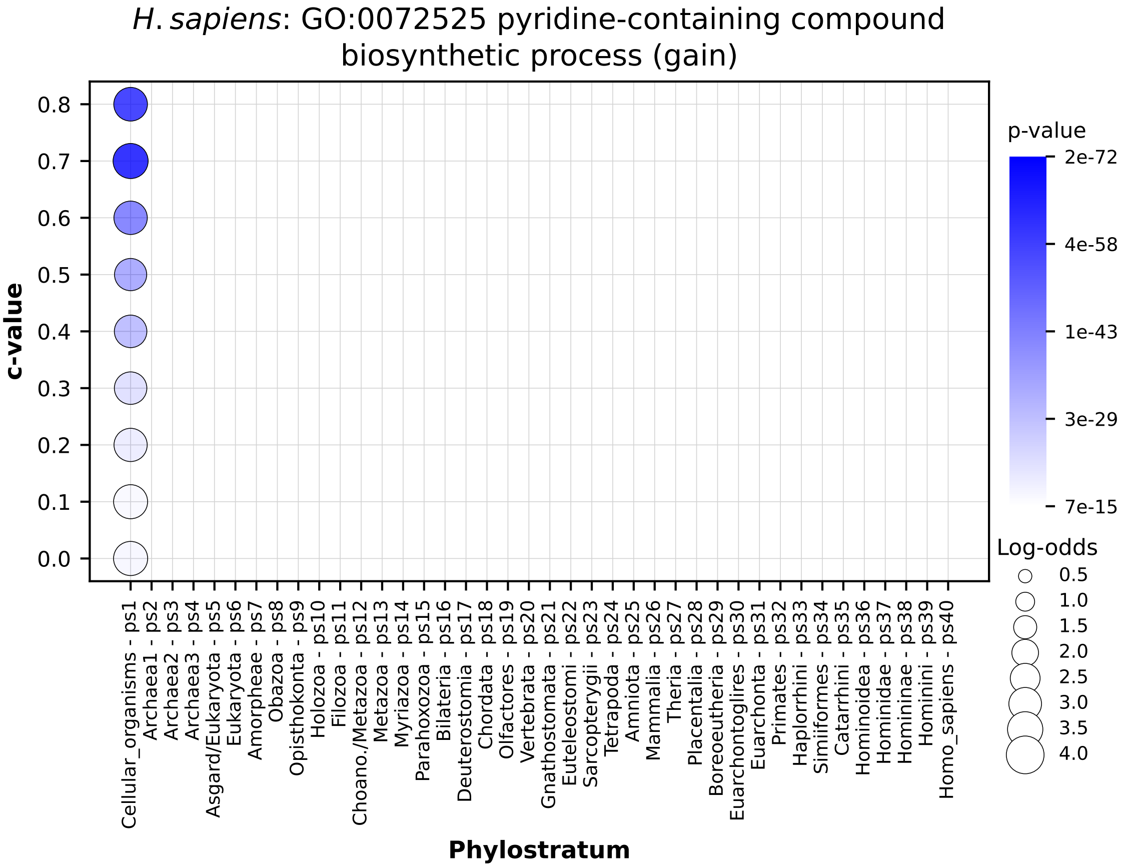

H. sapiens: GO:0072525 pyridine-containing compound biosynthetic process (gain)
c-value
0.8
0.7
0.6
0.5
0.4
0.3
0.2
0.1
0.0
p-value
2e-72
4e-58
1e-43
3e-29
7e-15
Log-odds
0.5
1.0
1.5
2.0
2.5
3.0
3.5
4.0
Cellular_organisms - ps1
Archaea1 - ps2
Archaea2 - ps3
Archaea3 - ps4
Asgard/Eukaryota - ps5
Eukaryota - ps6
Amorpheae - ps7
Obazoa - ps8
Opisthokonta - ps9
Holozoa - ps10
Filozoa - ps11
Choano./Metazoa - ps12
Metazoa - ps13
Myriazoa - ps14
Parahoxozoa - ps15
Bilateria - ps16
Deuterostomia - ps17
Chordata - ps18
Olfactores - ps19
Vertebrata - ps20
Gnathostomata - ps21
Euteleostomi - ps22
Sarcopterygii - ps23
Tetrapoda - ps24
Amniota - ps25
Mammalia - ps26
Theria - ps27
Placentalia - ps28
Boreoeutheria - ps29
Euarchontoglires - ps30
Euarchonta - ps31
Primates - ps32
Haplorrhini - ps33
Simiiformes - ps34
Catarrhini - ps35
Hominoidea - ps36
Hominidae - ps37
Homininae - ps38
Hominini - ps39
Homo_sapiens - ps40
Phylostratum

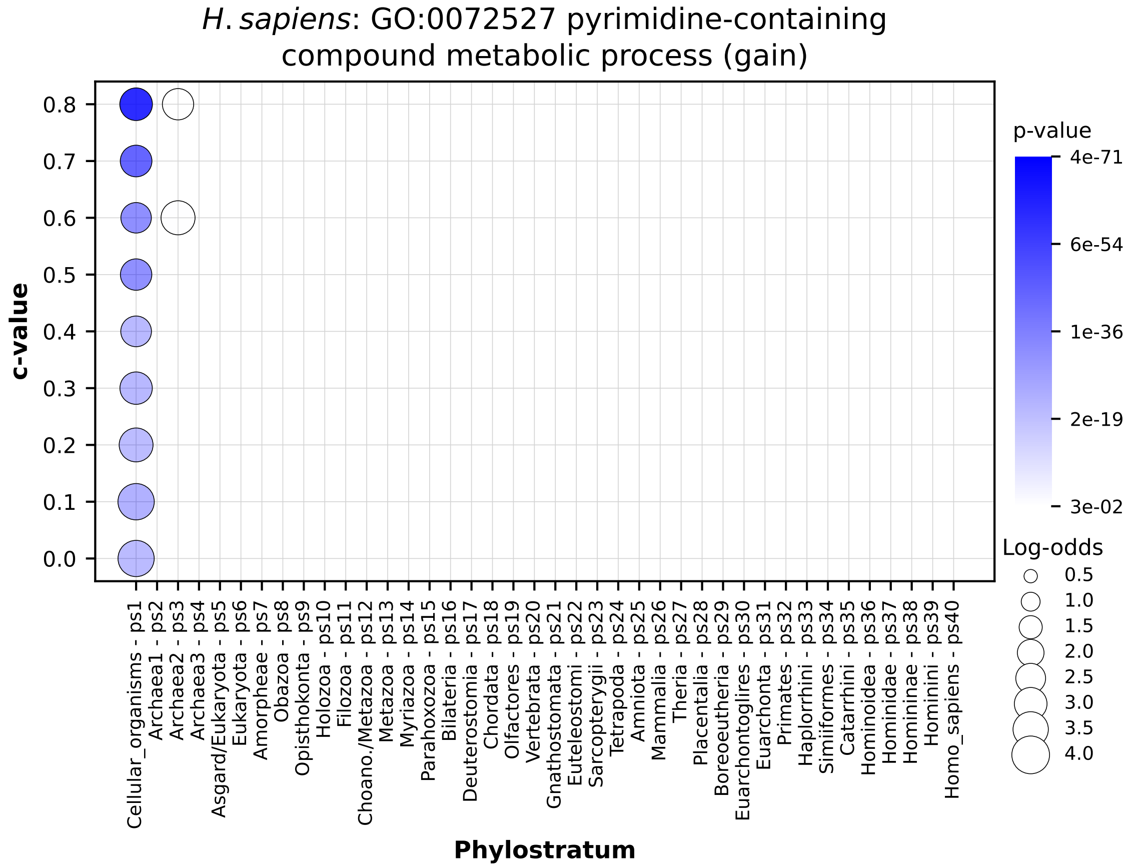
H. sapiens: GO:0072527 pyrimidine-containing compound metabolic process (gain)
c-value
0.8
0.7
0.6
0.5
0.4
0.3
0.2
0.1
0.0
Phylostratum
Cellular_organisms - ps1
Archaea1 - ps2
Archaea2 - ps3
Archaea3 - ps4
Asgard/Eukaryota - ps5
Eukaryota - ps6
Amorpheae - ps7
Obazoa - ps8
Opisthokonta - ps9
Holozoa - ps10
Filozoa - ps11
Choano./Metazoa - ps12
Metazoa - ps13
Myriazoa - ps14
Parahoxozoa - ps15
Bilateria - ps16
Deuterostomia - ps17
Chordata - ps18
Olfactores - ps19
Vertebrata - ps20
Gnathostomata - ps21
Euteleostomi - ps22
Sarcopterygii - ps23
Tetrapoda - ps24
Amniota - ps25
Mammalia - ps26
Theria - ps27
Placentalia - ps28
Boreoeutheria - ps29
Euarchontoglires - ps30
Euarchonta - ps31
Primates - ps32
Haplorrhini - ps33
Simiiformes - ps34
Catarrhini - ps35
Hominoidea - ps36
Hominidae - ps37
Homininae - ps38
Hominini - ps39
Homo_sapiens - ps40
p-value
4e-71
6e-54
1e-36
2e-19
3e-02
Log-odds
0.5
1.0
1.5
2.0
2.5
3.0
3.5
4.0

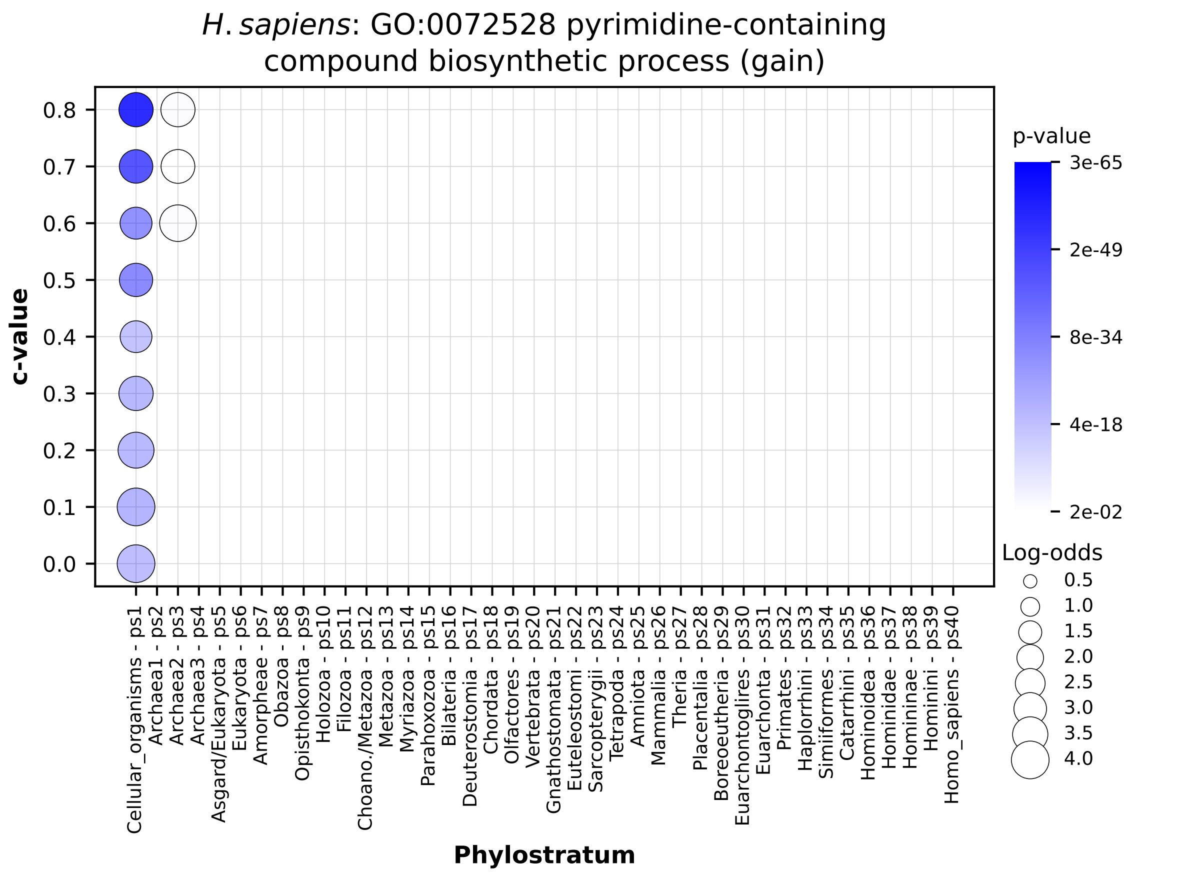

H. sapiens: GO:0072528 pyrimidine-containing compound biosynthetic process (gain)
c-value
0.8
0.7
0.6
0.5
0.4
0.3
0.2
0.1
0.0
Phylostratum
Cellular_organisms - ps1
Archaea1 - ps2
Archaea2 - ps3
Archaea3 - ps4
Asgard/Eukaryota - ps5
Eukaryota - ps6
Amorpheae - ps7
Obazoa - ps8
Opisthokonta - ps9
Holozoa - ps10
Filozoa - ps11
Choano./Metazoa - ps12
Metazoa - ps13
Myriazoa - ps14
Parahoxozoa - ps15
Bilateria - ps16
Deuterostomia - ps17
Chordata - ps18
Olfactores - ps19
Vertebrata - ps20
Gnathostomata - ps21
Euteleostomi - ps22
Sarcopterygii - ps23
Tetrapoda - ps24
Amniota - ps25
Mammalia - ps26
Theria - ps27
Placentalia - ps28
Boreoeutheria - ps29
Euarchontoglires - ps30
Euarchonta - ps31
Primates - ps32
Haplorrhini - ps33
Simiiformes - ps34
Catarrhini - ps35
Hominoidea - ps36
Hominidae - ps37
Homininae - ps38
Hominini - ps39
Homo_sapiens - ps40
p-value
3e-65
2e-49
8e-34
4e-18
2e-02
Log-odds
0.5
1.0
1.5
2.0
2.5
3.0
3.5
4.0

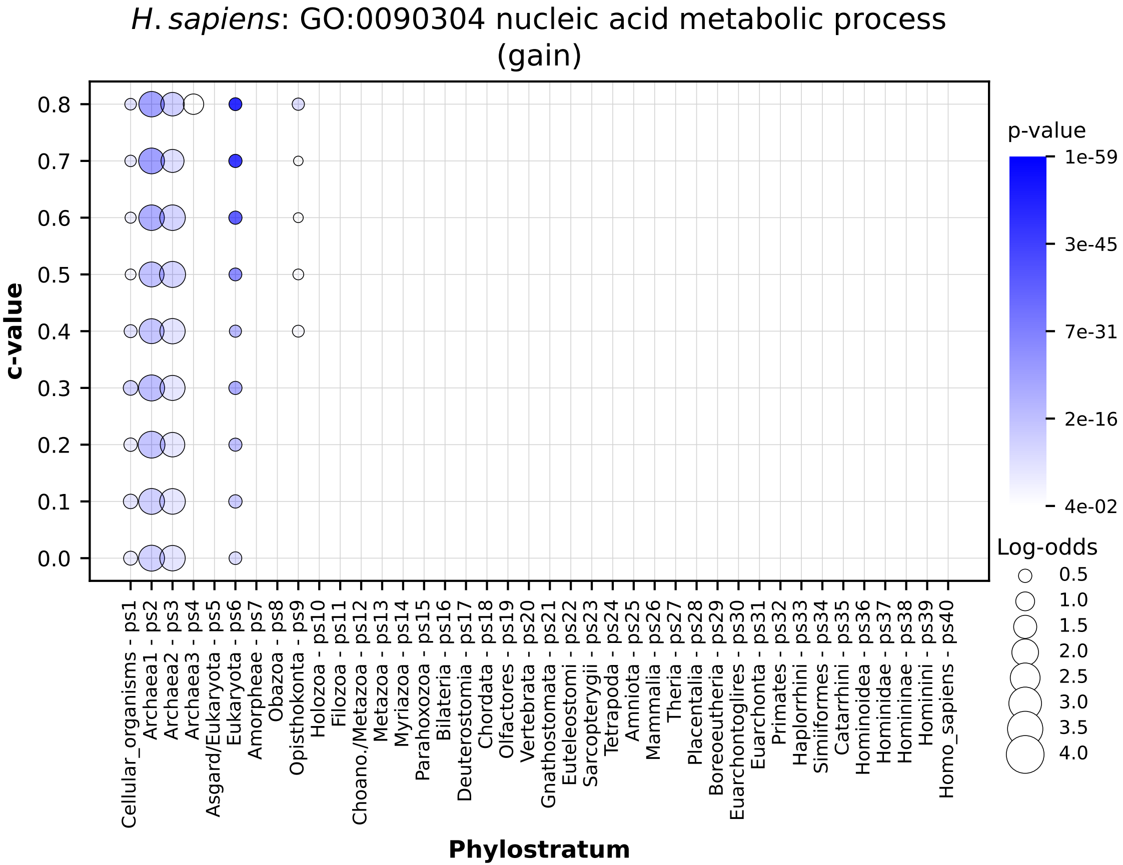

H. sapiens: GO:0090304 nucleic acid metabolic process
(gain)
c-value
0.8
0.7
0.6
0.5
0.4
0.3
0.2
0.1
0.0
p-value
1e-59
3e-45
7e-31
2e-16
4e-02
Log-odds
0.5
1.0
1.5
2.0
2.5
3.0
3.5
4.0
Cellular_organisms - ps1
Archaea1 - ps2
Archaea2 - ps3
Archaea3 - ps4
Asgard/Eukaryota - ps5
Eukaryota - ps6
Amorpheae - ps7
Obazoa - ps8
Opisthokonta - ps9
Holozoa - ps10
Filozoa - ps11
Choano./Metazoa - ps12
Metazoa - ps13
Myriazoa - ps14
Parahoxozoa - ps15
Bilateria - ps16
Deuterostomia - ps17
Chordata - ps18
Olfactores - ps19
Vertebrata - ps20
Gnathostomata - ps21
Euteleostomi - ps22
Sarcopterygii - ps23
Tetrapoda - ps24
Amniota - ps25
Mammalia - ps26
Theria - ps27
Placentalia - ps28
Boreoeutheria - ps29
Euarchontoglires - ps30
Euarchonta - ps31
Primates - ps32
Haplorrhini - ps33
Simiiformes - ps34
Catarrhini - ps35
Hominoidea - ps36
Hominidae - ps37
Homininae - ps38
Hominini - ps39
Homo_sapiens - ps40
Phylostratum

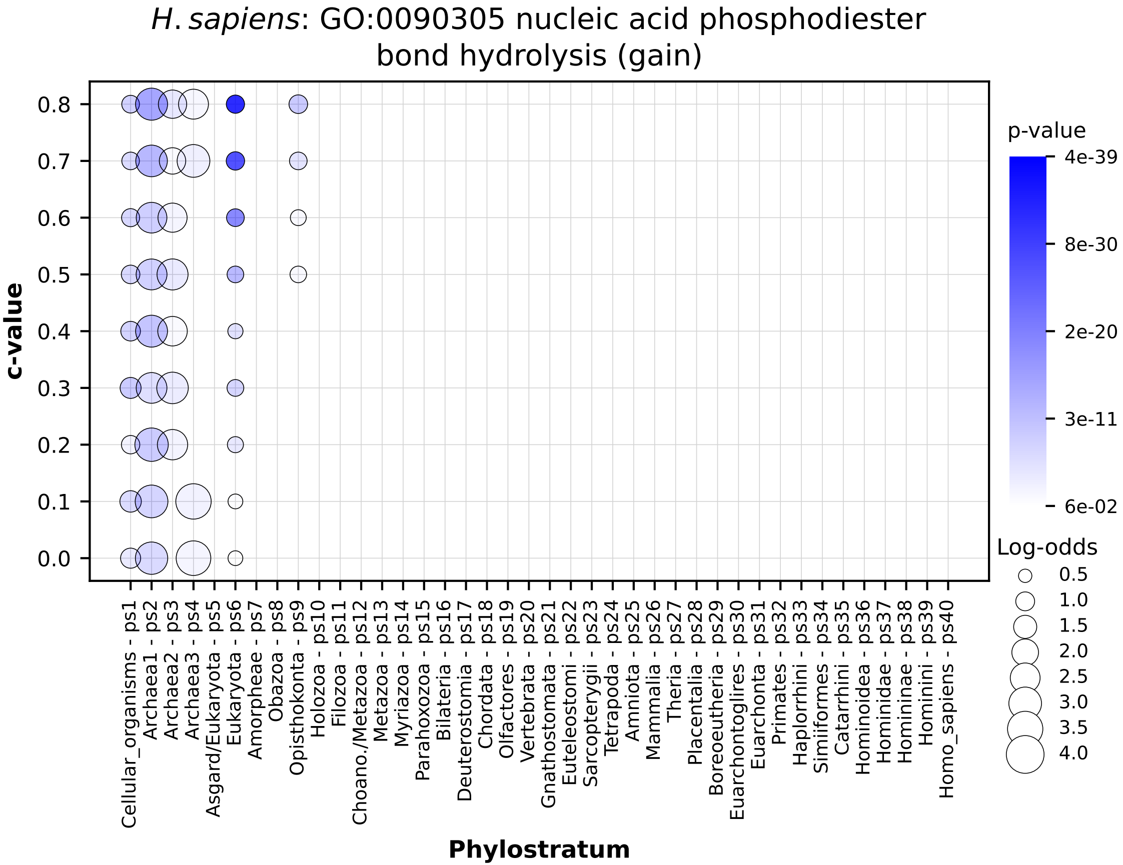
H. sapiens: GO:0090305 nucleic acid phosphodiester bond hydrolysis (gain)
c-value
0.8
0.7
0.6
0.5
0.4
0.3
0.2
0.1
0.0
p-value
4e-39
8e-30
2e-20
3e-11
6e-02
Log-odds
0.5
1.0
1.5
2.0
2.5
3.0
3.5
4.0
Cellular_organisms - ps1
Archaea1 - ps2
Archaea2 - ps3
Archaea3 - ps4
Asgard/Eukaryota - ps5
Eukaryota - ps6
Amorpheae - ps7
Obazoa - ps8
Opisthokonta - ps9
Holozoa - ps10
Filozoa - ps11
Choano./Metazoa - ps12
Metazoa - ps13
Myriazoa - ps14
Parahoxozoa - ps15
Bilateria - ps16
Deuterostomia - ps17
Chordata - ps18
Olfactores - ps19
Vertebrata - ps20
Gnathostomata - ps21
Euteleostomi - ps22
Sarcopterygii - ps23
Tetrapoda - ps24
Amniota - ps25
Mammalia - ps26
Theria - ps27
Placentalia - ps28
Boreoeutheria - ps29
Euarchontoglires - ps30
Euarchonta - ps31
Primates - ps32
Haplorrhini - ps33
Simiiformes - ps34
Catarrhini - ps35
Hominoidea - ps36
Hominidae - ps37
Homininae - ps38
Hominini - ps39
Homo_sapiens - ps40
Phylostratum

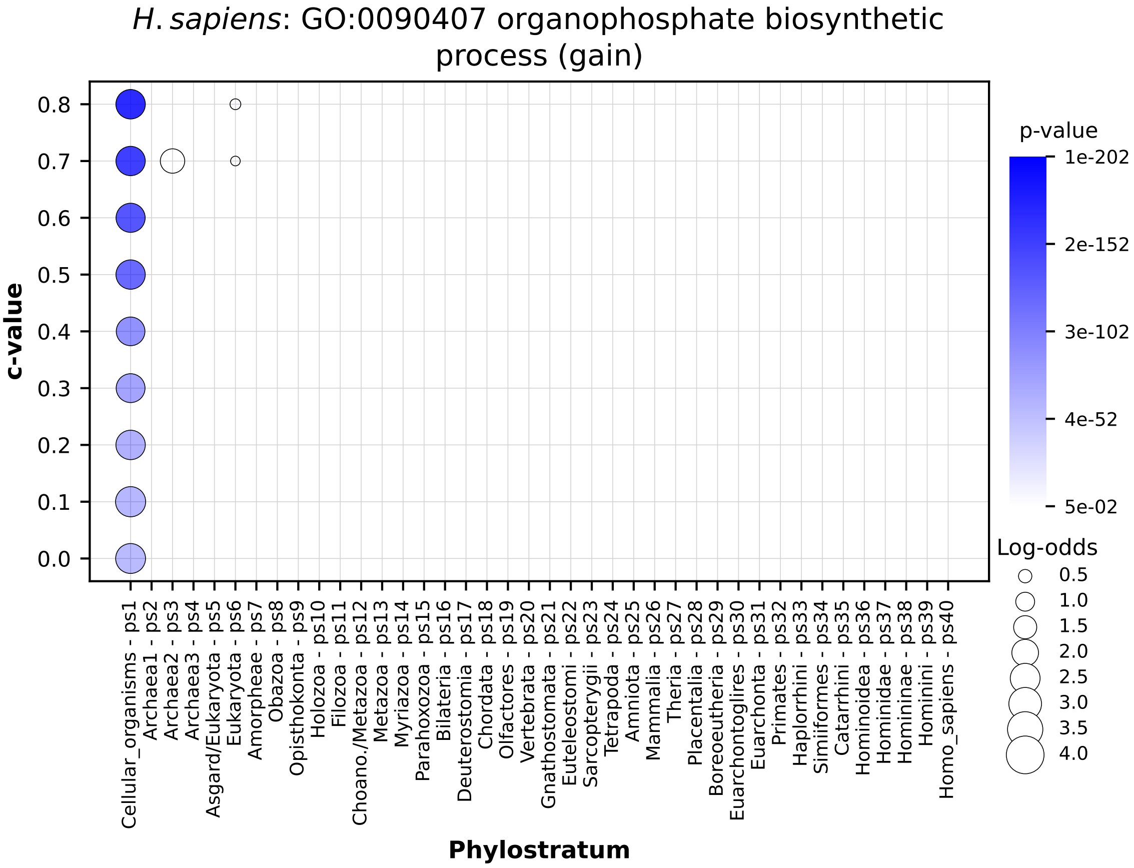

H. sapiens: GO:0090407 organophosphate biosynthetic process (gain)
c-value
0.8
0.7
0.6
0.5
0.4
0.3
0.2
0.1
0.0
Phylostratum
Cellular_organisms - ps1
Archaea1 - ps2
Archaea2 - ps3
Archaea3 - ps4
Asgard/Eukaryota - ps5
Eukaryota - ps6
Amorpheae - ps7
Obazoa - ps8
Opisthokonta - ps9
Holozoa - ps10
Filozoa - ps11
Choano./Metazoa - ps12
Metazoa - ps13
Myriazoa - ps14
Parahoxozoa - ps15
Bilateria - ps16
Deuterostomia - ps17
Chordata - ps18
Olfactores - ps19
Vertebrata - ps20
Gnathostomata - ps21
Euteleostomi - ps22
Sarcopterygii - ps23
Tetrapoda - ps24
Amniota - ps25
Mammalia - ps26
Theria - ps27
Placentalia - ps28
Boreoeutheria - ps29
Euarchontoglires - ps30
Euarchonta - ps31
Primates - ps32
Haplorrhini - ps33
Simiiformes - ps34
Catarrhini - ps35
Hominoidea - ps36
Hominidae - ps37
Homininae - ps38
Hominini - ps39
Homo_sapiens - ps40
p-value
1e-202
2e-152
3e-102
4e-52
5e-02
Log-odds
0.5
1.0
1.5
2.0
2.5
3.0
3.5
4.0

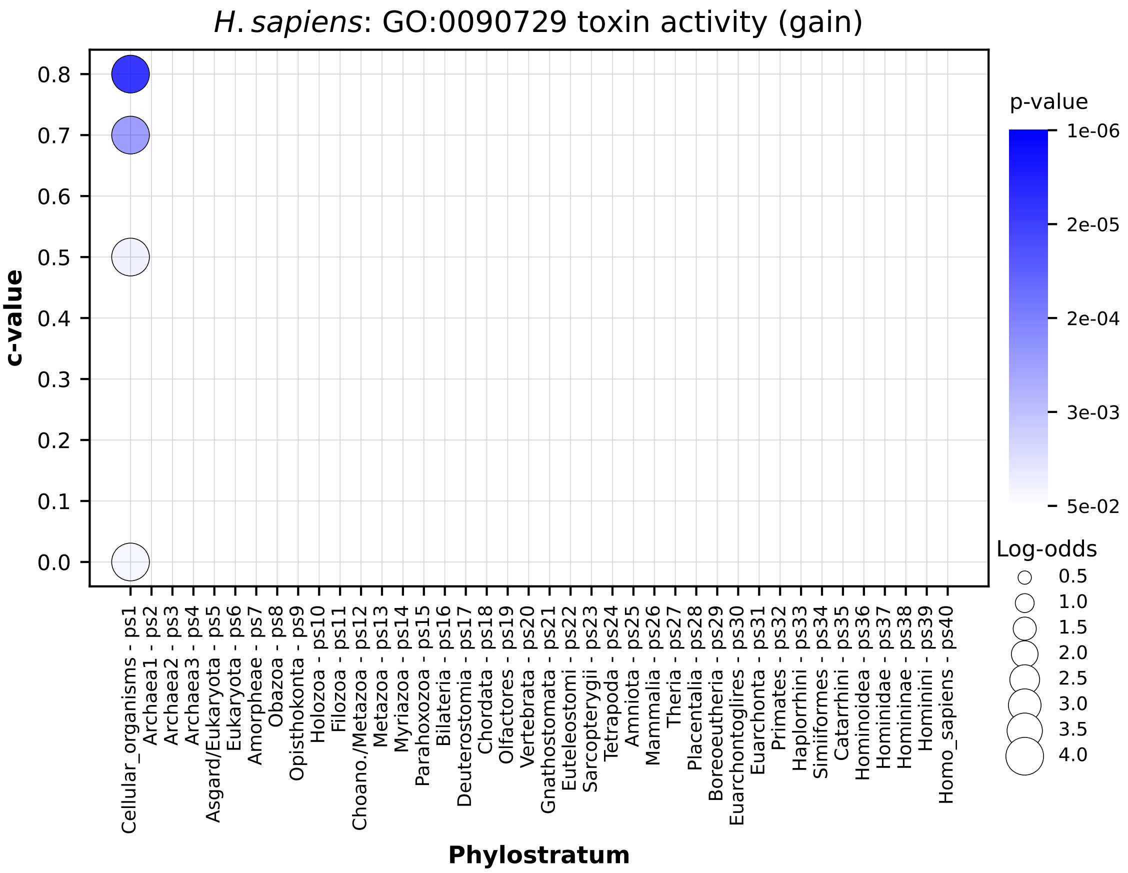
H. sapiens: GO:0090729 toxin activity (gain)
c-value
0.8
0.7
0.6
0.5
0.4
0.3
0.2
0.1
0.0
Phylostratum
Cellular_organisms - ps1
Archaea1 - ps2
Archaea2 - ps3
Archaea3 - ps4
Asgard/Eukaryota - ps5
Eukaryota - ps6
Amorpheae - ps7
Obazoa - ps8
Opisthokonta - ps9
Holozoa - ps10
Filozoa - ps11
Choano./Metazoa - ps12
Metazoa - ps13
Myriazoa - ps14
Parahoxozoa - ps15
Bilateria - ps16
Deuterostomia - ps17
Chordata - ps18
Olfactores - ps19
Vertebrata - ps20
Gnathostomata - ps21
Euteleostomi - ps22
Sarcopterygii - ps23
Tetrapoda - ps24
Amniota - ps25
Mammalia - ps26
Theria - ps27
Placentalia - ps28
Boreoeutheria - ps29
Euarchontoglires - ps30
Euarchonta - ps31
Primates - ps32
Haplorrhini - ps33
Simiiformes - ps34
Catarrhini - ps35
Hominoidea - ps36
Hominidae - ps37
Homininae - ps38
Hominini - ps39
Homo_sapiens - ps40
p-value
1e-06
2e-05
2e-04
3e-03
5e-02
Log-odds
0.5
1.0
1.5
2.0
2.5
3.0
3.5
4.0

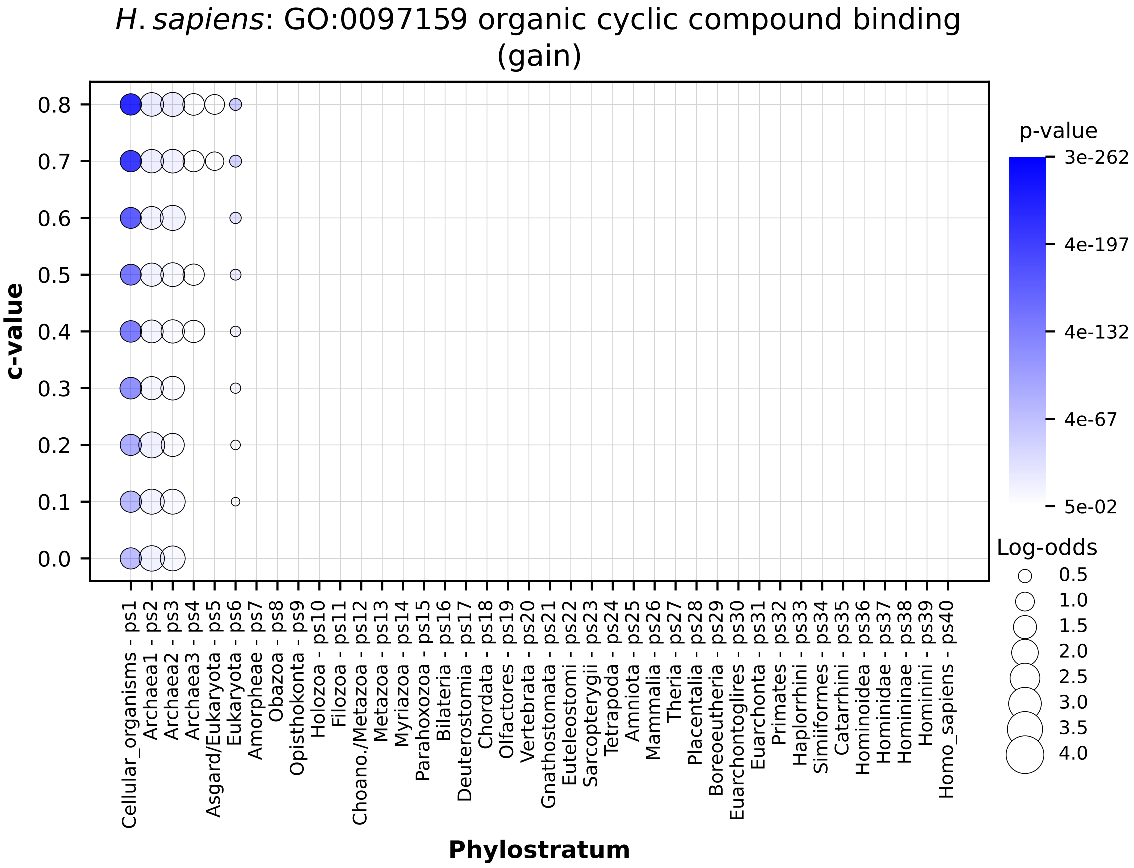

H. sapiens: GO:0097159 organic cyclic compound binding
(gain)
c-value
0.8
0.7
0.6
0.5
0.4
0.3
0.2
0.1
0.0
p-value
3e-262
4e-197
4e-132
4e-67
5e-02
Log-odds
0.5
1.0
1.5
2.0
2.5
3.0
3.5
4.0
Cellular_organisms - ps1
Archaea1 - ps2
Archaea2 - ps3
Archaea3 - ps4
Asgard/Eukaryota - ps5
Eukaryota - ps6
Amorpheae - ps7
Obazoa - ps8
Opisthokonta - ps9
Holozoa - ps10
Filozoa - ps11
Choano./Metazoa - ps12
Metazoa - ps13
Myriazoa - ps14
Parahoxozoa - ps15
Bilateria - ps16
Deuterostomia - ps17
Chordata - ps18
Olfactores - ps19
Vertebrata - ps20
Gnathostomata - ps21
Euteleostomi - ps22
Sarcopterygii - ps23
Tetrapoda - ps24
Amniota - ps25
Mammalia - ps26
Theria - ps27
Placentalia - ps28
Boreoeutheria - ps29
Euarchontoglires - ps30
Euarchonta - ps31
Primates - ps32
Haplorrhini - ps33
Simiiformes - ps34
Catarrhini - ps35
Hominoidea - ps36
Hominidae - ps37
Homininae - ps38
Hominini - ps39
Homo_sapiens - ps40
Phylostratum

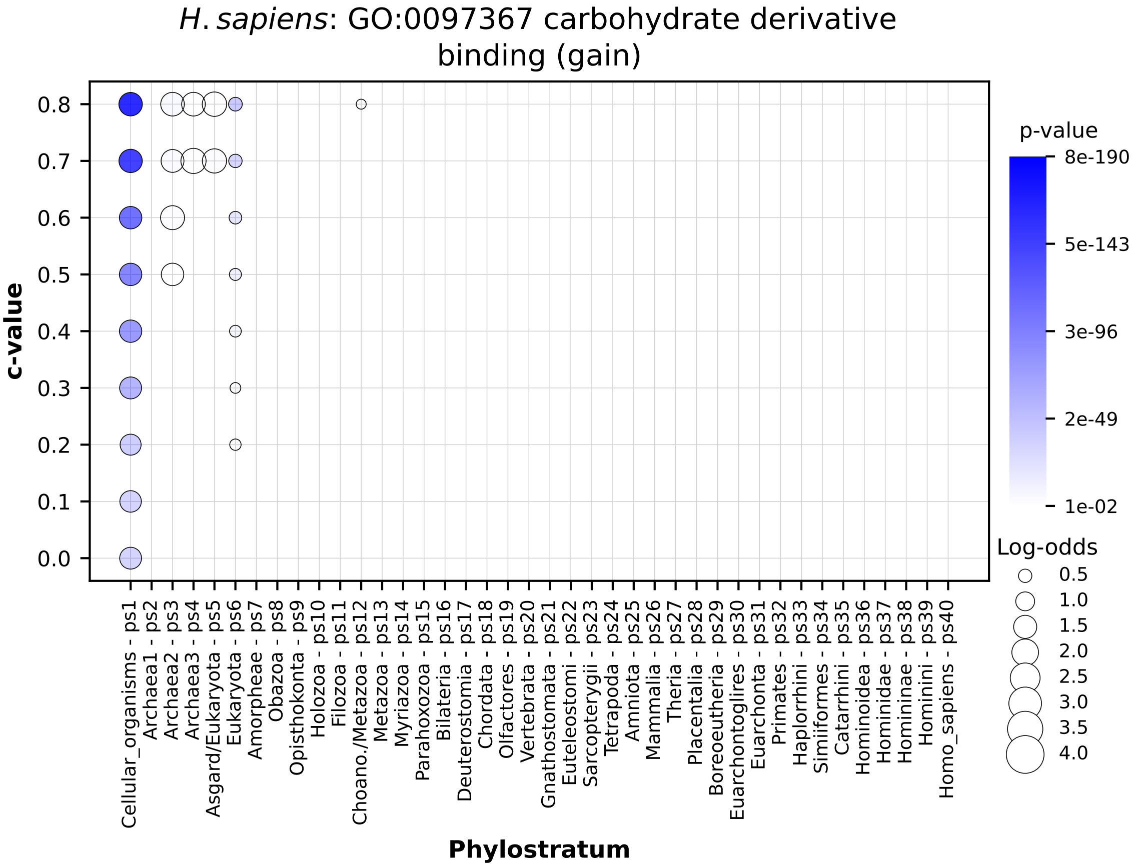

H. sapiens: GO:0097367 carbohydrate derivative binding (gain)
c-value
0.0
0.1
0.2
0.3
0.4
0.5
0.6
0.7
0.8
Phylostratum
Cellular_organisms - ps1
Archaea1 - ps2
Archaea2 - ps3
Archaea3 - ps4
Asgard/Eukaryota - ps5
Eukaryota - ps6
Amorpheae - ps7
Obazoa - ps8
Opisthokonta - ps9
Holozoa - ps10
Filozoa - ps11
Choano./Metazoa - ps12
Metazoa - ps13
Myriazoa - ps14
Parahoxozoa - ps15
Bilateria - ps16
Deuterostomia - ps17
Chordata - ps18
Olfactores - ps19
Vertebrata - ps20
Gnathostomata - ps21
Euteleostomi - ps22
Sarcopterygii - ps23
Tetrapoda - ps24
Amniota - ps25
Mammalia - ps26
Theria - ps27
Placentalia - ps28
Boreoeutheria - ps29
Euarchontoglires - ps30
Euarchonta - ps31
Primates - ps32
Haplorrhini - ps33
Simiiformes - ps34
Catarrhini - ps35
Hominoidea - ps36
Hominidae - ps37
Homininae - ps38
Hominini - ps39
Homo_sapiens - ps40
p-value
8e-190
5e-143
3e-96
2e-49
1e-02
Log-odds
0.5
1.0
1.5
2.0
2.5
3.0
3.5
4.0

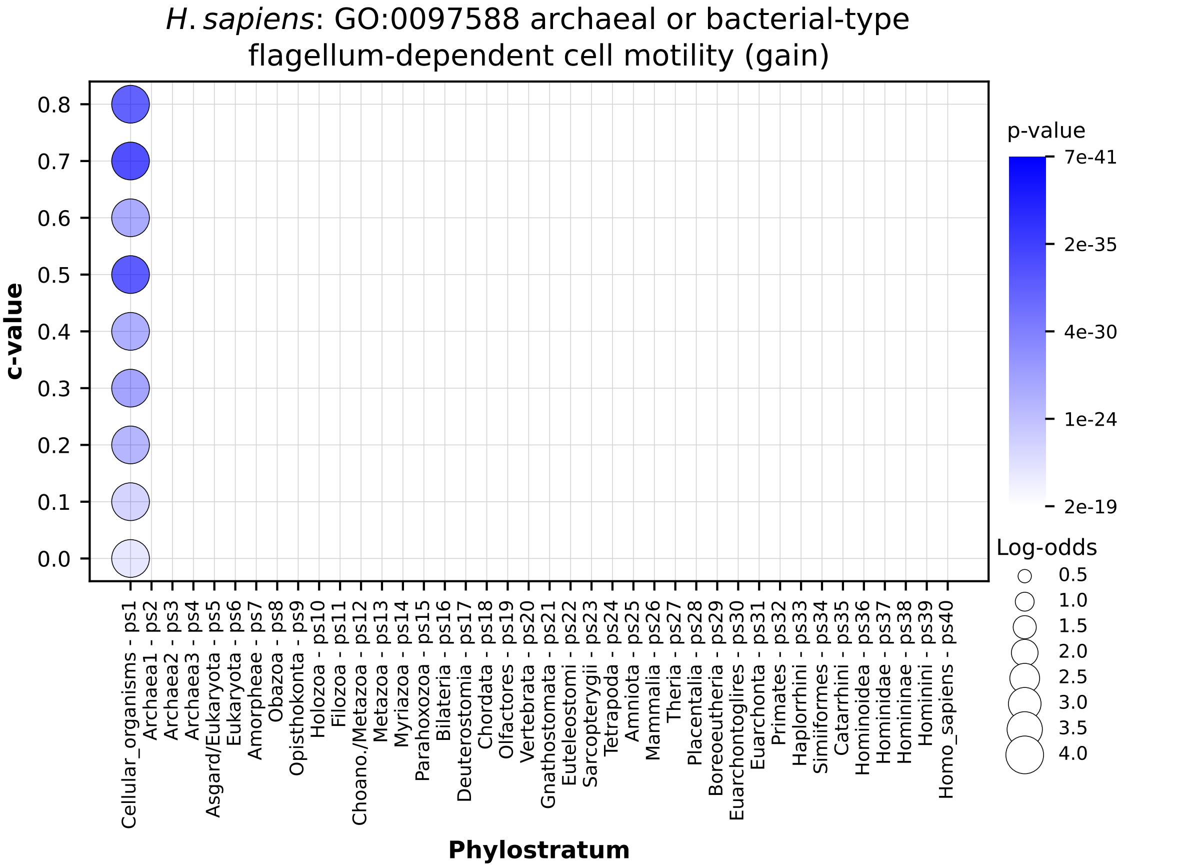
H. sapiens: GO:0097588 archaeal or bacterial-type flagellum-dependent cell motility (gain)
c-value
0.8
0.7
0.6
0.5
0.4
0.3
0.2
0.1
0.0
Phylostratum
Cellular_organisms - ps1
Archaea1 - ps2
Archaea2 - ps3
Archaea3 - ps4
Asgard/Eukaryota - ps5
Eukaryota - ps6
Amorpheae - ps7
Obazoa - ps8
Opisthokonta - ps9
Holozoa - ps10
Filozoa - ps11
Choano./Metazoa - ps12
Metazoa - ps13
Myriazoa - ps14
Parahoxozoa - ps15
Bilateria - ps16
Deuterostomia - ps17
Chordata - ps18
Olfactores - ps19
Vertebrata - ps20
Gnathostomata - ps21
Euteleostomi - ps22
Sarcopterygii - ps23
Tetrapoda - ps24
Amniota - ps25
Mammalia - ps26
Theria - ps27
Placentalia - ps28
Boreoeutheria - ps29
Euarchontoglires - ps30
Euarchonta - ps31
Primates - ps32
Haplorrhini - ps33
Simiiformes - ps34
Catarrhini - ps35
Hominoidea - ps36
Hominidae - ps37
Homininae - ps38
Hominini - ps39
Homo_sapiens - ps40
p-value
7e-41
2e-35
4e-30
1e-24
2e-19
Log-odds
0.5
1.0
1.5
2.0
2.5
3.0
3.5
4.0

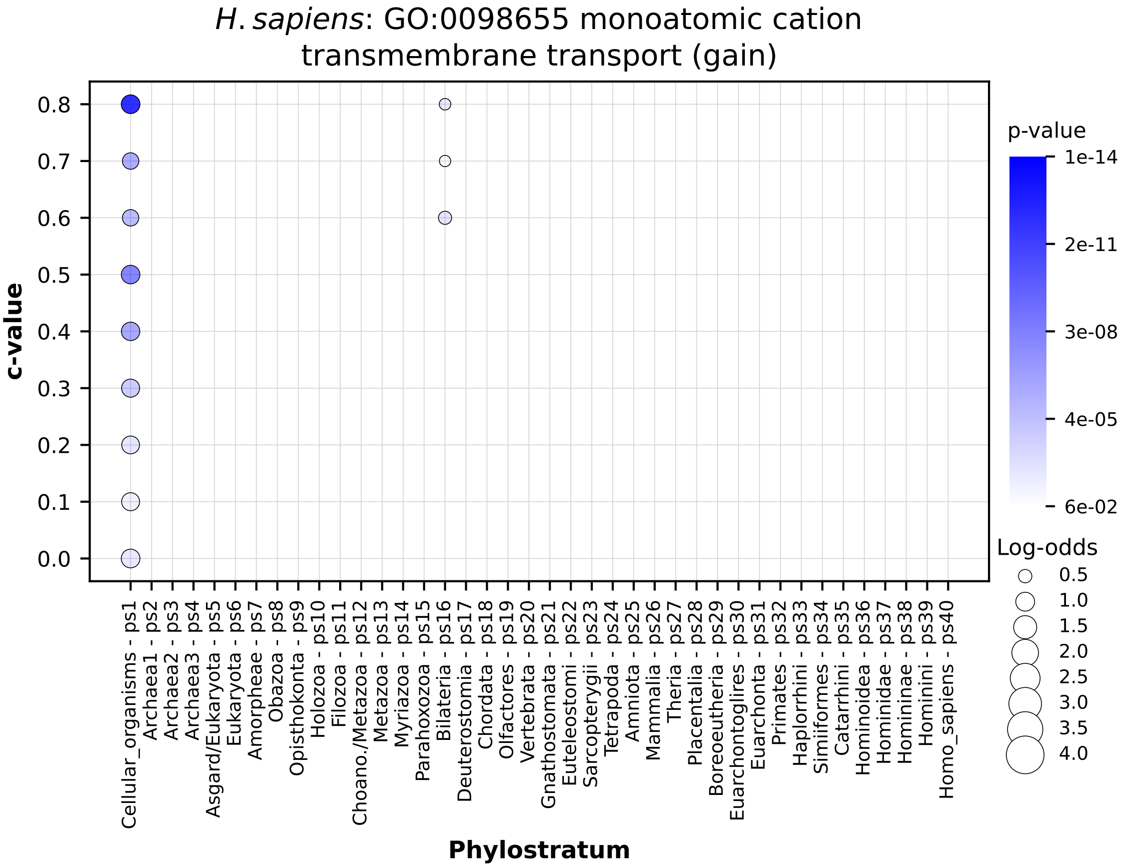

*H. sapiens*: GO:0098655 monoatomic cation transmembrane transport (gain)
c-value
0.8
0.7
0.6
0.5
0.4
0.3
0.2
0.1
0.0
Phylostratum
Cellular_organisms - ps1
Archaea1 - ps2
Archaea2 - ps3
Archaea3 - ps4
Asgard/Eukaryota - ps5
Eukaryota - ps6
Amorpheae - ps7
Obazoa - ps8
Opisthokonta - ps9
Holozoa - ps10
Filozoa - ps11
Choano./Metazoa - ps12
Metazoa - ps13
Myriazoa - ps14
Parahoxozoa - ps15
Bilateria - ps16
Deuterostomia - ps17
Chordata - ps18
Olfactores - ps19
Vertebrata - ps20
Gnathostomata - ps21
Euteleostomi - ps22
Sarcopterygii - ps23
Tetrapoda - ps24
Amniota - ps25
Mammalia - ps26
Theria - ps27
Placentalia - ps28
Boreoeutheria - ps29
Euarchontoglires - ps30
Euarchonta - ps31
Primates - ps32
Haplorrhini - ps33
Simiiformes - ps34
Catarrhini - ps35
Hominoidea - ps36
Hominidae - ps37
Homininae - ps38
Hominini - ps39
Homo_sapiens - ps40
p-value
1e-14
2e-11
3e-08
4e-05
6e-02
Log-odds
0.5
1.0
1.5
2.0
2.5
3.0
3.5
4.0

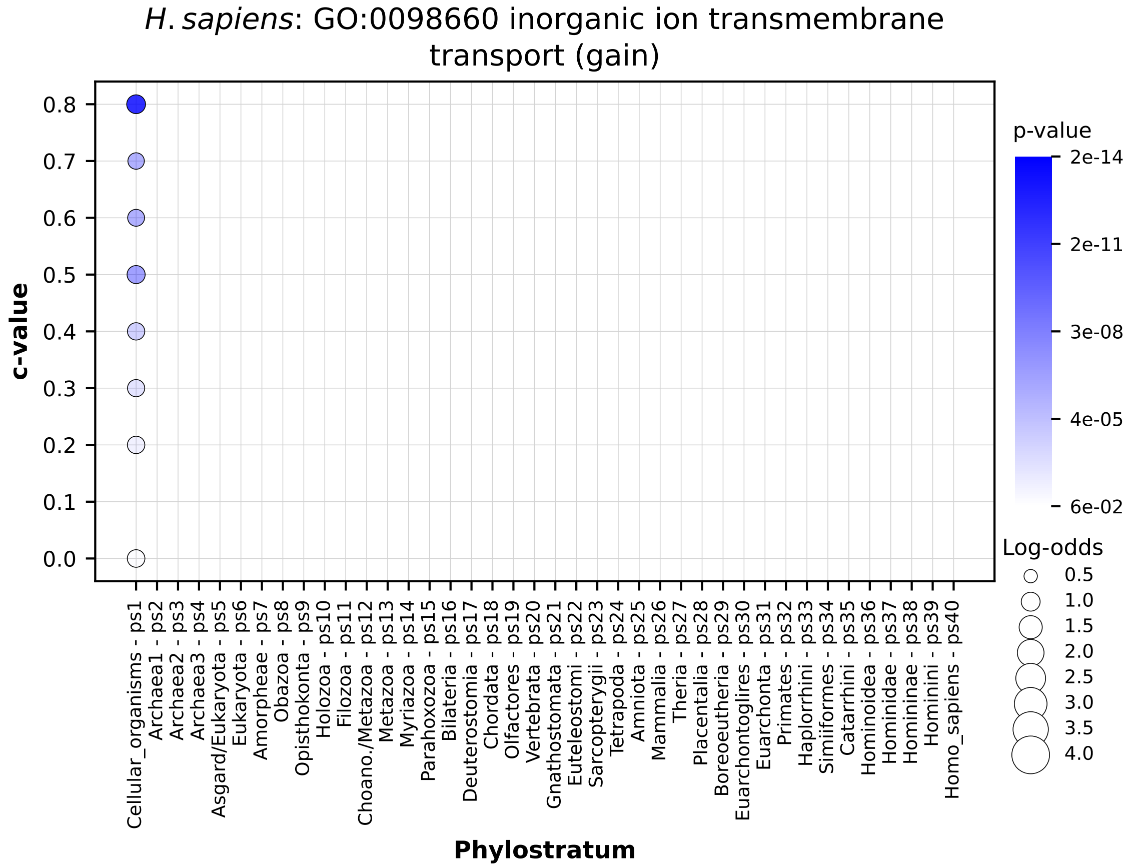
H. sapiens: GO:0098660 inorganic ion transmembrane transport (gain)
c-value
0.8
0.7
0.6
0.5
0.4
0.3
0.2
0.1
0.0
p-value
2e-14
2e-11
3e-08
4e-05
6e-02
Log-odds
0.5
1.0
1.5
2.0
2.5
3.0
3.5
4.0
Cellular_organisms - ps1
Archaea1 - ps2
Archaea2 - ps3
Archaea3 - ps4
Asgard/Eukaryota - ps5
Eukaryota - ps6
Amorpheae - ps7
Obazoa - ps8
Opisthokonta - ps9
Holozoa - ps10
Filozoa - ps11
Choano./Metazoa - ps12
Metazoa - ps13
Myriazoa - ps14
Parahoxozoa - ps15
Bilateria - ps16
Deuterostomia - ps17
Chordata - ps18
Olfactores - ps19
Vertebrata - ps20
Gnathostomata - ps21
Euteleostomi - ps22
Sarcopterygii - ps23
Tetrapoda - ps24
Amniota - ps25
Mammalia - ps26
Theria - ps27
Placentalia - ps28
Boreoeutheria - ps29
Euarchontoglires - ps30
Euarchonta - ps31
Primates - ps32
Haplorrhini - ps33
Simiiformes - ps34
Catarrhini - ps35
Hominoidea - ps36
Hominidae - ps37
Homininae - ps38
Hominini - ps39
Homo_sapiens - ps40
Phylostratum

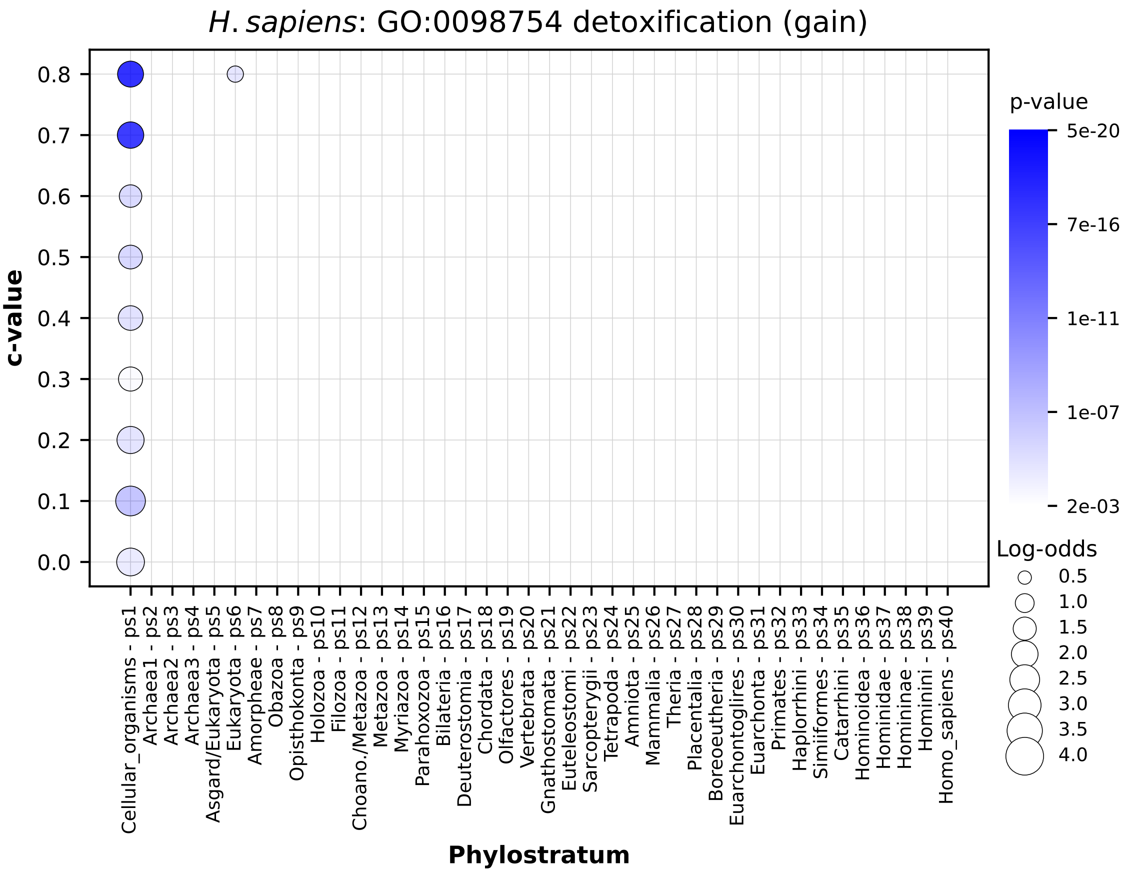
H. sapiens: GO:0098754 detoxification (gain)
c-value
0.0
0.1
0.2
0.3
0.4
0.5
0.6
0.7
0.8
Phylostratum
Cellular_organisms - ps1
Archaea1 - ps2
Archaea2 - ps3
Archaea3 - ps4
Asgard/Eukaryota - ps5
Eukaryota - ps6
Amorpheae - ps7
Obazoa - ps8
Opisthokonta - ps9
Holozoa - ps10
Filozoa - ps11
Choano./Metazoa - ps12
Metazoa - ps13
Myriazoa - ps14
Parahoxozoa - ps15
Bilateria - ps16
Deuterostomia - ps17
Chordata - ps18
Olfactores - ps19
Vertebrata - ps20
Gnathostomata - ps21
Euteleostomi - ps22
Sarcopterygii - ps23
Tetrapoda - ps24
Amniota - ps25
Mammalia - ps26
Theria - ps27
Placentalia - ps28
Boreoeutheria - ps29
Euarchontoglires - ps30
Euarchonta - ps31
Primates - ps32
Haplorrhini - ps33
Simiiformes - ps34
Catarrhini - ps35
Hominoidea - ps36
Hominidae - ps37
Homininae - ps38
Hominini - ps39
Homo_sapiens - ps40
p-value
5e-20
7e-16
1e-11
1e-07
2e-03
Log-odds
0.5
1.0
1.5
2.0
2.5
3.0
3.5
4.0

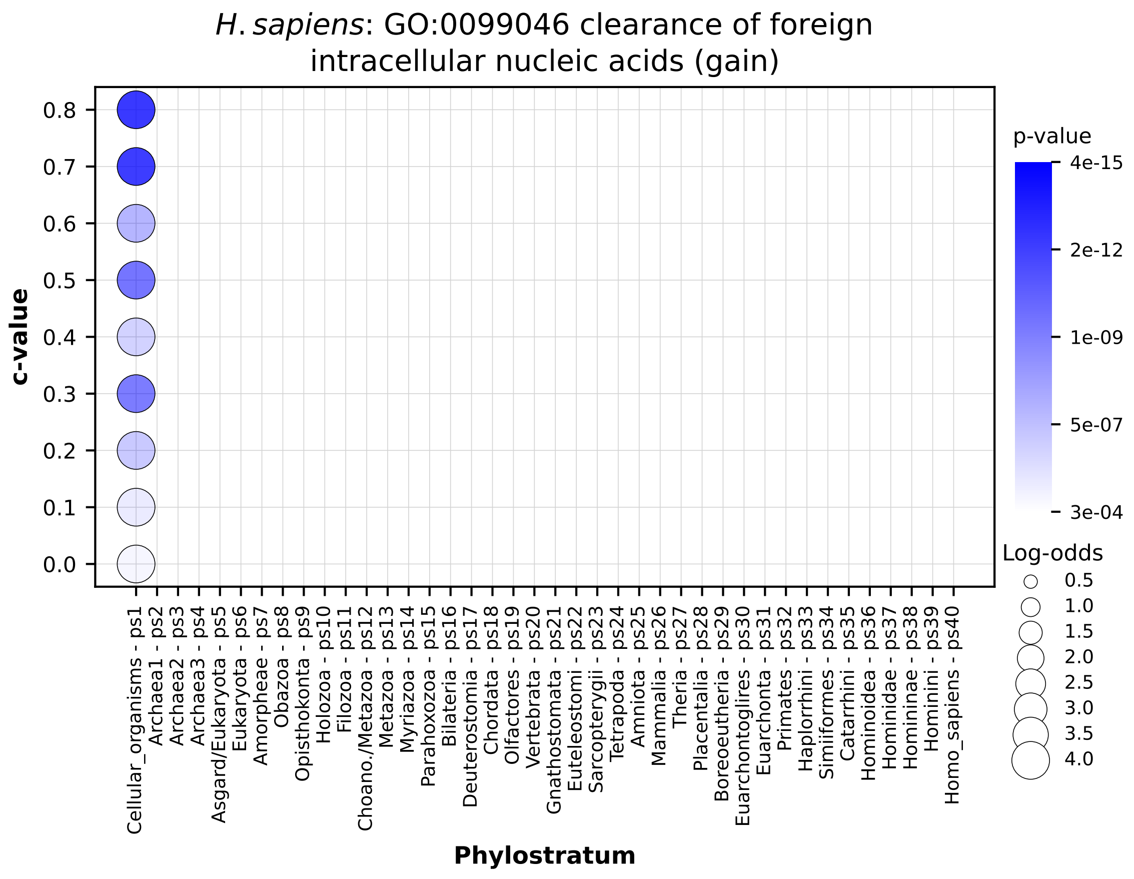
H. sapiens: GO:0099046 clearance of foreign intracellular nucleic acids (gain)
c-value
0.0
0.1
0.2
0.3
0.4
0.5
0.6
0.7
0.8
p-value
4e-15
2e-12
1e-09
5e-07
3e-04
Log-odds
0.5
1.0
1.5
2.0
2.5
3.0
3.5
4.0
Cellular_organisms - ps1
Archaea1 - ps2
Archaea2 - ps3
Archaea3 - ps4
Asgard/Eukaryota - ps5
Eukaryota - ps6
Amorpheae - ps7
Obazoa - ps8
Opisthokonta - ps9
Holozoa - ps10
Filozoa - ps11
Choano./Metazoa - ps12
Metazoa - ps13
Myriazoa - ps14
Parahoxozoa - ps15
Bilateria - ps16
Deuterostomia - ps17
Chordata - ps18
Olfactores - ps19
Vertebrata - ps20
Gnathostomata - ps21
Euteleostomi - ps22
Sarcopterygii - ps23
Tetrapoda - ps24
Amniota - ps25
Mammalia - ps26
Theria - ps27
Placentalia - ps28
Boreoeutheria - ps29
Euarchontoglires - ps30
Euarchonta - ps31
Primates - ps32
Haplorrhini - ps33
Simiiformes - ps34
Catarrhini - ps35
Hominoidea - ps36
Hominidae - ps37
Homininae - ps38
Hominini - ps39
Homo_sapiens - ps40
Phylostratum

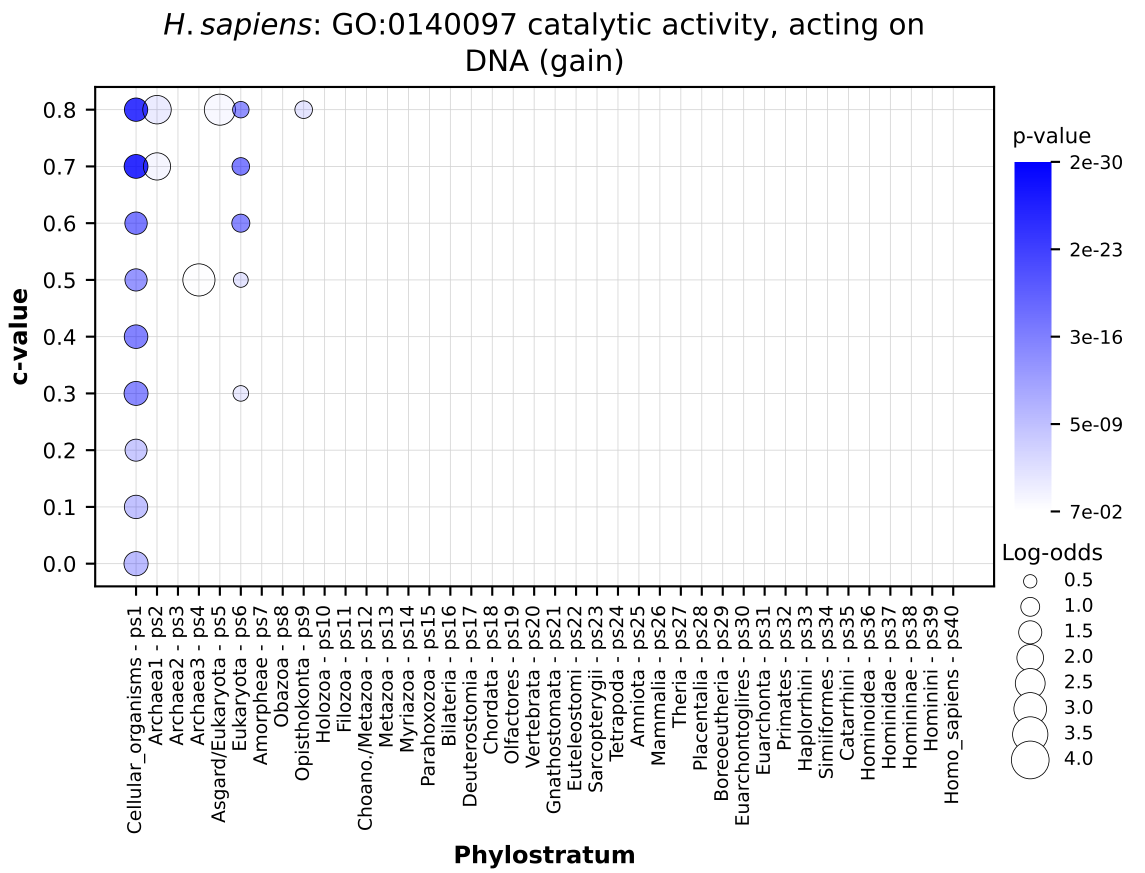
H. sapiens: GO:0140097 catalytic activity, acting on DNA (gain)
c-value
0.8
0.7
0.6
0.5
0.4
0.3
0.2
0.1
0.0
p-value
2e-30
2e-23
3e-16
5e-09
7e-02
Log-odds
0.5
1.0
1.5
2.0
2.5
3.0
3.5
4.0
Cellular_organisms - ps1
Archaea1 - ps2
Archaea2 - ps3
Archaea3 - ps4
Asgard/Eukaryota - ps5
Eukaryota - ps6
Amorpheae - ps7
Obazoa - ps8
Opisthokonta - ps9
Holozoa - ps10
Filozoa - ps11
Choano./Metazoa - ps12
Metazoa - ps13
Myriazoa - ps14
Parahoxozoa - ps15
Bilateria - ps16
Deuterostomia - ps17
Chordata - ps18
Olfactores - ps19
Vertebrata - ps20
Gnathostomata - ps21
Euteleostomi - ps22
Sarcopterygii - ps23
Tetrapoda - ps24
Amniota - ps25
Mammalia - ps26
Theria - ps27
Placentalia - ps28
Boreoeutheria - ps29
Euarchontoglires - ps30
Euarchonta - ps31
Primates - ps32
Haplorrhini - ps33
Simiiformes - ps34
Catarrhini - ps35
Hominoidea - ps36
Hominidae - ps37
Homininae - ps38
Hominini - ps39
Homo_sapiens - ps40
Phylostratum

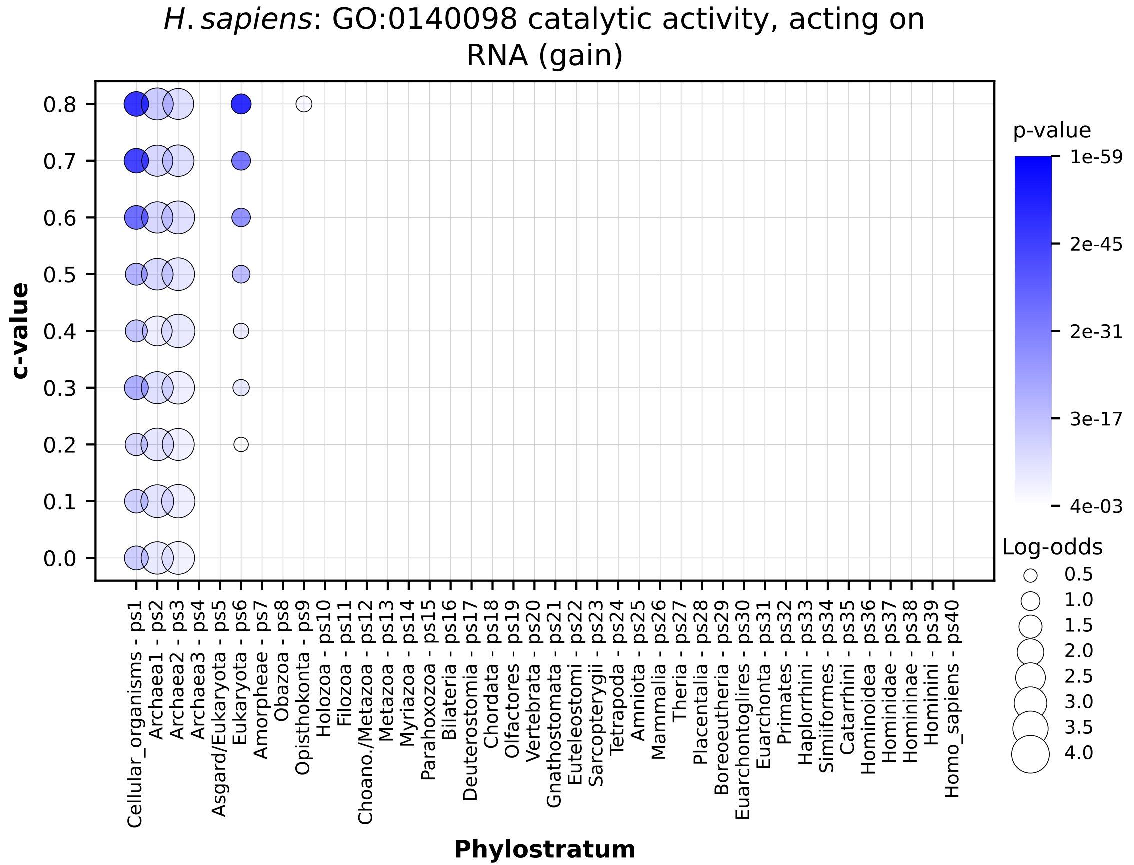
H. sapiens: GO:0140098 catalytic activity, acting on RNA (gain)
c-value
0.8
0.7
0.6
0.5
0.4
0.3
0.2
0.1
0.0
Phylostratum
Cellular_organisms - ps1
Archaea1 - ps2
Archaea2 - ps3
Archaea3 - ps4
Asgard/Eukaryota - ps5
Eukaryota - ps6
Amorpheae - ps7
Obazoa - ps8
Opisthokonta - ps9
Holozoa - ps10
Filozoa - ps11
Choano./Metazoa - ps12
Metazoa - ps13
Myriazoa - ps14
Parahoxozoa - ps15
Bilateria - ps16
Deuterostomia - ps17
Chordata - ps18
Olfactores - ps19
Vertebrata - ps20
Gnathostomata - ps21
Euteleostomi - ps22
Sarcopterygii - ps23
Tetrapoda - ps24
Amniota - ps25
Mammalia - ps26
Theria - ps27
Placentalia - ps28
Boreoeutheria - ps29
Euarchontoglires - ps30
Euarchonta - ps31
Primates - ps32
Haplorrhini - ps33
Simiiformes - ps34
Catarrhini - ps35
Hominoidea - ps36
Hominidae - ps37
Homininae - ps38
Hominini - ps39
Homo_sapiens - ps40
p-value
1e-59
2e-45
2e-31
3e-17
4e-03
Log-odds
0.5
1.0
1.5
2.0
2.5
3.0
3.5
4.0

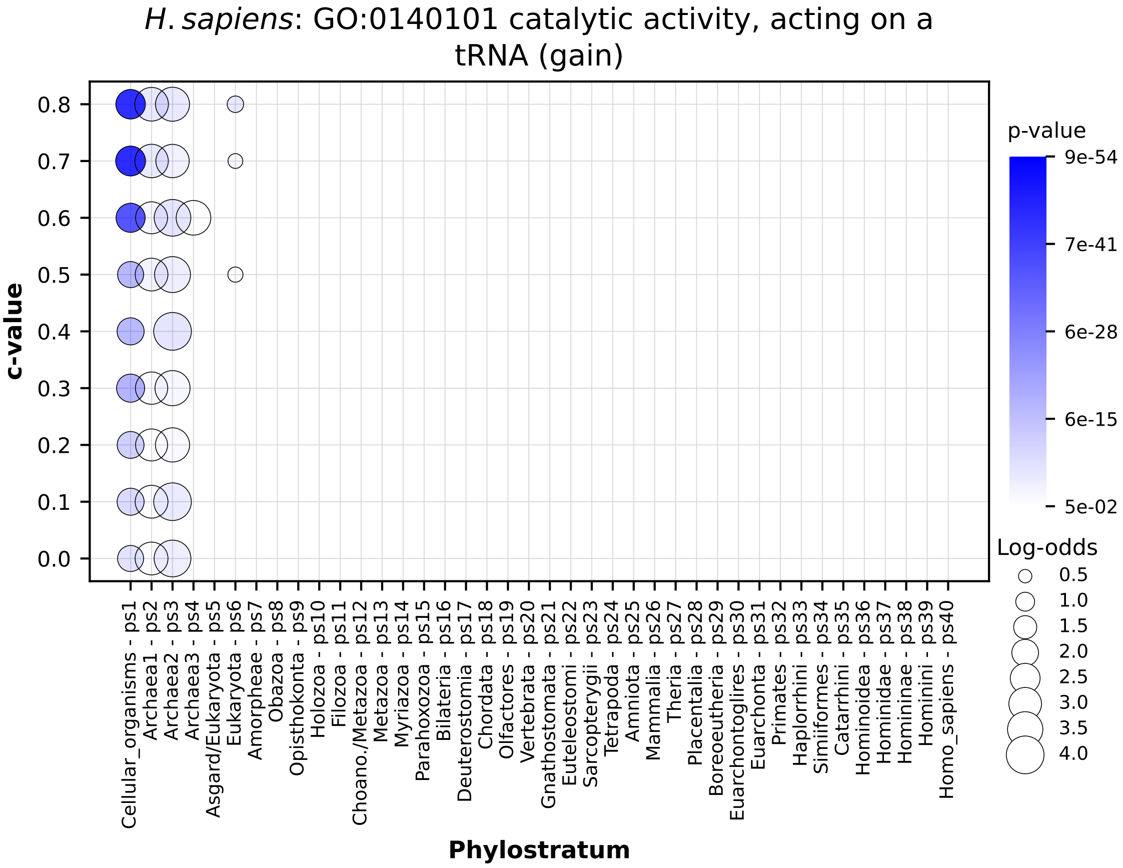

H. sapiens: GO:0140101 catalytic activity, acting on a tRNA (gain)
c-value
0.8
0.7
0.6
0.5
0.4
0.3
0.2
0.1
0.0
p-value
9e-54
7e-41
6e-28
6e-15
5e-02
Log-odds
0.5
1.0
1.5
2.0
2.5
3.0
3.5
4.0
Cellular_organisms - ps1
Archaea1 - ps2
Archaea2 - ps3
Archaea3 - ps4
Asgard/Eukaryota - ps5
Eukaryota - ps6
Amorpheae - ps7
Obazoa - ps8
Opisthokonta - ps9
Holozoa - ps10
Filozoa - ps11
Choano./Metazoa - ps12
Metazoa - ps13
Myriazoa - ps14
Parahoxozoa - ps15
Bilateria - ps16
Deuterostomia - ps17
Chordata - ps18
Olfactores - ps19
Vertebrata - ps20
Gnathostomata - ps21
Euteleostomi - ps22
Sarcopterygii - ps23
Tetrapoda - ps24
Amniota - ps25
Mammalia - ps26
Theria - ps27
Placentalia - ps28
Boreoeutheria - ps29
Euarchontoglires - ps30
Euarchonta - ps31
Primates - ps32
Haplorrhini - ps33
Simiiformes - ps34
Catarrhini - ps35
Hominoidea - ps36
Hominidae - ps37
Homininae - ps38
Hominini - ps39
Homo_sapiens - ps40
Phylostratum

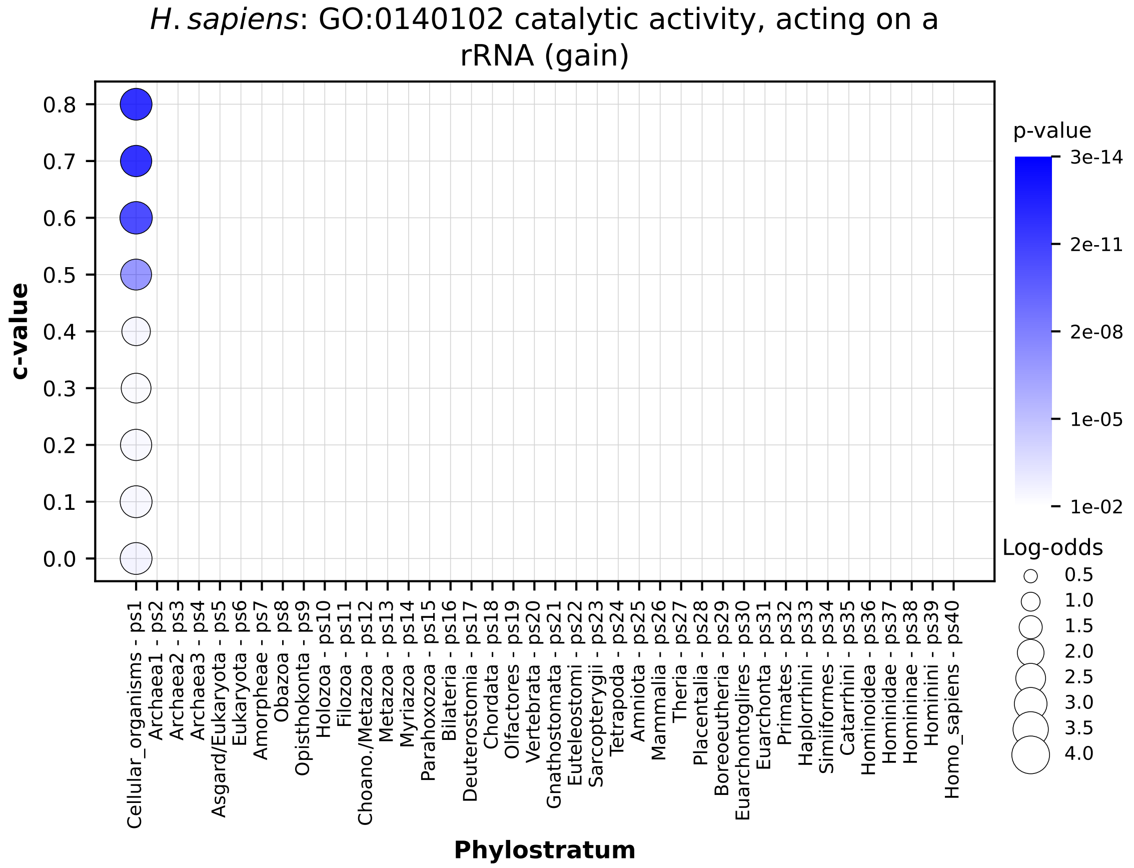

H. sapiens: GO:0140102 catalytic activity, acting on a rRNA (gain)
c-value
0.8
0.7
0.6
0.5
0.4
0.3
0.2
0.1
0.0
Phylostratum
Cellular_organisms - ps1
Archaea1 - ps2
Archaea2 - ps3
Archaea3 - ps4
Asgard/Eukaryota - ps5
Eukaryota - ps6
Amorpheae - ps7
Obazoa - ps8
Opisthokonta - ps9
Holozoa - ps10
Filozoa - ps11
Choano./Metazoa - ps12
Metazoa - ps13
Myriazoa - ps14
Parahoxozoa - ps15
Bilateria - ps16
Deuterostomia - ps17
Chordata - ps18
Olfactores - ps19
Vertebrata - ps20
Gnathostomata - ps21
Euteleostomi - ps22
Sarcopterygii - ps23
Tetrapoda - ps24
Amniota - ps25
Mammalia - ps26
Theria - ps27
Placentalia - ps28
Boreoeutheria - ps29
Euarchontoglires - ps30
Euarchonta - ps31
Primates - ps32
Haplorrhini - ps33
Simiiformes - ps34
Catarrhini - ps35
Hominoidea - ps36
Hominidae - ps37
Homininae - ps38
Hominini - ps39
Homo_sapiens - ps40
p-value
3e-14
2e-11
2e-08
1e-05
1e-02
Log-odds
0.5
1.0
1.5
2.0
2.5
3.0
3.5
4.0

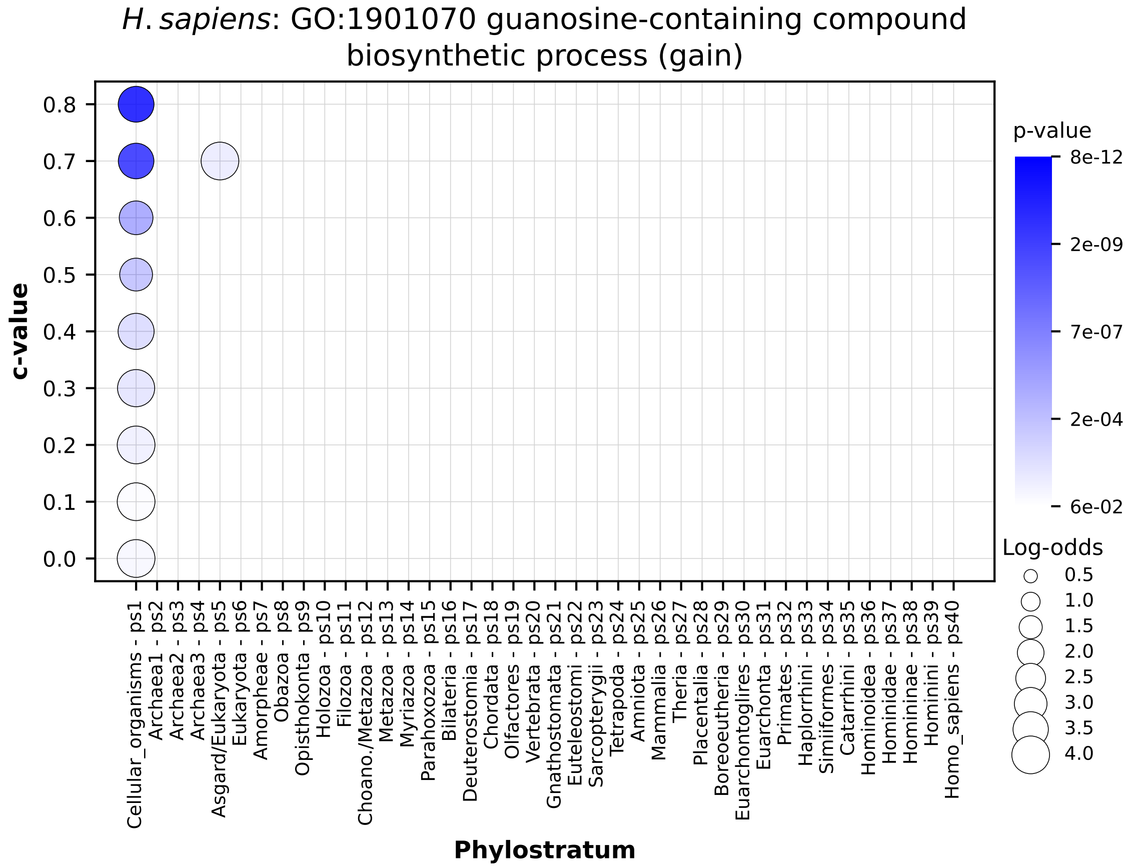

H. sapiens: GO:1901070 guanosine-containing compound biosynthetic process (gain)
c-value
0.0
0.1
0.2
0.3
0.4
0.5
0.6
0.7
0.8
Phylostratum
Cellular_organisms - ps1
Archaea1 - ps2
Archaea2 - ps3
Archaea3 - ps4
Asgard/Eukaryota - ps5
Eukaryota - ps6
Amorpheae - ps7
Obazoa - ps8
Opisthokonta - ps9
Holozoa - ps10
Filozoa - ps11
Choano./Metazoa - ps12
Metazoa - ps13
Myriazoa - ps14
Parahoxozoa - ps15
Bilateria - ps16
Deuterostomia - ps17
Chordata - ps18
Olfactores - ps19
Vertebrata - ps20
Gnathostomata - ps21
Euteleostomi - ps22
Sarcopterygii - ps23
Tetrapoda - ps24
Amniota - ps25
Mammalia - ps26
Theria - ps27
Placentalia - ps28
Boreoeutheria - ps29
Euarchontoglires - ps30
Euarchonta - ps31
Primates - ps32
Haplorrhini - ps33
Simiiformes - ps34
Catarrhini - ps35
Hominoidea - ps36
Hominidae - ps37
Homininae - ps38
Hominini - ps39
Homo_sapiens - ps40
p-value
8e-12
2e-09
7e-07
2e-04
6e-02
Log-odds
0.5
1.0
1.5
2.0
2.5
3.0
3.5
4.0

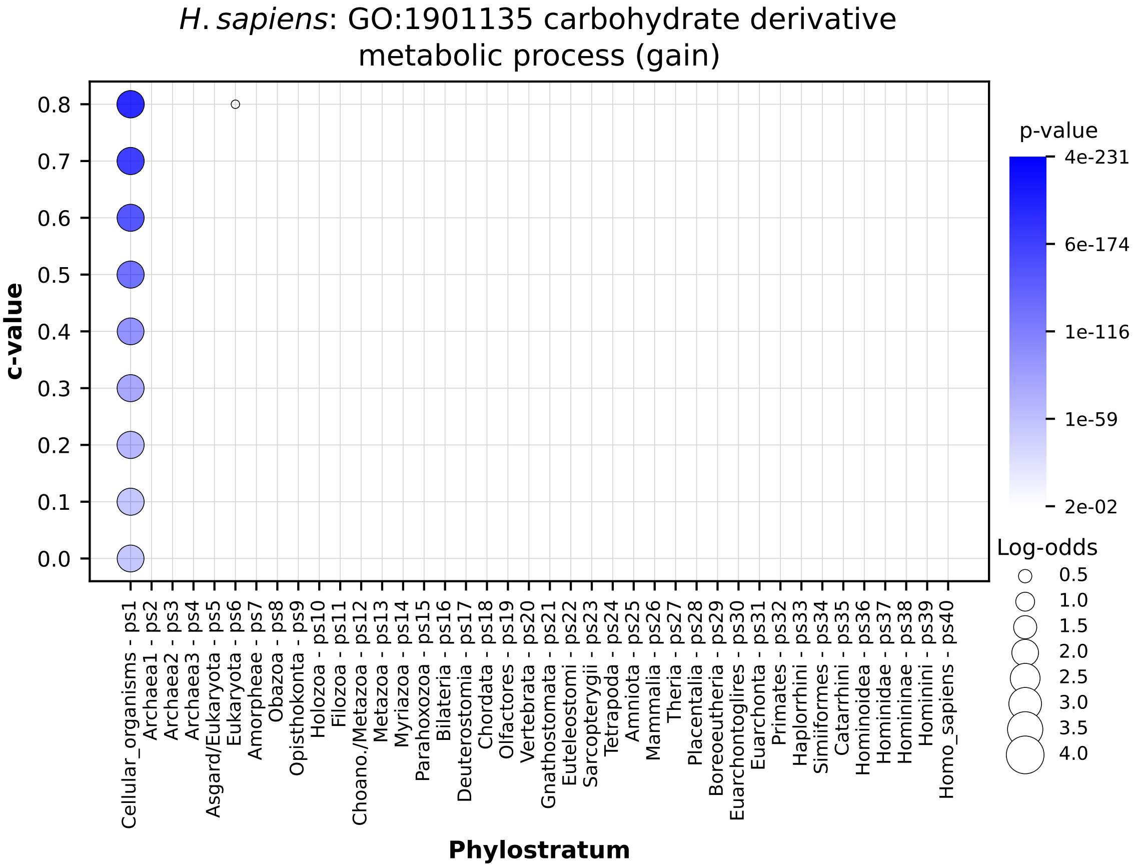

H. sapiens: GO:1901135 carbohydrate derivative metabolic process (gain)
c-value
0.8
0.7
0.6
0.5
0.4
0.3
0.2
0.1
0.0
p-value
4e-231
6e-174
1e-116
1e-59
2e-02
Log-odds
0.5
1.0
1.5
2.0
2.5
3.0
3.5
4.0
Cellular_organisms - ps1
Archaea1 - ps2
Archaea2 - ps3
Archaea3 - ps4
Asgard/Eukaryota - ps5
Eukaryota - ps6
Amorpheae - ps7
Obazoa - ps8
Opisthokonta - ps9
Holozoa - ps10
Filozoa - ps11
Choano./Metazoa - ps12
Metazoa - ps13
Myriazoa - ps14
Parahoxozoa - ps15
Bilateria - ps16
Deuterostomia - ps17
Chordata - ps18
Olfactores - ps19
Vertebrata - ps20
Gnathostomata - ps21
Euteleostomi - ps22
Sarcopterygii - ps23
Tetrapoda - ps24
Amniota - ps25
Mammalia - ps26
Theria - ps27
Placentalia - ps28
Boreoeutheria - ps29
Euarchontoglires - ps30
Euarchonta - ps31
Primates - ps32
Haplorrhini - ps33
Simiiformes - ps34
Catarrhini - ps35
Hominoidea - ps36
Hominidae - ps37
Homininae - ps38
Hominini - ps39
Homo_sapiens - ps40
Phylostratum

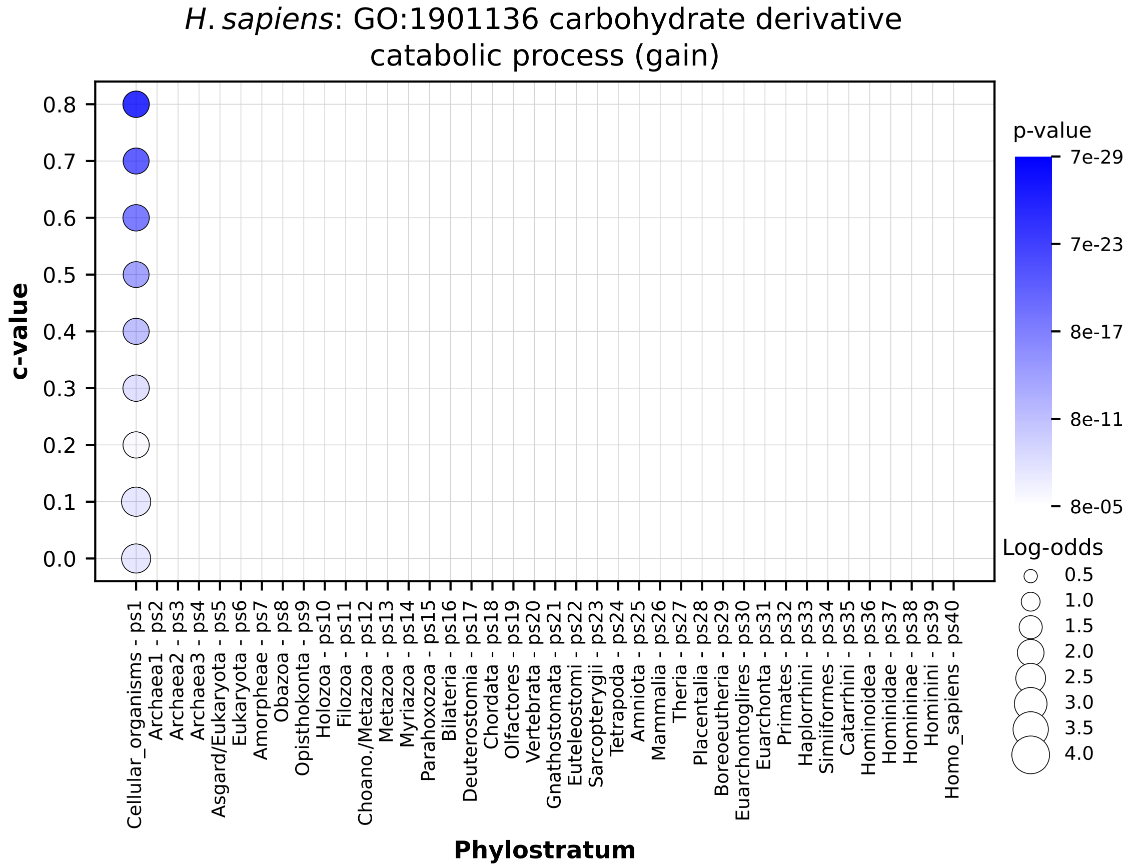

H. sapiens: GO:1901136 carbohydrate derivative catabolic process (gain)
c-value
0.8
0.7
0.6
0.5
0.4
0.3
0.2
0.1
0.0
p-value
7e-29
7e-23
8e-17
8e-11
8e-05
Log-odds
0.5
1.0
1.5
2.0
2.5
3.0
3.5
4.0
Cellular_organisms - ps1
Archaea1 - ps2
Archaea2 - ps3
Archaea3 - ps4
Asgard/Eukaryota - ps5
Eukaryota - ps6
Amorpheae - ps7
Obazoa - ps8
Opisthokonta - ps9
Holozoa - ps10
Filozoa - ps11
Choano./Metazoa - ps12
Metazoa - ps13
Myriazoa - ps14
Parahoxozoa - ps15
Bilateria - ps16
Deuterostomia - ps17
Chordata - ps18
Olfactores - ps19
Vertebrata - ps20
Gnathostomata - ps21
Euteleostomi - ps22
Sarcopterygii - ps23
Tetrapoda - ps24
Amniota - ps25
Mammalia - ps26
Theria - ps27
Placentalia - ps28
Boreoeutheria - ps29
Euarchontoglires - ps30
Euarchonta - ps31
Primates - ps32
Haplorrhini - ps33
Simiiformes - ps34
Catarrhini - ps35
Hominoidea - ps36
Hominidae - ps37
Homininae - ps38
Hominini - ps39
Homo_sapiens - ps40
Phylostratum

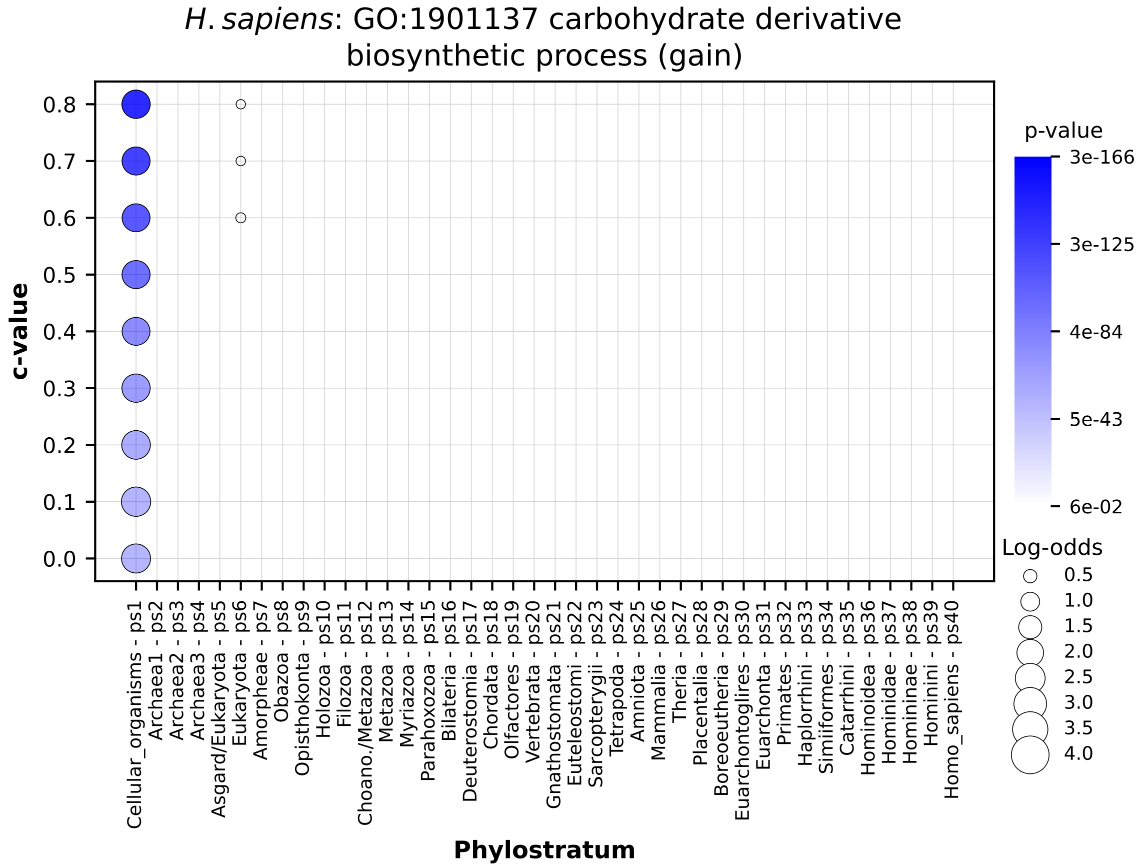

H. sapiens: GO:1901137 carbohydrate derivative biosynthetic process (gain)
c-value
0.8
0.7
0.6
0.5
0.4
0.3
0.2
0.1
0.0
Phylostratum
Cellular_organisms - ps1
Archaea1 - ps2
Archaea2 - ps3
Archaea3 - ps4
Asgard/Eukaryota - ps5
Eukaryota - ps6
Amorpheae - ps7
Obazoa - ps8
Opisthokonta - ps9
Holozoa - ps10
Filozoa - ps11
Choano./Metazoa - ps12
Metazoa - ps13
Myriazoa - ps14
Parahoxozoa - ps15
Bilateria - ps16
Deuterostomia - ps17
Chordata - ps18
Olfactores - ps19
Vertebrata - ps20
Gnathostomata - ps21
Euteleostomi - ps22
Sarcopterygii - ps23
Tetrapoda - ps24
Amniota - ps25
Mammalia - ps26
Theria - ps27
Placentalia - ps28
Boreoeutheria - ps29
Euarchontoglires - ps30
Euarchonta - ps31
Primates - ps32
Haplorrhini - ps33
Simiiformes - ps34
Catarrhini - ps35
Hominoidea - ps36
Hominidae - ps37
Homininae - ps38
Hominini - ps39
Homo_sapiens - ps40
p-value
3e-166
3e-125
4e-84
5e-43
6e-02
Log-odds
0.5
1.0
1.5
2.0
2.5
3.0
3.5
4.0

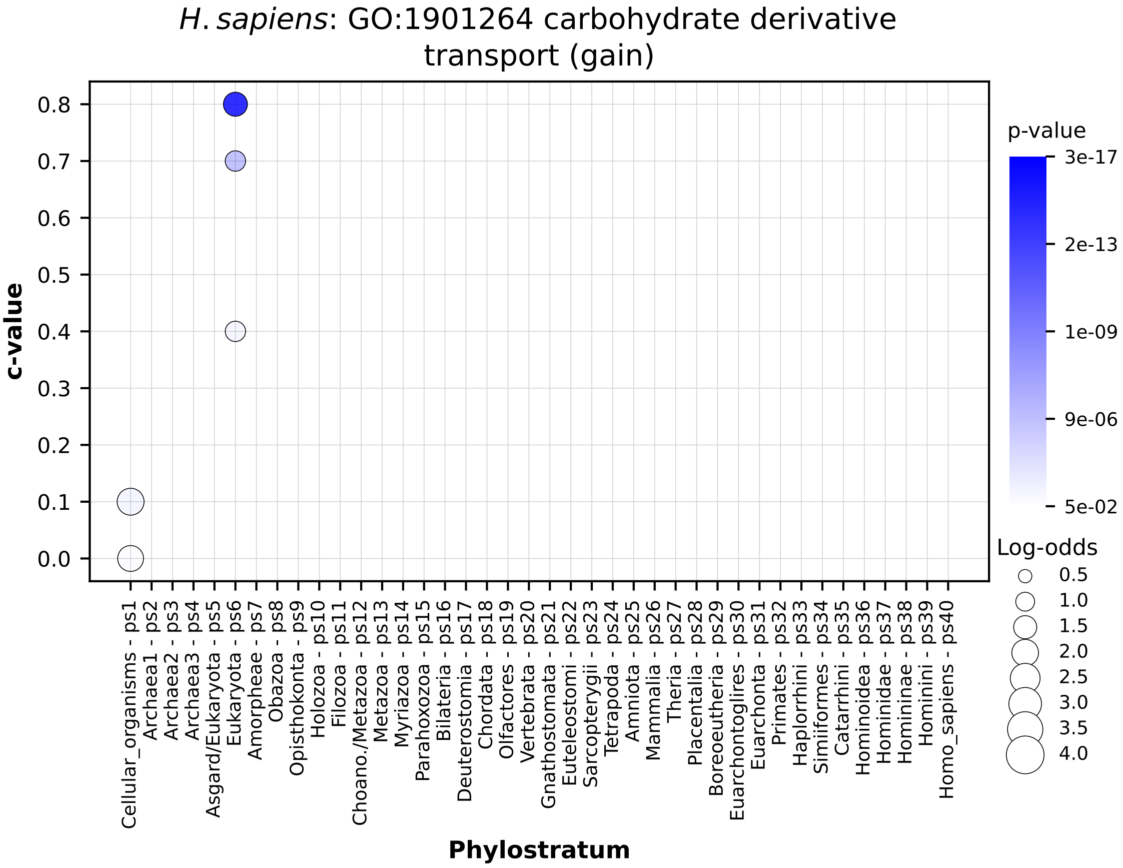

H. sapiens: GO:1901264 carbohydrate derivative transport (gain)
c-value
0.0
0.1
0.2
0.3
0.4
0.5
0.6
0.7
0.8
Phylostratum
Cellular_organisms - ps1
Archaea1 - ps2
Archaea2 - ps3
Archaea3 - ps4
Asgard/Eukaryota - ps5
Eukaryota - ps6
Amorpheae - ps7
Obazoa - ps8
Opisthokonta - ps9
Holozoa - ps10
Filozoa - ps11
Choano./Metazoa - ps12
Metazoa - ps13
Myriazoa - ps14
Parahoxozoa - ps15
Bilateria - ps16
Deuterostomia - ps17
Chordata - ps18
Olfactores - ps19
Vertebrata - ps20
Gnathostomata - ps21
Euteleostomi - ps22
Sarcopterygii - ps23
Tetrapoda - ps24
Amniota - ps25
Mammalia - ps26
Theria - ps27
Placentalia - ps28
Boreoeutheria - ps29
Euarchontoglires - ps30
Euarchonta - ps31
Primates - ps32
Haplorrhini - ps33
Simiiformes - ps34
Catarrhini - ps35
Hominoidea - ps36
Hominidae - ps37
Homininae - ps38
Hominini - ps39
Homo_sapiens - ps40
p-value
3e-17
2e-13
1e-09
9e-06
5e-02
Log-odds
0.5
1.0
1.5
2.0
2.5
3.0
3.5
4.0

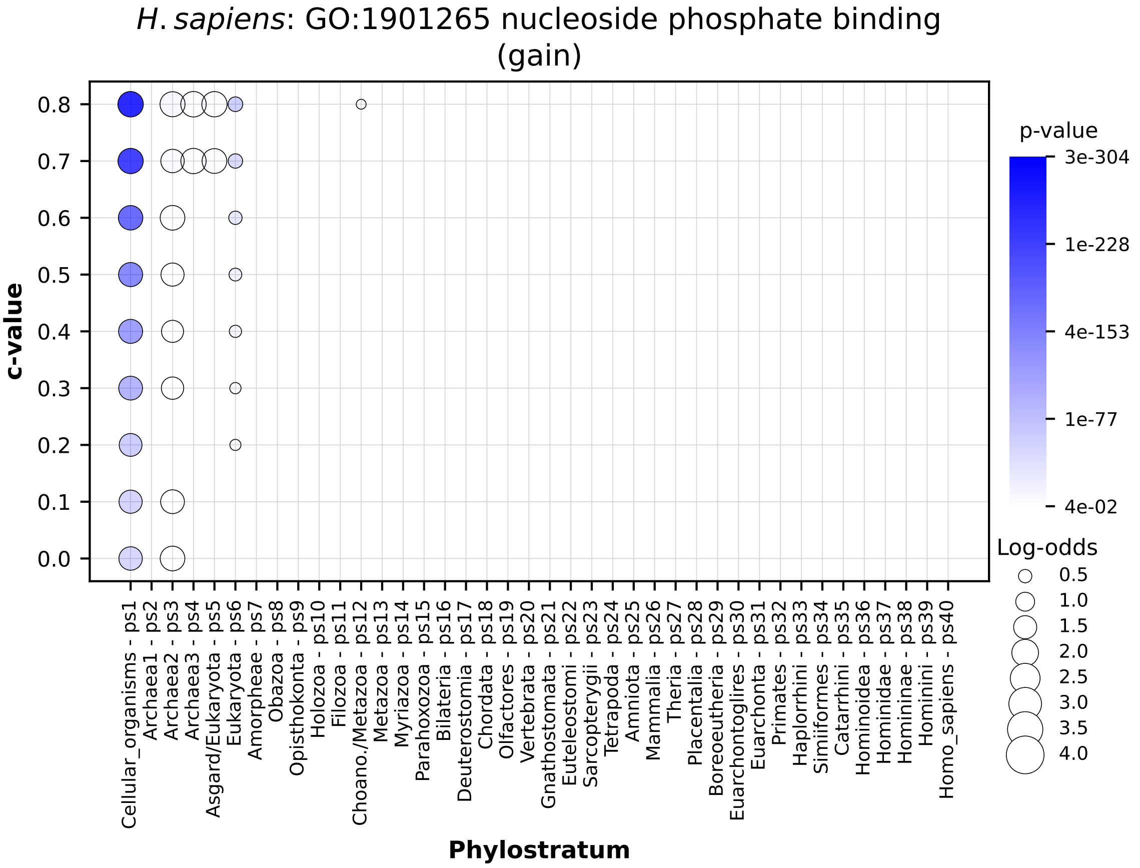
H. sapiens: GO:1901265 nucleoside phosphate binding
(gain)
c-value
0.8
0.7
0.6
0.5
0.4
0.3
0.2
0.1
0.0
Phylostratum
Cellular_organisms - ps1
Archaea1 - ps2
Archaea2 - ps3
Archaea3 - ps4
Asgard/Eukaryota - ps5
Eukaryota - ps6
Amorpheae - ps7
Obazoa - ps8
Opisthokonta - ps9
Holozoa - ps10
Filozoa - ps11
Choano./Metazoa - ps12
Metazoa - ps13
Myriazoa - ps14
Parahoxozoa - ps15
Bilateria - ps16
Deuterostomia - ps17
Chordata - ps18
Olfactores - ps19
Vertebrata - ps20
Gnathostomata - ps21
Euteleostomi - ps22
Sarcopterygii - ps23
Tetrapoda - ps24
Amniota - ps25
Mammalia - ps26
Theria - ps27
Placentalia - ps28
Boreoeutheria - ps29
Euarchontoglires - ps30
Euarchonta - ps31
Primates - ps32
Haplorrhini - ps33
Simiiformes - ps34
Catarrhini - ps35
Hominoidea - ps36
Hominidae - ps37
Homininae - ps38
Hominini - ps39
Homo_sapiens - ps40
p-value
3e-304
1e-228
4e-153
1e-77
4e-02
Log-odds
0.5
1.0
1.5
2.0
2.5
3.0
3.5
4.0

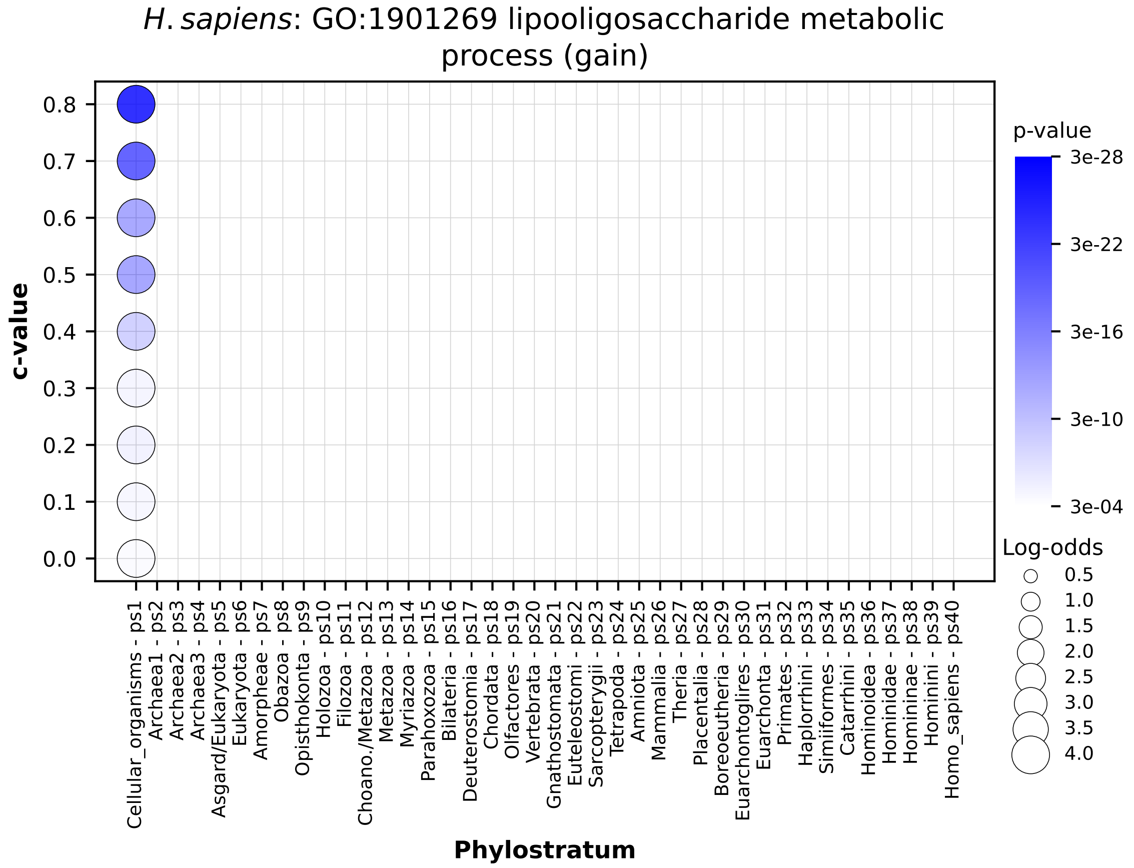

H. sapiens: GO:1901269 lipooligosaccharide metabolic process (gain)
c-value
0.8
0.7
0.6
0.5
0.4
0.3
0.2
0.1
0.0
p-value
3e-28
3e-22
3e-16
3e-10
3e-04
Log-odds
0.5
1.0
1.5
2.0
2.5
3.0
3.5
4.0
Cellular_organisms - ps1
Archaea1 - ps2
Archaea2 - ps3
Archaea3 - ps4
Asgard/Eukaryota - ps5
Eukaryota - ps6
Amorpheae - ps7
Obazoa - ps8
Opisthokonta - ps9
Holozoa - ps10
Filozoa - ps11
Choano./Metazoa - ps12
Metazoa - ps13
Myriazoa - ps14
Parahoxozoa - ps15
Bilateria - ps16
Deuterostomia - ps17
Chordata - ps18
Olfactores - ps19
Vertebrata - ps20
Gnathostomata - ps21
Euteleostomi - ps22
Sarcopterygii - ps23
Tetrapoda - ps24
Amniota - ps25
Mammalia - ps26
Theria - ps27
Placentalia - ps28
Boreoeutheria - ps29
Euarchontoglires - ps30
Euarchonta - ps31
Primates - ps32
Haplorrhini - ps33
Simiiformes - ps34
Catarrhini - ps35
Hominoidea - ps36
Hominidae - ps37
Homininae - ps38
Hominini - ps39
Homo_sapiens - ps40
Phylostratum

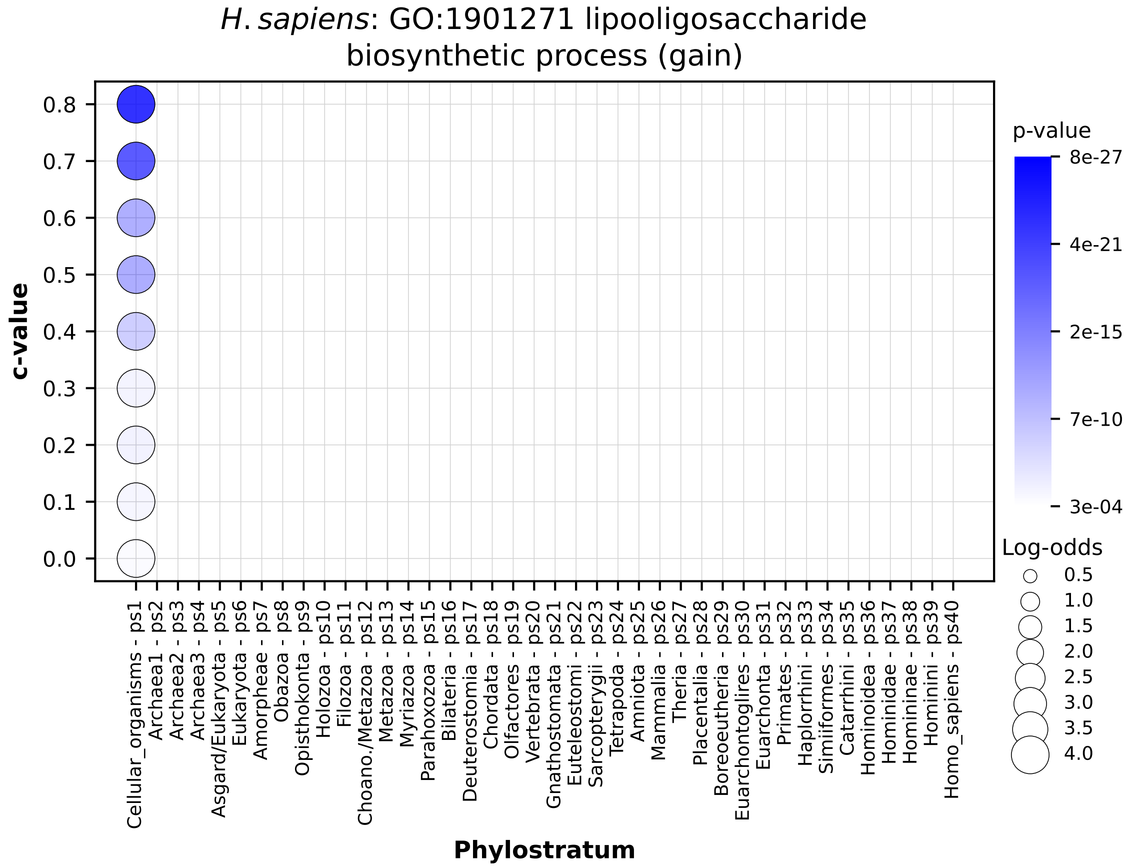

*H. sapiens*: GO:1901271 lipooligosaccharide biosynthetic process (gain)
c-value
0.8
0.7
0.6
0.5
0.4
0.3
0.2
0.1
0.0
p-value
8e-27
4e-21
2e-15
7e-10
3e-04
Log-odds
0.5
1.0
1.5
2.0
2.5
3.0
3.5
4.0
Cellular_organisms - ps1
Archaea1 - ps2
Archaea2 - ps3
Archaea3 - ps4
Asgard/Eukaryota - ps5
Eukaryota - ps6
Amorpheae - ps7
Obazoa - ps8
Opisthokonta - ps9
Holozoa - ps10
Filozoa - ps11
Choano./Metazoa - ps12
Metazoa - ps13
Myriazoa - ps14
Parahoxozoa - ps15
Bilateria - ps16
Deuterostomia - ps17
Chordata - ps18
Olfactores - ps19
Vertebrata - ps20
Gnathostomata - ps21
Euteleostomi - ps22
Sarcopterygii - ps23
Tetrapoda - ps24
Amniota - ps25
Mammalia - ps26
Theria - ps27
Placentalia - ps28
Boreoeutheria - ps29
Euarchontoglires - ps30
Euarchonta - ps31
Primates - ps32
Haplorrhini - ps33
Simiiformes - ps34
Catarrhini - ps35
Hominoidea - ps36
Hominidae - ps37
Homininae - ps38
Hominini - ps39
Homo_sapiens - ps40
Phylostratum

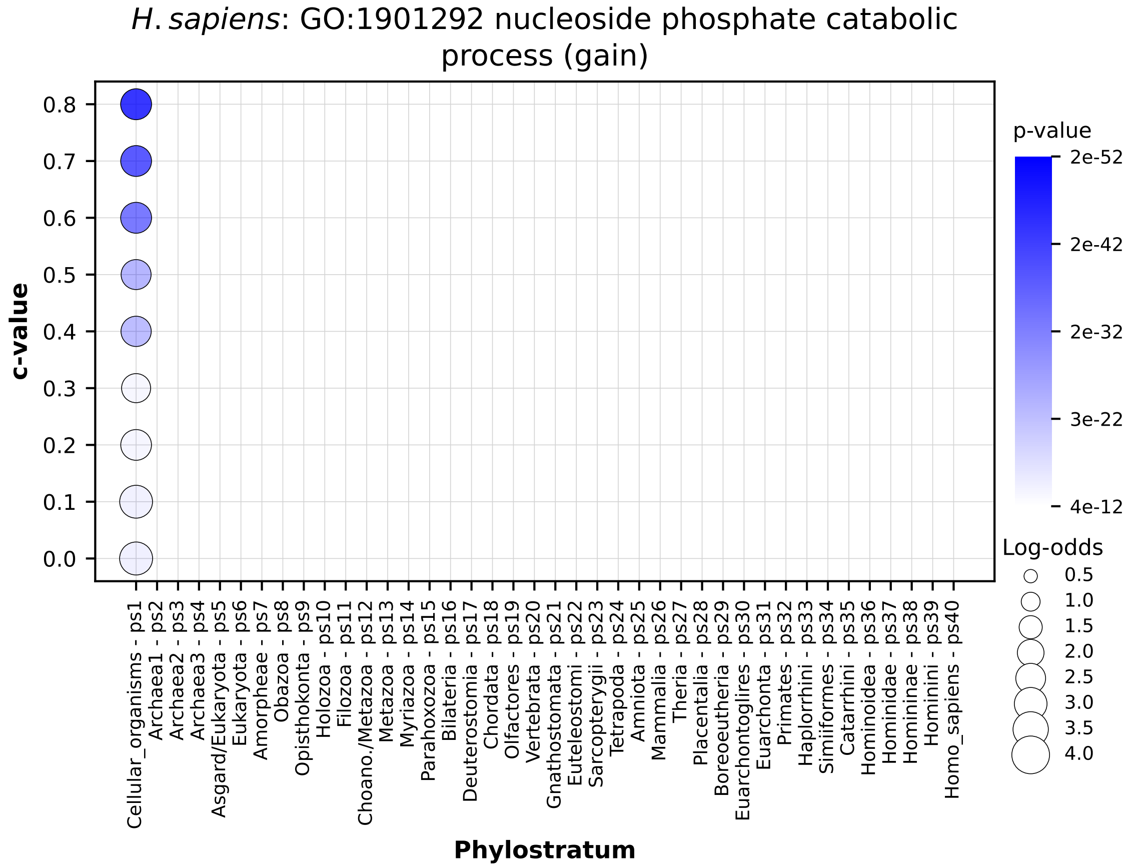

*H. sapiens*: GO:1901292 nucleoside phosphate catabolic process (gain)
c-value
0.8
0.7
0.6
0.5
0.4
0.3
0.2
0.1
0.0
p-value
2e-52
2e-42
2e-32
3e-22
4e-12
Log-odds
0.5
1.0
1.5
2.0
2.5
3.0
3.5
4.0
Cellular_organisms - ps1
Archaea1 - ps2
Archaea2 - ps3
Archaea3 - ps4
Asgard/Eukaryota - ps5
Eukaryota - ps6
Amorpheae - ps7
Obazoa - ps8
Opisthokonta - ps9
Holozoa - ps10
Filozoa - ps11
Choano./Metazoa - ps12
Metazoa - ps13
Myriazoa - ps14
Parahoxozoa - ps15
Bilateria - ps16
Deuterostomia - ps17
Chordata - ps18
Olfactores - ps19
Vertebrata - ps20
Gnathostomata - ps21
Euteleostomi - ps22
Sarcopterygii - ps23
Tetrapoda - ps24
Amniota - ps25
Mammalia - ps26
Theria - ps27
Placentalia - ps28
Boreoeutheria - ps29
Euarchontoglires - ps30
Euarchonta - ps31
Primates - ps32
Haplorrhini - ps33
Simiiformes - ps34
Catarrhini - ps35
Hominoidea - ps36
Hominidae - ps37
Homininae - ps38
Hominini - ps39
Homo_sapiens - ps40
Phylostratum

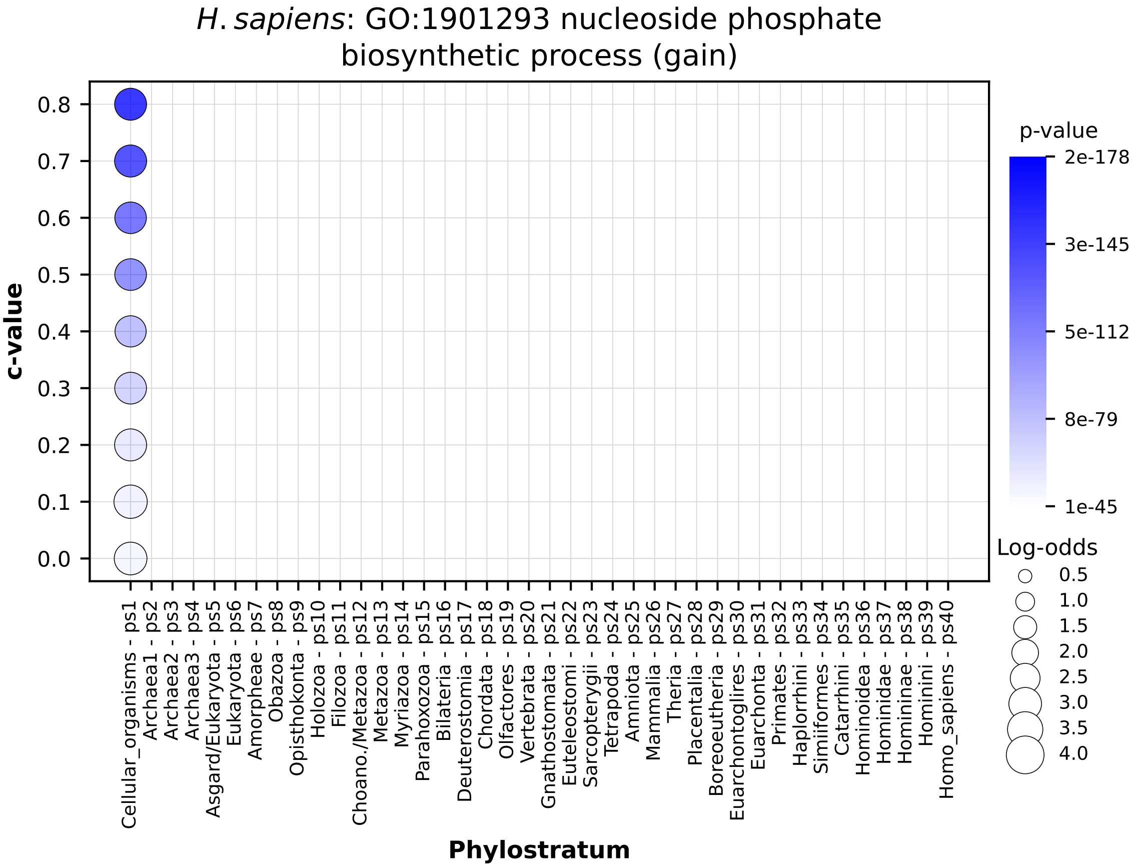

*H. sapiens*: GO:1901293 nucleoside phosphate biosynthetic process (gain)
c-value
0.8
0.7
0.6
0.5
0.4
0.3
0.2
0.1
0.0
p-value
2e-178
3e-145
5e-112
8e-79
1e-45
Log-odds
0.5
1.0
1.5
2.0
2.5
3.0
3.5
4.0
Cellular_organisms - ps1
Archaea1 - ps2
Archaea2 - ps3
Archaea3 - ps4
Asgard/Eukaryota - ps5
Eukaryota - ps6
Amorpheae - ps7
Obazoa - ps8
Opisthokonta - ps9
Holozoa - ps10
Filozoa - ps11
Choano./Metazoa - ps12
Metazoa - ps13
Myriazoa - ps14
Parahoxozoa - ps15
Bilateria - ps16
Deuterostomia - ps17
Chordata - ps18
Olfactores - ps19
Vertebrata - ps20
Gnathostomata - ps21
Euteleostomi - ps22
Sarcopterygii - ps23
Tetrapoda - ps24
Amniota - ps25
Mammalia - ps26
Theria - ps27
Placentalia - ps28
Boreoeutheria - ps29
Euarchontoglires - ps30
Euarchonta - ps31
Primates - ps32
Haplorrhini - ps33
Simiiformes - ps34
Catarrhini - ps35
Hominoidea - ps36
Hominidae - ps37
Homininae - ps38
Hominini - ps39
Homo_sapiens - ps40
Phylostratum

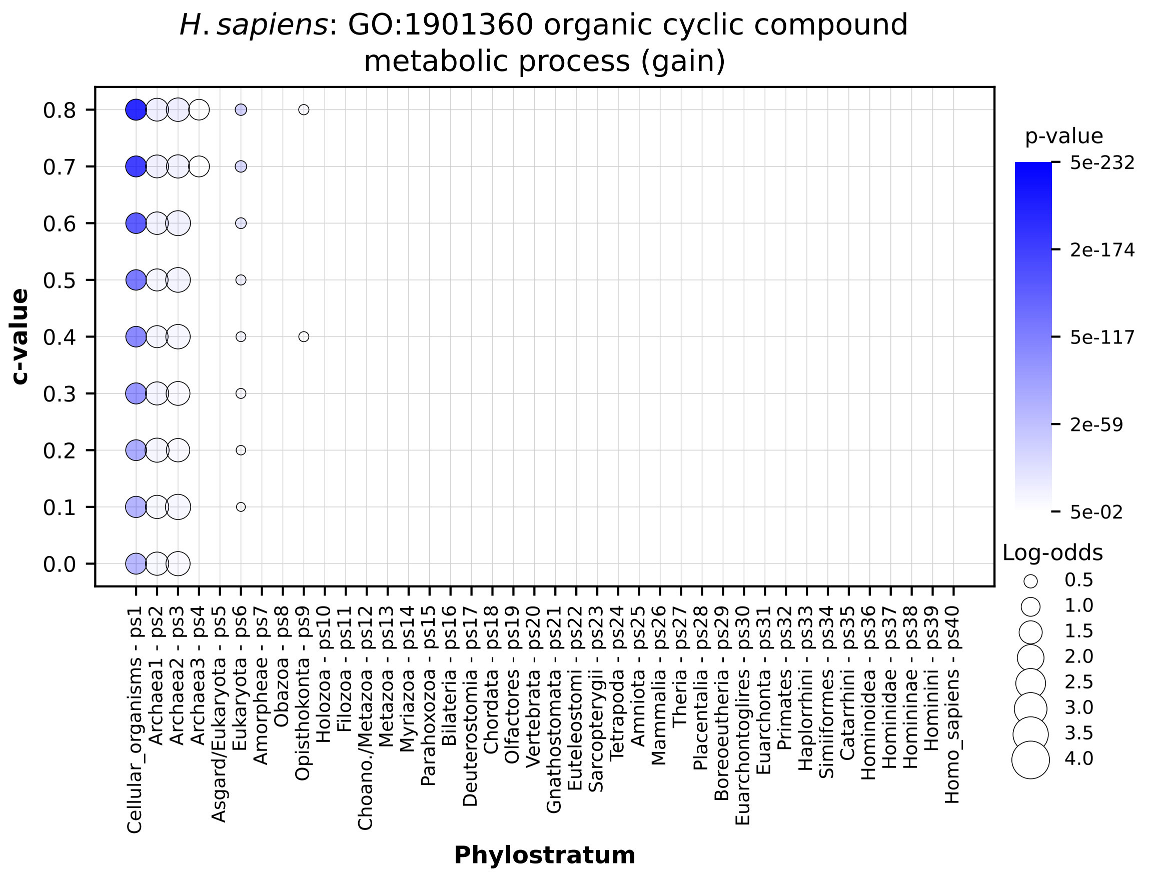

H. sapiens: GO:1901360 organic cyclic compound metabolic process (gain)
c-value
0.8
0.7
0.6
0.5
0.4
0.3
0.2
0.1
0.0
p-value
5e-232
2e-174
5e-117
2e-59
5e-02
Log-odds
0.5
1.0
1.5
2.0
2.5
3.0
3.5
4.0
Cellular_organisms - ps1
Archaea1 - ps2
Archaea2 - ps3
Archaea3 - ps4
Asgard/Eukaryota - ps5
Eukaryota - ps6
Amorpheae - ps7
Obazoa - ps8
Opisthokonta - ps9
Holozoa - ps10
Filozoa - ps11
Choano./Metazoa - ps12
Metazoa - ps13
Myriazoa - ps14
Parahoxozoa - ps15
Bilateria - ps16
Deuterostomia - ps17
Chordata - ps18
Olfactores - ps19
Vertebrata - ps20
Gnathostomata - ps21
Euteleostomi - ps22
Sarcopterygii - ps23
Tetrapoda - ps24
Amniota - ps25
Mammalia - ps26
Theria - ps27
Placentalia - ps28
Boreoeutheria - ps29
Euarchontoglires - ps30
Euarchonta - ps31
Primates - ps32
Haplorrhini - ps33
Simiiformes - ps34
Catarrhini - ps35
Hominoidea - ps36
Hominidae - ps37
Homininae - ps38
Hominini - ps39
Homo_sapiens - ps40
Phylostratum

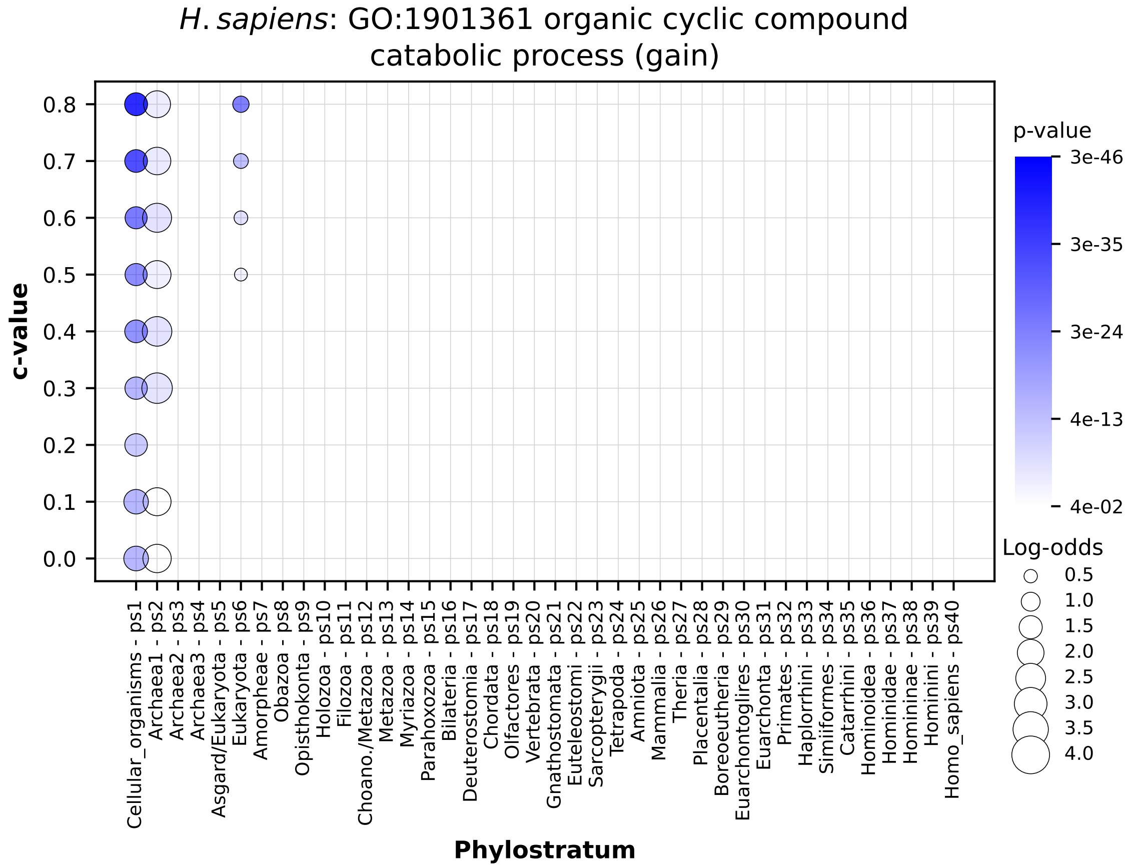

H. sapiens: GO:1901361 organic cyclic compound catabolic process (gain)
c-value
0.8
0.7
0.6
0.5
0.4
0.3
0.2
0.1
0.0
p-value
3e-46
3e-35
3e-24
4e-13
4e-02
Log-odds
0.5
1.0
1.5
2.0
2.5
3.0
3.5
4.0
Cellular_organisms - ps1
Archaea1 - ps2
Archaea2 - ps3
Archaea3 - ps4
Asgard/Eukaryota - ps5
Eukaryota - ps6
Amorpheae - ps7
Obazoa - ps8
Opisthokonta - ps9
Holozoa - ps10
Filozoa - ps11
Choano./Metazoa - ps12
Metazoa - ps13
Myriazoa - ps14
Parahoxozoa - ps15
Bilateria - ps16
Deuterostomia - ps17
Chordata - ps18
Olfactores - ps19
Vertebrata - ps20
Gnathostomata - ps21
Euteleostomi - ps22
Sarcopterygii - ps23
Tetrapoda - ps24
Amniota - ps25
Mammalia - ps26
Theria - ps27
Placentalia - ps28
Boreoeutheria - ps29
Euarchontoglires - ps30
Euarchonta - ps31
Primates - ps32
Haplorrhini - ps33
Simiiformes - ps34
Catarrhini - ps35
Hominoidea - ps36
Hominidae - ps37
Homininae - ps38
Hominini - ps39
Homo_sapiens - ps40
Phylostratum

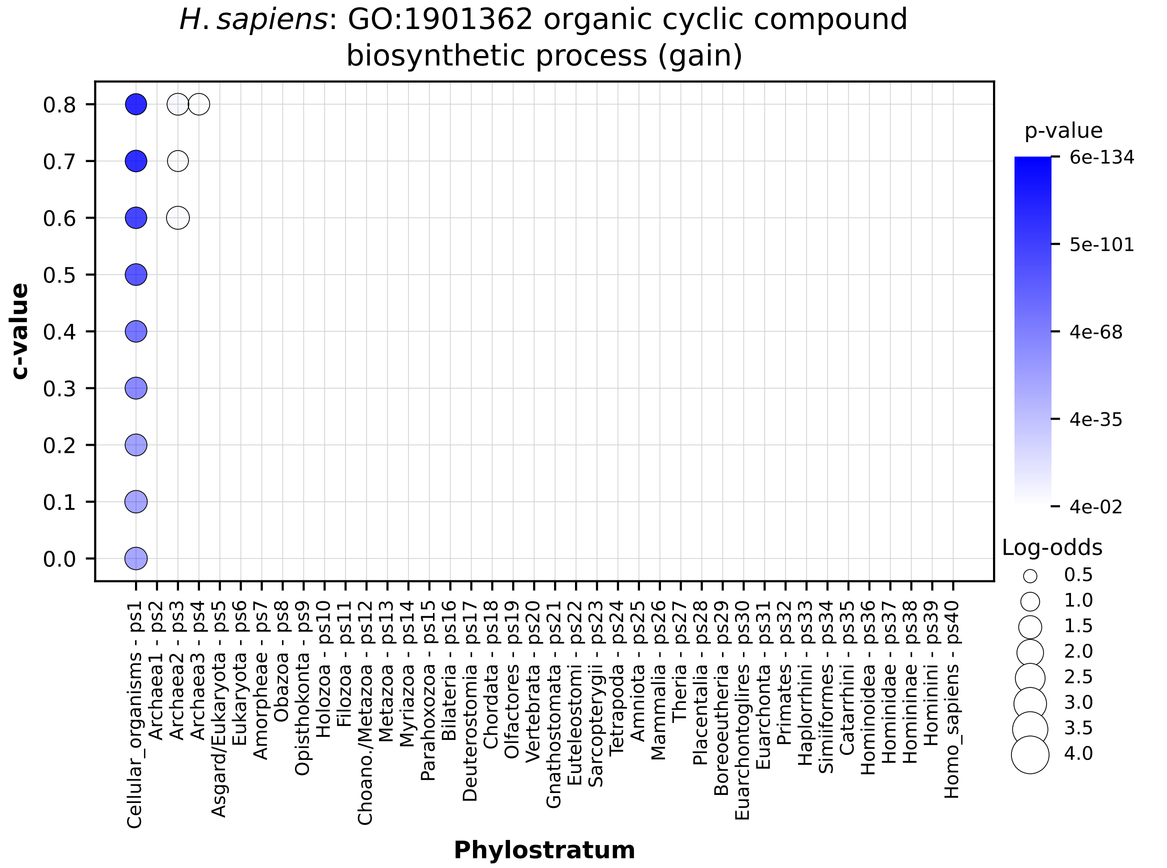
H. sapiens: GO:1901362 organic cyclic compound biosynthetic process (gain)
c-value
0.8
0.7
0.6
0.5
0.4
0.3
0.2
0.1
0.0
p-value
6e-134
5e-101
4e-68
4e-35
4e-02
Log-odds
0.5
1.0
1.5
2.0
2.5
3.0
3.5
4.0
Cellular_organisms - ps1
Archaea1 - ps2
Archaea2 - ps3
Archaea3 - ps4
Asgard/Eukaryota - ps5
Eukaryota - ps6
Amorpheae - ps7
Obazoa - ps8
Opisthokonta - ps9
Holozoa - ps10
Filozoa - ps11
Choano./Metazoa - ps12
Metazoa - ps13
Myriazoa - ps14
Parahoxozoa - ps15
Bilateria - ps16
Deuterostomia - ps17
Chordata - ps18
Olfactores - ps19
Vertebrata - ps20
Gnathostomata - ps21
Euteleostomi - ps22
Sarcopterygii - ps23
Tetrapoda - ps24
Amniota - ps25
Mammalia - ps26
Theria - ps27
Placentalia - ps28
Boreoeutheria - ps29
Euarchontoglires - ps30
Euarchonta - ps31
Primates - ps32
Haplorrhini - ps33
Simiiformes - ps34
Catarrhini - ps35
Hominoidea - ps36
Hominidae - ps37
Homininae - ps38
Hominini - ps39
Homo_sapiens - ps40
Phylostratum

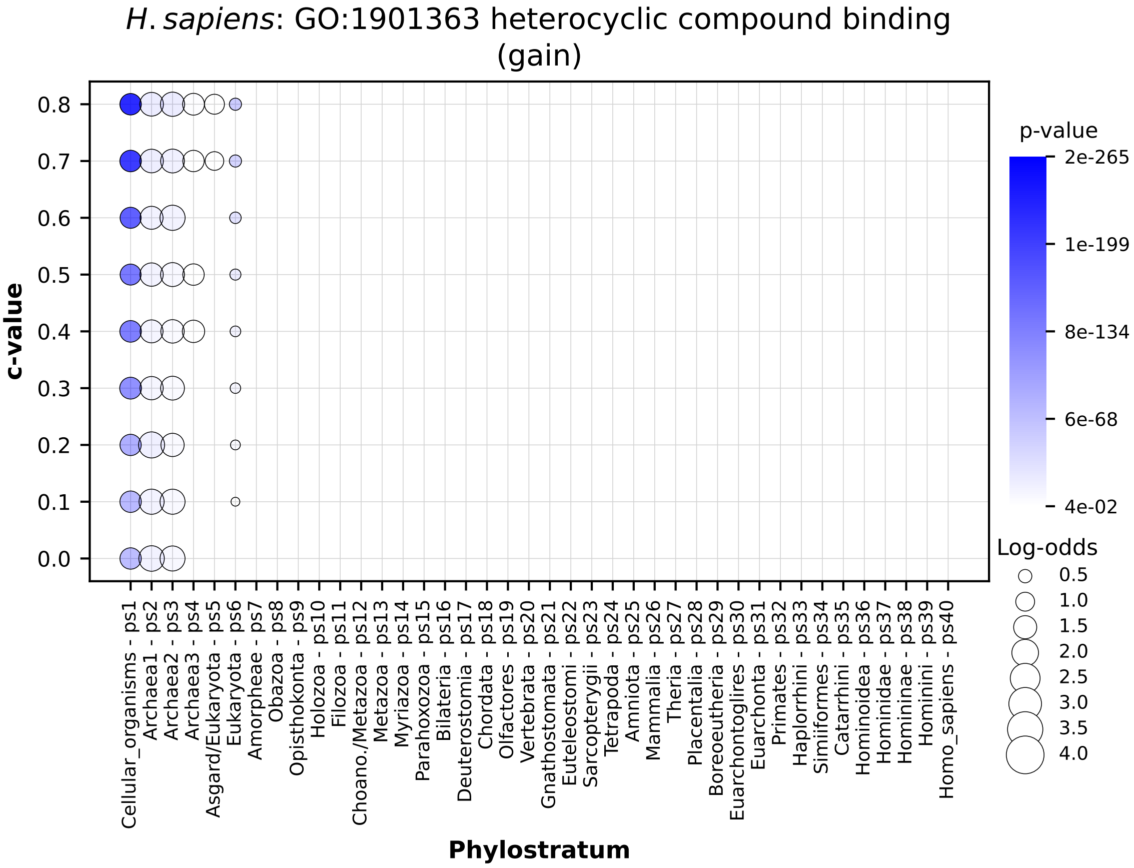
H. sapiens: GO:1901363 heterocyclic compound binding (gain)
c-value
0.8
0.7
0.6
0.5
0.4
0.3
0.2
0.1
0.0
Phylostratum
Cellular_organisms - ps1
Archaea1 - ps2
Archaea2 - ps3
Archaea3 - ps4
Asgard/Eukaryota - ps5
Eukaryota - ps6
Amorpheae - ps7
Obazoa - ps8
Opisthokonta - ps9
Holozoa - ps10
Filozoa - ps11
Choano./Metazoa - ps12
Metazoa - ps13
Myriazoa - ps14
Parahoxozoa - ps15
Bilateria - ps16
Deuterostomia - ps17
Chordata - ps18
Olfactores - ps19
Vertebrata - ps20
Gnathostomata - ps21
Euteleostomi - ps22
Sarcopterygii - ps23
Tetrapoda - ps24
Amniota - ps25
Mammalia - ps26
Theria - ps27
Placentalia - ps28
Boreoeutheria - ps29
Euarchontoglires - ps30
Euarchonta - ps31
Primates - ps32
Haplorrhini - ps33
Simiiformes - ps34
Catarrhini - ps35
Hominoidea - ps36
Hominidae - ps37
Homininae - ps38
Hominini - ps39
Homo_sapiens - ps40
p-value
2e-265
1e-199
8e-134
6e-68
4e-02
Log-odds
0.5
1.0
1.5
2.0
2.5
3.0
3.5
4.0

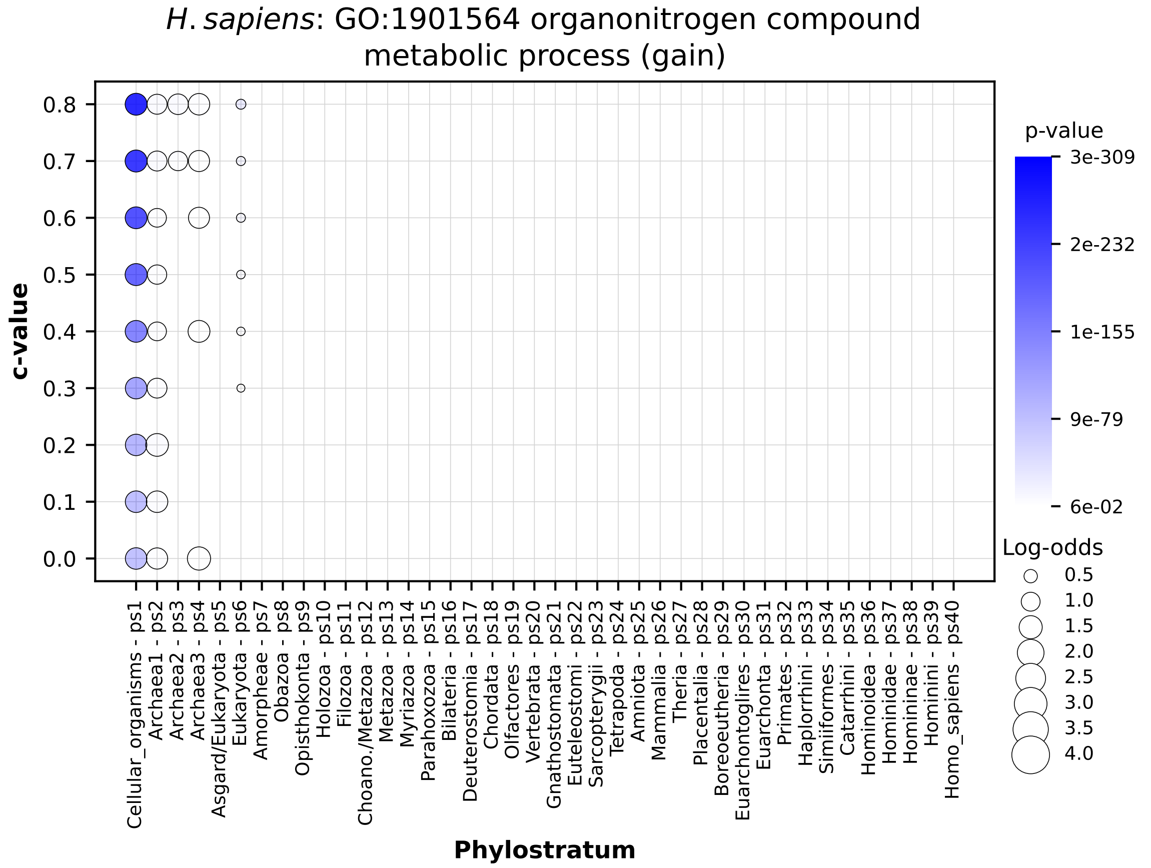

H. sapiens: GO:1901564 organonitrogen compound metabolic process (gain)
c-value
0.8
0.7
0.6
0.5
0.4
0.3
0.2
0.1
0.0
p-value
3e-309
2e-232
1e-155
9e-79
6e-02
Log-odds
0.5
1.0
1.5
2.0
2.5
3.0
3.5
4.0
Cellular_organisms - ps1
Archaea1 - ps2
Archaea2 - ps3
Archaea3 - ps4
Asgard/Eukaryota - ps5
Eukaryota - ps6
Amorpheae - ps7
Obazoa - ps8
Opisthokonta - ps9
Holozoa - ps10
Filozoa - ps11
Choano./Metazoa - ps12
Metazoa - ps13
Myriazoa - ps14
Parahoxozoa - ps15
Bilateria - ps16
Deuterostomia - ps17
Chordata - ps18
Olfactores - ps19
Vertebrata - ps20
Gnathostomata - ps21
Euteleostomi - ps22
Sarcopterygii - ps23
Tetrapoda - ps24
Amniota - ps25
Mammalia - ps26
Theria - ps27
Placentalia - ps28
Boreoeutheria - ps29
Euarchontoglires - ps30
Euarchonta - ps31
Primates - ps32
Haplorrhini - ps33
Simiiformes - ps34
Catarrhini - ps35
Hominoidea - ps36
Hominidae - ps37
Homininae - ps38
Hominini - ps39
Homo_sapiens - ps40
Phylostratum

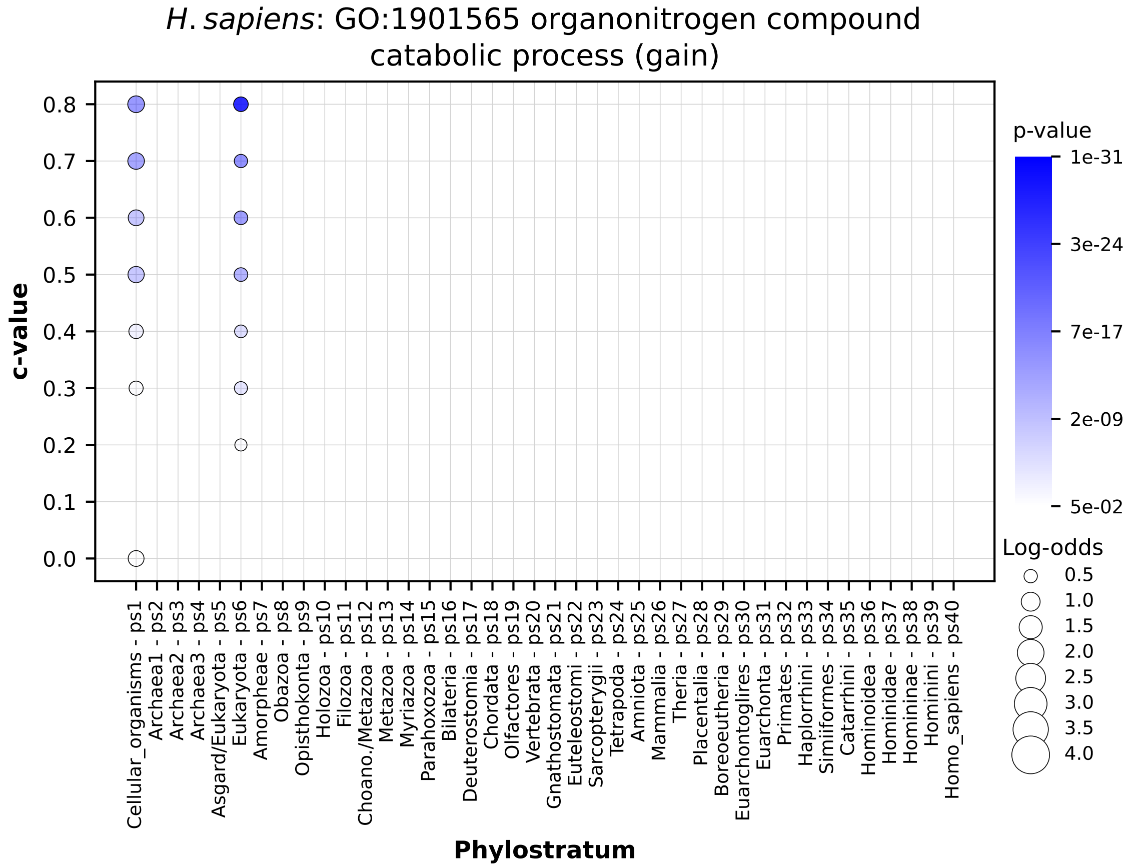
H. sapiens: GO:1901565 organonitrogen compound catabolic process (gain)
c-value
0.8
0.7
0.6
0.5
0.4
0.3
0.2
0.1
0.0
p-value
1e-31
3e-24
7e-17
2e-09
5e-02
Log-odds
0.5
1.0
1.5
2.0
2.5
3.0
3.5
4.0
Cellular_organisms - ps1
Archaea1 - ps2
Archaea2 - ps3
Archaea3 - ps4
Asgard/Eukaryota - ps5
Eukaryota - ps6
Amorpheae - ps7
Obazoa - ps8
Opisthokonta - ps9
Holozoa - ps10
Filozoa - ps11
Choano./Metazoa - ps12
Metazoa - ps13
Myriazoa - ps14
Parahoxozoa - ps15
Bilateria - ps16
Deuterostomia - ps17
Chordata - ps18
Olfactores - ps19
Vertebrata - ps20
Gnathostomata - ps21
Euteleostomi - ps22
Sarcopterygii - ps23
Tetrapoda - ps24
Amniota - ps25
Mammalia - ps26
Theria - ps27
Placentalia - ps28
Boreoeutheria - ps29
Euarchontoglires - ps30
Euarchonta - ps31
Primates - ps32
Haplorrhini - ps33
Simiiformes - ps34
Catarrhini - ps35
Hominoidea - ps36
Hominidae - ps37
Homininae - ps38
Hominini - ps39
Homo_sapiens - ps40
Phylostratum

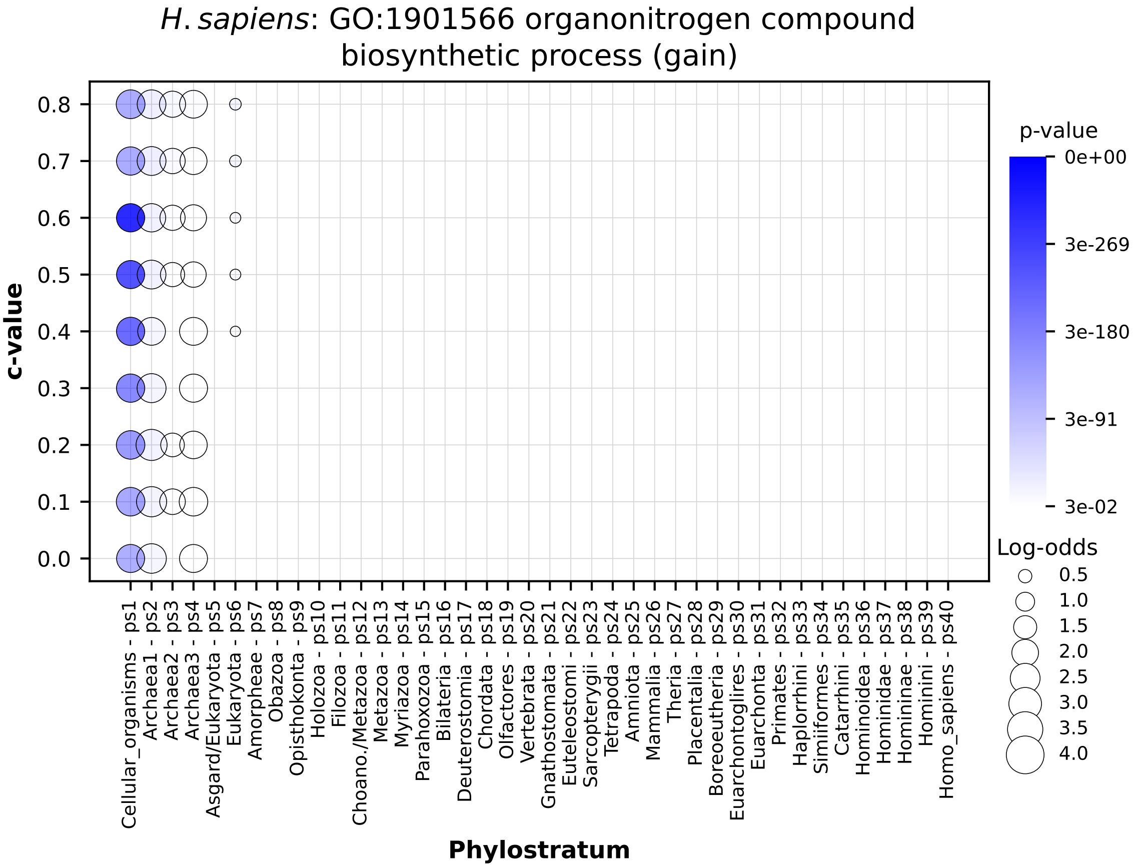

H. sapiens: GO:1901566 organonitrogen compound biosynthetic process (gain)
c-value
0.0
0.1
0.2
0.3
0.4
0.5
0.6
0.7
0.8
Phylostratum
Cellular_organisms - ps1
Archaea1 - ps2
Archaea2 - ps3
Archaea3 - ps4
Asgard/Eukaryota - ps5
Eukaryota - ps6
Amorpheae - ps7
Obazoa - ps8
Opisthokonta - ps9
Holozoa - ps10
Filozoa - ps11
Choano./Metazoa - ps12
Metazoa - ps13
Myriazoa - ps14
Parahoxozoa - ps15
Bilateria - ps16
Deuterostomia - ps17
Chordata - ps18
Olfactores - ps19
Vertebrata - ps20
Gnathostomata - ps21
Euteleostomi - ps22
Sarcopterygii - ps23
Tetrapoda - ps24
Amniota - ps25
Mammalia - ps26
Theria - ps27
Placentalia - ps28
Boreoeutheria - ps29
Euarchontoglires - ps30
Euarchonta - ps31
Primates - ps32
Haplorrhini - ps33
Simiiformes - ps34
Catarrhini - ps35
Hominoidea - ps36
Hominidae - ps37
Homininae - ps38
Hominini - ps39
Homo_sapiens - ps40
p-value
0e+00
3e-269
3e-180
3e-91
3e-02
Log-odds
0.5
1.0
1.5
2.0
2.5
3.0
3.5
4.0

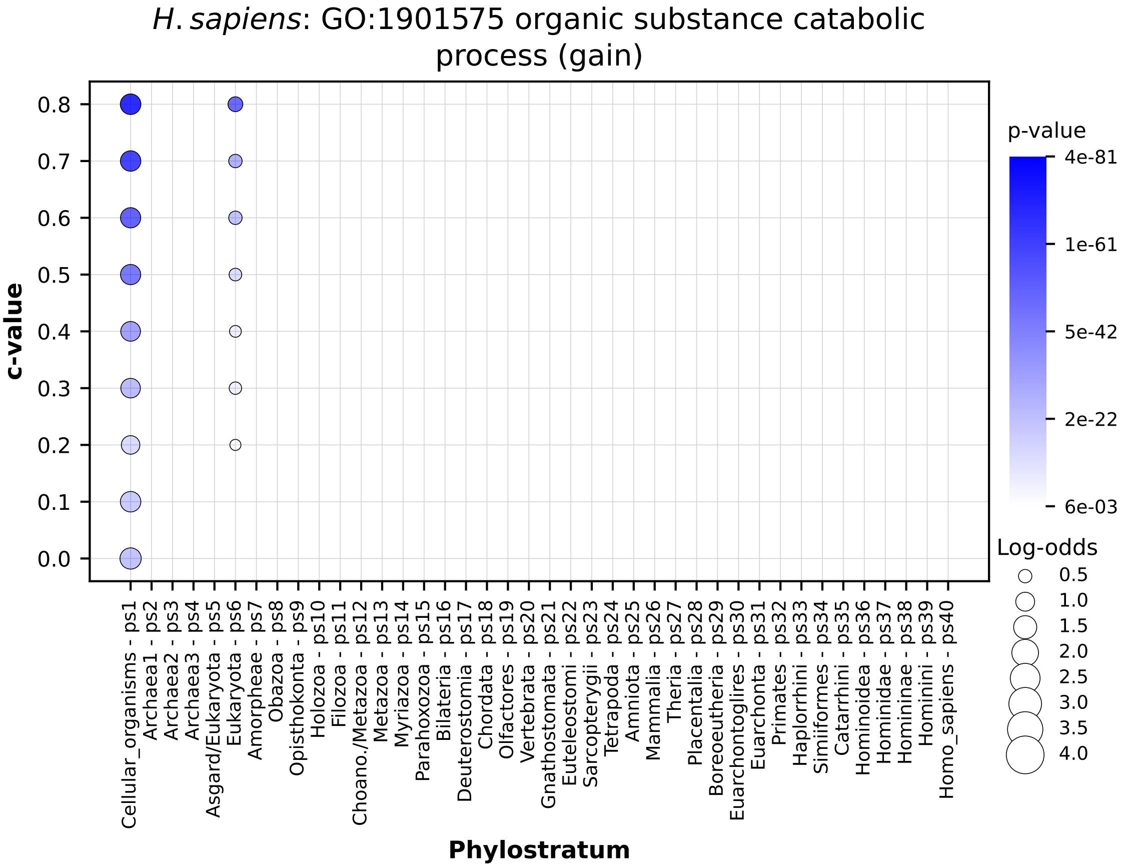
H. sapiens: GO:1901575 organic substance catabolic process (gain)
c-value
0.8
0.7
0.6
0.5
0.4
0.3
0.2
0.1
0.0
p-value
4e-81
1e-61
5e-42
2e-22
6e-03
Log-odds
0.5
1.0
1.5
2.0
2.5
3.0
3.5
4.0
Cellular_organisms - ps1
Archaea1 - ps2
Archaea2 - ps3
Archaea3 - ps4
Asgard/Eukaryota - ps5
Eukaryota - ps6
Amorpheae - ps7
Obazoa - ps8
Opisthokonta - ps9
Holozoa - ps10
Filozoa - ps11
Choano./Metazoa - ps12
Metazoa - ps13
Myriazoa - ps14
Parahoxozoa - ps15
Bilateria - ps16
Deuterostomia - ps17
Chordata - ps18
Olfactores - ps19
Vertebrata - ps20
Gnathostomata - ps21
Euteleostomi - ps22
Sarcopterygii - ps23
Tetrapoda - ps24
Amniota - ps25
Mammalia - ps26
Theria - ps27
Placentalia - ps28
Boreoeutheria - ps29
Euarchontoglires - ps30
Euarchonta - ps31
Primates - ps32
Haplorrhini - ps33
Simiiformes - ps34
Catarrhini - ps35
Hominoidea - ps36
Hominidae - ps37
Homininae - ps38
Hominini - ps39
Homo_sapiens - ps40
Phylostratum

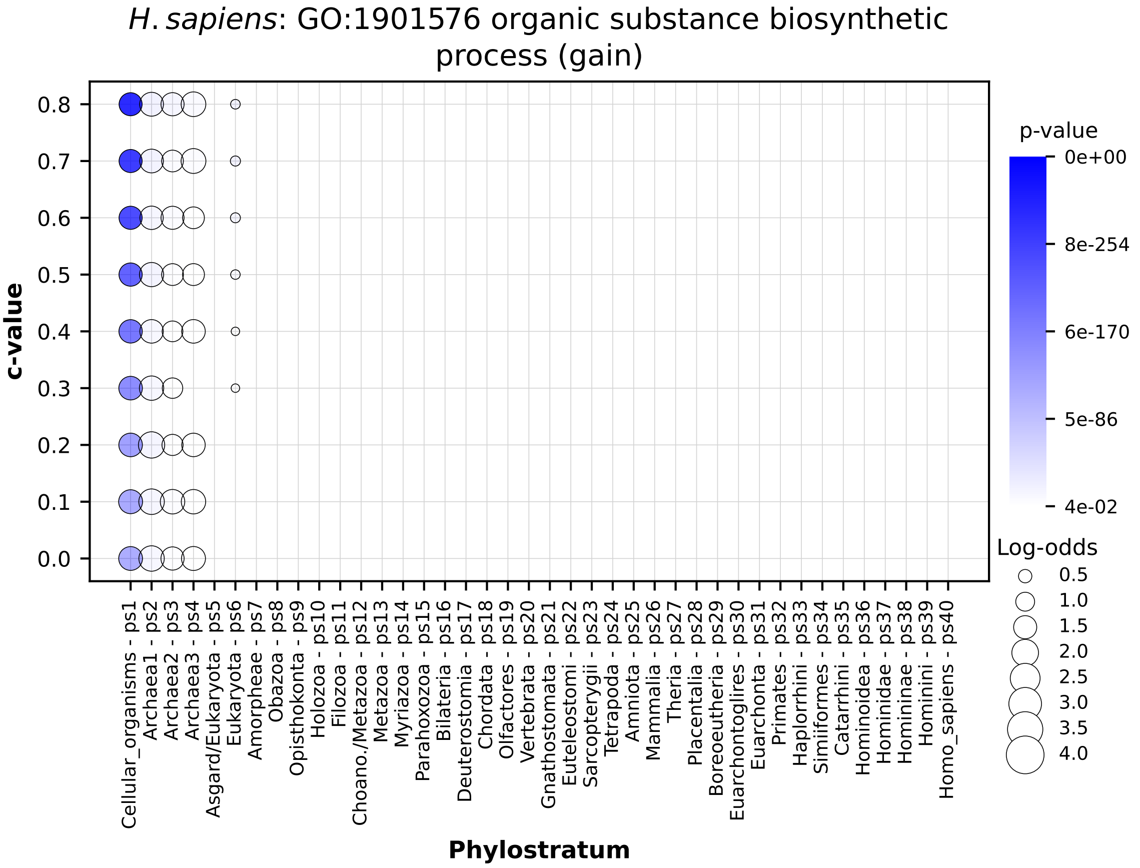
H. sapiens: GO:1901576 organic substance biosynthetic process (gain)
c-value
0.8
0.7
0.6
0.5
0.4
0.3
0.2
0.1
0.0
p-value
0e+00
8e-254
6e-170
5e-86
4e-02
Log-odds
0.5
1.0
1.5
2.0
2.5
3.0
3.5
4.0
Cellular_organisms - ps1
Archaea1 - ps2
Archaea2 - ps3
Archaea3 - ps4
Asgard/Eukaryota - ps5
Eukaryota - ps6
Amorpheae - ps7
Obazoa - ps8
Opisthokonta - ps9
Holozoa - ps10
Filozoa - ps11
Choano./Metazoa - ps12
Metazoa - ps13
Myriazoa - ps14
Parahoxozoa - ps15
Bilateria - ps16
Deuterostomia - ps17
Chordata - ps18
Olfactores - ps19
Vertebrata - ps20
Gnathostomata - ps21
Euteleostomi - ps22
Sarcopterygii - ps23
Tetrapoda - ps24
Amniota - ps25
Mammalia - ps26
Theria - ps27
Placentalia - ps28
Boreoeutheria - ps29
Euarchontoglires - ps30
Euarchonta - ps31
Primates - ps32
Haplorrhini - ps33
Simiiformes - ps34
Catarrhini - ps35
Hominoidea - ps36
Hominidae - ps37
Homininae - ps38
Hominini - ps39
Homo_sapiens - ps40
Phylostratum

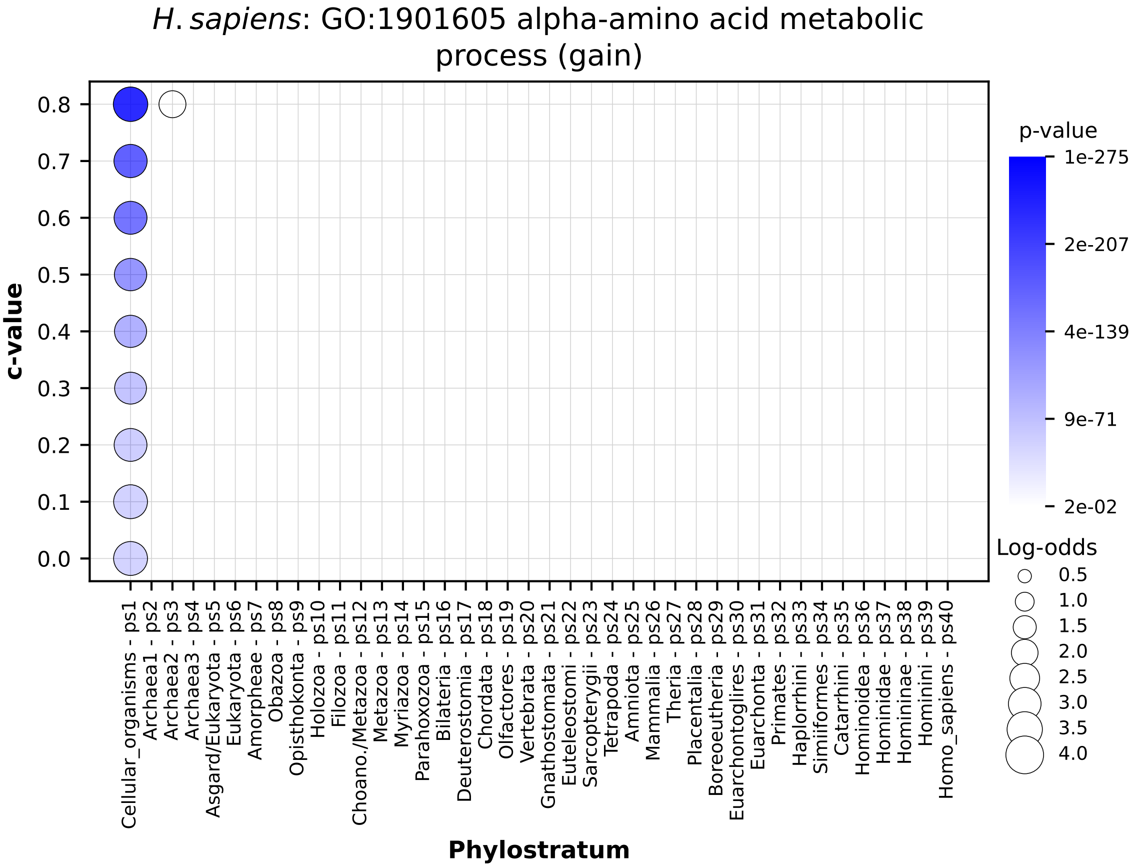
H. sapiens: GO:1901605 alpha-amino acid metabolic process (gain)
c-value
0.0
0.1
0.2
0.3
0.4
0.5
0.6
0.7
0.8
Phylostratum
Cellular_organisms - ps1
Archaea1 - ps2
Archaea2 - ps3
Archaea3 - ps4
Asgard/Eukaryota - ps5
Eukaryota - ps6
Amorpheae - ps7
Obazoa - ps8
Opisthokonta - ps9
Holozoa - ps10
Filozoa - ps11
Choano./Metazoa - ps12
Metazoa - ps13
Myriazoa - ps14
Parahoxozoa - ps15
Bilateria - ps16
Deuterostomia - ps17
Chordata - ps18
Olfactores - ps19
Vertebrata - ps20
Gnathostomata - ps21
Euteleostomi - ps22
Sarcopterygii - ps23
Tetrapoda - ps24
Amniota - ps25
Mammalia - ps26
Theria - ps27
Placentalia - ps28
Boreoeutheria - ps29
Euarchontoglires - ps30
Euarchonta - ps31
Primates - ps32
Haplorrhini - ps33
Simiiformes - ps34
Catarrhini - ps35
Hominoidea - ps36
Hominidae - ps37
Homininae - ps38
Hominini - ps39
Homo_sapiens - ps40
p-value
1e-275
2e-207
4e-139
9e-71
2e-02
Log-odds
0.5
1.0
1.5
2.0
2.5
3.0
3.5
4.0

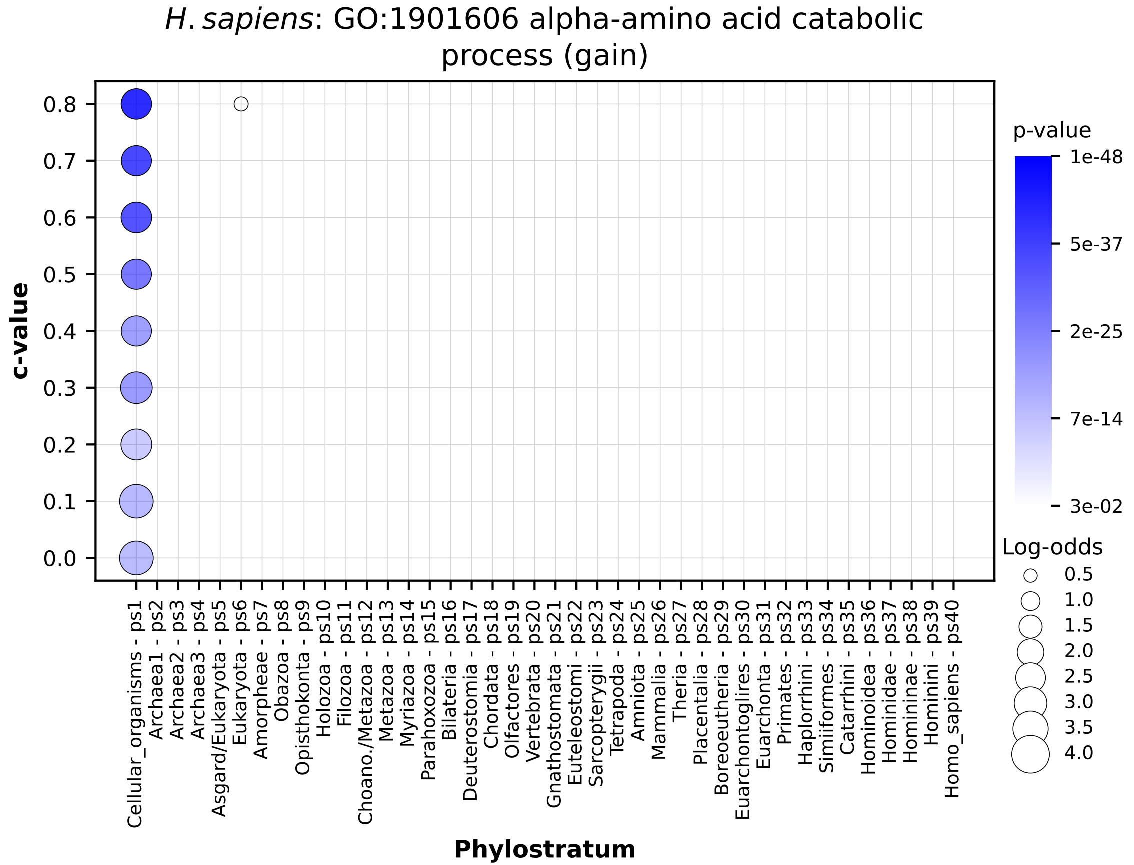

H. sapiens: GO:1901606 alpha-amino acid catabolic process (gain)
c-value
0.8
0.7
0.6
0.5
0.4
0.3
0.2
0.1
0.0
Phylostratum
Cellular_organisms - ps1
Archaea1 - ps2
Archaea2 - ps3
Archaea3 - ps4
Asgard/Eukaryota - ps5
Eukaryota - ps6
Amorpheae - ps7
Obazoa - ps8
Opisthokonta - ps9
Holozoa - ps10
Filozoa - ps11
Choano./Metazoa - ps12
Metazoa - ps13
Myriazoa - ps14
Parahoxozoa - ps15
Bilateria - ps16
Deuterostomia - ps17
Chordata - ps18
Olfactores - ps19
Vertebrata - ps20
Gnathostomata - ps21
Euteleostomi - ps22
Sarcopterygii - ps23
Tetrapoda - ps24
Amniota - ps25
Mammalia - ps26
Theria - ps27
Placentalia - ps28
Boreoeutheria - ps29
Euarchontoglires - ps30
Euarchonta - ps31
Primates - ps32
Haplorrhini - ps33
Simiiformes - ps34
Catarrhini - ps35
Hominoidea - ps36
Hominidae - ps37
Homininae - ps38
Hominini - ps39
Homo_sapiens - ps40
p-value
1e-48
5e-37
2e-25
7e-14
3e-02
Log-odds
0.5
1.0
1.5
2.0
2.5
3.0
3.5
4.0

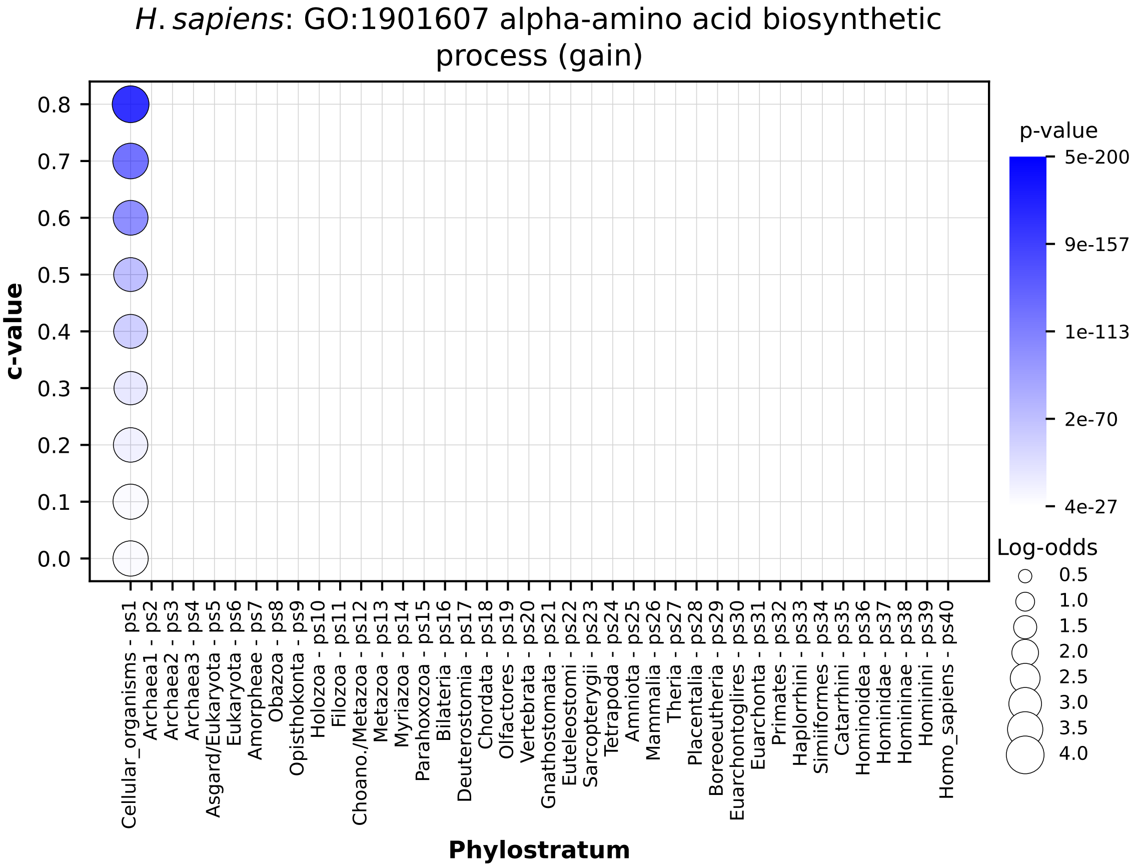
H. sapiens: GO:1901607 alpha-amino acid biosynthetic process (gain)
c-value
0.8
0.7
0.6
0.5
0.4
0.3
0.2
0.1
0.0
Phylostratum
Cellular_organisms - ps1
Archaea1 - ps2
Archaea2 - ps3
Archaea3 - ps4
Asgard/Eukaryota - ps5
Eukaryota - ps6
Amorpheae - ps7
Obazoa - ps8
Opisthokonta - ps9
Holozoa - ps10
Filozoa - ps11
Choano./Metazoa - ps12
Metazoa - ps13
Myriazoa - ps14
Parahoxozoa - ps15
Bilateria - ps16
Deuterostomia - ps17
Chordata - ps18
Olfactores - ps19
Vertebrata - ps20
Gnathostomata - ps21
Euteleostomi - ps22
Sarcopterygii - ps23
Tetrapoda - ps24
Amniota - ps25
Mammalia - ps26
Theria - ps27
Placentalia - ps28
Boreoeutheria - ps29
Euarchontoglires - ps30
Euarchonta - ps31
Primates - ps32
Haplorrhini - ps33
Simiiformes - ps34
Catarrhini - ps35
Hominoidea - ps36
Hominidae - ps37
Homininae - ps38
Hominini - ps39
Homo_sapiens - ps40
p-value
5e-200
9e-157
1e-113
2e-70
4e-27
Log-odds
0.5
1.0
1.5
2.0
2.5
3.0
3.5
4.0

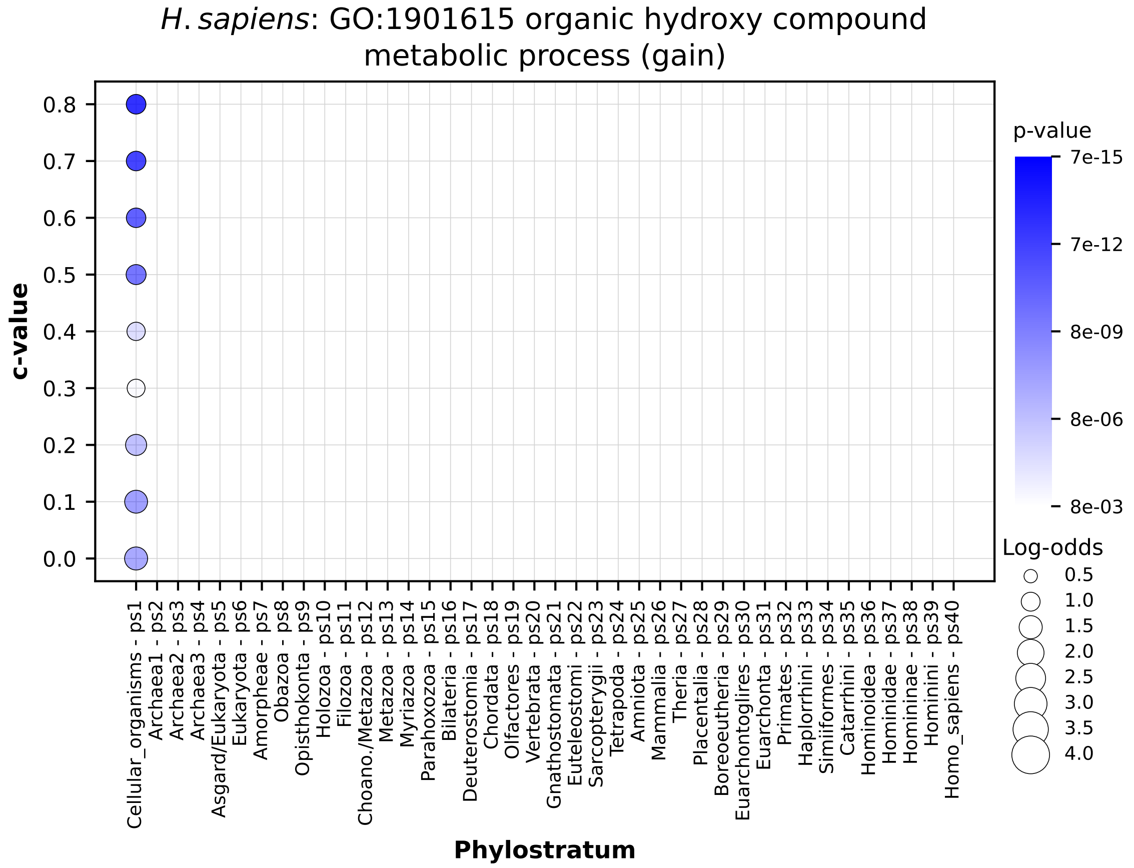
H. sapiens: GO:1901615 organic hydroxy compound metabolic process (gain)
c-value
0.0
0.1
0.2
0.3
0.4
0.5
0.6
0.7
0.8
p-value
7e-15
7e-12
8e-09
8e-06
8e-03
Log-odds
0.5
1.0
1.5
2.0
2.5
3.0
3.5
4.0
Cellular_organisms - ps1
Archaea1 - ps2
Archaea2 - ps3
Archaea3 - ps4
Asgard/Eukaryota - ps5
Eukaryota - ps6
Amorpheae - ps7
Obazoa - ps8
Opisthokonta - ps9
Holozoa - ps10
Filozoa - ps11
Choano./Metazoa - ps12
Metazoa - ps13
Myriazoa - ps14
Parahoxozoa - ps15
Bilateria - ps16
Deuterostomia - ps17
Chordata - ps18
Olfactores - ps19
Vertebrata - ps20
Gnathostomata - ps21
Euteleostomi - ps22
Sarcopterygii - ps23
Tetrapoda - ps24
Amniota - ps25
Mammalia - ps26
Theria - ps27
Placentalia - ps28
Boreoeutheria - ps29
Euarchontoglires - ps30
Euarchonta - ps31
Primates - ps32
Haplorrhini - ps33
Simiiformes - ps34
Catarrhini - ps35
Hominoidea - ps36
Hominidae - ps37
Homininae - ps38
Hominini - ps39
Homo_sapiens - ps40
Phylostratum

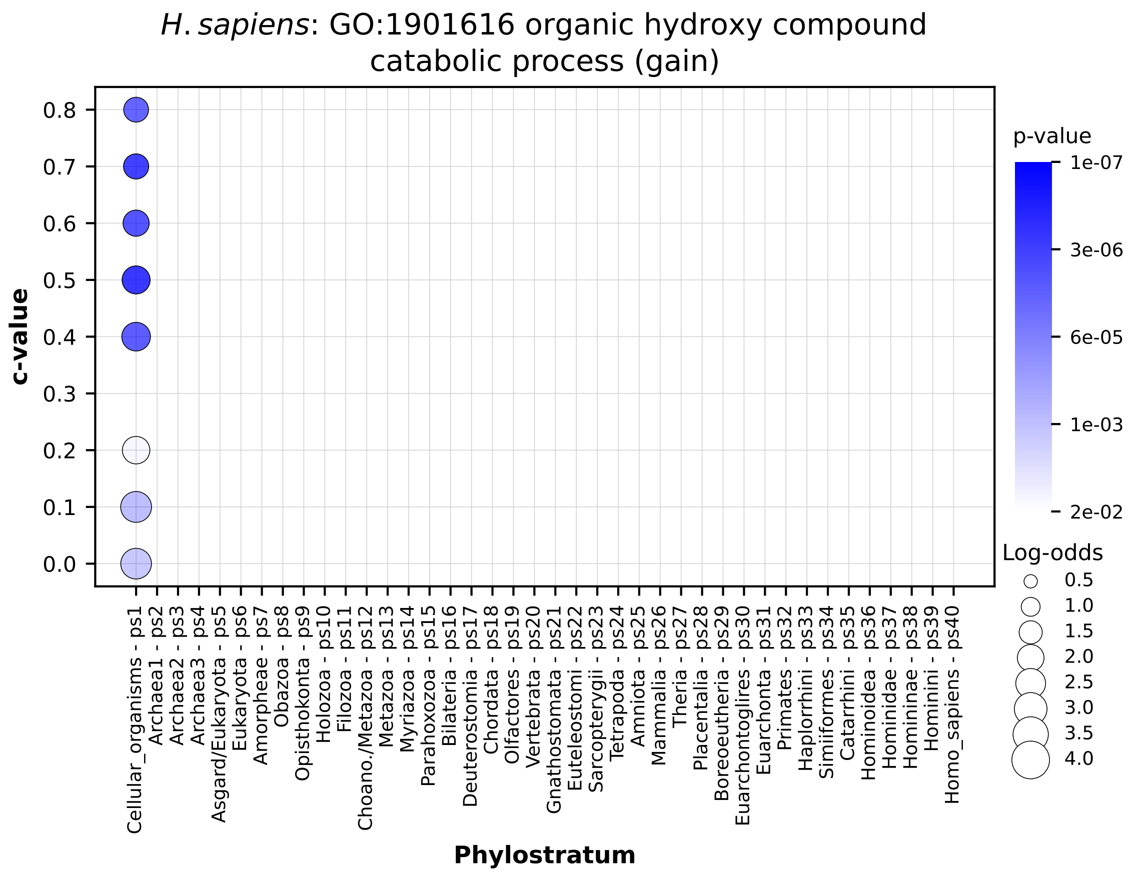

H. sapiens: GO:1901616 organic hydroxy compound catabolic process (gain)
c-value
0.8
0.7
0.6
0.5
0.4
0.3
0.2
0.1
0.0
p-value
1e-07
3e-06
6e-05
1e-03
2e-02
Log-odds
0.5
1.0
1.5
2.0
2.5
3.0
3.5
4.0
Cellular_organisms - ps1
Archaea1 - ps2
Archaea2 - ps3
Archaea3 - ps4
Asgard/Eukaryota - ps5
Eukaryota - ps6
Amorpheae - ps7
Obazoa - ps8
Opisthokonta - ps9
Holozoa - ps10
Filozoa - ps11
Choano./Metazoa - ps12
Metazoa - ps13
Myriazoa - ps14
Parahoxozoa - ps15
Bilateria - ps16
Deuterostomia - ps17
Chordata - ps18
Olfactores - ps19
Vertebrata - ps20
Gnathostomata - ps21
Euteleostomi - ps22
Sarcopterygii - ps23
Tetrapoda - ps24
Amniota - ps25
Mammalia - ps26
Theria - ps27
Placentalia - ps28
Boreoeutheria - ps29
Euarchontoglires - ps30
Euarchonta - ps31
Primates - ps32
Haplorrhini - ps33
Simiiformes - ps34
Catarrhini - ps35
Hominoidea - ps36
Hominidae - ps37
Homininae - ps38
Hominini - ps39
Homo_sapiens - ps40
Phylostratum

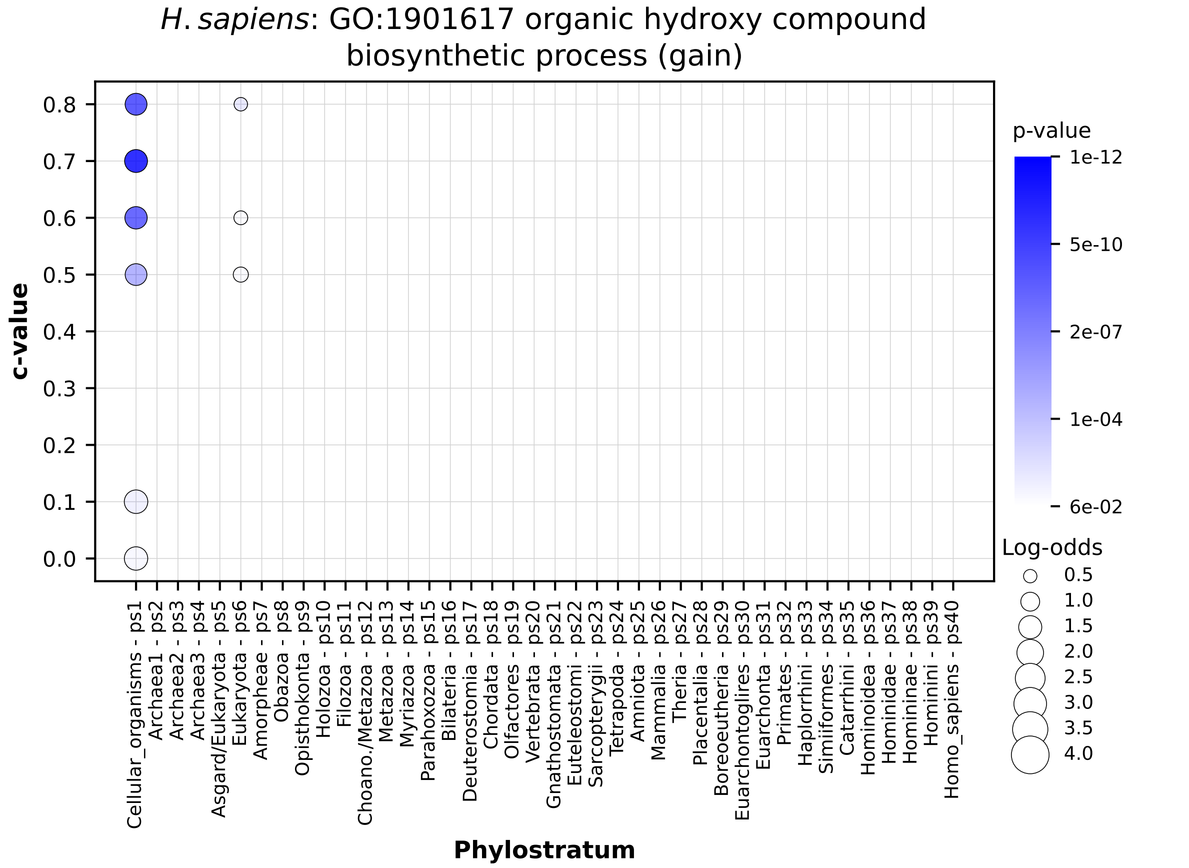

*H. sapiens*: GO:1901617 organic hydroxy compound biosynthetic process (gain)
c-value
0.8
0.7
0.6
0.5
0.4
0.3
0.2
0.1
0.0
p-value
1e-12
5e-10
2e-07
1e-04
6e-02
Log-odds
0.5
1.0
1.5
2.0
2.5
3.0
3.5
4.0
Cellular_organisms - ps1
Archaea1 - ps2
Archaea2 - ps3
Archaea3 - ps4
Asgard/Eukaryota - ps5
Eukaryota - ps6
Amorpheae - ps7
Obazoa - ps8
Opisthokonta - ps9
Holozoa - ps10
Filozoa - ps11
Choano./Metazoa - ps12
Metazoa - ps13
Myriazoa - ps14
Parahoxozoa - ps15
Bilateria - ps16
Deuterostomia - ps17
Chordata - ps18
Olfactores - ps19
Vertebrata - ps20
Gnathostomata - ps21
Euteleostomi - ps22
Sarcopterygii - ps23
Tetrapoda - ps24
Amniota - ps25
Mammalia - ps26
Theria - ps27
Placentalia - ps28
Boreoeutheria - ps29
Euarchontoglires - ps30
Euarchonta - ps31
Primates - ps32
Haplorrhini - ps33
Simiiformes - ps34
Catarrhini - ps35
Hominoidea - ps36
Hominidae - ps37
Homininae - ps38
Hominini - ps39
Homo_sapiens - ps40
Phylostratum

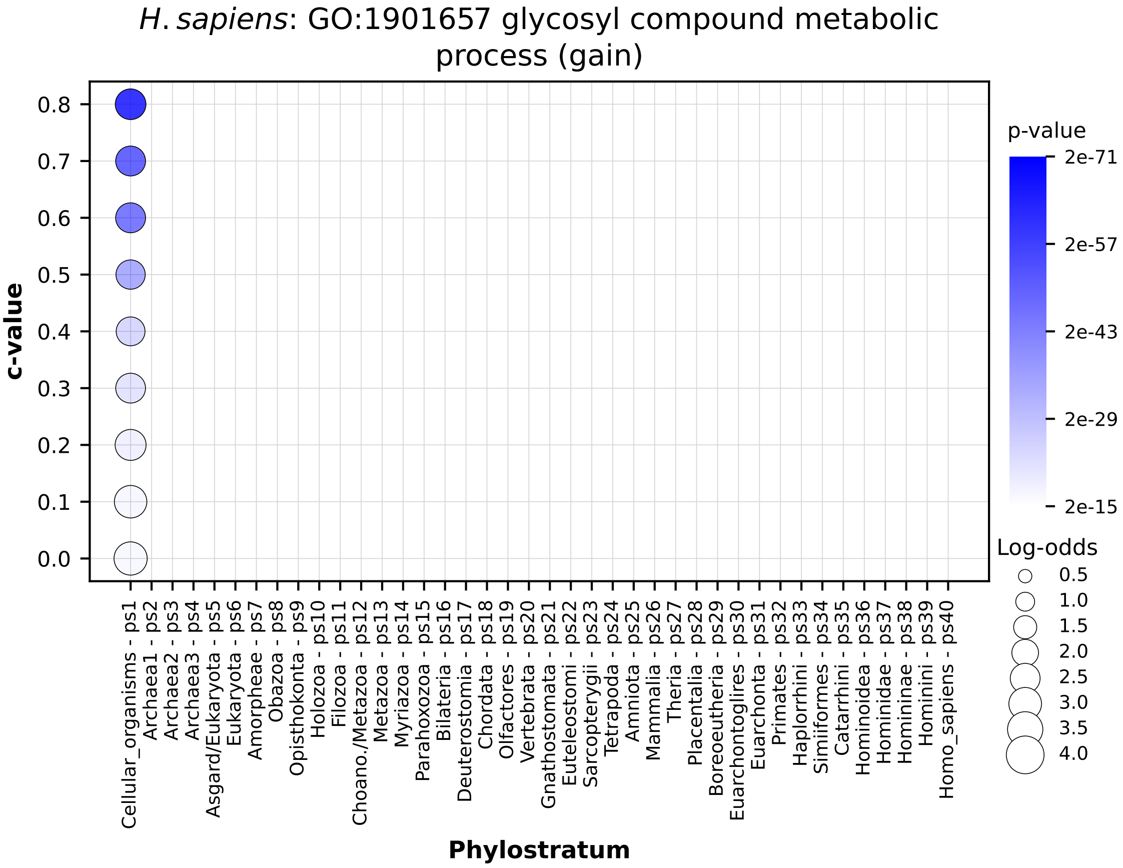

*H. sapiens*: GO:1901657 glycosyl compound metabolic process (gain)
c-value
0.8
0.7
0.6
0.5
0.4
0.3
0.2
0.1
0.0
p-value
2e-71
2e-57
2e-43
2e-29
2e-15
Log-odds
0.5
1.0
1.5
2.0
2.5
3.0
3.5
4.0
Cellular_organisms - ps1
Archaea1 - ps2
Archaea2 - ps3
Archaea3 - ps4
Asgard/Eukaryota - ps5
Eukaryota - ps6
Amorpheae - ps7
Obazoa - ps8
Opisthokonta - ps9
Holozoa - ps10
Filozoa - ps11
Choano./Metazoa - ps12
Metazoa - ps13
Myriazoa - ps14
Parahoxozoa - ps15
Bilateria - ps16
Deuterostomia - ps17
Chordata - ps18
Olfactores - ps19
Vertebrata - ps20
Gnathostomata - ps21
Euteleostomi - ps22
Sarcopterygii - ps23
Tetrapoda - ps24
Amniota - ps25
Mammalia - ps26
Theria - ps27
Placentalia - ps28
Boreoeutheria - ps29
Euarchontoglires - ps30
Euarchonta - ps31
Primates - ps32
Haplorrhini - ps33
Simiiformes - ps34
Catarrhini - ps35
Hominoidea - ps36
Hominidae - ps37
Homininae - ps38
Hominini - ps39
Homo_sapiens - ps40
Phylostratum

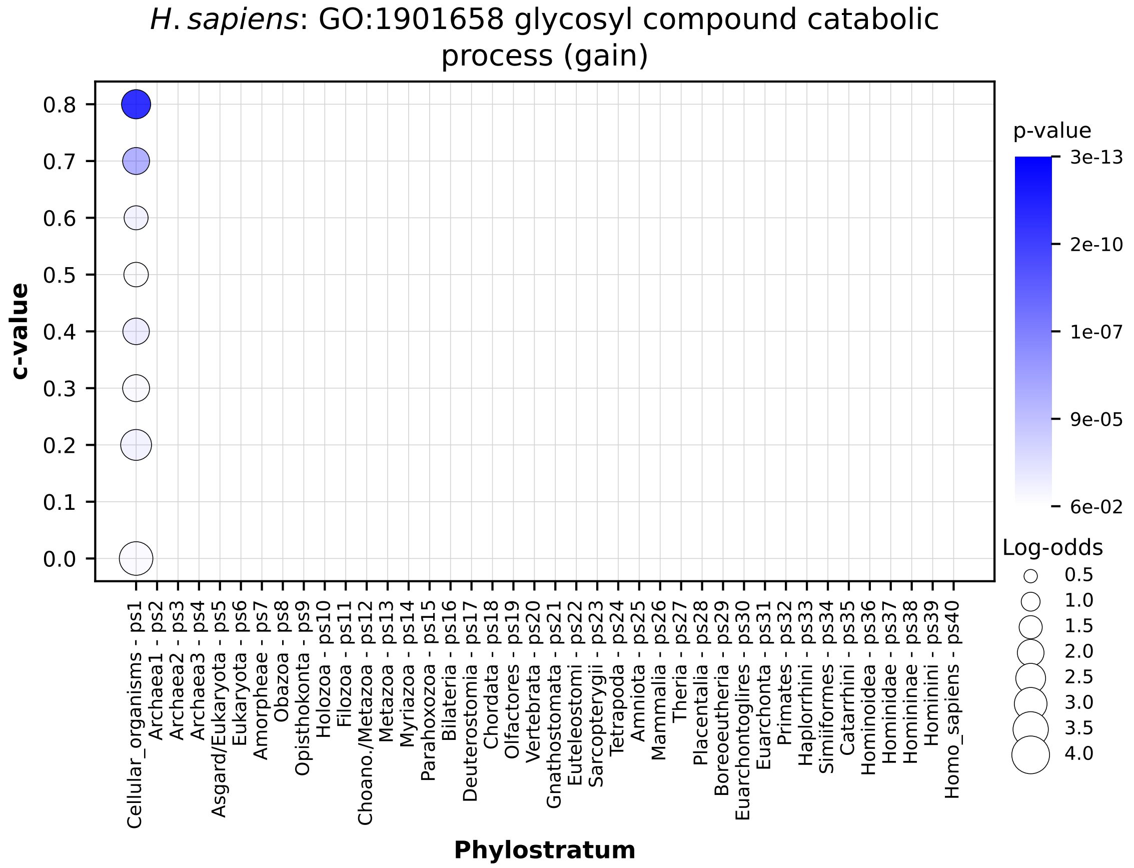
H. sapiens: GO:1901658 glycosyl compound catabolic process (gain)
c-value
0.8
0.7
0.6
0.5
0.4
0.3
0.2
0.1
0.0
Phylostratum
Cellular_organisms - ps1
Archaea1 - ps2
Archaea2 - ps3
Archaea3 - ps4
Asgard/Eukaryota - ps5
Eukaryota - ps6
Amorpheae - ps7
Obazoa - ps8
Opisthokonta - ps9
Holozoa - ps10
Filozoa - ps11
Choano./Metazoa - ps12
Metazoa - ps13
Myriazoa - ps14
Parahoxozoa - ps15
Bilateria - ps16
Deuterostomia - ps17
Chordata - ps18
Olfactores - ps19
Vertebrata - ps20
Gnathostomata - ps21
Euteleostomi - ps22
Sarcopterygii - ps23
Tetrapoda - ps24
Amniota - ps25
Mammalia - ps26
Theria - ps27
Placentalia - ps28
Boreoeutheria - ps29
Euarchontoglires - ps30
Euarchonta - ps31
Primates - ps32
Haplorrhini - ps33
Simiiformes - ps34
Catarrhini - ps35
Hominoidea - ps36
Hominidae - ps37
Homininae - ps38
Hominini - ps39
Homo_sapiens - ps40
p-value
3e-13
2e-10
1e-07
9e-05
6e-02
Log-odds
0.5
1.0
1.5
2.0
2.5
3.0
3.5
4.0

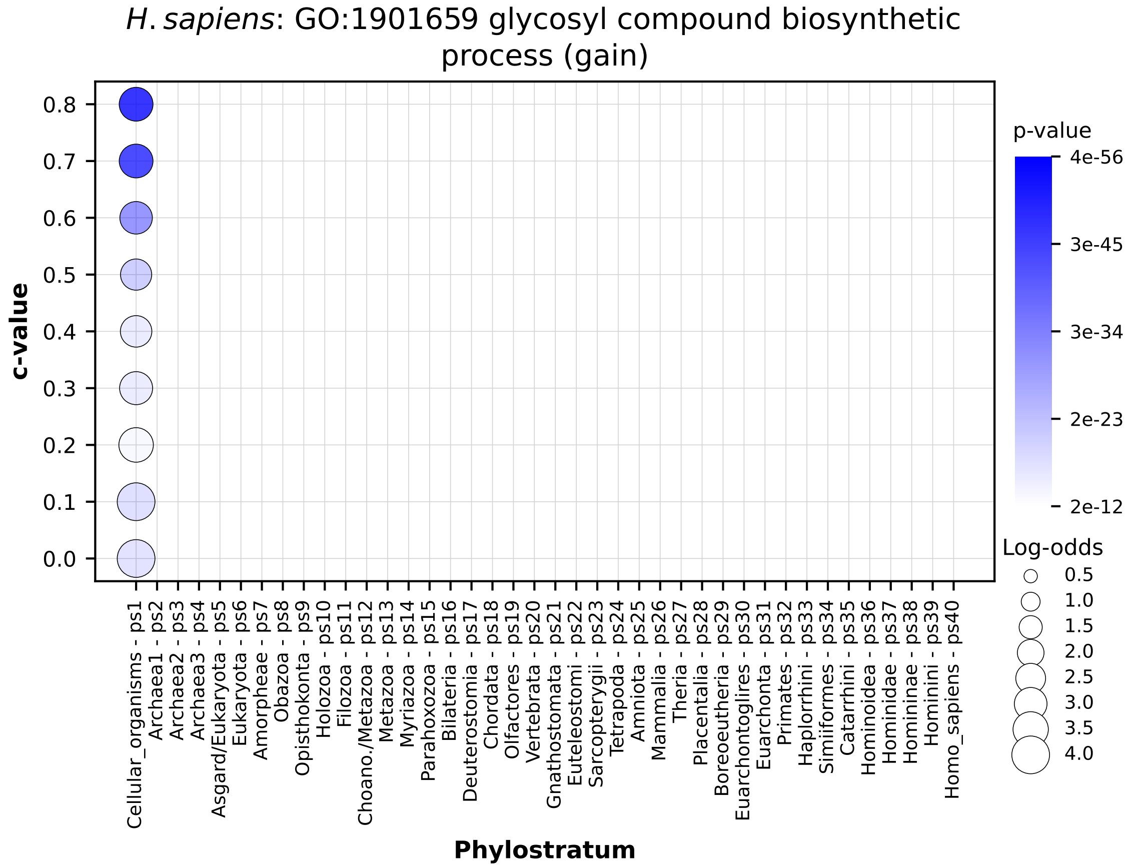

H. sapiens: GO:1901659 glycosyl compound biosynthetic process (gain)
c-value
0.8
0.7
0.6
0.5
0.4
0.3
0.2
0.1
0.0
p-value
4e-56
3e-45
3e-34
2e-23
2e-12
Log-odds
0.5
1.0
1.5
2.0
2.5
3.0
3.5
4.0
Cellular_organisms - ps1
Archaea1 - ps2
Archaea2 - ps3
Archaea3 - ps4
Asgard/Eukaryota - ps5
Eukaryota - ps6
Amorpheae - ps7
Obazoa - ps8
Opisthokonta - ps9
Holozoa - ps10
Filozoa - ps11
Choano./Metazoa - ps12
Metazoa - ps13
Myriazoa - ps14
Parahoxozoa - ps15
Bilateria - ps16
Deuterostomia - ps17
Chordata - ps18
Olfactores - ps19
Vertebrata - ps20
Gnathostomata - ps21
Euteleostomi - ps22
Sarcopterygii - ps23
Tetrapoda - ps24
Amniota - ps25
Mammalia - ps26
Theria - ps27
Placentalia - ps28
Boreoeutheria - ps29
Euarchontoglires - ps30
Euarchonta - ps31
Primates - ps32
Haplorrhini - ps33
Simiiformes - ps34
Catarrhini - ps35
Hominoidea - ps36
Hominidae - ps37
Homininae - ps38
Hominini - ps39
Homo_sapiens - ps40
Phylostratum

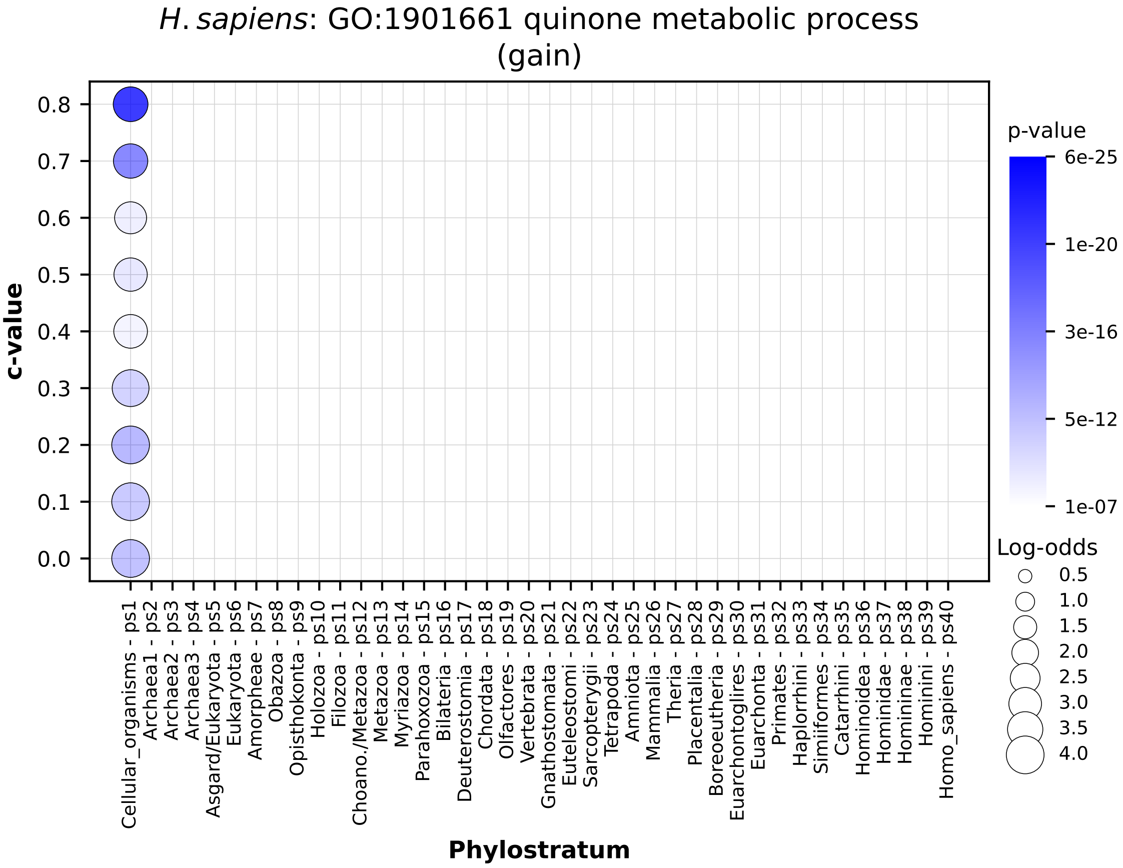

H. sapiens: GO:1901661 quinone metabolic process
(gain)
c-value
0.8
0.7
0.6
0.5
0.4
0.3
0.2
0.1
0.0
p-value
6e-25
1e-20
3e-16
5e-12
1e-07
Log-odds
0.5
1.0
1.5
2.0
2.5
3.0
3.5
4.0
Cellular_organisms - ps1
Archaea1 - ps2
Archaea2 - ps3
Archaea3 - ps4
Asgard/Eukaryota - ps5
Eukaryota - ps6
Amorpheae - ps7
Obazoa - ps8
Opisthokonta - ps9
Holozoa - ps10
Filozoa - ps11
Choano./Metazoa - ps12
Metazoa - ps13
Myriazoa - ps14
Parahoxozoa - ps15
Bilateria - ps16
Deuterostomia - ps17
Chordata - ps18
Olfactores - ps19
Vertebrata - ps20
Gnathostomata - ps21
Euteleostomi - ps22
Sarcopterygii - ps23
Tetrapoda - ps24
Amniota - ps25
Mammalia - ps26
Theria - ps27
Placentalia - ps28
Boreoeutheria - ps29
Euarchontoglires - ps30
Euarchonta - ps31
Primates - ps32
Haplorrhini - ps33
Simiiformes - ps34
Catarrhini - ps35
Hominoidea - ps36
Hominidae - ps37
Homininae - ps38
Hominini - ps39
Homo_sapiens - ps40
Phylostratum

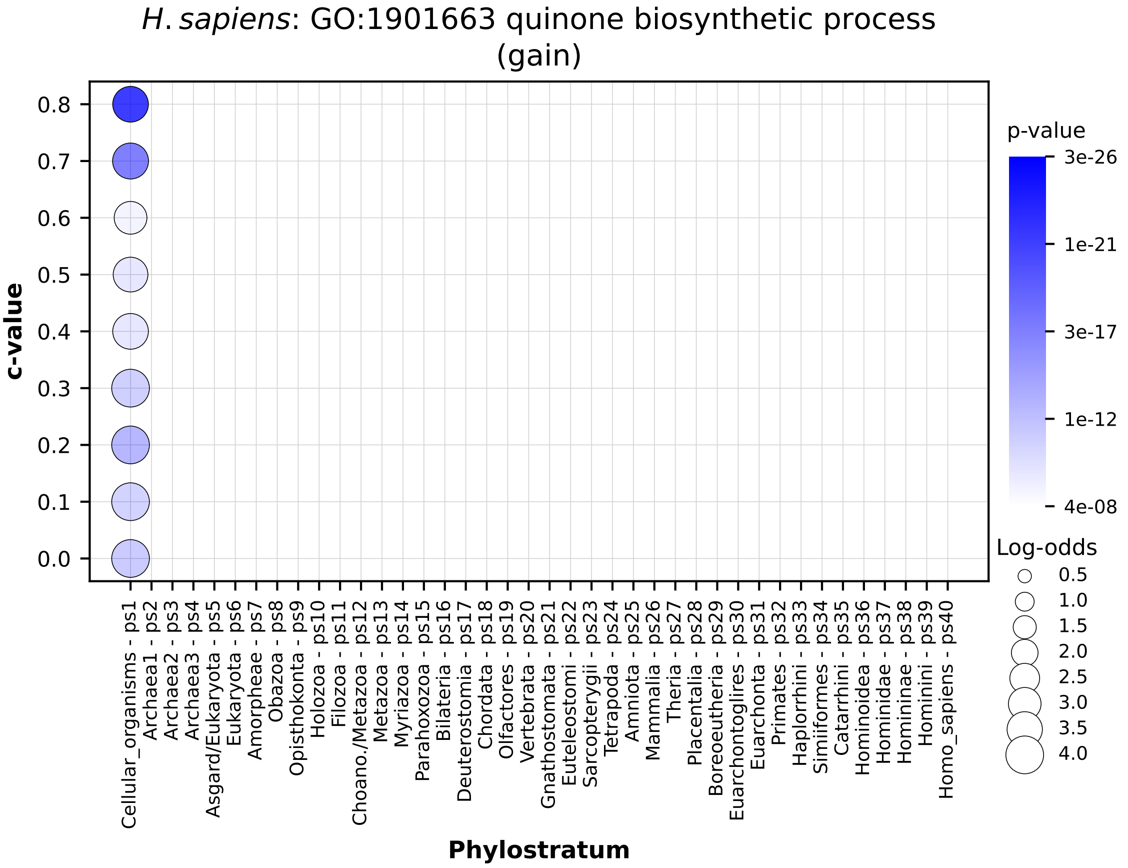
H. sapiens: GO:1901663 quinone biosynthetic process
(gain)
c-value
0.0
0.1
0.2
0.3
0.4
0.5
0.6
0.7
0.8
p-value
3e-26
1e-21
3e-17
1e-12
4e-08
Log-odds
0.5
1.0
1.5
2.0
2.5
3.0
3.5
4.0
Cellular_organisms - ps1
Archaea1 - ps2
Archaea2 - ps3
Archaea3 - ps4
Asgard/Eukaryota - ps5
Eukaryota - ps6
Amorpheae - ps7
Obazoa - ps8
Opisthokonta - ps9
Holozoa - ps10
Filozoa - ps11
Choano./Metazoa - ps12
Metazoa - ps13
Myriazoa - ps14
Parahoxozoa - ps15
Bilateria - ps16
Deuterostomia - ps17
Chordata - ps18
Olfactores - ps19
Vertebrata - ps20
Gnathostomata - ps21
Euteleostomi - ps22
Sarcopterygii - ps23
Tetrapoda - ps24
Amniota - ps25
Mammalia - ps26
Theria - ps27
Placentalia - ps28
Boreoeutheria - ps29
Euarchontoglires - ps30
Euarchonta - ps31
Primates - ps32
Haplorrhini - ps33
Simiiformes - ps34
Catarrhini - ps35
Hominoidea - ps36
Hominidae - ps37
Homininae - ps38
Hominini - ps39
Homo_sapiens - ps40
Phylostratum

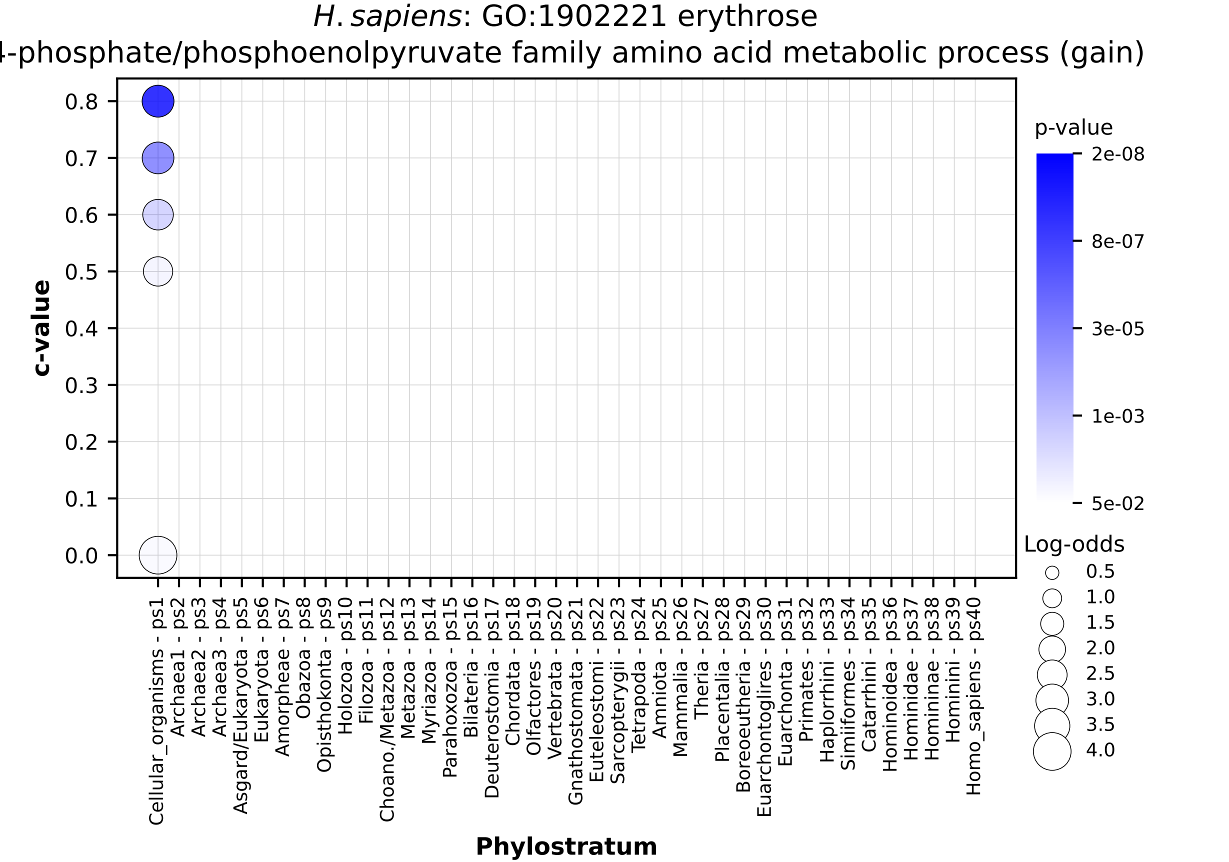

H. sapiens: GO:1902221 erythrose 4-phosphate/phosphoenolpyruvate family amino acid metabolic process (gain)
c-value
0.0
0.1
0.2
0.3
0.4
0.5
0.6
0.7
0.8
p-value
2e-08
8e-07
3e-05
1e-03
5e-02
Log-odds
0.5
1.0
1.5
2.0
2.5
3.0
3.5
4.0
Cellular_organisms - ps1
Archaea1 - ps2
Archaea2 - ps3
Archaea3 - ps4
Asgard/Eukaryota - ps5
Eukaryota - ps6
Amorpheae - ps7
Obazoa - ps8
Opisthokonta - ps9
Holozoa - ps10
Filozoa - ps11
Choano./Metazoa - ps12
Metazoa - ps13
Myriazoa - ps14
Parahoxozoa - ps15
Bilateria - ps16
Deuterostomia - ps17
Chordata - ps18
Olfactores - ps19
Vertebrata - ps20
Gnathostomata - ps21
Euteleostomi - ps22
Sarcopterygii - ps23
Tetrapoda - ps24
Amniota - ps25
Mammalia - ps26
Theria - ps27
Placentalia - ps28
Boreoeutheria - ps29
Euarchontoglires - ps30
Euarchonta - ps31
Primates - ps32
Haplorrhini - ps33
Simiiformes - ps34
Catarrhini - ps35
Hominoidea - ps36
Hominidae - ps37
Homininae - ps38
Hominini - ps39
Homo_sapiens - ps40
Phylostratum

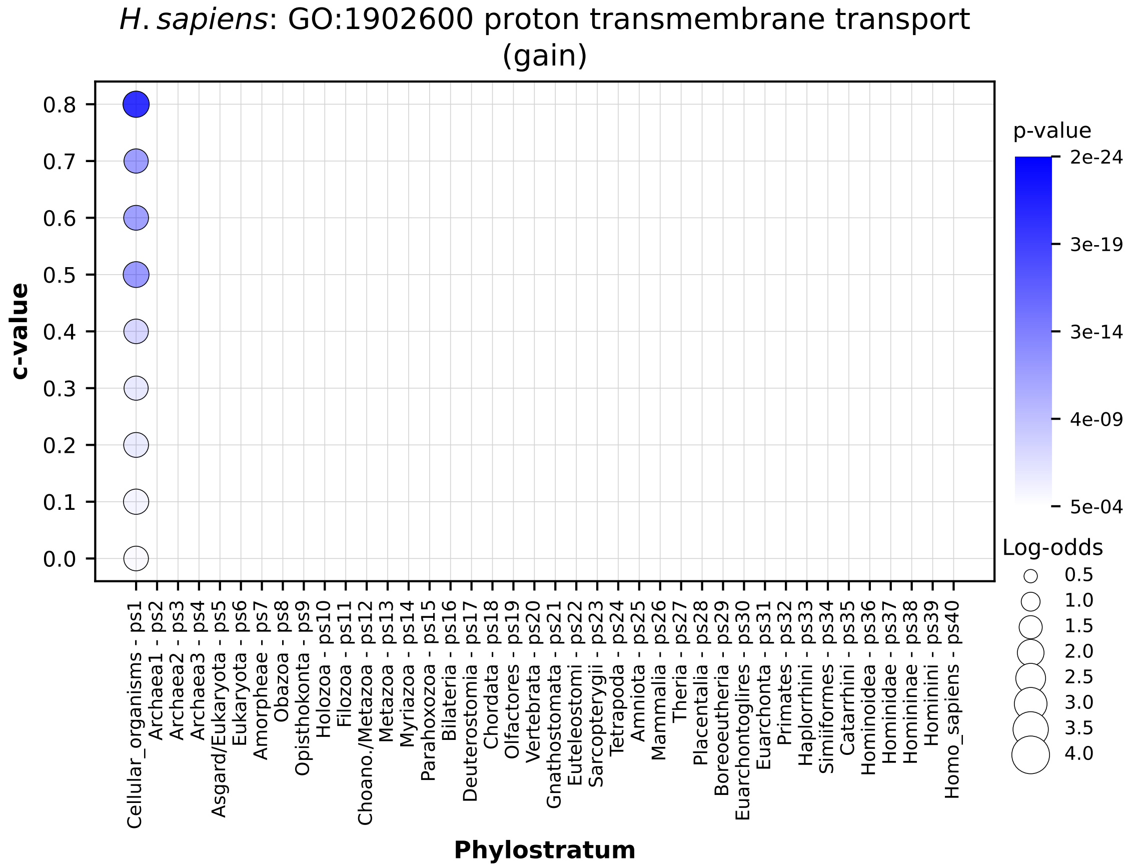

H. sapiens: GO:1902600 proton transmembrane transport
(gain)
c-value
0.8
0.7
0.6
0.5
0.4
0.3
0.2
0.1
0.0
p-value
2e-24
3e-19
3e-14
4e-09
5e-04
Log-odds
0.5
1.0
1.5
2.0
2.5
3.0
3.5
4.0
Cellular_organisms - ps1
Archaea1 - ps2
Archaea2 - ps3
Archaea3 - ps4
Asgard/Eukaryota - ps5
Eukaryota - ps6
Amorpheae - ps7
Obazoa - ps8
Opisthokonta - ps9
Holozoa - ps10
Filozoa - ps11
Choano./Metazoa - ps12
Metazoa - ps13
Myriazoa - ps14
Parahoxozoa - ps15
Bilateria - ps16
Deuterostomia - ps17
Chordata - ps18
Olfactores - ps19
Vertebrata - ps20
Gnathostomata - ps21
Euteleostomi - ps22
Sarcopterygii - ps23
Tetrapoda - ps24
Amniota - ps25
Mammalia - ps26
Theria - ps27
Placentalia - ps28
Boreoeutheria - ps29
Euarchontoglires - ps30
Euarchonta - ps31
Primates - ps32
Haplorrhini - ps33
Simiiformes - ps34
Catarrhini - ps35
Hominoidea - ps36
Hominidae - ps37
Homininae - ps38
Hominini - ps39
Homo_sapiens - ps40
Phylostratum

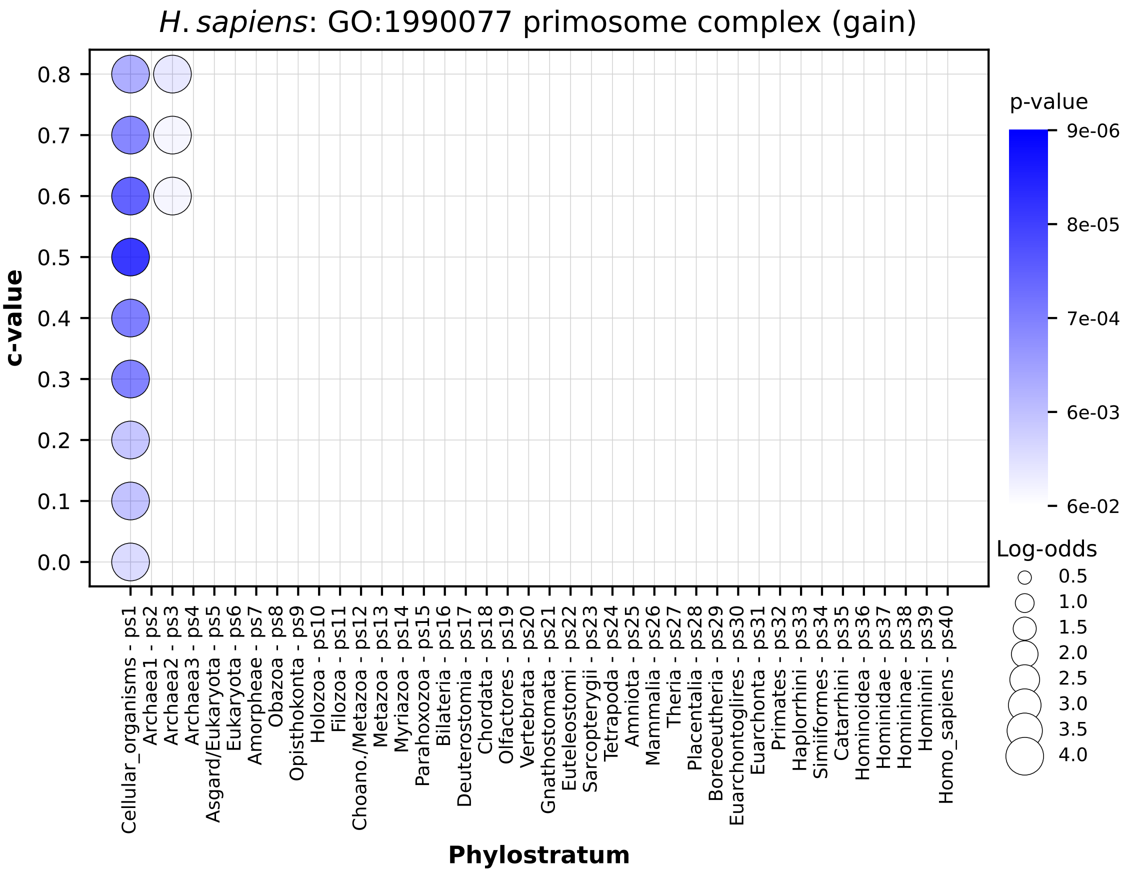

H. sapiens: GO:1990077 primosome complex (gain)
c-value
0.8
0.7
0.6
0.5
0.4
0.3
0.2
0.1
0.0
Phylostratum
Cellular_organisms - ps1
Archaea1 - ps2
Archaea2 - ps3
Archaea3 - ps4
Asgard/Eukaryota - ps5
Eukaryota - ps6
Amorpheae - ps7
Obazoa - ps8
Opisthokonta - ps9
Holozoa - ps10
Filozoa - ps11
Choano./Metazoa - ps12
Metazoa - ps13
Myriazoa - ps14
Parahoxozoa - ps15
Bilateria - ps16
Deuterostomia - ps17
Chordata - ps18
Olfactores - ps19
Vertebrata - ps20
Gnathostomata - ps21
Euteleostomi - ps22
Sarcopterygii - ps23
Tetrapoda - ps24
Amniota - ps25
Mammalia - ps26
Theria - ps27
Placentalia - ps28
Boreoeutheria - ps29
Euarchontoglires - ps30
Euarchonta - ps31
Primates - ps32
Haplorrhini - ps33
Simiiformes - ps34
Catarrhini - ps35
Hominoidea - ps36
Hominidae - ps37
Homininae - ps38
Hominini - ps39
Homo_sapiens - ps40
p-value
9e-06
8e-05
7e-04
6e-03
6e-02
Log-odds
0.5
1.0
1.5
2.0
2.5
3.0
3.5
4.0

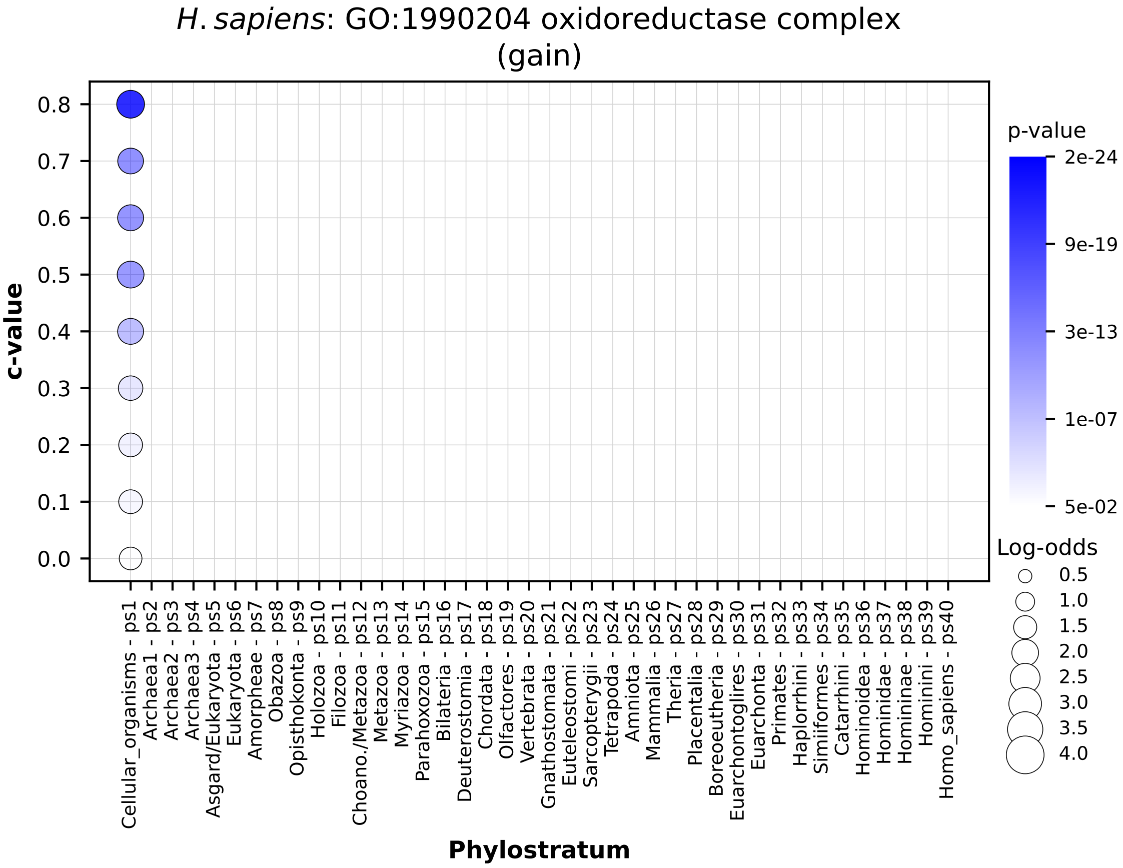
H. sapiens: GO:1990204 oxidoreductase complex
(gain)
c-value
0.0
0.1
0.2
0.3
0.4
0.5
0.6
0.7
0.8
p-value
2e-24
9e-19
3e-13
1e-07
5e-02
Log-odds
0.5
1.0
1.5
2.0
2.5
3.0
3.5
4.0
Cellular_organisms - ps1
Archaea1 - ps2
Archaea2 - ps3
Archaea3 - ps4
Asgard/Eukaryota - ps5
Eukaryota - ps6
Amorpheae - ps7
Obazoa - ps8
Opisthokonta - ps9
Holozoa - ps10
Filozoa - ps11
Choano./Metazoa - ps12
Metazoa - ps13
Myriazoa - ps14
Parahoxozoa - ps15
Bilateria - ps16
Deuterostomia - ps17
Chordata - ps18
Olfactores - ps19
Vertebrata - ps20
Gnathostomata - ps21
Euteleostomi - ps22
Sarcopterygii - ps23
Tetrapoda - ps24
Amniota - ps25
Mammalia - ps26
Theria - ps27
Placentalia - ps28
Boreoeutheria - ps29
Euarchontoglires - ps30
Euarchonta - ps31
Primates - ps32
Haplorrhini - ps33
Simiiformes - ps34
Catarrhini - ps35
Hominoidea - ps36
Hominidae - ps37
Homininae - ps38
Hominini - ps39
Homo_sapiens - ps40
Phylostratum

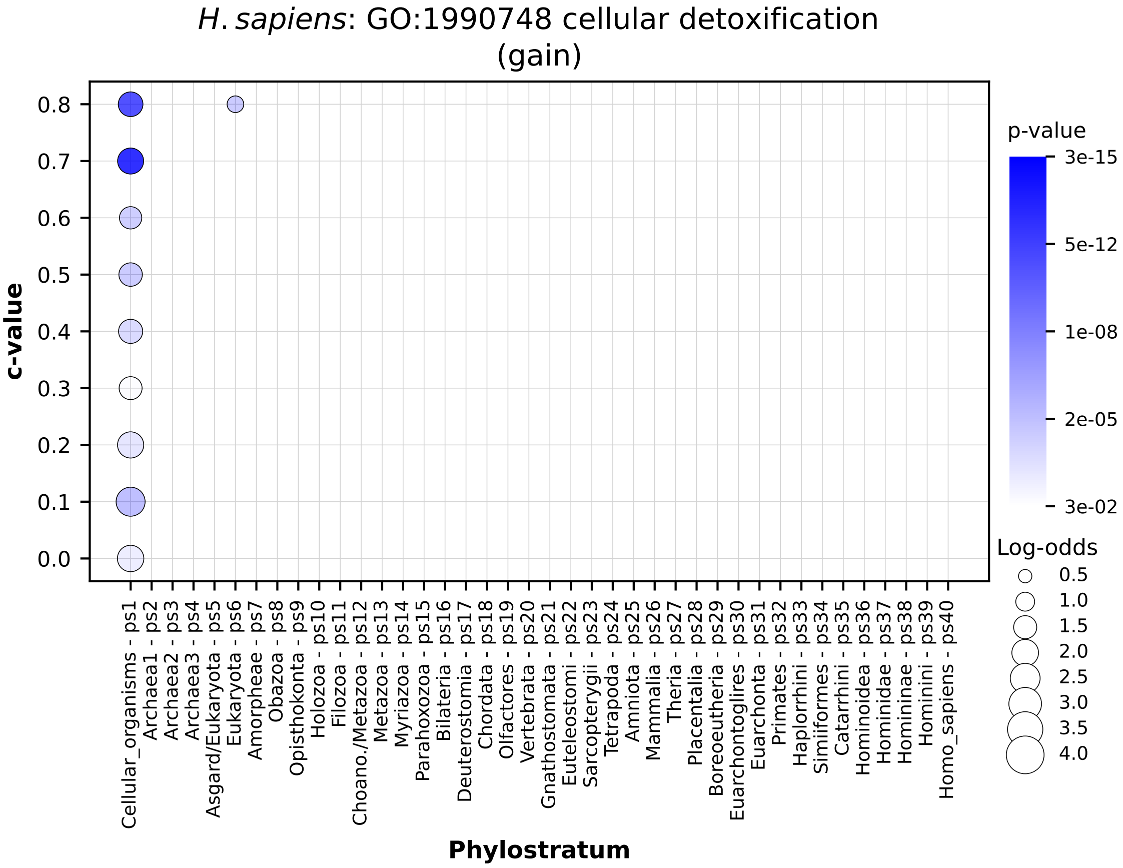

H. sapiens: GOSSIP:1990748 cellular detoxification

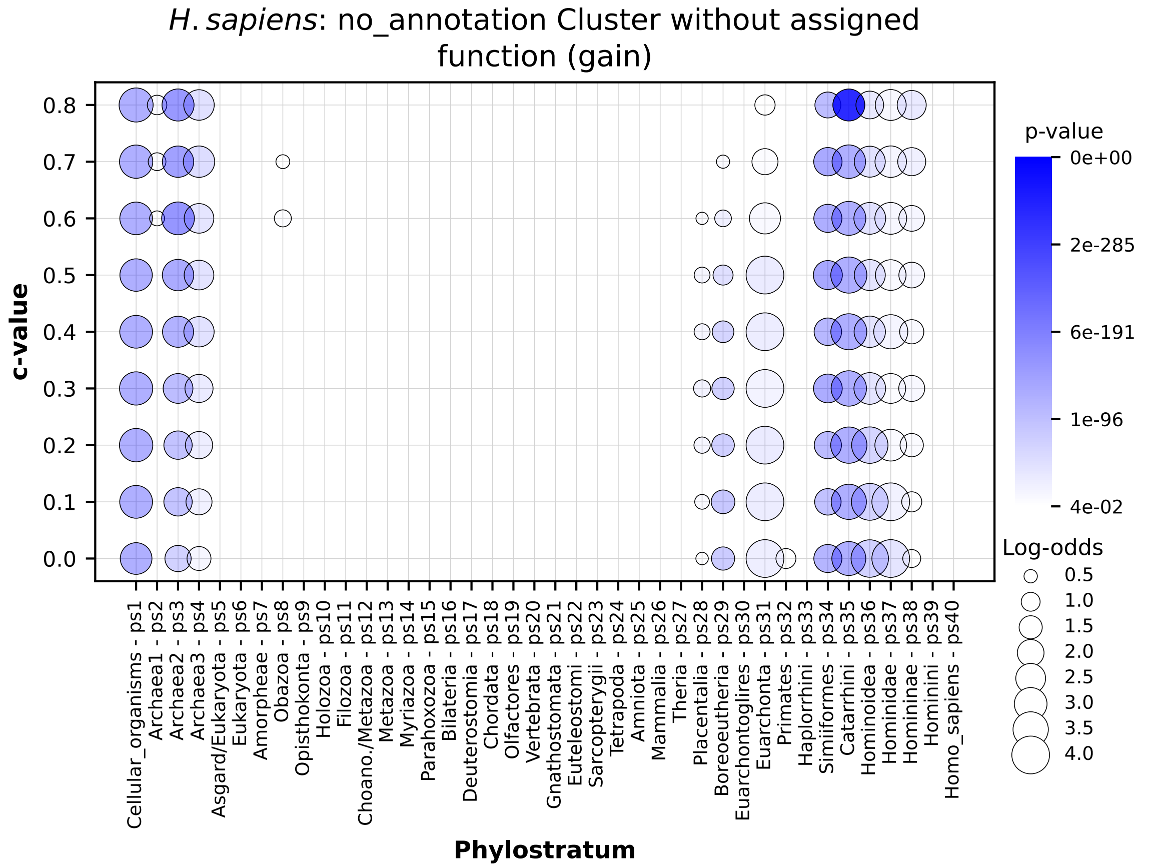
H. sapiens: no_annotation Cluster without assigned function (gain)
c-value
0.8
0.7
0.6
0.5
0.4
0.3
0.2
0.1
0.0
p-value
0e+00
2e-285
6e-191
1e-96
4e-02
Log-odds
0.5
1.0
1.5
2.0
2.5
3.0
3.5
4.0
Cellular_organisms - ps1
Archaea1 - ps2
Archaea2 - ps3
Archaea3 - ps4
Asgard/Eukaryota - ps5
Eukaryota - ps6
Amorpheae - ps7
Obazoa - ps8
Opisthokonta - ps9
Holozoa - ps10
Filozoa - ps11
Choano./Metazoa - ps12
Metazoa - ps13
Myriazoa - ps14
Parahoxozoa - ps15
Bilateria - ps16
Deuterostomia - ps17
Chordata - ps18
Olfactores - ps19
Vertebrata - ps20
Gnathostomata - ps21
Euteleostomi - ps22
Sarcopterygii - ps23
Tetrapoda - ps24
Amniota - ps25
Mammalia - ps26
Theria - ps27
Placentalia - ps28
Boreoeutheria - ps29
Euarchontoglires - ps30
Euarchonta - ps31
Primates - ps32
Haplorrhini - ps33
Simiiformes - ps34
Catarrhini - ps35
Hominoidea - ps36
Hominidae - ps37
Homininae - ps38
Hominini - ps39
Homo_sapiens - ps40
Phylostratum

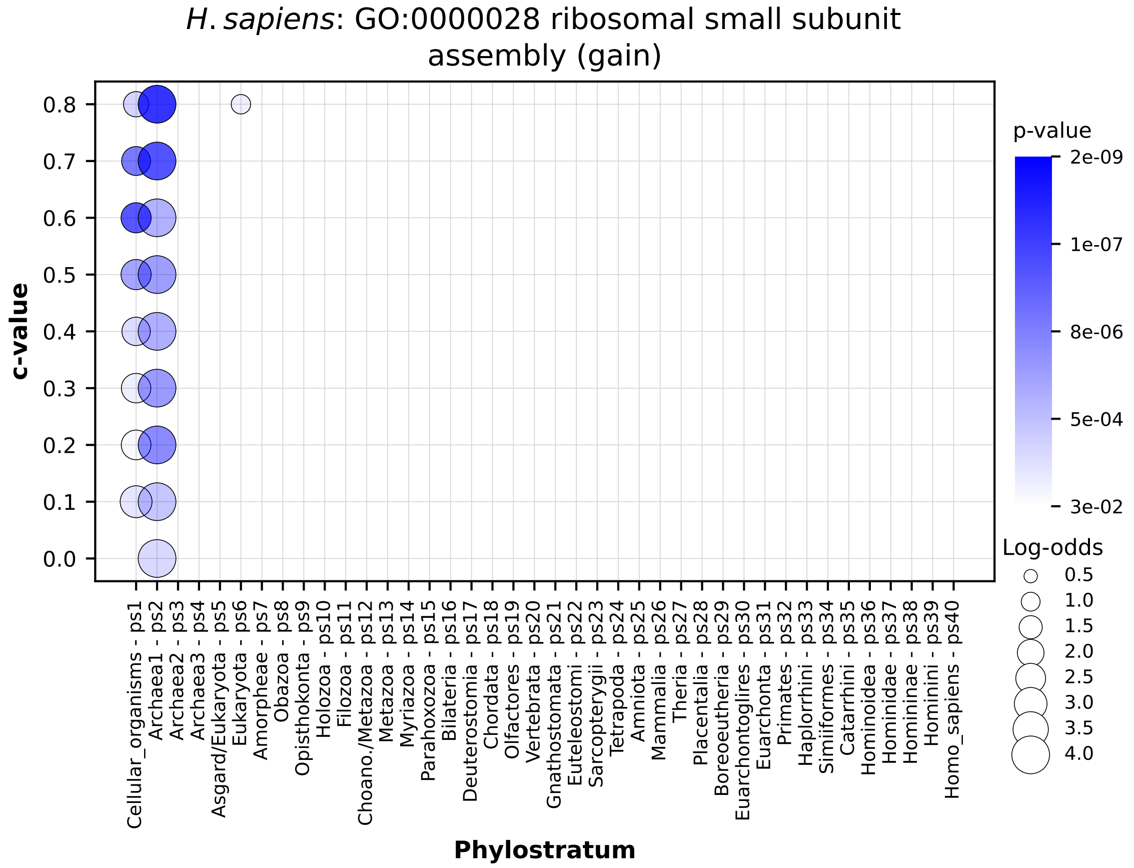
H. sapiens: GO:0000028 ribosomal small subunit assembly (gain)
c-value
0.8
0.7
0.6
0.5
0.4
0.3
0.2
0.1
0.0
p-value
2e-09
1e-07
8e-06
5e-04
3e-02
Log-odds
0.5
1.0
1.5
2.0
2.5
3.0
3.5
4.0
Cellular_organisms - ps1
Archaea1 - ps2
Archaea2 - ps3
Archaea3 - ps4
Asgard/Eukaryota - ps5
Eukaryota - ps6
Amorpheae - ps7
Obazoa - ps8
Opisthokonta - ps9
Holozoa - ps10
Filozoa - ps11
Choano./Metazoa - ps12
Metazoa - ps13
Myriazoa - ps14
Parahoxozoa - ps15
Bilateria - ps16
Deuterostomia - ps17
Chordata - ps18
Olfactores - ps19
Vertebrata - ps20
Gnathostomata - ps21
Euteleostomi - ps22
Sarcopterygii - ps23
Tetrapoda - ps24
Amniota - ps25
Mammalia - ps26
Theria - ps27
Placentalia - ps28
Boreoeutheria - ps29
Euarchontoglires - ps30
Euarchonta - ps31
Primates - ps32
Haplorrhini - ps33
Simiiformes - ps34
Catarrhini - ps35
Hominoidea - ps36
Hominidae - ps37
Homininae - ps38
Hominini - ps39
Homo_sapiens - ps40
Phylostratum

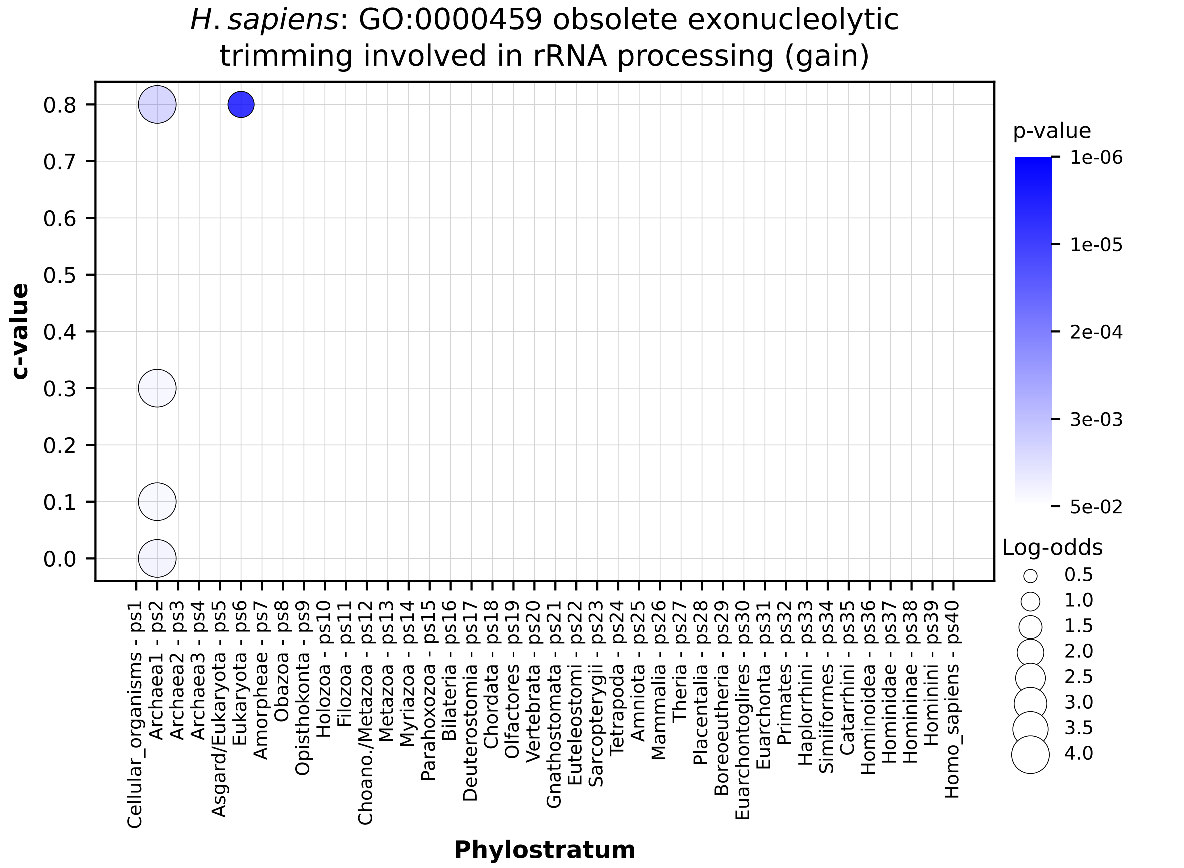

*H. sapiens*: GO:0000459 obsolete exonucleolytic trimming involved in rRNA processing (gain)
c-value
0.0
0.1
0.2
0.3
0.4
0.5
0.6
0.7
0.8
Phylostratum
Cellular_organisms - ps1
Archaea1 - ps2
Archaea2 - ps3
Archaea3 - ps4
Asgard/Eukaryota - ps5
Eukaryota - ps6
Amorpheae - ps7
Obazoa - ps8
Opisthokonta - ps9
Holozoa - ps10
Filozoa - ps11
Choano./Metazoa - ps12
Metazoa - ps13
Myriazoa - ps14
Parahoxozoa - ps15
Bilateria - ps16
Deuterostomia - ps17
Chordata - ps18
Olfactores - ps19
Vertebrata - ps20
Gnathostomata - ps21
Euteleostomi - ps22
Sarcopterygii - ps23
Tetrapoda - ps24
Amniota - ps25
Mammalia - ps26
Theria - ps27
Placentalia - ps28
Boreoeutheria - ps29
Euarchontoglires - ps30
Euarchonta - ps31
Primates - ps32
Haplorrhini - ps33
Simiiformes - ps34
Catarrhini - ps35
Hominoidea - ps36
Hominidae - ps37
Homininae - ps38
Hominini - ps39
Homo_sapiens - ps40
p-value
1e-06
1e-05
2e-04
3e-03
5e-02
Log-odds
0.5
1.0
1.5
2.0
2.5
3.0
3.5
4.0

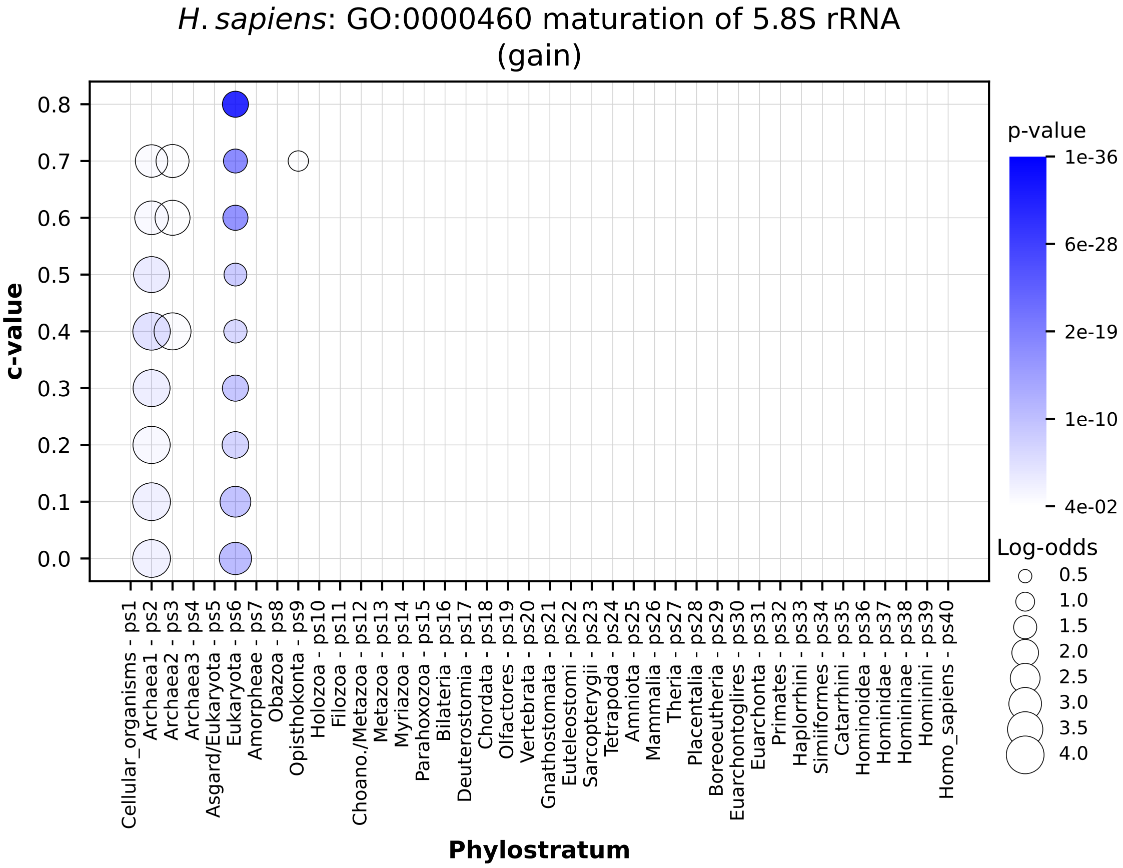
H. sapiens: GO:0000460 maturation of 5.8S rRNA
(gain)
c-value
0.8
0.7
0.6
0.5
0.4
0.3
0.2
0.1
0.0
p-value
1e-36
6e-28
2e-19
1e-10
4e-02
Log-odds
0.5
1.0
1.5
2.0
2.5
3.0
3.5
4.0
Cellular_organisms - ps1
Archaea1 - ps2
Archaea2 - ps3
Archaea3 - ps4
Asgard/Eukaryota - ps5
Eukaryota - ps6
Amorpheae - ps7
Obazoa - ps8
Opisthokonta - ps9
Holozoa - ps10
Filozoa - ps11
Choano./Metazoa - ps12
Metazoa - ps13
Myriazoa - ps14
Parahoxozoa - ps15
Bilateria - ps16
Deuterostomia - ps17
Chordata - ps18
Olfactores - ps19
Vertebrata - ps20
Gnathostomata - ps21
Euteleostomi - ps22
Sarcopterygii - ps23
Tetrapoda - ps24
Amniota - ps25
Mammalia - ps26
Theria - ps27
Placentalia - ps28
Boreoeutheria - ps29
Euarchontoglires - ps30
Euarchonta - ps31
Primates - ps32
Haplorrhini - ps33
Simiiformes - ps34
Catarrhini - ps35
Hominoidea - ps36
Hominidae - ps37
Homininae - ps38
Hominini - ps39
Homo_sapiens - ps40
Phylostratum

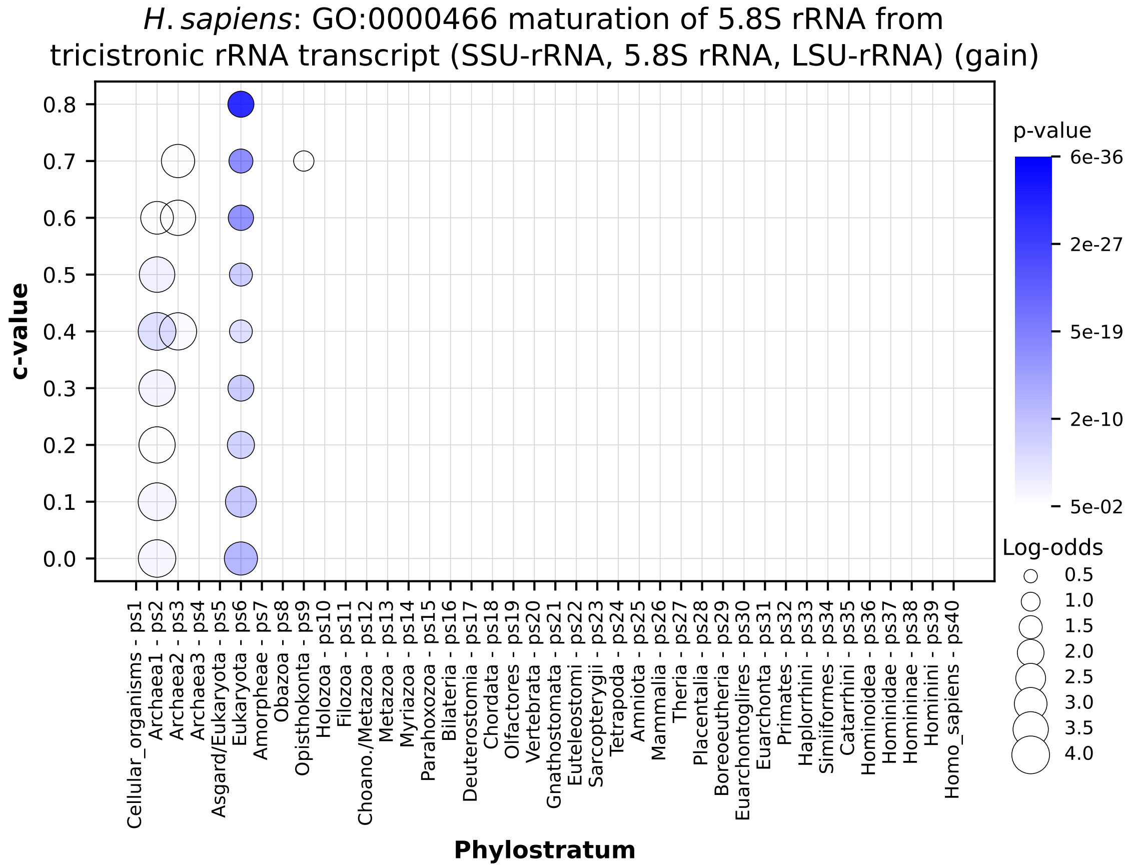

*H. sapiens*: GO:0000466 maturation of 5.8S rRNA from tricistronic rRNA transcript (SSU-rRNA, 5.8S rRNA, LSU-rRNA) (gain)
c-value
0.0
0.1
0.2
0.3
0.4
0.5
0.6
0.7
0.8
Phylostratum
Cellular_organisms - ps1
Archaea1 - ps2
Archaea2 - ps3
Archaea3 - ps4
Asgard/Eukaryota - ps5
Eukaryota - ps6
Amorpheae - ps7
Obazoa - ps8
Opisthokonta - ps9
Holozoa - ps10
Filozoa - ps11
Choano./Metazoa - ps12
Metazoa - ps13
Myriazoa - ps14
Parahoxozoa - ps15
Bilateria - ps16
Deuterostomia - ps17
Chordata - ps18
Olfactores - ps19
Vertebrata - ps20
Gnathostomata - ps21
Euteleostomi - ps22
Sarcopterygii - ps23
Tetrapoda - ps24
Amniota - ps25
Mammalia - ps26
Theria - ps27
Placentalia - ps28
Boreoeutheria - ps29
Euarchontoglires - ps30
Euarchonta - ps31
Primates - ps32
Haplorrhini - ps33
Simiiformes - ps34
Catarrhini - ps35
Hominoidea - ps36
Hominidae - ps37
Homininae - ps38
Hominini - ps39
Homo_sapiens - ps40
p-value
6e-36
2e-27
5e-19
2e-10
5e-02
Log-odds
0.5
1.0
1.5
2.0
2.5
3.0
3.5
4.0

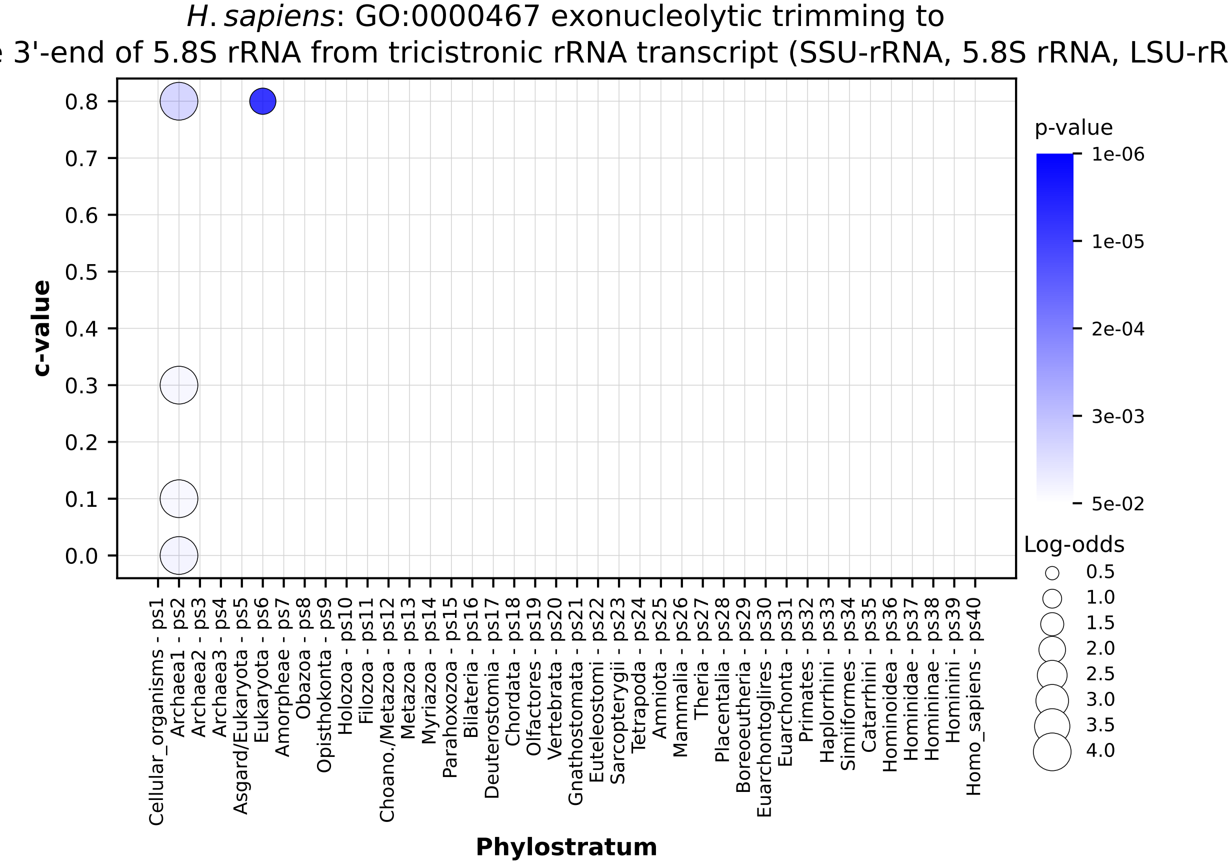

*H. sapiens*: GO:0000467 exonucleolytic trimming to
e 3'-end of 5.8S rRNA from tricistronic rRNA transcript (SSU-rRNA, 5.8S rRNA, LSU-rR
c-value
0.8
0.7
0.6
0.5
0.4
0.3
0.2
0.1
0.0
p-value
1e-06
1e-05
2e-04
3e-03
5e-02
Log-odds
0.5
1.0
1.5
2.0
2.5
3.0
3.5
4.0
Cellular_organisms - ps1
Archaea1 - ps2
Archaea2 - ps3
Archaea3 - ps4
Asgard/Eukaryota - ps5
Eukaryota - ps6
Amorpheae - ps7
Obazoa - ps8
Opisthokonta - ps9
Holozoa - ps10
Filozoa - ps11
Choano./Metazoa - ps12
Metazoa - ps13
Myriazoa - ps14
Parahoxozoa - ps15
Bilateria - ps16
Deuterostomia - ps17
Chordata - ps18
Olfactores - ps19
Vertebrata - ps20
Gnathostomata - ps21
Euteleostomi - ps22
Sarcopterygii - ps23
Tetrapoda - ps24
Amniota - ps25
Mammalia - ps26
Theria - ps27
Placentalia - ps28
Boreoeutheria - ps29
Euarchontoglires - ps30
Euarchonta - ps31
Primates - ps32
Haplorrhini - ps33
Simiiformes - ps34
Catarrhini - ps35
Hominoidea - ps36
Hominidae - ps37
Homininae - ps38
Hominini - ps39
Homo_sapiens - ps40
Phylostratum

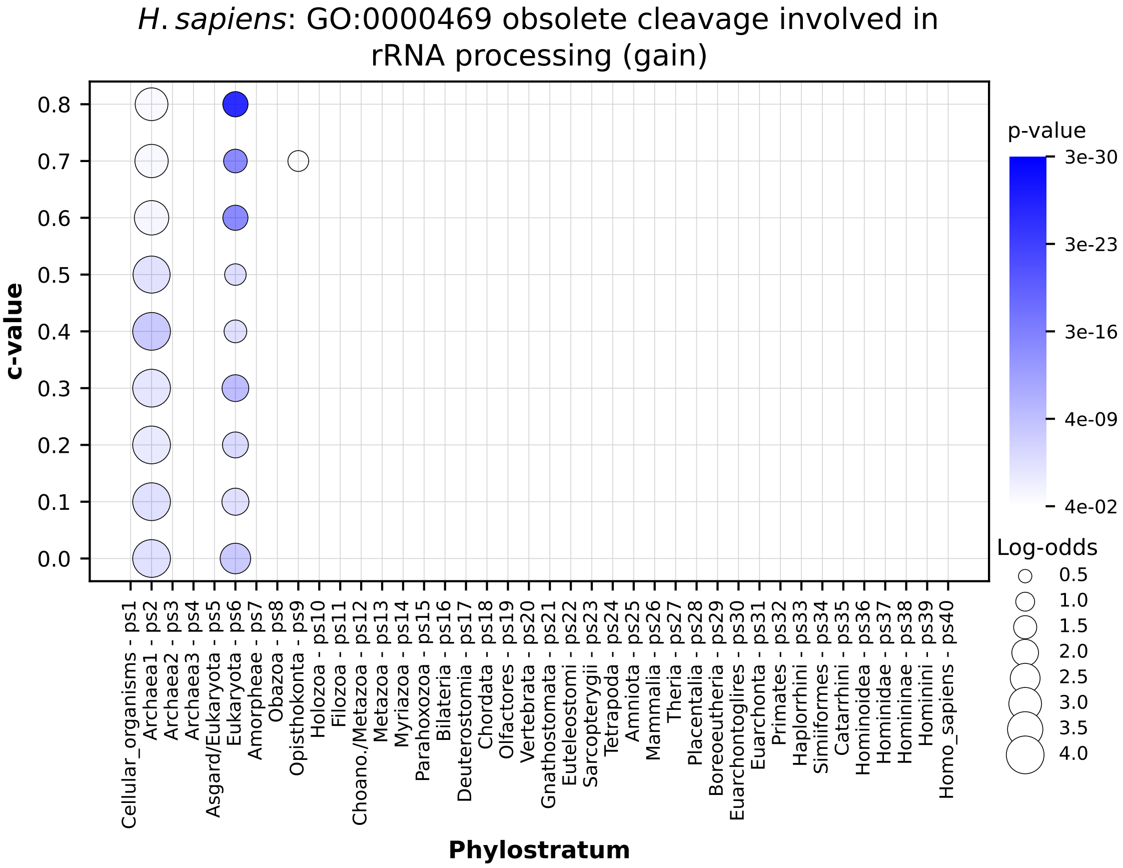
*H. sapiens*: GO:0000469 obsolete cleavage involved in rRNA processing (gain)
c-value
0.8
0.7
0.6
0.5
0.4
0.3
0.2
0.1
0.0
p-value
3e-30
3e-23
3e-16
4e-09
4e-02
Log-odds
0.5
1.0
1.5
2.0
2.5
3.0
3.5
4.0
Cellular_organisms - ps1
Archaea1 - ps2
Archaea2 - ps3
Archaea3 - ps4
Asgard/Eukaryota - ps5
Eukaryota - ps6
Amorpheae - ps7
Obazoa - ps8
Opisthokonta - ps9
Holozoa - ps10
Filozoa - ps11
Choano./Metazoa - ps12
Metazoa - ps13
Myriazoa - ps14
Parahoxozoa - ps15
Bilateria - ps16
Deuterostomia - ps17
Chordata - ps18
Olfactores - ps19
Vertebrata - ps20
Gnathostomata - ps21
Euteleostomi - ps22
Sarcopterygii - ps23
Tetrapoda - ps24
Amniota - ps25
Mammalia - ps26
Theria - ps27
Placentalia - ps28
Boreoeutheria - ps29
Euarchontoglires - ps30
Euarchonta - ps31
Primates - ps32
Haplorrhini - ps33
Simiiformes - ps34
Catarrhini - ps35
Hominoidea - ps36
Hominidae - ps37
Homininae - ps38
Hominini - ps39
Homo_sapiens - ps40
Phylostratum

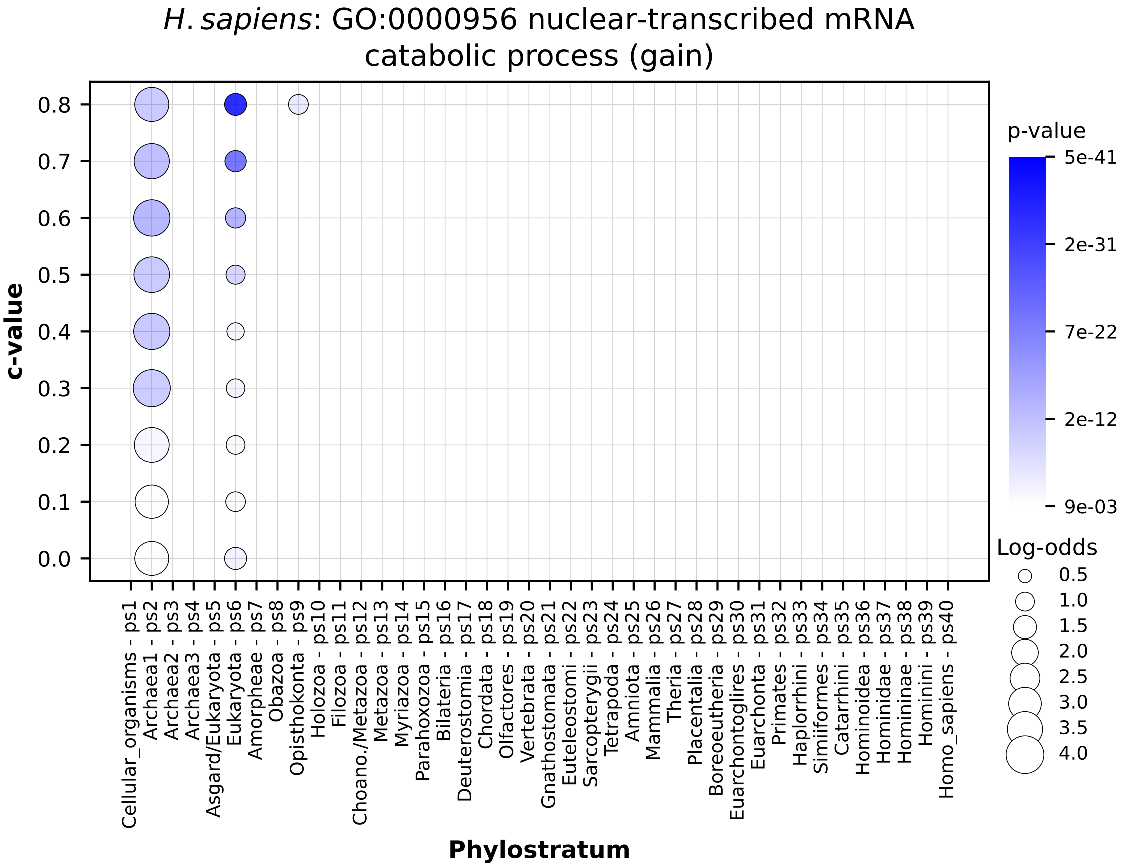

H. sapiens: GO:0000956 nuclear-transcribed mRNA catabolic process (gain)
c-value
0.8
0.7
0.6
0.5
0.4
0.3
0.2
0.1
0.0
p-value
5e-41
2e-31
7e-22
2e-12
9e-03
Log-odds
0.5
1.0
1.5
2.0
2.5
3.0
3.5
4.0
Cellular_organisms - ps1
Archaea1 - ps2
Archaea2 - ps3
Archaea3 - ps4
Asgard/Eukaryota - ps5
Eukaryota - ps6
Amorpheae - ps7
Obazoa - ps8
Opisthokonta - ps9
Holozoa - ps10
Filozoa - ps11
Choano./Metazoa - ps12
Metazoa - ps13
Myriazoa - ps14
Parahoxozoa - ps15
Bilateria - ps16
Deuterostomia - ps17
Chordata - ps18
Olfactores - ps19
Vertebrata - ps20
Gnathostomata - ps21
Euteleostomi - ps22
Sarcopterygii - ps23
Tetrapoda - ps24
Amniota - ps25
Mammalia - ps26
Theria - ps27
Placentalia - ps28
Boreoeutheria - ps29
Euarchontoglires - ps30
Euarchonta - ps31
Primates - ps32
Haplorrhini - ps33
Simiiformes - ps34
Catarrhini - ps35
Hominoidea - ps36
Hominidae - ps37
Homininae - ps38
Hominini - ps39
Homo_sapiens - ps40
Phylostratum

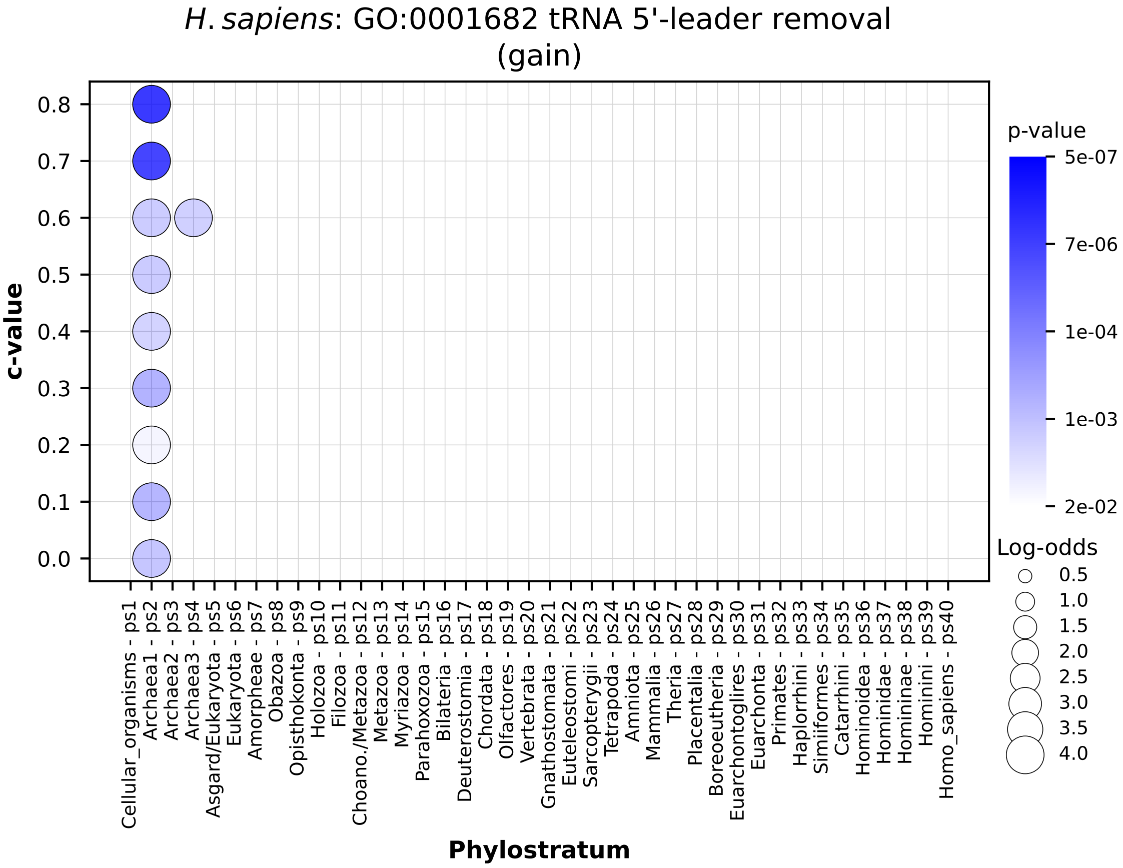

H. sapiens: GO:0001682 tRNA 5'-leader removal
(gain)
c-value
0.0
0.1
0.2
0.3
0.4
0.5
0.6
0.7
0.8
p-value
5e-07
7e-06
1e-04
1e-03
2e-02
Log-odds
0.5
1.0
1.5
2.0
2.5
3.0
3.5
4.0
Cellular_organisms - ps1
Archaea1 - ps2
Archaea2 - ps3
Archaea3 - ps4
Asgard/Eukaryota - ps5
Eukaryota - ps6
Amorpheae - ps7
Obazoa - ps8
Opisthokonta - ps9
Holozoa - ps10
Filozoa - ps11
Choano./Metazoa - ps12
Metazoa - ps13
Myriazoa - ps14
Parahoxozoa - ps15
Bilateria - ps16
Deuterostomia - ps17
Chordata - ps18
Olfactores - ps19
Vertebrata - ps20
Gnathostomata - ps21
Euteleostomi - ps22
Sarcopterygii - ps23
Tetrapoda - ps24
Amniota - ps25
Mammalia - ps26
Theria - ps27
Placentalia - ps28
Boreoeutheria - ps29
Euarchontoglires - ps30
Euarchonta - ps31
Primates - ps32
Haplorrhini - ps33
Simiiformes - ps34
Catarrhini - ps35
Hominoidea - ps36
Hominidae - ps37
Homininae - ps38
Hominini - ps39
Homo_sapiens - ps40
Phylostratum

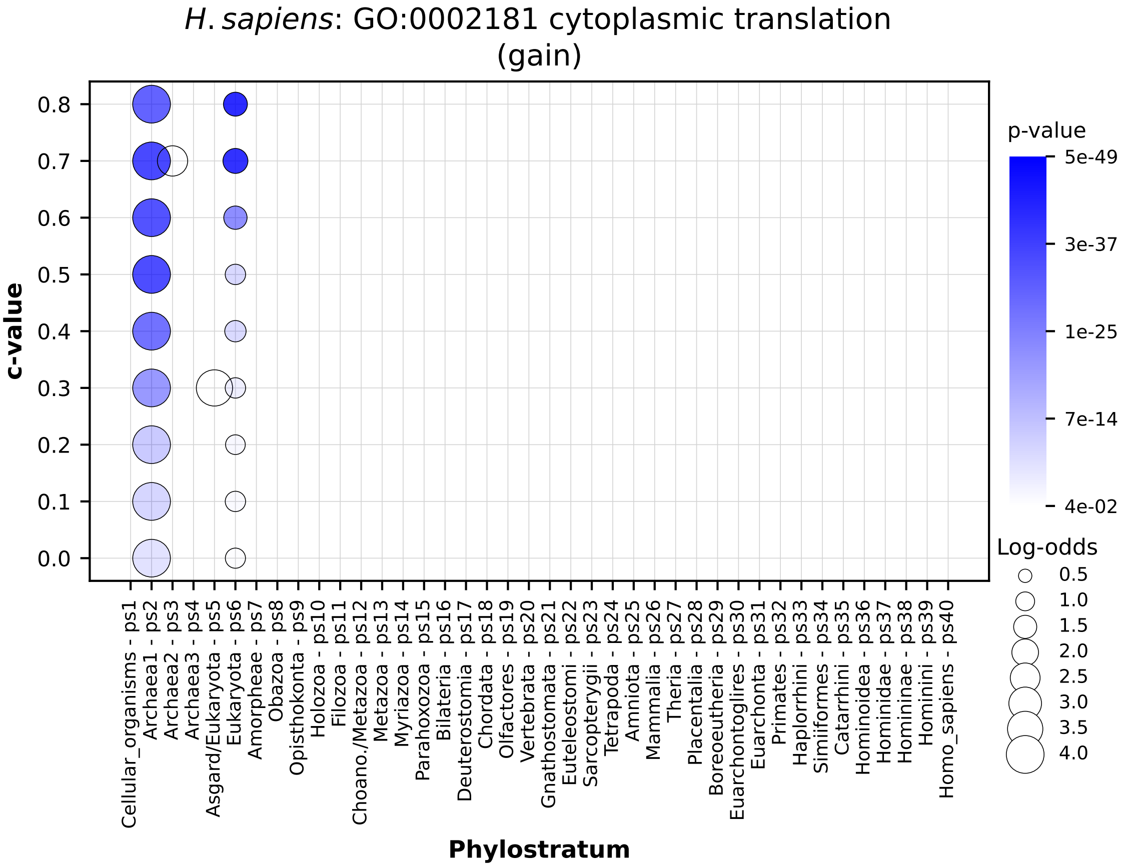

*H. sapiens*: GO:0002181 cytoplasmic translation
(gain)
c-value
0.8
0.7
0.6
0.5
0.4
0.3
0.2
0.1
0.0
Phylostratum
Cellular_organisms - ps1
Archaea1 - ps2
Archaea2 - ps3
Archaea3 - ps4
Asgard/Eukaryota - ps5
Eukaryota - ps6
Amorpheae - ps7
Obazoa - ps8
Opisthokonta - ps9
Holozoa - ps10
Filozoa - ps11
Choano./Metazoa - ps12
Metazoa - ps13
Myriazoa - ps14
Parahoxozoa - ps15
Bilateria - ps16
Deuterostomia - ps17
Chordata - ps18
Olfactores - ps19
Vertebrata - ps20
Gnathostomata - ps21
Euteleostomi - ps22
Sarcopterygii - ps23
Tetrapoda - ps24
Amniota - ps25
Mammalia - ps26
Theria - ps27
Placentalia - ps28
Boreoeutheria - ps29
Euarchontoglires - ps30
Euarchonta - ps31
Primates - ps32
Haplorrhini - ps33
Simiiformes - ps34
Catarrhini - ps35
Hominoidea - ps36
Hominidae - ps37
Homininae - ps38
Hominini - ps39
Homo_sapiens - ps40
p-value
5e-49
3e-37
1e-25
7e-14
4e-02
Log-odds
0.5
1.0
1.5
2.0
2.5
3.0
3.5
4.0

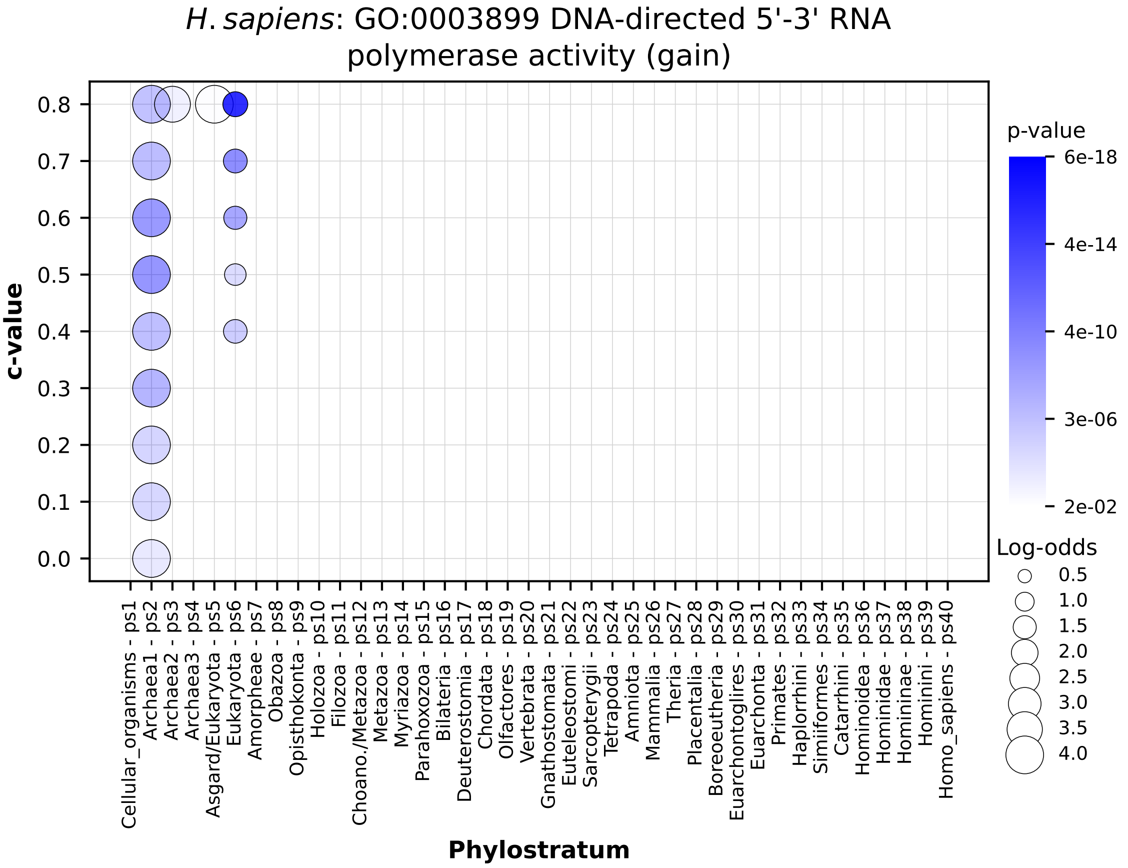
*H. sapiens*: GO:0003899 DNA-directed 5'-3' RNA polymerase activity (gain)
c-value
0.0
0.1
0.2
0.3
0.4
0.5
0.6
0.7
0.8
p-value
6e-18
4e-14
4e-10
3e-06
2e-02
Log-odds
0.5
1.0
1.5
2.0
2.5
3.0
3.5
4.0
Cellular_organisms - ps1
Archaea1 - ps2
Archaea2 - ps3
Archaea3 - ps4
Asgard/Eukaryota - ps5
Eukaryota - ps6
Amorpheae - ps7
Obazoa - ps8
Opisthokonta - ps9
Holozoa - ps10
Filozoa - ps11
Choano./Metazoa - ps12
Metazoa - ps13
Myriazoa - ps14
Parahoxozoa - ps15
Bilateria - ps16
Deuterostomia - ps17
Chordata - ps18
Olfactores - ps19
Vertebrata - ps20
Gnathostomata - ps21
Euteleostomi - ps22
Sarcopterygii - ps23
Tetrapoda - ps24
Amniota - ps25
Mammalia - ps26
Theria - ps27
Placentalia - ps28
Boreoeutheria - ps29
Euarchontoglires - ps30
Euarchonta - ps31
Primates - ps32
Haplorrhini - ps33
Simiiformes - ps34
Catarrhini - ps35
Hominoidea - ps36
Hominidae - ps37
Homininae - ps38
Hominini - ps39
Homo_sapiens - ps40
Phylostratum

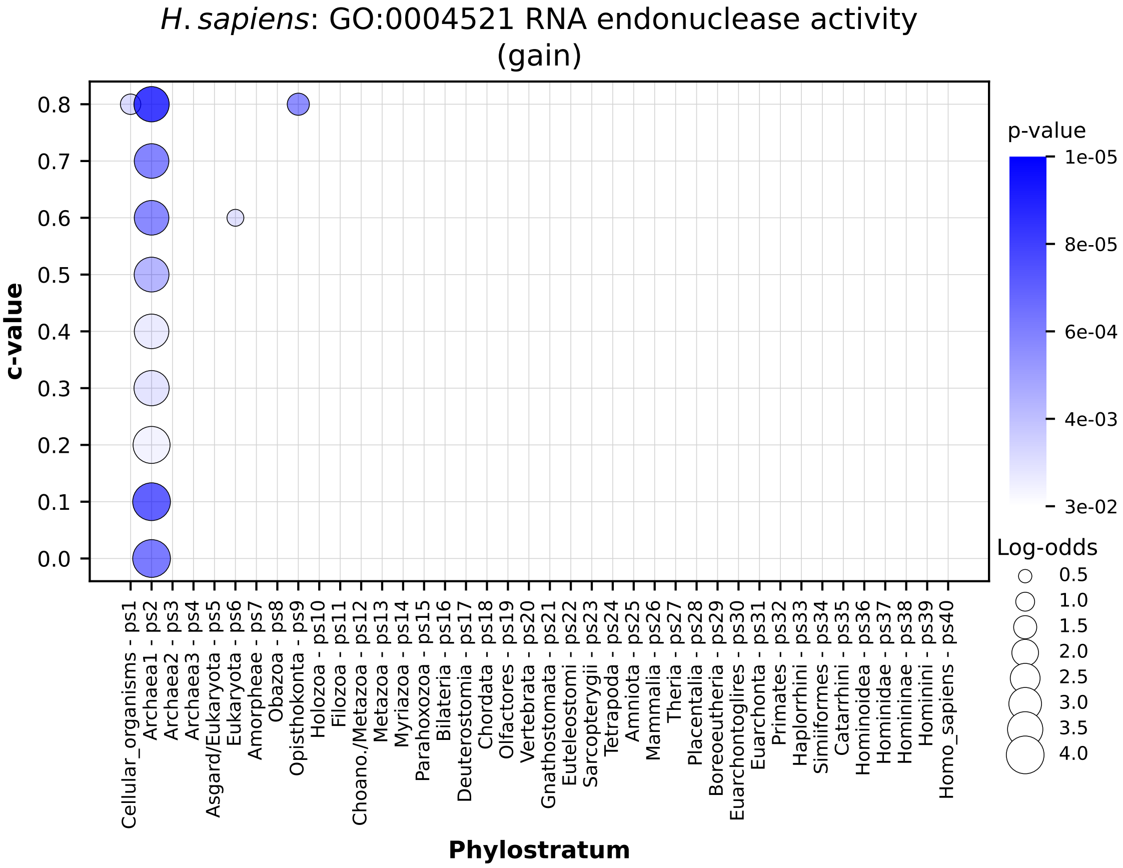

H. sapiens: GO:0004521 RNA endonuclease activity
(gain)
c-value
0.8
0.7
0.6
0.5
0.4
0.3
0.2
0.1
0.0
p-value
1e-05
8e-05
6e-04
4e-03
3e-02
Log-odds
0.5
1.0
1.5
2.0
2.5
3.0
3.5
4.0
Cellular_organisms - ps1
Archaea1 - ps2
Archaea2 - ps3
Archaea3 - ps4
Asgard/Eukaryota - ps5
Eukaryota - ps6
Amorpheae - ps7
Obazoa - ps8
Opisthokonta - ps9
Holozoa - ps10
Filozoa - ps11
Choano./Metazoa - ps12
Metazoa - ps13
Myriazoa - ps14
Parahoxozoa - ps15
Bilateria - ps16
Deuterostomia - ps17
Chordata - ps18
Olfactores - ps19
Vertebrata - ps20
Gnathostomata - ps21
Euteleostomi - ps22
Sarcopterygii - ps23
Tetrapoda - ps24
Amniota - ps25
Mammalia - ps26
Theria - ps27
Placentalia - ps28
Boreoeutheria - ps29
Euarchontoglires - ps30
Euarchonta - ps31
Primates - ps32
Haplorrhini - ps33
Simiiformes - ps34
Catarrhini - ps35
Hominoidea - ps36
Hominidae - ps37
Homininae - ps38
Hominini - ps39
Homo_sapiens - ps40
Phylostratum

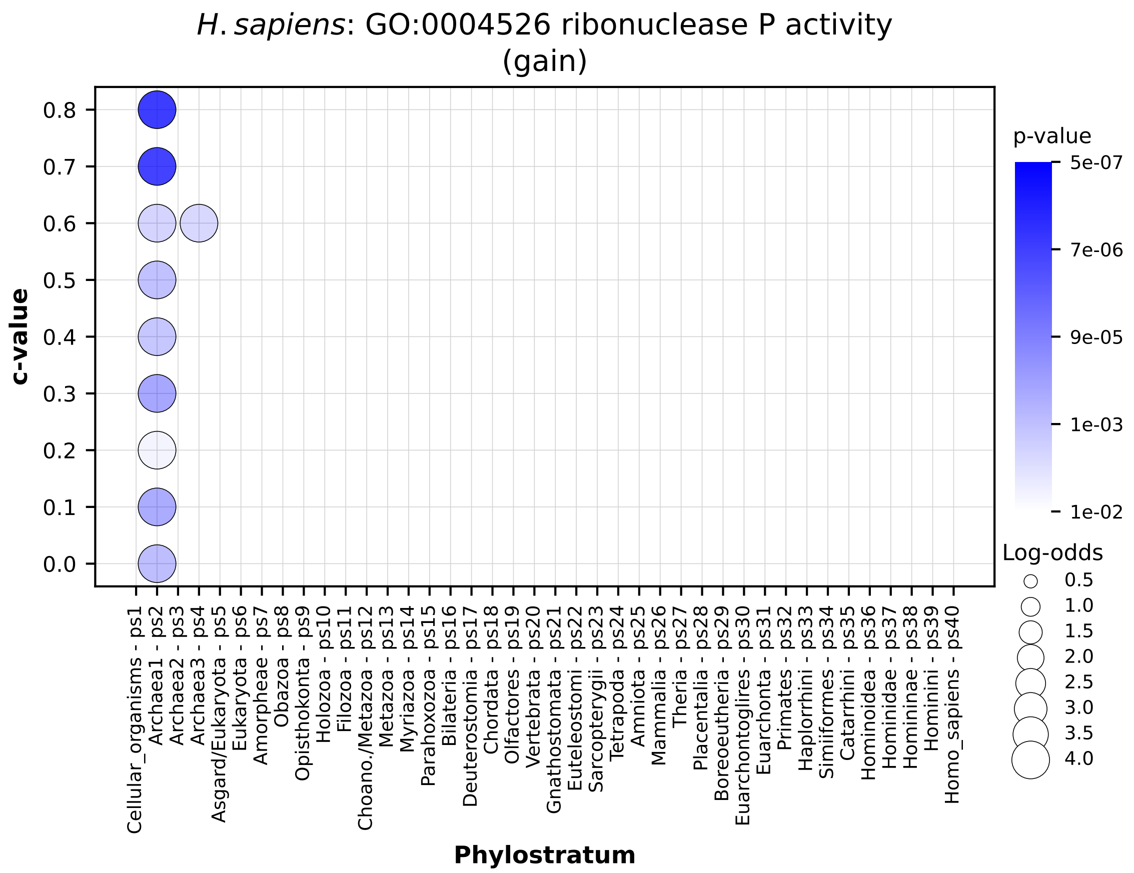

H. sapiens: GO:0004526 ribonuclease P activity
(gain)
c-value
0.8
0.7
0.6
0.5
0.4
0.3
0.2
0.1
0.0
p-value
5e-07
7e-06
9e-05
1e-03
1e-02
Log-odds
0.5
1.0
1.5
2.0
2.5
3.0
3.5
4.0
Cellular_organisms - ps1
Archaea1 - ps2
Archaea2 - ps3
Archaea3 - ps4
Asgard/Eukaryota - ps5
Eukaryota - ps6
Amorpheae - ps7
Obazoa - ps8
Opisthokonta - ps9
Holozoa - ps10
Filozoa - ps11
Choano./Metazoa - ps12
Metazoa - ps13
Myriazoa - ps14
Parahoxozoa - ps15
Bilateria - ps16
Deuterostomia - ps17
Chordata - ps18
Olfactores - ps19
Vertebrata - ps20
Gnathostomata - ps21
Euteleostomi - ps22
Sarcopterygii - ps23
Tetrapoda - ps24
Amniota - ps25
Mammalia - ps26
Theria - ps27
Placentalia - ps28
Boreoeutheria - ps29
Euarchontoglires - ps30
Euarchonta - ps31
Primates - ps32
Haplorrhini - ps33
Simiiformes - ps34
Catarrhini - ps35
Hominoidea - ps36
Hominidae - ps37
Homininae - ps38
Hominini - ps39
Homo_sapiens - ps40
Phylostratum

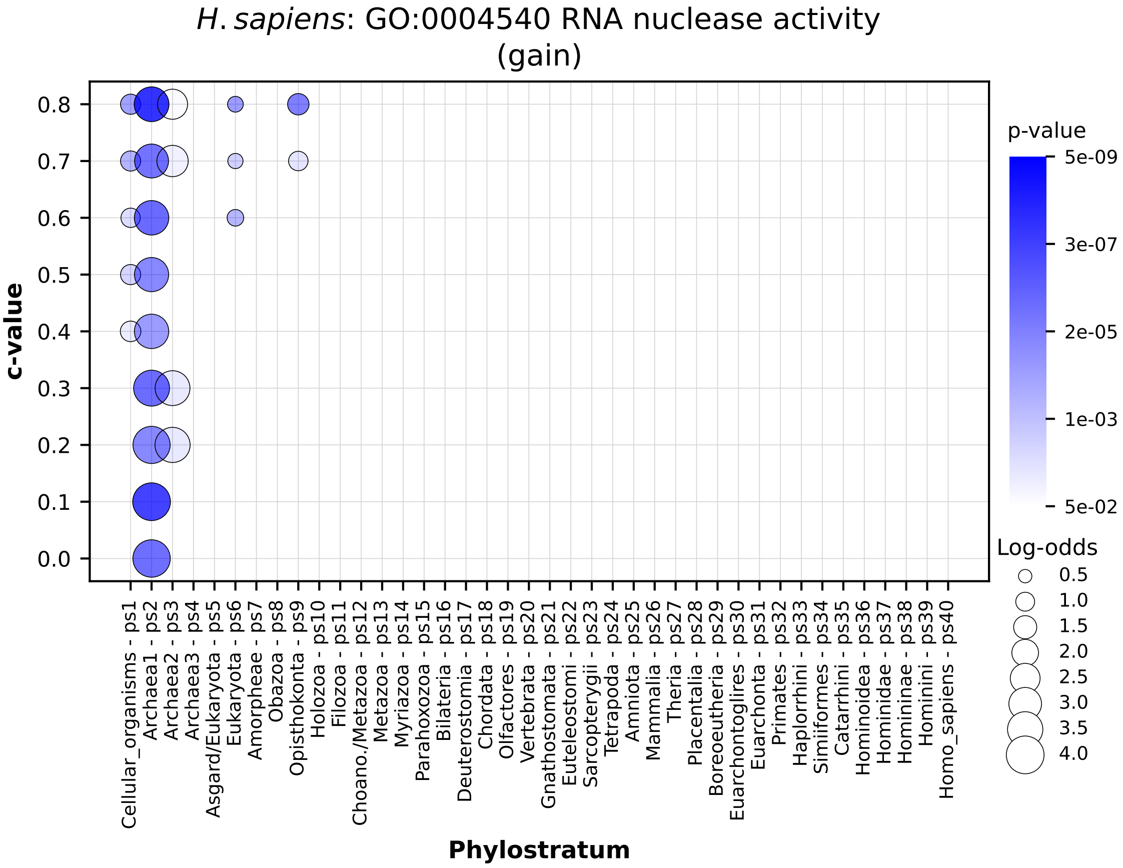

H. sapiens: GO:0004540 RNA nuclease activity
(gain)
c-value
0.8
0.7
0.6
0.5
0.4
0.3
0.2
0.1
0.0
p-value
5e-09
3e-07
2e-05
1e-03
5e-02
Log-odds
0.5
1.0
1.5
2.0
2.5
3.0
3.5
4.0
Cellular_organisms - ps1
Archaea1 - ps2
Archaea2 - ps3
Archaea3 - ps4
Asgard/Eukaryota - ps5
Eukaryota - ps6
Amorpheae - ps7
Obazoa - ps8
Opisthokonta - ps9
Holozoa - ps10
Filozoa - ps11
Choano./Metazoa - ps12
Metazoa - ps13
Myriazoa - ps14
Parahoxozoa - ps15
Bilateria - ps16
Deuterostomia - ps17
Chordata - ps18
Olfactores - ps19
Vertebrata - ps20
Gnathostomata - ps21
Euteleostomi - ps22
Sarcopterygii - ps23
Tetrapoda - ps24
Amniota - ps25
Mammalia - ps26
Theria - ps27
Placentalia - ps28
Boreoeutheria - ps29
Euarchontoglires - ps30
Euarchonta - ps31
Primates - ps32
Haplorrhini - ps33
Simiiformes - ps34
Catarrhini - ps35
Hominoidea - ps36
Hominidae - ps37
Homininae - ps38
Hominini - ps39
Homo_sapiens - ps40
Phylostratum

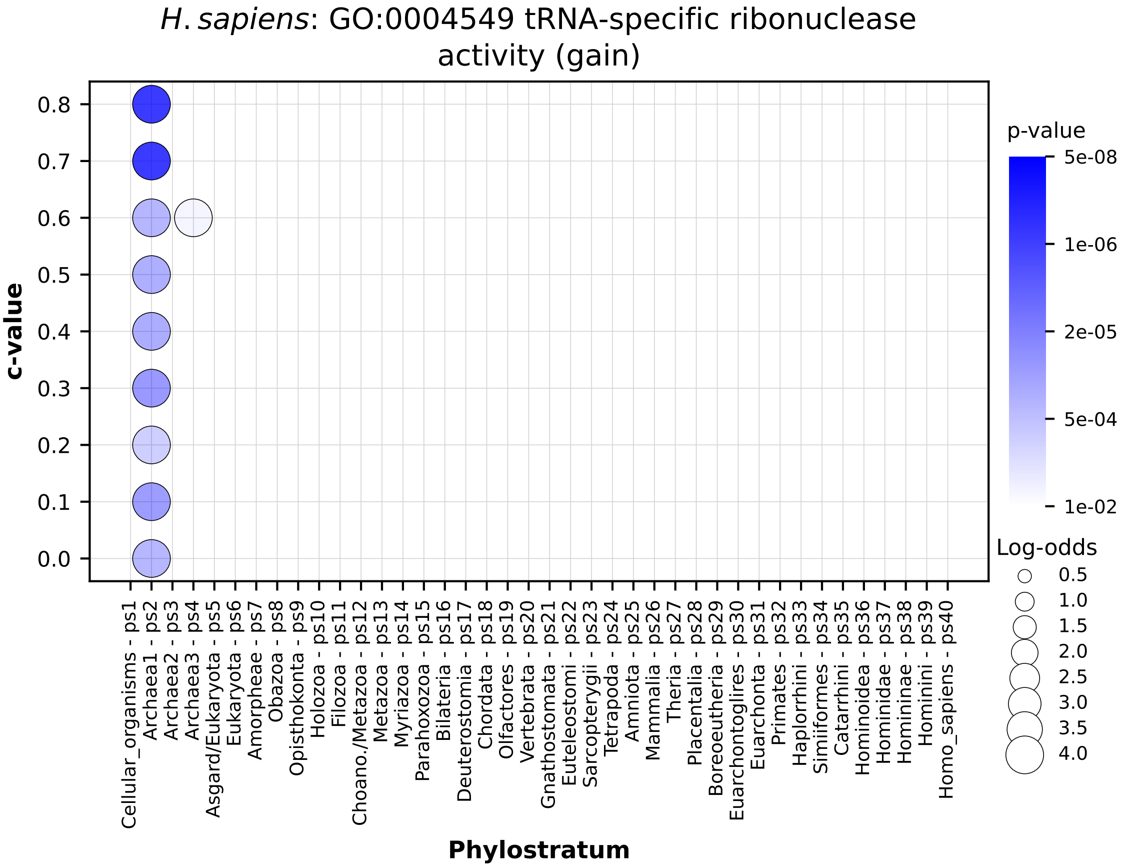

H. sapiens: GO:0004549 tRNA-specific ribonuclease activity (gain)
c-value
0.8
0.7
0.6
0.5
0.4
0.3
0.2
0.1
0.0
Phylostratum
Cellular_organisms - ps1
Archaea1 - ps2
Archaea2 - ps3
Archaea3 - ps4
Asgard/Eukaryota - ps5
Eukaryota - ps6
Amorpheae - ps7
Obazoa - ps8
Opisthokonta - ps9
Holozoa - ps10
Filozoa - ps11
Choano./Metazoa - ps12
Metazoa - ps13
Myriazoa - ps14
Parahoxozoa - ps15
Bilateria - ps16
Deuterostomia - ps17
Chordata - ps18
Olfactores - ps19
Vertebrata - ps20
Gnathostomata - ps21
Euteleostomi - ps22
Sarcopterygii - ps23
Tetrapoda - ps24
Amniota - ps25
Mammalia - ps26
Theria - ps27
Placentalia - ps28
Boreoeutheria - ps29
Euarchontoglires - ps30
Euarchonta - ps31
Primates - ps32
Haplorrhini - ps33
Simiiformes - ps34
Catarrhini - ps35
Hominoidea - ps36
Hominidae - ps37
Homininae - ps38
Hominini - ps39
Homo_sapiens - ps40
p-value
5e-08
1e-06
2e-05
5e-04
1e-02
Log-odds
0.5
1.0
1.5
2.0
2.5
3.0
3.5
4.0

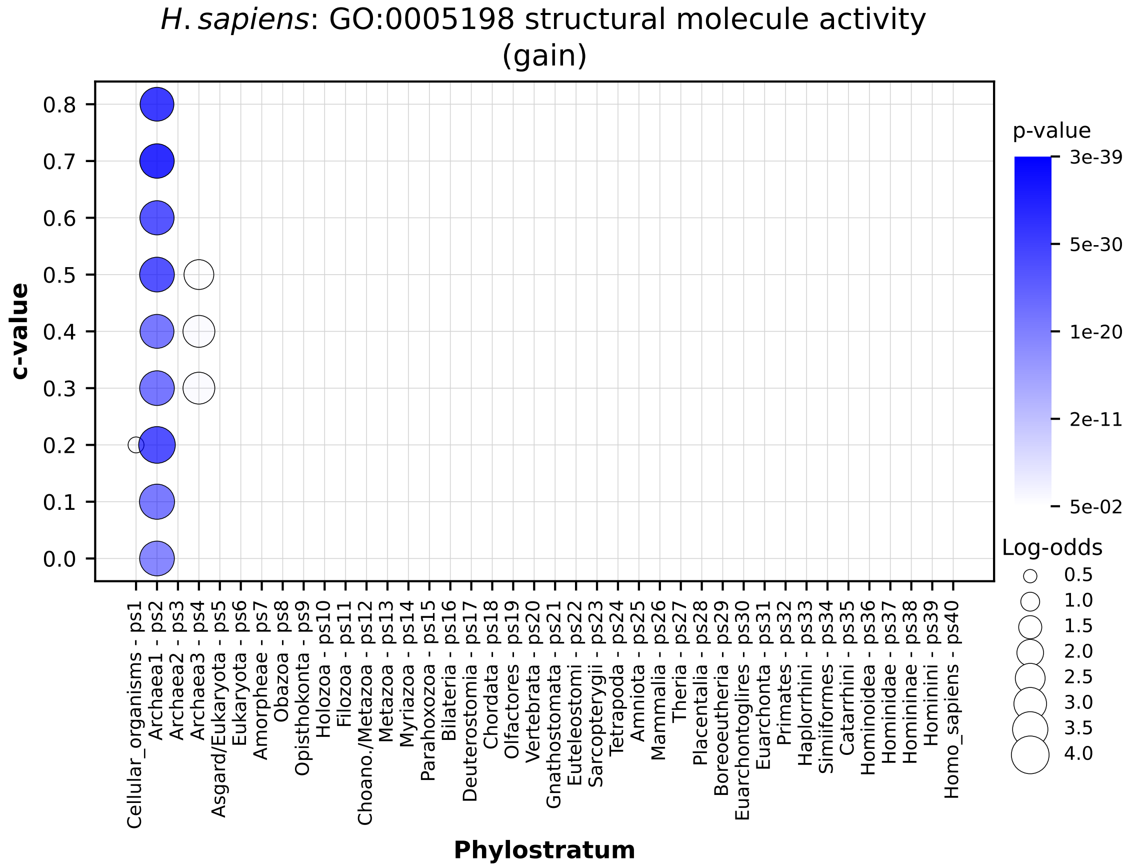

H. sapiens: GO:0005198 structural molecule activity
(gain)
c-value
0.8
0.7
0.6
0.5
0.4
0.3
0.2
0.1
0.0
p-value
3e-39
5e-30
1e-20
2e-11
5e-02
Log-odds
0.5
1.0
1.5
2.0
2.5
3.0
3.5
4.0
Cellular_organisms - ps1
Archaea1 - ps2
Archaea2 - ps3
Archaea3 - ps4
Asgard/Eukaryota - ps5
Eukaryota - ps6
Amorpheae - ps7
Obazoa - ps8
Opisthokonta - ps9
Holozoa - ps10
Filozoa - ps11
Choano./Metazoa - ps12
Metazoa - ps13
Myriazoa - ps14
Parahoxozoa - ps15
Bilateria - ps16
Deuterostomia - ps17
Chordata - ps18
Olfactores - ps19
Vertebrata - ps20
Gnathostomata - ps21
Euteleostomi - ps22
Sarcopterygii - ps23
Tetrapoda - ps24
Amniota - ps25
Mammalia - ps26
Theria - ps27
Placentalia - ps28
Boreoeutheria - ps29
Euarchontoglires - ps30
Euarchonta - ps31
Primates - ps32
Haplorrhini - ps33
Simiiformes - ps34
Catarrhini - ps35
Hominoidea - ps36
Hominidae - ps37
Homininae - ps38
Hominini - ps39
Homo_sapiens - ps40
Phylostratum

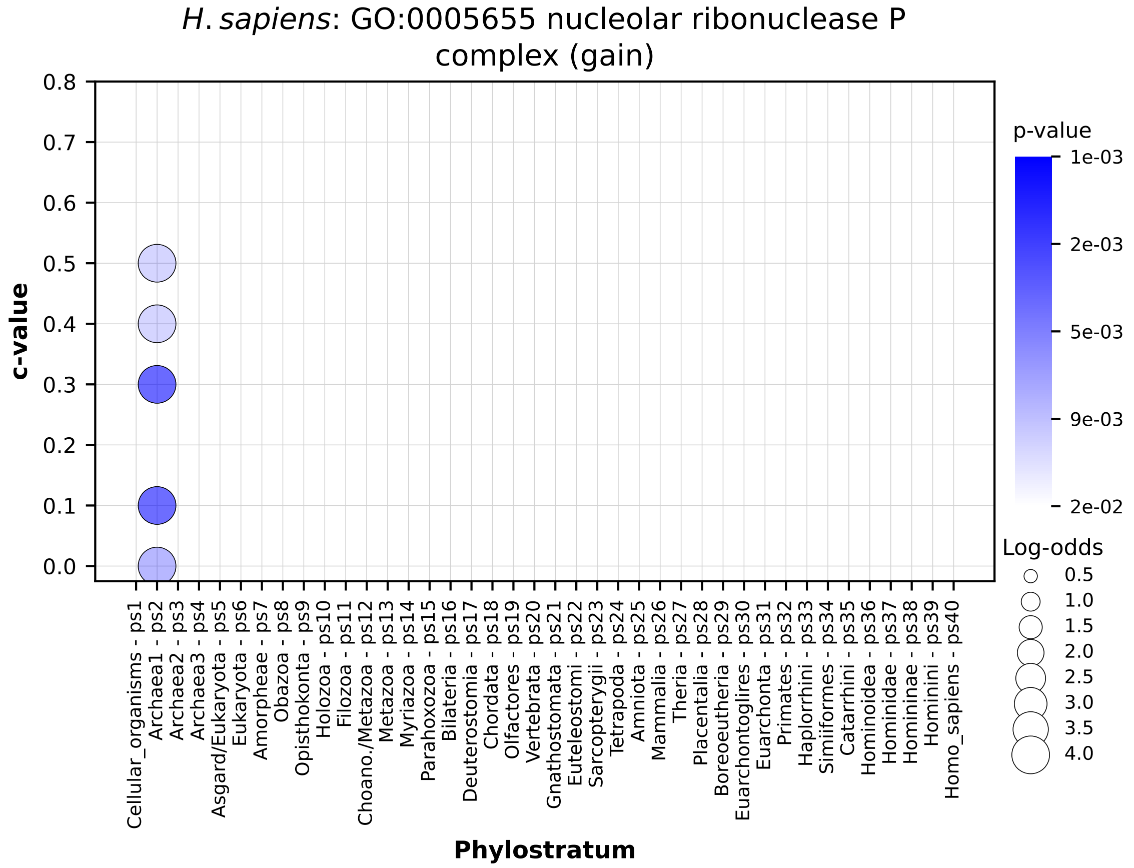

H. sapiens: GO:0005655 nucleolar ribonuclease P complex (gain)
c-value
0.0
0.1
0.2
0.3
0.4
0.5
0.6
0.7
0.8
Phylostratum
Cellular_organisms - ps1
Archaea1 - ps2
Archaea2 - ps3
Archaea3 - ps4
Asgard/Eukaryota - ps5
Eukaryota - ps6
Amorpheae - ps7
Obazoa - ps8
Opisthokonta - ps9
Holozoa - ps10
Filozoa - ps11
Choano./Metazoa - ps12
Metazoa - ps13
Myriazoa - ps14
Parahoxozoa - ps15
Bilateria - ps16
Deuterostomia - ps17
Chordata - ps18
Olfactores - ps19
Vertebrata - ps20
Gnathostomata - ps21
Euteleostomi - ps22
Sarcopterygii - ps23
Tetrapoda - ps24
Amniota - ps25
Mammalia - ps26
Theria - ps27
Placentalia - ps28
Boreoeutheria - ps29
Euarchontoglires - ps30
Euarchonta - ps31
Primates - ps32
Haplorrhini - ps33
Simiiformes - ps34
Catarrhini - ps35
Hominoidea - ps36
Hominidae - ps37
Homininae - ps38
Hominini - ps39
Homo_sapiens - ps40
p-value
1e-03
2e-03
5e-03
9e-03
2e-02
Log-odds
0.5
1.0
1.5
2.0
2.5
3.0
3.5
4.0

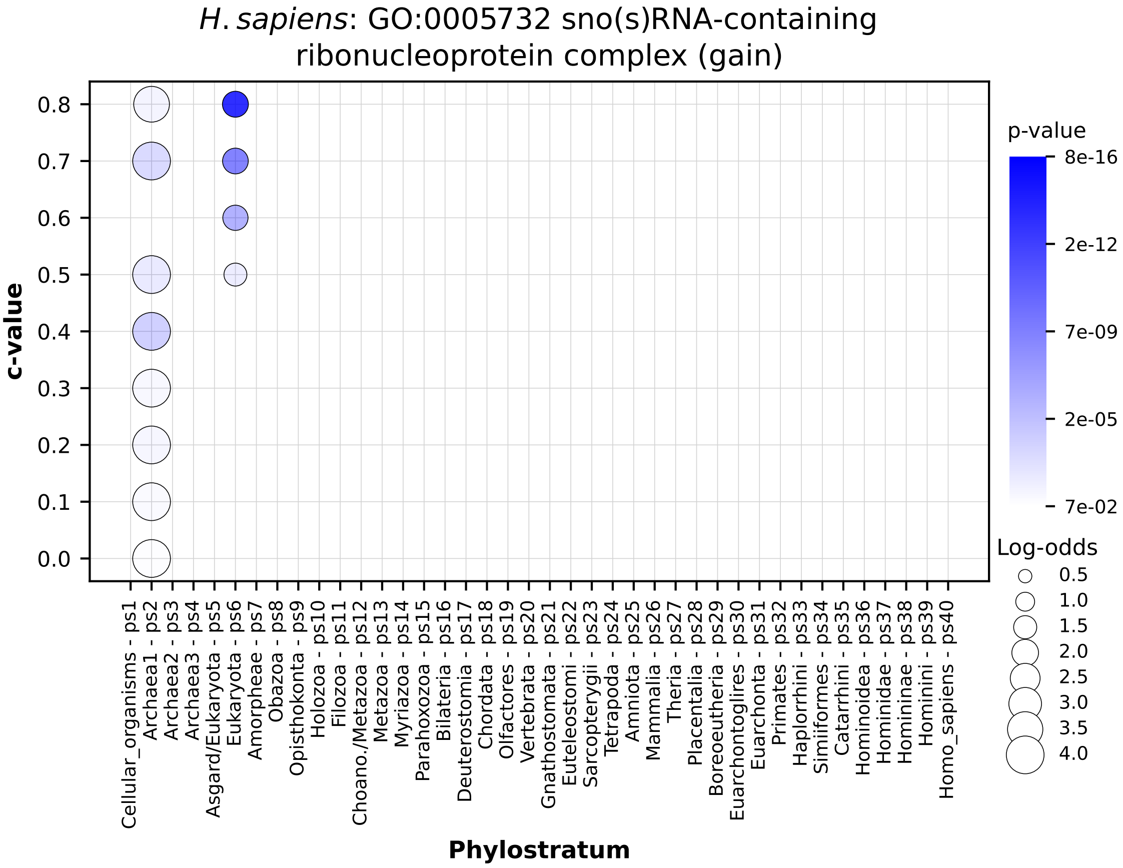
H. sapiens: GO:0005732 sno(s)RNA-containing ribonucleoprotein complex (gain)
c-value
0.8
0.7
0.6
0.5
0.4
0.3
0.2
0.1
0.0
p-value
8e-16
2e-12
7e-09
2e-05
7e-02
Log-odds
0.5
1.0
1.5
2.0
2.5
3.0
3.5
4.0
Cellular_organisms - ps1
Archaea1 - ps2
Archaea2 - ps3
Archaea3 - ps4
Asgard/Eukaryota - ps5
Eukaryota - ps6
Amorpheae - ps7
Obazoa - ps8
Opisthokonta - ps9
Holozoa - ps10
Filozoa - ps11
Choano./Metazoa - ps12
Metazoa - ps13
Myriazoa - ps14
Parahoxozoa - ps15
Bilateria - ps16
Deuterostomia - ps17
Chordata - ps18
Olfactores - ps19
Vertebrata - ps20
Gnathostomata - ps21
Euteleostomi - ps22
Sarcopterygii - ps23
Tetrapoda - ps24
Amniota - ps25
Mammalia - ps26
Theria - ps27
Placentalia - ps28
Boreoeutheria - ps29
Euarchontoglires - ps30
Euarchonta - ps31
Primates - ps32
Haplorrhini - ps33
Simiiformes - ps34
Catarrhini - ps35
Hominoidea - ps36
Hominidae - ps37
Homininae - ps38
Hominini - ps39
Homo_sapiens - ps40
Phylostratum

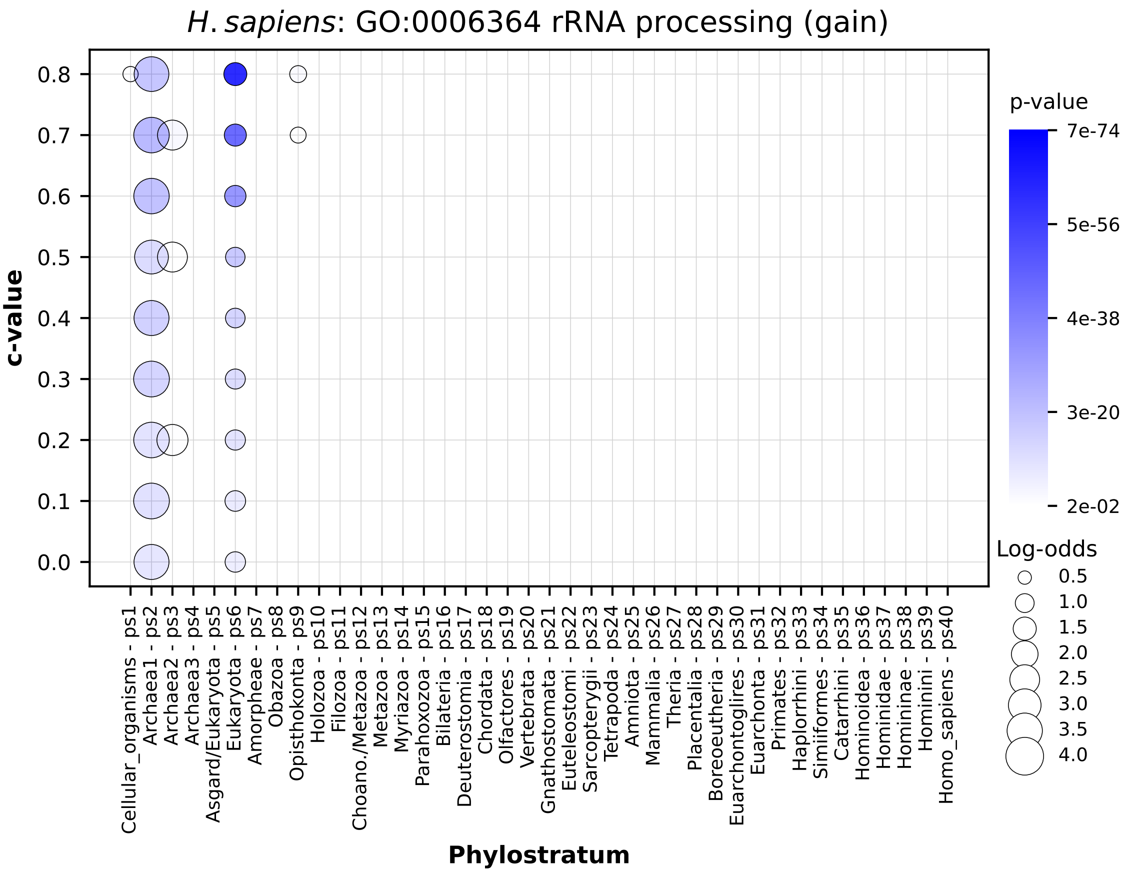

H. sapiens: GO:0006364 rRNA processing (gain)
c-value
0.0
0.1
0.2
0.3
0.4
0.5
0.6
0.7
0.8
Phylostratum
Cellular_organisms - ps1
Archaea1 - ps2
Archaea2 - ps3
Archaea3 - ps4
Asgard/Eukaryota - ps5
Eukaryota - ps6
Amorpheae - ps7
Obazoa - ps8
Opisthokonta - ps9
Holozoa - ps10
Filozoa - ps11
Choano./Metazoa - ps12
Metazoa - ps13
Myriazoa - ps14
Parahoxozoa - ps15
Bilateria - ps16
Deuterostomia - ps17
Chordata - ps18
Olfactores - ps19
Vertebrata - ps20
Gnathostomata - ps21
Euteleostomi - ps22
Sarcopterygii - ps23
Tetrapoda - ps24
Amniota - ps25
Mammalia - ps26
Theria - ps27
Placentalia - ps28
Boreoeutheria - ps29
Euarchontoglires - ps30
Euarchonta - ps31
Primates - ps32
Haplorrhini - ps33
Simiiformes - ps34
Catarrhini - ps35
Hominoidea - ps36
Hominidae - ps37
Homininae - ps38
Hominini - ps39
Homo_sapiens - ps40
p-value
7e-74
5e-56
4e-38
3e-20
2e-02
Log-odds
0.5
1.0
1.5
2.0
2.5
3.0
3.5
4.0

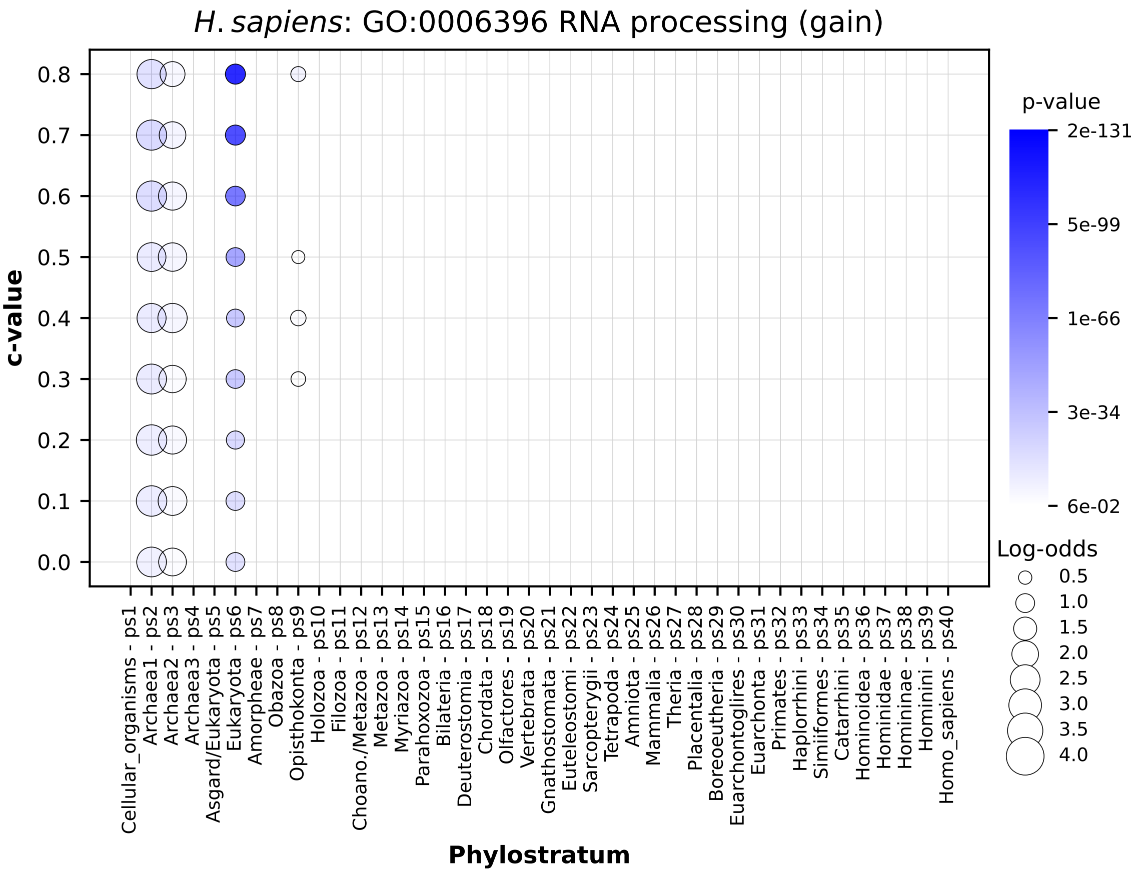

H. sapiens: GO:0006396 RNA processing (gain)
c-value
0.8
0.7
0.6
0.5
0.4
0.3
0.2
0.1
0.0
p-value
2e-131
5e-99
1e-66
3e-34
6e-02
Log-odds
0.5
1.0
1.5
2.0
2.5
3.0
3.5
4.0
Cellular_organisms - ps1
Archaea1 - ps2
Archaea2 - ps3
Archaea3 - ps4
Asgard/Eukaryota - ps5
Eukaryota - ps6
Amorpheae - ps7
Obazoa - ps8
Opisthokonta - ps9
Holozoa - ps10
Filozoa - ps11
Choano./Metazoa - ps12
Metazoa - ps13
Myriazoa - ps14
Parahoxozoa - ps15
Bilateria - ps16
Deuterostomia - ps17
Chordata - ps18
Olfactores - ps19
Vertebrata - ps20
Gnathostomata - ps21
Euteleostomi - ps22
Sarcopterygii - ps23
Tetrapoda - ps24
Amniota - ps25
Mammalia - ps26
Theria - ps27
Placentalia - ps28
Boreoeutheria - ps29
Euarchontoglires - ps30
Euarchonta - ps31
Primates - ps32
Haplorrhini - ps33
Simiiformes - ps34
Catarrhini - ps35
Hominoidea - ps36
Hominidae - ps37
Homininae - ps38
Hominini - ps39
Homo_sapiens - ps40
Phylostratum

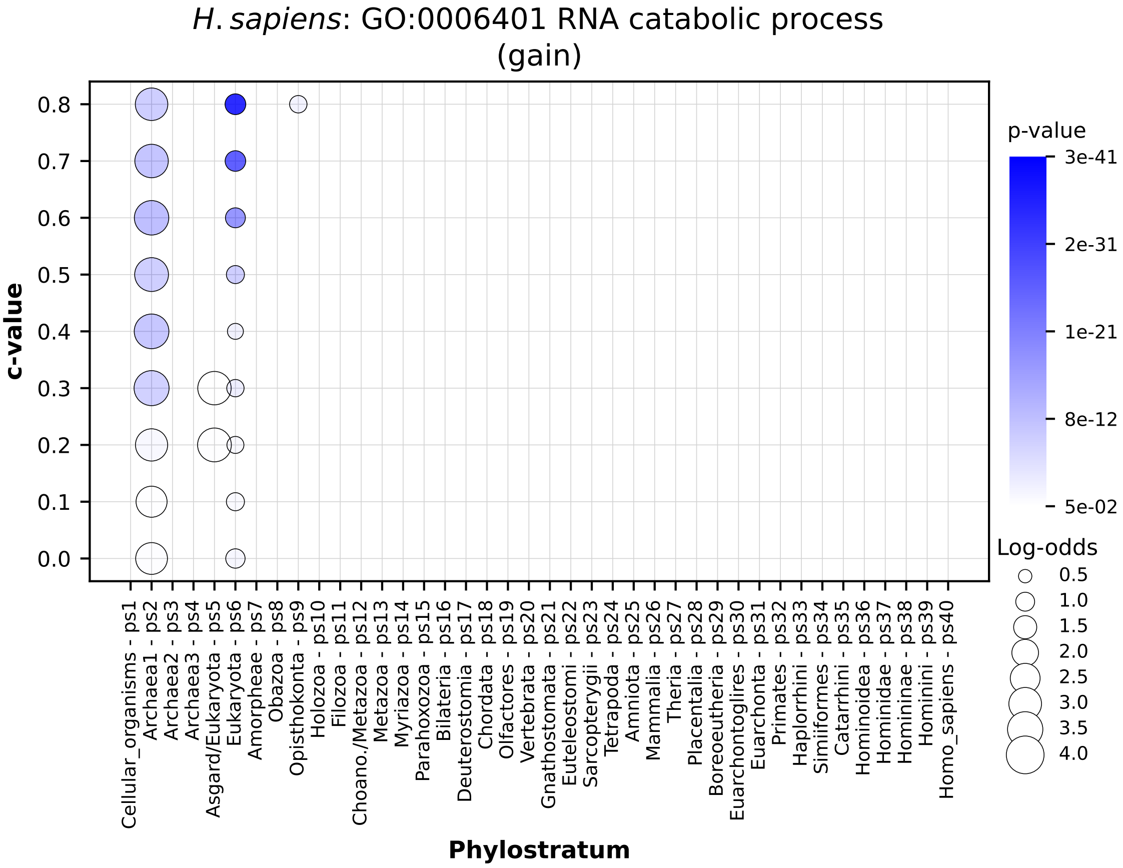

H. sapiens: GO:0006401 RNA catabolic process
(gain)
c-value
0.8
0.7
0.6
0.5
0.4
0.3
0.2
0.1
0.0
p-value
3e-41
2e-31
1e-21
8e-12
5e-02
Log-odds
0.5
1.0
1.5
2.0
2.5
3.0
3.5
4.0
Cellular_organisms - ps1
Archaea1 - ps2
Archaea2 - ps3
Archaea3 - ps4
Asgard/Eukaryota - ps5
Eukaryota - ps6
Amorpheae - ps7
Obazoa - ps8
Opisthokonta - ps9
Holozoa - ps10
Filozoa - ps11
Choano./Metazoa - ps12
Metazoa - ps13
Myriazoa - ps14
Parahoxozoa - ps15
Bilateria - ps16
Deuterostomia - ps17
Chordata - ps18
Olfactores - ps19
Vertebrata - ps20
Gnathostomata - ps21
Euteleostomi - ps22
Sarcopterygii - ps23
Tetrapoda - ps24
Amniota - ps25
Mammalia - ps26
Theria - ps27
Placentalia - ps28
Boreoeutheria - ps29
Euarchontoglires - ps30
Euarchonta - ps31
Primates - ps32
Haplorrhini - ps33
Simiiformes - ps34
Catarrhini - ps35
Hominoidea - ps36
Hominidae - ps37
Homininae - ps38
Hominini - ps39
Homo_sapiens - ps40
Phylostratum

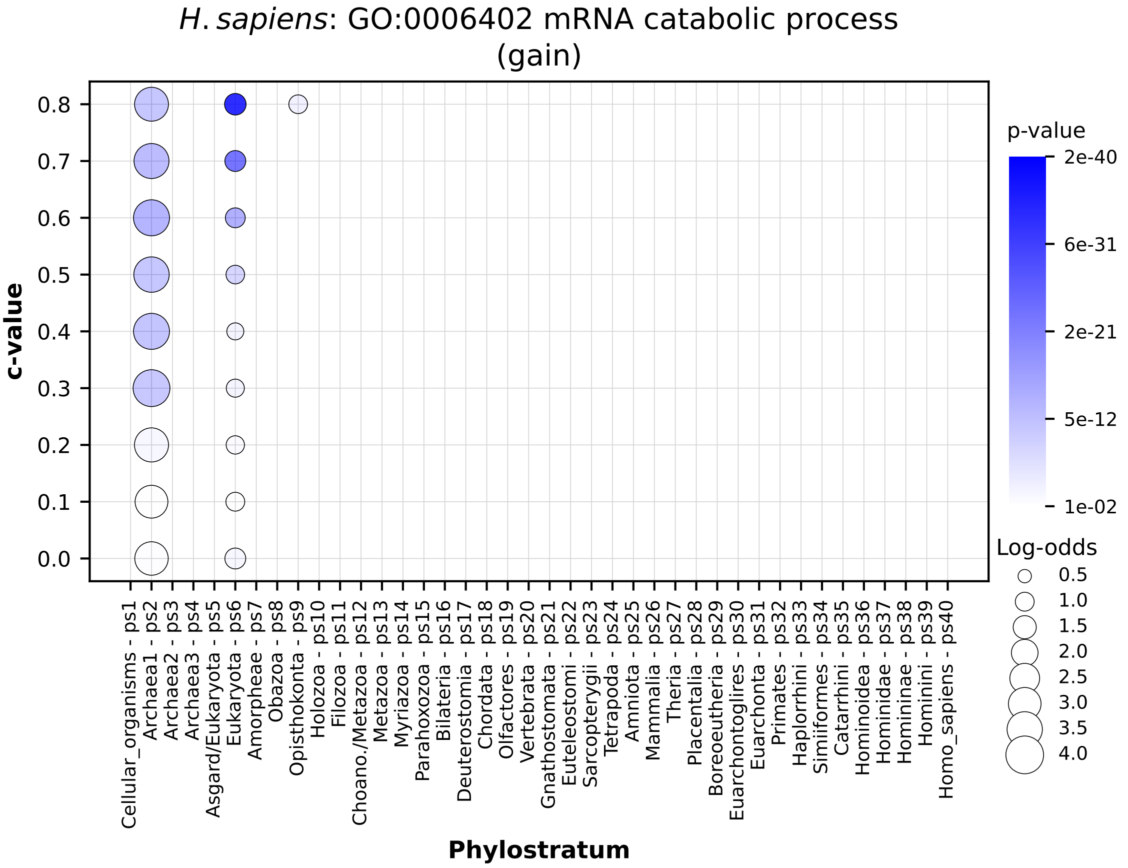

$H. sapiens$: GO:0006402 mRNA catabolic process
(gain)
c-value
0.8
0.7
0.6
0.5
0.4
0.3
0.2
0.1
0.0
p-value
2e-40
6e-31
2e-21
5e-12
1e-02
Log-odds
0.5
1.0
1.5
2.0
2.5
3.0
3.5
4.0
Cellular_organisms - ps1
Archaea1 - ps2
Archaea2 - ps3
Archaea3 - ps4
Asgard/Eukaryota - ps5
Eukaryota - ps6
Amorpheae - ps7
Obazoa - ps8
Opisthokonta - ps9
Holozoa - ps10
Filozoa - ps11
Choano./Metazoa - ps12
Metazoa - ps13
Myriazoa - ps14
Parahoxozoa - ps15
Bilateria - ps16
Deuterostomia - ps17
Chordata - ps18
Olfactores - ps19
Vertebrata - ps20
Gnathostomata - ps21
Euteleostomi - ps22
Sarcopterygii - ps23
Tetrapoda - ps24
Amniota - ps25
Mammalia - ps26
Theria - ps27
Placentalia - ps28
Boreoeutheria - ps29
Euarchontoglires - ps30
Euarchonta - ps31
Primates - ps32
Haplorrhini - ps33
Simiiformes - ps34
Catarrhini - ps35
Hominoidea - ps36
Hominidae - ps37
Homininae - ps38
Hominini - ps39
Homo_sapiens - ps40
Phylostratum

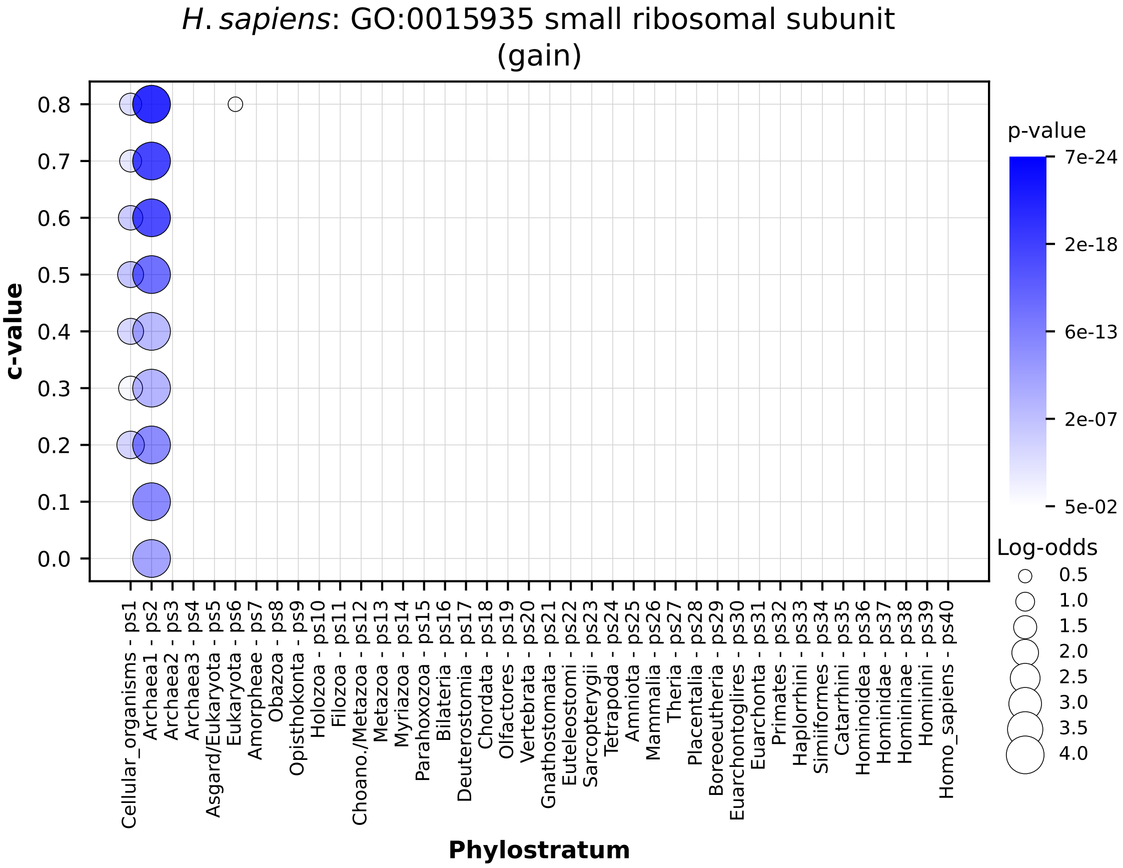

H. sapiens: GO:0015935 small ribosomal subunit
(gain)
c-value
0.8
0.7
0.6
0.5
0.4
0.3
0.2
0.1
0.0
p-value
7e-24
2e-18
6e-13
2e-07
5e-02
Log-odds
0.5
1.0
1.5
2.0
2.5
3.0
3.5
4.0
Cellular_organisms - ps1
Archaea1 - ps2
Archaea2 - ps3
Archaea3 - ps4
Asgard/Eukaryota - ps5
Eukaryota - ps6
Amorpheae - ps7
Obazoa - ps8
Opisthokonta - ps9
Holozoa - ps10
Filozoa - ps11
Choano./Metazoa - ps12
Metazoa - ps13
Myriazoa - ps14
Parahoxozoa - ps15
Bilateria - ps16
Deuterostomia - ps17
Chordata - ps18
Olfactores - ps19
Vertebrata - ps20
Gnathostomata - ps21
Euteleostomi - ps22
Sarcopterygii - ps23
Tetrapoda - ps24
Amniota - ps25
Mammalia - ps26
Theria - ps27
Placentalia - ps28
Boreoeutheria - ps29
Euarchontoglires - ps30
Euarchonta - ps31
Primates - ps32
Haplorrhini - ps33
Simiiformes - ps34
Catarrhini - ps35
Hominoidea - ps36
Hominidae - ps37
Homininae - ps38
Hominini - ps39
Homo_sapiens - ps40
Phylostratum

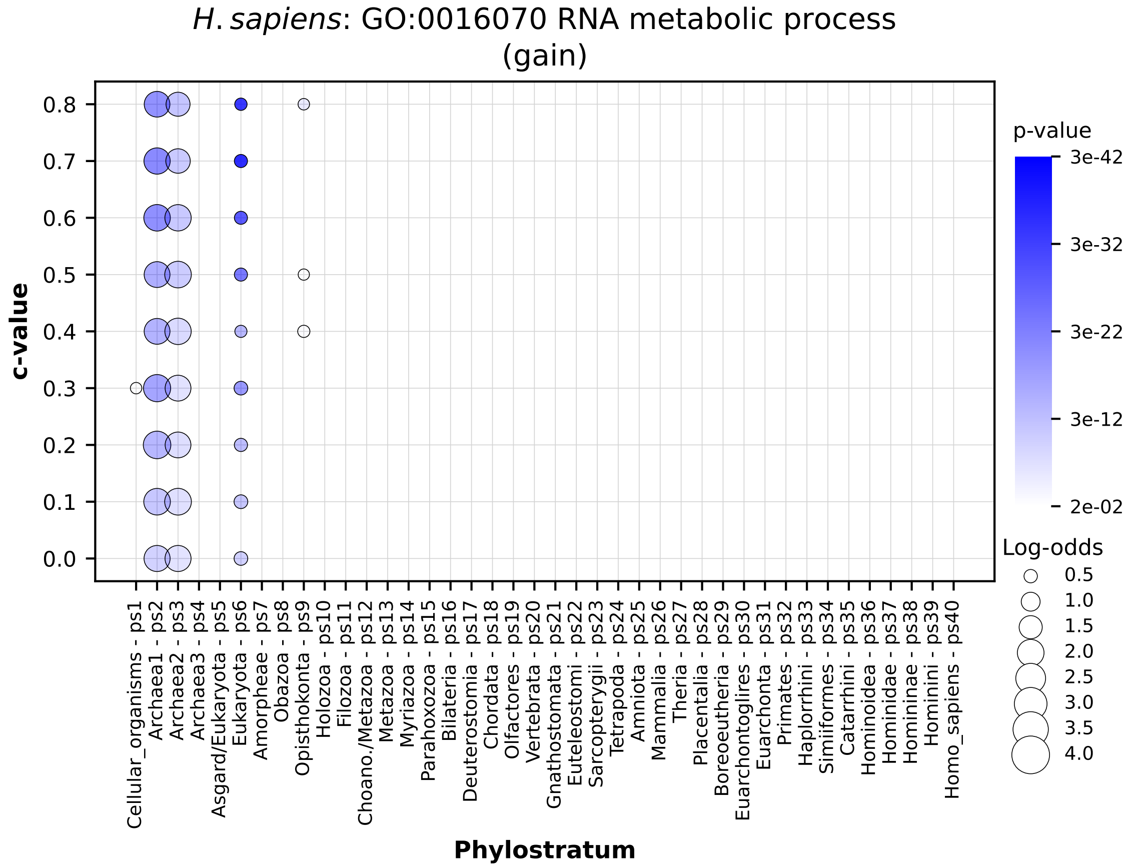

H. sapiens: GO:0016070 RNA metabolic process
(gain)
c-value
0.0
0.1
0.2
0.3
0.4
0.5
0.6
0.7
0.8
Phylostratum
Cellular_organisms - ps1
Archaea1 - ps2
Archaea2 - ps3
Archaea3 - ps4
Asgard/Eukaryota - ps5
Eukaryota - ps6
Amorpheae - ps7
Obazoa - ps8
Opisthokonta - ps9
Holozoa - ps10
Filozoa - ps11
Choano./Metazoa - ps12
Metazoa - ps13
Myriazoa - ps14
Parahoxozoa - ps15
Bilateria - ps16
Deuterostomia - ps17
Chordata - ps18
Olfactores - ps19
Vertebrata - ps20
Gnathostomata - ps21
Euteleostomi - ps22
Sarcopterygii - ps23
Tetrapoda - ps24
Amniota - ps25
Mammalia - ps26
Theria - ps27
Placentalia - ps28
Boreoeutheria - ps29
Euarchontoglires - ps30
Euarchonta - ps31
Primates - ps32
Haplorrhini - ps33
Simiiformes - ps34
Catarrhini - ps35
Hominoidea - ps36
Hominidae - ps37
Homininae - ps38
Hominini - ps39
Homo_sapiens - ps40
p-value
3e-42
3e-32
3e-22
3e-12
2e-02
Log-odds
0.5
1.0
1.5
2.0
2.5
3.0
3.5
4.0

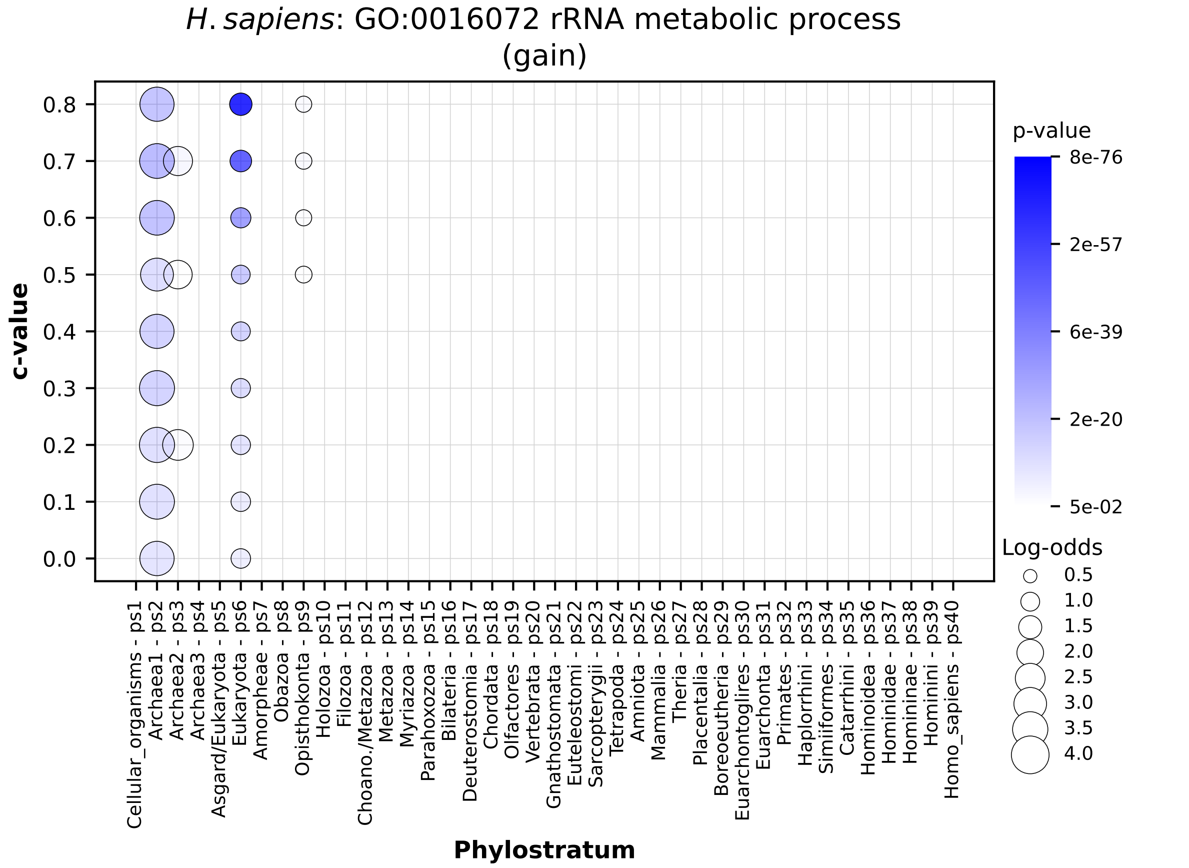

H. sapiens: GO:0016072 rRNA metabolic process
(gain)
c-value
0.8
0.7
0.6
0.5
0.4
0.3
0.2
0.1
0.0
Phylostratum
Cellular_organisms - ps1
Archaea1 - ps2
Archaea2 - ps3
Archaea3 - ps4
Asgard/Eukaryota - ps5
Eukaryota - ps6
Amorpheae - ps7
Obazoa - ps8
Opisthokonta - ps9
Holozoa - ps10
Filozoa - ps11
Choano./Metazoa - ps12
Metazoa - ps13
Myriazoa - ps14
Parahoxozoa - ps15
Bilateria - ps16
Deuterostomia - ps17
Chordata - ps18
Olfactores - ps19
Vertebrata - ps20
Gnathostomata - ps21
Euteleostomi - ps22
Sarcopterygii - ps23
Tetrapoda - ps24
Amniota - ps25
Mammalia - ps26
Theria - ps27
Placentalia - ps28
Boreoeutheria - ps29
Euarchontoglires - ps30
Euarchonta - ps31
Primates - ps32
Haplorrhini - ps33
Simiiformes - ps34
Catarrhini - ps35
Hominoidea - ps36
Hominidae - ps37
Homininae - ps38
Hominini - ps39
Homo_sapiens - ps40
p-value
8e-76
2e-57
6e-39
2e-20
5e-02
Log-odds
0.5
1.0
1.5
2.0
2.5
3.0
3.5
4.0

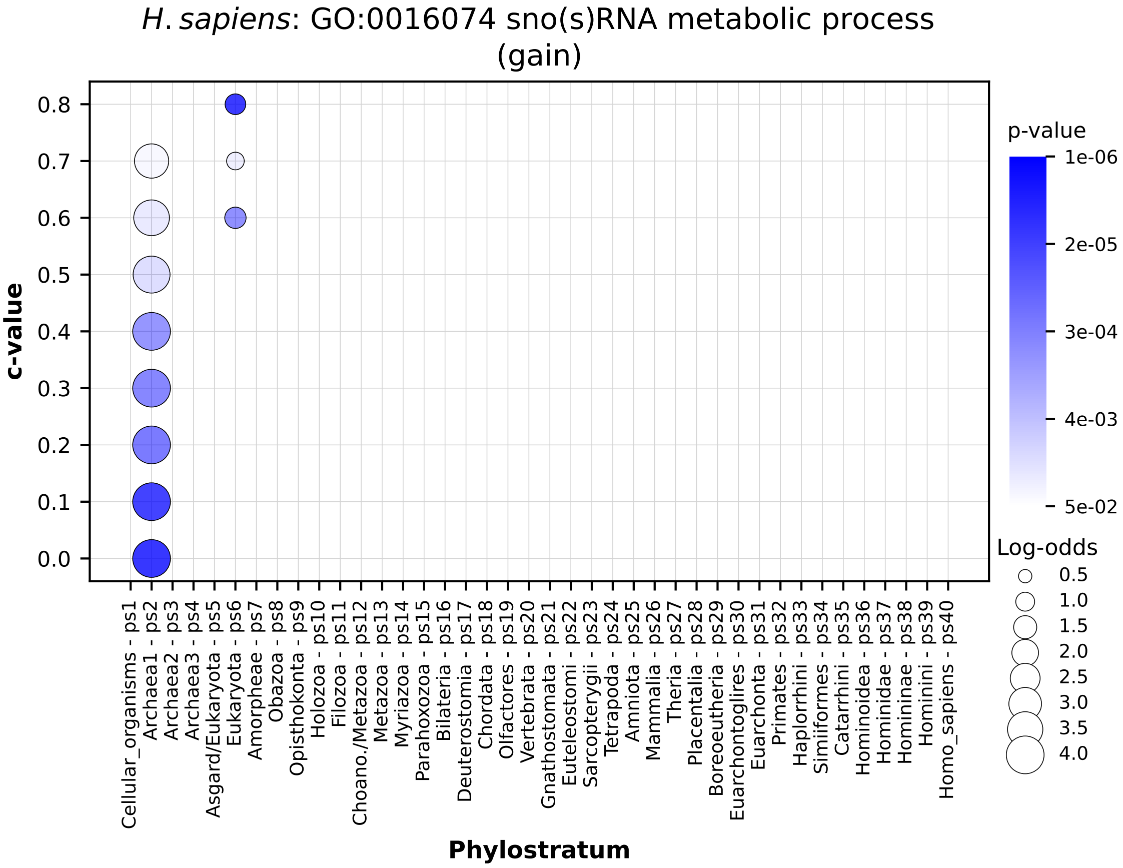

H. sapiens: GO:0016074 sno(s)RNA metabolic process
(gain)
c-value
0.8
0.7
0.6
0.5
0.4
0.3
0.2
0.1
0.0
p-value
1e-06
2e-05
3e-04
4e-03
5e-02
Log-odds
0.5
1.0
1.5
2.0
2.5
3.0
3.5
4.0
Cellular_organisms - ps1
Archaea1 - ps2
Archaea2 - ps3
Archaea3 - ps4
Asgard/Eukaryota - ps5
Eukaryota - ps6
Amorpheae - ps7
Obazoa - ps8
Opisthokonta - ps9
Holozoa - ps10
Filozoa - ps11
Choano./Metazoa - ps12
Metazoa - ps13
Myriazoa - ps14
Parahoxozoa - ps15
Bilateria - ps16
Deuterostomia - ps17
Chordata - ps18
Olfactores - ps19
Vertebrata - ps20
Gnathostomata - ps21
Euteleostomi - ps22
Sarcopterygii - ps23
Tetrapoda - ps24
Amniota - ps25
Mammalia - ps26
Theria - ps27
Placentalia - ps28
Boreoeutheria - ps29
Euarchontoglires - ps30
Euarchonta - ps31
Primates - ps32
Haplorrhini - ps33
Simiiformes - ps34
Catarrhini - ps35
Hominoidea - ps36
Hominidae - ps37
Homininae - ps38
Hominini - ps39
Homo_sapiens - ps40
Phylostratum

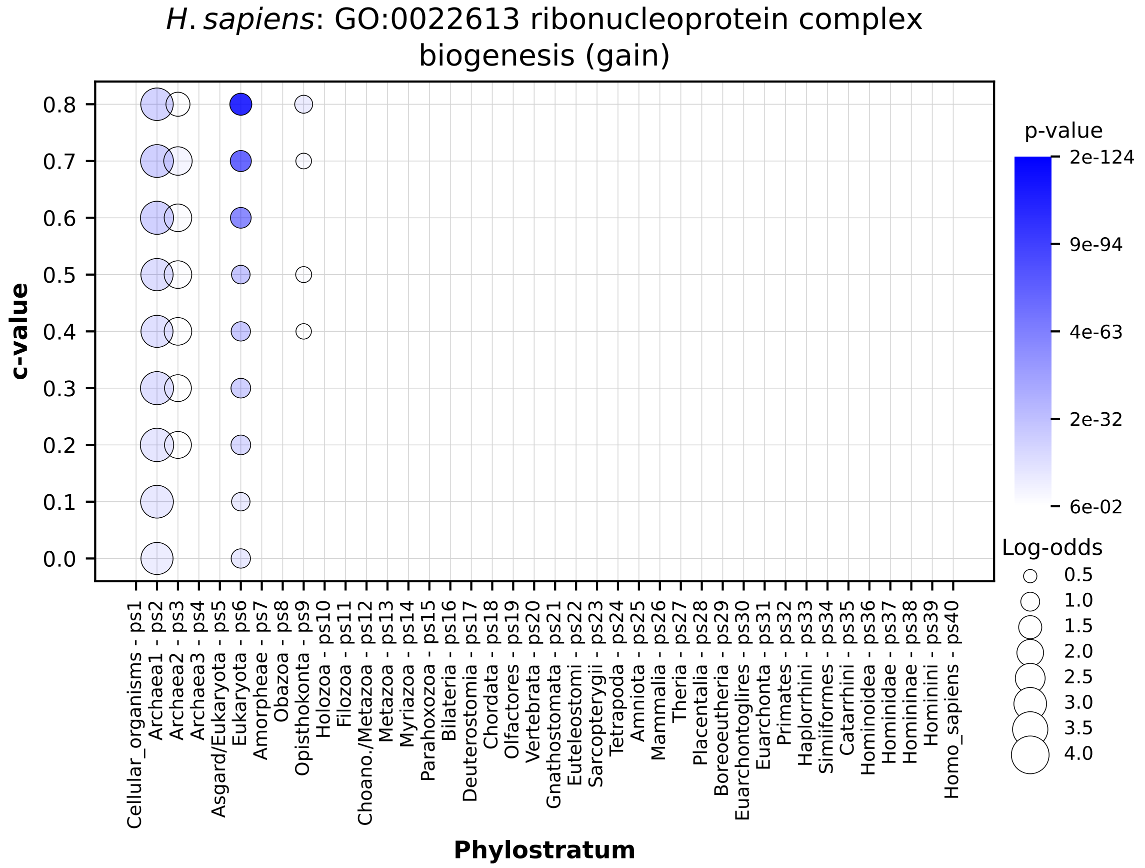

H. sapiens: GO:0022613 ribonucleoprotein complex biogenesis (gain)
c-value
0.0
0.1
0.2
0.3
0.4
0.5
0.6
0.7
0.8
Phylostratum
Cellular_organisms - ps1
Archaea1 - ps2
Archaea2 - ps3
Archaea3 - ps4
Asgard/Eukaryota - ps5
Eukaryota - ps6
Amorpheae - ps7
Obazoa - ps8
Opisthokonta - ps9
Holozoa - ps10
Filozoa - ps11
Choano./Metazoa - ps12
Metazoa - ps13
Myriazoa - ps14
Parahoxozoa - ps15
Bilateria - ps16
Deuterostomia - ps17
Chordata - ps18
Olfactores - ps19
Vertebrata - ps20
Gnathostomata - ps21
Euteleostomi - ps22
Sarcopterygii - ps23
Tetrapoda - ps24
Amniota - ps25
Mammalia - ps26
Theria - ps27
Placentalia - ps28
Boreoeutheria - ps29
Euarchontoglires - ps30
Euarchonta - ps31
Primates - ps32
Haplorrhini - ps33
Simiiformes - ps34
Catarrhini - ps35
Hominoidea - ps36
Hominidae - ps37
Homininae - ps38
Hominini - ps39
Homo_sapiens - ps40
p-value
2e-124
9e-94
4e-63
2e-32
6e-02
Log-odds
0.5
1.0
1.5
2.0
2.5
3.0
3.5
4.0

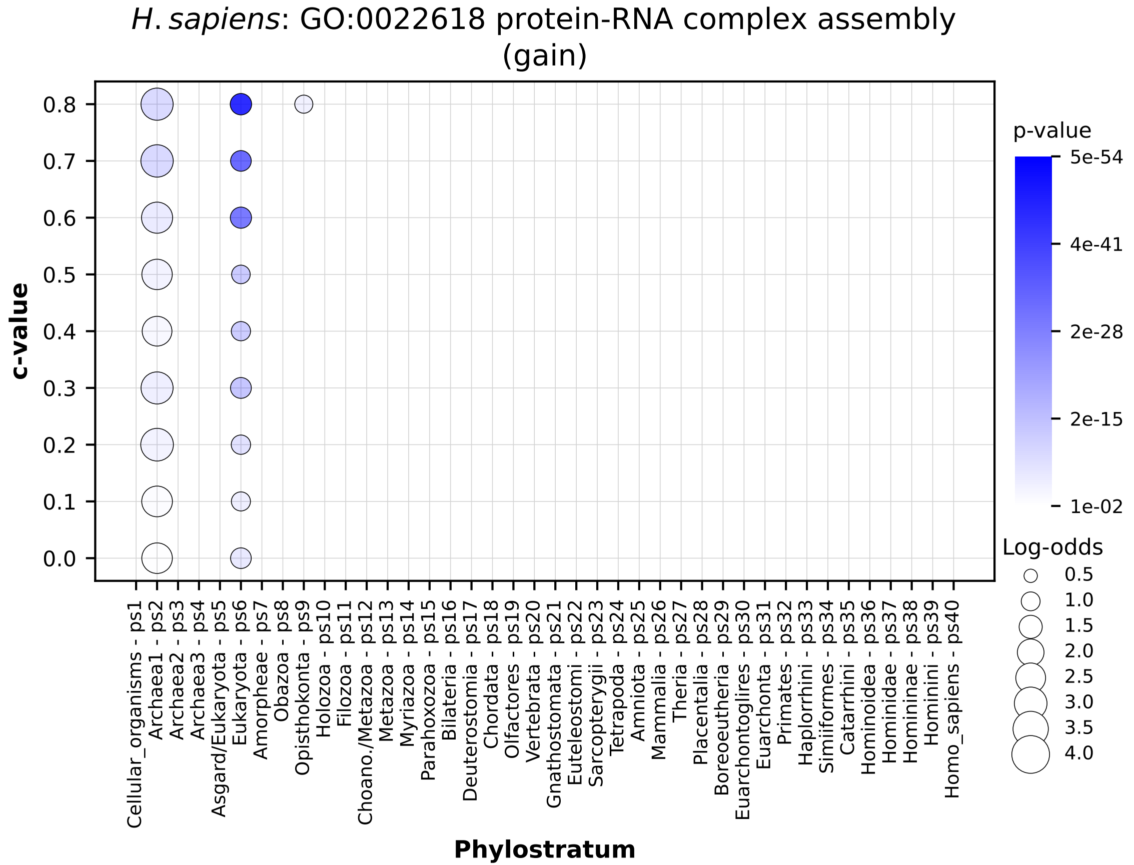

*H. sapiens*: GO:0022618 protein-RNA complex assembly
(gain)
c-value
0.8
0.7
0.6
0.5
0.4
0.3
0.2
0.1
0.0
p-value
5e-54
4e-41
2e-28
2e-15
1e-02
Log-odds
0.5
1.0
1.5
2.0
2.5
3.0
3.5
4.0
Cellular_organisms - ps1
Archaea1 - ps2
Archaea2 - ps3
Archaea3 - ps4
Asgard/Eukaryota - ps5
Eukaryota - ps6
Amorpheae - ps7
Obazoa - ps8
Opisthokonta - ps9
Holozoa - ps10
Filozoa - ps11
Choano./Metazoa - ps12
Metazoa - ps13
Myriazoa - ps14
Parahoxozoa - ps15
Bilateria - ps16
Deuterostomia - ps17
Chordata - ps18
Olfactores - ps19
Vertebrata - ps20
Gnathostomata - ps21
Euteleostomi - ps22
Sarcopterygii - ps23
Tetrapoda - ps24
Amniota - ps25
Mammalia - ps26
Theria - ps27
Placentalia - ps28
Boreoeutheria - ps29
Euarchontoglires - ps30
Euarchonta - ps31
Primates - ps32
Haplorrhini - ps33
Simiiformes - ps34
Catarrhini - ps35
Hominoidea - ps36
Hominidae - ps37
Homininae - ps38
Hominini - ps39
Homo_sapiens - ps40
Phylostratum

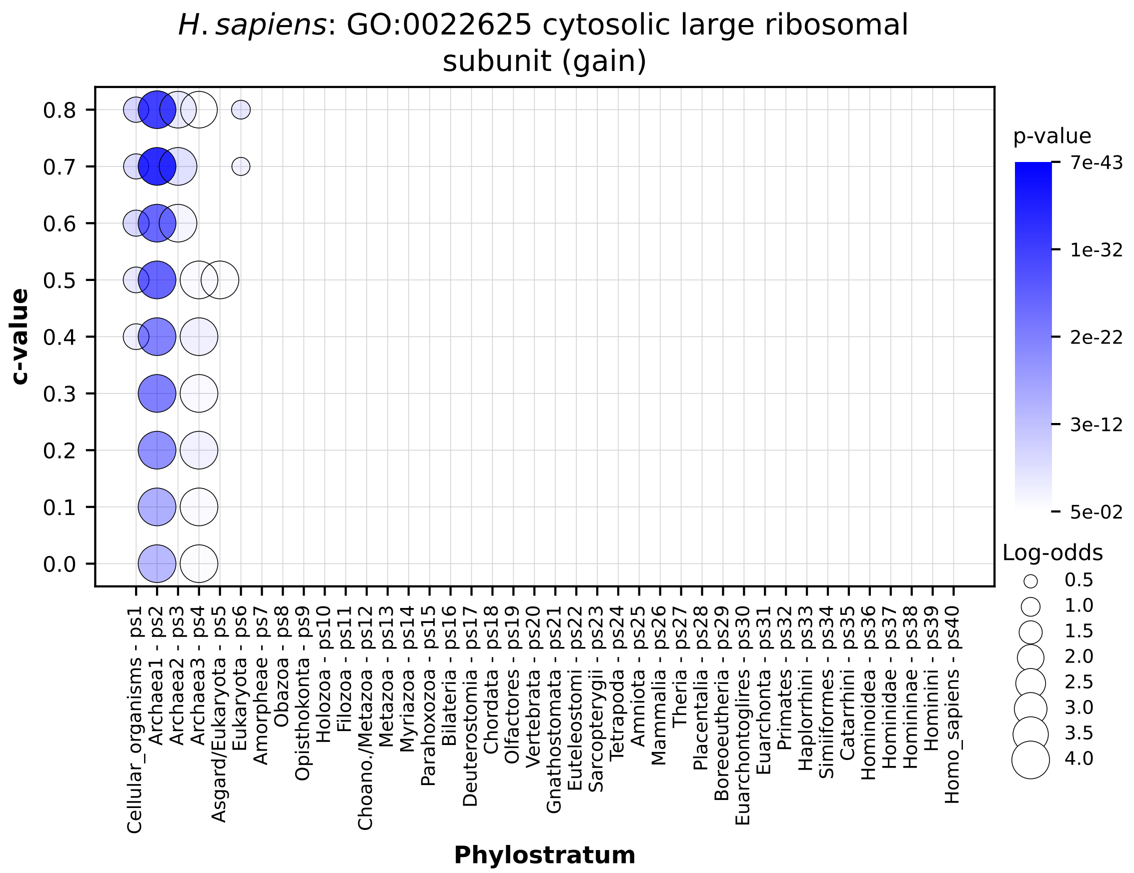

*H. sapiens*: GO:0022625 cytosolic large ribosomal subunit (gain)
c-value
0.8
0.7
0.6
0.5
0.4
0.3
0.2
0.1
0.0
Phylostratum
Cellular_organisms - ps1
Archaea1 - ps2
Archaea2 - ps3
Archaea3 - ps4
Asgard/Eukaryota - ps5
Eukaryota - ps6
Amorpheae - ps7
Obazoa - ps8
Opisthokonta - ps9
Holozoa - ps10
Filozoa - ps11
Choano./Metazoa - ps12
Metazoa - ps13
Myriazoa - ps14
Parahoxozoa - ps15
Bilateria - ps16
Deuterostomia - ps17
Chordata - ps18
Olfactores - ps19
Vertebrata - ps20
Gnathostomata - ps21
Euteleostomi - ps22
Sarcopterygii - ps23
Tetrapoda - ps24
Amniota - ps25
Mammalia - ps26
Theria - ps27
Placentalia - ps28
Boreoeutheria - ps29
Euarchontoglires - ps30
Euarchonta - ps31
Primates - ps32
Haplorrhini - ps33
Simiiformes - ps34
Catarrhini - ps35
Hominoidea - ps36
Hominidae - ps37
Homininae - ps38
Hominini - ps39
Homo_sapiens - ps40
p-value
7e-43
1e-32
2e-22
3e-12
5e-02
Log-odds
0.5
1.0
1.5
2.0
2.5
3.0
3.5
4.0

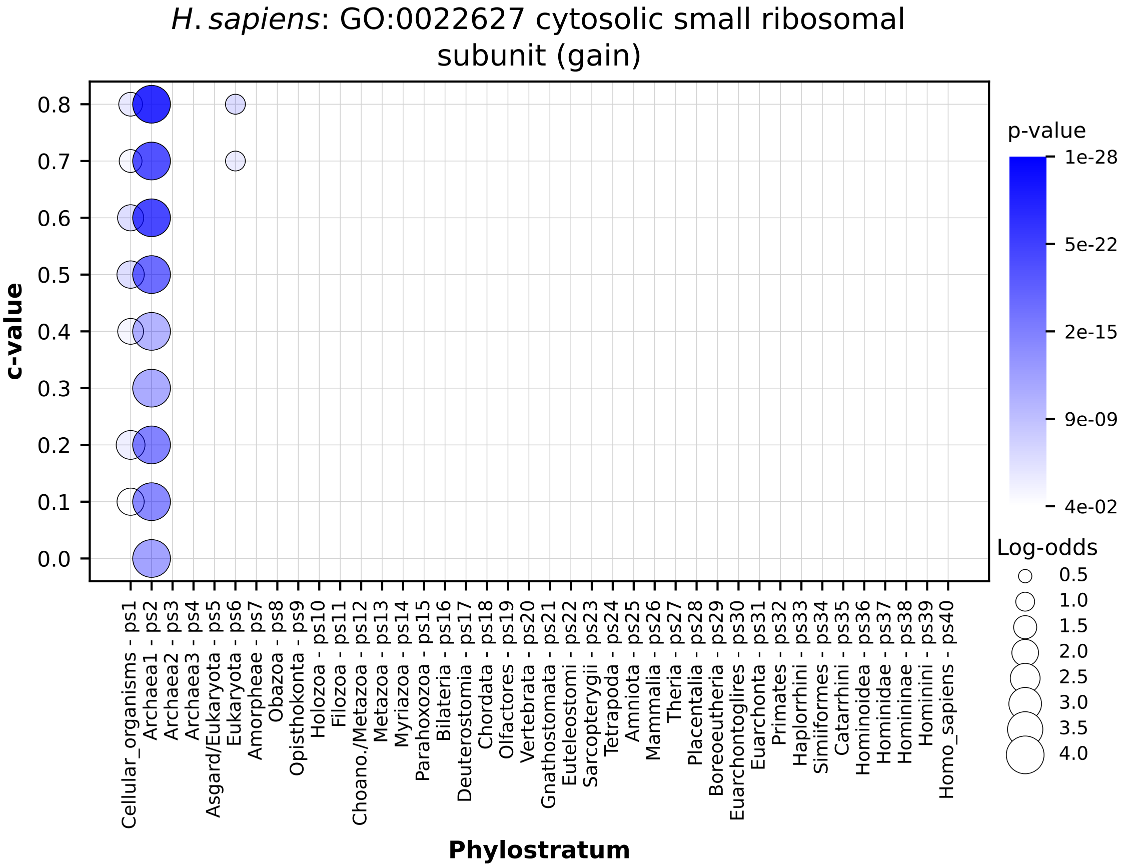

H. sapiens: GO:0022627 cytosolic small ribosomal subunit (gain)
c-value
0.8
0.7
0.6
0.5
0.4
0.3
0.2
0.1
0.0
Phylostratum
Cellular_organisms - ps1
Archaea1 - ps2
Archaea2 - ps3
Archaea3 - ps4
Asgard/Eukaryota - ps5
Eukaryota - ps6
Amorpheae - ps7
Obazoa - ps8
Opisthokonta - ps9
Holozoa - ps10
Filozoa - ps11
Choano./Metazoa - ps12
Metazoa - ps13
Myriazoa - ps14
Parahoxozoa - ps15
Bilateria - ps16
Deuterostomia - ps17
Chordata - ps18
Olfactores - ps19
Vertebrata - ps20
Gnathostomata - ps21
Euteleostomi - ps22
Sarcopterygii - ps23
Tetrapoda - ps24
Amniota - ps25
Mammalia - ps26
Theria - ps27
Placentalia - ps28
Boreoeutheria - ps29
Euarchontoglires - ps30
Euarchonta - ps31
Primates - ps32
Haplorrhini - ps33
Simiiformes - ps34
Catarrhini - ps35
Hominoidea - ps36
Hominidae - ps37
Homininae - ps38
Hominini - ps39
Homo_sapiens - ps40
p-value
1e-28
5e-22
2e-15
9e-09
4e-02
Log-odds
0.5
1.0
1.5
2.0
2.5
3.0
3.5
4.0

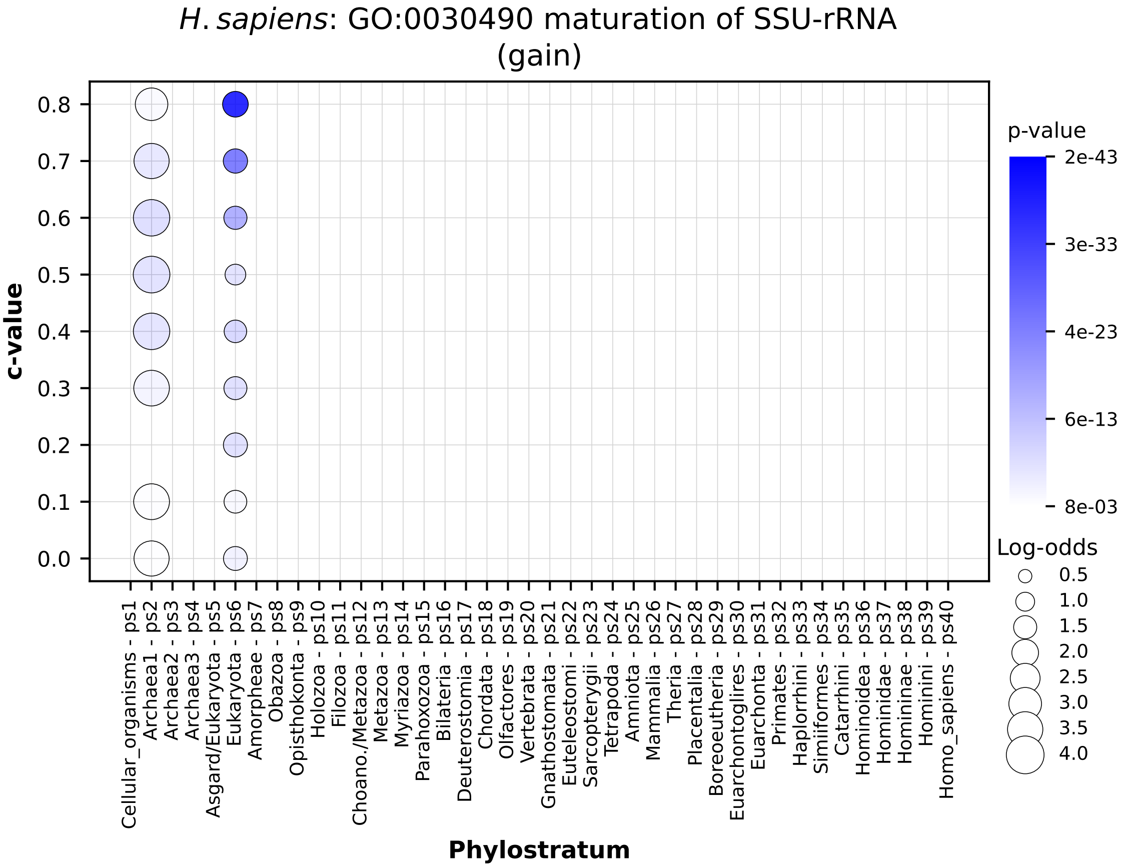

H. sapiens: GO:0030490 maturation of SSU-rRNA
(gain)
c-value
0.8
0.7
0.6
0.5
0.4
0.3
0.2
0.1
0.0
Phylostratum
Cellular_organisms - ps1
Archaea1 - ps2
Archaea2 - ps3
Archaea3 - ps4
Asgard/Eukaryota - ps5
Eukaryota - ps6
Amorpheae - ps7
Obazoa - ps8
Opisthokonta - ps9
Holozoa - ps10
Filozoa - ps11
Choano./Metazoa - ps12
Metazoa - ps13
Myriazoa - ps14
Parahoxozoa - ps15
Bilateria - ps16
Deuterostomia - ps17
Chordata - ps18
Olfactores - ps19
Vertebrata - ps20
Gnathostomata - ps21
Euteleostomi - ps22
Sarcopterygii - ps23
Tetrapoda - ps24
Amniota - ps25
Mammalia - ps26
Theria - ps27
Placentalia - ps28
Boreoeutheria - ps29
Euarchontoglires - ps30
Euarchonta - ps31
Primates - ps32
Haplorrhini - ps33
Simiiformes - ps34
Catarrhini - ps35
Hominoidea - ps36
Hominidae - ps37
Homininae - ps38
Hominini - ps39
Homo_sapiens - ps40
p-value
2e-43
3e-33
4e-23
6e-13
8e-03
Log-odds
0.5
1.0
1.5
2.0
2.5
3.0
3.5
4.0

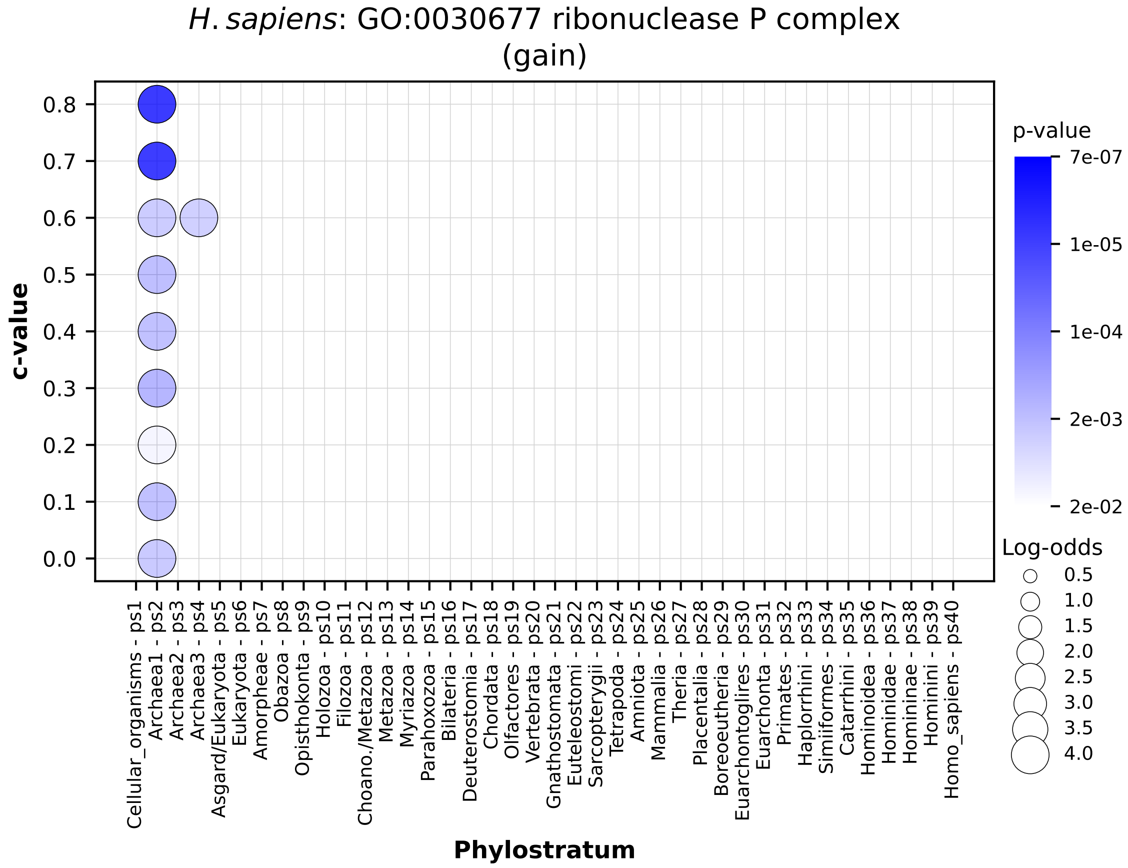

H. sapiens: GO:0030677 ribonuclease P complex
(gain)
c-value
0.8
0.7
0.6
0.5
0.4
0.3
0.2
0.1
0.0
p-value
7e-07
1e-05
1e-04
2e-03
2e-02
Log-odds
0.5
1.0
1.5
2.0
2.5
3.0
3.5
4.0
Cellular_organisms - ps1
Archaea1 - ps2
Archaea2 - ps3
Archaea3 - ps4
Asgard/Eukaryota - ps5
Eukaryota - ps6
Amorpheae - ps7
Obazoa - ps8
Opisthokonta - ps9
Holozoa - ps10
Filozoa - ps11
Choano./Metazoa - ps12
Metazoa - ps13
Myriazoa - ps14
Parahoxozoa - ps15
Bilateria - ps16
Deuterostomia - ps17
Chordata - ps18
Olfactores - ps19
Vertebrata - ps20
Gnathostomata - ps21
Euteleostomi - ps22
Sarcopterygii - ps23
Tetrapoda - ps24
Amniota - ps25
Mammalia - ps26
Theria - ps27
Placentalia - ps28
Boreoeutheria - ps29
Euarchontoglires - ps30
Euarchonta - ps31
Primates - ps32
Haplorrhini - ps33
Simiiformes - ps34
Catarrhini - ps35
Hominoidea - ps36
Hominidae - ps37
Homininae - ps38
Hominini - ps39
Homo_sapiens - ps40
Phylostratum

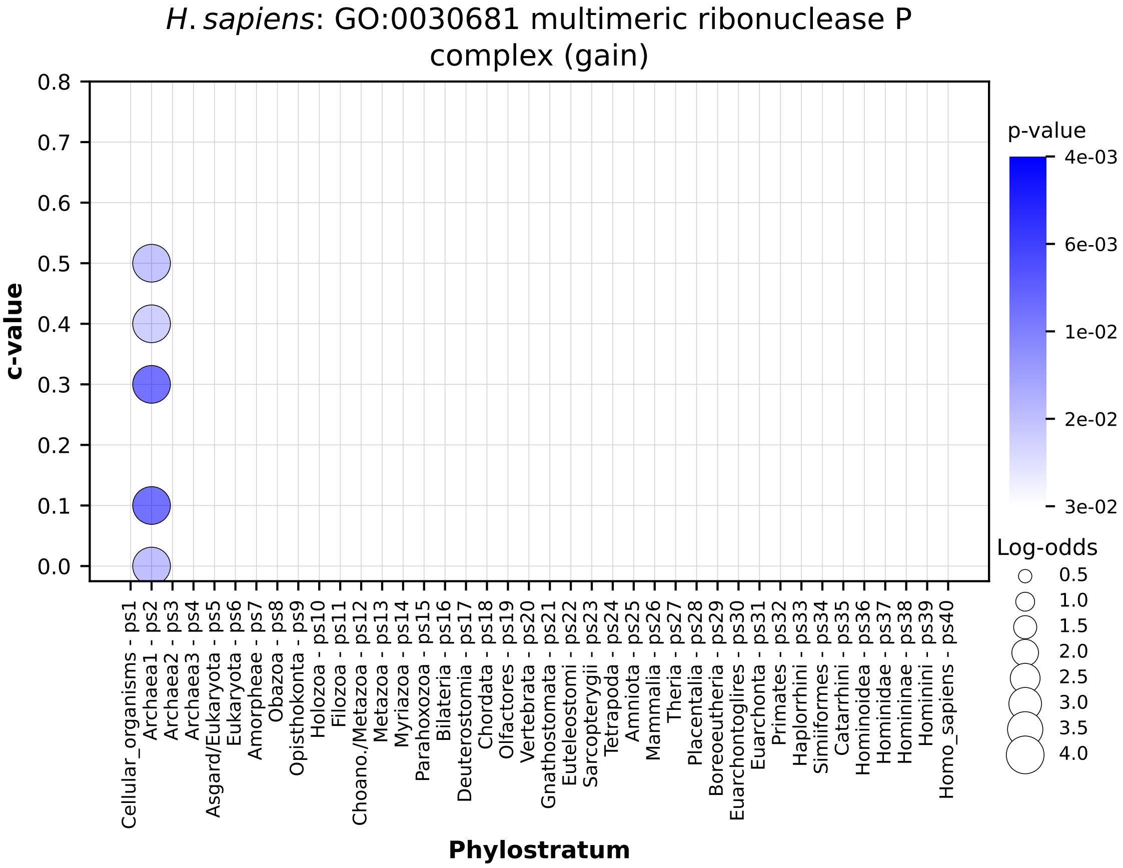
H. sapiens: GO:0030681 multimeric ribonuclease P complex (gain)
c-value
Phylostratum
p-value
4e-03
6e-03
1e-02
2e-02
3e-02
Log-odds
0.5
1.0
1.5
2.0
2.5
3.0
3.5
4.0
Cellular_organisms - ps1
Archaea1 - ps2
Archaea2 - ps3
Archaea3 - ps4
Asgard/Eukaryota - ps5
Eukaryota - ps6
Amorpheae - ps7
Obazoa - ps8
Opisthokonta - ps9
Holozoa - ps10
Filozoa - ps11
Choano./Metazoa - ps12
Metazoa - ps13
Myriazoa - ps14
Parahoxozoa - ps15
Bilateria - ps16
Deuterostomia - ps17
Chordata - ps18
Olfactores - ps19
Vertebrata - ps20
Gnathostomata - ps21
Euteleostomi - ps22
Sarcopterygii - ps23
Tetrapoda - ps24
Amniota - ps25
Mammalia - ps26
Theria - ps27
Placentalia - ps28
Boreoeutheria - ps29
Euarchontoglires - ps30
Euarchonta - ps31
Primates - ps32
Haplorrhini - ps33
Simiiformes - ps34
Catarrhini - ps35
Hominoidea - ps36
Hominidae - ps37
Homininae - ps38
Hominini - ps39
Homo_sapiens - ps40

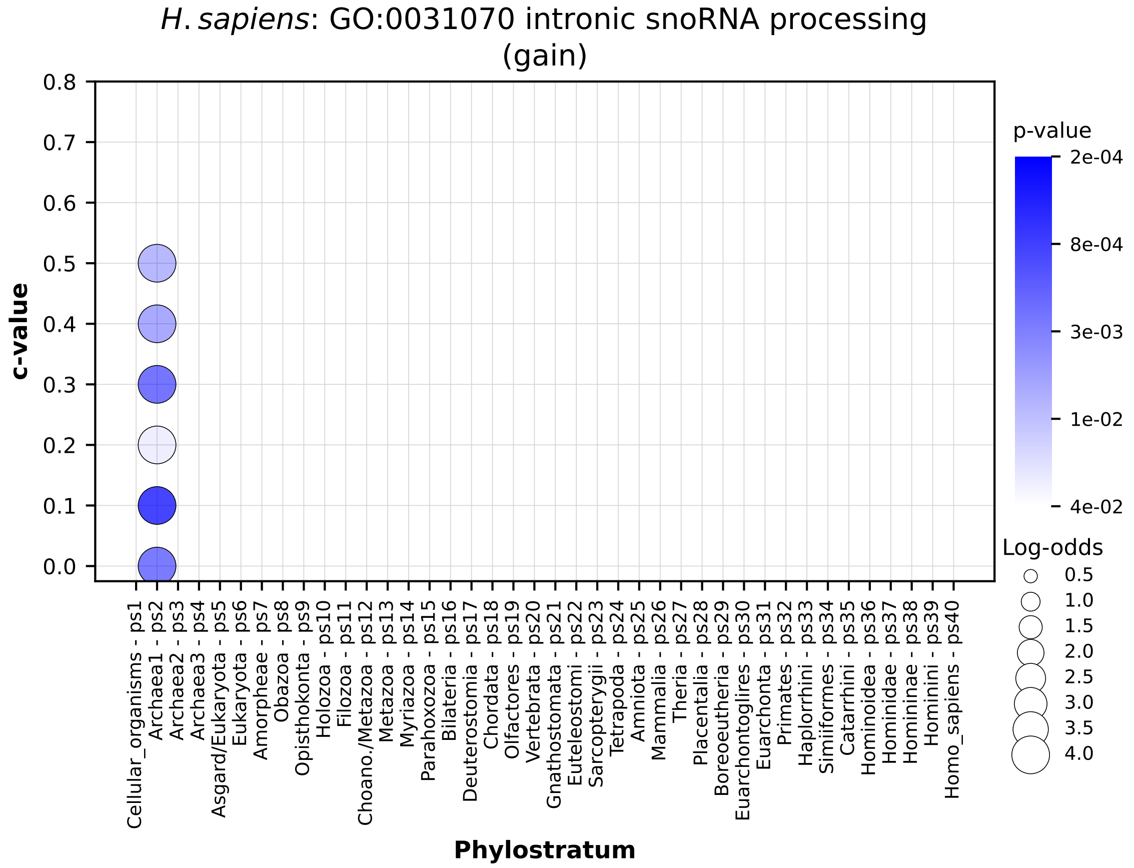

H. sapiens: GO:0031070 intronic snoRNA processing
(gain)
c-value
0.8
0.7
0.6
0.5
0.4
0.3
0.2
0.1
0.0
Phylostratum
Cellular_organisms - ps1
Archaea1 - ps2
Archaea2 - ps3
Archaea3 - ps4
Asgard/Eukaryota - ps5
Eukaryota - ps6
Amorpheae - ps7
Obazoa - ps8
Opisthokonta - ps9
Holozoa - ps10
Filozoa - ps11
Choano./Metazoa - ps12
Metazoa - ps13
Myriazoa - ps14
Parahoxozoa - ps15
Bilateria - ps16
Deuterostomia - ps17
Chordata - ps18
Olfactores - ps19
Vertebrata - ps20
Gnathostomata - ps21
Euteleostomi - ps22
Sarcopterygii - ps23
Tetrapoda - ps24
Amniota - ps25
Mammalia - ps26
Theria - ps27
Placentalia - ps28
Boreoeutheria - ps29
Euarchontoglires - ps30
Euarchonta - ps31
Primates - ps32
Haplorrhini - ps33
Simiiformes - ps34
Catarrhini - ps35
Hominoidea - ps36
Hominidae - ps37
Homininae - ps38
Hominini - ps39
Homo_sapiens - ps40
p-value
2e-04
8e-04
3e-03
1e-02
4e-02
Log-odds
0.5
1.0
1.5
2.0
2.5
3.0
3.5
4.0

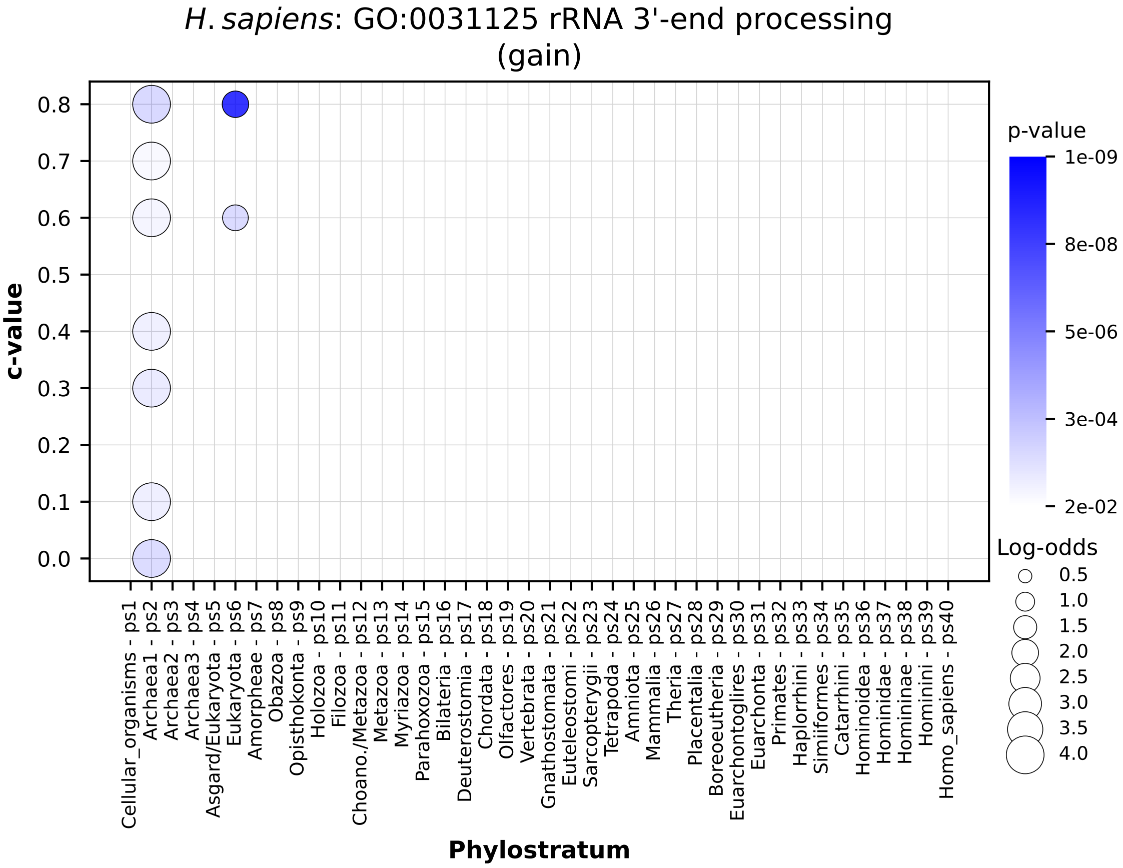

H. sapiens: GO:0031125 rRNA 3'-end processing
(gain)
c-value
0.8
0.7
0.6
0.5
0.4
0.3
0.2
0.1
0.0
p-value
1e-09
8e-08
5e-06
3e-04
2e-02
Log-odds
0.5
1.0
1.5
2.0
2.5
3.0
3.5
4.0
Cellular_organisms - ps1
Archaea1 - ps2
Archaea2 - ps3
Archaea3 - ps4
Asgard/Eukaryota - ps5
Eukaryota - ps6
Amorpheae - ps7
Obazoa - ps8
Opisthokonta - ps9
Holozoa - ps10
Filozoa - ps11
Choano./Metazoa - ps12
Metazoa - ps13
Myriazoa - ps14
Parahoxozoa - ps15
Bilateria - ps16
Deuterostomia - ps17
Chordata - ps18
Olfactores - ps19
Vertebrata - ps20
Gnathostomata - ps21
Euteleostomi - ps22
Sarcopterygii - ps23
Tetrapoda - ps24
Amniota - ps25
Mammalia - ps26
Theria - ps27
Placentalia - ps28
Boreoeutheria - ps29
Euarchontoglires - ps30
Euarchonta - ps31
Primates - ps32
Haplorrhini - ps33
Simiiformes - ps34
Catarrhini - ps35
Hominoidea - ps36
Hominidae - ps37
Homininae - ps38
Hominini - ps39
Homo_sapiens - ps40
Phylostratum

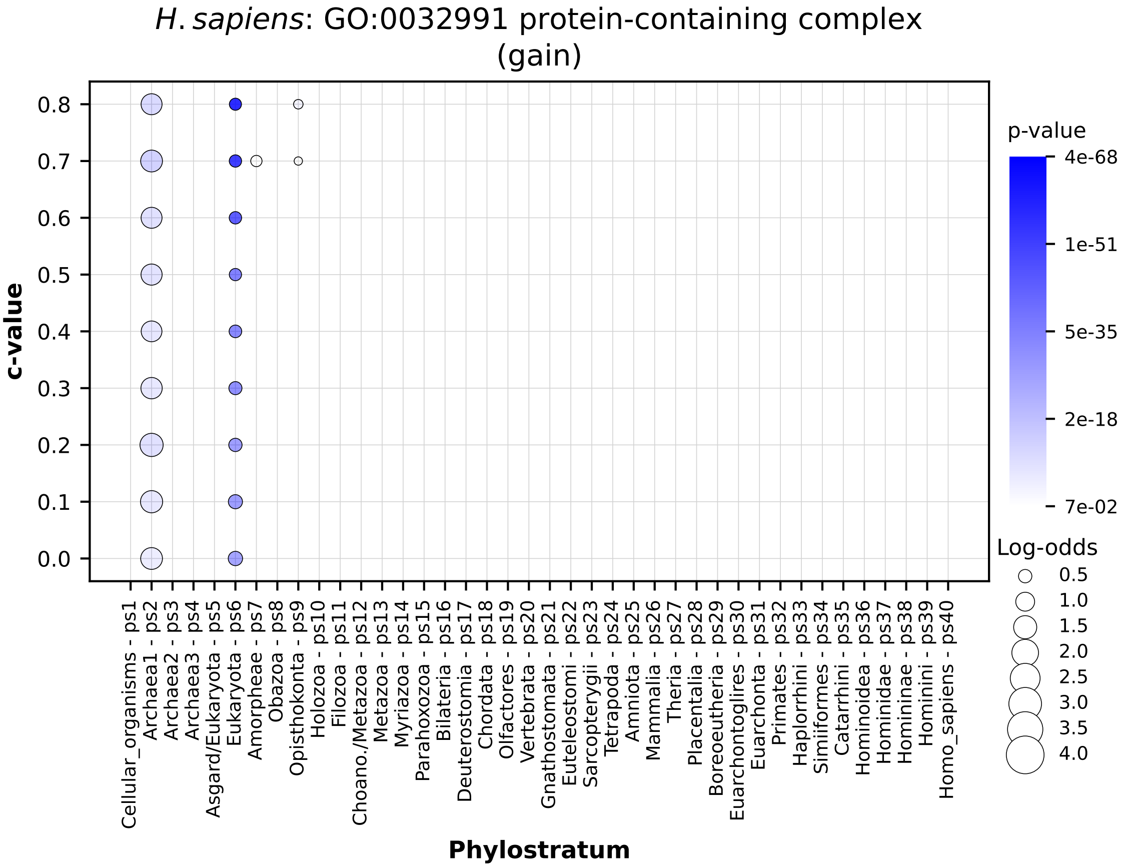

H. sapiens: GO:0032991 protein-containing complex
(gain)
c-value
0.8
0.7
0.6
0.5
0.4
0.3
0.2
0.1
0.0
p-value
4e-68
1e-51
5e-35
2e-18
7e-02
Log-odds
0.5
1.0
1.5
2.0
2.5
3.0
3.5
4.0
Cellular_organisms - ps1
Archaea1 - ps2
Archaea2 - ps3
Archaea3 - ps4
Asgard/Eukaryota - ps5
Eukaryota - ps6
Amorpheae - ps7
Obazoa - ps8
Opisthokonta - ps9
Holozoa - ps10
Filozoa - ps11
Choano./Metazoa - ps12
Metazoa - ps13
Myriazoa - ps14
Parahoxozoa - ps15
Bilateria - ps16
Deuterostomia - ps17
Chordata - ps18
Olfactores - ps19
Vertebrata - ps20
Gnathostomata - ps21
Euteleostomi - ps22
Sarcopterygii - ps23
Tetrapoda - ps24
Amniota - ps25
Mammalia - ps26
Theria - ps27
Placentalia - ps28
Boreoeutheria - ps29
Euarchontoglires - ps30
Euarchonta - ps31
Primates - ps32
Haplorrhini - ps33
Simiiformes - ps34
Catarrhini - ps35
Hominoidea - ps36
Hominidae - ps37
Homininae - ps38
Hominini - ps39
Homo_sapiens - ps40
Phylostratum

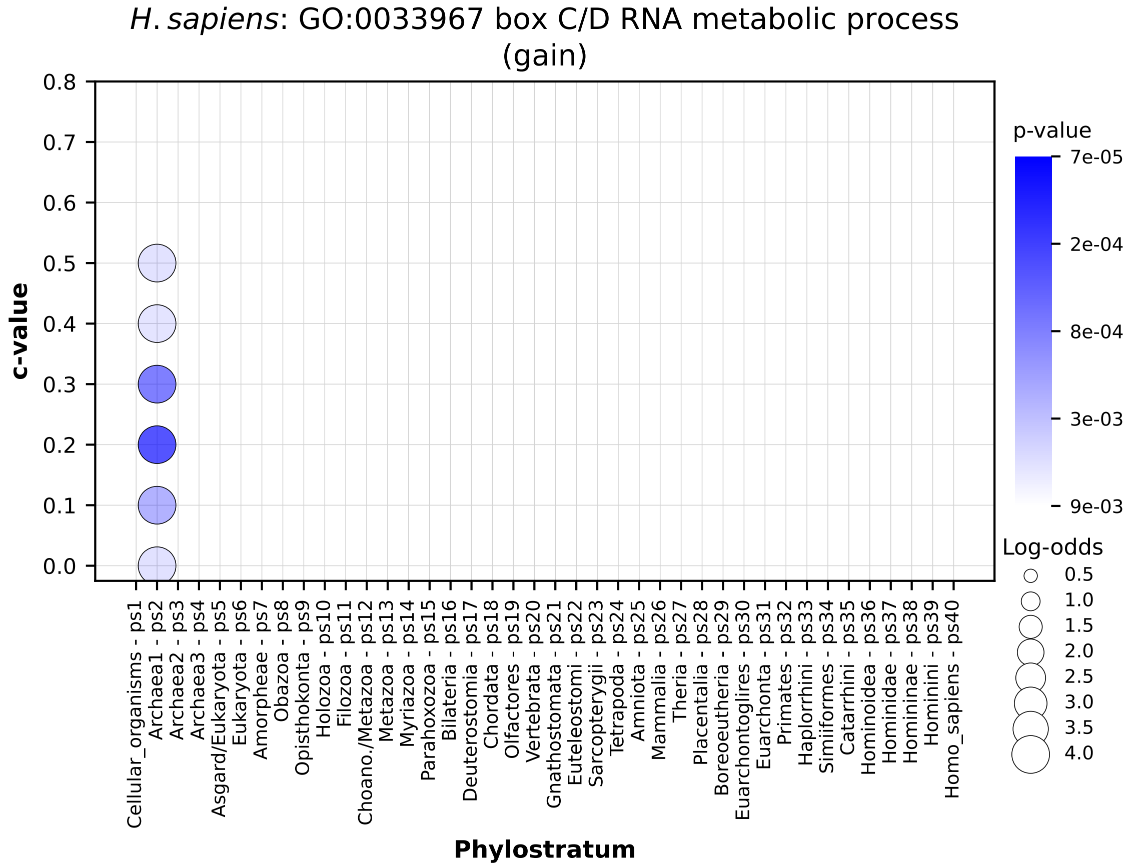
H. sapiens: GO:0033967 box C/D RNA metabolic process
(gain)
c-value
0.8
0.7
0.6
0.5
0.4
0.3
0.2
0.1
0.0
Phylostratum
Cellular_organisms - ps1
Archaea1 - ps2
Archaea2 - ps3
Archaea3 - ps4
Asgard/Eukaryota - ps5
Eukaryota - ps6
Amorpheae - ps7
Obazoa - ps8
Opisthokonta - ps9
Holozoa - ps10
Filozoa - ps11
Choano./Metazoa - ps12
Metazoa - ps13
Myriazoa - ps14
Parahoxozoa - ps15
Bilateria - ps16
Deuterostomia - ps17
Chordata - ps18
Olfactores - ps19
Vertebrata - ps20
Gnathostomata - ps21
Euteleostomi - ps22
Sarcopterygii - ps23
Tetrapoda - ps24
Amniota - ps25
Mammalia - ps26
Theria - ps27
Placentalia - ps28
Boreoeutheria - ps29
Euarchontoglires - ps30
Euarchonta - ps31
Primates - ps32
Haplorrhini - ps33
Simiiformes - ps34
Catarrhini - ps35
Hominoidea - ps36
Hominidae - ps37
Homininae - ps38
Hominini - ps39
Homo_sapiens - ps40
p-value
7e-05
2e-04
8e-04
3e-03
9e-03
Log-odds
0.5
1.0
1.5
2.0
2.5
3.0
3.5
4.0

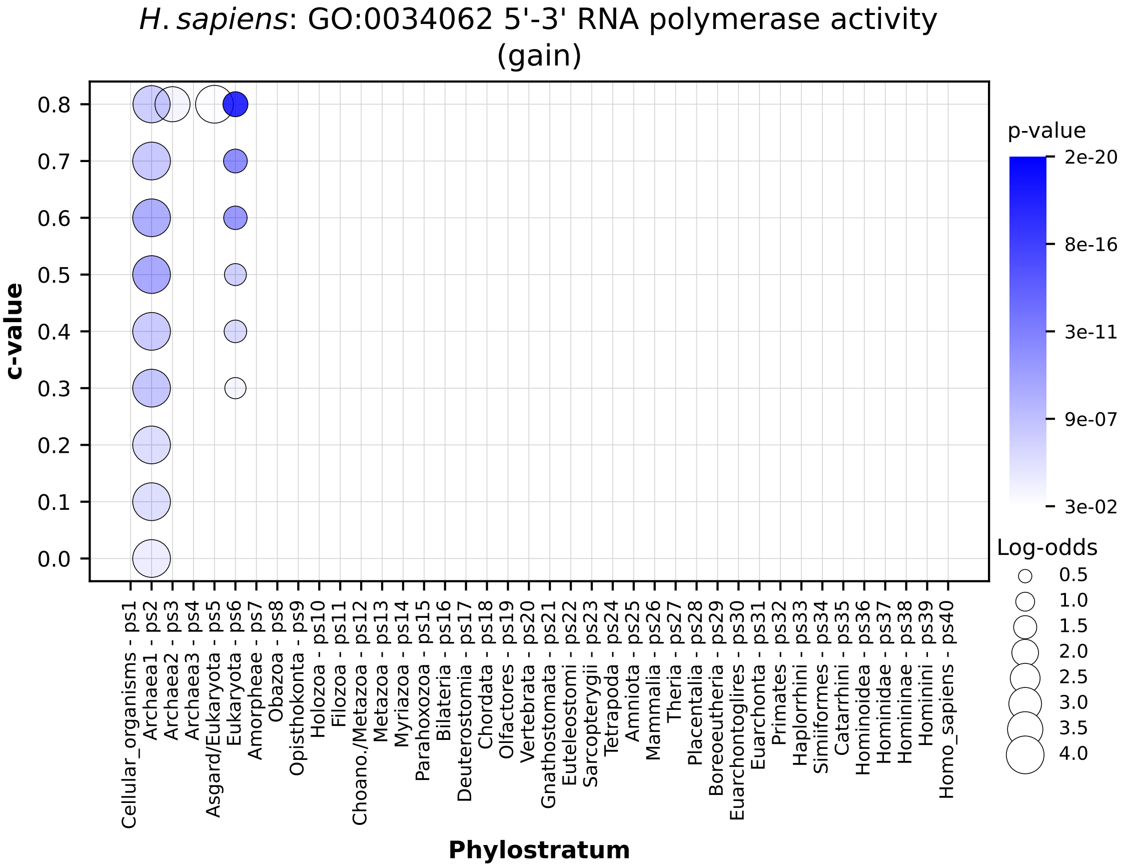

$H. sapiens$: GO:0034062 5'-3' RNA polymerase activity
(gain)
c-value
0.8
0.7
0.6
0.5
0.4
0.3
0.2
0.1
0.0
p-value
2e-20
8e-16
3e-11
9e-07
3e-02
Log-odds
0.5
1.0
1.5
2.0
2.5
3.0
3.5
4.0
Cellular_organisms - ps1
Archaea1 - ps2
Archaea2 - ps3
Archaea3 - ps4
Asgard/Eukaryota - ps5
Eukaryota - ps6
Amorpheae - ps7
Obazoa - ps8
Opisthokonta - ps9
Holozoa - ps10
Filozoa - ps11
Choano./Metazoa - ps12
Metazoa - ps13
Myriazoa - ps14
Parahoxozoa - ps15
Bilateria - ps16
Deuterostomia - ps17
Chordata - ps18
Olfactores - ps19
Vertebrata - ps20
Gnathostomata - ps21
Euteleostomi - ps22
Sarcopterygii - ps23
Tetrapoda - ps24
Amniota - ps25
Mammalia - ps26
Theria - ps27
Placentalia - ps28
Boreoeutheria - ps29
Euarchontoglires - ps30
Euarchonta - ps31
Primates - ps32
Haplorrhini - ps33
Simiiformes - ps34
Catarrhini - ps35
Hominoidea - ps36
Hominidae - ps37
Homininae - ps38
Hominini - ps39
Homo_sapiens - ps40
Phylostratum

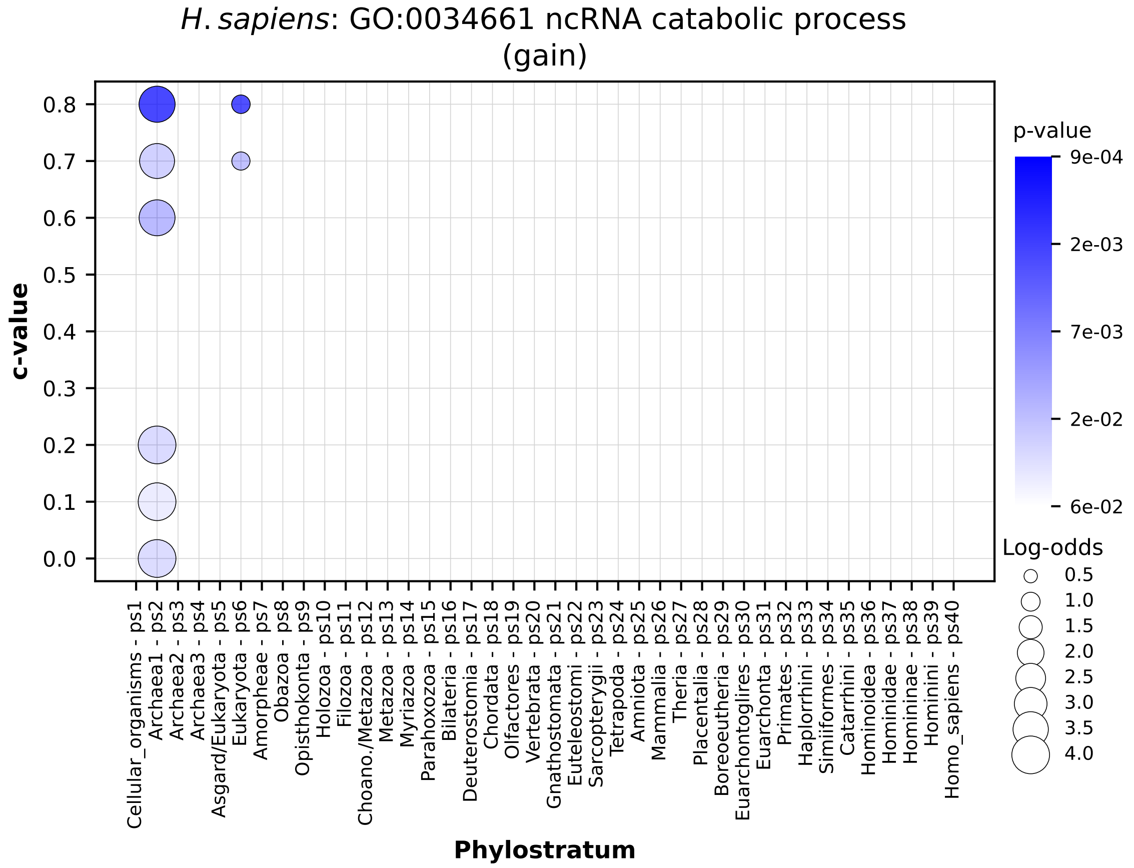
H. sapiens: GO:0034661 ncRNA catabolic process
(gain)
c-value
0.8
0.7
0.6
0.5
0.4
0.3
0.2
0.1
0.0
Phylostratum
Cellular_organisms - ps1
Archaea1 - ps2
Archaea2 - ps3
Archaea3 - ps4
Asgard/Eukaryota - ps5
Eukaryota - ps6
Amorpheae - ps7
Obazoa - ps8
Opisthokonta - ps9
Holozoa - ps10
Filozoa - ps11
Choano./Metazoa - ps12
Metazoa - ps13
Myriazoa - ps14
Parahoxozoa - ps15
Bilateria - ps16
Deuterostomia - ps17
Chordata - ps18
Olfactores - ps19
Vertebrata - ps20
Gnathostomata - ps21
Euteleostomi - ps22
Sarcopterygii - ps23
Tetrapoda - ps24
Amniota - ps25
Mammalia - ps26
Theria - ps27
Placentalia - ps28
Boreoeutheria - ps29
Euarchontoglires - ps30
Euarchonta - ps31
Primates - ps32
Haplorrhini - ps33
Simiiformes - ps34
Catarrhini - ps35
Hominoidea - ps36
Hominidae - ps37
Homininae - ps38
Hominini - ps39
Homo_sapiens - ps40
p-value
9e-04
2e-03
7e-03
2e-02
6e-02
Log-odds
0.5
1.0
1.5
2.0
2.5
3.0
3.5
4.0

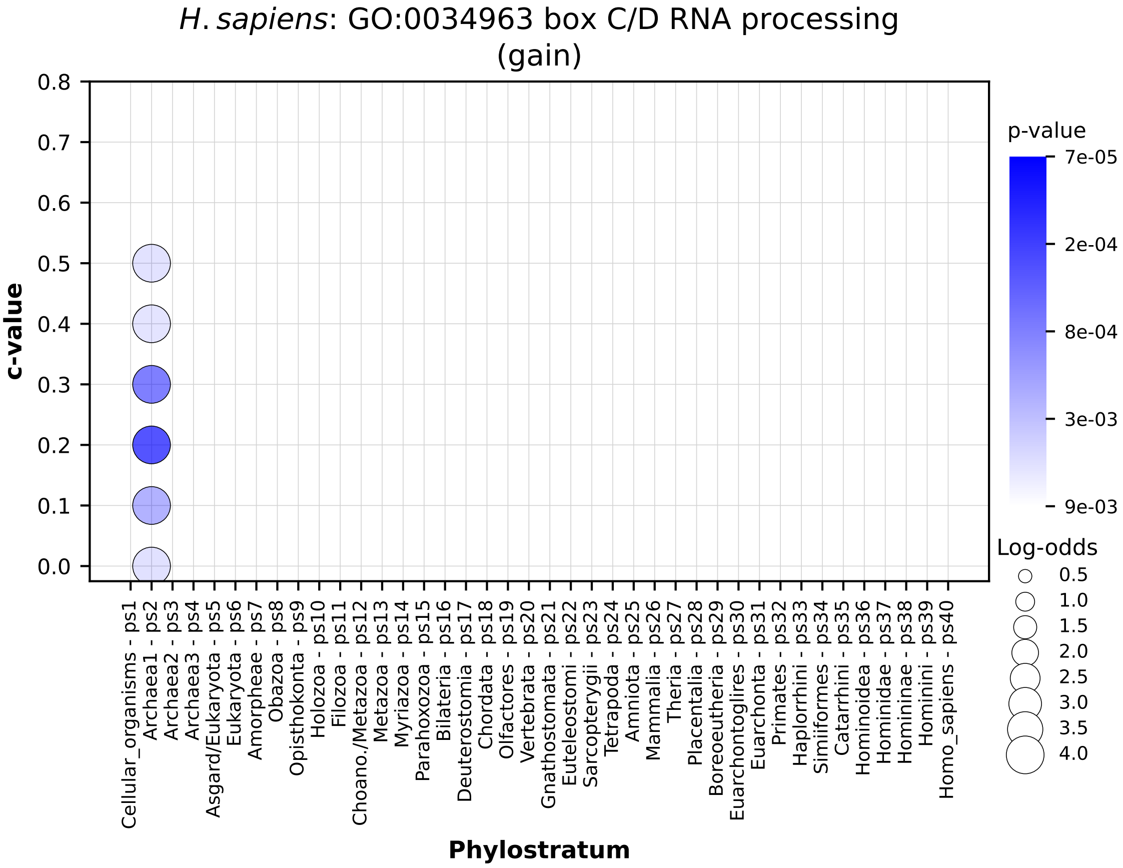

*H. sapiens*: GO:0034963 box C/D RNA processing
(gain)
c-value
0.8
0.7
0.6
0.5
0.4
0.3
0.2
0.1
0.0
Phylostratum
Cellular_organisms - ps1
Archaea1 - ps2
Archaea2 - ps3
Archaea3 - ps4
Asgard/Eukaryota - ps5
Eukaryota - ps6
Amorpheae - ps7
Obazoa - ps8
Opisthokonta - ps9
Holozoa - ps10
Filozoa - ps11
Choano./Metazoa - ps12
Metazoa - ps13
Myriazoa - ps14
Parahoxozoa - ps15
Bilateria - ps16
Deuterostomia - ps17
Chordata - ps18
Olfactores - ps19
Vertebrata - ps20
Gnathostomata - ps21
Euteleostomi - ps22
Sarcopterygii - ps23
Tetrapoda - ps24
Amniota - ps25
Mammalia - ps26
Theria - ps27
Placentalia - ps28
Boreoeutheria - ps29
Euarchontoglires - ps30
Euarchonta - ps31
Primates - ps32
Haplorrhini - ps33
Simiiformes - ps34
Catarrhini - ps35
Hominoidea - ps36
Hominidae - ps37
Homininae - ps38
Hominini - ps39
Homo_sapiens - ps40
p-value
7e-05
2e-04
8e-04
3e-03
9e-03
Log-odds
0.5
1.0
1.5
2.0
2.5
3.0
3.5
4.0

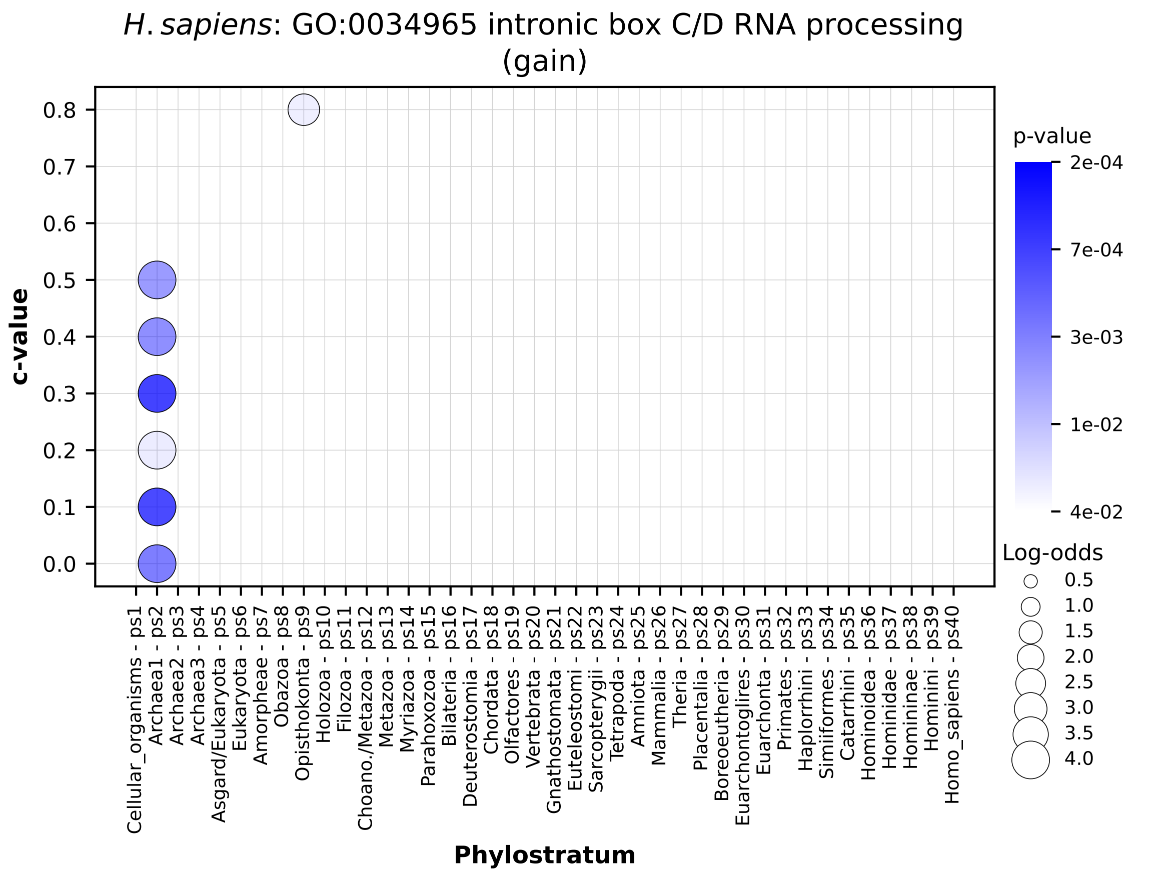
H. sapiens: GO:0034965 intronic box C/D RNA processing
(gain)
c-value
0.0
0.1
0.2
0.3
0.4
0.5
0.6
0.7
0.8
p-value
2e-04
7e-04
3e-03
1e-02
4e-02
Log-odds
0.5
1.0
1.5
2.0
2.5
3.0
3.5
4.0
Cellular_organisms - ps1
Archaea1 - ps2
Archaea2 - ps3
Archaea3 - ps4
Asgard/Eukaryota - ps5
Eukaryota - ps6
Amorpheae - ps7
Obazoa - ps8
Opisthokonta - ps9
Holozoa - ps10
Filozoa - ps11
Choano./Metazoa - ps12
Metazoa - ps13
Myriazoa - ps14
Parahoxozoa - ps15
Bilateria - ps16
Deuterostomia - ps17
Chordata - ps18
Olfactores - ps19
Vertebrata - ps20
Gnathostomata - ps21
Euteleostomi - ps22
Sarcopterygii - ps23
Tetrapoda - ps24
Amniota - ps25
Mammalia - ps26
Theria - ps27
Placentalia - ps28
Boreoeutheria - ps29
Euarchontoglires - ps30
Euarchonta - ps31
Primates - ps32
Haplorrhini - ps33
Simiiformes - ps34
Catarrhini - ps35
Hominoidea - ps36
Hominidae - ps37
Homininae - ps38
Hominini - ps39
Homo_sapiens - ps40
Phylostratum

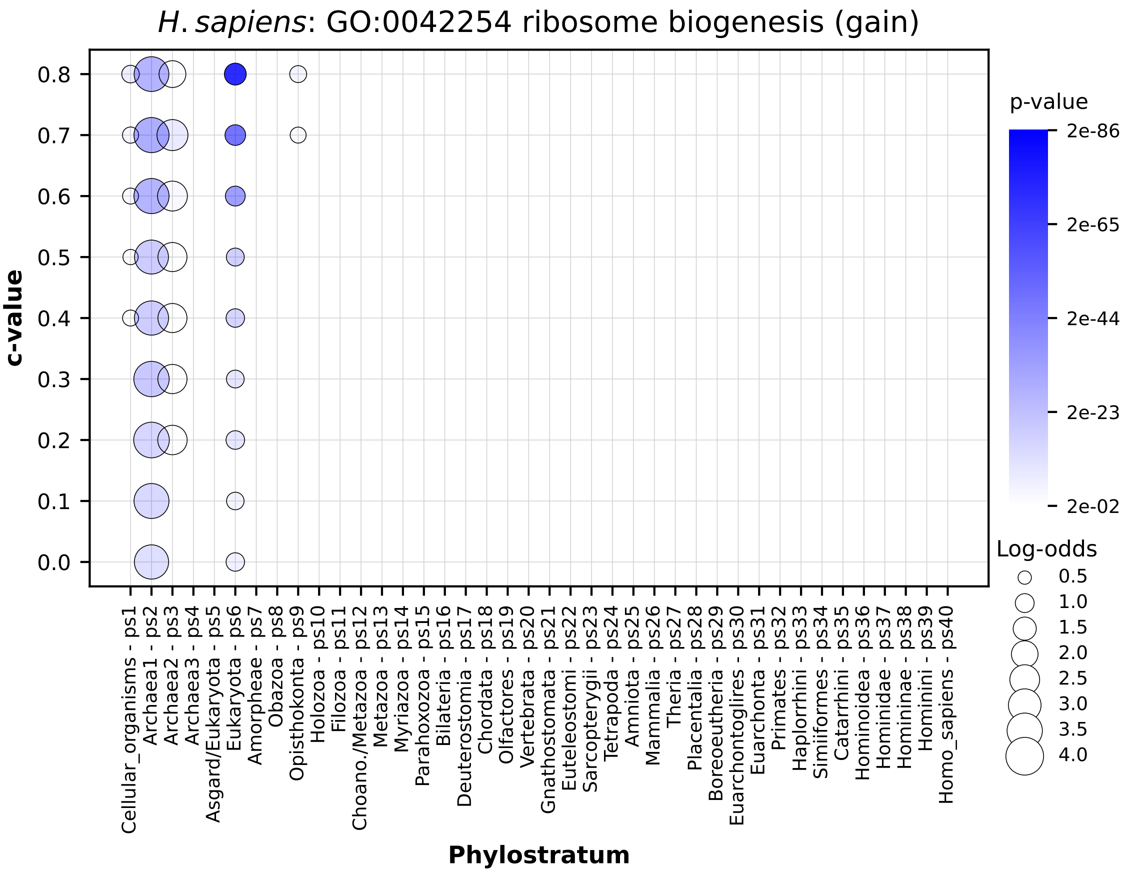
H. sapiens: GO:0042254 ribosome biogenesis (gain)
c-value
0.8
0.7
0.6
0.5
0.4
0.3
0.2
0.1
0.0
Phylostratum
Cellular_organisms - ps1
Archaea1 - ps2
Archaea2 - ps3
Archaea3 - ps4
Asgard/Eukaryota - ps5
Eukaryota - ps6
Amorpheae - ps7
Obazoa - ps8
Opisthokonta - ps9
Holozoa - ps10
Filozoa - ps11
Choano./Metazoa - ps12
Metazoa - ps13
Myriazoa - ps14
Parahoxozoa - ps15
Bilateria - ps16
Deuterostomia - ps17
Chordata - ps18
Olfactores - ps19
Vertebrata - ps20
Gnathostomata - ps21
Euteleostomi - ps22
Sarcopterygii - ps23
Tetrapoda - ps24
Amniota - ps25
Mammalia - ps26
Theria - ps27
Placentalia - ps28
Boreoeutheria - ps29
Euarchontoglires - ps30
Euarchonta - ps31
Primates - ps32
Haplorrhini - ps33
Simiiformes - ps34
Catarrhini - ps35
Hominoidea - ps36
Hominidae - ps37
Homininae - ps38
Hominini - ps39
Homo_sapiens - ps40
p-value
2e-86
2e-65
2e-44
2e-23
2e-02
Log-odds
0.5
1.0
1.5
2.0
2.5
3.0
3.5
4.0

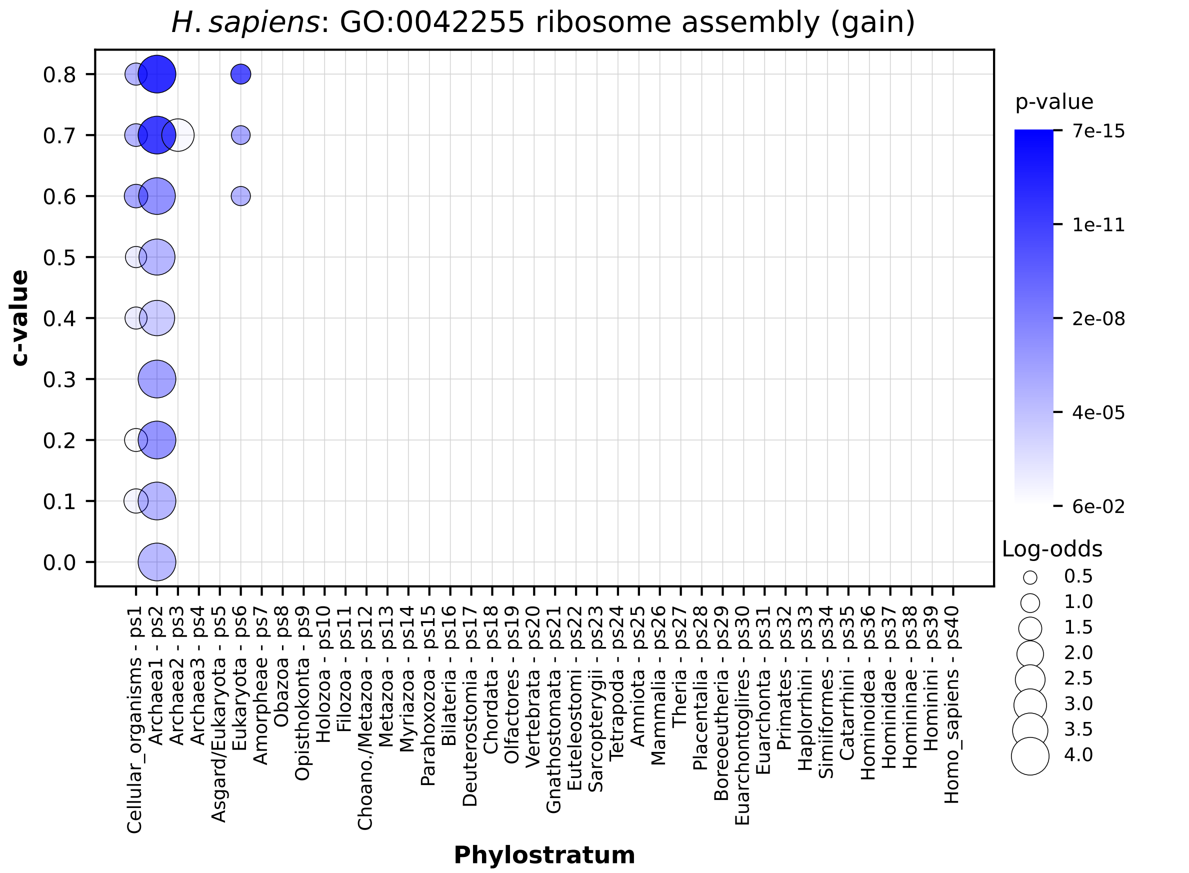

H. sapiens: GO:0042255 ribosome assembly (gain)
c-value
0.8
0.7
0.6
0.5
0.4
0.3
0.2
0.1
0.0
p-value
7e-15
1e-11
2e-08
4e-05
6e-02
Log-odds
0.5
1.0
1.5
2.0
2.5
3.0
3.5
4.0
Cellular_organisms - ps1
Archaea1 - ps2
Archaea2 - ps3
Archaea3 - ps4
Asgard/Eukaryota - ps5
Eukaryota - ps6
Amorpheae - ps7
Obazoa - ps8
Opisthokonta - ps9
Holozoa - ps10
Filozoa - ps11
Choano./Metazoa - ps12
Metazoa - ps13
Myriazoa - ps14
Parahoxozoa - ps15
Bilateria - ps16
Deuterostomia - ps17
Chordata - ps18
Olfactores - ps19
Vertebrata - ps20
Gnathostomata - ps21
Euteleostomi - ps22
Sarcopterygii - ps23
Tetrapoda - ps24
Amniota - ps25
Mammalia - ps26
Theria - ps27
Placentalia - ps28
Boreoeutheria - ps29
Euarchontoglires - ps30
Euarchonta - ps31
Primates - ps32
Haplorrhini - ps33
Simiiformes - ps34
Catarrhini - ps35
Hominoidea - ps36
Hominidae - ps37
Homininae - ps38
Hominini - ps39
Homo_sapiens - ps40
Phylostratum

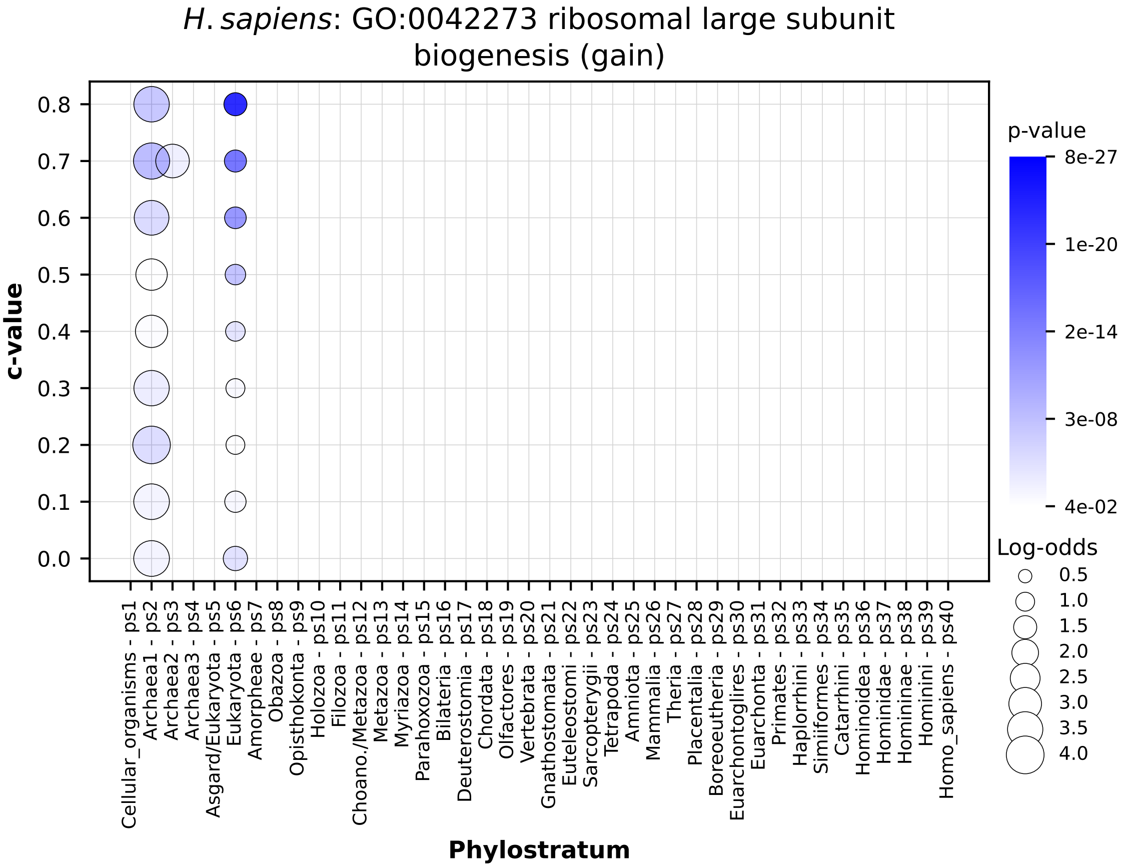
H. sapiens: GO:0042273 ribosomal large subunit biogenesis (gain)
c-value
0.8
0.7
0.6
0.5
0.4
0.3
0.2
0.1
0.0
p-value
8e-27
1e-20
2e-14
3e-08
4e-02
Log-odds
0.5
1.0
1.5
2.0
2.5
3.0
3.5
4.0
Cellular_organisms - ps1
Archaea1 - ps2
Archaea2 - ps3
Archaea3 - ps4
Asgard/Eukaryota - ps5
Eukaryota - ps6
Amorpheae - ps7
Obazoa - ps8
Opisthokonta - ps9
Holozoa - ps10
Filozoa - ps11
Choano./Metazoa - ps12
Metazoa - ps13
Myriazoa - ps14
Parahoxozoa - ps15
Bilateria - ps16
Deuterostomia - ps17
Chordata - ps18
Olfactores - ps19
Vertebrata - ps20
Gnathostomata - ps21
Euteleostomi - ps22
Sarcopterygii - ps23
Tetrapoda - ps24
Amniota - ps25
Mammalia - ps26
Theria - ps27
Placentalia - ps28
Boreoeutheria - ps29
Euarchontoglires - ps30
Euarchonta - ps31
Primates - ps32
Haplorrhini - ps33
Simiiformes - ps34
Catarrhini - ps35
Hominoidea - ps36
Hominidae - ps37
Homininae - ps38
Hominini - ps39
Homo_sapiens - ps40
Phylostratum

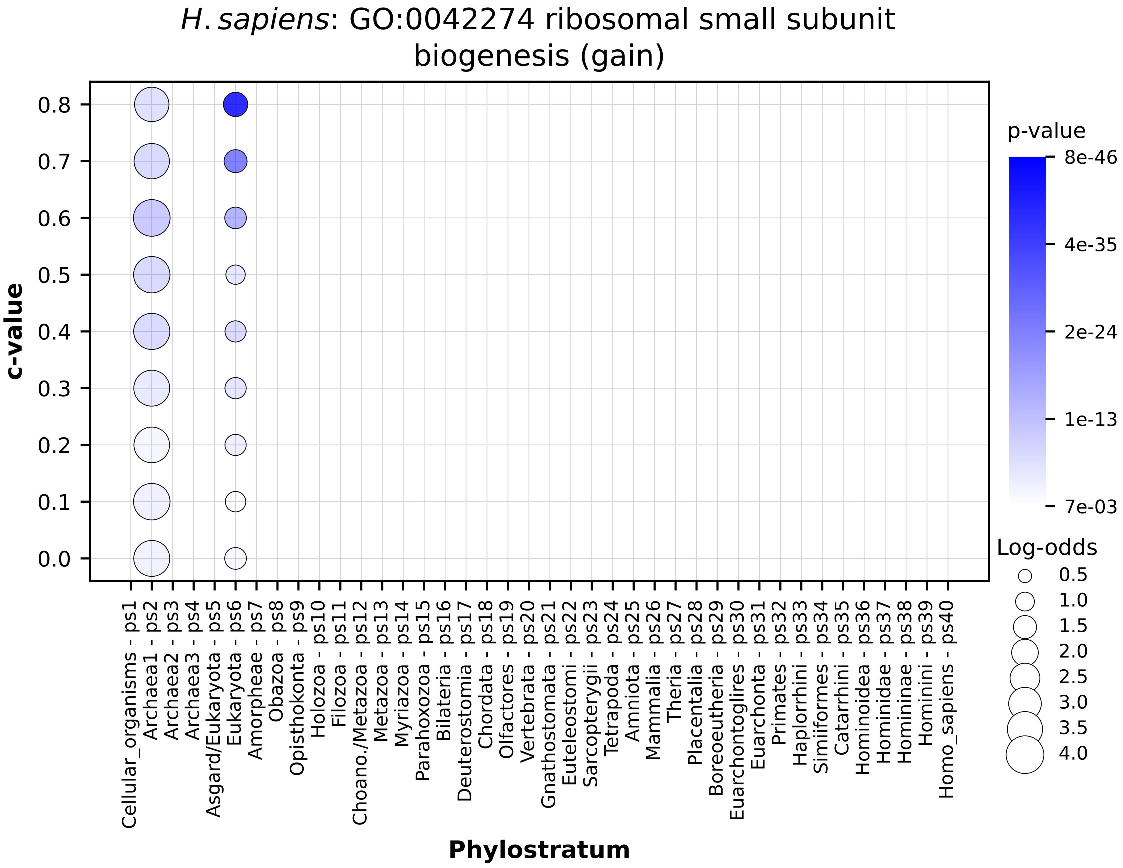

H. sapiens: GO:0042274 ribosomal small subunit biogenesis (gain)
c-value
0.8
0.7
0.6
0.5
0.4
0.3
0.2
0.1
0.0
p-value
8e-46
4e-35
2e-24
1e-13
7e-03
Log-odds
0.5
1.0
1.5
2.0
2.5
3.0
3.5
4.0
Cellular_organisms - ps1
Archaea1 - ps2
Archaea2 - ps3
Archaea3 - ps4
Asgard/Eukaryota - ps5
Eukaryota - ps6
Amorpheae - ps7
Obazoa - ps8
Opisthokonta - ps9
Holozoa - ps10
Filozoa - ps11
Choano./Metazoa - ps12
Metazoa - ps13
Myriazoa - ps14
Parahoxozoa - ps15
Bilateria - ps16
Deuterostomia - ps17
Chordata - ps18
Olfactores - ps19
Vertebrata - ps20
Gnathostomata - ps21
Euteleostomi - ps22
Sarcopterygii - ps23
Tetrapoda - ps24
Amniota - ps25
Mammalia - ps26
Theria - ps27
Placentalia - ps28
Boreoeutheria - ps29
Euarchontoglires - ps30
Euarchonta - ps31
Primates - ps32
Haplorrhini - ps33
Simiiformes - ps34
Catarrhini - ps35
Hominoidea - ps36
Hominidae - ps37
Homininae - ps38
Hominini - ps39
Homo_sapiens - ps40
Phylostratum

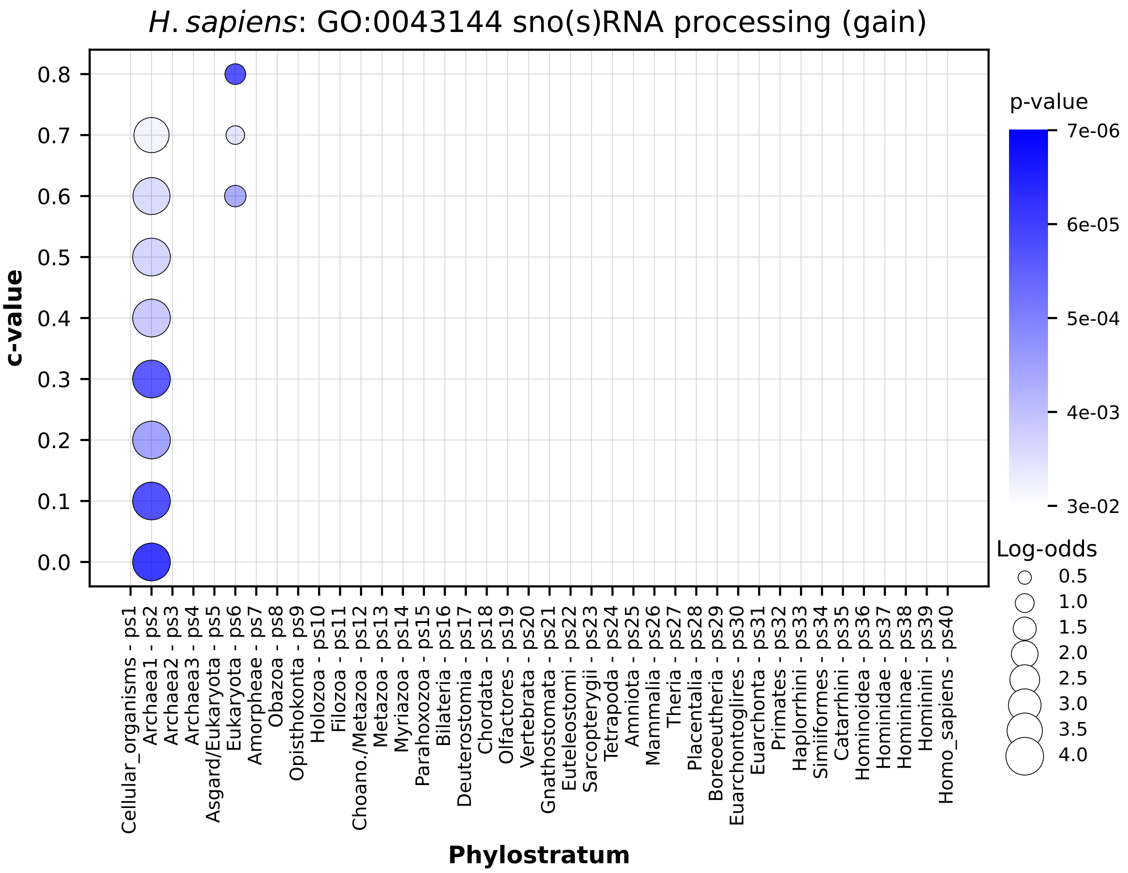

H. sapiens: GO:0043144 sno(s)RNA processing (gain)
c-value
0.0
0.1
0.2
0.3
0.4
0.5
0.6
0.7
0.8
Phylostratum
Cellular_organisms - ps1
Archaea1 - ps2
Archaea2 - ps3
Archaea3 - ps4
Asgard/Eukaryota - ps5
Eukaryota - ps6
Amorpheae - ps7
Obazoa - ps8
Opisthokonta - ps9
Holozoa - ps10
Filozoa - ps11
Choano./Metazoa - ps12
Metazoa - ps13
Myriazoa - ps14
Parahoxozoa - ps15
Bilateria - ps16
Deuterostomia - ps17
Chordata - ps18
Olfactores - ps19
Vertebrata - ps20
Gnathostomata - ps21
Euteleostomi - ps22
Sarcopterygii - ps23
Tetrapoda - ps24
Amniota - ps25
Mammalia - ps26
Theria - ps27
Placentalia - ps28
Boreoeutheria - ps29
Euarchontoglires - ps30
Euarchonta - ps31
Primates - ps32
Haplorrhini - ps33
Simiiformes - ps34
Catarrhini - ps35
Hominoidea - ps36
Hominidae - ps37
Homininae - ps38
Hominini - ps39
Homo_sapiens - ps40
p-value
7e-06
6e-05
5e-04
4e-03
3e-02
Log-odds
0.5
1.0
1.5
2.0
2.5
3.0
3.5
4.0

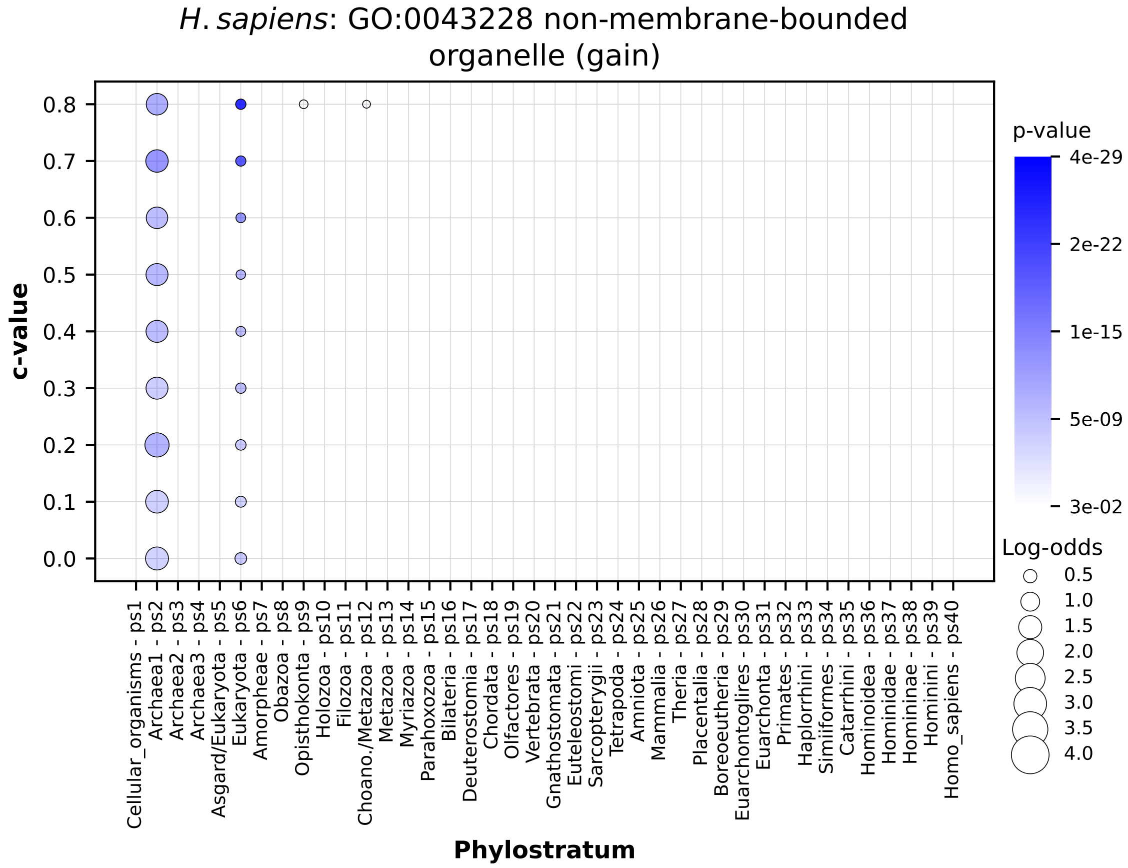
H. sapiens: GO:0043228 non-membrane-bounded organelle (gain)
c-value
0.8
0.7
0.6
0.5
0.4
0.3
0.2
0.1
0.0
Phylostratum
Cellular_organisms - ps1
Archaea1 - ps2
Archaea2 - ps3
Archaea3 - ps4
Asgard/Eukaryota - ps5
Eukaryota - ps6
Amorpheae - ps7
Obazoa - ps8
Opisthokonta - ps9
Holozoa - ps10
Filozoa - ps11
Choano./Metazoa - ps12
Metazoa - ps13
Myriazoa - ps14
Parahoxozoa - ps15
Bilateria - ps16
Deuterostomia - ps17
Chordata - ps18
Olfactores - ps19
Vertebrata - ps20
Gnathostomata - ps21
Euteleostomi - ps22
Sarcopterygii - ps23
Tetrapoda - ps24
Amniota - ps25
Mammalia - ps26
Theria - ps27
Placentalia - ps28
Boreoeutheria - ps29
Euarchontoglires - ps30
Euarchonta - ps31
Primates - ps32
Haplorrhini - ps33
Simiiformes - ps34
Catarrhini - ps35
Hominoidea - ps36
Hominidae - ps37
Homininae - ps38
Hominini - ps39
Homo_sapiens - ps40
p-value
4e-29
2e-22
1e-15
5e-09
3e-02
Log-odds
0.5
1.0
1.5
2.0
2.5
3.0
3.5
4.0

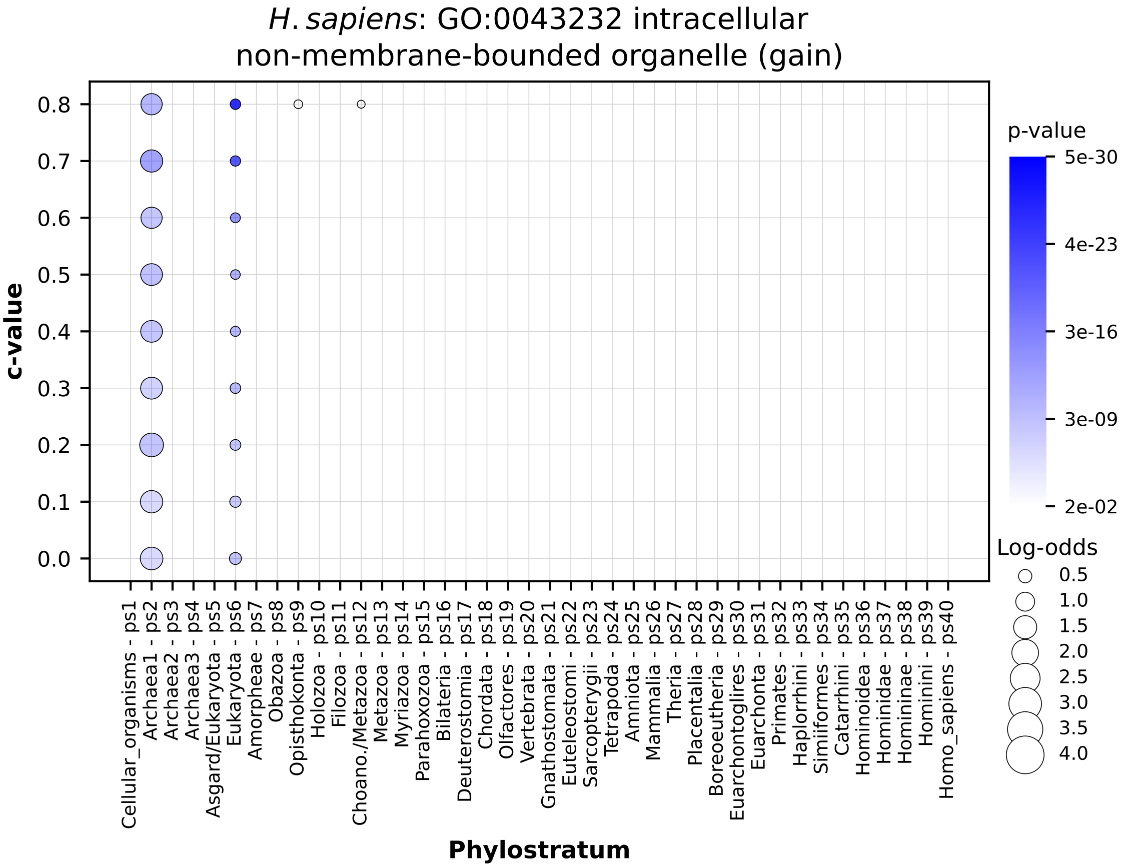
H. sapiens: GO:0043232 intracellular non-membrane-bounded organelle (gain)
c-value
0.0
0.1
0.2
0.3
0.4
0.5
0.6
0.7
0.8
Phylostratum
Cellular_organisms - ps1
Archaea1 - ps2
Archaea2 - ps3
Archaea3 - ps4
Asgard/Eukaryota - ps5
Eukaryota - ps6
Amorpheae - ps7
Obazoa - ps8
Opisthokonta - ps9
Holozoa - ps10
Filozoa - ps11
Choano./Metazoa - ps12
Metazoa - ps13
Myriazoa - ps14
Parahoxozoa - ps15
Bilateria - ps16
Deuterostomia - ps17
Chordata - ps18
Olfactores - ps19
Vertebrata - ps20
Gnathostomata - ps21
Euteleostomi - ps22
Sarcopterygii - ps23
Tetrapoda - ps24
Amniota - ps25
Mammalia - ps26
Theria - ps27
Placentalia - ps28
Boreoeutheria - ps29
Euarchontoglires - ps30
Euarchonta - ps31
Primates - ps32
Haplorrhini - ps33
Simiiformes - ps34
Catarrhini - ps35
Hominoidea - ps36
Hominidae - ps37
Homininae - ps38
Hominini - ps39
Homo_sapiens - ps40
p-value
5e-30
4e-23
3e-16
3e-09
2e-02
Log-odds
0.5
1.0
1.5
2.0
2.5
3.0
3.5
4.0

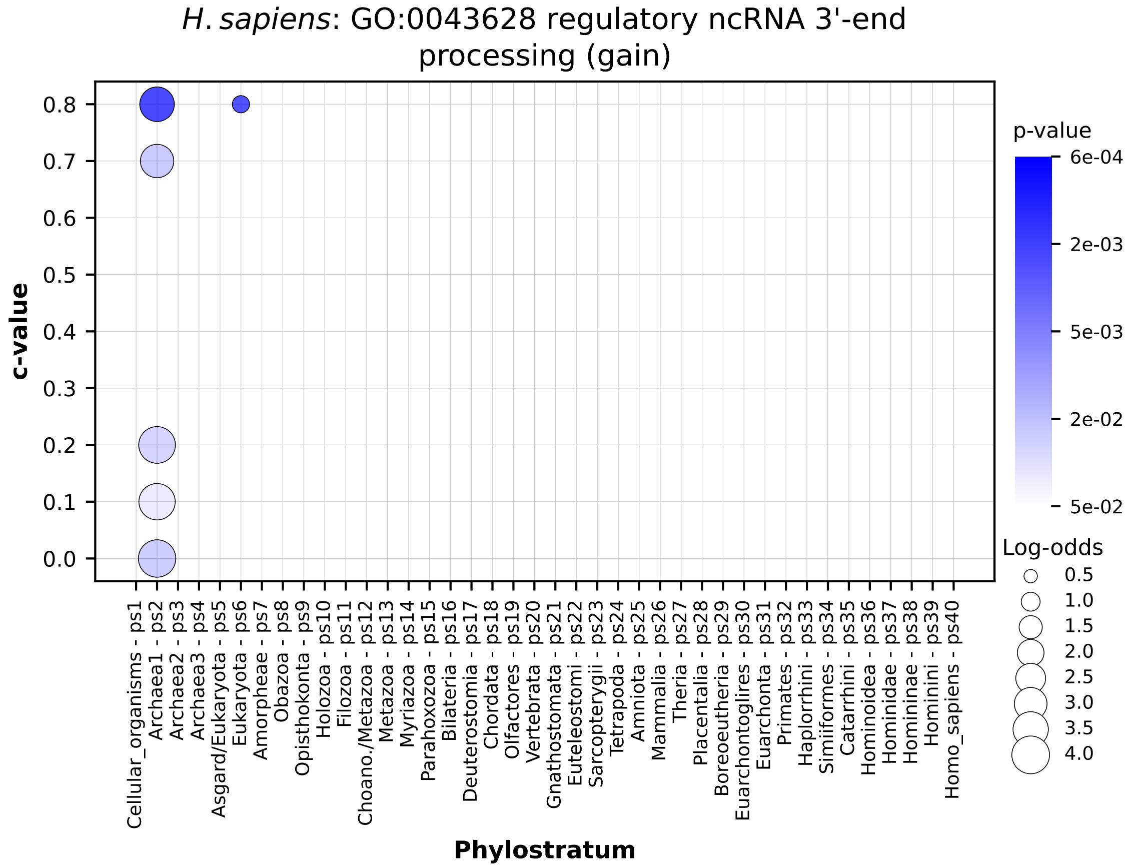
H. sapiens: GO:0043628 regulatory ncRNA 3'-end processing (gain)
c-value
0.0
0.1
0.2
0.3
0.4
0.5
0.6
0.7
0.8
Phylostratum
Cellular_organisms - ps1
Archaea1 - ps2
Archaea2 - ps3
Archaea3 - ps4
Asgard/Eukaryota - ps5
Eukaryota - ps6
Amorpheae - ps7
Obazoa - ps8
Opisthokonta - ps9
Holozoa - ps10
Filozoa - ps11
Choano./Metazoa - ps12
Metazoa - ps13
Myriazoa - ps14
Parahoxozoa - ps15
Bilateria - ps16
Deuterostomia - ps17
Chordata - ps18
Olfactores - ps19
Vertebrata - ps20
Gnathostomata - ps21
Euteleostomi - ps22
Sarcopterygii - ps23
Tetrapoda - ps24
Amniota - ps25
Mammalia - ps26
Theria - ps27
Placentalia - ps28
Boreoeutheria - ps29
Euarchontoglires - ps30
Euarchonta - ps31
Primates - ps32
Haplorrhini - ps33
Simiiformes - ps34
Catarrhini - ps35
Hominoidea - ps36
Hominidae - ps37
Homininae - ps38
Hominini - ps39
Homo_sapiens - ps40
p-value
6e-04
2e-03
5e-03
2e-02
5e-02
Log-odds
0.5
1.0
1.5
2.0
2.5
3.0
3.5
4.0

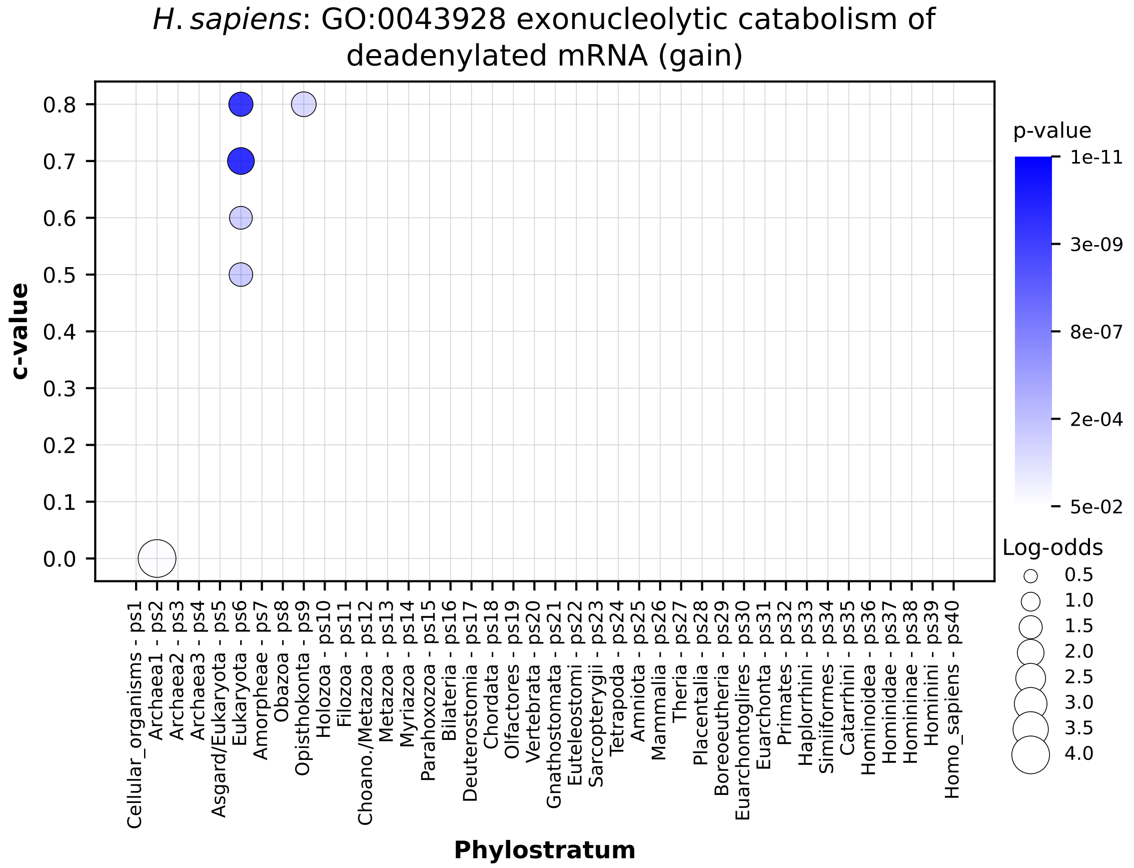

H. sapiens: GO:0043928 exonucleolytic catabolism of deadenylated mRNA (gain)
c-value
0.8
0.7
0.6
0.5
0.4
0.3
0.2
0.1
0.0
p-value
1e-11
3e-09
8e-07
2e-04
5e-02
Log-odds
0.5
1.0
1.5
2.0
2.5
3.0
3.5
4.0
Cellular_organisms - ps1
Archaea1 - ps2
Archaea2 - ps3
Archaea3 - ps4
Asgard/Eukaryota - ps5
Eukaryota - ps6
Amorpheae - ps7
Obazoa - ps8
Opisthokonta - ps9
Holozoa - ps10
Filozoa - ps11
Choano./Metazoa - ps12
Metazoa - ps13
Myriazoa - ps14
Parahoxozoa - ps15
Bilateria - ps16
Deuterostomia - ps17
Chordata - ps18
Olfactores - ps19
Vertebrata - ps20
Gnathostomata - ps21
Euteleostomi - ps22
Sarcopterygii - ps23
Tetrapoda - ps24
Amniota - ps25
Mammalia - ps26
Theria - ps27
Placentalia - ps28
Boreoeutheria - ps29
Euarchontoglires - ps30
Euarchonta - ps31
Primates - ps32
Haplorrhini - ps33
Simiiformes - ps34
Catarrhini - ps35
Hominoidea - ps36
Hominidae - ps37
Homininae - ps38
Hominini - ps39
Homo_sapiens - ps40
Phylostratum

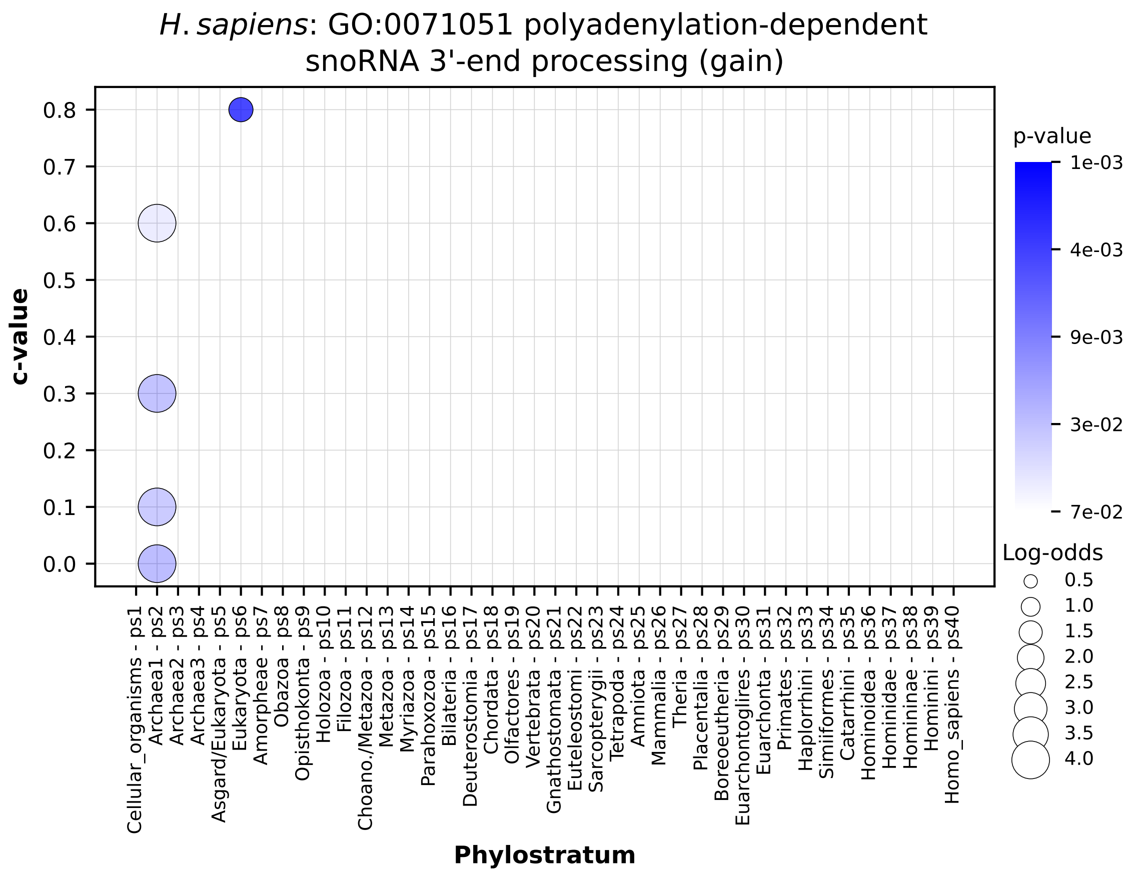

H. sapiens: GO:0071051 polyadenylation-dependent snoRNA 3'-end processing (gain)
c-value
0.0
0.1
0.2
0.3
0.4
0.5
0.6
0.7
0.8
Phylostratum
Cellular_organisms - ps1
Archaea1 - ps2
Archaea2 - ps3
Archaea3 - ps4
Asgard/Eukaryota - ps5
Eukaryota - ps6
Amorpheae - ps7
Obazoa - ps8
Opisthokonta - ps9
Holozoa - ps10
Filozoa - ps11
Choano./Metazoa - ps12
Metazoa - ps13
Myriazoa - ps14
Parahoxozoa - ps15
Bilateria - ps16
Deuterostomia - ps17
Chordata - ps18
Olfactores - ps19
Vertebrata - ps20
Gnathostomata - ps21
Euteleostomi - ps22
Sarcopterygii - ps23
Tetrapoda - ps24
Amniota - ps25
Mammalia - ps26
Theria - ps27
Placentalia - ps28
Boreoeutheria - ps29
Euarchontoglires - ps30
Euarchonta - ps31
Primates - ps32
Haplorrhini - ps33
Simiiformes - ps34
Catarrhini - ps35
Hominoidea - ps36
Hominidae - ps37
Homininae - ps38
Hominini - ps39
Homo_sapiens - ps40
p-value
1e-03
4e-03
9e-03
3e-02
7e-02
Log-odds
0.5
1.0
1.5
2.0
2.5
3.0
3.5
4.0

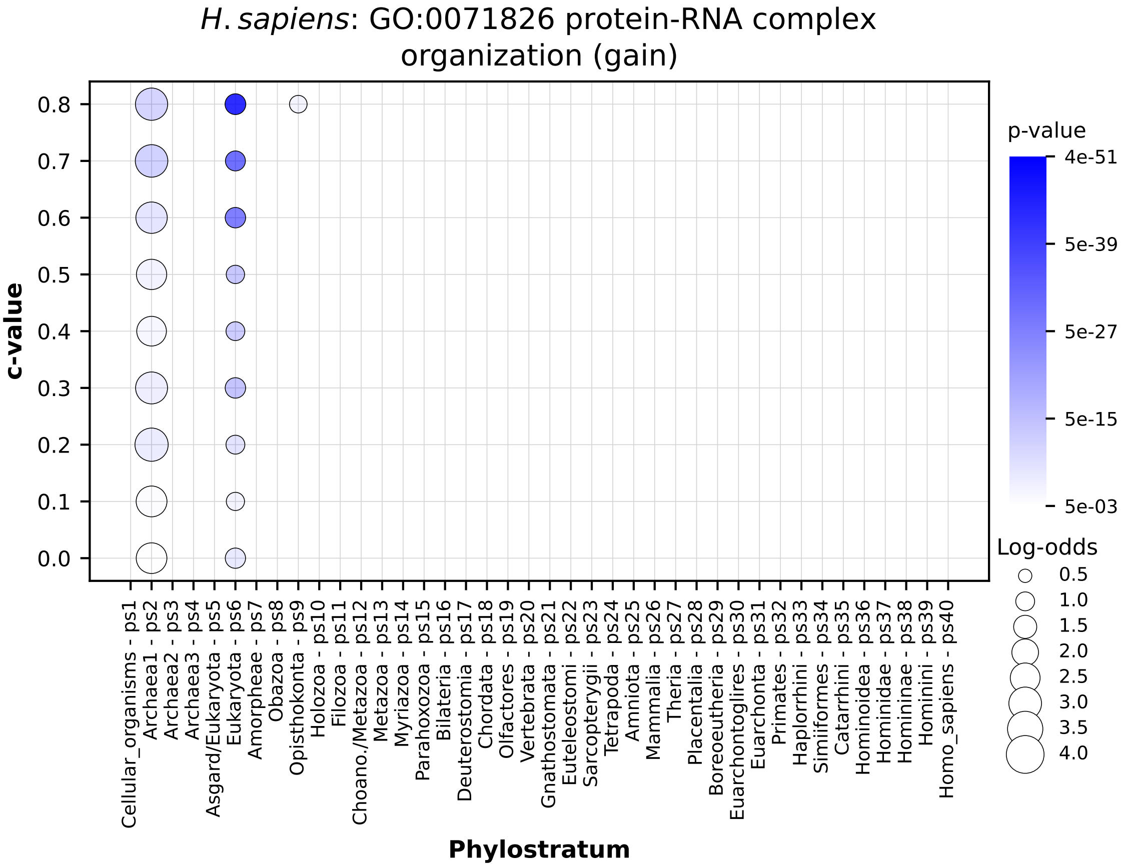

*H. sapiens*: GO:0071826 protein-RNA complex organization (gain)
c-value
0.8
0.7
0.6
0.5
0.4
0.3
0.2
0.1
0.0
p-value
4e-51
5e-39
5e-27
5e-15
5e-03
Log-odds
0.5
1.0
1.5
2.0
2.5
3.0
3.5
4.0
Cellular_organisms - ps1
Archaea1 - ps2
Archaea2 - ps3
Archaea3 - ps4
Asgard/Eukaryota - ps5
Eukaryota - ps6
Amorpheae - ps7
Obazoa - ps8
Opisthokonta - ps9
Holozoa - ps10
Filozoa - ps11
Choano./Metazoa - ps12
Metazoa - ps13
Myriazoa - ps14
Parahoxozoa - ps15
Bilateria - ps16
Deuterostomia - ps17
Chordata - ps18
Olfactores - ps19
Vertebrata - ps20
Gnathostomata - ps21
Euteleostomi - ps22
Sarcopterygii - ps23
Tetrapoda - ps24
Amniota - ps25
Mammalia - ps26
Theria - ps27
Placentalia - ps28
Boreoeutheria - ps29
Euarchontoglires - ps30
Euarchonta - ps31
Primates - ps32
Haplorrhini - ps33
Simiiformes - ps34
Catarrhini - ps35
Hominoidea - ps36
Hominidae - ps37
Homininae - ps38
Hominini - ps39
Homo_sapiens - ps40
Phylostratum

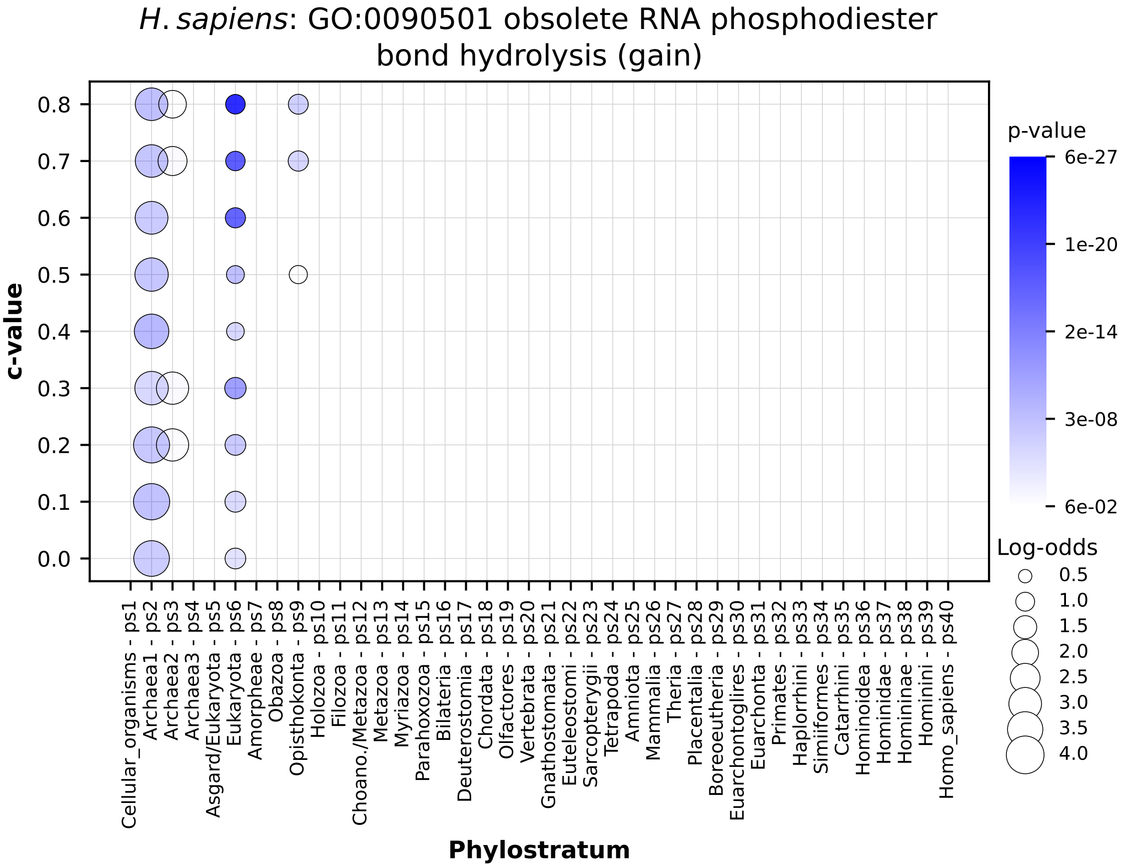

H. sapiens: GO:0090501 obsolete RNA phosphodiester bond hydrolysis (gain)
c-value
0.8
0.7
0.6
0.5
0.4
0.3
0.2
0.1
0.0
p-value
6e-27
1e-20
2e-14
3e-08
6e-02
Log-odds
0.5
1.0
1.5
2.0
2.5
3.0
3.5
4.0
Cellular_organisms - ps1
Archaea1 - ps2
Archaea2 - ps3
Archaea3 - ps4
Asgard/Eukaryota - ps5
Eukaryota - ps6
Amorpheae - ps7
Obazoa - ps8
Opisthokonta - ps9
Holozoa - ps10
Filozoa - ps11
Choano./Metazoa - ps12
Metazoa - ps13
Myriazoa - ps14
Parahoxozoa - ps15
Bilateria - ps16
Deuterostomia - ps17
Chordata - ps18
Olfactores - ps19
Vertebrata - ps20
Gnathostomata - ps21
Euteleostomi - ps22
Sarcopterygii - ps23
Tetrapoda - ps24
Amniota - ps25
Mammalia - ps26
Theria - ps27
Placentalia - ps28
Boreoeutheria - ps29
Euarchontoglires - ps30
Euarchonta - ps31
Primates - ps32
Haplorrhini - ps33
Simiiformes - ps34
Catarrhini - ps35
Hominoidea - ps36
Hominidae - ps37
Homininae - ps38
Hominini - ps39
Homo_sapiens - ps40
Phylostratum

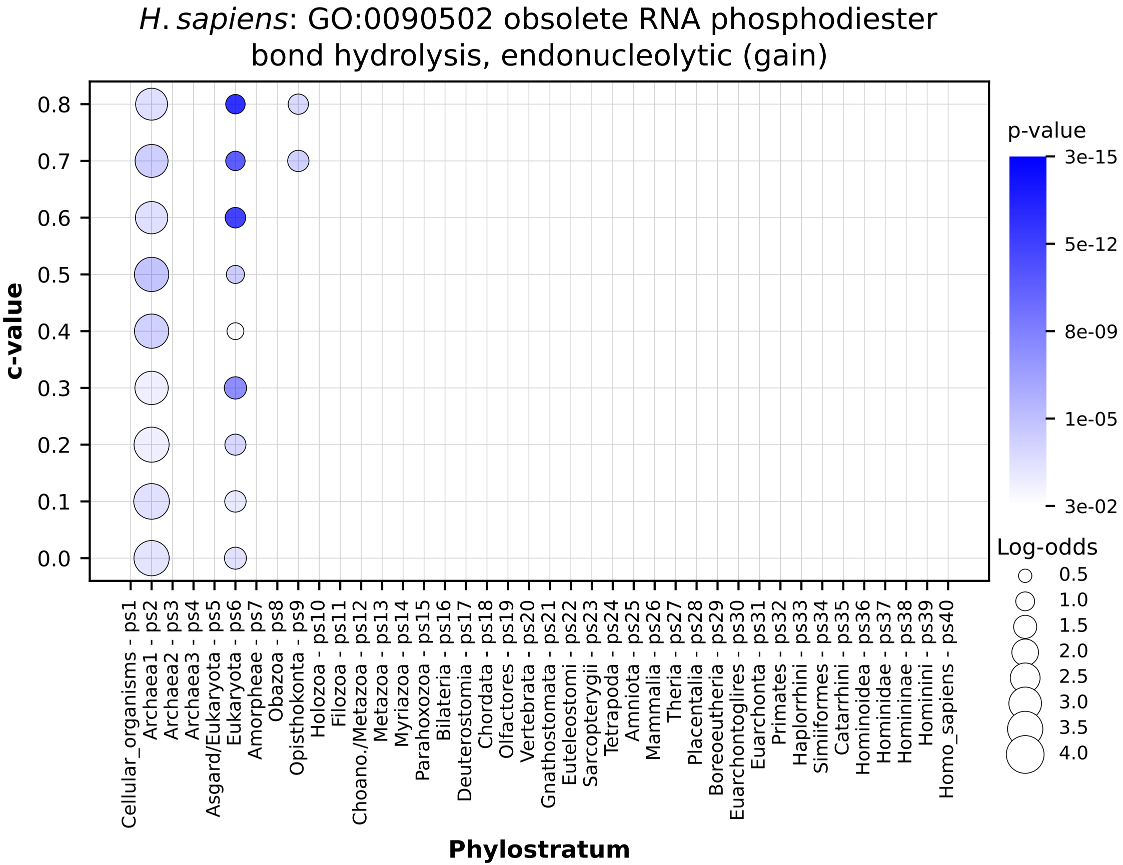
*H. sapiens*: GO:0090502 obsolete RNA phosphodiester bond hydrolysis, endonucleolytic (gain)
c-value
0.8
0.7
0.6
0.5
0.4
0.3
0.2
0.1
0.0
p-value
3e-15
5e-12
8e-09
1e-05
3e-02
Log-odds
0.5
1.0
1.5
2.0
2.5
3.0
3.5
4.0
Cellular_organisms - ps1
Archaea1 - ps2
Archaea2 - ps3
Archaea3 - ps4
Asgard/Eukaryota - ps5
Eukaryota - ps6
Amorpheae - ps7
Obazoa - ps8
Opisthokonta - ps9
Holozoa - ps10
Filozoa - ps11
Choano./Metazoa - ps12
Metazoa - ps13
Myriazoa - ps14
Parahoxozoa - ps15
Bilateria - ps16
Deuterostomia - ps17
Chordata - ps18
Olfactores - ps19
Vertebrata - ps20
Gnathostomata - ps21
Euteleostomi - ps22
Sarcopterygii - ps23
Tetrapoda - ps24
Amniota - ps25
Mammalia - ps26
Theria - ps27
Placentalia - ps28
Boreoeutheria - ps29
Euarchontoglires - ps30
Euarchonta - ps31
Primates - ps32
Haplorrhini - ps33
Simiiformes - ps34
Catarrhini - ps35
Hominoidea - ps36
Hominidae - ps37
Homininae - ps38
Hominini - ps39
Homo_sapiens - ps40
Phylostratum

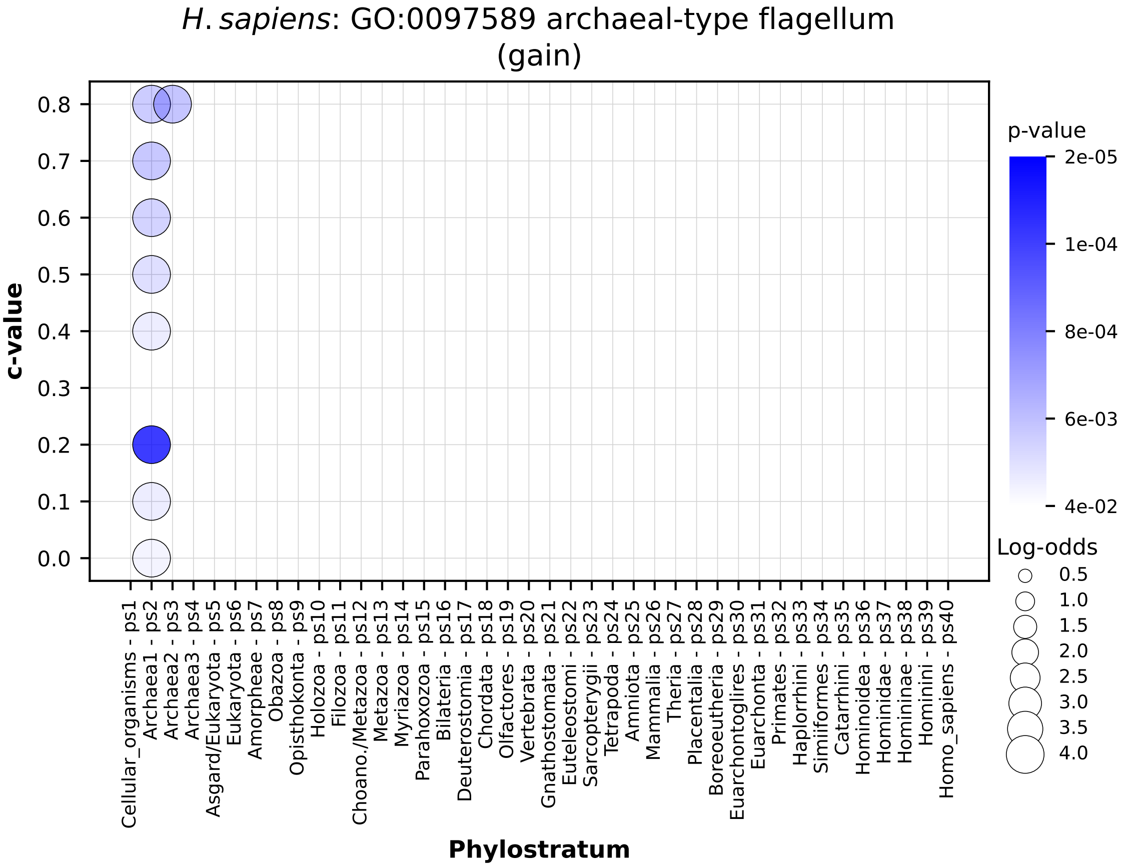

H. sapiens: GO:0097589 archaeal-type flagellum
(gain)
c-value
0.8
0.7
0.6
0.5
0.4
0.3
0.2
0.1
0.0
Phylostratum
Cellular_organisms - ps1
Archaea1 - ps2
Archaea2 - ps3
Archaea3 - ps4
Asgard/Eukaryota - ps5
Eukaryota - ps6
Amorpheae - ps7
Obazoa - ps8
Opisthokonta - ps9
Holozoa - ps10
Filozoa - ps11
Choano./Metazoa - ps12
Metazoa - ps13
Myriazoa - ps14
Parahoxozoa - ps15
Bilateria - ps16
Deuterostomia - ps17
Chordata - ps18
Olfactores - ps19
Vertebrata - ps20
Gnathostomata - ps21
Euteleostomi - ps22
Sarcopterygii - ps23
Tetrapoda - ps24
Amniota - ps25
Mammalia - ps26
Theria - ps27
Placentalia - ps28
Boreoeutheria - ps29
Euarchontoglires - ps30
Euarchonta - ps31
Primates - ps32
Haplorrhini - ps33
Simiiformes - ps34
Catarrhini - ps35
Hominoidea - ps36
Hominidae - ps37
Homininae - ps38
Hominini - ps39
Homo_sapiens - ps40
p-value
2e-05
1e-04
8e-04
6e-03
4e-02
Log-odds
0.5
1.0
1.5
2.0
2.5
3.0
3.5
4.0

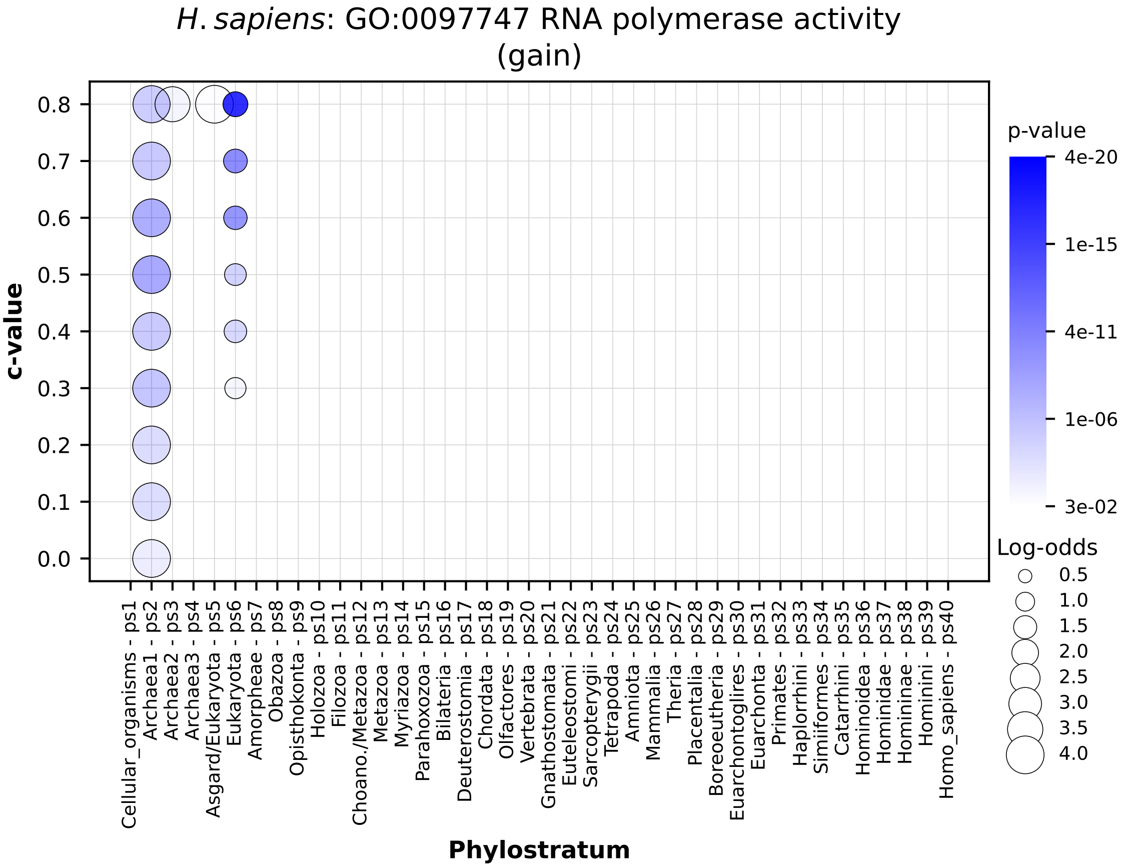

H. sapiens: GO:0097747 RNA polymerase activity
(gain)
c-value
0.0
0.1
0.2
0.3
0.4
0.5
0.6
0.7
0.8
p-value
4e-20
1e-15
4e-11
1e-06
3e-02
Log-odds
0.5
1.0
1.5
2.0
2.5
3.0
3.5
4.0
Cellular_organisms - ps1
Archaea1 - ps2
Archaea2 - ps3
Archaea3 - ps4
Asgard/Eukaryota - ps5
Eukaryota - ps6
Amorpheae - ps7
Obazoa - ps8
Opisthokonta - ps9
Holozoa - ps10
Filozoa - ps11
Choano./Metazoa - ps12
Metazoa - ps13
Myriazoa - ps14
Parahoxozoa - ps15
Bilateria - ps16
Deuterostomia - ps17
Chordata - ps18
Olfactores - ps19
Vertebrata - ps20
Gnathostomata - ps21
Euteleostomi - ps22
Sarcopterygii - ps23
Tetrapoda - ps24
Amniota - ps25
Mammalia - ps26
Theria - ps27
Placentalia - ps28
Boreoeutheria - ps29
Euarchontoglires - ps30
Euarchonta - ps31
Primates - ps32
Haplorrhini - ps33
Simiiformes - ps34
Catarrhini - ps35
Hominoidea - ps36
Hominidae - ps37
Homininae - ps38
Hominini - ps39
Homo_sapiens - ps40
Phylostratum

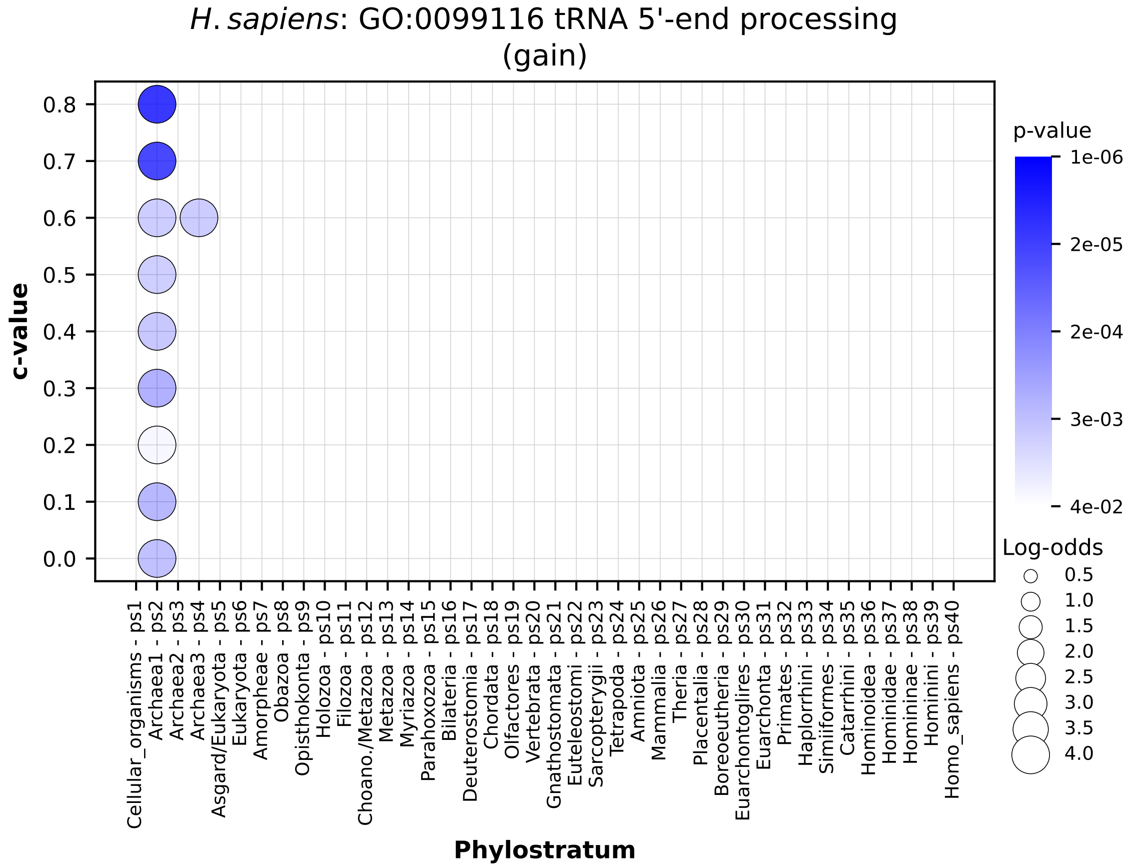
H. sapiens: GO:0099116 tRNA 5'-end processing
(gain)
c-value
0.8
0.7
0.6
0.5
0.4
0.3
0.2
0.1
0.0
p-value
1e-06
2e-05
2e-04
3e-03
4e-02
Log-odds
0.5
1.0
1.5
2.0
2.5
3.0
3.5
4.0
Cellular_organisms - ps1
Archaea1 - ps2
Archaea2 - ps3
Archaea3 - ps4
Asgard/Eukaryota - ps5
Eukaryota - ps6
Amorpheae - ps7
Obazoa - ps8
Opisthokonta - ps9
Holozoa - ps10
Filozoa - ps11
Choano./Metazoa - ps12
Metazoa - ps13
Myriazoa - ps14
Parahoxozoa - ps15
Bilateria - ps16
Deuterostomia - ps17
Chordata - ps18
Olfactores - ps19
Vertebrata - ps20
Gnathostomata - ps21
Euteleostomi - ps22
Sarcopterygii - ps23
Tetrapoda - ps24
Amniota - ps25
Mammalia - ps26
Theria - ps27
Placentalia - ps28
Boreoeutheria - ps29
Euarchontoglires - ps30
Euarchonta - ps31
Primates - ps32
Haplorrhini - ps33
Simiiformes - ps34
Catarrhini - ps35
Hominoidea - ps36
Hominidae - ps37
Homininae - ps38
Hominini - ps39
Homo_sapiens - ps40
Phylostratum

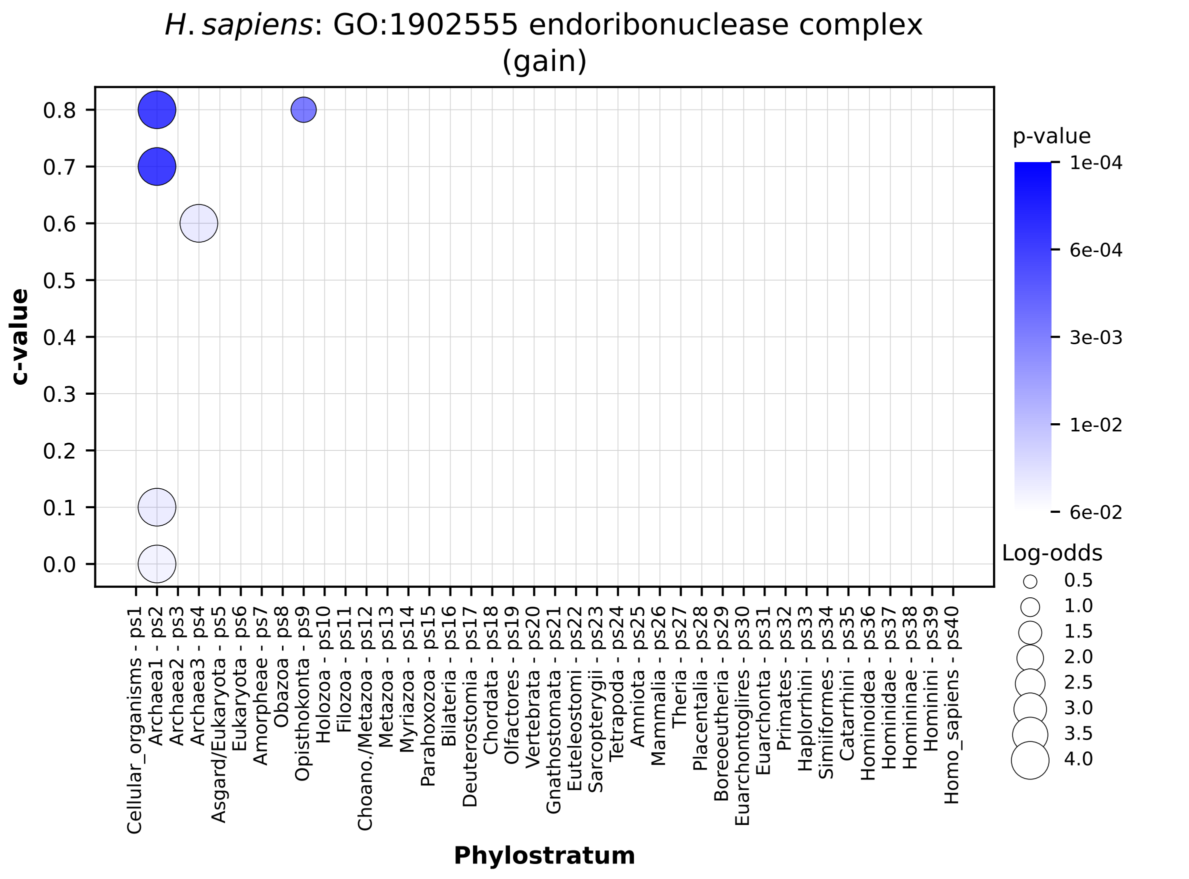

$H. sapiens$: GO:1902555 endoribonuclease complex
(gain)
c-value
0.0
0.1
0.2
0.3
0.4
0.5
0.6
0.7
0.8
p-value
1e-04
6e-04
3e-03
1e-02
6e-02
Log-odds
0.5
1.0
1.5
2.0
2.5
3.0
3.5
4.0
Cellular_organisms - ps1
Archaea1 - ps2
Archaea2 - ps3
Archaea3 - ps4
Asgard/Eukaryota - ps5
Eukaryota - ps6
Amorpheae - ps7
Obazoa - ps8
Opisthokonta - ps9
Holozoa - ps10
Filozoa - ps11
Choano./Metazoa - ps12
Metazoa - ps13
Myriazoa - ps14
Parahoxozoa - ps15
Bilateria - ps16
Deuterostomia - ps17
Chordata - ps18
Olfactores - ps19
Vertebrata - ps20
Gnathostomata - ps21
Euteleostomi - ps22
Sarcopterygii - ps23
Tetrapoda - ps24
Amniota - ps25
Mammalia - ps26
Theria - ps27
Placentalia - ps28
Boreoeutheria - ps29
Euarchontoglires - ps30
Euarchonta - ps31
Primates - ps32
Haplorrhini - ps33
Simiiformes - ps34
Catarrhini - ps35
Hominoidea - ps36
Hominidae - ps37
Homininae - ps38
Hominini - ps39
Homo_sapiens - ps40
Phylostratum

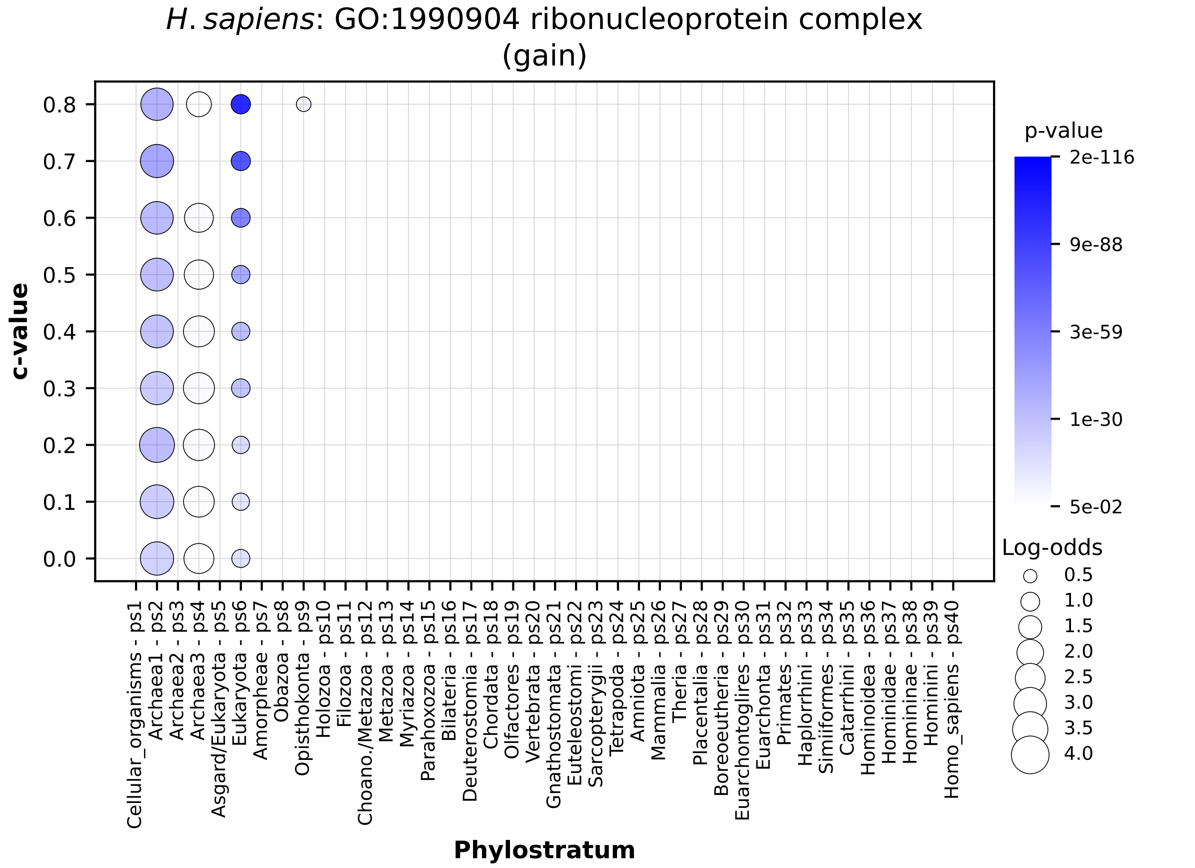

*H. sapiens*: GO:1990904 ribonucleoprotein complex
(gain)
c-value
0.8
0.7
0.6
0.5
0.4
0.3
0.2
0.1
0.0
p-value
2e-116
9e-88
3e-59
1e-30
5e-02
Log-odds
0.5
1.0
1.5
2.0
2.5
3.0
3.5
4.0
Cellular_organisms - ps1
Archaea1 - ps2
Archaea2 - ps3
Archaea3 - ps4
Asgard/Eukaryota - ps5
Eukaryota - ps6
Amorpheae - ps7
Obazoa - ps8
Opisthokonta - ps9
Holozoa - ps10
Filozoa - ps11
Choano./Metazoa - ps12
Metazoa - ps13
Myriazoa - ps14
Parahoxozoa - ps15
Bilateria - ps16
Deuterostomia - ps17
Chordata - ps18
Olfactores - ps19
Vertebrata - ps20
Gnathostomata - ps21
Euteleostomi - ps22
Sarcopterygii - ps23
Tetrapoda - ps24
Amniota - ps25
Mammalia - ps26
Theria - ps27
Placentalia - ps28
Boreoeutheria - ps29
Euarchontoglires - ps30
Euarchonta - ps31
Primates - ps32
Haplorrhini - ps33
Simiiformes - ps34
Catarrhini - ps35
Hominoidea - ps36
Hominidae - ps37
Homininae - ps38
Hominini - ps39
Homo_sapiens - ps40
Phylostratum

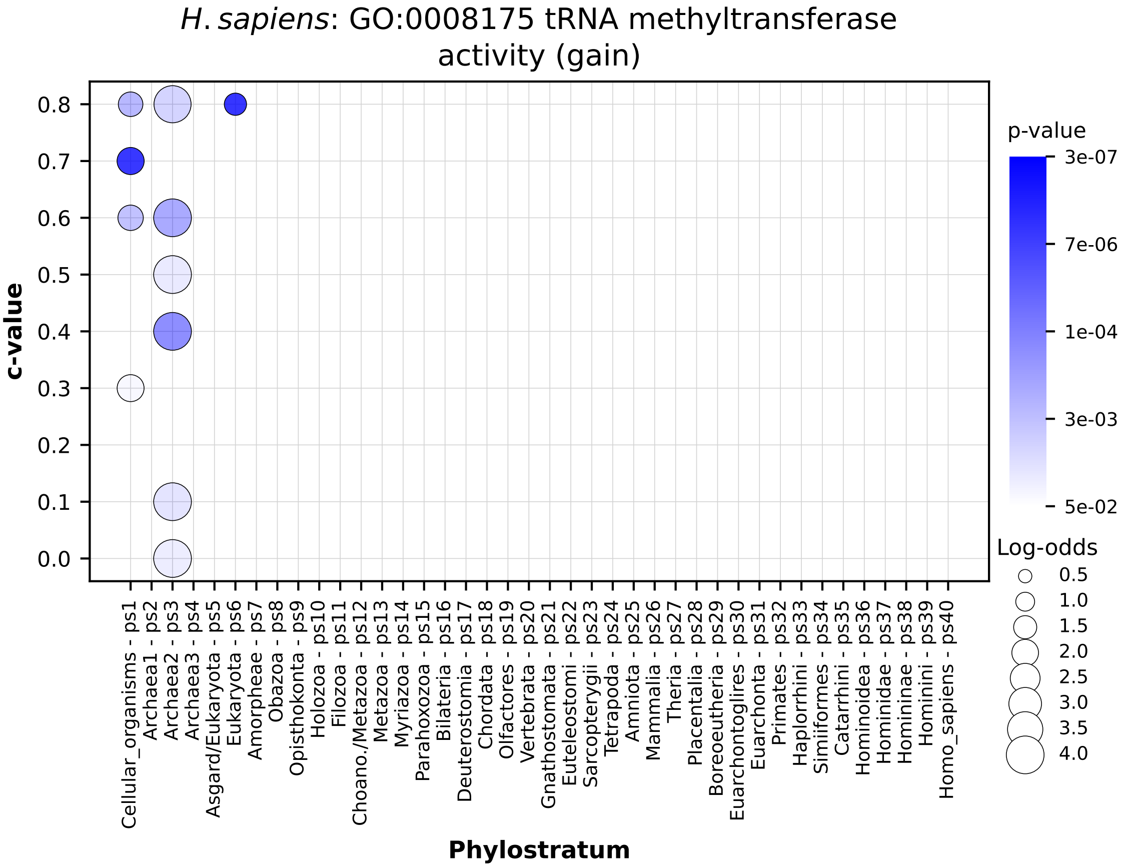
H. sapiens: GO:0008175 tRNA methyltransferase activity (gain)
c-value
0.8
0.7
0.6
0.5
0.4
0.3
0.2
0.1
0.0
Phylostratum
Cellular_organisms - ps1
Archaea1 - ps2
Archaea2 - ps3
Archaea3 - ps4
Asgard/Eukaryota - ps5
Eukaryota - ps6
Amorpheae - ps7
Obazoa - ps8
Opisthokonta - ps9
Holozoa - ps10
Filozoa - ps11
Choano./Metazoa - ps12
Metazoa - ps13
Myriazoa - ps14
Parahoxozoa - ps15
Bilateria - ps16
Deuterostomia - ps17
Chordata - ps18
Olfactores - ps19
Vertebrata - ps20
Gnathostomata - ps21
Euteleostomi - ps22
Sarcopterygii - ps23
Tetrapoda - ps24
Amniota - ps25
Mammalia - ps26
Theria - ps27
Placentalia - ps28
Boreoeutheria - ps29
Euarchontoglires - ps30
Euarchonta - ps31
Primates - ps32
Haplorrhini - ps33
Simiiformes - ps34
Catarrhini - ps35
Hominoidea - ps36
Hominidae - ps37
Homininae - ps38
Hominini - ps39
Homo_sapiens - ps40
p-value
3e-07
7e-06
1e-04
3e-03
5e-02
Log-odds
0.5
1.0
1.5
2.0
2.5
3.0
3.5
4.0

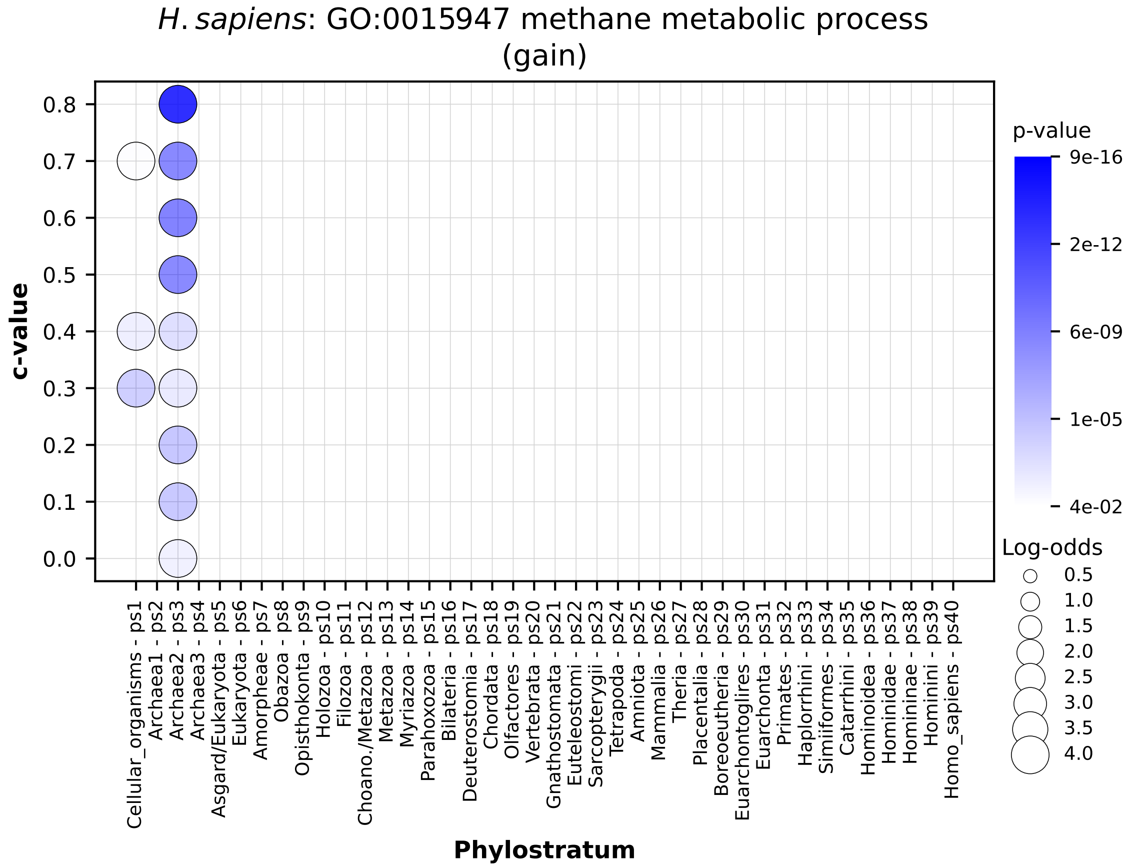

H. sapiens: GO:0015947 methane metabolic process
(gain)
c-value
0.8
0.7
0.6
0.5
0.4
0.3
0.2
0.1
0.0
p-value
9e-16
2e-12
6e-09
1e-05
4e-02
Log-odds
0.5
1.0
1.5
2.0
2.5
3.0
3.5
4.0
Cellular_organisms - ps1
Archaea1 - ps2
Archaea2 - ps3
Archaea3 - ps4
Asgard/Eukaryota - ps5
Eukaryota - ps6
Amorpheae - ps7
Obazoa - ps8
Opisthokonta - ps9
Holozoa - ps10
Filozoa - ps11
Choano./Metazoa - ps12
Metazoa - ps13
Myriazoa - ps14
Parahoxozoa - ps15
Bilateria - ps16
Deuterostomia - ps17
Chordata - ps18
Olfactores - ps19
Vertebrata - ps20
Gnathostomata - ps21
Euteleostomi - ps22
Sarcopterygii - ps23
Tetrapoda - ps24
Amniota - ps25
Mammalia - ps26
Theria - ps27
Placentalia - ps28
Boreoeutheria - ps29
Euarchontoglires - ps30
Euarchonta - ps31
Primates - ps32
Haplorrhini - ps33
Simiiformes - ps34
Catarrhini - ps35
Hominoidea - ps36
Hominidae - ps37
Homininae - ps38
Hominini - ps39
Homo_sapiens - ps40
Phylostratum

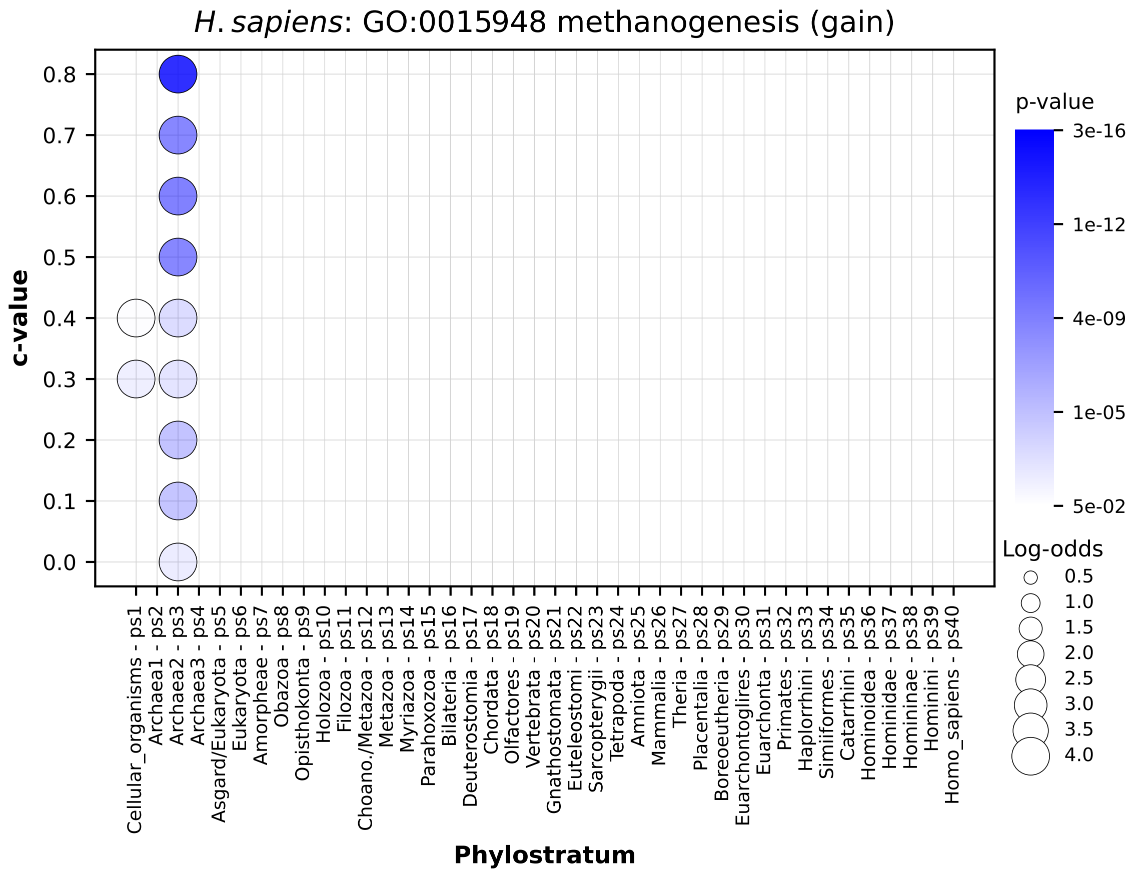

H. sapiens: GO:0015948 methanogenesis (gain)
c-value
0.8
0.7
0.6
0.5
0.4
0.3
0.2
0.1
0.0
Cellular_organisms - ps1
Archaea1 - ps2
Archaea2 - ps3
Archaea3 - ps4
Asgard/Eukaryota - ps5
Eukaryota - ps6
Amorpheae - ps7
Obazoa - ps8
Opisthokonta - ps9
Holozoa - ps10
Filozoa - ps11
Choano./Metazoa - ps12
Metazoa - ps13
Myriazoa - ps14
Parahoxozoa - ps15
Bilateria - ps16
Deuterostomia - ps17
Chordata - ps18
Olfactores - ps19
Vertebrata - ps20
Gnathostomata - ps21
Euteleostomi - ps22
Sarcopterygii - ps23
Tetrapoda - ps24
Amniota - ps25
Mammalia - ps26
Theria - ps27
Placentalia - ps28
Boreoeutheria - ps29
Euarchontoglires - ps30
Euarchonta - ps31
Primates - ps32
Haplorrhini - ps33
Simiiformes - ps34
Catarrhini - ps35
Hominoidea - ps36
Hominidae - ps37
Homininae - ps38
Hominini - ps39
Homo_sapiens - ps40
Phylostratum
p-value
3e-16
1e-12
4e-09
1e-05
5e-02
Log-odds
0.5
1.0
1.5
2.0
2.5
3.0
3.5
4.0

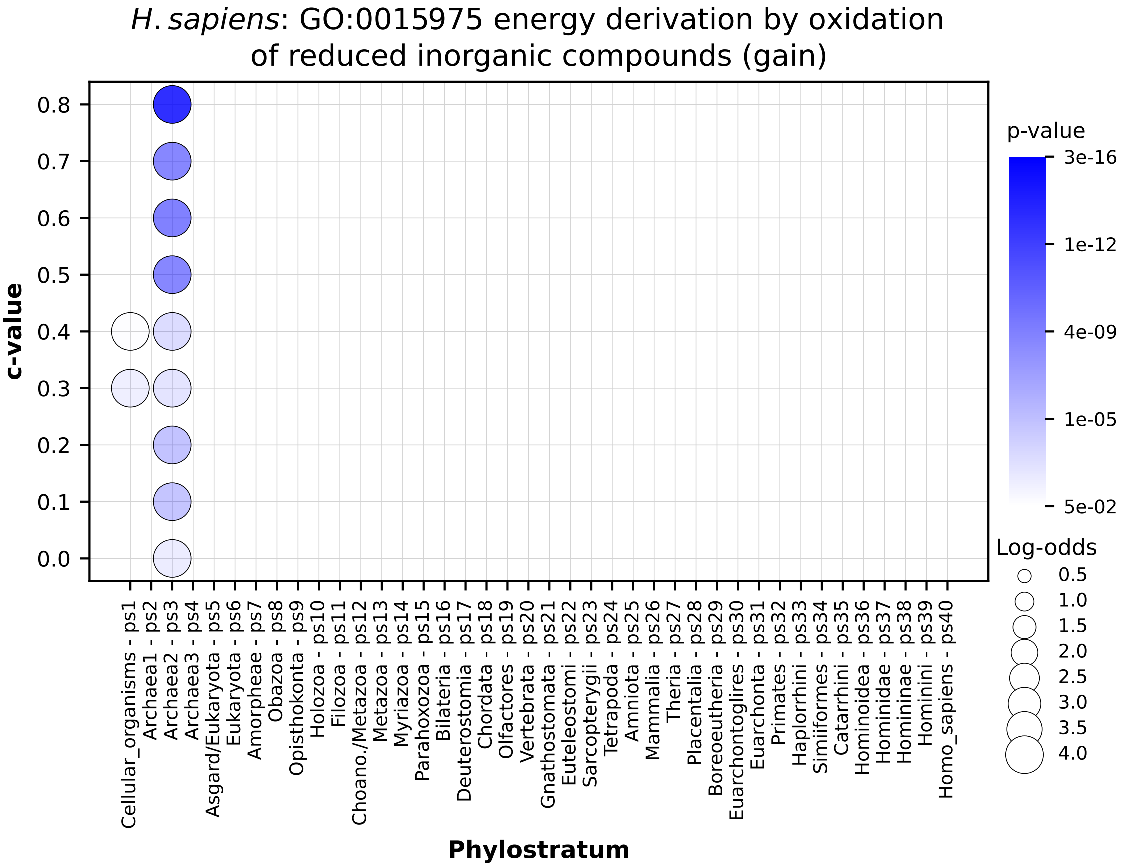

H. sapiens: GO:0015975 energy derivation by oxidation of reduced inorganic compounds (gain)
c-value
0.8
0.7
0.6
0.5
0.4
0.3
0.2
0.1
0.0
p-value
3e-16
1e-12
4e-09
1e-05
5e-02
Log-odds
0.5
1.0
1.5
2.0
2.5
3.0
3.5
4.0
Cellular_organisms - ps1
Archaea1 - ps2
Archaea2 - ps3
Archaea3 - ps4
Asgard/Eukaryota - ps5
Eukaryota - ps6
Amorpheae - ps7
Obazoa - ps8
Opisthokonta - ps9
Holozoa - ps10
Filozoa - ps11
Choano./Metazoa - ps12
Metazoa - ps13
Myriazoa - ps14
Parahoxozoa - ps15
Bilateria - ps16
Deuterostomia - ps17
Chordata - ps18
Olfactores - ps19
Vertebrata - ps20
Gnathostomata - ps21
Euteleostomi - ps22
Sarcopterygii - ps23
Tetrapoda - ps24
Amniota - ps25
Mammalia - ps26
Theria - ps27
Placentalia - ps28
Boreoeutheria - ps29
Euarchontoglires - ps30
Euarchonta - ps31
Primates - ps32
Haplorrhini - ps33
Simiiformes - ps34
Catarrhini - ps35
Hominoidea - ps36
Hominidae - ps37
Homininae - ps38
Hominini - ps39
Homo_sapiens - ps40
Phylostratum

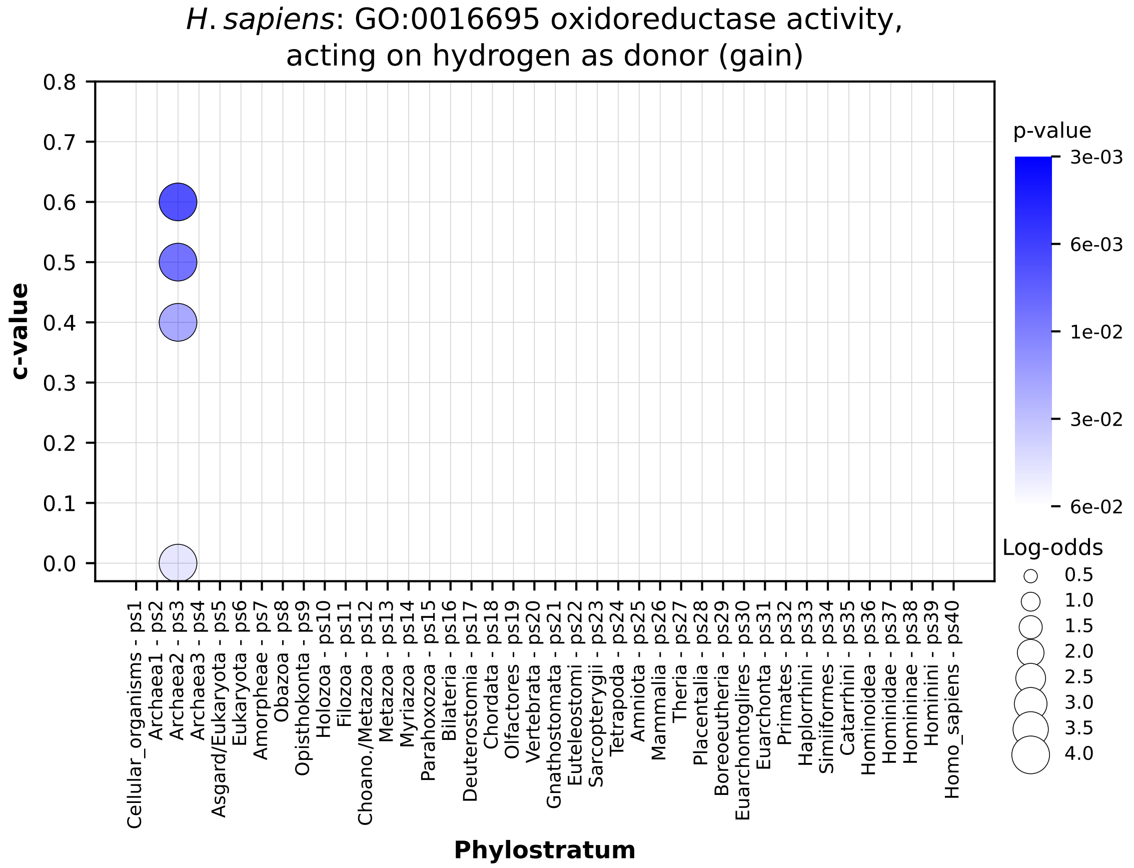

H. sapiens: GO:0016695 oxidoreductase activity, acting on hydrogen as donor (gain)
c-value
0.8
0.7
0.6
0.5
0.4
0.3
0.2
0.1
0.0
Phylostratum
Cellular_organisms - ps1
Archaea1 - ps2
Archaea2 - ps3
Archaea3 - ps4
Asgard/Eukaryota - ps5
Eukaryota - ps6
Amorpheae - ps7
Obazoa - ps8
Opisthokonta - ps9
Holozoa - ps10
Filozoa - ps11
Choano./Metazoa - ps12
Metazoa - ps13
Myriazoa - ps14
Parahoxozoa - ps15
Bilateria - ps16
Deuterostomia - ps17
Chordata - ps18
Olfactores - ps19
Vertebrata - ps20
Gnathostomata - ps21
Euteleostomi - ps22
Sarcopterygii - ps23
Tetrapoda - ps24
Amniota - ps25
Mammalia - ps26
Theria - ps27
Placentalia - ps28
Boreoeutheria - ps29
Euarchontoglires - ps30
Euarchonta - ps31
Primates - ps32
Haplorrhini - ps33
Simiiformes - ps34
Catarrhini - ps35
Hominoidea - ps36
Hominidae - ps37
Homininae - ps38
Hominini - ps39
Homo_sapiens - ps40
p-value
3e-03
6e-03
1e-02
3e-02
6e-02
Log-odds
0.5
1.0
1.5
2.0
2.5
3.0
3.5
4.0

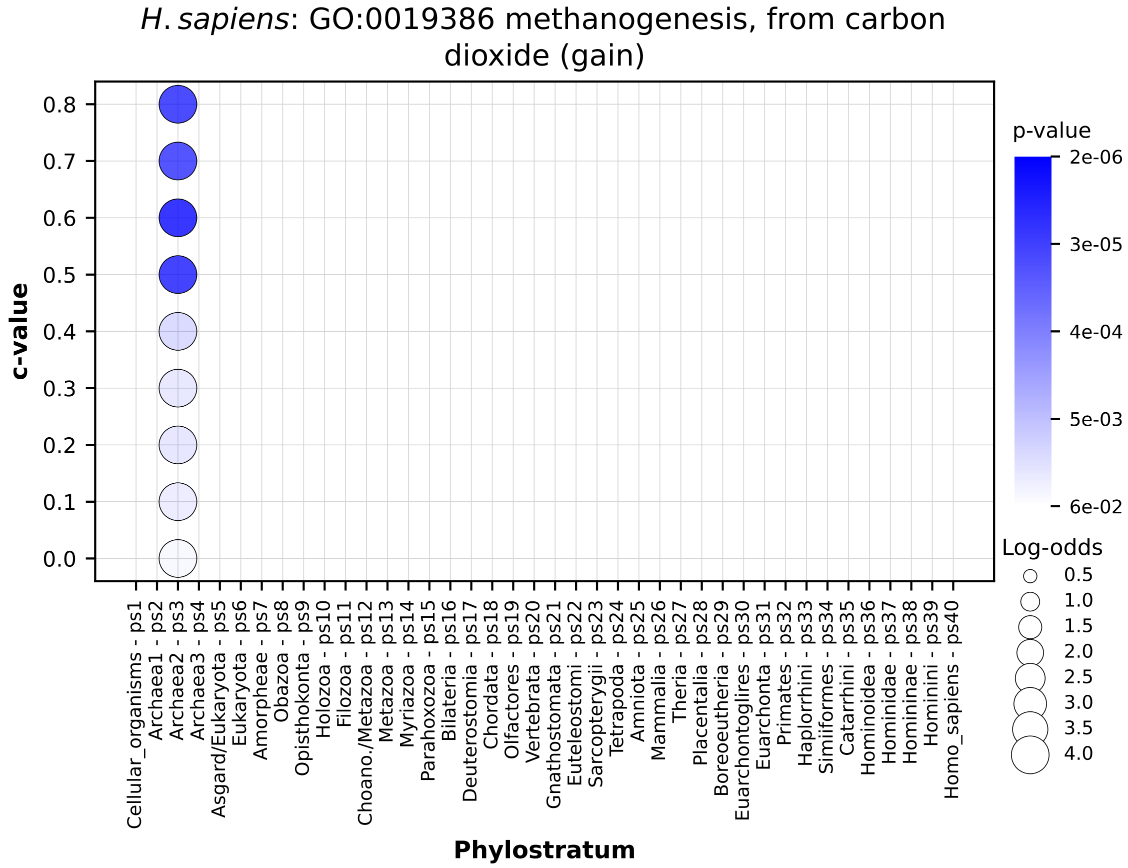
H. sapiens: GO:0019386 methanogenesis, from carbon dioxide (gain)
c-value
0.8
0.7
0.6
0.5
0.4
0.3
0.2
0.1
0.0
p-value
2e-06
3e-05
4e-04
5e-03
6e-02
Log-odds
0.5
1.0
1.5
2.0
2.5
3.0
3.5
4.0
Cellular_organisms - ps1
Archaea1 - ps2
Archaea2 - ps3
Archaea3 - ps4
Asgard/Eukaryota - ps5
Eukaryota - ps6
Amorpheae - ps7
Obazoa - ps8
Opisthokonta - ps9
Holozoa - ps10
Filozoa - ps11
Choano./Metazoa - ps12
Metazoa - ps13
Myriazoa - ps14
Parahoxozoa - ps15
Bilateria - ps16
Deuterostomia - ps17
Chordata - ps18
Olfactores - ps19
Vertebrata - ps20
Gnathostomata - ps21
Euteleostomi - ps22
Sarcopterygii - ps23
Tetrapoda - ps24
Amniota - ps25
Mammalia - ps26
Theria - ps27
Placentalia - ps28
Boreoeutheria - ps29
Euarchontoglires - ps30
Euarchonta - ps31
Primates - ps32
Haplorrhini - ps33
Simiiformes - ps34
Catarrhini - ps35
Hominoidea - ps36
Hominidae - ps37
Homininae - ps38
Hominini - ps39
Homo_sapiens - ps40
Phylostratum

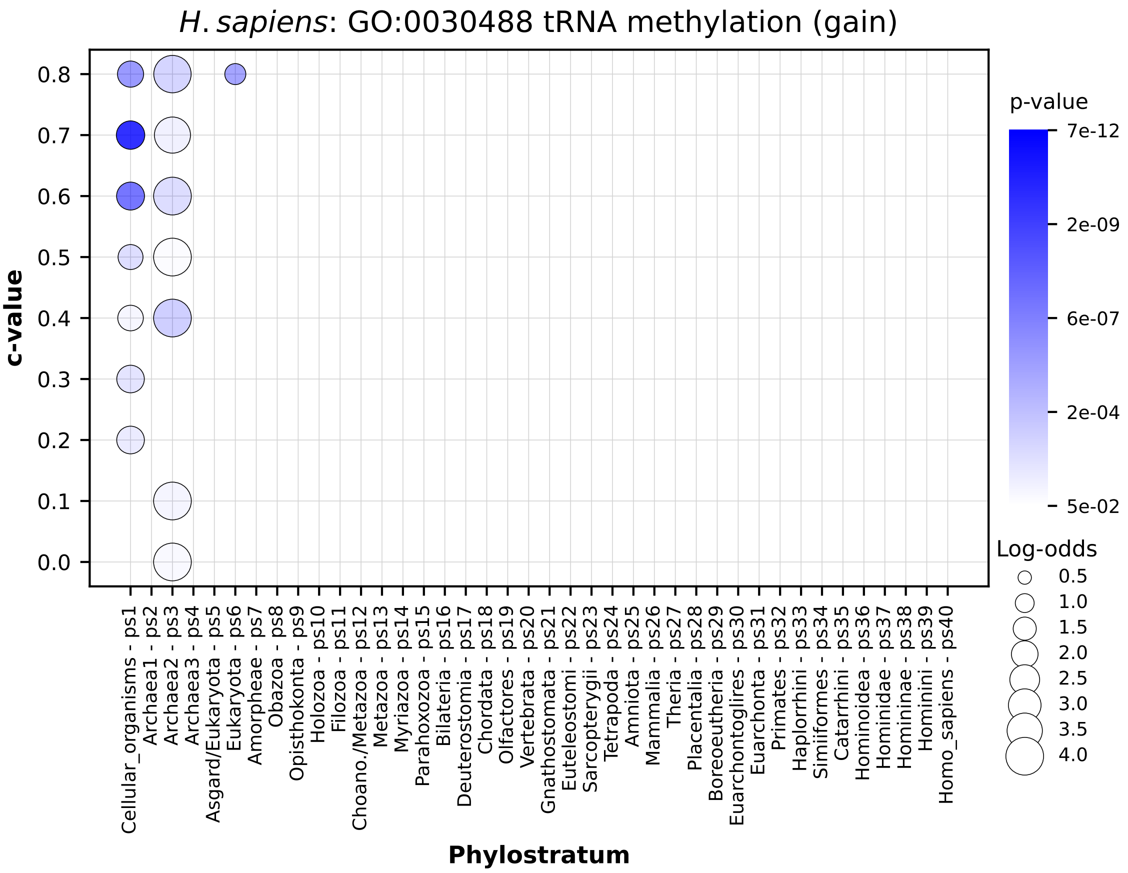

H. sapiens: GO:0030488 tRNA methylation (gain)
c-value
0.8
0.7
0.6
0.5
0.4
0.3
0.2
0.1
0.0
Phylostratum
Cellular_organisms - ps1
Archaea1 - ps2
Archaea2 - ps3
Archaea3 - ps4
Asgard/Eukaryota - ps5
Eukaryota - ps6
Amorpheae - ps7
Obazoa - ps8
Opisthokonta - ps9
Holozoa - ps10
Filozoa - ps11
Choano./Metazoa - ps12
Metazoa - ps13
Myriazoa - ps14
Parahoxozoa - ps15
Bilateria - ps16
Deuterostomia - ps17
Chordata - ps18
Olfactores - ps19
Vertebrata - ps20
Gnathostomata - ps21
Euteleostomi - ps22
Sarcopterygii - ps23
Tetrapoda - ps24
Amniota - ps25
Mammalia - ps26
Theria - ps27
Placentalia - ps28
Boreoeutheria - ps29
Euarchontoglires - ps30
Euarchonta - ps31
Primates - ps32
Haplorrhini - ps33
Simiiformes - ps34
Catarrhini - ps35
Hominoidea - ps36
Hominidae - ps37
Homininae - ps38
Hominini - ps39
Homo_sapiens - ps40
p-value
7e-12
2e-09
6e-07
2e-04
5e-02
Log-odds
0.5
1.0
1.5
2.0
2.5
3.0
3.5
4.0

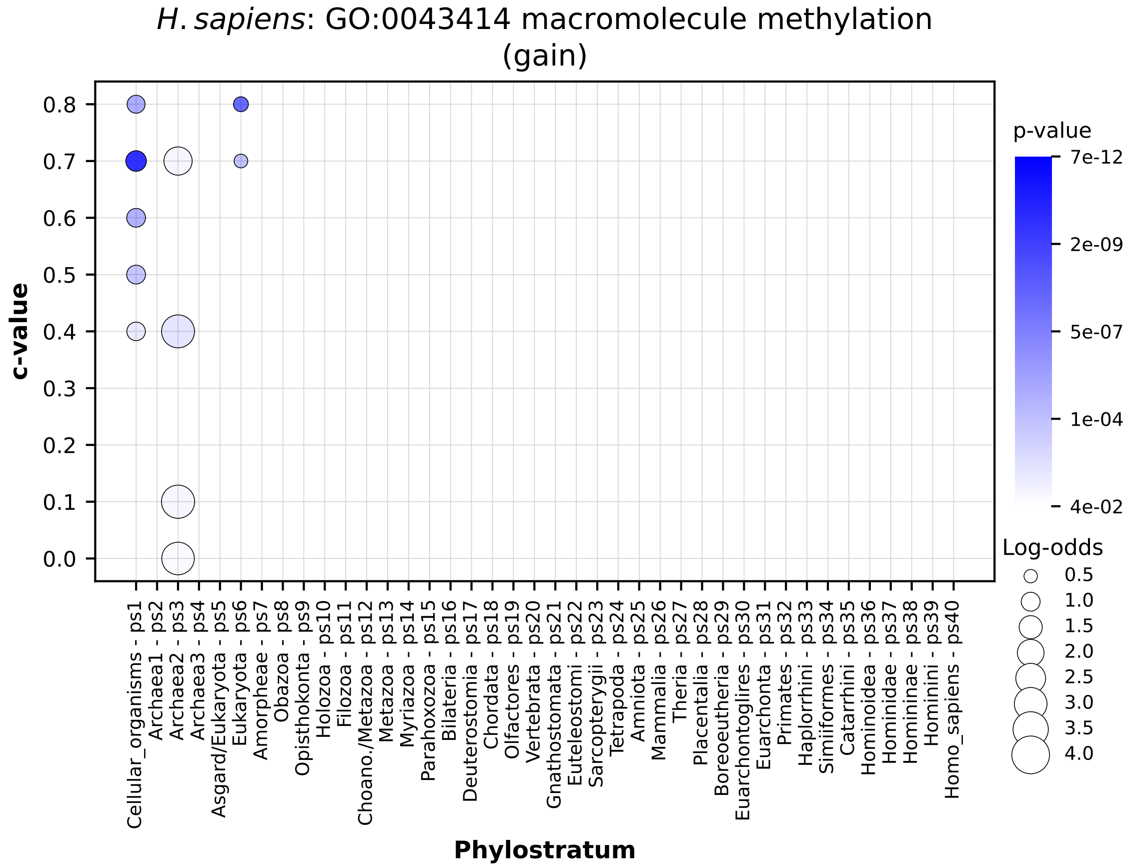

H. sapiens: GO:0043414 macromolecule methylation
(gain)
c-value
0.8
0.7
0.6
0.5
0.4
0.3
0.2
0.1
0.0
p-value
7e-12
2e-09
5e-07
1e-04
4e-02
Log-odds
0.5
1.0
1.5
2.0
2.5
3.0
3.5
4.0
Cellular_organisms - ps1
Archaea1 - ps2
Archaea2 - ps3
Archaea3 - ps4
Asgard/Eukaryota - ps5
Eukaryota - ps6
Amorpheae - ps7
Obazoa - ps8
Opisthokonta - ps9
Holozoa - ps10
Filozoa - ps11
Choano./Metazoa - ps12
Metazoa - ps13
Myriazoa - ps14
Parahoxozoa - ps15
Bilateria - ps16
Deuterostomia - ps17
Chordata - ps18
Olfactores - ps19
Vertebrata - ps20
Gnathostomata - ps21
Euteleostomi - ps22
Sarcopterygii - ps23
Tetrapoda - ps24
Amniota - ps25
Mammalia - ps26
Theria - ps27
Placentalia - ps28
Boreoeutheria - ps29
Euarchontoglires - ps30
Euarchonta - ps31
Primates - ps32
Haplorrhini - ps33
Simiiformes - ps34
Catarrhini - ps35
Hominoidea - ps36
Hominidae - ps37
Homininae - ps38
Hominini - ps39
Homo_sapiens - ps40
Phylostratum

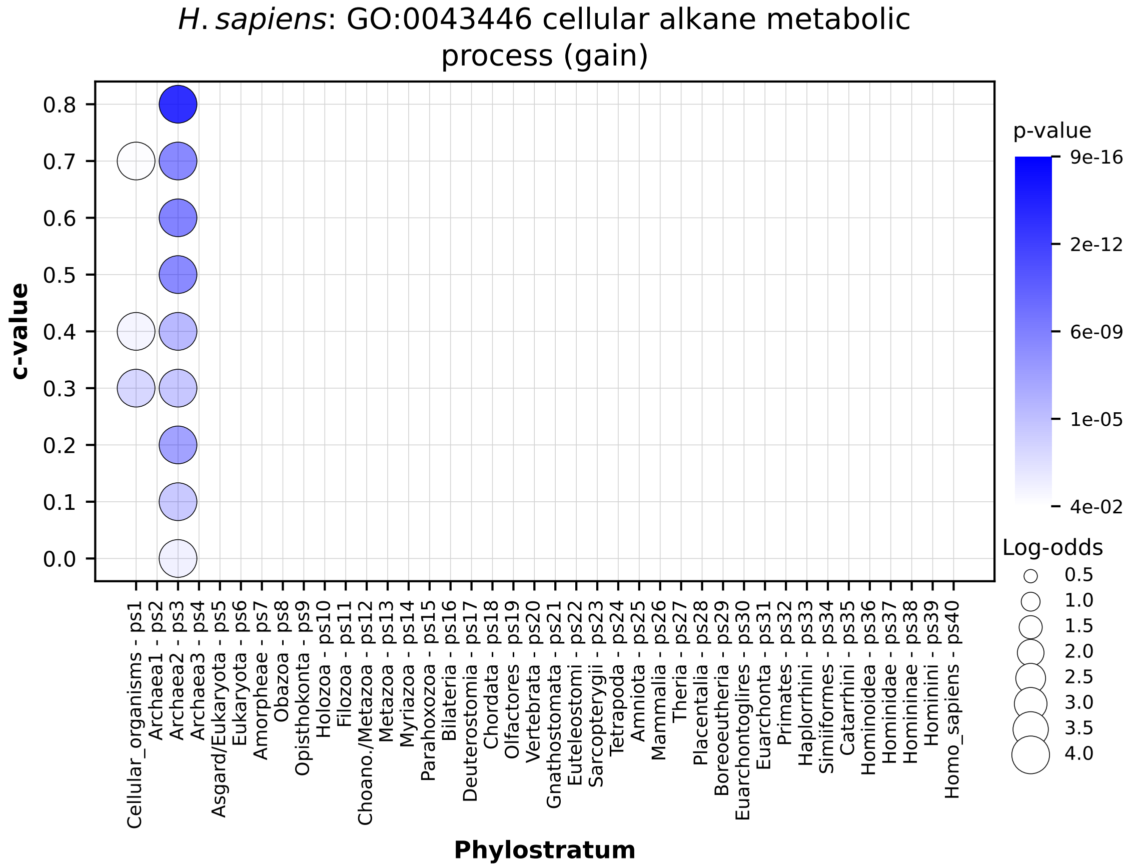

H. sapiens: GO:0043446 cellular alkane metabolic process (gain)
c-value
0.8
0.7
0.6
0.5
0.4
0.3
0.2
0.1
0.0
p-value
9e-16
2e-12
6e-09
1e-05
4e-02
Log-odds
0.5
1.0
1.5
2.0
2.5
3.0
3.5
4.0
Cellular_organisms - ps1
Archaea1 - ps2
Archaea2 - ps3
Archaea3 - ps4
Asgard/Eukaryota - ps5
Eukaryota - ps6
Amorpheae - ps7
Obazoa - ps8
Opisthokonta - ps9
Holozoa - ps10
Filozoa - ps11
Choano./Metazoa - ps12
Metazoa - ps13
Myriazoa - ps14
Parahoxozoa - ps15
Bilateria - ps16
Deuterostomia - ps17
Chordata - ps18
Olfactores - ps19
Vertebrata - ps20
Gnathostomata - ps21
Euteleostomi - ps22
Sarcopterygii - ps23
Tetrapoda - ps24
Amniota - ps25
Mammalia - ps26
Theria - ps27
Placentalia - ps28
Boreoeutheria - ps29
Euarchontoglires - ps30
Euarchonta - ps31
Primates - ps32
Haplorrhini - ps33
Simiiformes - ps34
Catarrhini - ps35
Hominoidea - ps36
Hominidae - ps37
Homininae - ps38
Hominini - ps39
Homo_sapiens - ps40
Phylostratum

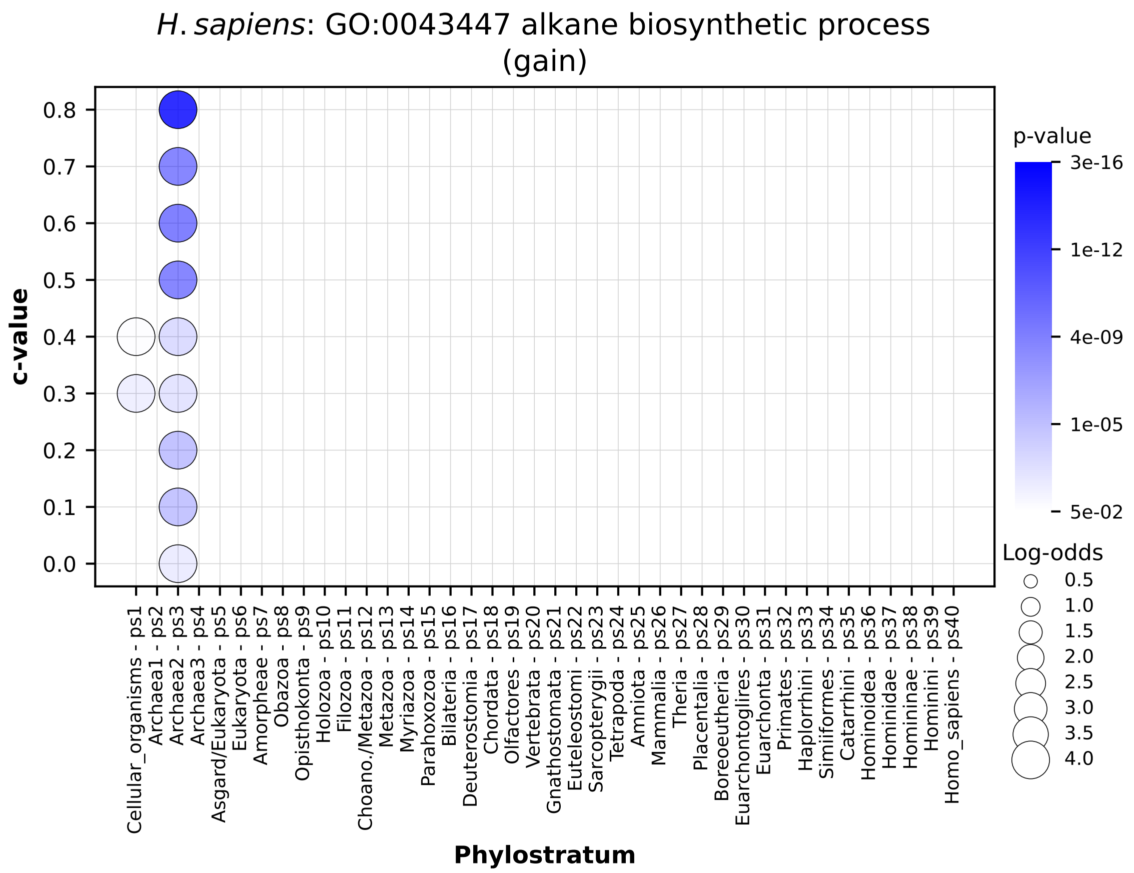

*H. sapiens*: GO:0043447 alkane biosynthetic process
(gain)
c-value
0.8
0.7
0.6
0.5
0.4
0.3
0.2
0.1
0.0
p-value
3e-16
1e-12
4e-09
1e-05
5e-02
Log-odds
0.5
1.0
1.5
2.0
2.5
3.0
3.5
4.0
Cellular_organisms - ps1
Archaea1 - ps2
Archaea2 - ps3
Archaea3 - ps4
Asgard/Eukaryota - ps5
Eukaryota - ps6
Amorpheae - ps7
Obazoa - ps8
Opisthokonta - ps9
Holozoa - ps10
Filozoa - ps11
Choano./Metazoa - ps12
Metazoa - ps13
Myriazoa - ps14
Parahoxozoa - ps15
Bilateria - ps16
Deuterostomia - ps17
Chordata - ps18
Olfactores - ps19
Vertebrata - ps20
Gnathostomata - ps21
Euteleostomi - ps22
Sarcopterygii - ps23
Tetrapoda - ps24
Amniota - ps25
Mammalia - ps26
Theria - ps27
Placentalia - ps28
Boreoeutheria - ps29
Euarchontoglires - ps30
Euarchonta - ps31
Primates - ps32
Haplorrhini - ps33
Simiiformes - ps34
Catarrhini - ps35
Hominoidea - ps36
Hominidae - ps37
Homininae - ps38
Hominini - ps39
Homo_sapiens - ps40
Phylostratum

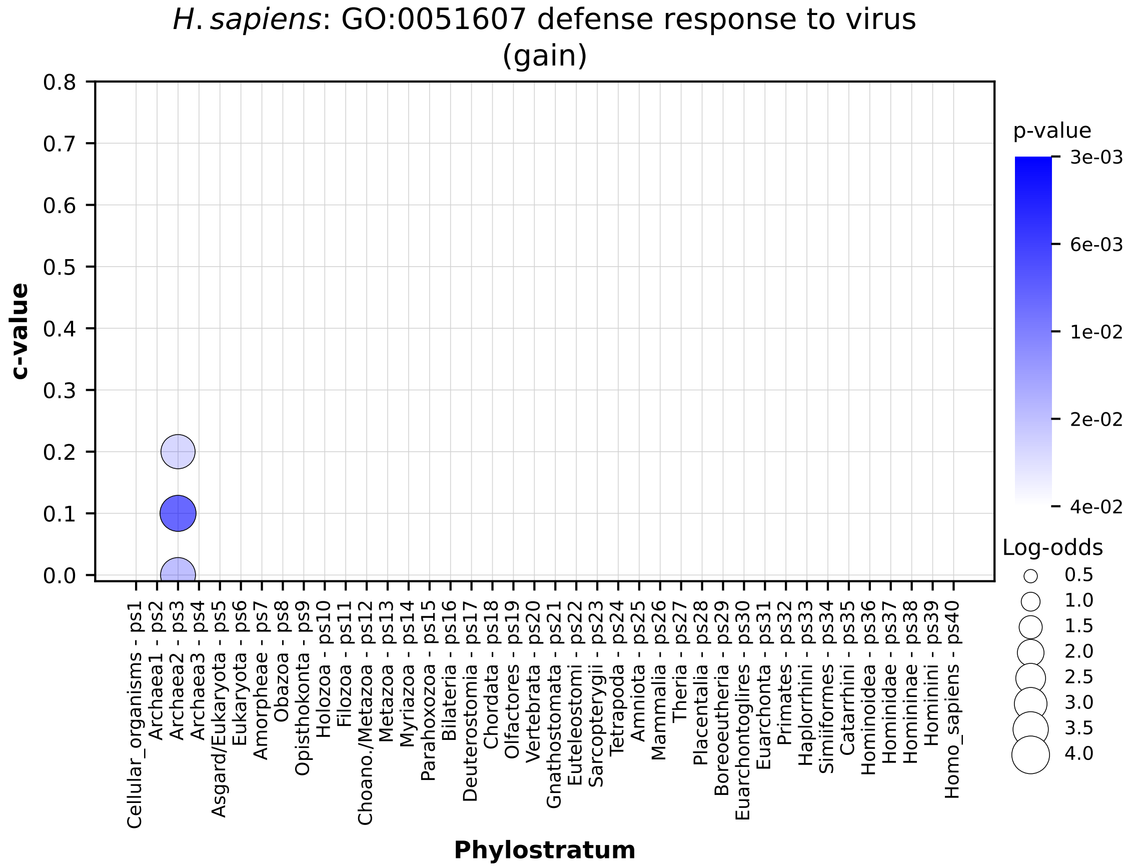

H. sapiens: GO:0051607 defense response to virus
(gain)
c-value
0.8
0.7
0.6
0.5
0.4
0.3
0.2
0.1
0.0
Phylostratum
Cellular_organisms - ps1
Archaea1 - ps2
Archaea2 - ps3
Archaea3 - ps4
Asgard/Eukaryota - ps5
Eukaryota - ps6
Amorpheae - ps7
Obazoa - ps8
Opisthokonta - ps9
Holozoa - ps10
Filozoa - ps11
Choano./Metazoa - ps12
Metazoa - ps13
Myriazoa - ps14
Parahoxozoa - ps15
Bilateria - ps16
Deuterostomia - ps17
Chordata - ps18
Olfactores - ps19
Vertebrata - ps20
Gnathostomata - ps21
Euteleostomi - ps22
Sarcopterygii - ps23
Tetrapoda - ps24
Amniota - ps25
Mammalia - ps26
Theria - ps27
Placentalia - ps28
Boreoeutheria - ps29
Euarchontoglires - ps30
Euarchonta - ps31
Primates - ps32
Haplorrhini - ps33
Simiiformes - ps34
Catarrhini - ps35
Hominoidea - ps36
Hominidae - ps37
Homininae - ps38
Hominini - ps39
Homo_sapiens - ps40
p-value
3e-03
6e-03
1e-02
2e-02
4e-02
Log-odds
0.5
1.0
1.5
2.0
2.5
3.0
3.5
4.0

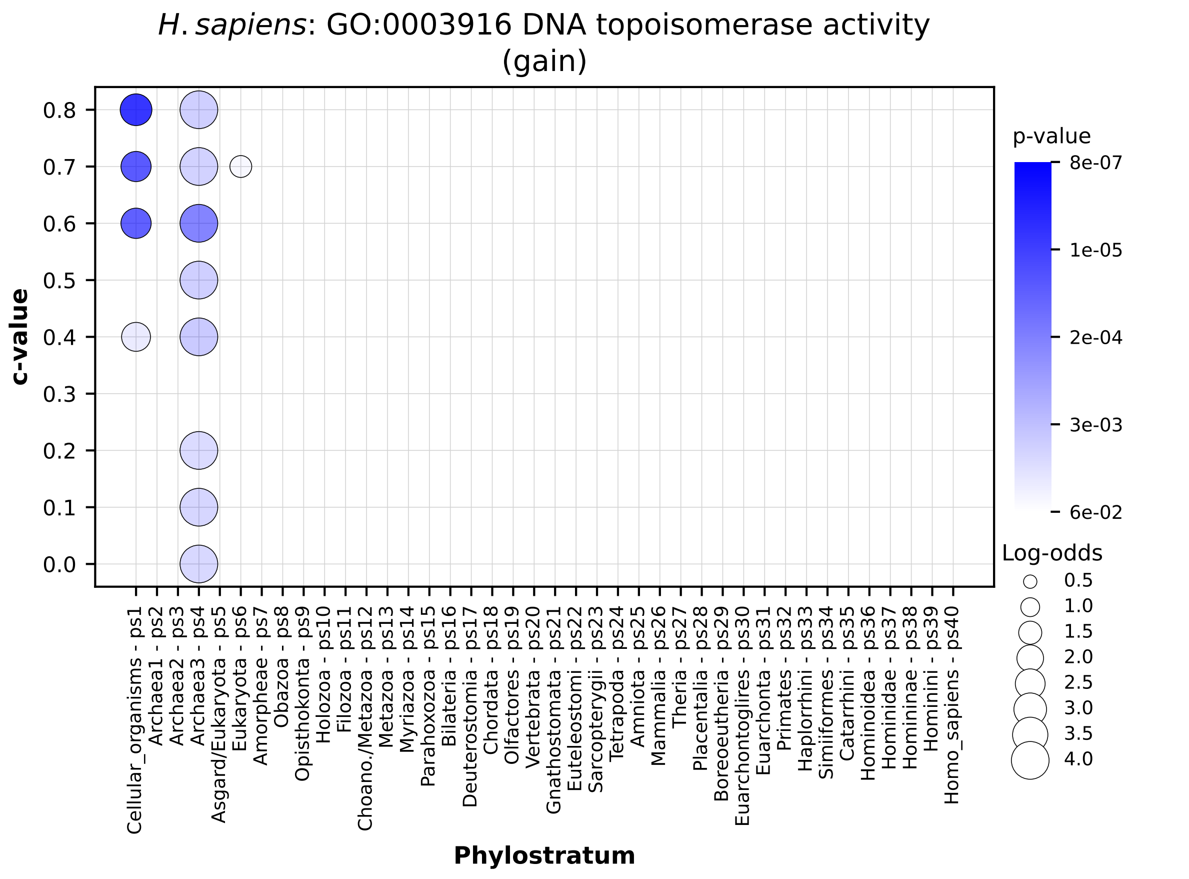

H. sapiens: GO:0003916 DNA topoisomerase activity
(gain)
c-value
0.0
0.1
0.2
0.3
0.4
0.5
0.6
0.7
0.8
p-value
8e-07
1e-05
2e-04
3e-03
6e-02
Log-odds
0.5
1.0
1.5
2.0
2.5
3.0
3.5
4.0
Cellular_organisms - ps1
Archaea1 - ps2
Archaea2 - ps3
Archaea3 - ps4
Asgard/Eukaryota - ps5
Eukaryota - ps6
Amorpheae - ps7
Obazoa - ps8
Opisthokonta - ps9
Holozoa - ps10
Filozoa - ps11
Choano./Metazoa - ps12
Metazoa - ps13
Myriazoa - ps14
Parahoxozoa - ps15
Bilateria - ps16
Deuterostomia - ps17
Chordata - ps18
Olfactores - ps19
Vertebrata - ps20
Gnathostomata - ps21
Euteleostomi - ps22
Sarcopterygii - ps23
Tetrapoda - ps24
Amniota - ps25
Mammalia - ps26
Theria - ps27
Placentalia - ps28
Boreoeutheria - ps29
Euarchontoglires - ps30
Euarchonta - ps31
Primates - ps32
Haplorrhini - ps33
Simiiformes - ps34
Catarrhini - ps35
Hominoidea - ps36
Hominidae - ps37
Homininae - ps38
Hominini - ps39
Homo_sapiens - ps40
Phylostratum

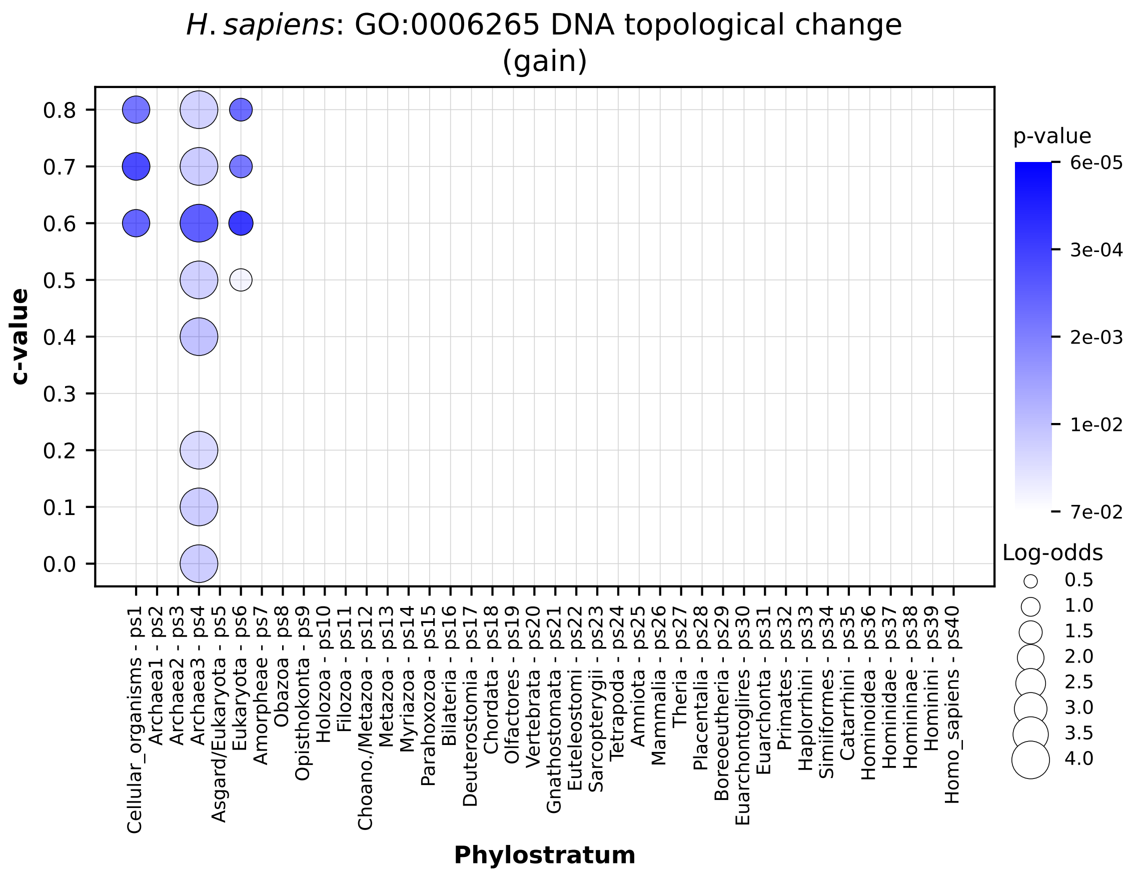

H. sapiens: GO:0006265 DNA topological change
(gain)
c-value
0.8
0.7
0.6
0.5
0.4
0.3
0.2
0.1
0.0
Phylostratum
Cellular_organisms - ps1
Archaea1 - ps2
Archaea2 - ps3
Archaea3 - ps4
Asgard/Eukaryota - ps5
Eukaryota - ps6
Amorpheae - ps7
Obazoa - ps8
Opisthokonta - ps9
Holozoa - ps10
Filozoa - ps11
Choano./Metazoa - ps12
Metazoa - ps13
Myriazoa - ps14
Parahoxozoa - ps15
Bilateria - ps16
Deuterostomia - ps17
Chordata - ps18
Olfactores - ps19
Vertebrata - ps20
Gnathostomata - ps21
Euteleostomi - ps22
Sarcopterygii - ps23
Tetrapoda - ps24
Amniota - ps25
Mammalia - ps26
Theria - ps27
Placentalia - ps28
Boreoeutheria - ps29
Euarchontoglires - ps30
Euarchonta - ps31
Primates - ps32
Haplorrhini - ps33
Simiiformes - ps34
Catarrhini - ps35
Hominoidea - ps36
Hominidae - ps37
Homininae - ps38
Hominini - ps39
Homo_sapiens - ps40
p-value
6e-05
3e-04
2e-03
1e-02
7e-02
Log-odds
0.5
1.0
1.5
2.0
2.5
3.0
3.5
4.0

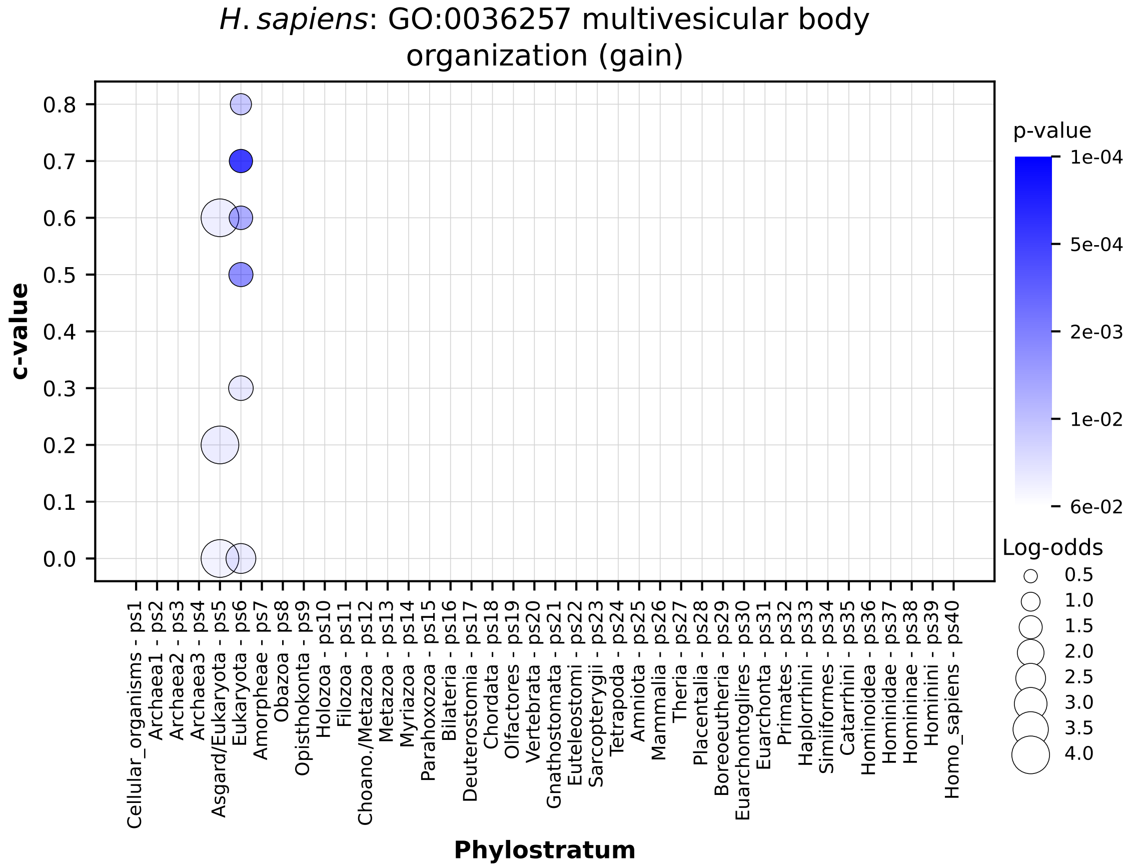

H. sapiens: GO:0036257 multivesicular body organization (gain)
c-value
0.8
0.7
0.6
0.5
0.4
0.3
0.2
0.1
0.0
p-value
1e-04
5e-04
2e-03
1e-02
6e-02
Log-odds
0.5
1.0
1.5
2.0
2.5
3.0
3.5
4.0
Cellular_organisms - ps1
Archaea1 - ps2
Archaea2 - ps3
Archaea3 - ps4
Asgard/Eukaryota - ps5
Eukaryota - ps6
Amorpheae - ps7
Obazoa - ps8
Opisthokonta - ps9
Holozoa - ps10
Filozoa - ps11
Choano./Metazoa - ps12
Metazoa - ps13
Myriazoa - ps14
Parahoxozoa - ps15
Bilateria - ps16
Deuterostomia - ps17
Chordata - ps18
Olfactores - ps19
Vertebrata - ps20
Gnathostomata - ps21
Euteleostomi - ps22
Sarcopterygii - ps23
Tetrapoda - ps24
Amniota - ps25
Mammalia - ps26
Theria - ps27
Placentalia - ps28
Boreoeutheria - ps29
Euarchontoglires - ps30
Euarchonta - ps31
Primates - ps32
Haplorrhini - ps33
Simiiformes - ps34
Catarrhini - ps35
Hominoidea - ps36
Hominidae - ps37
Homininae - ps38
Hominini - ps39
Homo_sapiens - ps40
Phylostratum

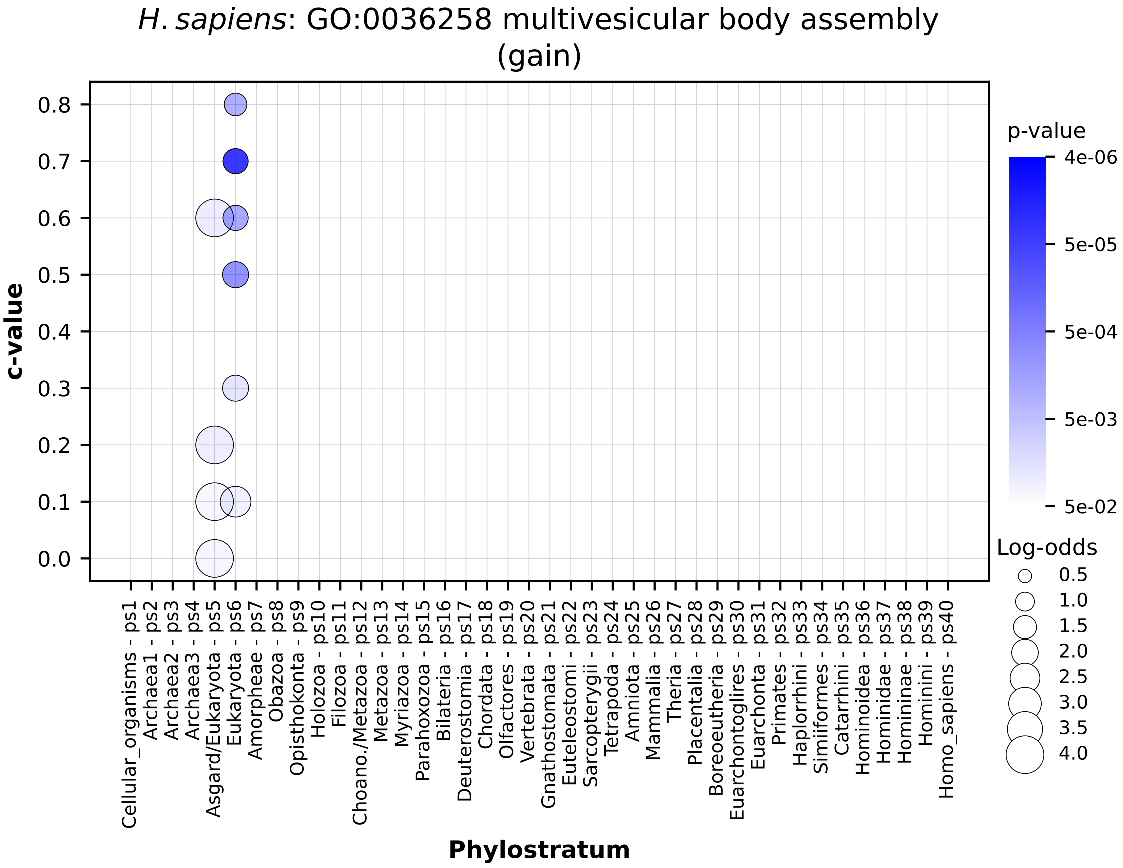

H. sapiens: GO:0036258 multivesicular body assembly (gain)
c-value
0.0
0.1
0.2
0.3
0.4
0.5
0.6
0.7
0.8
Phylostratum
Cellular_organisms - ps1
Archaea1 - ps2
Archaea2 - ps3
Archaea3 - ps4
Asgard/Eukaryota - ps5
Eukaryota - ps6
Amorpheae - ps7
Obazoa - ps8
Opisthokonta - ps9
Holozoa - ps10
Filozoa - ps11
Choano./Metazoa - ps12
Metazoa - ps13
Myriazoa - ps14
Parahoxozoa - ps15
Bilateria - ps16
Deuterostomia - ps17
Chordata - ps18
Olfactores - ps19
Vertebrata - ps20
Gnathostomata - ps21
Euteleostomi - ps22
Sarcopterygii - ps23
Tetrapoda - ps24
Amniota - ps25
Mammalia - ps26
Theria - ps27
Placentalia - ps28
Boreoeutheria - ps29
Euarchontoglires - ps30
Euarchonta - ps31
Primates - ps32
Haplorrhini - ps33
Simiiformes - ps34
Catarrhini - ps35
Hominoidea - ps36
Hominidae - ps37
Homininae - ps38
Hominini - ps39
Homo_sapiens - ps40
p-value
4e-06
5e-05
5e-04
5e-03
5e-02
Log-odds
0.5
1.0
1.5
2.0
2.5
3.0
3.5
4.0

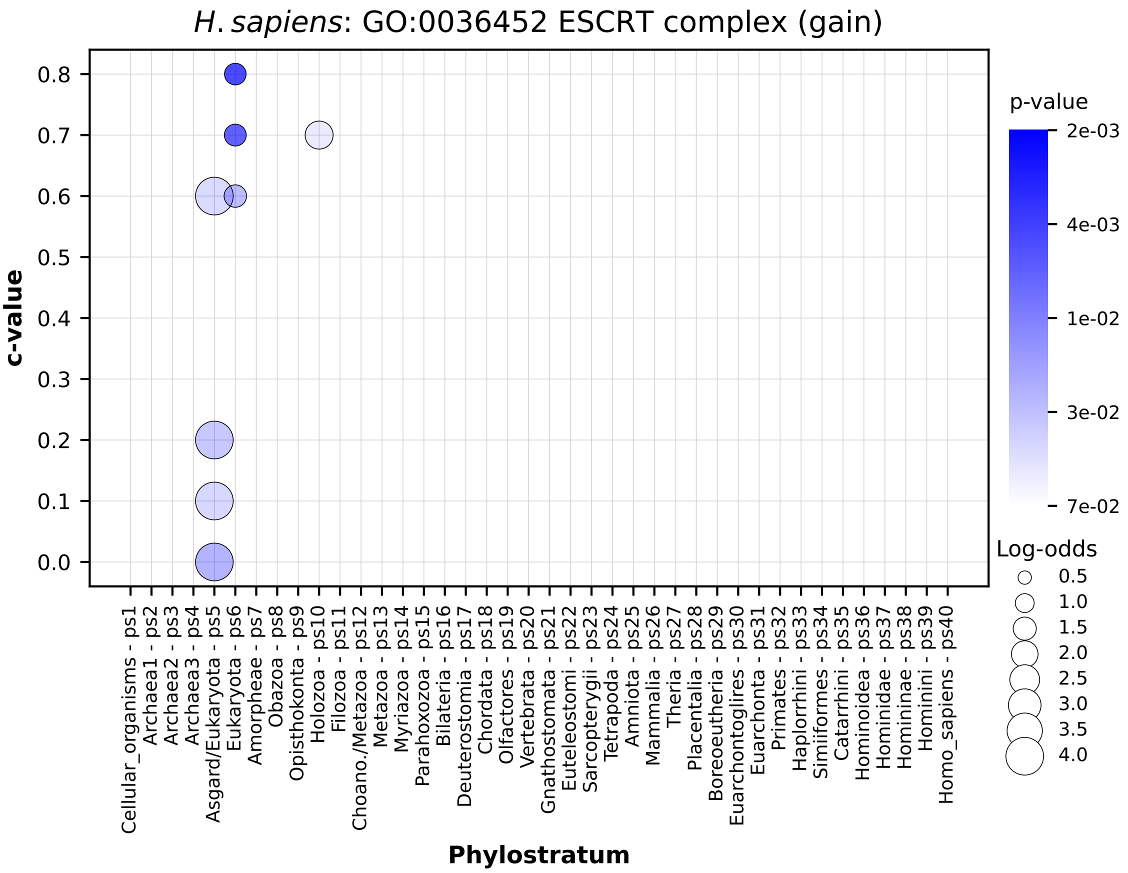
H. sapiens: GO:0036452 ESCRT complex (gain)
c-value
0.8
0.7
0.6
0.5
0.4
0.3
0.2
0.1
0.0
Phylostratum
Cellular_organisms - ps1
Archaea1 - ps2
Archaea2 - ps3
Archaea3 - ps4
Asgard/Eukaryota - ps5
Eukaryota - ps6
Amorpheae - ps7
Obazoa - ps8
Opisthokonta - ps9
Holozoa - ps10
Filozoa - ps11
Choano./Metazoa - ps12
Metazoa - ps13
Myriazoa - ps14
Parahoxozoa - ps15
Bilateria - ps16
Deuterostomia - ps17
Chordata - ps18
Olfactores - ps19
Vertebrata - ps20
Gnathostomata - ps21
Euteleostomi - ps22
Sarcopterygii - ps23
Tetrapoda - ps24
Amniota - ps25
Mammalia - ps26
Theria - ps27
Placentalia - ps28
Boreoeutheria - ps29
Euarchontoglires - ps30
Euarchonta - ps31
Primates - ps32
Haplorrhini - ps33
Simiiformes - ps34
Catarrhini - ps35
Hominoidea - ps36
Hominidae - ps37
Homininae - ps38
Hominini - ps39
Homo_sapiens - ps40
p-value
2e-03
4e-03
1e-02
3e-02
7e-02
Log-odds
0.5
1.0
1.5
2.0
2.5
3.0
3.5
4.0

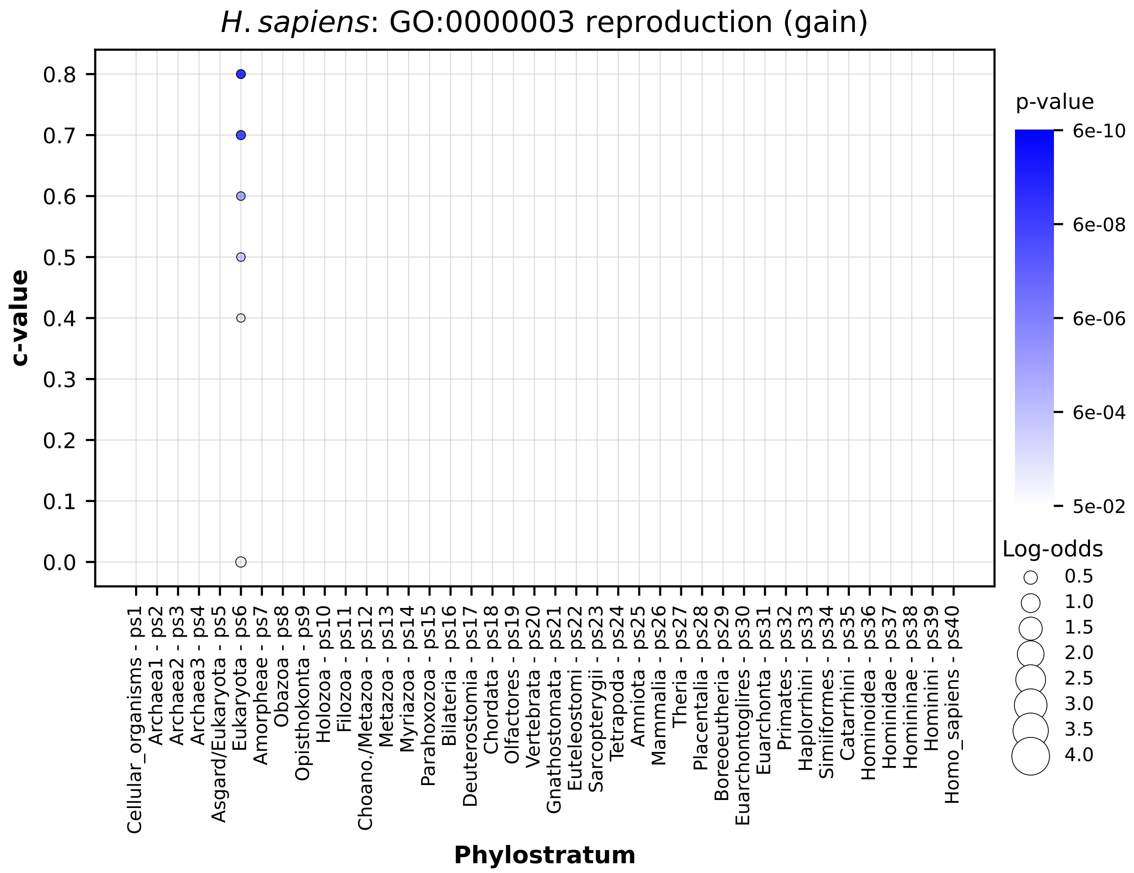

*H. sapiens*: GO:0000003 reproduction (gain)
c-value
0.8
0.7
0.6
0.5
0.4
0.3
0.2
0.1
0.0
Phylostratum
Cellular_organisms - ps1
Archaea1 - ps2
Archaea2 - ps3
Archaea3 - ps4
Asgard/Eukaryota - ps5
Eukaryota - ps6
Amorpheae - ps7
Obazoa - ps8
Opisthokonta - ps9
Holozoa - ps10
Filozoa - ps11
Choano./Metazoa - ps12
Metazoa - ps13
Myriazoa - ps14
Parahoxozoa - ps15
Bilateria - ps16
Deuterostomia - ps17
Chordata - ps18
Olfactores - ps19
Vertebrata - ps20
Gnathostomata - ps21
Euteleostomi - ps22
Sarcopterygii - ps23
Tetrapoda - ps24
Amniota - ps25
Mammalia - ps26
Theria - ps27
Placentalia - ps28
Boreoeutheria - ps29
Euarchontoglires - ps30
Euarchonta - ps31
Primates - ps32
Haplorrhini - ps33
Simiiformes - ps34
Catarrhini - ps35
Hominoidea - ps36
Hominidae - ps37
Homininae - ps38
Hominini - ps39
Homo_sapiens - ps40
p-value
6e-10
6e-08
6e-06
6e-04
5e-02
Log-odds
0.5
1.0
1.5
2.0
2.5
3.0
3.5
4.0

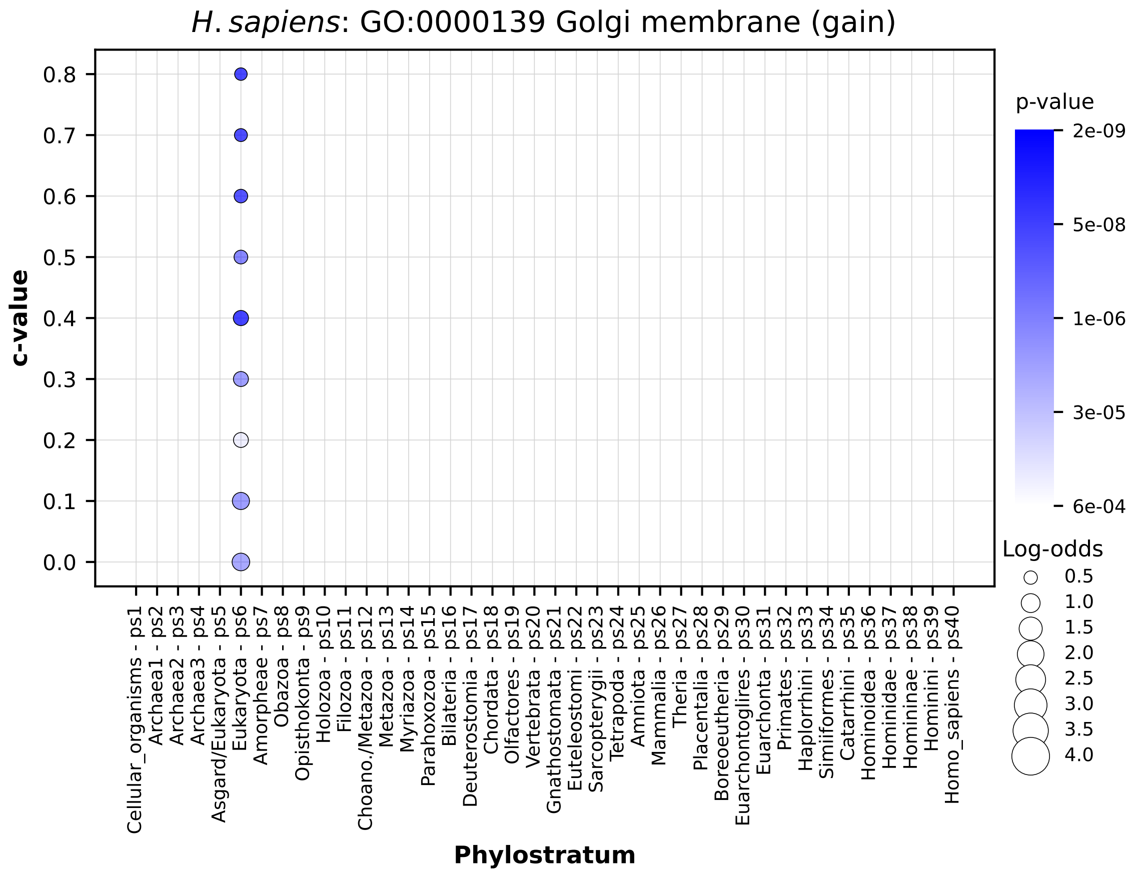
H. sapiens: GO:0000139 Golgi membrane (gain)
c-value
0.8
0.7
0.6
0.5
0.4
0.3
0.2
0.1
0.0
Phylostratum
Cellular_organisms - ps1
Archaea1 - ps2
Archaea2 - ps3
Archaea3 - ps4
Asgard/Eukaryota - ps5
Eukaryota - ps6
Amorpheae - ps7
Obazoa - ps8
Opisthokonta - ps9
Holozoa - ps10
Filozoa - ps11
Choano./Metazoa - ps12
Metazoa - ps13
Myriazoa - ps14
Parahoxozoa - ps15
Bilateria - ps16
Deuterostomia - ps17
Chordata - ps18
Olfactores - ps19
Vertebrata - ps20
Gnathostomata - ps21
Euteleostomi - ps22
Sarcopterygii - ps23
Tetrapoda - ps24
Amniota - ps25
Mammalia - ps26
Theria - ps27
Placentalia - ps28
Boreoeutheria - ps29
Euarchontoglires - ps30
Euarchonta - ps31
Primates - ps32
Haplorrhini - ps33
Simiiformes - ps34
Catarrhini - ps35
Hominoidea - ps36
Hominidae - ps37
Homininae - ps38
Hominini - ps39
Homo_sapiens - ps40
p-value
2e-09
5e-08
1e-06
3e-05
6e-04
Log-odds
0.5
1.0
1.5
2.0
2.5
3.0
3.5
4.0

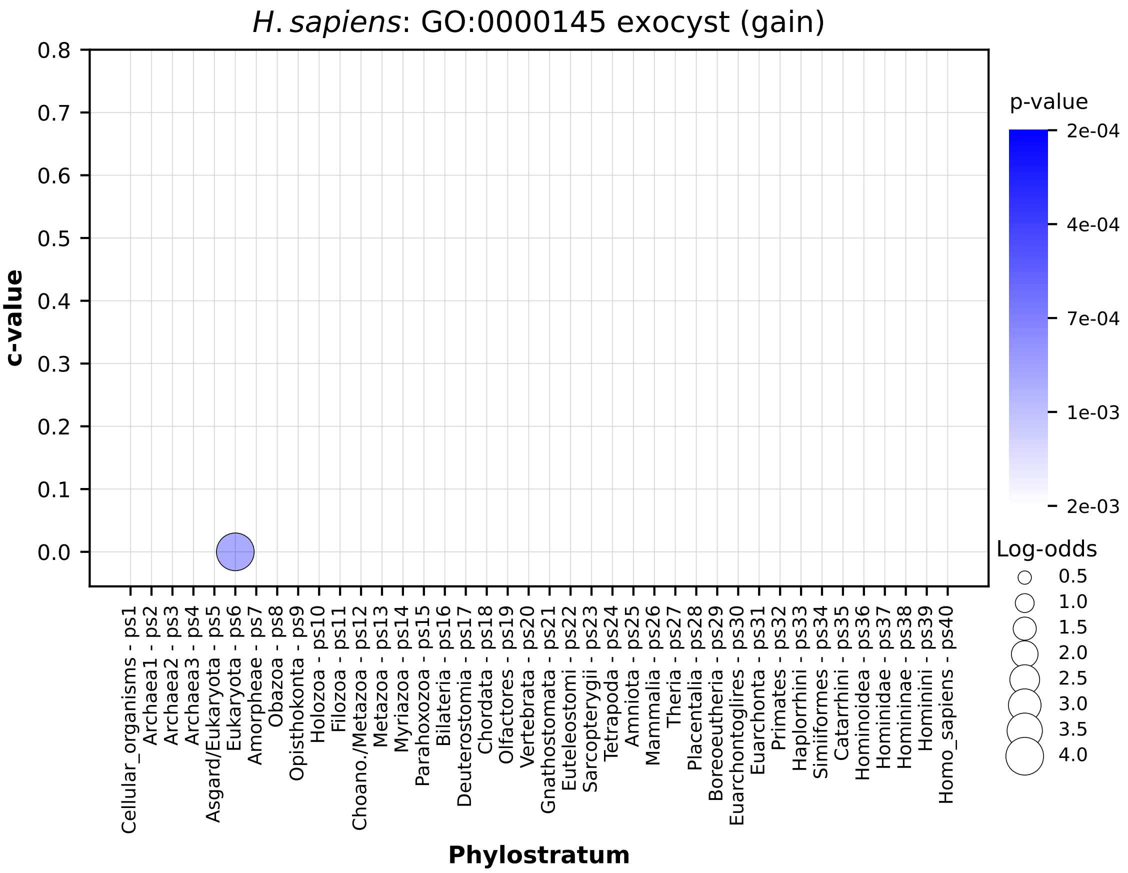

H. sapiens: GO:0000145 exocyst (gain)
c-value
0.8
0.7
0.6
0.5
0.4
0.3
0.2
0.1
0.0
Phylostratum
Cellular_organisms - ps1
Archaea1 - ps2
Archaea2 - ps3
Archaea3 - ps4
Asgard/Eukaryota - ps5
Eukaryota - ps6
Amorpheae - ps7
Obazoa - ps8
Opisthokonta - ps9
Holozoa - ps10
Filozoa - ps11
Choano./Metazoa - ps12
Metazoa - ps13
Myriazoa - ps14
Parahoxozoa - ps15
Bilateria - ps16
Deuterostomia - ps17
Chordata - ps18
Olfactores - ps19
Vertebrata - ps20
Gnathostomata - ps21
Euteleostomi - ps22
Sarcopterygii - ps23
Tetrapoda - ps24
Amniota - ps25
Mammalia - ps26
Theria - ps27
Placentalia - ps28
Boreoeutheria - ps29
Euarchontoglires - ps30
Euarchonta - ps31
Primates - ps32
Haplorrhini - ps33
Simiiformes - ps34
Catarrhini - ps35
Hominoidea - ps36
Hominidae - ps37
Homininae - ps38
Hominini - ps39
Homo_sapiens - ps40
p-value
2e-04
4e-04
7e-04
1e-03
2e-03
Log-odds
0.5
1.0
1.5
2.0
2.5
3.0
3.5
4.0

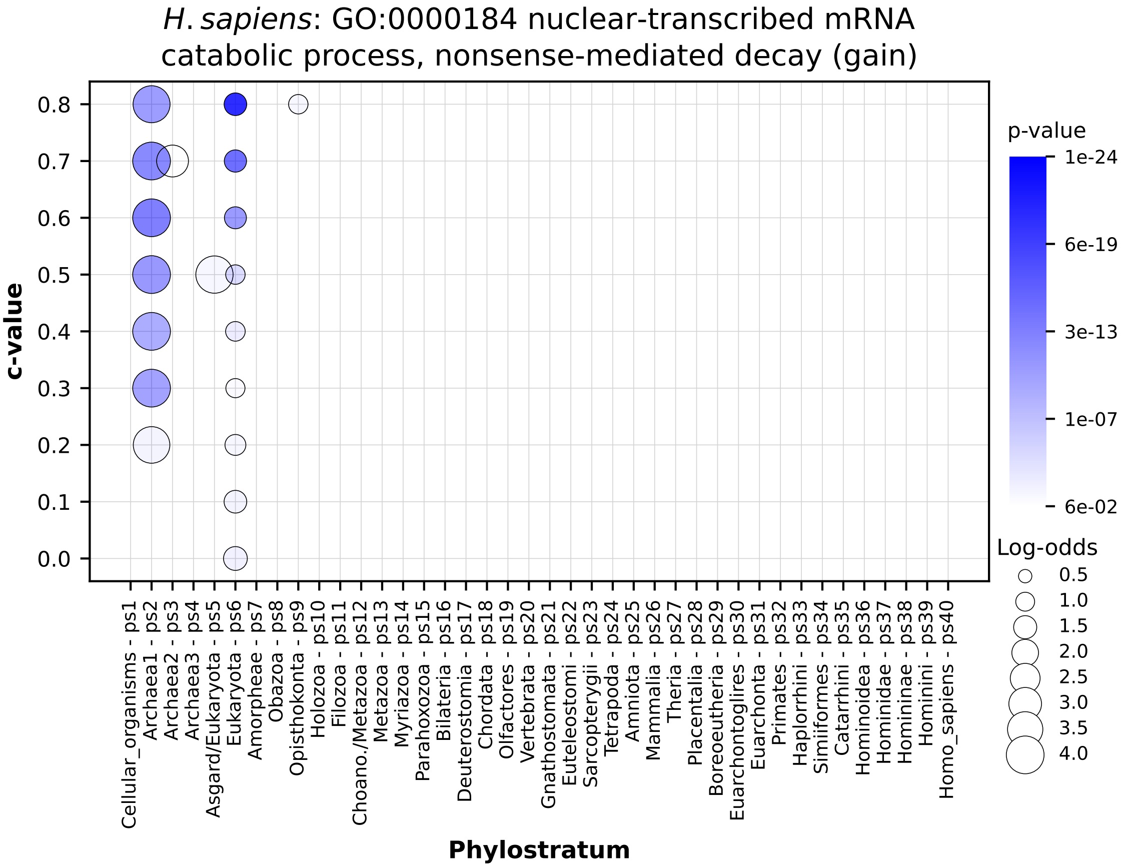

H. sapiens: GO:0000184 nuclear-transcribed mRNA catabolic process, nonsense-mediated decay (gain)
c-value
0.8
0.7
0.6
0.5
0.4
0.3
0.2
0.1
0.0
Phylostratum
Cellular_organisms - ps1
Archaea1 - ps2
Archaea2 - ps3
Archaea3 - ps4
Asgard/Eukaryota - ps5
Eukaryota - ps6
Amorpheae - ps7
Obazoa - ps8
Opisthokonta - ps9
Holozoa - ps10
Filozoa - ps11
Choano./Metazoa - ps12
Metazoa - ps13
Myriazoa - ps14
Parahoxozoa - ps15
Bilateria - ps16
Deuterostomia - ps17
Chordata - ps18
Olfactores - ps19
Vertebrata - ps20
Gnathostomata - ps21
Euteleostomi - ps22
Sarcopterygii - ps23
Tetrapoda - ps24
Amniota - ps25
Mammalia - ps26
Theria - ps27
Placentalia - ps28
Boreoeutheria - ps29
Euarchontoglires - ps30
Euarchonta - ps31
Primates - ps32
Haplorrhini - ps33
Simiiformes - ps34
Catarrhini - ps35
Hominoidea - ps36
Hominidae - ps37
Homininae - ps38
Hominini - ps39
Homo_sapiens - ps40
p-value
1e-24
6e-19
3e-13
1e-07
6e-02
Log-odds
0.5
1.0
1.5
2.0
2.5
3.0
3.5
4.0

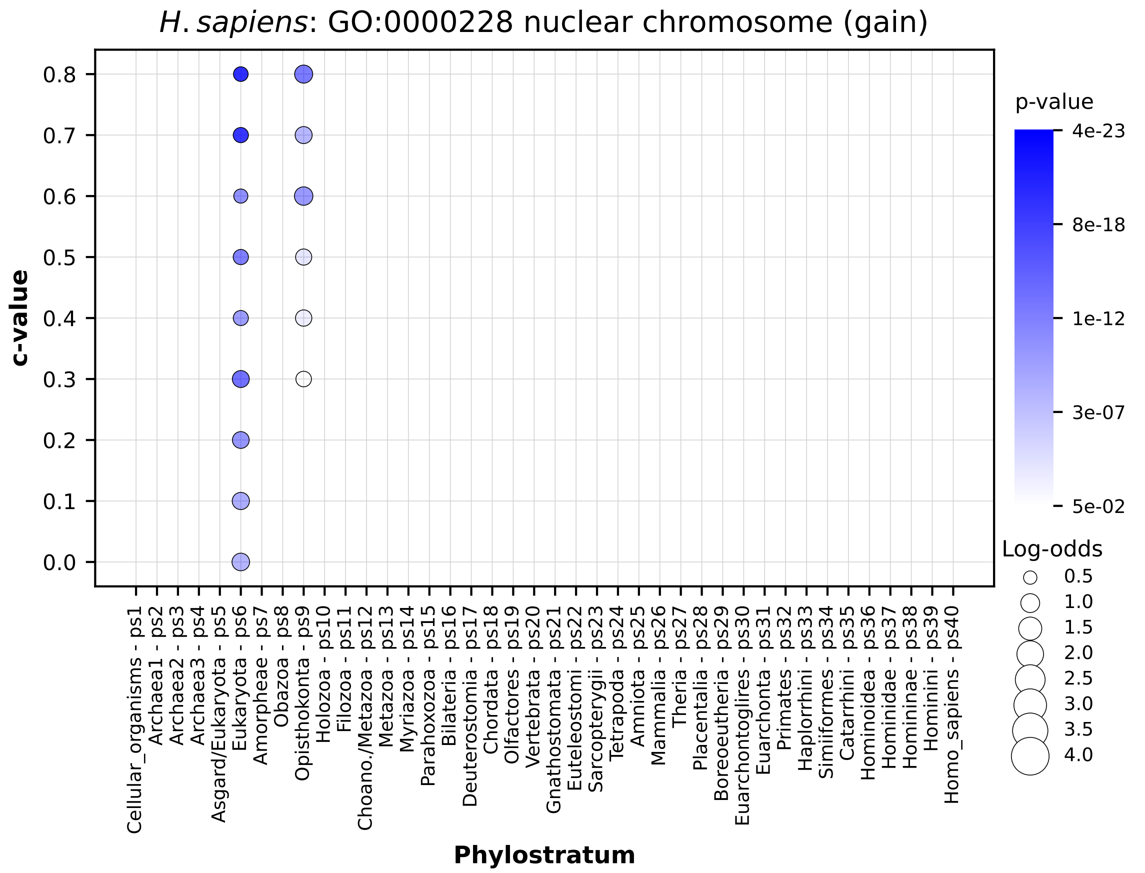

H. sapiens: GO:0000228 nuclear chromosome (gain)
c-value
0.0
0.1
0.2
0.3
0.4
0.5
0.6
0.7
0.8
Phylostratum
Cellular_organisms - ps1
Archaea1 - ps2
Archaea2 - ps3
Archaea3 - ps4
Asgard/Eukaryota - ps5
Eukaryota - ps6
Amorpheae - ps7
Obazoa - ps8
Opisthokonta - ps9
Holozoa - ps10
Filozoa - ps11
Choano./Metazoa - ps12
Metazoa - ps13
Myriazoa - ps14
Parahoxozoa - ps15
Bilateria - ps16
Deuterostomia - ps17
Chordata - ps18
Olfactores - ps19
Vertebrata - ps20
Gnathostomata - ps21
Euteleostomi - ps22
Sarcopterygii - ps23
Tetrapoda - ps24
Amniota - ps25
Mammalia - ps26
Theria - ps27
Placentalia - ps28
Boreoeutheria - ps29
Euarchontoglires - ps30
Euarchonta - ps31
Primates - ps32
Haplorrhini - ps33
Simiiformes - ps34
Catarrhini - ps35
Hominoidea - ps36
Hominidae - ps37
Homininae - ps38
Hominini - ps39
Homo_sapiens - ps40
p-value
4e-23
8e-18
1e-12
3e-07
5e-02
Log-odds
0.5
1.0
1.5
2.0
2.5
3.0
3.5
4.0

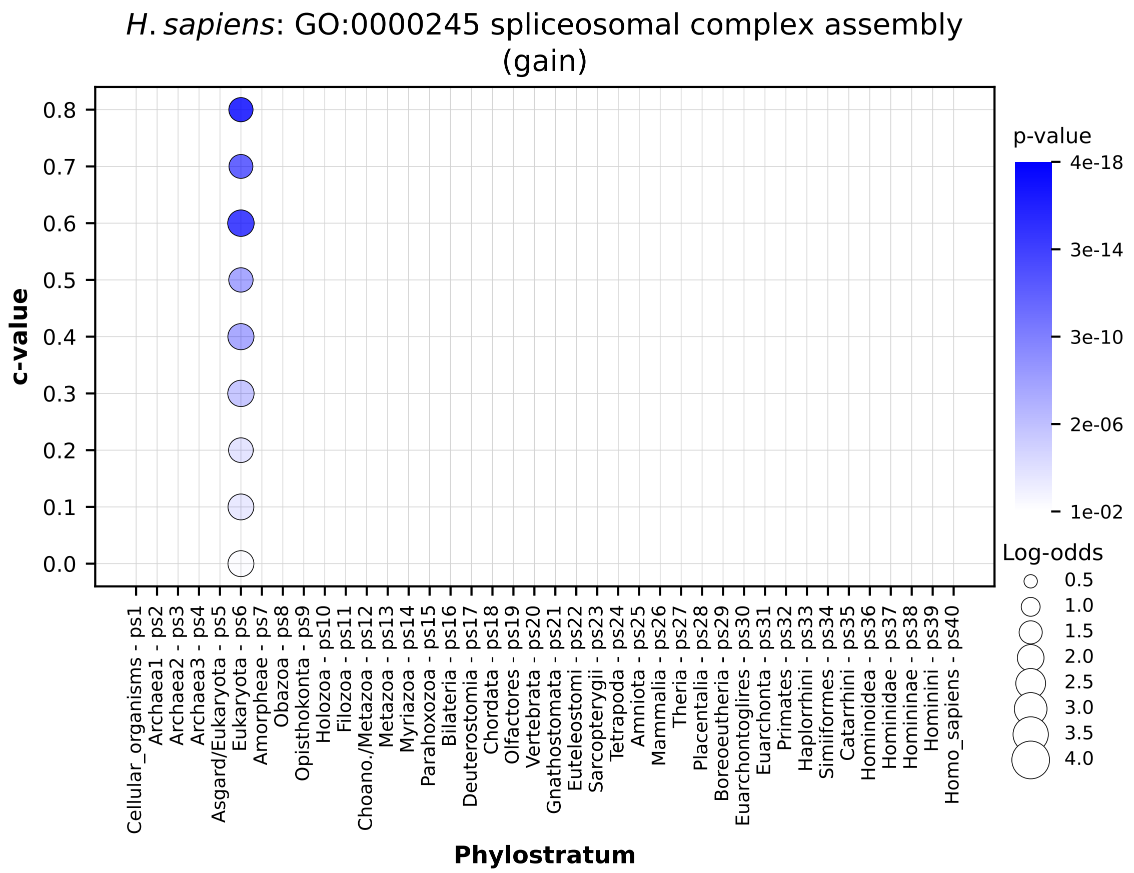
H. sapiens: GO:0000245 spliceosomal complex assembly
(gain)
c-value
0.8
0.7
0.6
0.5
0.4
0.3
0.2
0.1
0.0
p-value
4e-18
3e-14
3e-10
2e-06
1e-02
Log-odds
0.5
1.0
1.5
2.0
2.5
3.0
3.5
4.0
Cellular_organisms - ps1
Archaea1 - ps2
Archaea2 - ps3
Archaea3 - ps4
Asgard/Eukaryota - ps5
Eukaryota - ps6
Amorpheae - ps7
Obazoa - ps8
Opisthokonta - ps9
Holozoa - ps10
Filozoa - ps11
Choano./Metazoa - ps12
Metazoa - ps13
Myriazoa - ps14
Parahoxozoa - ps15
Bilateria - ps16
Deuterostomia - ps17
Chordata - ps18
Olfactores - ps19
Vertebrata - ps20
Gnathostomata - ps21
Euteleostomi - ps22
Sarcopterygii - ps23
Tetrapoda - ps24
Amniota - ps25
Mammalia - ps26
Theria - ps27
Placentalia - ps28
Boreoeutheria - ps29
Euarchontoglires - ps30
Euarchonta - ps31
Primates - ps32
Haplorrhini - ps33
Simiiformes - ps34
Catarrhini - ps35
Hominoidea - ps36
Hominidae - ps37
Homininae - ps38
Hominini - ps39
Homo_sapiens - ps40
Phylostratum

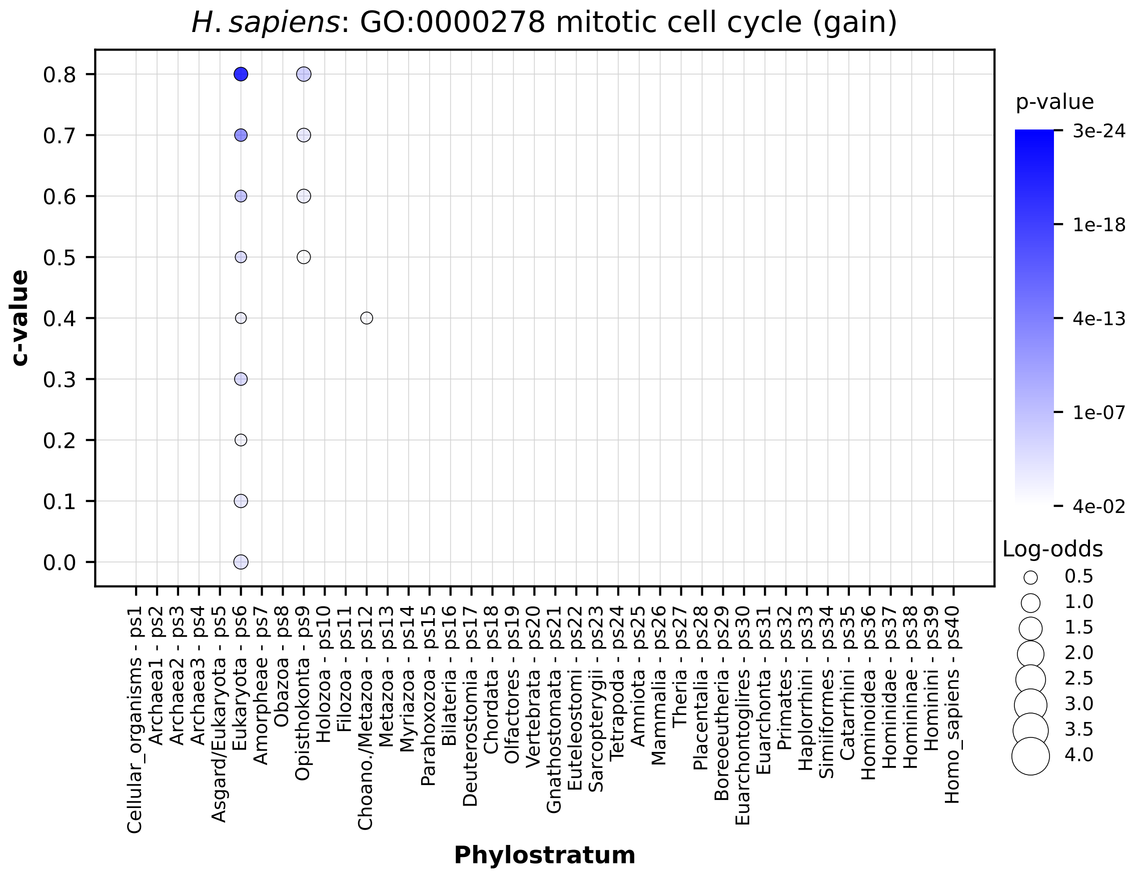

H. sapiens: GO:0000278 mitotic cell cycle (gain)
c-value
0.8
0.7
0.6
0.5
0.4
0.3
0.2
0.1
0.0
Phylostratum
Cellular_organisms - ps1
Archaea1 - ps2
Archaea2 - ps3
Archaea3 - ps4
Asgard/Eukaryota - ps5
Eukaryota - ps6
Amorpheae - ps7
Obazoa - ps8
Opisthokonta - ps9
Holozoa - ps10
Filozoa - ps11
Choano./Metazoa - ps12
Metazoa - ps13
Myriazoa - ps14
Parahoxozoa - ps15
Bilateria - ps16
Deuterostomia - ps17
Chordata - ps18
Olfactores - ps19
Vertebrata - ps20
Gnathostomata - ps21
Euteleostomi - ps22
Sarcopterygii - ps23
Tetrapoda - ps24
Amniota - ps25
Mammalia - ps26
Theria - ps27
Placentalia - ps28
Boreoeutheria - ps29
Euarchontoglires - ps30
Euarchonta - ps31
Primates - ps32
Haplorrhini - ps33
Simiiformes - ps34
Catarrhini - ps35
Hominoidea - ps36
Hominidae - ps37
Homininae - ps38
Hominini - ps39
Homo_sapiens - ps40
p-value
3e-24
1e-18
4e-13
1e-07
4e-02
Log-odds
0.5
1.0
1.5
2.0
2.5
3.0
3.5
4.0

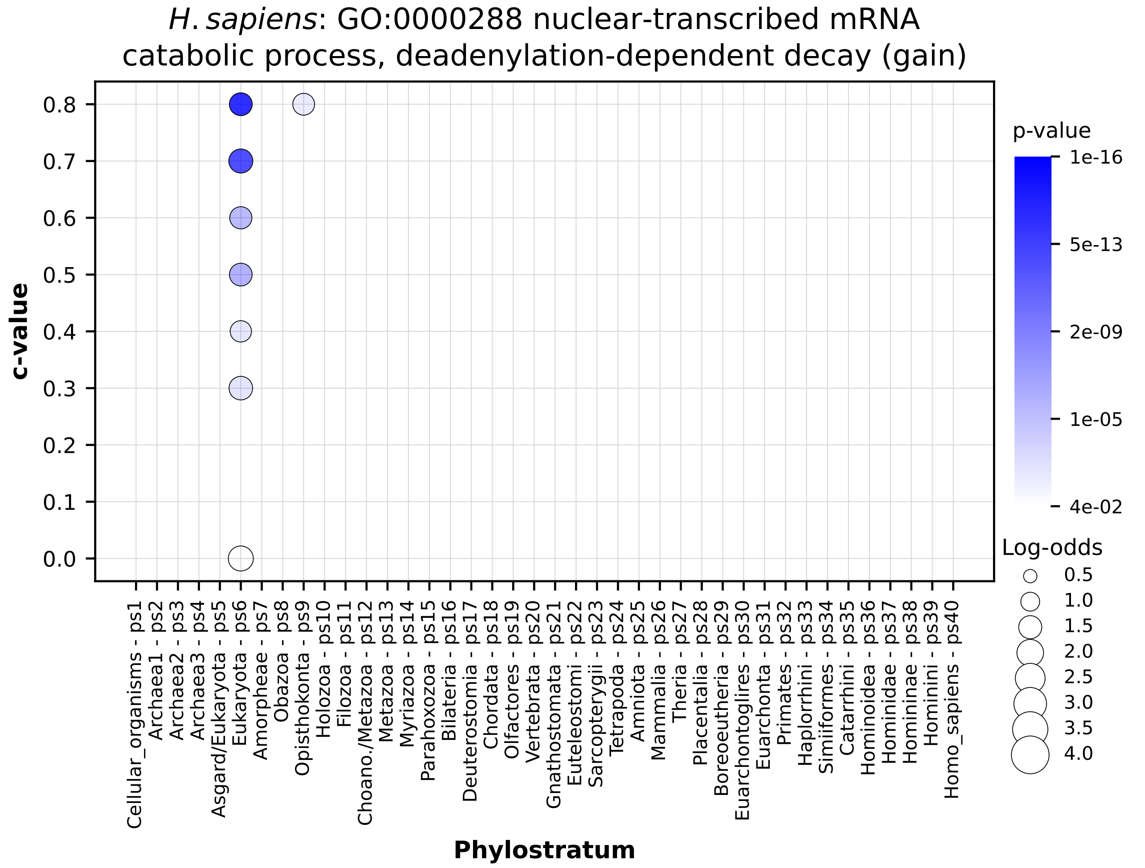

*H. sapiens*: GO:0000288 nuclear-transcribed mRNA catabolic process, deadenylation-dependent decay (gain)
c-value
0.8
0.7
0.6
0.5
0.4
0.3
0.2
0.1
0.0
Phylostratum
Cellular_organisms - ps1
Archaea1 - ps2
Archaea2 - ps3
Archaea3 - ps4
Asgard/Eukaryota - ps5
Eukaryota - ps6
Amorpheae - ps7
Obazoa - ps8
Opisthokonta - ps9
Holozoa - ps10
Filozoa - ps11
Choano./Metazoa - ps12
Metazoa - ps13
Myriazoa - ps14
Parahoxozoa - ps15
Bilateria - ps16
Deuterostomia - ps17
Chordata - ps18
Olfactores - ps19
Vertebrata - ps20
Gnathostomata - ps21
Euteleostomi - ps22
Sarcopterygii - ps23
Tetrapoda - ps24
Amniota - ps25
Mammalia - ps26
Theria - ps27
Placentalia - ps28
Boreoeutheria - ps29
Euarchontoglires - ps30
Euarchonta - ps31
Primates - ps32
Haplorrhini - ps33
Simiiformes - ps34
Catarrhini - ps35
Hominoidea - ps36
Hominidae - ps37
Homininae - ps38
Hominini - ps39
Homo_sapiens - ps40
p-value
1e-16
5e-13
2e-09
1e-05
4e-02
Log-odds
0.5
1.0
1.5
2.0
2.5
3.0
3.5
4.0

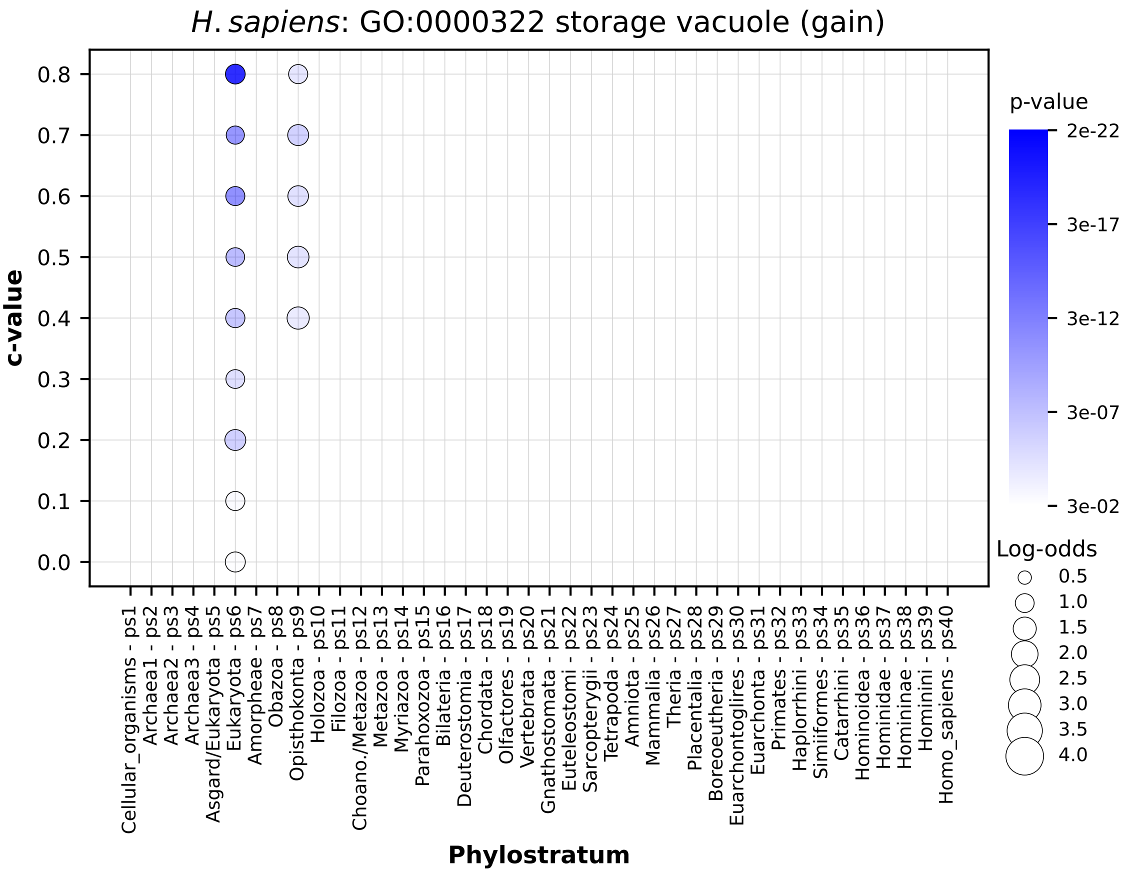
H. sapiens: GO:0000322 storage vacuole (gain)
c-value
0.8
0.7
0.6
0.5
0.4
0.3
0.2
0.1
0.0
p-value
2e-22
3e-17
3e-12
3e-07
3e-02
Log-odds
0.5
1.0
1.5
2.0
2.5
3.0
3.5
4.0
Cellular_organisms - ps1
Archaea1 - ps2
Archaea2 - ps3
Archaea3 - ps4
Asgard/Eukaryota - ps5
Eukaryota - ps6
Amorpheae - ps7
Obazoa - ps8
Opisthokonta - ps9
Holozoa - ps10
Filozoa - ps11
Choano./Metazoa - ps12
Metazoa - ps13
Myriazoa - ps14
Parahoxozoa - ps15
Bilateria - ps16
Deuterostomia - ps17
Chordata - ps18
Olfactores - ps19
Vertebrata - ps20
Gnathostomata - ps21
Euteleostomi - ps22
Sarcopterygii - ps23
Tetrapoda - ps24
Amniota - ps25
Mammalia - ps26
Theria - ps27
Placentalia - ps28
Boreoeutheria - ps29
Euarchontoglires - ps30
Euarchonta - ps31
Primates - ps32
Haplorrhini - ps33
Simiiformes - ps34
Catarrhini - ps35
Hominoidea - ps36
Hominidae - ps37
Homininae - ps38
Hominini - ps39
Homo_sapiens - ps40
Phylostratum

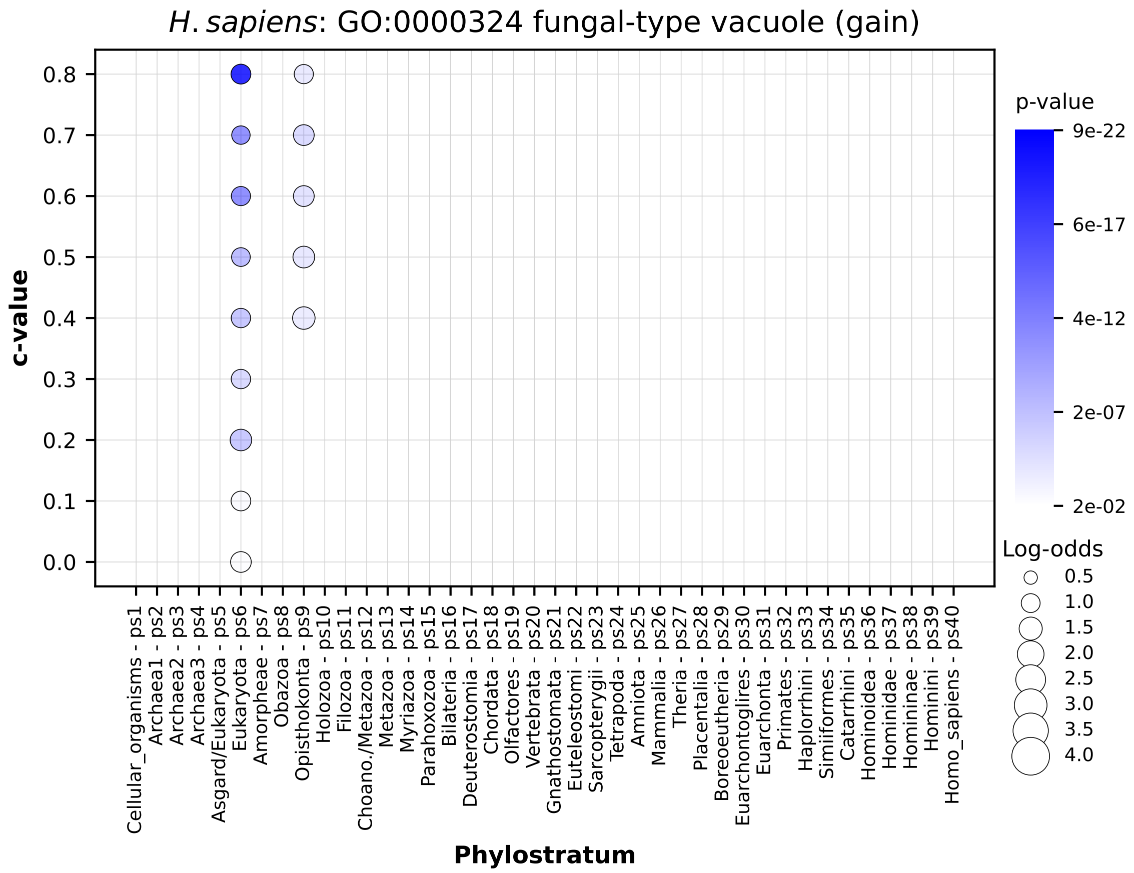

H. sapiens: GO:0000324 fungal-type vacuole (gain)
c-value
0.8
0.7
0.6
0.5
0.4
0.3
0.2
0.1
0.0
p-value
9e-22
6e-17
4e-12
2e-07
2e-02
Log-odds
0.5
1.0
1.5
2.0
2.5
3.0
3.5
4.0
Cellular_organisms - ps1
Archaea1 - ps2
Archaea2 - ps3
Archaea3 - ps4
Asgard/Eukaryota - ps5
Eukaryota - ps6
Amorpheae - ps7
Obazoa - ps8
Opisthokonta - ps9
Holozoa - ps10
Filozoa - ps11
Choano./Metazoa - ps12
Metazoa - ps13
Myriazoa - ps14
Parahoxozoa - ps15
Bilateria - ps16
Deuterostomia - ps17
Chordata - ps18
Olfactores - ps19
Vertebrata - ps20
Gnathostomata - ps21
Euteleostomi - ps22
Sarcopterygii - ps23
Tetrapoda - ps24
Amniota - ps25
Mammalia - ps26
Theria - ps27
Placentalia - ps28
Boreoeutheria - ps29
Euarchontoglires - ps30
Euarchonta - ps31
Primates - ps32
Haplorrhini - ps33
Simiiformes - ps34
Catarrhini - ps35
Hominoidea - ps36
Hominidae - ps37
Homininae - ps38
Hominini - ps39
Homo_sapiens - ps40
Phylostratum

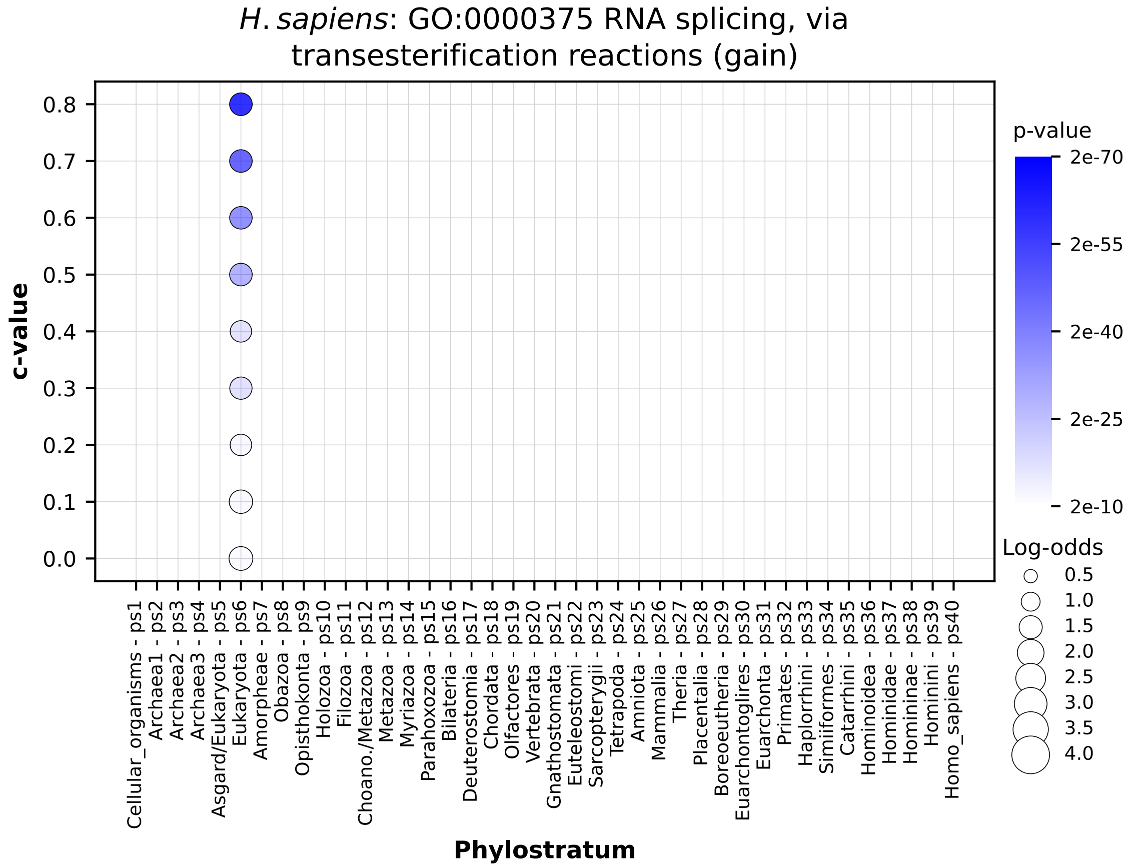
*H. sapiens*: GO:0000375 RNA splicing, via transesterification reactions (gain)
c-value
0.8
0.7
0.6
0.5
0.4
0.3
0.2
0.1
0.0
p-value
2e-70
2e-55
2e-40
2e-25
2e-10
Log-odds
0.5
1.0
1.5
2.0
2.5
3.0
3.5
4.0
Cellular_organisms - ps1
Archaea1 - ps2
Archaea2 - ps3
Archaea3 - ps4
Asgard/Eukaryota - ps5
Eukaryota - ps6
Amorpheae - ps7
Obazoa - ps8
Opisthokonta - ps9
Holozoa - ps10
Filozoa - ps11
Choano./Metazoa - ps12
Metazoa - ps13
Myriazoa - ps14
Parahoxozoa - ps15
Bilateria - ps16
Deuterostomia - ps17
Chordata - ps18
Olfactores - ps19
Vertebrata - ps20
Gnathostomata - ps21
Euteleostomi - ps22
Sarcopterygii - ps23
Tetrapoda - ps24
Amniota - ps25
Mammalia - ps26
Theria - ps27
Placentalia - ps28
Boreoeutheria - ps29
Euarchontoglires - ps30
Euarchonta - ps31
Primates - ps32
Haplorrhini - ps33
Simiiformes - ps34
Catarrhini - ps35
Hominoidea - ps36
Hominidae - ps37
Homininae - ps38
Hominini - ps39
Homo_sapiens - ps40
Phylostratum

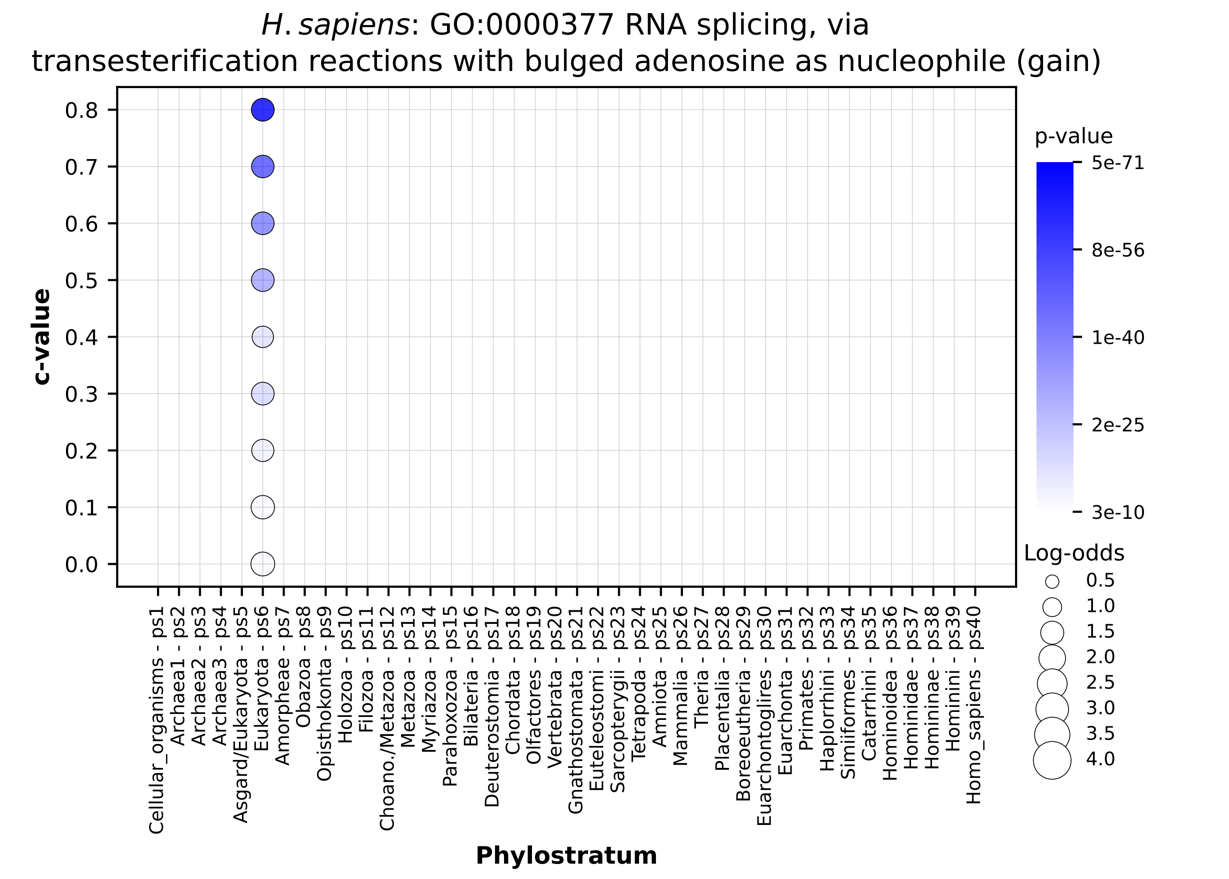
H. sapiens: GO:0000377 RNA splicing, via
transesterification reactions with bulged adenosine as nucleophile (gain)
c-value
0.8
0.7
0.6
0.5
0.4
0.3
0.2
0.1
0.0
Phylostratum
Cellular_organisms - ps1
Archaea1 - ps2
Archaea2 - ps3
Archaea3 - ps4
Asgard/Eukaryota - ps5
Eukaryota - ps6
Amorpheae - ps7
Obazoa - ps8
Opisthokonta - ps9
Holozoa - ps10
Filozoa - ps11
Choano./Metazoa - ps12
Metazoa - ps13
Myriazoa - ps14
Parahoxozoa - ps15
Bilateria - ps16
Deuterostomia - ps17
Chordata - ps18
Olfactores - ps19
Vertebrata - ps20
Gnathostomata - ps21
Euteleostomi - ps22
Sarcopterygii - ps23
Tetrapoda - ps24
Amniota - ps25
Mammalia - ps26
Theria - ps27
Placentalia - ps28
Boreoeutheria - ps29
Euarchontoglires - ps30
Euarchonta - ps31
Primates - ps32
Haplorrhini - ps33
Simiiformes - ps34
Catarrhini - ps35
Hominoidea - ps36
Hominidae - ps37
Homininae - ps38
Hominini - ps39
Homo_sapiens - ps40
p-value
5e-71
8e-56
1e-40
2e-25
3e-10
Log-odds
0.5
1.0
1.5
2.0
2.5
3.0
3.5
4.0

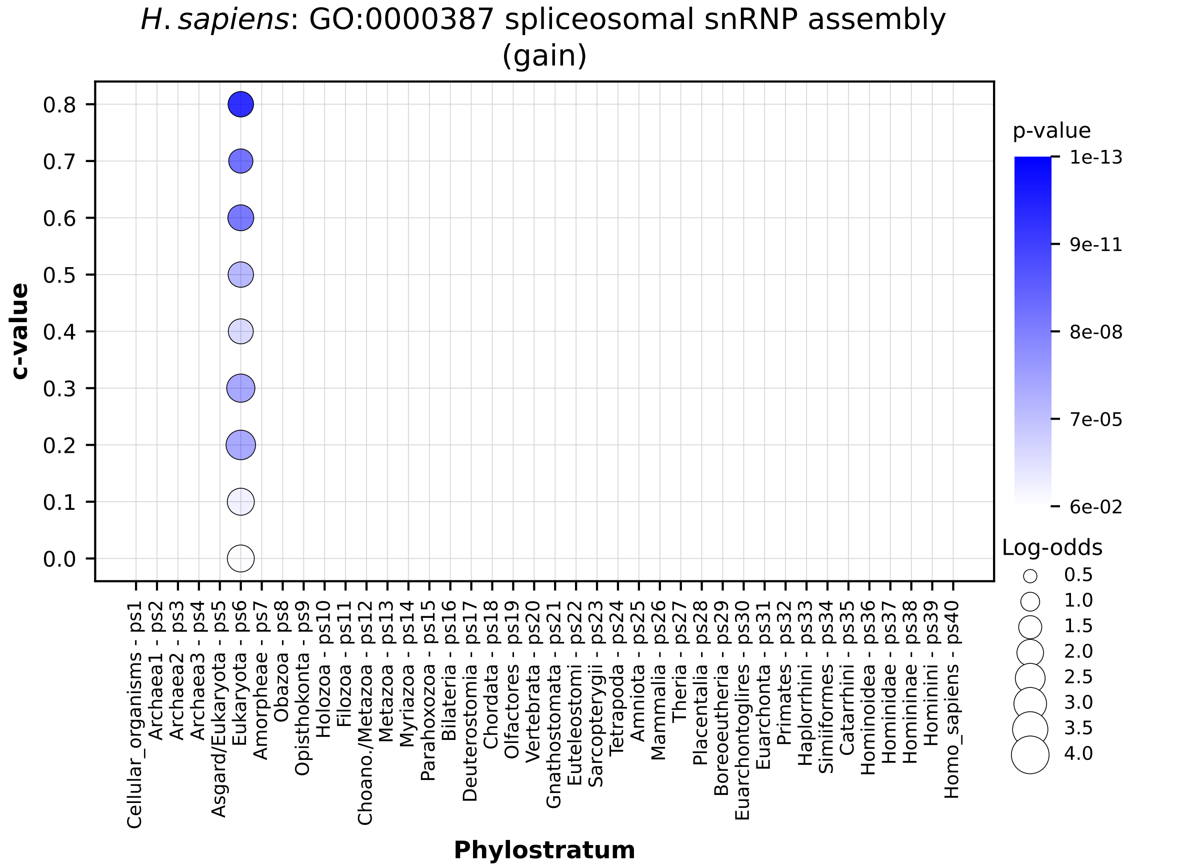

H. sapiens: GO:0000387 spliceosomal snRNP assembly
(gain)
c-value
0.8
0.7
0.6
0.5
0.4
0.3
0.2
0.1
0.0
p-value
1e-13
9e-11
8e-08
7e-05
6e-02
Log-odds
0.5
1.0
1.5
2.0
2.5
3.0
3.5
4.0
Cellular_organisms - ps1
Archaea1 - ps2
Archaea2 - ps3
Archaea3 - ps4
Asgard/Eukaryota - ps5
Eukaryota - ps6
Amorpheae - ps7
Obazoa - ps8
Opisthokonta - ps9
Holozoa - ps10
Filozoa - ps11
Choano./Metazoa - ps12
Metazoa - ps13
Myriazoa - ps14
Parahoxozoa - ps15
Bilateria - ps16
Deuterostomia - ps17
Chordata - ps18
Olfactores - ps19
Vertebrata - ps20
Gnathostomata - ps21
Euteleostomi - ps22
Sarcopterygii - ps23
Tetrapoda - ps24
Amniota - ps25
Mammalia - ps26
Theria - ps27
Placentalia - ps28
Boreoeutheria - ps29
Euarchontoglires - ps30
Euarchonta - ps31
Primates - ps32
Haplorrhini - ps33
Simiiformes - ps34
Catarrhini - ps35
Hominoidea - ps36
Hominidae - ps37
Homininae - ps38
Hominini - ps39
Homo_sapiens - ps40
Phylostratum

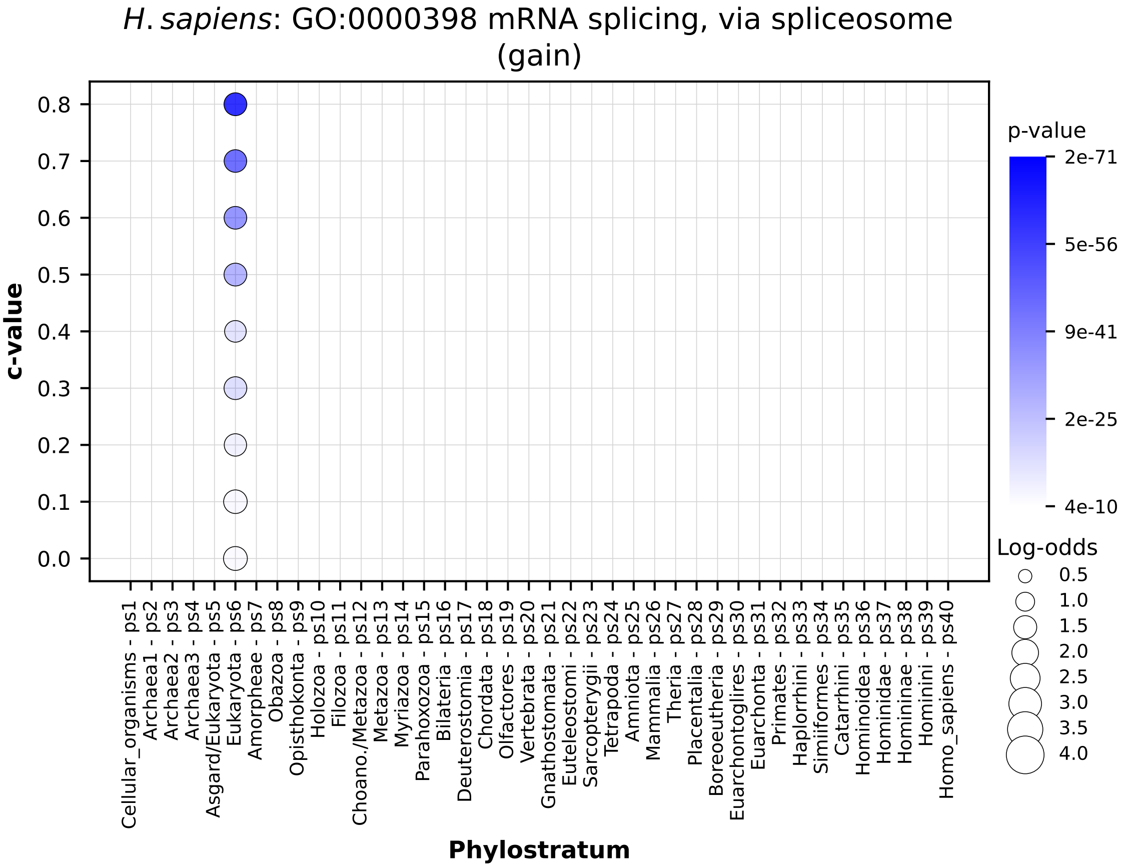

H. sapiens: GO:0000398 mRNA splicing, via spliceosome
(gain)
c-value
0.8
0.7
0.6
0.5
0.4
0.3
0.2
0.1
0.0
p-value
2e-71
5e-56
9e-41
2e-25
4e-10
Log-odds
0.5
1.0
1.5
2.0
2.5
3.0
3.5
4.0
Cellular_organisms - ps1
Archaea1 - ps2
Archaea2 - ps3
Archaea3 - ps4
Asgard/Eukaryota - ps5
Eukaryota - ps6
Amorpheae - ps7
Obazoa - ps8
Opisthokonta - ps9
Holozoa - ps10
Filozoa - ps11
Choano./Metazoa - ps12
Metazoa - ps13
Myriazoa - ps14
Parahoxozoa - ps15
Bilateria - ps16
Deuterostomia - ps17
Chordata - ps18
Olfactores - ps19
Vertebrata - ps20
Gnathostomata - ps21
Euteleostomi - ps22
Sarcopterygii - ps23
Tetrapoda - ps24
Amniota - ps25
Mammalia - ps26
Theria - ps27
Placentalia - ps28
Boreoeutheria - ps29
Euarchontoglires - ps30
Euarchonta - ps31
Primates - ps32
Haplorrhini - ps33
Simiiformes - ps34
Catarrhini - ps35
Hominoidea - ps36
Hominidae - ps37
Homininae - ps38
Hominini - ps39
Homo_sapiens - ps40
Phylostratum

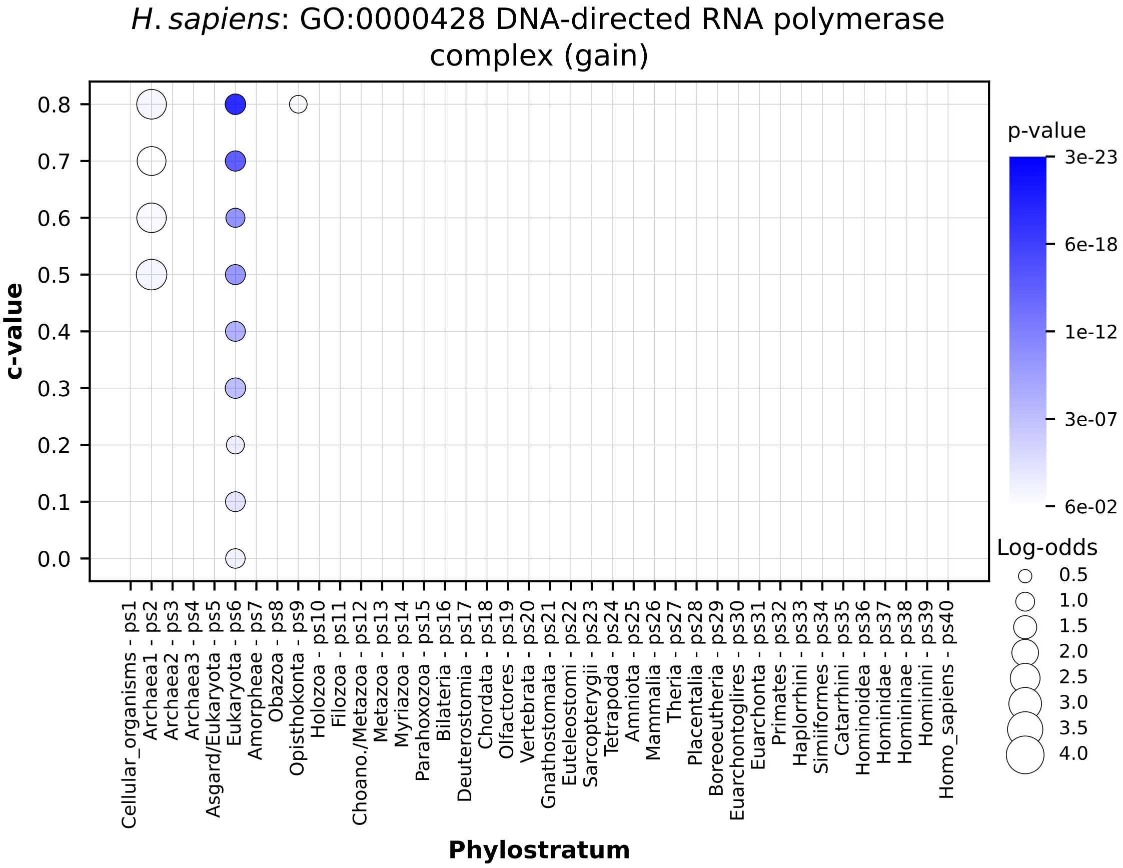

H. sapiens: GO:0000428 DNA-directed RNA polymerase complex (gain)
c-value
0.8
0.7
0.6
0.5
0.4
0.3
0.2
0.1
0.0
p-value
3e-23
6e-18
1e-12
3e-07
6e-02
Log-odds
0.5
1.0
1.5
2.0
2.5
3.0
3.5
4.0
Cellular_organisms - ps1
Archaea1 - ps2
Archaea2 - ps3
Archaea3 - ps4
Asgard/Eukaryota - ps5
Eukaryota - ps6
Amorpheae - ps7
Obazoa - ps8
Opisthokonta - ps9
Holozoa - ps10
Filozoa - ps11
Choano./Metazoa - ps12
Metazoa - ps13
Myriazoa - ps14
Parahoxozoa - ps15
Bilateria - ps16
Deuterostomia - ps17
Chordata - ps18
Olfactores - ps19
Vertebrata - ps20
Gnathostomata - ps21
Euteleostomi - ps22
Sarcopterygii - ps23
Tetrapoda - ps24
Amniota - ps25
Mammalia - ps26
Theria - ps27
Placentalia - ps28
Boreoeutheria - ps29
Euarchontoglires - ps30
Euarchonta - ps31
Primates - ps32
Haplorrhini - ps33
Simiiformes - ps34
Catarrhini - ps35
Hominoidea - ps36
Hominidae - ps37
Homininae - ps38
Hominini - ps39
Homo_sapiens - ps40
Phylostratum

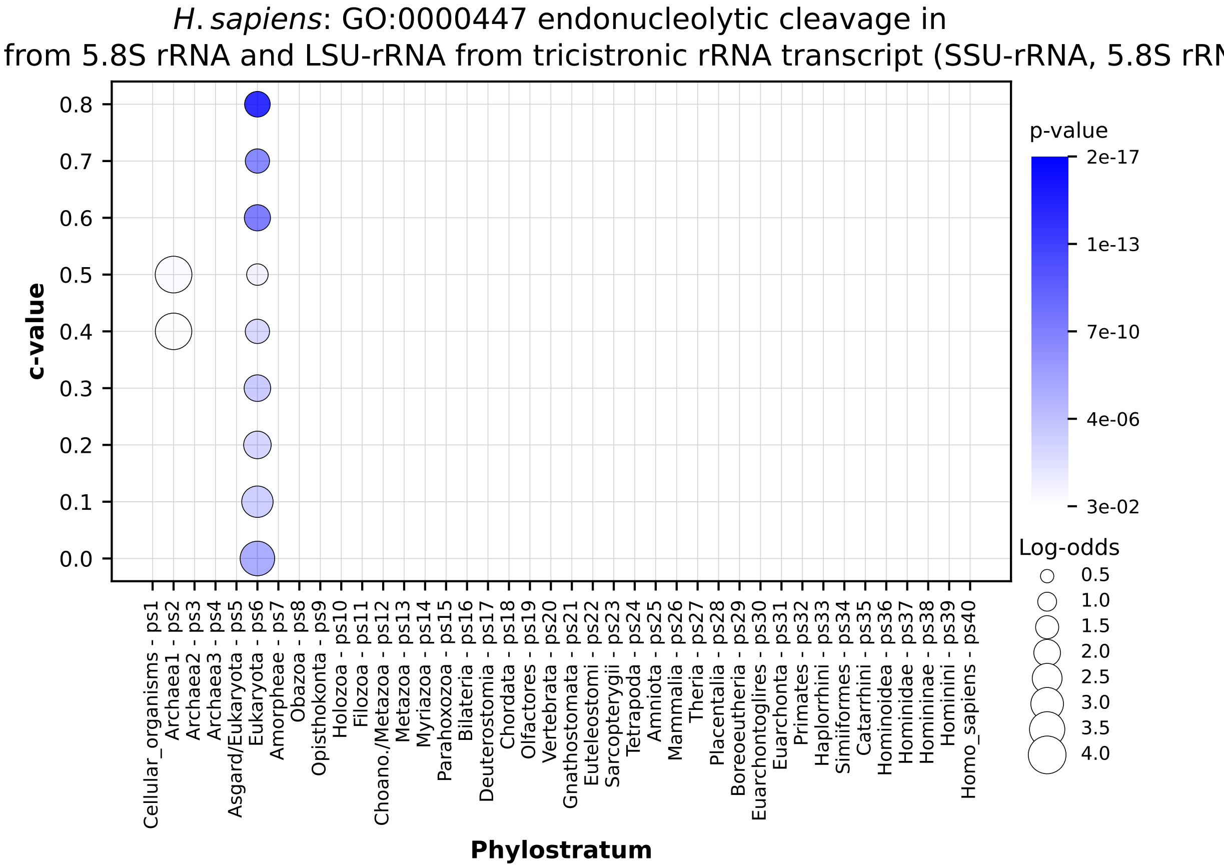
H. sapiens: GO:0000447 endonucleolytic cleavage in
from 5.8S rRNA and LSU-rRNA from tricistronic rRNA transcript (SSU-rRNA, 5.8S rRN
c-value
0.8
0.7
0.6
0.5
0.4
0.3
0.2
0.1
0.0
Phylostratum
Cellular_organisms - ps1
Archaea1 - ps2
Archaea2 - ps3
Archaea3 - ps4
Asgard/Eukaryota - ps5
Eukaryota - ps6
Amorpheae - ps7
Obazoa - ps8
Opisthokonta - ps9
Holozoa - ps10
Filozoa - ps11
Choano./Metazoa - ps12
Metazoa - ps13
Myriazoa - ps14
Parahoxozoa - ps15
Bilateria - ps16
Deuterostomia - ps17
Chordata - ps18
Olfactores - ps19
Vertebrata - ps20
Gnathostomata - ps21
Euteleostomi - ps22
Sarcopterygii - ps23
Tetrapoda - ps24
Amniota - ps25
Mammalia - ps26
Theria - ps27
Placentalia - ps28
Boreoeutheria - ps29
Euarchontoglires - ps30
Euarchonta - ps31
Primates - ps32
Haplorrhini - ps33
Simiiformes - ps34
Catarrhini - ps35
Hominoidea - ps36
Hominidae - ps37
Homininae - ps38
Hominini - ps39
Homo_sapiens - ps40
p-value
2e-17
1e-13
7e-10
4e-06
3e-02
Log-odds
0.5
1.0
1.5
2.0
2.5
3.0
3.5
4.0

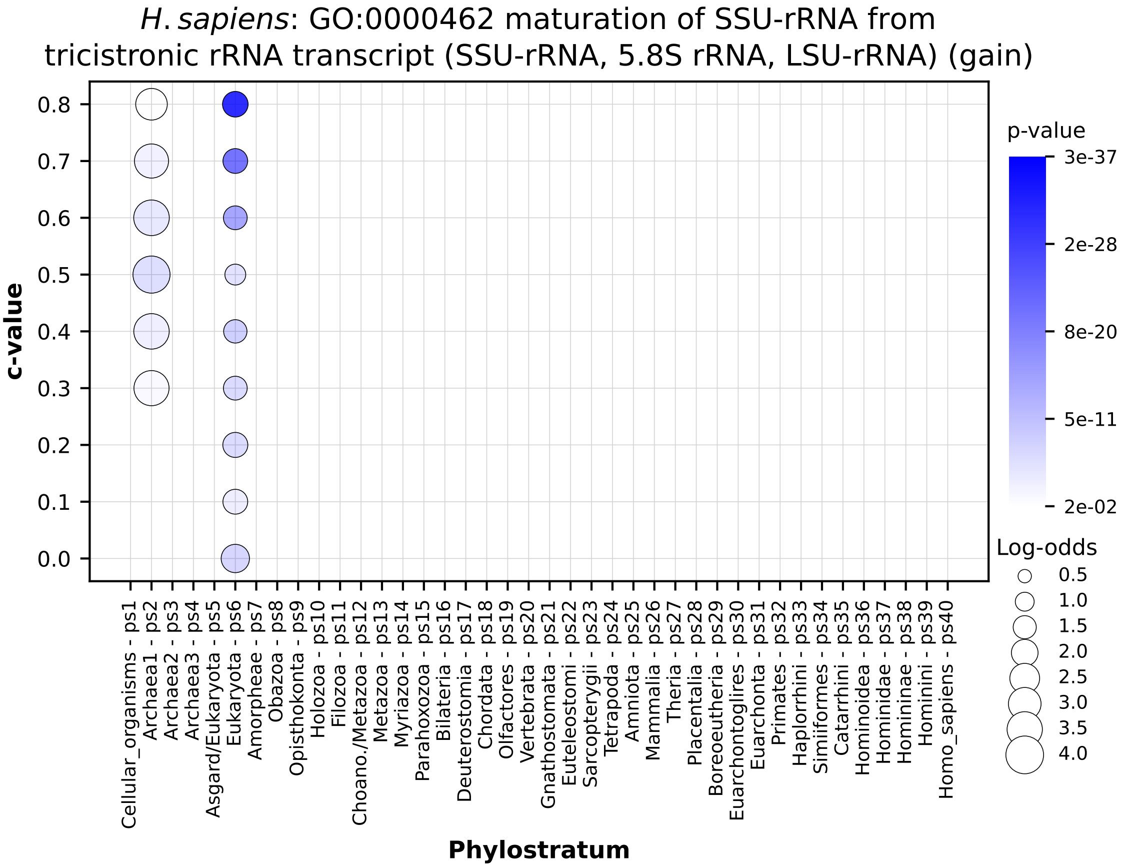

H. sapiens: GO:0000462 maturation of SSU-rRNA from tricistronic rRNA transcript (SSU-rRNA, 5.8S rRNA, LSU-rRNA) (gain)
c-value
0.8
0.7
0.6
0.5
0.4
0.3
0.2
0.1
0.0
p-value
3e-37
2e-28
8e-20
5e-11
2e-02
Log-odds
0.5
1.0
1.5
2.0
2.5
3.0
3.5
4.0
Cellular_organisms - ps1
Archaea1 - ps2
Archaea2 - ps3
Archaea3 - ps4
Asgard/Eukaryota - ps5
Eukaryota - ps6
Amorpheae - ps7
Obazoa - ps8
Opisthokonta - ps9
Holozoa - ps10
Filozoa - ps11
Choano./Metazoa - ps12
Metazoa - ps13
Myriazoa - ps14
Parahoxozoa - ps15
Bilateria - ps16
Deuterostomia - ps17
Chordata - ps18
Olfactores - ps19
Vertebrata - ps20
Gnathostomata - ps21
Euteleostomi - ps22
Sarcopterygii - ps23
Tetrapoda - ps24
Amniota - ps25
Mammalia - ps26
Theria - ps27
Placentalia - ps28
Boreoeutheria - ps29
Euarchontoglires - ps30
Euarchonta - ps31
Primates - ps32
Haplorrhini - ps33
Simiiformes - ps34
Catarrhini - ps35
Hominoidea - ps36
Hominidae - ps37
Homininae - ps38
Hominini - ps39
Homo_sapiens - ps40
Phylostratum

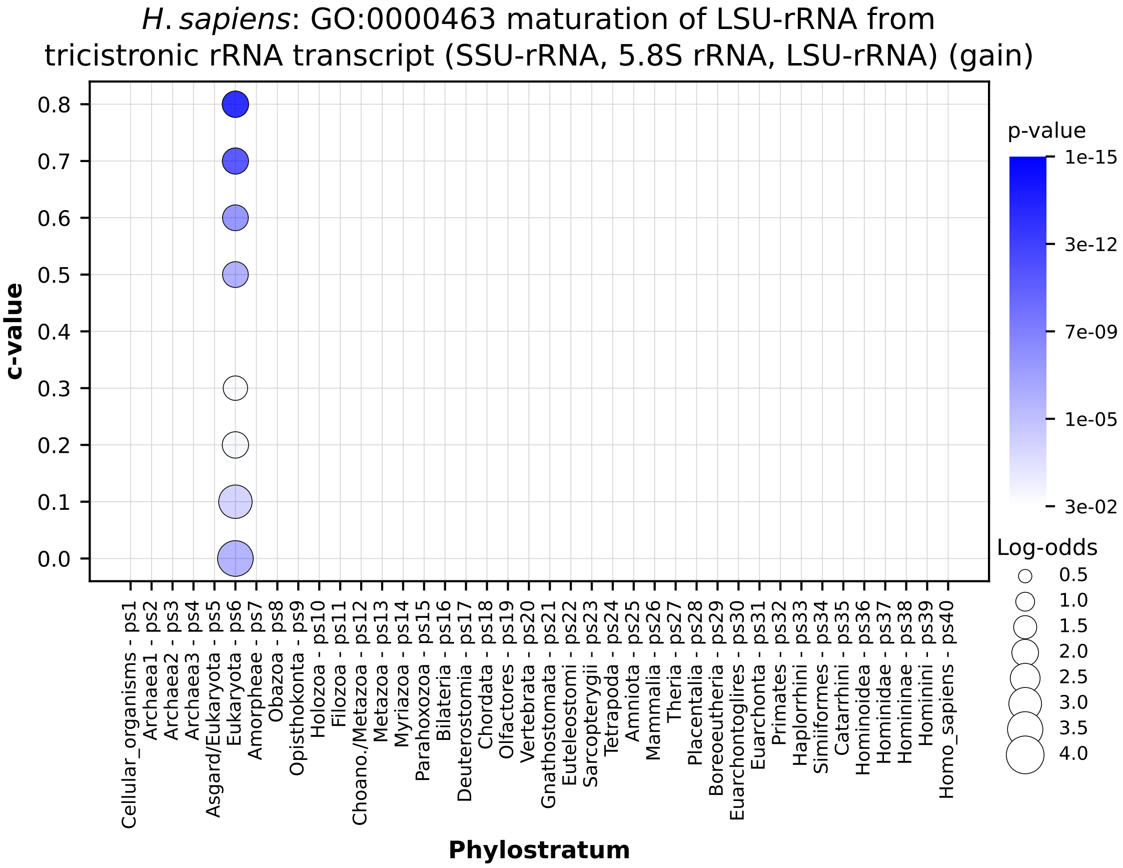

H. sapiens: GO:0000463 maturation of LSU-rRNA from tricistronic rRNA transcript (SSU-rRNA, 5.8S rRNA, LSU-rRNA) (gain)
c-value
0.8
0.7
0.6
0.5
0.4
0.3
0.2
0.1
0.0
p-value
1e-15
3e-12
7e-09
1e-05
3e-02
Log-odds
0.5
1.0
1.5
2.0
2.5
3.0
3.5
4.0
Cellular_organisms - ps1
Archaea1 - ps2
Archaea2 - ps3
Archaea3 - ps4
Asgard/Eukaryota - ps5
Eukaryota - ps6
Amorpheae - ps7
Obazoa - ps8
Opisthokonta - ps9
Holozoa - ps10
Filozoa - ps11
Choano./Metazoa - ps12
Metazoa - ps13
Myriazoa - ps14
Parahoxozoa - ps15
Bilateria - ps16
Deuterostomia - ps17
Chordata - ps18
Olfactores - ps19
Vertebrata - ps20
Gnathostomata - ps21
Euteleostomi - ps22
Sarcopterygii - ps23
Tetrapoda - ps24
Amniota - ps25
Mammalia - ps26
Theria - ps27
Placentalia - ps28
Boreoeutheria - ps29
Euarchontoglires - ps30
Euarchonta - ps31
Primates - ps32
Haplorrhini - ps33
Simiiformes - ps34
Catarrhini - ps35
Hominoidea - ps36
Hominidae - ps37
Homininae - ps38
Hominini - ps39
Homo_sapiens - ps40
Phylostratum

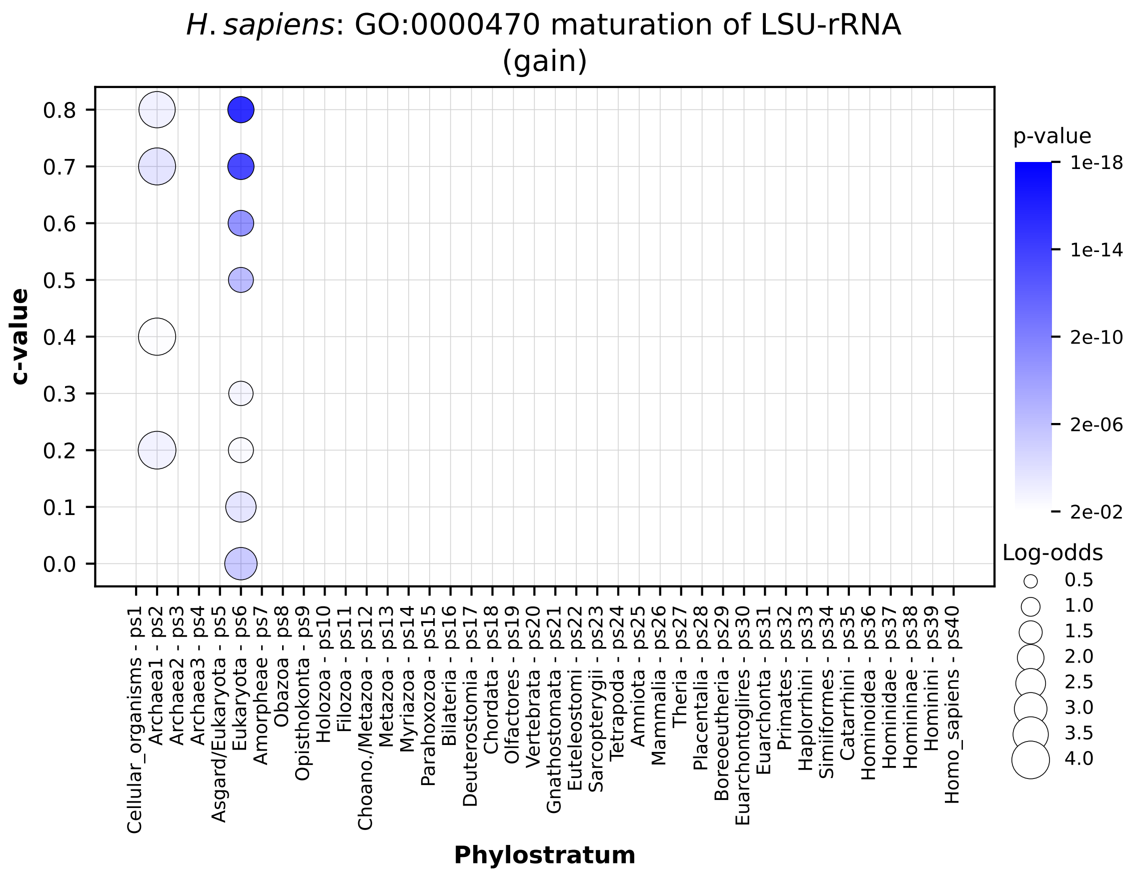

H. sapiens: GO:0000470 maturation of LSU-rRNA
(gain)
c-value
0.8
0.7
0.6
0.5
0.4
0.3
0.2
0.1
0.0
p-value
1e-18
1e-14
2e-10
2e-06
2e-02
Log-odds
0.5
1.0
1.5
2.0
2.5
3.0
3.5
4.0
Cellular_organisms - ps1
Archaea1 - ps2
Archaea2 - ps3
Archaea3 - ps4
Asgard/Eukaryota - ps5
Eukaryota - ps6
Amorpheae - ps7
Obazoa - ps8
Opisthokonta - ps9
Holozoa - ps10
Filozoa - ps11
Choano./Metazoa - ps12
Metazoa - ps13
Myriazoa - ps14
Parahoxozoa - ps15
Bilateria - ps16
Deuterostomia - ps17
Chordata - ps18
Olfactores - ps19
Vertebrata - ps20
Gnathostomata - ps21
Euteleostomi - ps22
Sarcopterygii - ps23
Tetrapoda - ps24
Amniota - ps25
Mammalia - ps26
Theria - ps27
Placentalia - ps28
Boreoeutheria - ps29
Euarchontoglires - ps30
Euarchonta - ps31
Primates - ps32
Haplorrhini - ps33
Simiiformes - ps34
Catarrhini - ps35
Hominoidea - ps36
Hominidae - ps37
Homininae - ps38
Hominini - ps39
Homo_sapiens - ps40
Phylostratum

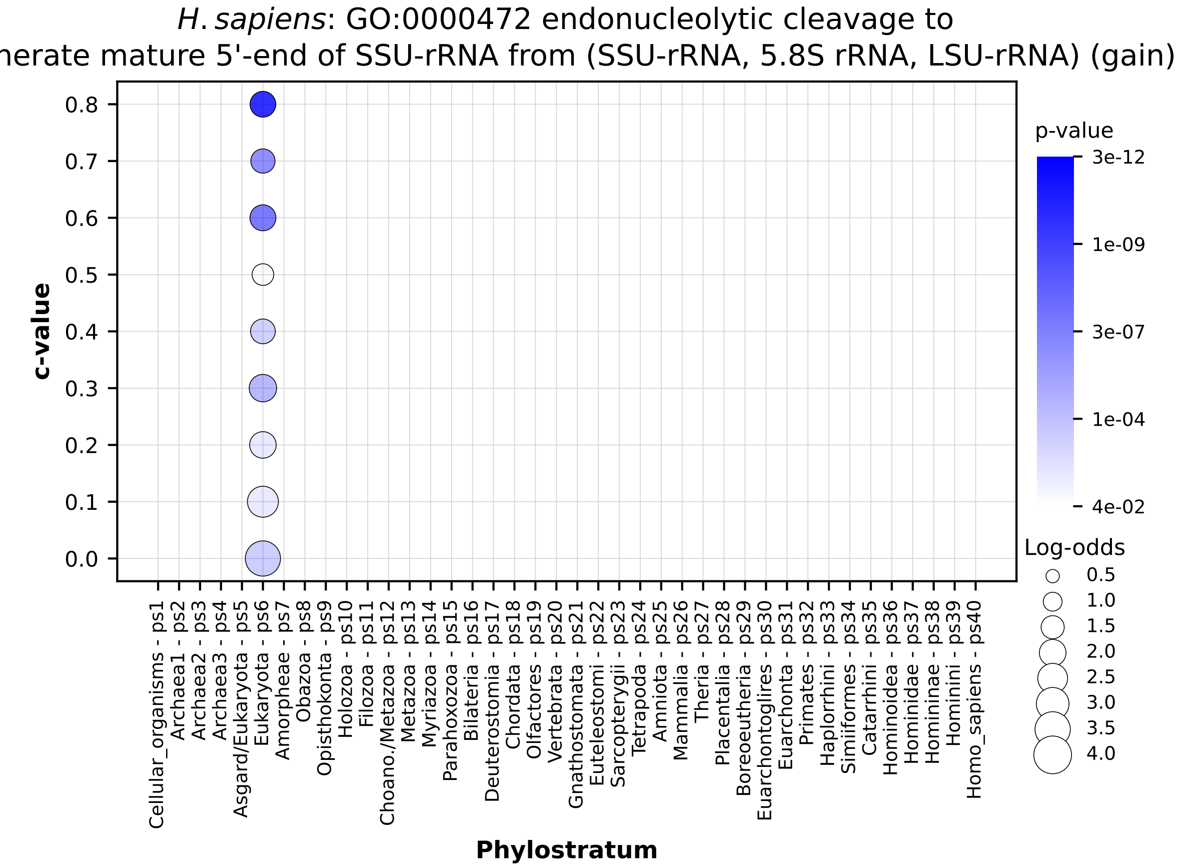

H. sapiens: GO:0000472 endonucleolytic cleavage to
nerate mature 5'-end of SSU-rRNA from (SSU-rRNA, 5.8S rRNA, LSU-rRNA) (gain)
c-value
0.8
0.7
0.6
0.5
0.4
0.3
0.2
0.1
0.0
Phylostratum
Cellular_organisms - ps1
Archaea1 - ps2
Archaea2 - ps3
Archaea3 - ps4
Asgard/Eukaryota - ps5
Eukaryota - ps6
Amorpheae - ps7
Obazoa - ps8
Opisthokonta - ps9
Holozoa - ps10
Filozoa - ps11
Choano./Metazoa - ps12
Metazoa - ps13
Myriazoa - ps14
Parahoxozoa - ps15
Bilateria - ps16
Deuterostomia - ps17
Chordata - ps18
Olfactores - ps19
Vertebrata - ps20
Gnathostomata - ps21
Euteleostomi - ps22
Sarcopterygii - ps23
Tetrapoda - ps24
Amniota - ps25
Mammalia - ps26
Theria - ps27
Placentalia - ps28
Boreoeutheria - ps29
Euarchontoglires - ps30
Euarchonta - ps31
Primates - ps32
Haplorrhini - ps33
Simiiformes - ps34
Catarrhini - ps35
Hominoidea - ps36
Hominidae - ps37
Homininae - ps38
Hominini - ps39
Homo_sapiens - ps40
p-value
3e-12
1e-09
3e-07
1e-04
4e-02
Log-odds
0.5
1.0
1.5
2.0
2.5
3.0
3.5
4.0

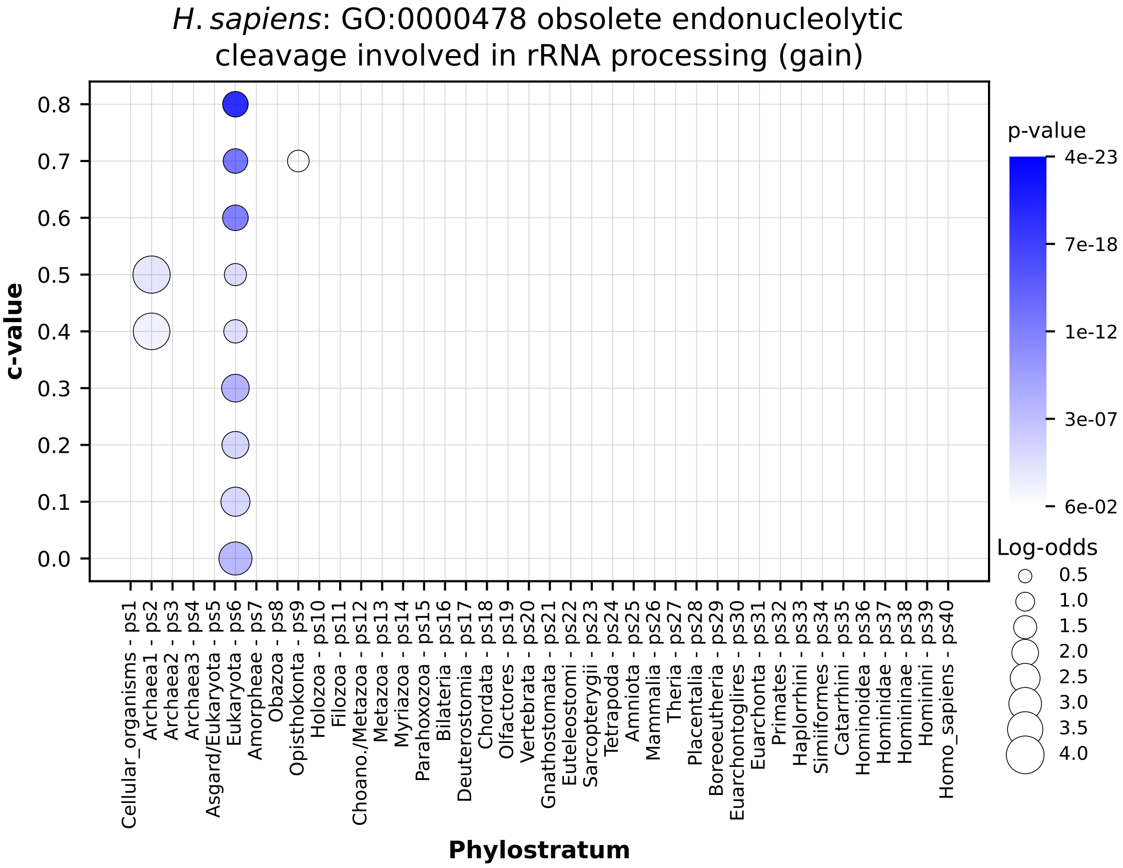

H. sapiens: GO:0000478 obsolete endonucleolytic cleavage involved in rRNA processing (gain)
c-value
0.8
0.7
0.6
0.5
0.4
0.3
0.2
0.1
0.0
Phylostratum
Cellular_organisms - ps1
Archaea1 - ps2
Archaea2 - ps3
Archaea3 - ps4
Asgard/Eukaryota - ps5
Eukaryota - ps6
Amorpheae - ps7
Obazoa - ps8
Opisthokonta - ps9
Holozoa - ps10
Filozoa - ps11
Choano./Metazoa - ps12
Metazoa - ps13
Myriazoa - ps14
Parahoxozoa - ps15
Bilateria - ps16
Deuterostomia - ps17
Chordata - ps18
Olfactores - ps19
Vertebrata - ps20
Gnathostomata - ps21
Euteleostomi - ps22
Sarcopterygii - ps23
Tetrapoda - ps24
Amniota - ps25
Mammalia - ps26
Theria - ps27
Placentalia - ps28
Boreoeutheria - ps29
Euarchontoglires - ps30
Euarchonta - ps31
Primates - ps32
Haplorrhini - ps33
Simiiformes - ps34
Catarrhini - ps35
Hominoidea - ps36
Hominidae - ps37
Homininae - ps38
Hominini - ps39
Homo_sapiens - ps40
p-value
4e-23
7e-18
1e-12
3e-07
6e-02
Log-odds
0.5
1.0
1.5
2.0
2.5
3.0
3.5
4.0

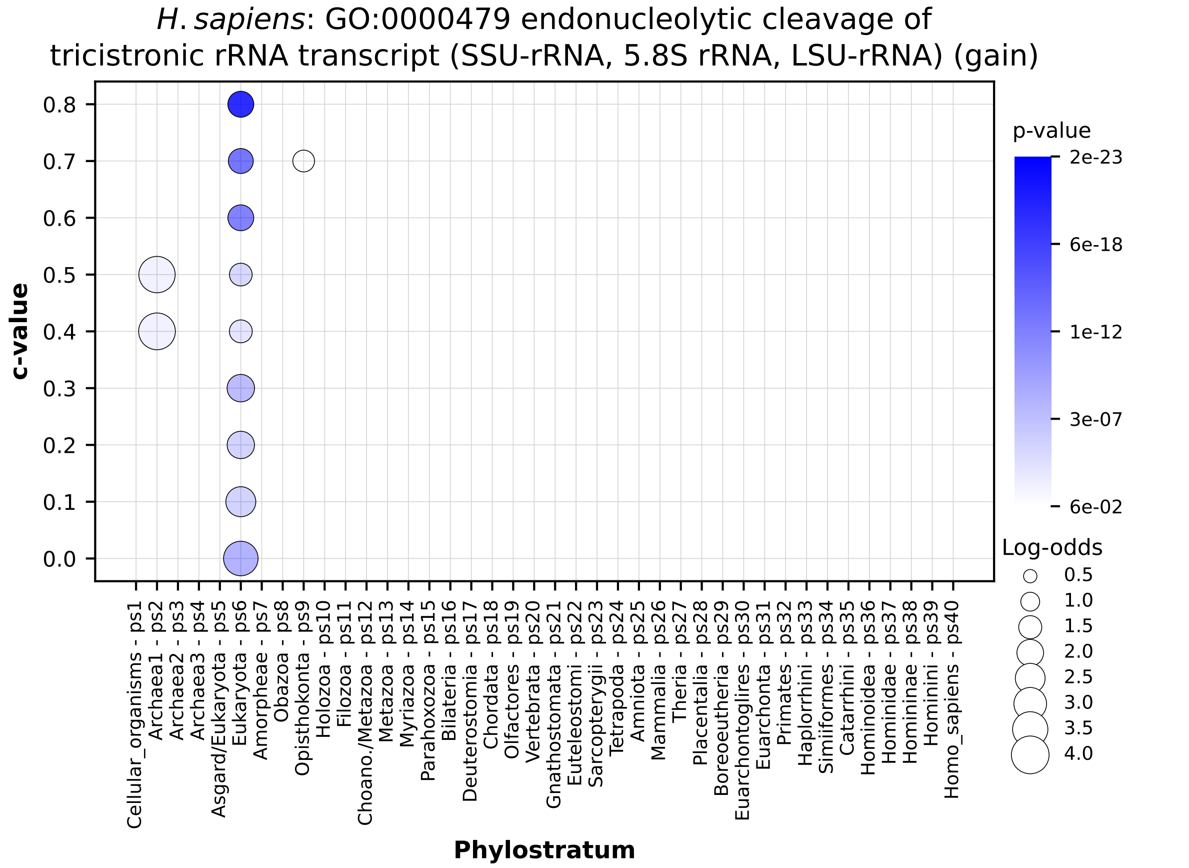
*H. sapiens*: GO:0000479 endonucleolytic cleavage of
tricistronic rRNA transcript (SSU-rRNA, 5.8S rRNA, LSU-rRNA) (gain)
c-value
0.8
0.7
0.6
0.5
0.4
0.3
0.2
0.1
0.0
p-value
2e-23
6e-18
1e-12
3e-07
6e-02
Log-odds
0.5
1.0
1.5
2.0
2.5
3.0
3.5
4.0
Cellular_organisms - ps1
Archaea1 - ps2
Archaea2 - ps3
Archaea3 - ps4
Asgard/Eukaryota - ps5
Eukaryota - ps6
Amorpheae - ps7
Obazoa - ps8
Opisthokonta - ps9
Holozoa - ps10
Filozoa - ps11
Choano./Metazoa - ps12
Metazoa - ps13
Myriazoa - ps14
Parahoxozoa - ps15
Bilateria - ps16
Deuterostomia - ps17
Chordata - ps18
Olfactores - ps19
Vertebrata - ps20
Gnathostomata - ps21
Euteleostomi - ps22
Sarcopterygii - ps23
Tetrapoda - ps24
Amniota - ps25
Mammalia - ps26
Theria - ps27
Placentalia - ps28
Boreoeutheria - ps29
Euarchontoglires - ps30
Euarchonta - ps31
Primates - ps32
Haplorrhini - ps33
Simiiformes - ps34
Catarrhini - ps35
Hominoidea - ps36
Hominidae - ps37
Homininae - ps38
Hominini - ps39
Homo_sapiens - ps40
Phylostratum

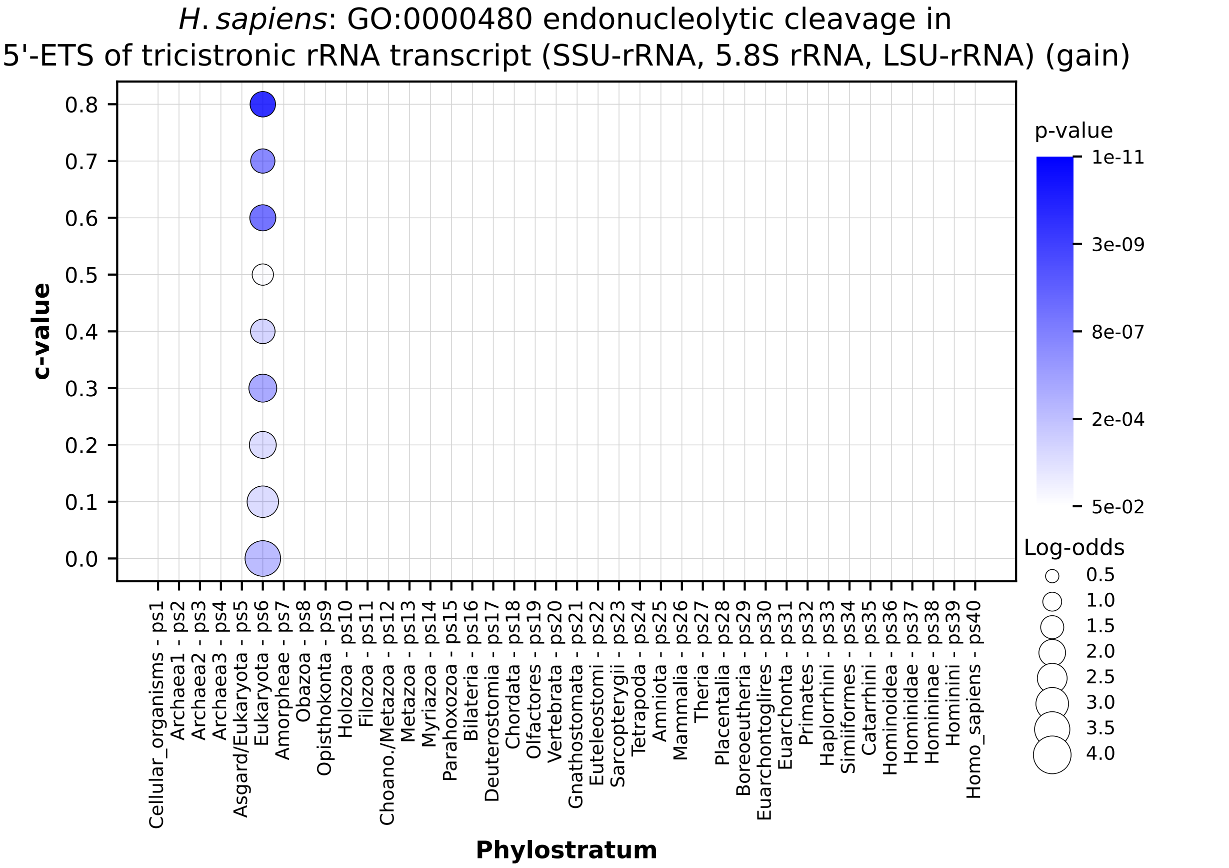

*H. sapiens*: GO:0000480 endonucleolytic cleavage in 5'-ETS of tricistronic rRNA transcript (SSU-rRNA, 5.8S rRNA, LSU-rRNA) (gain)
c-value
0.8
0.7
0.6
0.5
0.4
0.3
0.2
0.1
0.0
p-value
1e-11
3e-09
8e-07
2e-04
5e-02
Log-odds
0.5
1.0
1.5
2.0
2.5
3.0
3.5
4.0
Cellular_organisms - ps1
Archaea1 - ps2
Archaea2 - ps3
Archaea3 - ps4
Asgard/Eukaryota - ps5
Eukaryota - ps6
Amorpheae - ps7
Obazoa - ps8
Opisthokonta - ps9
Holozoa - ps10
Filozoa - ps11
Choano./Metazoa - ps12
Metazoa - ps13
Myriazoa - ps14
Parahoxozoa - ps15
Bilateria - ps16
Deuterostomia - ps17
Chordata - ps18
Olfactores - ps19
Vertebrata - ps20
Gnathostomata - ps21
Euteleostomi - ps22
Sarcopterygii - ps23
Tetrapoda - ps24
Amniota - ps25
Mammalia - ps26
Theria - ps27
Placentalia - ps28
Boreoeutheria - ps29
Euarchontoglires - ps30
Euarchonta - ps31
Primates - ps32
Haplorrhini - ps33
Simiiformes - ps34
Catarrhini - ps35
Hominoidea - ps36
Hominidae - ps37
Homininae - ps38
Hominini - ps39
Homo_sapiens - ps40
Phylostratum

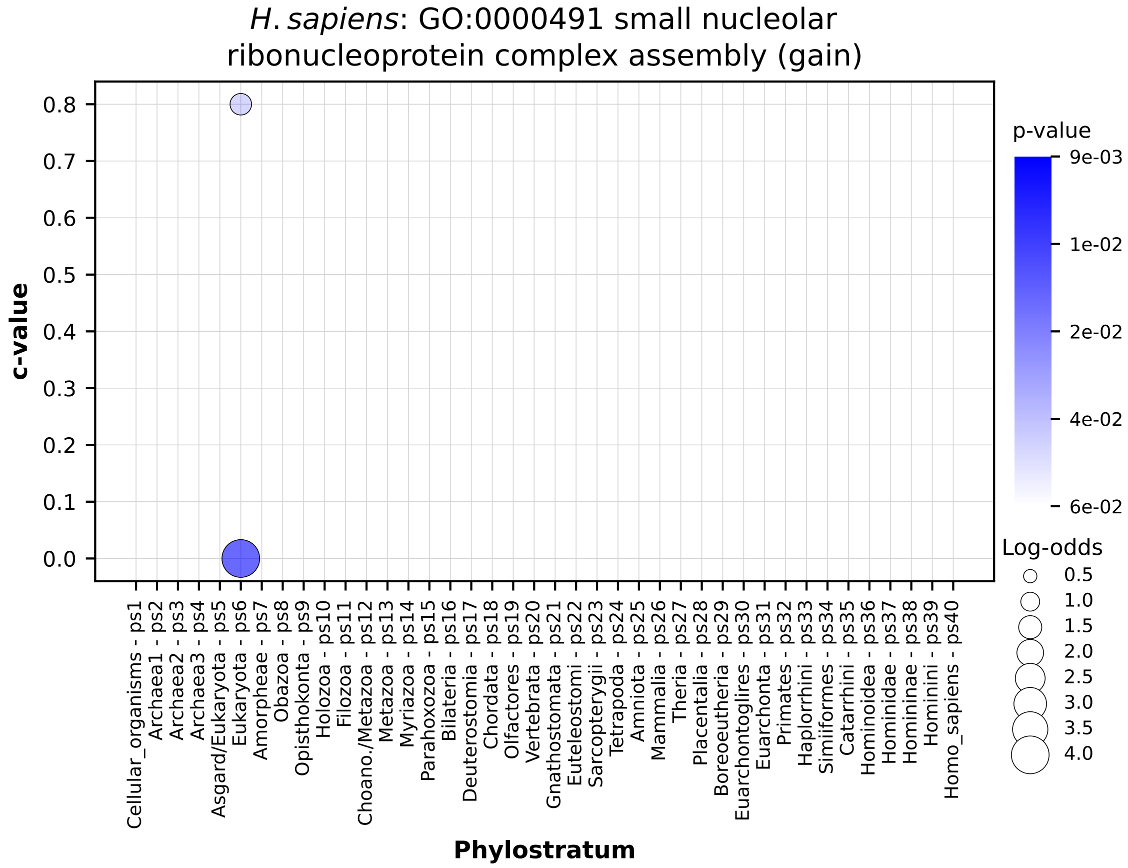

H. sapiens: GO:0000491 small nucleolar ribonucleoprotein complex assembly (gain)
c-value
0.8
0.7
0.6
0.5
0.4
0.3
0.2
0.1
0.0
p-value
9e-03
1e-02
2e-02
4e-02
6e-02
Log-odds
0.5
1.0
1.5
2.0
2.5
3.0
3.5
4.0
Cellular_organisms - ps1
Archaea1 - ps2
Archaea2 - ps3
Archaea3 - ps4
Asgard/Eukaryota - ps5
Eukaryota - ps6
Amorpheae - ps7
Obazoa - ps8
Opisthokonta - ps9
Holozoa - ps10
Filozoa - ps11
Choano./Metazoa - ps12
Metazoa - ps13
Myriazoa - ps14
Parahoxozoa - ps15
Bilateria - ps16
Deuterostomia - ps17
Chordata - ps18
Olfactores - ps19
Vertebrata - ps20
Gnathostomata - ps21
Euteleostomi - ps22
Sarcopterygii - ps23
Tetrapoda - ps24
Amniota - ps25
Mammalia - ps26
Theria - ps27
Placentalia - ps28
Boreoeutheria - ps29
Euarchontoglires - ps30
Euarchonta - ps31
Primates - ps32
Haplorrhini - ps33
Simiiformes - ps34
Catarrhini - ps35
Hominoidea - ps36
Hominidae - ps37
Homininae - ps38
Hominini - ps39
Homo_sapiens - ps40
Phylostratum

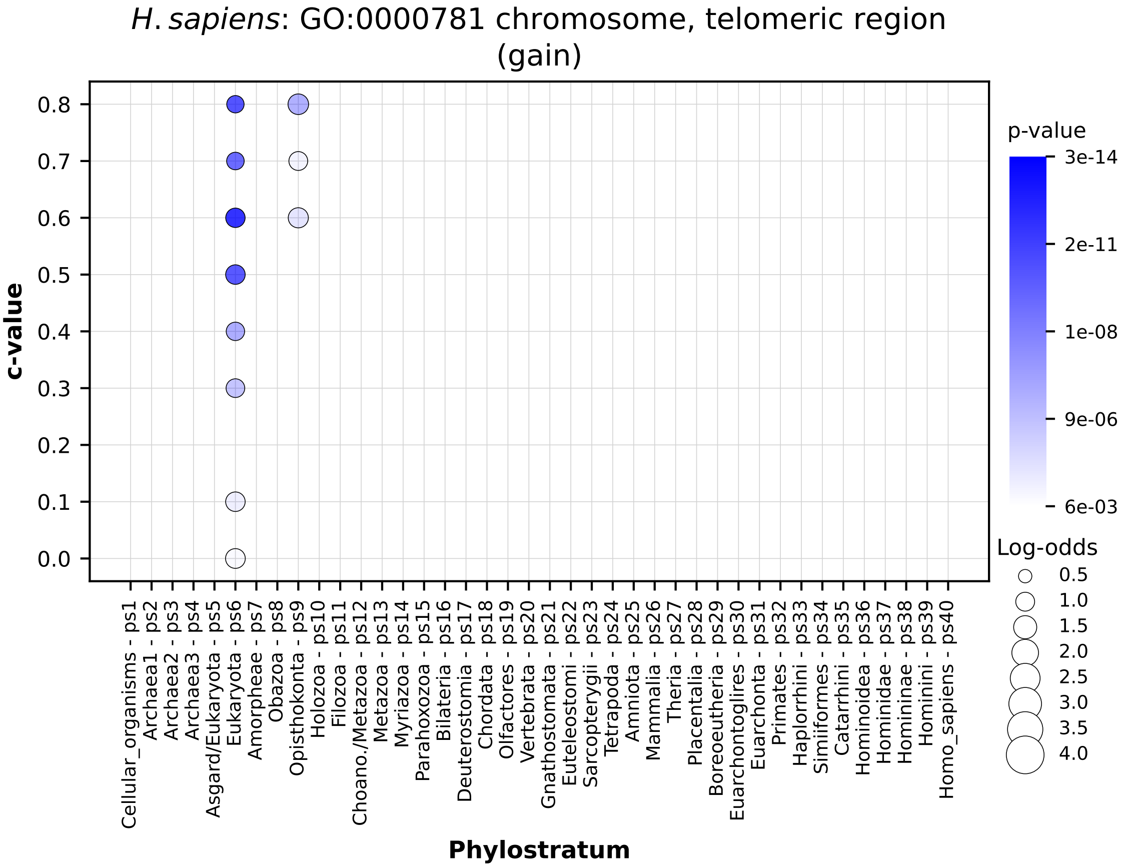
H. sapiens: GO:0000781 chromosome, telomeric region (gain)
c-value
0.0
0.1
0.2
0.3
0.4
0.5
0.6
0.7
0.8
p-value
3e-14
2e-11
1e-08
9e-06
6e-03
Log-odds
0.5
1.0
1.5
2.0
2.5
3.0
3.5
4.0
Cellular_organisms - ps1
Archaea1 - ps2
Archaea2 - ps3
Archaea3 - ps4
Asgard/Eukaryota - ps5
Eukaryota - ps6
Amorpheae - ps7
Obazoa - ps8
Opisthokonta - ps9
Holozoa - ps10
Filozoa - ps11
Choano./Metazoa - ps12
Metazoa - ps13
Myriazoa - ps14
Parahoxozoa - ps15
Bilateria - ps16
Deuterostomia - ps17
Chordata - ps18
Olfactores - ps19
Vertebrata - ps20
Gnathostomata - ps21
Euteleostomi - ps22
Sarcopterygii - ps23
Tetrapoda - ps24
Amniota - ps25
Mammalia - ps26
Theria - ps27
Placentalia - ps28
Boreoeutheria - ps29
Euarchontoglires - ps30
Euarchonta - ps31
Primates - ps32
Haplorrhini - ps33
Simiiformes - ps34
Catarrhini - ps35
Hominoidea - ps36
Hominidae - ps37
Homininae - ps38
Hominini - ps39
Homo_sapiens - ps40
Phylostratum

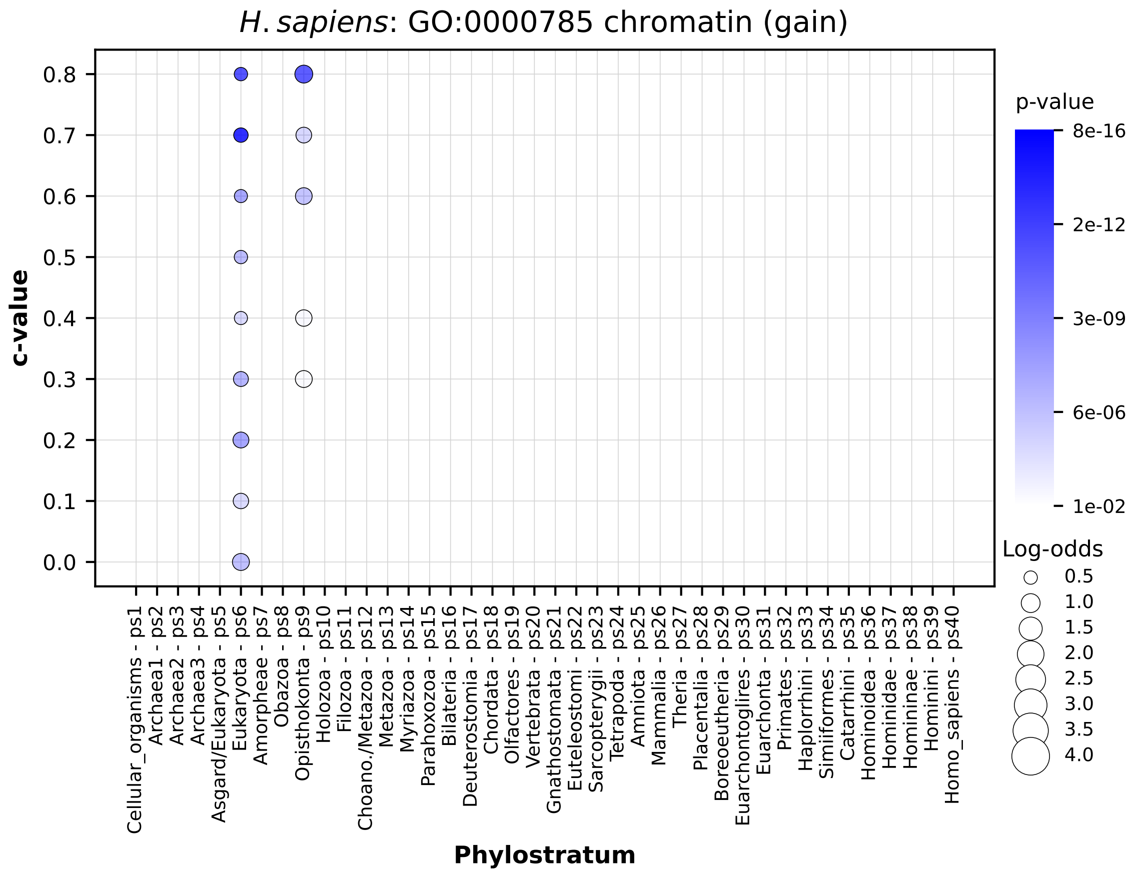
H. sapiens: GO:0000785 chromatin (gain)
c-value
0.8
0.7
0.6
0.5
0.4
0.3
0.2
0.1
0.0
Phylostratum
Cellular_organisms - ps1
Archaea1 - ps2
Archaea2 - ps3
Archaea3 - ps4
Asgard/Eukaryota - ps5
Eukaryota - ps6
Amorpheae - ps7
Obazoa - ps8
Opisthokonta - ps9
Holozoa - ps10
Filozoa - ps11
Choano./Metazoa - ps12
Metazoa - ps13
Myriazoa - ps14
Parahoxozoa - ps15
Bilateria - ps16
Deuterostomia - ps17
Chordata - ps18
Olfactores - ps19
Vertebrata - ps20
Gnathostomata - ps21
Euteleostomi - ps22
Sarcopterygii - ps23
Tetrapoda - ps24
Amniota - ps25
Mammalia - ps26
Theria - ps27
Placentalia - ps28
Boreoeutheria - ps29
Euarchontoglires - ps30
Euarchonta - ps31
Primates - ps32
Haplorrhini - ps33
Simiiformes - ps34
Catarrhini - ps35
Hominoidea - ps36
Hominidae - ps37
Homininae - ps38
Hominini - ps39
Homo_sapiens - ps40
p-value
8e-16
2e-12
3e-09
6e-06
1e-02
Log-odds
0.5
1.0
1.5
2.0
2.5
3.0
3.5
4.0

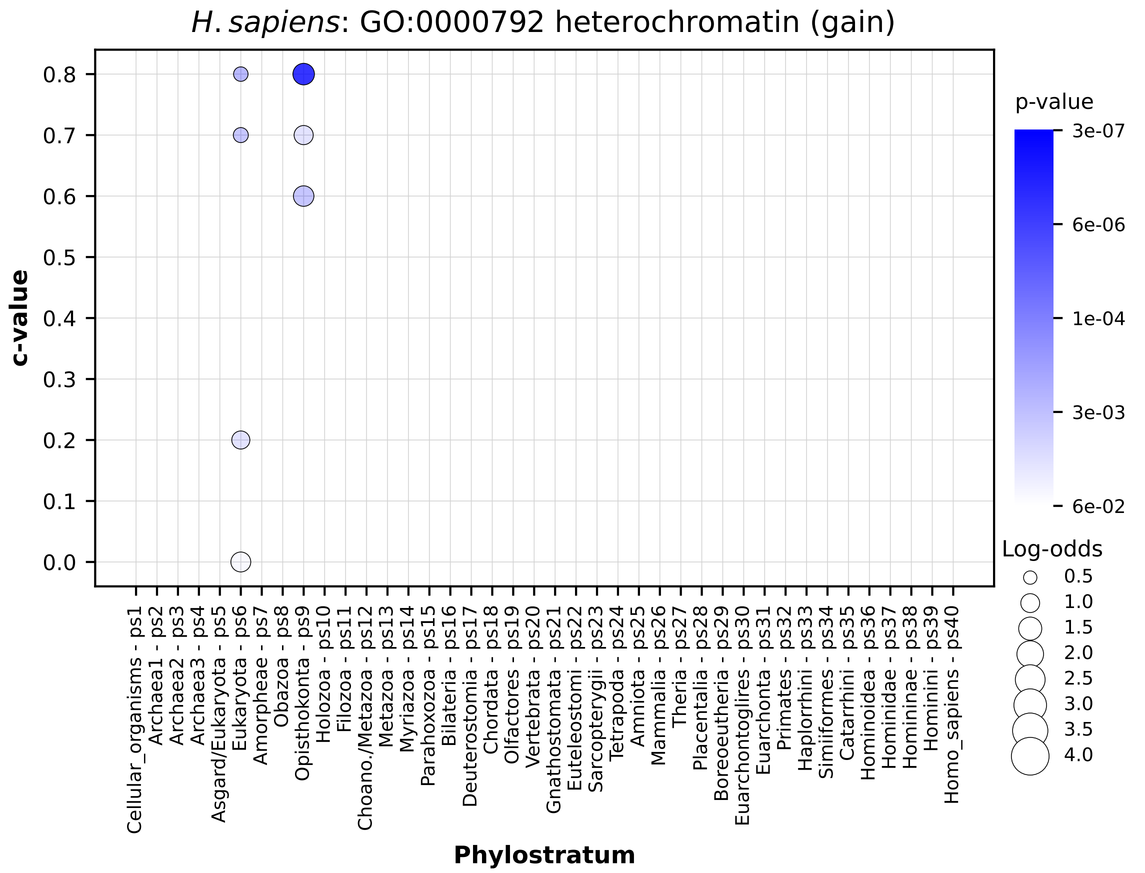
H. sapiens: GO:0000792 heterochromatin (gain)
c-value
0.8
0.7
0.6
0.5
0.4
0.3
0.2
0.1
0.0
Phylostratum
Cellular_organisms - ps1
Archaea1 - ps2
Archaea2 - ps3
Archaea3 - ps4
Asgard/Eukaryota - ps5
Eukaryota - ps6
Amorpheae - ps7
Obazoa - ps8
Opisthokonta - ps9
Holozoa - ps10
Filozoa - ps11
Choano./Metazoa - ps12
Metazoa - ps13
Myriazoa - ps14
Parahoxozoa - ps15
Bilateria - ps16
Deuterostomia - ps17
Chordata - ps18
Olfactores - ps19
Vertebrata - ps20
Gnathostomata - ps21
Euteleostomi - ps22
Sarcopterygii - ps23
Tetrapoda - ps24
Amniota - ps25
Mammalia - ps26
Theria - ps27
Placentalia - ps28
Boreoeutheria - ps29
Euarchontoglires - ps30
Euarchonta - ps31
Primates - ps32
Haplorrhini - ps33
Simiiformes - ps34
Catarrhini - ps35
Hominoidea - ps36
Hominidae - ps37
Homininae - ps38
Hominini - ps39
Homo_sapiens - ps40
p-value
3e-07
6e-06
1e-04
3e-03
6e-02
Log-odds
0.5
1.0
1.5
2.0
2.5
3.0
3.5
4.0

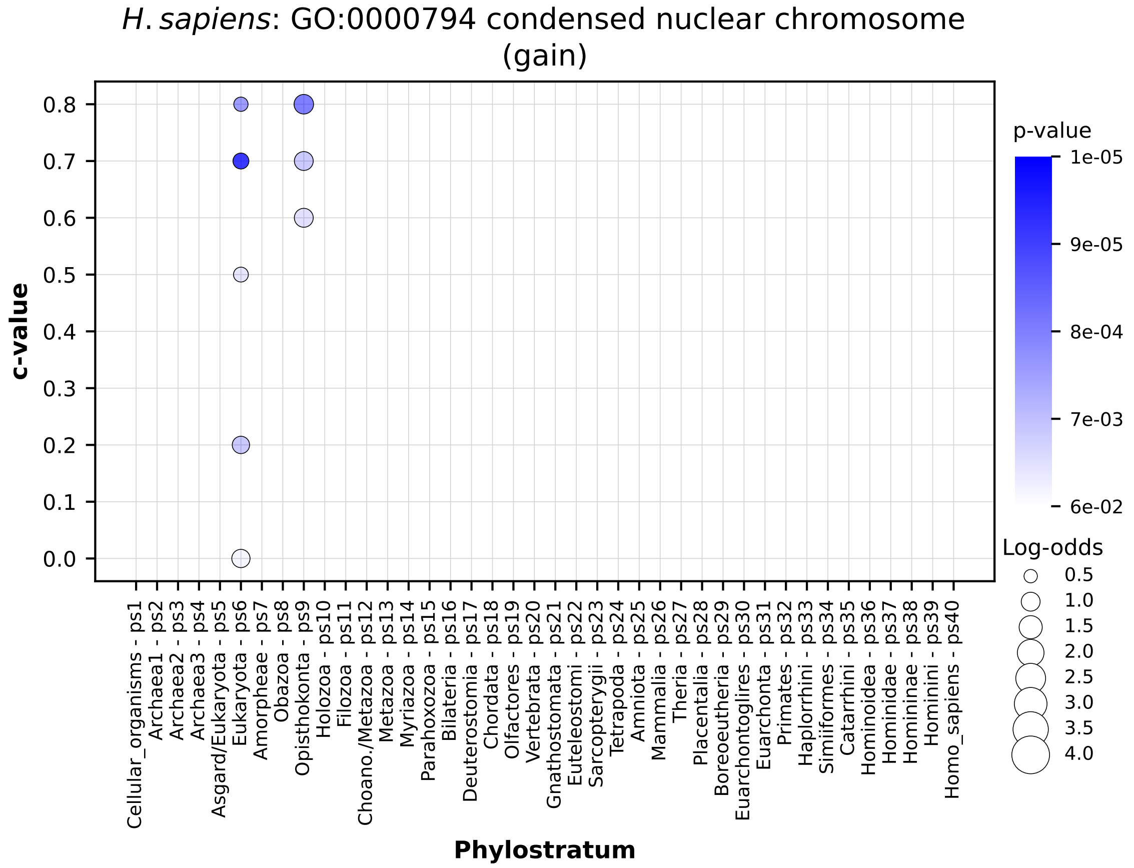

H. sapiens: GO:0000794 condensed nuclear chromosome (gain)
c-value
0.8
0.7
0.6
0.5
0.4
0.3
0.2
0.1
0.0
p-value
1e-05
9e-05
8e-04
7e-03
6e-02
Log-odds
0.5
1.0
1.5
2.0
2.5
3.0
3.5
4.0
Cellular_organisms - ps1
Archaea1 - ps2
Archaea2 - ps3
Archaea3 - ps4
Asgard/Eukaryota - ps5
Eukaryota - ps6
Amorpheae - ps7
Obazoa - ps8
Opisthokonta - ps9
Holozoa - ps10
Filozoa - ps11
Choano./Metazoa - ps12
Metazoa - ps13
Myriazoa - ps14
Parahoxozoa - ps15
Bilateria - ps16
Deuterostomia - ps17
Chordata - ps18
Olfactores - ps19
Vertebrata - ps20
Gnathostomata - ps21
Euteleostomi - ps22
Sarcopterygii - ps23
Tetrapoda - ps24
Amniota - ps25
Mammalia - ps26
Theria - ps27
Placentalia - ps28
Boreoeutheria - ps29
Euarchontoglires - ps30
Euarchonta - ps31
Primates - ps32
Haplorrhini - ps33
Simiiformes - ps34
Catarrhini - ps35
Hominoidea - ps36
Hominidae - ps37
Homininae - ps38
Hominini - ps39
Homo_sapiens - ps40
Phylostratum

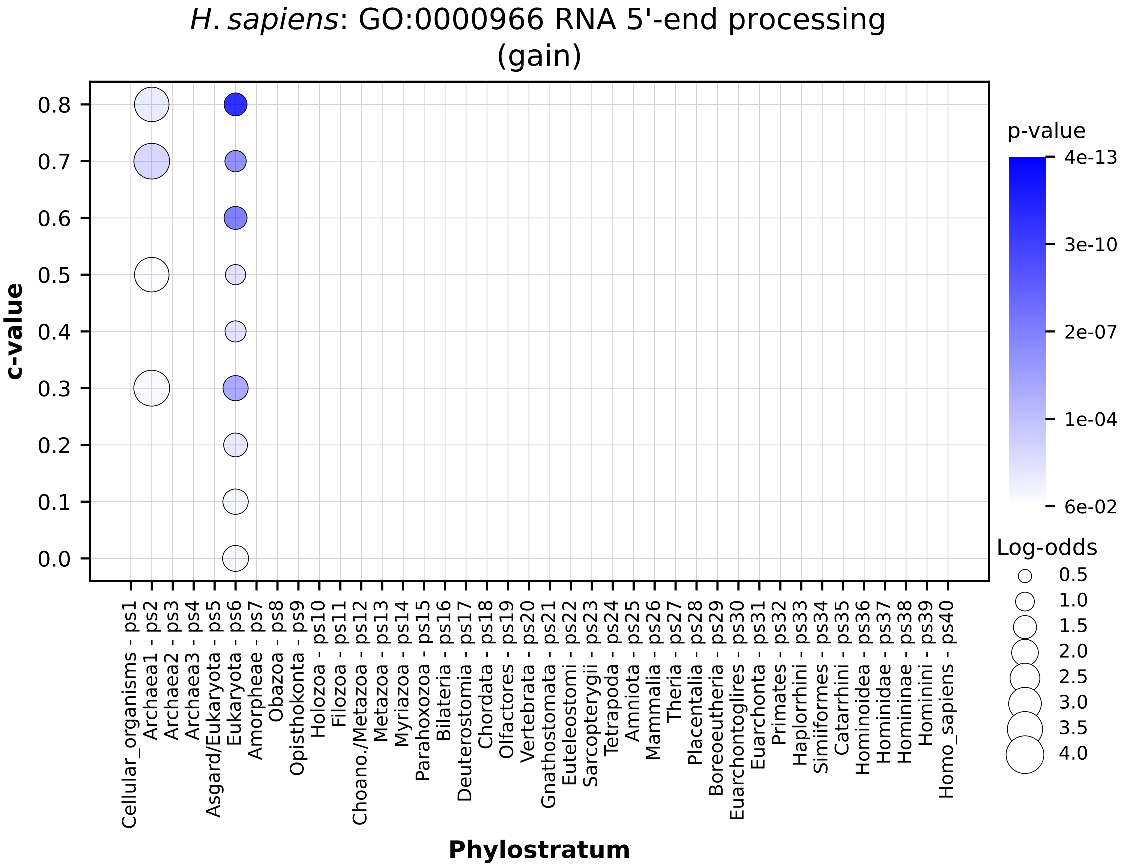
H. sapiens: GO:0000966 RNA 5'-end processing
(gain)
c-value
0.0
0.1
0.2
0.3
0.4
0.5
0.6
0.7
0.8
Phylostratum
Cellular_organisms - ps1
Archaea1 - ps2
Archaea2 - ps3
Archaea3 - ps4
Asgard/Eukaryota - ps5
Eukaryota - ps6
Amorpheae - ps7
Obazoa - ps8
Opisthokonta - ps9
Holozoa - ps10
Filozoa - ps11
Choano./Metazoa - ps12
Metazoa - ps13
Myriazoa - ps14
Parahoxozoa - ps15
Bilateria - ps16
Deuterostomia - ps17
Chordata - ps18
Olfactores - ps19
Vertebrata - ps20
Gnathostomata - ps21
Euteleostomi - ps22
Sarcopterygii - ps23
Tetrapoda - ps24
Amniota - ps25
Mammalia - ps26
Theria - ps27
Placentalia - ps28
Boreoeutheria - ps29
Euarchontoglires - ps30
Euarchonta - ps31
Primates - ps32
Haplorrhini - ps33
Simiiformes - ps34
Catarrhini - ps35
Hominoidea - ps36
Hominidae - ps37
Homininae - ps38
Hominini - ps39
Homo_sapiens - ps40
p-value
4e-13
3e-10
2e-07
1e-04
6e-02
Log-odds
0.5
1.0
1.5
2.0
2.5
3.0
3.5
4.0

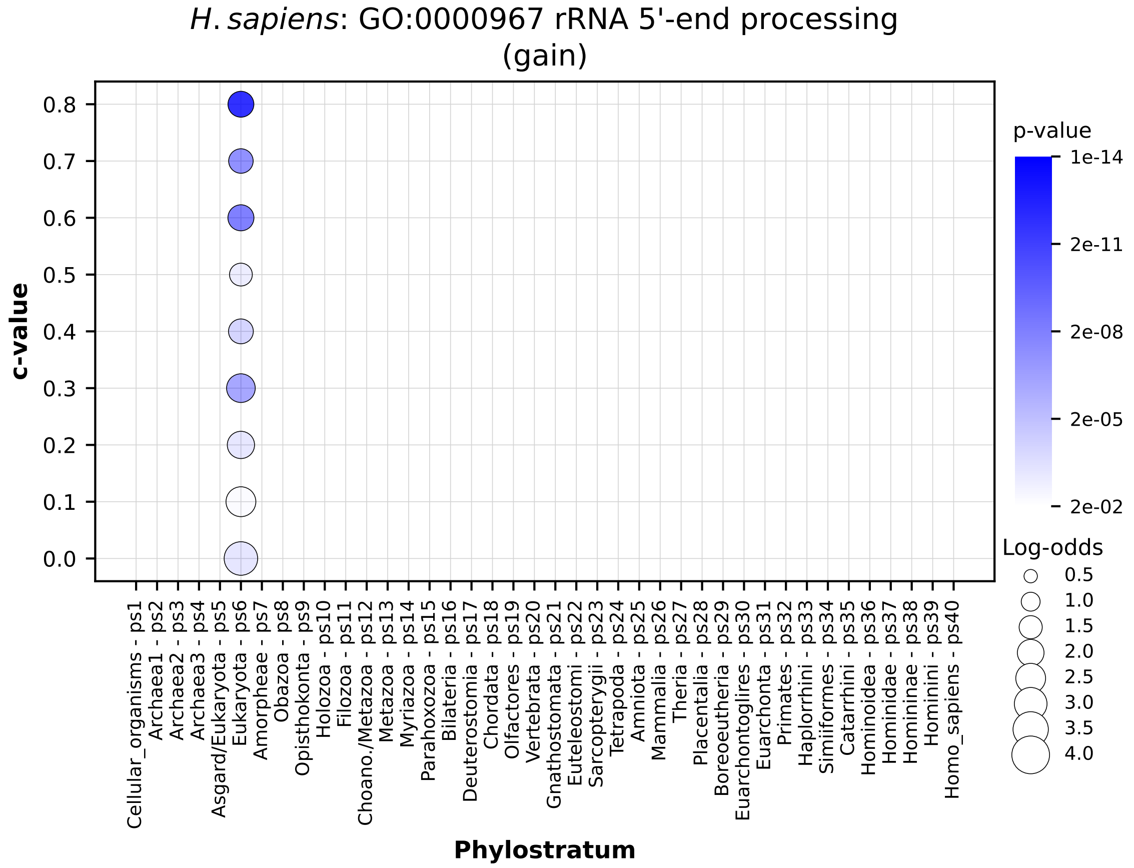

H. sapiens: GO:0000967 rRNA 5'-end processing (gain)
c-value
0.8
0.7
0.6
0.5
0.4
0.3
0.2
0.1
0.0
Phylostratum
Cellular_organisms - ps1
Archaea1 - ps2
Archaea2 - ps3
Archaea3 - ps4
Asgard/Eukaryota - ps5
Eukaryota - ps6
Amorpheae - ps7
Obazoa - ps8
Opisthokonta - ps9
Holozoa - ps10
Filozoa - ps11
Choano./Metazoa - ps12
Metazoa - ps13
Myriazoa - ps14
Parahoxozoa - ps15
Bilateria - ps16
Deuterostomia - ps17
Chordata - ps18
Olfactores - ps19
Vertebrata - ps20
Gnathostomata - ps21
Euteleostomi - ps22
Sarcopterygii - ps23
Tetrapoda - ps24
Amniota - ps25
Mammalia - ps26
Theria - ps27
Placentalia - ps28
Boreoeutheria - ps29
Euarchontoglires - ps30
Euarchonta - ps31
Primates - ps32
Haplorrhini - ps33
Simiiformes - ps34
Catarrhini - ps35
Hominoidea - ps36
Hominidae - ps37
Homininae - ps38
Hominini - ps39
Homo_sapiens - ps40
p-value
1e-14
2e-11
2e-08
2e-05
2e-02
Log-odds
0.5
1.0
1.5
2.0
2.5
3.0
3.5
4.0

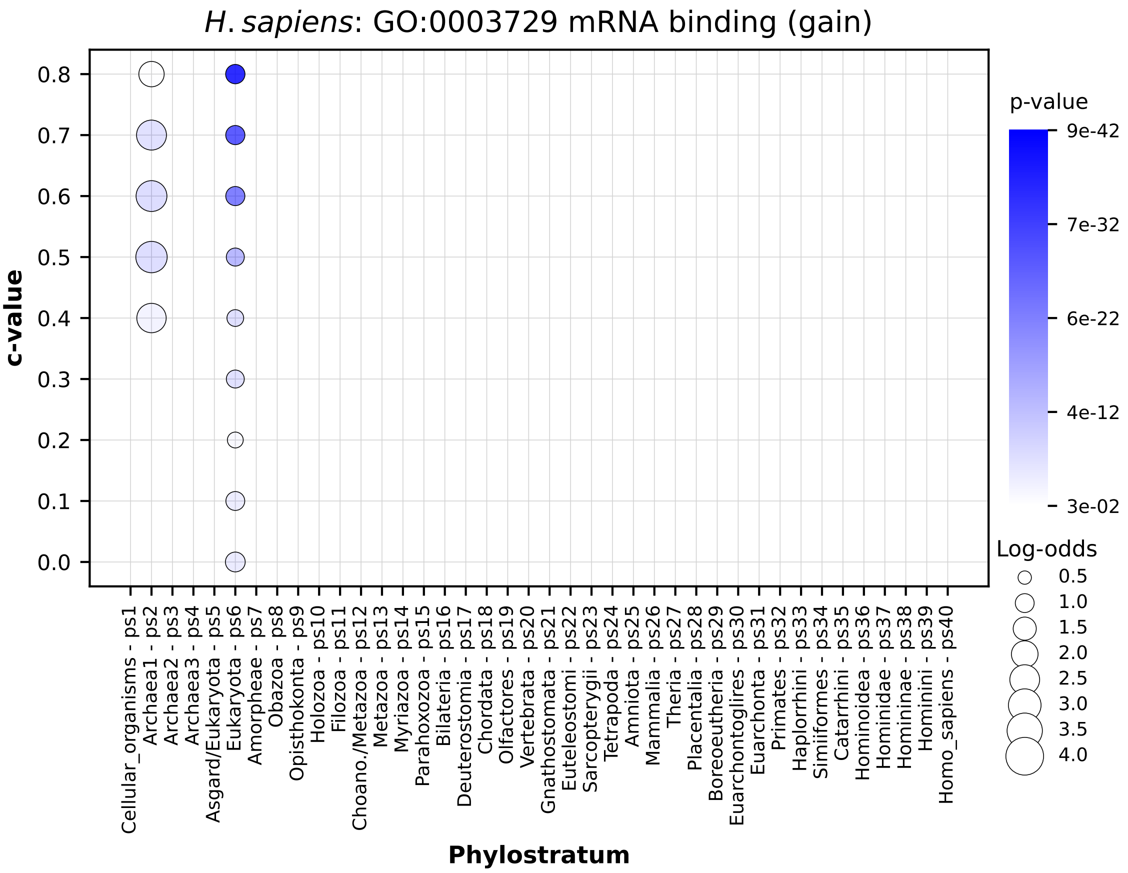

H. sapiens: GO:0003729 mRNA binding (gain)
c-value
0.0
0.1
0.2
0.3
0.4
0.5
0.6
0.7
0.8
p-value
9e-42
7e-32
6e-22
4e-12
3e-02
Log-odds
0.5
1.0
1.5
2.0
2.5
3.0
3.5
4.0
Cellular_organisms - ps1
Archaea1 - ps2
Archaea2 - ps3
Archaea3 - ps4
Asgard/Eukaryota - ps5
Eukaryota - ps6
Amorpheae - ps7
Obazoa - ps8
Opisthokonta - ps9
Holozoa - ps10
Filozoa - ps11
Choano./Metazoa - ps12
Metazoa - ps13
Myriazoa - ps14
Parahoxozoa - ps15
Bilateria - ps16
Deuterostomia - ps17
Chordata - ps18
Olfactores - ps19
Vertebrata - ps20
Gnathostomata - ps21
Euteleostomi - ps22
Sarcopterygii - ps23
Tetrapoda - ps24
Amniota - ps25
Mammalia - ps26
Theria - ps27
Placentalia - ps28
Boreoeutheria - ps29
Euarchontoglires - ps30
Euarchonta - ps31
Primates - ps32
Haplorrhini - ps33
Simiiformes - ps34
Catarrhini - ps35
Hominoidea - ps36
Hominidae - ps37
Homininae - ps38
Hominini - ps39
Homo_sapiens - ps40
Phylostratum

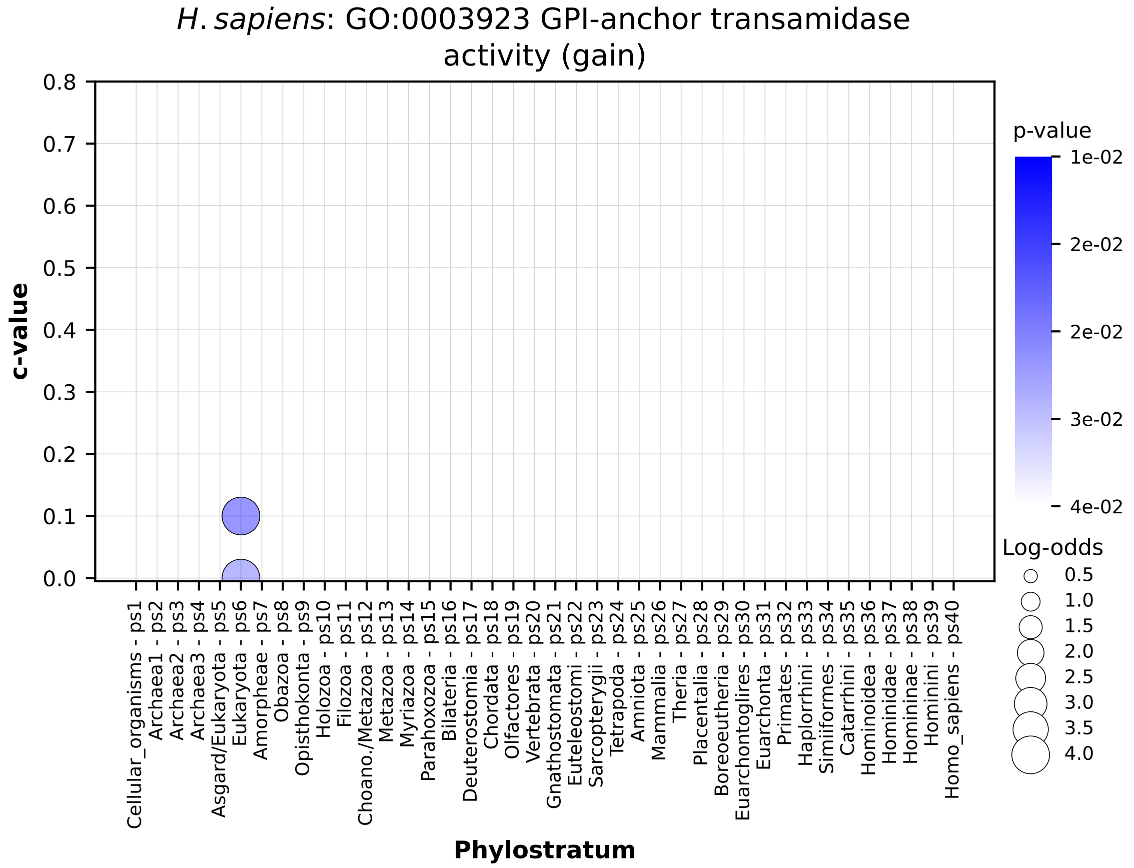

*H. sapiens*: GO:0003923 GPI-anchor transamidase activity (gain)
c-value
0.8
0.7
0.6
0.5
0.4
0.3
0.2
0.1
0.0
Phylostratum
Cellular_organisms - ps1
Archaea1 - ps2
Archaea2 - ps3
Archaea3 - ps4
Asgard/Eukaryota - ps5
Eukaryota - ps6
Amorpheae - ps7
Obazoa - ps8
Opisthokonta - ps9
Holozoa - ps10
Filozoa - ps11
Choano./Metazoa - ps12
Metazoa - ps13
Myriazoa - ps14
Parahoxozoa - ps15
Bilateria - ps16
Deuterostomia - ps17
Chordata - ps18
Olfactores - ps19
Vertebrata - ps20
Gnathostomata - ps21
Euteleostomi - ps22
Sarcopterygii - ps23
Tetrapoda - ps24
Amniota - ps25
Mammalia - ps26
Theria - ps27
Placentalia - ps28
Boreoeutheria - ps29
Euarchontoglires - ps30
Euarchonta - ps31
Primates - ps32
Haplorrhini - ps33
Simiiformes - ps34
Catarrhini - ps35
Hominoidea - ps36
Hominidae - ps37
Homininae - ps38
Hominini - ps39
Homo_sapiens - ps40
p-value
1e-02
2e-02
2e-02
3e-02
4e-02
Log-odds
0.5
1.0
1.5
2.0
2.5
3.0
3.5
4.0

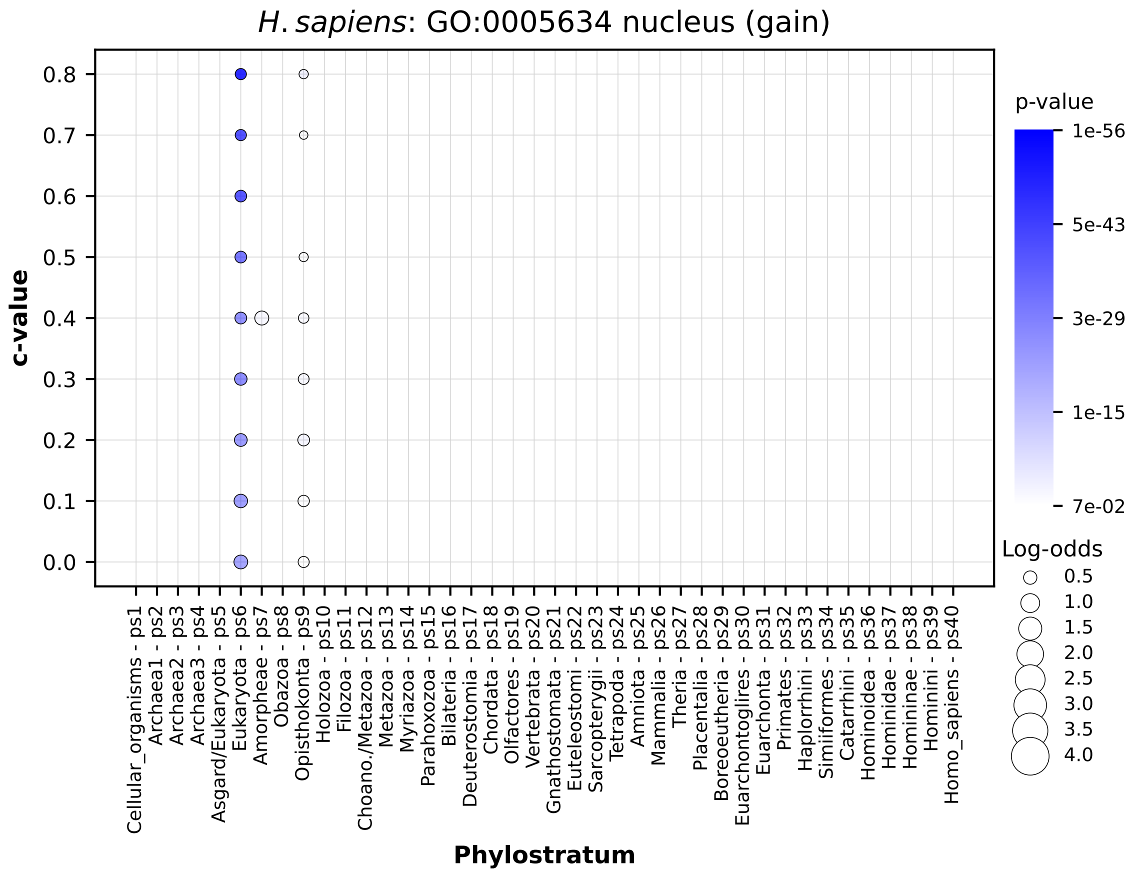

H. sapiens: GO:0005634 nucleus (gain)
c-value
0.8
0.7
0.6
0.5
0.4
0.3
0.2
0.1
0.0
Phylostratum
Cellular_organisms - ps1
Archaea1 - ps2
Archaea2 - ps3
Archaea3 - ps4
Asgard/Eukaryota - ps5
Eukaryota - ps6
Amorpheae - ps7
Obazoa - ps8
Opisthokonta - ps9
Holozoa - ps10
Filozoa - ps11
Choano./Metazoa - ps12
Metazoa - ps13
Myriazoa - ps14
Parahoxozoa - ps15
Bilateria - ps16
Deuterostomia - ps17
Chordata - ps18
Olfactores - ps19
Vertebrata - ps20
Gnathostomata - ps21
Euteleostomi - ps22
Sarcopterygii - ps23
Tetrapoda - ps24
Amniota - ps25
Mammalia - ps26
Theria - ps27
Placentalia - ps28
Boreoeutheria - ps29
Euarchontoglires - ps30
Euarchonta - ps31
Primates - ps32
Haplorrhini - ps33
Simiiformes - ps34
Catarrhini - ps35
Hominoidea - ps36
Hominidae - ps37
Homininae - ps38
Hominini - ps39
Homo_sapiens - ps40
p-value
1e-56
5e-43
3e-29
1e-15
7e-02
Log-odds
0.5
1.0
1.5
2.0
2.5
3.0
3.5
4.0

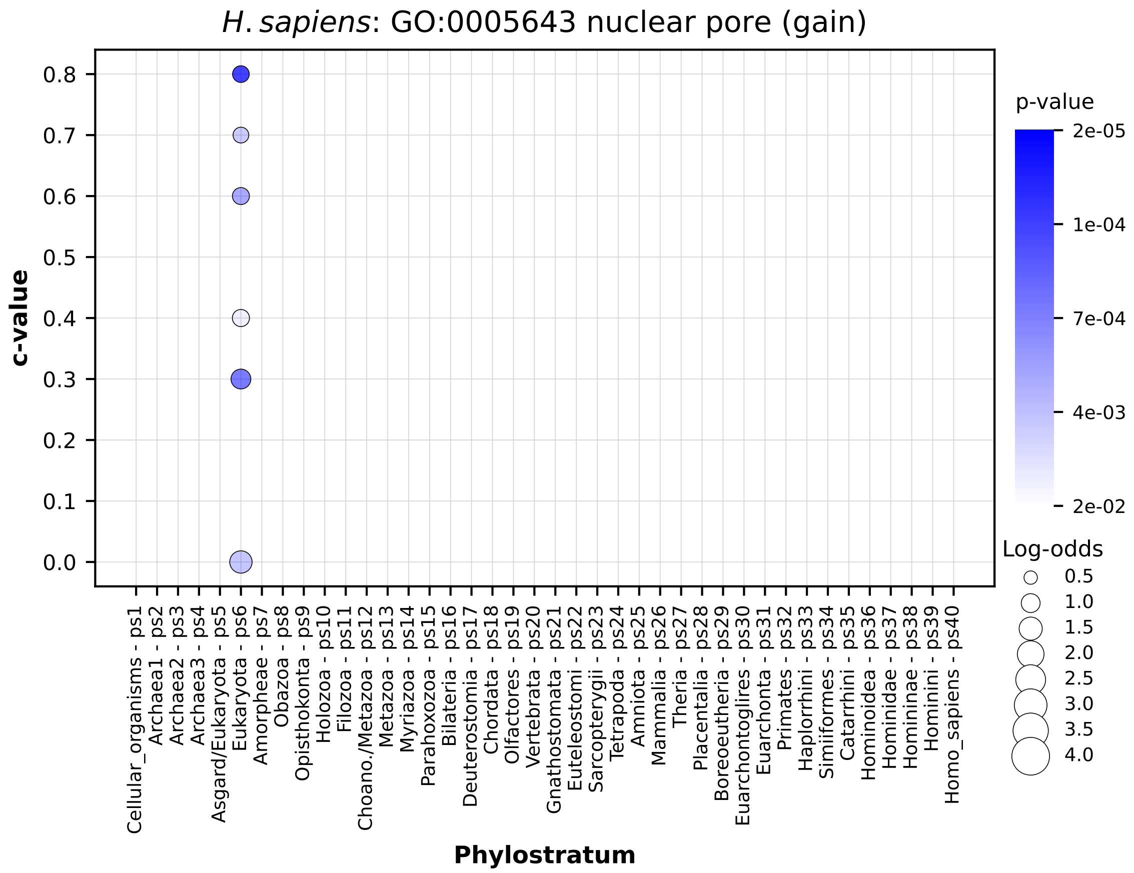

H. sapiens: GO:0005643 nuclear pore (gain)
c-value
0.0
0.1
0.2
0.3
0.4
0.5
0.6
0.7
0.8
Phylostratum
Cellular_organisms - ps1
Archaea1 - ps2
Archaea2 - ps3
Archaea3 - ps4
Asgard/Eukaryota - ps5
Eukaryota - ps6
Amorpheae - ps7
Obazoa - ps8
Opisthokonta - ps9
Holozoa - ps10
Filozoa - ps11
Choano./Metazoa - ps12
Metazoa - ps13
Myriazoa - ps14
Parahoxozoa - ps15
Bilateria - ps16
Deuterostomia - ps17
Chordata - ps18
Olfactores - ps19
Vertebrata - ps20
Gnathostomata - ps21
Euteleostomi - ps22
Sarcopterygii - ps23
Tetrapoda - ps24
Amniota - ps25
Mammalia - ps26
Theria - ps27
Placentalia - ps28
Boreoeutheria - ps29
Euarchontoglires - ps30
Euarchonta - ps31
Primates - ps32
Haplorrhini - ps33
Simiiformes - ps34
Catarrhini - ps35
Hominoidea - ps36
Hominidae - ps37
Homininae - ps38
Hominini - ps39
Homo_sapiens - ps40
p-value
2e-05
1e-04
7e-04
4e-03
2e-02
Log-odds
0.5
1.0
1.5
2.0
2.5
3.0
3.5
4.0

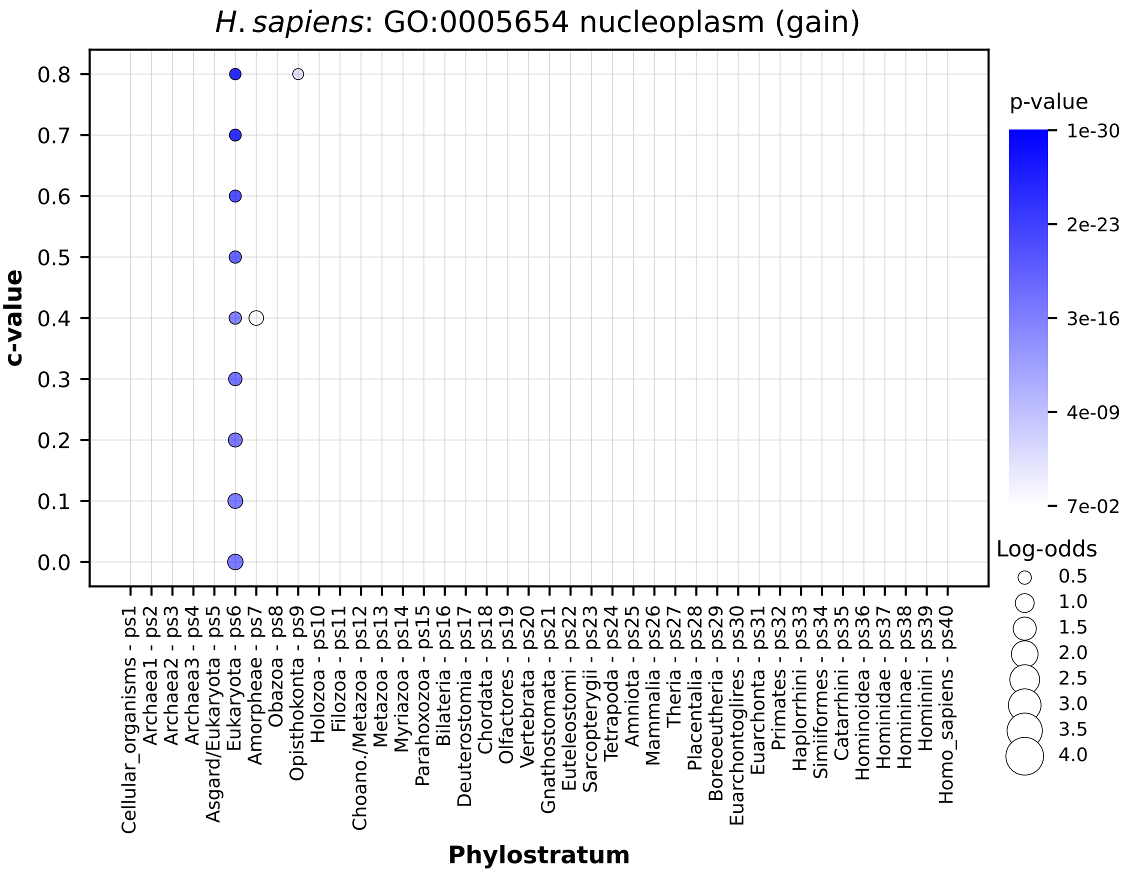

H. sapiens: GO:0005654 nucleoplasm (gain)
c-value
0.0
0.1
0.2
0.3
0.4
0.5
0.6
0.7
0.8
Phylostratum
Cellular_organisms - ps1
Archaea1 - ps2
Archaea2 - ps3
Archaea3 - ps4
Asgard/Eukaryota - ps5
Eukaryota - ps6
Amorpheae - ps7
Obazoa - ps8
Opisthokonta - ps9
Holozoa - ps10
Filozoa - ps11
Choano./Metazoa - ps12
Metazoa - ps13
Myriazoa - ps14
Parahoxozoa - ps15
Bilateria - ps16
Deuterostomia - ps17
Chordata - ps18
Olfactores - ps19
Vertebrata - ps20
Gnathostomata - ps21
Euteleostomi - ps22
Sarcopterygii - ps23
Tetrapoda - ps24
Amniota - ps25
Mammalia - ps26
Theria - ps27
Placentalia - ps28
Boreoeutheria - ps29
Euarchontoglires - ps30
Euarchonta - ps31
Primates - ps32
Haplorrhini - ps33
Simiiformes - ps34
Catarrhini - ps35
Hominoidea - ps36
Hominidae - ps37
Homininae - ps38
Hominini - ps39
Homo_sapiens - ps40
p-value
1e-30
2e-23
3e-16
4e-09
7e-02
Log-odds
0.5
1.0
1.5
2.0
2.5
3.0
3.5
4.0

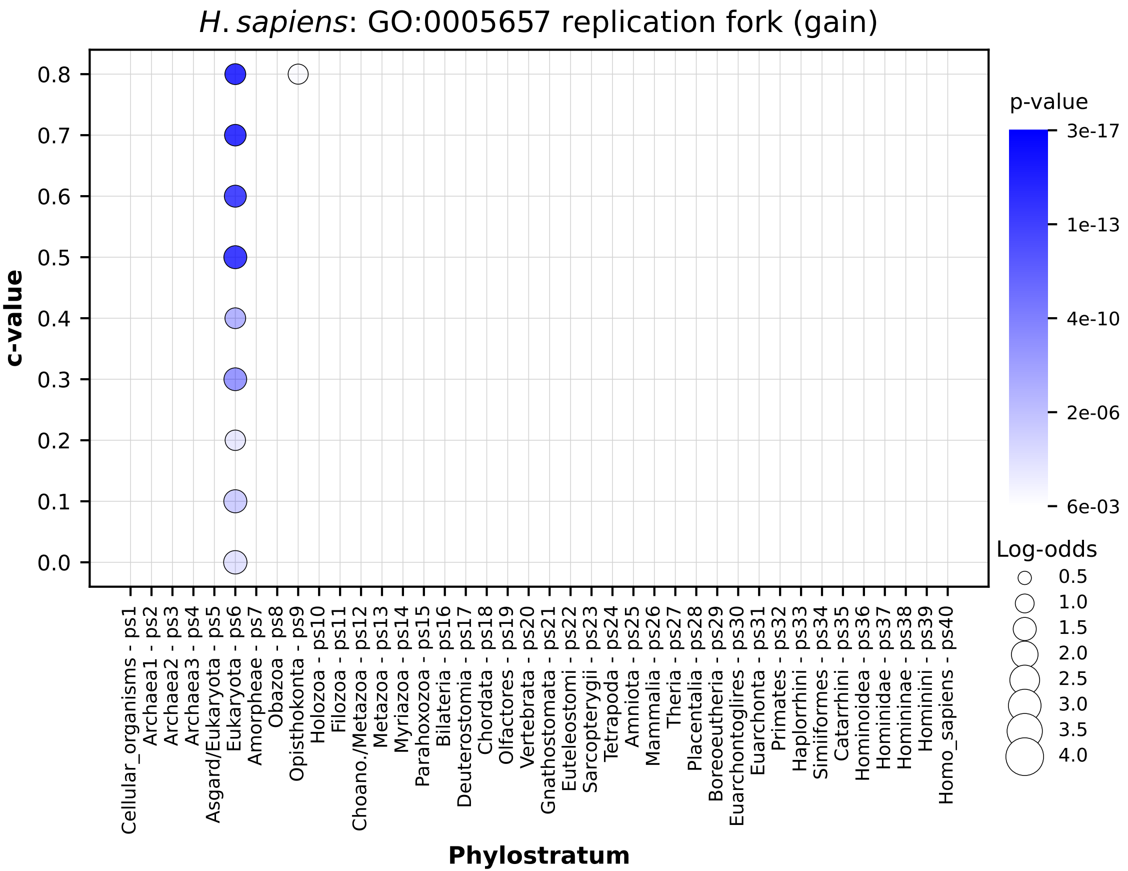

H. sapiens: GO:0005657 replication fork (gain)
c-value
0.8
0.7
0.6
0.5
0.4
0.3
0.2
0.1
0.0
Phylostratum
Cellular_organisms - ps1
Archaea1 - ps2
Archaea2 - ps3
Archaea3 - ps4
Asgard/Eukaryota - ps5
Eukaryota - ps6
Amorpheae - ps7
Obazoa - ps8
Opisthokonta - ps9
Holozoa - ps10
Filozoa - ps11
Choano./Metazoa - ps12
Metazoa - ps13
Myriazoa - ps14
Parahoxozoa - ps15
Bilateria - ps16
Deuterostomia - ps17
Chordata - ps18
Olfactores - ps19
Vertebrata - ps20
Gnathostomata - ps21
Euteleostomi - ps22
Sarcopterygii - ps23
Tetrapoda - ps24
Amniota - ps25
Mammalia - ps26
Theria - ps27
Placentalia - ps28
Boreoeutheria - ps29
Euarchontoglires - ps30
Euarchonta - ps31
Primates - ps32
Haplorrhini - ps33
Simiiformes - ps34
Catarrhini - ps35
Hominoidea - ps36
Hominidae - ps37
Homininae - ps38
Hominini - ps39
Homo_sapiens - ps40
p-value
3e-17
1e-13
4e-10
2e-06
6e-03
Log-odds
0.5
1.0
1.5
2.0
2.5
3.0
3.5
4.0

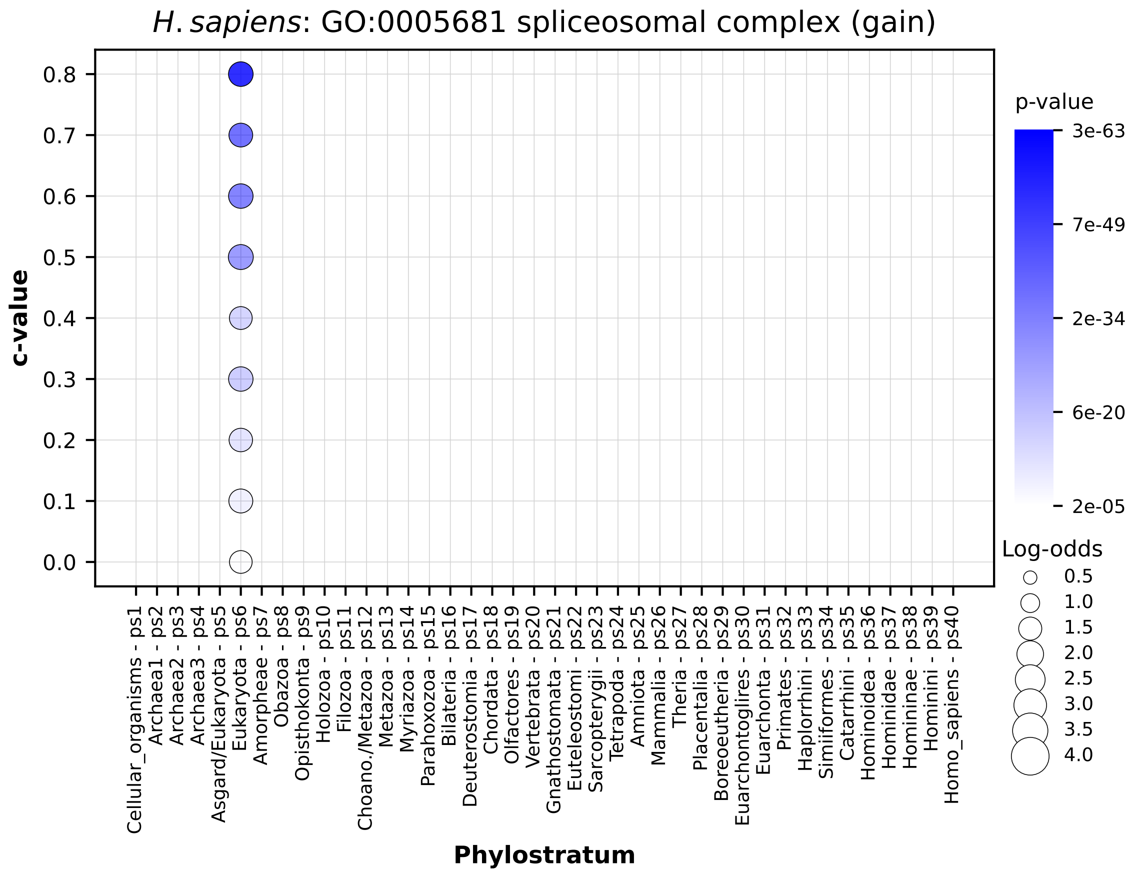
H. sapiens: GO:0005681 spliceosomal complex (gain)
c-value
0.0
0.1
0.2
0.3
0.4
0.5
0.6
0.7
0.8
Phylostratum
Cellular_organisms - ps1
Archaea1 - ps2
Archaea2 - ps3
Archaea3 - ps4
Asgard/Eukaryota - ps5
Eukaryota - ps6
Amorpheae - ps7
Obazoa - ps8
Opisthokonta - ps9
Holozoa - ps10
Filozoa - ps11
Choano./Metazoa - ps12
Metazoa - ps13
Myriazoa - ps14
Parahoxozoa - ps15
Bilateria - ps16
Deuterostomia - ps17
Chordata - ps18
Olfactores - ps19
Vertebrata - ps20
Gnathostomata - ps21
Euteleostomi - ps22
Sarcopterygii - ps23
Tetrapoda - ps24
Amniota - ps25
Mammalia - ps26
Theria - ps27
Placentalia - ps28
Boreoeutheria - ps29
Euarchontoglires - ps30
Euarchonta - ps31
Primates - ps32
Haplorrhini - ps33
Simiiformes - ps34
Catarrhini - ps35
Hominoidea - ps36
Hominidae - ps37
Homininae - ps38
Hominini - ps39
Homo_sapiens - ps40
p-value
3e-63
7e-49
2e-34
6e-20
2e-05
Log-odds
0.5
1.0
1.5
2.0
2.5
3.0
3.5
4.0

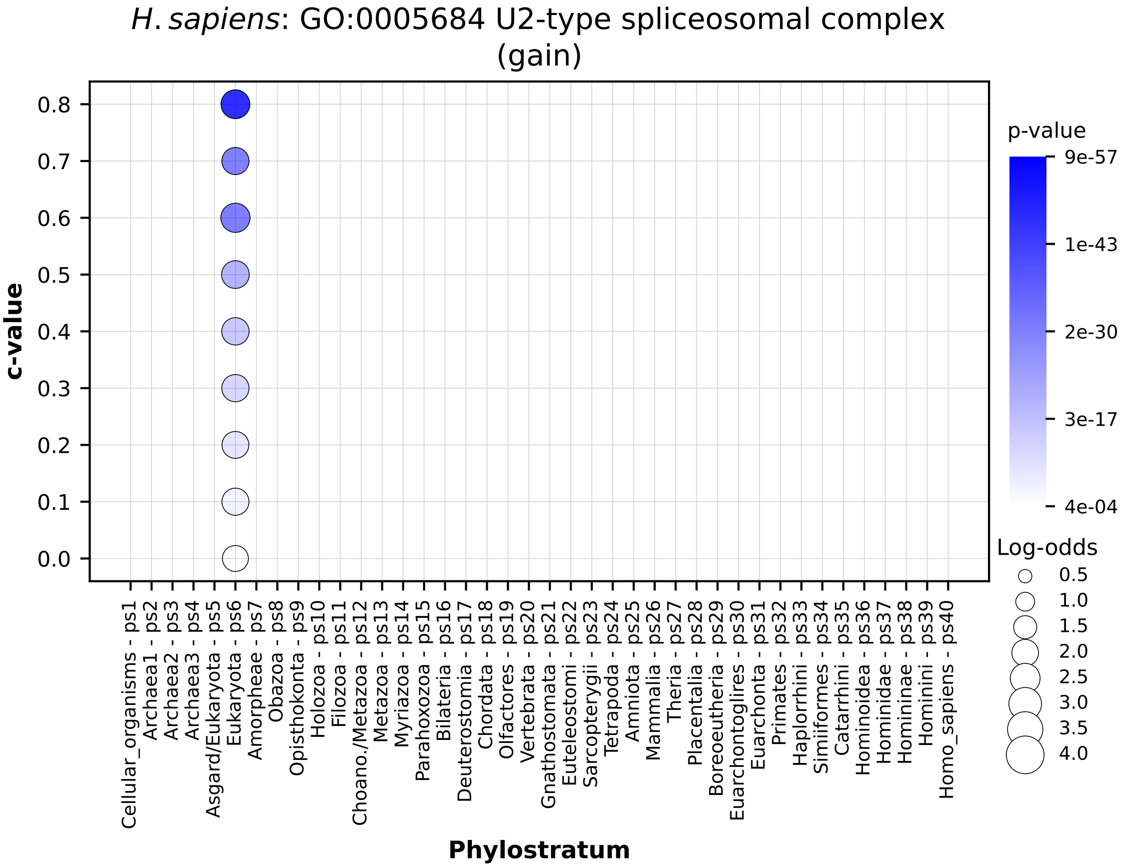
H. sapiens: GO:0005684 U2-type spliceosomal complex
(gain)
c-value
0.8
0.7
0.6
0.5
0.4
0.3
0.2
0.1
0.0
Phylostratum
Cellular_organisms - ps1
Archaea1 - ps2
Archaea2 - ps3
Archaea3 - ps4
Asgard/Eukaryota - ps5
Eukaryota - ps6
Amorpheae - ps7
Obazoa - ps8
Opisthokonta - ps9
Holozoa - ps10
Filozoa - ps11
Choano./Metazoa - ps12
Metazoa - ps13
Myriazoa - ps14
Parahoxozoa - ps15
Bilateria - ps16
Deuterostomia - ps17
Chordata - ps18
Olfactores - ps19
Vertebrata - ps20
Gnathostomata - ps21
Euteleostomi - ps22
Sarcopterygii - ps23
Tetrapoda - ps24
Amniota - ps25
Mammalia - ps26
Theria - ps27
Placentalia - ps28
Boreoeutheria - ps29
Euarchontoglires - ps30
Euarchonta - ps31
Primates - ps32
Haplorrhini - ps33
Simiiformes - ps34
Catarrhini - ps35
Hominoidea - ps36
Hominidae - ps37
Homininae - ps38
Hominini - ps39
Homo_sapiens - ps40
p-value
9e-57
1e-43
2e-30
3e-17
4e-04
Log-odds
0.5
1.0
1.5
2.0
2.5
3.0
3.5
4.0

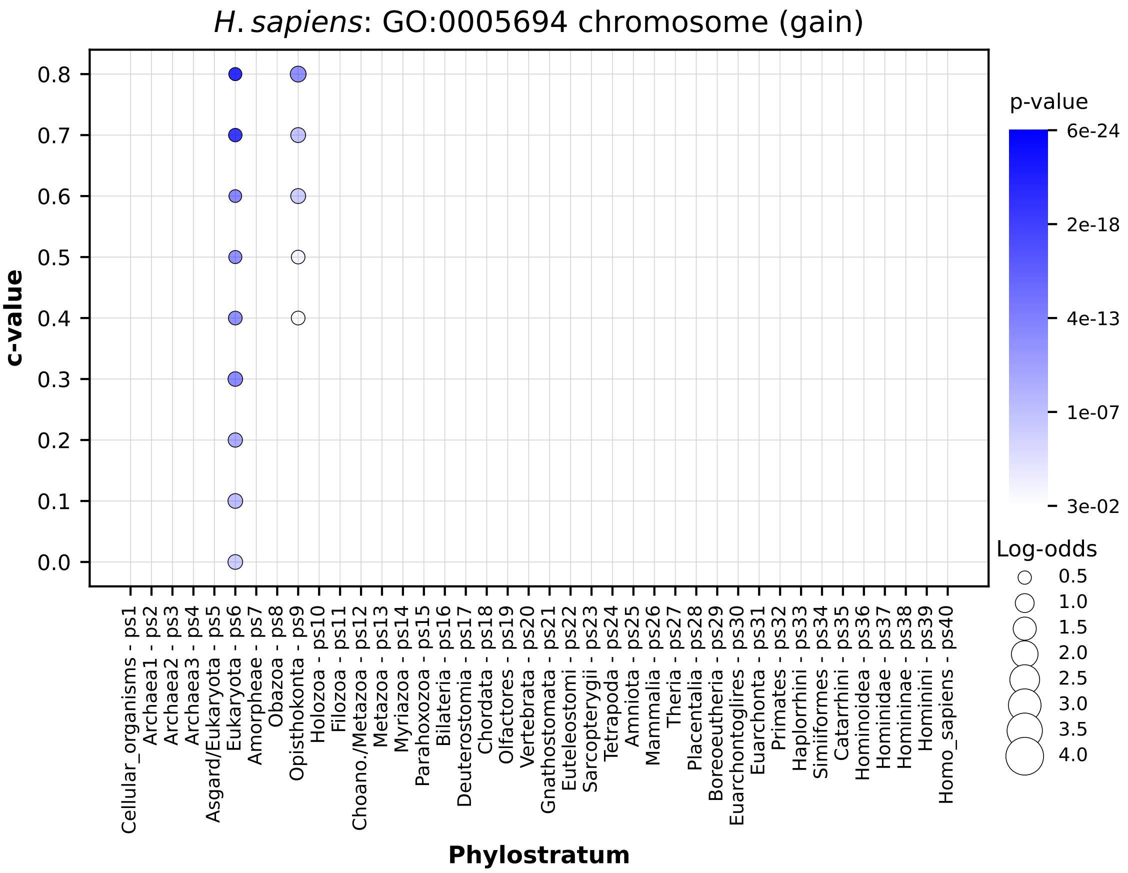

H. sapiens: GO:0005694 chromosome (gain)
c-value
0.0
0.1
0.2
0.3
0.4
0.5
0.6
0.7
0.8
Phylostratum
Cellular_organisms - ps1
Archaea1 - ps2
Archaea2 - ps3
Archaea3 - ps4
Asgard/Eukaryota - ps5
Eukaryota - ps6
Amorpheae - ps7
Obazoa - ps8
Opisthokonta - ps9
Holozoa - ps10
Filozoa - ps11
Choano./Metazoa - ps12
Metazoa - ps13
Myriazoa - ps14
Parahoxozoa - ps15
Bilateria - ps16
Deuterostomia - ps17
Chordata - ps18
Olfactores - ps19
Vertebrata - ps20
Gnathostomata - ps21
Euteleostomi - ps22
Sarcopterygii - ps23
Tetrapoda - ps24
Amniota - ps25
Mammalia - ps26
Theria - ps27
Placentalia - ps28
Boreoeutheria - ps29
Euarchontoglires - ps30
Euarchonta - ps31
Primates - ps32
Haplorrhini - ps33
Simiiformes - ps34
Catarrhini - ps35
Hominoidea - ps36
Hominidae - ps37
Homininae - ps38
Hominini - ps39
Homo_sapiens - ps40
p-value
6e-24
2e-18
4e-13
1e-07
3e-02
Log-odds
0.5
1.0
1.5
2.0
2.5
3.0
3.5
4.0

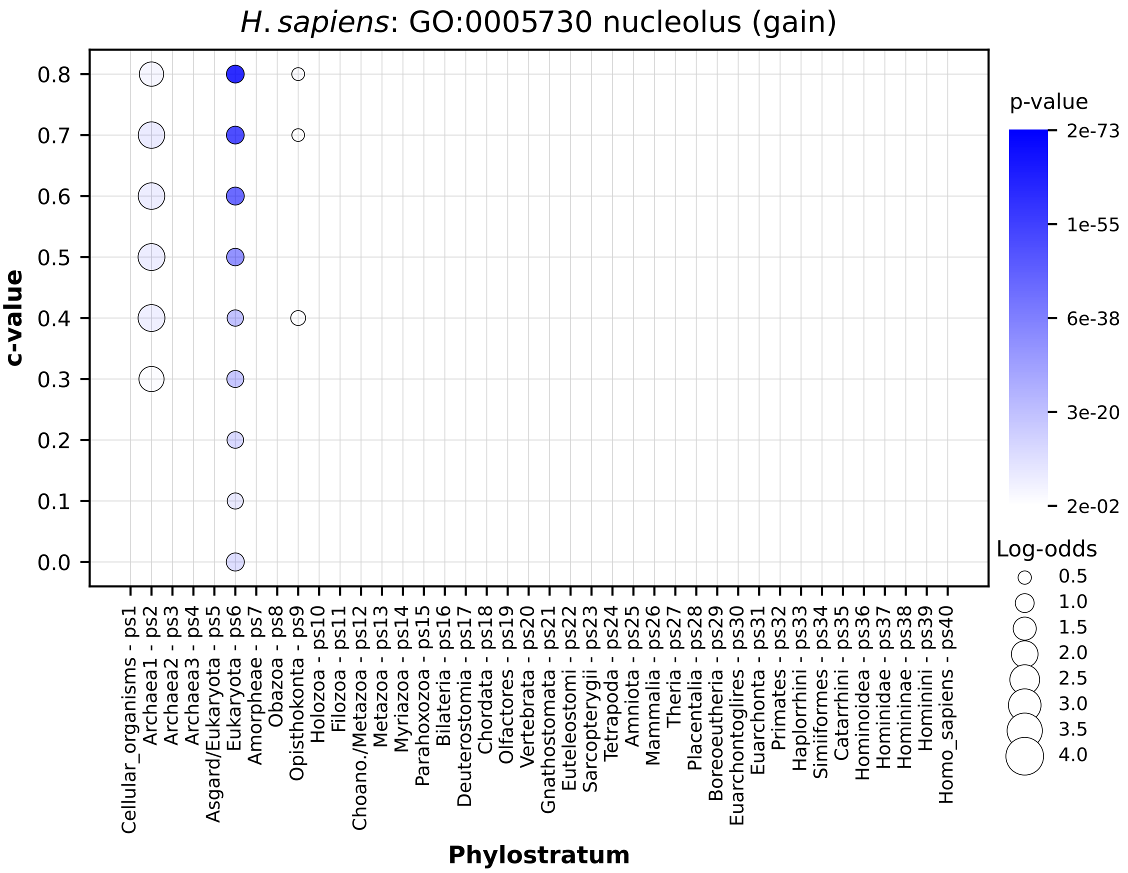
H. sapiens: GO:0005730 nucleolus (gain)
c-value
0.8
0.7
0.6
0.5
0.4
0.3
0.2
0.1
0.0
Phylostratum
Cellular_organisms - ps1
Archaea1 - ps2
Archaea2 - ps3
Archaea3 - ps4
Asgard/Eukaryota - ps5
Eukaryota - ps6
Amorpheae - ps7
Obazoa - ps8
Opisthokonta - ps9
Holozoa - ps10
Filozoa - ps11
Choano./Metazoa - ps12
Metazoa - ps13
Myriazoa - ps14
Parahoxozoa - ps15
Bilateria - ps16
Deuterostomia - ps17
Chordata - ps18
Olfactores - ps19
Vertebrata - ps20
Gnathostomata - ps21
Euteleostomi - ps22
Sarcopterygii - ps23
Tetrapoda - ps24
Amniota - ps25
Mammalia - ps26
Theria - ps27
Placentalia - ps28
Boreoeutheria - ps29
Euarchontoglires - ps30
Euarchonta - ps31
Primates - ps32
Haplorrhini - ps33
Simiiformes - ps34
Catarrhini - ps35
Hominoidea - ps36
Hominidae - ps37
Homininae - ps38
Hominini - ps39
Homo_sapiens - ps40
p-value
2e-73
1e-55
6e-38
3e-20
2e-02
Log-odds
0.5
1.0
1.5
2.0
2.5
3.0
3.5
4.0

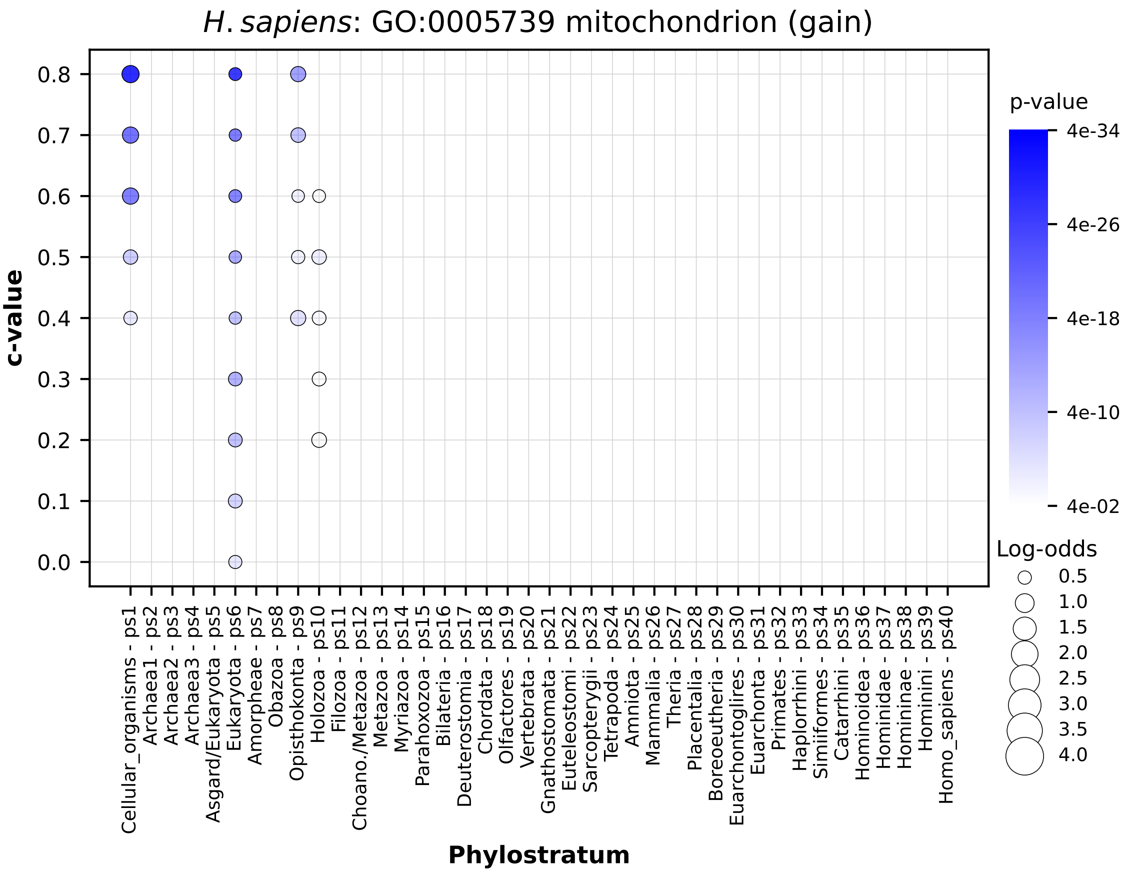

H. sapiens: GO:0005739 mitochondrion (gain)
c-value
0.8
0.7
0.6
0.5
0.4
0.3
0.2
0.1
0.0
Phylostratum
Cellular_organisms - ps1
Archaea1 - ps2
Archaea2 - ps3
Archaea3 - ps4
Asgard/Eukaryota - ps5
Eukaryota - ps6
Amorpheae - ps7
Obazoa - ps8
Opisthokonta - ps9
Holozoa - ps10
Filozoa - ps11
Choano./Metazoa - ps12
Metazoa - ps13
Myriazoa - ps14
Parahoxozoa - ps15
Bilateria - ps16
Deuterostomia - ps17
Chordata - ps18
Olfactores - ps19
Vertebrata - ps20
Gnathostomata - ps21
Euteleostomi - ps22
Sarcopterygii - ps23
Tetrapoda - ps24
Amniota - ps25
Mammalia - ps26
Theria - ps27
Placentalia - ps28
Boreoeutheria - ps29
Euarchontoglires - ps30
Euarchonta - ps31
Primates - ps32
Haplorrhini - ps33
Simiiformes - ps34
Catarrhini - ps35
Hominoidea - ps36
Hominidae - ps37
Homininae - ps38
Hominini - ps39
Homo_sapiens - ps40
p-value
4e-34
4e-26
4e-18
4e-10
4e-02
Log-odds
0.5
1.0
1.5
2.0
2.5
3.0
3.5
4.0

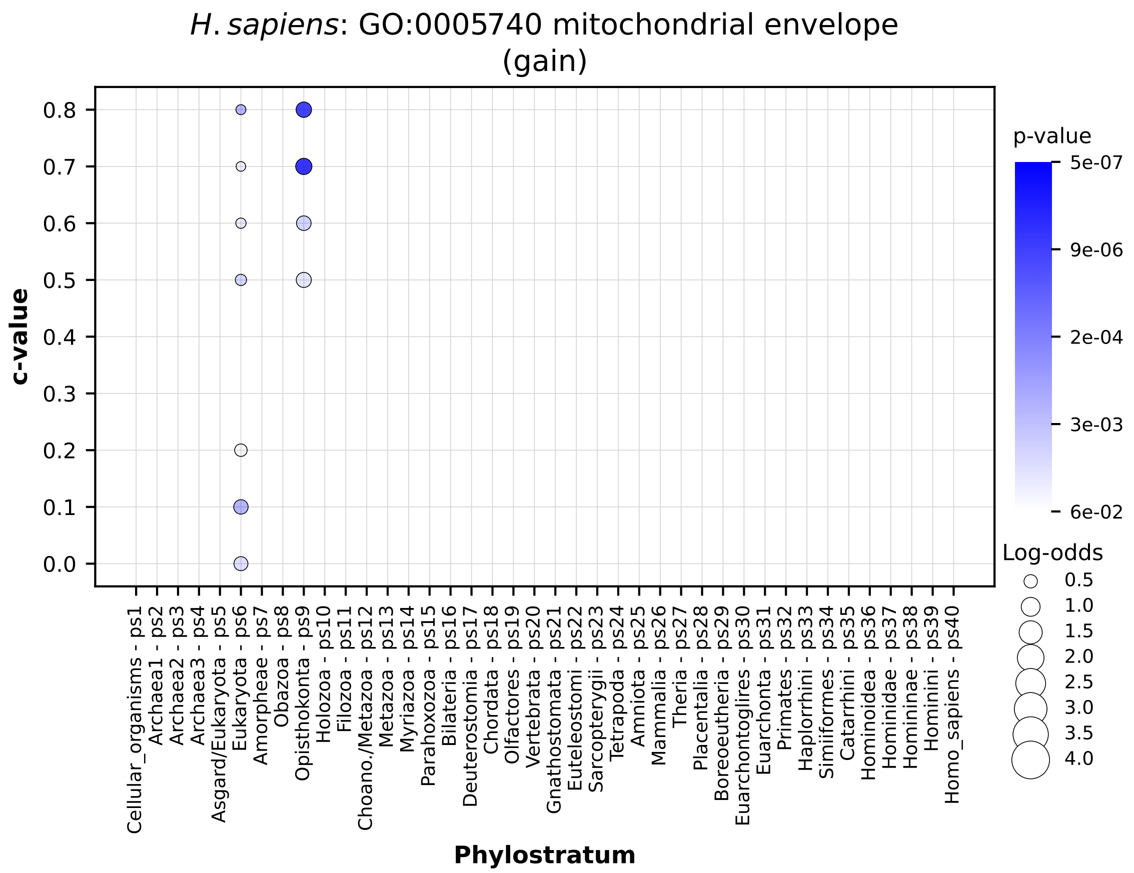

H. sapiens: GO:0005740 mitochondrial envelope
(gain)
c-value
0.8
0.7
0.6
0.5
0.4
0.3
0.2
0.1
0.0
Phylostratum
Cellular_organisms - ps1
Archaea1 - ps2
Archaea2 - ps3
Archaea3 - ps4
Asgard/Eukaryota - ps5
Eukaryota - ps6
Amorpheae - ps7
Obazoa - ps8
Opisthokonta - ps9
Holozoa - ps10
Filozoa - ps11
Choano./Metazoa - ps12
Metazoa - ps13
Myriazoa - ps14
Parahoxozoa - ps15
Bilateria - ps16
Deuterostomia - ps17
Chordata - ps18
Olfactores - ps19
Vertebrata - ps20
Gnathostomata - ps21
Euteleostomi - ps22
Sarcopterygii - ps23
Tetrapoda - ps24
Amniota - ps25
Mammalia - ps26
Theria - ps27
Placentalia - ps28
Boreoeutheria - ps29
Euarchontoglires - ps30
Euarchonta - ps31
Primates - ps32
Haplorrhini - ps33
Simiiformes - ps34
Catarrhini - ps35
Hominoidea - ps36
Hominidae - ps37
Homininae - ps38
Hominini - ps39
Homo_sapiens - ps40
p-value
5e-07
9e-06
2e-04
3e-03
6e-02
Log-odds
0.5
1.0
1.5
2.0
2.5
3.0
3.5
4.0

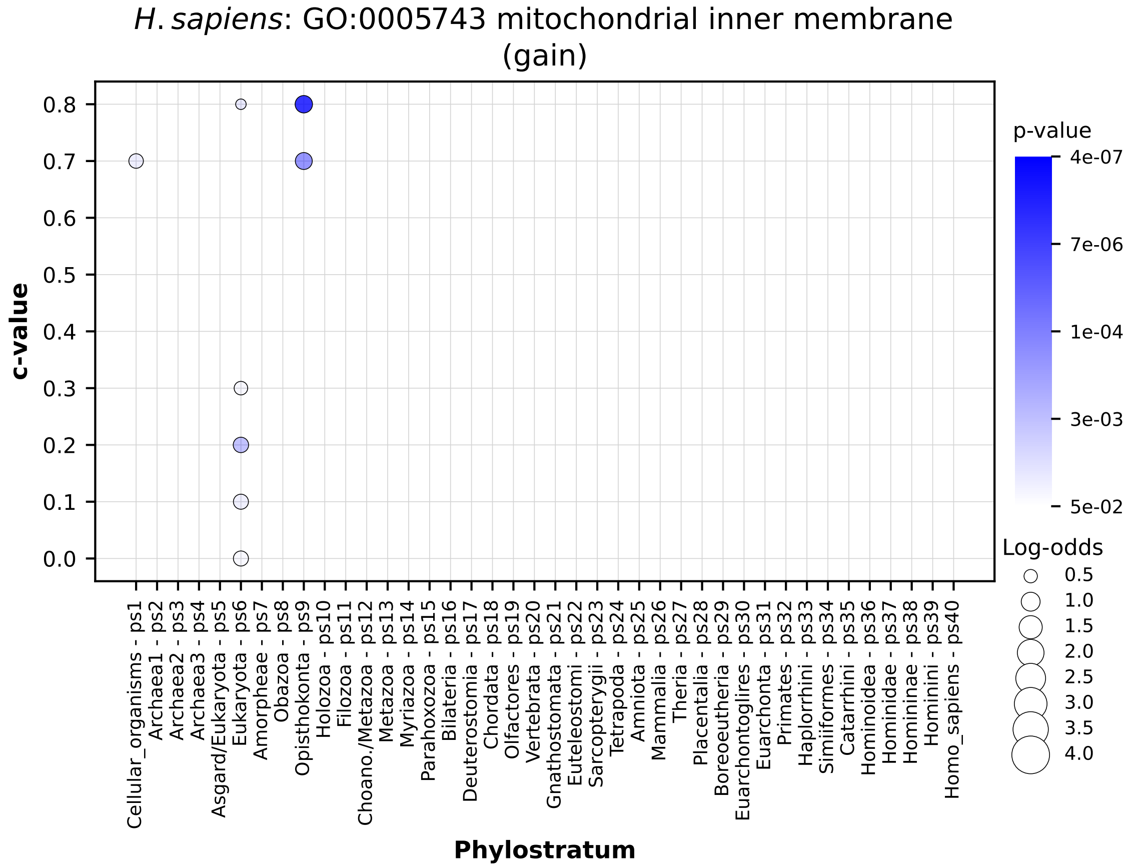

*H. sapiens*: GO:0005743 mitochondrial inner membrane (gain)
c-value
0.0
0.1
0.2
0.3
0.4
0.5
0.6
0.7
0.8
Phylostratum
Cellular_organisms - ps1
Archaea1 - ps2
Archaea2 - ps3
Archaea3 - ps4
Asgard/Eukaryota - ps5
Eukaryota - ps6
Amorpheae - ps7
Obazoa - ps8
Opisthokonta - ps9
Holozoa - ps10
Filozoa - ps11
Choano./Metazoa - ps12
Metazoa - ps13
Myriazoa - ps14
Parahoxozoa - ps15
Bilateria - ps16
Deuterostomia - ps17
Chordata - ps18
Olfactores - ps19
Vertebrata - ps20
Gnathostomata - ps21
Euteleostomi - ps22
Sarcopterygii - ps23
Tetrapoda - ps24
Amniota - ps25
Mammalia - ps26
Theria - ps27
Placentalia - ps28
Boreoeutheria - ps29
Euarchontoglires - ps30
Euarchonta - ps31
Primates - ps32
Haplorrhini - ps33
Simiiformes - ps34
Catarrhini - ps35
Hominoidea - ps36
Hominidae - ps37
Homininae - ps38
Hominini - ps39
Homo_sapiens - ps40
p-value
4e-07
7e-06
1e-04
3e-03
5e-02
Log-odds
0.5
1.0
1.5
2.0
2.5
3.0
3.5
4.0

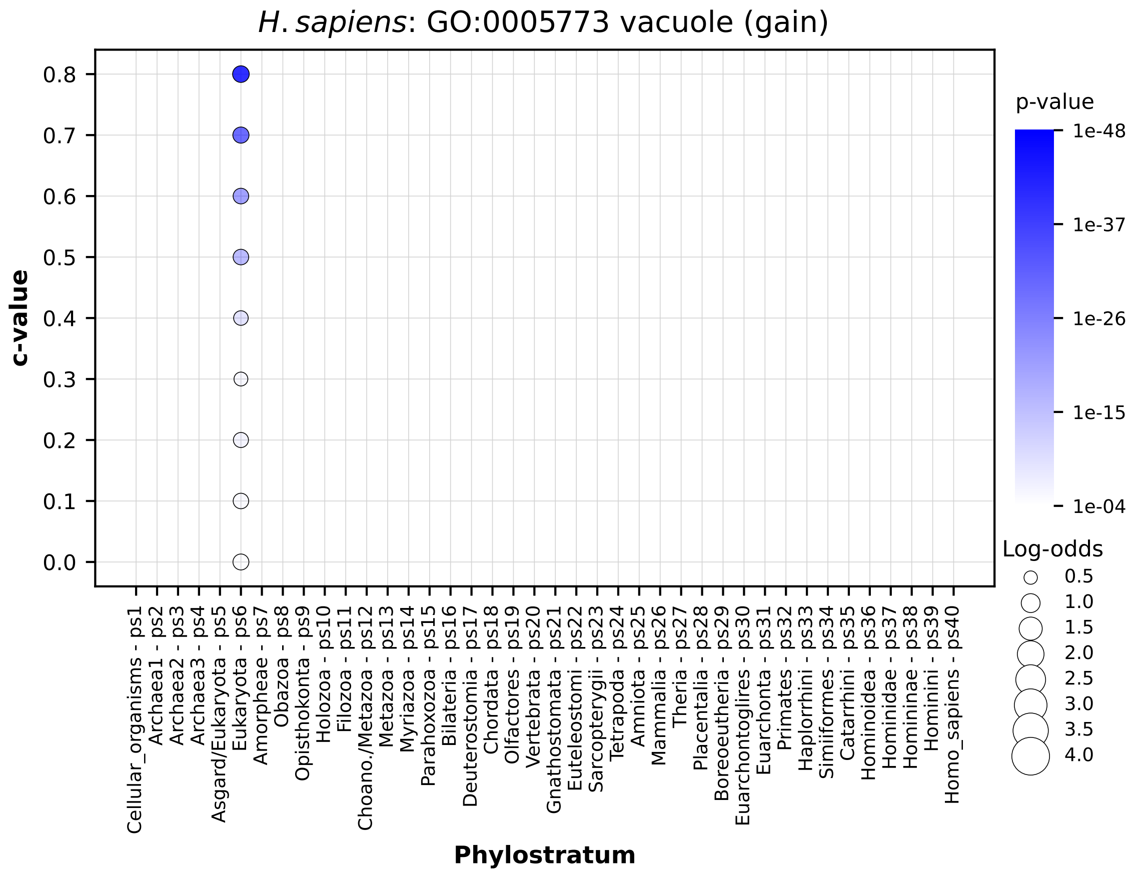

H. sapiens: GO:0005773 vacuole (gain)
c-value
0.0
0.1
0.2
0.3
0.4
0.5
0.6
0.7
0.8
p-value
1e-48
1e-37
1e-26
1e-15
1e-04
Log-odds
0.5
1.0
1.5
2.0
2.5
3.0
3.5
4.0
Cellular_organisms - ps1
Archaea1 - ps2
Archaea2 - ps3
Archaea3 - ps4
Asgard/Eukaryota - ps5
Eukaryota - ps6
Amorpheae - ps7
Obazoa - ps8
Opisthokonta - ps9
Holozoa - ps10
Filozoa - ps11
Choano./Metazoa - ps12
Metazoa - ps13
Myriazoa - ps14
Parahoxozoa - ps15
Bilateria - ps16
Deuterostomia - ps17
Chordata - ps18
Olfactores - ps19
Vertebrata - ps20
Gnathostomata - ps21
Euteleostomi - ps22
Sarcopterygii - ps23
Tetrapoda - ps24
Amniota - ps25
Mammalia - ps26
Theria - ps27
Placentalia - ps28
Boreoeutheria - ps29
Euarchontoglires - ps30
Euarchonta - ps31
Primates - ps32
Haplorrhini - ps33
Simiiformes - ps34
Catarrhini - ps35
Hominoidea - ps36
Hominidae - ps37
Homininae - ps38
Hominini - ps39
Homo_sapiens - ps40
Phylostratum

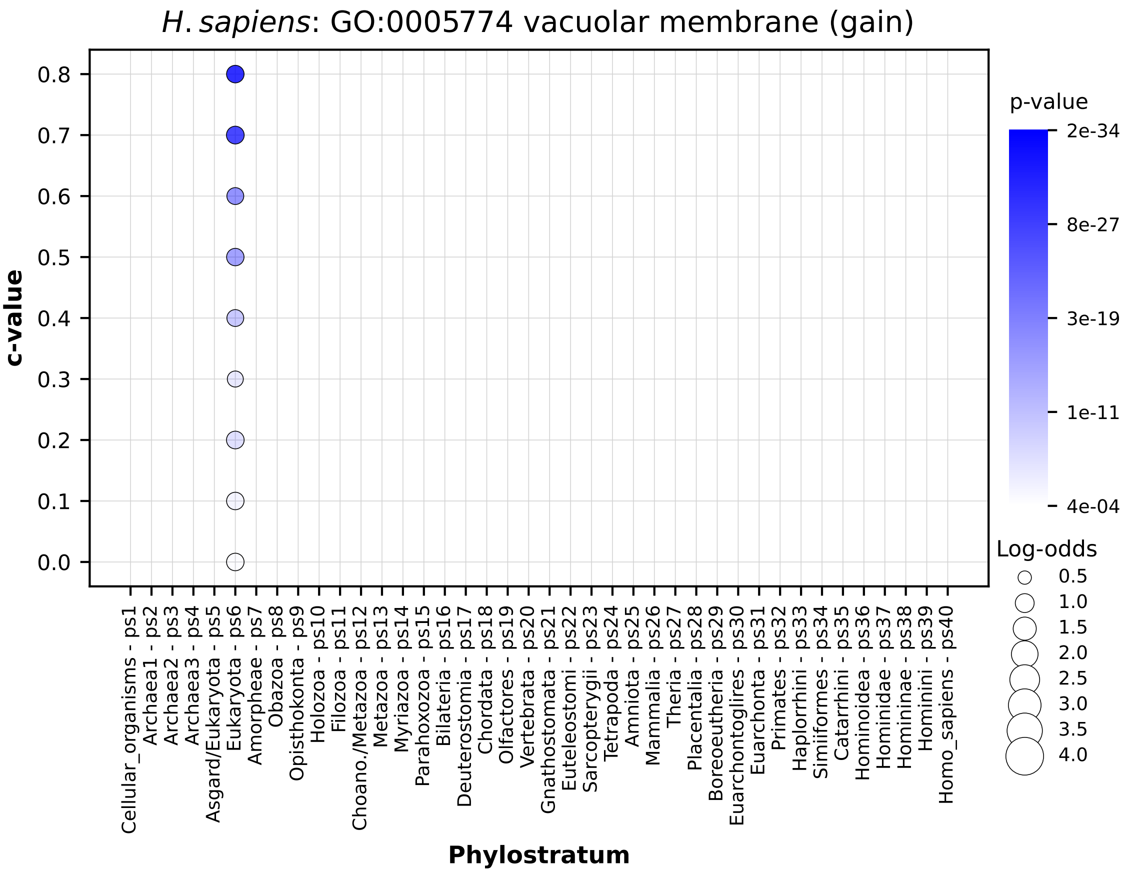

H. sapiens: GO:0005774 vacuolar membrane (gain)
c-value
0.8
0.7
0.6
0.5
0.4
0.3
0.2
0.1
0.0
Phylostratum
Cellular_organisms - ps1
Archaea1 - ps2
Archaea2 - ps3
Archaea3 - ps4
Asgard/Eukaryota - ps5
Eukaryota - ps6
Amorpheae - ps7
Obazoa - ps8
Opisthokonta - ps9
Holozoa - ps10
Filozoa - ps11
Choano./Metazoa - ps12
Metazoa - ps13
Myriazoa - ps14
Parahoxozoa - ps15
Bilateria - ps16
Deuterostomia - ps17
Chordata - ps18
Olfactores - ps19
Vertebrata - ps20
Gnathostomata - ps21
Euteleostomi - ps22
Sarcopterygii - ps23
Tetrapoda - ps24
Amniota - ps25
Mammalia - ps26
Theria - ps27
Placentalia - ps28
Boreoeutheria - ps29
Euarchontoglires - ps30
Euarchonta - ps31
Primates - ps32
Haplorrhini - ps33
Simiiformes - ps34
Catarrhini - ps35
Hominoidea - ps36
Hominidae - ps37
Homininae - ps38
Hominini - ps39
Homo_sapiens - ps40
p-value
2e-34
8e-27
3e-19
1e-11
4e-04
Log-odds
0.5
1.0
1.5
2.0
2.5
3.0
3.5
4.0

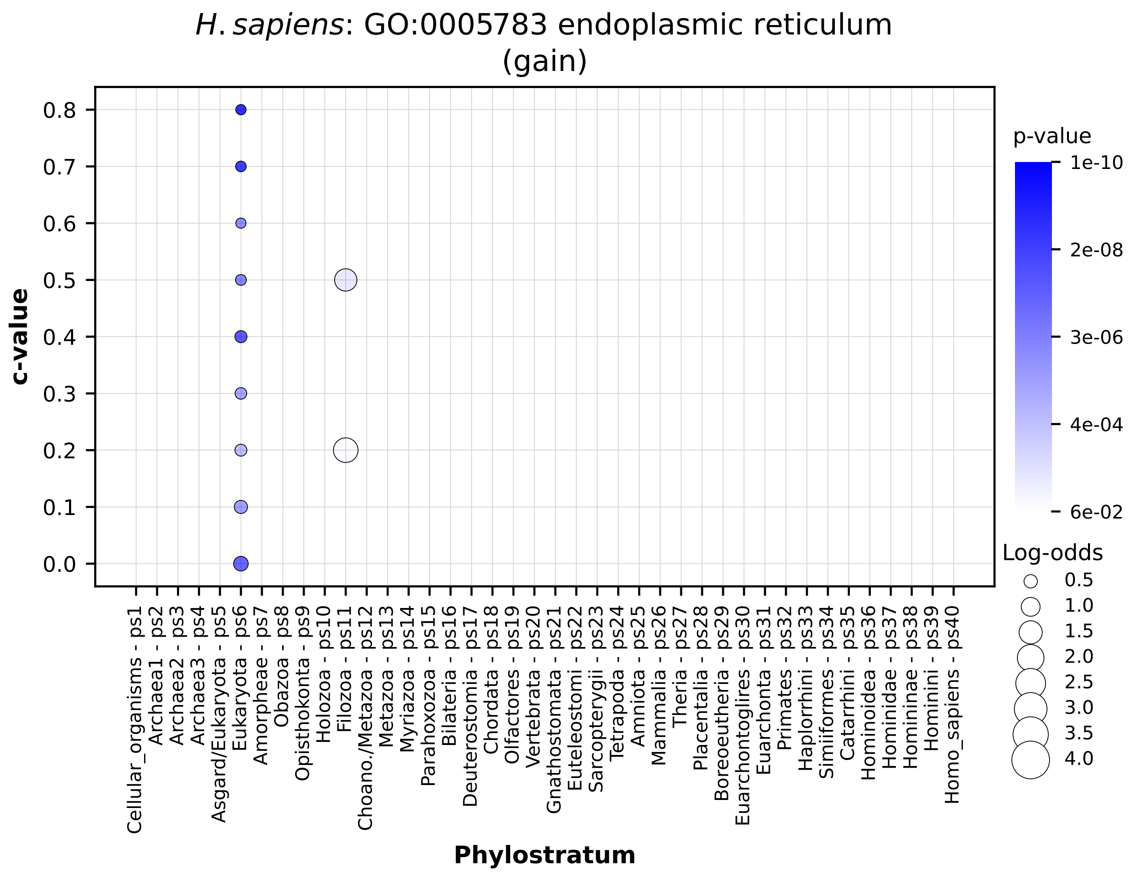
H. sapiens: GO:0005783 endoplasmic reticulum
(gain)
c-value
0.8
0.7
0.6
0.5
0.4
0.3
0.2
0.1
0.0
p-value
1e-10
2e-08
3e-06
4e-04
6e-02
Log-odds
0.5
1.0
1.5
2.0
2.5
3.0
3.5
4.0
Cellular_organisms - ps1
Archaea1 - ps2
Archaea2 - ps3
Archaea3 - ps4
Asgard/Eukaryota - ps5
Eukaryota - ps6
Amorpheae - ps7
Obazoa - ps8
Opisthokonta - ps9
Holozoa - ps10
Filozoa - ps11
Choano./Metazoa - ps12
Metazoa - ps13
Myriazoa - ps14
Parahoxozoa - ps15
Bilateria - ps16
Deuterostomia - ps17
Chordata - ps18
Olfactores - ps19
Vertebrata - ps20
Gnathostomata - ps21
Euteleostomi - ps22
Sarcopterygii - ps23
Tetrapoda - ps24
Amniota - ps25
Mammalia - ps26
Theria - ps27
Placentalia - ps28
Boreoeutheria - ps29
Euarchontoglires - ps30
Euarchonta - ps31
Primates - ps32
Haplorrhini - ps33
Simiiformes - ps34
Catarrhini - ps35
Hominoidea - ps36
Hominidae - ps37
Homininae - ps38
Hominini - ps39
Homo_sapiens - ps40
Phylostratum

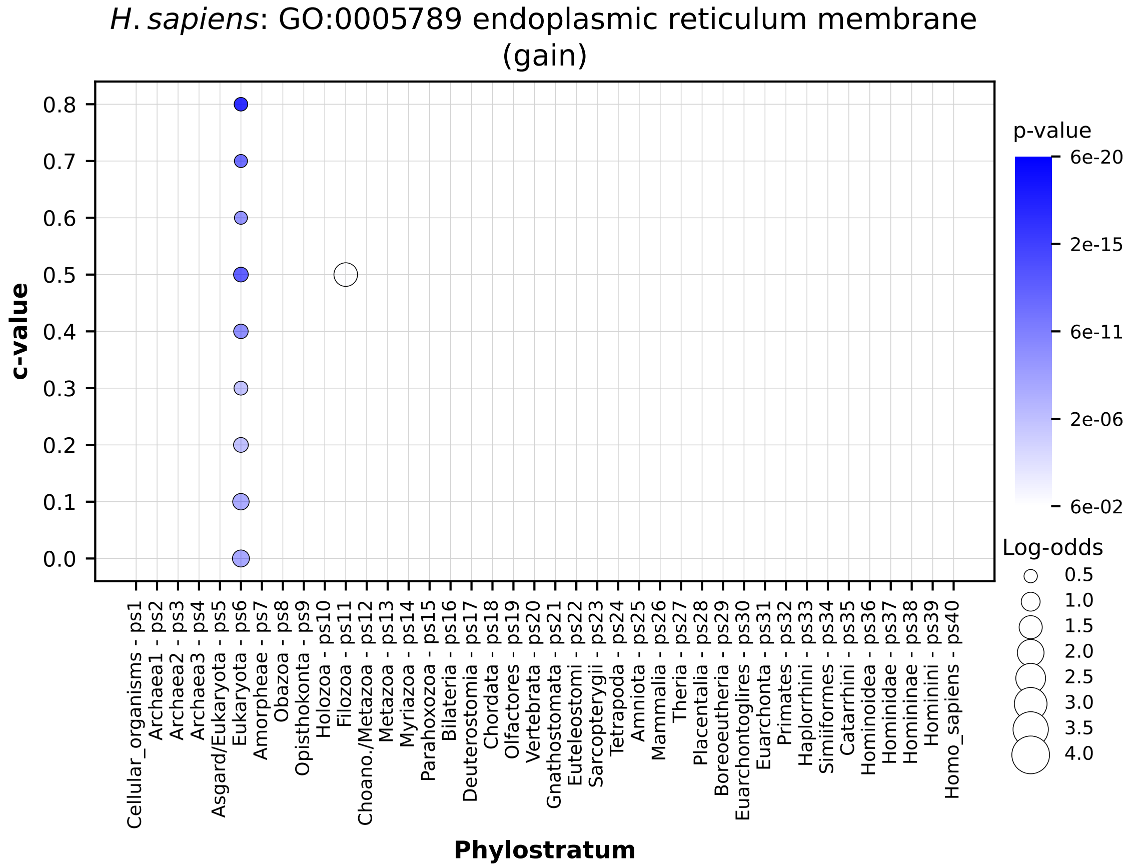
H. sapiens: GO:0005789 endoplasmic reticulum membrane
(gain)
c-value
0.8
0.7
0.6
0.5
0.4
0.3
0.2
0.1
0.0
Phylostratum
Cellular_organisms - ps1
Archaea1 - ps2
Archaea2 - ps3
Archaea3 - ps4
Asgard/Eukaryota - ps5
Eukaryota - ps6
Amorpheae - ps7
Obazoa - ps8
Opisthokonta - ps9
Holozoa - ps10
Filozoa - ps11
Choano./Metazoa - ps12
Metazoa - ps13
Myriazoa - ps14
Parahoxozoa - ps15
Bilateria - ps16
Deuterostomia - ps17
Chordata - ps18
Olfactores - ps19
Vertebrata - ps20
Gnathostomata - ps21
Euteleostomi - ps22
Sarcopterygii - ps23
Tetrapoda - ps24
Amniota - ps25
Mammalia - ps26
Theria - ps27
Placentalia - ps28
Boreoeutheria - ps29
Euarchontoglires - ps30
Euarchonta - ps31
Primates - ps32
Haplorrhini - ps33
Simiiformes - ps34
Catarrhini - ps35
Hominoidea - ps36
Hominidae - ps37
Homininae - ps38
Hominini - ps39
Homo_sapiens - ps40
p-value
6e-20
2e-15
6e-11
2e-06
6e-02
Log-odds
0.5
1.0
1.5
2.0
2.5
3.0
3.5
4.0

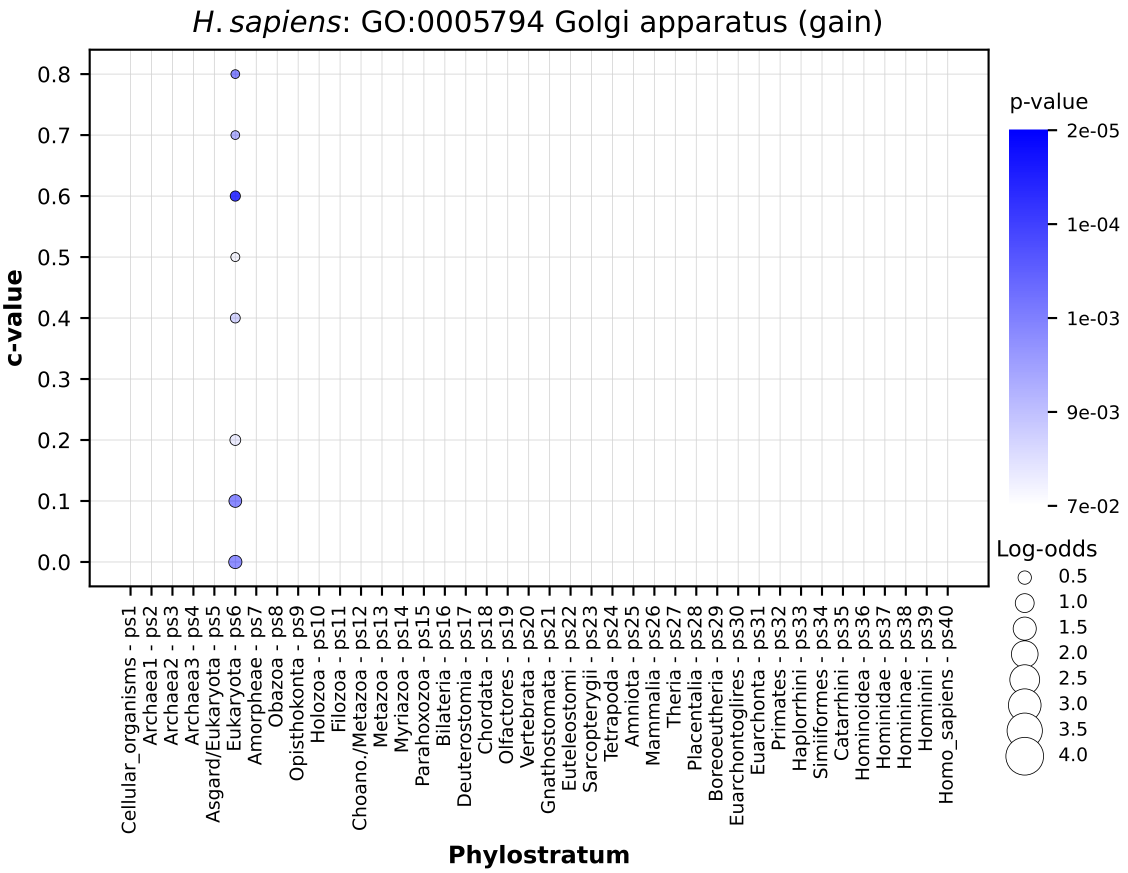
H. sapiens: GO:0005794 Golgi apparatus (gain)
c-value
0.8
0.7
0.6
0.5
0.4
0.3
0.2
0.1
0.0
Phylostratum
Cellular_organisms - ps1
Archaea1 - ps2
Archaea2 - ps3
Archaea3 - ps4
Asgard/Eukaryota - ps5
Eukaryota - ps6
Amorpheae - ps7
Obazoa - ps8
Opisthokonta - ps9
Holozoa - ps10
Filozoa - ps11
Choano./Metazoa - ps12
Metazoa - ps13
Myriazoa - ps14
Parahoxozoa - ps15
Bilateria - ps16
Deuterostomia - ps17
Chordata - ps18
Olfactores - ps19
Vertebrata - ps20
Gnathostomata - ps21
Euteleostomi - ps22
Sarcopterygii - ps23
Tetrapoda - ps24
Amniota - ps25
Mammalia - ps26
Theria - ps27
Placentalia - ps28
Boreoeutheria - ps29
Euarchontoglires - ps30
Euarchonta - ps31
Primates - ps32
Haplorrhini - ps33
Simiiformes - ps34
Catarrhini - ps35
Hominoidea - ps36
Hominidae - ps37
Homininae - ps38
Hominini - ps39
Homo_sapiens - ps40
p-value
2e-05
1e-04
1e-03
9e-03
7e-02
Log-odds
0.5
1.0
1.5
2.0
2.5
3.0
3.5
4.0

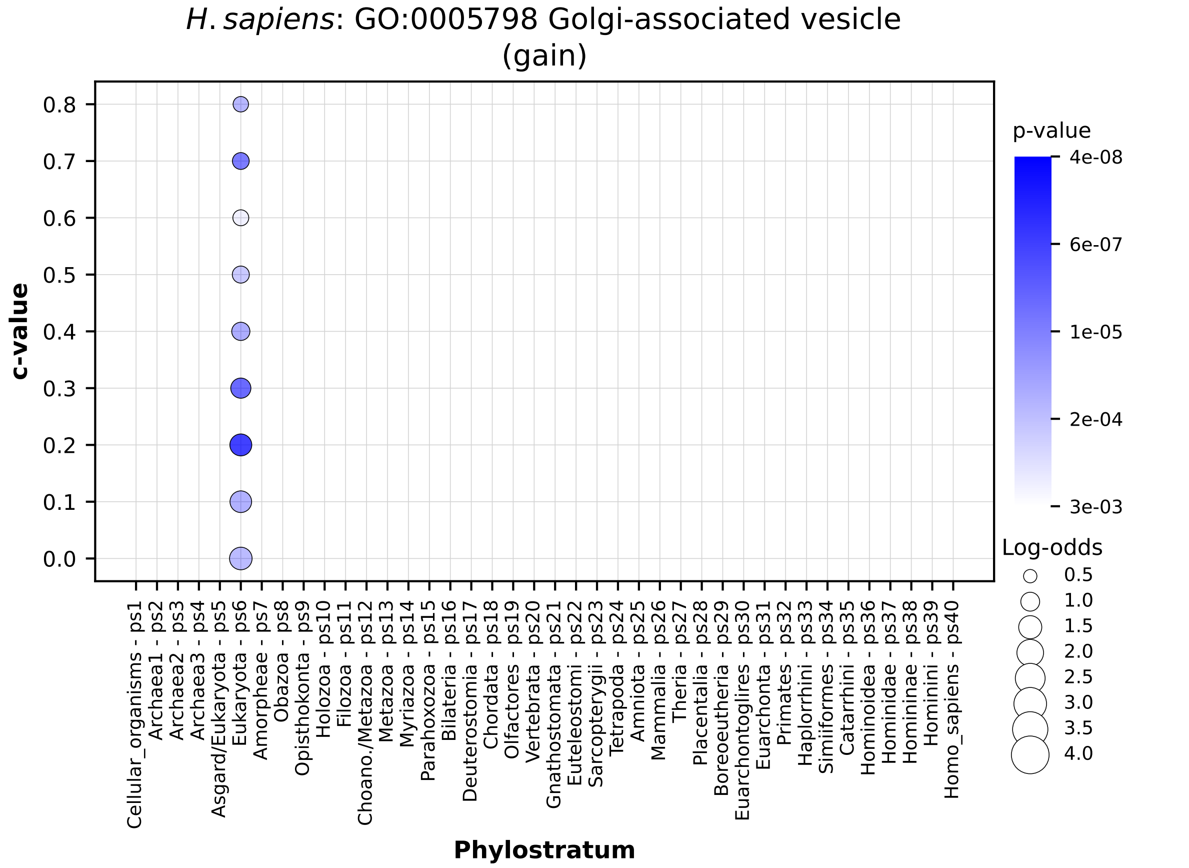

H. sapiens: GO:0005798 Golgi-associated vesicle
(gain)
c-value
0.0
0.1
0.2
0.3
0.4
0.5
0.6
0.7
0.8
Phylostratum
Cellular_organisms - ps1
Archaea1 - ps2
Archaea2 - ps3
Archaea3 - ps4
Asgard/Eukaryota - ps5
Eukaryota - ps6
Amorpheae - ps7
Obazoa - ps8
Opisthokonta - ps9
Holozoa - ps10
Filozoa - ps11
Choano./Metazoa - ps12
Metazoa - ps13
Myriazoa - ps14
Parahoxozoa - ps15
Bilateria - ps16
Deuterostomia - ps17
Chordata - ps18
Olfactores - ps19
Vertebrata - ps20
Gnathostomata - ps21
Euteleostomi - ps22
Sarcopterygii - ps23
Tetrapoda - ps24
Amniota - ps25
Mammalia - ps26
Theria - ps27
Placentalia - ps28
Boreoeutheria - ps29
Euarchontoglires - ps30
Euarchonta - ps31
Primates - ps32
Haplorrhini - ps33
Simiiformes - ps34
Catarrhini - ps35
Hominoidea - ps36
Hominidae - ps37
Homininae - ps38
Hominini - ps39
Homo_sapiens - ps40
p-value
4e-08
6e-07
1e-05
2e-04
3e-03
Log-odds
0.5
1.0
1.5
2.0
2.5
3.0
3.5
4.0

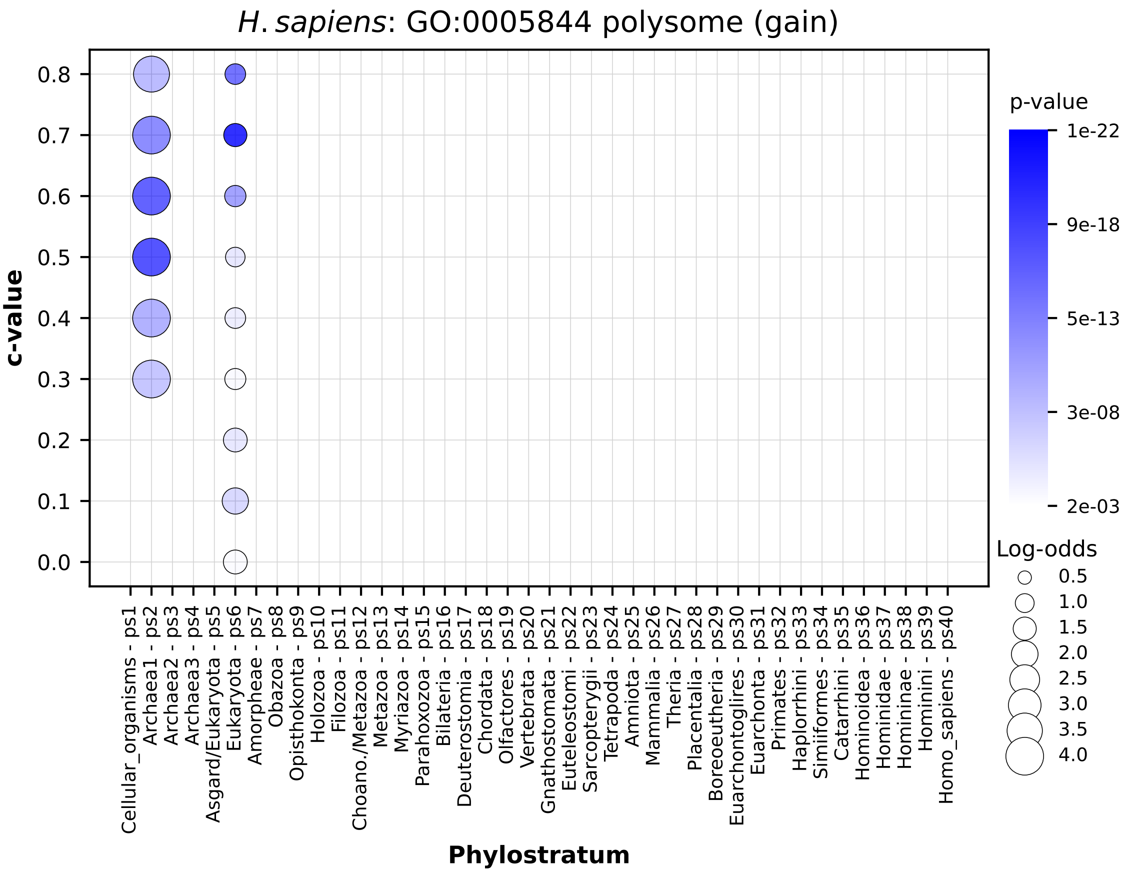

H. sapiens: GO:0005844 polysome (gain)
c-value
0.0
0.1
0.2
0.3
0.4
0.5
0.6
0.7
0.8
Phylostratum
Cellular_organisms - ps1
Archaea1 - ps2
Archaea2 - ps3
Archaea3 - ps4
Asgard/Eukaryota - ps5
Eukaryota - ps6
Amorpheae - ps7
Obazoa - ps8
Opisthokonta - ps9
Holozoa - ps10
Filozoa - ps11
Choano./Metazoa - ps12
Metazoa - ps13
Myriazoa - ps14
Parahoxozoa - ps15
Bilateria - ps16
Deuterostomia - ps17
Chordata - ps18
Olfactores - ps19
Vertebrata - ps20
Gnathostomata - ps21
Euteleostomi - ps22
Sarcopterygii - ps23
Tetrapoda - ps24
Amniota - ps25
Mammalia - ps26
Theria - ps27
Placentalia - ps28
Boreoeutheria - ps29
Euarchontoglires - ps30
Euarchonta - ps31
Primates - ps32
Haplorrhini - ps33
Simiiformes - ps34
Catarrhini - ps35
Hominoidea - ps36
Hominidae - ps37
Homininae - ps38
Hominini - ps39
Homo_sapiens - ps40
p-value
1e-22
9e-18
5e-13
3e-08
2e-03
Log-odds
0.5
1.0
1.5
2.0
2.5
3.0
3.5
4.0

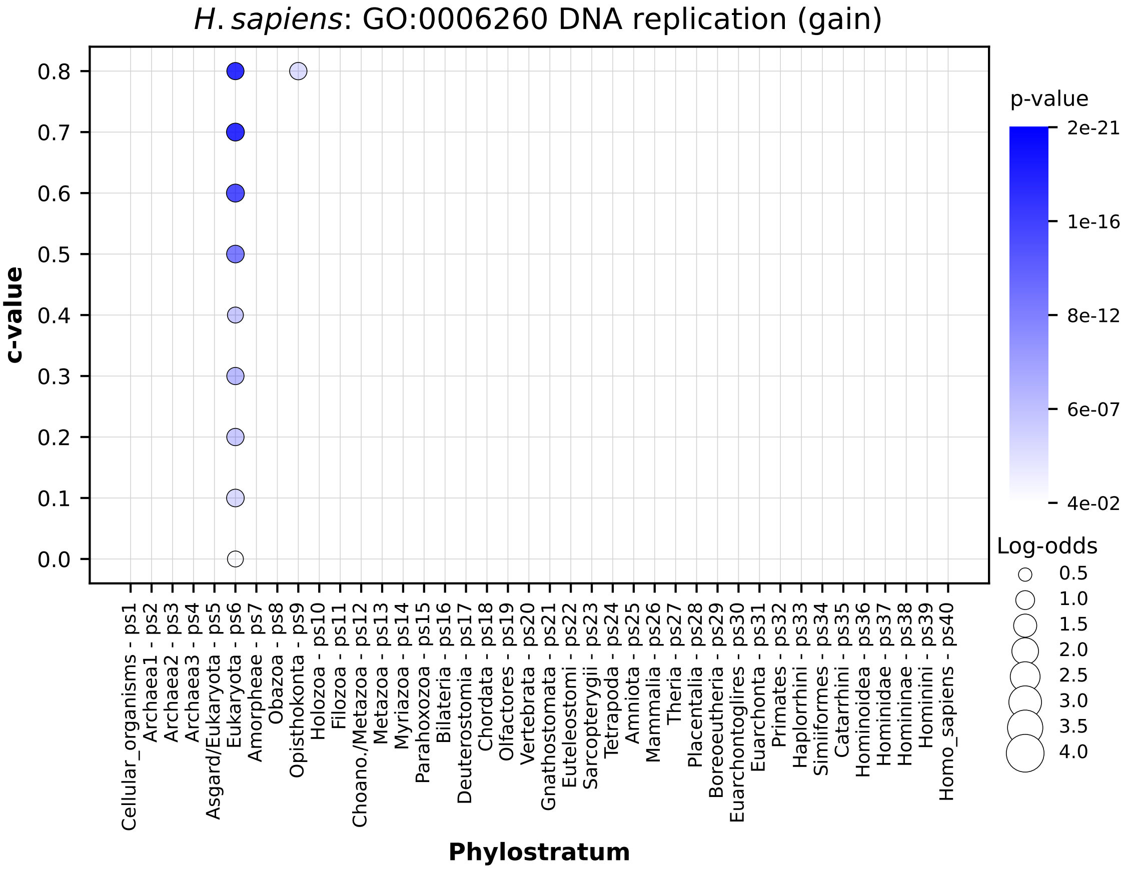

H. sapiens: GO:0006260 DNA replication (gain)
c-value
0.0
0.1
0.2
0.3
0.4
0.5
0.6
0.7
0.8
p-value
2e-21
1e-16
8e-12
6e-07
4e-02
Log-odds
0.5
1.0
1.5
2.0
2.5
3.0
3.5
4.0
Cellular_organisms - ps1
Archaea1 - ps2
Archaea2 - ps3
Archaea3 - ps4
Asgard/Eukaryota - ps5
Eukaryota - ps6
Amorpheae - ps7
Obazoa - ps8
Opisthokonta - ps9
Holozoa - ps10
Filozoa - ps11
Choano./Metazoa - ps12
Metazoa - ps13
Myriazoa - ps14
Parahoxozoa - ps15
Bilateria - ps16
Deuterostomia - ps17
Chordata - ps18
Olfactores - ps19
Vertebrata - ps20
Gnathostomata - ps21
Euteleostomi - ps22
Sarcopterygii - ps23
Tetrapoda - ps24
Amniota - ps25
Mammalia - ps26
Theria - ps27
Placentalia - ps28
Boreoeutheria - ps29
Euarchontoglires - ps30
Euarchonta - ps31
Primates - ps32
Haplorrhini - ps33
Simiiformes - ps34
Catarrhini - ps35
Hominoidea - ps36
Hominidae - ps37
Homininae - ps38
Hominini - ps39
Homo_sapiens - ps40
Phylostratum

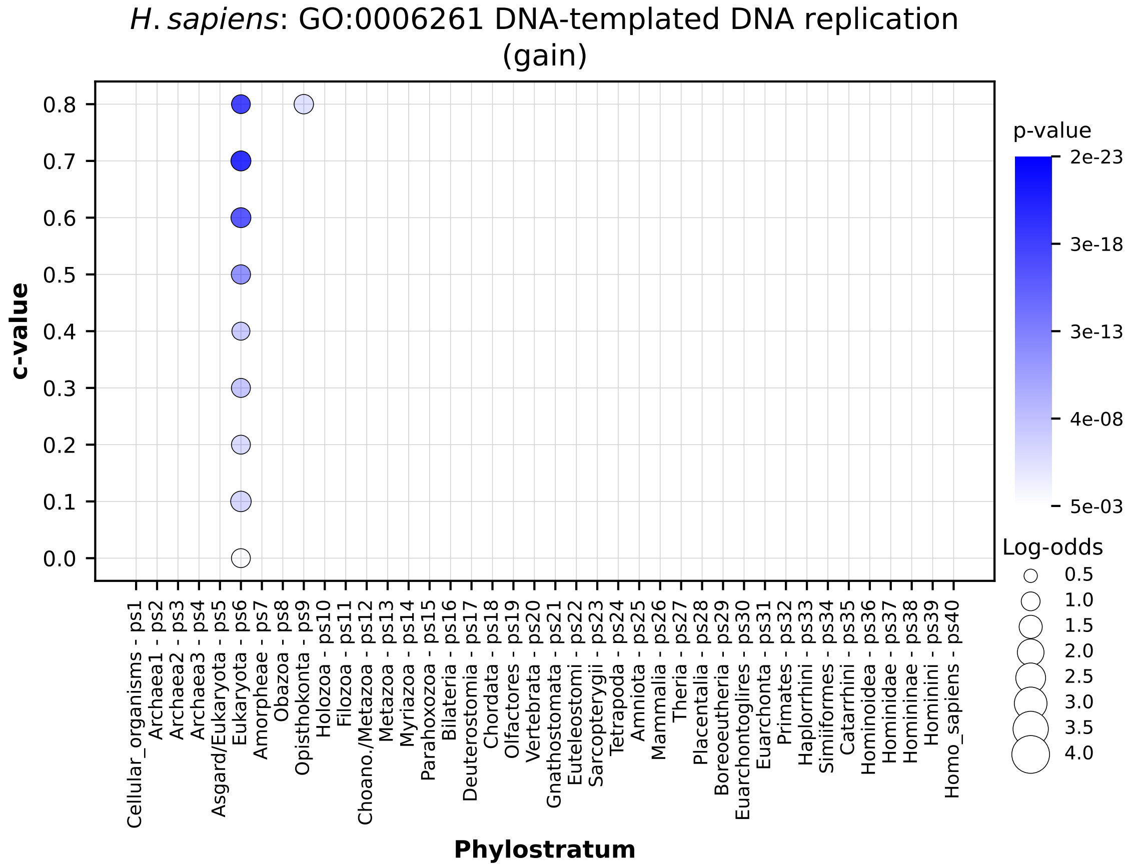

*H. sapiens*: GO:0006261 DNA-templated DNA replication (gain)
c-value
0.0
0.1
0.2
0.3
0.4
0.5
0.6
0.7
0.8
p-value
2e-23
3e-18
3e-13
4e-08
5e-03
Log-odds
0.5
1.0
1.5
2.0
2.5
3.0
3.5
4.0
Cellular_organisms - ps1
Archaea1 - ps2
Archaea2 - ps3
Archaea3 - ps4
Asgard/Eukaryota - ps5
Eukaryota - ps6
Amorpheae - ps7
Obazoa - ps8
Opisthokonta - ps9
Holozoa - ps10
Filozoa - ps11
Choano./Metazoa - ps12
Metazoa - ps13
Myriazoa - ps14
Parahoxozoa - ps15
Bilateria - ps16
Deuterostomia - ps17
Chordata - ps18
Olfactores - ps19
Vertebrata - ps20
Gnathostomata - ps21
Euteleostomi - ps22
Sarcopterygii - ps23
Tetrapoda - ps24
Amniota - ps25
Mammalia - ps26
Theria - ps27
Placentalia - ps28
Boreoeutheria - ps29
Euarchontoglires - ps30
Euarchonta - ps31
Primates - ps32
Haplorrhini - ps33
Simiiformes - ps34
Catarrhini - ps35
Hominoidea - ps36
Hominidae - ps37
Homininae - ps38
Hominini - ps39
Homo_sapiens - ps40
Phylostratum

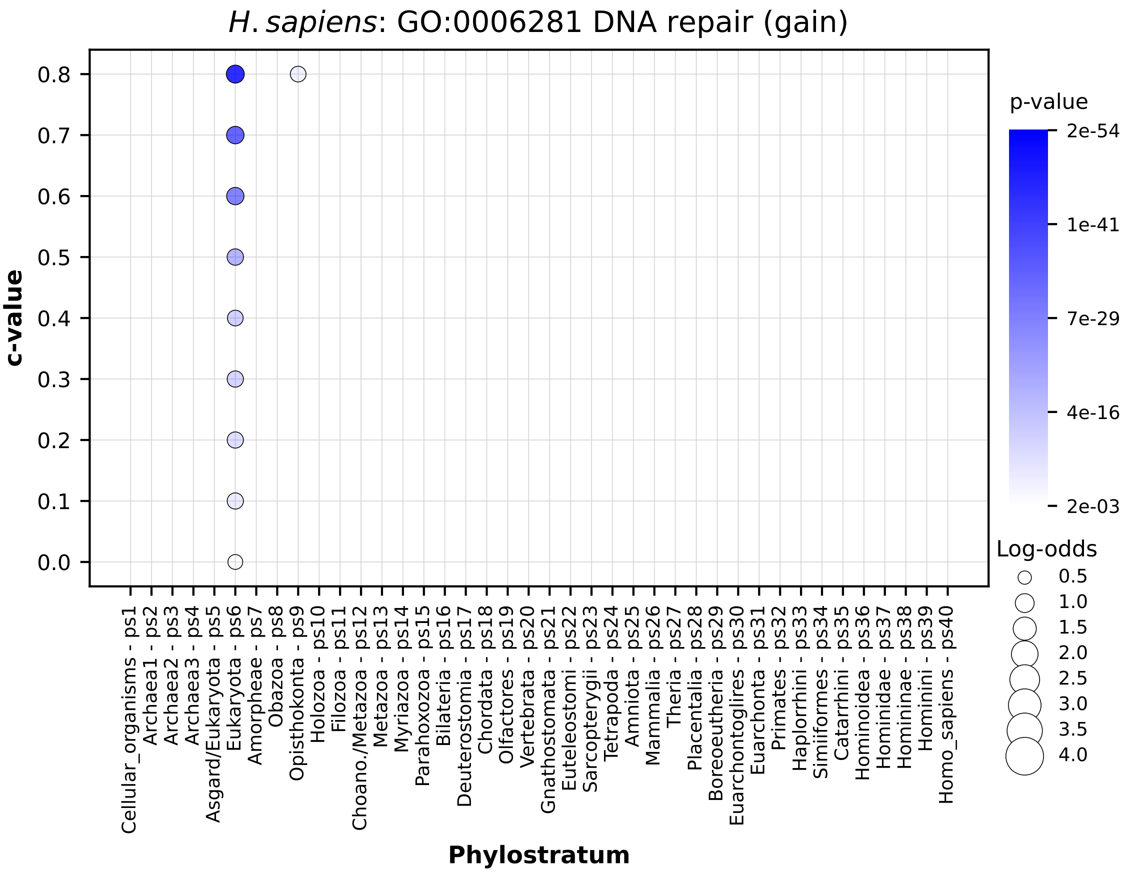
H. sapiens: GO:0006281 DNA repair (gain)
c-value
0.8
0.7
0.6
0.5
0.4
0.3
0.2
0.1
0.0
p-value
2e-54
1e-41
7e-29
4e-16
2e-03
Log-odds
0.5
1.0
1.5
2.0
2.5
3.0
3.5
4.0
Cellular_organisms - ps1
Archaea1 - ps2
Archaea2 - ps3
Archaea3 - ps4
Asgard/Eukaryota - ps5
Eukaryota - ps6
Amorpheae - ps7
Obazoa - ps8
Opisthokonta - ps9
Holozoa - ps10
Filozoa - ps11
Choano./Metazoa - ps12
Metazoa - ps13
Myriazoa - ps14
Parahoxozoa - ps15
Bilateria - ps16
Deuterostomia - ps17
Chordata - ps18
Olfactores - ps19
Vertebrata - ps20
Gnathostomata - ps21
Euteleostomi - ps22
Sarcopterygii - ps23
Tetrapoda - ps24
Amniota - ps25
Mammalia - ps26
Theria - ps27
Placentalia - ps28
Boreoeutheria - ps29
Euarchontoglires - ps30
Euarchonta - ps31
Primates - ps32
Haplorrhini - ps33
Simiiformes - ps34
Catarrhini - ps35
Hominoidea - ps36
Hominidae - ps37
Homininae - ps38
Hominini - ps39
Homo_sapiens - ps40
Phylostratum

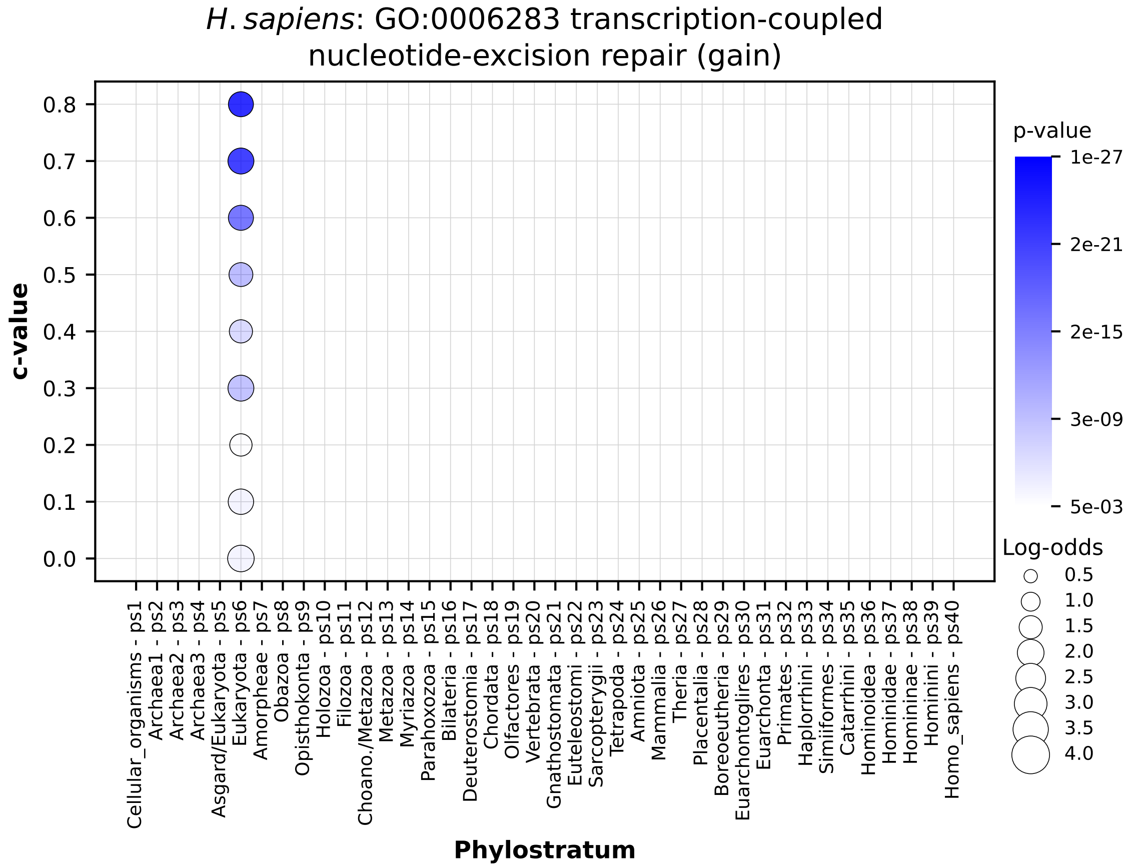

H. sapiens: GO:0006283 transcription-coupled nucleotide-excision repair (gain)
c-value
0.8
0.7
0.6
0.5
0.4
0.3
0.2
0.1
0.0
p-value
1e-27
2e-21
2e-15
3e-09
5e-03
Log-odds
0.5
1.0
1.5
2.0
2.5
3.0
3.5
4.0
Cellular_organisms - ps1
Archaea1 - ps2
Archaea2 - ps3
Archaea3 - ps4
Asgard/Eukaryota - ps5
Eukaryota - ps6
Amorpheae - ps7
Obazoa - ps8
Opisthokonta - ps9
Holozoa - ps10
Filozoa - ps11
Choano./Metazoa - ps12
Metazoa - ps13
Myriazoa - ps14
Parahoxozoa - ps15
Bilateria - ps16
Deuterostomia - ps17
Chordata - ps18
Olfactores - ps19
Vertebrata - ps20
Gnathostomata - ps21
Euteleostomi - ps22
Sarcopterygii - ps23
Tetrapoda - ps24
Amniota - ps25
Mammalia - ps26
Theria - ps27
Placentalia - ps28
Boreoeutheria - ps29
Euarchontoglires - ps30
Euarchonta - ps31
Primates - ps32
Haplorrhini - ps33
Simiiformes - ps34
Catarrhini - ps35
Hominoidea - ps36
Hominidae - ps37
Homininae - ps38
Hominini - ps39
Homo_sapiens - ps40
Phylostratum

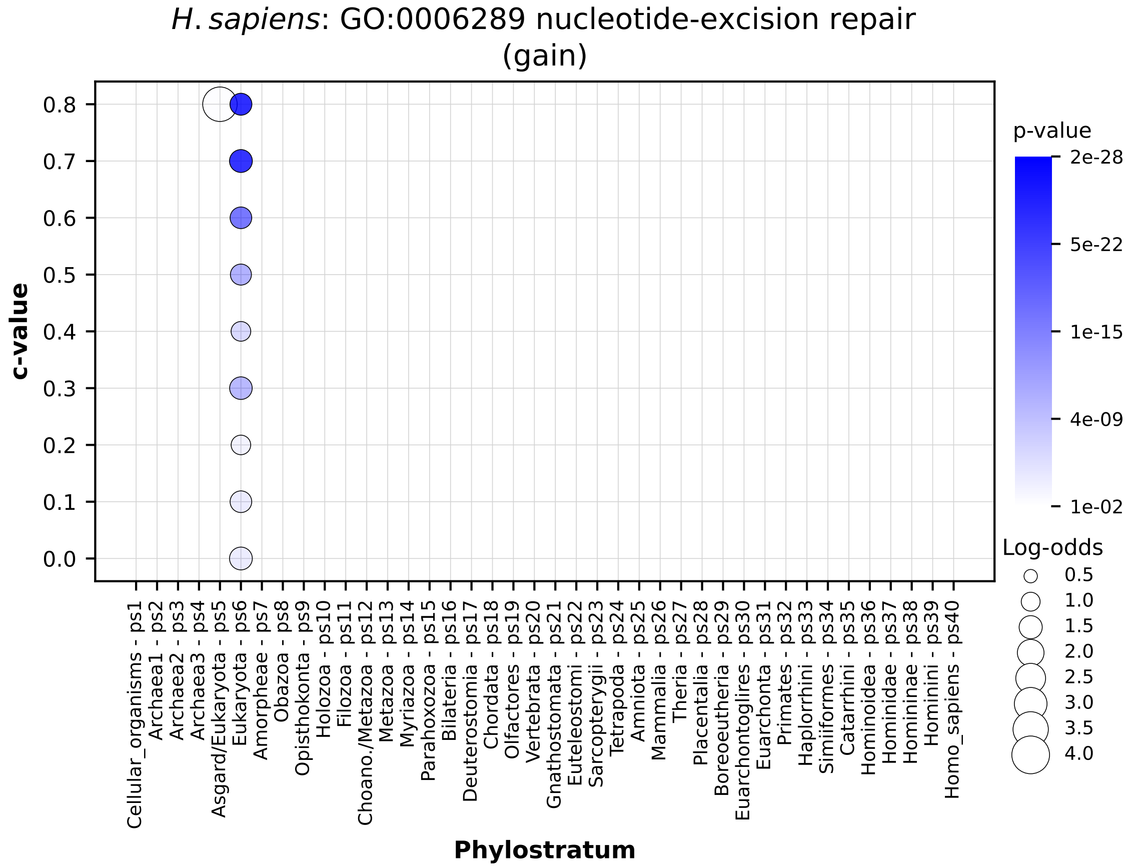

*H. sapiens*: GO:0006289 nucleotide-excision repair
(gain)
c-value
0.0
0.1
0.2
0.3
0.4
0.5
0.6
0.7
0.8
p-value
2e-28
5e-22
1e-15
4e-09
1e-02
Log-odds
0.5
1.0
1.5
2.0
2.5
3.0
3.5
4.0
Cellular_organisms - ps1
Archaea1 - ps2
Archaea2 - ps3
Archaea3 - ps4
Asgard/Eukaryota - ps5
Eukaryota - ps6
Amorpheae - ps7
Obazoa - ps8
Opisthokonta - ps9
Holozoa - ps10
Filozoa - ps11
Choano./Metazoa - ps12
Metazoa - ps13
Myriazoa - ps14
Parahoxozoa - ps15
Bilateria - ps16
Deuterostomia - ps17
Chordata - ps18
Olfactores - ps19
Vertebrata - ps20
Gnathostomata - ps21
Euteleostomi - ps22
Sarcopterygii - ps23
Tetrapoda - ps24
Amniota - ps25
Mammalia - ps26
Theria - ps27
Placentalia - ps28
Boreoeutheria - ps29
Euarchontoglires - ps30
Euarchonta - ps31
Primates - ps32
Haplorrhini - ps33
Simiiformes - ps34
Catarrhini - ps35
Hominoidea - ps36
Hominidae - ps37
Homininae - ps38
Hominini - ps39
Homo_sapiens - ps40
Phylostratum

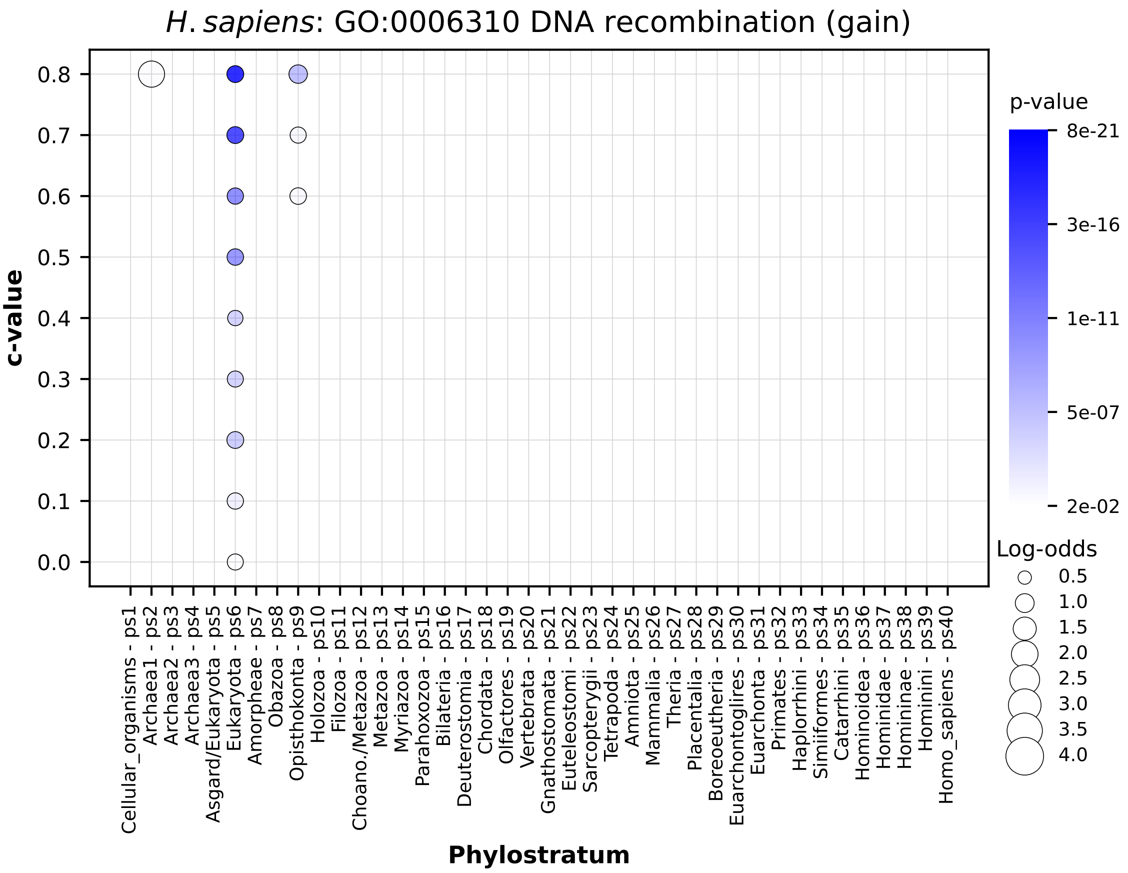

H. sapiens: GO:0006310 DNA recombination (gain)
c-value
0.8
0.7
0.6
0.5
0.4
0.3
0.2
0.1
0.0
Phylostratum
Cellular_organisms - ps1
Archaea1 - ps2
Archaea2 - ps3
Archaea3 - ps4
Asgard/Eukaryota - ps5
Eukaryota - ps6
Amorpheae - ps7
Obazoa - ps8
Opisthokonta - ps9
Holozoa - ps10
Filozoa - ps11
Choano./Metazoa - ps12
Metazoa - ps13
Myriazoa - ps14
Parahoxozoa - ps15
Bilateria - ps16
Deuterostomia - ps17
Chordata - ps18
Olfactores - ps19
Vertebrata - ps20
Gnathostomata - ps21
Euteleostomi - ps22
Sarcopterygii - ps23
Tetrapoda - ps24
Amniota - ps25
Mammalia - ps26
Theria - ps27
Placentalia - ps28
Boreoeutheria - ps29
Euarchontoglires - ps30
Euarchonta - ps31
Primates - ps32
Haplorrhini - ps33
Simiiformes - ps34
Catarrhini - ps35
Hominoidea - ps36
Hominidae - ps37
Homininae - ps38
Hominini - ps39
Homo_sapiens - ps40
p-value
8e-21
3e-16
1e-11
5e-07
2e-02
Log-odds
0.5
1.0
1.5
2.0
2.5
3.0
3.5
4.0

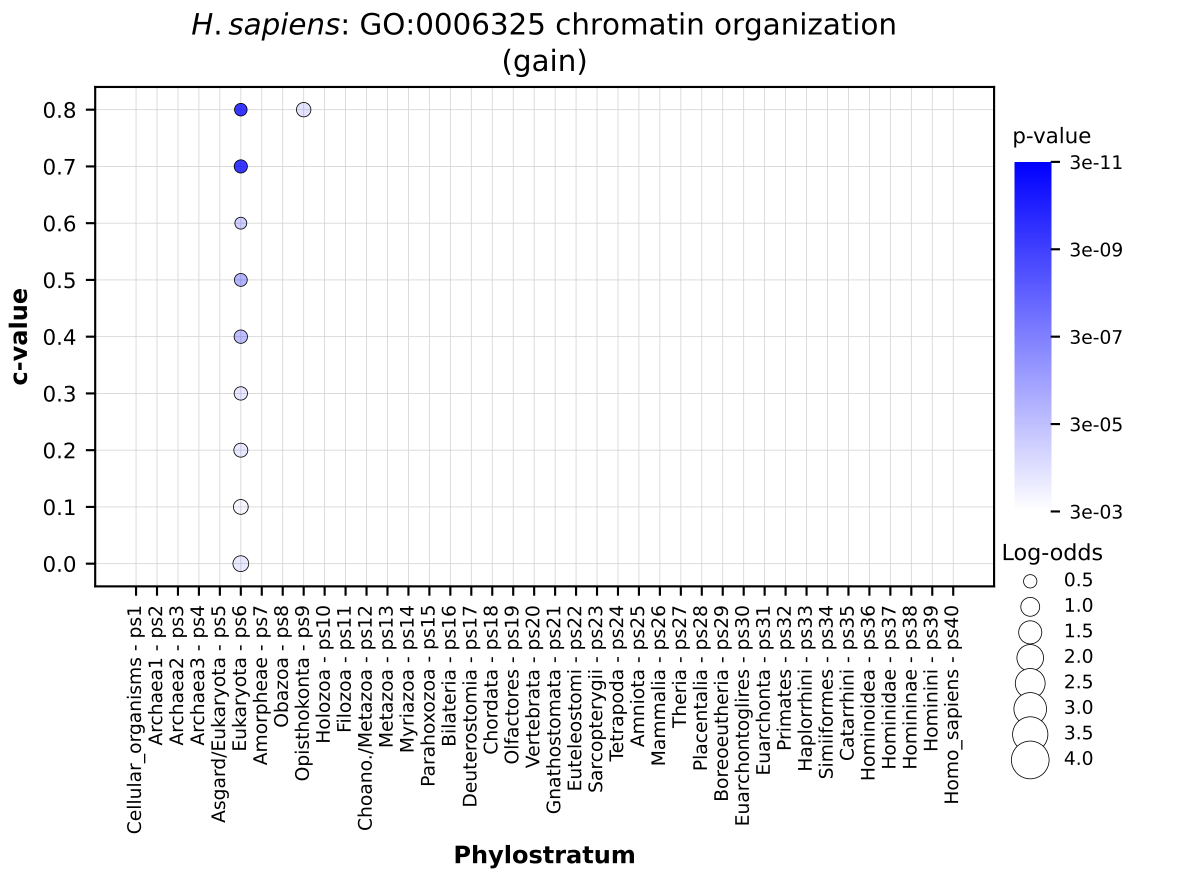

H. sapiens: GO:0006325 chromatin organization
(gain)
c-value
0.0
0.1
0.2
0.3
0.4
0.5
0.6
0.7
0.8
Phylostratum
Cellular_organisms - ps1
Archaea1 - ps2
Archaea2 - ps3
Archaea3 - ps4
Asgard/Eukaryota - ps5
Eukaryota - ps6
Amorpheae - ps7
Obazoa - ps8
Opisthokonta - ps9
Holozoa - ps10
Filozoa - ps11
Choano./Metazoa - ps12
Metazoa - ps13
Myriazoa - ps14
Parahoxozoa - ps15
Bilateria - ps16
Deuterostomia - ps17
Chordata - ps18
Olfactores - ps19
Vertebrata - ps20
Gnathostomata - ps21
Euteleostomi - ps22
Sarcopterygii - ps23
Tetrapoda - ps24
Amniota - ps25
Mammalia - ps26
Theria - ps27
Placentalia - ps28
Boreoeutheria - ps29
Euarchontoglires - ps30
Euarchonta - ps31
Primates - ps32
Haplorrhini - ps33
Simiiformes - ps34
Catarrhini - ps35
Hominoidea - ps36
Hominidae - ps37
Homininae - ps38
Hominini - ps39
Homo_sapiens - ps40
p-value
3e-11
3e-09
3e-07
3e-05
3e-03
Log-odds
0.5
1.0
1.5
2.0
2.5
3.0
3.5
4.0

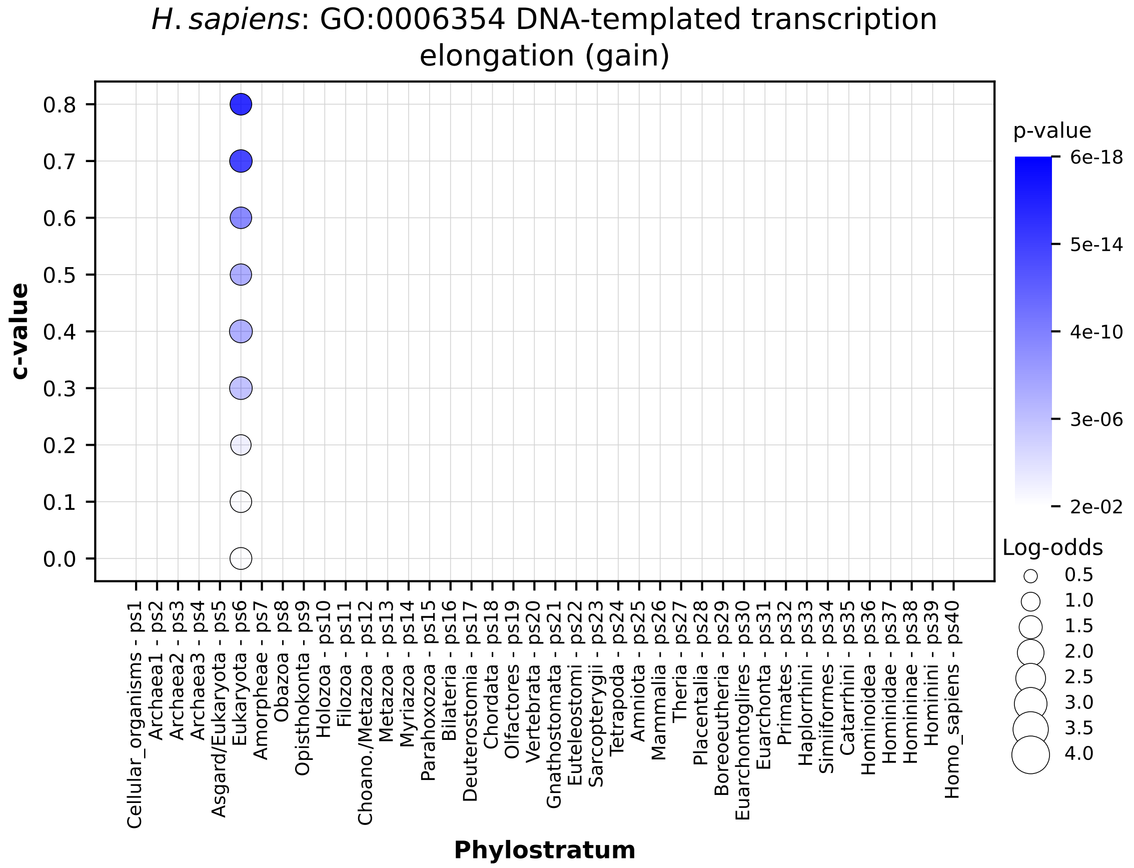
H. sapiens: GO:0006354 DNA-templated transcription elongation (gain)
c-value
0.0
0.1
0.2
0.3
0.4
0.5
0.6
0.7
0.8
Phylostratum
Cellular_organisms - ps1
Archaea1 - ps2
Archaea2 - ps3
Archaea3 - ps4
Asgard/Eukaryota - ps5
Eukaryota - ps6
Amorpheae - ps7
Obazoa - ps8
Opisthokonta - ps9
Holozoa - ps10
Filozoa - ps11
Choano./Metazoa - ps12
Metazoa - ps13
Myriazoa - ps14
Parahoxozoa - ps15
Bilateria - ps16
Deuterostomia - ps17
Chordata - ps18
Olfactores - ps19
Vertebrata - ps20
Gnathostomata - ps21
Euteleostomi - ps22
Sarcopterygii - ps23
Tetrapoda - ps24
Amniota - ps25
Mammalia - ps26
Theria - ps27
Placentalia - ps28
Boreoeutheria - ps29
Euarchontoglires - ps30
Euarchonta - ps31
Primates - ps32
Haplorrhini - ps33
Simiiformes - ps34
Catarrhini - ps35
Hominoidea - ps36
Hominidae - ps37
Homininae - ps38
Hominini - ps39
Homo_sapiens - ps40
p-value
6e-18
5e-14
4e-10
3e-06
2e-02
Log-odds
0.5
1.0
1.5
2.0
2.5
3.0
3.5
4.0

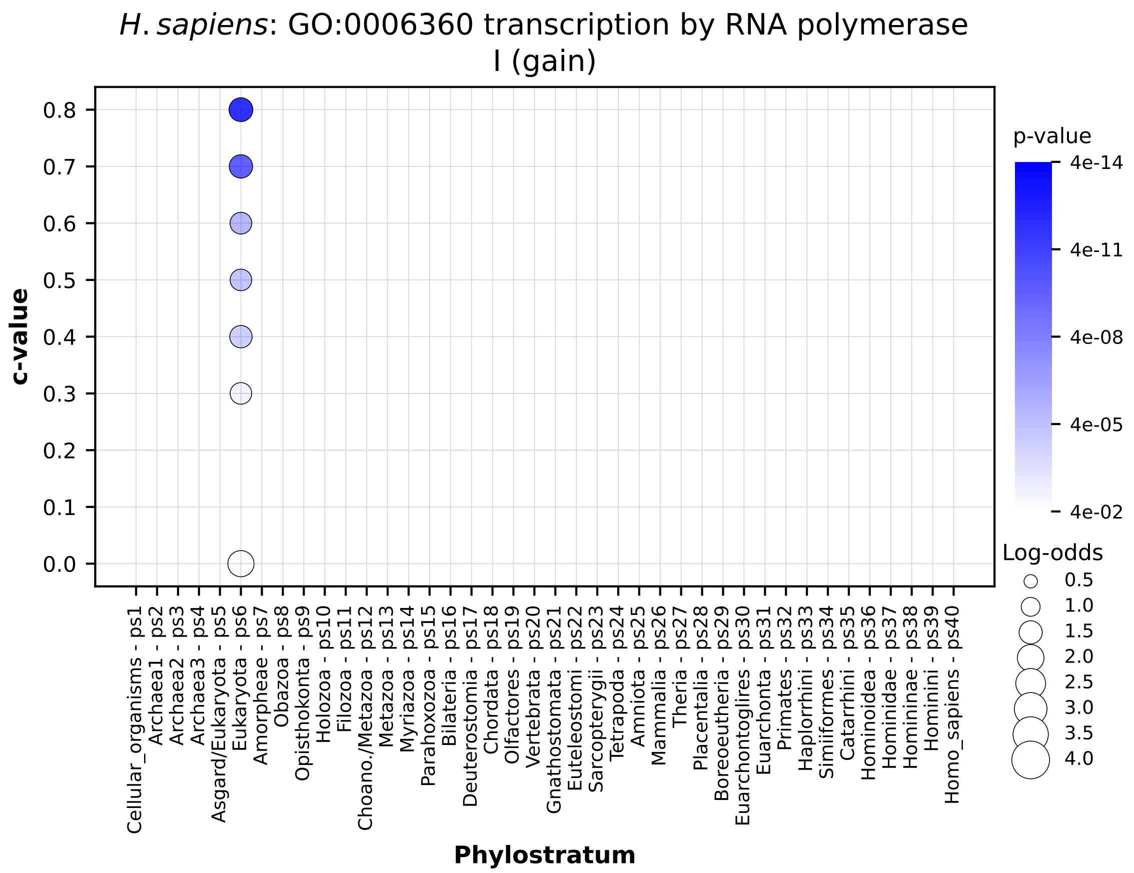
*H. sapiens*: GO:0006360 transcription by RNA polymerase I (gain)
c-value
0.0
0.1
0.2
0.3
0.4
0.5
0.6
0.7
0.8
p-value
4e-14
4e-11
4e-08
4e-05
4e-02
Log-odds
0.5
1.0
1.5
2.0
2.5
3.0
3.5
4.0
Cellular_organisms - ps1
Archaea1 - ps2
Archaea2 - ps3
Archaea3 - ps4
Asgard/Eukaryota - ps5
Eukaryota - ps6
Amorpheae - ps7
Obazoa - ps8
Opisthokonta - ps9
Holozoa - ps10
Filozoa - ps11
Choano./Metazoa - ps12
Metazoa - ps13
Myriazoa - ps14
Parahoxozoa - ps15
Bilateria - ps16
Deuterostomia - ps17
Chordata - ps18
Olfactores - ps19
Vertebrata - ps20
Gnathostomata - ps21
Euteleostomi - ps22
Sarcopterygii - ps23
Tetrapoda - ps24
Amniota - ps25
Mammalia - ps26
Theria - ps27
Placentalia - ps28
Boreoeutheria - ps29
Euarchontoglires - ps30
Euarchonta - ps31
Primates - ps32
Haplorrhini - ps33
Simiiformes - ps34
Catarrhini - ps35
Hominoidea - ps36
Hominidae - ps37
Homininae - ps38
Hominini - ps39
Homo_sapiens - ps40
Phylostratum

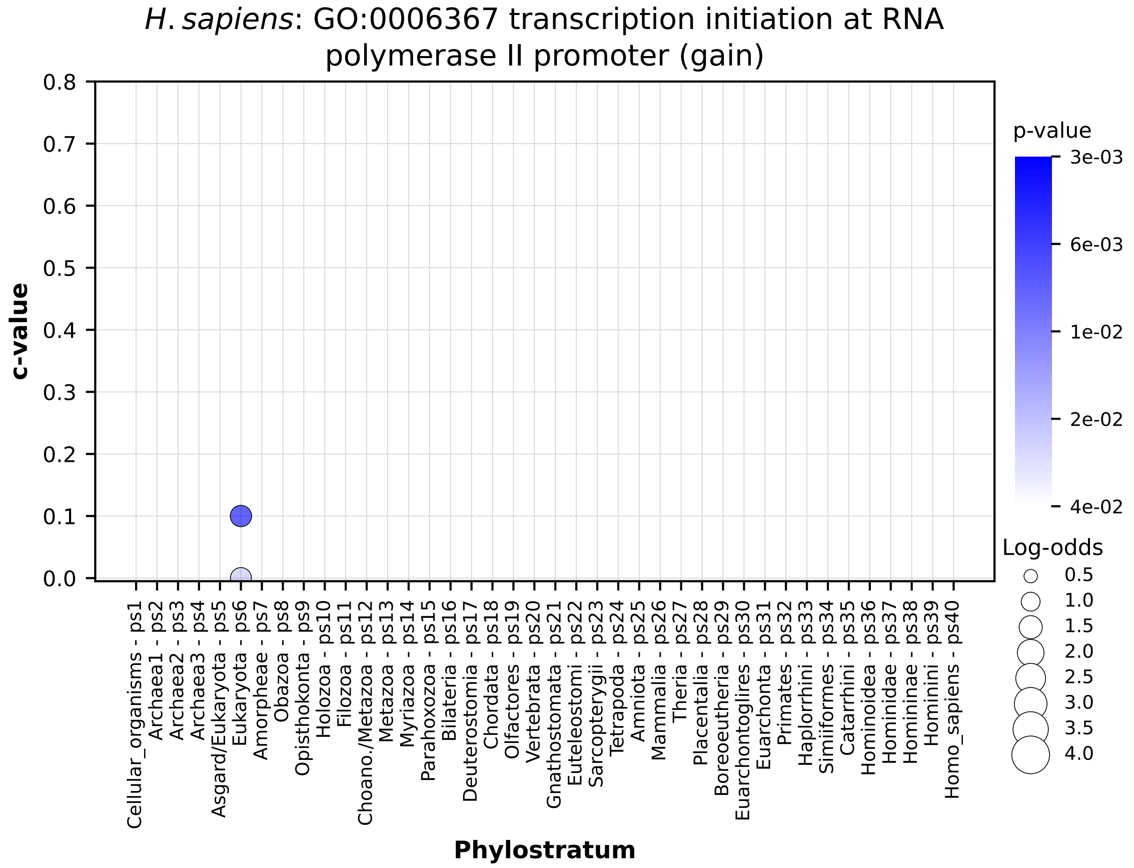

*H. sapiens*: GO:0006367 transcription initiation at RNA polymerase II promoter (gain)
c-value
0.8
0.7
0.6
0.5
0.4
0.3
0.2
0.1
0.0
Phylostratum
Cellular_organisms - ps1
Archaea1 - ps2
Archaea2 - ps3
Archaea3 - ps4
Asgard/Eukaryota - ps5
Eukaryota - ps6
Amorpheae - ps7
Obazoa - ps8
Opisthokonta - ps9
Holozoa - ps10
Filozoa - ps11
Choano./Metazoa - ps12
Metazoa - ps13
Myriazoa - ps14
Parahoxozoa - ps15
Bilateria - ps16
Deuterostomia - ps17
Chordata - ps18
Olfactores - ps19
Vertebrata - ps20
Gnathostomata - ps21
Euteleostomi - ps22
Sarcopterygii - ps23
Tetrapoda - ps24
Amniota - ps25
Mammalia - ps26
Theria - ps27
Placentalia - ps28
Boreoeutheria - ps29
Euarchontoglires - ps30
Euarchonta - ps31
Primates - ps32
Haplorrhini - ps33
Simiiformes - ps34
Catarrhini - ps35
Hominoidea - ps36
Hominidae - ps37
Homininae - ps38
Hominini - ps39
Homo_sapiens - ps40
p-value
3e-03
6e-03
1e-02
2e-02
4e-02
Log-odds
0.5
1.0
1.5
2.0
2.5
3.0
3.5
4.0

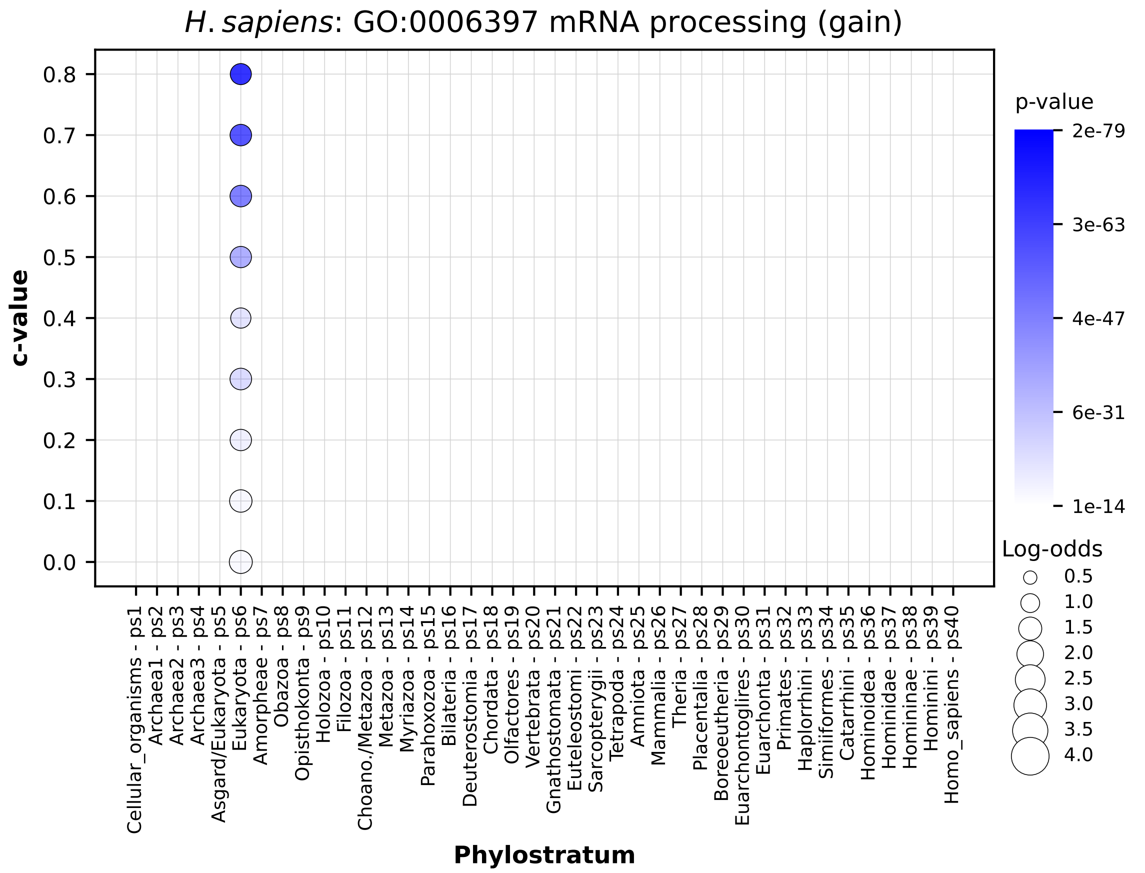

H. sapiens: GO:0006397 mRNA processing (gain)
c-value
0.8
0.7
0.6
0.5
0.4
0.3
0.2
0.1
0.0
Phylostratum
Cellular_organisms - ps1
Archaea1 - ps2
Archaea2 - ps3
Archaea3 - ps4
Asgard/Eukaryota - ps5
Eukaryota - ps6
Amorpheae - ps7
Obazoa - ps8
Opisthokonta - ps9
Holozoa - ps10
Filozoa - ps11
Choano./Metazoa - ps12
Metazoa - ps13
Myriazoa - ps14
Parahoxozoa - ps15
Bilateria - ps16
Deuterostomia - ps17
Chordata - ps18
Olfactores - ps19
Vertebrata - ps20
Gnathostomata - ps21
Euteleostomi - ps22
Sarcopterygii - ps23
Tetrapoda - ps24
Amniota - ps25
Mammalia - ps26
Theria - ps27
Placentalia - ps28
Boreoeutheria - ps29
Euarchontoglires - ps30
Euarchonta - ps31
Primates - ps32
Haplorrhini - ps33
Simiiformes - ps34
Catarrhini - ps35
Hominoidea - ps36
Hominidae - ps37
Homininae - ps38
Hominini - ps39
Homo_sapiens - ps40
p-value
2e-79
3e-63
4e-47
6e-31
1e-14
Log-odds
0.5
1.0
1.5
2.0
2.5
3.0
3.5
4.0

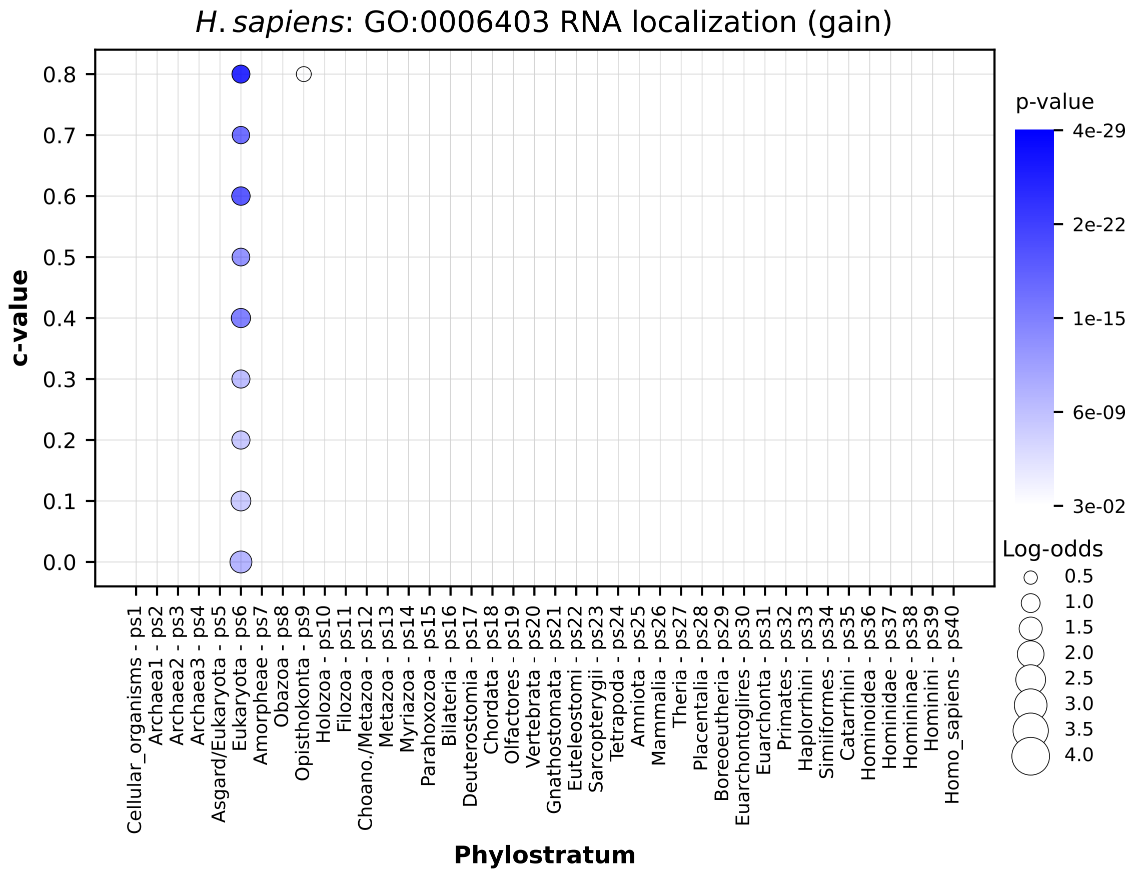

H. sapiens: GO:0006403 RNA localization (gain)
c-value
0.0
0.1
0.2
0.3
0.4
0.5
0.6
0.7
0.8
Phylostratum
Cellular_organisms - ps1
Archaea1 - ps2
Archaea2 - ps3
Archaea3 - ps4
Asgard/Eukaryota - ps5
Eukaryota - ps6
Amorpheae - ps7
Obazoa - ps8
Opisthokonta - ps9
Holozoa - ps10
Filozoa - ps11
Choano./Metazoa - ps12
Metazoa - ps13
Myriazoa - ps14
Parahoxozoa - ps15
Bilateria - ps16
Deuterostomia - ps17
Chordata - ps18
Olfactores - ps19
Vertebrata - ps20
Gnathostomata - ps21
Euteleostomi - ps22
Sarcopterygii - ps23
Tetrapoda - ps24
Amniota - ps25
Mammalia - ps26
Theria - ps27
Placentalia - ps28
Boreoeutheria - ps29
Euarchontoglires - ps30
Euarchonta - ps31
Primates - ps32
Haplorrhini - ps33
Simiiformes - ps34
Catarrhini - ps35
Hominoidea - ps36
Hominidae - ps37
Homininae - ps38
Hominini - ps39
Homo_sapiens - ps40
p-value
4e-29
2e-22
1e-15
6e-09
3e-02
Log-odds
0.5
1.0
1.5
2.0
2.5
3.0
3.5
4.0

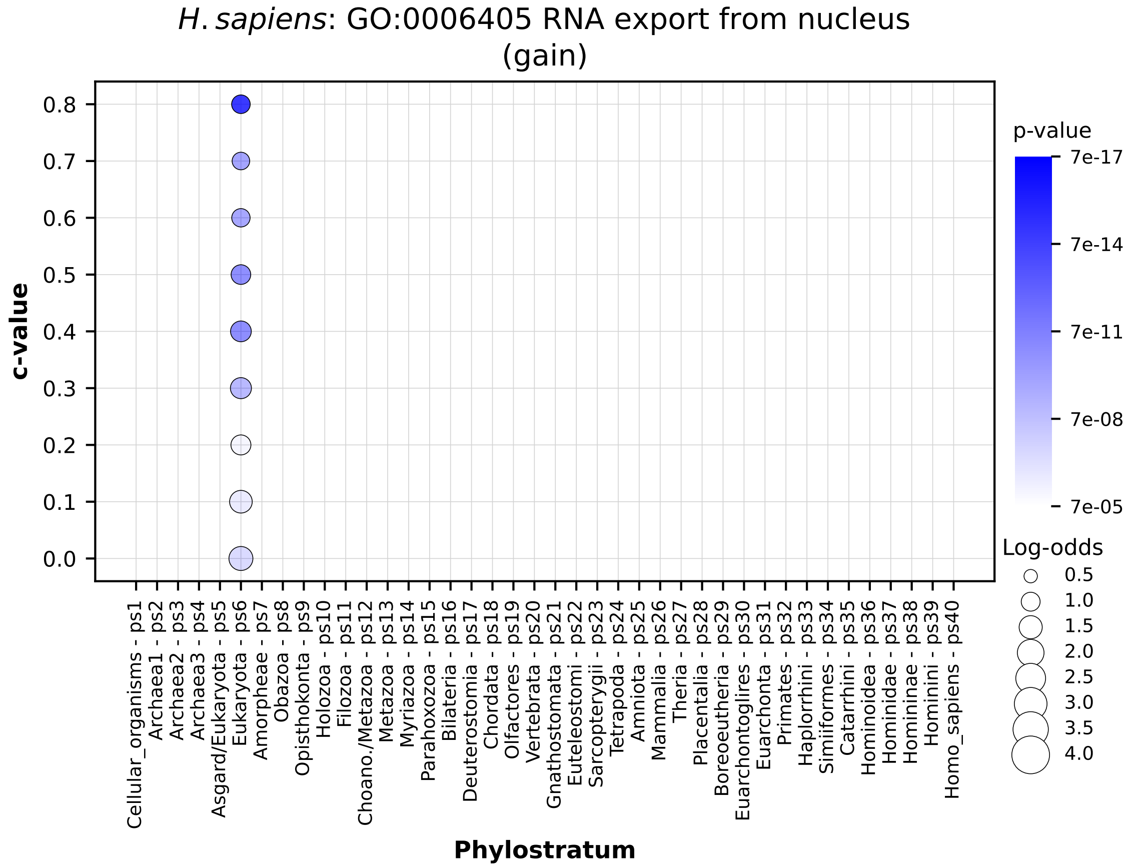

H. sapiens: GO:0006405 RNA export from nucleus
(gain)
c-value
0.8
0.7
0.6
0.5
0.4
0.3
0.2
0.1
0.0
Phylostratum
Cellular_organisms - ps1
Archaea1 - ps2
Archaea2 - ps3
Archaea3 - ps4
Asgard/Eukaryota - ps5
Eukaryota - ps6
Amorpheae - ps7
Obazoa - ps8
Opisthokonta - ps9
Holozoa - ps10
Filozoa - ps11
Choano./Metazoa - ps12
Metazoa - ps13
Myriazoa - ps14
Parahoxozoa - ps15
Bilateria - ps16
Deuterostomia - ps17
Chordata - ps18
Olfactores - ps19
Vertebrata - ps20
Gnathostomata - ps21
Euteleostomi - ps22
Sarcopterygii - ps23
Tetrapoda - ps24
Amniota - ps25
Mammalia - ps26
Theria - ps27
Placentalia - ps28
Boreoeutheria - ps29
Euarchontoglires - ps30
Euarchonta - ps31
Primates - ps32
Haplorrhini - ps33
Simiiformes - ps34
Catarrhini - ps35
Hominoidea - ps36
Hominidae - ps37
Homininae - ps38
Hominini - ps39
Homo_sapiens - ps40
p-value
7e-17
7e-14
7e-11
7e-08
7e-05
Log-odds
0.5
1.0
1.5
2.0
2.5
3.0
3.5
4.0

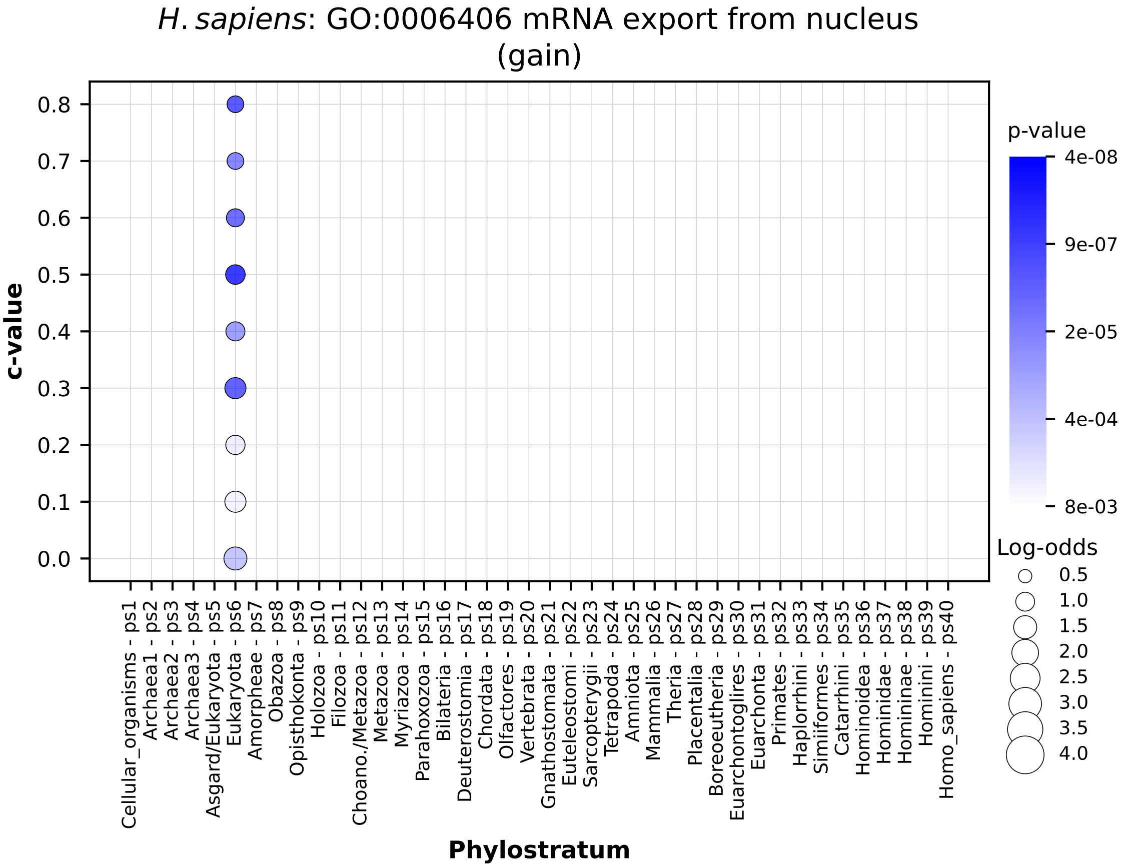

*H. sapiens*: GO:0006406 mRNA export from nucleus
(gain)
c-value
0.8
0.7
0.6
0.5
0.4
0.3
0.2
0.1
0.0
p-value
4e-08
9e-07
2e-05
4e-04
8e-03
Log-odds
0.5
1.0
1.5
2.0
2.5
3.0
3.5
4.0
Cellular_organisms - ps1
Archaea1 - ps2
Archaea2 - ps3
Archaea3 - ps4
Asgard/Eukaryota - ps5
Eukaryota - ps6
Amorpheae - ps7
Obazoa - ps8
Opisthokonta - ps9
Holozoa - ps10
Filozoa - ps11
Choano./Metazoa - ps12
Metazoa - ps13
Myriazoa - ps14
Parahoxozoa - ps15
Bilateria - ps16
Deuterostomia - ps17
Chordata - ps18
Olfactores - ps19
Vertebrata - ps20
Gnathostomata - ps21
Euteleostomi - ps22
Sarcopterygii - ps23
Tetrapoda - ps24
Amniota - ps25
Mammalia - ps26
Theria - ps27
Placentalia - ps28
Boreoeutheria - ps29
Euarchontoglires - ps30
Euarchonta - ps31
Primates - ps32
Haplorrhini - ps33
Simiiformes - ps34
Catarrhini - ps35
Hominoidea - ps36
Hominidae - ps37
Homininae - ps38
Hominini - ps39
Homo_sapiens - ps40
Phylostratum

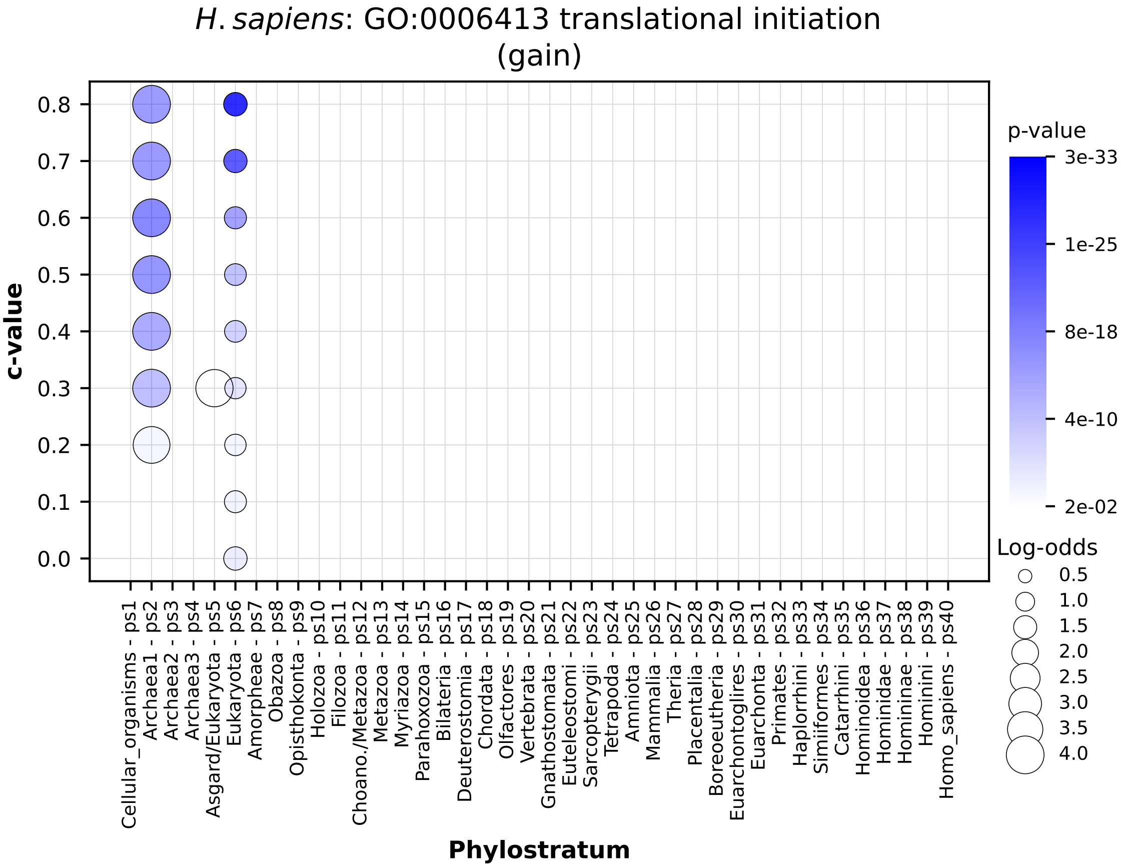

H. sapiens: GO:0006413 translational initiation
(gain)
c-value
0.0
0.1
0.2
0.3
0.4
0.5
0.6
0.7
0.8
Phylostratum
Cellular_organisms - ps1
Archaea1 - ps2
Archaea2 - ps3
Archaea3 - ps4
Asgard/Eukaryota - ps5
Eukaryota - ps6
Amorpheae - ps7
Obazoa - ps8
Opisthokonta - ps9
Holozoa - ps10
Filozoa - ps11
Choano./Metazoa - ps12
Metazoa - ps13
Myriazoa - ps14
Parahoxozoa - ps15
Bilateria - ps16
Deuterostomia - ps17
Chordata - ps18
Olfactores - ps19
Vertebrata - ps20
Gnathostomata - ps21
Euteleostomi - ps22
Sarcopterygii - ps23
Tetrapoda - ps24
Amniota - ps25
Mammalia - ps26
Theria - ps27
Placentalia - ps28
Boreoeutheria - ps29
Euarchontoglires - ps30
Euarchonta - ps31
Primates - ps32
Haplorrhini - ps33
Simiiformes - ps34
Catarrhini - ps35
Hominoidea - ps36
Hominidae - ps37
Homininae - ps38
Hominini - ps39
Homo_sapiens - ps40
p-value
3e-33
1e-25
8e-18
4e-10
2e-02
Log-odds
0.5
1.0
1.5
2.0
2.5
3.0
3.5
4.0

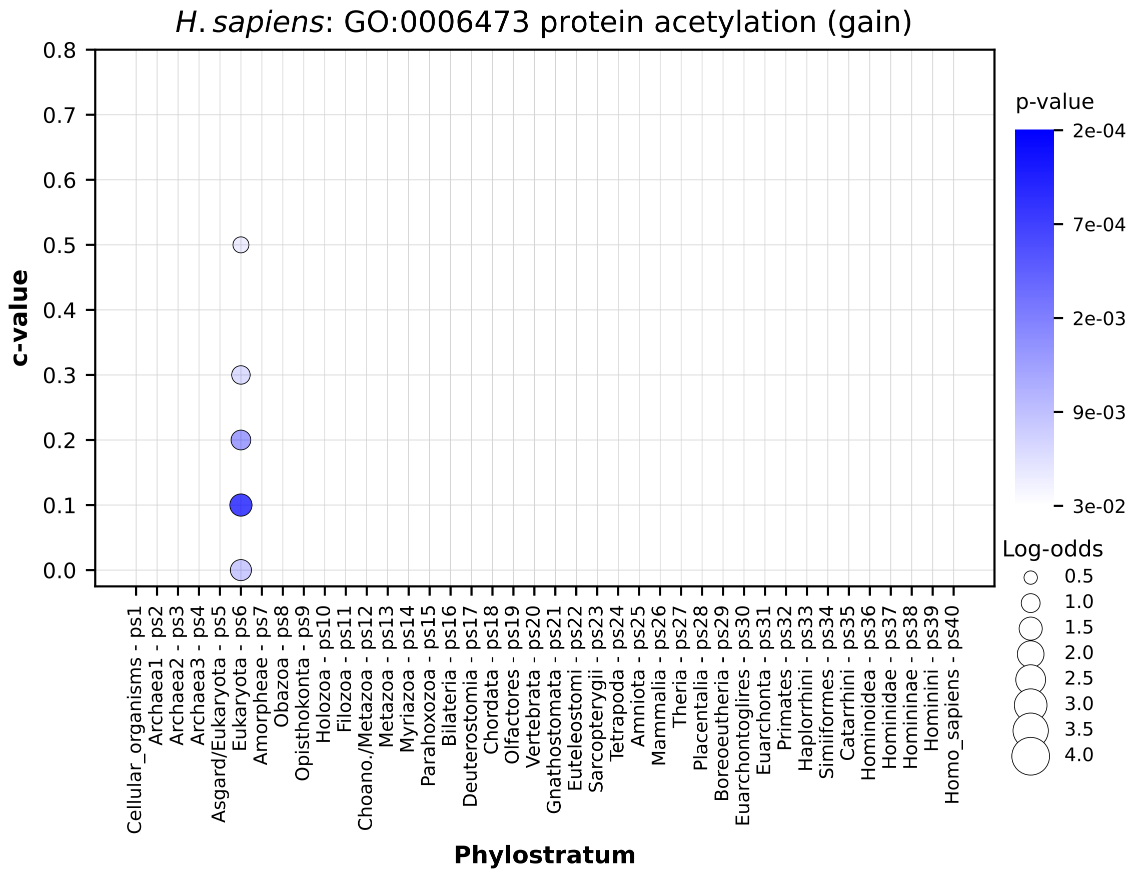

H. sapiens: GO:0006473 protein acetylation (gain)
c-value
0.8
0.7
0.6
0.5
0.4
0.3
0.2
0.1
0.0
Cellular_organisms - ps1
Archaea1 - ps2
Archaea2 - ps3
Archaea3 - ps4
Asgard/Eukaryota - ps5
Eukaryota - ps6
Amorpheae - ps7
Obazoa - ps8
Opisthokonta - ps9
Holozoa - ps10
Filozoa - ps11
Choano./Metazoa - ps12
Metazoa - ps13
Myriazoa - ps14
Parahoxozoa - ps15
Bilateria - ps16
Deuterostomia - ps17
Chordata - ps18
Olfactores - ps19
Vertebrata - ps20
Gnathostomata - ps21
Euteleostomi - ps22
Sarcopterygii - ps23
Tetrapoda - ps24
Amniota - ps25
Mammalia - ps26
Theria - ps27
Placentalia - ps28
Boreoeutheria - ps29
Euarchontoglires - ps30
Euarchonta - ps31
Primates - ps32
Haplorrhini - ps33
Simiiformes - ps34
Catarrhini - ps35
Hominoidea - ps36
Hominidae - ps37
Homininae - ps38
Hominini - ps39
Homo_sapiens - ps40
Phylostratum
p-value
2e-04
7e-04
2e-03
9e-03
3e-02
Log-odds
0.5
1.0
1.5
2.0
2.5
3.0
3.5
4.0

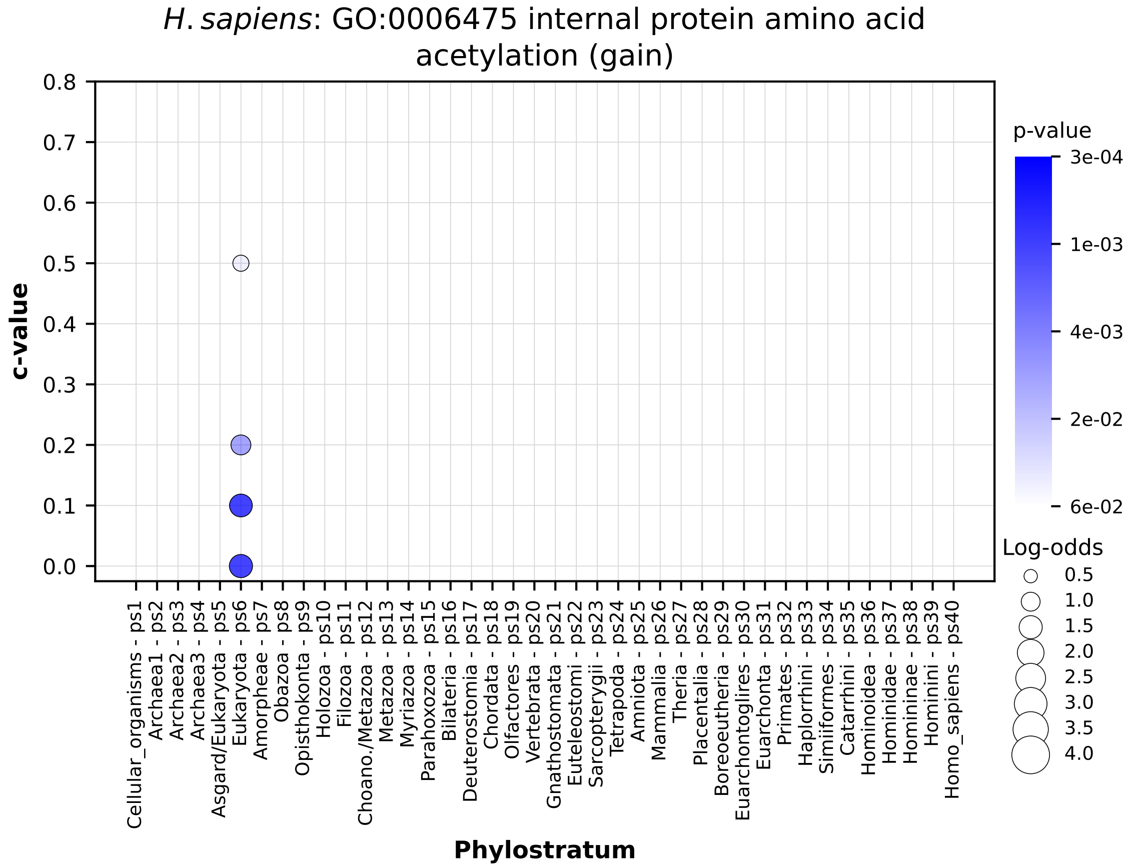

H. sapiens: GO:0006475 internal protein amino acid acetylation (gain)
c-value
0.8
0.7
0.6
0.5
0.4
0.3
0.2
0.1
0.0
p-value
3e-04
1e-03
4e-03
2e-02
6e-02
Log-odds
0.5
1.0
1.5
2.0
2.5
3.0
3.5
4.0
Cellular_organisms - ps1
Archaea1 - ps2
Archaea2 - ps3
Archaea3 - ps4
Asgard/Eukaryota - ps5
Eukaryota - ps6
Amorpheae - ps7
Obazoa - ps8
Opisthokonta - ps9
Holozoa - ps10
Filozoa - ps11
Choano./Metazoa - ps12
Metazoa - ps13
Myriazoa - ps14
Parahoxozoa - ps15
Bilateria - ps16
Deuterostomia - ps17
Chordata - ps18
Olfactores - ps19
Vertebrata - ps20
Gnathostomata - ps21
Euteleostomi - ps22
Sarcopterygii - ps23
Tetrapoda - ps24
Amniota - ps25
Mammalia - ps26
Theria - ps27
Placentalia - ps28
Boreoeutheria - ps29
Euarchontoglires - ps30
Euarchonta - ps31
Primates - ps32
Haplorrhini - ps33
Simiiformes - ps34
Catarrhini - ps35
Hominoidea - ps36
Hominidae - ps37
Homininae - ps38
Hominini - ps39
Homo_sapiens - ps40
Phylostratum

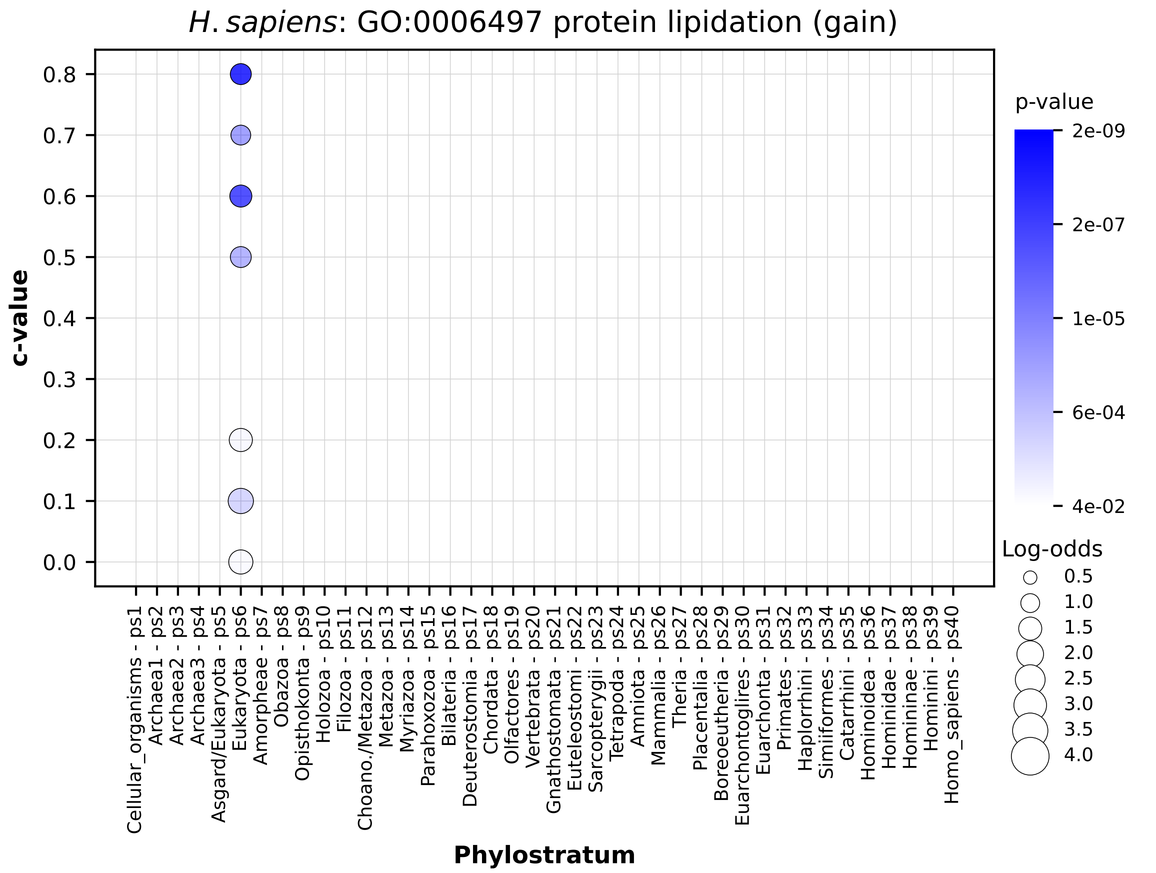

H. sapiens: GO:0006497 protein lipidation (gain)
c-value
0.8
0.7
0.6
0.5
0.4
0.3
0.2
0.1
0.0
p-value
2e-09
2e-07
1e-05
6e-04
4e-02
Log-odds
0.5
1.0
1.5
2.0
2.5
3.0
3.5
4.0
Cellular_organisms - ps1
Archaea1 - ps2
Archaea2 - ps3
Archaea3 - ps4
Asgard/Eukaryota - ps5
Eukaryota - ps6
Amorpheae - ps7
Obazoa - ps8
Opisthokonta - ps9
Holozoa - ps10
Filozoa - ps11
Choano./Metazoa - ps12
Metazoa - ps13
Myriazoa - ps14
Parahoxozoa - ps15
Bilateria - ps16
Deuterostomia - ps17
Chordata - ps18
Olfactores - ps19
Vertebrata - ps20
Gnathostomata - ps21
Euteleostomi - ps22
Sarcopterygii - ps23
Tetrapoda - ps24
Amniota - ps25
Mammalia - ps26
Theria - ps27
Placentalia - ps28
Boreoeutheria - ps29
Euarchontoglires - ps30
Euarchonta - ps31
Primates - ps32
Haplorrhini - ps33
Simiiformes - ps34
Catarrhini - ps35
Hominoidea - ps36
Hominidae - ps37
Homininae - ps38
Hominini - ps39
Homo_sapiens - ps40
Phylostratum

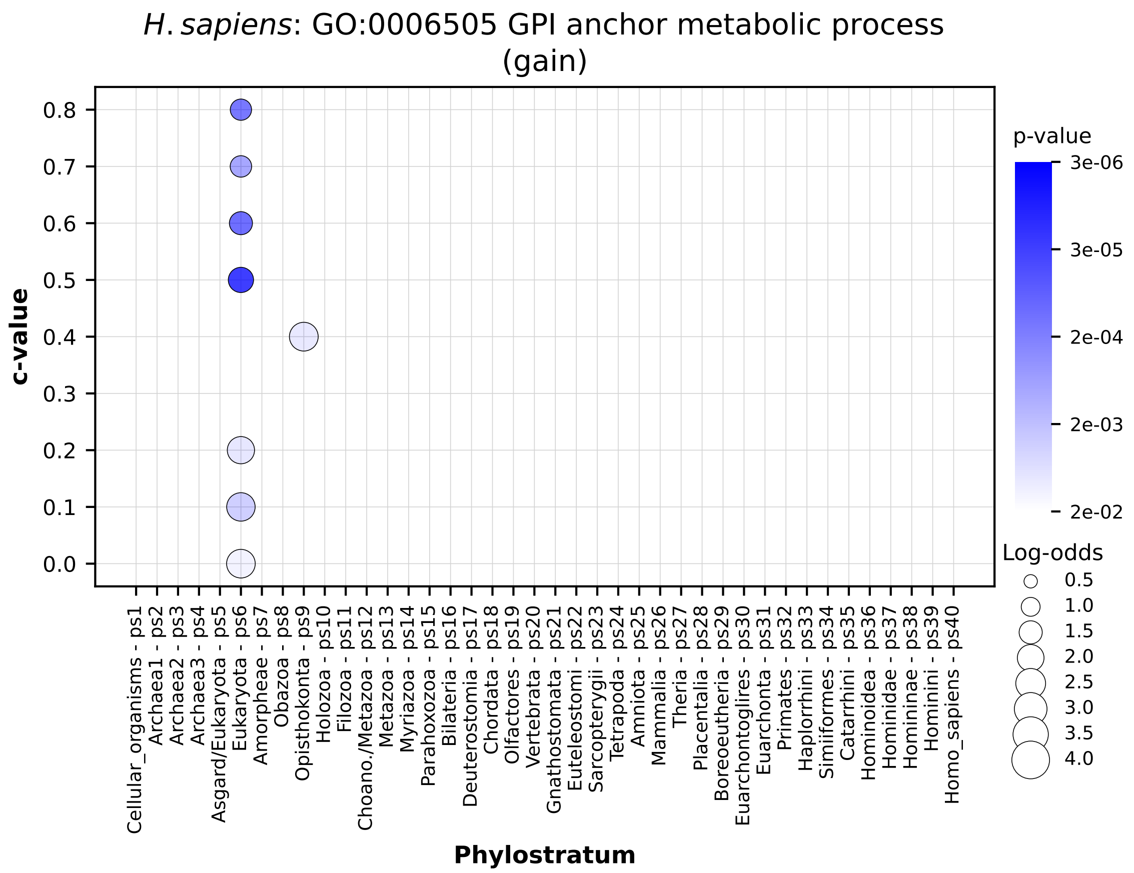

H. sapiens: GO:0006505 GPI anchor metabolic process (gain)
c-value
0.8
0.7
0.6
0.5
0.4
0.3
0.2
0.1
0.0
p-value
3e-06
3e-05
2e-04
2e-03
2e-02
Log-odds
0.5
1.0
1.5
2.0
2.5
3.0
3.5
4.0
Cellular_organisms - ps1
Archaea1 - ps2
Archaea2 - ps3
Archaea3 - ps4
Asgard/Eukaryota - ps5
Eukaryota - ps6
Amorpheae - ps7
Obazoa - ps8
Opisthokonta - ps9
Holozoa - ps10
Filozoa - ps11
Choano./Metazoa - ps12
Metazoa - ps13
Myriazoa - ps14
Parahoxozoa - ps15
Bilateria - ps16
Deuterostomia - ps17
Chordata - ps18
Olfactores - ps19
Vertebrata - ps20
Gnathostomata - ps21
Euteleostomi - ps22
Sarcopterygii - ps23
Tetrapoda - ps24
Amniota - ps25
Mammalia - ps26
Theria - ps27
Placentalia - ps28
Boreoeutheria - ps29
Euarchontoglires - ps30
Euarchonta - ps31
Primates - ps32
Haplorrhini - ps33
Simiiformes - ps34
Catarrhini - ps35
Hominoidea - ps36
Hominidae - ps37
Homininae - ps38
Hominini - ps39
Homo_sapiens - ps40
Phylostratum

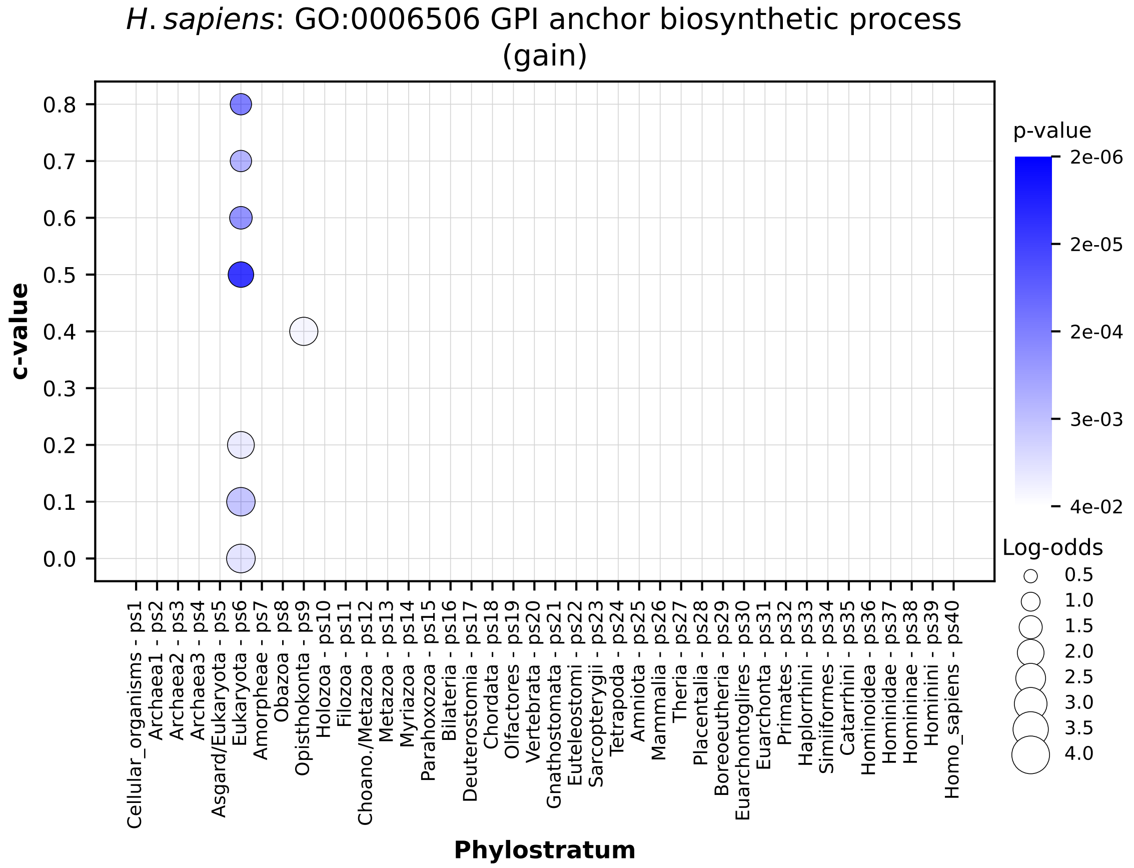

H. sapiens: GO:0006506 GPI anchor biosynthetic process
(gain)
c-value
0.0
0.1
0.2
0.3
0.4
0.5
0.6
0.7
0.8
Phylostratum
Cellular_organisms - ps1
Archaea1 - ps2
Archaea2 - ps3
Archaea3 - ps4
Asgard/Eukaryota - ps5
Eukaryota - ps6
Amorpheae - ps7
Obazoa - ps8
Opisthokonta - ps9
Holozoa - ps10
Filozoa - ps11
Choano./Metazoa - ps12
Metazoa - ps13
Myriazoa - ps14
Parahoxozoa - ps15
Bilateria - ps16
Deuterostomia - ps17
Chordata - ps18
Olfactores - ps19
Vertebrata - ps20
Gnathostomata - ps21
Euteleostomi - ps22
Sarcopterygii - ps23
Tetrapoda - ps24
Amniota - ps25
Mammalia - ps26
Theria - ps27
Placentalia - ps28
Boreoeutheria - ps29
Euarchontoglires - ps30
Euarchonta - ps31
Primates - ps32
Haplorrhini - ps33
Simiiformes - ps34
Catarrhini - ps35
Hominoidea - ps36
Hominidae - ps37
Homininae - ps38
Hominini - ps39
Homo_sapiens - ps40
p-value
2e-06
2e-05
2e-04
3e-03
4e-02
Log-odds
0.5
1.0
1.5
2.0
2.5
3.0
3.5
4.0

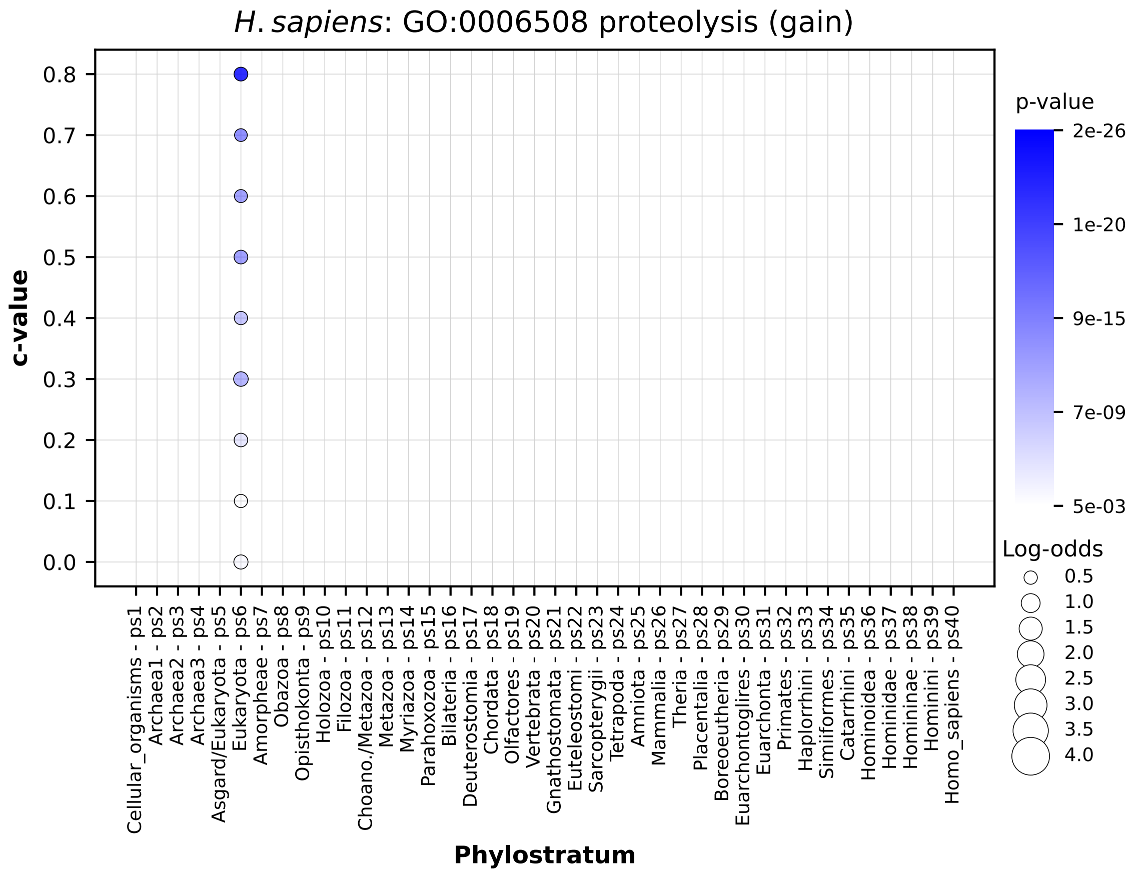

H. sapiens: GO:0006508 proteolysis (gain)
c-value
0.8
0.7
0.6
0.5
0.4
0.3
0.2
0.1
0.0
p-value
2e-26
1e-20
9e-15
7e-09
5e-03
Log-odds
0.5
1.0
1.5
2.0
2.5
3.0
3.5
4.0
Cellular_organisms - ps1
Archaea1 - ps2
Archaea2 - ps3
Archaea3 - ps4
Asgard/Eukaryota - ps5
Eukaryota - ps6
Amorpheae - ps7
Obazoa - ps8
Opisthokonta - ps9
Holozoa - ps10
Filozoa - ps11
Choano./Metazoa - ps12
Metazoa - ps13
Myriazoa - ps14
Parahoxozoa - ps15
Bilateria - ps16
Deuterostomia - ps17
Chordata - ps18
Olfactores - ps19
Vertebrata - ps20
Gnathostomata - ps21
Euteleostomi - ps22
Sarcopterygii - ps23
Tetrapoda - ps24
Amniota - ps25
Mammalia - ps26
Theria - ps27
Placentalia - ps28
Boreoeutheria - ps29
Euarchontoglires - ps30
Euarchonta - ps31
Primates - ps32
Haplorrhini - ps33
Simiiformes - ps34
Catarrhini - ps35
Hominoidea - ps36
Hominidae - ps37
Homininae - ps38
Hominini - ps39
Homo_sapiens - ps40
Phylostratum

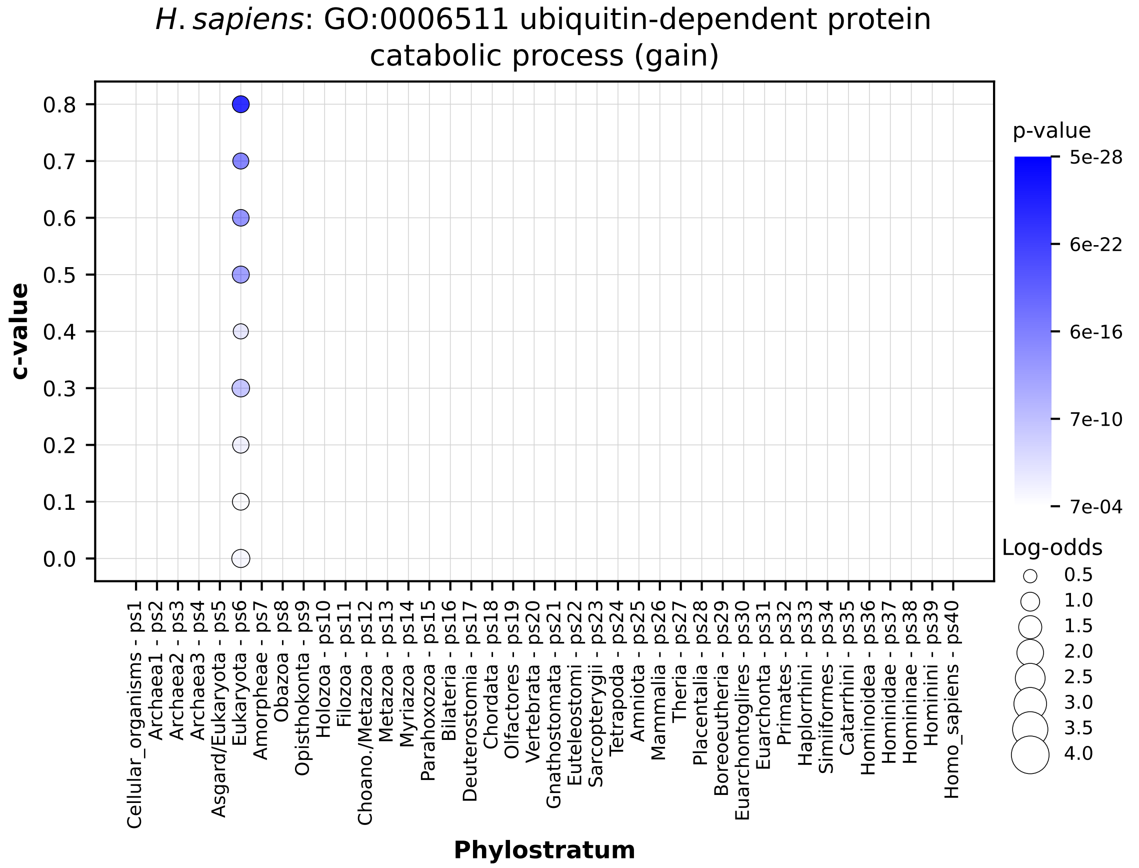

H. sapiens: GO:0006511 ubiquitin-dependent protein catabolic process (gain)
c-value
0.0
0.1
0.2
0.3
0.4
0.5
0.6
0.7
0.8
Phylostratum
Cellular_organisms - ps1
Archaea1 - ps2
Archaea2 - ps3
Archaea3 - ps4
Asgard/Eukaryota - ps5
Eukaryota - ps6
Amorpheae - ps7
Obazoa - ps8
Opisthokonta - ps9
Holozoa - ps10
Filozoa - ps11
Choano./Metazoa - ps12
Metazoa - ps13
Myriazoa - ps14
Parahoxozoa - ps15
Bilateria - ps16
Deuterostomia - ps17
Chordata - ps18
Olfactores - ps19
Vertebrata - ps20
Gnathostomata - ps21
Euteleostomi - ps22
Sarcopterygii - ps23
Tetrapoda - ps24
Amniota - ps25
Mammalia - ps26
Theria - ps27
Placentalia - ps28
Boreoeutheria - ps29
Euarchontoglires - ps30
Euarchonta - ps31
Primates - ps32
Haplorrhini - ps33
Simiiformes - ps34
Catarrhini - ps35
Hominoidea - ps36
Hominidae - ps37
Homininae - ps38
Hominini - ps39
Homo_sapiens - ps40
p-value
5e-28
6e-22
6e-16
7e-10
7e-04
Log-odds
0.5
1.0
1.5
2.0
2.5
3.0
3.5
4.0

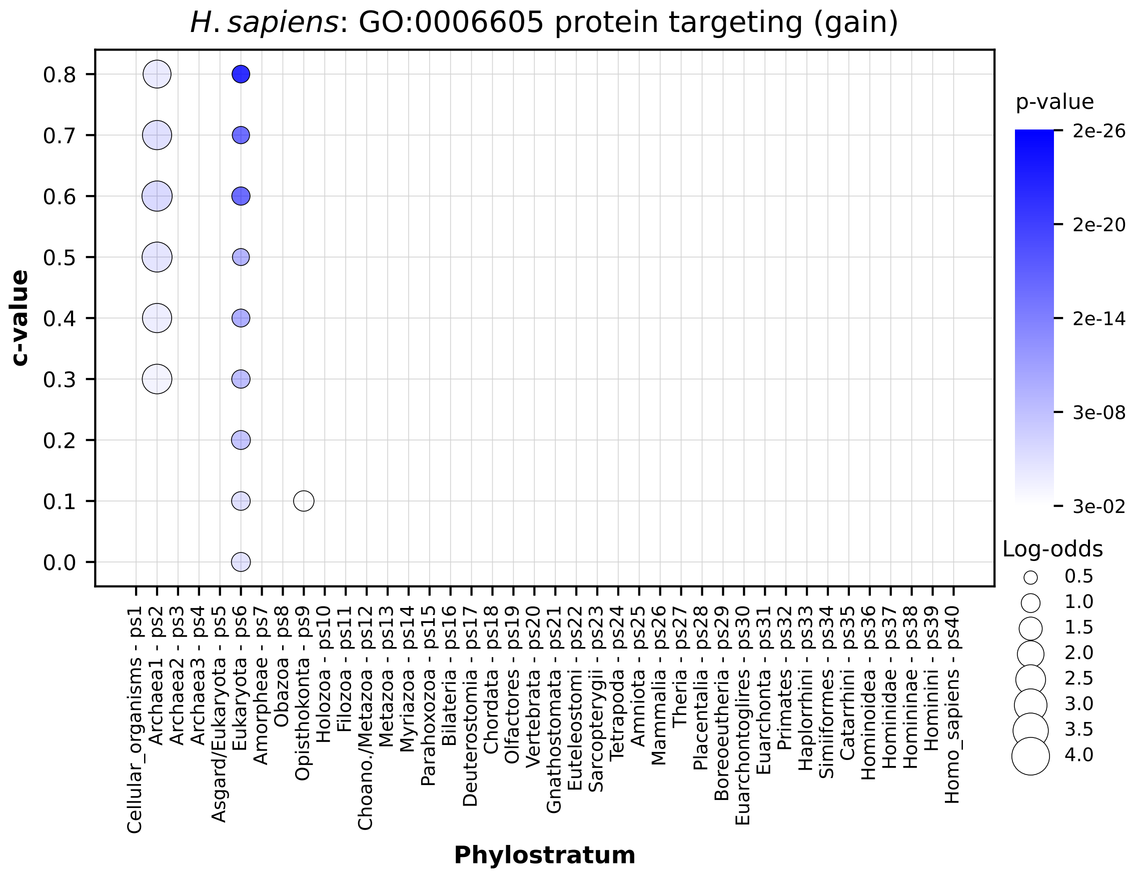

H. sapiens: GO:0006605 protein targeting (gain)
c-value
0.8
0.7
0.6
0.5
0.4
0.3
0.2
0.1
0.0
Phylostratum
Cellular_organisms - ps1
Archaea1 - ps2
Archaea2 - ps3
Archaea3 - ps4
Asgard/Eukaryota - ps5
Eukaryota - ps6
Amorpheae - ps7
Obazoa - ps8
Opisthokonta - ps9
Holozoa - ps10
Filozoa - ps11
Choano./Metazoa - ps12
Metazoa - ps13
Myriazoa - ps14
Parahoxozoa - ps15
Bilateria - ps16
Deuterostomia - ps17
Chordata - ps18
Olfactores - ps19
Vertebrata - ps20
Gnathostomata - ps21
Euteleostomi - ps22
Sarcopterygii - ps23
Tetrapoda - ps24
Amniota - ps25
Mammalia - ps26
Theria - ps27
Placentalia - ps28
Boreoeutheria - ps29
Euarchontoglires - ps30
Euarchonta - ps31
Primates - ps32
Haplorrhini - ps33
Simiiformes - ps34
Catarrhini - ps35
Hominoidea - ps36
Hominidae - ps37
Homininae - ps38
Hominini - ps39
Homo_sapiens - ps40
p-value
2e-26
2e-20
2e-14
3e-08
3e-02
Log-odds
0.5
1.0
1.5
2.0
2.5
3.0
3.5
4.0

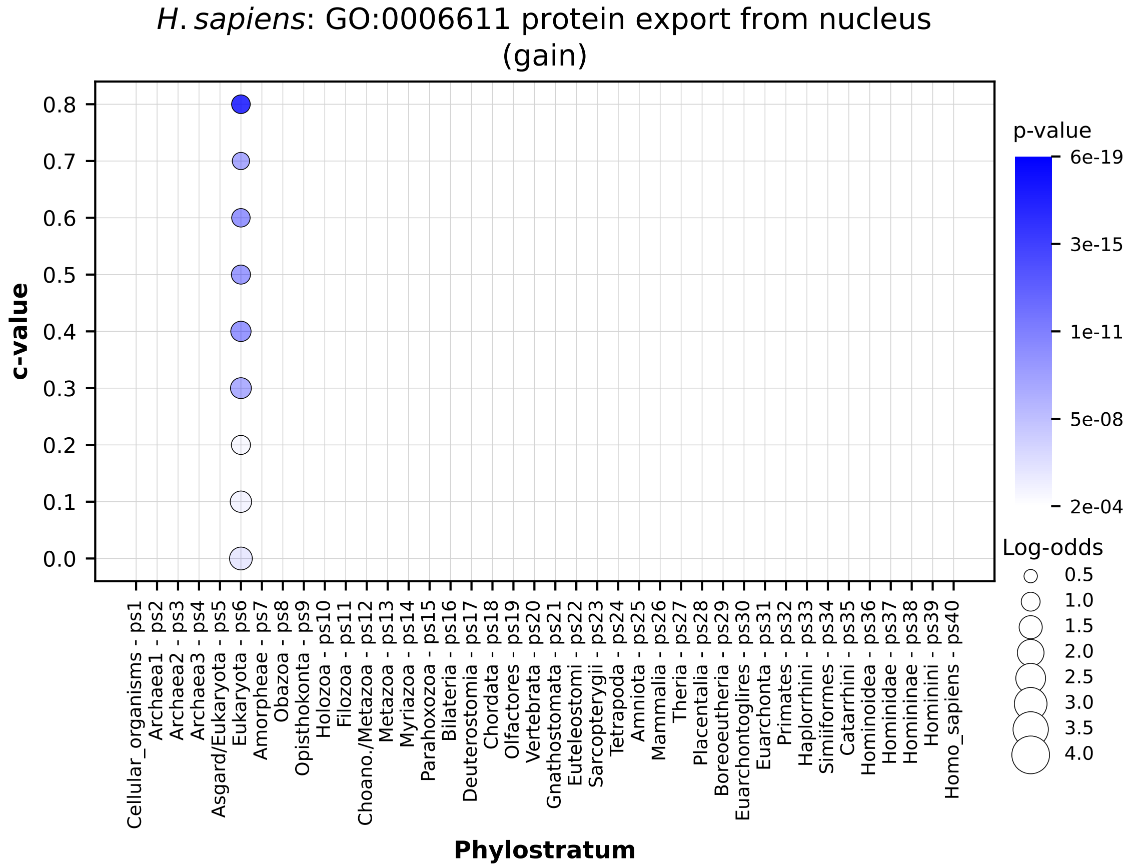

*H. sapiens*: GO:0006611 protein export from nucleus (gain)
c-value
0.8
0.7
0.6
0.5
0.4
0.3
0.2
0.1
0.0
p-value
6e-19
3e-15
1e-11
5e-08
2e-04
Log-odds
0.5
1.0
1.5
2.0
2.5
3.0
3.5
4.0
Cellular_organisms - ps1
Archaea1 - ps2
Archaea2 - ps3
Archaea3 - ps4
Asgard/Eukaryota - ps5
Eukaryota - ps6
Amorpheae - ps7
Obazoa - ps8
Opisthokonta - ps9
Holozoa - ps10
Filozoa - ps11
Choano./Metazoa - ps12
Metazoa - ps13
Myriazoa - ps14
Parahoxozoa - ps15
Bilateria - ps16
Deuterostomia - ps17
Chordata - ps18
Olfactores - ps19
Vertebrata - ps20
Gnathostomata - ps21
Euteleostomi - ps22
Sarcopterygii - ps23
Tetrapoda - ps24
Amniota - ps25
Mammalia - ps26
Theria - ps27
Placentalia - ps28
Boreoeutheria - ps29
Euarchontoglires - ps30
Euarchonta - ps31
Primates - ps32
Haplorrhini - ps33
Simiiformes - ps34
Catarrhini - ps35
Hominoidea - ps36
Hominidae - ps37
Homininae - ps38
Hominini - ps39
Homo_sapiens - ps40
Phylostratum

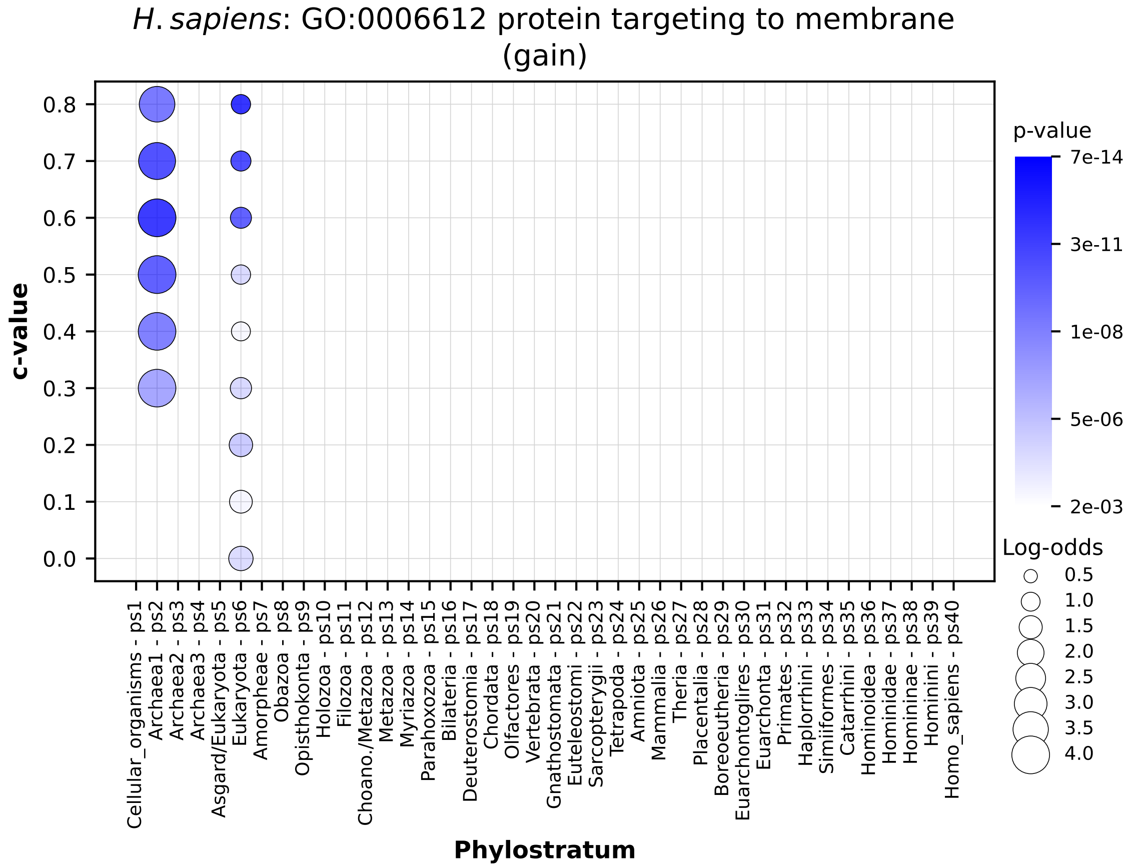
H. sapiens: GO:0006612 protein targeting to membrane
(gain)
c-value
0.8
0.7
0.6
0.5
0.4
0.3
0.2
0.1
0.0
p-value
7e-14
3e-11
1e-08
5e-06
2e-03
Log-odds
0.5
1.0
1.5
2.0
2.5
3.0
3.5
4.0
Cellular_organisms - ps1
Archaea1 - ps2
Archaea2 - ps3
Archaea3 - ps4
Asgard/Eukaryota - ps5
Eukaryota - ps6
Amorpheae - ps7
Obazoa - ps8
Opisthokonta - ps9
Holozoa - ps10
Filozoa - ps11
Choano./Metazoa - ps12
Metazoa - ps13
Myriazoa - ps14
Parahoxozoa - ps15
Bilateria - ps16
Deuterostomia - ps17
Chordata - ps18
Olfactores - ps19
Vertebrata - ps20
Gnathostomata - ps21
Euteleostomi - ps22
Sarcopterygii - ps23
Tetrapoda - ps24
Amniota - ps25
Mammalia - ps26
Theria - ps27
Placentalia - ps28
Boreoeutheria - ps29
Euarchontoglires - ps30
Euarchonta - ps31
Primates - ps32
Haplorrhini - ps33
Simiiformes - ps34
Catarrhini - ps35
Hominoidea - ps36
Hominidae - ps37
Homininae - ps38
Hominini - ps39
Homo_sapiens - ps40
Phylostratum

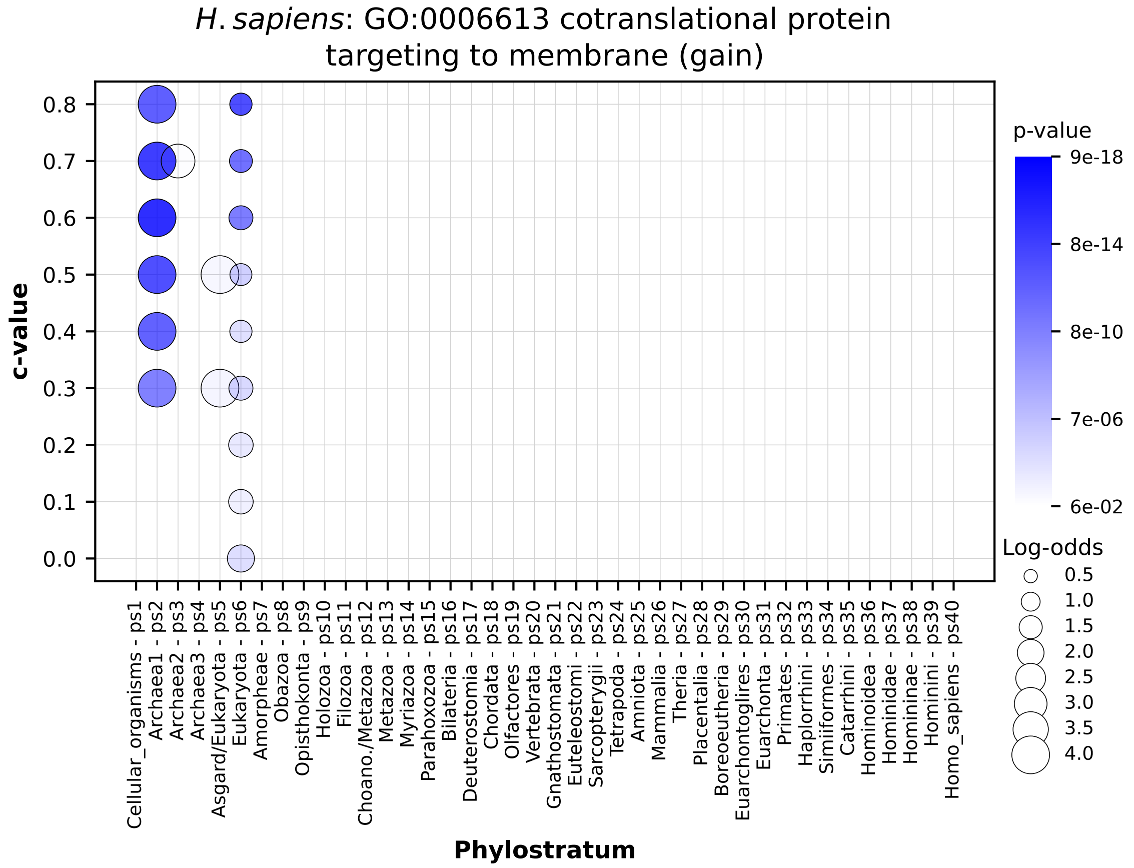

H. sapiens: GO:0006613 cotranslational protein targeting to membrane (gain)
c-value
0.8
0.7
0.6
0.5
0.4
0.3
0.2
0.1
0.0
p-value
9e-18
8e-14
8e-10
7e-06
6e-02
Log-odds
0.5
1.0
1.5
2.0
2.5
3.0
3.5
4.0
Cellular_organisms - ps1
Archaea1 - ps2
Archaea2 - ps3
Archaea3 - ps4
Asgard/Eukaryota - ps5
Eukaryota - ps6
Amorpheae - ps7
Obazoa - ps8
Opisthokonta - ps9
Holozoa - ps10
Filozoa - ps11
Choano./Metazoa - ps12
Metazoa - ps13
Myriazoa - ps14
Parahoxozoa - ps15
Bilateria - ps16
Deuterostomia - ps17
Chordata - ps18
Olfactores - ps19
Vertebrata - ps20
Gnathostomata - ps21
Euteleostomi - ps22
Sarcopterygii - ps23
Tetrapoda - ps24
Amniota - ps25
Mammalia - ps26
Theria - ps27
Placentalia - ps28
Boreoeutheria - ps29
Euarchontoglires - ps30
Euarchonta - ps31
Primates - ps32
Haplorrhini - ps33
Simiiformes - ps34
Catarrhini - ps35
Hominoidea - ps36
Hominidae - ps37
Homininae - ps38
Hominini - ps39
Homo_sapiens - ps40
Phylostratum

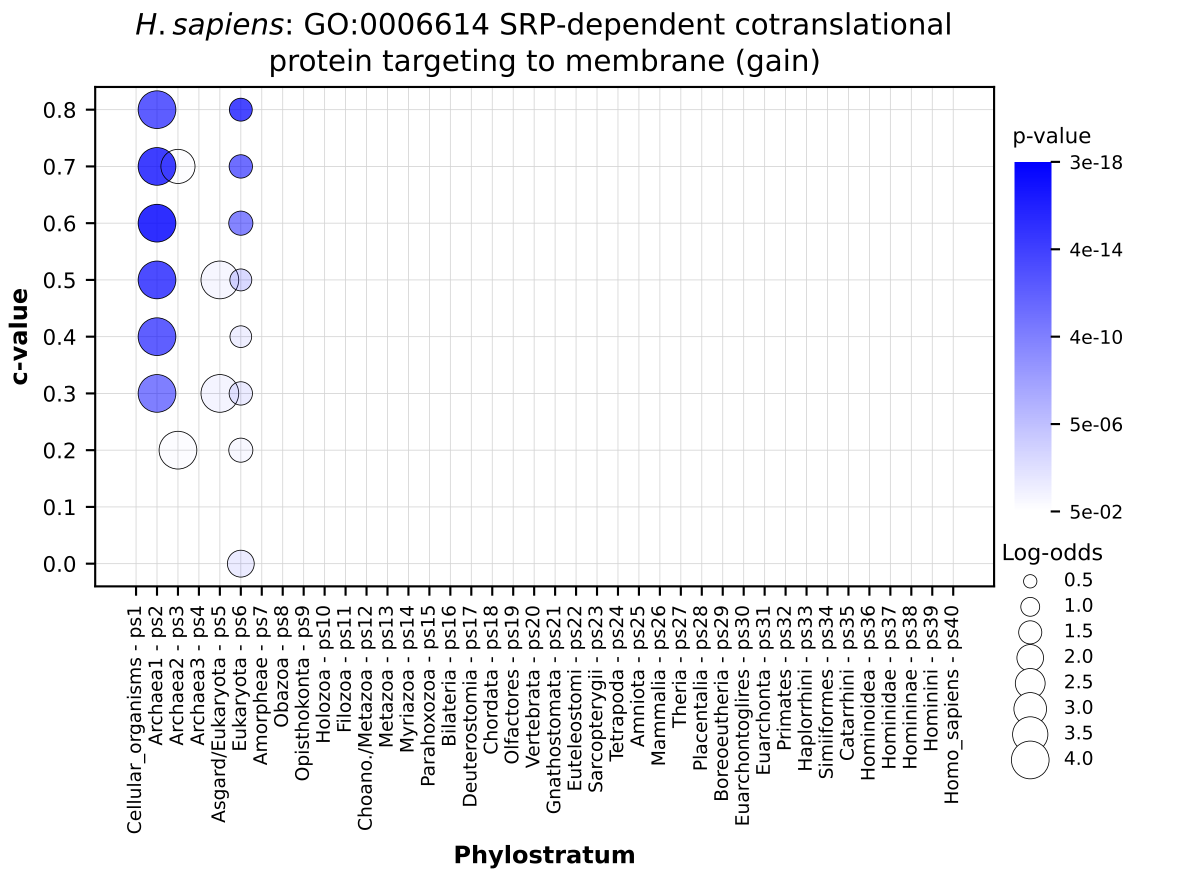

H. sapiens: GO:0006614 SRP-dependent cotranslational protein targeting to membrane (gain)
c-value
0.8
0.7
0.6
0.5
0.4
0.3
0.2
0.1
0.0
Phylostratum
Cellular_organisms - ps1
Archaea1 - ps2
Archaea2 - ps3
Archaea3 - ps4
Asgard/Eukaryota - ps5
Eukaryota - ps6
Amorpheae - ps7
Obazoa - ps8
Opisthokonta - ps9
Holozoa - ps10
Filozoa - ps11
Choano./Metazoa - ps12
Metazoa - ps13
Myriazoa - ps14
Parahoxozoa - ps15
Bilateria - ps16
Deuterostomia - ps17
Chordata - ps18
Olfactores - ps19
Vertebrata - ps20
Gnathostomata - ps21
Euteleostomi - ps22
Sarcopterygii - ps23
Tetrapoda - ps24
Amniota - ps25
Mammalia - ps26
Theria - ps27
Placentalia - ps28
Boreoeutheria - ps29
Euarchontoglires - ps30
Euarchonta - ps31
Primates - ps32
Haplorrhini - ps33
Simiiformes - ps34
Catarrhini - ps35
Hominoidea - ps36
Hominidae - ps37
Homininae - ps38
Hominini - ps39
Homo_sapiens - ps40
p-value
3e-18
4e-14
4e-10
5e-06
5e-02
Log-odds
0.5
1.0
1.5
2.0
2.5
3.0
3.5
4.0

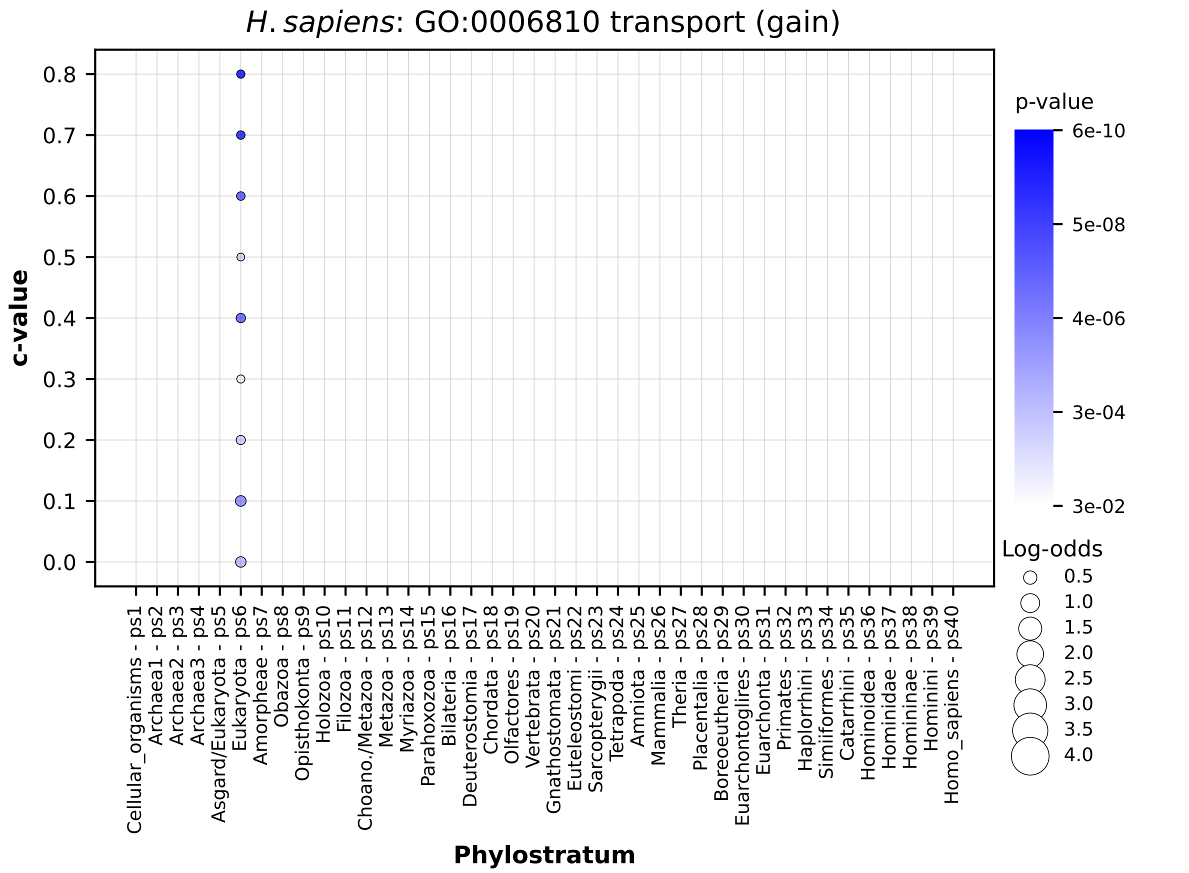
H. sapiens: GO:0006810 transport (gain)
c-value
0.8
0.7
0.6
0.5
0.4
0.3
0.2
0.1
0.0
p-value
6e-10
5e-08
4e-06
3e-04
3e-02
Log-odds
0.5
1.0
1.5
2.0
2.5
3.0
3.5
4.0
Cellular_organisms - ps1
Archaea1 - ps2
Archaea2 - ps3
Archaea3 - ps4
Asgard/Eukaryota - ps5
Eukaryota - ps6
Amorpheae - ps7
Obazoa - ps8
Opisthokonta - ps9
Holozoa - ps10
Filozoa - ps11
Choano./Metazoa - ps12
Metazoa - ps13
Myriazoa - ps14
Parahoxozoa - ps15
Bilateria - ps16
Deuterostomia - ps17
Chordata - ps18
Olfactores - ps19
Vertebrata - ps20
Gnathostomata - ps21
Euteleostomi - ps22
Sarcopterygii - ps23
Tetrapoda - ps24
Amniota - ps25
Mammalia - ps26
Theria - ps27
Placentalia - ps28
Boreoeutheria - ps29
Euarchontoglires - ps30
Euarchonta - ps31
Primates - ps32
Haplorrhini - ps33
Simiiformes - ps34
Catarrhini - ps35
Hominoidea - ps36
Hominidae - ps37
Homininae - ps38
Hominini - ps39
Homo_sapiens - ps40
Phylostratum

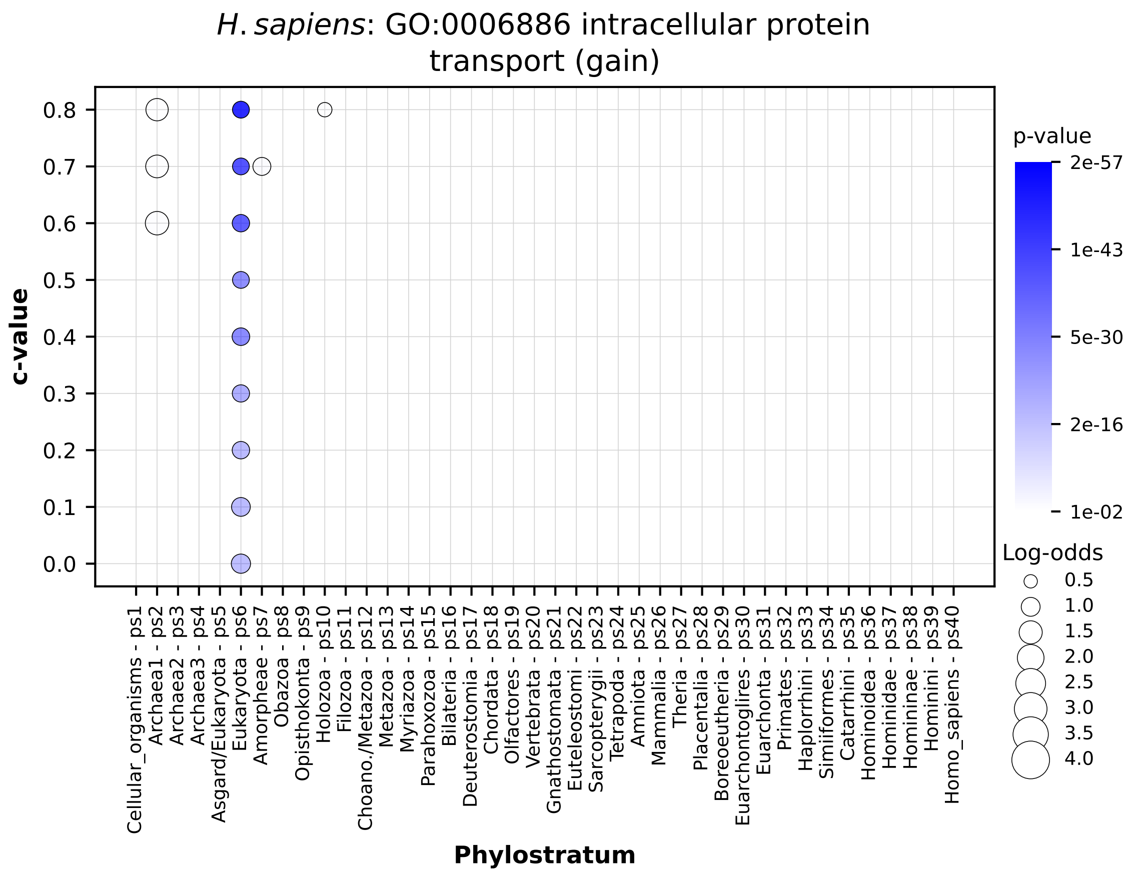

*H. sapiens*: GO:0006886 intracellular protein transport (gain)
c-value
0.8
0.7
0.6
0.5
0.4
0.3
0.2
0.1
0.0
Phylostratum
Cellular_organisms - ps1
Archaea1 - ps2
Archaea2 - ps3
Archaea3 - ps4
Asgard/Eukaryota - ps5
Eukaryota - ps6
Amorpheae - ps7
Obazoa - ps8
Opisthokonta - ps9
Holozoa - ps10
Filozoa - ps11
Choano./Metazoa - ps12
Metazoa - ps13
Myriazoa - ps14
Parahoxozoa - ps15
Bilateria - ps16
Deuterostomia - ps17
Chordata - ps18
Olfactores - ps19
Vertebrata - ps20
Gnathostomata - ps21
Euteleostomi - ps22
Sarcopterygii - ps23
Tetrapoda - ps24
Amniota - ps25
Mammalia - ps26
Theria - ps27
Placentalia - ps28
Boreoeutheria - ps29
Euarchontoglires - ps30
Euarchonta - ps31
Primates - ps32
Haplorrhini - ps33
Simiiformes - ps34
Catarrhini - ps35
Hominoidea - ps36
Hominidae - ps37
Homininae - ps38
Hominini - ps39
Homo_sapiens - ps40
p-value
2e-57
1e-43
5e-30
2e-16
1e-02
Log-odds
0.5
1.0
1.5
2.0
2.5
3.0
3.5
4.0

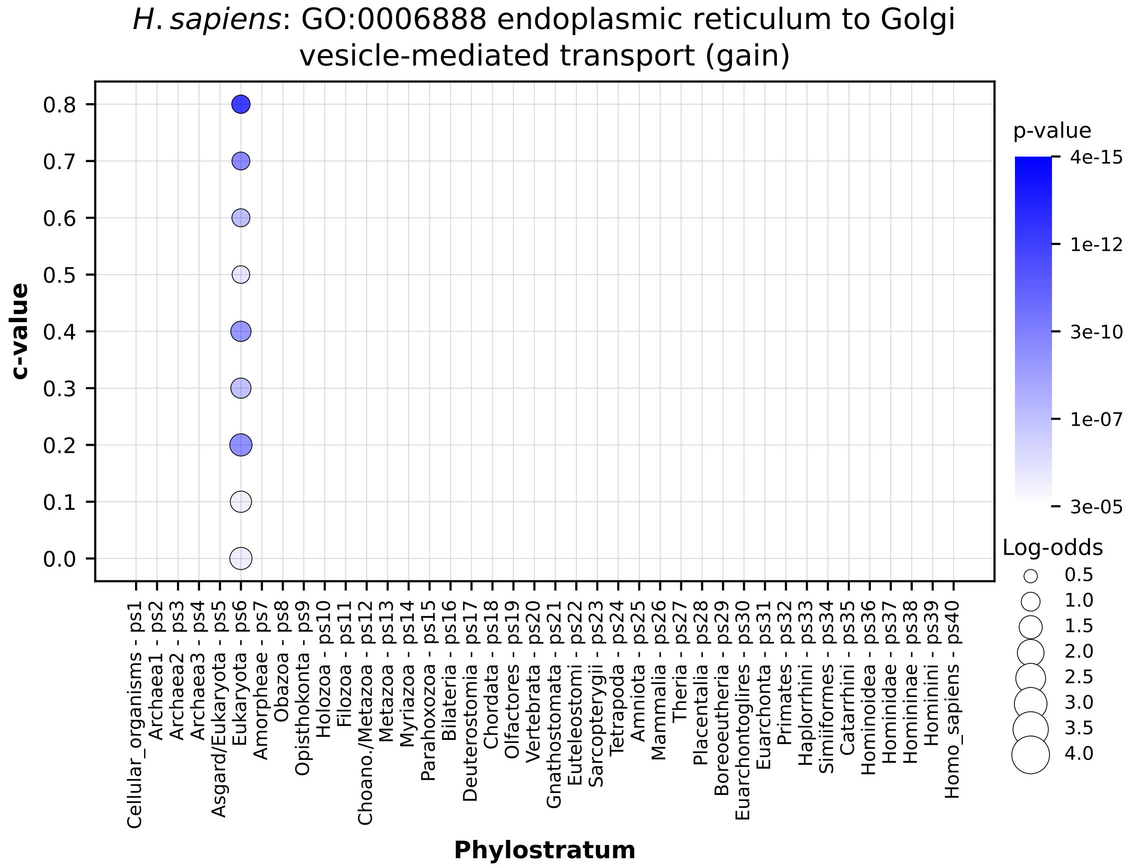

H. sapiens: GO:0006888 endoplasmic reticulum to Golgi vesicle-mediated transport (gain)
c-value
0.8
0.7
0.6
0.5
0.4
0.3
0.2
0.1
0.0
p-value
4e-15
1e-12
3e-10
1e-07
3e-05
Log-odds
0.5
1.0
1.5
2.0
2.5
3.0
3.5
4.0
Cellular_organisms - ps1
Archaea1 - ps2
Archaea2 - ps3
Archaea3 - ps4
Asgard/Eukaryota - ps5
Eukaryota - ps6
Amorpheae - ps7
Obazoa - ps8
Opisthokonta - ps9
Holozoa - ps10
Filozoa - ps11
Choano./Metazoa - ps12
Metazoa - ps13
Myriazoa - ps14
Parahoxozoa - ps15
Bilateria - ps16
Deuterostomia - ps17
Chordata - ps18
Olfactores - ps19
Vertebrata - ps20
Gnathostomata - ps21
Euteleostomi - ps22
Sarcopterygii - ps23
Tetrapoda - ps24
Amniota - ps25
Mammalia - ps26
Theria - ps27
Placentalia - ps28
Boreoeutheria - ps29
Euarchontoglires - ps30
Euarchonta - ps31
Primates - ps32
Haplorrhini - ps33
Simiiformes - ps34
Catarrhini - ps35
Hominoidea - ps36
Hominidae - ps37
Homininae - ps38
Hominini - ps39
Homo_sapiens - ps40
Phylostratum

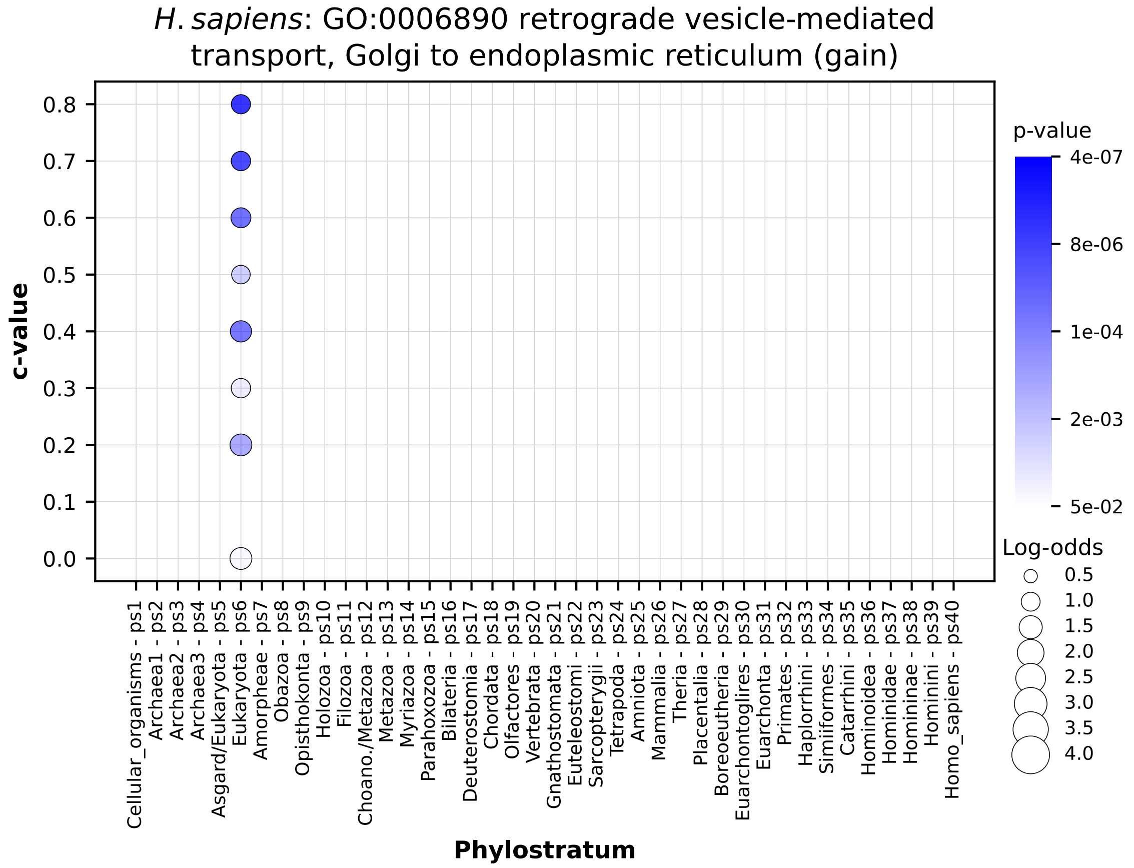

H. sapiens: GO:0006890 retrograde vesicle-mediated transport, Golgi to endoplasmic reticulum (gain)
c-value
0.8
0.7
0.6
0.5
0.4
0.3
0.2
0.1
0.0
Phylostratum
Cellular_organisms - ps1
Archaea1 - ps2
Archaea2 - ps3
Archaea3 - ps4
Asgard/Eukaryota - ps5
Eukaryota - ps6
Amorpheae - ps7
Obazoa - ps8
Opisthokonta - ps9
Holozoa - ps10
Filozoa - ps11
Choano./Metazoa - ps12
Metazoa - ps13
Myriazoa - ps14
Parahoxozoa - ps15
Bilateria - ps16
Deuterostomia - ps17
Chordata - ps18
Olfactores - ps19
Vertebrata - ps20
Gnathostomata - ps21
Euteleostomi - ps22
Sarcopterygii - ps23
Tetrapoda - ps24
Amniota - ps25
Mammalia - ps26
Theria - ps27
Placentalia - ps28
Boreoeutheria - ps29
Euarchontoglires - ps30
Euarchonta - ps31
Primates - ps32
Haplorrhini - ps33
Simiiformes - ps34
Catarrhini - ps35
Hominoidea - ps36
Hominidae - ps37
Homininae - ps38
Hominini - ps39
Homo_sapiens - ps40
p-value
4e-07
8e-06
1e-04
2e-03
5e-02
Log-odds
0.5
1.0
1.5
2.0
2.5
3.0
3.5
4.0

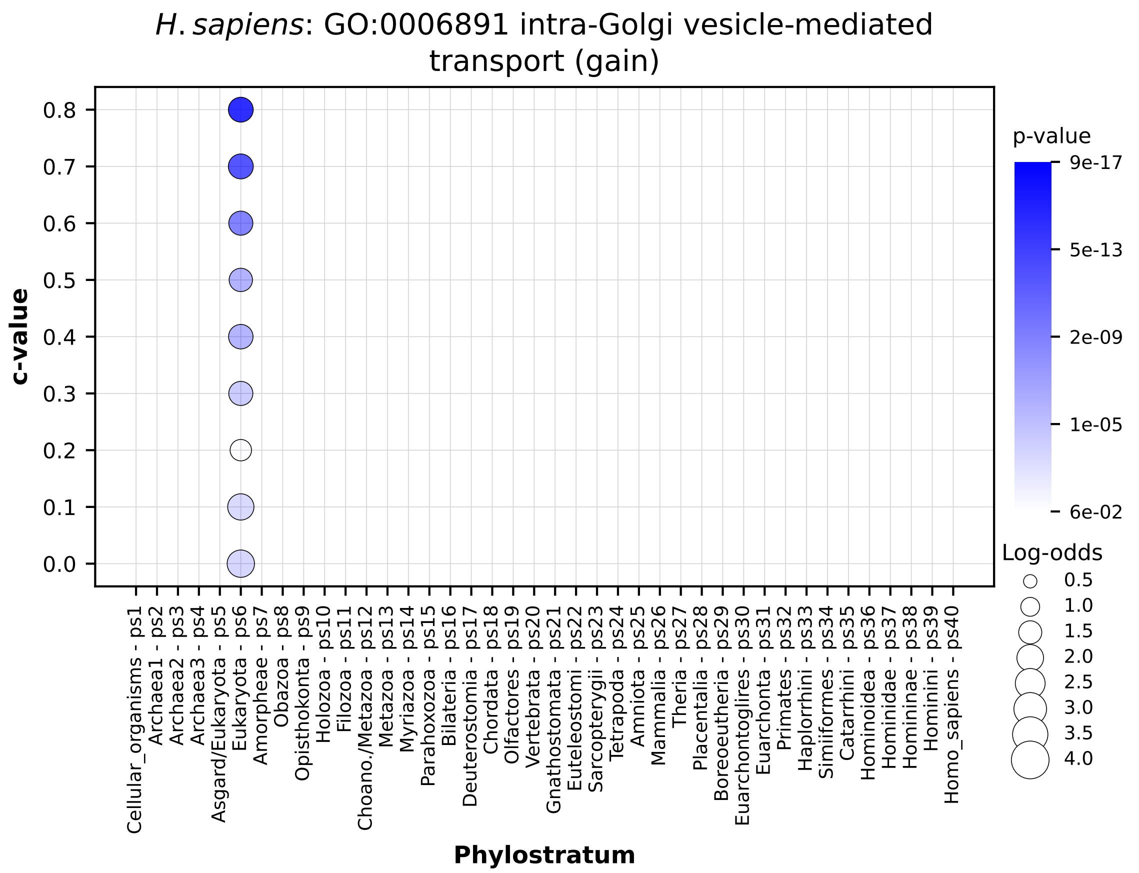

H. sapiens: GO:0006891 intra-Golgi vesicle-mediated transport (gain)
c-value
0.8
0.7
0.6
0.5
0.4
0.3
0.2
0.1
0.0
p-value
9e-17
5e-13
2e-09
1e-05
6e-02
Log-odds
0.5
1.0
1.5
2.0
2.5
3.0
3.5
4.0
Cellular_organisms - ps1
Archaea1 - ps2
Archaea2 - ps3
Archaea3 - ps4
Asgard/Eukaryota - ps5
Eukaryota - ps6
Amorpheae - ps7
Obazoa - ps8
Opisthokonta - ps9
Holozoa - ps10
Filozoa - ps11
Choano./Metazoa - ps12
Metazoa - ps13
Myriazoa - ps14
Parahoxozoa - ps15
Bilateria - ps16
Deuterostomia - ps17
Chordata - ps18
Olfactores - ps19
Vertebrata - ps20
Gnathostomata - ps21
Euteleostomi - ps22
Sarcopterygii - ps23
Tetrapoda - ps24
Amniota - ps25
Mammalia - ps26
Theria - ps27
Placentalia - ps28
Boreoeutheria - ps29
Euarchontoglires - ps30
Euarchonta - ps31
Primates - ps32
Haplorrhini - ps33
Simiiformes - ps34
Catarrhini - ps35
Hominoidea - ps36
Hominidae - ps37
Homininae - ps38
Hominini - ps39
Homo_sapiens - ps40
Phylostratum

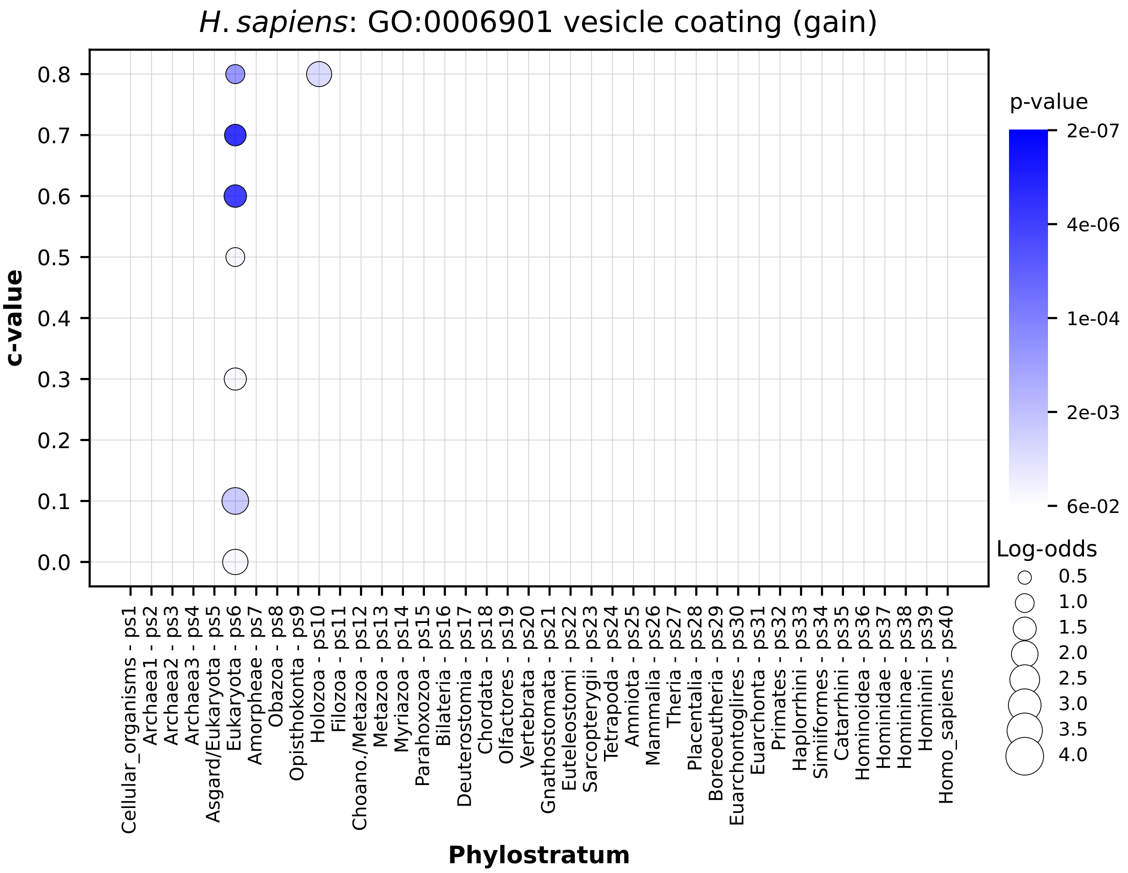

H. sapiens: GO:0006901 vesicle coating (gain)
c-value
0.0
0.1
0.2
0.3
0.4
0.5
0.6
0.7
0.8
Phylostratum
Cellular_organisms - ps1
Archaea1 - ps2
Archaea2 - ps3
Archaea3 - ps4
Asgard/Eukaryota - ps5
Eukaryota - ps6
Amorpheae - ps7
Obazoa - ps8
Opisthokonta - ps9
Holozoa - ps10
Filozoa - ps11
Choano./Metazoa - ps12
Metazoa - ps13
Myriazoa - ps14
Parahoxozoa - ps15
Bilateria - ps16
Deuterostomia - ps17
Chordata - ps18
Olfactores - ps19
Vertebrata - ps20
Gnathostomata - ps21
Euteleostomi - ps22
Sarcopterygii - ps23
Tetrapoda - ps24
Amniota - ps25
Mammalia - ps26
Theria - ps27
Placentalia - ps28
Boreoeutheria - ps29
Euarchontoglires - ps30
Euarchonta - ps31
Primates - ps32
Haplorrhini - ps33
Simiiformes - ps34
Catarrhini - ps35
Hominoidea - ps36
Hominidae - ps37
Homininae - ps38
Hominini - ps39
Homo_sapiens - ps40
p-value
2e-07
4e-06
1e-04
2e-03
6e-02
Log-odds
0.5
1.0
1.5
2.0
2.5
3.0
3.5
4.0

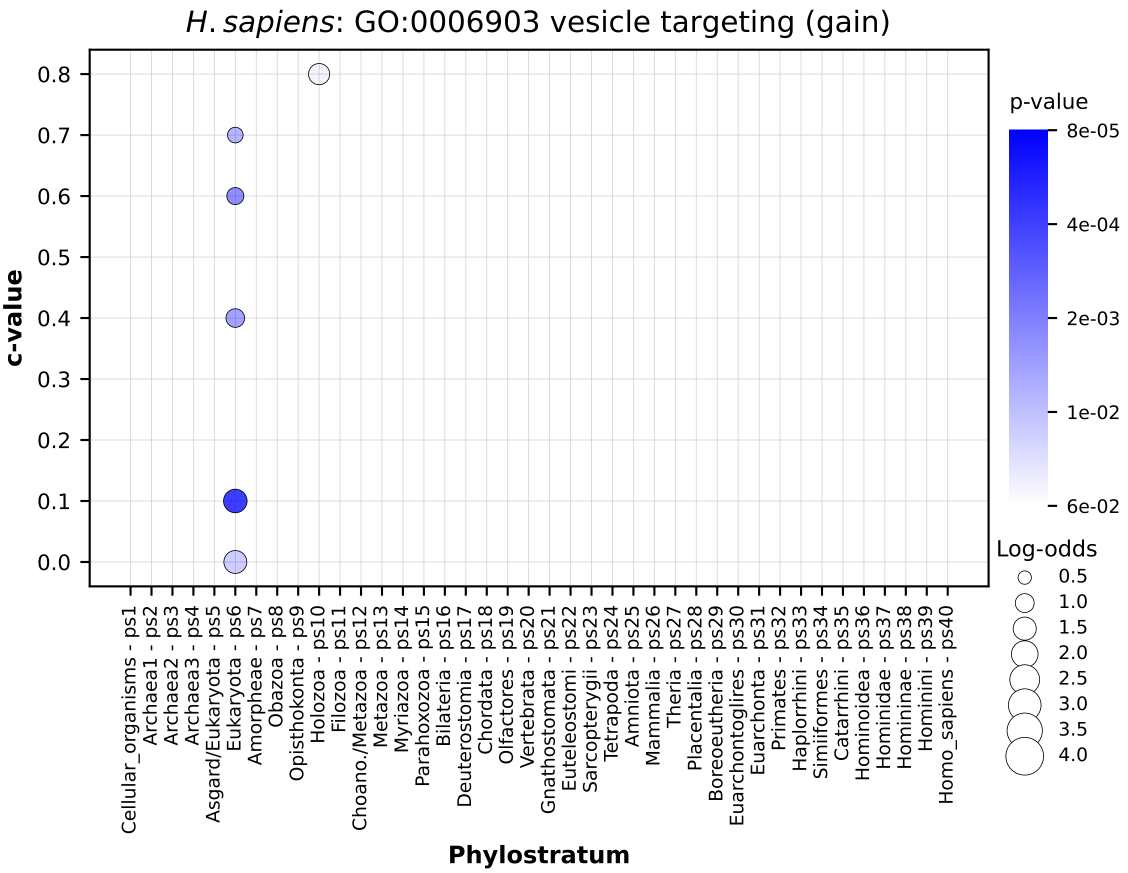
H. sapiens: GO:0006903 vesicle targeting (gain)
c-value
0.0
0.1
0.2
0.3
0.4
0.5
0.6
0.7
0.8
Phylostratum
Cellular_organisms - ps1
Archaea1 - ps2
Archaea2 - ps3
Archaea3 - ps4
Asgard/Eukaryota - ps5
Eukaryota - ps6
Amorpheae - ps7
Obazoa - ps8
Opisthokonta - ps9
Holozoa - ps10
Filozoa - ps11
Choano./Metazoa - ps12
Metazoa - ps13
Myriazoa - ps14
Parahoxozoa - ps15
Bilateria - ps16
Deuterostomia - ps17
Chordata - ps18
Olfactores - ps19
Vertebrata - ps20
Gnathostomata - ps21
Euteleostomi - ps22
Sarcopterygii - ps23
Tetrapoda - ps24
Amniota - ps25
Mammalia - ps26
Theria - ps27
Placentalia - ps28
Boreoeutheria - ps29
Euarchontoglires - ps30
Euarchonta - ps31
Primates - ps32
Haplorrhini - ps33
Simiiformes - ps34
Catarrhini - ps35
Hominoidea - ps36
Hominidae - ps37
Homininae - ps38
Hominini - ps39
Homo_sapiens - ps40
p-value
8e-05
4e-04
2e-03
1e-02
6e-02
Log-odds
0.5
1.0
1.5
2.0
2.5
3.0
3.5
4.0

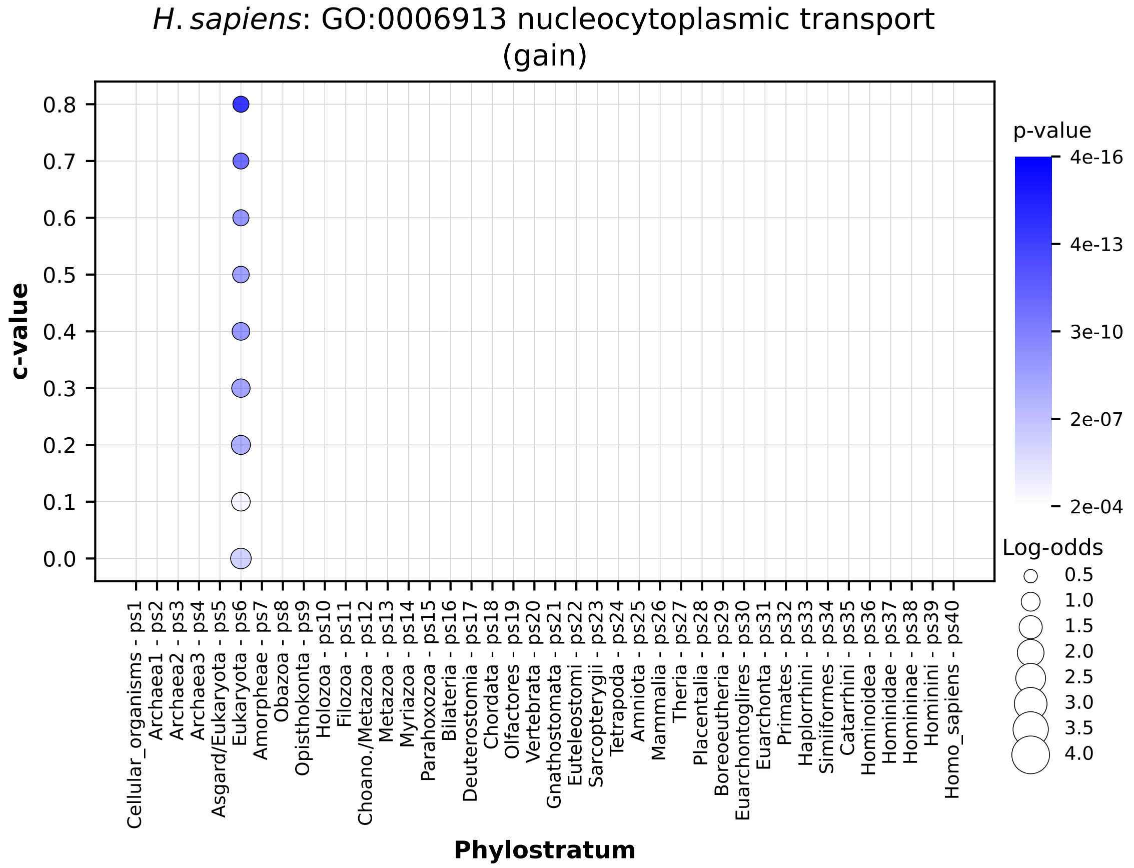
H. sapiens: GO:0006913 nucleocytoplasmic transport
(gain)
c-value
0.8
0.7
0.6
0.5
0.4
0.3
0.2
0.1
0.0
p-value
4e-16
4e-13
3e-10
2e-07
2e-04
Log-odds
0.5
1.0
1.5
2.0
2.5
3.0
3.5
4.0
Cellular_organisms - ps1
Archaea1 - ps2
Archaea2 - ps3
Archaea3 - ps4
Asgard/Eukaryota - ps5
Eukaryota - ps6
Amorpheae - ps7
Obazoa - ps8
Opisthokonta - ps9
Holozoa - ps10
Filozoa - ps11
Choano./Metazoa - ps12
Metazoa - ps13
Myriazoa - ps14
Parahoxozoa - ps15
Bilateria - ps16
Deuterostomia - ps17
Chordata - ps18
Olfactores - ps19
Vertebrata - ps20
Gnathostomata - ps21
Euteleostomi - ps22
Sarcopterygii - ps23
Tetrapoda - ps24
Amniota - ps25
Mammalia - ps26
Theria - ps27
Placentalia - ps28
Boreoeutheria - ps29
Euarchontoglires - ps30
Euarchonta - ps31
Primates - ps32
Haplorrhini - ps33
Simiiformes - ps34
Catarrhini - ps35
Hominoidea - ps36
Hominidae - ps37
Homininae - ps38
Hominini - ps39
Homo_sapiens - ps40
Phylostratum

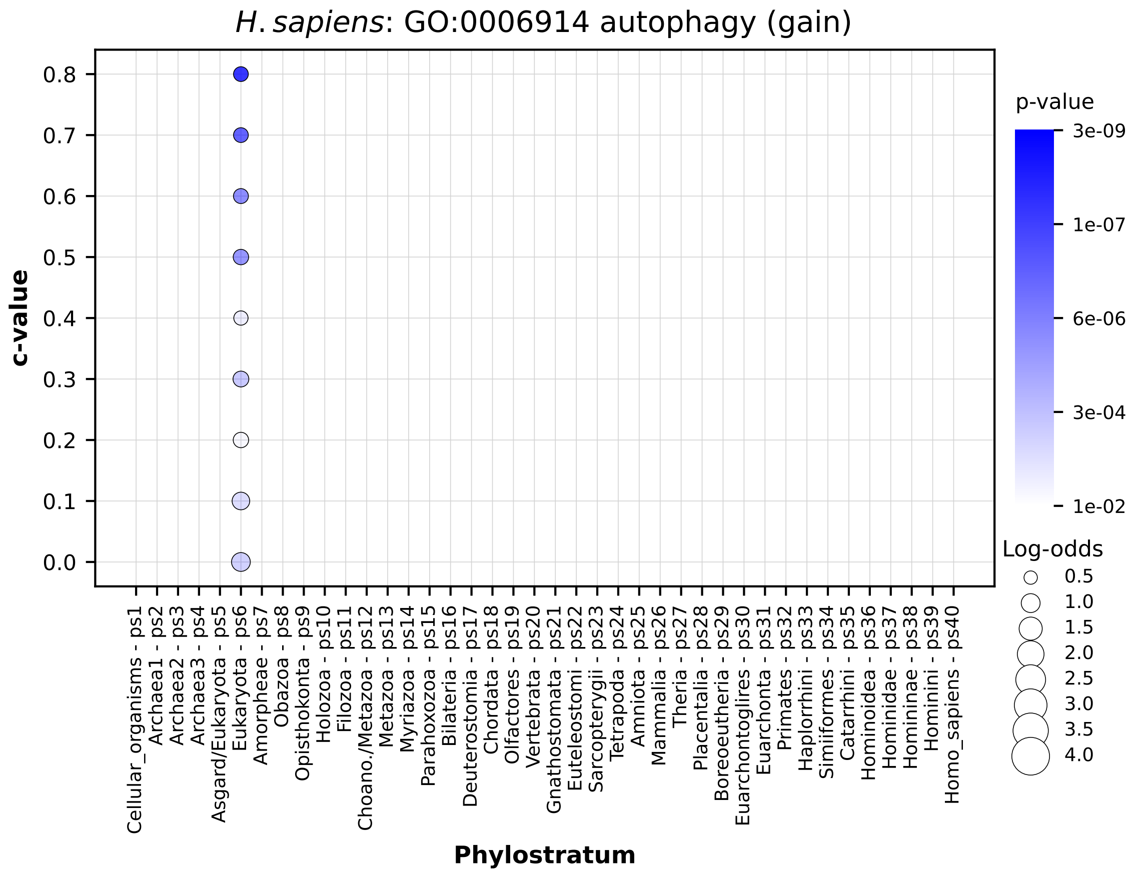
H. sapiens: GO:0006914 autophagy (gain)
c-value
0.8
0.7
0.6
0.5
0.4
0.3
0.2
0.1
0.0
p-value
3e-09
1e-07
6e-06
3e-04
1e-02
Log-odds
0.5
1.0
1.5
2.0
2.5
3.0
3.5
4.0
Cellular_organisms - ps1
Archaea1 - ps2
Archaea2 - ps3
Archaea3 - ps4
Asgard/Eukaryota - ps5
Eukaryota - ps6
Amorpheae - ps7
Obazoa - ps8
Opisthokonta - ps9
Holozoa - ps10
Filozoa - ps11
Choano./Metazoa - ps12
Metazoa - ps13
Myriazoa - ps14
Parahoxozoa - ps15
Bilateria - ps16
Deuterostomia - ps17
Chordata - ps18
Olfactores - ps19
Vertebrata - ps20
Gnathostomata - ps21
Euteleostomi - ps22
Sarcopterygii - ps23
Tetrapoda - ps24
Amniota - ps25
Mammalia - ps26
Theria - ps27
Placentalia - ps28
Boreoeutheria - ps29
Euarchontoglires - ps30
Euarchonta - ps31
Primates - ps32
Haplorrhini - ps33
Simiiformes - ps34
Catarrhini - ps35
Hominoidea - ps36
Hominidae - ps37
Homininae - ps38
Hominini - ps39
Homo_sapiens - ps40
Phylostratum

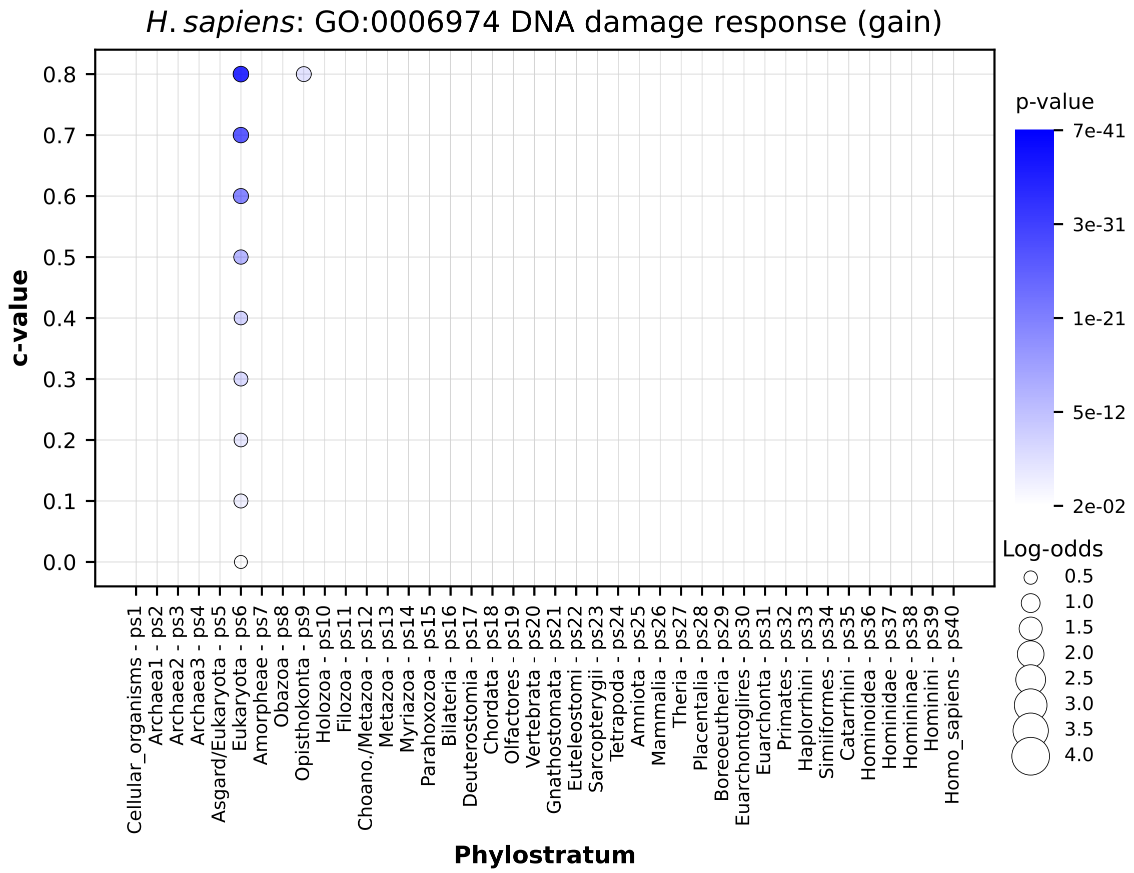

H. sapiens: GO:0006974 DNA damage response (gain)
c-value
0.8
0.7
0.6
0.5
0.4
0.3
0.2
0.1
0.0
p-value
7e-41
3e-31
1e-21
5e-12
2e-02
Log-odds
0.5
1.0
1.5
2.0
2.5
3.0
3.5
4.0
Cellular_organisms - ps1
Archaea1 - ps2
Archaea2 - ps3
Archaea3 - ps4
Asgard/Eukaryota - ps5
Eukaryota - ps6
Amorpheae - ps7
Obazoa - ps8
Opisthokonta - ps9
Holozoa - ps10
Filozoa - ps11
Choano./Metazoa - ps12
Metazoa - ps13
Myriazoa - ps14
Parahoxozoa - ps15
Bilateria - ps16
Deuterostomia - ps17
Chordata - ps18
Olfactores - ps19
Vertebrata - ps20
Gnathostomata - ps21
Euteleostomi - ps22
Sarcopterygii - ps23
Tetrapoda - ps24
Amniota - ps25
Mammalia - ps26
Theria - ps27
Placentalia - ps28
Boreoeutheria - ps29
Euarchontoglires - ps30
Euarchonta - ps31
Primates - ps32
Haplorrhini - ps33
Simiiformes - ps34
Catarrhini - ps35
Hominoidea - ps36
Hominidae - ps37
Homininae - ps38
Hominini - ps39
Homo_sapiens - ps40
Phylostratum

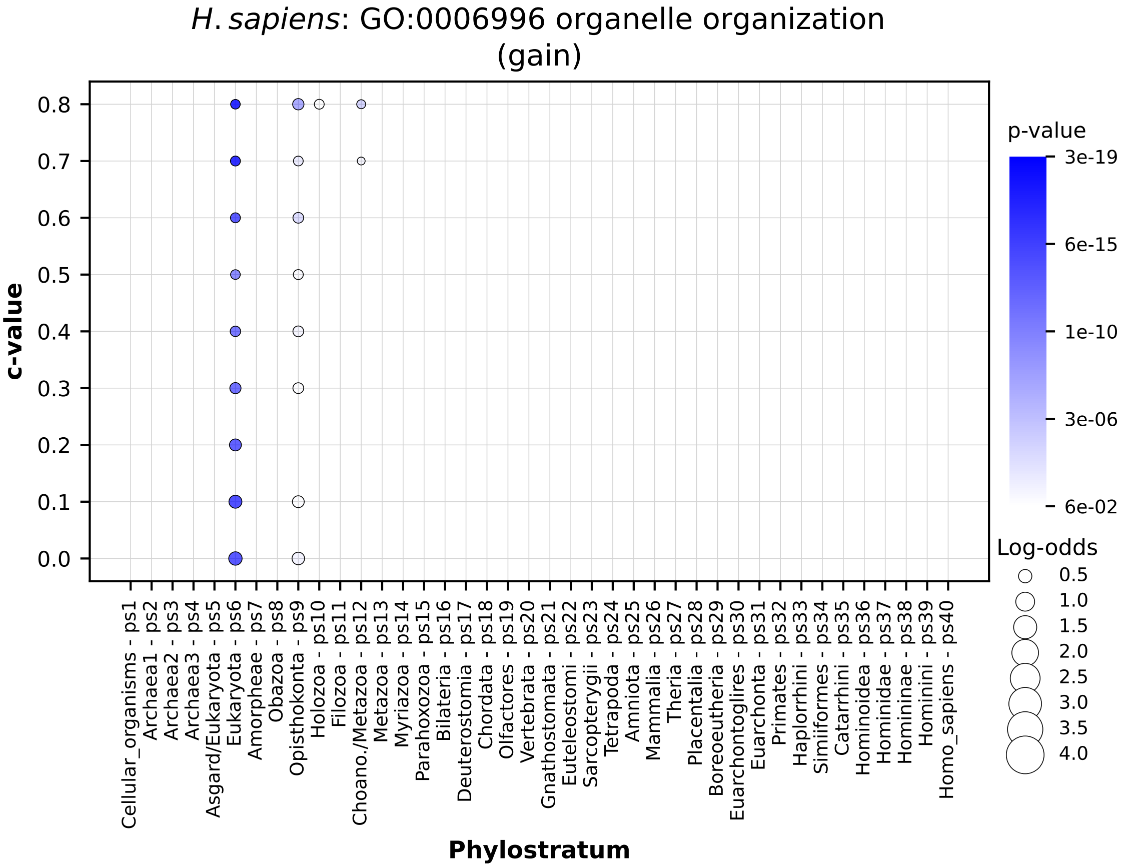

*H. sapiens*: GO:0006996 organelle organization
(gain)
c-value
0.8
0.7
0.6
0.5
0.4
0.3
0.2
0.1
0.0
Phylostratum
Cellular_organisms - ps1
Archaea1 - ps2
Archaea2 - ps3
Archaea3 - ps4
Asgard/Eukaryota - ps5
Eukaryota - ps6
Amorpheae - ps7
Obazoa - ps8
Opisthokonta - ps9
Holozoa - ps10
Filozoa - ps11
Choano./Metazoa - ps12
Metazoa - ps13
Myriazoa - ps14
Parahoxozoa - ps15
Bilateria - ps16
Deuterostomia - ps17
Chordata - ps18
Olfactores - ps19
Vertebrata - ps20
Gnathostomata - ps21
Euteleostomi - ps22
Sarcopterygii - ps23
Tetrapoda - ps24
Amniota - ps25
Mammalia - ps26
Theria - ps27
Placentalia - ps28
Boreoeutheria - ps29
Euarchontoglires - ps30
Euarchonta - ps31
Primates - ps32
Haplorrhini - ps33
Simiiformes - ps34
Catarrhini - ps35
Hominoidea - ps36
Hominidae - ps37
Homininae - ps38
Hominini - ps39
Homo_sapiens - ps40
p-value
3e-19
6e-15
1e-10
3e-06
6e-02
Log-odds
0.5
1.0
1.5
2.0
2.5
3.0
3.5
4.0

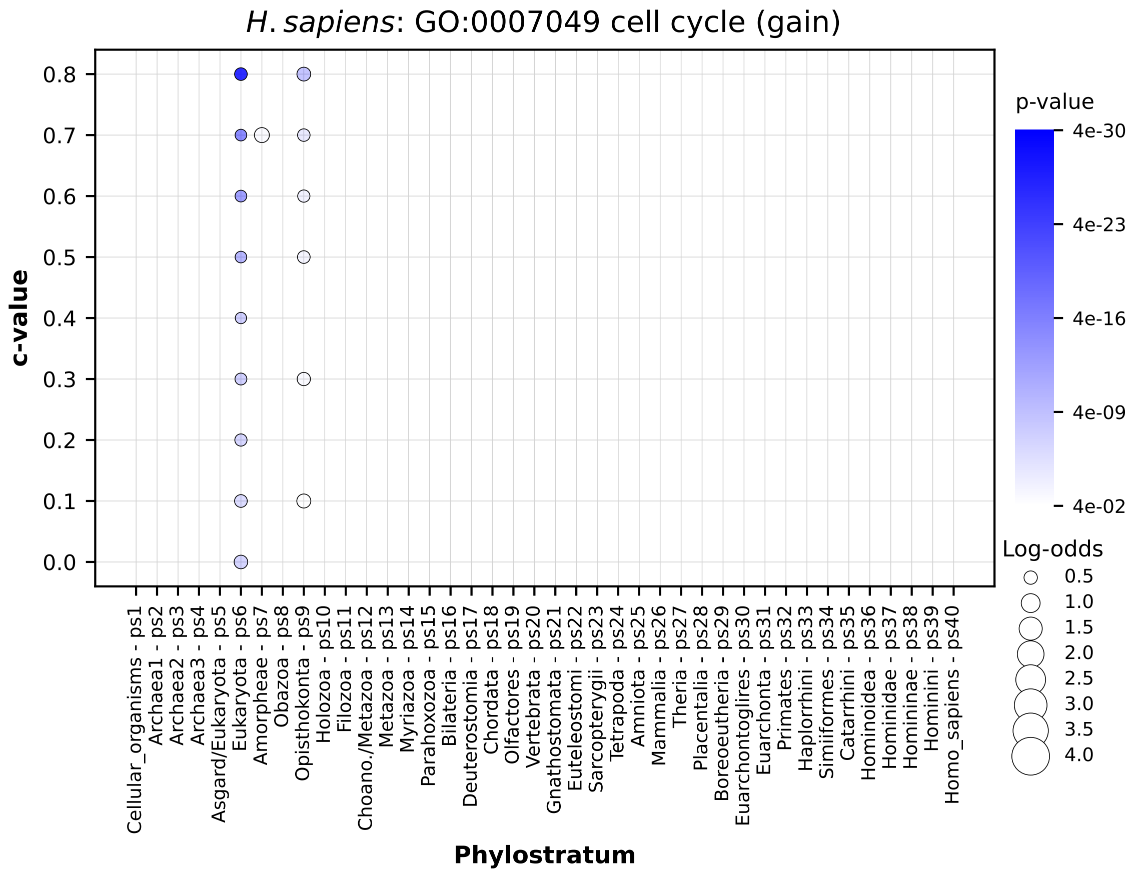

H. sapiens: GO:0007049 cell cycle (gain)
c-value
0.8
0.7
0.6
0.5
0.4
0.3
0.2
0.1
0.0
Phylostratum
Cellular_organisms - ps1
Archaea1 - ps2
Archaea2 - ps3
Archaea3 - ps4
Asgard/Eukaryota - ps5
Eukaryota - ps6
Amorpheae - ps7
Obazoa - ps8
Opisthokonta - ps9
Holozoa - ps10
Filozoa - ps11
Choano./Metazoa - ps12
Metazoa - ps13
Myriazoa - ps14
Parahoxozoa - ps15
Bilateria - ps16
Deuterostomia - ps17
Chordata - ps18
Olfactores - ps19
Vertebrata - ps20
Gnathostomata - ps21
Euteleostomi - ps22
Sarcopterygii - ps23
Tetrapoda - ps24
Amniota - ps25
Mammalia - ps26
Theria - ps27
Placentalia - ps28
Boreoeutheria - ps29
Euarchontoglires - ps30
Euarchonta - ps31
Primates - ps32
Haplorrhini - ps33
Simiiformes - ps34
Catarrhini - ps35
Hominoidea - ps36
Hominidae - ps37
Homininae - ps38
Hominini - ps39
Homo_sapiens - ps40
p-value
4e-30
4e-23
4e-16
4e-09
4e-02
Log-odds
0.5
1.0
1.5
2.0
2.5
3.0
3.5
4.0

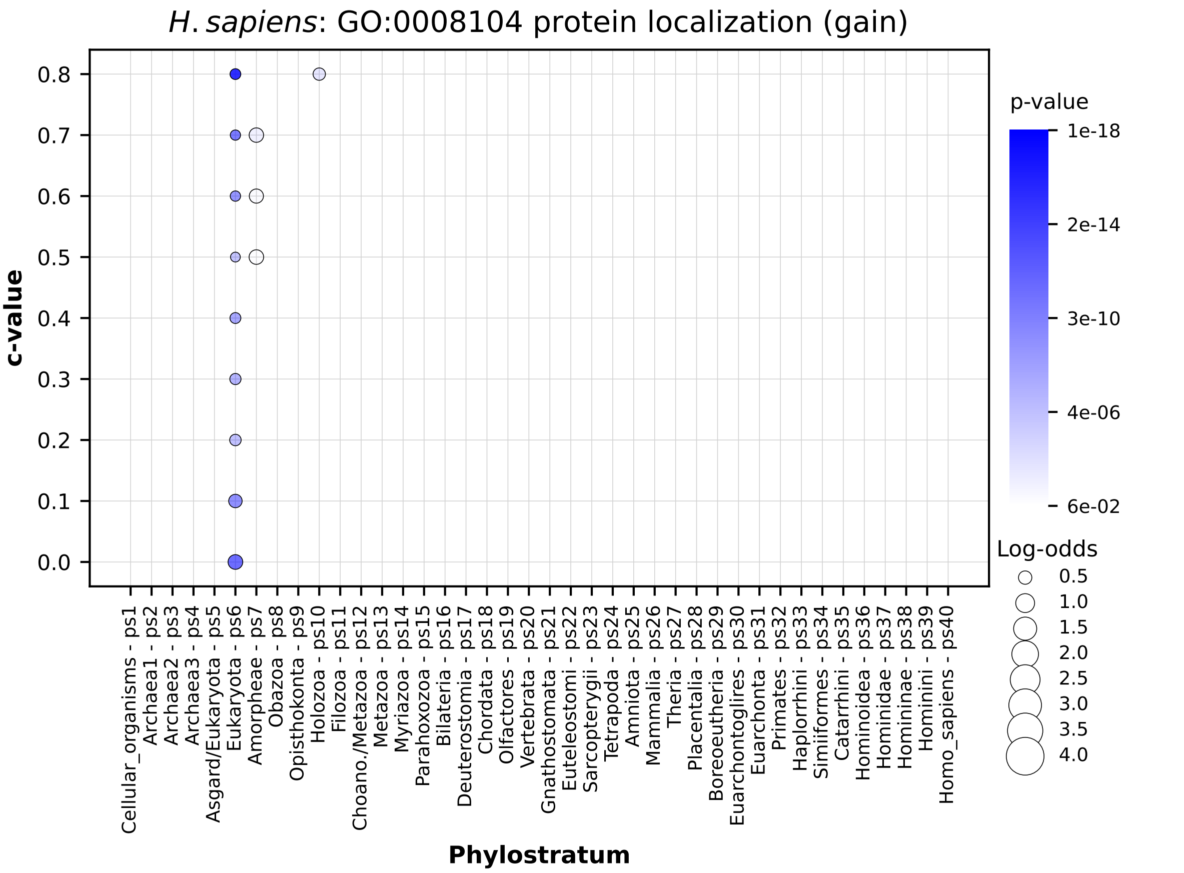

H. sapiens: GO:0008104 protein localization (gain)
c-value
0.0
0.1
0.2
0.3
0.4
0.5
0.6
0.7
0.8
p-value
1e-18
2e-14
3e-10
4e-06
6e-02
Log-odds
0.5
1.0
1.5
2.0
2.5
3.0
3.5
4.0
Cellular_organisms - ps1
Archaea1 - ps2
Archaea2 - ps3
Archaea3 - ps4
Asgard/Eukaryota - ps5
Eukaryota - ps6
Amorpheae - ps7
Obazoa - ps8
Opisthokonta - ps9
Holozoa - ps10
Filozoa - ps11
Choano./Metazoa - ps12
Metazoa - ps13
Myriazoa - ps14
Parahoxozoa - ps15
Bilateria - ps16
Deuterostomia - ps17
Chordata - ps18
Olfactores - ps19
Vertebrata - ps20
Gnathostomata - ps21
Euteleostomi - ps22
Sarcopterygii - ps23
Tetrapoda - ps24
Amniota - ps25
Mammalia - ps26
Theria - ps27
Placentalia - ps28
Boreoeutheria - ps29
Euarchontoglires - ps30
Euarchonta - ps31
Primates - ps32
Haplorrhini - ps33
Simiiformes - ps34
Catarrhini - ps35
Hominoidea - ps36
Hominidae - ps37
Homininae - ps38
Hominini - ps39
Homo_sapiens - ps40
Phylostratum

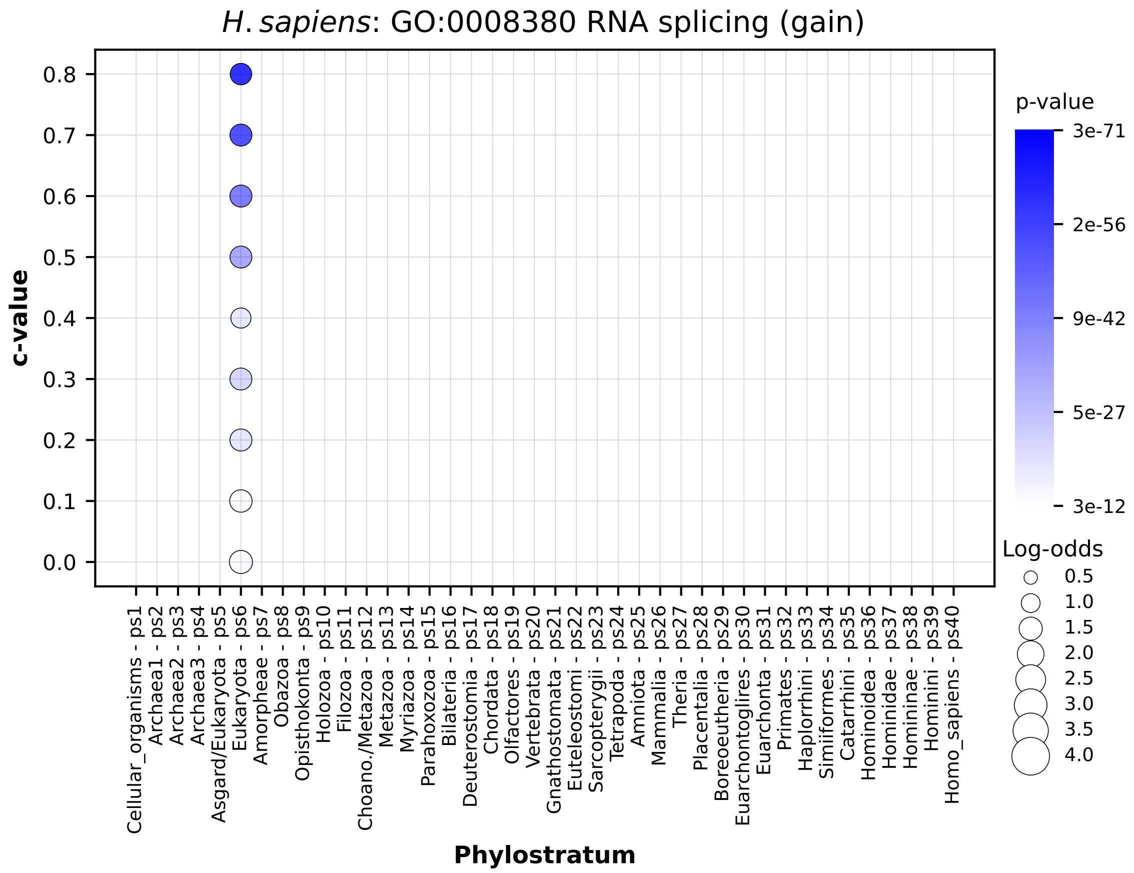

H. sapiens: GO:0008380 RNA splicing (gain)
c-value
0.0
0.1
0.2
0.3
0.4
0.5
0.6
0.7
0.8
Phylostratum
Cellular_organisms - ps1
Archaea1 - ps2
Archaea2 - ps3
Archaea3 - ps4
Asgard/Eukaryota - ps5
Eukaryota - ps6
Amorpheae - ps7
Obazoa - ps8
Opisthokonta - ps9
Holozoa - ps10
Filozoa - ps11
Choano./Metazoa - ps12
Metazoa - ps13
Myriazoa - ps14
Parahoxozoa - ps15
Bilateria - ps16
Deuterostomia - ps17
Chordata - ps18
Olfactores - ps19
Vertebrata - ps20
Gnathostomata - ps21
Euteleostomi - ps22
Sarcopterygii - ps23
Tetrapoda - ps24
Amniota - ps25
Mammalia - ps26
Theria - ps27
Placentalia - ps28
Boreoeutheria - ps29
Euarchontoglires - ps30
Euarchonta - ps31
Primates - ps32
Haplorrhini - ps33
Simiiformes - ps34
Catarrhini - ps35
Hominoidea - ps36
Hominidae - ps37
Homininae - ps38
Hominini - ps39
Homo_sapiens - ps40
p-value
3e-71
2e-56
9e-42
5e-27
3e-12
Log-odds
0.5
1.0
1.5
2.0
2.5
3.0
3.5
4.0

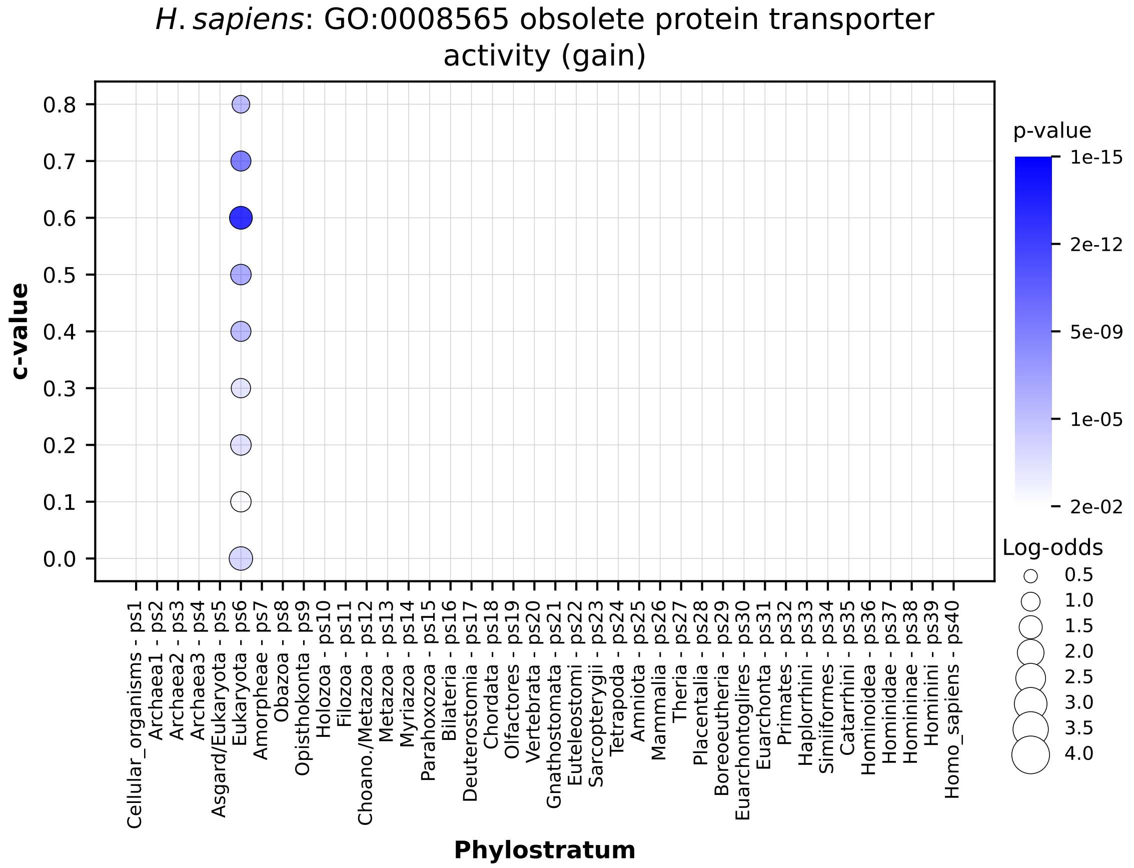

*H. sapiens*: GO:0008565 obsolete protein transporter activity (gain)
c-value
0.0
0.1
0.2
0.3
0.4
0.5
0.6
0.7
0.8
Phylostratum
Cellular_organisms - ps1
Archaea1 - ps2
Archaea2 - ps3
Archaea3 - ps4
Asgard/Eukaryota - ps5
Eukaryota - ps6
Amorpheae - ps7
Obazoa - ps8
Opisthokonta - ps9
Holozoa - ps10
Filozoa - ps11
Choano./Metazoa - ps12
Metazoa - ps13
Myriazoa - ps14
Parahoxozoa - ps15
Bilateria - ps16
Deuterostomia - ps17
Chordata - ps18
Olfactores - ps19
Vertebrata - ps20
Gnathostomata - ps21
Euteleostomi - ps22
Sarcopterygii - ps23
Tetrapoda - ps24
Amniota - ps25
Mammalia - ps26
Theria - ps27
Placentalia - ps28
Boreoeutheria - ps29
Euarchontoglires - ps30
Euarchonta - ps31
Primates - ps32
Haplorrhini - ps33
Simiiformes - ps34
Catarrhini - ps35
Hominoidea - ps36
Hominidae - ps37
Homininae - ps38
Hominini - ps39
Homo_sapiens - ps40
p-value
1e-15
2e-12
5e-09
1e-05
2e-02
Log-odds
0.5
1.0
1.5
2.0
2.5
3.0
3.5
4.0

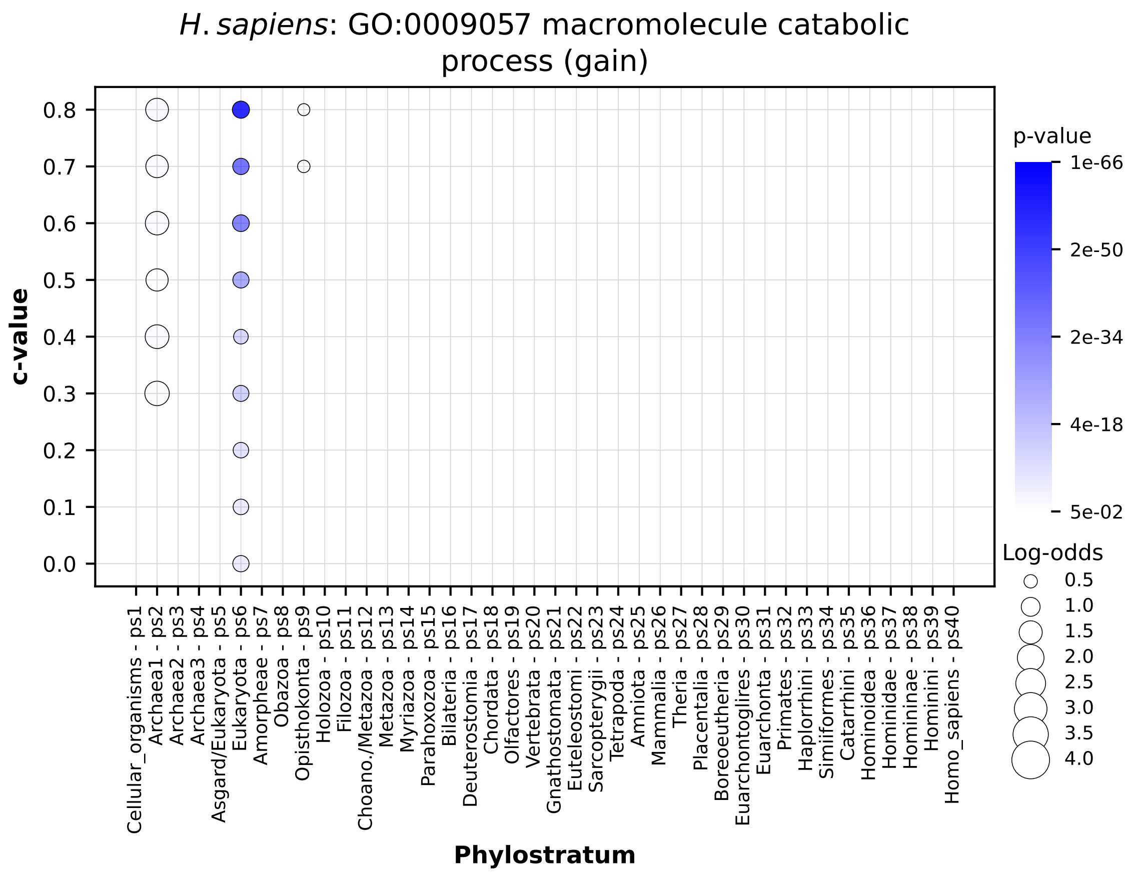

H. sapiens: GO:0009057 macromolecule catabolic process (gain)
c-value
0.0
0.1
0.2
0.3
0.4
0.5
0.6
0.7
0.8
Phylostratum
Cellular_organisms - ps1
Archaea1 - ps2
Archaea2 - ps3
Archaea3 - ps4
Asgard/Eukaryota - ps5
Eukaryota - ps6
Amorpheae - ps7
Obazoa - ps8
Opisthokonta - ps9
Holozoa - ps10
Filozoa - ps11
Choano./Metazoa - ps12
Metazoa - ps13
Myriazoa - ps14
Parahoxozoa - ps15
Bilateria - ps16
Deuterostomia - ps17
Chordata - ps18
Olfactores - ps19
Vertebrata - ps20
Gnathostomata - ps21
Euteleostomi - ps22
Sarcopterygii - ps23
Tetrapoda - ps24
Amniota - ps25
Mammalia - ps26
Theria - ps27
Placentalia - ps28
Boreoeutheria - ps29
Euarchontoglires - ps30
Euarchonta - ps31
Primates - ps32
Haplorrhini - ps33
Simiiformes - ps34
Catarrhini - ps35
Hominoidea - ps36
Hominidae - ps37
Homininae - ps38
Hominini - ps39
Homo_sapiens - ps40
p-value
1e-66
2e-50
2e-34
4e-18
5e-02
Log-odds
0.5
1.0
1.5
2.0
2.5
3.0
3.5
4.0

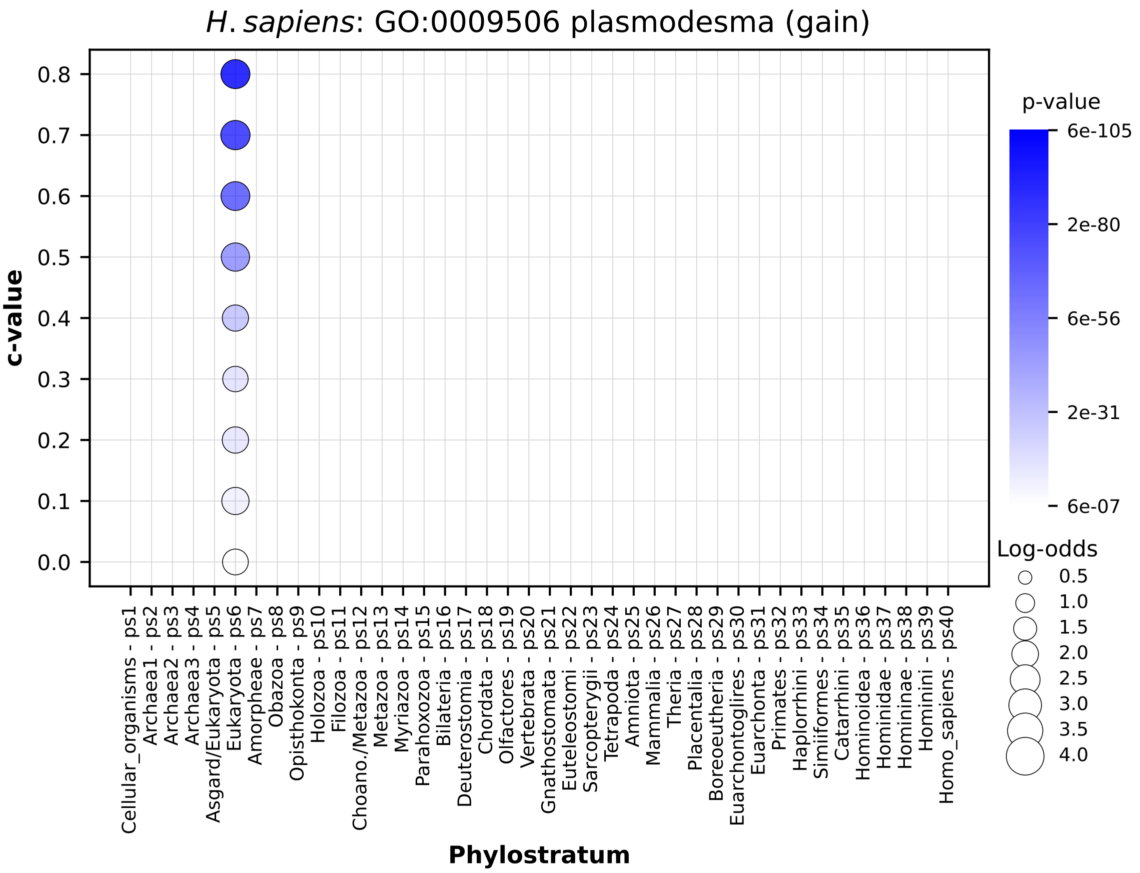

H. sapiens: GO:0009506 plasmodesma (gain)
c-value
0.0
0.1
0.2
0.3
0.4
0.5
0.6
0.7
0.8
Phylostratum
Cellular_organisms - ps1
Archaea1 - ps2
Archaea2 - ps3
Archaea3 - ps4
Asgard/Eukaryota - ps5
Eukaryota - ps6
Amorpheae - ps7
Obazoa - ps8
Opisthokonta - ps9
Holozoa - ps10
Filozoa - ps11
Choano./Metazoa - ps12
Metazoa - ps13
Myriazoa - ps14
Parahoxozoa - ps15
Bilateria - ps16
Deuterostomia - ps17
Chordata - ps18
Olfactores - ps19
Vertebrata - ps20
Gnathostomata - ps21
Euteleostomi - ps22
Sarcopterygii - ps23
Tetrapoda - ps24
Amniota - ps25
Mammalia - ps26
Theria - ps27
Placentalia - ps28
Boreoeutheria - ps29
Euarchontoglires - ps30
Euarchonta - ps31
Primates - ps32
Haplorrhini - ps33
Simiiformes - ps34
Catarrhini - ps35
Hominoidea - ps36
Hominidae - ps37
Homininae - ps38
Hominini - ps39
Homo_sapiens - ps40
p-value
6e-105
2e-80
6e-56
2e-31
6e-07
Log-odds
0.5
1.0
1.5
2.0
2.5
3.0
3.5
4.0

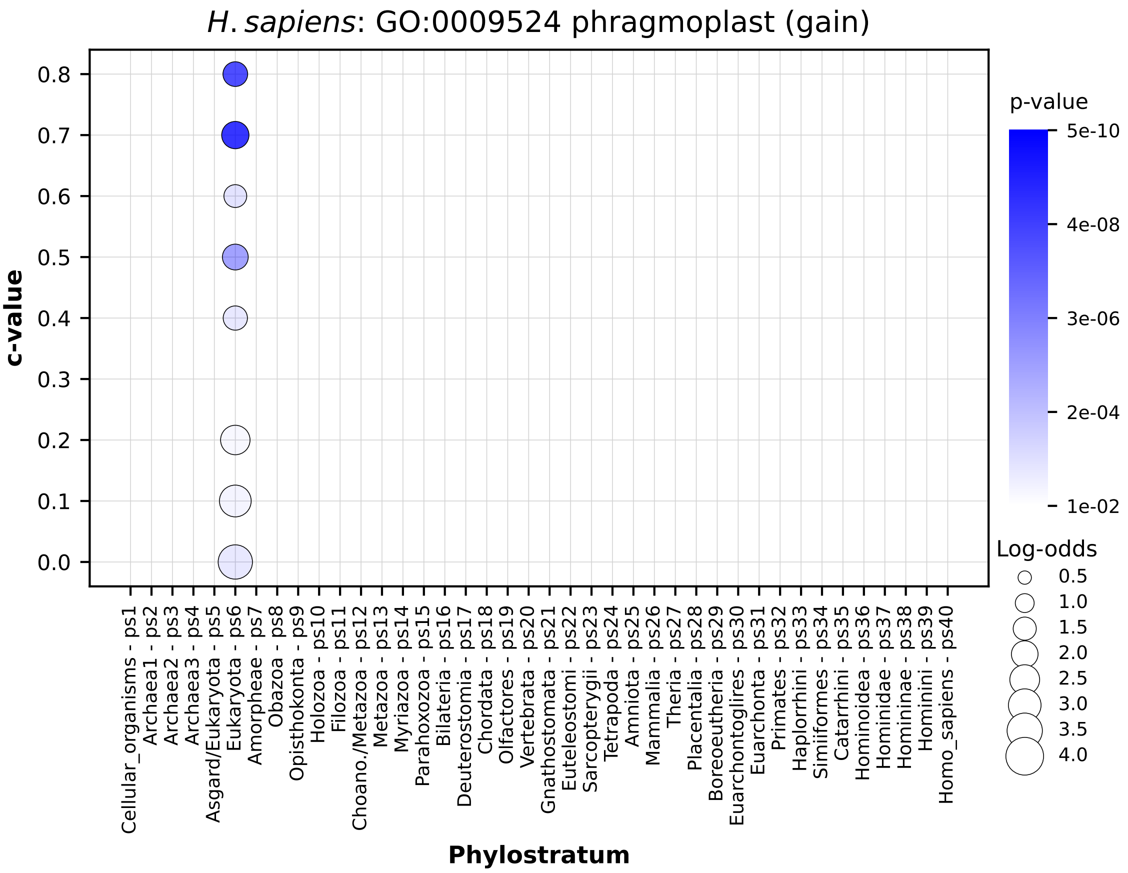

H. sapiens: GO:0009524 phragmoplast (gain)
c-value
0.0
0.1
0.2
0.3
0.4
0.5
0.6
0.7
0.8
Phylostratum
Cellular_organisms - ps1
Archaea1 - ps2
Archaea2 - ps3
Archaea3 - ps4
Asgard/Eukaryota - ps5
Eukaryota - ps6
Amorpheae - ps7
Obazoa - ps8
Opisthokonta - ps9
Holozoa - ps10
Filozoa - ps11
Choano./Metazoa - ps12
Metazoa - ps13
Myriazoa - ps14
Parahoxozoa - ps15
Bilateria - ps16
Deuterostomia - ps17
Chordata - ps18
Olfactores - ps19
Vertebrata - ps20
Gnathostomata - ps21
Euteleostomi - ps22
Sarcopterygii - ps23
Tetrapoda - ps24
Amniota - ps25
Mammalia - ps26
Theria - ps27
Placentalia - ps28
Boreoeutheria - ps29
Euarchontoglires - ps30
Euarchonta - ps31
Primates - ps32
Haplorrhini - ps33
Simiiformes - ps34
Catarrhini - ps35
Hominoidea - ps36
Hominidae - ps37
Homininae - ps38
Hominini - ps39
Homo_sapiens - ps40
p-value
5e-10
4e-08
3e-06
2e-04
1e-02
Log-odds
0.5
1.0
1.5
2.0
2.5
3.0
3.5
4.0

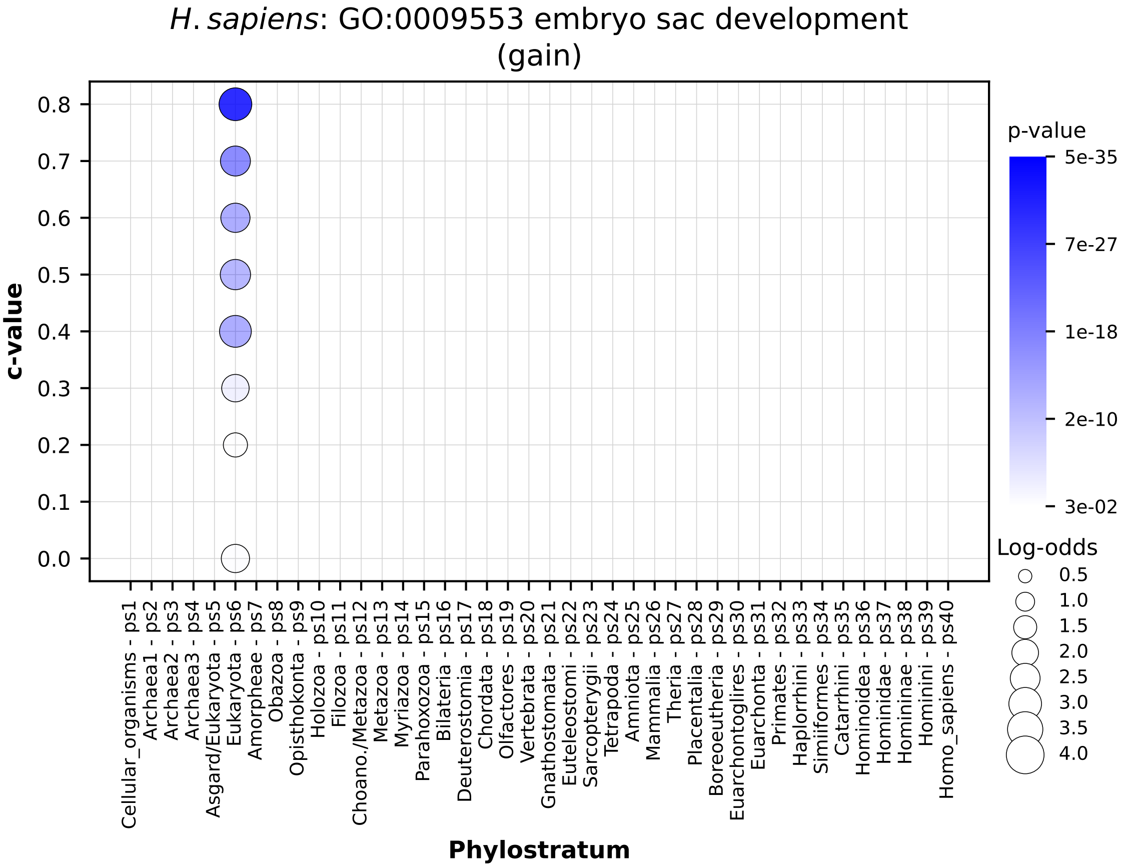

H. sapiens: GO:0009553 embryo sac development
(gain)
c-value
0.0
0.1
0.2
0.3
0.4
0.5
0.6
0.7
0.8
p-value
5e-35
7e-27
1e-18
2e-10
3e-02
Log-odds
0.5
1.0
1.5
2.0
2.5
3.0
3.5
4.0
Cellular_organisms - ps1
Archaea1 - ps2
Archaea2 - ps3
Archaea3 - ps4
Asgard/Eukaryota - ps5
Eukaryota - ps6
Amorpheae - ps7
Obazoa - ps8
Opisthokonta - ps9
Holozoa - ps10
Filozoa - ps11
Choano./Metazoa - ps12
Metazoa - ps13
Myriazoa - ps14
Parahoxozoa - ps15
Bilateria - ps16
Deuterostomia - ps17
Chordata - ps18
Olfactores - ps19
Vertebrata - ps20
Gnathostomata - ps21
Euteleostomi - ps22
Sarcopterygii - ps23
Tetrapoda - ps24
Amniota - ps25
Mammalia - ps26
Theria - ps27
Placentalia - ps28
Boreoeutheria - ps29
Euarchontoglires - ps30
Euarchonta - ps31
Primates - ps32
Haplorrhini - ps33
Simiiformes - ps34
Catarrhini - ps35
Hominoidea - ps36
Hominidae - ps37
Homininae - ps38
Hominini - ps39
Homo_sapiens - ps40
Phylostratum

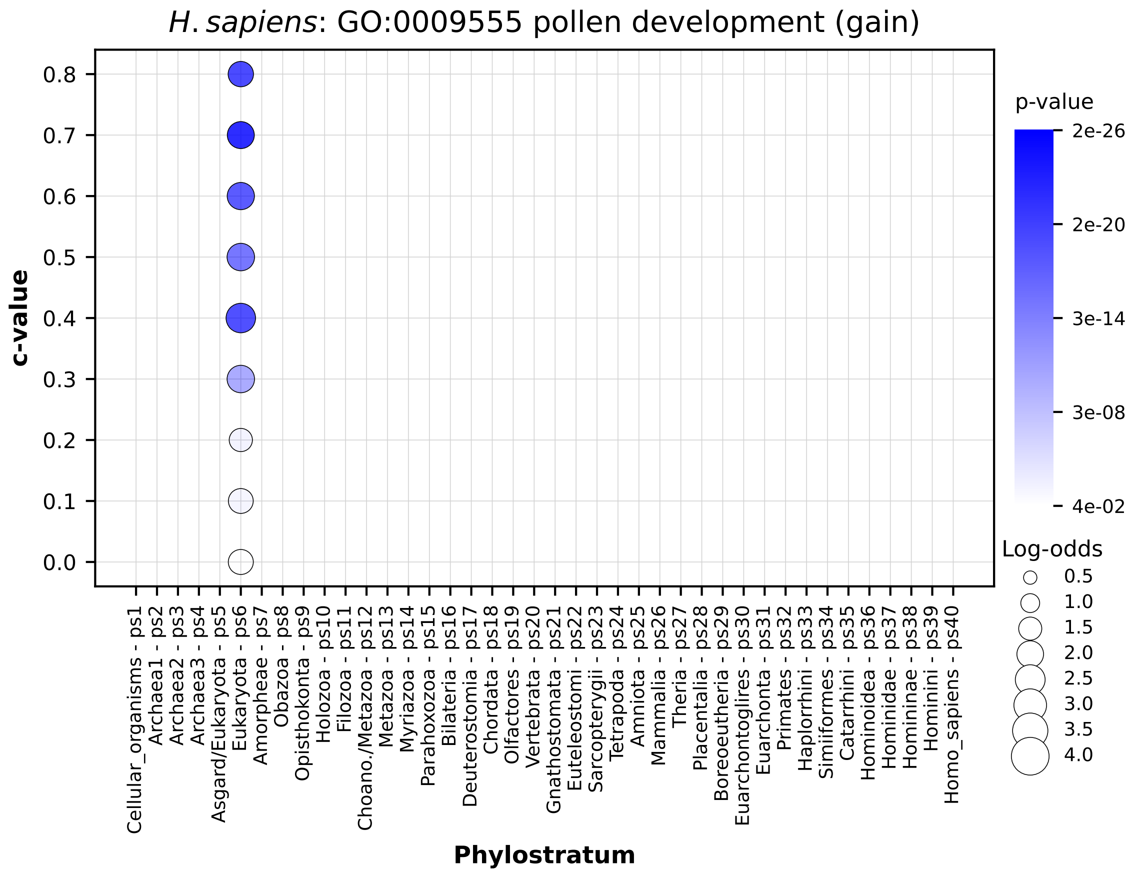
H. sapiens: GO:0009555 pollen development (gain)
c-value
0.8
0.7
0.6
0.5
0.4
0.3
0.2
0.1
0.0
p-value
2e-26
2e-20
3e-14
3e-08
4e-02
Log-odds
0.5
1.0
1.5
2.0
2.5
3.0
3.5
4.0
Cellular_organisms - ps1
Archaea1 - ps2
Archaea2 - ps3
Archaea3 - ps4
Asgard/Eukaryota - ps5
Eukaryota - ps6
Amorpheae - ps7
Obazoa - ps8
Opisthokonta - ps9
Holozoa - ps10
Filozoa - ps11
Choano./Metazoa - ps12
Metazoa - ps13
Myriazoa - ps14
Parahoxozoa - ps15
Bilateria - ps16
Deuterostomia - ps17
Chordata - ps18
Olfactores - ps19
Vertebrata - ps20
Gnathostomata - ps21
Euteleostomi - ps22
Sarcopterygii - ps23
Tetrapoda - ps24
Amniota - ps25
Mammalia - ps26
Theria - ps27
Placentalia - ps28
Boreoeutheria - ps29
Euarchontoglires - ps30
Euarchonta - ps31
Primates - ps32
Haplorrhini - ps33
Simiiformes - ps34
Catarrhini - ps35
Hominoidea - ps36
Hominidae - ps37
Homininae - ps38
Hominini - ps39
Homo_sapiens - ps40
Phylostratum

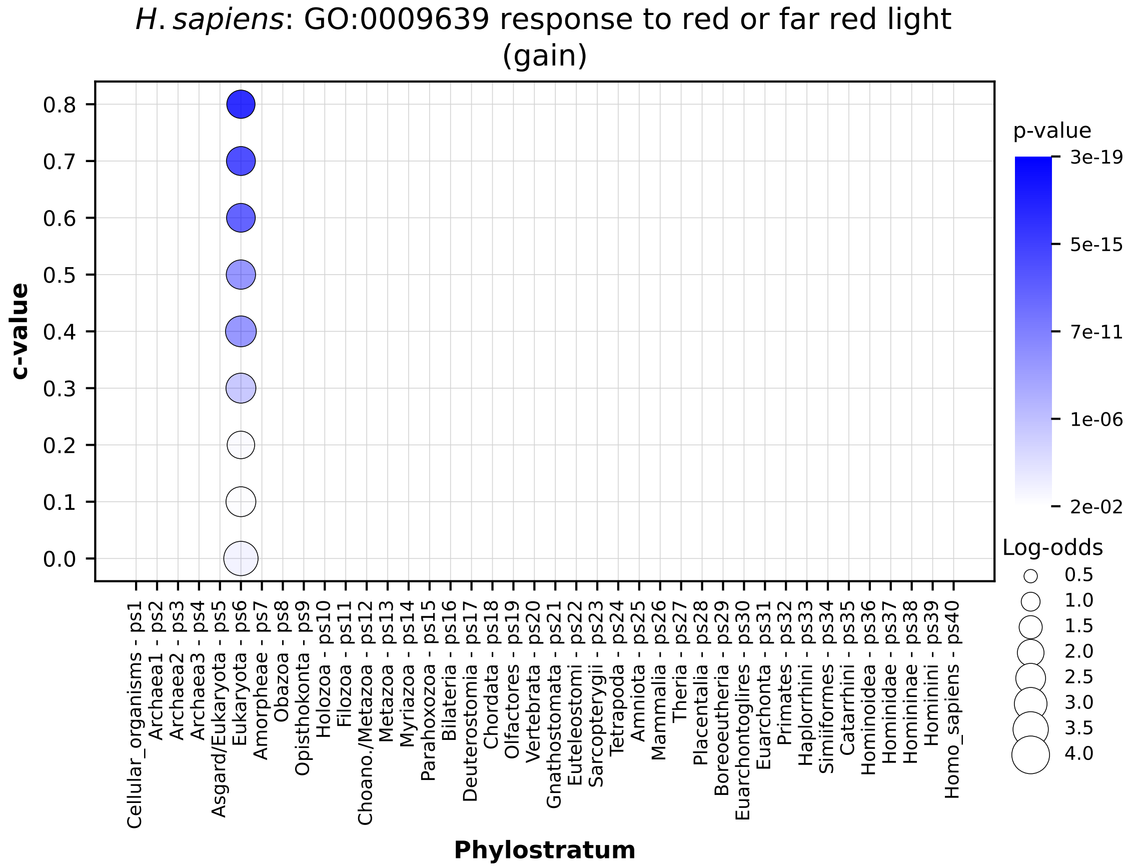
H. sapiens: GO:0009639 response to red or far red light
(gain)
c-value
0.8
0.7
0.6
0.5
0.4
0.3
0.2
0.1
0.0
p-value
3e-19
5e-15
7e-11
1e-06
2e-02
Log-odds
0.5
1.0
1.5
2.0
2.5
3.0
3.5
4.0
Cellular_organisms - ps1
Archaea1 - ps2
Archaea2 - ps3
Archaea3 - ps4
Asgard/Eukaryota - ps5
Eukaryota - ps6
Amorpheae - ps7
Obazoa - ps8
Opisthokonta - ps9
Holozoa - ps10
Filozoa - ps11
Choano./Metazoa - ps12
Metazoa - ps13
Myriazoa - ps14
Parahoxozoa - ps15
Bilateria - ps16
Deuterostomia - ps17
Chordata - ps18
Olfactores - ps19
Vertebrata - ps20
Gnathostomata - ps21
Euteleostomi - ps22
Sarcopterygii - ps23
Tetrapoda - ps24
Amniota - ps25
Mammalia - ps26
Theria - ps27
Placentalia - ps28
Boreoeutheria - ps29
Euarchontoglires - ps30
Euarchonta - ps31
Primates - ps32
Haplorrhini - ps33
Simiiformes - ps34
Catarrhini - ps35
Hominoidea - ps36
Hominidae - ps37
Homininae - ps38
Hominini - ps39
Homo_sapiens - ps40
Phylostratum

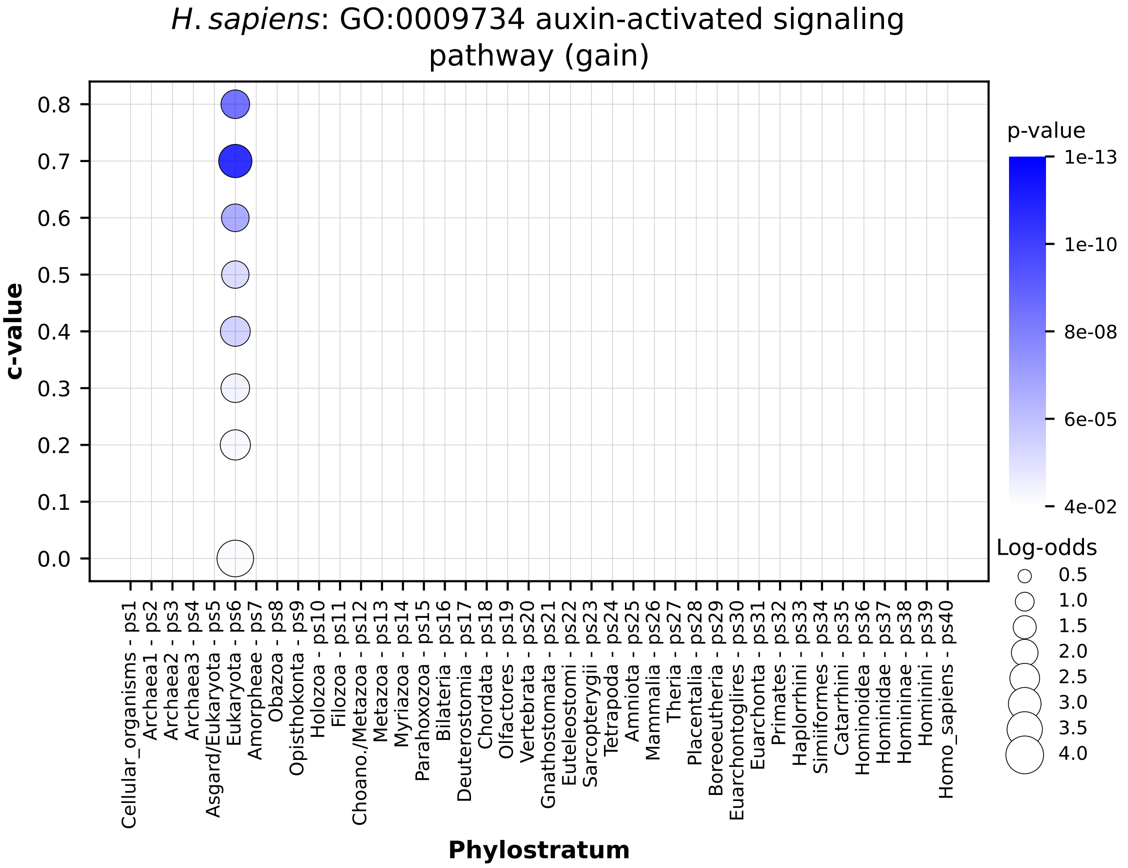

H. sapiens: GO:0009734 auxin-activated signaling pathway (gain)
c-value
0.8
0.7
0.6
0.5
0.4
0.3
0.2
0.1
0.0
Phylostratum
Cellular_organisms - ps1
Archaea1 - ps2
Archaea2 - ps3
Archaea3 - ps4
Asgard/Eukaryota - ps5
Eukaryota - ps6
Amorpheae - ps7
Obazoa - ps8
Opisthokonta - ps9
Holozoa - ps10
Filozoa - ps11
Choano./Metazoa - ps12
Metazoa - ps13
Myriazoa - ps14
Parahoxozoa - ps15
Bilateria - ps16
Deuterostomia - ps17
Chordata - ps18
Olfactores - ps19
Vertebrata - ps20
Gnathostomata - ps21
Euteleostomi - ps22
Sarcopterygii - ps23
Tetrapoda - ps24
Amniota - ps25
Mammalia - ps26
Theria - ps27
Placentalia - ps28
Boreoeutheria - ps29
Euarchontoglires - ps30
Euarchonta - ps31
Primates - ps32
Haplorrhini - ps33
Simiiformes - ps34
Catarrhini - ps35
Hominoidea - ps36
Hominidae - ps37
Homininae - ps38
Hominini - ps39
Homo_sapiens - ps40
p-value
1e-13
1e-10
8e-08
6e-05
4e-02
Log-odds
0.5
1.0
1.5
2.0
2.5
3.0
3.5
4.0

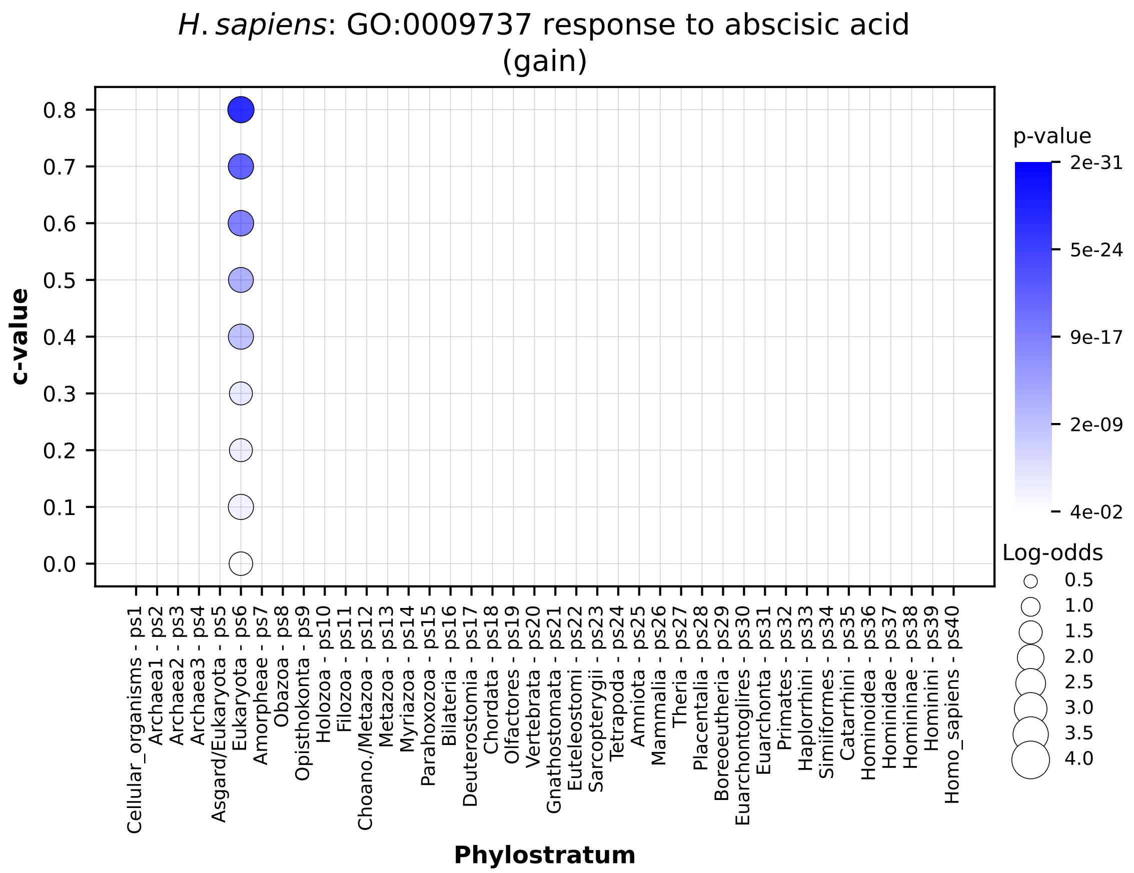

H. sapiens: GO:0009737 response to abscisic acid
(gain)
c-value
0.8
0.7
0.6
0.5
0.4
0.3
0.2
0.1
0.0
Phylostratum
Cellular_organisms - ps1
Archaea1 - ps2
Archaea2 - ps3
Archaea3 - ps4
Asgard/Eukaryota - ps5
Eukaryota - ps6
Amorpheae - ps7
Obazoa - ps8
Opisthokonta - ps9
Holozoa - ps10
Filozoa - ps11
Choano./Metazoa - ps12
Metazoa - ps13
Myriazoa - ps14
Parahoxozoa - ps15
Bilateria - ps16
Deuterostomia - ps17
Chordata - ps18
Olfactores - ps19
Vertebrata - ps20
Gnathostomata - ps21
Euteleostomi - ps22
Sarcopterygii - ps23
Tetrapoda - ps24
Amniota - ps25
Mammalia - ps26
Theria - ps27
Placentalia - ps28
Boreoeutheria - ps29
Euarchontoglires - ps30
Euarchonta - ps31
Primates - ps32
Haplorrhini - ps33
Simiiformes - ps34
Catarrhini - ps35
Hominoidea - ps36
Hominidae - ps37
Homininae - ps38
Hominini - ps39
Homo_sapiens - ps40
p-value
2e-31
5e-24
9e-17
2e-09
4e-02
Log-odds
0.5
1.0
1.5
2.0
2.5
3.0
3.5
4.0

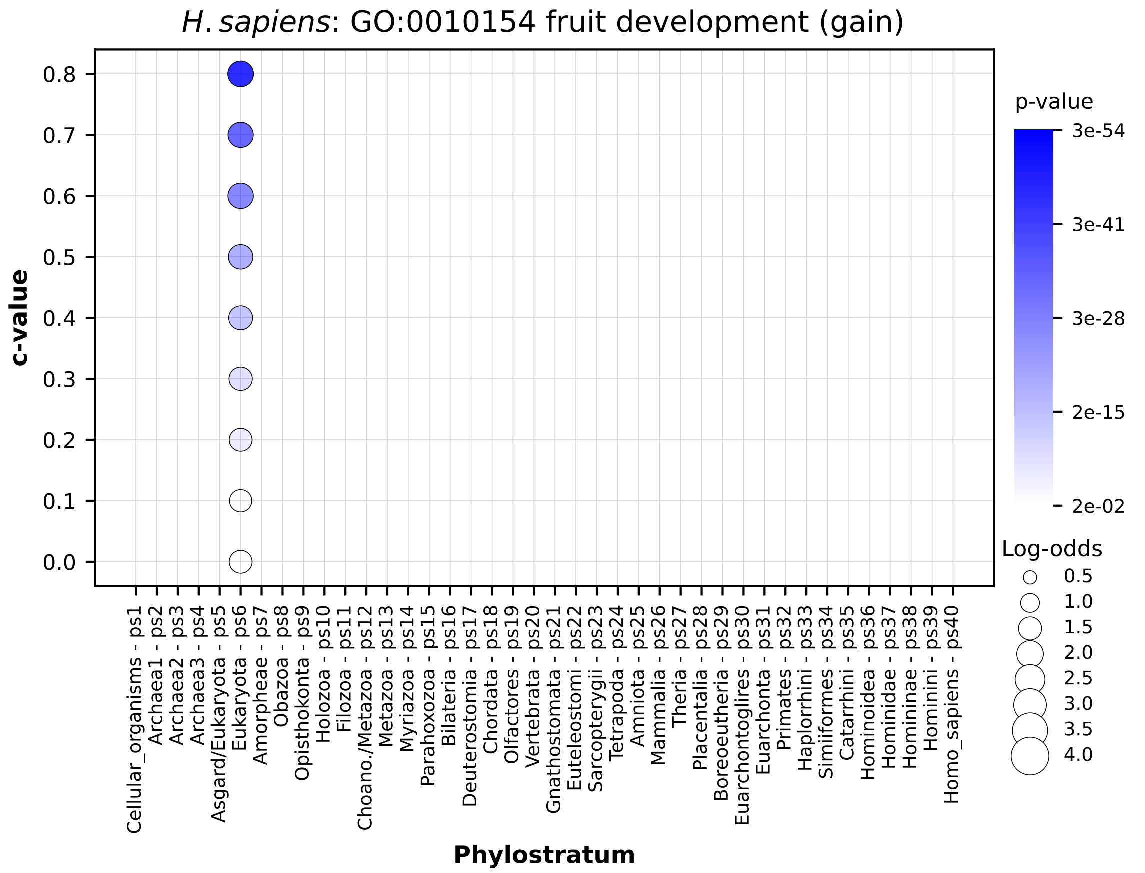
H. sapiens: GO:0010154 fruit development (gain)
c-value
0.8
0.7
0.6
0.5
0.4
0.3
0.2
0.1
0.0
p-value
3e-54
3e-41
3e-28
2e-15
2e-02
Log-odds
0.5
1.0
1.5
2.0
2.5
3.0
3.5
4.0
Cellular_organisms - ps1
Archaea1 - ps2
Archaea2 - ps3
Archaea3 - ps4
Asgard/Eukaryota - ps5
Eukaryota - ps6
Amorpheae - ps7
Obazoa - ps8
Opisthokonta - ps9
Holozoa - ps10
Filozoa - ps11
Choano./Metazoa - ps12
Metazoa - ps13
Myriazoa - ps14
Parahoxozoa - ps15
Bilateria - ps16
Deuterostomia - ps17
Chordata - ps18
Olfactores - ps19
Vertebrata - ps20
Gnathostomata - ps21
Euteleostomi - ps22
Sarcopterygii - ps23
Tetrapoda - ps24
Amniota - ps25
Mammalia - ps26
Theria - ps27
Placentalia - ps28
Boreoeutheria - ps29
Euarchontoglires - ps30
Euarchonta - ps31
Primates - ps32
Haplorrhini - ps33
Simiiformes - ps34
Catarrhini - ps35
Hominoidea - ps36
Hominidae - ps37
Homininae - ps38
Hominini - ps39
Homo_sapiens - ps40
Phylostratum

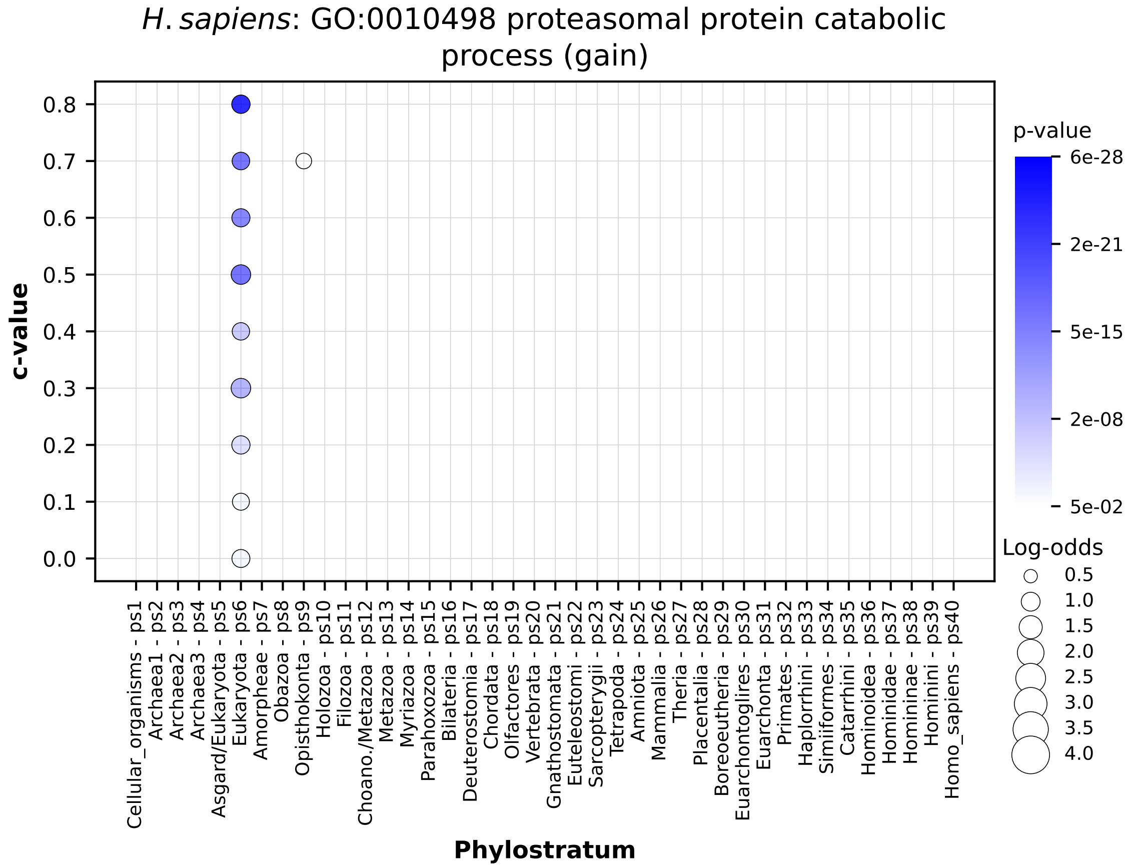

H. sapiens: GO:0010498 proteasomal protein catabolic process (gain)
c-value
0.8
0.7
0.6
0.5
0.4
0.3
0.2
0.1
0.0
p-value
6e-28
2e-21
5e-15
2e-08
5e-02
Log-odds
0.5
1.0
1.5
2.0
2.5
3.0
3.5
4.0
Cellular_organisms - ps1
Archaea1 - ps2
Archaea2 - ps3
Archaea3 - ps4
Asgard/Eukaryota - ps5
Eukaryota - ps6
Amorpheae - ps7
Obazoa - ps8
Opisthokonta - ps9
Holozoa - ps10
Filozoa - ps11
Choano./Metazoa - ps12
Metazoa - ps13
Myriazoa - ps14
Parahoxozoa - ps15
Bilateria - ps16
Deuterostomia - ps17
Chordata - ps18
Olfactores - ps19
Vertebrata - ps20
Gnathostomata - ps21
Euteleostomi - ps22
Sarcopterygii - ps23
Tetrapoda - ps24
Amniota - ps25
Mammalia - ps26
Theria - ps27
Placentalia - ps28
Boreoeutheria - ps29
Euarchontoglires - ps30
Euarchonta - ps31
Primates - ps32
Haplorrhini - ps33
Simiiformes - ps34
Catarrhini - ps35
Hominoidea - ps36
Hominidae - ps37
Homininae - ps38
Hominini - ps39
Homo_sapiens - ps40
Phylostratum

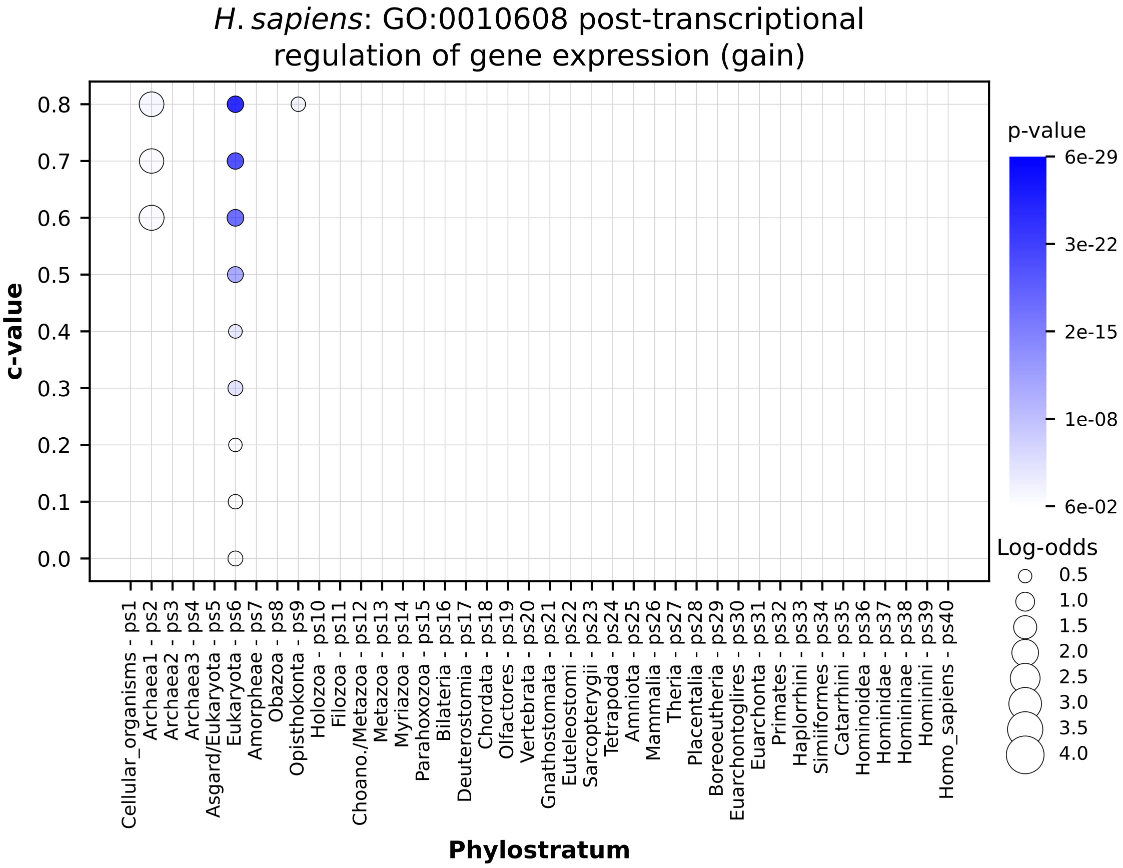

*H. sapiens*: GO:0010608 post-transcriptional regulation of gene expression (gain)
c-value
0.8
0.7
0.6
0.5
0.4
0.3
0.2
0.1
0.0
p-value
6e-29
3e-22
2e-15
1e-08
6e-02
Log-odds
0.5
1.0
1.5
2.0
2.5
3.0
3.5
4.0
Cellular_organisms - ps1
Archaea1 - ps2
Archaea2 - ps3
Archaea3 - ps4
Asgard/Eukaryota - ps5
Eukaryota - ps6
Amorpheae - ps7
Obazoa - ps8
Opisthokonta - ps9
Holozoa - ps10
Filozoa - ps11
Choano./Metazoa - ps12
Metazoa - ps13
Myriazoa - ps14
Parahoxozoa - ps15
Bilateria - ps16
Deuterostomia - ps17
Chordata - ps18
Olfactores - ps19
Vertebrata - ps20
Gnathostomata - ps21
Euteleostomi - ps22
Sarcopterygii - ps23
Tetrapoda - ps24
Amniota - ps25
Mammalia - ps26
Theria - ps27
Placentalia - ps28
Boreoeutheria - ps29
Euarchontoglires - ps30
Euarchonta - ps31
Primates - ps32
Haplorrhini - ps33
Simiiformes - ps34
Catarrhini - ps35
Hominoidea - ps36
Hominidae - ps37
Homininae - ps38
Hominini - ps39
Homo_sapiens - ps40
Phylostratum

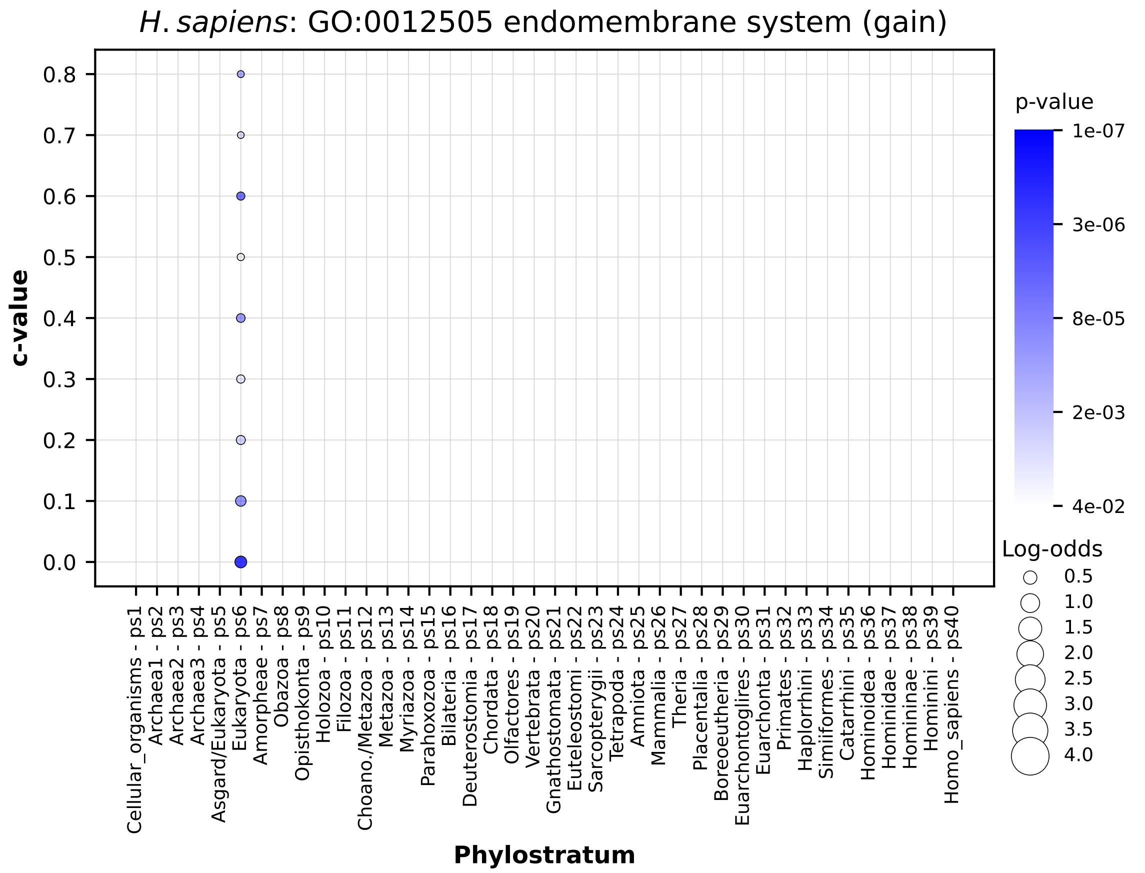
H. sapiens: GO:0012505 endomembrane system (gain)
c-value
0.8
0.7
0.6
0.5
0.4
0.3
0.2
0.1
0.0
p-value
1e-07
3e-06
8e-05
2e-03
4e-02
Log-odds
0.5
1.0
1.5
2.0
2.5
3.0
3.5
4.0
Cellular_organisms - ps1
Archaea1 - ps2
Archaea2 - ps3
Archaea3 - ps4
Asgard/Eukaryota - ps5
Eukaryota - ps6
Amorpheae - ps7
Obazoa - ps8
Opisthokonta - ps9
Holozoa - ps10
Filozoa - ps11
Choano./Metazoa - ps12
Metazoa - ps13
Myriazoa - ps14
Parahoxozoa - ps15
Bilateria - ps16
Deuterostomia - ps17
Chordata - ps18
Olfactores - ps19
Vertebrata - ps20
Gnathostomata - ps21
Euteleostomi - ps22
Sarcopterygii - ps23
Tetrapoda - ps24
Amniota - ps25
Mammalia - ps26
Theria - ps27
Placentalia - ps28
Boreoeutheria - ps29
Euarchontoglires - ps30
Euarchonta - ps31
Primates - ps32
Haplorrhini - ps33
Simiiformes - ps34
Catarrhini - ps35
Hominoidea - ps36
Hominidae - ps37
Homininae - ps38
Hominini - ps39
Homo_sapiens - ps40
Phylostratum

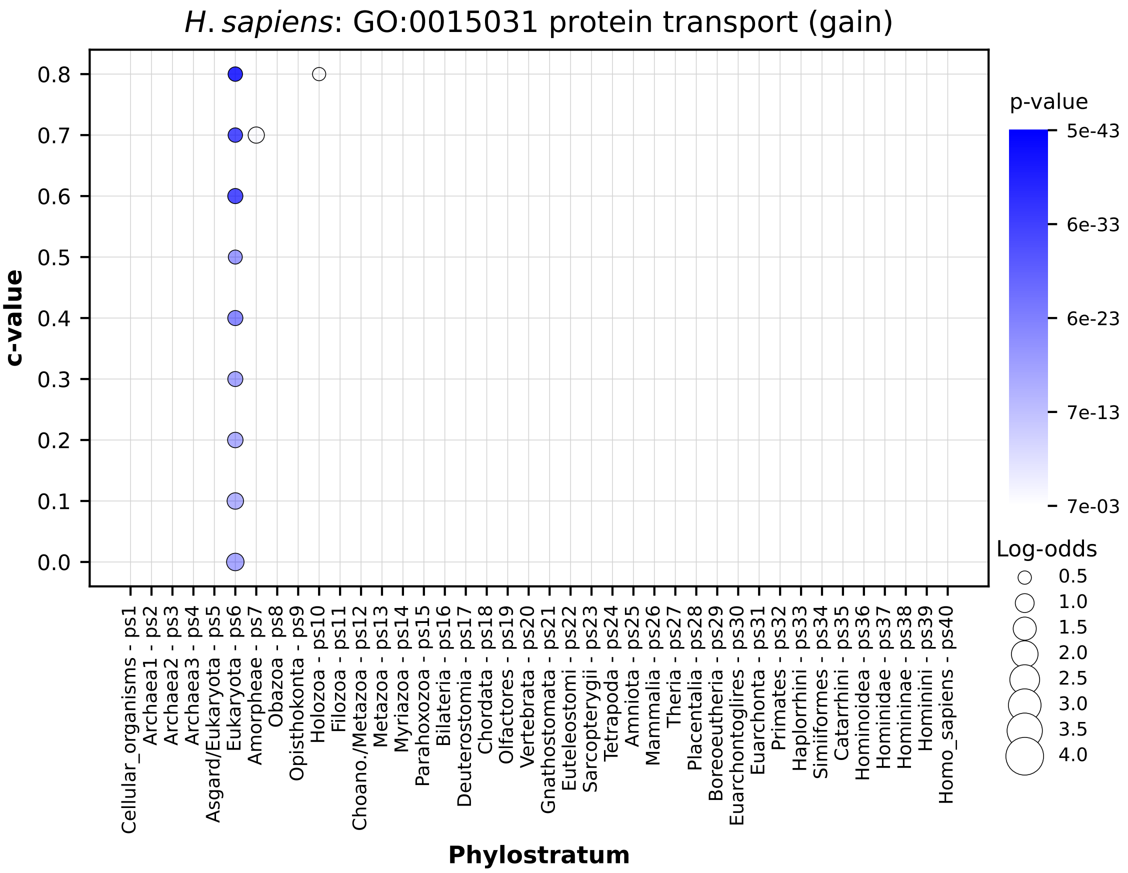

H. sapiens: GO:0015031 protein transport (gain)
c-value
0.8
0.7
0.6
0.5
0.4
0.3
0.2
0.1
0.0
Phylostratum
Cellular_organisms - ps1
Archaea1 - ps2
Archaea2 - ps3
Archaea3 - ps4
Asgard/Eukaryota - ps5
Eukaryota - ps6
Amorpheae - ps7
Obazoa - ps8
Opisthokonta - ps9
Holozoa - ps10
Filozoa - ps11
Choano./Metazoa - ps12
Metazoa - ps13
Myriazoa - ps14
Parahoxozoa - ps15
Bilateria - ps16
Deuterostomia - ps17
Chordata - ps18
Olfactores - ps19
Vertebrata - ps20
Gnathostomata - ps21
Euteleostomi - ps22
Sarcopterygii - ps23
Tetrapoda - ps24
Amniota - ps25
Mammalia - ps26
Theria - ps27
Placentalia - ps28
Boreoeutheria - ps29
Euarchontoglires - ps30
Euarchonta - ps31
Primates - ps32
Haplorrhini - ps33
Simiiformes - ps34
Catarrhini - ps35
Hominoidea - ps36
Hominidae - ps37
Homininae - ps38
Hominini - ps39
Homo_sapiens - ps40
p-value
5e-43
6e-33
6e-23
7e-13
7e-03
Log-odds
0.5
1.0
1.5
2.0
2.5
3.0
3.5
4.0

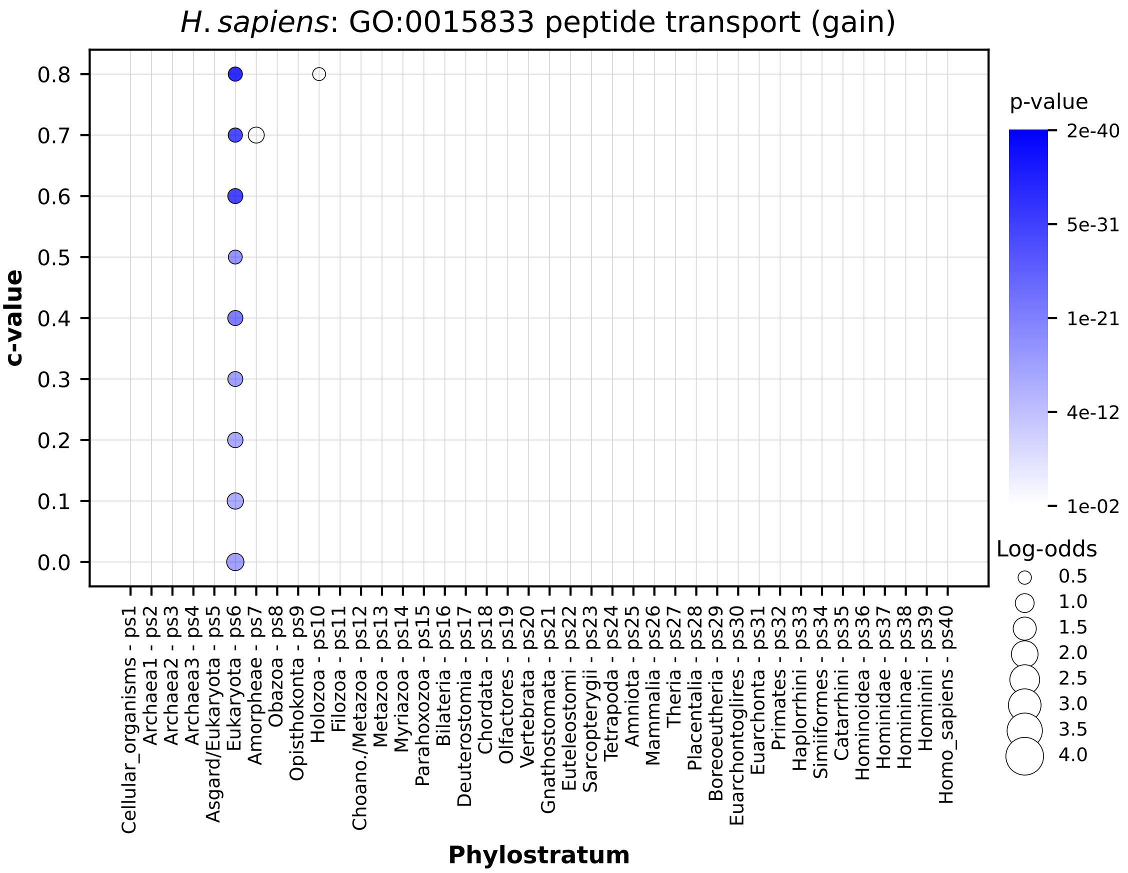

*H. sapiens*: GO:0015833 peptide transport (gain)
c-value
0.8
0.7
0.6
0.5
0.4
0.3
0.2
0.1
0.0
Phylostratum
Cellular_organisms - ps1
Archaea1 - ps2
Archaea2 - ps3
Archaea3 - ps4
Asgard/Eukaryota - ps5
Eukaryota - ps6
Amorpheae - ps7
Obazoa - ps8
Opisthokonta - ps9
Holozoa - ps10
Filozoa - ps11
Choano./Metazoa - ps12
Metazoa - ps13
Myriazoa - ps14
Parahoxozoa - ps15
Bilateria - ps16
Deuterostomia - ps17
Chordata - ps18
Olfactores - ps19
Vertebrata - ps20
Gnathostomata - ps21
Euteleostomi - ps22
Sarcopterygii - ps23
Tetrapoda - ps24
Amniota - ps25
Mammalia - ps26
Theria - ps27
Placentalia - ps28
Boreoeutheria - ps29
Euarchontoglires - ps30
Euarchonta - ps31
Primates - ps32
Haplorrhini - ps33
Simiiformes - ps34
Catarrhini - ps35
Hominoidea - ps36
Hominidae - ps37
Homininae - ps38
Hominini - ps39
Homo_sapiens - ps40
p-value
2e-40
5e-31
1e-21
4e-12
1e-02
Log-odds
0.5
1.0
1.5
2.0
2.5
3.0
3.5
4.0

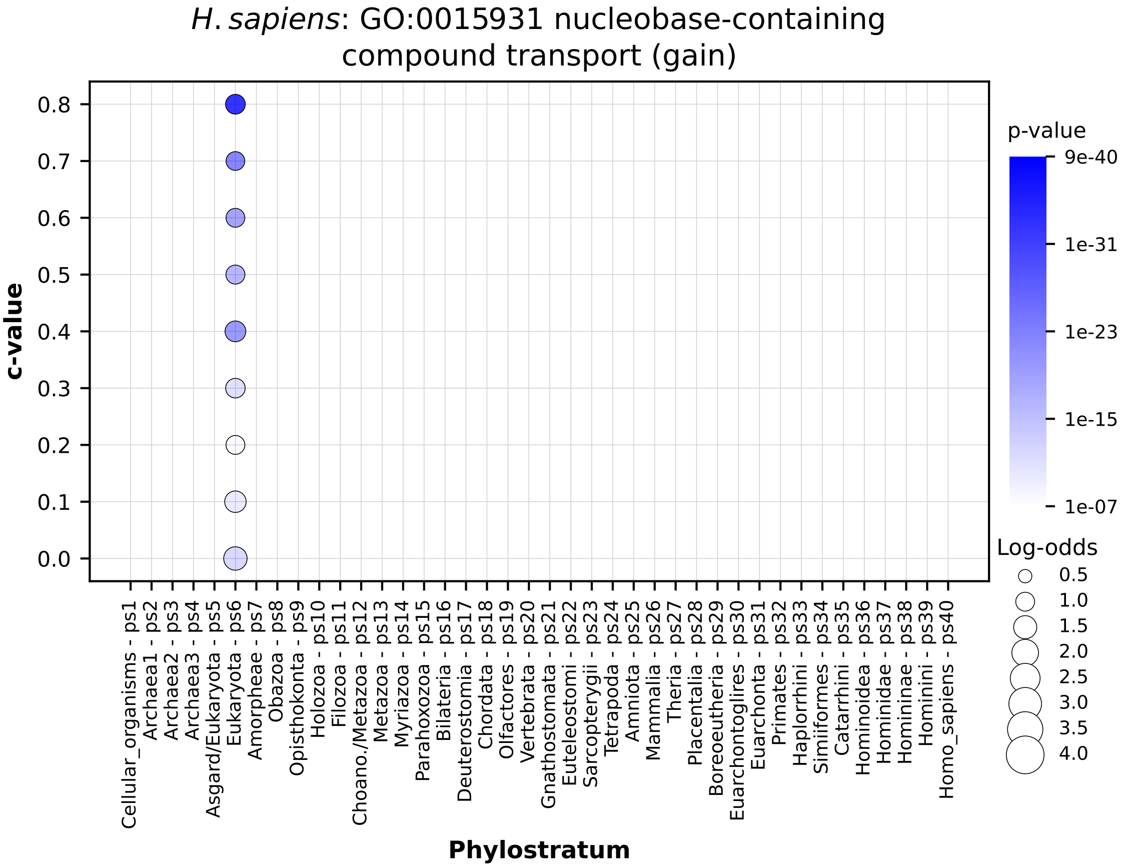

*H. sapiens*: GO:0015931 nucleobase-containing compound transport (gain)
c-value
0.8
0.7
0.6
0.5
0.4
0.3
0.2
0.1
0.0
Phylostratum
Cellular_organisms - ps1
Archaea1 - ps2
Archaea2 - ps3
Archaea3 - ps4
Asgard/Eukaryota - ps5
Eukaryota - ps6
Amorpheae - ps7
Obazoa - ps8
Opisthokonta - ps9
Holozoa - ps10
Filozoa - ps11
Choano./Metazoa - ps12
Metazoa - ps13
Myriazoa - ps14
Parahoxozoa - ps15
Bilateria - ps16
Deuterostomia - ps17
Chordata - ps18
Olfactores - ps19
Vertebrata - ps20
Gnathostomata - ps21
Euteleostomi - ps22
Sarcopterygii - ps23
Tetrapoda - ps24
Amniota - ps25
Mammalia - ps26
Theria - ps27
Placentalia - ps28
Boreoeutheria - ps29
Euarchontoglires - ps30
Euarchonta - ps31
Primates - ps32
Haplorrhini - ps33
Simiiformes - ps34
Catarrhini - ps35
Hominoidea - ps36
Hominidae - ps37
Homininae - ps38
Hominini - ps39
Homo_sapiens - ps40
p-value
9e-40
1e-31
1e-23
1e-15
1e-07
Log-odds
0.5
1.0
1.5
2.0
2.5
3.0
3.5
4.0

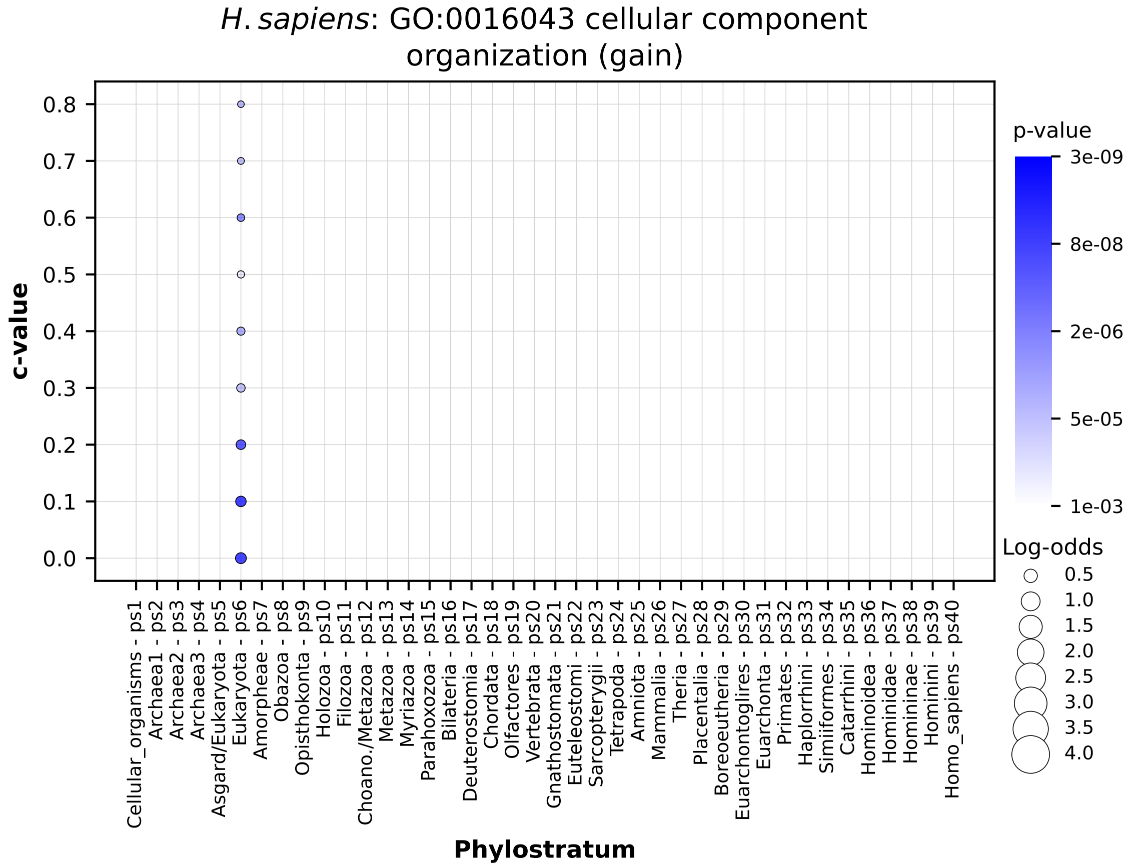

H. sapiens: GO:0016043 cellular component organization (gain)
c-value
0.8
0.7
0.6
0.5
0.4
0.3
0.2
0.1
0.0
p-value
3e-09
8e-08
2e-06
5e-05
1e-03
Log-odds
0.5
1.0
1.5
2.0
2.5
3.0
3.5
4.0
Cellular_organisms - ps1
Archaea1 - ps2
Archaea2 - ps3
Archaea3 - ps4
Asgard/Eukaryota - ps5
Eukaryota - ps6
Amorpheae - ps7
Obazoa - ps8
Opisthokonta - ps9
Holozoa - ps10
Filozoa - ps11
Choano./Metazoa - ps12
Metazoa - ps13
Myriazoa - ps14
Parahoxozoa - ps15
Bilateria - ps16
Deuterostomia - ps17
Chordata - ps18
Olfactores - ps19
Vertebrata - ps20
Gnathostomata - ps21
Euteleostomi - ps22
Sarcopterygii - ps23
Tetrapoda - ps24
Amniota - ps25
Mammalia - ps26
Theria - ps27
Placentalia - ps28
Boreoeutheria - ps29
Euarchontoglires - ps30
Euarchonta - ps31
Primates - ps32
Haplorrhini - ps33
Simiiformes - ps34
Catarrhini - ps35
Hominoidea - ps36
Hominidae - ps37
Homininae - ps38
Hominini - ps39
Homo_sapiens - ps40
Phylostratum

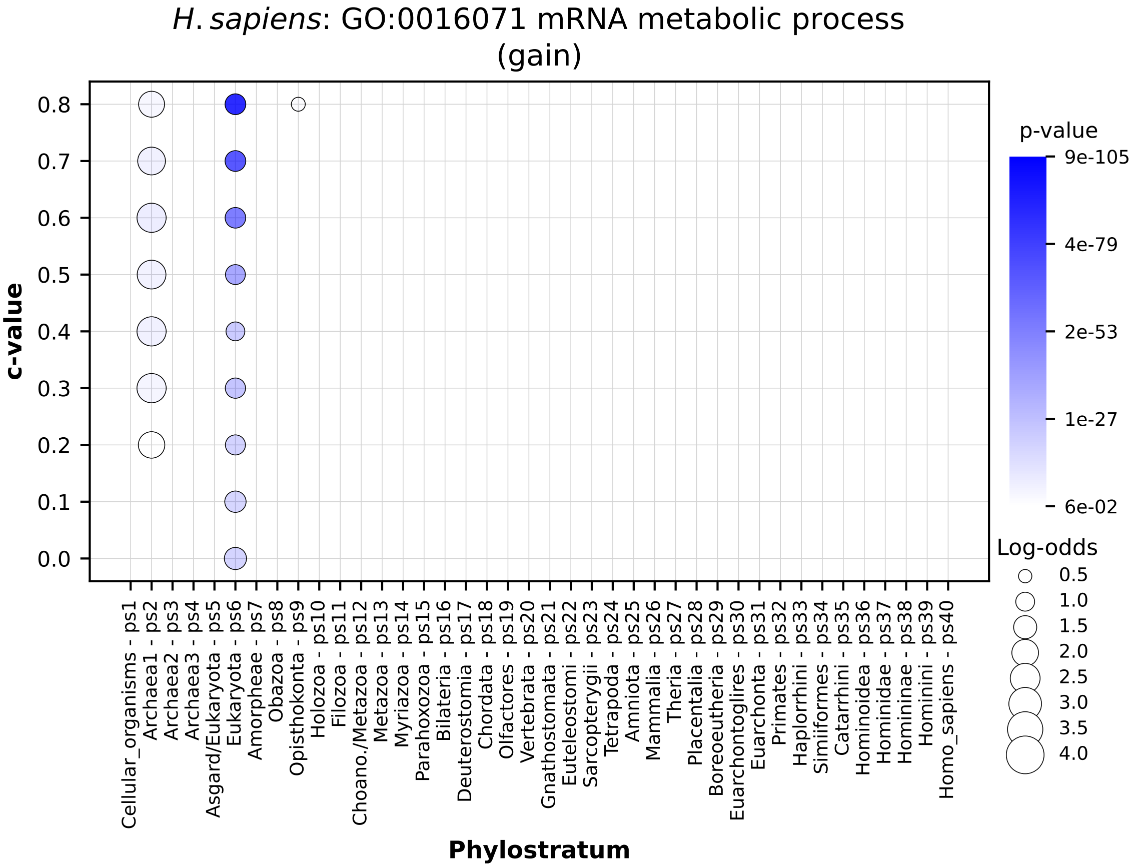

*H. sapiens*: GO:0016071 mRNA metabolic process
(gain)
c-value
0.0
0.1
0.2
0.3
0.4
0.5
0.6
0.7
0.8
p-value
9e-105
4e-79
2e-53
1e-27
6e-02
Log-odds
0.5
1.0
1.5
2.0
2.5
3.0
3.5
4.0
Cellular_organisms - ps1
Archaea1 - ps2
Archaea2 - ps3
Archaea3 - ps4
Asgard/Eukaryota - ps5
Eukaryota - ps6
Amorpheae - ps7
Obazoa - ps8
Opisthokonta - ps9
Holozoa - ps10
Filozoa - ps11
Choano./Metazoa - ps12
Metazoa - ps13
Myriazoa - ps14
Parahoxozoa - ps15
Bilateria - ps16
Deuterostomia - ps17
Chordata - ps18
Olfactores - ps19
Vertebrata - ps20
Gnathostomata - ps21
Euteleostomi - ps22
Sarcopterygii - ps23
Tetrapoda - ps24
Amniota - ps25
Mammalia - ps26
Theria - ps27
Placentalia - ps28
Boreoeutheria - ps29
Euarchontoglires - ps30
Euarchonta - ps31
Primates - ps32
Haplorrhini - ps33
Simiiformes - ps34
Catarrhini - ps35
Hominoidea - ps36
Hominidae - ps37
Homininae - ps38
Hominini - ps39
Homo_sapiens - ps40
Phylostratum

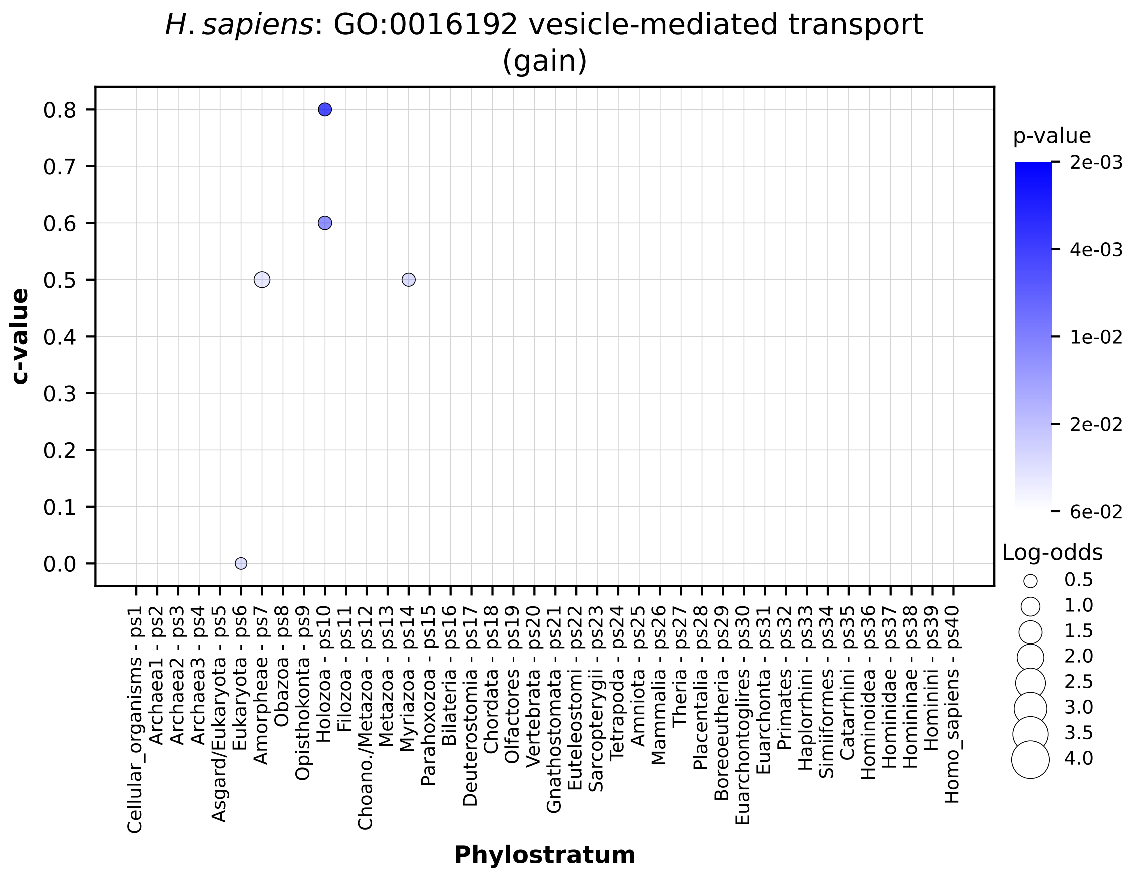

H. sapiens: GO:0016192 vesicle-mediated transport (gain)
c-value
0.0
0.1
0.2
0.3
0.4
0.5
0.6
0.7
0.8
Phylostratum
Cellular_organisms - ps1
Archaea1 - ps2
Archaea2 - ps3
Archaea3 - ps4
Asgard/Eukaryota - ps5
Eukaryota - ps6
Amorpheae - ps7
Obazoa - ps8
Opisthokonta - ps9
Holozoa - ps10
Filozoa - ps11
Choano./Metazoa - ps12
Metazoa - ps13
Myriazoa - ps14
Parahoxozoa - ps15
Bilateria - ps16
Deuterostomia - ps17
Chordata - ps18
Olfactores - ps19
Vertebrata - ps20
Gnathostomata - ps21
Euteleostomi - ps22
Sarcopterygii - ps23
Tetrapoda - ps24
Amniota - ps25
Mammalia - ps26
Theria - ps27
Placentalia - ps28
Boreoeutheria - ps29
Euarchontoglires - ps30
Euarchonta - ps31
Primates - ps32
Haplorrhini - ps33
Simiiformes - ps34
Catarrhini - ps35
Hominoidea - ps36
Hominidae - ps37
Homininae - ps38
Hominini - ps39
Homo_sapiens - ps40
p-value
2e-03
4e-03
1e-02
2e-02
6e-02
Log-odds
0.5
1.0
1.5
2.0
2.5
3.0
3.5
4.0

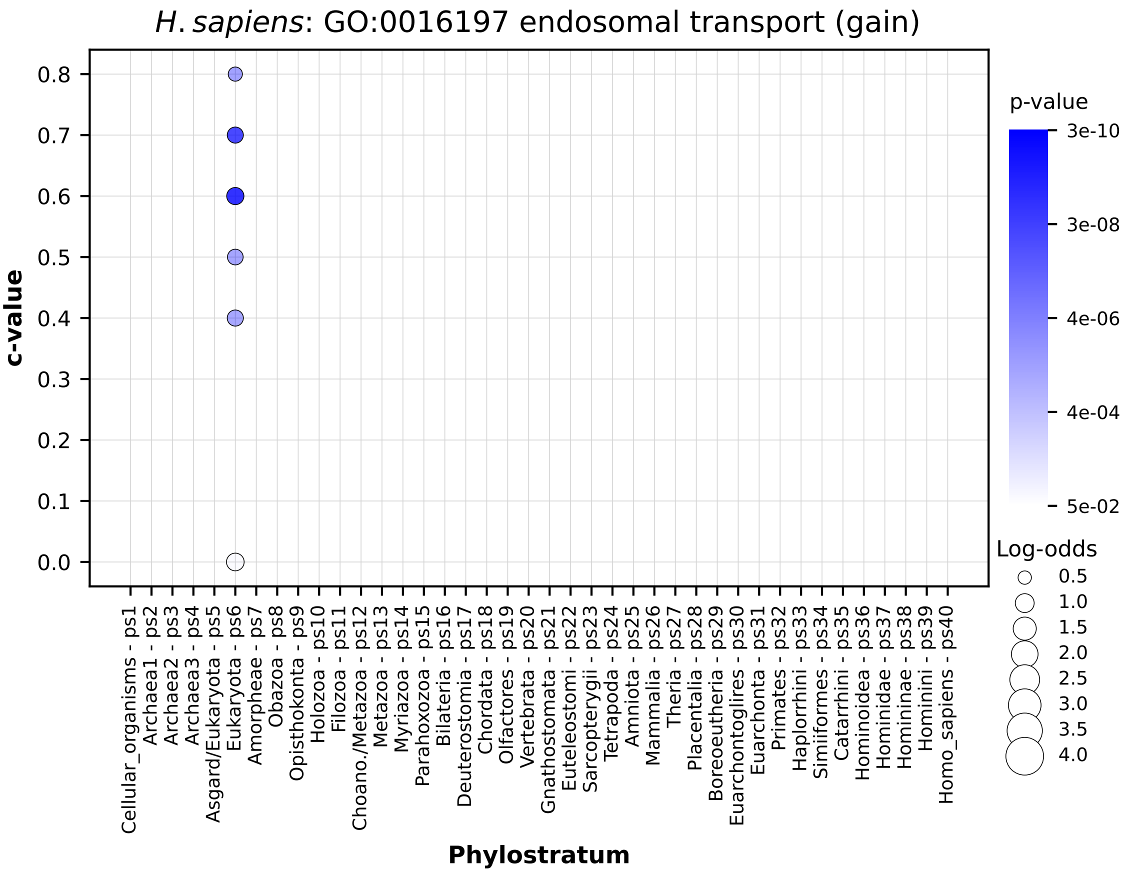
H. sapiens: GO:0016197 endosomal transport (gain)
c-value
0.0
0.1
0.2
0.3
0.4
0.5
0.6
0.7
0.8
p-value
3e-10
3e-08
4e-06
4e-04
5e-02
Log-odds
0.5
1.0
1.5
2.0
2.5
3.0
3.5
4.0
Cellular_organisms - ps1
Archaea1 - ps2
Archaea2 - ps3
Archaea3 - ps4
Asgard/Eukaryota - ps5
Eukaryota - ps6
Amorpheae - ps7
Obazoa - ps8
Opisthokonta - ps9
Holozoa - ps10
Filozoa - ps11
Choano./Metazoa - ps12
Metazoa - ps13
Myriazoa - ps14
Parahoxozoa - ps15
Bilateria - ps16
Deuterostomia - ps17
Chordata - ps18
Olfactores - ps19
Vertebrata - ps20
Gnathostomata - ps21
Euteleostomi - ps22
Sarcopterygii - ps23
Tetrapoda - ps24
Amniota - ps25
Mammalia - ps26
Theria - ps27
Placentalia - ps28
Boreoeutheria - ps29
Euarchontoglires - ps30
Euarchonta - ps31
Primates - ps32
Haplorrhini - ps33
Simiiformes - ps34
Catarrhini - ps35
Hominoidea - ps36
Hominidae - ps37
Homininae - ps38
Hominini - ps39
Homo_sapiens - ps40
Phylostratum

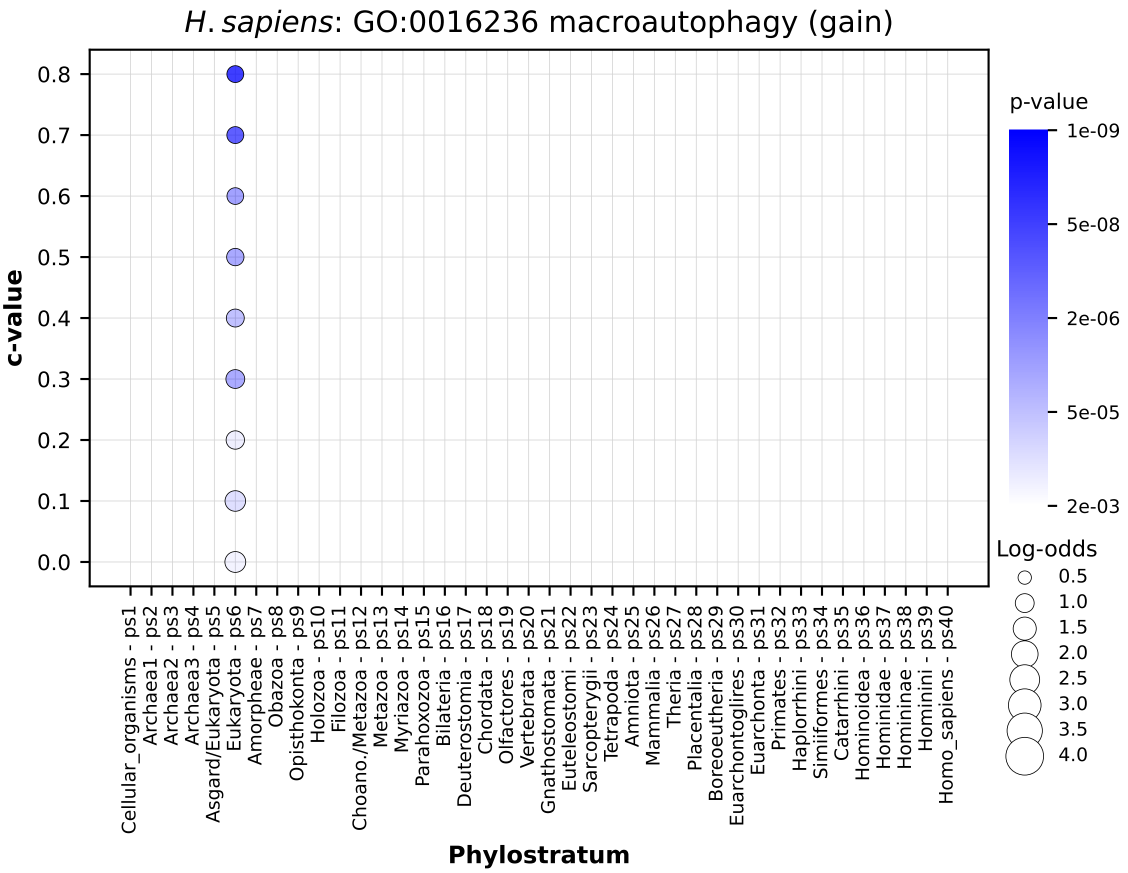

H. sapiens: GO:0016236 macroautophagy (gain)
c-value
0.8
0.7
0.6
0.5
0.4
0.3
0.2
0.1
0.0
Phylostratum
Cellular_organisms - ps1
Archaea1 - ps2
Archaea2 - ps3
Archaea3 - ps4
Asgard/Eukaryota - ps5
Eukaryota - ps6
Amorpheae - ps7
Obazoa - ps8
Opisthokonta - ps9
Holozoa - ps10
Filozoa - ps11
Choano./Metazoa - ps12
Metazoa - ps13
Myriazoa - ps14
Parahoxozoa - ps15
Bilateria - ps16
Deuterostomia - ps17
Chordata - ps18
Olfactores - ps19
Vertebrata - ps20
Gnathostomata - ps21
Euteleostomi - ps22
Sarcopterygii - ps23
Tetrapoda - ps24
Amniota - ps25
Mammalia - ps26
Theria - ps27
Placentalia - ps28
Boreoeutheria - ps29
Euarchontoglires - ps30
Euarchonta - ps31
Primates - ps32
Haplorrhini - ps33
Simiiformes - ps34
Catarrhini - ps35
Hominoidea - ps36
Hominidae - ps37
Homininae - ps38
Hominini - ps39
Homo_sapiens - ps40
p-value
1e-09
5e-08
2e-06
5e-05
2e-03
Log-odds
0.5
1.0
1.5
2.0
2.5
3.0
3.5
4.0

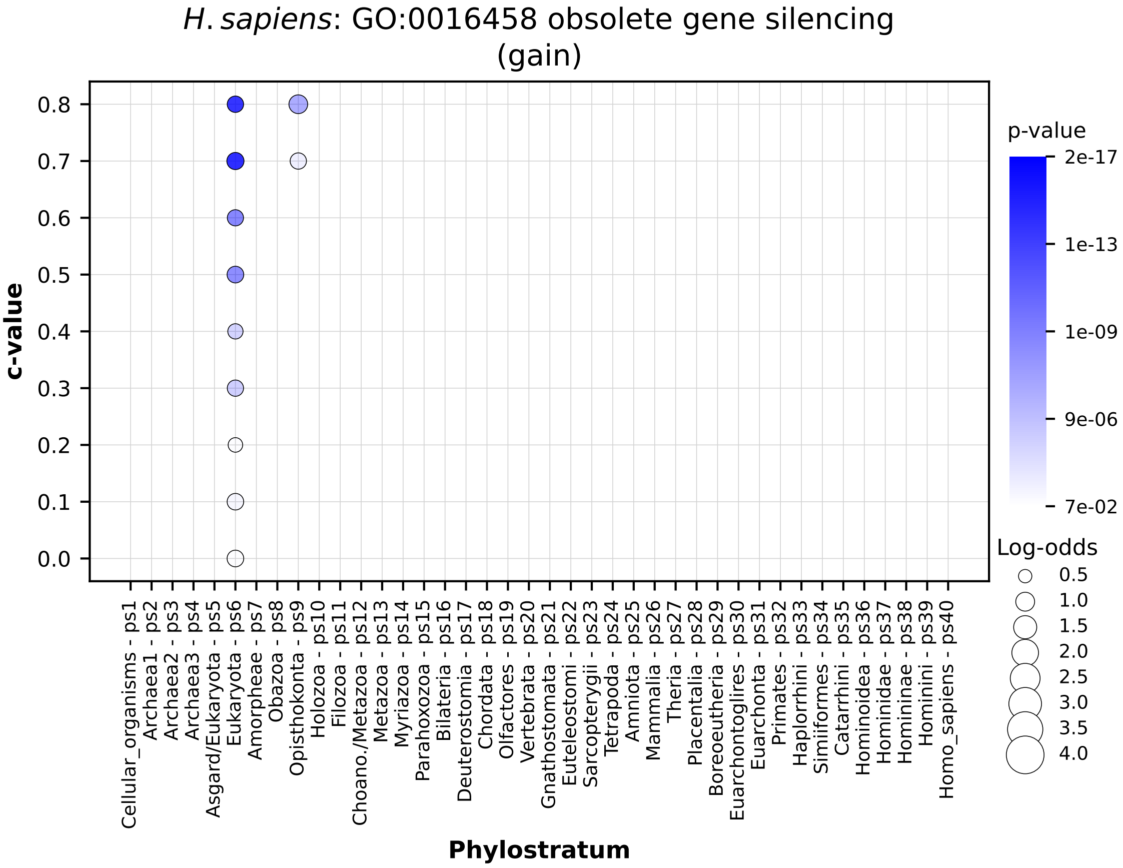
H. sapiens: GO:0016458 obsolete gene silencing
(gain)
c-value
0.0
0.1
0.2
0.3
0.4
0.5
0.6
0.7
0.8
Phylostratum
Cellular_organisms - ps1
Archaea1 - ps2
Archaea2 - ps3
Archaea3 - ps4
Asgard/Eukaryota - ps5
Eukaryota - ps6
Amorpheae - ps7
Obazoa - ps8
Opisthokonta - ps9
Holozoa - ps10
Filozoa - ps11
Choano./Metazoa - ps12
Metazoa - ps13
Myriazoa - ps14
Parahoxozoa - ps15
Bilateria - ps16
Deuterostomia - ps17
Chordata - ps18
Olfactores - ps19
Vertebrata - ps20
Gnathostomata - ps21
Euteleostomi - ps22
Sarcopterygii - ps23
Tetrapoda - ps24
Amniota - ps25
Mammalia - ps26
Theria - ps27
Placentalia - ps28
Boreoeutheria - ps29
Euarchontoglires - ps30
Euarchonta - ps31
Primates - ps32
Haplorrhini - ps33
Simiiformes - ps34
Catarrhini - ps35
Hominoidea - ps36
Hominidae - ps37
Homininae - ps38
Hominini - ps39
Homo_sapiens - ps40
p-value
2e-17
1e-13
1e-09
9e-06
7e-02
Log-odds
0.5
1.0
1.5
2.0
2.5
3.0
3.5
4.0

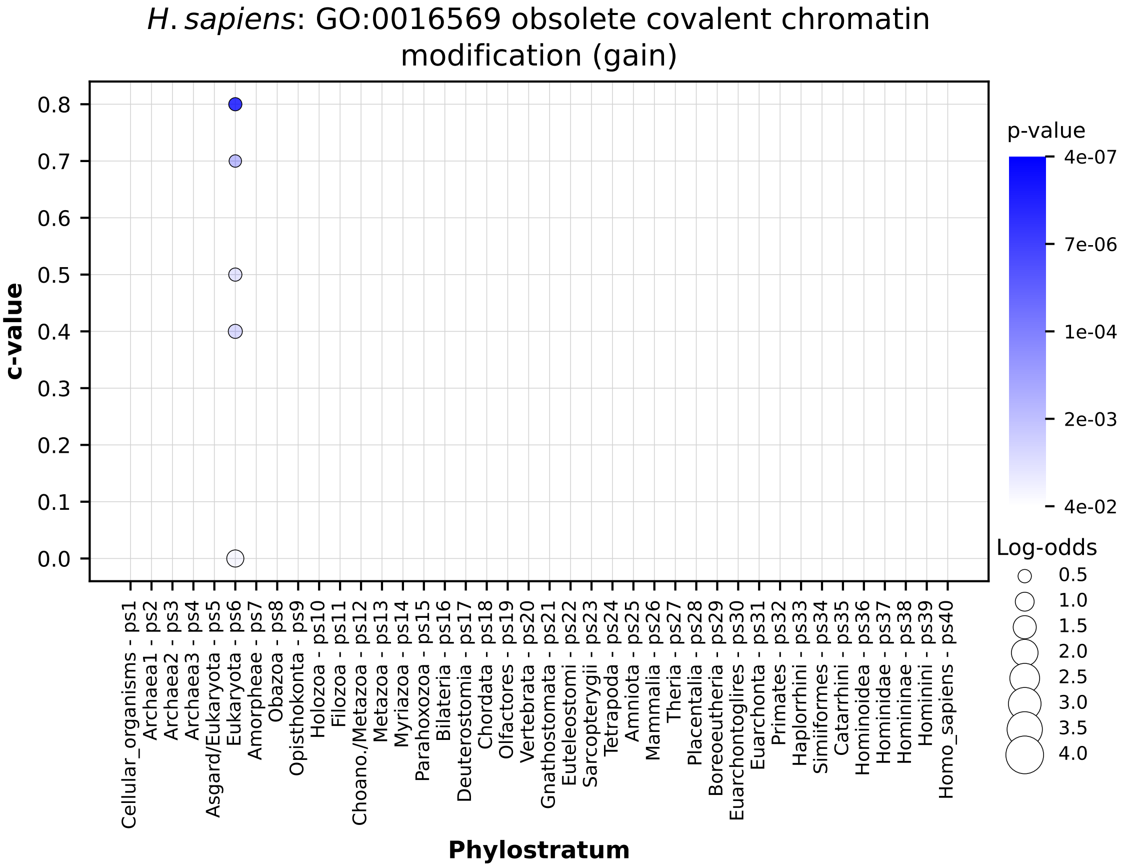

H. sapiens: GO:0016569 obsolete covalent chromatin modification (gain)
c-value
0.8
0.7
0.6
0.5
0.4
0.3
0.2
0.1
0.0
p-value
4e-07
7e-06
1e-04
2e-03
4e-02
Log-odds
0.5
1.0
1.5
2.0
2.5
3.0
3.5
4.0
Cellular_organisms - ps1
Archaea1 - ps2
Archaea2 - ps3
Archaea3 - ps4
Asgard/Eukaryota - ps5
Eukaryota - ps6
Amorpheae - ps7
Obazoa - ps8
Opisthokonta - ps9
Holozoa - ps10
Filozoa - ps11
Choano./Metazoa - ps12
Metazoa - ps13
Myriazoa - ps14
Parahoxozoa - ps15
Bilateria - ps16
Deuterostomia - ps17
Chordata - ps18
Olfactores - ps19
Vertebrata - ps20
Gnathostomata - ps21
Euteleostomi - ps22
Sarcopterygii - ps23
Tetrapoda - ps24
Amniota - ps25
Mammalia - ps26
Theria - ps27
Placentalia - ps28
Boreoeutheria - ps29
Euarchontoglires - ps30
Euarchonta - ps31
Primates - ps32
Haplorrhini - ps33
Simiiformes - ps34
Catarrhini - ps35
Hominoidea - ps36
Hominidae - ps37
Homininae - ps38
Hominini - ps39
Homo_sapiens - ps40
Phylostratum

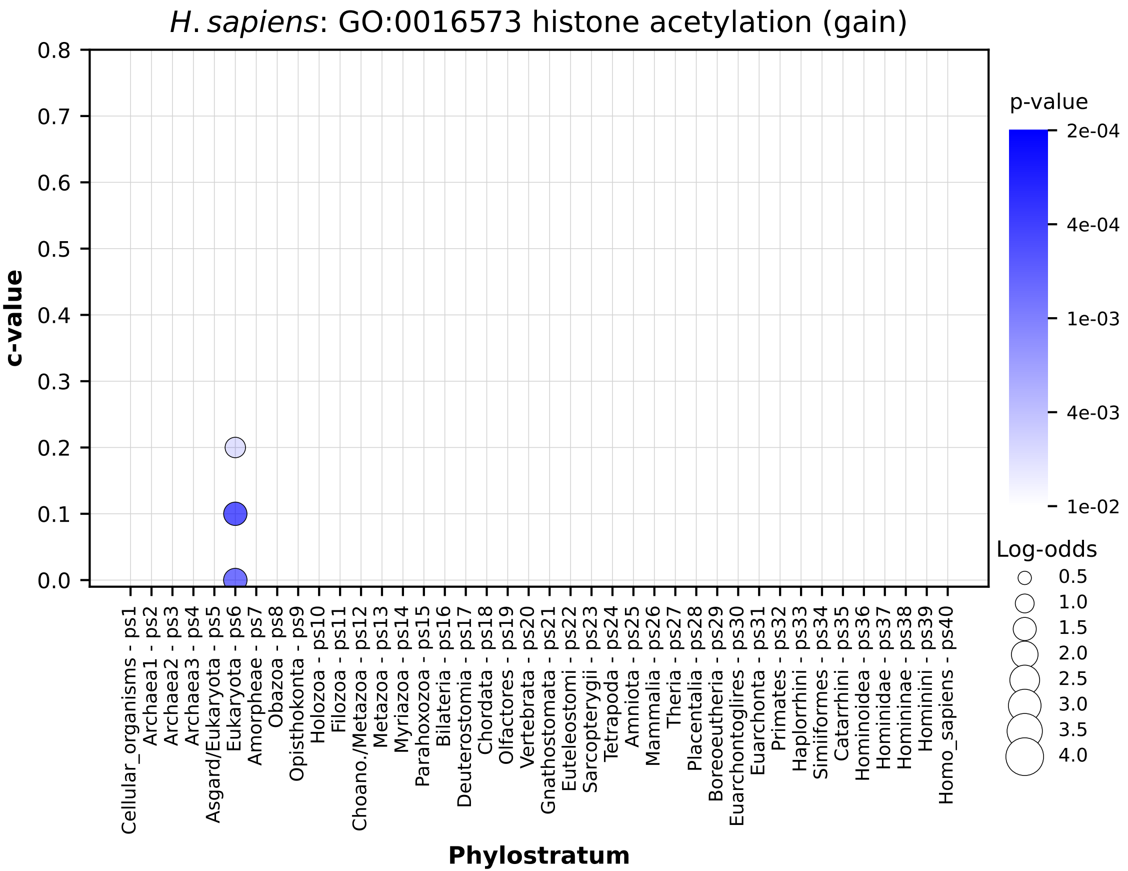
H. sapiens: GO:0016573 histone acetylation (gain)
c-value
0.8
0.7
0.6
0.5
0.4
0.3
0.2
0.1
0.0
Phylostratum
Cellular_organisms - ps1
Archaea1 - ps2
Archaea2 - ps3
Archaea3 - ps4
Asgard/Eukaryota - ps5
Eukaryota - ps6
Amorpheae - ps7
Obazoa - ps8
Opisthokonta - ps9
Holozoa - ps10
Filozoa - ps11
Choano./Metazoa - ps12
Metazoa - ps13
Myriazoa - ps14
Parahoxozoa - ps15
Bilateria - ps16
Deuterostomia - ps17
Chordata - ps18
Olfactores - ps19
Vertebrata - ps20
Gnathostomata - ps21
Euteleostomi - ps22
Sarcopterygii - ps23
Tetrapoda - ps24
Amniota - ps25
Mammalia - ps26
Theria - ps27
Placentalia - ps28
Boreoeutheria - ps29
Euarchontoglires - ps30
Euarchonta - ps31
Primates - ps32
Haplorrhini - ps33
Simiiformes - ps34
Catarrhini - ps35
Hominoidea - ps36
Hominidae - ps37
Homininae - ps38
Hominini - ps39
Homo_sapiens - ps40
p-value
2e-04
4e-04
1e-03
4e-03
1e-02
Log-odds
0.5
1.0
1.5
2.0
2.5
3.0
3.5
4.0

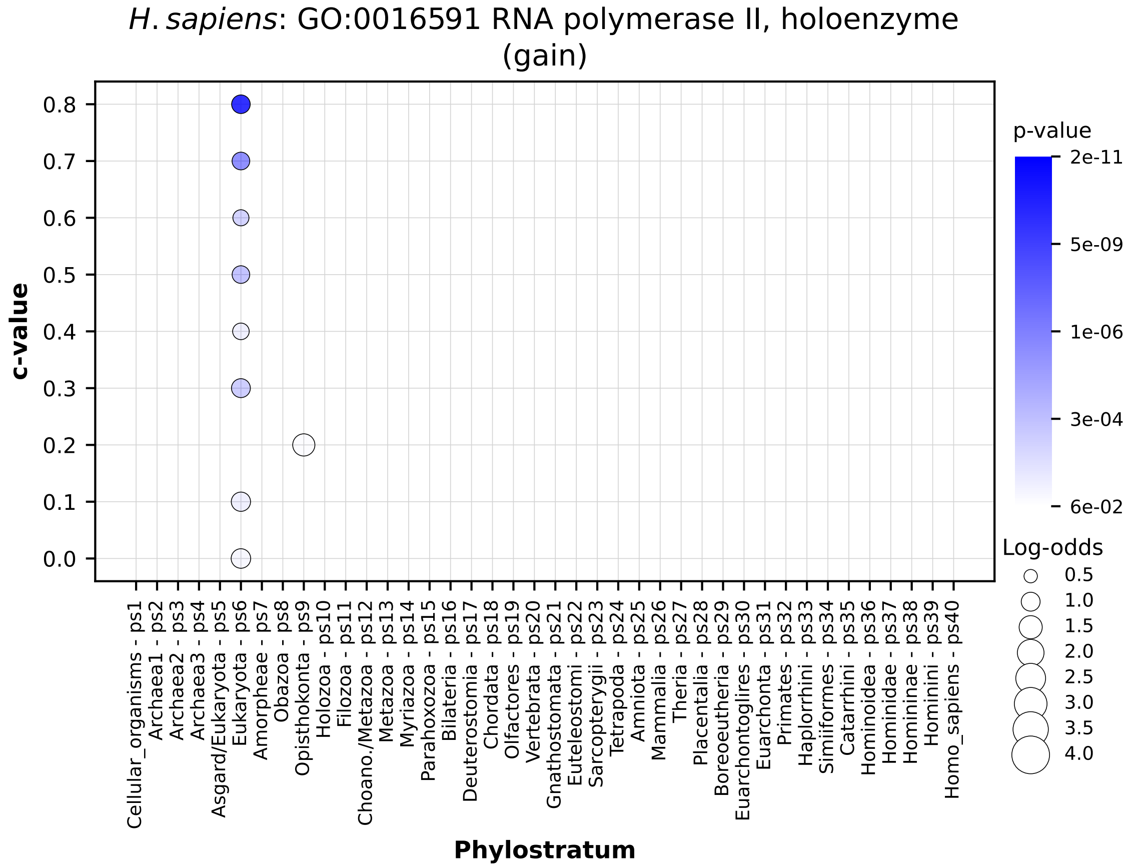

H. sapiens: GO:0016591 RNA polymerase II, holoenzyme (gain)
c-value
0.8
0.7
0.6
0.5
0.4
0.3
0.2
0.1
0.0
p-value
2e-11
5e-09
1e-06
3e-04
6e-02
Log-odds
0.5
1.0
1.5
2.0
2.5
3.0
3.5
4.0
Cellular_organisms - ps1
Archaea1 - ps2
Archaea2 - ps3
Archaea3 - ps4
Asgard/Eukaryota - ps5
Eukaryota - ps6
Amorpheae - ps7
Obazoa - ps8
Opisthokonta - ps9
Holozoa - ps10
Filozoa - ps11
Choano./Metazoa - ps12
Metazoa - ps13
Myriazoa - ps14
Parahoxozoa - ps15
Bilateria - ps16
Deuterostomia - ps17
Chordata - ps18
Olfactores - ps19
Vertebrata - ps20
Gnathostomata - ps21
Euteleostomi - ps22
Sarcopterygii - ps23
Tetrapoda - ps24
Amniota - ps25
Mammalia - ps26
Theria - ps27
Placentalia - ps28
Boreoeutheria - ps29
Euarchontoglires - ps30
Euarchonta - ps31
Primates - ps32
Haplorrhini - ps33
Simiiformes - ps34
Catarrhini - ps35
Hominoidea - ps36
Hominidae - ps37
Homininae - ps38
Hominini - ps39
Homo_sapiens - ps40
Phylostratum

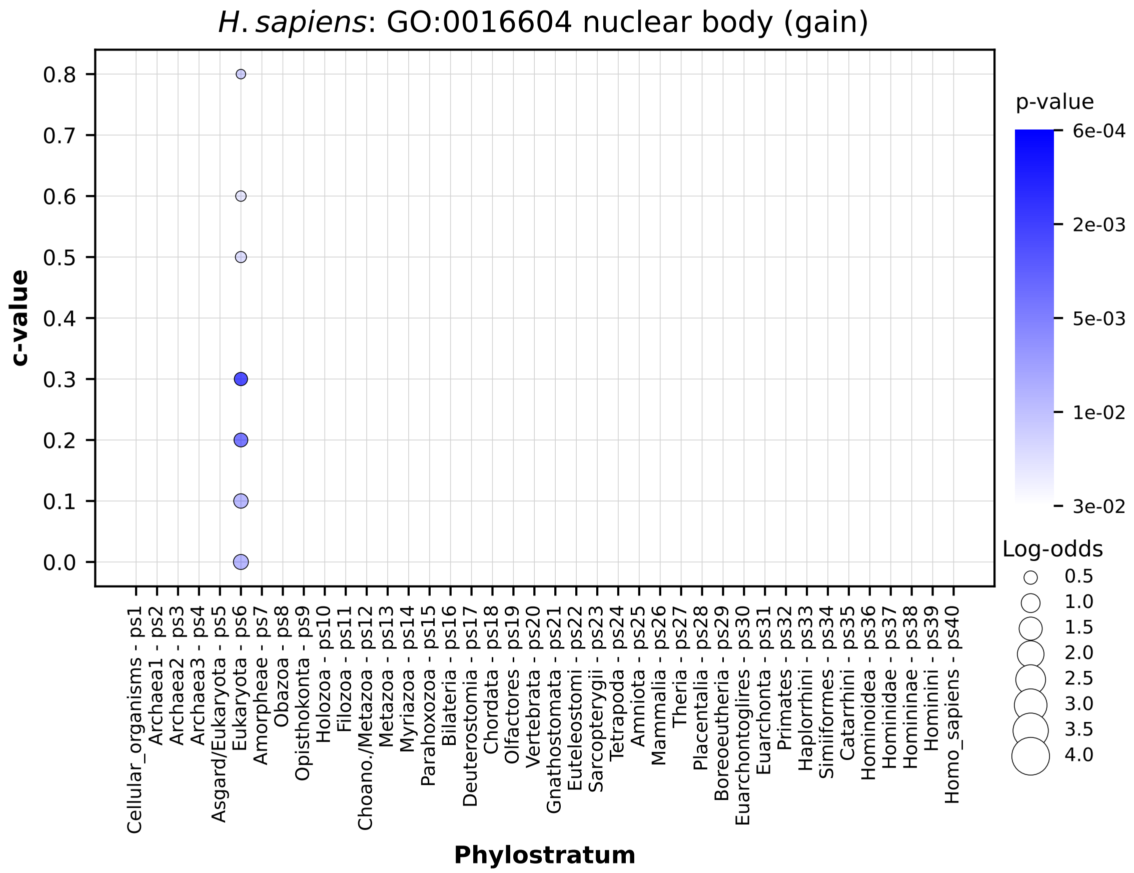

H. sapiens: GO:0016604 nuclear body (gain)
c-value
0.0
0.1
0.2
0.3
0.4
0.5
0.6
0.7
0.8
p-value
6e-04
2e-03
5e-03
1e-02
3e-02
Log-odds
0.5
1.0
1.5
2.0
2.5
3.0
3.5
4.0
Cellular_organisms - ps1
Archaea1 - ps2
Archaea2 - ps3
Archaea3 - ps4
Asgard/Eukaryota - ps5
Eukaryota - ps6
Amorpheae - ps7
Obazoa - ps8
Opisthokonta - ps9
Holozoa - ps10
Filozoa - ps11
Choano./Metazoa - ps12
Metazoa - ps13
Myriazoa - ps14
Parahoxozoa - ps15
Bilateria - ps16
Deuterostomia - ps17
Chordata - ps18
Olfactores - ps19
Vertebrata - ps20
Gnathostomata - ps21
Euteleostomi - ps22
Sarcopterygii - ps23
Tetrapoda - ps24
Amniota - ps25
Mammalia - ps26
Theria - ps27
Placentalia - ps28
Boreoeutheria - ps29
Euarchontoglires - ps30
Euarchonta - ps31
Primates - ps32
Haplorrhini - ps33
Simiiformes - ps34
Catarrhini - ps35
Hominoidea - ps36
Hominidae - ps37
Homininae - ps38
Hominini - ps39
Homo_sapiens - ps40
Phylostratum

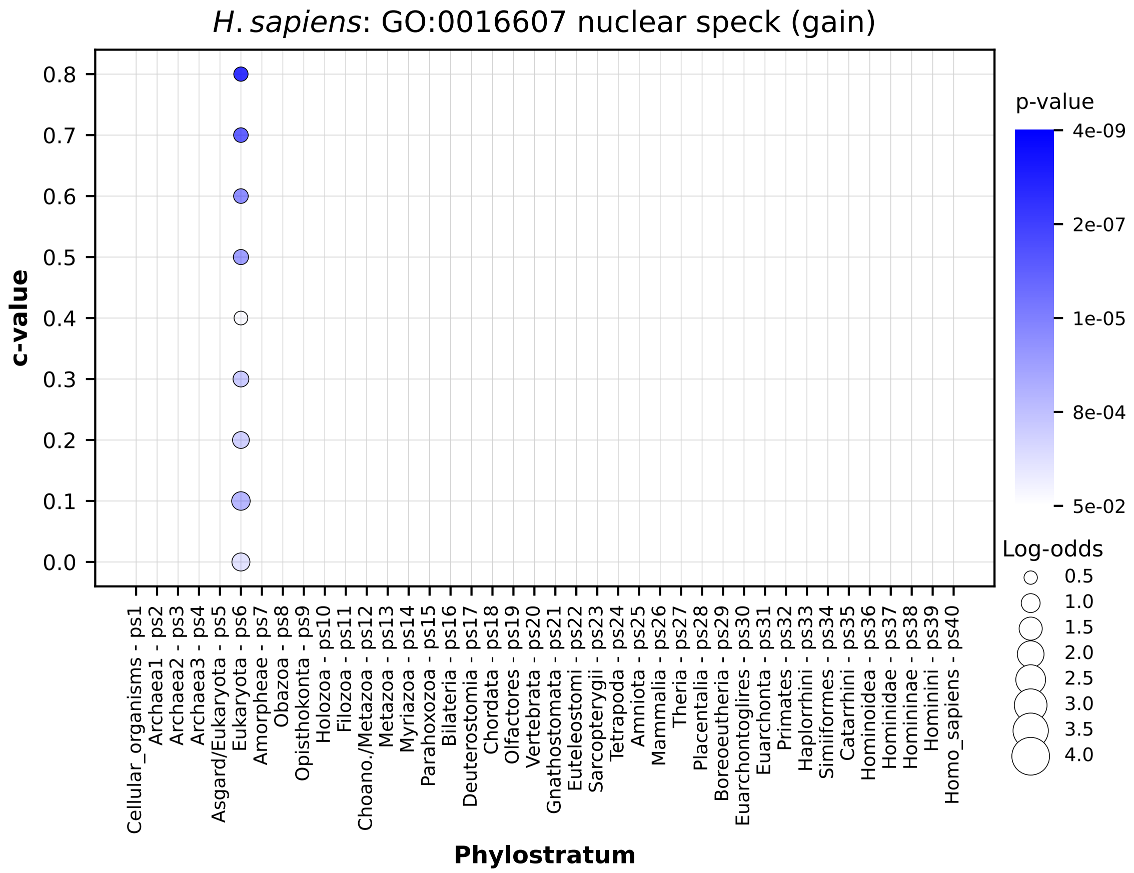

H. sapiens: GO:0016607 nuclear speck (gain)
c-value
0.8
0.7
0.6
0.5
0.4
0.3
0.2
0.1
0.0
Phylostratum
Cellular_organisms - ps1
Archaea1 - ps2
Archaea2 - ps3
Archaea3 - ps4
Asgard/Eukaryota - ps5
Eukaryota - ps6
Amorpheae - ps7
Obazoa - ps8
Opisthokonta - ps9
Holozoa - ps10
Filozoa - ps11
Choano./Metazoa - ps12
Metazoa - ps13
Myriazoa - ps14
Parahoxozoa - ps15
Bilateria - ps16
Deuterostomia - ps17
Chordata - ps18
Olfactores - ps19
Vertebrata - ps20
Gnathostomata - ps21
Euteleostomi - ps22
Sarcopterygii - ps23
Tetrapoda - ps24
Amniota - ps25
Mammalia - ps26
Theria - ps27
Placentalia - ps28
Boreoeutheria - ps29
Euarchontoglires - ps30
Euarchonta - ps31
Primates - ps32
Haplorrhini - ps33
Simiiformes - ps34
Catarrhini - ps35
Hominoidea - ps36
Hominidae - ps37
Homininae - ps38
Hominini - ps39
Homo_sapiens - ps40
p-value
4e-09
2e-07
1e-05
8e-04
5e-02
Log-odds
0.5
1.0
1.5
2.0
2.5
3.0
3.5
4.0

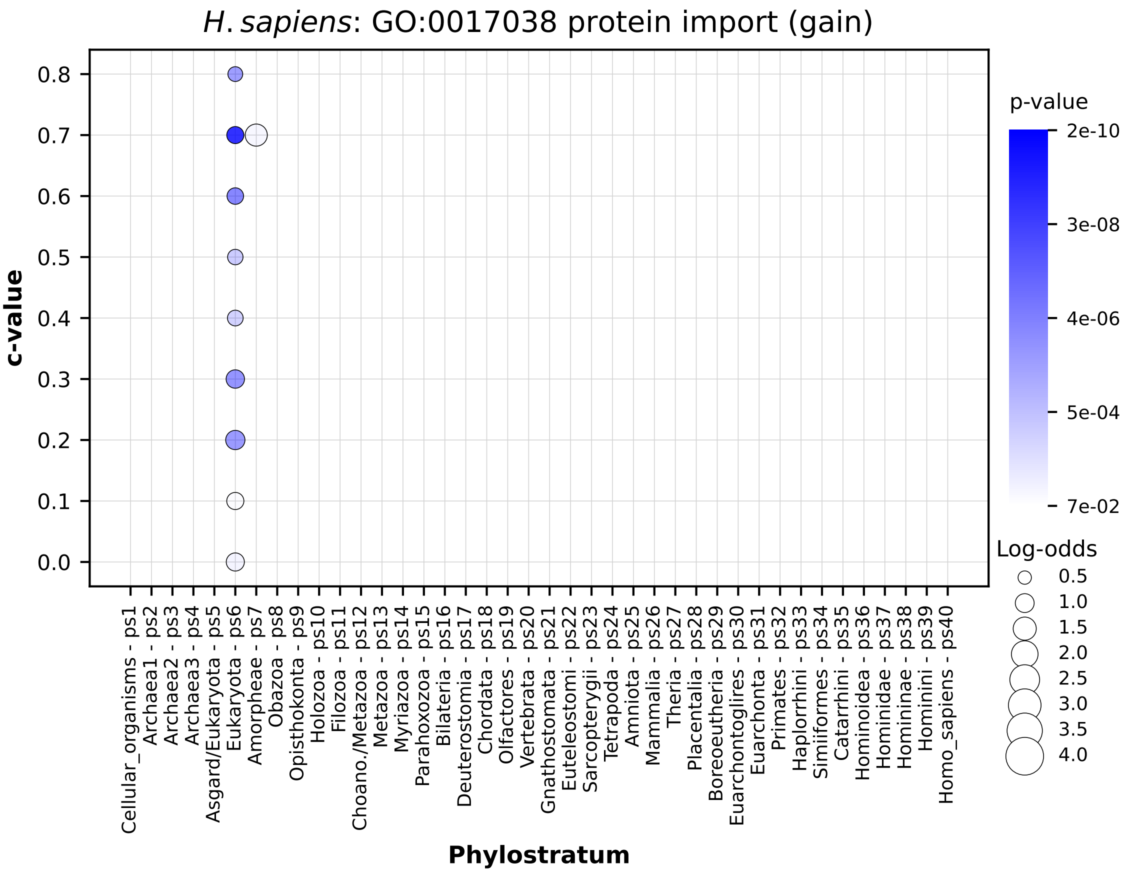

H. sapiens: GO:0017038 protein import (gain)
c-value
0.8
0.7
0.6
0.5
0.4
0.3
0.2
0.1
0.0
Phylostratum
Cellular_organisms - ps1
Archaea1 - ps2
Archaea2 - ps3
Archaea3 - ps4
Asgard/Eukaryota - ps5
Eukaryota - ps6
Amorpheae - ps7
Obazoa - ps8
Opisthokonta - ps9
Holozoa - ps10
Filozoa - ps11
Choano./Metazoa - ps12
Metazoa - ps13
Myriazoa - ps14
Parahoxozoa - ps15
Bilateria - ps16
Deuterostomia - ps17
Chordata - ps18
Olfactores - ps19
Vertebrata - ps20
Gnathostomata - ps21
Euteleostomi - ps22
Sarcopterygii - ps23
Tetrapoda - ps24
Amniota - ps25
Mammalia - ps26
Theria - ps27
Placentalia - ps28
Boreoeutheria - ps29
Euarchontoglires - ps30
Euarchonta - ps31
Primates - ps32
Haplorrhini - ps33
Simiiformes - ps34
Catarrhini - ps35
Hominoidea - ps36
Hominidae - ps37
Homininae - ps38
Hominini - ps39
Homo_sapiens - ps40
p-value
2e-10
3e-08
4e-06
5e-04
7e-02
Log-odds
0.5
1.0
1.5
2.0
2.5
3.0
3.5
4.0

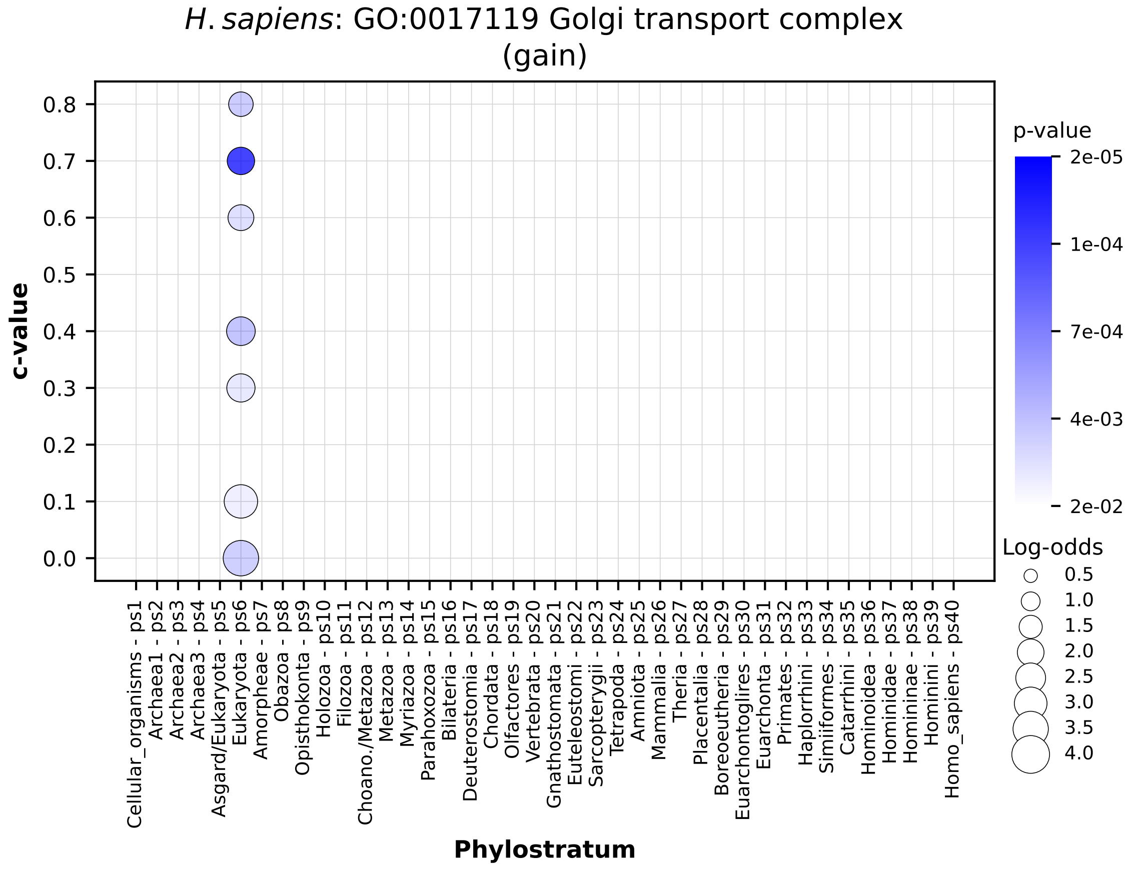

*H. sapiens*: GO:0017119 Golgi transport complex
(gain)
c-value
0.8
0.7
0.6
0.5
0.4
0.3
0.2
0.1
0.0
p-value
2e-05
1e-04
7e-04
4e-03
2e-02
Log-odds
0.5
1.0
1.5
2.0
2.5
3.0
3.5
4.0
Cellular_organisms - ps1
Archaea1 - ps2
Archaea2 - ps3
Archaea3 - ps4
Asgard/Eukaryota - ps5
Eukaryota - ps6
Amorpheae - ps7
Obazoa - ps8
Opisthokonta - ps9
Holozoa - ps10
Filozoa - ps11
Choano./Metazoa - ps12
Metazoa - ps13
Myriazoa - ps14
Parahoxozoa - ps15
Bilateria - ps16
Deuterostomia - ps17
Chordata - ps18
Olfactores - ps19
Vertebrata - ps20
Gnathostomata - ps21
Euteleostomi - ps22
Sarcopterygii - ps23
Tetrapoda - ps24
Amniota - ps25
Mammalia - ps26
Theria - ps27
Placentalia - ps28
Boreoeutheria - ps29
Euarchontoglires - ps30
Euarchonta - ps31
Primates - ps32
Haplorrhini - ps33
Simiiformes - ps34
Catarrhini - ps35
Hominoidea - ps36
Hominidae - ps37
Homininae - ps38
Hominini - ps39
Homo_sapiens - ps40
Phylostratum

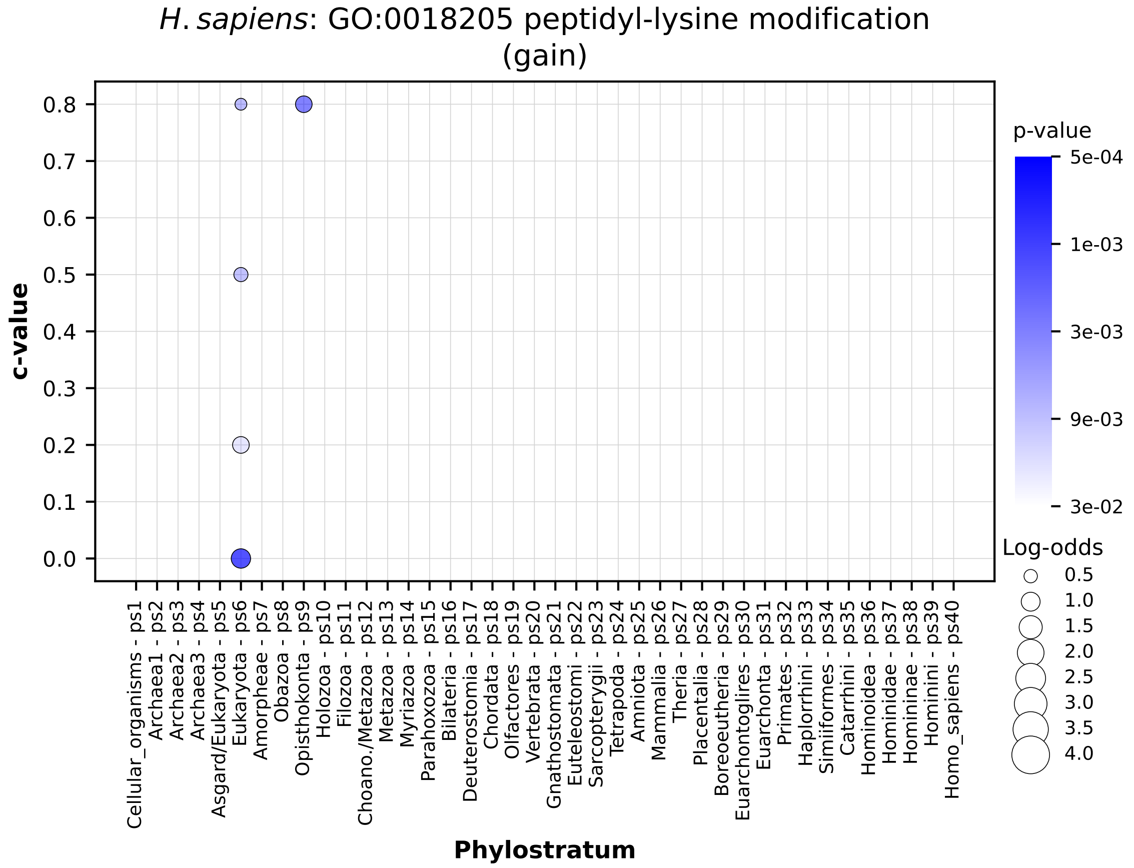

H. sapiens: GO:0018205 peptidyl-lysine modification
(gain)
c-value
0.0
0.1
0.2
0.3
0.4
0.5
0.6
0.7
0.8
Phylostratum
Cellular_organisms - ps1
Archaea1 - ps2
Archaea2 - ps3
Archaea3 - ps4
Asgard/Eukaryota - ps5
Eukaryota - ps6
Amorpheae - ps7
Obazoa - ps8
Opisthokonta - ps9
Holozoa - ps10
Filozoa - ps11
Choano./Metazoa - ps12
Metazoa - ps13
Myriazoa - ps14
Parahoxozoa - ps15
Bilateria - ps16
Deuterostomia - ps17
Chordata - ps18
Olfactores - ps19
Vertebrata - ps20
Gnathostomata - ps21
Euteleostomi - ps22
Sarcopterygii - ps23
Tetrapoda - ps24
Amniota - ps25
Mammalia - ps26
Theria - ps27
Placentalia - ps28
Boreoeutheria - ps29
Euarchontoglires - ps30
Euarchonta - ps31
Primates - ps32
Haplorrhini - ps33
Simiiformes - ps34
Catarrhini - ps35
Hominoidea - ps36
Hominidae - ps37
Homininae - ps38
Hominini - ps39
Homo_sapiens - ps40
p-value
5e-04
1e-03
3e-03
9e-03
3e-02
Log-odds
0.5
1.0
1.5
2.0
2.5
3.0
3.5
4.0

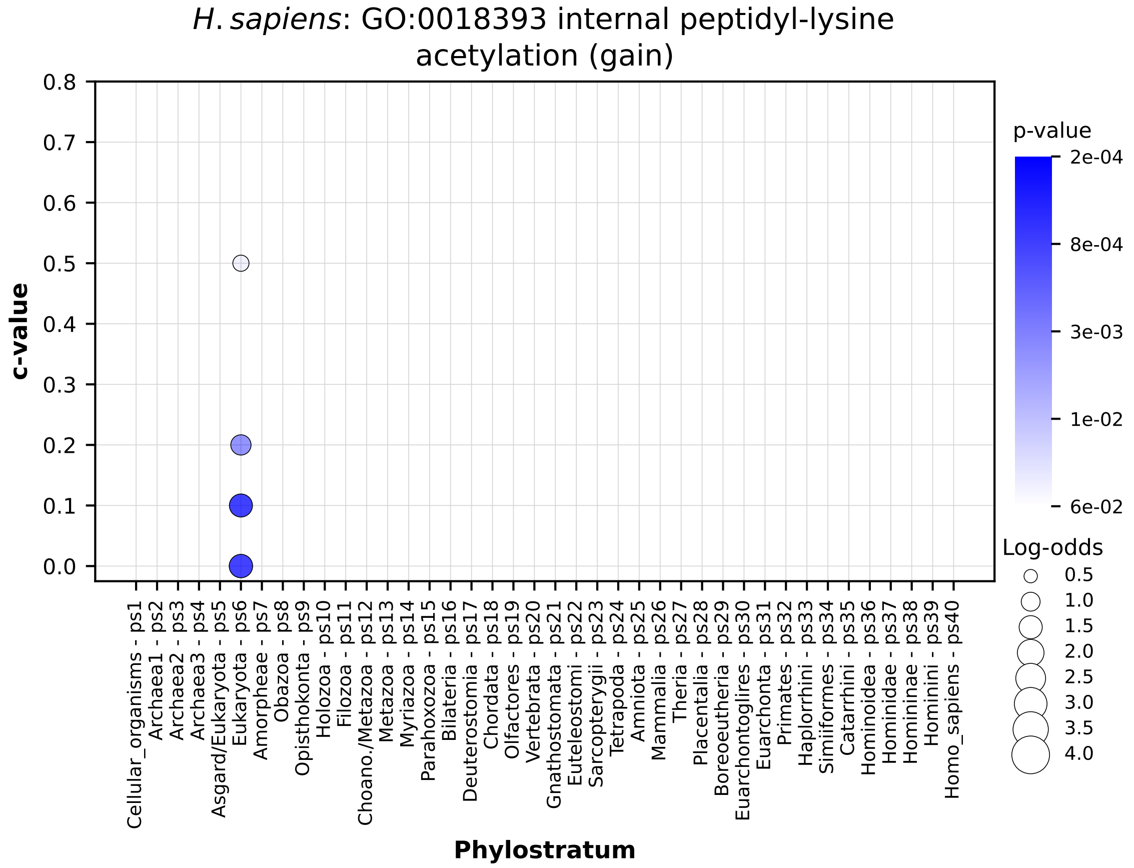

H. sapiens: GO:0018393 internal peptidyl-lysine acetylation (gain)
c-value
0.8
0.7
0.6
0.5
0.4
0.3
0.2
0.1
0.0
p-value
2e-04
8e-04
3e-03
1e-02
6e-02
Log-odds
0.5
1.0
1.5
2.0
2.5
3.0
3.5
4.0
Cellular_organisms - ps1
Archaea1 - ps2
Archaea2 - ps3
Archaea3 - ps4
Asgard/Eukaryota - ps5
Eukaryota - ps6
Amorpheae - ps7
Obazoa - ps8
Opisthokonta - ps9
Holozoa - ps10
Filozoa - ps11
Choano./Metazoa - ps12
Metazoa - ps13
Myriazoa - ps14
Parahoxozoa - ps15
Bilateria - ps16
Deuterostomia - ps17
Chordata - ps18
Olfactores - ps19
Vertebrata - ps20
Gnathostomata - ps21
Euteleostomi - ps22
Sarcopterygii - ps23
Tetrapoda - ps24
Amniota - ps25
Mammalia - ps26
Theria - ps27
Placentalia - ps28
Boreoeutheria - ps29
Euarchontoglires - ps30
Euarchonta - ps31
Primates - ps32
Haplorrhini - ps33
Simiiformes - ps34
Catarrhini - ps35
Hominoidea - ps36
Hominidae - ps37
Homininae - ps38
Hominini - ps39
Homo_sapiens - ps40
Phylostratum

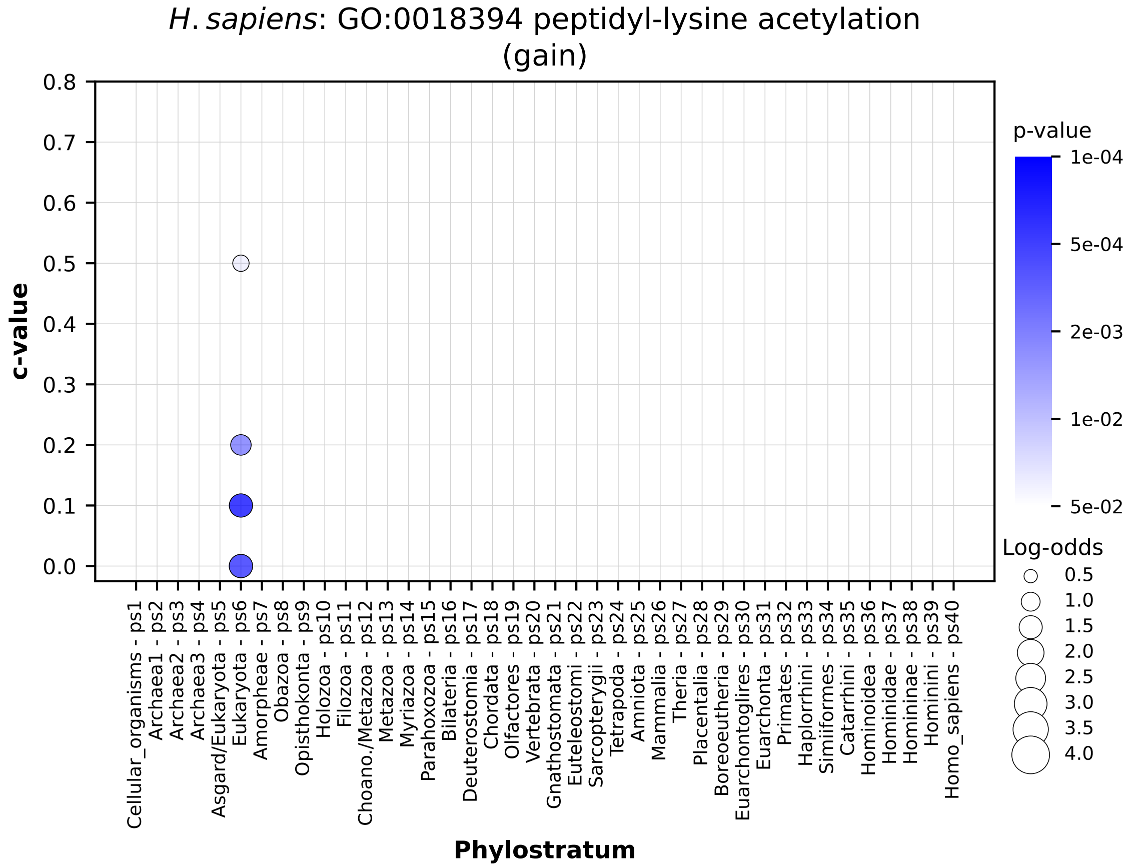

H. sapiens: GO:0018394 peptidyl-lysine acetylation
(gain)
c-value
0.0
0.1
0.2
0.3
0.4
0.5
0.6
0.7
0.8
p-value
1e-04
5e-04
2e-03
1e-02
5e-02
Log-odds
0.5
1.0
1.5
2.0
2.5
3.0
3.5
4.0
Cellular_organisms - ps1
Archaea1 - ps2
Archaea2 - ps3
Archaea3 - ps4
Asgard/Eukaryota - ps5
Eukaryota - ps6
Amorpheae - ps7
Obazoa - ps8
Opisthokonta - ps9
Holozoa - ps10
Filozoa - ps11
Choano./Metazoa - ps12
Metazoa - ps13
Myriazoa - ps14
Parahoxozoa - ps15
Bilateria - ps16
Deuterostomia - ps17
Chordata - ps18
Olfactores - ps19
Vertebrata - ps20
Gnathostomata - ps21
Euteleostomi - ps22
Sarcopterygii - ps23
Tetrapoda - ps24
Amniota - ps25
Mammalia - ps26
Theria - ps27
Placentalia - ps28
Boreoeutheria - ps29
Euarchontoglires - ps30
Euarchonta - ps31
Primates - ps32
Haplorrhini - ps33
Simiiformes - ps34
Catarrhini - ps35
Hominoidea - ps36
Hominidae - ps37
Homininae - ps38
Hominini - ps39
Homo_sapiens - ps40
Phylostratum

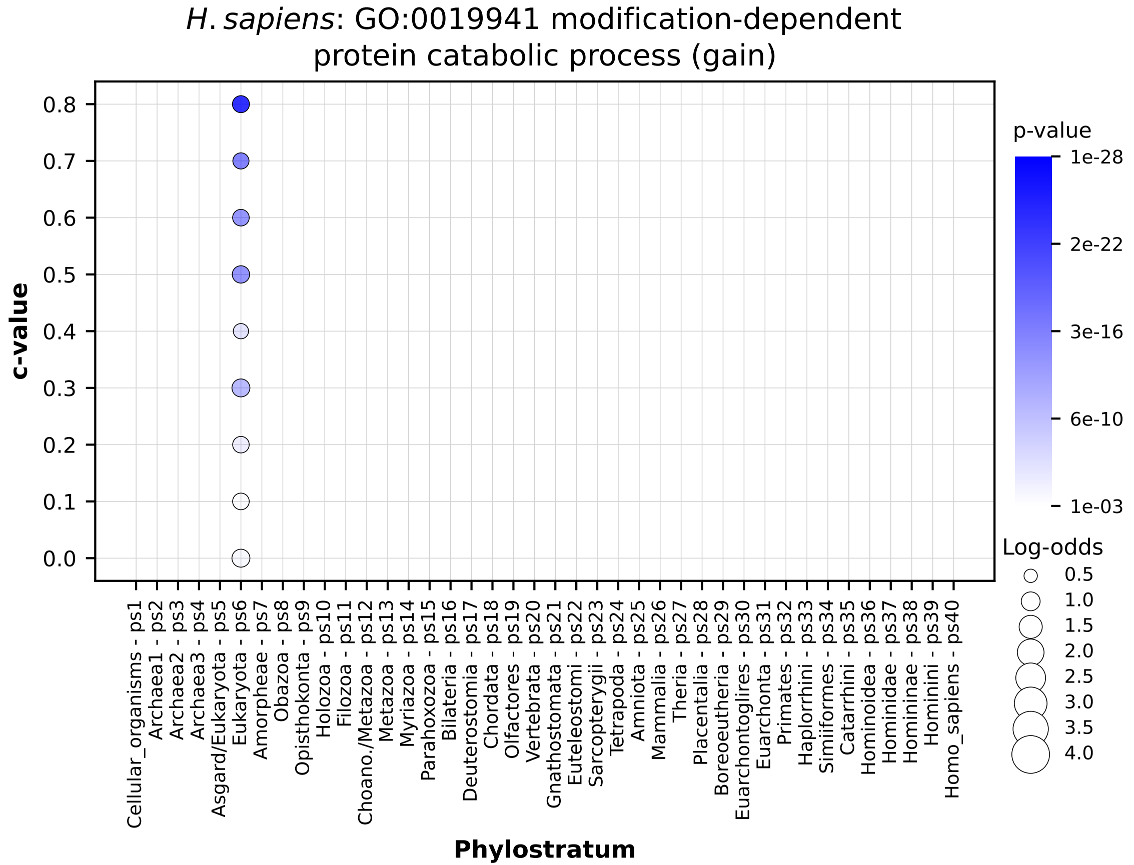

H. sapiens: GO:0019941 modification-dependent protein catabolic process (gain)
c-value
0.8
0.7
0.6
0.5
0.4
0.3
0.2
0.1
0.0
p-value
1e-28
2e-22
3e-16
6e-10
1e-03
Log-odds
0.5
1.0
1.5
2.0
2.5
3.0
3.5
4.0
Cellular_organisms - ps1
Archaea1 - ps2
Archaea2 - ps3
Archaea3 - ps4
Asgard/Eukaryota - ps5
Eukaryota - ps6
Amorpheae - ps7
Obazoa - ps8
Opisthokonta - ps9
Holozoa - ps10
Filozoa - ps11
Choano./Metazoa - ps12
Metazoa - ps13
Myriazoa - ps14
Parahoxozoa - ps15
Bilateria - ps16
Deuterostomia - ps17
Chordata - ps18
Olfactores - ps19
Vertebrata - ps20
Gnathostomata - ps21
Euteleostomi - ps22
Sarcopterygii - ps23
Tetrapoda - ps24
Amniota - ps25
Mammalia - ps26
Theria - ps27
Placentalia - ps28
Boreoeutheria - ps29
Euarchontoglires - ps30
Euarchonta - ps31
Primates - ps32
Haplorrhini - ps33
Simiiformes - ps34
Catarrhini - ps35
Hominoidea - ps36
Hominidae - ps37
Homininae - ps38
Hominini - ps39
Homo_sapiens - ps40
Phylostratum

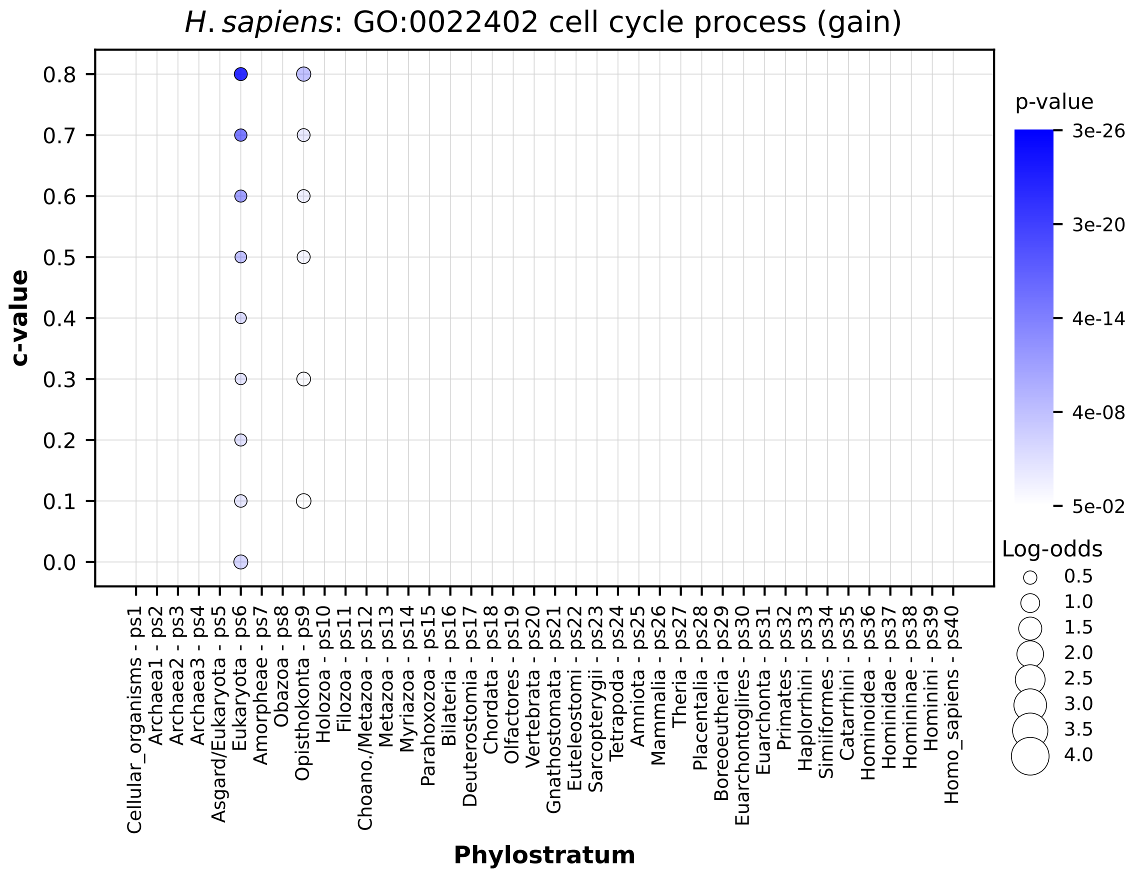
H. sapiens: GO:0022402 cell cycle process (gain)
c-value
0.8
0.7
0.6
0.5
0.4
0.3
0.2
0.1
0.0
p-value
3e-26
3e-20
4e-14
4e-08
5e-02
Log-odds
0.5
1.0
1.5
2.0
2.5
3.0
3.5
4.0
Cellular_organisms - ps1
Archaea1 - ps2
Archaea2 - ps3
Archaea3 - ps4
Asgard/Eukaryota - ps5
Eukaryota - ps6
Amorpheae - ps7
Obazoa - ps8
Opisthokonta - ps9
Holozoa - ps10
Filozoa - ps11
Choano./Metazoa - ps12
Metazoa - ps13
Myriazoa - ps14
Parahoxozoa - ps15
Bilateria - ps16
Deuterostomia - ps17
Chordata - ps18
Olfactores - ps19
Vertebrata - ps20
Gnathostomata - ps21
Euteleostomi - ps22
Sarcopterygii - ps23
Tetrapoda - ps24
Amniota - ps25
Mammalia - ps26
Theria - ps27
Placentalia - ps28
Boreoeutheria - ps29
Euarchontoglires - ps30
Euarchonta - ps31
Primates - ps32
Haplorrhini - ps33
Simiiformes - ps34
Catarrhini - ps35
Hominoidea - ps36
Hominidae - ps37
Homininae - ps38
Hominini - ps39
Homo_sapiens - ps40
Phylostratum

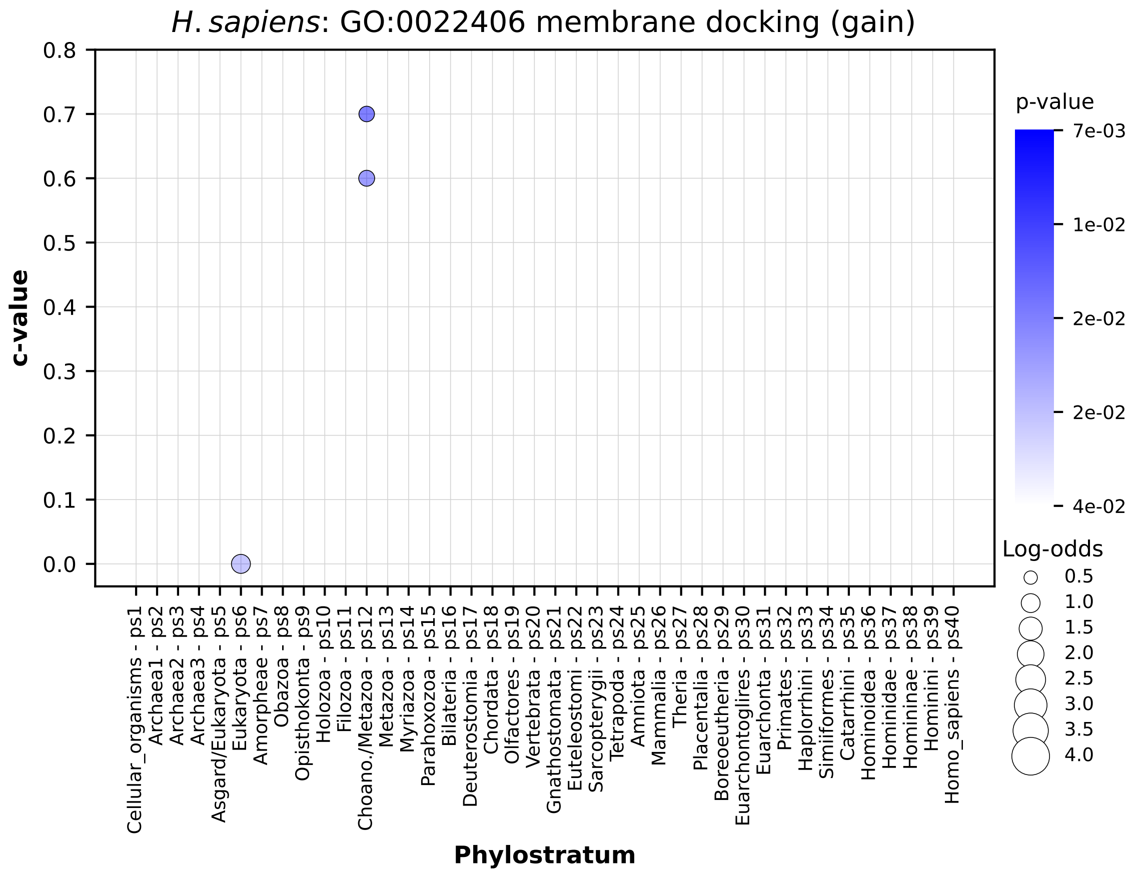

*H. sapiens*: GO:0022406 membrane docking (gain)
c-value
0.8
0.7
0.6
0.5
0.4
0.3
0.2
0.1
0.0
Phylostratum
Cellular_organisms - ps1
Archaea1 - ps2
Archaea2 - ps3
Archaea3 - ps4
Asgard/Eukaryota - ps5
Eukaryota - ps6
Amorpheae - ps7
Obazoa - ps8
Opisthokonta - ps9
Holozoa - ps10
Filozoa - ps11
Choano./Metazoa - ps12
Metazoa - ps13
Myriazoa - ps14
Parahoxozoa - ps15
Bilateria - ps16
Deuterostomia - ps17
Chordata - ps18
Olfactores - ps19
Vertebrata - ps20
Gnathostomata - ps21
Euteleostomi - ps22
Sarcopterygii - ps23
Tetrapoda - ps24
Amniota - ps25
Mammalia - ps26
Theria - ps27
Placentalia - ps28
Boreoeutheria - ps29
Euarchontoglires - ps30
Euarchonta - ps31
Primates - ps32
Haplorrhini - ps33
Simiiformes - ps34
Catarrhini - ps35
Hominoidea - ps36
Hominidae - ps37
Homininae - ps38
Hominini - ps39
Homo_sapiens - ps40
p-value
7e-03
1e-02
2e-02
2e-02
4e-02
Log-odds
0.5
1.0
1.5
2.0
2.5
3.0
3.5
4.0

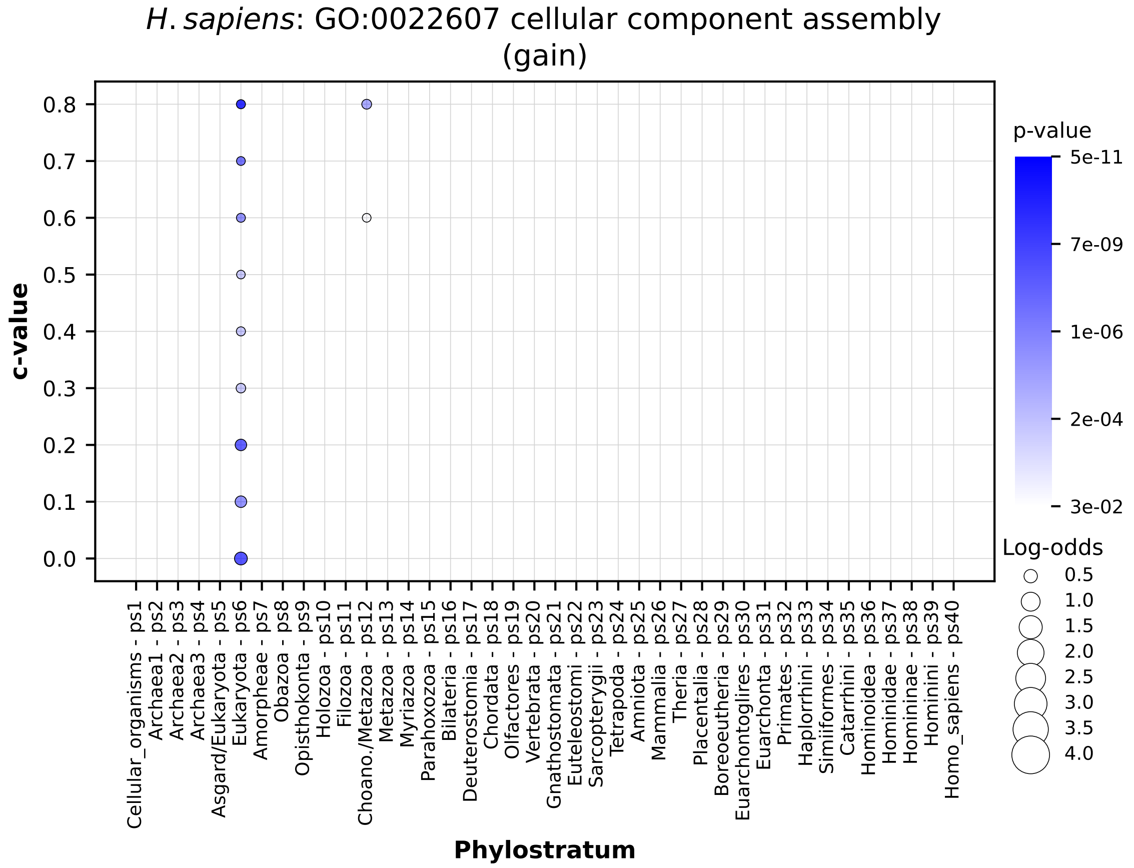

H. sapiens: GO:0022607 cellular component assembly
(gain)
c-value
0.8
0.7
0.6
0.5
0.4
0.3
0.2
0.1
0.0
p-value
5e-11
7e-09
1e-06
2e-04
3e-02
Log-odds
0.5
1.0
1.5
2.0
2.5
3.0
3.5
4.0
Cellular_organisms - ps1
Archaea1 - ps2
Archaea2 - ps3
Archaea3 - ps4
Asgard/Eukaryota - ps5
Eukaryota - ps6
Amorpheae - ps7
Obazoa - ps8
Opisthokonta - ps9
Holozoa - ps10
Filozoa - ps11
Choano./Metazoa - ps12
Metazoa - ps13
Myriazoa - ps14
Parahoxozoa - ps15
Bilateria - ps16
Deuterostomia - ps17
Chordata - ps18
Olfactores - ps19
Vertebrata - ps20
Gnathostomata - ps21
Euteleostomi - ps22
Sarcopterygii - ps23
Tetrapoda - ps24
Amniota - ps25
Mammalia - ps26
Theria - ps27
Placentalia - ps28
Boreoeutheria - ps29
Euarchontoglires - ps30
Euarchonta - ps31
Primates - ps32
Haplorrhini - ps33
Simiiformes - ps34
Catarrhini - ps35
Hominoidea - ps36
Hominidae - ps37
Homininae - ps38
Hominini - ps39
Homo_sapiens - ps40
Phylostratum

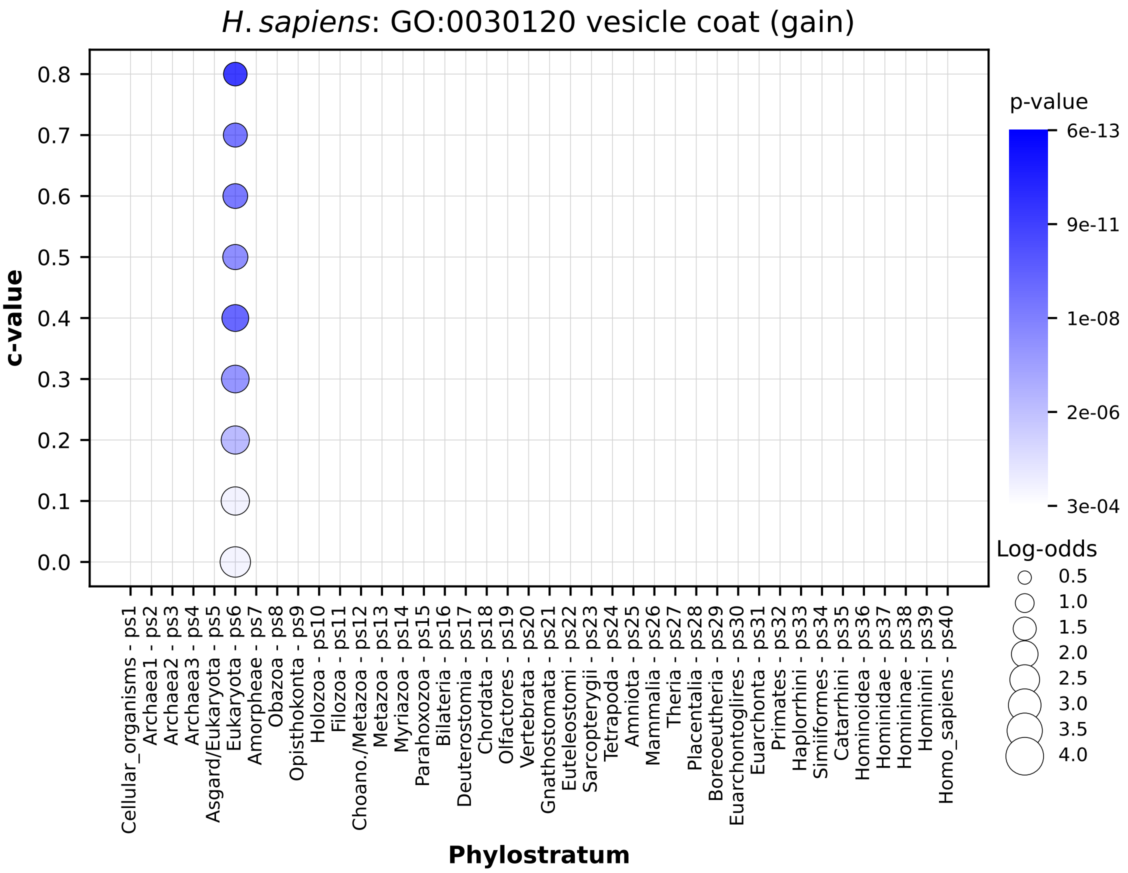
H. sapiens: GO:0030120 vesicle coat (gain)
c-value
0.0
0.1
0.2
0.3
0.4
0.5
0.6
0.7
0.8
Phylostratum
Cellular_organisms - ps1
Archaea1 - ps2
Archaea2 - ps3
Archaea3 - ps4
Asgard/Eukaryota - ps5
Eukaryota - ps6
Amorpheae - ps7
Obazoa - ps8
Opisthokonta - ps9
Holozoa - ps10
Filozoa - ps11
Choano./Metazoa - ps12
Metazoa - ps13
Myriazoa - ps14
Parahoxozoa - ps15
Bilateria - ps16
Deuterostomia - ps17
Chordata - ps18
Olfactores - ps19
Vertebrata - ps20
Gnathostomata - ps21
Euteleostomi - ps22
Sarcopterygii - ps23
Tetrapoda - ps24
Amniota - ps25
Mammalia - ps26
Theria - ps27
Placentalia - ps28
Boreoeutheria - ps29
Euarchontoglires - ps30
Euarchonta - ps31
Primates - ps32
Haplorrhini - ps33
Simiiformes - ps34
Catarrhini - ps35
Hominoidea - ps36
Hominidae - ps37
Homininae - ps38
Hominini - ps39
Homo_sapiens - ps40
p-value
6e-13
9e-11
1e-08
2e-06
3e-04
Log-odds
0.5
1.0
1.5
2.0
2.5
3.0
3.5
4.0

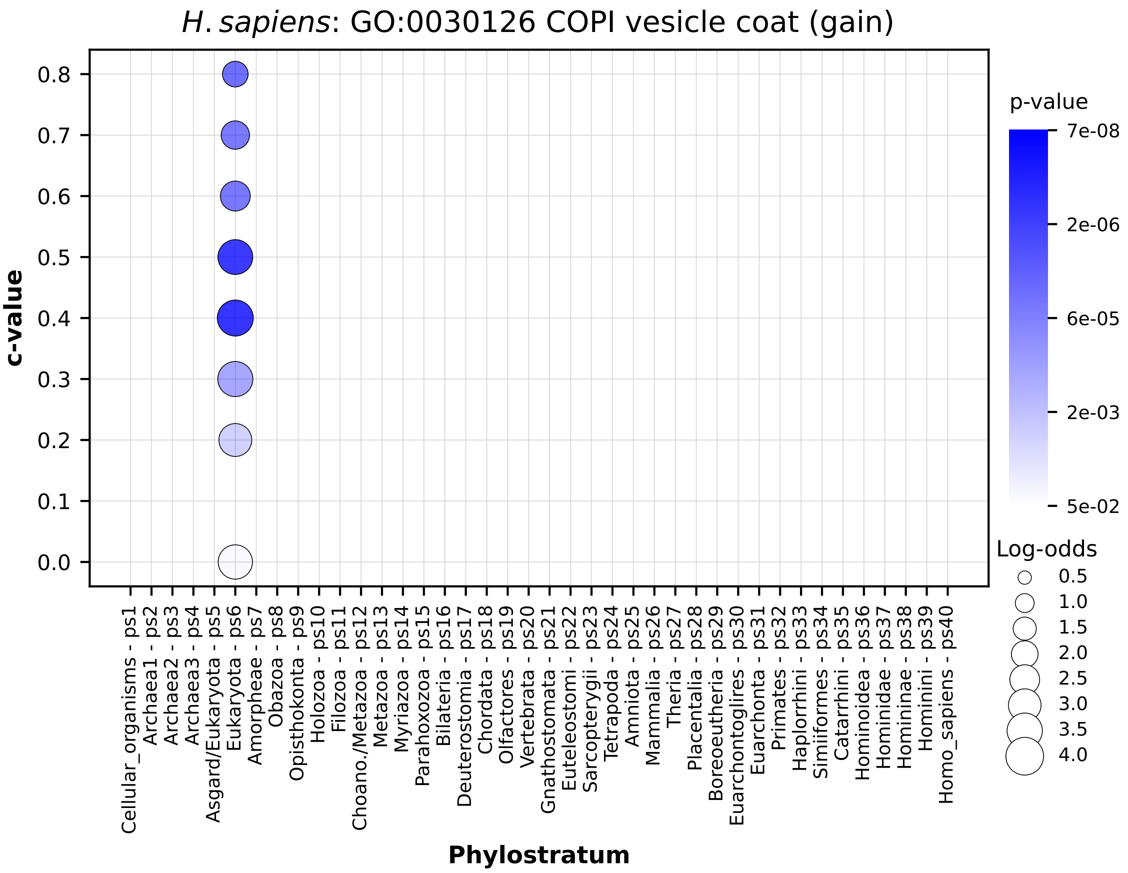
H. sapiens: GO:0030126 COPI vesicle coat (gain)
c-value
0.8
0.7
0.6
0.5
0.4
0.3
0.2
0.1
0.0
Phylostratum
Cellular_organisms - ps1
Archaea1 - ps2
Archaea2 - ps3
Archaea3 - ps4
Asgard/Eukaryota - ps5
Eukaryota - ps6
Amorpheae - ps7
Obazoa - ps8
Opisthokonta - ps9
Holozoa - ps10
Filozoa - ps11
Choano./Metazoa - ps12
Metazoa - ps13
Myriazoa - ps14
Parahoxozoa - ps15
Bilateria - ps16
Deuterostomia - ps17
Chordata - ps18
Olfactores - ps19
Vertebrata - ps20
Gnathostomata - ps21
Euteleostomi - ps22
Sarcopterygii - ps23
Tetrapoda - ps24
Amniota - ps25
Mammalia - ps26
Theria - ps27
Placentalia - ps28
Boreoeutheria - ps29
Euarchontoglires - ps30
Euarchonta - ps31
Primates - ps32
Haplorrhini - ps33
Simiiformes - ps34
Catarrhini - ps35
Hominoidea - ps36
Hominidae - ps37
Homininae - ps38
Hominini - ps39
Homo_sapiens - ps40
p-value
7e-08
2e-06
6e-05
2e-03
5e-02
Log-odds
0.5
1.0
1.5
2.0
2.5
3.0
3.5
4.0

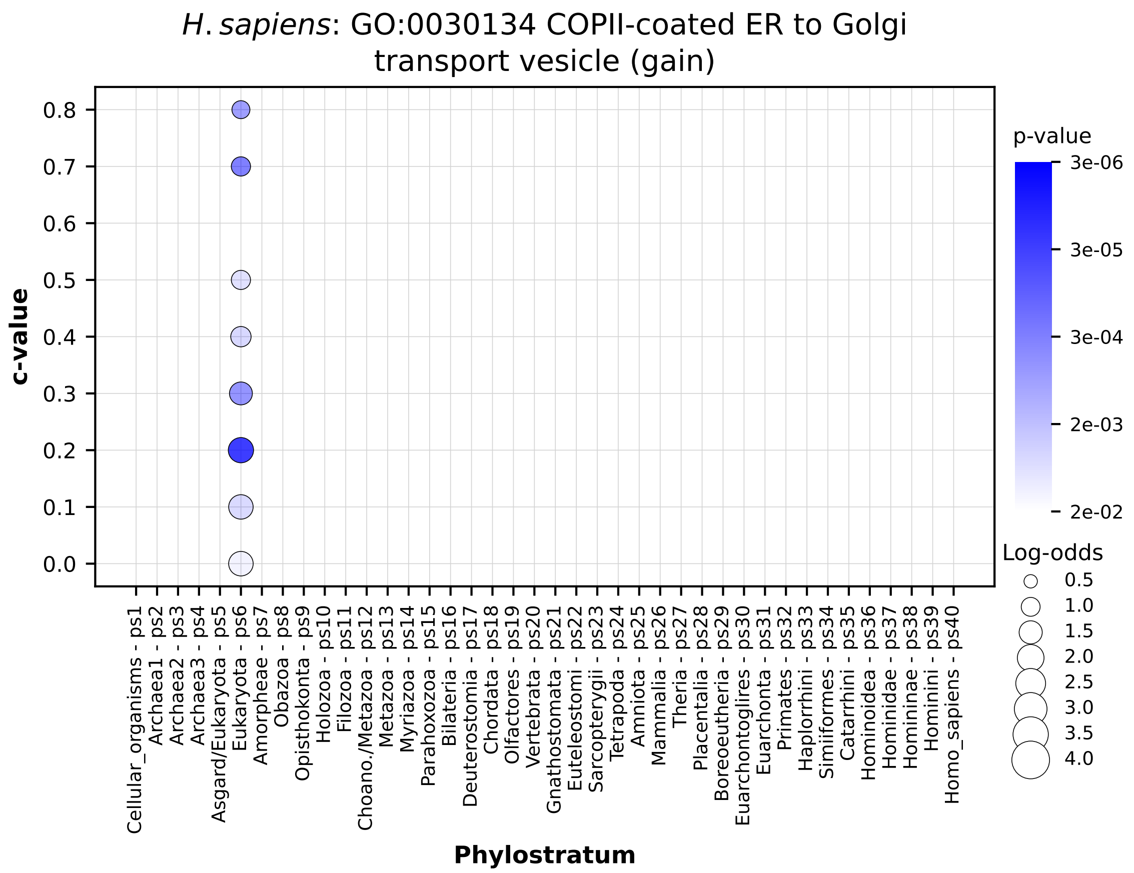

H. sapiens: GO:0030134 COPII-coated ER to Golgi transport vesicle (gain)
c-value
0.0
0.1
0.2
0.3
0.4
0.5
0.6
0.7
0.8
p-value
3e-06
3e-05
3e-04
2e-03
2e-02
Log-odds
0.5
1.0
1.5
2.0
2.5
3.0
3.5
4.0
Cellular_organisms - ps1
Archaea1 - ps2
Archaea2 - ps3
Archaea3 - ps4
Asgard/Eukaryota - ps5
Eukaryota - ps6
Amorpheae - ps7
Obazoa - ps8
Opisthokonta - ps9
Holozoa - ps10
Filozoa - ps11
Choano./Metazoa - ps12
Metazoa - ps13
Myriazoa - ps14
Parahoxozoa - ps15
Bilateria - ps16
Deuterostomia - ps17
Chordata - ps18
Olfactores - ps19
Vertebrata - ps20
Gnathostomata - ps21
Euteleostomi - ps22
Sarcopterygii - ps23
Tetrapoda - ps24
Amniota - ps25
Mammalia - ps26
Theria - ps27
Placentalia - ps28
Boreoeutheria - ps29
Euarchontoglires - ps30
Euarchonta - ps31
Primates - ps32
Haplorrhini - ps33
Simiiformes - ps34
Catarrhini - ps35
Hominoidea - ps36
Hominidae - ps37
Homininae - ps38
Hominini - ps39
Homo_sapiens - ps40
Phylostratum

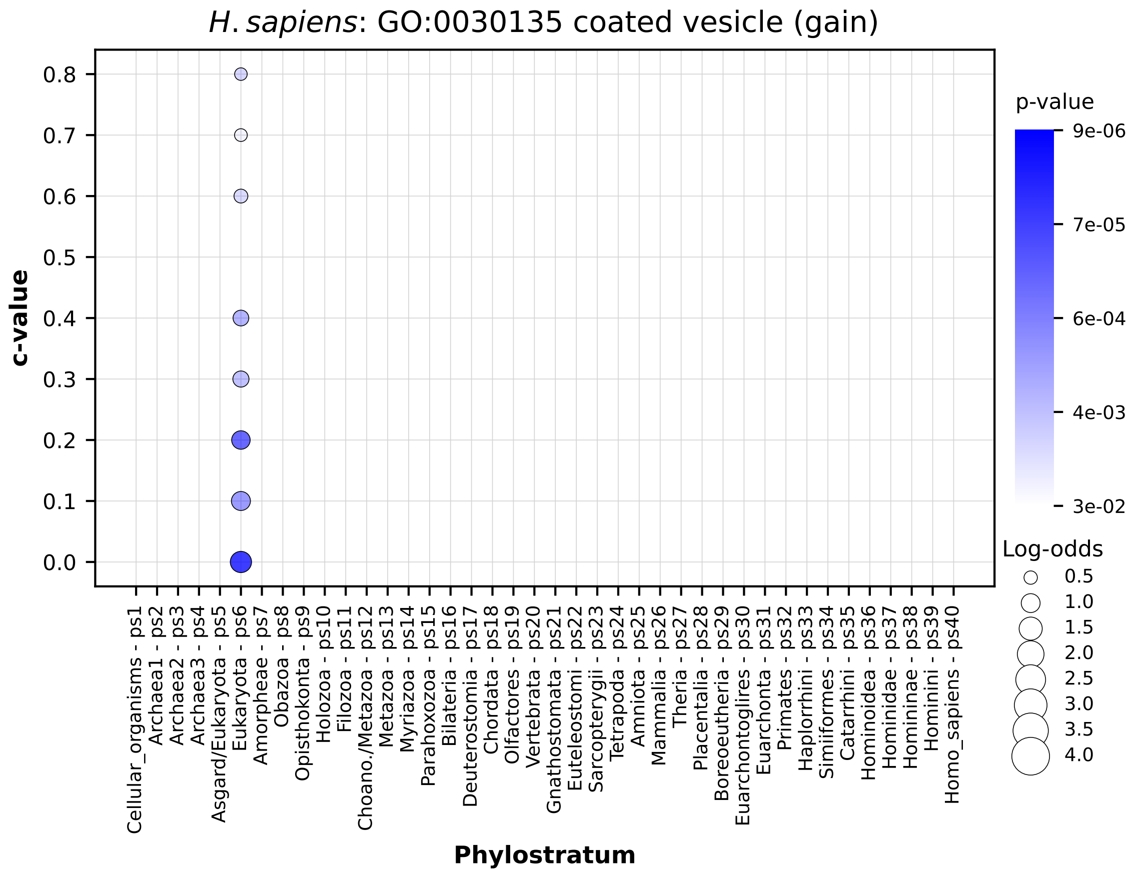
H. sapiens: GO:0030135 coated vesicle (gain)
c-value
0.8
0.7
0.6
0.5
0.4
0.3
0.2
0.1
0.0
Phylostratum
Cellular_organisms - ps1
Archaea1 - ps2
Archaea2 - ps3
Archaea3 - ps4
Asgard/Eukaryota - ps5
Eukaryota - ps6
Amorpheae - ps7
Obazoa - ps8
Opisthokonta - ps9
Holozoa - ps10
Filozoa - ps11
Choano./Metazoa - ps12
Metazoa - ps13
Myriazoa - ps14
Parahoxozoa - ps15
Bilateria - ps16
Deuterostomia - ps17
Chordata - ps18
Olfactores - ps19
Vertebrata - ps20
Gnathostomata - ps21
Euteleostomi - ps22
Sarcopterygii - ps23
Tetrapoda - ps24
Amniota - ps25
Mammalia - ps26
Theria - ps27
Placentalia - ps28
Boreoeutheria - ps29
Euarchontoglires - ps30
Euarchonta - ps31
Primates - ps32
Haplorrhini - ps33
Simiiformes - ps34
Catarrhini - ps35
Hominoidea - ps36
Hominidae - ps37
Homininae - ps38
Hominini - ps39
Homo_sapiens - ps40
p-value
9e-06
7e-05
6e-04
4e-03
3e-02
Log-odds
0.5
1.0
1.5
2.0
2.5
3.0
3.5
4.0

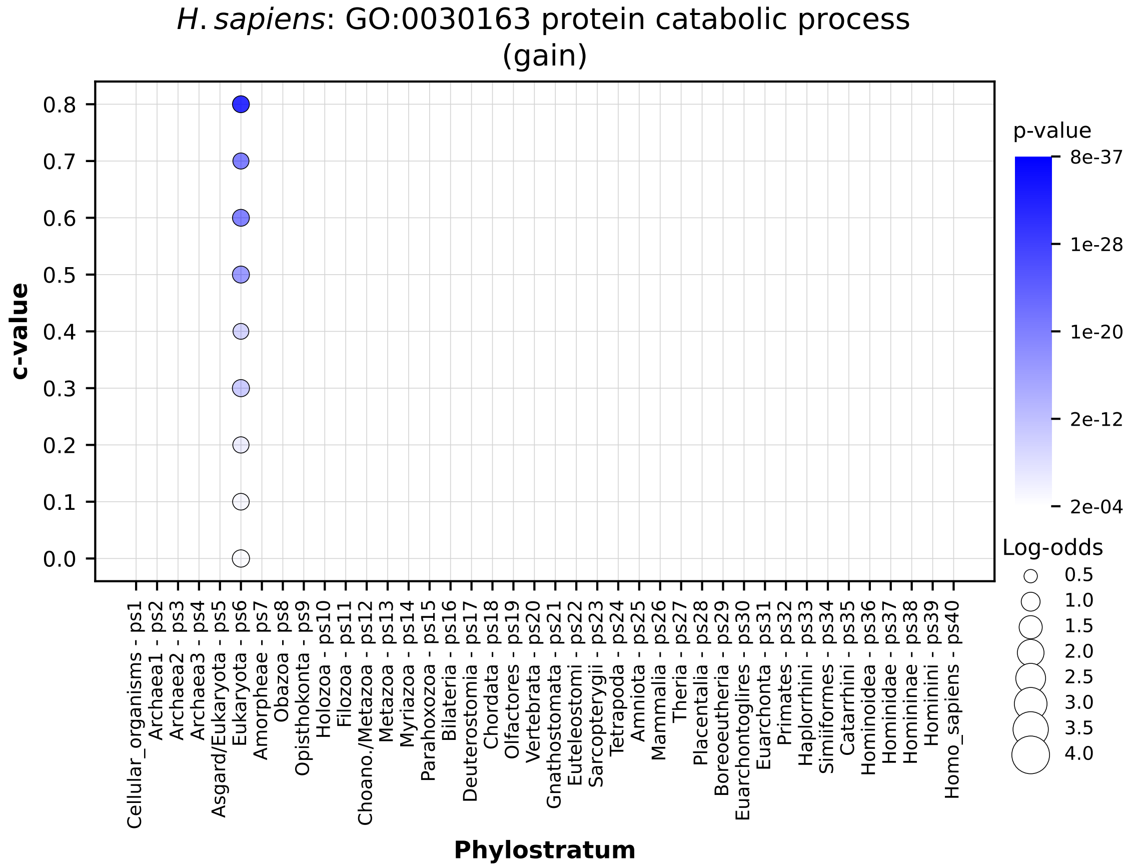
H. sapiens: GO:0030163 protein catabolic process
(gain)
c-value
0.8
0.7
0.6
0.5
0.4
0.3
0.2
0.1
0.0
p-value
8e-37
1e-28
1e-20
2e-12
2e-04
Log-odds
0.5
1.0
1.5
2.0
2.5
3.0
3.5
4.0
Cellular_organisms - ps1
Archaea1 - ps2
Archaea2 - ps3
Archaea3 - ps4
Asgard/Eukaryota - ps5
Eukaryota - ps6
Amorpheae - ps7
Obazoa - ps8
Opisthokonta - ps9
Holozoa - ps10
Filozoa - ps11
Choano./Metazoa - ps12
Metazoa - ps13
Myriazoa - ps14
Parahoxozoa - ps15
Bilateria - ps16
Deuterostomia - ps17
Chordata - ps18
Olfactores - ps19
Vertebrata - ps20
Gnathostomata - ps21
Euteleostomi - ps22
Sarcopterygii - ps23
Tetrapoda - ps24
Amniota - ps25
Mammalia - ps26
Theria - ps27
Placentalia - ps28
Boreoeutheria - ps29
Euarchontoglires - ps30
Euarchonta - ps31
Primates - ps32
Haplorrhini - ps33
Simiiformes - ps34
Catarrhini - ps35
Hominoidea - ps36
Hominidae - ps37
Homininae - ps38
Hominini - ps39
Homo_sapiens - ps40
Phylostratum

## *H. sapiens*: GO:0030176 obsolete integral component of endoplasmic reticulum membrane (gain)

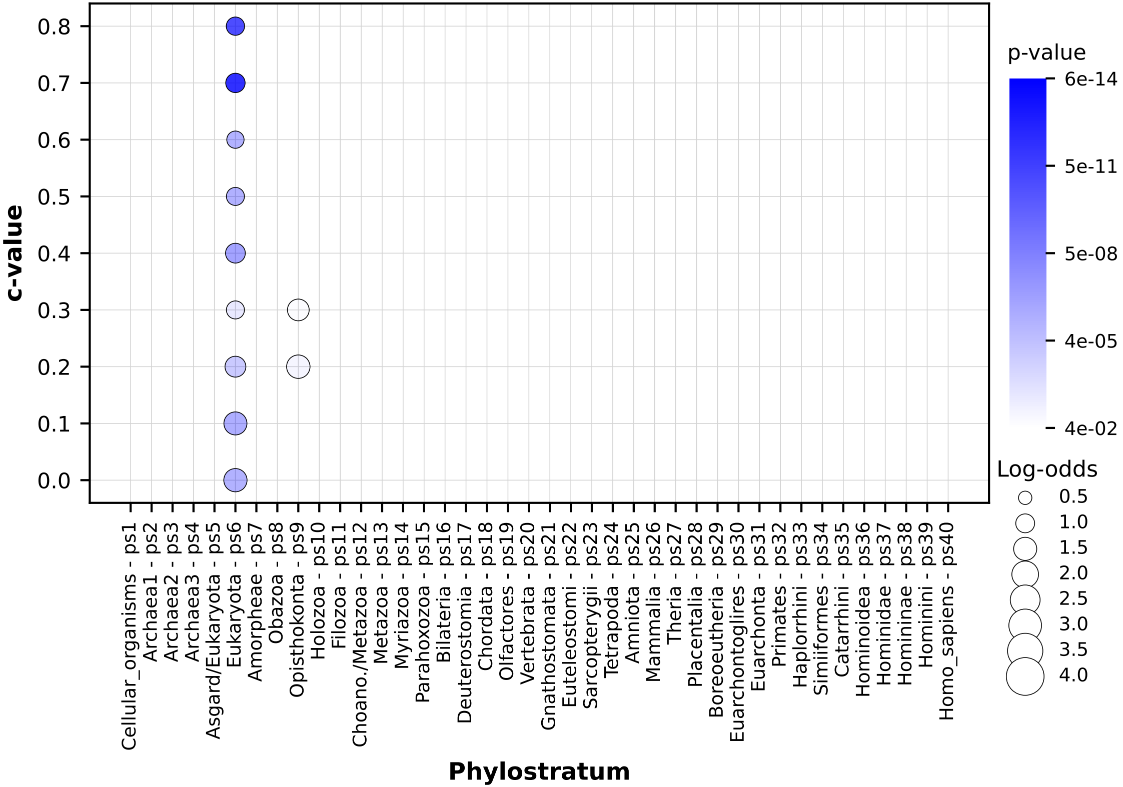

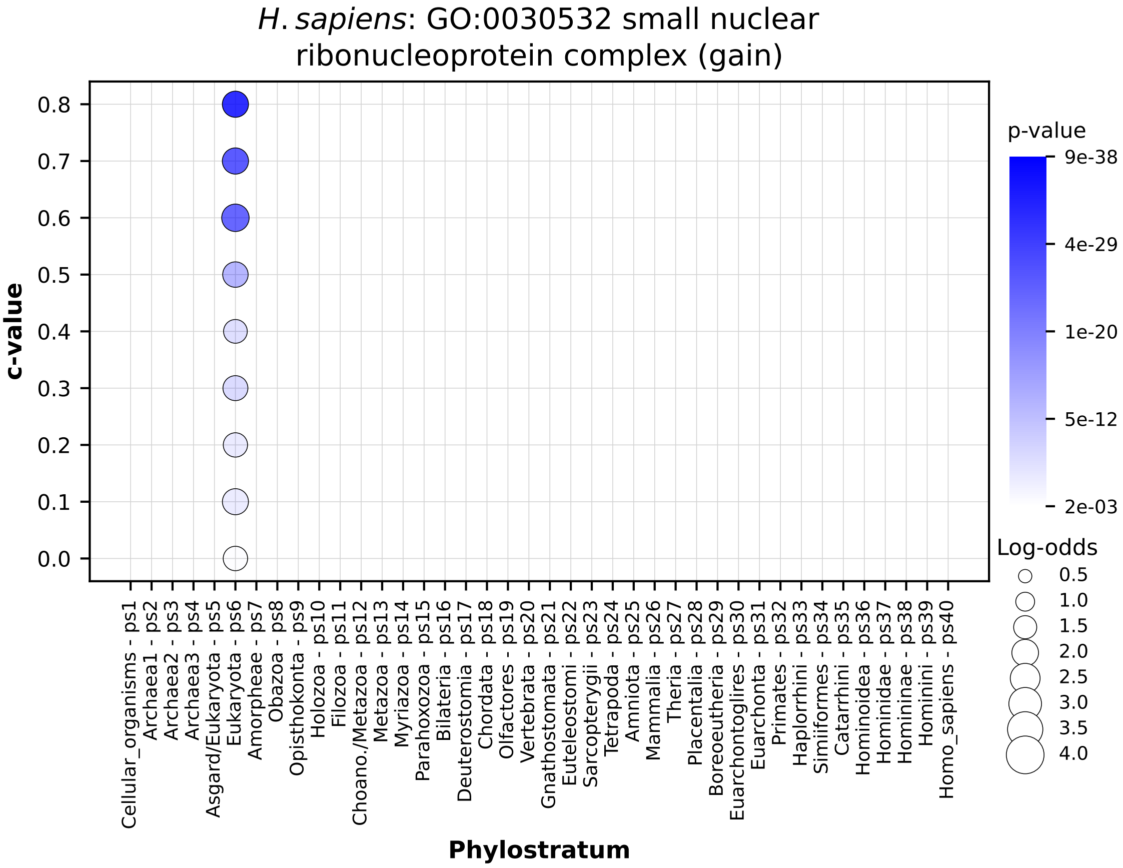
H. sapiens: GO:0030532 small nuclear ribonucleoprotein complex (gain)
c-value
0.8
0.7
0.6
0.5
0.4
0.3
0.2
0.1
0.0
Phylostratum
Cellular_organisms - ps1
Archaea1 - ps2
Archaea2 - ps3
Archaea3 - ps4
Asgard/Eukaryota - ps5
Eukaryota - ps6
Amorpheae - ps7
Obazoa - ps8
Opisthokonta - ps9
Holozoa - ps10
Filozoa - ps11
Choano./Metazoa - ps12
Metazoa - ps13
Myriazoa - ps14
Parahoxozoa - ps15
Bilateria - ps16
Deuterostomia - ps17
Chordata - ps18
Olfactores - ps19
Vertebrata - ps20
Gnathostomata - ps21
Euteleostomi - ps22
Sarcopterygii - ps23
Tetrapoda - ps24
Amniota - ps25
Mammalia - ps26
Theria - ps27
Placentalia - ps28
Boreoeutheria - ps29
Euarchontoglires - ps30
Euarchonta - ps31
Primates - ps32
Haplorrhini - ps33
Simiiformes - ps34
Catarrhini - ps35
Hominoidea - ps36
Hominidae - ps37
Homininae - ps38
Hominini - ps39
Homo_sapiens - ps40
p-value
9e-38
4e-29
1e-20
5e-12
2e-03
Log-odds
0.5
1.0
1.5
2.0
2.5
3.0
3.5
4.0

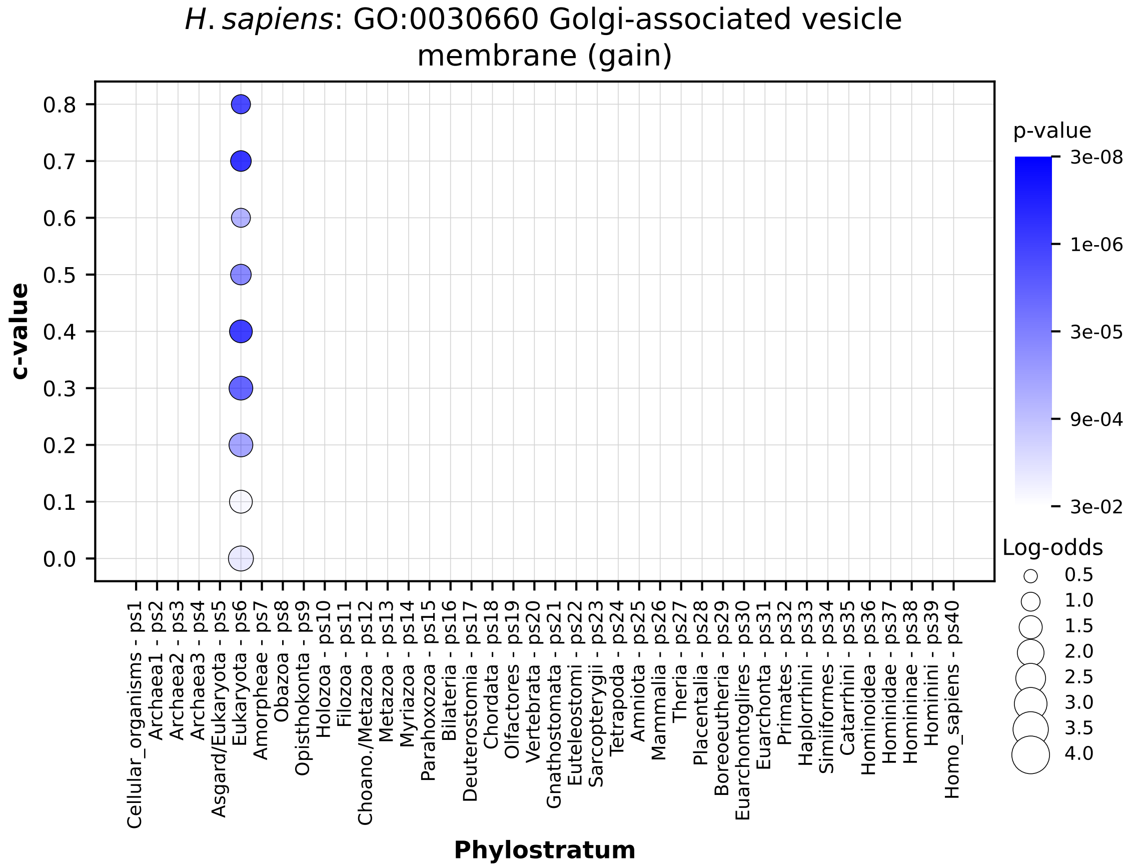

H. sapiens: GO:0030660 Golgi-associated vesicle membrane (gain)
c-value
0.8
0.7
0.6
0.5
0.4
0.3
0.2
0.1
0.0
p-value
3e-08
1e-06
3e-05
9e-04
3e-02
Log-odds
0.5
1.0
1.5
2.0
2.5
3.0
3.5
4.0
Cellular_organisms - ps1
Archaea1 - ps2
Archaea2 - ps3
Archaea3 - ps4
Asgard/Eukaryota - ps5
Eukaryota - ps6
Amorpheae - ps7
Obazoa - ps8
Opisthokonta - ps9
Holozoa - ps10
Filozoa - ps11
Choano./Metazoa - ps12
Metazoa - ps13
Myriazoa - ps14
Parahoxozoa - ps15
Bilateria - ps16
Deuterostomia - ps17
Chordata - ps18
Olfactores - ps19
Vertebrata - ps20
Gnathostomata - ps21
Euteleostomi - ps22
Sarcopterygii - ps23
Tetrapoda - ps24
Amniota - ps25
Mammalia - ps26
Theria - ps27
Placentalia - ps28
Boreoeutheria - ps29
Euarchontoglires - ps30
Euarchonta - ps31
Primates - ps32
Haplorrhini - ps33
Simiiformes - ps34
Catarrhini - ps35
Hominoidea - ps36
Hominidae - ps37
Homininae - ps38
Hominini - ps39
Homo_sapiens - ps40
Phylostratum

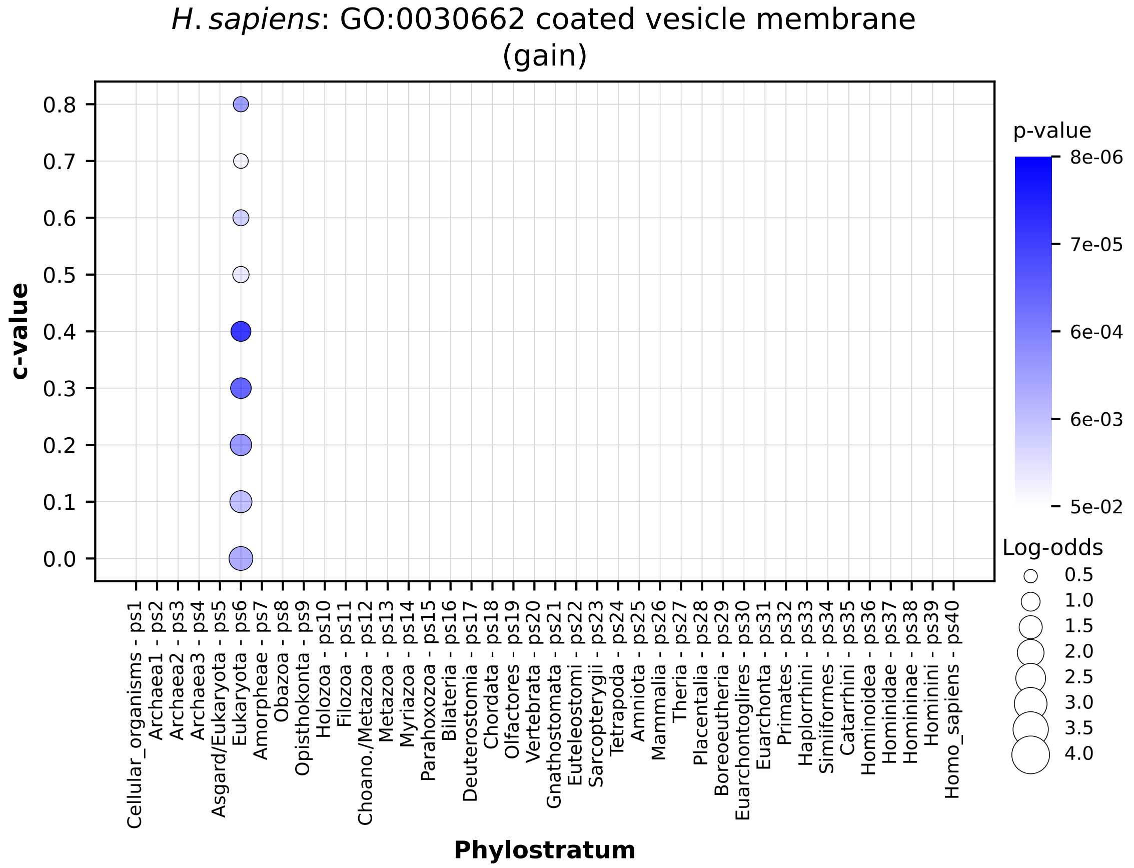
*H. sapiens*: GO:0030662 coated vesicle membrane
(gain)
c-value
0.0
0.1
0.2
0.3
0.4
0.5
0.6
0.7
0.8
p-value
8e-06
7e-05
6e-04
6e-03
5e-02
Log-odds
0.5
1.0
1.5
2.0
2.5
3.0
3.5
4.0
Cellular_organisms - ps1
Archaea1 - ps2
Archaea2 - ps3
Archaea3 - ps4
Asgard/Eukaryota - ps5
Eukaryota - ps6
Amorpheae - ps7
Obazoa - ps8
Opisthokonta - ps9
Holozoa - ps10
Filozoa - ps11
Choano./Metazoa - ps12
Metazoa - ps13
Myriazoa - ps14
Parahoxozoa - ps15
Bilateria - ps16
Deuterostomia - ps17
Chordata - ps18
Olfactores - ps19
Vertebrata - ps20
Gnathostomata - ps21
Euteleostomi - ps22
Sarcopterygii - ps23
Tetrapoda - ps24
Amniota - ps25
Mammalia - ps26
Theria - ps27
Placentalia - ps28
Boreoeutheria - ps29
Euarchontoglires - ps30
Euarchonta - ps31
Primates - ps32
Haplorrhini - ps33
Simiiformes - ps34
Catarrhini - ps35
Hominoidea - ps36
Hominidae - ps37
Homininae - ps38
Hominini - ps39
Homo_sapiens - ps40
Phylostratum

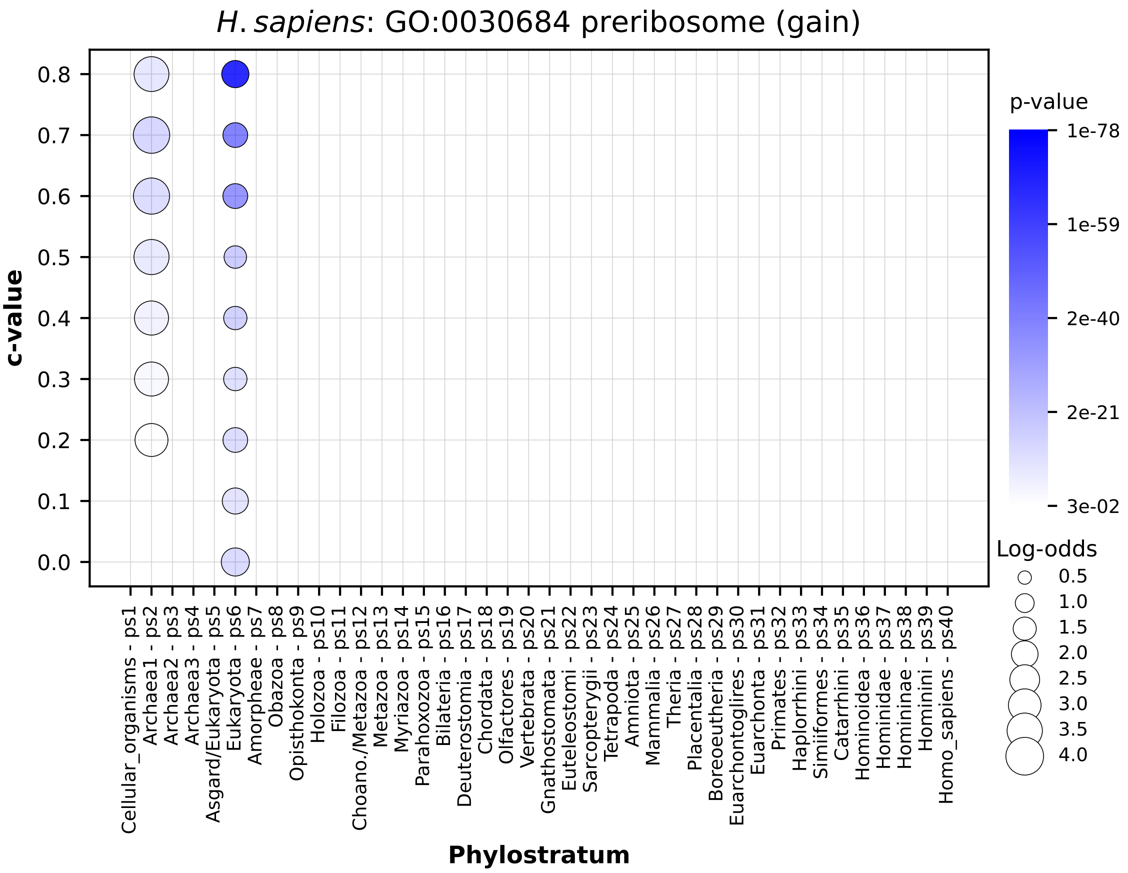

H. sapiens: GO:0030684 preribosome (gain)
c-value
0.8
0.7
0.6
0.5
0.4
0.3
0.2
0.1
0.0
p-value
1e-78
1e-59
2e-40
2e-21
3e-02
Log-odds
0.5
1.0
1.5
2.0
2.5
3.0
3.5
4.0
Cellular_organisms - ps1
Archaea1 - ps2
Archaea2 - ps3
Archaea3 - ps4
Asgard/Eukaryota - ps5
Eukaryota - ps6
Amorpheae - ps7
Obazoa - ps8
Opisthokonta - ps9
Holozoa - ps10
Filozoa - ps11
Choano./Metazoa - ps12
Metazoa - ps13
Myriazoa - ps14
Parahoxozoa - ps15
Bilateria - ps16
Deuterostomia - ps17
Chordata - ps18
Olfactores - ps19
Vertebrata - ps20
Gnathostomata - ps21
Euteleostomi - ps22
Sarcopterygii - ps23
Tetrapoda - ps24
Amniota - ps25
Mammalia - ps26
Theria - ps27
Placentalia - ps28
Boreoeutheria - ps29
Euarchontoglires - ps30
Euarchonta - ps31
Primates - ps32
Haplorrhini - ps33
Simiiformes - ps34
Catarrhini - ps35
Hominoidea - ps36
Hominidae - ps37
Homininae - ps38
Hominini - ps39
Homo_sapiens - ps40
Phylostratum

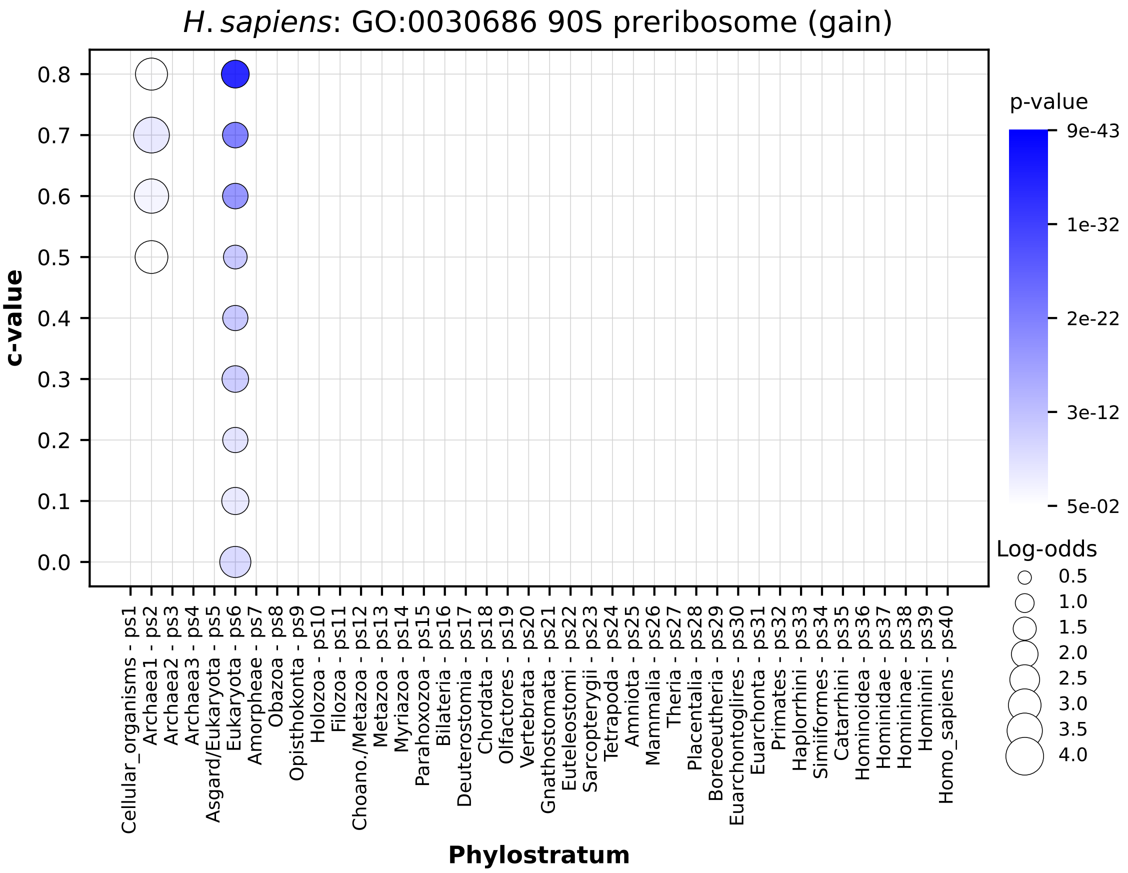

H. sapiens: GO:0030686 90S preribosome (gain)
c-value
0.8
0.7
0.6
0.5
0.4
0.3
0.2
0.1
0.0
p-value
9e-43
1e-32
2e-22
3e-12
5e-02
Log-odds
0.5
1.0
1.5
2.0
2.5
3.0
3.5
4.0
Cellular_organisms - ps1
Archaea1 - ps2
Archaea2 - ps3
Archaea3 - ps4
Asgard/Eukaryota - ps5
Eukaryota - ps6
Amorpheae - ps7
Obazoa - ps8
Opisthokonta - ps9
Holozoa - ps10
Filozoa - ps11
Choano./Metazoa - ps12
Metazoa - ps13
Myriazoa - ps14
Parahoxozoa - ps15
Bilateria - ps16
Deuterostomia - ps17
Chordata - ps18
Olfactores - ps19
Vertebrata - ps20
Gnathostomata - ps21
Euteleostomi - ps22
Sarcopterygii - ps23
Tetrapoda - ps24
Amniota - ps25
Mammalia - ps26
Theria - ps27
Placentalia - ps28
Boreoeutheria - ps29
Euarchontoglires - ps30
Euarchonta - ps31
Primates - ps32
Haplorrhini - ps33
Simiiformes - ps34
Catarrhini - ps35
Hominoidea - ps36
Hominidae - ps37
Homininae - ps38
Hominini - ps39
Homo_sapiens - ps40
Phylostratum

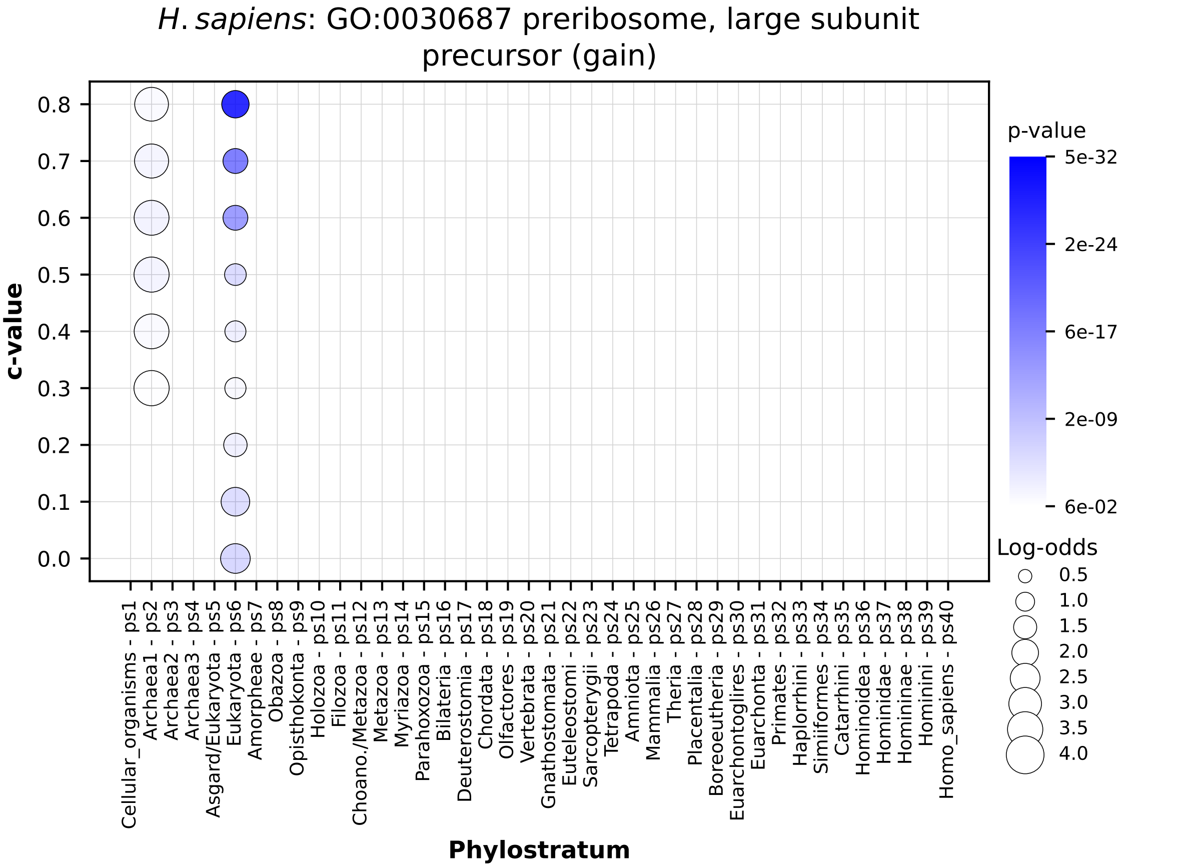
H. sapiens: GO:0030687 preribosome, large subunit precursor (gain)
c-value
0.8
0.7
0.6
0.5
0.4
0.3
0.2
0.1
0.0
Cellular_organisms - ps1
Archaea1 - ps2
Archaea2 - ps3
Archaea3 - ps4
Asgard/Eukaryota - ps5
Eukaryota - ps6
Amorpheae - ps7
Obazoa - ps8
Opisthokonta - ps9
Holozoa - ps10
Filozoa - ps11
Choano./Metazoa - ps12
Metazoa - ps13
Myriazoa - ps14
Parahoxozoa - ps15
Bilateria - ps16
Deuterostomia - ps17
Chordata - ps18
Olfactores - ps19
Vertebrata - ps20
Gnathostomata - ps21
Euteleostomi - ps22
Sarcopterygii - ps23
Tetrapoda - ps24
Amniota - ps25
Mammalia - ps26
Theria - ps27
Placentalia - ps28
Boreoeutheria - ps29
Euarchontoglires - ps30
Euarchonta - ps31
Primates - ps32
Haplorrhini - ps33
Simiiformes - ps34
Catarrhini - ps35
Hominoidea - ps36
Hominidae - ps37
Homininae - ps38
Hominini - ps39
Homo_sapiens - ps40
Phylostratum
p-value
5e-32
2e-24
6e-17
2e-09
6e-02
Log-odds
0.5
1.0
1.5
2.0
2.5
3.0
3.5
4.0

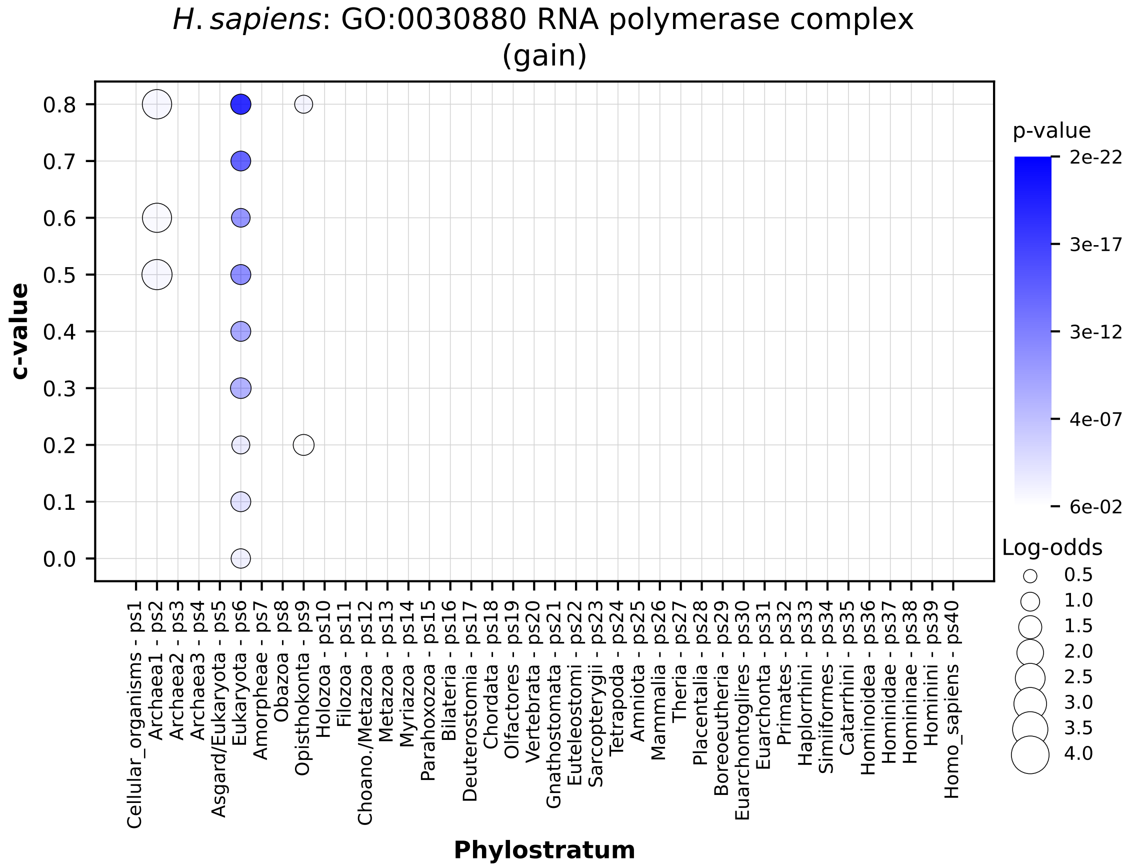

H. sapiens: GO:0030880 RNA polymerase complex
(gain)
c-value
0.8
0.7
0.6
0.5
0.4
0.3
0.2
0.1
0.0
p-value
2e-22
3e-17
3e-12
4e-07
6e-02
Log-odds
0.5
1.0
1.5
2.0
2.5
3.0
3.5
4.0
Cellular_organisms - ps1
Archaea1 - ps2
Archaea2 - ps3
Archaea3 - ps4
Asgard/Eukaryota - ps5
Eukaryota - ps6
Amorpheae - ps7
Obazoa - ps8
Opisthokonta - ps9
Holozoa - ps10
Filozoa - ps11
Choano./Metazoa - ps12
Metazoa - ps13
Myriazoa - ps14
Parahoxozoa - ps15
Bilateria - ps16
Deuterostomia - ps17
Chordata - ps18
Olfactores - ps19
Vertebrata - ps20
Gnathostomata - ps21
Euteleostomi - ps22
Sarcopterygii - ps23
Tetrapoda - ps24
Amniota - ps25
Mammalia - ps26
Theria - ps27
Placentalia - ps28
Boreoeutheria - ps29
Euarchontoglires - ps30
Euarchonta - ps31
Primates - ps32
Haplorrhini - ps33
Simiiformes - ps34
Catarrhini - ps35
Hominoidea - ps36
Hominidae - ps37
Homininae - ps38
Hominini - ps39
Homo_sapiens - ps40
Phylostratum

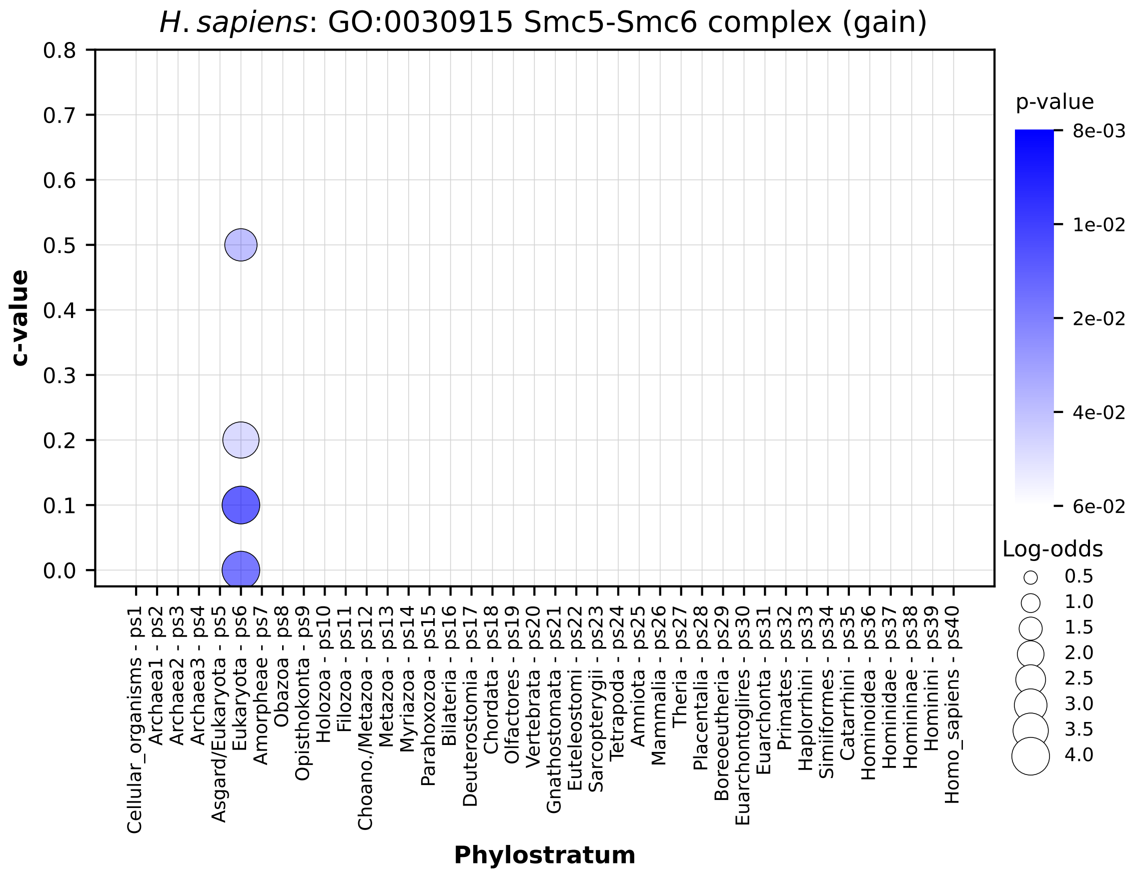

H. sapiens: GO:0030915 Smc5-Smc6 complex (gain)
c-value
0.8
0.7
0.6
0.5
0.4
0.3
0.2
0.1
0.0
Phylostratum
Cellular_organisms - ps1
Archaea1 - ps2
Archaea2 - ps3
Archaea3 - ps4
Asgard/Eukaryota - ps5
Eukaryota - ps6
Amorpheae - ps7
Obazoa - ps8
Opisthokonta - ps9
Holozoa - ps10
Filozoa - ps11
Choano./Metazoa - ps12
Metazoa - ps13
Myriazoa - ps14
Parahoxozoa - ps15
Bilateria - ps16
Deuterostomia - ps17
Chordata - ps18
Olfactores - ps19
Vertebrata - ps20
Gnathostomata - ps21
Euteleostomi - ps22
Sarcopterygii - ps23
Tetrapoda - ps24
Amniota - ps25
Mammalia - ps26
Theria - ps27
Placentalia - ps28
Boreoeutheria - ps29
Euarchontoglires - ps30
Euarchonta - ps31
Primates - ps32
Haplorrhini - ps33
Simiiformes - ps34
Catarrhini - ps35
Hominoidea - ps36
Hominidae - ps37
Homininae - ps38
Hominini - ps39
Homo_sapiens - ps40
p-value
8e-03
1e-02
2e-02
4e-02
6e-02
Log-odds
0.5
1.0
1.5
2.0
2.5
3.0
3.5
4.0

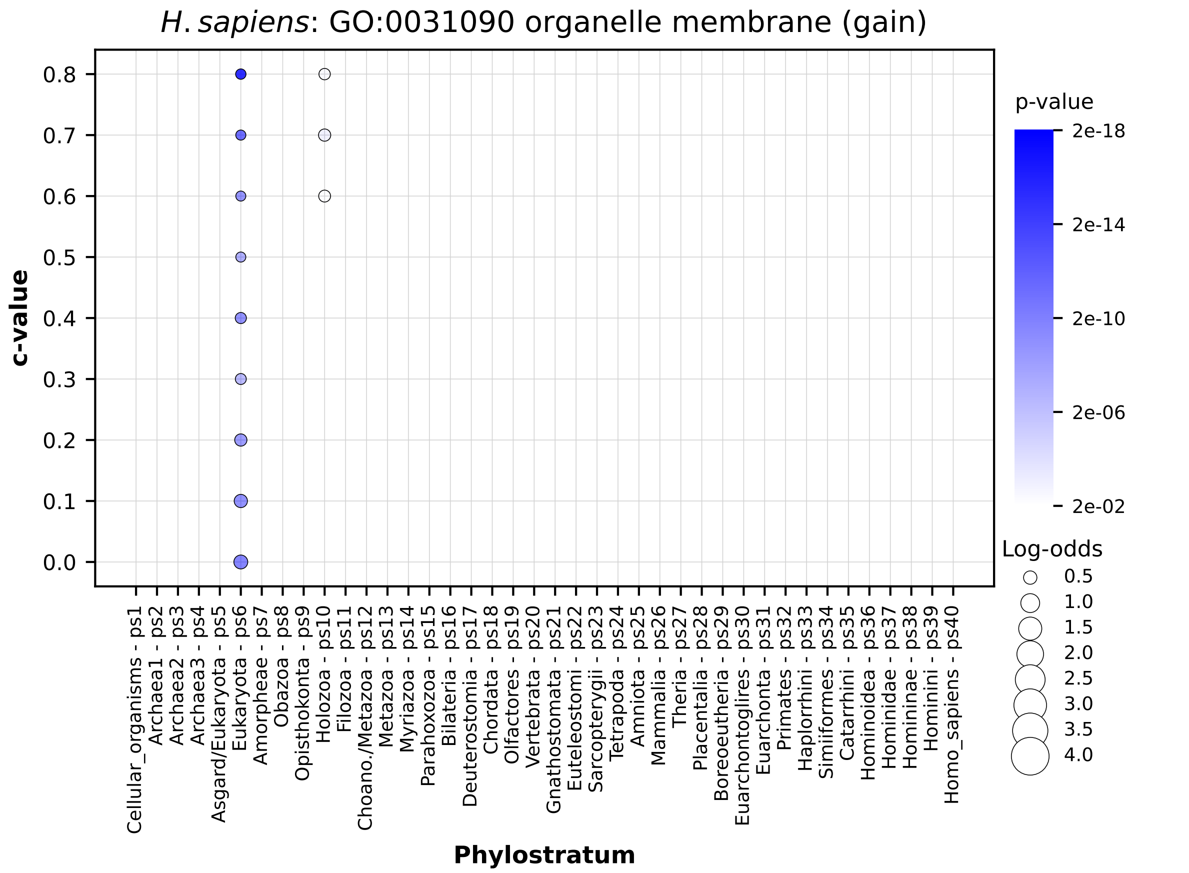
H. sapiens: GO:0031090 organelle membrane (gain)
c-value
0.0
0.1
0.2
0.3
0.4
0.5
0.6
0.7
0.8
Phylostratum
Cellular_organisms - ps1
Archaea1 - ps2
Archaea2 - ps3
Archaea3 - ps4
Asgard/Eukaryota - ps5
Eukaryota - ps6
Amorpheae - ps7
Obazoa - ps8
Opisthokonta - ps9
Holozoa - ps10
Filozoa - ps11
Choano./Metazoa - ps12
Metazoa - ps13
Myriazoa - ps14
Parahoxozoa - ps15
Bilateria - ps16
Deuterostomia - ps17
Chordata - ps18
Olfactores - ps19
Vertebrata - ps20
Gnathostomata - ps21
Euteleostomi - ps22
Sarcopterygii - ps23
Tetrapoda - ps24
Amniota - ps25
Mammalia - ps26
Theria - ps27
Placentalia - ps28
Boreoeutheria - ps29
Euarchontoglires - ps30
Euarchonta - ps31
Primates - ps32
Haplorrhini - ps33
Simiiformes - ps34
Catarrhini - ps35
Hominoidea - ps36
Hominidae - ps37
Homininae - ps38
Hominini - ps39
Homo_sapiens - ps40
p-value
2e-18
2e-14
2e-10
2e-06
2e-02
Log-odds
0.5
1.0
1.5
2.0
2.5
3.0
3.5
4.0

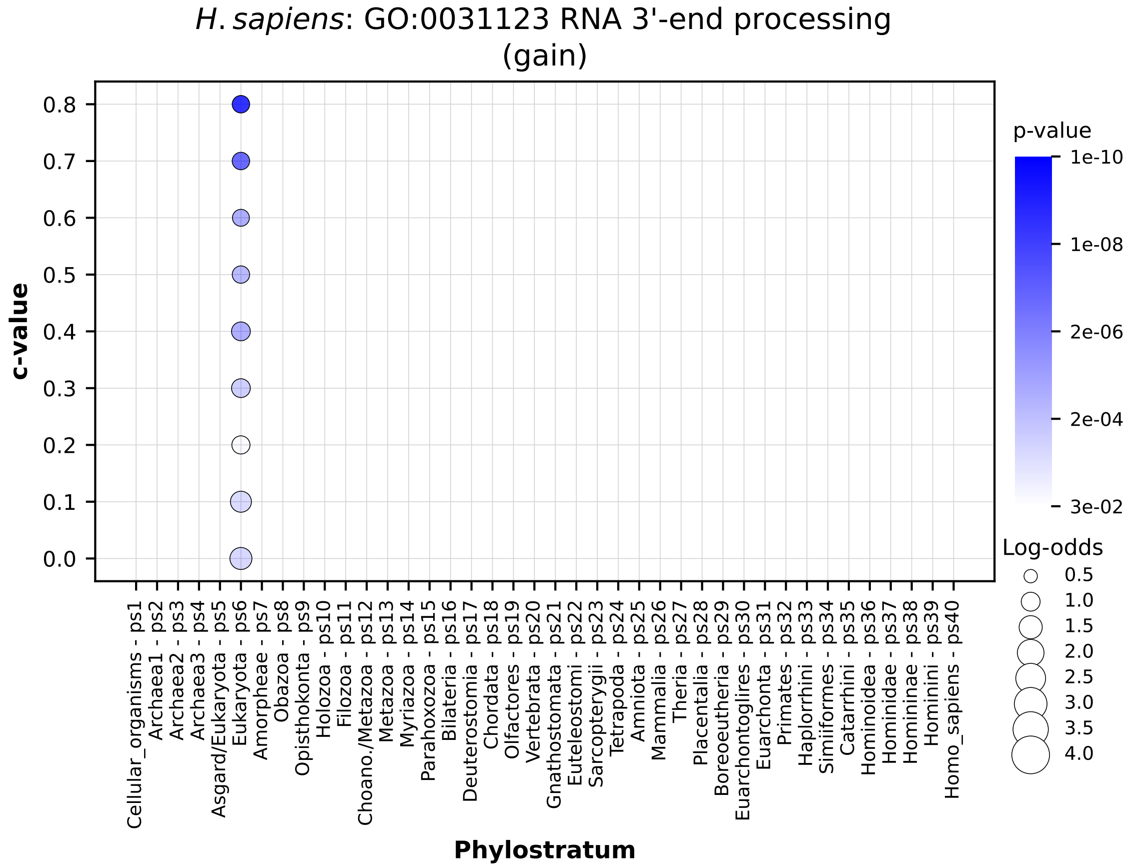
H. sapiens: GO:0031123 RNA 3'-end processing
(gain)
c-value
0.8
0.7
0.6
0.5
0.4
0.3
0.2
0.1
0.0
p-value
1e-10
1e-08
2e-06
2e-04
3e-02
Log-odds
0.5
1.0
1.5
2.0
2.5
3.0
3.5
4.0
Cellular_organisms - ps1
Archaea1 - ps2
Archaea2 - ps3
Archaea3 - ps4
Asgard/Eukaryota - ps5
Eukaryota - ps6
Amorpheae - ps7
Obazoa - ps8
Opisthokonta - ps9
Holozoa - ps10
Filozoa - ps11
Choano./Metazoa - ps12
Metazoa - ps13
Myriazoa - ps14
Parahoxozoa - ps15
Bilateria - ps16
Deuterostomia - ps17
Chordata - ps18
Olfactores - ps19
Vertebrata - ps20
Gnathostomata - ps21
Euteleostomi - ps22
Sarcopterygii - ps23
Tetrapoda - ps24
Amniota - ps25
Mammalia - ps26
Theria - ps27
Placentalia - ps28
Boreoeutheria - ps29
Euarchontoglires - ps30
Euarchonta - ps31
Primates - ps32
Haplorrhini - ps33
Simiiformes - ps34
Catarrhini - ps35
Hominoidea - ps36
Hominidae - ps37
Homininae - ps38
Hominini - ps39
Homo_sapiens - ps40
Phylostratum

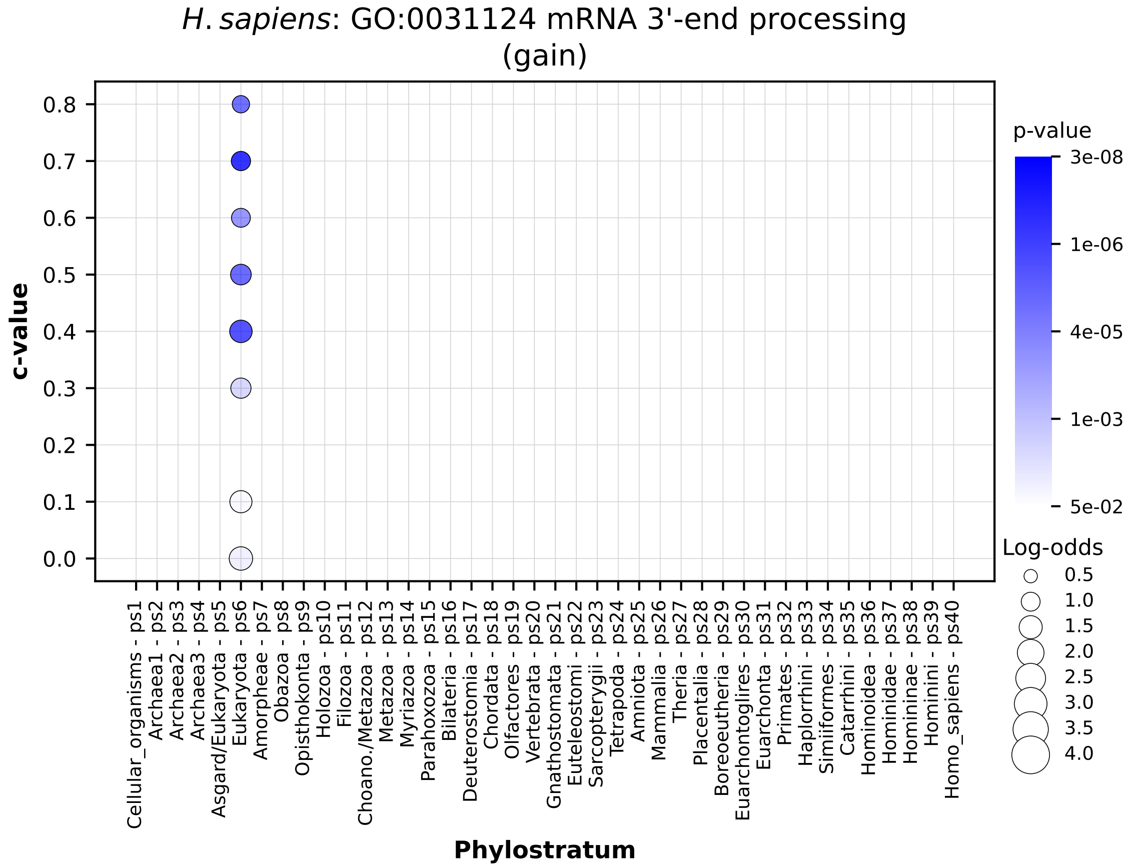
*H. sapiens*: GO:0031124 mRNA 3'-end processing
(gain)
c-value
0.8
0.7
0.6
0.5
0.4
0.3
0.2
0.1
0.0
p-value
3e-08
1e-06
4e-05
1e-03
5e-02
Log-odds
0.5
1.0
1.5
2.0
2.5
3.0
3.5
4.0
Cellular_organisms - ps1
Archaea1 - ps2
Archaea2 - ps3
Archaea3 - ps4
Asgard/Eukaryota - ps5
Eukaryota - ps6
Amorpheae - ps7
Obazoa - ps8
Opisthokonta - ps9
Holozoa - ps10
Filozoa - ps11
Choano./Metazoa - ps12
Metazoa - ps13
Myriazoa - ps14
Parahoxozoa - ps15
Bilateria - ps16
Deuterostomia - ps17
Chordata - ps18
Olfactores - ps19
Vertebrata - ps20
Gnathostomata - ps21
Euteleostomi - ps22
Sarcopterygii - ps23
Tetrapoda - ps24
Amniota - ps25
Mammalia - ps26
Theria - ps27
Placentalia - ps28
Boreoeutheria - ps29
Euarchontoglires - ps30
Euarchonta - ps31
Primates - ps32
Haplorrhini - ps33
Simiiformes - ps34
Catarrhini - ps35
Hominoidea - ps36
Hominidae - ps37
Homininae - ps38
Hominini - ps39
Homo_sapiens - ps40
Phylostratum

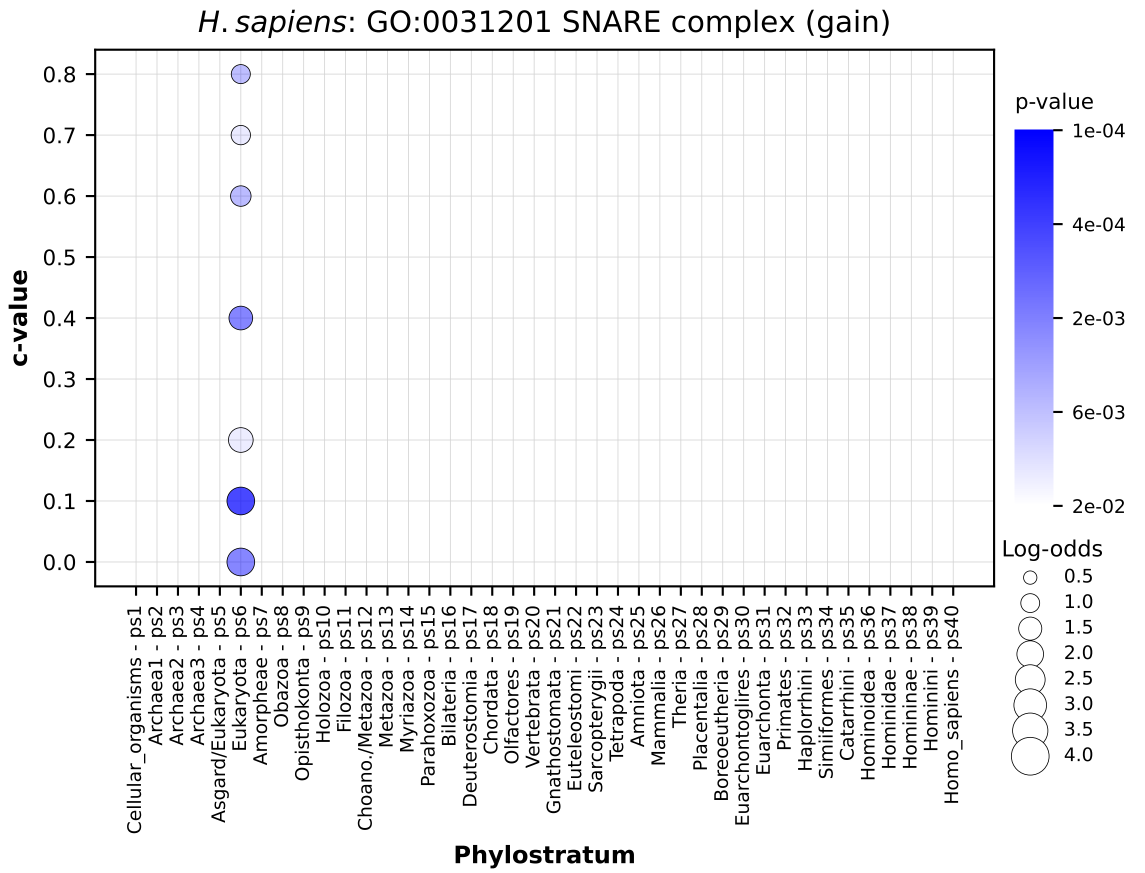

H. sapiens: GO:0031201 SNARE complex (gain)
c-value
0.8
0.7
0.6
0.5
0.4
0.3
0.2
0.1
0.0
Phylostratum
Cellular_organisms - ps1
Archaea1 - ps2
Archaea2 - ps3
Archaea3 - ps4
Asgard/Eukaryota - ps5
Eukaryota - ps6
Amorpheae - ps7
Obazoa - ps8
Opisthokonta - ps9
Holozoa - ps10
Filozoa - ps11
Choano./Metazoa - ps12
Metazoa - ps13
Myriazoa - ps14
Parahoxozoa - ps15
Bilateria - ps16
Deuterostomia - ps17
Chordata - ps18
Olfactores - ps19
Vertebrata - ps20
Gnathostomata - ps21
Euteleostomi - ps22
Sarcopterygii - ps23
Tetrapoda - ps24
Amniota - ps25
Mammalia - ps26
Theria - ps27
Placentalia - ps28
Boreoeutheria - ps29
Euarchontoglires - ps30
Euarchonta - ps31
Primates - ps32
Haplorrhini - ps33
Simiiformes - ps34
Catarrhini - ps35
Hominoidea - ps36
Hominidae - ps37
Homininae - ps38
Hominini - ps39
Homo_sapiens - ps40
p-value
1e-04
4e-04
2e-03
6e-03
2e-02
Log-odds
0.5
1.0
1.5
2.0
2.5
3.0
3.5
4.0

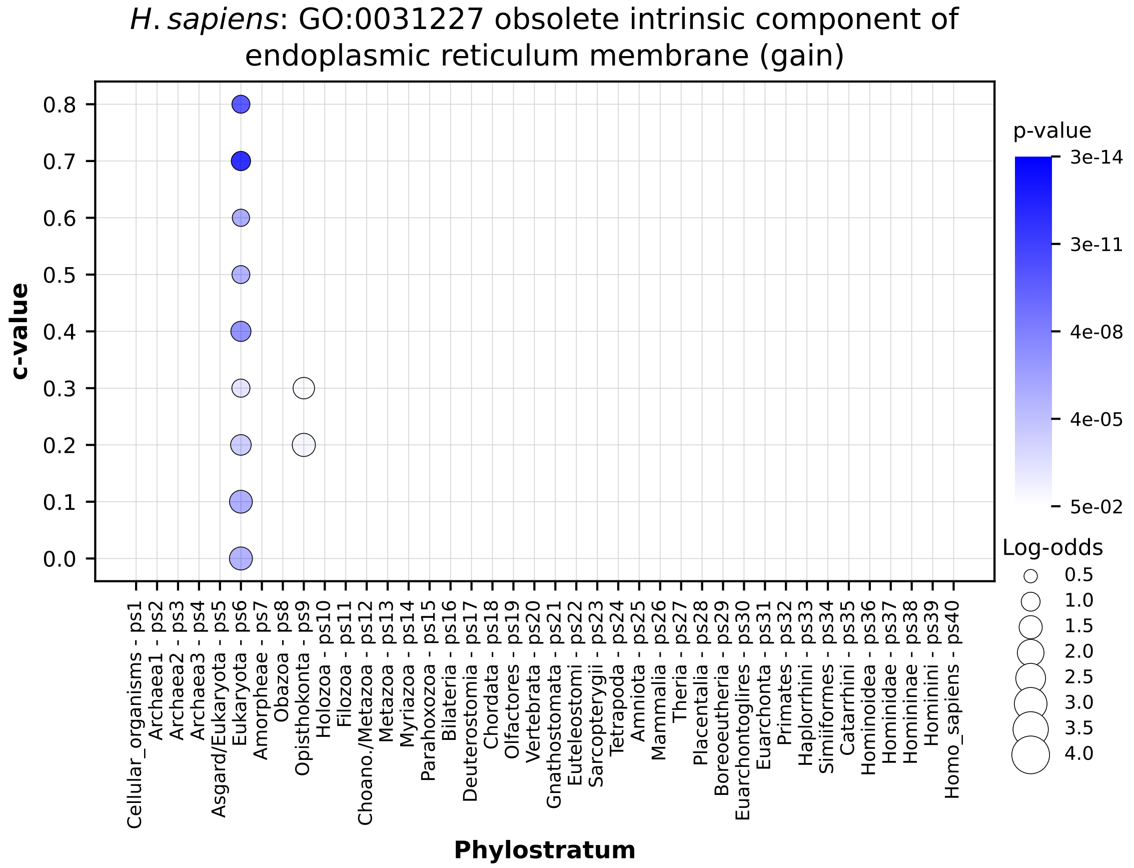
H. sapiens: GO:0031227 obsolete intrinsic component of endoplasmic reticulum membrane (gain)
c-value
0.8
0.7
0.6
0.5
0.4
0.3
0.2
0.1
0.0
p-value
3e-14
3e-11
4e-08
4e-05
5e-02
Log-odds
0.5
1.0
1.5
2.0
2.5
3.0
3.5
4.0
Cellular_organisms - ps1
Archaea1 - ps2
Archaea2 - ps3
Archaea3 - ps4
Asgard/Eukaryota - ps5
Eukaryota - ps6
Amorpheae - ps7
Obazoa - ps8
Opisthokonta - ps9
Holozoa - ps10
Filozoa - ps11
Choano./Metazoa - ps12
Metazoa - ps13
Myriazoa - ps14
Parahoxozoa - ps15
Bilateria - ps16
Deuterostomia - ps17
Chordata - ps18
Olfactores - ps19
Vertebrata - ps20
Gnathostomata - ps21
Euteleostomi - ps22
Sarcopterygii - ps23
Tetrapoda - ps24
Amniota - ps25
Mammalia - ps26
Theria - ps27
Placentalia - ps28
Boreoeutheria - ps29
Euarchontoglires - ps30
Euarchonta - ps31
Primates - ps32
Haplorrhini - ps33
Simiiformes - ps34
Catarrhini - ps35
Hominoidea - ps36
Hominidae - ps37
Homininae - ps38
Hominini - ps39
Homo_sapiens - ps40
Phylostratum

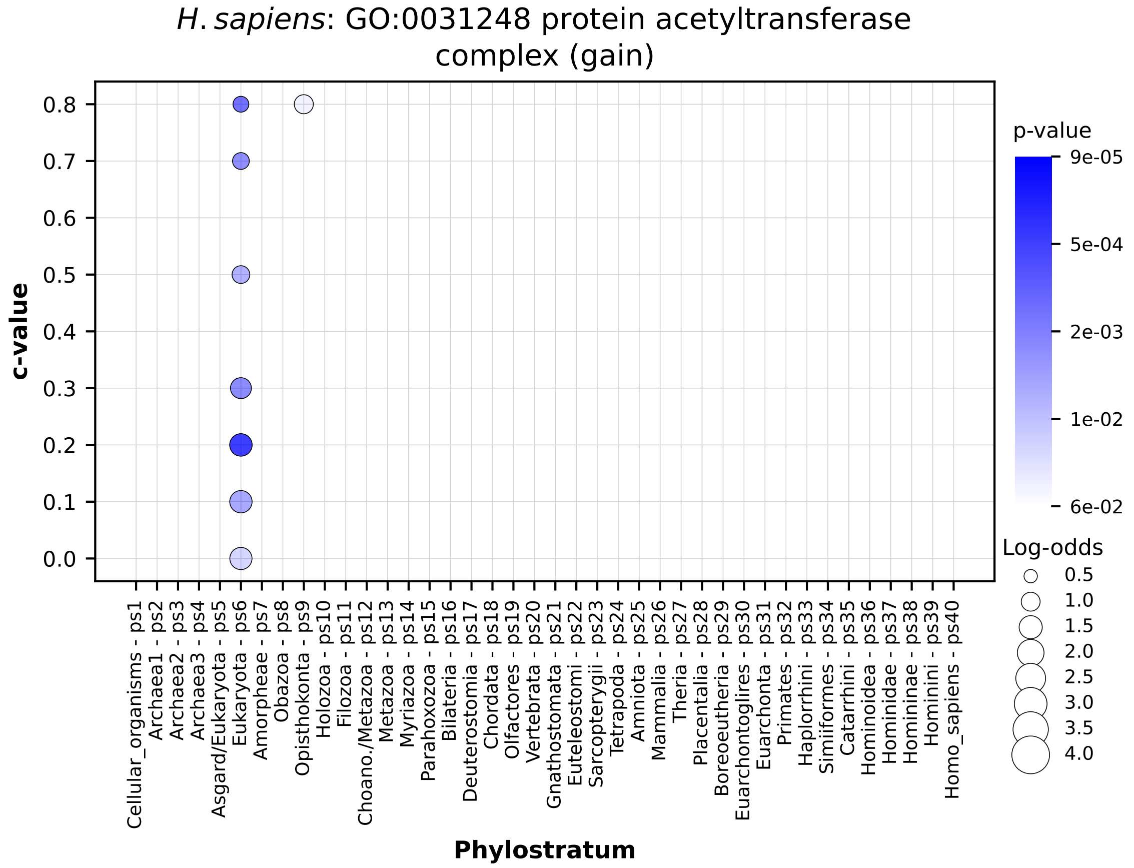

H. sapiens: GO:0031248 protein acetyltransferase complex (gain)
c-value
0.8
0.7
0.6
0.5
0.4
0.3
0.2
0.1
0.0
Phylostratum
Cellular_organisms - ps1
Archaea1 - ps2
Archaea2 - ps3
Archaea3 - ps4
Asgard/Eukaryota - ps5
Eukaryota - ps6
Amorpheae - ps7
Obazoa - ps8
Opisthokonta - ps9
Holozoa - ps10
Filozoa - ps11
Choano./Metazoa - ps12
Metazoa - ps13
Myriazoa - ps14
Parahoxozoa - ps15
Bilateria - ps16
Deuterostomia - ps17
Chordata - ps18
Olfactores - ps19
Vertebrata - ps20
Gnathostomata - ps21
Euteleostomi - ps22
Sarcopterygii - ps23
Tetrapoda - ps24
Amniota - ps25
Mammalia - ps26
Theria - ps27
Placentalia - ps28
Boreoeutheria - ps29
Euarchontoglires - ps30
Euarchonta - ps31
Primates - ps32
Haplorrhini - ps33
Simiiformes - ps34
Catarrhini - ps35
Hominoidea - ps36
Hominidae - ps37
Homininae - ps38
Hominini - ps39
Homo_sapiens - ps40
p-value
9e-05
5e-04
2e-03
1e-02
6e-02
Log-odds
0.5
1.0
1.5
2.0
2.5
3.0
3.5
4.0

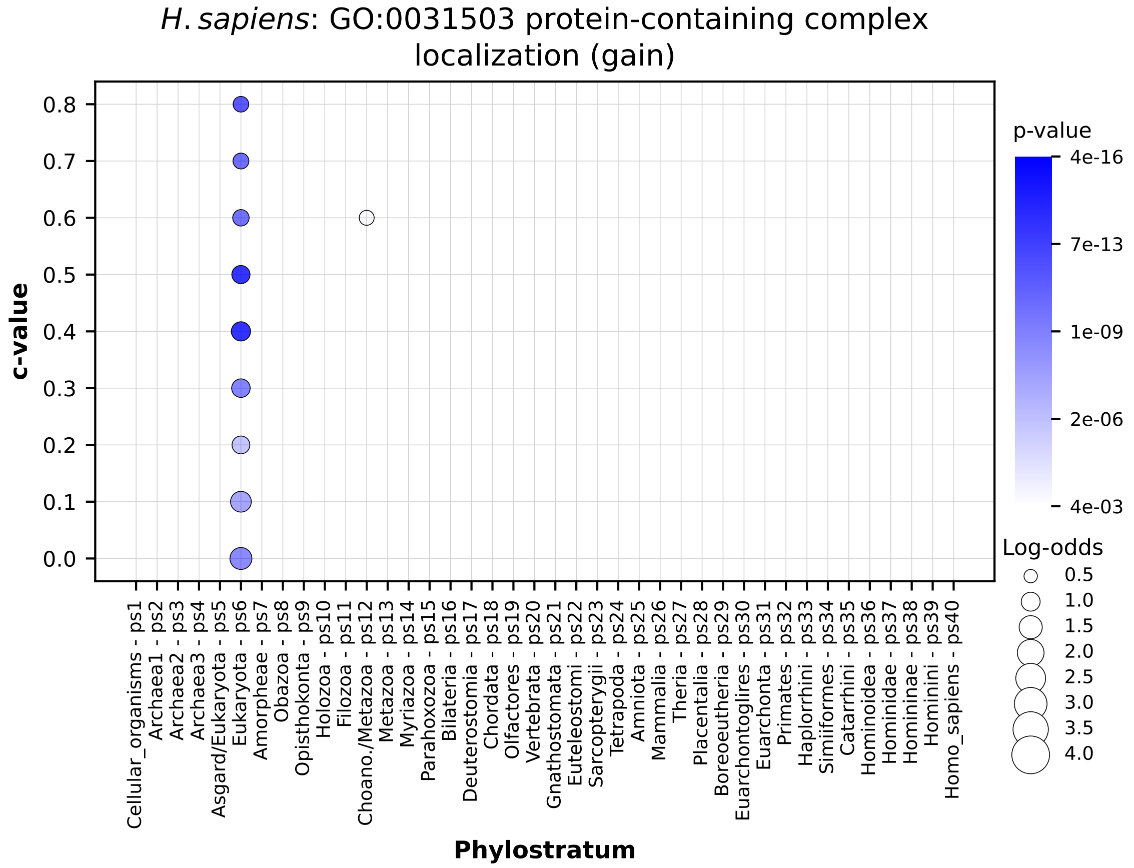

H. sapiens: GO:0031503 protein-containing complex localization (gain)
c-value
0.8
0.7
0.6
0.5
0.4
0.3
0.2
0.1
0.0
p-value
4e-16
7e-13
1e-09
2e-06
4e-03
Log-odds
0.5
1.0
1.5
2.0
2.5
3.0
3.5
4.0
Cellular_organisms - ps1
Archaea1 - ps2
Archaea2 - ps3
Archaea3 - ps4
Asgard/Eukaryota - ps5
Eukaryota - ps6
Amorpheae - ps7
Obazoa - ps8
Opisthokonta - ps9
Holozoa - ps10
Filozoa - ps11
Choano./Metazoa - ps12
Metazoa - ps13
Myriazoa - ps14
Parahoxozoa - ps15
Bilateria - ps16
Deuterostomia - ps17
Chordata - ps18
Olfactores - ps19
Vertebrata - ps20
Gnathostomata - ps21
Euteleostomi - ps22
Sarcopterygii - ps23
Tetrapoda - ps24
Amniota - ps25
Mammalia - ps26
Theria - ps27
Placentalia - ps28
Boreoeutheria - ps29
Euarchontoglires - ps30
Euarchonta - ps31
Primates - ps32
Haplorrhini - ps33
Simiiformes - ps34
Catarrhini - ps35
Hominoidea - ps36
Hominidae - ps37
Homininae - ps38
Hominini - ps39
Homo_sapiens - ps40
Phylostratum

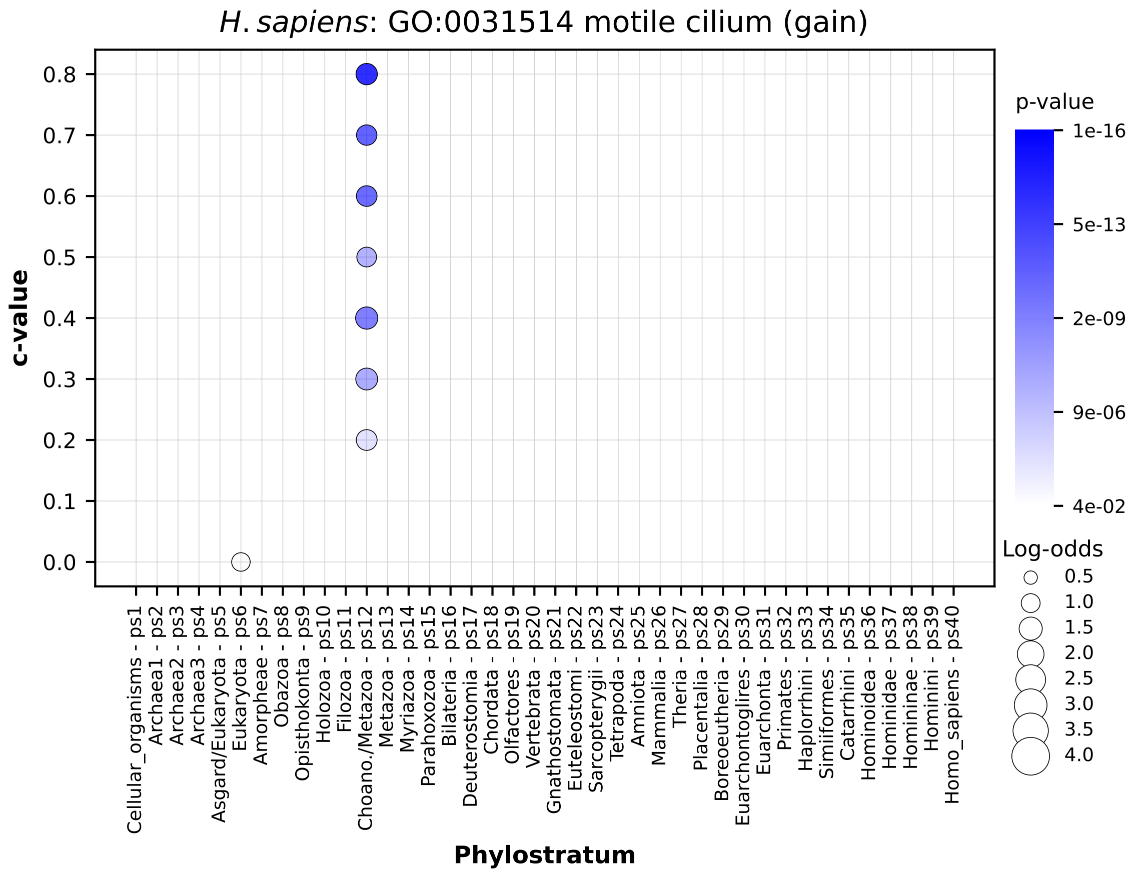
H. sapiens: GO:0031514 motile cilium (gain)
c-value
0.8
0.7
0.6
0.5
0.4
0.3
0.2
0.1
0.0
Phylostratum
Cellular_organisms - ps1
Archaea1 - ps2
Archaea2 - ps3
Archaea3 - ps4
Asgard/Eukaryota - ps5
Eukaryota - ps6
Amorpheae - ps7
Obazoa - ps8
Opisthokonta - ps9
Holozoa - ps10
Filozoa - ps11
Choano./Metazoa - ps12
Metazoa - ps13
Myriazoa - ps14
Parahoxozoa - ps15
Bilateria - ps16
Deuterostomia - ps17
Chordata - ps18
Olfactores - ps19
Vertebrata - ps20
Gnathostomata - ps21
Euteleostomi - ps22
Sarcopterygii - ps23
Tetrapoda - ps24
Amniota - ps25
Mammalia - ps26
Theria - ps27
Placentalia - ps28
Boreoeutheria - ps29
Euarchontoglires - ps30
Euarchonta - ps31
Primates - ps32
Haplorrhini - ps33
Simiiformes - ps34
Catarrhini - ps35
Hominoidea - ps36
Hominidae - ps37
Homininae - ps38
Hominini - ps39
Homo_sapiens - ps40
p-value
1e-16
5e-13
2e-09
9e-06
4e-02
Log-odds
0.5
1.0
1.5
2.0
2.5
3.0
3.5
4.0

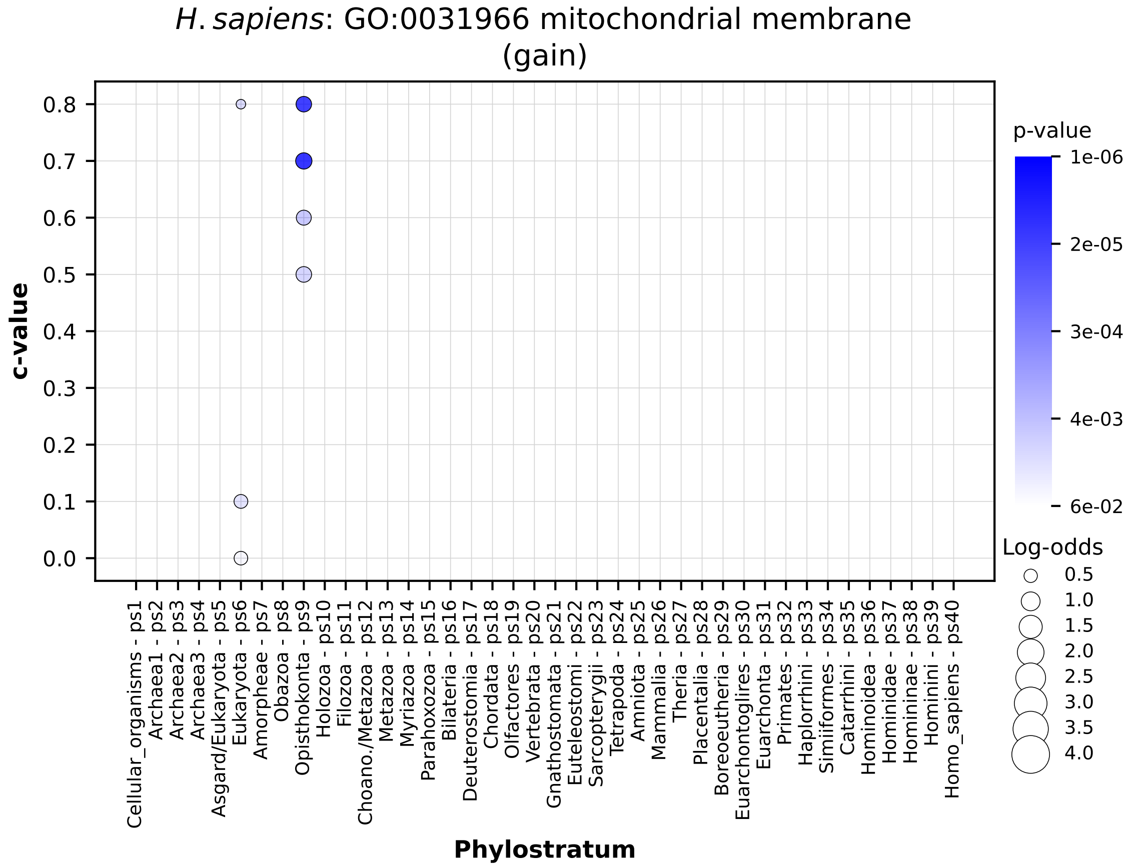

H. sapiens: GO:0031966 mitochondrial membrane
(gain)
c-value
0.8
0.7
0.6
0.5
0.4
0.3
0.2
0.1
0.0
p-value
1e-06
2e-05
3e-04
4e-03
6e-02
Log-odds
0.5
1.0
1.5
2.0
2.5
3.0
3.5
4.0
Cellular_organisms - ps1
Archaea1 - ps2
Archaea2 - ps3
Archaea3 - ps4
Asgard/Eukaryota - ps5
Eukaryota - ps6
Amorpheae - ps7
Obazoa - ps8
Opisthokonta - ps9
Holozoa - ps10
Filozoa - ps11
Choano./Metazoa - ps12
Metazoa - ps13
Myriazoa - ps14
Parahoxozoa - ps15
Bilateria - ps16
Deuterostomia - ps17
Chordata - ps18
Olfactores - ps19
Vertebrata - ps20
Gnathostomata - ps21
Euteleostomi - ps22
Sarcopterygii - ps23
Tetrapoda - ps24
Amniota - ps25
Mammalia - ps26
Theria - ps27
Placentalia - ps28
Boreoeutheria - ps29
Euarchontoglires - ps30
Euarchonta - ps31
Primates - ps32
Haplorrhini - ps33
Simiiformes - ps34
Catarrhini - ps35
Hominoidea - ps36
Hominidae - ps37
Homininae - ps38
Hominini - ps39
Homo_sapiens - ps40
Phylostratum

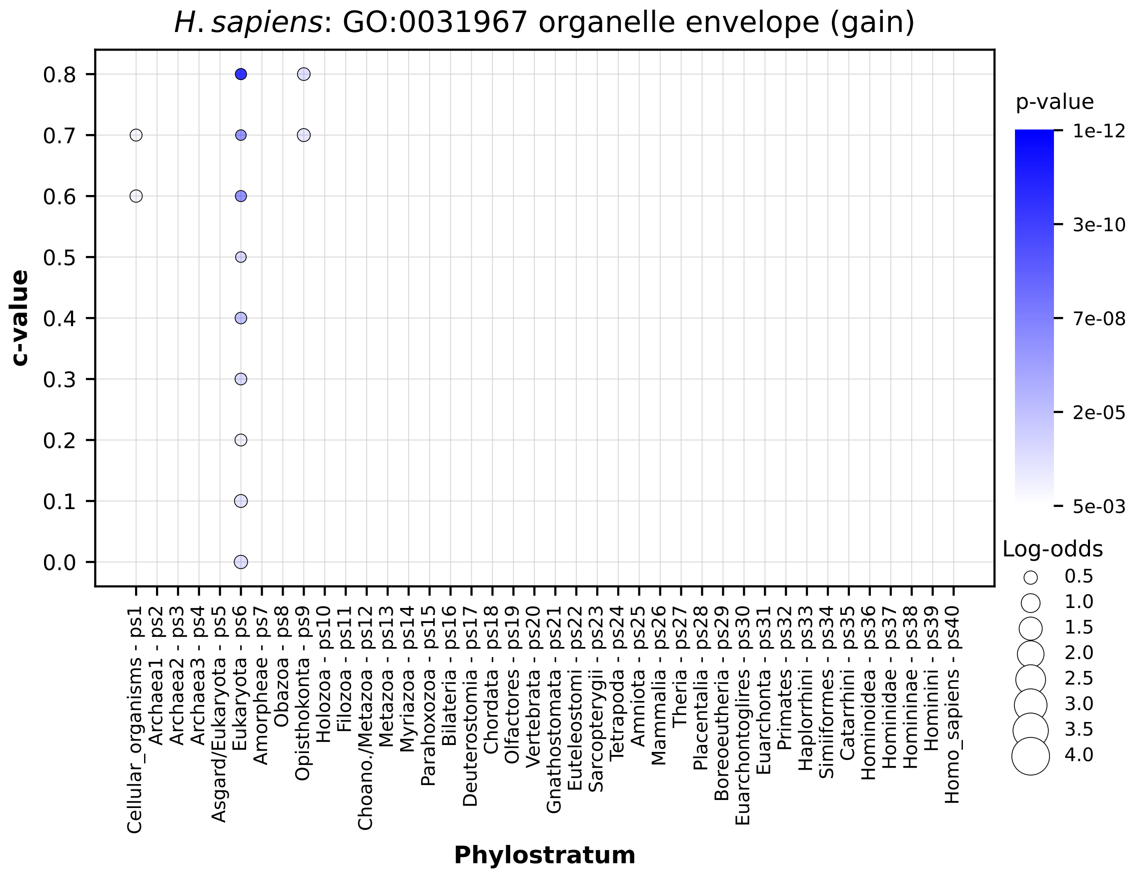

H. sapiens: GO:0031967 organelle envelope (gain)
c-value
0.0
0.1
0.2
0.3
0.4
0.5
0.6
0.7
0.8
Phylostratum
Cellular_organisms - ps1
Archaea1 - ps2
Archaea2 - ps3
Archaea3 - ps4
Asgard/Eukaryota - ps5
Eukaryota - ps6
Amorpheae - ps7
Obazoa - ps8
Opisthokonta - ps9
Holozoa - ps10
Filozoa - ps11
Choano./Metazoa - ps12
Metazoa - ps13
Myriazoa - ps14
Parahoxozoa - ps15
Bilateria - ps16
Deuterostomia - ps17
Chordata - ps18
Olfactores - ps19
Vertebrata - ps20
Gnathostomata - ps21
Euteleostomi - ps22
Sarcopterygii - ps23
Tetrapoda - ps24
Amniota - ps25
Mammalia - ps26
Theria - ps27
Placentalia - ps28
Boreoeutheria - ps29
Euarchontoglires - ps30
Euarchonta - ps31
Primates - ps32
Haplorrhini - ps33
Simiiformes - ps34
Catarrhini - ps35
Hominoidea - ps36
Hominidae - ps37
Homininae - ps38
Hominini - ps39
Homo_sapiens - ps40
p-value
1e-12
3e-10
7e-08
2e-05
5e-03
Log-odds
0.5
1.0
1.5
2.0
2.5
3.0
3.5
4.0

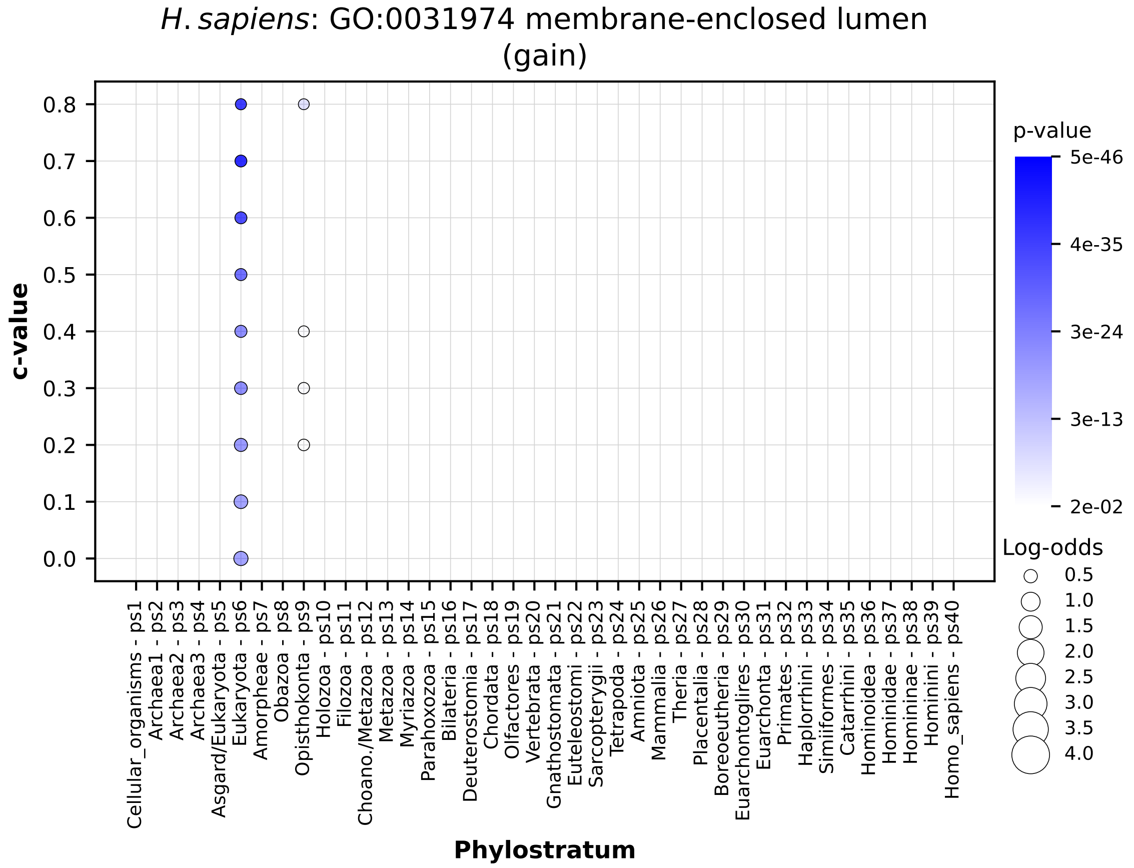

H. sapiens: GO:0031974 membrane-enclosed lumen
(gain)
c-value
0.8
0.7
0.6
0.5
0.4
0.3
0.2
0.1
0.0
p-value
5e-46
4e-35
3e-24
3e-13
2e-02
Log-odds
0.5
1.0
1.5
2.0
2.5
3.0
3.5
4.0
Cellular_organisms - ps1
Archaea1 - ps2
Archaea2 - ps3
Archaea3 - ps4
Asgard/Eukaryota - ps5
Eukaryota - ps6
Amorpheae - ps7
Obazoa - ps8
Opisthokonta - ps9
Holozoa - ps10
Filozoa - ps11
Choano./Metazoa - ps12
Metazoa - ps13
Myriazoa - ps14
Parahoxozoa - ps15
Bilateria - ps16
Deuterostomia - ps17
Chordata - ps18
Olfactores - ps19
Vertebrata - ps20
Gnathostomata - ps21
Euteleostomi - ps22
Sarcopterygii - ps23
Tetrapoda - ps24
Amniota - ps25
Mammalia - ps26
Theria - ps27
Placentalia - ps28
Boreoeutheria - ps29
Euarchontoglires - ps30
Euarchonta - ps31
Primates - ps32
Haplorrhini - ps33
Simiiformes - ps34
Catarrhini - ps35
Hominoidea - ps36
Hominidae - ps37
Homininae - ps38
Hominini - ps39
Homo_sapiens - ps40
Phylostratum

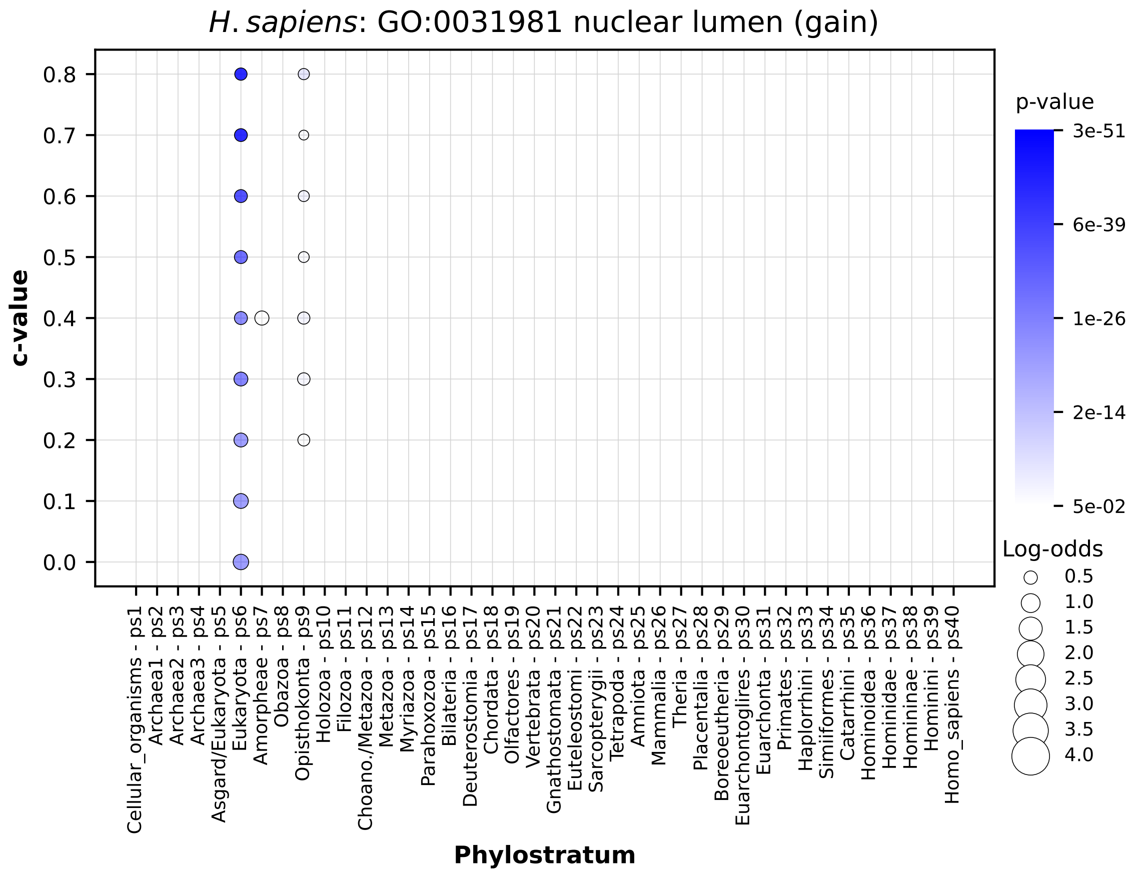

H. sapiens: GO:0031981 nuclear lumen (gain)
c-value
0.8
0.7
0.6
0.5
0.4
0.3
0.2
0.1
0.0
Phylostratum
Cellular_organisms - ps1
Archaea1 - ps2
Archaea2 - ps3
Archaea3 - ps4
Asgard/Eukaryota - ps5
Eukaryota - ps6
Amorpheae - ps7
Obazoa - ps8
Opisthokonta - ps9
Holozoa - ps10
Filozoa - ps11
Choano./Metazoa - ps12
Metazoa - ps13
Myriazoa - ps14
Parahoxozoa - ps15
Bilateria - ps16
Deuterostomia - ps17
Chordata - ps18
Olfactores - ps19
Vertebrata - ps20
Gnathostomata - ps21
Euteleostomi - ps22
Sarcopterygii - ps23
Tetrapoda - ps24
Amniota - ps25
Mammalia - ps26
Theria - ps27
Placentalia - ps28
Boreoeutheria - ps29
Euarchontoglires - ps30
Euarchonta - ps31
Primates - ps32
Haplorrhini - ps33
Simiiformes - ps34
Catarrhini - ps35
Hominoidea - ps36
Hominidae - ps37
Homininae - ps38
Hominini - ps39
Homo_sapiens - ps40
p-value
3e-51
6e-39
1e-26
2e-14
5e-02
Log-odds
0.5
1.0
1.5
2.0
2.5
3.0
3.5
4.0

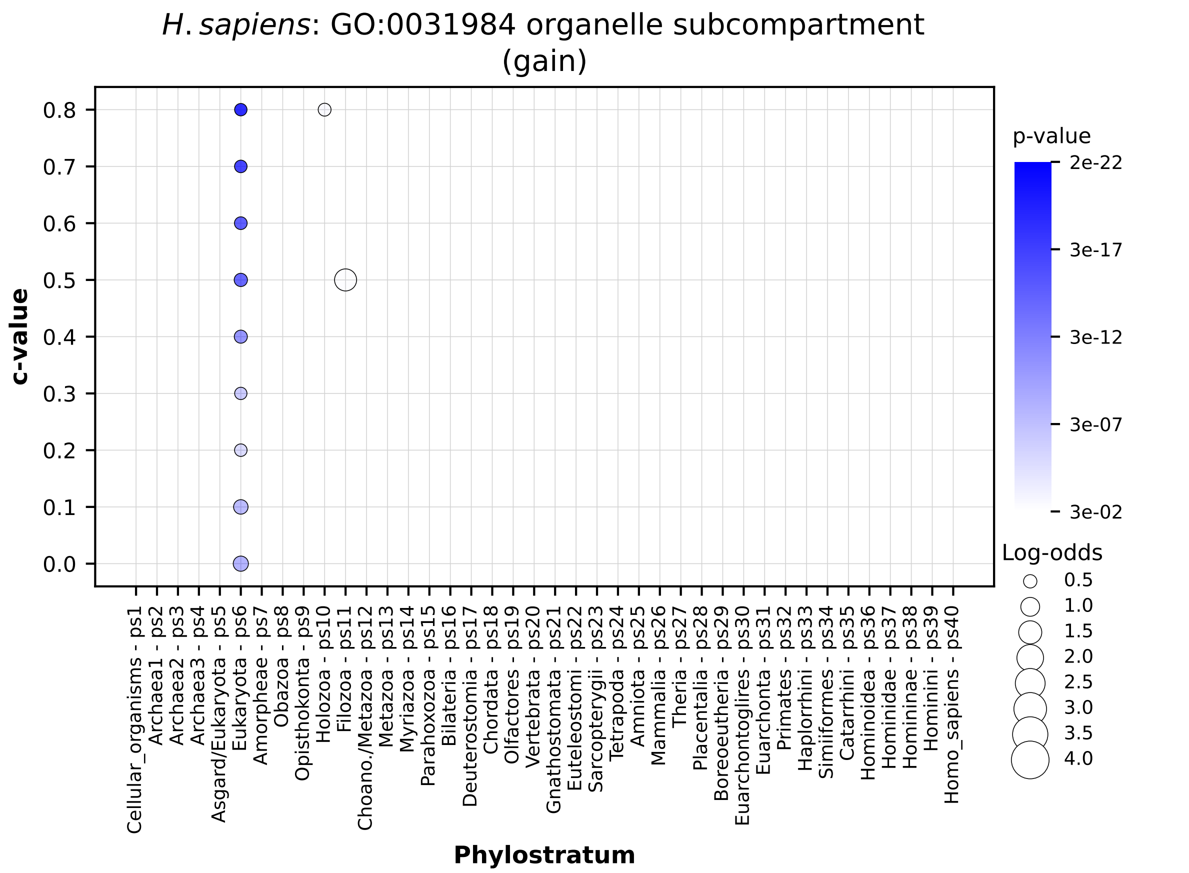

H. sapiens: GO:0031984 organelle subcompartment
(gain)
c-value
0.0
0.1
0.2
0.3
0.4
0.5
0.6
0.7
0.8
Phylostratum
Cellular_organisms - ps1
Archaea1 - ps2
Archaea2 - ps3
Archaea3 - ps4
Asgard/Eukaryota - ps5
Eukaryota - ps6
Amorpheae - ps7
Obazoa - ps8
Opisthokonta - ps9
Holozoa - ps10
Filozoa - ps11
Choano./Metazoa - ps12
Metazoa - ps13
Myriazoa - ps14
Parahoxozoa - ps15
Bilateria - ps16
Deuterostomia - ps17
Chordata - ps18
Olfactores - ps19
Vertebrata - ps20
Gnathostomata - ps21
Euteleostomi - ps22
Sarcopterygii - ps23
Tetrapoda - ps24
Amniota - ps25
Mammalia - ps26
Theria - ps27
Placentalia - ps28
Boreoeutheria - ps29
Euarchontoglires - ps30
Euarchonta - ps31
Primates - ps32
Haplorrhini - ps33
Simiiformes - ps34
Catarrhini - ps35
Hominoidea - ps36
Hominidae - ps37
Homininae - ps38
Hominini - ps39
Homo_sapiens - ps40
p-value
2e-22
3e-17
3e-12
3e-07
3e-02
Log-odds
0.5
1.0
1.5
2.0
2.5
3.0
3.5
4.0

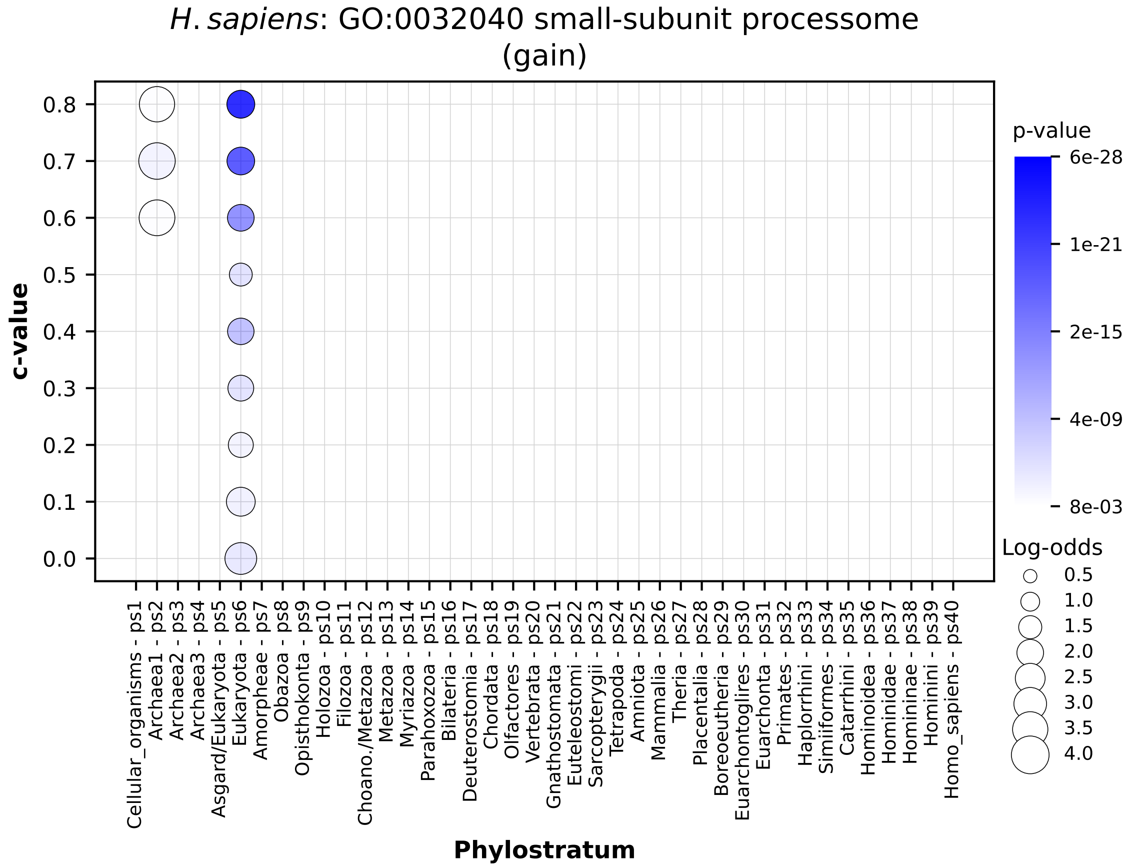

H. sapiens: GO:0032040 small-subunit processome
(gain)
c-value
0.8
0.7
0.6
0.5
0.4
0.3
0.2
0.1
0.0
p-value
6e-28
1e-21
2e-15
4e-09
8e-03
Log-odds
0.5
1.0
1.5
2.0
2.5
3.0
3.5
4.0
Cellular_organisms - ps1
Archaea1 - ps2
Archaea2 - ps3
Archaea3 - ps4
Asgard/Eukaryota - ps5
Eukaryota - ps6
Amorpheae - ps7
Obazoa - ps8
Opisthokonta - ps9
Holozoa - ps10
Filozoa - ps11
Choano./Metazoa - ps12
Metazoa - ps13
Myriazoa - ps14
Parahoxozoa - ps15
Bilateria - ps16
Deuterostomia - ps17
Chordata - ps18
Olfactores - ps19
Vertebrata - ps20
Gnathostomata - ps21
Euteleostomi - ps22
Sarcopterygii - ps23
Tetrapoda - ps24
Amniota - ps25
Mammalia - ps26
Theria - ps27
Placentalia - ps28
Boreoeutheria - ps29
Euarchontoglires - ps30
Euarchonta - ps31
Primates - ps32
Haplorrhini - ps33
Simiiformes - ps34
Catarrhini - ps35
Hominoidea - ps36
Hominidae - ps37
Homininae - ps38
Hominini - ps39
Homo_sapiens - ps40
Phylostratum

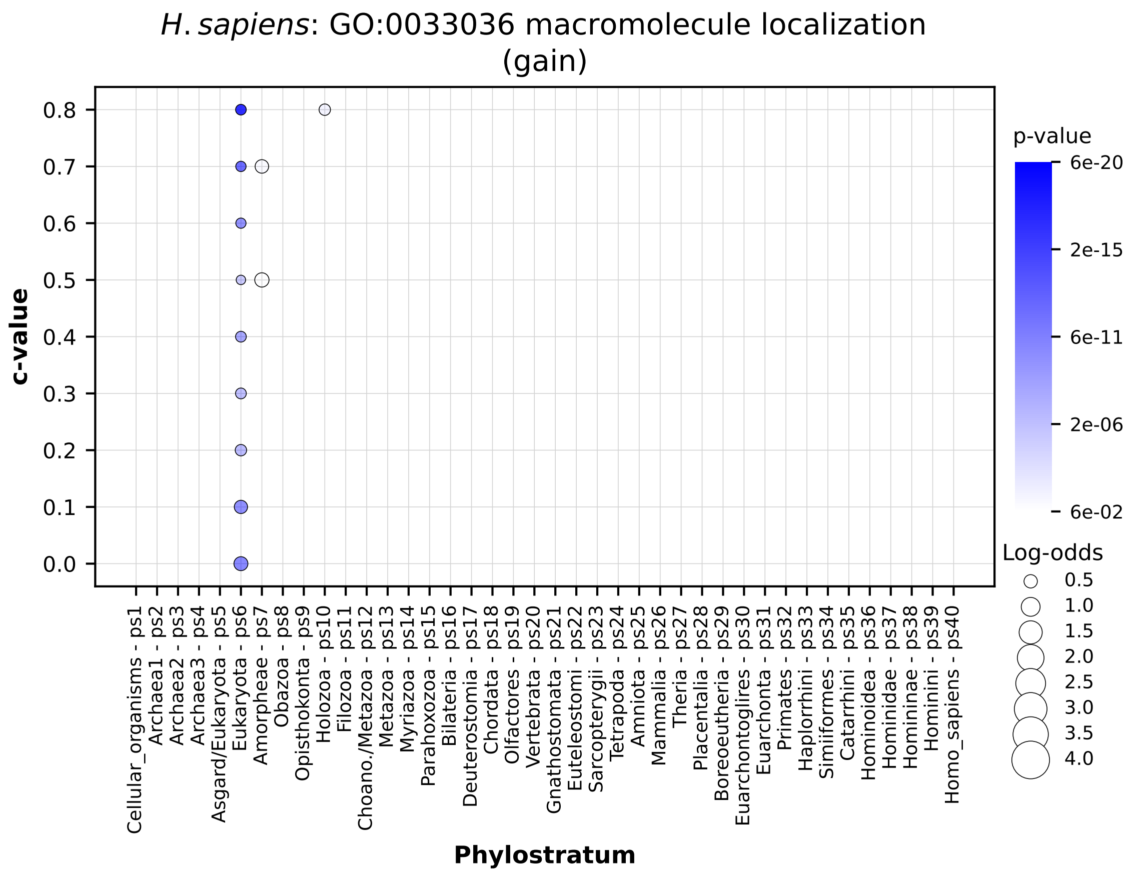
H. sapiens: GO:0033036 macromolecule localization
(gain)
c-value
0.8
0.7
0.6
0.5
0.4
0.3
0.2
0.1
0.0
Phylostratum
Cellular_organisms - ps1
Archaea1 - ps2
Archaea2 - ps3
Archaea3 - ps4
Asgard/Eukaryota - ps5
Eukaryota - ps6
Amorpheae - ps7
Obazoa - ps8
Opisthokonta - ps9
Holozoa - ps10
Filozoa - ps11
Choano./Metazoa - ps12
Metazoa - ps13
Myriazoa - ps14
Parahoxozoa - ps15
Bilateria - ps16
Deuterostomia - ps17
Chordata - ps18
Olfactores - ps19
Vertebrata - ps20
Gnathostomata - ps21
Euteleostomi - ps22
Sarcopterygii - ps23
Tetrapoda - ps24
Amniota - ps25
Mammalia - ps26
Theria - ps27
Placentalia - ps28
Boreoeutheria - ps29
Euarchontoglires - ps30
Euarchonta - ps31
Primates - ps32
Haplorrhini - ps33
Simiiformes - ps34
Catarrhini - ps35
Hominoidea - ps36
Hominidae - ps37
Homininae - ps38
Hominini - ps39
Homo_sapiens - ps40
p-value
6e-20
2e-15
6e-11
2e-06
6e-02
Log-odds
0.5
1.0
1.5
2.0
2.5
3.0
3.5
4.0

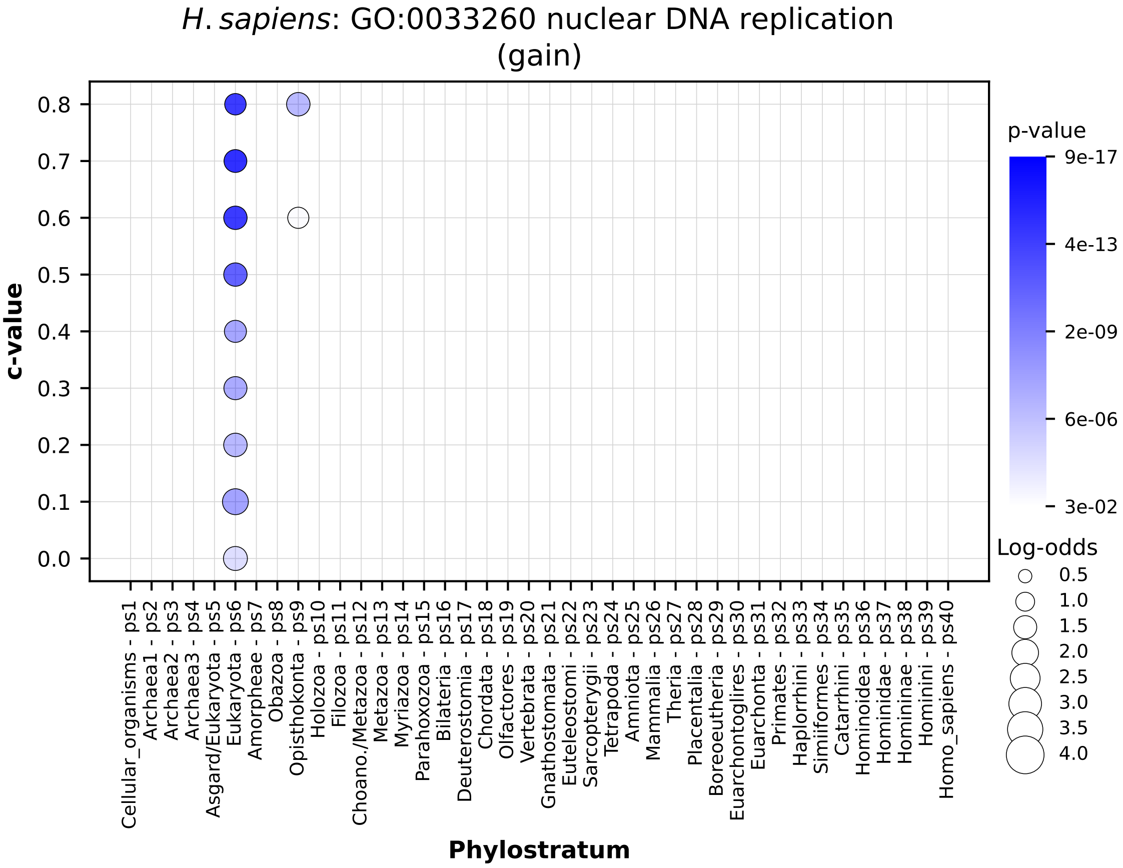

H. sapiens: GO:0033260 nuclear DNA replication
(gain)
c-value
0.8
0.7
0.6
0.5
0.4
0.3
0.2
0.1
0.0
p-value
9e-17
4e-13
2e-09
6e-06
3e-02
Log-odds
0.5
1.0
1.5
2.0
2.5
3.0
3.5
4.0
Cellular_organisms - ps1
Archaea1 - ps2
Archaea2 - ps3
Archaea3 - ps4
Asgard/Eukaryota - ps5
Eukaryota - ps6
Amorpheae - ps7
Obazoa - ps8
Opisthokonta - ps9
Holozoa - ps10
Filozoa - ps11
Choano./Metazoa - ps12
Metazoa - ps13
Myriazoa - ps14
Parahoxozoa - ps15
Bilateria - ps16
Deuterostomia - ps17
Chordata - ps18
Olfactores - ps19
Vertebrata - ps20
Gnathostomata - ps21
Euteleostomi - ps22
Sarcopterygii - ps23
Tetrapoda - ps24
Amniota - ps25
Mammalia - ps26
Theria - ps27
Placentalia - ps28
Boreoeutheria - ps29
Euarchontoglires - ps30
Euarchonta - ps31
Primates - ps32
Haplorrhini - ps33
Simiiformes - ps34
Catarrhini - ps35
Hominoidea - ps36
Hominidae - ps37
Homininae - ps38
Hominini - ps39
Homo_sapiens - ps40
Phylostratum

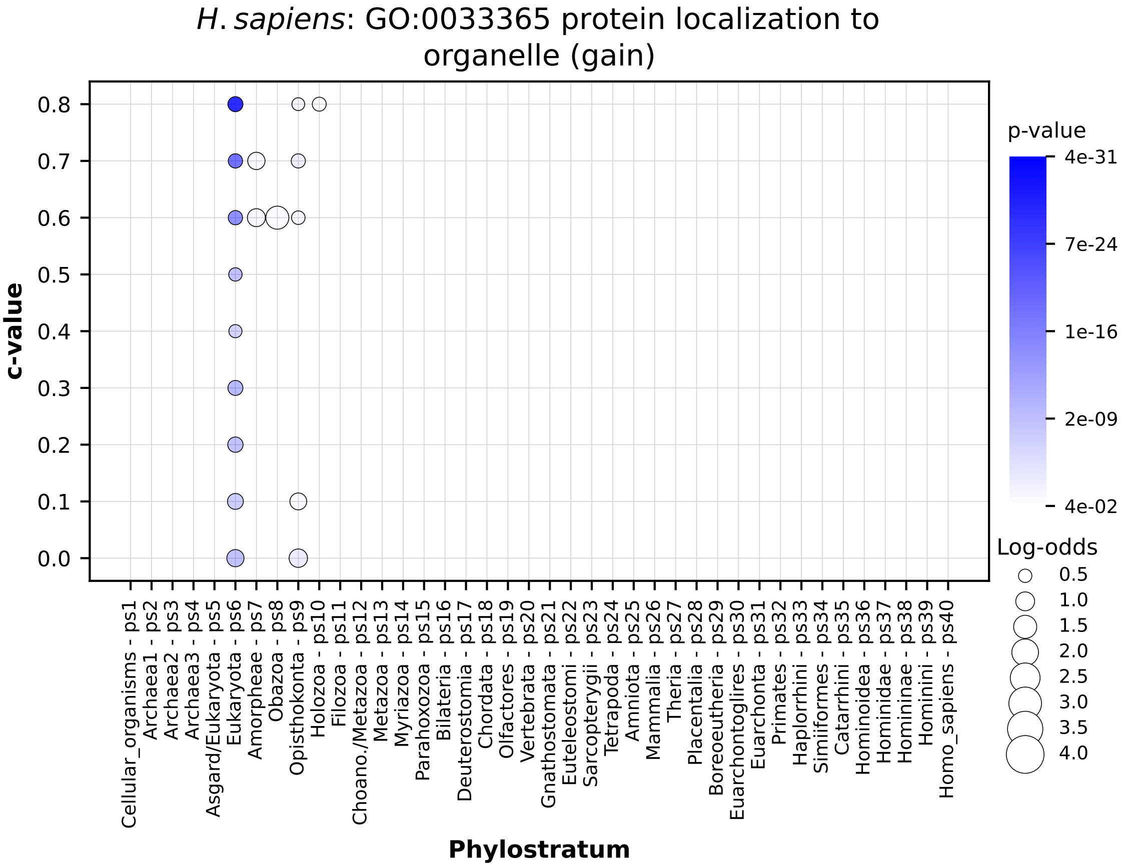

H. sapiens: GO:0033365 protein localization to organelle (gain)
c-value
0.8
0.7
0.6
0.5
0.4
0.3
0.2
0.1
0.0
Phylostratum
Cellular_organisms - ps1
Archaea1 - ps2
Archaea2 - ps3
Archaea3 - ps4
Asgard/Eukaryota - ps5
Eukaryota - ps6
Amorpheae - ps7
Obazoa - ps8
Opisthokonta - ps9
Holozoa - ps10
Filozoa - ps11
Choano./Metazoa - ps12
Metazoa - ps13
Myriazoa - ps14
Parahoxozoa - ps15
Bilateria - ps16
Deuterostomia - ps17
Chordata - ps18
Olfactores - ps19
Vertebrata - ps20
Gnathostomata - ps21
Euteleostomi - ps22
Sarcopterygii - ps23
Tetrapoda - ps24
Amniota - ps25
Mammalia - ps26
Theria - ps27
Placentalia - ps28
Boreoeutheria - ps29
Euarchontoglires - ps30
Euarchonta - ps31
Primates - ps32
Haplorrhini - ps33
Simiiformes - ps34
Catarrhini - ps35
Hominoidea - ps36
Hominidae - ps37
Homininae - ps38
Hominini - ps39
Homo_sapiens - ps40
p-value
4e-31
7e-24
1e-16
2e-09
4e-02
Log-odds
0.5
1.0
1.5
2.0
2.5
3.0
3.5
4.0

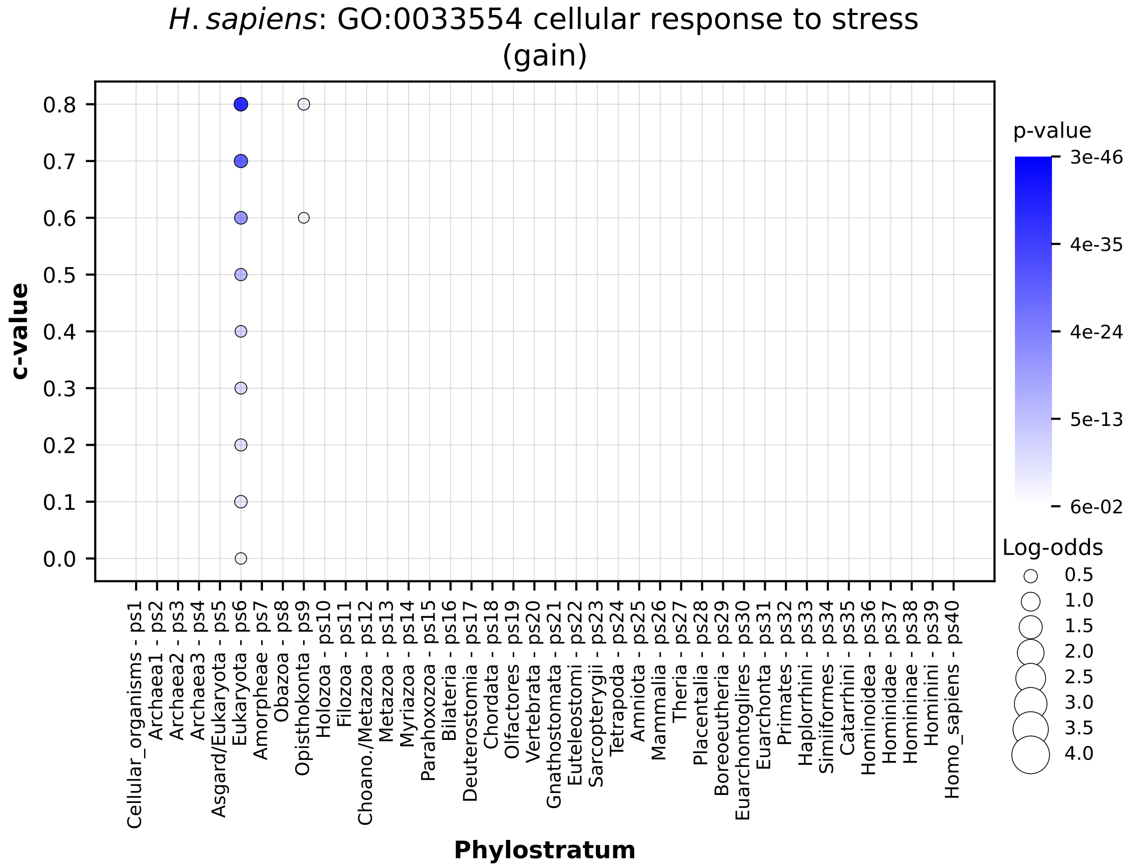

*H. sapiens*: GO:0033554 cellular response to stress
(gain)
c-value
0.8
0.7
0.6
0.5
0.4
0.3
0.2
0.1
0.0
p-value
3e-46
4e-35
4e-24
5e-13
6e-02
Log-odds
0.5
1.0
1.5
2.0
2.5
3.0
3.5
4.0
Cellular_organisms - ps1
Archaea1 - ps2
Archaea2 - ps3
Archaea3 - ps4
Asgard/Eukaryota - ps5
Eukaryota - ps6
Amorpheae - ps7
Obazoa - ps8
Opisthokonta - ps9
Holozoa - ps10
Filozoa - ps11
Choano./Metazoa - ps12
Metazoa - ps13
Myriazoa - ps14
Parahoxozoa - ps15
Bilateria - ps16
Deuterostomia - ps17
Chordata - ps18
Olfactores - ps19
Vertebrata - ps20
Gnathostomata - ps21
Euteleostomi - ps22
Sarcopterygii - ps23
Tetrapoda - ps24
Amniota - ps25
Mammalia - ps26
Theria - ps27
Placentalia - ps28
Boreoeutheria - ps29
Euarchontoglires - ps30
Euarchonta - ps31
Primates - ps32
Haplorrhini - ps33
Simiiformes - ps34
Catarrhini - ps35
Hominoidea - ps36
Hominidae - ps37
Homininae - ps38
Hominini - ps39
Homo_sapiens - ps40
Phylostratum

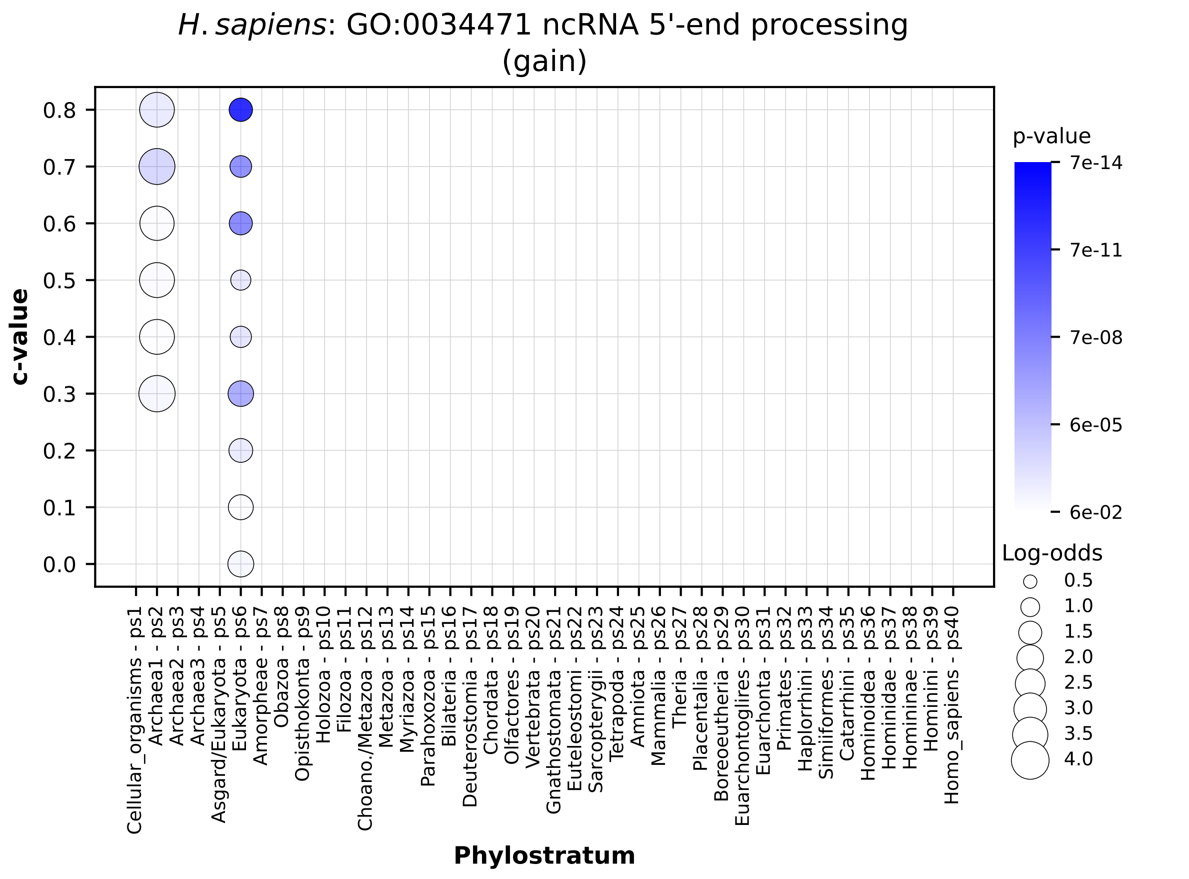
H. sapiens: GO:0034471 ncRNA 5'-end processing
(gain)
c-value
0.0
0.1
0.2
0.3
0.4
0.5
0.6
0.7
0.8
Phylostratum
Cellular_organisms - ps1
Archaea1 - ps2
Archaea2 - ps3
Archaea3 - ps4
Asgard/Eukaryota - ps5
Eukaryota - ps6
Amorpheae - ps7
Obazoa - ps8
Opisthokonta - ps9
Holozoa - ps10
Filozoa - ps11
Choano./Metazoa - ps12
Metazoa - ps13
Myriazoa - ps14
Parahoxozoa - ps15
Bilateria - ps16
Deuterostomia - ps17
Chordata - ps18
Olfactores - ps19
Vertebrata - ps20
Gnathostomata - ps21
Euteleostomi - ps22
Sarcopterygii - ps23
Tetrapoda - ps24
Amniota - ps25
Mammalia - ps26
Theria - ps27
Placentalia - ps28
Boreoeutheria - ps29
Euarchontoglires - ps30
Euarchonta - ps31
Primates - ps32
Haplorrhini - ps33
Simiiformes - ps34
Catarrhini - ps35
Hominoidea - ps36
Hominidae - ps37
Homininae - ps38
Hominini - ps39
Homo_sapiens - ps40
p-value
7e-14
7e-11
7e-08
6e-05
6e-02
Log-odds
0.5
1.0
1.5
2.0
2.5
3.0
3.5
4.0

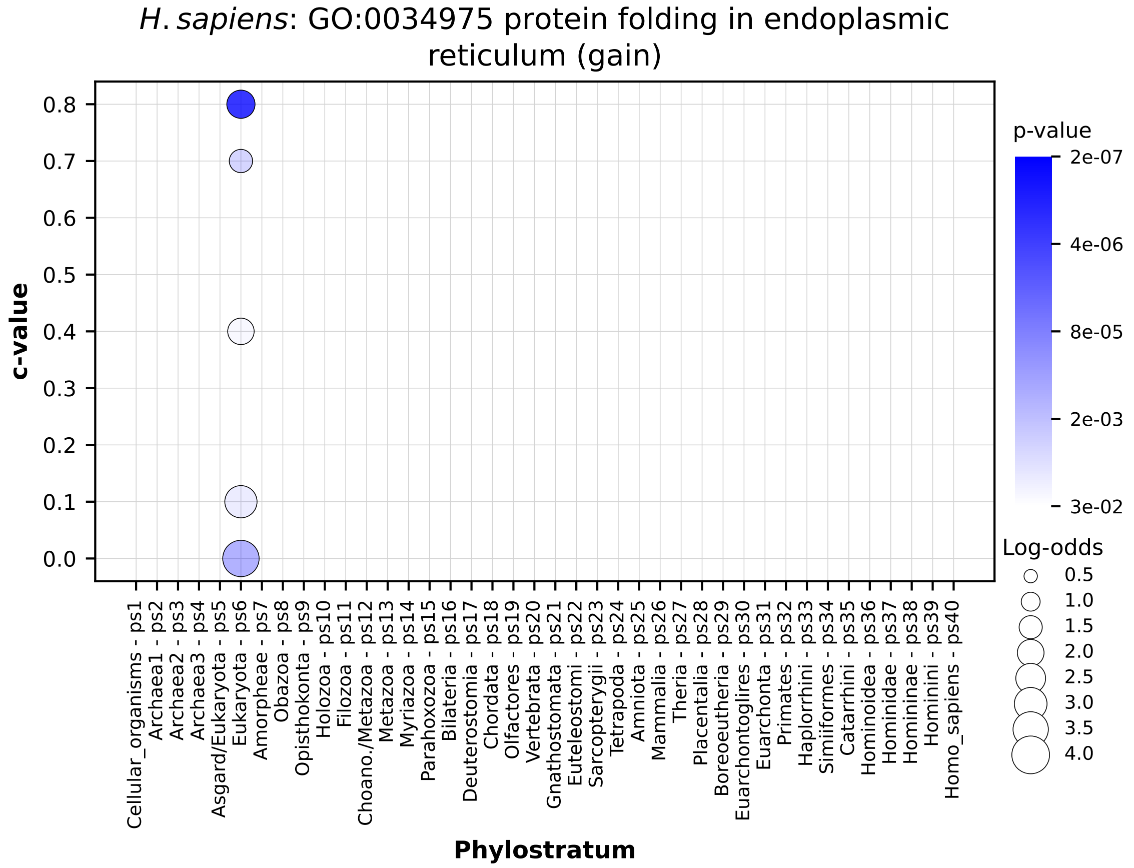

H. sapiens: GO:0034975 protein folding in endoplasmic reticulum (gain)
c-value
0.8
0.7
0.6
0.5
0.4
0.3
0.2
0.1
0.0
p-value
2e-07
4e-06
8e-05
2e-03
3e-02
Log-odds
0.5
1.0
1.5
2.0
2.5
3.0
3.5
4.0
Cellular_organisms - ps1
Archaea1 - ps2
Archaea2 - ps3
Archaea3 - ps4
Asgard/Eukaryota - ps5
Eukaryota - ps6
Amorpheae - ps7
Obazoa - ps8
Opisthokonta - ps9
Holozoa - ps10
Filozoa - ps11
Choano./Metazoa - ps12
Metazoa - ps13
Myriazoa - ps14
Parahoxozoa - ps15
Bilateria - ps16
Deuterostomia - ps17
Chordata - ps18
Olfactores - ps19
Vertebrata - ps20
Gnathostomata - ps21
Euteleostomi - ps22
Sarcopterygii - ps23
Tetrapoda - ps24
Amniota - ps25
Mammalia - ps26
Theria - ps27
Placentalia - ps28
Boreoeutheria - ps29
Euarchontoglires - ps30
Euarchonta - ps31
Primates - ps32
Haplorrhini - ps33
Simiiformes - ps34
Catarrhini - ps35
Hominoidea - ps36
Hominidae - ps37
Homininae - ps38
Hominini - ps39
Homo_sapiens - ps40
Phylostratum

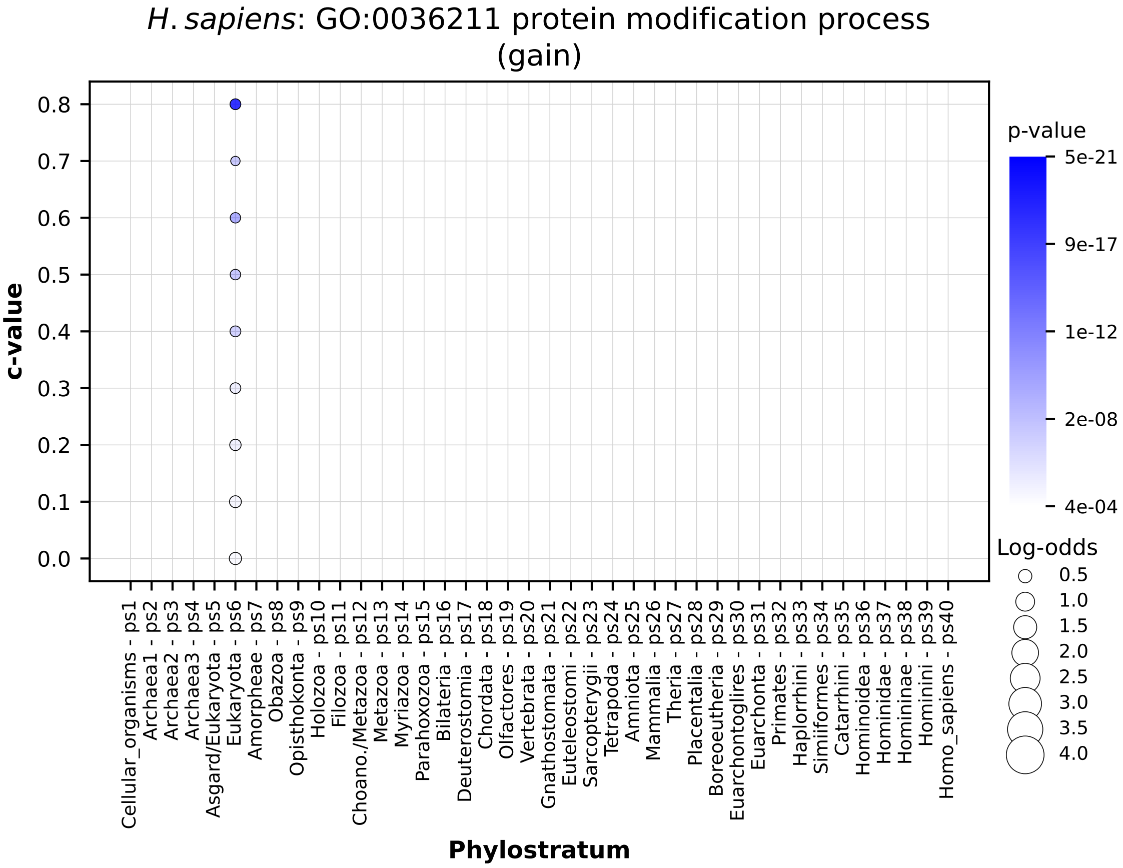
H. sapiens: GO:0036211 protein modification process
(gain)
c-value
0.8
0.7
0.6
0.5
0.4
0.3
0.2
0.1
0.0
p-value
5e-21
9e-17
1e-12
2e-08
4e-04
Log-odds
0.5
1.0
1.5
2.0
2.5
3.0
3.5
4.0
Cellular_organisms - ps1
Archaea1 - ps2
Archaea2 - ps3
Archaea3 - ps4
Asgard/Eukaryota - ps5
Eukaryota - ps6
Amorpheae - ps7
Obazoa - ps8
Opisthokonta - ps9
Holozoa - ps10
Filozoa - ps11
Choano./Metazoa - ps12
Metazoa - ps13
Myriazoa - ps14
Parahoxozoa - ps15
Bilateria - ps16
Deuterostomia - ps17
Chordata - ps18
Olfactores - ps19
Vertebrata - ps20
Gnathostomata - ps21
Euteleostomi - ps22
Sarcopterygii - ps23
Tetrapoda - ps24
Amniota - ps25
Mammalia - ps26
Theria - ps27
Placentalia - ps28
Boreoeutheria - ps29
Euarchontoglires - ps30
Euarchonta - ps31
Primates - ps32
Haplorrhini - ps33
Simiiformes - ps34
Catarrhini - ps35
Hominoidea - ps36
Hominidae - ps37
Homininae - ps38
Hominini - ps39
Homo_sapiens - ps40
Phylostratum

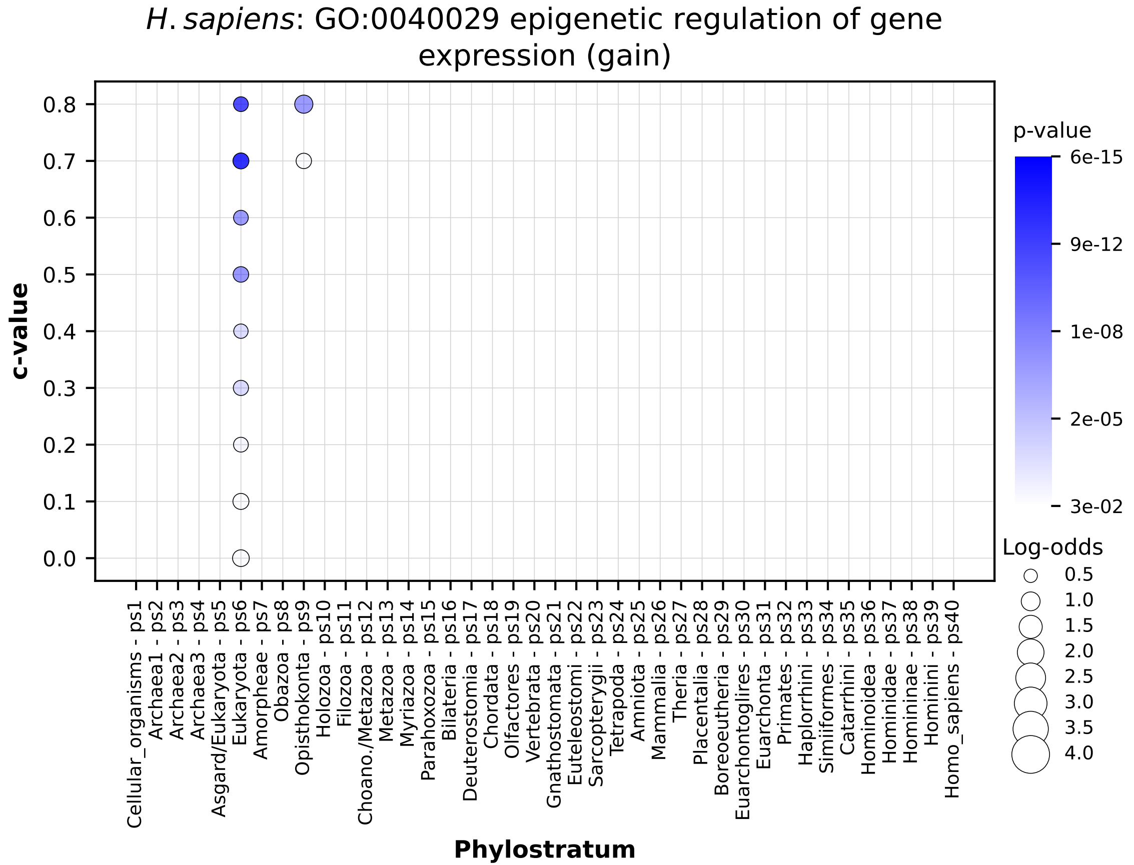
H. sapiens: GO:0040029 epigenetic regulation of gene expression (gain)
c-value
0.8
0.7
0.6
0.5
0.4
0.3
0.2
0.1
0.0
p-value
6e-15
9e-12
1e-08
2e-05
3e-02
Log-odds
0.5
1.0
1.5
2.0
2.5
3.0
3.5
4.0
Cellular_organisms - ps1
Archaea1 - ps2
Archaea2 - ps3
Archaea3 - ps4
Asgard/Eukaryota - ps5
Eukaryota - ps6
Amorpheae - ps7
Obazoa - ps8
Opisthokonta - ps9
Holozoa - ps10
Filozoa - ps11
Choano./Metazoa - ps12
Metazoa - ps13
Myriazoa - ps14
Parahoxozoa - ps15
Bilateria - ps16
Deuterostomia - ps17
Chordata - ps18
Olfactores - ps19
Vertebrata - ps20
Gnathostomata - ps21
Euteleostomi - ps22
Sarcopterygii - ps23
Tetrapoda - ps24
Amniota - ps25
Mammalia - ps26
Theria - ps27
Placentalia - ps28
Boreoeutheria - ps29
Euarchontoglires - ps30
Euarchonta - ps31
Primates - ps32
Haplorrhini - ps33
Simiiformes - ps34
Catarrhini - ps35
Hominoidea - ps36
Hominidae - ps37
Homininae - ps38
Hominini - ps39
Homo_sapiens - ps40
Phylostratum

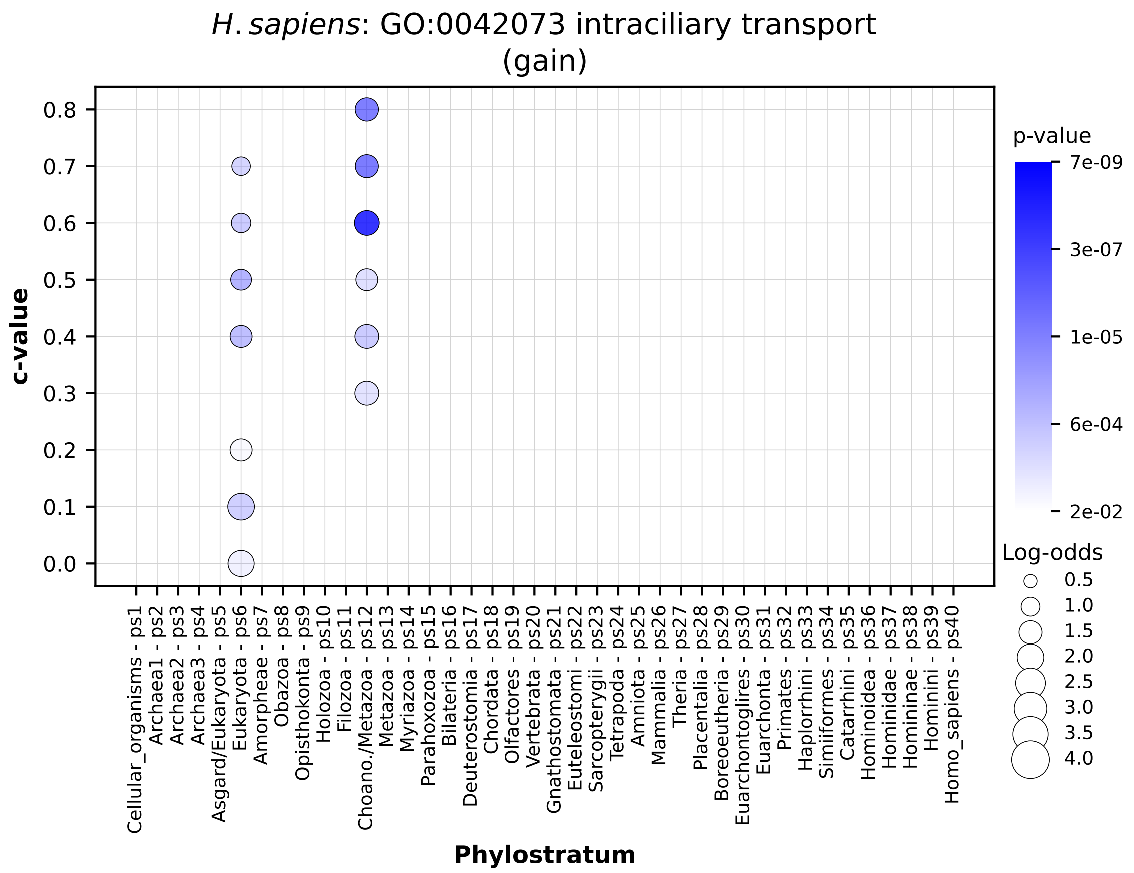

H. sapiens: GO:0042073 intraciliary transport
(gain)
c-value
0.0
0.1
0.2
0.3
0.4
0.5
0.6
0.7
0.8
p-value
7e-09
3e-07
1e-05
6e-04
2e-02
Log-odds
0.5
1.0
1.5
2.0
2.5
3.0
3.5
4.0
Cellular_organisms - ps1
Archaea1 - ps2
Archaea2 - ps3
Archaea3 - ps4
Asgard/Eukaryota - ps5
Eukaryota - ps6
Amorpheae - ps7
Obazoa - ps8
Opisthokonta - ps9
Holozoa - ps10
Filozoa - ps11
Choano./Metazoa - ps12
Metazoa - ps13
Myriazoa - ps14
Parahoxozoa - ps15
Bilateria - ps16
Deuterostomia - ps17
Chordata - ps18
Olfactores - ps19
Vertebrata - ps20
Gnathostomata - ps21
Euteleostomi - ps22
Sarcopterygii - ps23
Tetrapoda - ps24
Amniota - ps25
Mammalia - ps26
Theria - ps27
Placentalia - ps28
Boreoeutheria - ps29
Euarchontoglires - ps30
Euarchonta - ps31
Primates - ps32
Haplorrhini - ps33
Simiiformes - ps34
Catarrhini - ps35
Hominoidea - ps36
Hominidae - ps37
Homininae - ps38
Hominini - ps39
Homo_sapiens - ps40
Phylostratum

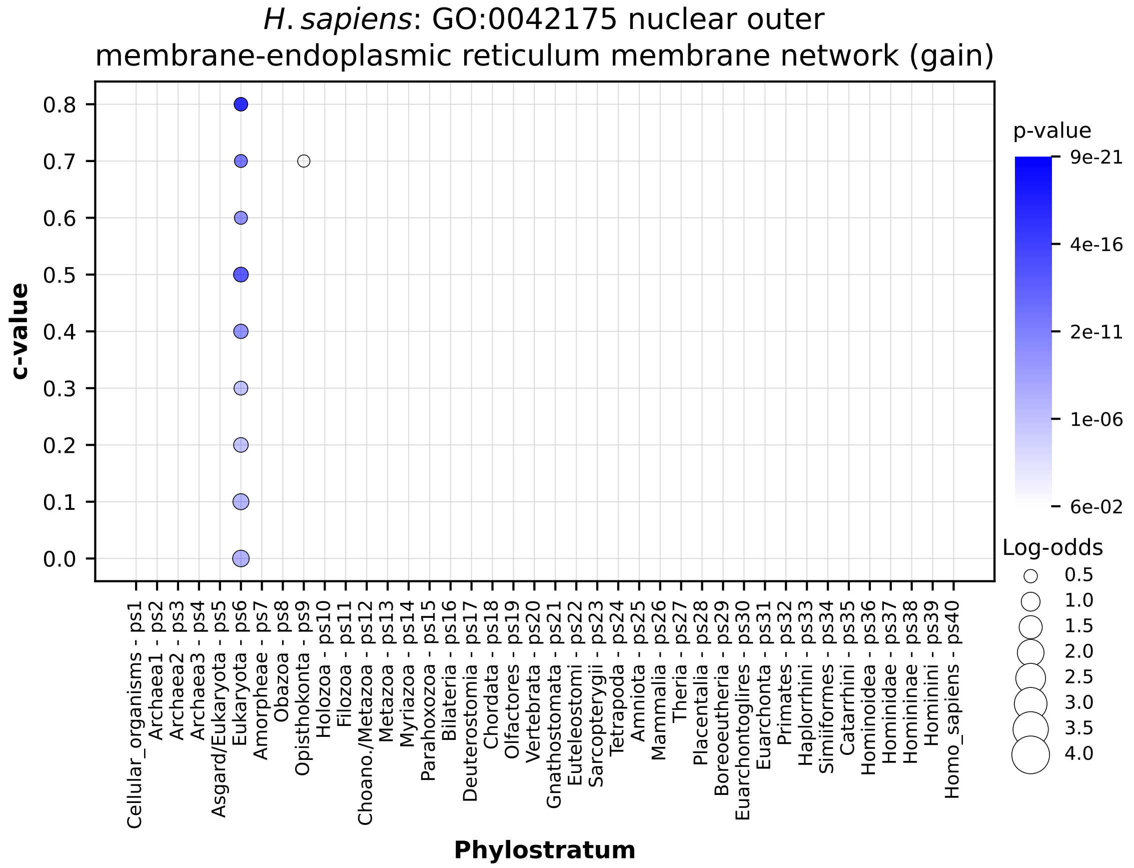

H. sapiens: GO:0042175 nuclear outer membrane-endoplasmic reticulum membrane network (gain)
c-value
0.8
0.7
0.6
0.5
0.4
0.3
0.2
0.1
0.0
Phylostratum
Cellular_organisms - ps1
Archaea1 - ps2
Archaea2 - ps3
Archaea3 - ps4
Asgard/Eukaryota - ps5
Eukaryota - ps6
Amorpheae - ps7
Obazoa - ps8
Opisthokonta - ps9
Holozoa - ps10
Filozoa - ps11
Choano./Metazoa - ps12
Metazoa - ps13
Myriazoa - ps14
Parahoxozoa - ps15
Bilateria - ps16
Deuterostomia - ps17
Chordata - ps18
Olfactores - ps19
Vertebrata - ps20
Gnathostomata - ps21
Euteleostomi - ps22
Sarcopterygii - ps23
Tetrapoda - ps24
Amniota - ps25
Mammalia - ps26
Theria - ps27
Placentalia - ps28
Boreoeutheria - ps29
Euarchontoglires - ps30
Euarchonta - ps31
Primates - ps32
Haplorrhini - ps33
Simiiformes - ps34
Catarrhini - ps35
Hominoidea - ps36
Hominidae - ps37
Homininae - ps38
Hominini - ps39
Homo_sapiens - ps40
p-value
9e-21
4e-16
2e-11
1e-06
6e-02
Log-odds
0.5
1.0
1.5
2.0
2.5
3.0
3.5
4.0

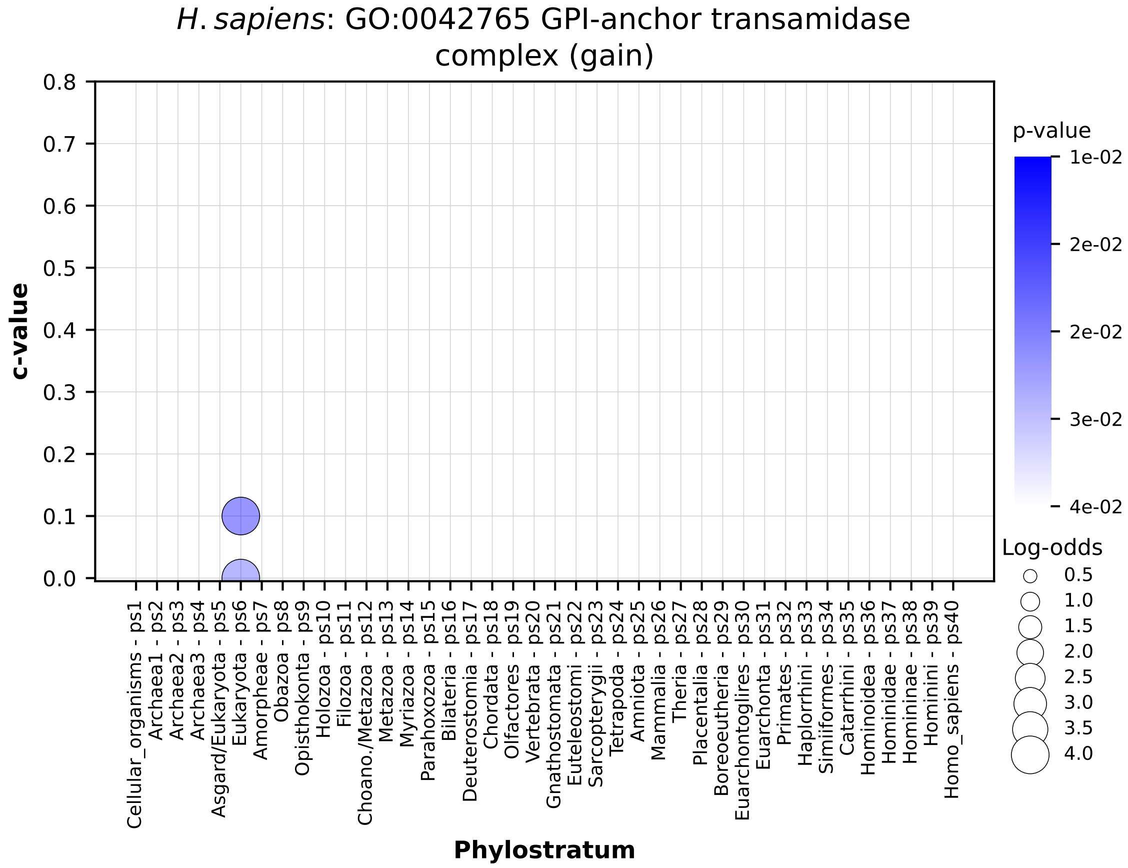
H. sapiens: GO:0042765 GPI-anchor transamidase complex (gain)
c-value
0.8
0.7
0.6
0.5
0.4
0.3
0.2
0.1
0.0
p-value
1e-02
2e-02
2e-02
3e-02
4e-02
Log-odds
0.5
1.0
1.5
2.0
2.5
3.0
3.5
4.0
Cellular_organisms - ps1
Archaea1 - ps2
Archaea2 - ps3
Archaea3 - ps4
Asgard/Eukaryota - ps5
Eukaryota - ps6
Amorpheae - ps7
Obazoa - ps8
Opisthokonta - ps9
Holozoa - ps10
Filozoa - ps11
Choano./Metazoa - ps12
Metazoa - ps13
Myriazoa - ps14
Parahoxozoa - ps15
Bilateria - ps16
Deuterostomia - ps17
Chordata - ps18
Olfactores - ps19
Vertebrata - ps20
Gnathostomata - ps21
Euteleostomi - ps22
Sarcopterygii - ps23
Tetrapoda - ps24
Amniota - ps25
Mammalia - ps26
Theria - ps27
Placentalia - ps28
Boreoeutheria - ps29
Euarchontoglires - ps30
Euarchonta - ps31
Primates - ps32
Haplorrhini - ps33
Simiiformes - ps34
Catarrhini - ps35
Hominoidea - ps36
Hominidae - ps37
Homininae - ps38
Hominini - ps39
Homo_sapiens - ps40
Phylostratum

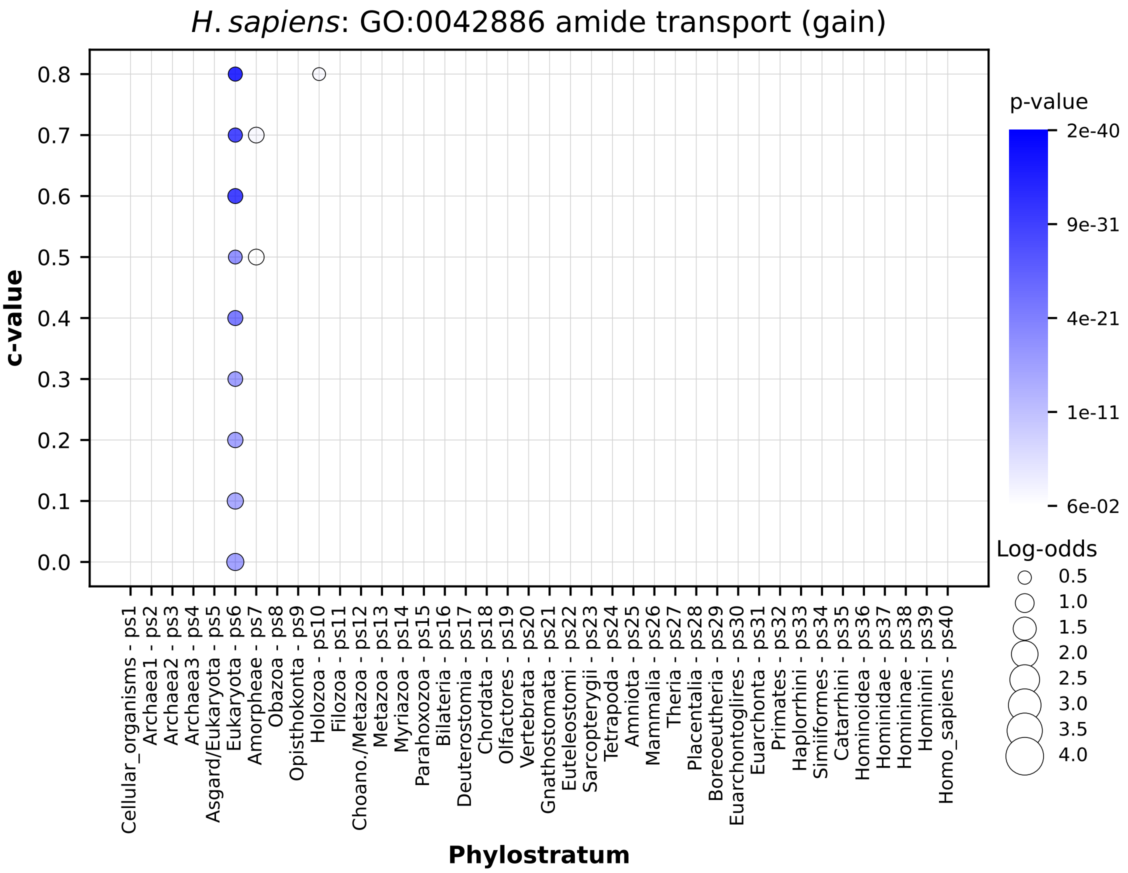
H. sapiens: GO:0042886 amide transport (gain)
c-value
0.0
0.1
0.2
0.3
0.4
0.5
0.6
0.7
0.8
Phylostratum
Cellular_organisms - ps1
Archaea1 - ps2
Archaea2 - ps3
Archaea3 - ps4
Asgard/Eukaryota - ps5
Eukaryota - ps6
Amorpheae - ps7
Obazoa - ps8
Opisthokonta - ps9
Holozoa - ps10
Filozoa - ps11
Choano./Metazoa - ps12
Metazoa - ps13
Myriazoa - ps14
Parahoxozoa - ps15
Bilateria - ps16
Deuterostomia - ps17
Chordata - ps18
Olfactores - ps19
Vertebrata - ps20
Gnathostomata - ps21
Euteleostomi - ps22
Sarcopterygii - ps23
Tetrapoda - ps24
Amniota - ps25
Mammalia - ps26
Theria - ps27
Placentalia - ps28
Boreoeutheria - ps29
Euarchontoglires - ps30
Euarchonta - ps31
Primates - ps32
Haplorrhini - ps33
Simiiformes - ps34
Catarrhini - ps35
Hominoidea - ps36
Hominidae - ps37
Homininae - ps38
Hominini - ps39
Homo_sapiens - ps40
p-value
2e-40
9e-31
4e-21
1e-11
6e-02
Log-odds
0.5
1.0
1.5
2.0
2.5
3.0
3.5
4.0

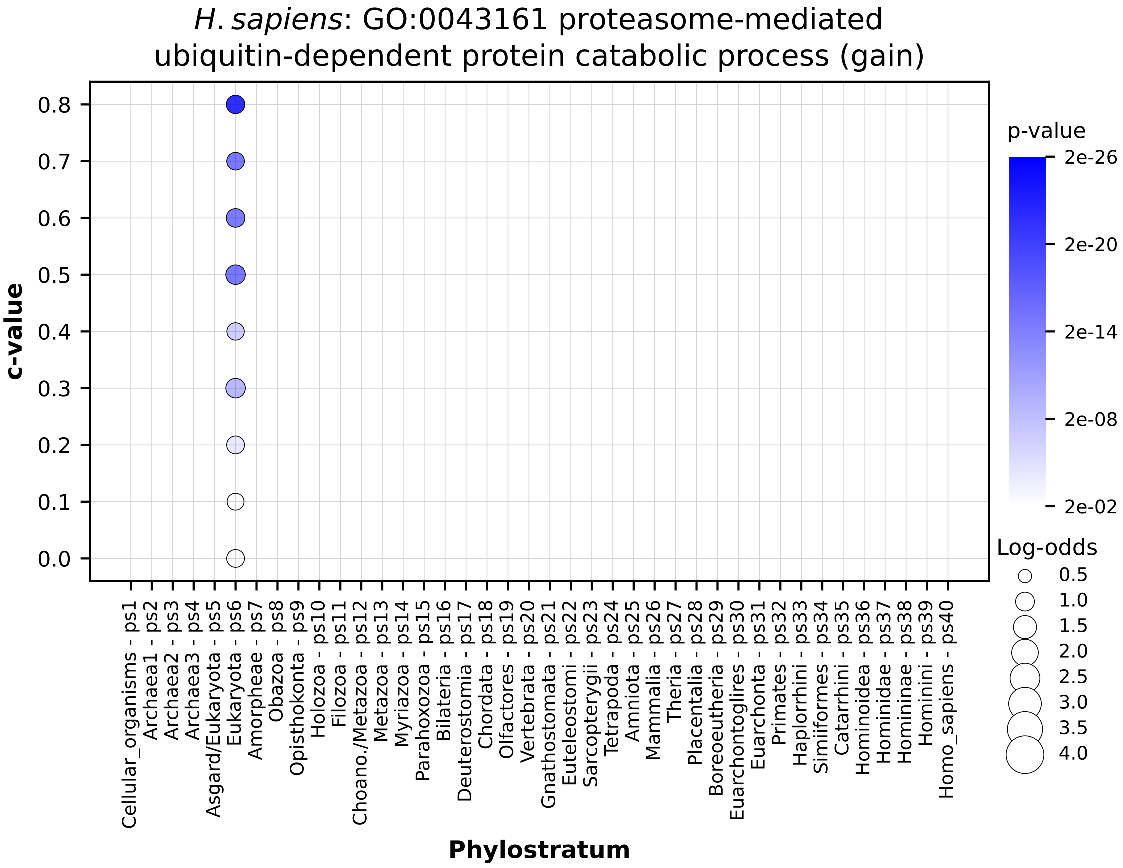

*H. sapiens*: GO:0043161 proteasome-mediated
ubiquitin-dependent protein catabolic process (gain)
c-value
0.8
0.7
0.6
0.5
0.4
0.3
0.2
0.1
0.0
Phylostratum
Cellular_organisms - ps1
Archaea1 - ps2
Archaea2 - ps3
Archaea3 - ps4
Asgard/Eukaryota - ps5
Eukaryota - ps6
Amorpheae - ps7
Obazoa - ps8
Opisthokonta - ps9
Holozoa - ps10
Filozoa - ps11
Choano./Metazoa - ps12
Metazoa - ps13
Myriazoa - ps14
Parahoxozoa - ps15
Bilateria - ps16
Deuterostomia - ps17
Chordata - ps18
Olfactores - ps19
Vertebrata - ps20
Gnathostomata - ps21
Euteleostomi - ps22
Sarcopterygii - ps23
Tetrapoda - ps24
Amniota - ps25
Mammalia - ps26
Theria - ps27
Placentalia - ps28
Boreoeutheria - ps29
Euarchontoglires - ps30
Euarchonta - ps31
Primates - ps32
Haplorrhini - ps33
Simiiformes - ps34
Catarrhini - ps35
Hominoidea - ps36
Hominidae - ps37
Homininae - ps38
Hominini - ps39
Homo_sapiens - ps40
p-value
2e-26
2e-20
2e-14
2e-08
2e-02
Log-odds
0.5
1.0
1.5
2.0
2.5
3.0
3.5
4.0

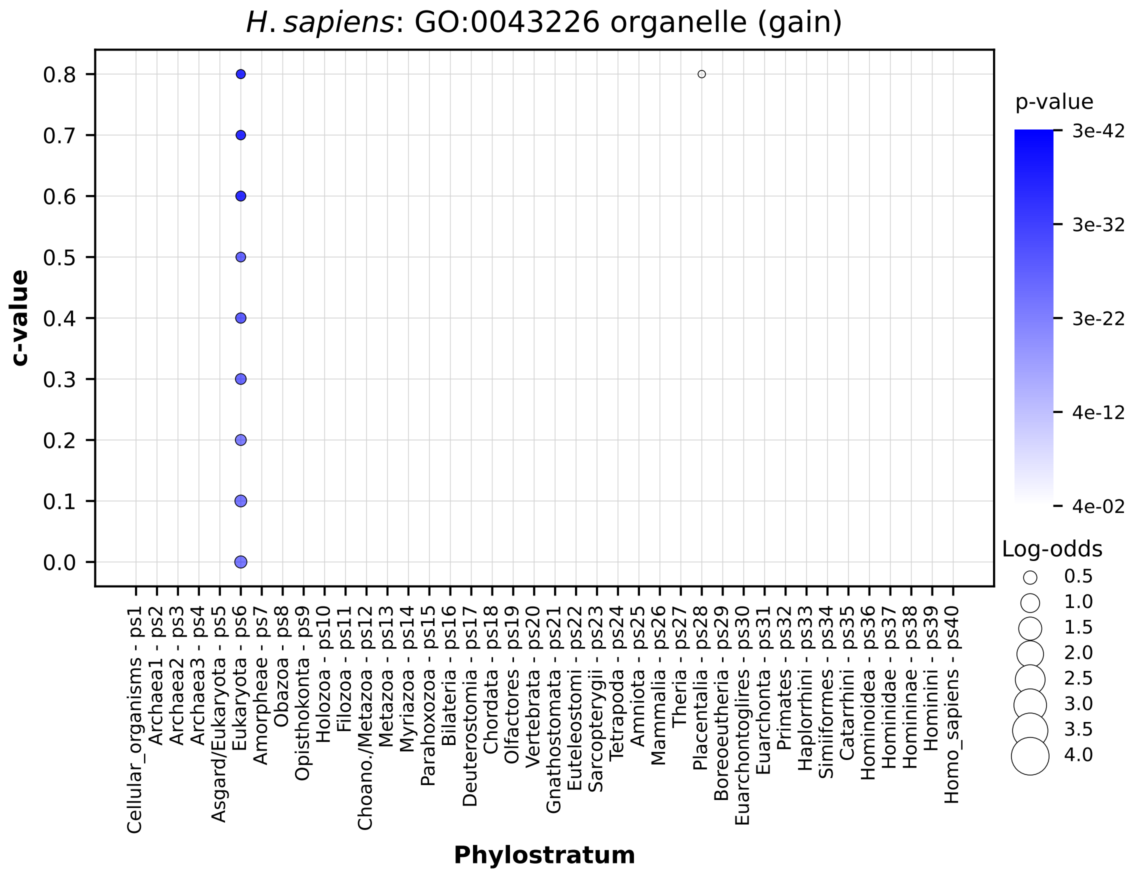

H. sapiens: GO:0043226 organelle (gain)
c-value
0.8
0.7
0.6
0.5
0.4
0.3
0.2
0.1
0.0
p-value
3e-42
3e-32
3e-22
4e-12
4e-02
Log-odds
0.5
1.0
1.5
2.0
2.5
3.0
3.5
4.0
Cellular_organisms - ps1
Archaea1 - ps2
Archaea2 - ps3
Archaea3 - ps4
Asgard/Eukaryota - ps5
Eukaryota - ps6
Amorpheae - ps7
Obazoa - ps8
Opisthokonta - ps9
Holozoa - ps10
Filozoa - ps11
Choano./Metazoa - ps12
Metazoa - ps13
Myriazoa - ps14
Parahoxozoa - ps15
Bilateria - ps16
Deuterostomia - ps17
Chordata - ps18
Olfactores - ps19
Vertebrata - ps20
Gnathostomata - ps21
Euteleostomi - ps22
Sarcopterygii - ps23
Tetrapoda - ps24
Amniota - ps25
Mammalia - ps26
Theria - ps27
Placentalia - ps28
Boreoeutheria - ps29
Euarchontoglires - ps30
Euarchonta - ps31
Primates - ps32
Haplorrhini - ps33
Simiiformes - ps34
Catarrhini - ps35
Hominoidea - ps36
Hominidae - ps37
Homininae - ps38
Hominini - ps39
Homo_sapiens - ps40
Phylostratum

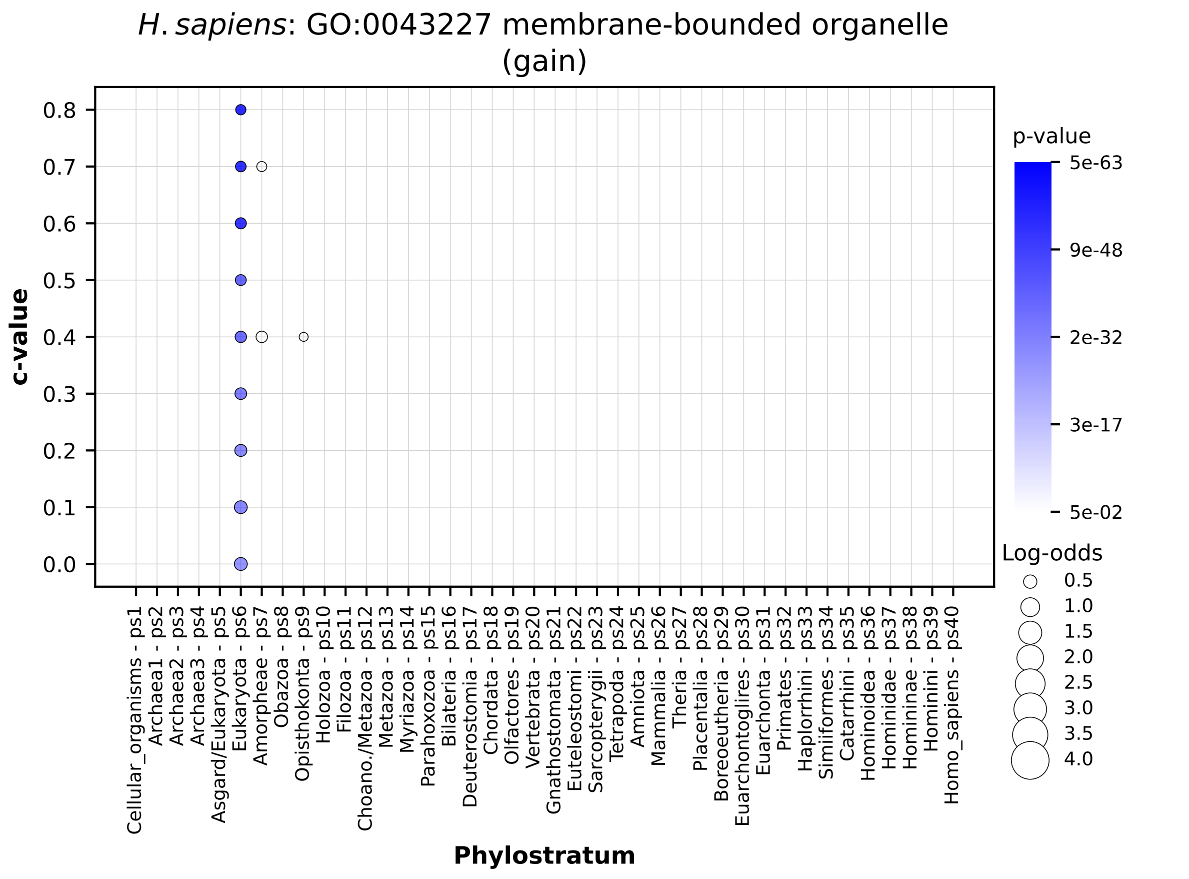

H. sapiens: GO:0043227 membrane-bounded organelle
(gain)
c-value
0.8
0.7
0.6
0.5
0.4
0.3
0.2
0.1
0.0
Phylostratum
Cellular_organisms - ps1
Archaea1 - ps2
Archaea2 - ps3
Archaea3 - ps4
Asgard/Eukaryota - ps5
Eukaryota - ps6
Amorpheae - ps7
Obazoa - ps8
Opisthokonta - ps9
Holozoa - ps10
Filozoa - ps11
Choano./Metazoa - ps12
Metazoa - ps13
Myriazoa - ps14
Parahoxozoa - ps15
Bilateria - ps16
Deuterostomia - ps17
Chordata - ps18
Olfactores - ps19
Vertebrata - ps20
Gnathostomata - ps21
Euteleostomi - ps22
Sarcopterygii - ps23
Tetrapoda - ps24
Amniota - ps25
Mammalia - ps26
Theria - ps27
Placentalia - ps28
Boreoeutheria - ps29
Euarchontoglires - ps30
Euarchonta - ps31
Primates - ps32
Haplorrhini - ps33
Simiiformes - ps34
Catarrhini - ps35
Hominoidea - ps36
Hominidae - ps37
Homininae - ps38
Hominini - ps39
Homo_sapiens - ps40
p-value
5e-63
9e-48
2e-32
3e-17
5e-02
Log-odds
0.5
1.0
1.5
2.0
2.5
3.0
3.5
4.0

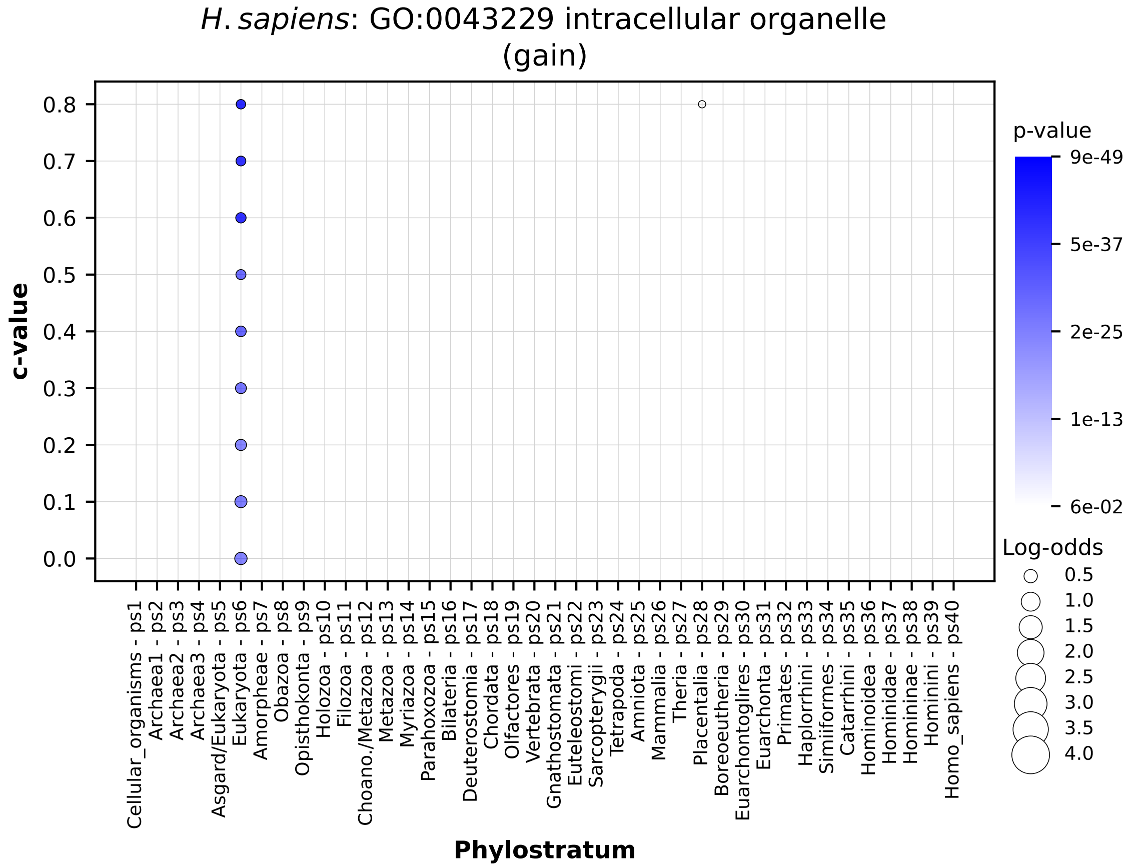

H. sapiens: GO:0043229 intracellular organelle
(gain)
c-value
0.8
0.7
0.6
0.5
0.4
0.3
0.2
0.1
0.0
p-value
9e-49
5e-37
2e-25
1e-13
6e-02
Log-odds
0.5
1.0
1.5
2.0
2.5
3.0
3.5
4.0
Cellular_organisms - ps1
Archaea1 - ps2
Archaea2 - ps3
Archaea3 - ps4
Asgard/Eukaryota - ps5
Eukaryota - ps6
Amorpheae - ps7
Obazoa - ps8
Opisthokonta - ps9
Holozoa - ps10
Filozoa - ps11
Choano./Metazoa - ps12
Metazoa - ps13
Myriazoa - ps14
Parahoxozoa - ps15
Bilateria - ps16
Deuterostomia - ps17
Chordata - ps18
Olfactores - ps19
Vertebrata - ps20
Gnathostomata - ps21
Euteleostomi - ps22
Sarcopterygii - ps23
Tetrapoda - ps24
Amniota - ps25
Mammalia - ps26
Theria - ps27
Placentalia - ps28
Boreoeutheria - ps29
Euarchontoglires - ps30
Euarchonta - ps31
Primates - ps32
Haplorrhini - ps33
Simiiformes - ps34
Catarrhini - ps35
Hominoidea - ps36
Hominidae - ps37
Homininae - ps38
Hominini - ps39
Homo_sapiens - ps40
Phylostratum

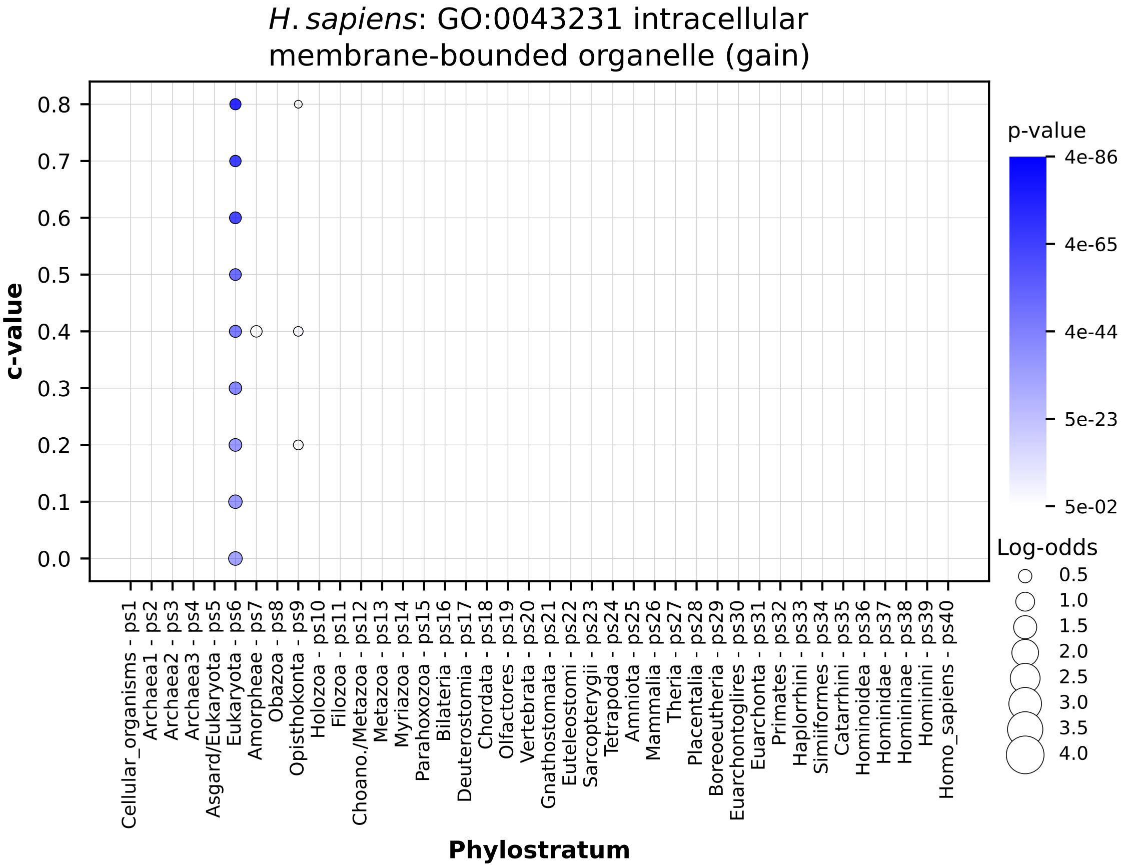
H. sapiens: GO:0043231 intracellular membrane-bounded organelle (gain)
c-value
0.8
0.7
0.6
0.5
0.4
0.3
0.2
0.1
0.0
Phylostratum
Cellular_organisms - ps1
Archaea1 - ps2
Archaea2 - ps3
Archaea3 - ps4
Asgard/Eukaryota - ps5
Eukaryota - ps6
Amorpheae - ps7
Obazoa - ps8
Opisthokonta - ps9
Holozoa - ps10
Filozoa - ps11
Choano./Metazoa - ps12
Metazoa - ps13
Myriazoa - ps14
Parahoxozoa - ps15
Bilateria - ps16
Deuterostomia - ps17
Chordata - ps18
Olfactores - ps19
Vertebrata - ps20
Gnathostomata - ps21
Euteleostomi - ps22
Sarcopterygii - ps23
Tetrapoda - ps24
Amniota - ps25
Mammalia - ps26
Theria - ps27
Placentalia - ps28
Boreoeutheria - ps29
Euarchontoglires - ps30
Euarchonta - ps31
Primates - ps32
Haplorrhini - ps33
Simiiformes - ps34
Catarrhini - ps35
Hominoidea - ps36
Hominidae - ps37
Homininae - ps38
Hominini - ps39
Homo_sapiens - ps40
p-value
4e-86
4e-65
4e-44
5e-23
5e-02
Log-odds
0.5
1.0
1.5
2.0
2.5
3.0
3.5
4.0

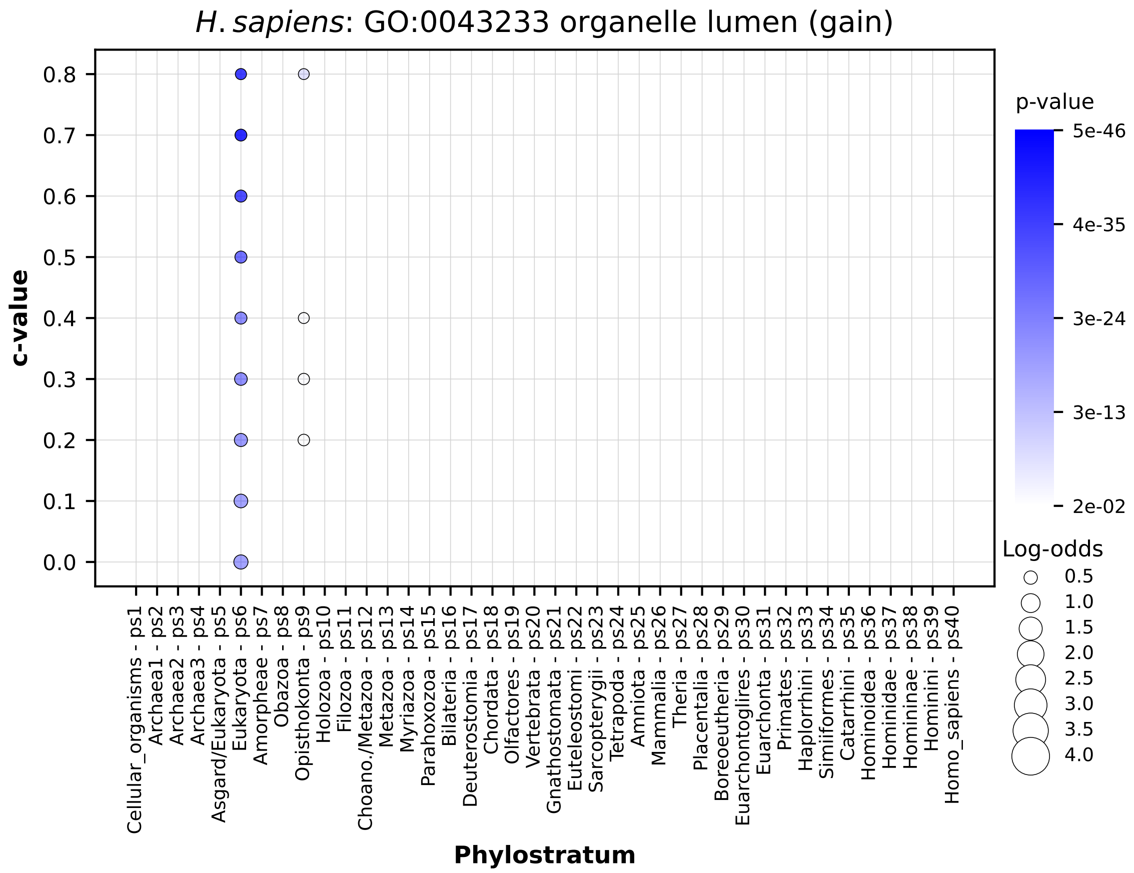
H. sapiens: GO:0043233 organelle lumen (gain)
c-value
0.8
0.7
0.6
0.5
0.4
0.3
0.2
0.1
0.0
Phylostratum
Cellular_organisms - ps1
Archaea1 - ps2
Archaea2 - ps3
Archaea3 - ps4
Asgard/Eukaryota - ps5
Eukaryota - ps6
Amorpheae - ps7
Obazoa - ps8
Opisthokonta - ps9
Holozoa - ps10
Filozoa - ps11
Choano./Metazoa - ps12
Metazoa - ps13
Myriazoa - ps14
Parahoxozoa - ps15
Bilateria - ps16
Deuterostomia - ps17
Chordata - ps18
Olfactores - ps19
Vertebrata - ps20
Gnathostomata - ps21
Euteleostomi - ps22
Sarcopterygii - ps23
Tetrapoda - ps24
Amniota - ps25
Mammalia - ps26
Theria - ps27
Placentalia - ps28
Boreoeutheria - ps29
Euarchontoglires - ps30
Euarchonta - ps31
Primates - ps32
Haplorrhini - ps33
Simiiformes - ps34
Catarrhini - ps35
Hominoidea - ps36
Hominidae - ps37
Homininae - ps38
Hominini - ps39
Homo_sapiens - ps40
p-value
5e-46
4e-35
3e-24
3e-13
2e-02
Log-odds
0.5
1.0
1.5
2.0
2.5
3.0
3.5
4.0

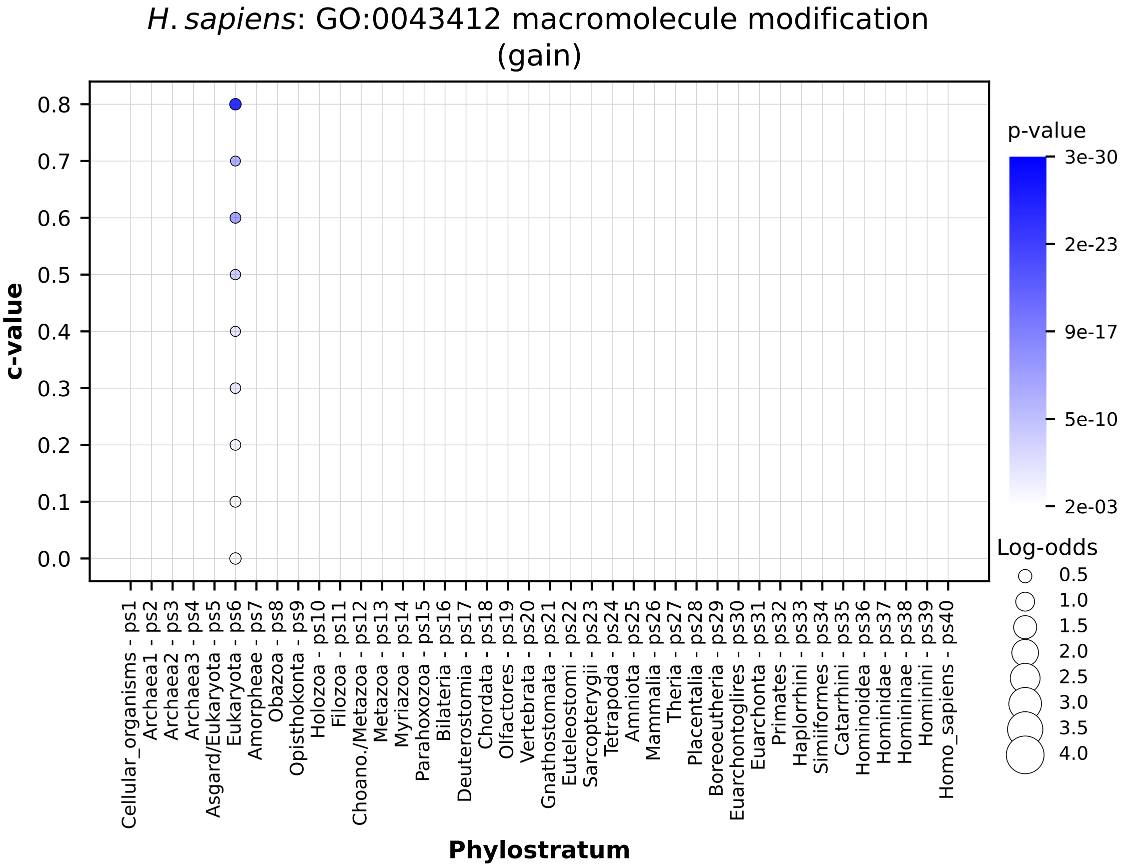

H. sapiens: GO:0043412 macromolecule modification
(gain)
c-value
0.0
0.1
0.2
0.3
0.4
0.5
0.6
0.7
0.8
Phylostratum
Cellular_organisms - ps1
Archaea1 - ps2
Archaea2 - ps3
Archaea3 - ps4
Asgard/Eukaryota - ps5
Eukaryota - ps6
Amorpheae - ps7
Obazoa - ps8
Opisthokonta - ps9
Holozoa - ps10
Filozoa - ps11
Choano./Metazoa - ps12
Metazoa - ps13
Myriazoa - ps14
Parahoxozoa - ps15
Bilateria - ps16
Deuterostomia - ps17
Chordata - ps18
Olfactores - ps19
Vertebrata - ps20
Gnathostomata - ps21
Euteleostomi - ps22
Sarcopterygii - ps23
Tetrapoda - ps24
Amniota - ps25
Mammalia - ps26
Theria - ps27
Placentalia - ps28
Boreoeutheria - ps29
Euarchontoglires - ps30
Euarchonta - ps31
Primates - ps32
Haplorrhini - ps33
Simiiformes - ps34
Catarrhini - ps35
Hominoidea - ps36
Hominidae - ps37
Homininae - ps38
Hominini - ps39
Homo_sapiens - ps40
p-value
3e-30
2e-23
9e-17
5e-10
2e-03
Log-odds
0.5
1.0
1.5
2.0
2.5
3.0
3.5
4.0

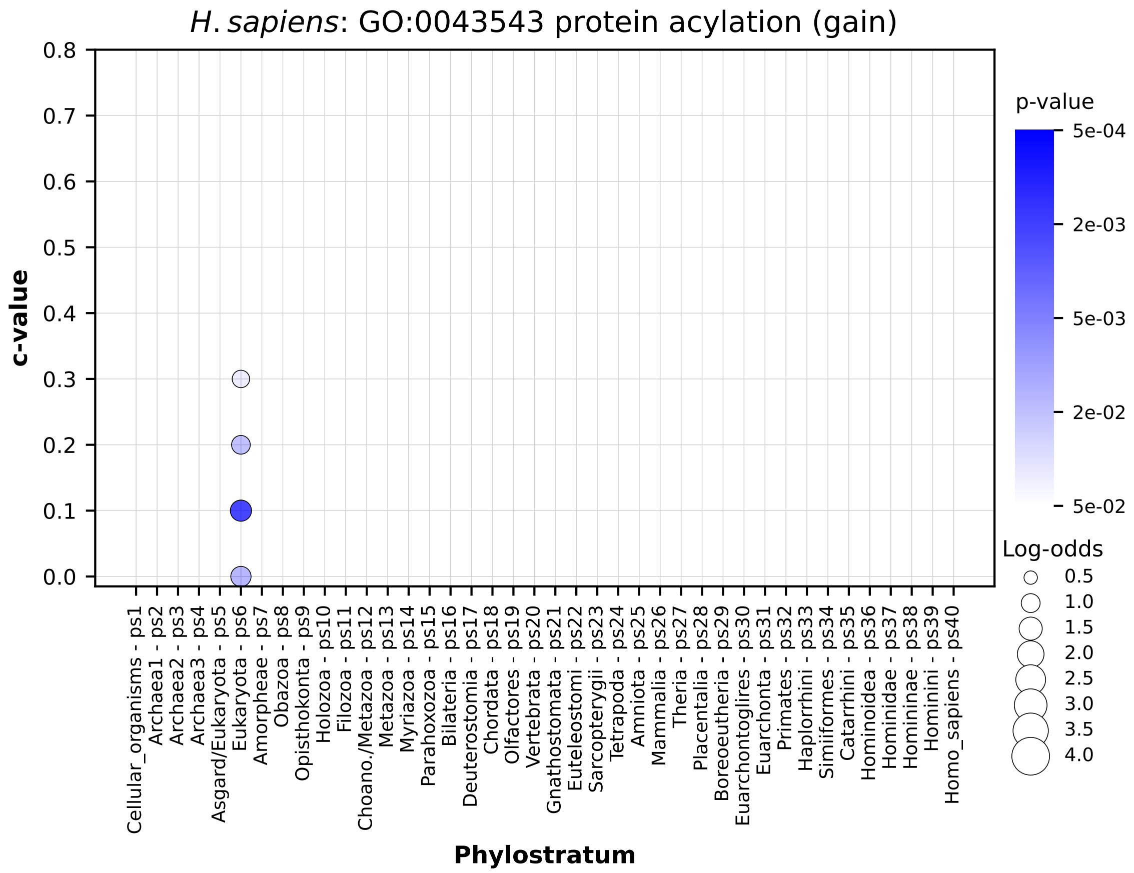

H. sapiens: GO:0043543 protein acylation (gain)
c-value
0.0
0.1
0.2
0.3
0.4
0.5
0.6
0.7
0.8
Phylostratum
Cellular_organisms - ps1
Archaea1 - ps2
Archaea2 - ps3
Archaea3 - ps4
Asgard/Eukaryota - ps5
Eukaryota - ps6
Amorpheae - ps7
Obazoa - ps8
Opisthokonta - ps9
Holozoa - ps10
Filozoa - ps11
Choano./Metazoa - ps12
Metazoa - ps13
Myriazoa - ps14
Parahoxozoa - ps15
Bilateria - ps16
Deuterostomia - ps17
Chordata - ps18
Olfactores - ps19
Vertebrata - ps20
Gnathostomata - ps21
Euteleostomi - ps22
Sarcopterygii - ps23
Tetrapoda - ps24
Amniota - ps25
Mammalia - ps26
Theria - ps27
Placentalia - ps28
Boreoeutheria - ps29
Euarchontoglires - ps30
Euarchonta - ps31
Primates - ps32
Haplorrhini - ps33
Simiiformes - ps34
Catarrhini - ps35
Hominoidea - ps36
Hominidae - ps37
Homininae - ps38
Hominini - ps39
Homo_sapiens - ps40
p-value
5e-04
2e-03
5e-03
2e-02
5e-02
Log-odds
0.5
1.0
1.5
2.0
2.5
3.0
3.5
4.0

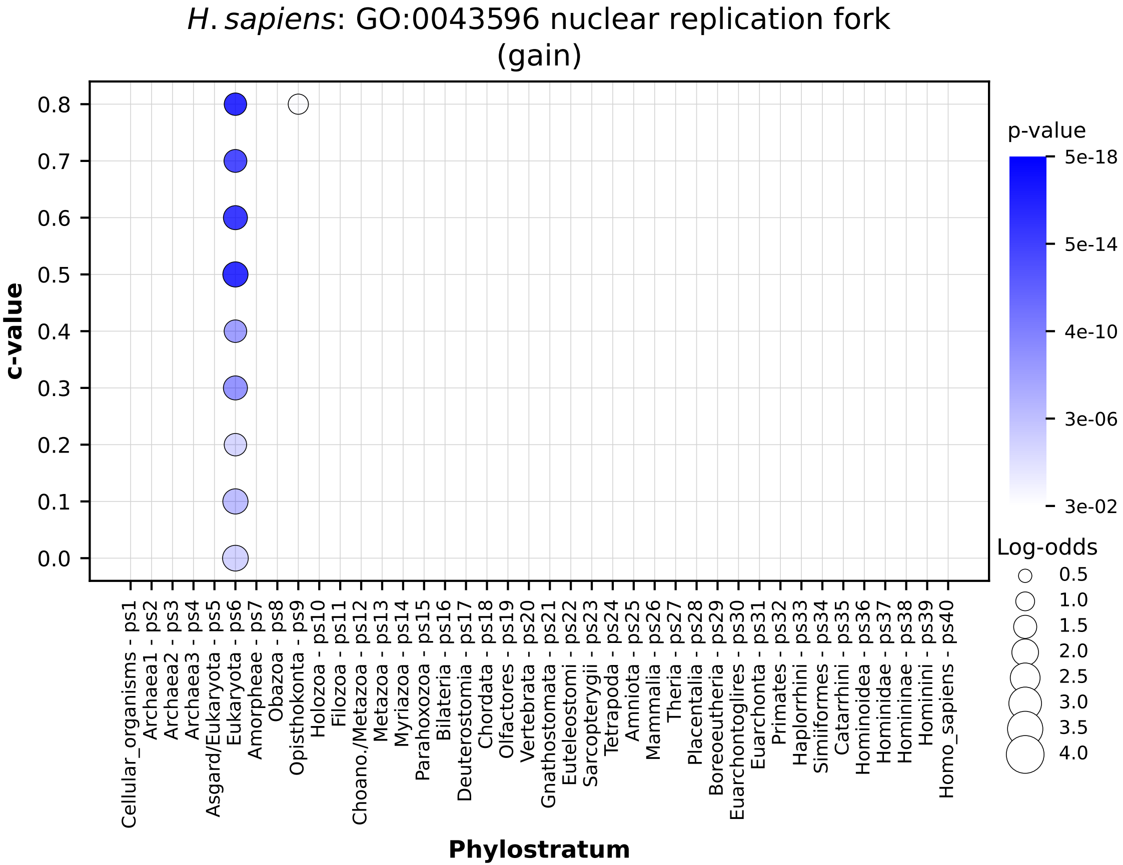
H. sapiens: GO:0043596 nuclear replication fork
(gain)
c-value
0.8
0.7
0.6
0.5
0.4
0.3
0.2
0.1
0.0
Phylostratum
Cellular_organisms - ps1
Archaea1 - ps2
Archaea2 - ps3
Archaea3 - ps4
Asgard/Eukaryota - ps5
Eukaryota - ps6
Amorpheae - ps7
Obazoa - ps8
Opisthokonta - ps9
Holozoa - ps10
Filozoa - ps11
Choano./Metazoa - ps12
Metazoa - ps13
Myriazoa - ps14
Parahoxozoa - ps15
Bilateria - ps16
Deuterostomia - ps17
Chordata - ps18
Olfactores - ps19
Vertebrata - ps20
Gnathostomata - ps21
Euteleostomi - ps22
Sarcopterygii - ps23
Tetrapoda - ps24
Amniota - ps25
Mammalia - ps26
Theria - ps27
Placentalia - ps28
Boreoeutheria - ps29
Euarchontoglires - ps30
Euarchonta - ps31
Primates - ps32
Haplorrhini - ps33
Simiiformes - ps34
Catarrhini - ps35
Hominoidea - ps36
Hominidae - ps37
Homininae - ps38
Hominini - ps39
Homo_sapiens - ps40
p-value
5e-18
5e-14
4e-10
3e-06
3e-02
Log-odds
0.5
1.0
1.5
2.0
2.5
3.0
3.5
4.0

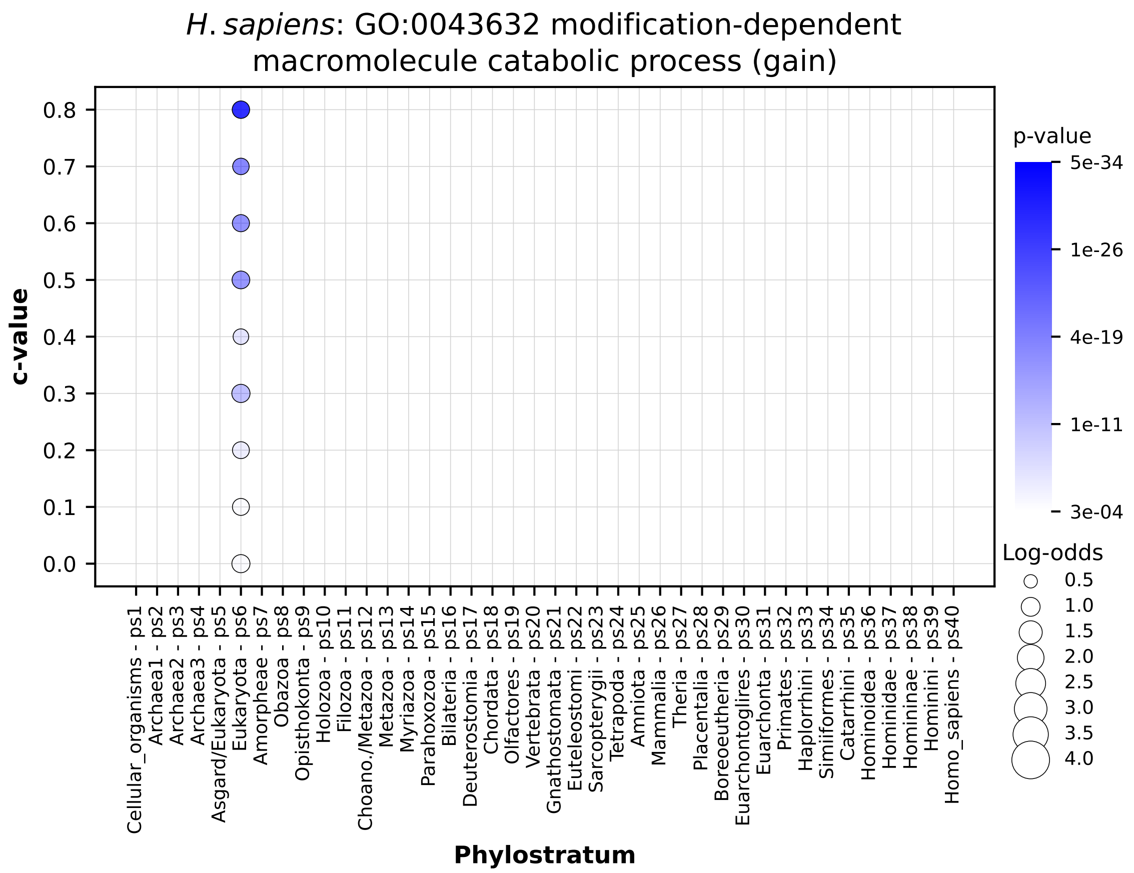

H. sapiens: GO:0043632 modification-dependent macromolecule catabolic process (gain)
c-value
0.0
0.1
0.2
0.3
0.4
0.5
0.6
0.7
0.8
p-value
5e-34
1e-26
4e-19
1e-11
3e-04
Log-odds
0.5
1.0
1.5
2.0
2.5
3.0
3.5
4.0
Cellular_organisms - ps1
Archaea1 - ps2
Archaea2 - ps3
Archaea3 - ps4
Asgard/Eukaryota - ps5
Eukaryota - ps6
Amorpheae - ps7
Obazoa - ps8
Opisthokonta - ps9
Holozoa - ps10
Filozoa - ps11
Choano./Metazoa - ps12
Metazoa - ps13
Myriazoa - ps14
Parahoxozoa - ps15
Bilateria - ps16
Deuterostomia - ps17
Chordata - ps18
Olfactores - ps19
Vertebrata - ps20
Gnathostomata - ps21
Euteleostomi - ps22
Sarcopterygii - ps23
Tetrapoda - ps24
Amniota - ps25
Mammalia - ps26
Theria - ps27
Placentalia - ps28
Boreoeutheria - ps29
Euarchontoglires - ps30
Euarchonta - ps31
Primates - ps32
Haplorrhini - ps33
Simiiformes - ps34
Catarrhini - ps35
Hominoidea - ps36
Hominidae - ps37
Homininae - ps38
Hominini - ps39
Homo_sapiens - ps40
Phylostratum

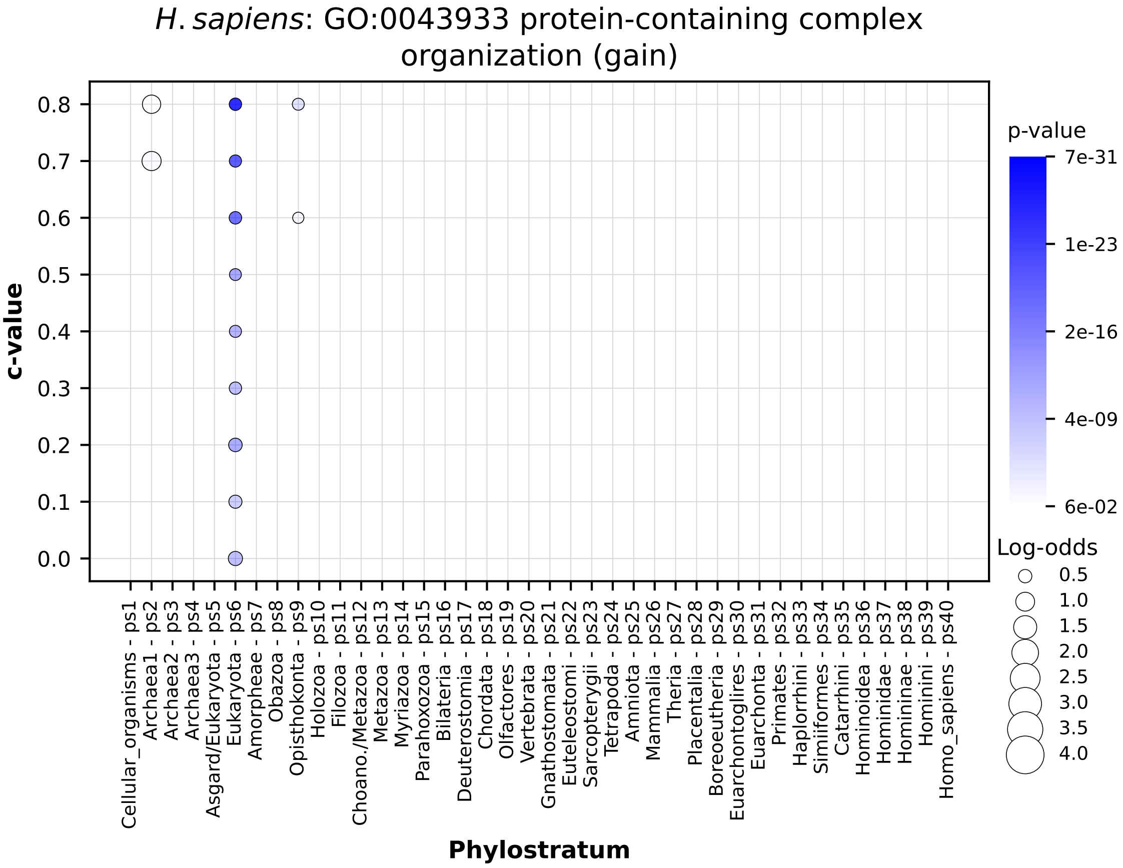

H. sapiens: GO:0043933 protein-containing complex organization (gain)
c-value
0.8
0.7
0.6
0.5
0.4
0.3
0.2
0.1
0.0
Phylostratum
Cellular_organisms - ps1
Archaea1 - ps2
Archaea2 - ps3
Archaea3 - ps4
Asgard/Eukaryota - ps5
Eukaryota - ps6
Amorpheae - ps7
Obazoa - ps8
Opisthokonta - ps9
Holozoa - ps10
Filozoa - ps11
Choano./Metazoa - ps12
Metazoa - ps13
Myriazoa - ps14
Parahoxozoa - ps15
Bilateria - ps16
Deuterostomia - ps17
Chordata - ps18
Olfactores - ps19
Vertebrata - ps20
Gnathostomata - ps21
Euteleostomi - ps22
Sarcopterygii - ps23
Tetrapoda - ps24
Amniota - ps25
Mammalia - ps26
Theria - ps27
Placentalia - ps28
Boreoeutheria - ps29
Euarchontoglires - ps30
Euarchonta - ps31
Primates - ps32
Haplorrhini - ps33
Simiiformes - ps34
Catarrhini - ps35
Hominoidea - ps36
Hominidae - ps37
Homininae - ps38
Hominini - ps39
Homo_sapiens - ps40
p-value
7e-31
1e-23
2e-16
4e-09
6e-02
Log-odds
0.5
1.0
1.5
2.0
2.5
3.0
3.5
4.0

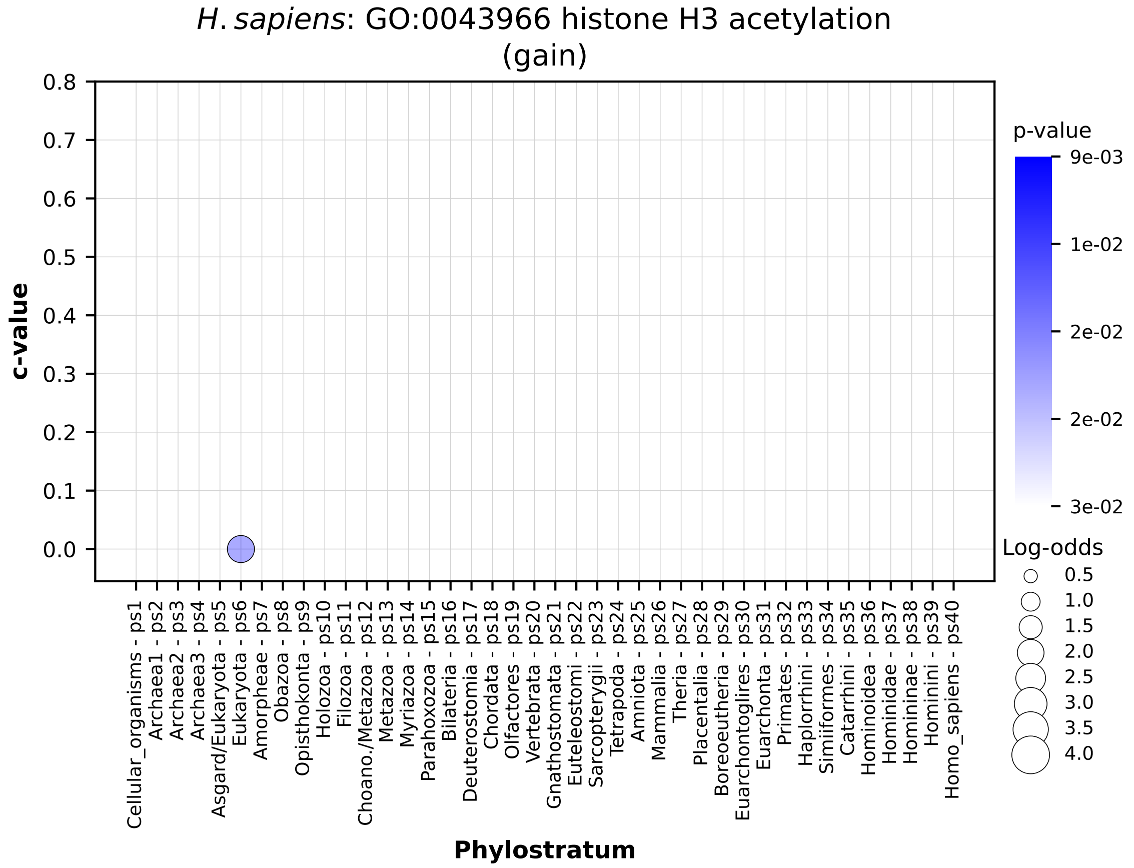

H. sapiens: GO:0043966 histone H3 acetylation
(gain)
c-value
0.8
0.7
0.6
0.5
0.4
0.3
0.2
0.1
0.0
p-value
9e-03
1e-02
2e-02
2e-02
3e-02
Log-odds
0.5
1.0
1.5
2.0
2.5
3.0
3.5
4.0
Cellular_organisms - ps1
Archaea1 - ps2
Archaea2 - ps3
Archaea3 - ps4
Asgard/Eukaryota - ps5
Eukaryota - ps6
Amorpheae - ps7
Obazoa - ps8
Opisthokonta - ps9
Holozoa - ps10
Filozoa - ps11
Choano./Metazoa - ps12
Metazoa - ps13
Myriazoa - ps14
Parahoxozoa - ps15
Bilateria - ps16
Deuterostomia - ps17
Chordata - ps18
Olfactores - ps19
Vertebrata - ps20
Gnathostomata - ps21
Euteleostomi - ps22
Sarcopterygii - ps23
Tetrapoda - ps24
Amniota - ps25
Mammalia - ps26
Theria - ps27
Placentalia - ps28
Boreoeutheria - ps29
Euarchontoglires - ps30
Euarchonta - ps31
Primates - ps32
Haplorrhini - ps33
Simiiformes - ps34
Catarrhini - ps35
Hominoidea - ps36
Hominidae - ps37
Homininae - ps38
Hominini - ps39
Homo_sapiens - ps40
Phylostratum

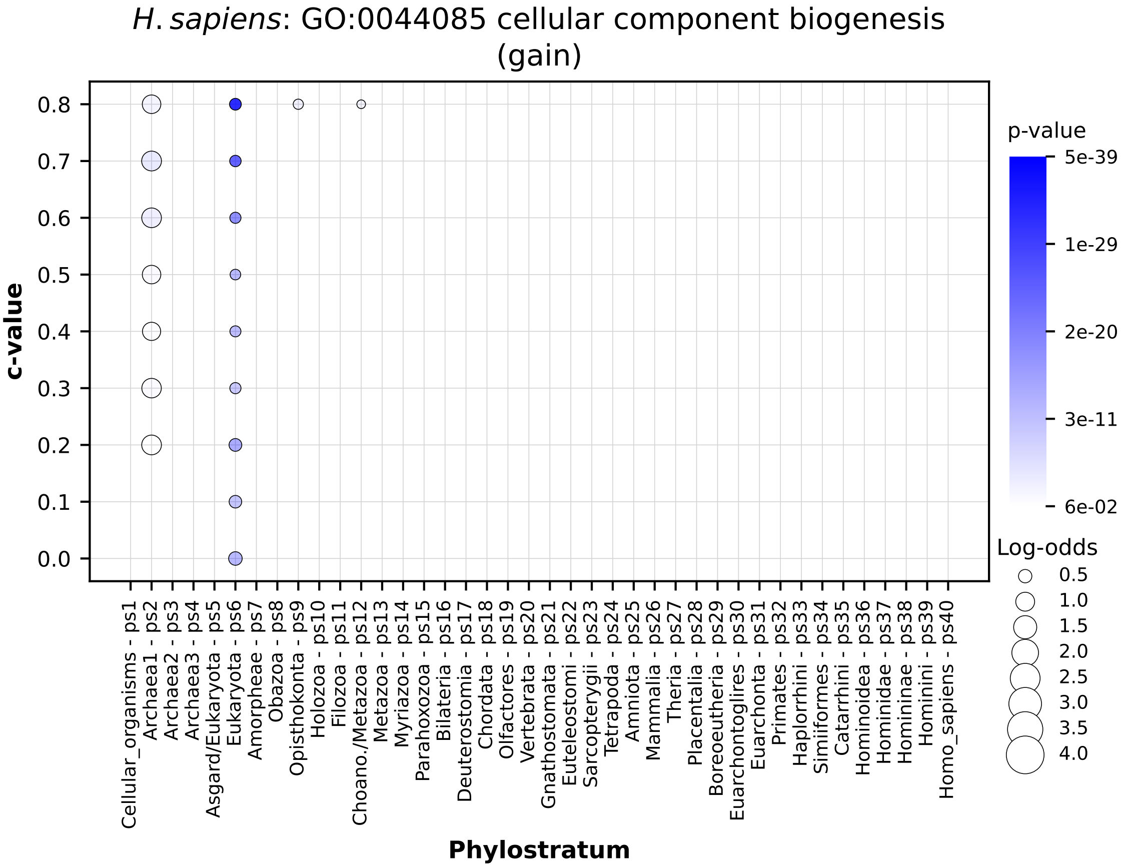
H. sapiens: GO:0044085 cellular component biogenesis
(gain)
c-value
0.8
0.7
0.6
0.5
0.4
0.3
0.2
0.1
0.0
p-value
5e-39
1e-29
2e-20
3e-11
6e-02
Log-odds
0.5
1.0
1.5
2.0
2.5
3.0
3.5
4.0
Cellular_organisms - ps1
Archaea1 - ps2
Archaea2 - ps3
Archaea3 - ps4
Asgard/Eukaryota - ps5
Eukaryota - ps6
Amorpheae - ps7
Obazoa - ps8
Opisthokonta - ps9
Holozoa - ps10
Filozoa - ps11
Choano./Metazoa - ps12
Metazoa - ps13
Myriazoa - ps14
Parahoxozoa - ps15
Bilateria - ps16
Deuterostomia - ps17
Chordata - ps18
Olfactores - ps19
Vertebrata - ps20
Gnathostomata - ps21
Euteleostomi - ps22
Sarcopterygii - ps23
Tetrapoda - ps24
Amniota - ps25
Mammalia - ps26
Theria - ps27
Placentalia - ps28
Boreoeutheria - ps29
Euarchontoglires - ps30
Euarchonta - ps31
Primates - ps32
Haplorrhini - ps33
Simiiformes - ps34
Catarrhini - ps35
Hominoidea - ps36
Hominidae - ps37
Homininae - ps38
Hominini - ps39
Homo_sapiens - ps40
Phylostratum

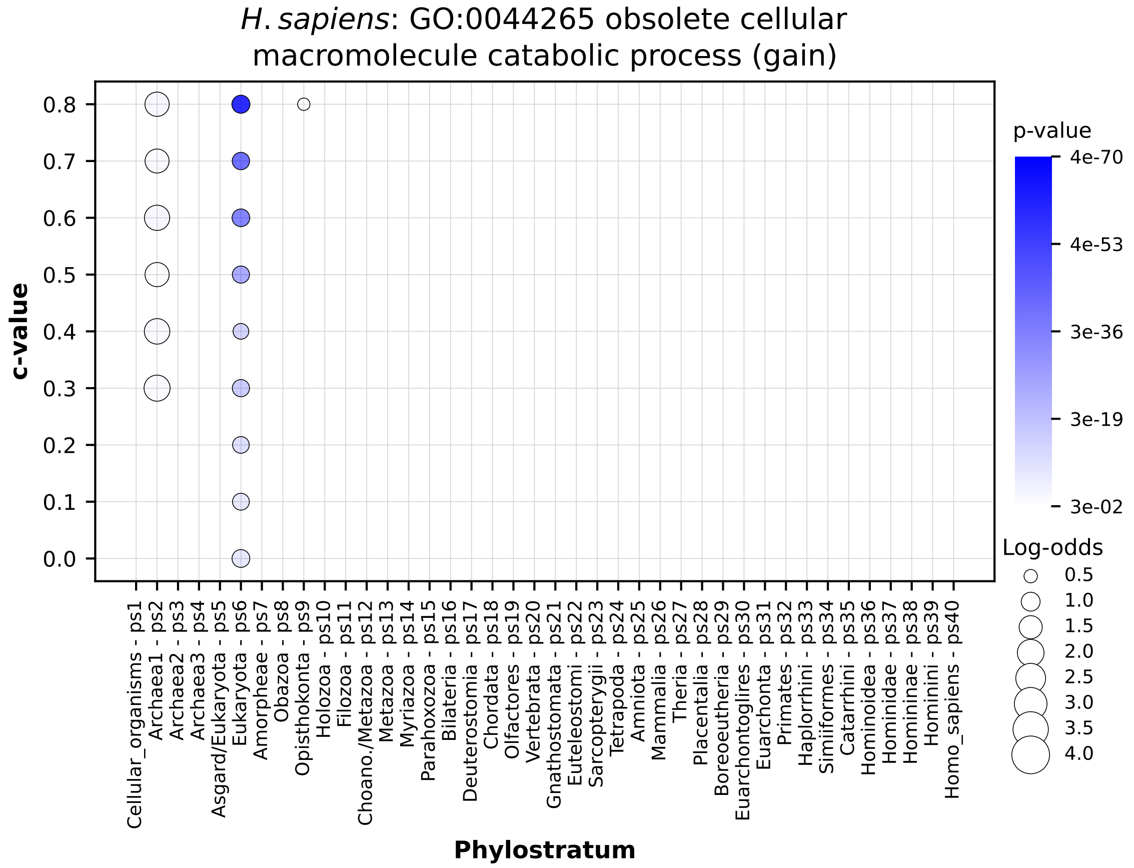

H. sapiens: GO:0044265 obsolete cellular macromolecule catabolic process (gain)
c-value
0.8
0.7
0.6
0.5
0.4
0.3
0.2
0.1
0.0
p-value
4e-70
4e-53
3e-36
3e-19
3e-02
Log-odds
0.5
1.0
1.5
2.0
2.5
3.0
3.5
4.0
Cellular_organisms - ps1
Archaea1 - ps2
Archaea2 - ps3
Archaea3 - ps4
Asgard/Eukaryota - ps5
Eukaryota - ps6
Amorpheae - ps7
Obazoa - ps8
Opisthokonta - ps9
Holozoa - ps10
Filozoa - ps11
Choano./Metazoa - ps12
Metazoa - ps13
Myriazoa - ps14
Parahoxozoa - ps15
Bilateria - ps16
Deuterostomia - ps17
Chordata - ps18
Olfactores - ps19
Vertebrata - ps20
Gnathostomata - ps21
Euteleostomi - ps22
Sarcopterygii - ps23
Tetrapoda - ps24
Amniota - ps25
Mammalia - ps26
Theria - ps27
Placentalia - ps28
Boreoeutheria - ps29
Euarchontoglires - ps30
Euarchonta - ps31
Primates - ps32
Haplorrhini - ps33
Simiiformes - ps34
Catarrhini - ps35
Hominoidea - ps36
Hominidae - ps37
Homininae - ps38
Hominini - ps39
Homo_sapiens - ps40
Phylostratum

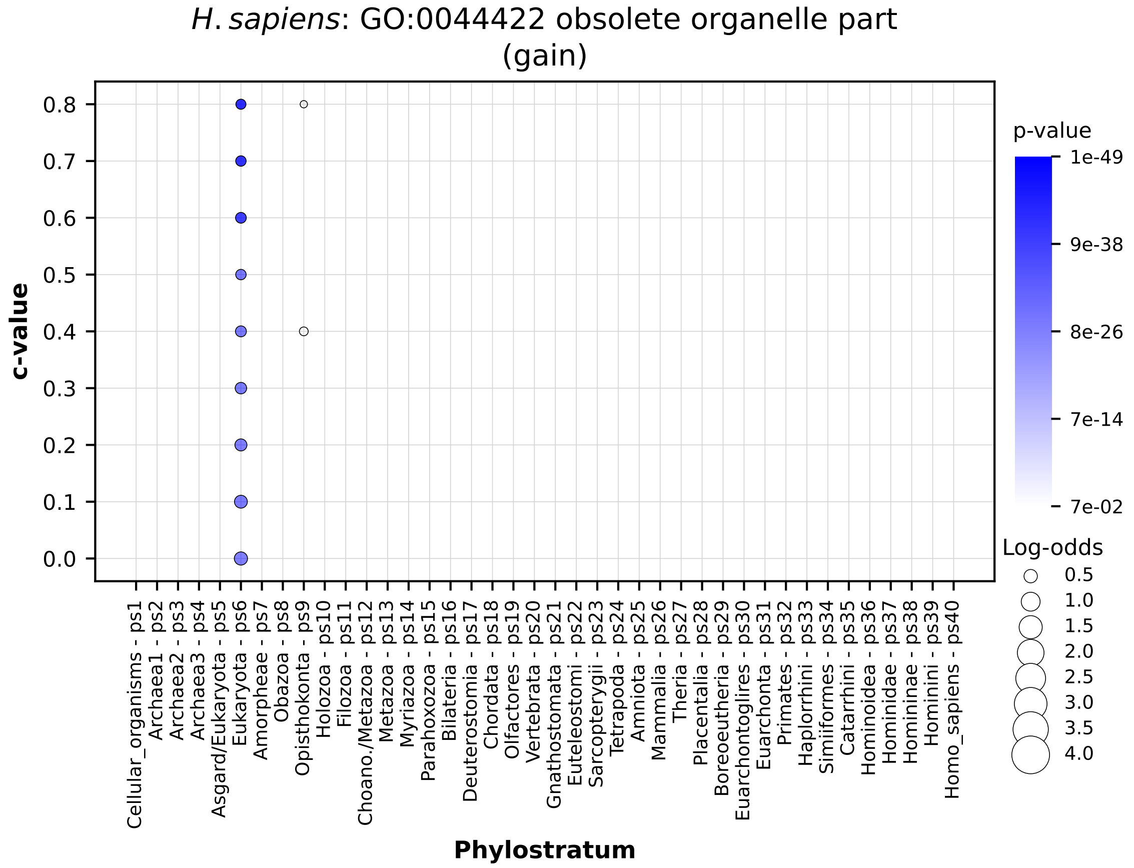

H. sapiens: GO:0044422 obsolete organelle part
(gain)
c-value
0.0
0.1
0.2
0.3
0.4
0.5
0.6
0.7
0.8
p-value
1e-49
9e-38
8e-26
7e-14
7e-02
Log-odds
0.5
1.0
1.5
2.0
2.5
3.0
3.5
4.0
Cellular_organisms - ps1
Archaea1 - ps2
Archaea2 - ps3
Archaea3 - ps4
Asgard/Eukaryota - ps5
Eukaryota - ps6
Amorpheae - ps7
Obazoa - ps8
Opisthokonta - ps9
Holozoa - ps10
Filozoa - ps11
Choano./Metazoa - ps12
Metazoa - ps13
Myriazoa - ps14
Parahoxozoa - ps15
Bilateria - ps16
Deuterostomia - ps17
Chordata - ps18
Olfactores - ps19
Vertebrata - ps20
Gnathostomata - ps21
Euteleostomi - ps22
Sarcopterygii - ps23
Tetrapoda - ps24
Amniota - ps25
Mammalia - ps26
Theria - ps27
Placentalia - ps28
Boreoeutheria - ps29
Euarchontoglires - ps30
Euarchonta - ps31
Primates - ps32
Haplorrhini - ps33
Simiiformes - ps34
Catarrhini - ps35
Hominoidea - ps36
Hominidae - ps37
Homininae - ps38
Hominini - ps39
Homo_sapiens - ps40
Phylostratum

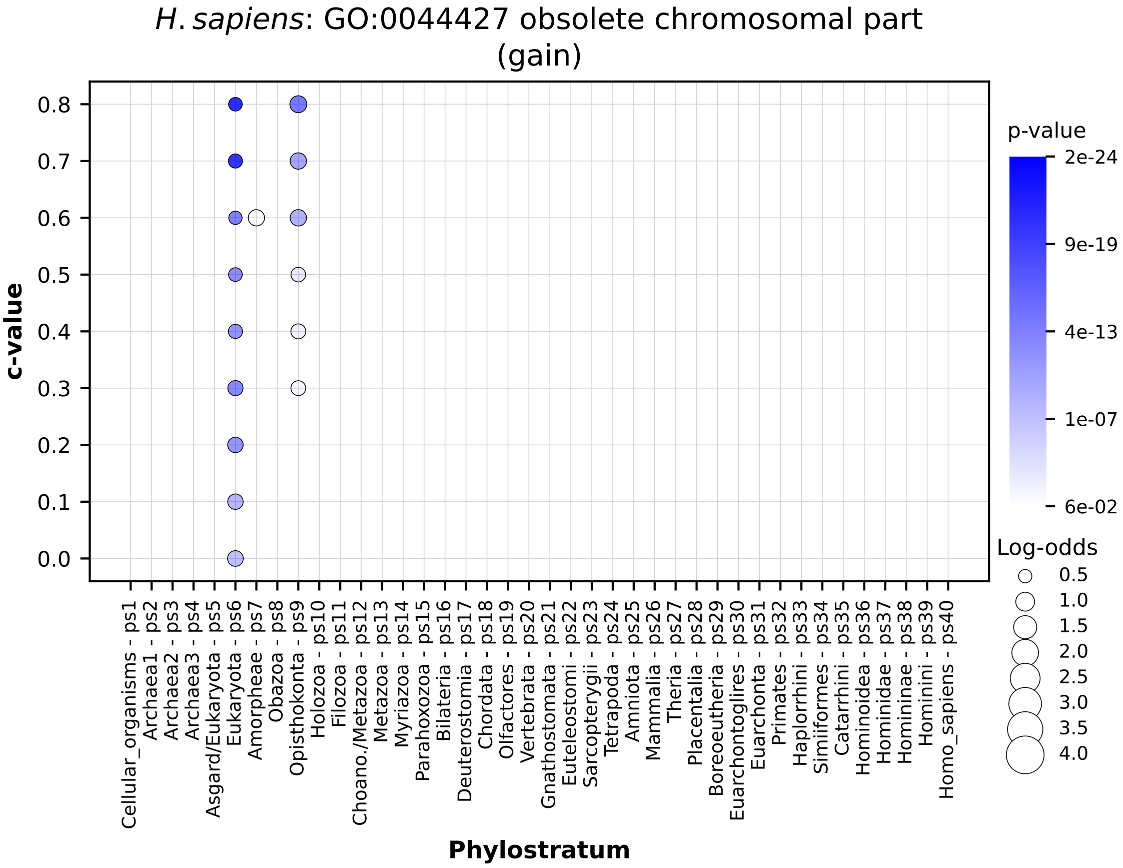

H. sapiens: GO:0044427 obsolete chromosomal part
(gain)
c-value
0.0
0.1
0.2
0.3
0.4
0.5
0.6
0.7
0.8
p-value
2e-24
9e-19
4e-13
1e-07
6e-02
Log-odds
0.5
1.0
1.5
2.0
2.5
3.0
3.5
4.0
Phylostratum
Cellular_organisms - ps1
Archaea1 - ps2
Archaea2 - ps3
Archaea3 - ps4
Asgard/Eukaryota - ps5
Eukaryota - ps6
Amorpheae - ps7
Obazoa - ps8
Opisthokonta - ps9
Holozoa - ps10
Filozoa - ps11
Choano./Metazoa - ps12
Metazoa - ps13
Myriazoa - ps14
Parahoxozoa - ps15
Bilateria - ps16
Deuterostomia - ps17
Chordata - ps18
Olfactores - ps19
Vertebrata - ps20
Gnathostomata - ps21
Euteleostomi - ps22
Sarcopterygii - ps23
Tetrapoda - ps24
Amniota - ps25
Mammalia - ps26
Theria - ps27
Placentalia - ps28
Boreoeutheria - ps29
Euarchontoglires - ps30
Euarchonta - ps31
Primates - ps32
Haplorrhini - ps33
Simiiformes - ps34
Catarrhini - ps35
Hominoidea - ps36
Hominidae - ps37
Homininae - ps38
Hominini - ps39
Homo_sapiens - ps40

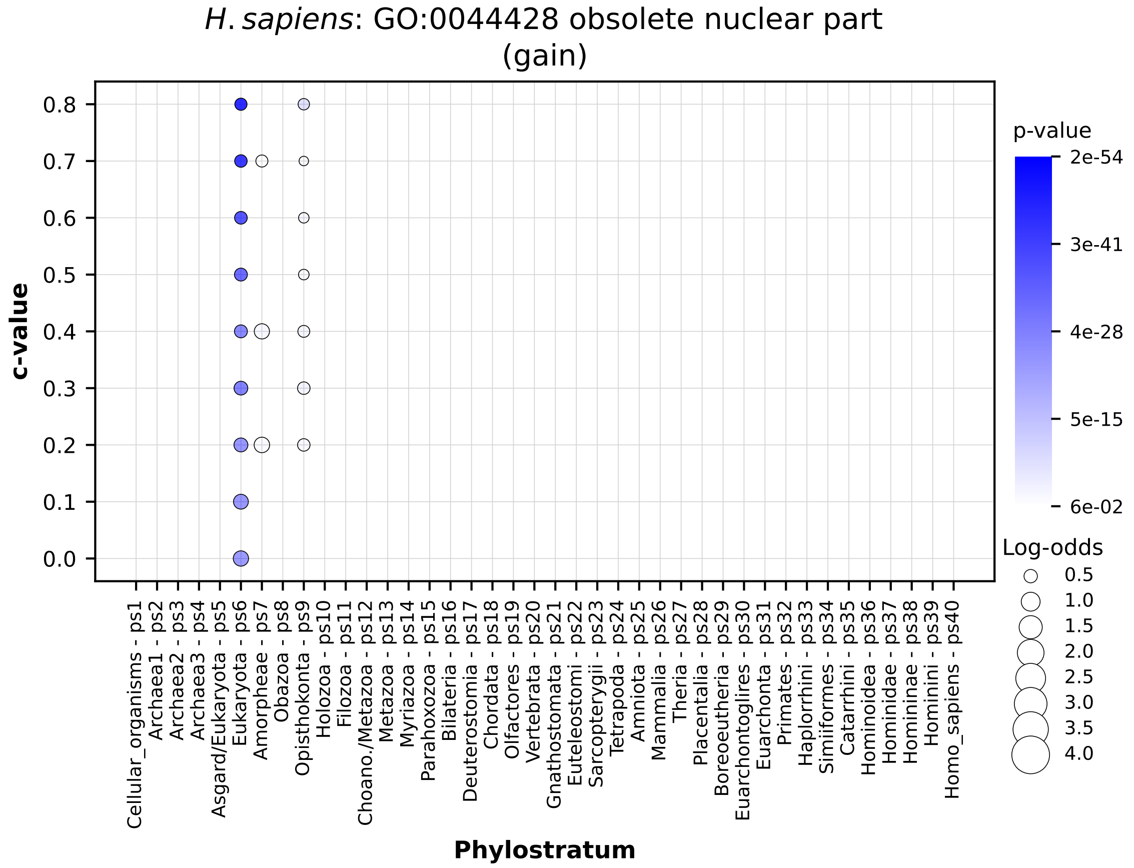

H. sapiens: GO:0044428 obsolete nuclear part
(gain)
c-value
0.8
0.7
0.6
0.5
0.4
0.3
0.2
0.1
0.0
p-value
2e-54
3e-41
4e-28
5e-15
6e-02
Log-odds
0.5
1.0
1.5
2.0
2.5
3.0
3.5
4.0
Cellular_organisms - ps1
Archaea1 - ps2
Archaea2 - ps3
Archaea3 - ps4
Asgard/Eukaryota - ps5
Eukaryota - ps6
Amorpheae - ps7
Obazoa - ps8
Opisthokonta - ps9
Holozoa - ps10
Filozoa - ps11
Choano./Metazoa - ps12
Metazoa - ps13
Myriazoa - ps14
Parahoxozoa - ps15
Bilateria - ps16
Deuterostomia - ps17
Chordata - ps18
Olfactores - ps19
Vertebrata - ps20
Gnathostomata - ps21
Euteleostomi - ps22
Sarcopterygii - ps23
Tetrapoda - ps24
Amniota - ps25
Mammalia - ps26
Theria - ps27
Placentalia - ps28
Boreoeutheria - ps29
Euarchontoglires - ps30
Euarchonta - ps31
Primates - ps32
Haplorrhini - ps33
Simiiformes - ps34
Catarrhini - ps35
Hominoidea - ps36
Hominidae - ps37
Homininae - ps38
Hominini - ps39
Homo_sapiens - ps40
Phylostratum

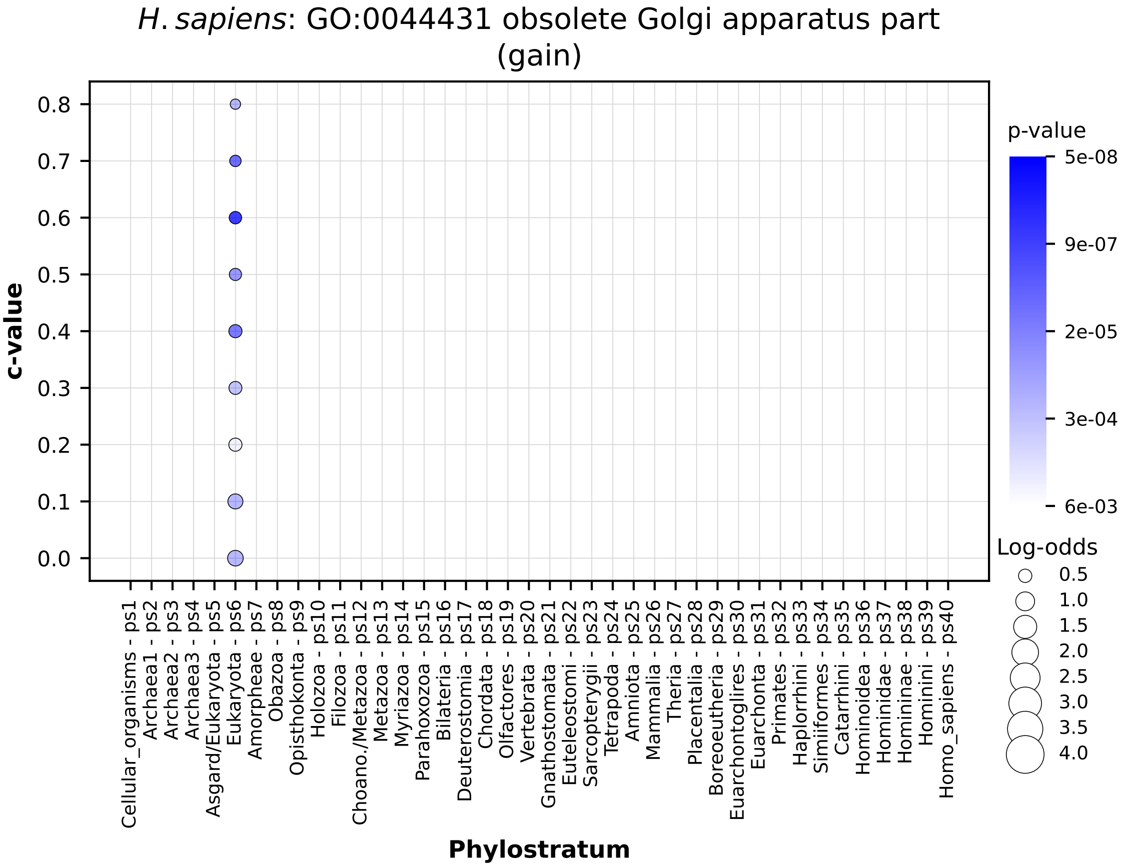
H. sapiens: GO:0044431 obsolete Golgi apparatus part (gain)
c-value
0.8
0.7
0.6
0.5
0.4
0.3
0.2
0.1
0.0
p-value
5e-08
9e-07
2e-05
3e-04
6e-03
Log-odds
0.5
1.0
1.5
2.0
2.5
3.0
3.5
4.0
Cellular_organisms - ps1
Archaea1 - ps2
Archaea2 - ps3
Archaea3 - ps4
Asgard/Eukaryota - ps5
Eukaryota - ps6
Amorpheae - ps7
Obazoa - ps8
Opisthokonta - ps9
Holozoa - ps10
Filozoa - ps11
Choano./Metazoa - ps12
Metazoa - ps13
Myriazoa - ps14
Parahoxozoa - ps15
Bilateria - ps16
Deuterostomia - ps17
Chordata - ps18
Olfactores - ps19
Vertebrata - ps20
Gnathostomata - ps21
Euteleostomi - ps22
Sarcopterygii - ps23
Tetrapoda - ps24
Amniota - ps25
Mammalia - ps26
Theria - ps27
Placentalia - ps28
Boreoeutheria - ps29
Euarchontoglires - ps30
Euarchonta - ps31
Primates - ps32
Haplorrhini - ps33
Simiiformes - ps34
Catarrhini - ps35
Hominoidea - ps36
Hominidae - ps37
Homininae - ps38
Hominini - ps39
Homo_sapiens - ps40
Phylostratum

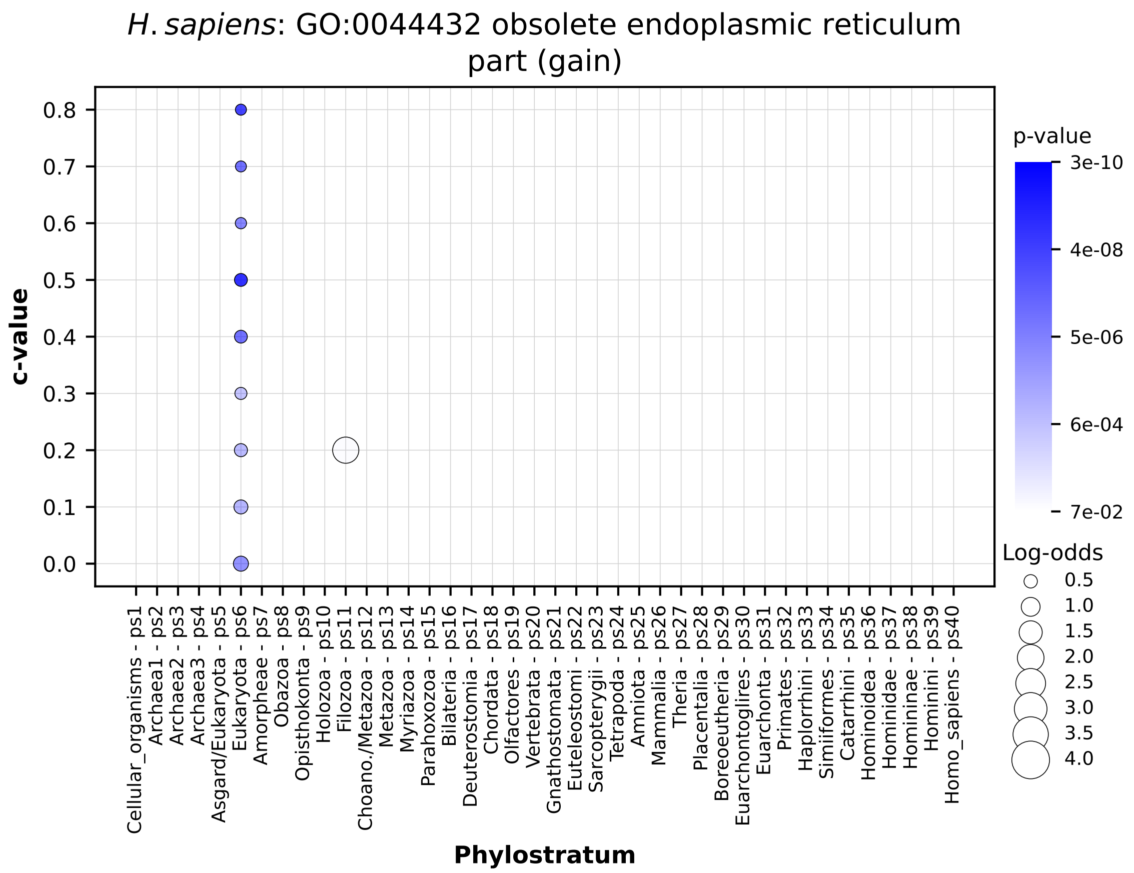

H. sapiens: GO:0044432 obsolete endoplasmic reticulum part (gain)
c-value
0.0
0.1
0.2
0.3
0.4
0.5
0.6
0.7
0.8
p-value
3e-10
4e-08
5e-06
6e-04
7e-02
Log-odds
0.5
1.0
1.5
2.0
2.5
3.0
3.5
4.0
Cellular_organisms - ps1
Archaea1 - ps2
Archaea2 - ps3
Archaea3 - ps4
Asgard/Eukaryota - ps5
Eukaryota - ps6
Amorpheae - ps7
Obazoa - ps8
Opisthokonta - ps9
Holozoa - ps10
Filozoa - ps11
Choano./Metazoa - ps12
Metazoa - ps13
Myriazoa - ps14
Parahoxozoa - ps15
Bilateria - ps16
Deuterostomia - ps17
Chordata - ps18
Olfactores - ps19
Vertebrata - ps20
Gnathostomata - ps21
Euteleostomi - ps22
Sarcopterygii - ps23
Tetrapoda - ps24
Amniota - ps25
Mammalia - ps26
Theria - ps27
Placentalia - ps28
Boreoeutheria - ps29
Euarchontoglires - ps30
Euarchonta - ps31
Primates - ps32
Haplorrhini - ps33
Simiiformes - ps34
Catarrhini - ps35
Hominoidea - ps36
Hominidae - ps37
Homininae - ps38
Hominini - ps39
Homo_sapiens - ps40
Phylostratum

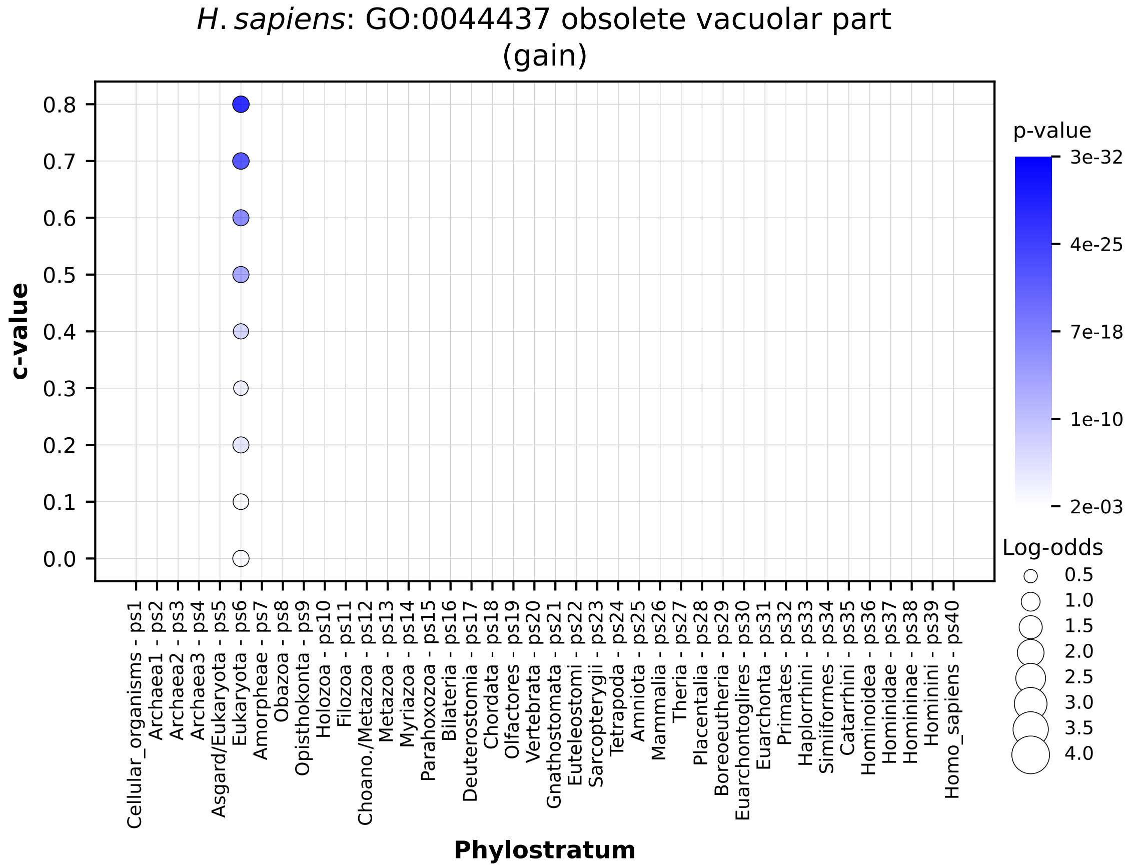

*H. sapiens*: GO:0044437 obsolete vacuolar part
(gain)
c-value
0.8
0.7
0.6
0.5
0.4
0.3
0.2
0.1
0.0
p-value
3e-32
4e-25
7e-18
1e-10
2e-03
Log-odds
0.5
1.0
1.5
2.0
2.5
3.0
3.5
4.0
Cellular_organisms - ps1
Archaea1 - ps2
Archaea2 - ps3
Archaea3 - ps4
Asgard/Eukaryota - ps5
Eukaryota - ps6
Amorpheae - ps7
Obazoa - ps8
Opisthokonta - ps9
Holozoa - ps10
Filozoa - ps11
Choano./Metazoa - ps12
Metazoa - ps13
Myriazoa - ps14
Parahoxozoa - ps15
Bilateria - ps16
Deuterostomia - ps17
Chordata - ps18
Olfactores - ps19
Vertebrata - ps20
Gnathostomata - ps21
Euteleostomi - ps22
Sarcopterygii - ps23
Tetrapoda - ps24
Amniota - ps25
Mammalia - ps26
Theria - ps27
Placentalia - ps28
Boreoeutheria - ps29
Euarchontoglires - ps30
Euarchonta - ps31
Primates - ps32
Haplorrhini - ps33
Simiiformes - ps34
Catarrhini - ps35
Hominoidea - ps36
Hominidae - ps37
Homininae - ps38
Hominini - ps39
Homo_sapiens - ps40
Phylostratum

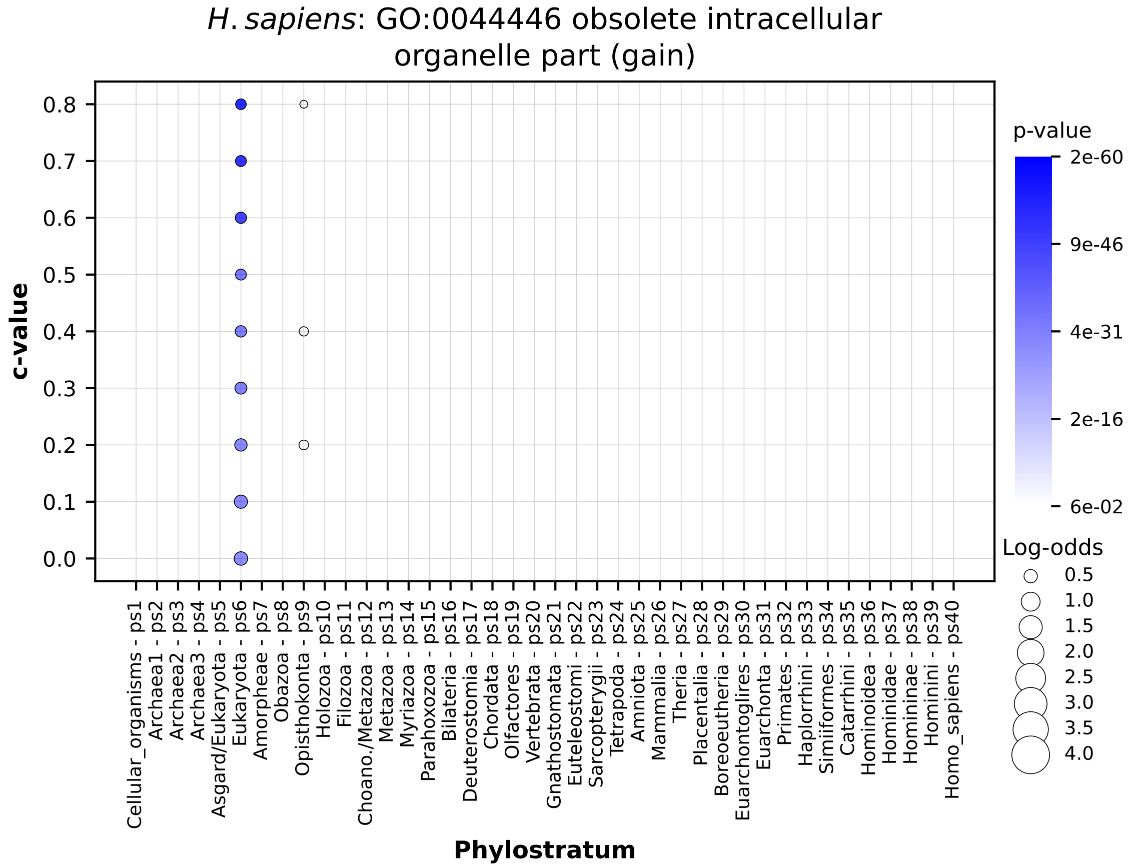

H. sapiens: GO:0044446 obsolete intracellular organelle part (gain)
c-value
0.8
0.7
0.6
0.5
0.4
0.3
0.2
0.1
0.0
Phylostratum
Cellular_organisms - ps1
Archaea1 - ps2
Archaea2 - ps3
Archaea3 - ps4
Asgard/Eukaryota - ps5
Eukaryota - ps6
Amorpheae - ps7
Obazoa - ps8
Opisthokonta - ps9
Holozoa - ps10
Filozoa - ps11
Choano./Metazoa - ps12
Metazoa - ps13
Myriazoa - ps14
Parahoxozoa - ps15
Bilateria - ps16
Deuterostomia - ps17
Chordata - ps18
Olfactores - ps19
Vertebrata - ps20
Gnathostomata - ps21
Euteleostomi - ps22
Sarcopterygii - ps23
Tetrapoda - ps24
Amniota - ps25
Mammalia - ps26
Theria - ps27
Placentalia - ps28
Boreoeutheria - ps29
Euarchontoglires - ps30
Euarchonta - ps31
Primates - ps32
Haplorrhini - ps33
Simiiformes - ps34
Catarrhini - ps35
Hominoidea - ps36
Hominidae - ps37
Homininae - ps38
Hominini - ps39
Homo_sapiens - ps40
p-value
2e-60
9e-46
4e-31
2e-16
6e-02
Log-odds
0.5
1.0
1.5
2.0
2.5
3.0
3.5
4.0

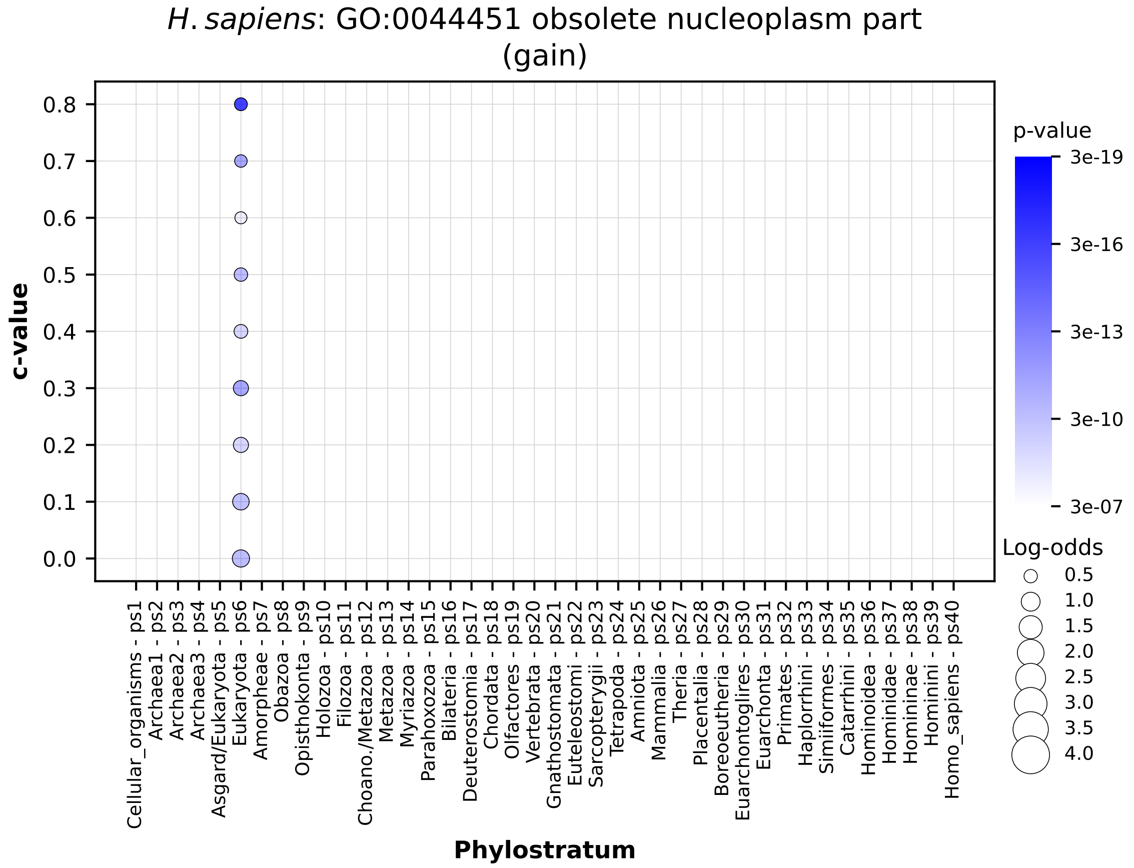

H. sapiens: GO:0044451 obsolete nucleoplasm part
(gain)
c-value
0.8
0.7
0.6
0.5
0.4
0.3
0.2
0.1
0.0
p-value
3e-19
3e-16
3e-13
3e-10
3e-07
Log-odds
0.5
1.0
1.5
2.0
2.5
3.0
3.5
4.0
Cellular_organisms - ps1
Archaea1 - ps2
Archaea2 - ps3
Archaea3 - ps4
Asgard/Eukaryota - ps5
Eukaryota - ps6
Amorpheae - ps7
Obazoa - ps8
Opisthokonta - ps9
Holozoa - ps10
Filozoa - ps11
Choano./Metazoa - ps12
Metazoa - ps13
Myriazoa - ps14
Parahoxozoa - ps15
Bilateria - ps16
Deuterostomia - ps17
Chordata - ps18
Olfactores - ps19
Vertebrata - ps20
Gnathostomata - ps21
Euteleostomi - ps22
Sarcopterygii - ps23
Tetrapoda - ps24
Amniota - ps25
Mammalia - ps26
Theria - ps27
Placentalia - ps28
Boreoeutheria - ps29
Euarchontoglires - ps30
Euarchonta - ps31
Primates - ps32
Haplorrhini - ps33
Simiiformes - ps34
Catarrhini - ps35
Hominoidea - ps36
Hominidae - ps37
Homininae - ps38
Hominini - ps39
Homo_sapiens - ps40
Phylostratum

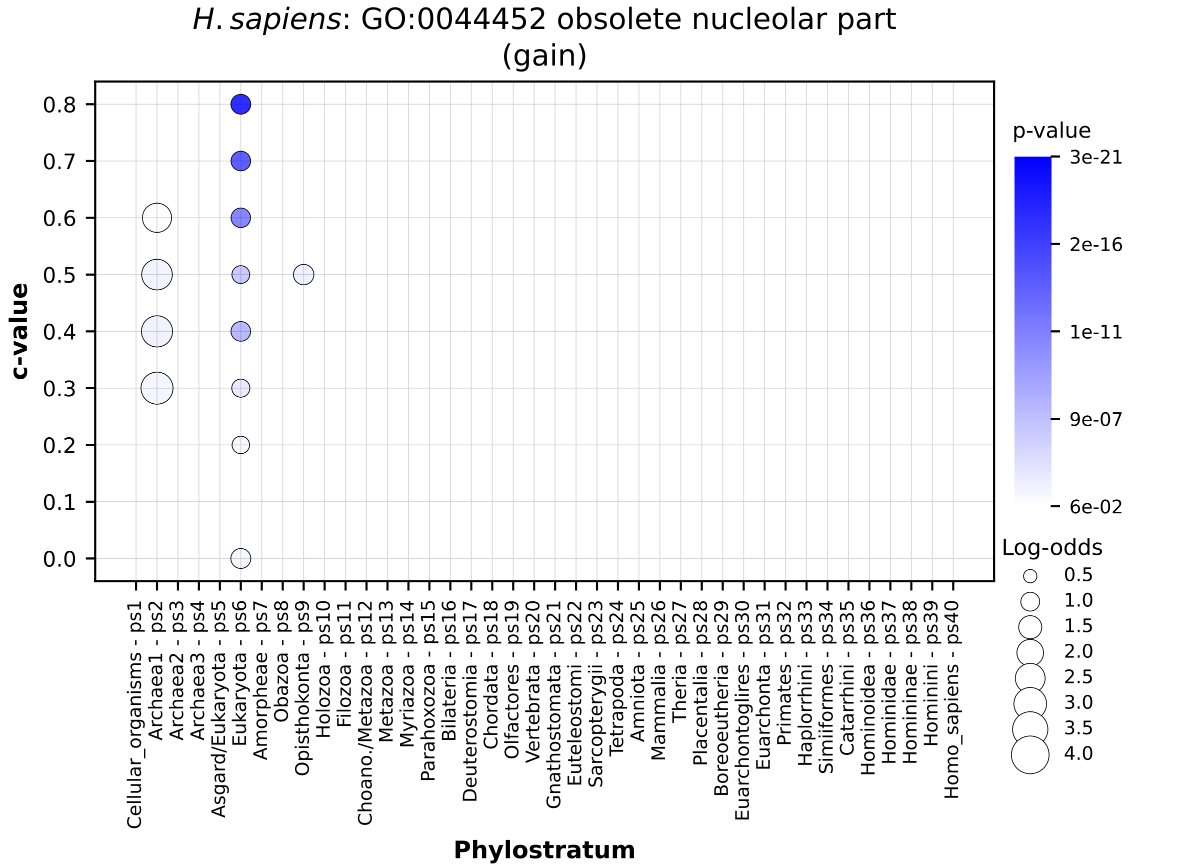
H. sapiens: GO:0044452 obsolete nucleolar part
(gain)
c-value
0.8
0.7
0.6
0.5
0.4
0.3
0.2
0.1
0.0
p-value
3e-21
2e-16
1e-11
9e-07
6e-02
Log-odds
0.5
1.0
1.5
2.0
2.5
3.0
3.5
4.0
Cellular_organisms - ps1
Archaea1 - ps2
Archaea2 - ps3
Archaea3 - ps4
Asgard/Eukaryota - ps5
Eukaryota - ps6
Amorpheae - ps7
Obazoa - ps8
Opisthokonta - ps9
Holozoa - ps10
Filozoa - ps11
Choano./Metazoa - ps12
Metazoa - ps13
Myriazoa - ps14
Parahoxozoa - ps15
Bilateria - ps16
Deuterostomia - ps17
Chordata - ps18
Olfactores - ps19
Vertebrata - ps20
Gnathostomata - ps21
Euteleostomi - ps22
Sarcopterygii - ps23
Tetrapoda - ps24
Amniota - ps25
Mammalia - ps26
Theria - ps27
Placentalia - ps28
Boreoeutheria - ps29
Euarchontoglires - ps30
Euarchonta - ps31
Primates - ps32
Haplorrhini - ps33
Simiiformes - ps34
Catarrhini - ps35
Hominoidea - ps36
Hominidae - ps37
Homininae - ps38
Hominini - ps39
Homo_sapiens - ps40
Phylostratum

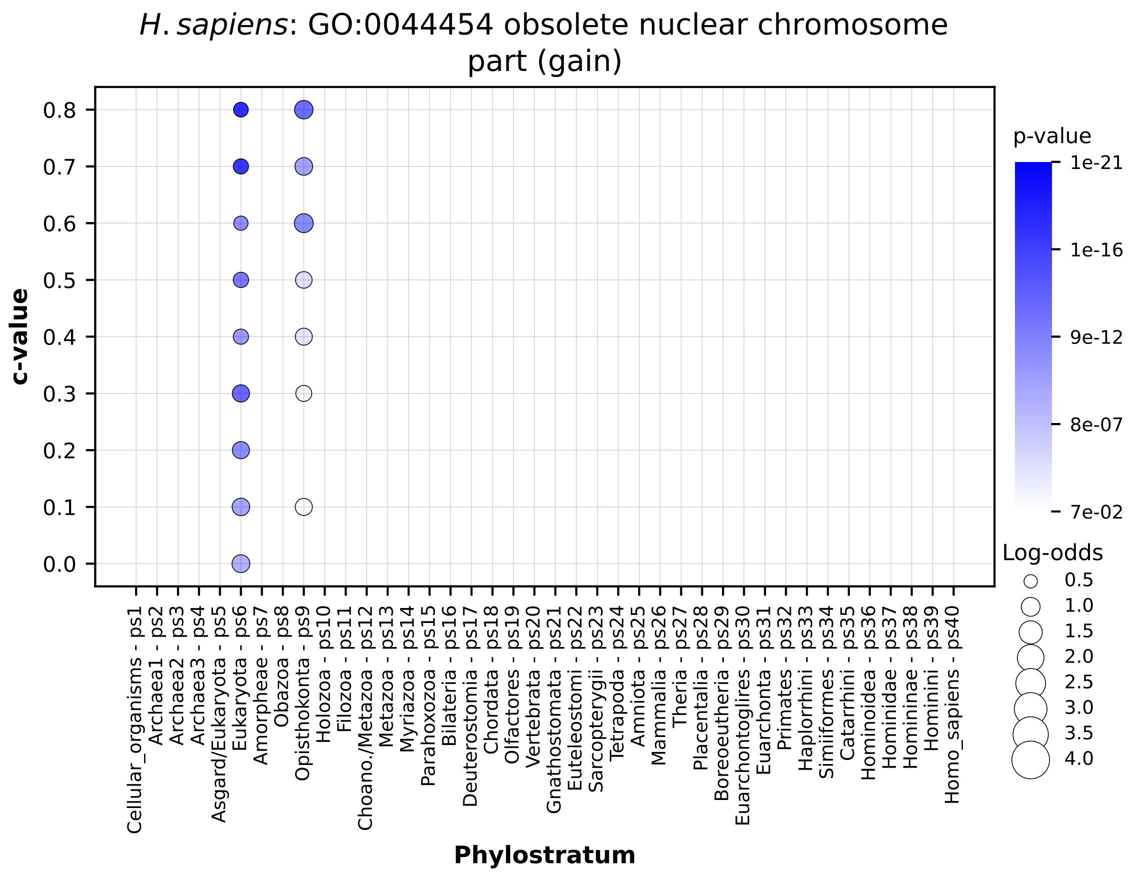

H. sapiens: GO:0044454 obsolete nuclear chromosome part (gain)
c-value
0.0
0.1
0.2
0.3
0.4
0.5
0.6
0.7
0.8
Phylostratum
Cellular_organisms - ps1
Archaea1 - ps2
Archaea2 - ps3
Archaea3 - ps4
Asgard/Eukaryota - ps5
Eukaryota - ps6
Amorpheae - ps7
Obazoa - ps8
Opisthokonta - ps9
Holozoa - ps10
Filozoa - ps11
Choano./Metazoa - ps12
Metazoa - ps13
Myriazoa - ps14
Parahoxozoa - ps15
Bilateria - ps16
Deuterostomia - ps17
Chordata - ps18
Olfactores - ps19
Vertebrata - ps20
Gnathostomata - ps21
Euteleostomi - ps22
Sarcopterygii - ps23
Tetrapoda - ps24
Amniota - ps25
Mammalia - ps26
Theria - ps27
Placentalia - ps28
Boreoeutheria - ps29
Euarchontoglires - ps30
Euarchonta - ps31
Primates - ps32
Haplorrhini - ps33
Simiiformes - ps34
Catarrhini - ps35
Hominoidea - ps36
Hominidae - ps37
Homininae - ps38
Hominini - ps39
Homo_sapiens - ps40
p-value
1e-21
1e-16
9e-12
8e-07
7e-02
Log-odds
0.5
1.0
1.5
2.0
2.5
3.0
3.5
4.0

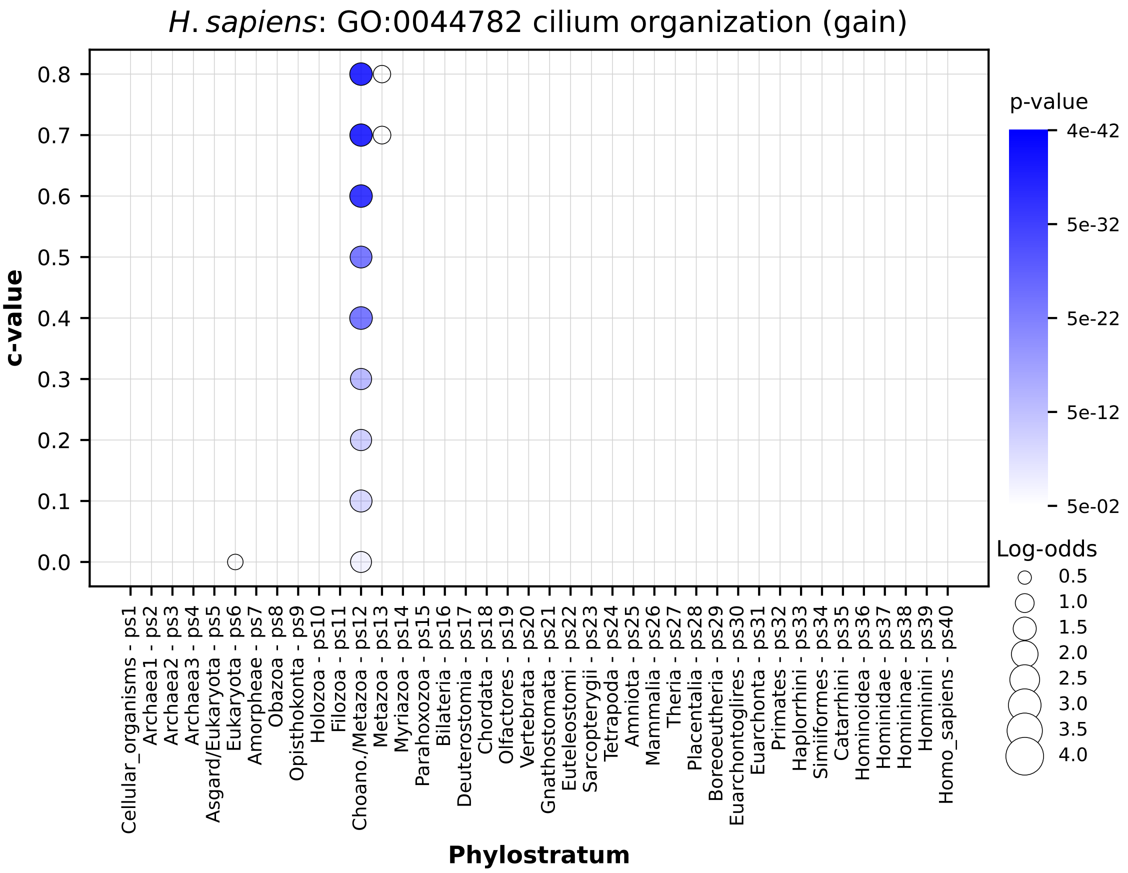

H. sapiens: GO:0044782 cilium organization (gain)
c-value
0.8
0.7
0.6
0.5
0.4
0.3
0.2
0.1
0.0
p-value
4e-42
5e-32
5e-22
5e-12
5e-02
Log-odds
0.5
1.0
1.5
2.0
2.5
3.0
3.5
4.0
Cellular_organisms - ps1
Archaea1 - ps2
Archaea2 - ps3
Archaea3 - ps4
Asgard/Eukaryota - ps5
Eukaryota - ps6
Amorpheae - ps7
Obazoa - ps8
Opisthokonta - ps9
Holozoa - ps10
Filozoa - ps11
Choano./Metazoa - ps12
Metazoa - ps13
Myriazoa - ps14
Parahoxozoa - ps15
Bilateria - ps16
Deuterostomia - ps17
Chordata - ps18
Olfactores - ps19
Vertebrata - ps20
Gnathostomata - ps21
Euteleostomi - ps22
Sarcopterygii - ps23
Tetrapoda - ps24
Amniota - ps25
Mammalia - ps26
Theria - ps27
Placentalia - ps28
Boreoeutheria - ps29
Euarchontoglires - ps30
Euarchonta - ps31
Primates - ps32
Haplorrhini - ps33
Simiiformes - ps34
Catarrhini - ps35
Hominoidea - ps36
Hominidae - ps37
Homininae - ps38
Hominini - ps39
Homo_sapiens - ps40
Phylostratum

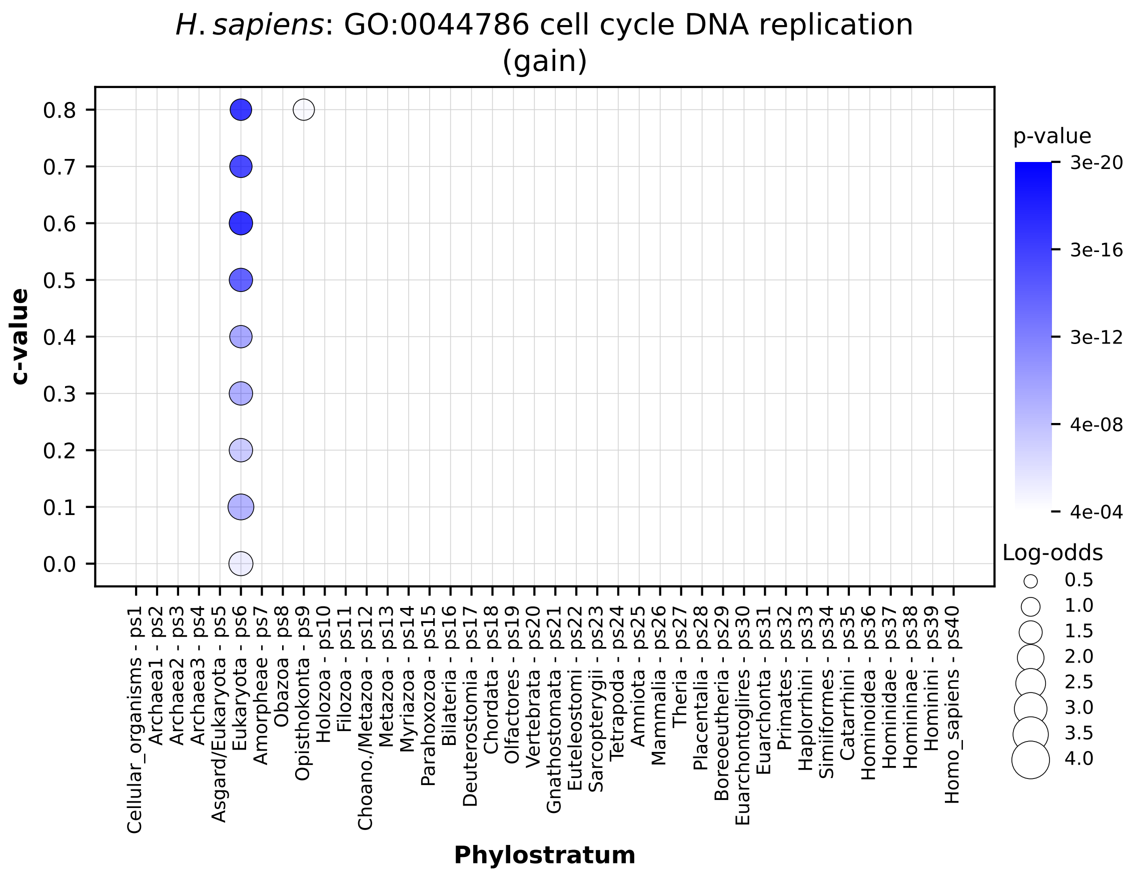
H. sapiens: GO:0044786 cell cycle DNA replication
(gain)
c-value
0.0
0.1
0.2
0.3
0.4
0.5
0.6
0.7
0.8
p-value
3e-20
3e-16
3e-12
4e-08
4e-04
Log-odds
0.5
1.0
1.5
2.0
2.5
3.0
3.5
4.0
Cellular_organisms - ps1
Archaea1 - ps2
Archaea2 - ps3
Archaea3 - ps4
Asgard/Eukaryota - ps5
Eukaryota - ps6
Amorpheae - ps7
Obazoa - ps8
Opisthokonta - ps9
Holozoa - ps10
Filozoa - ps11
Choano./Metazoa - ps12
Metazoa - ps13
Myriazoa - ps14
Parahoxozoa - ps15
Bilateria - ps16
Deuterostomia - ps17
Chordata - ps18
Olfactores - ps19
Vertebrata - ps20
Gnathostomata - ps21
Euteleostomi - ps22
Sarcopterygii - ps23
Tetrapoda - ps24
Amniota - ps25
Mammalia - ps26
Theria - ps27
Placentalia - ps28
Boreoeutheria - ps29
Euarchontoglires - ps30
Euarchonta - ps31
Primates - ps32
Haplorrhini - ps33
Simiiformes - ps34
Catarrhini - ps35
Hominoidea - ps36
Hominidae - ps37
Homininae - ps38
Hominini - ps39
Homo_sapiens - ps40
Phylostratum

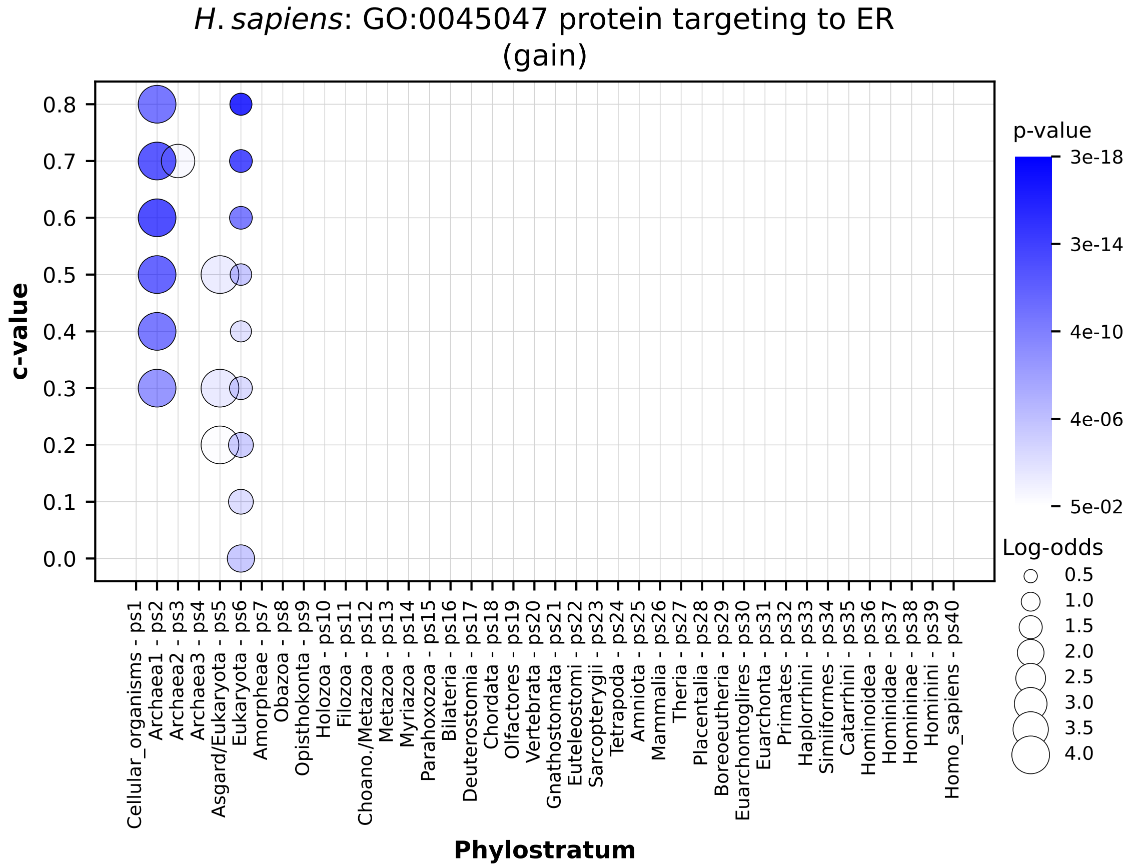

H. sapiens: GO:0045047 protein targeting to ER
(gain)
c-value
0.8
0.7
0.6
0.5
0.4
0.3
0.2
0.1
0.0
p-value
3e-18
3e-14
4e-10
4e-06
5e-02
Log-odds
0.5
1.0
1.5
2.0
2.5
3.0
3.5
4.0
Cellular_organisms - ps1
Archaea1 - ps2
Archaea2 - ps3
Archaea3 - ps4
Asgard/Eukaryota - ps5
Eukaryota - ps6
Amorpheae - ps7
Obazoa - ps8
Opisthokonta - ps9
Holozoa - ps10
Filozoa - ps11
Choano./Metazoa - ps12
Metazoa - ps13
Myriazoa - ps14
Parahoxozoa - ps15
Bilateria - ps16
Deuterostomia - ps17
Chordata - ps18
Olfactores - ps19
Vertebrata - ps20
Gnathostomata - ps21
Euteleostomi - ps22
Sarcopterygii - ps23
Tetrapoda - ps24
Amniota - ps25
Mammalia - ps26
Theria - ps27
Placentalia - ps28
Boreoeutheria - ps29
Euarchontoglires - ps30
Euarchonta - ps31
Primates - ps32
Haplorrhini - ps33
Simiiformes - ps34
Catarrhini - ps35
Hominoidea - ps36
Hominidae - ps37
Homininae - ps38
Hominini - ps39
Homo_sapiens - ps40
Phylostratum

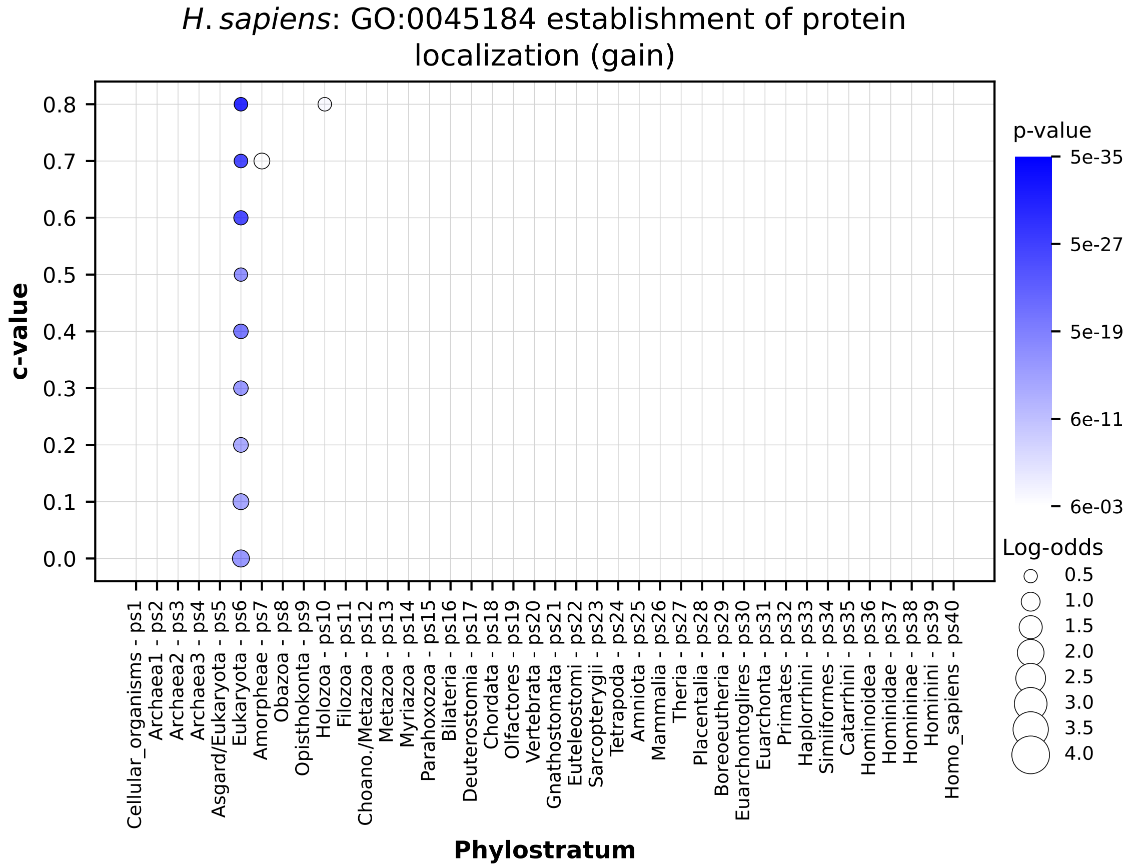

H. sapiens: GO:0045184 establishment of protein localization (gain)
c-value
0.8
0.7
0.6
0.5
0.4
0.3
0.2
0.1
0.0
Phylostratum
Cellular_organisms - ps1
Archaea1 - ps2
Archaea2 - ps3
Archaea3 - ps4
Asgard/Eukaryota - ps5
Eukaryota - ps6
Amorpheae - ps7
Obazoa - ps8
Opisthokonta - ps9
Holozoa - ps10
Filozoa - ps11
Choano./Metazoa - ps12
Metazoa - ps13
Myriazoa - ps14
Parahoxozoa - ps15
Bilateria - ps16
Deuterostomia - ps17
Chordata - ps18
Olfactores - ps19
Vertebrata - ps20
Gnathostomata - ps21
Euteleostomi - ps22
Sarcopterygii - ps23
Tetrapoda - ps24
Amniota - ps25
Mammalia - ps26
Theria - ps27
Placentalia - ps28
Boreoeutheria - ps29
Euarchontoglires - ps30
Euarchonta - ps31
Primates - ps32
Haplorrhini - ps33
Simiiformes - ps34
Catarrhini - ps35
Hominoidea - ps36
Hominidae - ps37
Homininae - ps38
Hominini - ps39
Homo_sapiens - ps40
p-value
5e-35
5e-27
5e-19
6e-11
6e-03
Log-odds
0.5
1.0
1.5
2.0
2.5
3.0
3.5
4.0

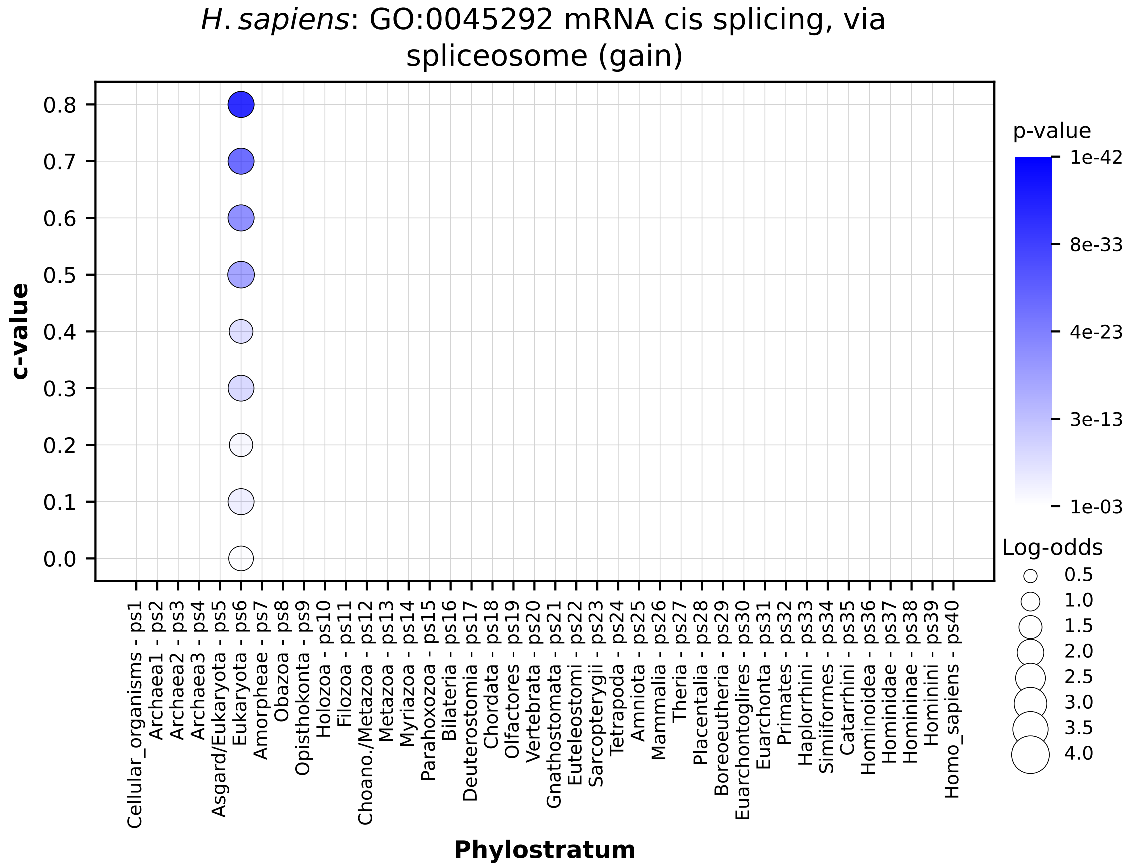

H. sapiens: GO:0045292 mRNA cis splicing, via spliceosome (gain)
c-value
0.8
0.7
0.6
0.5
0.4
0.3
0.2
0.1
0.0
Phylostratum
Cellular_organisms - ps1
Archaea1 - ps2
Archaea2 - ps3
Archaea3 - ps4
Asgard/Eukaryota - ps5
Eukaryota - ps6
Amorpheae - ps7
Obazoa - ps8
Opisthokonta - ps9
Holozoa - ps10
Filozoa - ps11
Choano./Metazoa - ps12
Metazoa - ps13
Myriazoa - ps14
Parahoxozoa - ps15
Bilateria - ps16
Deuterostomia - ps17
Chordata - ps18
Olfactores - ps19
Vertebrata - ps20
Gnathostomata - ps21
Euteleostomi - ps22
Sarcopterygii - ps23
Tetrapoda - ps24
Amniota - ps25
Mammalia - ps26
Theria - ps27
Placentalia - ps28
Boreoeutheria - ps29
Euarchontoglires - ps30
Euarchonta - ps31
Primates - ps32
Haplorrhini - ps33
Simiiformes - ps34
Catarrhini - ps35
Hominoidea - ps36
Hominidae - ps37
Homininae - ps38
Hominini - ps39
Homo_sapiens - ps40
p-value
1e-42
8e-33
4e-23
3e-13
1e-03
Log-odds
0.5
1.0
1.5
2.0
2.5
3.0
3.5
4.0

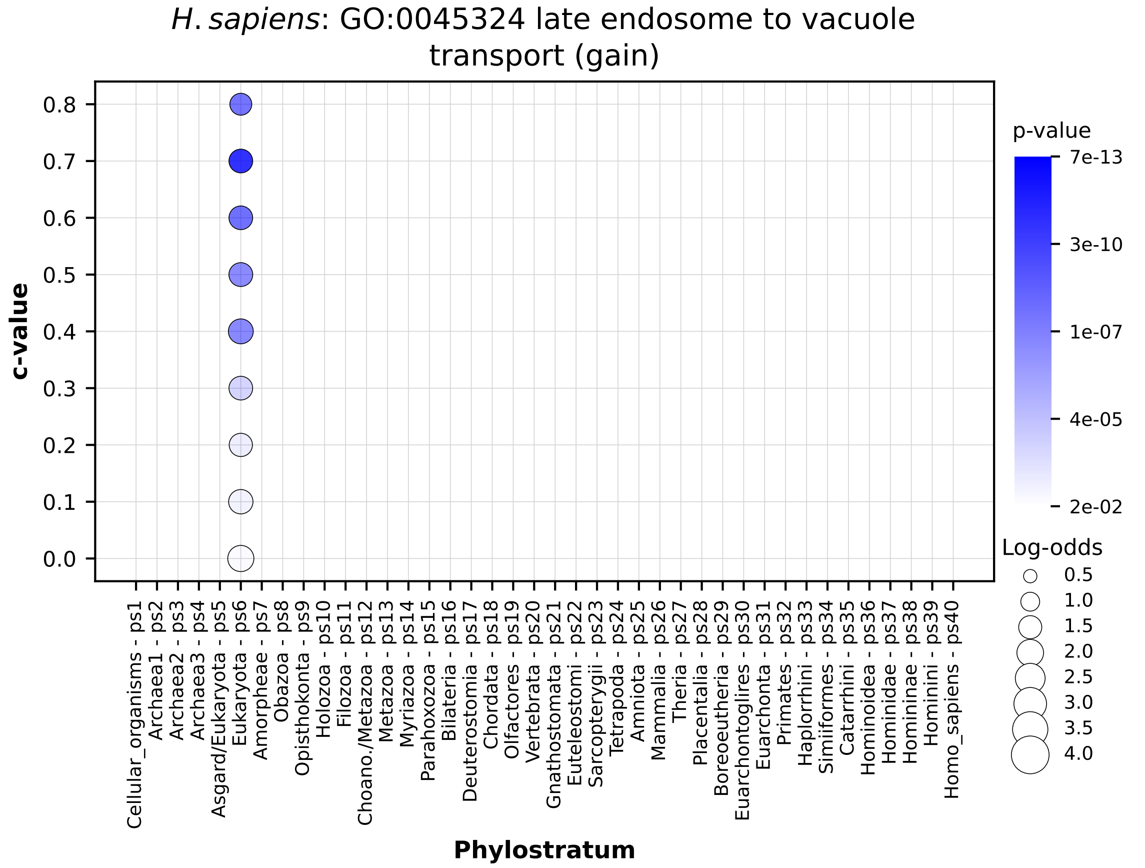
H. sapiens: GO:0045324 late endosome to vacuole transport (gain)
c-value
0.8
0.7
0.6
0.5
0.4
0.3
0.2
0.1
0.0
p-value
7e-13
3e-10
1e-07
4e-05
2e-02
Log-odds
0.5
1.0
1.5
2.0
2.5
3.0
3.5
4.0
Cellular_organisms - ps1
Archaea1 - ps2
Archaea2 - ps3
Archaea3 - ps4
Asgard/Eukaryota - ps5
Eukaryota - ps6
Amorpheae - ps7
Obazoa - ps8
Opisthokonta - ps9
Holozoa - ps10
Filozoa - ps11
Choano./Metazoa - ps12
Metazoa - ps13
Myriazoa - ps14
Parahoxozoa - ps15
Bilateria - ps16
Deuterostomia - ps17
Chordata - ps18
Olfactores - ps19
Vertebrata - ps20
Gnathostomata - ps21
Euteleostomi - ps22
Sarcopterygii - ps23
Tetrapoda - ps24
Amniota - ps25
Mammalia - ps26
Theria - ps27
Placentalia - ps28
Boreoeutheria - ps29
Euarchontoglires - ps30
Euarchonta - ps31
Primates - ps32
Haplorrhini - ps33
Simiiformes - ps34
Catarrhini - ps35
Hominoidea - ps36
Hominidae - ps37
Homininae - ps38
Hominini - ps39
Homo_sapiens - ps40
Phylostratum

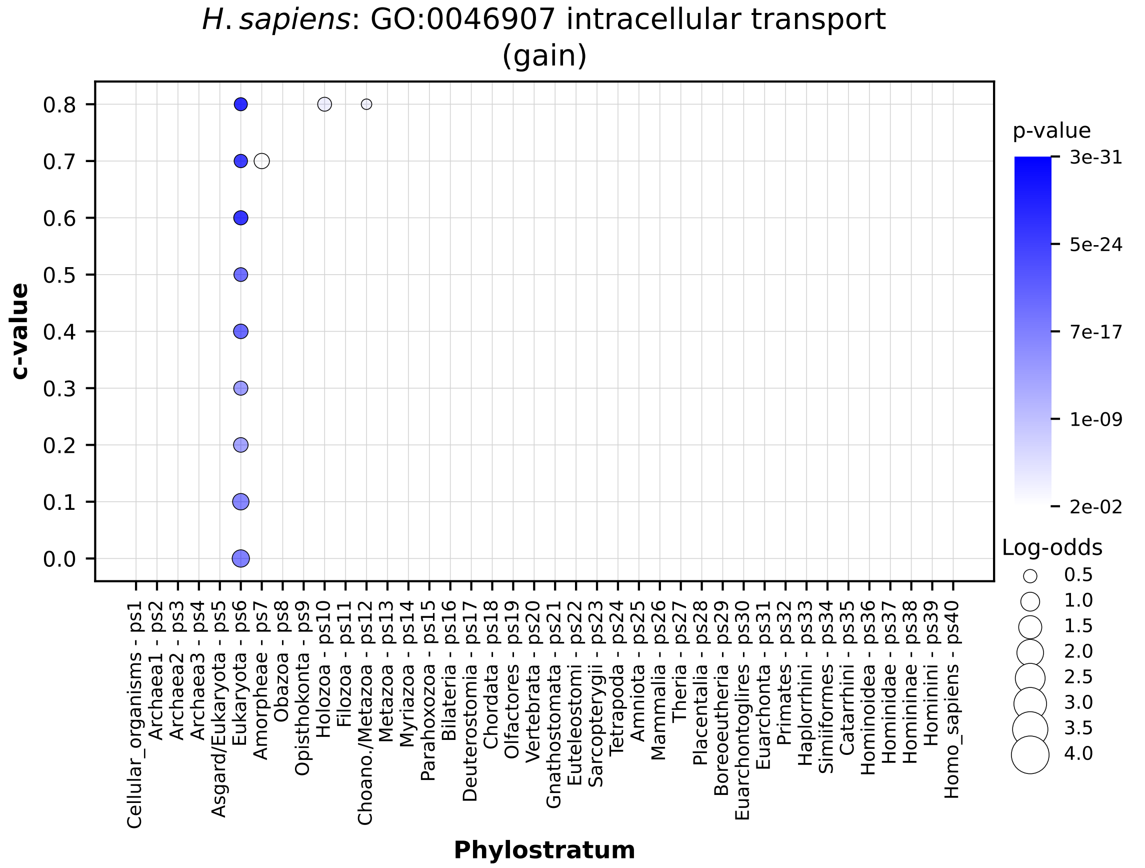

H. sapiens: GO:0046907 intracellular transport
(gain)
c-value
0.0
0.1
0.2
0.3
0.4
0.5
0.6
0.7
0.8
Phylostratum
Cellular_organisms - ps1
Archaea1 - ps2
Archaea2 - ps3
Archaea3 - ps4
Asgard/Eukaryota - ps5
Eukaryota - ps6
Amorpheae - ps7
Obazoa - ps8
Opisthokonta - ps9
Holozoa - ps10
Filozoa - ps11
Choano./Metazoa - ps12
Metazoa - ps13
Myriazoa - ps14
Parahoxozoa - ps15
Bilateria - ps16
Deuterostomia - ps17
Chordata - ps18
Olfactores - ps19
Vertebrata - ps20
Gnathostomata - ps21
Euteleostomi - ps22
Sarcopterygii - ps23
Tetrapoda - ps24
Amniota - ps25
Mammalia - ps26
Theria - ps27
Placentalia - ps28
Boreoeutheria - ps29
Euarchontoglires - ps30
Euarchonta - ps31
Primates - ps32
Haplorrhini - ps33
Simiiformes - ps34
Catarrhini - ps35
Hominoidea - ps36
Hominidae - ps37
Homininae - ps38
Hominini - ps39
Homo_sapiens - ps40
p-value
3e-31
5e-24
7e-17
1e-09
2e-02
Log-odds
0.5
1.0
1.5
2.0
2.5
3.0
3.5
4.0

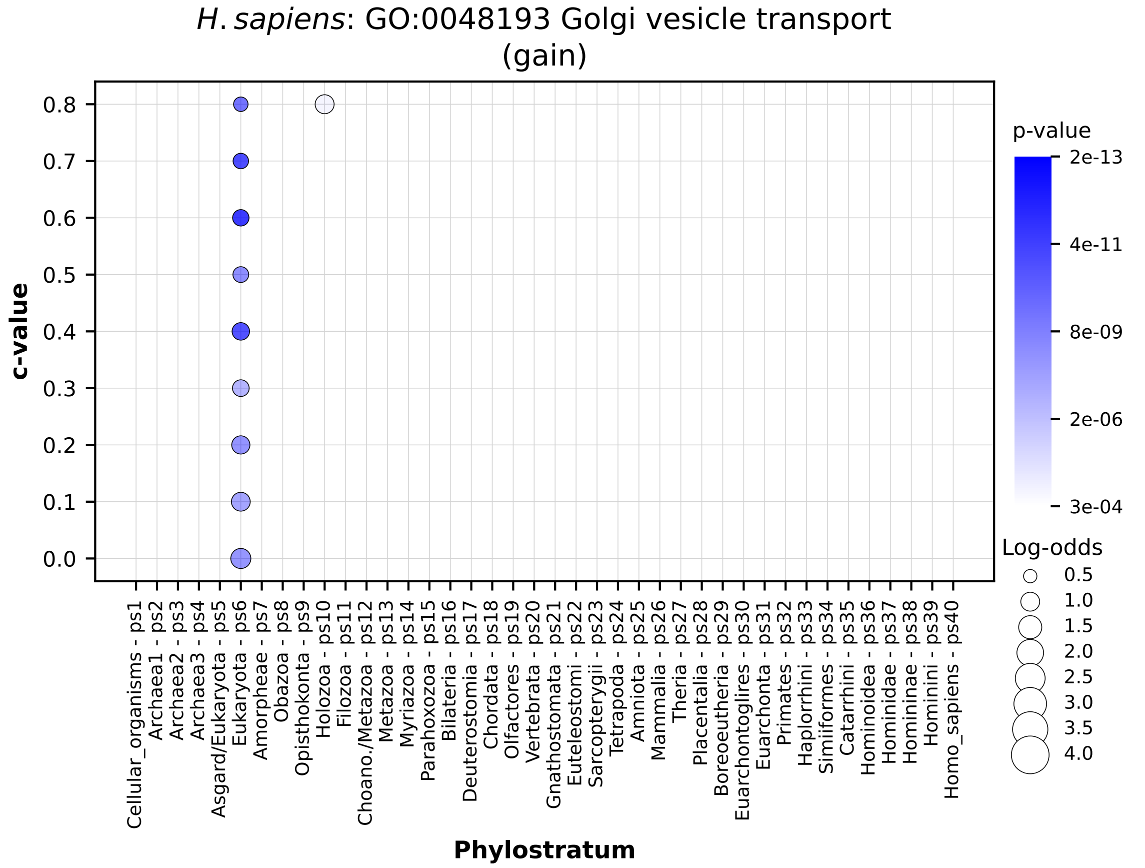
H. sapiens: GO:0048193 Golgi vesicle transport
(gain)
c-value
0.0
0.1
0.2
0.3
0.4
0.5
0.6
0.7
0.8
Phylostratum
Cellular_organisms - ps1
Archaea1 - ps2
Archaea2 - ps3
Archaea3 - ps4
Asgard/Eukaryota - ps5
Eukaryota - ps6
Amorpheae - ps7
Obazoa - ps8
Opisthokonta - ps9
Holozoa - ps10
Filozoa - ps11
Choano./Metazoa - ps12
Metazoa - ps13
Myriazoa - ps14
Parahoxozoa - ps15
Bilateria - ps16
Deuterostomia - ps17
Chordata - ps18
Olfactores - ps19
Vertebrata - ps20
Gnathostomata - ps21
Euteleostomi - ps22
Sarcopterygii - ps23
Tetrapoda - ps24
Amniota - ps25
Mammalia - ps26
Theria - ps27
Placentalia - ps28
Boreoeutheria - ps29
Euarchontoglires - ps30
Euarchonta - ps31
Primates - ps32
Haplorrhini - ps33
Simiiformes - ps34
Catarrhini - ps35
Hominoidea - ps36
Hominidae - ps37
Homininae - ps38
Hominini - ps39
Homo_sapiens - ps40
p-value
2e-13
4e-11
8e-09
2e-06
3e-04
Log-odds
0.5
1.0
1.5
2.0
2.5
3.0
3.5
4.0

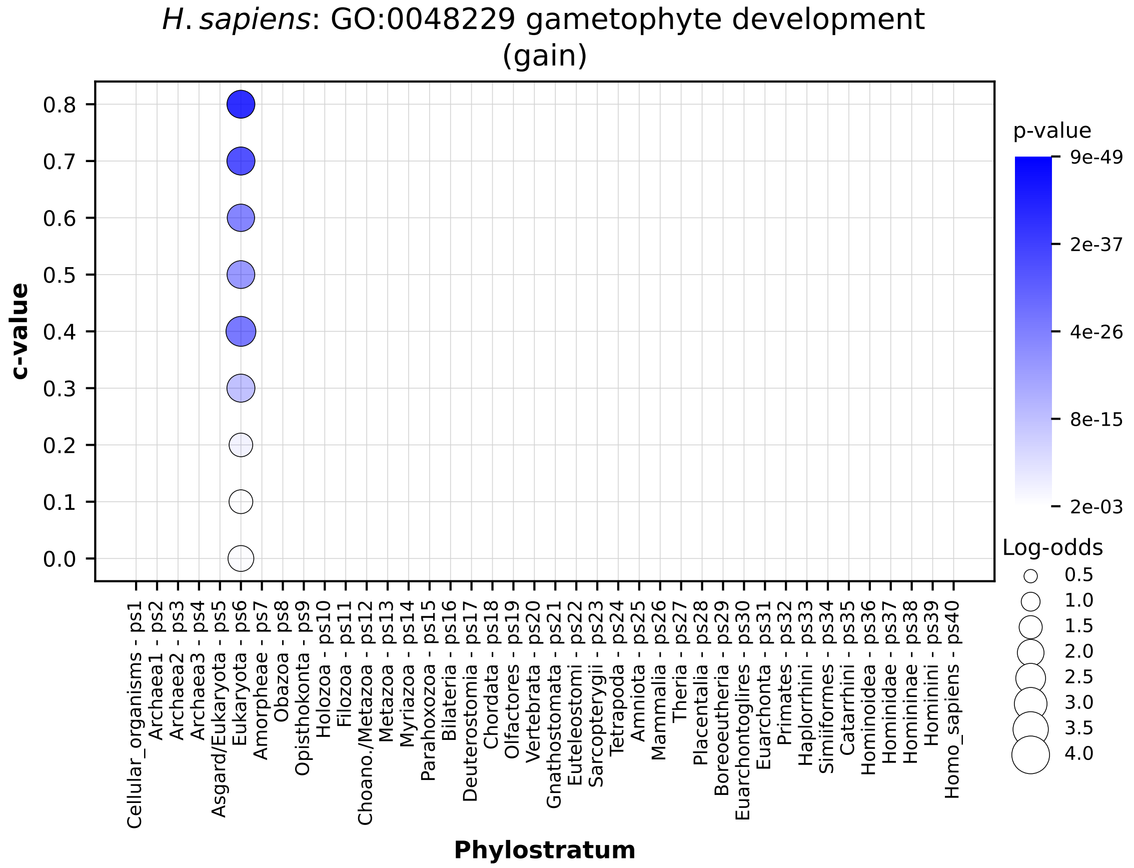

H. sapiens: GO:0048229 gametophyte development (gain)
c-value
0.0
0.1
0.2
0.3
0.4
0.5
0.6
0.7
0.8
p-value
9e-49
2e-37
4e-26
8e-15
2e-03
Log-odds
0.5
1.0
1.5
2.0
2.5
3.0
3.5
4.0
Cellular_organisms - ps1
Archaea1 - ps2
Archaea2 - ps3
Archaea3 - ps4
Asgard/Eukaryota - ps5
Eukaryota - ps6
Amorpheae - ps7
Obazoa - ps8
Opisthokonta - ps9
Holozoa - ps10
Filozoa - ps11
Choano./Metazoa - ps12
Metazoa - ps13
Myriazoa - ps14
Parahoxozoa - ps15
Bilateria - ps16
Deuterostomia - ps17
Chordata - ps18
Olfactores - ps19
Vertebrata - ps20
Gnathostomata - ps21
Euteleostomi - ps22
Sarcopterygii - ps23
Tetrapoda - ps24
Amniota - ps25
Mammalia - ps26
Theria - ps27
Placentalia - ps28
Boreoeutheria - ps29
Euarchontoglires - ps30
Euarchonta - ps31
Primates - ps32
Haplorrhini - ps33
Simiiformes - ps34
Catarrhini - ps35
Hominoidea - ps36
Hominidae - ps37
Homininae - ps38
Hominini - ps39
Homo_sapiens - ps40
Phylostratum

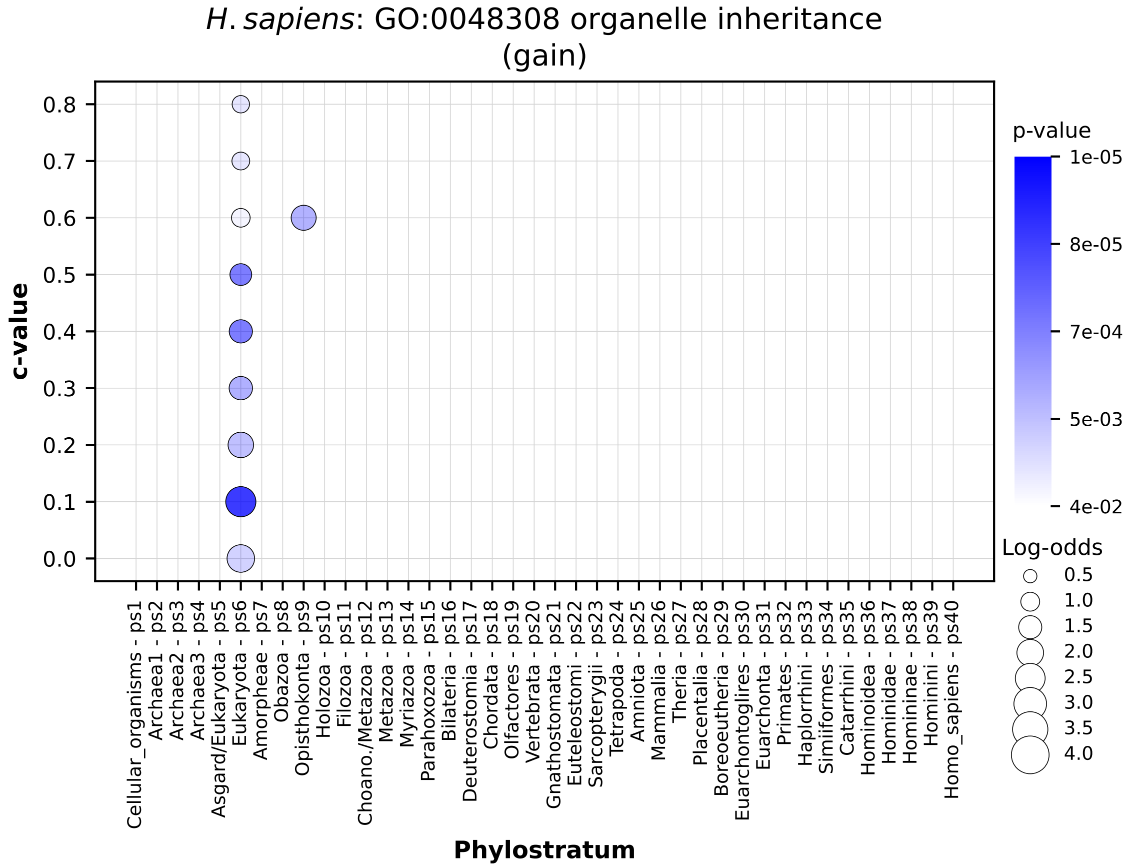

*H. sapiens*: GO:0048308 organelle inheritance
(gain)
c-value
0.8
0.7
0.6
0.5
0.4
0.3
0.2
0.1
0.0
p-value
1e-05
8e-05
7e-04
5e-03
4e-02
Log-odds
0.5
1.0
1.5
2.0
2.5
3.0
3.5
4.0
Cellular_organisms - ps1
Archaea1 - ps2
Archaea2 - ps3
Archaea3 - ps4
Asgard/Eukaryota - ps5
Eukaryota - ps6
Amorpheae - ps7
Obazoa - ps8
Opisthokonta - ps9
Holozoa - ps10
Filozoa - ps11
Choano./Metazoa - ps12
Metazoa - ps13
Myriazoa - ps14
Parahoxozoa - ps15
Bilateria - ps16
Deuterostomia - ps17
Chordata - ps18
Olfactores - ps19
Vertebrata - ps20
Gnathostomata - ps21
Euteleostomi - ps22
Sarcopterygii - ps23
Tetrapoda - ps24
Amniota - ps25
Mammalia - ps26
Theria - ps27
Placentalia - ps28
Boreoeutheria - ps29
Euarchontoglires - ps30
Euarchonta - ps31
Primates - ps32
Haplorrhini - ps33
Simiiformes - ps34
Catarrhini - ps35
Hominoidea - ps36
Hominidae - ps37
Homininae - ps38
Hominini - ps39
Homo_sapiens - ps40
Phylostratum

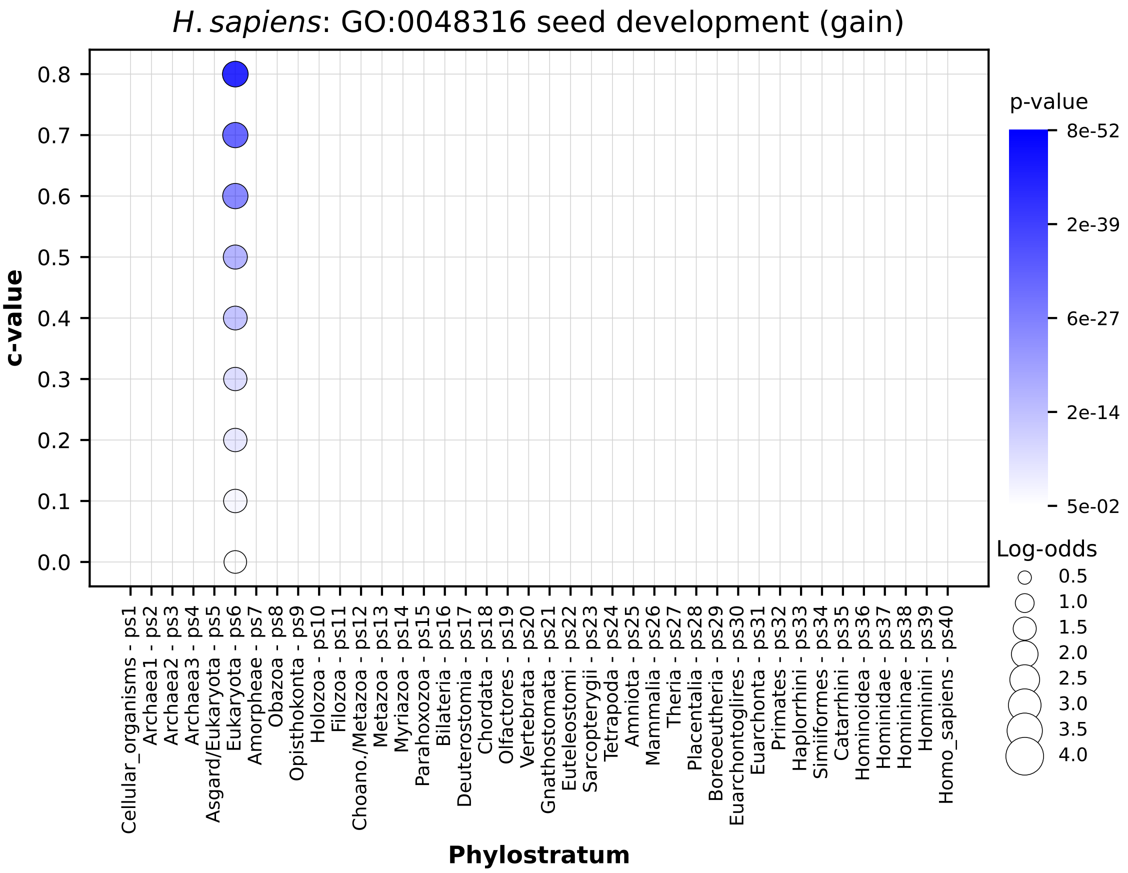

H. sapiens: GO:0048316 seed development (gain)
c-value
0.8
0.7
0.6
0.5
0.4
0.3
0.2
0.1
0.0
Phylostratum
Cellular_organisms - ps1
Archaea1 - ps2
Archaea2 - ps3
Archaea3 - ps4
Asgard/Eukaryota - ps5
Eukaryota - ps6
Amorpheae - ps7
Obazoa - ps8
Opisthokonta - ps9
Holozoa - ps10
Filozoa - ps11
Choano./Metazoa - ps12
Metazoa - ps13
Myriazoa - ps14
Parahoxozoa - ps15
Bilateria - ps16
Deuterostomia - ps17
Chordata - ps18
Olfactores - ps19
Vertebrata - ps20
Gnathostomata - ps21
Euteleostomi - ps22
Sarcopterygii - ps23
Tetrapoda - ps24
Amniota - ps25
Mammalia - ps26
Theria - ps27
Placentalia - ps28
Boreoeutheria - ps29
Euarchontoglires - ps30
Euarchonta - ps31
Primates - ps32
Haplorrhini - ps33
Simiiformes - ps34
Catarrhini - ps35
Hominoidea - ps36
Hominidae - ps37
Homininae - ps38
Hominini - ps39
Homo_sapiens - ps40
p-value
8e-52
2e-39
6e-27
2e-14
5e-02
Log-odds
0.5
1.0
1.5
2.0
2.5
3.0
3.5
4.0

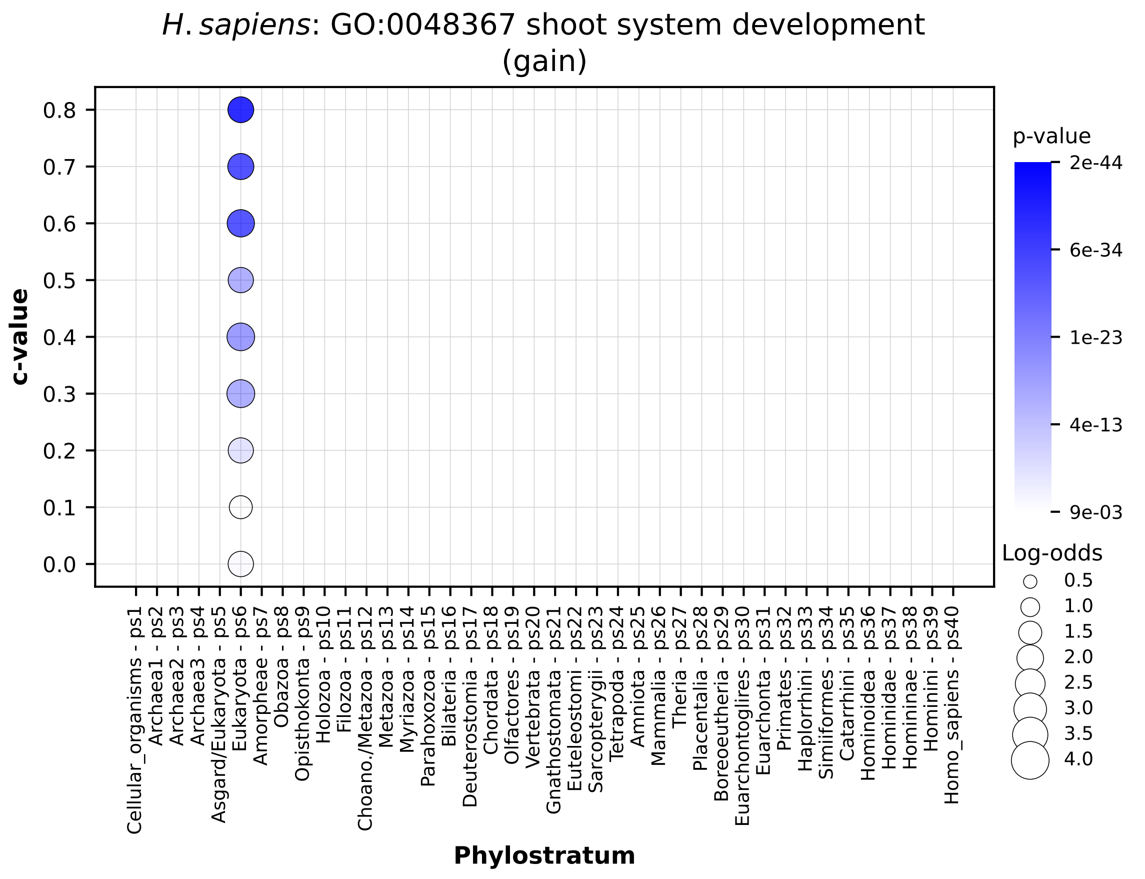

*H. sapiens*: GO:0048367 shoot system development
(gain)
c-value
0.0
0.1
0.2
0.3
0.4
0.5
0.6
0.7
0.8
p-value
2e-44
6e-34
1e-23
4e-13
9e-03
Log-odds
0.5
1.0
1.5
2.0
2.5
3.0
3.5
4.0
Cellular_organisms - ps1
Archaea1 - ps2
Archaea2 - ps3
Archaea3 - ps4
Asgard/Eukaryota - ps5
Eukaryota - ps6
Amorpheae - ps7
Obazoa - ps8
Opisthokonta - ps9
Holozoa - ps10
Filozoa - ps11
Choano./Metazoa - ps12
Metazoa - ps13
Myriazoa - ps14
Parahoxozoa - ps15
Bilateria - ps16
Deuterostomia - ps17
Chordata - ps18
Olfactores - ps19
Vertebrata - ps20
Gnathostomata - ps21
Euteleostomi - ps22
Sarcopterygii - ps23
Tetrapoda - ps24
Amniota - ps25
Mammalia - ps26
Theria - ps27
Placentalia - ps28
Boreoeutheria - ps29
Euarchontoglires - ps30
Euarchonta - ps31
Primates - ps32
Haplorrhini - ps33
Simiiformes - ps34
Catarrhini - ps35
Hominoidea - ps36
Hominidae - ps37
Homininae - ps38
Hominini - ps39
Homo_sapiens - ps40
Phylostratum

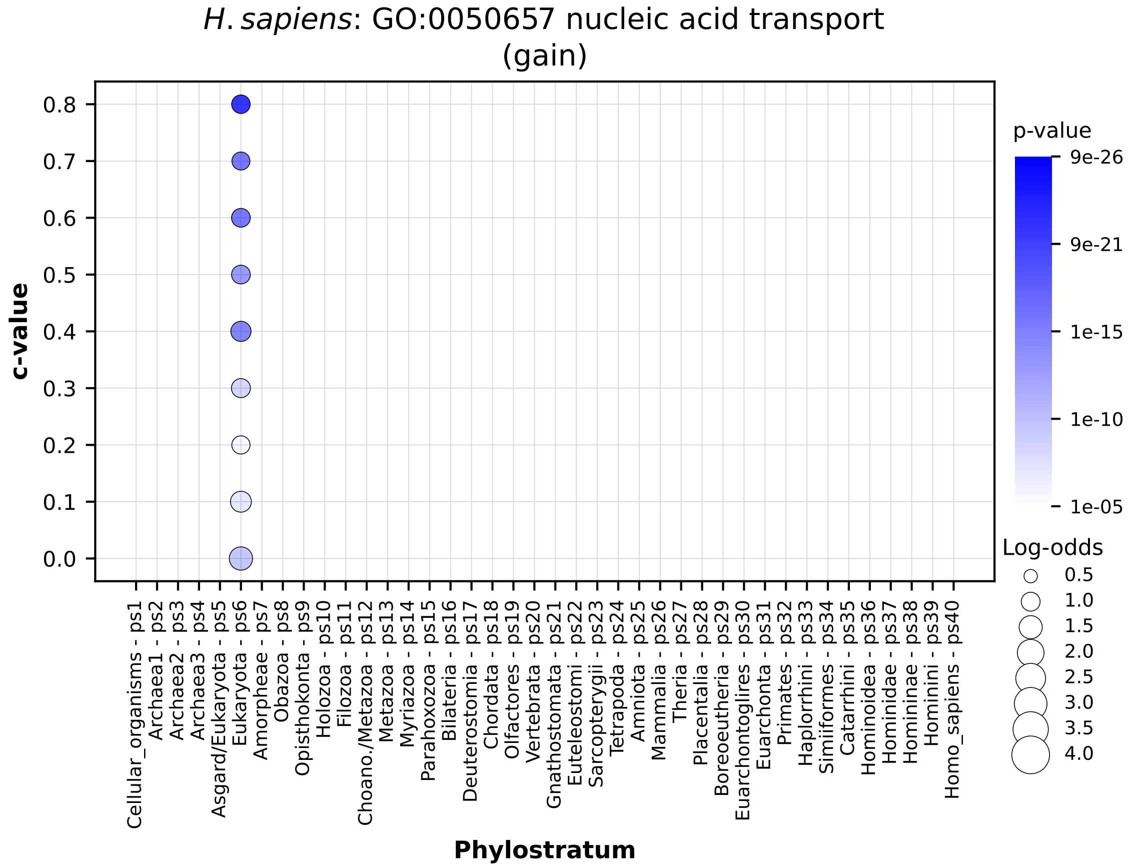

H. sapiens: GO:0050657 nucleic acid transport (gain)
c-value
0.8
0.7
0.6
0.5
0.4
0.3
0.2
0.1
0.0
Phylostratum
Cellular_organisms - ps1
Archaea1 - ps2
Archaea2 - ps3
Archaea3 - ps4
Asgard/Eukaryota - ps5
Eukaryota - ps6
Amorpheae - ps7
Obazoa - ps8
Opisthokonta - ps9
Holozoa - ps10
Filozoa - ps11
Choano./Metazoa - ps12
Metazoa - ps13
Myriazoa - ps14
Parahoxozoa - ps15
Bilateria - ps16
Deuterostomia - ps17
Chordata - ps18
Olfactores - ps19
Vertebrata - ps20
Gnathostomata - ps21
Euteleostomi - ps22
Sarcopterygii - ps23
Tetrapoda - ps24
Amniota - ps25
Mammalia - ps26
Theria - ps27
Placentalia - ps28
Boreoeutheria - ps29
Euarchontoglires - ps30
Euarchonta - ps31
Primates - ps32
Haplorrhini - ps33
Simiiformes - ps34
Catarrhini - ps35
Hominoidea - ps36
Hominidae - ps37
Homininae - ps38
Hominini - ps39
Homo_sapiens - ps40
p-value
9e-26
9e-21
1e-15
1e-10
1e-05
Log-odds
0.5
1.0
1.5
2.0
2.5
3.0
3.5
4.0

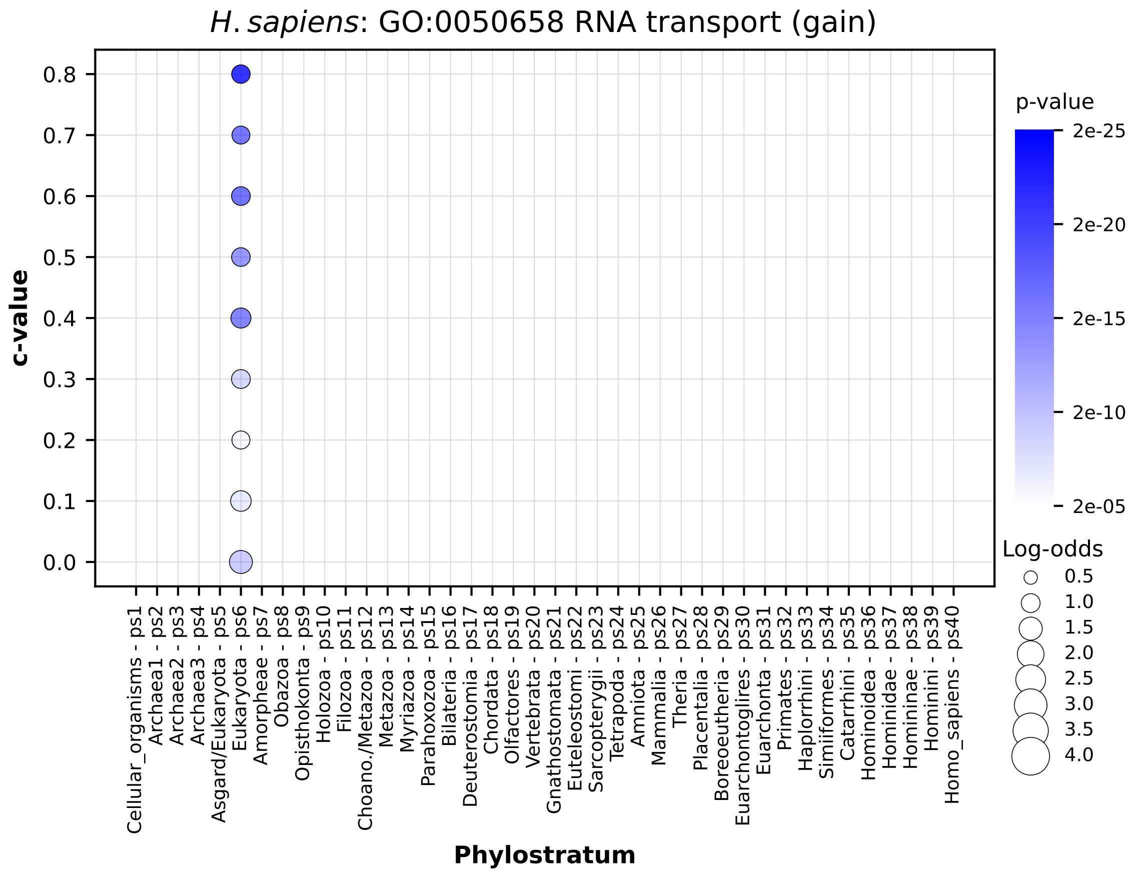
H. sapiens: GO:0050658 RNA transport (gain)
c-value
0.0
0.1
0.2
0.3
0.4
0.5
0.6
0.7
0.8
Phylostratum
Cellular_organisms - ps1
Archaea1 - ps2
Archaea2 - ps3
Archaea3 - ps4
Asgard/Eukaryota - ps5
Eukaryota - ps6
Amorpheae - ps7
Obazoa - ps8
Opisthokonta - ps9
Holozoa - ps10
Filozoa - ps11
Choano./Metazoa - ps12
Metazoa - ps13
Myriazoa - ps14
Parahoxozoa - ps15
Bilateria - ps16
Deuterostomia - ps17
Chordata - ps18
Olfactores - ps19
Vertebrata - ps20
Gnathostomata - ps21
Euteleostomi - ps22
Sarcopterygii - ps23
Tetrapoda - ps24
Amniota - ps25
Mammalia - ps26
Theria - ps27
Placentalia - ps28
Boreoeutheria - ps29
Euarchontoglires - ps30
Euarchonta - ps31
Primates - ps32
Haplorrhini - ps33
Simiiformes - ps34
Catarrhini - ps35
Hominoidea - ps36
Hominidae - ps37
Homininae - ps38
Hominini - ps39
Homo_sapiens - ps40
p-value
2e-25
2e-20
2e-15
2e-10
2e-05
Log-odds
0.5
1.0
1.5
2.0
2.5
3.0
3.5
4.0

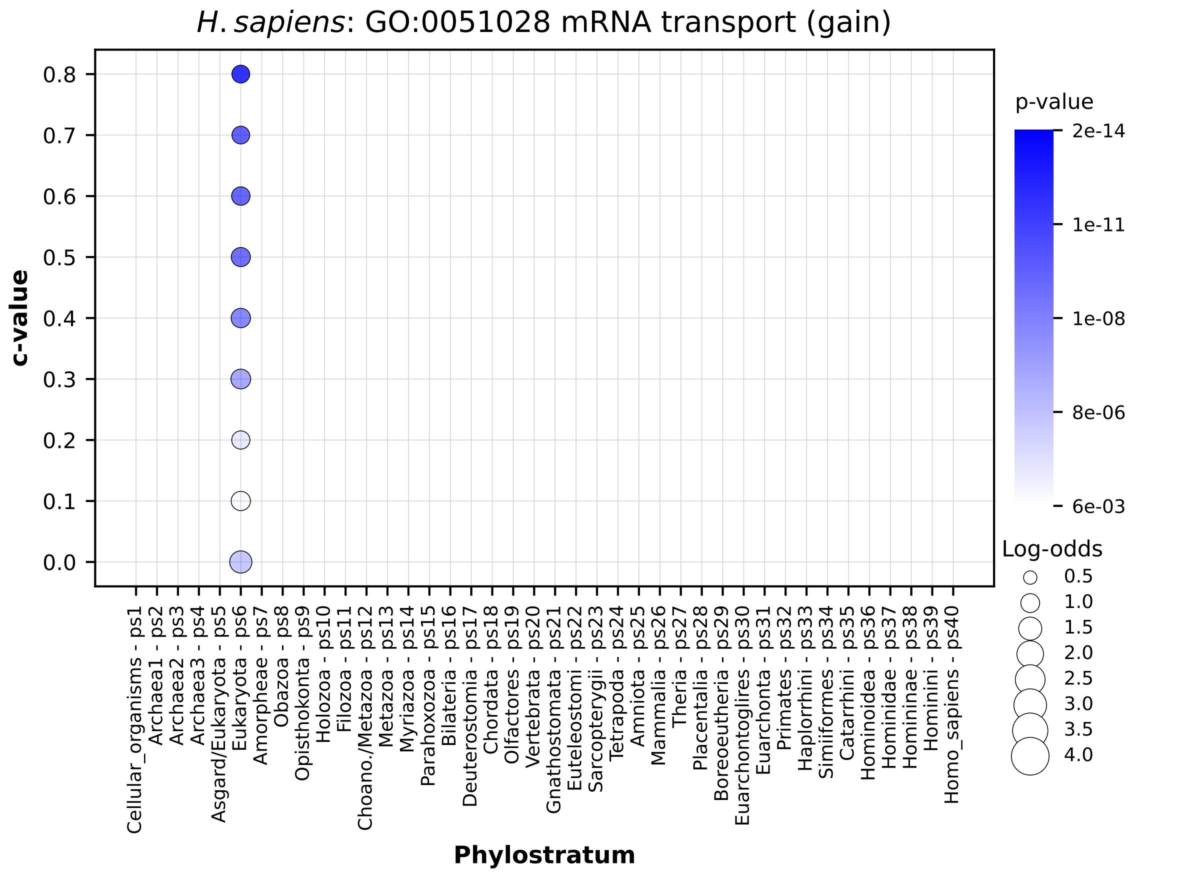
H. sapiens: GO:0051028 mRNA transport (gain)
c-value
0.8
0.7
0.6
0.5
0.4
0.3
0.2
0.1
0.0
Phylostratum
Cellular_organisms - ps1
Archaea1 - ps2
Archaea2 - ps3
Archaea3 - ps4
Asgard/Eukaryota - ps5
Eukaryota - ps6
Amorpheae - ps7
Obazoa - ps8
Opisthokonta - ps9
Holozoa - ps10
Filozoa - ps11
Choano./Metazoa - ps12
Metazoa - ps13
Myriazoa - ps14
Parahoxozoa - ps15
Bilateria - ps16
Deuterostomia - ps17
Chordata - ps18
Olfactores - ps19
Vertebrata - ps20
Gnathostomata - ps21
Euteleostomi - ps22
Sarcopterygii - ps23
Tetrapoda - ps24
Amniota - ps25
Mammalia - ps26
Theria - ps27
Placentalia - ps28
Boreoeutheria - ps29
Euarchontoglires - ps30
Euarchonta - ps31
Primates - ps32
Haplorrhini - ps33
Simiiformes - ps34
Catarrhini - ps35
Hominoidea - ps36
Hominidae - ps37
Homininae - ps38
Hominini - ps39
Homo_sapiens - ps40
p-value
2e-14
1e-11
1e-08
8e-06
6e-03
Log-odds
0.5
1.0
1.5
2.0
2.5
3.0
3.5
4.0

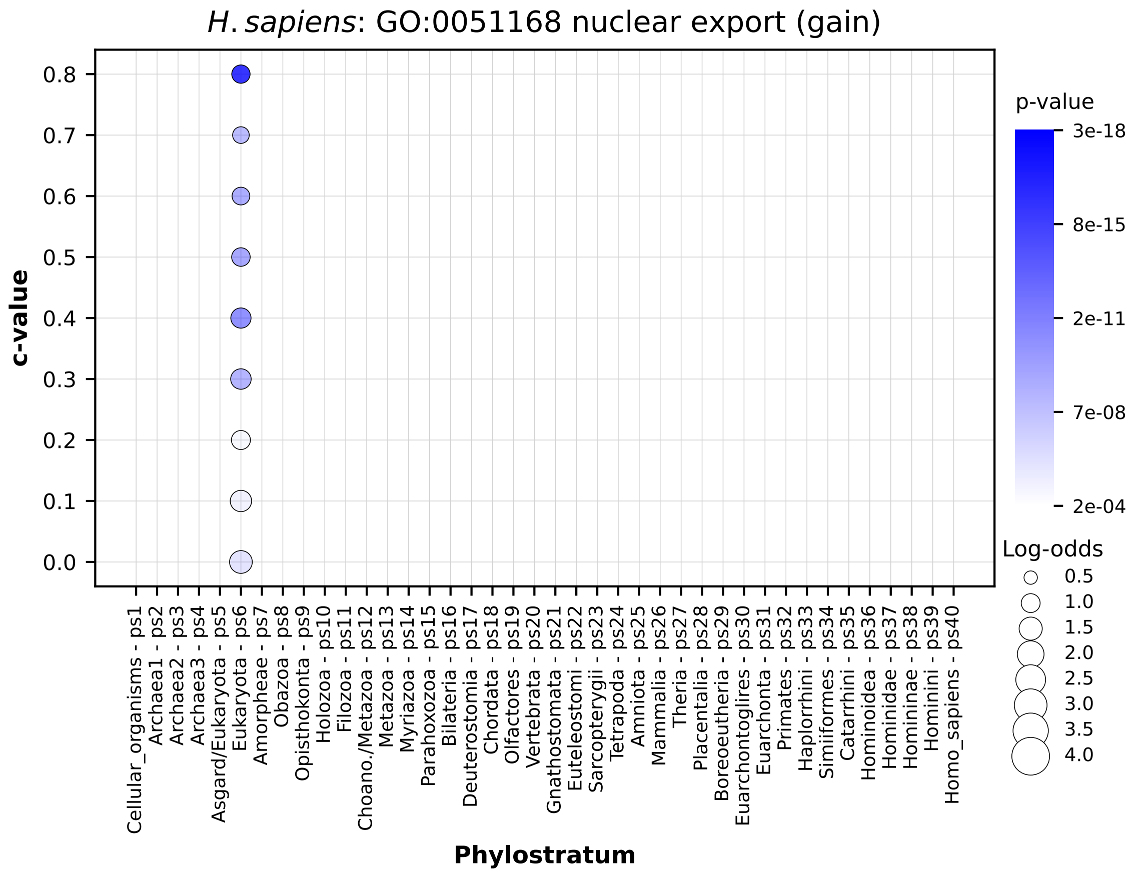
H. sapiens: GO:0051168 nuclear export (gain)
c-value
0.8
0.7
0.6
0.5
0.4
0.3
0.2
0.1
0.0
p-value
3e-18
8e-15
2e-11
7e-08
2e-04
Log-odds
0.5
1.0
1.5
2.0
2.5
3.0
3.5
4.0
Cellular_organisms - ps1
Archaea1 - ps2
Archaea2 - ps3
Archaea3 - ps4
Asgard/Eukaryota - ps5
Eukaryota - ps6
Amorpheae - ps7
Obazoa - ps8
Opisthokonta - ps9
Holozoa - ps10
Filozoa - ps11
Choano./Metazoa - ps12
Metazoa - ps13
Myriazoa - ps14
Parahoxozoa - ps15
Bilateria - ps16
Deuterostomia - ps17
Chordata - ps18
Olfactores - ps19
Vertebrata - ps20
Gnathostomata - ps21
Euteleostomi - ps22
Sarcopterygii - ps23
Tetrapoda - ps24
Amniota - ps25
Mammalia - ps26
Theria - ps27
Placentalia - ps28
Boreoeutheria - ps29
Euarchontoglires - ps30
Euarchonta - ps31
Primates - ps32
Haplorrhini - ps33
Simiiformes - ps34
Catarrhini - ps35
Hominoidea - ps36
Hominidae - ps37
Homininae - ps38
Hominini - ps39
Homo_sapiens - ps40
Phylostratum

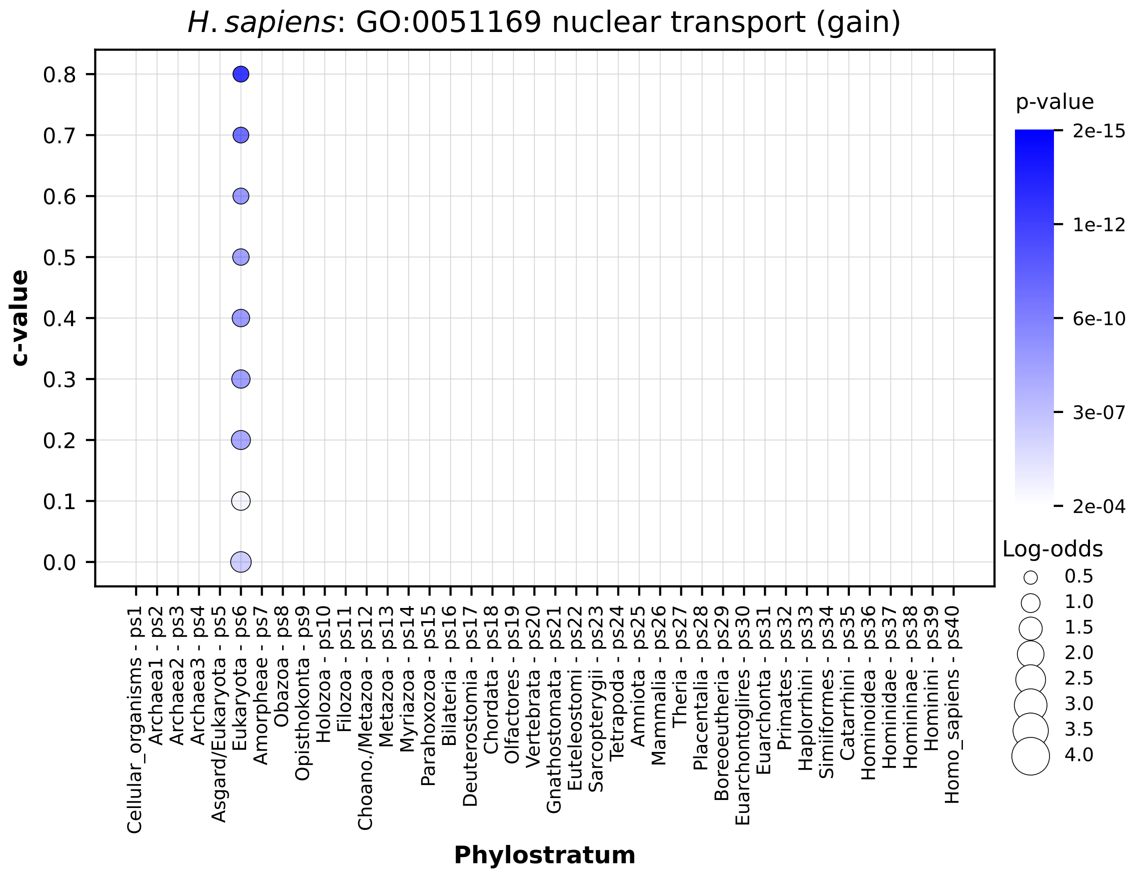

H. sapiens: GO:0051169 nuclear transport (gain)
c-value
0.8
0.7
0.6
0.5
0.4
0.3
0.2
0.1
0.0
Phylostratum
Cellular_organisms - ps1
Archaea1 - ps2
Archaea2 - ps3
Archaea3 - ps4
Asgard/Eukaryota - ps5
Eukaryota - ps6
Amorpheae - ps7
Obazoa - ps8
Opisthokonta - ps9
Holozoa - ps10
Filozoa - ps11
Choano./Metazoa - ps12
Metazoa - ps13
Myriazoa - ps14
Parahoxozoa - ps15
Bilateria - ps16
Deuterostomia - ps17
Chordata - ps18
Olfactores - ps19
Vertebrata - ps20
Gnathostomata - ps21
Euteleostomi - ps22
Sarcopterygii - ps23
Tetrapoda - ps24
Amniota - ps25
Mammalia - ps26
Theria - ps27
Placentalia - ps28
Boreoeutheria - ps29
Euarchontoglires - ps30
Euarchonta - ps31
Primates - ps32
Haplorrhini - ps33
Simiiformes - ps34
Catarrhini - ps35
Hominoidea - ps36
Hominidae - ps37
Homininae - ps38
Hominini - ps39
Homo_sapiens - ps40
p-value
2e-15
1e-12
6e-10
3e-07
2e-04
Log-odds
0.5
1.0
1.5
2.0
2.5
3.0
3.5
4.0

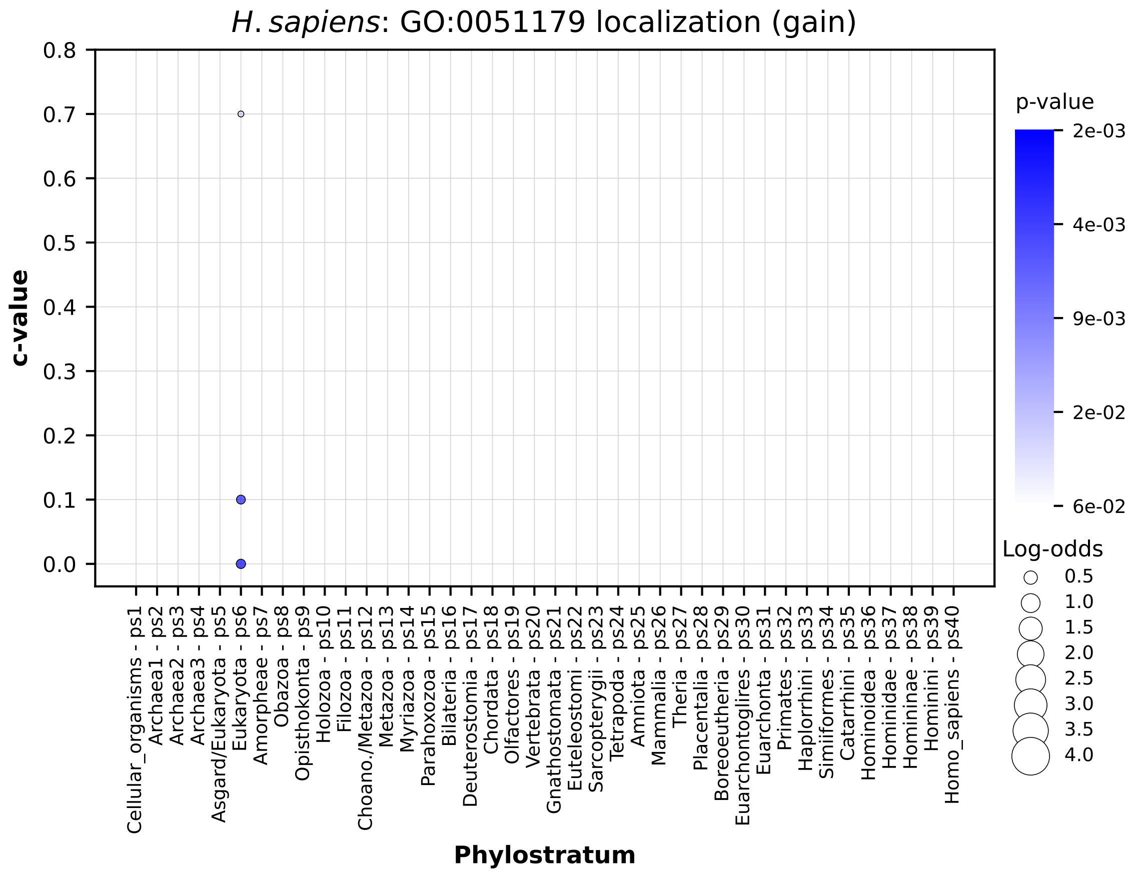

*H. sapiens*: GO:0051179 localization (gain)
c-value
0.0
0.1
0.2
0.3
0.4
0.5
0.6
0.7
0.8
p-value
2e-03
4e-03
9e-03
2e-02
6e-02
Log-odds
0.5
1.0
1.5
2.0
2.5
3.0
3.5
4.0
Cellular_organisms - ps1
Archaea1 - ps2
Archaea2 - ps3
Archaea3 - ps4
Asgard/Eukaryota - ps5
Eukaryota - ps6
Amorpheae - ps7
Obazoa - ps8
Opisthokonta - ps9
Holozoa - ps10
Filozoa - ps11
Choano./Metazoa - ps12
Metazoa - ps13
Myriazoa - ps14
Parahoxozoa - ps15
Bilateria - ps16
Deuterostomia - ps17
Chordata - ps18
Olfactores - ps19
Vertebrata - ps20
Gnathostomata - ps21
Euteleostomi - ps22
Sarcopterygii - ps23
Tetrapoda - ps24
Amniota - ps25
Mammalia - ps26
Theria - ps27
Placentalia - ps28
Boreoeutheria - ps29
Euarchontoglires - ps30
Euarchonta - ps31
Primates - ps32
Haplorrhini - ps33
Simiiformes - ps34
Catarrhini - ps35
Hominoidea - ps36
Hominidae - ps37
Homininae - ps38
Hominini - ps39
Homo_sapiens - ps40
Phylostratum

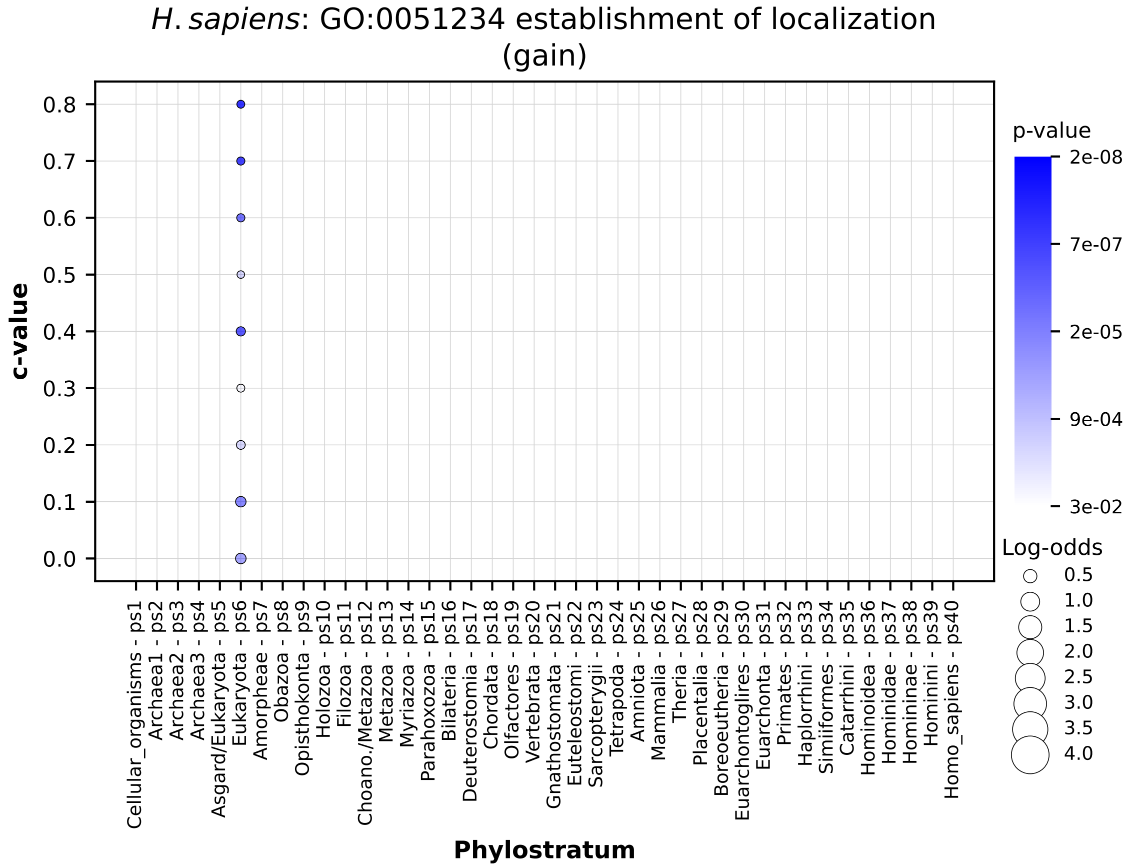

H. sapiens: GO:0051234 establishment of localization (gain)
c-value
0.8
0.7
0.6
0.5
0.4
0.3
0.2
0.1
0.0
p-value
2e-08
7e-07
2e-05
9e-04
3e-02
Log-odds
0.5
1.0
1.5
2.0
2.5
3.0
3.5
4.0
Cellular_organisms - ps1
Archaea1 - ps2
Archaea2 - ps3
Archaea3 - ps4
Asgard/Eukaryota - ps5
Eukaryota - ps6
Amorpheae - ps7
Obazoa - ps8
Opisthokonta - ps9
Holozoa - ps10
Filozoa - ps11
Choano./Metazoa - ps12
Metazoa - ps13
Myriazoa - ps14
Parahoxozoa - ps15
Bilateria - ps16
Deuterostomia - ps17
Chordata - ps18
Olfactores - ps19
Vertebrata - ps20
Gnathostomata - ps21
Euteleostomi - ps22
Sarcopterygii - ps23
Tetrapoda - ps24
Amniota - ps25
Mammalia - ps26
Theria - ps27
Placentalia - ps28
Boreoeutheria - ps29
Euarchontoglires - ps30
Euarchonta - ps31
Primates - ps32
Haplorrhini - ps33
Simiiformes - ps34
Catarrhini - ps35
Hominoidea - ps36
Hominidae - ps37
Homininae - ps38
Hominini - ps39
Homo_sapiens - ps40
Phylostratum

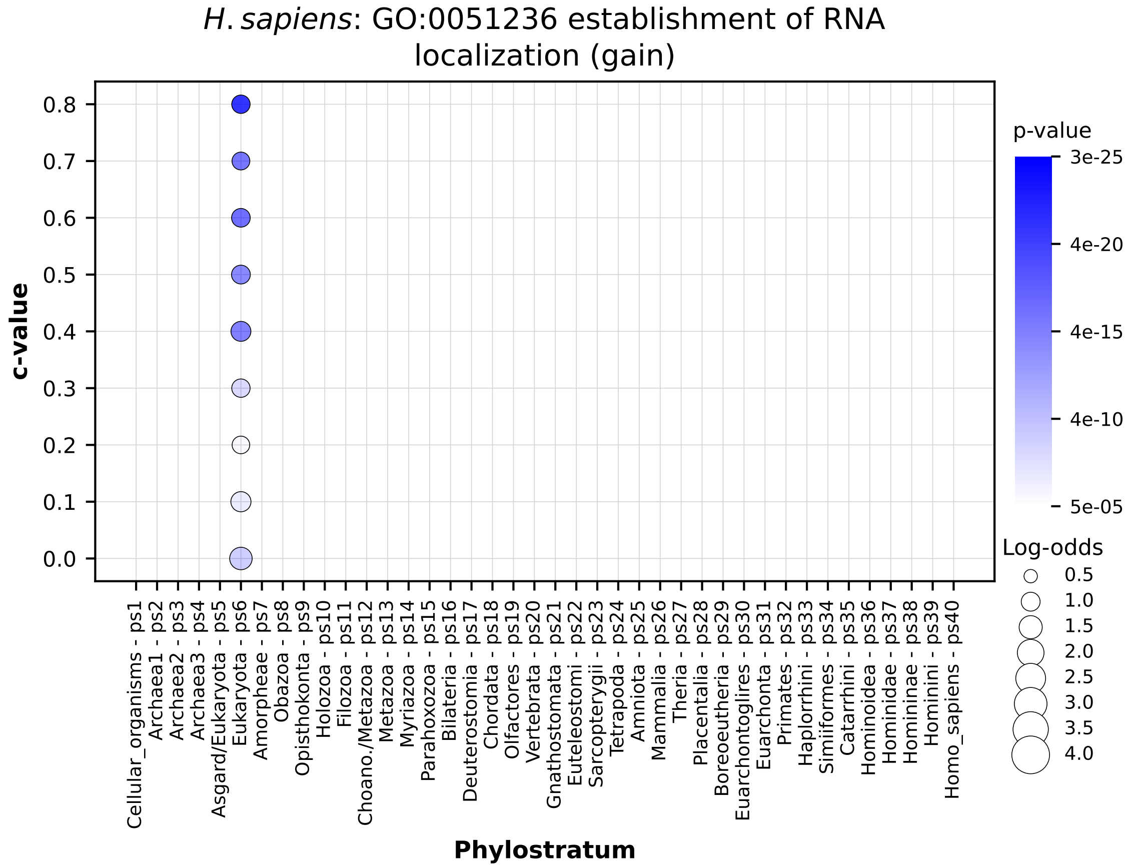
H. sapiens: GO:0051236 establishment of RNA localization (gain)
c-value
0.8
0.7
0.6
0.5
0.4
0.3
0.2
0.1
0.0
Phylostratum
Cellular_organisms - ps1
Archaea1 - ps2
Archaea2 - ps3
Archaea3 - ps4
Asgard/Eukaryota - ps5
Eukaryota - ps6
Amorpheae - ps7
Obazoa - ps8
Opisthokonta - ps9
Holozoa - ps10
Filozoa - ps11
Choano./Metazoa - ps12
Metazoa - ps13
Myriazoa - ps14
Parahoxozoa - ps15
Bilateria - ps16
Deuterostomia - ps17
Chordata - ps18
Olfactores - ps19
Vertebrata - ps20
Gnathostomata - ps21
Euteleostomi - ps22
Sarcopterygii - ps23
Tetrapoda - ps24
Amniota - ps25
Mammalia - ps26
Theria - ps27
Placentalia - ps28
Boreoeutheria - ps29
Euarchontoglires - ps30
Euarchonta - ps31
Primates - ps32
Haplorrhini - ps33
Simiiformes - ps34
Catarrhini - ps35
Hominoidea - ps36
Hominidae - ps37
Homininae - ps38
Hominini - ps39
Homo_sapiens - ps40
p-value
3e-25
4e-20
4e-15
4e-10
5e-05
Log-odds
0.5
1.0
1.5
2.0
2.5
3.0
3.5
4.0

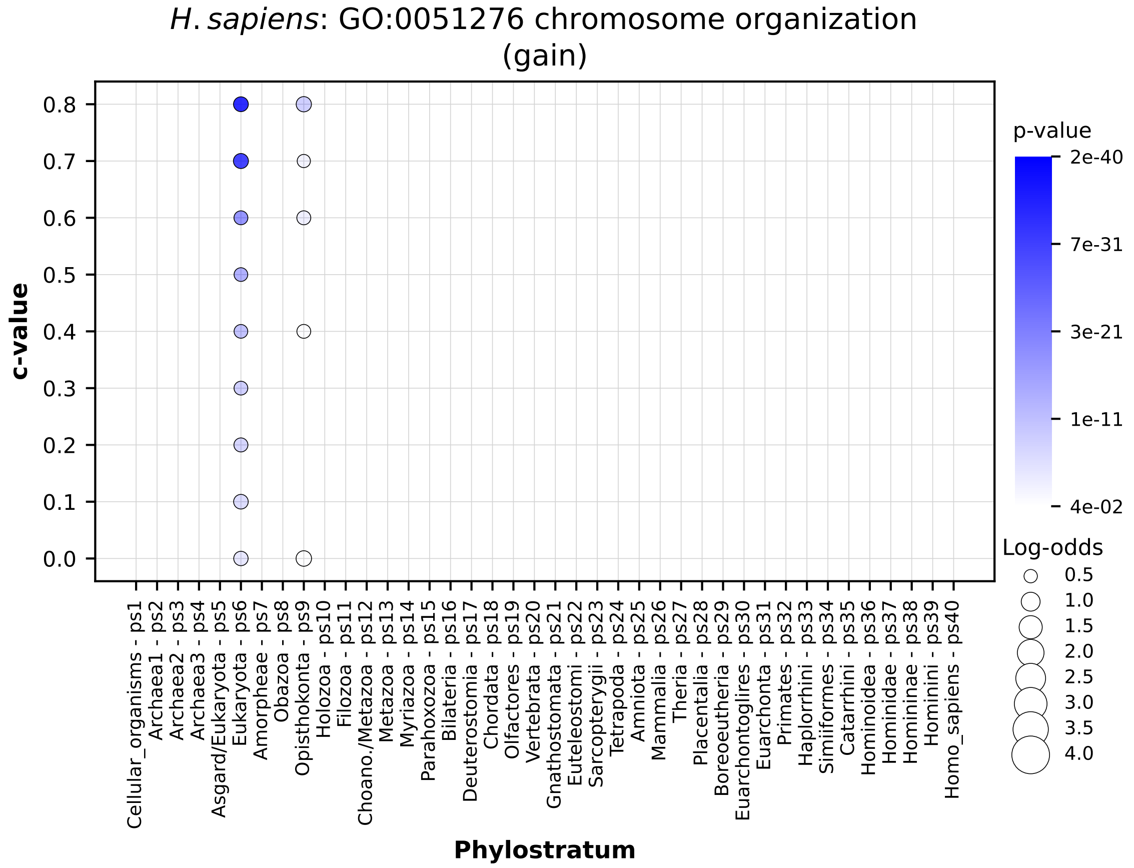
*H. sapiens*: GO:0051276 chromosome organization
(gain)
c-value
0.8
0.7
0.6
0.5
0.4
0.3
0.2
0.1
0.0
p-value
2e-40
7e-31
3e-21
1e-11
4e-02
Log-odds
0.5
1.0
1.5
2.0
2.5
3.0
3.5
4.0
Cellular_organisms - ps1
Archaea1 - ps2
Archaea2 - ps3
Archaea3 - ps4
Asgard/Eukaryota - ps5
Eukaryota - ps6
Amorpheae - ps7
Obazoa - ps8
Opisthokonta - ps9
Holozoa - ps10
Filozoa - ps11
Choano./Metazoa - ps12
Metazoa - ps13
Myriazoa - ps14
Parahoxozoa - ps15
Bilateria - ps16
Deuterostomia - ps17
Chordata - ps18
Olfactores - ps19
Vertebrata - ps20
Gnathostomata - ps21
Euteleostomi - ps22
Sarcopterygii - ps23
Tetrapoda - ps24
Amniota - ps25
Mammalia - ps26
Theria - ps27
Placentalia - ps28
Boreoeutheria - ps29
Euarchontoglires - ps30
Euarchonta - ps31
Primates - ps32
Haplorrhini - ps33
Simiiformes - ps34
Catarrhini - ps35
Hominoidea - ps36
Hominidae - ps37
Homininae - ps38
Hominini - ps39
Homo_sapiens - ps40
Phylostratum

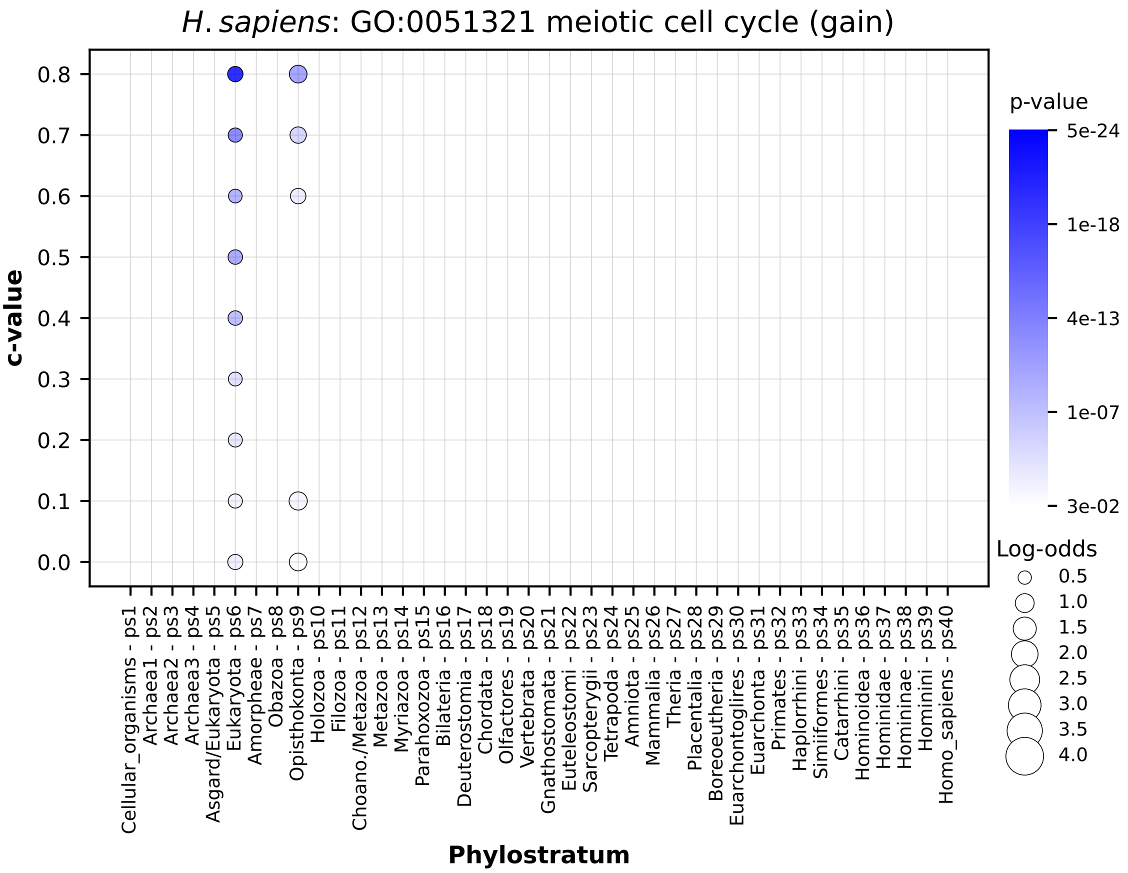

H. sapiens: GO:0051321 meiotic cell cycle (gain)
c-value
0.0
0.1
0.2
0.3
0.4
0.5
0.6
0.7
0.8
Phylostratum
Cellular_organisms - ps1
Archaea1 - ps2
Archaea2 - ps3
Archaea3 - ps4
Asgard/Eukaryota - ps5
Eukaryota - ps6
Amorpheae - ps7
Obazoa - ps8
Opisthokonta - ps9
Holozoa - ps10
Filozoa - ps11
Choano./Metazoa - ps12
Metazoa - ps13
Myriazoa - ps14
Parahoxozoa - ps15
Bilateria - ps16
Deuterostomia - ps17
Chordata - ps18
Olfactores - ps19
Vertebrata - ps20
Gnathostomata - ps21
Euteleostomi - ps22
Sarcopterygii - ps23
Tetrapoda - ps24
Amniota - ps25
Mammalia - ps26
Theria - ps27
Placentalia - ps28
Boreoeutheria - ps29
Euarchontoglires - ps30
Euarchonta - ps31
Primates - ps32
Haplorrhini - ps33
Simiiformes - ps34
Catarrhini - ps35
Hominoidea - ps36
Hominidae - ps37
Homininae - ps38
Hominini - ps39
Homo_sapiens - ps40
p-value
5e-24
1e-18
4e-13
1e-07
3e-02
Log-odds
0.5
1.0
1.5
2.0
2.5
3.0
3.5
4.0

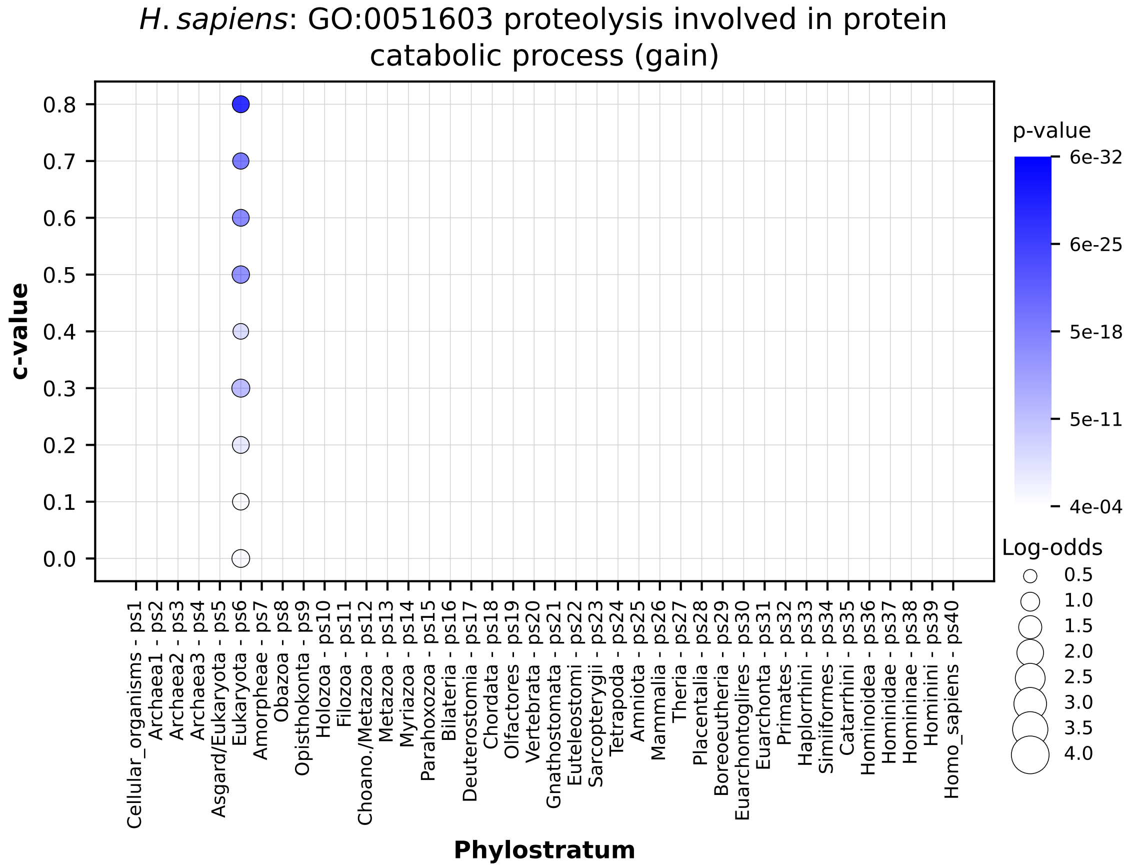

H. sapiens: GO:0051603 proteolysis involved in protein catabolic process (gain)
c-value
0.8
0.7
0.6
0.5
0.4
0.3
0.2
0.1
0.0
p-value
6e-32
6e-25
5e-18
5e-11
4e-04
Log-odds
0.5
1.0
1.5
2.0
2.5
3.0
3.5
4.0
Cellular_organisms - ps1
Archaea1 - ps2
Archaea2 - ps3
Archaea3 - ps4
Asgard/Eukaryota - ps5
Eukaryota - ps6
Amorpheae - ps7
Obazoa - ps8
Opisthokonta - ps9
Holozoa - ps10
Filozoa - ps11
Choano./Metazoa - ps12
Metazoa - ps13
Myriazoa - ps14
Parahoxozoa - ps15
Bilateria - ps16
Deuterostomia - ps17
Chordata - ps18
Olfactores - ps19
Vertebrata - ps20
Gnathostomata - ps21
Euteleostomi - ps22
Sarcopterygii - ps23
Tetrapoda - ps24
Amniota - ps25
Mammalia - ps26
Theria - ps27
Placentalia - ps28
Boreoeutheria - ps29
Euarchontoglires - ps30
Euarchonta - ps31
Primates - ps32
Haplorrhini - ps33
Simiiformes - ps34
Catarrhini - ps35
Hominoidea - ps36
Hominidae - ps37
Homininae - ps38
Hominini - ps39
Homo_sapiens - ps40
Phylostratum

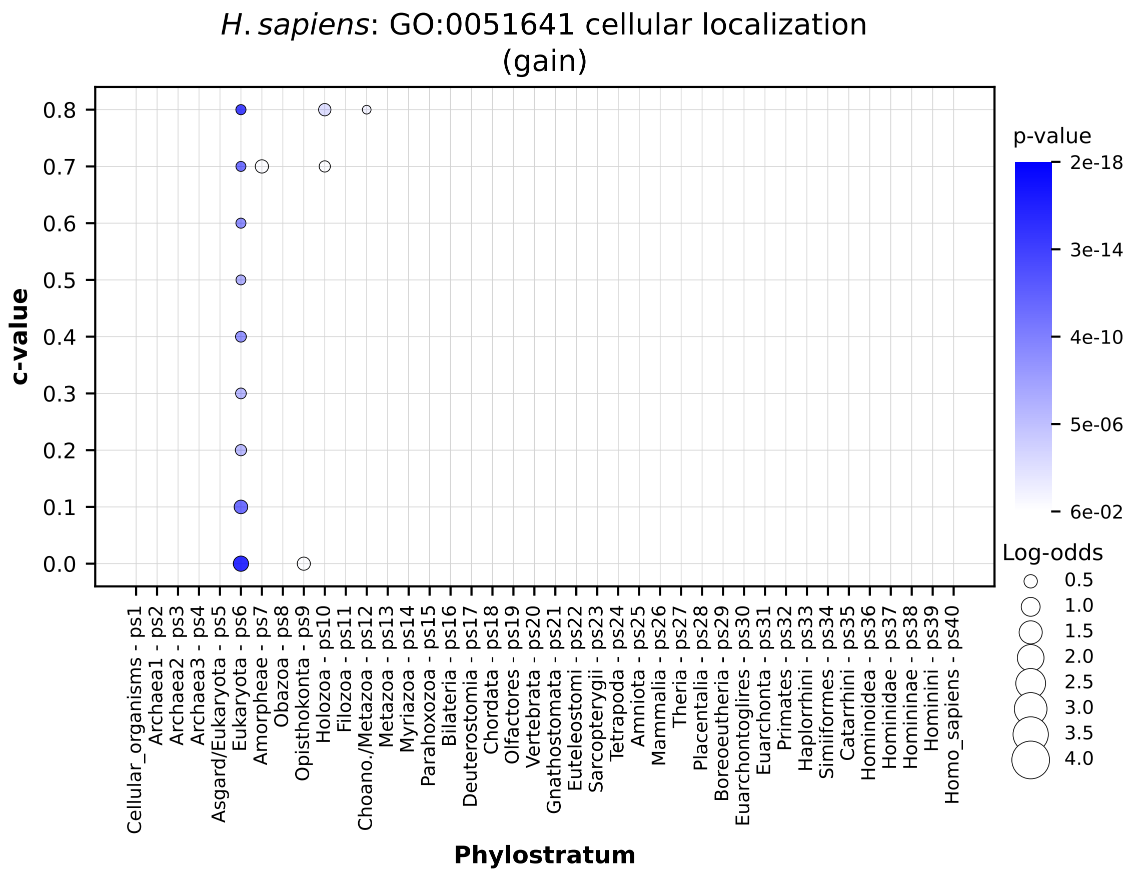

*H. sapiens*: GO:0051641 cellular localization
(gain)
c-value
0.8
0.7
0.6
0.5
0.4
0.3
0.2
0.1
0.0
p-value
2e-18
3e-14
4e-10
5e-06
6e-02
Log-odds
0.5
1.0
1.5
2.0
2.5
3.0
3.5
4.0
Cellular_organisms - ps1
Archaea1 - ps2
Archaea2 - ps3
Archaea3 - ps4
Asgard/Eukaryota - ps5
Eukaryota - ps6
Amorpheae - ps7
Obazoa - ps8
Opisthokonta - ps9
Holozoa - ps10
Filozoa - ps11
Choano./Metazoa - ps12
Metazoa - ps13
Myriazoa - ps14
Parahoxozoa - ps15
Bilateria - ps16
Deuterostomia - ps17
Chordata - ps18
Olfactores - ps19
Vertebrata - ps20
Gnathostomata - ps21
Euteleostomi - ps22
Sarcopterygii - ps23
Tetrapoda - ps24
Amniota - ps25
Mammalia - ps26
Theria - ps27
Placentalia - ps28
Boreoeutheria - ps29
Euarchontoglires - ps30
Euarchonta - ps31
Primates - ps32
Haplorrhini - ps33
Simiiformes - ps34
Catarrhini - ps35
Hominoidea - ps36
Hominidae - ps37
Homininae - ps38
Hominini - ps39
Homo_sapiens - ps40
Phylostratum

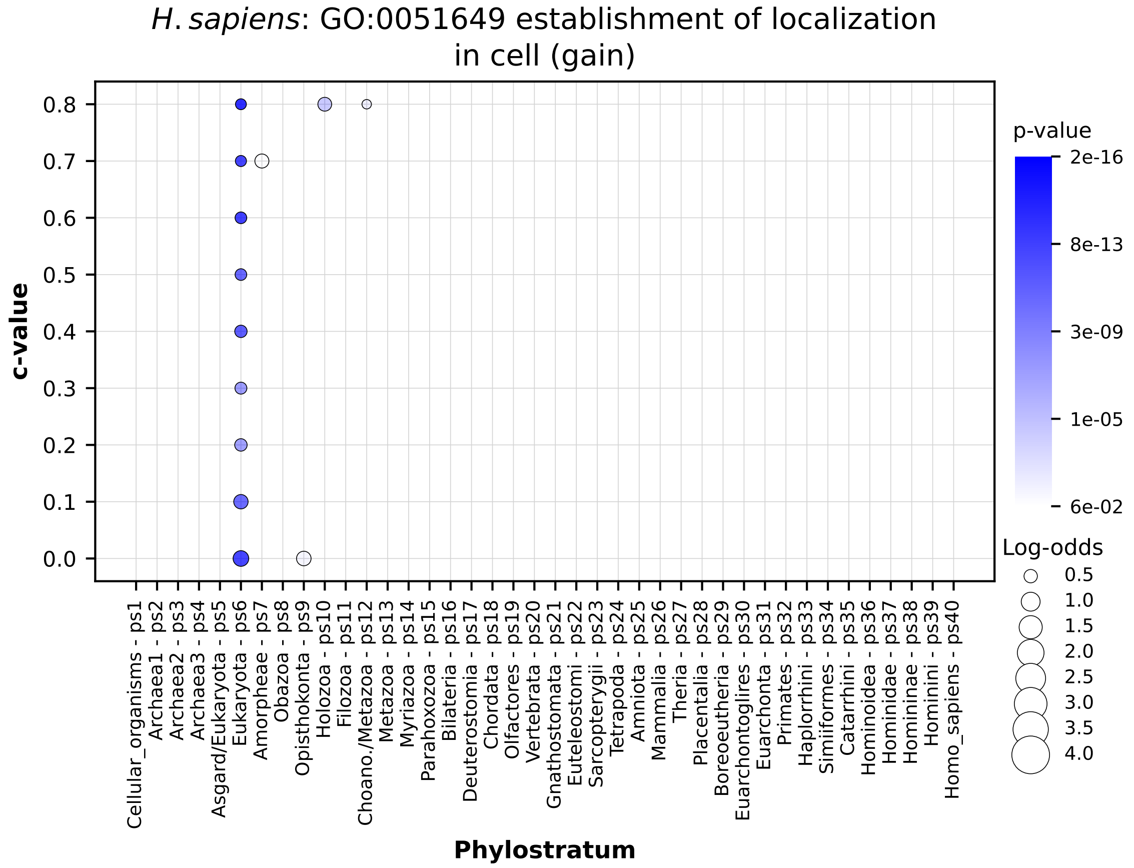

H. sapiens: GO:0051649 establishment of localization in cell (gain)
c-value
0.8
0.7
0.6
0.5
0.4
0.3
0.2
0.1
0.0
Phylostratum
Cellular_organisms - ps1
Archaea1 - ps2
Archaea2 - ps3
Archaea3 - ps4
Asgard/Eukaryota - ps5
Eukaryota - ps6
Amorpheae - ps7
Obazoa - ps8
Opisthokonta - ps9
Holozoa - ps10
Filozoa - ps11
Choano./Metazoa - ps12
Metazoa - ps13
Myriazoa - ps14
Parahoxozoa - ps15
Bilateria - ps16
Deuterostomia - ps17
Chordata - ps18
Olfactores - ps19
Vertebrata - ps20
Gnathostomata - ps21
Euteleostomi - ps22
Sarcopterygii - ps23
Tetrapoda - ps24
Amniota - ps25
Mammalia - ps26
Theria - ps27
Placentalia - ps28
Boreoeutheria - ps29
Euarchontoglires - ps30
Euarchonta - ps31
Primates - ps32
Haplorrhini - ps33
Simiiformes - ps34
Catarrhini - ps35
Hominoidea - ps36
Hominidae - ps37
Homininae - ps38
Hominini - ps39
Homo_sapiens - ps40
p-value
2e-16
8e-13
3e-09
1e-05
6e-02
Log-odds
0.5
1.0
1.5
2.0
2.5
3.0
3.5
4.0

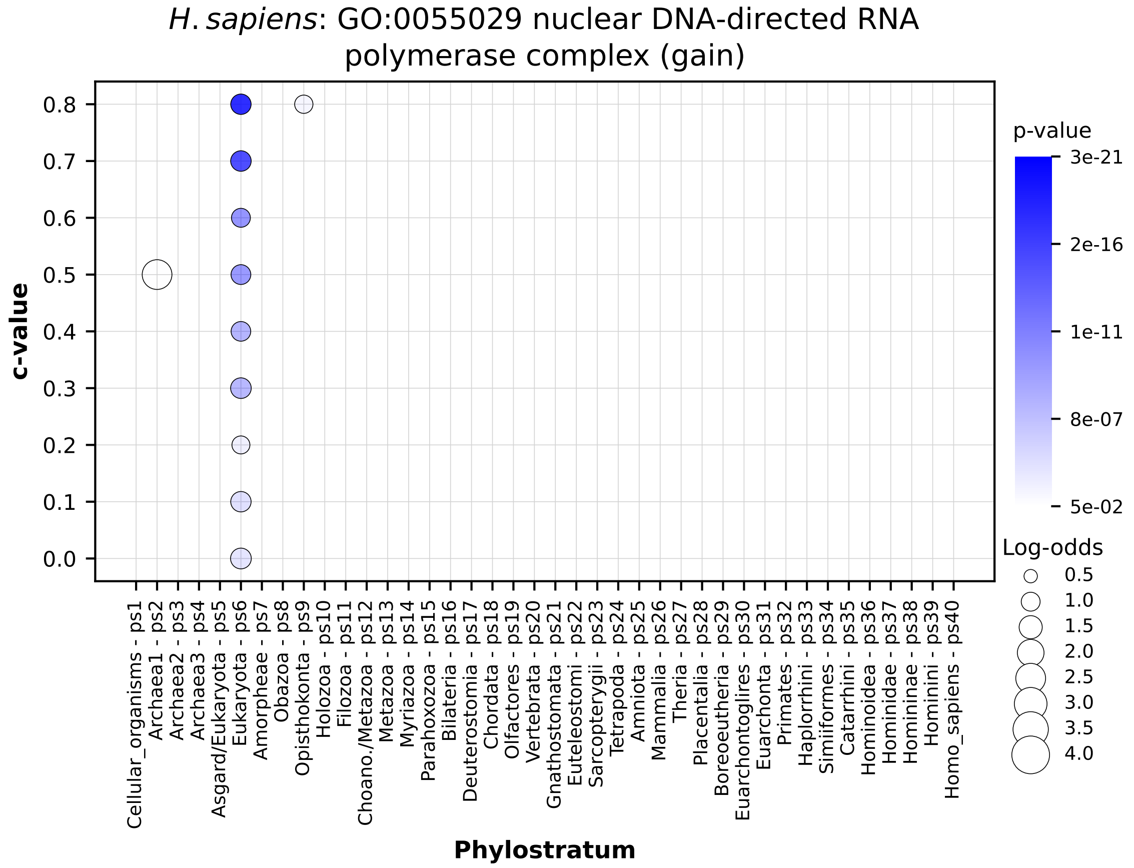

H. sapiens: GO:0055029 nuclear DNA-directed RNA polymerase complex (gain)
c-value
0.8
0.7
0.6
0.5
0.4
0.3
0.2
0.1
0.0
p-value
3e-21
2e-16
1e-11
8e-07
5e-02
Log-odds
0.5
1.0
1.5
2.0
2.5
3.0
3.5
4.0
Cellular_organisms - ps1
Archaea1 - ps2
Archaea2 - ps3
Archaea3 - ps4
Asgard/Eukaryota - ps5
Eukaryota - ps6
Amorpheae - ps7
Obazoa - ps8
Opisthokonta - ps9
Holozoa - ps10
Filozoa - ps11
Choano./Metazoa - ps12
Metazoa - ps13
Myriazoa - ps14
Parahoxozoa - ps15
Bilateria - ps16
Deuterostomia - ps17
Chordata - ps18
Olfactores - ps19
Vertebrata - ps20
Gnathostomata - ps21
Euteleostomi - ps22
Sarcopterygii - ps23
Tetrapoda - ps24
Amniota - ps25
Mammalia - ps26
Theria - ps27
Placentalia - ps28
Boreoeutheria - ps29
Euarchontoglires - ps30
Euarchonta - ps31
Primates - ps32
Haplorrhini - ps33
Simiiformes - ps34
Catarrhini - ps35
Hominoidea - ps36
Hominidae - ps37
Homininae - ps38
Hominini - ps39
Homo_sapiens - ps40
Phylostratum

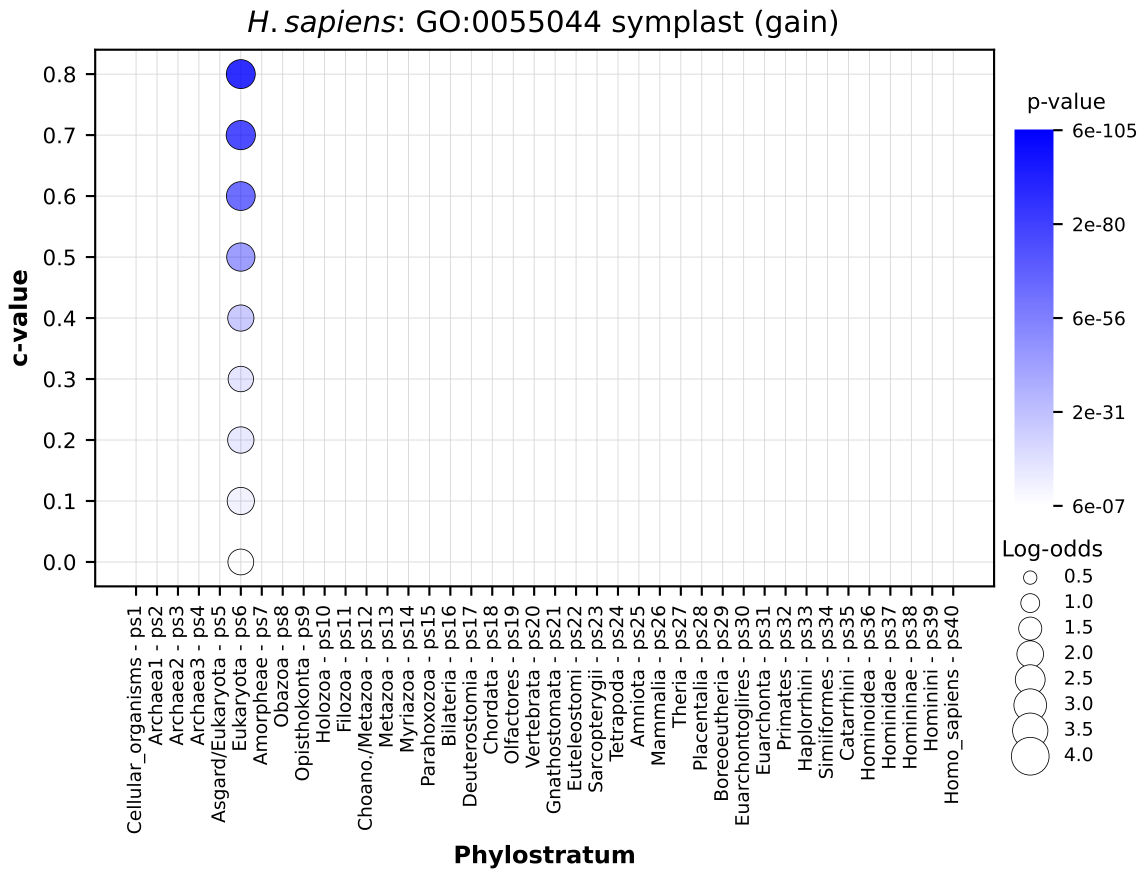

*H. sapiens*: GO:0055044 symplast (gain)
c-value
0.8
0.7
0.6
0.5
0.4
0.3
0.2
0.1
0.0
p-value
6e-105
2e-80
6e-56
2e-31
6e-07
Log-odds
0.5
1.0
1.5
2.0
2.5
3.0
3.5
4.0
Cellular_organisms - ps1
Archaea1 - ps2
Archaea2 - ps3
Archaea3 - ps4
Asgard/Eukaryota - ps5
Eukaryota - ps6
Amorpheae - ps7
Obazoa - ps8
Opisthokonta - ps9
Holozoa - ps10
Filozoa - ps11
Choano./Metazoa - ps12
Metazoa - ps13
Myriazoa - ps14
Parahoxozoa - ps15
Bilateria - ps16
Deuterostomia - ps17
Chordata - ps18
Olfactores - ps19
Vertebrata - ps20
Gnathostomata - ps21
Euteleostomi - ps22
Sarcopterygii - ps23
Tetrapoda - ps24
Amniota - ps25
Mammalia - ps26
Theria - ps27
Placentalia - ps28
Boreoeutheria - ps29
Euarchontoglires - ps30
Euarchonta - ps31
Primates - ps32
Haplorrhini - ps33
Simiiformes - ps34
Catarrhini - ps35
Hominoidea - ps36
Hominidae - ps37
Homininae - ps38
Hominini - ps39
Homo_sapiens - ps40
Phylostratum

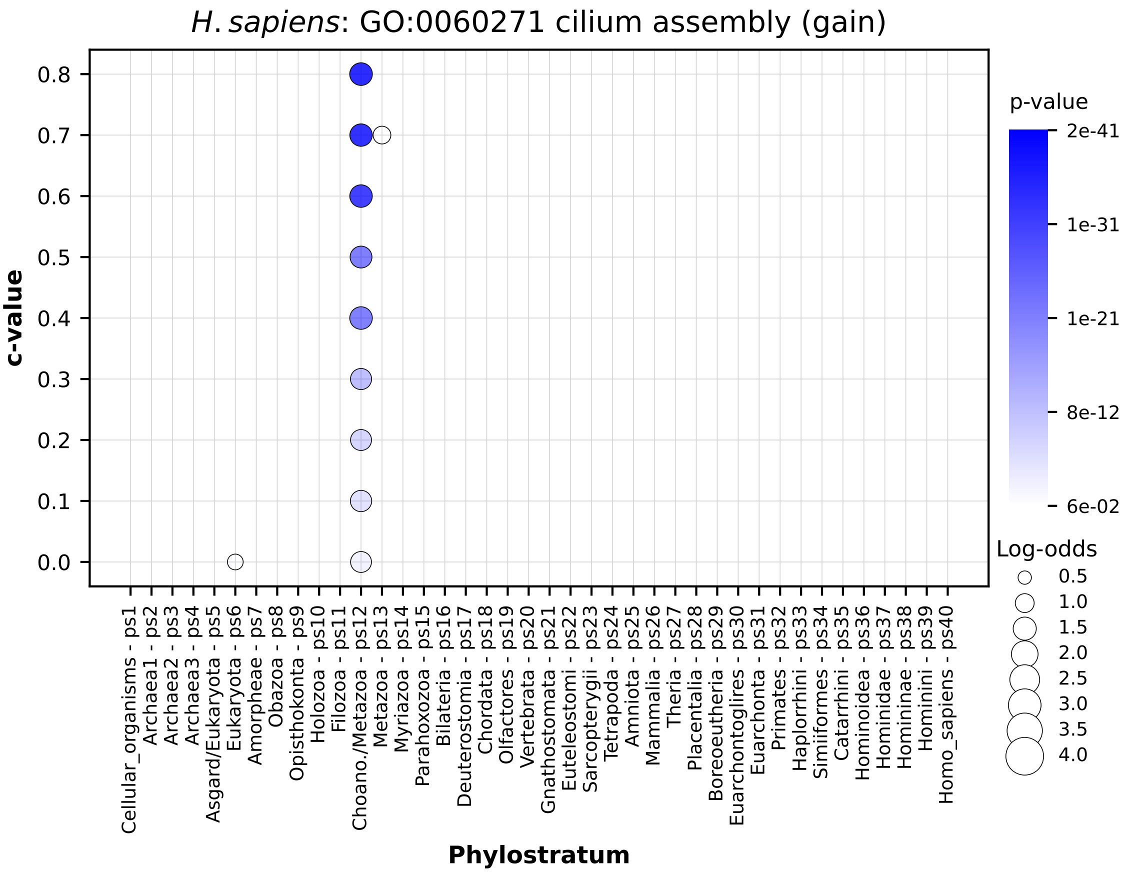
H. sapiens: GO:0060271 cilium assembly (gain)
c-value
0.8
0.7
0.6
0.5
0.4
0.3
0.2
0.1
0.0
p-value
2e-41
1e-31
1e-21
8e-12
6e-02
Log-odds
0.5
1.0
1.5
2.0
2.5
3.0
3.5
4.0
Cellular_organisms - ps1
Archaea1 - ps2
Archaea2 - ps3
Archaea3 - ps4
Asgard/Eukaryota - ps5
Eukaryota - ps6
Amorpheae - ps7
Obazoa - ps8
Opisthokonta - ps9
Holozoa - ps10
Filozoa - ps11
Choano./Metazoa - ps12
Metazoa - ps13
Myriazoa - ps14
Parahoxozoa - ps15
Bilateria - ps16
Deuterostomia - ps17
Chordata - ps18
Olfactores - ps19
Vertebrata - ps20
Gnathostomata - ps21
Euteleostomi - ps22
Sarcopterygii - ps23
Tetrapoda - ps24
Amniota - ps25
Mammalia - ps26
Theria - ps27
Placentalia - ps28
Boreoeutheria - ps29
Euarchontoglires - ps30
Euarchonta - ps31
Primates - ps32
Haplorrhini - ps33
Simiiformes - ps34
Catarrhini - ps35
Hominoidea - ps36
Hominidae - ps37
Homininae - ps38
Hominini - ps39
Homo_sapiens - ps40
Phylostratum

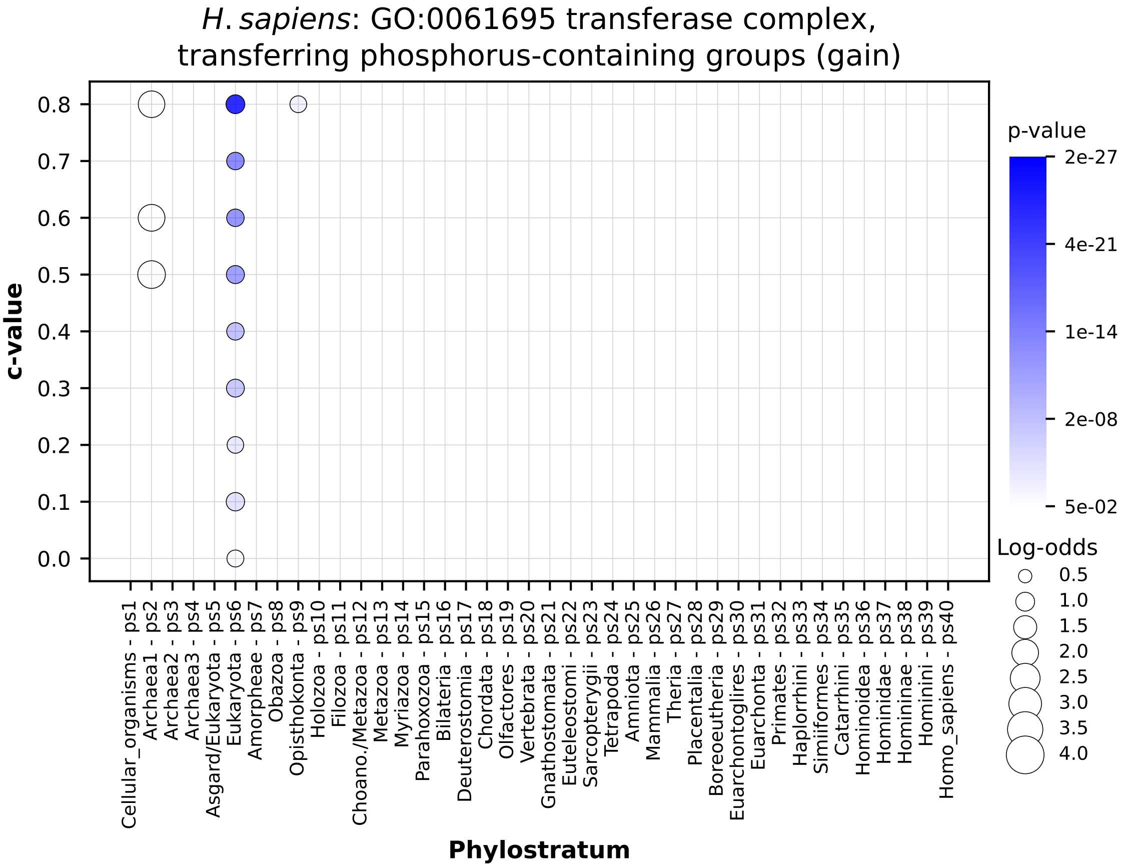

*H. sapiens*: GO:0061695 transferase complex, transferring phosphorus-containing groups (gain)
c-value
0.8
0.7
0.6
0.5
0.4
0.3
0.2
0.1
0.0
Phylostratum
Cellular_organisms - ps1
Archaea1 - ps2
Archaea2 - ps3
Archaea3 - ps4
Asgard/Eukaryota - ps5
Eukaryota - ps6
Amorpheae - ps7
Obazoa - ps8
Opisthokonta - ps9
Holozoa - ps10
Filozoa - ps11
Choano./Metazoa - ps12
Metazoa - ps13
Myriazoa - ps14
Parahoxozoa - ps15
Bilateria - ps16
Deuterostomia - ps17
Chordata - ps18
Olfactores - ps19
Vertebrata - ps20
Gnathostomata - ps21
Euteleostomi - ps22
Sarcopterygii - ps23
Tetrapoda - ps24
Amniota - ps25
Mammalia - ps26
Theria - ps27
Placentalia - ps28
Boreoeutheria - ps29
Euarchontoglires - ps30
Euarchonta - ps31
Primates - ps32
Haplorrhini - ps33
Simiiformes - ps34
Catarrhini - ps35
Hominoidea - ps36
Hominidae - ps37
Homininae - ps38
Hominini - ps39
Homo_sapiens - ps40
p-value
2e-27
4e-21
1e-14
2e-08
5e-02
Log-odds
0.5
1.0
1.5
2.0
2.5
3.0
3.5
4.0

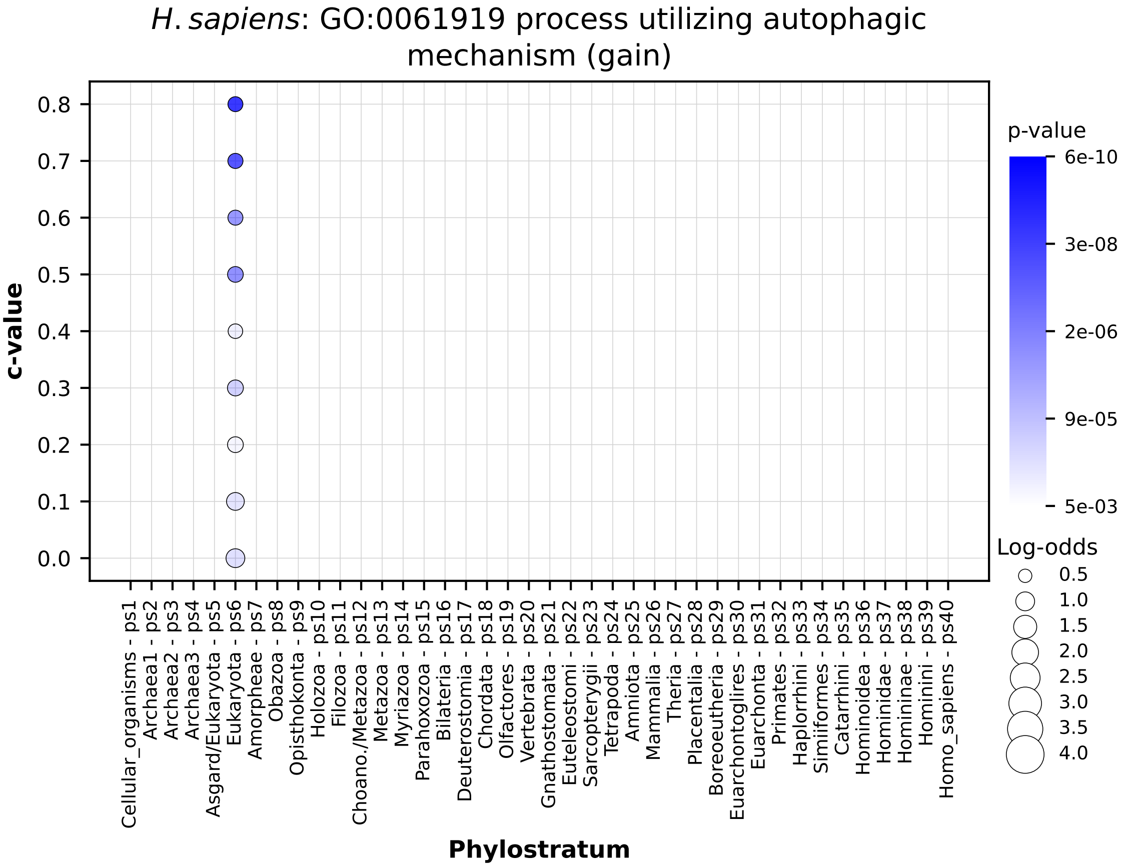

H. sapiens: GO:0061919 process utilizing autophagic mechanism (gain)
c-value
0.8
0.7
0.6
0.5
0.4
0.3
0.2
0.1
0.0
Phylostratum
Cellular_organisms - ps1
Archaea1 - ps2
Archaea2 - ps3
Archaea3 - ps4
Asgard/Eukaryota - ps5
Eukaryota - ps6
Amorpheae - ps7
Obazoa - ps8
Opisthokonta - ps9
Holozoa - ps10
Filozoa - ps11
Choano./Metazoa - ps12
Metazoa - ps13
Myriazoa - ps14
Parahoxozoa - ps15
Bilateria - ps16
Deuterostomia - ps17
Chordata - ps18
Olfactores - ps19
Vertebrata - ps20
Gnathostomata - ps21
Euteleostomi - ps22
Sarcopterygii - ps23
Tetrapoda - ps24
Amniota - ps25
Mammalia - ps26
Theria - ps27
Placentalia - ps28
Boreoeutheria - ps29
Euarchontoglires - ps30
Euarchonta - ps31
Primates - ps32
Haplorrhini - ps33
Simiiformes - ps34
Catarrhini - ps35
Hominoidea - ps36
Hominidae - ps37
Homininae - ps38
Hominini - ps39
Homo_sapiens - ps40
p-value
6e-10
3e-08
2e-06
9e-05
5e-03
Log-odds
0.5
1.0
1.5
2.0
2.5
3.0
3.5
4.0

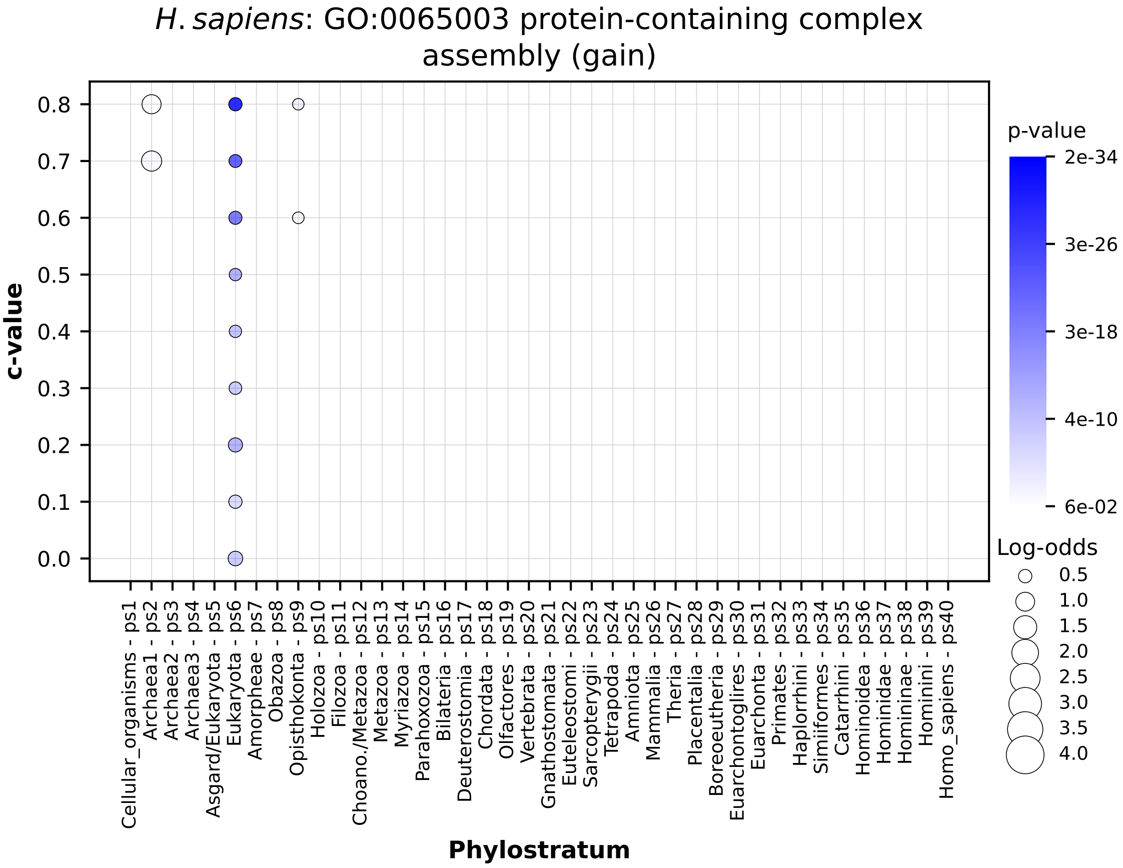

H. sapiens: GO:0065003 protein-containing complex assembly (gain)
c-value
0.0
0.1
0.2
0.3
0.4
0.5
0.6
0.7
0.8
Phylostratum
Cellular_organisms - ps1
Archaea1 - ps2
Archaea2 - ps3
Archaea3 - ps4
Asgard/Eukaryota - ps5
Eukaryota - ps6
Amorpheae - ps7
Obazoa - ps8
Opisthokonta - ps9
Holozoa - ps10
Filozoa - ps11
Choano./Metazoa - ps12
Metazoa - ps13
Myriazoa - ps14
Parahoxozoa - ps15
Bilateria - ps16
Deuterostomia - ps17
Chordata - ps18
Olfactores - ps19
Vertebrata - ps20
Gnathostomata - ps21
Euteleostomi - ps22
Sarcopterygii - ps23
Tetrapoda - ps24
Amniota - ps25
Mammalia - ps26
Theria - ps27
Placentalia - ps28
Boreoeutheria - ps29
Euarchontoglires - ps30
Euarchonta - ps31
Primates - ps32
Haplorrhini - ps33
Simiiformes - ps34
Catarrhini - ps35
Hominoidea - ps36
Hominidae - ps37
Homininae - ps38
Hominini - ps39
Homo_sapiens - ps40
p-value
2e-34
3e-26
3e-18
4e-10
6e-02
Log-odds
0.5
1.0
1.5
2.0
2.5
3.0
3.5
4.0

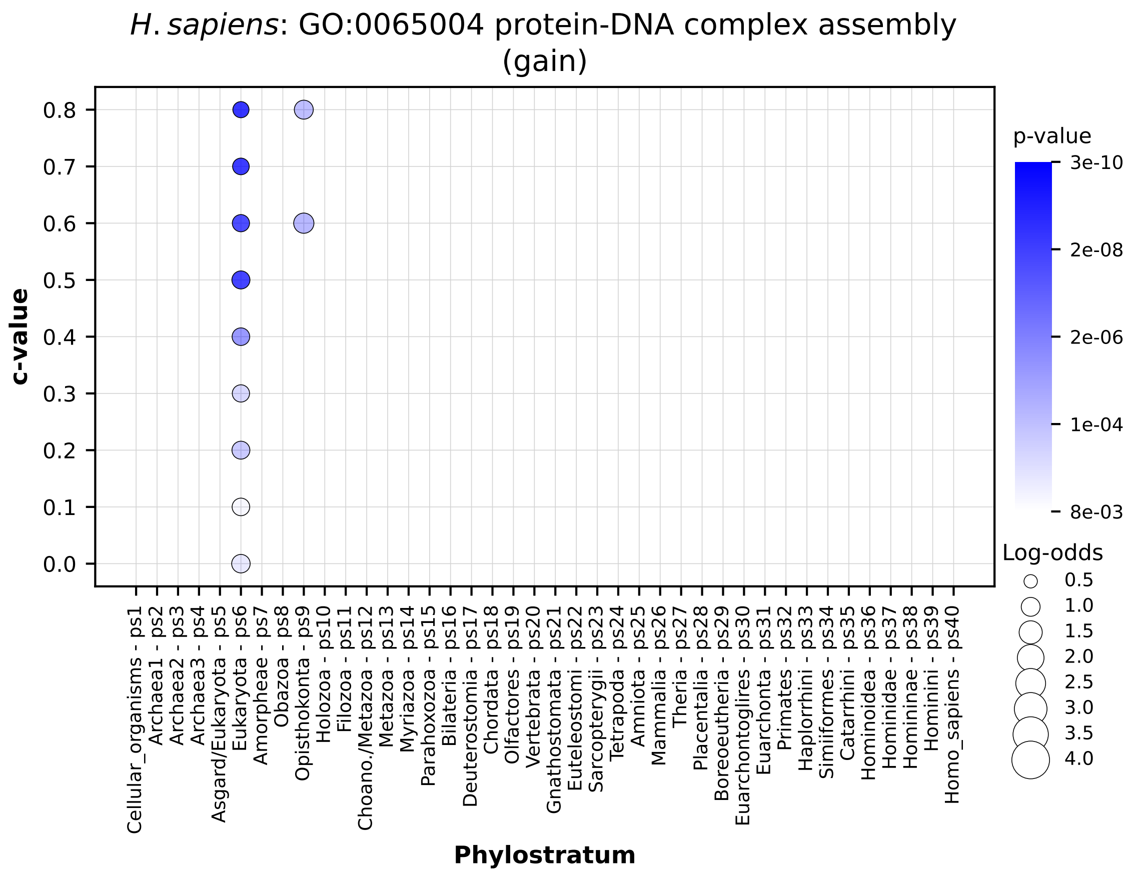
H. sapiens: GO:0065004 protein-DNA complex assembly (gain)
c-value
0.8
0.7
0.6
0.5
0.4
0.3
0.2
0.1
0.0
Phylostratum
Cellular_organisms - ps1
Archaea1 - ps2
Archaea2 - ps3
Archaea3 - ps4
Asgard/Eukaryota - ps5
Eukaryota - ps6
Amorpheae - ps7
Obazoa - ps8
Opisthokonta - ps9
Holozoa - ps10
Filozoa - ps11
Choano./Metazoa - ps12
Metazoa - ps13
Myriazoa - ps14
Parahoxozoa - ps15
Bilateria - ps16
Deuterostomia - ps17
Chordata - ps18
Olfactores - ps19
Vertebrata - ps20
Gnathostomata - ps21
Euteleostomi - ps22
Sarcopterygii - ps23
Tetrapoda - ps24
Amniota - ps25
Mammalia - ps26
Theria - ps27
Placentalia - ps28
Boreoeutheria - ps29
Euarchontoglires - ps30
Euarchonta - ps31
Primates - ps32
Haplorrhini - ps33
Simiiformes - ps34
Catarrhini - ps35
Hominoidea - ps36
Hominidae - ps37
Homininae - ps38
Hominini - ps39
Homo_sapiens - ps40
p-value
3e-10
2e-08
2e-06
1e-04
8e-03
Log-odds
0.5
1.0
1.5
2.0
2.5
3.0
3.5
4.0

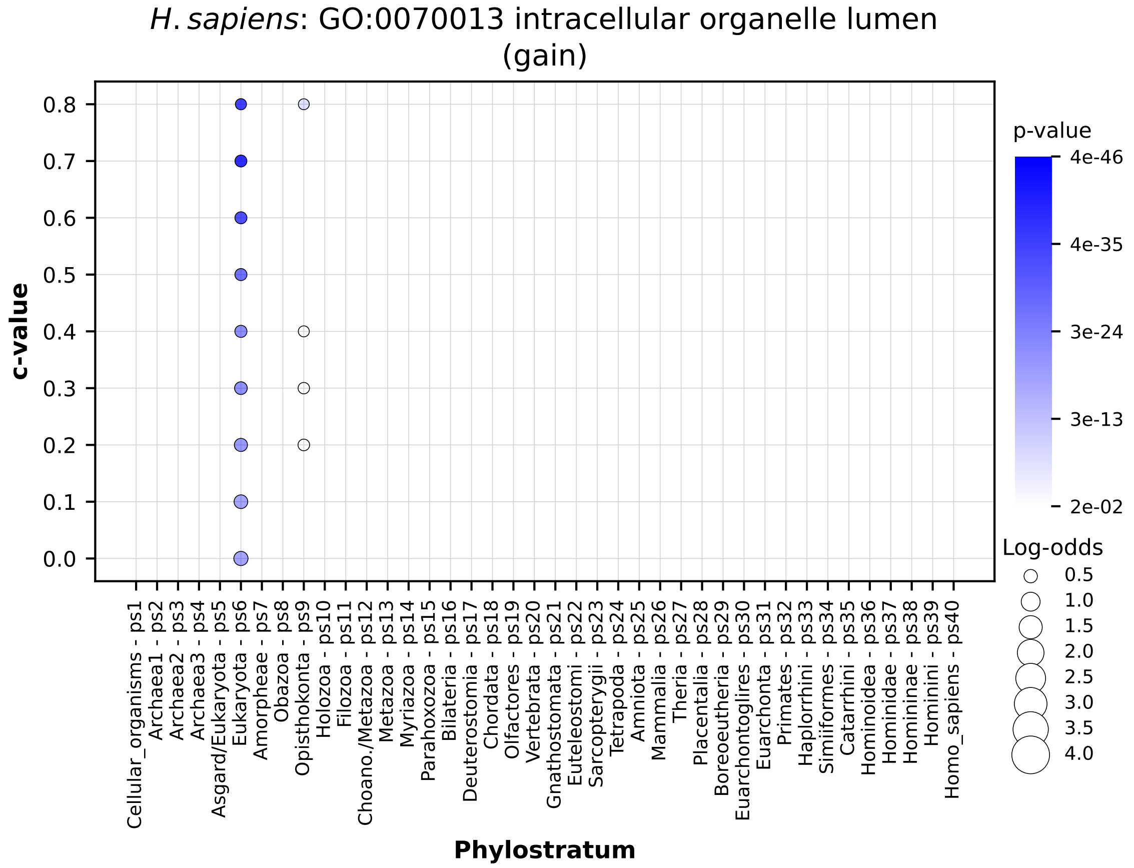
H. sapiens: GO:0070013 intracellular organelle lumen
(gain)
c-value
0.8
0.7
0.6
0.5
0.4
0.3
0.2
0.1
0.0
p-value
4e-46
4e-35
3e-24
3e-13
2e-02
Log-odds
0.5
1.0
1.5
2.0
2.5
3.0
3.5
4.0
Cellular_organisms - ps1
Archaea1 - ps2
Archaea2 - ps3
Archaea3 - ps4
Asgard/Eukaryota - ps5
Eukaryota - ps6
Amorpheae - ps7
Obazoa - ps8
Opisthokonta - ps9
Holozoa - ps10
Filozoa - ps11
Choano./Metazoa - ps12
Metazoa - ps13
Myriazoa - ps14
Parahoxozoa - ps15
Bilateria - ps16
Deuterostomia - ps17
Chordata - ps18
Olfactores - ps19
Vertebrata - ps20
Gnathostomata - ps21
Euteleostomi - ps22
Sarcopterygii - ps23
Tetrapoda - ps24
Amniota - ps25
Mammalia - ps26
Theria - ps27
Placentalia - ps28
Boreoeutheria - ps29
Euarchontoglires - ps30
Euarchonta - ps31
Primates - ps32
Haplorrhini - ps33
Simiiformes - ps34
Catarrhini - ps35
Hominoidea - ps36
Hominidae - ps37
Homininae - ps38
Hominini - ps39
Homo_sapiens - ps40
Phylostratum

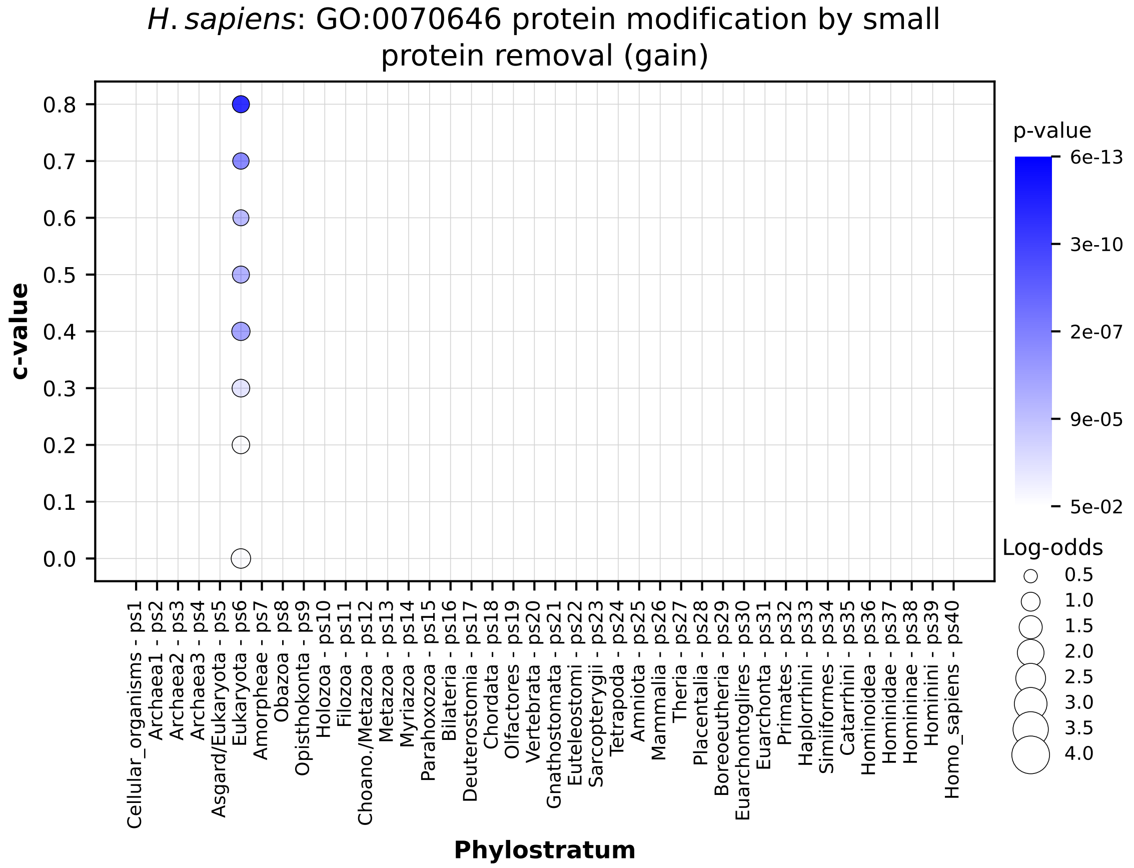

H. sapiens: GO:0070646 protein modification by small protein removal (gain)
c-value
0.0
0.1
0.2
0.3
0.4
0.5
0.6
0.7
0.8
p-value
6e-13
3e-10
2e-07
9e-05
5e-02
Log-odds
0.5
1.0
1.5
2.0
2.5
3.0
3.5
4.0
Cellular_organisms - ps1
Archaea1 - ps2
Archaea2 - ps3
Archaea3 - ps4
Asgard/Eukaryota - ps5
Eukaryota - ps6
Amorpheae - ps7
Obazoa - ps8
Opisthokonta - ps9
Holozoa - ps10
Filozoa - ps11
Choano./Metazoa - ps12
Metazoa - ps13
Myriazoa - ps14
Parahoxozoa - ps15
Bilateria - ps16
Deuterostomia - ps17
Chordata - ps18
Olfactores - ps19
Vertebrata - ps20
Gnathostomata - ps21
Euteleostomi - ps22
Sarcopterygii - ps23
Tetrapoda - ps24
Amniota - ps25
Mammalia - ps26
Theria - ps27
Placentalia - ps28
Boreoeutheria - ps29
Euarchontoglires - ps30
Euarchonta - ps31
Primates - ps32
Haplorrhini - ps33
Simiiformes - ps34
Catarrhini - ps35
Hominoidea - ps36
Hominidae - ps37
Homininae - ps38
Hominini - ps39
Homo_sapiens - ps40
Phylostratum

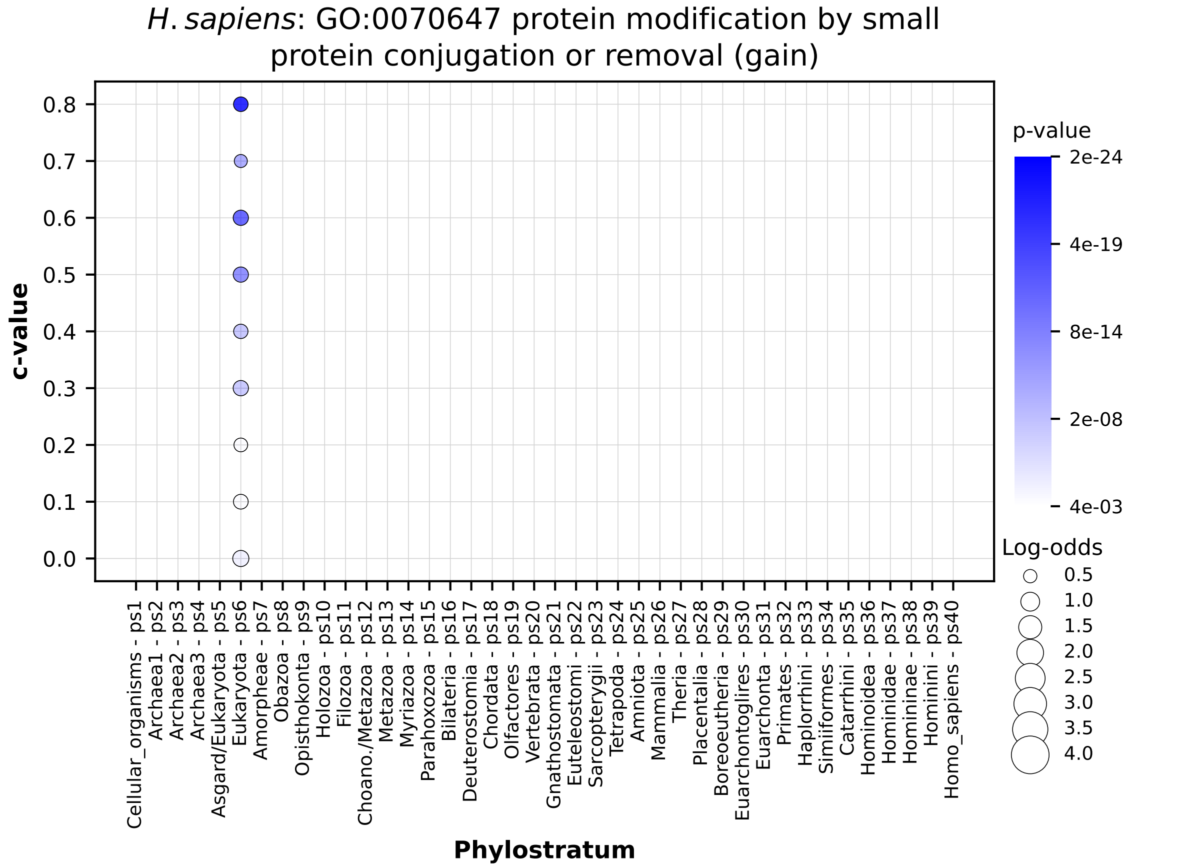

H. sapiens: GO:0070647 protein modification by small protein conjugation or removal (gain)
c-value
0.8
0.7
0.6
0.5
0.4
0.3
0.2
0.1
0.0
Phylostratum
Cellular_organisms - ps1
Archaea1 - ps2
Archaea2 - ps3
Archaea3 - ps4
Asgard/Eukaryota - ps5
Eukaryota - ps6
Amorpheae - ps7
Obazoa - ps8
Opisthokonta - ps9
Holozoa - ps10
Filozoa - ps11
Choano./Metazoa - ps12
Metazoa - ps13
Myriazoa - ps14
Parahoxozoa - ps15
Bilateria - ps16
Deuterostomia - ps17
Chordata - ps18
Olfactores - ps19
Vertebrata - ps20
Gnathostomata - ps21
Euteleostomi - ps22
Sarcopterygii - ps23
Tetrapoda - ps24
Amniota - ps25
Mammalia - ps26
Theria - ps27
Placentalia - ps28
Boreoeutheria - ps29
Euarchontoglires - ps30
Euarchonta - ps31
Primates - ps32
Haplorrhini - ps33
Simiiformes - ps34
Catarrhini - ps35
Hominoidea - ps36
Hominidae - ps37
Homininae - ps38
Hominini - ps39
Homo_sapiens - ps40
p-value
2e-24
4e-19
8e-14
2e-08
4e-03
Log-odds
0.5
1.0
1.5
2.0
2.5
3.0
3.5
4.0

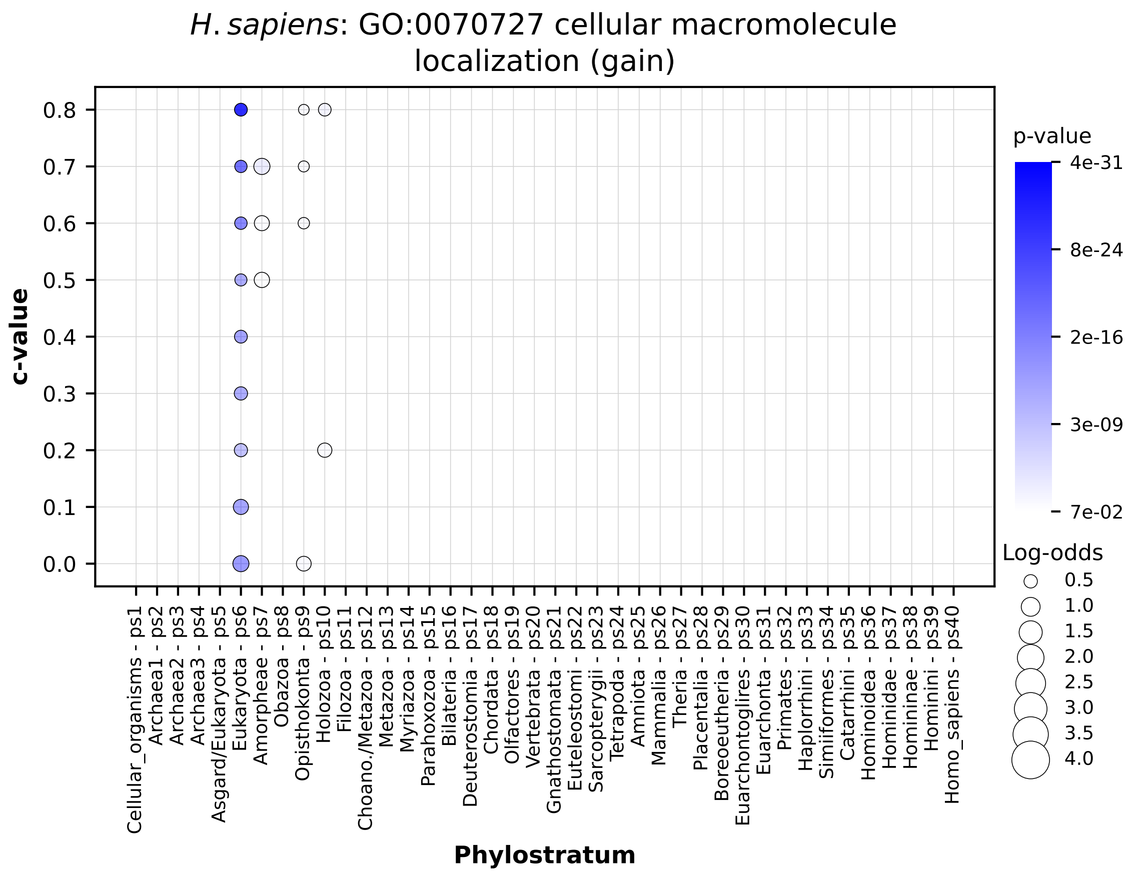
H. sapiens: GO:0070727 cellular macromolecule localization (gain)
c-value
0.8
0.7
0.6
0.5
0.4
0.3
0.2
0.1
0.0
p-value
4e-31
8e-24
2e-16
3e-09
7e-02
Log-odds
0.5
1.0
1.5
2.0
2.5
3.0
3.5
4.0
Cellular_organisms - ps1
Archaea1 - ps2
Archaea2 - ps3
Archaea3 - ps4
Asgard/Eukaryota - ps5
Eukaryota - ps6
Amorpheae - ps7
Obazoa - ps8
Opisthokonta - ps9
Holozoa - ps10
Filozoa - ps11
Choano./Metazoa - ps12
Metazoa - ps13
Myriazoa - ps14
Parahoxozoa - ps15
Bilateria - ps16
Deuterostomia - ps17
Chordata - ps18
Olfactores - ps19
Vertebrata - ps20
Gnathostomata - ps21
Euteleostomi - ps22
Sarcopterygii - ps23
Tetrapoda - ps24
Amniota - ps25
Mammalia - ps26
Theria - ps27
Placentalia - ps28
Boreoeutheria - ps29
Euarchontoglires - ps30
Euarchonta - ps31
Primates - ps32
Haplorrhini - ps33
Simiiformes - ps34
Catarrhini - ps35
Hominoidea - ps36
Hominidae - ps37
Homininae - ps38
Hominini - ps39
Homo_sapiens - ps40
Phylostratum

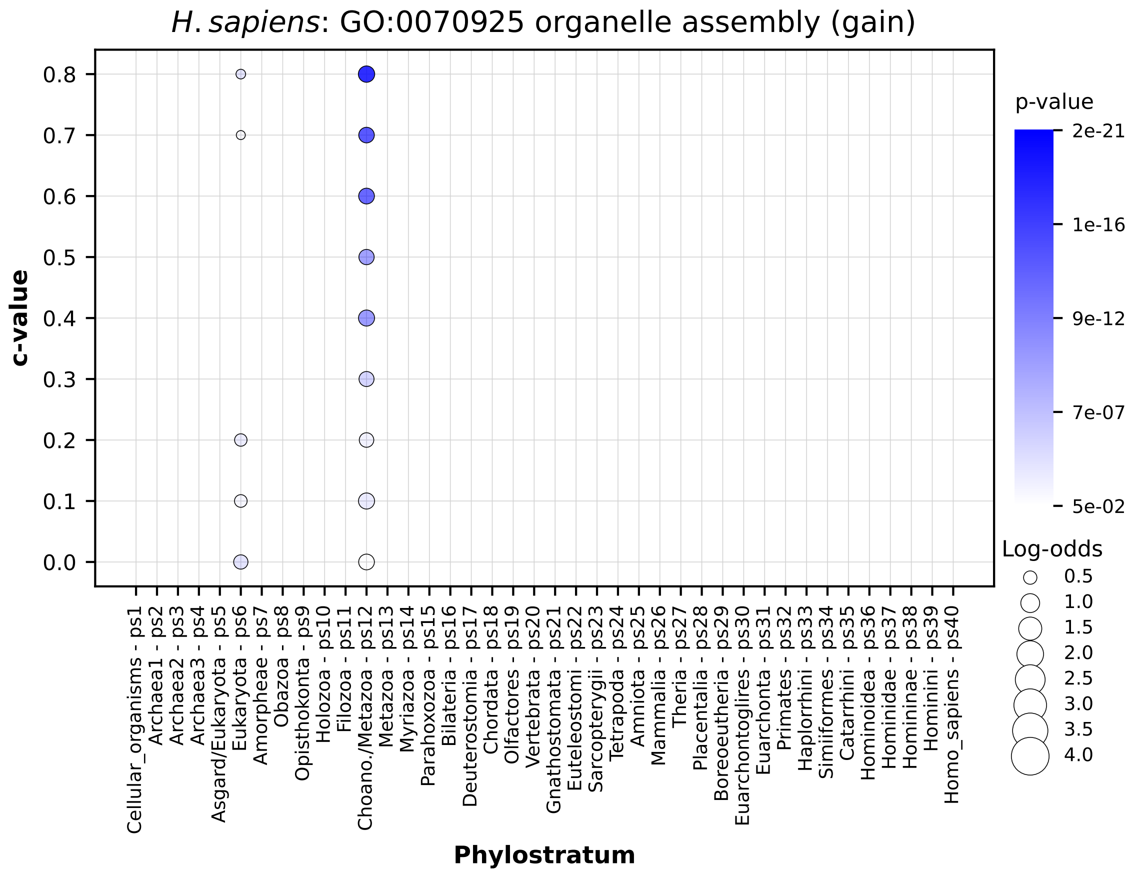
H. sapiens: GO:0070925 organelle assembly (gain)
c-value
0.0
0.1
0.2
0.3
0.4
0.5
0.6
0.7
0.8
Phylostratum
Cellular_organisms - ps1
Archaea1 - ps2
Archaea2 - ps3
Archaea3 - ps4
Asgard/Eukaryota - ps5
Eukaryota - ps6
Amorpheae - ps7
Obazoa - ps8
Opisthokonta - ps9
Holozoa - ps10
Filozoa - ps11
Choano./Metazoa - ps12
Metazoa - ps13
Myriazoa - ps14
Parahoxozoa - ps15
Bilateria - ps16
Deuterostomia - ps17
Chordata - ps18
Olfactores - ps19
Vertebrata - ps20
Gnathostomata - ps21
Euteleostomi - ps22
Sarcopterygii - ps23
Tetrapoda - ps24
Amniota - ps25
Mammalia - ps26
Theria - ps27
Placentalia - ps28
Boreoeutheria - ps29
Euarchontoglires - ps30
Euarchonta - ps31
Primates - ps32
Haplorrhini - ps33
Simiiformes - ps34
Catarrhini - ps35
Hominoidea - ps36
Hominidae - ps37
Homininae - ps38
Hominini - ps39
Homo_sapiens - ps40
p-value
2e-21
1e-16
9e-12
7e-07
5e-02
Log-odds
0.5
1.0
1.5
2.0
2.5
3.0
3.5
4.0

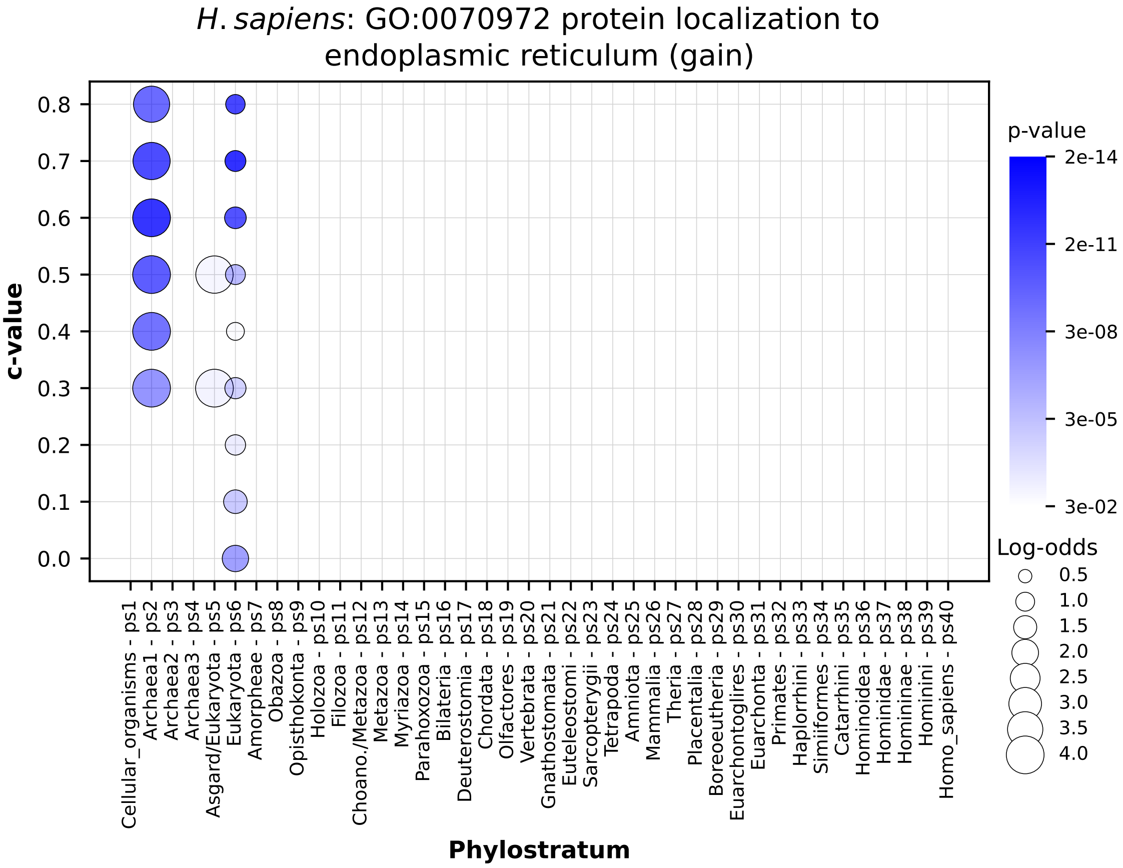

H. sapiens: GO:0070972 protein localization to endoplasmic reticulum (gain)
c-value
0.8
0.7
0.6
0.5
0.4
0.3
0.2
0.1
0.0
p-value
2e-14
2e-11
3e-08
3e-05
3e-02
Log-odds
0.5
1.0
1.5
2.0
2.5
3.0
3.5
4.0
Cellular_organisms - ps1
Archaea1 - ps2
Archaea2 - ps3
Archaea3 - ps4
Asgard/Eukaryota - ps5
Eukaryota - ps6
Amorpheae - ps7
Obazoa - ps8
Opisthokonta - ps9
Holozoa - ps10
Filozoa - ps11
Choano./Metazoa - ps12
Metazoa - ps13
Myriazoa - ps14
Parahoxozoa - ps15
Bilateria - ps16
Deuterostomia - ps17
Chordata - ps18
Olfactores - ps19
Vertebrata - ps20
Gnathostomata - ps21
Euteleostomi - ps22
Sarcopterygii - ps23
Tetrapoda - ps24
Amniota - ps25
Mammalia - ps26
Theria - ps27
Placentalia - ps28
Boreoeutheria - ps29
Euarchontoglires - ps30
Euarchonta - ps31
Primates - ps32
Haplorrhini - ps33
Simiiformes - ps34
Catarrhini - ps35
Hominoidea - ps36
Hominidae - ps37
Homininae - ps38
Hominini - ps39
Homo_sapiens - ps40
Phylostratum

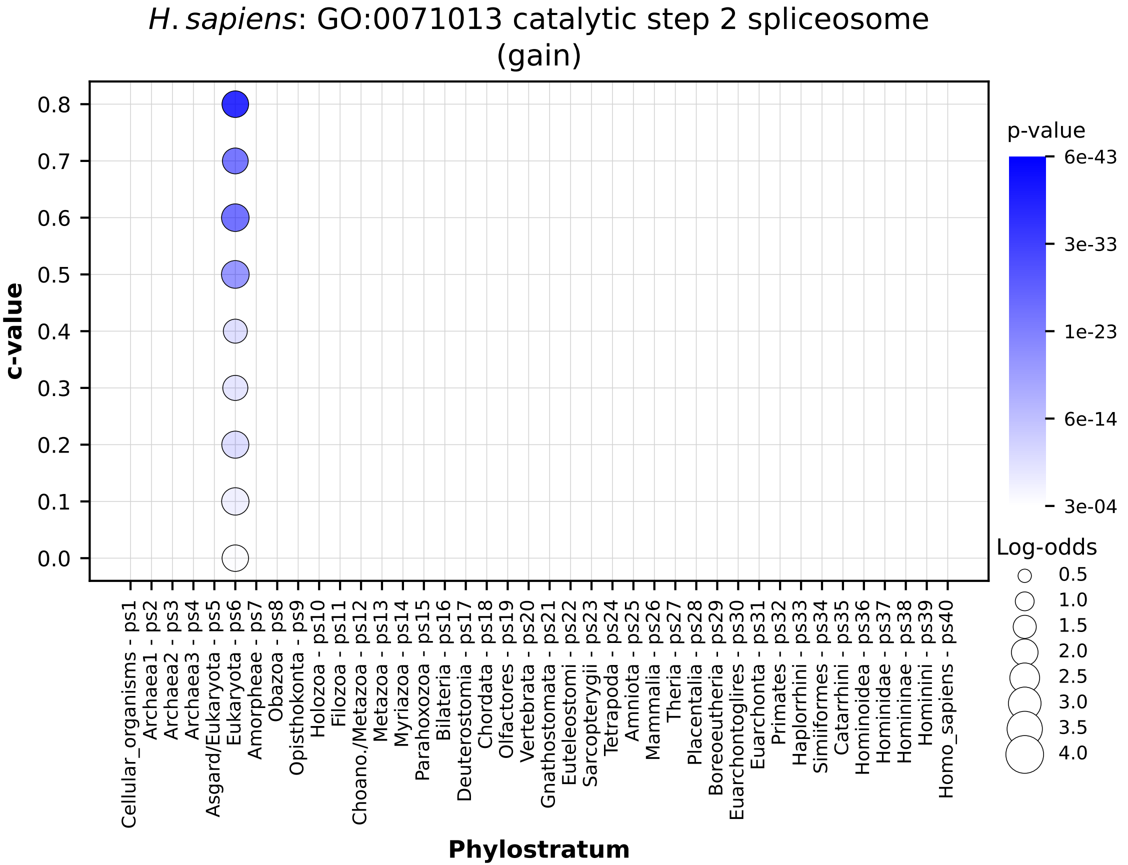

H. sapiens: GO:0071013 catalytic step 2 spliceosome
(gain)
c-value
0.8
0.7
0.6
0.5
0.4
0.3
0.2
0.1
0.0
Phylostratum
Cellular_organisms - ps1
Archaea1 - ps2
Archaea2 - ps3
Archaea3 - ps4
Asgard/Eukaryota - ps5
Eukaryota - ps6
Amorpheae - ps7
Obazoa - ps8
Opisthokonta - ps9
Holozoa - ps10
Filozoa - ps11
Choano./Metazoa - ps12
Metazoa - ps13
Myriazoa - ps14
Parahoxozoa - ps15
Bilateria - ps16
Deuterostomia - ps17
Chordata - ps18
Olfactores - ps19
Vertebrata - ps20
Gnathostomata - ps21
Euteleostomi - ps22
Sarcopterygii - ps23
Tetrapoda - ps24
Amniota - ps25
Mammalia - ps26
Theria - ps27
Placentalia - ps28
Boreoeutheria - ps29
Euarchontoglires - ps30
Euarchonta - ps31
Primates - ps32
Haplorrhini - ps33
Simiiformes - ps34
Catarrhini - ps35
Hominoidea - ps36
Hominidae - ps37
Homininae - ps38
Hominini - ps39
Homo_sapiens - ps40
p-value
6e-43
3e-33
1e-23
6e-14
3e-04
Log-odds
0.5
1.0
1.5
2.0
2.5
3.0
3.5
4.0

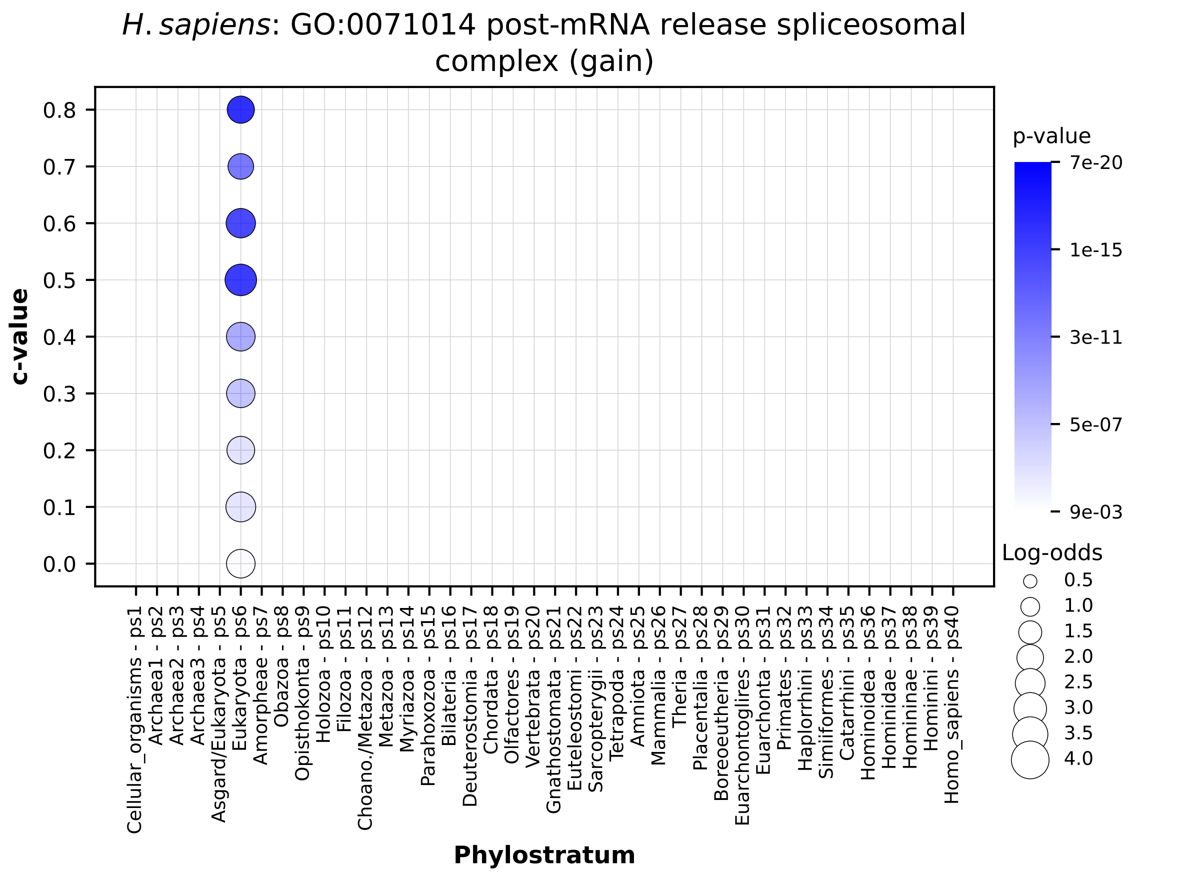

H. sapiens: GO:0071014 post-mRNA release spliceosomal complex (gain)
c-value
0.8
0.7
0.6
0.5
0.4
0.3
0.2
0.1
0.0
p-value
7e-20
1e-15
3e-11
5e-07
9e-03
Log-odds
0.5
1.0
1.5
2.0
2.5
3.0
3.5
4.0
Cellular_organisms - ps1
Archaea1 - ps2
Archaea2 - ps3
Archaea3 - ps4
Asgard/Eukaryota - ps5
Eukaryota - ps6
Amorpheae - ps7
Obazoa - ps8
Opisthokonta - ps9
Holozoa - ps10
Filozoa - ps11
Choano./Metazoa - ps12
Metazoa - ps13
Myriazoa - ps14
Parahoxozoa - ps15
Bilateria - ps16
Deuterostomia - ps17
Chordata - ps18
Olfactores - ps19
Vertebrata - ps20
Gnathostomata - ps21
Euteleostomi - ps22
Sarcopterygii - ps23
Tetrapoda - ps24
Amniota - ps25
Mammalia - ps26
Theria - ps27
Placentalia - ps28
Boreoeutheria - ps29
Euarchontoglires - ps30
Euarchonta - ps31
Primates - ps32
Haplorrhini - ps33
Simiiformes - ps34
Catarrhini - ps35
Hominoidea - ps36
Hominidae - ps37
Homininae - ps38
Hominini - ps39
Homo_sapiens - ps40
Phylostratum

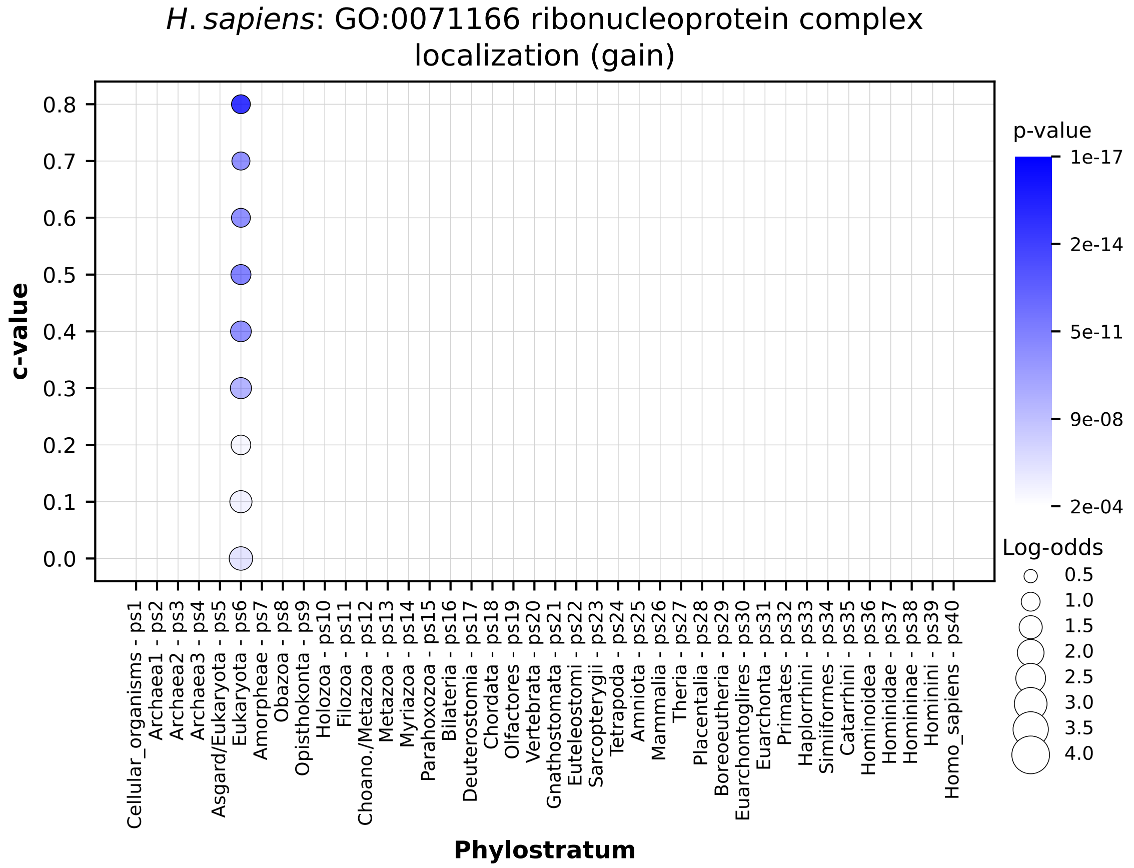

$H. sapiens$: GO:0071166 ribonucleoprotein complex localization (gain)
c-value
0.8
0.7
0.6
0.5
0.4
0.3
0.2
0.1
0.0
p-value
1e-17
2e-14
5e-11
9e-08
2e-04
Log-odds
0.5
1.0
1.5
2.0
2.5
3.0
3.5
4.0
Cellular_organisms - ps1
Archaea1 - ps2
Archaea2 - ps3
Archaea3 - ps4
Asgard/Eukaryota - ps5
Eukaryota - ps6
Amorpheae - ps7
Obazoa - ps8
Opisthokonta - ps9
Holozoa - ps10
Filozoa - ps11
Choano./Metazoa - ps12
Metazoa - ps13
Myriazoa - ps14
Parahoxozoa - ps15
Bilateria - ps16
Deuterostomia - ps17
Chordata - ps18
Olfactores - ps19
Vertebrata - ps20
Gnathostomata - ps21
Euteleostomi - ps22
Sarcopterygii - ps23
Tetrapoda - ps24
Amniota - ps25
Mammalia - ps26
Theria - ps27
Placentalia - ps28
Boreoeutheria - ps29
Euarchontoglires - ps30
Euarchonta - ps31
Primates - ps32
Haplorrhini - ps33
Simiiformes - ps34
Catarrhini - ps35
Hominoidea - ps36
Hominidae - ps37
Homininae - ps38
Hominini - ps39
Homo_sapiens - ps40
Phylostratum

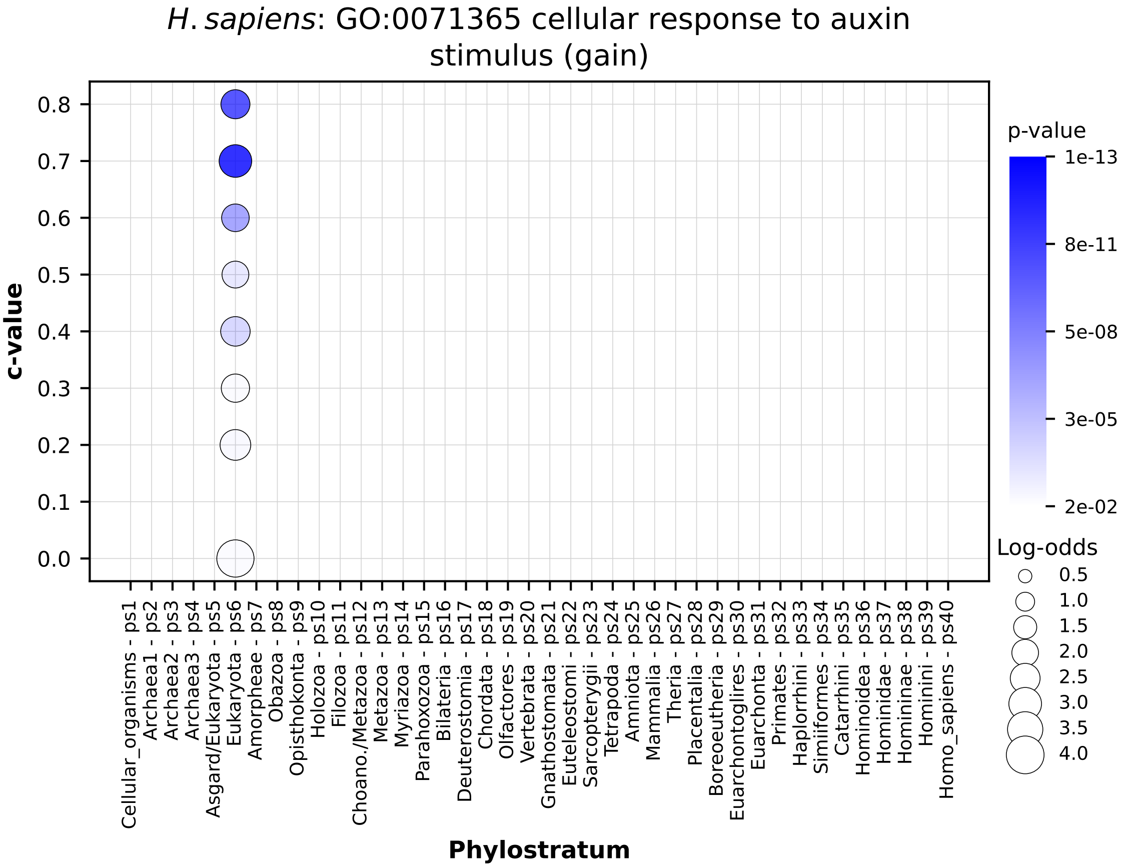

H. sapiens: GO:0071365 cellular response to auxin stimulus (gain)
c-value
0.0
0.1
0.2
0.3
0.4
0.5
0.6
0.7
0.8
Phylostratum
Cellular_organisms - ps1
Archaea1 - ps2
Archaea2 - ps3
Archaea3 - ps4
Asgard/Eukaryota - ps5
Eukaryota - ps6
Amorpheae - ps7
Obazoa - ps8
Opisthokonta - ps9
Holozoa - ps10
Filozoa - ps11
Choano./Metazoa - ps12
Metazoa - ps13
Myriazoa - ps14
Parahoxozoa - ps15
Bilateria - ps16
Deuterostomia - ps17
Chordata - ps18
Olfactores - ps19
Vertebrata - ps20
Gnathostomata - ps21
Euteleostomi - ps22
Sarcopterygii - ps23
Tetrapoda - ps24
Amniota - ps25
Mammalia - ps26
Theria - ps27
Placentalia - ps28
Boreoeutheria - ps29
Euarchontoglires - ps30
Euarchonta - ps31
Primates - ps32
Haplorrhini - ps33
Simiiformes - ps34
Catarrhini - ps35
Hominoidea - ps36
Hominidae - ps37
Homininae - ps38
Hominini - ps39
Homo_sapiens - ps40
p-value
1e-13
8e-11
5e-08
3e-05
2e-02
Log-odds
0.5
1.0
1.5
2.0
2.5
3.0
3.5
4.0

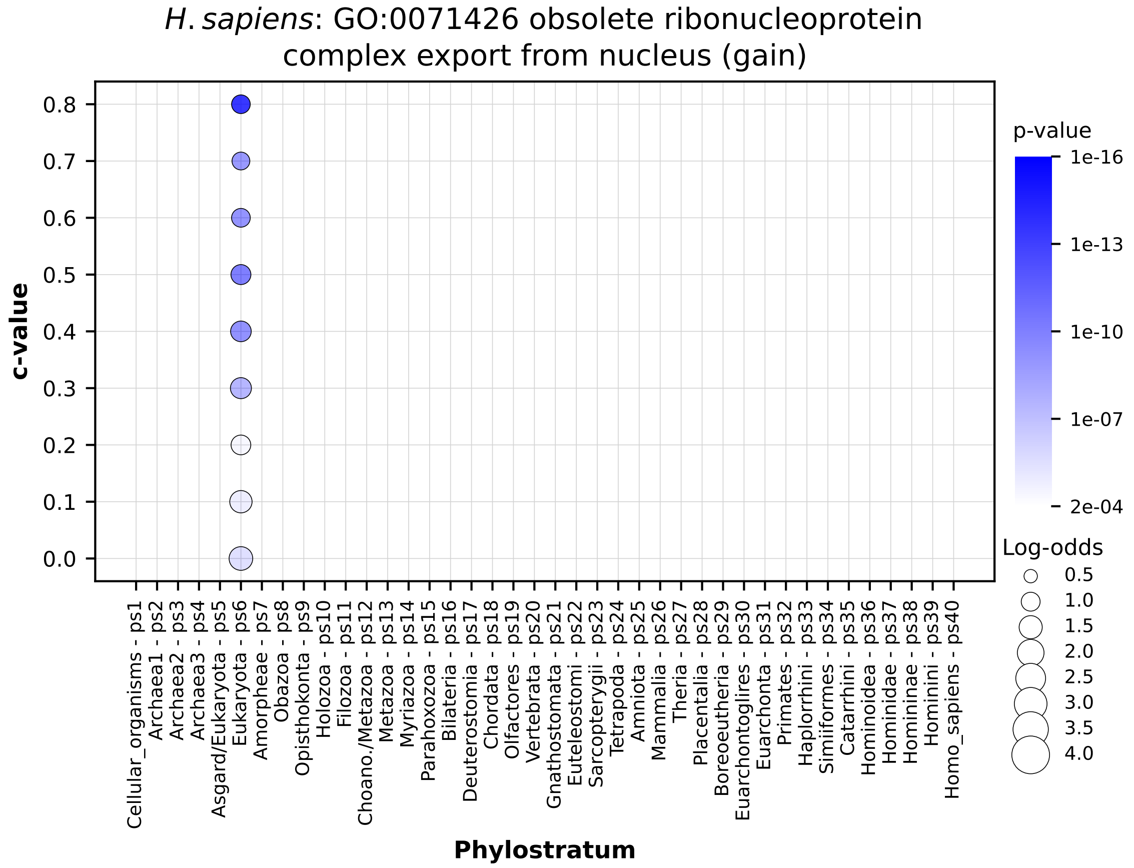

H. sapiens: GO:0071426 obsolete ribonucleoprotein complex export from nucleus (gain)
c-value
0.8
0.7
0.6
0.5
0.4
0.3
0.2
0.1
0.0
p-value
1e-16
1e-13
1e-10
1e-07
2e-04
Log-odds
0.5
1.0
1.5
2.0
2.5
3.0
3.5
4.0
Cellular_organisms - ps1
Archaea1 - ps2
Archaea2 - ps3
Archaea3 - ps4
Asgard/Eukaryota - ps5
Eukaryota - ps6
Amorpheae - ps7
Obazoa - ps8
Opisthokonta - ps9
Holozoa - ps10
Filozoa - ps11
Choano./Metazoa - ps12
Metazoa - ps13
Myriazoa - ps14
Parahoxozoa - ps15
Bilateria - ps16
Deuterostomia - ps17
Chordata - ps18
Olfactores - ps19
Vertebrata - ps20
Gnathostomata - ps21
Euteleostomi - ps22
Sarcopterygii - ps23
Tetrapoda - ps24
Amniota - ps25
Mammalia - ps26
Theria - ps27
Placentalia - ps28
Boreoeutheria - ps29
Euarchontoglires - ps30
Euarchonta - ps31
Primates - ps32
Haplorrhini - ps33
Simiiformes - ps34
Catarrhini - ps35
Hominoidea - ps36
Hominidae - ps37
Homininae - ps38
Hominini - ps39
Homo_sapiens - ps40
Phylostratum

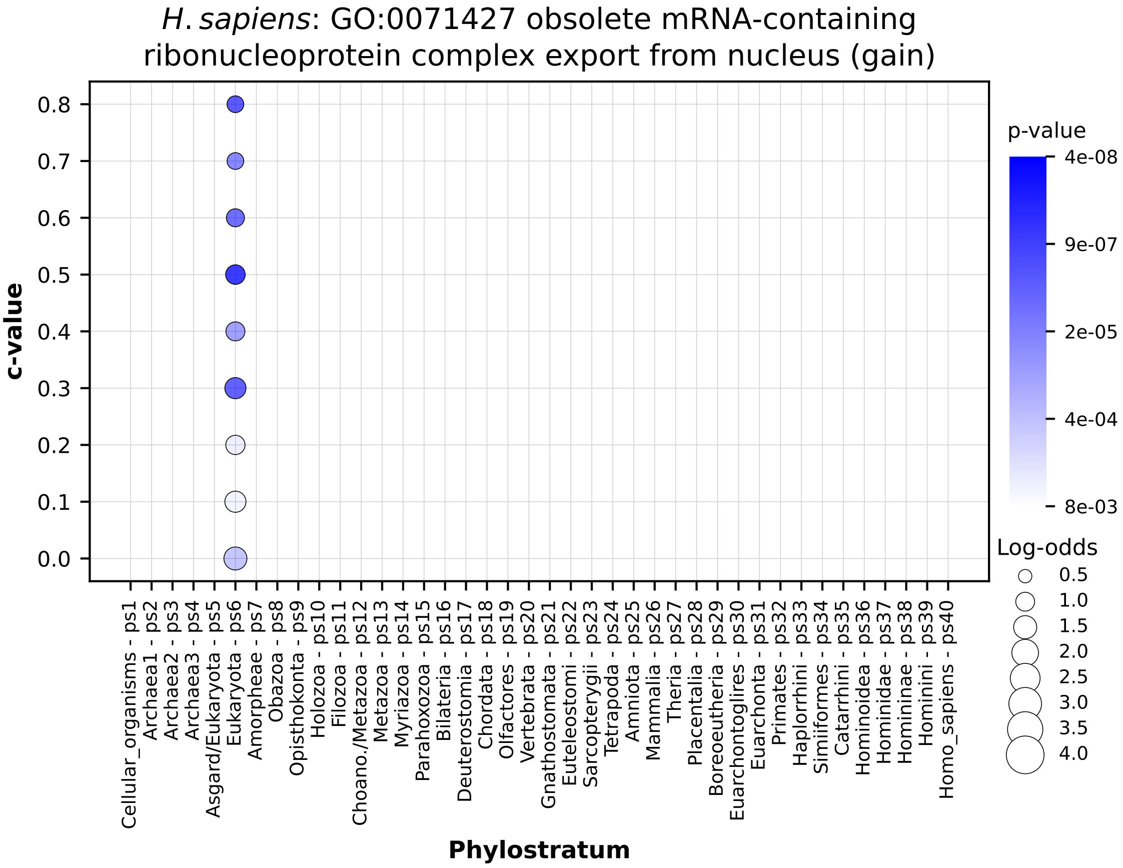

*H. sapiens*: GO:0071427 obsolete mRNA-containing ribonucleoprotein complex export from nucleus (gain)
c-value
0.8
0.7
0.6
0.5
0.4
0.3
0.2
0.1
0.0
p-value
4e-08
9e-07
2e-05
4e-04
8e-03
Log-odds
0.5
1.0
1.5
2.0
2.5
3.0
3.5
4.0
Cellular_organisms - ps1
Archaea1 - ps2
Archaea2 - ps3
Archaea3 - ps4
Asgard/Eukaryota - ps5
Eukaryota - ps6
Amorpheae - ps7
Obazoa - ps8
Opisthokonta - ps9
Holozoa - ps10
Filozoa - ps11
Choano./Metazoa - ps12
Metazoa - ps13
Myriazoa - ps14
Parahoxozoa - ps15
Bilateria - ps16
Deuterostomia - ps17
Chordata - ps18
Olfactores - ps19
Vertebrata - ps20
Gnathostomata - ps21
Euteleostomi - ps22
Sarcopterygii - ps23
Tetrapoda - ps24
Amniota - ps25
Mammalia - ps26
Theria - ps27
Placentalia - ps28
Boreoeutheria - ps29
Euarchontoglires - ps30
Euarchonta - ps31
Primates - ps32
Haplorrhini - ps33
Simiiformes - ps34
Catarrhini - ps35
Hominoidea - ps36
Hominidae - ps37
Homininae - ps38
Hominini - ps39
Homo_sapiens - ps40
Phylostratum

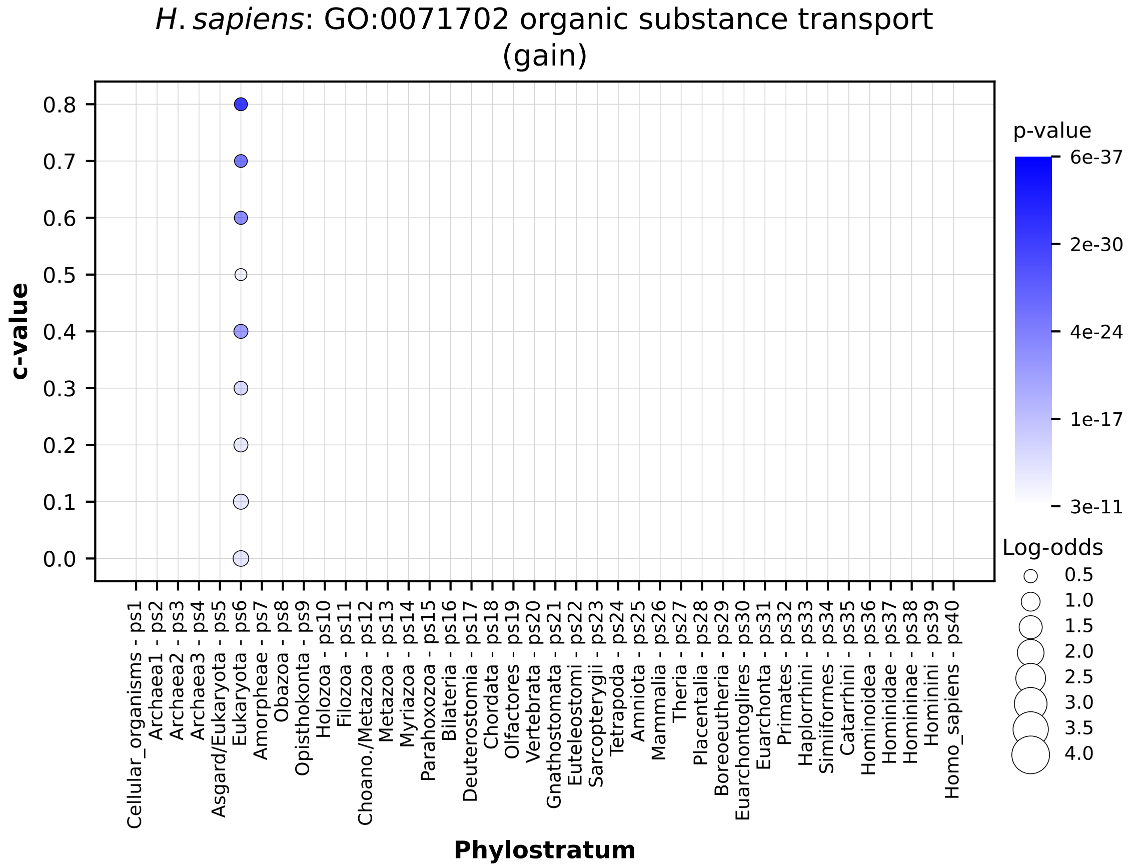
H. sapiens: GO:0071702 organic substance transport
(gain)
c-value
0.8
0.7
0.6
0.5
0.4
0.3
0.2
0.1
0.0
p-value
6e-37
2e-30
4e-24
1e-17
3e-11
Log-odds
0.5
1.0
1.5
2.0
2.5
3.0
3.5
4.0
Cellular_organisms - ps1
Archaea1 - ps2
Archaea2 - ps3
Archaea3 - ps4
Asgard/Eukaryota - ps5
Eukaryota - ps6
Amorpheae - ps7
Obazoa - ps8
Opisthokonta - ps9
Holozoa - ps10
Filozoa - ps11
Choano./Metazoa - ps12
Metazoa - ps13
Myriazoa - ps14
Parahoxozoa - ps15
Bilateria - ps16
Deuterostomia - ps17
Chordata - ps18
Olfactores - ps19
Vertebrata - ps20
Gnathostomata - ps21
Euteleostomi - ps22
Sarcopterygii - ps23
Tetrapoda - ps24
Amniota - ps25
Mammalia - ps26
Theria - ps27
Placentalia - ps28
Boreoeutheria - ps29
Euarchontoglires - ps30
Euarchonta - ps31
Primates - ps32
Haplorrhini - ps33
Simiiformes - ps34
Catarrhini - ps35
Hominoidea - ps36
Hominidae - ps37
Homininae - ps38
Hominini - ps39
Homo_sapiens - ps40
Phylostratum

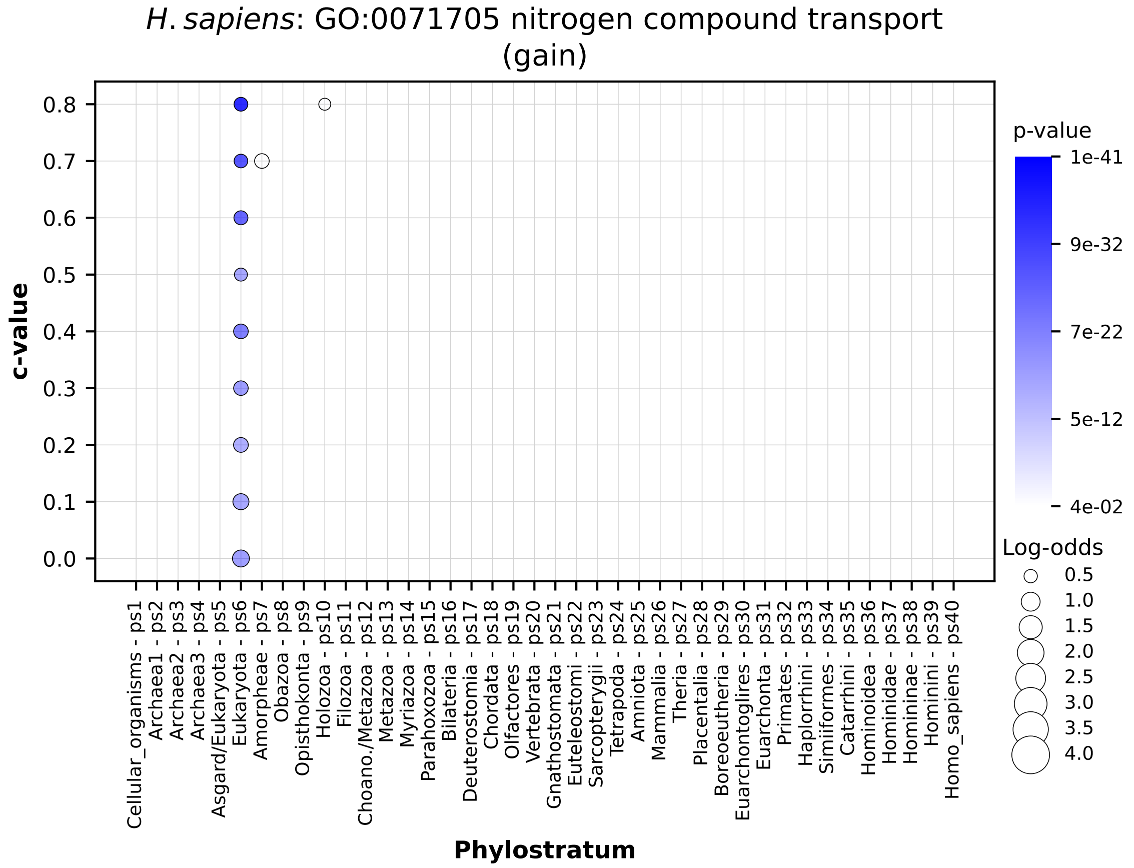

H. sapiens: GO:0071705 nitrogen compound transport
(gain)
c-value
0.8
0.7
0.6
0.5
0.4
0.3
0.2
0.1
0.0
Phylostratum
Cellular_organisms - ps1
Archaea1 - ps2
Archaea2 - ps3
Archaea3 - ps4
Asgard/Eukaryota - ps5
Eukaryota - ps6
Amorpheae - ps7
Obazoa - ps8
Opisthokonta - ps9
Holozoa - ps10
Filozoa - ps11
Choano./Metazoa - ps12
Metazoa - ps13
Myriazoa - ps14
Parahoxozoa - ps15
Bilateria - ps16
Deuterostomia - ps17
Chordata - ps18
Olfactores - ps19
Vertebrata - ps20
Gnathostomata - ps21
Euteleostomi - ps22
Sarcopterygii - ps23
Tetrapoda - ps24
Amniota - ps25
Mammalia - ps26
Theria - ps27
Placentalia - ps28
Boreoeutheria - ps29
Euarchontoglires - ps30
Euarchonta - ps31
Primates - ps32
Haplorrhini - ps33
Simiiformes - ps34
Catarrhini - ps35
Hominoidea - ps36
Hominidae - ps37
Homininae - ps38
Hominini - ps39
Homo_sapiens - ps40
p-value
1e-41
9e-32
7e-22
5e-12
4e-02
Log-odds
0.5
1.0
1.5
2.0
2.5
3.0
3.5
4.0

*H. sapiens*: GO:0071824 protein-DNA complex organization (gain)

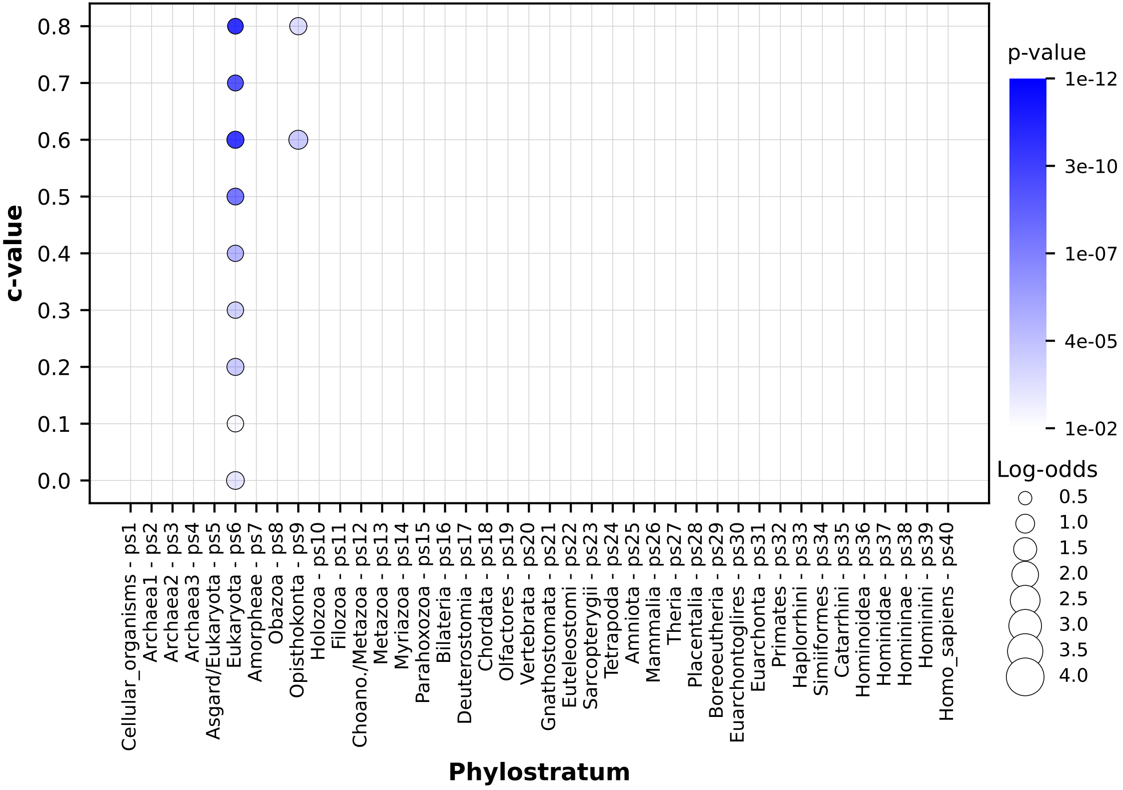

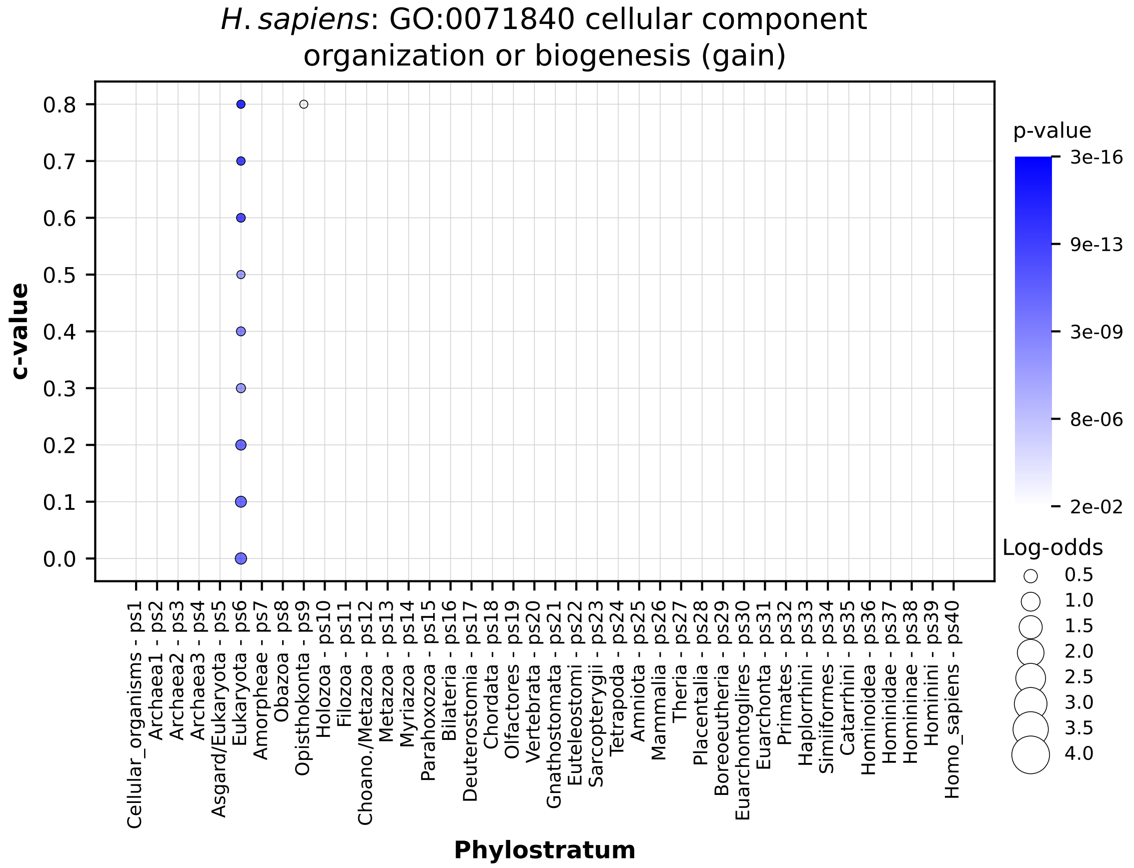
H. sapiens: GO:0071840 cellular component organization or biogenesis (gain)
c-value
0.8
0.7
0.6
0.5
0.4
0.3
0.2
0.1
0.0
p-value
3e-16
9e-13
3e-09
8e-06
2e-02
Log-odds
0.5
1.0
1.5
2.0
2.5
3.0
3.5
4.0
Cellular_organisms - ps1
Archaea1 - ps2
Archaea2 - ps3
Archaea3 - ps4
Asgard/Eukaryota - ps5
Eukaryota - ps6
Amorpheae - ps7
Obazoa - ps8
Opisthokonta - ps9
Holozoa - ps10
Filozoa - ps11
Choano./Metazoa - ps12
Metazoa - ps13
Myriazoa - ps14
Parahoxozoa - ps15
Bilateria - ps16
Deuterostomia - ps17
Chordata - ps18
Olfactores - ps19
Vertebrata - ps20
Gnathostomata - ps21
Euteleostomi - ps22
Sarcopterygii - ps23
Tetrapoda - ps24
Amniota - ps25
Mammalia - ps26
Theria - ps27
Placentalia - ps28
Boreoeutheria - ps29
Euarchontoglires - ps30
Euarchonta - ps31
Primates - ps32
Haplorrhini - ps33
Simiiformes - ps34
Catarrhini - ps35
Hominoidea - ps36
Hominidae - ps37
Homininae - ps38
Hominini - ps39
Homo_sapiens - ps40
Phylostratum

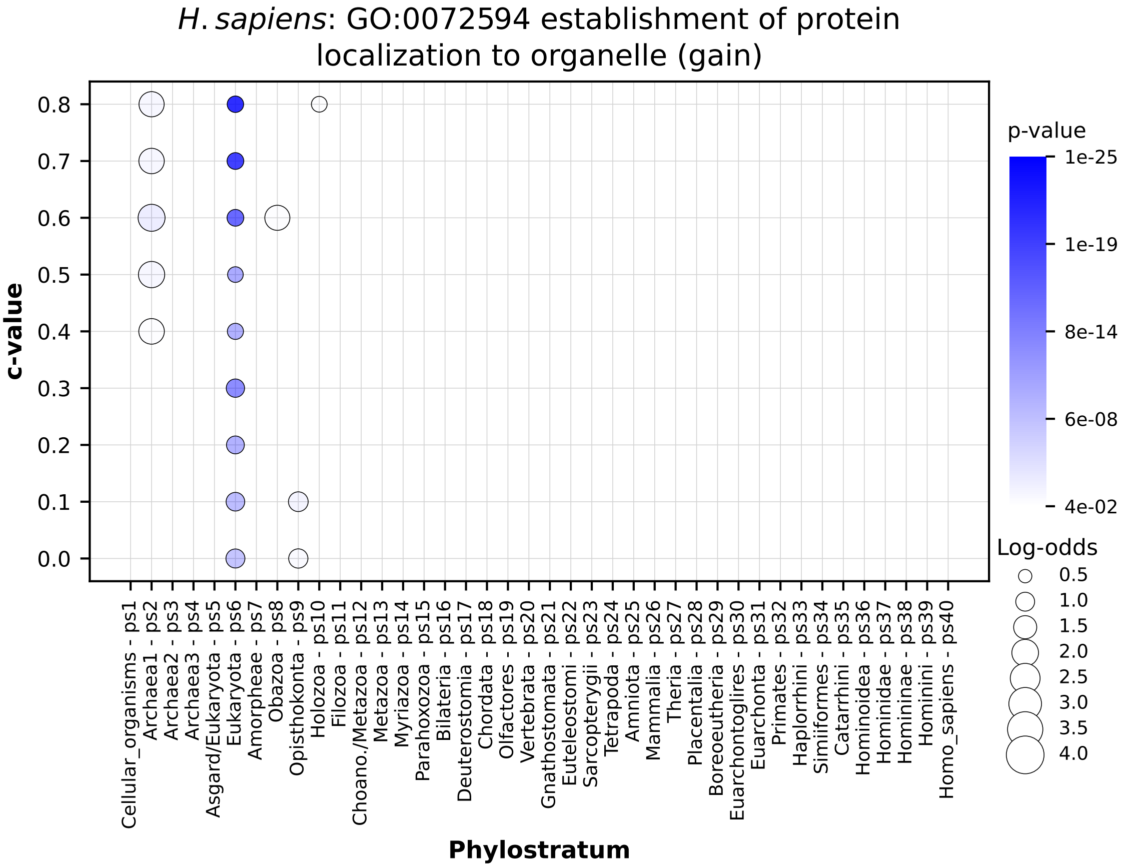

H. sapiens: GO:0072594 establishment of protein localization to organelle (gain)
c-value
0.8
0.7
0.6
0.5
0.4
0.3
0.2
0.1
0.0
Phylostratum
Cellular_organisms - ps1
Archaea1 - ps2
Archaea2 - ps3
Archaea3 - ps4
Asgard/Eukaryota - ps5
Eukaryota - ps6
Amorpheae - ps7
Obazoa - ps8
Opisthokonta - ps9
Holozoa - ps10
Filozoa - ps11
Choano./Metazoa - ps12
Metazoa - ps13
Myriazoa - ps14
Parahoxozoa - ps15
Bilateria - ps16
Deuterostomia - ps17
Chordata - ps18
Olfactores - ps19
Vertebrata - ps20
Gnathostomata - ps21
Euteleostomi - ps22
Sarcopterygii - ps23
Tetrapoda - ps24
Amniota - ps25
Mammalia - ps26
Theria - ps27
Placentalia - ps28
Boreoeutheria - ps29
Euarchontoglires - ps30
Euarchonta - ps31
Primates - ps32
Haplorrhini - ps33
Simiiformes - ps34
Catarrhini - ps35
Hominoidea - ps36
Hominidae - ps37
Homininae - ps38
Hominini - ps39
Homo_sapiens - ps40
p-value
1e-25
1e-19
8e-14
6e-08
4e-02
Log-odds
0.5
1.0
1.5
2.0
2.5
3.0
3.5
4.0

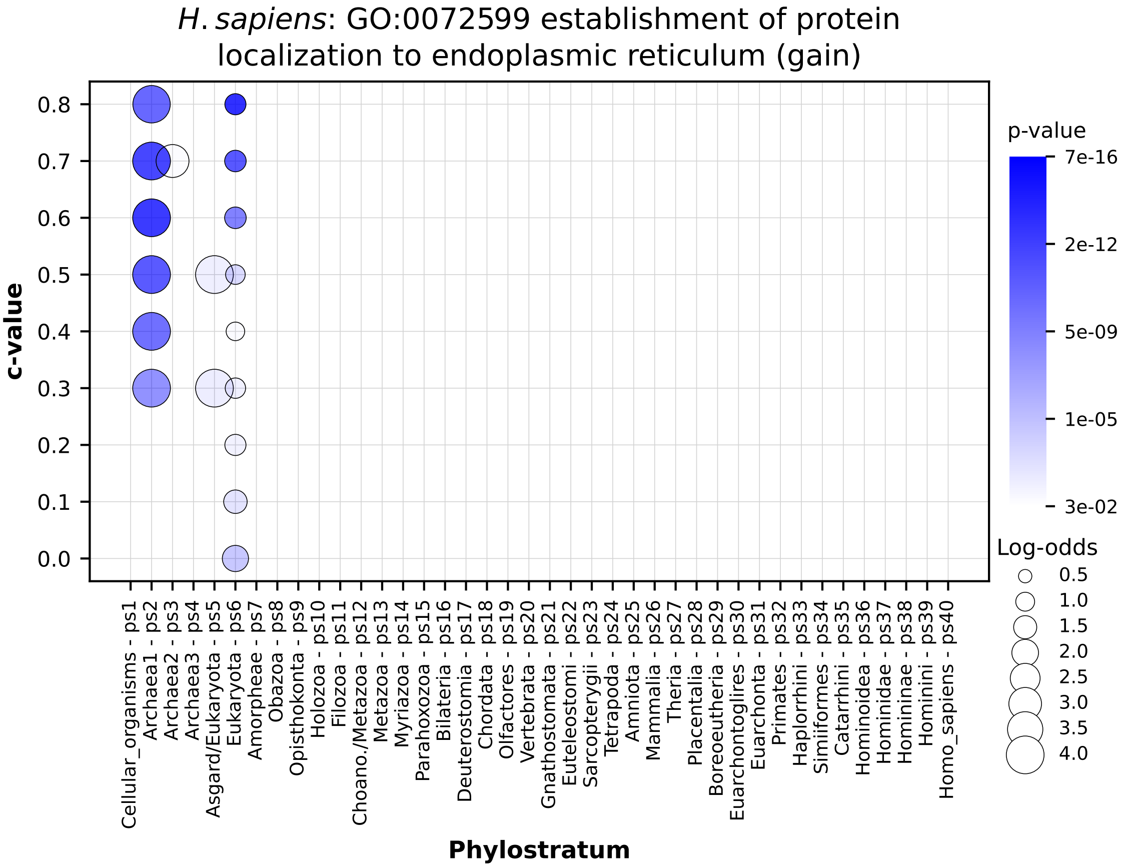

H. sapiens: GO:0072599 establishment of protein localization to endoplasmic reticulum (gain)
c-value
0.8
0.7
0.6
0.5
0.4
0.3
0.2
0.1
0.0
Phylostratum
Cellular_organisms - ps1
Archaea1 - ps2
Archaea2 - ps3
Archaea3 - ps4
Asgard/Eukaryota - ps5
Eukaryota - ps6
Amorpheae - ps7
Obazoa - ps8
Opisthokonta - ps9
Holozoa - ps10
Filozoa - ps11
Choano./Metazoa - ps12
Metazoa - ps13
Myriazoa - ps14
Parahoxozoa - ps15
Bilateria - ps16
Deuterostomia - ps17
Chordata - ps18
Olfactores - ps19
Vertebrata - ps20
Gnathostomata - ps21
Euteleostomi - ps22
Sarcopterygii - ps23
Tetrapoda - ps24
Amniota - ps25
Mammalia - ps26
Theria - ps27
Placentalia - ps28
Boreoeutheria - ps29
Euarchontoglires - ps30
Euarchonta - ps31
Primates - ps32
Haplorrhini - ps33
Simiiformes - ps34
Catarrhini - ps35
Hominoidea - ps36
Hominidae - ps37
Homininae - ps38
Hominini - ps39
Homo_sapiens - ps40
p-value
7e-16
2e-12
5e-09
1e-05
3e-02
Log-odds
0.5
1.0
1.5
2.0
2.5
3.0
3.5
4.0

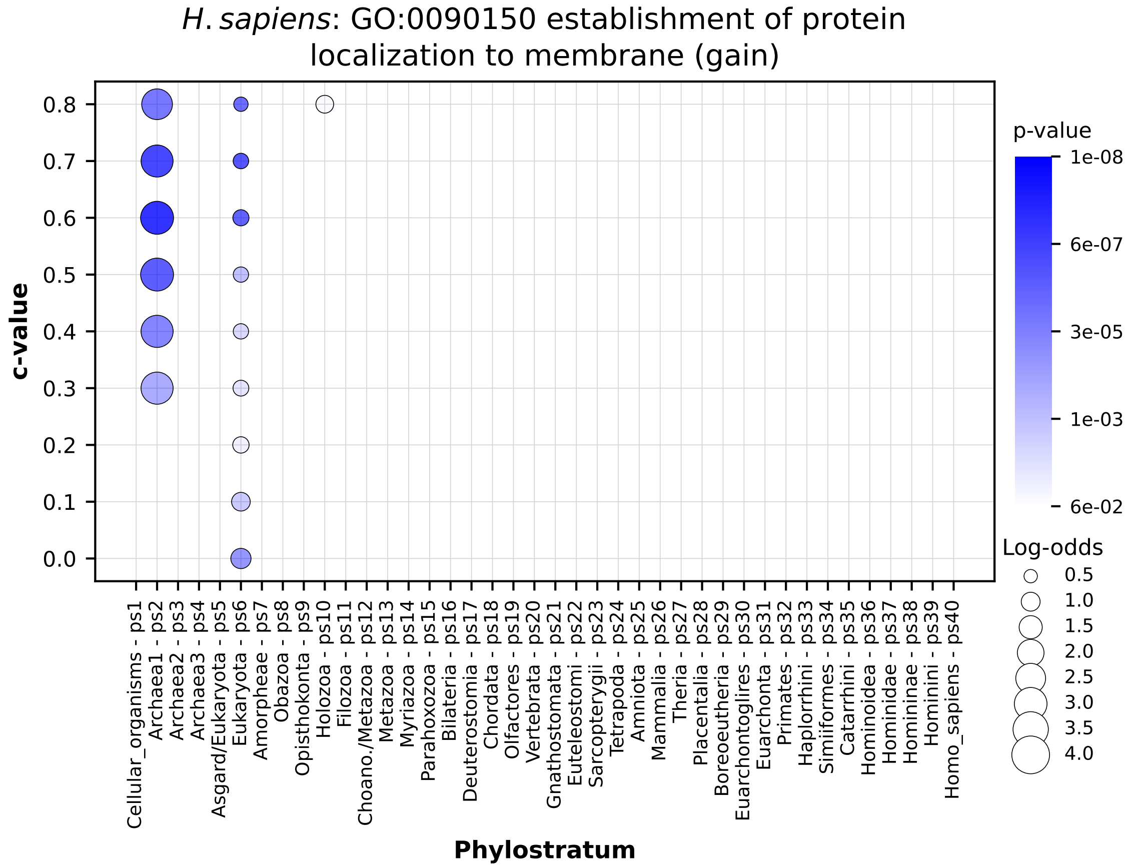

H. sapiens: GO:0090150 establishment of protein localization to membrane (gain)
c-value
0.8
0.7
0.6
0.5
0.4
0.3
0.2
0.1
0.0
p-value
1e-08
6e-07
3e-05
1e-03
6e-02
Log-odds
0.5
1.0
1.5
2.0
2.5
3.0
3.5
4.0
Cellular_organisms - ps1
Archaea1 - ps2
Archaea2 - ps3
Archaea3 - ps4
Asgard/Eukaryota - ps5
Eukaryota - ps6
Amorpheae - ps7
Obazoa - ps8
Opisthokonta - ps9
Holozoa - ps10
Filozoa - ps11
Choano./Metazoa - ps12
Metazoa - ps13
Myriazoa - ps14
Parahoxozoa - ps15
Bilateria - ps16
Deuterostomia - ps17
Chordata - ps18
Olfactores - ps19
Vertebrata - ps20
Gnathostomata - ps21
Euteleostomi - ps22
Sarcopterygii - ps23
Tetrapoda - ps24
Amniota - ps25
Mammalia - ps26
Theria - ps27
Placentalia - ps28
Boreoeutheria - ps29
Euarchontoglires - ps30
Euarchonta - ps31
Primates - ps32
Haplorrhini - ps33
Simiiformes - ps34
Catarrhini - ps35
Hominoidea - ps36
Hominidae - ps37
Homininae - ps38
Hominini - ps39
Homo_sapiens - ps40
Phylostratum

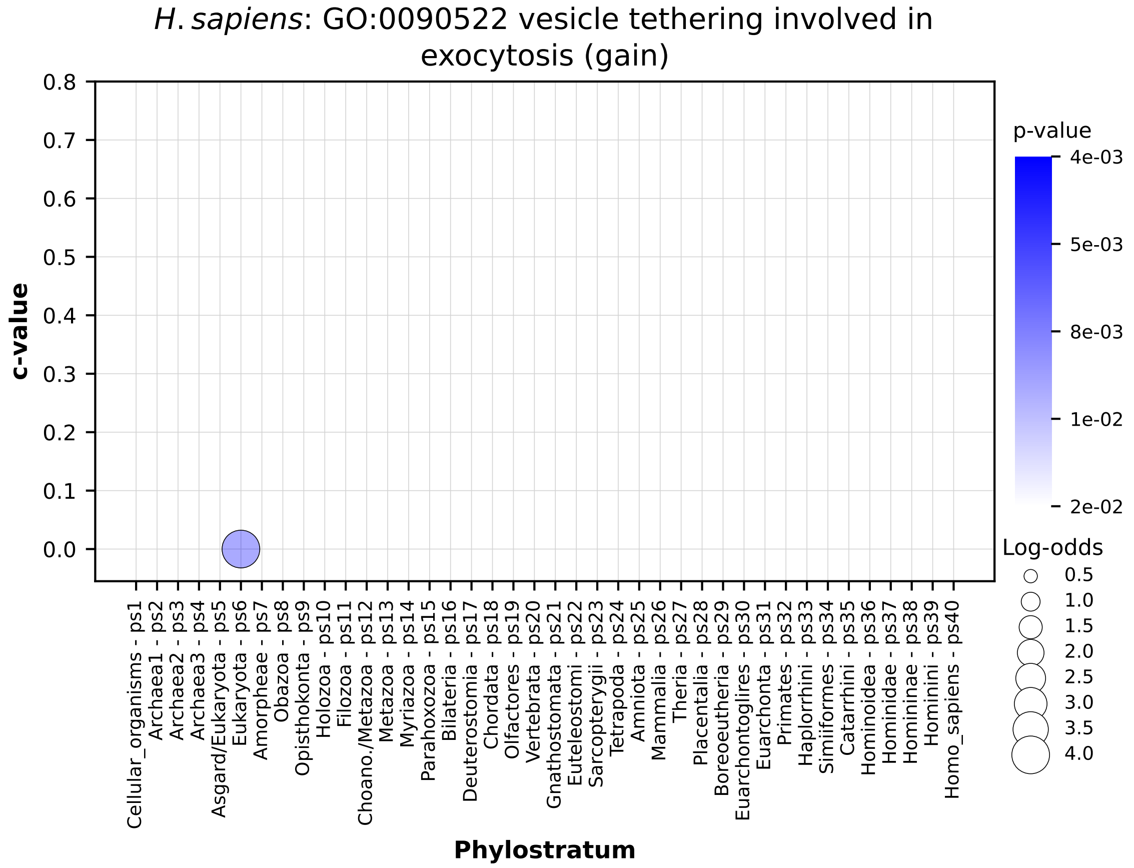

*H. sapiens*: GO:0090522 vesicle tethering involved in exocytosis (gain)
c-value
0.8
0.7
0.6
0.5
0.4
0.3
0.2
0.1
0.0
Phylostratum
Cellular_organisms - ps1
Archaea1 - ps2
Archaea2 - ps3
Archaea3 - ps4
Asgard/Eukaryota - ps5
Eukaryota - ps6
Amorpheae - ps7
Obazoa - ps8
Opisthokonta - ps9
Holozoa - ps10
Filozoa - ps11
Choano./Metazoa - ps12
Metazoa - ps13
Myriazoa - ps14
Parahoxozoa - ps15
Bilateria - ps16
Deuterostomia - ps17
Chordata - ps18
Olfactores - ps19
Vertebrata - ps20
Gnathostomata - ps21
Euteleostomi - ps22
Sarcopterygii - ps23
Tetrapoda - ps24
Amniota - ps25
Mammalia - ps26
Theria - ps27
Placentalia - ps28
Boreoeutheria - ps29
Euarchontoglires - ps30
Euarchonta - ps31
Primates - ps32
Haplorrhini - ps33
Simiiformes - ps34
Catarrhini - ps35
Hominoidea - ps36
Hominidae - ps37
Homininae - ps38
Hominini - ps39
Homo_sapiens - ps40
p-value
4e-03
5e-03
8e-03
1e-02
2e-02
Log-odds
0.5
1.0
1.5
2.0
2.5
3.0
3.5
4.0

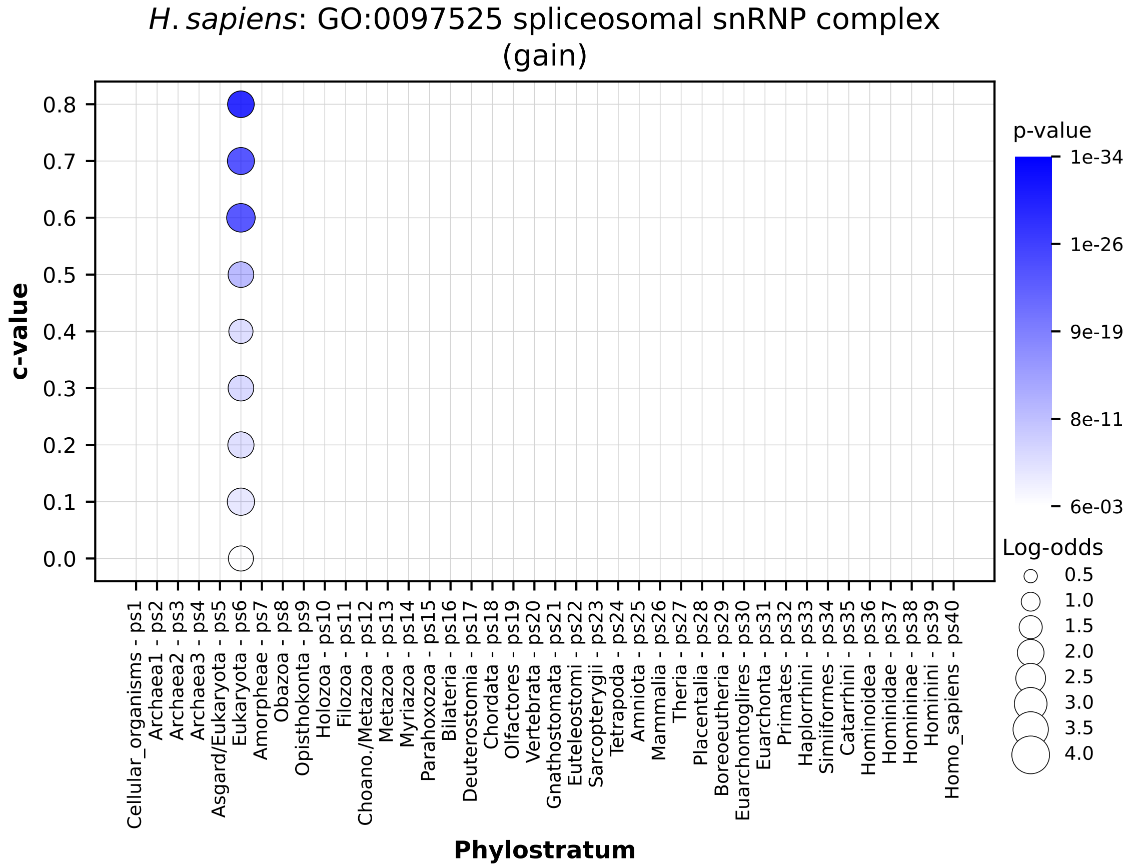

H. sapiens: GO:0097525 spliceosomal snRNP complex
(gain)
c-value
0.0
0.1
0.2
0.3
0.4
0.5
0.6
0.7
0.8
Phylostratum
Cellular_organisms - ps1
Archaea1 - ps2
Archaea2 - ps3
Archaea3 - ps4
Asgard/Eukaryota - ps5
Eukaryota - ps6
Amorpheae - ps7
Obazoa - ps8
Opisthokonta - ps9
Holozoa - ps10
Filozoa - ps11
Choano./Metazoa - ps12
Metazoa - ps13
Myriazoa - ps14
Parahoxozoa - ps15
Bilateria - ps16
Deuterostomia - ps17
Chordata - ps18
Olfactores - ps19
Vertebrata - ps20
Gnathostomata - ps21
Euteleostomi - ps22
Sarcopterygii - ps23
Tetrapoda - ps24
Amniota - ps25
Mammalia - ps26
Theria - ps27
Placentalia - ps28
Boreoeutheria - ps29
Euarchontoglires - ps30
Euarchonta - ps31
Primates - ps32
Haplorrhini - ps33
Simiiformes - ps34
Catarrhini - ps35
Hominoidea - ps36
Hominidae - ps37
Homininae - ps38
Hominini - ps39
Homo_sapiens - ps40
p-value
1e-34
1e-26
9e-19
8e-11
6e-03
Log-odds
0.5
1.0
1.5
2.0
2.5
3.0
3.5
4.0

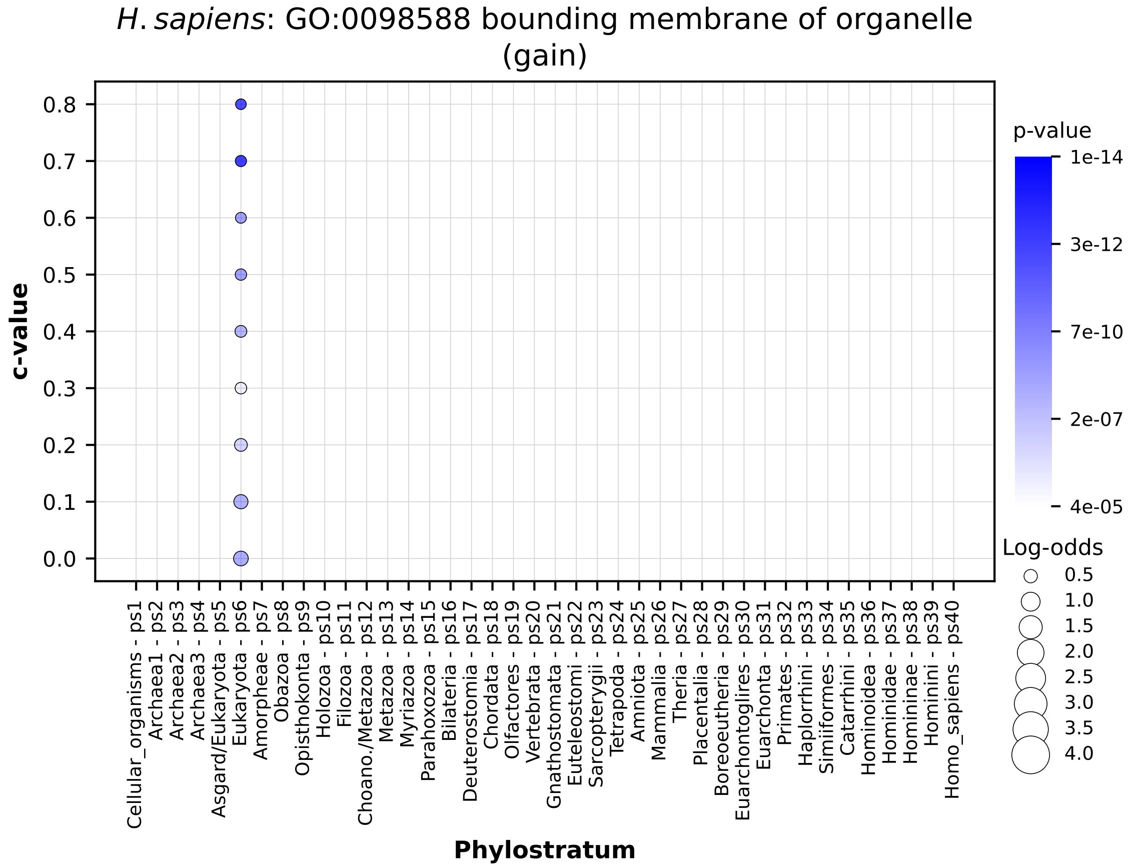
H. sapiens: GO:0098588 bounding membrane of organelle (gain)
c-value
0.0
0.1
0.2
0.3
0.4
0.5
0.6
0.7
0.8
Phylostratum
Cellular_organisms - ps1
Archaea1 - ps2
Archaea2 - ps3
Archaea3 - ps4
Asgard/Eukaryota - ps5
Eukaryota - ps6
Amorpheae - ps7
Obazoa - ps8
Opisthokonta - ps9
Holozoa - ps10
Filozoa - ps11
Choano./Metazoa - ps12
Metazoa - ps13
Myriazoa - ps14
Parahoxozoa - ps15
Bilateria - ps16
Deuterostomia - ps17
Chordata - ps18
Olfactores - ps19
Vertebrata - ps20
Gnathostomata - ps21
Euteleostomi - ps22
Sarcopterygii - ps23
Tetrapoda - ps24
Amniota - ps25
Mammalia - ps26
Theria - ps27
Placentalia - ps28
Boreoeutheria - ps29
Euarchontoglires - ps30
Euarchonta - ps31
Primates - ps32
Haplorrhini - ps33
Simiiformes - ps34
Catarrhini - ps35
Hominoidea - ps36
Hominidae - ps37
Homininae - ps38
Hominini - ps39
Homo_sapiens - ps40
p-value
1e-14
3e-12
7e-10
2e-07
4e-05
Log-odds
0.5
1.0
1.5
2.0
2.5
3.0
3.5
4.0

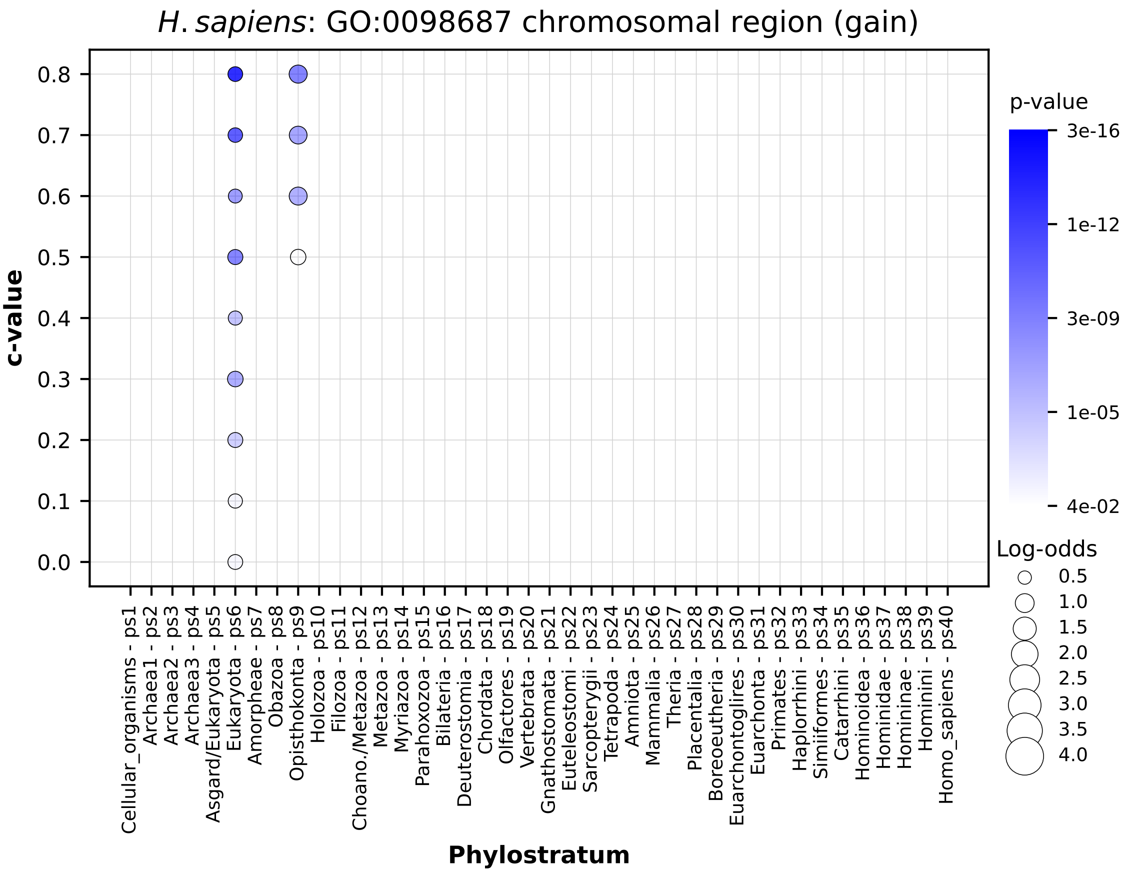
H. sapiens: GO:0098687 chromosomal region (gain)
c-value
0.8
0.7
0.6
0.5
0.4
0.3
0.2
0.1
0.0
p-value
3e-16
1e-12
3e-09
1e-05
4e-02
Log-odds
0.5
1.0
1.5
2.0
2.5
3.0
3.5
4.0
Cellular_organisms - ps1
Archaea1 - ps2
Archaea2 - ps3
Archaea3 - ps4
Asgard/Eukaryota - ps5
Eukaryota - ps6
Amorpheae - ps7
Obazoa - ps8
Opisthokonta - ps9
Holozoa - ps10
Filozoa - ps11
Choano./Metazoa - ps12
Metazoa - ps13
Myriazoa - ps14
Parahoxozoa - ps15
Bilateria - ps16
Deuterostomia - ps17
Chordata - ps18
Olfactores - ps19
Vertebrata - ps20
Gnathostomata - ps21
Euteleostomi - ps22
Sarcopterygii - ps23
Tetrapoda - ps24
Amniota - ps25
Mammalia - ps26
Theria - ps27
Placentalia - ps28
Boreoeutheria - ps29
Euarchontoglires - ps30
Euarchonta - ps31
Primates - ps32
Haplorrhini - ps33
Simiiformes - ps34
Catarrhini - ps35
Hominoidea - ps36
Hominidae - ps37
Homininae - ps38
Hominini - ps39
Homo_sapiens - ps40
Phylostratum

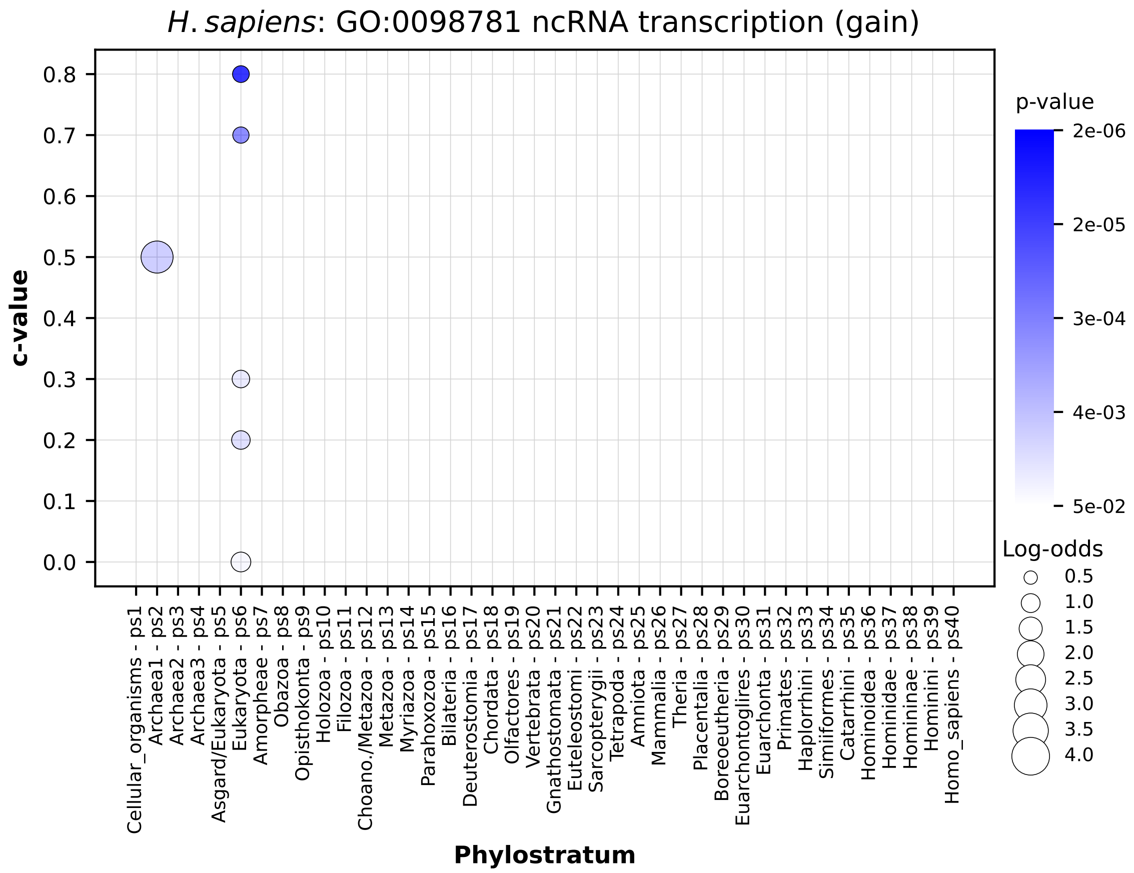

H. sapiens: GO:0098781 ncRNA transcription (gain)
c-value
0.8
0.7
0.6
0.5
0.4
0.3
0.2
0.1
0.0
p-value
2e-06
2e-05
3e-04
4e-03
5e-02
Log-odds
0.5
1.0
1.5
2.0
2.5
3.0
3.5
4.0
Cellular_organisms - ps1
Archaea1 - ps2
Archaea2 - ps3
Archaea3 - ps4
Asgard/Eukaryota - ps5
Eukaryota - ps6
Amorpheae - ps7
Obazoa - ps8
Opisthokonta - ps9
Holozoa - ps10
Filozoa - ps11
Choano./Metazoa - ps12
Metazoa - ps13
Myriazoa - ps14
Parahoxozoa - ps15
Bilateria - ps16
Deuterostomia - ps17
Chordata - ps18
Olfactores - ps19
Vertebrata - ps20
Gnathostomata - ps21
Euteleostomi - ps22
Sarcopterygii - ps23
Tetrapoda - ps24
Amniota - ps25
Mammalia - ps26
Theria - ps27
Placentalia - ps28
Boreoeutheria - ps29
Euarchontoglires - ps30
Euarchonta - ps31
Primates - ps32
Haplorrhini - ps33
Simiiformes - ps34
Catarrhini - ps35
Hominoidea - ps36
Hominidae - ps37
Homininae - ps38
Hominini - ps39
Homo_sapiens - ps40
Phylostratum

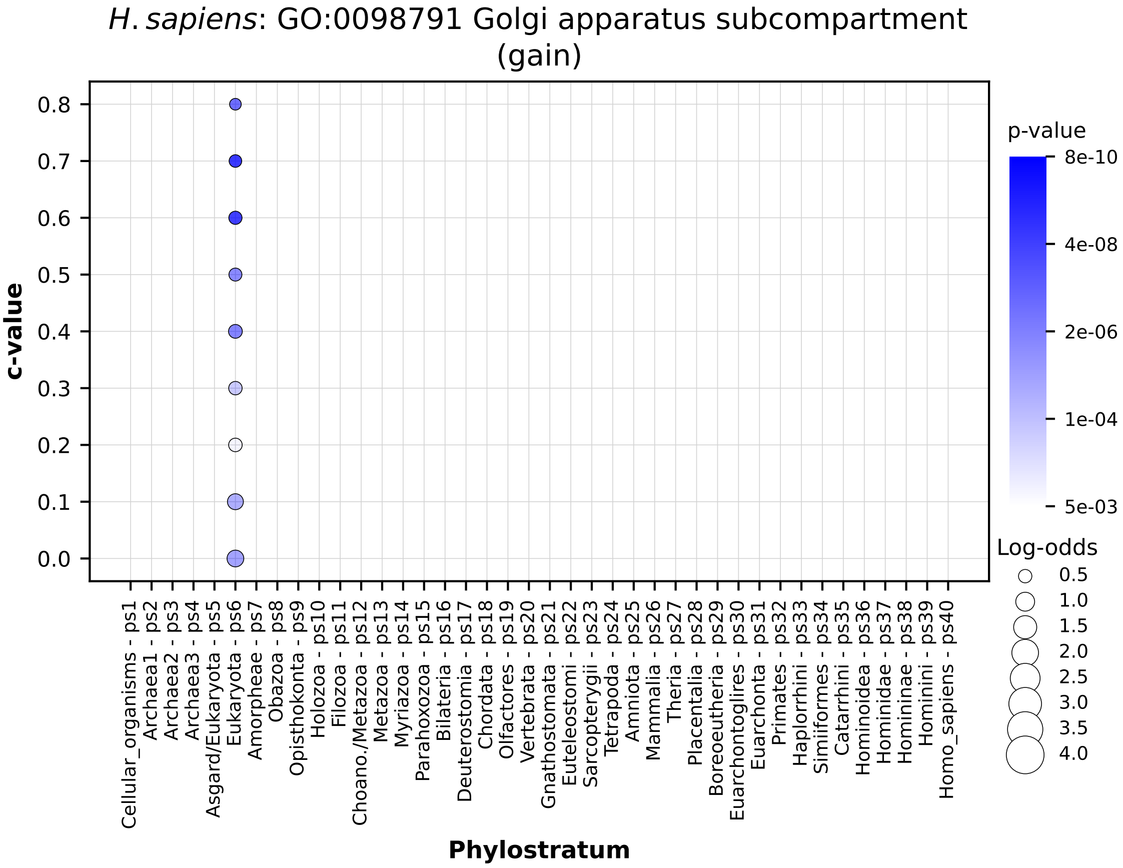
H. sapiens: GO:0098791 Golgi apparatus subcompartment (gain)
c-value
0.8
0.7
0.6
0.5
0.4
0.3
0.2
0.1
0.0
p-value
8e-10
4e-08
2e-06
1e-04
5e-03
Log-odds
0.5
1.0
1.5
2.0
2.5
3.0
3.5
4.0
Cellular_organisms - ps1
Archaea1 - ps2
Archaea2 - ps3
Archaea3 - ps4
Asgard/Eukaryota - ps5
Eukaryota - ps6
Amorpheae - ps7
Obazoa - ps8
Opisthokonta - ps9
Holozoa - ps10
Filozoa - ps11
Choano./Metazoa - ps12
Metazoa - ps13
Myriazoa - ps14
Parahoxozoa - ps15
Bilateria - ps16
Deuterostomia - ps17
Chordata - ps18
Olfactores - ps19
Vertebrata - ps20
Gnathostomata - ps21
Euteleostomi - ps22
Sarcopterygii - ps23
Tetrapoda - ps24
Amniota - ps25
Mammalia - ps26
Theria - ps27
Placentalia - ps28
Boreoeutheria - ps29
Euarchontoglires - ps30
Euarchonta - ps31
Primates - ps32
Haplorrhini - ps33
Simiiformes - ps34
Catarrhini - ps35
Hominoidea - ps36
Hominidae - ps37
Homininae - ps38
Hominini - ps39
Homo_sapiens - ps40
Phylostratum

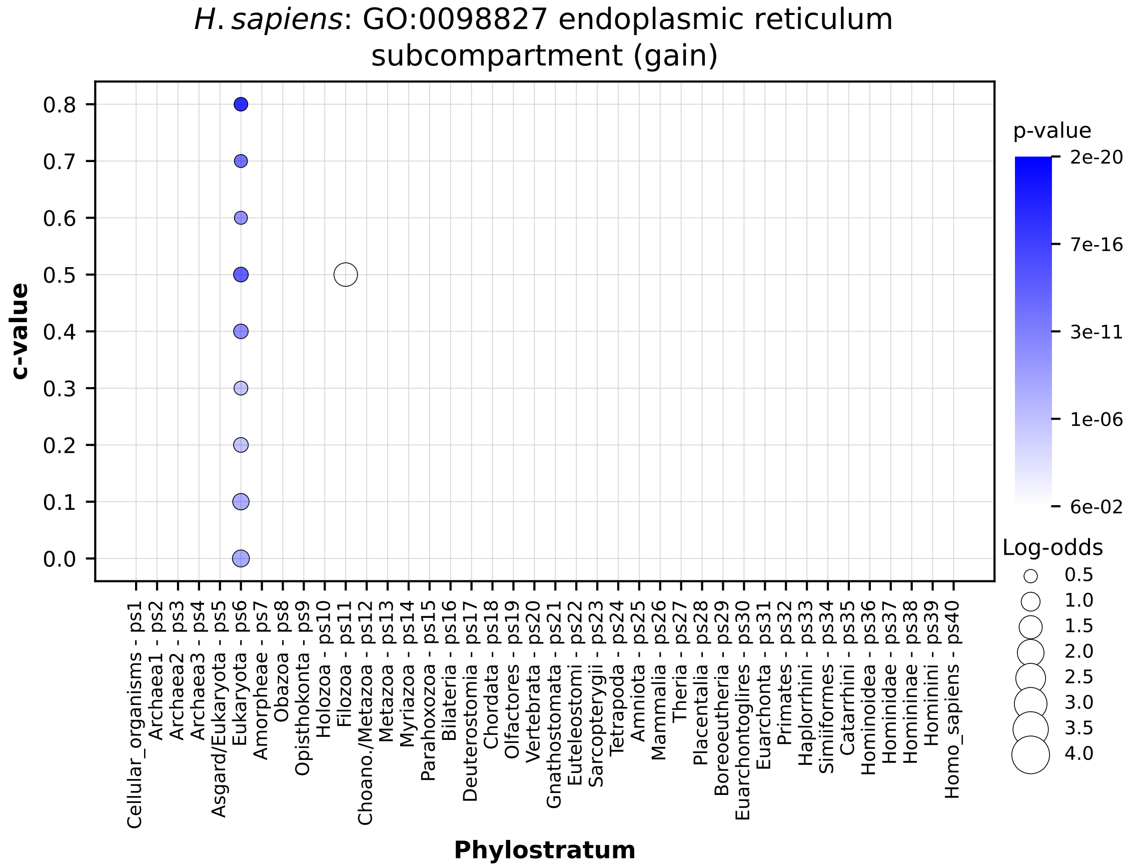

H. sapiens: GO:0098827 endoplasmic reticulum subcompartment (gain)
c-value
0.8
0.7
0.6
0.5
0.4
0.3
0.2
0.1
0.0
p-value
2e-20
7e-16
3e-11
1e-06
6e-02
Log-odds
0.5
1.0
1.5
2.0
2.5
3.0
3.5
4.0
Cellular_organisms - ps1
Archaea1 - ps2
Archaea2 - ps3
Archaea3 - ps4
Asgard/Eukaryota - ps5
Eukaryota - ps6
Amorpheae - ps7
Obazoa - ps8
Opisthokonta - ps9
Holozoa - ps10
Filozoa - ps11
Choano./Metazoa - ps12
Metazoa - ps13
Myriazoa - ps14
Parahoxozoa - ps15
Bilateria - ps16
Deuterostomia - ps17
Chordata - ps18
Olfactores - ps19
Vertebrata - ps20
Gnathostomata - ps21
Euteleostomi - ps22
Sarcopterygii - ps23
Tetrapoda - ps24
Amniota - ps25
Mammalia - ps26
Theria - ps27
Placentalia - ps28
Boreoeutheria - ps29
Euarchontoglires - ps30
Euarchonta - ps31
Primates - ps32
Haplorrhini - ps33
Simiiformes - ps34
Catarrhini - ps35
Hominoidea - ps36
Hominidae - ps37
Homininae - ps38
Hominini - ps39
Homo_sapiens - ps40
Phylostratum

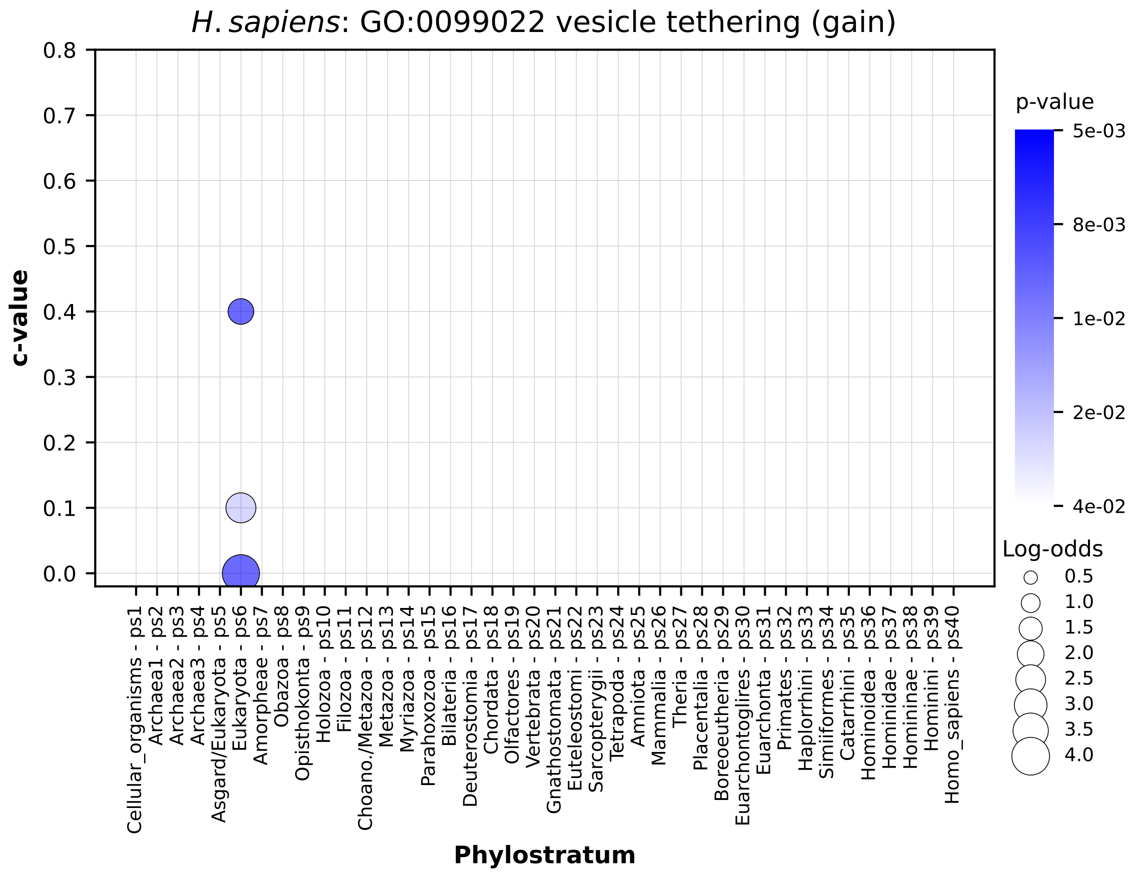

H. sapiens: GO:0099022 vesicle tethering (gain)
c-value
0.8
0.7
0.6
0.5
0.4
0.3
0.2
0.1
0.0
Phylostratum
Cellular_organisms - ps1
Archaea1 - ps2
Archaea2 - ps3
Archaea3 - ps4
Asgard/Eukaryota - ps5
Eukaryota - ps6
Amorpheae - ps7
Obazoa - ps8
Opisthokonta - ps9
Holozoa - ps10
Filozoa - ps11
Choano./Metazoa - ps12
Metazoa - ps13
Myriazoa - ps14
Parahoxozoa - ps15
Bilateria - ps16
Deuterostomia - ps17
Chordata - ps18
Olfactores - ps19
Vertebrata - ps20
Gnathostomata - ps21
Euteleostomi - ps22
Sarcopterygii - ps23
Tetrapoda - ps24
Amniota - ps25
Mammalia - ps26
Theria - ps27
Placentalia - ps28
Boreoeutheria - ps29
Euarchontoglires - ps30
Euarchonta - ps31
Primates - ps32
Haplorrhini - ps33
Simiiformes - ps34
Catarrhini - ps35
Hominoidea - ps36
Hominidae - ps37
Homininae - ps38
Hominini - ps39
Homo_sapiens - ps40
p-value
5e-03
8e-03
1e-02
2e-02
4e-02
Log-odds
0.5
1.0
1.5
2.0
2.5
3.0
3.5
4.0

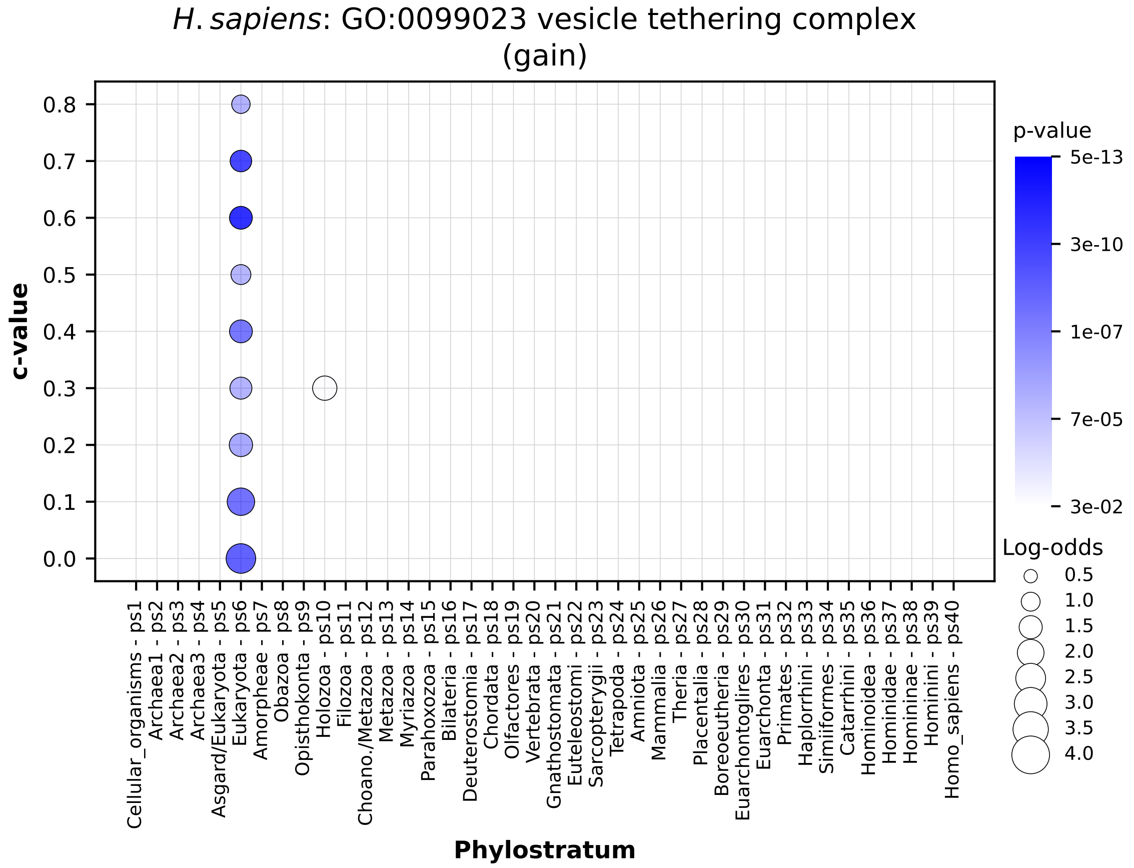

*H. sapiens*: GO:0099023 vesicle tethering complex
(gain)
c-value
0.0
0.1
0.2
0.3
0.4
0.5
0.6
0.7
0.8
Phylostratum
Cellular_organisms - ps1
Archaea1 - ps2
Archaea2 - ps3
Archaea3 - ps4
Asgard/Eukaryota - ps5
Eukaryota - ps6
Amorpheae - ps7
Obazoa - ps8
Opisthokonta - ps9
Holozoa - ps10
Filozoa - ps11
Choano./Metazoa - ps12
Metazoa - ps13
Myriazoa - ps14
Parahoxozoa - ps15
Bilateria - ps16
Deuterostomia - ps17
Chordata - ps18
Olfactores - ps19
Vertebrata - ps20
Gnathostomata - ps21
Euteleostomi - ps22
Sarcopterygii - ps23
Tetrapoda - ps24
Amniota - ps25
Mammalia - ps26
Theria - ps27
Placentalia - ps28
Boreoeutheria - ps29
Euarchontoglires - ps30
Euarchonta - ps31
Primates - ps32
Haplorrhini - ps33
Simiiformes - ps34
Catarrhini - ps35
Hominoidea - ps36
Hominidae - ps37
Homininae - ps38
Hominini - ps39
Homo_sapiens - ps40
p-value
5e-13
3e-10
1e-07
7e-05
3e-02
Log-odds
0.5
1.0
1.5
2.0
2.5
3.0
3.5
4.0

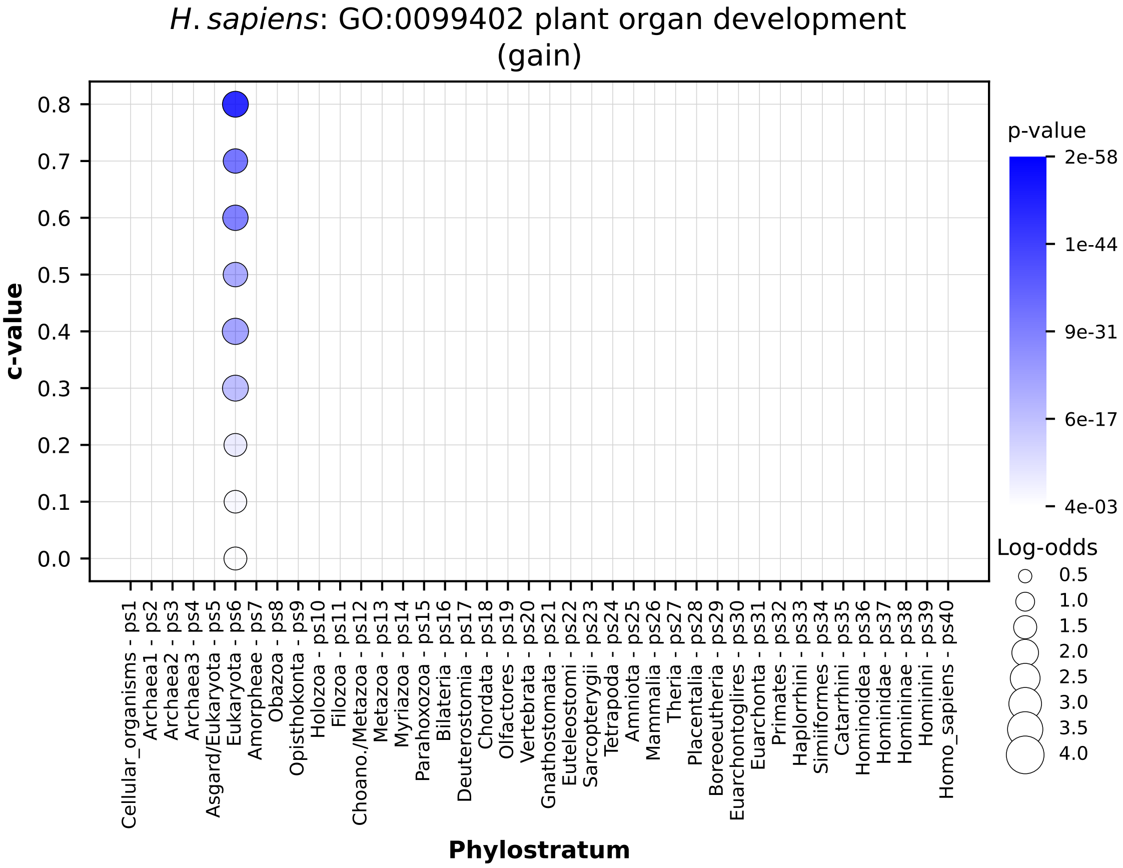
H. sapiens: GO:0099402 plant organ development
(gain)
c-value
0.8
0.7
0.6
0.5
0.4
0.3
0.2
0.1
0.0
Phylostratum
Cellular_organisms - ps1
Archaea1 - ps2
Archaea2 - ps3
Archaea3 - ps4
Asgard/Eukaryota - ps5
Eukaryota - ps6
Amorpheae - ps7
Obazoa - ps8
Opisthokonta - ps9
Holozoa - ps10
Filozoa - ps11
Choano./Metazoa - ps12
Metazoa - ps13
Myriazoa - ps14
Parahoxozoa - ps15
Bilateria - ps16
Deuterostomia - ps17
Chordata - ps18
Olfactores - ps19
Vertebrata - ps20
Gnathostomata - ps21
Euteleostomi - ps22
Sarcopterygii - ps23
Tetrapoda - ps24
Amniota - ps25
Mammalia - ps26
Theria - ps27
Placentalia - ps28
Boreoeutheria - ps29
Euarchontoglires - ps30
Euarchonta - ps31
Primates - ps32
Haplorrhini - ps33
Simiiformes - ps34
Catarrhini - ps35
Hominoidea - ps36
Hominidae - ps37
Homininae - ps38
Hominini - ps39
Homo_sapiens - ps40
p-value
2e-58
1e-44
9e-31
6e-17
4e-03
Log-odds
0.5
1.0
1.5
2.0
2.5
3.0
3.5
4.0

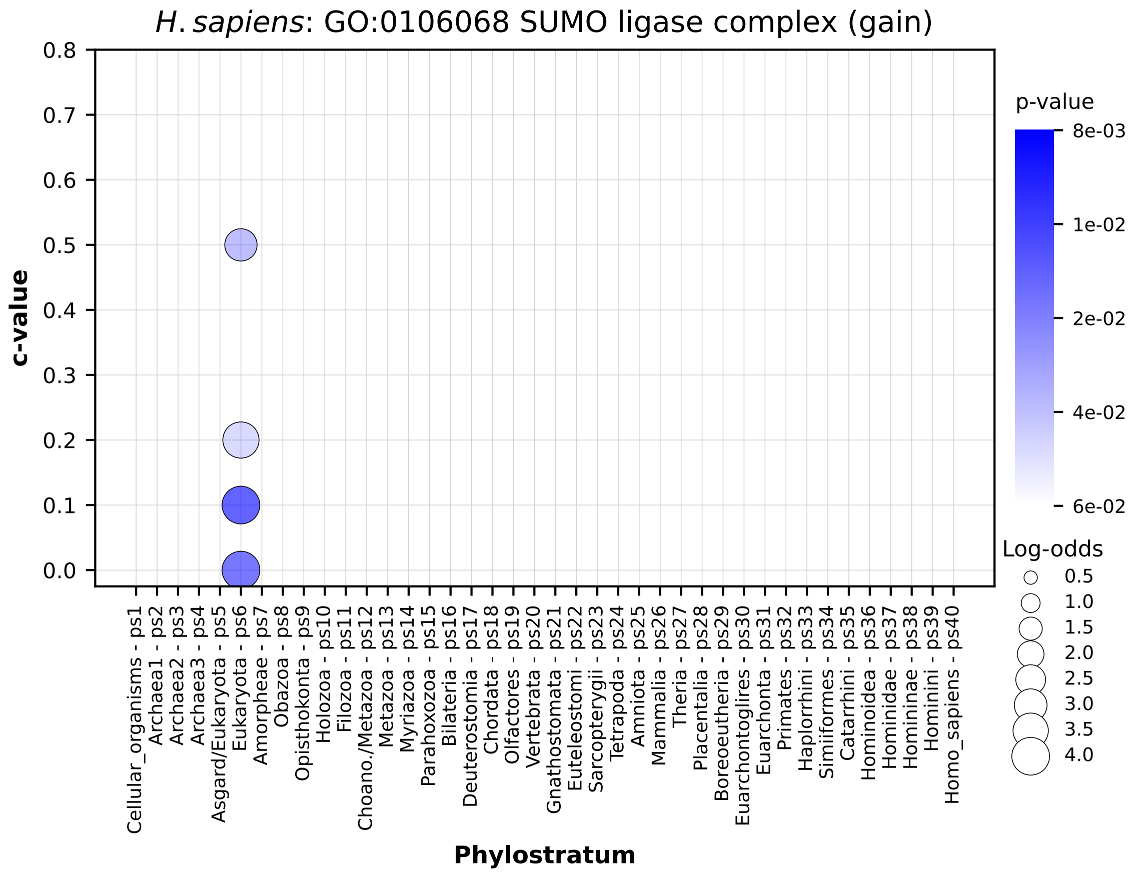
H. sapiens: GO:0106068 SUMO ligase complex (gain)
c-value
0.8
0.7
0.6
0.5
0.4
0.3
0.2
0.1
0.0
Phylostratum
Cellular_organisms - ps1
Archaea1 - ps2
Archaea2 - ps3
Archaea3 - ps4
Asgard/Eukaryota - ps5
Eukaryota - ps6
Amorpheae - ps7
Obazoa - ps8
Opisthokonta - ps9
Holozoa - ps10
Filozoa - ps11
Choano./Metazoa - ps12
Metazoa - ps13
Myriazoa - ps14
Parahoxozoa - ps15
Bilateria - ps16
Deuterostomia - ps17
Chordata - ps18
Olfactores - ps19
Vertebrata - ps20
Gnathostomata - ps21
Euteleostomi - ps22
Sarcopterygii - ps23
Tetrapoda - ps24
Amniota - ps25
Mammalia - ps26
Theria - ps27
Placentalia - ps28
Boreoeutheria - ps29
Euarchontoglires - ps30
Euarchonta - ps31
Primates - ps32
Haplorrhini - ps33
Simiiformes - ps34
Catarrhini - ps35
Hominoidea - ps36
Hominidae - ps37
Homininae - ps38
Hominini - ps39
Homo_sapiens - ps40
p-value
8e-03
1e-02
2e-02
4e-02
6e-02
Log-odds
0.5
1.0
1.5
2.0
2.5
3.0
3.5
4.0

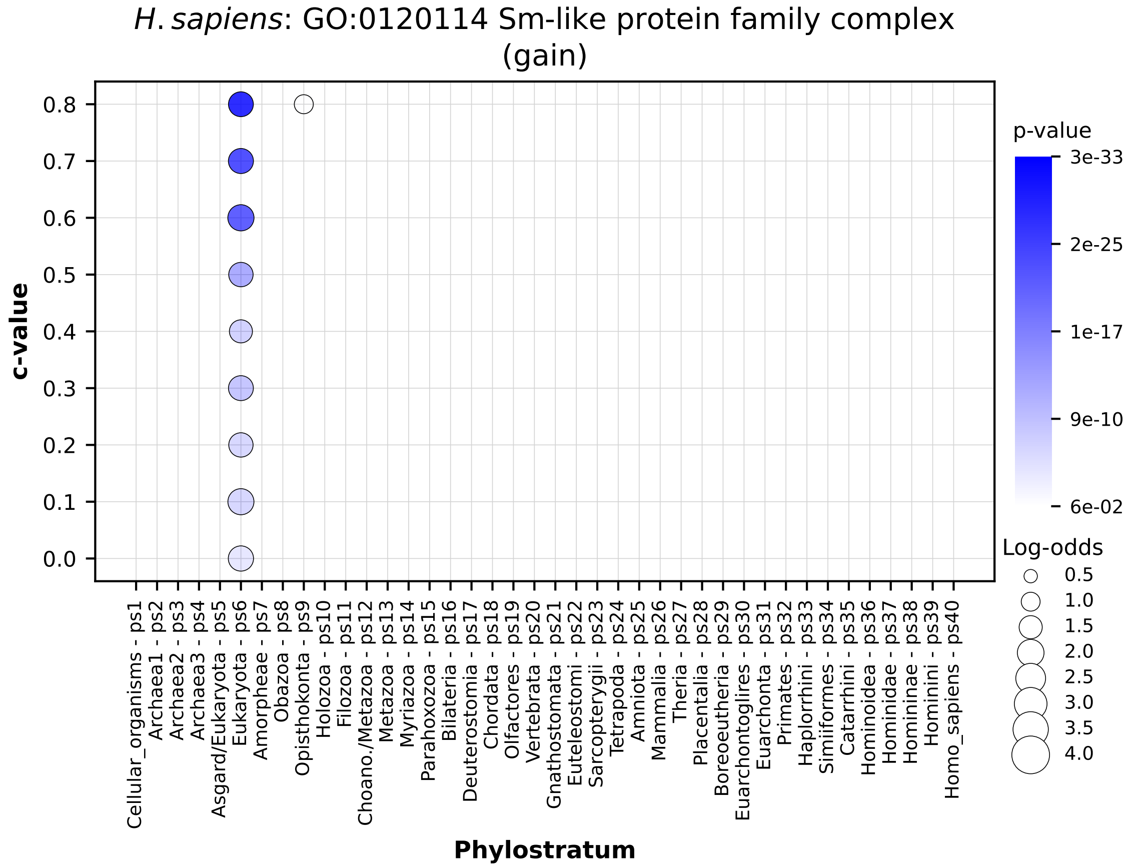

H. sapiens: GO:0120114 Sm-like protein family complex
(gain)
c-value
0.8
0.7
0.6
0.5
0.4
0.3
0.2
0.1
0.0
p-value
3e-33
2e-25
1e-17
9e-10
6e-02
Log-odds
0.5
1.0
1.5
2.0
2.5
3.0
3.5
4.0
Cellular_organisms - ps1
Archaea1 - ps2
Archaea2 - ps3
Archaea3 - ps4
Asgard/Eukaryota - ps5
Eukaryota - ps6
Amorpheae - ps7
Obazoa - ps8
Opisthokonta - ps9
Holozoa - ps10
Filozoa - ps11
Choano./Metazoa - ps12
Metazoa - ps13
Myriazoa - ps14
Parahoxozoa - ps15
Bilateria - ps16
Deuterostomia - ps17
Chordata - ps18
Olfactores - ps19
Vertebrata - ps20
Gnathostomata - ps21
Euteleostomi - ps22
Sarcopterygii - ps23
Tetrapoda - ps24
Amniota - ps25
Mammalia - ps26
Theria - ps27
Placentalia - ps28
Boreoeutheria - ps29
Euarchontoglires - ps30
Euarchonta - ps31
Primates - ps32
Haplorrhini - ps33
Simiiformes - ps34
Catarrhini - ps35
Hominoidea - ps36
Hominidae - ps37
Homininae - ps38
Hominini - ps39
Homo_sapiens - ps40
Phylostratum

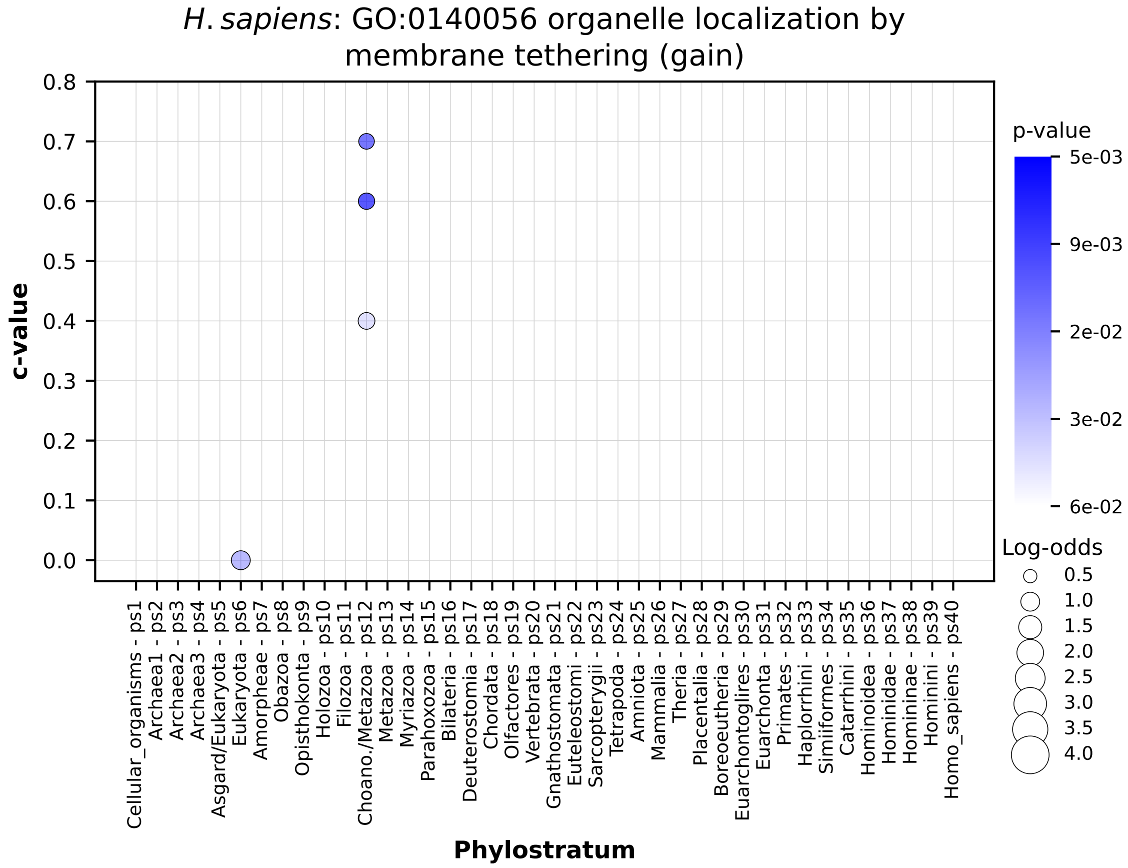

H. sapiens: GO:0140056 organelle localization by membrane tethering (gain)
c-value
0.8
0.7
0.6
0.5
0.4
0.3
0.2
0.1
0.0
p-value
5e-03
9e-03
2e-02
3e-02
6e-02
Log-odds
0.5
1.0
1.5
2.0
2.5
3.0
3.5
4.0
Cellular_organisms - ps1
Archaea1 - ps2
Archaea2 - ps3
Archaea3 - ps4
Asgard/Eukaryota - ps5
Eukaryota - ps6
Amorpheae - ps7
Obazoa - ps8
Opisthokonta - ps9
Holozoa - ps10
Filozoa - ps11
Choano./Metazoa - ps12
Metazoa - ps13
Myriazoa - ps14
Parahoxozoa - ps15
Bilateria - ps16
Deuterostomia - ps17
Chordata - ps18
Olfactores - ps19
Vertebrata - ps20
Gnathostomata - ps21
Euteleostomi - ps22
Sarcopterygii - ps23
Tetrapoda - ps24
Amniota - ps25
Mammalia - ps26
Theria - ps27
Placentalia - ps28
Boreoeutheria - ps29
Euarchontoglires - ps30
Euarchonta - ps31
Primates - ps32
Haplorrhini - ps33
Simiiformes - ps34
Catarrhini - ps35
Hominoidea - ps36
Hominidae - ps37
Homininae - ps38
Hominini - ps39
Homo_sapiens - ps40
Phylostratum

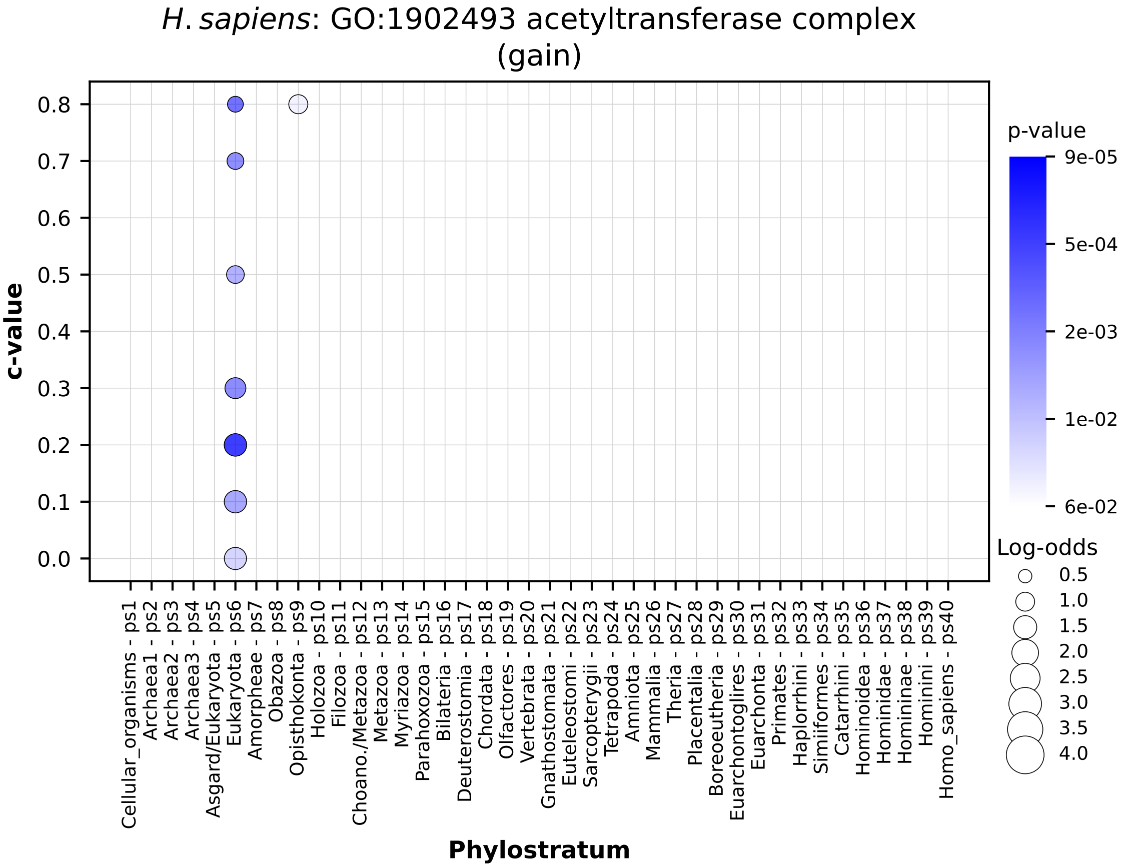

*H. sapiens*: GO:1902493 acetyltransferase complex (gain)
c-value
0.8
0.7
0.6
0.5
0.4
0.3
0.2
0.1
0.0
Phylostratum
Cellular_organisms - ps1
Archaea1 - ps2
Archaea2 - ps3
Archaea3 - ps4
Asgard/Eukaryota - ps5
Eukaryota - ps6
Amorpheae - ps7
Obazoa - ps8
Opisthokonta - ps9
Holozoa - ps10
Filozoa - ps11
Choano./Metazoa - ps12
Metazoa - ps13
Myriazoa - ps14
Parahoxozoa - ps15
Bilateria - ps16
Deuterostomia - ps17
Chordata - ps18
Olfactores - ps19
Vertebrata - ps20
Gnathostomata - ps21
Euteleostomi - ps22
Sarcopterygii - ps23
Tetrapoda - ps24
Amniota - ps25
Mammalia - ps26
Theria - ps27
Placentalia - ps28
Boreoeutheria - ps29
Euarchontoglires - ps30
Euarchonta - ps31
Primates - ps32
Haplorrhini - ps33
Simiiformes - ps34
Catarrhini - ps35
Hominoidea - ps36
Hominidae - ps37
Homininae - ps38
Hominini - ps39
Homo_sapiens - ps40
p-value
9e-05
5e-04
2e-03
1e-02
6e-02
Log-odds
0.5
1.0
1.5
2.0
2.5
3.0
3.5
4.0

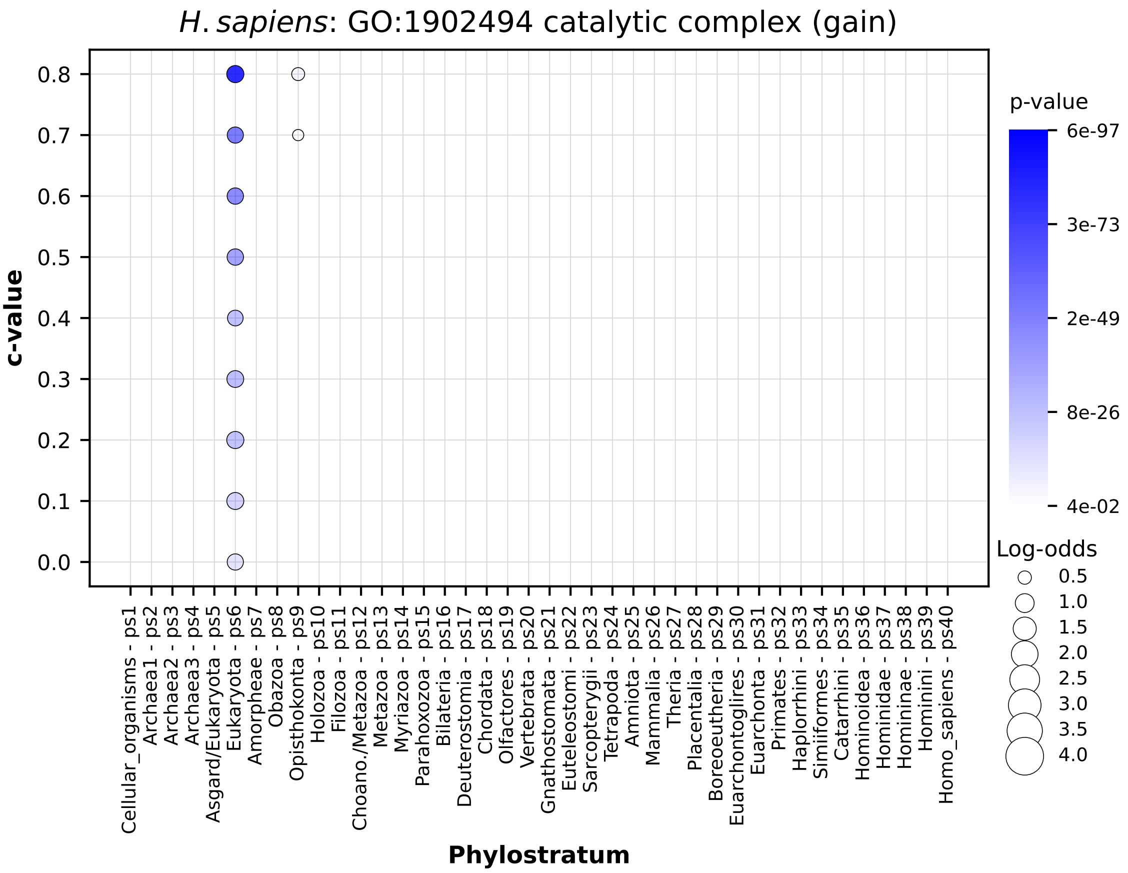
H. sapiens: GO:1902494 catalytic complex (gain)
c-value
0.8
0.7
0.6
0.5
0.4
0.3
0.2
0.1
0.0
p-value
6e-97
3e-73
2e-49
8e-26
4e-02
Log-odds
0.5
1.0
1.5
2.0
2.5
3.0
3.5
4.0
Cellular_organisms - ps1
Archaea1 - ps2
Archaea2 - ps3
Archaea3 - ps4
Asgard/Eukaryota - ps5
Eukaryota - ps6
Amorpheae - ps7
Obazoa - ps8
Opisthokonta - ps9
Holozoa - ps10
Filozoa - ps11
Choano./Metazoa - ps12
Metazoa - ps13
Myriazoa - ps14
Parahoxozoa - ps15
Bilateria - ps16
Deuterostomia - ps17
Chordata - ps18
Olfactores - ps19
Vertebrata - ps20
Gnathostomata - ps21
Euteleostomi - ps22
Sarcopterygii - ps23
Tetrapoda - ps24
Amniota - ps25
Mammalia - ps26
Theria - ps27
Placentalia - ps28
Boreoeutheria - ps29
Euarchontoglires - ps30
Euarchonta - ps31
Primates - ps32
Haplorrhini - ps33
Simiiformes - ps34
Catarrhini - ps35
Hominoidea - ps36
Hominidae - ps37
Homininae - ps38
Hominini - ps39
Homo_sapiens - ps40
Phylostratum

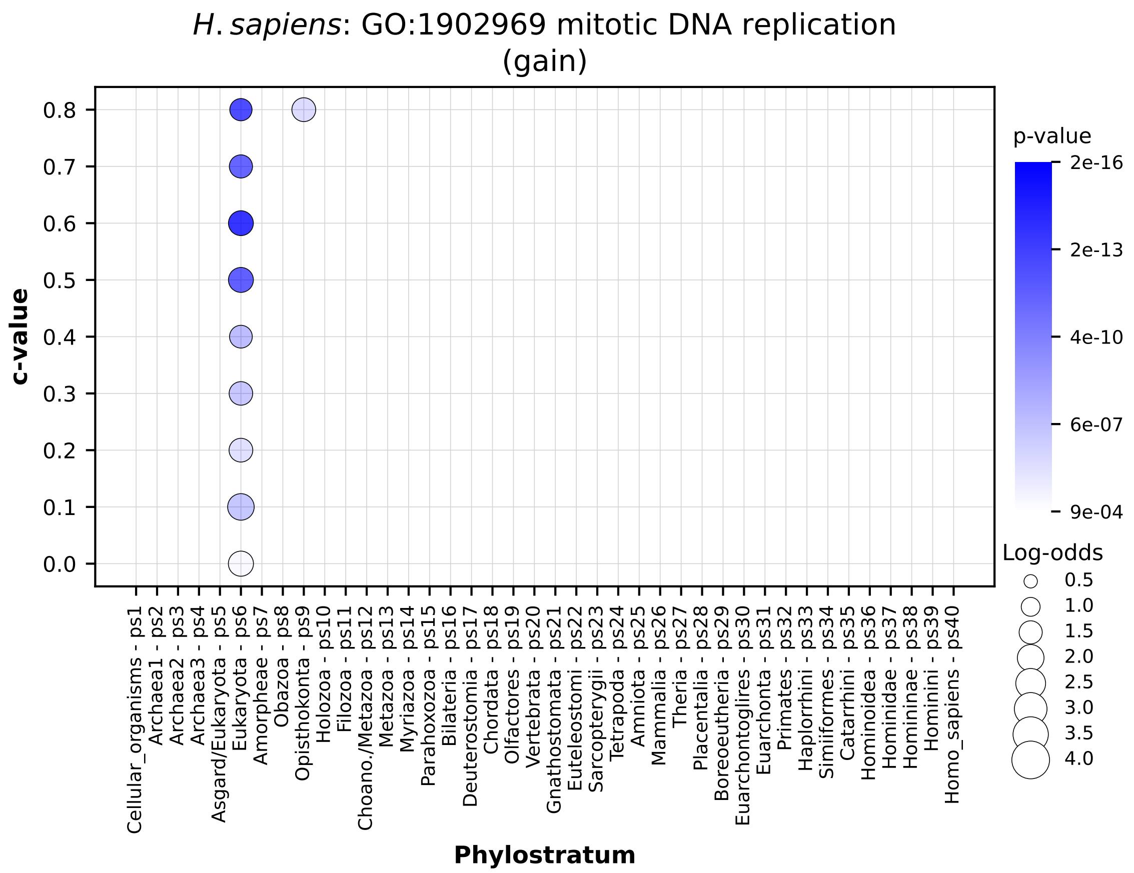

H. sapiens: GO:1902969 mitotic DNA replication
(gain)
c-value
0.8
0.7
0.6
0.5
0.4
0.3
0.2
0.1
0.0
p-value
2e-16
2e-13
4e-10
6e-07
9e-04
Log-odds
0.5
1.0
1.5
2.0
2.5
3.0
3.5
4.0
Cellular_organisms - ps1
Archaea1 - ps2
Archaea2 - ps3
Archaea3 - ps4
Asgard/Eukaryota - ps5
Eukaryota - ps6
Amorpheae - ps7
Obazoa - ps8
Opisthokonta - ps9
Holozoa - ps10
Filozoa - ps11
Choano./Metazoa - ps12
Metazoa - ps13
Myriazoa - ps14
Parahoxozoa - ps15
Bilateria - ps16
Deuterostomia - ps17
Chordata - ps18
Olfactores - ps19
Vertebrata - ps20
Gnathostomata - ps21
Euteleostomi - ps22
Sarcopterygii - ps23
Tetrapoda - ps24
Amniota - ps25
Mammalia - ps26
Theria - ps27
Placentalia - ps28
Boreoeutheria - ps29
Euarchontoglires - ps30
Euarchonta - ps31
Primates - ps32
Haplorrhini - ps33
Simiiformes - ps34
Catarrhini - ps35
Hominoidea - ps36
Hominidae - ps37
Homininae - ps38
Hominini - ps39
Homo_sapiens - ps40
Phylostratum

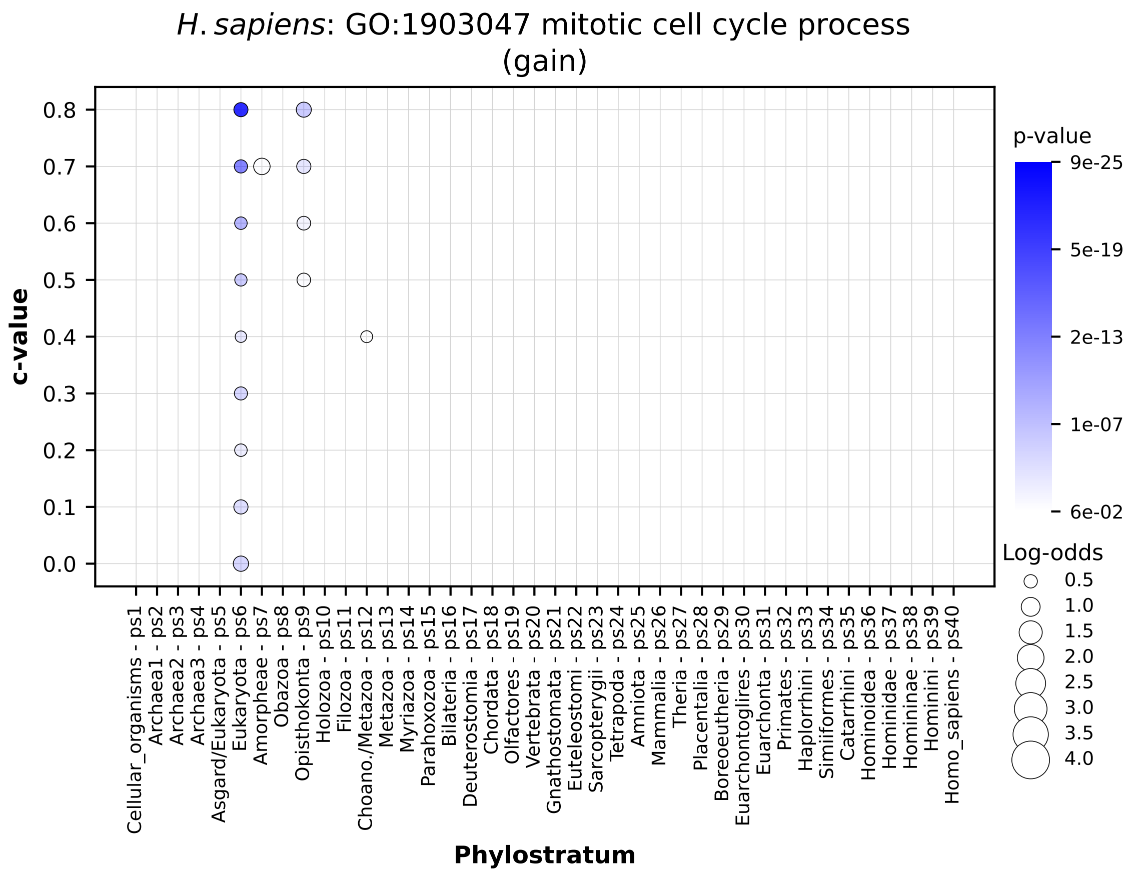

H. sapiens: GO:1903047 mitotic cell cycle process
(gain)
c-value
0.8
0.7
0.6
0.5
0.4
0.3
0.2
0.1
0.0
Phylostratum
Cellular_organisms - ps1
Archaea1 - ps2
Archaea2 - ps3
Archaea3 - ps4
Asgard/Eukaryota - ps5
Eukaryota - ps6
Amorpheae - ps7
Obazoa - ps8
Opisthokonta - ps9
Holozoa - ps10
Filozoa - ps11
Choano./Metazoa - ps12
Metazoa - ps13
Myriazoa - ps14
Parahoxozoa - ps15
Bilateria - ps16
Deuterostomia - ps17
Chordata - ps18
Olfactores - ps19
Vertebrata - ps20
Gnathostomata - ps21
Euteleostomi - ps22
Sarcopterygii - ps23
Tetrapoda - ps24
Amniota - ps25
Mammalia - ps26
Theria - ps27
Placentalia - ps28
Boreoeutheria - ps29
Euarchontoglires - ps30
Euarchonta - ps31
Primates - ps32
Haplorrhini - ps33
Simiiformes - ps34
Catarrhini - ps35
Hominoidea - ps36
Hominidae - ps37
Homininae - ps38
Hominini - ps39
Homo_sapiens - ps40
p-value
9e-25
5e-19
2e-13
1e-07
6e-02
Log-odds
0.5
1.0
1.5
2.0
2.5
3.0
3.5
4.0

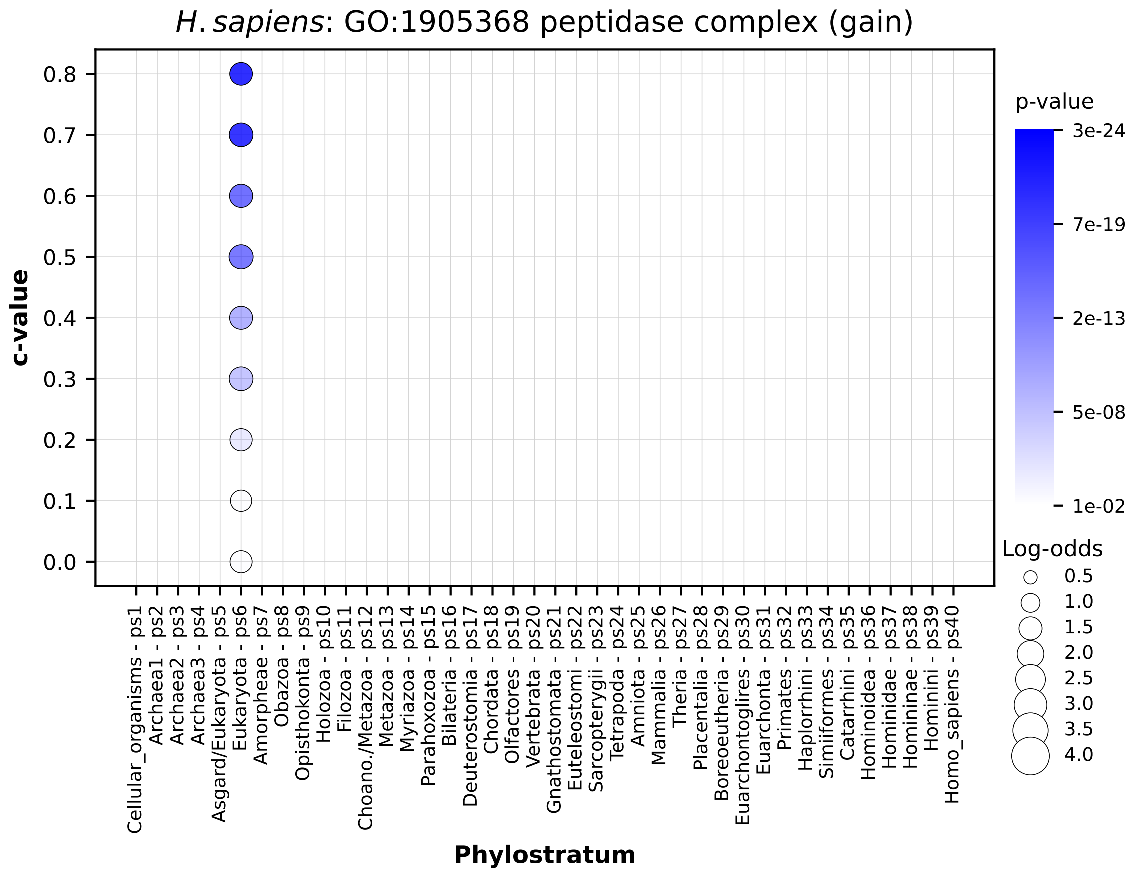

H. sapiens: GO:1905368 peptidase complex (gain)
c-value
0.8
0.7
0.6
0.5
0.4
0.3
0.2
0.1
0.0
p-value
3e-24
7e-19
2e-13
5e-08
1e-02
Log-odds
0.5
1.0
1.5
2.0
2.5
3.0
3.5
4.0
Cellular_organisms - ps1
Archaea1 - ps2
Archaea2 - ps3
Archaea3 - ps4
Asgard/Eukaryota - ps5
Eukaryota - ps6
Amorpheae - ps7
Obazoa - ps8
Opisthokonta - ps9
Holozoa - ps10
Filozoa - ps11
Choano./Metazoa - ps12
Metazoa - ps13
Myriazoa - ps14
Parahoxozoa - ps15
Bilateria - ps16
Deuterostomia - ps17
Chordata - ps18
Olfactores - ps19
Vertebrata - ps20
Gnathostomata - ps21
Euteleostomi - ps22
Sarcopterygii - ps23
Tetrapoda - ps24
Amniota - ps25
Mammalia - ps26
Theria - ps27
Placentalia - ps28
Boreoeutheria - ps29
Euarchontoglires - ps30
Euarchonta - ps31
Primates - ps32
Haplorrhini - ps33
Simiiformes - ps34
Catarrhini - ps35
Hominoidea - ps36
Hominidae - ps37
Homininae - ps38
Hominini - ps39
Homo_sapiens - ps40
Phylostratum

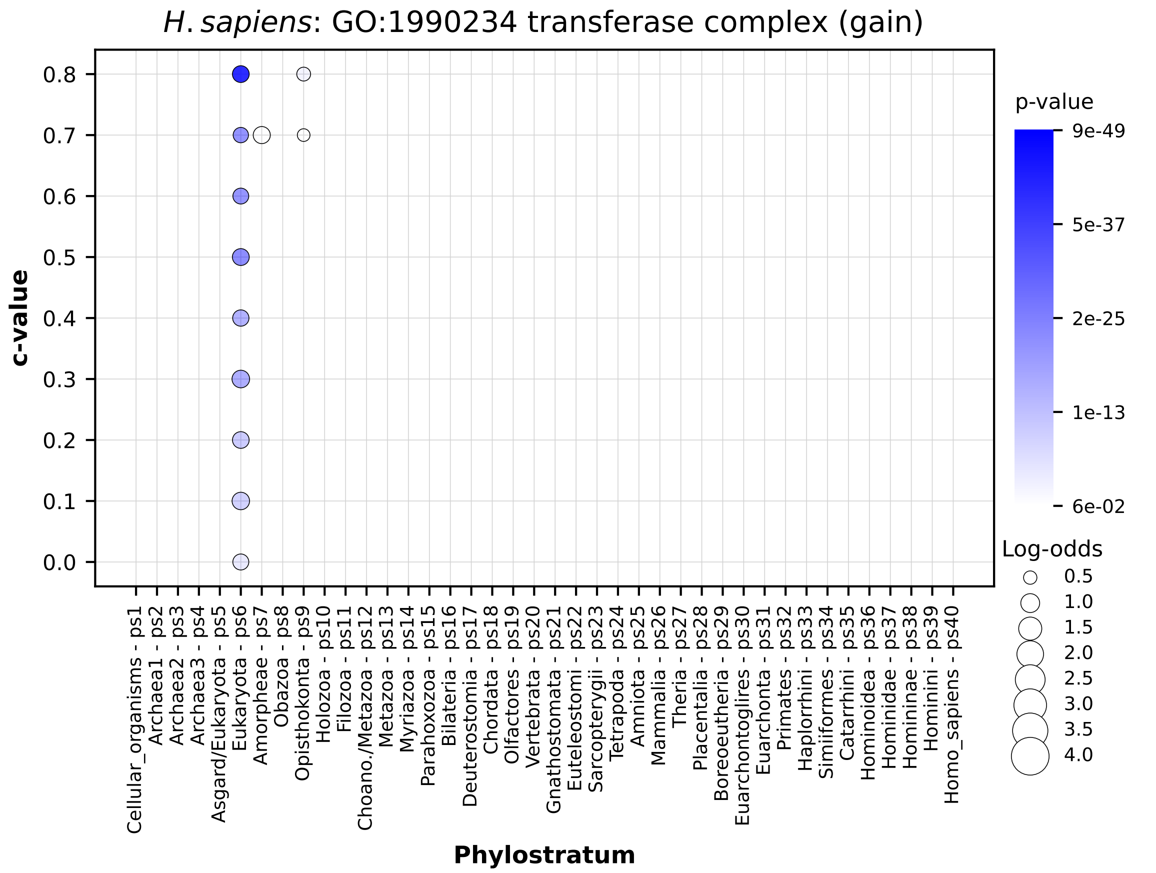

H. sapiens: GO:1990234 transferase complex (gain)
c-value
0.8
0.7
0.6
0.5
0.4
0.3
0.2
0.1
0.0
p-value
9e-49
5e-37
2e-25
1e-13
6e-02
Log-odds
0.5
1.0
1.5
2.0
2.5
3.0
3.5
4.0
Cellular_organisms - ps1
Archaea1 - ps2
Archaea2 - ps3
Archaea3 - ps4
Asgard/Eukaryota - ps5
Eukaryota - ps6
Amorpheae - ps7
Obazoa - ps8
Opisthokonta - ps9
Holozoa - ps10
Filozoa - ps11
Choano./Metazoa - ps12
Metazoa - ps13
Myriazoa - ps14
Parahoxozoa - ps15
Bilateria - ps16
Deuterostomia - ps17
Chordata - ps18
Olfactores - ps19
Vertebrata - ps20
Gnathostomata - ps21
Euteleostomi - ps22
Sarcopterygii - ps23
Tetrapoda - ps24
Amniota - ps25
Mammalia - ps26
Theria - ps27
Placentalia - ps28
Boreoeutheria - ps29
Euarchontoglires - ps30
Euarchonta - ps31
Primates - ps32
Haplorrhini - ps33
Simiiformes - ps34
Catarrhini - ps35
Hominoidea - ps36
Hominidae - ps37
Homininae - ps38
Hominini - ps39
Homo_sapiens - ps40
Phylostratum

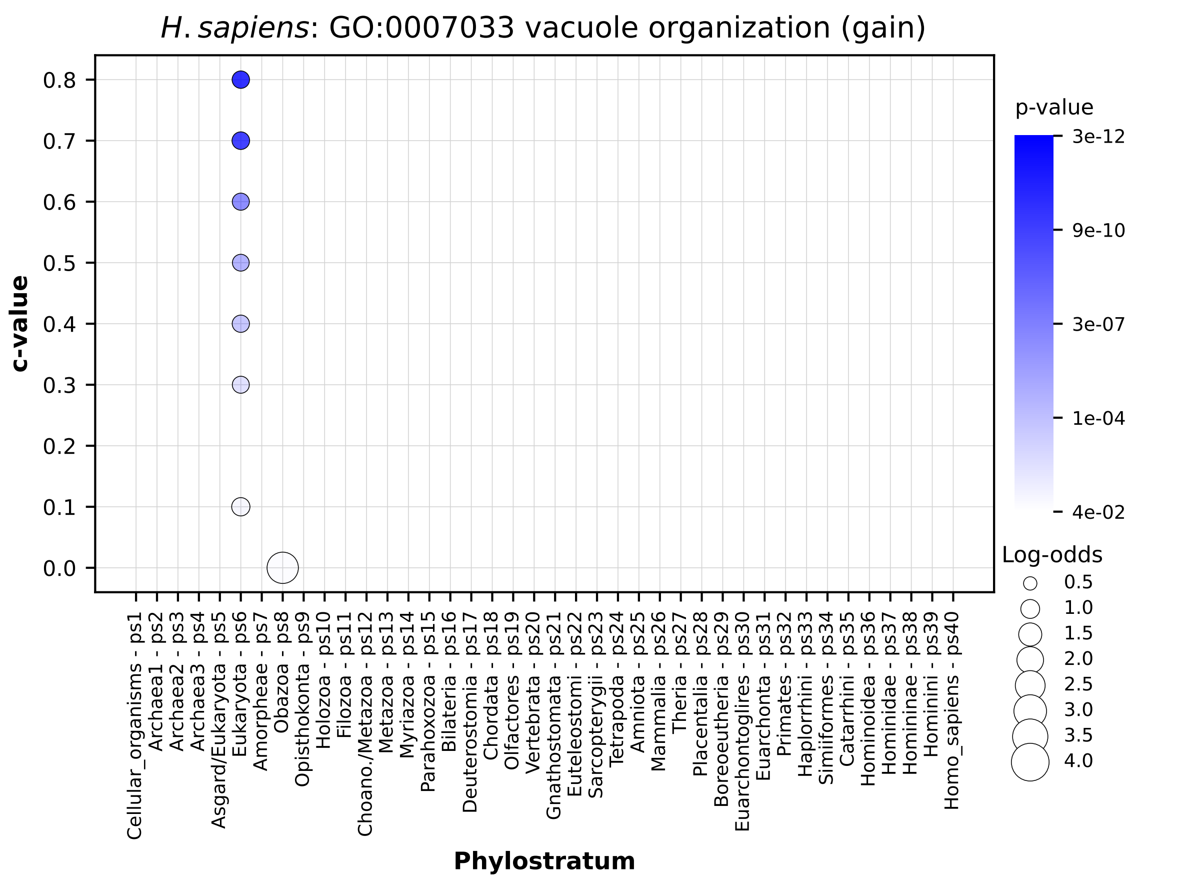
H. sapiens: GO:0007033 vacuole organization (gain)
c-value
0.8
0.7
0.6
0.5
0.4
0.3
0.2
0.1
0.0
p-value
3e-12
9e-10
3e-07
1e-04
4e-02
Log-odds
0.5
1.0
1.5
2.0
2.5
3.0
3.5
4.0
Cellular_organisms - ps1
Archaea1 - ps2
Archaea2 - ps3
Archaea3 - ps4
Asgard/Eukaryota - ps5
Eukaryota - ps6
Amorpheae - ps7
Obazoa - ps8
Opisthokonta - ps9
Holozoa - ps10
Filozoa - ps11
Choano./Metazoa - ps12
Metazoa - ps13
Myriazoa - ps14
Parahoxozoa - ps15
Bilateria - ps16
Deuterostomia - ps17
Chordata - ps18
Olfactores - ps19
Vertebrata - ps20
Gnathostomata - ps21
Euteleostomi - ps22
Sarcopterygii - ps23
Tetrapoda - ps24
Amniota - ps25
Mammalia - ps26
Theria - ps27
Placentalia - ps28
Boreoeutheria - ps29
Euarchontoglires - ps30
Euarchonta - ps31
Primates - ps32
Haplorrhini - ps33
Simiiformes - ps34
Catarrhini - ps35
Hominoidea - ps36
Hominidae - ps37
Homininae - ps38
Hominini - ps39
Homo_sapiens - ps40
Phylostratum

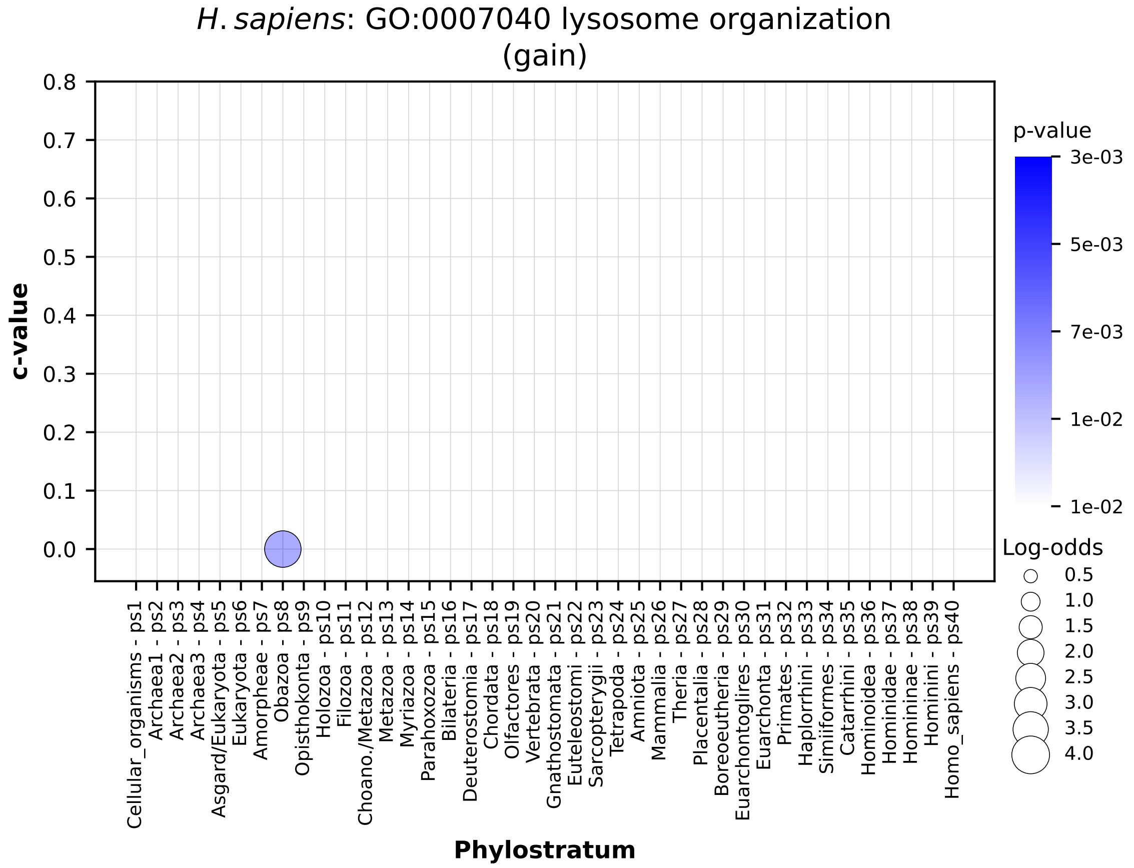
*H. sapiens*: GO:0007040 lysosome organization
(gain)
c-value
0.8
0.7
0.6
0.5
0.4
0.3
0.2
0.1
0.0
Phylostratum
Cellular_organisms - ps1
Archaea1 - ps2
Archaea2 - ps3
Archaea3 - ps4
Asgard/Eukaryota - ps5
Eukaryota - ps6
Amorpheae - ps7
Obazoa - ps8
Opisthokonta - ps9
Holozoa - ps10
Filozoa - ps11
Choano./Metazoa - ps12
Metazoa - ps13
Myriazoa - ps14
Parahoxozoa - ps15
Bilateria - ps16
Deuterostomia - ps17
Chordata - ps18
Olfactores - ps19
Vertebrata - ps20
Gnathostomata - ps21
Euteleostomi - ps22
Sarcopterygii - ps23
Tetrapoda - ps24
Amniota - ps25
Mammalia - ps26
Theria - ps27
Placentalia - ps28
Boreoeutheria - ps29
Euarchontoglires - ps30
Euarchonta - ps31
Primates - ps32
Haplorrhini - ps33
Simiiformes - ps34
Catarrhini - ps35
Hominoidea - ps36
Hominidae - ps37
Homininae - ps38
Hominini - ps39
Homo_sapiens - ps40
p-value
3e-03
5e-03
7e-03
1e-02
1e-02
Log-odds
0.5
1.0
1.5
2.0
2.5
3.0
3.5
4.0

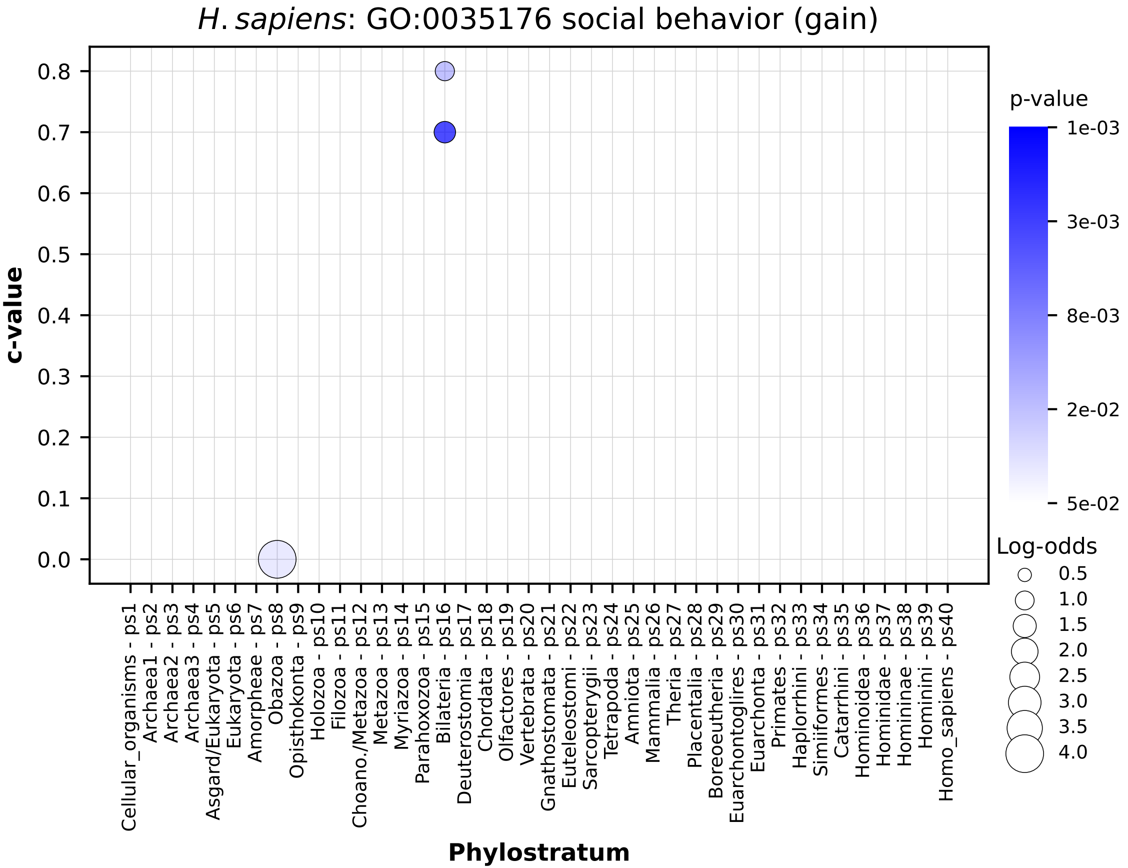
H. sapiens: GO:0035176 social behavior (gain)
c-value
0.0
0.1
0.2
0.3
0.4
0.5
0.6
0.7
0.8
Phylostratum
Cellular_organisms - ps1
Archaea1 - ps2
Archaea2 - ps3
Archaea3 - ps4
Asgard/Eukaryota - ps5
Eukaryota - ps6
Amorpheae - ps7
Obazoa - ps8
Opisthokonta - ps9
Holozoa - ps10
Filozoa - ps11
Choano./Metazoa - ps12
Metazoa - ps13
Myriazoa - ps14
Parahoxozoa - ps15
Bilateria - ps16
Deuterostomia - ps17
Chordata - ps18
Olfactores - ps19
Vertebrata - ps20
Gnathostomata - ps21
Euteleostomi - ps22
Sarcopterygii - ps23
Tetrapoda - ps24
Amniota - ps25
Mammalia - ps26
Theria - ps27
Placentalia - ps28
Boreoeutheria - ps29
Euarchontoglires - ps30
Euarchonta - ps31
Primates - ps32
Haplorrhini - ps33
Simiiformes - ps34
Catarrhini - ps35
Hominoidea - ps36
Hominidae - ps37
Homininae - ps38
Hominini - ps39
Homo_sapiens - ps40
p-value
1e-03
3e-03
8e-03
2e-02
5e-02
Log-odds
0.5
1.0
1.5
2.0
2.5
3.0
3.5
4.0

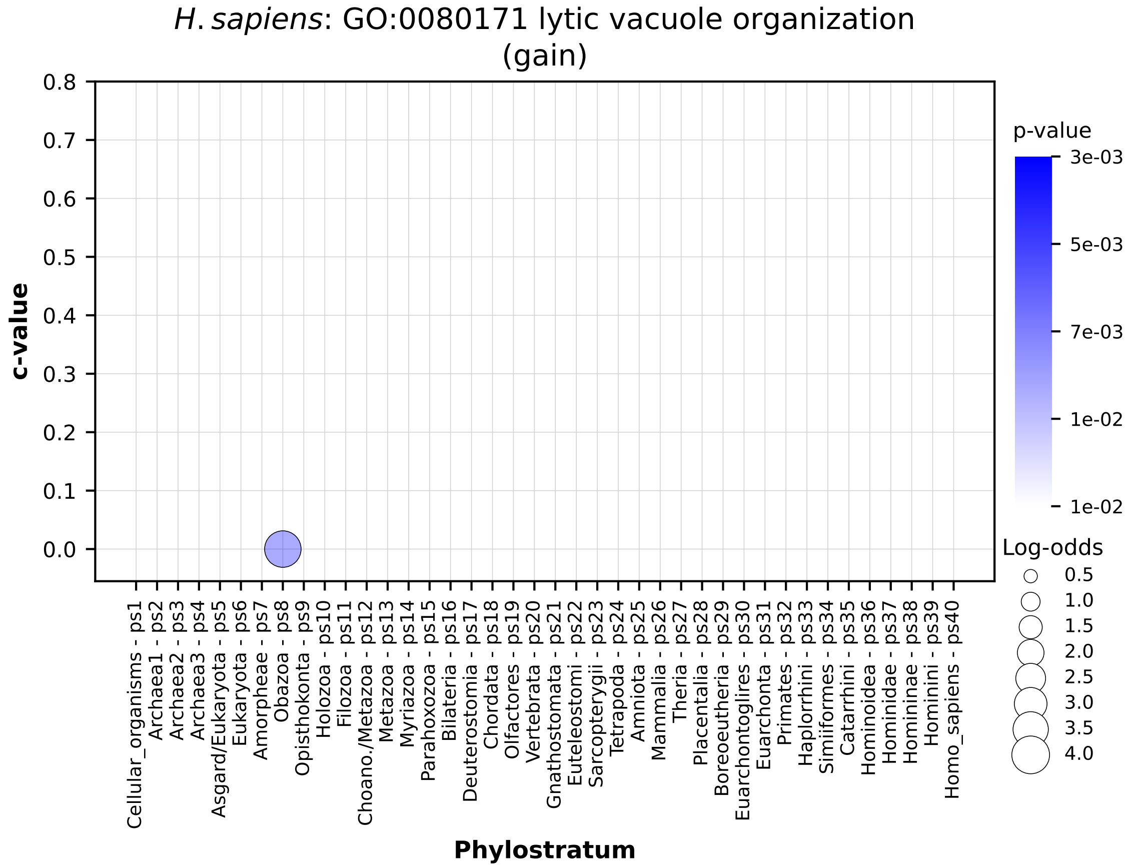

H. sapiens: GO:0080171 lytic vacuole organization
(gain)
c-value
0.8
0.7
0.6
0.5
0.4
0.3
0.2
0.1
0.0
Phylostratum
Cellular_organisms - ps1
Archaea1 - ps2
Archaea2 - ps3
Archaea3 - ps4
Asgard/Eukaryota - ps5
Eukaryota - ps6
Amorpheae - ps7
Obazoa - ps8
Opisthokonta - ps9
Holozoa - ps10
Filozoa - ps11
Choano./Metazoa - ps12
Metazoa - ps13
Myriazoa - ps14
Parahoxozoa - ps15
Bilateria - ps16
Deuterostomia - ps17
Chordata - ps18
Olfactores - ps19
Vertebrata - ps20
Gnathostomata - ps21
Euteleostomi - ps22
Sarcopterygii - ps23
Tetrapoda - ps24
Amniota - ps25
Mammalia - ps26
Theria - ps27
Placentalia - ps28
Boreoeutheria - ps29
Euarchontoglires - ps30
Euarchonta - ps31
Primates - ps32
Haplorrhini - ps33
Simiiformes - ps34
Catarrhini - ps35
Hominoidea - ps36
Hominidae - ps37
Homininae - ps38
Hominini - ps39
Homo_sapiens - ps40
p-value
3e-03
5e-03
7e-03
1e-02
1e-02
Log-odds
0.5
1.0
1.5
2.0
2.5
3.0
3.5
4.0

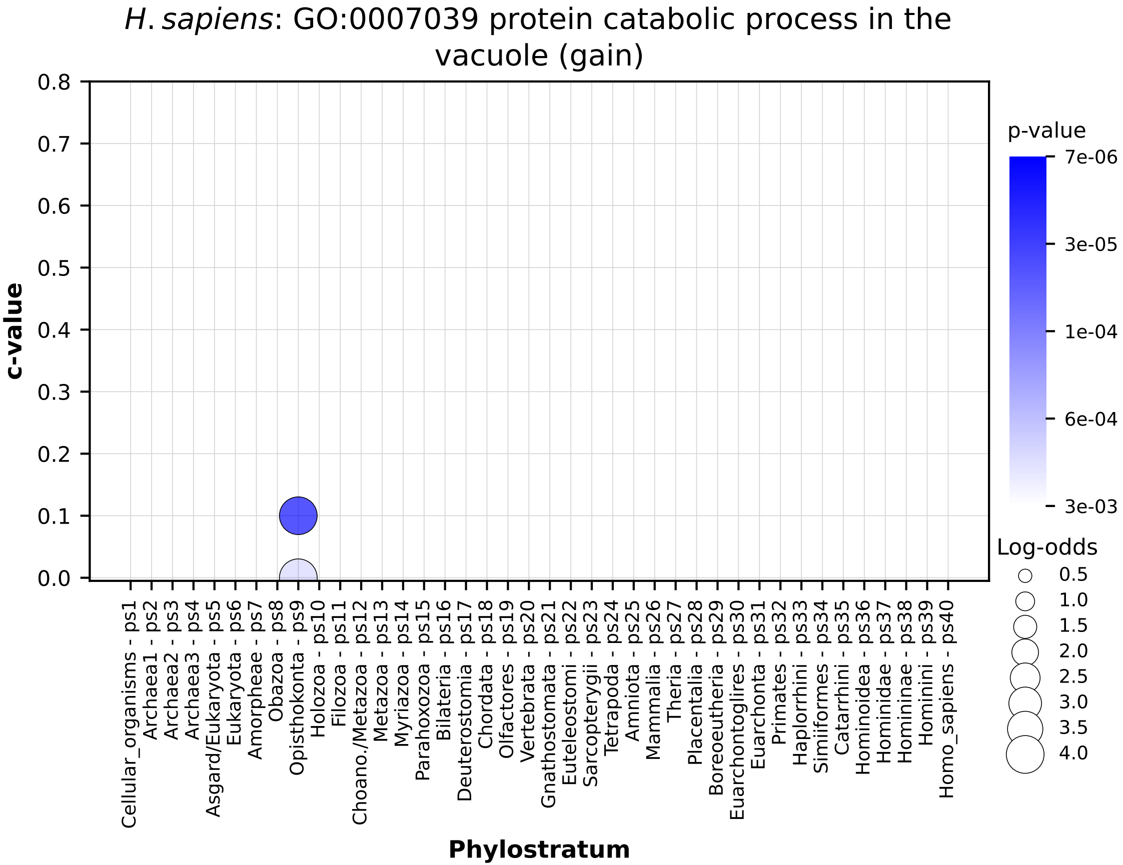

H. sapiens: GO:0007039 protein catabolic process in the vacuole (gain)
c-value
0.8
0.7
0.6
0.5
0.4
0.3
0.2
0.1
0.0
Phylostratum
Cellular_organisms - ps1
Archaea1 - ps2
Archaea2 - ps3
Archaea3 - ps4
Asgard/Eukaryota - ps5
Eukaryota - ps6
Amorpheae - ps7
Obazoa - ps8
Opisthokonta - ps9
Holozoa - ps10
Filozoa - ps11
Choano./Metazoa - ps12
Metazoa - ps13
Myriazoa - ps14
Parahoxozoa - ps15
Bilateria - ps16
Deuterostomia - ps17
Chordata - ps18
Olfactores - ps19
Vertebrata - ps20
Gnathostomata - ps21
Euteleostomi - ps22
Sarcopterygii - ps23
Tetrapoda - ps24
Amniota - ps25
Mammalia - ps26
Theria - ps27
Placentalia - ps28
Boreoeutheria - ps29
Euarchontoglires - ps30
Euarchonta - ps31
Primates - ps32
Haplorrhini - ps33
Simiiformes - ps34
Catarrhini - ps35
Hominoidea - ps36
Hominidae - ps37
Homininae - ps38
Hominini - ps39
Homo_sapiens - ps40
p-value
7e-06
3e-05
1e-04
6e-04
3e-03
Log-odds
0.5
1.0
1.5
2.0
2.5
3.0
3.5
4.0

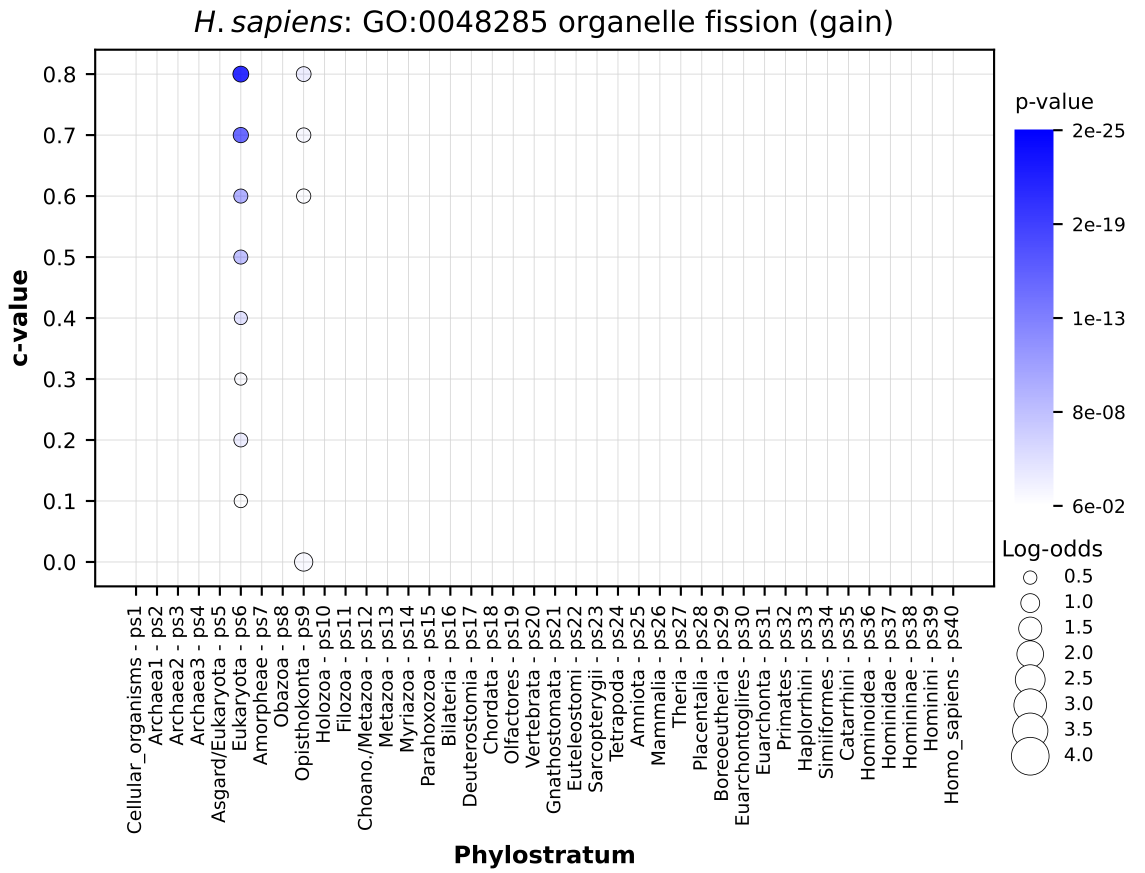

*H. sapiens*: GO:0048285 organelle fission (gain)
c-value
0.8
0.7
0.6
0.5
0.4
0.3
0.2
0.1
0.0
Phylostratum
Cellular_organisms - ps1
Archaea1 - ps2
Archaea2 - ps3
Archaea3 - ps4
Asgard/Eukaryota - ps5
Eukaryota - ps6
Amorpheae - ps7
Obazoa - ps8
Opisthokonta - ps9
Holozoa - ps10
Filozoa - ps11
Choano./Metazoa - ps12
Metazoa - ps13
Myriazoa - ps14
Parahoxozoa - ps15
Bilateria - ps16
Deuterostomia - ps17
Chordata - ps18
Olfactores - ps19
Vertebrata - ps20
Gnathostomata - ps21
Euteleostomi - ps22
Sarcopterygii - ps23
Tetrapoda - ps24
Amniota - ps25
Mammalia - ps26
Theria - ps27
Placentalia - ps28
Boreoeutheria - ps29
Euarchontoglires - ps30
Euarchonta - ps31
Primates - ps32
Haplorrhini - ps33
Simiiformes - ps34
Catarrhini - ps35
Hominoidea - ps36
Hominidae - ps37
Homininae - ps38
Hominini - ps39
Homo_sapiens - ps40
p-value
2e-25
2e-19
1e-13
8e-08
6e-02
Log-odds
0.5
1.0
1.5
2.0
2.5
3.0
3.5
4.0

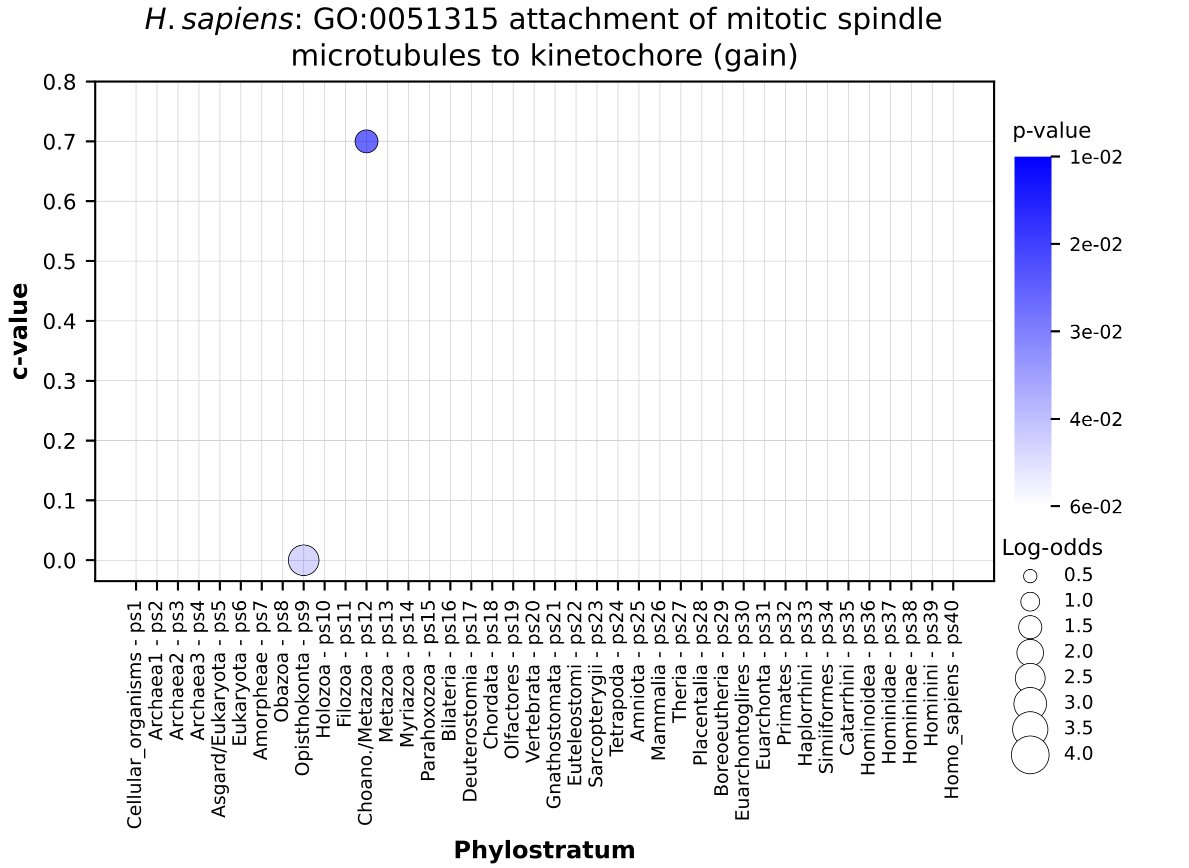

H. sapiens: GO:0051315 attachment of mitotic spindle microtubules to kinetochore (gain)
c-value
0.0
0.1
0.2
0.3
0.4
0.5
0.6
0.7
0.8
p-value
1e-02
2e-02
3e-02
4e-02
6e-02
Log-odds
0.5
1.0
1.5
2.0
2.5
3.0
3.5
4.0
Cellular_organisms - ps1
Archaea1 - ps2
Archaea2 - ps3
Archaea3 - ps4
Asgard/Eukaryota - ps5
Eukaryota - ps6
Amorpheae - ps7
Obazoa - ps8
Opisthokonta - ps9
Holozoa - ps10
Filozoa - ps11
Choano./Metazoa - ps12
Metazoa - ps13
Myriazoa - ps14
Parahoxozoa - ps15
Bilateria - ps16
Deuterostomia - ps17
Chordata - ps18
Olfactores - ps19
Vertebrata - ps20
Gnathostomata - ps21
Euteleostomi - ps22
Sarcopterygii - ps23
Tetrapoda - ps24
Amniota - ps25
Mammalia - ps26
Theria - ps27
Placentalia - ps28
Boreoeutheria - ps29
Euarchontoglires - ps30
Euarchonta - ps31
Primates - ps32
Haplorrhini - ps33
Simiiformes - ps34
Catarrhini - ps35
Hominoidea - ps36
Hominidae - ps37
Homininae - ps38
Hominini - ps39
Homo_sapiens - ps40
Phylostratum

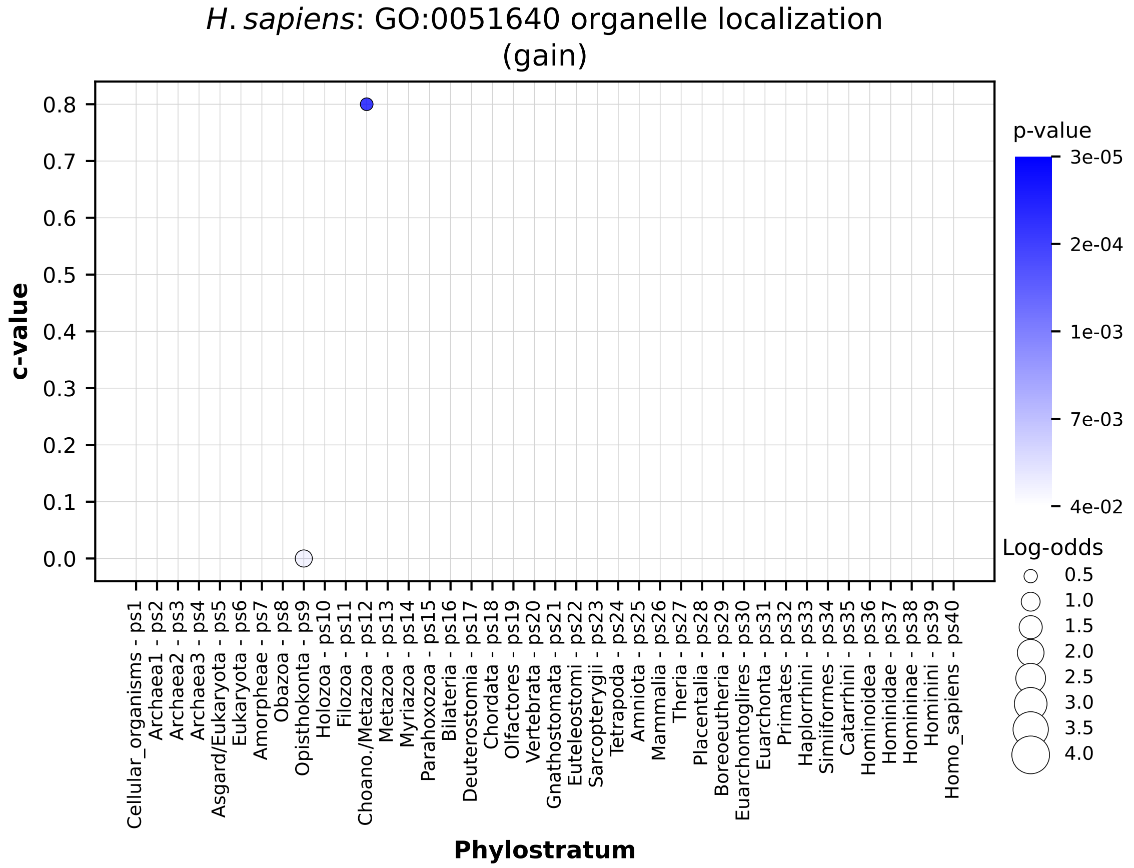

*H. sapiens*: GO:0051640 organelle localization
(gain)
c-value
0.8
0.7
0.6
0.5
0.4
0.3
0.2
0.1
0.0
p-value
3e-05
2e-04
1e-03
7e-03
4e-02
Log-odds
0.5
1.0
1.5
2.0
2.5
3.0
3.5
4.0
Cellular_organisms - ps1
Archaea1 - ps2
Archaea2 - ps3
Archaea3 - ps4
Asgard/Eukaryota - ps5
Eukaryota - ps6
Amorpheae - ps7
Obazoa - ps8
Opisthokonta - ps9
Holozoa - ps10
Filozoa - ps11
Choano./Metazoa - ps12
Metazoa - ps13
Myriazoa - ps14
Parahoxozoa - ps15
Bilateria - ps16
Deuterostomia - ps17
Chordata - ps18
Olfactores - ps19
Vertebrata - ps20
Gnathostomata - ps21
Euteleostomi - ps22
Sarcopterygii - ps23
Tetrapoda - ps24
Amniota - ps25
Mammalia - ps26
Theria - ps27
Placentalia - ps28
Boreoeutheria - ps29
Euarchontoglires - ps30
Euarchonta - ps31
Primates - ps32
Haplorrhini - ps33
Simiiformes - ps34
Catarrhini - ps35
Hominoidea - ps36
Hominidae - ps37
Homininae - ps38
Hominini - ps39
Homo_sapiens - ps40
Phylostratum

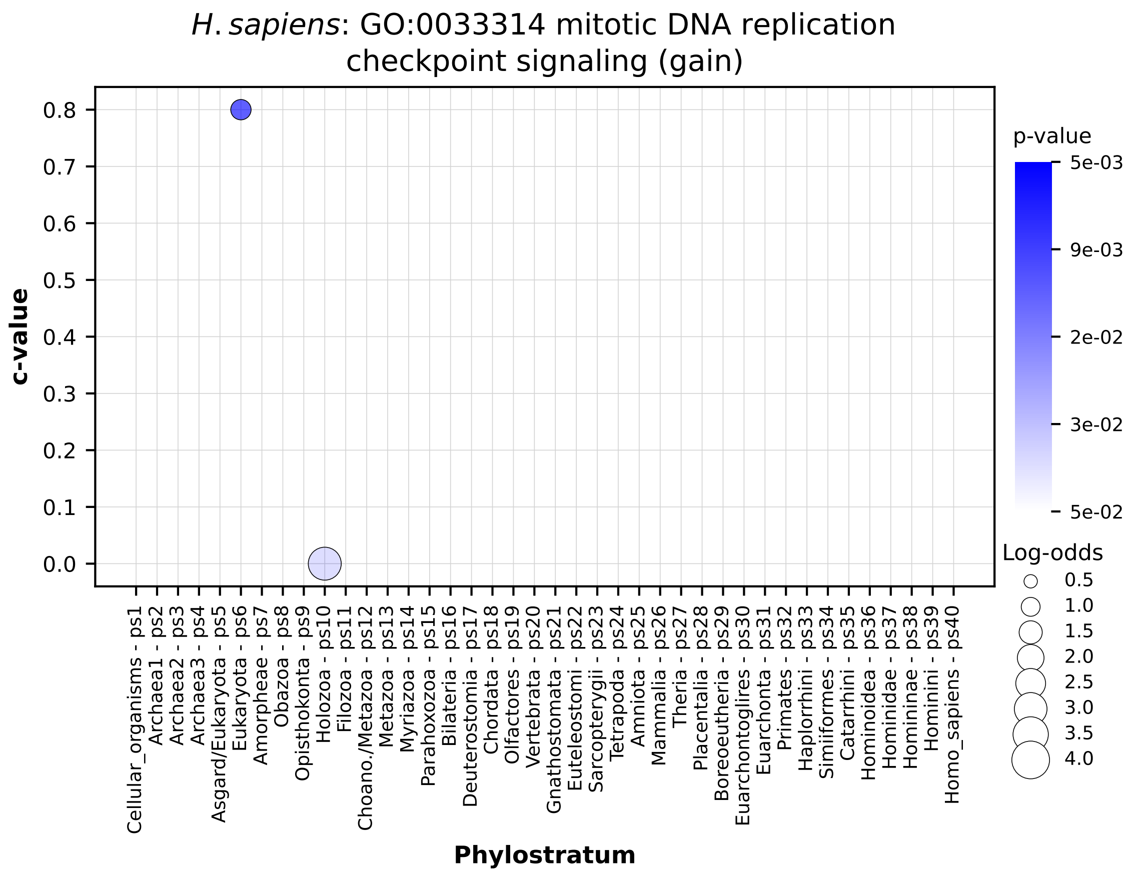

H. sapiens: GO:0033314 mitotic DNA replication checkpoint signaling (gain)
c-value
0.8
0.7
0.6
0.5
0.4
0.3
0.2
0.1
0.0
p-value
5e-03
9e-03
2e-02
3e-02
5e-02
Log-odds
0.5
1.0
1.5
2.0
2.5
3.0
3.5
4.0
Cellular_organisms - ps1
Archaea1 - ps2
Archaea2 - ps3
Archaea3 - ps4
Asgard/Eukaryota - ps5
Eukaryota - ps6
Amorpheae - ps7
Obazoa - ps8
Opisthokonta - ps9
Holozoa - ps10
Filozoa - ps11
Choano./Metazoa - ps12
Metazoa - ps13
Myriazoa - ps14
Parahoxozoa - ps15
Bilateria - ps16
Deuterostomia - ps17
Chordata - ps18
Olfactores - ps19
Vertebrata - ps20
Gnathostomata - ps21
Euteleostomi - ps22
Sarcopterygii - ps23
Tetrapoda - ps24
Amniota - ps25
Mammalia - ps26
Theria - ps27
Placentalia - ps28
Boreoeutheria - ps29
Euarchontoglires - ps30
Euarchonta - ps31
Primates - ps32
Haplorrhini - ps33
Simiiformes - ps34
Catarrhini - ps35
Hominoidea - ps36
Hominidae - ps37
Homininae - ps38
Hominini - ps39
Homo_sapiens - ps40
Phylostratum

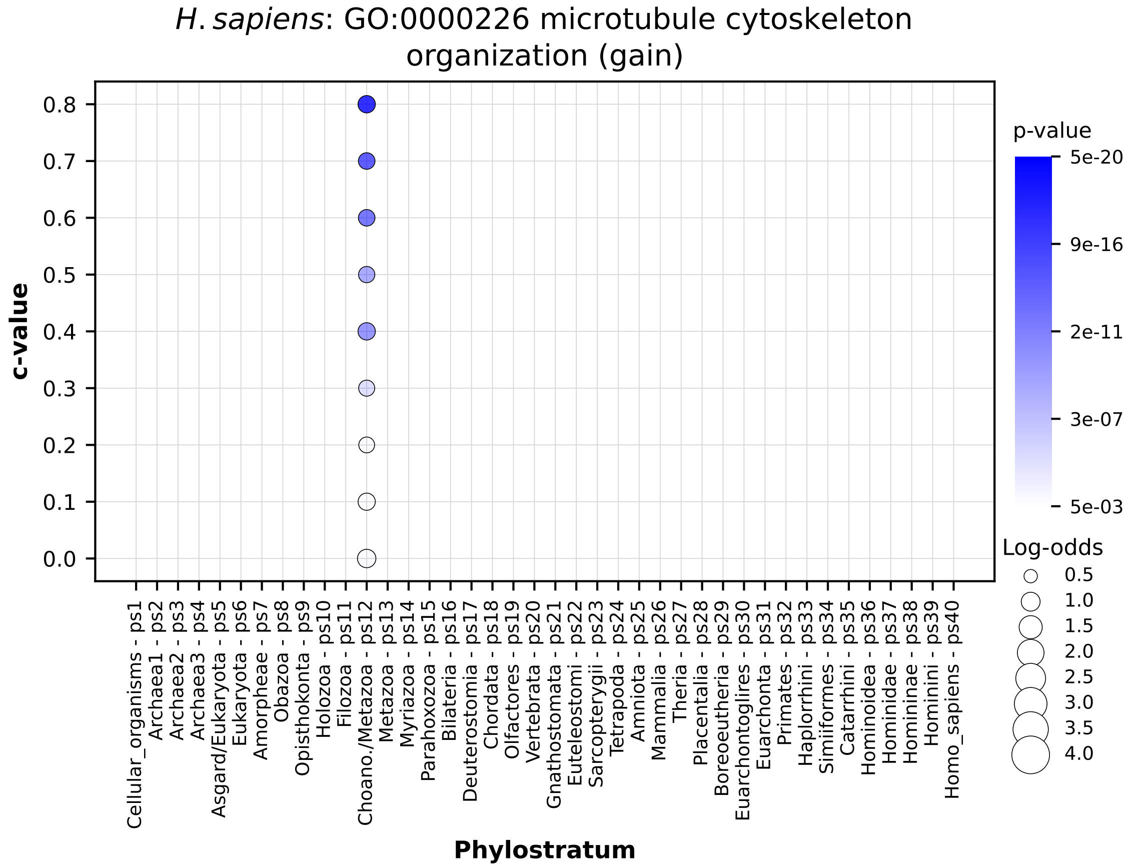

H. sapiens: GO:0000226 microtubule cytoskeleton organization (gain)
c-value
0.8
0.7
0.6
0.5
0.4
0.3
0.2
0.1
0.0
p-value
5e-20
9e-16
2e-11
3e-07
5e-03
Log-odds
0.5
1.0
1.5
2.0
2.5
3.0
3.5
4.0
Cellular_organisms - ps1
Archaea1 - ps2
Archaea2 - ps3
Archaea3 - ps4
Asgard/Eukaryota - ps5
Eukaryota - ps6
Amorpheae - ps7
Obazoa - ps8
Opisthokonta - ps9
Holozoa - ps10
Filozoa - ps11
Choano./Metazoa - ps12
Metazoa - ps13
Myriazoa - ps14
Parahoxozoa - ps15
Bilateria - ps16
Deuterostomia - ps17
Chordata - ps18
Olfactores - ps19
Vertebrata - ps20
Gnathostomata - ps21
Euteleostomi - ps22
Sarcopterygii - ps23
Tetrapoda - ps24
Amniota - ps25
Mammalia - ps26
Theria - ps27
Placentalia - ps28
Boreoeutheria - ps29
Euarchontoglires - ps30
Euarchonta - ps31
Primates - ps32
Haplorrhini - ps33
Simiiformes - ps34
Catarrhini - ps35
Hominoidea - ps36
Hominidae - ps37
Homininae - ps38
Hominini - ps39
Homo_sapiens - ps40
Phylostratum

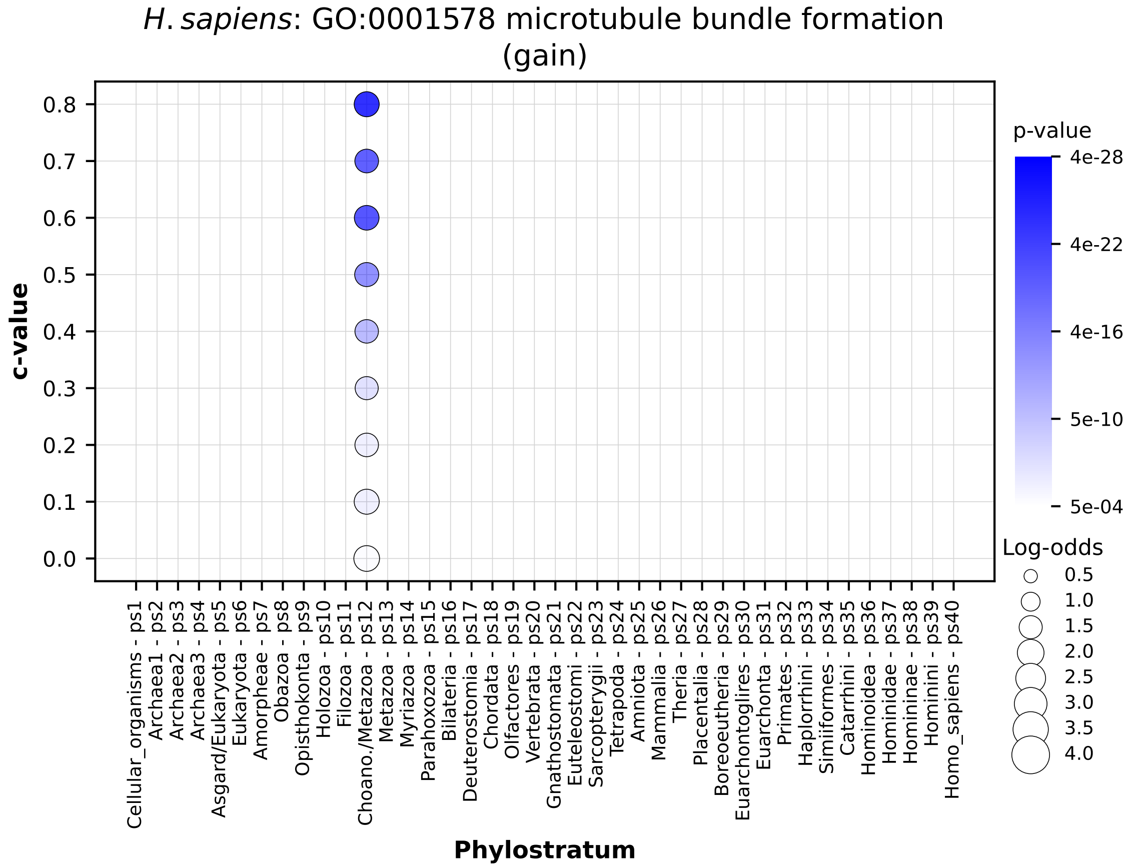

*H. sapiens*: GO:0001578 microtubule bundle formation
(gain)
c-value
0.8
0.7
0.6
0.5
0.4
0.3
0.2
0.1
0.0
p-value
4e-28
4e-22
4e-16
5e-10
5e-04
Log-odds
0.5
1.0
1.5
2.0
2.5
3.0
3.5
4.0
Cellular_organisms - ps1
Archaea1 - ps2
Archaea2 - ps3
Archaea3 - ps4
Asgard/Eukaryota - ps5
Eukaryota - ps6
Amorpheae - ps7
Obazoa - ps8
Opisthokonta - ps9
Holozoa - ps10
Filozoa - ps11
Choano./Metazoa - ps12
Metazoa - ps13
Myriazoa - ps14
Parahoxozoa - ps15
Bilateria - ps16
Deuterostomia - ps17
Chordata - ps18
Olfactores - ps19
Vertebrata - ps20
Gnathostomata - ps21
Euteleostomi - ps22
Sarcopterygii - ps23
Tetrapoda - ps24
Amniota - ps25
Mammalia - ps26
Theria - ps27
Placentalia - ps28
Boreoeutheria - ps29
Euarchontoglires - ps30
Euarchonta - ps31
Primates - ps32
Haplorrhini - ps33
Simiiformes - ps34
Catarrhini - ps35
Hominoidea - ps36
Hominidae - ps37
Homininae - ps38
Hominini - ps39
Homo_sapiens - ps40
Phylostratum

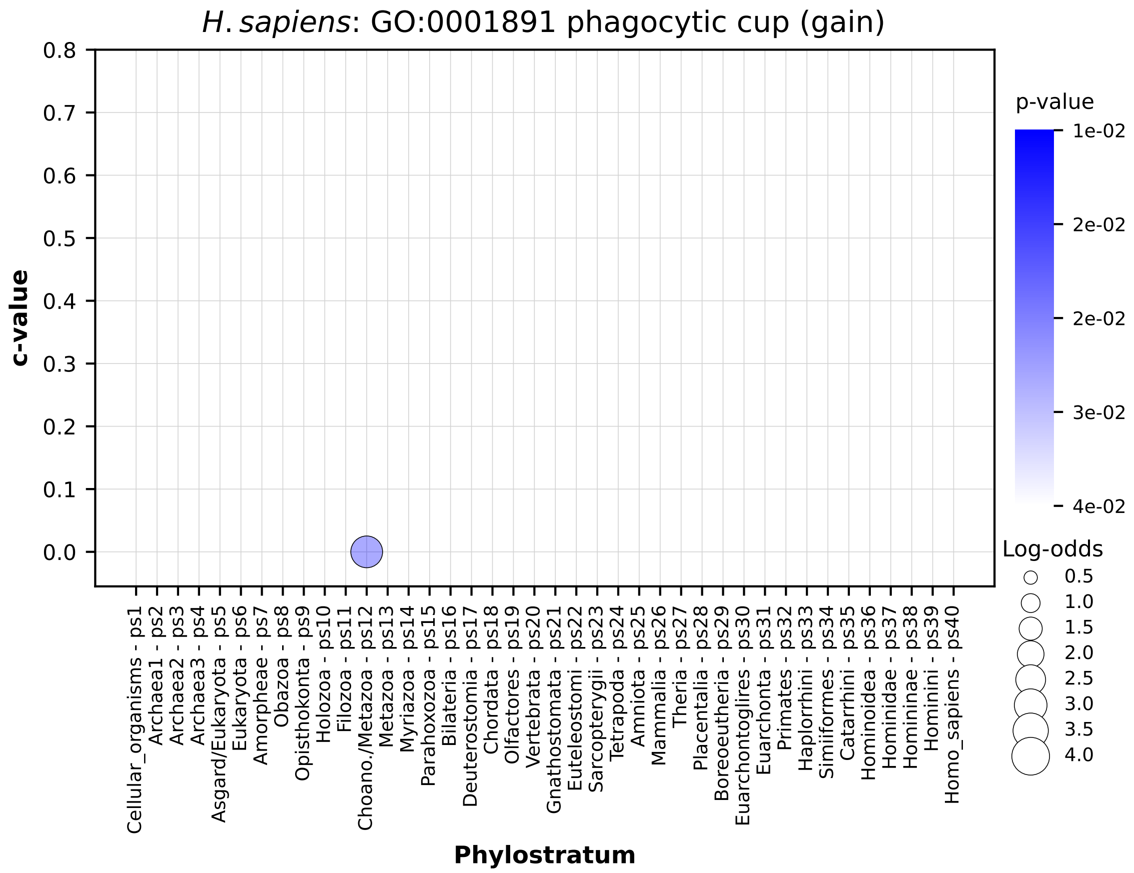

H. sapiens: GO:0001891 phagocytic cup (gain)
c-value
0.8
0.7
0.6
0.5
0.4
0.3
0.2
0.1
0.0
Phylostratum
Cellular_organisms - ps1
Archaea1 - ps2
Archaea2 - ps3
Archaea3 - ps4
Asgard/Eukaryota - ps5
Eukaryota - ps6
Amorpheae - ps7
Obazoa - ps8
Opisthokonta - ps9
Holozoa - ps10
Filozoa - ps11
Choano./Metazoa - ps12
Metazoa - ps13
Myriazoa - ps14
Parahoxozoa - ps15
Bilateria - ps16
Deuterostomia - ps17
Chordata - ps18
Olfactores - ps19
Vertebrata - ps20
Gnathostomata - ps21
Euteleostomi - ps22
Sarcopterygii - ps23
Tetrapoda - ps24
Amniota - ps25
Mammalia - ps26
Theria - ps27
Placentalia - ps28
Boreoeutheria - ps29
Euarchontoglires - ps30
Euarchonta - ps31
Primates - ps32
Haplorrhini - ps33
Simiiformes - ps34
Catarrhini - ps35
Hominoidea - ps36
Hominidae - ps37
Homininae - ps38
Hominini - ps39
Homo_sapiens - ps40
p-value
1e-02
2e-02
2e-02
3e-02
4e-02
Log-odds
0.5
1.0
1.5
2.0
2.5
3.0
3.5
4.0

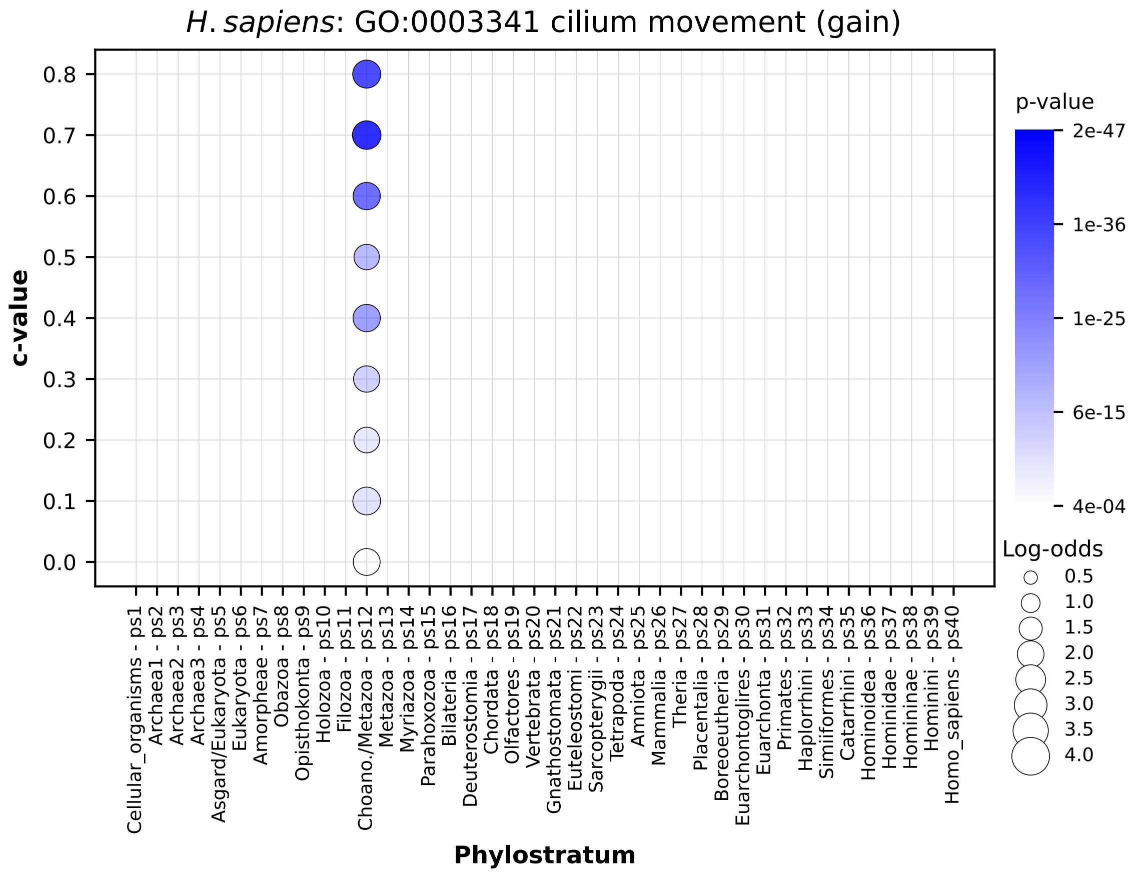

*H. sapiens*: GO:0003341 cilium movement (gain)
c-value
0.8
0.7
0.6
0.5
0.4
0.3
0.2
0.1
0.0
p-value
2e-47
1e-36
1e-25
6e-15
4e-04
Log-odds
0.5
1.0
1.5
2.0
2.5
3.0
3.5
4.0
Cellular_organisms - ps1
Archaea1 - ps2
Archaea2 - ps3
Archaea3 - ps4
Asgard/Eukaryota - ps5
Eukaryota - ps6
Amorpheae - ps7
Obazoa - ps8
Opisthokonta - ps9
Holozoa - ps10
Filozoa - ps11
Choano./Metazoa - ps12
Metazoa - ps13
Myriazoa - ps14
Parahoxozoa - ps15
Bilateria - ps16
Deuterostomia - ps17
Chordata - ps18
Olfactores - ps19
Vertebrata - ps20
Gnathostomata - ps21
Euteleostomi - ps22
Sarcopterygii - ps23
Tetrapoda - ps24
Amniota - ps25
Mammalia - ps26
Theria - ps27
Placentalia - ps28
Boreoeutheria - ps29
Euarchontoglires - ps30
Euarchonta - ps31
Primates - ps32
Haplorrhini - ps33
Simiiformes - ps34
Catarrhini - ps35
Hominoidea - ps36
Hominidae - ps37
Homininae - ps38
Hominini - ps39
Homo_sapiens - ps40
Phylostratum

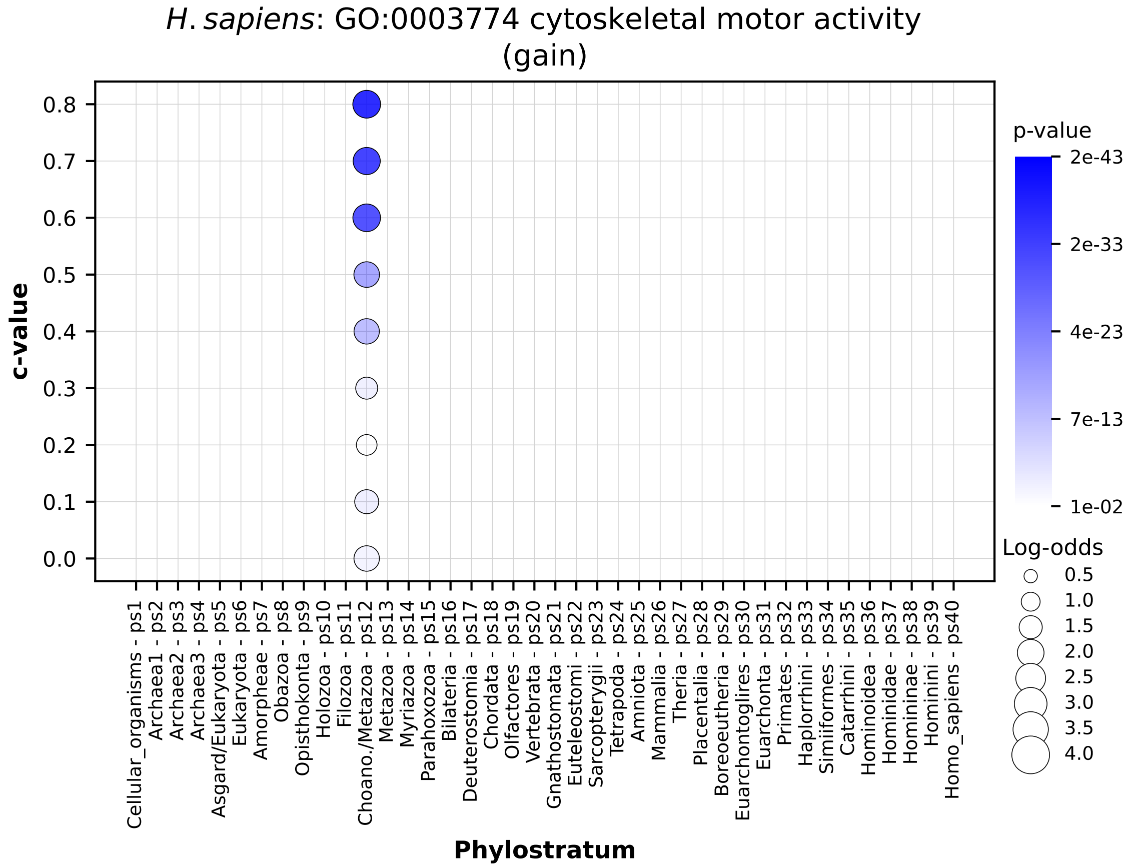

H. sapiens: GO:0003774 cytoskeletal motor activity
(gain)
c-value
0.8
0.7
0.6
0.5
0.4
0.3
0.2
0.1
0.0
p-value
2e-43
2e-33
4e-23
7e-13
1e-02
Log-odds
0.5
1.0
1.5
2.0
2.5
3.0
3.5
4.0
Cellular_organisms - ps1
Archaea1 - ps2
Archaea2 - ps3
Archaea3 - ps4
Asgard/Eukaryota - ps5
Eukaryota - ps6
Amorpheae - ps7
Obazoa - ps8
Opisthokonta - ps9
Holozoa - ps10
Filozoa - ps11
Choano./Metazoa - ps12
Metazoa - ps13
Myriazoa - ps14
Parahoxozoa - ps15
Bilateria - ps16
Deuterostomia - ps17
Chordata - ps18
Olfactores - ps19
Vertebrata - ps20
Gnathostomata - ps21
Euteleostomi - ps22
Sarcopterygii - ps23
Tetrapoda - ps24
Amniota - ps25
Mammalia - ps26
Theria - ps27
Placentalia - ps28
Boreoeutheria - ps29
Euarchontoglires - ps30
Euarchonta - ps31
Primates - ps32
Haplorrhini - ps33
Simiiformes - ps34
Catarrhini - ps35
Hominoidea - ps36
Hominidae - ps37
Homininae - ps38
Hominini - ps39
Homo_sapiens - ps40
Phylostratum

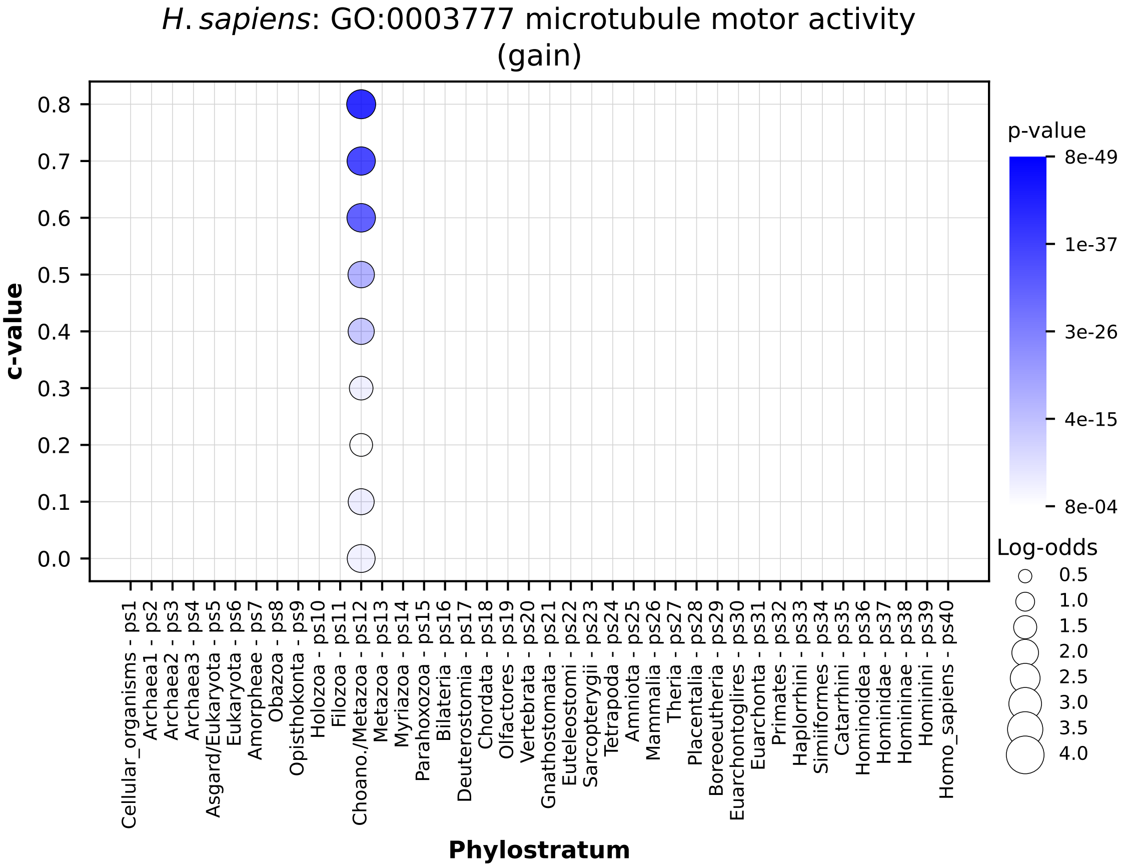

H. sapiens: GO:0003777 microtubule motor activity
(gain)
c-value
0.8
0.7
0.6
0.5
0.4
0.3
0.2
0.1
0.0
Phylostratum
Cellular_organisms - ps1
Archaea1 - ps2
Archaea2 - ps3
Archaea3 - ps4
Asgard/Eukaryota - ps5
Eukaryota - ps6
Amorpheae - ps7
Obazoa - ps8
Opisthokonta - ps9
Holozoa - ps10
Filozoa - ps11
Choano./Metazoa - ps12
Metazoa - ps13
Myriazoa - ps14
Parahoxozoa - ps15
Bilateria - ps16
Deuterostomia - ps17
Chordata - ps18
Olfactores - ps19
Vertebrata - ps20
Gnathostomata - ps21
Euteleostomi - ps22
Sarcopterygii - ps23
Tetrapoda - ps24
Amniota - ps25
Mammalia - ps26
Theria - ps27
Placentalia - ps28
Boreoeutheria - ps29
Euarchontoglires - ps30
Euarchonta - ps31
Primates - ps32
Haplorrhini - ps33
Simiiformes - ps34
Catarrhini - ps35
Hominoidea - ps36
Hominidae - ps37
Homininae - ps38
Hominini - ps39
Homo_sapiens - ps40
p-value
8e-49
1e-37
3e-26
4e-15
8e-04
Log-odds
0.5
1.0
1.5
2.0
2.5
3.0
3.5
4.0

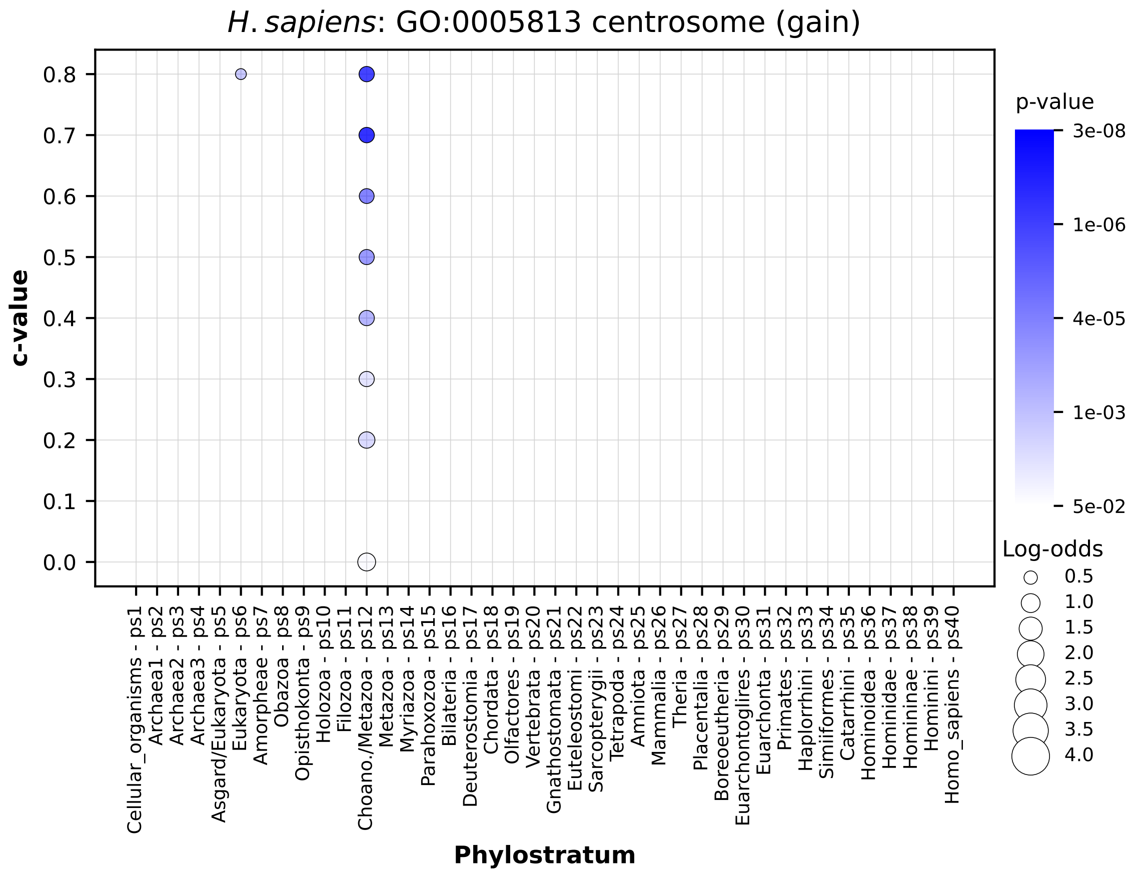
*H. sapiens*: GO:0005813 centrosome (gain)
c-value
0.8
0.7
0.6
0.5
0.4
0.3
0.2
0.1
0.0
Phylostratum
Cellular_organisms - ps1
Archaea1 - ps2
Archaea2 - ps3
Archaea3 - ps4
Asgard/Eukaryota - ps5
Eukaryota - ps6
Amorpheae - ps7
Obazoa - ps8
Opisthokonta - ps9
Holozoa - ps10
Filozoa - ps11
Choano./Metazoa - ps12
Metazoa - ps13
Myriazoa - ps14
Parahoxozoa - ps15
Bilateria - ps16
Deuterostomia - ps17
Chordata - ps18
Olfactores - ps19
Vertebrata - ps20
Gnathostomata - ps21
Euteleostomi - ps22
Sarcopterygii - ps23
Tetrapoda - ps24
Amniota - ps25
Mammalia - ps26
Theria - ps27
Placentalia - ps28
Boreoeutheria - ps29
Euarchontoglires - ps30
Euarchonta - ps31
Primates - ps32
Haplorrhini - ps33
Simiiformes - ps34
Catarrhini - ps35
Hominoidea - ps36
Hominidae - ps37
Homininae - ps38
Hominini - ps39
Homo_sapiens - ps40
p-value
3e-08
1e-06
4e-05
1e-03
5e-02
Log-odds
0.5
1.0
1.5
2.0
2.5
3.0
3.5
4.0

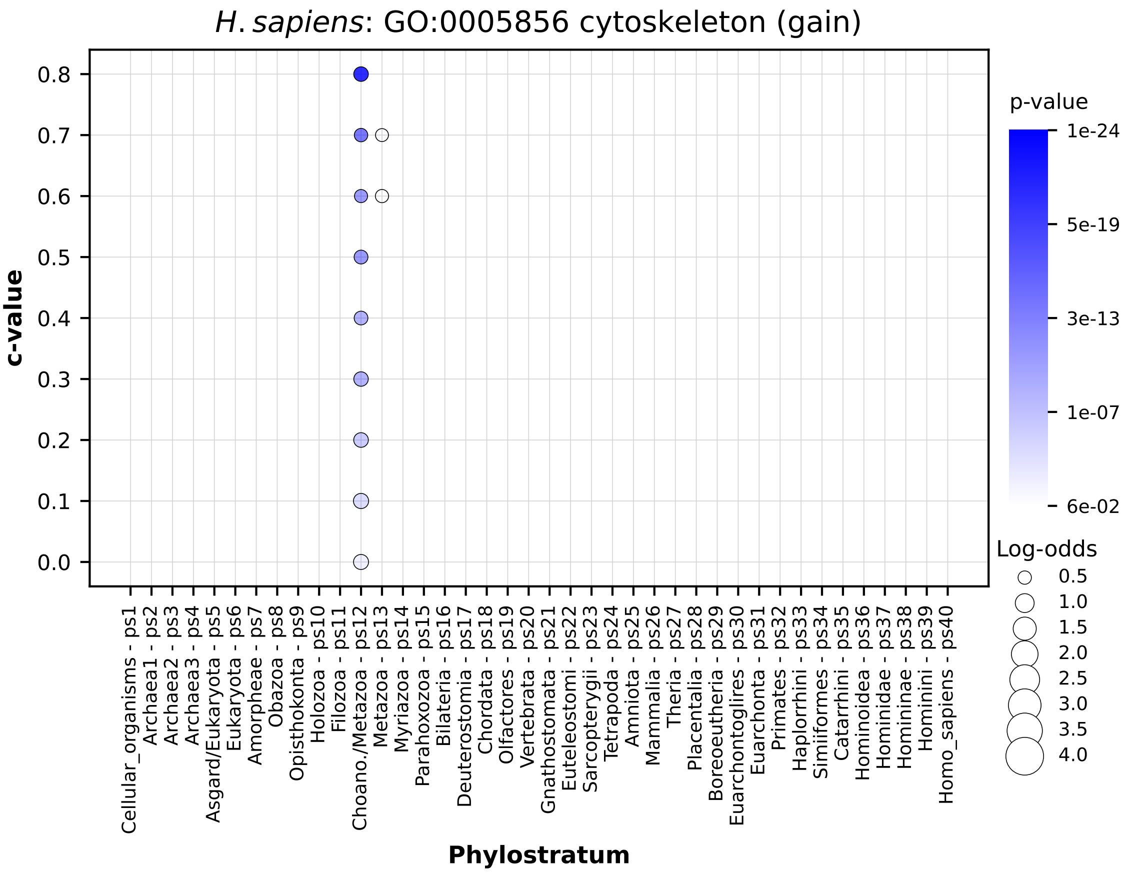
H. sapiens: GO:0005856 cytoskeleton (gain)
c-value
0.8
0.7
0.6
0.5
0.4
0.3
0.2
0.1
0.0
p-value
1e-24
5e-19
3e-13
1e-07
6e-02
Log-odds
0.5
1.0
1.5
2.0
2.5
3.0
3.5
4.0
Cellular_organisms - ps1
Archaea1 - ps2
Archaea2 - ps3
Archaea3 - ps4
Asgard/Eukaryota - ps5
Eukaryota - ps6
Amorpheae - ps7
Obazoa - ps8
Opisthokonta - ps9
Holozoa - ps10
Filozoa - ps11
Choano./Metazoa - ps12
Metazoa - ps13
Myriazoa - ps14
Parahoxozoa - ps15
Bilateria - ps16
Deuterostomia - ps17
Chordata - ps18
Olfactores - ps19
Vertebrata - ps20
Gnathostomata - ps21
Euteleostomi - ps22
Sarcopterygii - ps23
Tetrapoda - ps24
Amniota - ps25
Mammalia - ps26
Theria - ps27
Placentalia - ps28
Boreoeutheria - ps29
Euarchontoglires - ps30
Euarchonta - ps31
Primates - ps32
Haplorrhini - ps33
Simiiformes - ps34
Catarrhini - ps35
Hominoidea - ps36
Hominidae - ps37
Homininae - ps38
Hominini - ps39
Homo_sapiens - ps40
Phylostratum

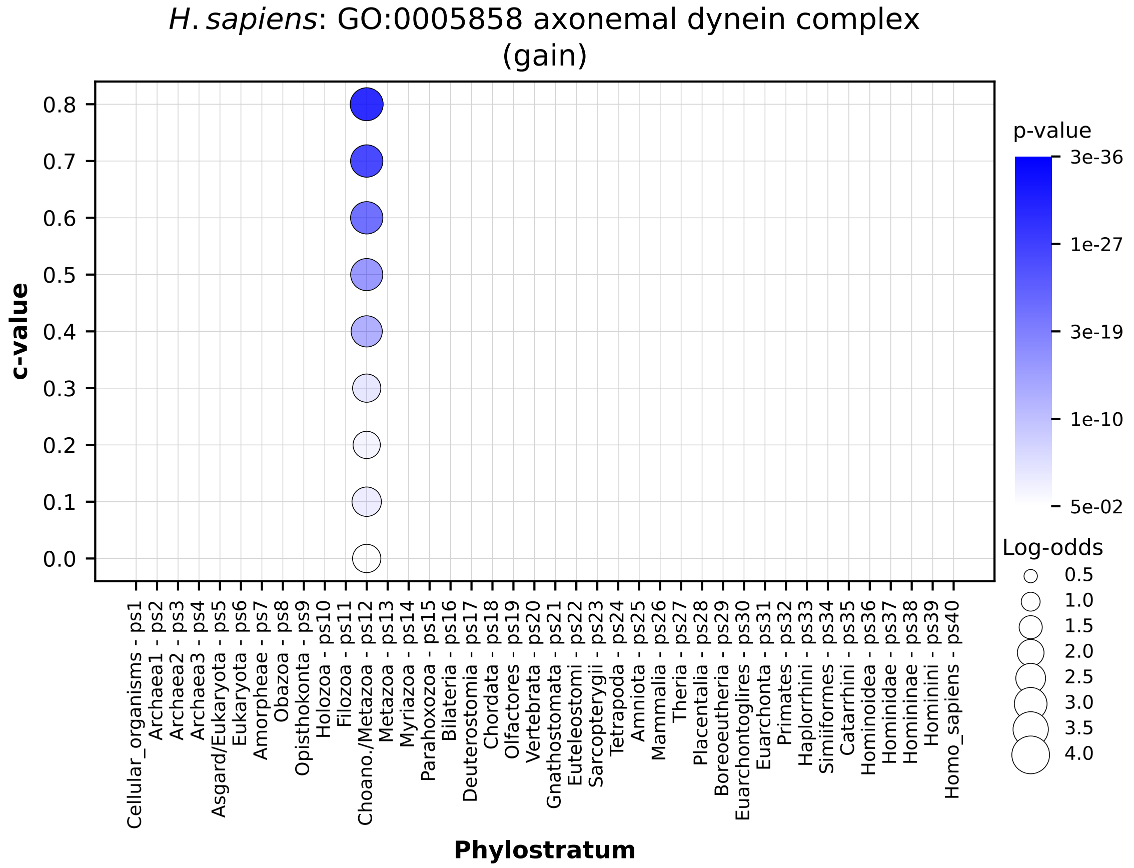

H. sapiens: GO:0005858 axonemal dynein complex
(gain)
c-value
0.8
0.7
0.6
0.5
0.4
0.3
0.2
0.1
0.0
p-value
3e-36
1e-27
3e-19
1e-10
5e-02
Log-odds
0.5
1.0
1.5
2.0
2.5
3.0
3.5
4.0
Cellular_organisms - ps1
Archaea1 - ps2
Archaea2 - ps3
Archaea3 - ps4
Asgard/Eukaryota - ps5
Eukaryota - ps6
Amorpheae - ps7
Obazoa - ps8
Opisthokonta - ps9
Holozoa - ps10
Filozoa - ps11
Choano./Metazoa - ps12
Metazoa - ps13
Myriazoa - ps14
Parahoxozoa - ps15
Bilateria - ps16
Deuterostomia - ps17
Chordata - ps18
Olfactores - ps19
Vertebrata - ps20
Gnathostomata - ps21
Euteleostomi - ps22
Sarcopterygii - ps23
Tetrapoda - ps24
Amniota - ps25
Mammalia - ps26
Theria - ps27
Placentalia - ps28
Boreoeutheria - ps29
Euarchontoglires - ps30
Euarchonta - ps31
Primates - ps32
Haplorrhini - ps33
Simiiformes - ps34
Catarrhini - ps35
Hominoidea - ps36
Hominidae - ps37
Homininae - ps38
Hominini - ps39
Homo_sapiens - ps40
Phylostratum

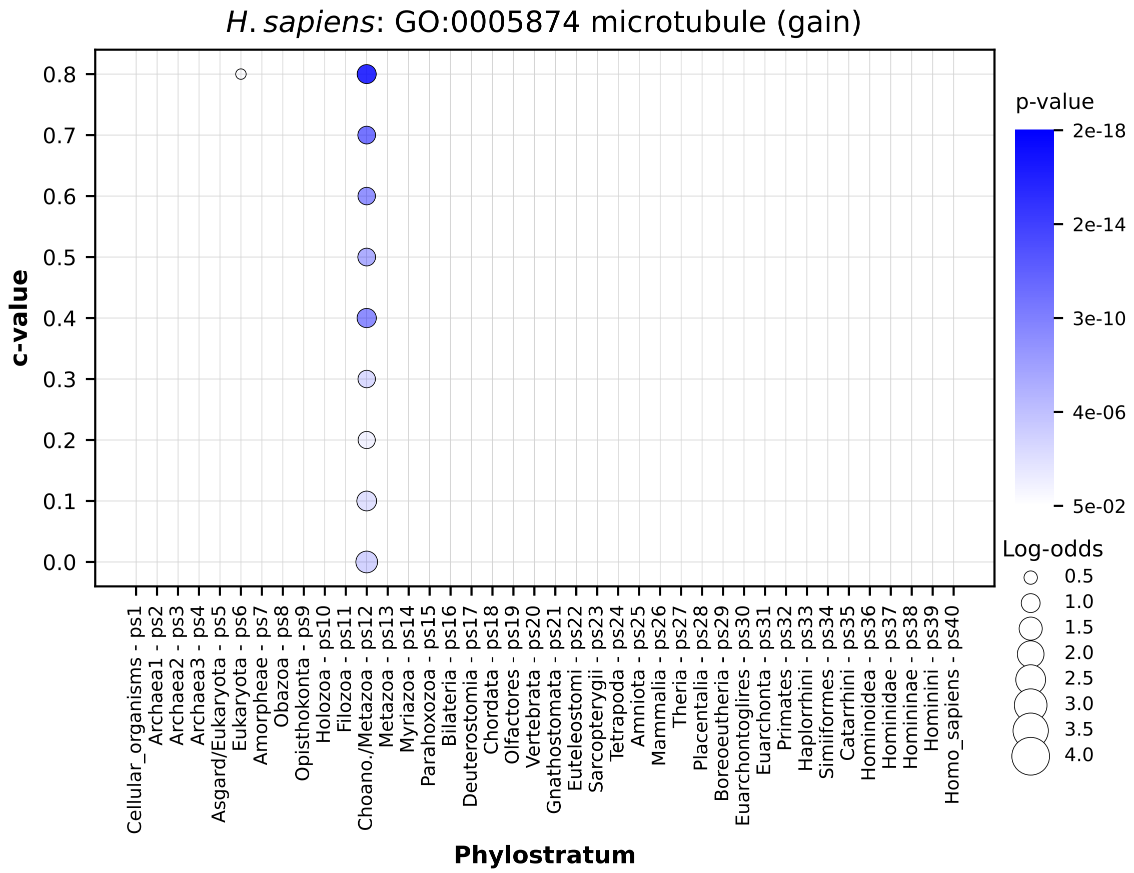

*H. sapiens*: GO:0005874 microtubule (gain)
c-value
0.8
0.7
0.6
0.5
0.4
0.3
0.2
0.1
0.0
Phylostratum
Cellular_organisms - ps1
Archaea1 - ps2
Archaea2 - ps3
Archaea3 - ps4
Asgard/Eukaryota - ps5
Eukaryota - ps6
Amorpheae - ps7
Obazoa - ps8
Opisthokonta - ps9
Holozoa - ps10
Filozoa - ps11
Choano./Metazoa - ps12
Metazoa - ps13
Myriazoa - ps14
Parahoxozoa - ps15
Bilateria - ps16
Deuterostomia - ps17
Chordata - ps18
Olfactores - ps19
Vertebrata - ps20
Gnathostomata - ps21
Euteleostomi - ps22
Sarcopterygii - ps23
Tetrapoda - ps24
Amniota - ps25
Mammalia - ps26
Theria - ps27
Placentalia - ps28
Boreoeutheria - ps29
Euarchontoglires - ps30
Euarchonta - ps31
Primates - ps32
Haplorrhini - ps33
Simiiformes - ps34
Catarrhini - ps35
Hominoidea - ps36
Hominidae - ps37
Homininae - ps38
Hominini - ps39
Homo_sapiens - ps40
p-value
2e-18
2e-14
3e-10
4e-06
5e-02
Log-odds
0.5
1.0
1.5
2.0
2.5
3.0
3.5
4.0

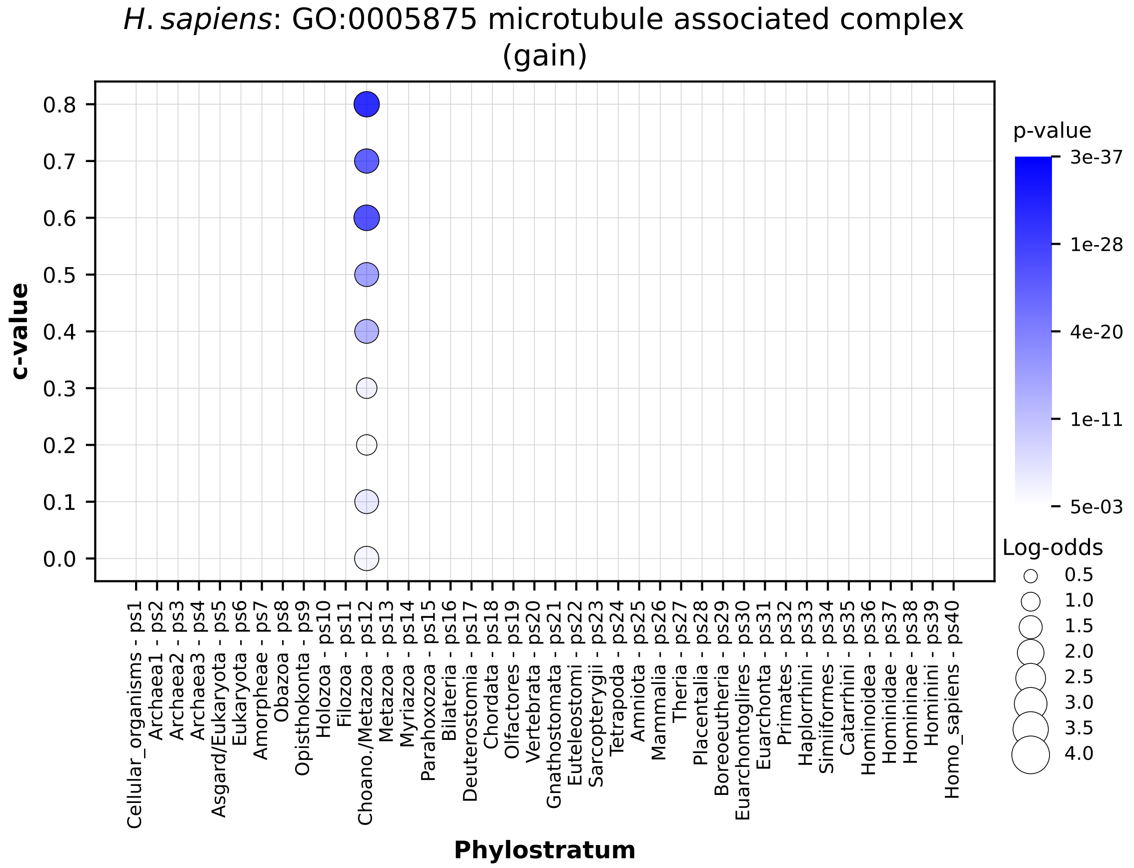

H. sapiens: GO:0005875 microtubule associated complex
(gain)
c-value
0.8
0.7
0.6
0.5
0.4
0.3
0.2
0.1
0.0
p-value
3e-37
1e-28
4e-20
1e-11
5e-03
Log-odds
0.5
1.0
1.5
2.0
2.5
3.0
3.5
4.0
Cellular_organisms - ps1
Archaea1 - ps2
Archaea2 - ps3
Archaea3 - ps4
Asgard/Eukaryota - ps5
Eukaryota - ps6
Amorpheae - ps7
Obazoa - ps8
Opisthokonta - ps9
Holozoa - ps10
Filozoa - ps11
Choano./Metazoa - ps12
Metazoa - ps13
Myriazoa - ps14
Parahoxozoa - ps15
Bilateria - ps16
Deuterostomia - ps17
Chordata - ps18
Olfactores - ps19
Vertebrata - ps20
Gnathostomata - ps21
Euteleostomi - ps22
Sarcopterygii - ps23
Tetrapoda - ps24
Amniota - ps25
Mammalia - ps26
Theria - ps27
Placentalia - ps28
Boreoeutheria - ps29
Euarchontoglires - ps30
Euarchonta - ps31
Primates - ps32
Haplorrhini - ps33
Simiiformes - ps34
Catarrhini - ps35
Hominoidea - ps36
Hominidae - ps37
Homininae - ps38
Hominini - ps39
Homo_sapiens - ps40
Phylostratum

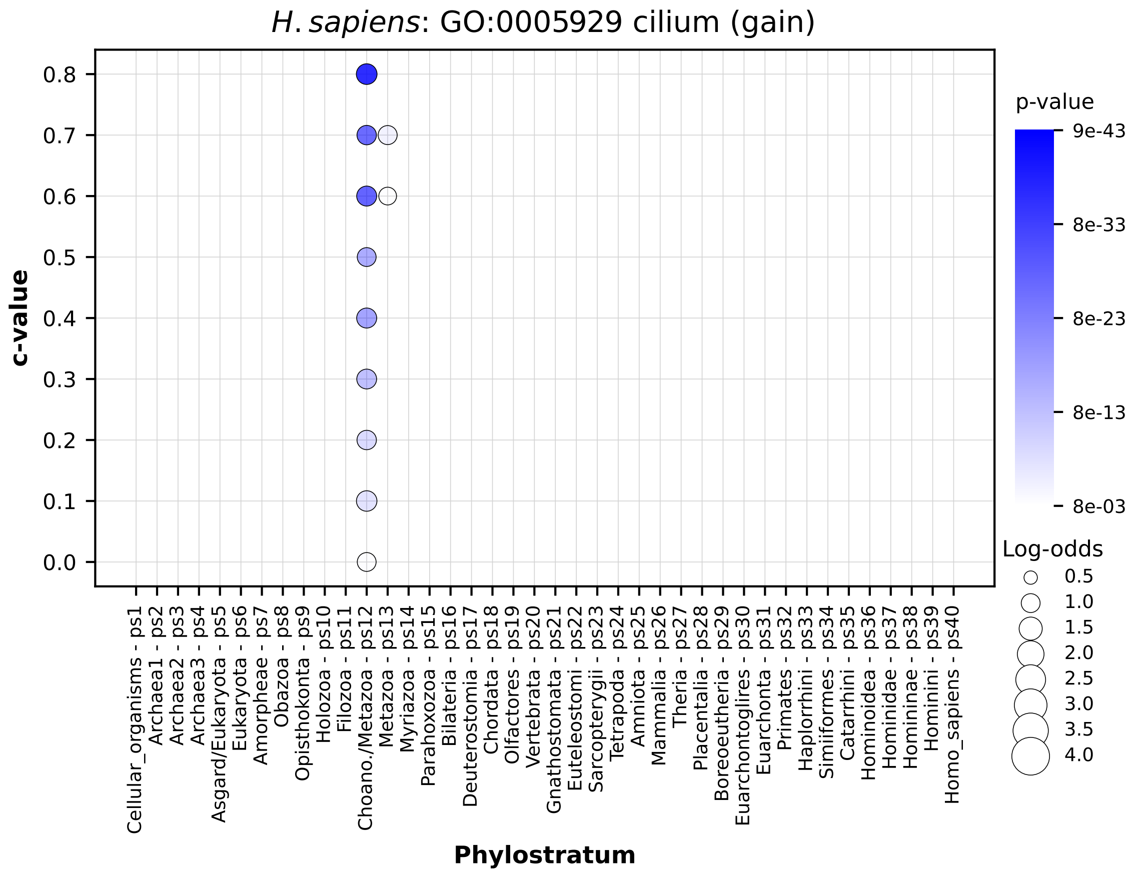
H. sapiens: GO:0005929 cilium (gain)
c-value
0.0
0.1
0.2
0.3
0.4
0.5
0.6
0.7
0.8
p-value
9e-43
8e-33
8e-23
8e-13
8e-03
Log-odds
0.5
1.0
1.5
2.0
2.5
3.0
3.5
4.0
Cellular_organisms - ps1
Archaea1 - ps2
Archaea2 - ps3
Archaea3 - ps4
Asgard/Eukaryota - ps5
Eukaryota - ps6
Amorpheae - ps7
Obazoa - ps8
Opisthokonta - ps9
Holozoa - ps10
Filozoa - ps11
Choano./Metazoa - ps12
Metazoa - ps13
Myriazoa - ps14
Parahoxozoa - ps15
Bilateria - ps16
Deuterostomia - ps17
Chordata - ps18
Olfactores - ps19
Vertebrata - ps20
Gnathostomata - ps21
Euteleostomi - ps22
Sarcopterygii - ps23
Tetrapoda - ps24
Amniota - ps25
Mammalia - ps26
Theria - ps27
Placentalia - ps28
Boreoeutheria - ps29
Euarchontoglires - ps30
Euarchonta - ps31
Primates - ps32
Haplorrhini - ps33
Simiiformes - ps34
Catarrhini - ps35
Hominoidea - ps36
Hominidae - ps37
Homininae - ps38
Hominini - ps39
Homo_sapiens - ps40
Phylostratum

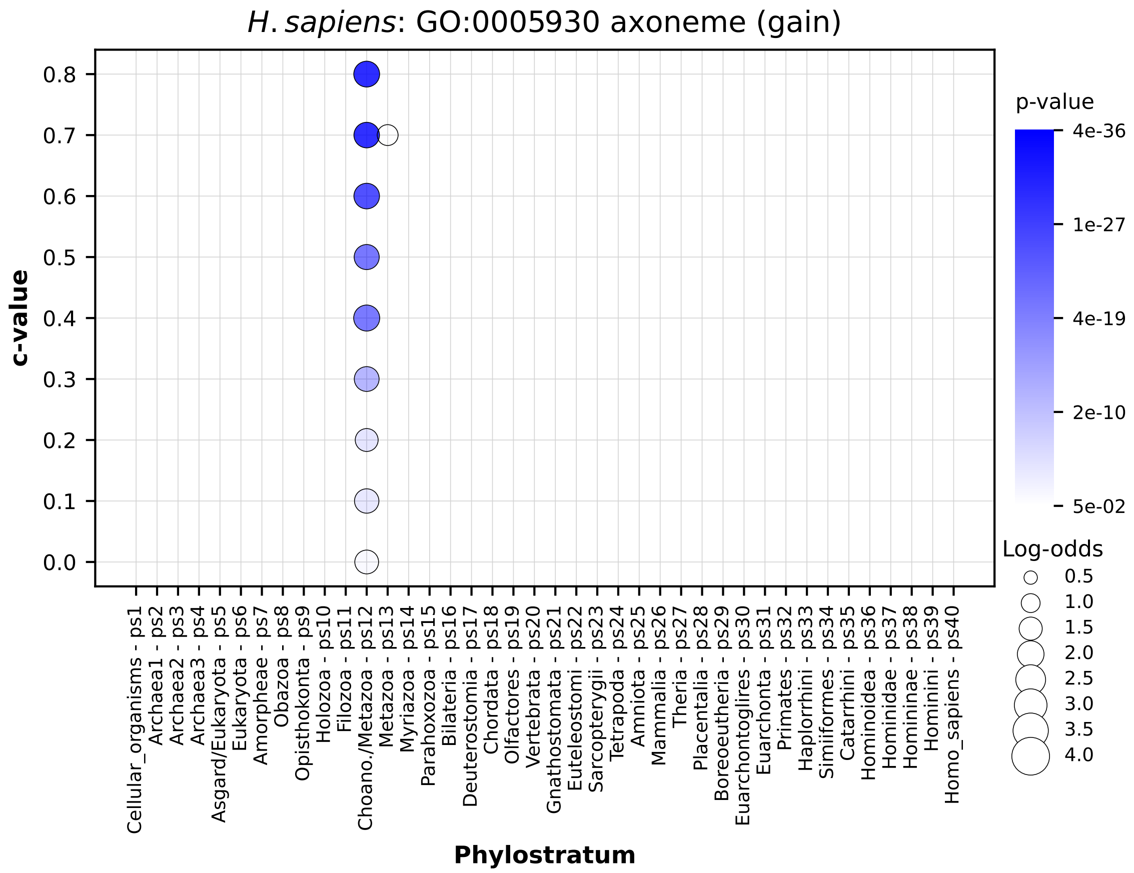
$H. sapiens$: GO:0005930 axoneme (gain)
c-value
0.8
0.7
0.6
0.5
0.4
0.3
0.2
0.1
0.0
Phylostratum
Cellular_organisms - ps1
Archaea1 - ps2
Archaea2 - ps3
Archaea3 - ps4
Asgard/Eukaryota - ps5
Eukaryota - ps6
Amorpheae - ps7
Obazoa - ps8
Opisthokonta - ps9
Holozoa - ps10
Filozoa - ps11
Choano./Metazoa - ps12
Metazoa - ps13
Myriazoa - ps14
Parahoxozoa - ps15
Bilateria - ps16
Deuterostomia - ps17
Chordata - ps18
Olfactores - ps19
Vertebrata - ps20
Gnathostomata - ps21
Euteleostomi - ps22
Sarcopterygii - ps23
Tetrapoda - ps24
Amniota - ps25
Mammalia - ps26
Theria - ps27
Placentalia - ps28
Boreoeutheria - ps29
Euarchontoglires - ps30
Euarchonta - ps31
Primates - ps32
Haplorrhini - ps33
Simiiformes - ps34
Catarrhini - ps35
Hominoidea - ps36
Hominidae - ps37
Homininae - ps38
Hominini - ps39
Homo_sapiens - ps40
p-value
4e-36
1e-27
4e-19
2e-10
5e-02
Log-odds
0.5
1.0
1.5
2.0
2.5
3.0
3.5
4.0

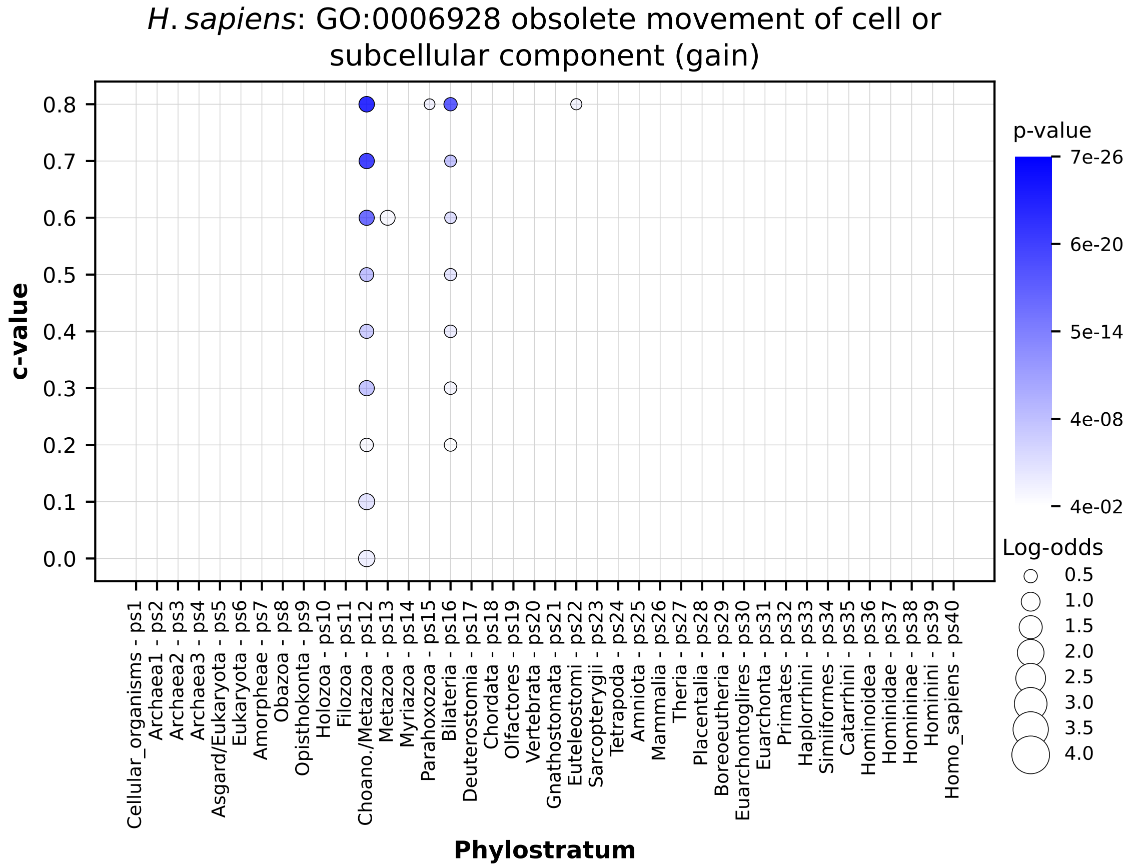

*H. sapiens*: GO:0006928 obsolete movement of cell or subcellular component (gain)
c-value
0.8
0.7
0.6
0.5
0.4
0.3
0.2
0.1
0.0
p-value
7e-26
6e-20
5e-14
4e-08
4e-02
Log-odds
0.5
1.0
1.5
2.0
2.5
3.0
3.5
4.0
Cellular_organisms - ps1
Archaea1 - ps2
Archaea2 - ps3
Archaea3 - ps4
Asgard/Eukaryota - ps5
Eukaryota - ps6
Amorpheae - ps7
Obazoa - ps8
Opisthokonta - ps9
Holozoa - ps10
Filozoa - ps11
Choano./Metazoa - ps12
Metazoa - ps13
Myriazoa - ps14
Parahoxozoa - ps15
Bilateria - ps16
Deuterostomia - ps17
Chordata - ps18
Olfactores - ps19
Vertebrata - ps20
Gnathostomata - ps21
Euteleostomi - ps22
Sarcopterygii - ps23
Tetrapoda - ps24
Amniota - ps25
Mammalia - ps26
Theria - ps27
Placentalia - ps28
Boreoeutheria - ps29
Euarchontoglires - ps30
Euarchonta - ps31
Primates - ps32
Haplorrhini - ps33
Simiiformes - ps34
Catarrhini - ps35
Hominoidea - ps36
Hominidae - ps37
Homininae - ps38
Hominini - ps39
Homo_sapiens - ps40
Phylostratum

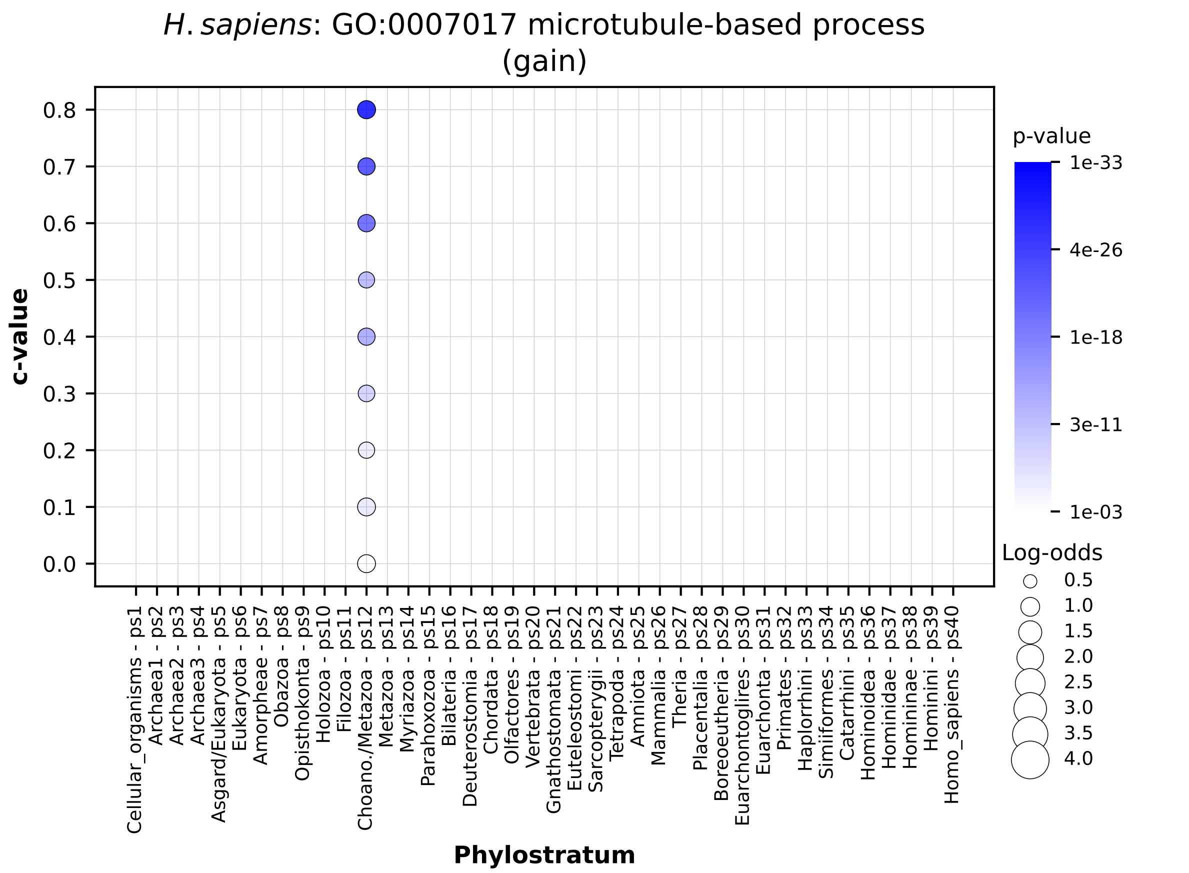

H. sapiens: GO:0007017 microtubule-based process
(gain)
c-value
0.8
0.7
0.6
0.5
0.4
0.3
0.2
0.1
0.0
p-value
1e-33
4e-26
1e-18
3e-11
1e-03
Log-odds
0.5
1.0
1.5
2.0
2.5
3.0
3.5
4.0
Cellular_organisms - ps1
Archaea1 - ps2
Archaea2 - ps3
Archaea3 - ps4
Asgard/Eukaryota - ps5
Eukaryota - ps6
Amorpheae - ps7
Obazoa - ps8
Opisthokonta - ps9
Holozoa - ps10
Filozoa - ps11
Choano./Metazoa - ps12
Metazoa - ps13
Myriazoa - ps14
Parahoxozoa - ps15
Bilateria - ps16
Deuterostomia - ps17
Chordata - ps18
Olfactores - ps19
Vertebrata - ps20
Gnathostomata - ps21
Euteleostomi - ps22
Sarcopterygii - ps23
Tetrapoda - ps24
Amniota - ps25
Mammalia - ps26
Theria - ps27
Placentalia - ps28
Boreoeutheria - ps29
Euarchontoglires - ps30
Euarchonta - ps31
Primates - ps32
Haplorrhini - ps33
Simiiformes - ps34
Catarrhini - ps35
Hominoidea - ps36
Hominidae - ps37
Homininae - ps38
Hominini - ps39
Homo_sapiens - ps40
Phylostratum

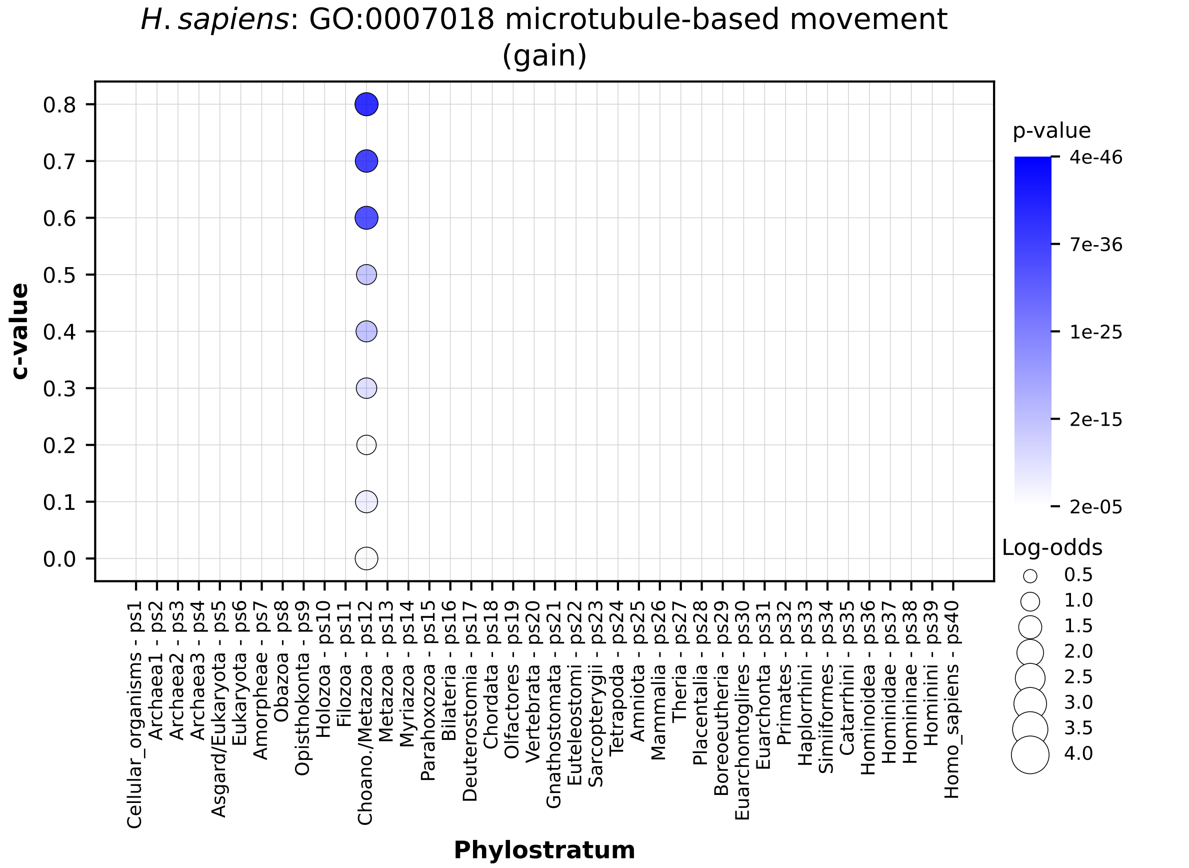

H. sapiens: GO:0007018 microtubule-based movement
(gain)
c-value
0.8
0.7
0.6
0.5
0.4
0.3
0.2
0.1
0.0
p-value
4e-46
7e-36
1e-25
2e-15
2e-05
Log-odds
0.5
1.0
1.5
2.0
2.5
3.0
3.5
4.0
Cellular_organisms - ps1
Archaea1 - ps2
Archaea2 - ps3
Archaea3 - ps4
Asgard/Eukaryota - ps5
Eukaryota - ps6
Amorpheae - ps7
Obazoa - ps8
Opisthokonta - ps9
Holozoa - ps10
Filozoa - ps11
Choano./Metazoa - ps12
Metazoa - ps13
Myriazoa - ps14
Parahoxozoa - ps15
Bilateria - ps16
Deuterostomia - ps17
Chordata - ps18
Olfactores - ps19
Vertebrata - ps20
Gnathostomata - ps21
Euteleostomi - ps22
Sarcopterygii - ps23
Tetrapoda - ps24
Amniota - ps25
Mammalia - ps26
Theria - ps27
Placentalia - ps28
Boreoeutheria - ps29
Euarchontoglires - ps30
Euarchonta - ps31
Primates - ps32
Haplorrhini - ps33
Simiiformes - ps34
Catarrhini - ps35
Hominoidea - ps36
Hominidae - ps37
Homininae - ps38
Hominini - ps39
Homo_sapiens - ps40
Phylostratum

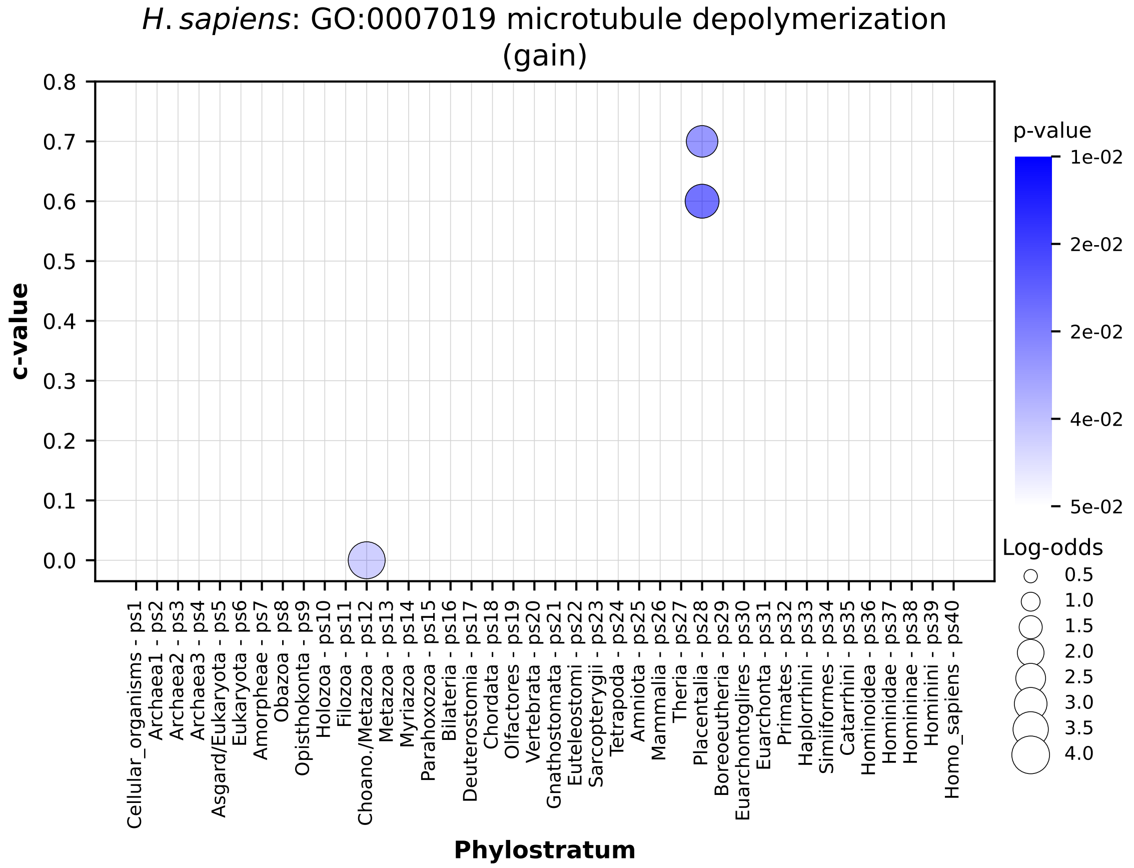

*H. sapiens*: GO:0007019 microtubule depolymerization (gain)
c-value
0.8
0.7
0.6
0.5
0.4
0.3
0.2
0.1
0.0
Phylostratum
Cellular_organisms - ps1
Archaea1 - ps2
Archaea2 - ps3
Archaea3 - ps4
Asgard/Eukaryota - ps5
Eukaryota - ps6
Amorpheae - ps7
Obazoa - ps8
Opisthokonta - ps9
Holozoa - ps10
Filozoa - ps11
Choano./Metazoa - ps12
Metazoa - ps13
Myriazoa - ps14
Parahoxozoa - ps15
Bilateria - ps16
Deuterostomia - ps17
Chordata - ps18
Olfactores - ps19
Vertebrata - ps20
Gnathostomata - ps21
Euteleostomi - ps22
Sarcopterygii - ps23
Tetrapoda - ps24
Amniota - ps25
Mammalia - ps26
Theria - ps27
Placentalia - ps28
Boreoeutheria - ps29
Euarchontoglires - ps30
Euarchonta - ps31
Primates - ps32
Haplorrhini - ps33
Simiiformes - ps34
Catarrhini - ps35
Hominoidea - ps36
Hominidae - ps37
Homininae - ps38
Hominini - ps39
Homo_sapiens - ps40
p-value
1e-02
2e-02
2e-02
4e-02
5e-02
Log-odds
0.5
1.0
1.5
2.0
2.5
3.0
3.5
4.0

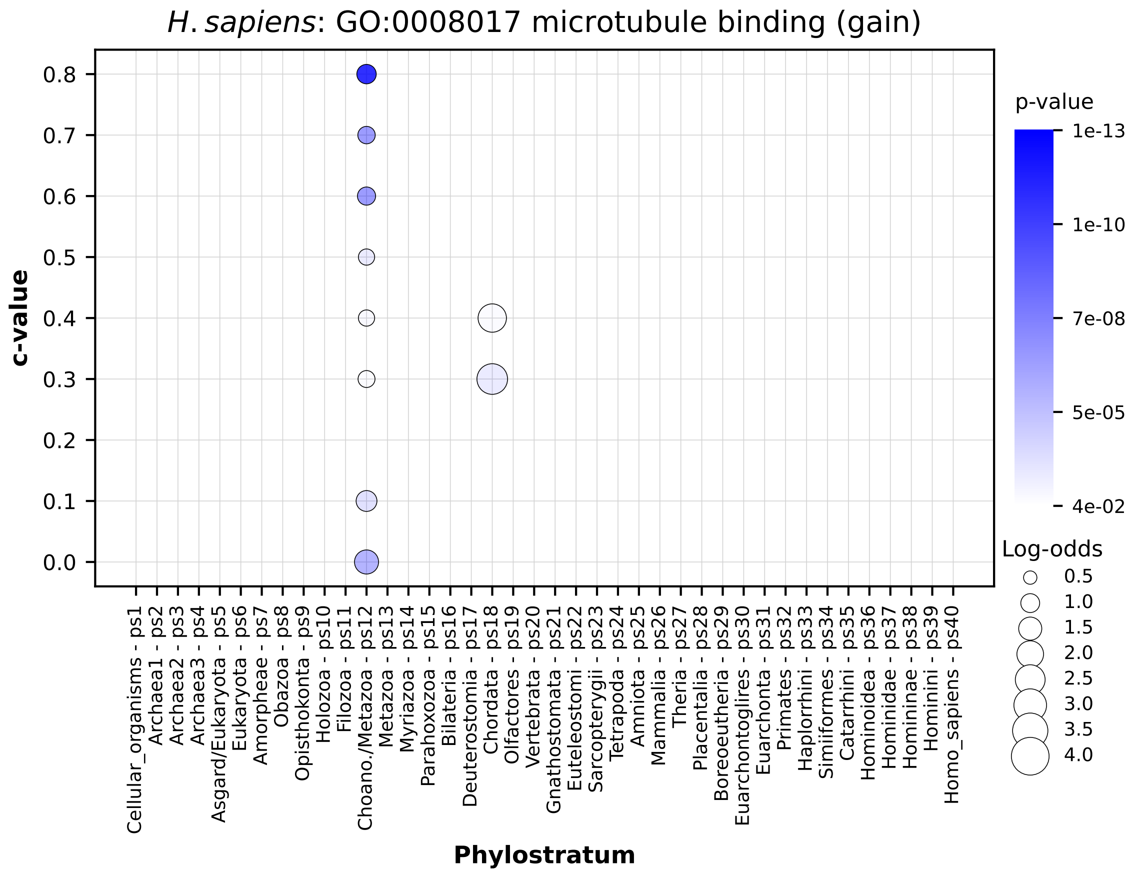
H. sapiens: GO:0008017 microtubule binding (gain)
c-value
0.8
0.7
0.6
0.5
0.4
0.3
0.2
0.1
0.0
p-value
1e-13
1e-10
7e-08
5e-05
4e-02
Log-odds
0.5
1.0
1.5
2.0
2.5
3.0
3.5
4.0
Cellular_organisms - ps1
Archaea1 - ps2
Archaea2 - ps3
Archaea3 - ps4
Asgard/Eukaryota - ps5
Eukaryota - ps6
Amorpheae - ps7
Obazoa - ps8
Opisthokonta - ps9
Holozoa - ps10
Filozoa - ps11
Choano./Metazoa - ps12
Metazoa - ps13
Myriazoa - ps14
Parahoxozoa - ps15
Bilateria - ps16
Deuterostomia - ps17
Chordata - ps18
Olfactores - ps19
Vertebrata - ps20
Gnathostomata - ps21
Euteleostomi - ps22
Sarcopterygii - ps23
Tetrapoda - ps24
Amniota - ps25
Mammalia - ps26
Theria - ps27
Placentalia - ps28
Boreoeutheria - ps29
Euarchontoglires - ps30
Euarchonta - ps31
Primates - ps32
Haplorrhini - ps33
Simiiformes - ps34
Catarrhini - ps35
Hominoidea - ps36
Hominidae - ps37
Homininae - ps38
Hominini - ps39
Homo_sapiens - ps40
Phylostratum

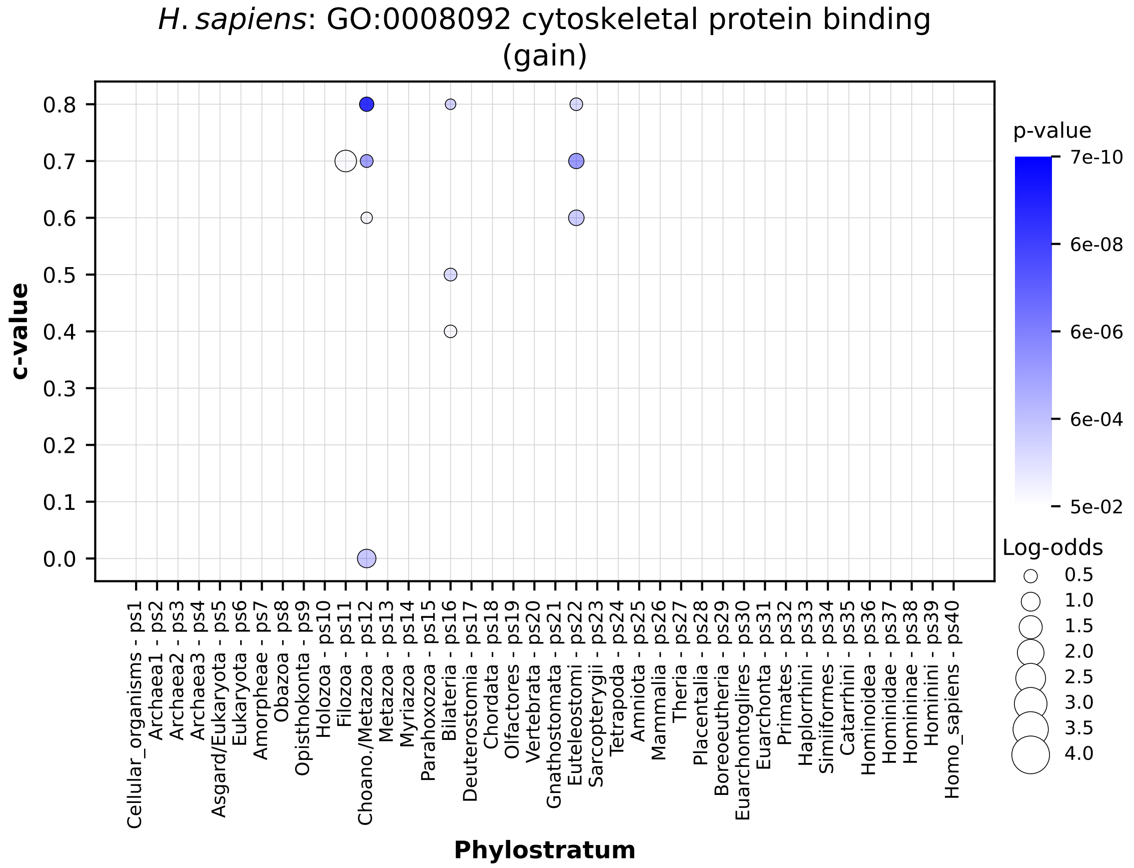

*H. sapiens*: GO:0008092 cytoskeletal protein binding
(gain)
c-value
0.0
0.1
0.2
0.3
0.4
0.5
0.6
0.7
0.8
Phylostratum
Cellular_organisms - ps1
Archaea1 - ps2
Archaea2 - ps3
Archaea3 - ps4
Asgard/Eukaryota - ps5
Eukaryota - ps6
Amorpheae - ps7
Obazoa - ps8
Opisthokonta - ps9
Holozoa - ps10
Filozoa - ps11
Choano./Metazoa - ps12
Metazoa - ps13
Myriazoa - ps14
Parahoxozoa - ps15
Bilateria - ps16
Deuterostomia - ps17
Chordata - ps18
Olfactores - ps19
Vertebrata - ps20
Gnathostomata - ps21
Euteleostomi - ps22
Sarcopterygii - ps23
Tetrapoda - ps24
Amniota - ps25
Mammalia - ps26
Theria - ps27
Placentalia - ps28
Boreoeutheria - ps29
Euarchontoglires - ps30
Euarchonta - ps31
Primates - ps32
Haplorrhini - ps33
Simiiformes - ps34
Catarrhini - ps35
Hominoidea - ps36
Hominidae - ps37
Homininae - ps38
Hominini - ps39
Homo_sapiens - ps40
p-value
7e-10
6e-08
6e-06
6e-04
5e-02
Log-odds
0.5
1.0
1.5
2.0
2.5
3.0
3.5
4.0

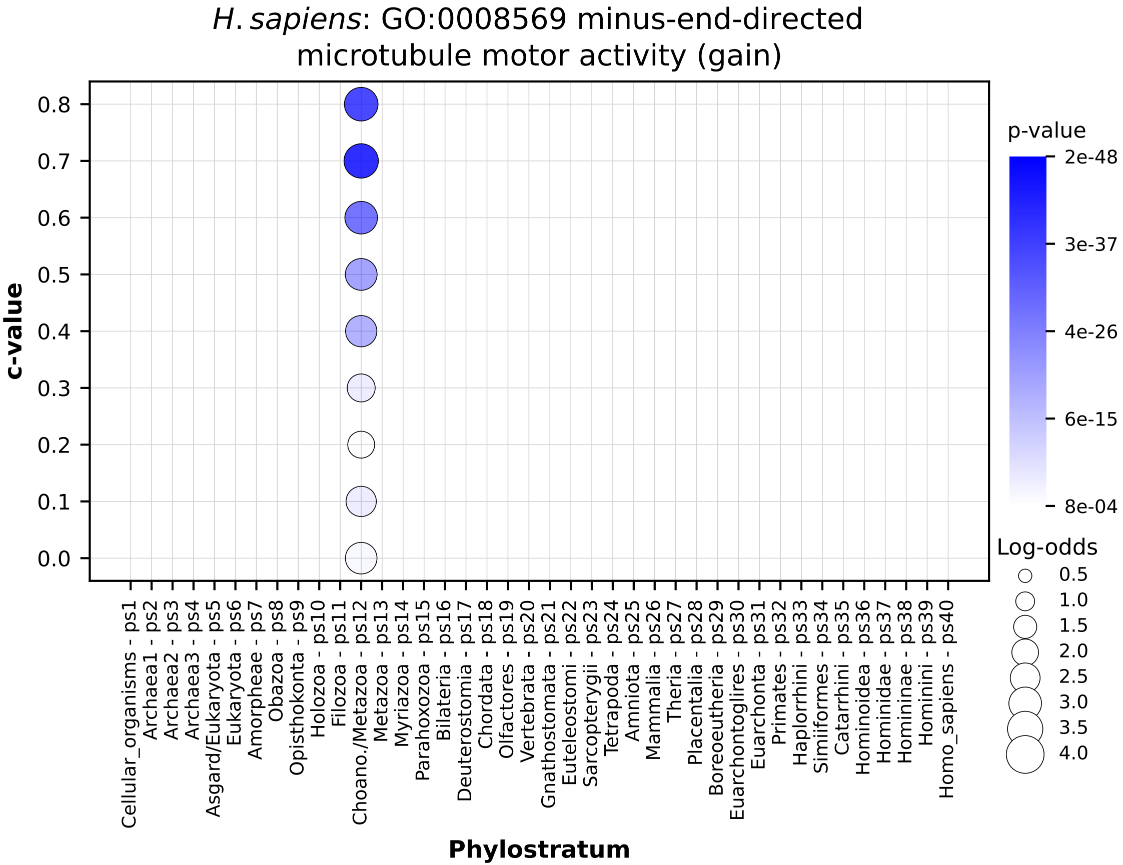

H. sapiens: GO:0008569 minus-end-directed microtubule motor activity (gain)
c-value
0.0
0.1
0.2
0.3
0.4
0.5
0.6
0.7
0.8
p-value
2e-48
3e-37
4e-26
6e-15
8e-04
Log-odds
0.5
1.0
1.5
2.0
2.5
3.0
3.5
4.0
Cellular_organisms - ps1
Archaea1 - ps2
Archaea2 - ps3
Archaea3 - ps4
Asgard/Eukaryota - ps5
Eukaryota - ps6
Amorpheae - ps7
Obazoa - ps8
Opisthokonta - ps9
Holozoa - ps10
Filozoa - ps11
Choano./Metazoa - ps12
Metazoa - ps13
Myriazoa - ps14
Parahoxozoa - ps15
Bilateria - ps16
Deuterostomia - ps17
Chordata - ps18
Olfactores - ps19
Vertebrata - ps20
Gnathostomata - ps21
Euteleostomi - ps22
Sarcopterygii - ps23
Tetrapoda - ps24
Amniota - ps25
Mammalia - ps26
Theria - ps27
Placentalia - ps28
Boreoeutheria - ps29
Euarchontoglires - ps30
Euarchonta - ps31
Primates - ps32
Haplorrhini - ps33
Simiiformes - ps34
Catarrhini - ps35
Hominoidea - ps36
Hominidae - ps37
Homininae - ps38
Hominini - ps39
Homo_sapiens - ps40
Phylostratum

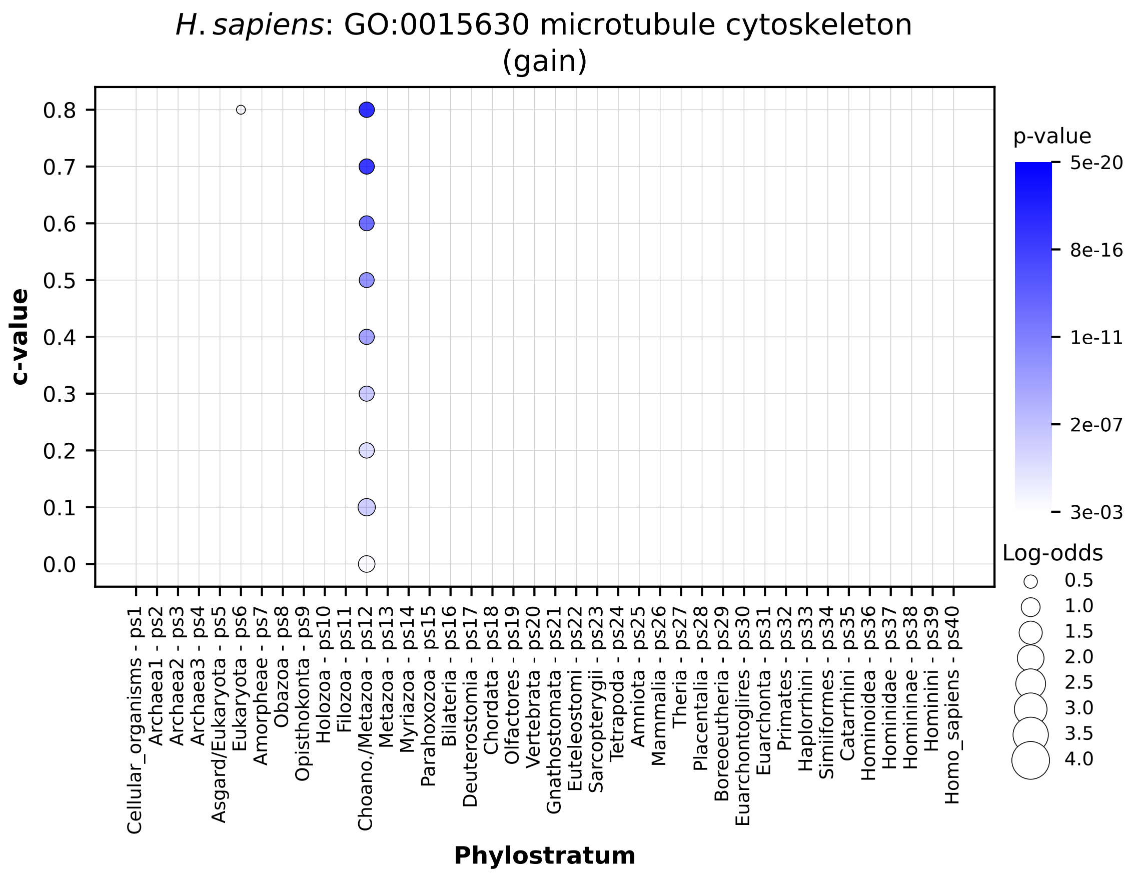

H. sapiens: GO:0015630 microtubule cytoskeleton
(gain)
c-value
0.8
0.7
0.6
0.5
0.4
0.3
0.2
0.1
0.0
p-value
5e-20
8e-16
1e-11
2e-07
3e-03
Log-odds
0.5
1.0
1.5
2.0
2.5
3.0
3.5
4.0
Cellular_organisms - ps1
Archaea1 - ps2
Archaea2 - ps3
Archaea3 - ps4
Asgard/Eukaryota - ps5
Eukaryota - ps6
Amorpheae - ps7
Obazoa - ps8
Opisthokonta - ps9
Holozoa - ps10
Filozoa - ps11
Choano./Metazoa - ps12
Metazoa - ps13
Myriazoa - ps14
Parahoxozoa - ps15
Bilateria - ps16
Deuterostomia - ps17
Chordata - ps18
Olfactores - ps19
Vertebrata - ps20
Gnathostomata - ps21
Euteleostomi - ps22
Sarcopterygii - ps23
Tetrapoda - ps24
Amniota - ps25
Mammalia - ps26
Theria - ps27
Placentalia - ps28
Boreoeutheria - ps29
Euarchontoglires - ps30
Euarchonta - ps31
Primates - ps32
Haplorrhini - ps33
Simiiformes - ps34
Catarrhini - ps35
Hominoidea - ps36
Hominidae - ps37
Homininae - ps38
Hominini - ps39
Homo_sapiens - ps40
Phylostratum

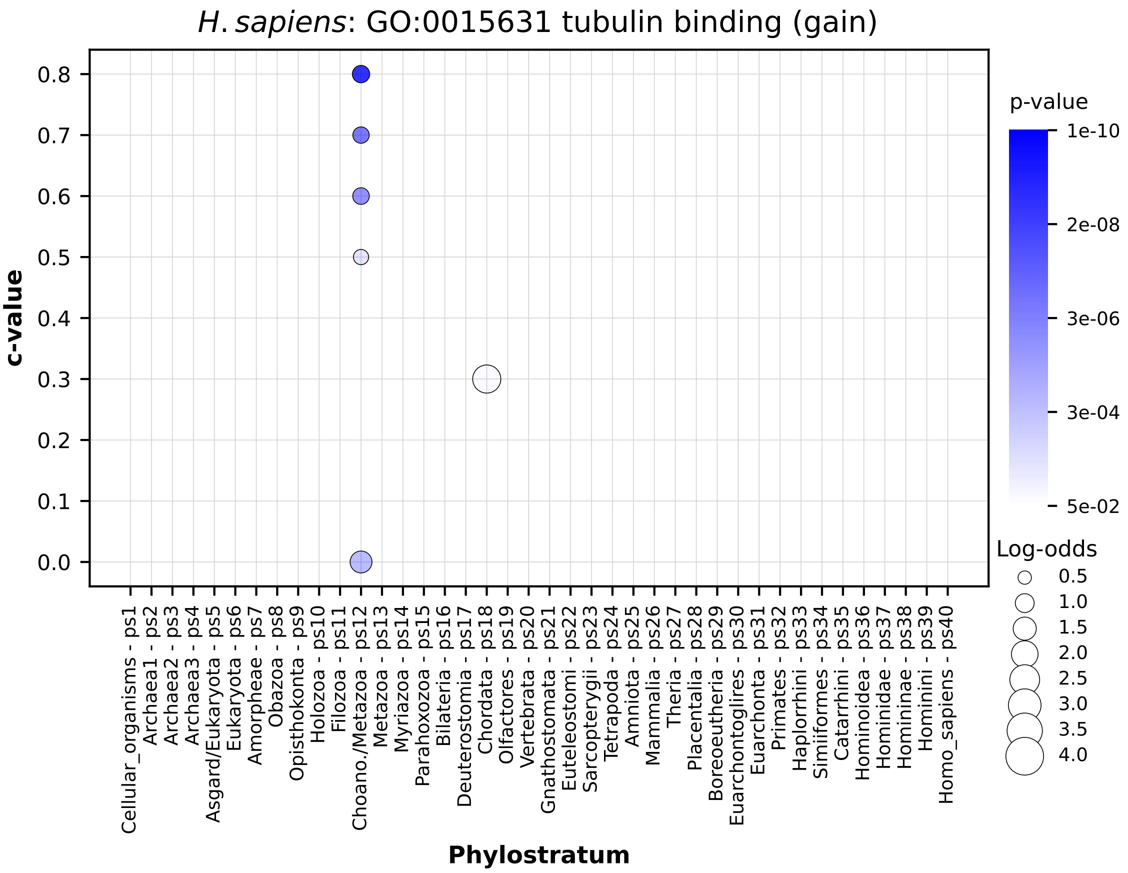

H. sapiens: GO:0015631 tubulin binding (gain)
c-value
0.8
0.7
0.6
0.5
0.4
0.3
0.2
0.1
0.0
Cellular_organisms - ps1
Archaea1 - ps2
Archaea2 - ps3
Archaea3 - ps4
Asgard/Eukaryota - ps5
Eukaryota - ps6
Amorpheae - ps7
Obazoa - ps8
Opisthokonta - ps9
Holozoa - ps10
Filozoa - ps11
Choano./Metazoa - ps12
Metazoa - ps13
Myriazoa - ps14
Parahoxozoa - ps15
Bilateria - ps16
Deuterostomia - ps17
Chordata - ps18
Olfactores - ps19
Vertebrata - ps20
Gnathostomata - ps21
Euteleostomi - ps22
Sarcopterygii - ps23
Tetrapoda - ps24
Amniota - ps25
Mammalia - ps26
Theria - ps27
Placentalia - ps28
Boreoeutheria - ps29
Euarchontoglires - ps30
Euarchonta - ps31
Primates - ps32
Haplorrhini - ps33
Simiiformes - ps34
Catarrhini - ps35
Hominoidea - ps36
Hominidae - ps37
Homininae - ps38
Hominini - ps39
Homo_sapiens - ps40
Phylostratum
p-value
1e-10
2e-08
3e-06
3e-04
5e-02
Log-odds
0.5
1.0
1.5
2.0
2.5
3.0
3.5
4.0

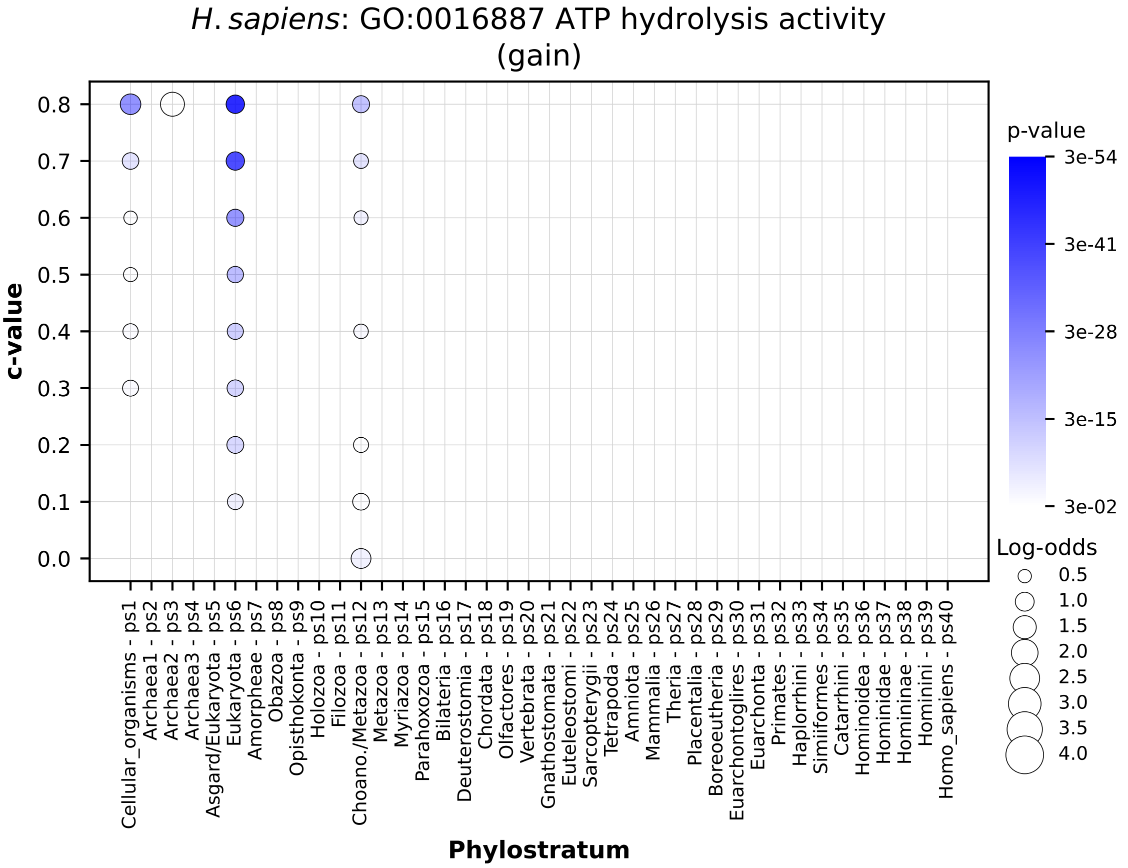
H. sapiens: GO:0016887 ATP hydrolysis activity
(gain)
c-value
0.0
0.1
0.2
0.3
0.4
0.5
0.6
0.7
0.8
Phylostratum
Cellular_organisms - ps1
Archaea1 - ps2
Archaea2 - ps3
Archaea3 - ps4
Asgard/Eukaryota - ps5
Eukaryota - ps6
Amorpheae - ps7
Obazoa - ps8
Opisthokonta - ps9
Holozoa - ps10
Filozoa - ps11
Choano./Metazoa - ps12
Metazoa - ps13
Myriazoa - ps14
Parahoxozoa - ps15
Bilateria - ps16
Deuterostomia - ps17
Chordata - ps18
Olfactores - ps19
Vertebrata - ps20
Gnathostomata - ps21
Euteleostomi - ps22
Sarcopterygii - ps23
Tetrapoda - ps24
Amniota - ps25
Mammalia - ps26
Theria - ps27
Placentalia - ps28
Boreoeutheria - ps29
Euarchontoglires - ps30
Euarchonta - ps31
Primates - ps32
Haplorrhini - ps33
Simiiformes - ps34
Catarrhini - ps35
Hominoidea - ps36
Hominidae - ps37
Homininae - ps38
Hominini - ps39
Homo_sapiens - ps40
p-value
3e-54
3e-41
3e-28
3e-15
3e-02
Log-odds
0.5
1.0
1.5
2.0
2.5
3.0
3.5
4.0

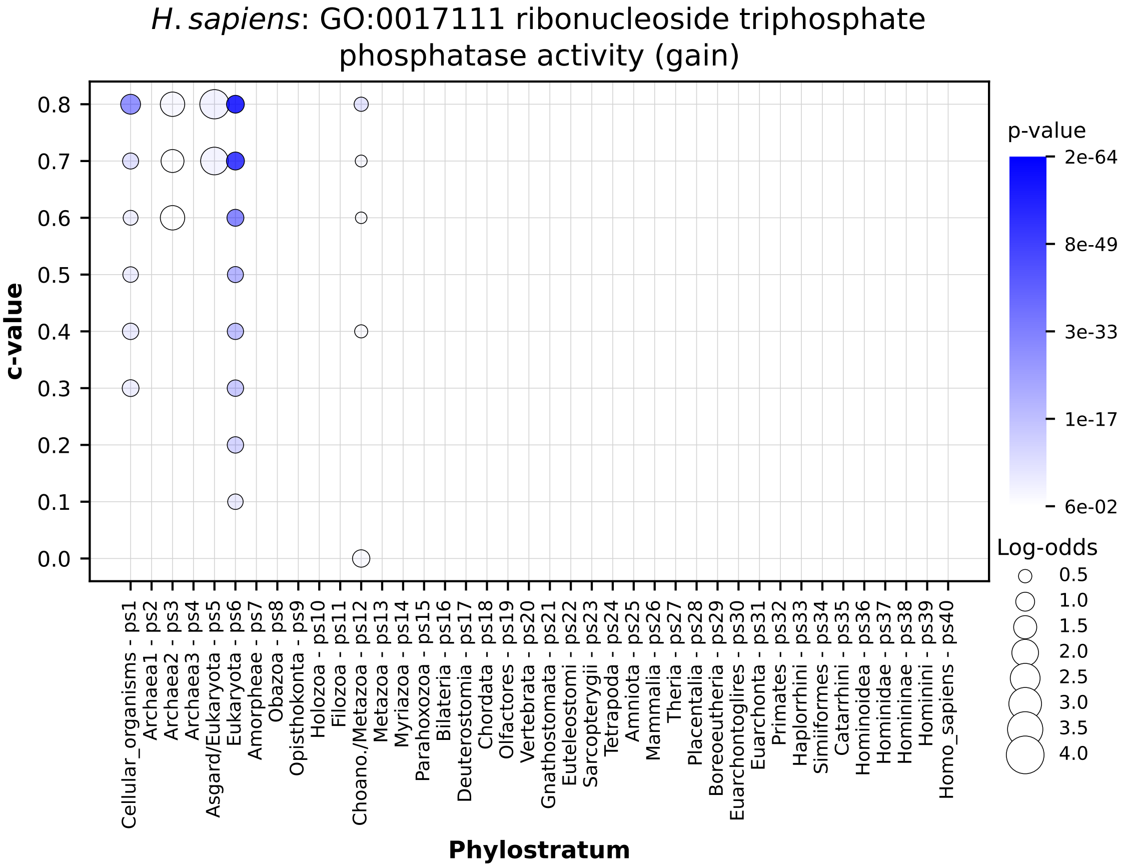

*H. sapiens*: GO:0017111 ribonucleoside triphosphate phosphatase activity (gain)
c-value
0.8
0.7
0.6
0.5
0.4
0.3
0.2
0.1
0.0
Phylostratum
Cellular_organisms - ps1
Archaea1 - ps2
Archaea2 - ps3
Archaea3 - ps4
Asgard/Eukaryota - ps5
Eukaryota - ps6
Amorpheae - ps7
Obazoa - ps8
Opisthokonta - ps9
Holozoa - ps10
Filozoa - ps11
Choano./Metazoa - ps12
Metazoa - ps13
Myriazoa - ps14
Parahoxozoa - ps15
Bilateria - ps16
Deuterostomia - ps17
Chordata - ps18
Olfactores - ps19
Vertebrata - ps20
Gnathostomata - ps21
Euteleostomi - ps22
Sarcopterygii - ps23
Tetrapoda - ps24
Amniota - ps25
Mammalia - ps26
Theria - ps27
Placentalia - ps28
Boreoeutheria - ps29
Euarchontoglires - ps30
Euarchonta - ps31
Primates - ps32
Haplorrhini - ps33
Simiiformes - ps34
Catarrhini - ps35
Hominoidea - ps36
Hominidae - ps37
Homininae - ps38
Hominini - ps39
Homo_sapiens - ps40
p-value
2e-64
8e-49
3e-33
1e-17
6e-02
Log-odds
0.5
1.0
1.5
2.0
2.5
3.0
3.5
4.0

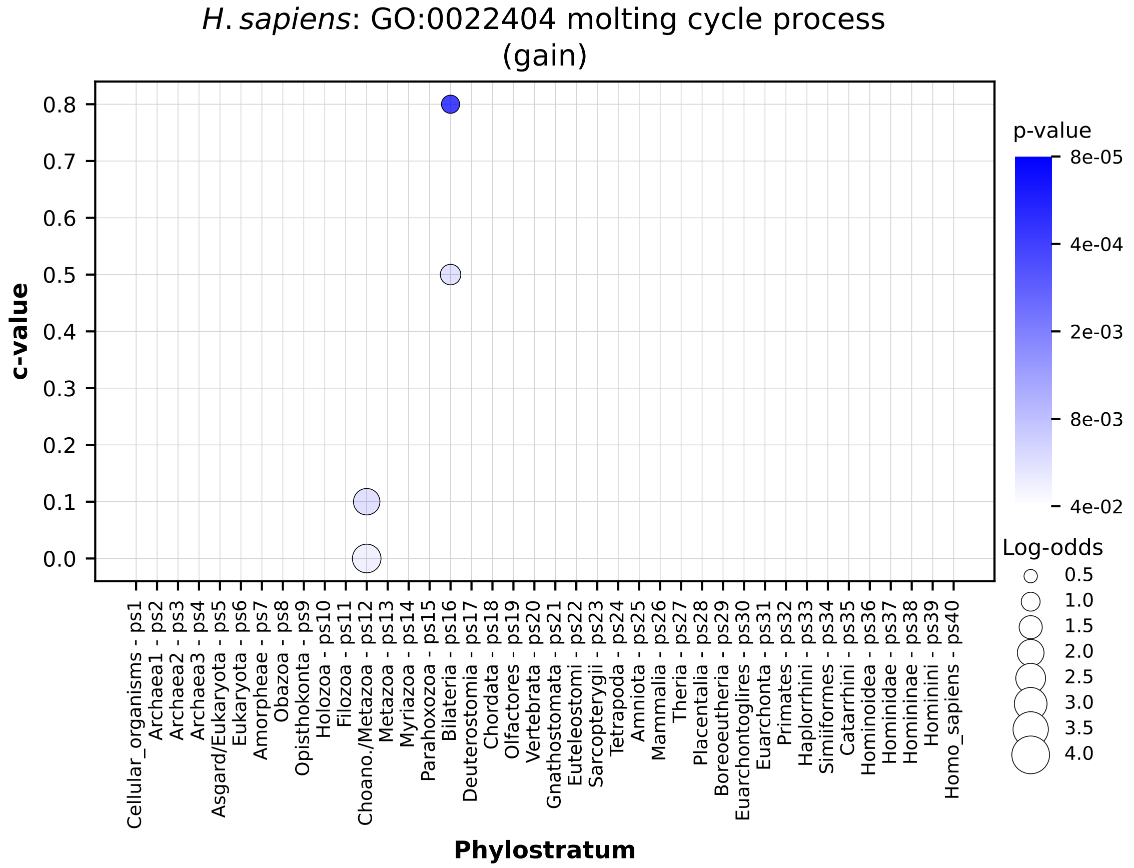

*H. sapiens*: GO:0022404 molting cycle process
(gain)
c-value
0.8
0.7
0.6
0.5
0.4
0.3
0.2
0.1
0.0
p-value
8e-05
4e-04
2e-03
8e-03
4e-02
Log-odds
0.5
1.0
1.5
2.0
2.5
3.0
3.5
4.0
Cellular_organisms - ps1
Archaea1 - ps2
Archaea2 - ps3
Archaea3 - ps4
Asgard/Eukaryota - ps5
Eukaryota - ps6
Amorpheae - ps7
Obazoa - ps8
Opisthokonta - ps9
Holozoa - ps10
Filozoa - ps11
Choano./Metazoa - ps12
Metazoa - ps13
Myriazoa - ps14
Parahoxozoa - ps15
Bilateria - ps16
Deuterostomia - ps17
Chordata - ps18
Olfactores - ps19
Vertebrata - ps20
Gnathostomata - ps21
Euteleostomi - ps22
Sarcopterygii - ps23
Tetrapoda - ps24
Amniota - ps25
Mammalia - ps26
Theria - ps27
Placentalia - ps28
Boreoeutheria - ps29
Euarchontoglires - ps30
Euarchonta - ps31
Primates - ps32
Haplorrhini - ps33
Simiiformes - ps34
Catarrhini - ps35
Hominoidea - ps36
Hominidae - ps37
Homininae - ps38
Hominini - ps39
Homo_sapiens - ps40
Phylostratum

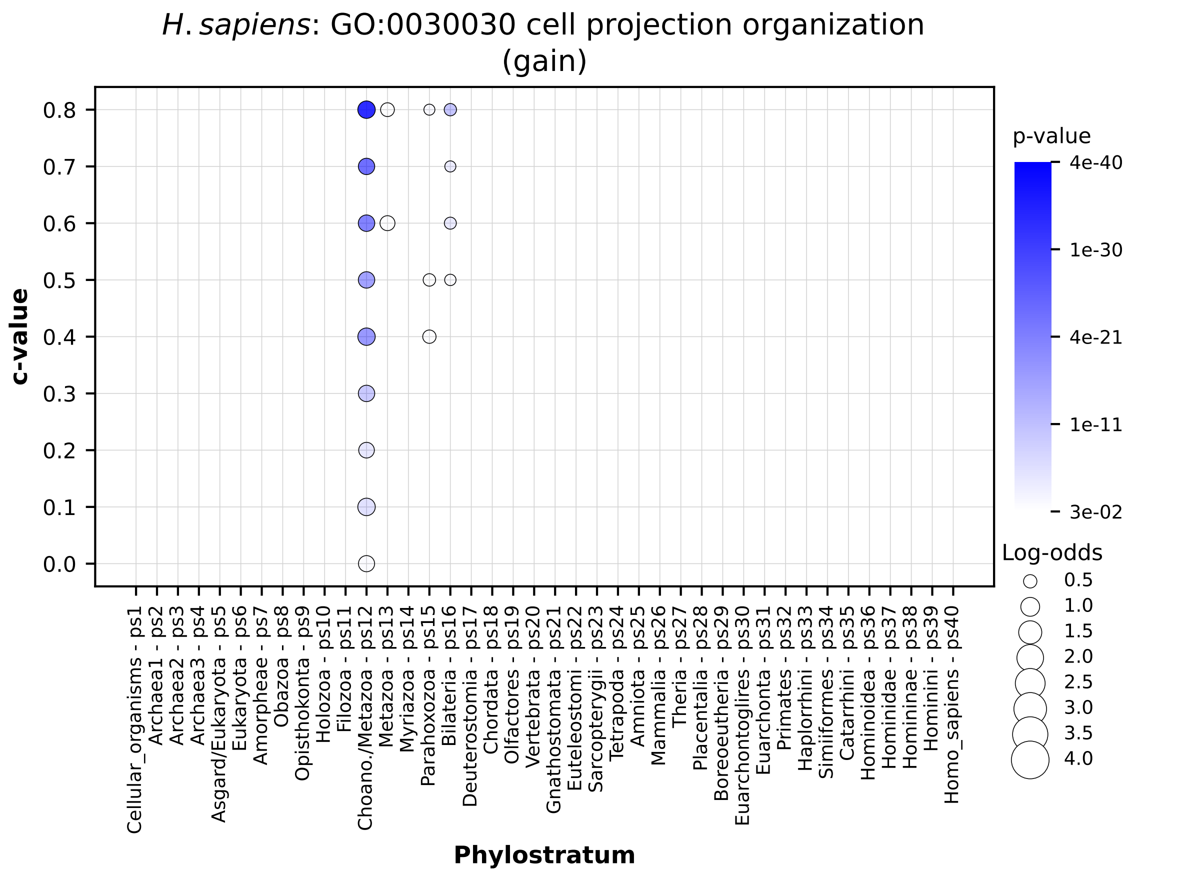

H. sapiens: GO:0030030 cell projection organization (gain)
c-value
0.0
0.1
0.2
0.3
0.4
0.5
0.6
0.7
0.8
Phylostratum
Cellular_organisms - ps1
Archaea1 - ps2
Archaea2 - ps3
Archaea3 - ps4
Asgard/Eukaryota - ps5
Eukaryota - ps6
Amorpheae - ps7
Obazoa - ps8
Opisthokonta - ps9
Holozoa - ps10
Filozoa - ps11
Choano./Metazoa - ps12
Metazoa - ps13
Myriazoa - ps14
Parahoxozoa - ps15
Bilateria - ps16
Deuterostomia - ps17
Chordata - ps18
Olfactores - ps19
Vertebrata - ps20
Gnathostomata - ps21
Euteleostomi - ps22
Sarcopterygii - ps23
Tetrapoda - ps24
Amniota - ps25
Mammalia - ps26
Theria - ps27
Placentalia - ps28
Boreoeutheria - ps29
Euarchontoglires - ps30
Euarchonta - ps31
Primates - ps32
Haplorrhini - ps33
Simiiformes - ps34
Catarrhini - ps35
Hominoidea - ps36
Hominidae - ps37
Homininae - ps38
Hominini - ps39
Homo_sapiens - ps40
p-value
4e-40
1e-30
4e-21
1e-11
3e-02
Log-odds
0.5
1.0
1.5
2.0
2.5
3.0
3.5
4.0

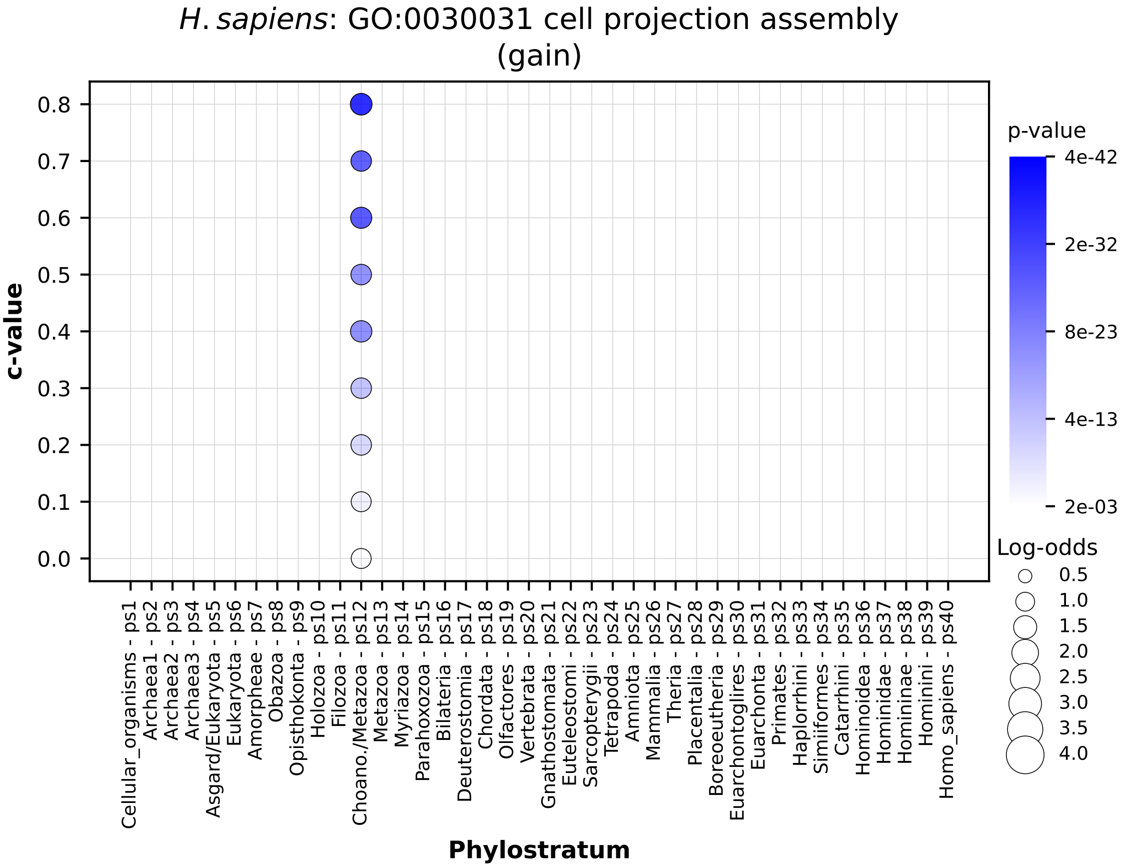

H. sapiens: GO:0030031 cell projection assembly
(gain)
c-value
0.8
0.7
0.6
0.5
0.4
0.3
0.2
0.1
0.0
p-value
4e-42
2e-32
8e-23
4e-13
2e-03
Log-odds
0.5
1.0
1.5
2.0
2.5
3.0
3.5
4.0
Cellular_organisms - ps1
Archaea1 - ps2
Archaea2 - ps3
Archaea3 - ps4
Asgard/Eukaryota - ps5
Eukaryota - ps6
Amorpheae - ps7
Obazoa - ps8
Opisthokonta - ps9
Holozoa - ps10
Filozoa - ps11
Choano./Metazoa - ps12
Metazoa - ps13
Myriazoa - ps14
Parahoxozoa - ps15
Bilateria - ps16
Deuterostomia - ps17
Chordata - ps18
Olfactores - ps19
Vertebrata - ps20
Gnathostomata - ps21
Euteleostomi - ps22
Sarcopterygii - ps23
Tetrapoda - ps24
Amniota - ps25
Mammalia - ps26
Theria - ps27
Placentalia - ps28
Boreoeutheria - ps29
Euarchontoglires - ps30
Euarchonta - ps31
Primates - ps32
Haplorrhini - ps33
Simiiformes - ps34
Catarrhini - ps35
Hominoidea - ps36
Hominidae - ps37
Homininae - ps38
Hominini - ps39
Homo_sapiens - ps40
Phylostratum

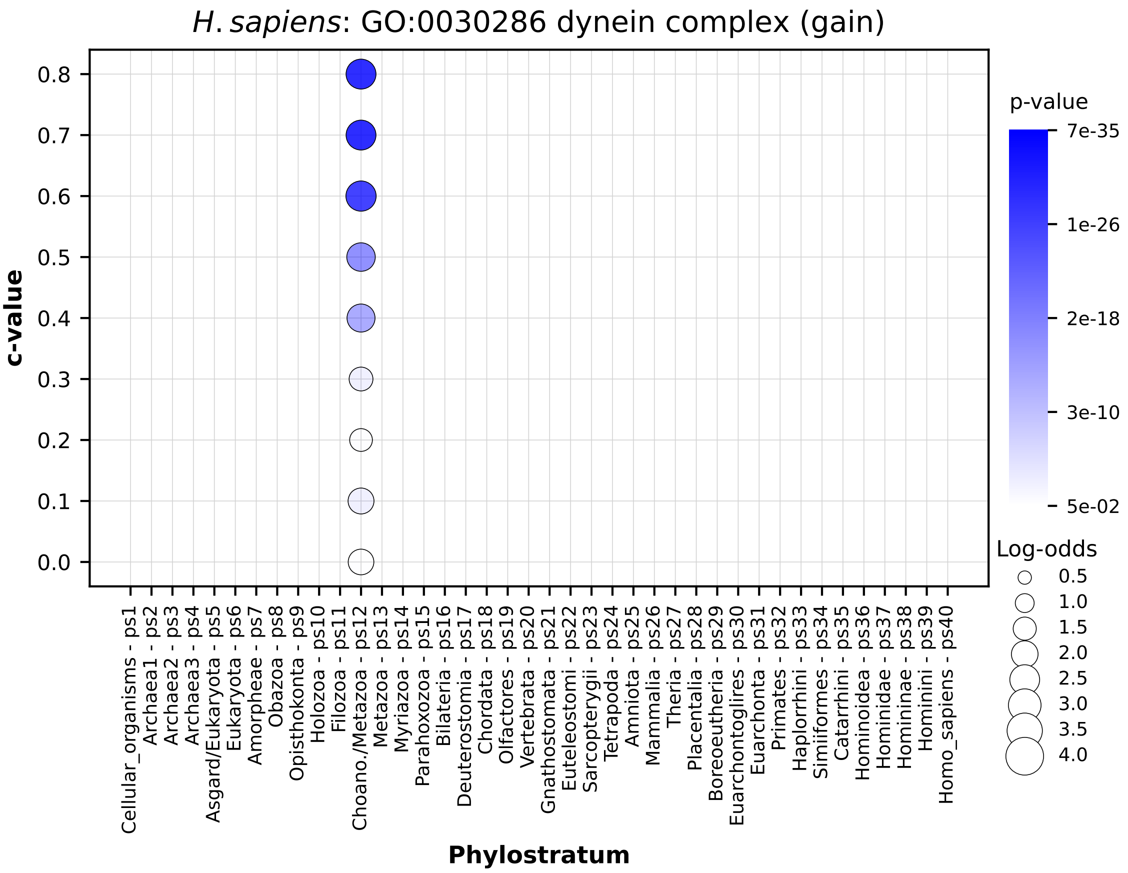

H. sapiens: GO:0030286 dynein complex (gain)
c-value
0.8
0.7
0.6
0.5
0.4
0.3
0.2
0.1
0.0
Phylostratum
Cellular_organisms - ps1
Archaea1 - ps2
Archaea2 - ps3
Archaea3 - ps4
Asgard/Eukaryota - ps5
Eukaryota - ps6
Amorpheae - ps7
Obazoa - ps8
Opisthokonta - ps9
Holozoa - ps10
Filozoa - ps11
Choano./Metazoa - ps12
Metazoa - ps13
Myriazoa - ps14
Parahoxozoa - ps15
Bilateria - ps16
Deuterostomia - ps17
Chordata - ps18
Olfactores - ps19
Vertebrata - ps20
Gnathostomata - ps21
Euteleostomi - ps22
Sarcopterygii - ps23
Tetrapoda - ps24
Amniota - ps25
Mammalia - ps26
Theria - ps27
Placentalia - ps28
Boreoeutheria - ps29
Euarchontoglires - ps30
Euarchonta - ps31
Primates - ps32
Haplorrhini - ps33
Simiiformes - ps34
Catarrhini - ps35
Hominoidea - ps36
Hominidae - ps37
Homininae - ps38
Hominini - ps39
Homo_sapiens - ps40
p-value
7e-35
1e-26
2e-18
3e-10
5e-02
Log-odds
0.5
1.0
1.5
2.0
2.5
3.0
3.5
4.0

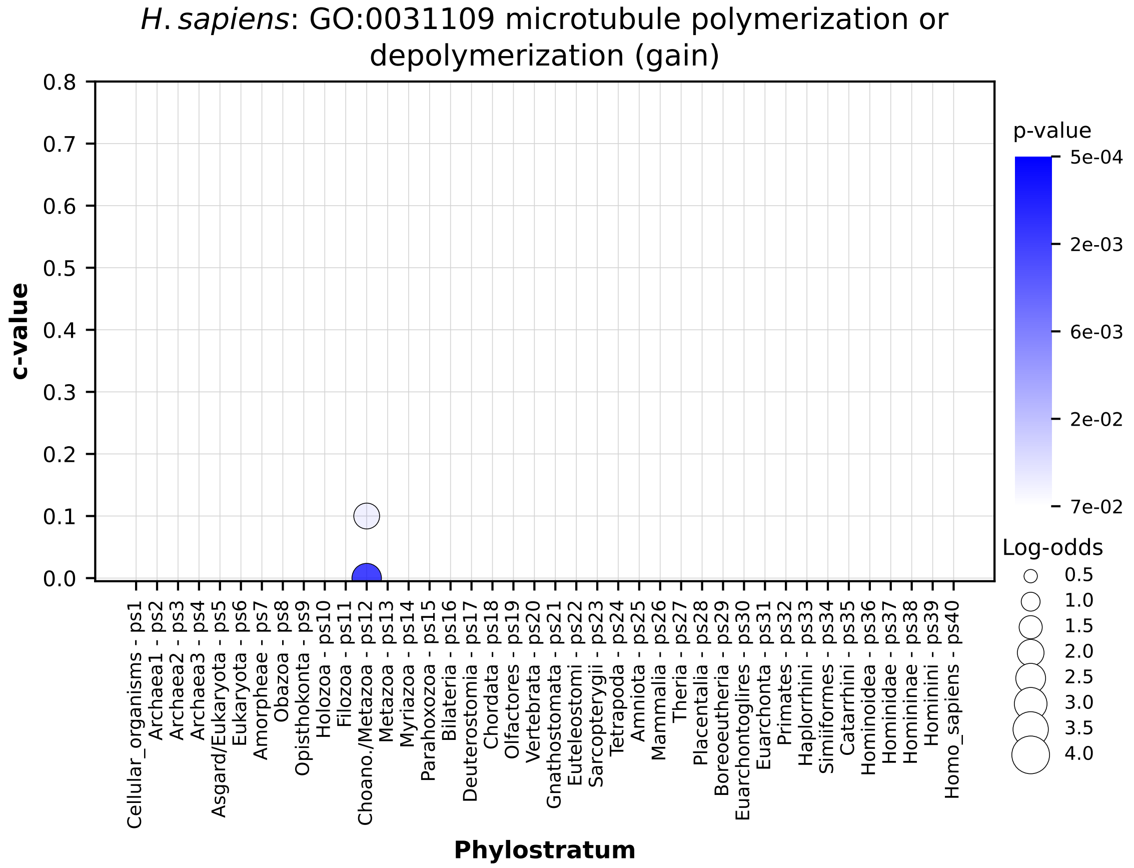

*H. sapiens*: GO:0031109 microtubule polymerization or depolymerization (gain)
c-value
0.8
0.7
0.6
0.5
0.4
0.3
0.2
0.1
0.0
Phylostratum
Cellular_organisms - ps1
Archaea1 - ps2
Archaea2 - ps3
Archaea3 - ps4
Asgard/Eukaryota - ps5
Eukaryota - ps6
Amorpheae - ps7
Obazoa - ps8
Opisthokonta - ps9
Holozoa - ps10
Filozoa - ps11
Choano./Metazoa - ps12
Metazoa - ps13
Myriazoa - ps14
Parahoxozoa - ps15
Bilateria - ps16
Deuterostomia - ps17
Chordata - ps18
Olfactores - ps19
Vertebrata - ps20
Gnathostomata - ps21
Euteleostomi - ps22
Sarcopterygii - ps23
Tetrapoda - ps24
Amniota - ps25
Mammalia - ps26
Theria - ps27
Placentalia - ps28
Boreoeutheria - ps29
Euarchontoglires - ps30
Euarchonta - ps31
Primates - ps32
Haplorrhini - ps33
Simiiformes - ps34
Catarrhini - ps35
Hominoidea - ps36
Hominidae - ps37
Homininae - ps38
Hominini - ps39
Homo_sapiens - ps40
p-value
5e-04
2e-03
6e-03
2e-02
7e-02
Log-odds
0.5
1.0
1.5
2.0
2.5
3.0
3.5
4.0

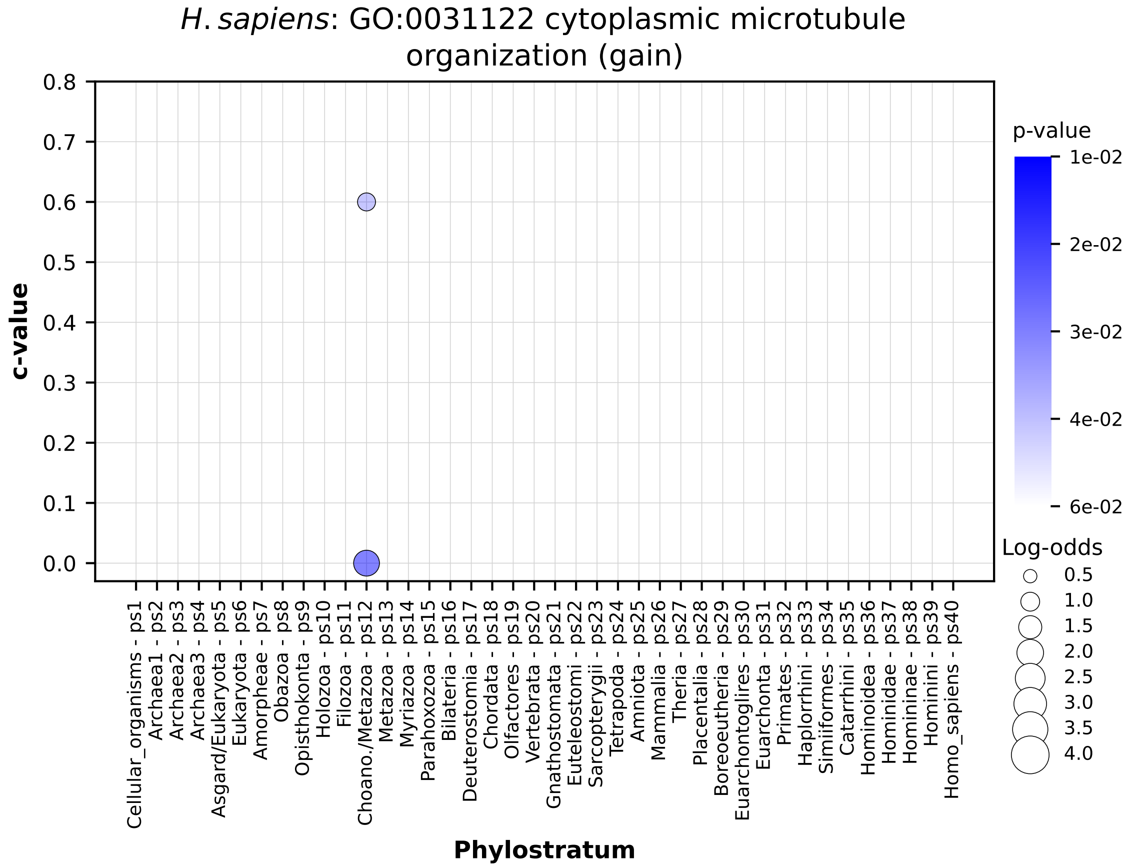

H. sapiens: GO:0031122 cytoplasmic microtubule organization (gain)
c-value
0.0
0.1
0.2
0.3
0.4
0.5
0.6
0.7
0.8
Phylostratum
Cellular_organisms - ps1
Archaea1 - ps2
Archaea2 - ps3
Archaea3 - ps4
Asgard/Eukaryota - ps5
Eukaryota - ps6
Amorpheae - ps7
Obazoa - ps8
Opisthokonta - ps9
Holozoa - ps10
Filozoa - ps11
Choano./Metazoa - ps12
Metazoa - ps13
Myriazoa - ps14
Parahoxozoa - ps15
Bilateria - ps16
Deuterostomia - ps17
Chordata - ps18
Olfactores - ps19
Vertebrata - ps20
Gnathostomata - ps21
Euteleostomi - ps22
Sarcopterygii - ps23
Tetrapoda - ps24
Amniota - ps25
Mammalia - ps26
Theria - ps27
Placentalia - ps28
Boreoeutheria - ps29
Euarchontoglires - ps30
Euarchonta - ps31
Primates - ps32
Haplorrhini - ps33
Simiiformes - ps34
Catarrhini - ps35
Hominoidea - ps36
Hominidae - ps37
Homininae - ps38
Hominini - ps39
Homo_sapiens - ps40
p-value
1e-02
2e-02
3e-02
4e-02
6e-02
Log-odds
0.5
1.0
1.5
2.0
2.5
3.0
3.5
4.0

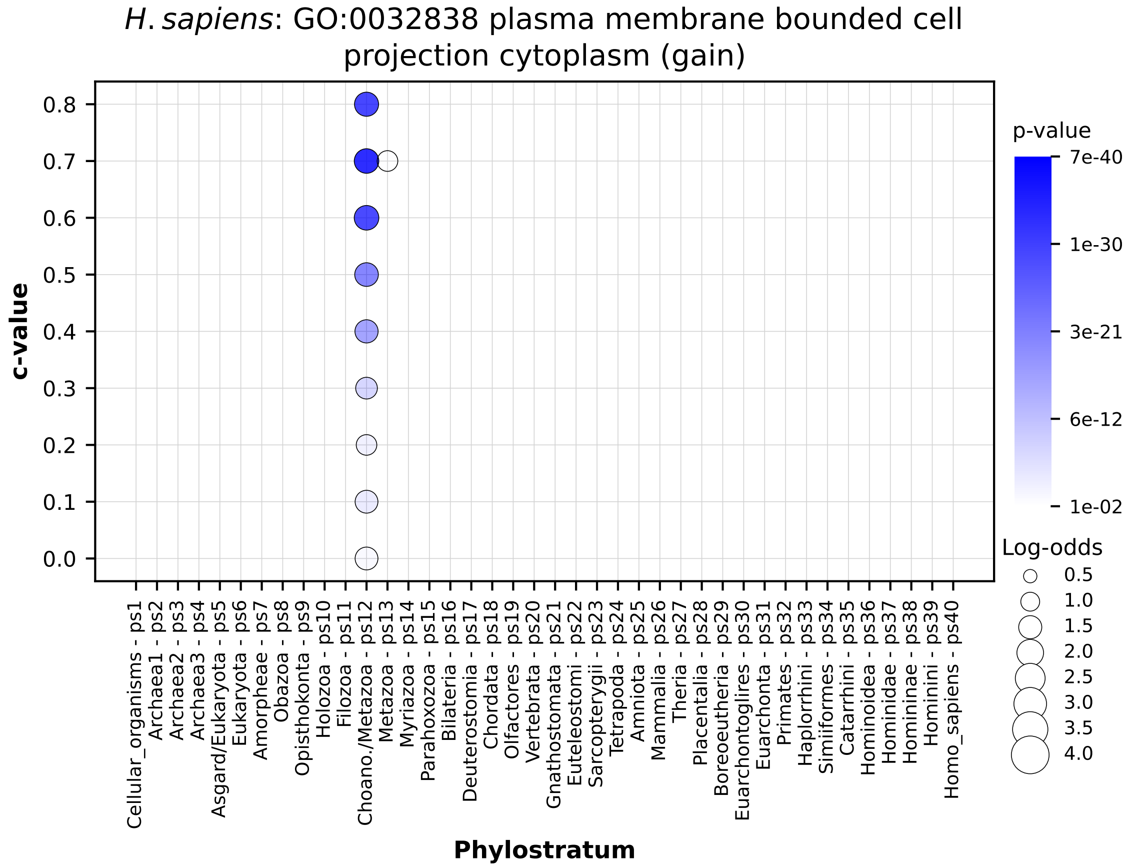
H. sapiens: GO:0032838 plasma membrane bounded cell projection cytoplasm (gain)
c-value
0.0
0.1
0.2
0.3
0.4
0.5
0.6
0.7
0.8
p-value
7e-40
1e-30
3e-21
6e-12
1e-02
Log-odds
0.5
1.0
1.5
2.0
2.5
3.0
3.5
4.0
Cellular_organisms - ps1
Archaea1 - ps2
Archaea2 - ps3
Archaea3 - ps4
Asgard/Eukaryota - ps5
Eukaryota - ps6
Amorpheae - ps7
Obazoa - ps8
Opisthokonta - ps9
Holozoa - ps10
Filozoa - ps11
Choano./Metazoa - ps12
Metazoa - ps13
Myriazoa - ps14
Parahoxozoa - ps15
Bilateria - ps16
Deuterostomia - ps17
Chordata - ps18
Olfactores - ps19
Vertebrata - ps20
Gnathostomata - ps21
Euteleostomi - ps22
Sarcopterygii - ps23
Tetrapoda - ps24
Amniota - ps25
Mammalia - ps26
Theria - ps27
Placentalia - ps28
Boreoeutheria - ps29
Euarchontoglires - ps30
Euarchonta - ps31
Primates - ps32
Haplorrhini - ps33
Simiiformes - ps34
Catarrhini - ps35
Hominoidea - ps36
Hominidae - ps37
Homininae - ps38
Hominini - ps39
Homo_sapiens - ps40
Phylostratum

# *H. sapiens*: GO:0035082 axoneme assembly (gain)

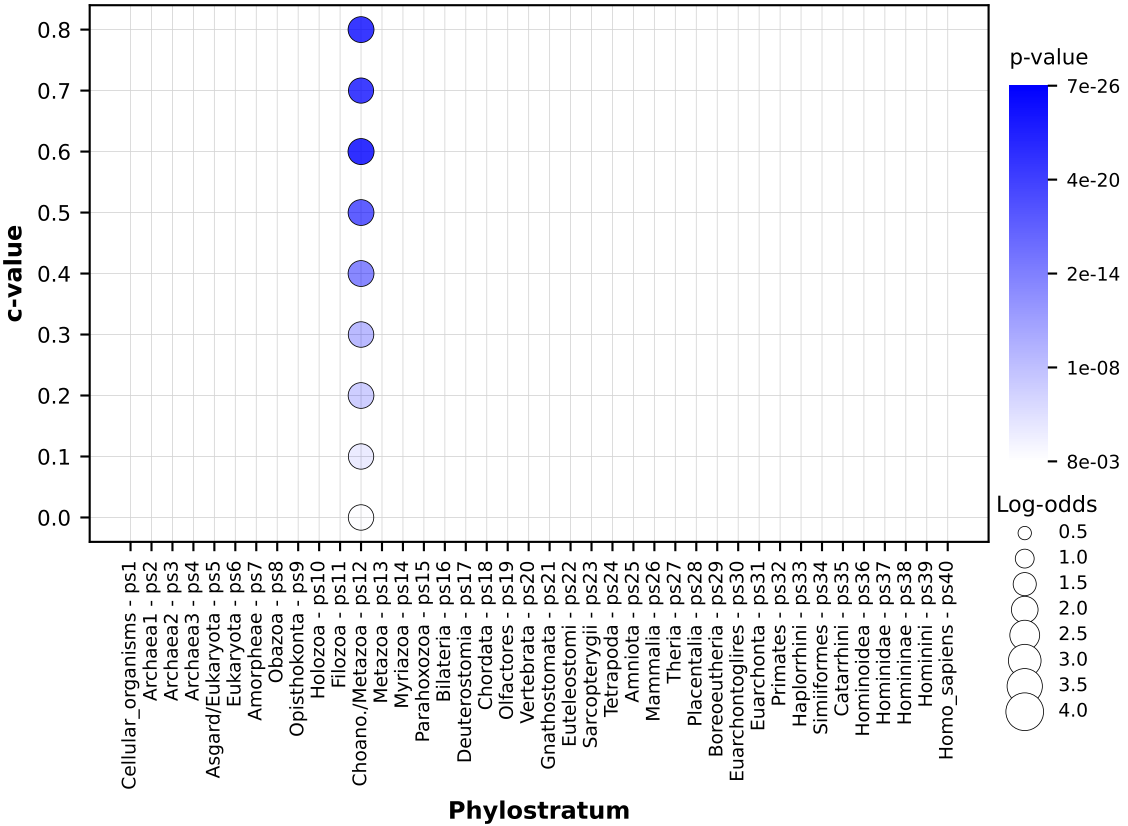

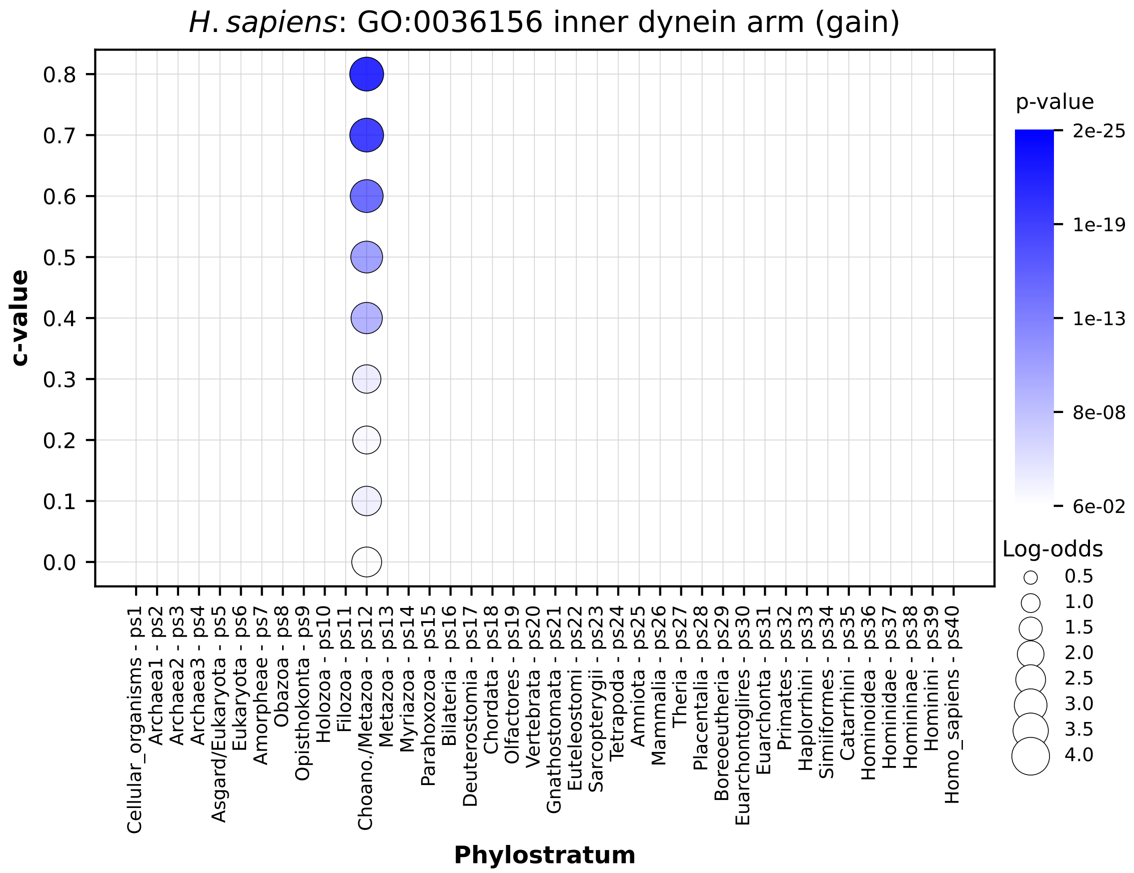

H. sapiens: GO:0036156 inner dynein arm (gain)
c-value
0.8
0.7
0.6
0.5
0.4
0.3
0.2
0.1
0.0
Phylostratum
Cellular_organisms - ps1
Archaea1 - ps2
Archaea2 - ps3
Archaea3 - ps4
Asgard/Eukaryota - ps5
Eukaryota - ps6
Amorpheae - ps7
Obazoa - ps8
Opisthokonta - ps9
Holozoa - ps10
Filozoa - ps11
Choano./Metazoa - ps12
Metazoa - ps13
Myriazoa - ps14
Parahoxozoa - ps15
Bilateria - ps16
Deuterostomia - ps17
Chordata - ps18
Olfactores - ps19
Vertebrata - ps20
Gnathostomata - ps21
Euteleostomi - ps22
Sarcopterygii - ps23
Tetrapoda - ps24
Amniota - ps25
Mammalia - ps26
Theria - ps27
Placentalia - ps28
Boreoeutheria - ps29
Euarchontoglires - ps30
Euarchonta - ps31
Primates - ps32
Haplorrhini - ps33
Simiiformes - ps34
Catarrhini - ps35
Hominoidea - ps36
Hominidae - ps37
Homininae - ps38
Hominini - ps39
Homo_sapiens - ps40
p-value
2e-25
1e-19
1e-13
8e-08
6e-02
Log-odds
0.5
1.0
1.5
2.0
2.5
3.0
3.5
4.0

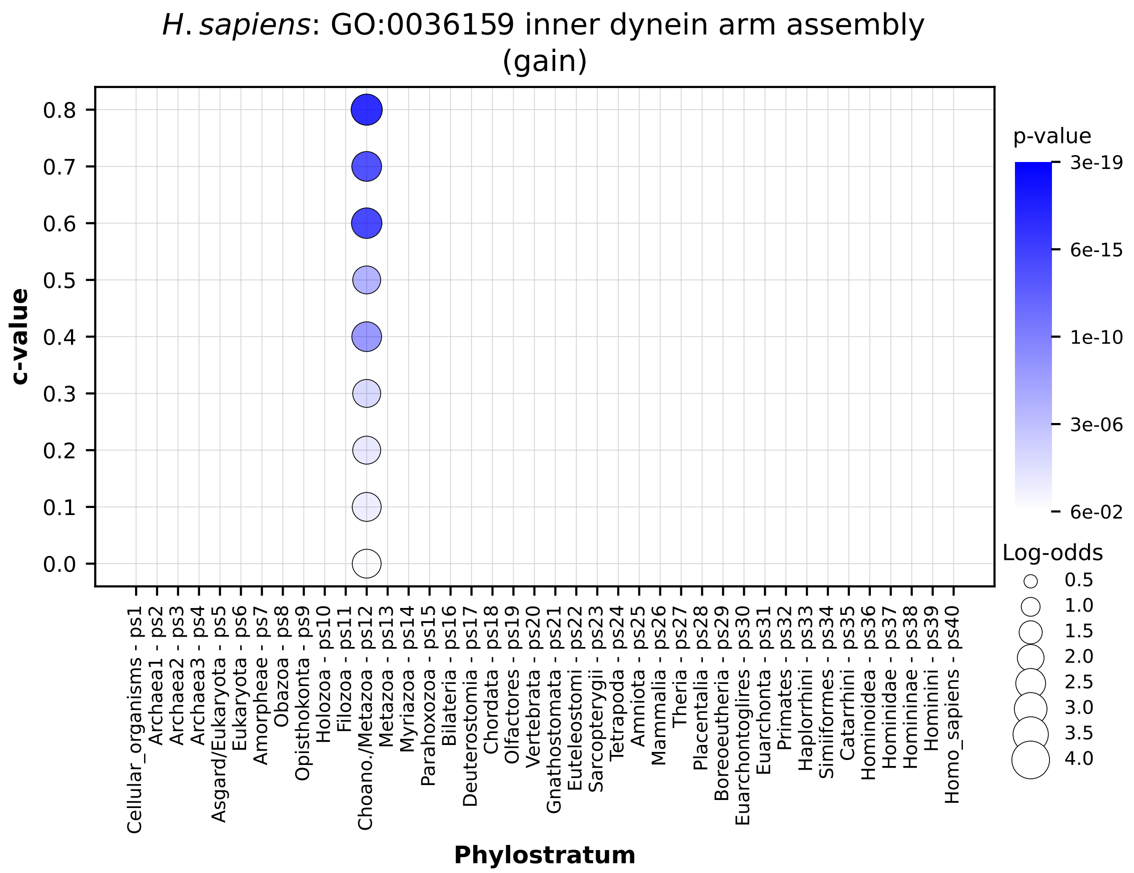
H. sapiens: GO:0036159 inner dynein arm assembly
(gain)
c-value
0.8
0.7
0.6
0.5
0.4
0.3
0.2
0.1
0.0
p-value
3e-19
6e-15
1e-10
3e-06
6e-02
Log-odds
0.5
1.0
1.5
2.0
2.5
3.0
3.5
4.0
Cellular_organisms - ps1
Archaea1 - ps2
Archaea2 - ps3
Archaea3 - ps4
Asgard/Eukaryota - ps5
Eukaryota - ps6
Amorpheae - ps7
Obazoa - ps8
Opisthokonta - ps9
Holozoa - ps10
Filozoa - ps11
Choano./Metazoa - ps12
Metazoa - ps13
Myriazoa - ps14
Parahoxozoa - ps15
Bilateria - ps16
Deuterostomia - ps17
Chordata - ps18
Olfactores - ps19
Vertebrata - ps20
Gnathostomata - ps21
Euteleostomi - ps22
Sarcopterygii - ps23
Tetrapoda - ps24
Amniota - ps25
Mammalia - ps26
Theria - ps27
Placentalia - ps28
Boreoeutheria - ps29
Euarchontoglires - ps30
Euarchonta - ps31
Primates - ps32
Haplorrhini - ps33
Simiiformes - ps34
Catarrhini - ps35
Hominoidea - ps36
Hominidae - ps37
Homininae - ps38
Hominini - ps39
Homo_sapiens - ps40
Phylostratum

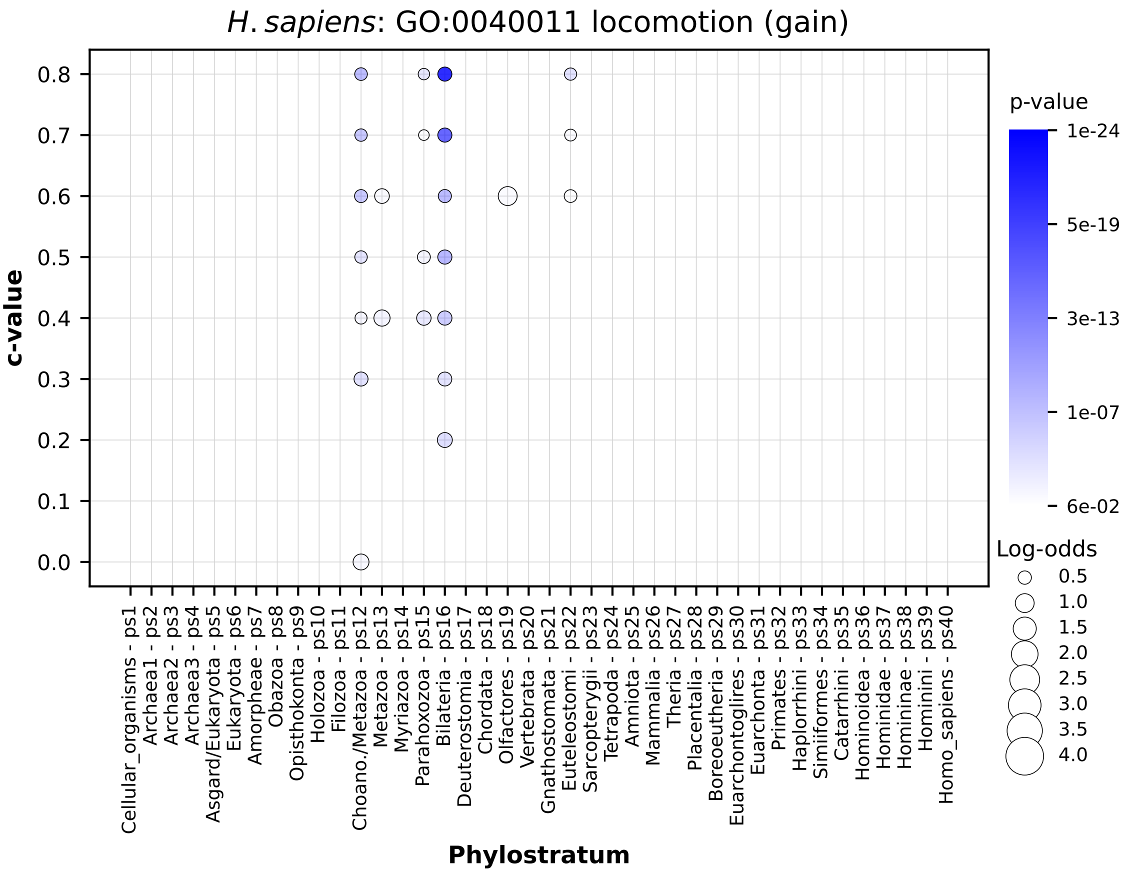

$H. sapiens$: GO:0040011 locomotion (gain)
c-value
0.0
0.1
0.2
0.3
0.4
0.5
0.6
0.7
0.8
Phylostratum
Cellular_organisms - ps1
Archaea1 - ps2
Archaea2 - ps3
Archaea3 - ps4
Asgard/Eukaryota - ps5
Eukaryota - ps6
Amorpheae - ps7
Obazoa - ps8
Opisthokonta - ps9
Holozoa - ps10
Filozoa - ps11
Choano./Metazoa - ps12
Metazoa - ps13
Myriazoa - ps14
Parahoxozoa - ps15
Bilateria - ps16
Deuterostomia - ps17
Chordata - ps18
Olfactores - ps19
Vertebrata - ps20
Gnathostomata - ps21
Euteleostomi - ps22
Sarcopterygii - ps23
Tetrapoda - ps24
Amniota - ps25
Mammalia - ps26
Theria - ps27
Placentalia - ps28
Boreoeutheria - ps29
Euarchontoglires - ps30
Euarchonta - ps31
Primates - ps32
Haplorrhini - ps33
Simiiformes - ps34
Catarrhini - ps35
Hominoidea - ps36
Hominidae - ps37
Homininae - ps38
Hominini - ps39
Homo_sapiens - ps40
p-value
1e-24
5e-19
3e-13
1e-07
6e-02
Log-odds
0.5
1.0
1.5
2.0
2.5
3.0
3.5
4.0

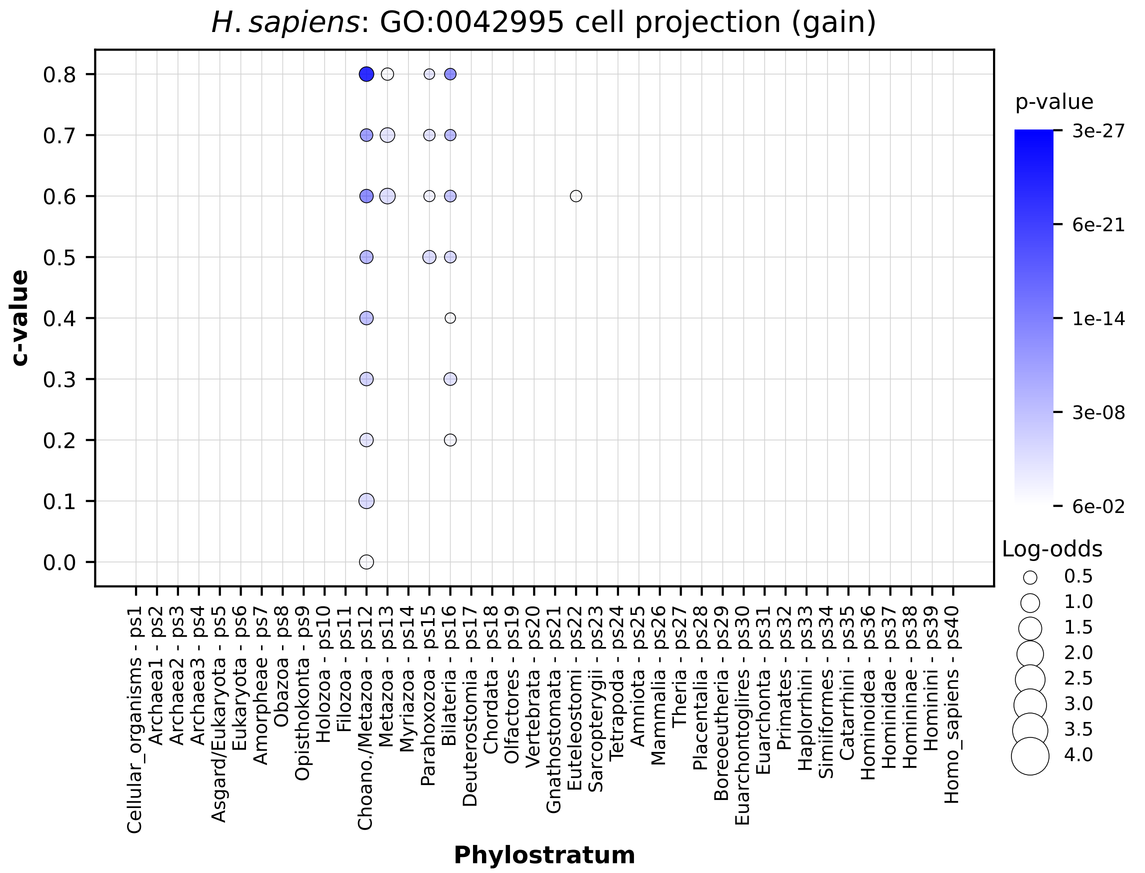

H. sapiens: GO:0042995 cell projection (gain)
c-value
0.0
0.1
0.2
0.3
0.4
0.5
0.6
0.7
0.8
Phylostratum
Cellular_organisms - ps1
Archaea1 - ps2
Archaea2 - ps3
Archaea3 - ps4
Asgard/Eukaryota - ps5
Eukaryota - ps6
Amorpheae - ps7
Obazoa - ps8
Opisthokonta - ps9
Holozoa - ps10
Filozoa - ps11
Choano./Metazoa - ps12
Metazoa - ps13
Myriazoa - ps14
Parahoxozoa - ps15
Bilateria - ps16
Deuterostomia - ps17
Chordata - ps18
Olfactores - ps19
Vertebrata - ps20
Gnathostomata - ps21
Euteleostomi - ps22
Sarcopterygii - ps23
Tetrapoda - ps24
Amniota - ps25
Mammalia - ps26
Theria - ps27
Placentalia - ps28
Boreoeutheria - ps29
Euarchontoglires - ps30
Euarchonta - ps31
Primates - ps32
Haplorrhini - ps33
Simiiformes - ps34
Catarrhini - ps35
Hominoidea - ps36
Hominidae - ps37
Homininae - ps38
Hominini - ps39
Homo_sapiens - ps40
p-value
3e-27
6e-21
1e-14
3e-08
6e-02
Log-odds
0.5
1.0
1.5
2.0
2.5
3.0
3.5
4.0

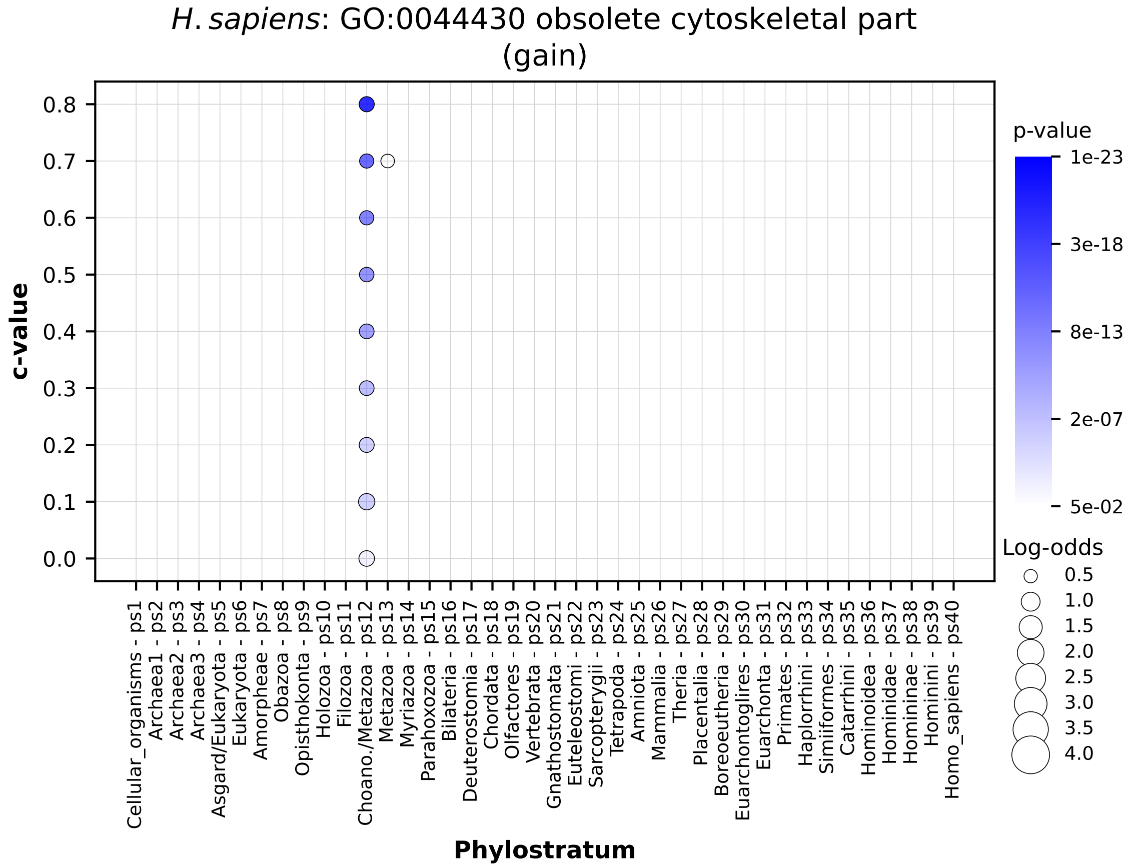

H. sapiens: GO:0044430 obsolete cytoskeletal part
(gain)
c-value
0.0
0.1
0.2
0.3
0.4
0.5
0.6
0.7
0.8
Phylostratum
Cellular_organisms - ps1
Archaea1 - ps2
Archaea2 - ps3
Archaea3 - ps4
Asgard/Eukaryota - ps5
Eukaryota - ps6
Amorpheae - ps7
Obazoa - ps8
Opisthokonta - ps9
Holozoa - ps10
Filozoa - ps11
Choano./Metazoa - ps12
Metazoa - ps13
Myriazoa - ps14
Parahoxozoa - ps15
Bilateria - ps16
Deuterostomia - ps17
Chordata - ps18
Olfactores - ps19
Vertebrata - ps20
Gnathostomata - ps21
Euteleostomi - ps22
Sarcopterygii - ps23
Tetrapoda - ps24
Amniota - ps25
Mammalia - ps26
Theria - ps27
Placentalia - ps28
Boreoeutheria - ps29
Euarchontoglires - ps30
Euarchonta - ps31
Primates - ps32
Haplorrhini - ps33
Simiiformes - ps34
Catarrhini - ps35
Hominoidea - ps36
Hominidae - ps37
Homininae - ps38
Hominini - ps39
Homo_sapiens - ps40
p-value
1e-23
3e-18
8e-13
2e-07
5e-02
Log-odds
0.5
1.0
1.5
2.0
2.5
3.0
3.5
4.0

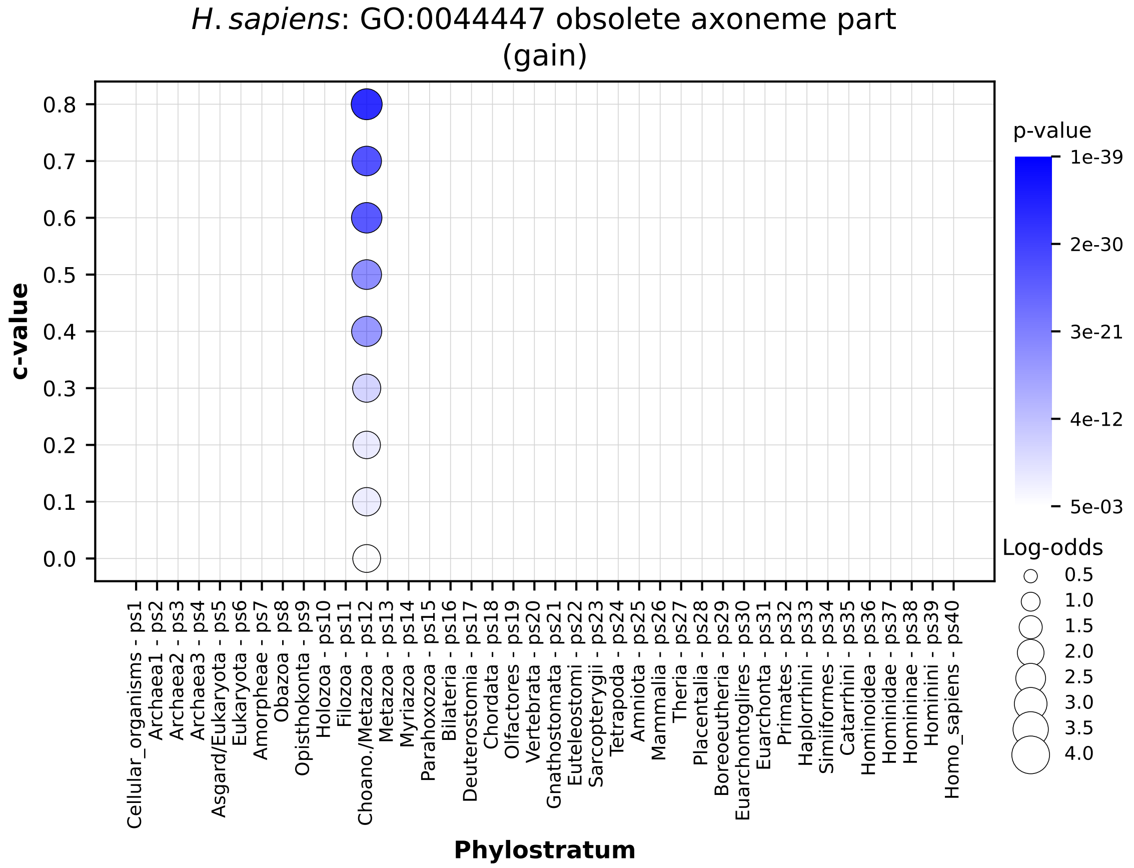

H. sapiens: GO:0044447 obsolete axoneme part
(gain)
c-value
0.8
0.7
0.6
0.5
0.4
0.3
0.2
0.1
0.0
p-value
1e-39
2e-30
3e-21
4e-12
5e-03
Log-odds
0.5
1.0
1.5
2.0
2.5
3.0
3.5
4.0
Cellular_organisms - ps1
Archaea1 - ps2
Archaea2 - ps3
Archaea3 - ps4
Asgard/Eukaryota - ps5
Eukaryota - ps6
Amorpheae - ps7
Obazoa - ps8
Opisthokonta - ps9
Holozoa - ps10
Filozoa - ps11
Choano./Metazoa - ps12
Metazoa - ps13
Myriazoa - ps14
Parahoxozoa - ps15
Bilateria - ps16
Deuterostomia - ps17
Chordata - ps18
Olfactores - ps19
Vertebrata - ps20
Gnathostomata - ps21
Euteleostomi - ps22
Sarcopterygii - ps23
Tetrapoda - ps24
Amniota - ps25
Mammalia - ps26
Theria - ps27
Placentalia - ps28
Boreoeutheria - ps29
Euarchontoglires - ps30
Euarchonta - ps31
Primates - ps32
Haplorrhini - ps33
Simiiformes - ps34
Catarrhini - ps35
Hominoidea - ps36
Hominidae - ps37
Homininae - ps38
Hominini - ps39
Homo_sapiens - ps40
Phylostratum

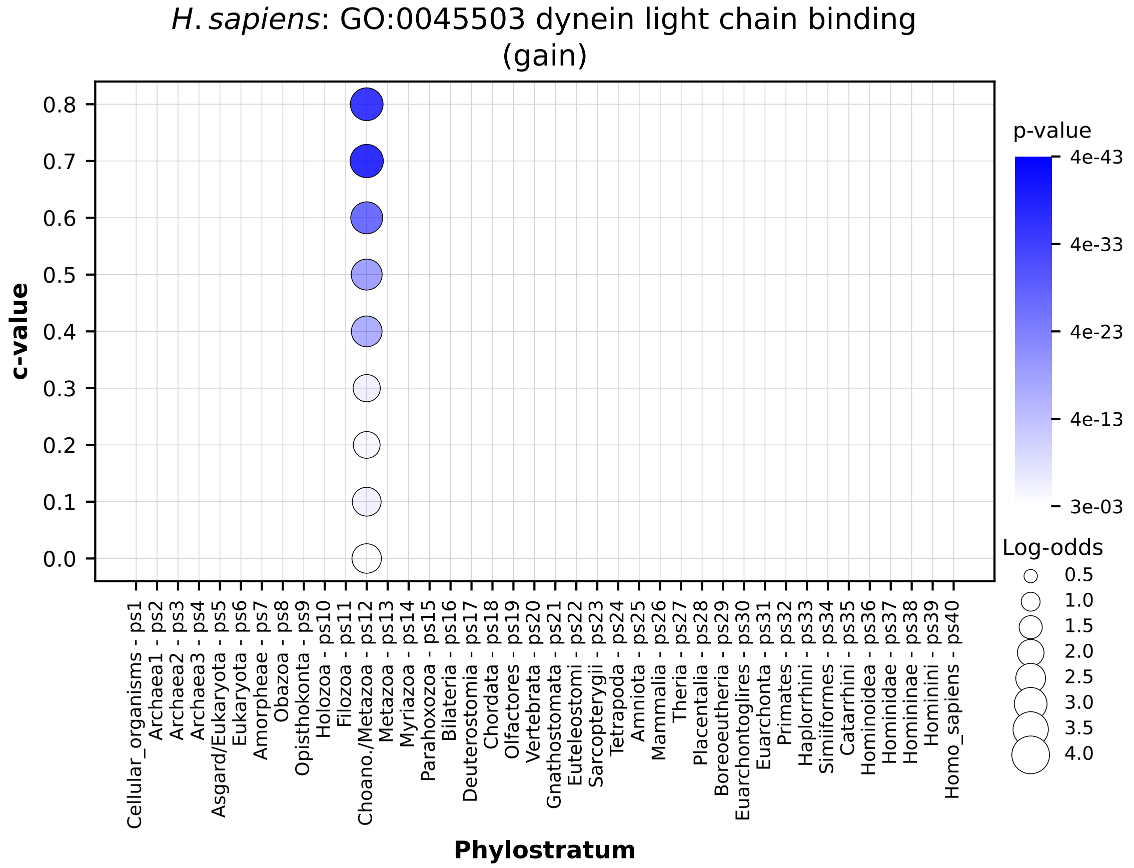
H. sapiens: GO:0045503 dynein light chain binding
(gain)
c-value
0.8
0.7
0.6
0.5
0.4
0.3
0.2
0.1
0.0
p-value
4e-43
4e-33
4e-23
4e-13
3e-03
Log-odds
0.5
1.0
1.5
2.0
2.5
3.0
3.5
4.0
Cellular_organisms - ps1
Archaea1 - ps2
Archaea2 - ps3
Archaea3 - ps4
Asgard/Eukaryota - ps5
Eukaryota - ps6
Amorpheae - ps7
Obazoa - ps8
Opisthokonta - ps9
Holozoa - ps10
Filozoa - ps11
Choano./Metazoa - ps12
Metazoa - ps13
Myriazoa - ps14
Parahoxozoa - ps15
Bilateria - ps16
Deuterostomia - ps17
Chordata - ps18
Olfactores - ps19
Vertebrata - ps20
Gnathostomata - ps21
Euteleostomi - ps22
Sarcopterygii - ps23
Tetrapoda - ps24
Amniota - ps25
Mammalia - ps26
Theria - ps27
Placentalia - ps28
Boreoeutheria - ps29
Euarchontoglires - ps30
Euarchonta - ps31
Primates - ps32
Haplorrhini - ps33
Simiiformes - ps34
Catarrhini - ps35
Hominoidea - ps36
Hominidae - ps37
Homininae - ps38
Hominini - ps39
Homo_sapiens - ps40
Phylostratum

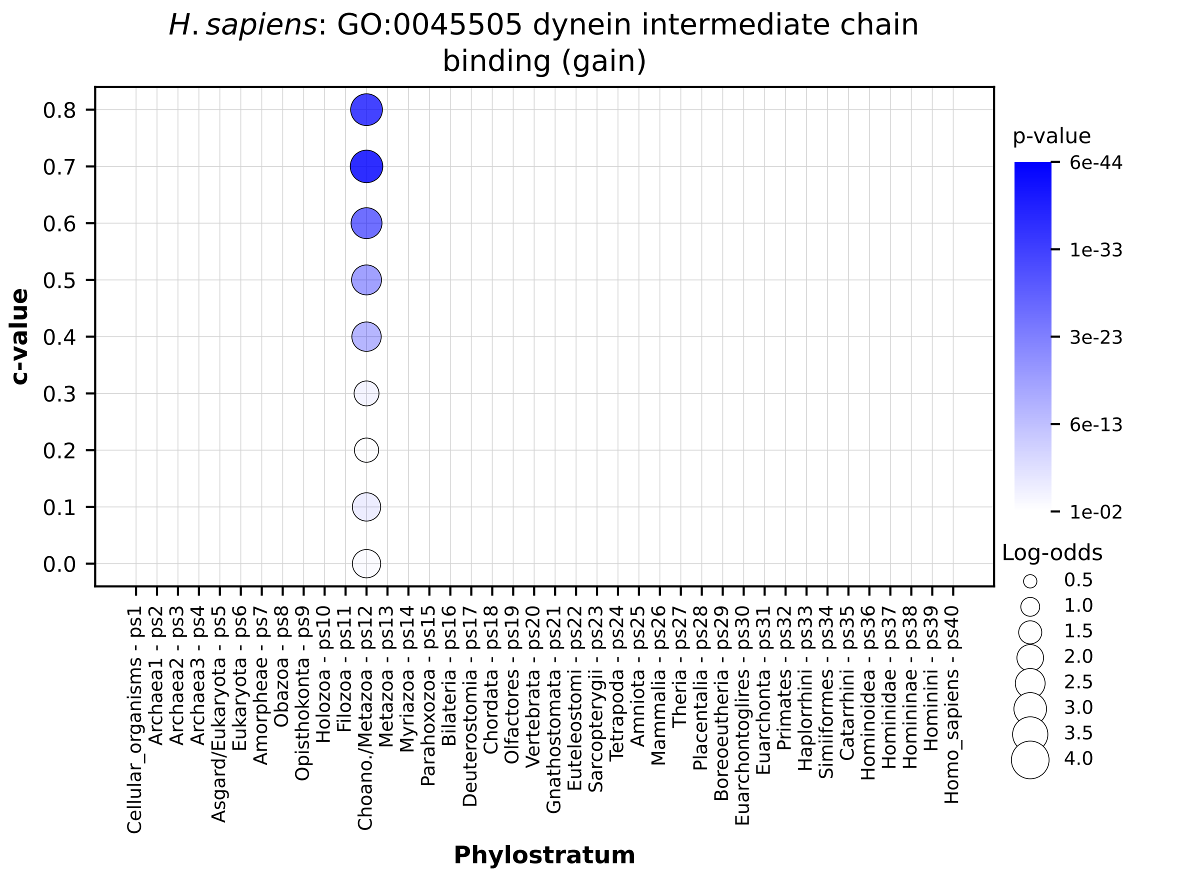

*H. sapiens*: GO:0045505 dynein intermediate chain binding (gain)
c-value
0.8
0.7
0.6
0.5
0.4
0.3
0.2
0.1
0.0
p-value
6e-44
1e-33
3e-23
6e-13
1e-02
Log-odds
0.5
1.0
1.5
2.0
2.5
3.0
3.5
4.0
Cellular_organisms - ps1
Archaea1 - ps2
Archaea2 - ps3
Archaea3 - ps4
Asgard/Eukaryota - ps5
Eukaryota - ps6
Amorpheae - ps7
Obazoa - ps8
Opisthokonta - ps9
Holozoa - ps10
Filozoa - ps11
Choano./Metazoa - ps12
Metazoa - ps13
Myriazoa - ps14
Parahoxozoa - ps15
Bilateria - ps16
Deuterostomia - ps17
Chordata - ps18
Olfactores - ps19
Vertebrata - ps20
Gnathostomata - ps21
Euteleostomi - ps22
Sarcopterygii - ps23
Tetrapoda - ps24
Amniota - ps25
Mammalia - ps26
Theria - ps27
Placentalia - ps28
Boreoeutheria - ps29
Euarchontoglires - ps30
Euarchonta - ps31
Primates - ps32
Haplorrhini - ps33
Simiiformes - ps34
Catarrhini - ps35
Hominoidea - ps36
Hominidae - ps37
Homininae - ps38
Hominini - ps39
Homo_sapiens - ps40
Phylostratum

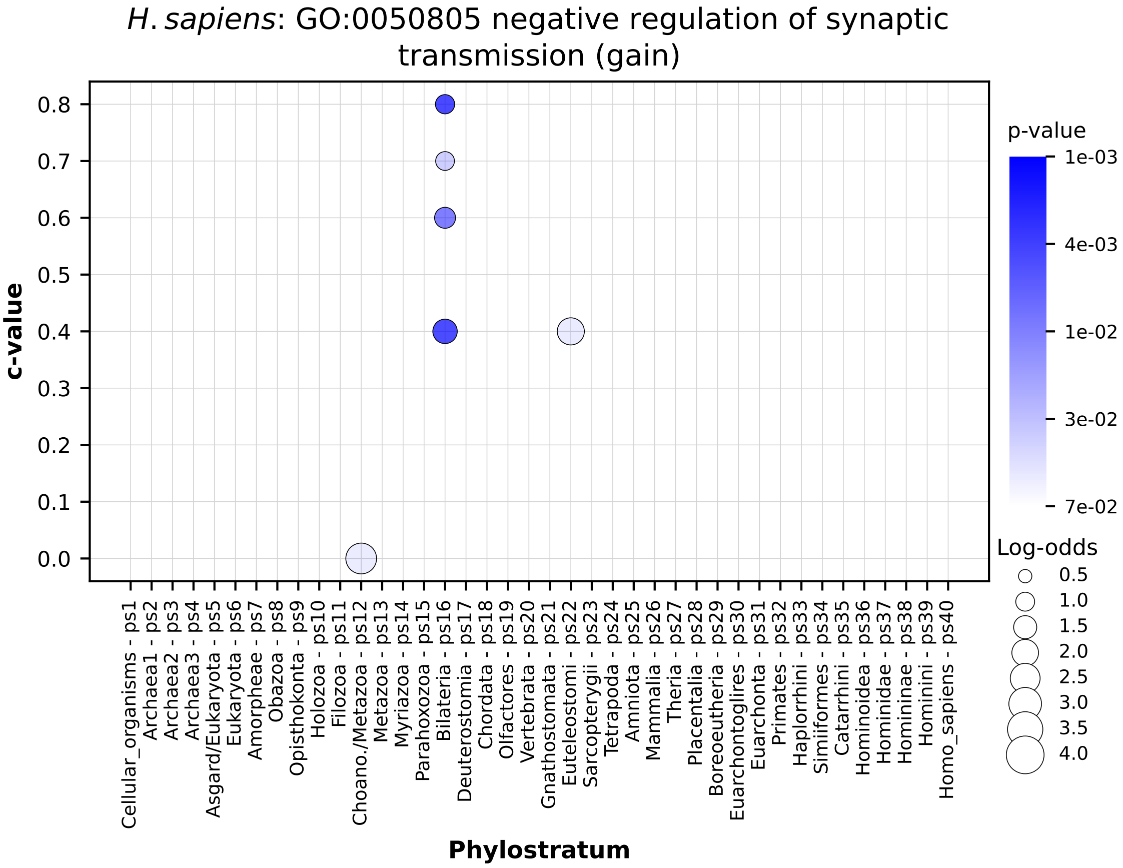

*H. sapiens*: GO:0050805 negative regulation of synaptic transmission (gain)
c-value
0.8
0.7
0.6
0.5
0.4
0.3
0.2
0.1
0.0
Phylostratum
Cellular_organisms - ps1
Archaea1 - ps2
Archaea2 - ps3
Archaea3 - ps4
Asgard/Eukaryota - ps5
Eukaryota - ps6
Amorpheae - ps7
Obazoa - ps8
Opisthokonta - ps9
Holozoa - ps10
Filozoa - ps11
Choano./Metazoa - ps12
Metazoa - ps13
Myriazoa - ps14
Parahoxozoa - ps15
Bilateria - ps16
Deuterostomia - ps17
Chordata - ps18
Olfactores - ps19
Vertebrata - ps20
Gnathostomata - ps21
Euteleostomi - ps22
Sarcopterygii - ps23
Tetrapoda - ps24
Amniota - ps25
Mammalia - ps26
Theria - ps27
Placentalia - ps28
Boreoeutheria - ps29
Euarchontoglires - ps30
Euarchonta - ps31
Primates - ps32
Haplorrhini - ps33
Simiiformes - ps34
Catarrhini - ps35
Hominoidea - ps36
Hominidae - ps37
Homininae - ps38
Hominini - ps39
Homo_sapiens - ps40
p-value
1e-03
4e-03
1e-02
3e-02
7e-02
Log-odds
0.5
1.0
1.5
2.0
2.5
3.0
3.5
4.0

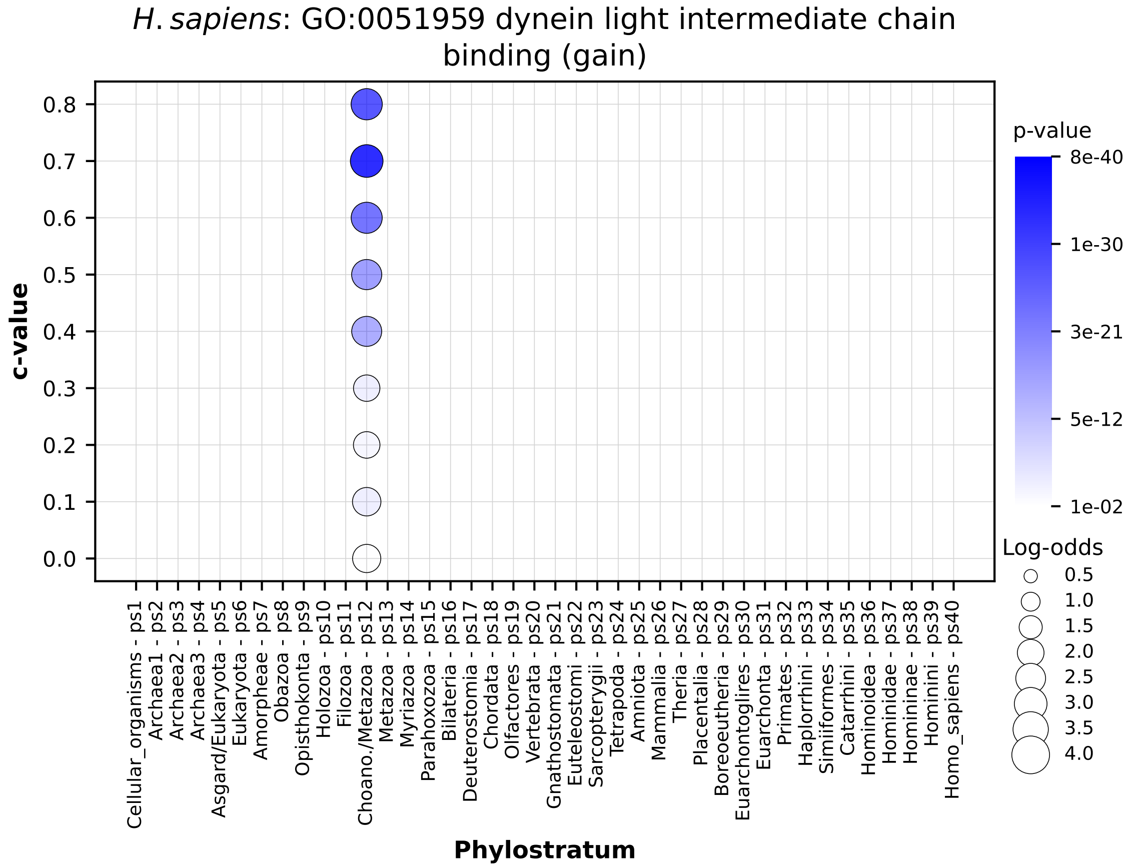

*H. sapiens*: GO:0051959 dynein light intermediate chain binding (gain)
c-value
0.8
0.7
0.6
0.5
0.4
0.3
0.2
0.1
0.0
p-value
8e-40
1e-30
3e-21
5e-12
1e-02
Log-odds
0.5
1.0
1.5
2.0
2.5
3.0
3.5
4.0
Cellular_organisms - ps1
Archaea1 - ps2
Archaea2 - ps3
Archaea3 - ps4
Asgard/Eukaryota - ps5
Eukaryota - ps6
Amorpheae - ps7
Obazoa - ps8
Opisthokonta - ps9
Holozoa - ps10
Filozoa - ps11
Choano./Metazoa - ps12
Metazoa - ps13
Myriazoa - ps14
Parahoxozoa - ps15
Bilateria - ps16
Deuterostomia - ps17
Chordata - ps18
Olfactores - ps19
Vertebrata - ps20
Gnathostomata - ps21
Euteleostomi - ps22
Sarcopterygii - ps23
Tetrapoda - ps24
Amniota - ps25
Mammalia - ps26
Theria - ps27
Placentalia - ps28
Boreoeutheria - ps29
Euarchontoglires - ps30
Euarchonta - ps31
Primates - ps32
Haplorrhini - ps33
Simiiformes - ps34
Catarrhini - ps35
Hominoidea - ps36
Hominidae - ps37
Homininae - ps38
Hominini - ps39
Homo_sapiens - ps40
Phylostratum

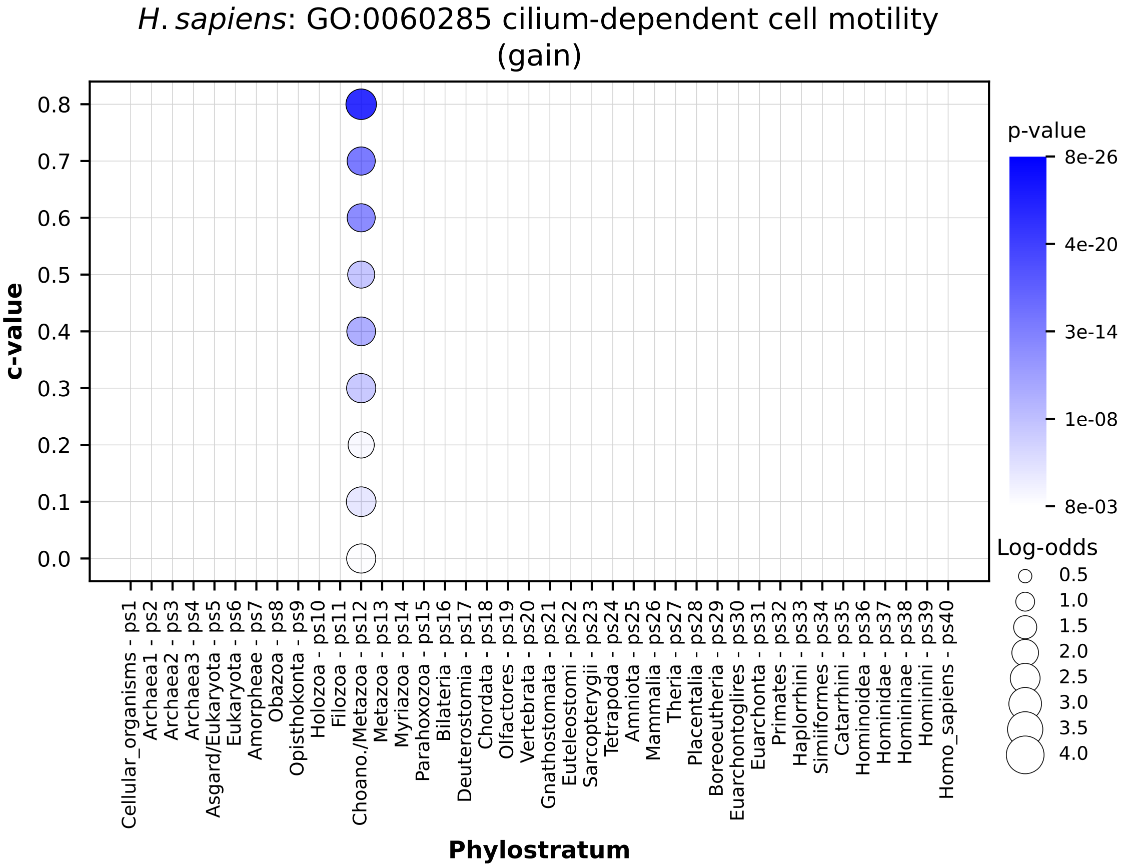

H. sapiens: GO:0060285 cilium-dependent cell motility
(gain)
c-value
0.8
0.7
0.6
0.5
0.4
0.3
0.2
0.1
0.0
p-value
8e-26
4e-20
3e-14
1e-08
8e-03
Log-odds
0.5
1.0
1.5
2.0
2.5
3.0
3.5
4.0
Cellular_organisms - ps1
Archaea1 - ps2
Archaea2 - ps3
Archaea3 - ps4
Asgard/Eukaryota - ps5
Eukaryota - ps6
Amorpheae - ps7
Obazoa - ps8
Opisthokonta - ps9
Holozoa - ps10
Filozoa - ps11
Choano./Metazoa - ps12
Metazoa - ps13
Myriazoa - ps14
Parahoxozoa - ps15
Bilateria - ps16
Deuterostomia - ps17
Chordata - ps18
Olfactores - ps19
Vertebrata - ps20
Gnathostomata - ps21
Euteleostomi - ps22
Sarcopterygii - ps23
Tetrapoda - ps24
Amniota - ps25
Mammalia - ps26
Theria - ps27
Placentalia - ps28
Boreoeutheria - ps29
Euarchontoglires - ps30
Euarchonta - ps31
Primates - ps32
Haplorrhini - ps33
Simiiformes - ps34
Catarrhini - ps35
Hominoidea - ps36
Hominidae - ps37
Homininae - ps38
Hominini - ps39
Homo_sapiens - ps40
Phylostratum

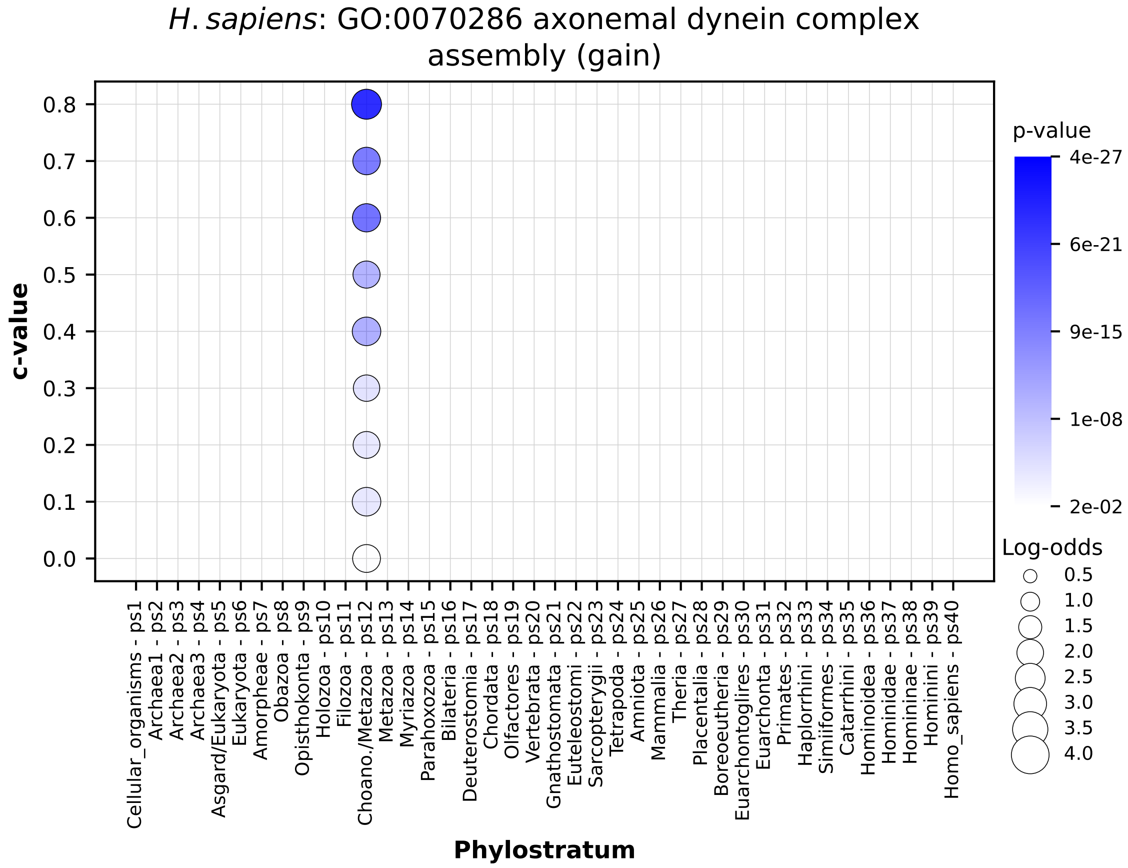

H. sapiens: GO:0070286 axonemal dynein complex assembly (gain)
c-value
0.0
0.1
0.2
0.3
0.4
0.5
0.6
0.7
0.8
Phylostratum
Cellular_organisms - ps1
Archaea1 - ps2
Archaea2 - ps3
Archaea3 - ps4
Asgard/Eukaryota - ps5
Eukaryota - ps6
Amorpheae - ps7
Obazoa - ps8
Opisthokonta - ps9
Holozoa - ps10
Filozoa - ps11
Choano./Metazoa - ps12
Metazoa - ps13
Myriazoa - ps14
Parahoxozoa - ps15
Bilateria - ps16
Deuterostomia - ps17
Chordata - ps18
Olfactores - ps19
Vertebrata - ps20
Gnathostomata - ps21
Euteleostomi - ps22
Sarcopterygii - ps23
Tetrapoda - ps24
Amniota - ps25
Mammalia - ps26
Theria - ps27
Placentalia - ps28
Boreoeutheria - ps29
Euarchontoglires - ps30
Euarchonta - ps31
Primates - ps32
Haplorrhini - ps33
Simiiformes - ps34
Catarrhini - ps35
Hominoidea - ps36
Hominidae - ps37
Homininae - ps38
Hominini - ps39
Homo_sapiens - ps40
p-value
4e-27
6e-21
9e-15
1e-08
2e-02
Log-odds
0.5
1.0
1.5
2.0
2.5
3.0
3.5
4.0

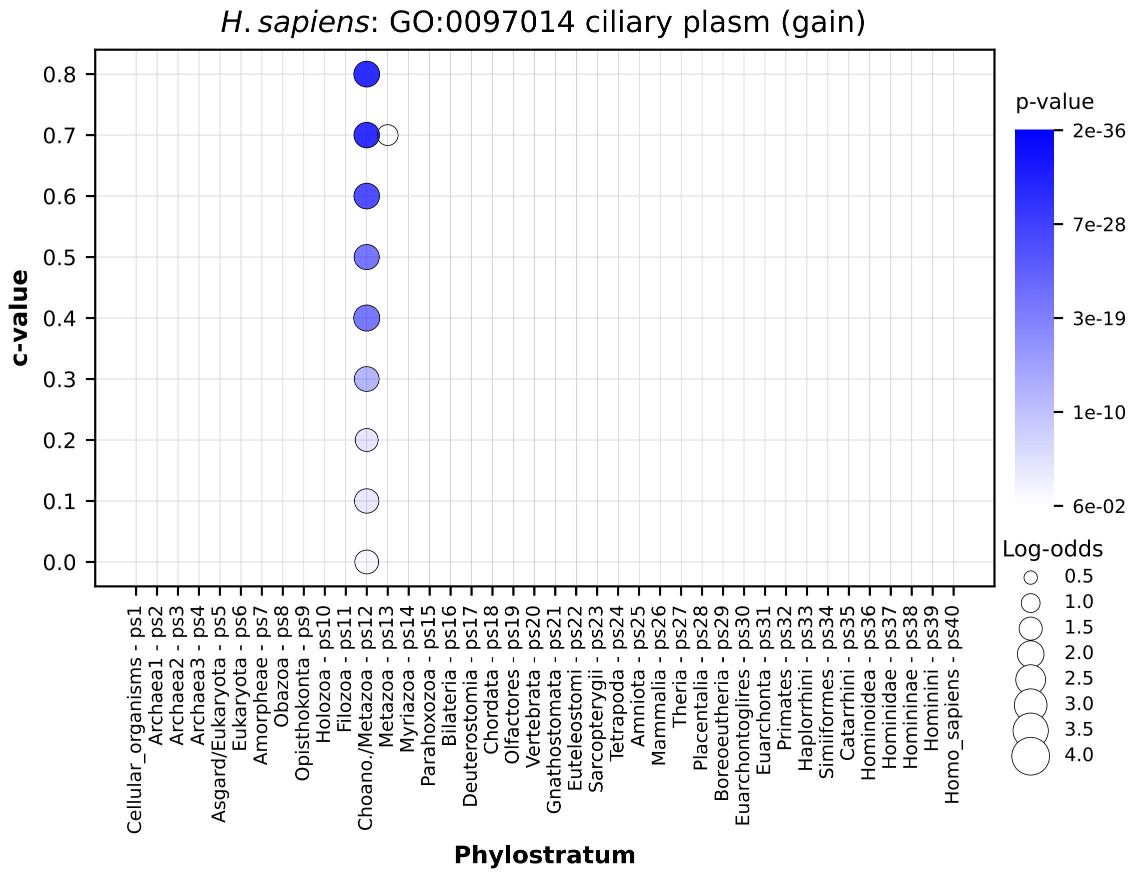
H. sapiens: GO:0097014 ciliary plasm (gain)
c-value
0.8
0.7
0.6
0.5
0.4
0.3
0.2
0.1
0.0
Phylostratum
Cellular_organisms - ps1
Archaea1 - ps2
Archaea2 - ps3
Archaea3 - ps4
Asgard/Eukaryota - ps5
Eukaryota - ps6
Amorpheae - ps7
Obazoa - ps8
Opisthokonta - ps9
Holozoa - ps10
Filozoa - ps11
Choano./Metazoa - ps12
Metazoa - ps13
Myriazoa - ps14
Parahoxozoa - ps15
Bilateria - ps16
Deuterostomia - ps17
Chordata - ps18
Olfactores - ps19
Vertebrata - ps20
Gnathostomata - ps21
Euteleostomi - ps22
Sarcopterygii - ps23
Tetrapoda - ps24
Amniota - ps25
Mammalia - ps26
Theria - ps27
Placentalia - ps28
Boreoeutheria - ps29
Euarchontoglires - ps30
Euarchonta - ps31
Primates - ps32
Haplorrhini - ps33
Simiiformes - ps34
Catarrhini - ps35
Hominoidea - ps36
Hominidae - ps37
Homininae - ps38
Hominini - ps39
Homo_sapiens - ps40
p-value
2e-36
7e-28
3e-19
1e-10
6e-02
Log-odds
0.5
1.0
1.5
2.0
2.5
3.0
3.5
4.0

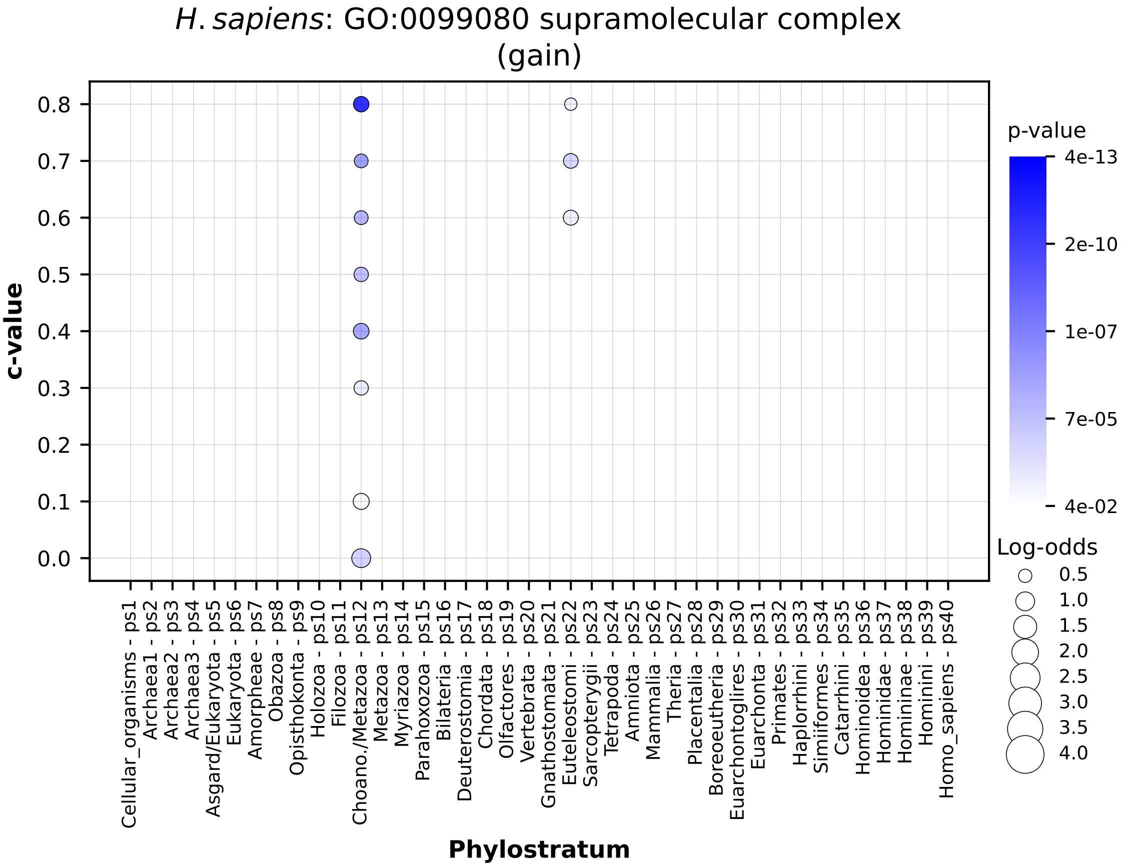

H. sapiens: GO:0099080 supramolecular complex
(gain)
c-value
0.0
0.1
0.2
0.3
0.4
0.5
0.6
0.7
0.8
Phylostratum
Cellular_organisms - ps1
Archaea1 - ps2
Archaea2 - ps3
Archaea3 - ps4
Asgard/Eukaryota - ps5
Eukaryota - ps6
Amorpheae - ps7
Obazoa - ps8
Opisthokonta - ps9
Holozoa - ps10
Filozoa - ps11
Choano./Metazoa - ps12
Metazoa - ps13
Myriazoa - ps14
Parahoxozoa - ps15
Bilateria - ps16
Deuterostomia - ps17
Chordata - ps18
Olfactores - ps19
Vertebrata - ps20
Gnathostomata - ps21
Euteleostomi - ps22
Sarcopterygii - ps23
Tetrapoda - ps24
Amniota - ps25
Mammalia - ps26
Theria - ps27
Placentalia - ps28
Boreoeutheria - ps29
Euarchontoglires - ps30
Euarchonta - ps31
Primates - ps32
Haplorrhini - ps33
Simiiformes - ps34
Catarrhini - ps35
Hominoidea - ps36
Hominidae - ps37
Homininae - ps38
Hominini - ps39
Homo_sapiens - ps40
p-value
4e-13
2e-10
1e-07
7e-05
4e-02
Log-odds
0.5
1.0
1.5
2.0
2.5
3.0
3.5
4.0

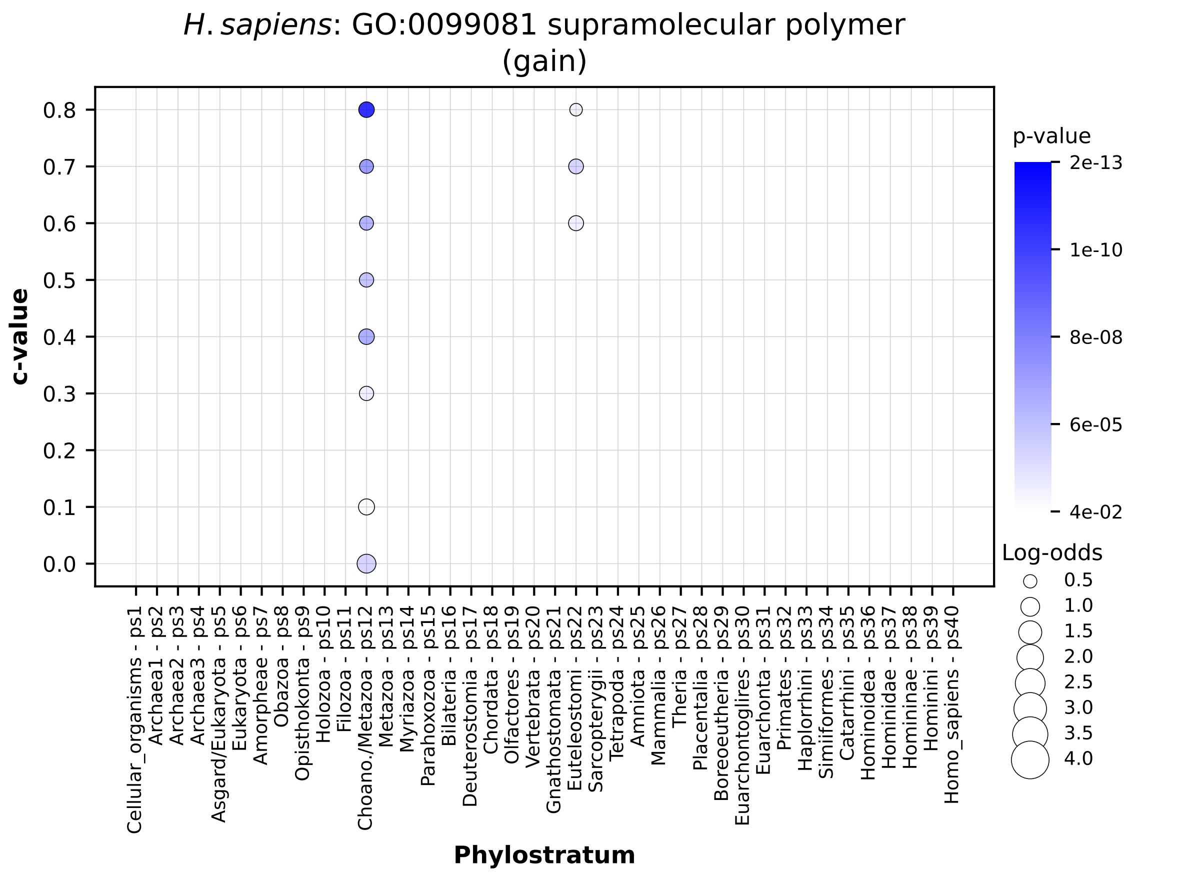

H. sapiens: GO:0099081 supramolecular polymer
(gain)
c-value
0.8
0.7
0.6
0.5
0.4
0.3
0.2
0.1
0.0
p-value
2e-13
1e-10
8e-08
6e-05
4e-02
Log-odds
0.5
1.0
1.5
2.0
2.5
3.0
3.5
4.0
Cellular_organisms - ps1
Archaea1 - ps2
Archaea2 - ps3
Archaea3 - ps4
Asgard/Eukaryota - ps5
Eukaryota - ps6
Amorpheae - ps7
Obazoa - ps8
Opisthokonta - ps9
Holozoa - ps10
Filozoa - ps11
Choano./Metazoa - ps12
Metazoa - ps13
Myriazoa - ps14
Parahoxozoa - ps15
Bilateria - ps16
Deuterostomia - ps17
Chordata - ps18
Olfactores - ps19
Vertebrata - ps20
Gnathostomata - ps21
Euteleostomi - ps22
Sarcopterygii - ps23
Tetrapoda - ps24
Amniota - ps25
Mammalia - ps26
Theria - ps27
Placentalia - ps28
Boreoeutheria - ps29
Euarchontoglires - ps30
Euarchonta - ps31
Primates - ps32
Haplorrhini - ps33
Simiiformes - ps34
Catarrhini - ps35
Hominoidea - ps36
Hominidae - ps37
Homininae - ps38
Hominini - ps39
Homo_sapiens - ps40
Phylostratum

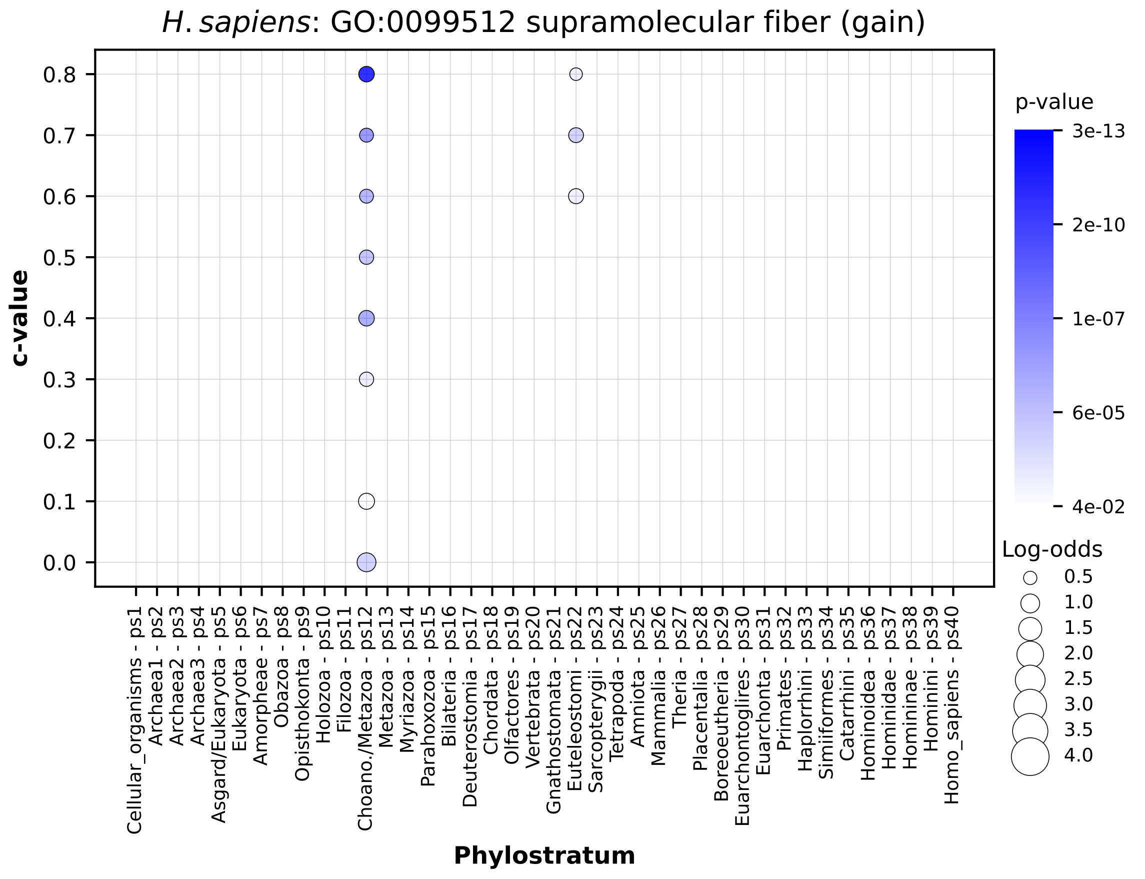
H. sapiens: GO:0099512 supramolecular fiber (gain)
c-value
0.8
0.7
0.6
0.5
0.4
0.3
0.2
0.1
0.0
Phylostratum
Cellular_organisms - ps1
Archaea1 - ps2
Archaea2 - ps3
Archaea3 - ps4
Asgard/Eukaryota - ps5
Eukaryota - ps6
Amorpheae - ps7
Obazoa - ps8
Opisthokonta - ps9
Holozoa - ps10
Filozoa - ps11
Choano./Metazoa - ps12
Metazoa - ps13
Myriazoa - ps14
Parahoxozoa - ps15
Bilateria - ps16
Deuterostomia - ps17
Chordata - ps18
Olfactores - ps19
Vertebrata - ps20
Gnathostomata - ps21
Euteleostomi - ps22
Sarcopterygii - ps23
Tetrapoda - ps24
Amniota - ps25
Mammalia - ps26
Theria - ps27
Placentalia - ps28
Boreoeutheria - ps29
Euarchontoglires - ps30
Euarchonta - ps31
Primates - ps32
Haplorrhini - ps33
Simiiformes - ps34
Catarrhini - ps35
Hominoidea - ps36
Hominidae - ps37
Homininae - ps38
Hominini - ps39
Homo_sapiens - ps40
p-value
3e-13
2e-10
1e-07
6e-05
4e-02
Log-odds
0.5
1.0
1.5
2.0
2.5
3.0
3.5
4.0

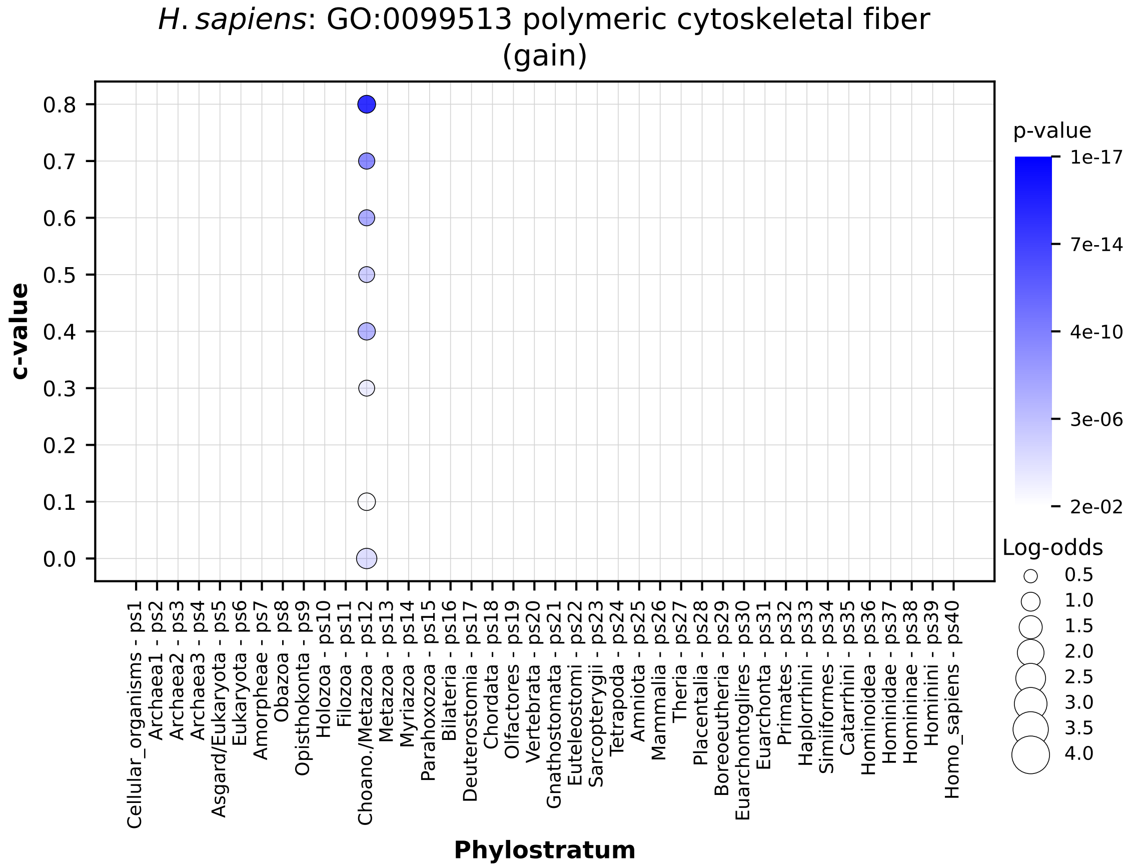

*H. sapiens*: GO:0099513 polymeric cytoskeletal fiber
(gain)
c-value
0.8
0.7
0.6
0.5
0.4
0.3
0.2
0.1
0.0
p-value
1e-17
7e-14
4e-10
3e-06
2e-02
Log-odds
0.5
1.0
1.5
2.0
2.5
3.0
3.5
4.0
Cellular_organisms - ps1
Archaea1 - ps2
Archaea2 - ps3
Archaea3 - ps4
Asgard/Eukaryota - ps5
Eukaryota - ps6
Amorpheae - ps7
Obazoa - ps8
Opisthokonta - ps9
Holozoa - ps10
Filozoa - ps11
Choano./Metazoa - ps12
Metazoa - ps13
Myriazoa - ps14
Parahoxozoa - ps15
Bilateria - ps16
Deuterostomia - ps17
Chordata - ps18
Olfactores - ps19
Vertebrata - ps20
Gnathostomata - ps21
Euteleostomi - ps22
Sarcopterygii - ps23
Tetrapoda - ps24
Amniota - ps25
Mammalia - ps26
Theria - ps27
Placentalia - ps28
Boreoeutheria - ps29
Euarchontoglires - ps30
Euarchonta - ps31
Primates - ps32
Haplorrhini - ps33
Simiiformes - ps34
Catarrhini - ps35
Hominoidea - ps36
Hominidae - ps37
Homininae - ps38
Hominini - ps39
Homo_sapiens - ps40
Phylostratum

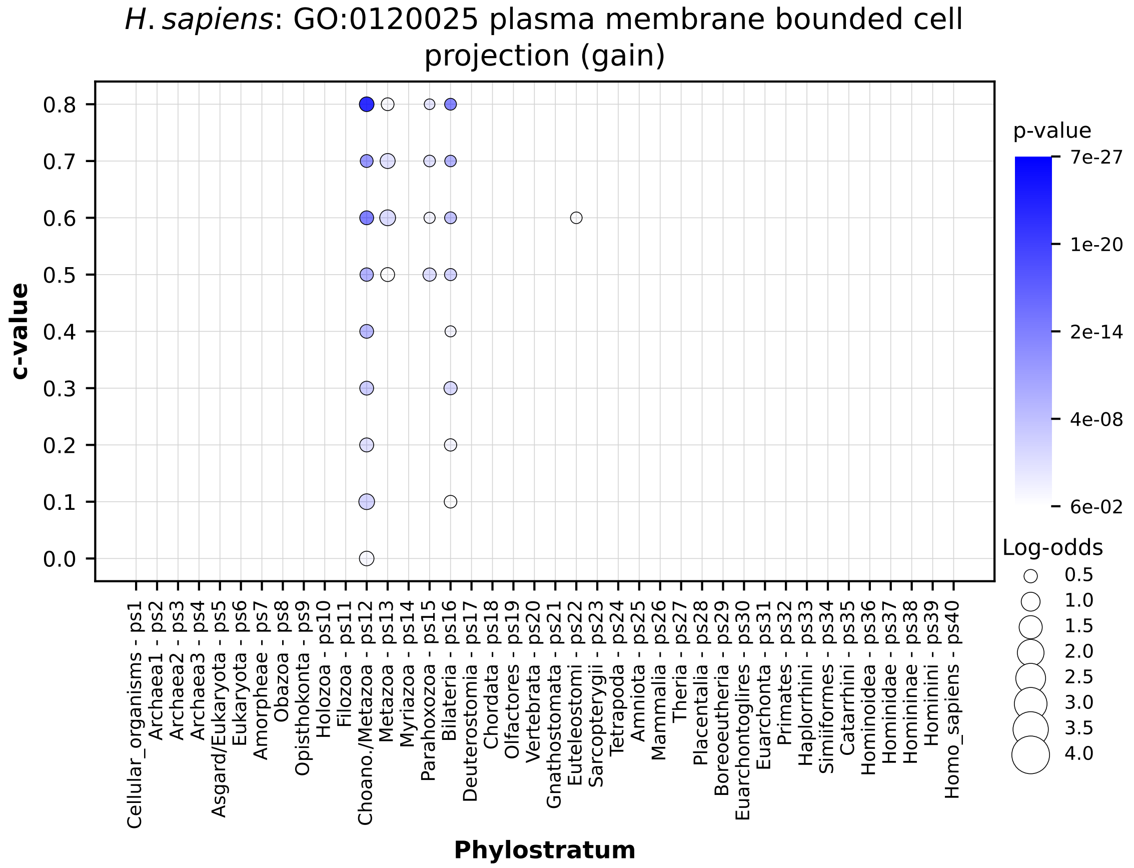

H. sapiens: GO:0120025 plasma membrane bounded cell projection (gain)
c-value
0.8
0.7
0.6
0.5
0.4
0.3
0.2
0.1
0.0
Phylostratum
Cellular_organisms - ps1
Archaea1 - ps2
Archaea2 - ps3
Archaea3 - ps4
Asgard/Eukaryota - ps5
Eukaryota - ps6
Amorpheae - ps7
Obazoa - ps8
Opisthokonta - ps9
Holozoa - ps10
Filozoa - ps11
Choano./Metazoa - ps12
Metazoa - ps13
Myriazoa - ps14
Parahoxozoa - ps15
Bilateria - ps16
Deuterostomia - ps17
Chordata - ps18
Olfactores - ps19
Vertebrata - ps20
Gnathostomata - ps21
Euteleostomi - ps22
Sarcopterygii - ps23
Tetrapoda - ps24
Amniota - ps25
Mammalia - ps26
Theria - ps27
Placentalia - ps28
Boreoeutheria - ps29
Euarchontoglires - ps30
Euarchonta - ps31
Primates - ps32
Haplorrhini - ps33
Simiiformes - ps34
Catarrhini - ps35
Hominoidea - ps36
Hominidae - ps37
Homininae - ps38
Hominini - ps39
Homo_sapiens - ps40
p-value
7e-27
1e-20
2e-14
4e-08
6e-02
Log-odds
0.5
1.0
1.5
2.0
2.5
3.0
3.5
4.0

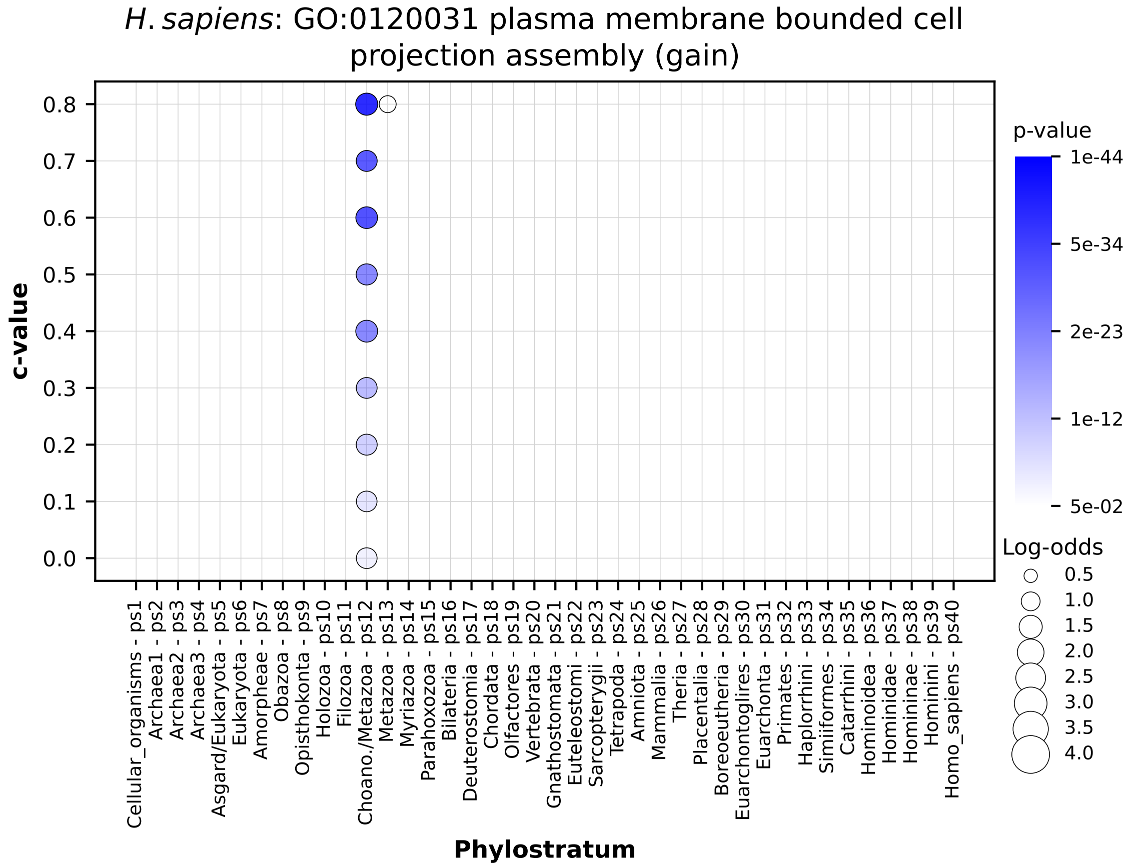
H. sapiens: GO:0120031 plasma membrane bounded cell projection assembly (gain)
c-value
0.8
0.7
0.6
0.5
0.4
0.3
0.2
0.1
0.0
Phylostratum
Cellular_organisms - ps1
Archaea1 - ps2
Archaea2 - ps3
Archaea3 - ps4
Asgard/Eukaryota - ps5
Eukaryota - ps6
Amorpheae - ps7
Obazoa - ps8
Opisthokonta - ps9
Holozoa - ps10
Filozoa - ps11
Choano./Metazoa - ps12
Metazoa - ps13
Myriazoa - ps14
Parahoxozoa - ps15
Bilateria - ps16
Deuterostomia - ps17
Chordata - ps18
Olfactores - ps19
Vertebrata - ps20
Gnathostomata - ps21
Euteleostomi - ps22
Sarcopterygii - ps23
Tetrapoda - ps24
Amniota - ps25
Mammalia - ps26
Theria - ps27
Placentalia - ps28
Boreoeutheria - ps29
Euarchontoglires - ps30
Euarchonta - ps31
Primates - ps32
Haplorrhini - ps33
Simiiformes - ps34
Catarrhini - ps35
Hominoidea - ps36
Hominidae - ps37
Homininae - ps38
Hominini - ps39
Homo_sapiens - ps40
p-value
1e-44
5e-34
2e-23
1e-12
5e-02
Log-odds
0.5
1.0
1.5
2.0
2.5
3.0
3.5
4.0

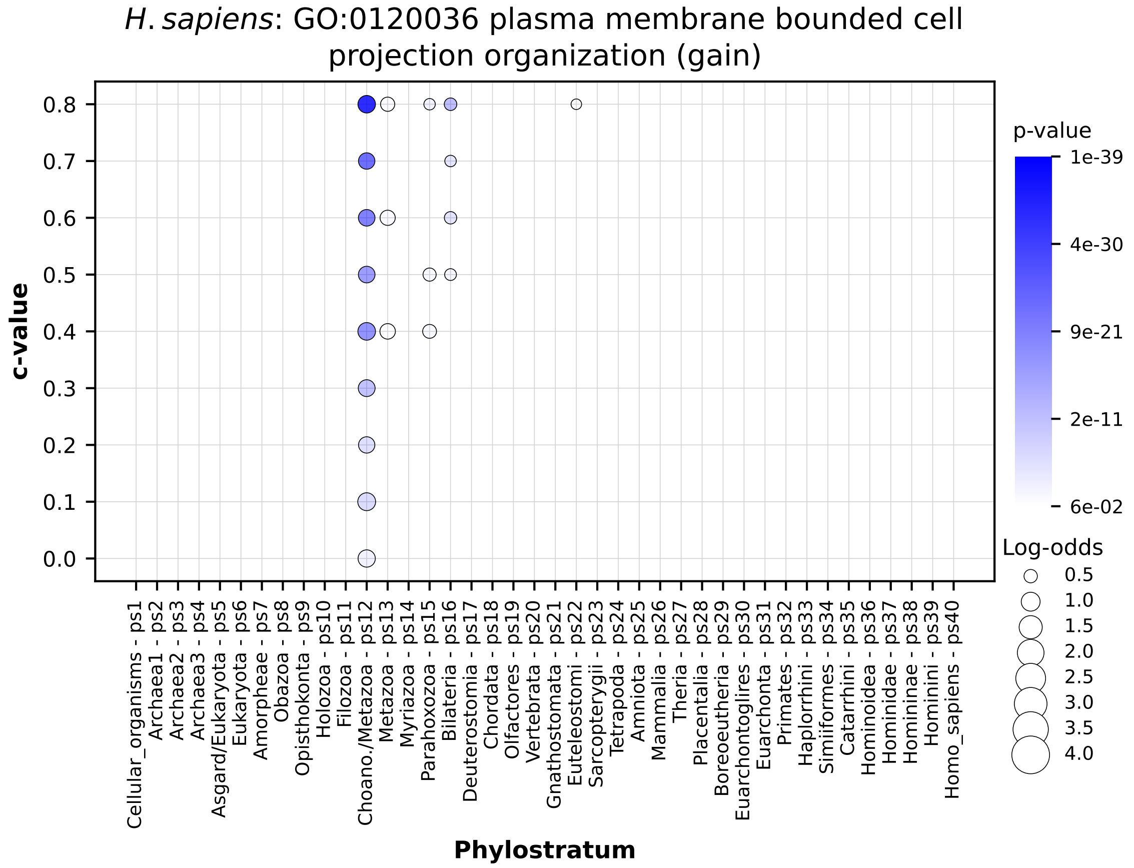

H. sapiens: GO:0120036 plasma membrane bounded cell projection organization (gain)
c-value
0.8
0.7
0.6
0.5
0.4
0.3
0.2
0.1
0.0
Phylostratum
Cellular_organisms - ps1
Archaea1 - ps2
Archaea2 - ps3
Archaea3 - ps4
Asgard/Eukaryota - ps5
Eukaryota - ps6
Amorpheae - ps7
Obazoa - ps8
Opisthokonta - ps9
Holozoa - ps10
Filozoa - ps11
Choano./Metazoa - ps12
Metazoa - ps13
Myriazoa - ps14
Parahoxozoa - ps15
Bilateria - ps16
Deuterostomia - ps17
Chordata - ps18
Olfactores - ps19
Vertebrata - ps20
Gnathostomata - ps21
Euteleostomi - ps22
Sarcopterygii - ps23
Tetrapoda - ps24
Amniota - ps25
Mammalia - ps26
Theria - ps27
Placentalia - ps28
Boreoeutheria - ps29
Euarchontoglires - ps30
Euarchonta - ps31
Primates - ps32
Haplorrhini - ps33
Simiiformes - ps34
Catarrhini - ps35
Hominoidea - ps36
Hominidae - ps37
Homininae - ps38
Hominini - ps39
Homo_sapiens - ps40
p-value
1e-39
4e-30
9e-21
2e-11
6e-02
Log-odds
0.5
1.0
1.5
2.0
2.5
3.0
3.5
4.0

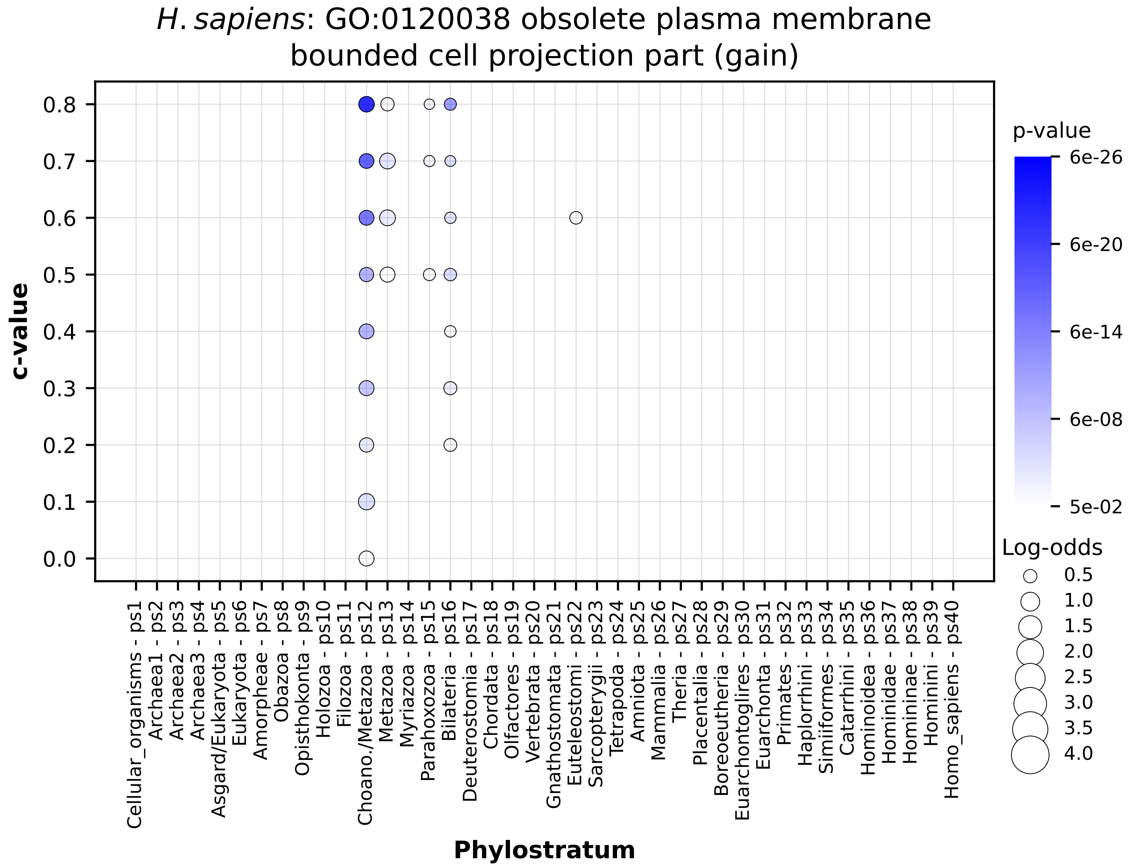

H. sapiens: GO:0120038 obsolete plasma membrane bounded cell projection part (gain)
c-value
0.8
0.7
0.6
0.5
0.4
0.3
0.2
0.1
0.0
p-value
6e-26
6e-20
6e-14
6e-08
5e-02
Log-odds
0.5
1.0
1.5
2.0
2.5
3.0
3.5
4.0
Cellular_organisms - ps1
Archaea1 - ps2
Archaea2 - ps3
Archaea3 - ps4
Asgard/Eukaryota - ps5
Eukaryota - ps6
Amorpheae - ps7
Obazoa - ps8
Opisthokonta - ps9
Holozoa - ps10
Filozoa - ps11
Choano./Metazoa - ps12
Metazoa - ps13
Myriazoa - ps14
Parahoxozoa - ps15
Bilateria - ps16
Deuterostomia - ps17
Chordata - ps18
Olfactores - ps19
Vertebrata - ps20
Gnathostomata - ps21
Euteleostomi - ps22
Sarcopterygii - ps23
Tetrapoda - ps24
Amniota - ps25
Mammalia - ps26
Theria - ps27
Placentalia - ps28
Boreoeutheria - ps29
Euarchontoglires - ps30
Euarchonta - ps31
Primates - ps32
Haplorrhini - ps33
Simiiformes - ps34
Catarrhini - ps35
Hominoidea - ps36
Hominidae - ps37
Homininae - ps38
Hominini - ps39
Homo_sapiens - ps40
Phylostratum

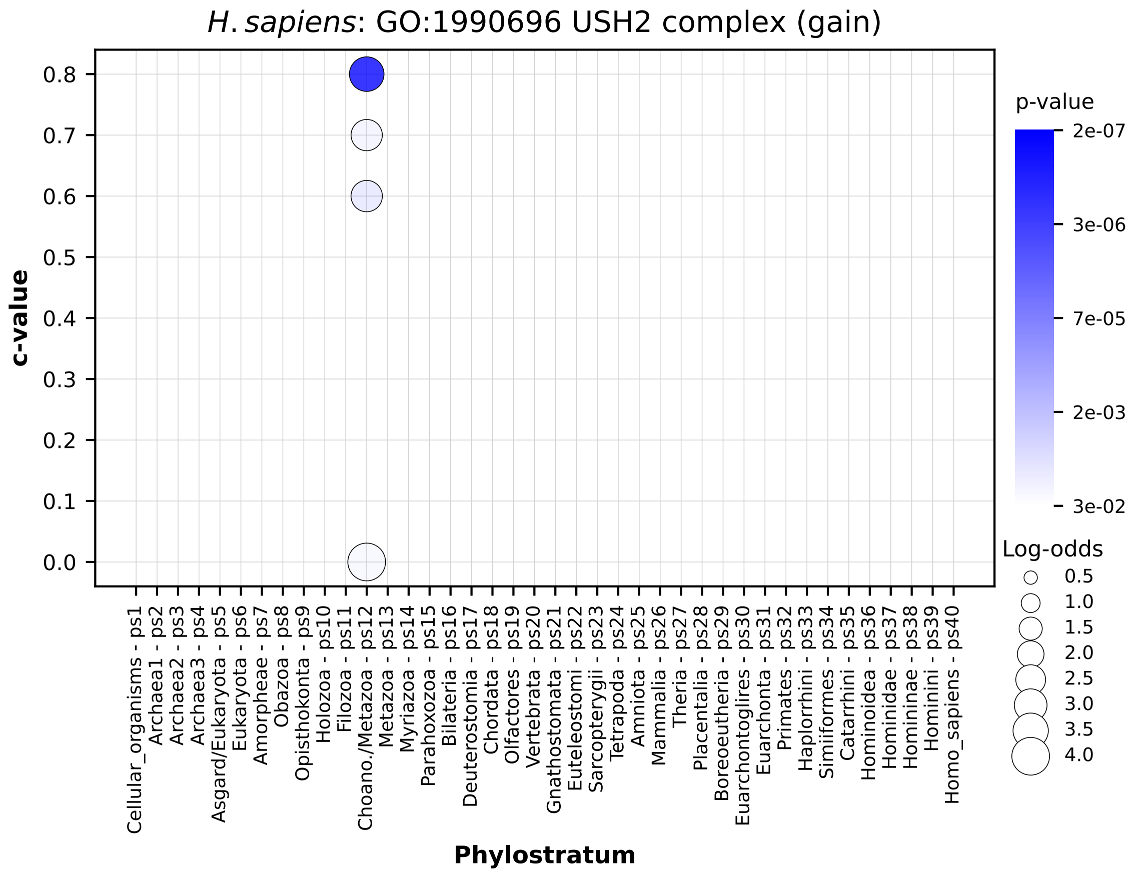

H. sapiens: GO:1990696 USH2 complex (gain)
c-value
0.8
0.7
0.6
0.5
0.4
0.3
0.2
0.1
0.0
p-value
2e-07
3e-06
7e-05
2e-03
3e-02
Log-odds
0.5
1.0
1.5
2.0
2.5
3.0
3.5
4.0
Cellular_organisms - ps1
Archaea1 - ps2
Archaea2 - ps3
Archaea3 - ps4
Asgard/Eukaryota - ps5
Eukaryota - ps6
Amorpheae - ps7
Obazoa - ps8
Opisthokonta - ps9
Holozoa - ps10
Filozoa - ps11
Choano./Metazoa - ps12
Metazoa - ps13
Myriazoa - ps14
Parahoxozoa - ps15
Bilateria - ps16
Deuterostomia - ps17
Chordata - ps18
Olfactores - ps19
Vertebrata - ps20
Gnathostomata - ps21
Euteleostomi - ps22
Sarcopterygii - ps23
Tetrapoda - ps24
Amniota - ps25
Mammalia - ps26
Theria - ps27
Placentalia - ps28
Boreoeutheria - ps29
Euarchontoglires - ps30
Euarchonta - ps31
Primates - ps32
Haplorrhini - ps33
Simiiformes - ps34
Catarrhini - ps35
Hominoidea - ps36
Hominidae - ps37
Homininae - ps38
Hominini - ps39
Homo_sapiens - ps40
Phylostratum

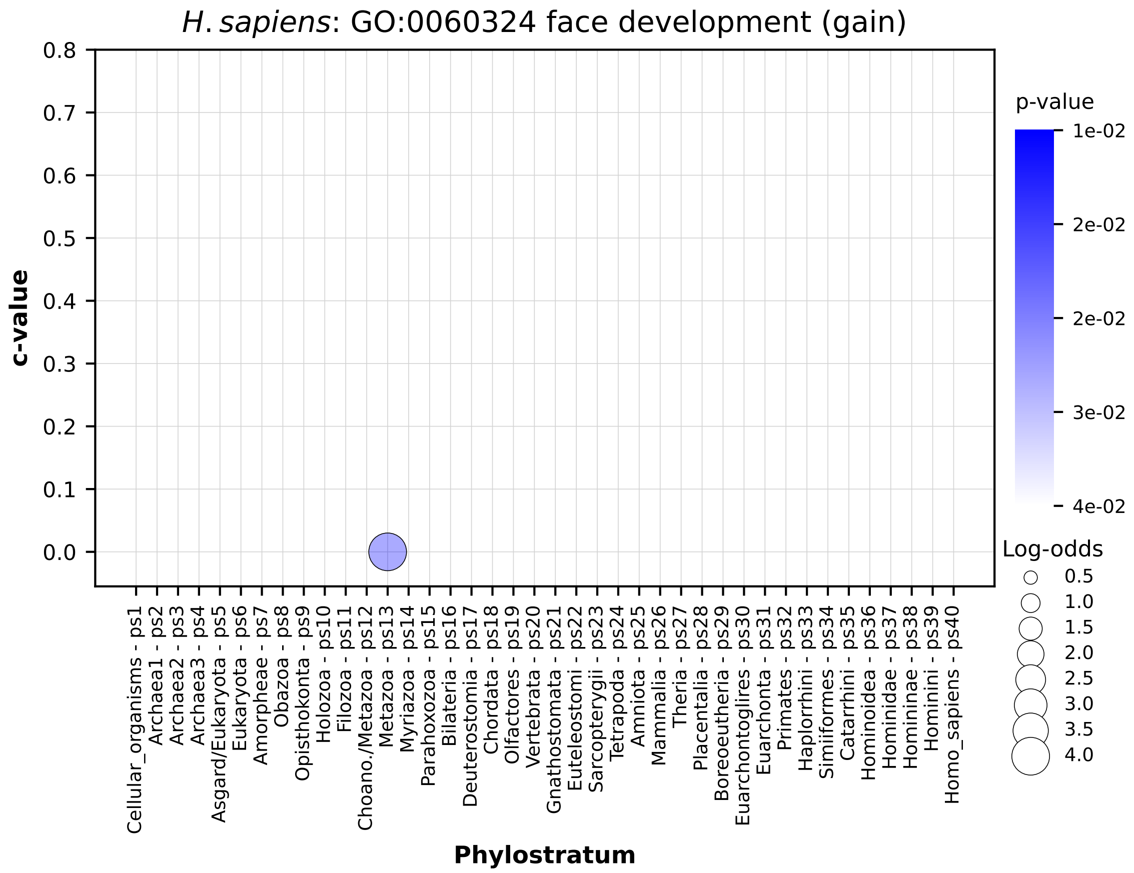

H. sapiens: GO:0060324 face development (gain)
c-value
0.8
0.7
0.6
0.5
0.4
0.3
0.2
0.1
0.0
p-value
1e-02
2e-02
2e-02
3e-02
4e-02
Log-odds
0.5
1.0
1.5
2.0
2.5
3.0
3.5
4.0
Cellular_organisms - ps1
Archaea1 - ps2
Archaea2 - ps3
Archaea3 - ps4
Asgard/Eukaryota - ps5
Eukaryota - ps6
Amorpheae - ps7
Obazoa - ps8
Opisthokonta - ps9
Holozoa - ps10
Filozoa - ps11
Choano./Metazoa - ps12
Metazoa - ps13
Myriazoa - ps14
Parahoxozoa - ps15
Bilateria - ps16
Deuterostomia - ps17
Chordata - ps18
Olfactores - ps19
Vertebrata - ps20
Gnathostomata - ps21
Euteleostomi - ps22
Sarcopterygii - ps23
Tetrapoda - ps24
Amniota - ps25
Mammalia - ps26
Theria - ps27
Placentalia - ps28
Boreoeutheria - ps29
Euarchontoglires - ps30
Euarchonta - ps31
Primates - ps32
Haplorrhini - ps33
Simiiformes - ps34
Catarrhini - ps35
Hominoidea - ps36
Hominidae - ps37
Homininae - ps38
Hominini - ps39
Homo_sapiens - ps40
Phylostratum

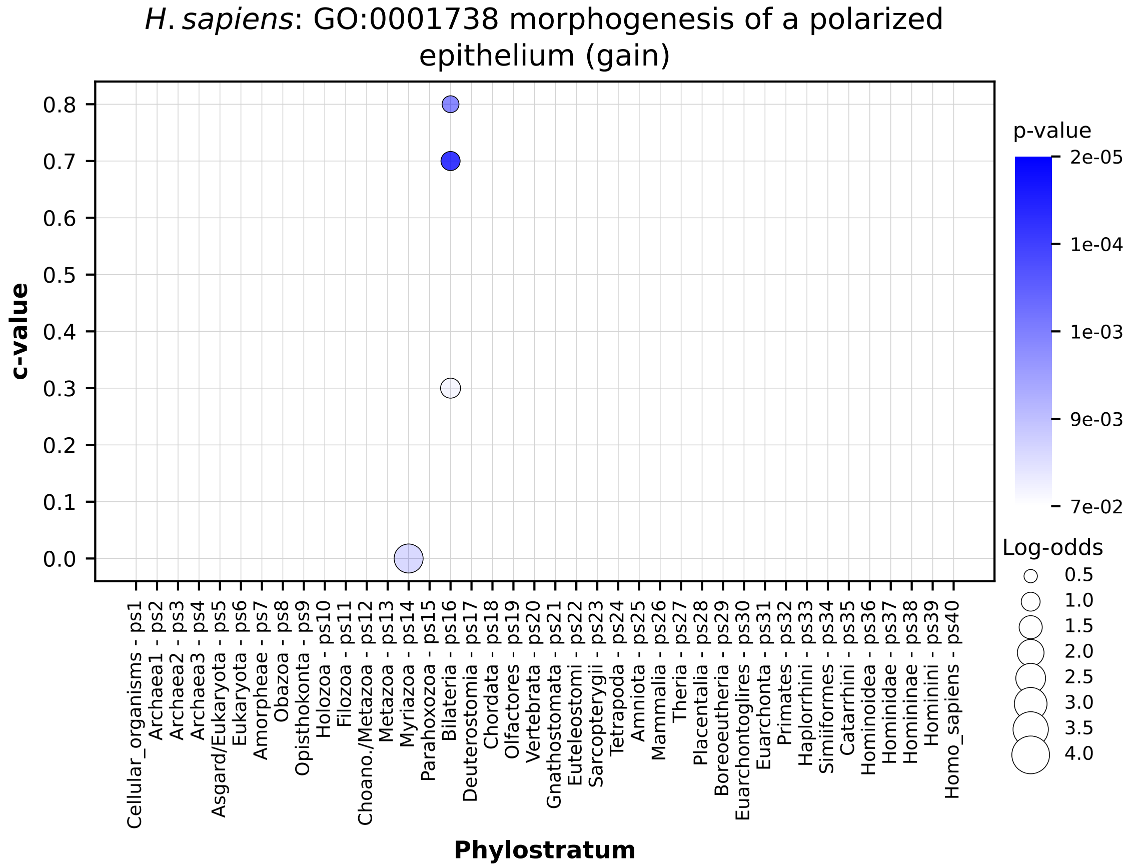

*H. sapiens*: GO:0001738 morphogenesis of a polarized epithelium (gain)
c-value
0.0
0.1
0.2
0.3
0.4
0.5
0.6
0.7
0.8
Phylostratum
Cellular_organisms - ps1
Archaea1 - ps2
Archaea2 - ps3
Archaea3 - ps4
Asgard/Eukaryota - ps5
Eukaryota - ps6
Amorpheae - ps7
Obazoa - ps8
Opisthokonta - ps9
Holozoa - ps10
Filozoa - ps11
Choano./Metazoa - ps12
Metazoa - ps13
Myriazoa - ps14
Parahoxozoa - ps15
Bilateria - ps16
Deuterostomia - ps17
Chordata - ps18
Olfactores - ps19
Vertebrata - ps20
Gnathostomata - ps21
Euteleostomi - ps22
Sarcopterygii - ps23
Tetrapoda - ps24
Amniota - ps25
Mammalia - ps26
Theria - ps27
Placentalia - ps28
Boreoeutheria - ps29
Euarchontoglires - ps30
Euarchonta - ps31
Primates - ps32
Haplorrhini - ps33
Simiiformes - ps34
Catarrhini - ps35
Hominoidea - ps36
Hominidae - ps37
Homininae - ps38
Hominini - ps39
Homo_sapiens - ps40
p-value
2e-05
1e-04
1e-03
9e-03
7e-02
Log-odds
0.5
1.0
1.5
2.0
2.5
3.0
3.5
4.0

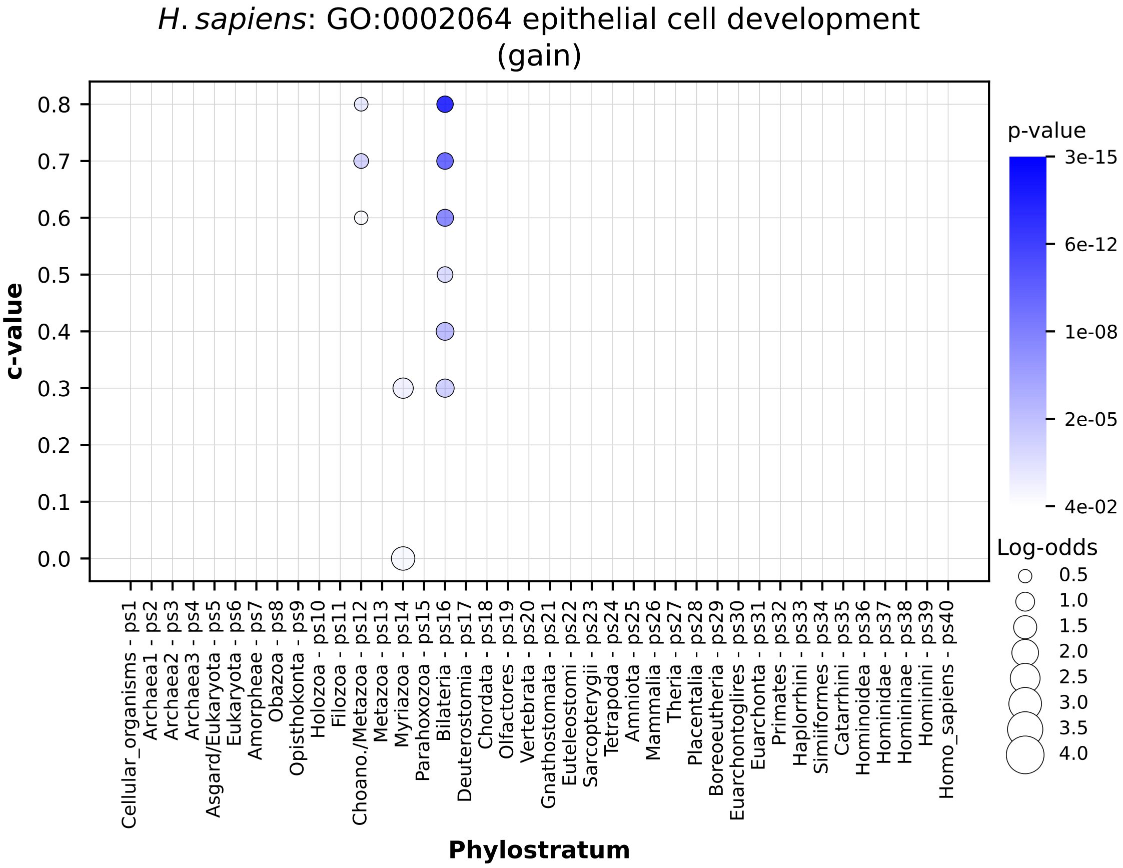

H. sapiens: GO:0002064 epithelial cell development
(gain)
c-value
0.8
0.7
0.6
0.5
0.4
0.3
0.2
0.1
0.0
Phylostratum
Cellular_organisms - ps1
Archaea1 - ps2
Archaea2 - ps3
Archaea3 - ps4
Asgard/Eukaryota - ps5
Eukaryota - ps6
Amorpheae - ps7
Obazoa - ps8
Opisthokonta - ps9
Holozoa - ps10
Filozoa - ps11
Choano./Metazoa - ps12
Metazoa - ps13
Myriazoa - ps14
Parahoxozoa - ps15
Bilateria - ps16
Deuterostomia - ps17
Chordata - ps18
Olfactores - ps19
Vertebrata - ps20
Gnathostomata - ps21
Euteleostomi - ps22
Sarcopterygii - ps23
Tetrapoda - ps24
Amniota - ps25
Mammalia - ps26
Theria - ps27
Placentalia - ps28
Boreoeutheria - ps29
Euarchontoglires - ps30
Euarchonta - ps31
Primates - ps32
Haplorrhini - ps33
Simiiformes - ps34
Catarrhini - ps35
Hominoidea - ps36
Hominidae - ps37
Homininae - ps38
Hominini - ps39
Homo_sapiens - ps40
p-value
3e-15
6e-12
1e-08
2e-05
4e-02
Log-odds
0.5
1.0
1.5
2.0
2.5
3.0
3.5
4.0

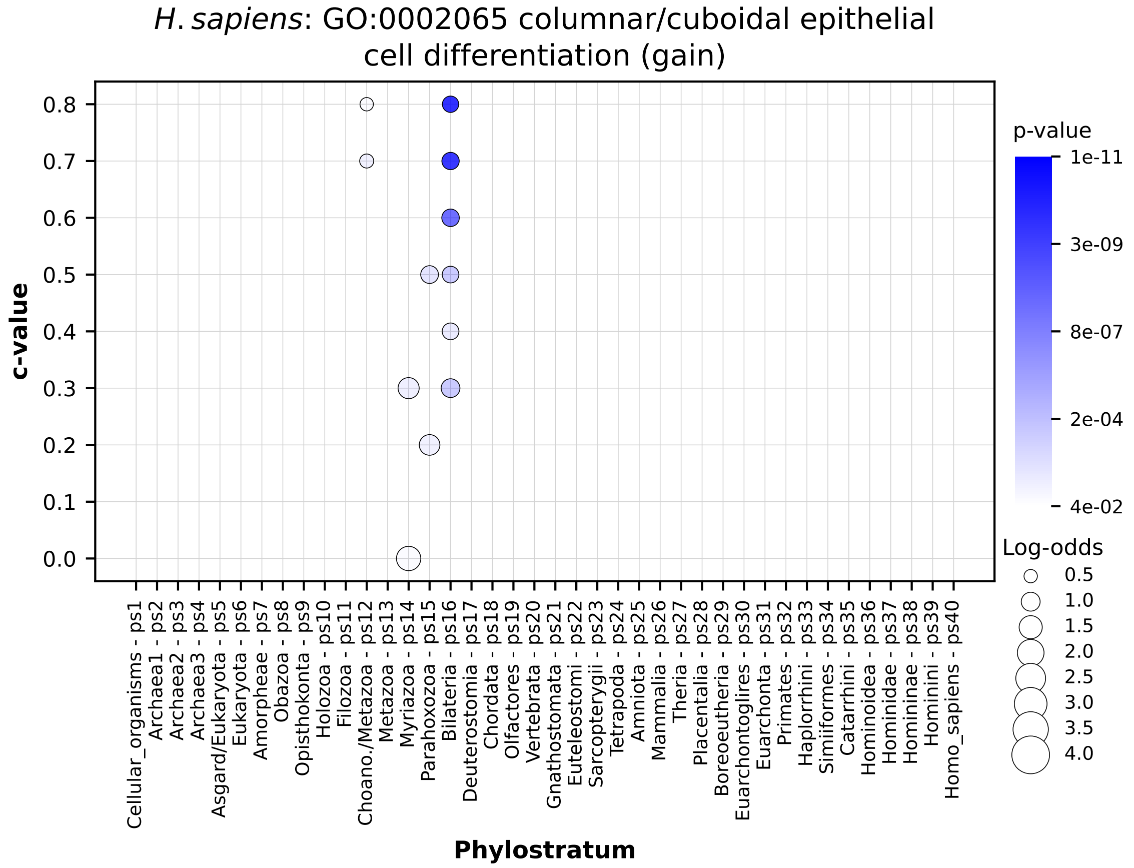
H. sapiens: GO:0002065 columnar/cuboidal epithelial cell differentiation (gain)
c-value
0.8
0.7
0.6
0.5
0.4
0.3
0.2
0.1
0.0
p-value
1e-11
3e-09
8e-07
2e-04
4e-02
Log-odds
0.5
1.0
1.5
2.0
2.5
3.0
3.5
4.0
Cellular_organisms - ps1
Archaea1 - ps2
Archaea2 - ps3
Archaea3 - ps4
Asgard/Eukaryota - ps5
Eukaryota - ps6
Amorpheae - ps7
Obazoa - ps8
Opisthokonta - ps9
Holozoa - ps10
Filozoa - ps11
Choano./Metazoa - ps12
Metazoa - ps13
Myriazoa - ps14
Parahoxozoa - ps15
Bilateria - ps16
Deuterostomia - ps17
Chordata - ps18
Olfactores - ps19
Vertebrata - ps20
Gnathostomata - ps21
Euteleostomi - ps22
Sarcopterygii - ps23
Tetrapoda - ps24
Amniota - ps25
Mammalia - ps26
Theria - ps27
Placentalia - ps28
Boreoeutheria - ps29
Euarchontoglires - ps30
Euarchonta - ps31
Primates - ps32
Haplorrhini - ps33
Simiiformes - ps34
Catarrhini - ps35
Hominoidea - ps36
Hominidae - ps37
Homininae - ps38
Hominini - ps39
Homo_sapiens - ps40
Phylostratum

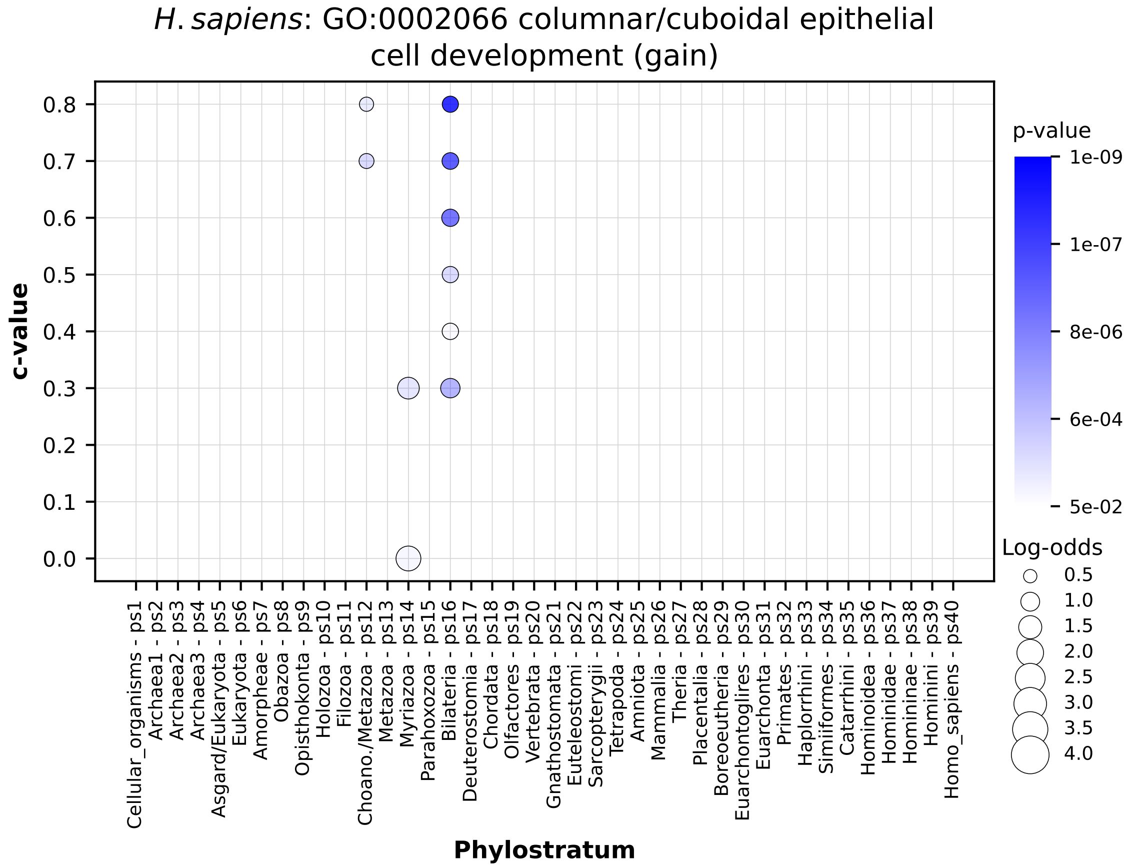

*H. sapiens*: GO:0002066 columnar/cuboidal epithelial cell development (gain)
c-value
0.8
0.7
0.6
0.5
0.4
0.3
0.2
0.1
0.0
p-value
1e-09
1e-07
8e-06
6e-04
5e-02
Log-odds
0.5
1.0
1.5
2.0
2.5
3.0
3.5
4.0
Cellular_organisms - ps1
Archaea1 - ps2
Archaea2 - ps3
Archaea3 - ps4
Asgard/Eukaryota - ps5
Eukaryota - ps6
Amorpheae - ps7
Obazoa - ps8
Opisthokonta - ps9
Holozoa - ps10
Filozoa - ps11
Choano./Metazoa - ps12
Metazoa - ps13
Myriazoa - ps14
Parahoxozoa - ps15
Bilateria - ps16
Deuterostomia - ps17
Chordata - ps18
Olfactores - ps19
Vertebrata - ps20
Gnathostomata - ps21
Euteleostomi - ps22
Sarcopterygii - ps23
Tetrapoda - ps24
Amniota - ps25
Mammalia - ps26
Theria - ps27
Placentalia - ps28
Boreoeutheria - ps29
Euarchontoglires - ps30
Euarchonta - ps31
Primates - ps32
Haplorrhini - ps33
Simiiformes - ps34
Catarrhini - ps35
Hominoidea - ps36
Hominidae - ps37
Homininae - ps38
Hominini - ps39
Homo_sapiens - ps40
Phylostratum

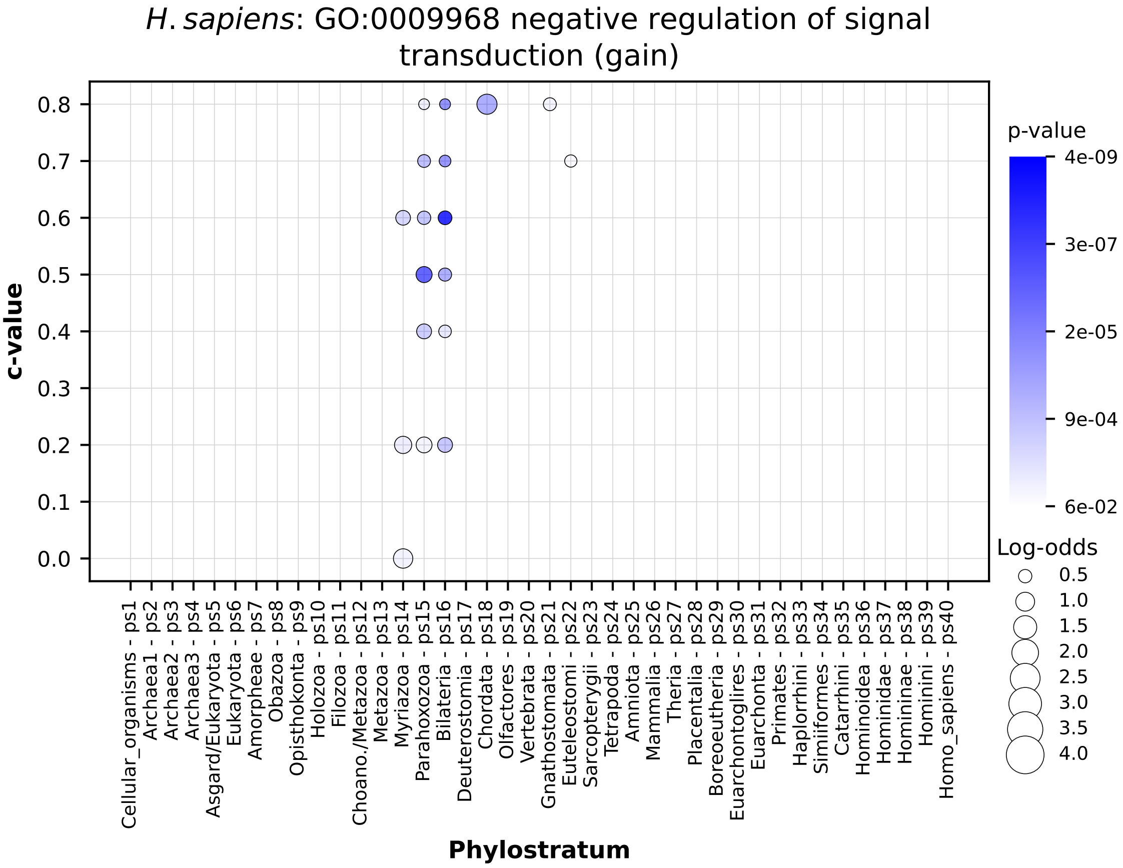

H. sapiens: GO:0009968 negative regulation of signal transduction (gain)
c-value
0.8
0.7
0.6
0.5
0.4
0.3
0.2
0.1
0.0
Phylostratum
Cellular_organisms - ps1
Archaea1 - ps2
Archaea2 - ps3
Archaea3 - ps4
Asgard/Eukaryota - ps5
Eukaryota - ps6
Amorpheae - ps7
Obazoa - ps8
Opisthokonta - ps9
Holozoa - ps10
Filozoa - ps11
Choano./Metazoa - ps12
Metazoa - ps13
Myriazoa - ps14
Parahoxozoa - ps15
Bilateria - ps16
Deuterostomia - ps17
Chordata - ps18
Olfactores - ps19
Vertebrata - ps20
Gnathostomata - ps21
Euteleostomi - ps22
Sarcopterygii - ps23
Tetrapoda - ps24
Amniota - ps25
Mammalia - ps26
Theria - ps27
Placentalia - ps28
Boreoeutheria - ps29
Euarchontoglires - ps30
Euarchonta - ps31
Primates - ps32
Haplorrhini - ps33
Simiiformes - ps34
Catarrhini - ps35
Hominoidea - ps36
Hominidae - ps37
Homininae - ps38
Hominini - ps39
Homo_sapiens - ps40
p-value
4e-09
3e-07
2e-05
9e-04
6e-02
Log-odds
0.5
1.0
1.5
2.0
2.5
3.0
3.5
4.0

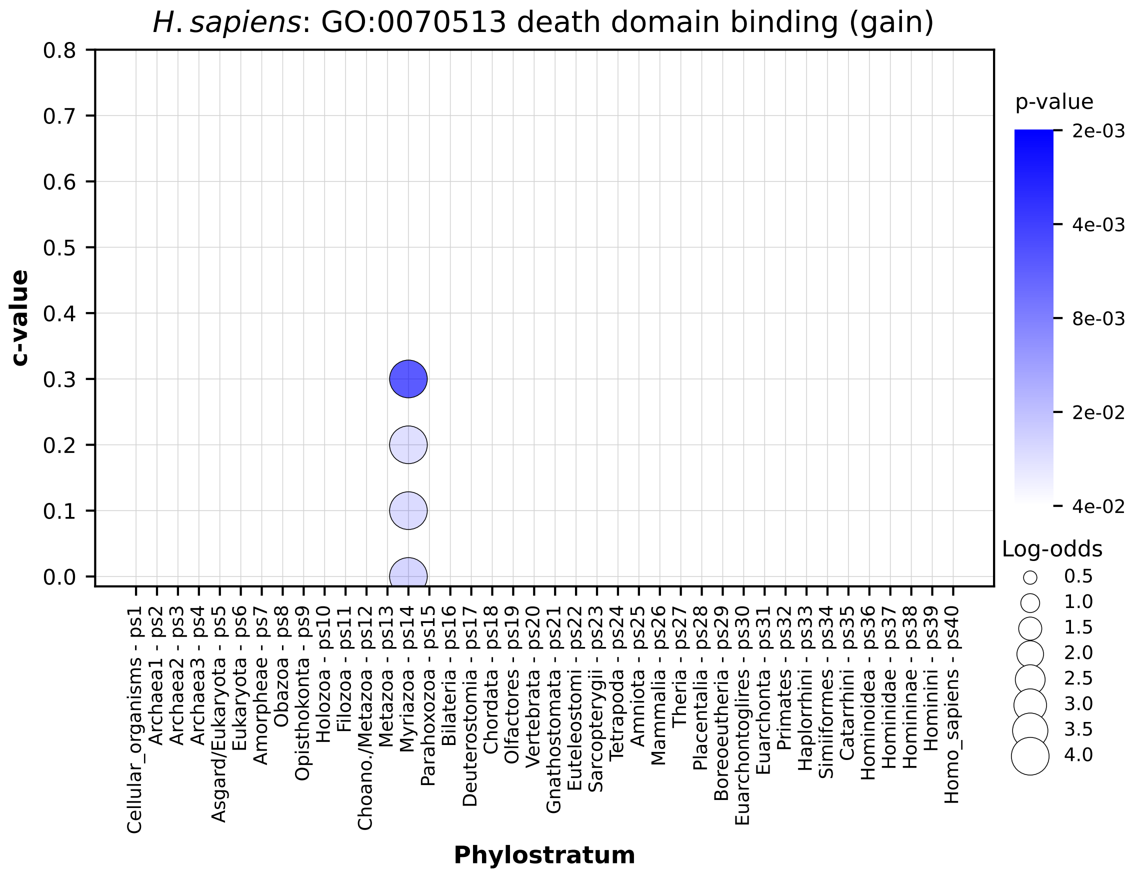

H. sapiens: GO:0070513 death domain binding (gain)
c-value
0.8
0.7
0.6
0.5
0.4
0.3
0.2
0.1
0.0
p-value
2e-03
4e-03
8e-03
2e-02
4e-02
Log-odds
0.5
1.0
1.5
2.0
2.5
3.0
3.5
4.0
Cellular_organisms - ps1
Archaea1 - ps2
Archaea2 - ps3
Archaea3 - ps4
Asgard/Eukaryota - ps5
Eukaryota - ps6
Amorpheae - ps7
Obazoa - ps8
Opisthokonta - ps9
Holozoa - ps10
Filozoa - ps11
Choano./Metazoa - ps12
Metazoa - ps13
Myriazoa - ps14
Parahoxozoa - ps15
Bilateria - ps16
Deuterostomia - ps17
Chordata - ps18
Olfactores - ps19
Vertebrata - ps20
Gnathostomata - ps21
Euteleostomi - ps22
Sarcopterygii - ps23
Tetrapoda - ps24
Amniota - ps25
Mammalia - ps26
Theria - ps27
Placentalia - ps28
Boreoeutheria - ps29
Euarchontoglires - ps30
Euarchonta - ps31
Primates - ps32
Haplorrhini - ps33
Simiiformes - ps34
Catarrhini - ps35
Hominoidea - ps36
Hominidae - ps37
Homininae - ps38
Hominini - ps39
Homo_sapiens - ps40
Phylostratum

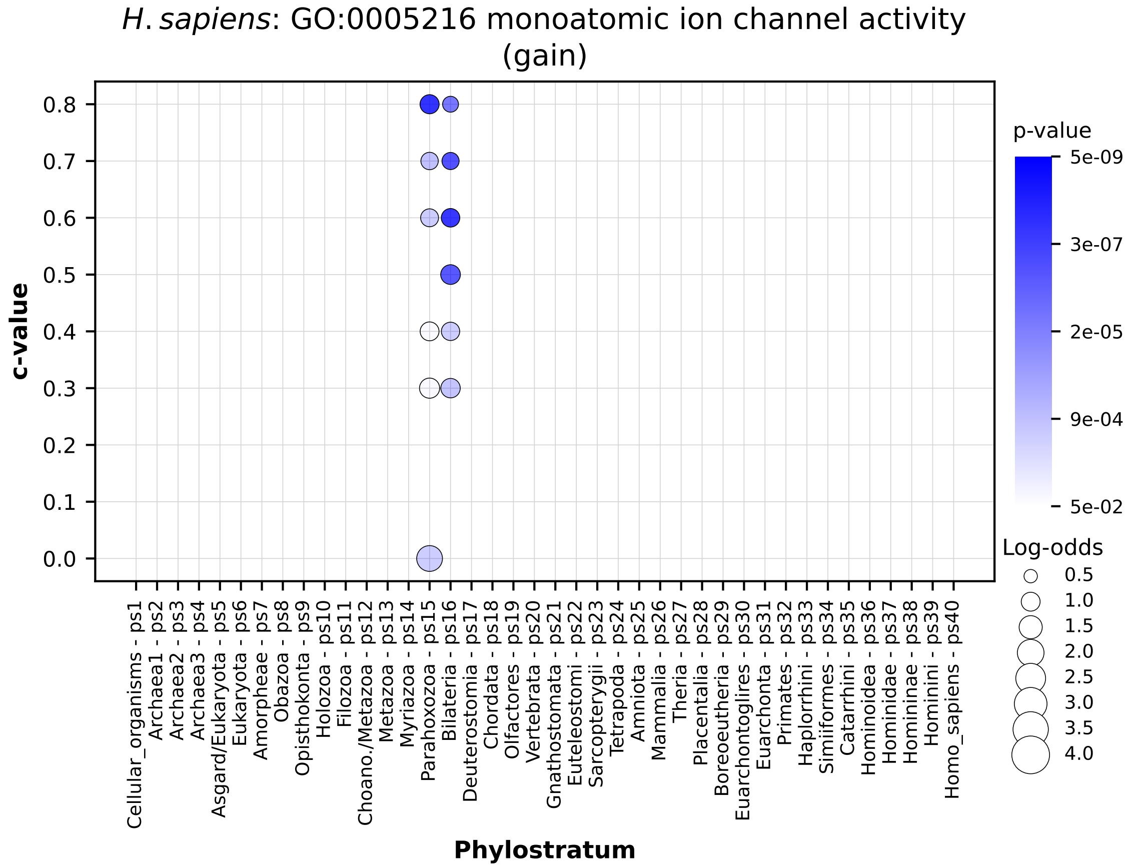

H. sapiens: GO:0005216 monoatomic ion channel activity
(gain)
c-value
0.8
0.7
0.6
0.5
0.4
0.3
0.2
0.1
0.0
p-value
5e-09
3e-07
2e-05
9e-04
5e-02
Log-odds
0.5
1.0
1.5
2.0
2.5
3.0
3.5
4.0
Cellular_organisms - ps1
Archaea1 - ps2
Archaea2 - ps3
Archaea3 - ps4
Asgard/Eukaryota - ps5
Eukaryota - ps6
Amorpheae - ps7
Obazoa - ps8
Opisthokonta - ps9
Holozoa - ps10
Filozoa - ps11
Choano./Metazoa - ps12
Metazoa - ps13
Myriazoa - ps14
Parahoxozoa - ps15
Bilateria - ps16
Deuterostomia - ps17
Chordata - ps18
Olfactores - ps19
Vertebrata - ps20
Gnathostomata - ps21
Euteleostomi - ps22
Sarcopterygii - ps23
Tetrapoda - ps24
Amniota - ps25
Mammalia - ps26
Theria - ps27
Placentalia - ps28
Boreoeutheria - ps29
Euarchontoglires - ps30
Euarchonta - ps31
Primates - ps32
Haplorrhini - ps33
Simiiformes - ps34
Catarrhini - ps35
Hominoidea - ps36
Hominidae - ps37
Homininae - ps38
Hominini - ps39
Homo_sapiens - ps40
Phylostratum

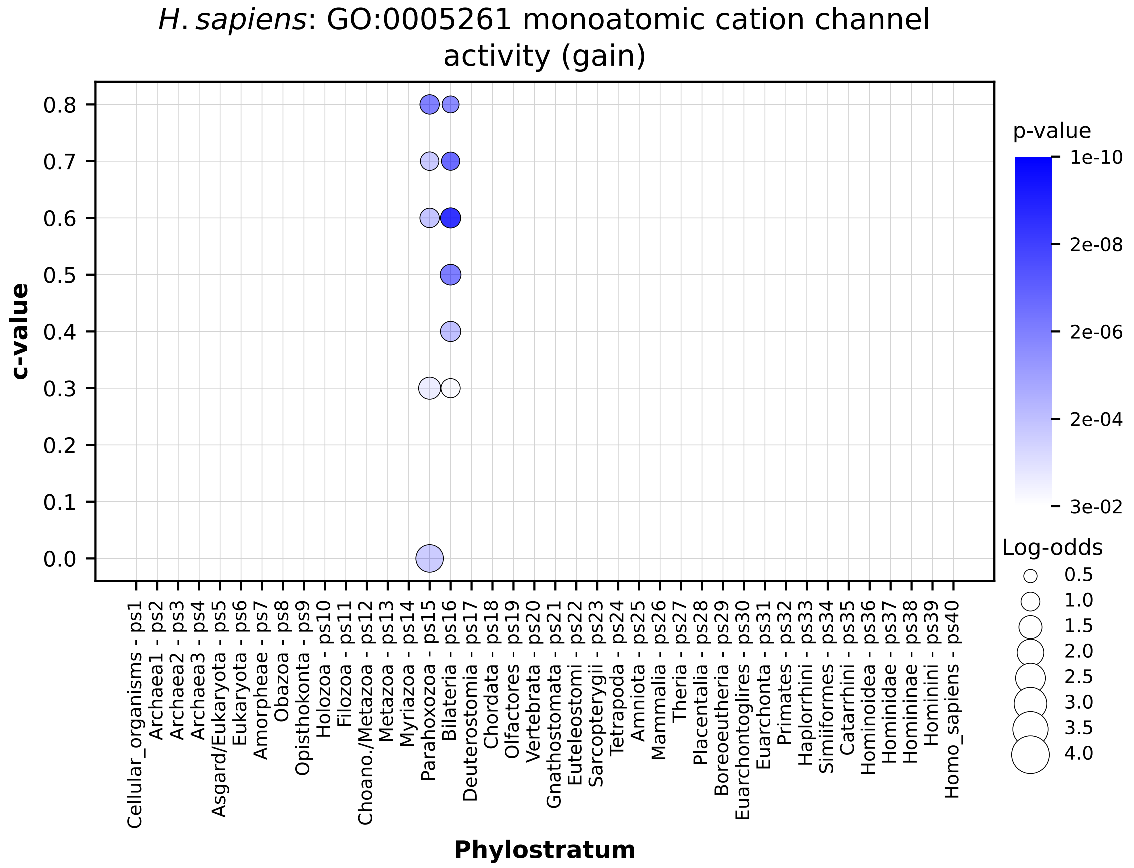

H. sapiens: GO:0005261 monoatomic cation channel activity (gain)
c-value
0.8
0.7
0.6
0.5
0.4
0.3
0.2
0.1
0.0
Phylostratum
Cellular_organisms - ps1
Archaea1 - ps2
Archaea2 - ps3
Archaea3 - ps4
Asgard/Eukaryota - ps5
Eukaryota - ps6
Amorpheae - ps7
Obazoa - ps8
Opisthokonta - ps9
Holozoa - ps10
Filozoa - ps11
Choano./Metazoa - ps12
Metazoa - ps13
Myriazoa - ps14
Parahoxozoa - ps15
Bilateria - ps16
Deuterostomia - ps17
Chordata - ps18
Olfactores - ps19
Vertebrata - ps20
Gnathostomata - ps21
Euteleostomi - ps22
Sarcopterygii - ps23
Tetrapoda - ps24
Amniota - ps25
Mammalia - ps26
Theria - ps27
Placentalia - ps28
Boreoeutheria - ps29
Euarchontoglires - ps30
Euarchonta - ps31
Primates - ps32
Haplorrhini - ps33
Simiiformes - ps34
Catarrhini - ps35
Hominoidea - ps36
Hominidae - ps37
Homininae - ps38
Hominini - ps39
Homo_sapiens - ps40
p-value
1e-10
2e-08
2e-06
2e-04
3e-02
Log-odds
0.5
1.0
1.5
2.0
2.5
3.0
3.5
4.0

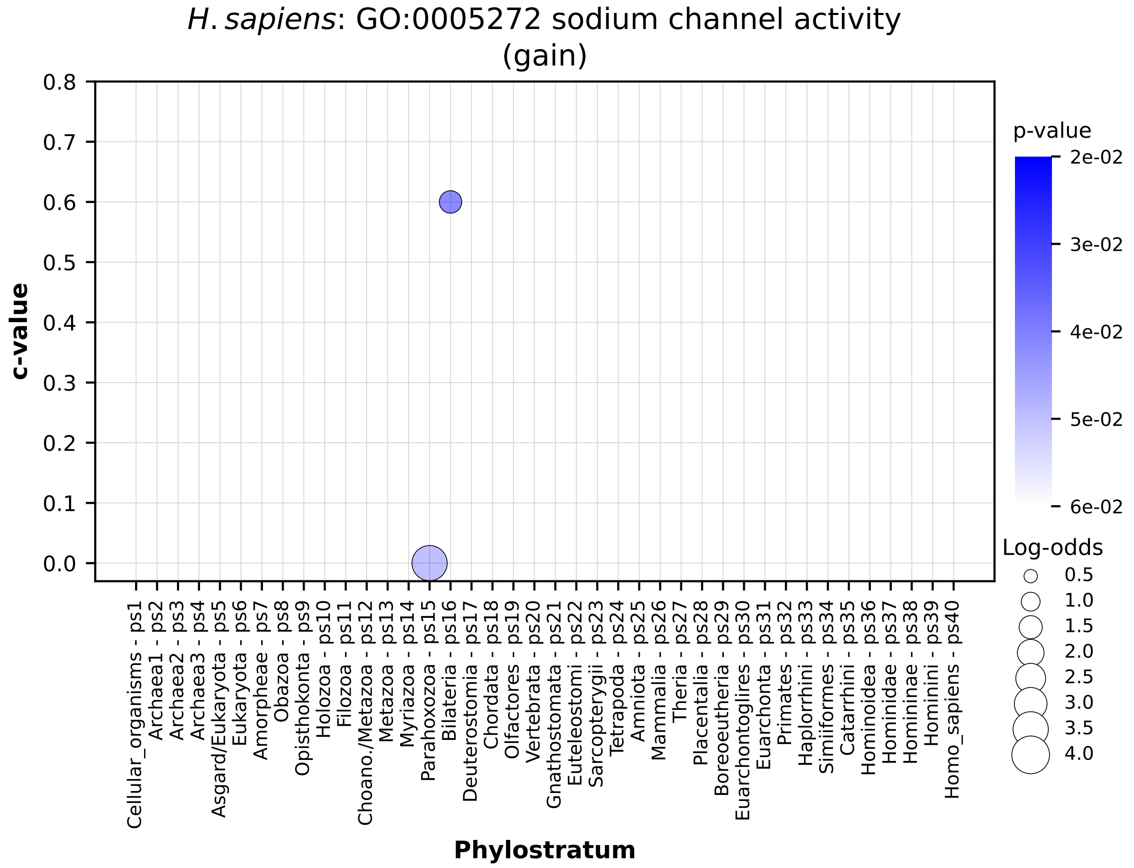

H. sapiens: GO:0005272 sodium channel activity
(gain)
c-value
0.8
0.7
0.6
0.5
0.4
0.3
0.2
0.1
0.0
p-value
2e-02
3e-02
4e-02
5e-02
6e-02
Log-odds
0.5
1.0
1.5
2.0
2.5
3.0
3.5
4.0
Cellular_organisms - ps1
Archaea1 - ps2
Archaea2 - ps3
Archaea3 - ps4
Asgard/Eukaryota - ps5
Eukaryota - ps6
Amorpheae - ps7
Obazoa - ps8
Opisthokonta - ps9
Holozoa - ps10
Filozoa - ps11
Choano./Metazoa - ps12
Metazoa - ps13
Myriazoa - ps14
Parahoxozoa - ps15
Bilateria - ps16
Deuterostomia - ps17
Chordata - ps18
Olfactores - ps19
Vertebrata - ps20
Gnathostomata - ps21
Euteleostomi - ps22
Sarcopterygii - ps23
Tetrapoda - ps24
Amniota - ps25
Mammalia - ps26
Theria - ps27
Placentalia - ps28
Boreoeutheria - ps29
Euarchontoglires - ps30
Euarchonta - ps31
Primates - ps32
Haplorrhini - ps33
Simiiformes - ps34
Catarrhini - ps35
Hominoidea - ps36
Hominidae - ps37
Homininae - ps38
Hominini - ps39
Homo_sapiens - ps40
Phylostratum

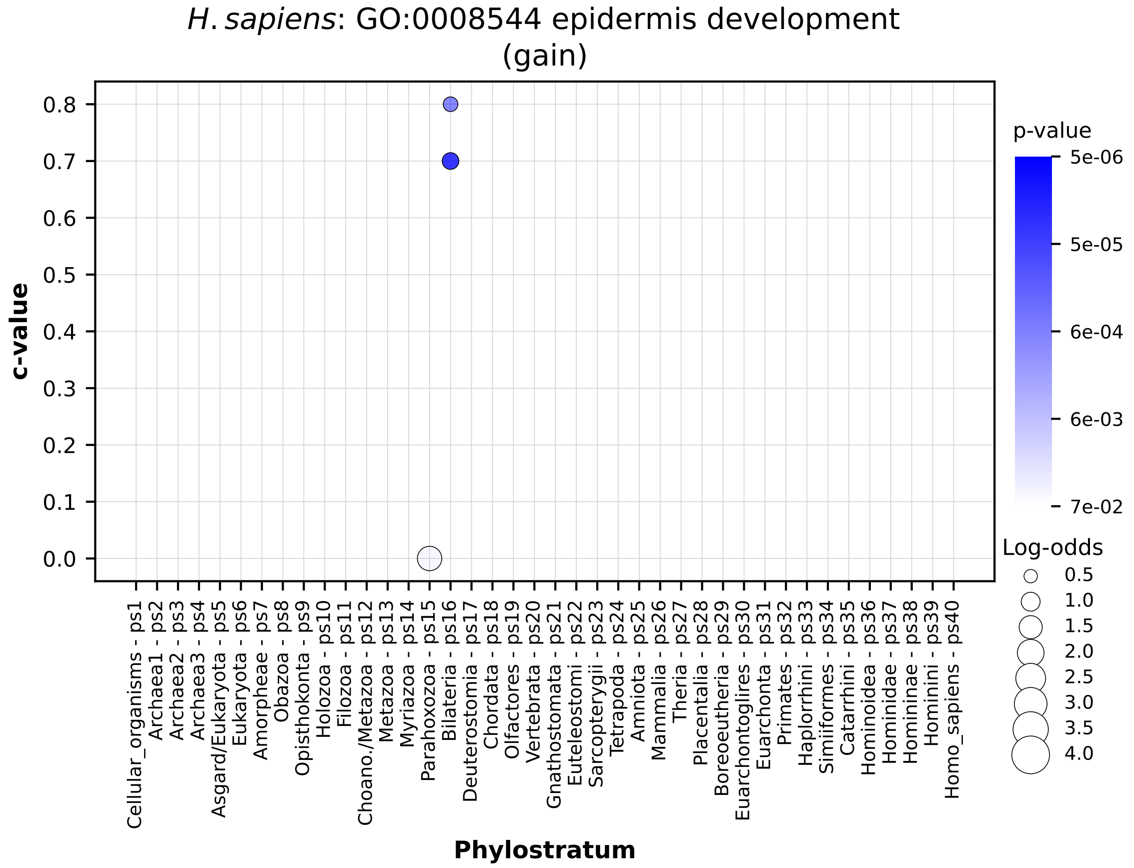

H. sapiens: GO:0008544 epidermis development
(gain)
c-value
0.8
0.7
0.6
0.5
0.4
0.3
0.2
0.1
0.0
p-value
5e-06
5e-05
6e-04
6e-03
7e-02
Log-odds
0.5
1.0
1.5
2.0
2.5
3.0
3.5
4.0
Cellular_organisms - ps1
Archaea1 - ps2
Archaea2 - ps3
Archaea3 - ps4
Asgard/Eukaryota - ps5
Eukaryota - ps6
Amorpheae - ps7
Obazoa - ps8
Opisthokonta - ps9
Holozoa - ps10
Filozoa - ps11
Choano./Metazoa - ps12
Metazoa - ps13
Myriazoa - ps14
Parahoxozoa - ps15
Bilateria - ps16
Deuterostomia - ps17
Chordata - ps18
Olfactores - ps19
Vertebrata - ps20
Gnathostomata - ps21
Euteleostomi - ps22
Sarcopterygii - ps23
Tetrapoda - ps24
Amniota - ps25
Mammalia - ps26
Theria - ps27
Placentalia - ps28
Boreoeutheria - ps29
Euarchontoglires - ps30
Euarchonta - ps31
Primates - ps32
Haplorrhini - ps33
Simiiformes - ps34
Catarrhini - ps35
Hominoidea - ps36
Hominidae - ps37
Homininae - ps38
Hominini - ps39
Homo_sapiens - ps40
Phylostratum

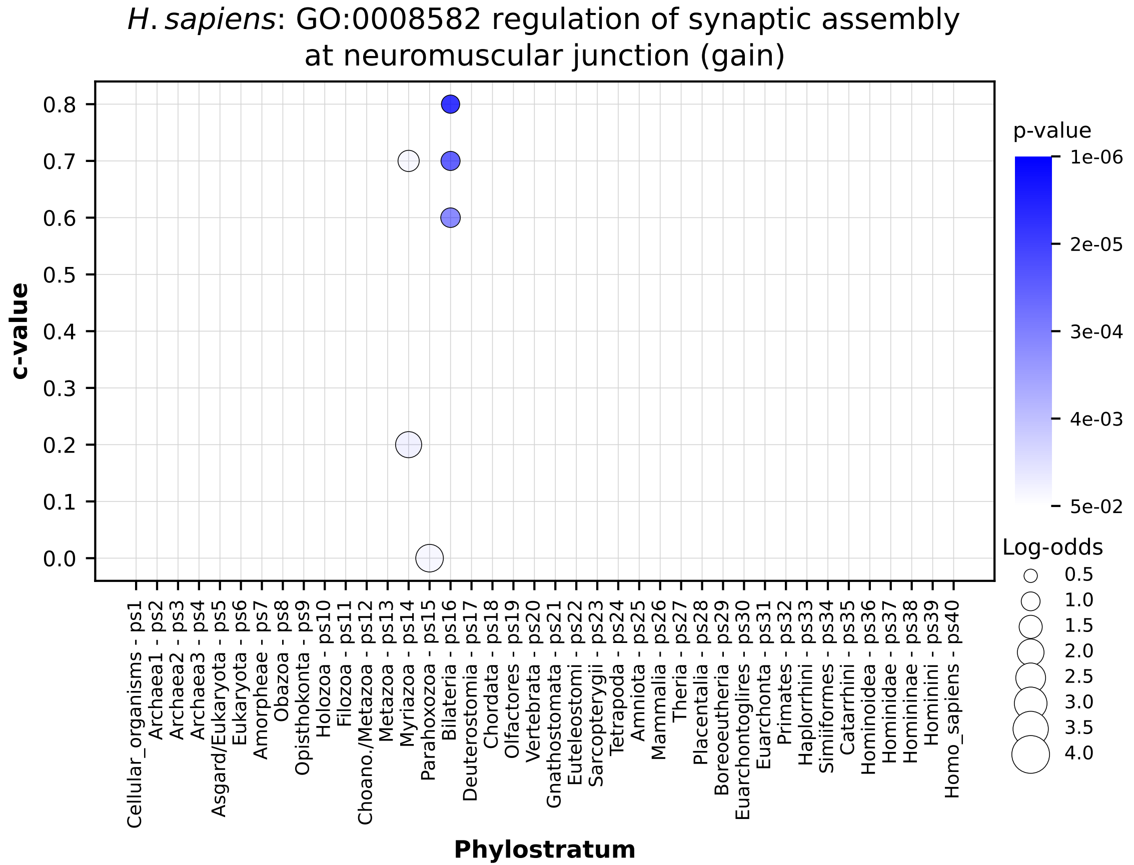

H. sapiens: GO:0008582 regulation of synaptic assembly at neuromuscular junction (gain)
c-value
0.8
0.7
0.6
0.5
0.4
0.3
0.2
0.1
0.0
p-value
1e-06
2e-05
3e-04
4e-03
5e-02
Log-odds
0.5
1.0
1.5
2.0
2.5
3.0
3.5
4.0
Cellular_organisms - ps1
Archaea1 - ps2
Archaea2 - ps3
Archaea3 - ps4
Asgard/Eukaryota - ps5
Eukaryota - ps6
Amorpheae - ps7
Obazoa - ps8
Opisthokonta - ps9
Holozoa - ps10
Filozoa - ps11
Choano./Metazoa - ps12
Metazoa - ps13
Myriazoa - ps14
Parahoxozoa - ps15
Bilateria - ps16
Deuterostomia - ps17
Chordata - ps18
Olfactores - ps19
Vertebrata - ps20
Gnathostomata - ps21
Euteleostomi - ps22
Sarcopterygii - ps23
Tetrapoda - ps24
Amniota - ps25
Mammalia - ps26
Theria - ps27
Placentalia - ps28
Boreoeutheria - ps29
Euarchontoglires - ps30
Euarchonta - ps31
Primates - ps32
Haplorrhini - ps33
Simiiformes - ps34
Catarrhini - ps35
Hominoidea - ps36
Hominidae - ps37
Homininae - ps38
Hominini - ps39
Homo_sapiens - ps40
Phylostratum

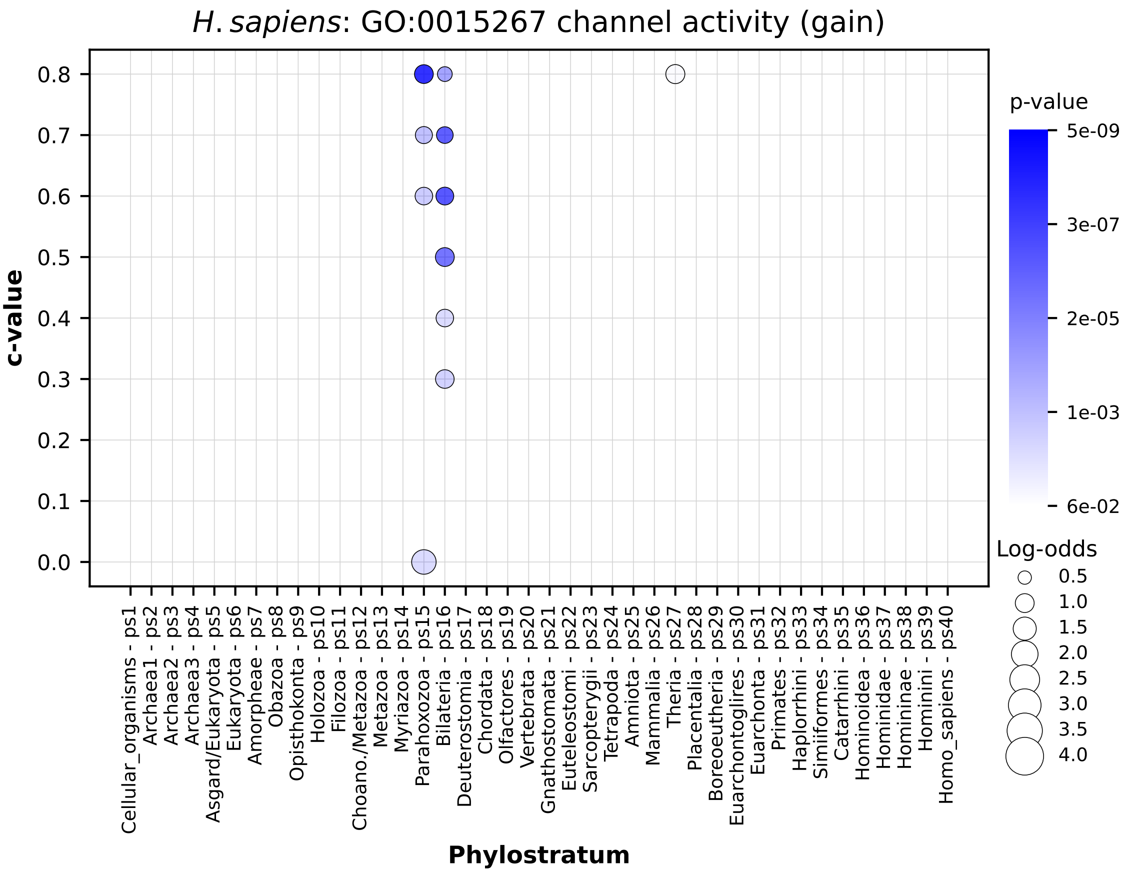

H. sapiens: GO:0015267 channel activity (gain)
c-value
0.0
0.1
0.2
0.3
0.4
0.5
0.6
0.7
0.8
Phylostratum
Cellular_organisms - ps1
Archaea1 - ps2
Archaea2 - ps3
Archaea3 - ps4
Asgard/Eukaryota - ps5
Eukaryota - ps6
Amorpheae - ps7
Obazoa - ps8
Opisthokonta - ps9
Holozoa - ps10
Filozoa - ps11
Choano./Metazoa - ps12
Metazoa - ps13
Myriazoa - ps14
Parahoxozoa - ps15
Bilateria - ps16
Deuterostomia - ps17
Chordata - ps18
Olfactores - ps19
Vertebrata - ps20
Gnathostomata - ps21
Euteleostomi - ps22
Sarcopterygii - ps23
Tetrapoda - ps24
Amniota - ps25
Mammalia - ps26
Theria - ps27
Placentalia - ps28
Boreoeutheria - ps29
Euarchontoglires - ps30
Euarchonta - ps31
Primates - ps32
Haplorrhini - ps33
Simiiformes - ps34
Catarrhini - ps35
Hominoidea - ps36
Hominidae - ps37
Homininae - ps38
Hominini - ps39
Homo_sapiens - ps40
p-value
5e-09
3e-07
2e-05
1e-03
6e-02
Log-odds
0.5
1.0
1.5
2.0
2.5
3.0
3.5
4.0

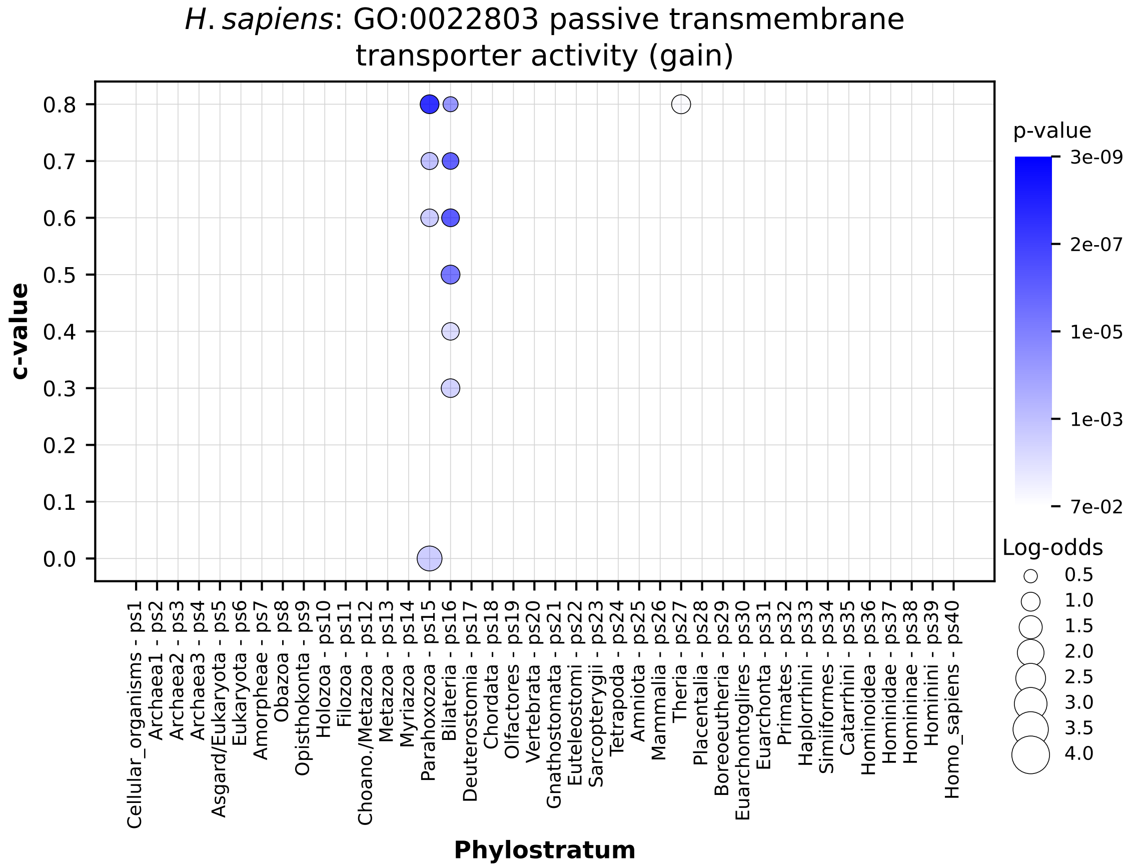

H. sapiens: GO:0022803 passive transmembrane transporter activity (gain)
c-value
0.8
0.7
0.6
0.5
0.4
0.3
0.2
0.1
0.0
p-value
3e-09
2e-07
1e-05
1e-03
7e-02
Log-odds
0.5
1.0
1.5
2.0
2.5
3.0
3.5
4.0
Cellular_organisms - ps1
Archaea1 - ps2
Archaea2 - ps3
Archaea3 - ps4
Asgard/Eukaryota - ps5
Eukaryota - ps6
Amorpheae - ps7
Obazoa - ps8
Opisthokonta - ps9
Holozoa - ps10
Filozoa - ps11
Choano./Metazoa - ps12
Metazoa - ps13
Myriazoa - ps14
Parahoxozoa - ps15
Bilateria - ps16
Deuterostomia - ps17
Chordata - ps18
Olfactores - ps19
Vertebrata - ps20
Gnathostomata - ps21
Euteleostomi - ps22
Sarcopterygii - ps23
Tetrapoda - ps24
Amniota - ps25
Mammalia - ps26
Theria - ps27
Placentalia - ps28
Boreoeutheria - ps29
Euarchontoglires - ps30
Euarchonta - ps31
Primates - ps32
Haplorrhini - ps33
Simiiformes - ps34
Catarrhini - ps35
Hominoidea - ps36
Hominidae - ps37
Homininae - ps38
Hominini - ps39
Homo_sapiens - ps40
Phylostratum

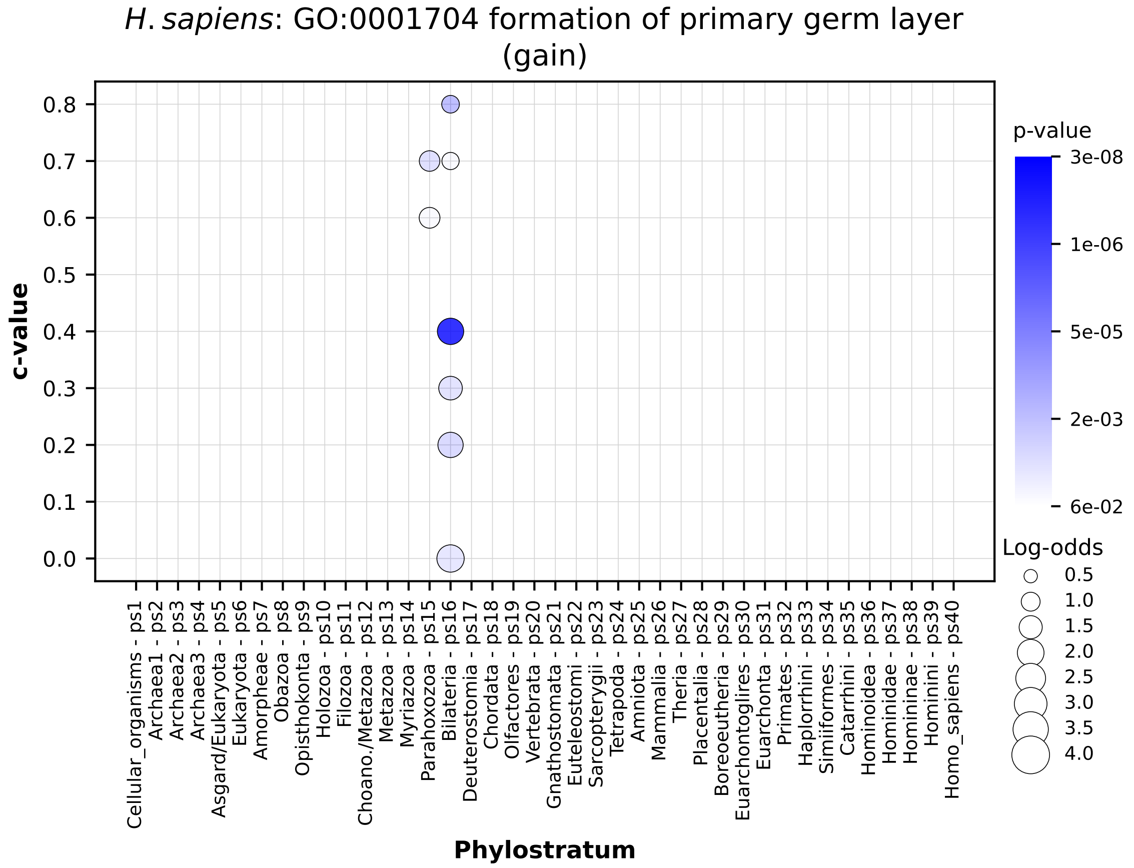

H. sapiens: GO:0001704 formation of primary germ layer
(gain)
c-value
0.8
0.7
0.6
0.5
0.4
0.3
0.2
0.1
0.0
Phylostratum
Cellular_organisms - ps1
Archaea1 - ps2
Archaea2 - ps3
Archaea3 - ps4
Asgard/Eukaryota - ps5
Eukaryota - ps6
Amorpheae - ps7
Obazoa - ps8
Opisthokonta - ps9
Holozoa - ps10
Filozoa - ps11
Choano./Metazoa - ps12
Metazoa - ps13
Myriazoa - ps14
Parahoxozoa - ps15
Bilateria - ps16
Deuterostomia - ps17
Chordata - ps18
Olfactores - ps19
Vertebrata - ps20
Gnathostomata - ps21
Euteleostomi - ps22
Sarcopterygii - ps23
Tetrapoda - ps24
Amniota - ps25
Mammalia - ps26
Theria - ps27
Placentalia - ps28
Boreoeutheria - ps29
Euarchontoglires - ps30
Euarchonta - ps31
Primates - ps32
Haplorrhini - ps33
Simiiformes - ps34
Catarrhini - ps35
Hominoidea - ps36
Hominidae - ps37
Homininae - ps38
Hominini - ps39
Homo_sapiens - ps40
p-value
3e-08
1e-06
5e-05
2e-03
6e-02
Log-odds
0.5
1.0
1.5
2.0
2.5
3.0
3.5
4.0

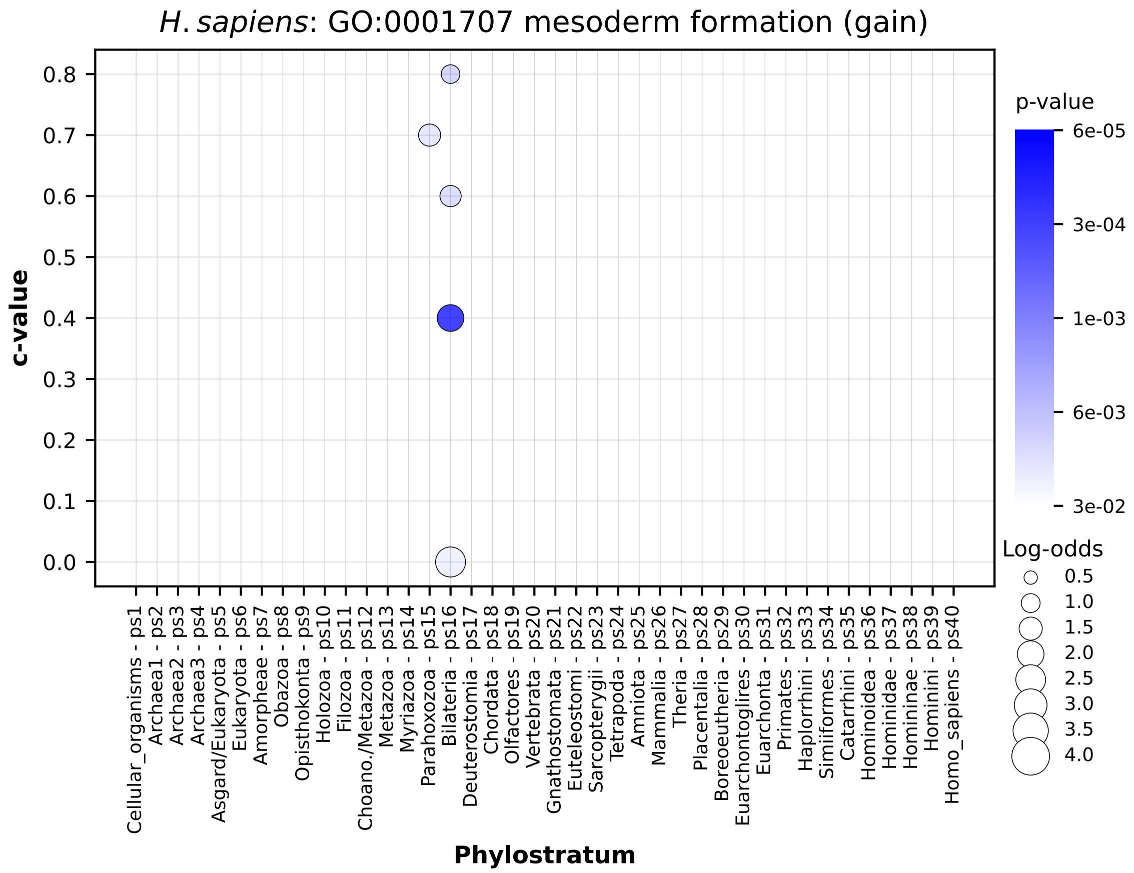

H. sapiens: GO:0001707 mesoderm formation (gain)
c-value
0.8
0.7
0.6
0.5
0.4
0.3
0.2
0.1
0.0
p-value
6e-05
3e-04
1e-03
6e-03
3e-02
Log-odds
0.5
1.0
1.5
2.0
2.5
3.0
3.5
4.0
Cellular_organisms - ps1
Archaea1 - ps2
Archaea2 - ps3
Archaea3 - ps4
Asgard/Eukaryota - ps5
Eukaryota - ps6
Amorpheae - ps7
Obazoa - ps8
Opisthokonta - ps9
Holozoa - ps10
Filozoa - ps11
Choano./Metazoa - ps12
Metazoa - ps13
Myriazoa - ps14
Parahoxozoa - ps15
Bilateria - ps16
Deuterostomia - ps17
Chordata - ps18
Olfactores - ps19
Vertebrata - ps20
Gnathostomata - ps21
Euteleostomi - ps22
Sarcopterygii - ps23
Tetrapoda - ps24
Amniota - ps25
Mammalia - ps26
Theria - ps27
Placentalia - ps28
Boreoeutheria - ps29
Euarchontoglires - ps30
Euarchonta - ps31
Primates - ps32
Haplorrhini - ps33
Simiiformes - ps34
Catarrhini - ps35
Hominoidea - ps36
Hominidae - ps37
Homininae - ps38
Hominini - ps39
Homo_sapiens - ps40
Phylostratum

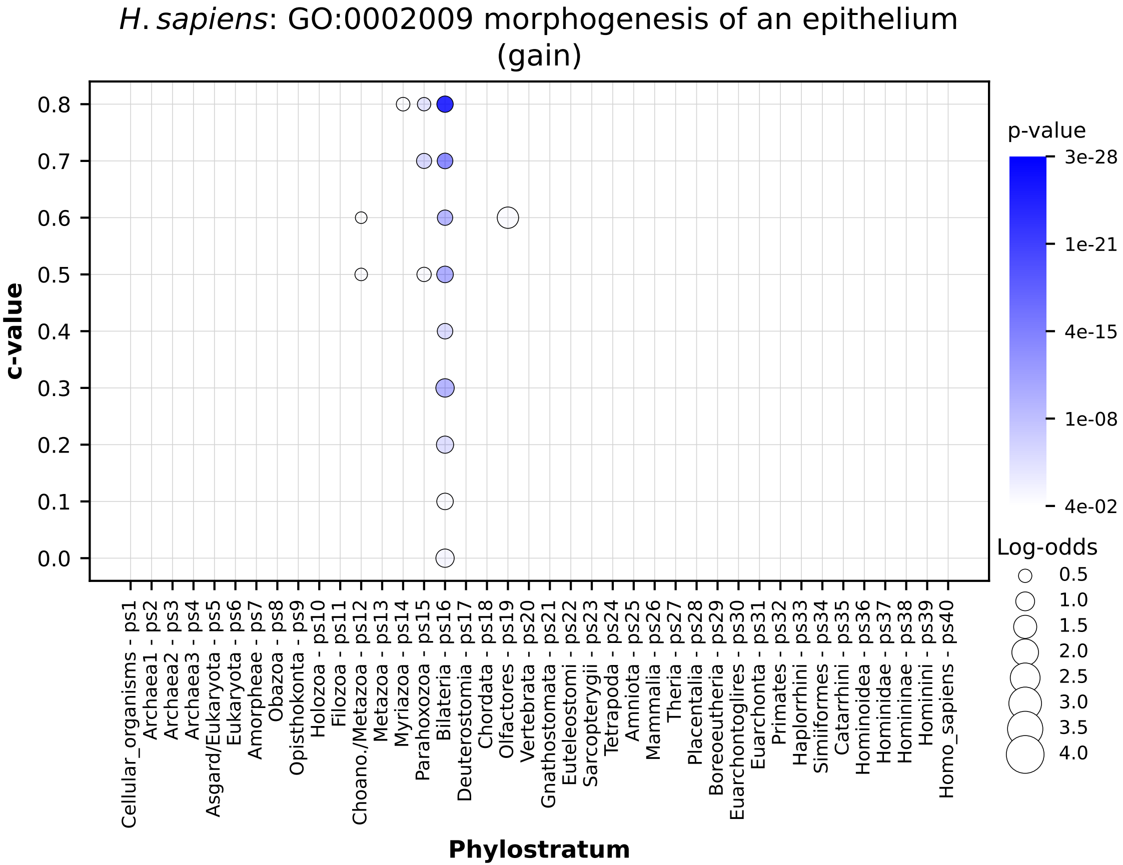

H. sapiens: GO:0002009 morphogenesis of an epithelium
(gain)
c-value
0.8
0.7
0.6
0.5
0.4
0.3
0.2
0.1
0.0
Phylostratum
Cellular_organisms - ps1
Archaea1 - ps2
Archaea2 - ps3
Archaea3 - ps4
Asgard/Eukaryota - ps5
Eukaryota - ps6
Amorpheae - ps7
Obazoa - ps8
Opisthokonta - ps9
Holozoa - ps10
Filozoa - ps11
Choano./Metazoa - ps12
Metazoa - ps13
Myriazoa - ps14
Parahoxozoa - ps15
Bilateria - ps16
Deuterostomia - ps17
Chordata - ps18
Olfactores - ps19
Vertebrata - ps20
Gnathostomata - ps21
Euteleostomi - ps22
Sarcopterygii - ps23
Tetrapoda - ps24
Amniota - ps25
Mammalia - ps26
Theria - ps27
Placentalia - ps28
Boreoeutheria - ps29
Euarchontoglires - ps30
Euarchonta - ps31
Primates - ps32
Haplorrhini - ps33
Simiiformes - ps34
Catarrhini - ps35
Hominoidea - ps36
Hominidae - ps37
Homininae - ps38
Hominini - ps39
Homo_sapiens - ps40
p-value
3e-28
1e-21
4e-15
1e-08
4e-02
Log-odds
0.5
1.0
1.5
2.0
2.5
3.0
3.5
4.0

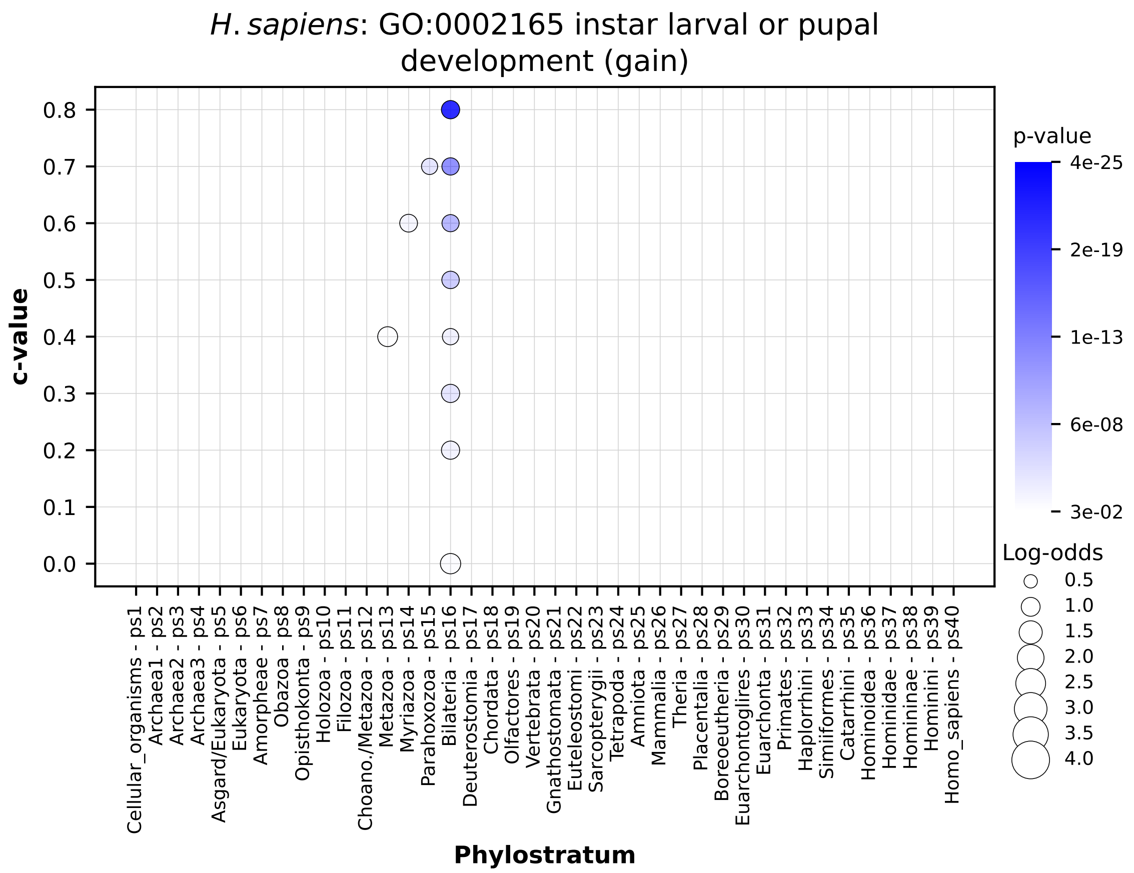

H. sapiens: GO:0002165 instar larval or pupal development (gain)
c-value
0.8
0.7
0.6
0.5
0.4
0.3
0.2
0.1
0.0
Phylostratum
Cellular_organisms - ps1
Archaea1 - ps2
Archaea2 - ps3
Archaea3 - ps4
Asgard/Eukaryota - ps5
Eukaryota - ps6
Amorpheae - ps7
Obazoa - ps8
Opisthokonta - ps9
Holozoa - ps10
Filozoa - ps11
Choano./Metazoa - ps12
Metazoa - ps13
Myriazoa - ps14
Parahoxozoa - ps15
Bilateria - ps16
Deuterostomia - ps17
Chordata - ps18
Olfactores - ps19
Vertebrata - ps20
Gnathostomata - ps21
Euteleostomi - ps22
Sarcopterygii - ps23
Tetrapoda - ps24
Amniota - ps25
Mammalia - ps26
Theria - ps27
Placentalia - ps28
Boreoeutheria - ps29
Euarchontoglires - ps30
Euarchonta - ps31
Primates - ps32
Haplorrhini - ps33
Simiiformes - ps34
Catarrhini - ps35
Hominoidea - ps36
Hominidae - ps37
Homininae - ps38
Hominini - ps39
Homo_sapiens - ps40
p-value
4e-25
2e-19
1e-13
6e-08
3e-02
Log-odds
0.5
1.0
1.5
2.0
2.5
3.0
3.5
4.0

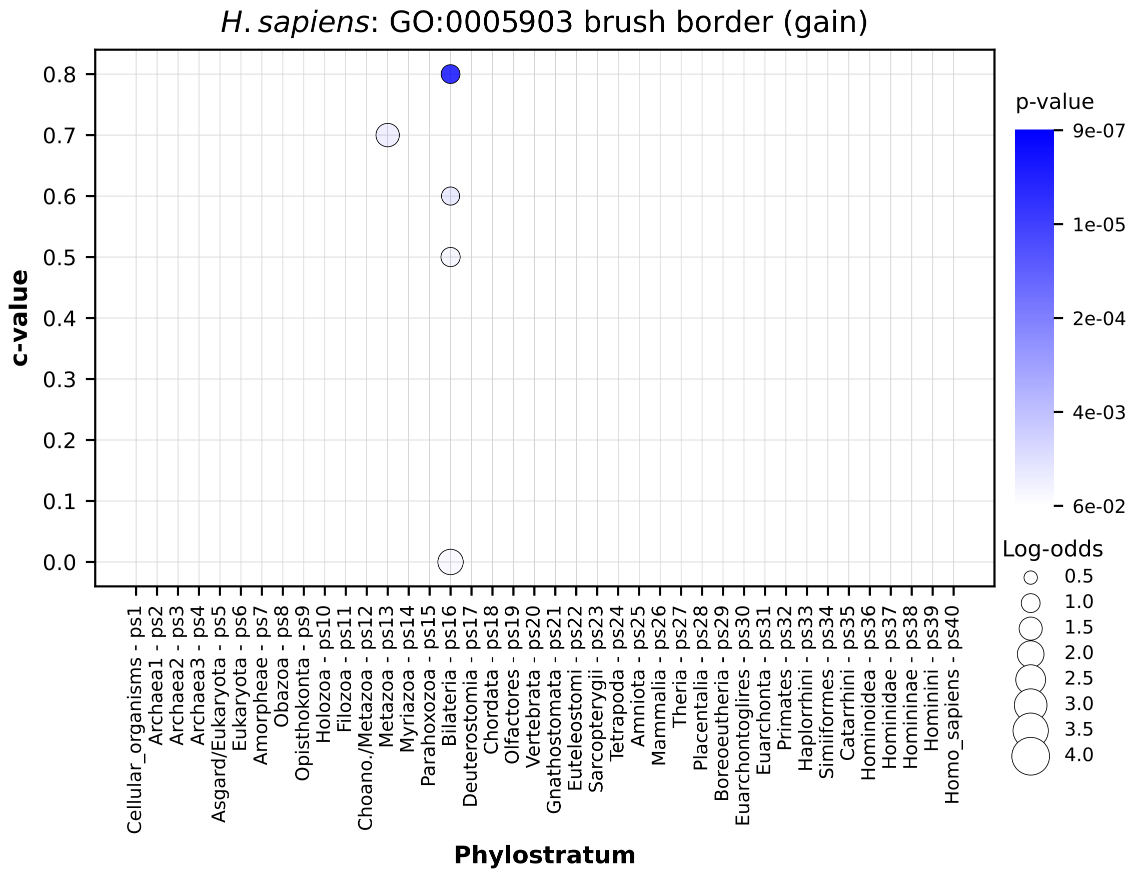

H. sapiens: GO:0005903 brush border (gain)
c-value
0.0
0.1
0.2
0.3
0.4
0.5
0.6
0.7
0.8
Phylostratum
Cellular_organisms - ps1
Archaea1 - ps2
Archaea2 - ps3
Archaea3 - ps4
Asgard/Eukaryota - ps5
Eukaryota - ps6
Amorpheae - ps7
Obazoa - ps8
Opisthokonta - ps9
Holozoa - ps10
Filozoa - ps11
Choano./Metazoa - ps12
Metazoa - ps13
Myriazoa - ps14
Parahoxozoa - ps15
Bilateria - ps16
Deuterostomia - ps17
Chordata - ps18
Olfactores - ps19
Vertebrata - ps20
Gnathostomata - ps21
Euteleostomi - ps22
Sarcopterygii - ps23
Tetrapoda - ps24
Amniota - ps25
Mammalia - ps26
Theria - ps27
Placentalia - ps28
Boreoeutheria - ps29
Euarchontoglires - ps30
Euarchonta - ps31
Primates - ps32
Haplorrhini - ps33
Simiiformes - ps34
Catarrhini - ps35
Hominoidea - ps36
Hominidae - ps37
Homininae - ps38
Hominini - ps39
Homo_sapiens - ps40
p-value
9e-07
1e-05
2e-04
4e-03
6e-02
Log-odds
0.5
1.0
1.5
2.0
2.5
3.0
3.5
4.0

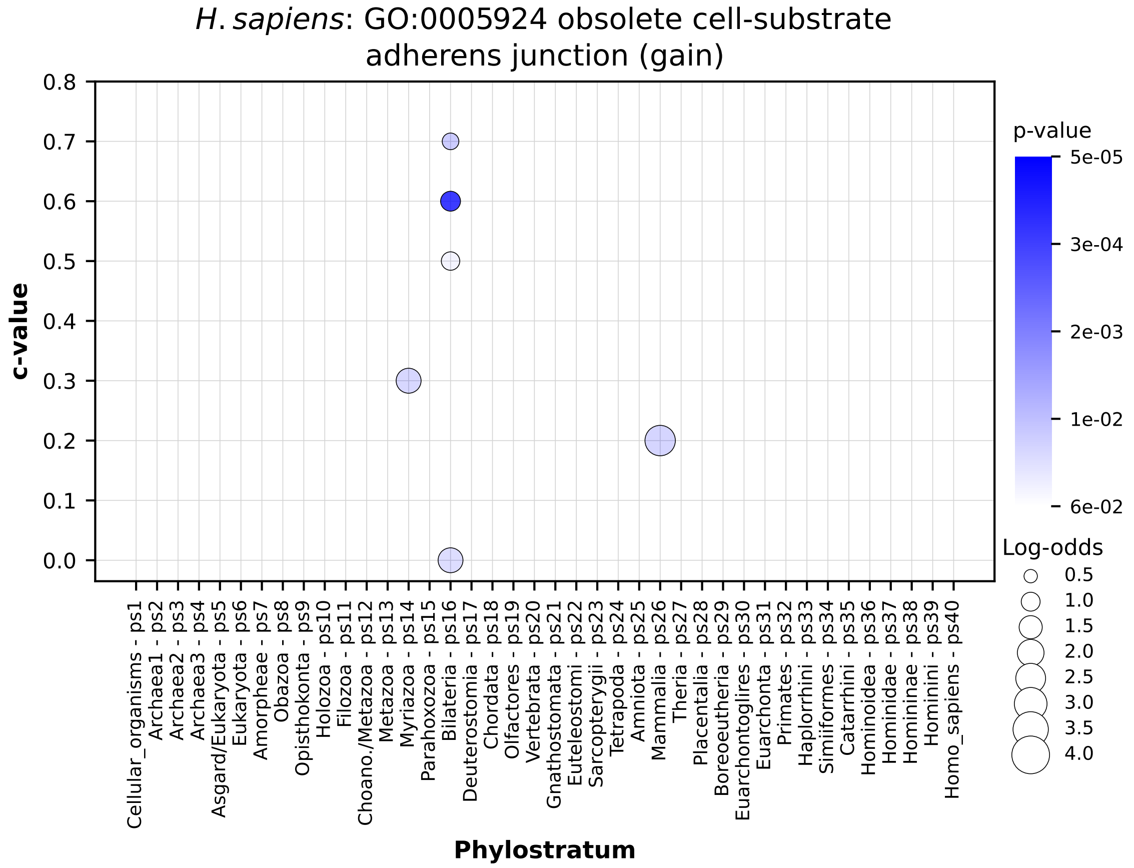
H. sapiens: GO:0005924 obsolete cell-substrate adherens junction (gain)
c-value
0.8
0.7
0.6
0.5
0.4
0.3
0.2
0.1
0.0
p-value
5e-05
3e-04
2e-03
1e-02
6e-02
Log-odds
0.5
1.0
1.5
2.0
2.5
3.0
3.5
4.0
Cellular_organisms - ps1
Archaea1 - ps2
Archaea2 - ps3
Archaea3 - ps4
Asgard/Eukaryota - ps5
Eukaryota - ps6
Amorpheae - ps7
Obazoa - ps8
Opisthokonta - ps9
Holozoa - ps10
Filozoa - ps11
Choano./Metazoa - ps12
Metazoa - ps13
Myriazoa - ps14
Parahoxozoa - ps15
Bilateria - ps16
Deuterostomia - ps17
Chordata - ps18
Olfactores - ps19
Vertebrata - ps20
Gnathostomata - ps21
Euteleostomi - ps22
Sarcopterygii - ps23
Tetrapoda - ps24
Amniota - ps25
Mammalia - ps26
Theria - ps27
Placentalia - ps28
Boreoeutheria - ps29
Euarchontoglires - ps30
Euarchonta - ps31
Primates - ps32
Haplorrhini - ps33
Simiiformes - ps34
Catarrhini - ps35
Hominoidea - ps36
Hominidae - ps37
Homininae - ps38
Hominini - ps39
Homo_sapiens - ps40
Phylostratum

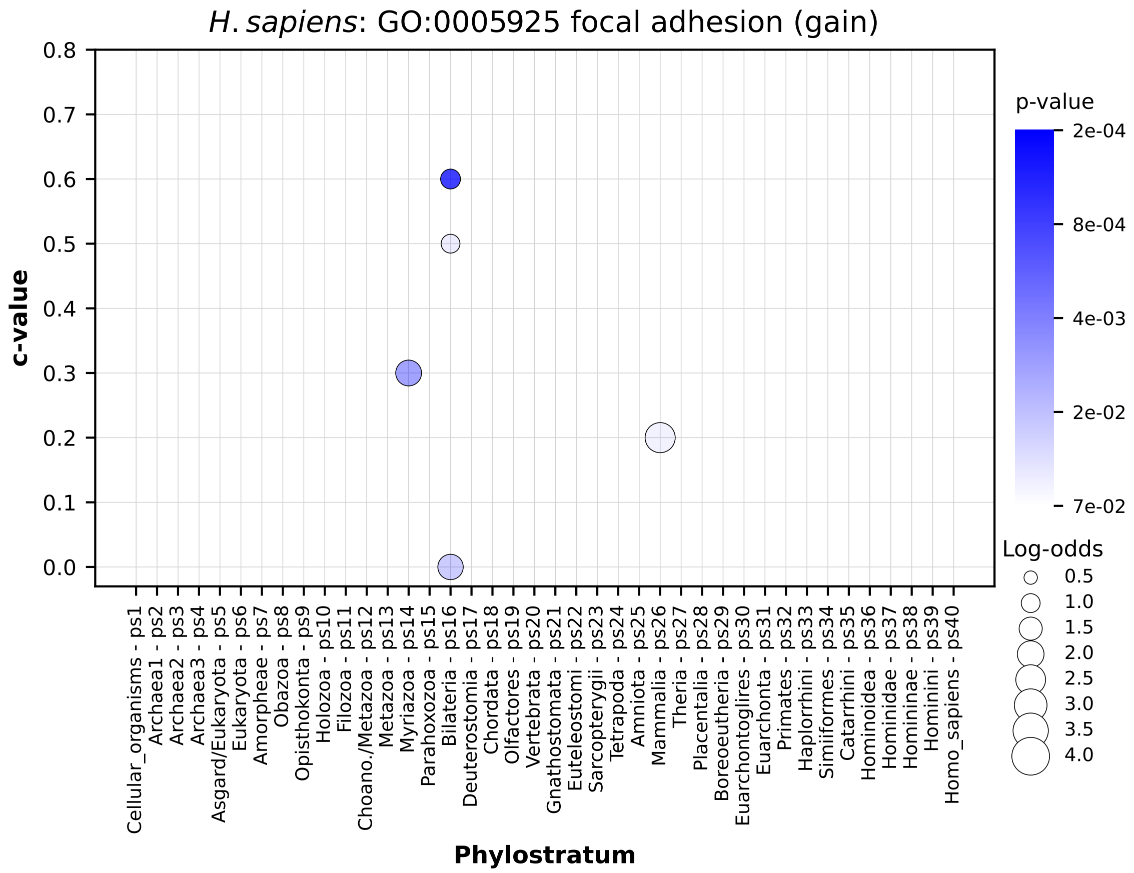

H. sapiens: GO:0005925 focal adhesion (gain)
c-value
0.8
0.7
0.6
0.5
0.4
0.3
0.2
0.1
0.0
Phylostratum
Cellular_organisms - ps1
Archaea1 - ps2
Archaea2 - ps3
Archaea3 - ps4
Asgard/Eukaryota - ps5
Eukaryota - ps6
Amorpheae - ps7
Obazoa - ps8
Opisthokonta - ps9
Holozoa - ps10
Filozoa - ps11
Choano./Metazoa - ps12
Metazoa - ps13
Myriazoa - ps14
Parahoxozoa - ps15
Bilateria - ps16
Deuterostomia - ps17
Chordata - ps18
Olfactores - ps19
Vertebrata - ps20
Gnathostomata - ps21
Euteleostomi - ps22
Sarcopterygii - ps23
Tetrapoda - ps24
Amniota - ps25
Mammalia - ps26
Theria - ps27
Placentalia - ps28
Boreoeutheria - ps29
Euarchontoglires - ps30
Euarchonta - ps31
Primates - ps32
Haplorrhini - ps33
Simiiformes - ps34
Catarrhini - ps35
Hominoidea - ps36
Hominidae - ps37
Homininae - ps38
Hominini - ps39
Homo_sapiens - ps40
p-value
2e-04
8e-04
4e-03
2e-02
7e-02
Log-odds
0.5
1.0
1.5
2.0
2.5
3.0
3.5
4.0

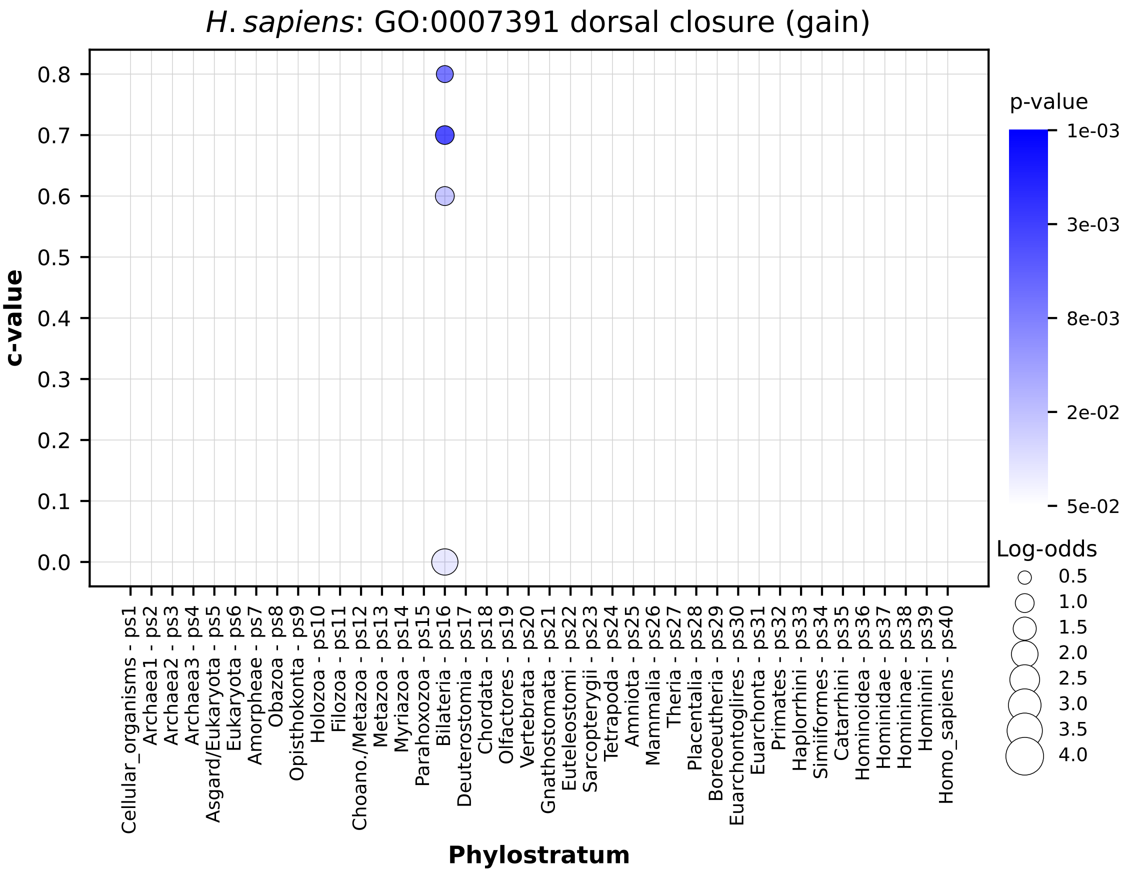

*H. sapiens*: GO:0007391 dorsal closure (gain)
c-value
0.8
0.7
0.6
0.5
0.4
0.3
0.2
0.1
0.0
Phylostratum
Cellular_organisms - ps1
Archaea1 - ps2
Archaea2 - ps3
Archaea3 - ps4
Asgard/Eukaryota - ps5
Eukaryota - ps6
Amorpheae - ps7
Obazoa - ps8
Opisthokonta - ps9
Holozoa - ps10
Filozoa - ps11
Choano./Metazoa - ps12
Metazoa - ps13
Myriazoa - ps14
Parahoxozoa - ps15
Bilateria - ps16
Deuterostomia - ps17
Chordata - ps18
Olfactores - ps19
Vertebrata - ps20
Gnathostomata - ps21
Euteleostomi - ps22
Sarcopterygii - ps23
Tetrapoda - ps24
Amniota - ps25
Mammalia - ps26
Theria - ps27
Placentalia - ps28
Boreoeutheria - ps29
Euarchontoglires - ps30
Euarchonta - ps31
Primates - ps32
Haplorrhini - ps33
Simiiformes - ps34
Catarrhini - ps35
Hominoidea - ps36
Hominidae - ps37
Homininae - ps38
Hominini - ps39
Homo_sapiens - ps40
p-value
1e-03
3e-03
8e-03
2e-02
5e-02
Log-odds
0.5
1.0
1.5
2.0
2.5
3.0
3.5
4.0

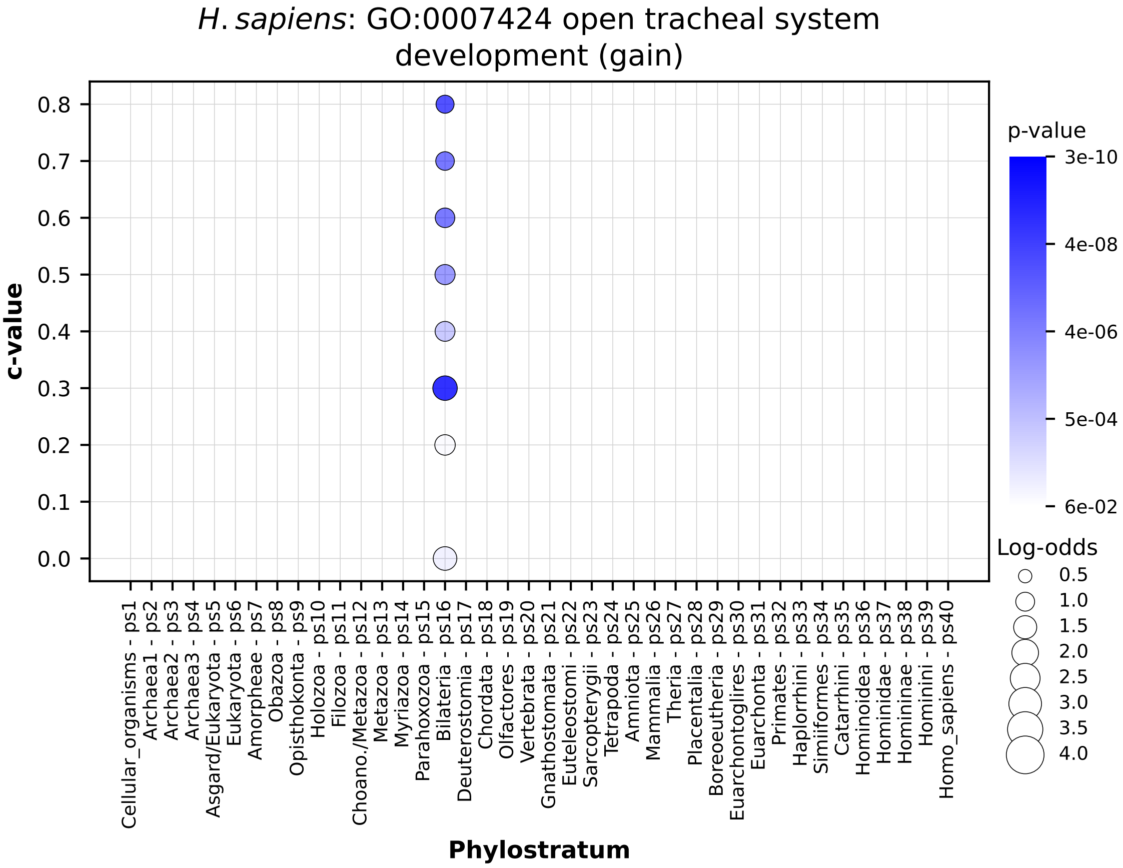
H. sapiens: GO:0007424 open tracheal system development (gain)
c-value
0.0
0.1
0.2
0.3
0.4
0.5
0.6
0.7
0.8
Phylostratum
Cellular_organisms - ps1
Archaea1 - ps2
Archaea2 - ps3
Archaea3 - ps4
Asgard/Eukaryota - ps5
Eukaryota - ps6
Amorpheae - ps7
Obazoa - ps8
Opisthokonta - ps9
Holozoa - ps10
Filozoa - ps11
Choano./Metazoa - ps12
Metazoa - ps13
Myriazoa - ps14
Parahoxozoa - ps15
Bilateria - ps16
Deuterostomia - ps17
Chordata - ps18
Olfactores - ps19
Vertebrata - ps20
Gnathostomata - ps21
Euteleostomi - ps22
Sarcopterygii - ps23
Tetrapoda - ps24
Amniota - ps25
Mammalia - ps26
Theria - ps27
Placentalia - ps28
Boreoeutheria - ps29
Euarchontoglires - ps30
Euarchonta - ps31
Primates - ps32
Haplorrhini - ps33
Simiiformes - ps34
Catarrhini - ps35
Hominoidea - ps36
Hominidae - ps37
Homininae - ps38
Hominini - ps39
Homo_sapiens - ps40
p-value
3e-10
4e-08
4e-06
5e-04
6e-02
Log-odds
0.5
1.0
1.5
2.0
2.5
3.0
3.5
4.0

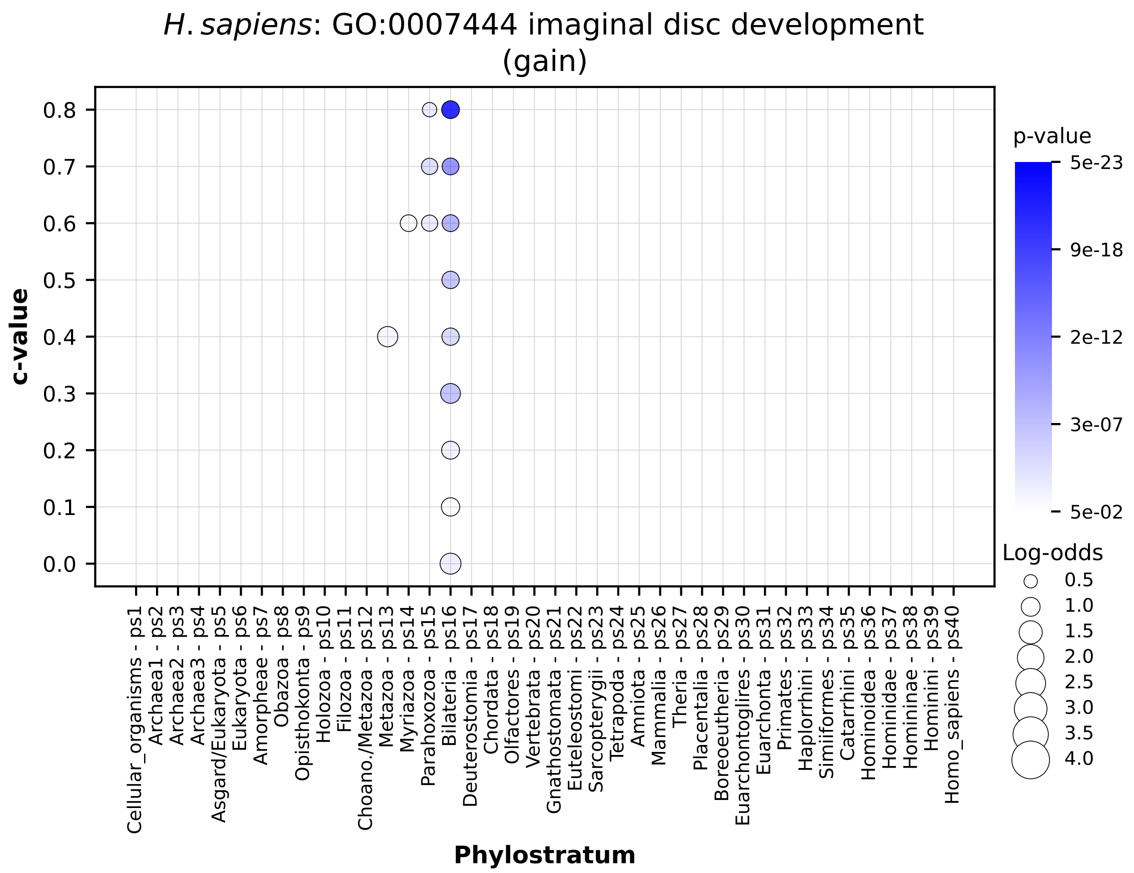

H. sapiens: GO:0007444 imaginal disc development
(gain)
c-value
0.8
0.7
0.6
0.5
0.4
0.3
0.2
0.1
0.0
Phylostratum
Cellular_organisms - ps1
Archaea1 - ps2
Archaea2 - ps3
Archaea3 - ps4
Asgard/Eukaryota - ps5
Eukaryota - ps6
Amorpheae - ps7
Obazoa - ps8
Opisthokonta - ps9
Holozoa - ps10
Filozoa - ps11
Choano./Metazoa - ps12
Metazoa - ps13
Myriazoa - ps14
Parahoxozoa - ps15
Bilateria - ps16
Deuterostomia - ps17
Chordata - ps18
Olfactores - ps19
Vertebrata - ps20
Gnathostomata - ps21
Euteleostomi - ps22
Sarcopterygii - ps23
Tetrapoda - ps24
Amniota - ps25
Mammalia - ps26
Theria - ps27
Placentalia - ps28
Boreoeutheria - ps29
Euarchontoglires - ps30
Euarchonta - ps31
Primates - ps32
Haplorrhini - ps33
Simiiformes - ps34
Catarrhini - ps35
Hominoidea - ps36
Hominidae - ps37
Homininae - ps38
Hominini - ps39
Homo_sapiens - ps40
p-value
5e-23
9e-18
2e-12
3e-07
5e-02
Log-odds
0.5
1.0
1.5
2.0
2.5
3.0
3.5
4.0

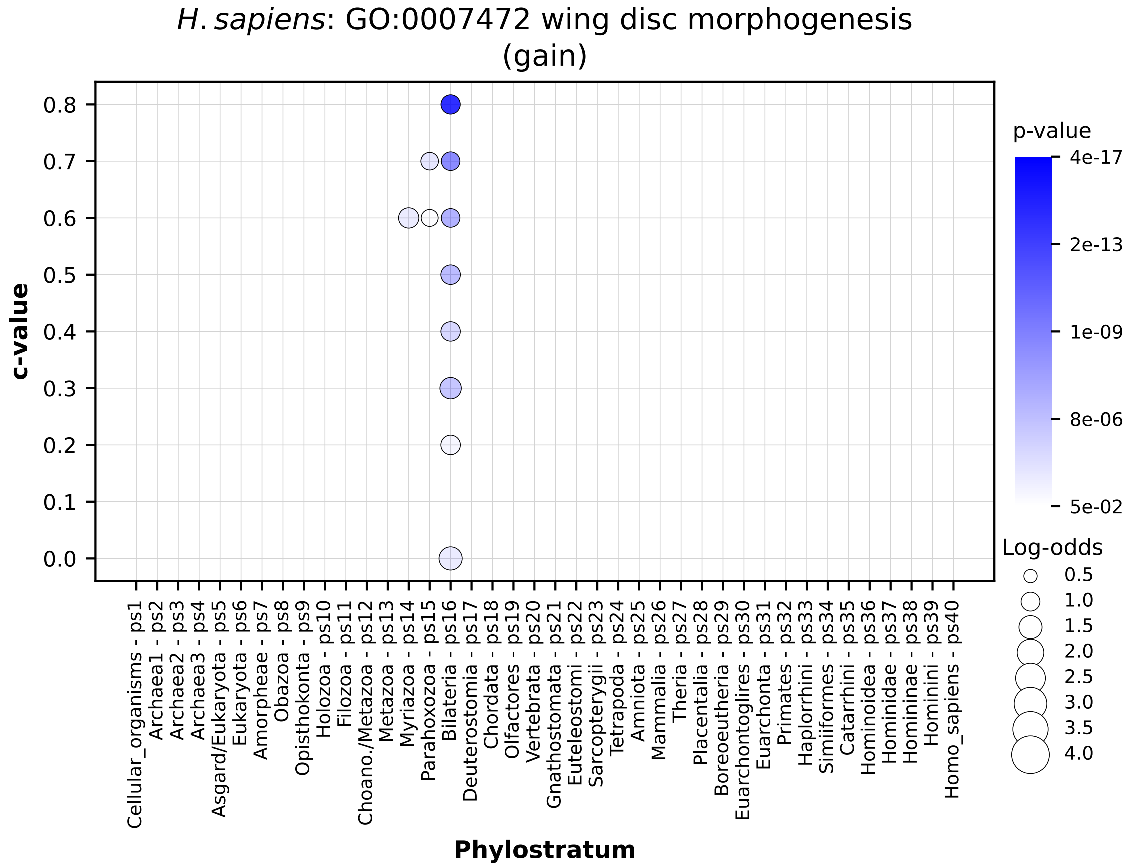

H. sapiens: GO:0007472 wing disc morphogenesis (gain)
c-value
0.0
0.1
0.2
0.3
0.4
0.5
0.6
0.7
0.8
Phylostratum
Cellular_organisms - ps1
Archaea1 - ps2
Archaea2 - ps3
Archaea3 - ps4
Asgard/Eukaryota - ps5
Eukaryota - ps6
Amorpheae - ps7
Obazoa - ps8
Opisthokonta - ps9
Holozoa - ps10
Filozoa - ps11
Choano./Metazoa - ps12
Metazoa - ps13
Myriazoa - ps14
Parahoxozoa - ps15
Bilateria - ps16
Deuterostomia - ps17
Chordata - ps18
Olfactores - ps19
Vertebrata - ps20
Gnathostomata - ps21
Euteleostomi - ps22
Sarcopterygii - ps23
Tetrapoda - ps24
Amniota - ps25
Mammalia - ps26
Theria - ps27
Placentalia - ps28
Boreoeutheria - ps29
Euarchontoglires - ps30
Euarchonta - ps31
Primates - ps32
Haplorrhini - ps33
Simiiformes - ps34
Catarrhini - ps35
Hominoidea - ps36
Hominidae - ps37
Homininae - ps38
Hominini - ps39
Homo_sapiens - ps40
p-value
4e-17
2e-13
1e-09
8e-06
5e-02
Log-odds
0.5
1.0
1.5
2.0
2.5
3.0
3.5
4.0

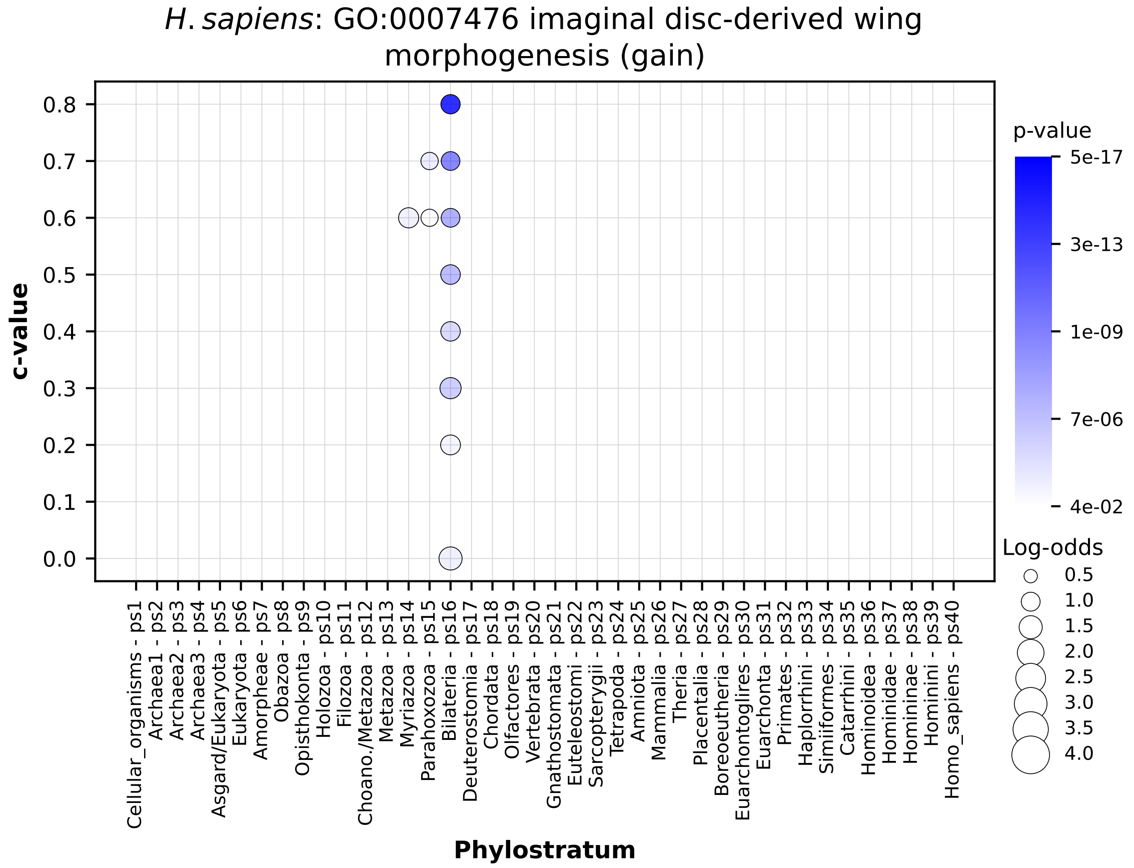

H. sapiens: GO:0007476 imaginal disc-derived wing morphogenesis (gain)
c-value
0.8
0.7
0.6
0.5
0.4
0.3
0.2
0.1
0.0
p-value
5e-17
3e-13
1e-09
7e-06
4e-02
Log-odds
0.5
1.0
1.5
2.0
2.5
3.0
3.5
4.0
Cellular_organisms - ps1
Archaea1 - ps2
Archaea2 - ps3
Archaea3 - ps4
Asgard/Eukaryota - ps5
Eukaryota - ps6
Amorpheae - ps7
Obazoa - ps8
Opisthokonta - ps9
Holozoa - ps10
Filozoa - ps11
Choano./Metazoa - ps12
Metazoa - ps13
Myriazoa - ps14
Parahoxozoa - ps15
Bilateria - ps16
Deuterostomia - ps17
Chordata - ps18
Olfactores - ps19
Vertebrata - ps20
Gnathostomata - ps21
Euteleostomi - ps22
Sarcopterygii - ps23
Tetrapoda - ps24
Amniota - ps25
Mammalia - ps26
Theria - ps27
Placentalia - ps28
Boreoeutheria - ps29
Euarchontoglires - ps30
Euarchonta - ps31
Primates - ps32
Haplorrhini - ps33
Simiiformes - ps34
Catarrhini - ps35
Hominoidea - ps36
Hominidae - ps37
Homininae - ps38
Hominini - ps39
Homo_sapiens - ps40
Phylostratum

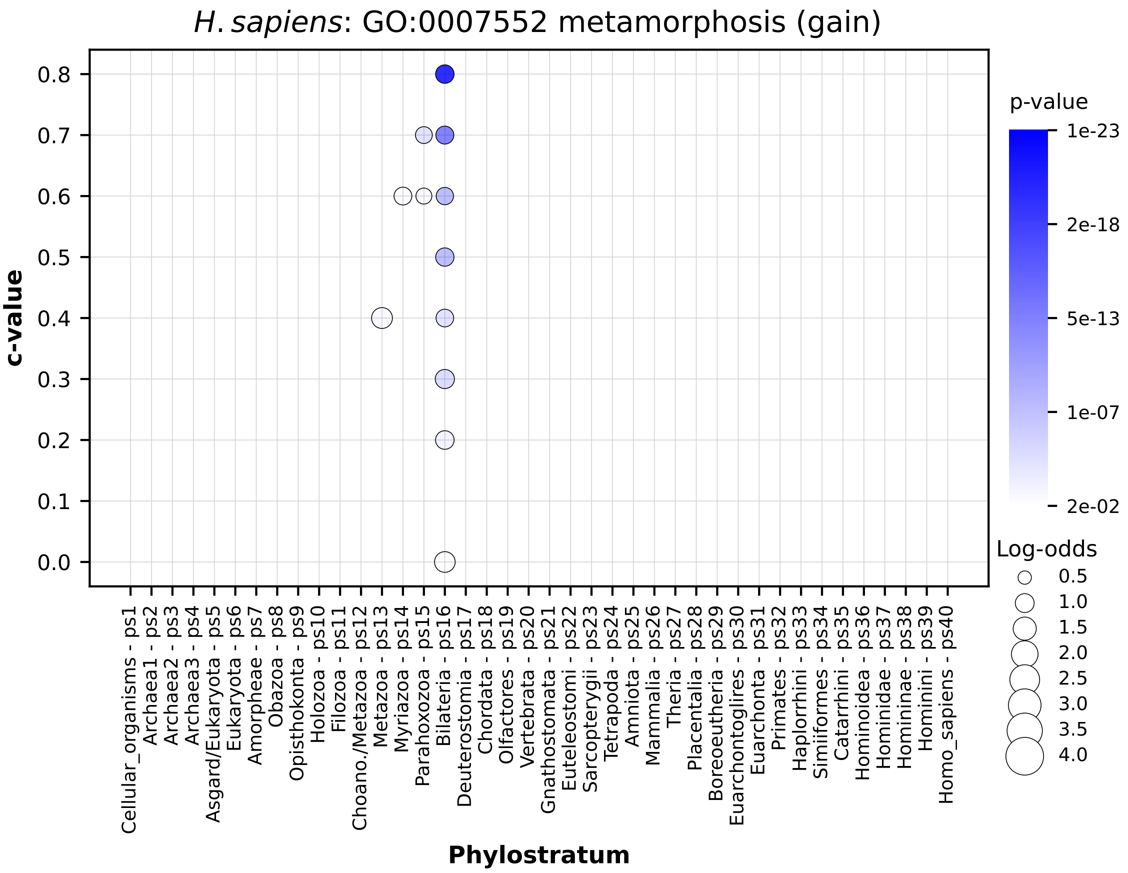

*H. sapiens*: GO:0007552 metamorphosis (gain)
c-value
0.8
0.7
0.6
0.5
0.4
0.3
0.2
0.1
0.0
Phylostratum
Cellular_organisms - ps1
Archaea1 - ps2
Archaea2 - ps3
Archaea3 - ps4
Asgard/Eukaryota - ps5
Eukaryota - ps6
Amorpheae - ps7
Obazoa - ps8
Opisthokonta - ps9
Holozoa - ps10
Filozoa - ps11
Choano./Metazoa - ps12
Metazoa - ps13
Myriazoa - ps14
Parahoxozoa - ps15
Bilateria - ps16
Deuterostomia - ps17
Chordata - ps18
Olfactores - ps19
Vertebrata - ps20
Gnathostomata - ps21
Euteleostomi - ps22
Sarcopterygii - ps23
Tetrapoda - ps24
Amniota - ps25
Mammalia - ps26
Theria - ps27
Placentalia - ps28
Boreoeutheria - ps29
Euarchontoglires - ps30
Euarchonta - ps31
Primates - ps32
Haplorrhini - ps33
Simiiformes - ps34
Catarrhini - ps35
Hominoidea - ps36
Hominidae - ps37
Homininae - ps38
Hominini - ps39
Homo_sapiens - ps40
p-value
1e-23
2e-18
5e-13
1e-07
2e-02
Log-odds
0.5
1.0
1.5
2.0
2.5
3.0
3.5
4.0

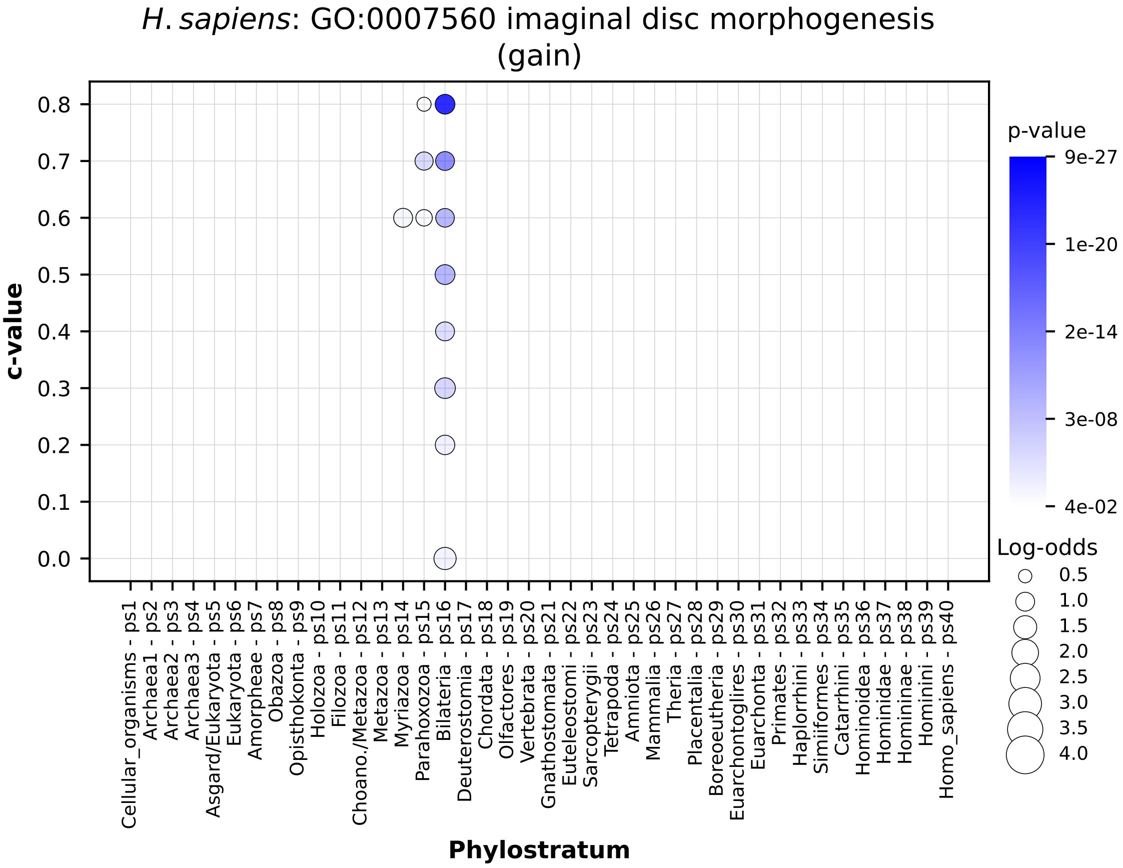

H. sapiens: GO:0007560 imaginal disc morphogenesis
(gain)
c-value
0.8
0.7
0.6
0.5
0.4
0.3
0.2
0.1
0.0
Phylostratum
Cellular_organisms - ps1
Archaea1 - ps2
Archaea2 - ps3
Archaea3 - ps4
Asgard/Eukaryota - ps5
Eukaryota - ps6
Amorpheae - ps7
Obazoa - ps8
Opisthokonta - ps9
Holozoa - ps10
Filozoa - ps11
Choano./Metazoa - ps12
Metazoa - ps13
Myriazoa - ps14
Parahoxozoa - ps15
Bilateria - ps16
Deuterostomia - ps17
Chordata - ps18
Olfactores - ps19
Vertebrata - ps20
Gnathostomata - ps21
Euteleostomi - ps22
Sarcopterygii - ps23
Tetrapoda - ps24
Amniota - ps25
Mammalia - ps26
Theria - ps27
Placentalia - ps28
Boreoeutheria - ps29
Euarchontoglires - ps30
Euarchonta - ps31
Primates - ps32
Haplorrhini - ps33
Simiiformes - ps34
Catarrhini - ps35
Hominoidea - ps36
Hominidae - ps37
Homininae - ps38
Hominini - ps39
Homo_sapiens - ps40
p-value
9e-27
1e-20
2e-14
3e-08
4e-02
Log-odds
0.5
1.0
1.5
2.0
2.5
3.0
3.5
4.0

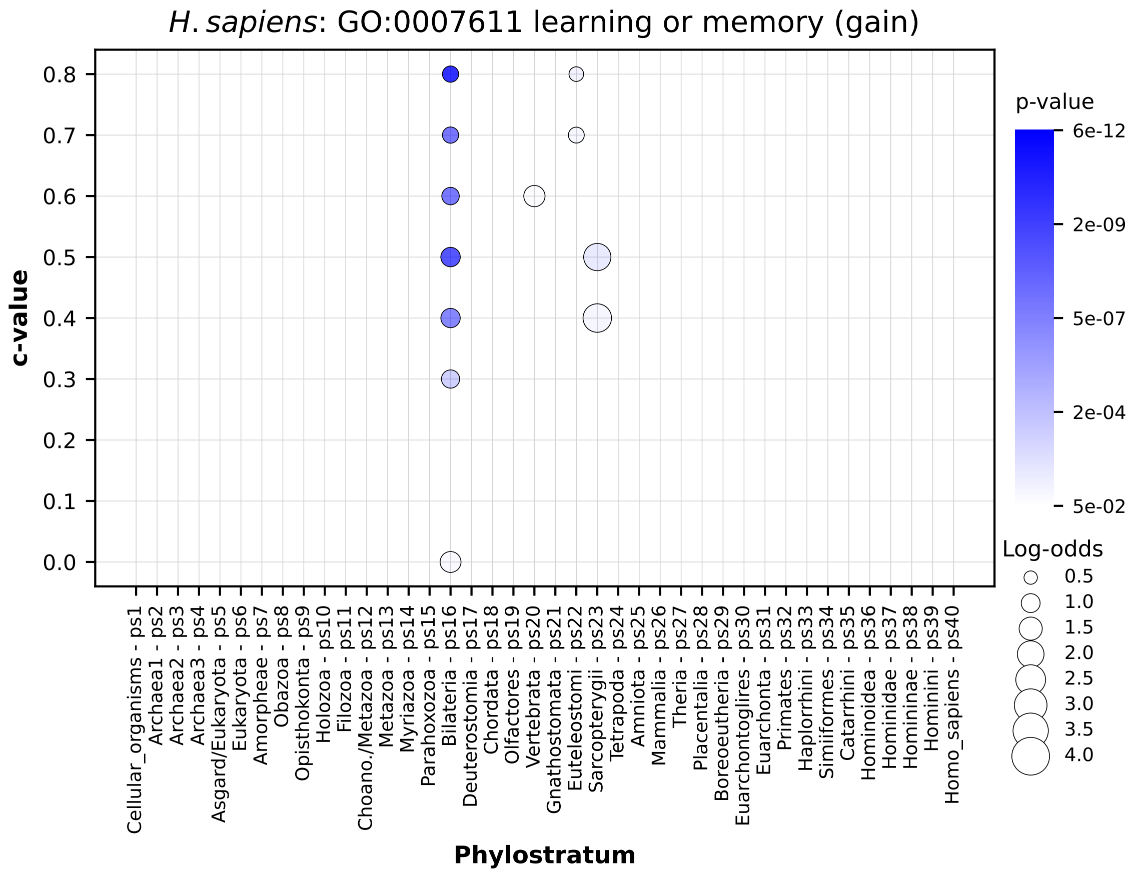

*H. sapiens*: GO:0007611 learning or memory (gain)
c-value
0.8
0.7
0.6
0.5
0.4
0.3
0.2
0.1
0.0
p-value
6e-12
2e-09
5e-07
2e-04
5e-02
Log-odds
0.5
1.0
1.5
2.0
2.5
3.0
3.5
4.0
Cellular_organisms - ps1
Archaea1 - ps2
Archaea2 - ps3
Archaea3 - ps4
Asgard/Eukaryota - ps5
Eukaryota - ps6
Amorpheae - ps7
Obazoa - ps8
Opisthokonta - ps9
Holozoa - ps10
Filozoa - ps11
Choano./Metazoa - ps12
Metazoa - ps13
Myriazoa - ps14
Parahoxozoa - ps15
Bilateria - ps16
Deuterostomia - ps17
Chordata - ps18
Olfactores - ps19
Vertebrata - ps20
Gnathostomata - ps21
Euteleostomi - ps22
Sarcopterygii - ps23
Tetrapoda - ps24
Amniota - ps25
Mammalia - ps26
Theria - ps27
Placentalia - ps28
Boreoeutheria - ps29
Euarchontoglires - ps30
Euarchonta - ps31
Primates - ps32
Haplorrhini - ps33
Simiiformes - ps34
Catarrhini - ps35
Hominoidea - ps36
Hominidae - ps37
Homininae - ps38
Hominini - ps39
Homo_sapiens - ps40
Phylostratum

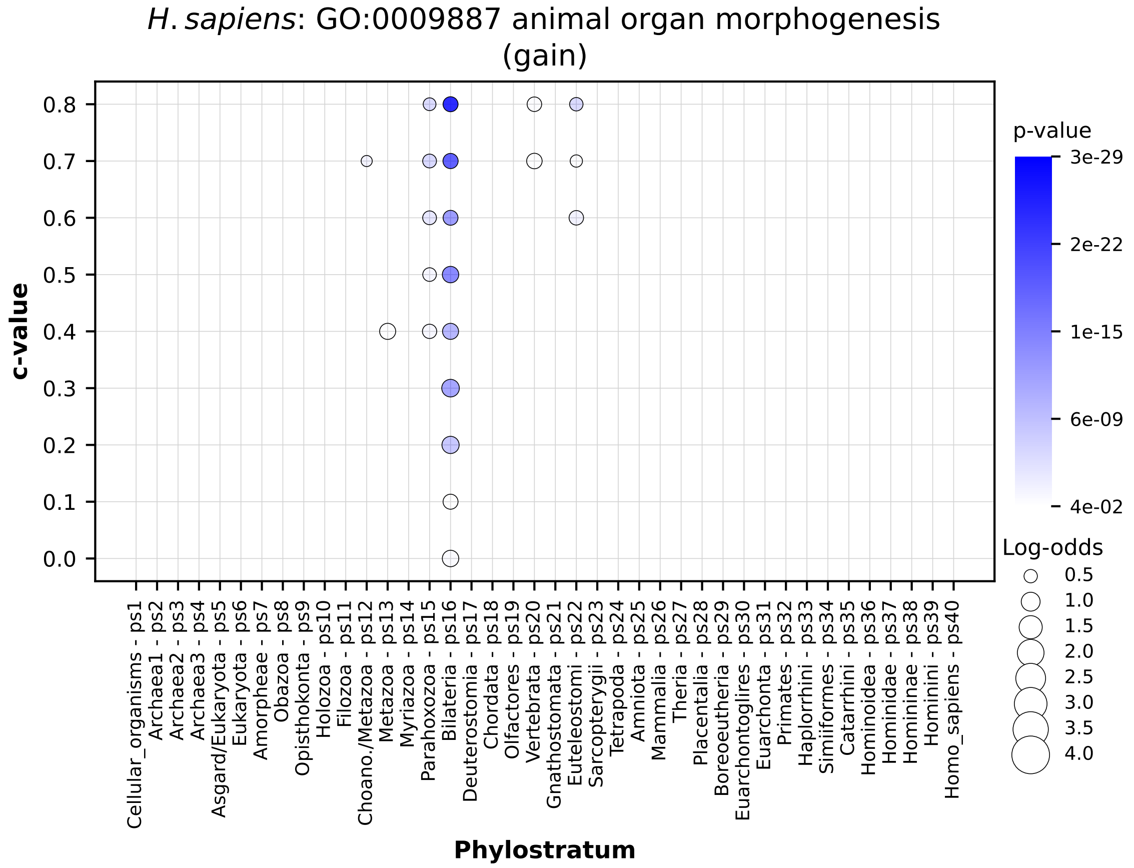

H. sapiens: GO:0009887 animal organ morphogenesis
(gain)
c-value
0.8
0.7
0.6
0.5
0.4
0.3
0.2
0.1
0.0
Phylostratum
Cellular_organisms - ps1
Archaea1 - ps2
Archaea2 - ps3
Archaea3 - ps4
Asgard/Eukaryota - ps5
Eukaryota - ps6
Amorpheae - ps7
Obazoa - ps8
Opisthokonta - ps9
Holozoa - ps10
Filozoa - ps11
Choano./Metazoa - ps12
Metazoa - ps13
Myriazoa - ps14
Parahoxozoa - ps15
Bilateria - ps16
Deuterostomia - ps17
Chordata - ps18
Olfactores - ps19
Vertebrata - ps20
Gnathostomata - ps21
Euteleostomi - ps22
Sarcopterygii - ps23
Tetrapoda - ps24
Amniota - ps25
Mammalia - ps26
Theria - ps27
Placentalia - ps28
Boreoeutheria - ps29
Euarchontoglires - ps30
Euarchonta - ps31
Primates - ps32
Haplorrhini - ps33
Simiiformes - ps34
Catarrhini - ps35
Hominoidea - ps36
Hominidae - ps37
Homininae - ps38
Hominini - ps39
Homo_sapiens - ps40
p-value
3e-29
2e-22
1e-15
6e-09
4e-02
Log-odds
0.5
1.0
1.5
2.0
2.5
3.0
3.5
4.0

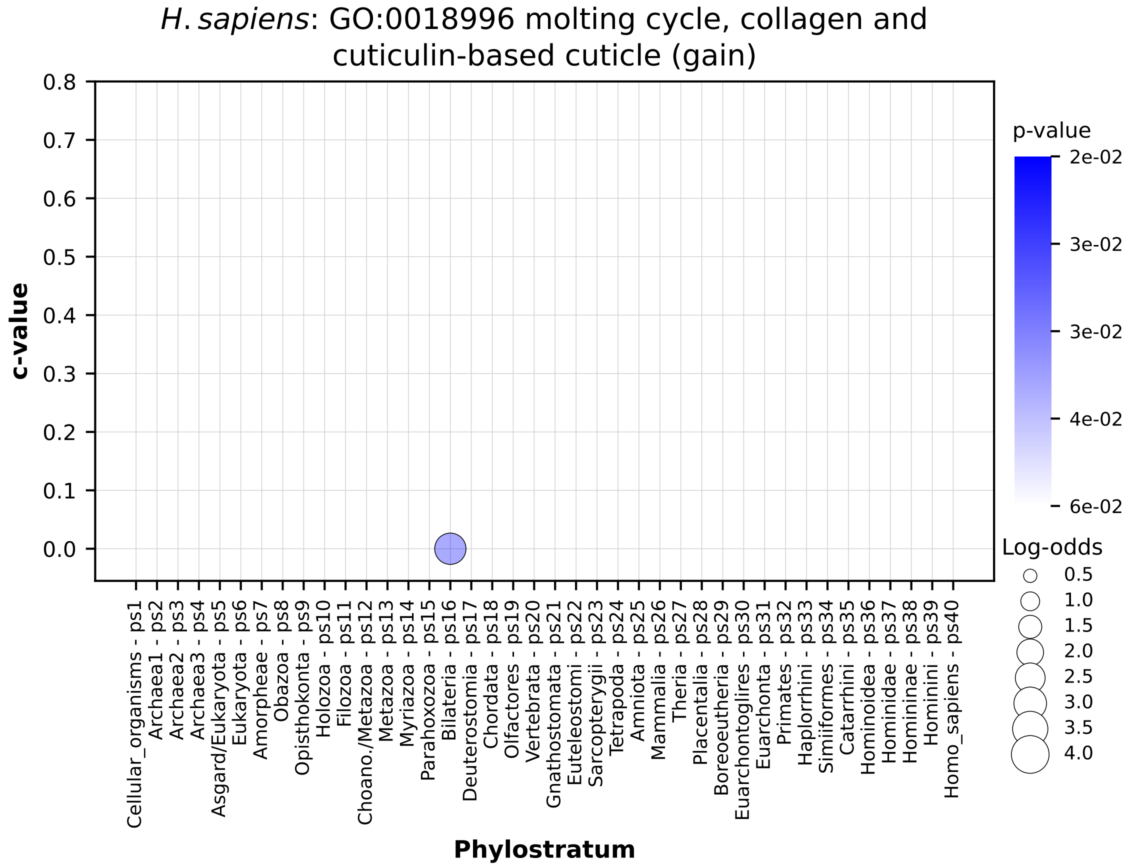

H. sapiens: GO:0018996 molting cycle, collagen and cuticulin-based cuticle (gain)
c-value
0.8
0.7
0.6
0.5
0.4
0.3
0.2
0.1
0.0
Phylostratum
Cellular_organisms - ps1
Archaea1 - ps2
Archaea2 - ps3
Archaea3 - ps4
Asgard/Eukaryota - ps5
Eukaryota - ps6
Amorpheae - ps7
Obazoa - ps8
Opisthokonta - ps9
Holozoa - ps10
Filozoa - ps11
Choano./Metazoa - ps12
Metazoa - ps13
Myriazoa - ps14
Parahoxozoa - ps15
Bilateria - ps16
Deuterostomia - ps17
Chordata - ps18
Olfactores - ps19
Vertebrata - ps20
Gnathostomata - ps21
Euteleostomi - ps22
Sarcopterygii - ps23
Tetrapoda - ps24
Amniota - ps25
Mammalia - ps26
Theria - ps27
Placentalia - ps28
Boreoeutheria - ps29
Euarchontoglires - ps30
Euarchonta - ps31
Primates - ps32
Haplorrhini - ps33
Simiiformes - ps34
Catarrhini - ps35
Hominoidea - ps36
Hominidae - ps37
Homininae - ps38
Hominini - ps39
Homo_sapiens - ps40
p-value
2e-02
3e-02
3e-02
4e-02
6e-02
Log-odds
0.5
1.0
1.5
2.0
2.5
3.0
3.5
4.0

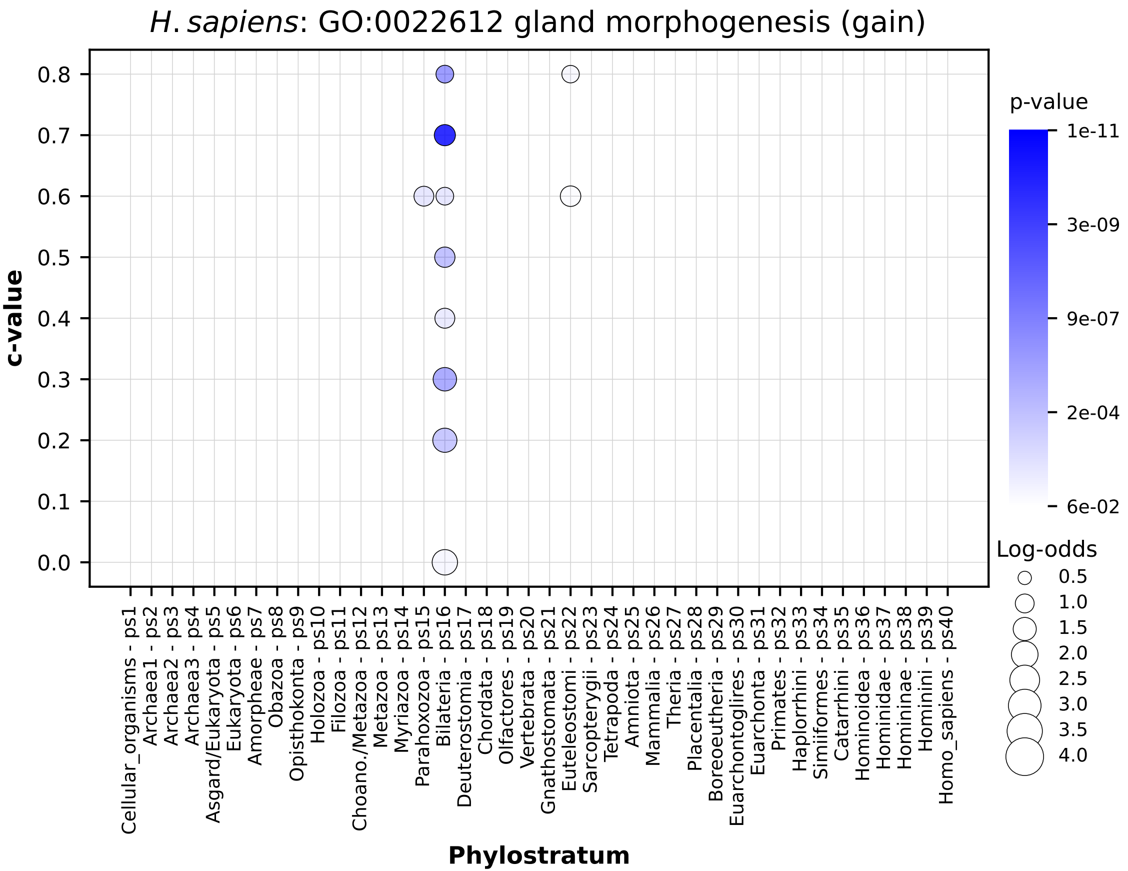

H. sapiens: GO:0022612 gland morphogenesis (gain)
c-value
0.8
0.7
0.6
0.5
0.4
0.3
0.2
0.1
0.0
Phylostratum
Cellular_organisms - ps1
Archaea1 - ps2
Archaea2 - ps3
Archaea3 - ps4
Asgard/Eukaryota - ps5
Eukaryota - ps6
Amorpheae - ps7
Obazoa - ps8
Opisthokonta - ps9
Holozoa - ps10
Filozoa - ps11
Choano./Metazoa - ps12
Metazoa - ps13
Myriazoa - ps14
Parahoxozoa - ps15
Bilateria - ps16
Deuterostomia - ps17
Chordata - ps18
Olfactores - ps19
Vertebrata - ps20
Gnathostomata - ps21
Euteleostomi - ps22
Sarcopterygii - ps23
Tetrapoda - ps24
Amniota - ps25
Mammalia - ps26
Theria - ps27
Placentalia - ps28
Boreoeutheria - ps29
Euarchontoglires - ps30
Euarchonta - ps31
Primates - ps32
Haplorrhini - ps33
Simiiformes - ps34
Catarrhini - ps35
Hominoidea - ps36
Hominidae - ps37
Homininae - ps38
Hominini - ps39
Homo_sapiens - ps40
p-value
1e-11
3e-09
9e-07
2e-04
6e-02
Log-odds
0.5
1.0
1.5
2.0
2.5
3.0
3.5
4.0

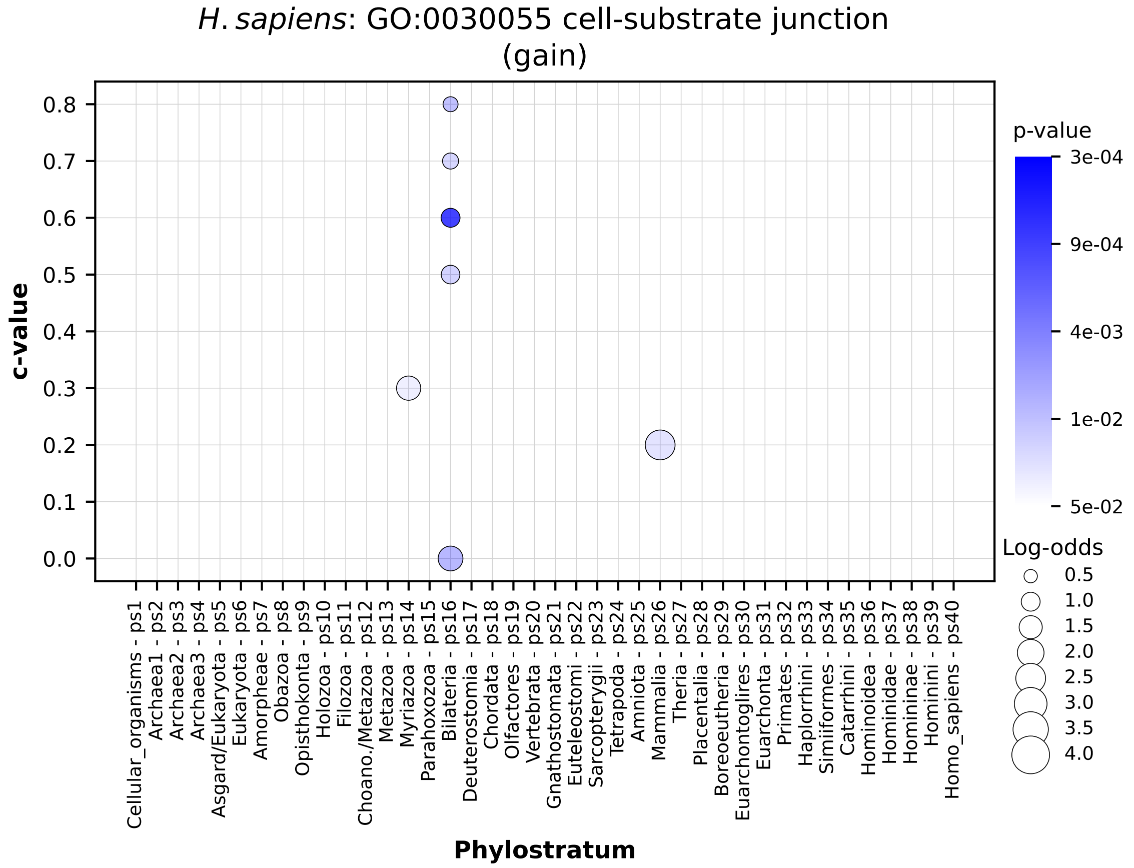
*H. sapiens*: GO:0030055 cell-substrate junction
(gain)
c-value
0.0
0.1
0.2
0.3
0.4
0.5
0.6
0.7
0.8
Phylostratum
Cellular_organisms - ps1
Archaea1 - ps2
Archaea2 - ps3
Archaea3 - ps4
Asgard/Eukaryota - ps5
Eukaryota - ps6
Amorpheae - ps7
Obazoa - ps8
Opisthokonta - ps9
Holozoa - ps10
Filozoa - ps11
Choano./Metazoa - ps12
Metazoa - ps13
Myriazoa - ps14
Parahoxozoa - ps15
Bilateria - ps16
Deuterostomia - ps17
Chordata - ps18
Olfactores - ps19
Vertebrata - ps20
Gnathostomata - ps21
Euteleostomi - ps22
Sarcopterygii - ps23
Tetrapoda - ps24
Amniota - ps25
Mammalia - ps26
Theria - ps27
Placentalia - ps28
Boreoeutheria - ps29
Euarchontoglires - ps30
Euarchonta - ps31
Primates - ps32
Haplorrhini - ps33
Simiiformes - ps34
Catarrhini - ps35
Hominoidea - ps36
Hominidae - ps37
Homininae - ps38
Hominini - ps39
Homo_sapiens - ps40
p-value
3e-04
9e-04
4e-03
1e-02
5e-02
Log-odds
0.5
1.0
1.5
2.0
2.5
3.0
3.5
4.0

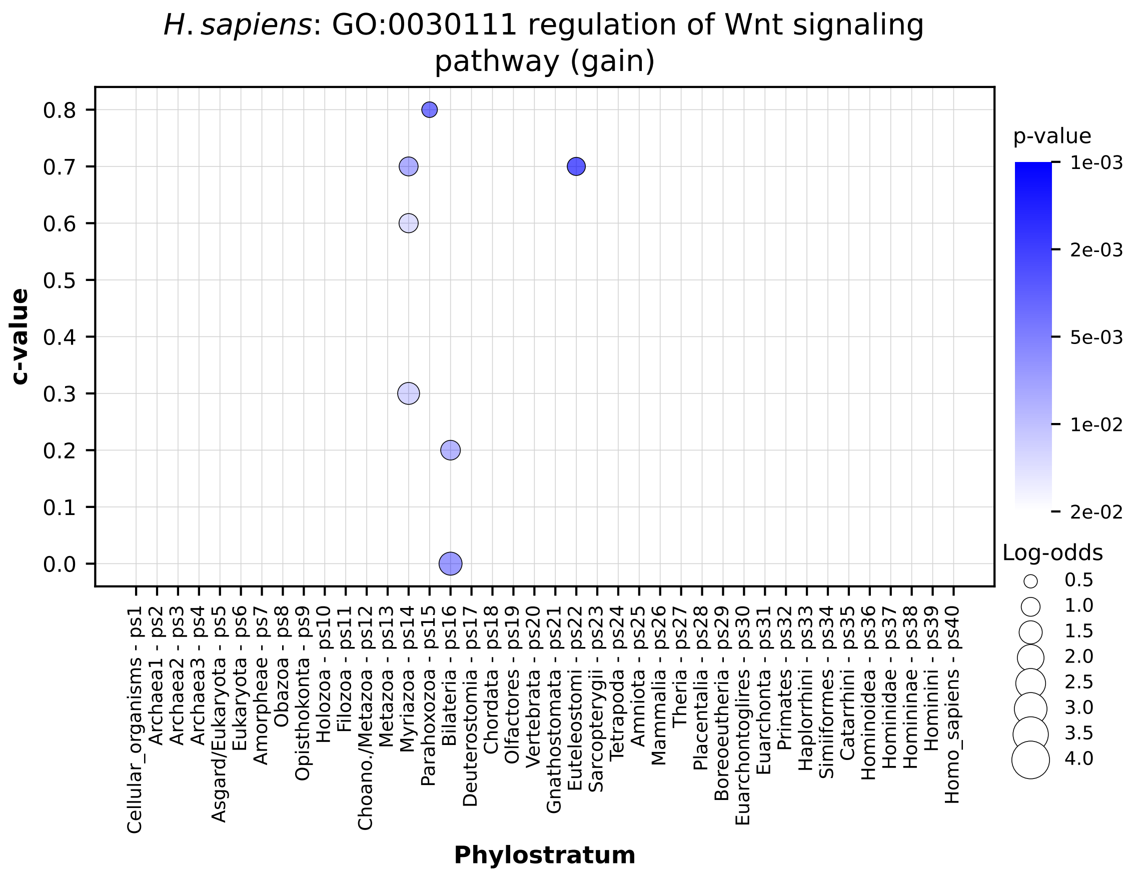

H. sapiens: GO:0030111 regulation of Wnt signaling pathway (gain)
c-value
0.8
0.7
0.6
0.5
0.4
0.3
0.2
0.1
0.0
Phylostratum
Cellular_organisms - ps1
Archaea1 - ps2
Archaea2 - ps3
Archaea3 - ps4
Asgard/Eukaryota - ps5
Eukaryota - ps6
Amorpheae - ps7
Obazoa - ps8
Opisthokonta - ps9
Holozoa - ps10
Filozoa - ps11
Choano./Metazoa - ps12
Metazoa - ps13
Myriazoa - ps14
Parahoxozoa - ps15
Bilateria - ps16
Deuterostomia - ps17
Chordata - ps18
Olfactores - ps19
Vertebrata - ps20
Gnathostomata - ps21
Euteleostomi - ps22
Sarcopterygii - ps23
Tetrapoda - ps24
Amniota - ps25
Mammalia - ps26
Theria - ps27
Placentalia - ps28
Boreoeutheria - ps29
Euarchontoglires - ps30
Euarchonta - ps31
Primates - ps32
Haplorrhini - ps33
Simiiformes - ps34
Catarrhini - ps35
Hominoidea - ps36
Hominidae - ps37
Homininae - ps38
Hominini - ps39
Homo_sapiens - ps40
p-value
1e-03
2e-03
5e-03
1e-02
2e-02
Log-odds
0.5
1.0
1.5
2.0
2.5
3.0
3.5
4.0

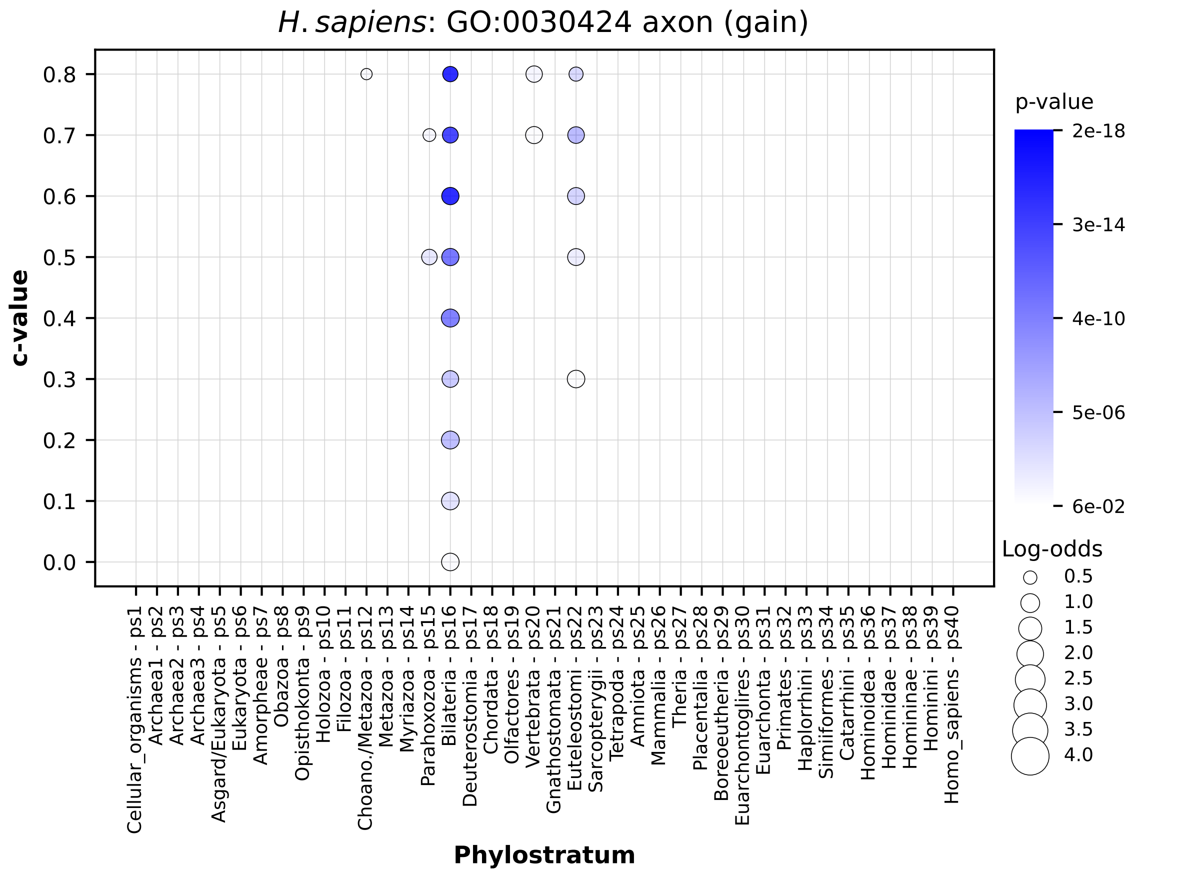

H. sapiens: GO:0030424 axon (gain)
c-value
0.0
0.1
0.2
0.3
0.4
0.5
0.6
0.7
0.8
p-value
2e-18
3e-14
4e-10
5e-06
6e-02
Log-odds
0.5
1.0
1.5
2.0
2.5
3.0
3.5
4.0
Cellular_organisms - ps1
Archaea1 - ps2
Archaea2 - ps3
Archaea3 - ps4
Asgard/Eukaryota - ps5
Eukaryota - ps6
Amorpheae - ps7
Obazoa - ps8
Opisthokonta - ps9
Holozoa - ps10
Filozoa - ps11
Choano./Metazoa - ps12
Metazoa - ps13
Myriazoa - ps14
Parahoxozoa - ps15
Bilateria - ps16
Deuterostomia - ps17
Chordata - ps18
Olfactores - ps19
Vertebrata - ps20
Gnathostomata - ps21
Euteleostomi - ps22
Sarcopterygii - ps23
Tetrapoda - ps24
Amniota - ps25
Mammalia - ps26
Theria - ps27
Placentalia - ps28
Boreoeutheria - ps29
Euarchontoglires - ps30
Euarchonta - ps31
Primates - ps32
Haplorrhini - ps33
Simiiformes - ps34
Catarrhini - ps35
Hominoidea - ps36
Hominidae - ps37
Homininae - ps38
Hominini - ps39
Homo_sapiens - ps40
Phylostratum

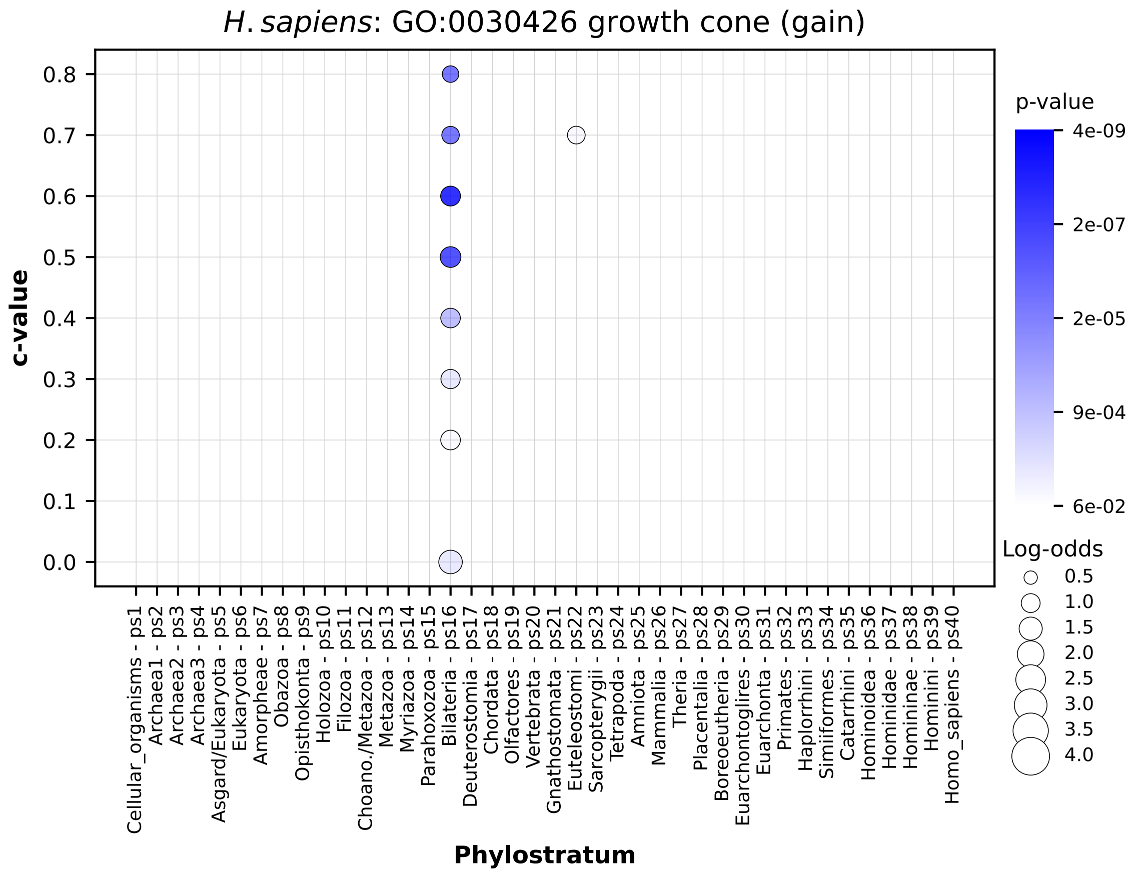
H. sapiens: GO:0030426 growth cone (gain)
c-value
0.8
0.7
0.6
0.5
0.4
0.3
0.2
0.1
0.0
Phylostratum
Cellular_organisms - ps1
Archaea1 - ps2
Archaea2 - ps3
Archaea3 - ps4
Asgard/Eukaryota - ps5
Eukaryota - ps6
Amorpheae - ps7
Obazoa - ps8
Opisthokonta - ps9
Holozoa - ps10
Filozoa - ps11
Choano./Metazoa - ps12
Metazoa - ps13
Myriazoa - ps14
Parahoxozoa - ps15
Bilateria - ps16
Deuterostomia - ps17
Chordata - ps18
Olfactores - ps19
Vertebrata - ps20
Gnathostomata - ps21
Euteleostomi - ps22
Sarcopterygii - ps23
Tetrapoda - ps24
Amniota - ps25
Mammalia - ps26
Theria - ps27
Placentalia - ps28
Boreoeutheria - ps29
Euarchontoglires - ps30
Euarchonta - ps31
Primates - ps32
Haplorrhini - ps33
Simiiformes - ps34
Catarrhini - ps35
Hominoidea - ps36
Hominidae - ps37
Homininae - ps38
Hominini - ps39
Homo_sapiens - ps40
p-value
4e-09
2e-07
2e-05
9e-04
6e-02
Log-odds
0.5
1.0
1.5
2.0
2.5
3.0
3.5
4.0

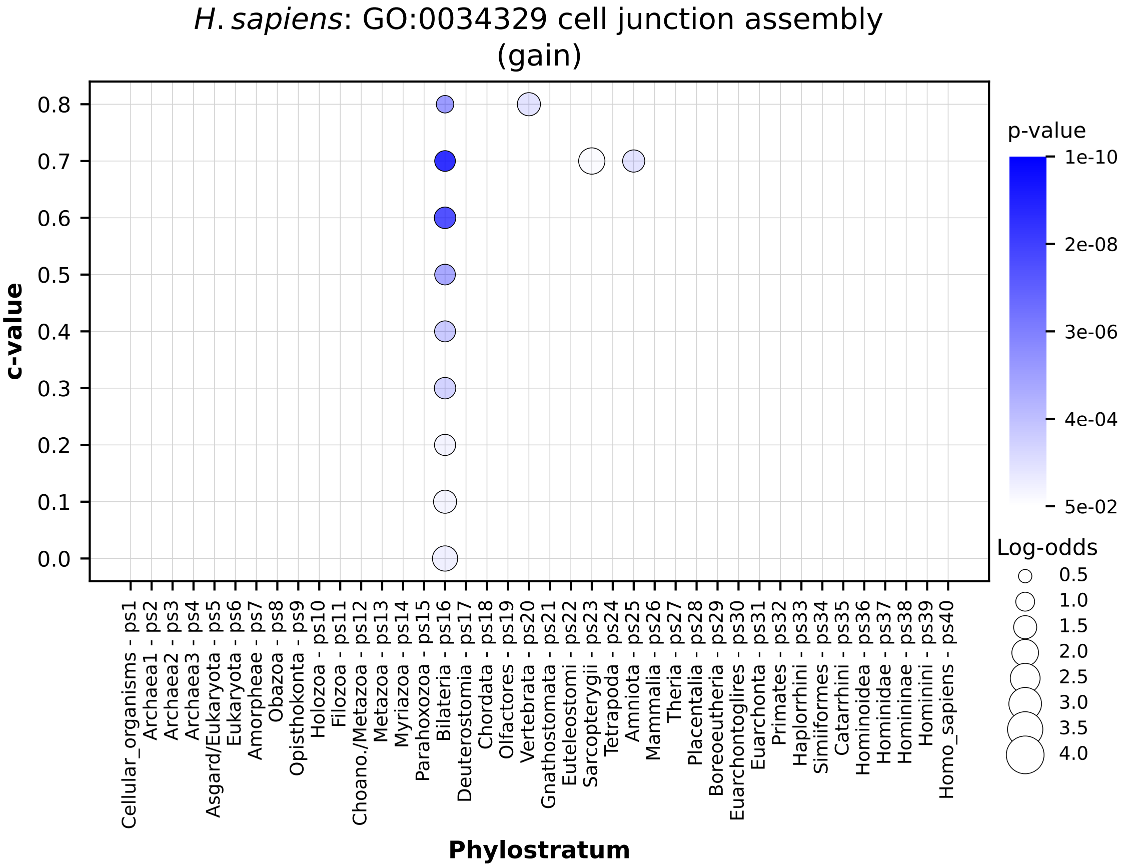

H. sapiens: GO:0034329 cell junction assembly
(gain)
c-value
0.8
0.7
0.6
0.5
0.4
0.3
0.2
0.1
0.0
Phylostratum
Cellular_organisms - ps1
Archaea1 - ps2
Archaea2 - ps3
Archaea3 - ps4
Asgard/Eukaryota - ps5
Eukaryota - ps6
Amorpheae - ps7
Obazoa - ps8
Opisthokonta - ps9
Holozoa - ps10
Filozoa - ps11
Choano./Metazoa - ps12
Metazoa - ps13
Myriazoa - ps14
Parahoxozoa - ps15
Bilateria - ps16
Deuterostomia - ps17
Chordata - ps18
Olfactores - ps19
Vertebrata - ps20
Gnathostomata - ps21
Euteleostomi - ps22
Sarcopterygii - ps23
Tetrapoda - ps24
Amniota - ps25
Mammalia - ps26
Theria - ps27
Placentalia - ps28
Boreoeutheria - ps29
Euarchontoglires - ps30
Euarchonta - ps31
Primates - ps32
Haplorrhini - ps33
Simiiformes - ps34
Catarrhini - ps35
Hominoidea - ps36
Hominidae - ps37
Homininae - ps38
Hominini - ps39
Homo_sapiens - ps40
p-value
1e-10
2e-08
3e-06
4e-04
5e-02
Log-odds
0.5
1.0
1.5
2.0
2.5
3.0
3.5
4.0

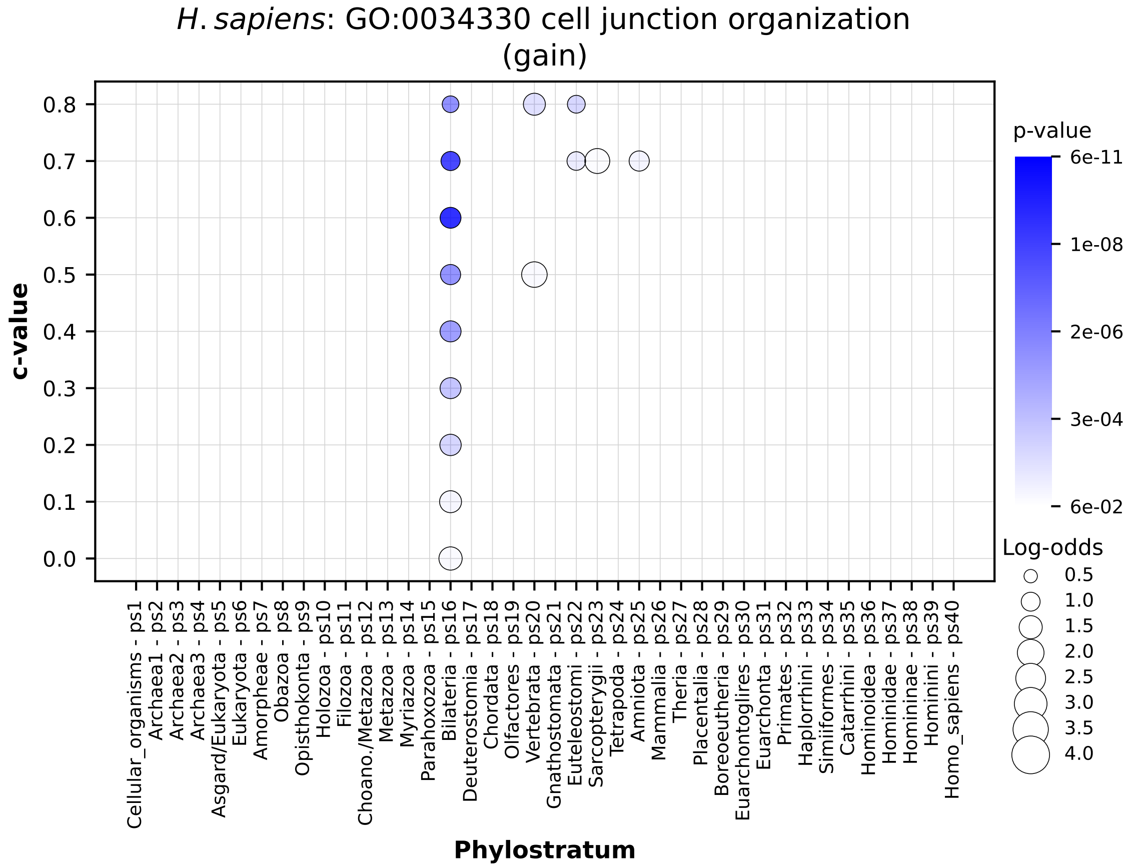

*H. sapiens*: GO:0034330 cell junction organization (gain)
c-value
0.8
0.7
0.6
0.5
0.4
0.3
0.2
0.1
0.0
p-value
6e-11
1e-08
2e-06
3e-04
6e-02
Log-odds
0.5
1.0
1.5
2.0
2.5
3.0
3.5
4.0
Cellular_organisms - ps1
Archaea1 - ps2
Archaea2 - ps3
Archaea3 - ps4
Asgard/Eukaryota - ps5
Eukaryota - ps6
Amorpheae - ps7
Obazoa - ps8
Opisthokonta - ps9
Holozoa - ps10
Filozoa - ps11
Choano./Metazoa - ps12
Metazoa - ps13
Myriazoa - ps14
Parahoxozoa - ps15
Bilateria - ps16
Deuterostomia - ps17
Chordata - ps18
Olfactores - ps19
Vertebrata - ps20
Gnathostomata - ps21
Euteleostomi - ps22
Sarcopterygii - ps23
Tetrapoda - ps24
Amniota - ps25
Mammalia - ps26
Theria - ps27
Placentalia - ps28
Boreoeutheria - ps29
Euarchontoglires - ps30
Euarchonta - ps31
Primates - ps32
Haplorrhini - ps33
Simiiformes - ps34
Catarrhini - ps35
Hominoidea - ps36
Hominidae - ps37
Homininae - ps38
Hominini - ps39
Homo_sapiens - ps40
Phylostratum

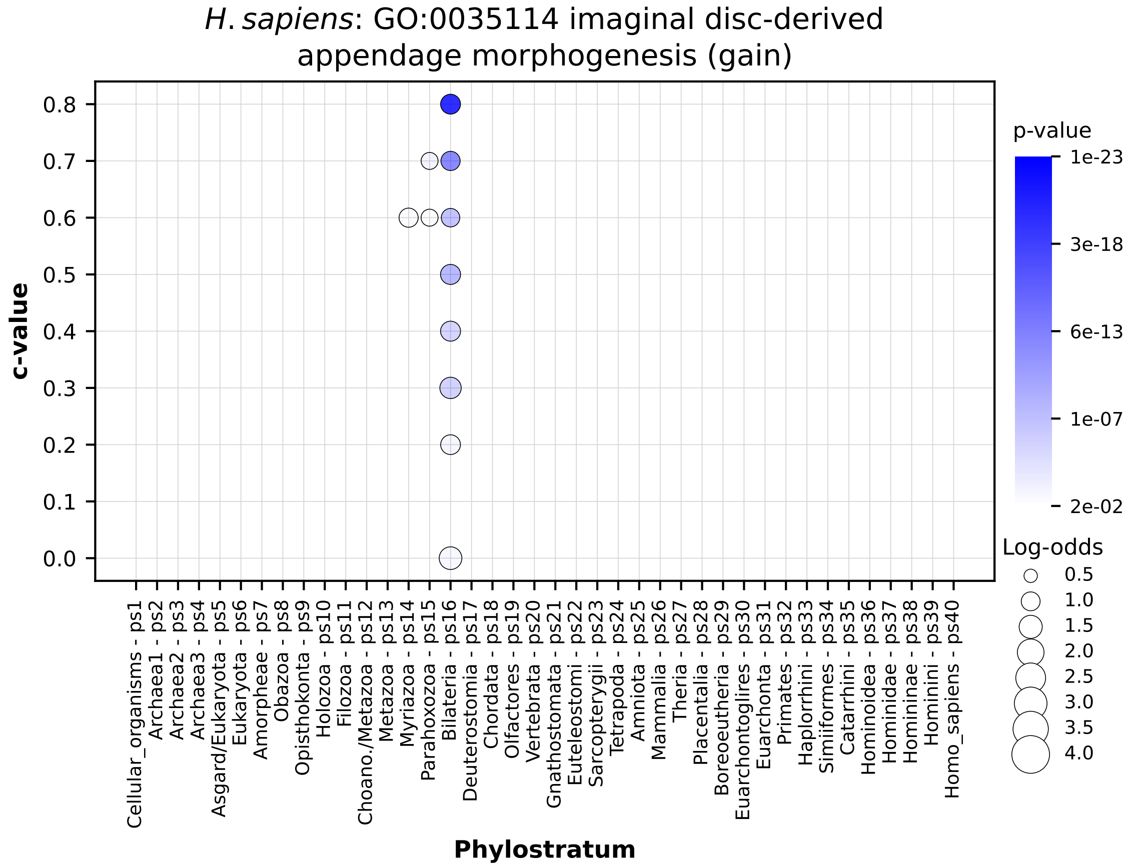

*H. sapiens*: GO:0035114 imaginal disc-derived appendage morphogenesis (gain)
c-value
0.8
0.7
0.6
0.5
0.4
0.3
0.2
0.1
0.0
Phylostratum
Cellular_organisms - ps1
Archaea1 - ps2
Archaea2 - ps3
Archaea3 - ps4
Asgard/Eukaryota - ps5
Eukaryota - ps6
Amorpheae - ps7
Obazoa - ps8
Opisthokonta - ps9
Holozoa - ps10
Filozoa - ps11
Choano./Metazoa - ps12
Metazoa - ps13
Myriazoa - ps14
Parahoxozoa - ps15
Bilateria - ps16
Deuterostomia - ps17
Chordata - ps18
Olfactores - ps19
Vertebrata - ps20
Gnathostomata - ps21
Euteleostomi - ps22
Sarcopterygii - ps23
Tetrapoda - ps24
Amniota - ps25
Mammalia - ps26
Theria - ps27
Placentalia - ps28
Boreoeutheria - ps29
Euarchontoglires - ps30
Euarchonta - ps31
Primates - ps32
Haplorrhini - ps33
Simiiformes - ps34
Catarrhini - ps35
Hominoidea - ps36
Hominidae - ps37
Homininae - ps38
Hominini - ps39
Homo_sapiens - ps40
p-value
1e-23
3e-18
6e-13
1e-07
2e-02
Log-odds
0.5
1.0
1.5
2.0
2.5
3.0
3.5
4.0

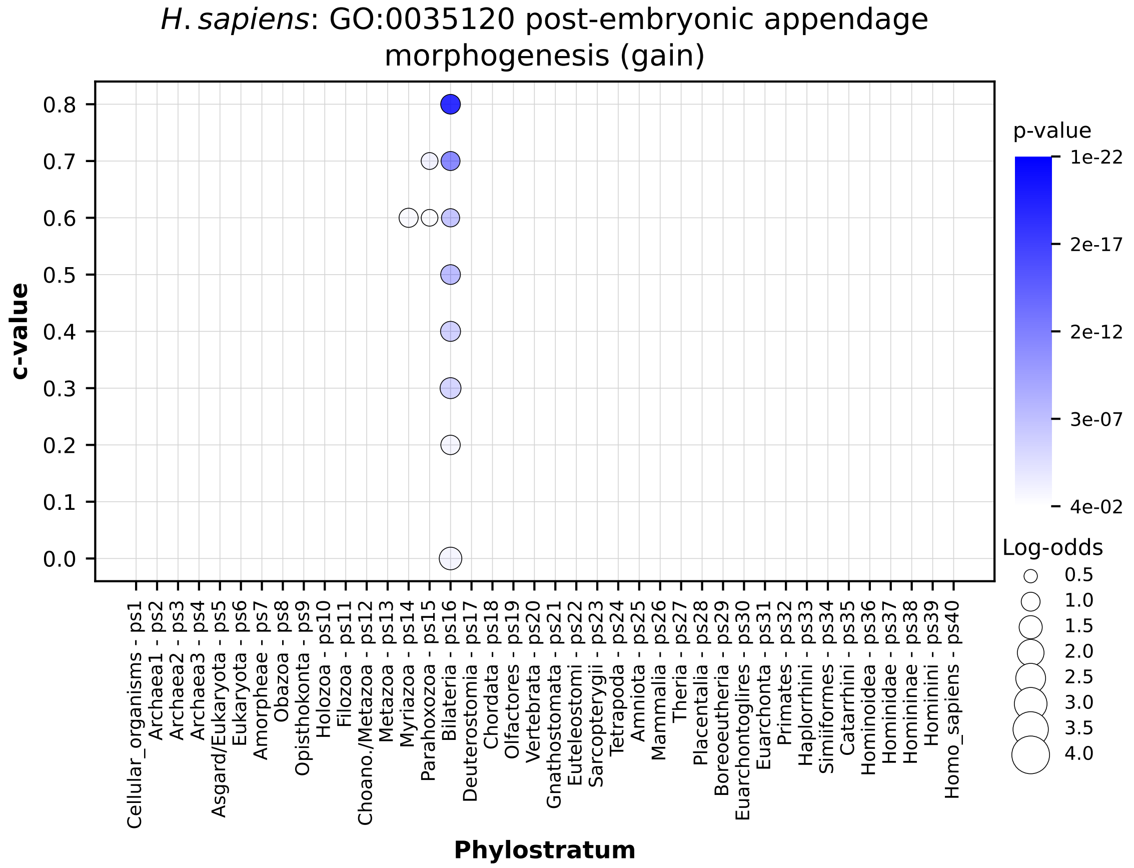

H. sapiens: GO:0035120 post-embryonic appendage morphogenesis (gain)
c-value
0.8
0.7
0.6
0.5
0.4
0.3
0.2
0.1
0.0
Phylostratum
Cellular_organisms - ps1
Archaea1 - ps2
Archaea2 - ps3
Archaea3 - ps4
Asgard/Eukaryota - ps5
Eukaryota - ps6
Amorpheae - ps7
Obazoa - ps8
Opisthokonta - ps9
Holozoa - ps10
Filozoa - ps11
Choano./Metazoa - ps12
Metazoa - ps13
Myriazoa - ps14
Parahoxozoa - ps15
Bilateria - ps16
Deuterostomia - ps17
Chordata - ps18
Olfactores - ps19
Vertebrata - ps20
Gnathostomata - ps21
Euteleostomi - ps22
Sarcopterygii - ps23
Tetrapoda - ps24
Amniota - ps25
Mammalia - ps26
Theria - ps27
Placentalia - ps28
Boreoeutheria - ps29
Euarchontoglires - ps30
Euarchonta - ps31
Primates - ps32
Haplorrhini - ps33
Simiiformes - ps34
Catarrhini - ps35
Hominoidea - ps36
Hominidae - ps37
Homininae - ps38
Hominini - ps39
Homo_sapiens - ps40
p-value
1e-22
2e-17
2e-12
3e-07
4e-02
Log-odds
0.5
1.0
1.5
2.0
2.5
3.0
3.5
4.0

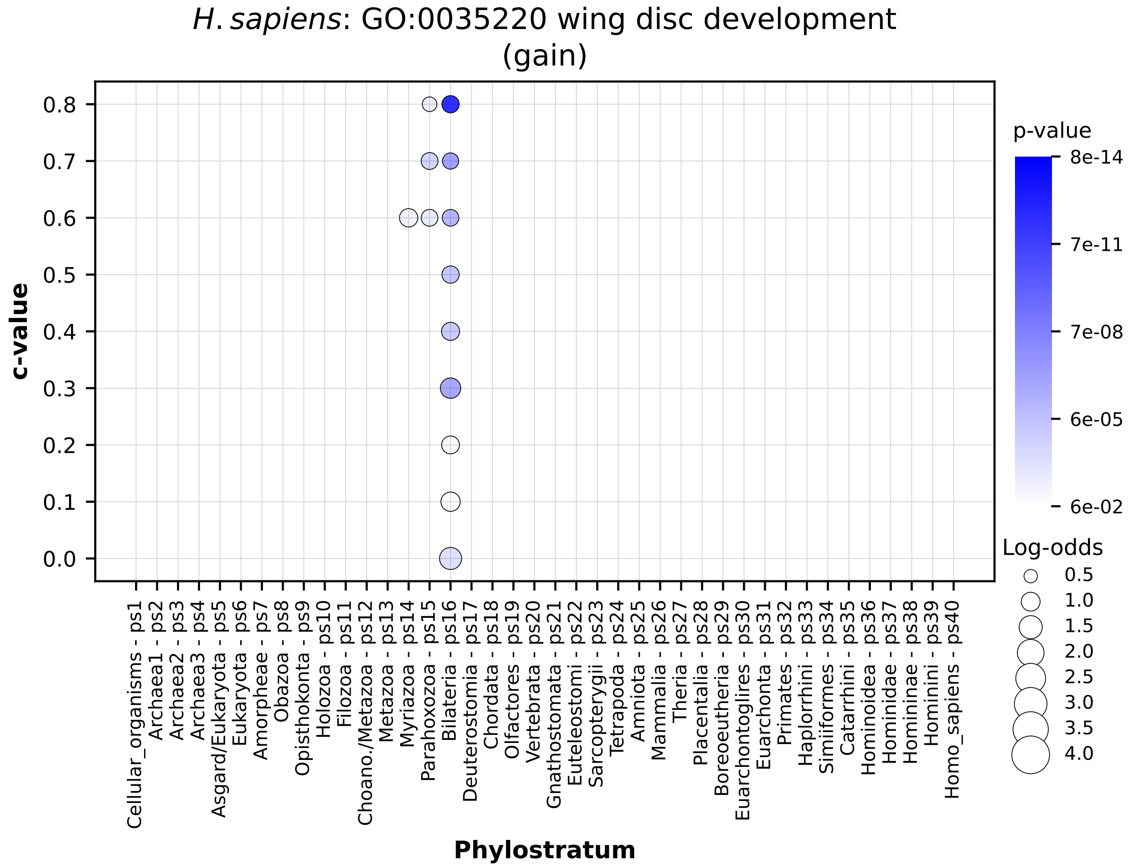

*H. sapiens*: GO:0035220 wing disc development
(gain)
c-value
0.8
0.7
0.6
0.5
0.4
0.3
0.2
0.1
0.0
Phylostratum
Cellular_organisms - ps1
Archaea1 - ps2
Archaea2 - ps3
Archaea3 - ps4
Asgard/Eukaryota - ps5
Eukaryota - ps6
Amorpheae - ps7
Obazoa - ps8
Opisthokonta - ps9
Holozoa - ps10
Filozoa - ps11
Choano./Metazoa - ps12
Metazoa - ps13
Myriazoa - ps14
Parahoxozoa - ps15
Bilateria - ps16
Deuterostomia - ps17
Chordata - ps18
Olfactores - ps19
Vertebrata - ps20
Gnathostomata - ps21
Euteleostomi - ps22
Sarcopterygii - ps23
Tetrapoda - ps24
Amniota - ps25
Mammalia - ps26
Theria - ps27
Placentalia - ps28
Boreoeutheria - ps29
Euarchontoglires - ps30
Euarchonta - ps31
Primates - ps32
Haplorrhini - ps33
Simiiformes - ps34
Catarrhini - ps35
Hominoidea - ps36
Hominidae - ps37
Homininae - ps38
Hominini - ps39
Homo_sapiens - ps40
p-value
8e-14
7e-11
7e-08
6e-05
6e-02
Log-odds
0.5
1.0
1.5
2.0
2.5
3.0
3.5
4.0

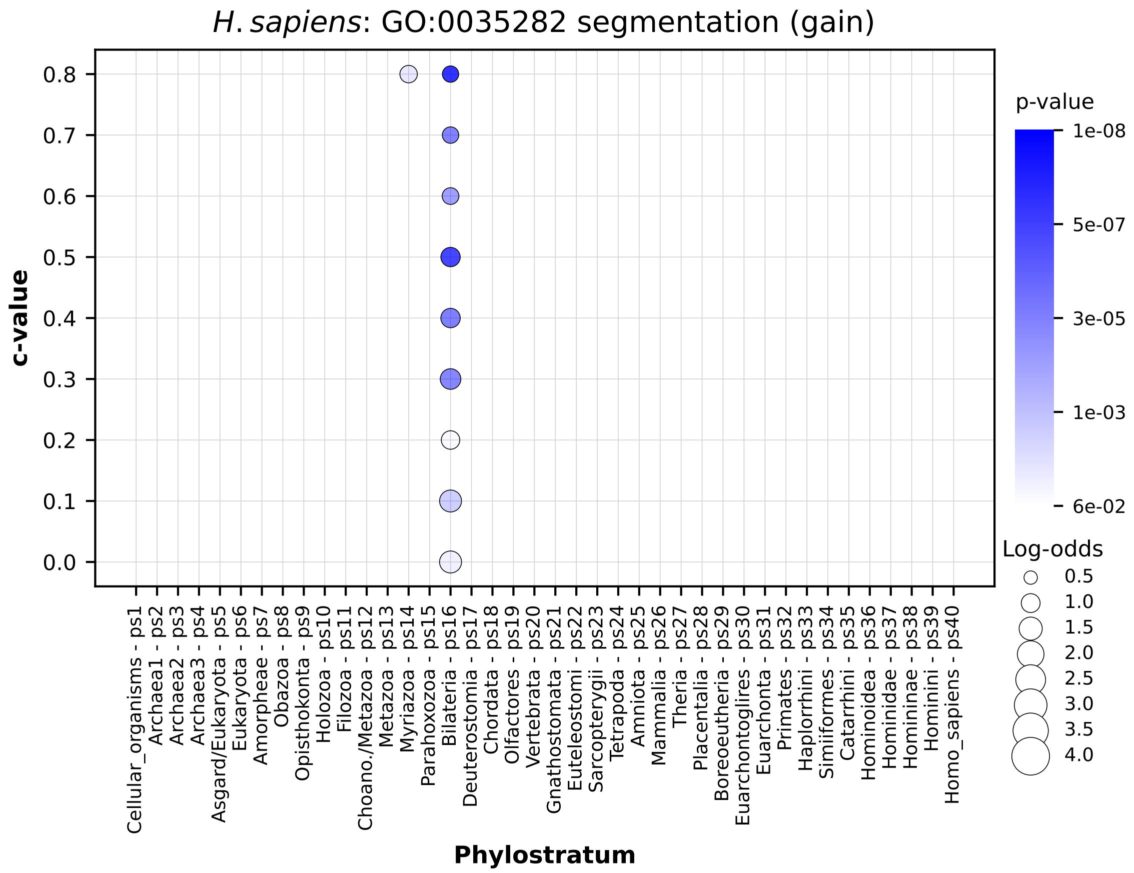

H. sapiens: GO:0035282 segmentation (gain)
c-value
0.8
0.7
0.6
0.5
0.4
0.3
0.2
0.1
0.0
p-value
1e-08
5e-07
3e-05
1e-03
6e-02
Log-odds
0.5
1.0
1.5
2.0
2.5
3.0
3.5
4.0
Cellular_organisms - ps1
Archaea1 - ps2
Archaea2 - ps3
Archaea3 - ps4
Asgard/Eukaryota - ps5
Eukaryota - ps6
Amorpheae - ps7
Obazoa - ps8
Opisthokonta - ps9
Holozoa - ps10
Filozoa - ps11
Choano./Metazoa - ps12
Metazoa - ps13
Myriazoa - ps14
Parahoxozoa - ps15
Bilateria - ps16
Deuterostomia - ps17
Chordata - ps18
Olfactores - ps19
Vertebrata - ps20
Gnathostomata - ps21
Euteleostomi - ps22
Sarcopterygii - ps23
Tetrapoda - ps24
Amniota - ps25
Mammalia - ps26
Theria - ps27
Placentalia - ps28
Boreoeutheria - ps29
Euarchontoglires - ps30
Euarchonta - ps31
Primates - ps32
Haplorrhini - ps33
Simiiformes - ps34
Catarrhini - ps35
Hominoidea - ps36
Hominidae - ps37
Homininae - ps38
Hominini - ps39
Homo_sapiens - ps40
Phylostratum

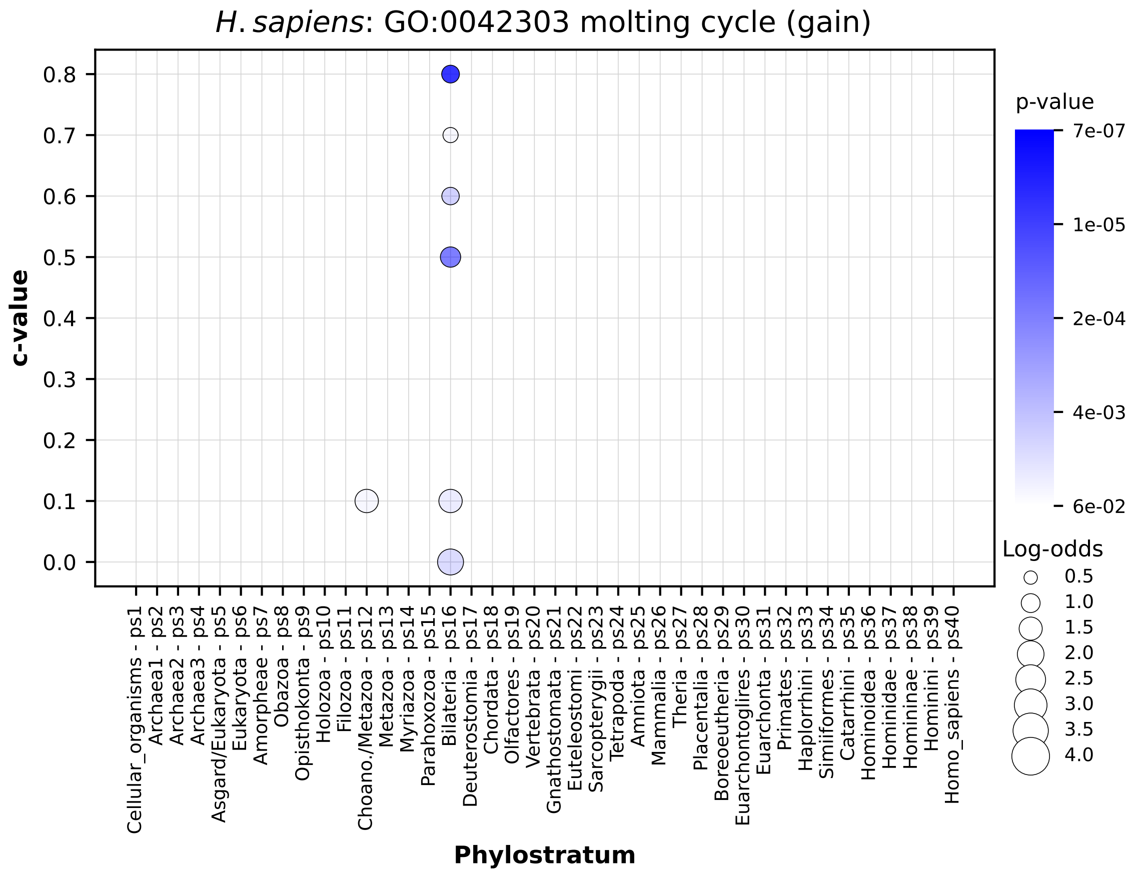
H. sapiens: GO:0042303 molting cycle (gain)
c-value
0.8
0.7
0.6
0.5
0.4
0.3
0.2
0.1
0.0
p-value
7e-07
1e-05
2e-04
4e-03
6e-02
Log-odds
0.5
1.0
1.5
2.0
2.5
3.0
3.5
4.0
Cellular_organisms - ps1
Archaea1 - ps2
Archaea2 - ps3
Archaea3 - ps4
Asgard/Eukaryota - ps5
Eukaryota - ps6
Amorpheae - ps7
Obazoa - ps8
Opisthokonta - ps9
Holozoa - ps10
Filozoa - ps11
Choano./Metazoa - ps12
Metazoa - ps13
Myriazoa - ps14
Parahoxozoa - ps15
Bilateria - ps16
Deuterostomia - ps17
Chordata - ps18
Olfactores - ps19
Vertebrata - ps20
Gnathostomata - ps21
Euteleostomi - ps22
Sarcopterygii - ps23
Tetrapoda - ps24
Amniota - ps25
Mammalia - ps26
Theria - ps27
Placentalia - ps28
Boreoeutheria - ps29
Euarchontoglires - ps30
Euarchonta - ps31
Primates - ps32
Haplorrhini - ps33
Simiiformes - ps34
Catarrhini - ps35
Hominoidea - ps36
Hominidae - ps37
Homininae - ps38
Hominini - ps39
Homo_sapiens - ps40
Phylostratum

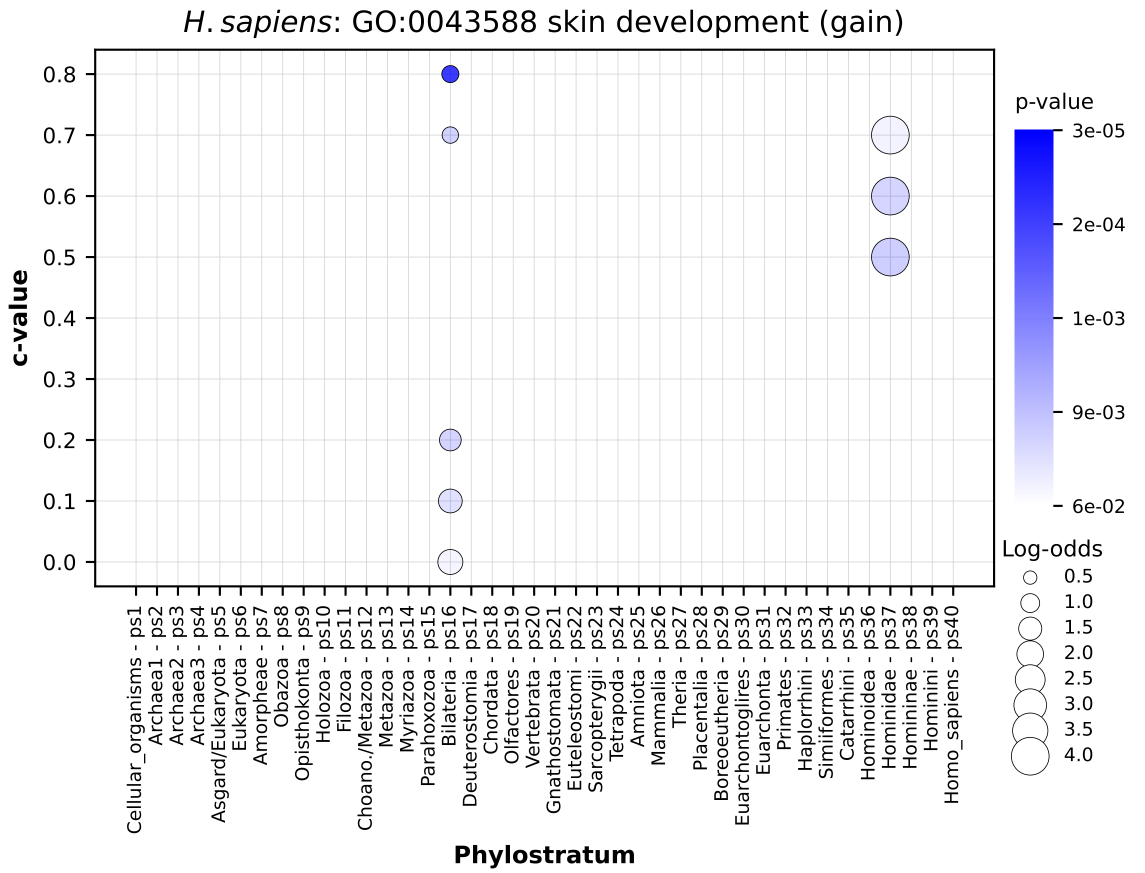

H. sapiens: GO:0043588 skin development (gain)
c-value
0.0
0.1
0.2
0.3
0.4
0.5
0.6
0.7
0.8
Phylostratum
Cellular_organisms - ps1
Archaea1 - ps2
Archaea2 - ps3
Archaea3 - ps4
Asgard/Eukaryota - ps5
Eukaryota - ps6
Amorpheae - ps7
Obazoa - ps8
Opisthokonta - ps9
Holozoa - ps10
Filozoa - ps11
Choano./Metazoa - ps12
Metazoa - ps13
Myriazoa - ps14
Parahoxozoa - ps15
Bilateria - ps16
Deuterostomia - ps17
Chordata - ps18
Olfactores - ps19
Vertebrata - ps20
Gnathostomata - ps21
Euteleostomi - ps22
Sarcopterygii - ps23
Tetrapoda - ps24
Amniota - ps25
Mammalia - ps26
Theria - ps27
Placentalia - ps28
Boreoeutheria - ps29
Euarchontoglires - ps30
Euarchonta - ps31
Primates - ps32
Haplorrhini - ps33
Simiiformes - ps34
Catarrhini - ps35
Hominoidea - ps36
Hominidae - ps37
Homininae - ps38
Hominini - ps39
Homo_sapiens - ps40
p-value
3e-05
2e-04
1e-03
9e-03
6e-02
Log-odds
0.5
1.0
1.5
2.0
2.5
3.0
3.5
4.0

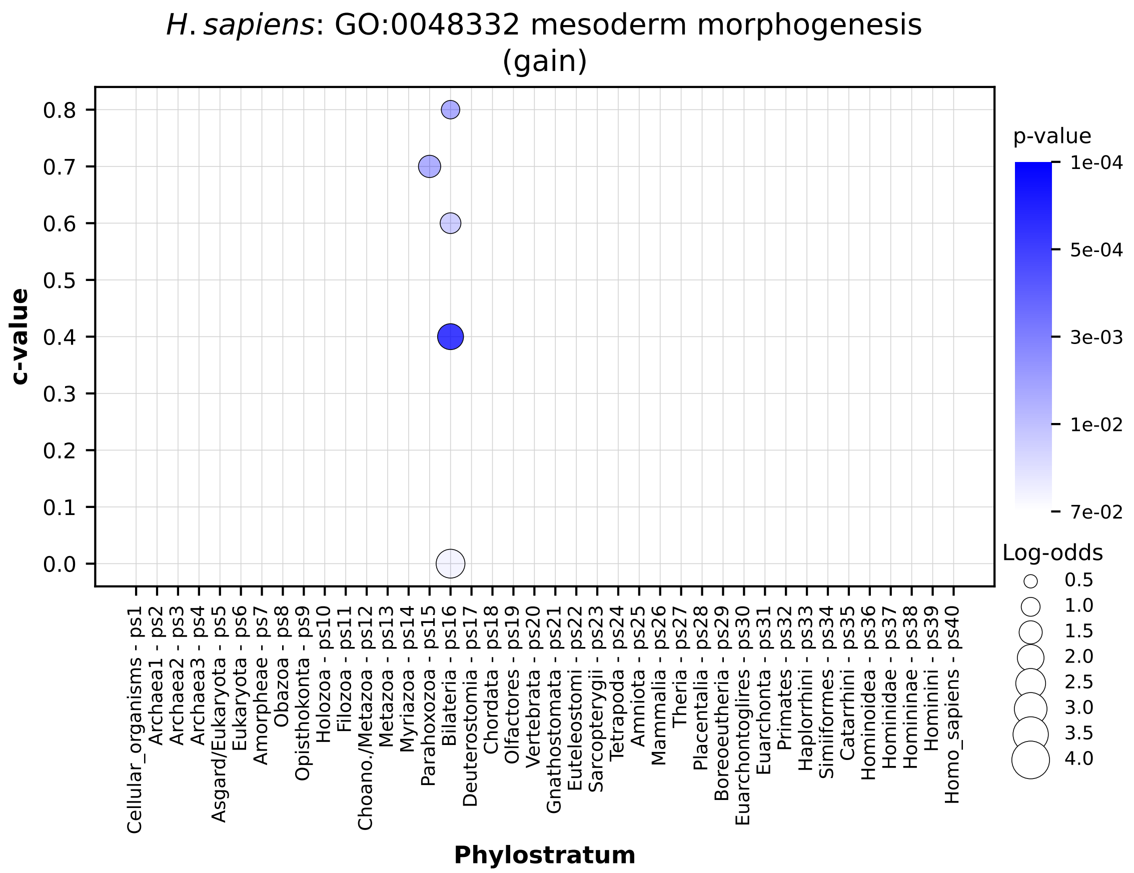

H. sapiens: GO:0048332 mesoderm morphogenesis
(gain)
c-value
0.8
0.7
0.6
0.5
0.4
0.3
0.2
0.1
0.0
p-value
1e-04
5e-04
3e-03
1e-02
7e-02
Log-odds
0.5
1.0
1.5
2.0
2.5
3.0
3.5
4.0
Cellular_organisms - ps1
Archaea1 - ps2
Archaea2 - ps3
Archaea3 - ps4
Asgard/Eukaryota - ps5
Eukaryota - ps6
Amorpheae - ps7
Obazoa - ps8
Opisthokonta - ps9
Holozoa - ps10
Filozoa - ps11
Choano./Metazoa - ps12
Metazoa - ps13
Myriazoa - ps14
Parahoxozoa - ps15
Bilateria - ps16
Deuterostomia - ps17
Chordata - ps18
Olfactores - ps19
Vertebrata - ps20
Gnathostomata - ps21
Euteleostomi - ps22
Sarcopterygii - ps23
Tetrapoda - ps24
Amniota - ps25
Mammalia - ps26
Theria - ps27
Placentalia - ps28
Boreoeutheria - ps29
Euarchontoglires - ps30
Euarchonta - ps31
Primates - ps32
Haplorrhini - ps33
Simiiformes - ps34
Catarrhini - ps35
Hominoidea - ps36
Hominidae - ps37
Homininae - ps38
Hominini - ps39
Homo_sapiens - ps40
Phylostratum

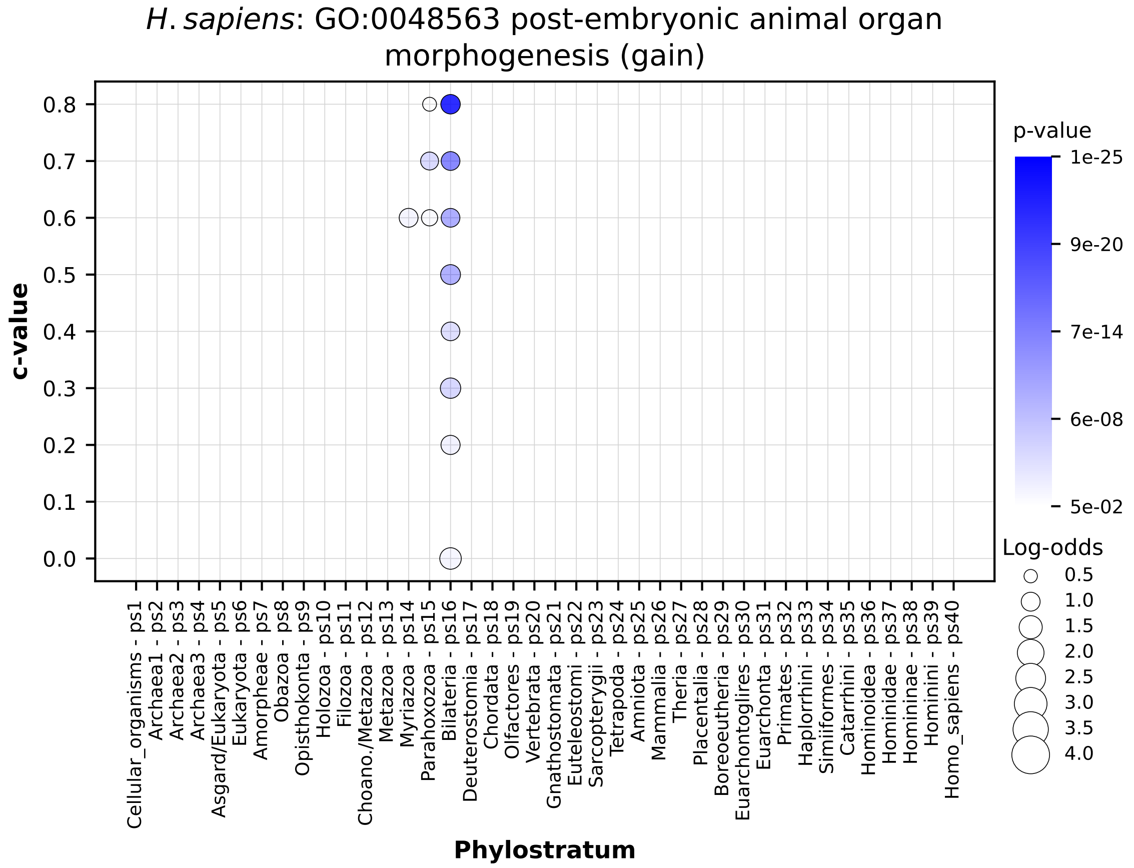

H. sapiens: GO:0048563 post-embryonic animal organ morphogenesis (gain)
c-value
0.8
0.7
0.6
0.5
0.4
0.3
0.2
0.1
0.0
Phylostratum
Cellular_organisms - ps1
Archaea1 - ps2
Archaea2 - ps3
Archaea3 - ps4
Asgard/Eukaryota - ps5
Eukaryota - ps6
Amorpheae - ps7
Obazoa - ps8
Opisthokonta - ps9
Holozoa - ps10
Filozoa - ps11
Choano./Metazoa - ps12
Metazoa - ps13
Myriazoa - ps14
Parahoxozoa - ps15
Bilateria - ps16
Deuterostomia - ps17
Chordata - ps18
Olfactores - ps19
Vertebrata - ps20
Gnathostomata - ps21
Euteleostomi - ps22
Sarcopterygii - ps23
Tetrapoda - ps24
Amniota - ps25
Mammalia - ps26
Theria - ps27
Placentalia - ps28
Boreoeutheria - ps29
Euarchontoglires - ps30
Euarchonta - ps31
Primates - ps32
Haplorrhini - ps33
Simiiformes - ps34
Catarrhini - ps35
Hominoidea - ps36
Hominidae - ps37
Homininae - ps38
Hominini - ps39
Homo_sapiens - ps40
p-value
1e-25
9e-20
7e-14
6e-08
5e-02
Log-odds
0.5
1.0
1.5
2.0
2.5
3.0
3.5
4.0

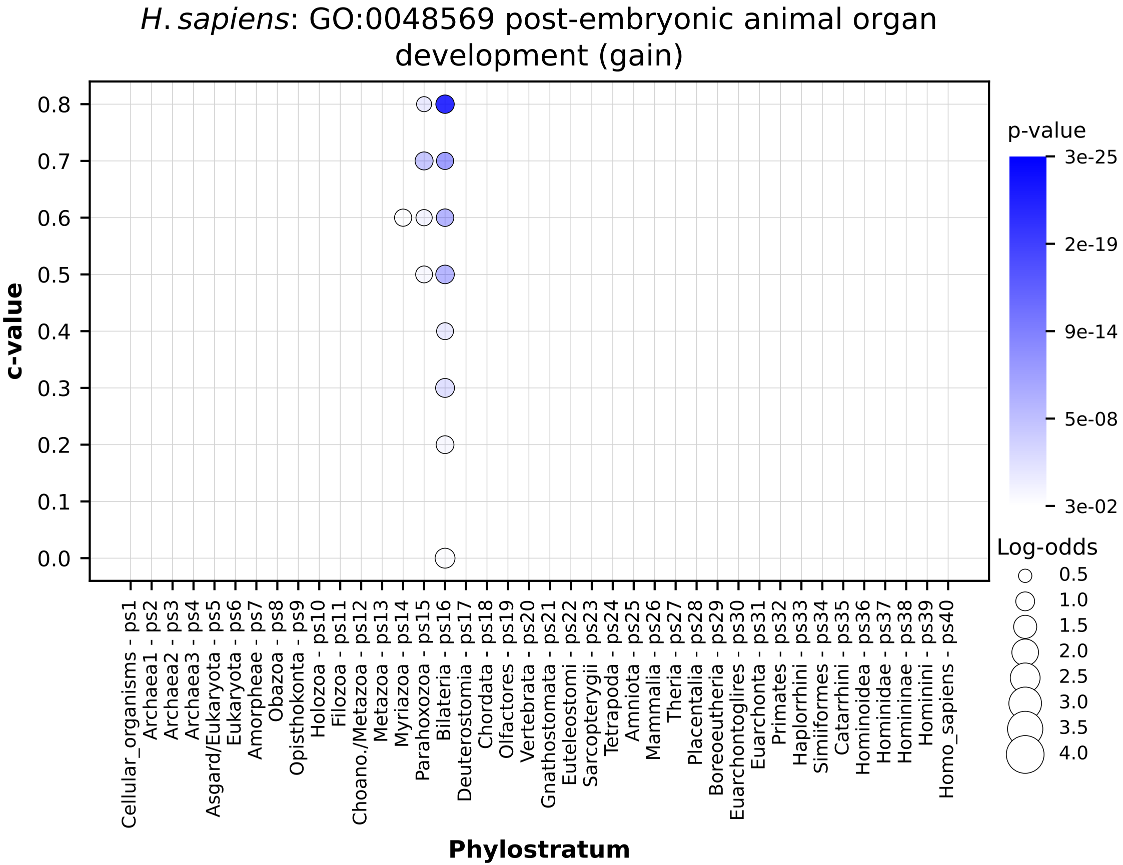

*H. sapiens*: GO:0048569 post-embryonic animal organ development (gain)
c-value
0.8
0.7
0.6
0.5
0.4
0.3
0.2
0.1
0.0
Phylostratum
Cellular_organisms - ps1
Archaea1 - ps2
Archaea2 - ps3
Archaea3 - ps4
Asgard/Eukaryota - ps5
Eukaryota - ps6
Amorpheae - ps7
Obazoa - ps8
Opisthokonta - ps9
Holozoa - ps10
Filozoa - ps11
Choano./Metazoa - ps12
Metazoa - ps13
Myriazoa - ps14
Parahoxozoa - ps15
Bilateria - ps16
Deuterostomia - ps17
Chordata - ps18
Olfactores - ps19
Vertebrata - ps20
Gnathostomata - ps21
Euteleostomi - ps22
Sarcopterygii - ps23
Tetrapoda - ps24
Amniota - ps25
Mammalia - ps26
Theria - ps27
Placentalia - ps28
Boreoeutheria - ps29
Euarchontoglires - ps30
Euarchonta - ps31
Primates - ps32
Haplorrhini - ps33
Simiiformes - ps34
Catarrhini - ps35
Hominoidea - ps36
Hominidae - ps37
Homininae - ps38
Hominini - ps39
Homo_sapiens - ps40
p-value
3e-25
2e-19
9e-14
5e-08
3e-02
Log-odds
0.5
1.0
1.5
2.0
2.5
3.0
3.5
4.0

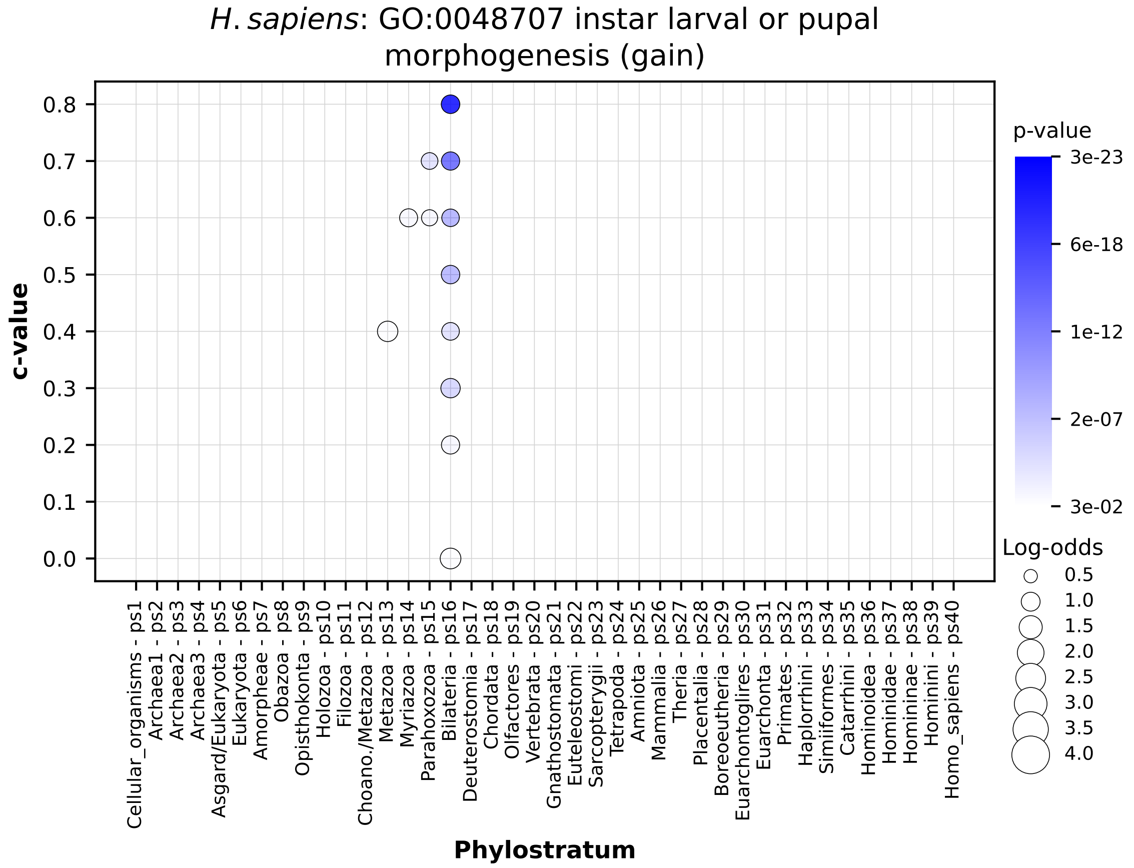
H. sapiens: GO:0048707 instar larval or pupal morphogenesis (gain)
c-value
0.8
0.7
0.6
0.5
0.4
0.3
0.2
0.1
0.0
p-value
3e-23
6e-18
1e-12
2e-07
3e-02
Log-odds
0.5
1.0
1.5
2.0
2.5
3.0
3.5
4.0
Cellular_organisms - ps1
Archaea1 - ps2
Archaea2 - ps3
Archaea3 - ps4
Asgard/Eukaryota - ps5
Eukaryota - ps6
Amorpheae - ps7
Obazoa - ps8
Opisthokonta - ps9
Holozoa - ps10
Filozoa - ps11
Choano./Metazoa - ps12
Metazoa - ps13
Myriazoa - ps14
Parahoxozoa - ps15
Bilateria - ps16
Deuterostomia - ps17
Chordata - ps18
Olfactores - ps19
Vertebrata - ps20
Gnathostomata - ps21
Euteleostomi - ps22
Sarcopterygii - ps23
Tetrapoda - ps24
Amniota - ps25
Mammalia - ps26
Theria - ps27
Placentalia - ps28
Boreoeutheria - ps29
Euarchontoglires - ps30
Euarchonta - ps31
Primates - ps32
Haplorrhini - ps33
Simiiformes - ps34
Catarrhini - ps35
Hominoidea - ps36
Hominidae - ps37
Homininae - ps38
Hominini - ps39
Homo_sapiens - ps40
Phylostratum

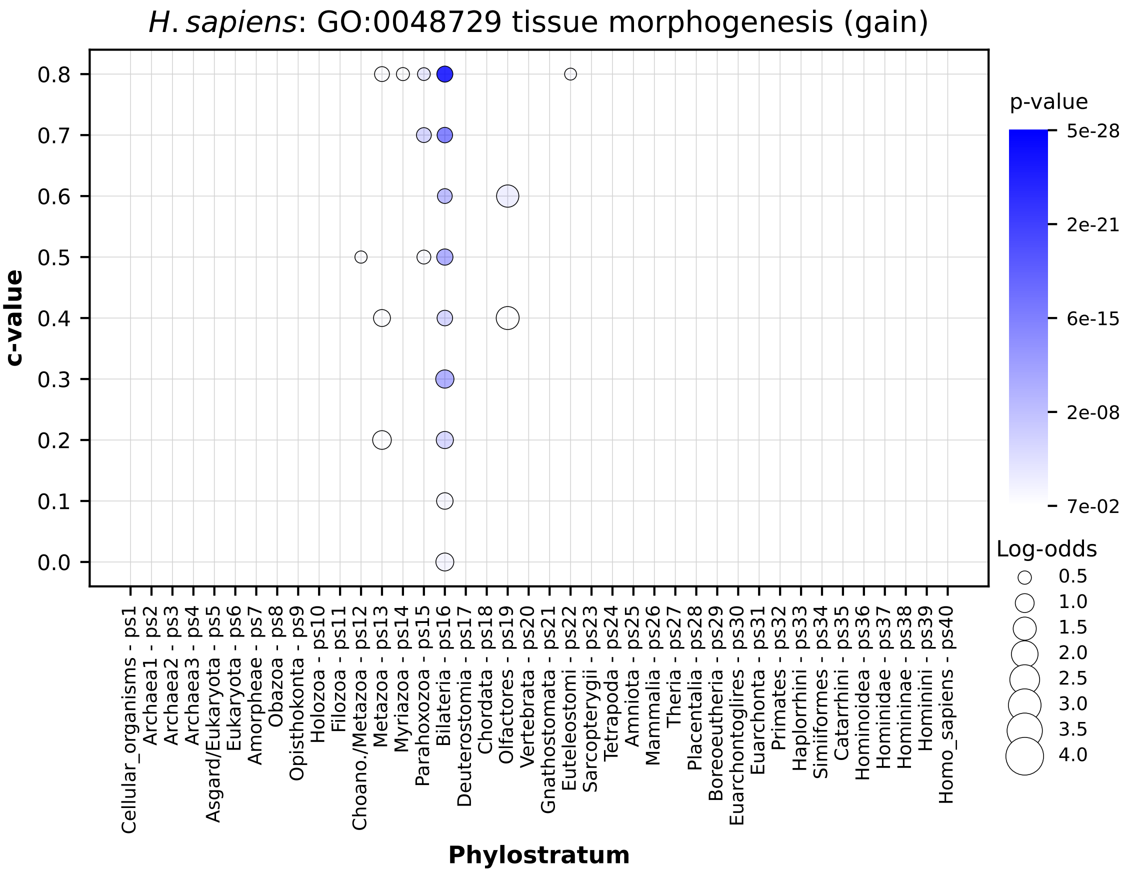
H. sapiens: GO:0048729 tissue morphogenesis (gain)
c-value
0.8
0.7
0.6
0.5
0.4
0.3
0.2
0.1
0.0
p-value
5e-28
2e-21
6e-15
2e-08
7e-02
Log-odds
0.5
1.0
1.5
2.0
2.5
3.0
3.5
4.0
Cellular_organisms - ps1
Archaea1 - ps2
Archaea2 - ps3
Archaea3 - ps4
Asgard/Eukaryota - ps5
Eukaryota - ps6
Amorpheae - ps7
Obazoa - ps8
Opisthokonta - ps9
Holozoa - ps10
Filozoa - ps11
Choano./Metazoa - ps12
Metazoa - ps13
Myriazoa - ps14
Parahoxozoa - ps15
Bilateria - ps16
Deuterostomia - ps17
Chordata - ps18
Olfactores - ps19
Vertebrata - ps20
Gnathostomata - ps21
Euteleostomi - ps22
Sarcopterygii - ps23
Tetrapoda - ps24
Amniota - ps25
Mammalia - ps26
Theria - ps27
Placentalia - ps28
Boreoeutheria - ps29
Euarchontoglires - ps30
Euarchonta - ps31
Primates - ps32
Haplorrhini - ps33
Simiiformes - ps34
Catarrhini - ps35
Hominoidea - ps36
Hominidae - ps37
Homininae - ps38
Hominini - ps39
Homo_sapiens - ps40
Phylostratum

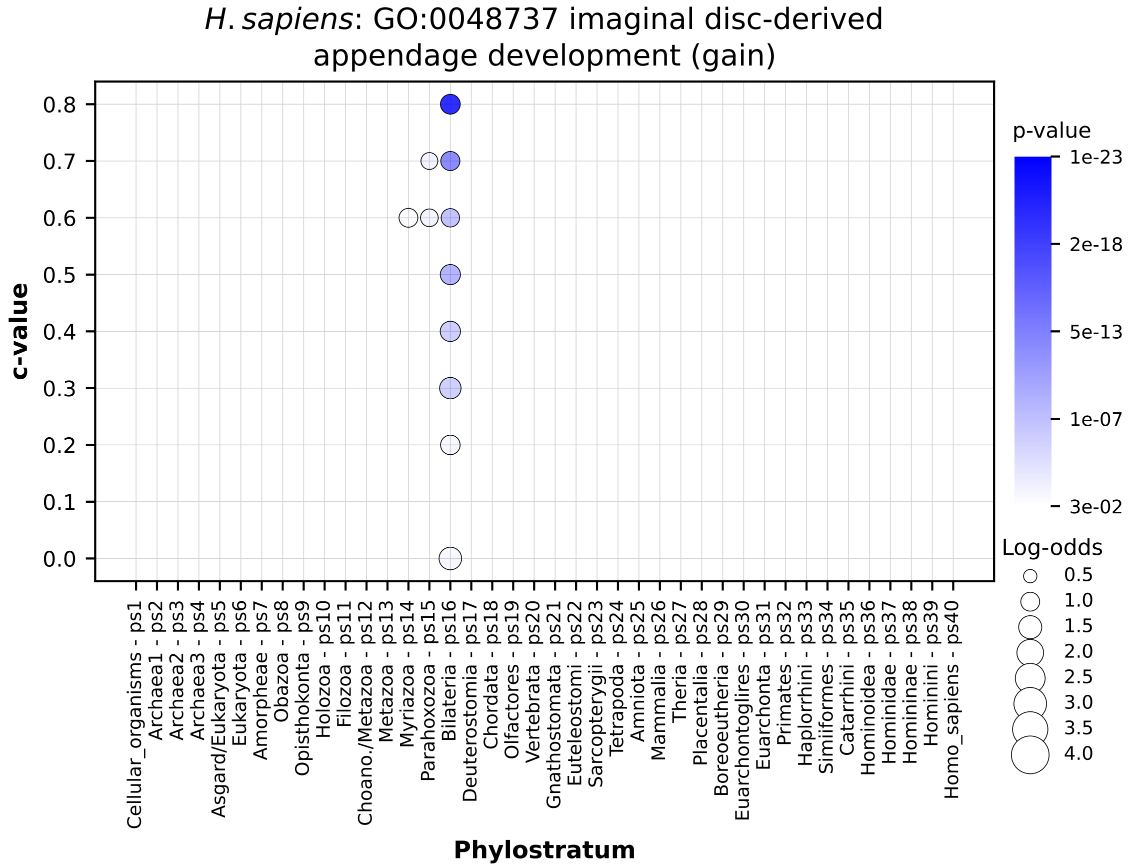

H. sapiens: GO:0048737 imaginal disc-derived appendage development (gain)
c-value
0.8
0.7
0.6
0.5
0.4
0.3
0.2
0.1
0.0
p-value
1e-23
2e-18
5e-13
1e-07
3e-02
Log-odds
0.5
1.0
1.5
2.0
2.5
3.0
3.5
4.0
Cellular_organisms - ps1
Archaea1 - ps2
Archaea2 - ps3
Archaea3 - ps4
Asgard/Eukaryota - ps5
Eukaryota - ps6
Amorpheae - ps7
Obazoa - ps8
Opisthokonta - ps9
Holozoa - ps10
Filozoa - ps11
Choano./Metazoa - ps12
Metazoa - ps13
Myriazoa - ps14
Parahoxozoa - ps15
Bilateria - ps16
Deuterostomia - ps17
Chordata - ps18
Olfactores - ps19
Vertebrata - ps20
Gnathostomata - ps21
Euteleostomi - ps22
Sarcopterygii - ps23
Tetrapoda - ps24
Amniota - ps25
Mammalia - ps26
Theria - ps27
Placentalia - ps28
Boreoeutheria - ps29
Euarchontoglires - ps30
Euarchonta - ps31
Primates - ps32
Haplorrhini - ps33
Simiiformes - ps34
Catarrhini - ps35
Hominoidea - ps36
Hominidae - ps37
Homininae - ps38
Hominini - ps39
Homo_sapiens - ps40
Phylostratum

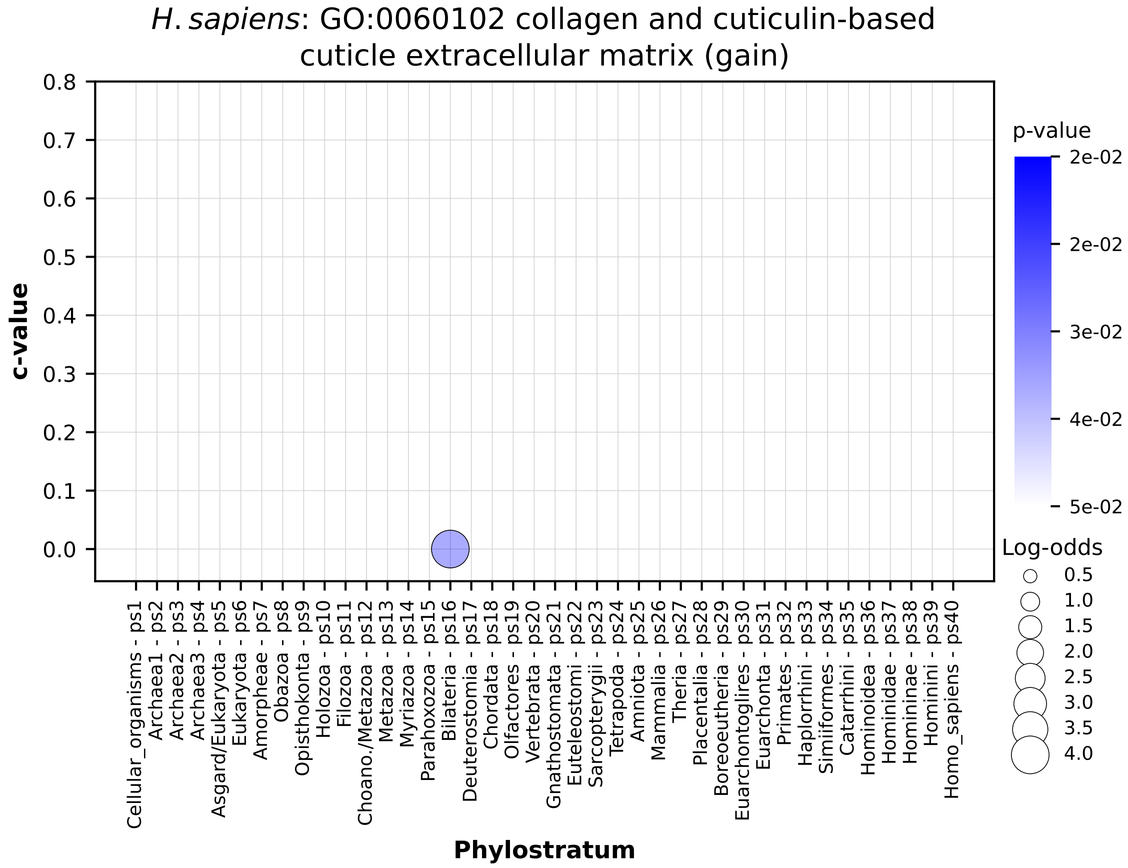

*H. sapiens*: GO:0060102 collagen and cuticulin-based cuticle extracellular matrix (gain)
c-value
0.8
0.7
0.6
0.5
0.4
0.3
0.2
0.1
0.0
p-value
2e-02
2e-02
3e-02
4e-02
5e-02
Log-odds
0.5
1.0
1.5
2.0
2.5
3.0
3.5
4.0
Cellular_organisms - ps1
Archaea1 - ps2
Archaea2 - ps3
Archaea3 - ps4
Asgard/Eukaryota - ps5
Eukaryota - ps6
Amorpheae - ps7
Obazoa - ps8
Opisthokonta - ps9
Holozoa - ps10
Filozoa - ps11
Choano./Metazoa - ps12
Metazoa - ps13
Myriazoa - ps14
Parahoxozoa - ps15
Bilateria - ps16
Deuterostomia - ps17
Chordata - ps18
Olfactores - ps19
Vertebrata - ps20
Gnathostomata - ps21
Euteleostomi - ps22
Sarcopterygii - ps23
Tetrapoda - ps24
Amniota - ps25
Mammalia - ps26
Theria - ps27
Placentalia - ps28
Boreoeutheria - ps29
Euarchontoglires - ps30
Euarchonta - ps31
Primates - ps32
Haplorrhini - ps33
Simiiformes - ps34
Catarrhini - ps35
Hominoidea - ps36
Hominidae - ps37
Homininae - ps38
Hominini - ps39
Homo_sapiens - ps40
Phylostratum

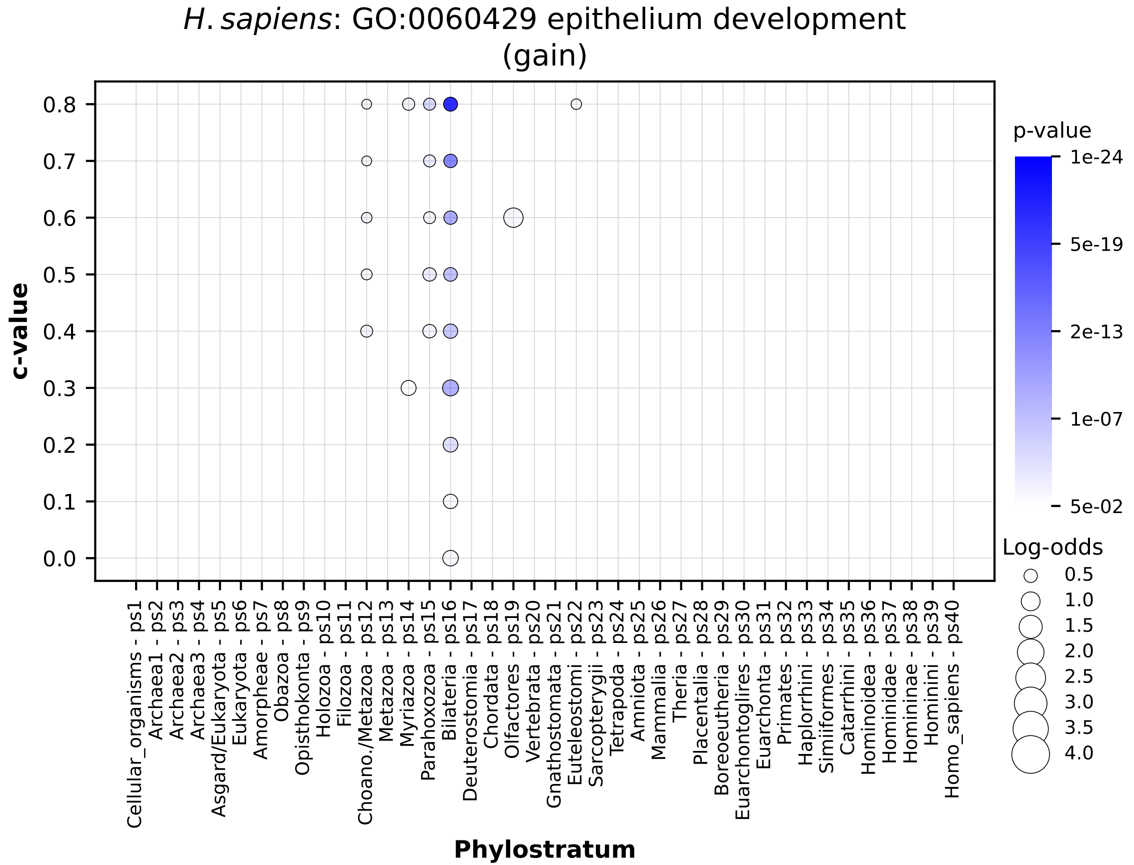

H. sapiens: GO:0060429 epithelium development
(gain)
c-value
0.8
0.7
0.6
0.5
0.4
0.3
0.2
0.1
0.0
Phylostratum
Cellular_organisms - ps1
Archaea1 - ps2
Archaea2 - ps3
Archaea3 - ps4
Asgard/Eukaryota - ps5
Eukaryota - ps6
Amorpheae - ps7
Obazoa - ps8
Opisthokonta - ps9
Holozoa - ps10
Filozoa - ps11
Choano./Metazoa - ps12
Metazoa - ps13
Myriazoa - ps14
Parahoxozoa - ps15
Bilateria - ps16
Deuterostomia - ps17
Chordata - ps18
Olfactores - ps19
Vertebrata - ps20
Gnathostomata - ps21
Euteleostomi - ps22
Sarcopterygii - ps23
Tetrapoda - ps24
Amniota - ps25
Mammalia - ps26
Theria - ps27
Placentalia - ps28
Boreoeutheria - ps29
Euarchontoglires - ps30
Euarchonta - ps31
Primates - ps32
Haplorrhini - ps33
Simiiformes - ps34
Catarrhini - ps35
Hominoidea - ps36
Hominidae - ps37
Homininae - ps38
Hominini - ps39
Homo_sapiens - ps40
p-value
1e-24
5e-19
2e-13
1e-07
5e-02
Log-odds
0.5
1.0
1.5
2.0
2.5
3.0
3.5
4.0

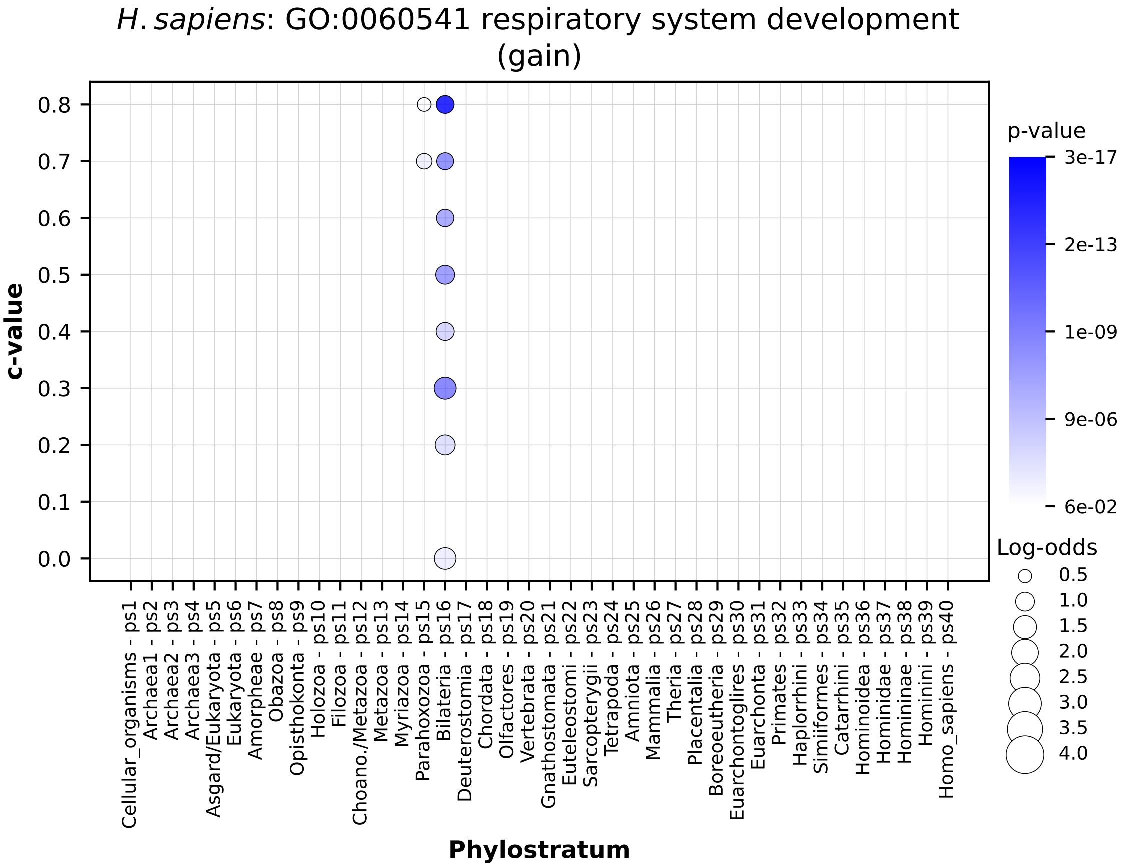

H. sapiens: GO:0060541 respiratory system development (gain)
c-value
0.8
0.7
0.6
0.5
0.4
0.3
0.2
0.1
0.0
p-value
3e-17
2e-13
1e-09
9e-06
6e-02
Log-odds
0.5
1.0
1.5
2.0
2.5
3.0
3.5
4.0
Cellular_organisms - ps1
Archaea1 - ps2
Archaea2 - ps3
Archaea3 - ps4
Asgard/Eukaryota - ps5
Eukaryota - ps6
Amorpheae - ps7
Obazoa - ps8
Opisthokonta - ps9
Holozoa - ps10
Filozoa - ps11
Choano./Metazoa - ps12
Metazoa - ps13
Myriazoa - ps14
Parahoxozoa - ps15
Bilateria - ps16
Deuterostomia - ps17
Chordata - ps18
Olfactores - ps19
Vertebrata - ps20
Gnathostomata - ps21
Euteleostomi - ps22
Sarcopterygii - ps23
Tetrapoda - ps24
Amniota - ps25
Mammalia - ps26
Theria - ps27
Placentalia - ps28
Boreoeutheria - ps29
Euarchontoglires - ps30
Euarchonta - ps31
Primates - ps32
Haplorrhini - ps33
Simiiformes - ps34
Catarrhini - ps35
Hominoidea - ps36
Hominidae - ps37
Homininae - ps38
Hominini - ps39
Homo_sapiens - ps40
Phylostratum

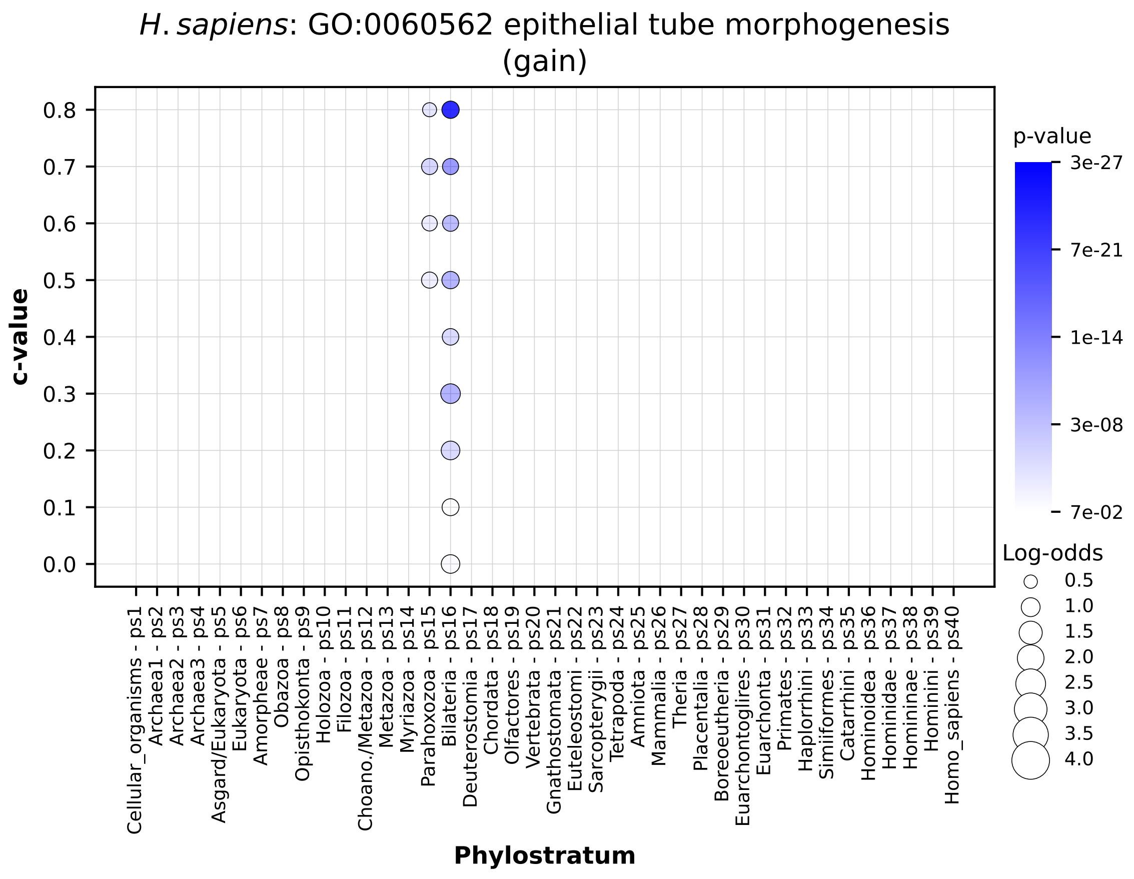

H. sapiens: GO:0060562 epithelial tube morphogenesis
(gain)
c-value
0.8
0.7
0.6
0.5
0.4
0.3
0.2
0.1
0.0
Phylostratum
Cellular_organisms - ps1
Archaea1 - ps2
Archaea2 - ps3
Archaea3 - ps4
Asgard/Eukaryota - ps5
Eukaryota - ps6
Amorpheae - ps7
Obazoa - ps8
Opisthokonta - ps9
Holozoa - ps10
Filozoa - ps11
Choano./Metazoa - ps12
Metazoa - ps13
Myriazoa - ps14
Parahoxozoa - ps15
Bilateria - ps16
Deuterostomia - ps17
Chordata - ps18
Olfactores - ps19
Vertebrata - ps20
Gnathostomata - ps21
Euteleostomi - ps22
Sarcopterygii - ps23
Tetrapoda - ps24
Amniota - ps25
Mammalia - ps26
Theria - ps27
Placentalia - ps28
Boreoeutheria - ps29
Euarchontoglires - ps30
Euarchonta - ps31
Primates - ps32
Haplorrhini - ps33
Simiiformes - ps34
Catarrhini - ps35
Hominoidea - ps36
Hominidae - ps37
Homininae - ps38
Hominini - ps39
Homo_sapiens - ps40
p-value
3e-27
7e-21
1e-14
3e-08
7e-02
Log-odds
0.5
1.0
1.5
2.0
2.5
3.0
3.5
4.0

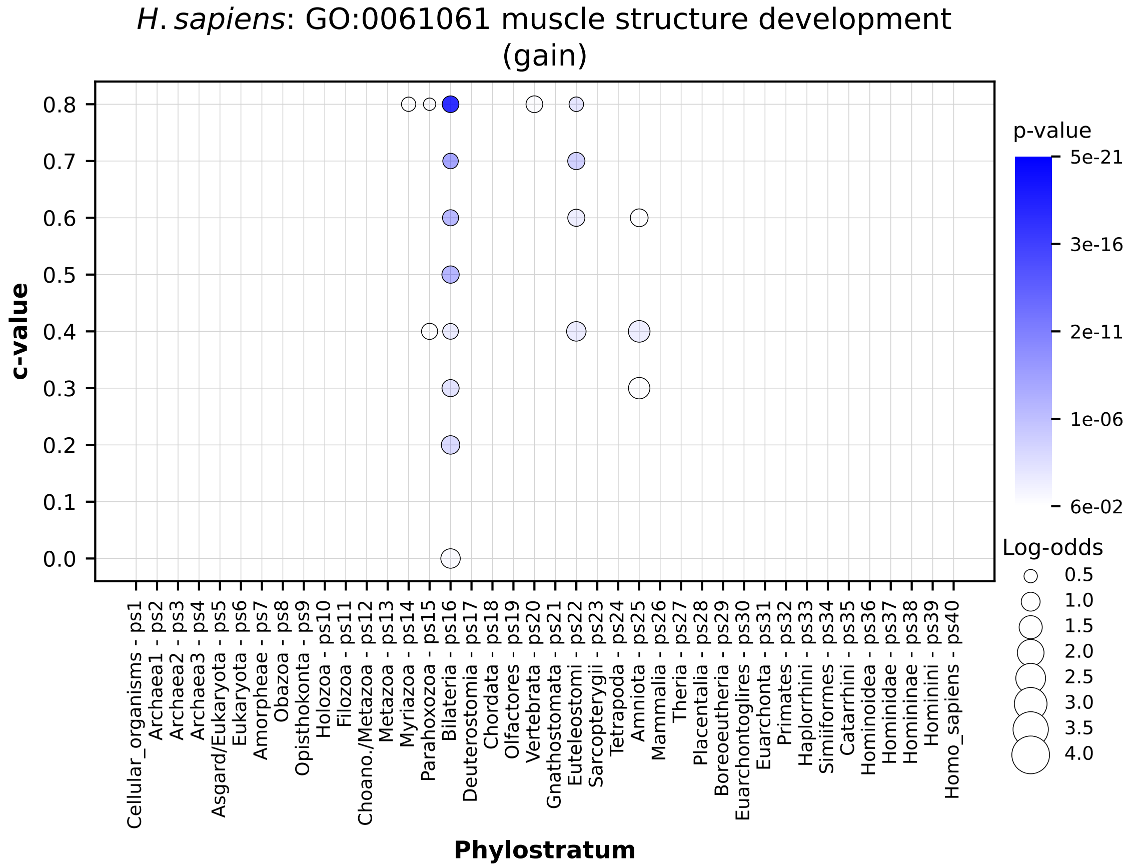
H. sapiens: GO:0061061 muscle structure development
(gain)
c-value
0.8
0.7
0.6
0.5
0.4
0.3
0.2
0.1
0.0
p-value
5e-21
3e-16
2e-11
1e-06
6e-02
Log-odds
0.5
1.0
1.5
2.0
2.5
3.0
3.5
4.0
Cellular_organisms - ps1
Archaea1 - ps2
Archaea2 - ps3
Archaea3 - ps4
Asgard/Eukaryota - ps5
Eukaryota - ps6
Amorpheae - ps7
Obazoa - ps8
Opisthokonta - ps9
Holozoa - ps10
Filozoa - ps11
Choano./Metazoa - ps12
Metazoa - ps13
Myriazoa - ps14
Parahoxozoa - ps15
Bilateria - ps16
Deuterostomia - ps17
Chordata - ps18
Olfactores - ps19
Vertebrata - ps20
Gnathostomata - ps21
Euteleostomi - ps22
Sarcopterygii - ps23
Tetrapoda - ps24
Amniota - ps25
Mammalia - ps26
Theria - ps27
Placentalia - ps28
Boreoeutheria - ps29
Euarchontoglires - ps30
Euarchonta - ps31
Primates - ps32
Haplorrhini - ps33
Simiiformes - ps34
Catarrhini - ps35
Hominoidea - ps36
Hominidae - ps37
Homininae - ps38
Hominini - ps39
Homo_sapiens - ps40
Phylostratum

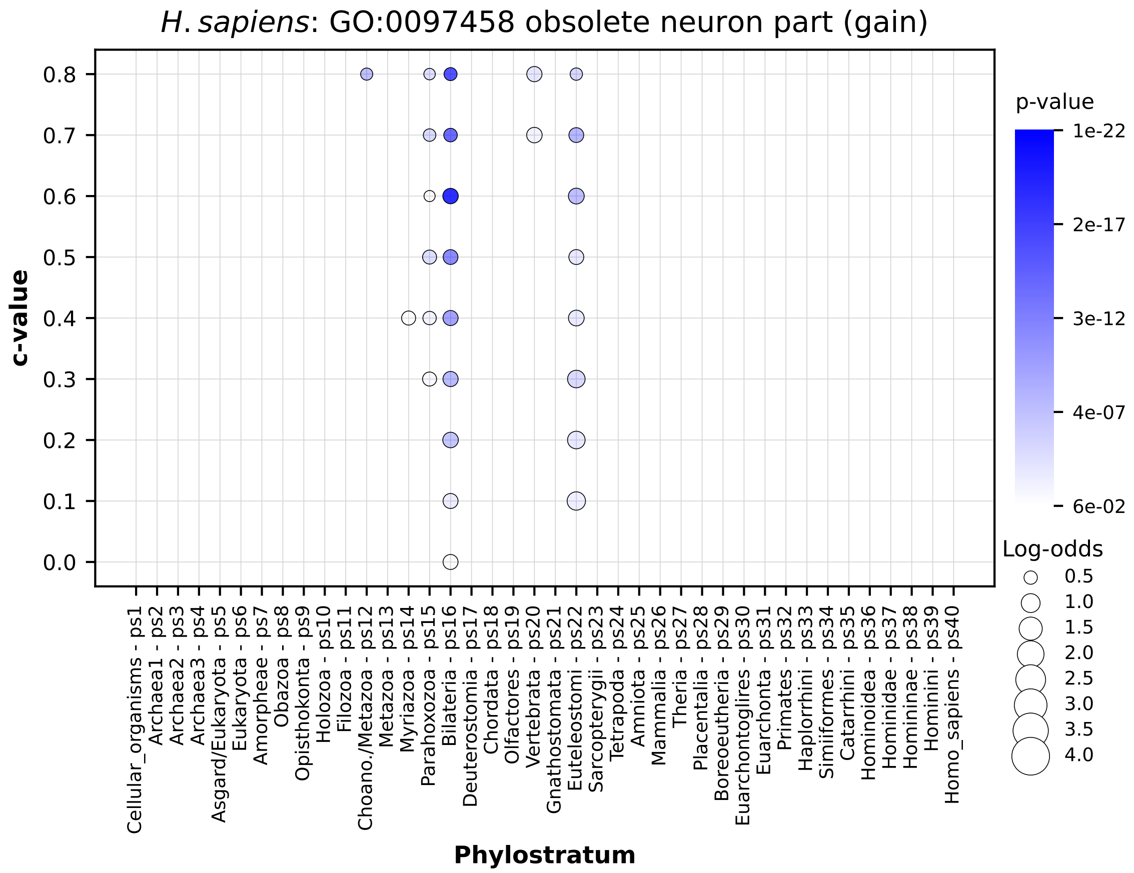

H. sapiens: GO:0097458 obsolete neuron part (gain)
c-value
0.8
0.7
0.6
0.5
0.4
0.3
0.2
0.1
0.0
Phylostratum
Cellular_organisms - ps1
Archaea1 - ps2
Archaea2 - ps3
Archaea3 - ps4
Asgard/Eukaryota - ps5
Eukaryota - ps6
Amorpheae - ps7
Obazoa - ps8
Opisthokonta - ps9
Holozoa - ps10
Filozoa - ps11
Choano./Metazoa - ps12
Metazoa - ps13
Myriazoa - ps14
Parahoxozoa - ps15
Bilateria - ps16
Deuterostomia - ps17
Chordata - ps18
Olfactores - ps19
Vertebrata - ps20
Gnathostomata - ps21
Euteleostomi - ps22
Sarcopterygii - ps23
Tetrapoda - ps24
Amniota - ps25
Mammalia - ps26
Theria - ps27
Placentalia - ps28
Boreoeutheria - ps29
Euarchontoglires - ps30
Euarchonta - ps31
Primates - ps32
Haplorrhini - ps33
Simiiformes - ps34
Catarrhini - ps35
Hominoidea - ps36
Hominidae - ps37
Homininae - ps38
Hominini - ps39
Homo_sapiens - ps40
p-value
1e-22
2e-17
3e-12
4e-07
6e-02
Log-odds
0.5
1.0
1.5
2.0
2.5
3.0
3.5
4.0

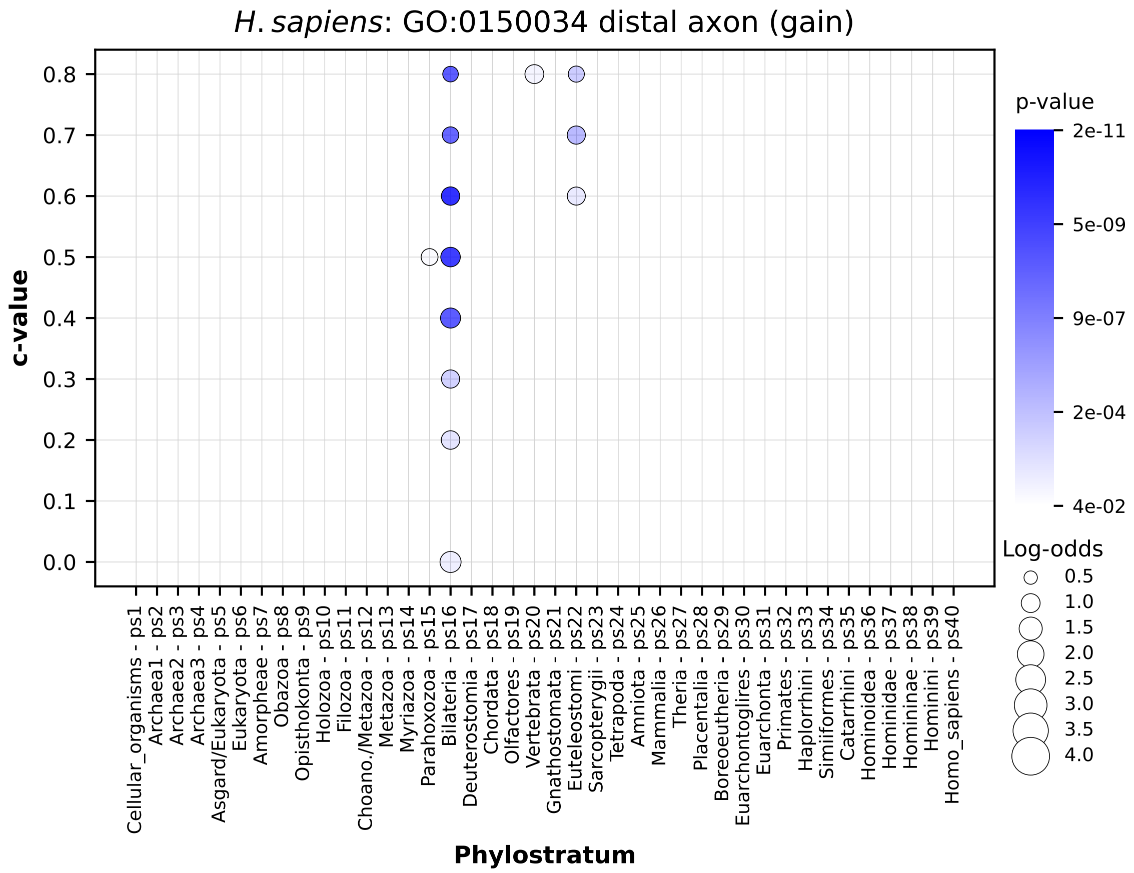

H. sapiens: GO:0150034 distal axon (gain)
c-value
0.8
0.7
0.6
0.5
0.4
0.3
0.2
0.1
0.0
p-value
2e-11
5e-09
9e-07
2e-04
4e-02
Log-odds
0.5
1.0
1.5
2.0
2.5
3.0
3.5
4.0
Cellular_organisms - ps1
Archaea1 - ps2
Archaea2 - ps3
Archaea3 - ps4
Asgard/Eukaryota - ps5
Eukaryota - ps6
Amorpheae - ps7
Obazoa - ps8
Opisthokonta - ps9
Holozoa - ps10
Filozoa - ps11
Choano./Metazoa - ps12
Metazoa - ps13
Myriazoa - ps14
Parahoxozoa - ps15
Bilateria - ps16
Deuterostomia - ps17
Chordata - ps18
Olfactores - ps19
Vertebrata - ps20
Gnathostomata - ps21
Euteleostomi - ps22
Sarcopterygii - ps23
Tetrapoda - ps24
Amniota - ps25
Mammalia - ps26
Theria - ps27
Placentalia - ps28
Boreoeutheria - ps29
Euarchontoglires - ps30
Euarchonta - ps31
Primates - ps32
Haplorrhini - ps33
Simiiformes - ps34
Catarrhini - ps35
Hominoidea - ps36
Hominidae - ps37
Homininae - ps38
Hominini - ps39
Homo_sapiens - ps40
Phylostratum

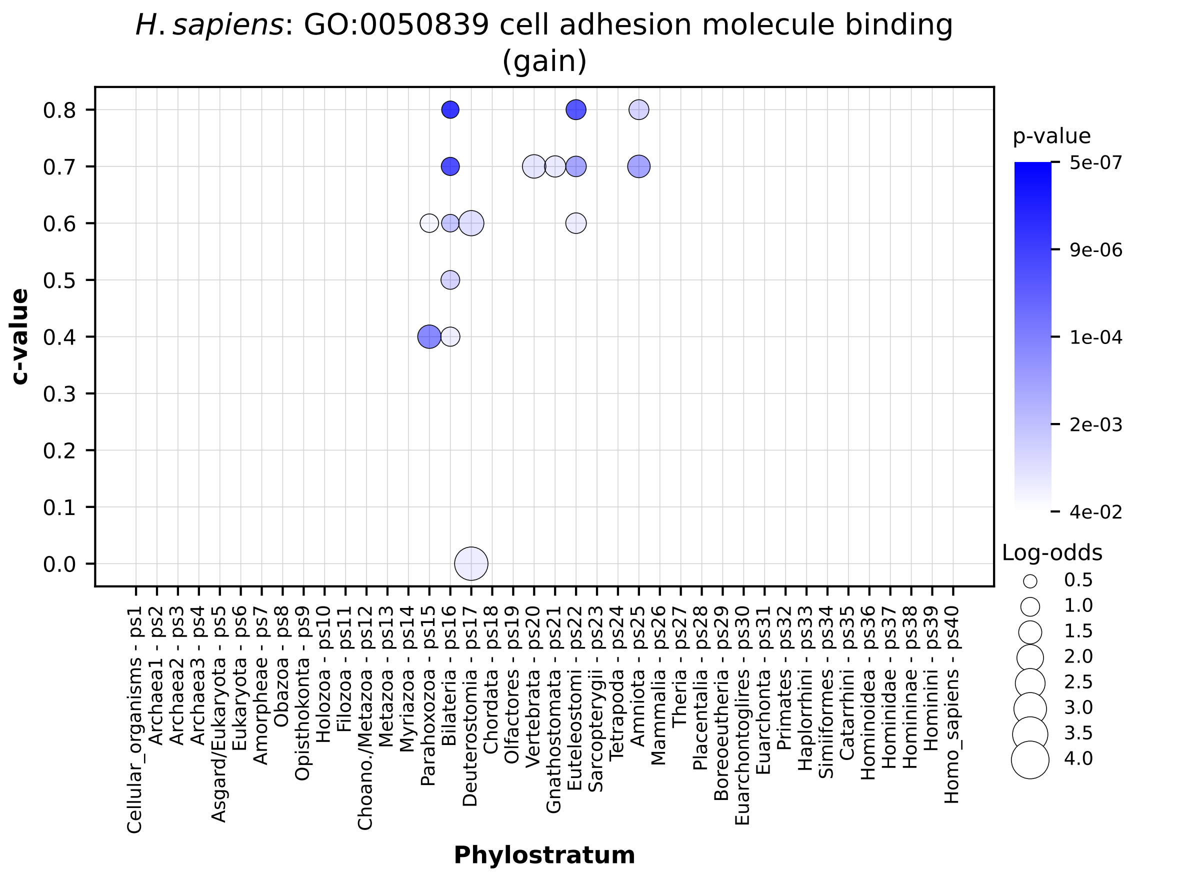

H. sapiens: GO:0050839 cell adhesion molecule binding (gain)
c-value
0.0
0.1
0.2
0.3
0.4
0.5
0.6
0.7
0.8
p-value
5e-07
9e-06
1e-04
2e-03
4e-02
Log-odds
0.5
1.0
1.5
2.0
2.5
3.0
3.5
4.0
Cellular_organisms - ps1
Archaea1 - ps2
Archaea2 - ps3
Archaea3 - ps4
Asgard/Eukaryota - ps5
Eukaryota - ps6
Amorpheae - ps7
Obazoa - ps8
Opisthokonta - ps9
Holozoa - ps10
Filozoa - ps11
Choano./Metazoa - ps12
Metazoa - ps13
Myriazoa - ps14
Parahoxozoa - ps15
Bilateria - ps16
Deuterostomia - ps17
Chordata - ps18
Olfactores - ps19
Vertebrata - ps20
Gnathostomata - ps21
Euteleostomi - ps22
Sarcopterygii - ps23
Tetrapoda - ps24
Amniota - ps25
Mammalia - ps26
Theria - ps27
Placentalia - ps28
Boreoeutheria - ps29
Euarchontoglires - ps30
Euarchonta - ps31
Primates - ps32
Haplorrhini - ps33
Simiiformes - ps34
Catarrhini - ps35
Hominoidea - ps36
Hominidae - ps37
Homininae - ps38
Hominini - ps39
Homo_sapiens - ps40
Phylostratum

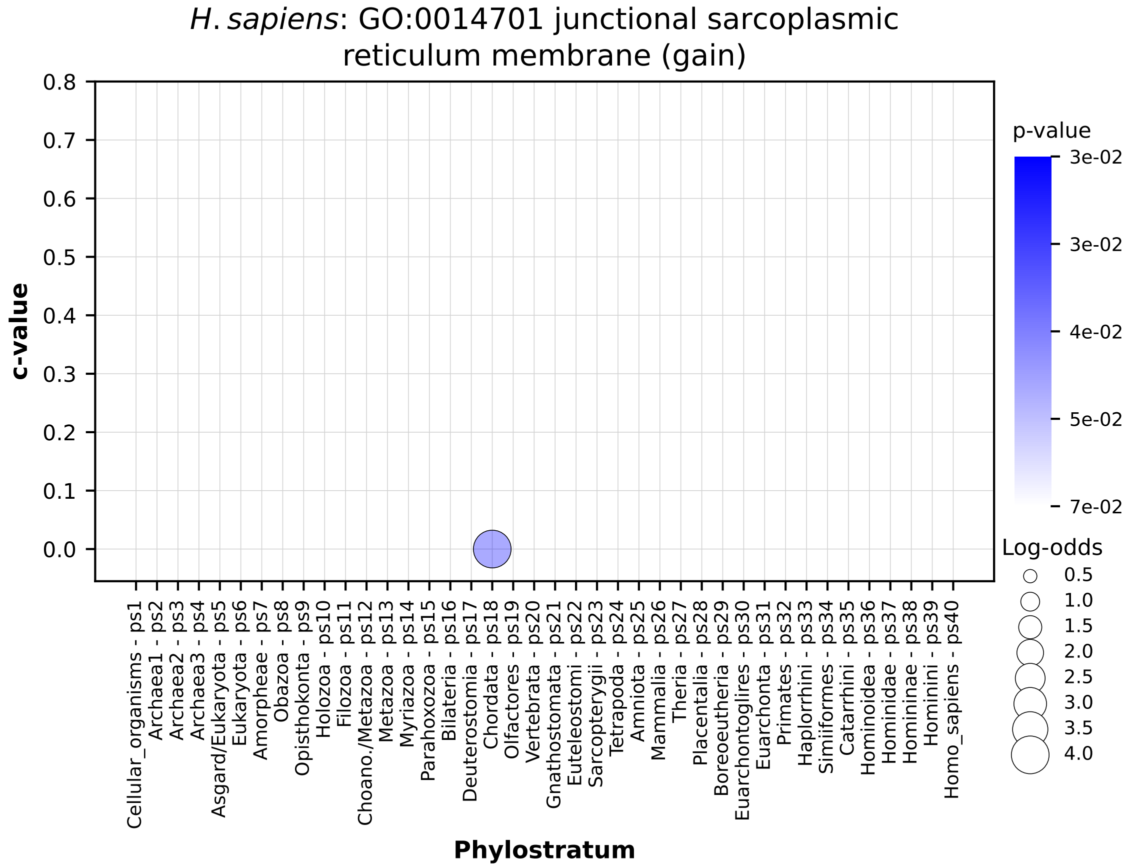

*H. sapiens*: GO:0014701 junctional sarcoplasmic reticulum membrane (gain)
c-value
0.8
0.7
0.6
0.5
0.4
0.3
0.2
0.1
0.0
Phylostratum
Cellular_organisms - ps1
Archaea1 - ps2
Archaea2 - ps3
Archaea3 - ps4
Asgard/Eukaryota - ps5
Eukaryota - ps6
Amorpheae - ps7
Obazoa - ps8
Opisthokonta - ps9
Holozoa - ps10
Filozoa - ps11
Choano./Metazoa - ps12
Metazoa - ps13
Myriazoa - ps14
Parahoxozoa - ps15
Bilateria - ps16
Deuterostomia - ps17
Chordata - ps18
Olfactores - ps19
Vertebrata - ps20
Gnathostomata - ps21
Euteleostomi - ps22
Sarcopterygii - ps23
Tetrapoda - ps24
Amniota - ps25
Mammalia - ps26
Theria - ps27
Placentalia - ps28
Boreoeutheria - ps29
Euarchontoglires - ps30
Euarchonta - ps31
Primates - ps32
Haplorrhini - ps33
Simiiformes - ps34
Catarrhini - ps35
Hominoidea - ps36
Hominidae - ps37
Homininae - ps38
Hominini - ps39
Homo_sapiens - ps40
p-value
3e-02
3e-02
4e-02
5e-02
7e-02
Log-odds
0.5
1.0
1.5
2.0
2.5
3.0
3.5
4.0

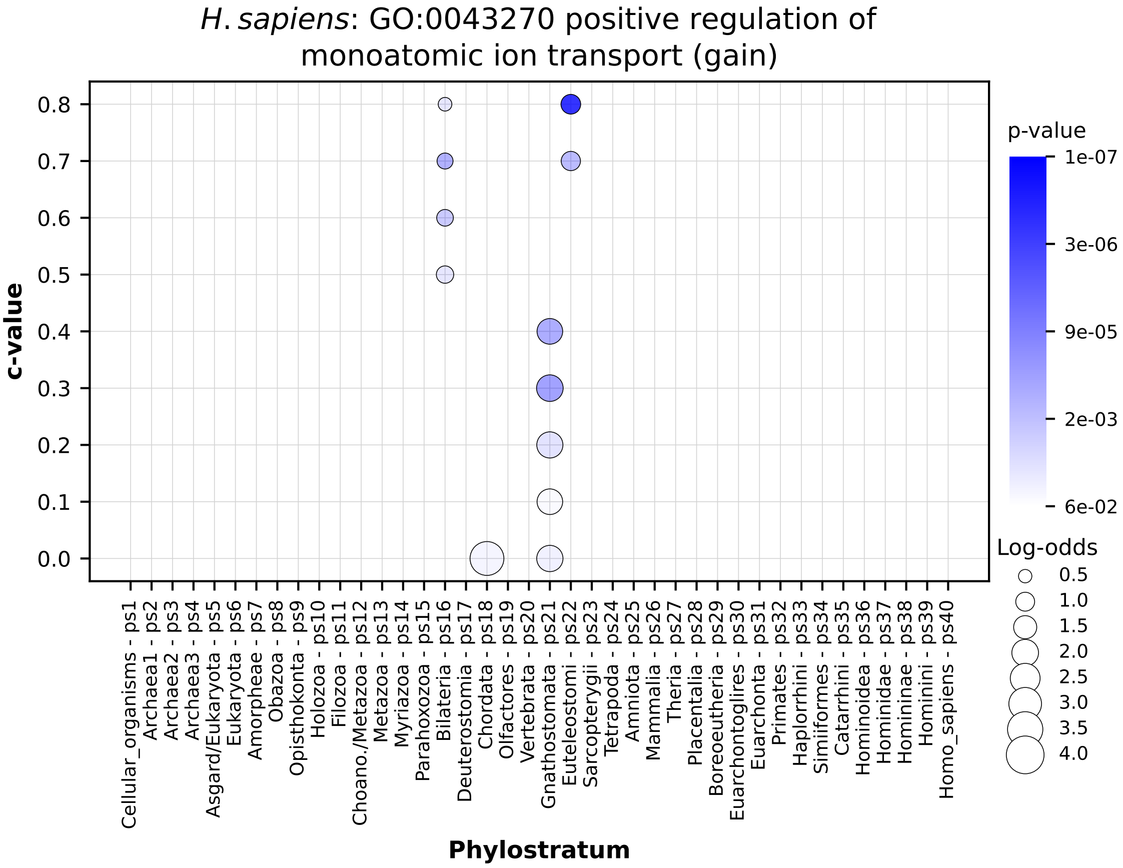

H. sapiens: GO:0043270 positive regulation of monoatomic ion transport (gain)
c-value
0.8
0.7
0.6
0.5
0.4
0.3
0.2
0.1
0.0
p-value
1e-07
3e-06
9e-05
2e-03
6e-02
Log-odds
0.5
1.0
1.5
2.0
2.5
3.0
3.5
4.0
Cellular_organisms - ps1
Archaea1 - ps2
Archaea2 - ps3
Archaea3 - ps4
Asgard/Eukaryota - ps5
Eukaryota - ps6
Amorpheae - ps7
Obazoa - ps8
Opisthokonta - ps9
Holozoa - ps10
Filozoa - ps11
Choano./Metazoa - ps12
Metazoa - ps13
Myriazoa - ps14
Parahoxozoa - ps15
Bilateria - ps16
Deuterostomia - ps17
Chordata - ps18
Olfactores - ps19
Vertebrata - ps20
Gnathostomata - ps21
Euteleostomi - ps22
Sarcopterygii - ps23
Tetrapoda - ps24
Amniota - ps25
Mammalia - ps26
Theria - ps27
Placentalia - ps28
Boreoeutheria - ps29
Euarchontoglires - ps30
Euarchonta - ps31
Primates - ps32
Haplorrhini - ps33
Simiiformes - ps34
Catarrhini - ps35
Hominoidea - ps36
Hominidae - ps37
Homininae - ps38
Hominini - ps39
Homo_sapiens - ps40
Phylostratum

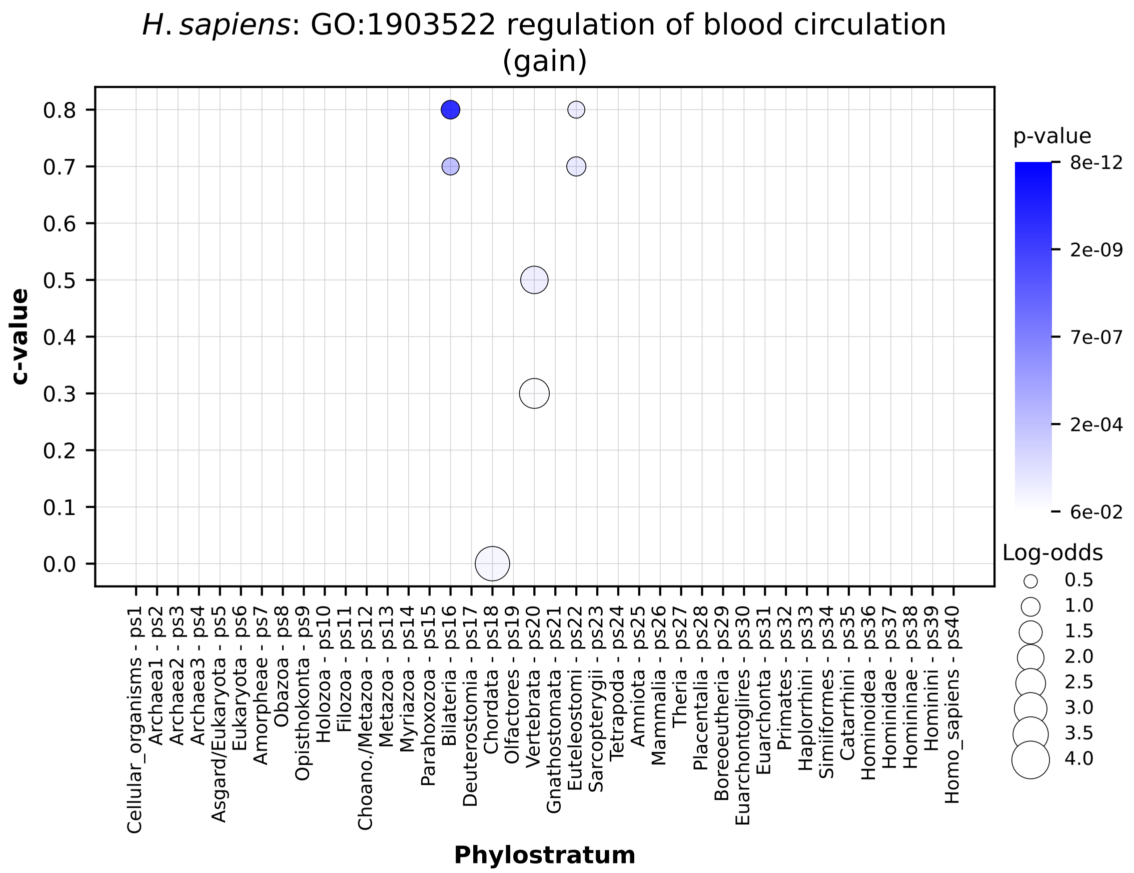
H. sapiens: GO:1903522 regulation of blood circulation (gain)
c-value
0.8
0.7
0.6
0.5
0.4
0.3
0.2
0.1
0.0
p-value
8e-12
2e-09
7e-07
2e-04
6e-02
Log-odds
0.5
1.0
1.5
2.0
2.5
3.0
3.5
4.0
Cellular_organisms - ps1
Archaea1 - ps2
Archaea2 - ps3
Archaea3 - ps4
Asgard/Eukaryota - ps5
Eukaryota - ps6
Amorpheae - ps7
Obazoa - ps8
Opisthokonta - ps9
Holozoa - ps10
Filozoa - ps11
Choano./Metazoa - ps12
Metazoa - ps13
Myriazoa - ps14
Parahoxozoa - ps15
Bilateria - ps16
Deuterostomia - ps17
Chordata - ps18
Olfactores - ps19
Vertebrata - ps20
Gnathostomata - ps21
Euteleostomi - ps22
Sarcopterygii - ps23
Tetrapoda - ps24
Amniota - ps25
Mammalia - ps26
Theria - ps27
Placentalia - ps28
Boreoeutheria - ps29
Euarchontoglires - ps30
Euarchonta - ps31
Primates - ps32
Haplorrhini - ps33
Simiiformes - ps34
Catarrhini - ps35
Hominoidea - ps36
Hominidae - ps37
Homininae - ps38
Hominini - ps39
Homo_sapiens - ps40
Phylostratum

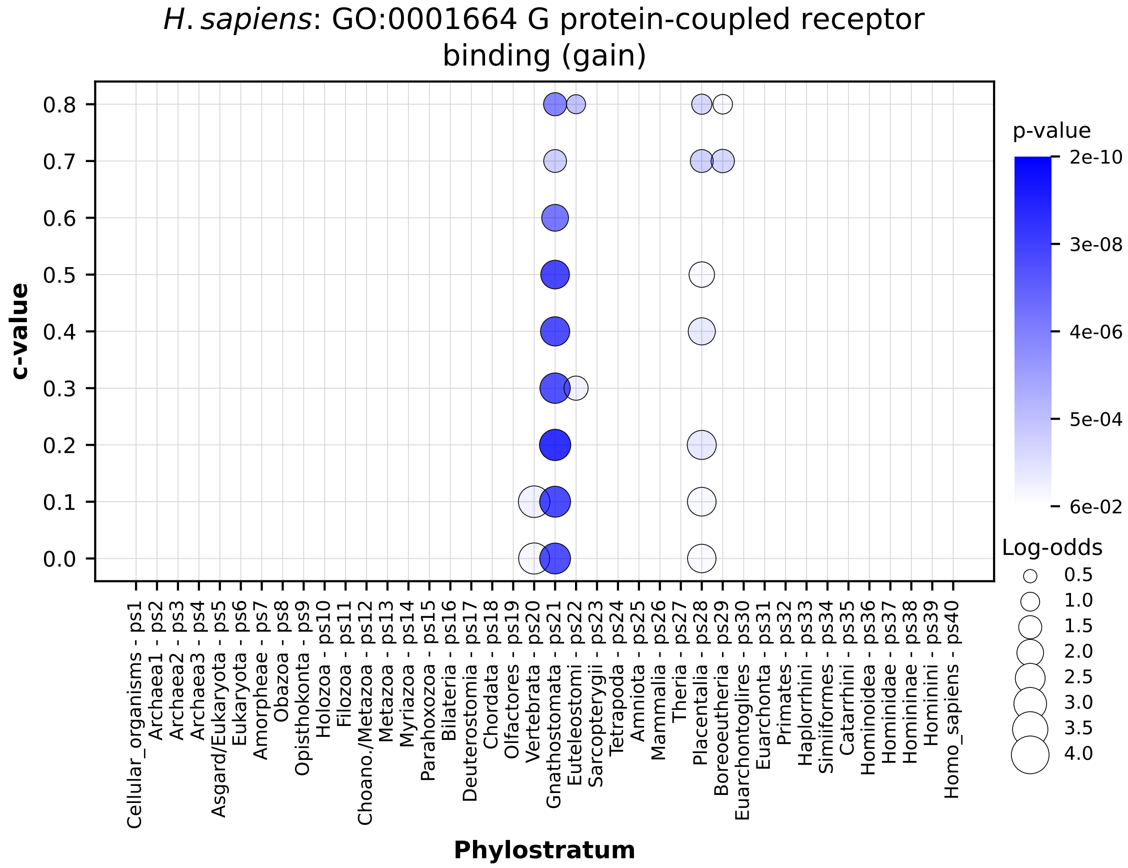
*H. sapiens*: GO:0001664 G protein-coupled receptor binding (gain)
c-value
0.8
0.7
0.6
0.5
0.4
0.3
0.2
0.1
0.0
Phylostratum
Cellular_organisms - ps1
Archaea1 - ps2
Archaea2 - ps3
Archaea3 - ps4
Asgard/Eukaryota - ps5
Eukaryota - ps6
Amorpheae - ps7
Obazoa - ps8
Opisthokonta - ps9
Holozoa - ps10
Filozoa - ps11
Choano./Metazoa - ps12
Metazoa - ps13
Myriazoa - ps14
Parahoxozoa - ps15
Bilateria - ps16
Deuterostomia - ps17
Chordata - ps18
Olfactores - ps19
Vertebrata - ps20
Gnathostomata - ps21
Euteleostomi - ps22
Sarcopterygii - ps23
Tetrapoda - ps24
Amniota - ps25
Mammalia - ps26
Theria - ps27
Placentalia - ps28
Boreoeutheria - ps29
Euarchontoglires - ps30
Euarchonta - ps31
Primates - ps32
Haplorrhini - ps33
Simiiformes - ps34
Catarrhini - ps35
Hominoidea - ps36
Hominidae - ps37
Homininae - ps38
Hominini - ps39
Homo_sapiens - ps40
p-value
2e-10
3e-08
4e-06
5e-04
6e-02
Log-odds
0.5
1.0
1.5
2.0
2.5
3.0
3.5
4.0

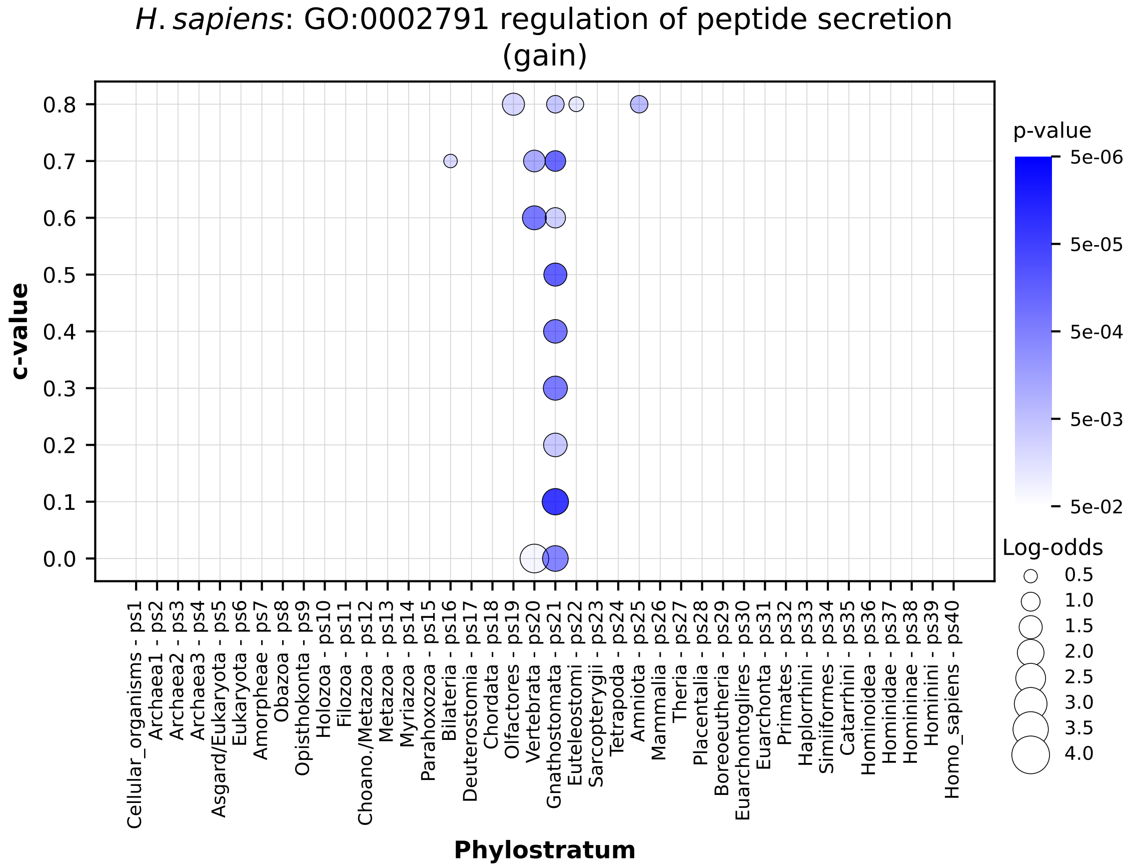

H. sapiens: GO:0002791 regulation of peptide secretion
(gain)
c-value
0.8
0.7
0.6
0.5
0.4
0.3
0.2
0.1
0.0
Phylostratum
Cellular_organisms - ps1
Archaea1 - ps2
Archaea2 - ps3
Archaea3 - ps4
Asgard/Eukaryota - ps5
Eukaryota - ps6
Amorpheae - ps7
Obazoa - ps8
Opisthokonta - ps9
Holozoa - ps10
Filozoa - ps11
Choano./Metazoa - ps12
Metazoa - ps13
Myriazoa - ps14
Parahoxozoa - ps15
Bilateria - ps16
Deuterostomia - ps17
Chordata - ps18
Olfactores - ps19
Vertebrata - ps20
Gnathostomata - ps21
Euteleostomi - ps22
Sarcopterygii - ps23
Tetrapoda - ps24
Amniota - ps25
Mammalia - ps26
Theria - ps27
Placentalia - ps28
Boreoeutheria - ps29
Euarchontoglires - ps30
Euarchonta - ps31
Primates - ps32
Haplorrhini - ps33
Simiiformes - ps34
Catarrhini - ps35
Hominoidea - ps36
Hominidae - ps37
Homininae - ps38
Hominini - ps39
Homo_sapiens - ps40
p-value
5e-06
5e-05
5e-04
5e-03
5e-02
Log-odds
0.5
1.0
1.5
2.0
2.5
3.0
3.5
4.0

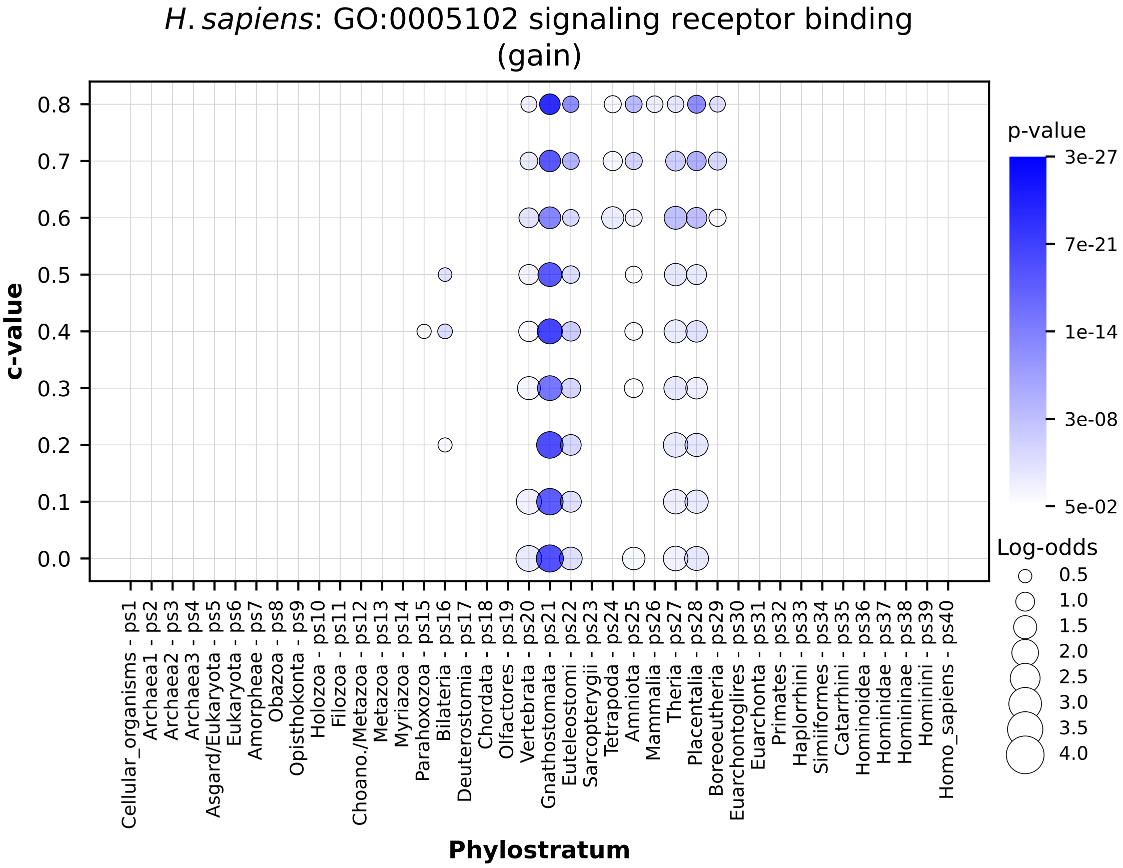
H. sapiens: GO:0005102 signaling receptor binding
(gain)
c-value
0.8
0.7
0.6
0.5
0.4
0.3
0.2
0.1
0.0
p-value
3e-27
7e-21
1e-14
3e-08
5e-02
Log-odds
0.5
1.0
1.5
2.0
2.5
3.0
3.5
4.0
Cellular_organisms - ps1
Archaea1 - ps2
Archaea2 - ps3
Archaea3 - ps4
Asgard/Eukaryota - ps5
Eukaryota - ps6
Amorpheae - ps7
Obazoa - ps8
Opisthokonta - ps9
Holozoa - ps10
Filozoa - ps11
Choano./Metazoa - ps12
Metazoa - ps13
Myriazoa - ps14
Parahoxozoa - ps15
Bilateria - ps16
Deuterostomia - ps17
Chordata - ps18
Olfactores - ps19
Vertebrata - ps20
Gnathostomata - ps21
Euteleostomi - ps22
Sarcopterygii - ps23
Tetrapoda - ps24
Amniota - ps25
Mammalia - ps26
Theria - ps27
Placentalia - ps28
Boreoeutheria - ps29
Euarchontoglires - ps30
Euarchonta - ps31
Primates - ps32
Haplorrhini - ps33
Simiiformes - ps34
Catarrhini - ps35
Hominoidea - ps36
Hominidae - ps37
Homininae - ps38
Hominini - ps39
Homo_sapiens - ps40
Phylostratum

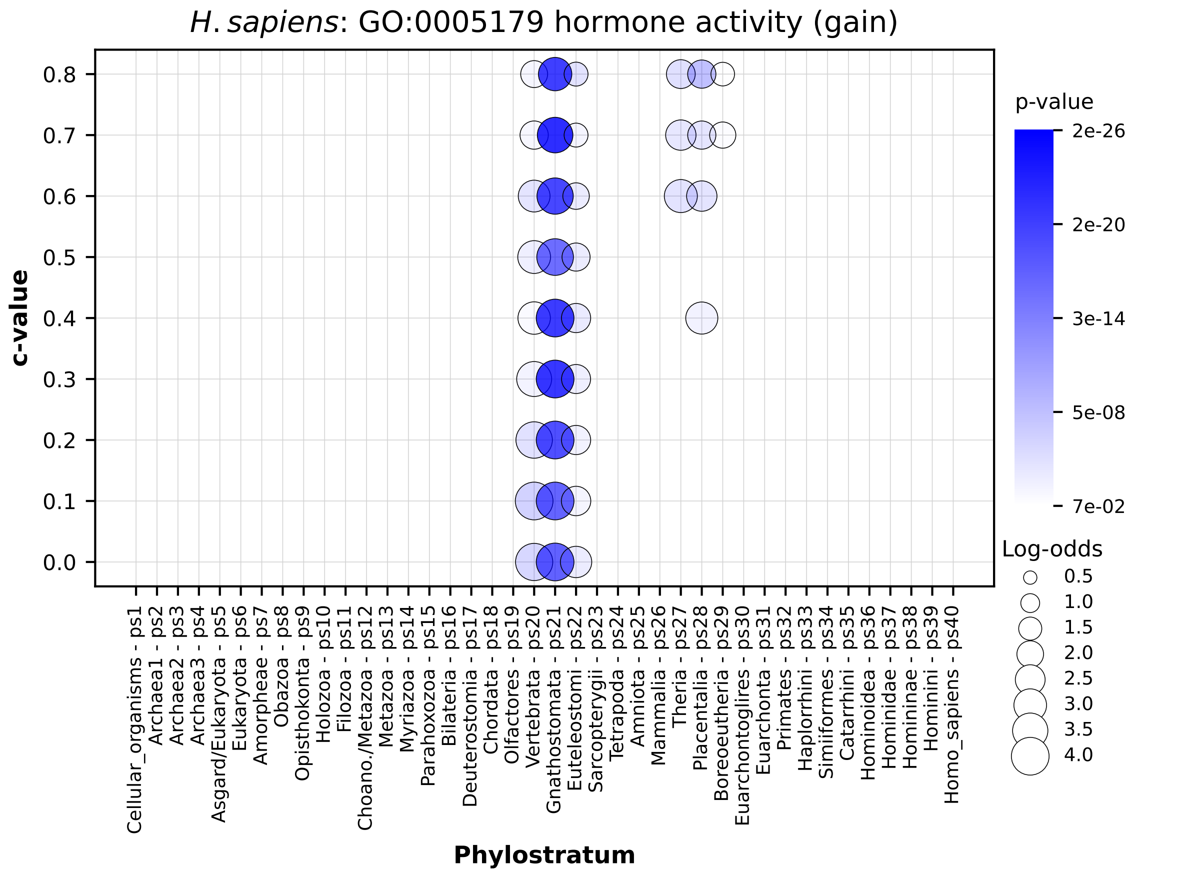

*H. sapiens*: GO:0005179 hormone activity (gain)
c-value
0.8
0.7
0.6
0.5
0.4
0.3
0.2
0.1
0.0
p-value
2e-26
2e-20
3e-14
5e-08
7e-02
Log-odds
0.5
1.0
1.5
2.0
2.5
3.0
3.5
4.0
Cellular_organisms - ps1
Archaea1 - ps2
Archaea2 - ps3
Archaea3 - ps4
Asgard/Eukaryota - ps5
Eukaryota - ps6
Amorpheae - ps7
Obazoa - ps8
Opisthokonta - ps9
Holozoa - ps10
Filozoa - ps11
Choano./Metazoa - ps12
Metazoa - ps13
Myriazoa - ps14
Parahoxozoa - ps15
Bilateria - ps16
Deuterostomia - ps17
Chordata - ps18
Olfactores - ps19
Vertebrata - ps20
Gnathostomata - ps21
Euteleostomi - ps22
Sarcopterygii - ps23
Tetrapoda - ps24
Amniota - ps25
Mammalia - ps26
Theria - ps27
Placentalia - ps28
Boreoeutheria - ps29
Euarchontoglires - ps30
Euarchonta - ps31
Primates - ps32
Haplorrhini - ps33
Simiiformes - ps34
Catarrhini - ps35
Hominoidea - ps36
Hominidae - ps37
Homininae - ps38
Hominini - ps39
Homo_sapiens - ps40
Phylostratum

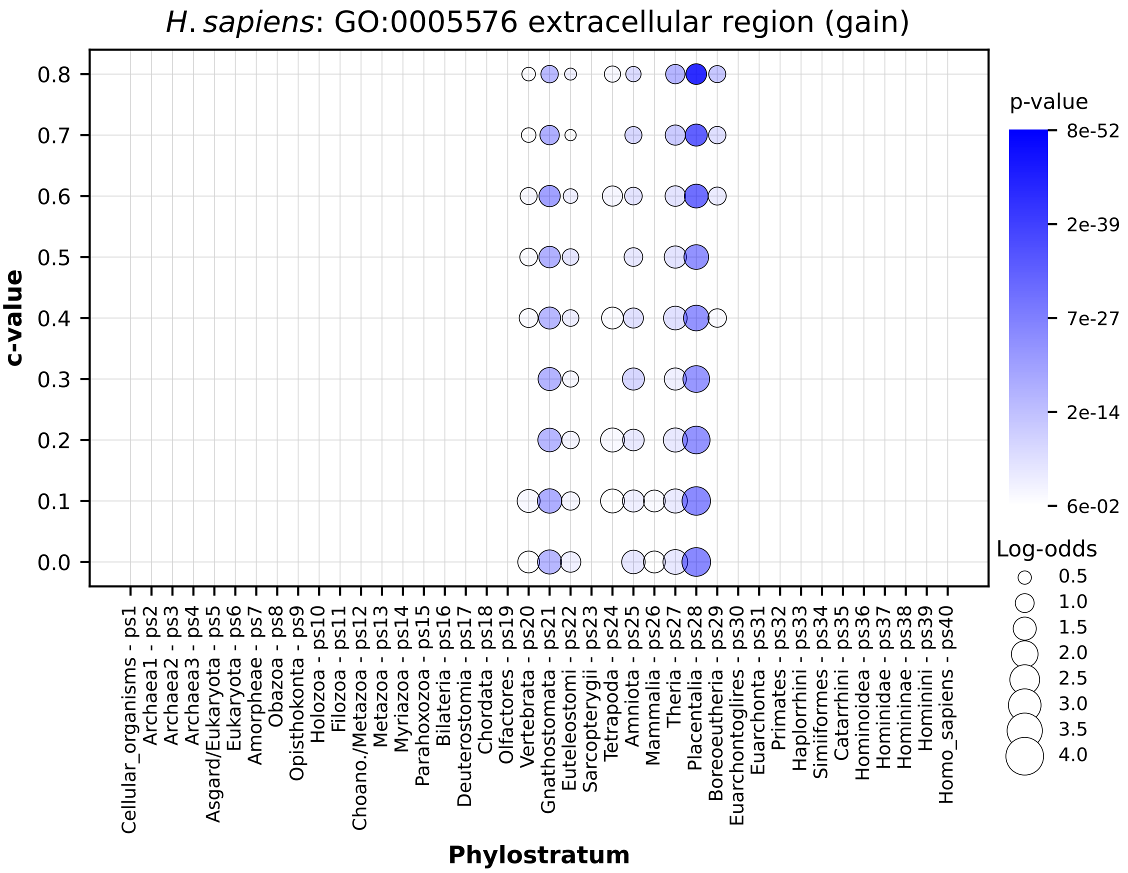

H. sapiens: GO:0005576 extracellular region (gain)
c-value
0.8
0.7
0.6
0.5
0.4
0.3
0.2
0.1
0.0
Phylostratum
Cellular_organisms - ps1
Archaea1 - ps2
Archaea2 - ps3
Archaea3 - ps4
Asgard/Eukaryota - ps5
Eukaryota - ps6
Amorpheae - ps7
Obazoa - ps8
Opisthokonta - ps9
Holozoa - ps10
Filozoa - ps11
Choano./Metazoa - ps12
Metazoa - ps13
Myriazoa - ps14
Parahoxozoa - ps15
Bilateria - ps16
Deuterostomia - ps17
Chordata - ps18
Olfactores - ps19
Vertebrata - ps20
Gnathostomata - ps21
Euteleostomi - ps22
Sarcopterygii - ps23
Tetrapoda - ps24
Amniota - ps25
Mammalia - ps26
Theria - ps27
Placentalia - ps28
Boreoeutheria - ps29
Euarchontoglires - ps30
Euarchonta - ps31
Primates - ps32
Haplorrhini - ps33
Simiiformes - ps34
Catarrhini - ps35
Hominoidea - ps36
Hominidae - ps37
Homininae - ps38
Hominini - ps39
Homo_sapiens - ps40
p-value
8e-52
2e-39
7e-27
2e-14
6e-02
Log-odds
0.5
1.0
1.5
2.0
2.5
3.0
3.5
4.0

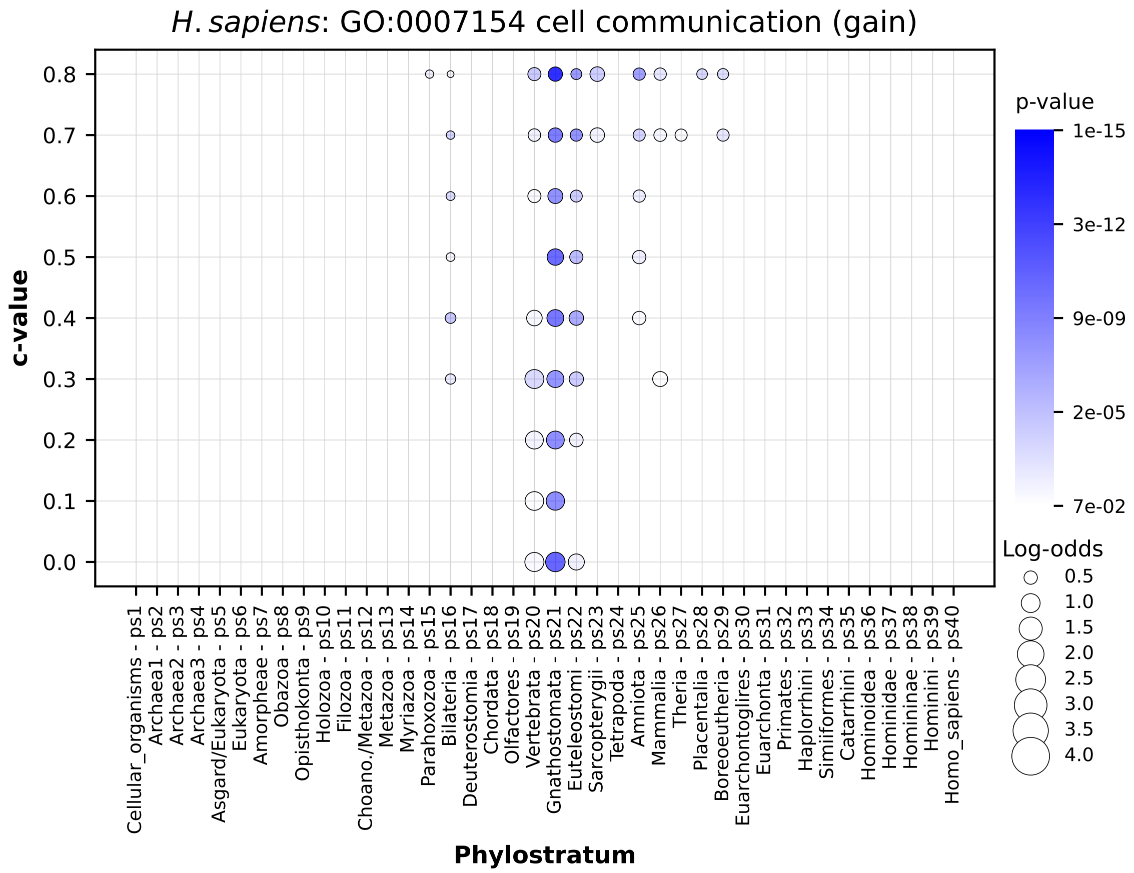

H. sapiens: GO:0007154 cell communication (gain)
c-value
0.8
0.7
0.6
0.5
0.4
0.3
0.2
0.1
0.0
Phylostratum
Cellular_organisms - ps1
Archaea1 - ps2
Archaea2 - ps3
Archaea3 - ps4
Asgard/Eukaryota - ps5
Eukaryota - ps6
Amorpheae - ps7
Obazoa - ps8
Opisthokonta - ps9
Holozoa - ps10
Filozoa - ps11
Choano./Metazoa - ps12
Metazoa - ps13
Myriazoa - ps14
Parahoxozoa - ps15
Bilateria - ps16
Deuterostomia - ps17
Chordata - ps18
Olfactores - ps19
Vertebrata - ps20
Gnathostomata - ps21
Euteleostomi - ps22
Sarcopterygii - ps23
Tetrapoda - ps24
Amniota - ps25
Mammalia - ps26
Theria - ps27
Placentalia - ps28
Boreoeutheria - ps29
Euarchontoglires - ps30
Euarchonta - ps31
Primates - ps32
Haplorrhini - ps33
Simiiformes - ps34
Catarrhini - ps35
Hominoidea - ps36
Hominidae - ps37
Homininae - ps38
Hominini - ps39
Homo_sapiens - ps40
p-value
1e-15
3e-12
9e-09
2e-05
7e-02
Log-odds
0.5
1.0
1.5
2.0
2.5
3.0
3.5
4.0

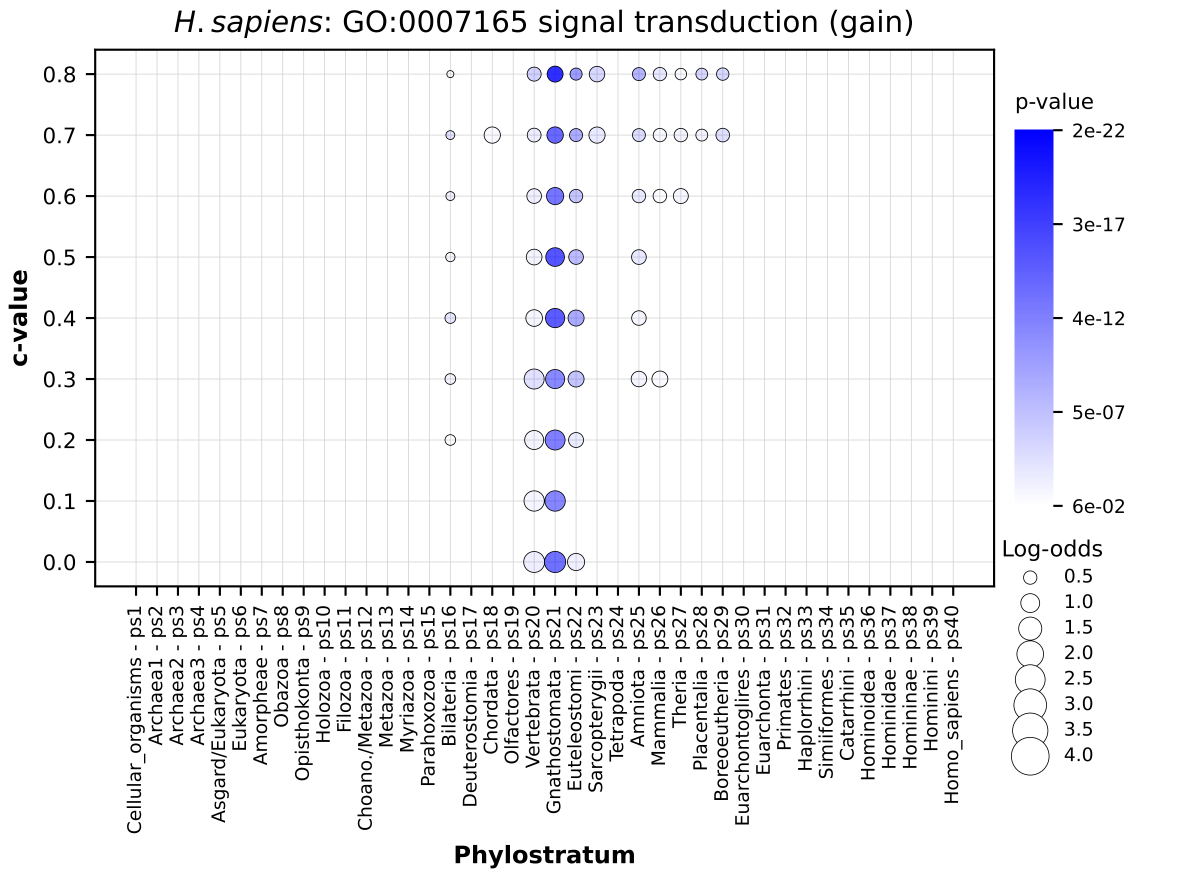

H. sapiens: GO:0007165 signal transduction (gain)
c-value
0.8
0.7
0.6
0.5
0.4
0.3
0.2
0.1
0.0
p-value
2e-22
3e-17
4e-12
5e-07
6e-02
Log-odds
0.5
1.0
1.5
2.0
2.5
3.0
3.5
4.0
Cellular_organisms - ps1
Archaea1 - ps2
Archaea2 - ps3
Archaea3 - ps4
Asgard/Eukaryota - ps5
Eukaryota - ps6
Amorpheae - ps7
Obazoa - ps8
Opisthokonta - ps9
Holozoa - ps10
Filozoa - ps11
Choano./Metazoa - ps12
Metazoa - ps13
Myriazoa - ps14
Parahoxozoa - ps15
Bilateria - ps16
Deuterostomia - ps17
Chordata - ps18
Olfactores - ps19
Vertebrata - ps20
Gnathostomata - ps21
Euteleostomi - ps22
Sarcopterygii - ps23
Tetrapoda - ps24
Amniota - ps25
Mammalia - ps26
Theria - ps27
Placentalia - ps28
Boreoeutheria - ps29
Euarchontoglires - ps30
Euarchonta - ps31
Primates - ps32
Haplorrhini - ps33
Simiiformes - ps34
Catarrhini - ps35
Hominoidea - ps36
Hominidae - ps37
Homininae - ps38
Hominini - ps39
Homo_sapiens - ps40
Phylostratum

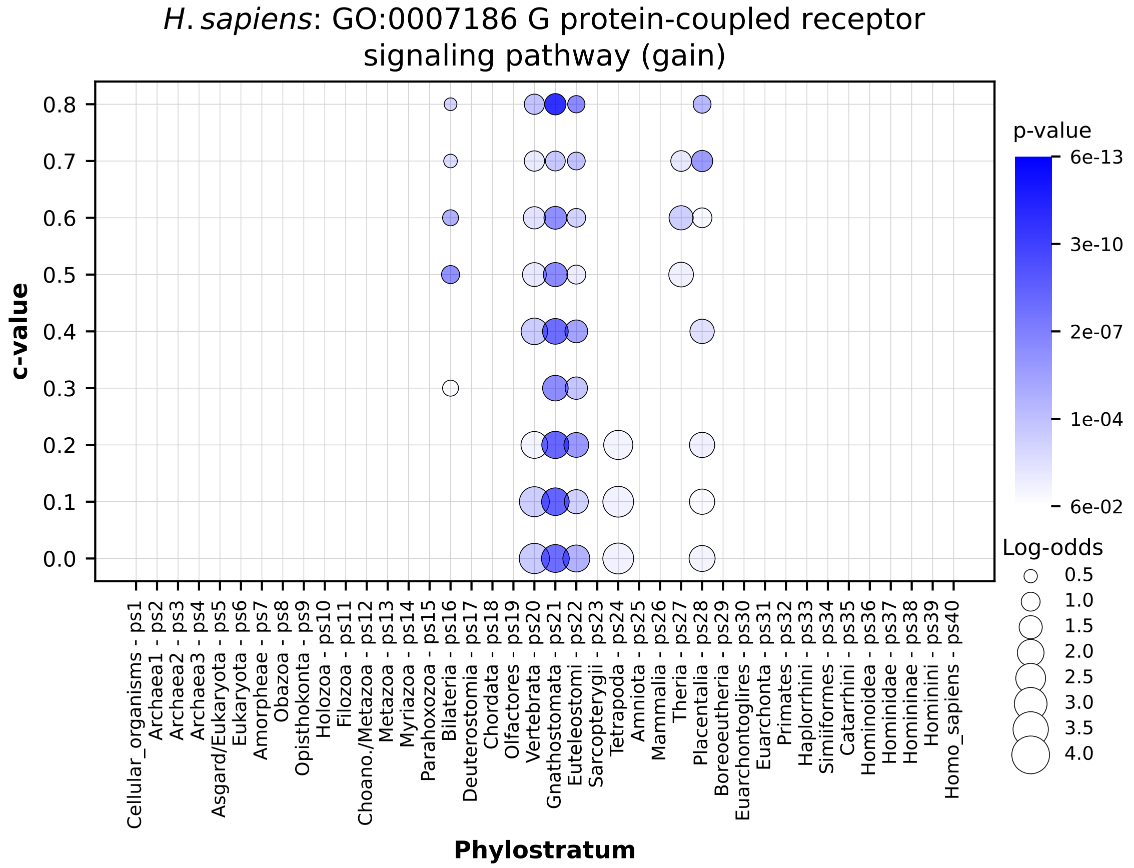

H. sapiens: GO:0007186 G protein-coupled receptor signaling pathway (gain)
c-value
0.8
0.7
0.6
0.5
0.4
0.3
0.2
0.1
0.0
p-value
6e-13
3e-10
2e-07
1e-04
6e-02
Log-odds
0.5
1.0
1.5
2.0
2.5
3.0
3.5
4.0
Cellular_organisms - ps1
Archaea1 - ps2
Archaea2 - ps3
Archaea3 - ps4
Asgard/Eukaryota - ps5
Eukaryota - ps6
Amorpheae - ps7
Obazoa - ps8
Opisthokonta - ps9
Holozoa - ps10
Filozoa - ps11
Choano./Metazoa - ps12
Metazoa - ps13
Myriazoa - ps14
Parahoxozoa - ps15
Bilateria - ps16
Deuterostomia - ps17
Chordata - ps18
Olfactores - ps19
Vertebrata - ps20
Gnathostomata - ps21
Euteleostomi - ps22
Sarcopterygii - ps23
Tetrapoda - ps24
Amniota - ps25
Mammalia - ps26
Theria - ps27
Placentalia - ps28
Boreoeutheria - ps29
Euarchontoglires - ps30
Euarchonta - ps31
Primates - ps32
Haplorrhini - ps33
Simiiformes - ps34
Catarrhini - ps35
Hominoidea - ps36
Hominidae - ps37
Homininae - ps38
Hominini - ps39
Homo_sapiens - ps40
Phylostratum

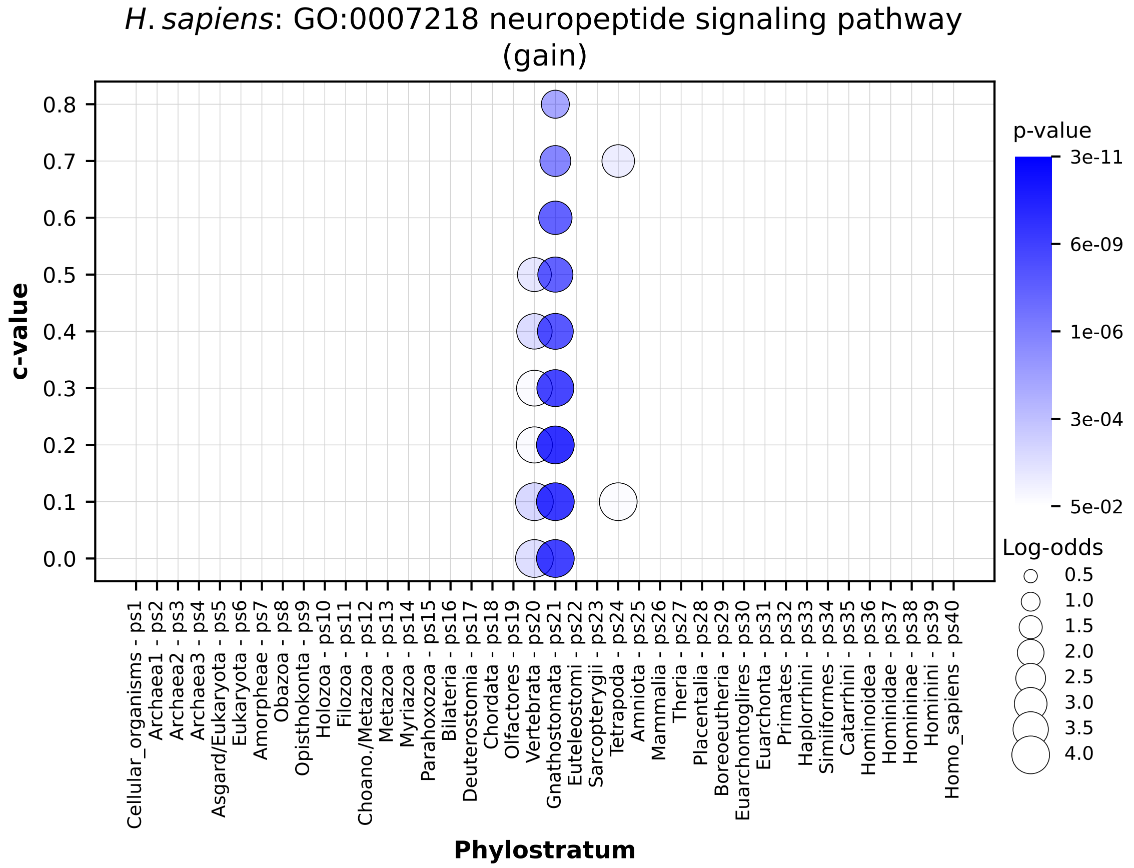

H. sapiens: GO:0007218 neuropeptide signaling pathway
(gain)
c-value
0.0
0.1
0.2
0.3
0.4
0.5
0.6
0.7
0.8
Phylostratum
Cellular_organisms - ps1
Archaea1 - ps2
Archaea2 - ps3
Archaea3 - ps4
Asgard/Eukaryota - ps5
Eukaryota - ps6
Amorpheae - ps7
Obazoa - ps8
Opisthokonta - ps9
Holozoa - ps10
Filozoa - ps11
Choano./Metazoa - ps12
Metazoa - ps13
Myriazoa - ps14
Parahoxozoa - ps15
Bilateria - ps16
Deuterostomia - ps17
Chordata - ps18
Olfactores - ps19
Vertebrata - ps20
Gnathostomata - ps21
Euteleostomi - ps22
Sarcopterygii - ps23
Tetrapoda - ps24
Amniota - ps25
Mammalia - ps26
Theria - ps27
Placentalia - ps28
Boreoeutheria - ps29
Euarchontoglires - ps30
Euarchonta - ps31
Primates - ps32
Haplorrhini - ps33
Simiiformes - ps34
Catarrhini - ps35
Hominoidea - ps36
Hominidae - ps37
Homininae - ps38
Hominini - ps39
Homo_sapiens - ps40
p-value
3e-11
6e-09
1e-06
3e-04
5e-02
Log-odds
0.5
1.0
1.5
2.0
2.5
3.0
3.5
4.0

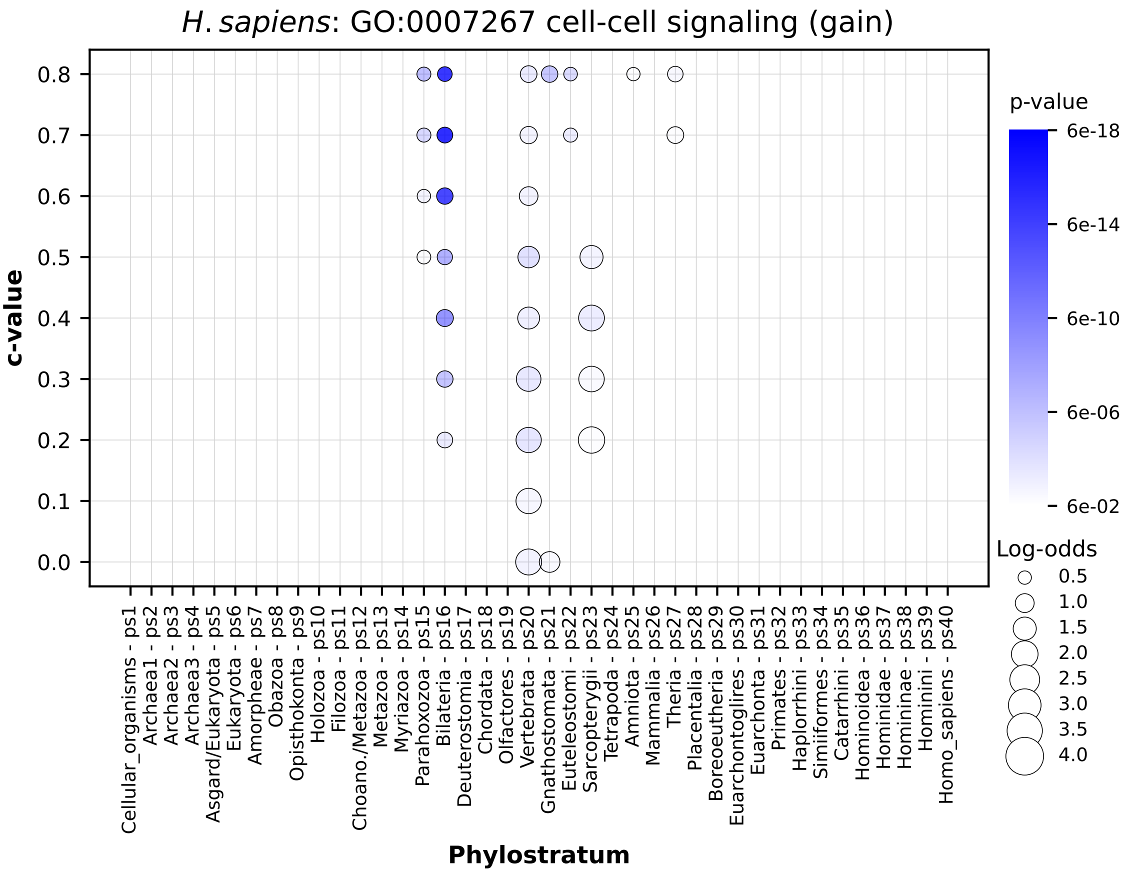

H. sapiens: GO:0007267 cell-cell signaling (gain)
c-value
0.8
0.7
0.6
0.5
0.4
0.3
0.2
0.1
0.0
Phylostratum
Cellular_organisms - ps1
Archaea1 - ps2
Archaea2 - ps3
Archaea3 - ps4
Asgard/Eukaryota - ps5
Eukaryota - ps6
Amorpheae - ps7
Obazoa - ps8
Opisthokonta - ps9
Holozoa - ps10
Filozoa - ps11
Choano./Metazoa - ps12
Metazoa - ps13
Myriazoa - ps14
Parahoxozoa - ps15
Bilateria - ps16
Deuterostomia - ps17
Chordata - ps18
Olfactores - ps19
Vertebrata - ps20
Gnathostomata - ps21
Euteleostomi - ps22
Sarcopterygii - ps23
Tetrapoda - ps24
Amniota - ps25
Mammalia - ps26
Theria - ps27
Placentalia - ps28
Boreoeutheria - ps29
Euarchontoglires - ps30
Euarchonta - ps31
Primates - ps32
Haplorrhini - ps33
Simiiformes - ps34
Catarrhini - ps35
Hominoidea - ps36
Hominidae - ps37
Homininae - ps38
Hominini - ps39
Homo_sapiens - ps40
p-value
6e-18
6e-14
6e-10
6e-06
6e-02
Log-odds
0.5
1.0
1.5
2.0
2.5
3.0
3.5
4.0

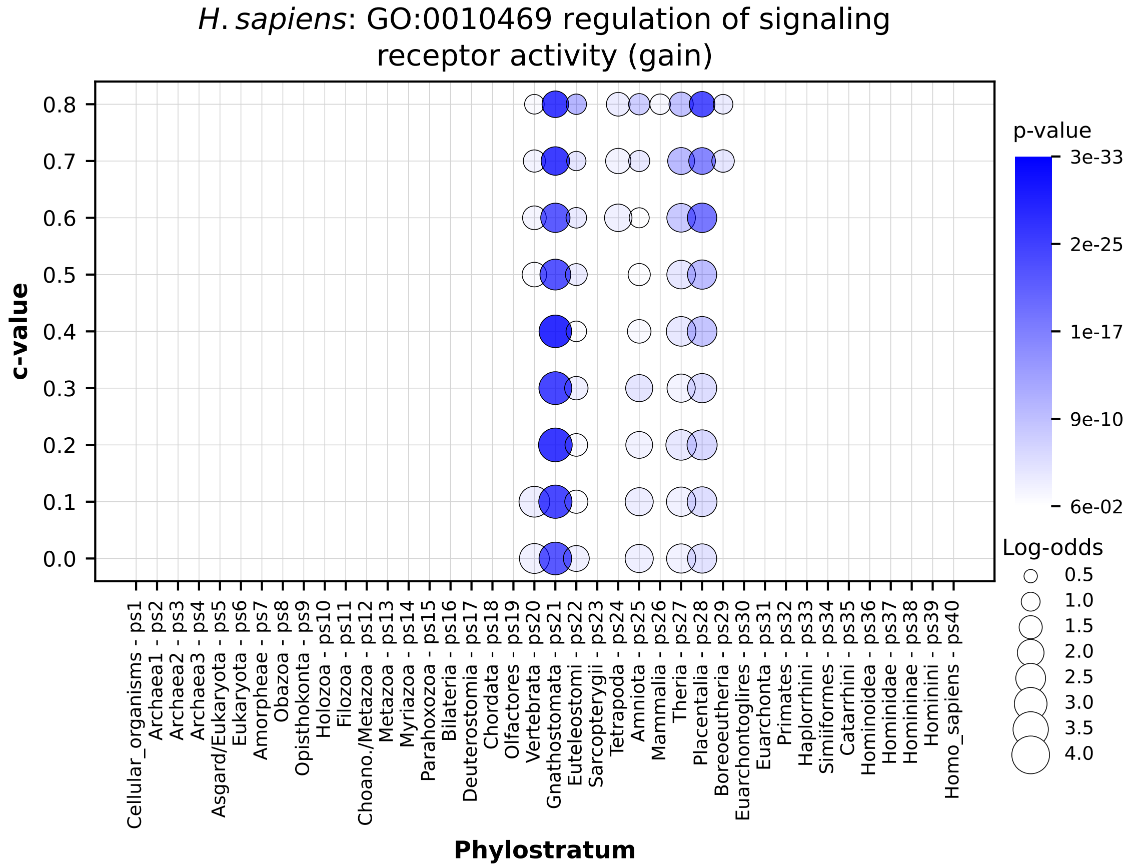

H. sapiens: GO:0010469 regulation of signaling receptor activity (gain)
c-value
0.8
0.7
0.6
0.5
0.4
0.3
0.2
0.1
0.0
p-value
3e-33
2e-25
1e-17
9e-10
6e-02
Log-odds
0.5
1.0
1.5
2.0
2.5
3.0
3.5
4.0
Cellular_organisms - ps1
Archaea1 - ps2
Archaea2 - ps3
Archaea3 - ps4
Asgard/Eukaryota - ps5
Eukaryota - ps6
Amorpheae - ps7
Obazoa - ps8
Opisthokonta - ps9
Holozoa - ps10
Filozoa - ps11
Choano./Metazoa - ps12
Metazoa - ps13
Myriazoa - ps14
Parahoxozoa - ps15
Bilateria - ps16
Deuterostomia - ps17
Chordata - ps18
Olfactores - ps19
Vertebrata - ps20
Gnathostomata - ps21
Euteleostomi - ps22
Sarcopterygii - ps23
Tetrapoda - ps24
Amniota - ps25
Mammalia - ps26
Theria - ps27
Placentalia - ps28
Boreoeutheria - ps29
Euarchontoglires - ps30
Euarchonta - ps31
Primates - ps32
Haplorrhini - ps33
Simiiformes - ps34
Catarrhini - ps35
Hominoidea - ps36
Hominidae - ps37
Homininae - ps38
Hominini - ps39
Homo_sapiens - ps40
Phylostratum

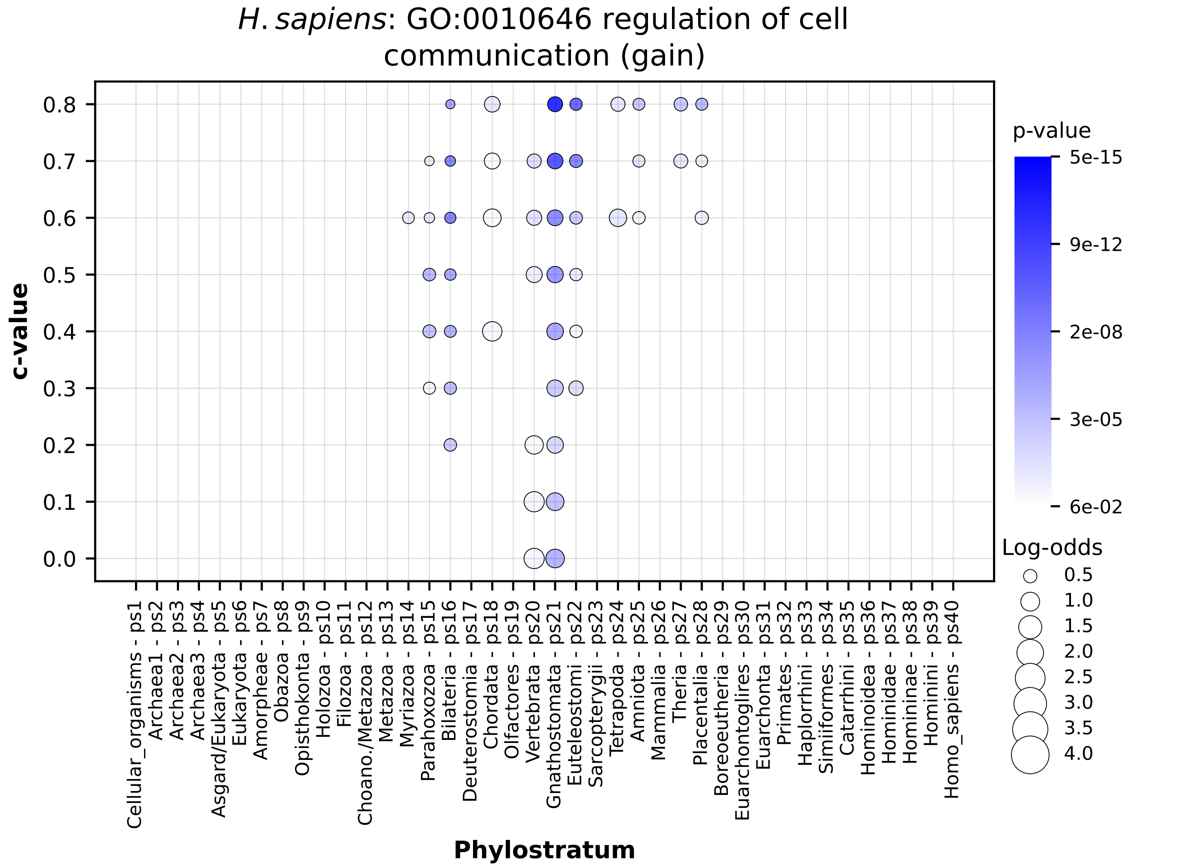

H. sapiens: GO:0010646 regulation of cell communication (gain)
c-value
0.8
0.7
0.6
0.5
0.4
0.3
0.2
0.1
0.0
Phylostratum
Cellular_organisms - ps1
Archaea1 - ps2
Archaea2 - ps3
Archaea3 - ps4
Asgard/Eukaryota - ps5
Eukaryota - ps6
Amorpheae - ps7
Obazoa - ps8
Opisthokonta - ps9
Holozoa - ps10
Filozoa - ps11
Choano./Metazoa - ps12
Metazoa - ps13
Myriazoa - ps14
Parahoxozoa - ps15
Bilateria - ps16
Deuterostomia - ps17
Chordata - ps18
Olfactores - ps19
Vertebrata - ps20
Gnathostomata - ps21
Euteleostomi - ps22
Sarcopterygii - ps23
Tetrapoda - ps24
Amniota - ps25
Mammalia - ps26
Theria - ps27
Placentalia - ps28
Boreoeutheria - ps29
Euarchontoglires - ps30
Euarchonta - ps31
Primates - ps32
Haplorrhini - ps33
Simiiformes - ps34
Catarrhini - ps35
Hominoidea - ps36
Hominidae - ps37
Homininae - ps38
Hominini - ps39
Homo_sapiens - ps40
p-value
5e-15
9e-12
2e-08
3e-05
6e-02
Log-odds
0.5
1.0
1.5
2.0
2.5
3.0
3.5
4.0

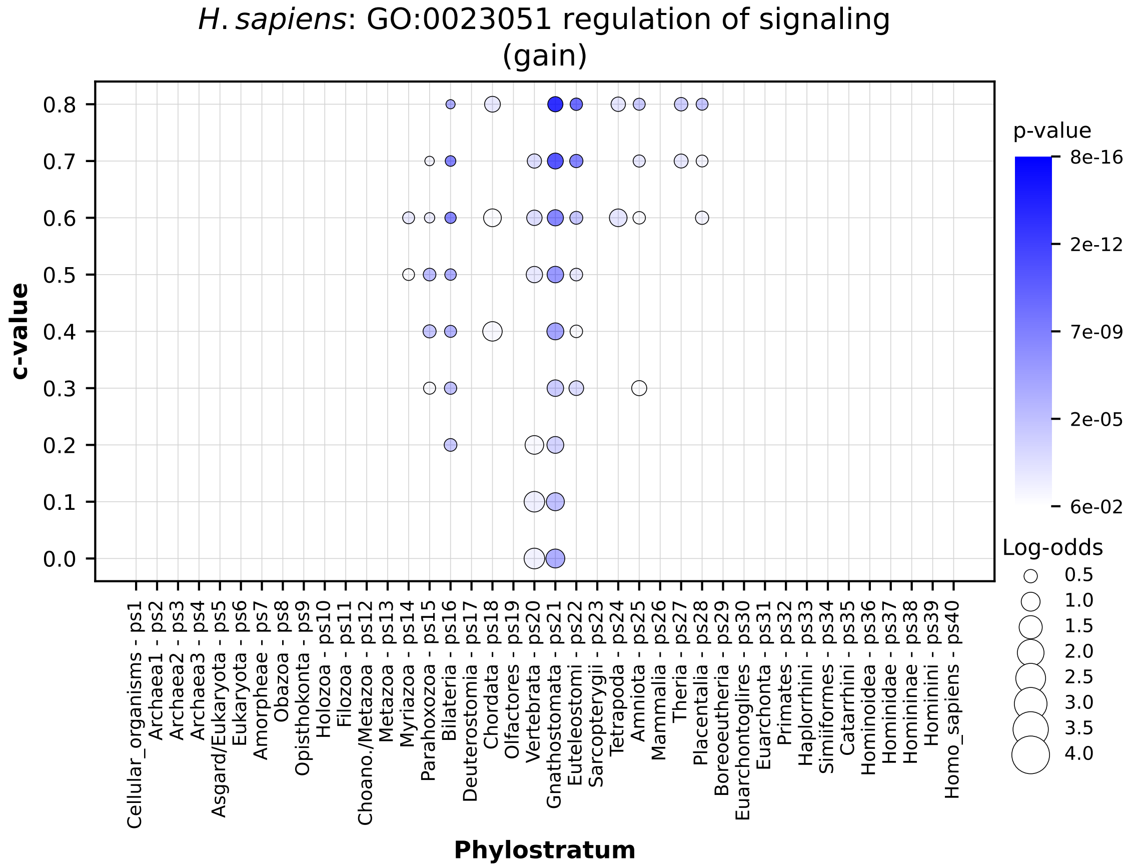

H. sapiens: GO:0023051 regulation of signaling
(gain)
c-value
0.8
0.7
0.6
0.5
0.4
0.3
0.2
0.1
0.0
p-value
8e-16
2e-12
7e-09
2e-05
6e-02
Log-odds
0.5
1.0
1.5
2.0
2.5
3.0
3.5
4.0
Cellular_organisms - ps1
Archaea1 - ps2
Archaea2 - ps3
Archaea3 - ps4
Asgard/Eukaryota - ps5
Eukaryota - ps6
Amorpheae - ps7
Obazoa - ps8
Opisthokonta - ps9
Holozoa - ps10
Filozoa - ps11
Choano./Metazoa - ps12
Metazoa - ps13
Myriazoa - ps14
Parahoxozoa - ps15
Bilateria - ps16
Deuterostomia - ps17
Chordata - ps18
Olfactores - ps19
Vertebrata - ps20
Gnathostomata - ps21
Euteleostomi - ps22
Sarcopterygii - ps23
Tetrapoda - ps24
Amniota - ps25
Mammalia - ps26
Theria - ps27
Placentalia - ps28
Boreoeutheria - ps29
Euarchontoglires - ps30
Euarchonta - ps31
Primates - ps32
Haplorrhini - ps33
Simiiformes - ps34
Catarrhini - ps35
Hominoidea - ps36
Hominidae - ps37
Homininae - ps38
Hominini - ps39
Homo_sapiens - ps40
Phylostratum

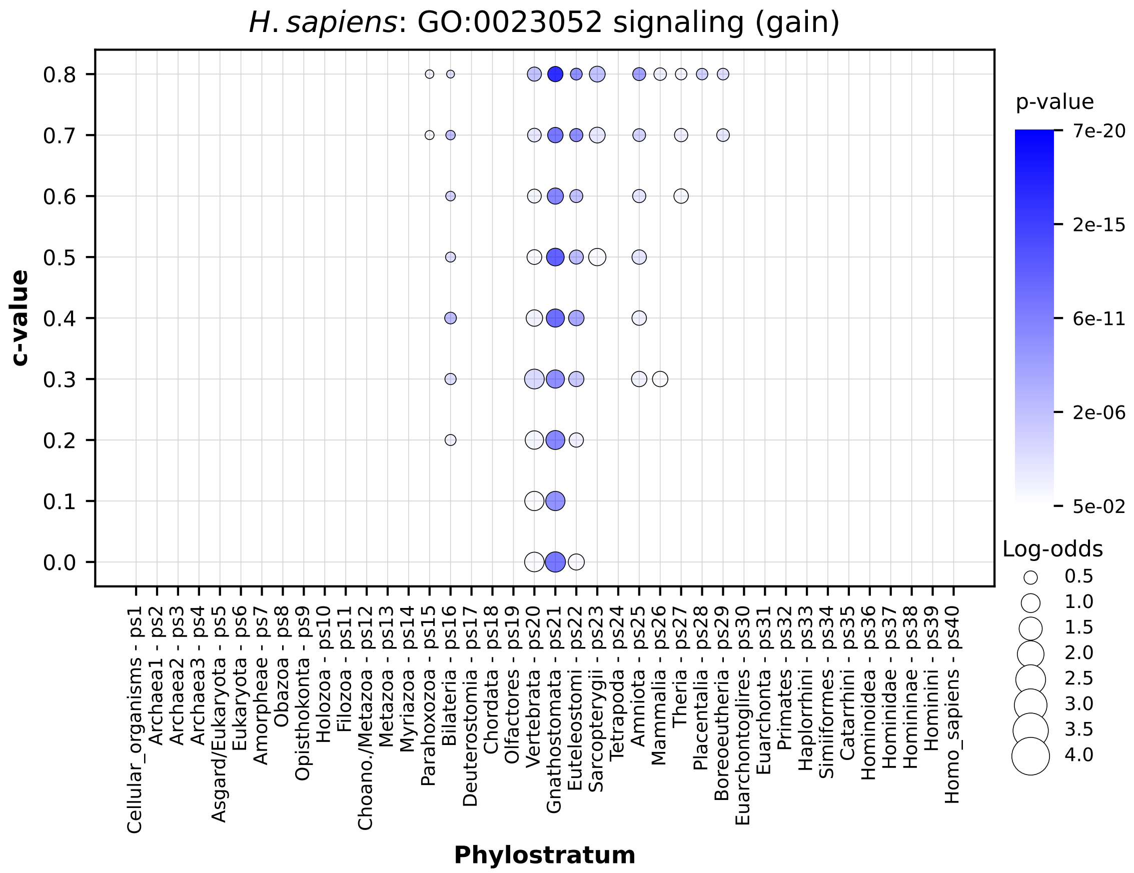

H. sapiens: GO:0023052 signaling (gain)
c-value
0.8
0.7
0.6
0.5
0.4
0.3
0.2
0.1
0.0
Phylostratum
Cellular_organisms - ps1
Archaea1 - ps2
Archaea2 - ps3
Archaea3 - ps4
Asgard/Eukaryota - ps5
Eukaryota - ps6
Amorpheae - ps7
Obazoa - ps8
Opisthokonta - ps9
Holozoa - ps10
Filozoa - ps11
Choano./Metazoa - ps12
Metazoa - ps13
Myriazoa - ps14
Parahoxozoa - ps15
Bilateria - ps16
Deuterostomia - ps17
Chordata - ps18
Olfactores - ps19
Vertebrata - ps20
Gnathostomata - ps21
Euteleostomi - ps22
Sarcopterygii - ps23
Tetrapoda - ps24
Amniota - ps25
Mammalia - ps26
Theria - ps27
Placentalia - ps28
Boreoeutheria - ps29
Euarchontoglires - ps30
Euarchonta - ps31
Primates - ps32
Haplorrhini - ps33
Simiiformes - ps34
Catarrhini - ps35
Hominoidea - ps36
Hominidae - ps37
Homininae - ps38
Hominini - ps39
Homo_sapiens - ps40
p-value
7e-20
2e-15
6e-11
2e-06
5e-02
Log-odds
0.5
1.0
1.5
2.0
2.5
3.0
3.5
4.0

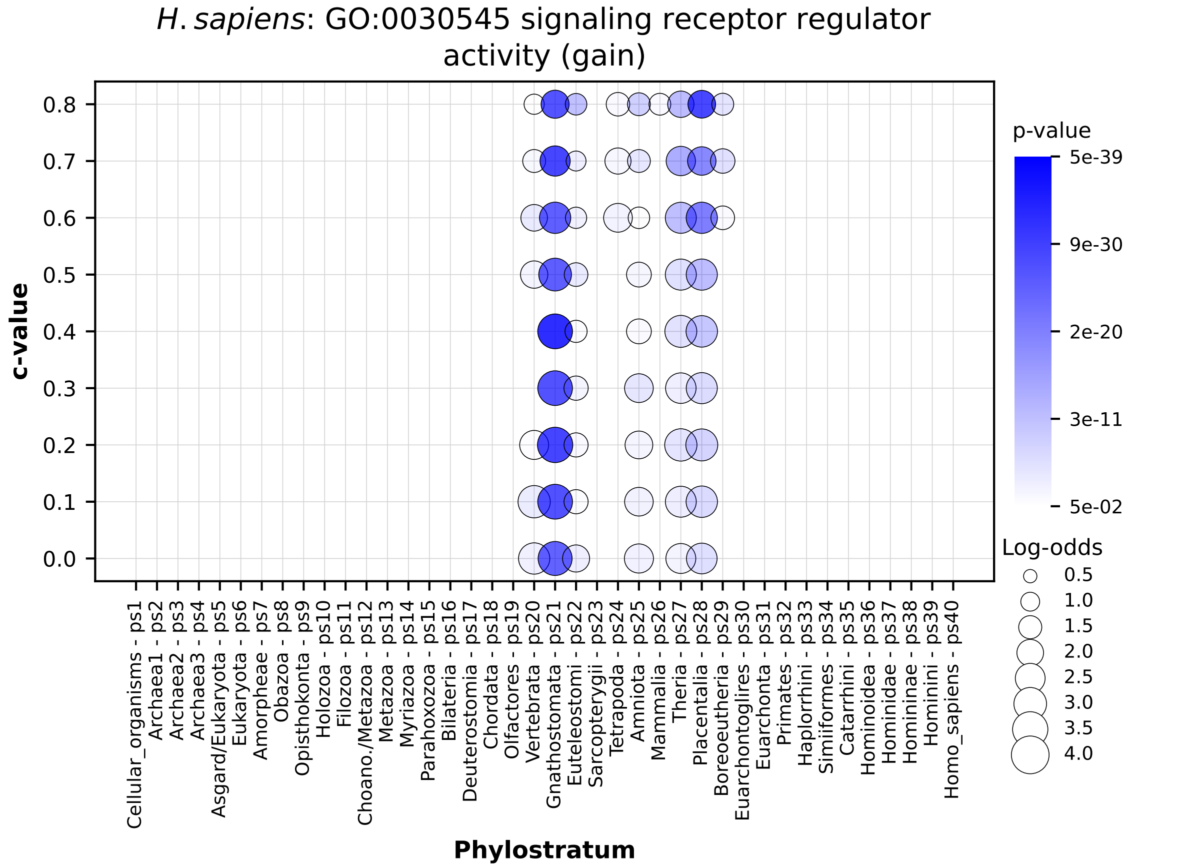

H. sapiens: GO:0030545 signaling receptor regulator activity (gain)
c-value
0.8
0.7
0.6
0.5
0.4
0.3
0.2
0.1
0.0
p-value
5e-39
9e-30
2e-20
3e-11
5e-02
Log-odds
0.5
1.0
1.5
2.0
2.5
3.0
3.5
4.0
Cellular_organisms - ps1
Archaea1 - ps2
Archaea2 - ps3
Archaea3 - ps4
Asgard/Eukaryota - ps5
Eukaryota - ps6
Amorpheae - ps7
Obazoa - ps8
Opisthokonta - ps9
Holozoa - ps10
Filozoa - ps11
Choano./Metazoa - ps12
Metazoa - ps13
Myriazoa - ps14
Parahoxozoa - ps15
Bilateria - ps16
Deuterostomia - ps17
Chordata - ps18
Olfactores - ps19
Vertebrata - ps20
Gnathostomata - ps21
Euteleostomi - ps22
Sarcopterygii - ps23
Tetrapoda - ps24
Amniota - ps25
Mammalia - ps26
Theria - ps27
Placentalia - ps28
Boreoeutheria - ps29
Euarchontoglires - ps30
Euarchonta - ps31
Primates - ps32
Haplorrhini - ps33
Simiiformes - ps34
Catarrhini - ps35
Hominoidea - ps36
Hominidae - ps37
Homininae - ps38
Hominini - ps39
Homo_sapiens - ps40
Phylostratum

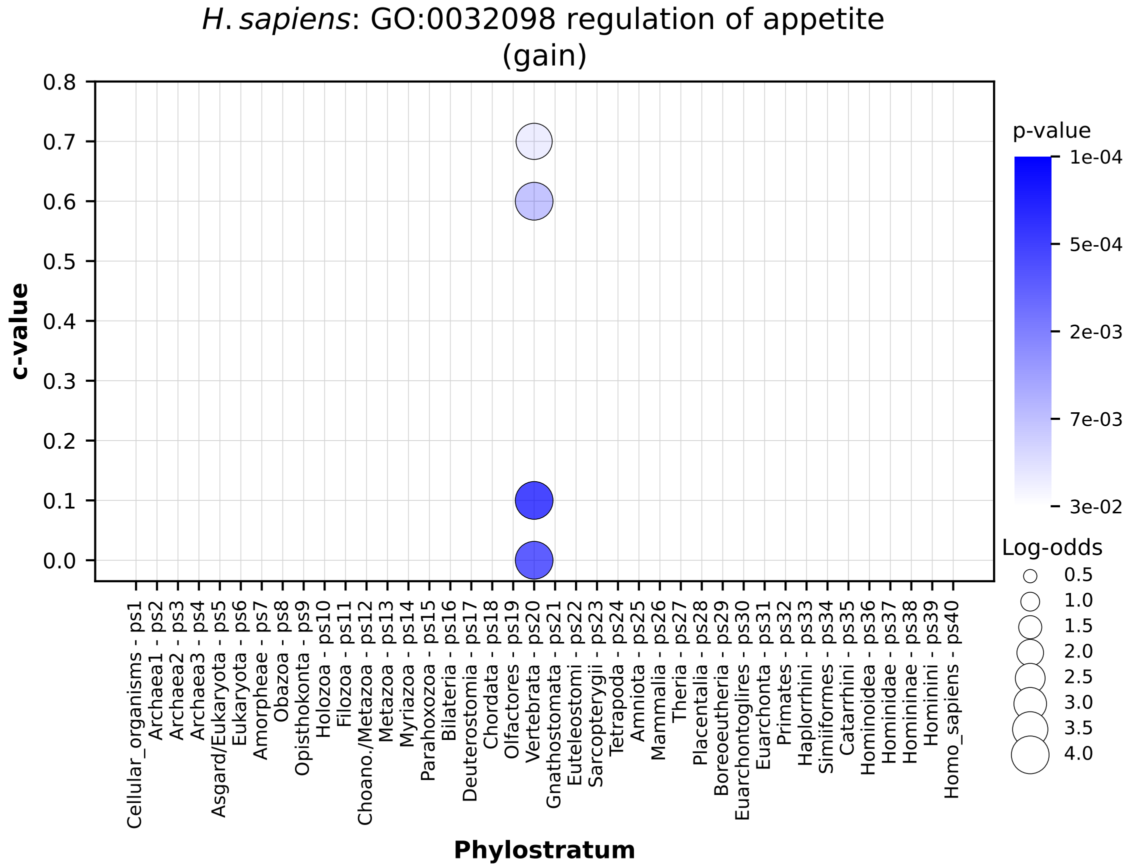
*H. sapiens*: GO:0032098 regulation of appetite
(gain)
c-value
0.8
0.7
0.6
0.5
0.4
0.3
0.2
0.1
0.0
Phylostratum
Cellular_organisms - ps1
Archaea1 - ps2
Archaea2 - ps3
Archaea3 - ps4
Asgard/Eukaryota - ps5
Eukaryota - ps6
Amorpheae - ps7
Obazoa - ps8
Opisthokonta - ps9
Holozoa - ps10
Filozoa - ps11
Choano./Metazoa - ps12
Metazoa - ps13
Myriazoa - ps14
Parahoxozoa - ps15
Bilateria - ps16
Deuterostomia - ps17
Chordata - ps18
Olfactores - ps19
Vertebrata - ps20
Gnathostomata - ps21
Euteleostomi - ps22
Sarcopterygii - ps23
Tetrapoda - ps24
Amniota - ps25
Mammalia - ps26
Theria - ps27
Placentalia - ps28
Boreoeutheria - ps29
Euarchontoglires - ps30
Euarchonta - ps31
Primates - ps32
Haplorrhini - ps33
Simiiformes - ps34
Catarrhini - ps35
Hominoidea - ps36
Hominidae - ps37
Homininae - ps38
Hominini - ps39
Homo_sapiens - ps40
p-value
1e-04
5e-04
2e-03
7e-03
3e-02
Log-odds
0.5
1.0
1.5
2.0
2.5
3.0
3.5
4.0

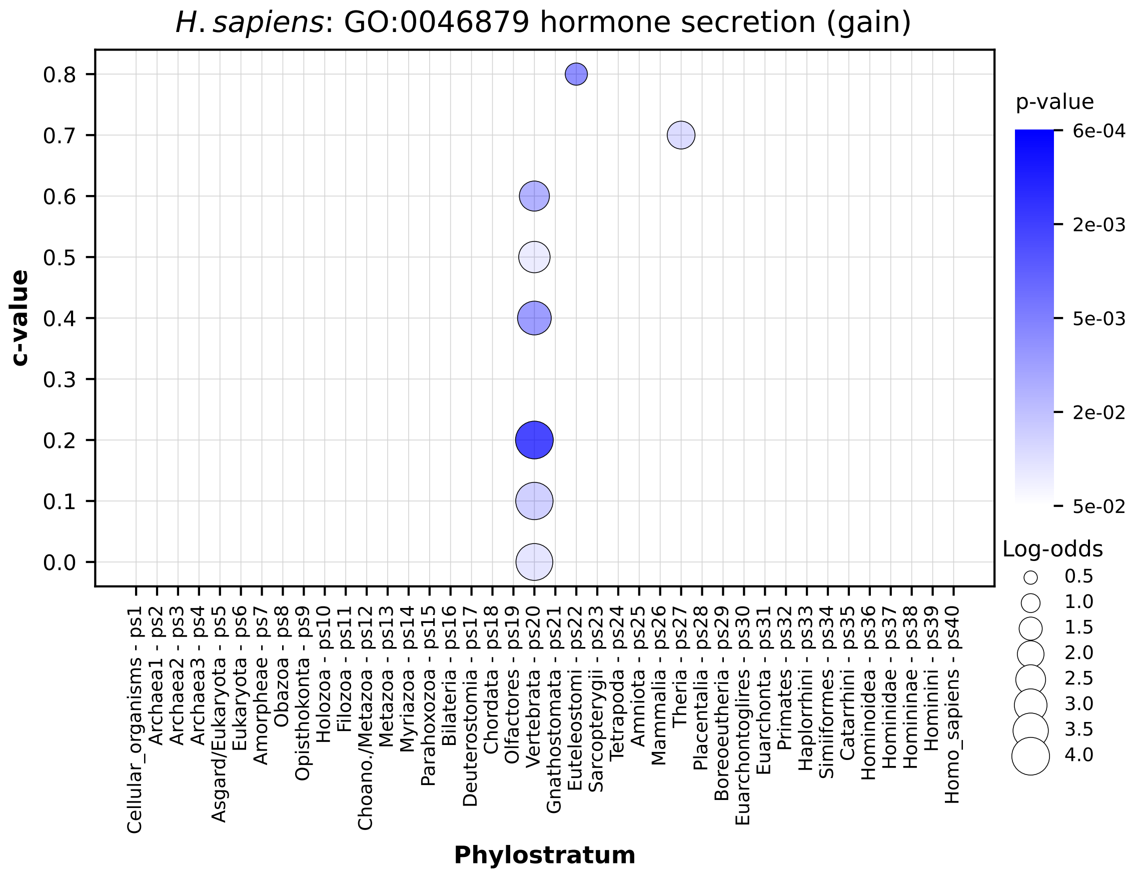
H. sapiens: GO:0046879 hormone secretion (gain)
c-value
0.0
0.1
0.2
0.3
0.4
0.5
0.6
0.7
0.8
p-value
6e-04
2e-03
5e-03
2e-02
5e-02
Log-odds
0.5
1.0
1.5
2.0
2.5
3.0
3.5
4.0
Cellular_organisms - ps1
Archaea1 - ps2
Archaea2 - ps3
Archaea3 - ps4
Asgard/Eukaryota - ps5
Eukaryota - ps6
Amorpheae - ps7
Obazoa - ps8
Opisthokonta - ps9
Holozoa - ps10
Filozoa - ps11
Choano./Metazoa - ps12
Metazoa - ps13
Myriazoa - ps14
Parahoxozoa - ps15
Bilateria - ps16
Deuterostomia - ps17
Chordata - ps18
Olfactores - ps19
Vertebrata - ps20
Gnathostomata - ps21
Euteleostomi - ps22
Sarcopterygii - ps23
Tetrapoda - ps24
Amniota - ps25
Mammalia - ps26
Theria - ps27
Placentalia - ps28
Boreoeutheria - ps29
Euarchontoglires - ps30
Euarchonta - ps31
Primates - ps32
Haplorrhini - ps33
Simiiformes - ps34
Catarrhini - ps35
Hominoidea - ps36
Hominidae - ps37
Homininae - ps38
Hominini - ps39
Homo_sapiens - ps40
Phylostratum

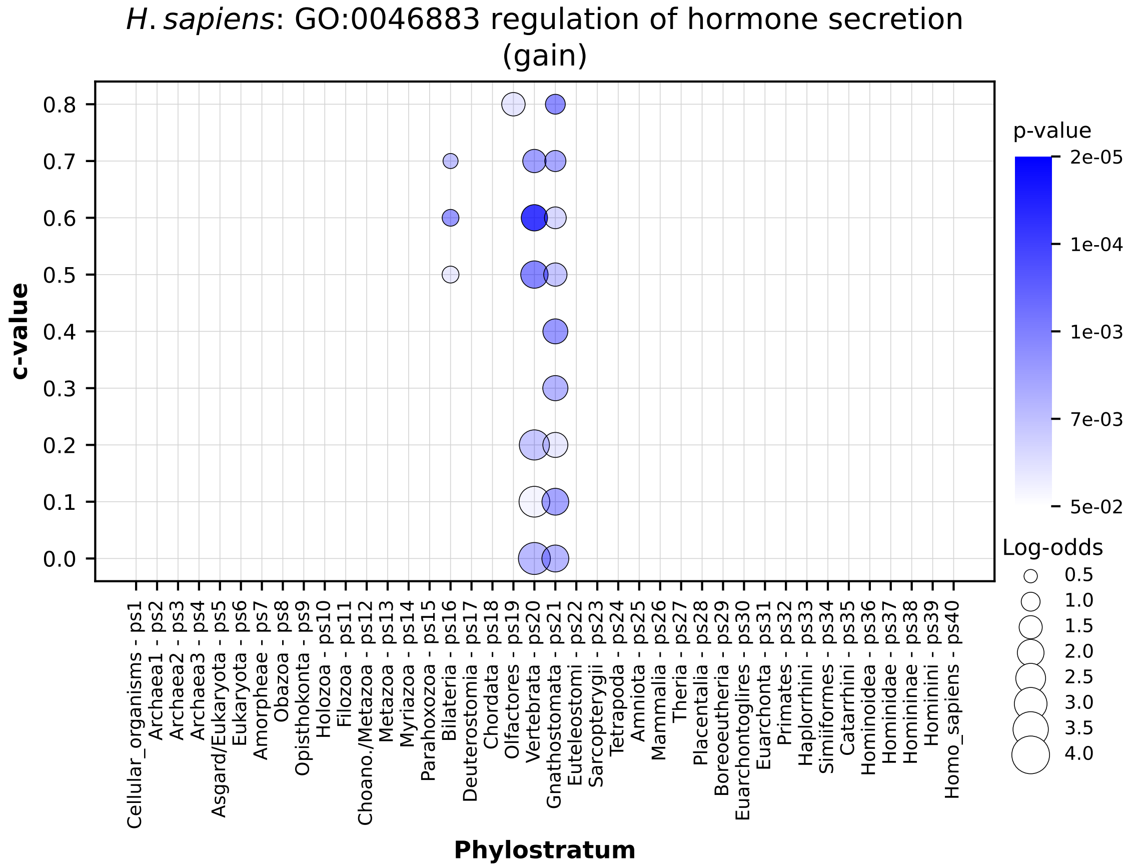

*H. sapiens*: GO:0046883 regulation of hormone secretion
(gain)
c-value
0.8
0.7
0.6
0.5
0.4
0.3
0.2
0.1
0.0
Phylostratum
Cellular_organisms - ps1
Archaea1 - ps2
Archaea2 - ps3
Archaea3 - ps4
Asgard/Eukaryota - ps5
Eukaryota - ps6
Amorpheae - ps7
Obazoa - ps8
Opisthokonta - ps9
Holozoa - ps10
Filozoa - ps11
Choano./Metazoa - ps12
Metazoa - ps13
Myriazoa - ps14
Parahoxozoa - ps15
Bilateria - ps16
Deuterostomia - ps17
Chordata - ps18
Olfactores - ps19
Vertebrata - ps20
Gnathostomata - ps21
Euteleostomi - ps22
Sarcopterygii - ps23
Tetrapoda - ps24
Amniota - ps25
Mammalia - ps26
Theria - ps27
Placentalia - ps28
Boreoeutheria - ps29
Euarchontoglires - ps30
Euarchonta - ps31
Primates - ps32
Haplorrhini - ps33
Simiiformes - ps34
Catarrhini - ps35
Hominoidea - ps36
Hominidae - ps37
Homininae - ps38
Hominini - ps39
Homo_sapiens - ps40
p-value
2e-05
1e-04
1e-03
7e-03
5e-02
Log-odds
0.5
1.0
1.5
2.0
2.5
3.0
3.5
4.0

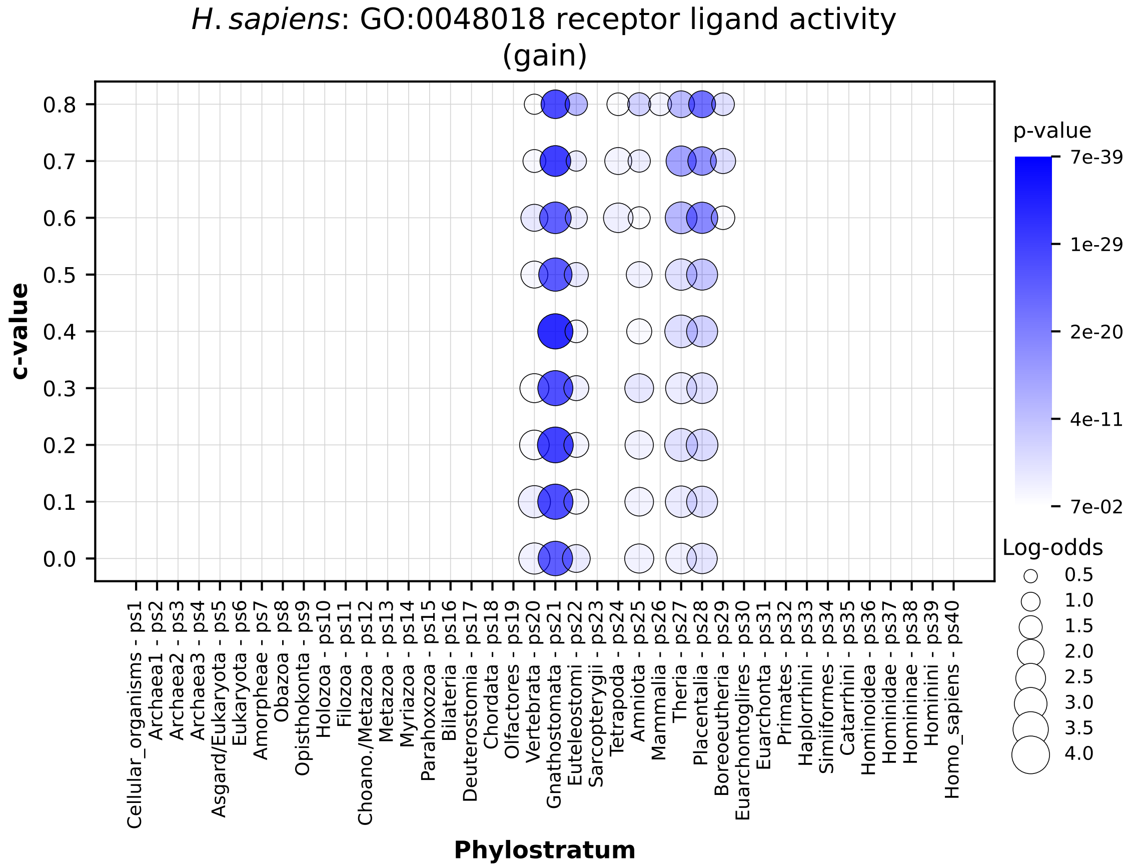

H. sapiens: GO:0048018 receptor ligand activity
(gain)
c-value
0.8
0.7
0.6
0.5
0.4
0.3
0.2
0.1
0.0
Phylostratum
Cellular_organisms - ps1
Archaea1 - ps2
Archaea2 - ps3
Archaea3 - ps4
Asgard/Eukaryota - ps5
Eukaryota - ps6
Amorpheae - ps7
Obazoa - ps8
Opisthokonta - ps9
Holozoa - ps10
Filozoa - ps11
Choano./Metazoa - ps12
Metazoa - ps13
Myriazoa - ps14
Parahoxozoa - ps15
Bilateria - ps16
Deuterostomia - ps17
Chordata - ps18
Olfactores - ps19
Vertebrata - ps20
Gnathostomata - ps21
Euteleostomi - ps22
Sarcopterygii - ps23
Tetrapoda - ps24
Amniota - ps25
Mammalia - ps26
Theria - ps27
Placentalia - ps28
Boreoeutheria - ps29
Euarchontoglires - ps30
Euarchonta - ps31
Primates - ps32
Haplorrhini - ps33
Simiiformes - ps34
Catarrhini - ps35
Hominoidea - ps36
Hominidae - ps37
Homininae - ps38
Hominini - ps39
Homo_sapiens - ps40
p-value
7e-39
1e-29
2e-20
4e-11
7e-02
Log-odds
0.5
1.0
1.5
2.0
2.5
3.0
3.5
4.0

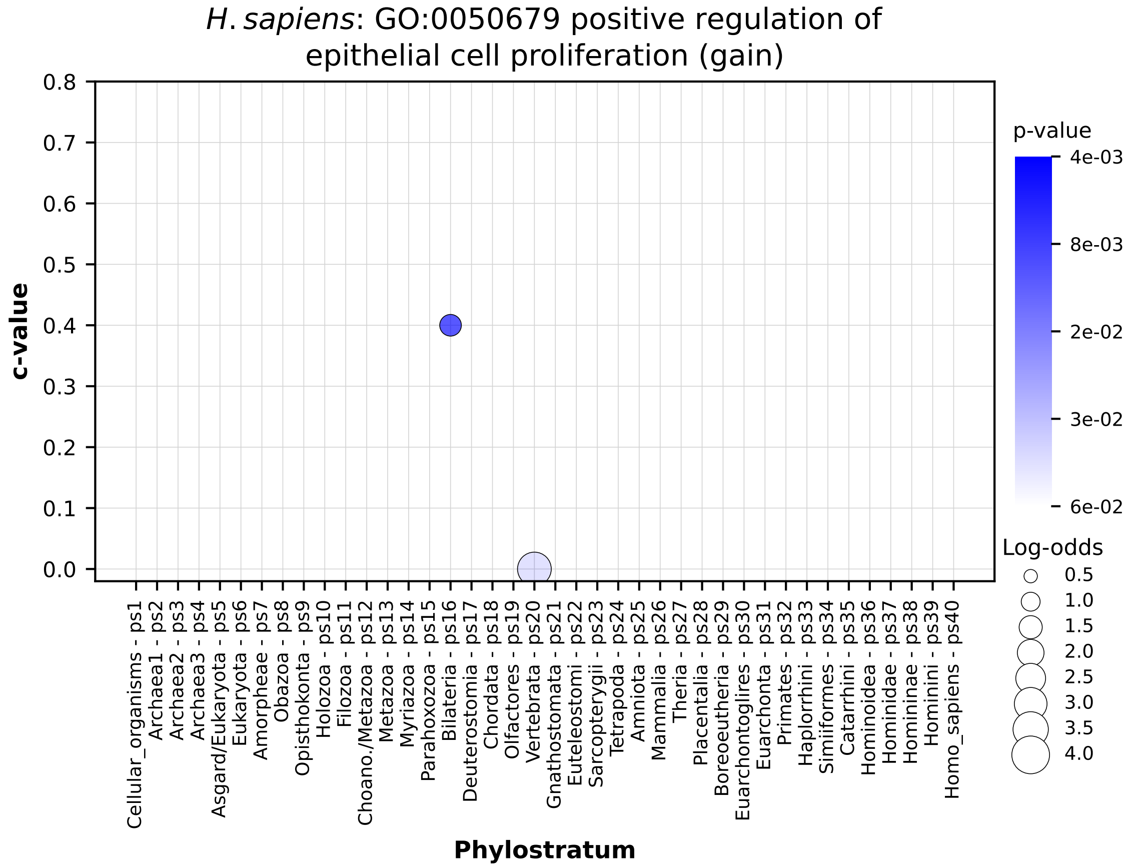

*H. sapiens*: GO:0050679 positive regulation of epithelial cell proliferation (gain)
c-value
0.8
0.7
0.6
0.5
0.4
0.3
0.2
0.1
0.0
p-value
4e-03
8e-03
2e-02
3e-02
6e-02
Log-odds
0.5
1.0
1.5
2.0
2.5
3.0
3.5
4.0
Cellular_organisms - ps1
Archaea1 - ps2
Archaea2 - ps3
Archaea3 - ps4
Asgard/Eukaryota - ps5
Eukaryota - ps6
Amorpheae - ps7
Obazoa - ps8
Opisthokonta - ps9
Holozoa - ps10
Filozoa - ps11
Choano./Metazoa - ps12
Metazoa - ps13
Myriazoa - ps14
Parahoxozoa - ps15
Bilateria - ps16
Deuterostomia - ps17
Chordata - ps18
Olfactores - ps19
Vertebrata - ps20
Gnathostomata - ps21
Euteleostomi - ps22
Sarcopterygii - ps23
Tetrapoda - ps24
Amniota - ps25
Mammalia - ps26
Theria - ps27
Placentalia - ps28
Boreoeutheria - ps29
Euarchontoglires - ps30
Euarchonta - ps31
Primates - ps32
Haplorrhini - ps33
Simiiformes - ps34
Catarrhini - ps35
Hominoidea - ps36
Hominidae - ps37
Homininae - ps38
Hominini - ps39
Homo_sapiens - ps40
Phylostratum

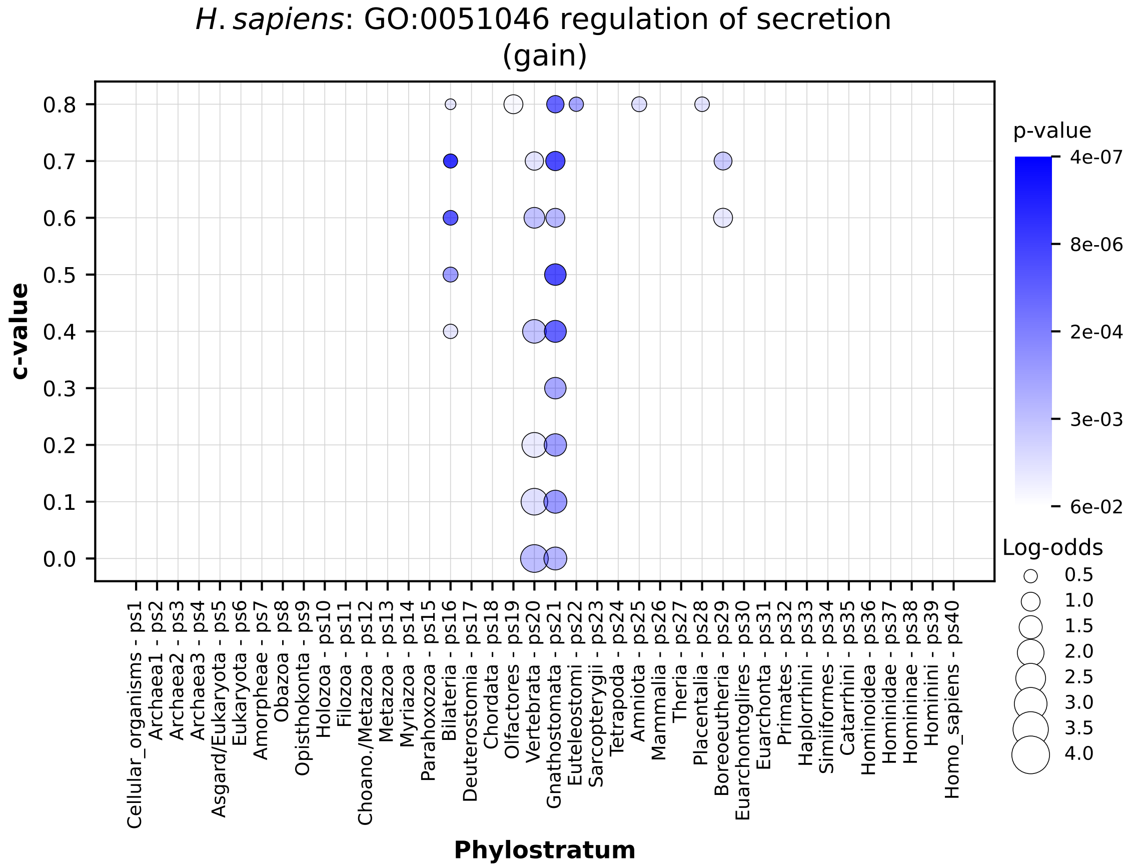

*H. sapiens*: GO:0051046 regulation of secretion (gain)
c-value
0.8
0.7
0.6
0.5
0.4
0.3
0.2
0.1
0.0
p-value
4e-07
8e-06
2e-04
3e-03
6e-02
Log-odds
0.5
1.0
1.5
2.0
2.5
3.0
3.5
4.0
Cellular_organisms - ps1
Archaea1 - ps2
Archaea2 - ps3
Archaea3 - ps4
Asgard/Eukaryota - ps5
Eukaryota - ps6
Amorpheae - ps7
Obazoa - ps8
Opisthokonta - ps9
Holozoa - ps10
Filozoa - ps11
Choano./Metazoa - ps12
Metazoa - ps13
Myriazoa - ps14
Parahoxozoa - ps15
Bilateria - ps16
Deuterostomia - ps17
Chordata - ps18
Olfactores - ps19
Vertebrata - ps20
Gnathostomata - ps21
Euteleostomi - ps22
Sarcopterygii - ps23
Tetrapoda - ps24
Amniota - ps25
Mammalia - ps26
Theria - ps27
Placentalia - ps28
Boreoeutheria - ps29
Euarchontoglires - ps30
Euarchonta - ps31
Primates - ps32
Haplorrhini - ps33
Simiiformes - ps34
Catarrhini - ps35
Hominoidea - ps36
Hominidae - ps37
Homininae - ps38
Hominini - ps39
Homo_sapiens - ps40
Phylostratum

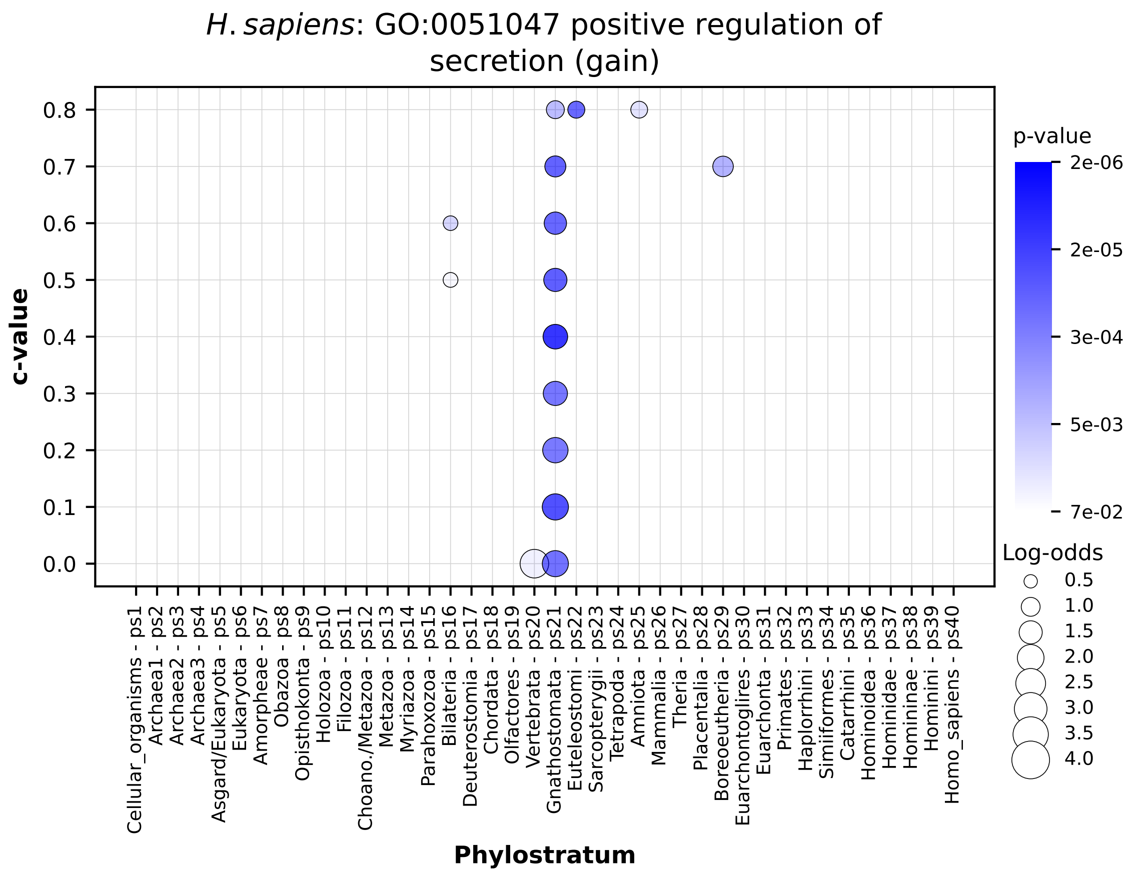

H. sapiens: GO:0051047 positive regulation of secretion (gain)
c-value
0.8
0.7
0.6
0.5
0.4
0.3
0.2
0.1
0.0
p-value
2e-06
2e-05
3e-04
5e-03
7e-02
Log-odds
0.5
1.0
1.5
2.0
2.5
3.0
3.5
4.0
Cellular_organisms - ps1
Archaea1 - ps2
Archaea2 - ps3
Archaea3 - ps4
Asgard/Eukaryota - ps5
Eukaryota - ps6
Amorpheae - ps7
Obazoa - ps8
Opisthokonta - ps9
Holozoa - ps10
Filozoa - ps11
Choano./Metazoa - ps12
Metazoa - ps13
Myriazoa - ps14
Parahoxozoa - ps15
Bilateria - ps16
Deuterostomia - ps17
Chordata - ps18
Olfactores - ps19
Vertebrata - ps20
Gnathostomata - ps21
Euteleostomi - ps22
Sarcopterygii - ps23
Tetrapoda - ps24
Amniota - ps25
Mammalia - ps26
Theria - ps27
Placentalia - ps28
Boreoeutheria - ps29
Euarchontoglires - ps30
Euarchonta - ps31
Primates - ps32
Haplorrhini - ps33
Simiiformes - ps34
Catarrhini - ps35
Hominoidea - ps36
Hominidae - ps37
Homininae - ps38
Hominini - ps39
Homo_sapiens - ps40
Phylostratum

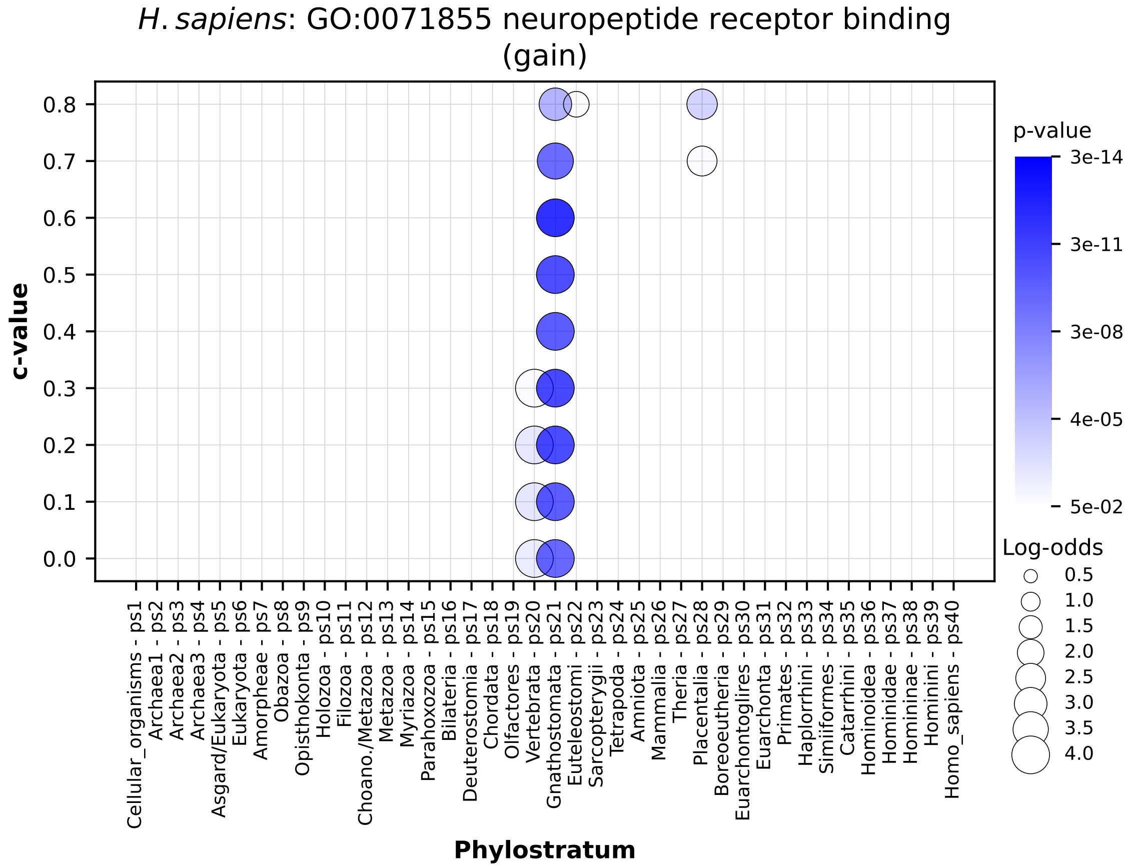

H. sapiens: GO:0071855 neuropeptide receptor binding
(gain)
c-value
0.0
0.1
0.2
0.3
0.4
0.5
0.6
0.7
0.8
Phylostratum
Cellular_organisms - ps1
Archaea1 - ps2
Archaea2 - ps3
Archaea3 - ps4
Asgard/Eukaryota - ps5
Eukaryota - ps6
Amorpheae - ps7
Obazoa - ps8
Opisthokonta - ps9
Holozoa - ps10
Filozoa - ps11
Choano./Metazoa - ps12
Metazoa - ps13
Myriazoa - ps14
Parahoxozoa - ps15
Bilateria - ps16
Deuterostomia - ps17
Chordata - ps18
Olfactores - ps19
Vertebrata - ps20
Gnathostomata - ps21
Euteleostomi - ps22
Sarcopterygii - ps23
Tetrapoda - ps24
Amniota - ps25
Mammalia - ps26
Theria - ps27
Placentalia - ps28
Boreoeutheria - ps29
Euarchontoglires - ps30
Euarchonta - ps31
Primates - ps32
Haplorrhini - ps33
Simiiformes - ps34
Catarrhini - ps35
Hominoidea - ps36
Hominidae - ps37
Homininae - ps38
Hominini - ps39
Homo_sapiens - ps40
p-value
3e-14
3e-11
3e-08
4e-05
5e-02
Log-odds
0.5
1.0
1.5
2.0
2.5
3.0
3.5
4.0

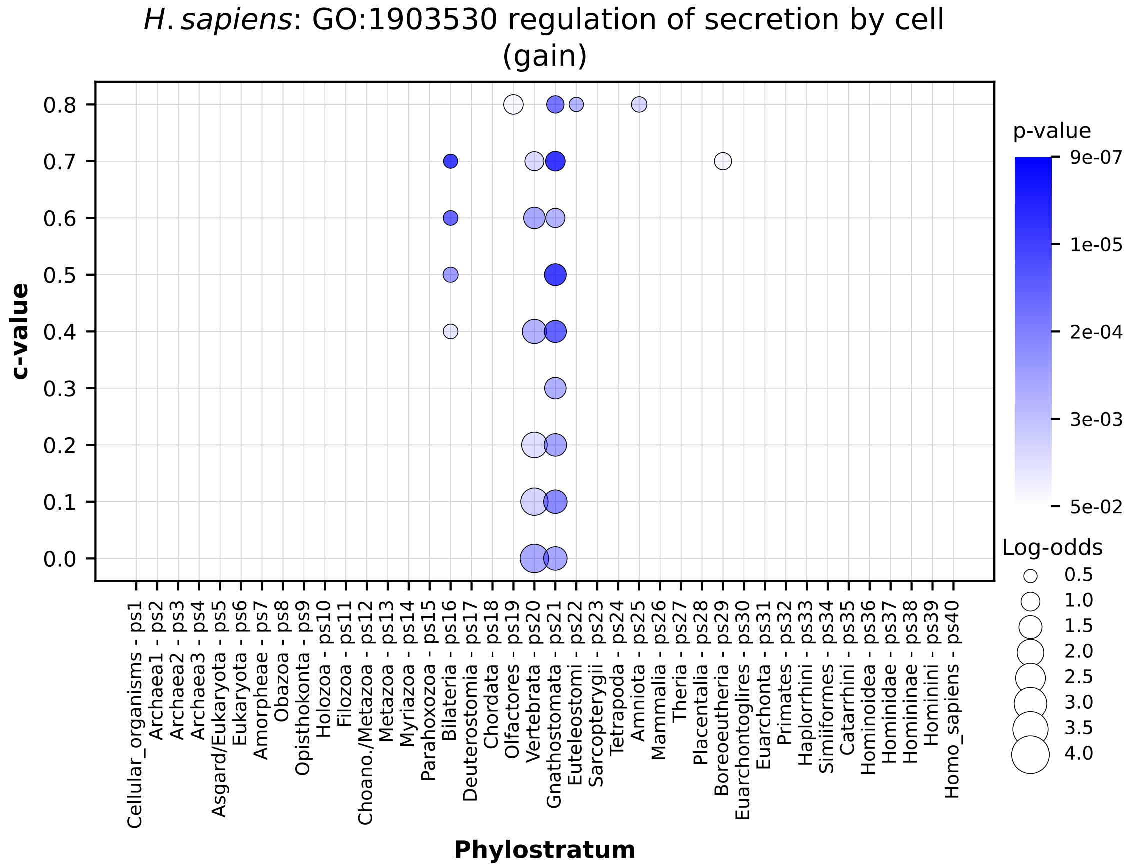

*H. sapiens*: GO:1903530 regulation of secretion by cell (gain)
c-value
0.8
0.7
0.6
0.5
0.4
0.3
0.2
0.1
0.0
p-value
9e-07
1e-05
2e-04
3e-03
5e-02
Log-odds
0.5
1.0
1.5
2.0
2.5
3.0
3.5
4.0
Cellular_organisms - ps1
Archaea1 - ps2
Archaea2 - ps3
Archaea3 - ps4
Asgard/Eukaryota - ps5
Eukaryota - ps6
Amorpheae - ps7
Obazoa - ps8
Opisthokonta - ps9
Holozoa - ps10
Filozoa - ps11
Choano./Metazoa - ps12
Metazoa - ps13
Myriazoa - ps14
Parahoxozoa - ps15
Bilateria - ps16
Deuterostomia - ps17
Chordata - ps18
Olfactores - ps19
Vertebrata - ps20
Gnathostomata - ps21
Euteleostomi - ps22
Sarcopterygii - ps23
Tetrapoda - ps24
Amniota - ps25
Mammalia - ps26
Theria - ps27
Placentalia - ps28
Boreoeutheria - ps29
Euarchontoglires - ps30
Euarchonta - ps31
Primates - ps32
Haplorrhini - ps33
Simiiformes - ps34
Catarrhini - ps35
Hominoidea - ps36
Hominidae - ps37
Homininae - ps38
Hominini - ps39
Homo_sapiens - ps40
Phylostratum

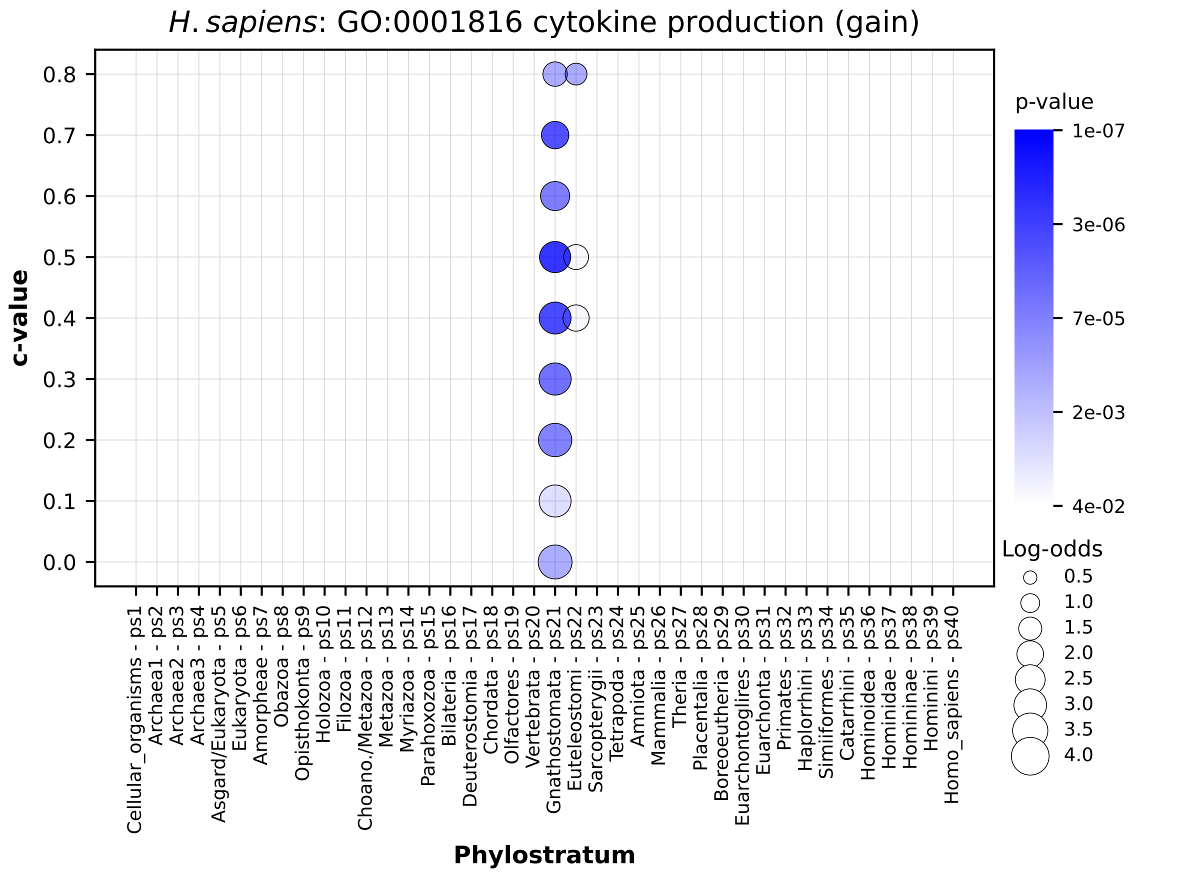

H. sapiens: GO:0001816 cytokine production (gain)
c-value
0.8
0.7
0.6
0.5
0.4
0.3
0.2
0.1
0.0
p-value
1e-07
3e-06
7e-05
2e-03
4e-02
Log-odds
0.5
1.0
1.5
2.0
2.5
3.0
3.5
4.0
Cellular_organisms - ps1
Archaea1 - ps2
Archaea2 - ps3
Archaea3 - ps4
Asgard/Eukaryota - ps5
Eukaryota - ps6
Amorpheae - ps7
Obazoa - ps8
Opisthokonta - ps9
Holozoa - ps10
Filozoa - ps11
Choano./Metazoa - ps12
Metazoa - ps13
Myriazoa - ps14
Parahoxozoa - ps15
Bilateria - ps16
Deuterostomia - ps17
Chordata - ps18
Olfactores - ps19
Vertebrata - ps20
Gnathostomata - ps21
Euteleostomi - ps22
Sarcopterygii - ps23
Tetrapoda - ps24
Amniota - ps25
Mammalia - ps26
Theria - ps27
Placentalia - ps28
Boreoeutheria - ps29
Euarchontoglires - ps30
Euarchonta - ps31
Primates - ps32
Haplorrhini - ps33
Simiiformes - ps34
Catarrhini - ps35
Hominoidea - ps36
Hominidae - ps37
Homininae - ps38
Hominini - ps39
Homo_sapiens - ps40
Phylostratum

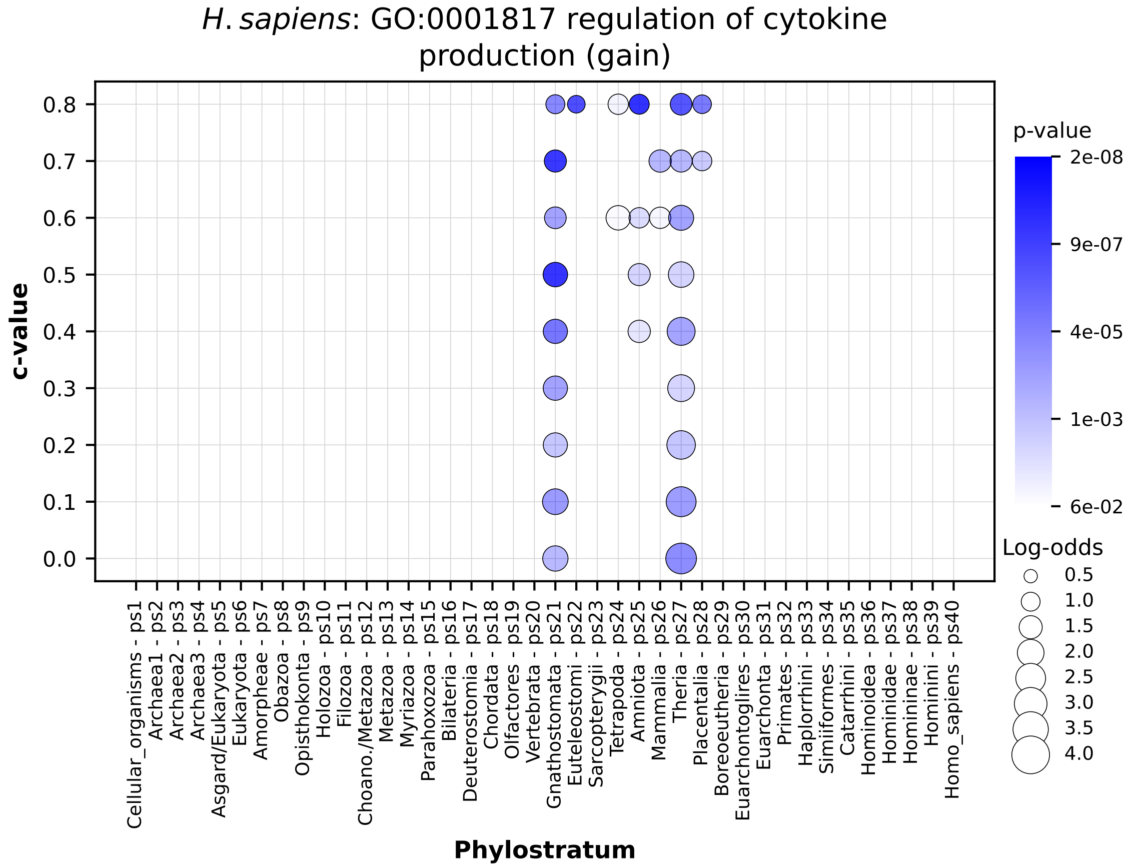
H. sapiens: GO:0001817 regulation of cytokine production (gain)
c-value
0.8
0.7
0.6
0.5
0.4
0.3
0.2
0.1
0.0
p-value
2e-08
9e-07
4e-05
1e-03
6e-02
Log-odds
0.5
1.0
1.5
2.0
2.5
3.0
3.5
4.0
Cellular_organisms - ps1
Archaea1 - ps2
Archaea2 - ps3
Archaea3 - ps4
Asgard/Eukaryota - ps5
Eukaryota - ps6
Amorpheae - ps7
Obazoa - ps8
Opisthokonta - ps9
Holozoa - ps10
Filozoa - ps11
Choano./Metazoa - ps12
Metazoa - ps13
Myriazoa - ps14
Parahoxozoa - ps15
Bilateria - ps16
Deuterostomia - ps17
Chordata - ps18
Olfactores - ps19
Vertebrata - ps20
Gnathostomata - ps21
Euteleostomi - ps22
Sarcopterygii - ps23
Tetrapoda - ps24
Amniota - ps25
Mammalia - ps26
Theria - ps27
Placentalia - ps28
Boreoeutheria - ps29
Euarchontoglires - ps30
Euarchonta - ps31
Primates - ps32
Haplorrhini - ps33
Simiiformes - ps34
Catarrhini - ps35
Hominoidea - ps36
Hominidae - ps37
Homininae - ps38
Hominini - ps39
Homo_sapiens - ps40
Phylostratum

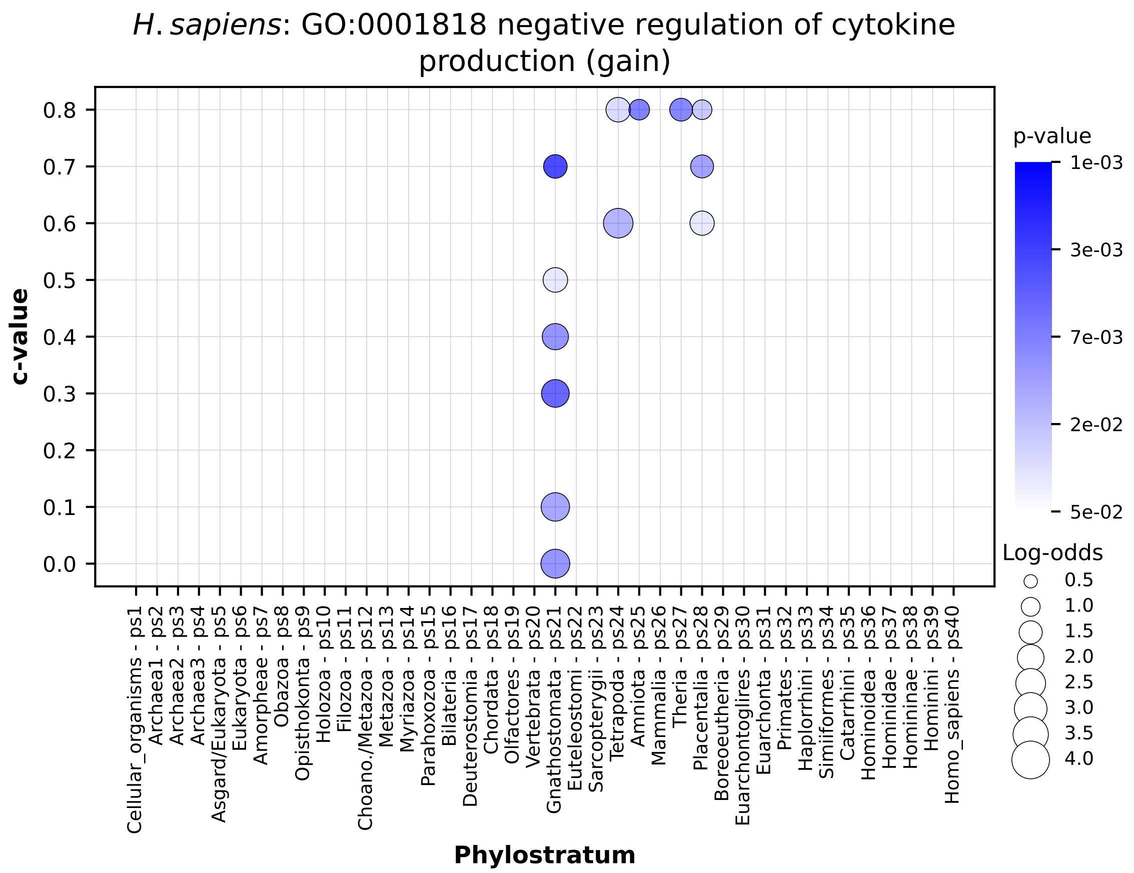

H. sapiens: GO:0001818 negative regulation of cytokine production (gain)
c-value
0.0
0.1
0.2
0.3
0.4
0.5
0.6
0.7
0.8
p-value
1e-03
3e-03
7e-03
2e-02
5e-02
Log-odds
0.5
1.0
1.5
2.0
2.5
3.0
3.5
4.0
Cellular_organisms - ps1
Archaea1 - ps2
Archaea2 - ps3
Archaea3 - ps4
Asgard/Eukaryota - ps5
Eukaryota - ps6
Amorpheae - ps7
Obazoa - ps8
Opisthokonta - ps9
Holozoa - ps10
Filozoa - ps11
Choano./Metazoa - ps12
Metazoa - ps13
Myriazoa - ps14
Parahoxozoa - ps15
Bilateria - ps16
Deuterostomia - ps17
Chordata - ps18
Olfactores - ps19
Vertebrata - ps20
Gnathostomata - ps21
Euteleostomi - ps22
Sarcopterygii - ps23
Tetrapoda - ps24
Amniota - ps25
Mammalia - ps26
Theria - ps27
Placentalia - ps28
Boreoeutheria - ps29
Euarchontoglires - ps30
Euarchonta - ps31
Primates - ps32
Haplorrhini - ps33
Simiiformes - ps34
Catarrhini - ps35
Hominoidea - ps36
Hominidae - ps37
Homininae - ps38
Hominini - ps39
Homo_sapiens - ps40
Phylostratum

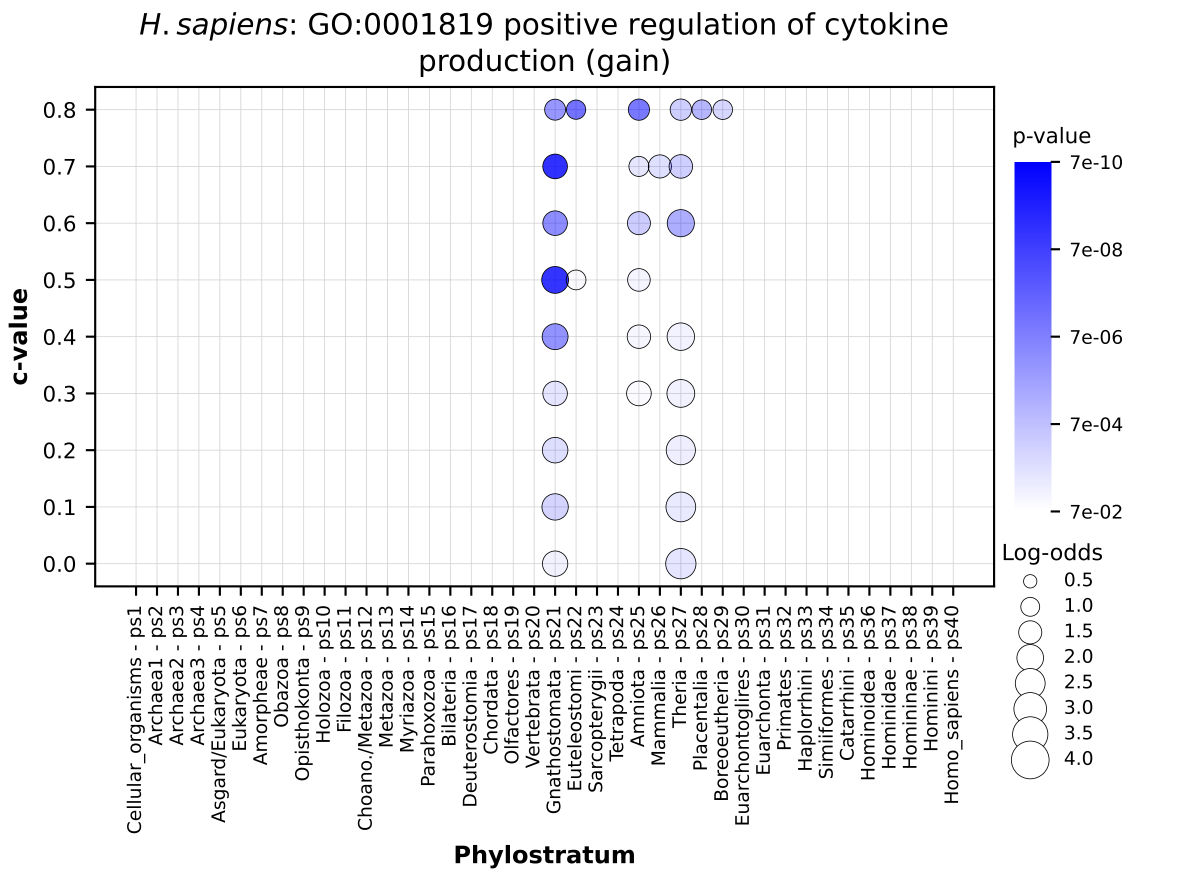

H. sapiens: GO:0001819 positive regulation of cytokine production (gain)
c-value
0.8
0.7
0.6
0.5
0.4
0.3
0.2
0.1
0.0
p-value
7e-10
7e-08
7e-06
7e-04
7e-02
Log-odds
0.5
1.0
1.5
2.0
2.5
3.0
3.5
4.0
Cellular_organisms - ps1
Archaea1 - ps2
Archaea2 - ps3
Archaea3 - ps4
Asgard/Eukaryota - ps5
Eukaryota - ps6
Amorpheae - ps7
Obazoa - ps8
Opisthokonta - ps9
Holozoa - ps10
Filozoa - ps11
Choano./Metazoa - ps12
Metazoa - ps13
Myriazoa - ps14
Parahoxozoa - ps15
Bilateria - ps16
Deuterostomia - ps17
Chordata - ps18
Olfactores - ps19
Vertebrata - ps20
Gnathostomata - ps21
Euteleostomi - ps22
Sarcopterygii - ps23
Tetrapoda - ps24
Amniota - ps25
Mammalia - ps26
Theria - ps27
Placentalia - ps28
Boreoeutheria - ps29
Euarchontoglires - ps30
Euarchonta - ps31
Primates - ps32
Haplorrhini - ps33
Simiiformes - ps34
Catarrhini - ps35
Hominoidea - ps36
Hominidae - ps37
Homininae - ps38
Hominini - ps39
Homo_sapiens - ps40
Phylostratum

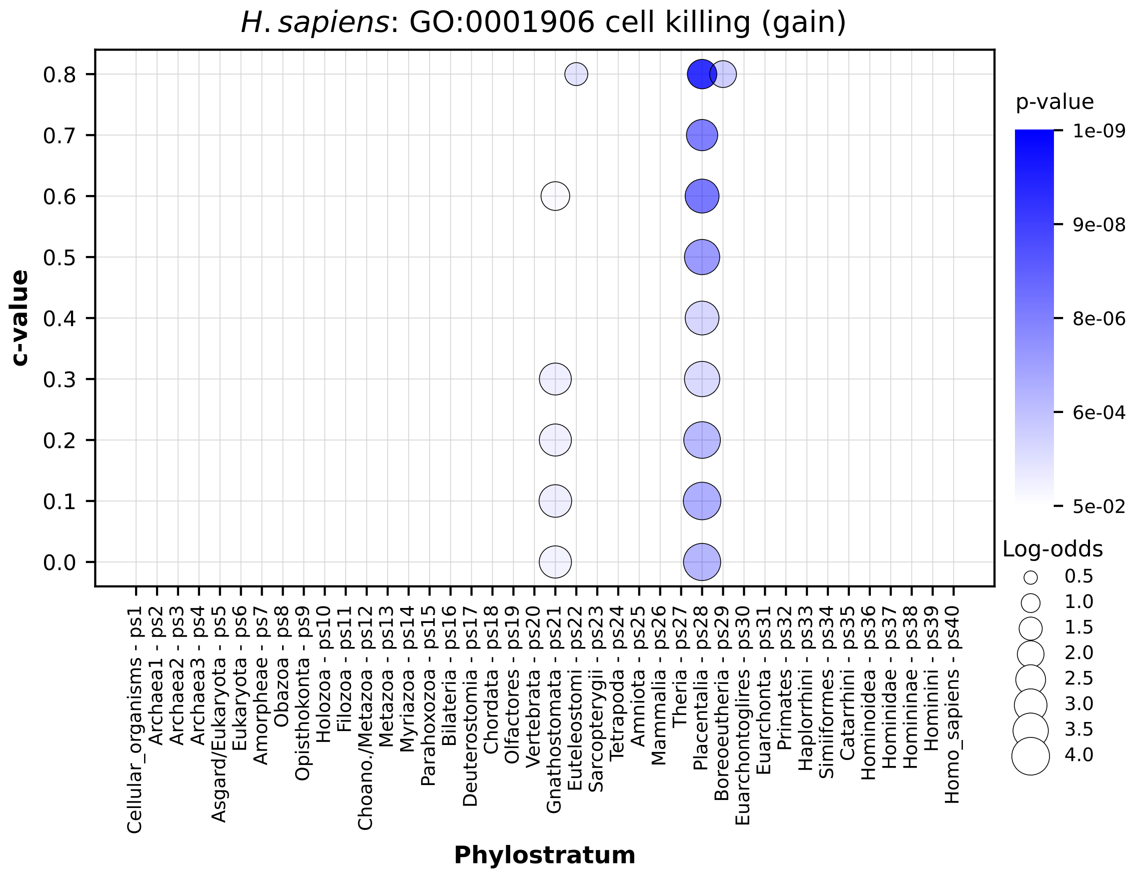
H. sapiens: GO:0001906 cell killing (gain)
c-value
0.8
0.7
0.6
0.5
0.4
0.3
0.2
0.1
0.0
p-value
1e-09
9e-08
8e-06
6e-04
5e-02
Log-odds
0.5
1.0
1.5
2.0
2.5
3.0
3.5
4.0
Cellular_organisms - ps1
Archaea1 - ps2
Archaea2 - ps3
Archaea3 - ps4
Asgard/Eukaryota - ps5
Eukaryota - ps6
Amorpheae - ps7
Obazoa - ps8
Opisthokonta - ps9
Holozoa - ps10
Filozoa - ps11
Choano./Metazoa - ps12
Metazoa - ps13
Myriazoa - ps14
Parahoxozoa - ps15
Bilateria - ps16
Deuterostomia - ps17
Chordata - ps18
Olfactores - ps19
Vertebrata - ps20
Gnathostomata - ps21
Euteleostomi - ps22
Sarcopterygii - ps23
Tetrapoda - ps24
Amniota - ps25
Mammalia - ps26
Theria - ps27
Placentalia - ps28
Boreoeutheria - ps29
Euarchontoglires - ps30
Euarchonta - ps31
Primates - ps32
Haplorrhini - ps33
Simiiformes - ps34
Catarrhini - ps35
Hominoidea - ps36
Hominidae - ps37
Homininae - ps38
Hominini - ps39
Homo_sapiens - ps40
Phylostratum

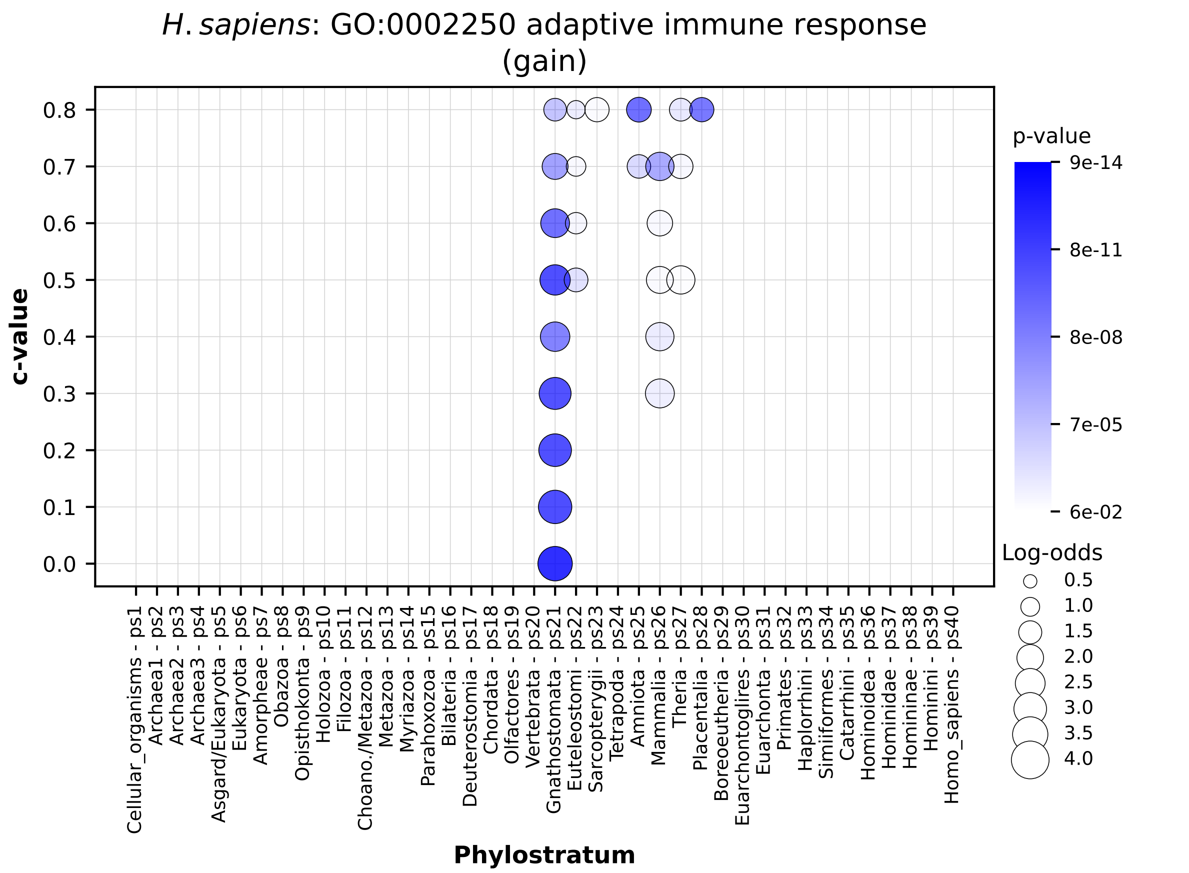

H. sapiens: GO:0002250 adaptive immune response
(gain)
c-value
0.8
0.7
0.6
0.5
0.4
0.3
0.2
0.1
0.0
Phylostratum
Cellular_organisms - ps1
Archaea1 - ps2
Archaea2 - ps3
Archaea3 - ps4
Asgard/Eukaryota - ps5
Eukaryota - ps6
Amorpheae - ps7
Obazoa - ps8
Opisthokonta - ps9
Holozoa - ps10
Filozoa - ps11
Choano./Metazoa - ps12
Metazoa - ps13
Myriazoa - ps14
Parahoxozoa - ps15
Bilateria - ps16
Deuterostomia - ps17
Chordata - ps18
Olfactores - ps19
Vertebrata - ps20
Gnathostomata - ps21
Euteleostomi - ps22
Sarcopterygii - ps23
Tetrapoda - ps24
Amniota - ps25
Mammalia - ps26
Theria - ps27
Placentalia - ps28
Boreoeutheria - ps29
Euarchontoglires - ps30
Euarchonta - ps31
Primates - ps32
Haplorrhini - ps33
Simiiformes - ps34
Catarrhini - ps35
Hominoidea - ps36
Hominidae - ps37
Homininae - ps38
Hominini - ps39
Homo_sapiens - ps40
p-value
9e-14
8e-11
8e-08
7e-05
6e-02
Log-odds
0.5
1.0
1.5
2.0
2.5
3.0
3.5
4.0

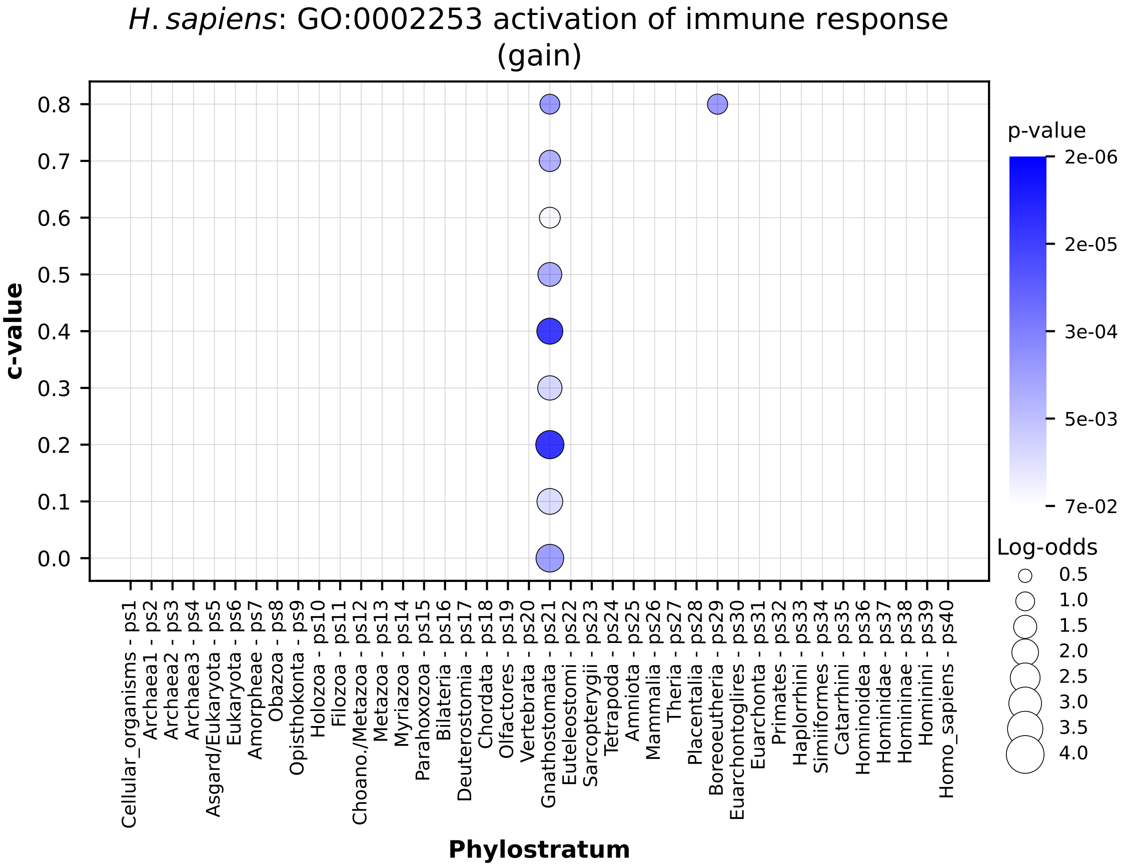

H. sapiens: GO:0002253 activation of immune response
(gain)
c-value
0.8
0.7
0.6
0.5
0.4
0.3
0.2
0.1
0.0
p-value
2e-06
2e-05
3e-04
5e-03
7e-02
Log-odds
0.5
1.0
1.5
2.0
2.5
3.0
3.5
4.0
Cellular_organisms - ps1
Archaea1 - ps2
Archaea2 - ps3
Archaea3 - ps4
Asgard/Eukaryota - ps5
Eukaryota - ps6
Amorpheae - ps7
Obazoa - ps8
Opisthokonta - ps9
Holozoa - ps10
Filozoa - ps11
Choano./Metazoa - ps12
Metazoa - ps13
Myriazoa - ps14
Parahoxozoa - ps15
Bilateria - ps16
Deuterostomia - ps17
Chordata - ps18
Olfactores - ps19
Vertebrata - ps20
Gnathostomata - ps21
Euteleostomi - ps22
Sarcopterygii - ps23
Tetrapoda - ps24
Amniota - ps25
Mammalia - ps26
Theria - ps27
Placentalia - ps28
Boreoeutheria - ps29
Euarchontoglires - ps30
Euarchonta - ps31
Primates - ps32
Haplorrhini - ps33
Simiiformes - ps34
Catarrhini - ps35
Hominoidea - ps36
Hominidae - ps37
Homininae - ps38
Hominini - ps39
Homo_sapiens - ps40
Phylostratum

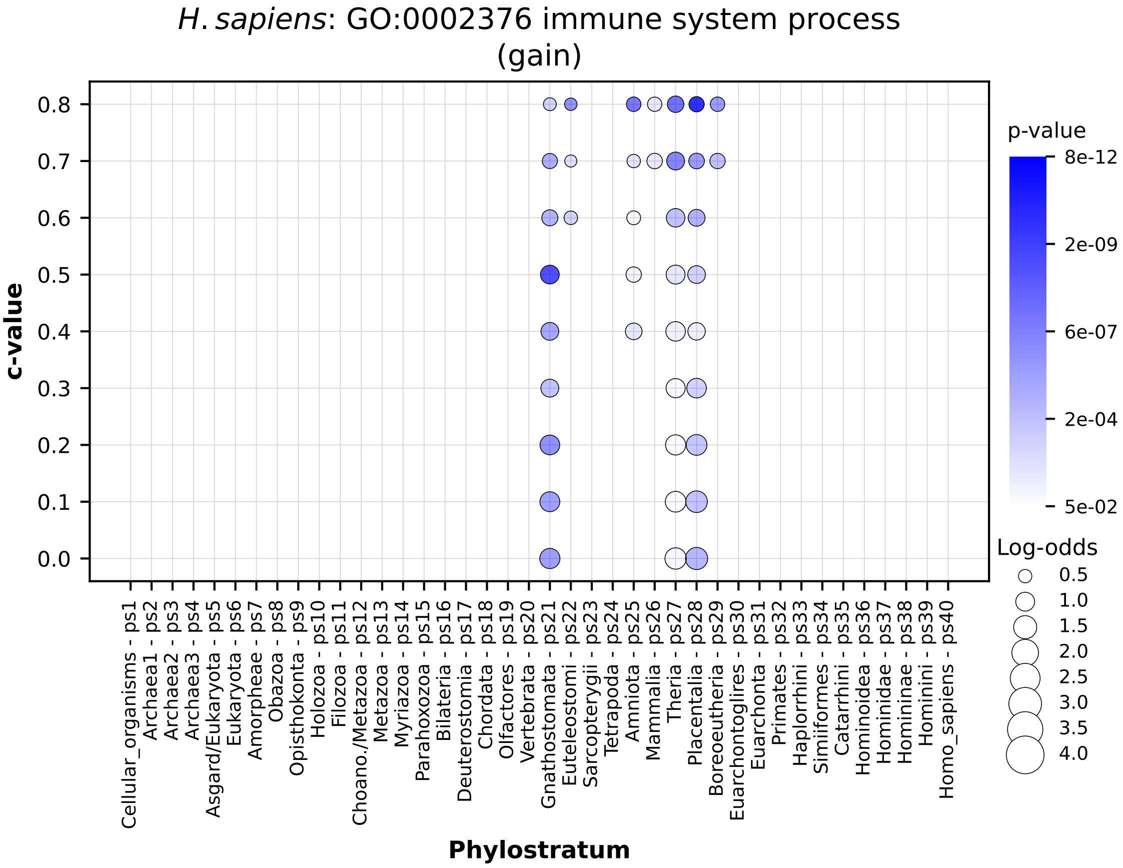

*H. sapiens*: GO:0002376 immune system process
(gain)
c-value
0.8
0.7
0.6
0.5
0.4
0.3
0.2
0.1
0.0
p-value
8e-12
2e-09
6e-07
2e-04
5e-02
Log-odds
0.5
1.0
1.5
2.0
2.5
3.0
3.5
4.0
Cellular_organisms - ps1
Archaea1 - ps2
Archaea2 - ps3
Archaea3 - ps4
Asgard/Eukaryota - ps5
Eukaryota - ps6
Amorpheae - ps7
Obazoa - ps8
Opisthokonta - ps9
Holozoa - ps10
Filozoa - ps11
Choano./Metazoa - ps12
Metazoa - ps13
Myriazoa - ps14
Parahoxozoa - ps15
Bilateria - ps16
Deuterostomia - ps17
Chordata - ps18
Olfactores - ps19
Vertebrata - ps20
Gnathostomata - ps21
Euteleostomi - ps22
Sarcopterygii - ps23
Tetrapoda - ps24
Amniota - ps25
Mammalia - ps26
Theria - ps27
Placentalia - ps28
Boreoeutheria - ps29
Euarchontoglires - ps30
Euarchonta - ps31
Primates - ps32
Haplorrhini - ps33
Simiiformes - ps34
Catarrhini - ps35
Hominoidea - ps36
Hominidae - ps37
Homininae - ps38
Hominini - ps39
Homo_sapiens - ps40
Phylostratum

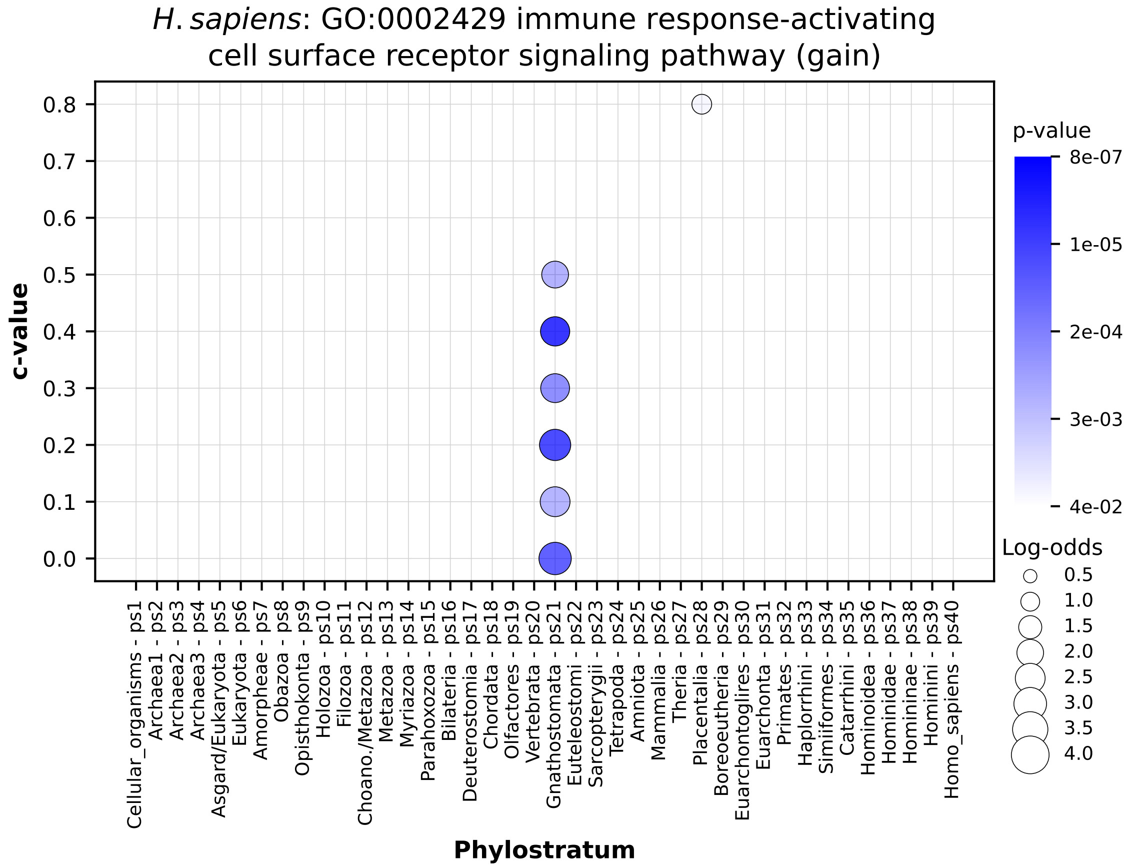
H. sapiens: GO:0002429 immune response-activating cell surface receptor signaling pathway (gain)
c-value
0.0
0.1
0.2
0.3
0.4
0.5
0.6
0.7
0.8
p-value
8e-07
1e-05
2e-04
3e-03
4e-02
Log-odds
0.5
1.0
1.5
2.0
2.5
3.0
3.5
4.0
Cellular_organisms - ps1
Archaea1 - ps2
Archaea2 - ps3
Archaea3 - ps4
Asgard/Eukaryota - ps5
Eukaryota - ps6
Amorpheae - ps7
Obazoa - ps8
Opisthokonta - ps9
Holozoa - ps10
Filozoa - ps11
Choano./Metazoa - ps12
Metazoa - ps13
Myriazoa - ps14
Parahoxozoa - ps15
Bilateria - ps16
Deuterostomia - ps17
Chordata - ps18
Olfactores - ps19
Vertebrata - ps20
Gnathostomata - ps21
Euteleostomi - ps22
Sarcopterygii - ps23
Tetrapoda - ps24
Amniota - ps25
Mammalia - ps26
Theria - ps27
Placentalia - ps28
Boreoeutheria - ps29
Euarchontoglires - ps30
Euarchonta - ps31
Primates - ps32
Haplorrhini - ps33
Simiiformes - ps34
Catarrhini - ps35
Hominoidea - ps36
Hominidae - ps37
Homininae - ps38
Hominini - ps39
Homo_sapiens - ps40
Phylostratum

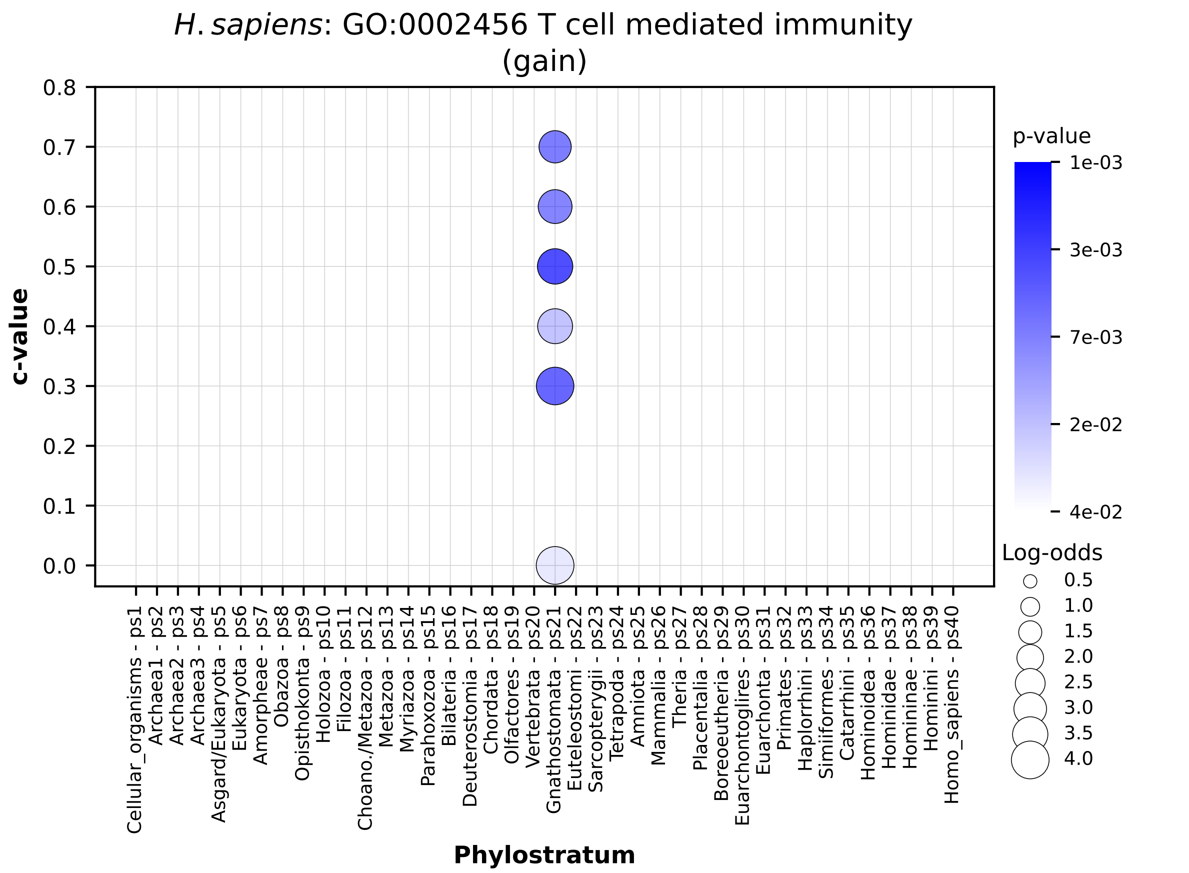

H. sapiens: GO:0002456 T cell mediated immunity
(gain)
c-value
0.8
0.7
0.6
0.5
0.4
0.3
0.2
0.1
0.0
p-value
1e-03
3e-03
7e-03
2e-02
4e-02
Log-odds
0.5
1.0
1.5
2.0
2.5
3.0
3.5
4.0
Cellular_organisms - ps1
Archaea1 - ps2
Archaea2 - ps3
Archaea3 - ps4
Asgard/Eukaryota - ps5
Eukaryota - ps6
Amorpheae - ps7
Obazoa - ps8
Opisthokonta - ps9
Holozoa - ps10
Filozoa - ps11
Choano./Metazoa - ps12
Metazoa - ps13
Myriazoa - ps14
Parahoxozoa - ps15
Bilateria - ps16
Deuterostomia - ps17
Chordata - ps18
Olfactores - ps19
Vertebrata - ps20
Gnathostomata - ps21
Euteleostomi - ps22
Sarcopterygii - ps23
Tetrapoda - ps24
Amniota - ps25
Mammalia - ps26
Theria - ps27
Placentalia - ps28
Boreoeutheria - ps29
Euarchontoglires - ps30
Euarchonta - ps31
Primates - ps32
Haplorrhini - ps33
Simiiformes - ps34
Catarrhini - ps35
Hominoidea - ps36
Hominidae - ps37
Homininae - ps38
Hominini - ps39
Homo_sapiens - ps40
Phylostratum

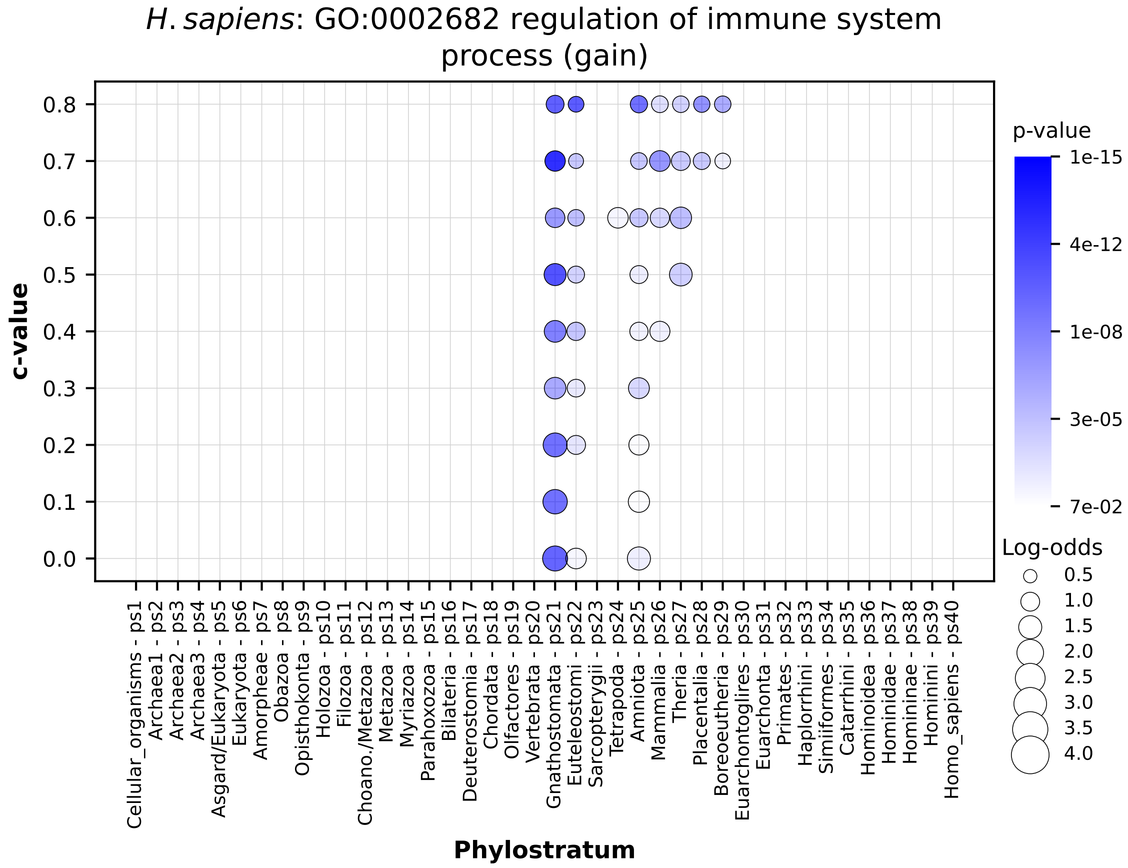
H. sapiens: GO:0002682 regulation of immune system process (gain)
c-value
0.8
0.7
0.6
0.5
0.4
0.3
0.2
0.1
0.0
p-value
1e-15
4e-12
1e-08
3e-05
7e-02
Log-odds
0.5
1.0
1.5
2.0
2.5
3.0
3.5
4.0
Cellular_organisms - ps1
Archaea1 - ps2
Archaea2 - ps3
Archaea3 - ps4
Asgard/Eukaryota - ps5
Eukaryota - ps6
Amorpheae - ps7
Obazoa - ps8
Opisthokonta - ps9
Holozoa - ps10
Filozoa - ps11
Choano./Metazoa - ps12
Metazoa - ps13
Myriazoa - ps14
Parahoxozoa - ps15
Bilateria - ps16
Deuterostomia - ps17
Chordata - ps18
Olfactores - ps19
Vertebrata - ps20
Gnathostomata - ps21
Euteleostomi - ps22
Sarcopterygii - ps23
Tetrapoda - ps24
Amniota - ps25
Mammalia - ps26
Theria - ps27
Placentalia - ps28
Boreoeutheria - ps29
Euarchontoglires - ps30
Euarchonta - ps31
Primates - ps32
Haplorrhini - ps33
Simiiformes - ps34
Catarrhini - ps35
Hominoidea - ps36
Hominidae - ps37
Homininae - ps38
Hominini - ps39
Homo_sapiens - ps40
Phylostratum

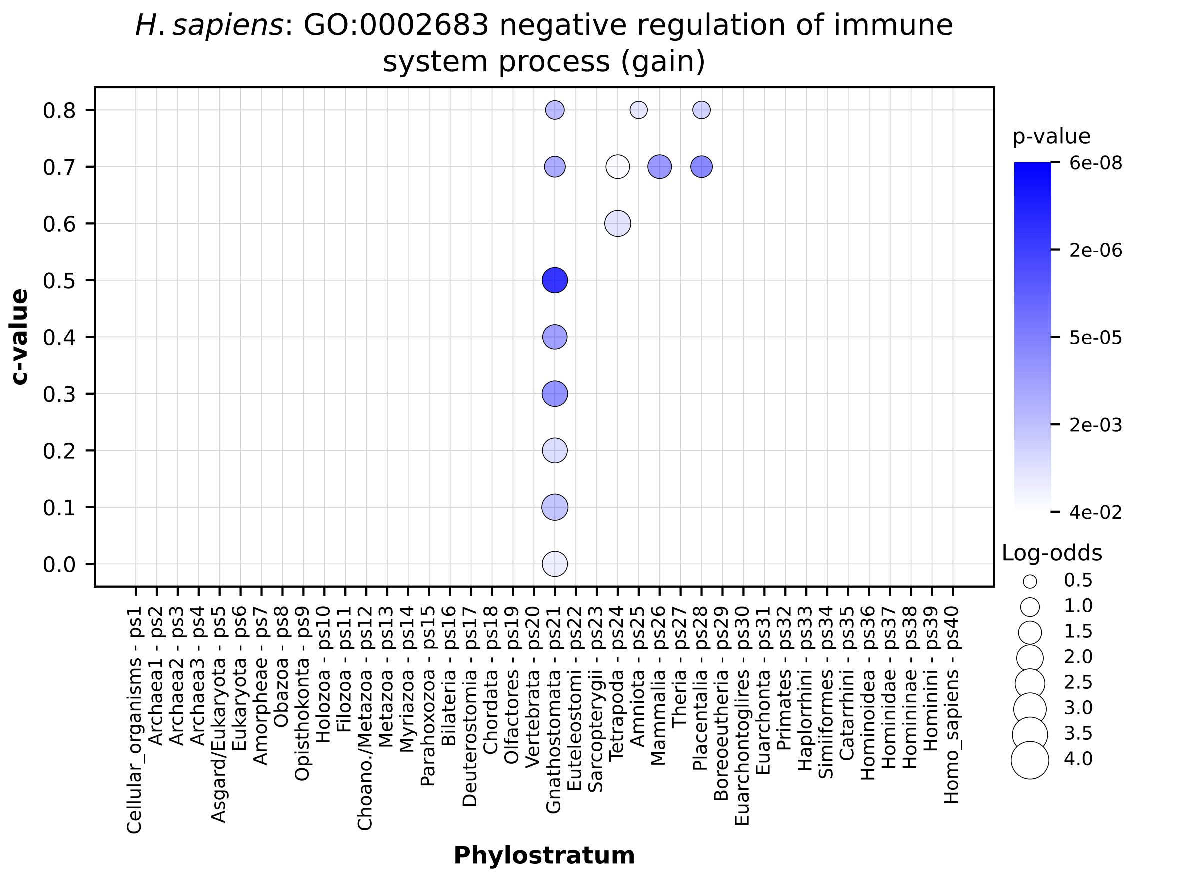

*H. sapiens*: GO:0002683 negative regulation of immune system process (gain)
c-value
0.8
0.7
0.6
0.5
0.4
0.3
0.2
0.1
0.0
Phylostratum
Cellular_organisms - ps1
Archaea1 - ps2
Archaea2 - ps3
Archaea3 - ps4
Asgard/Eukaryota - ps5
Eukaryota - ps6
Amorpheae - ps7
Obazoa - ps8
Opisthokonta - ps9
Holozoa - ps10
Filozoa - ps11
Choano./Metazoa - ps12
Metazoa - ps13
Myriazoa - ps14
Parahoxozoa - ps15
Bilateria - ps16
Deuterostomia - ps17
Chordata - ps18
Olfactores - ps19
Vertebrata - ps20
Gnathostomata - ps21
Euteleostomi - ps22
Sarcopterygii - ps23
Tetrapoda - ps24
Amniota - ps25
Mammalia - ps26
Theria - ps27
Placentalia - ps28
Boreoeutheria - ps29
Euarchontoglires - ps30
Euarchonta - ps31
Primates - ps32
Haplorrhini - ps33
Simiiformes - ps34
Catarrhini - ps35
Hominoidea - ps36
Hominidae - ps37
Homininae - ps38
Hominini - ps39
Homo_sapiens - ps40
p-value
6e-08
2e-06
5e-05
2e-03
4e-02
Log-odds
0.5
1.0
1.5
2.0
2.5
3.0
3.5
4.0

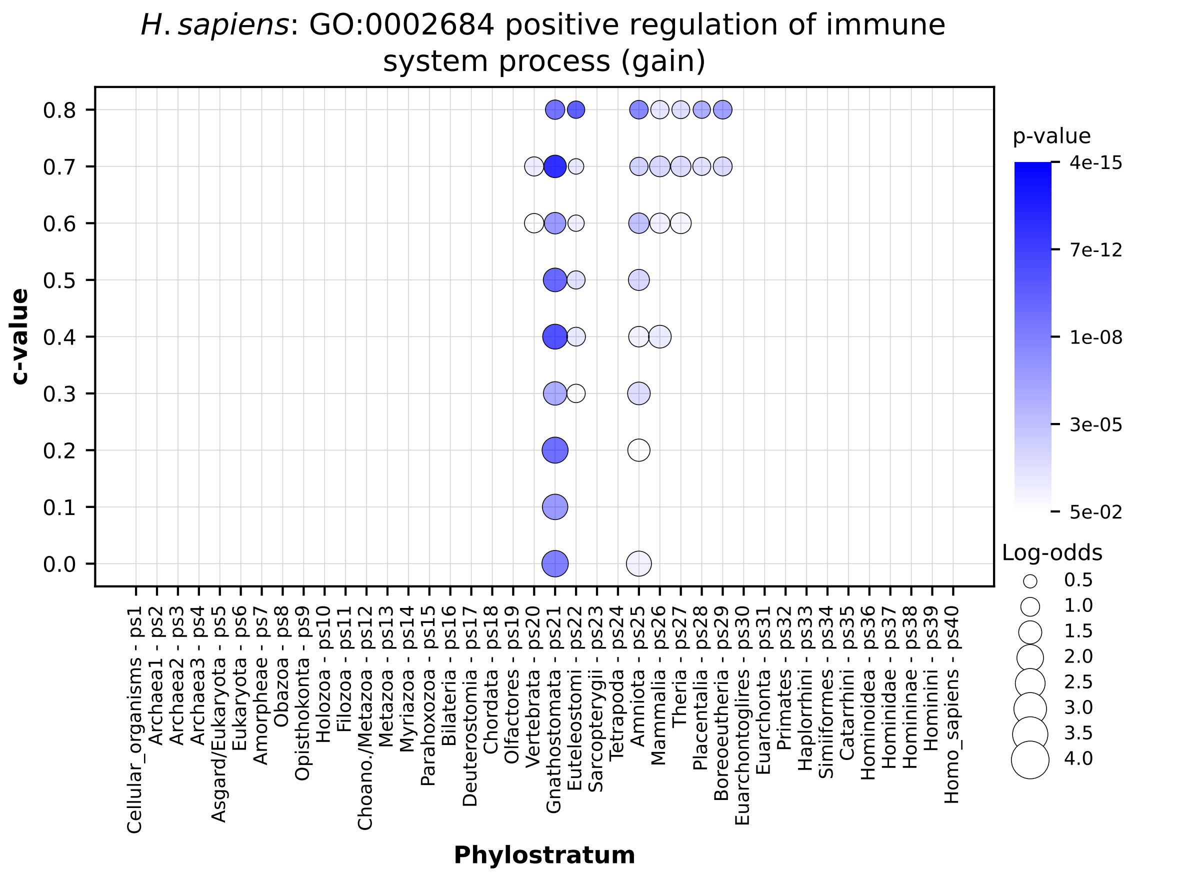
H. sapiens: GO:0002684 positive regulation of immune system process (gain)
c-value
0.0
0.1
0.2
0.3
0.4
0.5
0.6
0.7
0.8
Phylostratum
Cellular_organisms - ps1
Archaea1 - ps2
Archaea2 - ps3
Archaea3 - ps4
Asgard/Eukaryota - ps5
Eukaryota - ps6
Amorpheae - ps7
Obazoa - ps8
Opisthokonta - ps9
Holozoa - ps10
Filozoa - ps11
Choano./Metazoa - ps12
Metazoa - ps13
Myriazoa - ps14
Parahoxozoa - ps15
Bilateria - ps16
Deuterostomia - ps17
Chordata - ps18
Olfactores - ps19
Vertebrata - ps20
Gnathostomata - ps21
Euteleostomi - ps22
Sarcopterygii - ps23
Tetrapoda - ps24
Amniota - ps25
Mammalia - ps26
Theria - ps27
Placentalia - ps28
Boreoeutheria - ps29
Euarchontoglires - ps30
Euarchonta - ps31
Primates - ps32
Haplorrhini - ps33
Simiiformes - ps34
Catarrhini - ps35
Hominoidea - ps36
Hominidae - ps37
Homininae - ps38
Hominini - ps39
Homo_sapiens - ps40
p-value
4e-15
7e-12
1e-08
3e-05
5e-02
Log-odds
0.5
1.0
1.5
2.0
2.5
3.0
3.5
4.0

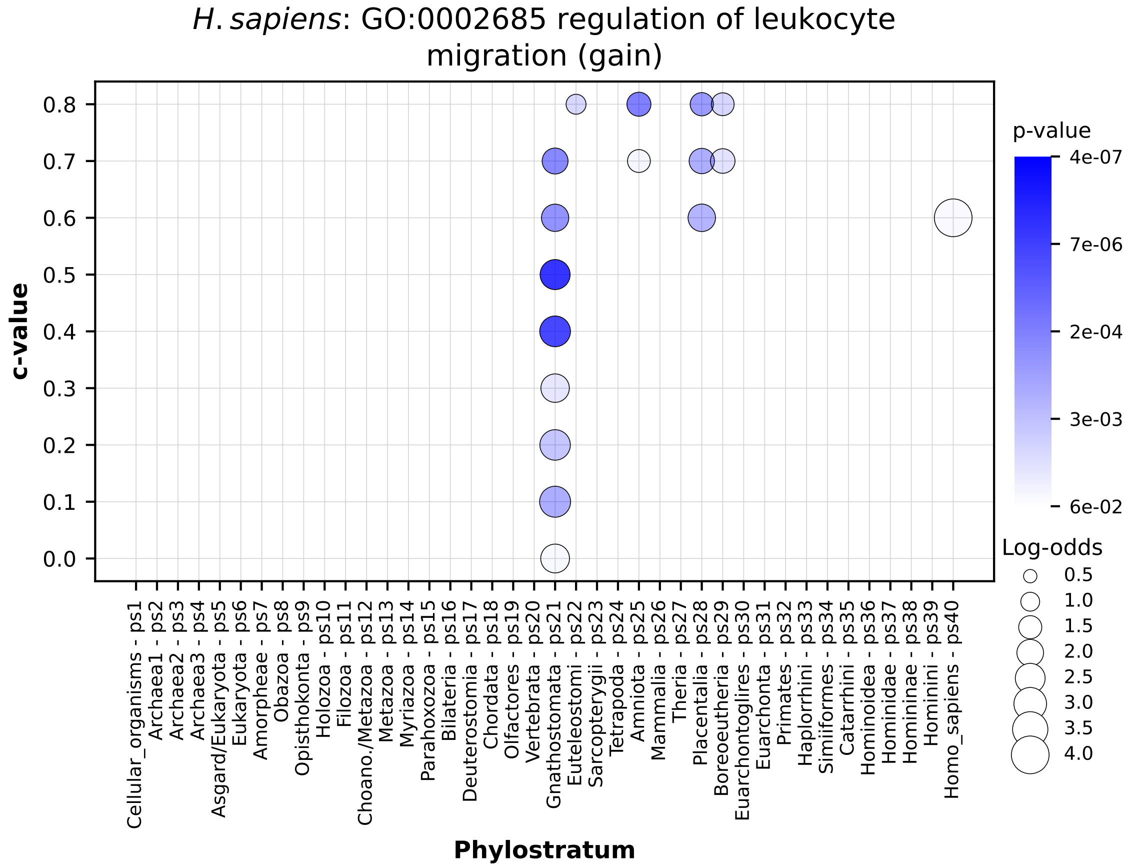

H. sapiens: GO:0002685 regulation of leukocyte migration (gain)
c-value
0.0
0.1
0.2
0.3
0.4
0.5
0.6
0.7
0.8
Phylostratum
Cellular_organisms - ps1
Archaea1 - ps2
Archaea2 - ps3
Archaea3 - ps4
Asgard/Eukaryota - ps5
Eukaryota - ps6
Amorpheae - ps7
Obazoa - ps8
Opisthokonta - ps9
Holozoa - ps10
Filozoa - ps11
Choano./Metazoa - ps12
Metazoa - ps13
Myriazoa - ps14
Parahoxozoa - ps15
Bilateria - ps16
Deuterostomia - ps17
Chordata - ps18
Olfactores - ps19
Vertebrata - ps20
Gnathostomata - ps21
Euteleostomi - ps22
Sarcopterygii - ps23
Tetrapoda - ps24
Amniota - ps25
Mammalia - ps26
Theria - ps27
Placentalia - ps28
Boreoeutheria - ps29
Euarchontoglires - ps30
Euarchonta - ps31
Primates - ps32
Haplorrhini - ps33
Simiiformes - ps34
Catarrhini - ps35
Hominoidea - ps36
Hominidae - ps37
Homininae - ps38
Hominini - ps39
Homo_sapiens - ps40
p-value
4e-07
7e-06
2e-04
3e-03
6e-02
Log-odds
0.5
1.0
1.5
2.0
2.5
3.0
3.5
4.0

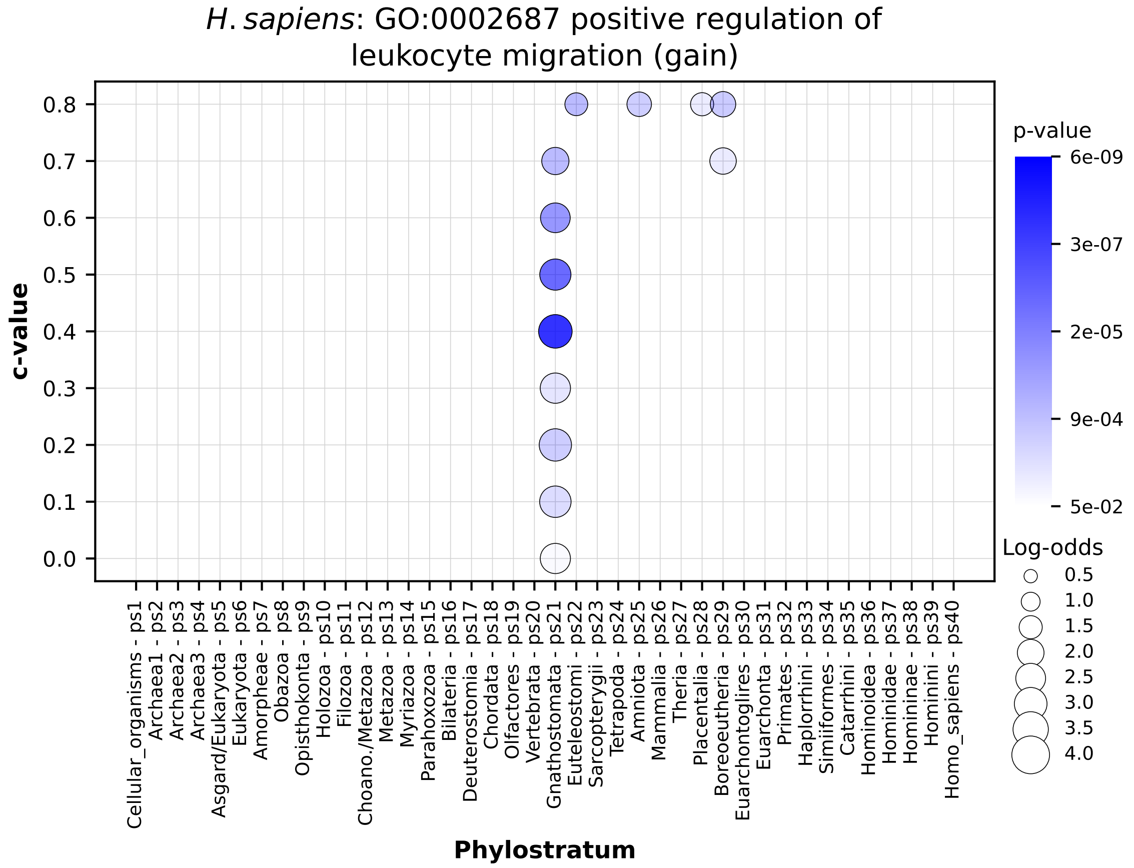
H. sapiens: GO:0002687 positive regulation of leukocyte migration (gain)
c-value
0.0
0.1
0.2
0.3
0.4
0.5
0.6
0.7
0.8
Phylostratum
Cellular_organisms - ps1
Archaea1 - ps2
Archaea2 - ps3
Archaea3 - ps4
Asgard/Eukaryota - ps5
Eukaryota - ps6
Amorpheae - ps7
Obazoa - ps8
Opisthokonta - ps9
Holozoa - ps10
Filozoa - ps11
Choano./Metazoa - ps12
Metazoa - ps13
Myriazoa - ps14
Parahoxozoa - ps15
Bilateria - ps16
Deuterostomia - ps17
Chordata - ps18
Olfactores - ps19
Vertebrata - ps20
Gnathostomata - ps21
Euteleostomi - ps22
Sarcopterygii - ps23
Tetrapoda - ps24
Amniota - ps25
Mammalia - ps26
Theria - ps27
Placentalia - ps28
Boreoeutheria - ps29
Euarchontoglires - ps30
Euarchonta - ps31
Primates - ps32
Haplorrhini - ps33
Simiiformes - ps34
Catarrhini - ps35
Hominoidea - ps36
Hominidae - ps37
Homininae - ps38
Hominini - ps39
Homo_sapiens - ps40
p-value
6e-09
3e-07
2e-05
9e-04
5e-02
Log-odds
0.5
1.0
1.5
2.0
2.5
3.0
3.5
4.0

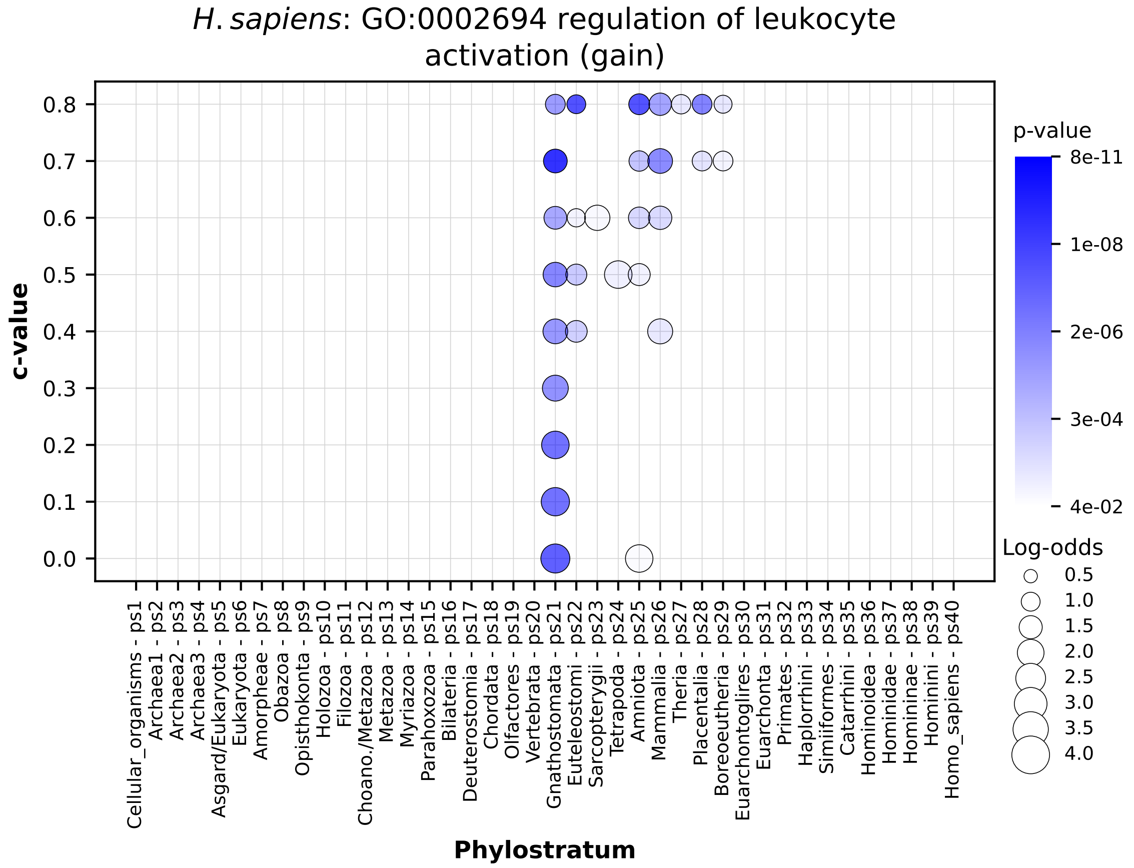

H. sapiens: GO:0002694 regulation of leukocyte activation (gain)
c-value
0.8
0.7
0.6
0.5
0.4
0.3
0.2
0.1
0.0
p-value
8e-11
1e-08
2e-06
3e-04
4e-02
Log-odds
0.5
1.0
1.5
2.0
2.5
3.0
3.5
4.0
Cellular_organisms - ps1
Archaea1 - ps2
Archaea2 - ps3
Archaea3 - ps4
Asgard/Eukaryota - ps5
Eukaryota - ps6
Amorpheae - ps7
Obazoa - ps8
Opisthokonta - ps9
Holozoa - ps10
Filozoa - ps11
Choano./Metazoa - ps12
Metazoa - ps13
Myriazoa - ps14
Parahoxozoa - ps15
Bilateria - ps16
Deuterostomia - ps17
Chordata - ps18
Olfactores - ps19
Vertebrata - ps20
Gnathostomata - ps21
Euteleostomi - ps22
Sarcopterygii - ps23
Tetrapoda - ps24
Amniota - ps25
Mammalia - ps26
Theria - ps27
Placentalia - ps28
Boreoeutheria - ps29
Euarchontoglires - ps30
Euarchonta - ps31
Primates - ps32
Haplorrhini - ps33
Simiiformes - ps34
Catarrhini - ps35
Hominoidea - ps36
Hominidae - ps37
Homininae - ps38
Hominini - ps39
Homo_sapiens - ps40
Phylostratum

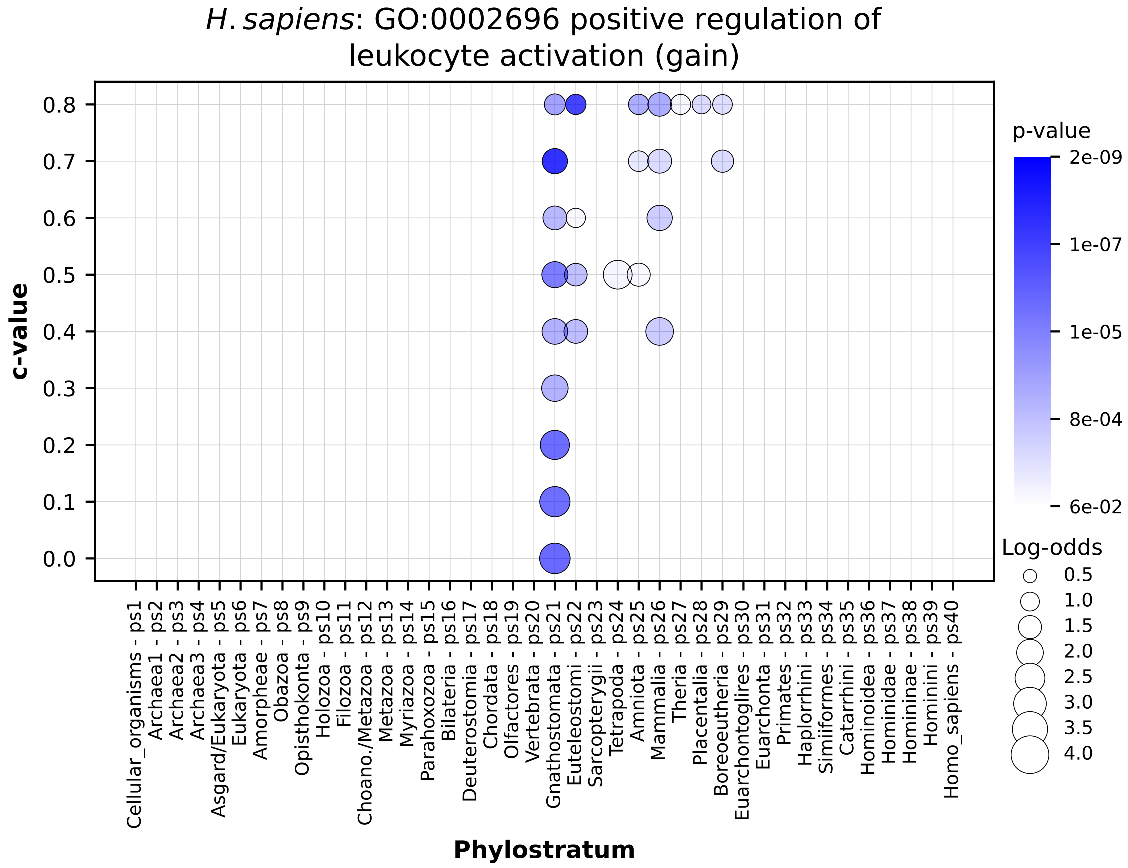

H. sapiens: GO:0002696 positive regulation of leukocyte activation (gain)
c-value
0.0
0.1
0.2
0.3
0.4
0.5
0.6
0.7
0.8
Phylostratum
Cellular_organisms - ps1
Archaea1 - ps2
Archaea2 - ps3
Archaea3 - ps4
Asgard/Eukaryota - ps5
Eukaryota - ps6
Amorpheae - ps7
Obazoa - ps8
Opisthokonta - ps9
Holozoa - ps10
Filozoa - ps11
Choano./Metazoa - ps12
Metazoa - ps13
Myriazoa - ps14
Parahoxozoa - ps15
Bilateria - ps16
Deuterostomia - ps17
Chordata - ps18
Olfactores - ps19
Vertebrata - ps20
Gnathostomata - ps21
Euteleostomi - ps22
Sarcopterygii - ps23
Tetrapoda - ps24
Amniota - ps25
Mammalia - ps26
Theria - ps27
Placentalia - ps28
Boreoeutheria - ps29
Euarchontoglires - ps30
Euarchonta - ps31
Primates - ps32
Haplorrhini - ps33
Simiiformes - ps34
Catarrhini - ps35
Hominoidea - ps36
Hominidae - ps37
Homininae - ps38
Hominini - ps39
Homo_sapiens - ps40
p-value
2e-09
1e-07
1e-05
8e-04
6e-02
Log-odds
0.5
1.0
1.5
2.0
2.5
3.0
3.5
4.0

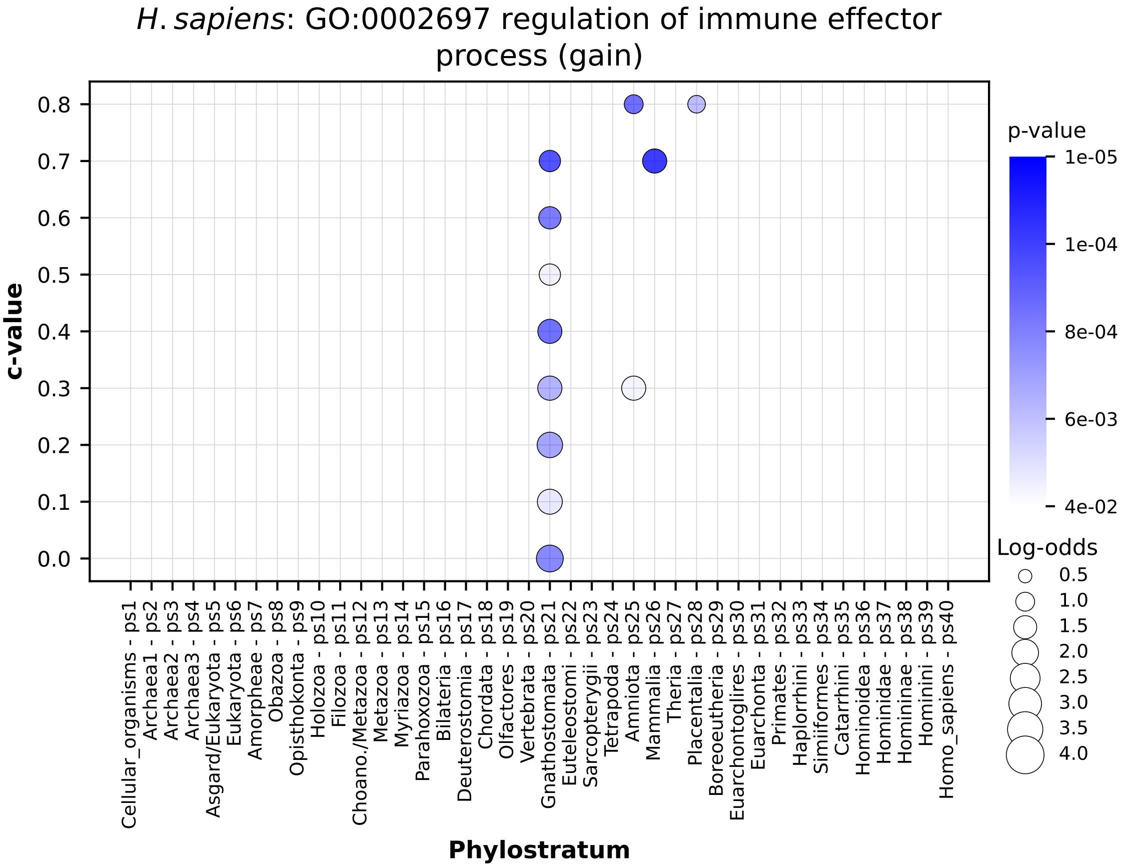

H. sapiens: GO:0002697 regulation of immune effector process (gain)
c-value
0.8
0.7
0.6
0.5
0.4
0.3
0.2
0.1
0.0
Phylostratum
Cellular_organisms - ps1
Archaea1 - ps2
Archaea2 - ps3
Archaea3 - ps4
Asgard/Eukaryota - ps5
Eukaryota - ps6
Amorpheae - ps7
Obazoa - ps8
Opisthokonta - ps9
Holozoa - ps10
Filozoa - ps11
Choano./Metazoa - ps12
Metazoa - ps13
Myriazoa - ps14
Parahoxozoa - ps15
Bilateria - ps16
Deuterostomia - ps17
Chordata - ps18
Olfactores - ps19
Vertebrata - ps20
Gnathostomata - ps21
Euteleostomi - ps22
Sarcopterygii - ps23
Tetrapoda - ps24
Amniota - ps25
Mammalia - ps26
Theria - ps27
Placentalia - ps28
Boreoeutheria - ps29
Euarchontoglires - ps30
Euarchonta - ps31
Primates - ps32
Haplorrhini - ps33
Simiiformes - ps34
Catarrhini - ps35
Hominoidea - ps36
Hominidae - ps37
Homininae - ps38
Hominini - ps39
Homo_sapiens - ps40
p-value
1e-05
1e-04
8e-04
6e-03
4e-02
Log-odds
0.5
1.0
1.5
2.0
2.5
3.0
3.5
4.0

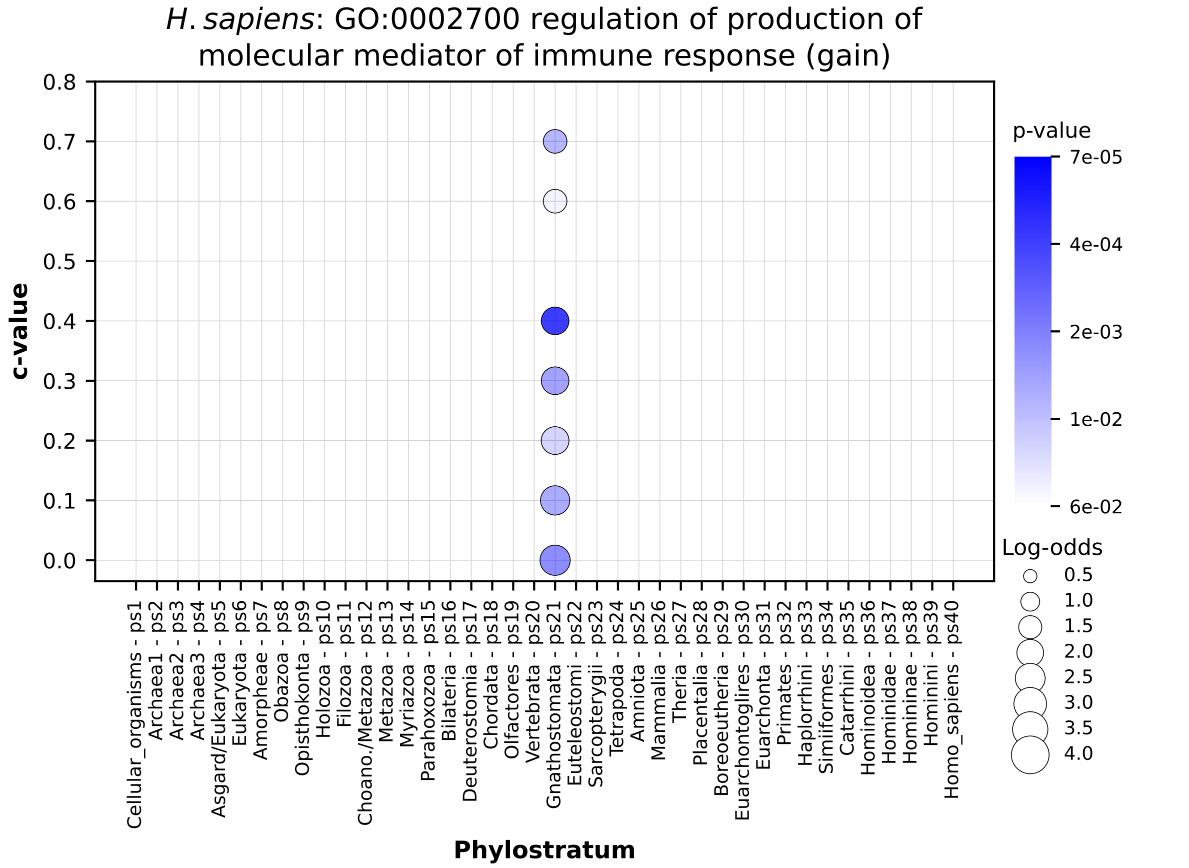

H. sapiens: GO:0002700 regulation of production of molecular mediator of immune response (gain)
c-value
0.0
0.1
0.2
0.3
0.4
0.5
0.6
0.7
0.8
Phylostratum
Cellular_organisms - ps1
Archaea1 - ps2
Archaea2 - ps3
Archaea3 - ps4
Asgard/Eukaryota - ps5
Eukaryota - ps6
Amorpheae - ps7
Obazoa - ps8
Opisthokonta - ps9
Holozoa - ps10
Filozoa - ps11
Choano./Metazoa - ps12
Metazoa - ps13
Myriazoa - ps14
Parahoxozoa - ps15
Bilateria - ps16
Deuterostomia - ps17
Chordata - ps18
Olfactores - ps19
Vertebrata - ps20
Gnathostomata - ps21
Euteleostomi - ps22
Sarcopterygii - ps23
Tetrapoda - ps24
Amniota - ps25
Mammalia - ps26
Theria - ps27
Placentalia - ps28
Boreoeutheria - ps29
Euarchontoglires - ps30
Euarchonta - ps31
Primates - ps32
Haplorrhini - ps33
Simiiformes - ps34
Catarrhini - ps35
Hominoidea - ps36
Hominidae - ps37
Homininae - ps38
Hominini - ps39
Homo_sapiens - ps40
p-value
7e-05
4e-04
2e-03
1e-02
6e-02
Log-odds
0.5
1.0
1.5
2.0
2.5
3.0
3.5
4.0

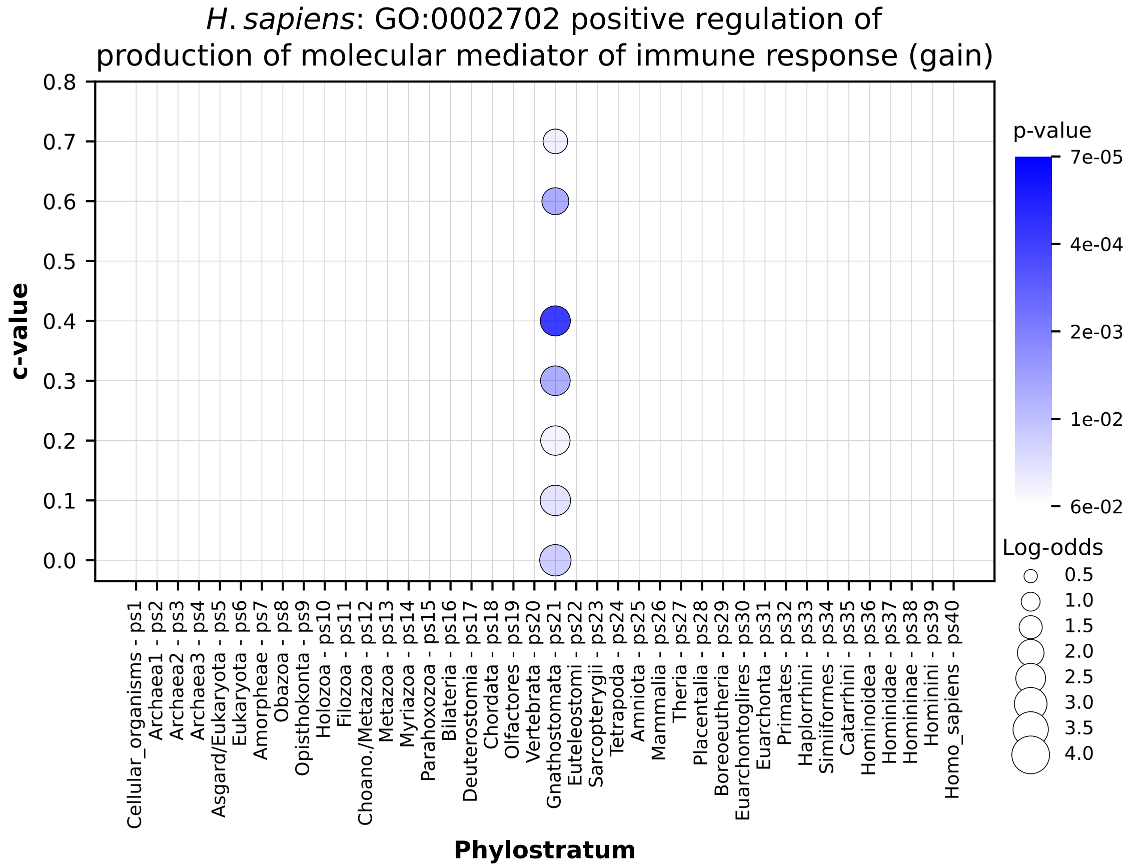

*H. sapiens*: GO:0002702 positive regulation of production of molecular mediator of immune response (gain)
c-value
0.8
0.7
0.6
0.5
0.4
0.3
0.2
0.1
0.0
Phylostratum
Cellular_organisms - ps1
Archaea1 - ps2
Archaea2 - ps3
Archaea3 - ps4
Asgard/Eukaryota - ps5
Eukaryota - ps6
Amorpheae - ps7
Obazoa - ps8
Opisthokonta - ps9
Holozoa - ps10
Filozoa - ps11
Choano./Metazoa - ps12
Metazoa - ps13
Myriazoa - ps14
Parahoxozoa - ps15
Bilateria - ps16
Deuterostomia - ps17
Chordata - ps18
Olfactores - ps19
Vertebrata - ps20
Gnathostomata - ps21
Euteleostomi - ps22
Sarcopterygii - ps23
Tetrapoda - ps24
Amniota - ps25
Mammalia - ps26
Theria - ps27
Placentalia - ps28
Boreoeutheria - ps29
Euarchontoglires - ps30
Euarchonta - ps31
Primates - ps32
Haplorrhini - ps33
Simiiformes - ps34
Catarrhini - ps35
Hominoidea - ps36
Hominidae - ps37
Homininae - ps38
Hominini - ps39
Homo_sapiens - ps40
p-value
7e-05
4e-04
2e-03
1e-02
6e-02
Log-odds
0.5
1.0
1.5
2.0
2.5
3.0
3.5
4.0

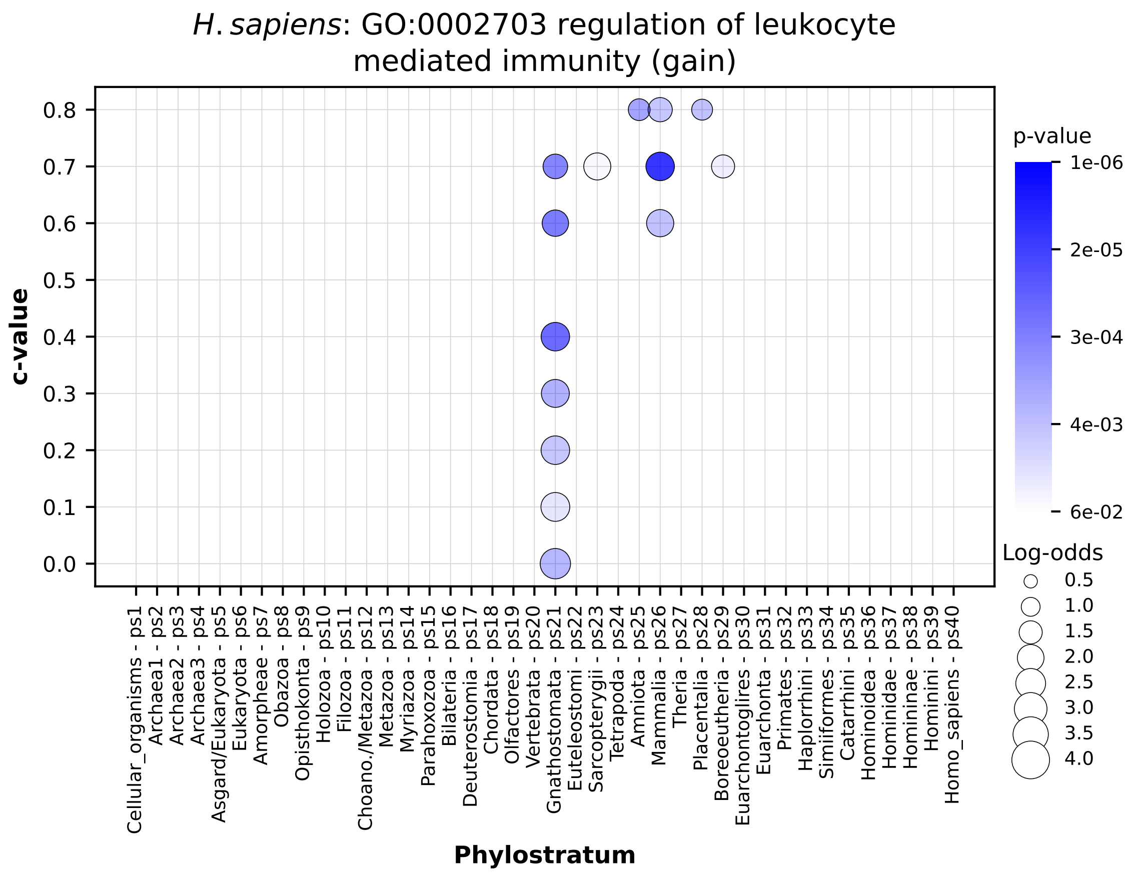

H. sapiens: GO:0002703 regulation of leukocyte mediated immunity (gain)
c-value
0.0
0.1
0.2
0.3
0.4
0.5
0.6
0.7
0.8
Phylostratum
Cellular_organisms - ps1
Archaea1 - ps2
Archaea2 - ps3
Archaea3 - ps4
Asgard/Eukaryota - ps5
Eukaryota - ps6
Amorpheae - ps7
Obazoa - ps8
Opisthokonta - ps9
Holozoa - ps10
Filozoa - ps11
Choano./Metazoa - ps12
Metazoa - ps13
Myriazoa - ps14
Parahoxozoa - ps15
Bilateria - ps16
Deuterostomia - ps17
Chordata - ps18
Olfactores - ps19
Vertebrata - ps20
Gnathostomata - ps21
Euteleostomi - ps22
Sarcopterygii - ps23
Tetrapoda - ps24
Amniota - ps25
Mammalia - ps26
Theria - ps27
Placentalia - ps28
Boreoeutheria - ps29
Euarchontoglires - ps30
Euarchonta - ps31
Primates - ps32
Haplorrhini - ps33
Simiiformes - ps34
Catarrhini - ps35
Hominoidea - ps36
Hominidae - ps37
Homininae - ps38
Hominini - ps39
Homo_sapiens - ps40
p-value
1e-06
2e-05
3e-04
4e-03
6e-02
Log-odds
0.5
1.0
1.5
2.0
2.5
3.0
3.5
4.0

*H. sapiens*: GO:0002705 positive regulation of leukocyte mediated immunity (gain)

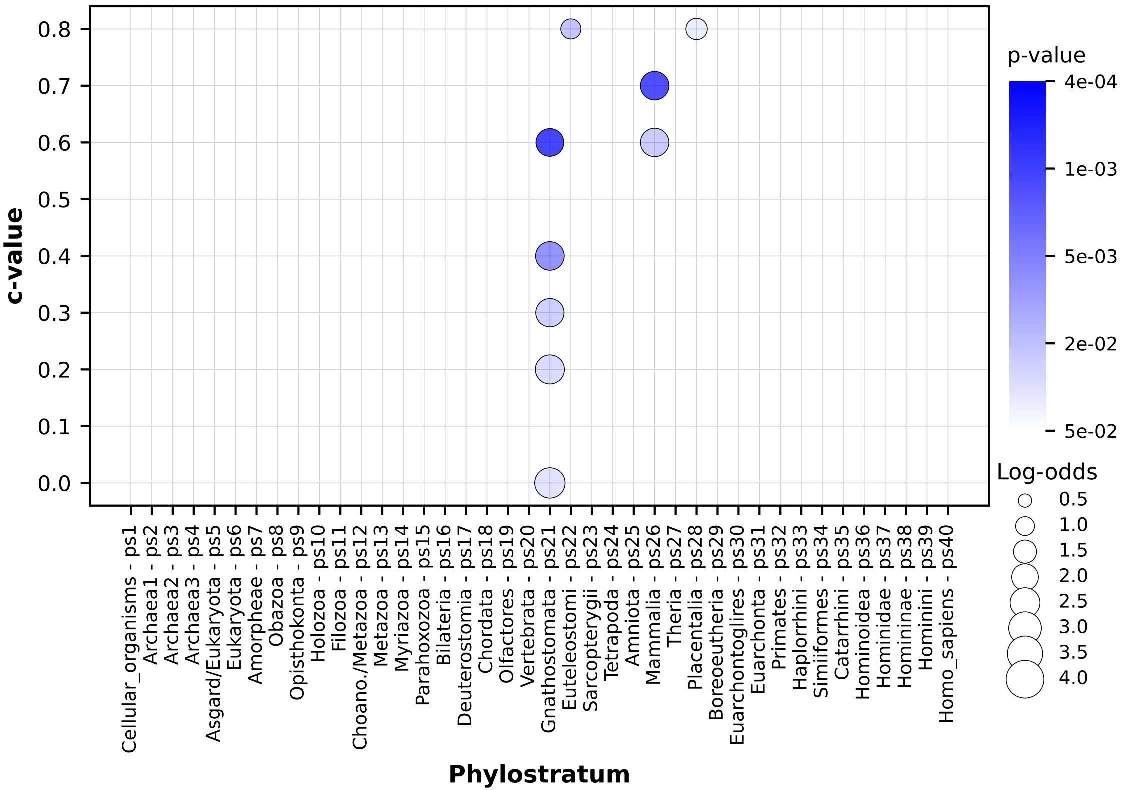

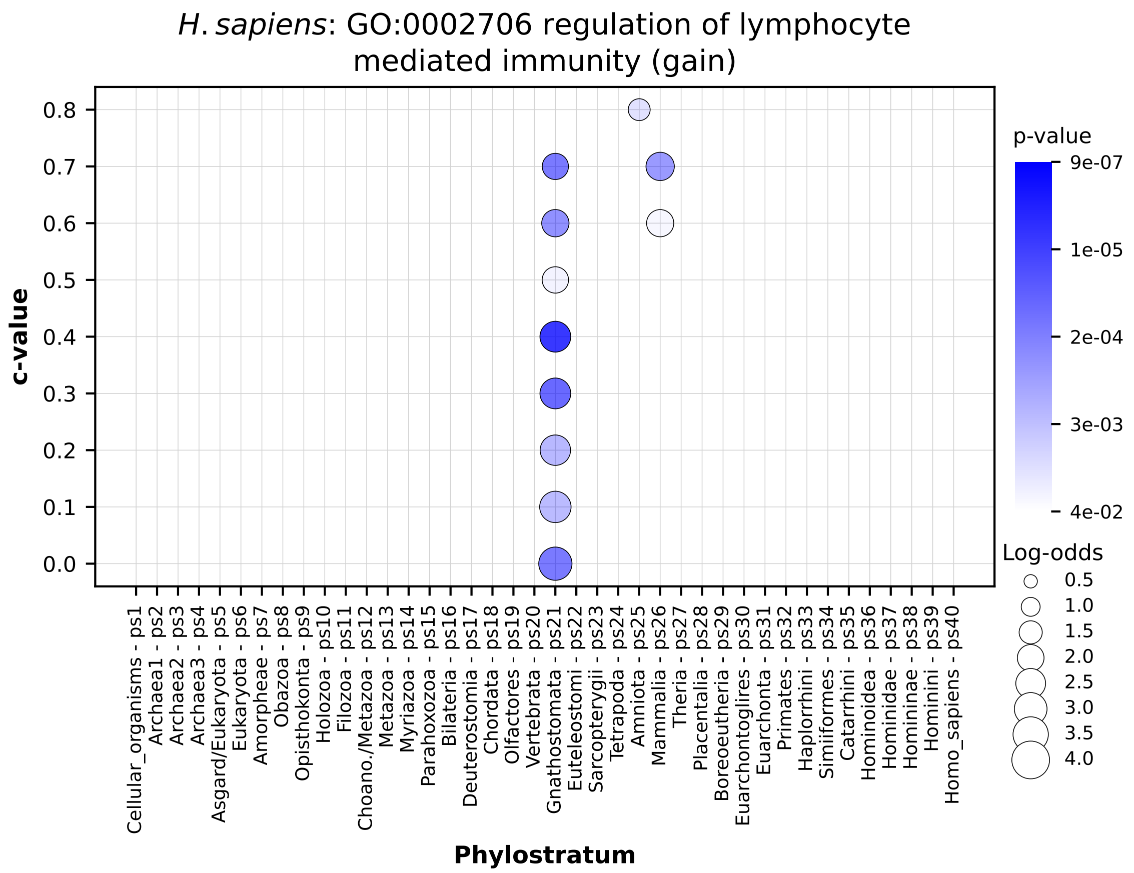

H. sapiens: GO:0002706 regulation of lymphocyte mediated immunity (gain)
c-value
0.0
0.1
0.2
0.3
0.4
0.5
0.6
0.7
0.8
p-value
9e-07
1e-05
2e-04
3e-03
4e-02
Log-odds
0.5
1.0
1.5
2.0
2.5
3.0
3.5
4.0
Cellular_organisms - ps1
Archaea1 - ps2
Archaea2 - ps3
Archaea3 - ps4
Asgard/Eukaryota - ps5
Eukaryota - ps6
Amorpheae - ps7
Obazoa - ps8
Opisthokonta - ps9
Holozoa - ps10
Filozoa - ps11
Choano./Metazoa - ps12
Metazoa - ps13
Myriazoa - ps14
Parahoxozoa - ps15
Bilateria - ps16
Deuterostomia - ps17
Chordata - ps18
Olfactores - ps19
Vertebrata - ps20
Gnathostomata - ps21
Euteleostomi - ps22
Sarcopterygii - ps23
Tetrapoda - ps24
Amniota - ps25
Mammalia - ps26
Theria - ps27
Placentalia - ps28
Boreoeutheria - ps29
Euarchontoglires - ps30
Euarchonta - ps31
Primates - ps32
Haplorrhini - ps33
Simiiformes - ps34
Catarrhini - ps35
Hominoidea - ps36
Hominidae - ps37
Homininae - ps38
Hominini - ps39
Homo_sapiens - ps40
Phylostratum

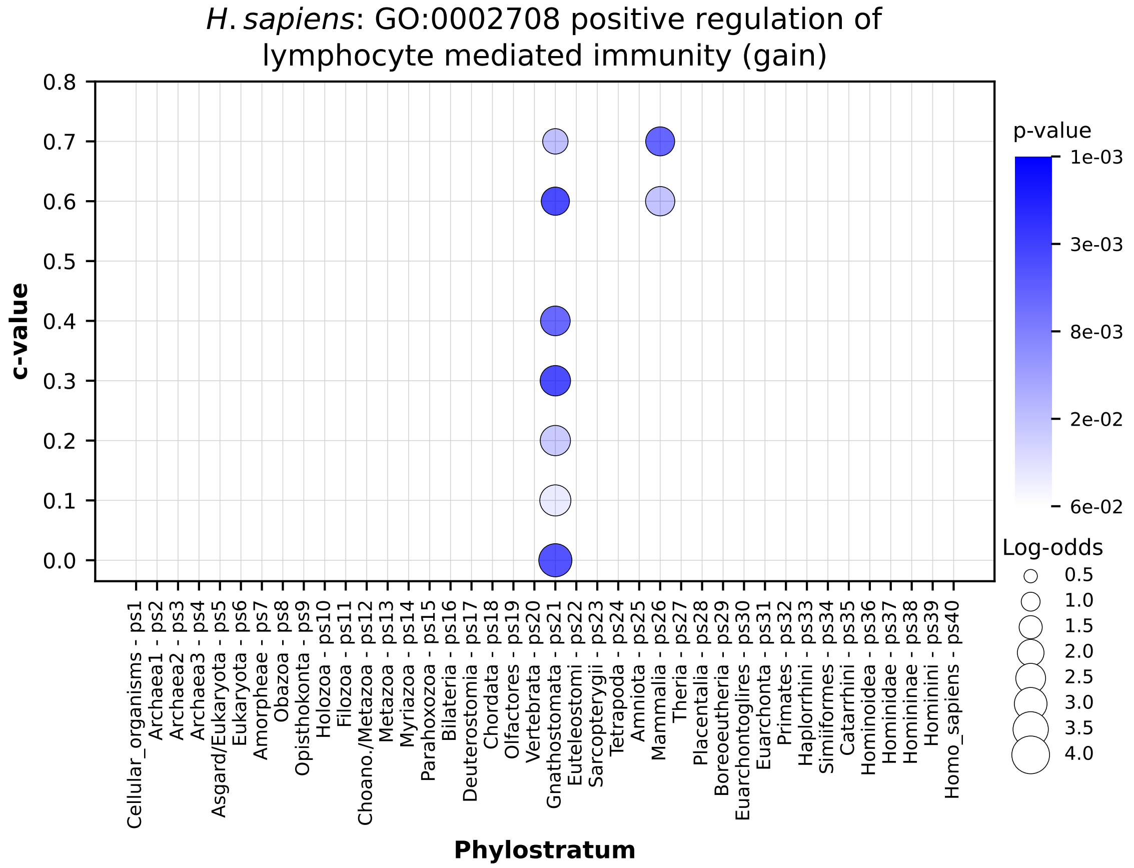

H. sapiens: GO:0002708 positive regulation of lymphocyte mediated immunity (gain)
c-value
0.8
0.7
0.6
0.5
0.4
0.3
0.2
0.1
0.0
Phylostratum
Cellular_organisms - ps1
Archaea1 - ps2
Archaea2 - ps3
Archaea3 - ps4
Asgard/Eukaryota - ps5
Eukaryota - ps6
Amorpheae - ps7
Obazoa - ps8
Opisthokonta - ps9
Holozoa - ps10
Filozoa - ps11
Choano./Metazoa - ps12
Metazoa - ps13
Myriazoa - ps14
Parahoxozoa - ps15
Bilateria - ps16
Deuterostomia - ps17
Chordata - ps18
Olfactores - ps19
Vertebrata - ps20
Gnathostomata - ps21
Euteleostomi - ps22
Sarcopterygii - ps23
Tetrapoda - ps24
Amniota - ps25
Mammalia - ps26
Theria - ps27
Placentalia - ps28
Boreoeutheria - ps29
Euarchontoglires - ps30
Euarchonta - ps31
Primates - ps32
Haplorrhini - ps33
Simiiformes - ps34
Catarrhini - ps35
Hominoidea - ps36
Hominidae - ps37
Homininae - ps38
Hominini - ps39
Homo_sapiens - ps40
p-value
1e-03
3e-03
8e-03
2e-02
6e-02
Log-odds
0.5
1.0
1.5
2.0
2.5
3.0
3.5
4.0

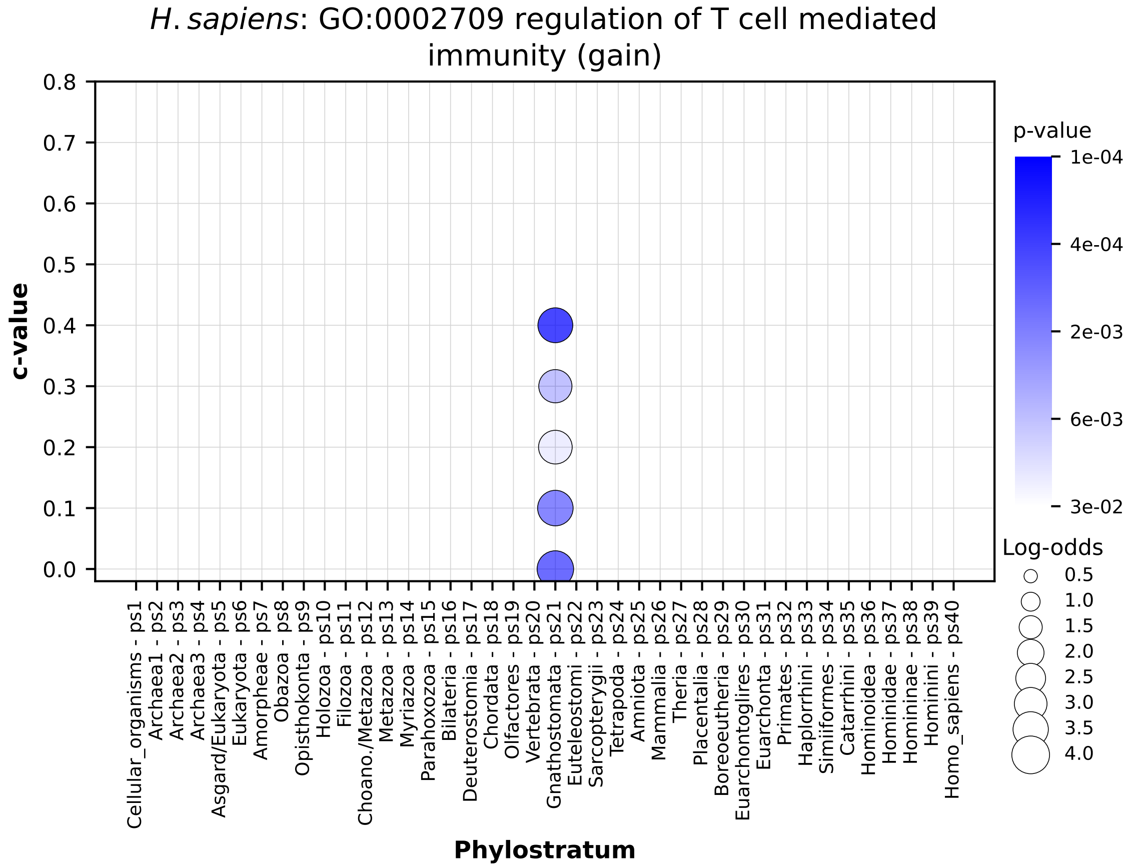
H. sapiens: GO:0002709 regulation of T cell mediated immunity (gain)
c-value
0.0
0.1
0.2
0.3
0.4
0.5
0.6
0.7
0.8
Phylostratum
Cellular_organisms - ps1
Archaea1 - ps2
Archaea2 - ps3
Archaea3 - ps4
Asgard/Eukaryota - ps5
Eukaryota - ps6
Amorpheae - ps7
Obazoa - ps8
Opisthokonta - ps9
Holozoa - ps10
Filozoa - ps11
Choano./Metazoa - ps12
Metazoa - ps13
Myriazoa - ps14
Parahoxozoa - ps15
Bilateria - ps16
Deuterostomia - ps17
Chordata - ps18
Olfactores - ps19
Vertebrata - ps20
Gnathostomata - ps21
Euteleostomi - ps22
Sarcopterygii - ps23
Tetrapoda - ps24
Amniota - ps25
Mammalia - ps26
Theria - ps27
Placentalia - ps28
Boreoeutheria - ps29
Euarchontoglires - ps30
Euarchonta - ps31
Primates - ps32
Haplorrhini - ps33
Simiiformes - ps34
Catarrhini - ps35
Hominoidea - ps36
Hominidae - ps37
Homininae - ps38
Hominini - ps39
Homo_sapiens - ps40
p-value
1e-04
4e-04
2e-03
6e-03
3e-02
Log-odds
0.5
1.0
1.5
2.0
2.5
3.0
3.5
4.0

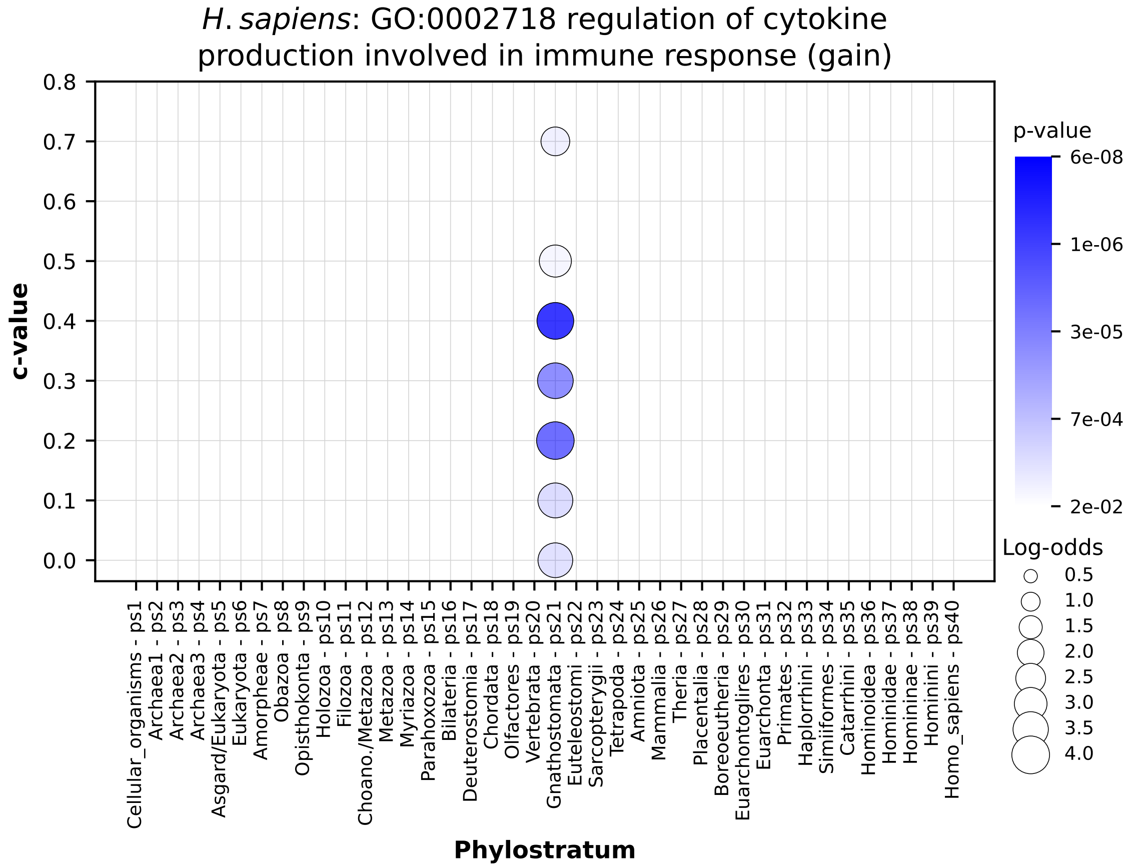

H. sapiens: GO:0002718 regulation of cytokine production involved in immune response (gain)
c-value
0.0
0.1
0.2
0.3
0.4
0.5
0.6
0.7
0.8
Phylostratum
Cellular_organisms - ps1
Archaea1 - ps2
Archaea2 - ps3
Archaea3 - ps4
Asgard/Eukaryota - ps5
Eukaryota - ps6
Amorpheae - ps7
Obazoa - ps8
Opisthokonta - ps9
Holozoa - ps10
Filozoa - ps11
Choano./Metazoa - ps12
Metazoa - ps13
Myriazoa - ps14
Parahoxozoa - ps15
Bilateria - ps16
Deuterostomia - ps17
Chordata - ps18
Olfactores - ps19
Vertebrata - ps20
Gnathostomata - ps21
Euteleostomi - ps22
Sarcopterygii - ps23
Tetrapoda - ps24
Amniota - ps25
Mammalia - ps26
Theria - ps27
Placentalia - ps28
Boreoeutheria - ps29
Euarchontoglires - ps30
Euarchonta - ps31
Primates - ps32
Haplorrhini - ps33
Simiiformes - ps34
Catarrhini - ps35
Hominoidea - ps36
Hominidae - ps37
Homininae - ps38
Hominini - ps39
Homo_sapiens - ps40
p-value
6e-08
1e-06
3e-05
7e-04
2e-02
Log-odds
0.5
1.0
1.5
2.0
2.5
3.0
3.5
4.0

# *H. sapiens*: GO:0002720 positive regulation of cytokine production involved in immune response (gain)

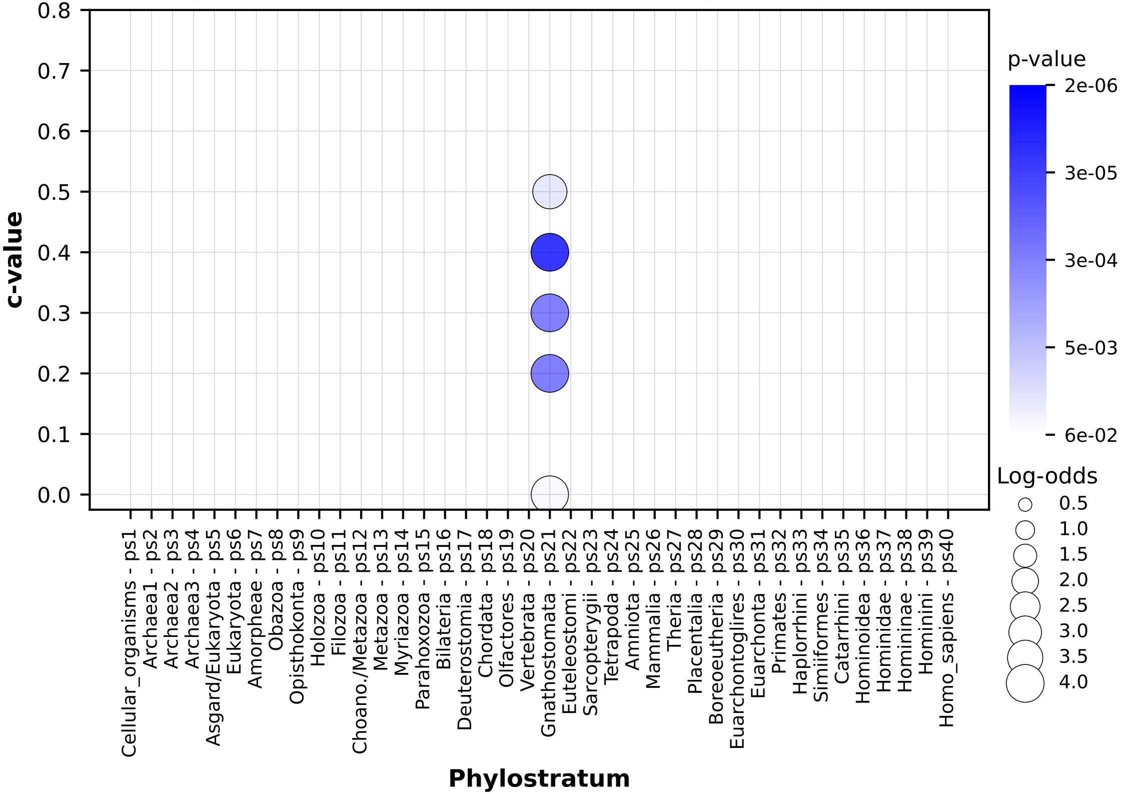

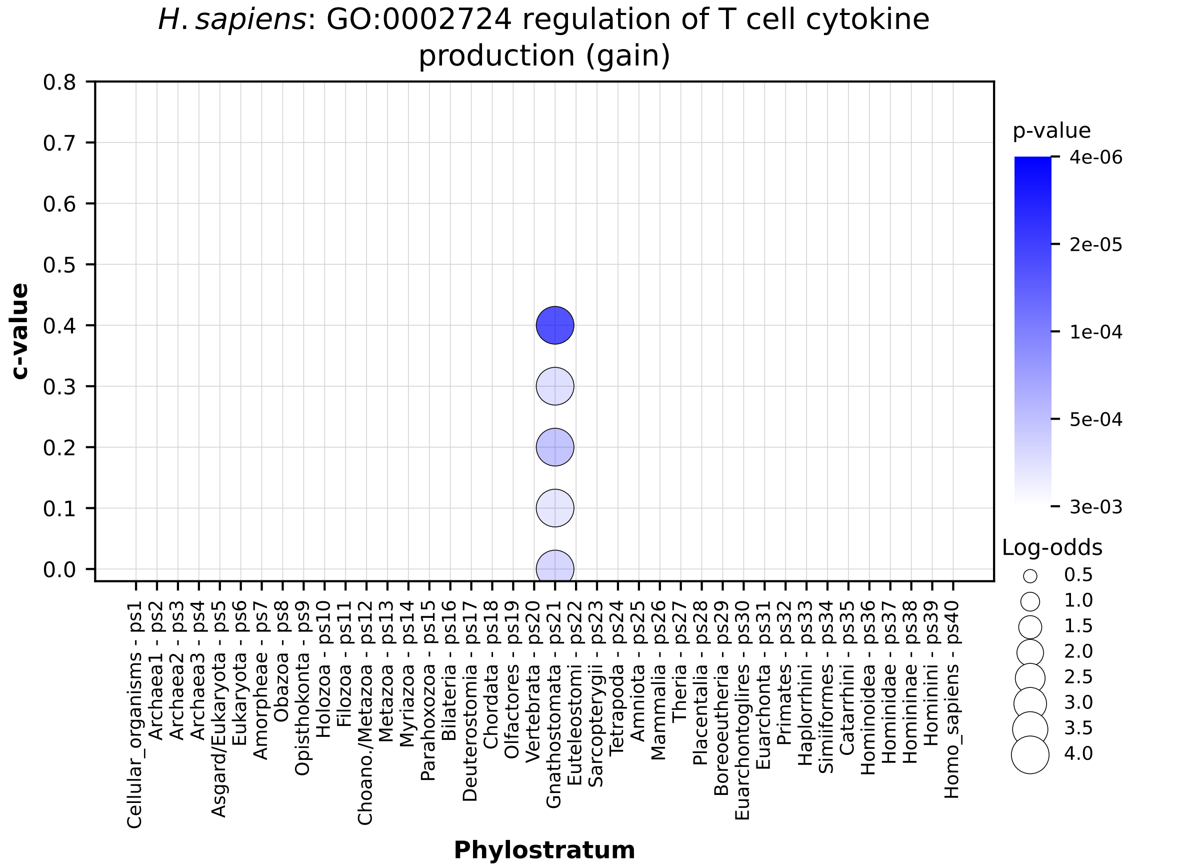

H. sapiens: GO:0002724 regulation of T cell cytokine production (gain)
c-value
0.0
0.1
0.2
0.3
0.4
0.5
0.6
0.7
0.8
Phylostratum
Cellular_organisms - ps1
Archaea1 - ps2
Archaea2 - ps3
Archaea3 - ps4
Asgard/Eukaryota - ps5
Eukaryota - ps6
Amorpheae - ps7
Obazoa - ps8
Opisthokonta - ps9
Holozoa - ps10
Filozoa - ps11
Choano./Metazoa - ps12
Metazoa - ps13
Myriazoa - ps14
Parahoxozoa - ps15
Bilateria - ps16
Deuterostomia - ps17
Chordata - ps18
Olfactores - ps19
Vertebrata - ps20
Gnathostomata - ps21
Euteleostomi - ps22
Sarcopterygii - ps23
Tetrapoda - ps24
Amniota - ps25
Mammalia - ps26
Theria - ps27
Placentalia - ps28
Boreoeutheria - ps29
Euarchontoglires - ps30
Euarchonta - ps31
Primates - ps32
Haplorrhini - ps33
Simiiformes - ps34
Catarrhini - ps35
Hominoidea - ps36
Hominidae - ps37
Homininae - ps38
Hominini - ps39
Homo_sapiens - ps40
p-value
4e-06
2e-05
1e-04
5e-04
3e-03
Log-odds
0.5
1.0
1.5
2.0
2.5
3.0
3.5
4.0

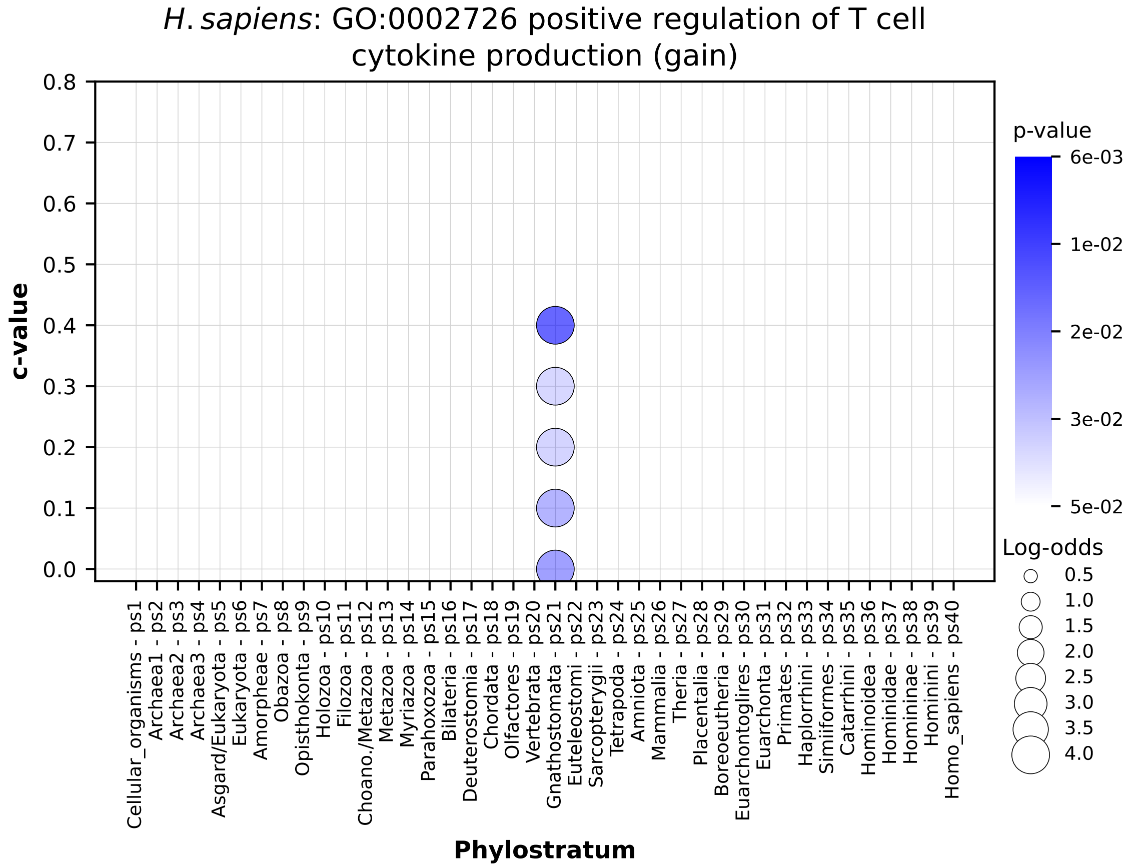

H. sapiens: GO:0002726 positive regulation of T cell cytokine production (gain)
c-value
0.8
0.7
0.6
0.5
0.4
0.3
0.2
0.1
0.0
Phylostratum
Cellular_organisms - ps1
Archaea1 - ps2
Archaea2 - ps3
Archaea3 - ps4
Asgard/Eukaryota - ps5
Eukaryota - ps6
Amorpheae - ps7
Obazoa - ps8
Opisthokonta - ps9
Holozoa - ps10
Filozoa - ps11
Choano./Metazoa - ps12
Metazoa - ps13
Myriazoa - ps14
Parahoxozoa - ps15
Bilateria - ps16
Deuterostomia - ps17
Chordata - ps18
Olfactores - ps19
Vertebrata - ps20
Gnathostomata - ps21
Euteleostomi - ps22
Sarcopterygii - ps23
Tetrapoda - ps24
Amniota - ps25
Mammalia - ps26
Theria - ps27
Placentalia - ps28
Boreoeutheria - ps29
Euarchontoglires - ps30
Euarchonta - ps31
Primates - ps32
Haplorrhini - ps33
Simiiformes - ps34
Catarrhini - ps35
Hominoidea - ps36
Hominidae - ps37
Homininae - ps38
Hominini - ps39
Homo_sapiens - ps40
p-value
6e-03
1e-02
2e-02
3e-02
5e-02
Log-odds
0.5
1.0
1.5
2.0
2.5
3.0
3.5
4.0

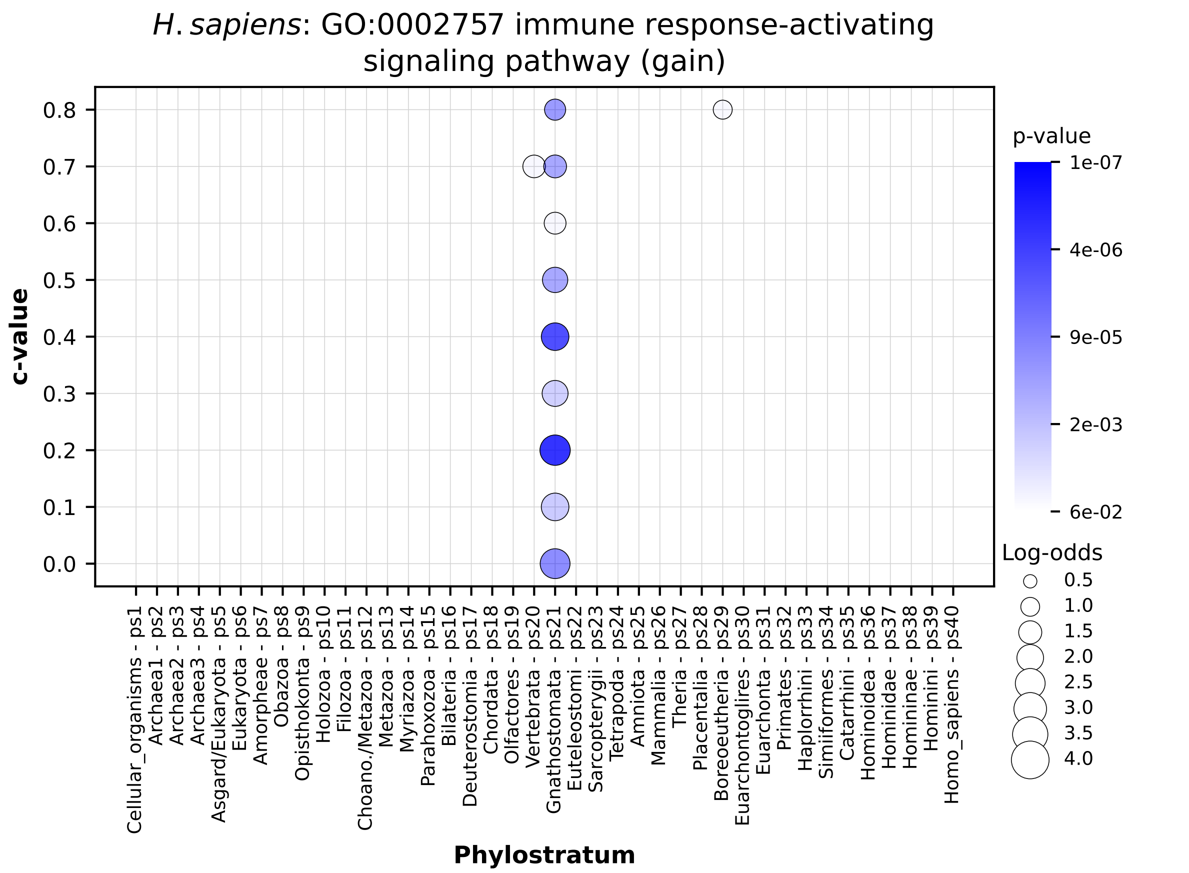
H. sapiens: GO:0002757 immune response-activating signaling pathway (gain)
c-value
0.8
0.7
0.6
0.5
0.4
0.3
0.2
0.1
0.0
p-value
1e-07
4e-06
9e-05
2e-03
6e-02
Log-odds
0.5
1.0
1.5
2.0
2.5
3.0
3.5
4.0
Cellular_organisms - ps1
Archaea1 - ps2
Archaea2 - ps3
Archaea3 - ps4
Asgard/Eukaryota - ps5
Eukaryota - ps6
Amorpheae - ps7
Obazoa - ps8
Opisthokonta - ps9
Holozoa - ps10
Filozoa - ps11
Choano./Metazoa - ps12
Metazoa - ps13
Myriazoa - ps14
Parahoxozoa - ps15
Bilateria - ps16
Deuterostomia - ps17
Chordata - ps18
Olfactores - ps19
Vertebrata - ps20
Gnathostomata - ps21
Euteleostomi - ps22
Sarcopterygii - ps23
Tetrapoda - ps24
Amniota - ps25
Mammalia - ps26
Theria - ps27
Placentalia - ps28
Boreoeutheria - ps29
Euarchontoglires - ps30
Euarchonta - ps31
Primates - ps32
Haplorrhini - ps33
Simiiformes - ps34
Catarrhini - ps35
Hominoidea - ps36
Hominidae - ps37
Homininae - ps38
Hominini - ps39
Homo_sapiens - ps40
Phylostratum

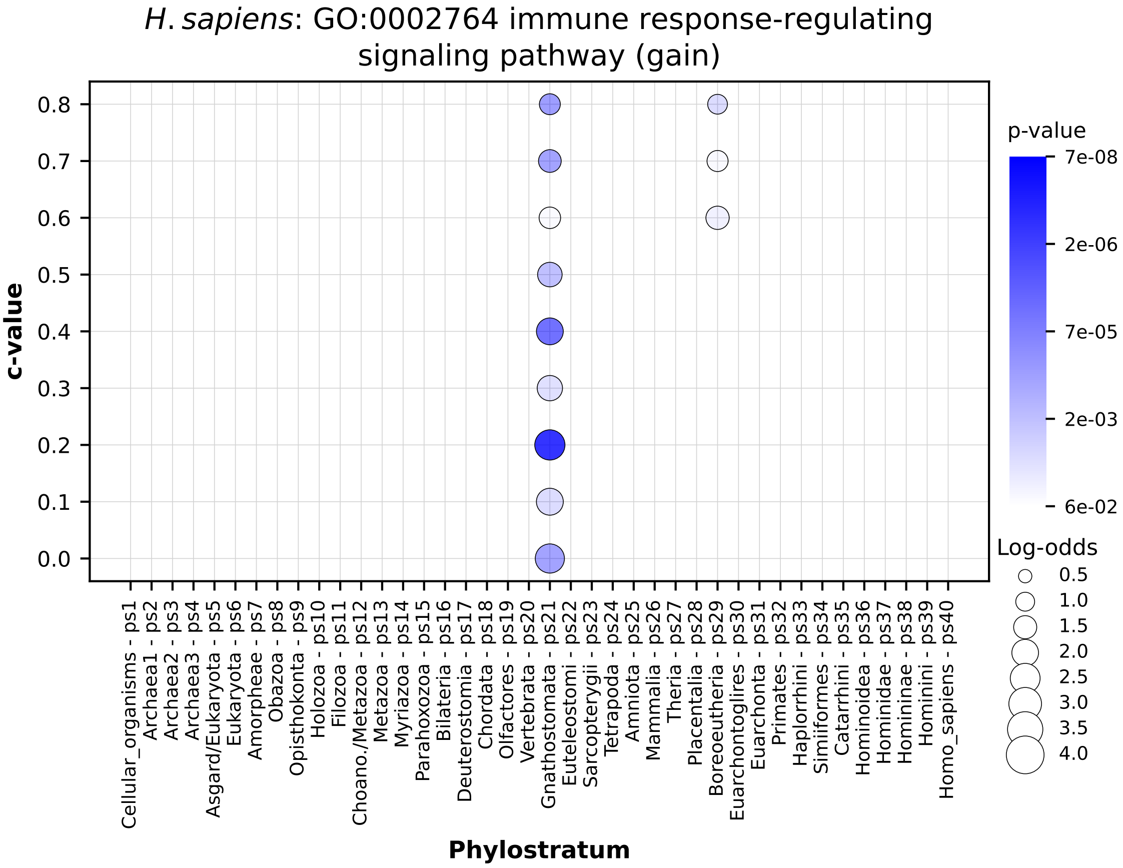

*H. sapiens*: GO:0002764 immune response-regulating signaling pathway (gain)
c-value
0.8
0.7
0.6
0.5
0.4
0.3
0.2
0.1
0.0
Phylostratum
Cellular_organisms - ps1
Archaea1 - ps2
Archaea2 - ps3
Archaea3 - ps4
Asgard/Eukaryota - ps5
Eukaryota - ps6
Amorpheae - ps7
Obazoa - ps8
Opisthokonta - ps9
Holozoa - ps10
Filozoa - ps11
Choano./Metazoa - ps12
Metazoa - ps13
Myriazoa - ps14
Parahoxozoa - ps15
Bilateria - ps16
Deuterostomia - ps17
Chordata - ps18
Olfactores - ps19
Vertebrata - ps20
Gnathostomata - ps21
Euteleostomi - ps22
Sarcopterygii - ps23
Tetrapoda - ps24
Amniota - ps25
Mammalia - ps26
Theria - ps27
Placentalia - ps28
Boreoeutheria - ps29
Euarchontoglires - ps30
Euarchonta - ps31
Primates - ps32
Haplorrhini - ps33
Simiiformes - ps34
Catarrhini - ps35
Hominoidea - ps36
Hominidae - ps37
Homininae - ps38
Hominini - ps39
Homo_sapiens - ps40
p-value
7e-08
2e-06
7e-05
2e-03
6e-02
Log-odds
0.5
1.0
1.5
2.0
2.5
3.0
3.5
4.0

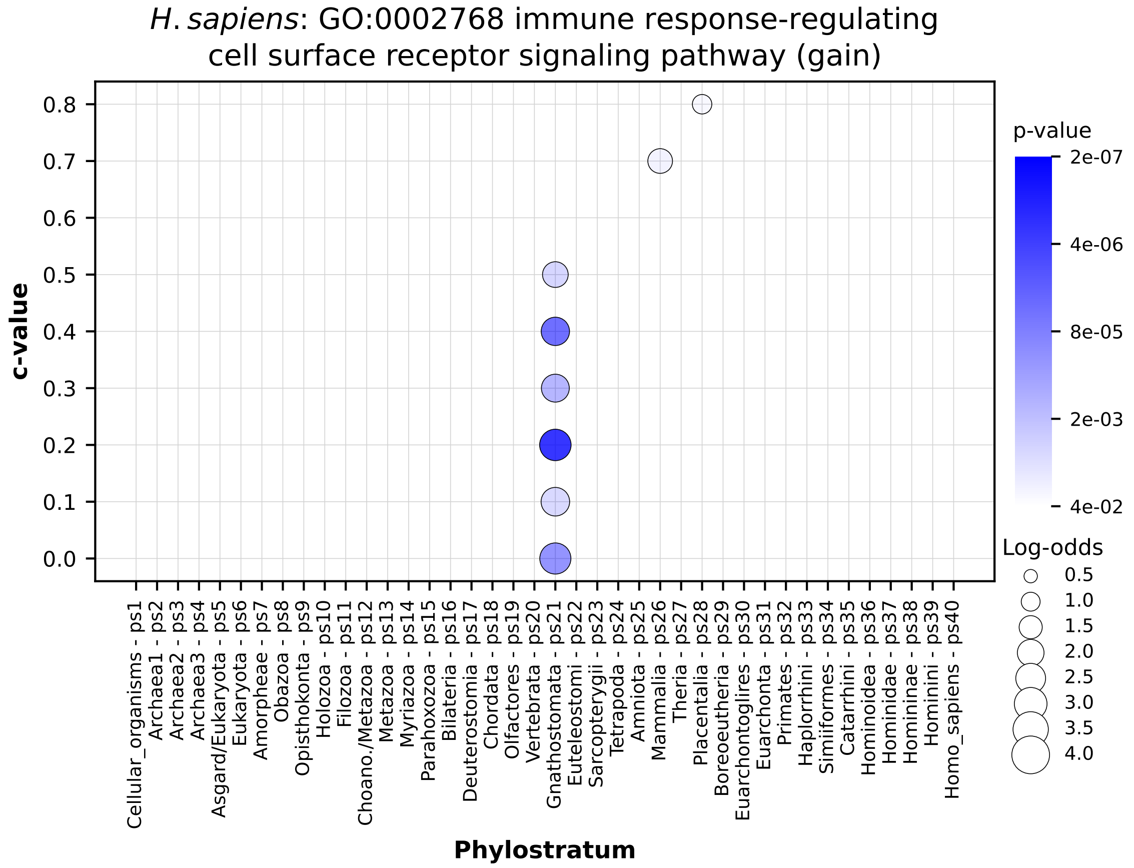

H. sapiens: GO:0002768 immune response-regulating cell surface receptor signaling pathway (gain)
c-value
0.8
0.7
0.6
0.5
0.4
0.3
0.2
0.1
0.0
Phylostratum
Cellular_organisms - ps1
Archaea1 - ps2
Archaea2 - ps3
Archaea3 - ps4
Asgard/Eukaryota - ps5
Eukaryota - ps6
Amorpheae - ps7
Obazoa - ps8
Opisthokonta - ps9
Holozoa - ps10
Filozoa - ps11
Choano./Metazoa - ps12
Metazoa - ps13
Myriazoa - ps14
Parahoxozoa - ps15
Bilateria - ps16
Deuterostomia - ps17
Chordata - ps18
Olfactores - ps19
Vertebrata - ps20
Gnathostomata - ps21
Euteleostomi - ps22
Sarcopterygii - ps23
Tetrapoda - ps24
Amniota - ps25
Mammalia - ps26
Theria - ps27
Placentalia - ps28
Boreoeutheria - ps29
Euarchontoglires - ps30
Euarchonta - ps31
Primates - ps32
Haplorrhini - ps33
Simiiformes - ps34
Catarrhini - ps35
Hominoidea - ps36
Hominidae - ps37
Homininae - ps38
Hominini - ps39
Homo_sapiens - ps40
p-value
2e-07
4e-06
8e-05
2e-03
4e-02
Log-odds
0.5
1.0
1.5
2.0
2.5
3.0
3.5
4.0

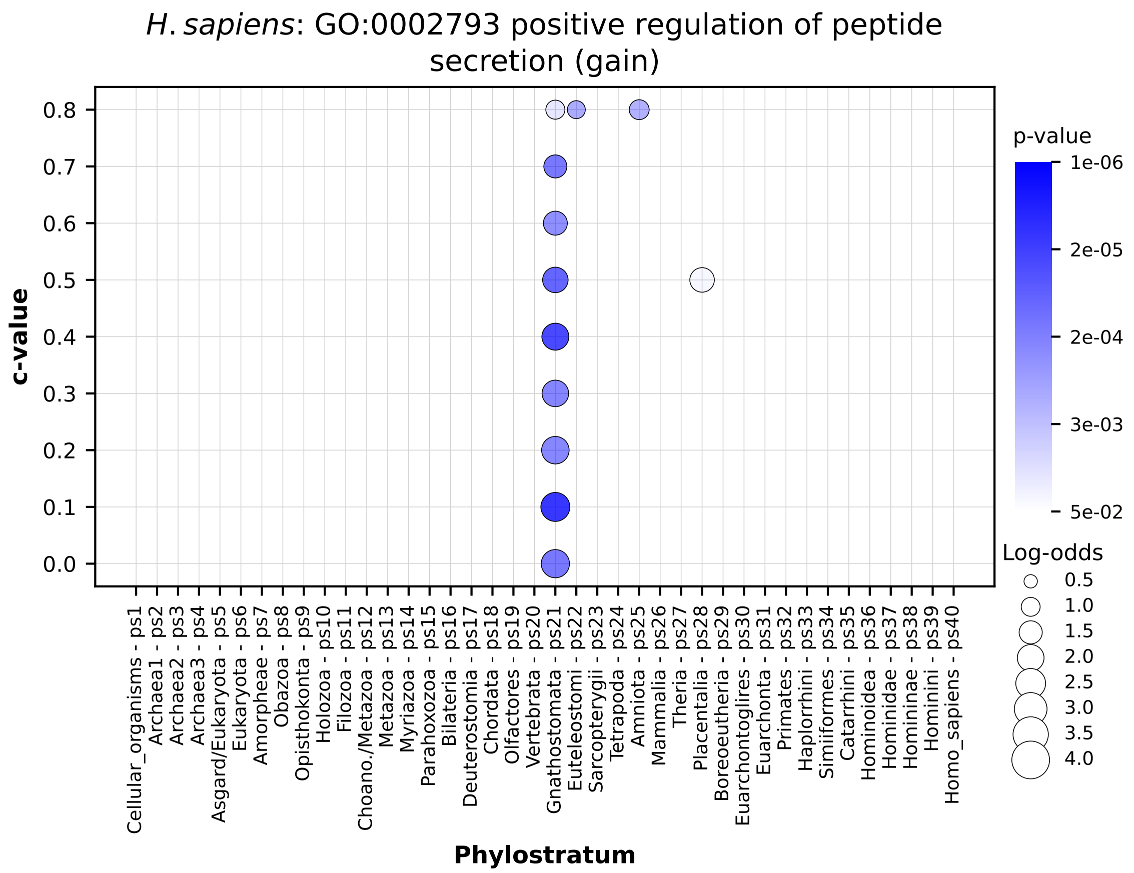

H. sapiens: GO:0002793 positive regulation of peptide secretion (gain)
c-value
0.8
0.7
0.6
0.5
0.4
0.3
0.2
0.1
0.0
Phylostratum
Cellular_organisms - ps1
Archaea1 - ps2
Archaea2 - ps3
Archaea3 - ps4
Asgard/Eukaryota - ps5
Eukaryota - ps6
Amorpheae - ps7
Obazoa - ps8
Opisthokonta - ps9
Holozoa - ps10
Filozoa - ps11
Choano./Metazoa - ps12
Metazoa - ps13
Myriazoa - ps14
Parahoxozoa - ps15
Bilateria - ps16
Deuterostomia - ps17
Chordata - ps18
Olfactores - ps19
Vertebrata - ps20
Gnathostomata - ps21
Euteleostomi - ps22
Sarcopterygii - ps23
Tetrapoda - ps24
Amniota - ps25
Mammalia - ps26
Theria - ps27
Placentalia - ps28
Boreoeutheria - ps29
Euarchontoglires - ps30
Euarchonta - ps31
Primates - ps32
Haplorrhini - ps33
Simiiformes - ps34
Catarrhini - ps35
Hominoidea - ps36
Hominidae - ps37
Homininae - ps38
Hominini - ps39
Homo_sapiens - ps40
p-value
1e-06
2e-05
2e-04
3e-03
5e-02
Log-odds
0.5
1.0
1.5
2.0
2.5
3.0
3.5
4.0

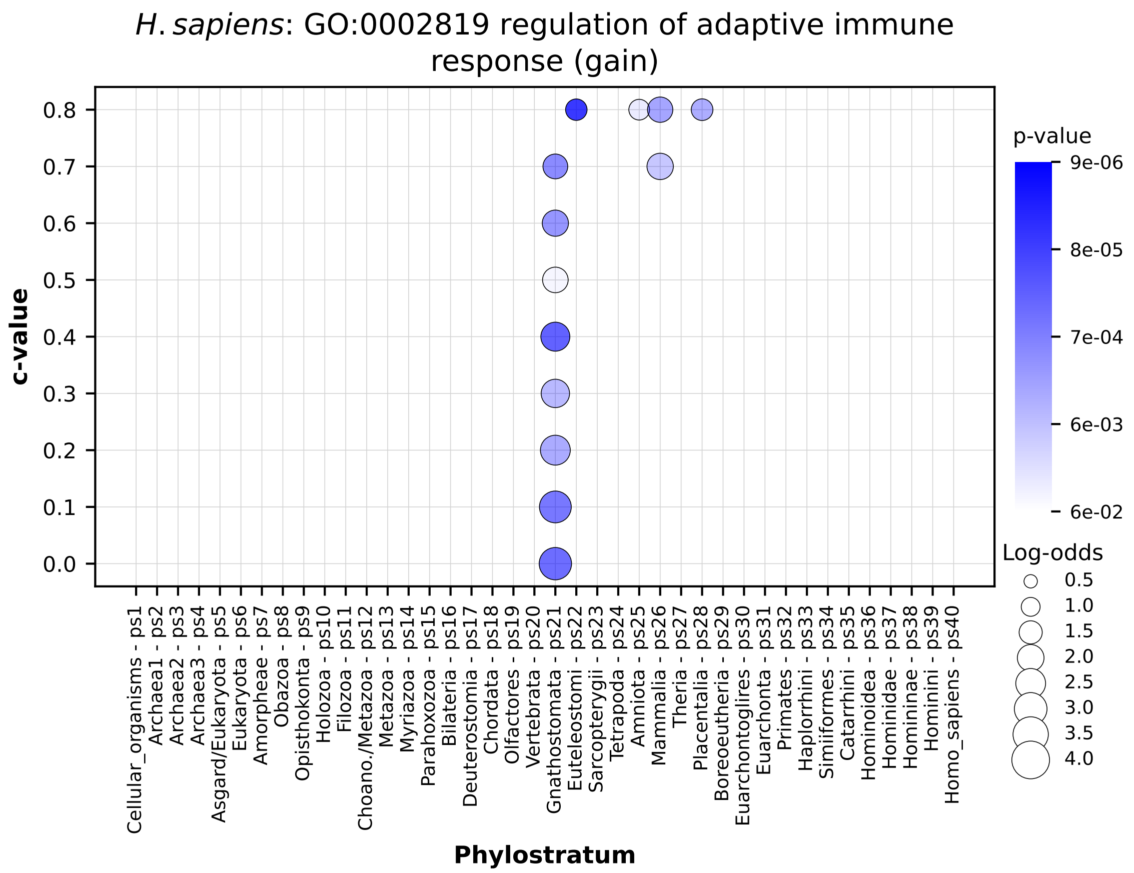
H. sapiens: GO:0002819 regulation of adaptive immune response (gain)
c-value
0.8
0.7
0.6
0.5
0.4
0.3
0.2
0.1
0.0
p-value
9e-06
8e-05
7e-04
6e-03
6e-02
Log-odds
0.5
1.0
1.5
2.0
2.5
3.0
3.5
4.0
Cellular_organisms - ps1
Archaea1 - ps2
Archaea2 - ps3
Archaea3 - ps4
Asgard/Eukaryota - ps5
Eukaryota - ps6
Amorpheae - ps7
Obazoa - ps8
Opisthokonta - ps9
Holozoa - ps10
Filozoa - ps11
Choano./Metazoa - ps12
Metazoa - ps13
Myriazoa - ps14
Parahoxozoa - ps15
Bilateria - ps16
Deuterostomia - ps17
Chordata - ps18
Olfactores - ps19
Vertebrata - ps20
Gnathostomata - ps21
Euteleostomi - ps22
Sarcopterygii - ps23
Tetrapoda - ps24
Amniota - ps25
Mammalia - ps26
Theria - ps27
Placentalia - ps28
Boreoeutheria - ps29
Euarchontoglires - ps30
Euarchonta - ps31
Primates - ps32
Haplorrhini - ps33
Simiiformes - ps34
Catarrhini - ps35
Hominoidea - ps36
Hominidae - ps37
Homininae - ps38
Hominini - ps39
Homo_sapiens - ps40
Phylostratum

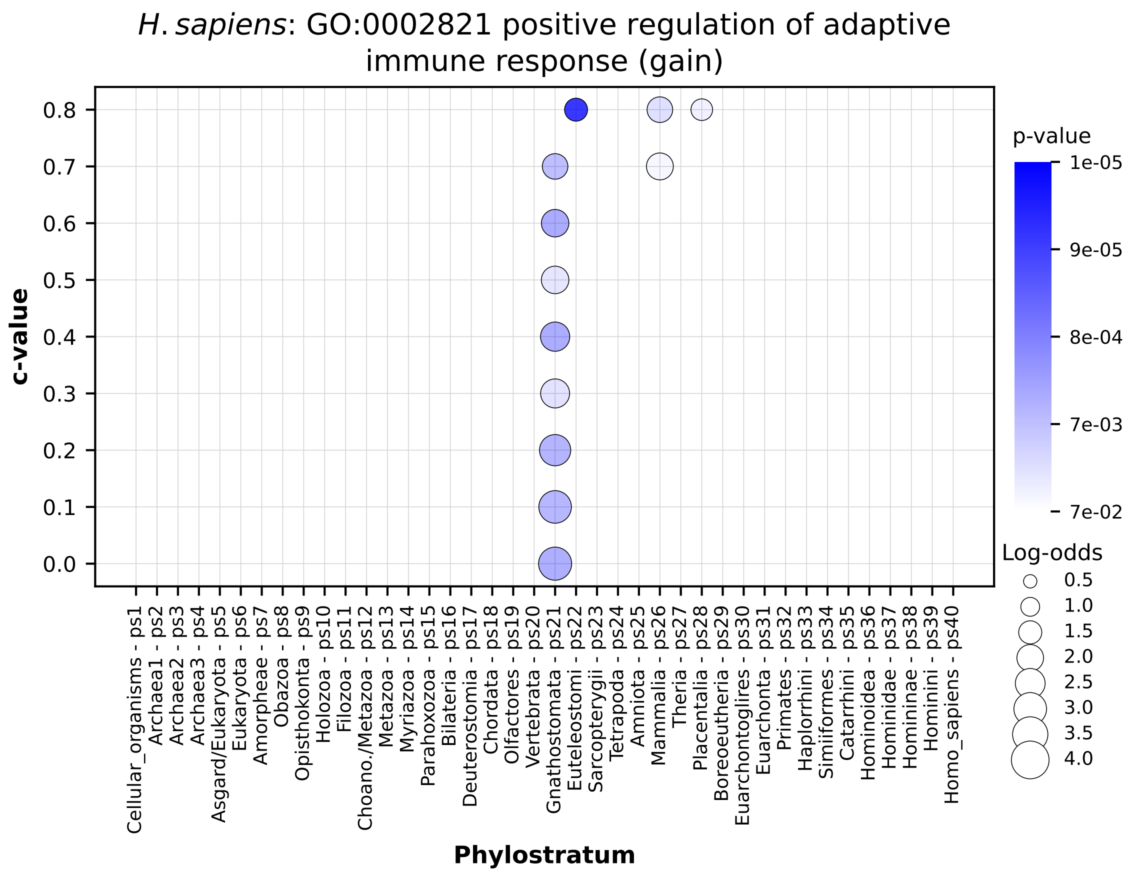

H. sapiens: GO:0002821 positive regulation of adaptive immune response (gain)
c-value
0.8
0.7
0.6
0.5
0.4
0.3
0.2
0.1
0.0
p-value
1e-05
9e-05
8e-04
7e-03
7e-02
Log-odds
0.5
1.0
1.5
2.0
2.5
3.0
3.5
4.0
Cellular_organisms - ps1
Archaea1 - ps2
Archaea2 - ps3
Archaea3 - ps4
Asgard/Eukaryota - ps5
Eukaryota - ps6
Amorpheae - ps7
Obazoa - ps8
Opisthokonta - ps9
Holozoa - ps10
Filozoa - ps11
Choano./Metazoa - ps12
Metazoa - ps13
Myriazoa - ps14
Parahoxozoa - ps15
Bilateria - ps16
Deuterostomia - ps17
Chordata - ps18
Olfactores - ps19
Vertebrata - ps20
Gnathostomata - ps21
Euteleostomi - ps22
Sarcopterygii - ps23
Tetrapoda - ps24
Amniota - ps25
Mammalia - ps26
Theria - ps27
Placentalia - ps28
Boreoeutheria - ps29
Euarchontoglires - ps30
Euarchonta - ps31
Primates - ps32
Haplorrhini - ps33
Simiiformes - ps34
Catarrhini - ps35
Hominoidea - ps36
Hominidae - ps37
Homininae - ps38
Hominini - ps39
Homo_sapiens - ps40
Phylostratum

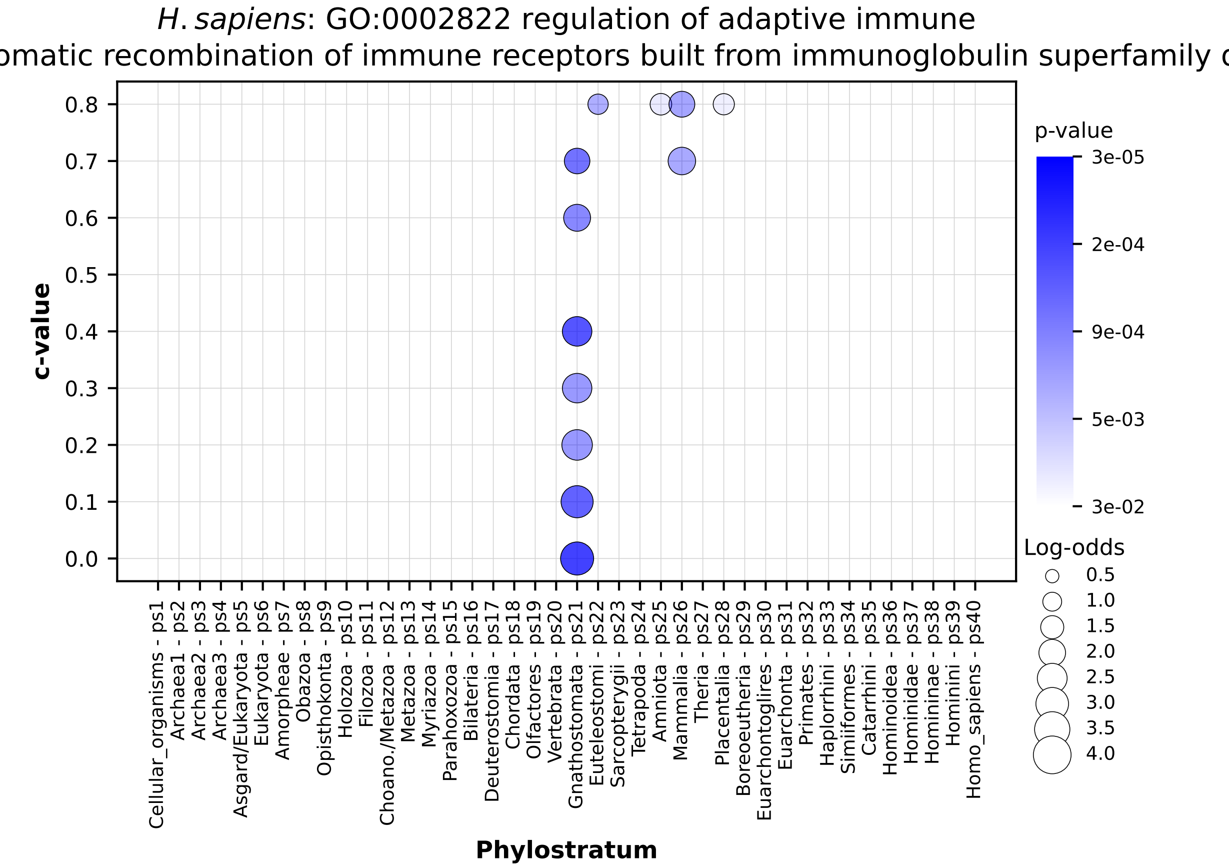

H. sapiens: GO:0002822 regulation of adaptive immune
omatic recombination of immune receptors built from immunoglobulin superfamily d
c-value
0.0
0.1
0.2
0.3
0.4
0.5
0.6
0.7
0.8
Phylostratum
Cellular_organisms - ps1
Archaea1 - ps2
Archaea2 - ps3
Archaea3 - ps4
Asgard/Eukaryota - ps5
Eukaryota - ps6
Amorpheae - ps7
Obazoa - ps8
Opisthokonta - ps9
Holozoa - ps10
Filozoa - ps11
Choano./Metazoa - ps12
Metazoa - ps13
Myriazoa - ps14
Parahoxozoa - ps15
Bilateria - ps16
Deuterostomia - ps17
Chordata - ps18
Olfactores - ps19
Vertebrata - ps20
Gnathostomata - ps21
Euteleostomi - ps22
Sarcopterygii - ps23
Tetrapoda - ps24
Amniota - ps25
Mammalia - ps26
Theria - ps27
Placentalia - ps28
Boreoeutheria - ps29
Euarchontoglires - ps30
Euarchonta - ps31
Primates - ps32
Haplorrhini - ps33
Simiiformes - ps34
Catarrhini - ps35
Hominoidea - ps36
Hominidae - ps37
Homininae - ps38
Hominini - ps39
Homo_sapiens - ps40
p-value
3e-05
2e-04
9e-04
5e-03
3e-02
Log-odds
0.5
1.0
1.5
2.0
2.5
3.0
3.5
4.0

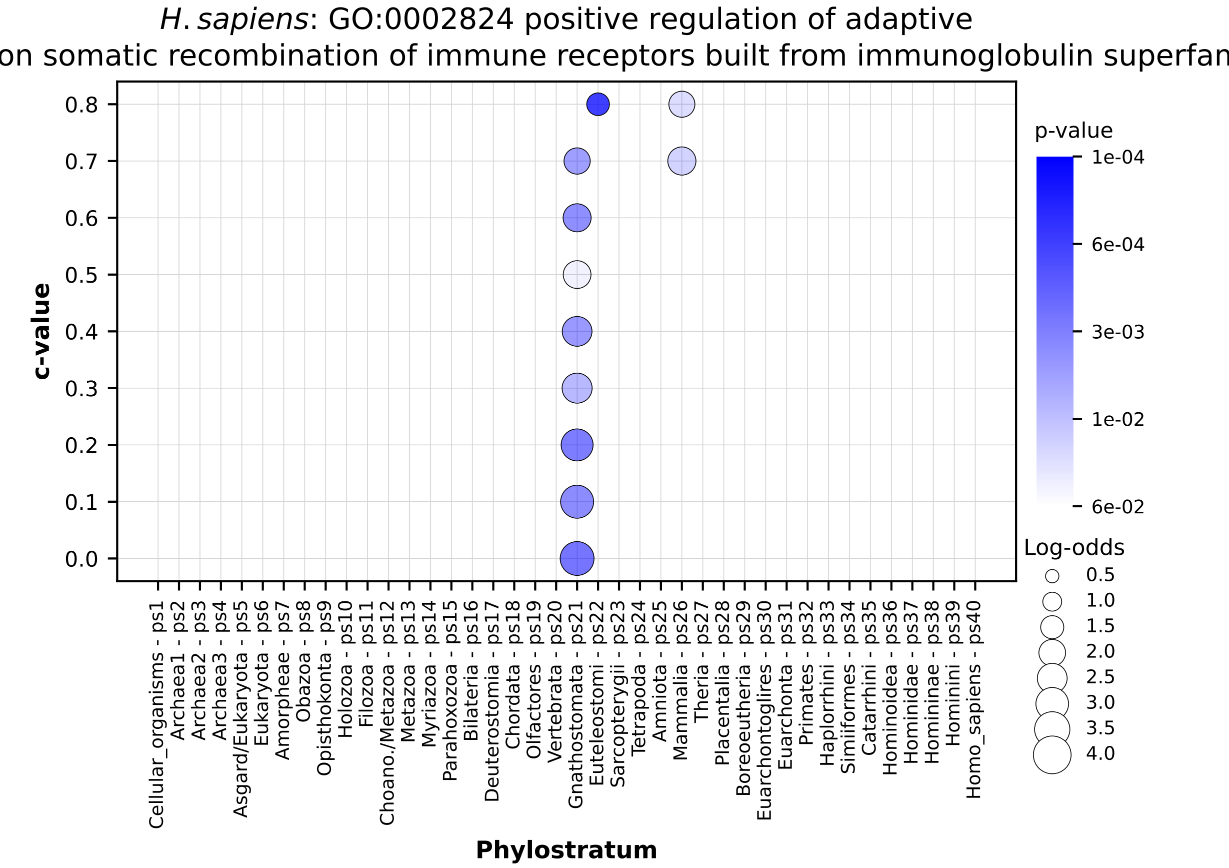

H. sapiens: GO:0002824 positive regulation of adaptive
on somatic recombination of immune receptors built from immunoglobulin superfam
c-value
0.0
0.1
0.2
0.3
0.4
0.5
0.6
0.7
0.8
Phylostratum
Cellular_organisms - ps1
Archaea1 - ps2
Archaea2 - ps3
Archaea3 - ps4
Asgard/Eukaryota - ps5
Eukaryota - ps6
Amorpheae - ps7
Obazoa - ps8
Opisthokonta - ps9
Holozoa - ps10
Filozoa - ps11
Choano./Metazoa - ps12
Metazoa - ps13
Myriazoa - ps14
Parahoxozoa - ps15
Bilateria - ps16
Deuterostomia - ps17
Chordata - ps18
Olfactores - ps19
Vertebrata - ps20
Gnathostomata - ps21
Euteleostomi - ps22
Sarcopterygii - ps23
Tetrapoda - ps24
Amniota - ps25
Mammalia - ps26
Theria - ps27
Placentalia - ps28
Boreoeutheria - ps29
Euarchontoglires - ps30
Euarchonta - ps31
Primates - ps32
Haplorrhini - ps33
Simiiformes - ps34
Catarrhini - ps35
Hominoidea - ps36
Hominidae - ps37
Homininae - ps38
Hominini - ps39
Homo_sapiens - ps40
p-value
1e-04
6e-04
3e-03
1e-02
6e-02
Log-odds
0.5
1.0
1.5
2.0
2.5
3.0
3.5
4.0

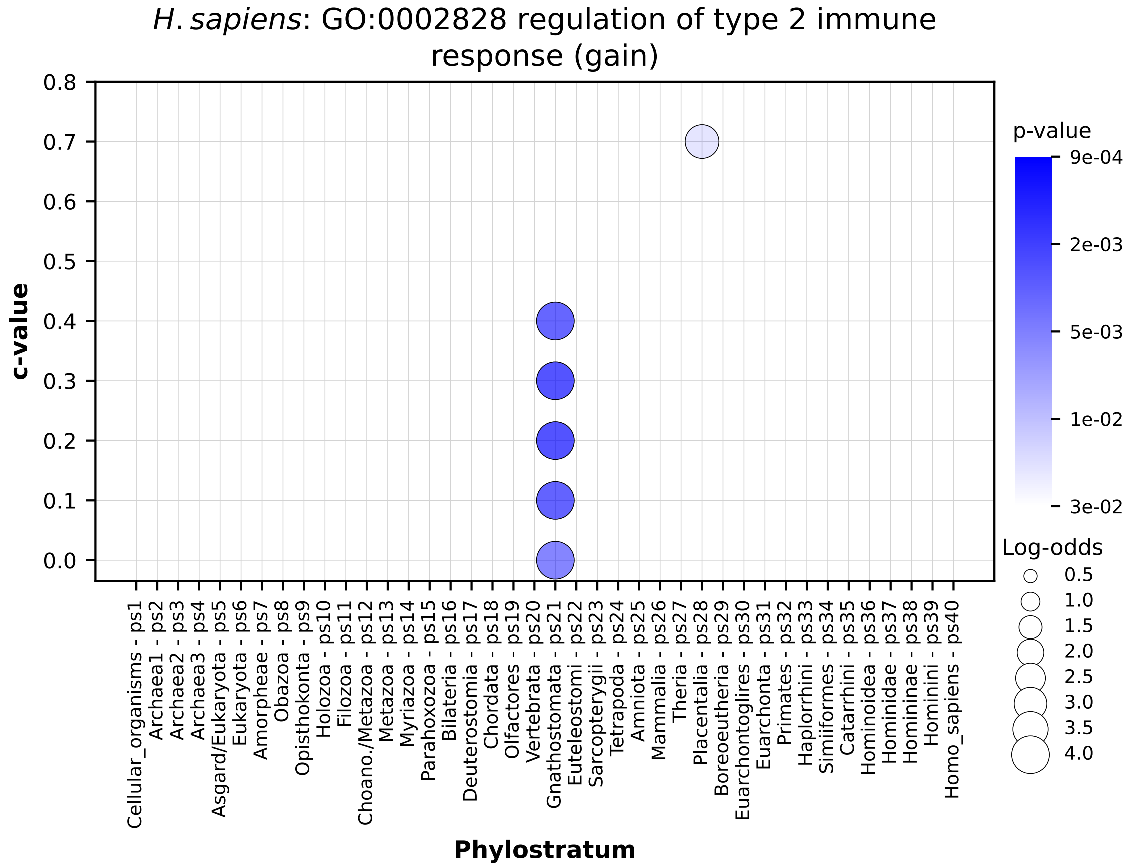

*H. sapiens*: GO:0002828 regulation of type 2 immune response (gain)
c-value
0.8
0.7
0.6
0.5
0.4
0.3
0.2
0.1
0.0
p-value
9e-04
2e-03
5e-03
1e-02
3e-02
Log-odds
0.5
1.0
1.5
2.0
2.5
3.0
3.5
4.0
Cellular_organisms - ps1
Archaea1 - ps2
Archaea2 - ps3
Archaea3 - ps4
Asgard/Eukaryota - ps5
Eukaryota - ps6
Amorpheae - ps7
Obazoa - ps8
Opisthokonta - ps9
Holozoa - ps10
Filozoa - ps11
Choano./Metazoa - ps12
Metazoa - ps13
Myriazoa - ps14
Parahoxozoa - ps15
Bilateria - ps16
Deuterostomia - ps17
Chordata - ps18
Olfactores - ps19
Vertebrata - ps20
Gnathostomata - ps21
Euteleostomi - ps22
Sarcopterygii - ps23
Tetrapoda - ps24
Amniota - ps25
Mammalia - ps26
Theria - ps27
Placentalia - ps28
Boreoeutheria - ps29
Euarchontoglires - ps30
Euarchonta - ps31
Primates - ps32
Haplorrhini - ps33
Simiiformes - ps34
Catarrhini - ps35
Hominoidea - ps36
Hominidae - ps37
Homininae - ps38
Hominini - ps39
Homo_sapiens - ps40
Phylostratum

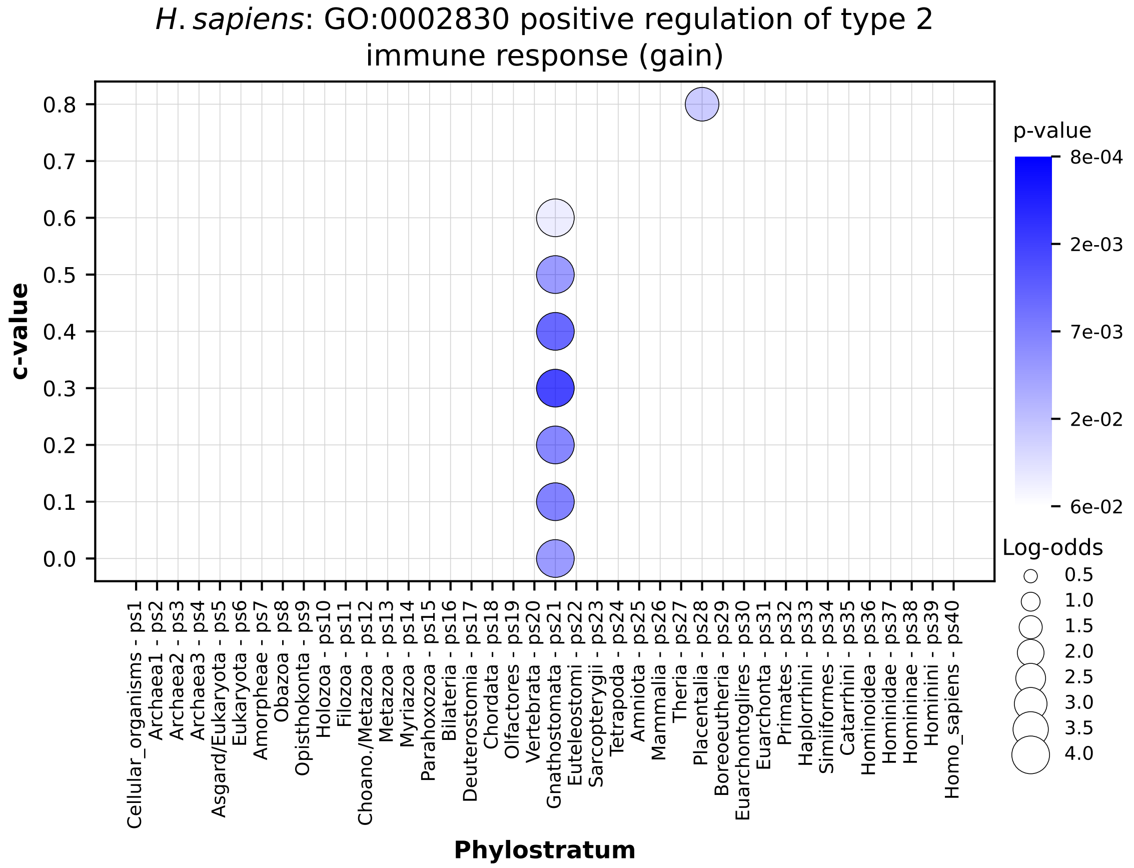

H. sapiens: GO:0002830 positive regulation of type 2 immune response (gain)
c-value
0.8
0.7
0.6
0.5
0.4
0.3
0.2
0.1
0.0
p-value
8e-04
2e-03
7e-03
2e-02
6e-02
Log-odds
0.5
1.0
1.5
2.0
2.5
3.0
3.5
4.0
Cellular_organisms - ps1
Archaea1 - ps2
Archaea2 - ps3
Archaea3 - ps4
Asgard/Eukaryota - ps5
Eukaryota - ps6
Amorpheae - ps7
Obazoa - ps8
Opisthokonta - ps9
Holozoa - ps10
Filozoa - ps11
Choano./Metazoa - ps12
Metazoa - ps13
Myriazoa - ps14
Parahoxozoa - ps15
Bilateria - ps16
Deuterostomia - ps17
Chordata - ps18
Olfactores - ps19
Vertebrata - ps20
Gnathostomata - ps21
Euteleostomi - ps22
Sarcopterygii - ps23
Tetrapoda - ps24
Amniota - ps25
Mammalia - ps26
Theria - ps27
Placentalia - ps28
Boreoeutheria - ps29
Euarchontoglires - ps30
Euarchonta - ps31
Primates - ps32
Haplorrhini - ps33
Simiiformes - ps34
Catarrhini - ps35
Hominoidea - ps36
Hominidae - ps37
Homininae - ps38
Hominini - ps39
Homo_sapiens - ps40
Phylostratum

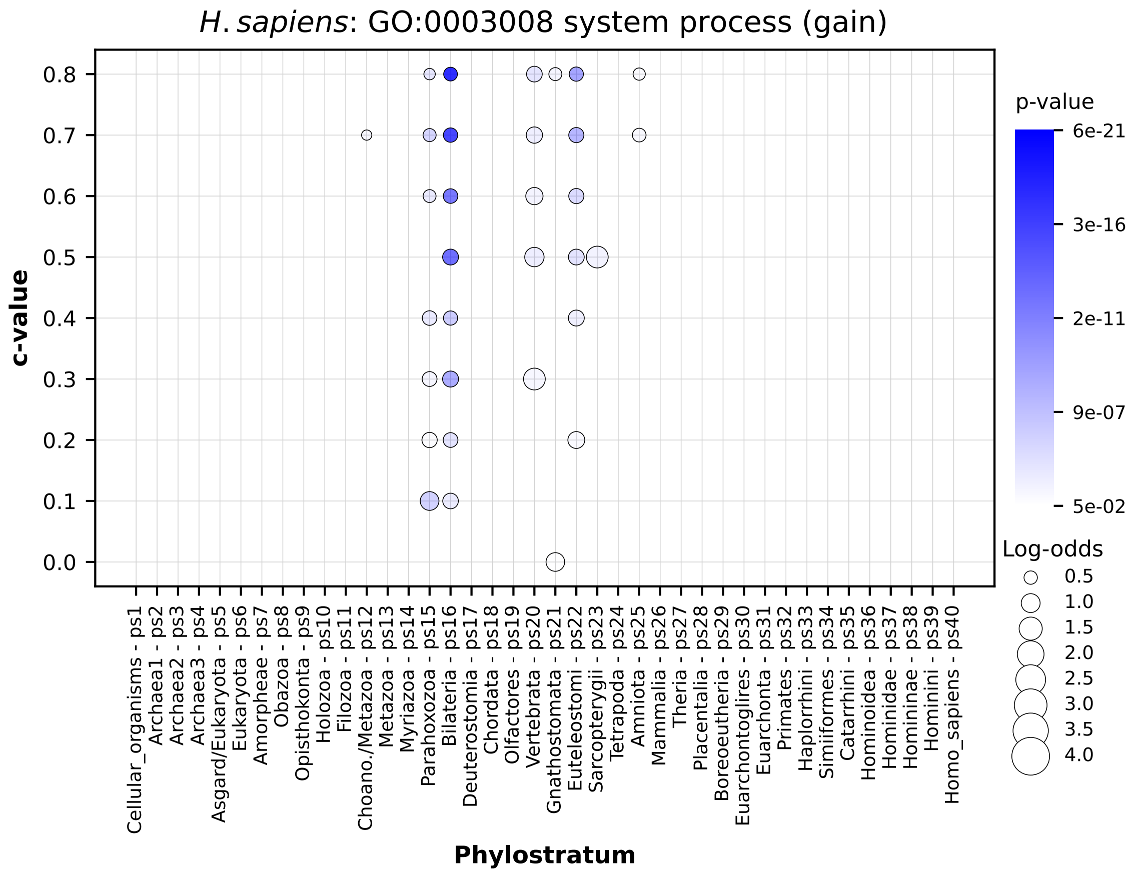

H. sapiens: GO:0003008 system process (gain)
c-value
0.8
0.7
0.6
0.5
0.4
0.3
0.2
0.1
0.0
p-value
6e-21
3e-16
2e-11
9e-07
5e-02
Log-odds
0.5
1.0
1.5
2.0
2.5
3.0
3.5
4.0
Cellular_organisms - ps1
Archaea1 - ps2
Archaea2 - ps3
Archaea3 - ps4
Asgard/Eukaryota - ps5
Eukaryota - ps6
Amorpheae - ps7
Obazoa - ps8
Opisthokonta - ps9
Holozoa - ps10
Filozoa - ps11
Choano./Metazoa - ps12
Metazoa - ps13
Myriazoa - ps14
Parahoxozoa - ps15
Bilateria - ps16
Deuterostomia - ps17
Chordata - ps18
Olfactores - ps19
Vertebrata - ps20
Gnathostomata - ps21
Euteleostomi - ps22
Sarcopterygii - ps23
Tetrapoda - ps24
Amniota - ps25
Mammalia - ps26
Theria - ps27
Placentalia - ps28
Boreoeutheria - ps29
Euarchontoglires - ps30
Euarchonta - ps31
Primates - ps32
Haplorrhini - ps33
Simiiformes - ps34
Catarrhini - ps35
Hominoidea - ps36
Hominidae - ps37
Homininae - ps38
Hominini - ps39
Homo_sapiens - ps40
Phylostratum

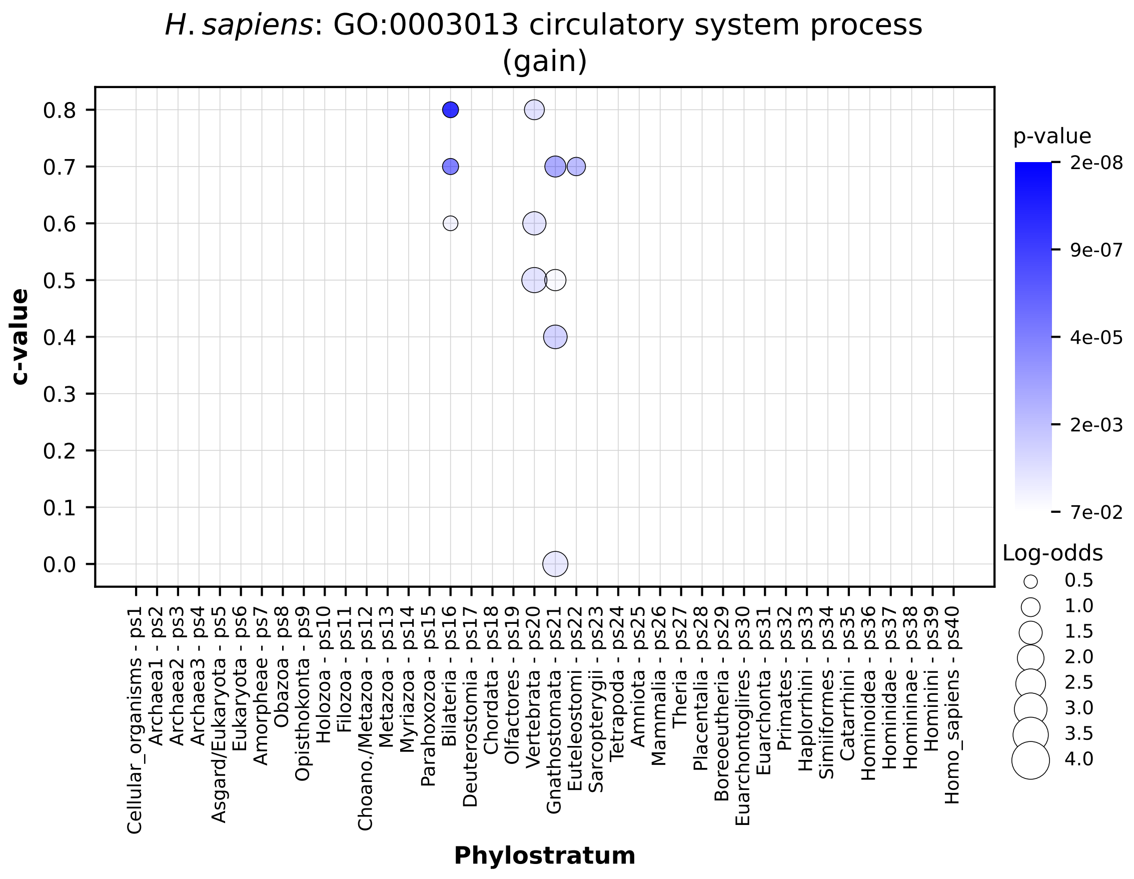

*H. sapiens*: GO:0003013 circulatory system process
(gain)
c-value
0.8
0.7
0.6
0.5
0.4
0.3
0.2
0.1
0.0
p-value
2e-08
9e-07
4e-05
2e-03
7e-02
Log-odds
0.5
1.0
1.5
2.0
2.5
3.0
3.5
4.0
Cellular_organisms - ps1
Archaea1 - ps2
Archaea2 - ps3
Archaea3 - ps4
Asgard/Eukaryota - ps5
Eukaryota - ps6
Amorpheae - ps7
Obazoa - ps8
Opisthokonta - ps9
Holozoa - ps10
Filozoa - ps11
Choano./Metazoa - ps12
Metazoa - ps13
Myriazoa - ps14
Parahoxozoa - ps15
Bilateria - ps16
Deuterostomia - ps17
Chordata - ps18
Olfactores - ps19
Vertebrata - ps20
Gnathostomata - ps21
Euteleostomi - ps22
Sarcopterygii - ps23
Tetrapoda - ps24
Amniota - ps25
Mammalia - ps26
Theria - ps27
Placentalia - ps28
Boreoeutheria - ps29
Euarchontoglires - ps30
Euarchonta - ps31
Primates - ps32
Haplorrhini - ps33
Simiiformes - ps34
Catarrhini - ps35
Hominoidea - ps36
Hominidae - ps37
Homininae - ps38
Hominini - ps39
Homo_sapiens - ps40
Phylostratum

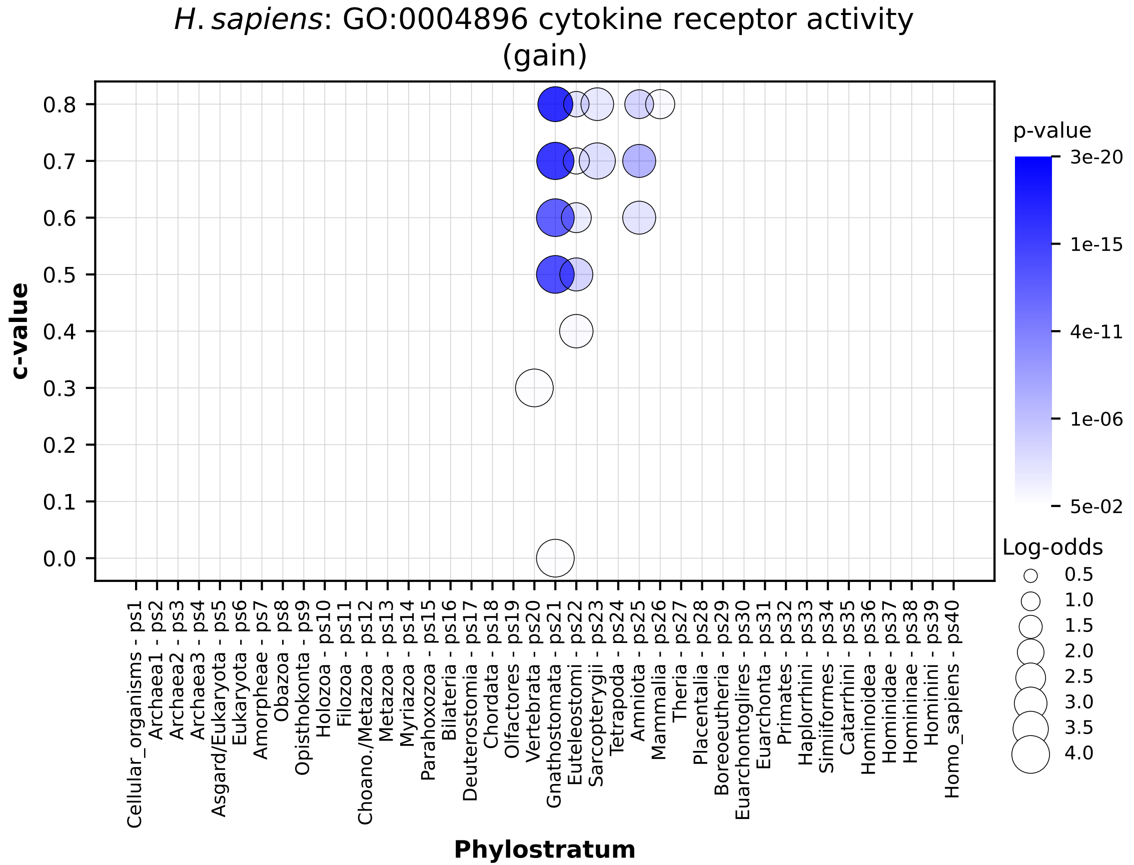

H. sapiens: GO:0004896 cytokine receptor activity
(gain)
c-value
0.8
0.7
0.6
0.5
0.4
0.3
0.2
0.1
0.0
p-value
3e-20
1e-15
4e-11
1e-06
5e-02
Log-odds
0.5
1.0
1.5
2.0
2.5
3.0
3.5
4.0
Cellular_organisms - ps1
Archaea1 - ps2
Archaea2 - ps3
Archaea3 - ps4
Asgard/Eukaryota - ps5
Eukaryota - ps6
Amorpheae - ps7
Obazoa - ps8
Opisthokonta - ps9
Holozoa - ps10
Filozoa - ps11
Choano./Metazoa - ps12
Metazoa - ps13
Myriazoa - ps14
Parahoxozoa - ps15
Bilateria - ps16
Deuterostomia - ps17
Chordata - ps18
Olfactores - ps19
Vertebrata - ps20
Gnathostomata - ps21
Euteleostomi - ps22
Sarcopterygii - ps23
Tetrapoda - ps24
Amniota - ps25
Mammalia - ps26
Theria - ps27
Placentalia - ps28
Boreoeutheria - ps29
Euarchontoglires - ps30
Euarchonta - ps31
Primates - ps32
Haplorrhini - ps33
Simiiformes - ps34
Catarrhini - ps35
Hominoidea - ps36
Hominidae - ps37
Homininae - ps38
Hominini - ps39
Homo_sapiens - ps40
Phylostratum

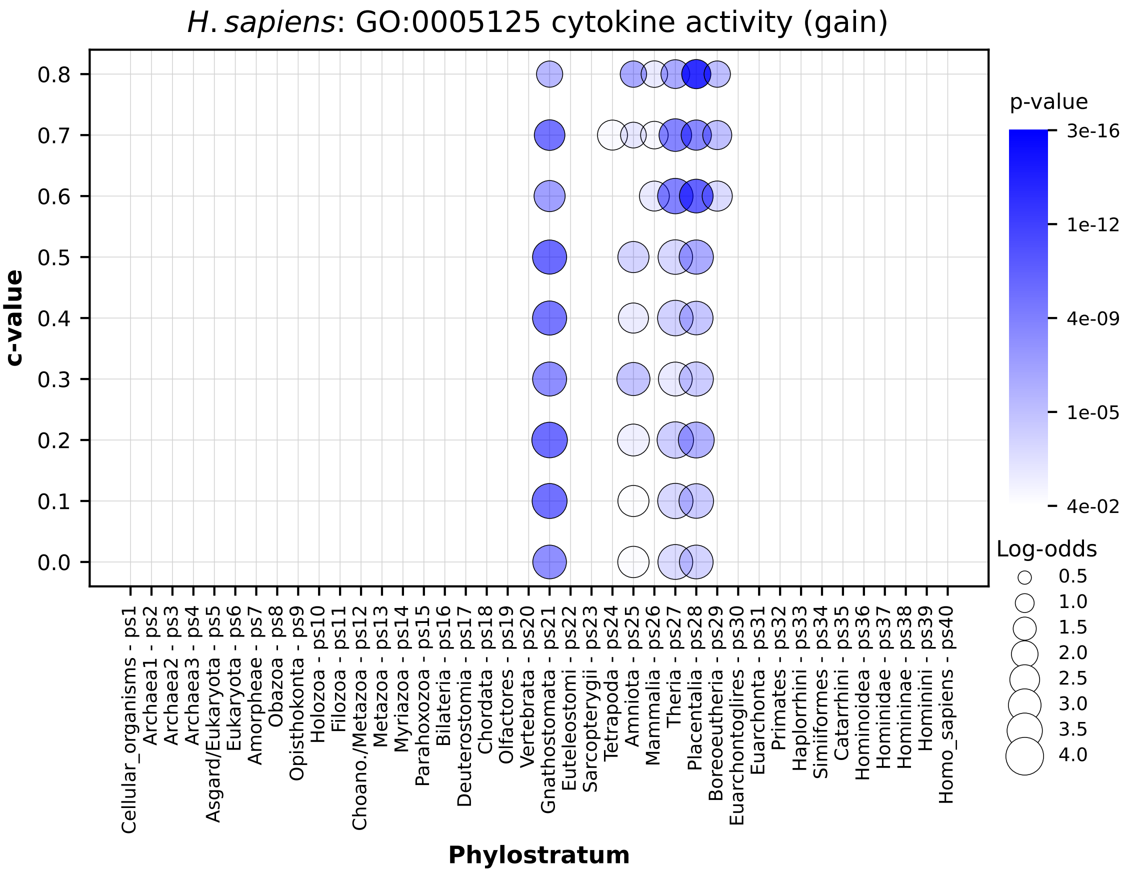

H. sapiens: GO:0005125 cytokine activity (gain)
c-value
0.8
0.7
0.6
0.5
0.4
0.3
0.2
0.1
0.0
p-value
3e-16
1e-12
4e-09
1e-05
4e-02
Log-odds
0.5
1.0
1.5
2.0
2.5
3.0
3.5
4.0
Cellular_organisms - ps1
Archaea1 - ps2
Archaea2 - ps3
Archaea3 - ps4
Asgard/Eukaryota - ps5
Eukaryota - ps6
Amorpheae - ps7
Obazoa - ps8
Opisthokonta - ps9
Holozoa - ps10
Filozoa - ps11
Choano./Metazoa - ps12
Metazoa - ps13
Myriazoa - ps14
Parahoxozoa - ps15
Bilateria - ps16
Deuterostomia - ps17
Chordata - ps18
Olfactores - ps19
Vertebrata - ps20
Gnathostomata - ps21
Euteleostomi - ps22
Sarcopterygii - ps23
Tetrapoda - ps24
Amniota - ps25
Mammalia - ps26
Theria - ps27
Placentalia - ps28
Boreoeutheria - ps29
Euarchontoglires - ps30
Euarchonta - ps31
Primates - ps32
Haplorrhini - ps33
Simiiformes - ps34
Catarrhini - ps35
Hominoidea - ps36
Hominidae - ps37
Homininae - ps38
Hominini - ps39
Homo_sapiens - ps40
Phylostratum

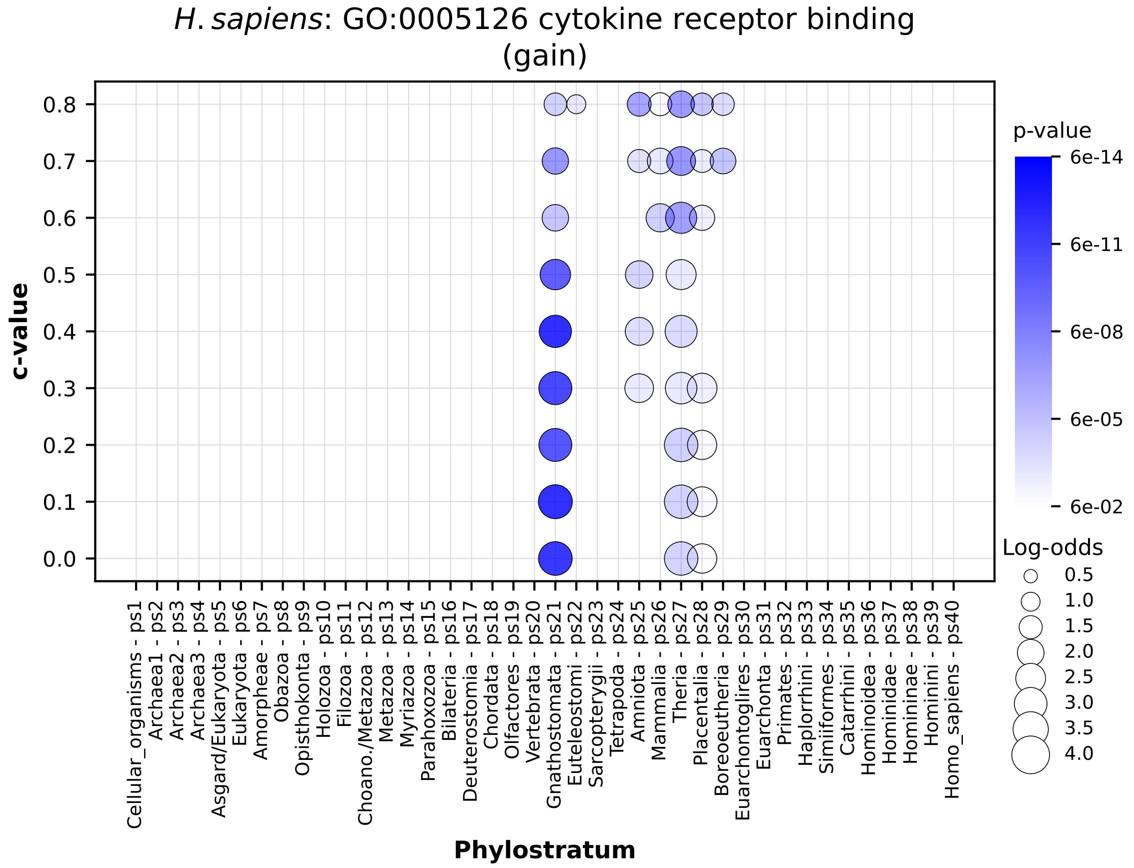

*H. sapiens*: GO:0005126 cytokine receptor binding
(gain)
c-value
0.0
0.1
0.2
0.3
0.4
0.5
0.6
0.7
0.8
Phylostratum
Cellular_organisms - ps1
Archaea1 - ps2
Archaea2 - ps3
Archaea3 - ps4
Asgard/Eukaryota - ps5
Eukaryota - ps6
Amorpheae - ps7
Obazoa - ps8
Opisthokonta - ps9
Holozoa - ps10
Filozoa - ps11
Choano./Metazoa - ps12
Metazoa - ps13
Myriazoa - ps14
Parahoxozoa - ps15
Bilateria - ps16
Deuterostomia - ps17
Chordata - ps18
Olfactores - ps19
Vertebrata - ps20
Gnathostomata - ps21
Euteleostomi - ps22
Sarcopterygii - ps23
Tetrapoda - ps24
Amniota - ps25
Mammalia - ps26
Theria - ps27
Placentalia - ps28
Boreoeutheria - ps29
Euarchontoglires - ps30
Euarchonta - ps31
Primates - ps32
Haplorrhini - ps33
Simiiformes - ps34
Catarrhini - ps35
Hominoidea - ps36
Hominidae - ps37
Homininae - ps38
Hominini - ps39
Homo_sapiens - ps40
p-value
6e-14
6e-11
6e-08
6e-05
6e-02
Log-odds
0.5
1.0
1.5
2.0
2.5
3.0
3.5
4.0

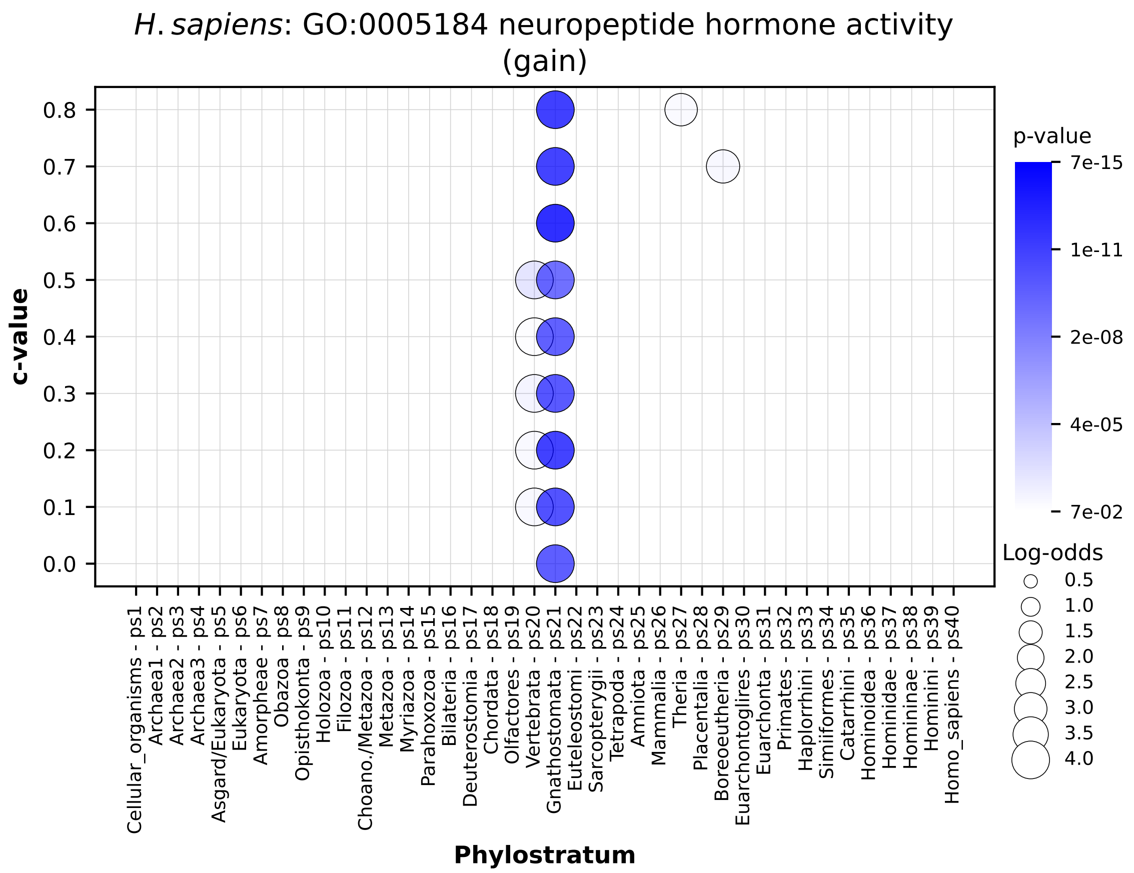

H. sapiens: GO:0005184 neuropeptide hormone activity
(gain)
c-value
0.8
0.7
0.6
0.5
0.4
0.3
0.2
0.1
0.0
Phylostratum
Cellular_organisms - ps1
Archaea1 - ps2
Archaea2 - ps3
Archaea3 - ps4
Asgard/Eukaryota - ps5
Eukaryota - ps6
Amorpheae - ps7
Obazoa - ps8
Opisthokonta - ps9
Holozoa - ps10
Filozoa - ps11
Choano./Metazoa - ps12
Metazoa - ps13
Myriazoa - ps14
Parahoxozoa - ps15
Bilateria - ps16
Deuterostomia - ps17
Chordata - ps18
Olfactores - ps19
Vertebrata - ps20
Gnathostomata - ps21
Euteleostomi - ps22
Sarcopterygii - ps23
Tetrapoda - ps24
Amniota - ps25
Mammalia - ps26
Theria - ps27
Placentalia - ps28
Boreoeutheria - ps29
Euarchontoglires - ps30
Euarchonta - ps31
Primates - ps32
Haplorrhini - ps33
Simiiformes - ps34
Catarrhini - ps35
Hominoidea - ps36
Hominidae - ps37
Homininae - ps38
Hominini - ps39
Homo_sapiens - ps40
p-value
7e-15
1e-11
2e-08
4e-05
7e-02
Log-odds
0.5
1.0
1.5
2.0
2.5
3.0
3.5
4.0

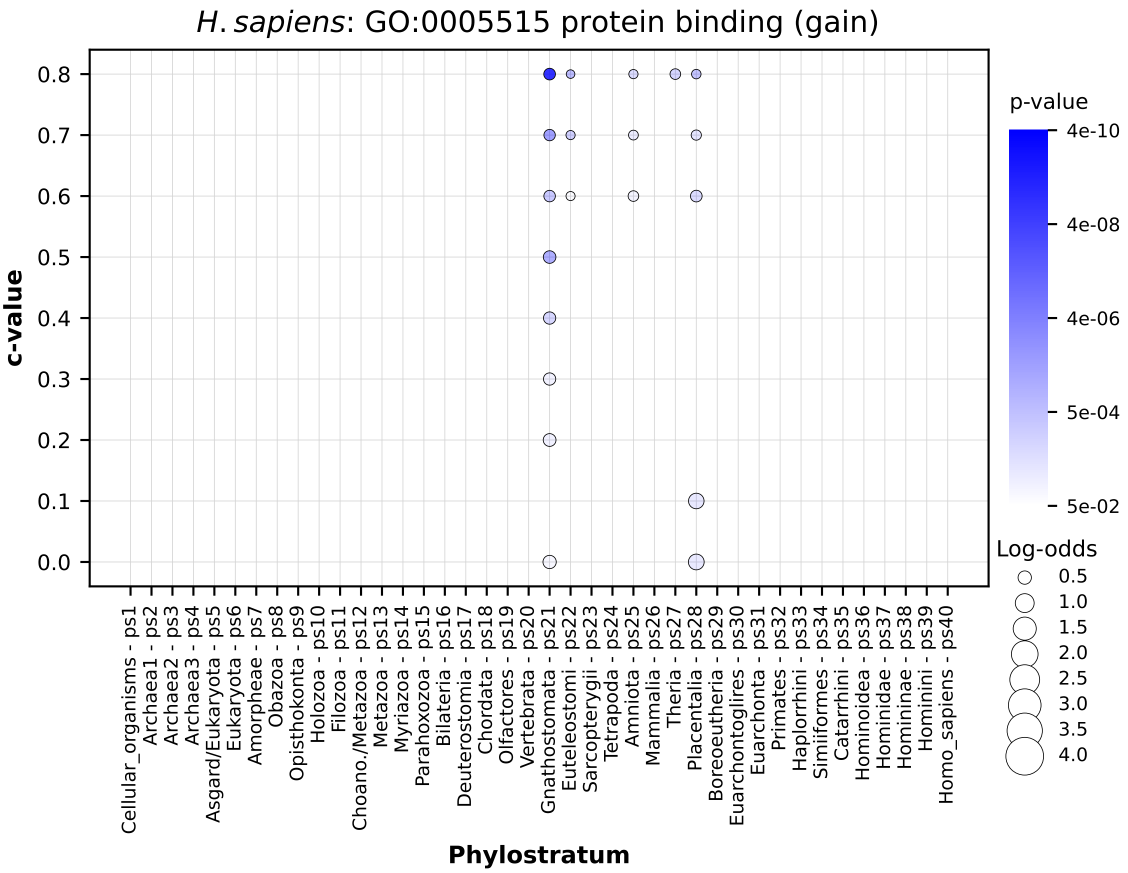

*H. sapiens*: GO:0005515 protein binding (gain)
c-value
0.8
0.7
0.6
0.5
0.4
0.3
0.2
0.1
0.0
p-value
4e-10
4e-08
4e-06
5e-04
5e-02
Log-odds
0.5
1.0
1.5
2.0
2.5
3.0
3.5
4.0
Cellular_organisms - ps1
Archaea1 - ps2
Archaea2 - ps3
Archaea3 - ps4
Asgard/Eukaryota - ps5
Eukaryota - ps6
Amorpheae - ps7
Obazoa - ps8
Opisthokonta - ps9
Holozoa - ps10
Filozoa - ps11
Choano./Metazoa - ps12
Metazoa - ps13
Myriazoa - ps14
Parahoxozoa - ps15
Bilateria - ps16
Deuterostomia - ps17
Chordata - ps18
Olfactores - ps19
Vertebrata - ps20
Gnathostomata - ps21
Euteleostomi - ps22
Sarcopterygii - ps23
Tetrapoda - ps24
Amniota - ps25
Mammalia - ps26
Theria - ps27
Placentalia - ps28
Boreoeutheria - ps29
Euarchontoglires - ps30
Euarchonta - ps31
Primates - ps32
Haplorrhini - ps33
Simiiformes - ps34
Catarrhini - ps35
Hominoidea - ps36
Hominidae - ps37
Homininae - ps38
Hominini - ps39
Homo_sapiens - ps40
Phylostratum

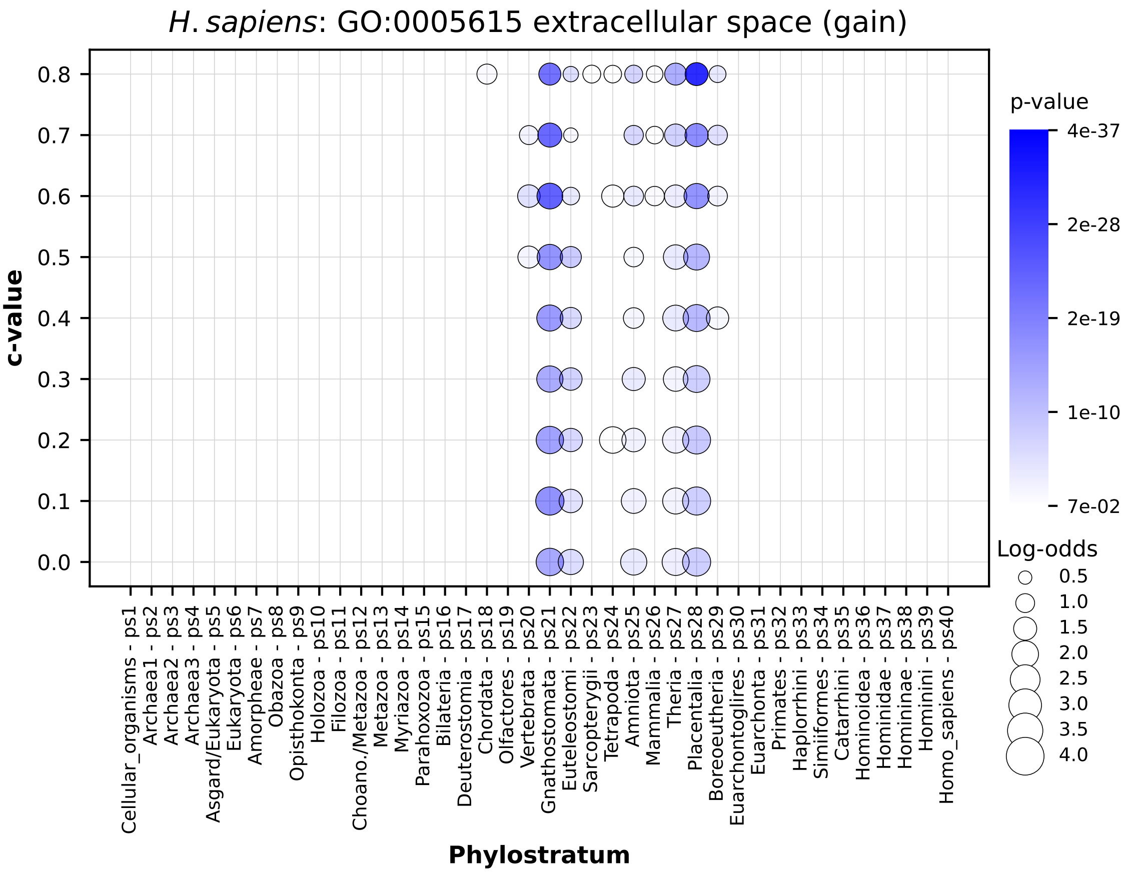

*H. sapiens*: GO:0005615 extracellular space (gain)
c-value
0.8
0.7
0.6
0.5
0.4
0.3
0.2
0.1
0.0
p-value
4e-37
2e-28
2e-19
1e-10
7e-02
Log-odds
0.5
1.0
1.5
2.0
2.5
3.0
3.5
4.0
Cellular_organisms - ps1
Archaea1 - ps2
Archaea2 - ps3
Archaea3 - ps4
Asgard/Eukaryota - ps5
Eukaryota - ps6
Amorpheae - ps7
Obazoa - ps8
Opisthokonta - ps9
Holozoa - ps10
Filozoa - ps11
Choano./Metazoa - ps12
Metazoa - ps13
Myriazoa - ps14
Parahoxozoa - ps15
Bilateria - ps16
Deuterostomia - ps17
Chordata - ps18
Olfactores - ps19
Vertebrata - ps20
Gnathostomata - ps21
Euteleostomi - ps22
Sarcopterygii - ps23
Tetrapoda - ps24
Amniota - ps25
Mammalia - ps26
Theria - ps27
Placentalia - ps28
Boreoeutheria - ps29
Euarchontoglires - ps30
Euarchonta - ps31
Primates - ps32
Haplorrhini - ps33
Simiiformes - ps34
Catarrhini - ps35
Hominoidea - ps36
Hominidae - ps37
Homininae - ps38
Hominini - ps39
Homo_sapiens - ps40
Phylostratum

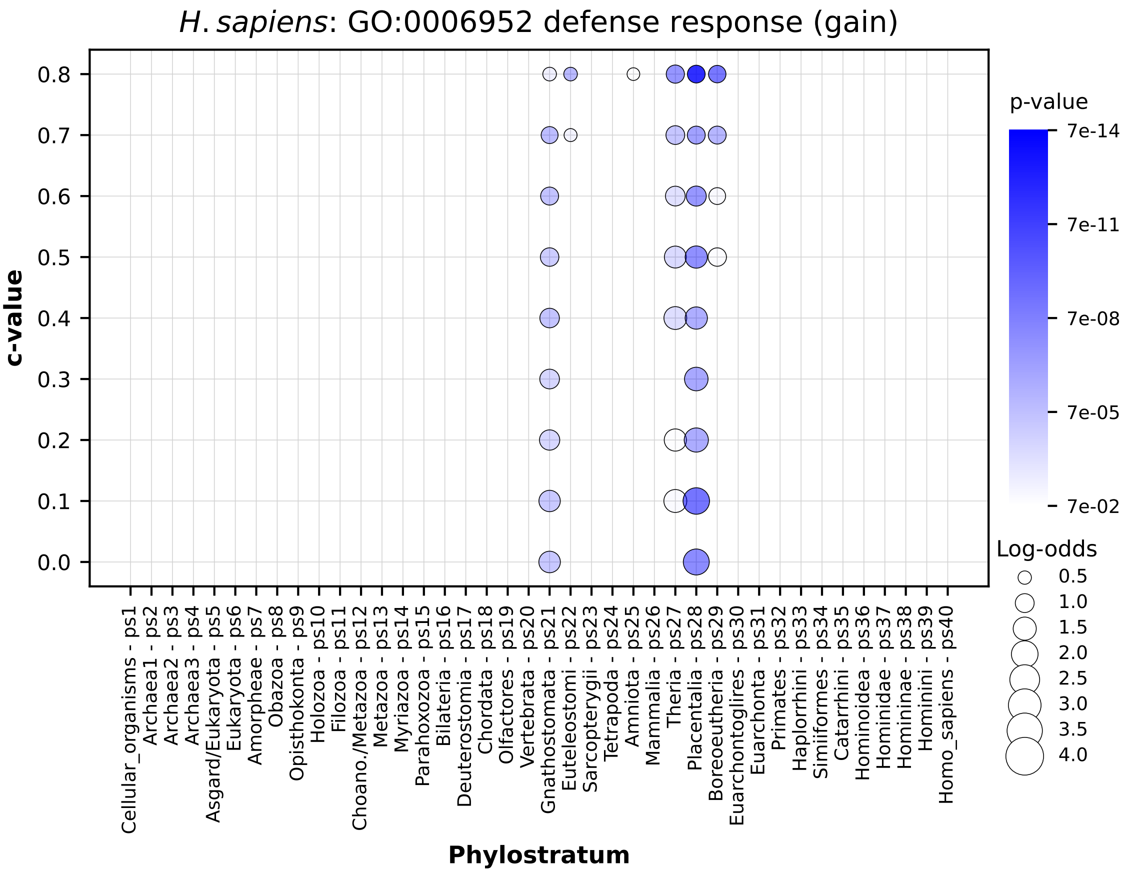

H. sapiens: GO:0006952 defense response (gain)
c-value
0.8
0.7
0.6
0.5
0.4
0.3
0.2
0.1
0.0
Phylostratum
Cellular_organisms - ps1
Archaea1 - ps2
Archaea2 - ps3
Archaea3 - ps4
Asgard/Eukaryota - ps5
Eukaryota - ps6
Amorpheae - ps7
Obazoa - ps8
Opisthokonta - ps9
Holozoa - ps10
Filozoa - ps11
Choano./Metazoa - ps12
Metazoa - ps13
Myriazoa - ps14
Parahoxozoa - ps15
Bilateria - ps16
Deuterostomia - ps17
Chordata - ps18
Olfactores - ps19
Vertebrata - ps20
Gnathostomata - ps21
Euteleostomi - ps22
Sarcopterygii - ps23
Tetrapoda - ps24
Amniota - ps25
Mammalia - ps26
Theria - ps27
Placentalia - ps28
Boreoeutheria - ps29
Euarchontoglires - ps30
Euarchonta - ps31
Primates - ps32
Haplorrhini - ps33
Simiiformes - ps34
Catarrhini - ps35
Hominoidea - ps36
Hominidae - ps37
Homininae - ps38
Hominini - ps39
Homo_sapiens - ps40
p-value
7e-14
7e-11
7e-08
7e-05
7e-02
Log-odds
0.5
1.0
1.5
2.0
2.5
3.0
3.5
4.0

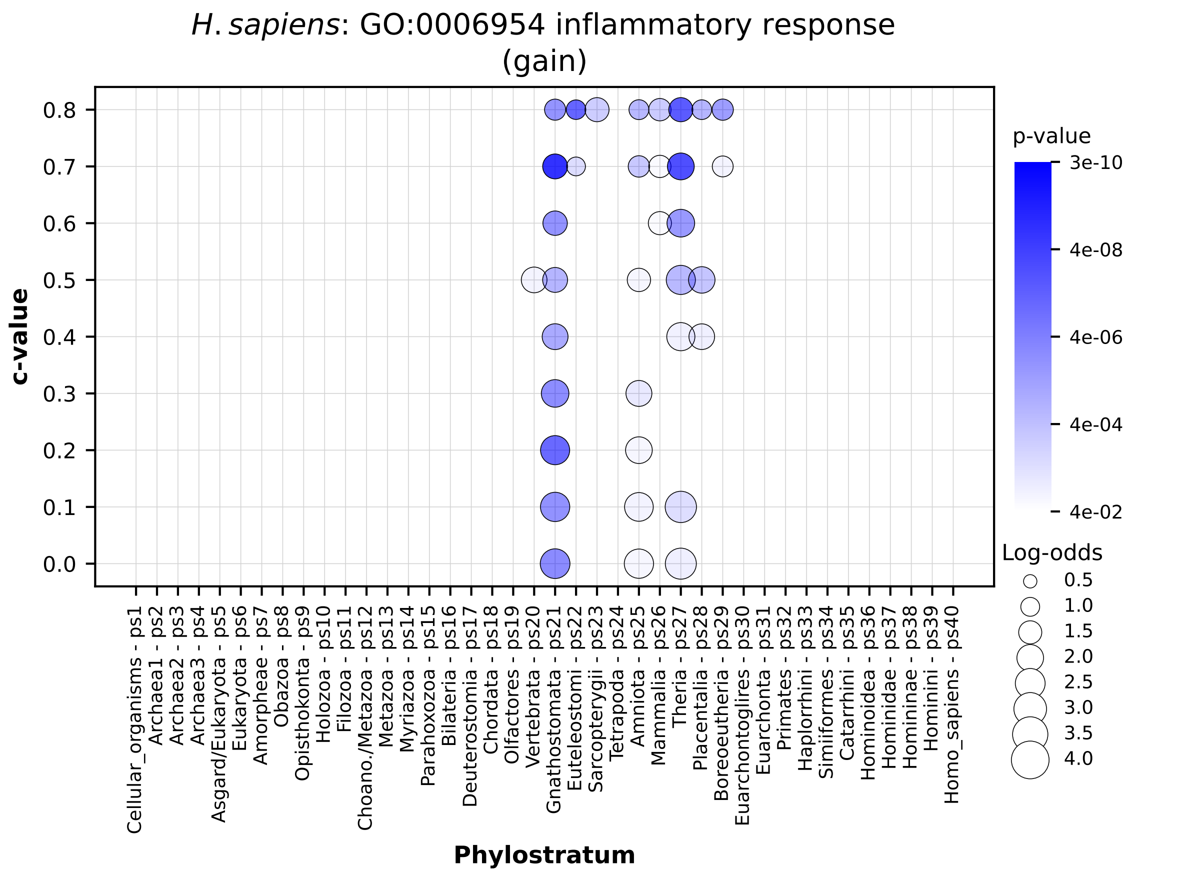

H. sapiens: GO:0006954 inflammatory response
(gain)
c-value
0.8
0.7
0.6
0.5
0.4
0.3
0.2
0.1
0.0
p-value
3e-10
4e-08
4e-06
4e-04
4e-02
Log-odds
0.5
1.0
1.5
2.0
2.5
3.0
3.5
4.0
Cellular_organisms - ps1
Archaea1 - ps2
Archaea2 - ps3
Archaea3 - ps4
Asgard/Eukaryota - ps5
Eukaryota - ps6
Amorpheae - ps7
Obazoa - ps8
Opisthokonta - ps9
Holozoa - ps10
Filozoa - ps11
Choano./Metazoa - ps12
Metazoa - ps13
Myriazoa - ps14
Parahoxozoa - ps15
Bilateria - ps16
Deuterostomia - ps17
Chordata - ps18
Olfactores - ps19
Vertebrata - ps20
Gnathostomata - ps21
Euteleostomi - ps22
Sarcopterygii - ps23
Tetrapoda - ps24
Amniota - ps25
Mammalia - ps26
Theria - ps27
Placentalia - ps28
Boreoeutheria - ps29
Euarchontoglires - ps30
Euarchonta - ps31
Primates - ps32
Haplorrhini - ps33
Simiiformes - ps34
Catarrhini - ps35
Hominoidea - ps36
Hominidae - ps37
Homininae - ps38
Hominini - ps39
Homo_sapiens - ps40
Phylostratum

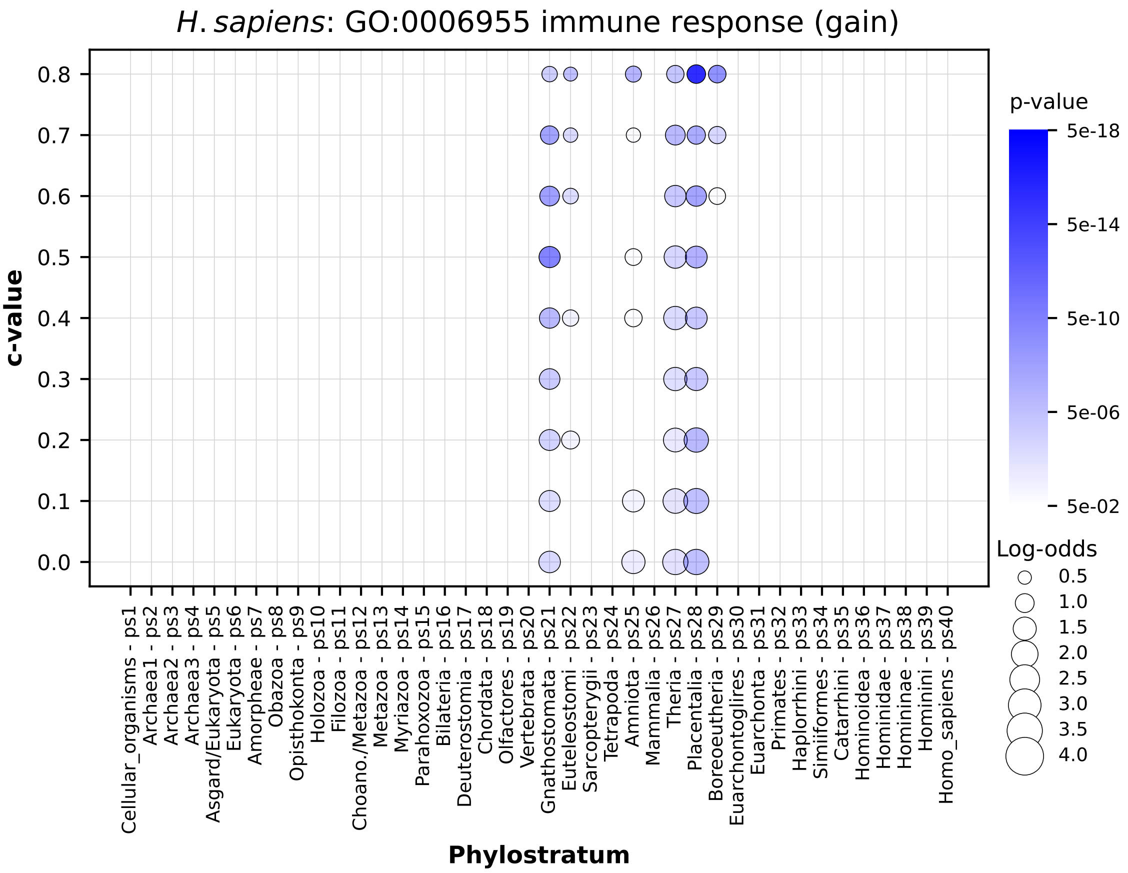
H. sapiens: GO:0006955 immune response (gain)
c-value
0.0
0.1
0.2
0.3
0.4
0.5
0.6
0.7
0.8
p-value
5e-18
5e-14
5e-10
5e-06
5e-02
Log-odds
0.5
1.0
1.5
2.0
2.5
3.0
3.5
4.0
Cellular_organisms - ps1
Archaea1 - ps2
Archaea2 - ps3
Archaea3 - ps4
Asgard/Eukaryota - ps5
Eukaryota - ps6
Amorpheae - ps7
Obazoa - ps8
Opisthokonta - ps9
Holozoa - ps10
Filozoa - ps11
Choano./Metazoa - ps12
Metazoa - ps13
Myriazoa - ps14
Parahoxozoa - ps15
Bilateria - ps16
Deuterostomia - ps17
Chordata - ps18
Olfactores - ps19
Vertebrata - ps20
Gnathostomata - ps21
Euteleostomi - ps22
Sarcopterygii - ps23
Tetrapoda - ps24
Amniota - ps25
Mammalia - ps26
Theria - ps27
Placentalia - ps28
Boreoeutheria - ps29
Euarchontoglires - ps30
Euarchonta - ps31
Primates - ps32
Haplorrhini - ps33
Simiiformes - ps34
Catarrhini - ps35
Hominoidea - ps36
Hominidae - ps37
Homininae - ps38
Hominini - ps39
Homo_sapiens - ps40
Phylostratum

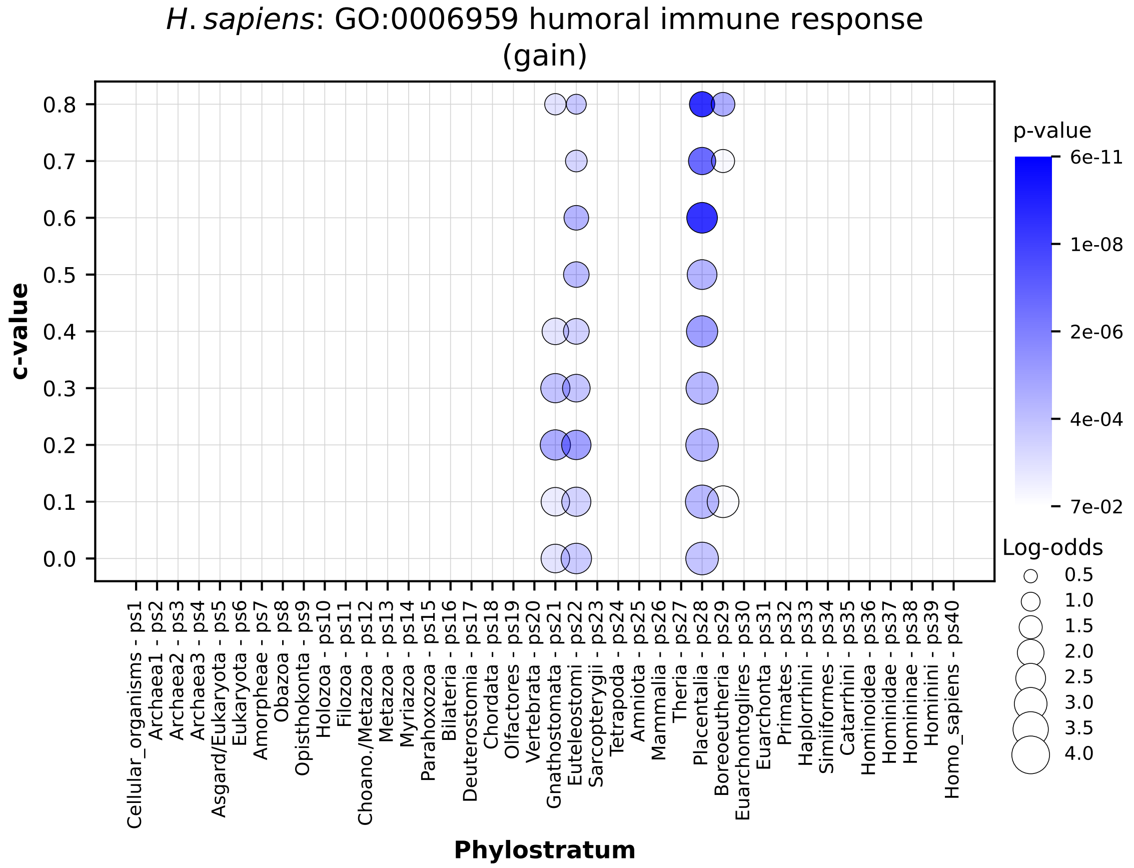

H. sapiens: GO:0006959 humoral immune response
(gain)
c-value
0.8
0.7
0.6
0.5
0.4
0.3
0.2
0.1
0.0
Cellular_organisms - ps1
Archaea1 - ps2
Archaea2 - ps3
Archaea3 - ps4
Asgard/Eukaryota - ps5
Eukaryota - ps6
Amorpheae - ps7
Obazoa - ps8
Opisthokonta - ps9
Holozoa - ps10
Filozoa - ps11
Choano./Metazoa - ps12
Metazoa - ps13
Myriazoa - ps14
Parahoxozoa - ps15
Bilateria - ps16
Deuterostomia - ps17
Chordata - ps18
Olfactores - ps19
Vertebrata - ps20
Gnathostomata - ps21
Euteleostomi - ps22
Sarcopterygii - ps23
Tetrapoda - ps24
Amniota - ps25
Mammalia - ps26
Theria - ps27
Placentalia - ps28
Boreoeutheria - ps29
Euarchontoglires - ps30
Euarchonta - ps31
Primates - ps32
Haplorrhini - ps33
Simiiformes - ps34
Catarrhini - ps35
Hominoidea - ps36
Hominidae - ps37
Homininae - ps38
Hominini - ps39
Homo_sapiens - ps40
Phylostratum
p-value
6e-11
1e-08
2e-06
4e-04
7e-02
Log-odds
0.5
1.0
1.5
2.0
2.5
3.0
3.5
4.0

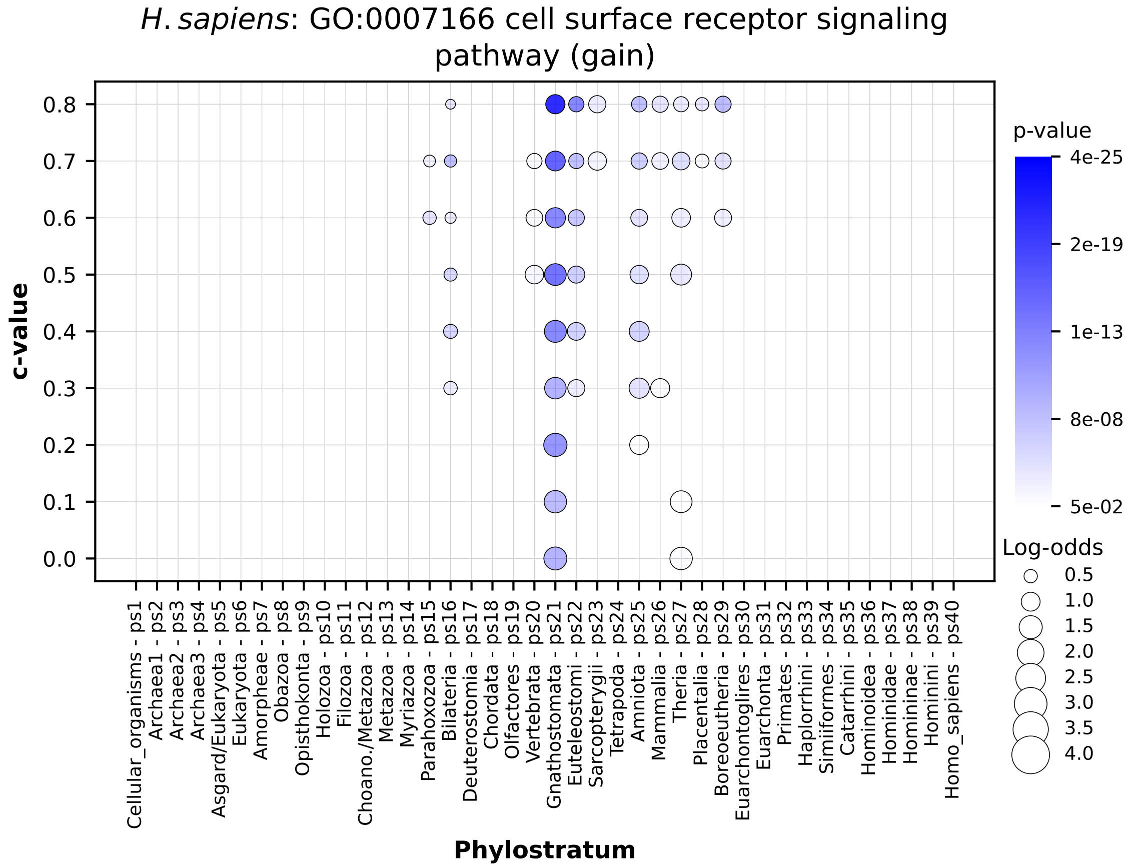

H. sapiens: GO:0007166 cell surface receptor signaling pathway (gain)
c-value
0.8
0.7
0.6
0.5
0.4
0.3
0.2
0.1
0.0
p-value
4e-25
2e-19
1e-13
8e-08
5e-02
Log-odds
0.5
1.0
1.5
2.0
2.5
3.0
3.5
4.0
Cellular_organisms - ps1
Archaea1 - ps2
Archaea2 - ps3
Archaea3 - ps4
Asgard/Eukaryota - ps5
Eukaryota - ps6
Amorpheae - ps7
Obazoa - ps8
Opisthokonta - ps9
Holozoa - ps10
Filozoa - ps11
Choano./Metazoa - ps12
Metazoa - ps13
Myriazoa - ps14
Parahoxozoa - ps15
Bilateria - ps16
Deuterostomia - ps17
Chordata - ps18
Olfactores - ps19
Vertebrata - ps20
Gnathostomata - ps21
Euteleostomi - ps22
Sarcopterygii - ps23
Tetrapoda - ps24
Amniota - ps25
Mammalia - ps26
Theria - ps27
Placentalia - ps28
Boreoeutheria - ps29
Euarchontoglires - ps30
Euarchonta - ps31
Primates - ps32
Haplorrhini - ps33
Simiiformes - ps34
Catarrhini - ps35
Hominoidea - ps36
Hominidae - ps37
Homininae - ps38
Hominini - ps39
Homo_sapiens - ps40
Phylostratum

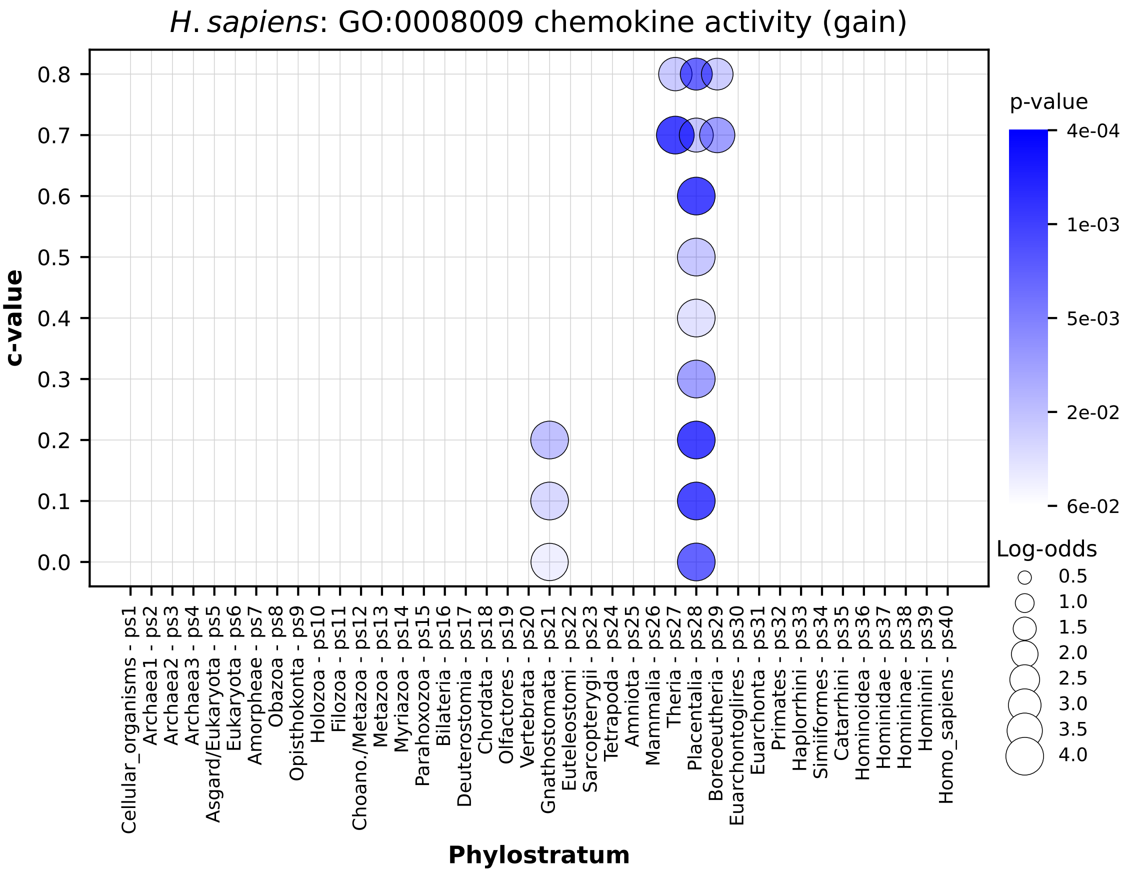

H. sapiens: GO:0008009 chemokine activity (gain)
c-value
0.0
0.1
0.2
0.3
0.4
0.5
0.6
0.7
0.8
Phylostratum
Cellular_organisms - ps1
Archaea1 - ps2
Archaea2 - ps3
Archaea3 - ps4
Asgard/Eukaryota - ps5
Eukaryota - ps6
Amorpheae - ps7
Obazoa - ps8
Opisthokonta - ps9
Holozoa - ps10
Filozoa - ps11
Choano./Metazoa - ps12
Metazoa - ps13
Myriazoa - ps14
Parahoxozoa - ps15
Bilateria - ps16
Deuterostomia - ps17
Chordata - ps18
Olfactores - ps19
Vertebrata - ps20
Gnathostomata - ps21
Euteleostomi - ps22
Sarcopterygii - ps23
Tetrapoda - ps24
Amniota - ps25
Mammalia - ps26
Theria - ps27
Placentalia - ps28
Boreoeutheria - ps29
Euarchontoglires - ps30
Euarchonta - ps31
Primates - ps32
Haplorrhini - ps33
Simiiformes - ps34
Catarrhini - ps35
Hominoidea - ps36
Hominidae - ps37
Homininae - ps38
Hominini - ps39
Homo_sapiens - ps40
p-value
4e-04
1e-03
5e-03
2e-02
6e-02
Log-odds
0.5
1.0
1.5
2.0
2.5
3.0
3.5
4.0

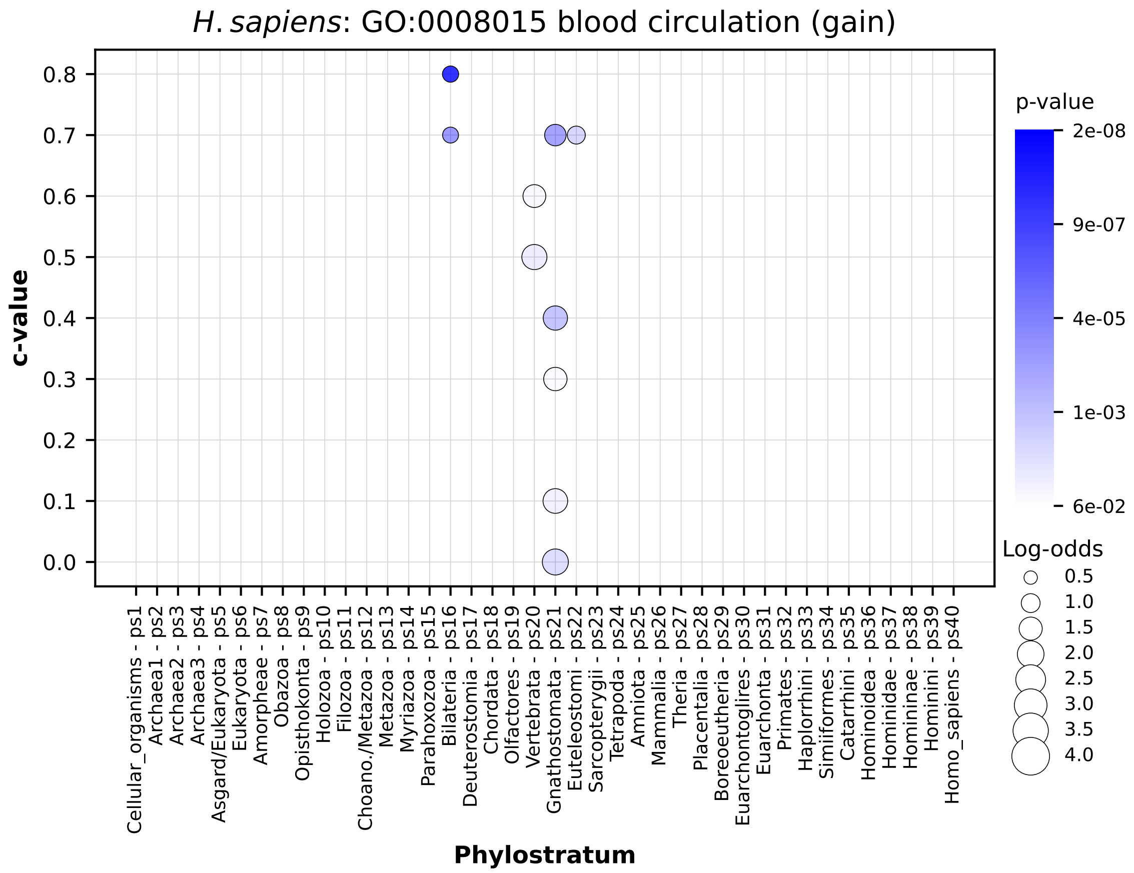

*H. sapiens*: GO:0008015 blood circulation (gain)
c-value
0.8
0.7
0.6
0.5
0.4
0.3
0.2
0.1
0.0
Phylostratum
Cellular_organisms - ps1
Archaea1 - ps2
Archaea2 - ps3
Archaea3 - ps4
Asgard/Eukaryota - ps5
Eukaryota - ps6
Amorpheae - ps7
Obazoa - ps8
Opisthokonta - ps9
Holozoa - ps10
Filozoa - ps11
Choano./Metazoa - ps12
Metazoa - ps13
Myriazoa - ps14
Parahoxozoa - ps15
Bilateria - ps16
Deuterostomia - ps17
Chordata - ps18
Olfactores - ps19
Vertebrata - ps20
Gnathostomata - ps21
Euteleostomi - ps22
Sarcopterygii - ps23
Tetrapoda - ps24
Amniota - ps25
Mammalia - ps26
Theria - ps27
Placentalia - ps28
Boreoeutheria - ps29
Euarchontoglires - ps30
Euarchonta - ps31
Primates - ps32
Haplorrhini - ps33
Simiiformes - ps34
Catarrhini - ps35
Hominoidea - ps36
Hominidae - ps37
Homininae - ps38
Hominini - ps39
Homo_sapiens - ps40
p-value
2e-08
9e-07
4e-05
1e-03
6e-02
Log-odds
0.5
1.0
1.5
2.0
2.5
3.0
3.5
4.0

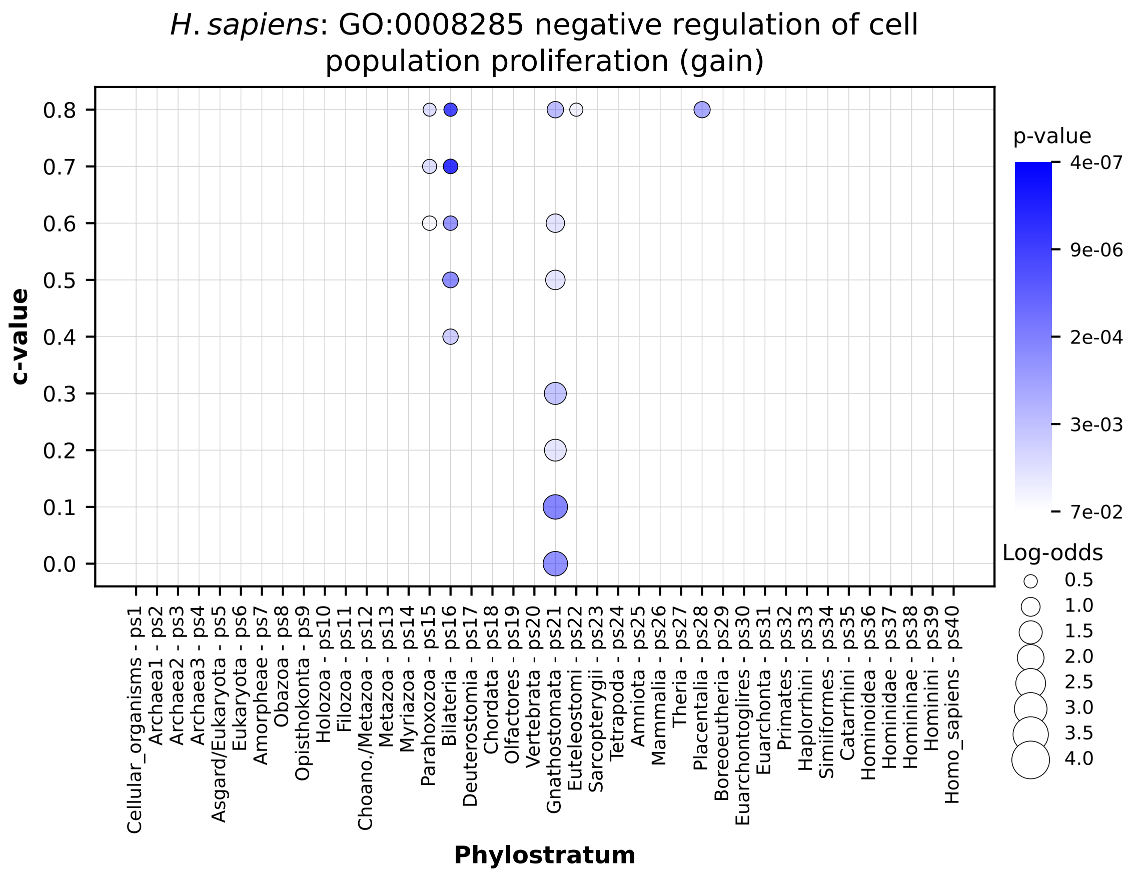

H. sapiens: GO:0008285 negative regulation of cell population proliferation (gain)
c-value
0.0
0.1
0.2
0.3
0.4
0.5
0.6
0.7
0.8
Phylostratum
Cellular_organisms - ps1
Archaea1 - ps2
Archaea2 - ps3
Archaea3 - ps4
Asgard/Eukaryota - ps5
Eukaryota - ps6
Amorpheae - ps7
Obazoa - ps8
Opisthokonta - ps9
Holozoa - ps10
Filozoa - ps11
Choano./Metazoa - ps12
Metazoa - ps13
Myriazoa - ps14
Parahoxozoa - ps15
Bilateria - ps16
Deuterostomia - ps17
Chordata - ps18
Olfactores - ps19
Vertebrata - ps20
Gnathostomata - ps21
Euteleostomi - ps22
Sarcopterygii - ps23
Tetrapoda - ps24
Amniota - ps25
Mammalia - ps26
Theria - ps27
Placentalia - ps28
Boreoeutheria - ps29
Euarchontoglires - ps30
Euarchonta - ps31
Primates - ps32
Haplorrhini - ps33
Simiiformes - ps34
Catarrhini - ps35
Hominoidea - ps36
Hominidae - ps37
Homininae - ps38
Hominini - ps39
Homo_sapiens - ps40
p-value
4e-07
9e-06
2e-04
3e-03
7e-02
Log-odds
0.5
1.0
1.5
2.0
2.5
3.0
3.5
4.0

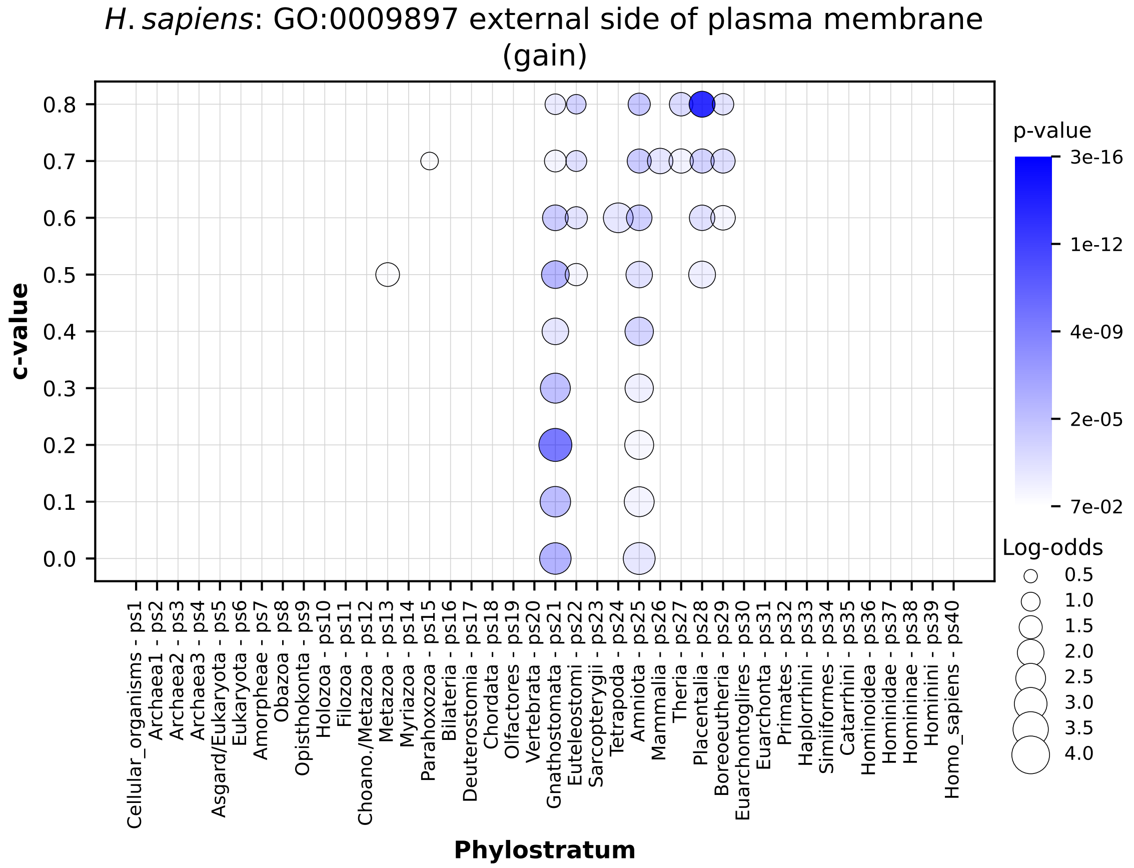
*H. sapiens*: GO:0009897 external side of plasma membrane
(gain)
c-value
0.8
0.7
0.6
0.5
0.4
0.3
0.2
0.1
0.0
Phylostratum
Cellular_organisms - ps1
Archaea1 - ps2
Archaea2 - ps3
Archaea3 - ps4
Asgard/Eukaryota - ps5
Eukaryota - ps6
Amorpheae - ps7
Obazoa - ps8
Opisthokonta - ps9
Holozoa - ps10
Filozoa - ps11
Choano./Metazoa - ps12
Metazoa - ps13
Myriazoa - ps14
Parahoxozoa - ps15
Bilateria - ps16
Deuterostomia - ps17
Chordata - ps18
Olfactores - ps19
Vertebrata - ps20
Gnathostomata - ps21
Euteleostomi - ps22
Sarcopterygii - ps23
Tetrapoda - ps24
Amniota - ps25
Mammalia - ps26
Theria - ps27
Placentalia - ps28
Boreoeutheria - ps29
Euarchontoglires - ps30
Euarchonta - ps31
Primates - ps32
Haplorrhini - ps33
Simiiformes - ps34
Catarrhini - ps35
Hominoidea - ps36
Hominidae - ps37
Homininae - ps38
Hominini - ps39
Homo_sapiens - ps40
p-value
3e-16
1e-12
4e-09
2e-05
7e-02
Log-odds
0.5
1.0
1.5
2.0
2.5
3.0
3.5
4.0

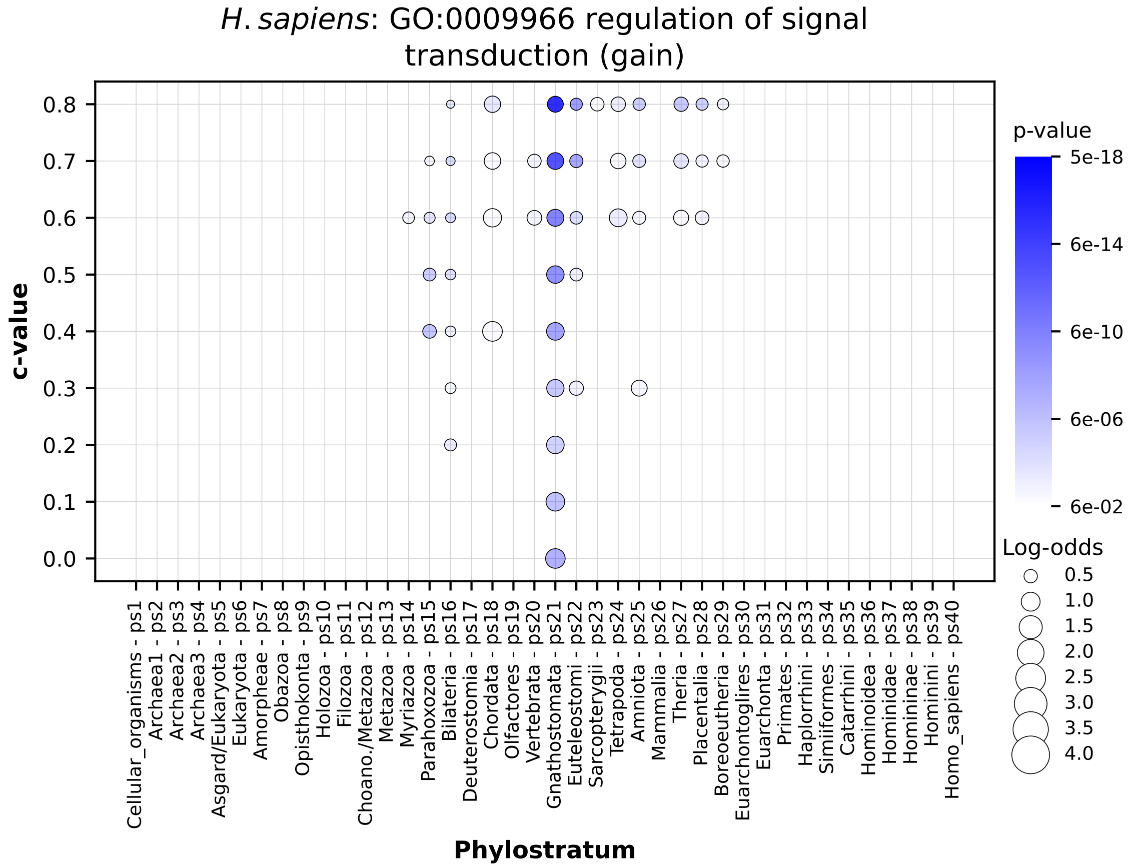

H. sapiens: GO:0009966 regulation of signal transduction (gain)
c-value
0.8
0.7
0.6
0.5
0.4
0.3
0.2
0.1
0.0
p-value
5e-18
6e-14
6e-10
6e-06
6e-02
Log-odds
0.5
1.0
1.5
2.0
2.5
3.0
3.5
4.0
Cellular_organisms - ps1
Archaea1 - ps2
Archaea2 - ps3
Archaea3 - ps4
Asgard/Eukaryota - ps5
Eukaryota - ps6
Amorpheae - ps7
Obazoa - ps8
Opisthokonta - ps9
Holozoa - ps10
Filozoa - ps11
Choano./Metazoa - ps12
Metazoa - ps13
Myriazoa - ps14
Parahoxozoa - ps15
Bilateria - ps16
Deuterostomia - ps17
Chordata - ps18
Olfactores - ps19
Vertebrata - ps20
Gnathostomata - ps21
Euteleostomi - ps22
Sarcopterygii - ps23
Tetrapoda - ps24
Amniota - ps25
Mammalia - ps26
Theria - ps27
Placentalia - ps28
Boreoeutheria - ps29
Euarchontoglires - ps30
Euarchonta - ps31
Primates - ps32
Haplorrhini - ps33
Simiiformes - ps34
Catarrhini - ps35
Hominoidea - ps36
Hominidae - ps37
Homininae - ps38
Hominini - ps39
Homo_sapiens - ps40
Phylostratum

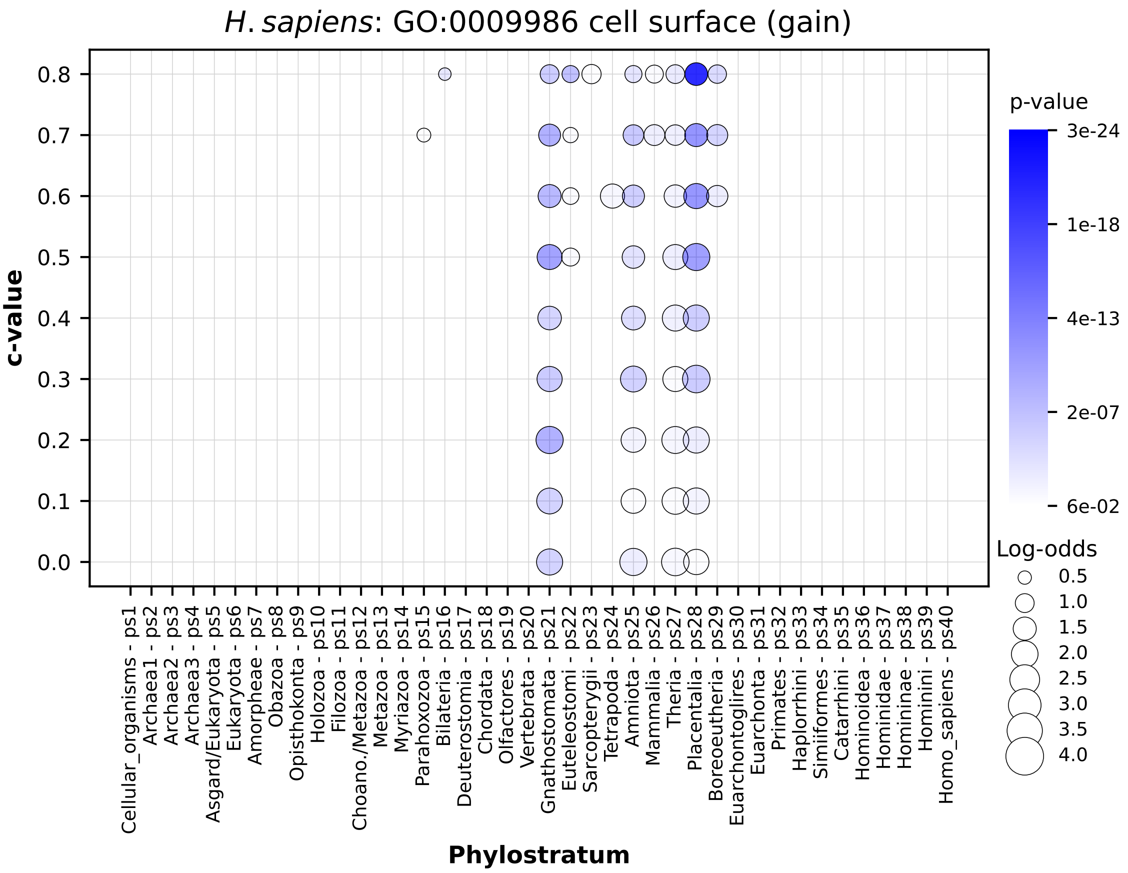

H. sapiens: GO:0009986 cell surface (gain)
c-value
0.8
0.7
0.6
0.5
0.4
0.3
0.2
0.1
0.0
Phylostratum
Cellular_organisms - ps1
Archaea1 - ps2
Archaea2 - ps3
Archaea3 - ps4
Asgard/Eukaryota - ps5
Eukaryota - ps6
Amorpheae - ps7
Obazoa - ps8
Opisthokonta - ps9
Holozoa - ps10
Filozoa - ps11
Choano./Metazoa - ps12
Metazoa - ps13
Myriazoa - ps14
Parahoxozoa - ps15
Bilateria - ps16
Deuterostomia - ps17
Chordata - ps18
Olfactores - ps19
Vertebrata - ps20
Gnathostomata - ps21
Euteleostomi - ps22
Sarcopterygii - ps23
Tetrapoda - ps24
Amniota - ps25
Mammalia - ps26
Theria - ps27
Placentalia - ps28
Boreoeutheria - ps29
Euarchontoglires - ps30
Euarchonta - ps31
Primates - ps32
Haplorrhini - ps33
Simiiformes - ps34
Catarrhini - ps35
Hominoidea - ps36
Hominidae - ps37
Homininae - ps38
Hominini - ps39
Homo_sapiens - ps40
p-value
3e-24
1e-18
4e-13
2e-07
6e-02
Log-odds
0.5
1.0
1.5
2.0
2.5
3.0
3.5
4.0

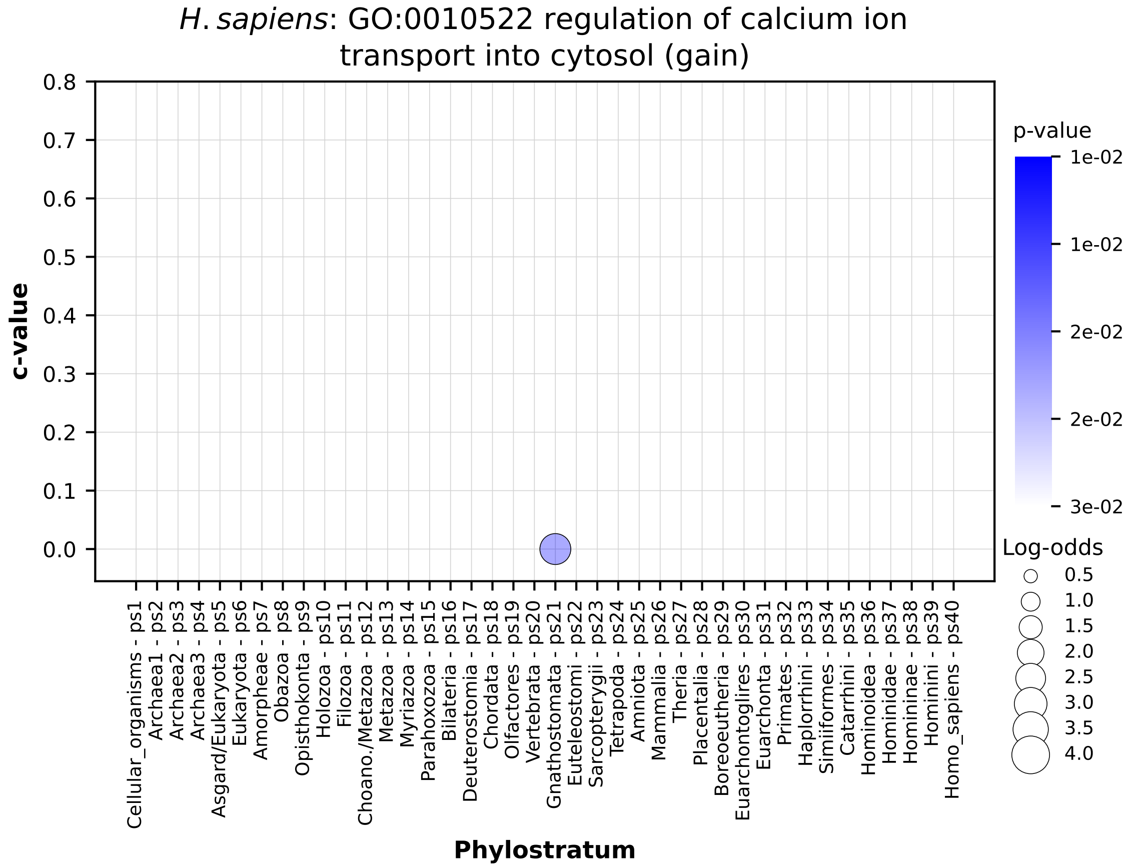

*H. sapiens*: GO:0010522 regulation of calcium ion transport into cytosol (gain)
c-value
0.8
0.7
0.6
0.5
0.4
0.3
0.2
0.1
0.0
Phylostratum
Cellular_organisms - ps1
Archaea1 - ps2
Archaea2 - ps3
Archaea3 - ps4
Asgard/Eukaryota - ps5
Eukaryota - ps6
Amorpheae - ps7
Obazoa - ps8
Opisthokonta - ps9
Holozoa - ps10
Filozoa - ps11
Choano./Metazoa - ps12
Metazoa - ps13
Myriazoa - ps14
Parahoxozoa - ps15
Bilateria - ps16
Deuterostomia - ps17
Chordata - ps18
Olfactores - ps19
Vertebrata - ps20
Gnathostomata - ps21
Euteleostomi - ps22
Sarcopterygii - ps23
Tetrapoda - ps24
Amniota - ps25
Mammalia - ps26
Theria - ps27
Placentalia - ps28
Boreoeutheria - ps29
Euarchontoglires - ps30
Euarchonta - ps31
Primates - ps32
Haplorrhini - ps33
Simiiformes - ps34
Catarrhini - ps35
Hominoidea - ps36
Hominidae - ps37
Homininae - ps38
Hominini - ps39
Homo_sapiens - ps40
p-value
1e-02
1e-02
2e-02
2e-02
3e-02
Log-odds
0.5
1.0
1.5
2.0
2.5
3.0
3.5
4.0

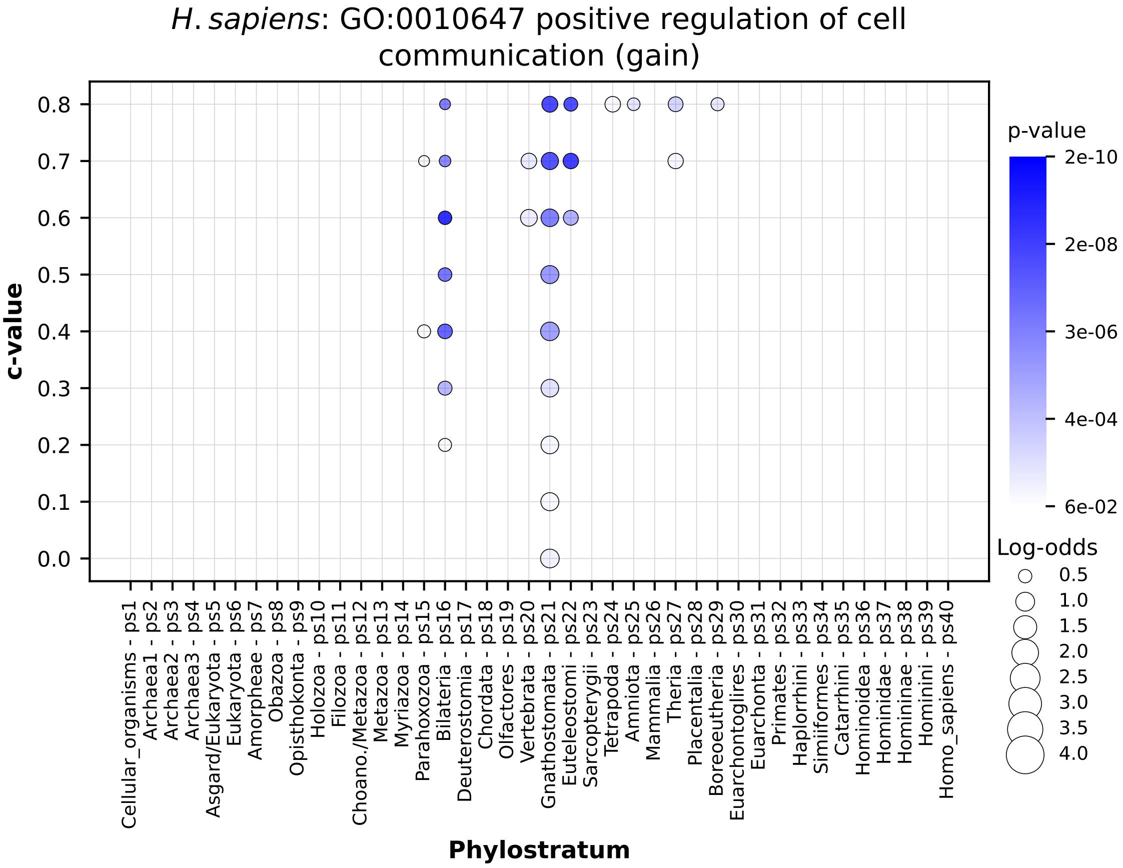
*H. sapiens*: GO:0010647 positive regulation of cell communication (gain)
c-value
0.8
0.7
0.6
0.5
0.4
0.3
0.2
0.1
0.0
p-value
2e-10
2e-08
3e-06
4e-04
6e-02
Log-odds
0.5
1.0
1.5
2.0
2.5
3.0
3.5
4.0
Cellular_organisms - ps1
Archaea1 - ps2
Archaea2 - ps3
Archaea3 - ps4
Asgard/Eukaryota - ps5
Eukaryota - ps6
Amorpheae - ps7
Obazoa - ps8
Opisthokonta - ps9
Holozoa - ps10
Filozoa - ps11
Choano./Metazoa - ps12
Metazoa - ps13
Myriazoa - ps14
Parahoxozoa - ps15
Bilateria - ps16
Deuterostomia - ps17
Chordata - ps18
Olfactores - ps19
Vertebrata - ps20
Gnathostomata - ps21
Euteleostomi - ps22
Sarcopterygii - ps23
Tetrapoda - ps24
Amniota - ps25
Mammalia - ps26
Theria - ps27
Placentalia - ps28
Boreoeutheria - ps29
Euarchontoglires - ps30
Euarchonta - ps31
Primates - ps32
Haplorrhini - ps33
Simiiformes - ps34
Catarrhini - ps35
Hominoidea - ps36
Hominidae - ps37
Homininae - ps38
Hominini - ps39
Homo_sapiens - ps40
Phylostratum

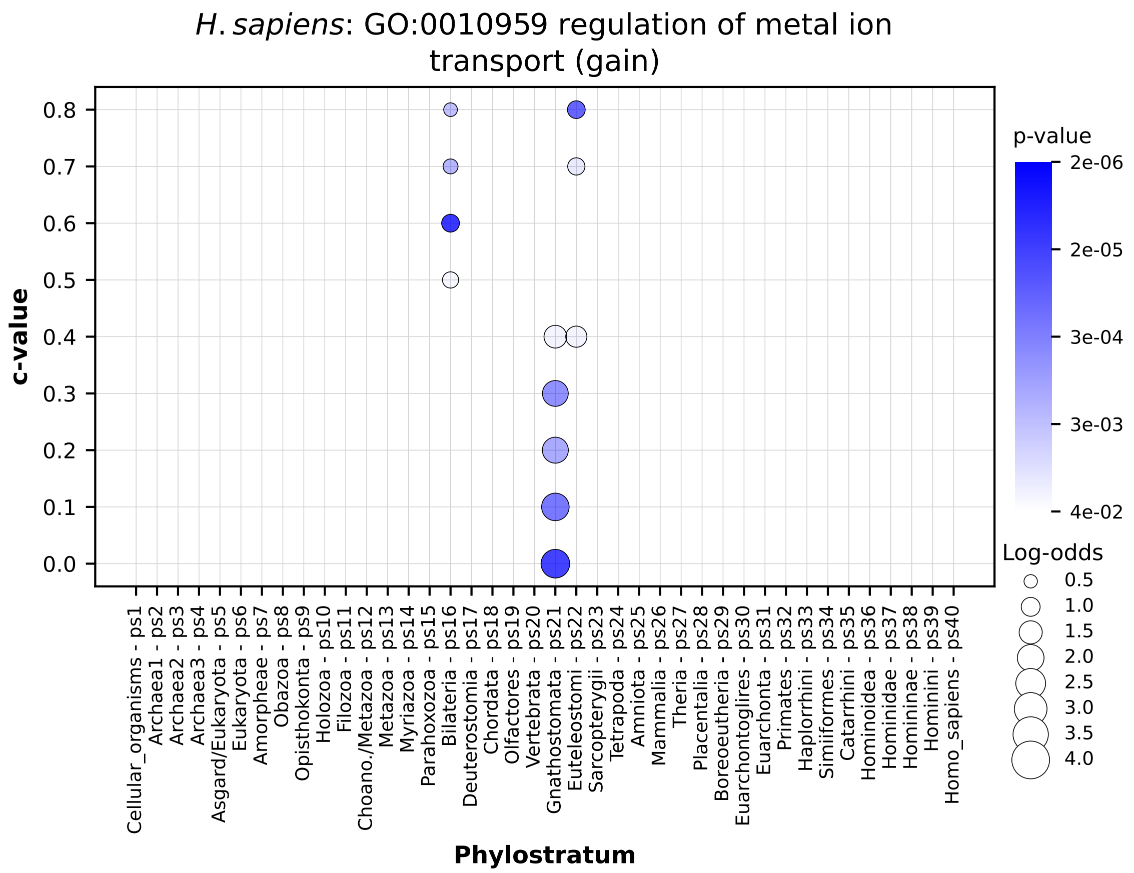

*H. sapiens*: GO:0010959 regulation of metal ion transport (gain)
c-value
0.8
0.7
0.6
0.5
0.4
0.3
0.2
0.1
0.0
p-value
2e-06
2e-05
3e-04
3e-03
4e-02
Log-odds
0.5
1.0
1.5
2.0
2.5
3.0
3.5
4.0
Cellular_organisms - ps1
Archaea1 - ps2
Archaea2 - ps3
Archaea3 - ps4
Asgard/Eukaryota - ps5
Eukaryota - ps6
Amorpheae - ps7
Obazoa - ps8
Opisthokonta - ps9
Holozoa - ps10
Filozoa - ps11
Choano./Metazoa - ps12
Metazoa - ps13
Myriazoa - ps14
Parahoxozoa - ps15
Bilateria - ps16
Deuterostomia - ps17
Chordata - ps18
Olfactores - ps19
Vertebrata - ps20
Gnathostomata - ps21
Euteleostomi - ps22
Sarcopterygii - ps23
Tetrapoda - ps24
Amniota - ps25
Mammalia - ps26
Theria - ps27
Placentalia - ps28
Boreoeutheria - ps29
Euarchontoglires - ps30
Euarchonta - ps31
Primates - ps32
Haplorrhini - ps33
Simiiformes - ps34
Catarrhini - ps35
Hominoidea - ps36
Hominidae - ps37
Homininae - ps38
Hominini - ps39
Homo_sapiens - ps40
Phylostratum

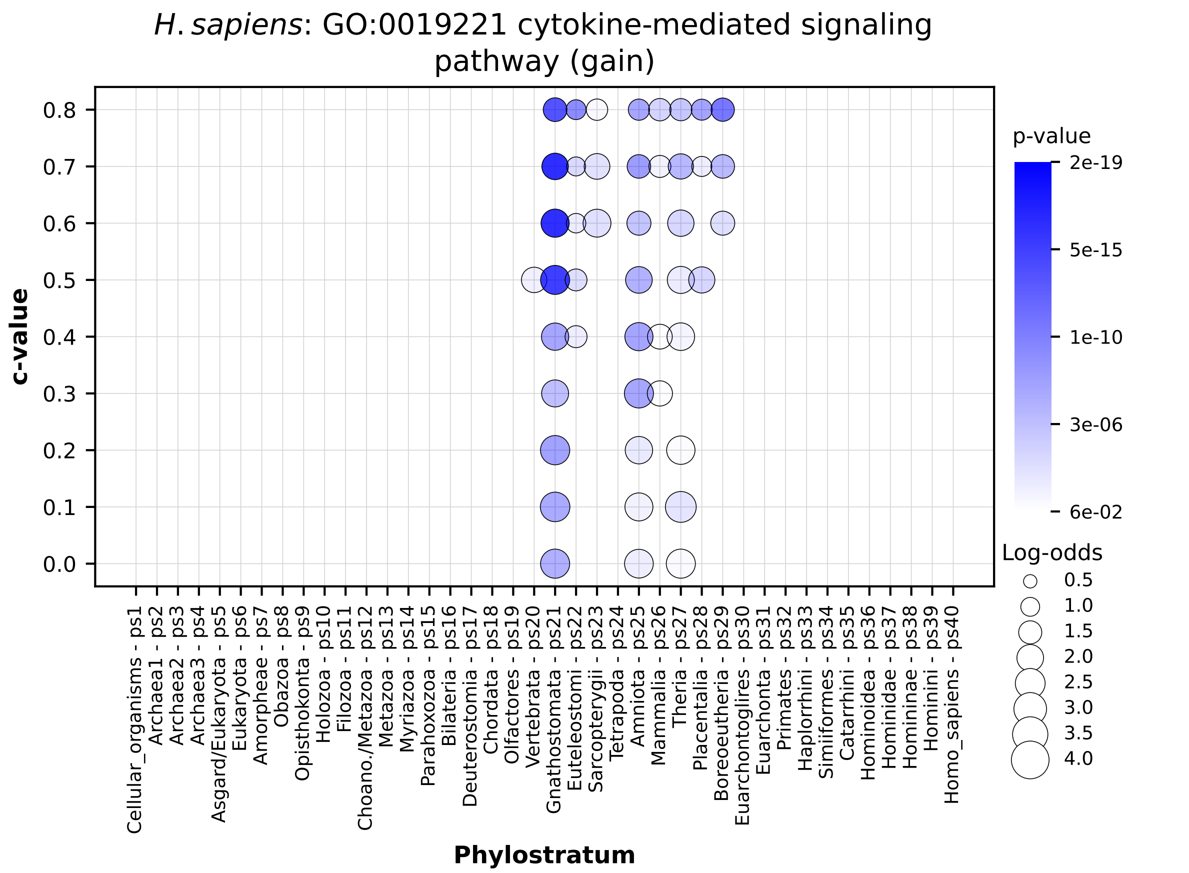

H. sapiens: GO:0019221 cytokine-mediated signaling pathway (gain)
c-value
0.8
0.7
0.6
0.5
0.4
0.3
0.2
0.1
0.0
p-value
2e-19
5e-15
1e-10
3e-06
6e-02
Log-odds
0.5
1.0
1.5
2.0
2.5
3.0
3.5
4.0
Cellular_organisms - ps1
Archaea1 - ps2
Archaea2 - ps3
Archaea3 - ps4
Asgard/Eukaryota - ps5
Eukaryota - ps6
Amorpheae - ps7
Obazoa - ps8
Opisthokonta - ps9
Holozoa - ps10
Filozoa - ps11
Choano./Metazoa - ps12
Metazoa - ps13
Myriazoa - ps14
Parahoxozoa - ps15
Bilateria - ps16
Deuterostomia - ps17
Chordata - ps18
Olfactores - ps19
Vertebrata - ps20
Gnathostomata - ps21
Euteleostomi - ps22
Sarcopterygii - ps23
Tetrapoda - ps24
Amniota - ps25
Mammalia - ps26
Theria - ps27
Placentalia - ps28
Boreoeutheria - ps29
Euarchontoglires - ps30
Euarchonta - ps31
Primates - ps32
Haplorrhini - ps33
Simiiformes - ps34
Catarrhini - ps35
Hominoidea - ps36
Hominidae - ps37
Homininae - ps38
Hominini - ps39
Homo_sapiens - ps40
Phylostratum

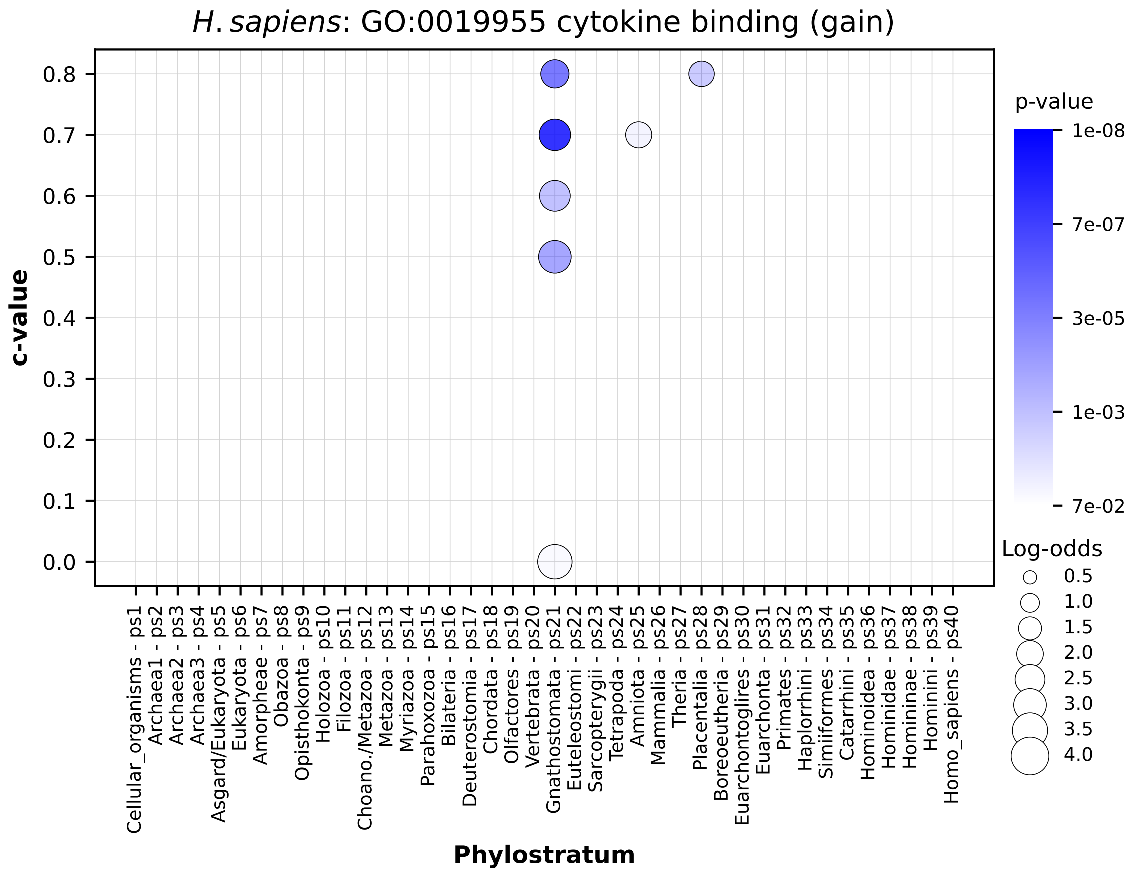
H. sapiens: GO:0019955 cytokine binding (gain)
c-value
0.8
0.7
0.6
0.5
0.4
0.3
0.2
0.1
0.0
p-value
1e-08
7e-07
3e-05
1e-03
7e-02
Log-odds
0.5
1.0
1.5
2.0
2.5
3.0
3.5
4.0
Cellular_organisms - ps1
Archaea1 - ps2
Archaea2 - ps3
Archaea3 - ps4
Asgard/Eukaryota - ps5
Eukaryota - ps6
Amorpheae - ps7
Obazoa - ps8
Opisthokonta - ps9
Holozoa - ps10
Filozoa - ps11
Choano./Metazoa - ps12
Metazoa - ps13
Myriazoa - ps14
Parahoxozoa - ps15
Bilateria - ps16
Deuterostomia - ps17
Chordata - ps18
Olfactores - ps19
Vertebrata - ps20
Gnathostomata - ps21
Euteleostomi - ps22
Sarcopterygii - ps23
Tetrapoda - ps24
Amniota - ps25
Mammalia - ps26
Theria - ps27
Placentalia - ps28
Boreoeutheria - ps29
Euarchontoglires - ps30
Euarchonta - ps31
Primates - ps32
Haplorrhini - ps33
Simiiformes - ps34
Catarrhini - ps35
Hominoidea - ps36
Hominidae - ps37
Homininae - ps38
Hominini - ps39
Homo_sapiens - ps40
Phylostratum

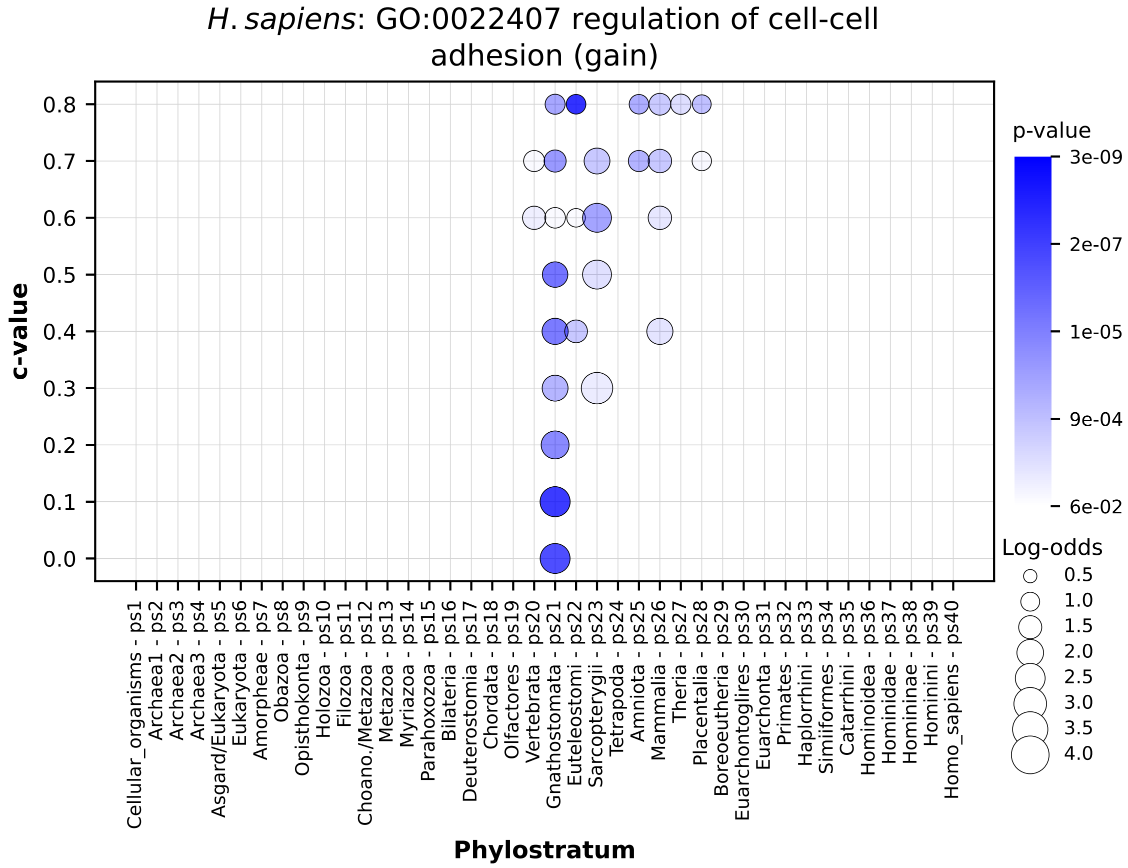

H. sapiens: GO:0022407 regulation of cell-cell adhesion (gain)
c-value
0.0
0.1
0.2
0.3
0.4
0.5
0.6
0.7
0.8
Phylostratum
Cellular_organisms - ps1
Archaea1 - ps2
Archaea2 - ps3
Archaea3 - ps4
Asgard/Eukaryota - ps5
Eukaryota - ps6
Amorpheae - ps7
Obazoa - ps8
Opisthokonta - ps9
Holozoa - ps10
Filozoa - ps11
Choano./Metazoa - ps12
Metazoa - ps13
Myriazoa - ps14
Parahoxozoa - ps15
Bilateria - ps16
Deuterostomia - ps17
Chordata - ps18
Olfactores - ps19
Vertebrata - ps20
Gnathostomata - ps21
Euteleostomi - ps22
Sarcopterygii - ps23
Tetrapoda - ps24
Amniota - ps25
Mammalia - ps26
Theria - ps27
Placentalia - ps28
Boreoeutheria - ps29
Euarchontoglires - ps30
Euarchonta - ps31
Primates - ps32
Haplorrhini - ps33
Simiiformes - ps34
Catarrhini - ps35
Hominoidea - ps36
Hominidae - ps37
Homininae - ps38
Hominini - ps39
Homo_sapiens - ps40
p-value
3e-09
2e-07
1e-05
9e-04
6e-02
Log-odds
0.5
1.0
1.5
2.0
2.5
3.0
3.5
4.0

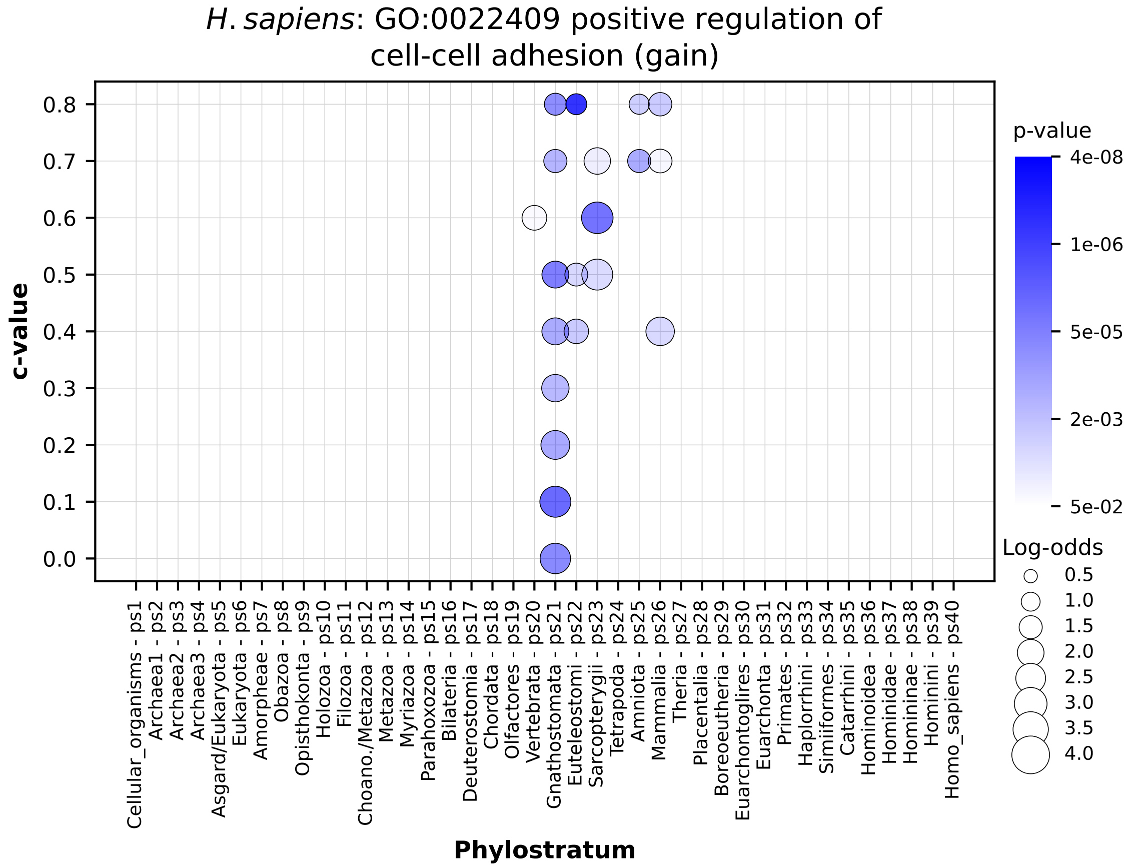

H. sapiens: GO:0022409 positive regulation of cell-cell adhesion (gain)
c-value
0.8
0.7
0.6
0.5
0.4
0.3
0.2
0.1
0.0
Phylostratum
Cellular_organisms - ps1
Archaea1 - ps2
Archaea2 - ps3
Archaea3 - ps4
Asgard/Eukaryota - ps5
Eukaryota - ps6
Amorpheae - ps7
Obazoa - ps8
Opisthokonta - ps9
Holozoa - ps10
Filozoa - ps11
Choano./Metazoa - ps12
Metazoa - ps13
Myriazoa - ps14
Parahoxozoa - ps15
Bilateria - ps16
Deuterostomia - ps17
Chordata - ps18
Olfactores - ps19
Vertebrata - ps20
Gnathostomata - ps21
Euteleostomi - ps22
Sarcopterygii - ps23
Tetrapoda - ps24
Amniota - ps25
Mammalia - ps26
Theria - ps27
Placentalia - ps28
Boreoeutheria - ps29
Euarchontoglires - ps30
Euarchonta - ps31
Primates - ps32
Haplorrhini - ps33
Simiiformes - ps34
Catarrhini - ps35
Hominoidea - ps36
Hominidae - ps37
Homininae - ps38
Hominini - ps39
Homo_sapiens - ps40
p-value
4e-08
1e-06
5e-05
2e-03
5e-02
Log-odds
0.5
1.0
1.5
2.0
2.5
3.0
3.5
4.0

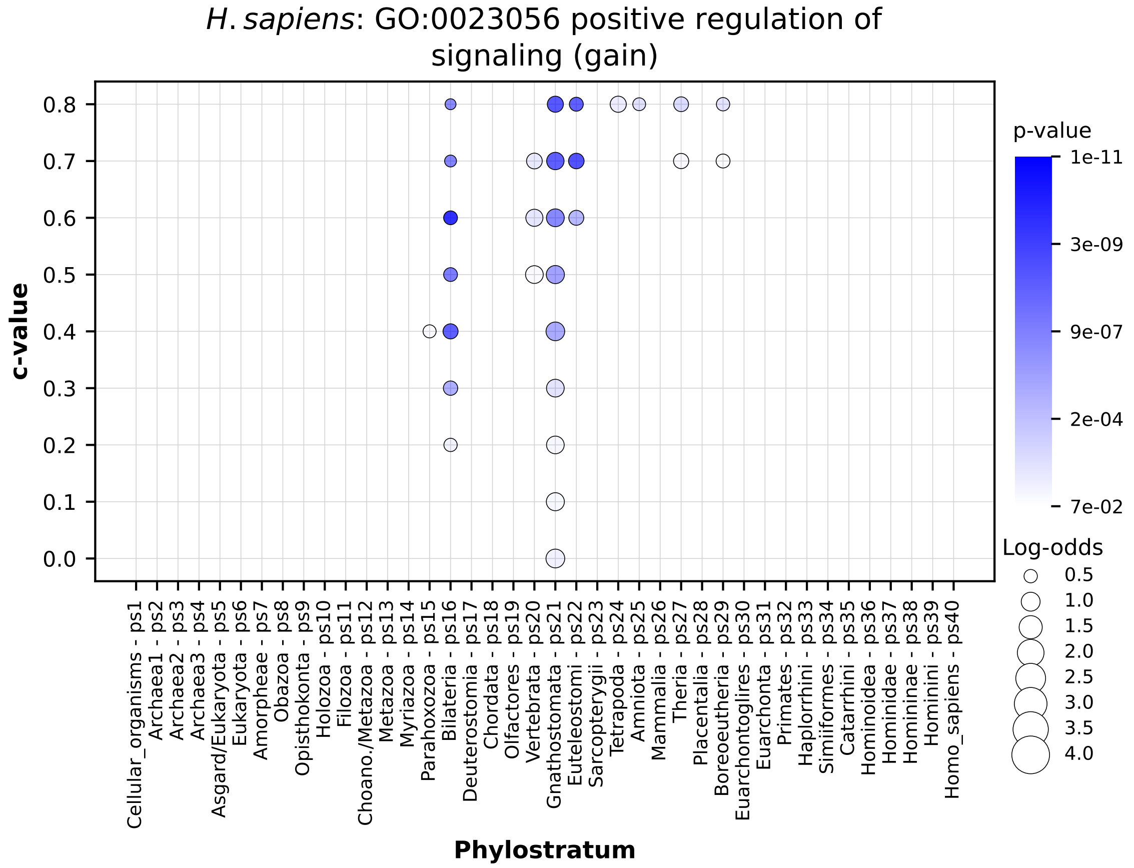

H. sapiens: GO:0023056 positive regulation of signaling (gain)
c-value
0.8
0.7
0.6
0.5
0.4
0.3
0.2
0.1
0.0
Phylostratum
Cellular_organisms - ps1
Archaea1 - ps2
Archaea2 - ps3
Archaea3 - ps4
Asgard/Eukaryota - ps5
Eukaryota - ps6
Amorpheae - ps7
Obazoa - ps8
Opisthokonta - ps9
Holozoa - ps10
Filozoa - ps11
Choano./Metazoa - ps12
Metazoa - ps13
Myriazoa - ps14
Parahoxozoa - ps15
Bilateria - ps16
Deuterostomia - ps17
Chordata - ps18
Olfactores - ps19
Vertebrata - ps20
Gnathostomata - ps21
Euteleostomi - ps22
Sarcopterygii - ps23
Tetrapoda - ps24
Amniota - ps25
Mammalia - ps26
Theria - ps27
Placentalia - ps28
Boreoeutheria - ps29
Euarchontoglires - ps30
Euarchonta - ps31
Primates - ps32
Haplorrhini - ps33
Simiiformes - ps34
Catarrhini - ps35
Hominoidea - ps36
Hominidae - ps37
Homininae - ps38
Hominini - ps39
Homo_sapiens - ps40
p-value
1e-11
3e-09
9e-07
2e-04
7e-02
Log-odds
0.5
1.0
1.5
2.0
2.5
3.0
3.5
4.0

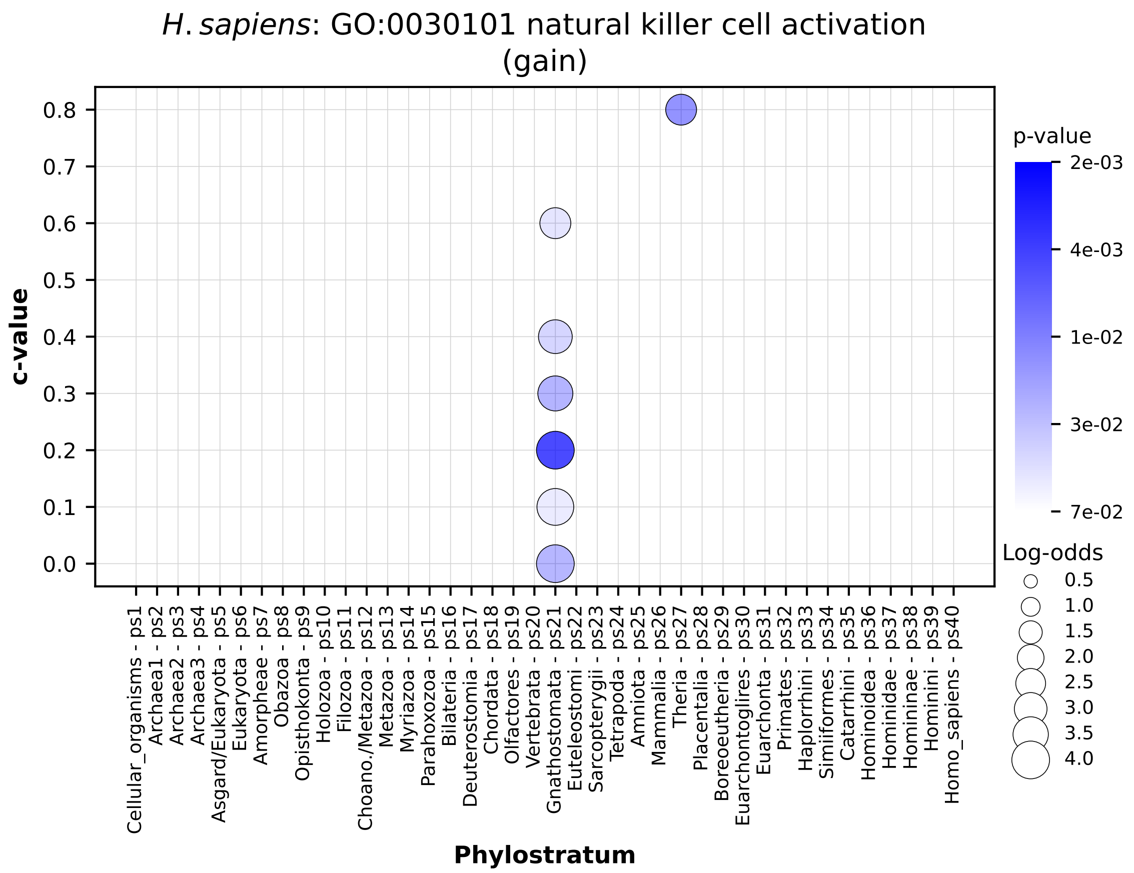
H. sapiens: GO:0030101 natural killer cell activation (gain)
c-value
0.8
0.7
0.6
0.5
0.4
0.3
0.2
0.1
0.0
Phylostratum
Cellular_organisms - ps1
Archaea1 - ps2
Archaea2 - ps3
Archaea3 - ps4
Asgard/Eukaryota - ps5
Eukaryota - ps6
Amorpheae - ps7
Obazoa - ps8
Opisthokonta - ps9
Holozoa - ps10
Filozoa - ps11
Choano./Metazoa - ps12
Metazoa - ps13
Myriazoa - ps14
Parahoxozoa - ps15
Bilateria - ps16
Deuterostomia - ps17
Chordata - ps18
Olfactores - ps19
Vertebrata - ps20
Gnathostomata - ps21
Euteleostomi - ps22
Sarcopterygii - ps23
Tetrapoda - ps24
Amniota - ps25
Mammalia - ps26
Theria - ps27
Placentalia - ps28
Boreoeutheria - ps29
Euarchontoglires - ps30
Euarchonta - ps31
Primates - ps32
Haplorrhini - ps33
Simiiformes - ps34
Catarrhini - ps35
Hominoidea - ps36
Hominidae - ps37
Homininae - ps38
Hominini - ps39
Homo_sapiens - ps40
p-value
2e-03
4e-03
1e-02
3e-02
7e-02
Log-odds
0.5
1.0
1.5
2.0
2.5
3.0
3.5
4.0

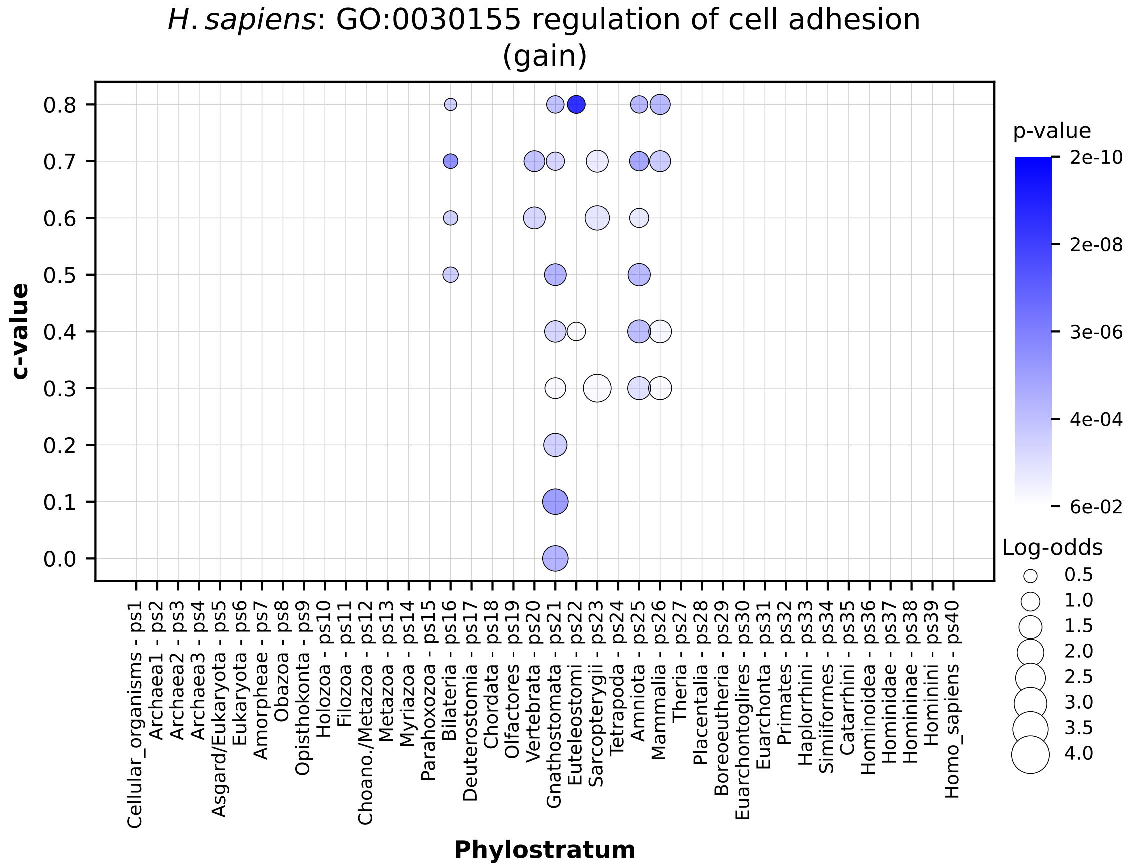

H. sapiens: GO:0030155 regulation of cell adhesion
(gain)
c-value
0.8
0.7
0.6
0.5
0.4
0.3
0.2
0.1
0.0
Phylostratum
Cellular_organisms - ps1
Archaea1 - ps2
Archaea2 - ps3
Archaea3 - ps4
Asgard/Eukaryota - ps5
Eukaryota - ps6
Amorpheae - ps7
Obazoa - ps8
Opisthokonta - ps9
Holozoa - ps10
Filozoa - ps11
Choano./Metazoa - ps12
Metazoa - ps13
Myriazoa - ps14
Parahoxozoa - ps15
Bilateria - ps16
Deuterostomia - ps17
Chordata - ps18
Olfactores - ps19
Vertebrata - ps20
Gnathostomata - ps21
Euteleostomi - ps22
Sarcopterygii - ps23
Tetrapoda - ps24
Amniota - ps25
Mammalia - ps26
Theria - ps27
Placentalia - ps28
Boreoeutheria - ps29
Euarchontoglires - ps30
Euarchonta - ps31
Primates - ps32
Haplorrhini - ps33
Simiiformes - ps34
Catarrhini - ps35
Hominoidea - ps36
Hominidae - ps37
Homininae - ps38
Hominini - ps39
Homo_sapiens - ps40
p-value
2e-10
2e-08
3e-06
4e-04
6e-02
Log-odds
0.5
1.0
1.5
2.0
2.5
3.0
3.5
4.0

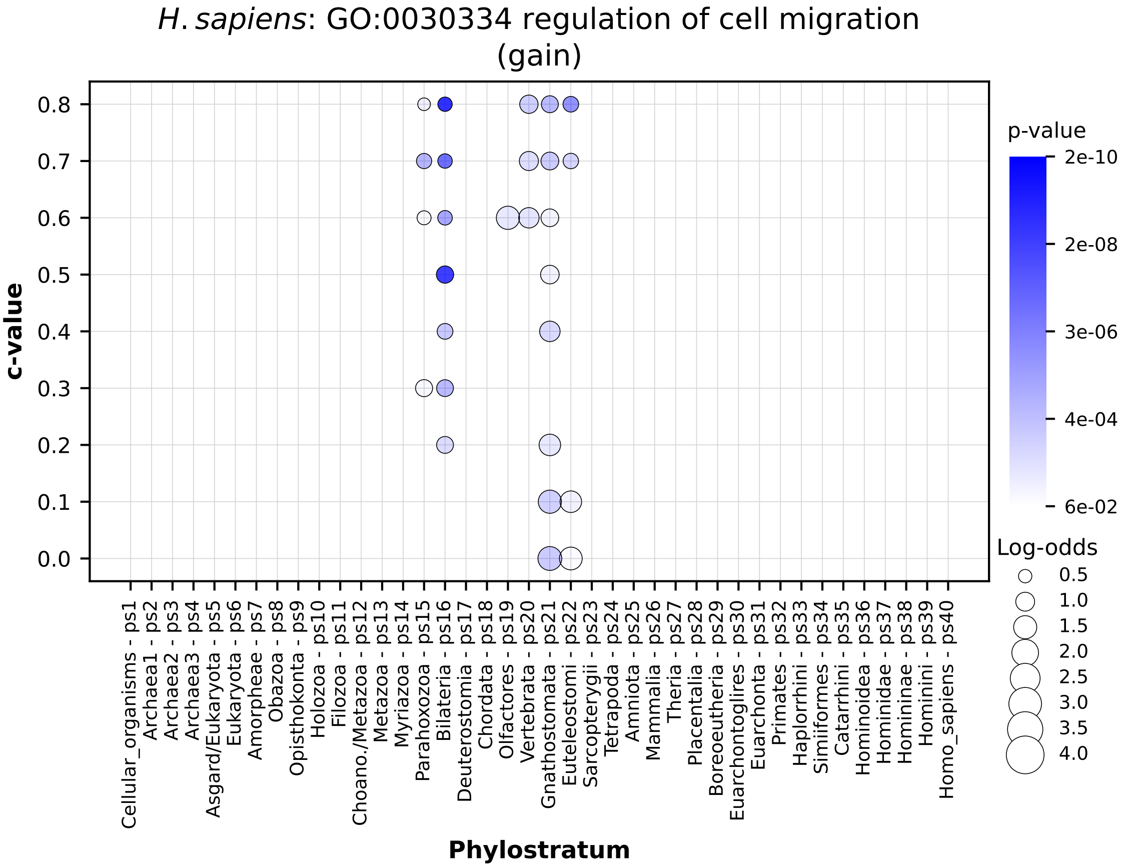

H. sapiens: GO:0030334 regulation of cell migration (gain)
c-value
0.0
0.1
0.2
0.3
0.4
0.5
0.6
0.7
0.8
Phylostratum
Cellular_organisms - ps1
Archaea1 - ps2
Archaea2 - ps3
Archaea3 - ps4
Asgard/Eukaryota - ps5
Eukaryota - ps6
Amorpheae - ps7
Obazoa - ps8
Opisthokonta - ps9
Holozoa - ps10
Filozoa - ps11
Choano./Metazoa - ps12
Metazoa - ps13
Myriazoa - ps14
Parahoxozoa - ps15
Bilateria - ps16
Deuterostomia - ps17
Chordata - ps18
Olfactores - ps19
Vertebrata - ps20
Gnathostomata - ps21
Euteleostomi - ps22
Sarcopterygii - ps23
Tetrapoda - ps24
Amniota - ps25
Mammalia - ps26
Theria - ps27
Placentalia - ps28
Boreoeutheria - ps29
Euarchontoglires - ps30
Euarchonta - ps31
Primates - ps32
Haplorrhini - ps33
Simiiformes - ps34
Catarrhini - ps35
Hominoidea - ps36
Hominidae - ps37
Homininae - ps38
Hominini - ps39
Homo_sapiens - ps40
p-value
2e-10
2e-08
3e-06
4e-04
6e-02
Log-odds
0.5
1.0
1.5
2.0
2.5
3.0
3.5
4.0

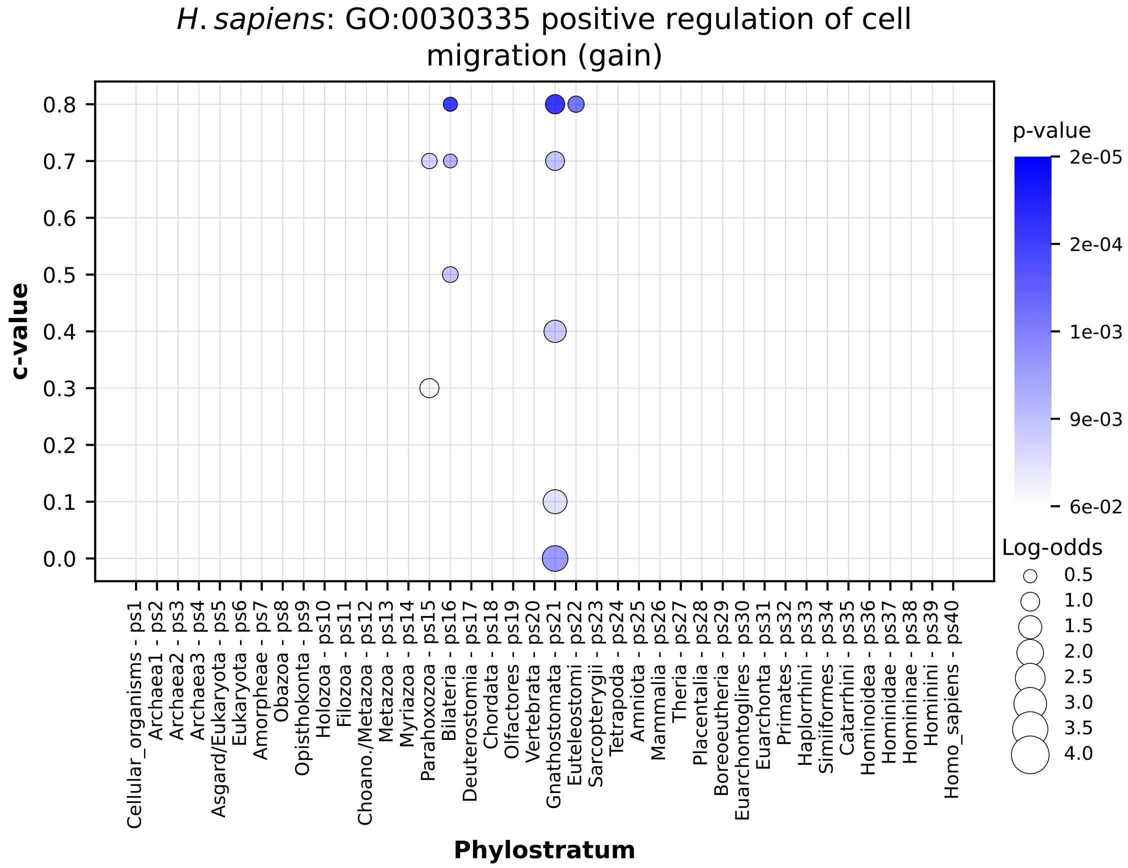
H. sapiens: GO:0030335 positive regulation of cell migration (gain)
c-value
0.8
0.7
0.6
0.5
0.4
0.3
0.2
0.1
0.0
Phylostratum
Cellular_organisms - ps1
Archaea1 - ps2
Archaea2 - ps3
Archaea3 - ps4
Asgard/Eukaryota - ps5
Eukaryota - ps6
Amorpheae - ps7
Obazoa - ps8
Opisthokonta - ps9
Holozoa - ps10
Filozoa - ps11
Choano./Metazoa - ps12
Metazoa - ps13
Myriazoa - ps14
Parahoxozoa - ps15
Bilateria - ps16
Deuterostomia - ps17
Chordata - ps18
Olfactores - ps19
Vertebrata - ps20
Gnathostomata - ps21
Euteleostomi - ps22
Sarcopterygii - ps23
Tetrapoda - ps24
Amniota - ps25
Mammalia - ps26
Theria - ps27
Placentalia - ps28
Boreoeutheria - ps29
Euarchontoglires - ps30
Euarchonta - ps31
Primates - ps32
Haplorrhini - ps33
Simiiformes - ps34
Catarrhini - ps35
Hominoidea - ps36
Hominidae - ps37
Homininae - ps38
Hominini - ps39
Homo_sapiens - ps40
p-value
2e-05
2e-04
1e-03
9e-03
6e-02
Log-odds
0.5
1.0
1.5
2.0
2.5
3.0
3.5
4.0

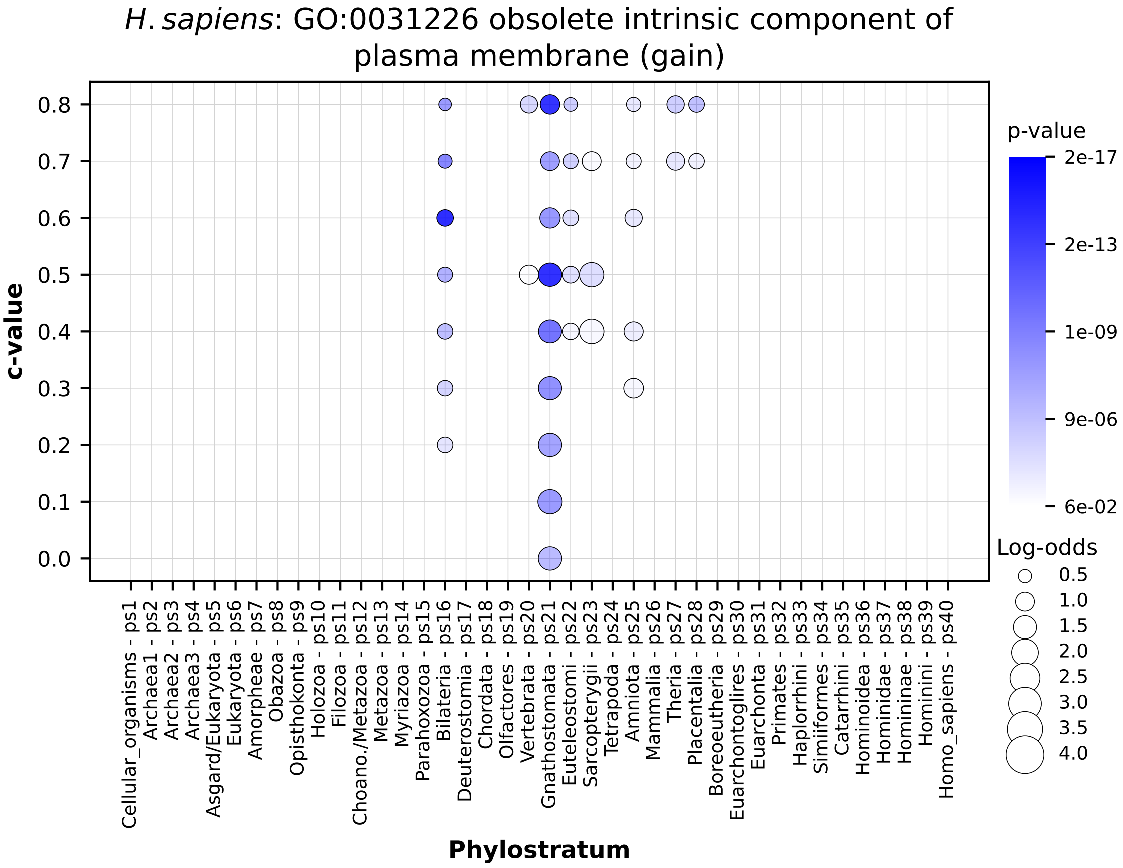

*H. sapiens*: GO:0031226 obsolete intrinsic component of plasma membrane (gain)
c-value
0.8
0.7
0.6
0.5
0.4
0.3
0.2
0.1
0.0
p-value
2e-17
2e-13
1e-09
9e-06
6e-02
Log-odds
0.5
1.0
1.5
2.0
2.5
3.0
3.5
4.0
Cellular_organisms - ps1
Archaea1 - ps2
Archaea2 - ps3
Archaea3 - ps4
Asgard/Eukaryota - ps5
Eukaryota - ps6
Amorpheae - ps7
Obazoa - ps8
Opisthokonta - ps9
Holozoa - ps10
Filozoa - ps11
Choano./Metazoa - ps12
Metazoa - ps13
Myriazoa - ps14
Parahoxozoa - ps15
Bilateria - ps16
Deuterostomia - ps17
Chordata - ps18
Olfactores - ps19
Vertebrata - ps20
Gnathostomata - ps21
Euteleostomi - ps22
Sarcopterygii - ps23
Tetrapoda - ps24
Amniota - ps25
Mammalia - ps26
Theria - ps27
Placentalia - ps28
Boreoeutheria - ps29
Euarchontoglires - ps30
Euarchonta - ps31
Primates - ps32
Haplorrhini - ps33
Simiiformes - ps34
Catarrhini - ps35
Hominoidea - ps36
Hominidae - ps37
Homininae - ps38
Hominini - ps39
Homo_sapiens - ps40
Phylostratum

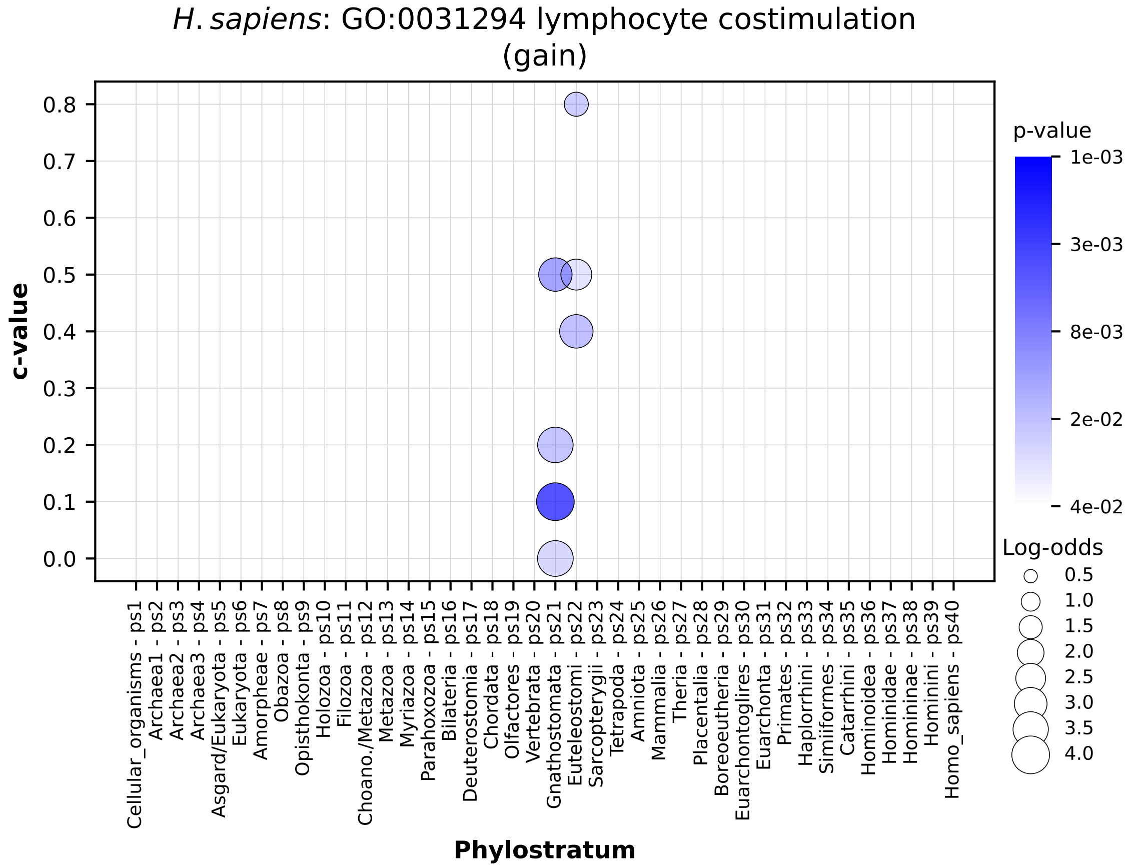

H. sapiens: GO:0031294 lymphocyte costimulation
(gain)
c-value
0.8
0.7
0.6
0.5
0.4
0.3
0.2
0.1
0.0
p-value
1e-03
3e-03
8e-03
2e-02
4e-02
Log-odds
0.5
1.0
1.5
2.0
2.5
3.0
3.5
4.0
Cellular_organisms - ps1
Archaea1 - ps2
Archaea2 - ps3
Archaea3 - ps4
Asgard/Eukaryota - ps5
Eukaryota - ps6
Amorpheae - ps7
Obazoa - ps8
Opisthokonta - ps9
Holozoa - ps10
Filozoa - ps11
Choano./Metazoa - ps12
Metazoa - ps13
Myriazoa - ps14
Parahoxozoa - ps15
Bilateria - ps16
Deuterostomia - ps17
Chordata - ps18
Olfactores - ps19
Vertebrata - ps20
Gnathostomata - ps21
Euteleostomi - ps22
Sarcopterygii - ps23
Tetrapoda - ps24
Amniota - ps25
Mammalia - ps26
Theria - ps27
Placentalia - ps28
Boreoeutheria - ps29
Euarchontoglires - ps30
Euarchonta - ps31
Primates - ps32
Haplorrhini - ps33
Simiiformes - ps34
Catarrhini - ps35
Hominoidea - ps36
Hominidae - ps37
Homininae - ps38
Hominini - ps39
Homo_sapiens - ps40
Phylostratum

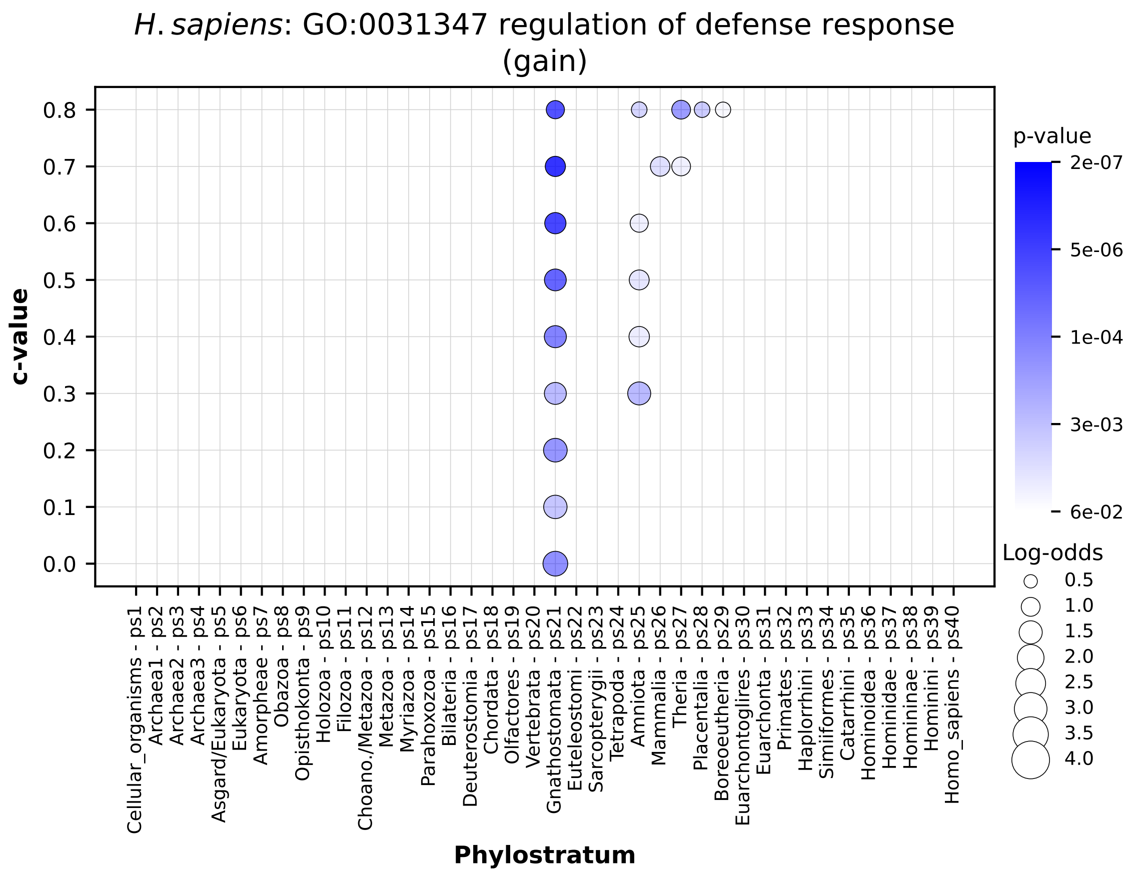

H. sapiens: GO:0031347 regulation of defense response
(gain)
c-value
0.0
0.1
0.2
0.3
0.4
0.5
0.6
0.7
0.8
Phylostratum
Cellular_organisms - ps1
Archaea1 - ps2
Archaea2 - ps3
Archaea3 - ps4
Asgard/Eukaryota - ps5
Eukaryota - ps6
Amorpheae - ps7
Obazoa - ps8
Opisthokonta - ps9
Holozoa - ps10
Filozoa - ps11
Choano./Metazoa - ps12
Metazoa - ps13
Myriazoa - ps14
Parahoxozoa - ps15
Bilateria - ps16
Deuterostomia - ps17
Chordata - ps18
Olfactores - ps19
Vertebrata - ps20
Gnathostomata - ps21
Euteleostomi - ps22
Sarcopterygii - ps23
Tetrapoda - ps24
Amniota - ps25
Mammalia - ps26
Theria - ps27
Placentalia - ps28
Boreoeutheria - ps29
Euarchontoglires - ps30
Euarchonta - ps31
Primates - ps32
Haplorrhini - ps33
Simiiformes - ps34
Catarrhini - ps35
Hominoidea - ps36
Hominidae - ps37
Homininae - ps38
Hominini - ps39
Homo_sapiens - ps40
p-value
2e-07
5e-06
1e-04
3e-03
6e-02
Log-odds
0.5
1.0
1.5
2.0
2.5
3.0
3.5
4.0

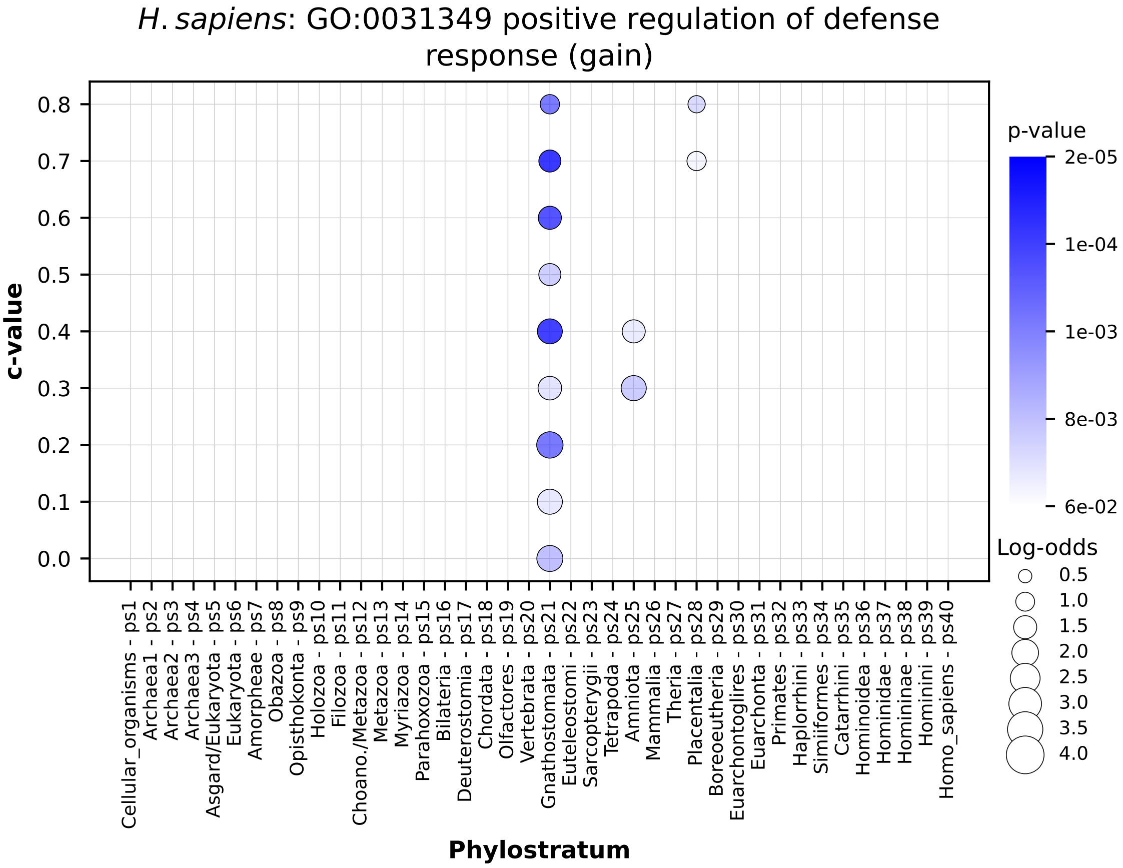

H. sapiens: GO:0031349 positive regulation of defense response (gain)
c-value
0.8
0.7
0.6
0.5
0.4
0.3
0.2
0.1
0.0
p-value
2e-05
1e-04
1e-03
8e-03
6e-02
Log-odds
0.5
1.0
1.5
2.0
2.5
3.0
3.5
4.0
Cellular_organisms - ps1
Archaea1 - ps2
Archaea2 - ps3
Archaea3 - ps4
Asgard/Eukaryota - ps5
Eukaryota - ps6
Amorpheae - ps7
Obazoa - ps8
Opisthokonta - ps9
Holozoa - ps10
Filozoa - ps11
Choano./Metazoa - ps12
Metazoa - ps13
Myriazoa - ps14
Parahoxozoa - ps15
Bilateria - ps16
Deuterostomia - ps17
Chordata - ps18
Olfactores - ps19
Vertebrata - ps20
Gnathostomata - ps21
Euteleostomi - ps22
Sarcopterygii - ps23
Tetrapoda - ps24
Amniota - ps25
Mammalia - ps26
Theria - ps27
Placentalia - ps28
Boreoeutheria - ps29
Euarchontoglires - ps30
Euarchonta - ps31
Primates - ps32
Haplorrhini - ps33
Simiiformes - ps34
Catarrhini - ps35
Hominoidea - ps36
Hominidae - ps37
Homininae - ps38
Hominini - ps39
Homo_sapiens - ps40
Phylostratum

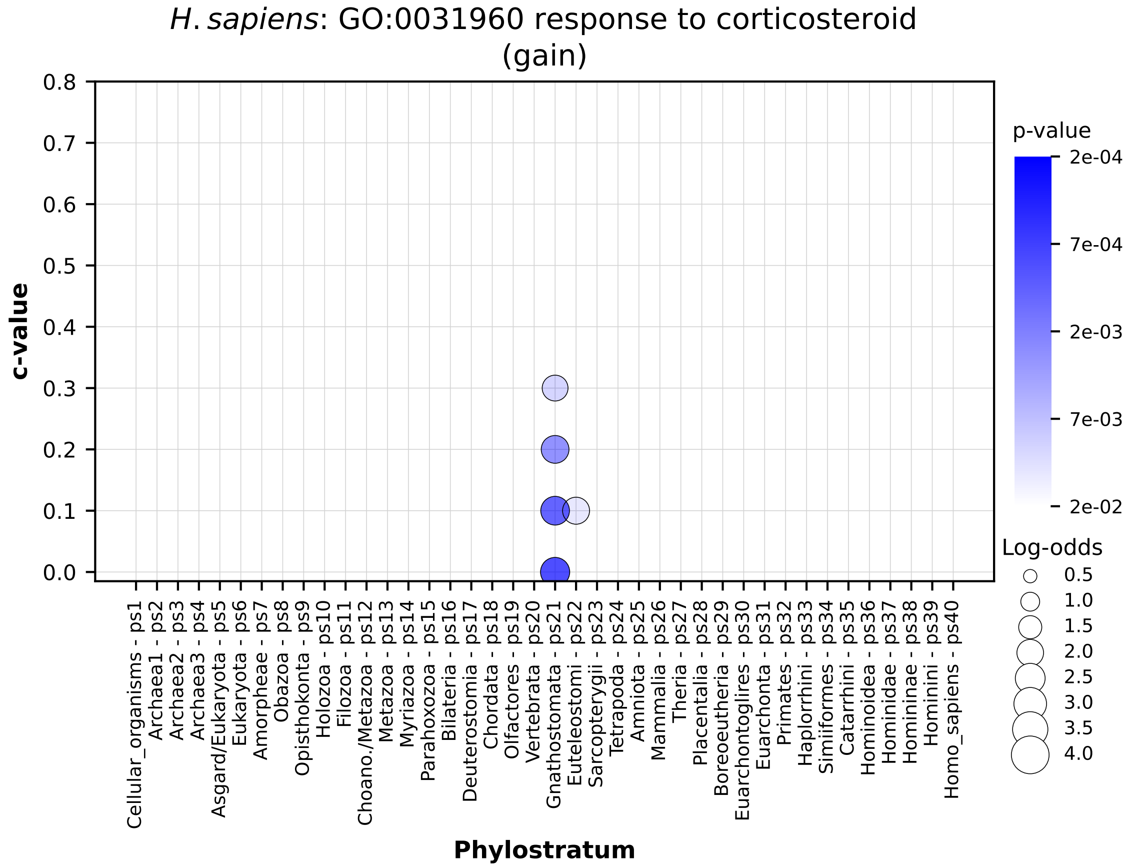

H. sapiens: GO:0031960 response to corticosteroid
(gain)
c-value
0.8
0.7
0.6
0.5
0.4
0.3
0.2
0.1
0.0
p-value
2e-04
7e-04
2e-03
7e-03
2e-02
Log-odds
0.5
1.0
1.5
2.0
2.5
3.0
3.5
4.0
Cellular_organisms - ps1
Archaea1 - ps2
Archaea2 - ps3
Archaea3 - ps4
Asgard/Eukaryota - ps5
Eukaryota - ps6
Amorpheae - ps7
Obazoa - ps8
Opisthokonta - ps9
Holozoa - ps10
Filozoa - ps11
Choano./Metazoa - ps12
Metazoa - ps13
Myriazoa - ps14
Parahoxozoa - ps15
Bilateria - ps16
Deuterostomia - ps17
Chordata - ps18
Olfactores - ps19
Vertebrata - ps20
Gnathostomata - ps21
Euteleostomi - ps22
Sarcopterygii - ps23
Tetrapoda - ps24
Amniota - ps25
Mammalia - ps26
Theria - ps27
Placentalia - ps28
Boreoeutheria - ps29
Euarchontoglires - ps30
Euarchonta - ps31
Primates - ps32
Haplorrhini - ps33
Simiiformes - ps34
Catarrhini - ps35
Hominoidea - ps36
Hominidae - ps37
Homininae - ps38
Hominini - ps39
Homo_sapiens - ps40
Phylostratum

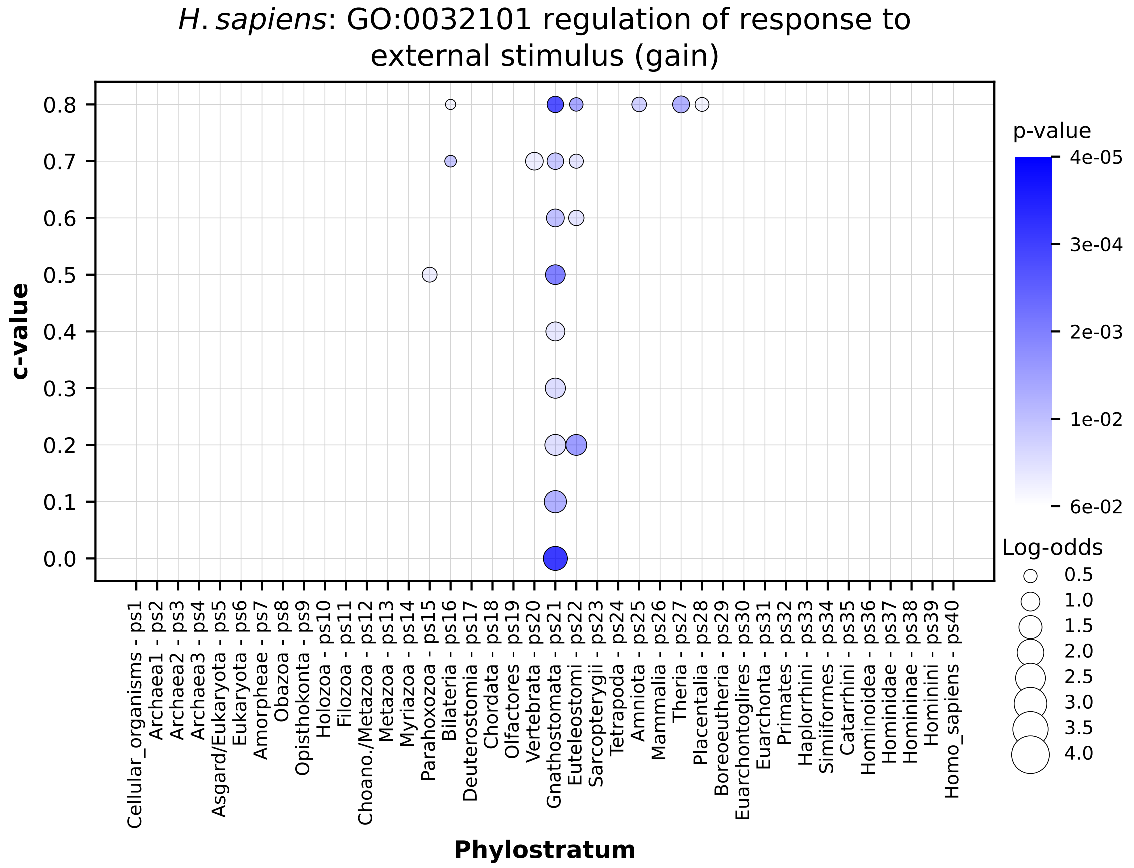

H. sapiens: GO:0032101 regulation of response to external stimulus (gain)
c-value
0.8
0.7
0.6
0.5
0.4
0.3
0.2
0.1
0.0
p-value
4e-05
3e-04
2e-03
1e-02
6e-02
Log-odds
0.5
1.0
1.5
2.0
2.5
3.0
3.5
4.0
Cellular_organisms - ps1
Archaea1 - ps2
Archaea2 - ps3
Archaea3 - ps4
Asgard/Eukaryota - ps5
Eukaryota - ps6
Amorpheae - ps7
Obazoa - ps8
Opisthokonta - ps9
Holozoa - ps10
Filozoa - ps11
Choano./Metazoa - ps12
Metazoa - ps13
Myriazoa - ps14
Parahoxozoa - ps15
Bilateria - ps16
Deuterostomia - ps17
Chordata - ps18
Olfactores - ps19
Vertebrata - ps20
Gnathostomata - ps21
Euteleostomi - ps22
Sarcopterygii - ps23
Tetrapoda - ps24
Amniota - ps25
Mammalia - ps26
Theria - ps27
Placentalia - ps28
Boreoeutheria - ps29
Euarchontoglires - ps30
Euarchonta - ps31
Primates - ps32
Haplorrhini - ps33
Simiiformes - ps34
Catarrhini - ps35
Hominoidea - ps36
Hominidae - ps37
Homininae - ps38
Hominini - ps39
Homo_sapiens - ps40
Phylostratum

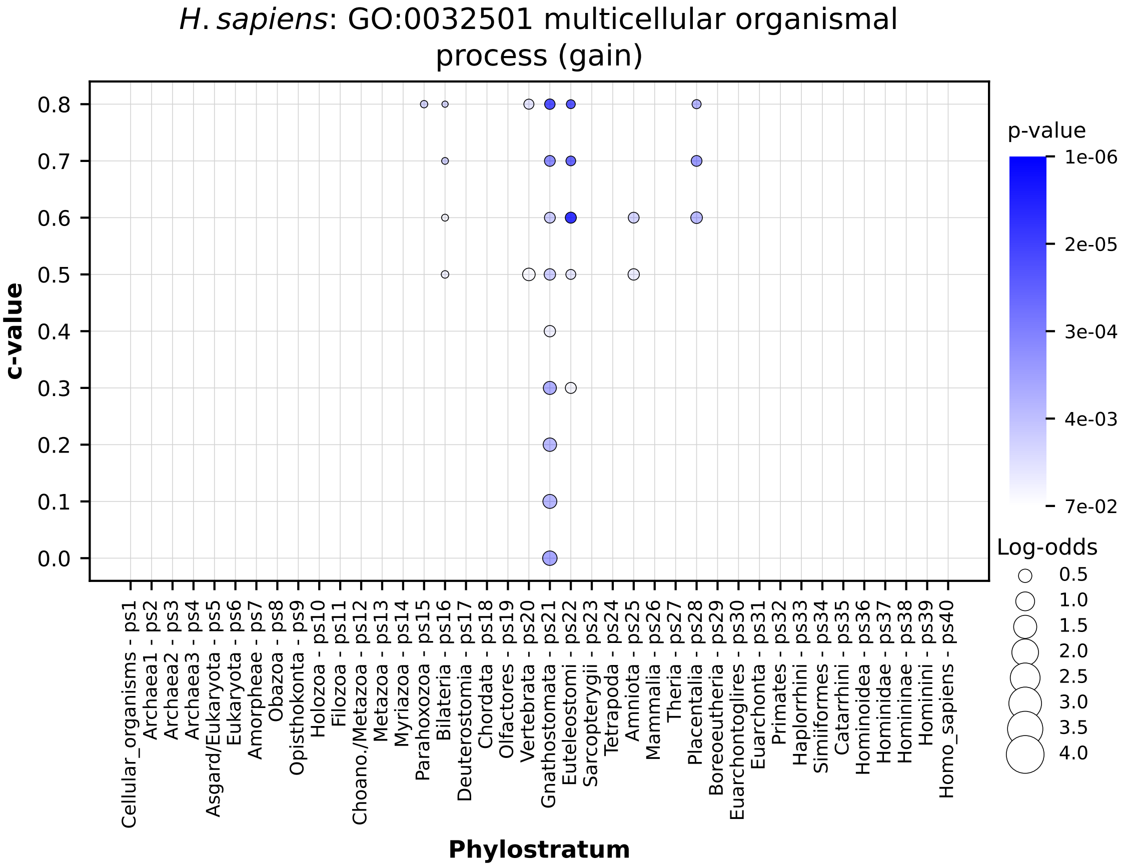
H. sapiens: GO:0032501 multicellular organismal process (gain)
c-value
0.8
0.7
0.6
0.5
0.4
0.3
0.2
0.1
0.0
Phylostratum
Cellular_organisms - ps1
Archaea1 - ps2
Archaea2 - ps3
Archaea3 - ps4
Asgard/Eukaryota - ps5
Eukaryota - ps6
Amorpheae - ps7
Obazoa - ps8
Opisthokonta - ps9
Holozoa - ps10
Filozoa - ps11
Choano./Metazoa - ps12
Metazoa - ps13
Myriazoa - ps14
Parahoxozoa - ps15
Bilateria - ps16
Deuterostomia - ps17
Chordata - ps18
Olfactores - ps19
Vertebrata - ps20
Gnathostomata - ps21
Euteleostomi - ps22
Sarcopterygii - ps23
Tetrapoda - ps24
Amniota - ps25
Mammalia - ps26
Theria - ps27
Placentalia - ps28
Boreoeutheria - ps29
Euarchontoglires - ps30
Euarchonta - ps31
Primates - ps32
Haplorrhini - ps33
Simiiformes - ps34
Catarrhini - ps35
Hominoidea - ps36
Hominidae - ps37
Homininae - ps38
Hominini - ps39
Homo_sapiens - ps40
p-value
1e-06
2e-05
3e-04
4e-03
7e-02
Log-odds
0.5
1.0
1.5
2.0
2.5
3.0
3.5
4.0

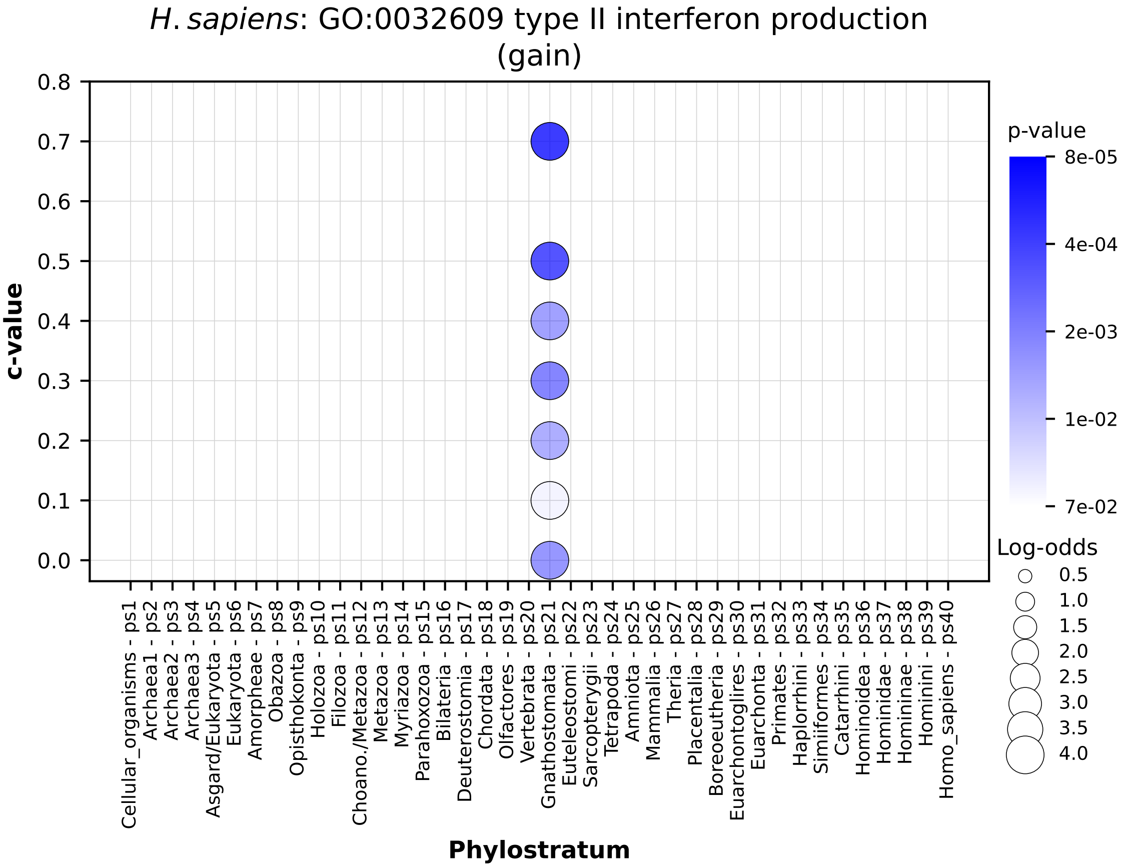

H. sapiens: GO:0032609 type II interferon production
(gain)
c-value
0.0
0.1
0.2
0.3
0.4
0.5
0.6
0.7
0.8
Phylostratum
Cellular_organisms - ps1
Archaea1 - ps2
Archaea2 - ps3
Archaea3 - ps4
Asgard/Eukaryota - ps5
Eukaryota - ps6
Amorpheae - ps7
Obazoa - ps8
Opisthokonta - ps9
Holozoa - ps10
Filozoa - ps11
Choano./Metazoa - ps12
Metazoa - ps13
Myriazoa - ps14
Parahoxozoa - ps15
Bilateria - ps16
Deuterostomia - ps17
Chordata - ps18
Olfactores - ps19
Vertebrata - ps20
Gnathostomata - ps21
Euteleostomi - ps22
Sarcopterygii - ps23
Tetrapoda - ps24
Amniota - ps25
Mammalia - ps26
Theria - ps27
Placentalia - ps28
Boreoeutheria - ps29
Euarchontoglires - ps30
Euarchonta - ps31
Primates - ps32
Haplorrhini - ps33
Simiiformes - ps34
Catarrhini - ps35
Hominoidea - ps36
Hominidae - ps37
Homininae - ps38
Hominini - ps39
Homo_sapiens - ps40
p-value
8e-05
4e-04
2e-03
1e-02
7e-02
Log-odds
0.5
1.0
1.5
2.0
2.5
3.0
3.5
4.0

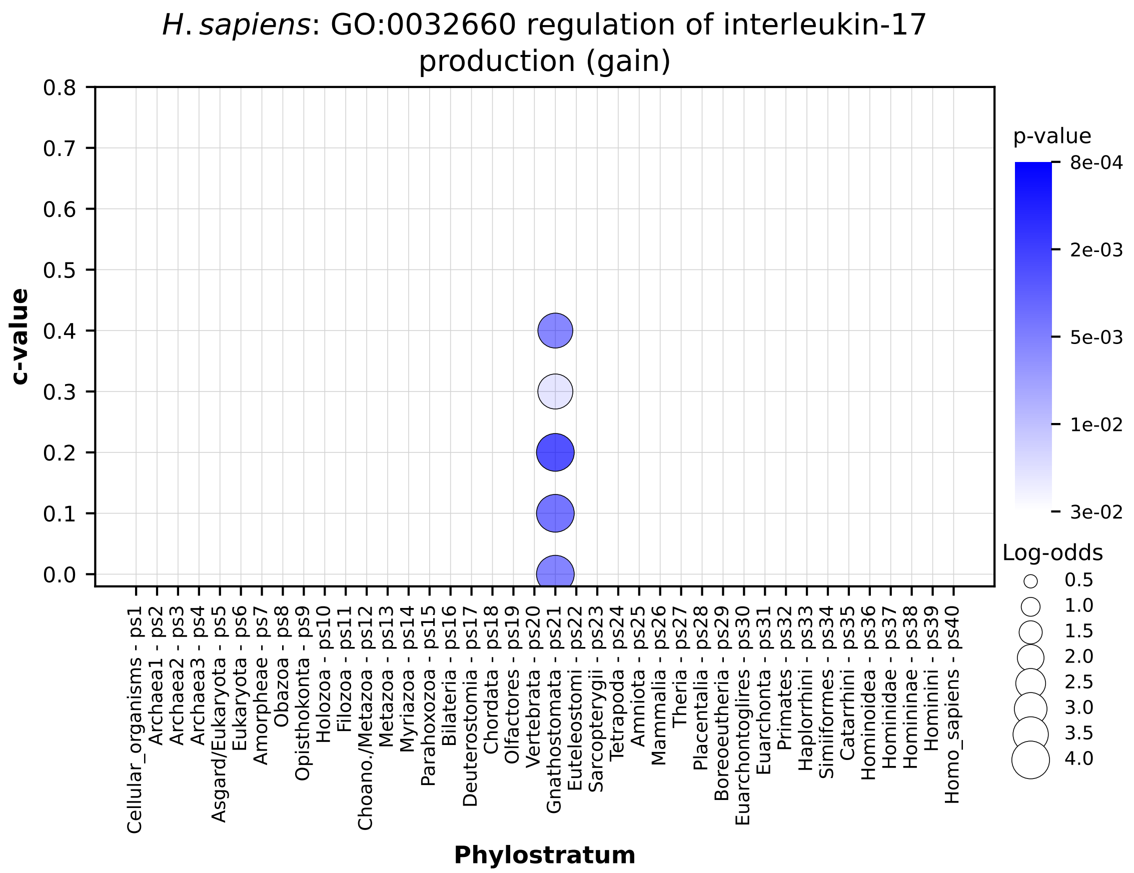

H. sapiens: GO:0032660 regulation of interleukin-17 production (gain)
c-value
0.8
0.7
0.6
0.5
0.4
0.3
0.2
0.1
0.0
p-value
8e-04
2e-03
5e-03
1e-02
3e-02
Log-odds
0.5
1.0
1.5
2.0
2.5
3.0
3.5
4.0
Cellular_organisms - ps1
Archaea1 - ps2
Archaea2 - ps3
Archaea3 - ps4
Asgard/Eukaryota - ps5
Eukaryota - ps6
Amorpheae - ps7
Obazoa - ps8
Opisthokonta - ps9
Holozoa - ps10
Filozoa - ps11
Choano./Metazoa - ps12
Metazoa - ps13
Myriazoa - ps14
Parahoxozoa - ps15
Bilateria - ps16
Deuterostomia - ps17
Chordata - ps18
Olfactores - ps19
Vertebrata - ps20
Gnathostomata - ps21
Euteleostomi - ps22
Sarcopterygii - ps23
Tetrapoda - ps24
Amniota - ps25
Mammalia - ps26
Theria - ps27
Placentalia - ps28
Boreoeutheria - ps29
Euarchontoglires - ps30
Euarchonta - ps31
Primates - ps32
Haplorrhini - ps33
Simiiformes - ps34
Catarrhini - ps35
Hominoidea - ps36
Hominidae - ps37
Homininae - ps38
Hominini - ps39
Homo_sapiens - ps40
Phylostratum

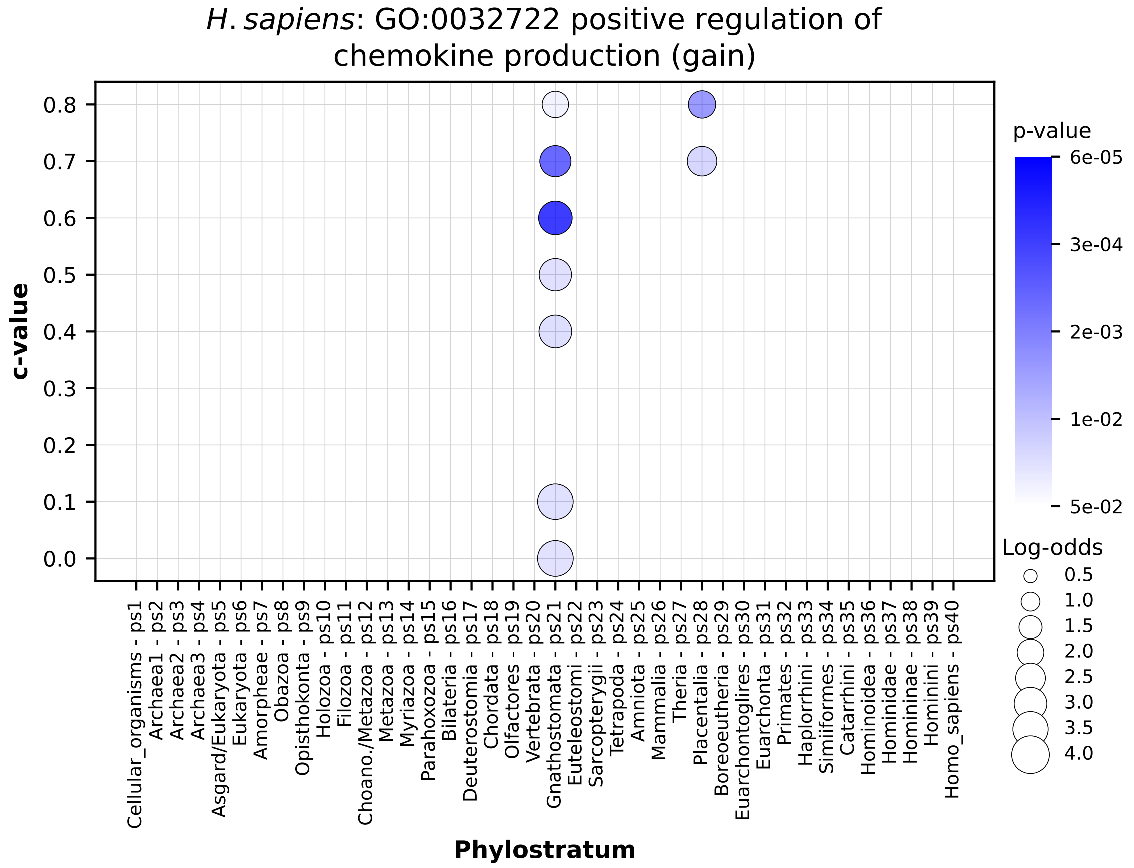

H. sapiens: GO:0032722 positive regulation of chemokine production (gain)
c-value
0.8
0.7
0.6
0.5
0.4
0.3
0.2
0.1
0.0
Phylostratum
Cellular_organisms - ps1
Archaea1 - ps2
Archaea2 - ps3
Archaea3 - ps4
Asgard/Eukaryota - ps5
Eukaryota - ps6
Amorpheae - ps7
Obazoa - ps8
Opisthokonta - ps9
Holozoa - ps10
Filozoa - ps11
Choano./Metazoa - ps12
Metazoa - ps13
Myriazoa - ps14
Parahoxozoa - ps15
Bilateria - ps16
Deuterostomia - ps17
Chordata - ps18
Olfactores - ps19
Vertebrata - ps20
Gnathostomata - ps21
Euteleostomi - ps22
Sarcopterygii - ps23
Tetrapoda - ps24
Amniota - ps25
Mammalia - ps26
Theria - ps27
Placentalia - ps28
Boreoeutheria - ps29
Euarchontoglires - ps30
Euarchonta - ps31
Primates - ps32
Haplorrhini - ps33
Simiiformes - ps34
Catarrhini - ps35
Hominoidea - ps36
Hominidae - ps37
Homininae - ps38
Hominini - ps39
Homo_sapiens - ps40
p-value
6e-05
3e-04
2e-03
1e-02
5e-02
Log-odds
0.5
1.0
1.5
2.0
2.5
3.0
3.5
4.0

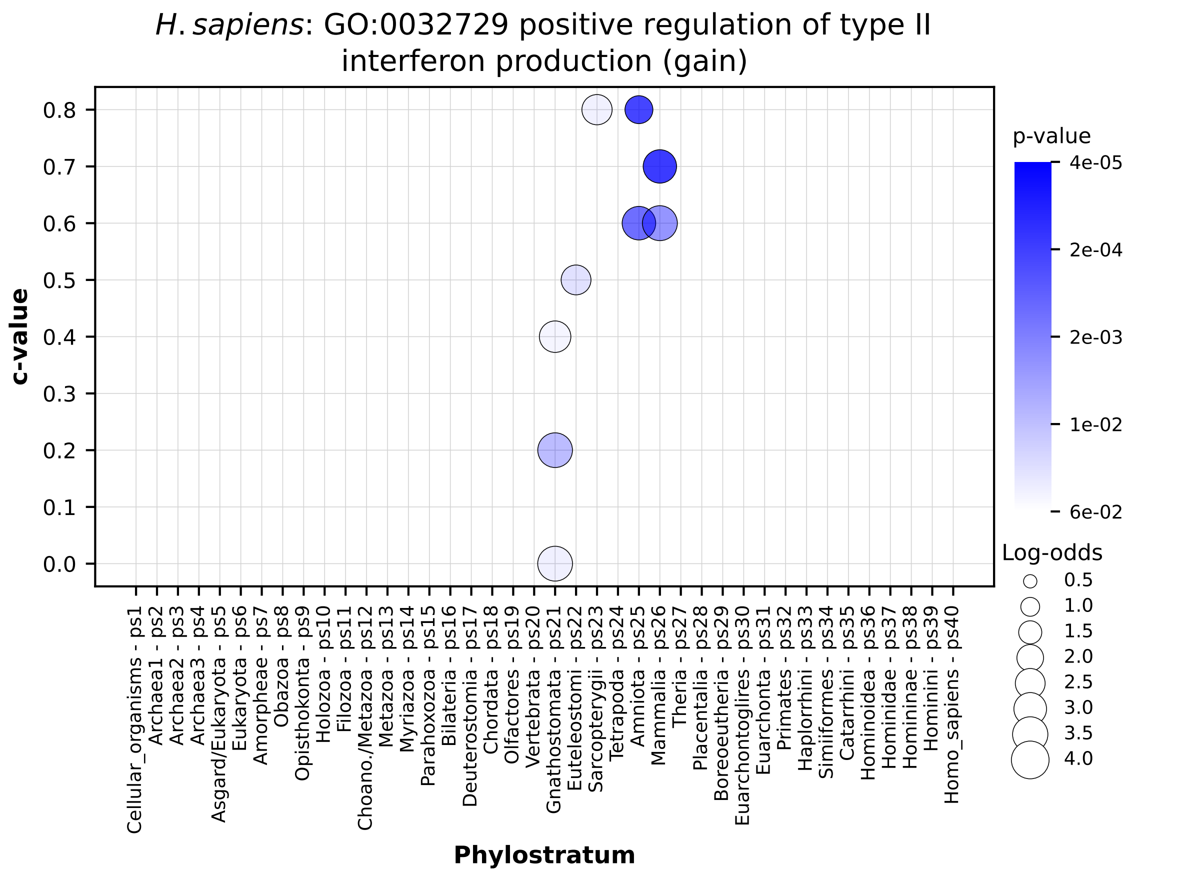

H. sapiens: GO:0032729 positive regulation of type II interferon production (gain)
c-value
0.8
0.7
0.6
0.5
0.4
0.3
0.2
0.1
0.0
p-value
4e-05
2e-04
2e-03
1e-02
6e-02
Log-odds
0.5
1.0
1.5
2.0
2.5
3.0
3.5
4.0
Cellular_organisms - ps1
Archaea1 - ps2
Archaea2 - ps3
Archaea3 - ps4
Asgard/Eukaryota - ps5
Eukaryota - ps6
Amorpheae - ps7
Obazoa - ps8
Opisthokonta - ps9
Holozoa - ps10
Filozoa - ps11
Choano./Metazoa - ps12
Metazoa - ps13
Myriazoa - ps14
Parahoxozoa - ps15
Bilateria - ps16
Deuterostomia - ps17
Chordata - ps18
Olfactores - ps19
Vertebrata - ps20
Gnathostomata - ps21
Euteleostomi - ps22
Sarcopterygii - ps23
Tetrapoda - ps24
Amniota - ps25
Mammalia - ps26
Theria - ps27
Placentalia - ps28
Boreoeutheria - ps29
Euarchontoglires - ps30
Euarchonta - ps31
Primates - ps32
Haplorrhini - ps33
Simiiformes - ps34
Catarrhini - ps35
Hominoidea - ps36
Hominidae - ps37
Homininae - ps38
Hominini - ps39
Homo_sapiens - ps40
Phylostratum

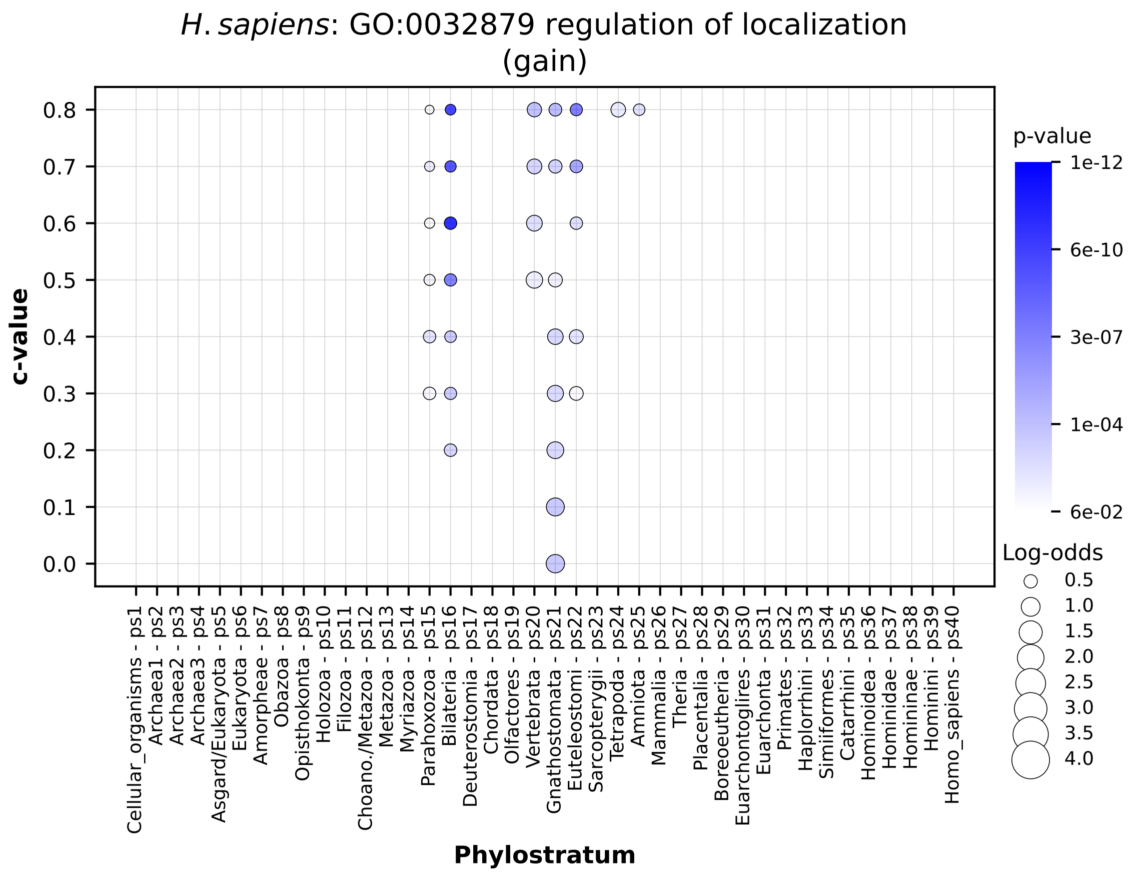

*H. sapiens*: GO:0032879 regulation of localization (gain)
c-value
0.8
0.7
0.6
0.5
0.4
0.3
0.2
0.1
0.0
Phylostratum
Cellular_organisms - ps1
Archaea1 - ps2
Archaea2 - ps3
Archaea3 - ps4
Asgard/Eukaryota - ps5
Eukaryota - ps6
Amorpheae - ps7
Obazoa - ps8
Opisthokonta - ps9
Holozoa - ps10
Filozoa - ps11
Choano./Metazoa - ps12
Metazoa - ps13
Myriazoa - ps14
Parahoxozoa - ps15
Bilateria - ps16
Deuterostomia - ps17
Chordata - ps18
Olfactores - ps19
Vertebrata - ps20
Gnathostomata - ps21
Euteleostomi - ps22
Sarcopterygii - ps23
Tetrapoda - ps24
Amniota - ps25
Mammalia - ps26
Theria - ps27
Placentalia - ps28
Boreoeutheria - ps29
Euarchontoglires - ps30
Euarchonta - ps31
Primates - ps32
Haplorrhini - ps33
Simiiformes - ps34
Catarrhini - ps35
Hominoidea - ps36
Hominidae - ps37
Homininae - ps38
Hominini - ps39
Homo_sapiens - ps40
p-value
1e-12
6e-10
3e-07
1e-04
6e-02
Log-odds
0.5
1.0
1.5
2.0
2.5
3.0
3.5
4.0

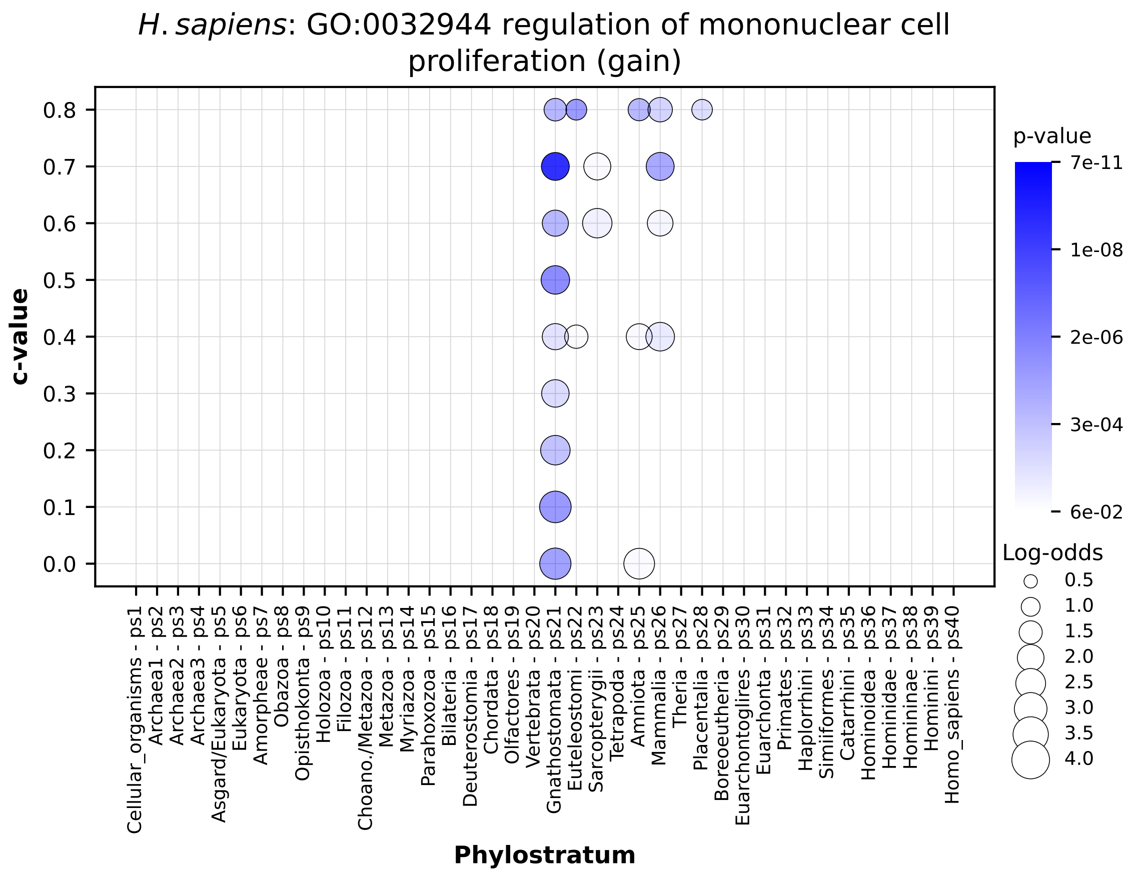

H. sapiens: GO:0032944 regulation of mononuclear cell proliferation (gain)
c-value
0.8
0.7
0.6
0.5
0.4
0.3
0.2
0.1
0.0
Phylostratum
Cellular_organisms - ps1
Archaea1 - ps2
Archaea2 - ps3
Archaea3 - ps4
Asgard/Eukaryota - ps5
Eukaryota - ps6
Amorpheae - ps7
Obazoa - ps8
Opisthokonta - ps9
Holozoa - ps10
Filozoa - ps11
Choano./Metazoa - ps12
Metazoa - ps13
Myriazoa - ps14
Parahoxozoa - ps15
Bilateria - ps16
Deuterostomia - ps17
Chordata - ps18
Olfactores - ps19
Vertebrata - ps20
Gnathostomata - ps21
Euteleostomi - ps22
Sarcopterygii - ps23
Tetrapoda - ps24
Amniota - ps25
Mammalia - ps26
Theria - ps27
Placentalia - ps28
Boreoeutheria - ps29
Euarchontoglires - ps30
Euarchonta - ps31
Primates - ps32
Haplorrhini - ps33
Simiiformes - ps34
Catarrhini - ps35
Hominoidea - ps36
Hominidae - ps37
Homininae - ps38
Hominini - ps39
Homo_sapiens - ps40
p-value
7e-11
1e-08
2e-06
3e-04
6e-02
Log-odds
0.5
1.0
1.5
2.0
2.5
3.0
3.5
4.0

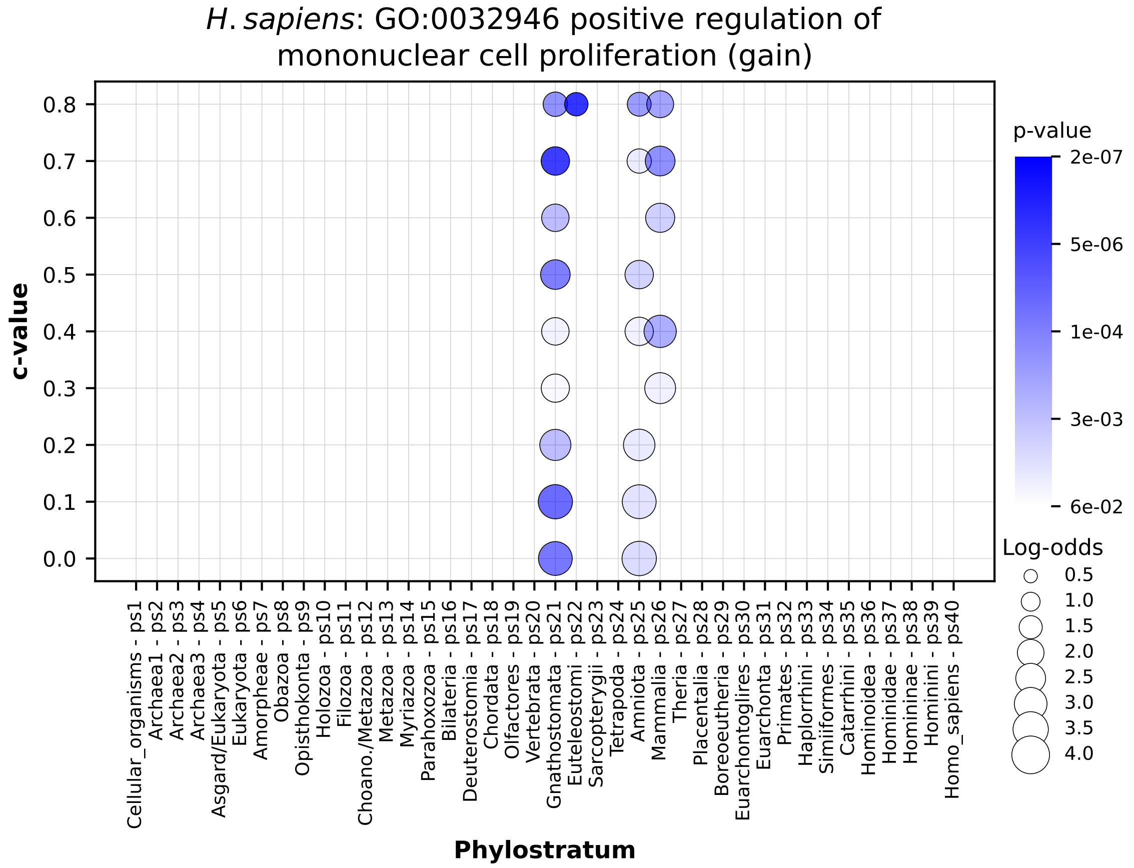

H. sapiens: GO:0032946 positive regulation of mononuclear cell proliferation (gain)
c-value
0.8
0.7
0.6
0.5
0.4
0.3
0.2
0.1
0.0
Phylostratum
Cellular_organisms - ps1
Archaea1 - ps2
Archaea2 - ps3
Archaea3 - ps4
Asgard/Eukaryota - ps5
Eukaryota - ps6
Amorpheae - ps7
Obazoa - ps8
Opisthokonta - ps9
Holozoa - ps10
Filozoa - ps11
Choano./Metazoa - ps12
Metazoa - ps13
Myriazoa - ps14
Parahoxozoa - ps15
Bilateria - ps16
Deuterostomia - ps17
Chordata - ps18
Olfactores - ps19
Vertebrata - ps20
Gnathostomata - ps21
Euteleostomi - ps22
Sarcopterygii - ps23
Tetrapoda - ps24
Amniota - ps25
Mammalia - ps26
Theria - ps27
Placentalia - ps28
Boreoeutheria - ps29
Euarchontoglires - ps30
Euarchonta - ps31
Primates - ps32
Haplorrhini - ps33
Simiiformes - ps34
Catarrhini - ps35
Hominoidea - ps36
Hominidae - ps37
Homininae - ps38
Hominini - ps39
Homo_sapiens - ps40
p-value
2e-07
5e-06
1e-04
3e-03
6e-02
Log-odds
0.5
1.0
1.5
2.0
2.5
3.0
3.5
4.0

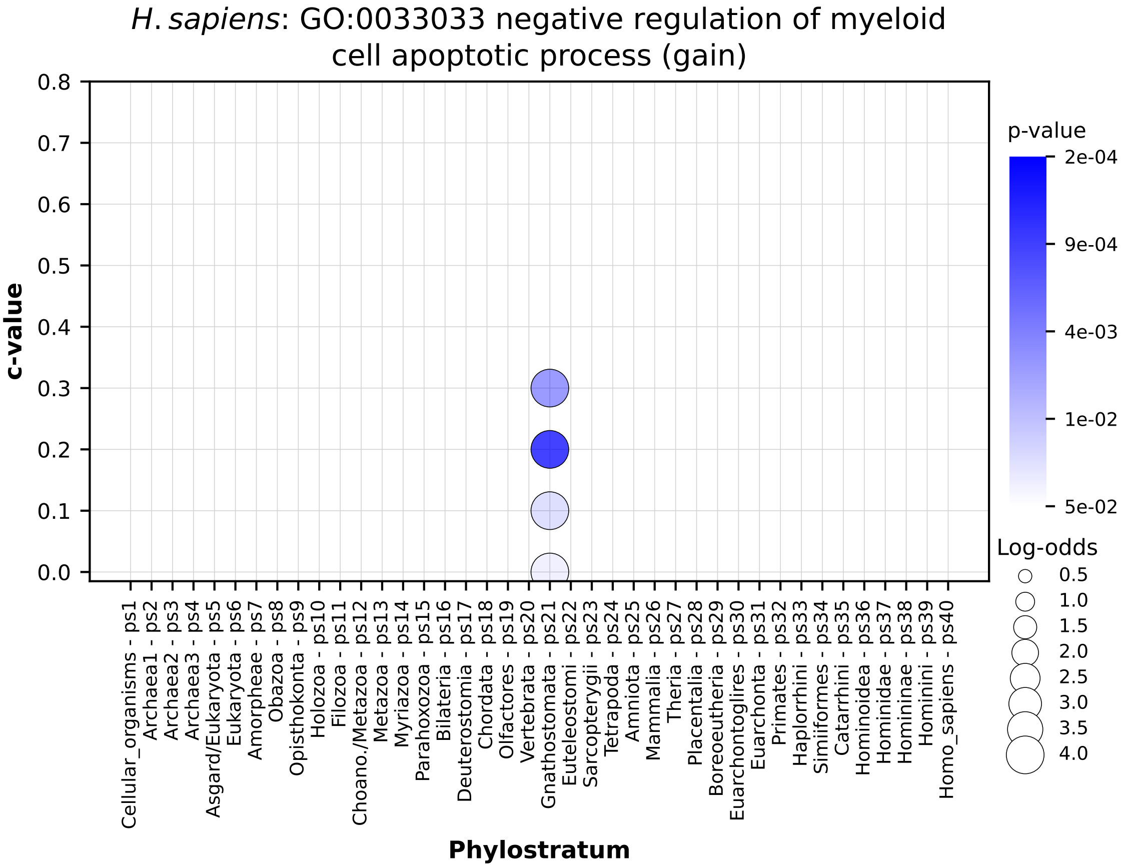
H. sapiens: GO:0033033 negative regulation of myeloid cell apoptotic process (gain)
c-value
0.8
0.7
0.6
0.5
0.4
0.3
0.2
0.1
0.0
Phylostratum
Cellular_organisms - ps1
Archaea1 - ps2
Archaea2 - ps3
Archaea3 - ps4
Asgard/Eukaryota - ps5
Eukaryota - ps6
Amorpheae - ps7
Obazoa - ps8
Opisthokonta - ps9
Holozoa - ps10
Filozoa - ps11
Choano./Metazoa - ps12
Metazoa - ps13
Myriazoa - ps14
Parahoxozoa - ps15
Bilateria - ps16
Deuterostomia - ps17
Chordata - ps18
Olfactores - ps19
Vertebrata - ps20
Gnathostomata - ps21
Euteleostomi - ps22
Sarcopterygii - ps23
Tetrapoda - ps24
Amniota - ps25
Mammalia - ps26
Theria - ps27
Placentalia - ps28
Boreoeutheria - ps29
Euarchontoglires - ps30
Euarchonta - ps31
Primates - ps32
Haplorrhini - ps33
Simiiformes - ps34
Catarrhini - ps35
Hominoidea - ps36
Hominidae - ps37
Homininae - ps38
Hominini - ps39
Homo_sapiens - ps40
p-value
2e-04
9e-04
4e-03
1e-02
5e-02
Log-odds
0.5
1.0
1.5
2.0
2.5
3.0
3.5
4.0

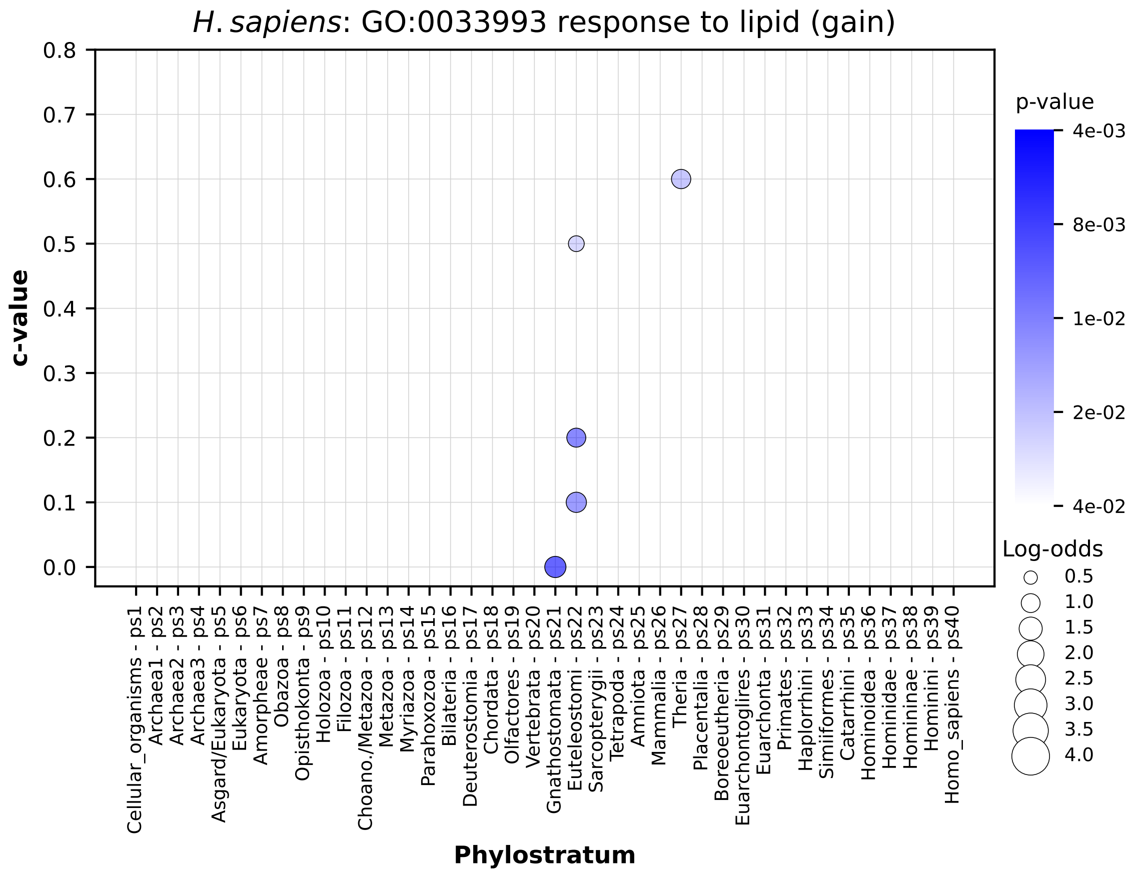

H. sapiens: GO:0033993 response to lipid (gain)
c-value
0.8
0.7
0.6
0.5
0.4
0.3
0.2
0.1
0.0
Phylostratum
Cellular_organisms - ps1
Archaea1 - ps2
Archaea2 - ps3
Archaea3 - ps4
Asgard/Eukaryota - ps5
Eukaryota - ps6
Amorpheae - ps7
Obazoa - ps8
Opisthokonta - ps9
Holozoa - ps10
Filozoa - ps11
Choano./Metazoa - ps12
Metazoa - ps13
Myriazoa - ps14
Parahoxozoa - ps15
Bilateria - ps16
Deuterostomia - ps17
Chordata - ps18
Olfactores - ps19
Vertebrata - ps20
Gnathostomata - ps21
Euteleostomi - ps22
Sarcopterygii - ps23
Tetrapoda - ps24
Amniota - ps25
Mammalia - ps26
Theria - ps27
Placentalia - ps28
Boreoeutheria - ps29
Euarchontoglires - ps30
Euarchonta - ps31
Primates - ps32
Haplorrhini - ps33
Simiiformes - ps34
Catarrhini - ps35
Hominoidea - ps36
Hominidae - ps37
Homininae - ps38
Hominini - ps39
Homo_sapiens - ps40
p-value
4e-03
8e-03
1e-02
2e-02
4e-02
Log-odds
0.5
1.0
1.5
2.0
2.5
3.0
3.5
4.0

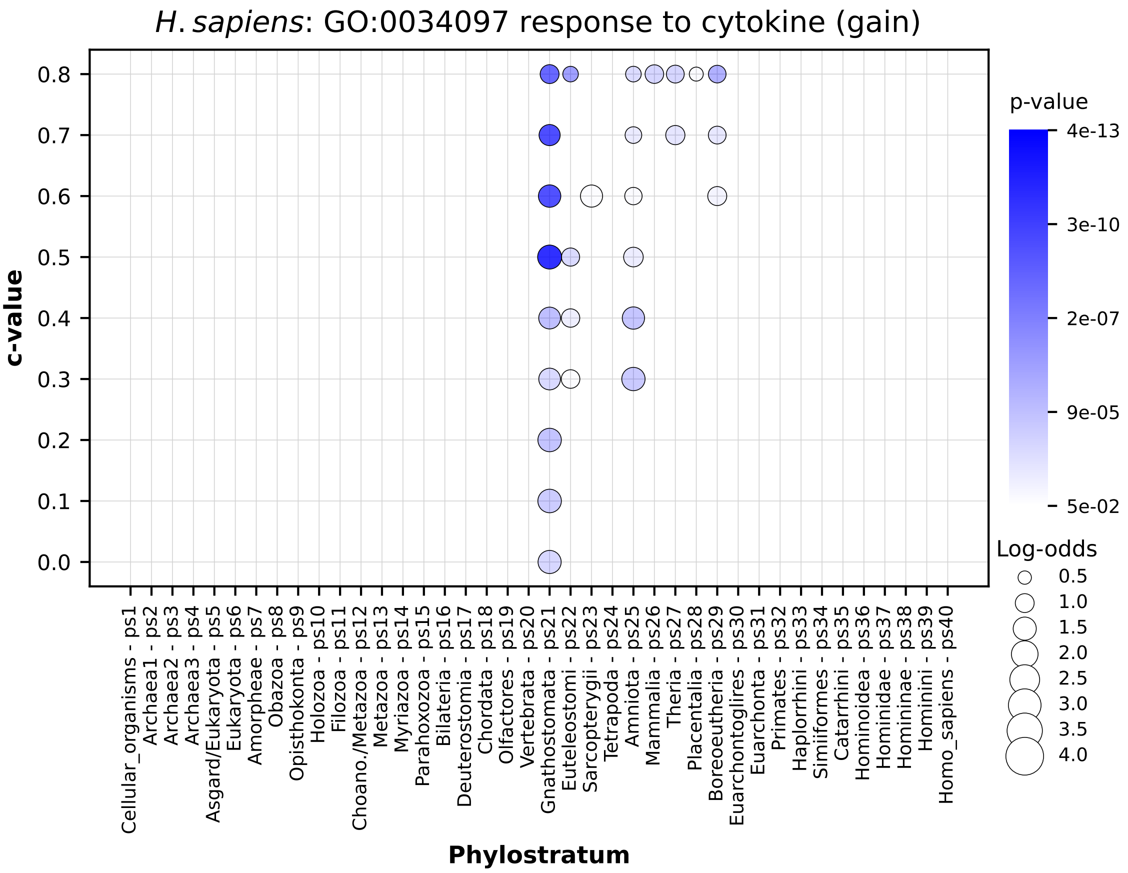
H. sapiens: GO:0034097 response to cytokine (gain)
c-value
0.8
0.7
0.6
0.5
0.4
0.3
0.2
0.1
0.0
Phylostratum
Cellular_organisms - ps1
Archaea1 - ps2
Archaea2 - ps3
Archaea3 - ps4
Asgard/Eukaryota - ps5
Eukaryota - ps6
Amorpheae - ps7
Obazoa - ps8
Opisthokonta - ps9
Holozoa - ps10
Filozoa - ps11
Choano./Metazoa - ps12
Metazoa - ps13
Myriazoa - ps14
Parahoxozoa - ps15
Bilateria - ps16
Deuterostomia - ps17
Chordata - ps18
Olfactores - ps19
Vertebrata - ps20
Gnathostomata - ps21
Euteleostomi - ps22
Sarcopterygii - ps23
Tetrapoda - ps24
Amniota - ps25
Mammalia - ps26
Theria - ps27
Placentalia - ps28
Boreoeutheria - ps29
Euarchontoglires - ps30
Euarchonta - ps31
Primates - ps32
Haplorrhini - ps33
Simiiformes - ps34
Catarrhini - ps35
Hominoidea - ps36
Hominidae - ps37
Homininae - ps38
Hominini - ps39
Homo_sapiens - ps40
p-value
4e-13
3e-10
2e-07
9e-05
5e-02
Log-odds
0.5
1.0
1.5
2.0
2.5
3.0
3.5
4.0

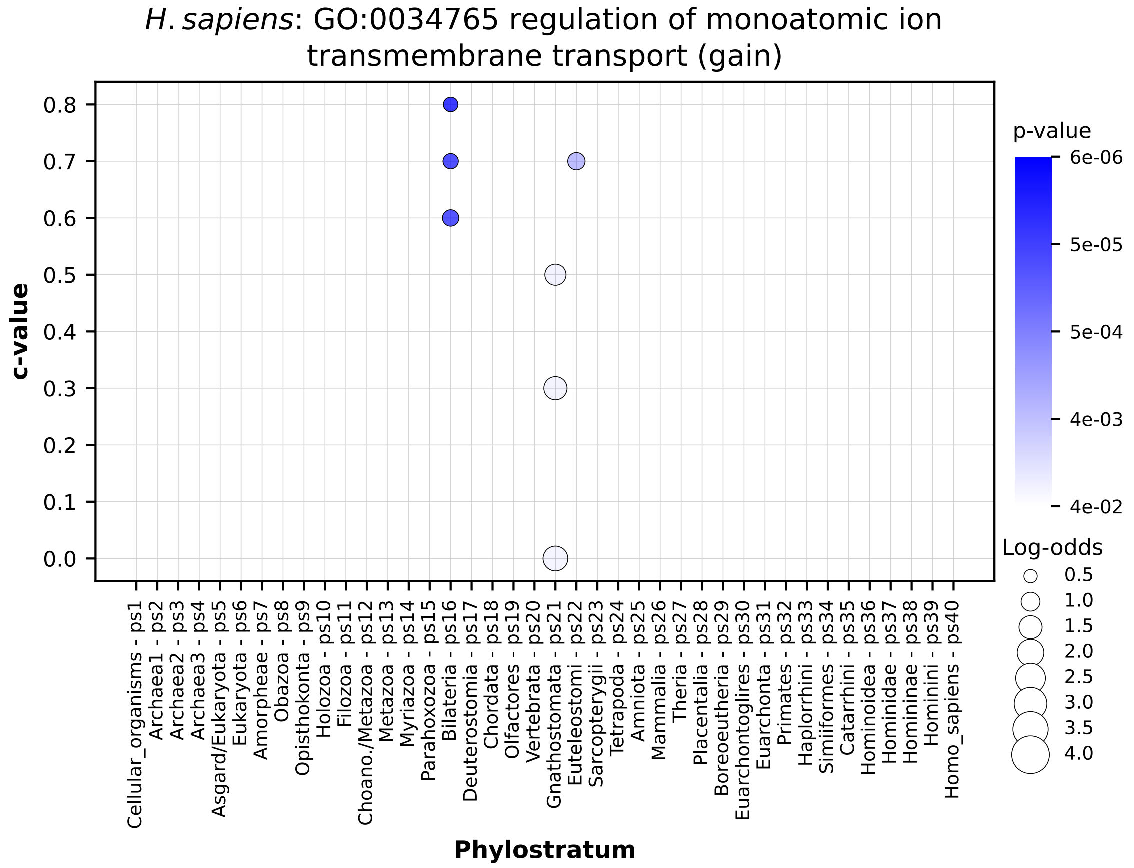

*H. sapiens*: GO:0034765 regulation of monoatomic ion transmembrane transport (gain)
c-value
0.8
0.7
0.6
0.5
0.4
0.3
0.2
0.1
0.0
Phylostratum
Cellular_organisms - ps1
Archaea1 - ps2
Archaea2 - ps3
Archaea3 - ps4
Asgard/Eukaryota - ps5
Eukaryota - ps6
Amorpheae - ps7
Obazoa - ps8
Opisthokonta - ps9
Holozoa - ps10
Filozoa - ps11
Choano./Metazoa - ps12
Metazoa - ps13
Myriazoa - ps14
Parahoxozoa - ps15
Bilateria - ps16
Deuterostomia - ps17
Chordata - ps18
Olfactores - ps19
Vertebrata - ps20
Gnathostomata - ps21
Euteleostomi - ps22
Sarcopterygii - ps23
Tetrapoda - ps24
Amniota - ps25
Mammalia - ps26
Theria - ps27
Placentalia - ps28
Boreoeutheria - ps29
Euarchontoglires - ps30
Euarchonta - ps31
Primates - ps32
Haplorrhini - ps33
Simiiformes - ps34
Catarrhini - ps35
Hominoidea - ps36
Hominidae - ps37
Homininae - ps38
Hominini - ps39
Homo_sapiens - ps40
p-value
6e-06
5e-05
5e-04
4e-03
4e-02
Log-odds
0.5
1.0
1.5
2.0
2.5
3.0
3.5
4.0

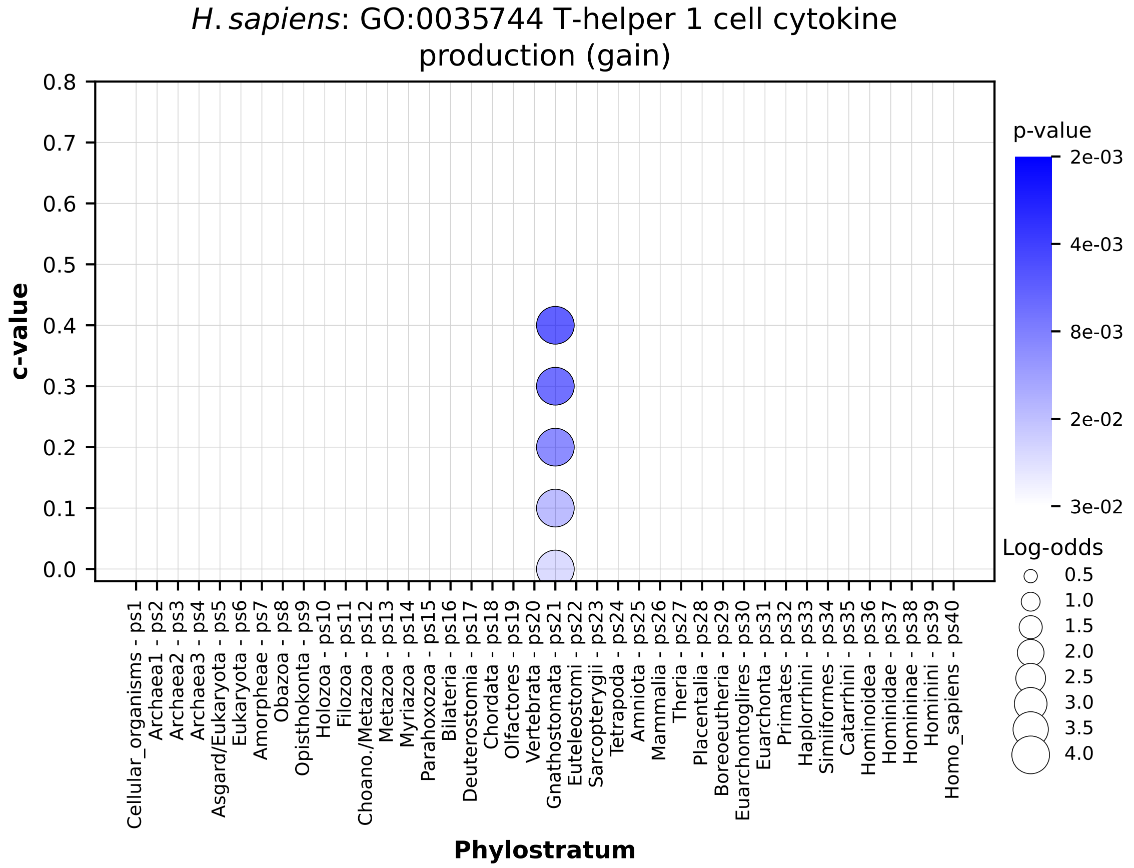

*H. sapiens*: GO:0035744 T-helper 1 cell cytokine production (gain)
c-value
0.8
0.7
0.6
0.5
0.4
0.3
0.2
0.1
0.0
p-value
2e-03
4e-03
8e-03
2e-02
3e-02
Log-odds
0.5
1.0
1.5
2.0
2.5
3.0
3.5
4.0
Cellular_organisms - ps1
Archaea1 - ps2
Archaea2 - ps3
Archaea3 - ps4
Asgard/Eukaryota - ps5
Eukaryota - ps6
Amorpheae - ps7
Obazoa - ps8
Opisthokonta - ps9
Holozoa - ps10
Filozoa - ps11
Choano./Metazoa - ps12
Metazoa - ps13
Myriazoa - ps14
Parahoxozoa - ps15
Bilateria - ps16
Deuterostomia - ps17
Chordata - ps18
Olfactores - ps19
Vertebrata - ps20
Gnathostomata - ps21
Euteleostomi - ps22
Sarcopterygii - ps23
Tetrapoda - ps24
Amniota - ps25
Mammalia - ps26
Theria - ps27
Placentalia - ps28
Boreoeutheria - ps29
Euarchontoglires - ps30
Euarchonta - ps31
Primates - ps32
Haplorrhini - ps33
Simiiformes - ps34
Catarrhini - ps35
Hominoidea - ps36
Hominidae - ps37
Homininae - ps38
Hominini - ps39
Homo_sapiens - ps40
Phylostratum

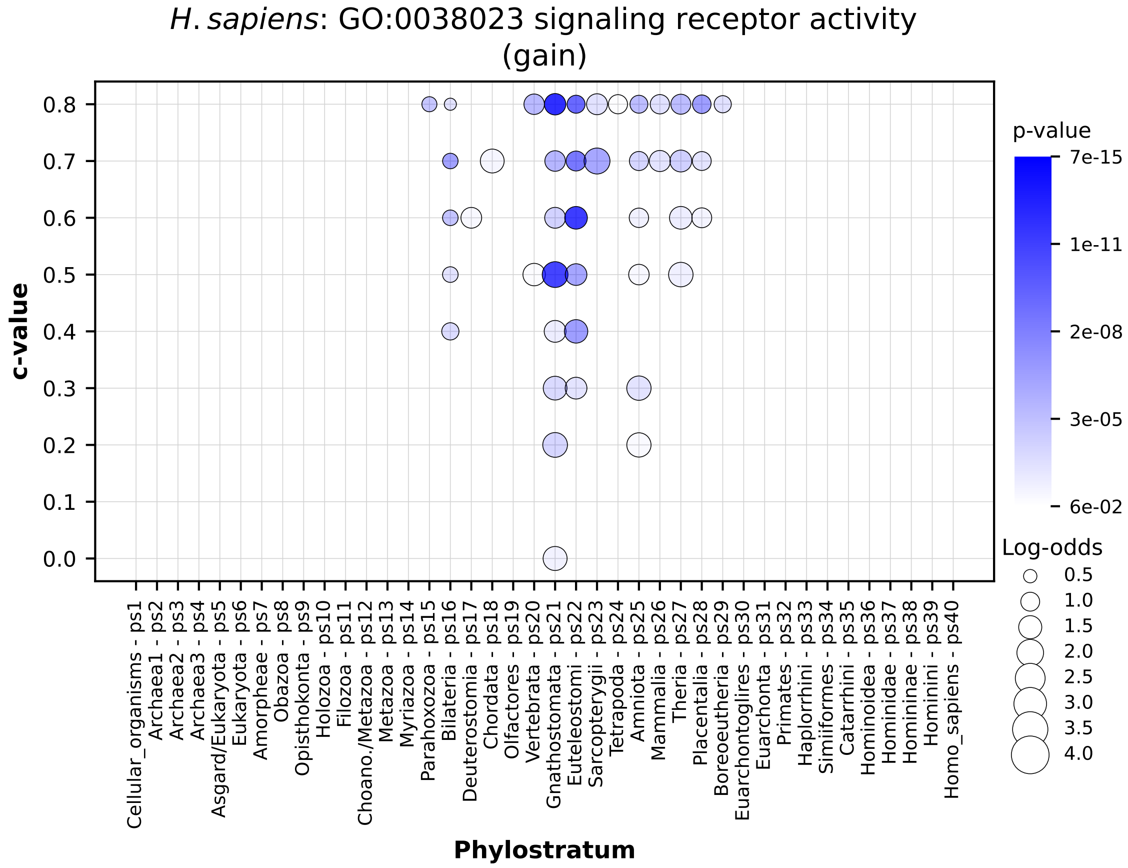

H. sapiens: GO:0038023 signaling receptor activity
(gain)
c-value
0.8
0.7
0.6
0.5
0.4
0.3
0.2
0.1
0.0
Phylostratum
Cellular_organisms - ps1
Archaea1 - ps2
Archaea2 - ps3
Archaea3 - ps4
Asgard/Eukaryota - ps5
Eukaryota - ps6
Amorpheae - ps7
Obazoa - ps8
Opisthokonta - ps9
Holozoa - ps10
Filozoa - ps11
Choano./Metazoa - ps12
Metazoa - ps13
Myriazoa - ps14
Parahoxozoa - ps15
Bilateria - ps16
Deuterostomia - ps17
Chordata - ps18
Olfactores - ps19
Vertebrata - ps20
Gnathostomata - ps21
Euteleostomi - ps22
Sarcopterygii - ps23
Tetrapoda - ps24
Amniota - ps25
Mammalia - ps26
Theria - ps27
Placentalia - ps28
Boreoeutheria - ps29
Euarchontoglires - ps30
Euarchonta - ps31
Primates - ps32
Haplorrhini - ps33
Simiiformes - ps34
Catarrhini - ps35
Hominoidea - ps36
Hominidae - ps37
Homininae - ps38
Hominini - ps39
Homo_sapiens - ps40
p-value
7e-15
1e-11
2e-08
3e-05
6e-02
Log-odds
0.5
1.0
1.5
2.0
2.5
3.0
3.5
4.0

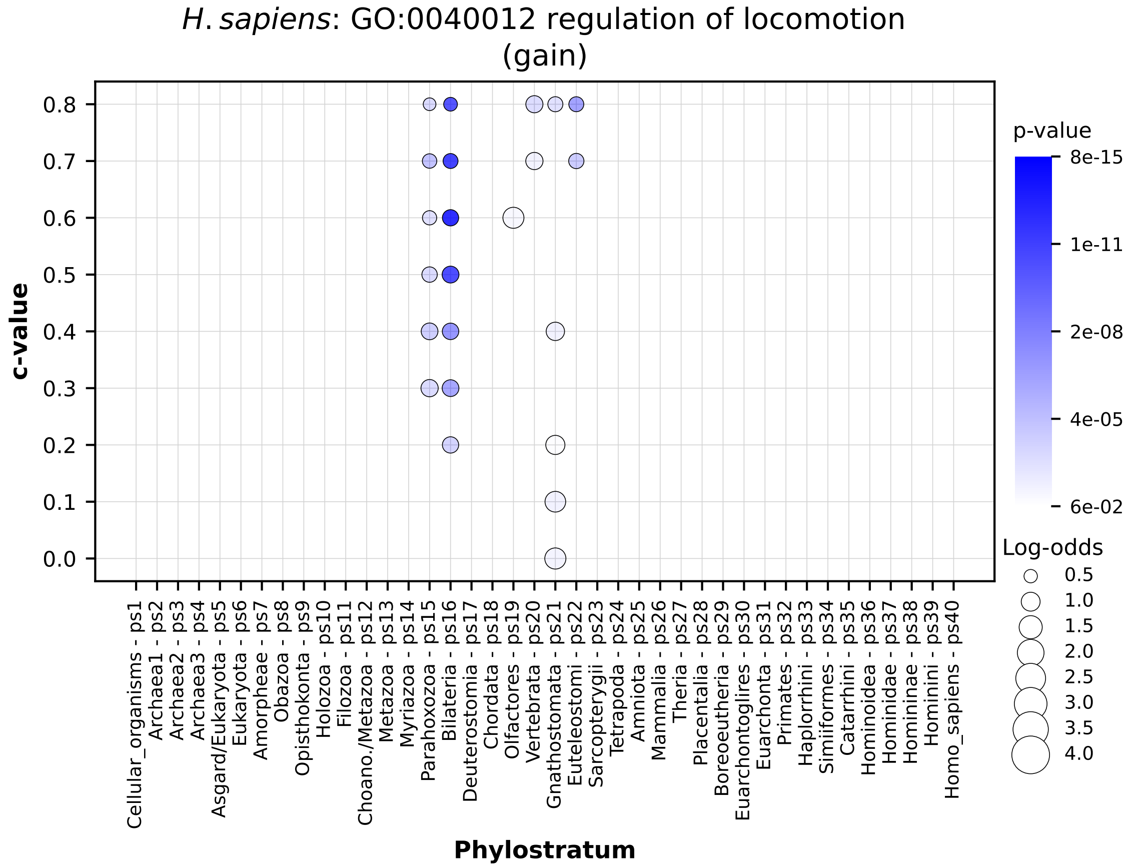

*H. sapiens*: GO:0040012 regulation of locomotion
(gain)
c-value
0.8
0.7
0.6
0.5
0.4
0.3
0.2
0.1
0.0
p-value
8e-15
1e-11
2e-08
4e-05
6e-02
Log-odds
0.5
1.0
1.5
2.0
2.5
3.0
3.5
4.0
Cellular_organisms - ps1
Archaea1 - ps2
Archaea2 - ps3
Archaea3 - ps4
Asgard/Eukaryota - ps5
Eukaryota - ps6
Amorpheae - ps7
Obazoa - ps8
Opisthokonta - ps9
Holozoa - ps10
Filozoa - ps11
Choano./Metazoa - ps12
Metazoa - ps13
Myriazoa - ps14
Parahoxozoa - ps15
Bilateria - ps16
Deuterostomia - ps17
Chordata - ps18
Olfactores - ps19
Vertebrata - ps20
Gnathostomata - ps21
Euteleostomi - ps22
Sarcopterygii - ps23
Tetrapoda - ps24
Amniota - ps25
Mammalia - ps26
Theria - ps27
Placentalia - ps28
Boreoeutheria - ps29
Euarchontoglires - ps30
Euarchonta - ps31
Primates - ps32
Haplorrhini - ps33
Simiiformes - ps34
Catarrhini - ps35
Hominoidea - ps36
Hominidae - ps37
Homininae - ps38
Hominini - ps39
Homo_sapiens - ps40
Phylostratum

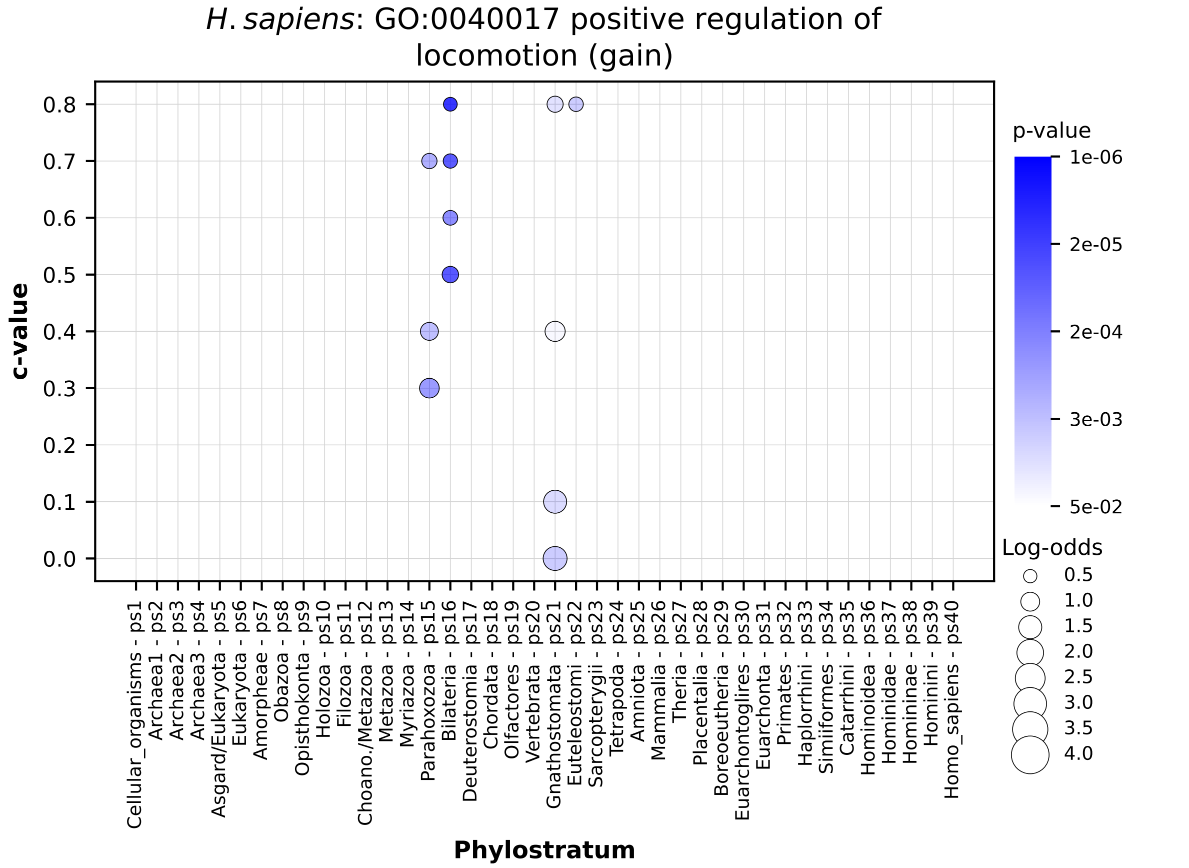
H. sapiens: GO:0040017 positive regulation of locomotion (gain)
c-value
0.8
0.7
0.6
0.5
0.4
0.3
0.2
0.1
0.0
Phylostratum
Cellular_organisms - ps1
Archaea1 - ps2
Archaea2 - ps3
Archaea3 - ps4
Asgard/Eukaryota - ps5
Eukaryota - ps6
Amorpheae - ps7
Obazoa - ps8
Opisthokonta - ps9
Holozoa - ps10
Filozoa - ps11
Choano./Metazoa - ps12
Metazoa - ps13
Myriazoa - ps14
Parahoxozoa - ps15
Bilateria - ps16
Deuterostomia - ps17
Chordata - ps18
Olfactores - ps19
Vertebrata - ps20
Gnathostomata - ps21
Euteleostomi - ps22
Sarcopterygii - ps23
Tetrapoda - ps24
Amniota - ps25
Mammalia - ps26
Theria - ps27
Placentalia - ps28
Boreoeutheria - ps29
Euarchontoglires - ps30
Euarchonta - ps31
Primates - ps32
Haplorrhini - ps33
Simiiformes - ps34
Catarrhini - ps35
Hominoidea - ps36
Hominidae - ps37
Homininae - ps38
Hominini - ps39
Homo_sapiens - ps40
p-value
1e-06
2e-05
2e-04
3e-03
5e-02
Log-odds
0.5
1.0
1.5
2.0
2.5
3.0
3.5
4.0

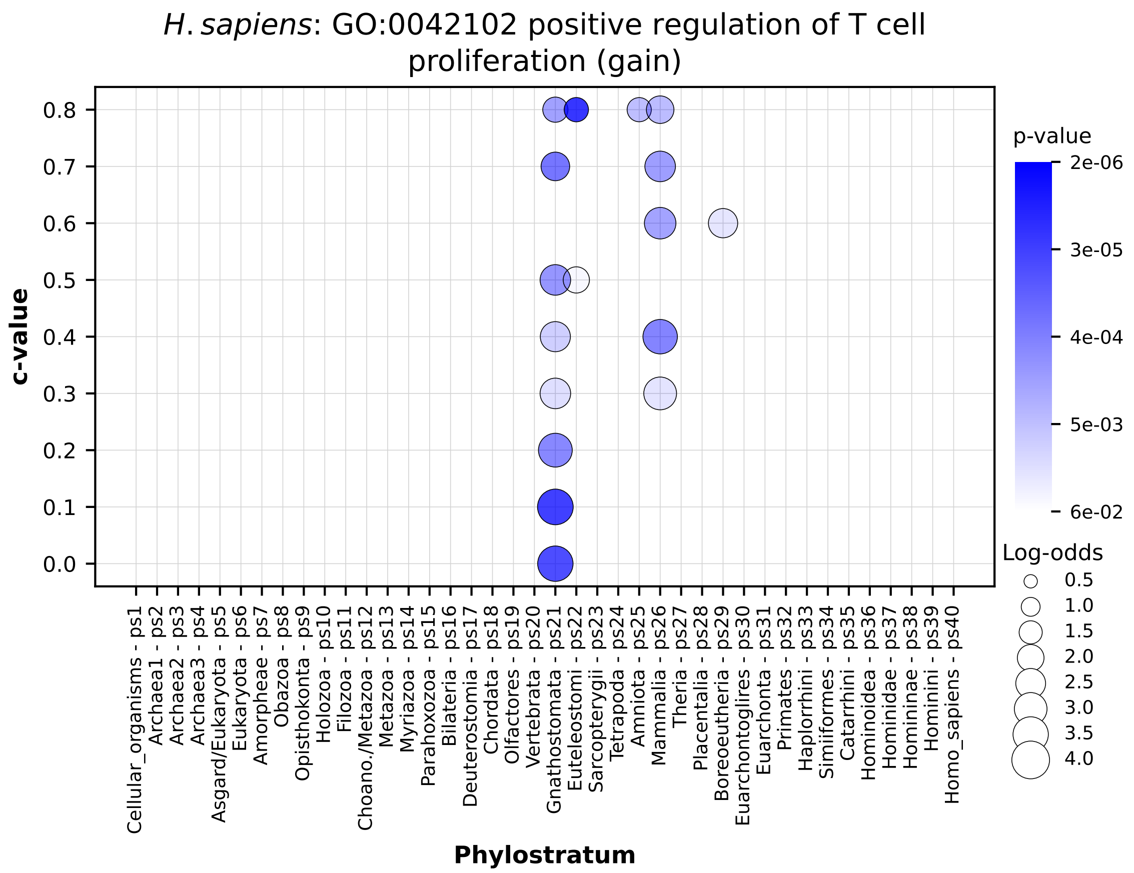

H. sapiens: GO:0042102 positive regulation of T cell proliferation (gain)
c-value
0.8
0.7
0.6
0.5
0.4
0.3
0.2
0.1
0.0
p-value
2e-06
3e-05
4e-04
5e-03
6e-02
Log-odds
0.5
1.0
1.5
2.0
2.5
3.0
3.5
4.0
Cellular_organisms - ps1
Archaea1 - ps2
Archaea2 - ps3
Archaea3 - ps4
Asgard/Eukaryota - ps5
Eukaryota - ps6
Amorpheae - ps7
Obazoa - ps8
Opisthokonta - ps9
Holozoa - ps10
Filozoa - ps11
Choano./Metazoa - ps12
Metazoa - ps13
Myriazoa - ps14
Parahoxozoa - ps15
Bilateria - ps16
Deuterostomia - ps17
Chordata - ps18
Olfactores - ps19
Vertebrata - ps20
Gnathostomata - ps21
Euteleostomi - ps22
Sarcopterygii - ps23
Tetrapoda - ps24
Amniota - ps25
Mammalia - ps26
Theria - ps27
Placentalia - ps28
Boreoeutheria - ps29
Euarchontoglires - ps30
Euarchonta - ps31
Primates - ps32
Haplorrhini - ps33
Simiiformes - ps34
Catarrhini - ps35
Hominoidea - ps36
Hominidae - ps37
Homininae - ps38
Hominini - ps39
Homo_sapiens - ps40
Phylostratum

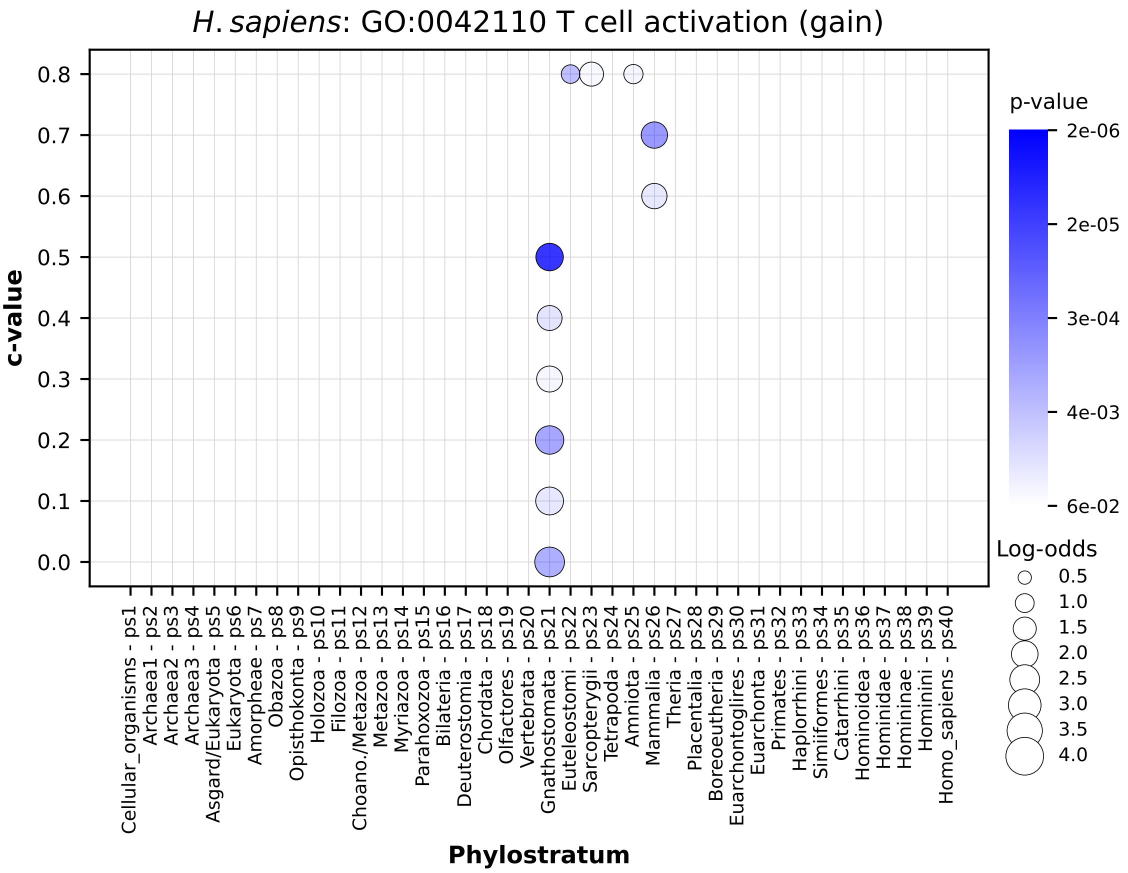

*H. sapiens*: GO:0042110 T cell activation (gain)
c-value
0.8
0.7
0.6
0.5
0.4
0.3
0.2
0.1
0.0
Phylostratum
Cellular_organisms - ps1
Archaea1 - ps2
Archaea2 - ps3
Archaea3 - ps4
Asgard/Eukaryota - ps5
Eukaryota - ps6
Amorpheae - ps7
Obazoa - ps8
Opisthokonta - ps9
Holozoa - ps10
Filozoa - ps11
Choano./Metazoa - ps12
Metazoa - ps13
Myriazoa - ps14
Parahoxozoa - ps15
Bilateria - ps16
Deuterostomia - ps17
Chordata - ps18
Olfactores - ps19
Vertebrata - ps20
Gnathostomata - ps21
Euteleostomi - ps22
Sarcopterygii - ps23
Tetrapoda - ps24
Amniota - ps25
Mammalia - ps26
Theria - ps27
Placentalia - ps28
Boreoeutheria - ps29
Euarchontoglires - ps30
Euarchonta - ps31
Primates - ps32
Haplorrhini - ps33
Simiiformes - ps34
Catarrhini - ps35
Hominoidea - ps36
Hominidae - ps37
Homininae - ps38
Hominini - ps39
Homo_sapiens - ps40
p-value
2e-06
2e-05
3e-04
4e-03
6e-02
Log-odds
0.5
1.0
1.5
2.0
2.5
3.0
3.5
4.0

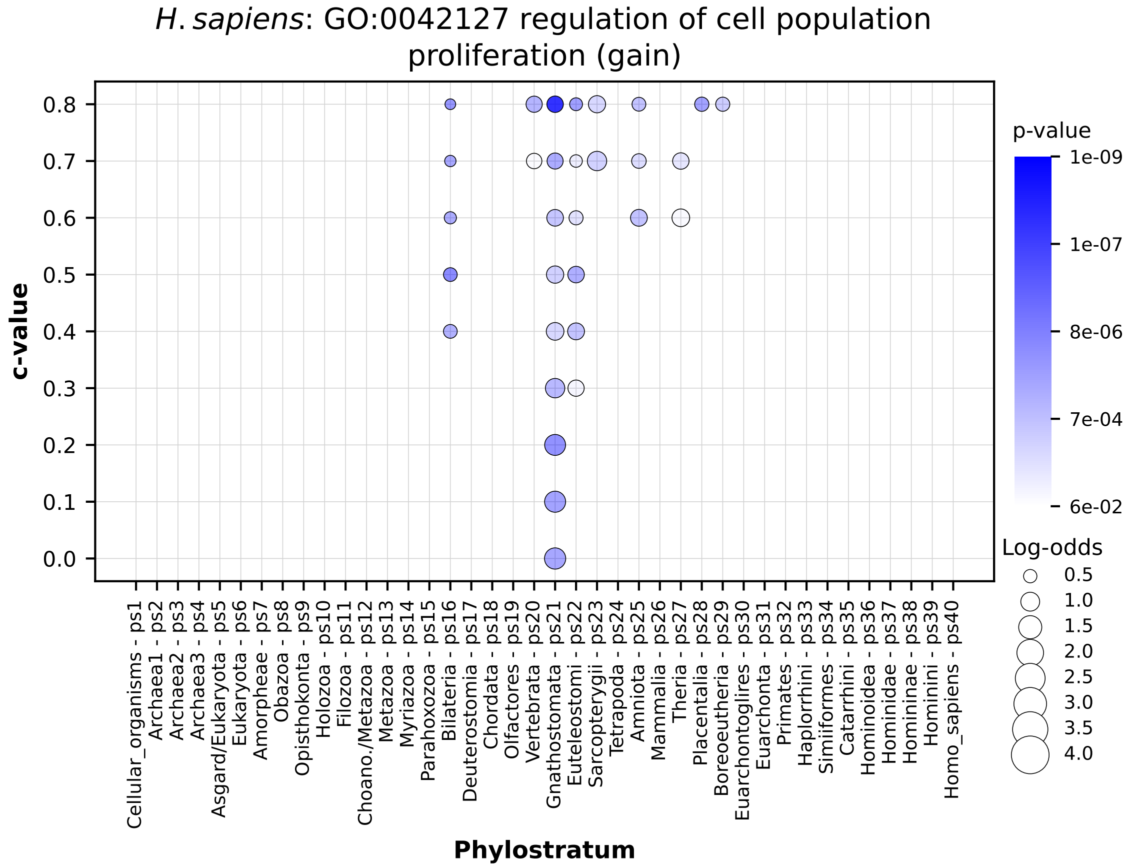

*H. sapiens*: GO:0042127 regulation of cell population proliferation (gain)
c-value
0.8
0.7
0.6
0.5
0.4
0.3
0.2
0.1
0.0
p-value
1e-09
1e-07
8e-06
7e-04
6e-02
Log-odds
0.5
1.0
1.5
2.0
2.5
3.0
3.5
4.0
Cellular_organisms - ps1
Archaea1 - ps2
Archaea2 - ps3
Archaea3 - ps4
Asgard/Eukaryota - ps5
Eukaryota - ps6
Amorpheae - ps7
Obazoa - ps8
Opisthokonta - ps9
Holozoa - ps10
Filozoa - ps11
Choano./Metazoa - ps12
Metazoa - ps13
Myriazoa - ps14
Parahoxozoa - ps15
Bilateria - ps16
Deuterostomia - ps17
Chordata - ps18
Olfactores - ps19
Vertebrata - ps20
Gnathostomata - ps21
Euteleostomi - ps22
Sarcopterygii - ps23
Tetrapoda - ps24
Amniota - ps25
Mammalia - ps26
Theria - ps27
Placentalia - ps28
Boreoeutheria - ps29
Euarchontoglires - ps30
Euarchonta - ps31
Primates - ps32
Haplorrhini - ps33
Simiiformes - ps34
Catarrhini - ps35
Hominoidea - ps36
Hominidae - ps37
Homininae - ps38
Hominini - ps39
Homo_sapiens - ps40
Phylostratum

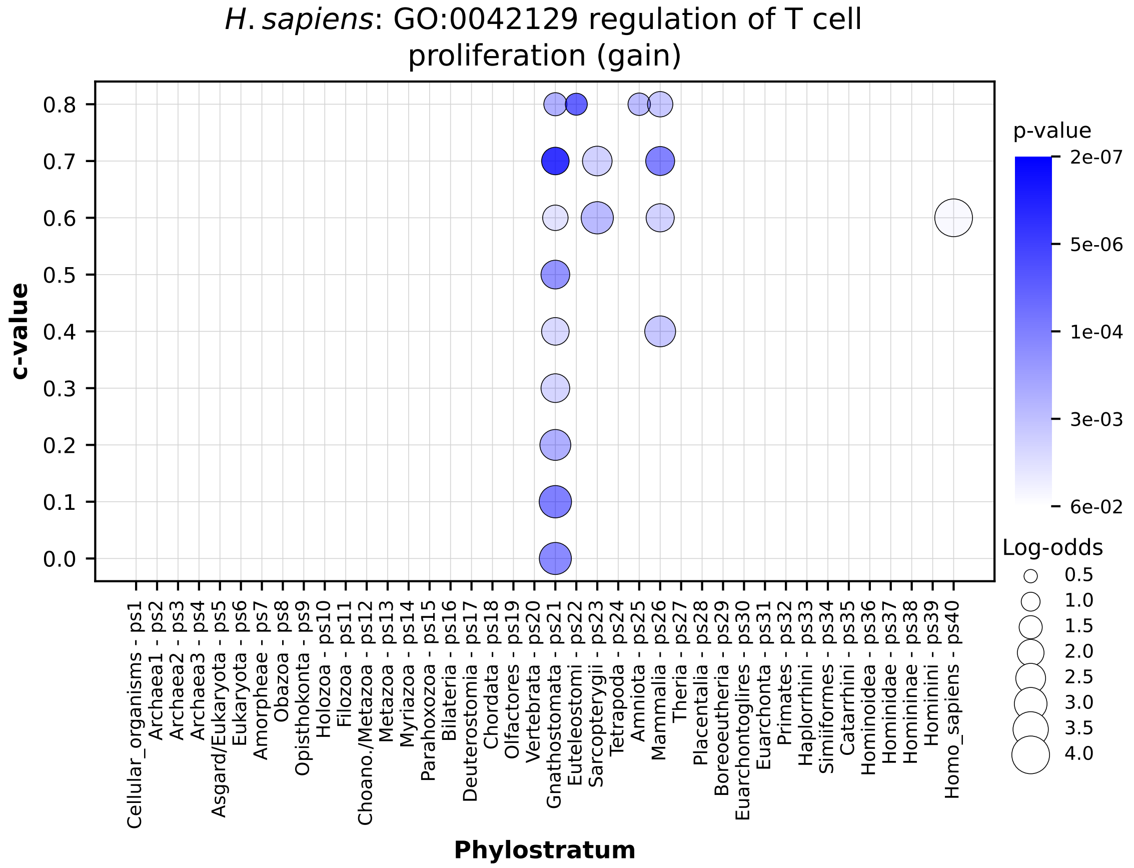

*H. sapiens*: GO:0042129 regulation of T cell proliferation (gain)
c-value
0.8
0.7
0.6
0.5
0.4
0.3
0.2
0.1
0.0
Phylostratum
Cellular_organisms - ps1
Archaea1 - ps2
Archaea2 - ps3
Archaea3 - ps4
Asgard/Eukaryota - ps5
Eukaryota - ps6
Amorpheae - ps7
Obazoa - ps8
Opisthokonta - ps9
Holozoa - ps10
Filozoa - ps11
Choano./Metazoa - ps12
Metazoa - ps13
Myriazoa - ps14
Parahoxozoa - ps15
Bilateria - ps16
Deuterostomia - ps17
Chordata - ps18
Olfactores - ps19
Vertebrata - ps20
Gnathostomata - ps21
Euteleostomi - ps22
Sarcopterygii - ps23
Tetrapoda - ps24
Amniota - ps25
Mammalia - ps26
Theria - ps27
Placentalia - ps28
Boreoeutheria - ps29
Euarchontoglires - ps30
Euarchonta - ps31
Primates - ps32
Haplorrhini - ps33
Simiiformes - ps34
Catarrhini - ps35
Hominoidea - ps36
Hominidae - ps37
Homininae - ps38
Hominini - ps39
Homo_sapiens - ps40
p-value
2e-07
5e-06
1e-04
3e-03
6e-02
Log-odds
0.5
1.0
1.5
2.0
2.5
3.0
3.5
4.0

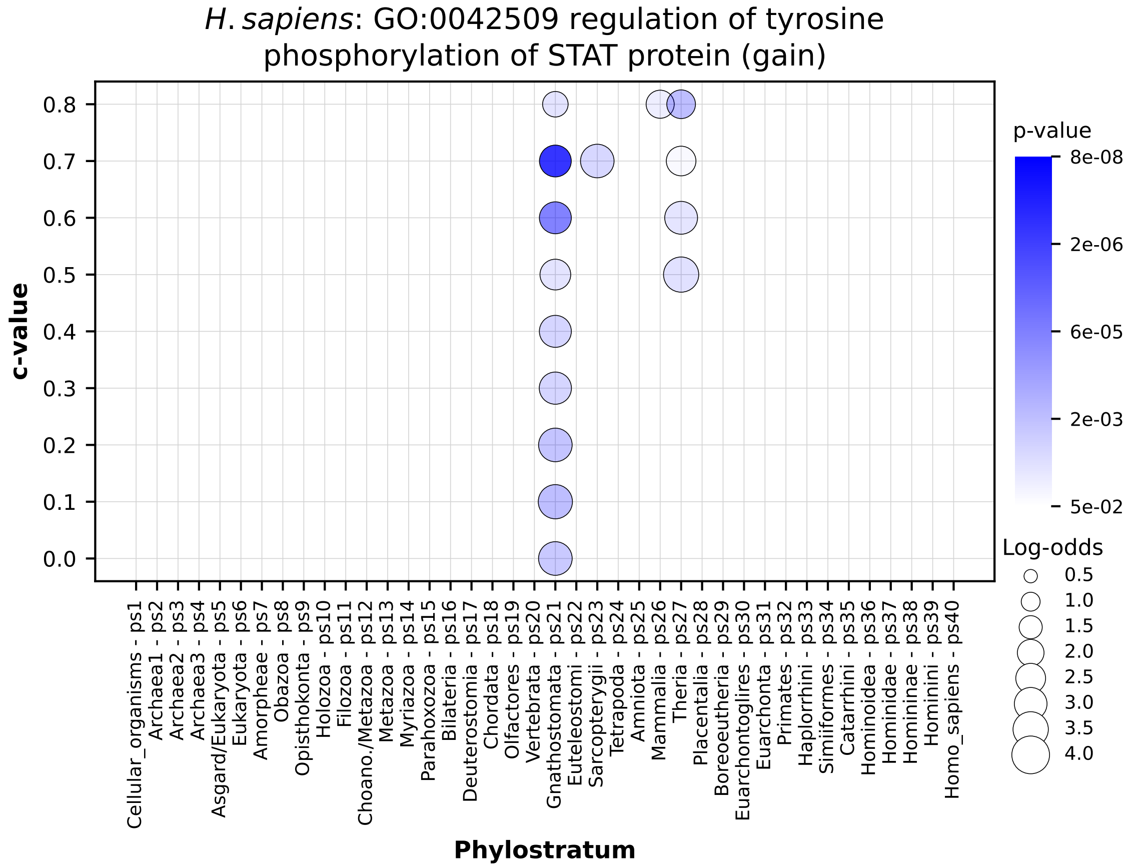

H. sapiens: GO:0042509 regulation of tyrosine phosphorylation of STAT protein (gain)
c-value
0.8
0.7
0.6
0.5
0.4
0.3
0.2
0.1
0.0
p-value
8e-08
2e-06
6e-05
2e-03
5e-02
Log-odds
0.5
1.0
1.5
2.0
2.5
3.0
3.5
4.0
Cellular_organisms - ps1
Archaea1 - ps2
Archaea2 - ps3
Archaea3 - ps4
Asgard/Eukaryota - ps5
Eukaryota - ps6
Amorpheae - ps7
Obazoa - ps8
Opisthokonta - ps9
Holozoa - ps10
Filozoa - ps11
Choano./Metazoa - ps12
Metazoa - ps13
Myriazoa - ps14
Parahoxozoa - ps15
Bilateria - ps16
Deuterostomia - ps17
Chordata - ps18
Olfactores - ps19
Vertebrata - ps20
Gnathostomata - ps21
Euteleostomi - ps22
Sarcopterygii - ps23
Tetrapoda - ps24
Amniota - ps25
Mammalia - ps26
Theria - ps27
Placentalia - ps28
Boreoeutheria - ps29
Euarchontoglires - ps30
Euarchonta - ps31
Primates - ps32
Haplorrhini - ps33
Simiiformes - ps34
Catarrhini - ps35
Hominoidea - ps36
Hominidae - ps37
Homininae - ps38
Hominini - ps39
Homo_sapiens - ps40
Phylostratum

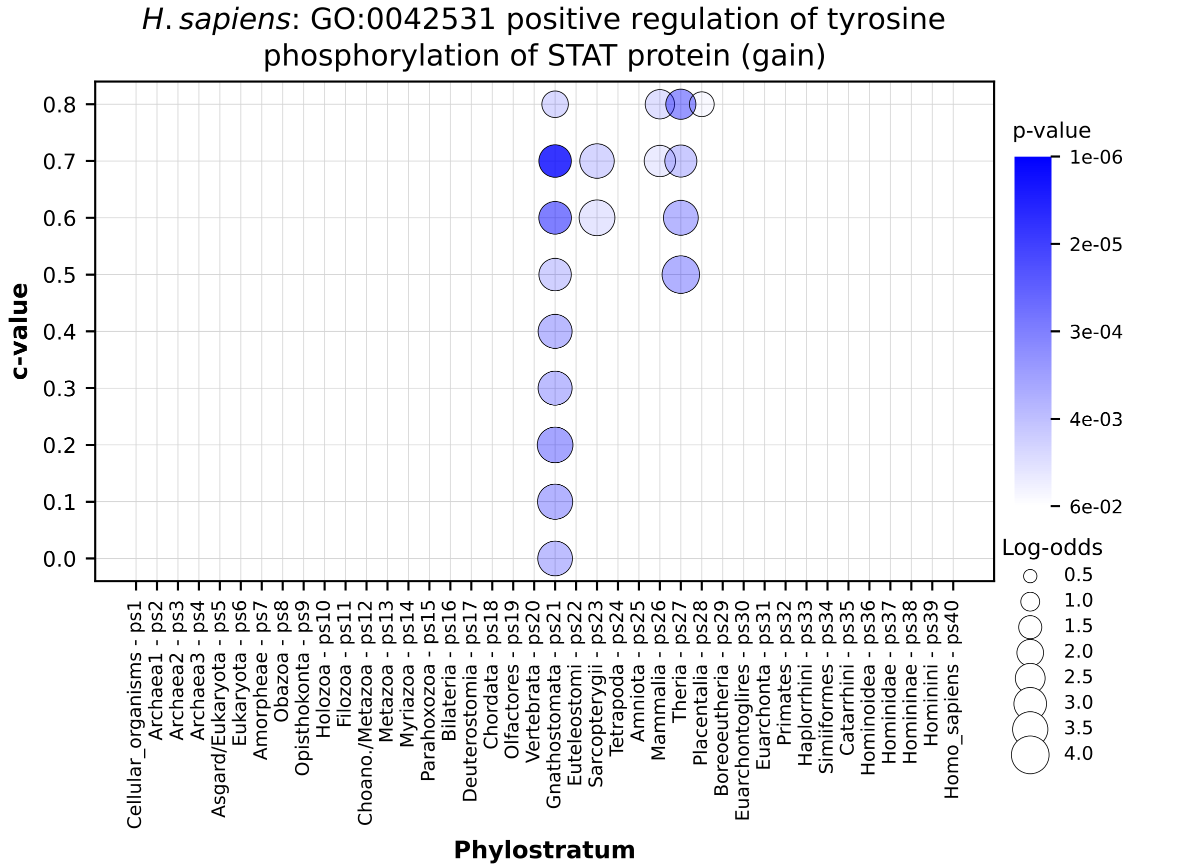

H. sapiens: GO:0042531 positive regulation of tyrosine phosphorylation of STAT protein (gain)
c-value
0.0
0.1
0.2
0.3
0.4
0.5
0.6
0.7
0.8
p-value
1e-06
2e-05
3e-04
4e-03
6e-02
Log-odds
0.5
1.0
1.5
2.0
2.5
3.0
3.5
4.0
Cellular_organisms - ps1
Archaea1 - ps2
Archaea2 - ps3
Archaea3 - ps4
Asgard/Eukaryota - ps5
Eukaryota - ps6
Amorpheae - ps7
Obazoa - ps8
Opisthokonta - ps9
Holozoa - ps10
Filozoa - ps11
Choano./Metazoa - ps12
Metazoa - ps13
Myriazoa - ps14
Parahoxozoa - ps15
Bilateria - ps16
Deuterostomia - ps17
Chordata - ps18
Olfactores - ps19
Vertebrata - ps20
Gnathostomata - ps21
Euteleostomi - ps22
Sarcopterygii - ps23
Tetrapoda - ps24
Amniota - ps25
Mammalia - ps26
Theria - ps27
Placentalia - ps28
Boreoeutheria - ps29
Euarchontoglires - ps30
Euarchonta - ps31
Primates - ps32
Haplorrhini - ps33
Simiiformes - ps34
Catarrhini - ps35
Hominoidea - ps36
Hominidae - ps37
Homininae - ps38
Hominini - ps39
Homo_sapiens - ps40
Phylostratum

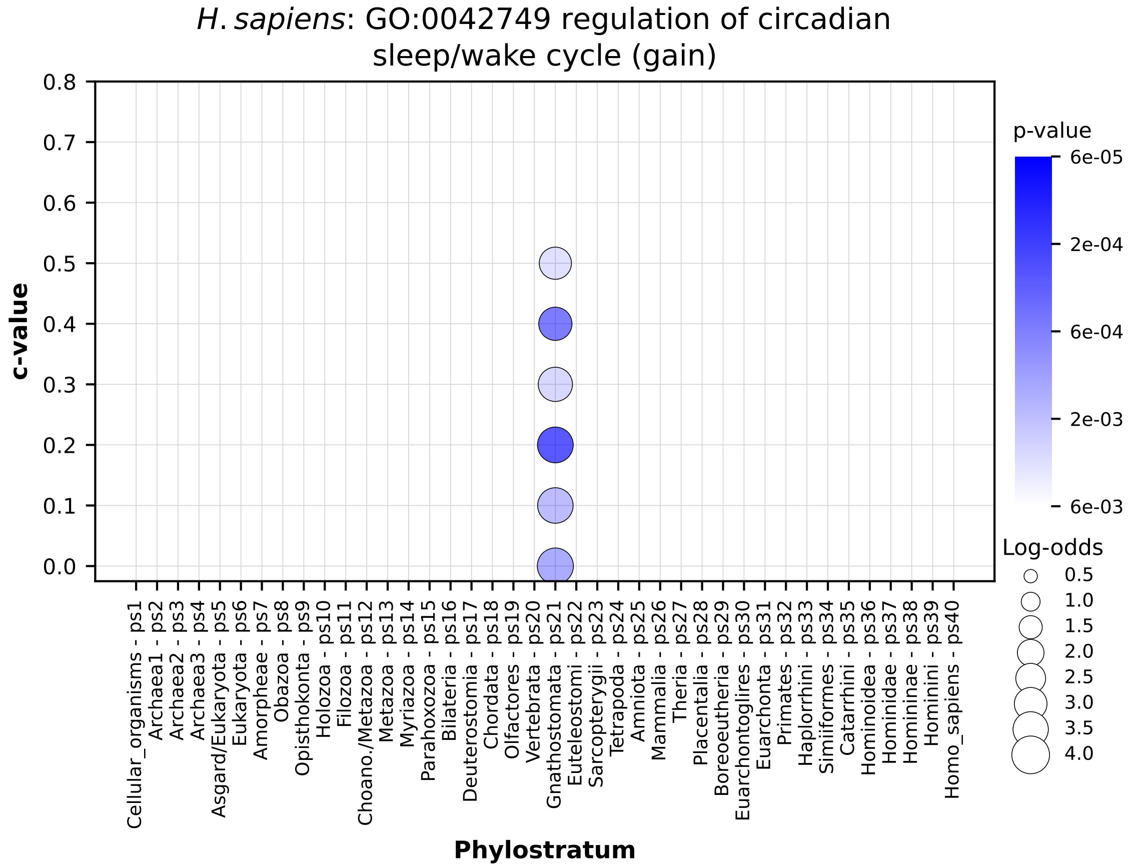
H. sapiens: GO:0042749 regulation of circadian sleep/wake cycle (gain)
c-value
0.0
0.1
0.2
0.3
0.4
0.5
0.6
0.7
0.8
Phylostratum
Cellular_organisms - ps1
Archaea1 - ps2
Archaea2 - ps3
Archaea3 - ps4
Asgard/Eukaryota - ps5
Eukaryota - ps6
Amorpheae - ps7
Obazoa - ps8
Opisthokonta - ps9
Holozoa - ps10
Filozoa - ps11
Choano./Metazoa - ps12
Metazoa - ps13
Myriazoa - ps14
Parahoxozoa - ps15
Bilateria - ps16
Deuterostomia - ps17
Chordata - ps18
Olfactores - ps19
Vertebrata - ps20
Gnathostomata - ps21
Euteleostomi - ps22
Sarcopterygii - ps23
Tetrapoda - ps24
Amniota - ps25
Mammalia - ps26
Theria - ps27
Placentalia - ps28
Boreoeutheria - ps29
Euarchontoglires - ps30
Euarchonta - ps31
Primates - ps32
Haplorrhini - ps33
Simiiformes - ps34
Catarrhini - ps35
Hominoidea - ps36
Hominidae - ps37
Homininae - ps38
Hominini - ps39
Homo_sapiens - ps40
p-value
6e-05
2e-04
6e-04
2e-03
6e-03
Log-odds
0.5
1.0
1.5
2.0
2.5
3.0
3.5
4.0

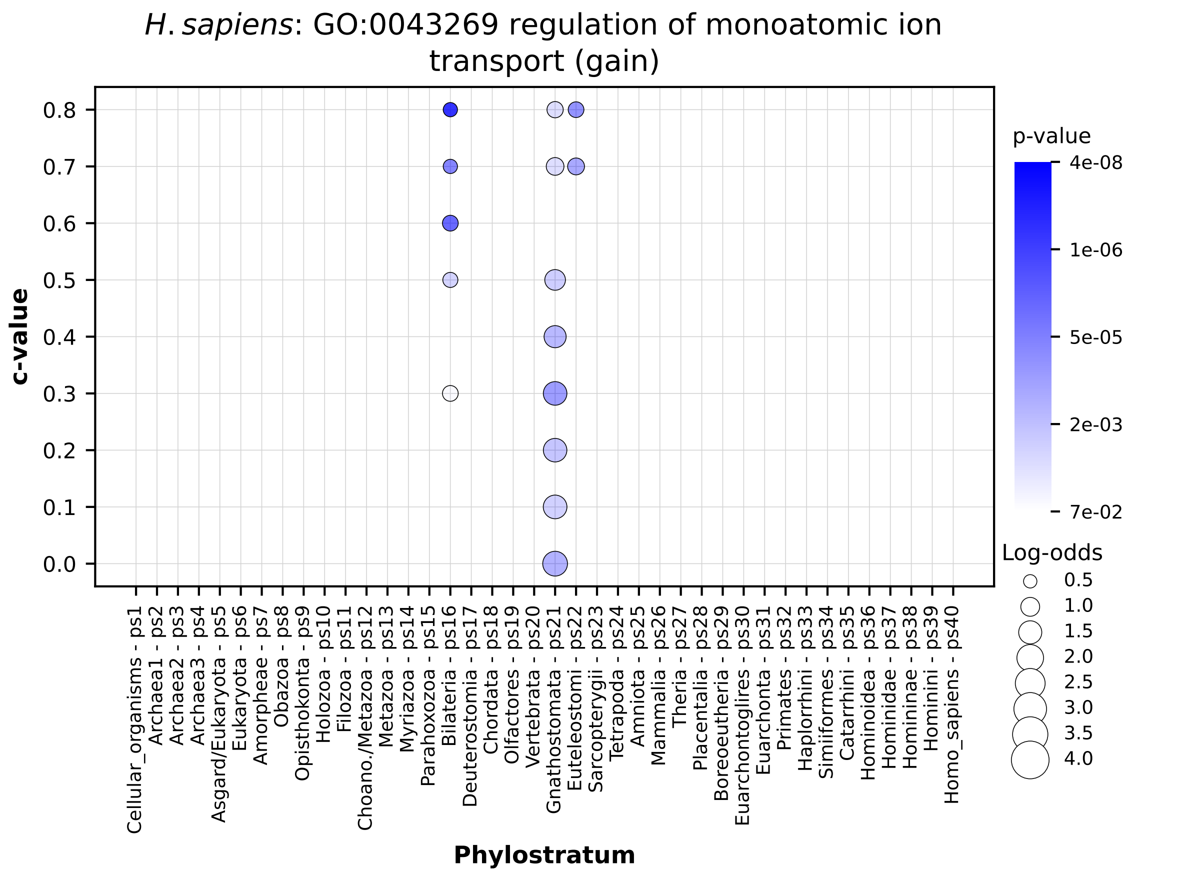

H. sapiens: GO:0043269 regulation of monoatomic ion transport (gain)
c-value
0.8
0.7
0.6
0.5
0.4
0.3
0.2
0.1
0.0
Phylostratum
Cellular_organisms - ps1
Archaea1 - ps2
Archaea2 - ps3
Archaea3 - ps4
Asgard/Eukaryota - ps5
Eukaryota - ps6
Amorpheae - ps7
Obazoa - ps8
Opisthokonta - ps9
Holozoa - ps10
Filozoa - ps11
Choano./Metazoa - ps12
Metazoa - ps13
Myriazoa - ps14
Parahoxozoa - ps15
Bilateria - ps16
Deuterostomia - ps17
Chordata - ps18
Olfactores - ps19
Vertebrata - ps20
Gnathostomata - ps21
Euteleostomi - ps22
Sarcopterygii - ps23
Tetrapoda - ps24
Amniota - ps25
Mammalia - ps26
Theria - ps27
Placentalia - ps28
Boreoeutheria - ps29
Euarchontoglires - ps30
Euarchonta - ps31
Primates - ps32
Haplorrhini - ps33
Simiiformes - ps34
Catarrhini - ps35
Hominoidea - ps36
Hominidae - ps37
Homininae - ps38
Hominini - ps39
Homo_sapiens - ps40
p-value
4e-08
1e-06
5e-05
2e-03
7e-02
Log-odds
0.5
1.0
1.5
2.0
2.5
3.0
3.5
4.0

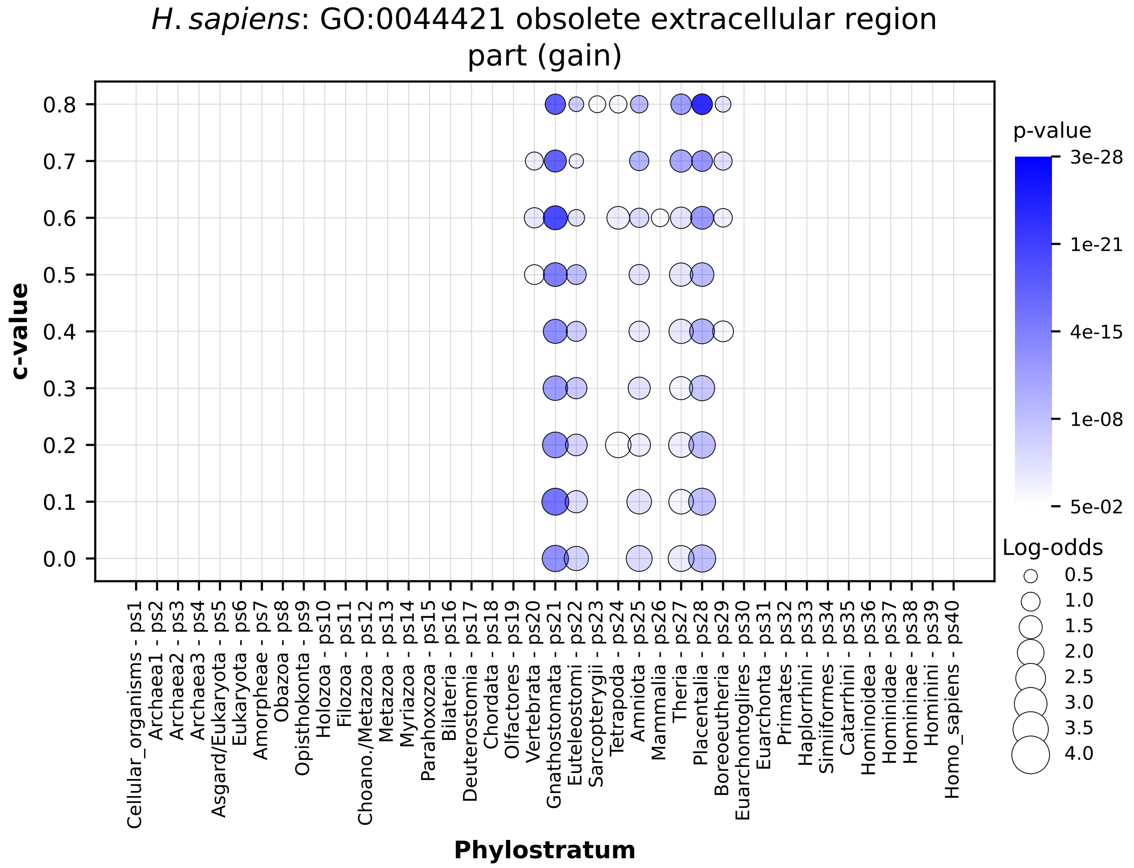
H. sapiens: GO:0044421 obsolete extracellular region part (gain)
c-value
0.8
0.7
0.6
0.5
0.4
0.3
0.2
0.1
0.0
p-value
3e-28
1e-21
4e-15
1e-08
5e-02
Log-odds
0.5
1.0
1.5
2.0
2.5
3.0
3.5
4.0
Cellular_organisms - ps1
Archaea1 - ps2
Archaea2 - ps3
Archaea3 - ps4
Asgard/Eukaryota - ps5
Eukaryota - ps6
Amorpheae - ps7
Obazoa - ps8
Opisthokonta - ps9
Holozoa - ps10
Filozoa - ps11
Choano./Metazoa - ps12
Metazoa - ps13
Myriazoa - ps14
Parahoxozoa - ps15
Bilateria - ps16
Deuterostomia - ps17
Chordata - ps18
Olfactores - ps19
Vertebrata - ps20
Gnathostomata - ps21
Euteleostomi - ps22
Sarcopterygii - ps23
Tetrapoda - ps24
Amniota - ps25
Mammalia - ps26
Theria - ps27
Placentalia - ps28
Boreoeutheria - ps29
Euarchontoglires - ps30
Euarchonta - ps31
Primates - ps32
Haplorrhini - ps33
Simiiformes - ps34
Catarrhini - ps35
Hominoidea - ps36
Hominidae - ps37
Homininae - ps38
Hominini - ps39
Homo_sapiens - ps40
Phylostratum

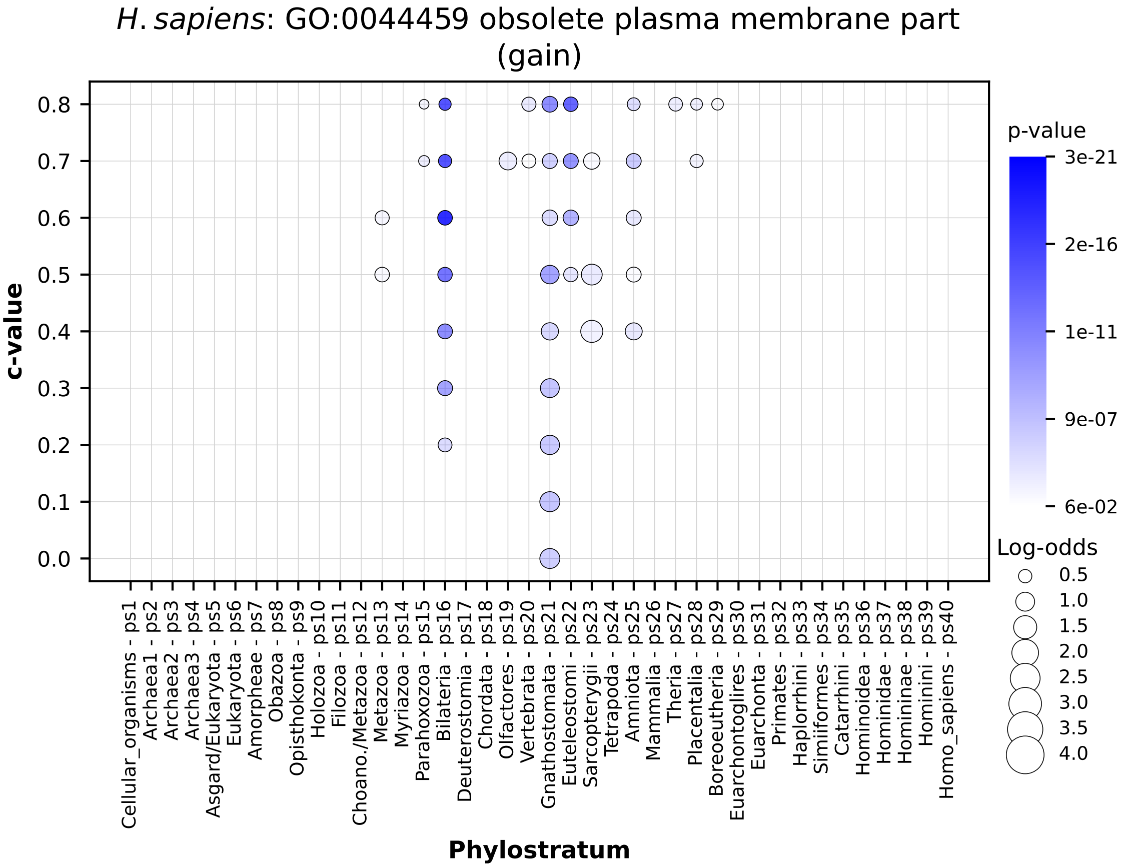

*H. sapiens*: GO:0044459 obsolete plasma membrane part
(gain)
c-value
0.8
0.7
0.6
0.5
0.4
0.3
0.2
0.1
0.0
Phylostratum
Cellular_organisms - ps1
Archaea1 - ps2
Archaea2 - ps3
Archaea3 - ps4
Asgard/Eukaryota - ps5
Eukaryota - ps6
Amorpheae - ps7
Obazoa - ps8
Opisthokonta - ps9
Holozoa - ps10
Filozoa - ps11
Choano./Metazoa - ps12
Metazoa - ps13
Myriazoa - ps14
Parahoxozoa - ps15
Bilateria - ps16
Deuterostomia - ps17
Chordata - ps18
Olfactores - ps19
Vertebrata - ps20
Gnathostomata - ps21
Euteleostomi - ps22
Sarcopterygii - ps23
Tetrapoda - ps24
Amniota - ps25
Mammalia - ps26
Theria - ps27
Placentalia - ps28
Boreoeutheria - ps29
Euarchontoglires - ps30
Euarchonta - ps31
Primates - ps32
Haplorrhini - ps33
Simiiformes - ps34
Catarrhini - ps35
Hominoidea - ps36
Hominidae - ps37
Homininae - ps38
Hominini - ps39
Homo_sapiens - ps40
p-value
3e-21
2e-16
1e-11
9e-07
6e-02
Log-odds
0.5
1.0
1.5
2.0
2.5
3.0
3.5
4.0

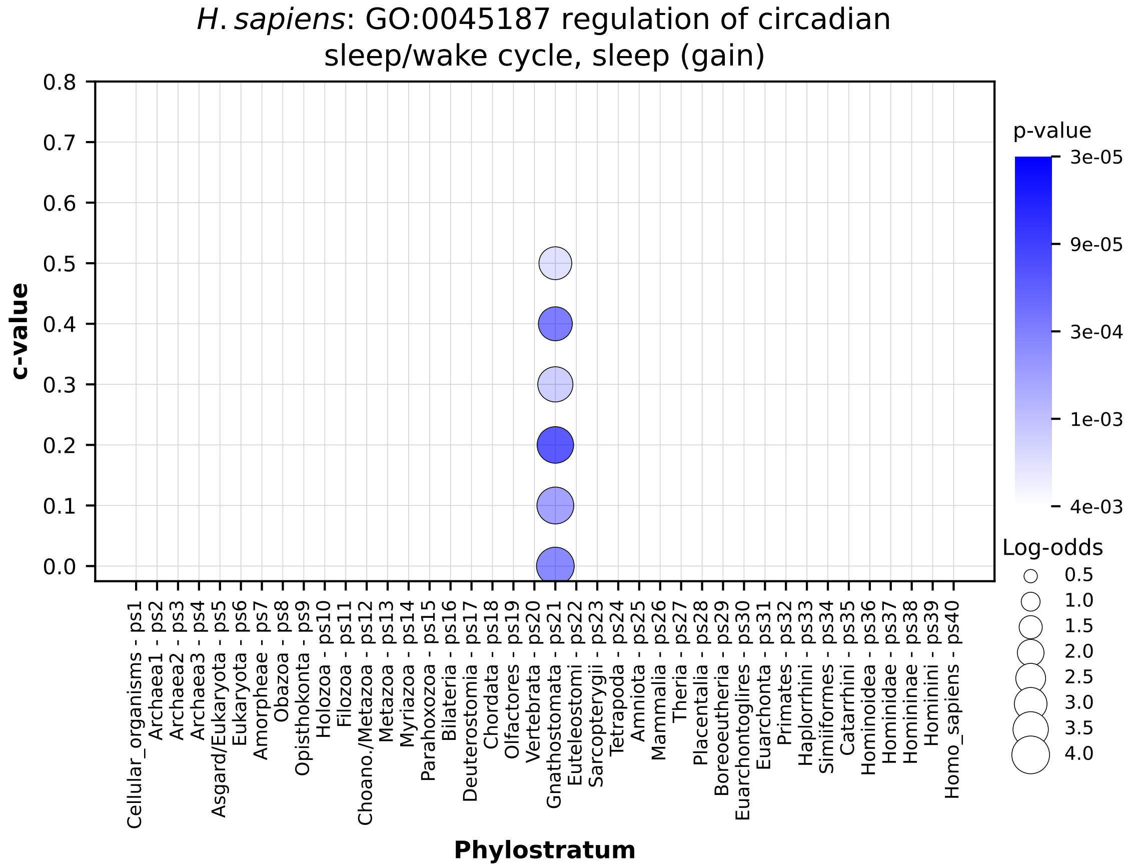

H. sapiens: GO:0045187 regulation of circadian sleep/wake cycle, sleep (gain)
c-value
0.8
0.7
0.6
0.5
0.4
0.3
0.2
0.1
0.0
p-value
3e-05
9e-05
3e-04
1e-03
4e-03
Log-odds
0.5
1.0
1.5
2.0
2.5
3.0
3.5
4.0
Cellular_organisms - ps1
Archaea1 - ps2
Archaea2 - ps3
Archaea3 - ps4
Asgard/Eukaryota - ps5
Eukaryota - ps6
Amorpheae - ps7
Obazoa - ps8
Opisthokonta - ps9
Holozoa - ps10
Filozoa - ps11
Choano./Metazoa - ps12
Metazoa - ps13
Myriazoa - ps14
Parahoxozoa - ps15
Bilateria - ps16
Deuterostomia - ps17
Chordata - ps18
Olfactores - ps19
Vertebrata - ps20
Gnathostomata - ps21
Euteleostomi - ps22
Sarcopterygii - ps23
Tetrapoda - ps24
Amniota - ps25
Mammalia - ps26
Theria - ps27
Placentalia - ps28
Boreoeutheria - ps29
Euarchontoglires - ps30
Euarchonta - ps31
Primates - ps32
Haplorrhini - ps33
Simiiformes - ps34
Catarrhini - ps35
Hominoidea - ps36
Hominidae - ps37
Homininae - ps38
Hominini - ps39
Homo_sapiens - ps40
Phylostratum

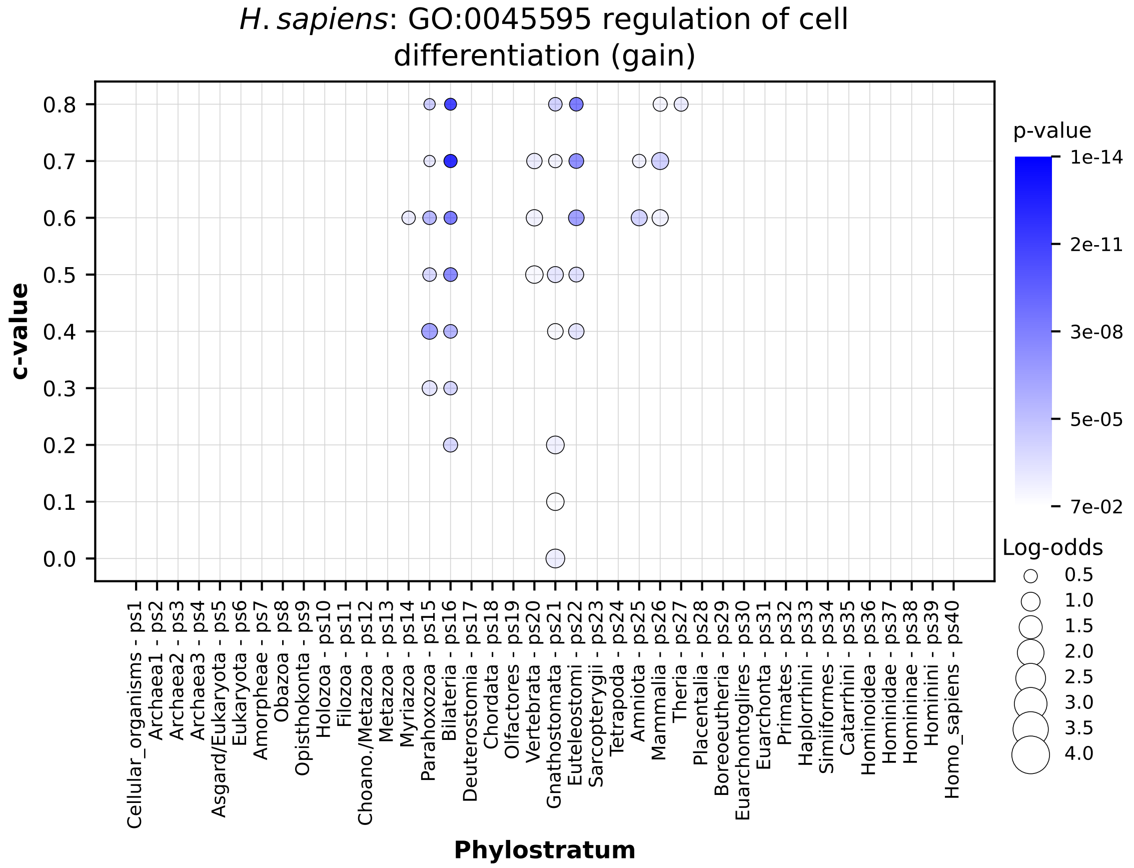
*H. sapiens*: GO:0045595 regulation of cell differentiation (gain)
c-value
0.8
0.7
0.6
0.5
0.4
0.3
0.2
0.1
0.0
p-value
1e-14
2e-11
3e-08
5e-05
7e-02
Log-odds
0.5
1.0
1.5
2.0
2.5
3.0
3.5
4.0
Cellular_organisms - ps1
Archaea1 - ps2
Archaea2 - ps3
Archaea3 - ps4
Asgard/Eukaryota - ps5
Eukaryota - ps6
Amorpheae - ps7
Obazoa - ps8
Opisthokonta - ps9
Holozoa - ps10
Filozoa - ps11
Choano./Metazoa - ps12
Metazoa - ps13
Myriazoa - ps14
Parahoxozoa - ps15
Bilateria - ps16
Deuterostomia - ps17
Chordata - ps18
Olfactores - ps19
Vertebrata - ps20
Gnathostomata - ps21
Euteleostomi - ps22
Sarcopterygii - ps23
Tetrapoda - ps24
Amniota - ps25
Mammalia - ps26
Theria - ps27
Placentalia - ps28
Boreoeutheria - ps29
Euarchontoglires - ps30
Euarchonta - ps31
Primates - ps32
Haplorrhini - ps33
Simiiformes - ps34
Catarrhini - ps35
Hominoidea - ps36
Hominidae - ps37
Homininae - ps38
Hominini - ps39
Homo_sapiens - ps40
Phylostratum

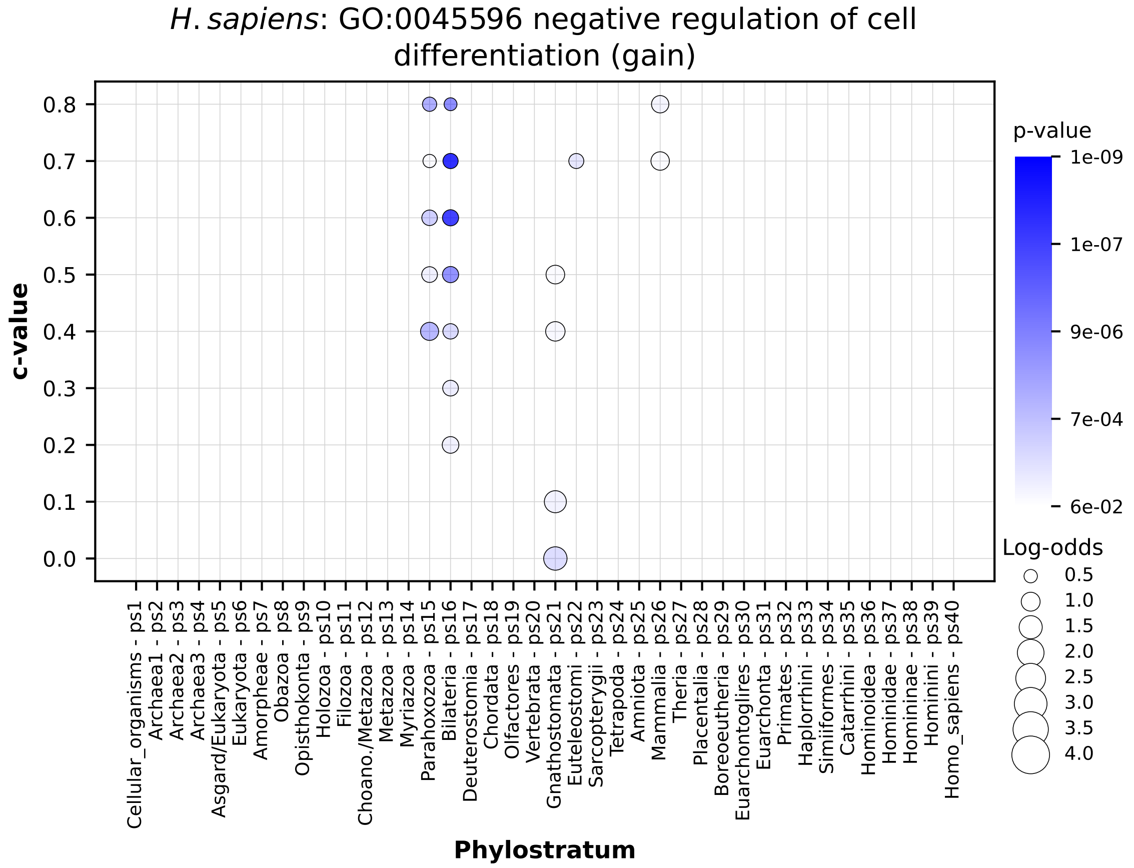

H. sapiens: GO:0045596 negative regulation of cell differentiation (gain)
c-value
0.8
0.7
0.6
0.5
0.4
0.3
0.2
0.1
0.0
Phylostratum
Cellular_organisms - ps1
Archaea1 - ps2
Archaea2 - ps3
Archaea3 - ps4
Asgard/Eukaryota - ps5
Eukaryota - ps6
Amorpheae - ps7
Obazoa - ps8
Opisthokonta - ps9
Holozoa - ps10
Filozoa - ps11
Choano./Metazoa - ps12
Metazoa - ps13
Myriazoa - ps14
Parahoxozoa - ps15
Bilateria - ps16
Deuterostomia - ps17
Chordata - ps18
Olfactores - ps19
Vertebrata - ps20
Gnathostomata - ps21
Euteleostomi - ps22
Sarcopterygii - ps23
Tetrapoda - ps24
Amniota - ps25
Mammalia - ps26
Theria - ps27
Placentalia - ps28
Boreoeutheria - ps29
Euarchontoglires - ps30
Euarchonta - ps31
Primates - ps32
Haplorrhini - ps33
Simiiformes - ps34
Catarrhini - ps35
Hominoidea - ps36
Hominidae - ps37
Homininae - ps38
Hominini - ps39
Homo_sapiens - ps40
p-value
1e-09
1e-07
9e-06
7e-04
6e-02
Log-odds
0.5
1.0
1.5
2.0
2.5
3.0
3.5
4.0

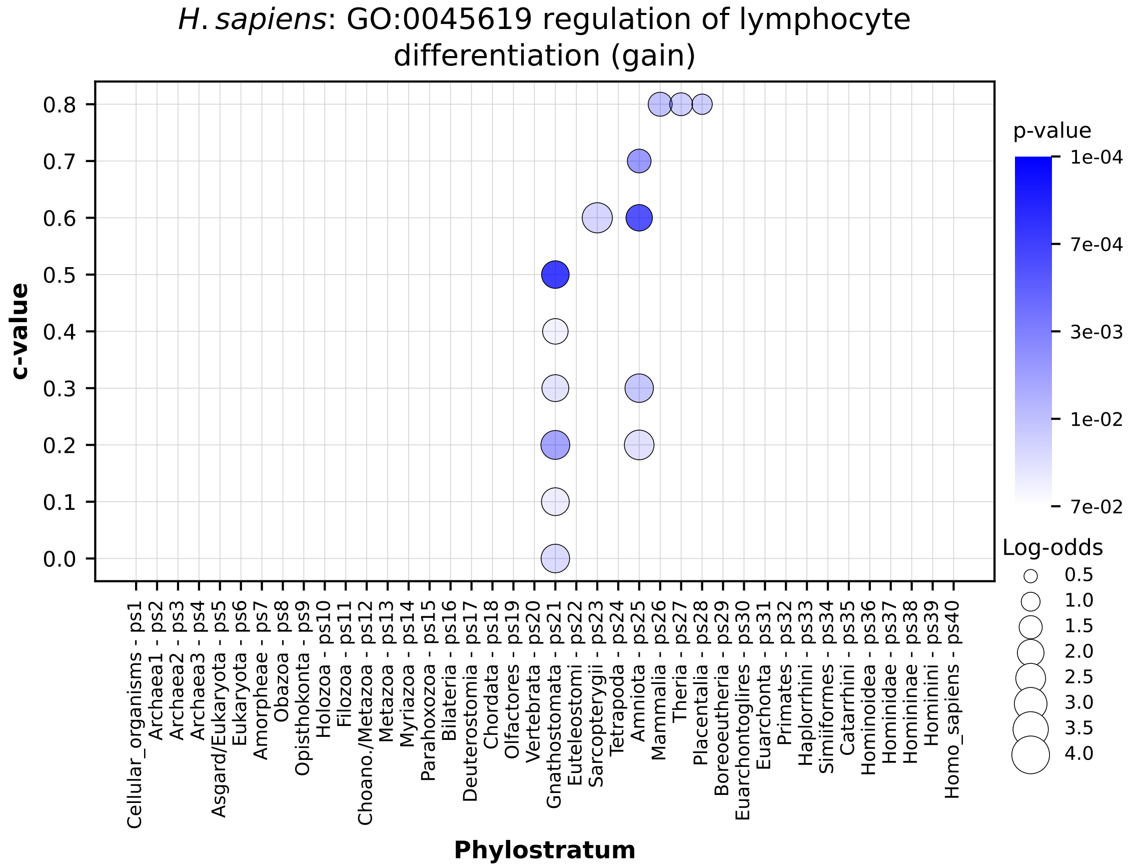

H. sapiens: GO:0045619 regulation of lymphocyte differentiation (gain)
c-value
0.8
0.7
0.6
0.5
0.4
0.3
0.2
0.1
0.0
Phylostratum
Cellular_organisms - ps1
Archaea1 - ps2
Archaea2 - ps3
Archaea3 - ps4
Asgard/Eukaryota - ps5
Eukaryota - ps6
Amorpheae - ps7
Obazoa - ps8
Opisthokonta - ps9
Holozoa - ps10
Filozoa - ps11
Choano./Metazoa - ps12
Metazoa - ps13
Myriazoa - ps14
Parahoxozoa - ps15
Bilateria - ps16
Deuterostomia - ps17
Chordata - ps18
Olfactores - ps19
Vertebrata - ps20
Gnathostomata - ps21
Euteleostomi - ps22
Sarcopterygii - ps23
Tetrapoda - ps24
Amniota - ps25
Mammalia - ps26
Theria - ps27
Placentalia - ps28
Boreoeutheria - ps29
Euarchontoglires - ps30
Euarchonta - ps31
Primates - ps32
Haplorrhini - ps33
Simiiformes - ps34
Catarrhini - ps35
Hominoidea - ps36
Hominidae - ps37
Homininae - ps38
Hominini - ps39
Homo_sapiens - ps40
p-value
1e-04
7e-04
3e-03
1e-02
7e-02
Log-odds
0.5
1.0
1.5
2.0
2.5
3.0
3.5
4.0

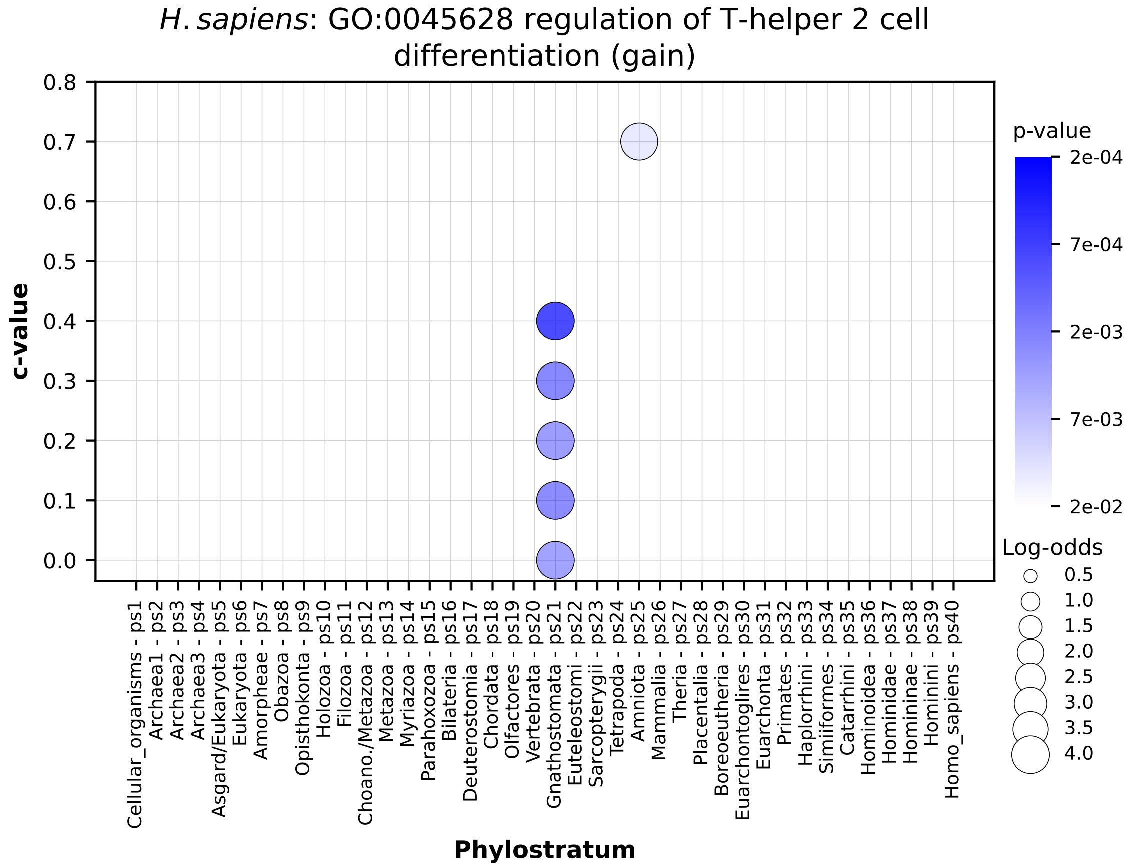

H. sapiens: GO:0045628 regulation of T-helper 2 cell differentiation (gain)
c-value
0.8
0.7
0.6
0.5
0.4
0.3
0.2
0.1
0.0
Phylostratum
Cellular_organisms - ps1
Archaea1 - ps2
Archaea2 - ps3
Archaea3 - ps4
Asgard/Eukaryota - ps5
Eukaryota - ps6
Amorpheae - ps7
Obazoa - ps8
Opisthokonta - ps9
Holozoa - ps10
Filozoa - ps11
Choano./Metazoa - ps12
Metazoa - ps13
Myriazoa - ps14
Parahoxozoa - ps15
Bilateria - ps16
Deuterostomia - ps17
Chordata - ps18
Olfactores - ps19
Vertebrata - ps20
Gnathostomata - ps21
Euteleostomi - ps22
Sarcopterygii - ps23
Tetrapoda - ps24
Amniota - ps25
Mammalia - ps26
Theria - ps27
Placentalia - ps28
Boreoeutheria - ps29
Euarchontoglires - ps30
Euarchonta - ps31
Primates - ps32
Haplorrhini - ps33
Simiiformes - ps34
Catarrhini - ps35
Hominoidea - ps36
Hominidae - ps37
Homininae - ps38
Hominini - ps39
Homo_sapiens - ps40
p-value
2e-04
7e-04
2e-03
7e-03
2e-02
Log-odds
0.5
1.0
1.5
2.0
2.5
3.0
3.5
4.0

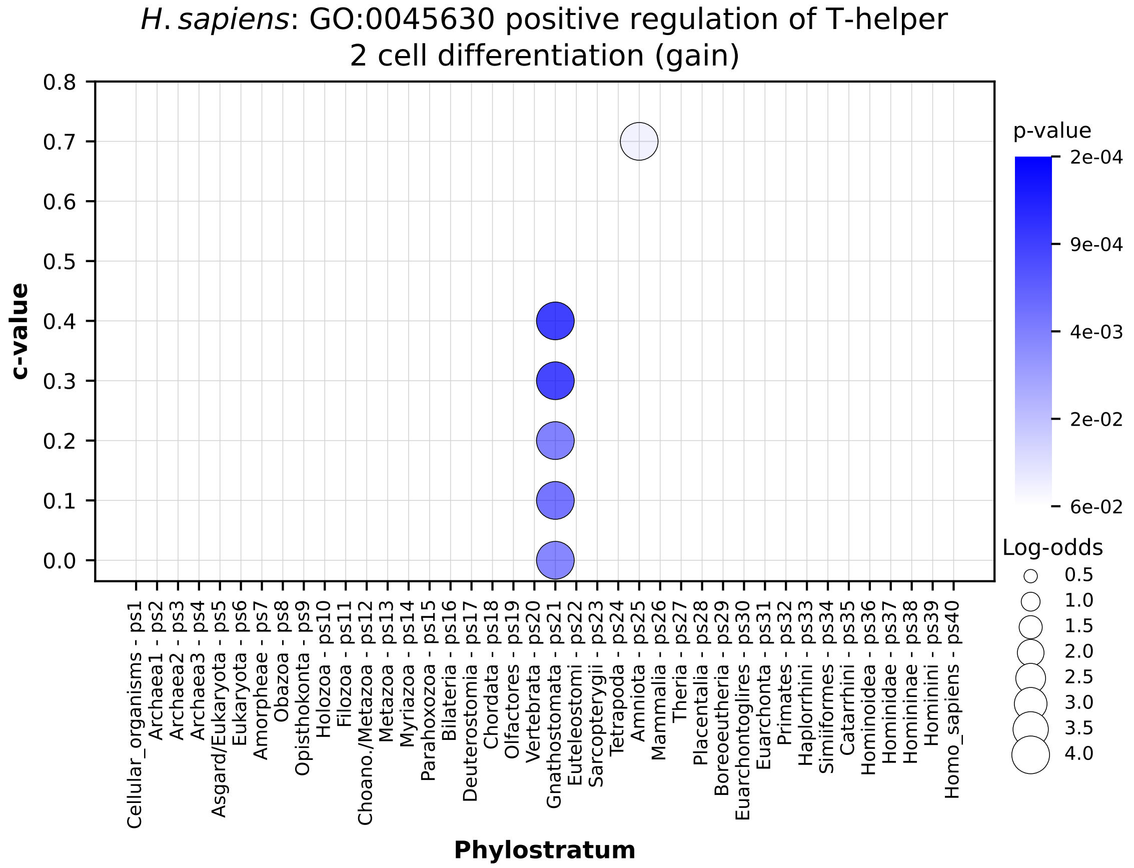
H. sapiens: GO:0045630 positive regulation of T-helper 2 cell differentiation (gain)
c-value
0.8
0.7
0.6
0.5
0.4
0.3
0.2
0.1
0.0
p-value
2e-04
9e-04
4e-03
2e-02
6e-02
Log-odds
0.5
1.0
1.5
2.0
2.5
3.0
3.5
4.0
Cellular_organisms - ps1
Archaea1 - ps2
Archaea2 - ps3
Archaea3 - ps4
Asgard/Eukaryota - ps5
Eukaryota - ps6
Amorpheae - ps7
Obazoa - ps8
Opisthokonta - ps9
Holozoa - ps10
Filozoa - ps11
Choano./Metazoa - ps12
Metazoa - ps13
Myriazoa - ps14
Parahoxozoa - ps15
Bilateria - ps16
Deuterostomia - ps17
Chordata - ps18
Olfactores - ps19
Vertebrata - ps20
Gnathostomata - ps21
Euteleostomi - ps22
Sarcopterygii - ps23
Tetrapoda - ps24
Amniota - ps25
Mammalia - ps26
Theria - ps27
Placentalia - ps28
Boreoeutheria - ps29
Euarchontoglires - ps30
Euarchonta - ps31
Primates - ps32
Haplorrhini - ps33
Simiiformes - ps34
Catarrhini - ps35
Hominoidea - ps36
Hominidae - ps37
Homininae - ps38
Hominini - ps39
Homo_sapiens - ps40
Phylostratum

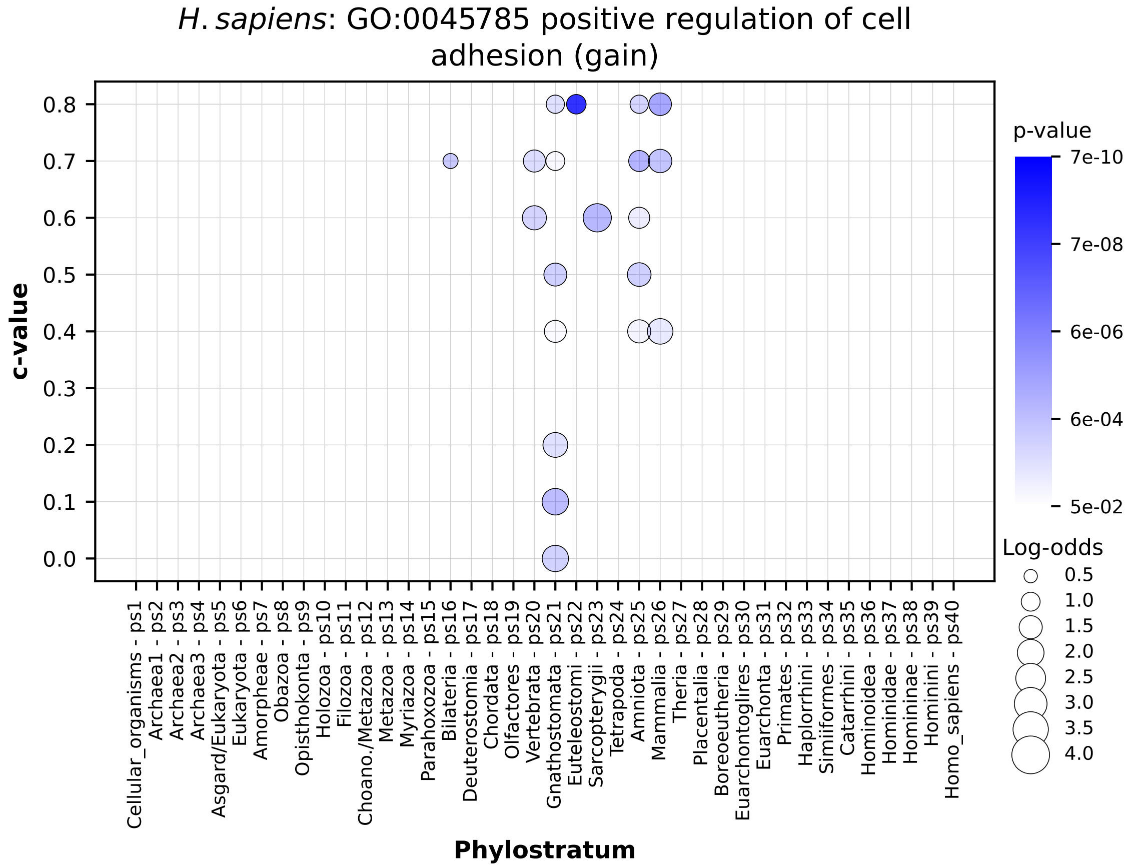

*H. sapiens*: GO:0045785 positive regulation of cell adhesion (gain)
c-value
0.8
0.7
0.6
0.5
0.4
0.3
0.2
0.1
0.0
p-value
7e-10
7e-08
6e-06
6e-04
5e-02
Log-odds
0.5
1.0
1.5
2.0
2.5
3.0
3.5
4.0
Cellular_organisms - ps1
Archaea1 - ps2
Archaea2 - ps3
Archaea3 - ps4
Asgard/Eukaryota - ps5
Eukaryota - ps6
Amorpheae - ps7
Obazoa - ps8
Opisthokonta - ps9
Holozoa - ps10
Filozoa - ps11
Choano./Metazoa - ps12
Metazoa - ps13
Myriazoa - ps14
Parahoxozoa - ps15
Bilateria - ps16
Deuterostomia - ps17
Chordata - ps18
Olfactores - ps19
Vertebrata - ps20
Gnathostomata - ps21
Euteleostomi - ps22
Sarcopterygii - ps23
Tetrapoda - ps24
Amniota - ps25
Mammalia - ps26
Theria - ps27
Placentalia - ps28
Boreoeutheria - ps29
Euarchontoglires - ps30
Euarchonta - ps31
Primates - ps32
Haplorrhini - ps33
Simiiformes - ps34
Catarrhini - ps35
Hominoidea - ps36
Hominidae - ps37
Homininae - ps38
Hominini - ps39
Homo_sapiens - ps40
Phylostratum

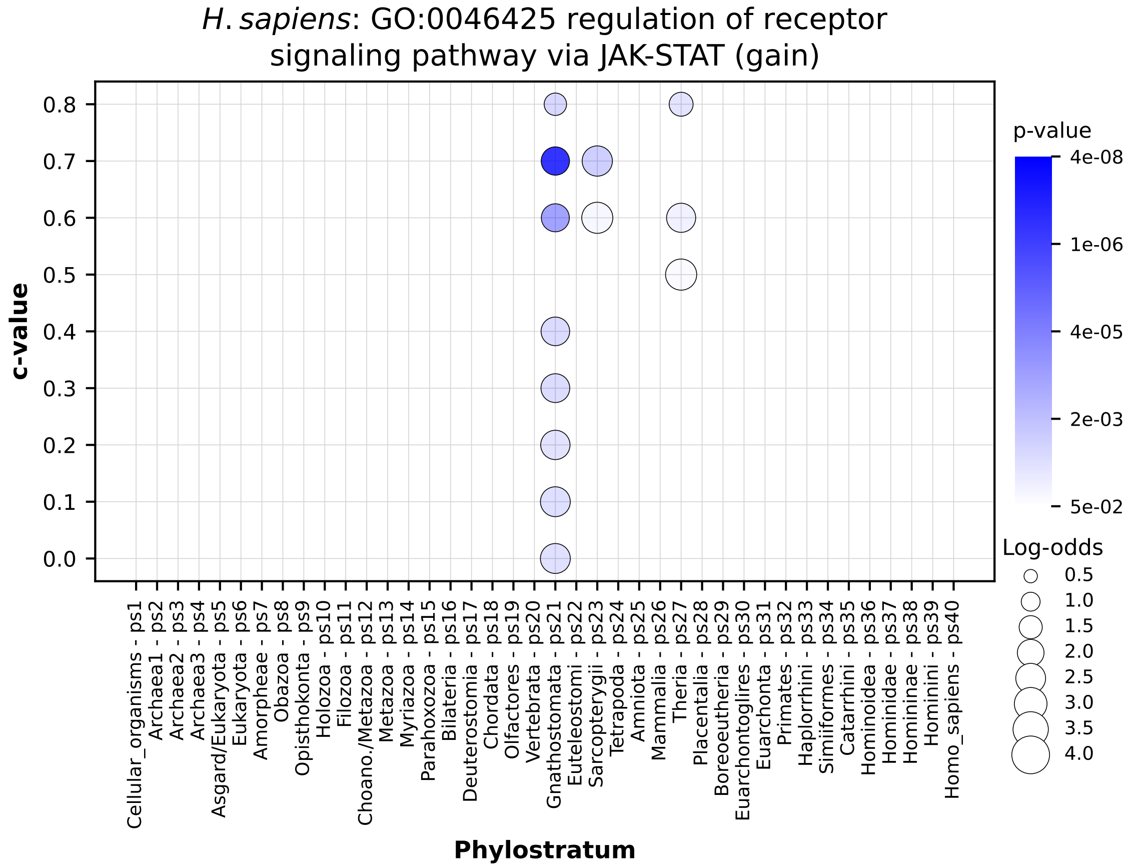

H. sapiens: GO:0046425 regulation of receptor signaling pathway via JAK-STAT (gain)
c-value
0.8
0.7
0.6
0.5
0.4
0.3
0.2
0.1
0.0
Phylostratum
Cellular_organisms - ps1
Archaea1 - ps2
Archaea2 - ps3
Archaea3 - ps4
Asgard/Eukaryota - ps5
Eukaryota - ps6
Amorpheae - ps7
Obazoa - ps8
Opisthokonta - ps9
Holozoa - ps10
Filozoa - ps11
Choano./Metazoa - ps12
Metazoa - ps13
Myriazoa - ps14
Parahoxozoa - ps15
Bilateria - ps16
Deuterostomia - ps17
Chordata - ps18
Olfactores - ps19
Vertebrata - ps20
Gnathostomata - ps21
Euteleostomi - ps22
Sarcopterygii - ps23
Tetrapoda - ps24
Amniota - ps25
Mammalia - ps26
Theria - ps27
Placentalia - ps28
Boreoeutheria - ps29
Euarchontoglires - ps30
Euarchonta - ps31
Primates - ps32
Haplorrhini - ps33
Simiiformes - ps34
Catarrhini - ps35
Hominoidea - ps36
Hominidae - ps37
Homininae - ps38
Hominini - ps39
Homo_sapiens - ps40
p-value
4e-08
1e-06
4e-05
2e-03
5e-02
Log-odds
0.5
1.0
1.5
2.0
2.5
3.0
3.5
4.0

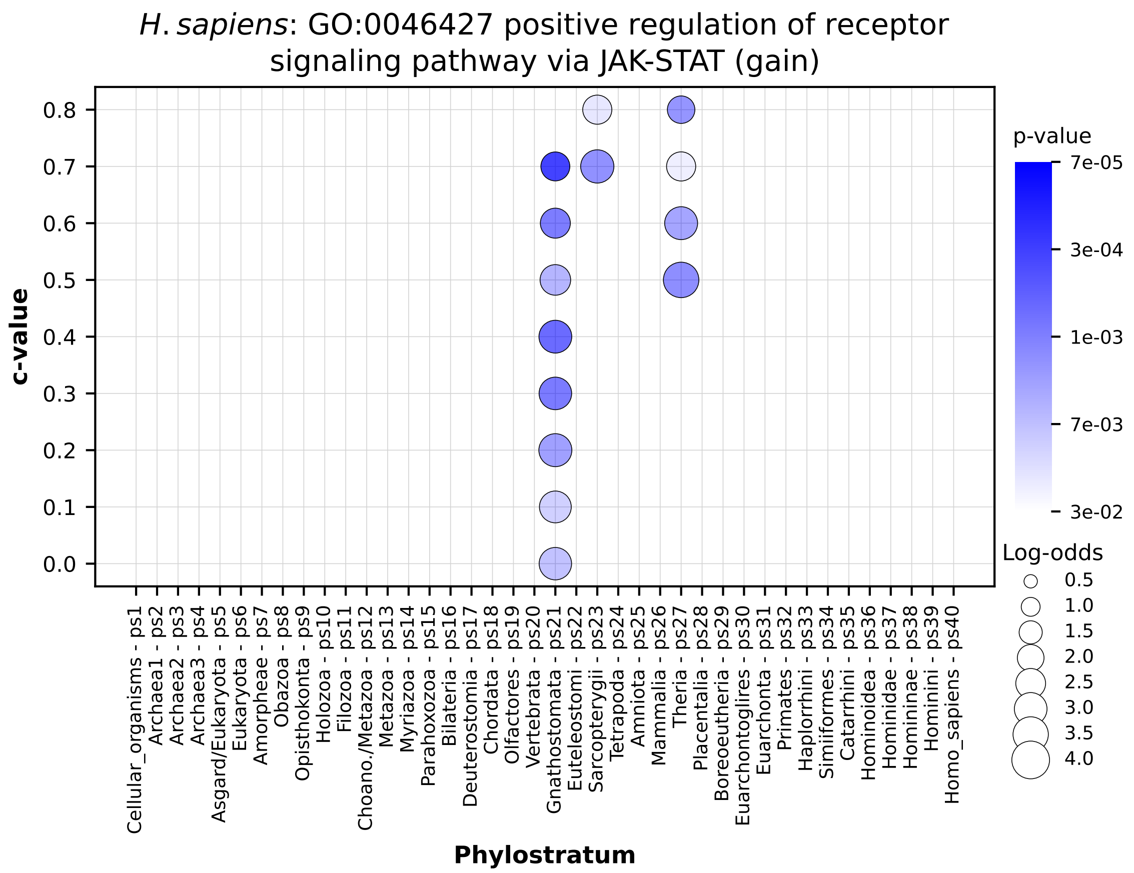

H. sapiens: GO:0046427 positive regulation of receptor signaling pathway via JAK-STAT (gain)
c-value
0.0
0.1
0.2
0.3
0.4
0.5
0.6
0.7
0.8
p-value
7e-05
3e-04
1e-03
7e-03
3e-02
Log-odds
0.5
1.0
1.5
2.0
2.5
3.0
3.5
4.0
Cellular_organisms - ps1
Archaea1 - ps2
Archaea2 - ps3
Archaea3 - ps4
Asgard/Eukaryota - ps5
Eukaryota - ps6
Amorpheae - ps7
Obazoa - ps8
Opisthokonta - ps9
Holozoa - ps10
Filozoa - ps11
Choano./Metazoa - ps12
Metazoa - ps13
Myriazoa - ps14
Parahoxozoa - ps15
Bilateria - ps16
Deuterostomia - ps17
Chordata - ps18
Olfactores - ps19
Vertebrata - ps20
Gnathostomata - ps21
Euteleostomi - ps22
Sarcopterygii - ps23
Tetrapoda - ps24
Amniota - ps25
Mammalia - ps26
Theria - ps27
Placentalia - ps28
Boreoeutheria - ps29
Euarchontoglires - ps30
Euarchonta - ps31
Primates - ps32
Haplorrhini - ps33
Simiiformes - ps34
Catarrhini - ps35
Hominoidea - ps36
Hominidae - ps37
Homininae - ps38
Hominini - ps39
Homo_sapiens - ps40
Phylostratum

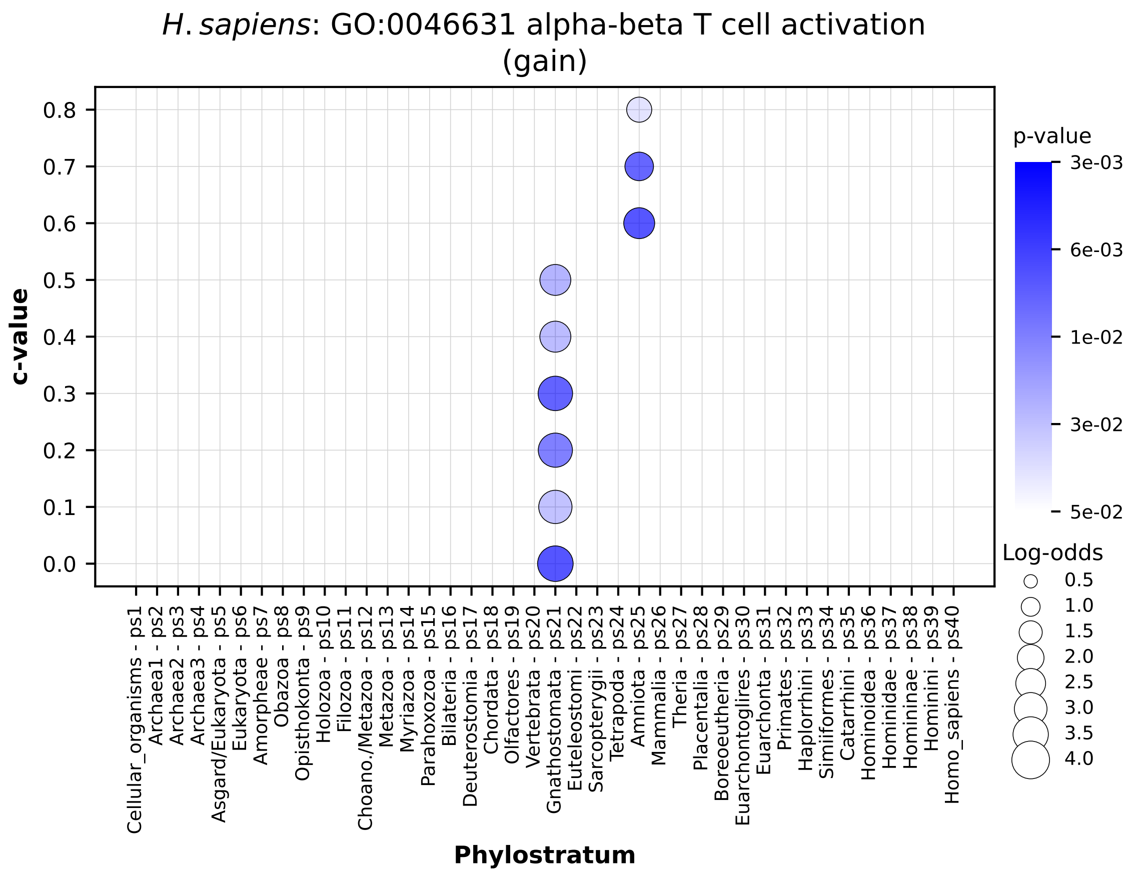

*H. sapiens*: GO:0046631 alpha-beta T cell activation
(gain)
c-value
0.0
0.1
0.2
0.3
0.4
0.5
0.6
0.7
0.8
Phylostratum
Cellular_organisms - ps1
Archaea1 - ps2
Archaea2 - ps3
Archaea3 - ps4
Asgard/Eukaryota - ps5
Eukaryota - ps6
Amorpheae - ps7
Obazoa - ps8
Opisthokonta - ps9
Holozoa - ps10
Filozoa - ps11
Choano./Metazoa - ps12
Metazoa - ps13
Myriazoa - ps14
Parahoxozoa - ps15
Bilateria - ps16
Deuterostomia - ps17
Chordata - ps18
Olfactores - ps19
Vertebrata - ps20
Gnathostomata - ps21
Euteleostomi - ps22
Sarcopterygii - ps23
Tetrapoda - ps24
Amniota - ps25
Mammalia - ps26
Theria - ps27
Placentalia - ps28
Boreoeutheria - ps29
Euarchontoglires - ps30
Euarchonta - ps31
Primates - ps32
Haplorrhini - ps33
Simiiformes - ps34
Catarrhini - ps35
Hominoidea - ps36
Hominidae - ps37
Homininae - ps38
Hominini - ps39
Homo_sapiens - ps40
p-value
3e-03
6e-03
1e-02
3e-02
5e-02
Log-odds
0.5
1.0
1.5
2.0
2.5
3.0
3.5
4.0

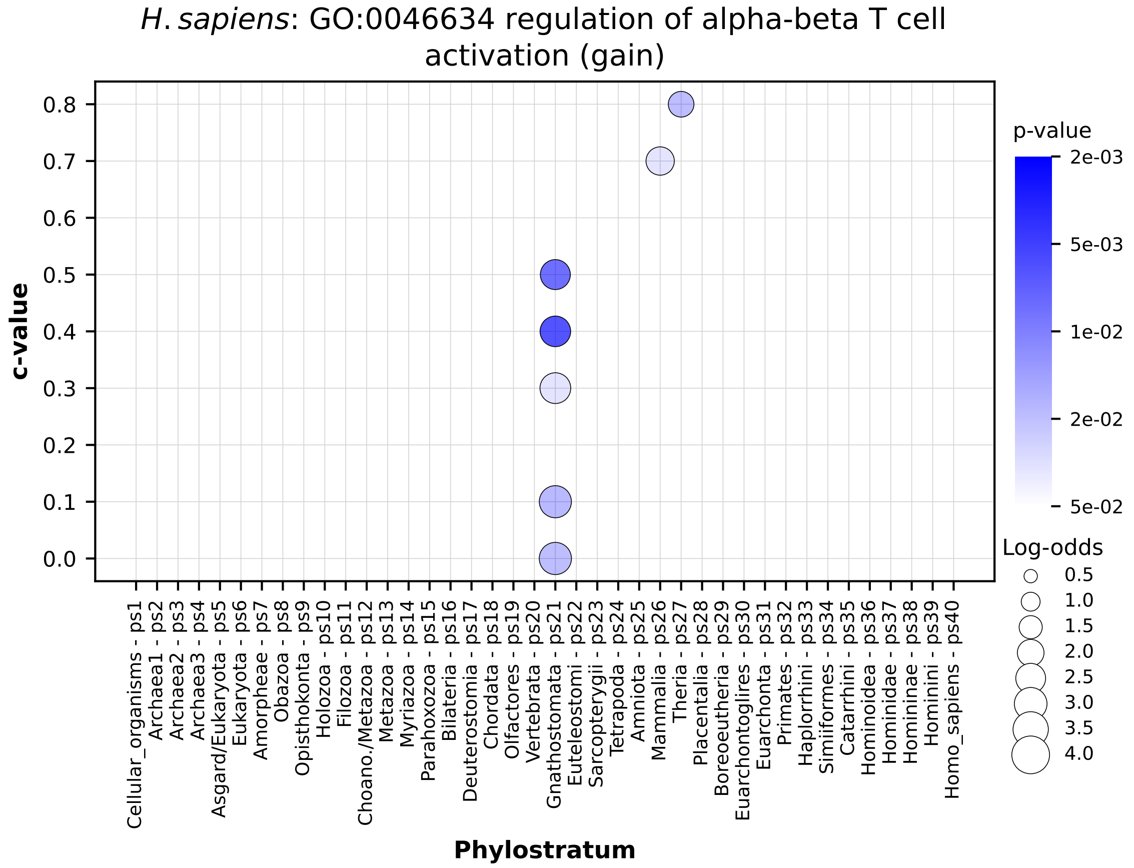

H. sapiens: GO:0046634 regulation of alpha-beta T cell activation (gain)
c-value
0.0
0.1
0.2
0.3
0.4
0.5
0.6
0.7
0.8
p-value
2e-03
5e-03
1e-02
2e-02
5e-02
Log-odds
0.5
1.0
1.5
2.0
2.5
3.0
3.5
4.0
Cellular_organisms - ps1
Archaea1 - ps2
Archaea2 - ps3
Archaea3 - ps4
Asgard/Eukaryota - ps5
Eukaryota - ps6
Amorpheae - ps7
Obazoa - ps8
Opisthokonta - ps9
Holozoa - ps10
Filozoa - ps11
Choano./Metazoa - ps12
Metazoa - ps13
Myriazoa - ps14
Parahoxozoa - ps15
Bilateria - ps16
Deuterostomia - ps17
Chordata - ps18
Olfactores - ps19
Vertebrata - ps20
Gnathostomata - ps21
Euteleostomi - ps22
Sarcopterygii - ps23
Tetrapoda - ps24
Amniota - ps25
Mammalia - ps26
Theria - ps27
Placentalia - ps28
Boreoeutheria - ps29
Euarchontoglires - ps30
Euarchonta - ps31
Primates - ps32
Haplorrhini - ps33
Simiiformes - ps34
Catarrhini - ps35
Hominoidea - ps36
Hominidae - ps37
Homininae - ps38
Hominini - ps39
Homo_sapiens - ps40
Phylostratum

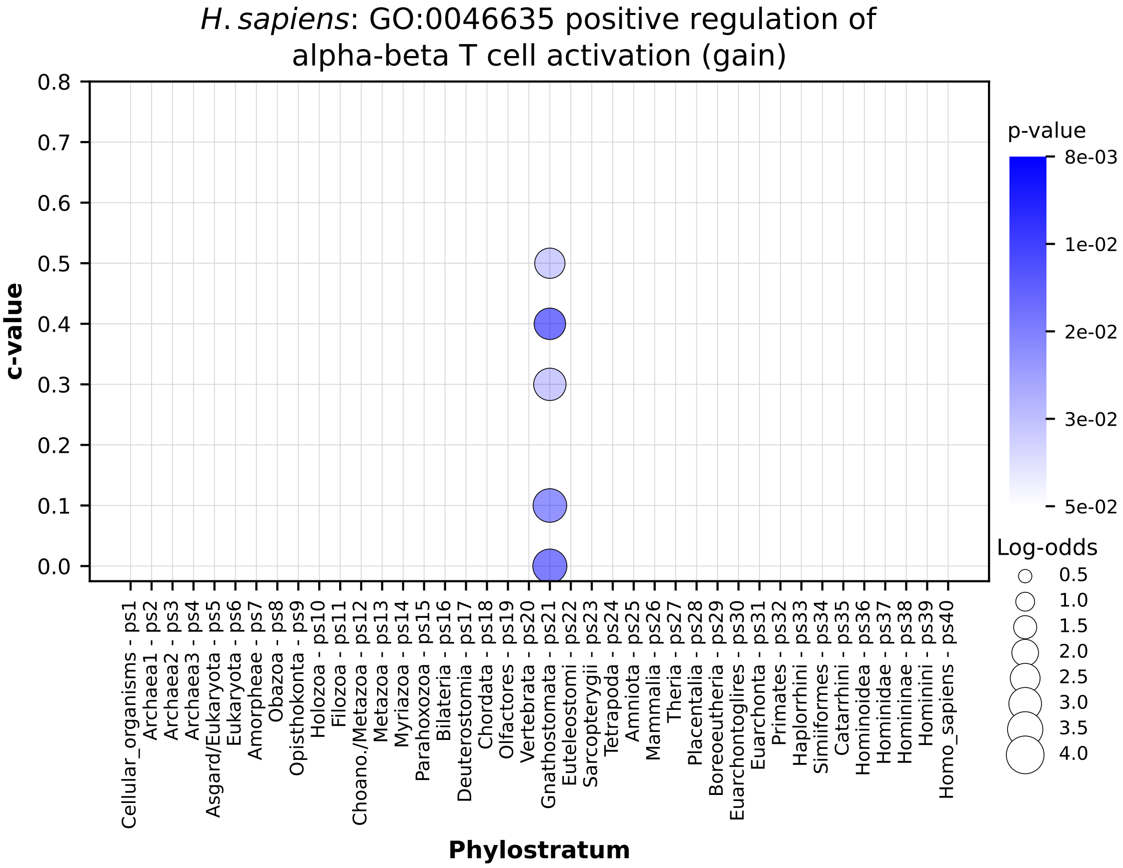

*H. sapiens*: GO:0046635 positive regulation of alpha-beta T cell activation (gain)
c-value
0.8
0.7
0.6
0.5
0.4
0.3
0.2
0.1
0.0
Phylostratum
Cellular_organisms - ps1
Archaea1 - ps2
Archaea2 - ps3
Archaea3 - ps4
Asgard/Eukaryota - ps5
Eukaryota - ps6
Amorpheae - ps7
Obazoa - ps8
Opisthokonta - ps9
Holozoa - ps10
Filozoa - ps11
Choano./Metazoa - ps12
Metazoa - ps13
Myriazoa - ps14
Parahoxozoa - ps15
Bilateria - ps16
Deuterostomia - ps17
Chordata - ps18
Olfactores - ps19
Vertebrata - ps20
Gnathostomata - ps21
Euteleostomi - ps22
Sarcopterygii - ps23
Tetrapoda - ps24
Amniota - ps25
Mammalia - ps26
Theria - ps27
Placentalia - ps28
Boreoeutheria - ps29
Euarchontoglires - ps30
Euarchonta - ps31
Primates - ps32
Haplorrhini - ps33
Simiiformes - ps34
Catarrhini - ps35
Hominoidea - ps36
Hominidae - ps37
Homininae - ps38
Hominini - ps39
Homo_sapiens - ps40
p-value
8e-03
1e-02
2e-02
3e-02
5e-02
Log-odds
0.5
1.0
1.5
2.0
2.5
3.0
3.5
4.0

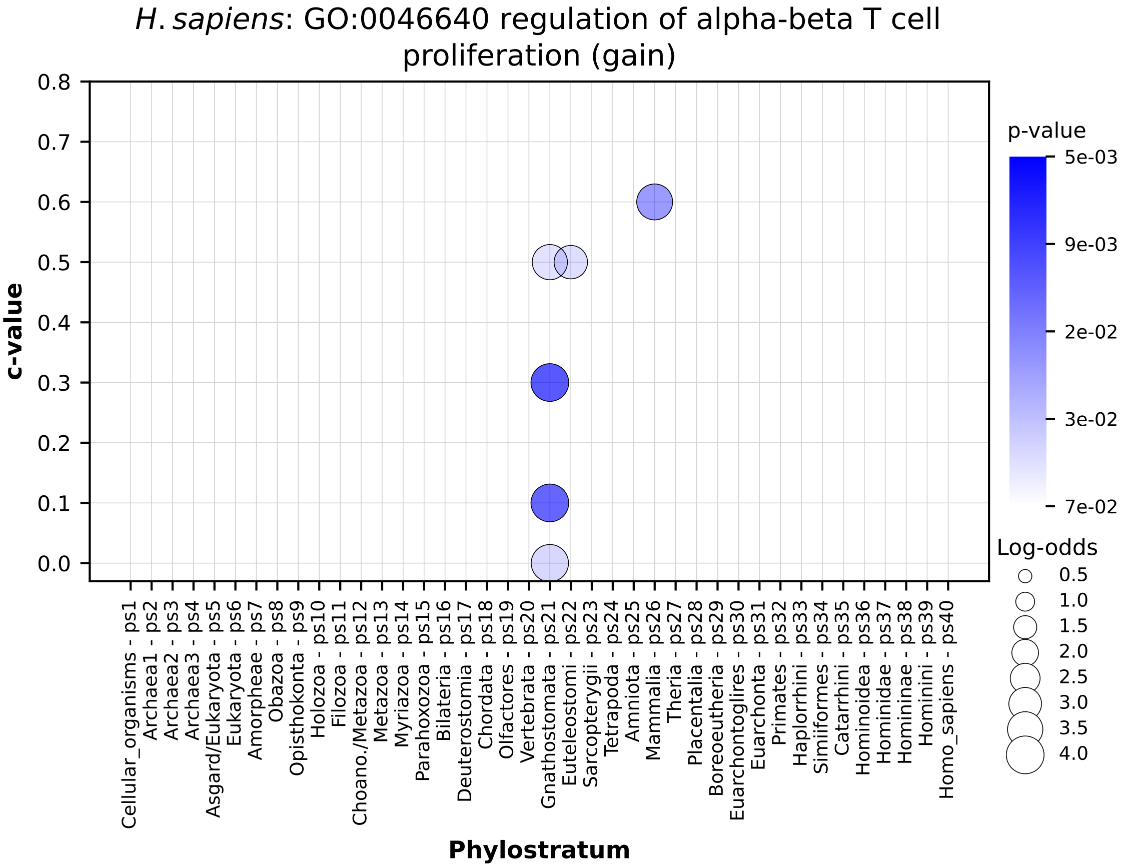

*H. sapiens*: GO:0046640 regulation of alpha-beta T cell proliferation (gain)
c-value
0.8
0.7
0.6
0.5
0.4
0.3
0.2
0.1
0.0
p-value
5e-03
9e-03
2e-02
3e-02
7e-02
Log-odds
0.5
1.0
1.5
2.0
2.5
3.0
3.5
4.0
Cellular_organisms - ps1
Archaea1 - ps2
Archaea2 - ps3
Archaea3 - ps4
Asgard/Eukaryota - ps5
Eukaryota - ps6
Amorpheae - ps7
Obazoa - ps8
Opisthokonta - ps9
Holozoa - ps10
Filozoa - ps11
Choano./Metazoa - ps12
Metazoa - ps13
Myriazoa - ps14
Parahoxozoa - ps15
Bilateria - ps16
Deuterostomia - ps17
Chordata - ps18
Olfactores - ps19
Vertebrata - ps20
Gnathostomata - ps21
Euteleostomi - ps22
Sarcopterygii - ps23
Tetrapoda - ps24
Amniota - ps25
Mammalia - ps26
Theria - ps27
Placentalia - ps28
Boreoeutheria - ps29
Euarchontoglires - ps30
Euarchonta - ps31
Primates - ps32
Haplorrhini - ps33
Simiiformes - ps34
Catarrhini - ps35
Hominoidea - ps36
Hominidae - ps37
Homininae - ps38
Hominini - ps39
Homo_sapiens - ps40
Phylostratum

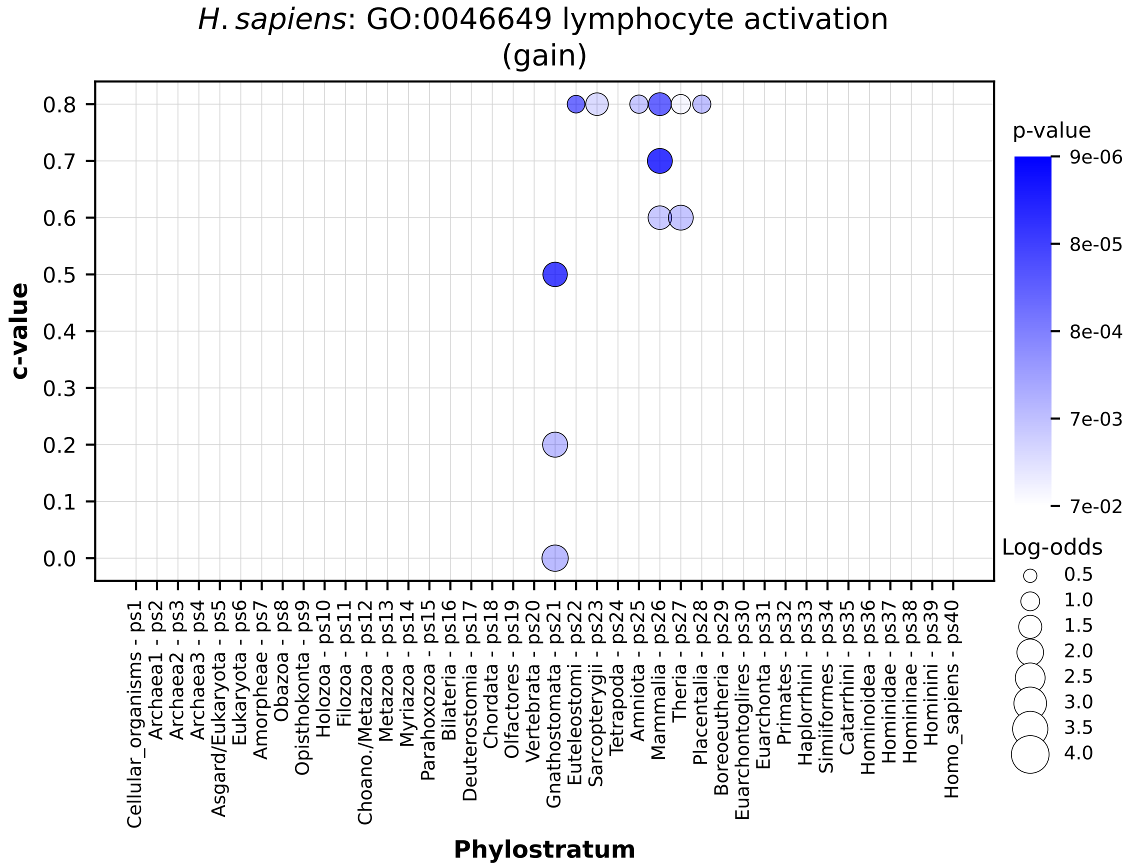

*H. sapiens*: GO:0046649 lymphocyte activation (gain)
c-value
0.8
0.7
0.6
0.5
0.4
0.3
0.2
0.1
0.0
p-value
9e-06
8e-05
8e-04
7e-03
7e-02
Log-odds
0.5
1.0
1.5
2.0
2.5
3.0
3.5
4.0
Cellular_organisms - ps1
Archaea1 - ps2
Archaea2 - ps3
Archaea3 - ps4
Asgard/Eukaryota - ps5
Eukaryota - ps6
Amorpheae - ps7
Obazoa - ps8
Opisthokonta - ps9
Holozoa - ps10
Filozoa - ps11
Choano./Metazoa - ps12
Metazoa - ps13
Myriazoa - ps14
Parahoxozoa - ps15
Bilateria - ps16
Deuterostomia - ps17
Chordata - ps18
Olfactores - ps19
Vertebrata - ps20
Gnathostomata - ps21
Euteleostomi - ps22
Sarcopterygii - ps23
Tetrapoda - ps24
Amniota - ps25
Mammalia - ps26
Theria - ps27
Placentalia - ps28
Boreoeutheria - ps29
Euarchontoglires - ps30
Euarchonta - ps31
Primates - ps32
Haplorrhini - ps33
Simiiformes - ps34
Catarrhini - ps35
Hominoidea - ps36
Hominidae - ps37
Homininae - ps38
Hominini - ps39
Homo_sapiens - ps40
Phylostratum

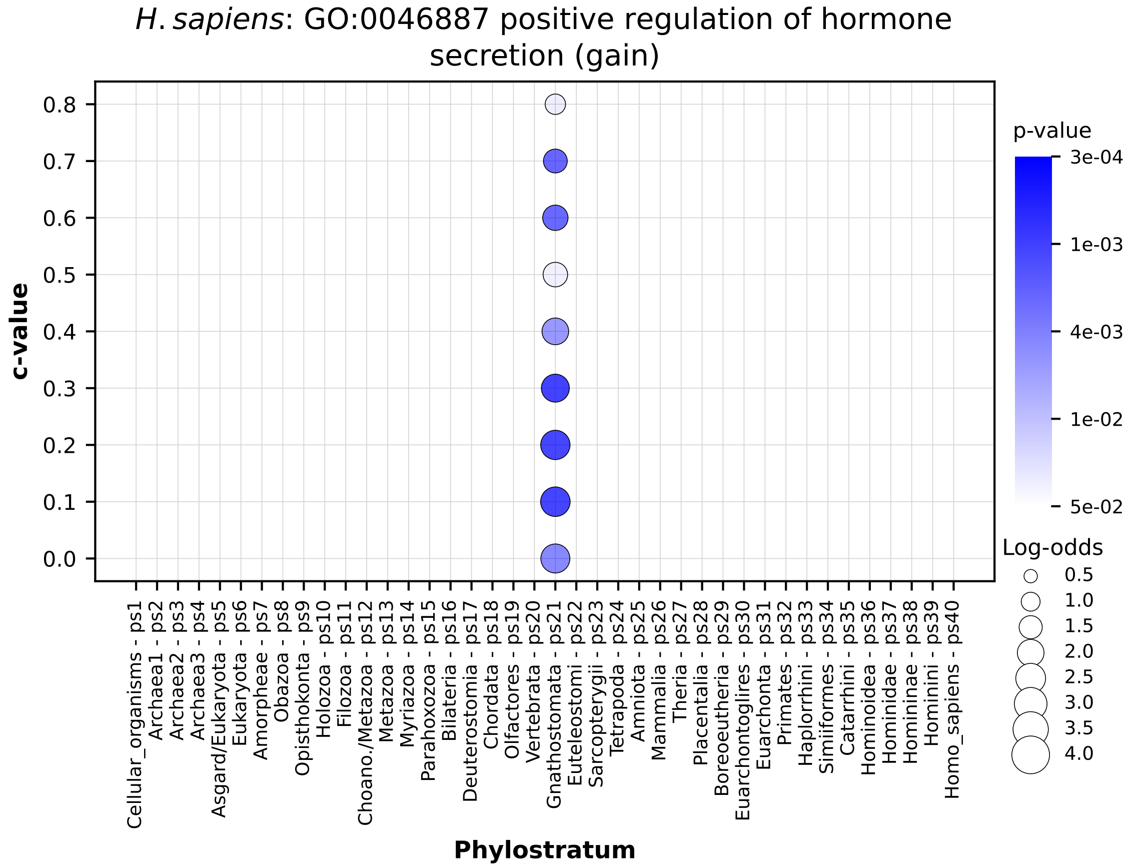

H. sapiens: GO:0046887 positive regulation of hormone secretion (gain)
c-value
0.8
0.7
0.6
0.5
0.4
0.3
0.2
0.1
0.0
Phylostratum
Cellular_organisms - ps1
Archaea1 - ps2
Archaea2 - ps3
Archaea3 - ps4
Asgard/Eukaryota - ps5
Eukaryota - ps6
Amorpheae - ps7
Obazoa - ps8
Opisthokonta - ps9
Holozoa - ps10
Filozoa - ps11
Choano./Metazoa - ps12
Metazoa - ps13
Myriazoa - ps14
Parahoxozoa - ps15
Bilateria - ps16
Deuterostomia - ps17
Chordata - ps18
Olfactores - ps19
Vertebrata - ps20
Gnathostomata - ps21
Euteleostomi - ps22
Sarcopterygii - ps23
Tetrapoda - ps24
Amniota - ps25
Mammalia - ps26
Theria - ps27
Placentalia - ps28
Boreoeutheria - ps29
Euarchontoglires - ps30
Euarchonta - ps31
Primates - ps32
Haplorrhini - ps33
Simiiformes - ps34
Catarrhini - ps35
Hominoidea - ps36
Hominidae - ps37
Homininae - ps38
Hominini - ps39
Homo_sapiens - ps40
p-value
3e-04
1e-03
4e-03
1e-02
5e-02
Log-odds
0.5
1.0
1.5
2.0
2.5
3.0
3.5
4.0

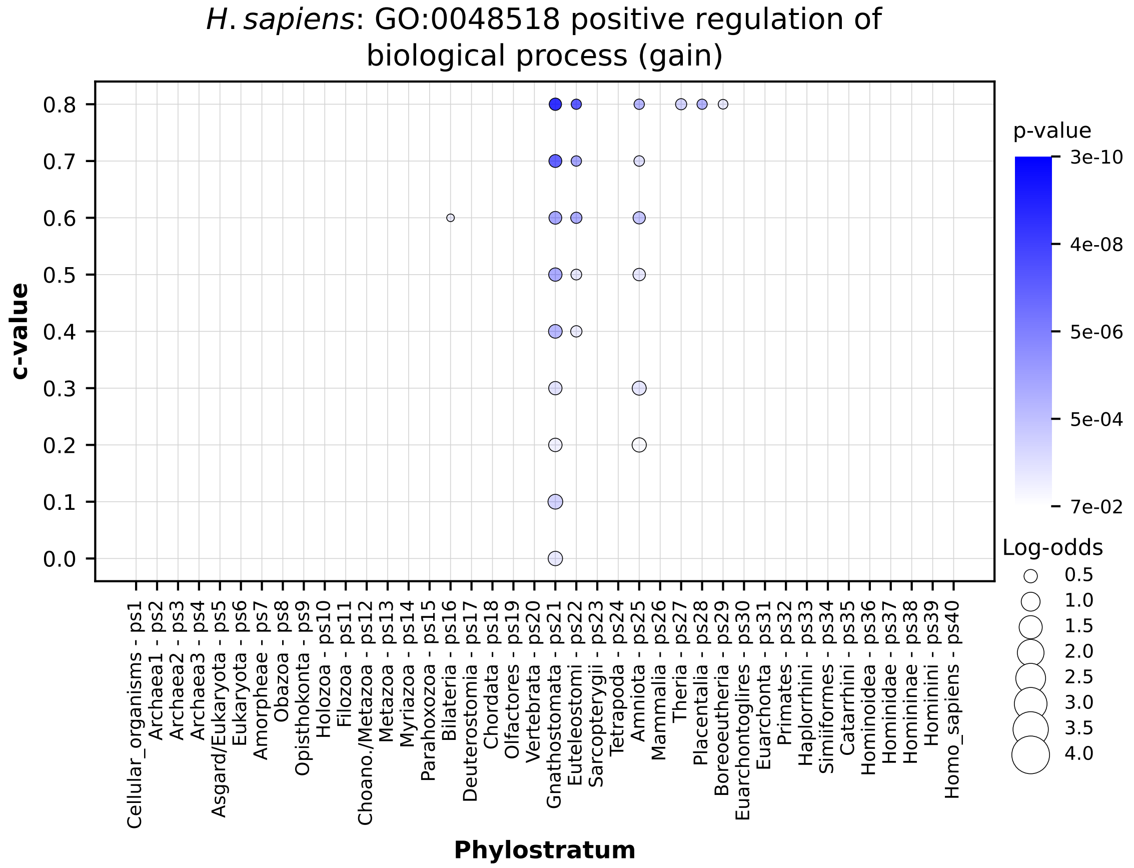

H. sapiens: GO:0048518 positive regulation of biological process (gain)
c-value
0.0
0.1
0.2
0.3
0.4
0.5
0.6
0.7
0.8
Phylostratum
Cellular_organisms - ps1
Archaea1 - ps2
Archaea2 - ps3
Archaea3 - ps4
Asgard/Eukaryota - ps5
Eukaryota - ps6
Amorpheae - ps7
Obazoa - ps8
Opisthokonta - ps9
Holozoa - ps10
Filozoa - ps11
Choano./Metazoa - ps12
Metazoa - ps13
Myriazoa - ps14
Parahoxozoa - ps15
Bilateria - ps16
Deuterostomia - ps17
Chordata - ps18
Olfactores - ps19
Vertebrata - ps20
Gnathostomata - ps21
Euteleostomi - ps22
Sarcopterygii - ps23
Tetrapoda - ps24
Amniota - ps25
Mammalia - ps26
Theria - ps27
Placentalia - ps28
Boreoeutheria - ps29
Euarchontoglires - ps30
Euarchonta - ps31
Primates - ps32
Haplorrhini - ps33
Simiiformes - ps34
Catarrhini - ps35
Hominoidea - ps36
Hominidae - ps37
Homininae - ps38
Hominini - ps39
Homo_sapiens - ps40
p-value
3e-10
4e-08
5e-06
5e-04
7e-02
Log-odds
0.5
1.0
1.5
2.0
2.5
3.0
3.5
4.0

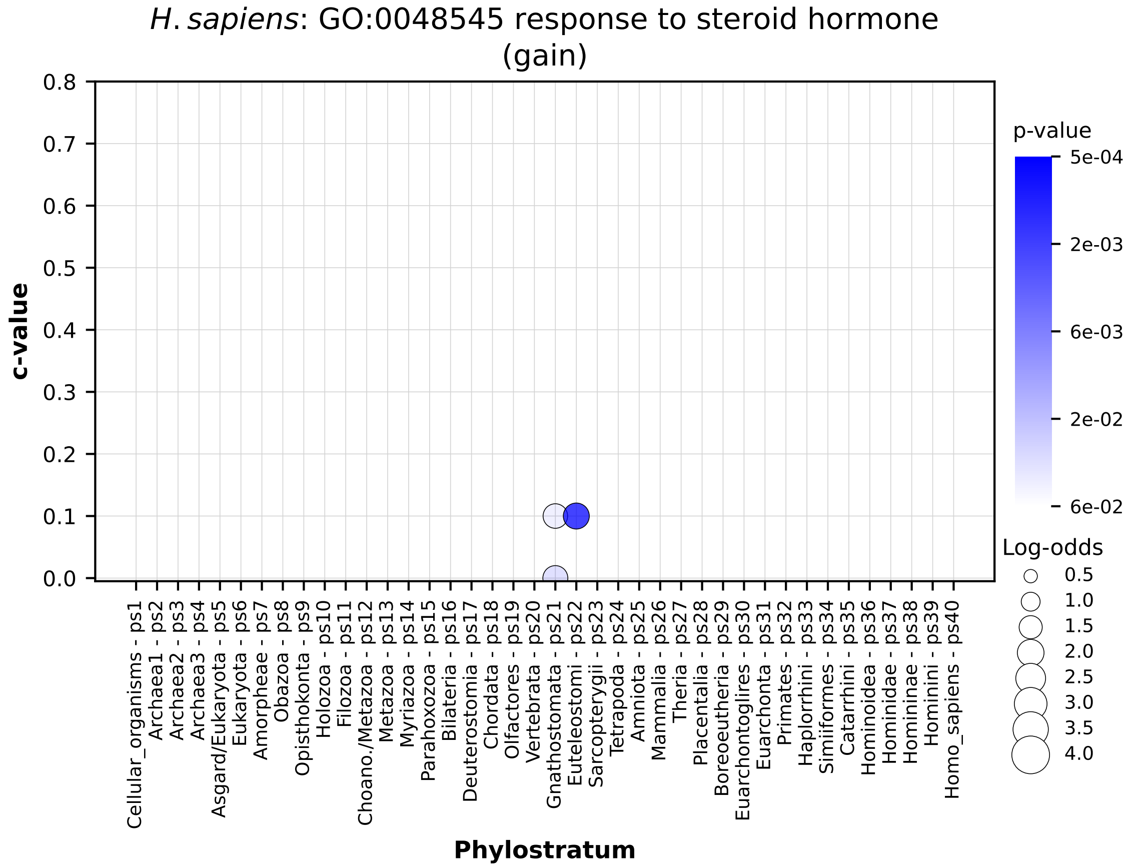

H. sapiens: GO:0048545 response to steroid hormone
(gain)
c-value
0.8
0.7
0.6
0.5
0.4
0.3
0.2
0.1
0.0
p-value
5e-04
2e-03
6e-03
2e-02
6e-02
Log-odds
0.5
1.0
1.5
2.0
2.5
3.0
3.5
4.0
Cellular_organisms - ps1
Archaea1 - ps2
Archaea2 - ps3
Archaea3 - ps4
Asgard/Eukaryota - ps5
Eukaryota - ps6
Amorpheae - ps7
Obazoa - ps8
Opisthokonta - ps9
Holozoa - ps10
Filozoa - ps11
Choano./Metazoa - ps12
Metazoa - ps13
Myriazoa - ps14
Parahoxozoa - ps15
Bilateria - ps16
Deuterostomia - ps17
Chordata - ps18
Olfactores - ps19
Vertebrata - ps20
Gnathostomata - ps21
Euteleostomi - ps22
Sarcopterygii - ps23
Tetrapoda - ps24
Amniota - ps25
Mammalia - ps26
Theria - ps27
Placentalia - ps28
Boreoeutheria - ps29
Euarchontoglires - ps30
Euarchonta - ps31
Primates - ps32
Haplorrhini - ps33
Simiiformes - ps34
Catarrhini - ps35
Hominoidea - ps36
Hominidae - ps37
Homininae - ps38
Hominini - ps39
Homo_sapiens - ps40
Phylostratum

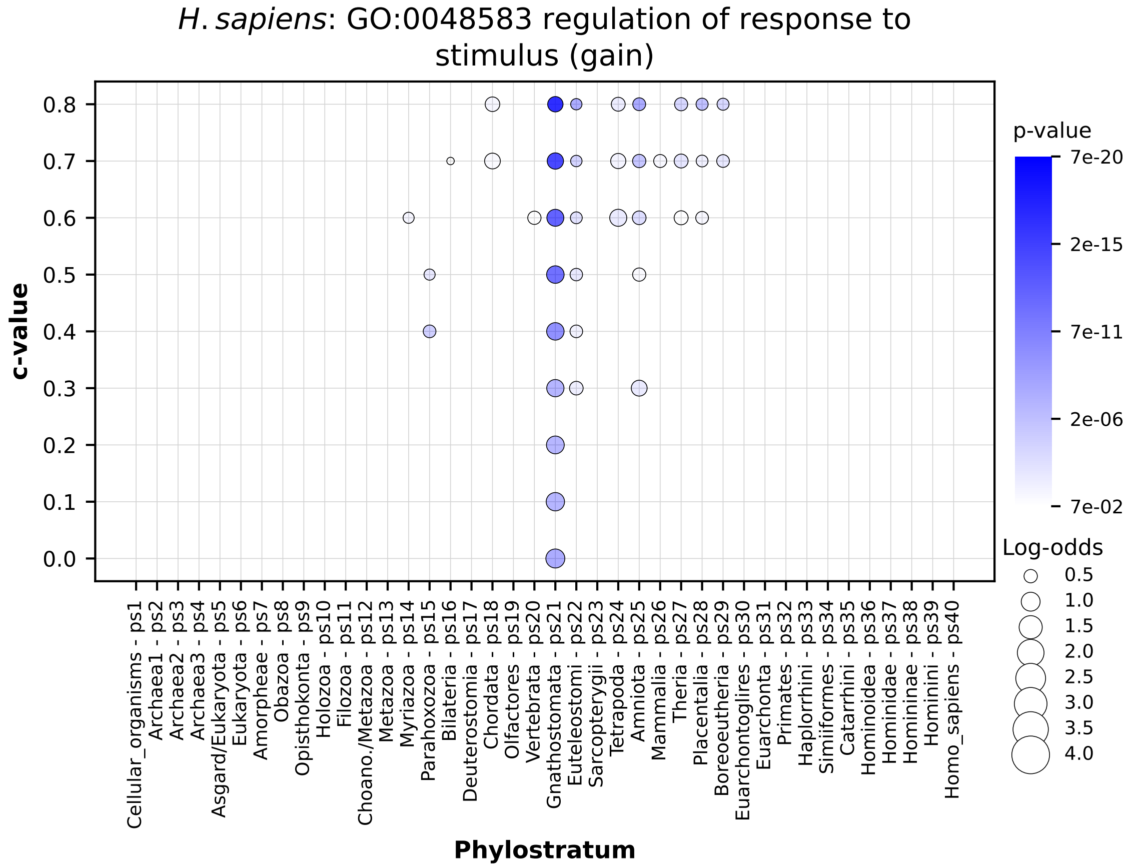

H. sapiens: GO:0048583 regulation of response to stimulus (gain)
c-value
0.0
0.1
0.2
0.3
0.4
0.5
0.6
0.7
0.8
Phylostratum
Cellular_organisms - ps1
Archaea1 - ps2
Archaea2 - ps3
Archaea3 - ps4
Asgard/Eukaryota - ps5
Eukaryota - ps6
Amorpheae - ps7
Obazoa - ps8
Opisthokonta - ps9
Holozoa - ps10
Filozoa - ps11
Choano./Metazoa - ps12
Metazoa - ps13
Myriazoa - ps14
Parahoxozoa - ps15
Bilateria - ps16
Deuterostomia - ps17
Chordata - ps18
Olfactores - ps19
Vertebrata - ps20
Gnathostomata - ps21
Euteleostomi - ps22
Sarcopterygii - ps23
Tetrapoda - ps24
Amniota - ps25
Mammalia - ps26
Theria - ps27
Placentalia - ps28
Boreoeutheria - ps29
Euarchontoglires - ps30
Euarchonta - ps31
Primates - ps32
Haplorrhini - ps33
Simiiformes - ps34
Catarrhini - ps35
Hominoidea - ps36
Hominidae - ps37
Homininae - ps38
Hominini - ps39
Homo_sapiens - ps40
p-value
7e-20
2e-15
7e-11
2e-06
7e-02
Log-odds
0.5
1.0
1.5
2.0
2.5
3.0
3.5
4.0

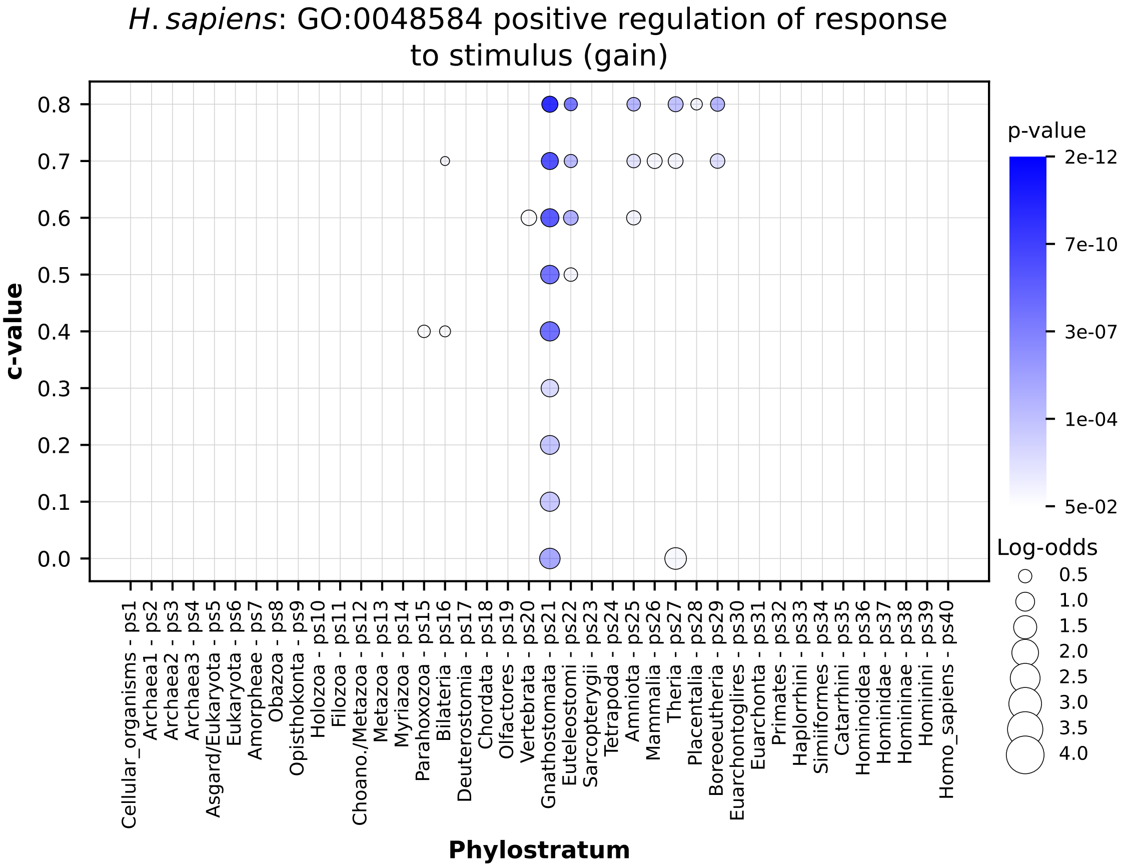

*H. sapiens*: GO:0048584 positive regulation of response to stimulus (gain)
c-value
0.8
0.7
0.6
0.5
0.4
0.3
0.2
0.1
0.0
p-value
2e-12
7e-10
3e-07
1e-04
5e-02
Log-odds
0.5
1.0
1.5
2.0
2.5
3.0
3.5
4.0
Cellular_organisms - ps1
Archaea1 - ps2
Archaea2 - ps3
Archaea3 - ps4
Asgard/Eukaryota - ps5
Eukaryota - ps6
Amorpheae - ps7
Obazoa - ps8
Opisthokonta - ps9
Holozoa - ps10
Filozoa - ps11
Choano./Metazoa - ps12
Metazoa - ps13
Myriazoa - ps14
Parahoxozoa - ps15
Bilateria - ps16
Deuterostomia - ps17
Chordata - ps18
Olfactores - ps19
Vertebrata - ps20
Gnathostomata - ps21
Euteleostomi - ps22
Sarcopterygii - ps23
Tetrapoda - ps24
Amniota - ps25
Mammalia - ps26
Theria - ps27
Placentalia - ps28
Boreoeutheria - ps29
Euarchontoglires - ps30
Euarchonta - ps31
Primates - ps32
Haplorrhini - ps33
Simiiformes - ps34
Catarrhini - ps35
Hominoidea - ps36
Hominidae - ps37
Homininae - ps38
Hominini - ps39
Homo_sapiens - ps40
Phylostratum

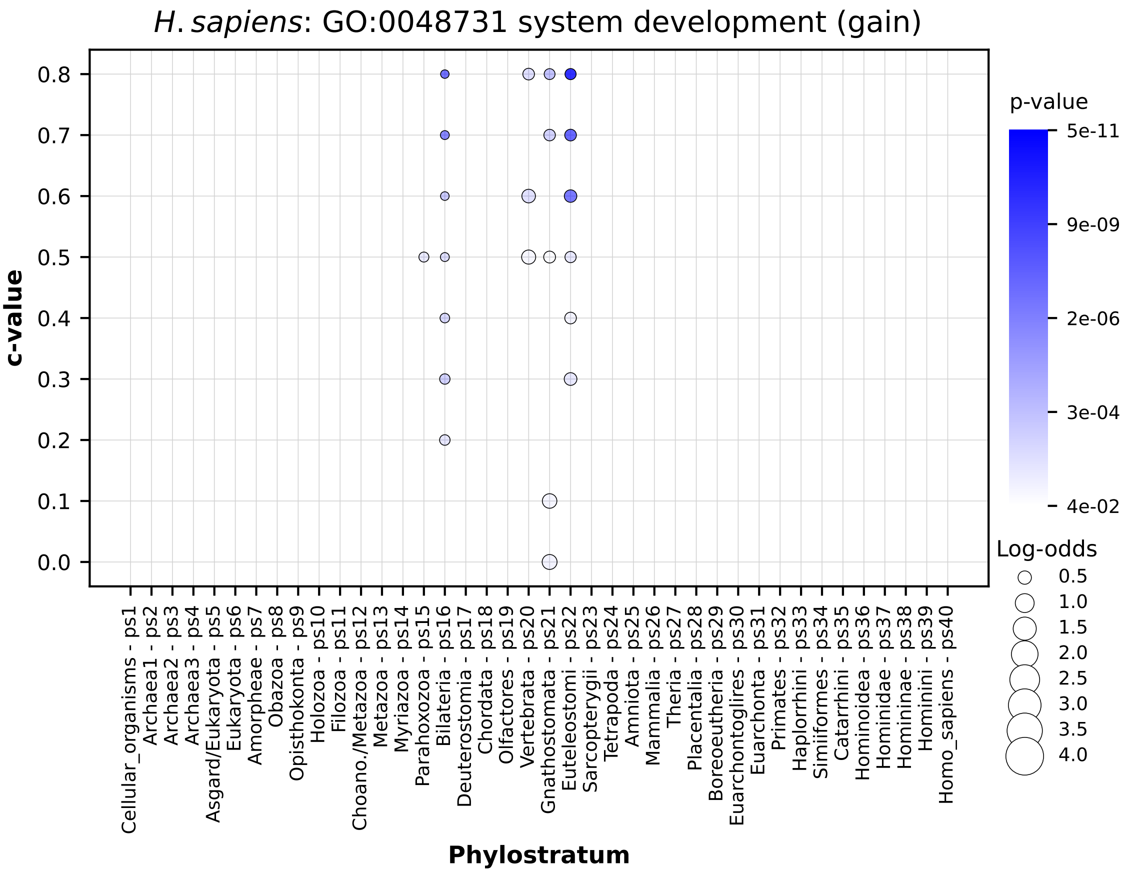
H. sapiens: GO:0048731 system development (gain)
c-value
0.8
0.7
0.6
0.5
0.4
0.3
0.2
0.1
0.0
p-value
5e-11
9e-09
2e-06
3e-04
4e-02
Log-odds
0.5
1.0
1.5
2.0
2.5
3.0
3.5
4.0
Cellular_organisms - ps1
Archaea1 - ps2
Archaea2 - ps3
Archaea3 - ps4
Asgard/Eukaryota - ps5
Eukaryota - ps6
Amorpheae - ps7
Obazoa - ps8
Opisthokonta - ps9
Holozoa - ps10
Filozoa - ps11
Choano./Metazoa - ps12
Metazoa - ps13
Myriazoa - ps14
Parahoxozoa - ps15
Bilateria - ps16
Deuterostomia - ps17
Chordata - ps18
Olfactores - ps19
Vertebrata - ps20
Gnathostomata - ps21
Euteleostomi - ps22
Sarcopterygii - ps23
Tetrapoda - ps24
Amniota - ps25
Mammalia - ps26
Theria - ps27
Placentalia - ps28
Boreoeutheria - ps29
Euarchontoglires - ps30
Euarchonta - ps31
Primates - ps32
Haplorrhini - ps33
Simiiformes - ps34
Catarrhini - ps35
Hominoidea - ps36
Hominidae - ps37
Homininae - ps38
Hominini - ps39
Homo_sapiens - ps40
Phylostratum

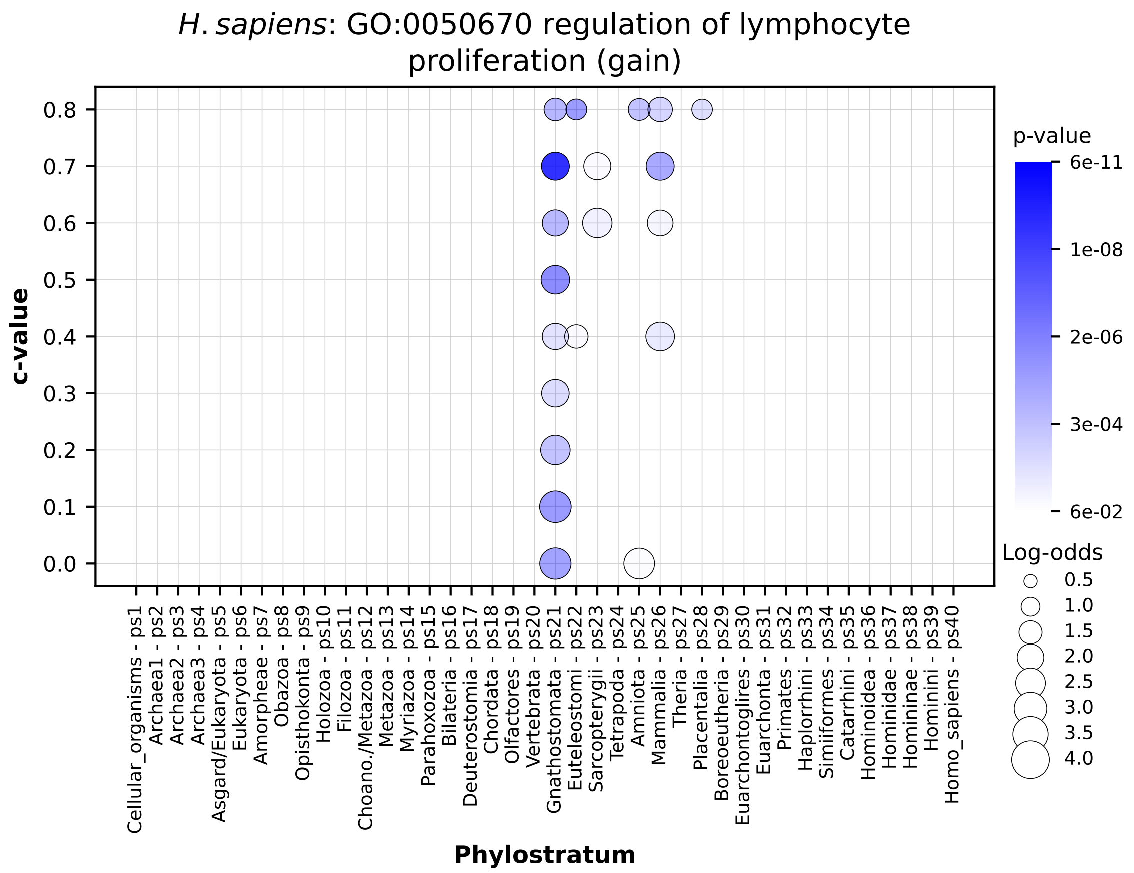
H. sapiens: GO:0050670 regulation of lymphocyte proliferation (gain)
c-value
0.8
0.7
0.6
0.5
0.4
0.3
0.2
0.1
0.0
p-value
6e-11
1e-08
2e-06
3e-04
6e-02
Log-odds
0.5
1.0
1.5
2.0
2.5
3.0
3.5
4.0
Cellular_organisms - ps1
Archaea1 - ps2
Archaea2 - ps3
Archaea3 - ps4
Asgard/Eukaryota - ps5
Eukaryota - ps6
Amorpheae - ps7
Obazoa - ps8
Opisthokonta - ps9
Holozoa - ps10
Filozoa - ps11
Choano./Metazoa - ps12
Metazoa - ps13
Myriazoa - ps14
Parahoxozoa - ps15
Bilateria - ps16
Deuterostomia - ps17
Chordata - ps18
Olfactores - ps19
Vertebrata - ps20
Gnathostomata - ps21
Euteleostomi - ps22
Sarcopterygii - ps23
Tetrapoda - ps24
Amniota - ps25
Mammalia - ps26
Theria - ps27
Placentalia - ps28
Boreoeutheria - ps29
Euarchontoglires - ps30
Euarchonta - ps31
Primates - ps32
Haplorrhini - ps33
Simiiformes - ps34
Catarrhini - ps35
Hominoidea - ps36
Hominidae - ps37
Homininae - ps38
Hominini - ps39
Homo_sapiens - ps40
Phylostratum

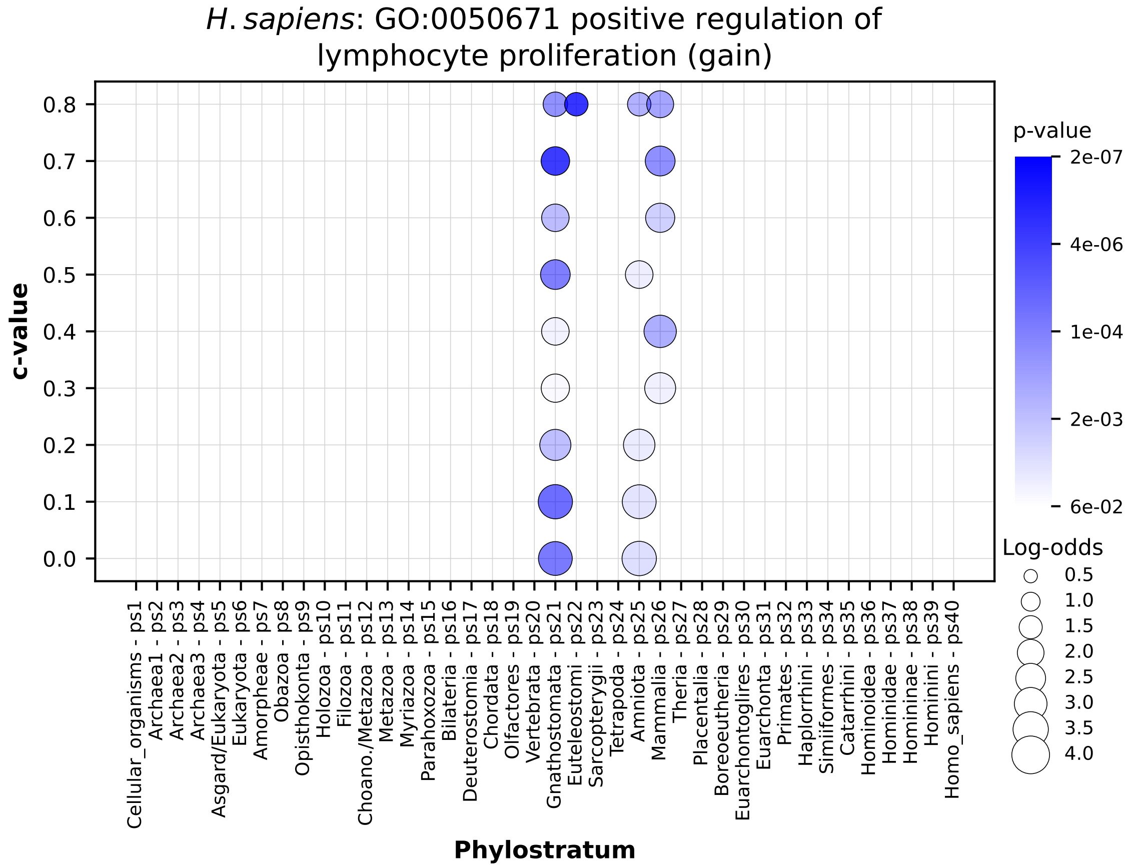

*H. sapiens*: GO:0050671 positive regulation of lymphocyte proliferation (gain)
c-value
0.8
0.7
0.6
0.5
0.4
0.3
0.2
0.1
0.0
Phylostratum
Cellular_organisms - ps1
Archaea1 - ps2
Archaea2 - ps3
Archaea3 - ps4
Asgard/Eukaryota - ps5
Eukaryota - ps6
Amorpheae - ps7
Obazoa - ps8
Opisthokonta - ps9
Holozoa - ps10
Filozoa - ps11
Choano./Metazoa - ps12
Metazoa - ps13
Myriazoa - ps14
Parahoxozoa - ps15
Bilateria - ps16
Deuterostomia - ps17
Chordata - ps18
Olfactores - ps19
Vertebrata - ps20
Gnathostomata - ps21
Euteleostomi - ps22
Sarcopterygii - ps23
Tetrapoda - ps24
Amniota - ps25
Mammalia - ps26
Theria - ps27
Placentalia - ps28
Boreoeutheria - ps29
Euarchontoglires - ps30
Euarchonta - ps31
Primates - ps32
Haplorrhini - ps33
Simiiformes - ps34
Catarrhini - ps35
Hominoidea - ps36
Hominidae - ps37
Homininae - ps38
Hominini - ps39
Homo_sapiens - ps40
p-value
2e-07
4e-06
1e-04
2e-03
6e-02
Log-odds
0.5
1.0
1.5
2.0
2.5
3.0
3.5
4.0

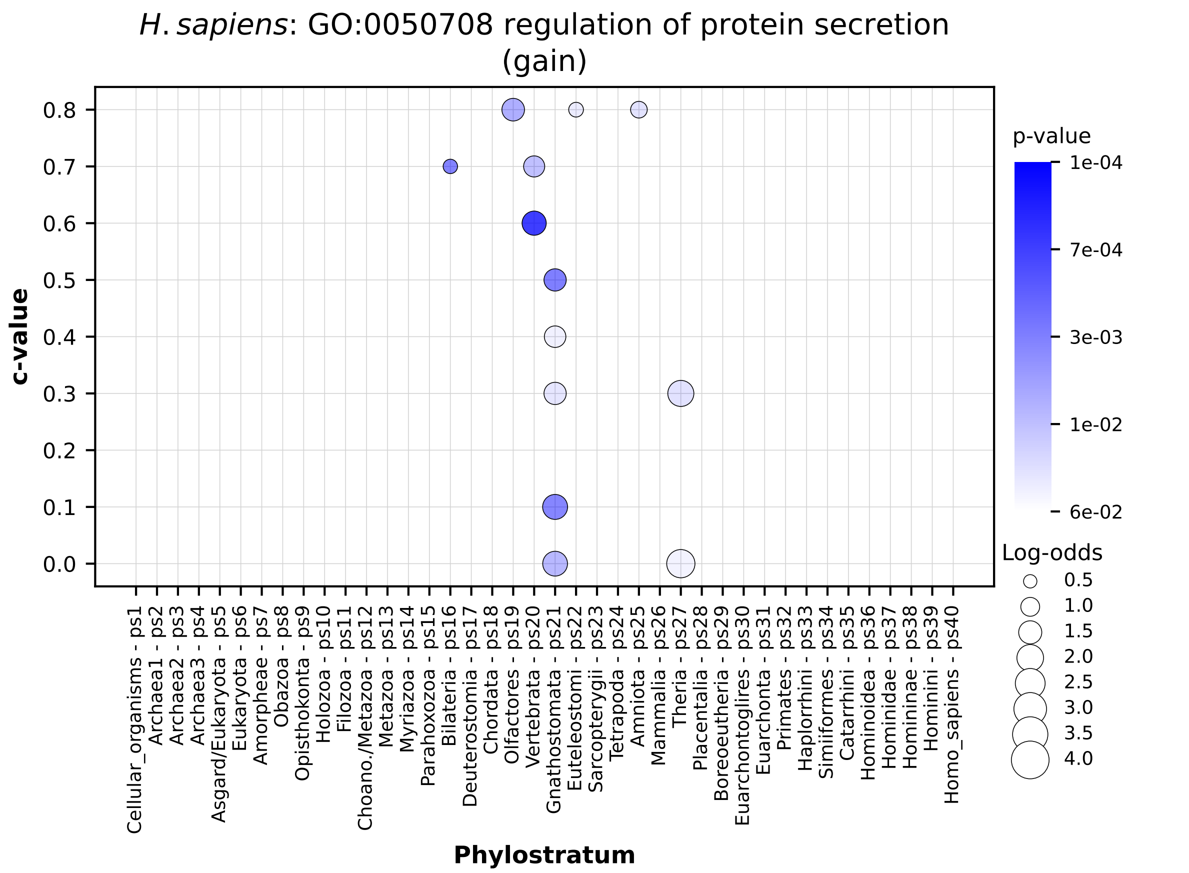

H. sapiens: GO:0050708 regulation of protein secretion (gain)
c-value
0.8
0.7
0.6
0.5
0.4
0.3
0.2
0.1
0.0
p-value
1e-04
7e-04
3e-03
1e-02
6e-02
Log-odds
0.5
1.0
1.5
2.0
2.5
3.0
3.5
4.0
Cellular_organisms - ps1
Archaea1 - ps2
Archaea2 - ps3
Archaea3 - ps4
Asgard/Eukaryota - ps5
Eukaryota - ps6
Amorpheae - ps7
Obazoa - ps8
Opisthokonta - ps9
Holozoa - ps10
Filozoa - ps11
Choano./Metazoa - ps12
Metazoa - ps13
Myriazoa - ps14
Parahoxozoa - ps15
Bilateria - ps16
Deuterostomia - ps17
Chordata - ps18
Olfactores - ps19
Vertebrata - ps20
Gnathostomata - ps21
Euteleostomi - ps22
Sarcopterygii - ps23
Tetrapoda - ps24
Amniota - ps25
Mammalia - ps26
Theria - ps27
Placentalia - ps28
Boreoeutheria - ps29
Euarchontoglires - ps30
Euarchonta - ps31
Primates - ps32
Haplorrhini - ps33
Simiiformes - ps34
Catarrhini - ps35
Hominoidea - ps36
Hominidae - ps37
Homininae - ps38
Hominini - ps39
Homo_sapiens - ps40
Phylostratum

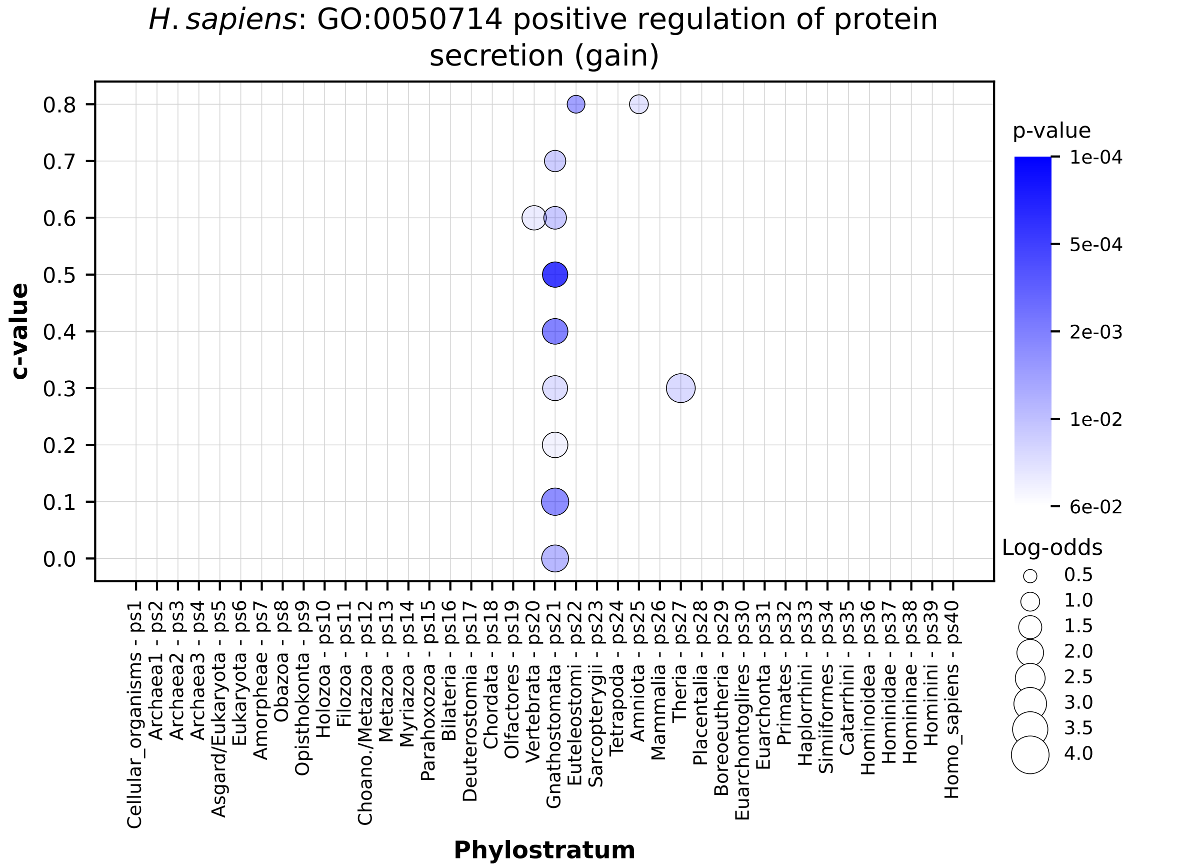

H. sapiens: GO:0050714 positive regulation of protein secretion (gain)
c-value
0.0
0.1
0.2
0.3
0.4
0.5
0.6
0.7
0.8
Phylostratum
Cellular_organisms - ps1
Archaea1 - ps2
Archaea2 - ps3
Archaea3 - ps4
Asgard/Eukaryota - ps5
Eukaryota - ps6
Amorpheae - ps7
Obazoa - ps8
Opisthokonta - ps9
Holozoa - ps10
Filozoa - ps11
Choano./Metazoa - ps12
Metazoa - ps13
Myriazoa - ps14
Parahoxozoa - ps15
Bilateria - ps16
Deuterostomia - ps17
Chordata - ps18
Olfactores - ps19
Vertebrata - ps20
Gnathostomata - ps21
Euteleostomi - ps22
Sarcopterygii - ps23
Tetrapoda - ps24
Amniota - ps25
Mammalia - ps26
Theria - ps27
Placentalia - ps28
Boreoeutheria - ps29
Euarchontoglires - ps30
Euarchonta - ps31
Primates - ps32
Haplorrhini - ps33
Simiiformes - ps34
Catarrhini - ps35
Hominoidea - ps36
Hominidae - ps37
Homininae - ps38
Hominini - ps39
Homo_sapiens - ps40
p-value
1e-04
5e-04
2e-03
1e-02
6e-02
Log-odds
0.5
1.0
1.5
2.0
2.5
3.0
3.5
4.0

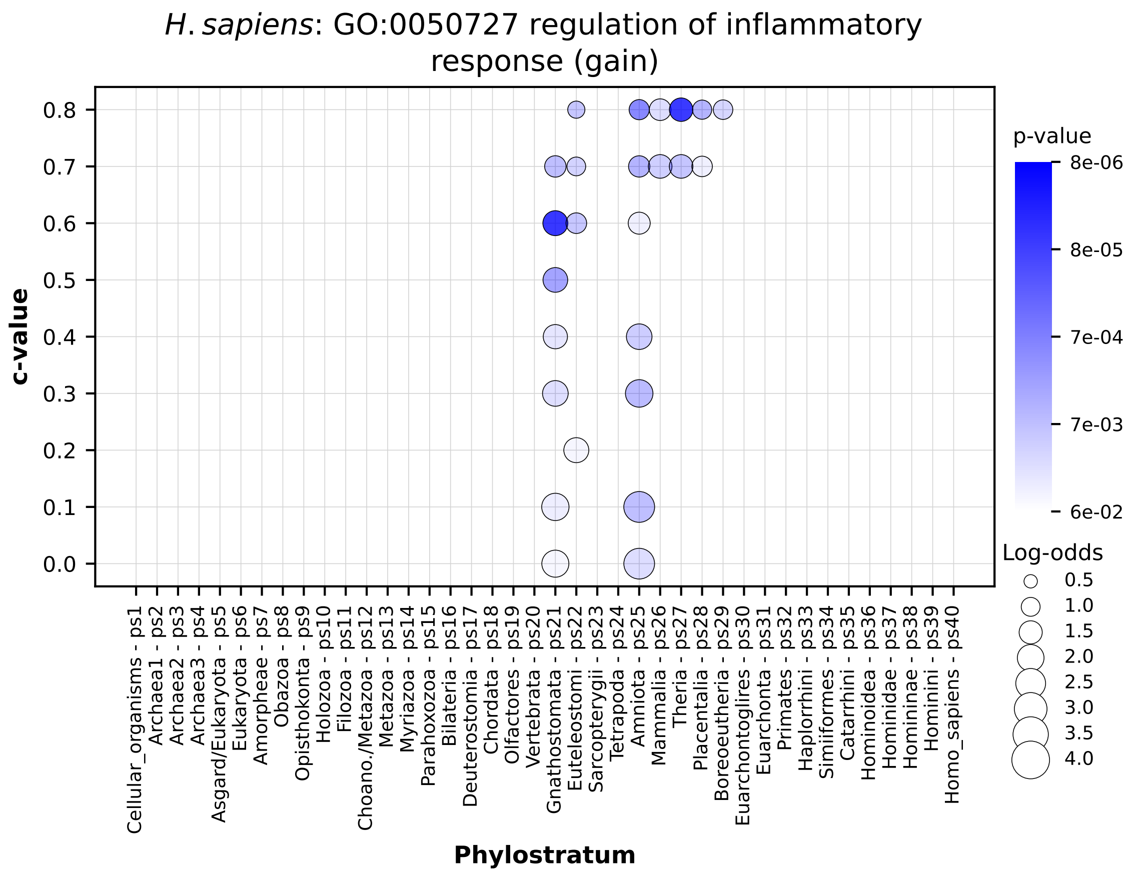

H. sapiens: GO:0050727 regulation of inflammatory response (gain)
c-value
0.8
0.7
0.6
0.5
0.4
0.3
0.2
0.1
0.0
p-value
8e-06
8e-05
7e-04
7e-03
6e-02
Log-odds
0.5
1.0
1.5
2.0
2.5
3.0
3.5
4.0
Cellular_organisms - ps1
Archaea1 - ps2
Archaea2 - ps3
Archaea3 - ps4
Asgard/Eukaryota - ps5
Eukaryota - ps6
Amorpheae - ps7
Obazoa - ps8
Opisthokonta - ps9
Holozoa - ps10
Filozoa - ps11
Choano./Metazoa - ps12
Metazoa - ps13
Myriazoa - ps14
Parahoxozoa - ps15
Bilateria - ps16
Deuterostomia - ps17
Chordata - ps18
Olfactores - ps19
Vertebrata - ps20
Gnathostomata - ps21
Euteleostomi - ps22
Sarcopterygii - ps23
Tetrapoda - ps24
Amniota - ps25
Mammalia - ps26
Theria - ps27
Placentalia - ps28
Boreoeutheria - ps29
Euarchontoglires - ps30
Euarchonta - ps31
Primates - ps32
Haplorrhini - ps33
Simiiformes - ps34
Catarrhini - ps35
Hominoidea - ps36
Hominidae - ps37
Homininae - ps38
Hominini - ps39
Homo_sapiens - ps40
Phylostratum

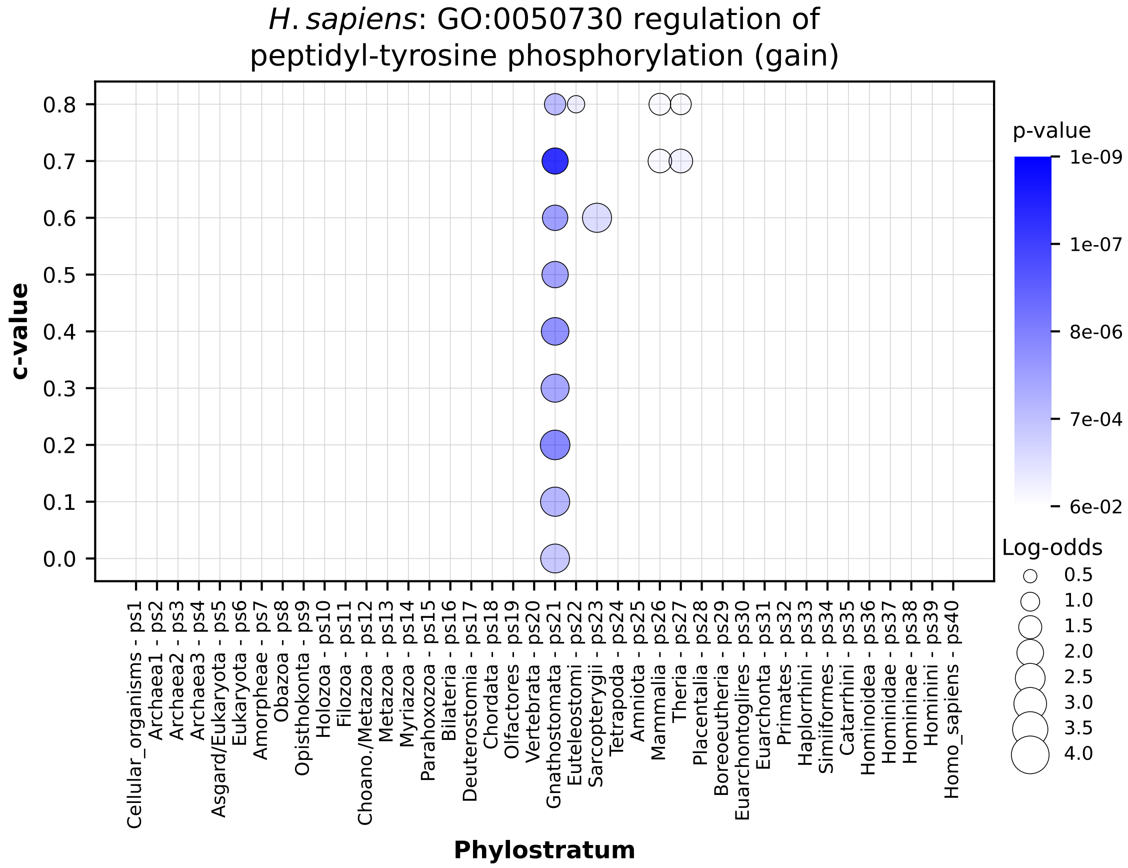

H. sapiens: GO:0050730 regulation of peptidyl-tyrosine phosphorylation (gain)
c-value
0.8
0.7
0.6
0.5
0.4
0.3
0.2
0.1
0.0
Phylostratum
Cellular_organisms - ps1
Archaea1 - ps2
Archaea2 - ps3
Archaea3 - ps4
Asgard/Eukaryota - ps5
Eukaryota - ps6
Amorpheae - ps7
Obazoa - ps8
Opisthokonta - ps9
Holozoa - ps10
Filozoa - ps11
Choano./Metazoa - ps12
Metazoa - ps13
Myriazoa - ps14
Parahoxozoa - ps15
Bilateria - ps16
Deuterostomia - ps17
Chordata - ps18
Olfactores - ps19
Vertebrata - ps20
Gnathostomata - ps21
Euteleostomi - ps22
Sarcopterygii - ps23
Tetrapoda - ps24
Amniota - ps25
Mammalia - ps26
Theria - ps27
Placentalia - ps28
Boreoeutheria - ps29
Euarchontoglires - ps30
Euarchonta - ps31
Primates - ps32
Haplorrhini - ps33
Simiiformes - ps34
Catarrhini - ps35
Hominoidea - ps36
Hominidae - ps37
Homininae - ps38
Hominini - ps39
Homo_sapiens - ps40
p-value
1e-09
1e-07
8e-06
7e-04
6e-02
Log-odds
0.5
1.0
1.5
2.0
2.5
3.0
3.5
4.0

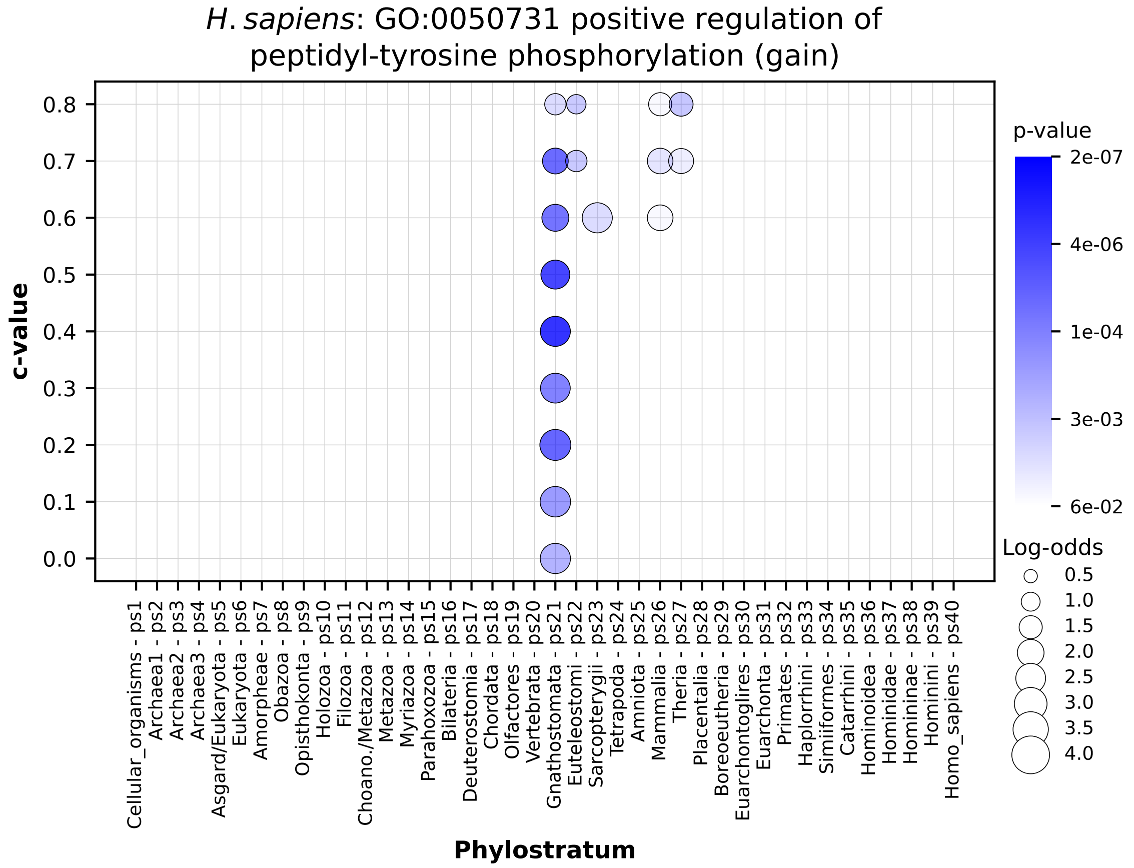

H. sapiens: GO:0050731 positive regulation of peptidyl-tyrosine phosphorylation (gain)
c-value
0.8
0.7
0.6
0.5
0.4
0.3
0.2
0.1
0.0
p-value
2e-07
4e-06
1e-04
3e-03
6e-02
Log-odds
0.5
1.0
1.5
2.0
2.5
3.0
3.5
4.0
Cellular_organisms - ps1
Archaea1 - ps2
Archaea2 - ps3
Archaea3 - ps4
Asgard/Eukaryota - ps5
Eukaryota - ps6
Amorpheae - ps7
Obazoa - ps8
Opisthokonta - ps9
Holozoa - ps10
Filozoa - ps11
Choano./Metazoa - ps12
Metazoa - ps13
Myriazoa - ps14
Parahoxozoa - ps15
Bilateria - ps16
Deuterostomia - ps17
Chordata - ps18
Olfactores - ps19
Vertebrata - ps20
Gnathostomata - ps21
Euteleostomi - ps22
Sarcopterygii - ps23
Tetrapoda - ps24
Amniota - ps25
Mammalia - ps26
Theria - ps27
Placentalia - ps28
Boreoeutheria - ps29
Euarchontoglires - ps30
Euarchonta - ps31
Primates - ps32
Haplorrhini - ps33
Simiiformes - ps34
Catarrhini - ps35
Hominoidea - ps36
Hominidae - ps37
Homininae - ps38
Hominini - ps39
Homo_sapiens - ps40
Phylostratum

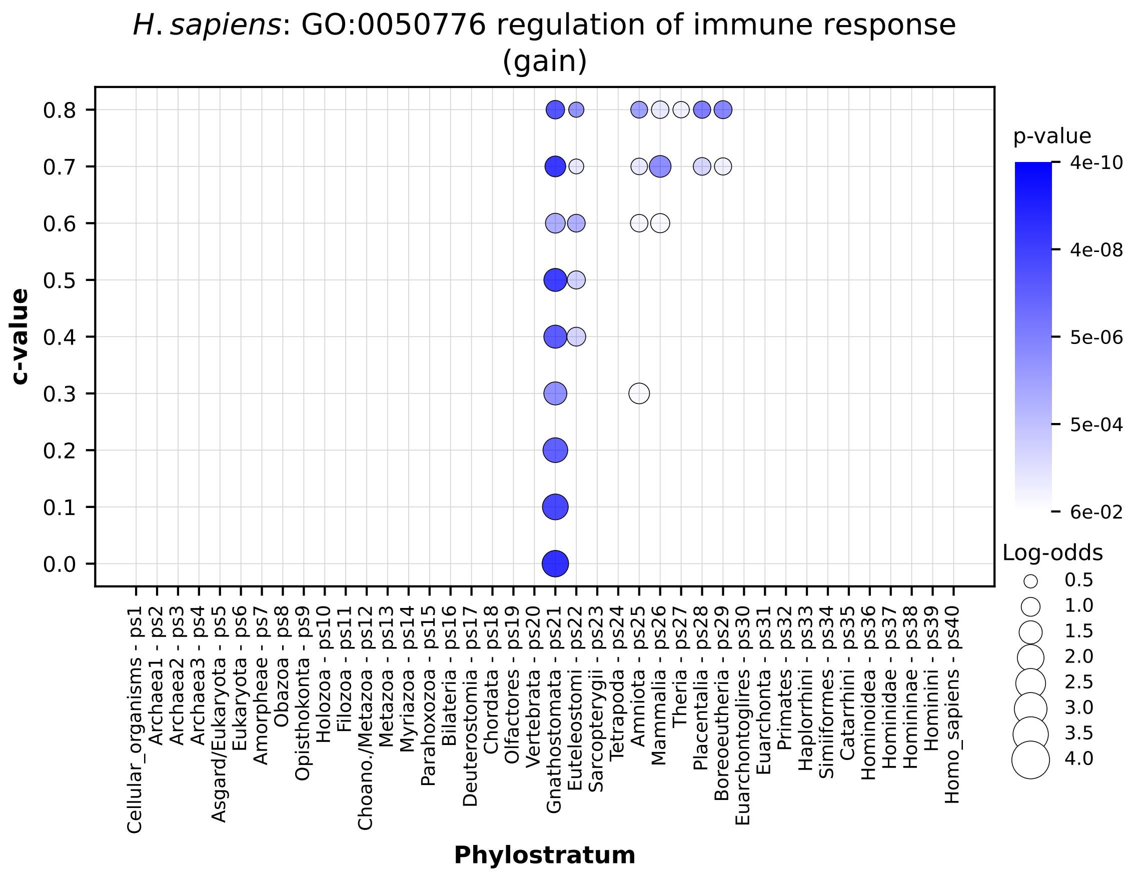

H. sapiens: GO:0050776 regulation of immune response
(gain)
c-value
0.0
0.1
0.2
0.3
0.4
0.5
0.6
0.7
0.8
Phylostratum
Cellular_organisms - ps1
Archaea1 - ps2
Archaea2 - ps3
Archaea3 - ps4
Asgard/Eukaryota - ps5
Eukaryota - ps6
Amorpheae - ps7
Obazoa - ps8
Opisthokonta - ps9
Holozoa - ps10
Filozoa - ps11
Choano./Metazoa - ps12
Metazoa - ps13
Myriazoa - ps14
Parahoxozoa - ps15
Bilateria - ps16
Deuterostomia - ps17
Chordata - ps18
Olfactores - ps19
Vertebrata - ps20
Gnathostomata - ps21
Euteleostomi - ps22
Sarcopterygii - ps23
Tetrapoda - ps24
Amniota - ps25
Mammalia - ps26
Theria - ps27
Placentalia - ps28
Boreoeutheria - ps29
Euarchontoglires - ps30
Euarchonta - ps31
Primates - ps32
Haplorrhini - ps33
Simiiformes - ps34
Catarrhini - ps35
Hominoidea - ps36
Hominidae - ps37
Homininae - ps38
Hominini - ps39
Homo_sapiens - ps40
p-value
4e-10
4e-08
5e-06
5e-04
6e-02
Log-odds
0.5
1.0
1.5
2.0
2.5
3.0
3.5
4.0

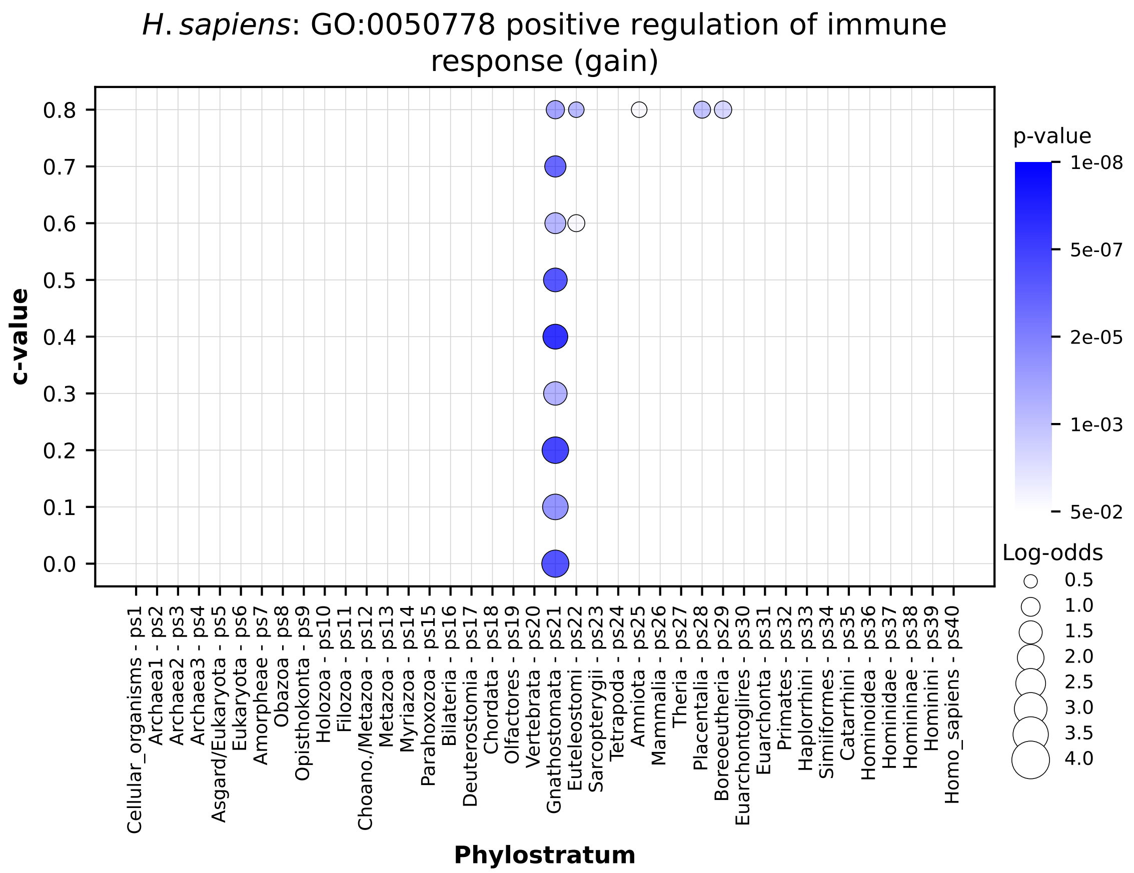

*H. sapiens*: GO:0050778 positive regulation of immune response (gain)
c-value
0.8
0.7
0.6
0.5
0.4
0.3
0.2
0.1
0.0
p-value
1e-08
5e-07
2e-05
1e-03
5e-02
Log-odds
0.5
1.0
1.5
2.0
2.5
3.0
3.5
4.0
Cellular_organisms - ps1
Archaea1 - ps2
Archaea2 - ps3
Archaea3 - ps4
Asgard/Eukaryota - ps5
Eukaryota - ps6
Amorpheae - ps7
Obazoa - ps8
Opisthokonta - ps9
Holozoa - ps10
Filozoa - ps11
Choano./Metazoa - ps12
Metazoa - ps13
Myriazoa - ps14
Parahoxozoa - ps15
Bilateria - ps16
Deuterostomia - ps17
Chordata - ps18
Olfactores - ps19
Vertebrata - ps20
Gnathostomata - ps21
Euteleostomi - ps22
Sarcopterygii - ps23
Tetrapoda - ps24
Amniota - ps25
Mammalia - ps26
Theria - ps27
Placentalia - ps28
Boreoeutheria - ps29
Euarchontoglires - ps30
Euarchonta - ps31
Primates - ps32
Haplorrhini - ps33
Simiiformes - ps34
Catarrhini - ps35
Hominoidea - ps36
Hominidae - ps37
Homininae - ps38
Hominini - ps39
Homo_sapiens - ps40
Phylostratum

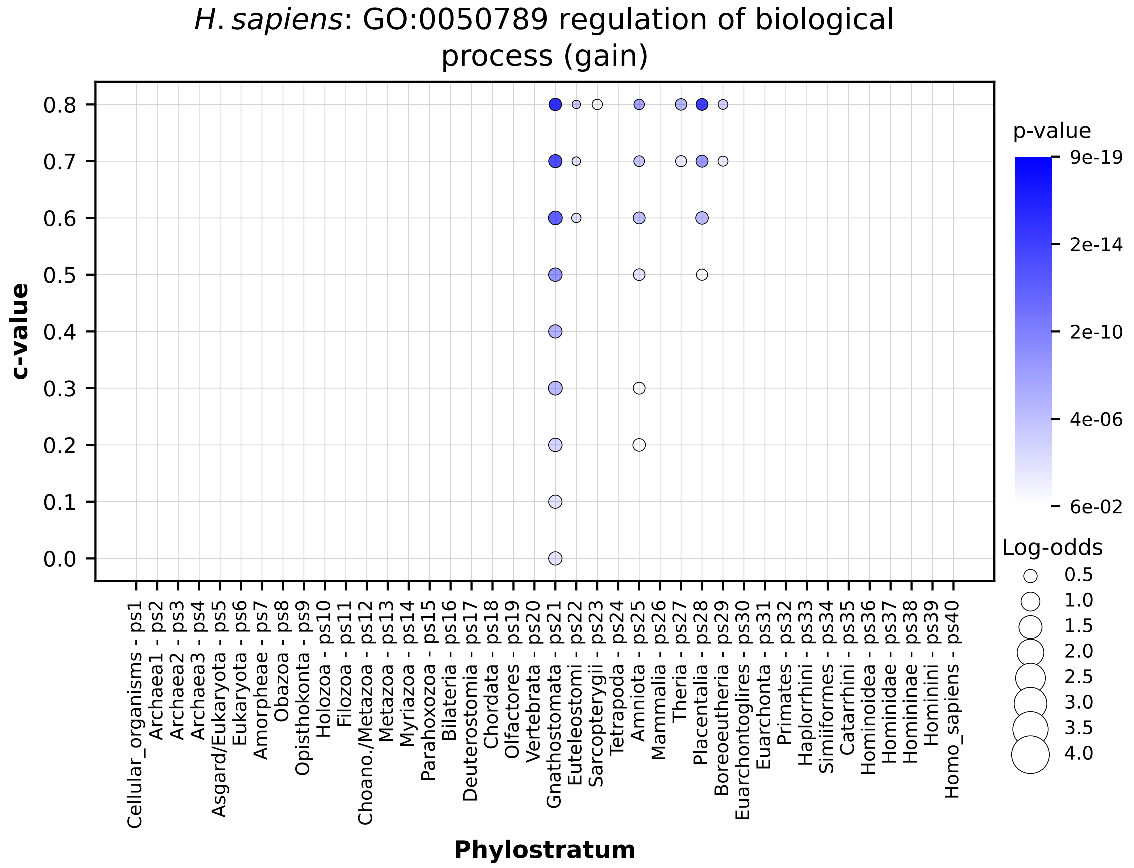

H. sapiens: GO:0050789 regulation of biological process (gain)
c-value
0.0
0.1
0.2
0.3
0.4
0.5
0.6
0.7
0.8
Phylostratum
Cellular_organisms - ps1
Archaea1 - ps2
Archaea2 - ps3
Archaea3 - ps4
Asgard/Eukaryota - ps5
Eukaryota - ps6
Amorpheae - ps7
Obazoa - ps8
Opisthokonta - ps9
Holozoa - ps10
Filozoa - ps11
Choano./Metazoa - ps12
Metazoa - ps13
Myriazoa - ps14
Parahoxozoa - ps15
Bilateria - ps16
Deuterostomia - ps17
Chordata - ps18
Olfactores - ps19
Vertebrata - ps20
Gnathostomata - ps21
Euteleostomi - ps22
Sarcopterygii - ps23
Tetrapoda - ps24
Amniota - ps25
Mammalia - ps26
Theria - ps27
Placentalia - ps28
Boreoeutheria - ps29
Euarchontoglires - ps30
Euarchonta - ps31
Primates - ps32
Haplorrhini - ps33
Simiiformes - ps34
Catarrhini - ps35
Hominoidea - ps36
Hominidae - ps37
Homininae - ps38
Hominini - ps39
Homo_sapiens - ps40
p-value
9e-19
2e-14
2e-10
4e-06
6e-02
Log-odds
0.5
1.0
1.5
2.0
2.5
3.0
3.5
4.0

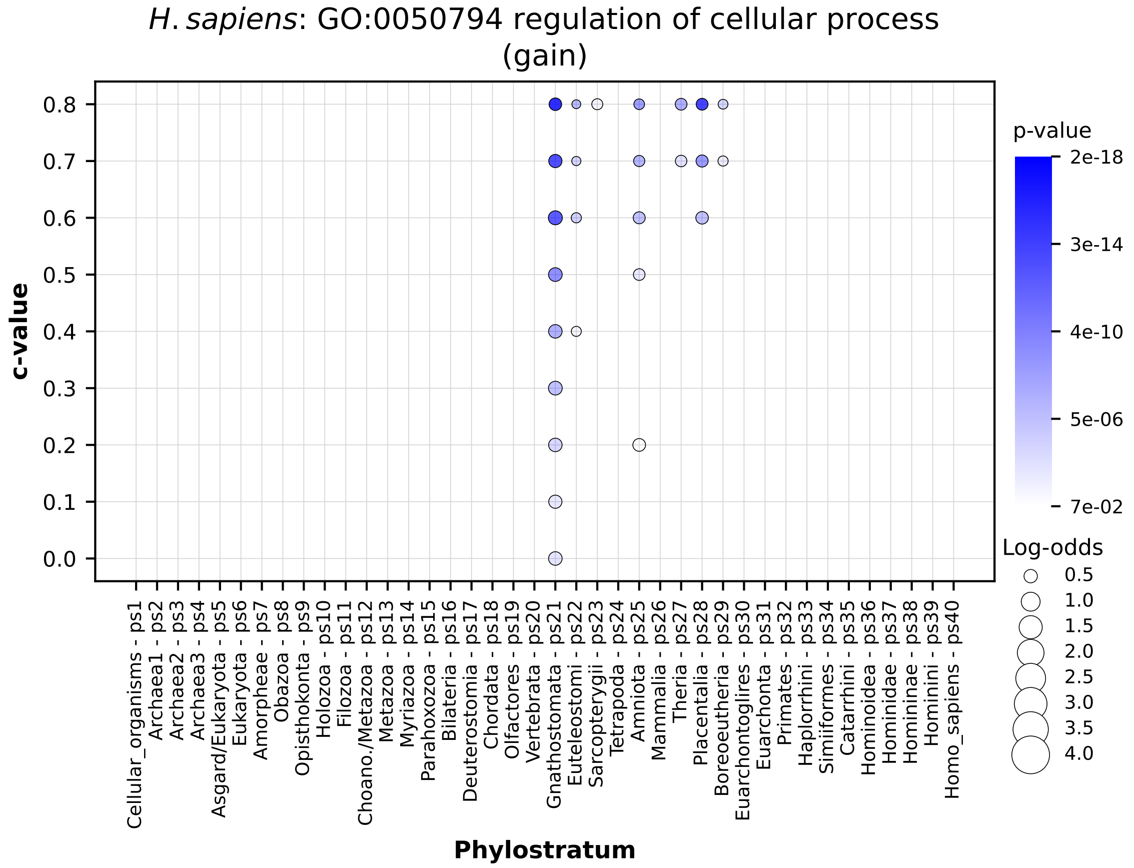

H. sapiens: GO:0050794 regulation of cellular process
(gain)
c-value
0.8
0.7
0.6
0.5
0.4
0.3
0.2
0.1
0.0
p-value
2e-18
3e-14
4e-10
5e-06
7e-02
Log-odds
0.5
1.0
1.5
2.0
2.5
3.0
3.5
4.0
Cellular_organisms - ps1
Archaea1 - ps2
Archaea2 - ps3
Archaea3 - ps4
Asgard/Eukaryota - ps5
Eukaryota - ps6
Amorpheae - ps7
Obazoa - ps8
Opisthokonta - ps9
Holozoa - ps10
Filozoa - ps11
Choano./Metazoa - ps12
Metazoa - ps13
Myriazoa - ps14
Parahoxozoa - ps15
Bilateria - ps16
Deuterostomia - ps17
Chordata - ps18
Olfactores - ps19
Vertebrata - ps20
Gnathostomata - ps21
Euteleostomi - ps22
Sarcopterygii - ps23
Tetrapoda - ps24
Amniota - ps25
Mammalia - ps26
Theria - ps27
Placentalia - ps28
Boreoeutheria - ps29
Euarchontoglires - ps30
Euarchonta - ps31
Primates - ps32
Haplorrhini - ps33
Simiiformes - ps34
Catarrhini - ps35
Hominoidea - ps36
Hominidae - ps37
Homininae - ps38
Hominini - ps39
Homo_sapiens - ps40
Phylostratum

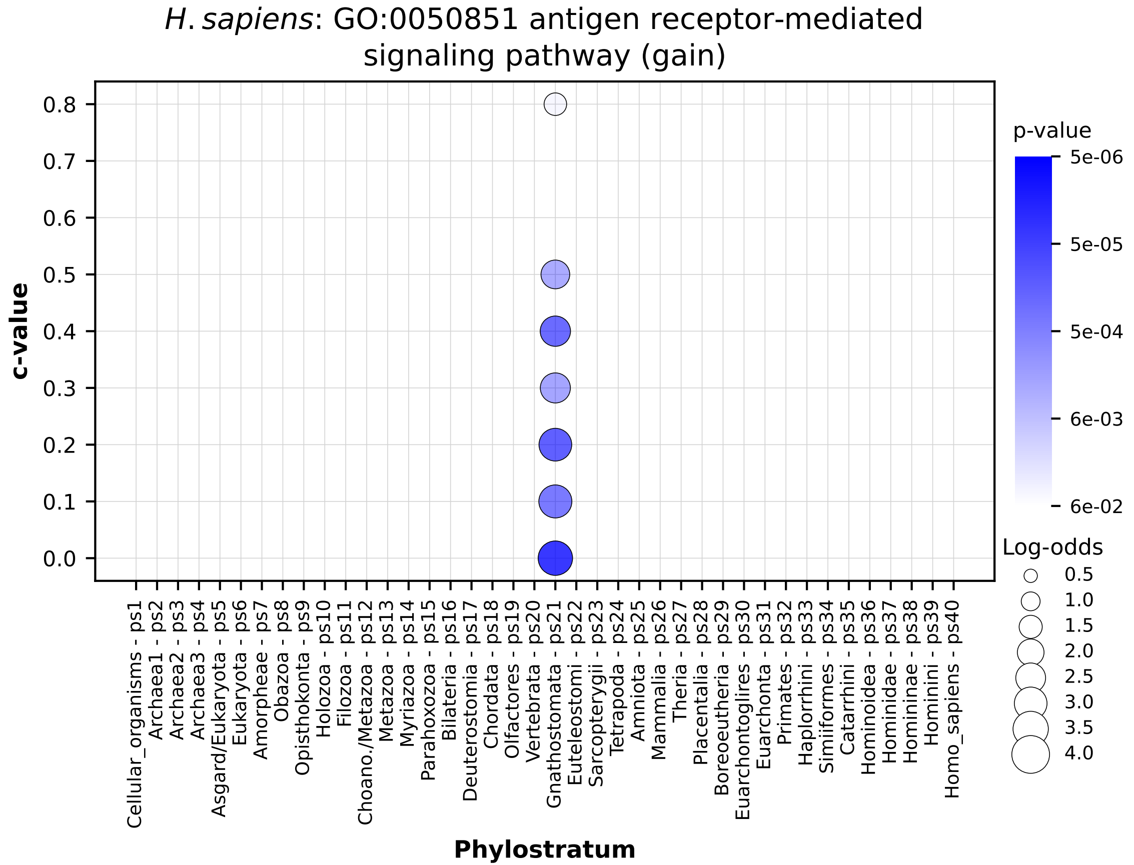

*H. sapiens*: GO:0050851 antigen receptor-mediated signaling pathway (gain)
c-value
0.0
0.1
0.2
0.3
0.4
0.5
0.6
0.7
0.8
p-value
5e-06
5e-05
5e-04
6e-03
6e-02
Log-odds
0.5
1.0
1.5
2.0
2.5
3.0
3.5
4.0
Cellular_organisms - ps1
Archaea1 - ps2
Archaea2 - ps3
Archaea3 - ps4
Asgard/Eukaryota - ps5
Eukaryota - ps6
Amorpheae - ps7
Obazoa - ps8
Opisthokonta - ps9
Holozoa - ps10
Filozoa - ps11
Choano./Metazoa - ps12
Metazoa - ps13
Myriazoa - ps14
Parahoxozoa - ps15
Bilateria - ps16
Deuterostomia - ps17
Chordata - ps18
Olfactores - ps19
Vertebrata - ps20
Gnathostomata - ps21
Euteleostomi - ps22
Sarcopterygii - ps23
Tetrapoda - ps24
Amniota - ps25
Mammalia - ps26
Theria - ps27
Placentalia - ps28
Boreoeutheria - ps29
Euarchontoglires - ps30
Euarchonta - ps31
Primates - ps32
Haplorrhini - ps33
Simiiformes - ps34
Catarrhini - ps35
Hominoidea - ps36
Hominidae - ps37
Homininae - ps38
Hominini - ps39
Homo_sapiens - ps40
Phylostratum

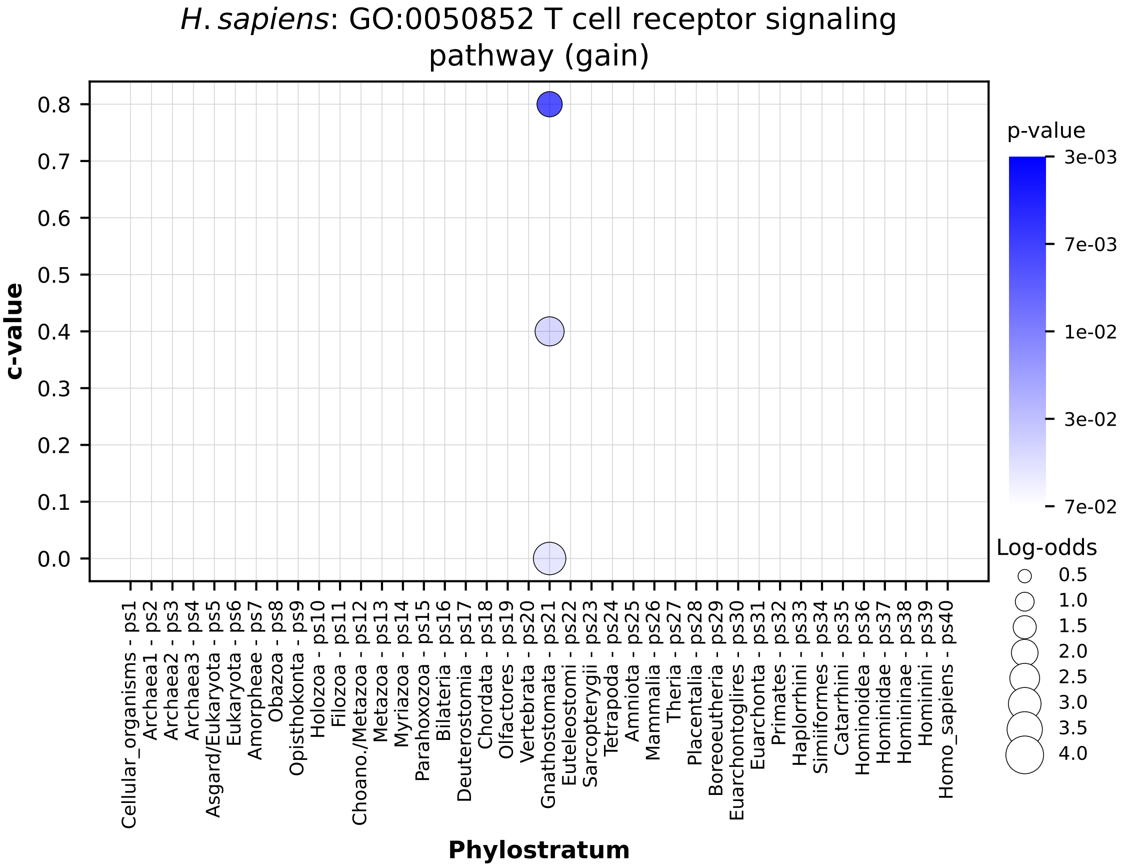

H. sapiens: GO:0050852 T cell receptor signaling pathway (gain)
c-value
0.0
0.1
0.2
0.3
0.4
0.5
0.6
0.7
0.8
Phylostratum
Cellular_organisms - ps1
Archaea1 - ps2
Archaea2 - ps3
Archaea3 - ps4
Asgard/Eukaryota - ps5
Eukaryota - ps6
Amorpheae - ps7
Obazoa - ps8
Opisthokonta - ps9
Holozoa - ps10
Filozoa - ps11
Choano./Metazoa - ps12
Metazoa - ps13
Myriazoa - ps14
Parahoxozoa - ps15
Bilateria - ps16
Deuterostomia - ps17
Chordata - ps18
Olfactores - ps19
Vertebrata - ps20
Gnathostomata - ps21
Euteleostomi - ps22
Sarcopterygii - ps23
Tetrapoda - ps24
Amniota - ps25
Mammalia - ps26
Theria - ps27
Placentalia - ps28
Boreoeutheria - ps29
Euarchontoglires - ps30
Euarchonta - ps31
Primates - ps32
Haplorrhini - ps33
Simiiformes - ps34
Catarrhini - ps35
Hominoidea - ps36
Hominidae - ps37
Homininae - ps38
Hominini - ps39
Homo_sapiens - ps40
p-value
3e-03
7e-03
1e-02
3e-02
7e-02
Log-odds
0.5
1.0
1.5
2.0
2.5
3.0
3.5
4.0

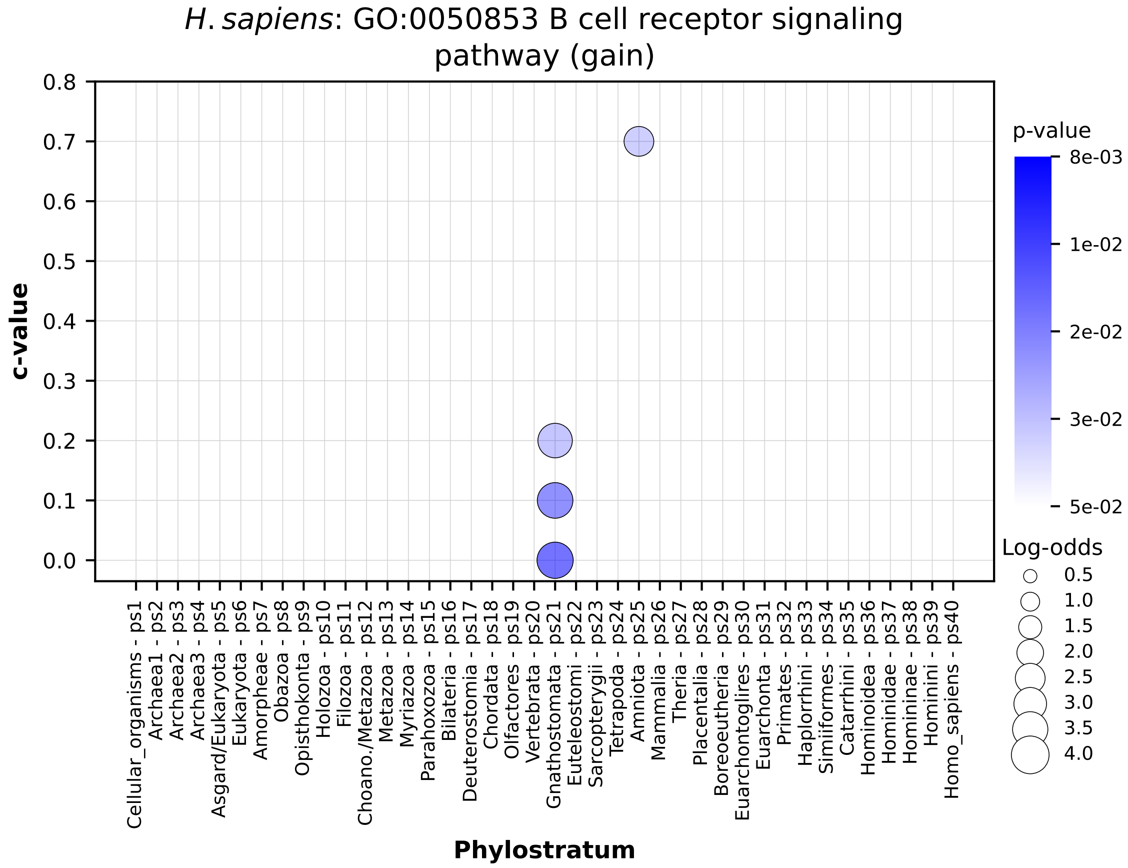

$H. sapiens$: GO:0050853 B cell receptor signaling pathway (gain)
c-value
0.8
0.7
0.6
0.5
0.4
0.3
0.2
0.1
0.0
Phylostratum
Cellular_organisms - ps1
Archaea1 - ps2
Archaea2 - ps3
Archaea3 - ps4
Asgard/Eukaryota - ps5
Eukaryota - ps6
Amorpheae - ps7
Obazoa - ps8
Opisthokonta - ps9
Holozoa - ps10
Filozoa - ps11
Choano./Metazoa - ps12
Metazoa - ps13
Myriazoa - ps14
Parahoxozoa - ps15
Bilateria - ps16
Deuterostomia - ps17
Chordata - ps18
Olfactores - ps19
Vertebrata - ps20
Gnathostomata - ps21
Euteleostomi - ps22
Sarcopterygii - ps23
Tetrapoda - ps24
Amniota - ps25
Mammalia - ps26
Theria - ps27
Placentalia - ps28
Boreoeutheria - ps29
Euarchontoglires - ps30
Euarchonta - ps31
Primates - ps32
Haplorrhini - ps33
Simiiformes - ps34
Catarrhini - ps35
Hominoidea - ps36
Hominidae - ps37
Homininae - ps38
Hominini - ps39
Homo_sapiens - ps40
p-value
8e-03
1e-02
2e-02
3e-02
5e-02
Log-odds
0.5
1.0
1.5
2.0
2.5
3.0
3.5
4.0

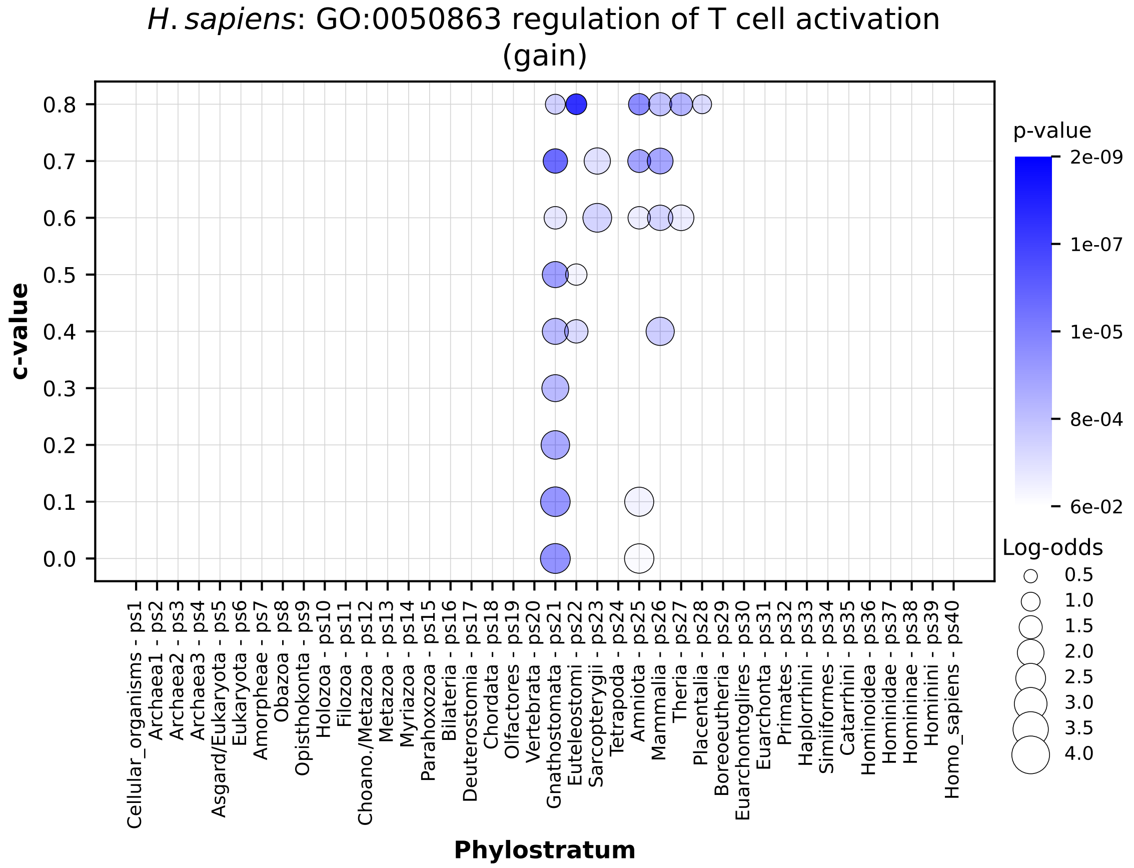

H. sapiens: GO:0050863 regulation of T cell activation (gain)
c-value
0.0
0.1
0.2
0.3
0.4
0.5
0.6
0.7
0.8
Phylostratum
Cellular_organisms - ps1
Archaea1 - ps2
Archaea2 - ps3
Archaea3 - ps4
Asgard/Eukaryota - ps5
Eukaryota - ps6
Amorpheae - ps7
Obazoa - ps8
Opisthokonta - ps9
Holozoa - ps10
Filozoa - ps11
Choano./Metazoa - ps12
Metazoa - ps13
Myriazoa - ps14
Parahoxozoa - ps15
Bilateria - ps16
Deuterostomia - ps17
Chordata - ps18
Olfactores - ps19
Vertebrata - ps20
Gnathostomata - ps21
Euteleostomi - ps22
Sarcopterygii - ps23
Tetrapoda - ps24
Amniota - ps25
Mammalia - ps26
Theria - ps27
Placentalia - ps28
Boreoeutheria - ps29
Euarchontoglires - ps30
Euarchonta - ps31
Primates - ps32
Haplorrhini - ps33
Simiiformes - ps34
Catarrhini - ps35
Hominoidea - ps36
Hominidae - ps37
Homininae - ps38
Hominini - ps39
Homo_sapiens - ps40
p-value
2e-09
1e-07
1e-05
8e-04
6e-02
Log-odds
0.5
1.0
1.5
2.0
2.5
3.0
3.5
4.0

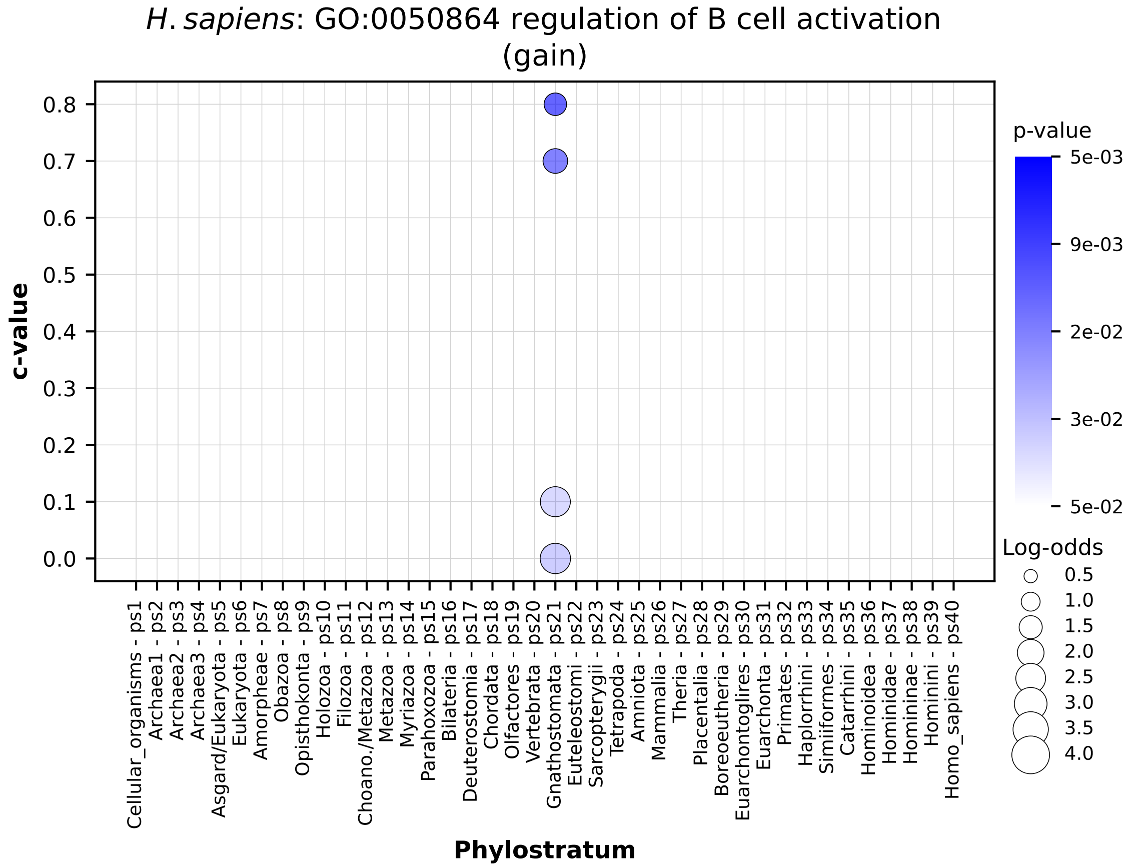
H. sapiens: GO:0050864 regulation of B cell activation (gain)
c-value
0.8
0.7
0.6
0.5
0.4
0.3
0.2
0.1
0.0
p-value
5e-03
9e-03
2e-02
3e-02
5e-02
Log-odds
0.5
1.0
1.5
2.0
2.5
3.0
3.5
4.0
Cellular_organisms - ps1
Archaea1 - ps2
Archaea2 - ps3
Archaea3 - ps4
Asgard/Eukaryota - ps5
Eukaryota - ps6
Amorpheae - ps7
Obazoa - ps8
Opisthokonta - ps9
Holozoa - ps10
Filozoa - ps11
Choano./Metazoa - ps12
Metazoa - ps13
Myriazoa - ps14
Parahoxozoa - ps15
Bilateria - ps16
Deuterostomia - ps17
Chordata - ps18
Olfactores - ps19
Vertebrata - ps20
Gnathostomata - ps21
Euteleostomi - ps22
Sarcopterygii - ps23
Tetrapoda - ps24
Amniota - ps25
Mammalia - ps26
Theria - ps27
Placentalia - ps28
Boreoeutheria - ps29
Euarchontoglires - ps30
Euarchonta - ps31
Primates - ps32
Haplorrhini - ps33
Simiiformes - ps34
Catarrhini - ps35
Hominoidea - ps36
Hominidae - ps37
Homininae - ps38
Hominini - ps39
Homo_sapiens - ps40
Phylostratum

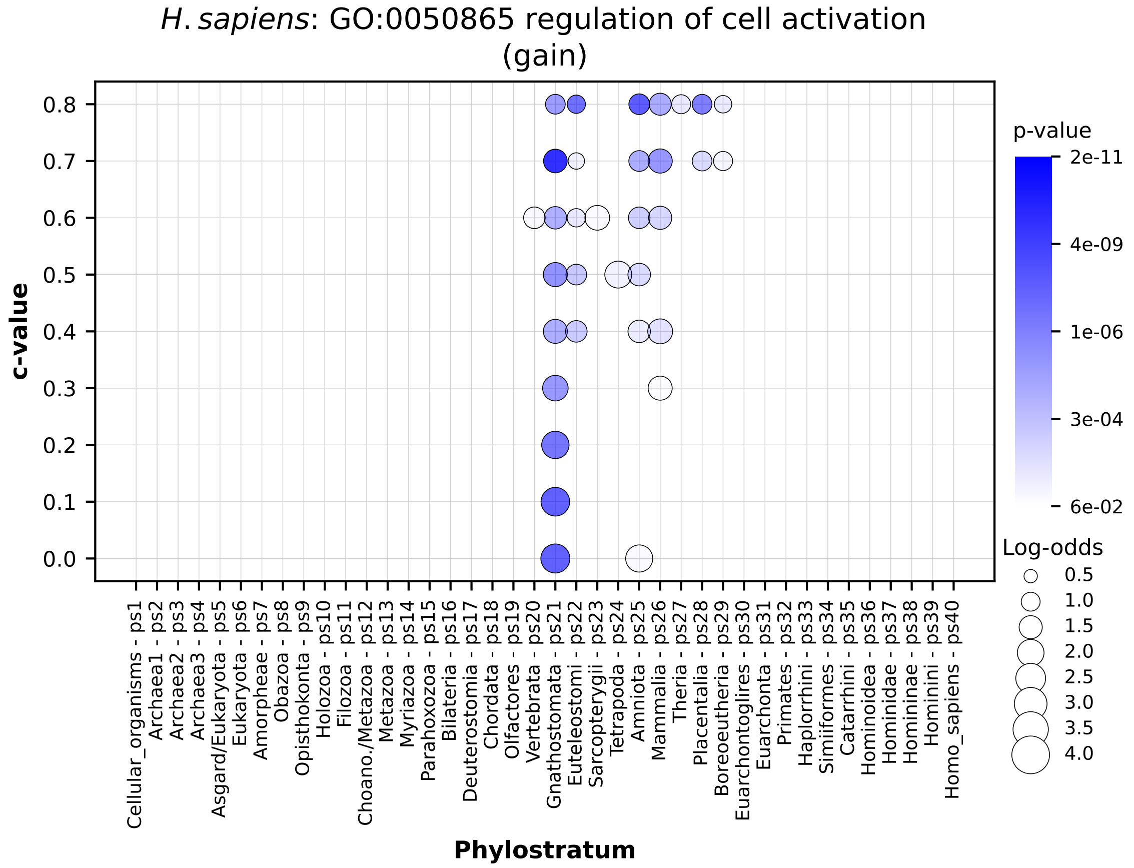

*H. sapiens*: GO:0050865 regulation of cell activation
(gain)
c-value
0.0
0.1
0.2
0.3
0.4
0.5
0.6
0.7
0.8
Phylostratum
Cellular_organisms - ps1
Archaea1 - ps2
Archaea2 - ps3
Archaea3 - ps4
Asgard/Eukaryota - ps5
Eukaryota - ps6
Amorpheae - ps7
Obazoa - ps8
Opisthokonta - ps9
Holozoa - ps10
Filozoa - ps11
Choano./Metazoa - ps12
Metazoa - ps13
Myriazoa - ps14
Parahoxozoa - ps15
Bilateria - ps16
Deuterostomia - ps17
Chordata - ps18
Olfactores - ps19
Vertebrata - ps20
Gnathostomata - ps21
Euteleostomi - ps22
Sarcopterygii - ps23
Tetrapoda - ps24
Amniota - ps25
Mammalia - ps26
Theria - ps27
Placentalia - ps28
Boreoeutheria - ps29
Euarchontoglires - ps30
Euarchonta - ps31
Primates - ps32
Haplorrhini - ps33
Simiiformes - ps34
Catarrhini - ps35
Hominoidea - ps36
Hominidae - ps37
Homininae - ps38
Hominini - ps39
Homo_sapiens - ps40
p-value
2e-11
4e-09
1e-06
3e-04
6e-02
Log-odds
0.5
1.0
1.5
2.0
2.5
3.0
3.5
4.0

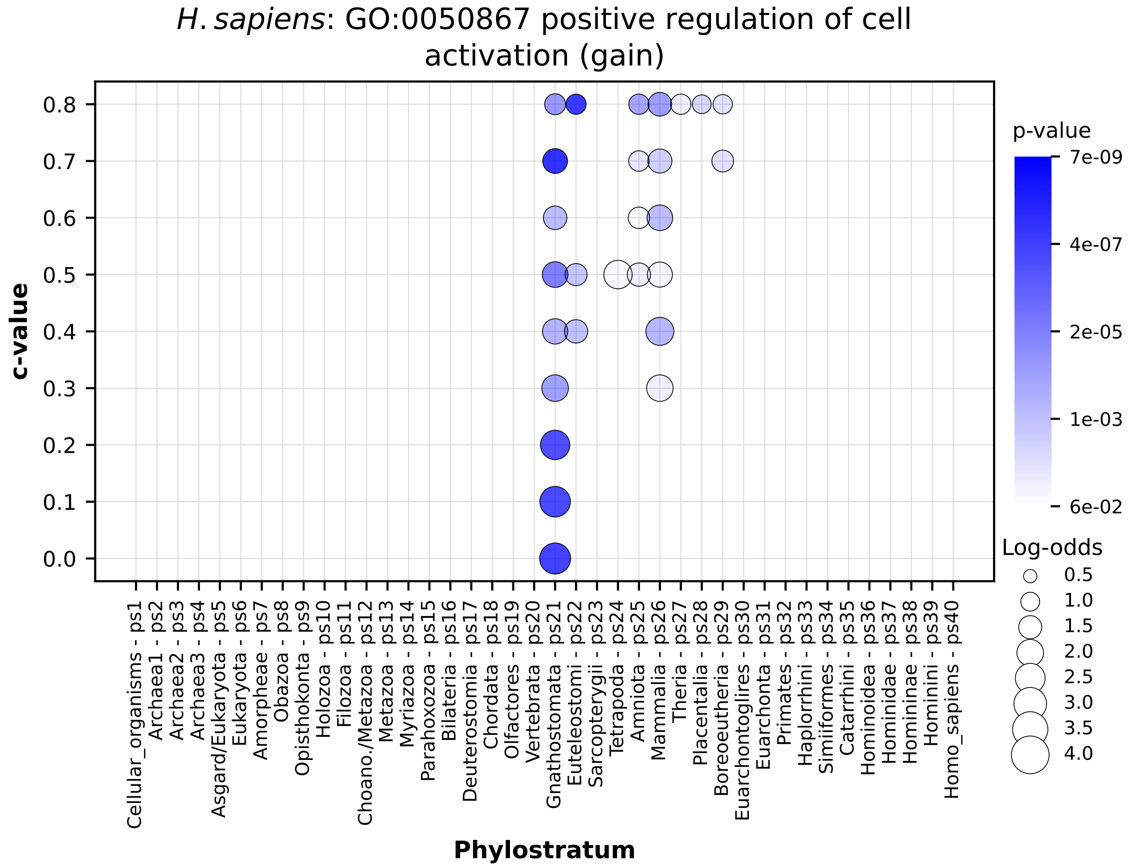
H. sapiens: GO:0050867 positive regulation of cell activation (gain)
c-value
0.0
0.1
0.2
0.3
0.4
0.5
0.6
0.7
0.8
Phylostratum
Cellular_organisms - ps1
Archaea1 - ps2
Archaea2 - ps3
Archaea3 - ps4
Asgard/Eukaryota - ps5
Eukaryota - ps6
Amorpheae - ps7
Obazoa - ps8
Opisthokonta - ps9
Holozoa - ps10
Filozoa - ps11
Choano./Metazoa - ps12
Metazoa - ps13
Myriazoa - ps14
Parahoxozoa - ps15
Bilateria - ps16
Deuterostomia - ps17
Chordata - ps18
Olfactores - ps19
Vertebrata - ps20
Gnathostomata - ps21
Euteleostomi - ps22
Sarcopterygii - ps23
Tetrapoda - ps24
Amniota - ps25
Mammalia - ps26
Theria - ps27
Placentalia - ps28
Boreoeutheria - ps29
Euarchontoglires - ps30
Euarchonta - ps31
Primates - ps32
Haplorrhini - ps33
Simiiformes - ps34
Catarrhini - ps35
Hominoidea - ps36
Hominidae - ps37
Homininae - ps38
Hominini - ps39
Homo_sapiens - ps40
p-value
7e-09
4e-07
2e-05
1e-03
6e-02
Log-odds
0.5
1.0
1.5
2.0
2.5
3.0
3.5
4.0

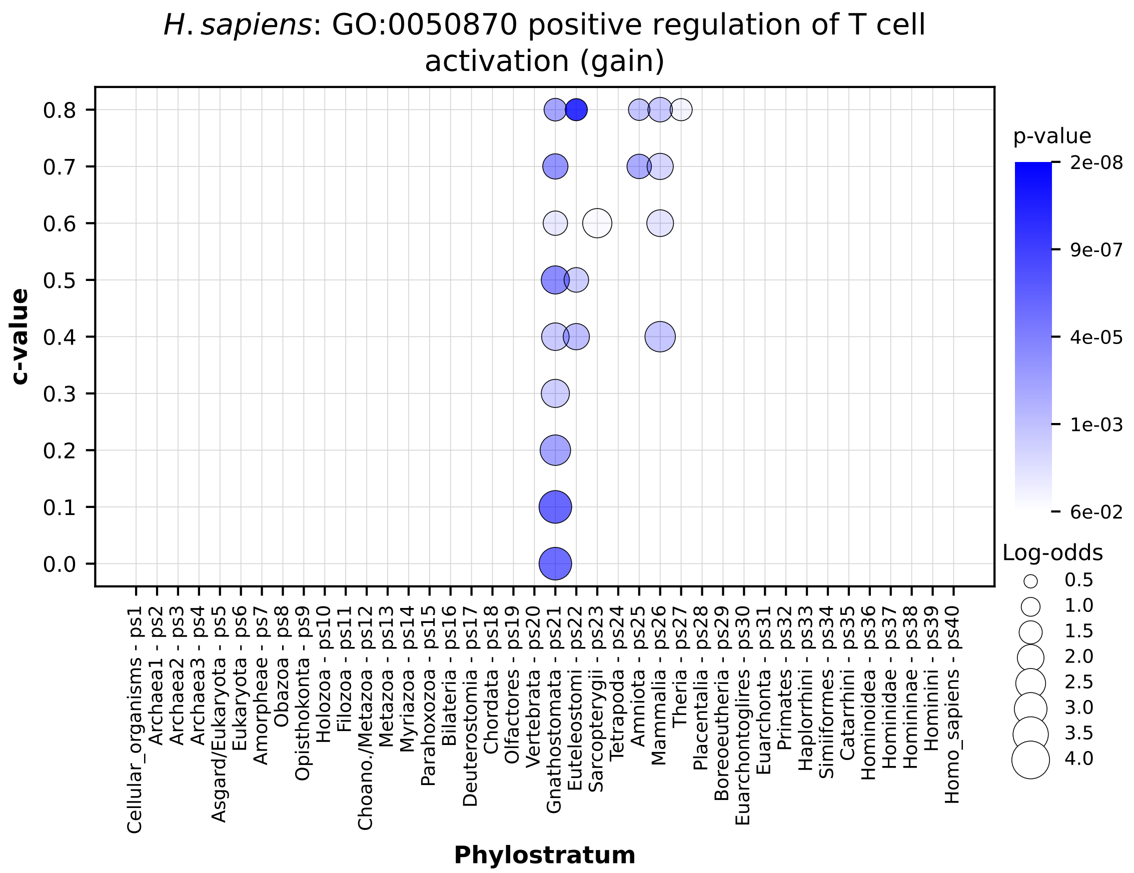

H. sapiens: GO:0050870 positive regulation of T cell activation (gain)
c-value
0.8
0.7
0.6
0.5
0.4
0.3
0.2
0.1
0.0
p-value
2e-08
9e-07
4e-05
1e-03
6e-02
Log-odds
0.5
1.0
1.5
2.0
2.5
3.0
3.5
4.0
Cellular_organisms - ps1
Archaea1 - ps2
Archaea2 - ps3
Archaea3 - ps4
Asgard/Eukaryota - ps5
Eukaryota - ps6
Amorpheae - ps7
Obazoa - ps8
Opisthokonta - ps9
Holozoa - ps10
Filozoa - ps11
Choano./Metazoa - ps12
Metazoa - ps13
Myriazoa - ps14
Parahoxozoa - ps15
Bilateria - ps16
Deuterostomia - ps17
Chordata - ps18
Olfactores - ps19
Vertebrata - ps20
Gnathostomata - ps21
Euteleostomi - ps22
Sarcopterygii - ps23
Tetrapoda - ps24
Amniota - ps25
Mammalia - ps26
Theria - ps27
Placentalia - ps28
Boreoeutheria - ps29
Euarchontoglires - ps30
Euarchonta - ps31
Primates - ps32
Haplorrhini - ps33
Simiiformes - ps34
Catarrhini - ps35
Hominoidea - ps36
Hominidae - ps37
Homininae - ps38
Hominini - ps39
Homo_sapiens - ps40
Phylostratum

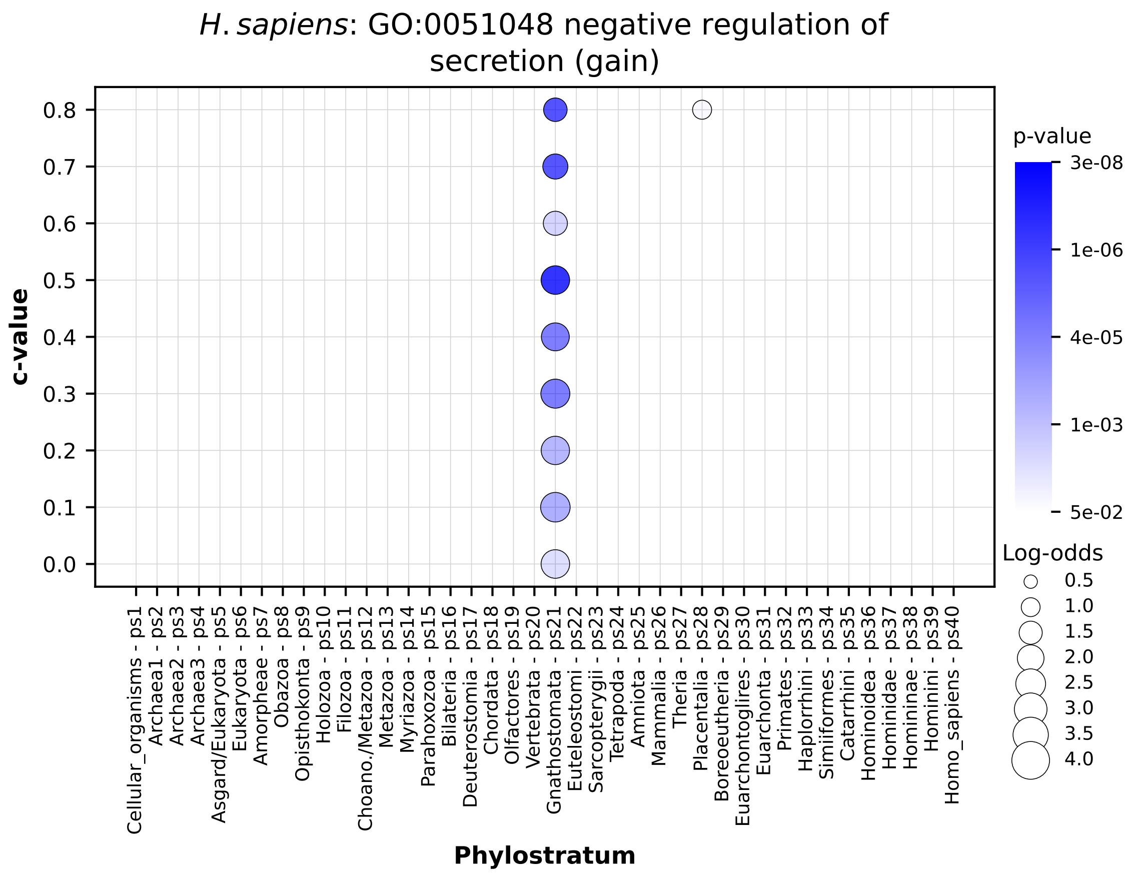

H. sapiens: GO:0051048 negative regulation of secretion (gain)
c-value
0.8
0.7
0.6
0.5
0.4
0.3
0.2
0.1
0.0
p-value
3e-08
1e-06
4e-05
1e-03
5e-02
Log-odds
0.5
1.0
1.5
2.0
2.5
3.0
3.5
4.0
Cellular_organisms - ps1
Archaea1 - ps2
Archaea2 - ps3
Archaea3 - ps4
Asgard/Eukaryota - ps5
Eukaryota - ps6
Amorpheae - ps7
Obazoa - ps8
Opisthokonta - ps9
Holozoa - ps10
Filozoa - ps11
Choano./Metazoa - ps12
Metazoa - ps13
Myriazoa - ps14
Parahoxozoa - ps15
Bilateria - ps16
Deuterostomia - ps17
Chordata - ps18
Olfactores - ps19
Vertebrata - ps20
Gnathostomata - ps21
Euteleostomi - ps22
Sarcopterygii - ps23
Tetrapoda - ps24
Amniota - ps25
Mammalia - ps26
Theria - ps27
Placentalia - ps28
Boreoeutheria - ps29
Euarchontoglires - ps30
Euarchonta - ps31
Primates - ps32
Haplorrhini - ps33
Simiiformes - ps34
Catarrhini - ps35
Hominoidea - ps36
Hominidae - ps37
Homininae - ps38
Hominini - ps39
Homo_sapiens - ps40
Phylostratum

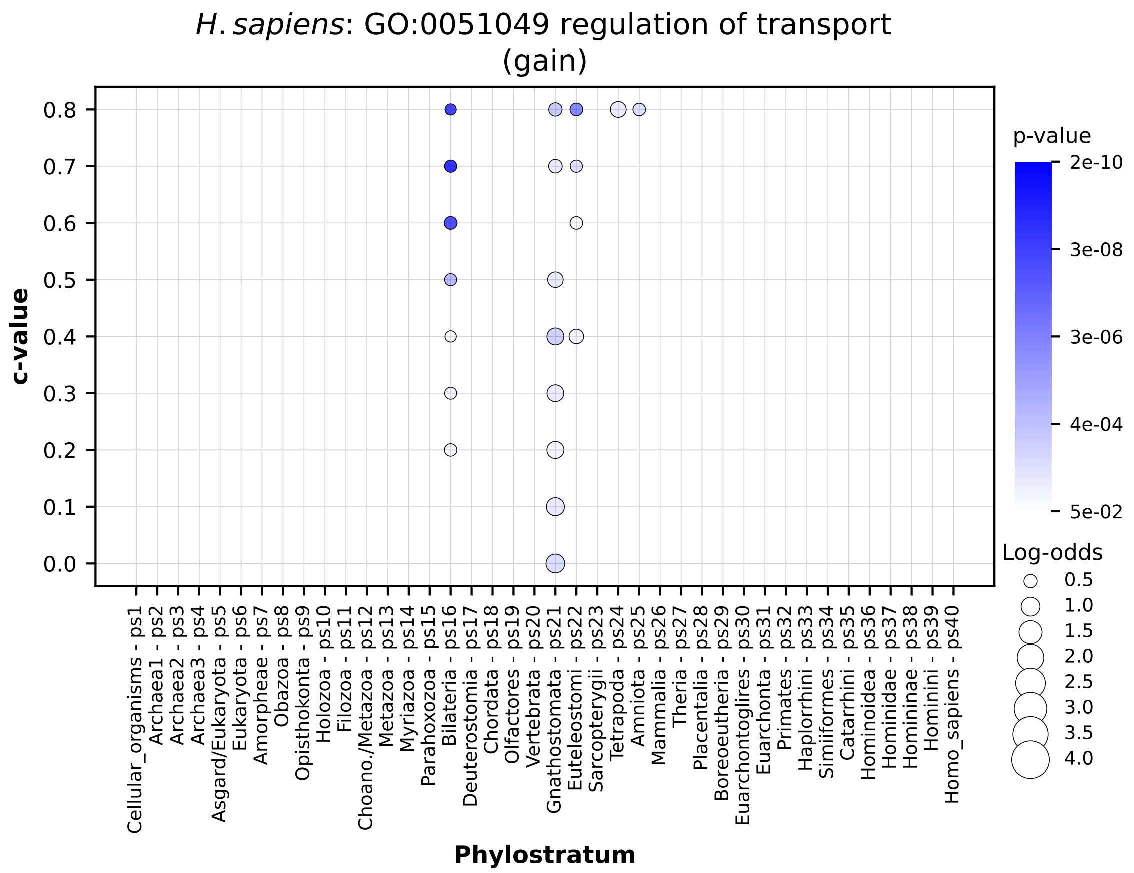

H. sapiens: GO:0051049 regulation of transport
(gain)
c-value
0.8
0.7
0.6
0.5
0.4
0.3
0.2
0.1
0.0
Phylostratum
Cellular_organisms - ps1
Archaea1 - ps2
Archaea2 - ps3
Archaea3 - ps4
Asgard/Eukaryota - ps5
Eukaryota - ps6
Amorpheae - ps7
Obazoa - ps8
Opisthokonta - ps9
Holozoa - ps10
Filozoa - ps11
Choano./Metazoa - ps12
Metazoa - ps13
Myriazoa - ps14
Parahoxozoa - ps15
Bilateria - ps16
Deuterostomia - ps17
Chordata - ps18
Olfactores - ps19
Vertebrata - ps20
Gnathostomata - ps21
Euteleostomi - ps22
Sarcopterygii - ps23
Tetrapoda - ps24
Amniota - ps25
Mammalia - ps26
Theria - ps27
Placentalia - ps28
Boreoeutheria - ps29
Euarchontoglires - ps30
Euarchonta - ps31
Primates - ps32
Haplorrhini - ps33
Simiiformes - ps34
Catarrhini - ps35
Hominoidea - ps36
Hominidae - ps37
Homininae - ps38
Hominini - ps39
Homo_sapiens - ps40
p-value
2e-10
3e-08
3e-06
4e-04
5e-02
Log-odds
0.5
1.0
1.5
2.0
2.5
3.0
3.5
4.0

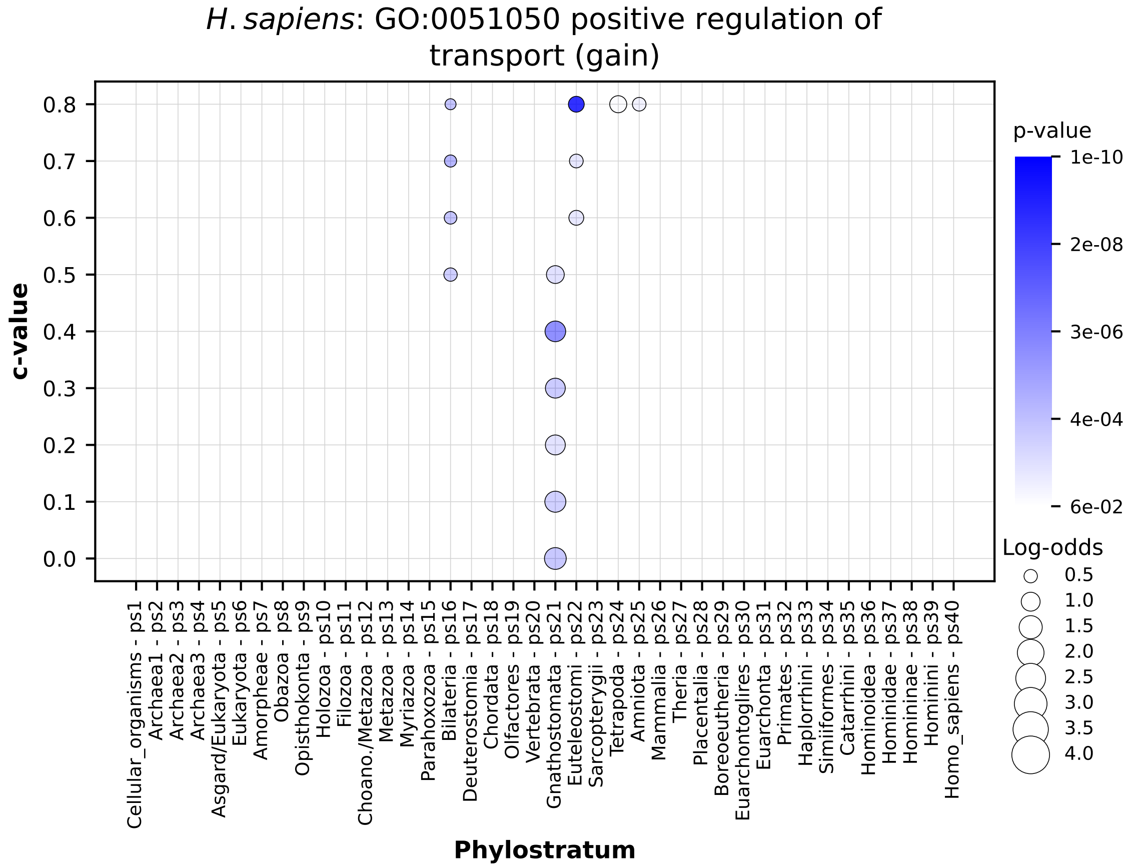

*H. sapiens*: GO:0051050 positive regulation of transport (gain)
c-value
0.8
0.7
0.6
0.5
0.4
0.3
0.2
0.1
0.0
p-value
1e-10
2e-08
3e-06
4e-04
6e-02
Log-odds
0.5
1.0
1.5
2.0
2.5
3.0
3.5
4.0
Cellular_organisms - ps1
Archaea1 - ps2
Archaea2 - ps3
Archaea3 - ps4
Asgard/Eukaryota - ps5
Eukaryota - ps6
Amorpheae - ps7
Obazoa - ps8
Opisthokonta - ps9
Holozoa - ps10
Filozoa - ps11
Choano./Metazoa - ps12
Metazoa - ps13
Myriazoa - ps14
Parahoxozoa - ps15
Bilateria - ps16
Deuterostomia - ps17
Chordata - ps18
Olfactores - ps19
Vertebrata - ps20
Gnathostomata - ps21
Euteleostomi - ps22
Sarcopterygii - ps23
Tetrapoda - ps24
Amniota - ps25
Mammalia - ps26
Theria - ps27
Placentalia - ps28
Boreoeutheria - ps29
Euarchontoglires - ps30
Euarchonta - ps31
Primates - ps32
Haplorrhini - ps33
Simiiformes - ps34
Catarrhini - ps35
Hominoidea - ps36
Hominidae - ps37
Homininae - ps38
Hominini - ps39
Homo_sapiens - ps40
Phylostratum

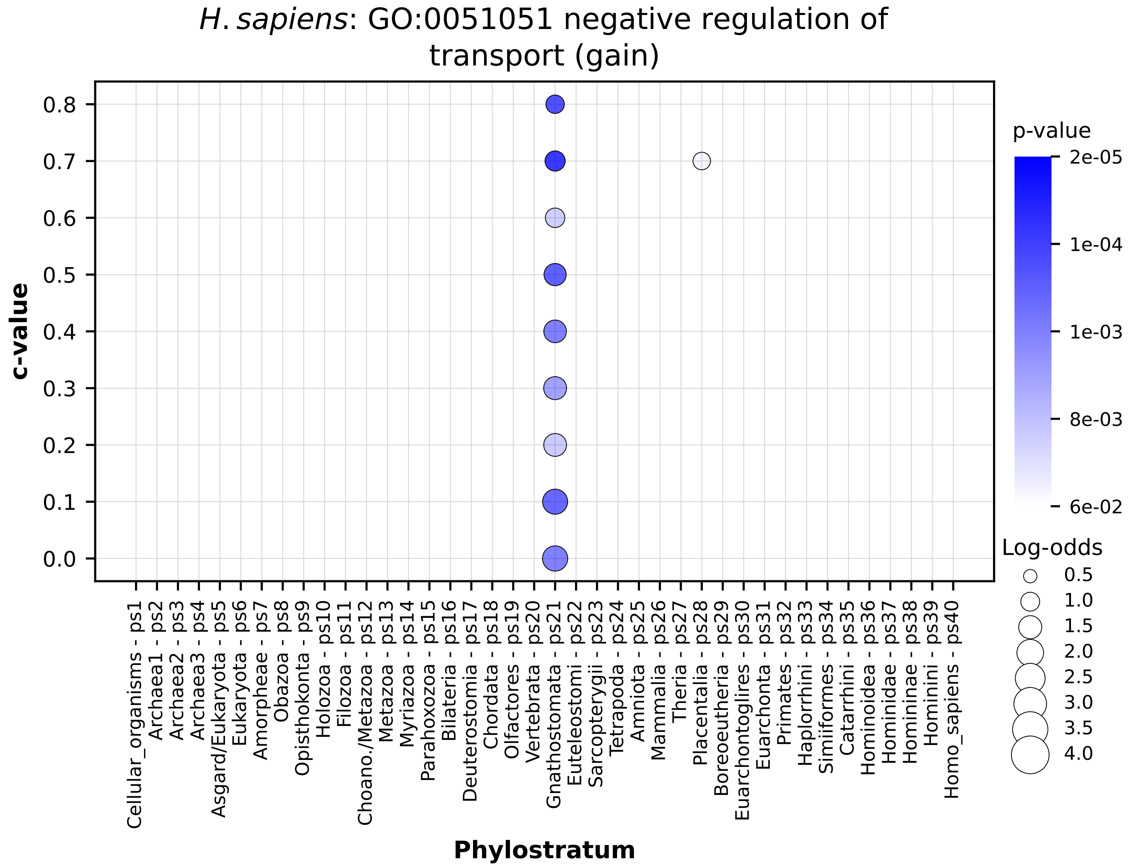

*H. sapiens*: GO:0051051 negative regulation of transport (gain)
c-value
0.8
0.7
0.6
0.5
0.4
0.3
0.2
0.1
0.0
p-value
2e-05
1e-04
1e-03
8e-03
6e-02
Log-odds
0.5
1.0
1.5
2.0
2.5
3.0
3.5
4.0
Cellular_organisms - ps1
Archaea1 - ps2
Archaea2 - ps3
Archaea3 - ps4
Asgard/Eukaryota - ps5
Eukaryota - ps6
Amorpheae - ps7
Obazoa - ps8
Opisthokonta - ps9
Holozoa - ps10
Filozoa - ps11
Choano./Metazoa - ps12
Metazoa - ps13
Myriazoa - ps14
Parahoxozoa - ps15
Bilateria - ps16
Deuterostomia - ps17
Chordata - ps18
Olfactores - ps19
Vertebrata - ps20
Gnathostomata - ps21
Euteleostomi - ps22
Sarcopterygii - ps23
Tetrapoda - ps24
Amniota - ps25
Mammalia - ps26
Theria - ps27
Placentalia - ps28
Boreoeutheria - ps29
Euarchontoglires - ps30
Euarchonta - ps31
Primates - ps32
Haplorrhini - ps33
Simiiformes - ps34
Catarrhini - ps35
Hominoidea - ps36
Hominidae - ps37
Homininae - ps38
Hominini - ps39
Homo_sapiens - ps40
Phylostratum

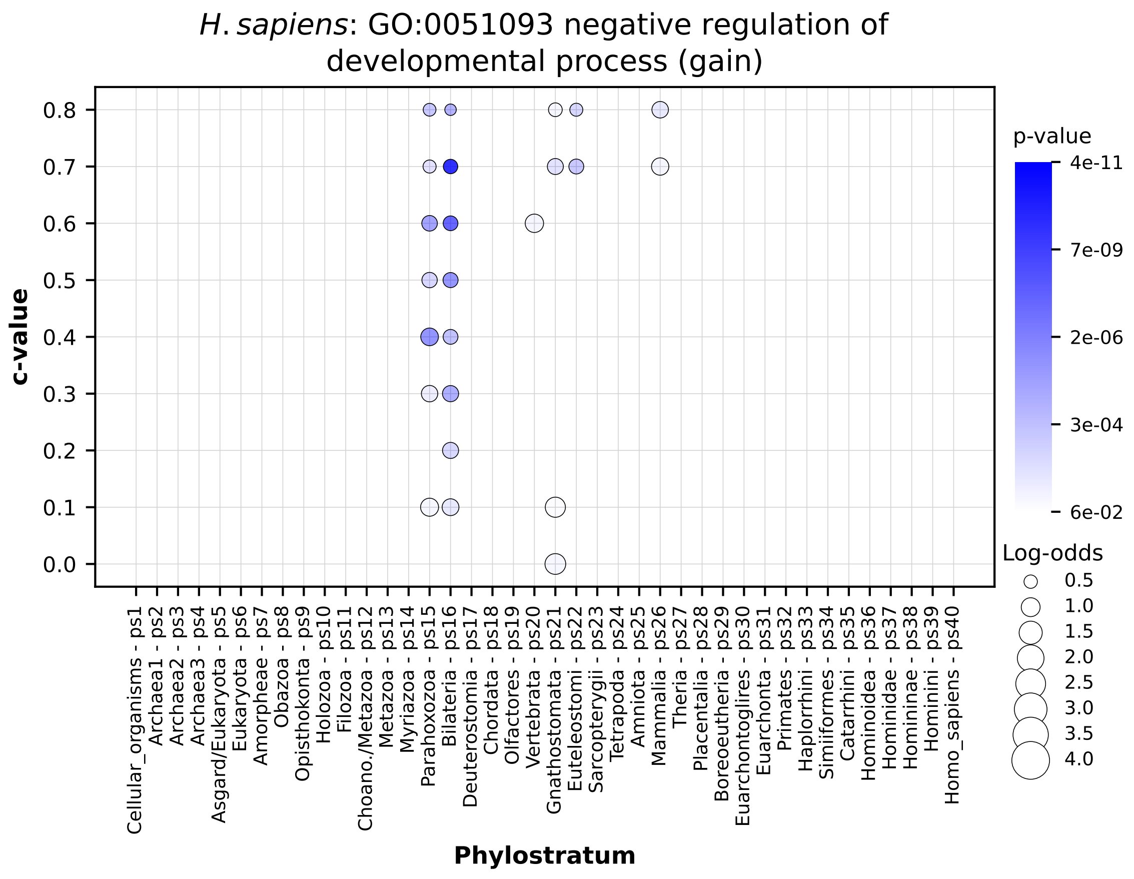
H. sapiens: GO:0051093 negative regulation of developmental process (gain)
c-value
0.8
0.7
0.6
0.5
0.4
0.3
0.2
0.1
0.0
Phylostratum
Cellular_organisms - ps1
Archaea1 - ps2
Archaea2 - ps3
Archaea3 - ps4
Asgard/Eukaryota - ps5
Eukaryota - ps6
Amorpheae - ps7
Obazoa - ps8
Opisthokonta - ps9
Holozoa - ps10
Filozoa - ps11
Choano./Metazoa - ps12
Metazoa - ps13
Myriazoa - ps14
Parahoxozoa - ps15
Bilateria - ps16
Deuterostomia - ps17
Chordata - ps18
Olfactores - ps19
Vertebrata - ps20
Gnathostomata - ps21
Euteleostomi - ps22
Sarcopterygii - ps23
Tetrapoda - ps24
Amniota - ps25
Mammalia - ps26
Theria - ps27
Placentalia - ps28
Boreoeutheria - ps29
Euarchontoglires - ps30
Euarchonta - ps31
Primates - ps32
Haplorrhini - ps33
Simiiformes - ps34
Catarrhini - ps35
Hominoidea - ps36
Hominidae - ps37
Homininae - ps38
Hominini - ps39
Homo_sapiens - ps40
p-value
4e-11
7e-09
2e-06
3e-04
6e-02
Log-odds
0.5
1.0
1.5
2.0
2.5
3.0
3.5
4.0

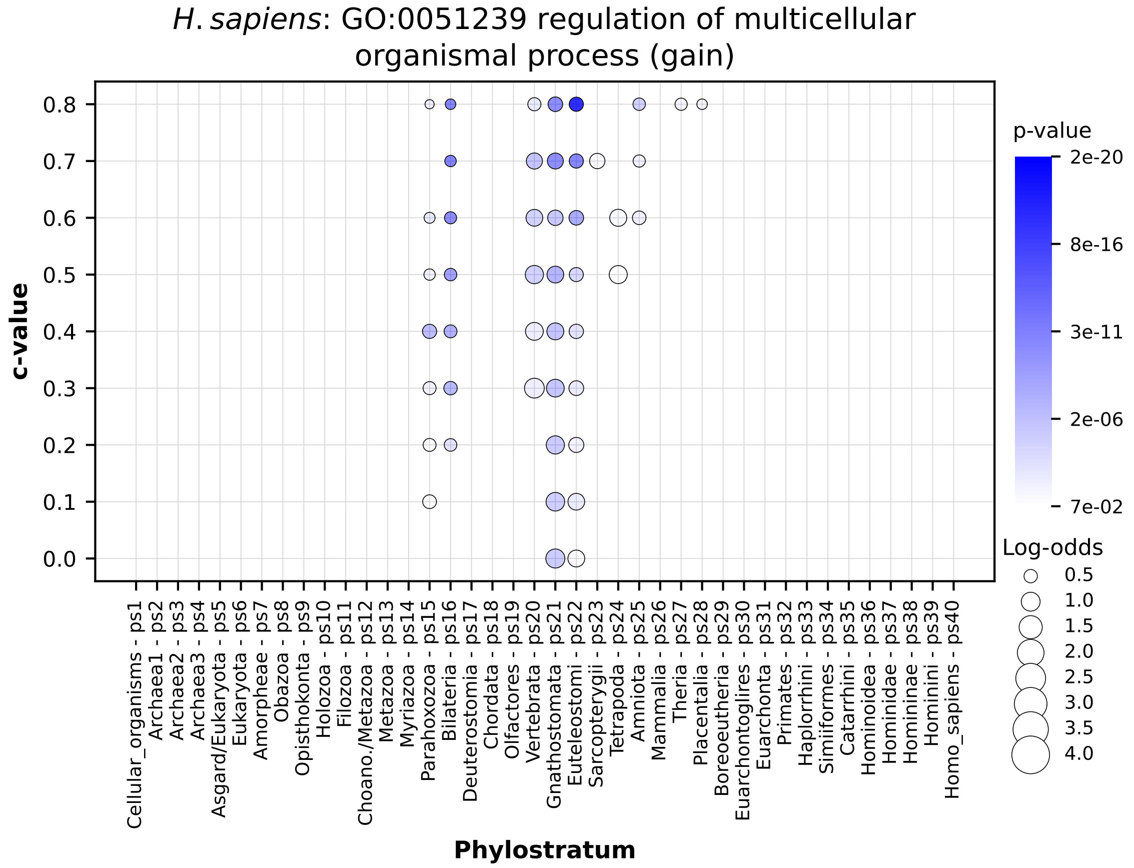

*H. sapiens*: GO:0051239 regulation of multicellular organismal process (gain)
c-value
0.8
0.7
0.6
0.5
0.4
0.3
0.2
0.1
0.0
Phylostratum
Cellular_organisms - ps1
Archaea1 - ps2
Archaea2 - ps3
Archaea3 - ps4
Asgard/Eukaryota - ps5
Eukaryota - ps6
Amorpheae - ps7
Obazoa - ps8
Opisthokonta - ps9
Holozoa - ps10
Filozoa - ps11
Choano./Metazoa - ps12
Metazoa - ps13
Myriazoa - ps14
Parahoxozoa - ps15
Bilateria - ps16
Deuterostomia - ps17
Chordata - ps18
Olfactores - ps19
Vertebrata - ps20
Gnathostomata - ps21
Euteleostomi - ps22
Sarcopterygii - ps23
Tetrapoda - ps24
Amniota - ps25
Mammalia - ps26
Theria - ps27
Placentalia - ps28
Boreoeutheria - ps29
Euarchontoglires - ps30
Euarchonta - ps31
Primates - ps32
Haplorrhini - ps33
Simiiformes - ps34
Catarrhini - ps35
Hominoidea - ps36
Hominidae - ps37
Homininae - ps38
Hominini - ps39
Homo_sapiens - ps40
p-value
2e-20
8e-16
3e-11
2e-06
7e-02
Log-odds
0.5
1.0
1.5
2.0
2.5
3.0
3.5
4.0

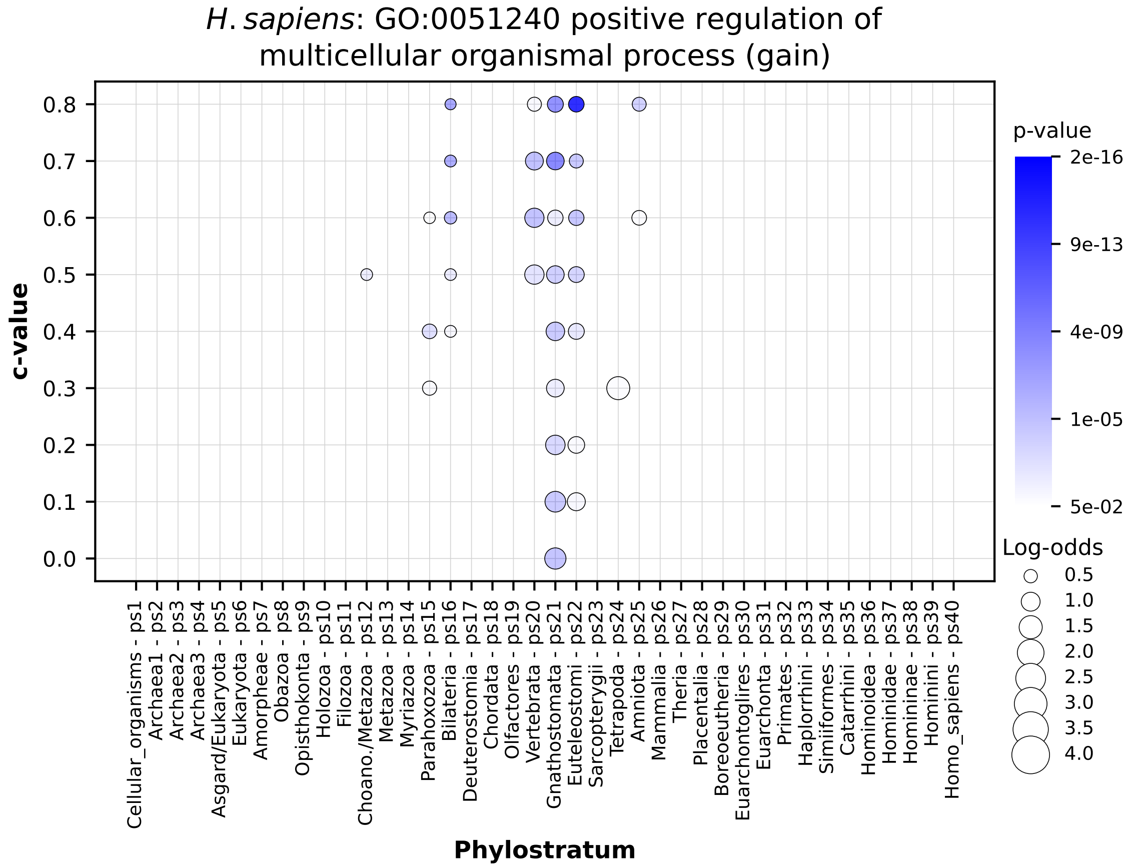

H. sapiens: GO:0051240 positive regulation of multicellular organismal process (gain)
c-value
0.0
0.1
0.2
0.3
0.4
0.5
0.6
0.7
0.8
Phylostratum
Cellular_organisms - ps1
Archaea1 - ps2
Archaea2 - ps3
Archaea3 - ps4
Asgard/Eukaryota - ps5
Eukaryota - ps6
Amorpheae - ps7
Obazoa - ps8
Opisthokonta - ps9
Holozoa - ps10
Filozoa - ps11
Choano./Metazoa - ps12
Metazoa - ps13
Myriazoa - ps14
Parahoxozoa - ps15
Bilateria - ps16
Deuterostomia - ps17
Chordata - ps18
Olfactores - ps19
Vertebrata - ps20
Gnathostomata - ps21
Euteleostomi - ps22
Sarcopterygii - ps23
Tetrapoda - ps24
Amniota - ps25
Mammalia - ps26
Theria - ps27
Placentalia - ps28
Boreoeutheria - ps29
Euarchontoglires - ps30
Euarchonta - ps31
Primates - ps32
Haplorrhini - ps33
Simiiformes - ps34
Catarrhini - ps35
Hominoidea - ps36
Hominidae - ps37
Homininae - ps38
Hominini - ps39
Homo_sapiens - ps40
p-value
2e-16
9e-13
4e-09
1e-05
5e-02
Log-odds
0.5
1.0
1.5
2.0
2.5
3.0
3.5
4.0

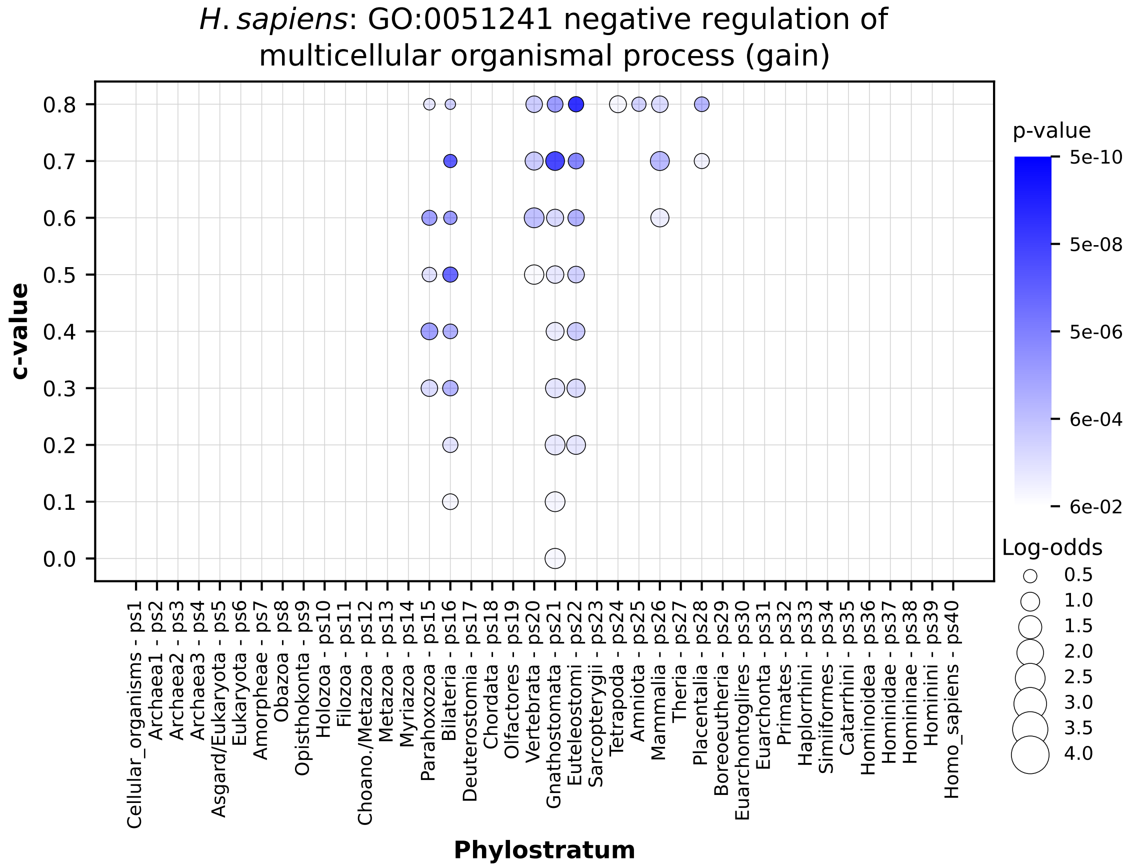

H. sapiens: GO:0051241 negative regulation of multicellular organismal process (gain)
c-value
0.8
0.7
0.6
0.5
0.4
0.3
0.2
0.1
0.0
p-value
5e-10
5e-08
5e-06
6e-04
6e-02
Log-odds
0.5
1.0
1.5
2.0
2.5
3.0
3.5
4.0
Cellular_organisms - ps1
Archaea1 - ps2
Archaea2 - ps3
Archaea3 - ps4
Asgard/Eukaryota - ps5
Eukaryota - ps6
Amorpheae - ps7
Obazoa - ps8
Opisthokonta - ps9
Holozoa - ps10
Filozoa - ps11
Choano./Metazoa - ps12
Metazoa - ps13
Myriazoa - ps14
Parahoxozoa - ps15
Bilateria - ps16
Deuterostomia - ps17
Chordata - ps18
Olfactores - ps19
Vertebrata - ps20
Gnathostomata - ps21
Euteleostomi - ps22
Sarcopterygii - ps23
Tetrapoda - ps24
Amniota - ps25
Mammalia - ps26
Theria - ps27
Placentalia - ps28
Boreoeutheria - ps29
Euarchontoglires - ps30
Euarchonta - ps31
Primates - ps32
Haplorrhini - ps33
Simiiformes - ps34
Catarrhini - ps35
Hominoidea - ps36
Hominidae - ps37
Homininae - ps38
Hominini - ps39
Homo_sapiens - ps40
Phylostratum

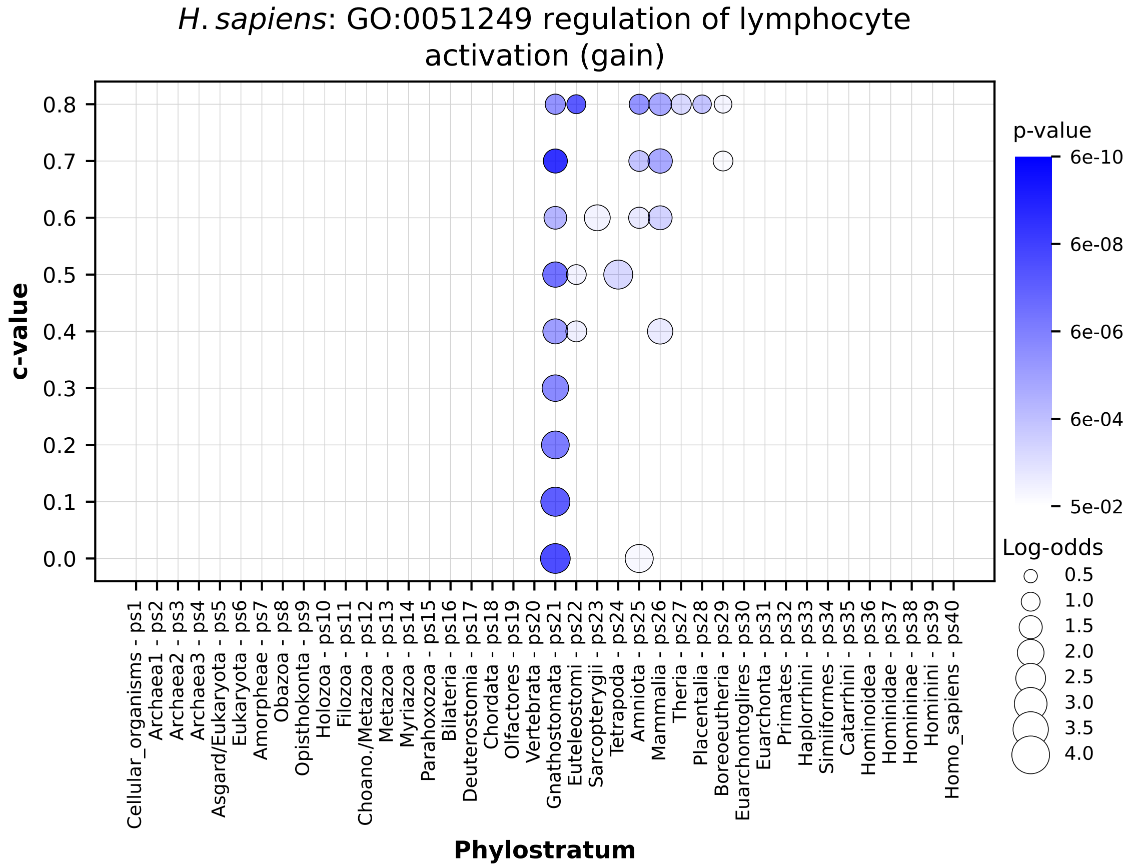

H. sapiens: GO:0051249 regulation of lymphocyte activation (gain)
c-value
0.8
0.7
0.6
0.5
0.4
0.3
0.2
0.1
0.0
p-value
6e-10
6e-08
6e-06
6e-04
5e-02
Log-odds
0.5
1.0
1.5
2.0
2.5
3.0
3.5
4.0
Cellular_organisms - ps1
Archaea1 - ps2
Archaea2 - ps3
Archaea3 - ps4
Asgard/Eukaryota - ps5
Eukaryota - ps6
Amorpheae - ps7
Obazoa - ps8
Opisthokonta - ps9
Holozoa - ps10
Filozoa - ps11
Choano./Metazoa - ps12
Metazoa - ps13
Myriazoa - ps14
Parahoxozoa - ps15
Bilateria - ps16
Deuterostomia - ps17
Chordata - ps18
Olfactores - ps19
Vertebrata - ps20
Gnathostomata - ps21
Euteleostomi - ps22
Sarcopterygii - ps23
Tetrapoda - ps24
Amniota - ps25
Mammalia - ps26
Theria - ps27
Placentalia - ps28
Boreoeutheria - ps29
Euarchontoglires - ps30
Euarchonta - ps31
Primates - ps32
Haplorrhini - ps33
Simiiformes - ps34
Catarrhini - ps35
Hominoidea - ps36
Hominidae - ps37
Homininae - ps38
Hominini - ps39
Homo_sapiens - ps40
Phylostratum

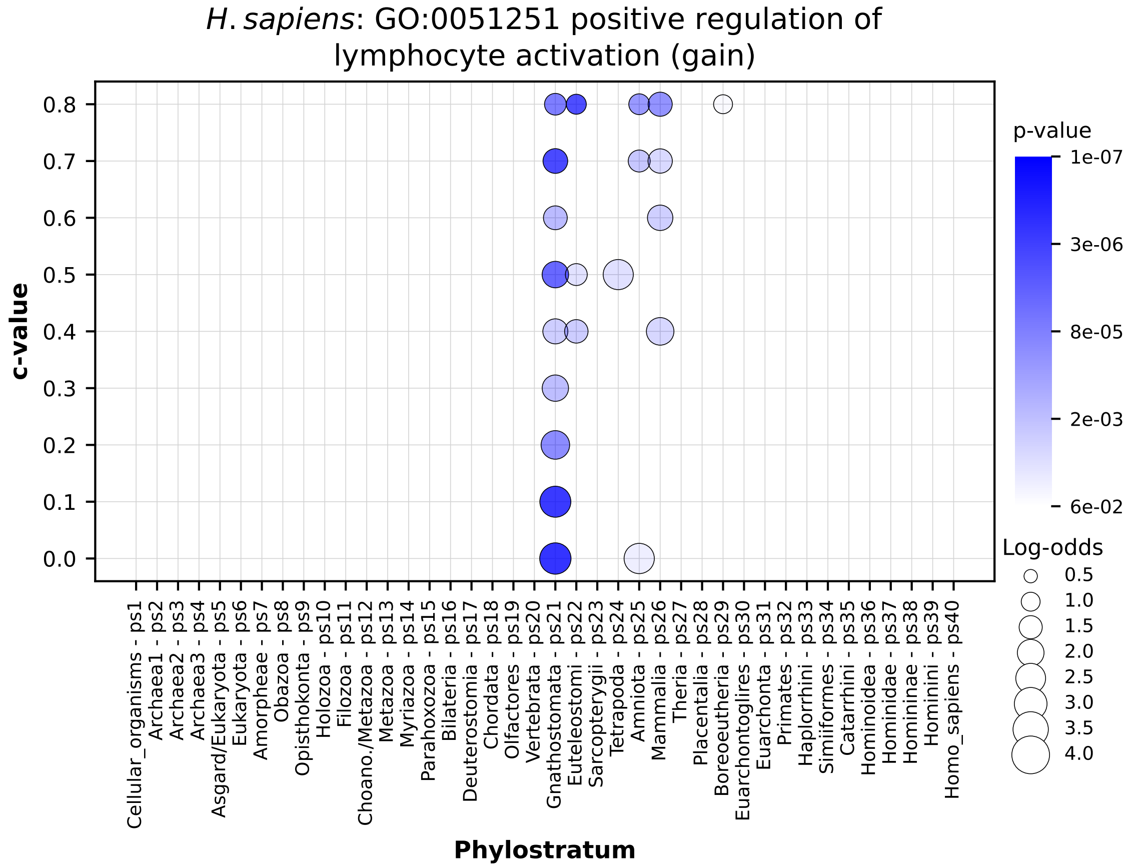

H. sapiens: GO:0051251 positive regulation of lymphocyte activation (gain)
c-value
0.8
0.7
0.6
0.5
0.4
0.3
0.2
0.1
0.0
p-value
1e-07
3e-06
8e-05
2e-03
6e-02
Log-odds
0.5
1.0
1.5
2.0
2.5
3.0
3.5
4.0
Cellular_organisms - ps1
Archaea1 - ps2
Archaea2 - ps3
Archaea3 - ps4
Asgard/Eukaryota - ps5
Eukaryota - ps6
Amorpheae - ps7
Obazoa - ps8
Opisthokonta - ps9
Holozoa - ps10
Filozoa - ps11
Choano./Metazoa - ps12
Metazoa - ps13
Myriazoa - ps14
Parahoxozoa - ps15
Bilateria - ps16
Deuterostomia - ps17
Chordata - ps18
Olfactores - ps19
Vertebrata - ps20
Gnathostomata - ps21
Euteleostomi - ps22
Sarcopterygii - ps23
Tetrapoda - ps24
Amniota - ps25
Mammalia - ps26
Theria - ps27
Placentalia - ps28
Boreoeutheria - ps29
Euarchontoglires - ps30
Euarchonta - ps31
Primates - ps32
Haplorrhini - ps33
Simiiformes - ps34
Catarrhini - ps35
Hominoidea - ps36
Hominidae - ps37
Homininae - ps38
Hominini - ps39
Homo_sapiens - ps40
Phylostratum

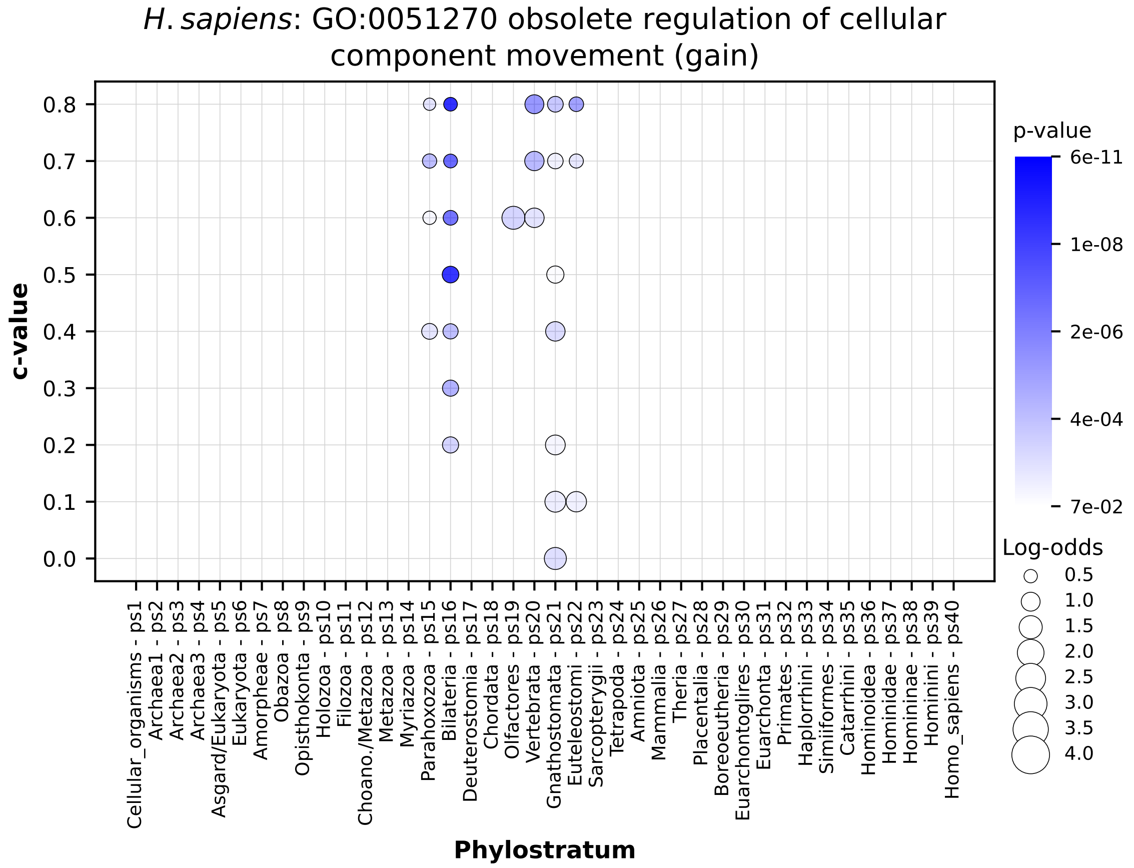

*H. sapiens*: GO:0051270 obsolete regulation of cellular component movement (gain)
c-value
0.8
0.7
0.6
0.5
0.4
0.3
0.2
0.1
0.0
p-value
6e-11
1e-08
2e-06
4e-04
7e-02
Log-odds
0.5
1.0
1.5
2.0
2.5
3.0
3.5
4.0
Cellular_organisms - ps1
Archaea1 - ps2
Archaea2 - ps3
Archaea3 - ps4
Asgard/Eukaryota - ps5
Eukaryota - ps6
Amorpheae - ps7
Obazoa - ps8
Opisthokonta - ps9
Holozoa - ps10
Filozoa - ps11
Choano./Metazoa - ps12
Metazoa - ps13
Myriazoa - ps14
Parahoxozoa - ps15
Bilateria - ps16
Deuterostomia - ps17
Chordata - ps18
Olfactores - ps19
Vertebrata - ps20
Gnathostomata - ps21
Euteleostomi - ps22
Sarcopterygii - ps23
Tetrapoda - ps24
Amniota - ps25
Mammalia - ps26
Theria - ps27
Placentalia - ps28
Boreoeutheria - ps29
Euarchontoglires - ps30
Euarchonta - ps31
Primates - ps32
Haplorrhini - ps33
Simiiformes - ps34
Catarrhini - ps35
Hominoidea - ps36
Hominidae - ps37
Homininae - ps38
Hominini - ps39
Homo_sapiens - ps40
Phylostratum

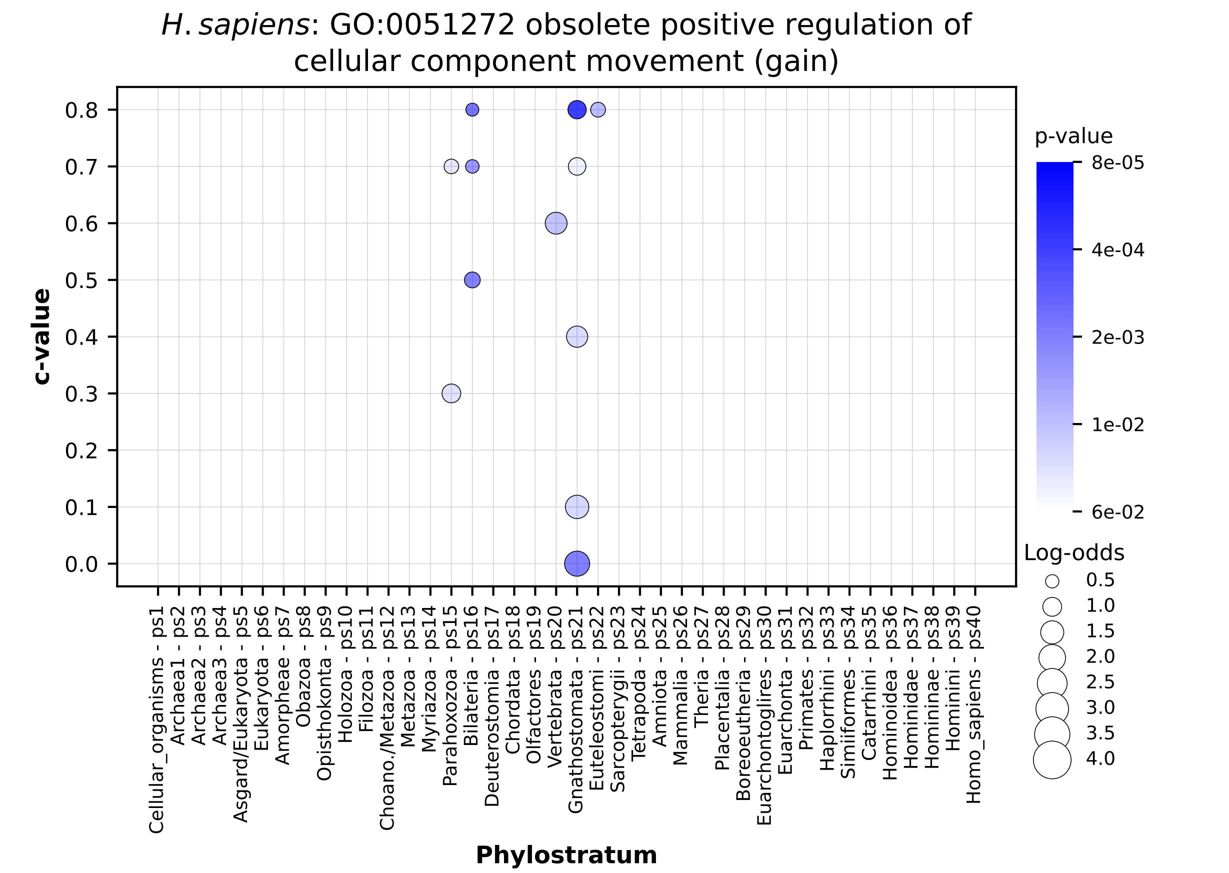

H. sapiens: GO:0051272 obsolete positive regulation of cellular component movement (gain)
c-value
0.8
0.7
0.6
0.5
0.4
0.3
0.2
0.1
0.0
Phylostratum
Cellular_organisms - ps1
Archaea1 - ps2
Archaea2 - ps3
Archaea3 - ps4
Asgard/Eukaryota - ps5
Eukaryota - ps6
Amorpheae - ps7
Obazoa - ps8
Opisthokonta - ps9
Holozoa - ps10
Filozoa - ps11
Choano./Metazoa - ps12
Metazoa - ps13
Myriazoa - ps14
Parahoxozoa - ps15
Bilateria - ps16
Deuterostomia - ps17
Chordata - ps18
Olfactores - ps19
Vertebrata - ps20
Gnathostomata - ps21
Euteleostomi - ps22
Sarcopterygii - ps23
Tetrapoda - ps24
Amniota - ps25
Mammalia - ps26
Theria - ps27
Placentalia - ps28
Boreoeutheria - ps29
Euarchontoglires - ps30
Euarchonta - ps31
Primates - ps32
Haplorrhini - ps33
Simiiformes - ps34
Catarrhini - ps35
Hominoidea - ps36
Hominidae - ps37
Homininae - ps38
Hominini - ps39
Homo_sapiens - ps40
p-value
8e-05
4e-04
2e-03
1e-02
6e-02
Log-odds
0.5
1.0
1.5
2.0
2.5
3.0
3.5
4.0

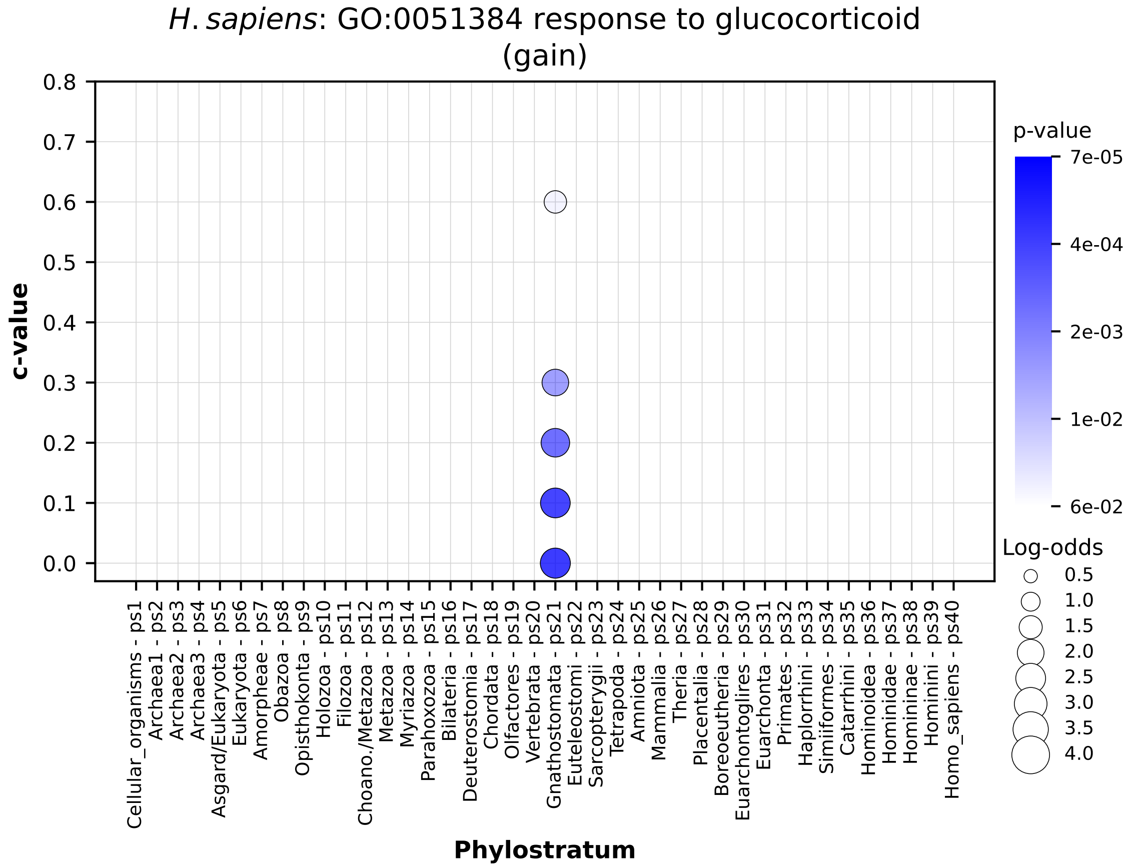

H. sapiens: GO:0051384 response to glucocorticoid
(gain)
c-value
0.8
0.7
0.6
0.5
0.4
0.3
0.2
0.1
0.0
p-value
7e-05
4e-04
2e-03
1e-02
6e-02
Log-odds
0.5
1.0
1.5
2.0
2.5
3.0
3.5
4.0
Cellular_organisms - ps1
Archaea1 - ps2
Archaea2 - ps3
Archaea3 - ps4
Asgard/Eukaryota - ps5
Eukaryota - ps6
Amorpheae - ps7
Obazoa - ps8
Opisthokonta - ps9
Holozoa - ps10
Filozoa - ps11
Choano./Metazoa - ps12
Metazoa - ps13
Myriazoa - ps14
Parahoxozoa - ps15
Bilateria - ps16
Deuterostomia - ps17
Chordata - ps18
Olfactores - ps19
Vertebrata - ps20
Gnathostomata - ps21
Euteleostomi - ps22
Sarcopterygii - ps23
Tetrapoda - ps24
Amniota - ps25
Mammalia - ps26
Theria - ps27
Placentalia - ps28
Boreoeutheria - ps29
Euarchontoglires - ps30
Euarchonta - ps31
Primates - ps32
Haplorrhini - ps33
Simiiformes - ps34
Catarrhini - ps35
Hominoidea - ps36
Hominidae - ps37
Homininae - ps38
Hominini - ps39
Homo_sapiens - ps40
Phylostratum

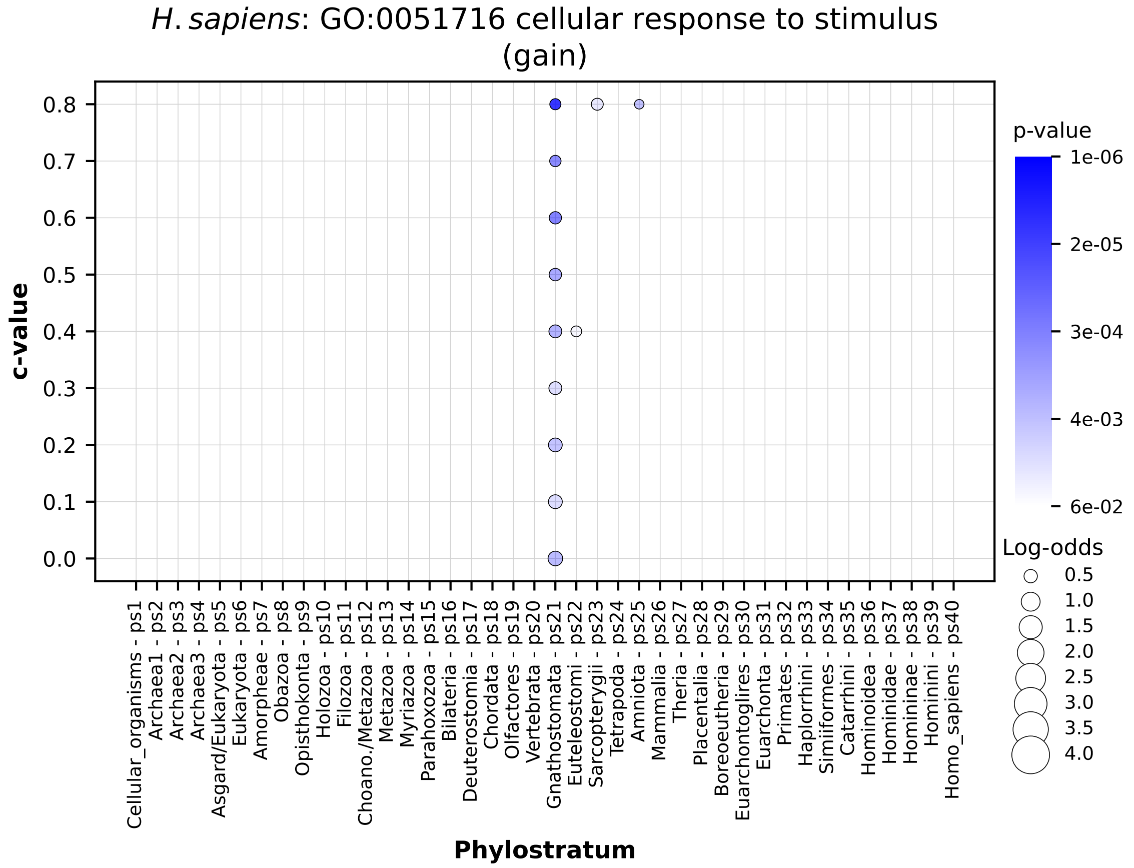

H. sapiens: GO:0051716 cellular response to stimulus
(gain)
c-value
0.8
0.7
0.6
0.5
0.4
0.3
0.2
0.1
0.0
Phylostratum
Cellular_organisms - ps1
Archaea1 - ps2
Archaea2 - ps3
Archaea3 - ps4
Asgard/Eukaryota - ps5
Eukaryota - ps6
Amorpheae - ps7
Obazoa - ps8
Opisthokonta - ps9
Holozoa - ps10
Filozoa - ps11
Choano./Metazoa - ps12
Metazoa - ps13
Myriazoa - ps14
Parahoxozoa - ps15
Bilateria - ps16
Deuterostomia - ps17
Chordata - ps18
Olfactores - ps19
Vertebrata - ps20
Gnathostomata - ps21
Euteleostomi - ps22
Sarcopterygii - ps23
Tetrapoda - ps24
Amniota - ps25
Mammalia - ps26
Theria - ps27
Placentalia - ps28
Boreoeutheria - ps29
Euarchontoglires - ps30
Euarchonta - ps31
Primates - ps32
Haplorrhini - ps33
Simiiformes - ps34
Catarrhini - ps35
Hominoidea - ps36
Hominidae - ps37
Homininae - ps38
Hominini - ps39
Homo_sapiens - ps40
p-value
1e-06
2e-05
3e-04
4e-03
6e-02
Log-odds
0.5
1.0
1.5
2.0
2.5
3.0
3.5
4.0

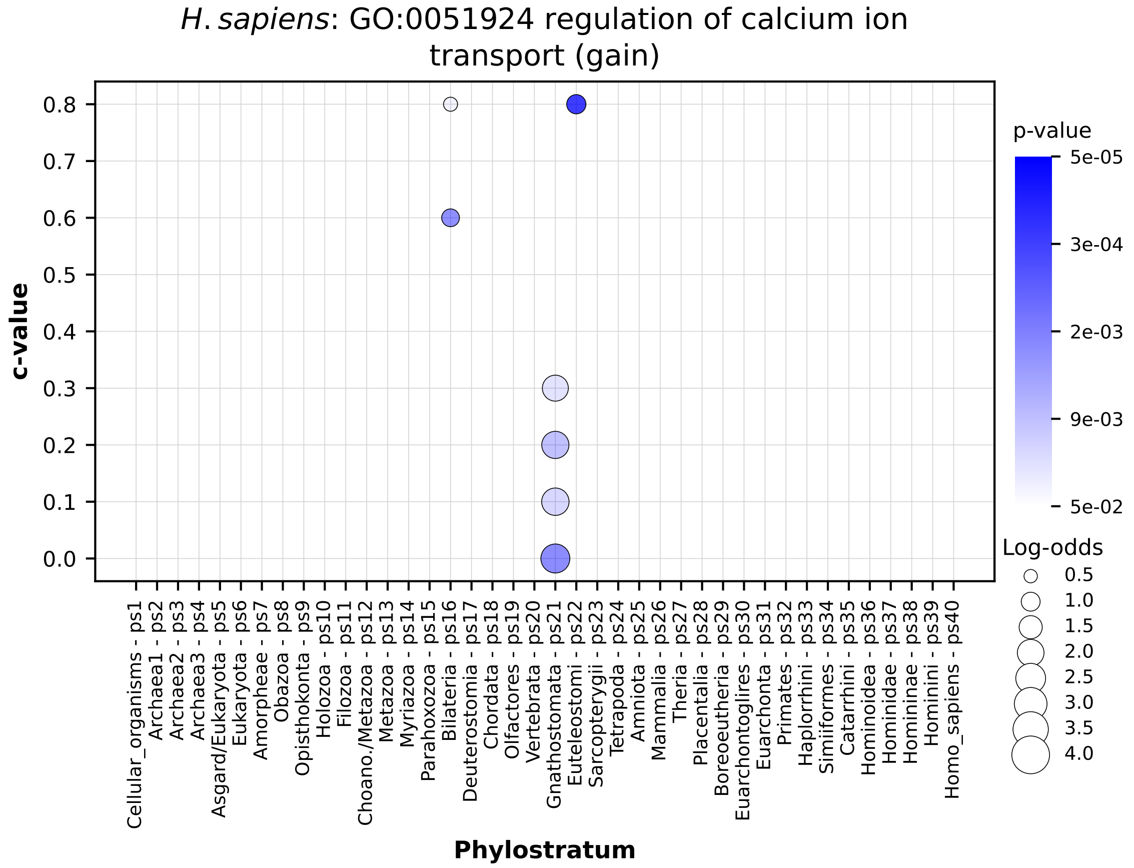

H. sapiens: GO:0051924 regulation of calcium ion transport (gain)
c-value
0.0
0.1
0.2
0.3
0.4
0.5
0.6
0.7
0.8
Phylostratum
Cellular_organisms - ps1
Archaea1 - ps2
Archaea2 - ps3
Archaea3 - ps4
Asgard/Eukaryota - ps5
Eukaryota - ps6
Amorpheae - ps7
Obazoa - ps8
Opisthokonta - ps9
Holozoa - ps10
Filozoa - ps11
Choano./Metazoa - ps12
Metazoa - ps13
Myriazoa - ps14
Parahoxozoa - ps15
Bilateria - ps16
Deuterostomia - ps17
Chordata - ps18
Olfactores - ps19
Vertebrata - ps20
Gnathostomata - ps21
Euteleostomi - ps22
Sarcopterygii - ps23
Tetrapoda - ps24
Amniota - ps25
Mammalia - ps26
Theria - ps27
Placentalia - ps28
Boreoeutheria - ps29
Euarchontoglires - ps30
Euarchonta - ps31
Primates - ps32
Haplorrhini - ps33
Simiiformes - ps34
Catarrhini - ps35
Hominoidea - ps36
Hominidae - ps37
Homininae - ps38
Hominini - ps39
Homo_sapiens - ps40
p-value
5e-05
3e-04
2e-03
9e-03
5e-02
Log-odds
0.5
1.0
1.5
2.0
2.5
3.0
3.5
4.0

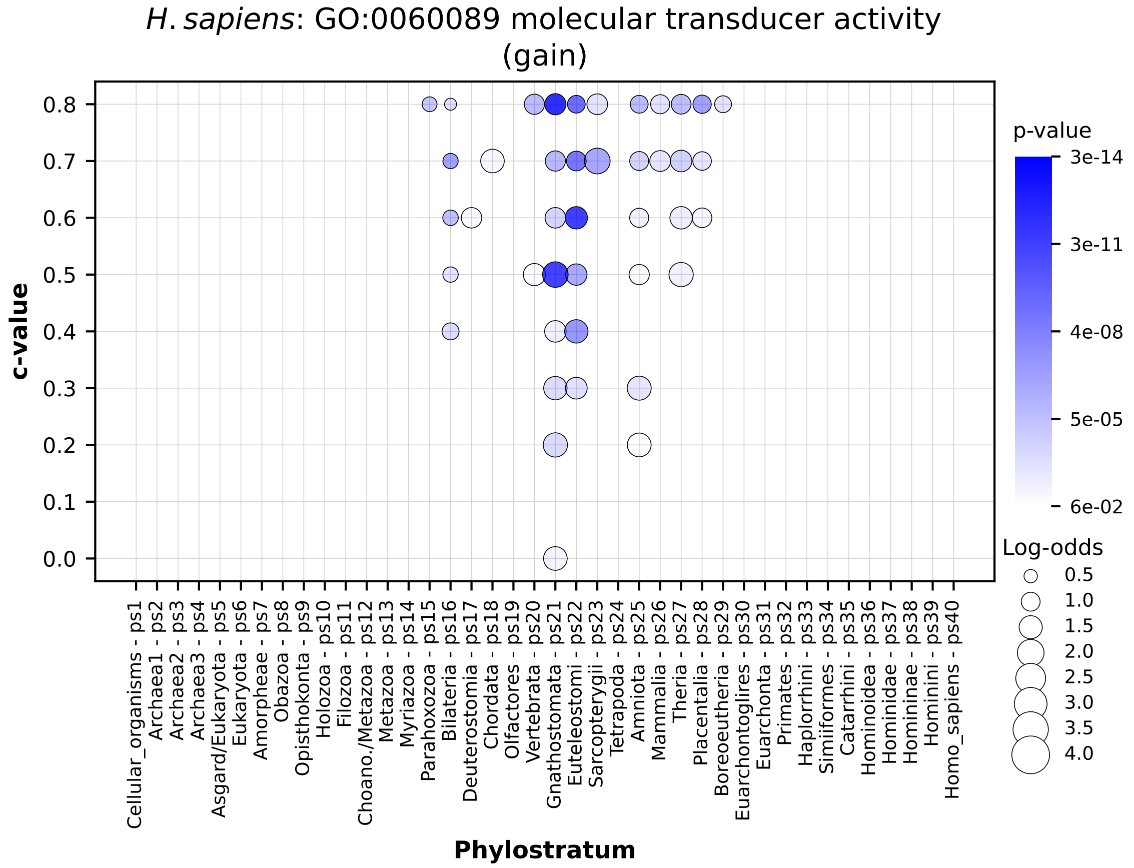
H. sapiens: GO:0060089 molecular transducer activity (gain)
c-value
0.0
0.1
0.2
0.3
0.4
0.5
0.6
0.7
0.8
p-value
3e-14
3e-11
4e-08
5e-05
6e-02
Log-odds
0.5
1.0
1.5
2.0
2.5
3.0
3.5
4.0
Cellular_organisms - ps1
Archaea1 - ps2
Archaea2 - ps3
Archaea3 - ps4
Asgard/Eukaryota - ps5
Eukaryota - ps6
Amorpheae - ps7
Obazoa - ps8
Opisthokonta - ps9
Holozoa - ps10
Filozoa - ps11
Choano./Metazoa - ps12
Metazoa - ps13
Myriazoa - ps14
Parahoxozoa - ps15
Bilateria - ps16
Deuterostomia - ps17
Chordata - ps18
Olfactores - ps19
Vertebrata - ps20
Gnathostomata - ps21
Euteleostomi - ps22
Sarcopterygii - ps23
Tetrapoda - ps24
Amniota - ps25
Mammalia - ps26
Theria - ps27
Placentalia - ps28
Boreoeutheria - ps29
Euarchontoglires - ps30
Euarchonta - ps31
Primates - ps32
Haplorrhini - ps33
Simiiformes - ps34
Catarrhini - ps35
Hominoidea - ps36
Hominidae - ps37
Homininae - ps38
Hominini - ps39
Homo_sapiens - ps40
Phylostratum

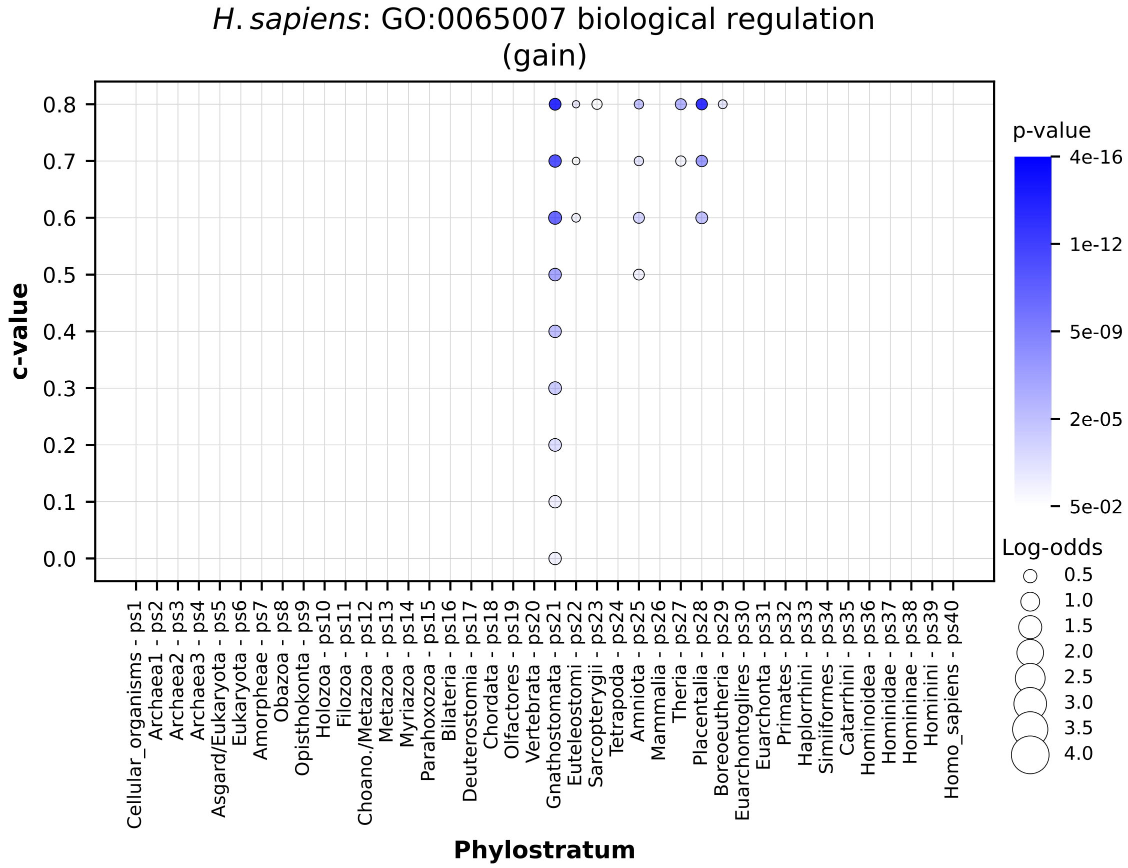

H. sapiens: GO:0065007 biological regulation
(gain)
c-value
0.8
0.7
0.6
0.5
0.4
0.3
0.2
0.1
0.0
Phylostratum
Cellular_organisms - ps1
Archaea1 - ps2
Archaea2 - ps3
Archaea3 - ps4
Asgard/Eukaryota - ps5
Eukaryota - ps6
Amorpheae - ps7
Obazoa - ps8
Opisthokonta - ps9
Holozoa - ps10
Filozoa - ps11
Choano./Metazoa - ps12
Metazoa - ps13
Myriazoa - ps14
Parahoxozoa - ps15
Bilateria - ps16
Deuterostomia - ps17
Chordata - ps18
Olfactores - ps19
Vertebrata - ps20
Gnathostomata - ps21
Euteleostomi - ps22
Sarcopterygii - ps23
Tetrapoda - ps24
Amniota - ps25
Mammalia - ps26
Theria - ps27
Placentalia - ps28
Boreoeutheria - ps29
Euarchontoglires - ps30
Euarchonta - ps31
Primates - ps32
Haplorrhini - ps33
Simiiformes - ps34
Catarrhini - ps35
Hominoidea - ps36
Hominidae - ps37
Homininae - ps38
Hominini - ps39
Homo_sapiens - ps40
p-value
4e-16
1e-12
5e-09
2e-05
5e-02
Log-odds
0.5
1.0
1.5
2.0
2.5
3.0
3.5
4.0

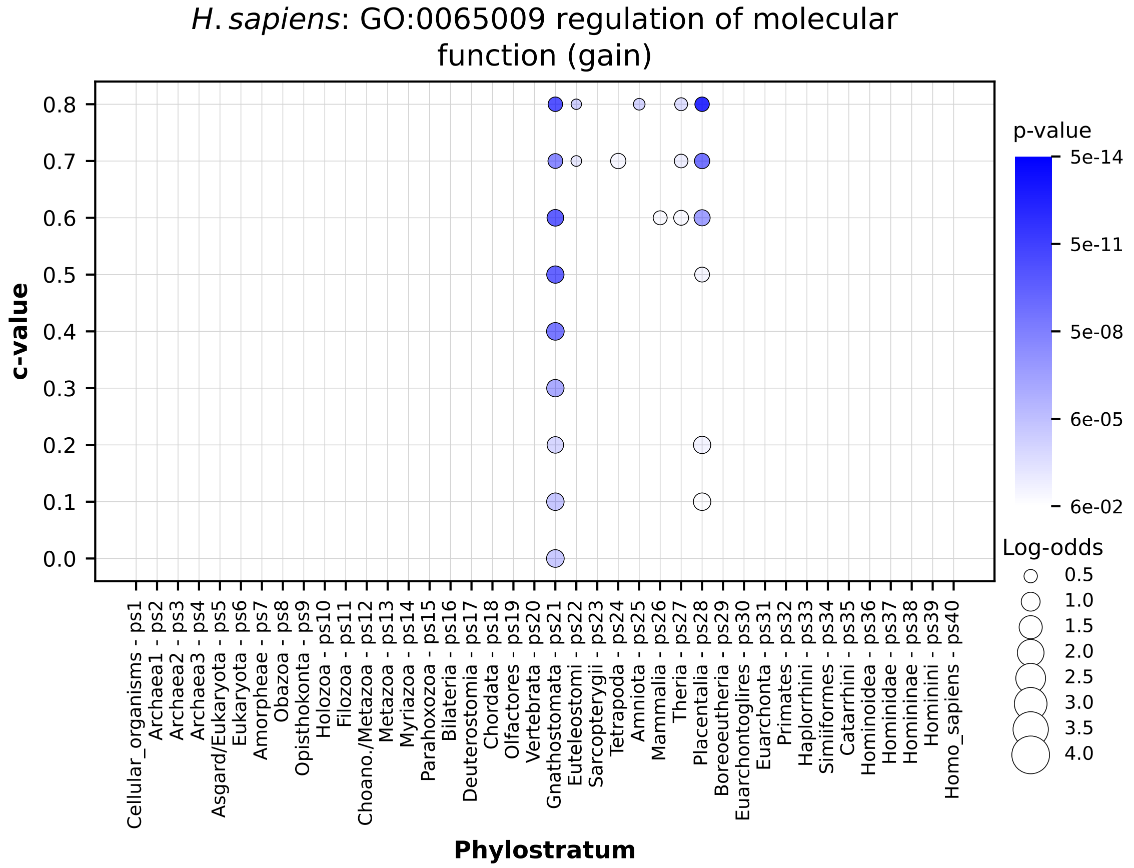

H. sapiens: GO:0065009 regulation of molecular function (gain)
c-value
0.8
0.7
0.6
0.5
0.4
0.3
0.2
0.1
0.0
p-value
5e-14
5e-11
5e-08
6e-05
6e-02
Log-odds
0.5
1.0
1.5
2.0
2.5
3.0
3.5
4.0
Cellular_organisms - ps1
Archaea1 - ps2
Archaea2 - ps3
Archaea3 - ps4
Asgard/Eukaryota - ps5
Eukaryota - ps6
Amorpheae - ps7
Obazoa - ps8
Opisthokonta - ps9
Holozoa - ps10
Filozoa - ps11
Choano./Metazoa - ps12
Metazoa - ps13
Myriazoa - ps14
Parahoxozoa - ps15
Bilateria - ps16
Deuterostomia - ps17
Chordata - ps18
Olfactores - ps19
Vertebrata - ps20
Gnathostomata - ps21
Euteleostomi - ps22
Sarcopterygii - ps23
Tetrapoda - ps24
Amniota - ps25
Mammalia - ps26
Theria - ps27
Placentalia - ps28
Boreoeutheria - ps29
Euarchontoglires - ps30
Euarchonta - ps31
Primates - ps32
Haplorrhini - ps33
Simiiformes - ps34
Catarrhini - ps35
Hominoidea - ps36
Hominidae - ps37
Homininae - ps38
Hominini - ps39
Homo_sapiens - ps40
Phylostratum

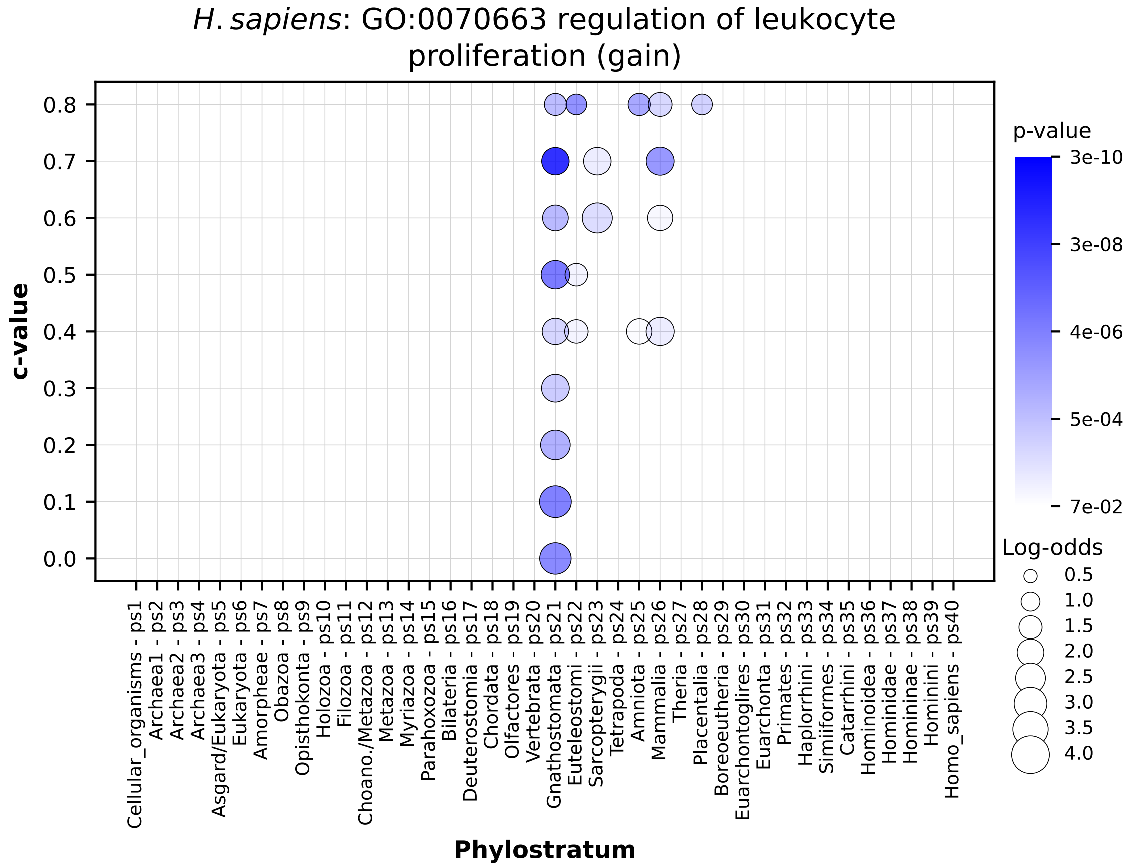

H. sapiens: GO:0070663 regulation of leukocyte proliferation (gain)
c-value
0.0
0.1
0.2
0.3
0.4
0.5
0.6
0.7
0.8
p-value
3e-10
3e-08
4e-06
5e-04
7e-02
Log-odds
0.5
1.0
1.5
2.0
2.5
3.0
3.5
4.0
Cellular_organisms - ps1
Archaea1 - ps2
Archaea2 - ps3
Archaea3 - ps4
Asgard/Eukaryota - ps5
Eukaryota - ps6
Amorpheae - ps7
Obazoa - ps8
Opisthokonta - ps9
Holozoa - ps10
Filozoa - ps11
Choano./Metazoa - ps12
Metazoa - ps13
Myriazoa - ps14
Parahoxozoa - ps15
Bilateria - ps16
Deuterostomia - ps17
Chordata - ps18
Olfactores - ps19
Vertebrata - ps20
Gnathostomata - ps21
Euteleostomi - ps22
Sarcopterygii - ps23
Tetrapoda - ps24
Amniota - ps25
Mammalia - ps26
Theria - ps27
Placentalia - ps28
Boreoeutheria - ps29
Euarchontoglires - ps30
Euarchonta - ps31
Primates - ps32
Haplorrhini - ps33
Simiiformes - ps34
Catarrhini - ps35
Hominoidea - ps36
Hominidae - ps37
Homininae - ps38
Hominini - ps39
Homo_sapiens - ps40
Phylostratum

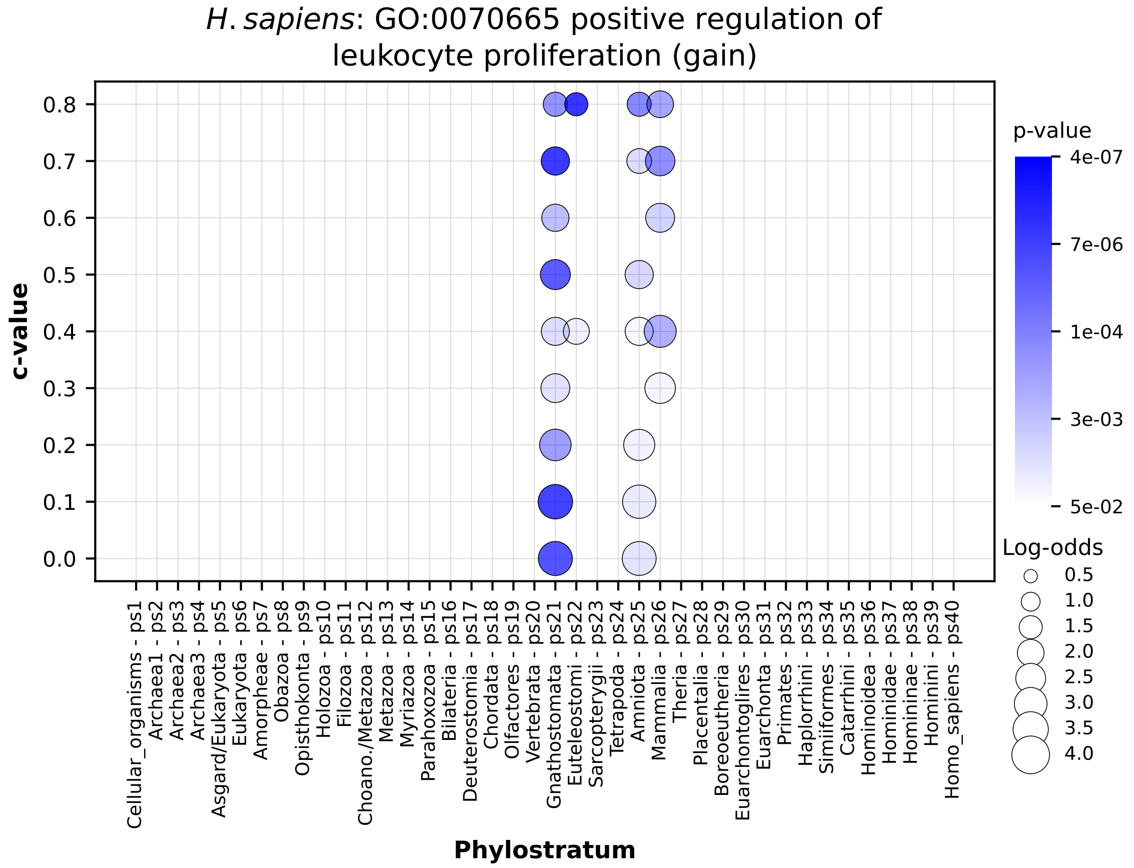

*H. sapiens*: GO:0070665 positive regulation of leukocyte proliferation (gain)
c-value
0.8
0.7
0.6
0.5
0.4
0.3
0.2
0.1
0.0
p-value
4e-07
7e-06
1e-04
3e-03
5e-02
Log-odds
0.5
1.0
1.5
2.0
2.5
3.0
3.5
4.0
Cellular_organisms - ps1
Archaea1 - ps2
Archaea2 - ps3
Archaea3 - ps4
Asgard/Eukaryota - ps5
Eukaryota - ps6
Amorpheae - ps7
Obazoa - ps8
Opisthokonta - ps9
Holozoa - ps10
Filozoa - ps11
Choano./Metazoa - ps12
Metazoa - ps13
Myriazoa - ps14
Parahoxozoa - ps15
Bilateria - ps16
Deuterostomia - ps17
Chordata - ps18
Olfactores - ps19
Vertebrata - ps20
Gnathostomata - ps21
Euteleostomi - ps22
Sarcopterygii - ps23
Tetrapoda - ps24
Amniota - ps25
Mammalia - ps26
Theria - ps27
Placentalia - ps28
Boreoeutheria - ps29
Euarchontoglires - ps30
Euarchonta - ps31
Primates - ps32
Haplorrhini - ps33
Simiiformes - ps34
Catarrhini - ps35
Hominoidea - ps36
Hominidae - ps37
Homininae - ps38
Hominini - ps39
Homo_sapiens - ps40
Phylostratum

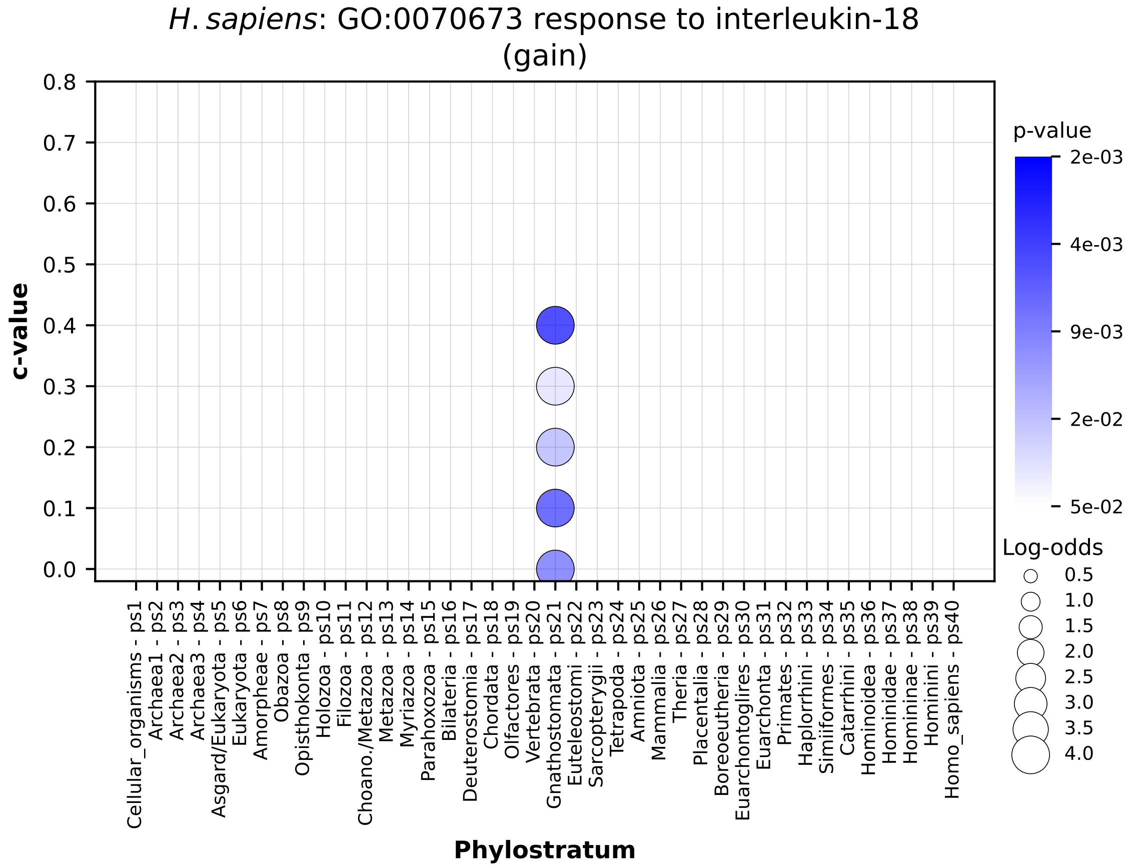

*H. sapiens*: GO:0070673 response to interleukin-18
(gain)
c-value
0.8
0.7
0.6
0.5
0.4
0.3
0.2
0.1
0.0
p-value
2e-03
4e-03
9e-03
2e-02
5e-02
Log-odds
0.5
1.0
1.5
2.0
2.5
3.0
3.5
4.0
Cellular_organisms - ps1
Archaea1 - ps2
Archaea2 - ps3
Archaea3 - ps4
Asgard/Eukaryota - ps5
Eukaryota - ps6
Amorpheae - ps7
Obazoa - ps8
Opisthokonta - ps9
Holozoa - ps10
Filozoa - ps11
Choano./Metazoa - ps12
Metazoa - ps13
Myriazoa - ps14
Parahoxozoa - ps15
Bilateria - ps16
Deuterostomia - ps17
Chordata - ps18
Olfactores - ps19
Vertebrata - ps20
Gnathostomata - ps21
Euteleostomi - ps22
Sarcopterygii - ps23
Tetrapoda - ps24
Amniota - ps25
Mammalia - ps26
Theria - ps27
Placentalia - ps28
Boreoeutheria - ps29
Euarchontoglires - ps30
Euarchonta - ps31
Primates - ps32
Haplorrhini - ps33
Simiiformes - ps34
Catarrhini - ps35
Hominoidea - ps36
Hominidae - ps37
Homininae - ps38
Hominini - ps39
Homo_sapiens - ps40
Phylostratum

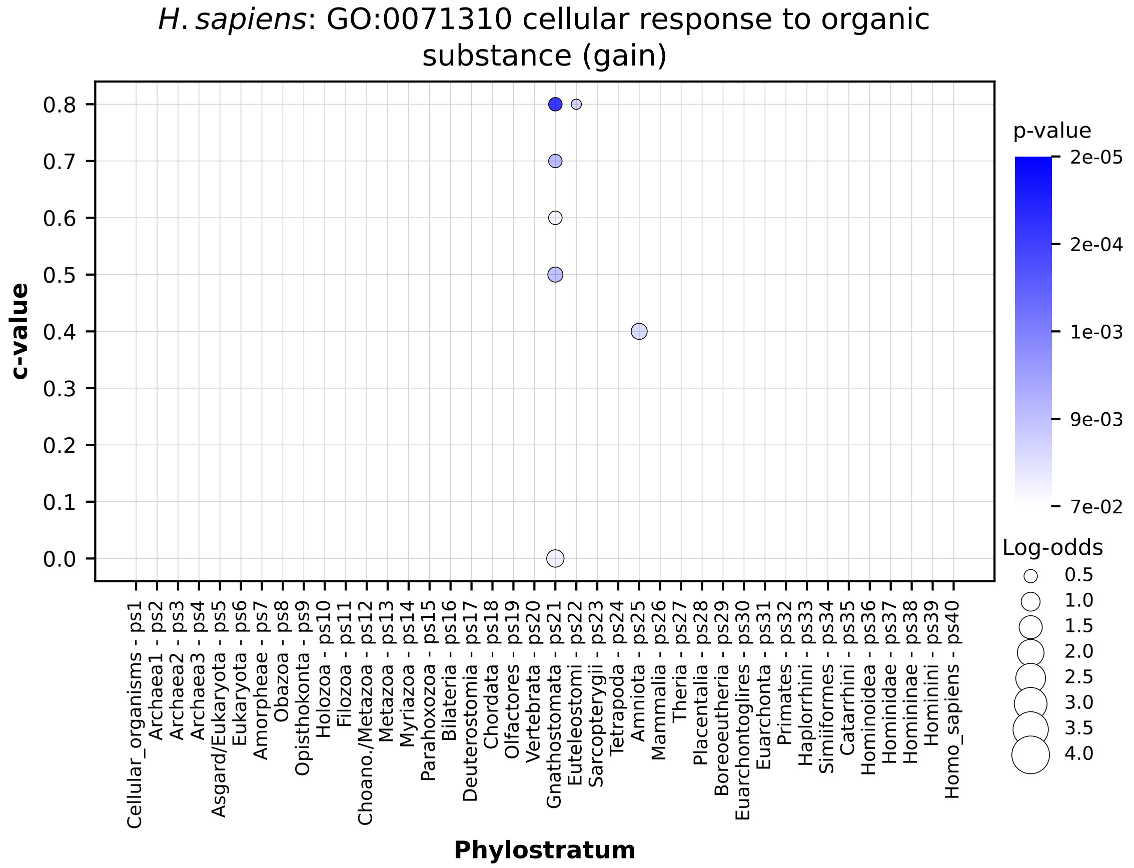
H. sapiens: GO:0071310 cellular response to organic substance (gain)
c-value
0.8
0.7
0.6
0.5
0.4
0.3
0.2
0.1
0.0
p-value
2e-05
2e-04
1e-03
9e-03
7e-02
Log-odds
0.5
1.0
1.5
2.0
2.5
3.0
3.5
4.0
Cellular_organisms - ps1
Archaea1 - ps2
Archaea2 - ps3
Archaea3 - ps4
Asgard/Eukaryota - ps5
Eukaryota - ps6
Amorpheae - ps7
Obazoa - ps8
Opisthokonta - ps9
Holozoa - ps10
Filozoa - ps11
Choano./Metazoa - ps12
Metazoa - ps13
Myriazoa - ps14
Parahoxozoa - ps15
Bilateria - ps16
Deuterostomia - ps17
Chordata - ps18
Olfactores - ps19
Vertebrata - ps20
Gnathostomata - ps21
Euteleostomi - ps22
Sarcopterygii - ps23
Tetrapoda - ps24
Amniota - ps25
Mammalia - ps26
Theria - ps27
Placentalia - ps28
Boreoeutheria - ps29
Euarchontoglires - ps30
Euarchonta - ps31
Primates - ps32
Haplorrhini - ps33
Simiiformes - ps34
Catarrhini - ps35
Hominoidea - ps36
Hominidae - ps37
Homininae - ps38
Hominini - ps39
Homo_sapiens - ps40
Phylostratum

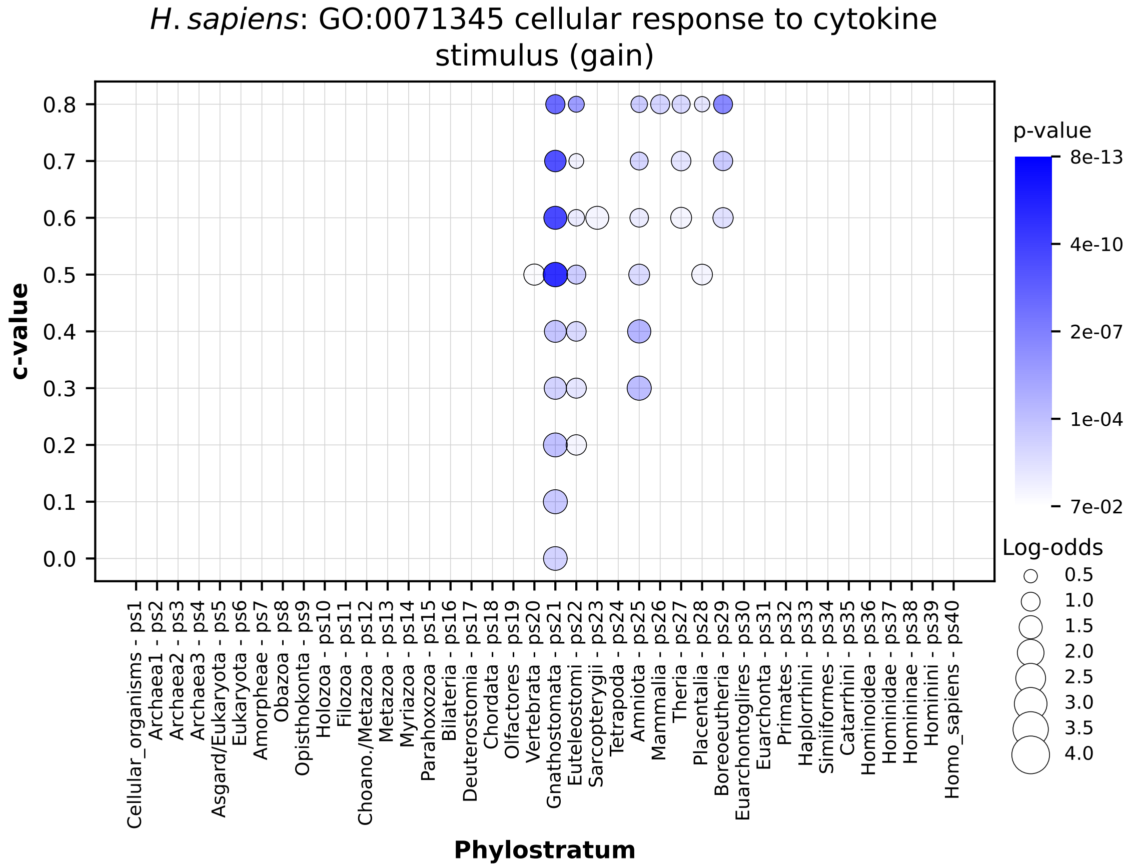
*H. sapiens*: GO:0071345 cellular response to cytokine stimulus (gain)
c-value
Phylostratum
p-value
8e-13
4e-10
2e-07
1e-04
7e-02
Log-odds
0.5
1.0
1.5
2.0
2.5
3.0
3.5
4.0
Cellular_organisms - ps1
Archaea1 - ps2
Archaea2 - ps3
Archaea3 - ps4
Asgard/Eukaryota - ps5
Eukaryota - ps6
Amorpheae - ps7
Obazoa - ps8
Opisthokonta - ps9
Holozoa - ps10
Filozoa - ps11
Choano./Metazoa - ps12
Metazoa - ps13
Myriazoa - ps14
Parahoxozoa - ps15
Bilateria - ps16
Deuterostomia - ps17
Chordata - ps18
Olfactores - ps19
Vertebrata - ps20
Gnathostomata - ps21
Euteleostomi - ps22
Sarcopterygii - ps23
Tetrapoda - ps24
Amniota - ps25
Mammalia - ps26
Theria - ps27
Placentalia - ps28
Boreoeutheria - ps29
Euarchontoglires - ps30
Euarchonta - ps31
Primates - ps32
Haplorrhini - ps33
Simiiformes - ps34
Catarrhini - ps35
Hominoidea - ps36
Hominidae - ps37
Homininae - ps38
Hominini - ps39
Homo_sapiens - ps40

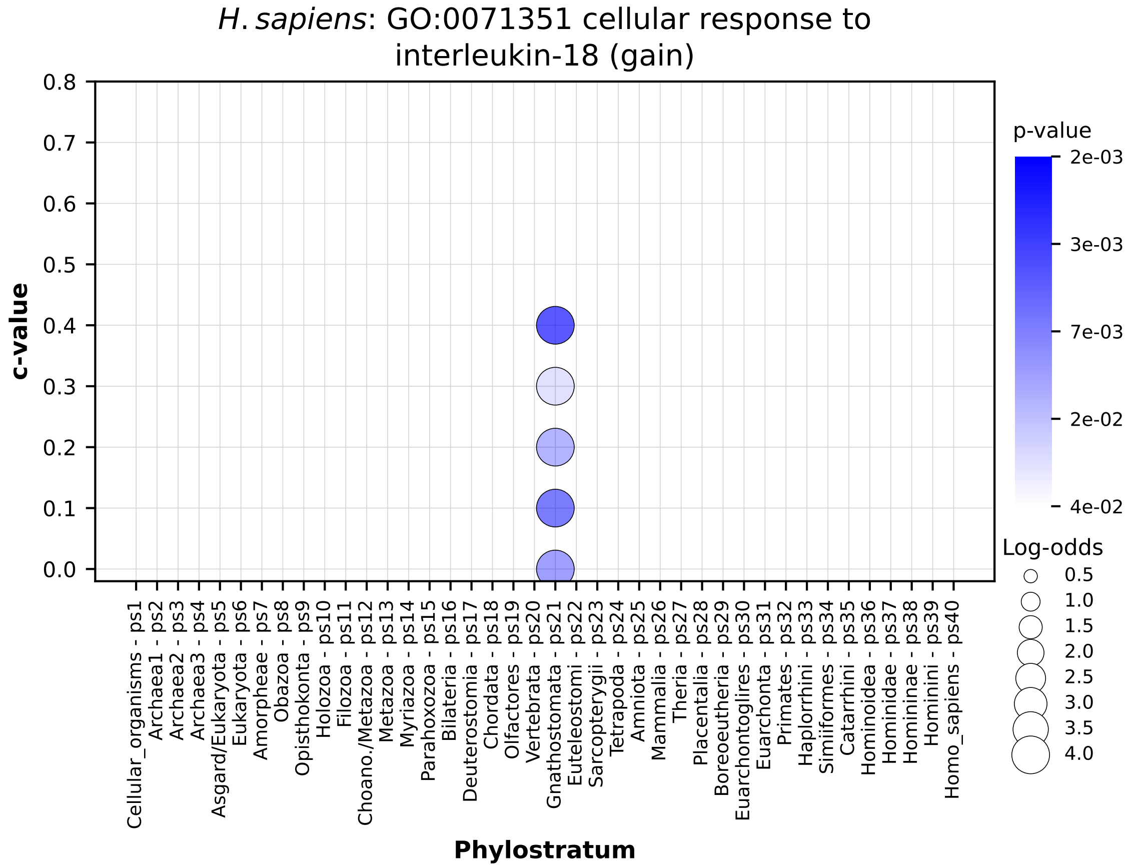

H. sapiens: GO:0071351 cellular response to interleukin-18 (gain)
c-value
0.8
0.7
0.6
0.5
0.4
0.3
0.2
0.1
0.0
Phylostratum
Cellular_organisms - ps1
Archaea1 - ps2
Archaea2 - ps3
Archaea3 - ps4
Asgard/Eukaryota - ps5
Eukaryota - ps6
Amorpheae - ps7
Obazoa - ps8
Opisthokonta - ps9
Holozoa - ps10
Filozoa - ps11
Choano./Metazoa - ps12
Metazoa - ps13
Myriazoa - ps14
Parahoxozoa - ps15
Bilateria - ps16
Deuterostomia - ps17
Chordata - ps18
Olfactores - ps19
Vertebrata - ps20
Gnathostomata - ps21
Euteleostomi - ps22
Sarcopterygii - ps23
Tetrapoda - ps24
Amniota - ps25
Mammalia - ps26
Theria - ps27
Placentalia - ps28
Boreoeutheria - ps29
Euarchontoglires - ps30
Euarchonta - ps31
Primates - ps32
Haplorrhini - ps33
Simiiformes - ps34
Catarrhini - ps35
Hominoidea - ps36
Hominidae - ps37
Homininae - ps38
Hominini - ps39
Homo_sapiens - ps40
p-value
2e-03
3e-03
7e-03
2e-02
4e-02
Log-odds
0.5
1.0
1.5
2.0
2.5
3.0
3.5
4.0

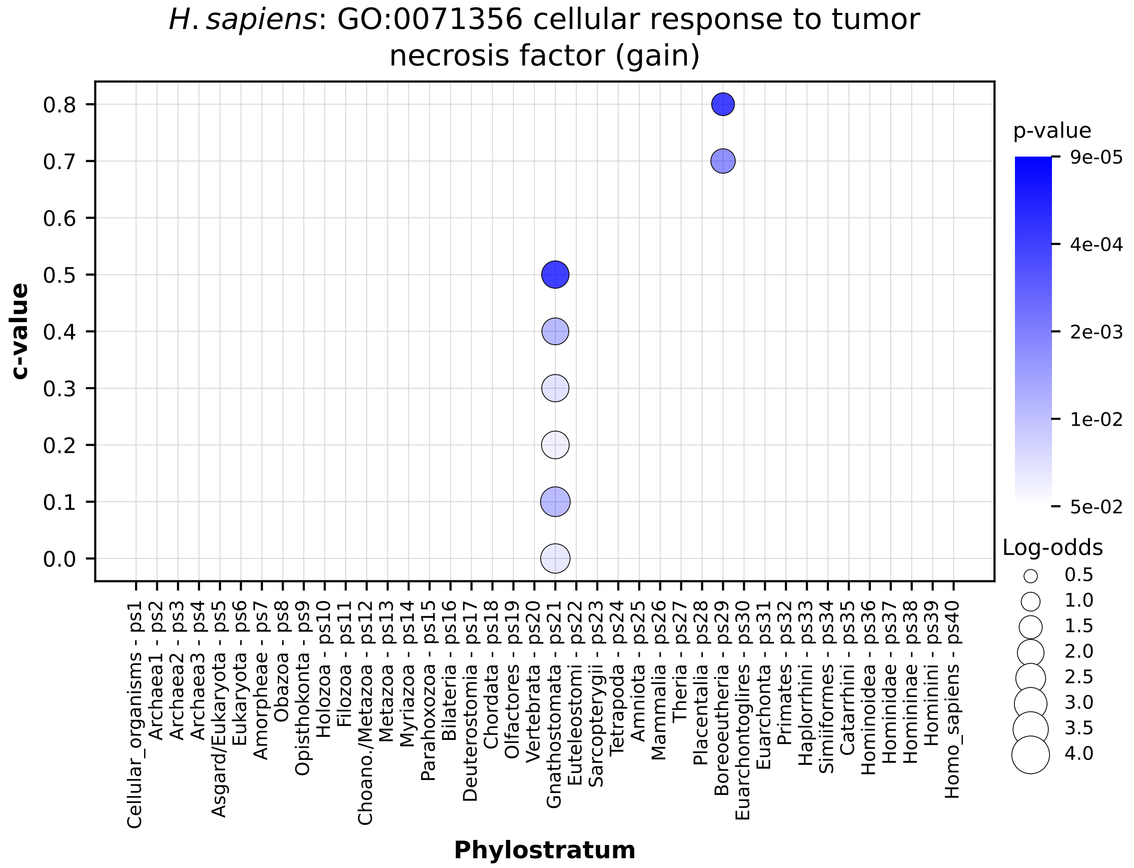

*H. sapiens*: GO:0071356 cellular response to tumor necrosis factor (gain)
c-value
0.8
0.7
0.6
0.5
0.4
0.3
0.2
0.1
0.0
p-value
9e-05
4e-04
2e-03
1e-02
5e-02
Log-odds
0.5
1.0
1.5
2.0
2.5
3.0
3.5
4.0
Cellular_organisms - ps1
Archaea1 - ps2
Archaea2 - ps3
Archaea3 - ps4
Asgard/Eukaryota - ps5
Eukaryota - ps6
Amorpheae - ps7
Obazoa - ps8
Opisthokonta - ps9
Holozoa - ps10
Filozoa - ps11
Choano./Metazoa - ps12
Metazoa - ps13
Myriazoa - ps14
Parahoxozoa - ps15
Bilateria - ps16
Deuterostomia - ps17
Chordata - ps18
Olfactores - ps19
Vertebrata - ps20
Gnathostomata - ps21
Euteleostomi - ps22
Sarcopterygii - ps23
Tetrapoda - ps24
Amniota - ps25
Mammalia - ps26
Theria - ps27
Placentalia - ps28
Boreoeutheria - ps29
Euarchontoglires - ps30
Euarchonta - ps31
Primates - ps32
Haplorrhini - ps33
Simiiformes - ps34
Catarrhini - ps35
Hominoidea - ps36
Hominidae - ps37
Homininae - ps38
Hominini - ps39
Homo_sapiens - ps40
Phylostratum

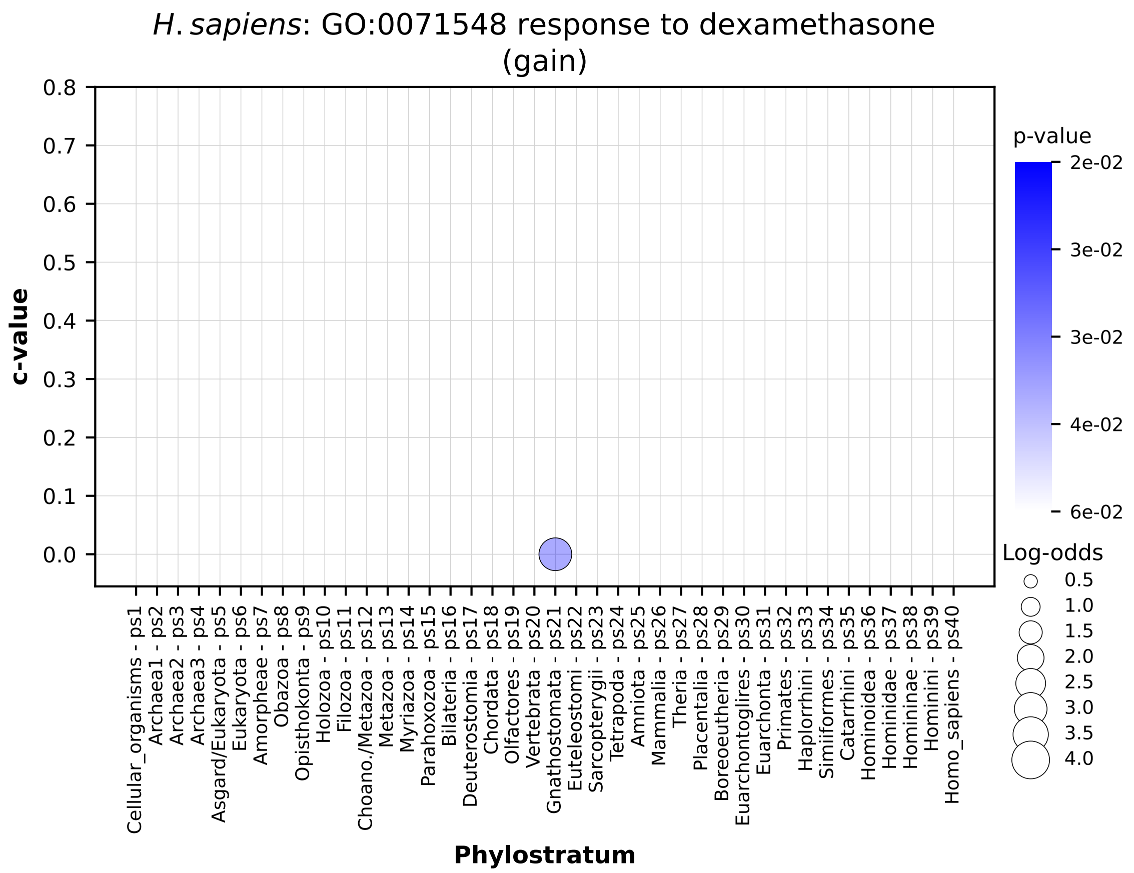

*H. sapiens*: GO:0071548 response to dexamethasone (gain)
c-value
0.8
0.7
0.6
0.5
0.4
0.3
0.2
0.1
0.0
p-value
2e-02
3e-02
3e-02
4e-02
6e-02
Log-odds
0.5
1.0
1.5
2.0
2.5
3.0
3.5
4.0
Cellular_organisms - ps1
Archaea1 - ps2
Archaea2 - ps3
Archaea3 - ps4
Asgard/Eukaryota - ps5
Eukaryota - ps6
Amorpheae - ps7
Obazoa - ps8
Opisthokonta - ps9
Holozoa - ps10
Filozoa - ps11
Choano./Metazoa - ps12
Metazoa - ps13
Myriazoa - ps14
Parahoxozoa - ps15
Bilateria - ps16
Deuterostomia - ps17
Chordata - ps18
Olfactores - ps19
Vertebrata - ps20
Gnathostomata - ps21
Euteleostomi - ps22
Sarcopterygii - ps23
Tetrapoda - ps24
Amniota - ps25
Mammalia - ps26
Theria - ps27
Placentalia - ps28
Boreoeutheria - ps29
Euarchontoglires - ps30
Euarchonta - ps31
Primates - ps32
Haplorrhini - ps33
Simiiformes - ps34
Catarrhini - ps35
Hominoidea - ps36
Hominidae - ps37
Homininae - ps38
Hominini - ps39
Homo_sapiens - ps40
Phylostratum

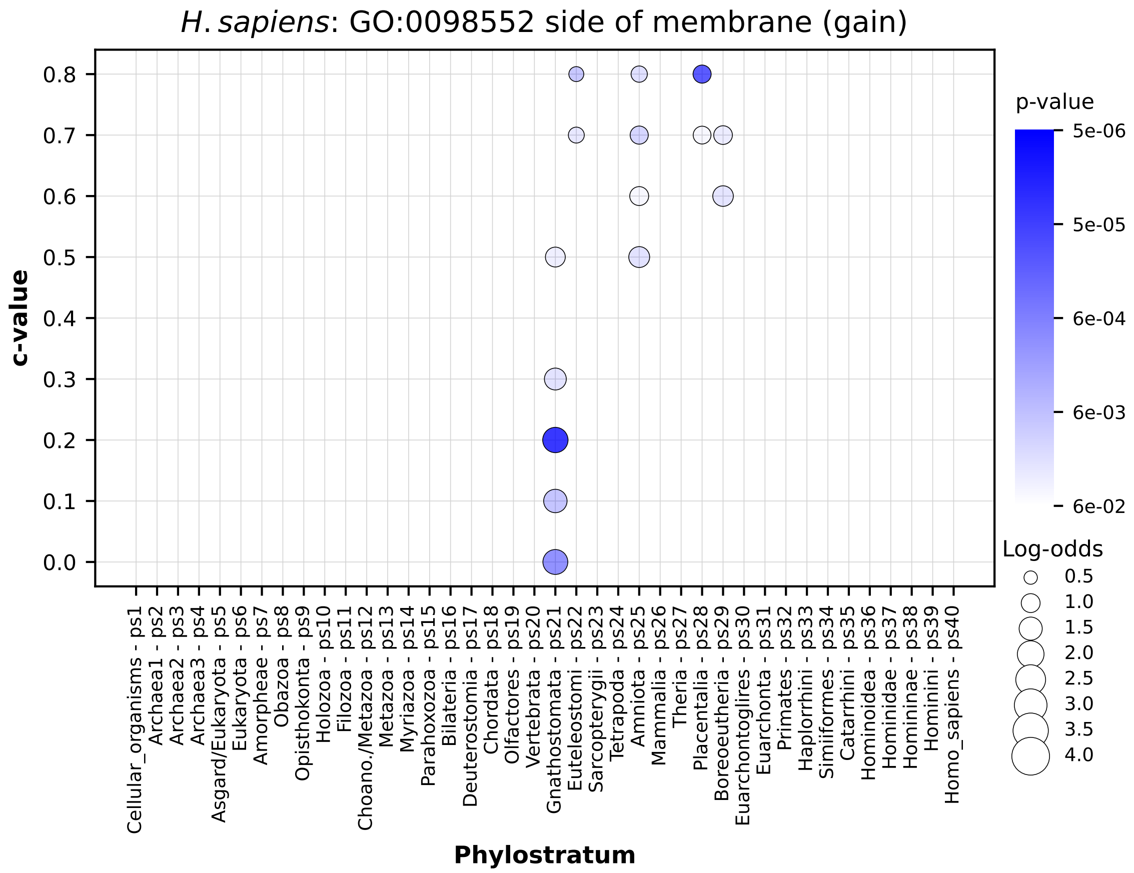

H. sapiens: GO:0098552 side of membrane (gain)
c-value
0.8
0.7
0.6
0.5
0.4
0.3
0.2
0.1
0.0
Phylostratum
Cellular_organisms - ps1
Archaea1 - ps2
Archaea2 - ps3
Archaea3 - ps4
Asgard/Eukaryota - ps5
Eukaryota - ps6
Amorpheae - ps7
Obazoa - ps8
Opisthokonta - ps9
Holozoa - ps10
Filozoa - ps11
Choano./Metazoa - ps12
Metazoa - ps13
Myriazoa - ps14
Parahoxozoa - ps15
Bilateria - ps16
Deuterostomia - ps17
Chordata - ps18
Olfactores - ps19
Vertebrata - ps20
Gnathostomata - ps21
Euteleostomi - ps22
Sarcopterygii - ps23
Tetrapoda - ps24
Amniota - ps25
Mammalia - ps26
Theria - ps27
Placentalia - ps28
Boreoeutheria - ps29
Euarchontoglires - ps30
Euarchonta - ps31
Primates - ps32
Haplorrhini - ps33
Simiiformes - ps34
Catarrhini - ps35
Hominoidea - ps36
Hominidae - ps37
Homininae - ps38
Hominini - ps39
Homo_sapiens - ps40
p-value
5e-06
5e-05
6e-04
6e-03
6e-02
Log-odds
0.5
1.0
1.5
2.0
2.5
3.0
3.5
4.0

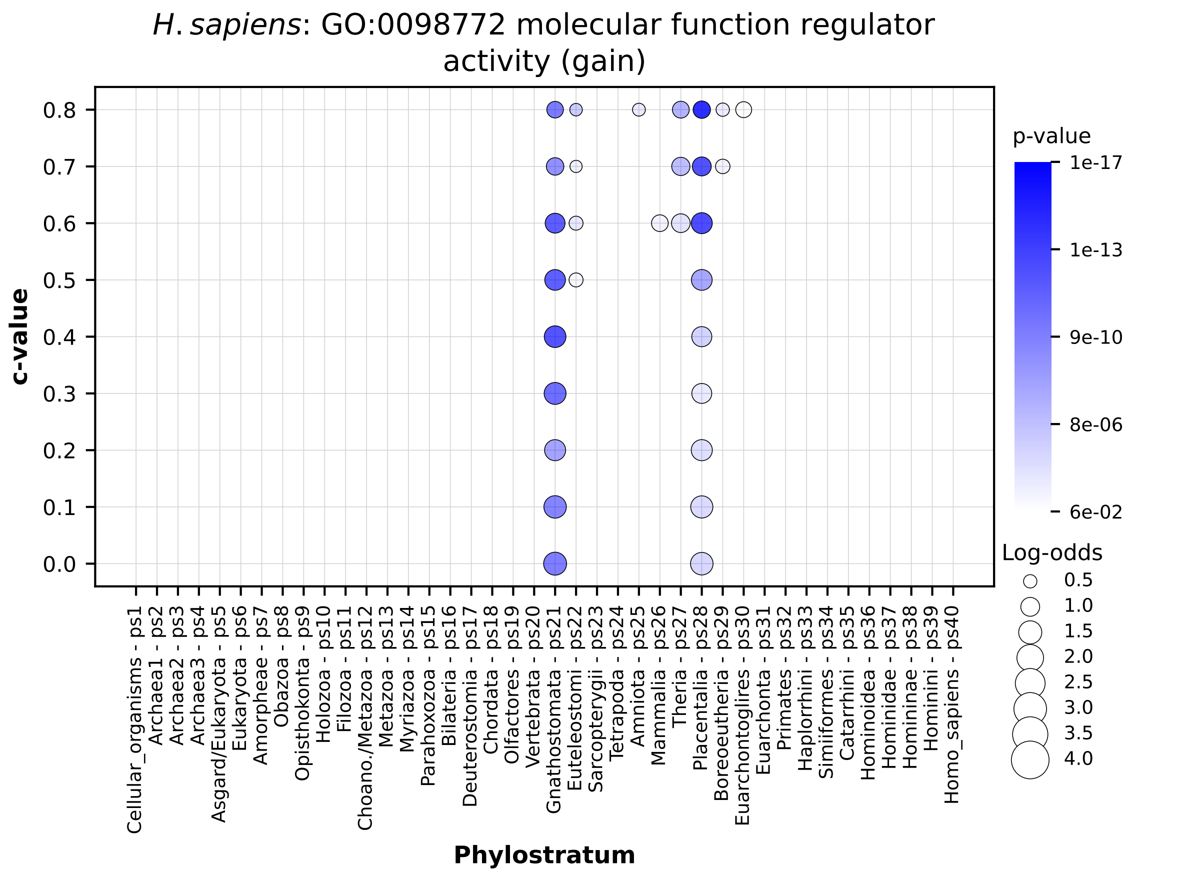

H. sapiens: GO:0098772 molecular function regulator activity (gain)
c-value
0.8
0.7
0.6
0.5
0.4
0.3
0.2
0.1
0.0
Phylostratum
Cellular_organisms - ps1
Archaea1 - ps2
Archaea2 - ps3
Archaea3 - ps4
Asgard/Eukaryota - ps5
Eukaryota - ps6
Amorpheae - ps7
Obazoa - ps8
Opisthokonta - ps9
Holozoa - ps10
Filozoa - ps11
Choano./Metazoa - ps12
Metazoa - ps13
Myriazoa - ps14
Parahoxozoa - ps15
Bilateria - ps16
Deuterostomia - ps17
Chordata - ps18
Olfactores - ps19
Vertebrata - ps20
Gnathostomata - ps21
Euteleostomi - ps22
Sarcopterygii - ps23
Tetrapoda - ps24
Amniota - ps25
Mammalia - ps26
Theria - ps27
Placentalia - ps28
Boreoeutheria - ps29
Euarchontoglires - ps30
Euarchonta - ps31
Primates - ps32
Haplorrhini - ps33
Simiiformes - ps34
Catarrhini - ps35
Hominoidea - ps36
Hominidae - ps37
Homininae - ps38
Hominini - ps39
Homo_sapiens - ps40
p-value
1e-17
1e-13
9e-10
8e-06
6e-02
Log-odds
0.5
1.0
1.5
2.0
2.5
3.0
3.5
4.0

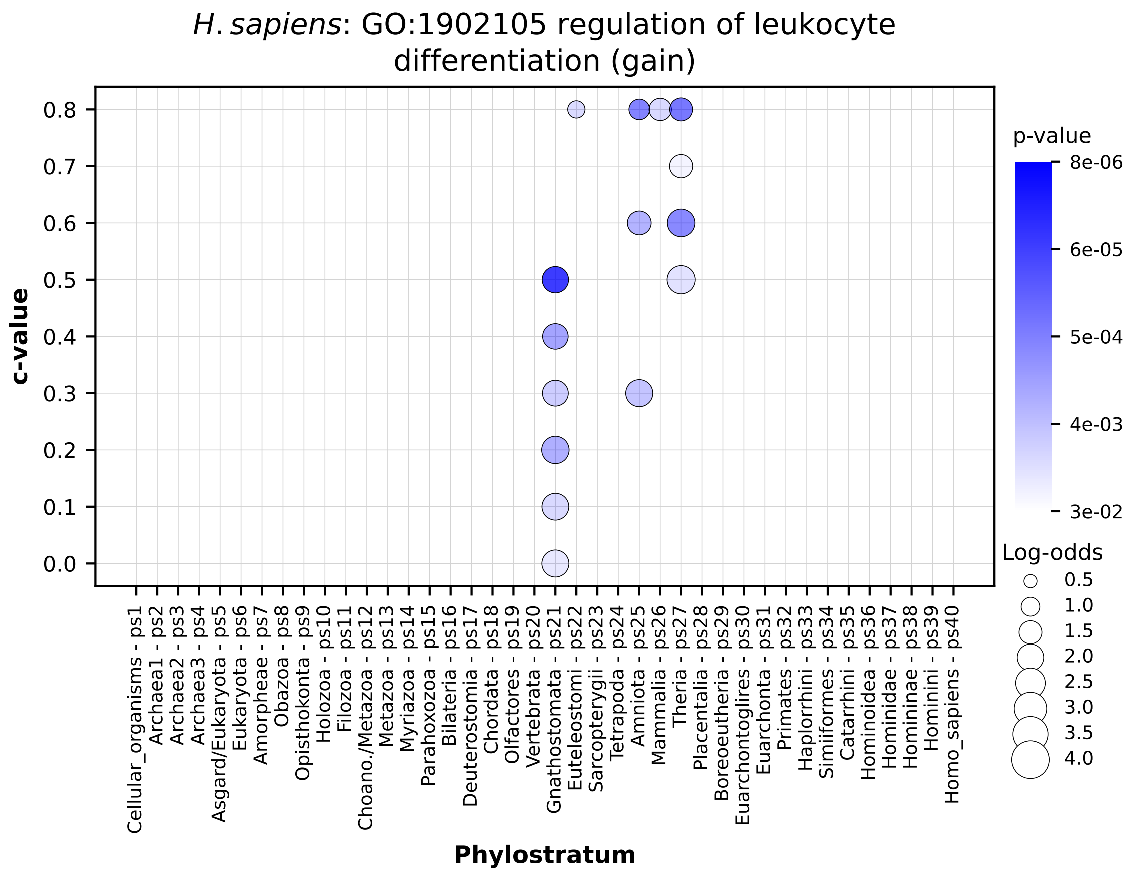
H. sapiens: GO:1902105 regulation of leukocyte differentiation (gain)
c-value
0.8
0.7
0.6
0.5
0.4
0.3
0.2
0.1
0.0
Phylostratum
Cellular_organisms - ps1
Archaea1 - ps2
Archaea2 - ps3
Archaea3 - ps4
Asgard/Eukaryota - ps5
Eukaryota - ps6
Amorpheae - ps7
Obazoa - ps8
Opisthokonta - ps9
Holozoa - ps10
Filozoa - ps11
Choano./Metazoa - ps12
Metazoa - ps13
Myriazoa - ps14
Parahoxozoa - ps15
Bilateria - ps16
Deuterostomia - ps17
Chordata - ps18
Olfactores - ps19
Vertebrata - ps20
Gnathostomata - ps21
Euteleostomi - ps22
Sarcopterygii - ps23
Tetrapoda - ps24
Amniota - ps25
Mammalia - ps26
Theria - ps27
Placentalia - ps28
Boreoeutheria - ps29
Euarchontoglires - ps30
Euarchonta - ps31
Primates - ps32
Haplorrhini - ps33
Simiiformes - ps34
Catarrhini - ps35
Hominoidea - ps36
Hominidae - ps37
Homininae - ps38
Hominini - ps39
Homo_sapiens - ps40
p-value
8e-06
6e-05
5e-04
4e-03
3e-02
Log-odds
0.5
1.0
1.5
2.0
2.5
3.0
3.5
4.0

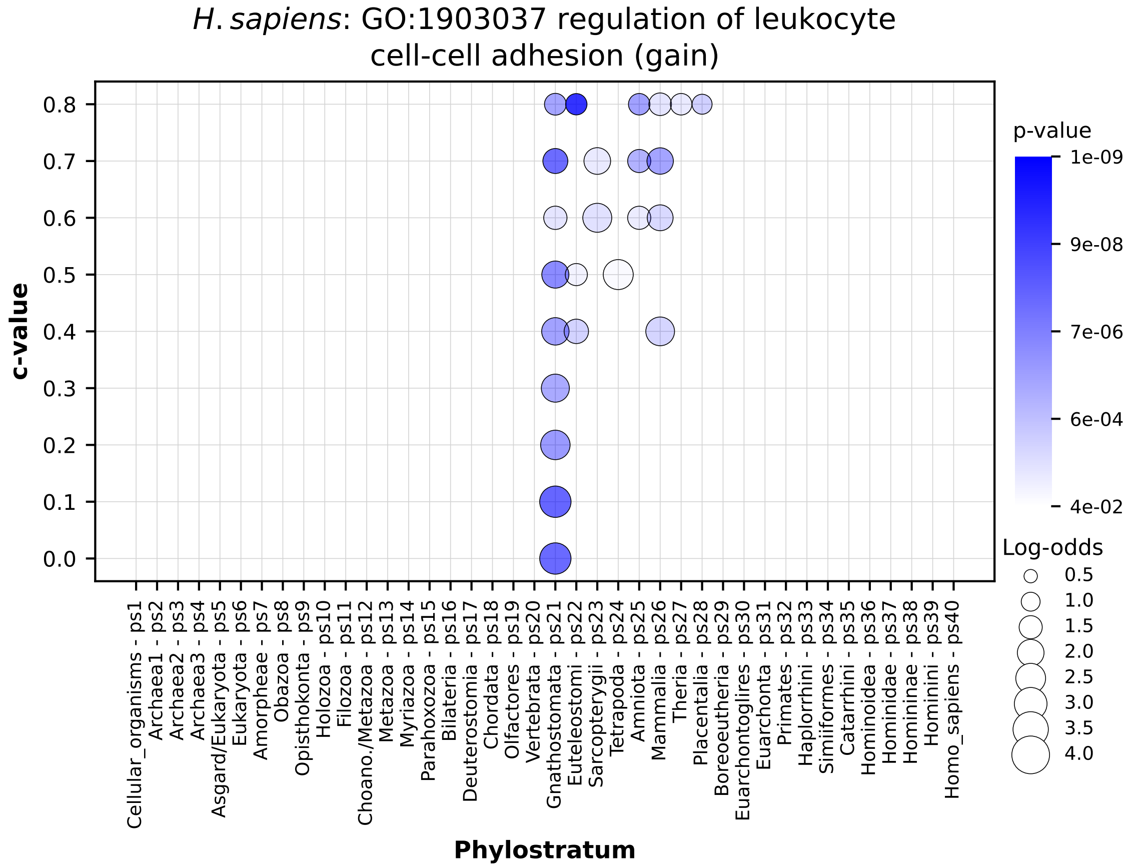

H. sapiens: GO:1903037 regulation of leukocyte cell-cell adhesion (gain)
c-value
0.8
0.7
0.6
0.5
0.4
0.3
0.2
0.1
0.0
Phylostratum
Cellular_organisms - ps1
Archaea1 - ps2
Archaea2 - ps3
Archaea3 - ps4
Asgard/Eukaryota - ps5
Eukaryota - ps6
Amorpheae - ps7
Obazoa - ps8
Opisthokonta - ps9
Holozoa - ps10
Filozoa - ps11
Choano./Metazoa - ps12
Metazoa - ps13
Myriazoa - ps14
Parahoxozoa - ps15
Bilateria - ps16
Deuterostomia - ps17
Chordata - ps18
Olfactores - ps19
Vertebrata - ps20
Gnathostomata - ps21
Euteleostomi - ps22
Sarcopterygii - ps23
Tetrapoda - ps24
Amniota - ps25
Mammalia - ps26
Theria - ps27
Placentalia - ps28
Boreoeutheria - ps29
Euarchontoglires - ps30
Euarchonta - ps31
Primates - ps32
Haplorrhini - ps33
Simiiformes - ps34
Catarrhini - ps35
Hominoidea - ps36
Hominidae - ps37
Homininae - ps38
Hominini - ps39
Homo_sapiens - ps40
p-value
1e-09
9e-08
7e-06
6e-04
4e-02
Log-odds
0.5
1.0
1.5
2.0
2.5
3.0
3.5
4.0

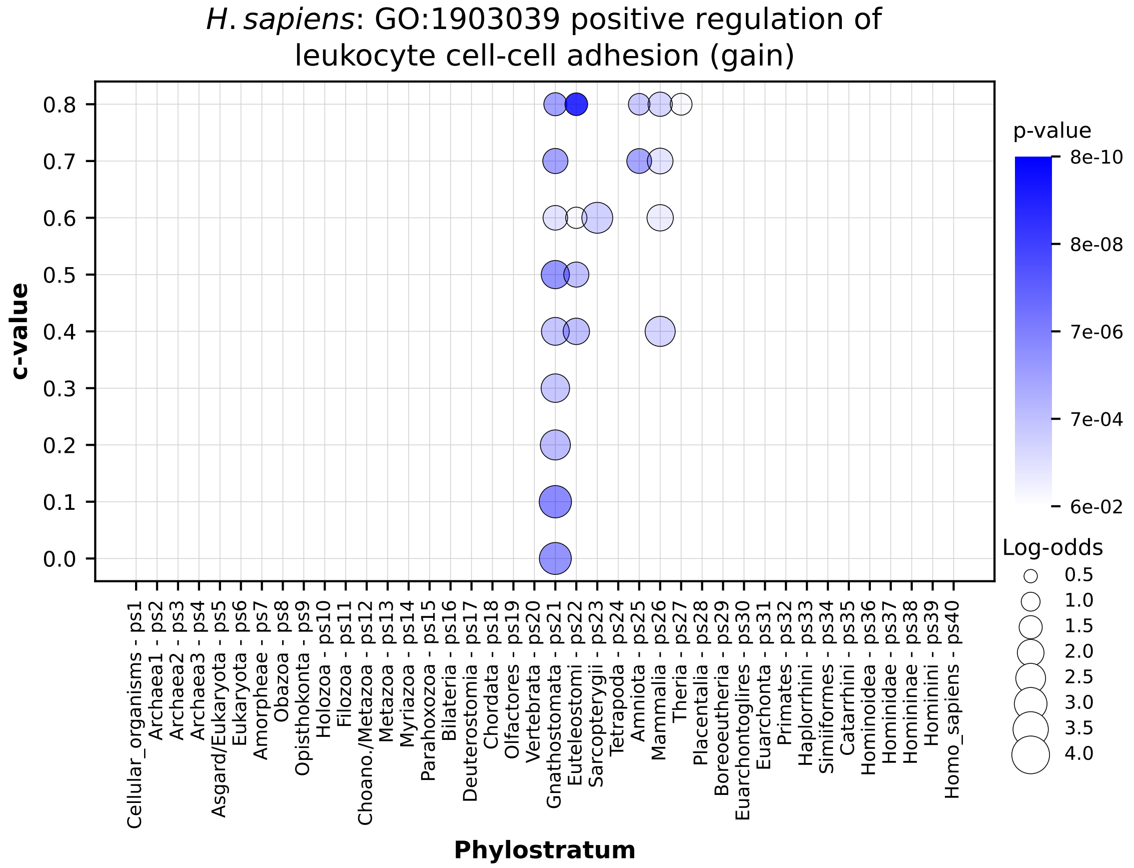

H. sapiens: GO:1903039 positive regulation of leukocyte cell-cell adhesion (gain)
c-value
0.8
0.7
0.6
0.5
0.4
0.3
0.2
0.1
0.0
p-value
8e-10
8e-08
7e-06
7e-04
6e-02
Log-odds
0.5
1.0
1.5
2.0
2.5
3.0
3.5
4.0
Cellular_organisms - ps1
Archaea1 - ps2
Archaea2 - ps3
Archaea3 - ps4
Asgard/Eukaryota - ps5
Eukaryota - ps6
Amorpheae - ps7
Obazoa - ps8
Opisthokonta - ps9
Holozoa - ps10
Filozoa - ps11
Choano./Metazoa - ps12
Metazoa - ps13
Myriazoa - ps14
Parahoxozoa - ps15
Bilateria - ps16
Deuterostomia - ps17
Chordata - ps18
Olfactores - ps19
Vertebrata - ps20
Gnathostomata - ps21
Euteleostomi - ps22
Sarcopterygii - ps23
Tetrapoda - ps24
Amniota - ps25
Mammalia - ps26
Theria - ps27
Placentalia - ps28
Boreoeutheria - ps29
Euarchontoglires - ps30
Euarchonta - ps31
Primates - ps32
Haplorrhini - ps33
Simiiformes - ps34
Catarrhini - ps35
Hominoidea - ps36
Hominidae - ps37
Homininae - ps38
Hominini - ps39
Homo_sapiens - ps40
Phylostratum

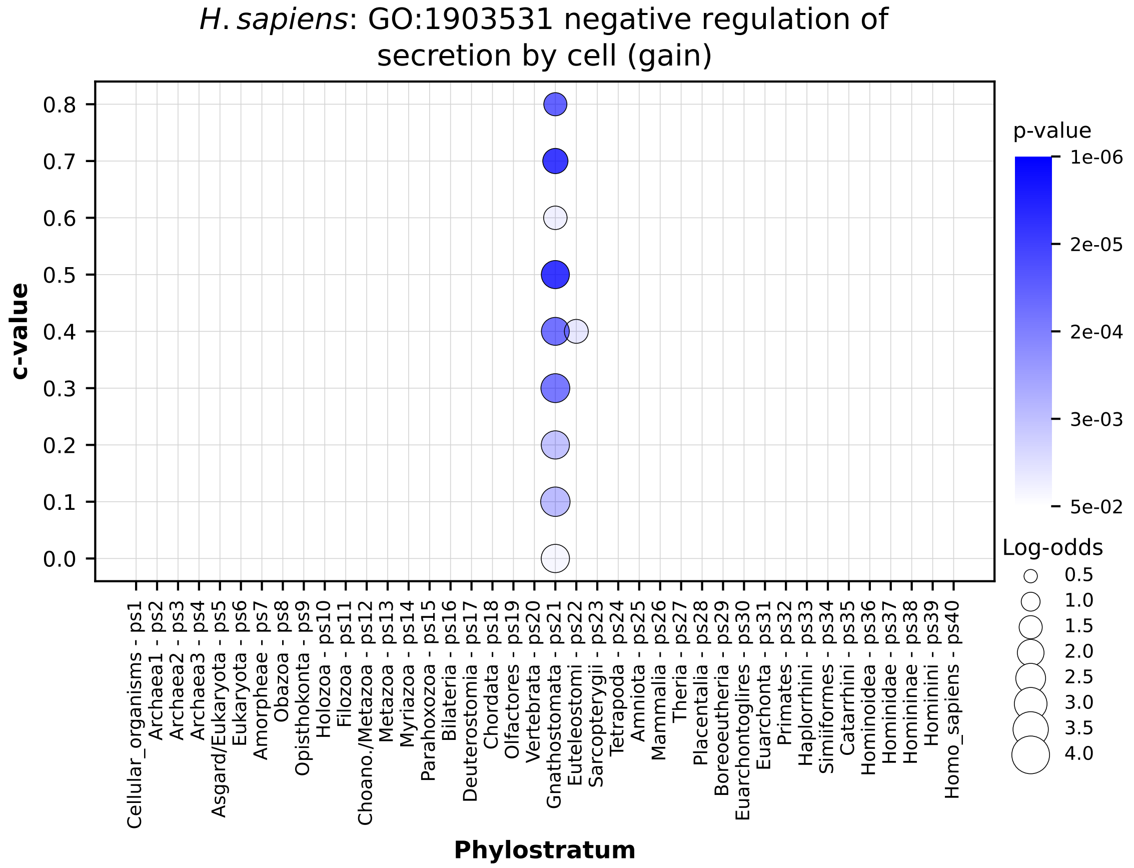
H. sapiens: GO:1903531 negative regulation of secretion by cell (gain)
c-value
0.8
0.7
0.6
0.5
0.4
0.3
0.2
0.1
0.0
Phylostratum
Cellular_organisms - ps1
Archaea1 - ps2
Archaea2 - ps3
Archaea3 - ps4
Asgard/Eukaryota - ps5
Eukaryota - ps6
Amorpheae - ps7
Obazoa - ps8
Opisthokonta - ps9
Holozoa - ps10
Filozoa - ps11
Choano./Metazoa - ps12
Metazoa - ps13
Myriazoa - ps14
Parahoxozoa - ps15
Bilateria - ps16
Deuterostomia - ps17
Chordata - ps18
Olfactores - ps19
Vertebrata - ps20
Gnathostomata - ps21
Euteleostomi - ps22
Sarcopterygii - ps23
Tetrapoda - ps24
Amniota - ps25
Mammalia - ps26
Theria - ps27
Placentalia - ps28
Boreoeutheria - ps29
Euarchontoglires - ps30
Euarchonta - ps31
Primates - ps32
Haplorrhini - ps33
Simiiformes - ps34
Catarrhini - ps35
Hominoidea - ps36
Hominidae - ps37
Homininae - ps38
Hominini - ps39
Homo_sapiens - ps40
p-value
1e-06
2e-05
2e-04
3e-03
5e-02
Log-odds
0.5
1.0
1.5
2.0
2.5
3.0
3.5
4.0

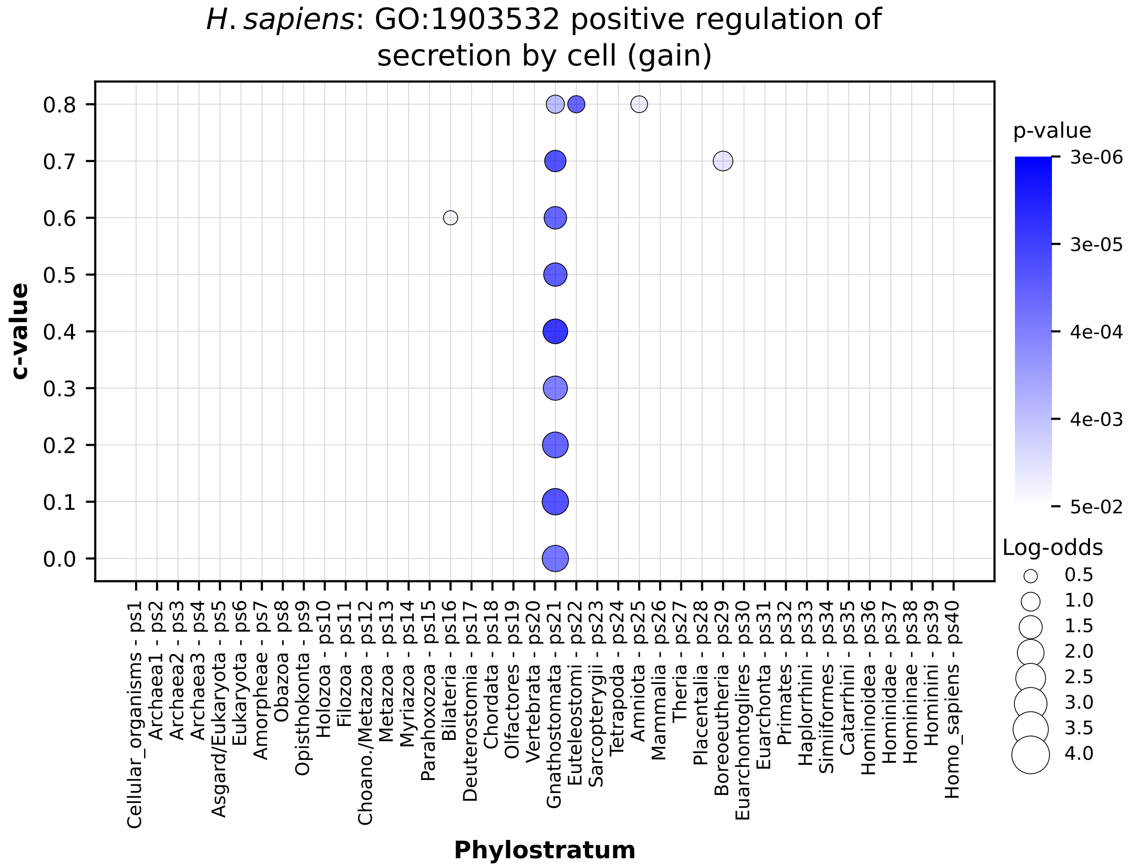

*H. sapiens*: GO:1903532 positive regulation of secretion by cell (gain)
c-value
0.8
0.7
0.6
0.5
0.4
0.3
0.2
0.1
0.0
Phylostratum
Cellular_organisms - ps1
Archaea1 - ps2
Archaea2 - ps3
Archaea3 - ps4
Asgard/Eukaryota - ps5
Eukaryota - ps6
Amorpheae - ps7
Obazoa - ps8
Opisthokonta - ps9
Holozoa - ps10
Filozoa - ps11
Choano./Metazoa - ps12
Metazoa - ps13
Myriazoa - ps14
Parahoxozoa - ps15
Bilateria - ps16
Deuterostomia - ps17
Chordata - ps18
Olfactores - ps19
Vertebrata - ps20
Gnathostomata - ps21
Euteleostomi - ps22
Sarcopterygii - ps23
Tetrapoda - ps24
Amniota - ps25
Mammalia - ps26
Theria - ps27
Placentalia - ps28
Boreoeutheria - ps29
Euarchontoglires - ps30
Euarchonta - ps31
Primates - ps32
Haplorrhini - ps33
Simiiformes - ps34
Catarrhini - ps35
Hominoidea - ps36
Hominidae - ps37
Homininae - ps38
Hominini - ps39
Homo_sapiens - ps40
p-value
3e-06
3e-05
4e-04
4e-03
5e-02
Log-odds
0.5
1.0
1.5
2.0
2.5
3.0
3.5
4.0

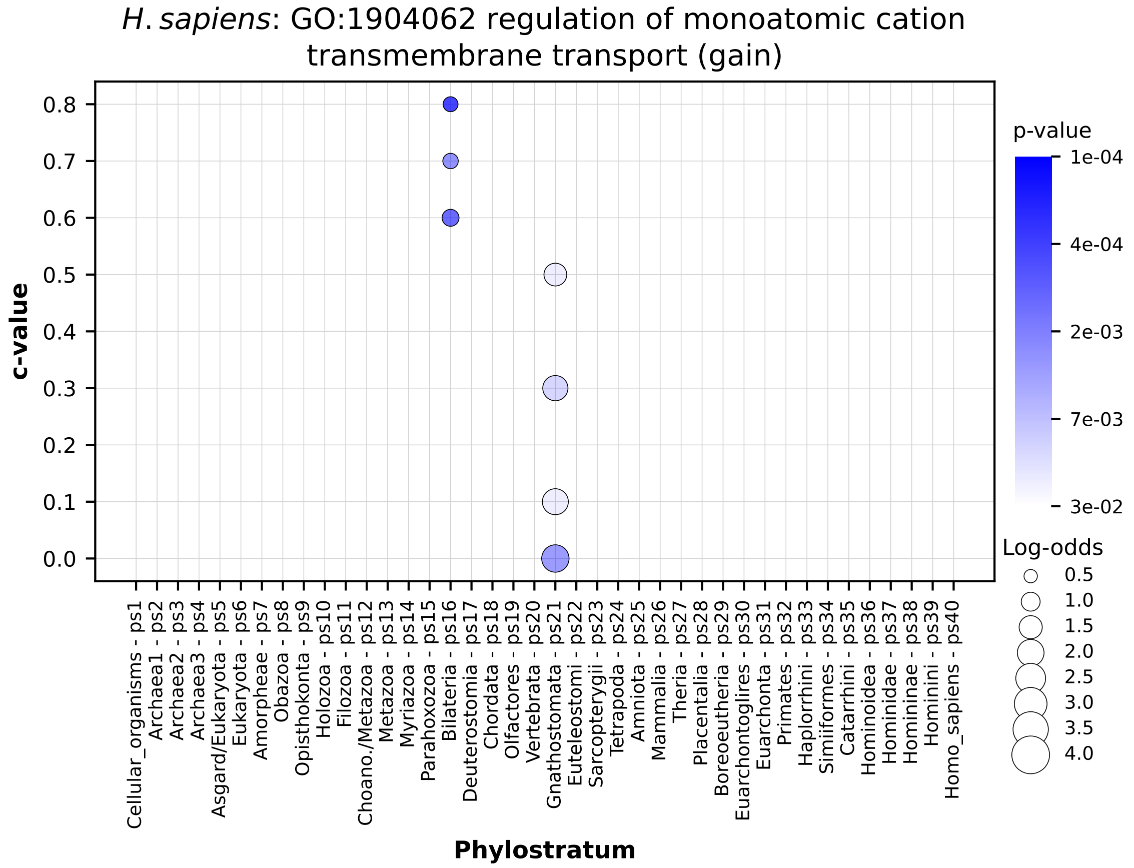

H. sapiens: GO:1904062 regulation of monoatomic cation transmembrane transport (gain)
c-value
0.8
0.7
0.6
0.5
0.4
0.3
0.2
0.1
0.0
Phylostratum
Cellular_organisms - ps1
Archaea1 - ps2
Archaea2 - ps3
Archaea3 - ps4
Asgard/Eukaryota - ps5
Eukaryota - ps6
Amorpheae - ps7
Obazoa - ps8
Opisthokonta - ps9
Holozoa - ps10
Filozoa - ps11
Choano./Metazoa - ps12
Metazoa - ps13
Myriazoa - ps14
Parahoxozoa - ps15
Bilateria - ps16
Deuterostomia - ps17
Chordata - ps18
Olfactores - ps19
Vertebrata - ps20
Gnathostomata - ps21
Euteleostomi - ps22
Sarcopterygii - ps23
Tetrapoda - ps24
Amniota - ps25
Mammalia - ps26
Theria - ps27
Placentalia - ps28
Boreoeutheria - ps29
Euarchontoglires - ps30
Euarchonta - ps31
Primates - ps32
Haplorrhini - ps33
Simiiformes - ps34
Catarrhini - ps35
Hominoidea - ps36
Hominidae - ps37
Homininae - ps38
Hominini - ps39
Homo_sapiens - ps40
p-value
1e-04
4e-04
2e-03
7e-03
3e-02
Log-odds
0.5
1.0
1.5
2.0
2.5
3.0
3.5
4.0

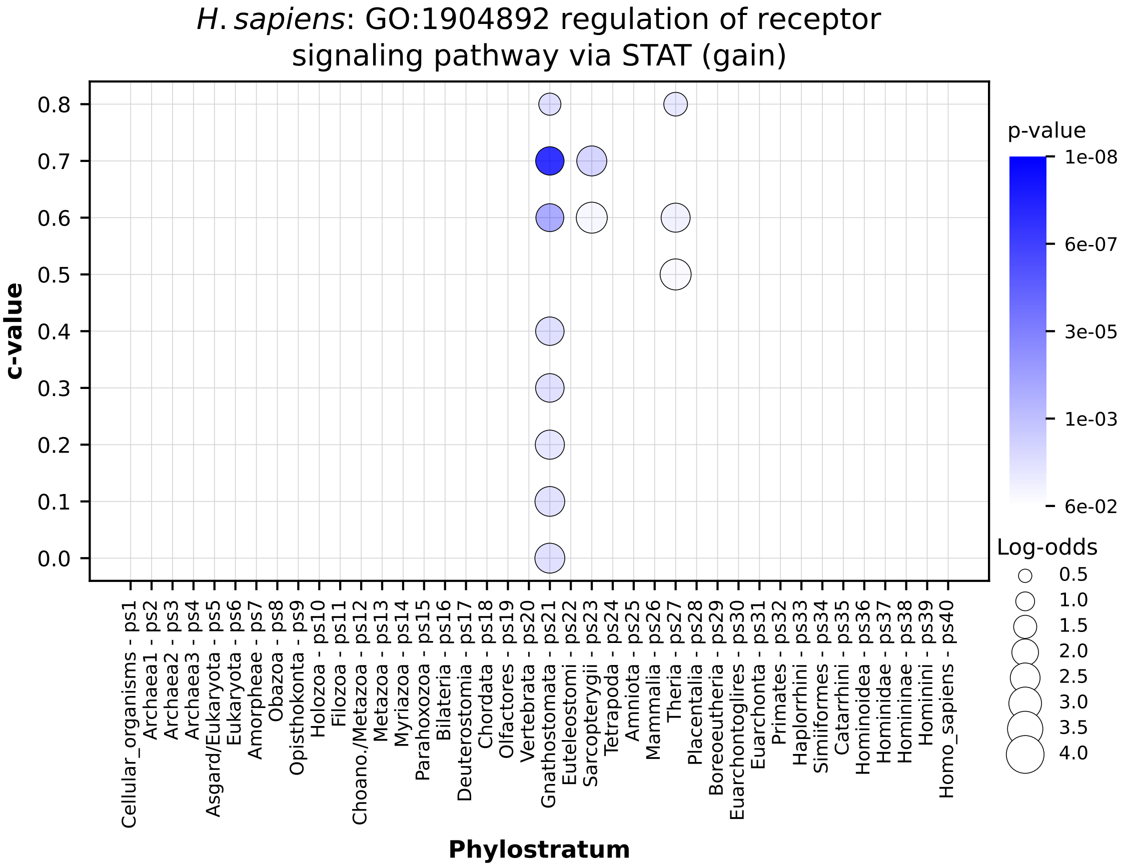

H. sapiens: GO:1904892 regulation of receptor signaling pathway via STAT (gain)
c-value
0.8
0.7
0.6
0.5
0.4
0.3
0.2
0.1
0.0
p-value
1e-08
6e-07
3e-05
1e-03
6e-02
Log-odds
0.5
1.0
1.5
2.0
2.5
3.0
3.5
4.0
Cellular_organisms - ps1
Archaea1 - ps2
Archaea2 - ps3
Archaea3 - ps4
Asgard/Eukaryota - ps5
Eukaryota - ps6
Amorpheae - ps7
Obazoa - ps8
Opisthokonta - ps9
Holozoa - ps10
Filozoa - ps11
Choano./Metazoa - ps12
Metazoa - ps13
Myriazoa - ps14
Parahoxozoa - ps15
Bilateria - ps16
Deuterostomia - ps17
Chordata - ps18
Olfactores - ps19
Vertebrata - ps20
Gnathostomata - ps21
Euteleostomi - ps22
Sarcopterygii - ps23
Tetrapoda - ps24
Amniota - ps25
Mammalia - ps26
Theria - ps27
Placentalia - ps28
Boreoeutheria - ps29
Euarchontoglires - ps30
Euarchonta - ps31
Primates - ps32
Haplorrhini - ps33
Simiiformes - ps34
Catarrhini - ps35
Hominoidea - ps36
Hominidae - ps37
Homininae - ps38
Hominini - ps39
Homo_sapiens - ps40
Phylostratum

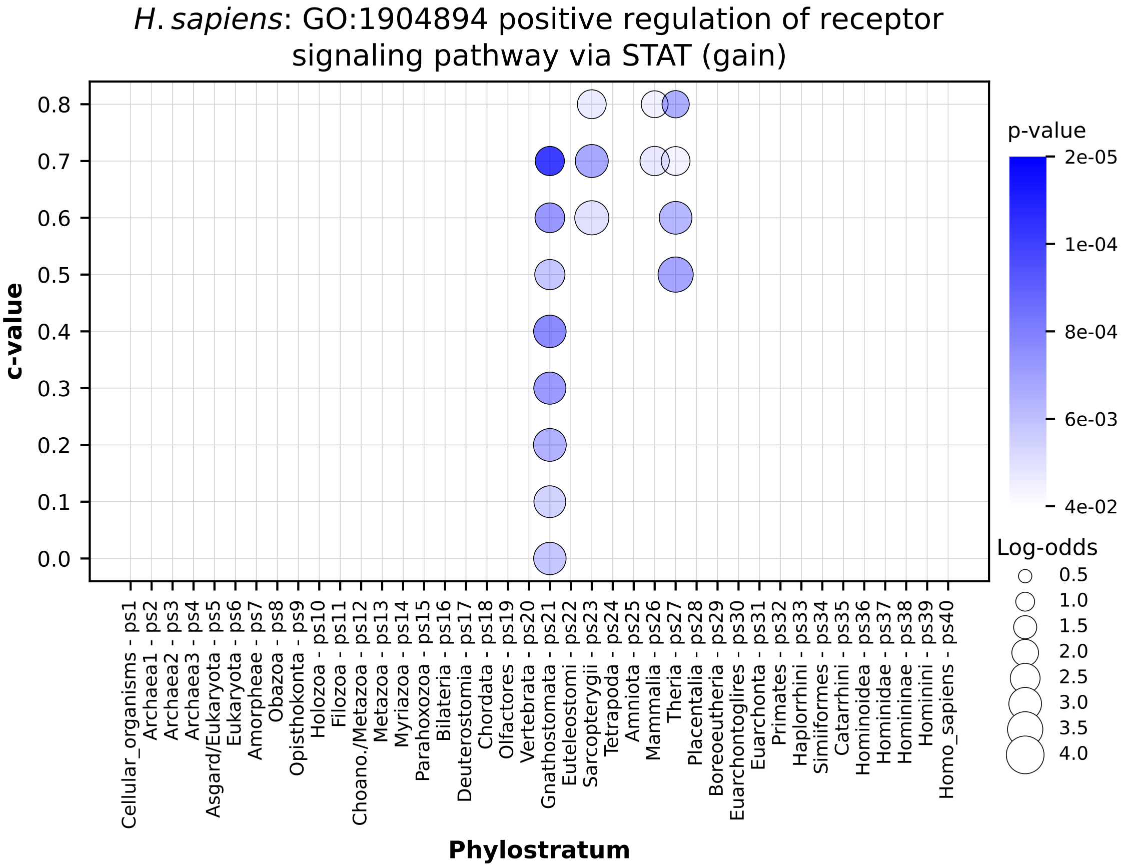

H. sapiens: GO:1904894 positive regulation of receptor signaling pathway via STAT (gain)
c-value
0.0
0.1
0.2
0.3
0.4
0.5
0.6
0.7
0.8
Phylostratum
Cellular_organisms - ps1
Archaea1 - ps2
Archaea2 - ps3
Archaea3 - ps4
Asgard/Eukaryota - ps5
Eukaryota - ps6
Amorpheae - ps7
Obazoa - ps8
Opisthokonta - ps9
Holozoa - ps10
Filozoa - ps11
Choano./Metazoa - ps12
Metazoa - ps13
Myriazoa - ps14
Parahoxozoa - ps15
Bilateria - ps16
Deuterostomia - ps17
Chordata - ps18
Olfactores - ps19
Vertebrata - ps20
Gnathostomata - ps21
Euteleostomi - ps22
Sarcopterygii - ps23
Tetrapoda - ps24
Amniota - ps25
Mammalia - ps26
Theria - ps27
Placentalia - ps28
Boreoeutheria - ps29
Euarchontoglires - ps30
Euarchonta - ps31
Primates - ps32
Haplorrhini - ps33
Simiiformes - ps34
Catarrhini - ps35
Hominoidea - ps36
Hominidae - ps37
Homininae - ps38
Hominini - ps39
Homo_sapiens - ps40
p-value
2e-05
1e-04
8e-04
6e-03
4e-02
Log-odds
0.5
1.0
1.5
2.0
2.5
3.0
3.5
4.0

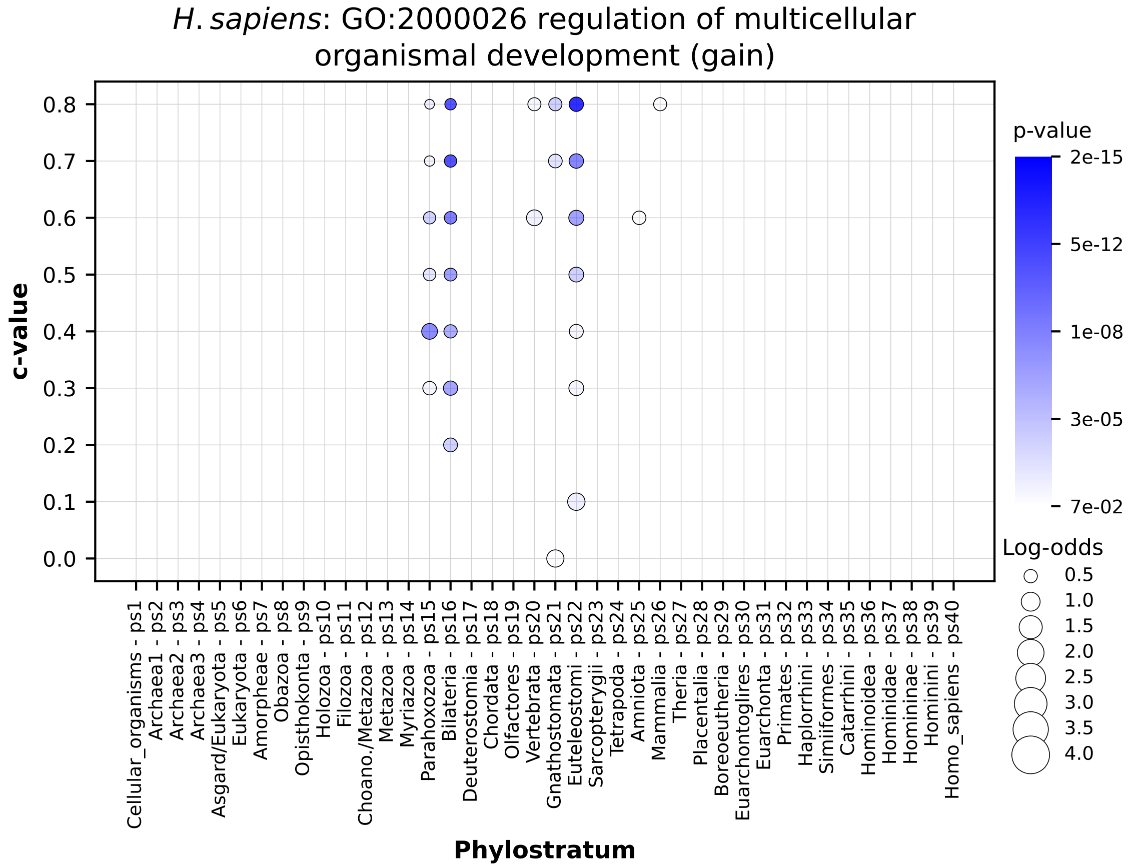

H. sapiens: GO:2000026 regulation of multicellular organismal development (gain)
c-value
0.8
0.7
0.6
0.5
0.4
0.3
0.2
0.1
0.0
Phylostratum
Cellular_organisms - ps1
Archaea1 - ps2
Archaea2 - ps3
Archaea3 - ps4
Asgard/Eukaryota - ps5
Eukaryota - ps6
Amorpheae - ps7
Obazoa - ps8
Opisthokonta - ps9
Holozoa - ps10
Filozoa - ps11
Choano./Metazoa - ps12
Metazoa - ps13
Myriazoa - ps14
Parahoxozoa - ps15
Bilateria - ps16
Deuterostomia - ps17
Chordata - ps18
Olfactores - ps19
Vertebrata - ps20
Gnathostomata - ps21
Euteleostomi - ps22
Sarcopterygii - ps23
Tetrapoda - ps24
Amniota - ps25
Mammalia - ps26
Theria - ps27
Placentalia - ps28
Boreoeutheria - ps29
Euarchontoglires - ps30
Euarchonta - ps31
Primates - ps32
Haplorrhini - ps33
Simiiformes - ps34
Catarrhini - ps35
Hominoidea - ps36
Hominidae - ps37
Homininae - ps38
Hominini - ps39
Homo_sapiens - ps40
p-value
2e-15
5e-12
1e-08
3e-05
7e-02
Log-odds
0.5
1.0
1.5
2.0
2.5
3.0
3.5
4.0

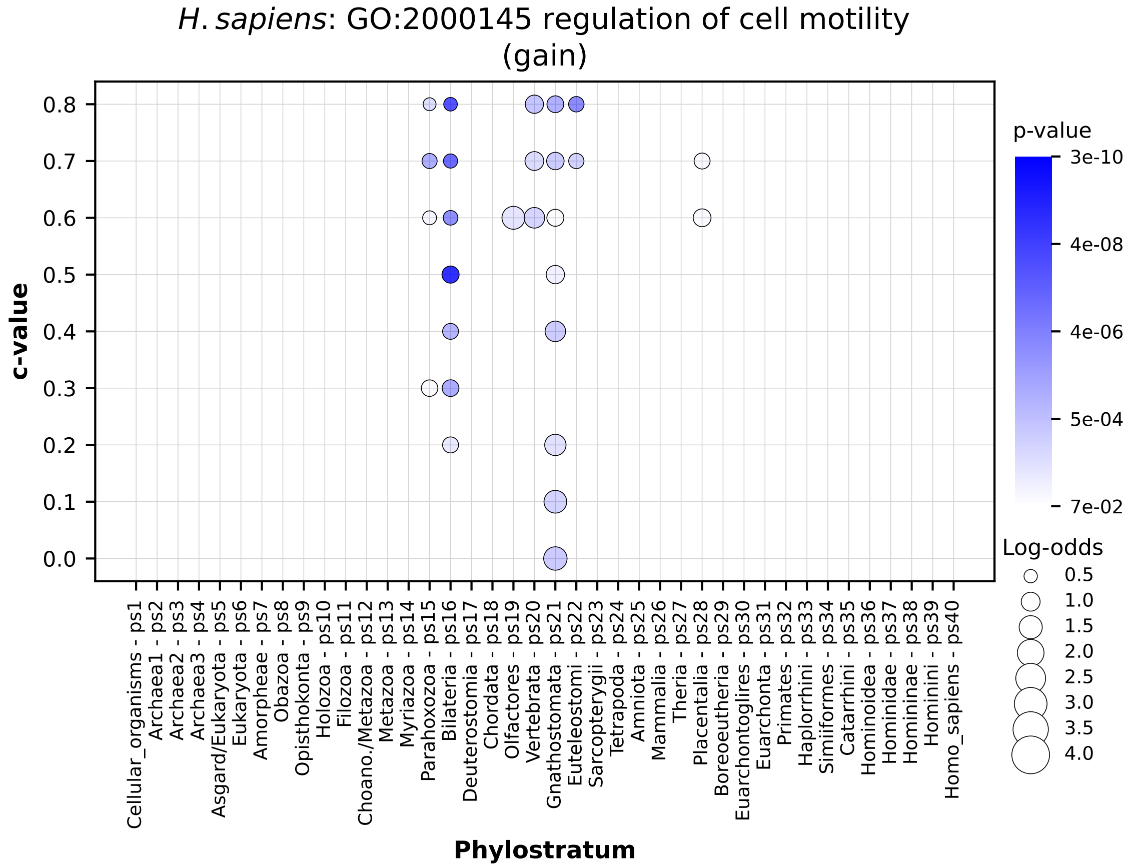
H. sapiens: GO:2000145 regulation of cell motility
(gain)
c-value
0.8
0.7
0.6
0.5
0.4
0.3
0.2
0.1
0.0
p-value
3e-10
4e-08
4e-06
5e-04
7e-02
Log-odds
0.5
1.0
1.5
2.0
2.5
3.0
3.5
4.0
Cellular_organisms - ps1
Archaea1 - ps2
Archaea2 - ps3
Archaea3 - ps4
Asgard/Eukaryota - ps5
Eukaryota - ps6
Amorpheae - ps7
Obazoa - ps8
Opisthokonta - ps9
Holozoa - ps10
Filozoa - ps11
Choano./Metazoa - ps12
Metazoa - ps13
Myriazoa - ps14
Parahoxozoa - ps15
Bilateria - ps16
Deuterostomia - ps17
Chordata - ps18
Olfactores - ps19
Vertebrata - ps20
Gnathostomata - ps21
Euteleostomi - ps22
Sarcopterygii - ps23
Tetrapoda - ps24
Amniota - ps25
Mammalia - ps26
Theria - ps27
Placentalia - ps28
Boreoeutheria - ps29
Euarchontoglires - ps30
Euarchonta - ps31
Primates - ps32
Haplorrhini - ps33
Simiiformes - ps34
Catarrhini - ps35
Hominoidea - ps36
Hominidae - ps37
Homininae - ps38
Hominini - ps39
Homo_sapiens - ps40
Phylostratum

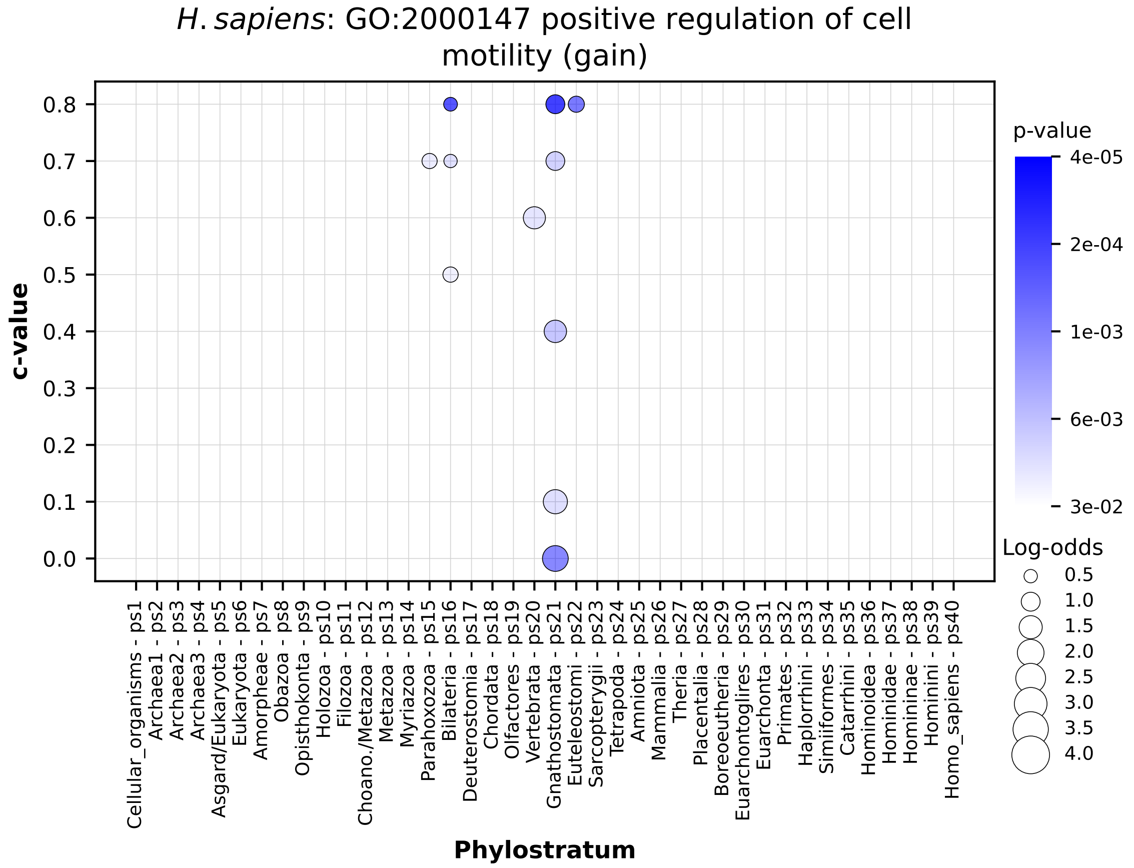

*H. sapiens*: GO:2000147 positive regulation of cell motility (gain)
c-value
0.8
0.7
0.6
0.5
0.4
0.3
0.2
0.1
0.0
p-value
4e-05
2e-04
1e-03
6e-03
3e-02
Log-odds
0.5
1.0
1.5
2.0
2.5
3.0
3.5
4.0
Cellular_organisms - ps1
Archaea1 - ps2
Archaea2 - ps3
Archaea3 - ps4
Asgard/Eukaryota - ps5
Eukaryota - ps6
Amorpheae - ps7
Obazoa - ps8
Opisthokonta - ps9
Holozoa - ps10
Filozoa - ps11
Choano./Metazoa - ps12
Metazoa - ps13
Myriazoa - ps14
Parahoxozoa - ps15
Bilateria - ps16
Deuterostomia - ps17
Chordata - ps18
Olfactores - ps19
Vertebrata - ps20
Gnathostomata - ps21
Euteleostomi - ps22
Sarcopterygii - ps23
Tetrapoda - ps24
Amniota - ps25
Mammalia - ps26
Theria - ps27
Placentalia - ps28
Boreoeutheria - ps29
Euarchontoglires - ps30
Euarchonta - ps31
Primates - ps32
Haplorrhini - ps33
Simiiformes - ps34
Catarrhini - ps35
Hominoidea - ps36
Hominidae - ps37
Homininae - ps38
Hominini - ps39
Homo_sapiens - ps40
Phylostratum

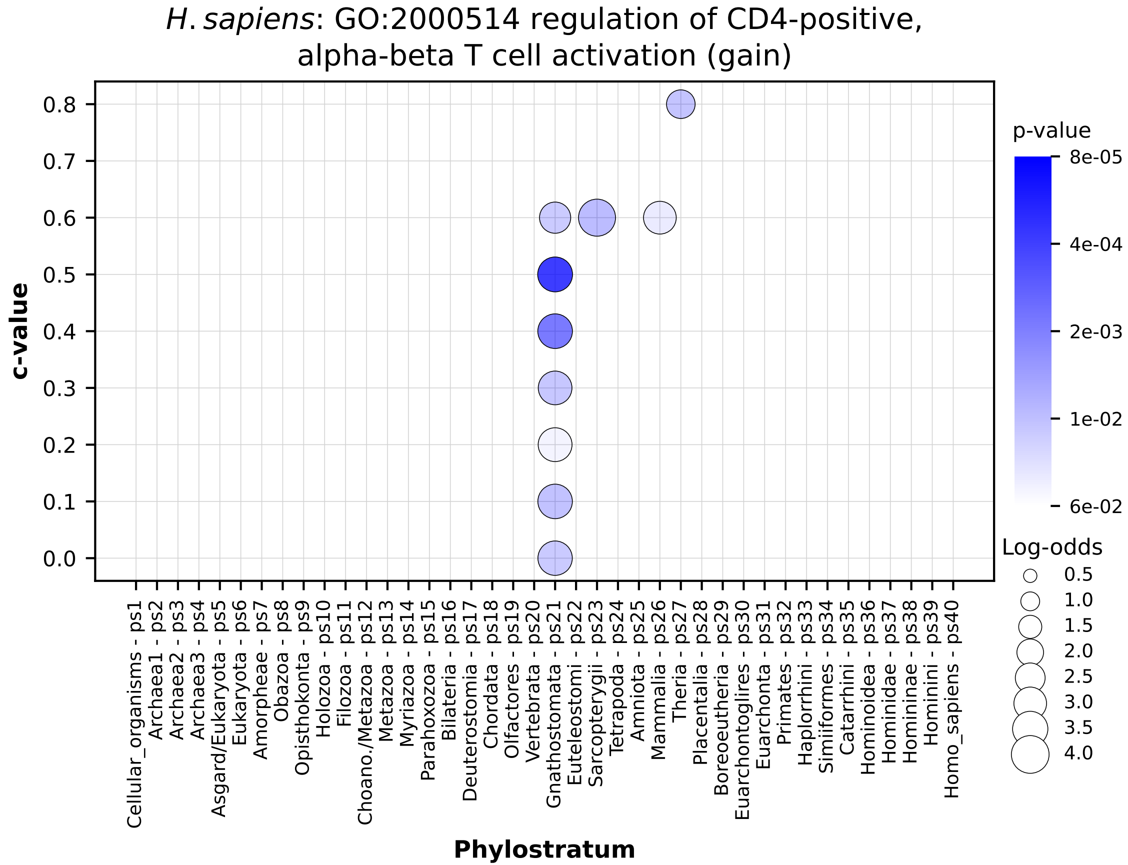
H. sapiens: GO:2000514 regulation of CD4-positive, alpha-beta T cell activation (gain)
c-value
0.0
0.1
0.2
0.3
0.4
0.5
0.6
0.7
0.8
Phylostratum
Cellular_organisms - ps1
Archaea1 - ps2
Archaea2 - ps3
Archaea3 - ps4
Asgard/Eukaryota - ps5
Eukaryota - ps6
Amorpheae - ps7
Obazoa - ps8
Opisthokonta - ps9
Holozoa - ps10
Filozoa - ps11
Choano./Metazoa - ps12
Metazoa - ps13
Myriazoa - ps14
Parahoxozoa - ps15
Bilateria - ps16
Deuterostomia - ps17
Chordata - ps18
Olfactores - ps19
Vertebrata - ps20
Gnathostomata - ps21
Euteleostomi - ps22
Sarcopterygii - ps23
Tetrapoda - ps24
Amniota - ps25
Mammalia - ps26
Theria - ps27
Placentalia - ps28
Boreoeutheria - ps29
Euarchontoglires - ps30
Euarchonta - ps31
Primates - ps32
Haplorrhini - ps33
Simiiformes - ps34
Catarrhini - ps35
Hominoidea - ps36
Hominidae - ps37
Homininae - ps38
Hominini - ps39
Homo_sapiens - ps40
p-value
8e-05
4e-04
2e-03
1e-02
6e-02
Log-odds
0.5
1.0
1.5
2.0
2.5
3.0
3.5
4.0

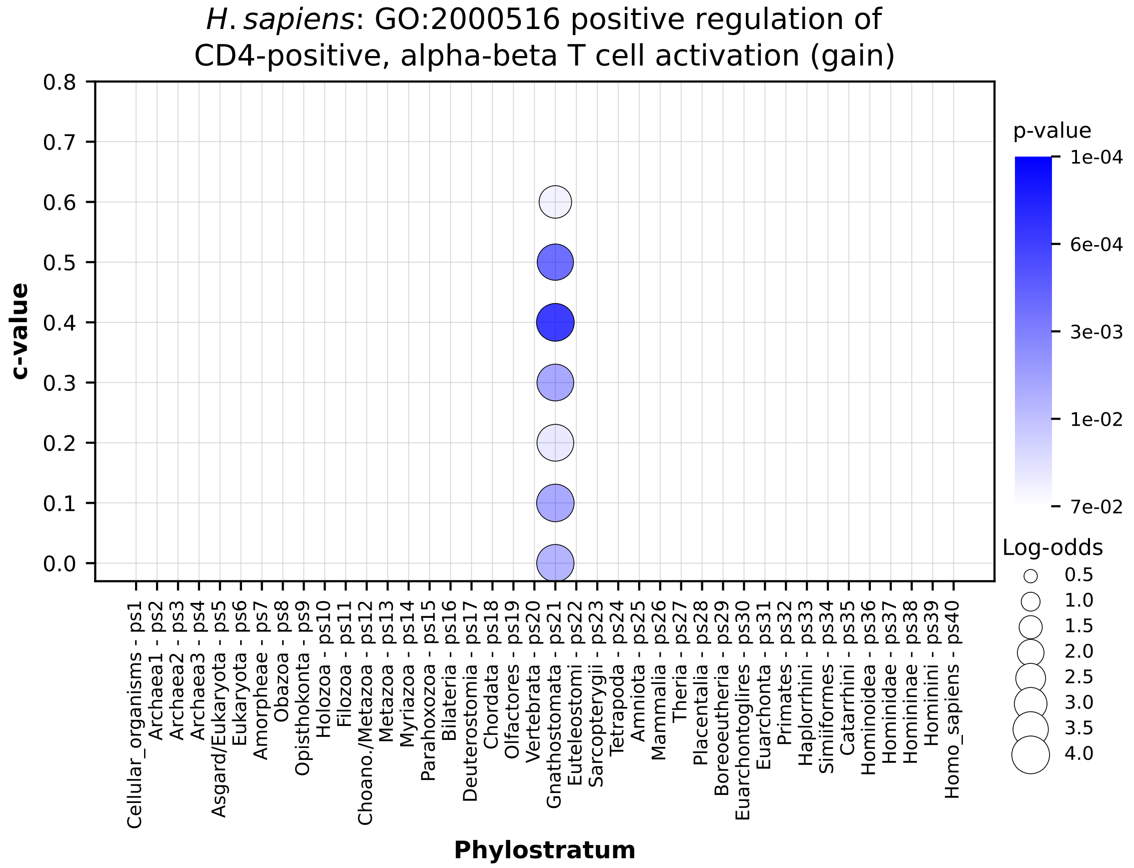

*H. sapiens*: GO:2000516 positive regulation of CD4-positive, alpha-beta T cell activation (gain)
c-value
0.8
0.7
0.6
0.5
0.4
0.3
0.2
0.1
0.0
Phylostratum
Cellular_organisms - ps1
Archaea1 - ps2
Archaea2 - ps3
Archaea3 - ps4
Asgard/Eukaryota - ps5
Eukaryota - ps6
Amorpheae - ps7
Obazoa - ps8
Opisthokonta - ps9
Holozoa - ps10
Filozoa - ps11
Choano./Metazoa - ps12
Metazoa - ps13
Myriazoa - ps14
Parahoxozoa - ps15
Bilateria - ps16
Deuterostomia - ps17
Chordata - ps18
Olfactores - ps19
Vertebrata - ps20
Gnathostomata - ps21
Euteleostomi - ps22
Sarcopterygii - ps23
Tetrapoda - ps24
Amniota - ps25
Mammalia - ps26
Theria - ps27
Placentalia - ps28
Boreoeutheria - ps29
Euarchontoglires - ps30
Euarchonta - ps31
Primates - ps32
Haplorrhini - ps33
Simiiformes - ps34
Catarrhini - ps35
Hominoidea - ps36
Hominidae - ps37
Homininae - ps38
Hominini - ps39
Homo_sapiens - ps40
p-value
1e-04
6e-04
3e-03
1e-02
7e-02
Log-odds
0.5
1.0
1.5
2.0
2.5
3.0
3.5
4.0

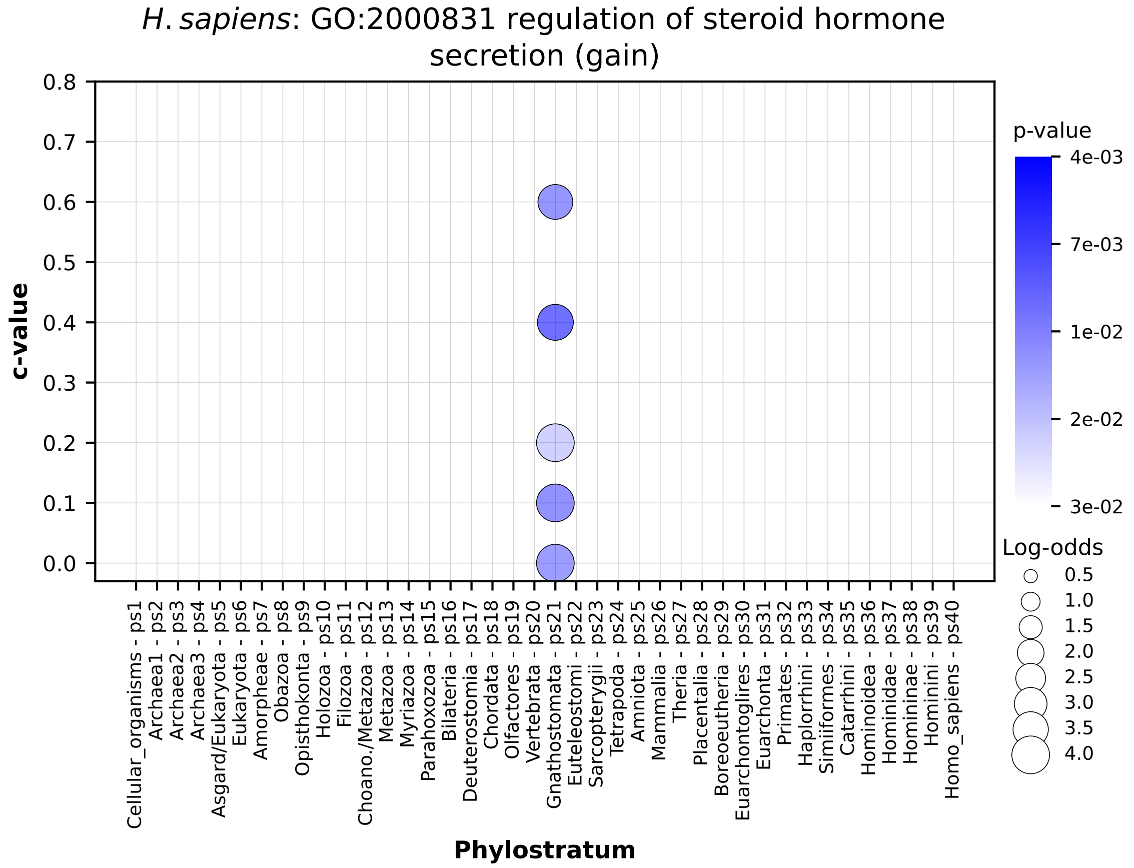

H. sapiens: GO:2000831 regulation of steroid hormone secretion (gain)
c-value
0.0
0.1
0.2
0.3
0.4
0.5
0.6
0.7
0.8
Phylostratum
Cellular_organisms - ps1
Archaea1 - ps2
Archaea2 - ps3
Archaea3 - ps4
Asgard/Eukaryota - ps5
Eukaryota - ps6
Amorpheae - ps7
Obazoa - ps8
Opisthokonta - ps9
Holozoa - ps10
Filozoa - ps11
Choano./Metazoa - ps12
Metazoa - ps13
Myriazoa - ps14
Parahoxozoa - ps15
Bilateria - ps16
Deuterostomia - ps17
Chordata - ps18
Olfactores - ps19
Vertebrata - ps20
Gnathostomata - ps21
Euteleostomi - ps22
Sarcopterygii - ps23
Tetrapoda - ps24
Amniota - ps25
Mammalia - ps26
Theria - ps27
Placentalia - ps28
Boreoeutheria - ps29
Euarchontoglires - ps30
Euarchonta - ps31
Primates - ps32
Haplorrhini - ps33
Simiiformes - ps34
Catarrhini - ps35
Hominoidea - ps36
Hominidae - ps37
Homininae - ps38
Hominini - ps39
Homo_sapiens - ps40
p-value
4e-03
7e-03
1e-02
2e-02
3e-02
Log-odds
0.5
1.0
1.5
2.0
2.5
3.0
3.5
4.0

## *H. sapiens*: GO:2000833 positive regulation of steroid hormone secretion (gain)

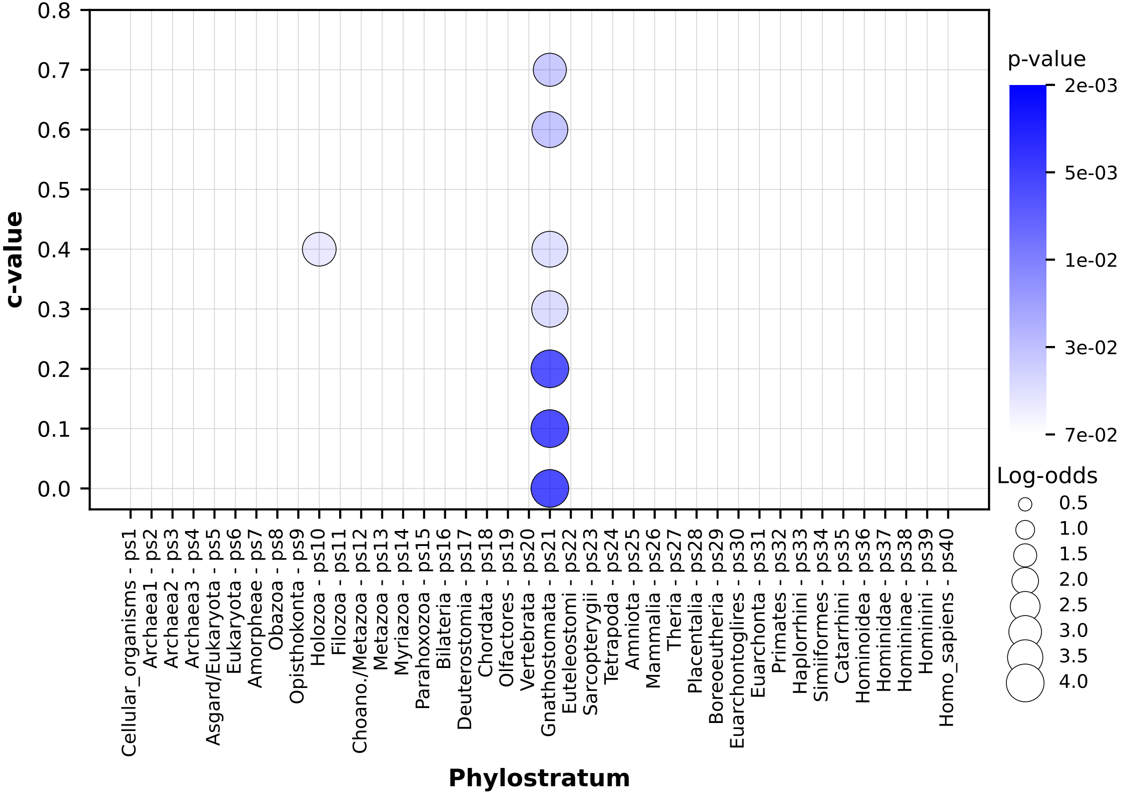

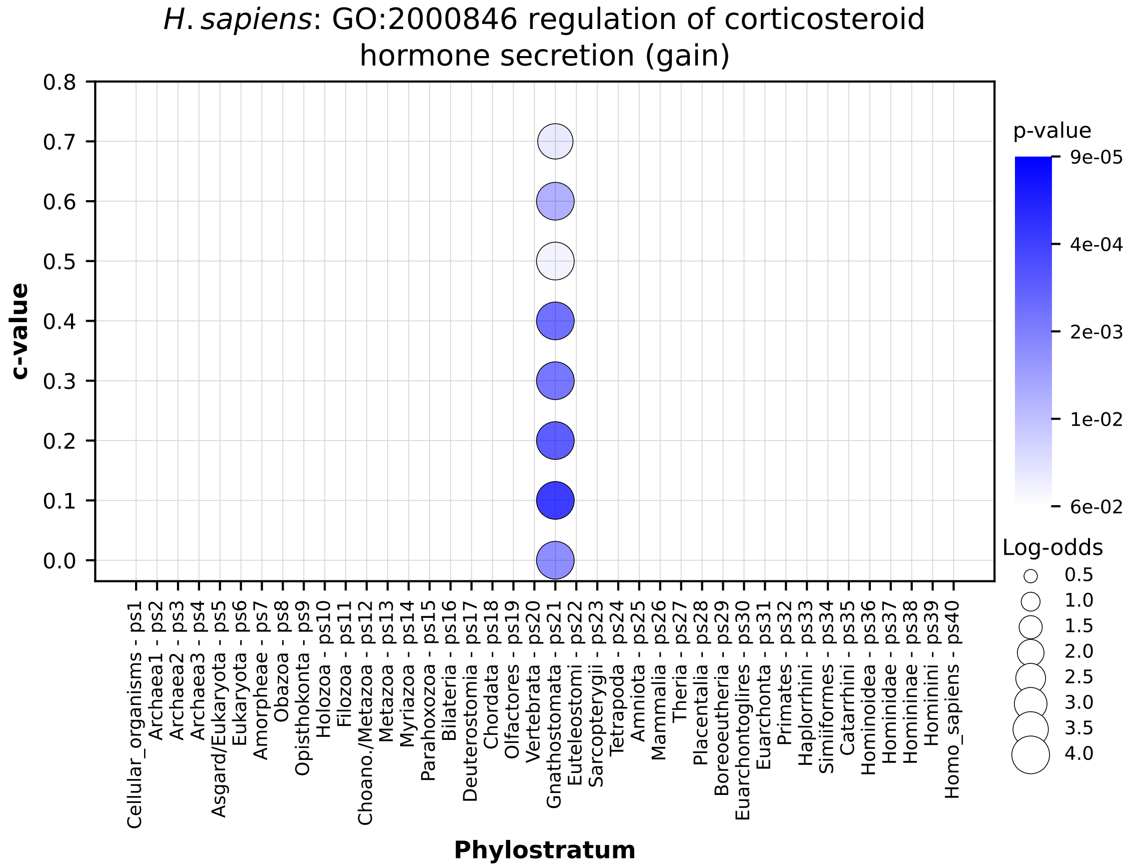

*H. sapiens*: GO:2000846 regulation of corticosteroid hormone secretion (gain)
c-value
0.8
0.7
0.6
0.5
0.4
0.3
0.2
0.1
0.0
Phylostratum
Cellular_organisms - ps1
Archaea1 - ps2
Archaea2 - ps3
Archaea3 - ps4
Asgard/Eukaryota - ps5
Eukaryota - ps6
Amorpheae - ps7
Obazoa - ps8
Opisthokonta - ps9
Holozoa - ps10
Filozoa - ps11
Choano./Metazoa - ps12
Metazoa - ps13
Myriazoa - ps14
Parahoxozoa - ps15
Bilateria - ps16
Deuterostomia - ps17
Chordata - ps18
Olfactores - ps19
Vertebrata - ps20
Gnathostomata - ps21
Euteleostomi - ps22
Sarcopterygii - ps23
Tetrapoda - ps24
Amniota - ps25
Mammalia - ps26
Theria - ps27
Placentalia - ps28
Boreoeutheria - ps29
Euarchontoglires - ps30
Euarchonta - ps31
Primates - ps32
Haplorrhini - ps33
Simiiformes - ps34
Catarrhini - ps35
Hominoidea - ps36
Hominidae - ps37
Homininae - ps38
Hominini - ps39
Homo_sapiens - ps40
p-value
9e-05
4e-04
2e-03
1e-02
6e-02
Log-odds
0.5
1.0
1.5
2.0
2.5
3.0
3.5
4.0

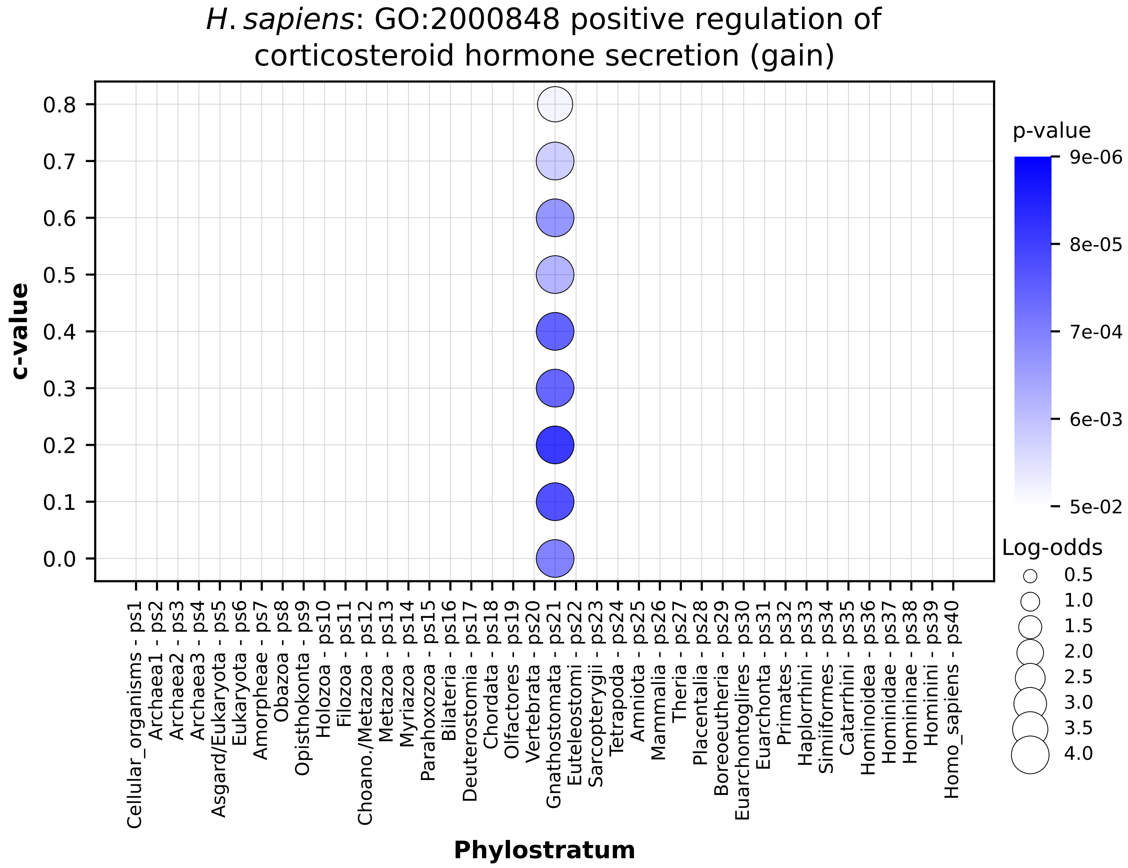

H. sapiens: GO:2000848 positive regulation of corticosteroid hormone secretion (gain)
c-value
0.8
0.7
0.6
0.5
0.4
0.3
0.2
0.1
0.0
Phylostratum
Cellular_organisms - ps1
Archaea1 - ps2
Archaea2 - ps3
Archaea3 - ps4
Asgard/Eukaryota - ps5
Eukaryota - ps6
Amorpheae - ps7
Obazoa - ps8
Opisthokonta - ps9
Holozoa - ps10
Filozoa - ps11
Choano./Metazoa - ps12
Metazoa - ps13
Myriazoa - ps14
Parahoxozoa - ps15
Bilateria - ps16
Deuterostomia - ps17
Chordata - ps18
Olfactores - ps19
Vertebrata - ps20
Gnathostomata - ps21
Euteleostomi - ps22
Sarcopterygii - ps23
Tetrapoda - ps24
Amniota - ps25
Mammalia - ps26
Theria - ps27
Placentalia - ps28
Boreoeutheria - ps29
Euarchontoglires - ps30
Euarchonta - ps31
Primates - ps32
Haplorrhini - ps33
Simiiformes - ps34
Catarrhini - ps35
Hominoidea - ps36
Hominidae - ps37
Homininae - ps38
Hominini - ps39
Homo_sapiens - ps40
p-value
9e-06
8e-05
7e-04
6e-03
5e-02
Log-odds
0.5
1.0
1.5
2.0
2.5
3.0
3.5
4.0

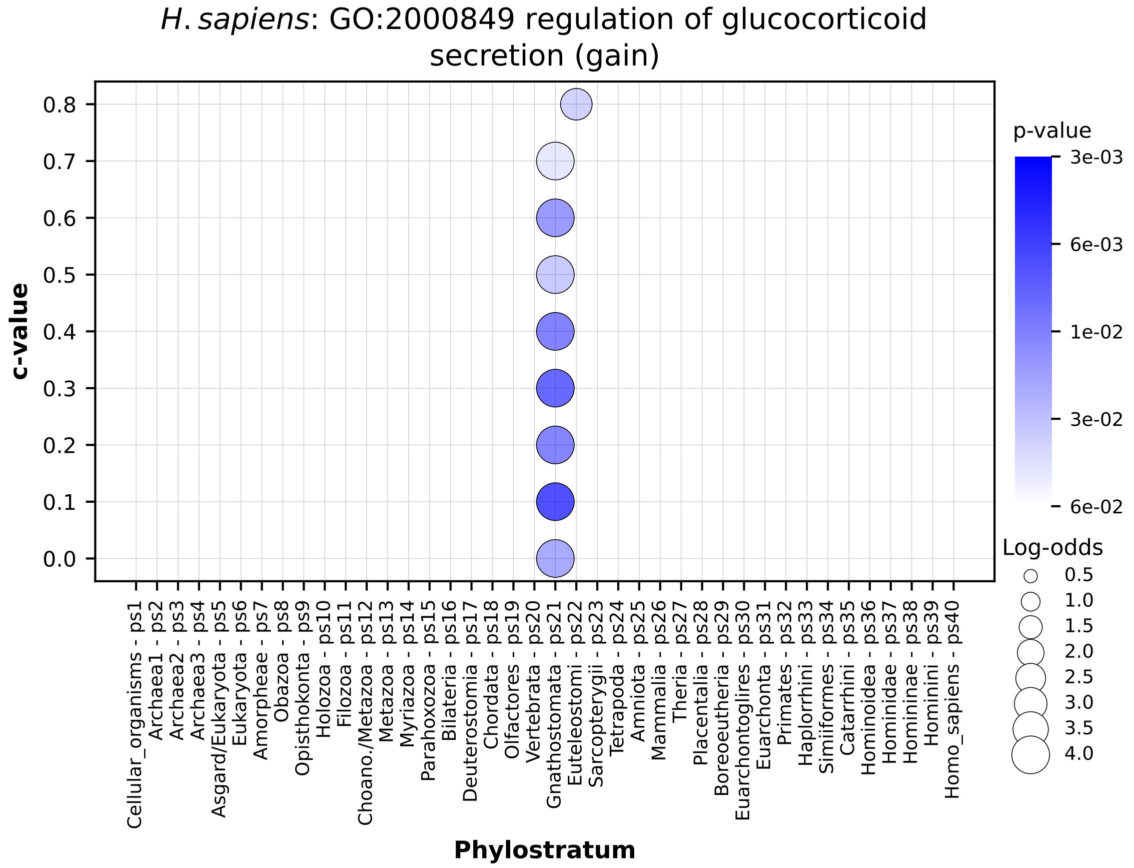

H. sapiens: GO:2000849 regulation of glucocorticoid secretion (gain)
c-value
0.8
0.7
0.6
0.5
0.4
0.3
0.2
0.1
0.0
p-value
3e-03
6e-03
1e-02
3e-02
6e-02
Log-odds
0.5
1.0
1.5
2.0
2.5
3.0
3.5
4.0
Cellular_organisms - ps1
Archaea1 - ps2
Archaea2 - ps3
Archaea3 - ps4
Asgard/Eukaryota - ps5
Eukaryota - ps6
Amorpheae - ps7
Obazoa - ps8
Opisthokonta - ps9
Holozoa - ps10
Filozoa - ps11
Choano./Metazoa - ps12
Metazoa - ps13
Myriazoa - ps14
Parahoxozoa - ps15
Bilateria - ps16
Deuterostomia - ps17
Chordata - ps18
Olfactores - ps19
Vertebrata - ps20
Gnathostomata - ps21
Euteleostomi - ps22
Sarcopterygii - ps23
Tetrapoda - ps24
Amniota - ps25
Mammalia - ps26
Theria - ps27
Placentalia - ps28
Boreoeutheria - ps29
Euarchontoglires - ps30
Euarchonta - ps31
Primates - ps32
Haplorrhini - ps33
Simiiformes - ps34
Catarrhini - ps35
Hominoidea - ps36
Hominidae - ps37
Homininae - ps38
Hominini - ps39
Homo_sapiens - ps40
Phylostratum

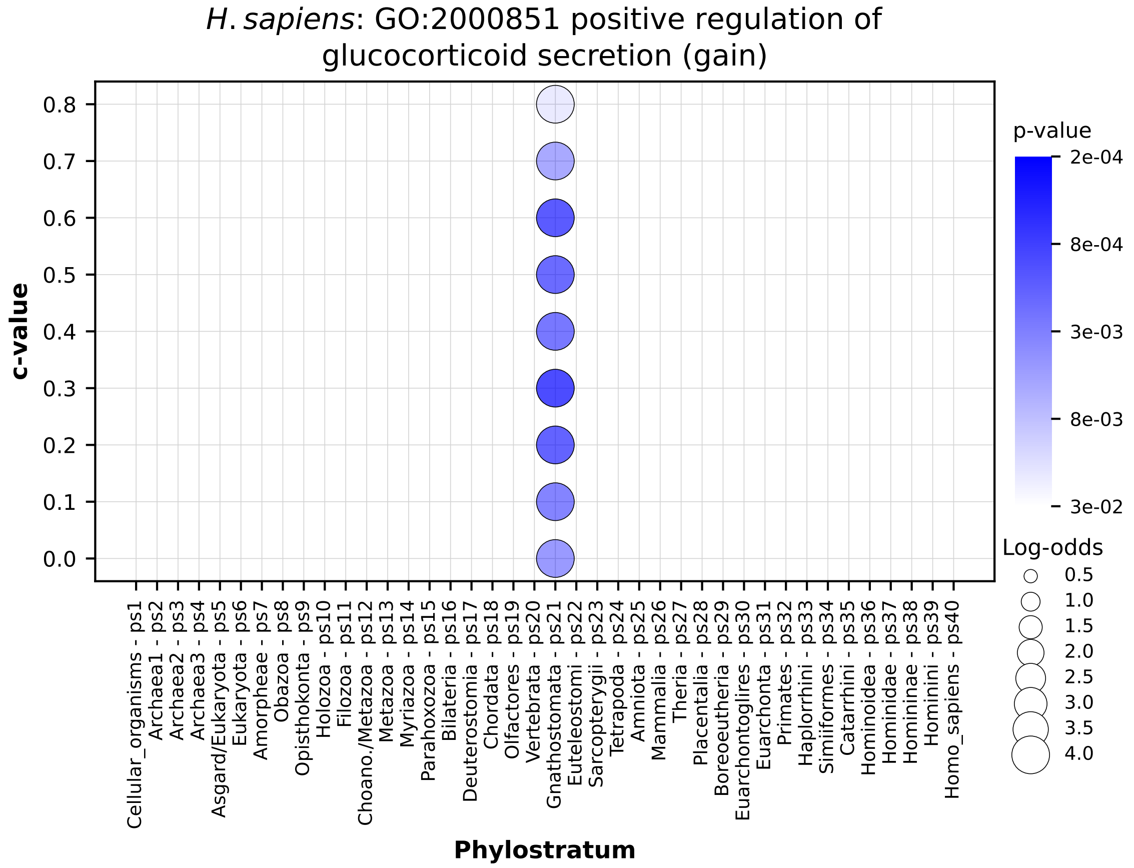

H. sapiens: GO:2000851 positive regulation of glucocorticoid secretion (gain)
c-value
0.8
0.7
0.6
0.5
0.4
0.3
0.2
0.1
0.0
p-value
2e-04
8e-04
3e-03
8e-03
3e-02
Log-odds
0.5
1.0
1.5
2.0
2.5
3.0
3.5
4.0
Cellular_organisms - ps1
Archaea1 - ps2
Archaea2 - ps3
Archaea3 - ps4
Asgard/Eukaryota - ps5
Eukaryota - ps6
Amorpheae - ps7
Obazoa - ps8
Opisthokonta - ps9
Holozoa - ps10
Filozoa - ps11
Choano./Metazoa - ps12
Metazoa - ps13
Myriazoa - ps14
Parahoxozoa - ps15
Bilateria - ps16
Deuterostomia - ps17
Chordata - ps18
Olfactores - ps19
Vertebrata - ps20
Gnathostomata - ps21
Euteleostomi - ps22
Sarcopterygii - ps23
Tetrapoda - ps24
Amniota - ps25
Mammalia - ps26
Theria - ps27
Placentalia - ps28
Boreoeutheria - ps29
Euarchontoglires - ps30
Euarchonta - ps31
Primates - ps32
Haplorrhini - ps33
Simiiformes - ps34
Catarrhini - ps35
Hominoidea - ps36
Hominidae - ps37
Homininae - ps38
Hominini - ps39
Homo_sapiens - ps40
Phylostratum

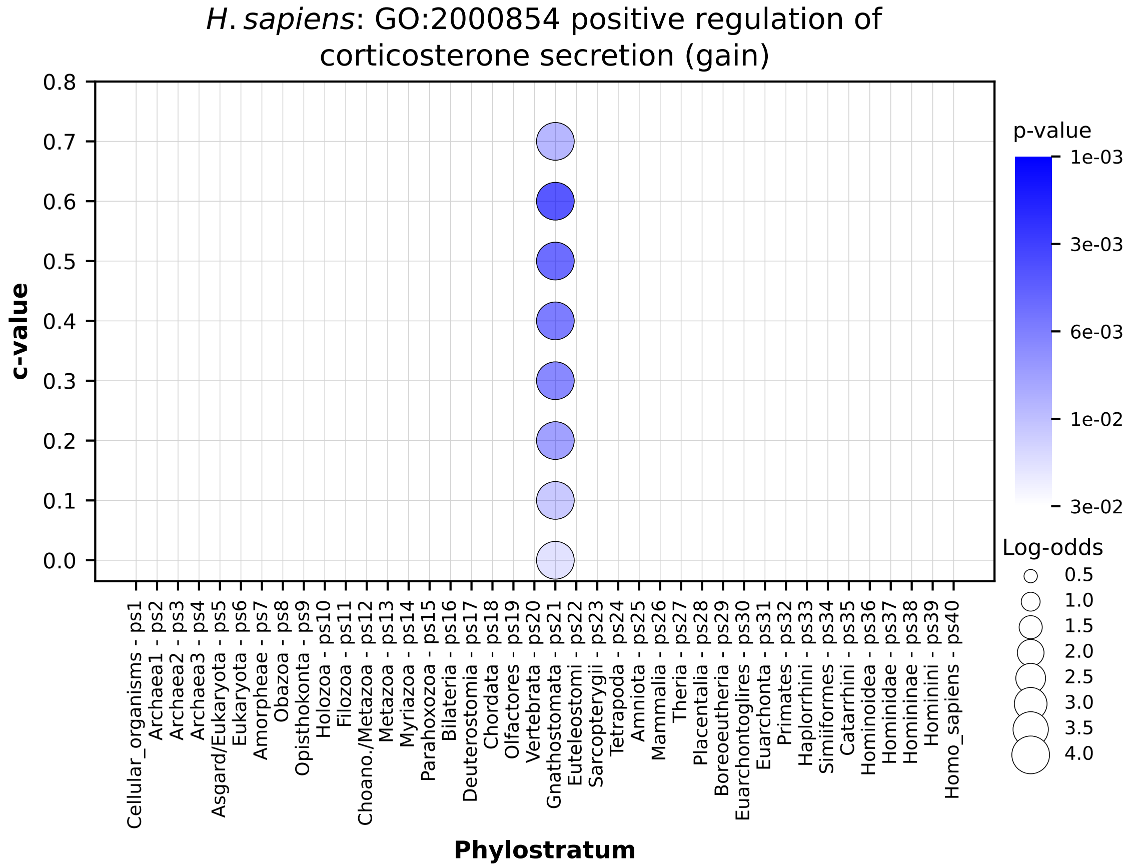

H. sapiens: GO:2000854 positive regulation of corticosterone secretion (gain)
c-value
0.0
0.1
0.2
0.3
0.4
0.5
0.6
0.7
0.8
Phylostratum
Cellular_organisms - ps1
Archaea1 - ps2
Archaea2 - ps3
Archaea3 - ps4
Asgard/Eukaryota - ps5
Eukaryota - ps6
Amorpheae - ps7
Obazoa - ps8
Opisthokonta - ps9
Holozoa - ps10
Filozoa - ps11
Choano./Metazoa - ps12
Metazoa - ps13
Myriazoa - ps14
Parahoxozoa - ps15
Bilateria - ps16
Deuterostomia - ps17
Chordata - ps18
Olfactores - ps19
Vertebrata - ps20
Gnathostomata - ps21
Euteleostomi - ps22
Sarcopterygii - ps23
Tetrapoda - ps24
Amniota - ps25
Mammalia - ps26
Theria - ps27
Placentalia - ps28
Boreoeutheria - ps29
Euarchontoglires - ps30
Euarchonta - ps31
Primates - ps32
Haplorrhini - ps33
Simiiformes - ps34
Catarrhini - ps35
Hominoidea - ps36
Hominidae - ps37
Homininae - ps38
Hominini - ps39
Homo_sapiens - ps40
p-value
1e-03
3e-03
6e-03
1e-02
3e-02
Log-odds
0.5
1.0
1.5
2.0
2.5
3.0
3.5
4.0

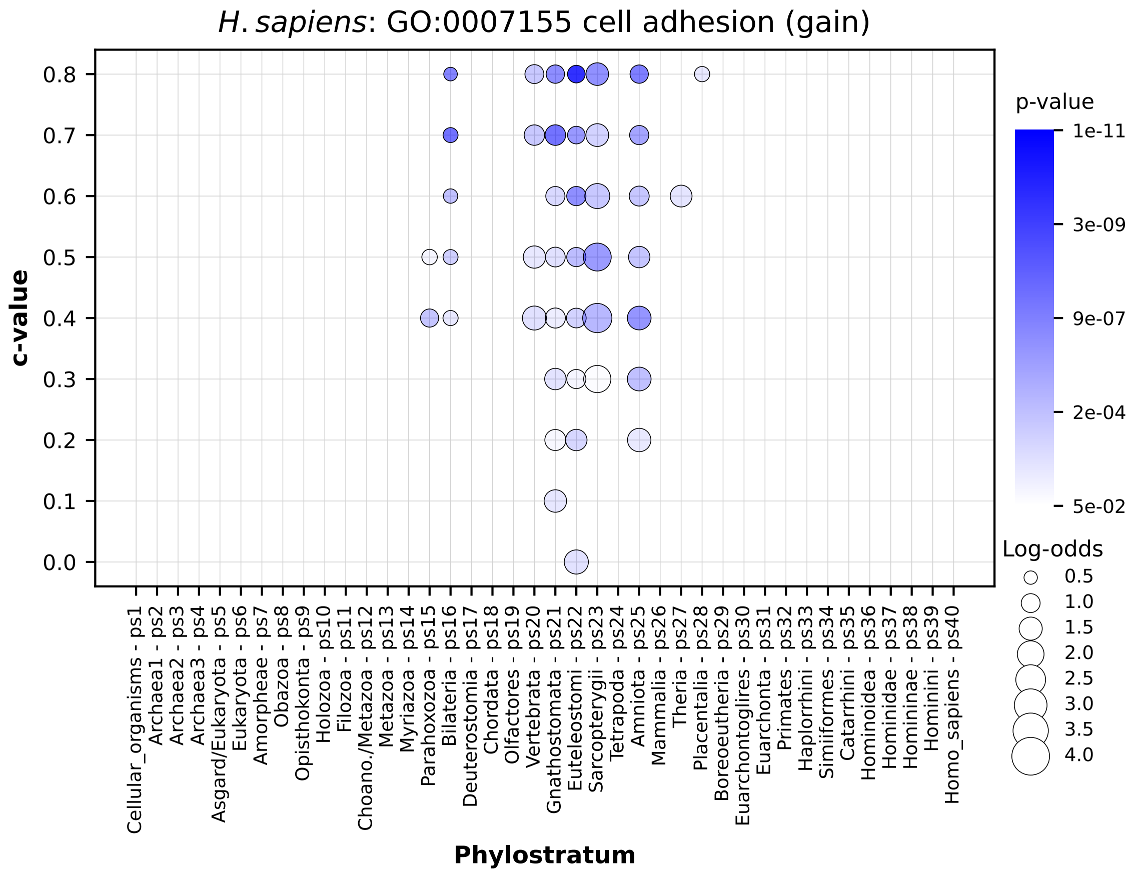
H. sapiens: GO:0007155 cell adhesion (gain)
c-value
0.8
0.7
0.6
0.5
0.4
0.3
0.2
0.1
0.0
p-value
1e-11
3e-09
9e-07
2e-04
5e-02
Log-odds
0.5
1.0
1.5
2.0
2.5
3.0
3.5
4.0
Cellular_organisms - ps1
Archaea1 - ps2
Archaea2 - ps3
Archaea3 - ps4
Asgard/Eukaryota - ps5
Eukaryota - ps6
Amorpheae - ps7
Obazoa - ps8
Opisthokonta - ps9
Holozoa - ps10
Filozoa - ps11
Choano./Metazoa - ps12
Metazoa - ps13
Myriazoa - ps14
Parahoxozoa - ps15
Bilateria - ps16
Deuterostomia - ps17
Chordata - ps18
Olfactores - ps19
Vertebrata - ps20
Gnathostomata - ps21
Euteleostomi - ps22
Sarcopterygii - ps23
Tetrapoda - ps24
Amniota - ps25
Mammalia - ps26
Theria - ps27
Placentalia - ps28
Boreoeutheria - ps29
Euarchontoglires - ps30
Euarchonta - ps31
Primates - ps32
Haplorrhini - ps33
Simiiformes - ps34
Catarrhini - ps35
Hominoidea - ps36
Hominidae - ps37
Homininae - ps38
Hominini - ps39
Homo_sapiens - ps40
Phylostratum

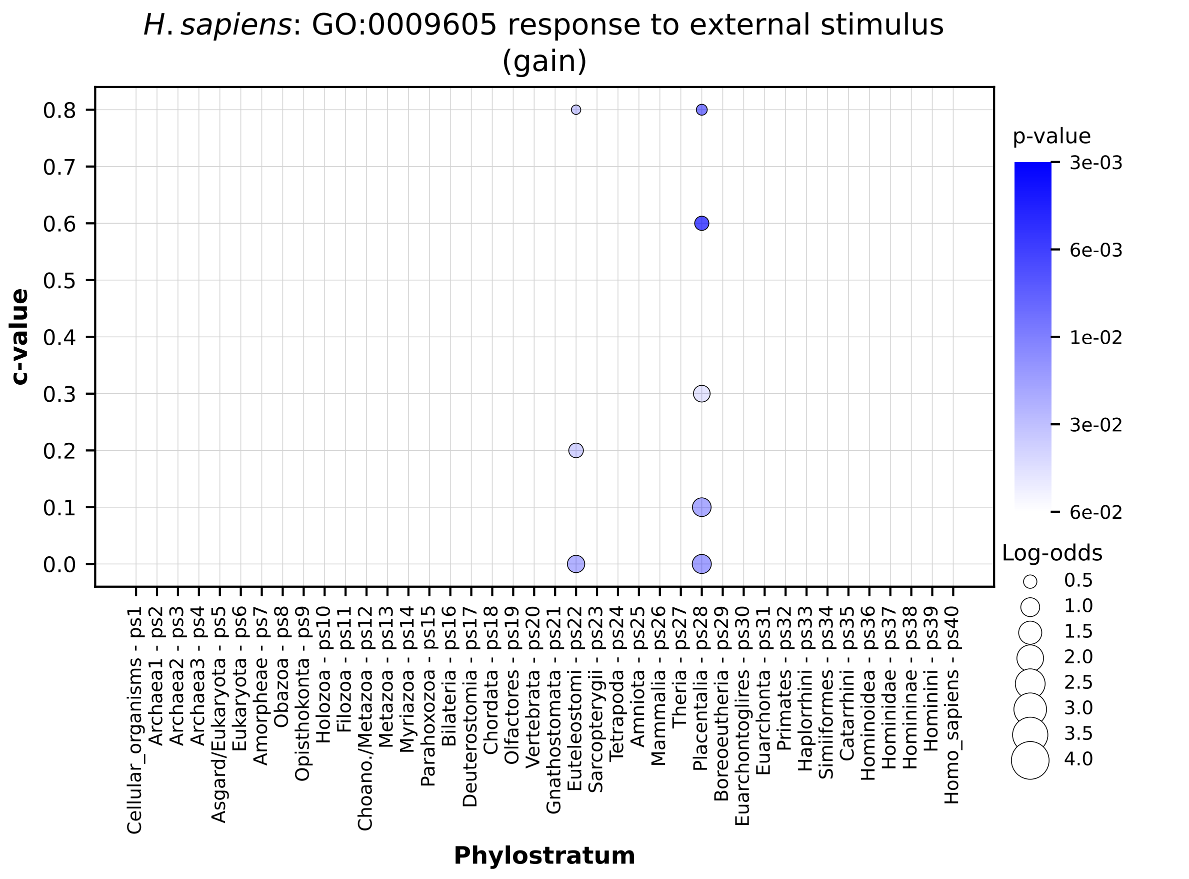

H. sapiens: GO:0009605 response to external stimulus
(gain)
c-value
0.0
0.1
0.2
0.3
0.4
0.5
0.6
0.7
0.8
p-value
3e-03
6e-03
1e-02
3e-02
6e-02
Log-odds
0.5
1.0
1.5
2.0
2.5
3.0
3.5
4.0
Cellular_organisms - ps1
Archaea1 - ps2
Archaea2 - ps3
Archaea3 - ps4
Asgard/Eukaryota - ps5
Eukaryota - ps6
Amorpheae - ps7
Obazoa - ps8
Opisthokonta - ps9
Holozoa - ps10
Filozoa - ps11
Choano./Metazoa - ps12
Metazoa - ps13
Myriazoa - ps14
Parahoxozoa - ps15
Bilateria - ps16
Deuterostomia - ps17
Chordata - ps18
Olfactores - ps19
Vertebrata - ps20
Gnathostomata - ps21
Euteleostomi - ps22
Sarcopterygii - ps23
Tetrapoda - ps24
Amniota - ps25
Mammalia - ps26
Theria - ps27
Placentalia - ps28
Boreoeutheria - ps29
Euarchontoglires - ps30
Euarchonta - ps31
Primates - ps32
Haplorrhini - ps33
Simiiformes - ps34
Catarrhini - ps35
Hominoidea - ps36
Hominidae - ps37
Homininae - ps38
Hominini - ps39
Homo_sapiens - ps40
Phylostratum

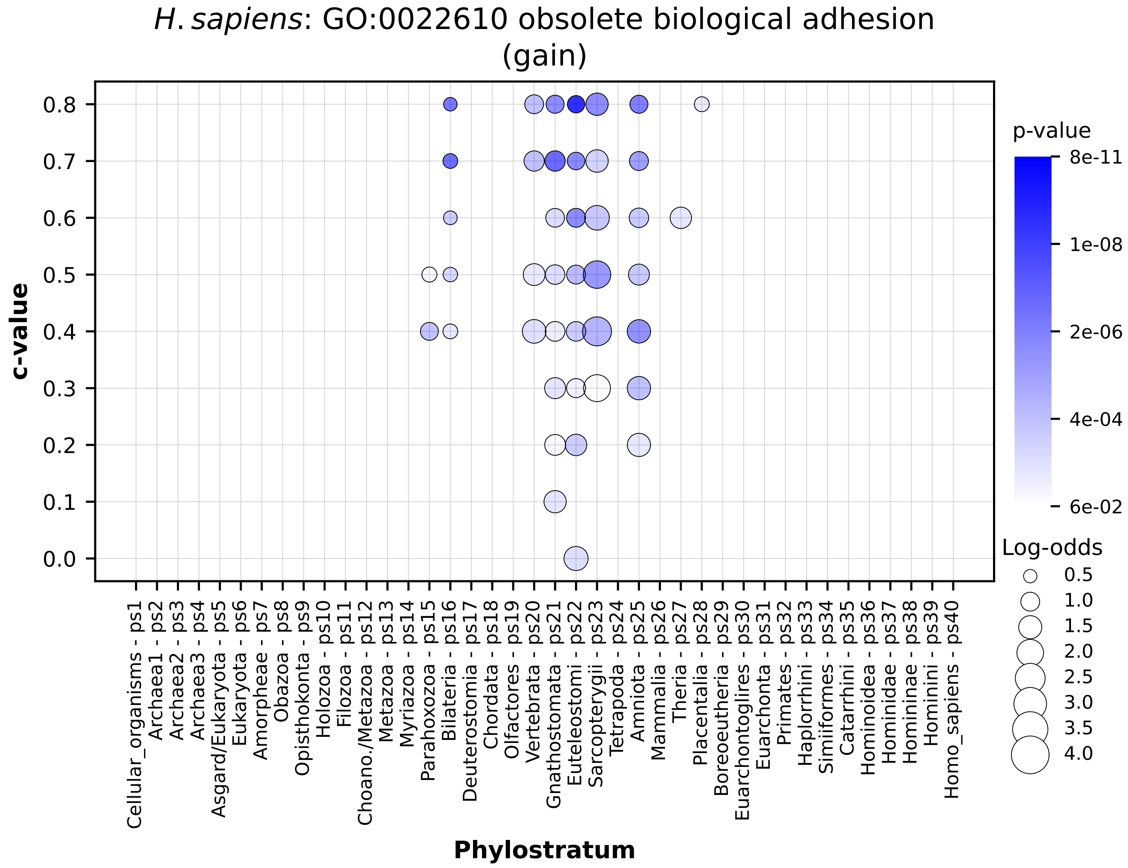

*H. sapiens*: GO:0022610 obsolete biological adhesion
(gain)
c-value
0.8
0.7
0.6
0.5
0.4
0.3
0.2
0.1
0.0
p-value
8e-11
1e-08
2e-06
4e-04
6e-02
Log-odds
0.5
1.0
1.5
2.0
2.5
3.0
3.5
4.0
Cellular_organisms - ps1
Archaea1 - ps2
Archaea2 - ps3
Archaea3 - ps4
Asgard/Eukaryota - ps5
Eukaryota - ps6
Amorpheae - ps7
Obazoa - ps8
Opisthokonta - ps9
Holozoa - ps10
Filozoa - ps11
Choano./Metazoa - ps12
Metazoa - ps13
Myriazoa - ps14
Parahoxozoa - ps15
Bilateria - ps16
Deuterostomia - ps17
Chordata - ps18
Olfactores - ps19
Vertebrata - ps20
Gnathostomata - ps21
Euteleostomi - ps22
Sarcopterygii - ps23
Tetrapoda - ps24
Amniota - ps25
Mammalia - ps26
Theria - ps27
Placentalia - ps28
Boreoeutheria - ps29
Euarchontoglires - ps30
Euarchonta - ps31
Primates - ps32
Haplorrhini - ps33
Simiiformes - ps34
Catarrhini - ps35
Hominoidea - ps36
Hominidae - ps37
Homininae - ps38
Hominini - ps39
Homo_sapiens - ps40
Phylostratum

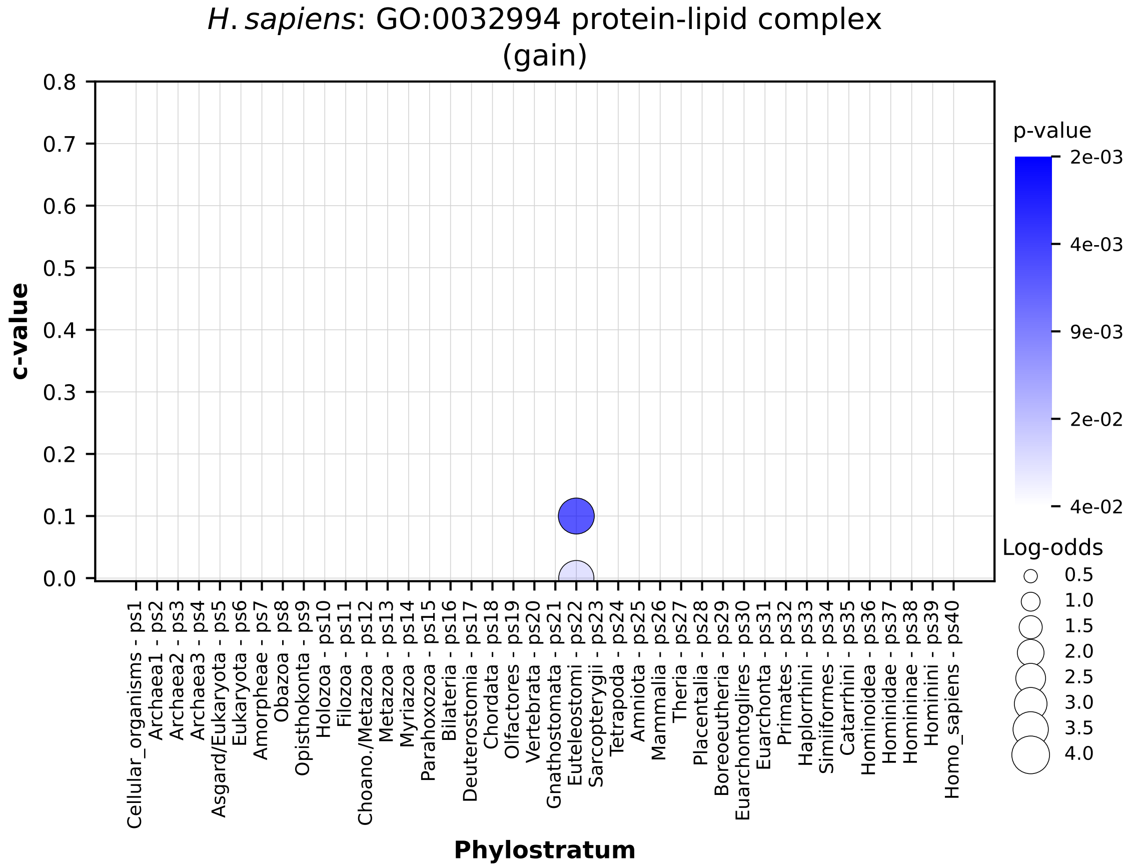

H. sapiens: GO:0032994 protein-lipid complex
(gain)
c-value
0.0
0.1
0.2
0.3
0.4
0.5
0.6
0.7
0.8
Phylostratum
Cellular_organisms - ps1
Archaea1 - ps2
Archaea2 - ps3
Archaea3 - ps4
Asgard/Eukaryota - ps5
Eukaryota - ps6
Amorpheae - ps7
Obazoa - ps8
Opisthokonta - ps9
Holozoa - ps10
Filozoa - ps11
Choano./Metazoa - ps12
Metazoa - ps13
Myriazoa - ps14
Parahoxozoa - ps15
Bilateria - ps16
Deuterostomia - ps17
Chordata - ps18
Olfactores - ps19
Vertebrata - ps20
Gnathostomata - ps21
Euteleostomi - ps22
Sarcopterygii - ps23
Tetrapoda - ps24
Amniota - ps25
Mammalia - ps26
Theria - ps27
Placentalia - ps28
Boreoeutheria - ps29
Euarchontoglires - ps30
Euarchonta - ps31
Primates - ps32
Haplorrhini - ps33
Simiiformes - ps34
Catarrhini - ps35
Hominoidea - ps36
Hominidae - ps37
Homininae - ps38
Hominini - ps39
Homo_sapiens - ps40
p-value
2e-03
4e-03
9e-03
2e-02
4e-02
Log-odds
0.5
1.0
1.5
2.0
2.5
3.0
3.5
4.0

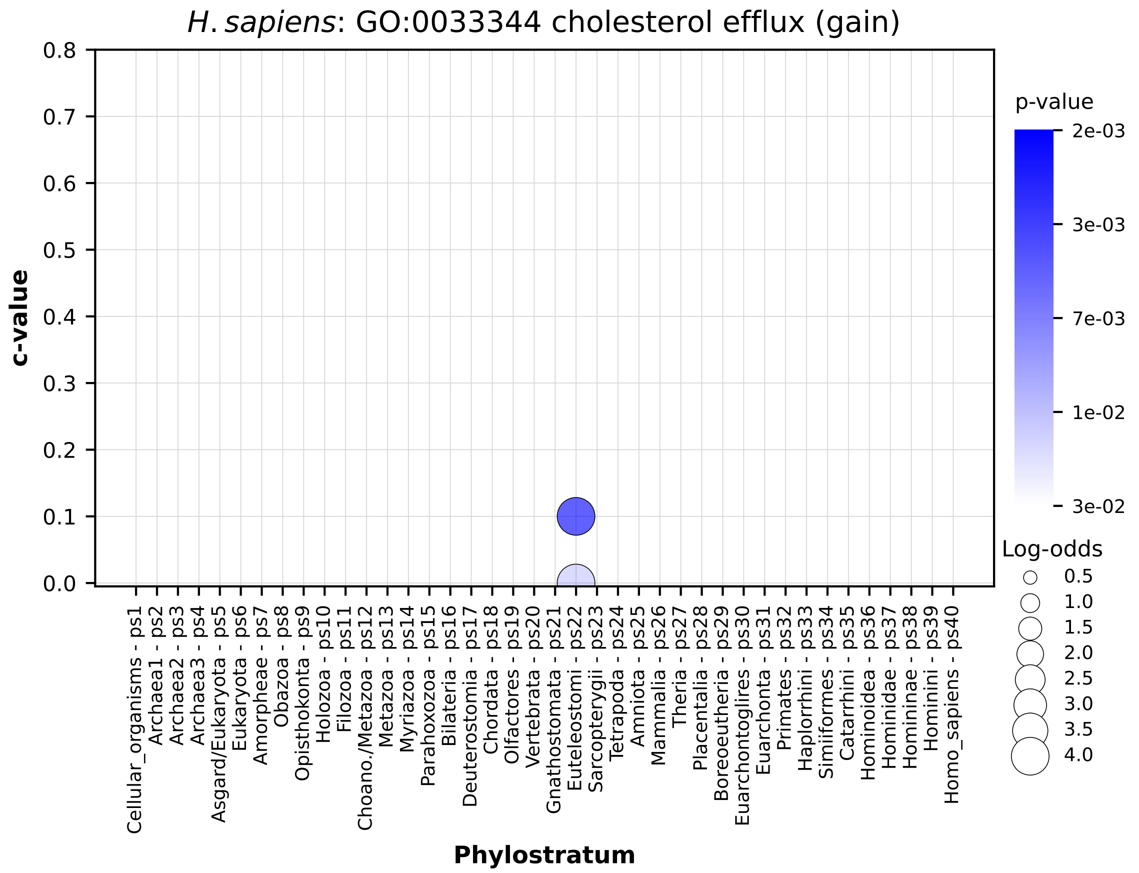
H. sapiens: GO:0033344 cholesterol efflux (gain)
c-value
0.8
0.7
0.6
0.5
0.4
0.3
0.2
0.1
0.0
p-value
2e-03
3e-03
7e-03
1e-02
3e-02
Log-odds
0.5
1.0
1.5
2.0
2.5
3.0
3.5
4.0
Cellular_organisms - ps1
Archaea1 - ps2
Archaea2 - ps3
Archaea3 - ps4
Asgard/Eukaryota - ps5
Eukaryota - ps6
Amorpheae - ps7
Obazoa - ps8
Opisthokonta - ps9
Holozoa - ps10
Filozoa - ps11
Choano./Metazoa - ps12
Metazoa - ps13
Myriazoa - ps14
Parahoxozoa - ps15
Bilateria - ps16
Deuterostomia - ps17
Chordata - ps18
Olfactores - ps19
Vertebrata - ps20
Gnathostomata - ps21
Euteleostomi - ps22
Sarcopterygii - ps23
Tetrapoda - ps24
Amniota - ps25
Mammalia - ps26
Theria - ps27
Placentalia - ps28
Boreoeutheria - ps29
Euarchontoglires - ps30
Euarchonta - ps31
Primates - ps32
Haplorrhini - ps33
Simiiformes - ps34
Catarrhini - ps35
Hominoidea - ps36
Hominidae - ps37
Homininae - ps38
Hominini - ps39
Homo_sapiens - ps40
Phylostratum

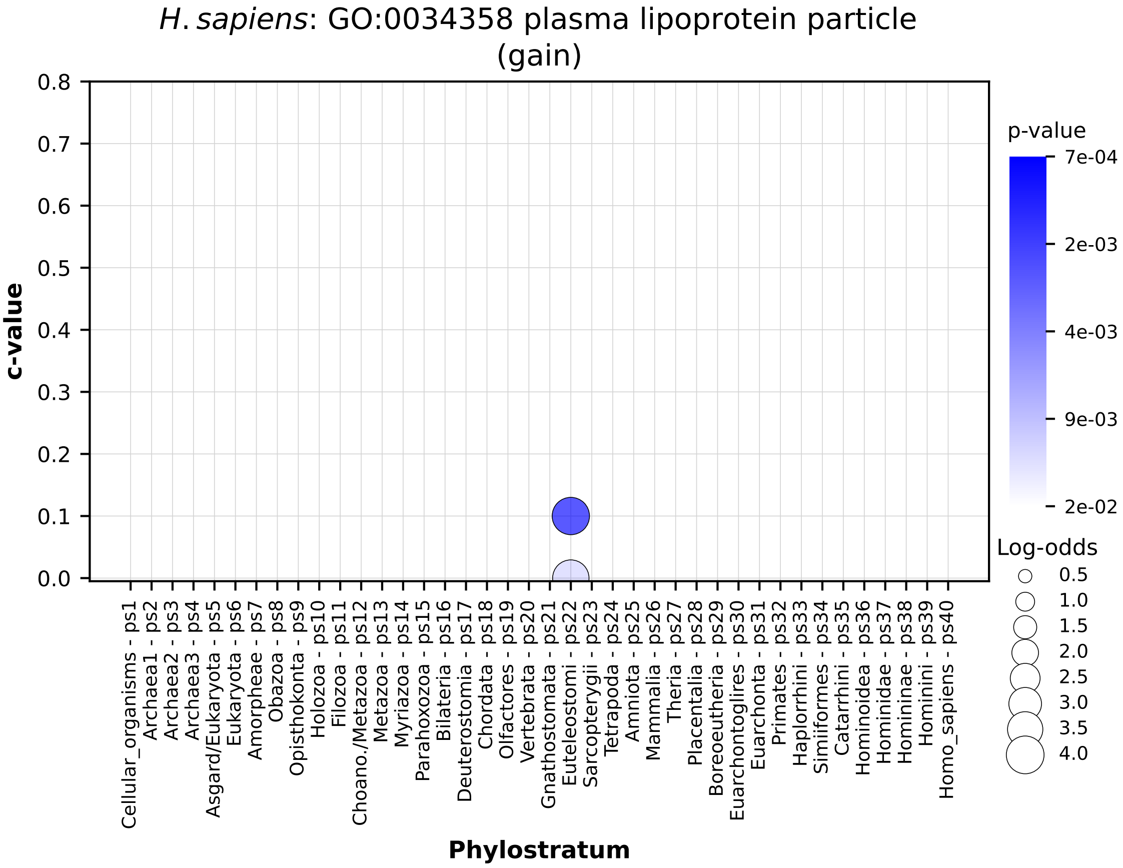

H. sapiens: GO:0034358 plasma lipoprotein particle
(gain)
c-value
0.8
0.7
0.6
0.5
0.4
0.3
0.2
0.1
0.0
Phylostratum
Cellular_organisms - ps1
Archaea1 - ps2
Archaea2 - ps3
Archaea3 - ps4
Asgard/Eukaryota - ps5
Eukaryota - ps6
Amorpheae - ps7
Obazoa - ps8
Opisthokonta - ps9
Holozoa - ps10
Filozoa - ps11
Choano./Metazoa - ps12
Metazoa - ps13
Myriazoa - ps14
Parahoxozoa - ps15
Bilateria - ps16
Deuterostomia - ps17
Chordata - ps18
Olfactores - ps19
Vertebrata - ps20
Gnathostomata - ps21
Euteleostomi - ps22
Sarcopterygii - ps23
Tetrapoda - ps24
Amniota - ps25
Mammalia - ps26
Theria - ps27
Placentalia - ps28
Boreoeutheria - ps29
Euarchontoglires - ps30
Euarchonta - ps31
Primates - ps32
Haplorrhini - ps33
Simiiformes - ps34
Catarrhini - ps35
Hominoidea - ps36
Hominidae - ps37
Homininae - ps38
Hominini - ps39
Homo_sapiens - ps40
p-value
7e-04
2e-03
4e-03
9e-03
2e-02
Log-odds
0.5
1.0
1.5
2.0
2.5
3.0
3.5
4.0

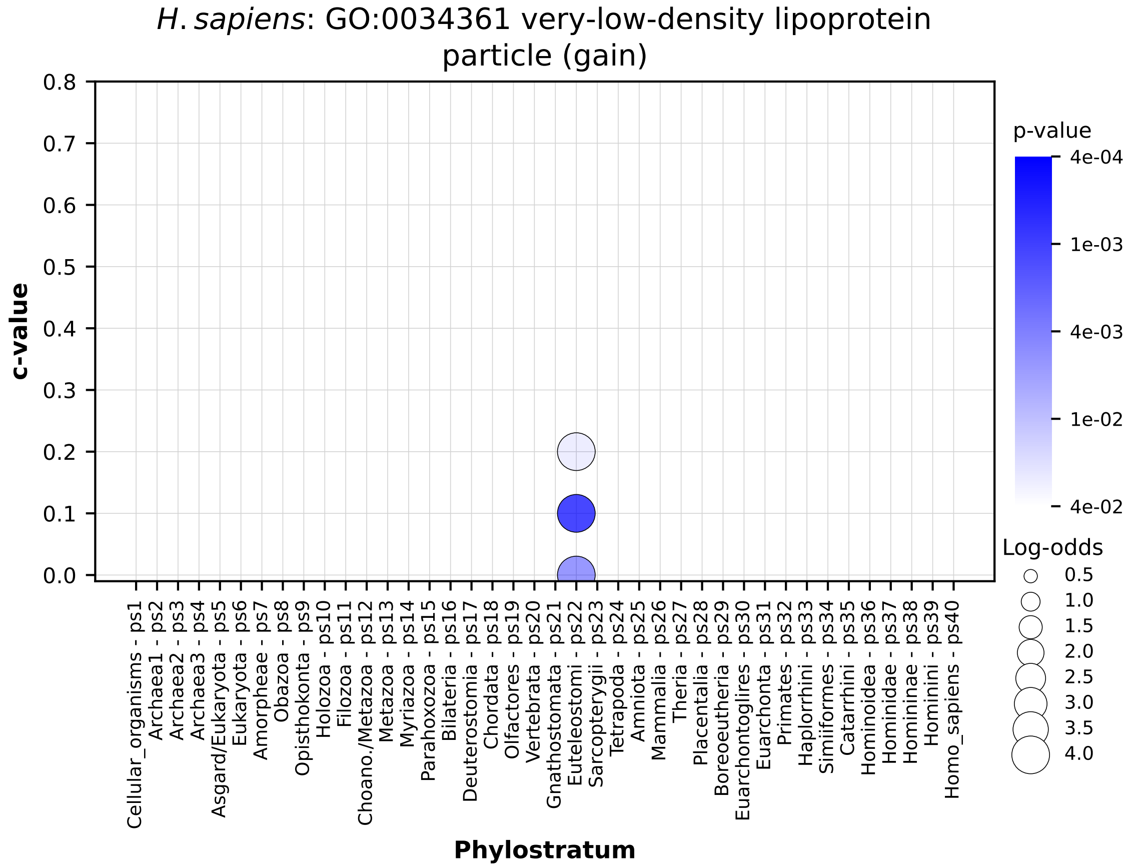
H. sapiens: GO:0034361 very-low-density lipoprotein particle (gain)
c-value
0.8
0.7
0.6
0.5
0.4
0.3
0.2
0.1
0.0
p-value
4e-04
1e-03
4e-03
1e-02
4e-02
Log-odds
0.5
1.0
1.5
2.0
2.5
3.0
3.5
4.0
Cellular_organisms - ps1
Archaea1 - ps2
Archaea2 - ps3
Archaea3 - ps4
Asgard/Eukaryota - ps5
Eukaryota - ps6
Amorpheae - ps7
Obazoa - ps8
Opisthokonta - ps9
Holozoa - ps10
Filozoa - ps11
Choano./Metazoa - ps12
Metazoa - ps13
Myriazoa - ps14
Parahoxozoa - ps15
Bilateria - ps16
Deuterostomia - ps17
Chordata - ps18
Olfactores - ps19
Vertebrata - ps20
Gnathostomata - ps21
Euteleostomi - ps22
Sarcopterygii - ps23
Tetrapoda - ps24
Amniota - ps25
Mammalia - ps26
Theria - ps27
Placentalia - ps28
Boreoeutheria - ps29
Euarchontoglires - ps30
Euarchonta - ps31
Primates - ps32
Haplorrhini - ps33
Simiiformes - ps34
Catarrhini - ps35
Hominoidea - ps36
Hominidae - ps37
Homininae - ps38
Hominini - ps39
Homo_sapiens - ps40
Phylostratum

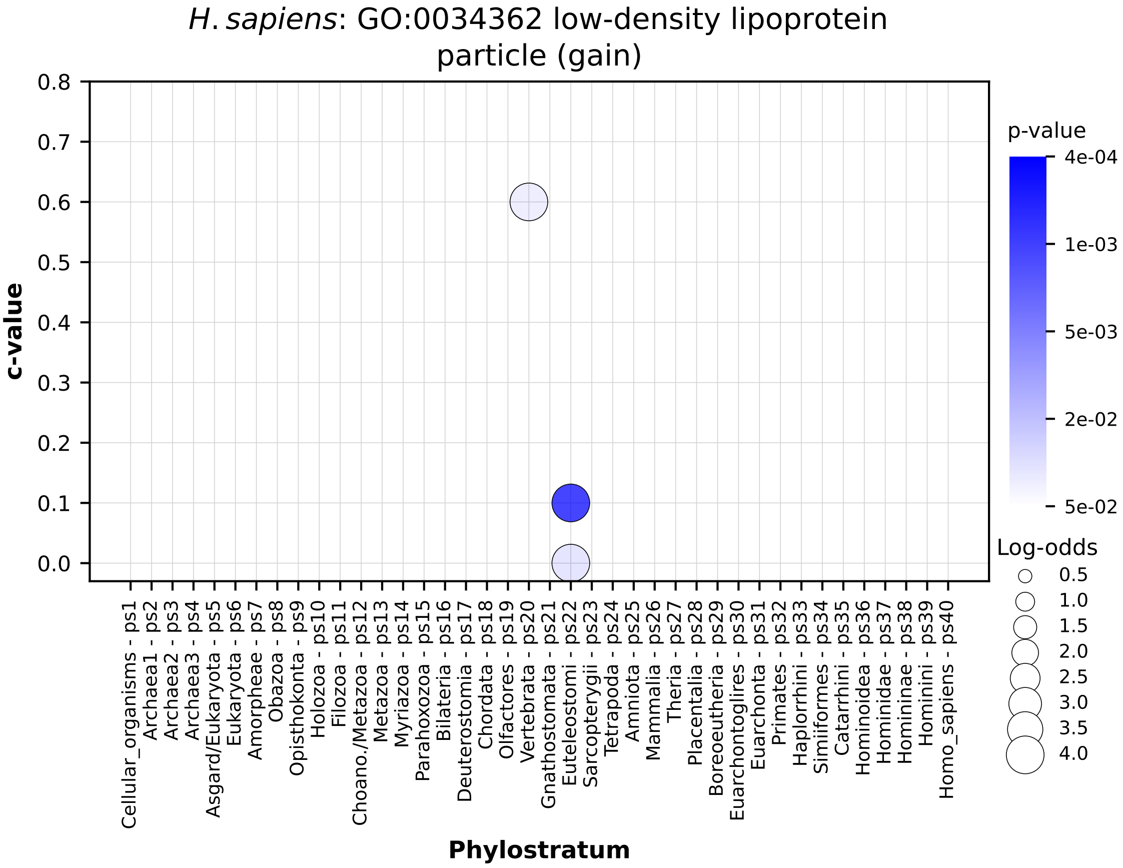

H. sapiens: GO:0034362 low-density lipoprotein particle (gain)
c-value
0.8
0.7
0.6
0.5
0.4
0.3
0.2
0.1
0.0
Phylostratum
Cellular_organisms - ps1
Archaea1 - ps2
Archaea2 - ps3
Archaea3 - ps4
Asgard/Eukaryota - ps5
Eukaryota - ps6
Amorpheae - ps7
Obazoa - ps8
Opisthokonta - ps9
Holozoa - ps10
Filozoa - ps11
Choano./Metazoa - ps12
Metazoa - ps13
Myriazoa - ps14
Parahoxozoa - ps15
Bilateria - ps16
Deuterostomia - ps17
Chordata - ps18
Olfactores - ps19
Vertebrata - ps20
Gnathostomata - ps21
Euteleostomi - ps22
Sarcopterygii - ps23
Tetrapoda - ps24
Amniota - ps25
Mammalia - ps26
Theria - ps27
Placentalia - ps28
Boreoeutheria - ps29
Euarchontoglires - ps30
Euarchonta - ps31
Primates - ps32
Haplorrhini - ps33
Simiiformes - ps34
Catarrhini - ps35
Hominoidea - ps36
Hominidae - ps37
Homininae - ps38
Hominini - ps39
Homo_sapiens - ps40
p-value
4e-04
1e-03
5e-03
2e-02
5e-02
Log-odds
0.5
1.0
1.5
2.0
2.5
3.0
3.5
4.0

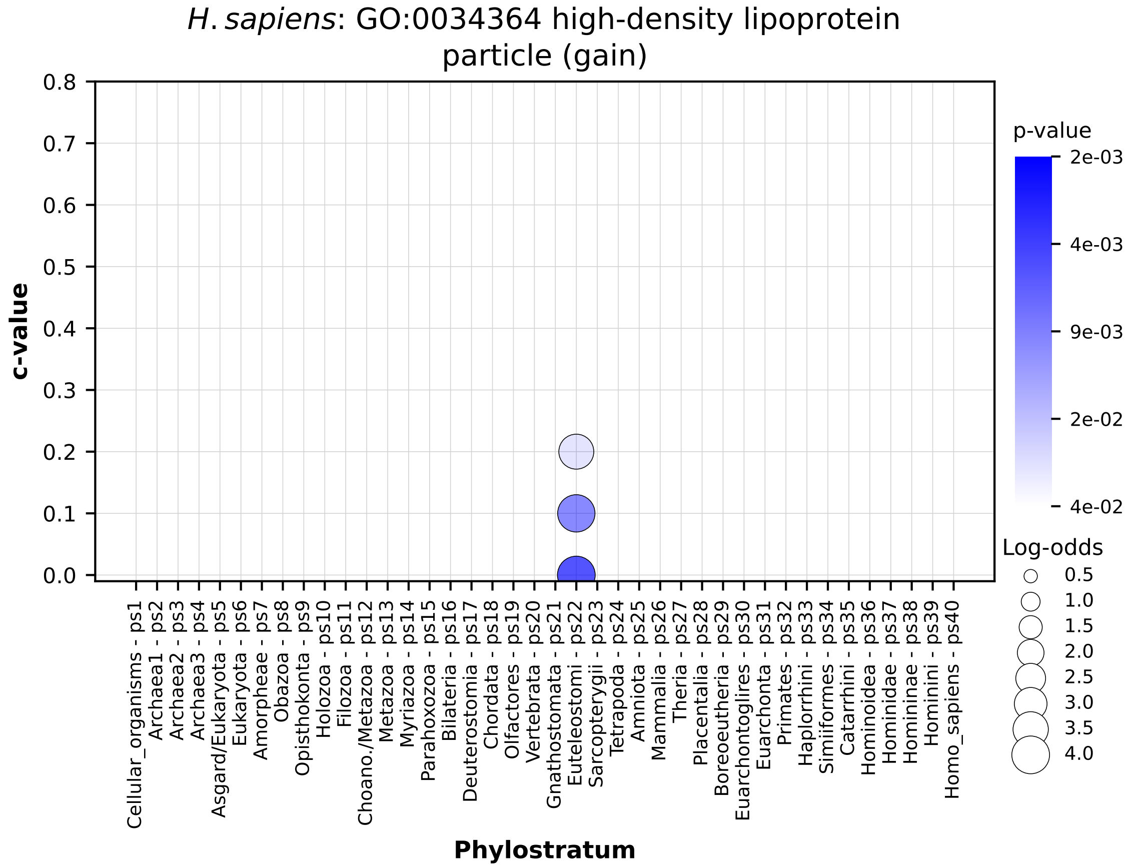

*H. sapiens*: GO:0034364 high-density lipoprotein particle (gain)
c-value
0.0
0.1
0.2
0.3
0.4
0.5
0.6
0.7
0.8
Phylostratum
Cellular_organisms - ps1
Archaea1 - ps2
Archaea2 - ps3
Archaea3 - ps4
Asgard/Eukaryota - ps5
Eukaryota - ps6
Amorpheae - ps7
Obazoa - ps8
Opisthokonta - ps9
Holozoa - ps10
Filozoa - ps11
Choano./Metazoa - ps12
Metazoa - ps13
Myriazoa - ps14
Parahoxozoa - ps15
Bilateria - ps16
Deuterostomia - ps17
Chordata - ps18
Olfactores - ps19
Vertebrata - ps20
Gnathostomata - ps21
Euteleostomi - ps22
Sarcopterygii - ps23
Tetrapoda - ps24
Amniota - ps25
Mammalia - ps26
Theria - ps27
Placentalia - ps28
Boreoeutheria - ps29
Euarchontoglires - ps30
Euarchonta - ps31
Primates - ps32
Haplorrhini - ps33
Simiiformes - ps34
Catarrhini - ps35
Hominoidea - ps36
Hominidae - ps37
Homininae - ps38
Hominini - ps39
Homo_sapiens - ps40
p-value
2e-03
4e-03
9e-03
2e-02
4e-02
Log-odds
0.5
1.0
1.5
2.0
2.5
3.0
3.5
4.0

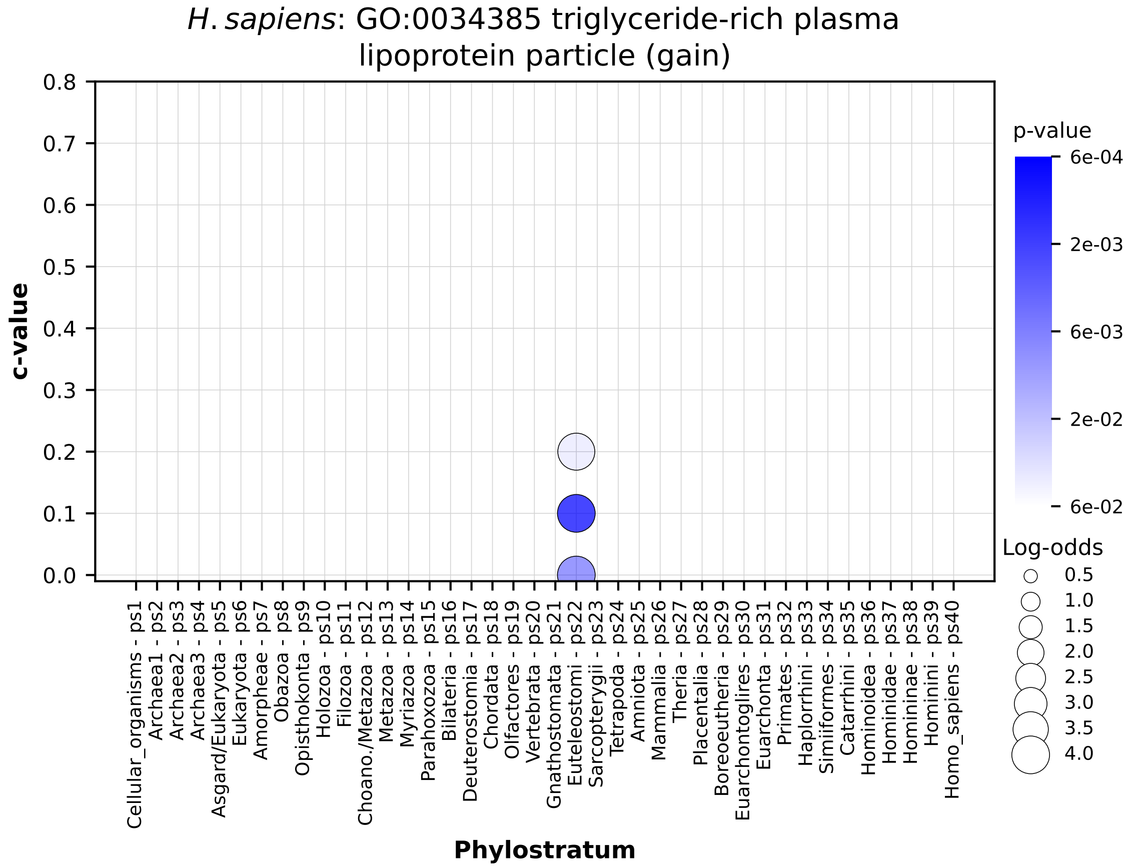

H. sapiens: GO:0034385 triglyceride-rich plasma lipoprotein particle (gain)
c-value
0.0
0.1
0.2
0.3
0.4
0.5
0.6
0.7
0.8
Phylostratum
Cellular_organisms - ps1
Archaea1 - ps2
Archaea2 - ps3
Archaea3 - ps4
Asgard/Eukaryota - ps5
Eukaryota - ps6
Amorpheae - ps7
Obazoa - ps8
Opisthokonta - ps9
Holozoa - ps10
Filozoa - ps11
Choano./Metazoa - ps12
Metazoa - ps13
Myriazoa - ps14
Parahoxozoa - ps15
Bilateria - ps16
Deuterostomia - ps17
Chordata - ps18
Olfactores - ps19
Vertebrata - ps20
Gnathostomata - ps21
Euteleostomi - ps22
Sarcopterygii - ps23
Tetrapoda - ps24
Amniota - ps25
Mammalia - ps26
Theria - ps27
Placentalia - ps28
Boreoeutheria - ps29
Euarchontoglires - ps30
Euarchonta - ps31
Primates - ps32
Haplorrhini - ps33
Simiiformes - ps34
Catarrhini - ps35
Hominoidea - ps36
Hominidae - ps37
Homininae - ps38
Hominini - ps39
Homo_sapiens - ps40
p-value
6e-04
2e-03
6e-03
2e-02
6e-02
Log-odds
0.5
1.0
1.5
2.0
2.5
3.0
3.5
4.0

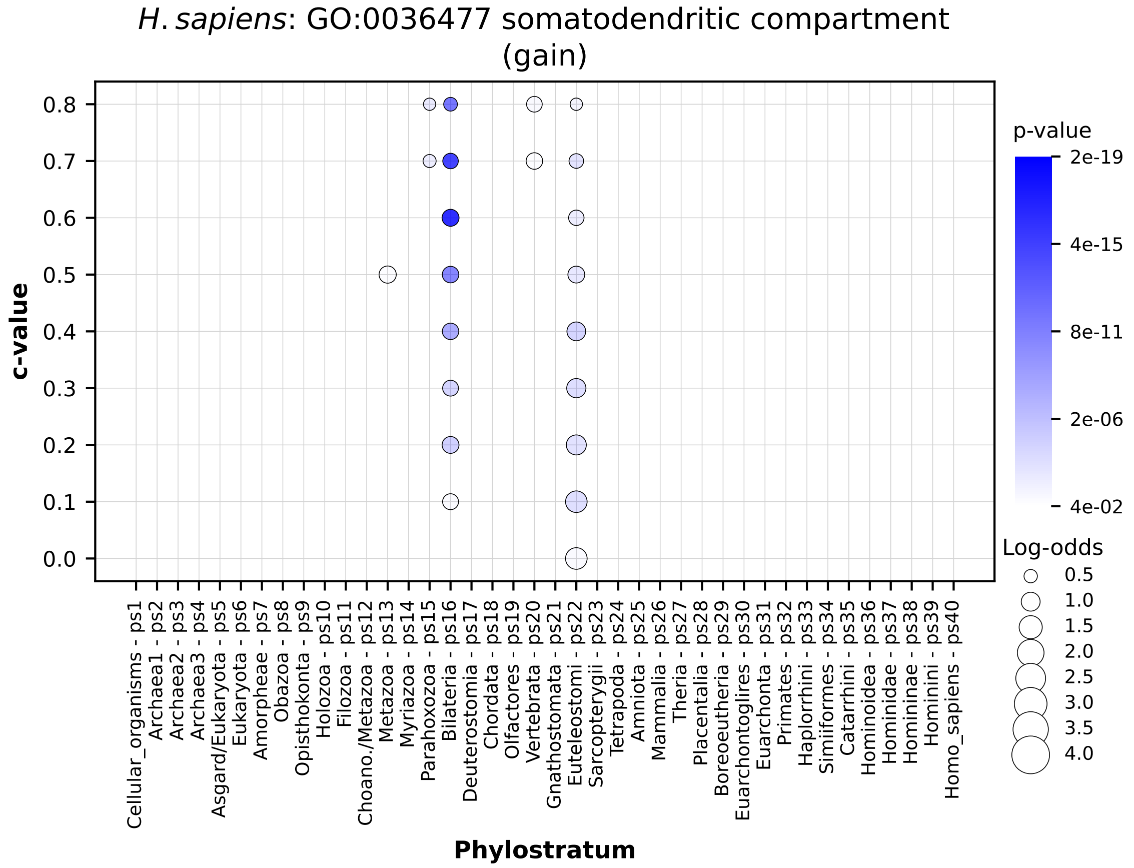

*H. sapiens*: GO:0036477 somatodendritic compartment
(gain)
c-value
0.8
0.7
0.6
0.5
0.4
0.3
0.2
0.1
0.0
p-value
2e-19
4e-15
8e-11
2e-06
4e-02
Log-odds
0.5
1.0
1.5
2.0
2.5
3.0
3.5
4.0
Cellular_organisms - ps1
Archaea1 - ps2
Archaea2 - ps3
Archaea3 - ps4
Asgard/Eukaryota - ps5
Eukaryota - ps6
Amorpheae - ps7
Obazoa - ps8
Opisthokonta - ps9
Holozoa - ps10
Filozoa - ps11
Choano./Metazoa - ps12
Metazoa - ps13
Myriazoa - ps14
Parahoxozoa - ps15
Bilateria - ps16
Deuterostomia - ps17
Chordata - ps18
Olfactores - ps19
Vertebrata - ps20
Gnathostomata - ps21
Euteleostomi - ps22
Sarcopterygii - ps23
Tetrapoda - ps24
Amniota - ps25
Mammalia - ps26
Theria - ps27
Placentalia - ps28
Boreoeutheria - ps29
Euarchontoglires - ps30
Euarchonta - ps31
Primates - ps32
Haplorrhini - ps33
Simiiformes - ps34
Catarrhini - ps35
Hominoidea - ps36
Hominidae - ps37
Homininae - ps38
Hominini - ps39
Homo_sapiens - ps40
Phylostratum

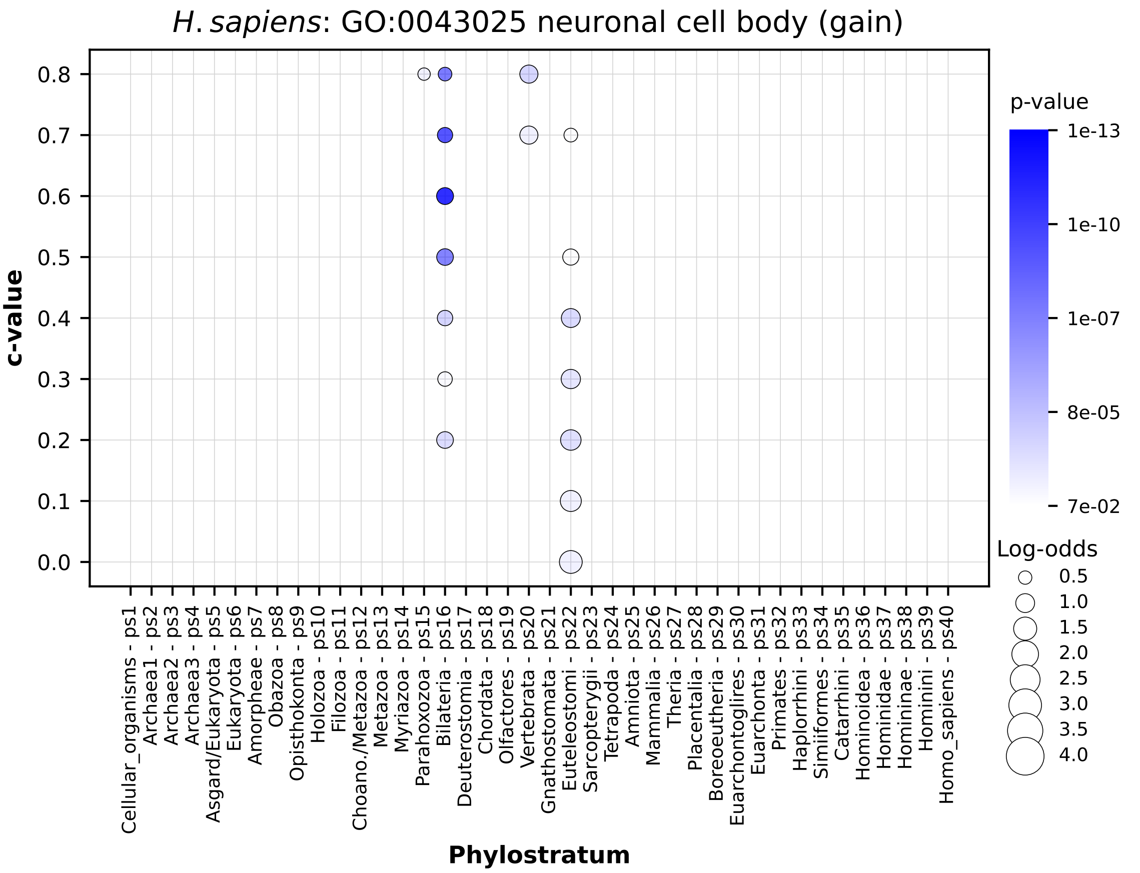

H. sapiens: GO:0043025 neuronal cell body (gain)
c-value
0.8
0.7
0.6
0.5
0.4
0.3
0.2
0.1
0.0
Phylostratum
Cellular_organisms - ps1
Archaea1 - ps2
Archaea2 - ps3
Archaea3 - ps4
Asgard/Eukaryota - ps5
Eukaryota - ps6
Amorpheae - ps7
Obazoa - ps8
Opisthokonta - ps9
Holozoa - ps10
Filozoa - ps11
Choano./Metazoa - ps12
Metazoa - ps13
Myriazoa - ps14
Parahoxozoa - ps15
Bilateria - ps16
Deuterostomia - ps17
Chordata - ps18
Olfactores - ps19
Vertebrata - ps20
Gnathostomata - ps21
Euteleostomi - ps22
Sarcopterygii - ps23
Tetrapoda - ps24
Amniota - ps25
Mammalia - ps26
Theria - ps27
Placentalia - ps28
Boreoeutheria - ps29
Euarchontoglires - ps30
Euarchonta - ps31
Primates - ps32
Haplorrhini - ps33
Simiiformes - ps34
Catarrhini - ps35
Hominoidea - ps36
Hominidae - ps37
Homininae - ps38
Hominini - ps39
Homo_sapiens - ps40
p-value
1e-13
1e-10
1e-07
8e-05
7e-02
Log-odds
0.5
1.0
1.5
2.0
2.5
3.0
3.5
4.0

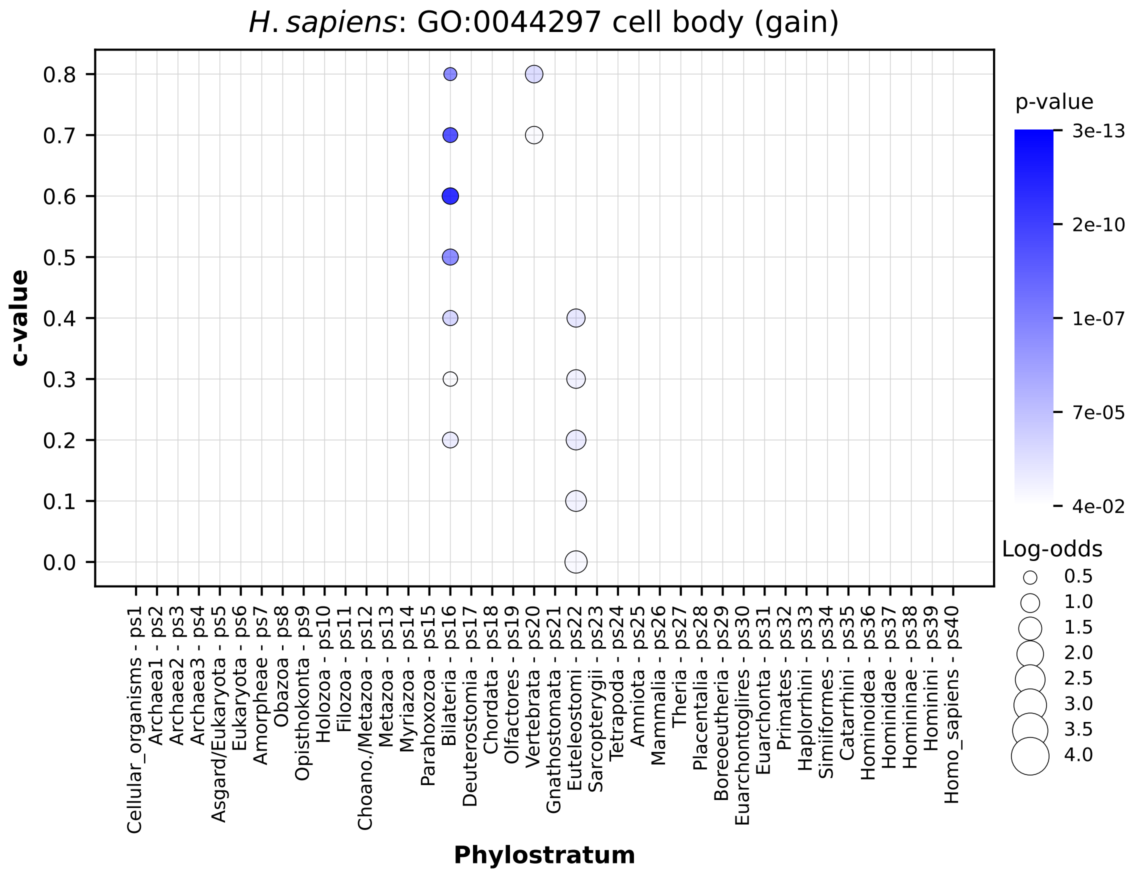

*H. sapiens*: GO:0044297 cell body (gain)
c-value
0.8
0.7
0.6
0.5
0.4
0.3
0.2
0.1
0.0
Phylostratum
Cellular_organisms - ps1
Archaea1 - ps2
Archaea2 - ps3
Archaea3 - ps4
Asgard/Eukaryota - ps5
Eukaryota - ps6
Amorpheae - ps7
Obazoa - ps8
Opisthokonta - ps9
Holozoa - ps10
Filozoa - ps11
Choano./Metazoa - ps12
Metazoa - ps13
Myriazoa - ps14
Parahoxozoa - ps15
Bilateria - ps16
Deuterostomia - ps17
Chordata - ps18
Olfactores - ps19
Vertebrata - ps20
Gnathostomata - ps21
Euteleostomi - ps22
Sarcopterygii - ps23
Tetrapoda - ps24
Amniota - ps25
Mammalia - ps26
Theria - ps27
Placentalia - ps28
Boreoeutheria - ps29
Euarchontoglires - ps30
Euarchonta - ps31
Primates - ps32
Haplorrhini - ps33
Simiiformes - ps34
Catarrhini - ps35
Hominoidea - ps36
Hominidae - ps37
Homininae - ps38
Hominini - ps39
Homo_sapiens - ps40
p-value
3e-13
2e-10
1e-07
7e-05
4e-02
Log-odds
0.5
1.0
1.5
2.0
2.5
3.0
3.5
4.0

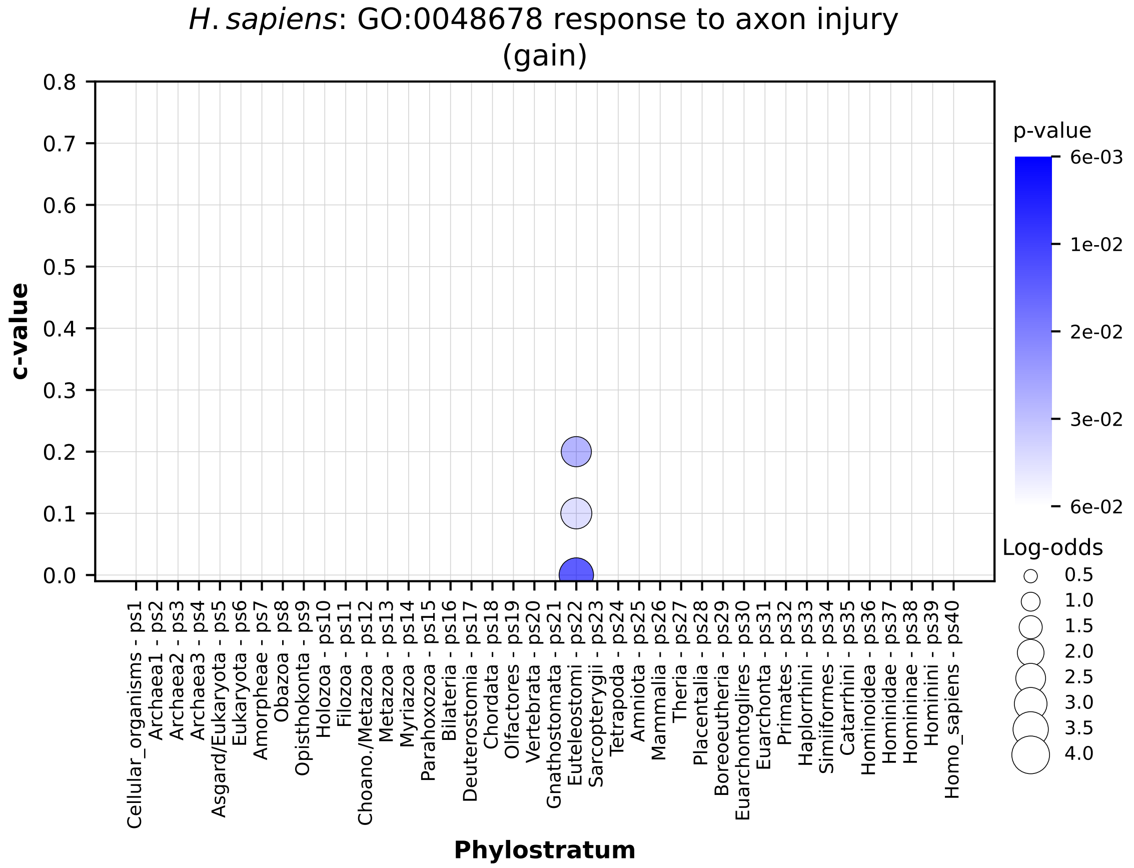

*H. sapiens*: GO:0048678 response to axon injury
(gain)
c-value
0.8
0.7
0.6
0.5
0.4
0.3
0.2
0.1
0.0
Phylostratum
Cellular_organisms - ps1
Archaea1 - ps2
Archaea2 - ps3
Archaea3 - ps4
Asgard/Eukaryota - ps5
Eukaryota - ps6
Amorpheae - ps7
Obazoa - ps8
Opisthokonta - ps9
Holozoa - ps10
Filozoa - ps11
Choano./Metazoa - ps12
Metazoa - ps13
Myriazoa - ps14
Parahoxozoa - ps15
Bilateria - ps16
Deuterostomia - ps17
Chordata - ps18
Olfactores - ps19
Vertebrata - ps20
Gnathostomata - ps21
Euteleostomi - ps22
Sarcopterygii - ps23
Tetrapoda - ps24
Amniota - ps25
Mammalia - ps26
Theria - ps27
Placentalia - ps28
Boreoeutheria - ps29
Euarchontoglires - ps30
Euarchonta - ps31
Primates - ps32
Haplorrhini - ps33
Simiiformes - ps34
Catarrhini - ps35
Hominoidea - ps36
Hominidae - ps37
Homininae - ps38
Hominini - ps39
Homo_sapiens - ps40
p-value
6e-03
1e-02
2e-02
3e-02
6e-02
Log-odds
0.5
1.0
1.5
2.0
2.5
3.0
3.5
4.0

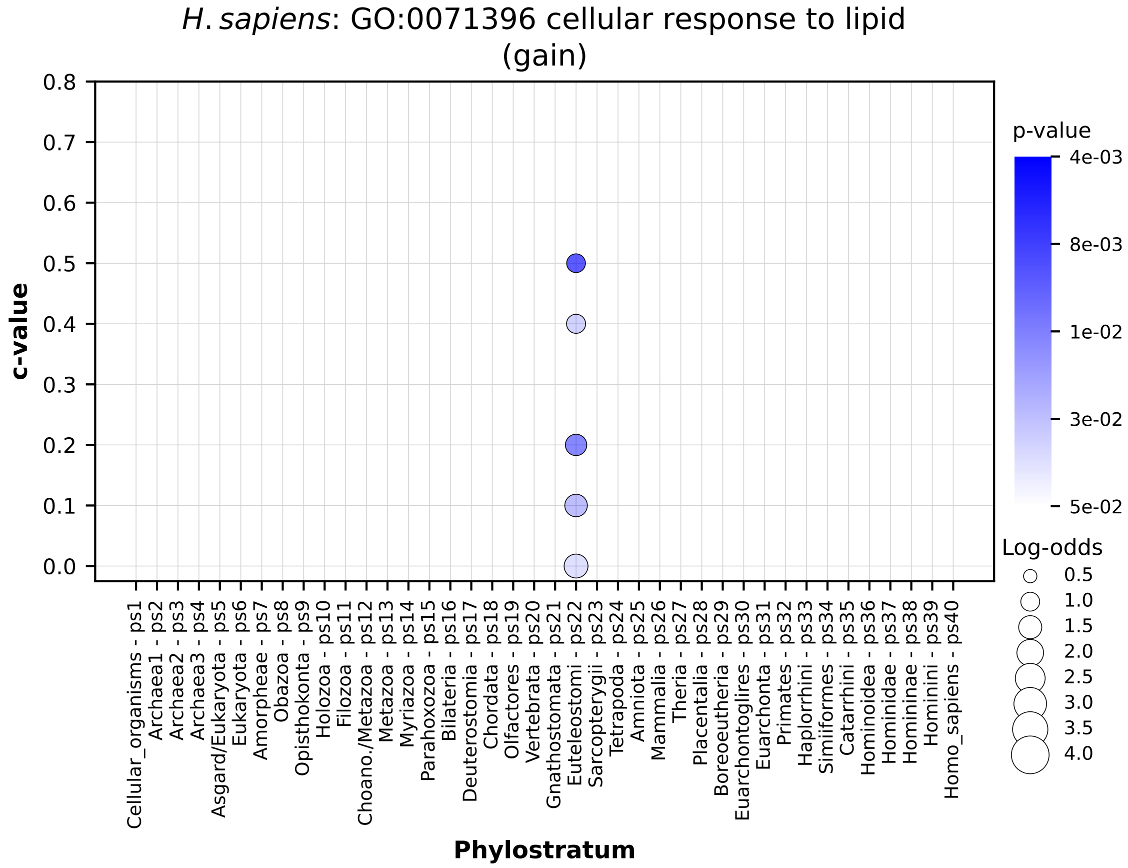

H. sapiens: GO:0071396 cellular response to lipid
(gain)
c-value
0.8
0.7
0.6
0.5
0.4
0.3
0.2
0.1
0.0
p-value
4e-03
8e-03
1e-02
3e-02
5e-02
Log-odds
0.5
1.0
1.5
2.0
2.5
3.0
3.5
4.0
Cellular_organisms - ps1
Archaea1 - ps2
Archaea2 - ps3
Archaea3 - ps4
Asgard/Eukaryota - ps5
Eukaryota - ps6
Amorpheae - ps7
Obazoa - ps8
Opisthokonta - ps9
Holozoa - ps10
Filozoa - ps11
Choano./Metazoa - ps12
Metazoa - ps13
Myriazoa - ps14
Parahoxozoa - ps15
Bilateria - ps16
Deuterostomia - ps17
Chordata - ps18
Olfactores - ps19
Vertebrata - ps20
Gnathostomata - ps21
Euteleostomi - ps22
Sarcopterygii - ps23
Tetrapoda - ps24
Amniota - ps25
Mammalia - ps26
Theria - ps27
Placentalia - ps28
Boreoeutheria - ps29
Euarchontoglires - ps30
Euarchonta - ps31
Primates - ps32
Haplorrhini - ps33
Simiiformes - ps34
Catarrhini - ps35
Hominoidea - ps36
Hominidae - ps37
Homininae - ps38
Hominini - ps39
Homo_sapiens - ps40
Phylostratum

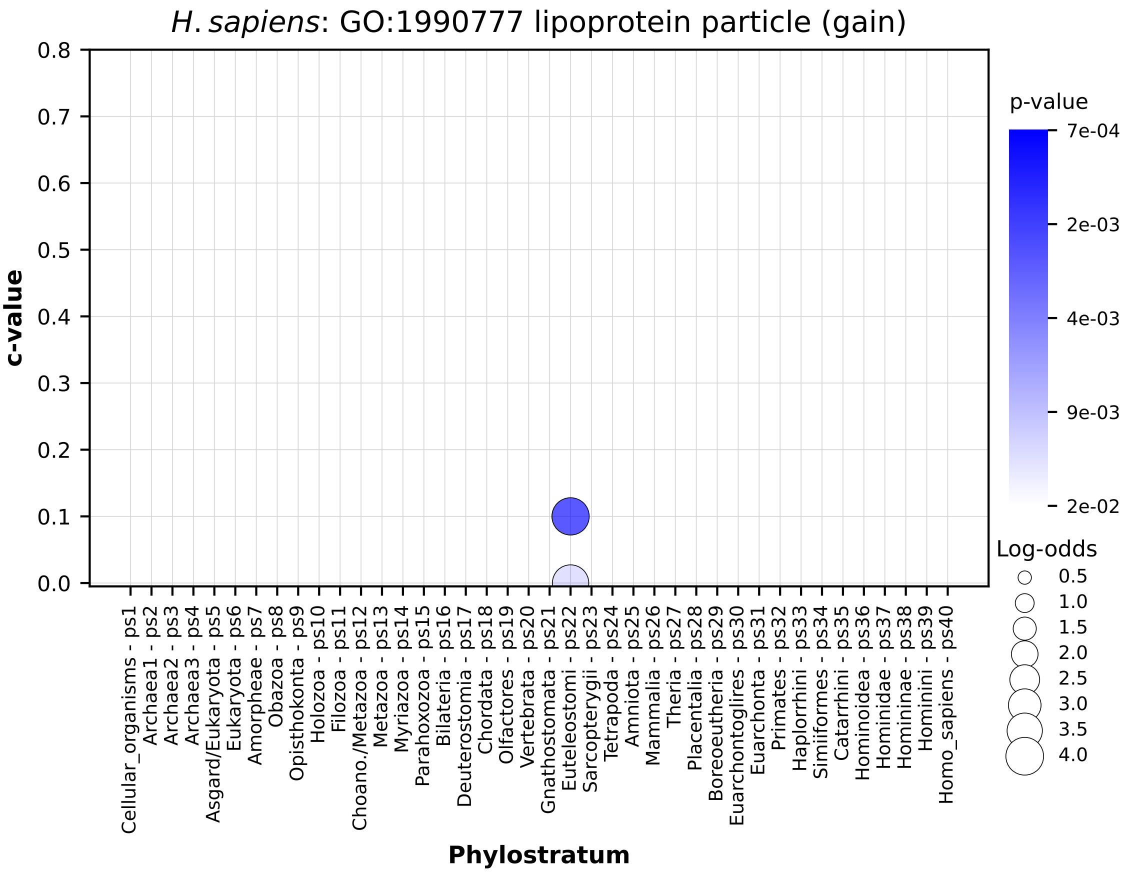

H. sapiens: GO:1990777 lipoprotein particle (gain)
c-value
0.8
0.7
0.6
0.5
0.4
0.3
0.2
0.1
0.0
Phylostratum
Cellular_organisms - ps1
Archaea1 - ps2
Archaea2 - ps3
Archaea3 - ps4
Asgard/Eukaryota - ps5
Eukaryota - ps6
Amorpheae - ps7
Obazoa - ps8
Opisthokonta - ps9
Holozoa - ps10
Filozoa - ps11
Choano./Metazoa - ps12
Metazoa - ps13
Myriazoa - ps14
Parahoxozoa - ps15
Bilateria - ps16
Deuterostomia - ps17
Chordata - ps18
Olfactores - ps19
Vertebrata - ps20
Gnathostomata - ps21
Euteleostomi - ps22
Sarcopterygii - ps23
Tetrapoda - ps24
Amniota - ps25
Mammalia - ps26
Theria - ps27
Placentalia - ps28
Boreoeutheria - ps29
Euarchontoglires - ps30
Euarchonta - ps31
Primates - ps32
Haplorrhini - ps33
Simiiformes - ps34
Catarrhini - ps35
Hominoidea - ps36
Hominidae - ps37
Homininae - ps38
Hominini - ps39
Homo_sapiens - ps40
p-value
7e-04
2e-03
4e-03
9e-03
2e-02
Log-odds
0.5
1.0
1.5
2.0
2.5
3.0
3.5
4.0

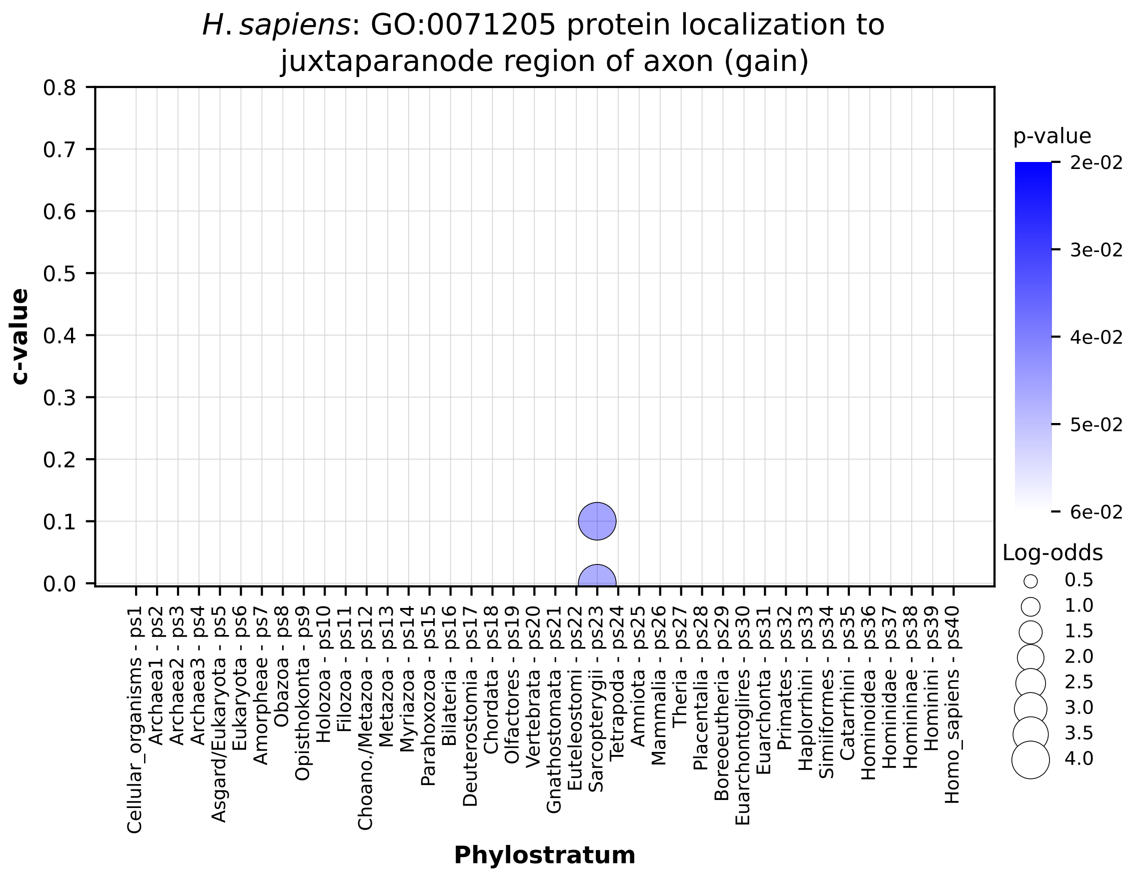

H. sapiens: GO:0071205 protein localization to juxtaparanode region of axon (gain)
c-value
0.8
0.7
0.6
0.5
0.4
0.3
0.2
0.1
0.0
Phylostratum
Cellular_organisms - ps1
Archaea1 - ps2
Archaea2 - ps3
Archaea3 - ps4
Asgard/Eukaryota - ps5
Eukaryota - ps6
Amorpheae - ps7
Obazoa - ps8
Opisthokonta - ps9
Holozoa - ps10
Filozoa - ps11
Choano./Metazoa - ps12
Metazoa - ps13
Myriazoa - ps14
Parahoxozoa - ps15
Bilateria - ps16
Deuterostomia - ps17
Chordata - ps18
Olfactores - ps19
Vertebrata - ps20
Gnathostomata - ps21
Euteleostomi - ps22
Sarcopterygii - ps23
Tetrapoda - ps24
Amniota - ps25
Mammalia - ps26
Theria - ps27
Placentalia - ps28
Boreoeutheria - ps29
Euarchontoglires - ps30
Euarchonta - ps31
Primates - ps32
Haplorrhini - ps33
Simiiformes - ps34
Catarrhini - ps35
Hominoidea - ps36
Hominidae - ps37
Homininae - ps38
Hominini - ps39
Homo_sapiens - ps40
p-value
2e-02
3e-02
4e-02
5e-02
6e-02
Log-odds
0.5
1.0
1.5
2.0
2.5
3.0
3.5
4.0

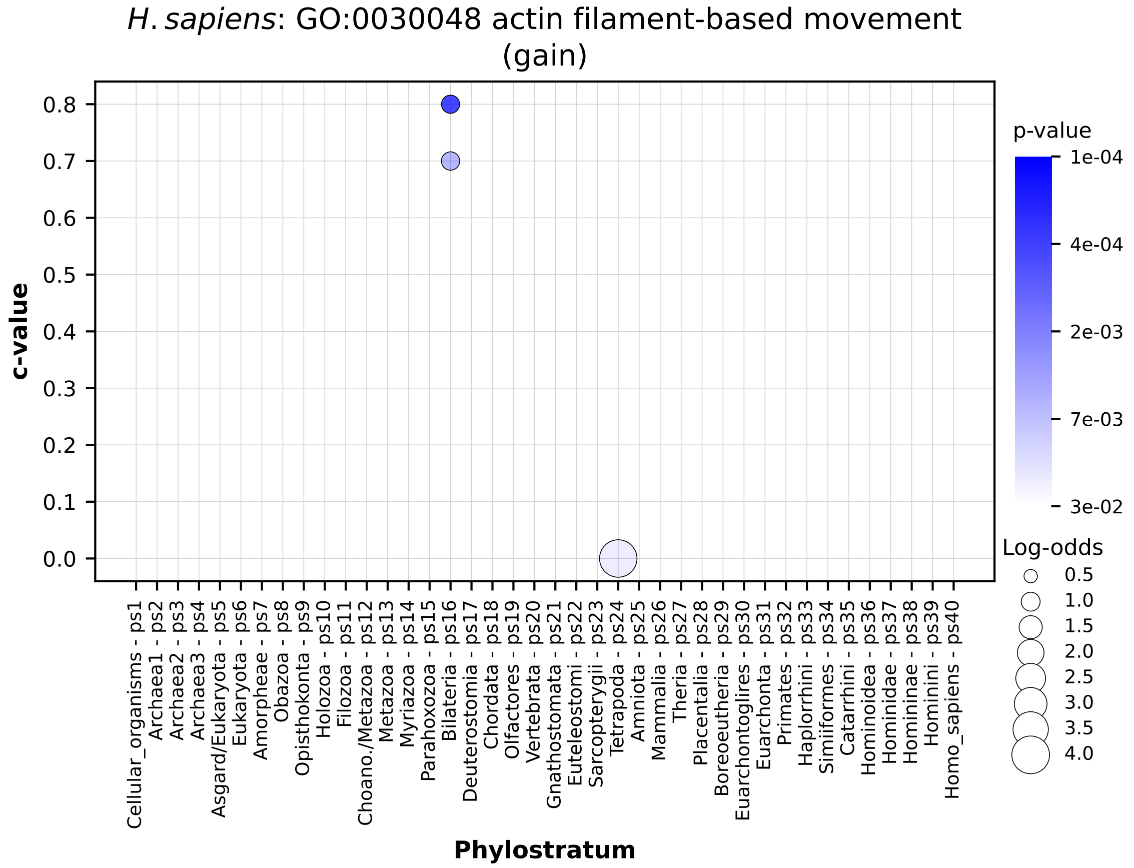

*H. sapiens*: GO:0030048 actin filament-based movement (gain)
c-value
0.8
0.7
0.6
0.5
0.4
0.3
0.2
0.1
0.0
p-value
1e-04
4e-04
2e-03
7e-03
3e-02
Log-odds
0.5
1.0
1.5
2.0
2.5
3.0
3.5
4.0
Cellular_organisms - ps1
Archaea1 - ps2
Archaea2 - ps3
Archaea3 - ps4
Asgard/Eukaryota - ps5
Eukaryota - ps6
Amorpheae - ps7
Obazoa - ps8
Opisthokonta - ps9
Holozoa - ps10
Filozoa - ps11
Choano./Metazoa - ps12
Metazoa - ps13
Myriazoa - ps14
Parahoxozoa - ps15
Bilateria - ps16
Deuterostomia - ps17
Chordata - ps18
Olfactores - ps19
Vertebrata - ps20
Gnathostomata - ps21
Euteleostomi - ps22
Sarcopterygii - ps23
Tetrapoda - ps24
Amniota - ps25
Mammalia - ps26
Theria - ps27
Placentalia - ps28
Boreoeutheria - ps29
Euarchontoglires - ps30
Euarchonta - ps31
Primates - ps32
Haplorrhini - ps33
Simiiformes - ps34
Catarrhini - ps35
Hominoidea - ps36
Hominidae - ps37
Homininae - ps38
Hominini - ps39
Homo_sapiens - ps40
Phylostratum

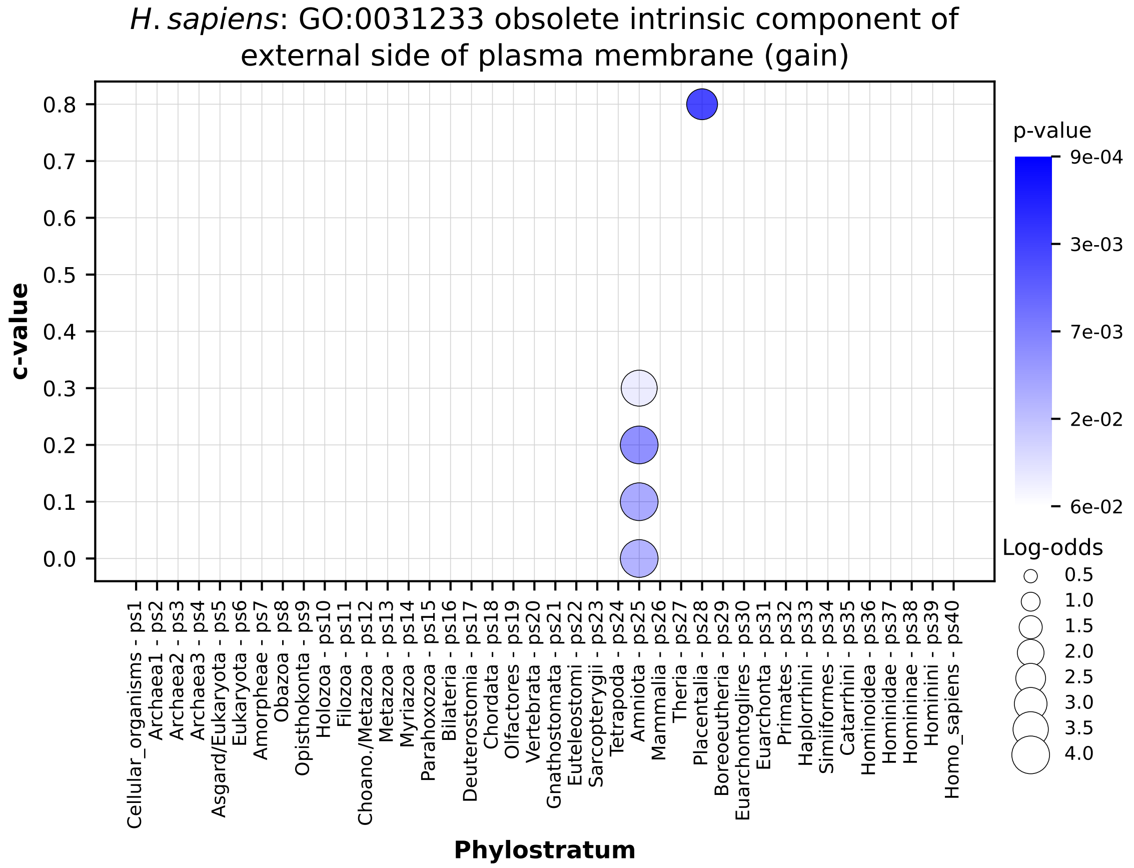
H. sapiens: GO:0031233 obsolete intrinsic component of external side of plasma membrane (gain)
c-value
0.0
0.1
0.2
0.3
0.4
0.5
0.6
0.7
0.8
p-value
9e-04
3e-03
7e-03
2e-02
6e-02
Log-odds
0.5
1.0
1.5
2.0
2.5
3.0
3.5
4.0
Cellular_organisms - ps1
Archaea1 - ps2
Archaea2 - ps3
Archaea3 - ps4
Asgard/Eukaryota - ps5
Eukaryota - ps6
Amorpheae - ps7
Obazoa - ps8
Opisthokonta - ps9
Holozoa - ps10
Filozoa - ps11
Choano./Metazoa - ps12
Metazoa - ps13
Myriazoa - ps14
Parahoxozoa - ps15
Bilateria - ps16
Deuterostomia - ps17
Chordata - ps18
Olfactores - ps19
Vertebrata - ps20
Gnathostomata - ps21
Euteleostomi - ps22
Sarcopterygii - ps23
Tetrapoda - ps24
Amniota - ps25
Mammalia - ps26
Theria - ps27
Placentalia - ps28
Boreoeutheria - ps29
Euarchontoglires - ps30
Euarchonta - ps31
Primates - ps32
Haplorrhini - ps33
Simiiformes - ps34
Catarrhini - ps35
Hominoidea - ps36
Hominidae - ps37
Homininae - ps38
Hominini - ps39
Homo_sapiens - ps40
Phylostratum

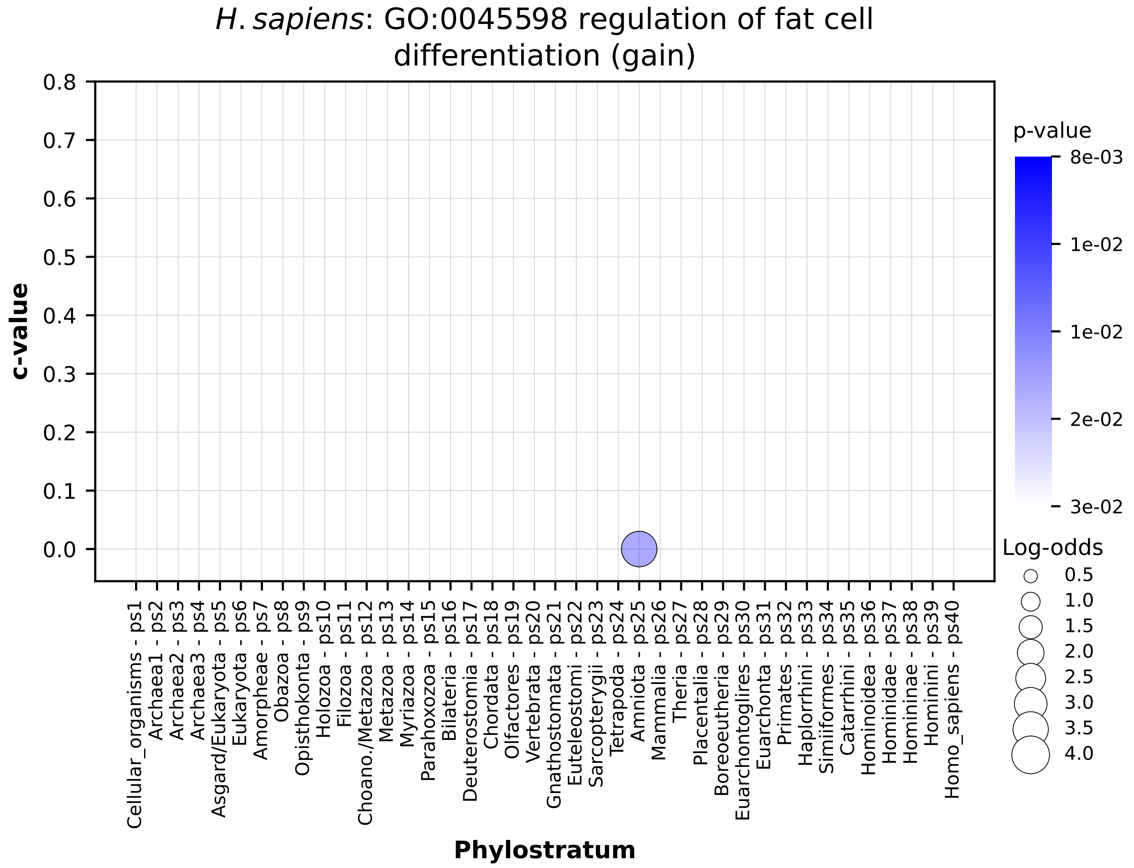

*H. sapiens*: GO:0045598 regulation of fat cell differentiation (gain)
c-value
0.8
0.7
0.6
0.5
0.4
0.3
0.2
0.1
0.0
p-value
8e-03
1e-02
1e-02
2e-02
3e-02
Log-odds
0.5
1.0
1.5
2.0
2.5
3.0
3.5
4.0
Cellular_organisms - ps1
Archaea1 - ps2
Archaea2 - ps3
Archaea3 - ps4
Asgard/Eukaryota - ps5
Eukaryota - ps6
Amorpheae - ps7
Obazoa - ps8
Opisthokonta - ps9
Holozoa - ps10
Filozoa - ps11
Choano./Metazoa - ps12
Metazoa - ps13
Myriazoa - ps14
Parahoxozoa - ps15
Bilateria - ps16
Deuterostomia - ps17
Chordata - ps18
Olfactores - ps19
Vertebrata - ps20
Gnathostomata - ps21
Euteleostomi - ps22
Sarcopterygii - ps23
Tetrapoda - ps24
Amniota - ps25
Mammalia - ps26
Theria - ps27
Placentalia - ps28
Boreoeutheria - ps29
Euarchontoglires - ps30
Euarchonta - ps31
Primates - ps32
Haplorrhini - ps33
Simiiformes - ps34
Catarrhini - ps35
Hominoidea - ps36
Hominidae - ps37
Homininae - ps38
Hominini - ps39
Homo_sapiens - ps40
Phylostratum

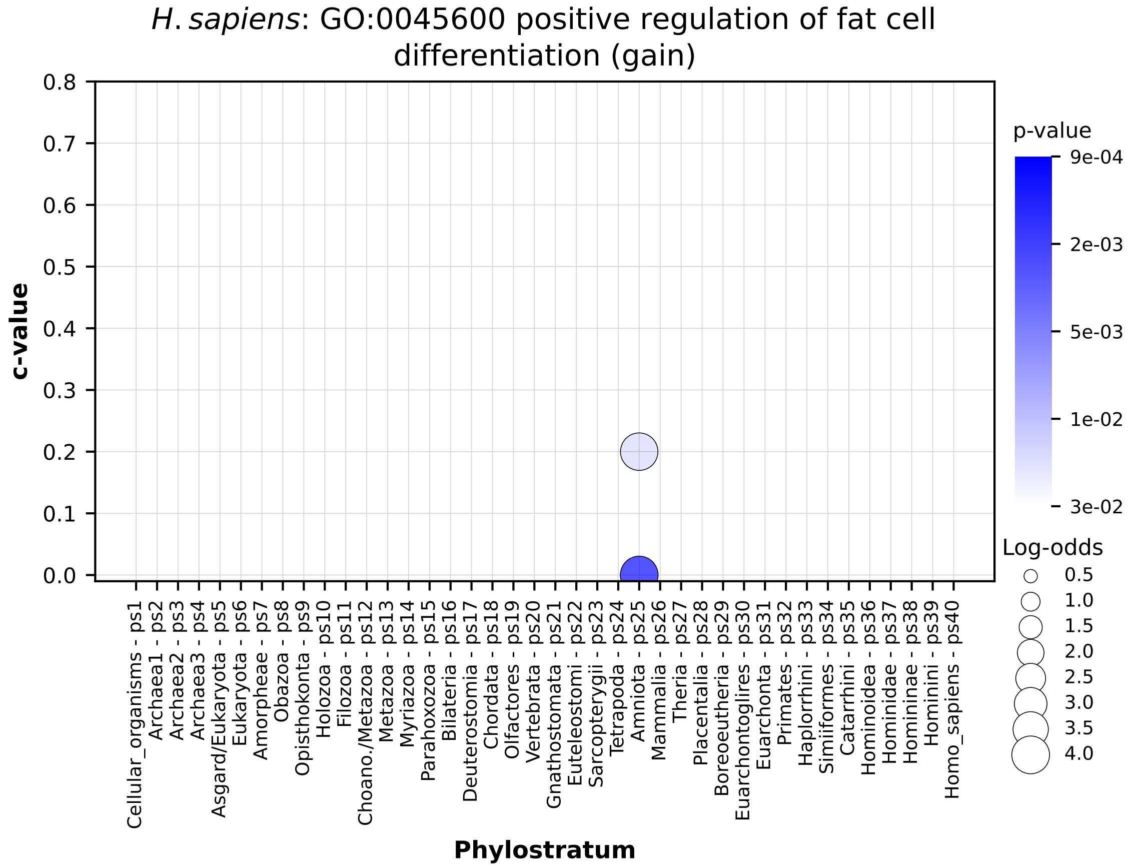

H. sapiens: GO:0045600 positive regulation of fat cell differentiation (gain)
c-value
0.8
0.7
0.6
0.5
0.4
0.3
0.2
0.1
0.0
Phylostratum
Cellular_organisms - ps1
Archaea1 - ps2
Archaea2 - ps3
Archaea3 - ps4
Asgard/Eukaryota - ps5
Eukaryota - ps6
Amorpheae - ps7
Obazoa - ps8
Opisthokonta - ps9
Holozoa - ps10
Filozoa - ps11
Choano./Metazoa - ps12
Metazoa - ps13
Myriazoa - ps14
Parahoxozoa - ps15
Bilateria - ps16
Deuterostomia - ps17
Chordata - ps18
Olfactores - ps19
Vertebrata - ps20
Gnathostomata - ps21
Euteleostomi - ps22
Sarcopterygii - ps23
Tetrapoda - ps24
Amniota - ps25
Mammalia - ps26
Theria - ps27
Placentalia - ps28
Boreoeutheria - ps29
Euarchontoglires - ps30
Euarchonta - ps31
Primates - ps32
Haplorrhini - ps33
Simiiformes - ps34
Catarrhini - ps35
Hominoidea - ps36
Hominidae - ps37
Homininae - ps38
Hominini - ps39
Homo_sapiens - ps40
p-value
9e-04
2e-03
5e-03
1e-02
3e-02
Log-odds
0.5
1.0
1.5
2.0
2.5
3.0
3.5
4.0

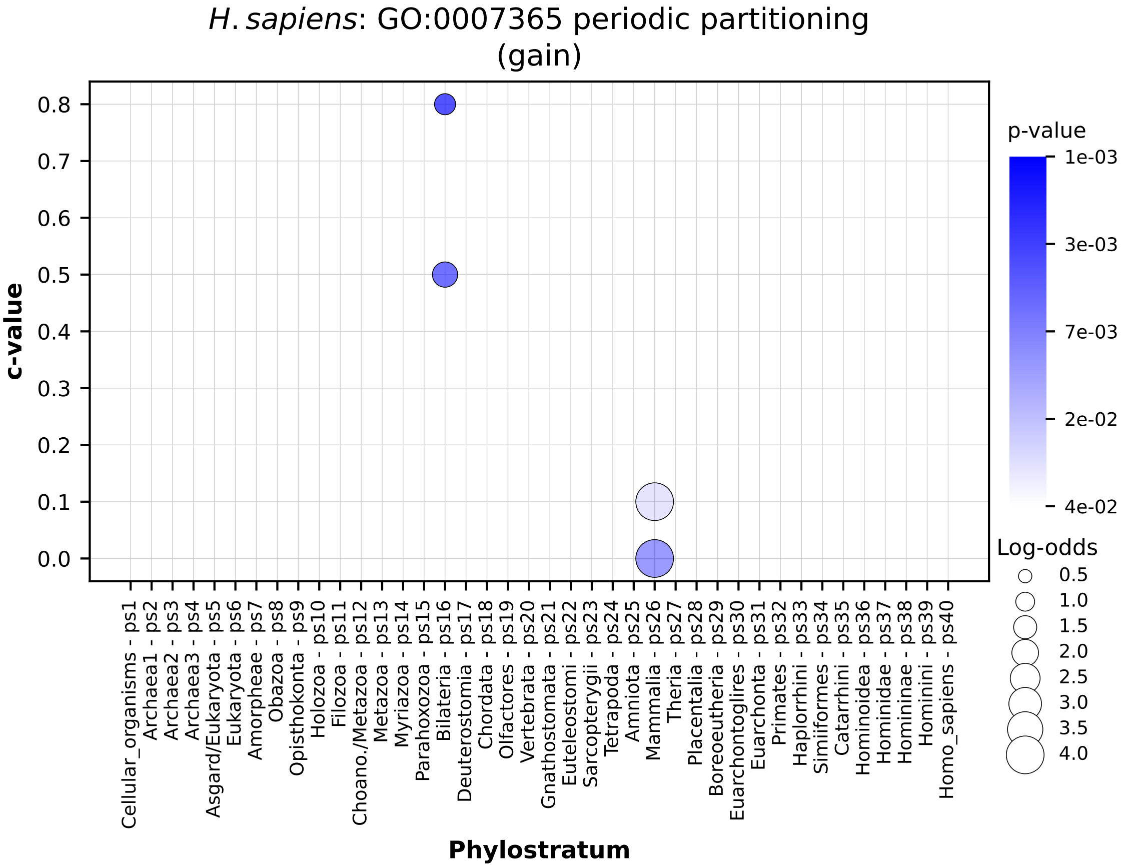

*H. sapiens*: GO:0007365 periodic partitioning
(gain)
c-value
0.8
0.7
0.6
0.5
0.4
0.3
0.2
0.1
0.0
p-value
1e-03
3e-03
7e-03
2e-02
4e-02
Log-odds
0.5
1.0
1.5
2.0
2.5
3.0
3.5
4.0
Cellular_organisms - ps1
Archaea1 - ps2
Archaea2 - ps3
Archaea3 - ps4
Asgard/Eukaryota - ps5
Eukaryota - ps6
Amorpheae - ps7
Obazoa - ps8
Opisthokonta - ps9
Holozoa - ps10
Filozoa - ps11
Choano./Metazoa - ps12
Metazoa - ps13
Myriazoa - ps14
Parahoxozoa - ps15
Bilateria - ps16
Deuterostomia - ps17
Chordata - ps18
Olfactores - ps19
Vertebrata - ps20
Gnathostomata - ps21
Euteleostomi - ps22
Sarcopterygii - ps23
Tetrapoda - ps24
Amniota - ps25
Mammalia - ps26
Theria - ps27
Placentalia - ps28
Boreoeutheria - ps29
Euarchontoglires - ps30
Euarchonta - ps31
Primates - ps32
Haplorrhini - ps33
Simiiformes - ps34
Catarrhini - ps35
Hominoidea - ps36
Hominidae - ps37
Homininae - ps38
Hominini - ps39
Homo_sapiens - ps40
Phylostratum

# *H. sapiens*: GO:0014068 obsolete positive regulation of phosphatidylinositol 3-kinase signaling (gain)

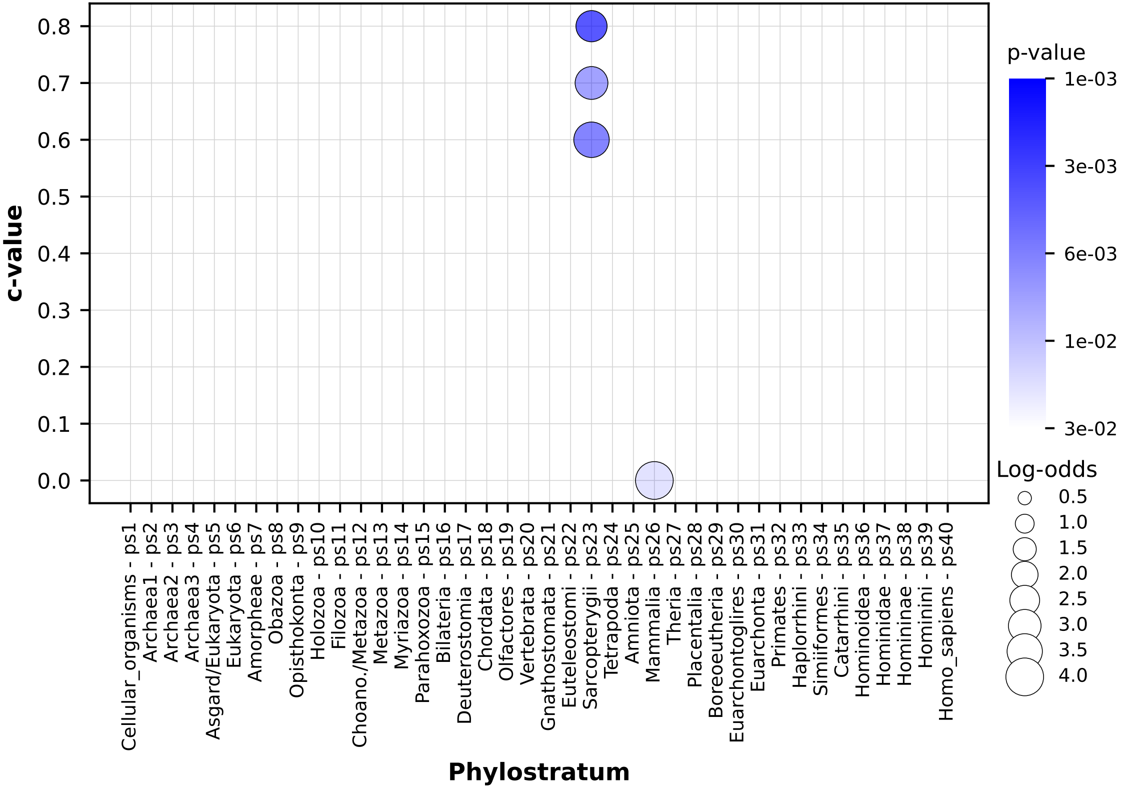

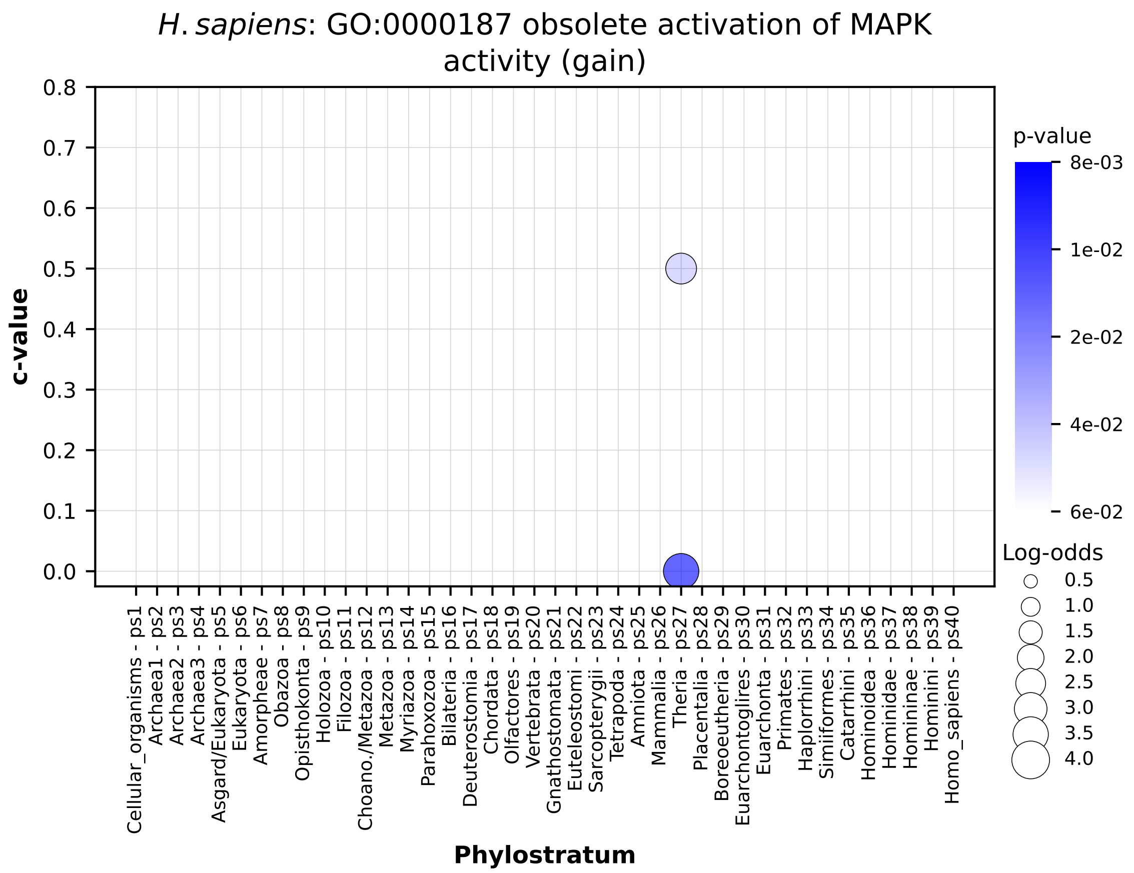

H. sapiens: GO:0000187 obsolete activation of MAPK activity (gain)
c-value
0.8
0.7
0.6
0.5
0.4
0.3
0.2
0.1
0.0
Phylostratum
Cellular_organisms - ps1
Archaea1 - ps2
Archaea2 - ps3
Archaea3 - ps4
Asgard/Eukaryota - ps5
Eukaryota - ps6
Amorpheae - ps7
Obazoa - ps8
Opisthokonta - ps9
Holozoa - ps10
Filozoa - ps11
Choano./Metazoa - ps12
Metazoa - ps13
Myriazoa - ps14
Parahoxozoa - ps15
Bilateria - ps16
Deuterostomia - ps17
Chordata - ps18
Olfactores - ps19
Vertebrata - ps20
Gnathostomata - ps21
Euteleostomi - ps22
Sarcopterygii - ps23
Tetrapoda - ps24
Amniota - ps25
Mammalia - ps26
Theria - ps27
Placentalia - ps28
Boreoeutheria - ps29
Euarchontoglires - ps30
Euarchonta - ps31
Primates - ps32
Haplorrhini - ps33
Simiiformes - ps34
Catarrhini - ps35
Hominoidea - ps36
Hominidae - ps37
Homininae - ps38
Hominini - ps39
Homo_sapiens - ps40
p-value
8e-03
1e-02
2e-02
4e-02
6e-02
Log-odds
0.5
1.0
1.5
2.0
2.5
3.0
3.5
4.0

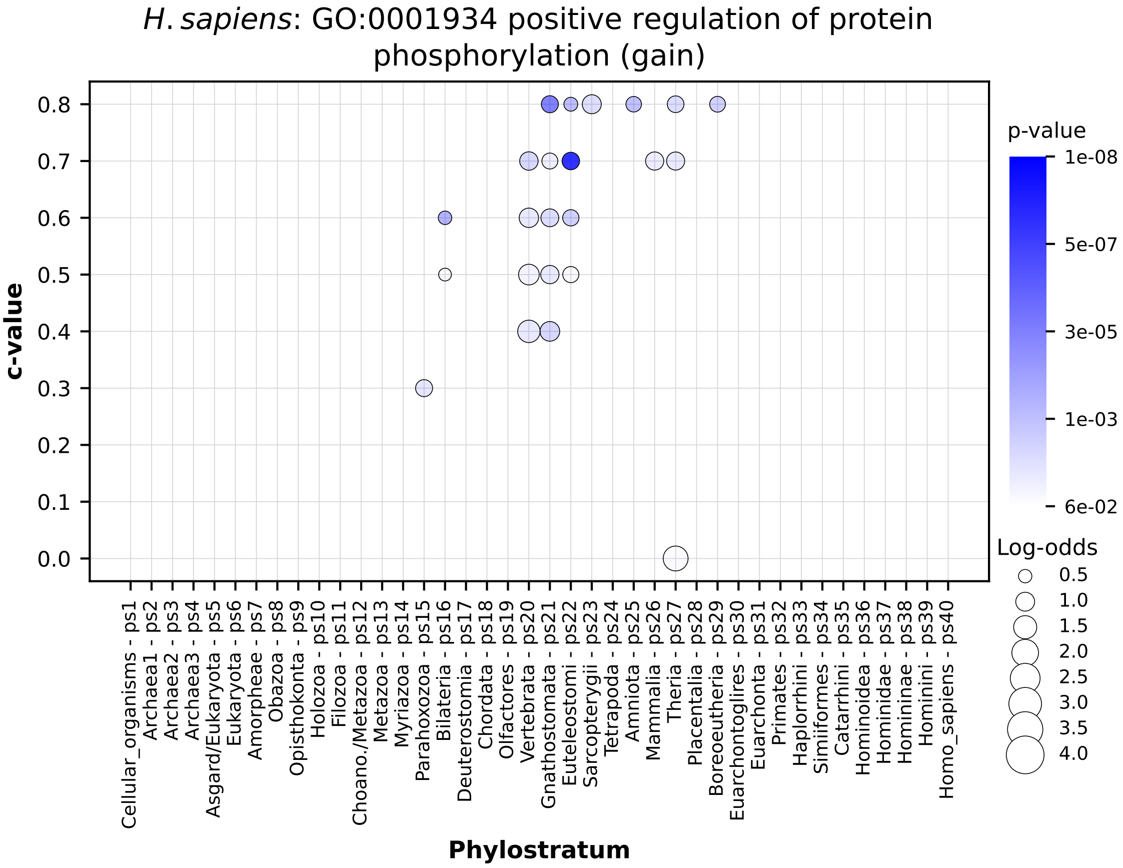
H. sapiens: GO:0001934 positive regulation of protein phosphorylation (gain)
c-value
0.8
0.7
0.6
0.5
0.4
0.3
0.2
0.1
0.0
p-value
1e-08
5e-07
3e-05
1e-03
6e-02
Log-odds
0.5
1.0
1.5
2.0
2.5
3.0
3.5
4.0
Cellular_organisms - ps1
Archaea1 - ps2
Archaea2 - ps3
Archaea3 - ps4
Asgard/Eukaryota - ps5
Eukaryota - ps6
Amorpheae - ps7
Obazoa - ps8
Opisthokonta - ps9
Holozoa - ps10
Filozoa - ps11
Choano./Metazoa - ps12
Metazoa - ps13
Myriazoa - ps14
Parahoxozoa - ps15
Bilateria - ps16
Deuterostomia - ps17
Chordata - ps18
Olfactores - ps19
Vertebrata - ps20
Gnathostomata - ps21
Euteleostomi - ps22
Sarcopterygii - ps23
Tetrapoda - ps24
Amniota - ps25
Mammalia - ps26
Theria - ps27
Placentalia - ps28
Boreoeutheria - ps29
Euarchontoglires - ps30
Euarchonta - ps31
Primates - ps32
Haplorrhini - ps33
Simiiformes - ps34
Catarrhini - ps35
Hominoidea - ps36
Hominidae - ps37
Homininae - ps38
Hominini - ps39
Homo_sapiens - ps40
Phylostratum

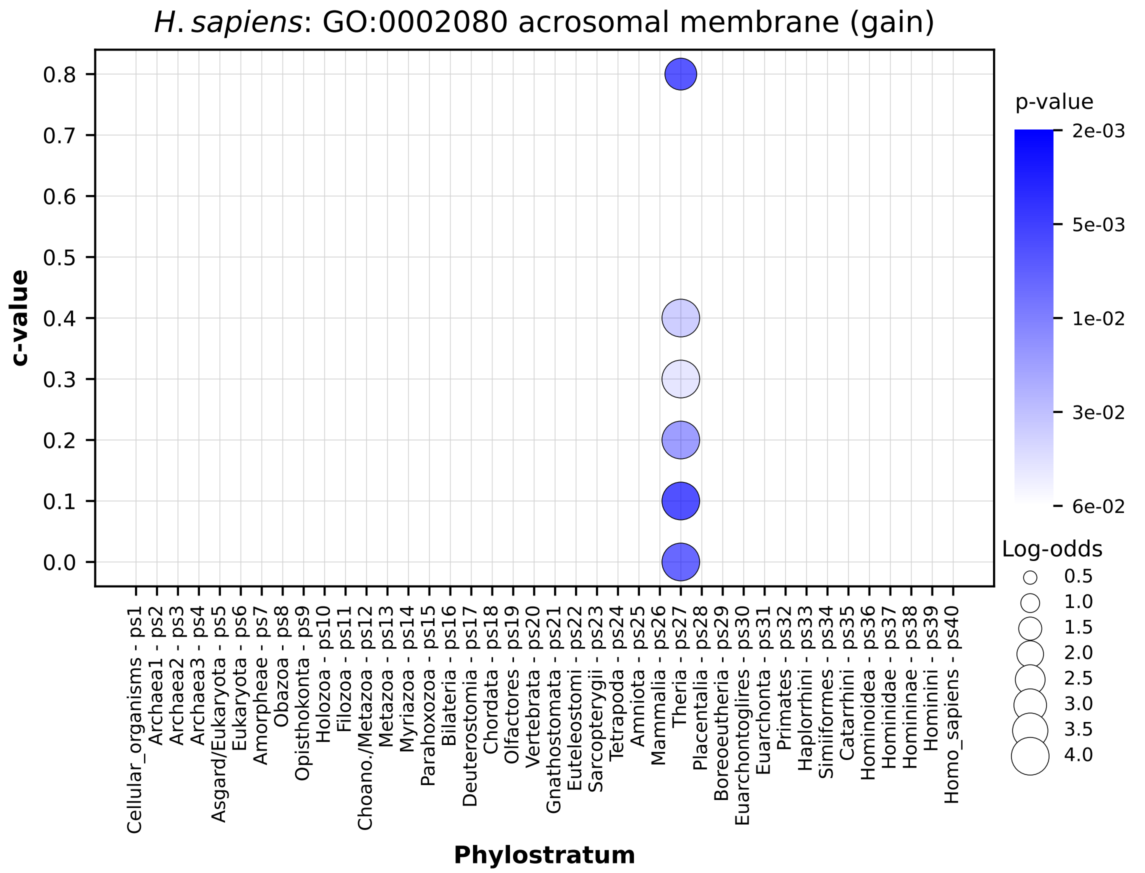
H. sapiens: GO:0002080 acrosomal membrane (gain)
c-value
0.0
0.1
0.2
0.3
0.4
0.5
0.6
0.7
0.8
Phylostratum
Cellular_organisms - ps1
Archaea1 - ps2
Archaea2 - ps3
Archaea3 - ps4
Asgard/Eukaryota - ps5
Eukaryota - ps6
Amorpheae - ps7
Obazoa - ps8
Opisthokonta - ps9
Holozoa - ps10
Filozoa - ps11
Choano./Metazoa - ps12
Metazoa - ps13
Myriazoa - ps14
Parahoxozoa - ps15
Bilateria - ps16
Deuterostomia - ps17
Chordata - ps18
Olfactores - ps19
Vertebrata - ps20
Gnathostomata - ps21
Euteleostomi - ps22
Sarcopterygii - ps23
Tetrapoda - ps24
Amniota - ps25
Mammalia - ps26
Theria - ps27
Placentalia - ps28
Boreoeutheria - ps29
Euarchontoglires - ps30
Euarchonta - ps31
Primates - ps32
Haplorrhini - ps33
Simiiformes - ps34
Catarrhini - ps35
Hominoidea - ps36
Hominidae - ps37
Homininae - ps38
Hominini - ps39
Homo_sapiens - ps40
p-value
2e-03
5e-03
1e-02
3e-02
6e-02
Log-odds
0.5
1.0
1.5
2.0
2.5
3.0
3.5
4.0

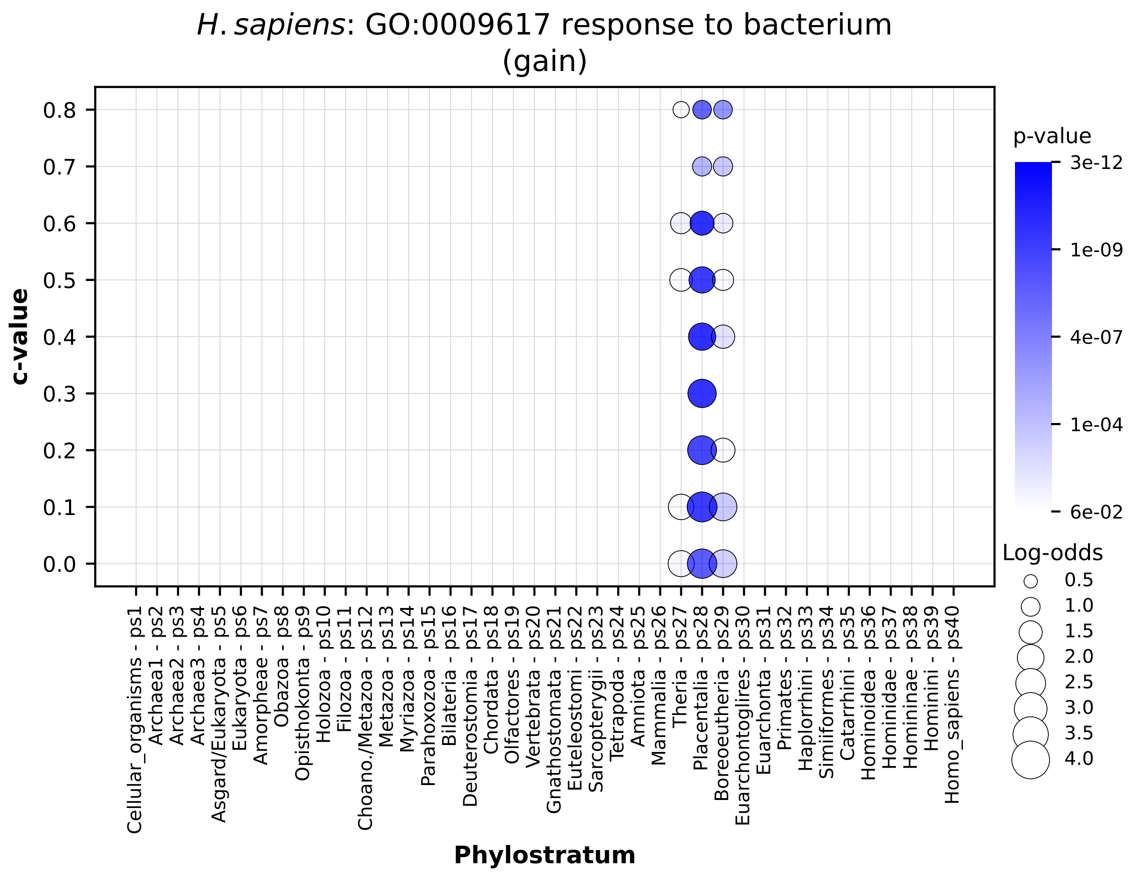
*H. sapiens*: GO:0009617 response to bacterium
(gain)
c-value
0.8
0.7
0.6
0.5
0.4
0.3
0.2
0.1
0.0
p-value
3e-12
1e-09
4e-07
1e-04
6e-02
Log-odds
0.5
1.0
1.5
2.0
2.5
3.0
3.5
4.0
Cellular_organisms - ps1
Archaea1 - ps2
Archaea2 - ps3
Archaea3 - ps4
Asgard/Eukaryota - ps5
Eukaryota - ps6
Amorpheae - ps7
Obazoa - ps8
Opisthokonta - ps9
Holozoa - ps10
Filozoa - ps11
Choano./Metazoa - ps12
Metazoa - ps13
Myriazoa - ps14
Parahoxozoa - ps15
Bilateria - ps16
Deuterostomia - ps17
Chordata - ps18
Olfactores - ps19
Vertebrata - ps20
Gnathostomata - ps21
Euteleostomi - ps22
Sarcopterygii - ps23
Tetrapoda - ps24
Amniota - ps25
Mammalia - ps26
Theria - ps27
Placentalia - ps28
Boreoeutheria - ps29
Euarchontoglires - ps30
Euarchonta - ps31
Primates - ps32
Haplorrhini - ps33
Simiiformes - ps34
Catarrhini - ps35
Hominoidea - ps36
Hominidae - ps37
Homininae - ps38
Hominini - ps39
Homo_sapiens - ps40
Phylostratum

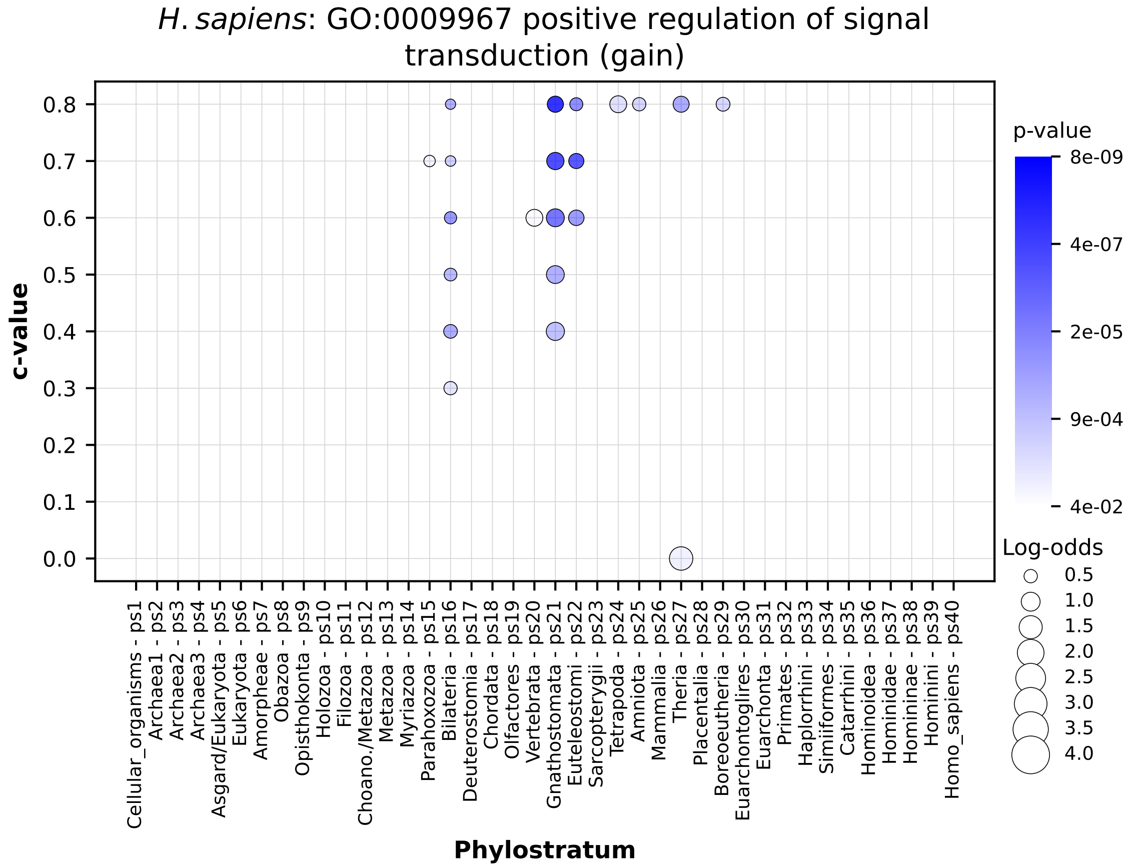

*H. sapiens*: GO:0009967 positive regulation of signal transduction (gain)
c-value
0.8
0.7
0.6
0.5
0.4
0.3
0.2
0.1
0.0
Phylostratum
Cellular_organisms - ps1
Archaea1 - ps2
Archaea2 - ps3
Archaea3 - ps4
Asgard/Eukaryota - ps5
Eukaryota - ps6
Amorpheae - ps7
Obazoa - ps8
Opisthokonta - ps9
Holozoa - ps10
Filozoa - ps11
Choano./Metazoa - ps12
Metazoa - ps13
Myriazoa - ps14
Parahoxozoa - ps15
Bilateria - ps16
Deuterostomia - ps17
Chordata - ps18
Olfactores - ps19
Vertebrata - ps20
Gnathostomata - ps21
Euteleostomi - ps22
Sarcopterygii - ps23
Tetrapoda - ps24
Amniota - ps25
Mammalia - ps26
Theria - ps27
Placentalia - ps28
Boreoeutheria - ps29
Euarchontoglires - ps30
Euarchonta - ps31
Primates - ps32
Haplorrhini - ps33
Simiiformes - ps34
Catarrhini - ps35
Hominoidea - ps36
Hominidae - ps37
Homininae - ps38
Hominini - ps39
Homo_sapiens - ps40
p-value
8e-09
4e-07
2e-05
9e-04
4e-02
Log-odds
0.5
1.0
1.5
2.0
2.5
3.0
3.5
4.0

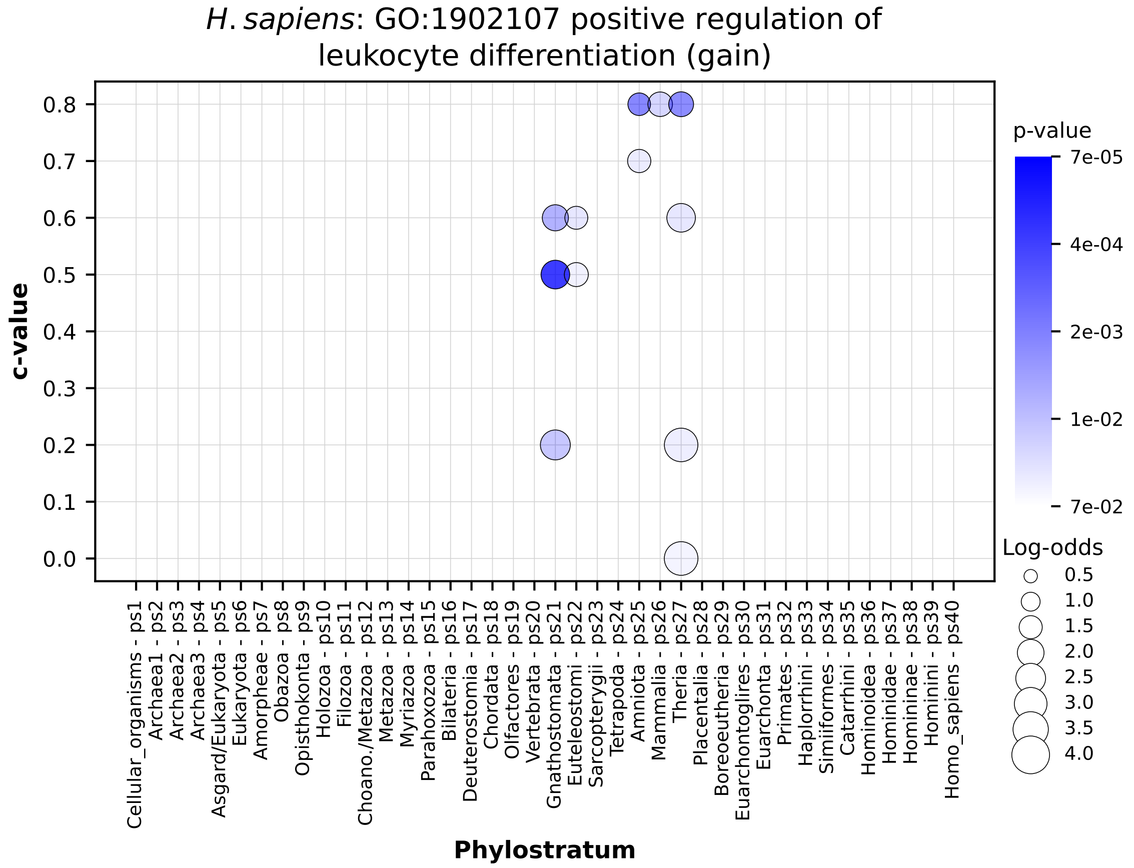

*H. sapiens*: GO:1902107 positive regulation of leukocyte differentiation (gain)
c-value
0.8
0.7
0.6
0.5
0.4
0.3
0.2
0.1
0.0
Phylostratum
Cellular_organisms - ps1
Archaea1 - ps2
Archaea2 - ps3
Archaea3 - ps4
Asgard/Eukaryota - ps5
Eukaryota - ps6
Amorpheae - ps7
Obazoa - ps8
Opisthokonta - ps9
Holozoa - ps10
Filozoa - ps11
Choano./Metazoa - ps12
Metazoa - ps13
Myriazoa - ps14
Parahoxozoa - ps15
Bilateria - ps16
Deuterostomia - ps17
Chordata - ps18
Olfactores - ps19
Vertebrata - ps20
Gnathostomata - ps21
Euteleostomi - ps22
Sarcopterygii - ps23
Tetrapoda - ps24
Amniota - ps25
Mammalia - ps26
Theria - ps27
Placentalia - ps28
Boreoeutheria - ps29
Euarchontoglires - ps30
Euarchonta - ps31
Primates - ps32
Haplorrhini - ps33
Simiiformes - ps34
Catarrhini - ps35
Hominoidea - ps36
Hominidae - ps37
Homininae - ps38
Hominini - ps39
Homo_sapiens - ps40
p-value
7e-05
4e-04
2e-03
1e-02
7e-02
Log-odds
0.5
1.0
1.5
2.0
2.5
3.0
3.5
4.0

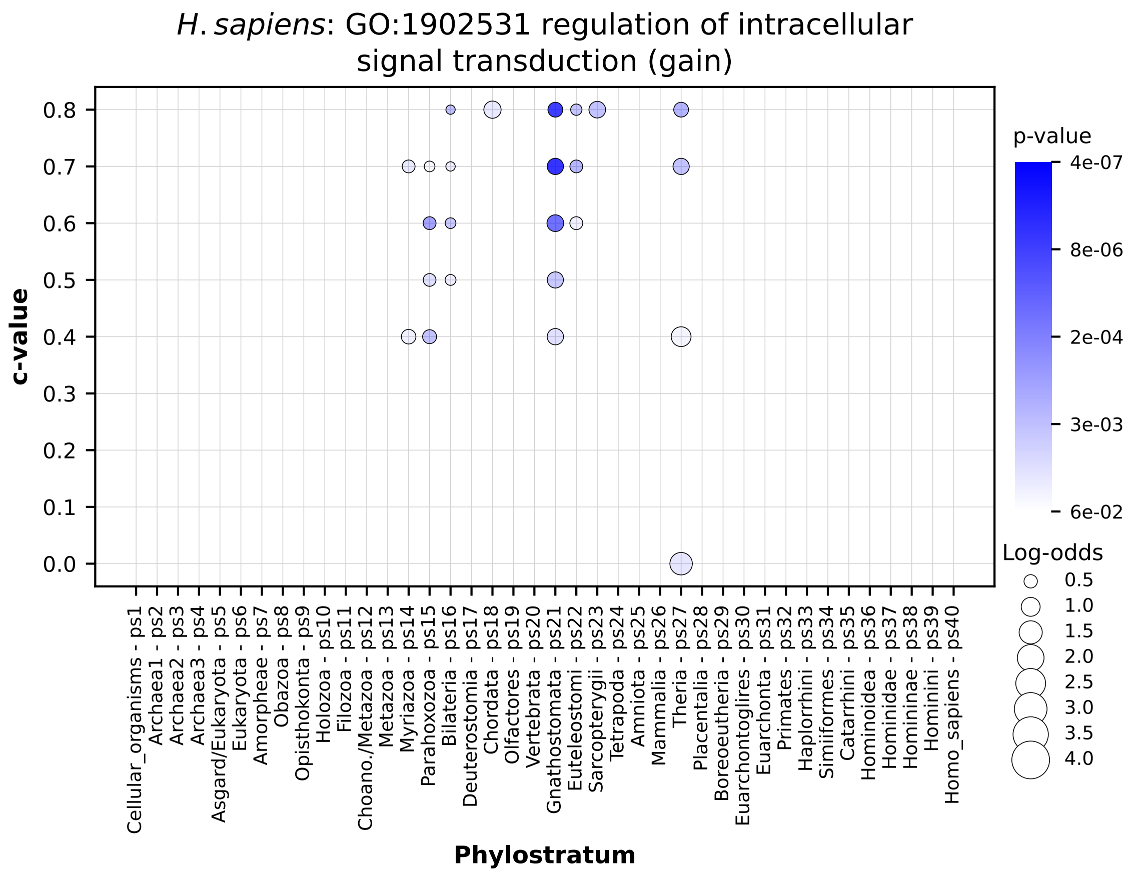

H. sapiens: GO:1902531 regulation of intracellular signal transduction (gain)
c-value
0.8
0.7
0.6
0.5
0.4
0.3
0.2
0.1
0.0
Phylostratum
Cellular_organisms - ps1
Archaea1 - ps2
Archaea2 - ps3
Archaea3 - ps4
Asgard/Eukaryota - ps5
Eukaryota - ps6
Amorpheae - ps7
Obazoa - ps8
Opisthokonta - ps9
Holozoa - ps10
Filozoa - ps11
Choano./Metazoa - ps12
Metazoa - ps13
Myriazoa - ps14
Parahoxozoa - ps15
Bilateria - ps16
Deuterostomia - ps17
Chordata - ps18
Olfactores - ps19
Vertebrata - ps20
Gnathostomata - ps21
Euteleostomi - ps22
Sarcopterygii - ps23
Tetrapoda - ps24
Amniota - ps25
Mammalia - ps26
Theria - ps27
Placentalia - ps28
Boreoeutheria - ps29
Euarchontoglires - ps30
Euarchonta - ps31
Primates - ps32
Haplorrhini - ps33
Simiiformes - ps34
Catarrhini - ps35
Hominoidea - ps36
Hominidae - ps37
Homininae - ps38
Hominini - ps39
Homo_sapiens - ps40
p-value
4e-07
8e-06
2e-04
3e-03
6e-02
Log-odds
0.5
1.0
1.5
2.0
2.5
3.0
3.5
4.0

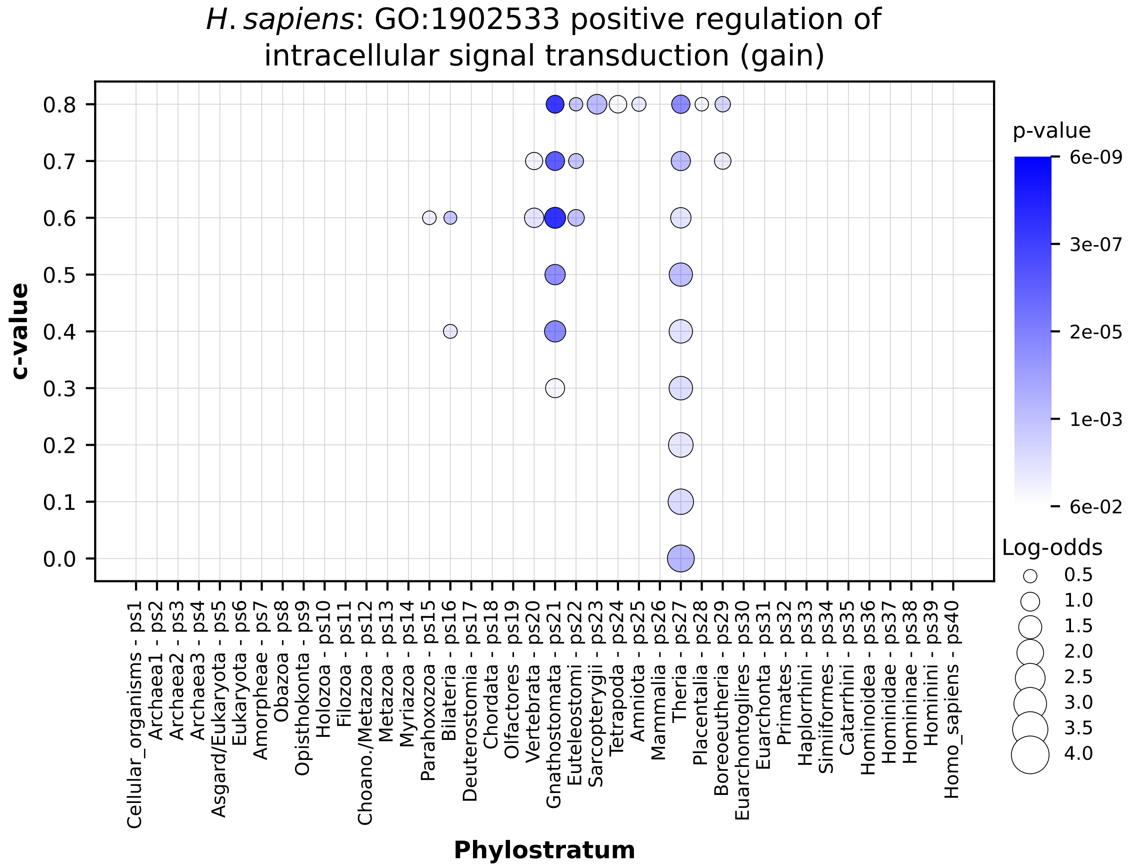
H. sapiens: GO:1902533 positive regulation of intracellular signal transduction (gain)
c-value
0.8
0.7
0.6
0.5
0.4
0.3
0.2
0.1
0.0
Phylostratum
Cellular_organisms - ps1
Archaea1 - ps2
Archaea2 - ps3
Archaea3 - ps4
Asgard/Eukaryota - ps5
Eukaryota - ps6
Amorpheae - ps7
Obazoa - ps8
Opisthokonta - ps9
Holozoa - ps10
Filozoa - ps11
Choano./Metazoa - ps12
Metazoa - ps13
Myriazoa - ps14
Parahoxozoa - ps15
Bilateria - ps16
Deuterostomia - ps17
Chordata - ps18
Olfactores - ps19
Vertebrata - ps20
Gnathostomata - ps21
Euteleostomi - ps22
Sarcopterygii - ps23
Tetrapoda - ps24
Amniota - ps25
Mammalia - ps26
Theria - ps27
Placentalia - ps28
Boreoeutheria - ps29
Euarchontoglires - ps30
Euarchonta - ps31
Primates - ps32
Haplorrhini - ps33
Simiiformes - ps34
Catarrhini - ps35
Hominoidea - ps36
Hominidae - ps37
Homininae - ps38
Hominini - ps39
Homo_sapiens - ps40
p-value
6e-09
3e-07
2e-05
1e-03
6e-02
Log-odds
0.5
1.0
1.5
2.0
2.5
3.0
3.5
4.0

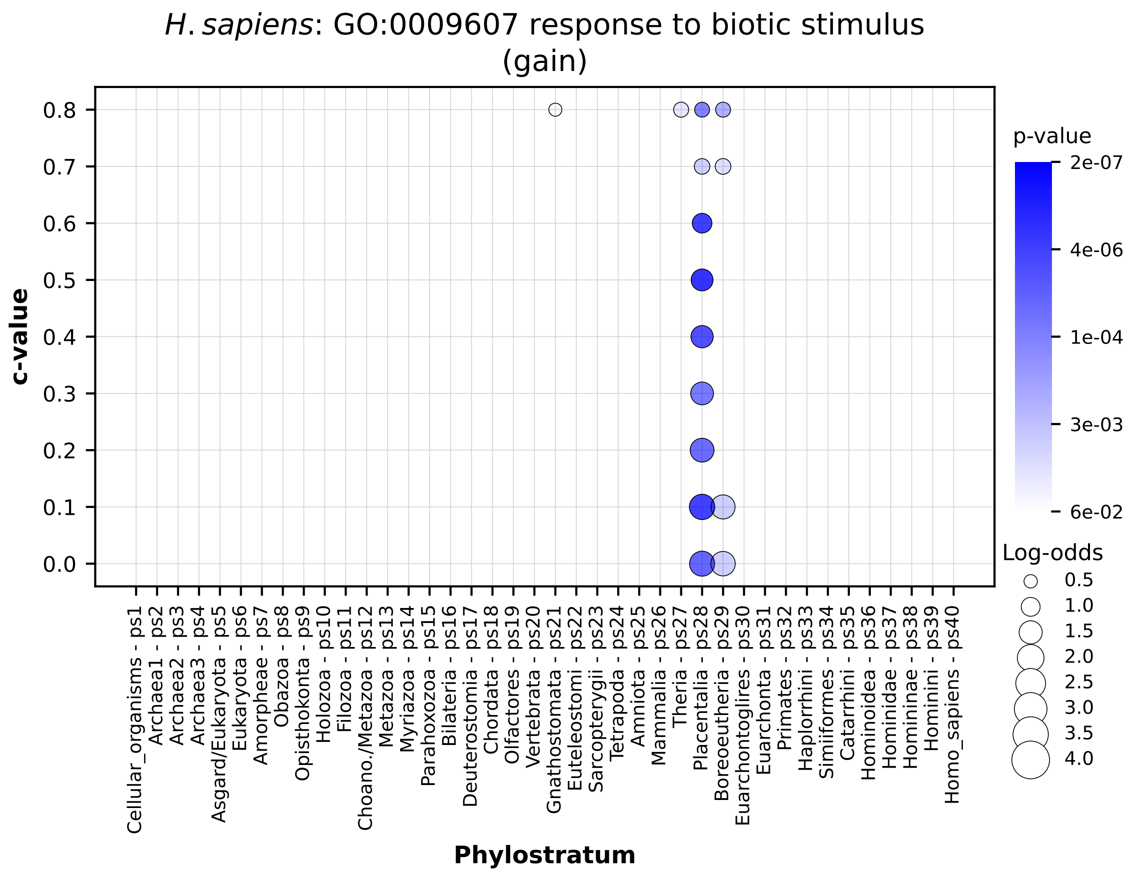
*H. sapiens*: GO:0009607 response to biotic stimulus (gain)
c-value
0.8
0.7
0.6
0.5
0.4
0.3
0.2
0.1
0.0
Phylostratum
Cellular_organisms - ps1
Archaea1 - ps2
Archaea2 - ps3
Archaea3 - ps4
Asgard/Eukaryota - ps5
Eukaryota - ps6
Amorpheae - ps7
Obazoa - ps8
Opisthokonta - ps9
Holozoa - ps10
Filozoa - ps11
Choano./Metazoa - ps12
Metazoa - ps13
Myriazoa - ps14
Parahoxozoa - ps15
Bilateria - ps16
Deuterostomia - ps17
Chordata - ps18
Olfactores - ps19
Vertebrata - ps20
Gnathostomata - ps21
Euteleostomi - ps22
Sarcopterygii - ps23
Tetrapoda - ps24
Amniota - ps25
Mammalia - ps26
Theria - ps27
Placentalia - ps28
Boreoeutheria - ps29
Euarchontoglires - ps30
Euarchonta - ps31
Primates - ps32
Haplorrhini - ps33
Simiiformes - ps34
Catarrhini - ps35
Hominoidea - ps36
Hominidae - ps37
Homininae - ps38
Hominini - ps39
Homo_sapiens - ps40
p-value
2e-07
4e-06
1e-04
3e-03
6e-02
Log-odds
0.5
1.0
1.5
2.0
2.5
3.0
3.5
4.0

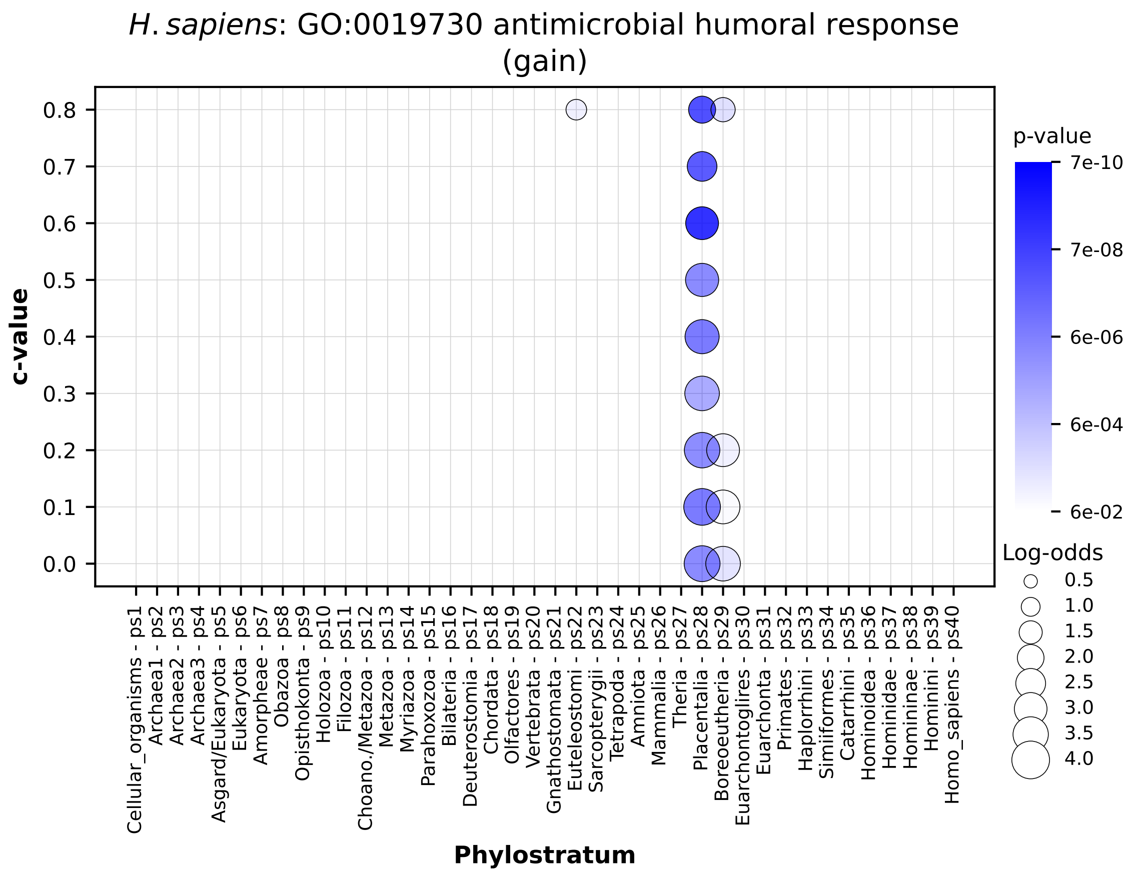
H. sapiens: GO:0019730 antimicrobial humoral response (gain)
c-value
0.8
0.7
0.6
0.5
0.4
0.3
0.2
0.1
0.0
p-value
7e-10
7e-08
6e-06
6e-04
6e-02
Log-odds
0.5
1.0
1.5
2.0
2.5
3.0
3.5
4.0
Cellular_organisms - ps1
Archaea1 - ps2
Archaea2 - ps3
Archaea3 - ps4
Asgard/Eukaryota - ps5
Eukaryota - ps6
Amorpheae - ps7
Obazoa - ps8
Opisthokonta - ps9
Holozoa - ps10
Filozoa - ps11
Choano./Metazoa - ps12
Metazoa - ps13
Myriazoa - ps14
Parahoxozoa - ps15
Bilateria - ps16
Deuterostomia - ps17
Chordata - ps18
Olfactores - ps19
Vertebrata - ps20
Gnathostomata - ps21
Euteleostomi - ps22
Sarcopterygii - ps23
Tetrapoda - ps24
Amniota - ps25
Mammalia - ps26
Theria - ps27
Placentalia - ps28
Boreoeutheria - ps29
Euarchontoglires - ps30
Euarchonta - ps31
Primates - ps32
Haplorrhini - ps33
Simiiformes - ps34
Catarrhini - ps35
Hominoidea - ps36
Hominidae - ps37
Homininae - ps38
Hominini - ps39
Homo_sapiens - ps40
Phylostratum

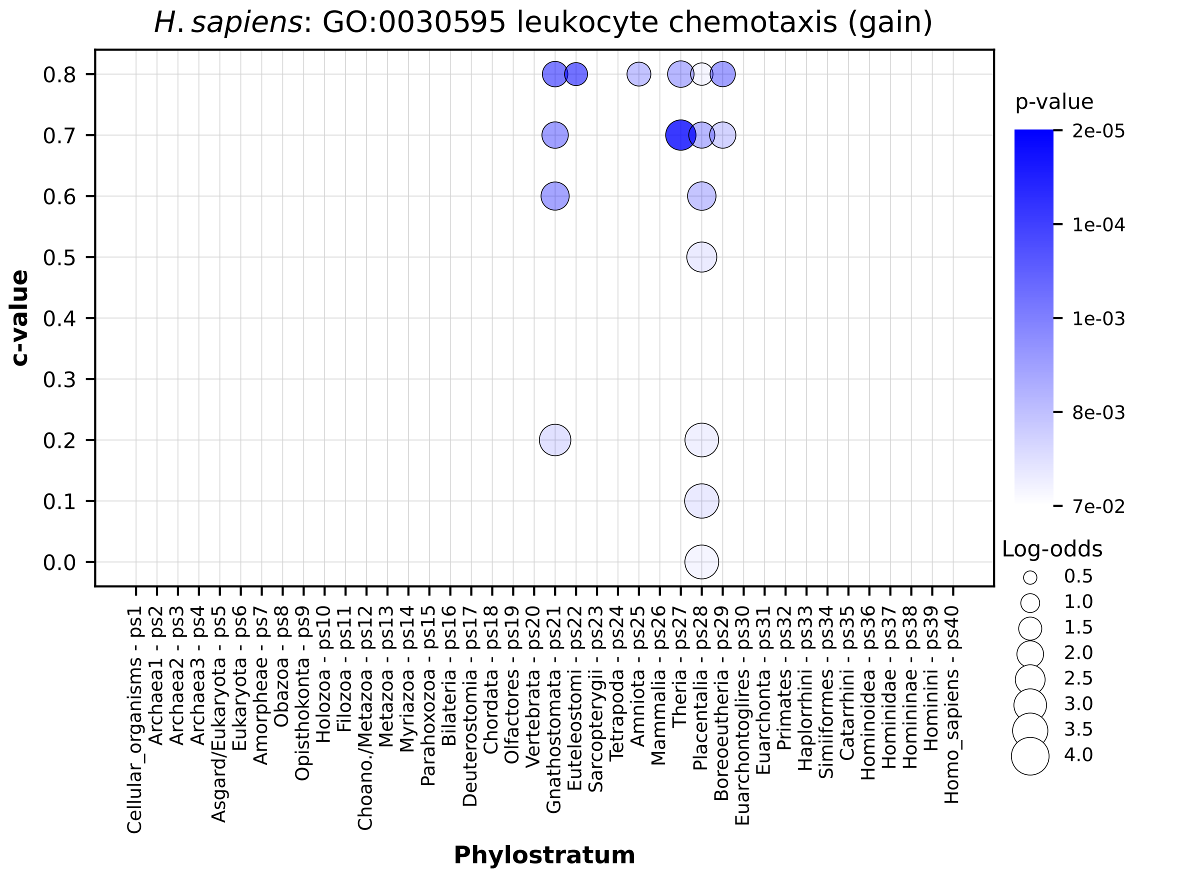
H. sapiens: GO:0030595 leukocyte chemotaxis (gain)
c-value
0.8
0.7
0.6
0.5
0.4
0.3
0.2
0.1
0.0
p-value
2e-05
1e-04
1e-03
8e-03
7e-02
Log-odds
0.5
1.0
1.5
2.0
2.5
3.0
3.5
4.0
Cellular_organisms - ps1
Archaea1 - ps2
Archaea2 - ps3
Archaea3 - ps4
Asgard/Eukaryota - ps5
Eukaryota - ps6
Amorpheae - ps7
Obazoa - ps8
Opisthokonta - ps9
Holozoa - ps10
Filozoa - ps11
Choano./Metazoa - ps12
Metazoa - ps13
Myriazoa - ps14
Parahoxozoa - ps15
Bilateria - ps16
Deuterostomia - ps17
Chordata - ps18
Olfactores - ps19
Vertebrata - ps20
Gnathostomata - ps21
Euteleostomi - ps22
Sarcopterygii - ps23
Tetrapoda - ps24
Amniota - ps25
Mammalia - ps26
Theria - ps27
Placentalia - ps28
Boreoeutheria - ps29
Euarchontoglires - ps30
Euarchonta - ps31
Primates - ps32
Haplorrhini - ps33
Simiiformes - ps34
Catarrhini - ps35
Hominoidea - ps36
Hominidae - ps37
Homininae - ps38
Hominini - ps39
Homo_sapiens - ps40
Phylostratum

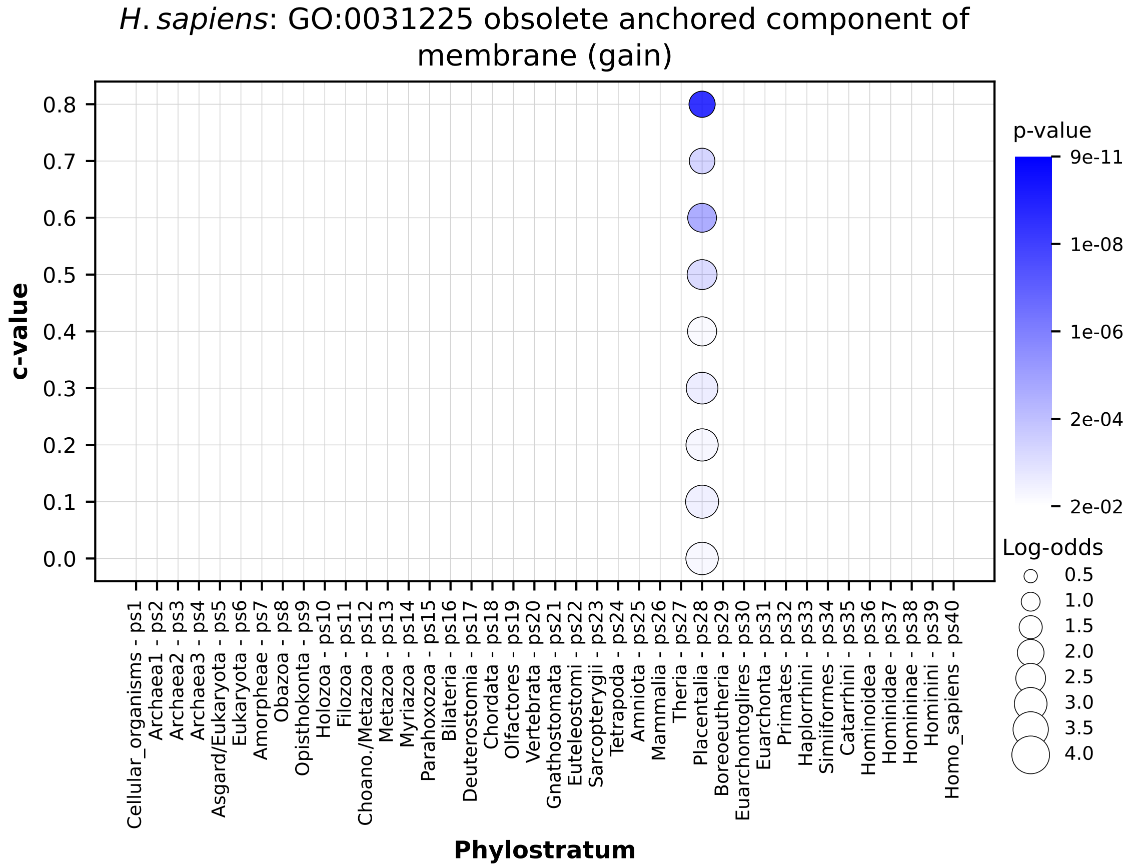

H. sapiens: GO:0031225 obsolete anchored component of membrane (gain)
c-value
0.8
0.7
0.6
0.5
0.4
0.3
0.2
0.1
0.0
p-value
9e-11
1e-08
1e-06
2e-04
2e-02
Log-odds
0.5
1.0
1.5
2.0
2.5
3.0
3.5
4.0
Cellular_organisms - ps1
Archaea1 - ps2
Archaea2 - ps3
Archaea3 - ps4
Asgard/Eukaryota - ps5
Eukaryota - ps6
Amorpheae - ps7
Obazoa - ps8
Opisthokonta - ps9
Holozoa - ps10
Filozoa - ps11
Choano./Metazoa - ps12
Metazoa - ps13
Myriazoa - ps14
Parahoxozoa - ps15
Bilateria - ps16
Deuterostomia - ps17
Chordata - ps18
Olfactores - ps19
Vertebrata - ps20
Gnathostomata - ps21
Euteleostomi - ps22
Sarcopterygii - ps23
Tetrapoda - ps24
Amniota - ps25
Mammalia - ps26
Theria - ps27
Placentalia - ps28
Boreoeutheria - ps29
Euarchontoglires - ps30
Euarchonta - ps31
Primates - ps32
Haplorrhini - ps33
Simiiformes - ps34
Catarrhini - ps35
Hominoidea - ps36
Hominidae - ps37
Homininae - ps38
Hominini - ps39
Homo_sapiens - ps40
Phylostratum

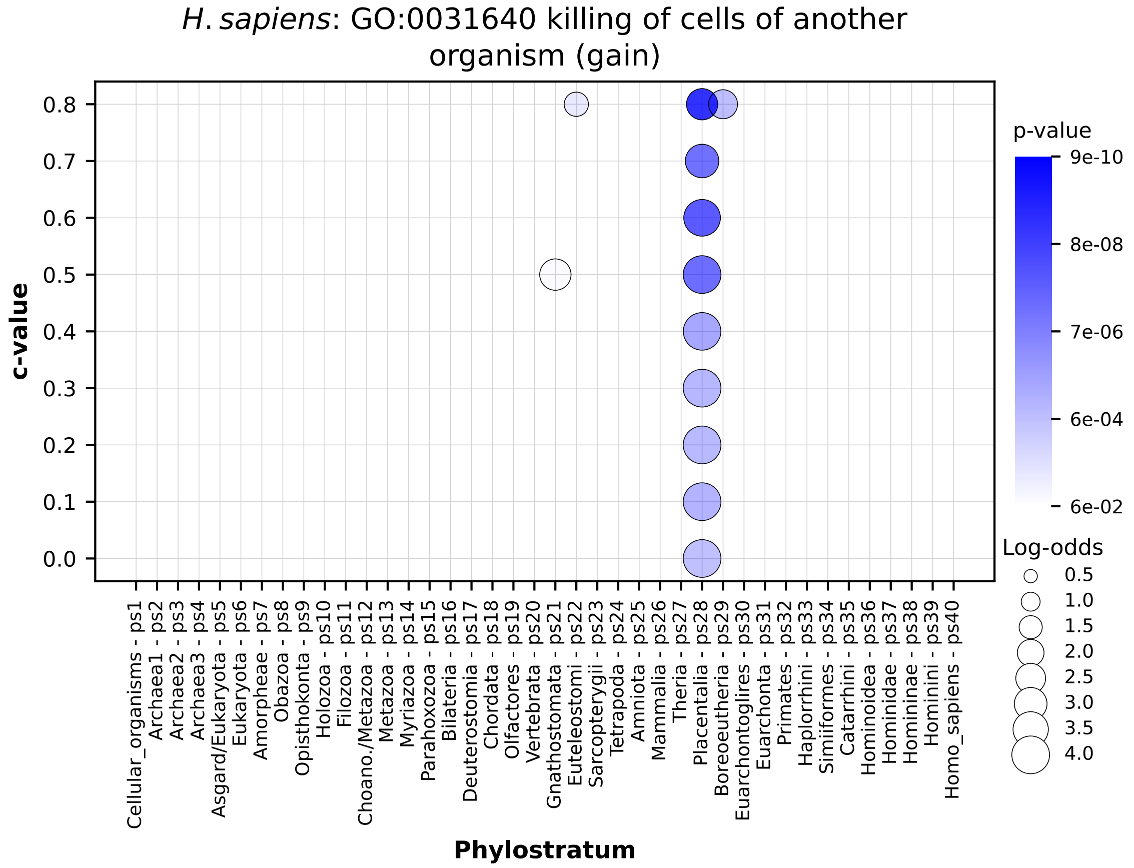

*H. sapiens*: GO:0031640 killing of cells of another organism (gain)
c-value
0.8
0.7
0.6
0.5
0.4
0.3
0.2
0.1
0.0
p-value
9e-10
8e-08
7e-06
6e-04
6e-02
Log-odds
0.5
1.0
1.5
2.0
2.5
3.0
3.5
4.0
Cellular_organisms - ps1
Archaea1 - ps2
Archaea2 - ps3
Archaea3 - ps4
Asgard/Eukaryota - ps5
Eukaryota - ps6
Amorpheae - ps7
Obazoa - ps8
Opisthokonta - ps9
Holozoa - ps10
Filozoa - ps11
Choano./Metazoa - ps12
Metazoa - ps13
Myriazoa - ps14
Parahoxozoa - ps15
Bilateria - ps16
Deuterostomia - ps17
Chordata - ps18
Olfactores - ps19
Vertebrata - ps20
Gnathostomata - ps21
Euteleostomi - ps22
Sarcopterygii - ps23
Tetrapoda - ps24
Amniota - ps25
Mammalia - ps26
Theria - ps27
Placentalia - ps28
Boreoeutheria - ps29
Euarchontoglires - ps30
Euarchonta - ps31
Primates - ps32
Haplorrhini - ps33
Simiiformes - ps34
Catarrhini - ps35
Hominoidea - ps36
Hominidae - ps37
Homininae - ps38
Hominini - ps39
Homo_sapiens - ps40
Phylostratum

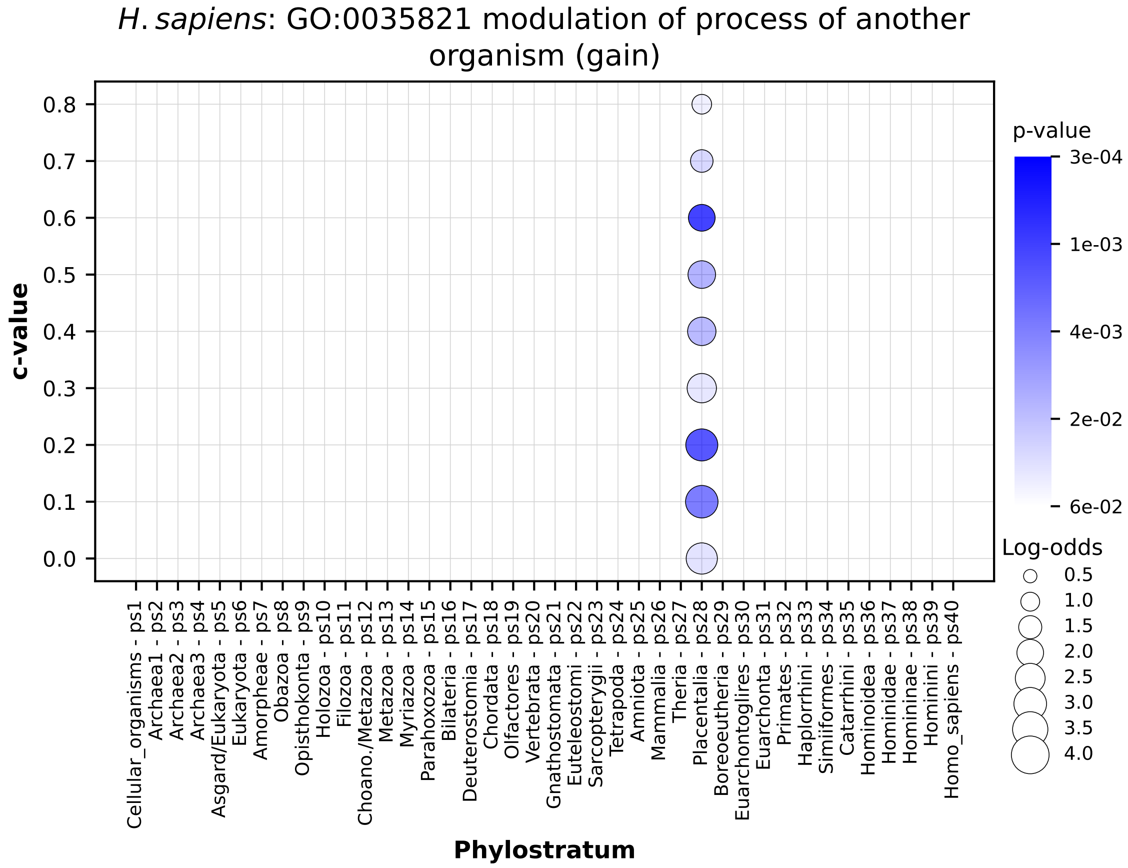
*H. sapiens*: GO:0035821 modulation of process of another organism (gain)
c-value
0.8
0.7
0.6
0.5
0.4
0.3
0.2
0.1
0.0
p-value
3e-04
1e-03
4e-03
2e-02
6e-02
Log-odds
0.5
1.0
1.5
2.0
2.5
3.0
3.5
4.0
Cellular_organisms - ps1
Archaea1 - ps2
Archaea2 - ps3
Archaea3 - ps4
Asgard/Eukaryota - ps5
Eukaryota - ps6
Amorpheae - ps7
Obazoa - ps8
Opisthokonta - ps9
Holozoa - ps10
Filozoa - ps11
Choano./Metazoa - ps12
Metazoa - ps13
Myriazoa - ps14
Parahoxozoa - ps15
Bilateria - ps16
Deuterostomia - ps17
Chordata - ps18
Olfactores - ps19
Vertebrata - ps20
Gnathostomata - ps21
Euteleostomi - ps22
Sarcopterygii - ps23
Tetrapoda - ps24
Amniota - ps25
Mammalia - ps26
Theria - ps27
Placentalia - ps28
Boreoeutheria - ps29
Euarchontoglires - ps30
Euarchonta - ps31
Primates - ps32
Haplorrhini - ps33
Simiiformes - ps34
Catarrhini - ps35
Hominoidea - ps36
Hominidae - ps37
Homininae - ps38
Hominini - ps39
Homo_sapiens - ps40
Phylostratum

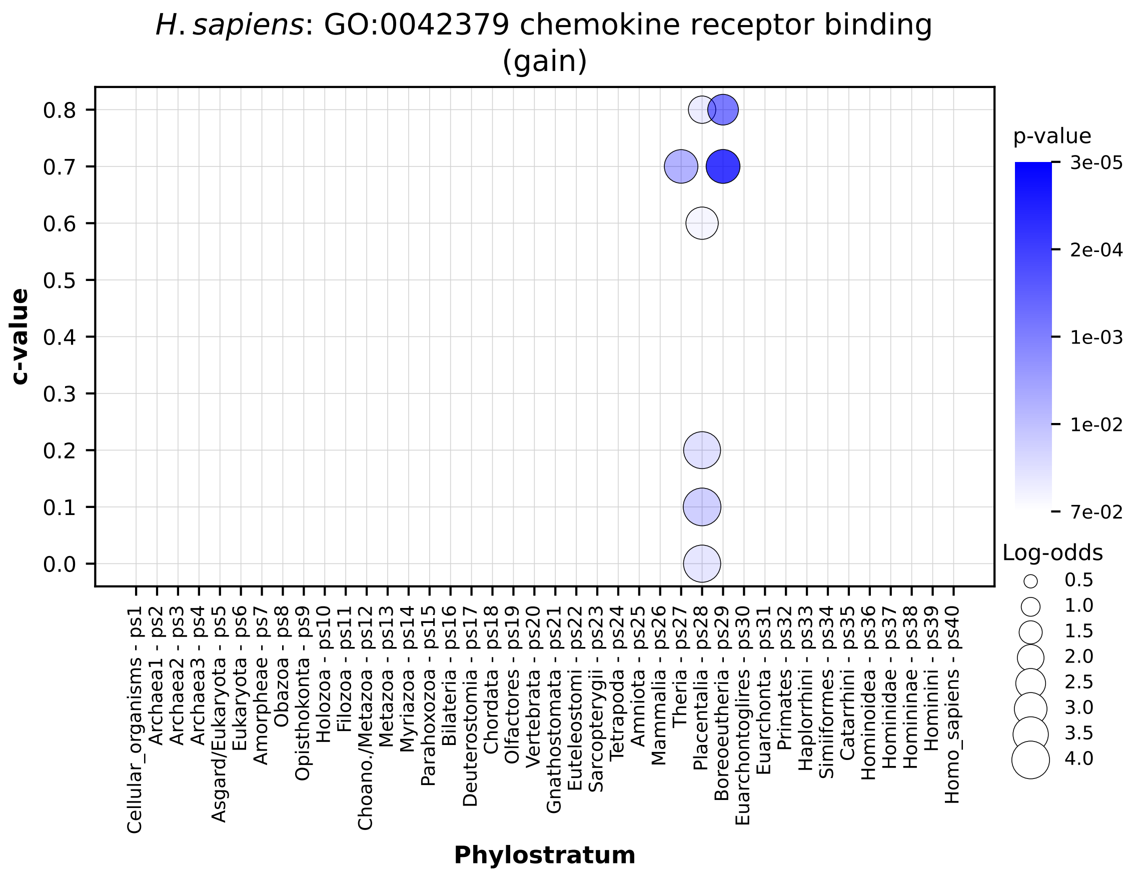
*H. sapiens*: GO:0042379 chemokine receptor binding (gain)
c-value
Phylostratum
p-value
3e-05
2e-04
1e-03
1e-02
7e-02
Log-odds
0.5
1.0
1.5
2.0
2.5
3.0
3.5
4.0
Cellular_organisms - ps1
Archaea1 - ps2
Archaea2 - ps3
Archaea3 - ps4
Asgard/Eukaryota - ps5
Eukaryota - ps6
Amorpheae - ps7
Obazoa - ps8
Opisthokonta - ps9
Holozoa - ps10
Filozoa - ps11
Choano./Metazoa - ps12
Metazoa - ps13
Myriazoa - ps14
Parahoxozoa - ps15
Bilateria - ps16
Deuterostomia - ps17
Chordata - ps18
Olfactores - ps19
Vertebrata - ps20
Gnathostomata - ps21
Euteleostomi - ps22
Sarcopterygii - ps23
Tetrapoda - ps24
Amniota - ps25
Mammalia - ps26
Theria - ps27
Placentalia - ps28
Boreoeutheria - ps29
Euarchontoglires - ps30
Euarchonta - ps31
Primates - ps32
Haplorrhini - ps33
Simiiformes - ps34
Catarrhini - ps35
Hominoidea - ps36
Hominidae - ps37
Homininae - ps38
Hominini - ps39
Homo_sapiens - ps40

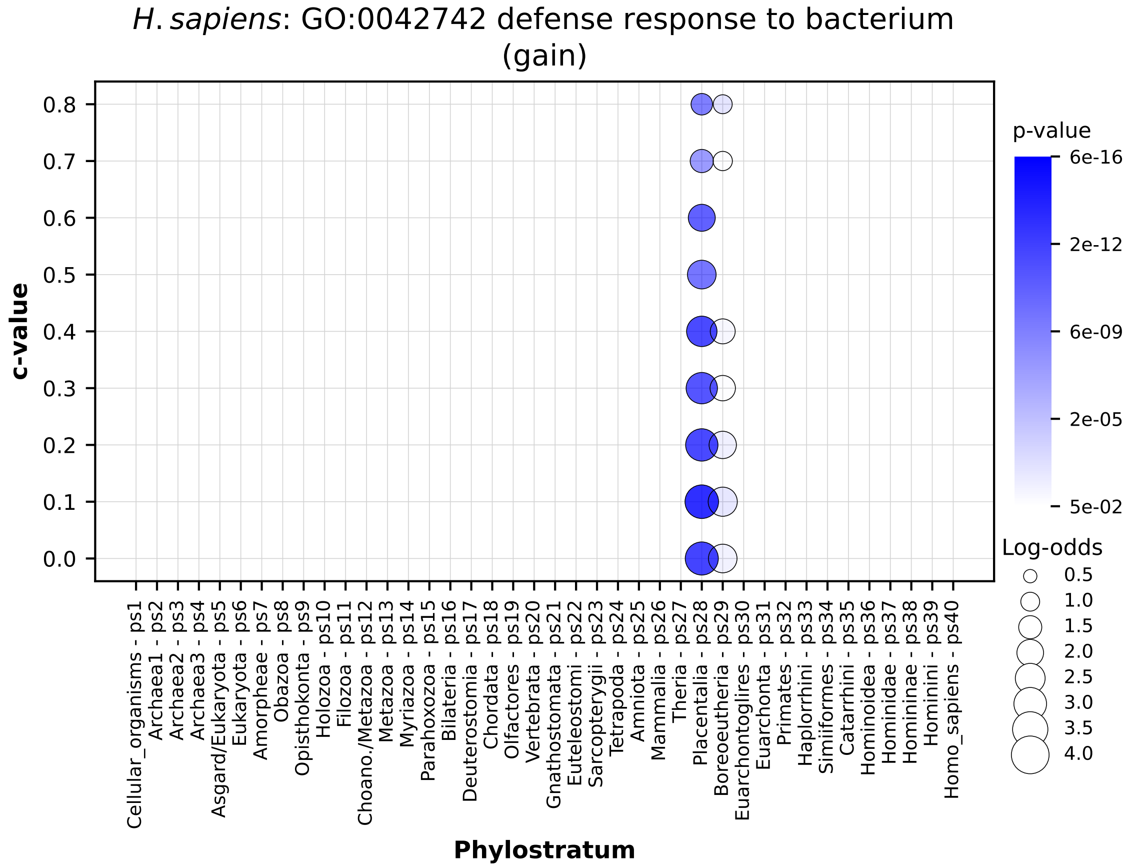
H. sapiens: GO:0042742 defense response to bacterium
(gain)
c-value
0.8
0.7
0.6
0.5
0.4
0.3
0.2
0.1
0.0
p-value
6e-16
2e-12
6e-09
2e-05
5e-02
Log-odds
0.5
1.0
1.5
2.0
2.5
3.0
3.5
4.0
Cellular_organisms - ps1
Archaea1 - ps2
Archaea2 - ps3
Archaea3 - ps4
Asgard/Eukaryota - ps5
Eukaryota - ps6
Amorpheae - ps7
Obazoa - ps8
Opisthokonta - ps9
Holozoa - ps10
Filozoa - ps11
Choano./Metazoa - ps12
Metazoa - ps13
Myriazoa - ps14
Parahoxozoa - ps15
Bilateria - ps16
Deuterostomia - ps17
Chordata - ps18
Olfactores - ps19
Vertebrata - ps20
Gnathostomata - ps21
Euteleostomi - ps22
Sarcopterygii - ps23
Tetrapoda - ps24
Amniota - ps25
Mammalia - ps26
Theria - ps27
Placentalia - ps28
Boreoeutheria - ps29
Euarchontoglires - ps30
Euarchonta - ps31
Primates - ps32
Haplorrhini - ps33
Simiiformes - ps34
Catarrhini - ps35
Hominoidea - ps36
Hominidae - ps37
Homininae - ps38
Hominini - ps39
Homo_sapiens - ps40
Phylostratum

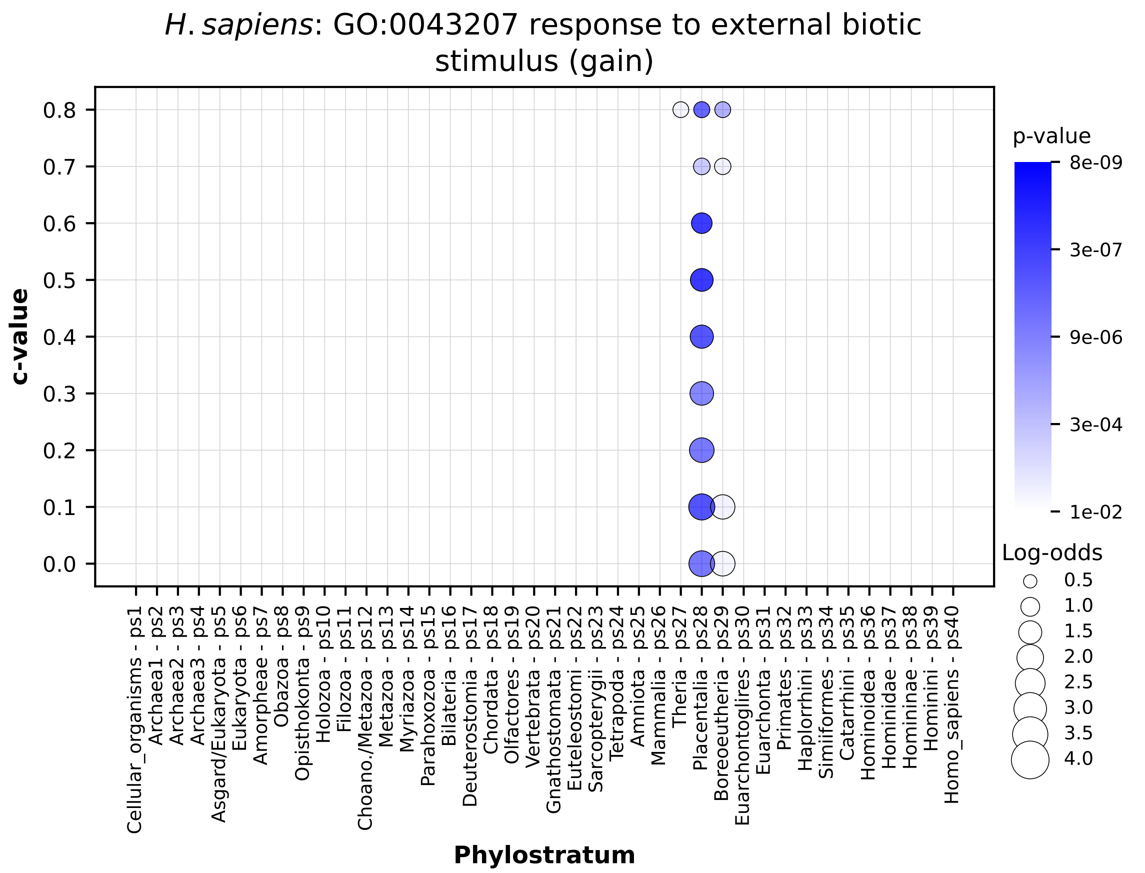

H. sapiens: GO:0043207 response to external biotic stimulus (gain)
c-value
0.0
0.1
0.2
0.3
0.4
0.5
0.6
0.7
0.8
Phylostratum
Cellular_organisms - ps1
Archaea1 - ps2
Archaea2 - ps3
Archaea3 - ps4
Asgard/Eukaryota - ps5
Eukaryota - ps6
Amorpheae - ps7
Obazoa - ps8
Opisthokonta - ps9
Holozoa - ps10
Filozoa - ps11
Choano./Metazoa - ps12
Metazoa - ps13
Myriazoa - ps14
Parahoxozoa - ps15
Bilateria - ps16
Deuterostomia - ps17
Chordata - ps18
Olfactores - ps19
Vertebrata - ps20
Gnathostomata - ps21
Euteleostomi - ps22
Sarcopterygii - ps23
Tetrapoda - ps24
Amniota - ps25
Mammalia - ps26
Theria - ps27
Placentalia - ps28
Boreoeutheria - ps29
Euarchontoglires - ps30
Euarchonta - ps31
Primates - ps32
Haplorrhini - ps33
Simiiformes - ps34
Catarrhini - ps35
Hominoidea - ps36
Hominidae - ps37
Homininae - ps38
Hominini - ps39
Homo_sapiens - ps40
p-value
8e-09
3e-07
9e-06
3e-04
1e-02
Log-odds
0.5
1.0
1.5
2.0
2.5
3.0
3.5
4.0

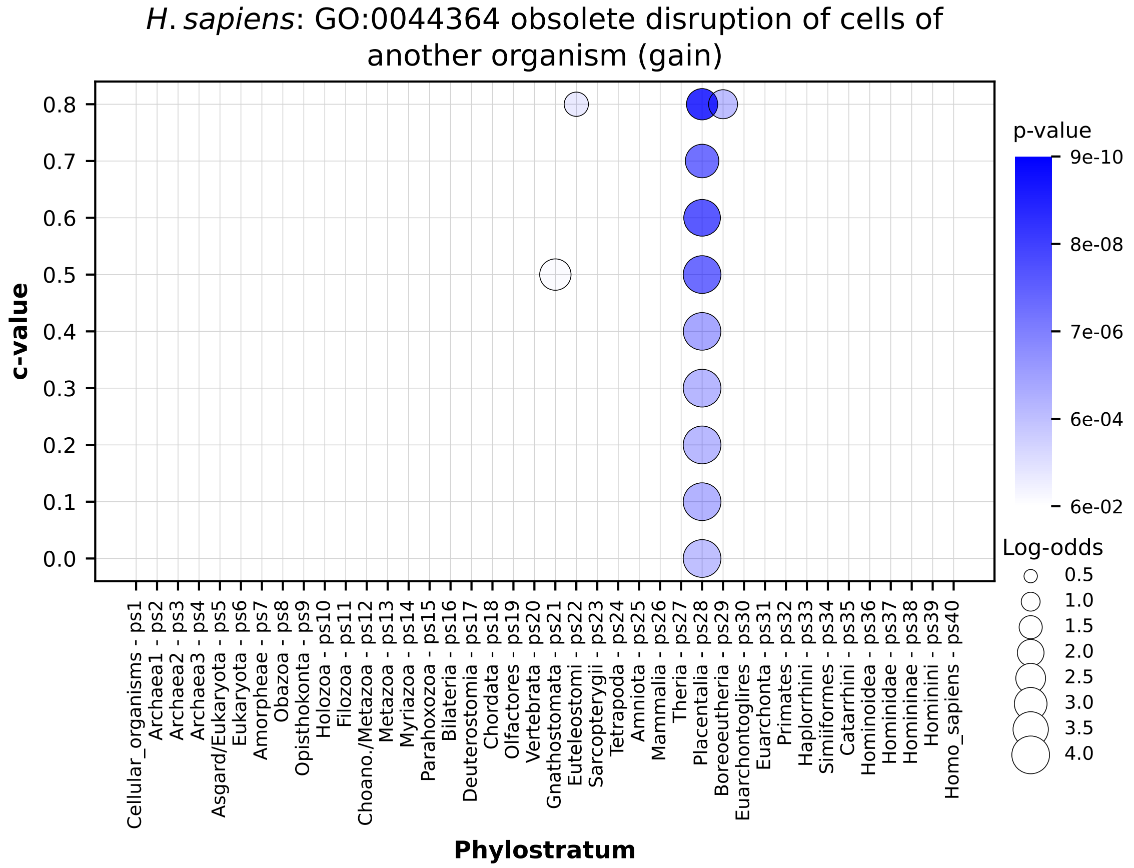
H. sapiens: GO:0044364 obsolete disruption of cells of another organism (gain)
c-value
0.0
0.1
0.2
0.3
0.4
0.5
0.6
0.7
0.8
Phylostratum
Cellular_organisms - ps1
Archaea1 - ps2
Archaea2 - ps3
Archaea3 - ps4
Asgard/Eukaryota - ps5
Eukaryota - ps6
Amorpheae - ps7
Obazoa - ps8
Opisthokonta - ps9
Holozoa - ps10
Filozoa - ps11
Choano./Metazoa - ps12
Metazoa - ps13
Myriazoa - ps14
Parahoxozoa - ps15
Bilateria - ps16
Deuterostomia - ps17
Chordata - ps18
Olfactores - ps19
Vertebrata - ps20
Gnathostomata - ps21
Euteleostomi - ps22
Sarcopterygii - ps23
Tetrapoda - ps24
Amniota - ps25
Mammalia - ps26
Theria - ps27
Placentalia - ps28
Boreoeutheria - ps29
Euarchontoglires - ps30
Euarchonta - ps31
Primates - ps32
Haplorrhini - ps33
Simiiformes - ps34
Catarrhini - ps35
Hominoidea - ps36
Hominidae - ps37
Homininae - ps38
Hominini - ps39
Homo_sapiens - ps40
p-value
9e-10
8e-08
7e-06
6e-04
6e-02
Log-odds
0.5
1.0
1.5
2.0
2.5
3.0
3.5
4.0

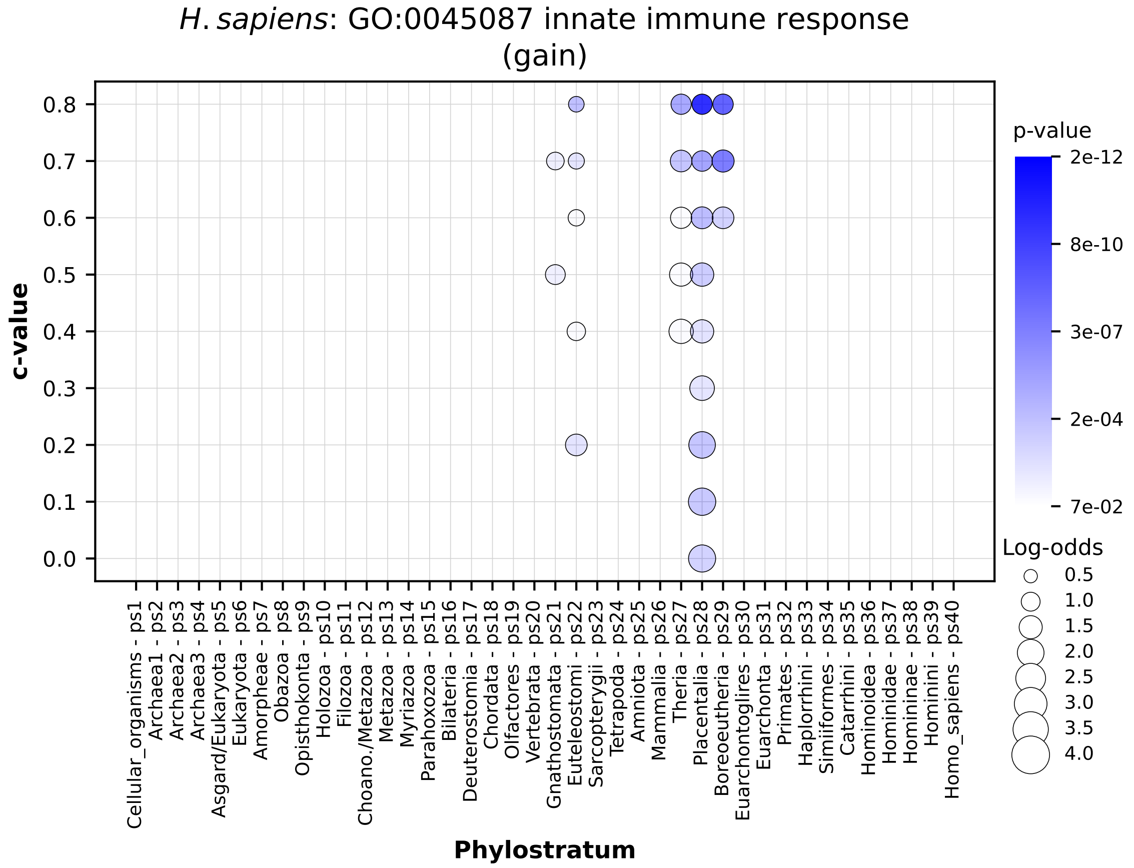
*H. sapiens*: GO:0045087 innate immune response
(gain)
c-value
0.0
0.1
0.2
0.3
0.4
0.5
0.6
0.7
0.8
p-value
2e-12
8e-10
3e-07
2e-04
7e-02
Log-odds
0.5
1.0
1.5
2.0
2.5
3.0
3.5
4.0
Cellular_organisms - ps1
Archaea1 - ps2
Archaea2 - ps3
Archaea3 - ps4
Asgard/Eukaryota - ps5
Eukaryota - ps6
Amorpheae - ps7
Obazoa - ps8
Opisthokonta - ps9
Holozoa - ps10
Filozoa - ps11
Choano./Metazoa - ps12
Metazoa - ps13
Myriazoa - ps14
Parahoxozoa - ps15
Bilateria - ps16
Deuterostomia - ps17
Chordata - ps18
Olfactores - ps19
Vertebrata - ps20
Gnathostomata - ps21
Euteleostomi - ps22
Sarcopterygii - ps23
Tetrapoda - ps24
Amniota - ps25
Mammalia - ps26
Theria - ps27
Placentalia - ps28
Boreoeutheria - ps29
Euarchontoglires - ps30
Euarchonta - ps31
Primates - ps32
Haplorrhini - ps33
Simiiformes - ps34
Catarrhini - ps35
Hominoidea - ps36
Hominidae - ps37
Homininae - ps38
Hominini - ps39
Homo_sapiens - ps40
Phylostratum

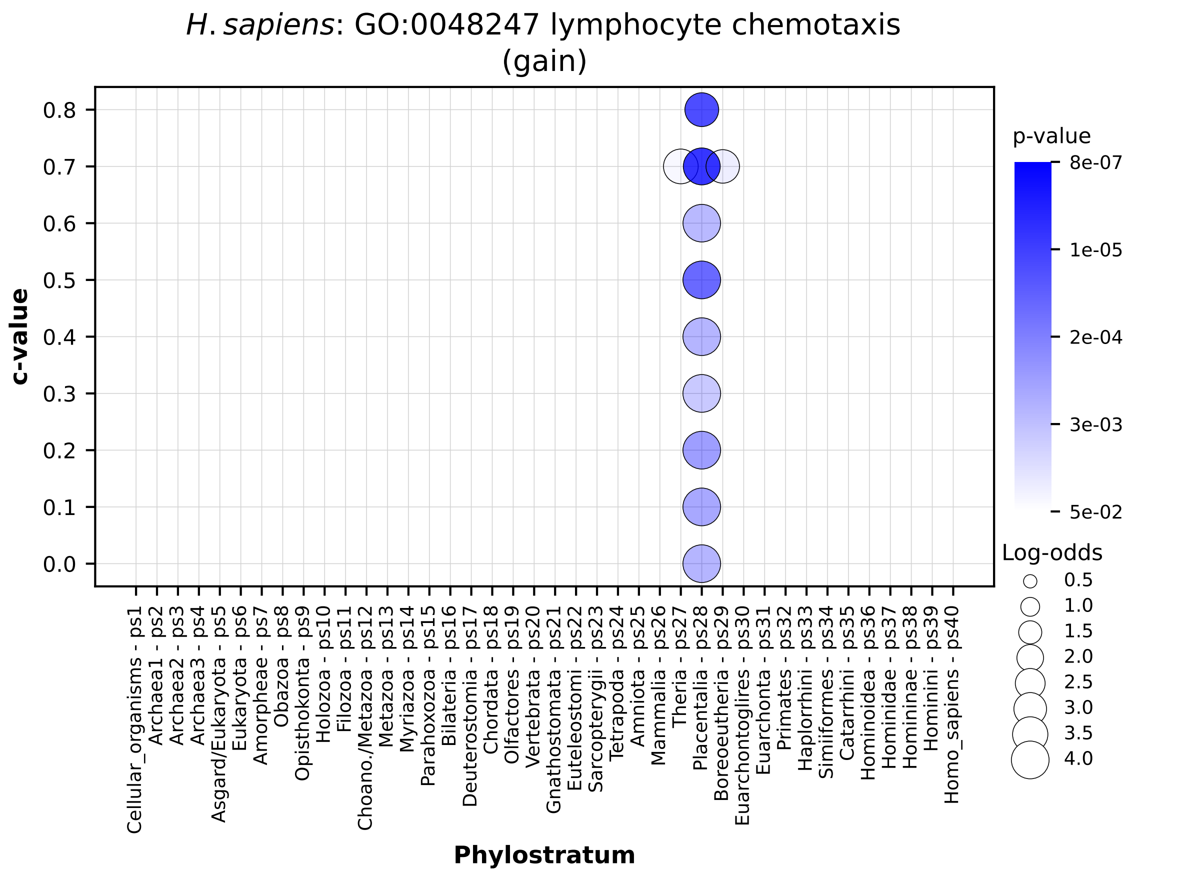

H. sapiens: GO:0048247 lymphocyte chemotaxis
(gain)
c-value
0.0
0.1
0.2
0.3
0.4
0.5
0.6
0.7
0.8
p-value
8e-07
1e-05
2e-04
3e-03
5e-02
Log-odds
0.5
1.0
1.5
2.0
2.5
3.0
3.5
4.0
Cellular_organisms - ps1
Archaea1 - ps2
Archaea2 - ps3
Archaea3 - ps4
Asgard/Eukaryota - ps5
Eukaryota - ps6
Amorpheae - ps7
Obazoa - ps8
Opisthokonta - ps9
Holozoa - ps10
Filozoa - ps11
Choano./Metazoa - ps12
Metazoa - ps13
Myriazoa - ps14
Parahoxozoa - ps15
Bilateria - ps16
Deuterostomia - ps17
Chordata - ps18
Olfactores - ps19
Vertebrata - ps20
Gnathostomata - ps21
Euteleostomi - ps22
Sarcopterygii - ps23
Tetrapoda - ps24
Amniota - ps25
Mammalia - ps26
Theria - ps27
Placentalia - ps28
Boreoeutheria - ps29
Euarchontoglires - ps30
Euarchonta - ps31
Primates - ps32
Haplorrhini - ps33
Simiiformes - ps34
Catarrhini - ps35
Hominoidea - ps36
Hominidae - ps37
Homininae - ps38
Hominini - ps39
Homo_sapiens - ps40
Phylostratum

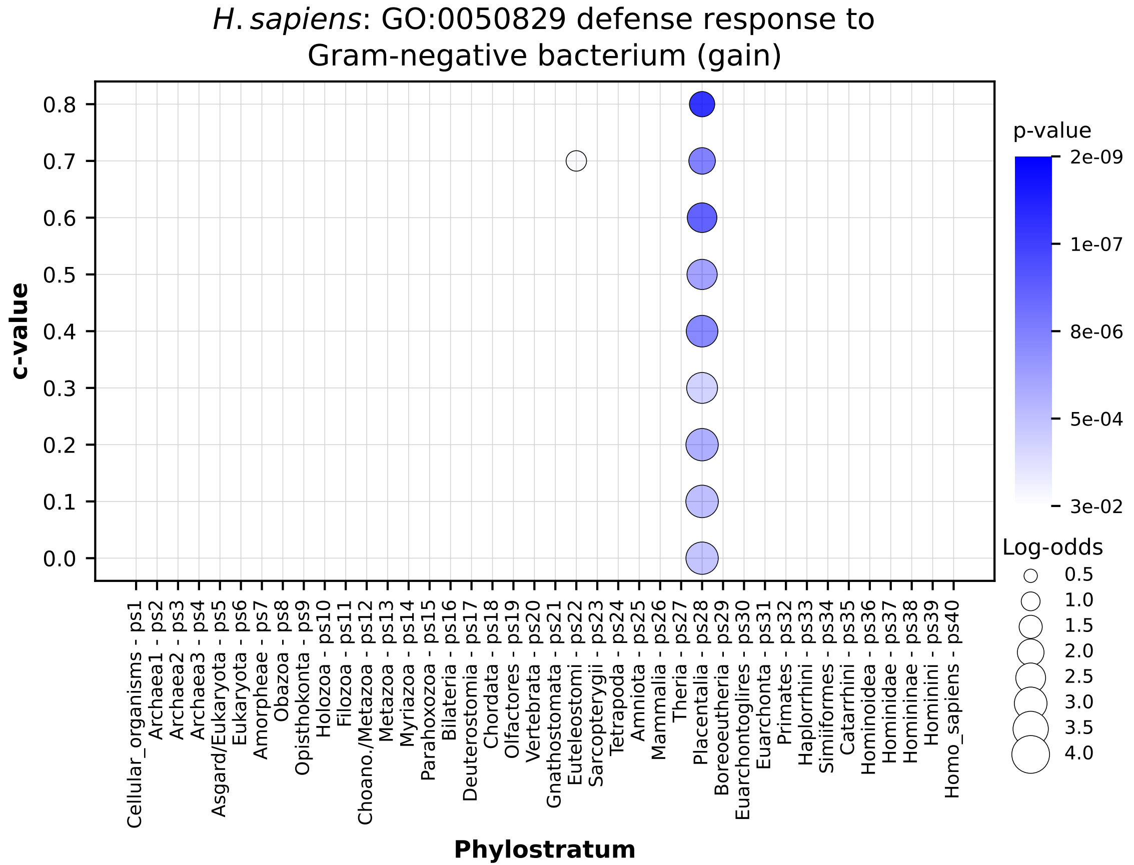

H. sapiens: GO:0050829 defense response to Gram-negative bacterium (gain)
c-value
0.8
0.7
0.6
0.5
0.4
0.3
0.2
0.1
0.0
Phylostratum
Cellular_organisms - ps1
Archaea1 - ps2
Archaea2 - ps3
Archaea3 - ps4
Asgard/Eukaryota - ps5
Eukaryota - ps6
Amorpheae - ps7
Obazoa - ps8
Opisthokonta - ps9
Holozoa - ps10
Filozoa - ps11
Choano./Metazoa - ps12
Metazoa - ps13
Myriazoa - ps14
Parahoxozoa - ps15
Bilateria - ps16
Deuterostomia - ps17
Chordata - ps18
Olfactores - ps19
Vertebrata - ps20
Gnathostomata - ps21
Euteleostomi - ps22
Sarcopterygii - ps23
Tetrapoda - ps24
Amniota - ps25
Mammalia - ps26
Theria - ps27
Placentalia - ps28
Boreoeutheria - ps29
Euarchontoglires - ps30
Euarchonta - ps31
Primates - ps32
Haplorrhini - ps33
Simiiformes - ps34
Catarrhini - ps35
Hominoidea - ps36
Hominidae - ps37
Homininae - ps38
Hominini - ps39
Homo_sapiens - ps40
p-value
2e-09
1e-07
8e-06
5e-04
3e-02
Log-odds
0.5
1.0
1.5
2.0
2.5
3.0
3.5
4.0

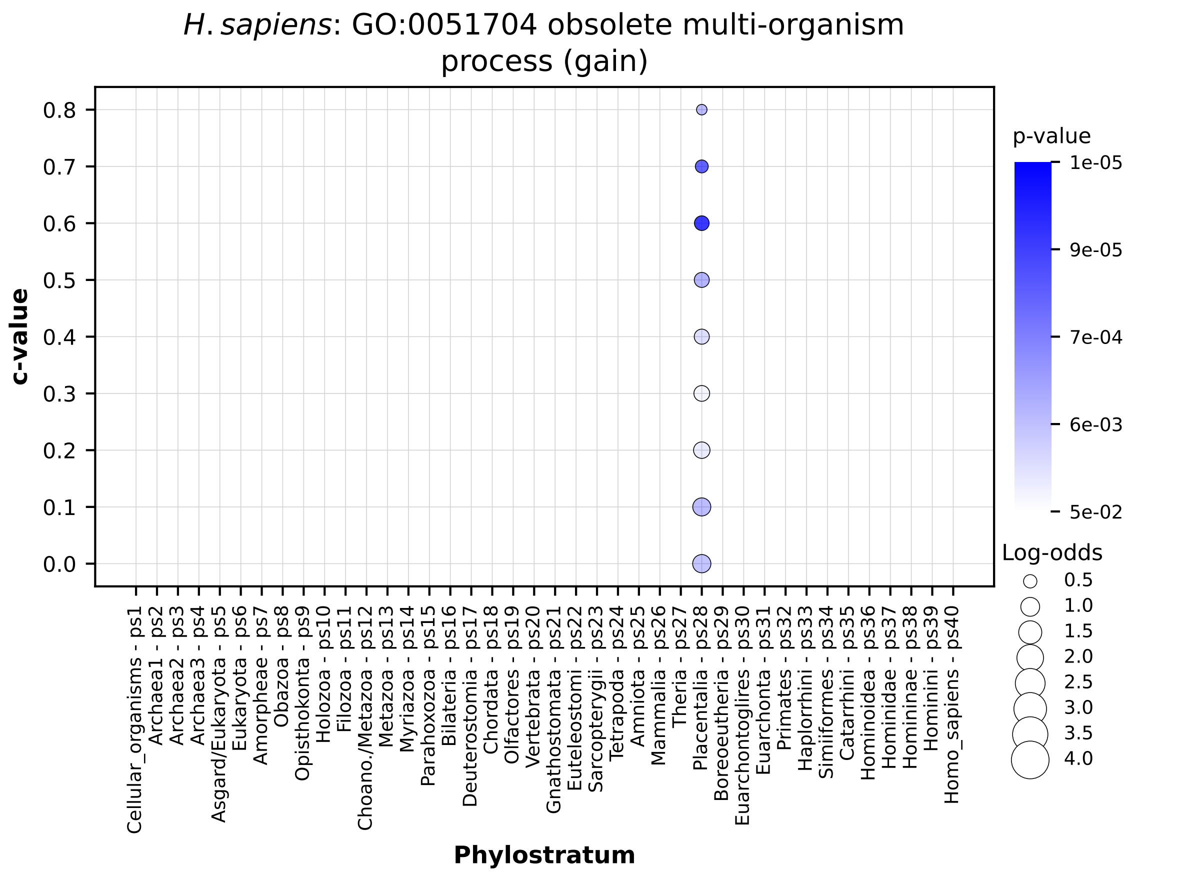

H. sapiens: GO:0051704 obsolete multi-organism process (gain)
c-value
0.8
0.7
0.6
0.5
0.4
0.3
0.2
0.1
0.0
Phylostratum
Cellular_organisms - ps1
Archaea1 - ps2
Archaea2 - ps3
Archaea3 - ps4
Asgard/Eukaryota - ps5
Eukaryota - ps6
Amorpheae - ps7
Obazoa - ps8
Opisthokonta - ps9
Holozoa - ps10
Filozoa - ps11
Choano./Metazoa - ps12
Metazoa - ps13
Myriazoa - ps14
Parahoxozoa - ps15
Bilateria - ps16
Deuterostomia - ps17
Chordata - ps18
Olfactores - ps19
Vertebrata - ps20
Gnathostomata - ps21
Euteleostomi - ps22
Sarcopterygii - ps23
Tetrapoda - ps24
Amniota - ps25
Mammalia - ps26
Theria - ps27
Placentalia - ps28
Boreoeutheria - ps29
Euarchontoglires - ps30
Euarchonta - ps31
Primates - ps32
Haplorrhini - ps33
Simiiformes - ps34
Catarrhini - ps35
Hominoidea - ps36
Hominidae - ps37
Homininae - ps38
Hominini - ps39
Homo_sapiens - ps40
p-value
1e-05
9e-05
7e-04
6e-03
5e-02
Log-odds
0.5
1.0
1.5
2.0
2.5
3.0
3.5
4.0

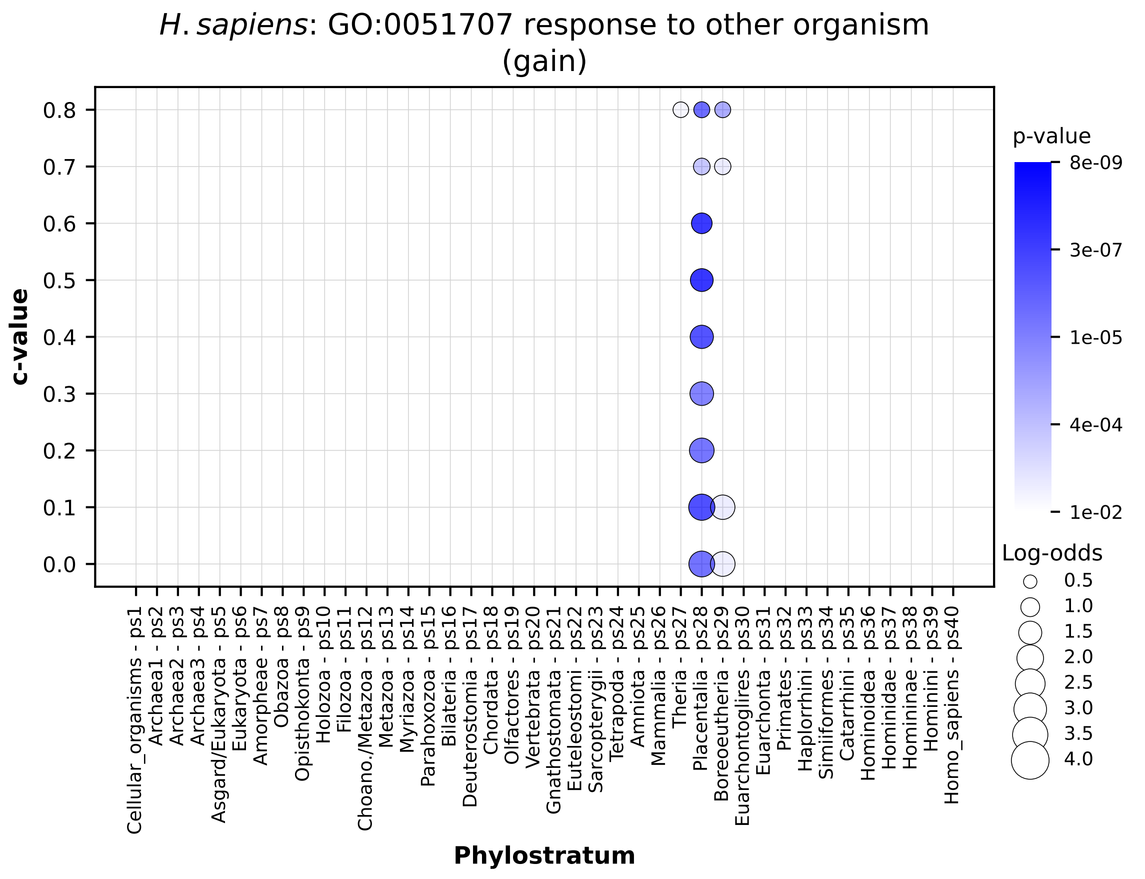

H. sapiens: GO:0051707 response to other organism
(gain)
c-value
0.8
0.7
0.6
0.5
0.4
0.3
0.2
0.1
0.0
p-value
8e-09
3e-07
1e-05
4e-04
1e-02
Log-odds
0.5
1.0
1.5
2.0
2.5
3.0
3.5
4.0
Cellular_organisms - ps1
Archaea1 - ps2
Archaea2 - ps3
Archaea3 - ps4
Asgard/Eukaryota - ps5
Eukaryota - ps6
Amorpheae - ps7
Obazoa - ps8
Opisthokonta - ps9
Holozoa - ps10
Filozoa - ps11
Choano./Metazoa - ps12
Metazoa - ps13
Myriazoa - ps14
Parahoxozoa - ps15
Bilateria - ps16
Deuterostomia - ps17
Chordata - ps18
Olfactores - ps19
Vertebrata - ps20
Gnathostomata - ps21
Euteleostomi - ps22
Sarcopterygii - ps23
Tetrapoda - ps24
Amniota - ps25
Mammalia - ps26
Theria - ps27
Placentalia - ps28
Boreoeutheria - ps29
Euarchontoglires - ps30
Euarchonta - ps31
Primates - ps32
Haplorrhini - ps33
Simiiformes - ps34
Catarrhini - ps35
Hominoidea - ps36
Hominidae - ps37
Homininae - ps38
Hominini - ps39
Homo_sapiens - ps40
Phylostratum

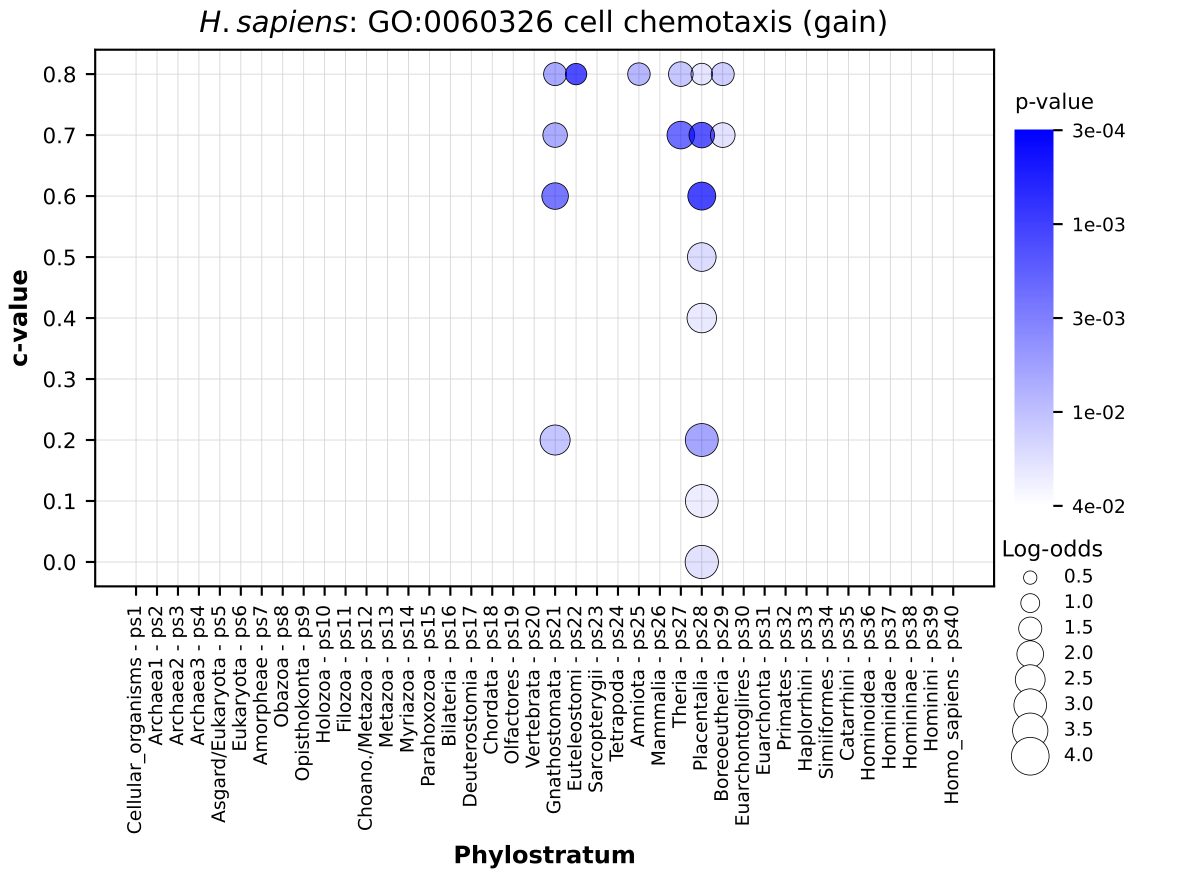

H. sapiens: GO:0060326 cell chemotaxis (gain)
c-value
0.8
0.7
0.6
0.5
0.4
0.3
0.2
0.1
0.0
Phylostratum
Cellular_organisms - ps1
Archaea1 - ps2
Archaea2 - ps3
Archaea3 - ps4
Asgard/Eukaryota - ps5
Eukaryota - ps6
Amorpheae - ps7
Obazoa - ps8
Opisthokonta - ps9
Holozoa - ps10
Filozoa - ps11
Choano./Metazoa - ps12
Metazoa - ps13
Myriazoa - ps14
Parahoxozoa - ps15
Bilateria - ps16
Deuterostomia - ps17
Chordata - ps18
Olfactores - ps19
Vertebrata - ps20
Gnathostomata - ps21
Euteleostomi - ps22
Sarcopterygii - ps23
Tetrapoda - ps24
Amniota - ps25
Mammalia - ps26
Theria - ps27
Placentalia - ps28
Boreoeutheria - ps29
Euarchontoglires - ps30
Euarchonta - ps31
Primates - ps32
Haplorrhini - ps33
Simiiformes - ps34
Catarrhini - ps35
Hominoidea - ps36
Hominidae - ps37
Homininae - ps38
Hominini - ps39
Homo_sapiens - ps40
p-value
3e-04
1e-03
3e-03
1e-02
4e-02
Log-odds
0.5
1.0
1.5
2.0
2.5
3.0
3.5
4.0

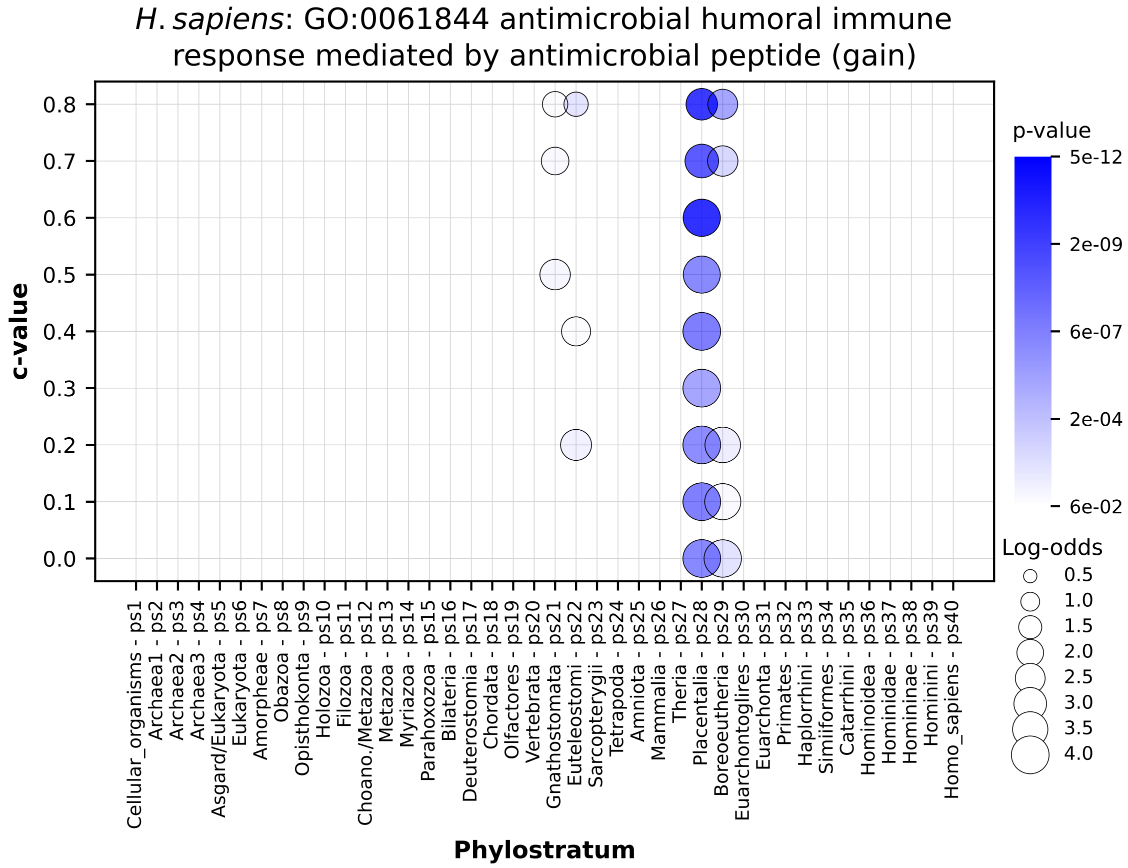

H. sapiens: GO:0061844 antimicrobial humoral immune response mediated by antimicrobial peptide (gain)
c-value
0.8
0.7
0.6
0.5
0.4
0.3
0.2
0.1
0.0
p-value
5e-12
2e-09
6e-07
2e-04
6e-02
Log-odds
0.5
1.0
1.5
2.0
2.5
3.0
3.5
4.0
Cellular_organisms - ps1
Archaea1 - ps2
Archaea2 - ps3
Archaea3 - ps4
Asgard/Eukaryota - ps5
Eukaryota - ps6
Amorpheae - ps7
Obazoa - ps8
Opisthokonta - ps9
Holozoa - ps10
Filozoa - ps11
Choano./Metazoa - ps12
Metazoa - ps13
Myriazoa - ps14
Parahoxozoa - ps15
Bilateria - ps16
Deuterostomia - ps17
Chordata - ps18
Olfactores - ps19
Vertebrata - ps20
Gnathostomata - ps21
Euteleostomi - ps22
Sarcopterygii - ps23
Tetrapoda - ps24
Amniota - ps25
Mammalia - ps26
Theria - ps27
Placentalia - ps28
Boreoeutheria - ps29
Euarchontoglires - ps30
Euarchonta - ps31
Primates - ps32
Haplorrhini - ps33
Simiiformes - ps34
Catarrhini - ps35
Hominoidea - ps36
Hominidae - ps37
Homininae - ps38
Hominini - ps39
Homo_sapiens - ps40
Phylostratum

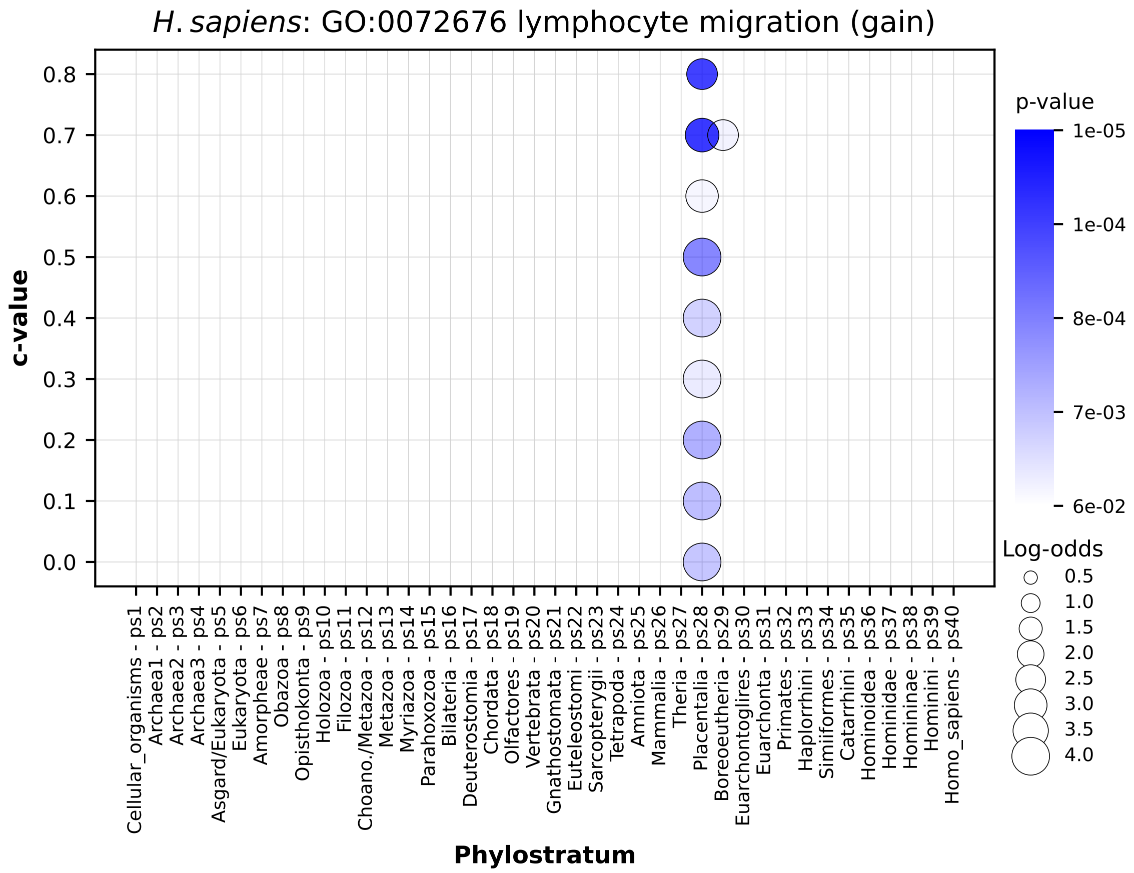
H. sapiens: GO:0072676 lymphocyte migration (gain)
c-value
0.8
0.7
0.6
0.5
0.4
0.3
0.2
0.1
0.0
p-value
1e-05
1e-04
8e-04
7e-03
6e-02
Log-odds
0.5
1.0
1.5
2.0
2.5
3.0
3.5
4.0
Cellular_organisms - ps1
Archaea1 - ps2
Archaea2 - ps3
Archaea3 - ps4
Asgard/Eukaryota - ps5
Eukaryota - ps6
Amorpheae - ps7
Obazoa - ps8
Opisthokonta - ps9
Holozoa - ps10
Filozoa - ps11
Choano./Metazoa - ps12
Metazoa - ps13
Myriazoa - ps14
Parahoxozoa - ps15
Bilateria - ps16
Deuterostomia - ps17
Chordata - ps18
Olfactores - ps19
Vertebrata - ps20
Gnathostomata - ps21
Euteleostomi - ps22
Sarcopterygii - ps23
Tetrapoda - ps24
Amniota - ps25
Mammalia - ps26
Theria - ps27
Placentalia - ps28
Boreoeutheria - ps29
Euarchontoglires - ps30
Euarchonta - ps31
Primates - ps32
Haplorrhini - ps33
Simiiformes - ps34
Catarrhini - ps35
Hominoidea - ps36
Hominidae - ps37
Homininae - ps38
Hominini - ps39
Homo_sapiens - ps40
Phylostratum

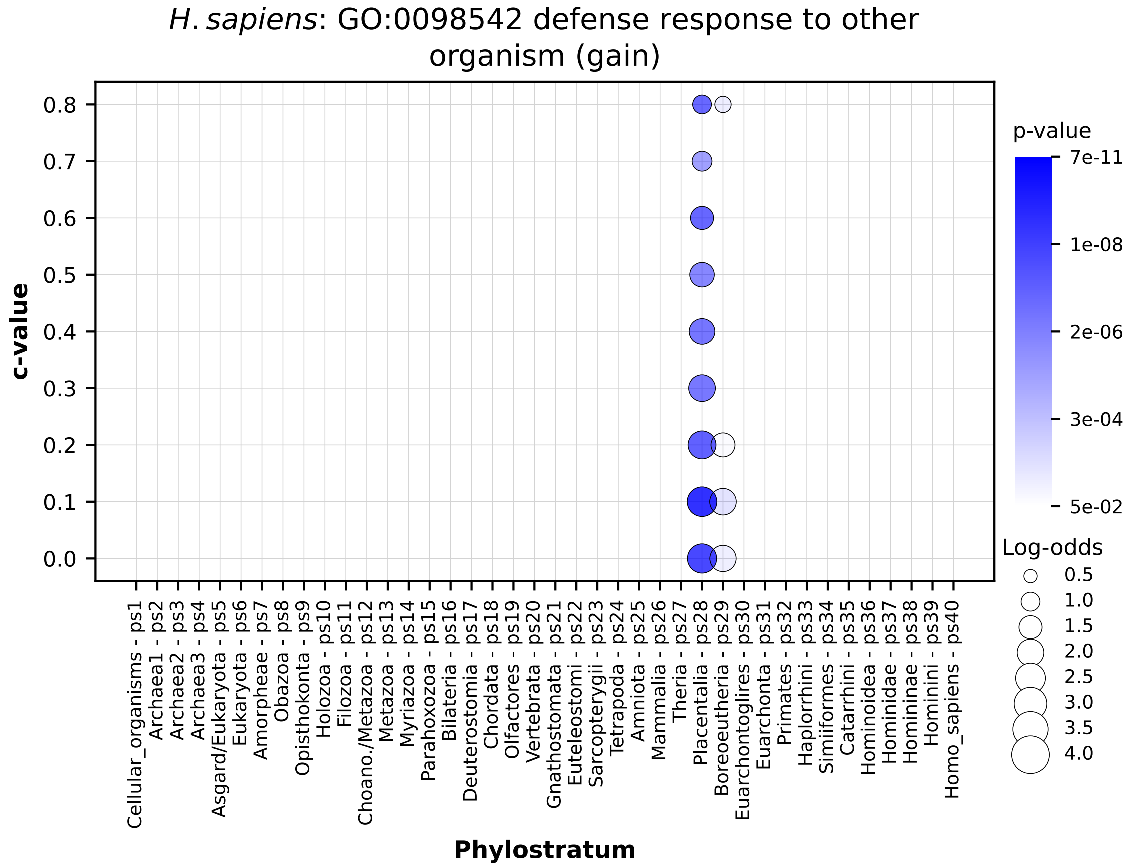

H. sapiens: GO:0098542 defense response to other organism (gain)
c-value
0.8
0.7
0.6
0.5
0.4
0.3
0.2
0.1
0.0
Phylostratum
Cellular_organisms - ps1
Archaea1 - ps2
Archaea2 - ps3
Archaea3 - ps4
Asgard/Eukaryota - ps5
Eukaryota - ps6
Amorpheae - ps7
Obazoa - ps8
Opisthokonta - ps9
Holozoa - ps10
Filozoa - ps11
Choano./Metazoa - ps12
Metazoa - ps13
Myriazoa - ps14
Parahoxozoa - ps15
Bilateria - ps16
Deuterostomia - ps17
Chordata - ps18
Olfactores - ps19
Vertebrata - ps20
Gnathostomata - ps21
Euteleostomi - ps22
Sarcopterygii - ps23
Tetrapoda - ps24
Amniota - ps25
Mammalia - ps26
Theria - ps27
Placentalia - ps28
Boreoeutheria - ps29
Euarchontoglires - ps30
Euarchonta - ps31
Primates - ps32
Haplorrhini - ps33
Simiiformes - ps34
Catarrhini - ps35
Hominoidea - ps36
Hominidae - ps37
Homininae - ps38
Hominini - ps39
Homo_sapiens - ps40
p-value
7e-11
1e-08
2e-06
3e-04
5e-02
Log-odds
0.5
1.0
1.5
2.0
2.5
3.0
3.5
4.0

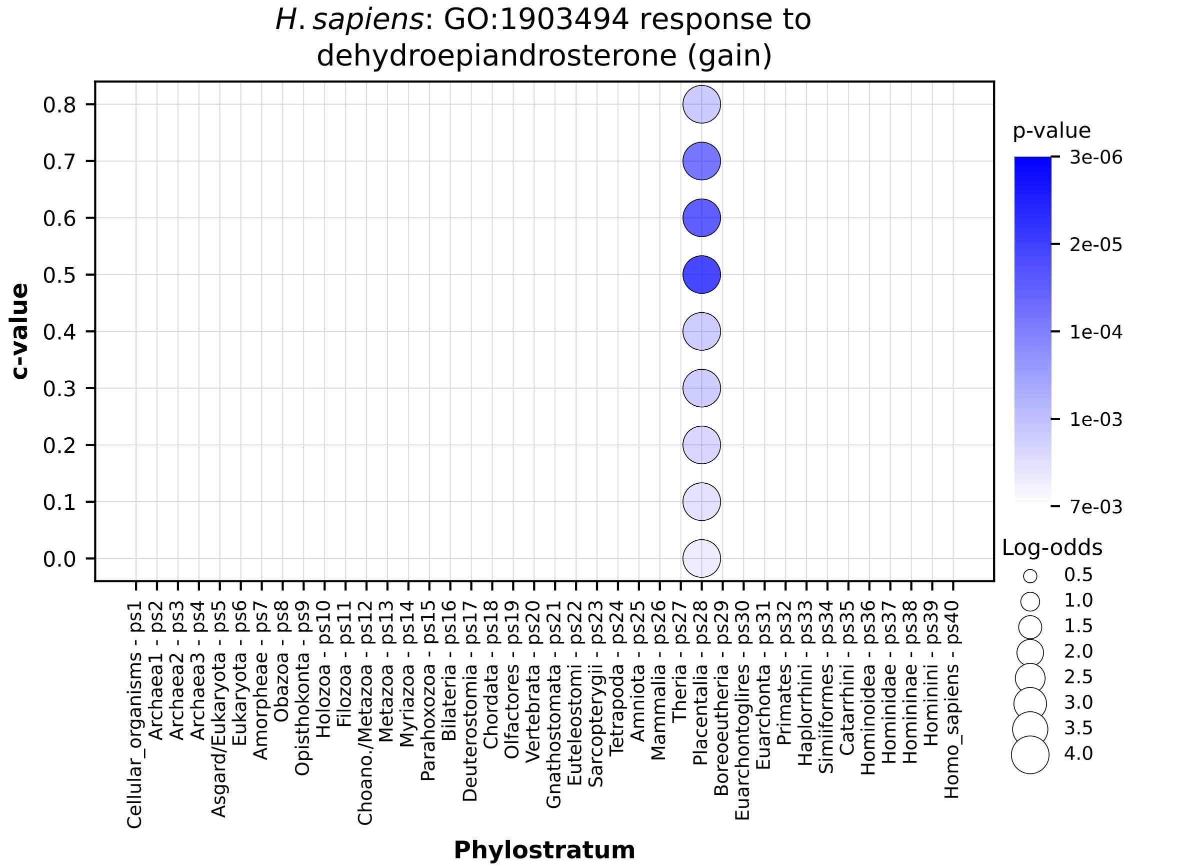
H. sapiens: GO:1903494 response to dehydroepiandrosterone (gain)
c-value
0.8
0.7
0.6
0.5
0.4
0.3
0.2
0.1
0.0
p-value
3e-06
2e-05
1e-04
1e-03
7e-03
Log-odds
0.5
1.0
1.5
2.0
2.5
3.0
3.5
4.0
Cellular_organisms - ps1
Archaea1 - ps2
Archaea2 - ps3
Archaea3 - ps4
Asgard/Eukaryota - ps5
Eukaryota - ps6
Amorpheae - ps7
Obazoa - ps8
Opisthokonta - ps9
Holozoa - ps10
Filozoa - ps11
Choano./Metazoa - ps12
Metazoa - ps13
Myriazoa - ps14
Parahoxozoa - ps15
Bilateria - ps16
Deuterostomia - ps17
Chordata - ps18
Olfactores - ps19
Vertebrata - ps20
Gnathostomata - ps21
Euteleostomi - ps22
Sarcopterygii - ps23
Tetrapoda - ps24
Amniota - ps25
Mammalia - ps26
Theria - ps27
Placentalia - ps28
Boreoeutheria - ps29
Euarchontoglires - ps30
Euarchonta - ps31
Primates - ps32
Haplorrhini - ps33
Simiiformes - ps34
Catarrhini - ps35
Hominoidea - ps36
Hominidae - ps37
Homininae - ps38
Hominini - ps39
Homo_sapiens - ps40
Phylostratum

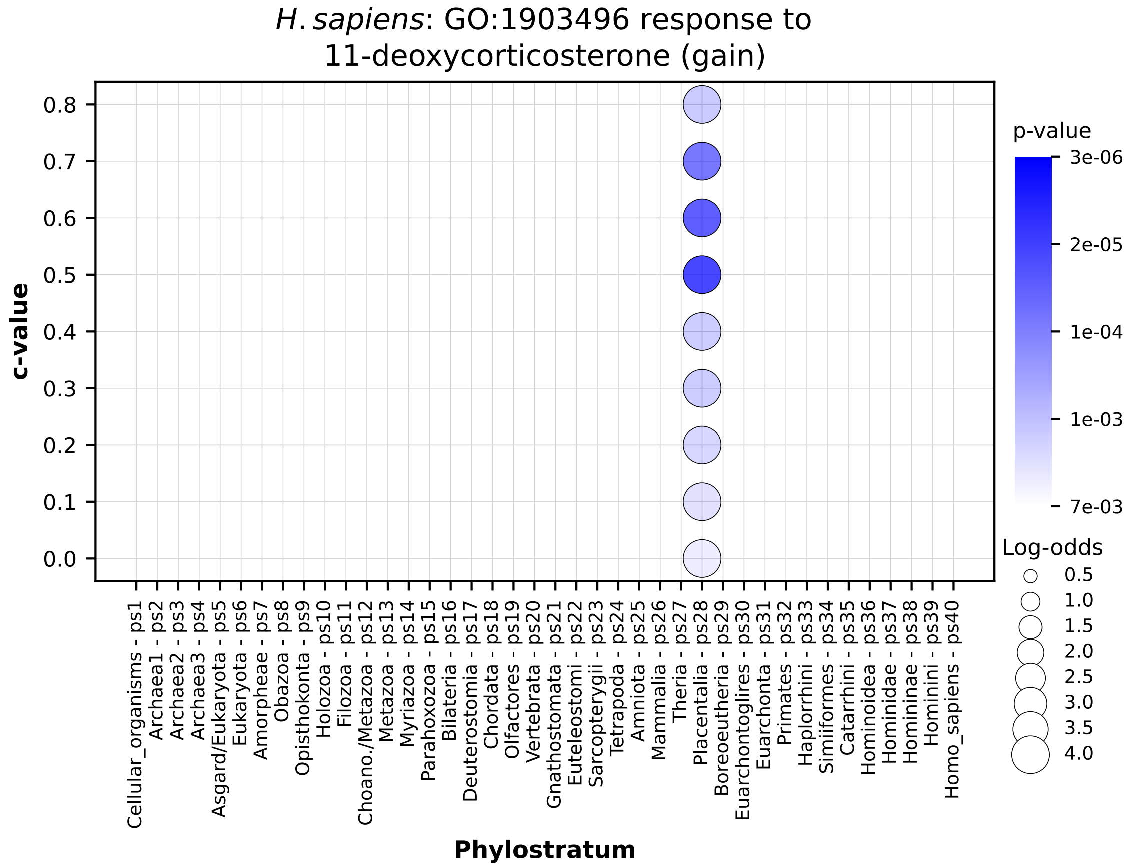

*H. sapiens*: GO:1903496 response to 11-deoxycorticosterone (gain)
c-value
0.8
0.7
0.6
0.5
0.4
0.3
0.2
0.1
0.0
p-value
3e-06
2e-05
1e-04
1e-03
7e-03
Log-odds
0.5
1.0
1.5
2.0
2.5
3.0
3.5
4.0
Cellular_organisms - ps1
Archaea1 - ps2
Archaea2 - ps3
Archaea3 - ps4
Asgard/Eukaryota - ps5
Eukaryota - ps6
Amorpheae - ps7
Obazoa - ps8
Opisthokonta - ps9
Holozoa - ps10
Filozoa - ps11
Choano./Metazoa - ps12
Metazoa - ps13
Myriazoa - ps14
Parahoxozoa - ps15
Bilateria - ps16
Deuterostomia - ps17
Chordata - ps18
Olfactores - ps19
Vertebrata - ps20
Gnathostomata - ps21
Euteleostomi - ps22
Sarcopterygii - ps23
Tetrapoda - ps24
Amniota - ps25
Mammalia - ps26
Theria - ps27
Placentalia - ps28
Boreoeutheria - ps29
Euarchontoglires - ps30
Euarchonta - ps31
Primates - ps32
Haplorrhini - ps33
Simiiformes - ps34
Catarrhini - ps35
Hominoidea - ps36
Hominidae - ps37
Homininae - ps38
Hominini - ps39
Homo_sapiens - ps40
Phylostratum

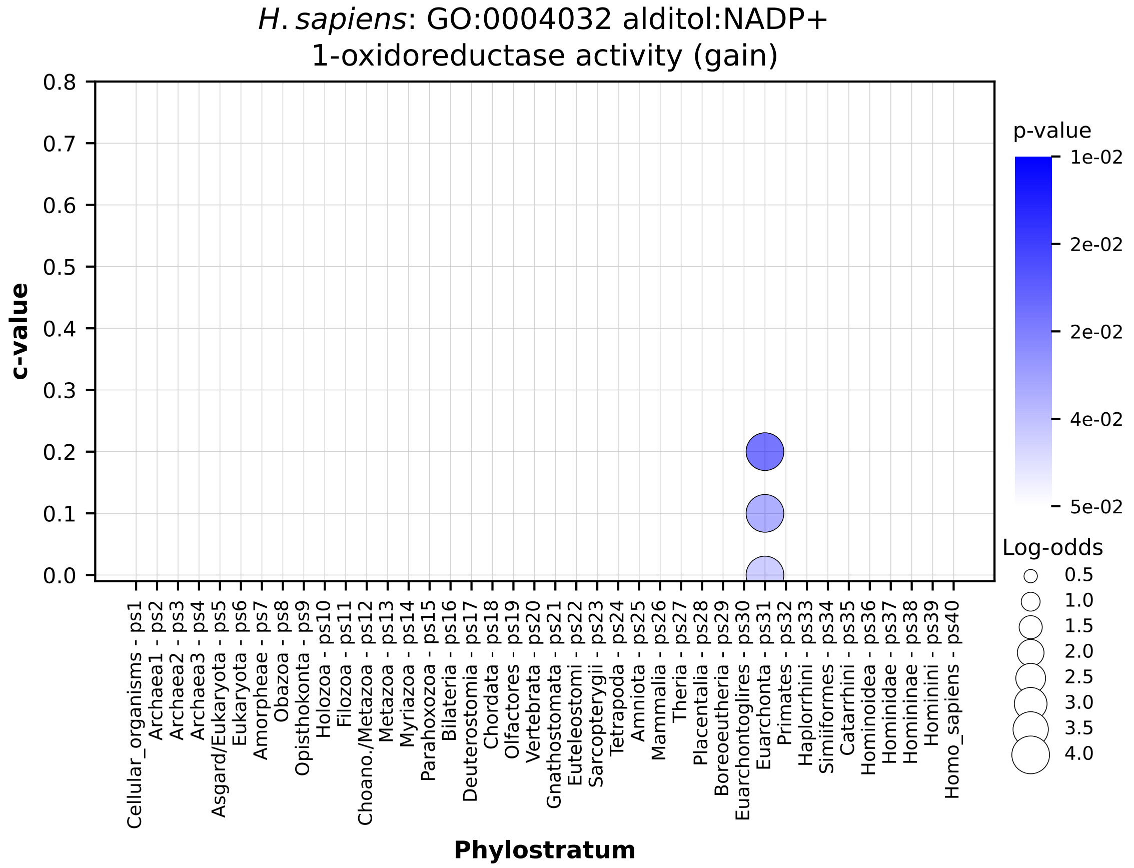

H. sapiens: GO:0004032 alditol:NADP+ 1-oxidoreductase activity (gain)
c-value
0.8
0.7
0.6
0.5
0.4
0.3
0.2
0.1
0.0
Phylostratum
Cellular_organisms - ps1
Archaea1 - ps2
Archaea2 - ps3
Archaea3 - ps4
Asgard/Eukaryota - ps5
Eukaryota - ps6
Amorpheae - ps7
Obazoa - ps8
Opisthokonta - ps9
Holozoa - ps10
Filozoa - ps11
Choano./Metazoa - ps12
Metazoa - ps13
Myriazoa - ps14
Parahoxozoa - ps15
Bilateria - ps16
Deuterostomia - ps17
Chordata - ps18
Olfactores - ps19
Vertebrata - ps20
Gnathostomata - ps21
Euteleostomi - ps22
Sarcopterygii - ps23
Tetrapoda - ps24
Amniota - ps25
Mammalia - ps26
Theria - ps27
Placentalia - ps28
Boreoeutheria - ps29
Euarchontoglires - ps30
Euarchonta - ps31
Primates - ps32
Haplorrhini - ps33
Simiiformes - ps34
Catarrhini - ps35
Hominoidea - ps36
Hominidae - ps37
Homininae - ps38
Hominini - ps39
Homo_sapiens - ps40
p-value
1e-02
2e-02
2e-02
4e-02
5e-02
Log-odds
0.5
1.0
1.5
2.0
2.5
3.0
3.5
4.0

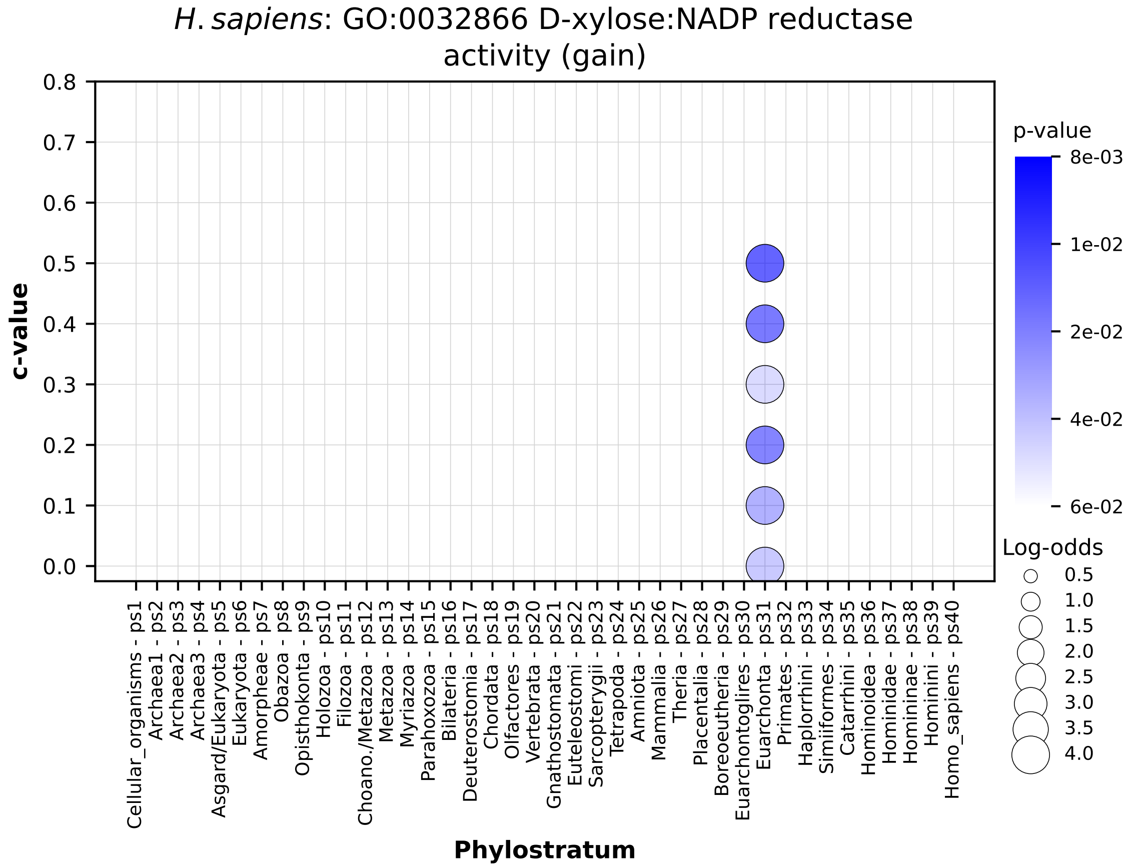

H. sapiens: GO:0032866 D-xylose:NADP reductase activity (gain)
c-value
0.8
0.7
0.6
0.5
0.4
0.3
0.2
0.1
0.0
Phylostratum
Cellular_organisms - ps1
Archaea1 - ps2
Archaea2 - ps3
Archaea3 - ps4
Asgard/Eukaryota - ps5
Eukaryota - ps6
Amorpheae - ps7
Obazoa - ps8
Opisthokonta - ps9
Holozoa - ps10
Filozoa - ps11
Choano./Metazoa - ps12
Metazoa - ps13
Myriazoa - ps14
Parahoxozoa - ps15
Bilateria - ps16
Deuterostomia - ps17
Chordata - ps18
Olfactores - ps19
Vertebrata - ps20
Gnathostomata - ps21
Euteleostomi - ps22
Sarcopterygii - ps23
Tetrapoda - ps24
Amniota - ps25
Mammalia - ps26
Theria - ps27
Placentalia - ps28
Boreoeutheria - ps29
Euarchontoglires - ps30
Euarchonta - ps31
Primates - ps32
Haplorrhini - ps33
Simiiformes - ps34
Catarrhini - ps35
Hominoidea - ps36
Hominidae - ps37
Homininae - ps38
Hominini - ps39
Homo_sapiens - ps40
p-value
8e-03
1e-02
2e-02
4e-02
6e-02
Log-odds
0.5
1.0
1.5
2.0
2.5
3.0
3.5
4.0

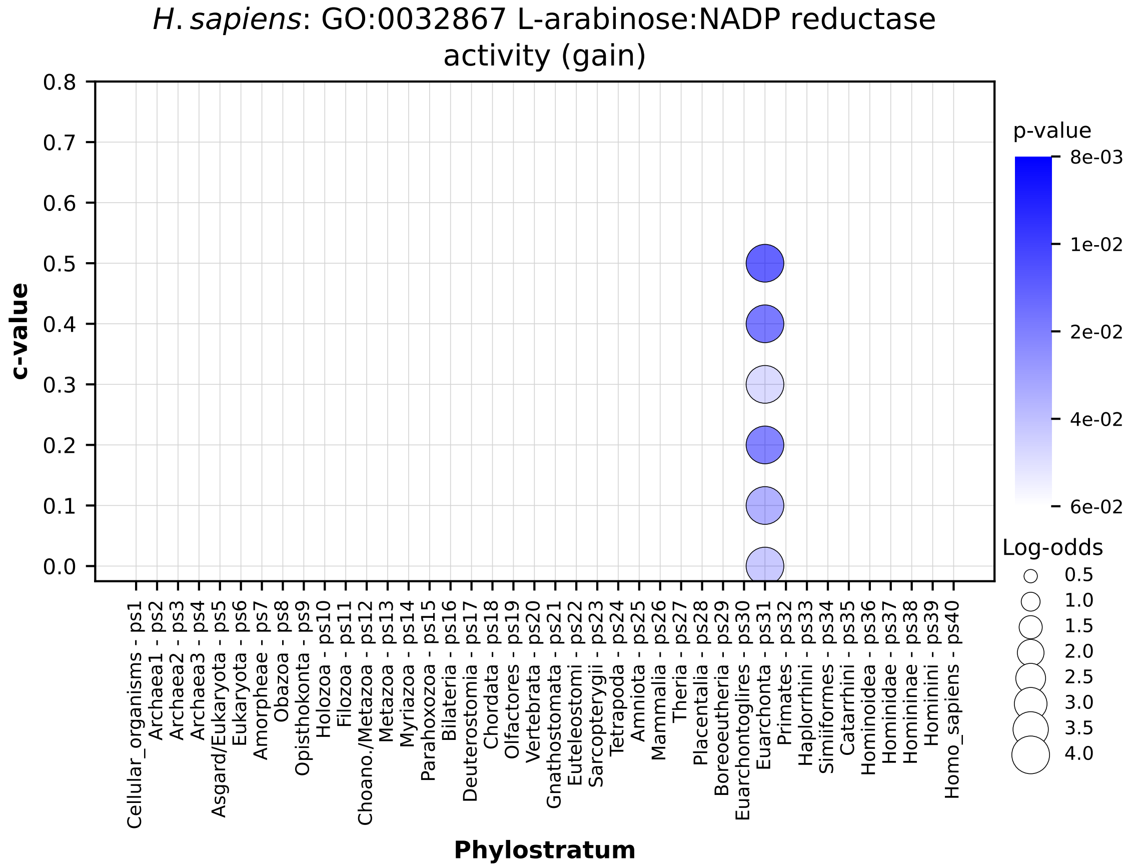

H. sapiens: GO:0032867 L-arabinose:NADP reductase activity (gain)
c-value
0.0
0.1
0.2
0.3
0.4
0.5
0.6
0.7
0.8
Phylostratum
Cellular_organisms - ps1
Archaea1 - ps2
Archaea2 - ps3
Archaea3 - ps4
Asgard/Eukaryota - ps5
Eukaryota - ps6
Amorpheae - ps7
Obazoa - ps8
Opisthokonta - ps9
Holozoa - ps10
Filozoa - ps11
Choano./Metazoa - ps12
Metazoa - ps13
Myriazoa - ps14
Parahoxozoa - ps15
Bilateria - ps16
Deuterostomia - ps17
Chordata - ps18
Olfactores - ps19
Vertebrata - ps20
Gnathostomata - ps21
Euteleostomi - ps22
Sarcopterygii - ps23
Tetrapoda - ps24
Amniota - ps25
Mammalia - ps26
Theria - ps27
Placentalia - ps28
Boreoeutheria - ps29
Euarchontoglires - ps30
Euarchonta - ps31
Primates - ps32
Haplorrhini - ps33
Simiiformes - ps34
Catarrhini - ps35
Hominoidea - ps36
Hominidae - ps37
Homininae - ps38
Hominini - ps39
Homo_sapiens - ps40
p-value
8e-03
1e-02
2e-02
4e-02
6e-02
Log-odds
0.5
1.0
1.5
2.0
2.5
3.0
3.5
4.0

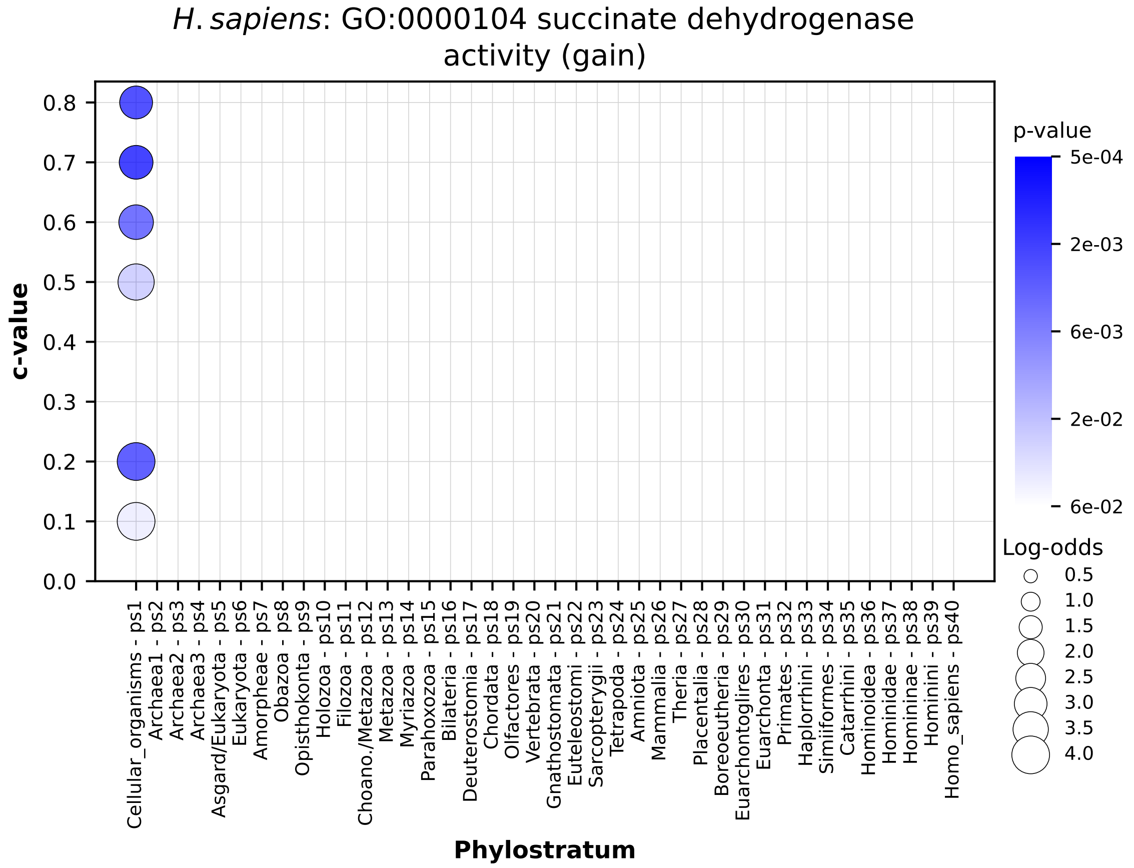

H. sapiens: GO:0000104 succinate dehydrogenase activity (gain)
c-value
0.0
0.1
0.2
0.3
0.4
0.5
0.6
0.7
0.8
Cellular_organisms - ps1
Archaea1 - ps2
Archaea2 - ps3
Archaea3 - ps4
Asgard/Eukaryota - ps5
Eukaryota - ps6
Amorpheae - ps7
Obazoa - ps8
Opisthokonta - ps9
Holozoa - ps10
Filozoa - ps11
Choano./Metazoa - ps12
Metazoa - ps13
Myriazoa - ps14
Parahoxozoa - ps15
Bilateria - ps16
Deuterostomia - ps17
Chordata - ps18
Olfactores - ps19
Vertebrata - ps20
Gnathostomata - ps21
Euteleostomi - ps22
Sarcopterygii - ps23
Tetrapoda - ps24
Amniota - ps25
Mammalia - ps26
Theria - ps27
Placentalia - ps28
Boreoeutheria - ps29
Euarchontoglires - ps30
Euarchonta - ps31
Primates - ps32
Haplorrhini - ps33
Simiiformes - ps34
Catarrhini - ps35
Hominoidea - ps36
Hominidae - ps37
Homininae - ps38
Hominini - ps39
Homo_sapiens - ps40
Phylostratum
p-value
5e-04
2e-03
6e-03
2e-02
6e-02
Log-odds
0.5
1.0
1.5
2.0
2.5
3.0
3.5
4.0

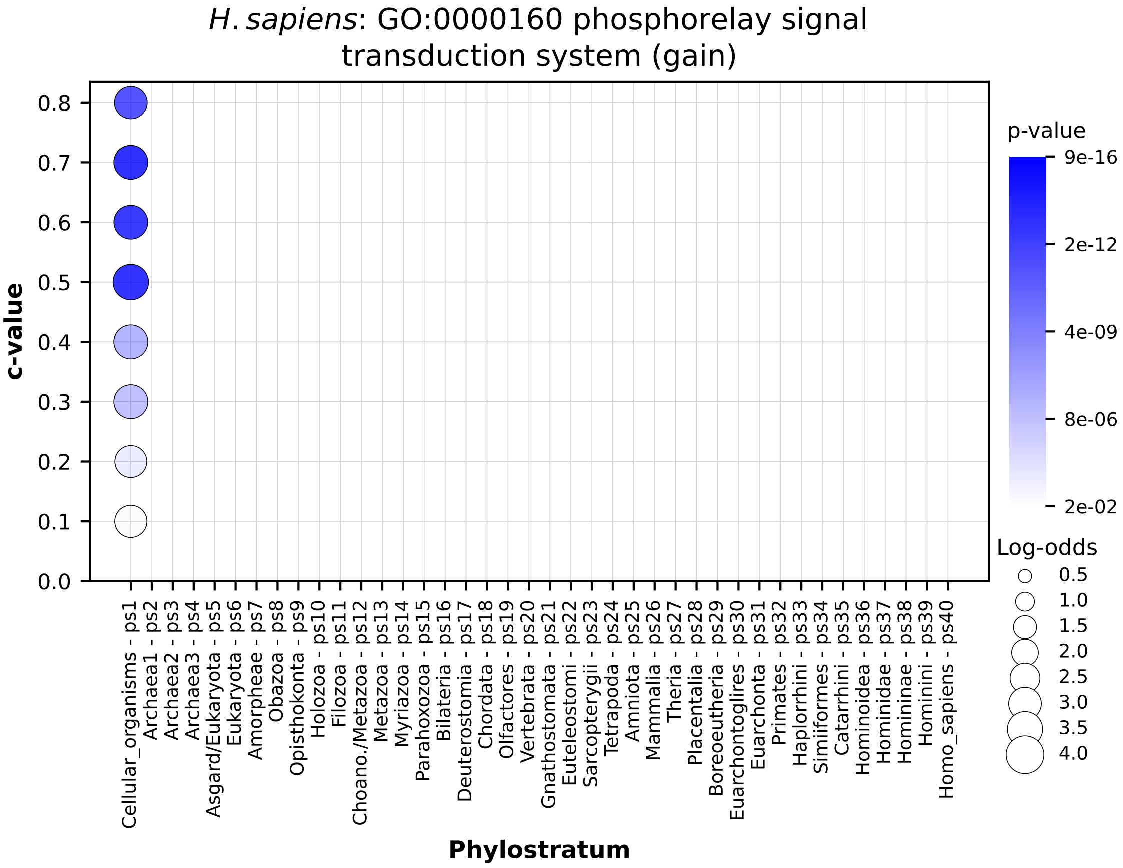

H. sapiens: GO:0000160 phosphorelay signal transduction system (gain)
c-value
0.0
0.1
0.2
0.3
0.4
0.5
0.6
0.7
0.8
Phylostratum
Cellular_organisms - ps1
Archaea1 - ps2
Archaea2 - ps3
Archaea3 - ps4
Asgard/Eukaryota - ps5
Eukaryota - ps6
Amorpheae - ps7
Obazoa - ps8
Opisthokonta - ps9
Holozoa - ps10
Filozoa - ps11
Choano./Metazoa - ps12
Metazoa - ps13
Myriazoa - ps14
Parahoxozoa - ps15
Bilateria - ps16
Deuterostomia - ps17
Chordata - ps18
Olfactores - ps19
Vertebrata - ps20
Gnathostomata - ps21
Euteleostomi - ps22
Sarcopterygii - ps23
Tetrapoda - ps24
Amniota - ps25
Mammalia - ps26
Theria - ps27
Placentalia - ps28
Boreoeutheria - ps29
Euarchontoglires - ps30
Euarchonta - ps31
Primates - ps32
Haplorrhini - ps33
Simiiformes - ps34
Catarrhini - ps35
Hominoidea - ps36
Hominidae - ps37
Homininae - ps38
Hominini - ps39
Homo_sapiens - ps40
p-value
9e-16
2e-12
4e-09
8e-06
2e-02
Log-odds
0.5
1.0
1.5
2.0
2.5
3.0
3.5
4.0

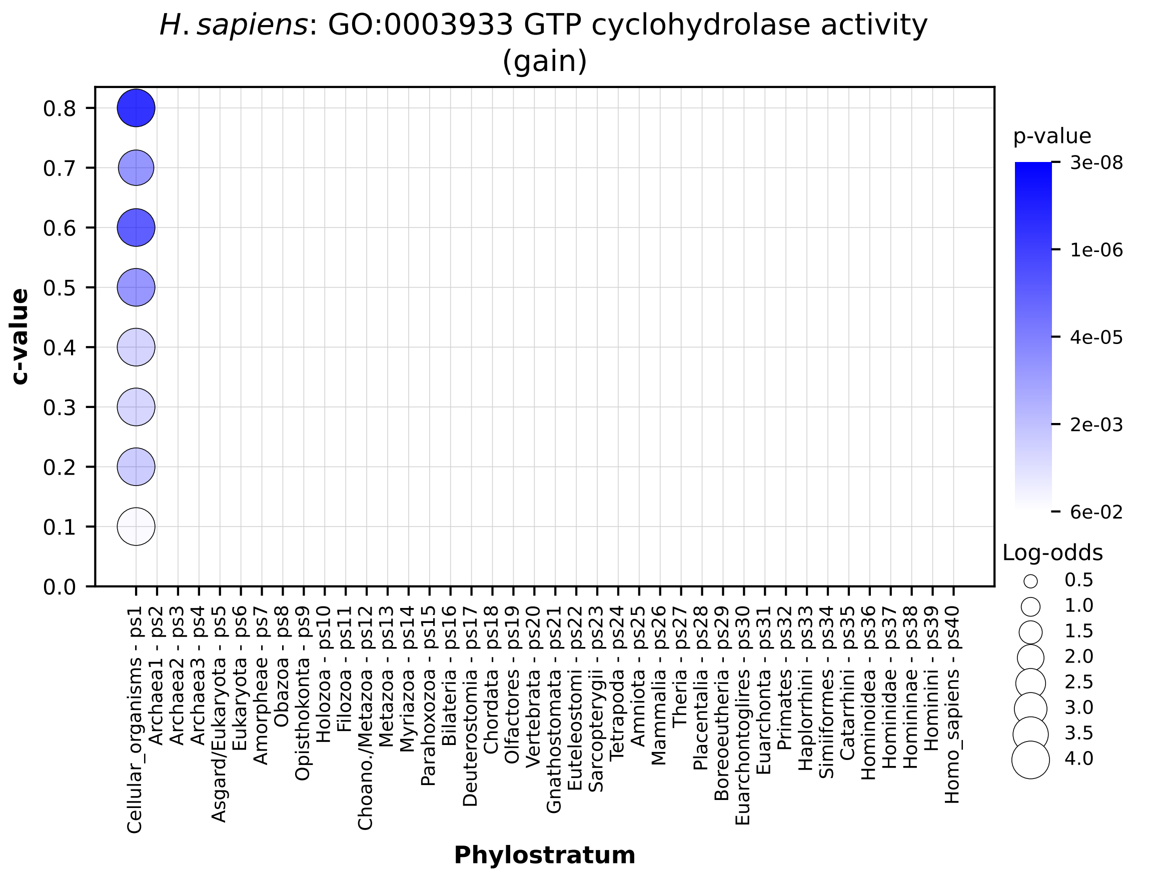

*H. sapiens*: GO:0003933 GTP cyclohydrolase activity
(gain)
c-value
0.0
0.1
0.2
0.3
0.4
0.5
0.6
0.7
0.8
Phylostratum
Cellular_organisms - ps1
Archaea1 - ps2
Archaea2 - ps3
Archaea3 - ps4
Asgard/Eukaryota - ps5
Eukaryota - ps6
Amorpheae - ps7
Obazoa - ps8
Opisthokonta - ps9
Holozoa - ps10
Filozoa - ps11
Choano./Metazoa - ps12
Metazoa - ps13
Myriazoa - ps14
Parahoxozoa - ps15
Bilateria - ps16
Deuterostomia - ps17
Chordata - ps18
Olfactores - ps19
Vertebrata - ps20
Gnathostomata - ps21
Euteleostomi - ps22
Sarcopterygii - ps23
Tetrapoda - ps24
Amniota - ps25
Mammalia - ps26
Theria - ps27
Placentalia - ps28
Boreoeutheria - ps29
Euarchontoglires - ps30
Euarchonta - ps31
Primates - ps32
Haplorrhini - ps33
Simiiformes - ps34
Catarrhini - ps35
Hominoidea - ps36
Hominidae - ps37
Homininae - ps38
Hominini - ps39
Homo_sapiens - ps40
p-value
3e-08
1e-06
4e-05
2e-03
6e-02
Log-odds
0.5
1.0
1.5
2.0
2.5
3.0
3.5
4.0

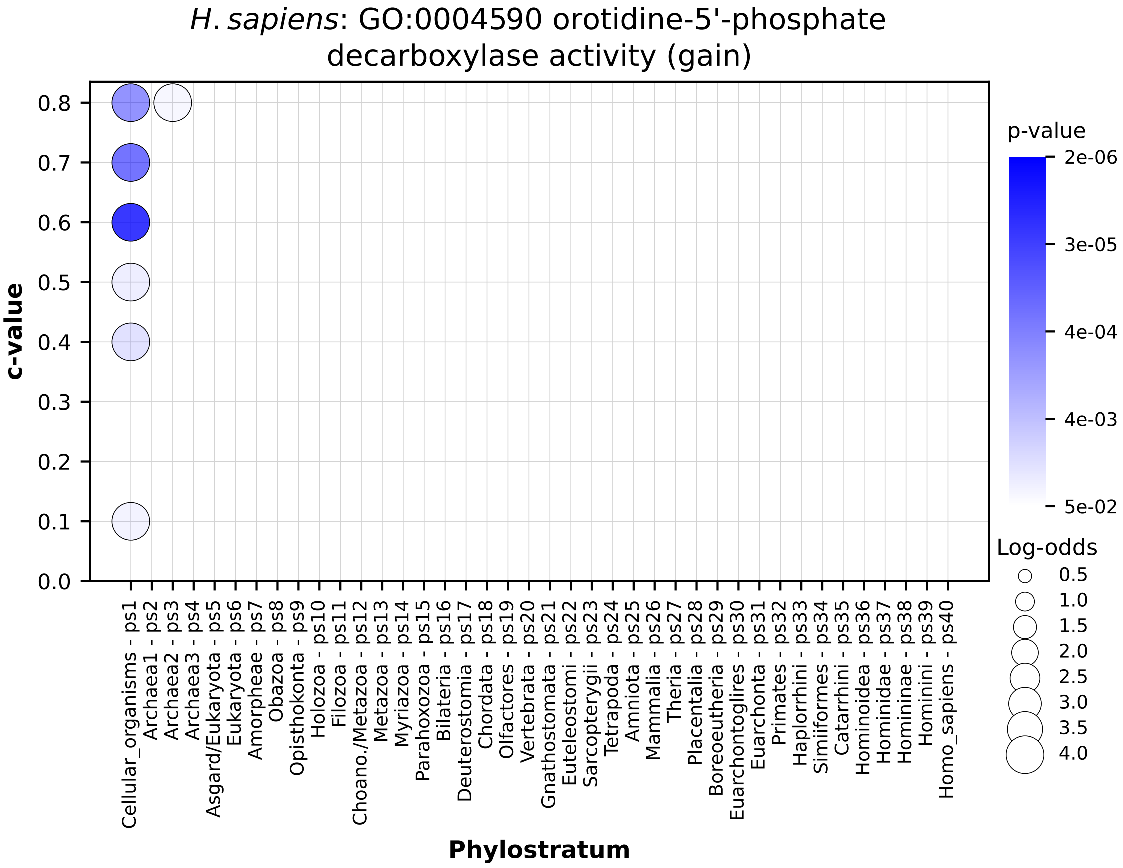
H. sapiens: GO:0004590 orotidine-5'-phosphate decarboxylase activity (gain)
c-value
0.0
0.1
0.2
0.3
0.4
0.5
0.6
0.7
0.8
Phylostratum
Cellular_organisms - ps1
Archaea1 - ps2
Archaea2 - ps3
Archaea3 - ps4
Asgard/Eukaryota - ps5
Eukaryota - ps6
Amorpheae - ps7
Obazoa - ps8
Opisthokonta - ps9
Holozoa - ps10
Filozoa - ps11
Choano./Metazoa - ps12
Metazoa - ps13
Myriazoa - ps14
Parahoxozoa - ps15
Bilateria - ps16
Deuterostomia - ps17
Chordata - ps18
Olfactores - ps19
Vertebrata - ps20
Gnathostomata - ps21
Euteleostomi - ps22
Sarcopterygii - ps23
Tetrapoda - ps24
Amniota - ps25
Mammalia - ps26
Theria - ps27
Placentalia - ps28
Boreoeutheria - ps29
Euarchontoglires - ps30
Euarchonta - ps31
Primates - ps32
Haplorrhini - ps33
Simiiformes - ps34
Catarrhini - ps35
Hominoidea - ps36
Hominidae - ps37
Homininae - ps38
Hominini - ps39
Homo_sapiens - ps40
p-value
2e-06
3e-05
4e-04
4e-03
5e-02
Log-odds
0.5
1.0
1.5
2.0
2.5
3.0
3.5
4.0

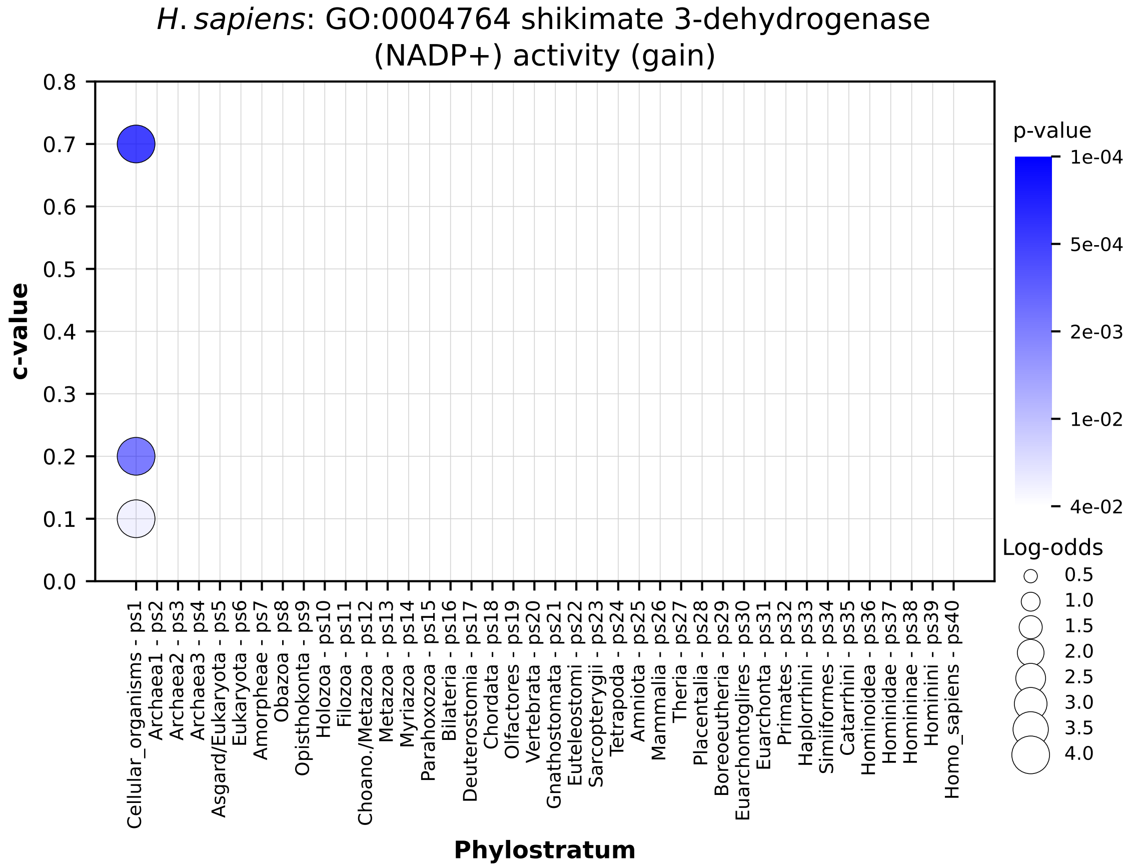

H. sapiens: GO:0004764 shikimate 3-dehydrogenase (NADP+) activity (gain)
c-value
0.0
0.1
0.2
0.3
0.4
0.5
0.6
0.7
0.8
Phylostratum
Cellular_organisms - ps1
Archaea1 - ps2
Archaea2 - ps3
Archaea3 - ps4
Asgard/Eukaryota - ps5
Eukaryota - ps6
Amorpheae - ps7
Obazoa - ps8
Opisthokonta - ps9
Holozoa - ps10
Filozoa - ps11
Choano./Metazoa - ps12
Metazoa - ps13
Myriazoa - ps14
Parahoxozoa - ps15
Bilateria - ps16
Deuterostomia - ps17
Chordata - ps18
Olfactores - ps19
Vertebrata - ps20
Gnathostomata - ps21
Euteleostomi - ps22
Sarcopterygii - ps23
Tetrapoda - ps24
Amniota - ps25
Mammalia - ps26
Theria - ps27
Placentalia - ps28
Boreoeutheria - ps29
Euarchontoglires - ps30
Euarchonta - ps31
Primates - ps32
Haplorrhini - ps33
Simiiformes - ps34
Catarrhini - ps35
Hominoidea - ps36
Hominidae - ps37
Homininae - ps38
Hominini - ps39
Homo_sapiens - ps40
p-value
1e-04
5e-04
2e-03
1e-02
4e-02
Log-odds
0.5
1.0
1.5
2.0
2.5
3.0
3.5
4.0

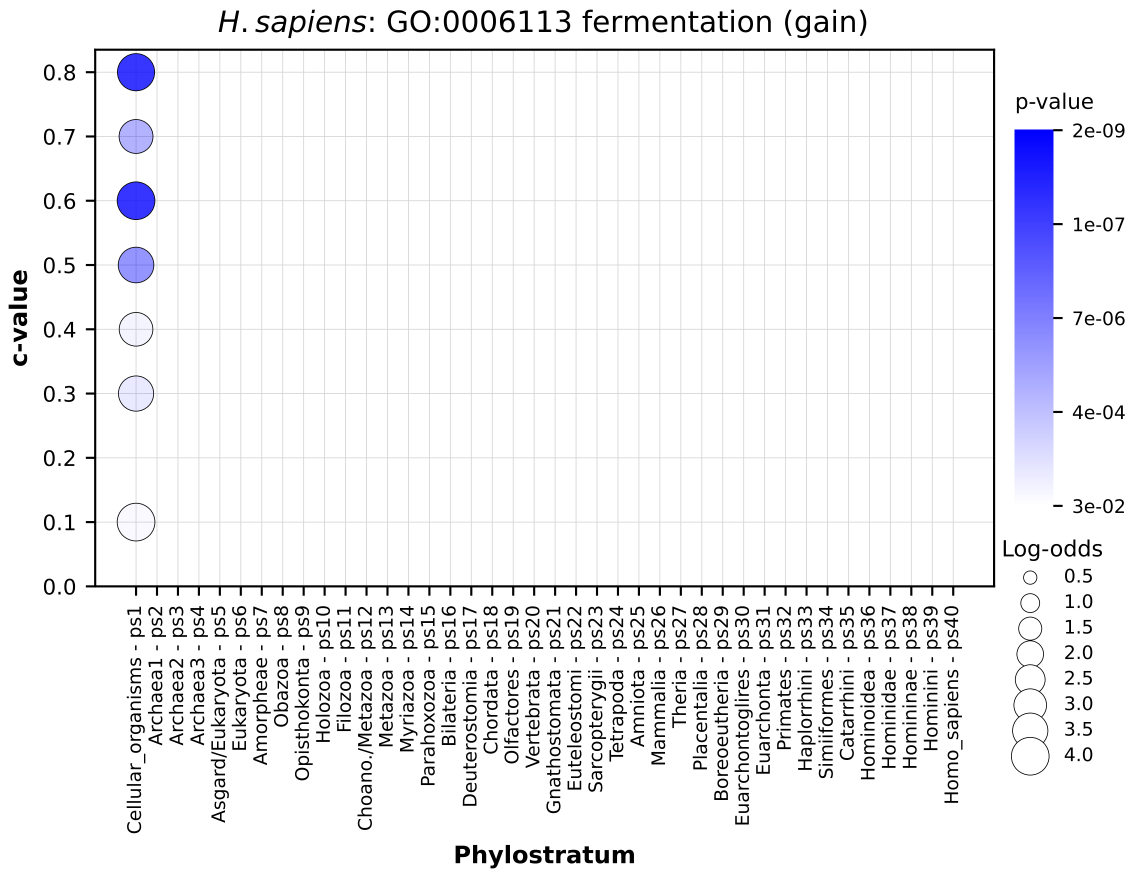

*H. sapiens*: GO:0006113 fermentation (gain)
c-value
0.0
0.1
0.2
0.3
0.4
0.5
0.6
0.7
0.8
Phylostratum
Cellular_organisms - ps1
Archaea1 - ps2
Archaea2 - ps3
Archaea3 - ps4
Asgard/Eukaryota - ps5
Eukaryota - ps6
Amorpheae - ps7
Obazoa - ps8
Opisthokonta - ps9
Holozoa - ps10
Filozoa - ps11
Choano./Metazoa - ps12
Metazoa - ps13
Myriazoa - ps14
Parahoxozoa - ps15
Bilateria - ps16
Deuterostomia - ps17
Chordata - ps18
Olfactores - ps19
Vertebrata - ps20
Gnathostomata - ps21
Euteleostomi - ps22
Sarcopterygii - ps23
Tetrapoda - ps24
Amniota - ps25
Mammalia - ps26
Theria - ps27
Placentalia - ps28
Boreoeutheria - ps29
Euarchontoglires - ps30
Euarchonta - ps31
Primates - ps32
Haplorrhini - ps33
Simiiformes - ps34
Catarrhini - ps35
Hominoidea - ps36
Hominidae - ps37
Homininae - ps38
Hominini - ps39
Homo_sapiens - ps40
p-value
2e-09
1e-07
7e-06
4e-04
3e-02
Log-odds
0.5
1.0
1.5
2.0
2.5
3.0
3.5
4.0

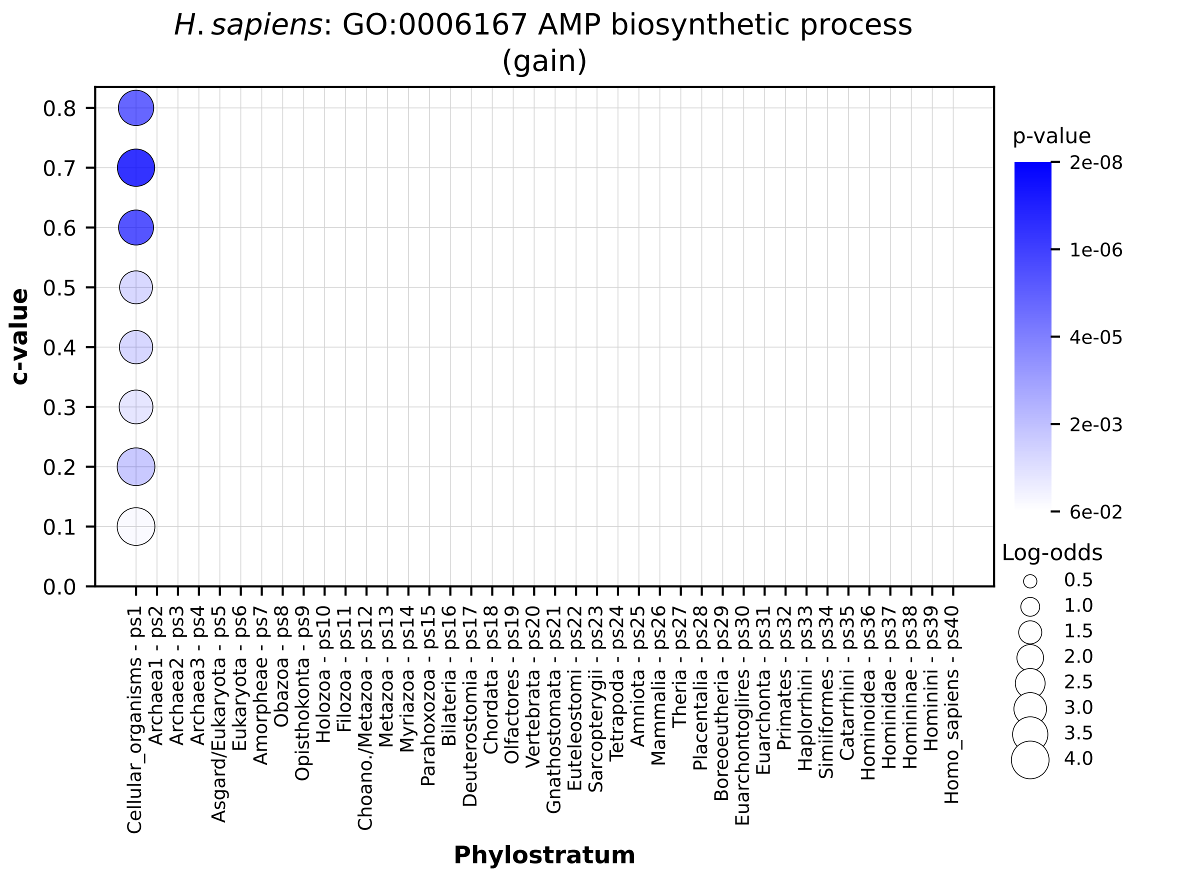

*H. sapiens*: GO:0006167 AMP biosynthetic process
(gain)
c-value
0.0
0.1
0.2
0.3
0.4
0.5
0.6
0.7
0.8
p-value
2e-08
1e-06
4e-05
2e-03
6e-02
Log-odds
0.5
1.0
1.5
2.0
2.5
3.0
3.5
4.0
Cellular_organisms - ps1
Archaea1 - ps2
Archaea2 - ps3
Archaea3 - ps4
Asgard/Eukaryota - ps5
Eukaryota - ps6
Amorpheae - ps7
Obazoa - ps8
Opisthokonta - ps9
Holozoa - ps10
Filozoa - ps11
Choano./Metazoa - ps12
Metazoa - ps13
Myriazoa - ps14
Parahoxozoa - ps15
Bilateria - ps16
Deuterostomia - ps17
Chordata - ps18
Olfactores - ps19
Vertebrata - ps20
Gnathostomata - ps21
Euteleostomi - ps22
Sarcopterygii - ps23
Tetrapoda - ps24
Amniota - ps25
Mammalia - ps26
Theria - ps27
Placentalia - ps28
Boreoeutheria - ps29
Euarchontoglires - ps30
Euarchonta - ps31
Primates - ps32
Haplorrhini - ps33
Simiiformes - ps34
Catarrhini - ps35
Hominoidea - ps36
Hominidae - ps37
Homininae - ps38
Hominini - ps39
Homo_sapiens - ps40
Phylostratum

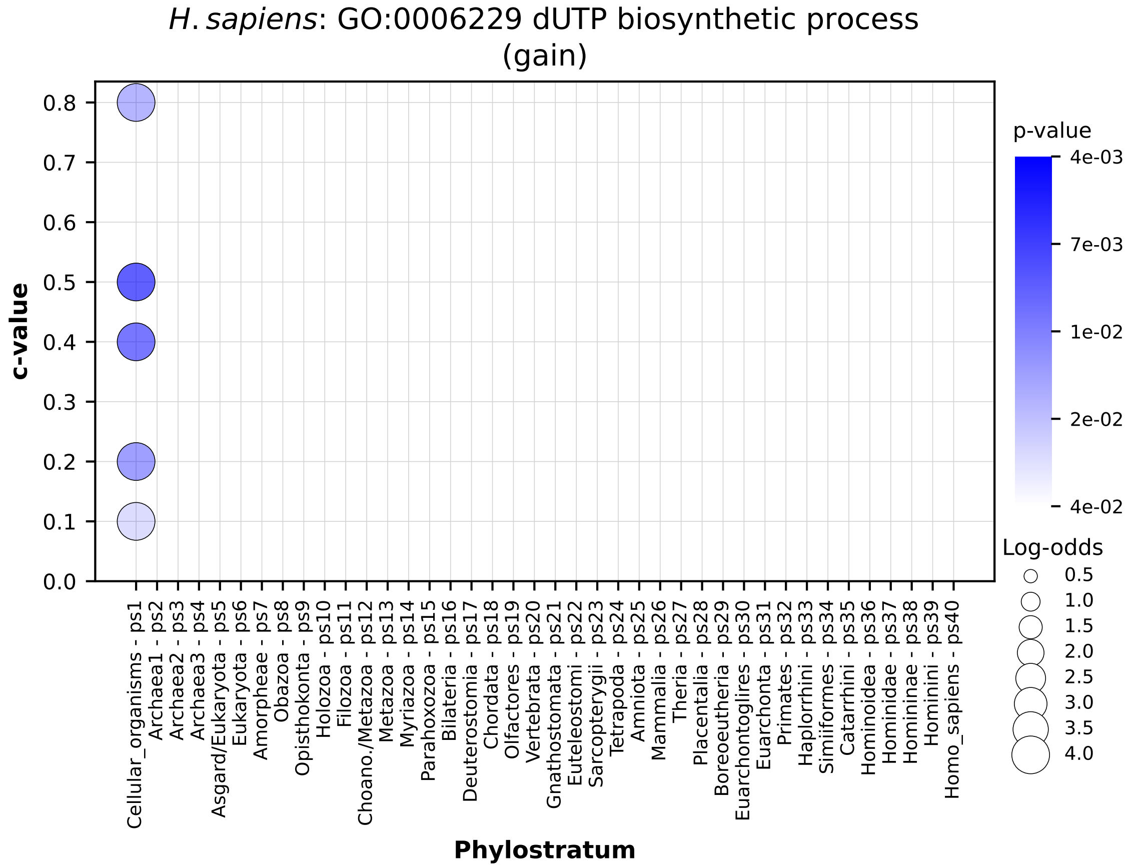
H. sapiens: GO:0006229 dUTP biosynthetic process
(gain)
c-value
0.0
0.1
0.2
0.3
0.4
0.5
0.6
0.7
0.8
Phylostratum
Cellular_organisms - ps1
Archaea1 - ps2
Archaea2 - ps3
Archaea3 - ps4
Asgard/Eukaryota - ps5
Eukaryota - ps6
Amorpheae - ps7
Obazoa - ps8
Opisthokonta - ps9
Holozoa - ps10
Filozoa - ps11
Choano./Metazoa - ps12
Metazoa - ps13
Myriazoa - ps14
Parahoxozoa - ps15
Bilateria - ps16
Deuterostomia - ps17
Chordata - ps18
Olfactores - ps19
Vertebrata - ps20
Gnathostomata - ps21
Euteleostomi - ps22
Sarcopterygii - ps23
Tetrapoda - ps24
Amniota - ps25
Mammalia - ps26
Theria - ps27
Placentalia - ps28
Boreoeutheria - ps29
Euarchontoglires - ps30
Euarchonta - ps31
Primates - ps32
Haplorrhini - ps33
Simiiformes - ps34
Catarrhini - ps35
Hominoidea - ps36
Hominidae - ps37
Homininae - ps38
Hominini - ps39
Homo_sapiens - ps40
p-value
4e-03
7e-03
1e-02
2e-02
4e-02
Log-odds
0.5
1.0
1.5
2.0
2.5
3.0
3.5
4.0

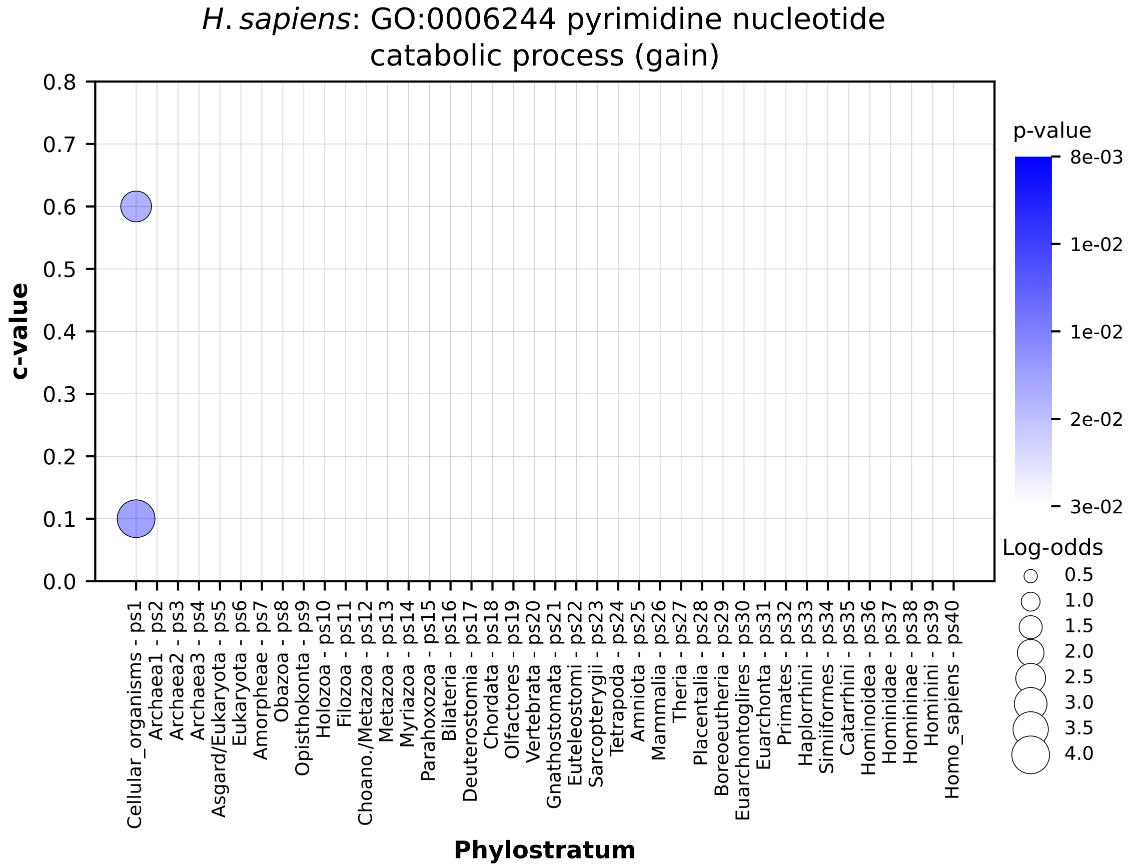

H. sapiens: GO:0006244 pyrimidine nucleotide catabolic process (gain)
c-value
0.0
0.1
0.2
0.3
0.4
0.5
0.6
0.7
0.8
Phylostratum
Cellular_organisms - ps1
Archaea1 - ps2
Archaea2 - ps3
Archaea3 - ps4
Asgard/Eukaryota - ps5
Eukaryota - ps6
Amorpheae - ps7
Obazoa - ps8
Opisthokonta - ps9
Holozoa - ps10
Filozoa - ps11
Choano./Metazoa - ps12
Metazoa - ps13
Myriazoa - ps14
Parahoxozoa - ps15
Bilateria - ps16
Deuterostomia - ps17
Chordata - ps18
Olfactores - ps19
Vertebrata - ps20
Gnathostomata - ps21
Euteleostomi - ps22
Sarcopterygii - ps23
Tetrapoda - ps24
Amniota - ps25
Mammalia - ps26
Theria - ps27
Placentalia - ps28
Boreoeutheria - ps29
Euarchontoglires - ps30
Euarchonta - ps31
Primates - ps32
Haplorrhini - ps33
Simiiformes - ps34
Catarrhini - ps35
Hominoidea - ps36
Hominidae - ps37
Homininae - ps38
Hominini - ps39
Homo_sapiens - ps40
p-value
8e-03
1e-02
1e-02
2e-02
3e-02
Log-odds
0.5
1.0
1.5
2.0
2.5
3.0
3.5
4.0

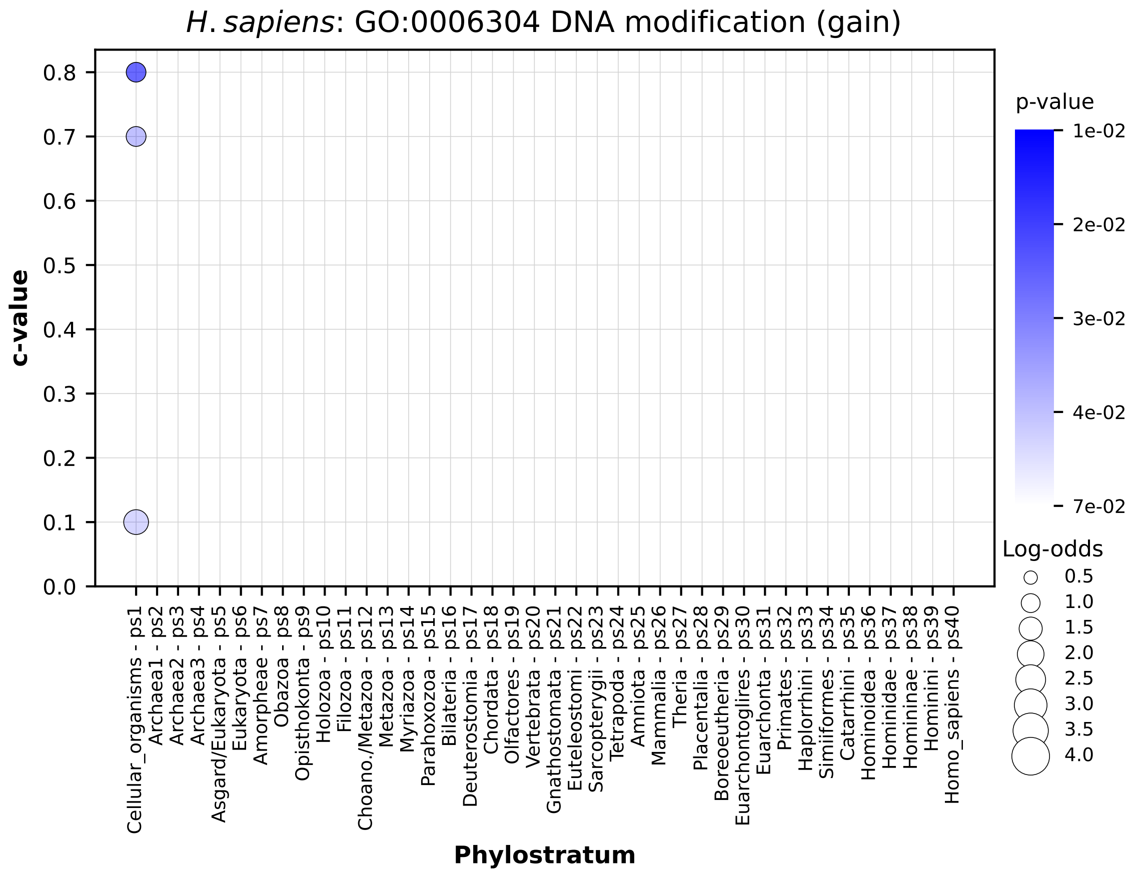

H. sapiens: GO:0006304 DNA modification (gain)
c-value
0.0
0.1
0.2
0.3
0.4
0.5
0.6
0.7
0.8
Phylostratum
Cellular_organisms - ps1
Archaea1 - ps2
Archaea2 - ps3
Archaea3 - ps4
Asgard/Eukaryota - ps5
Eukaryota - ps6
Amorpheae - ps7
Obazoa - ps8
Opisthokonta - ps9
Holozoa - ps10
Filozoa - ps11
Choano./Metazoa - ps12
Metazoa - ps13
Myriazoa - ps14
Parahoxozoa - ps15
Bilateria - ps16
Deuterostomia - ps17
Chordata - ps18
Olfactores - ps19
Vertebrata - ps20
Gnathostomata - ps21
Euteleostomi - ps22
Sarcopterygii - ps23
Tetrapoda - ps24
Amniota - ps25
Mammalia - ps26
Theria - ps27
Placentalia - ps28
Boreoeutheria - ps29
Euarchontoglires - ps30
Euarchonta - ps31
Primates - ps32
Haplorrhini - ps33
Simiiformes - ps34
Catarrhini - ps35
Hominoidea - ps36
Hominidae - ps37
Homininae - ps38
Hominini - ps39
Homo_sapiens - ps40
p-value
1e-02
2e-02
3e-02
4e-02
7e-02
Log-odds
0.5
1.0
1.5
2.0
2.5
3.0
3.5
4.0

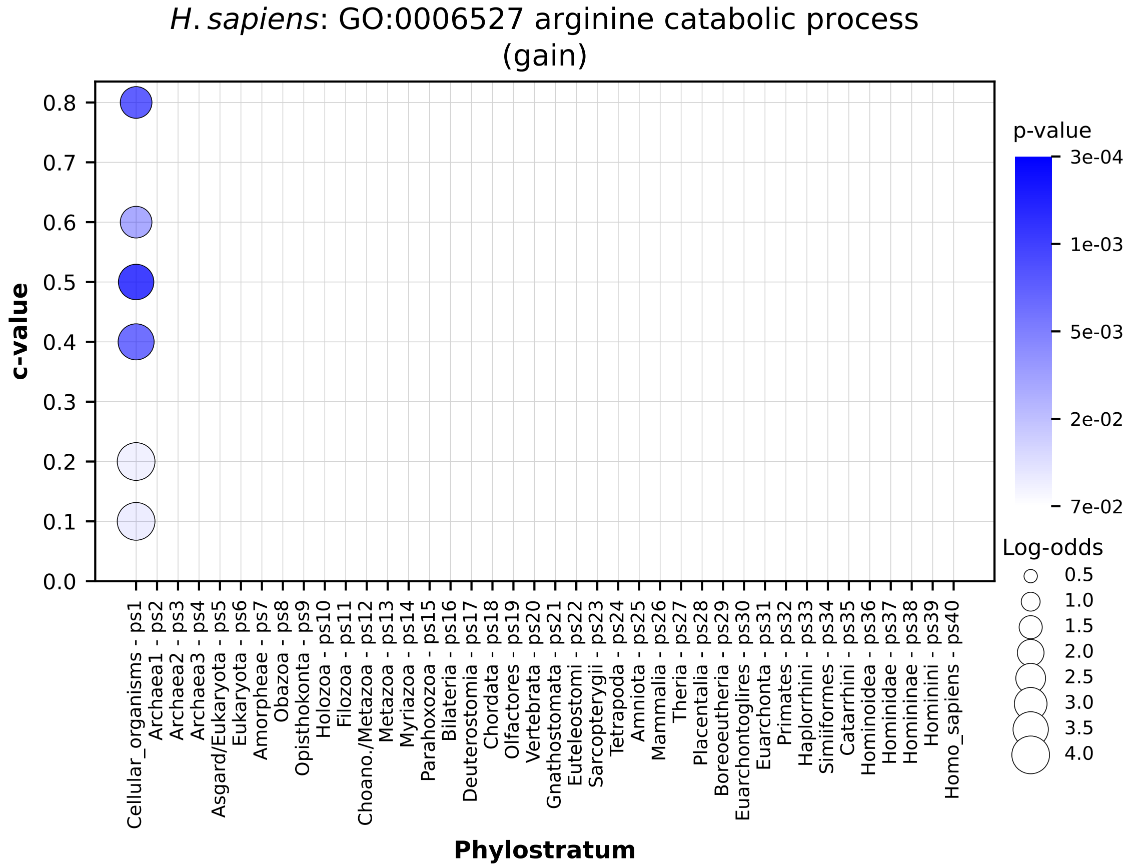
*H. sapiens*: GO:0006527 arginine catabolic process
(gain)
c-value
0.0
0.1
0.2
0.3
0.4
0.5
0.6
0.7
0.8
Phylostratum
Cellular_organisms - ps1
Archaea1 - ps2
Archaea2 - ps3
Archaea3 - ps4
Asgard/Eukaryota - ps5
Eukaryota - ps6
Amorpheae - ps7
Obazoa - ps8
Opisthokonta - ps9
Holozoa - ps10
Filozoa - ps11
Choano./Metazoa - ps12
Metazoa - ps13
Myriazoa - ps14
Parahoxozoa - ps15
Bilateria - ps16
Deuterostomia - ps17
Chordata - ps18
Olfactores - ps19
Vertebrata - ps20
Gnathostomata - ps21
Euteleostomi - ps22
Sarcopterygii - ps23
Tetrapoda - ps24
Amniota - ps25
Mammalia - ps26
Theria - ps27
Placentalia - ps28
Boreoeutheria - ps29
Euarchontoglires - ps30
Euarchonta - ps31
Primates - ps32
Haplorrhini - ps33
Simiiformes - ps34
Catarrhini - ps35
Hominoidea - ps36
Hominidae - ps37
Homininae - ps38
Hominini - ps39
Homo_sapiens - ps40
p-value
3e-04
1e-03
5e-03
2e-02
7e-02
Log-odds
0.5
1.0
1.5
2.0
2.5
3.0
3.5
4.0

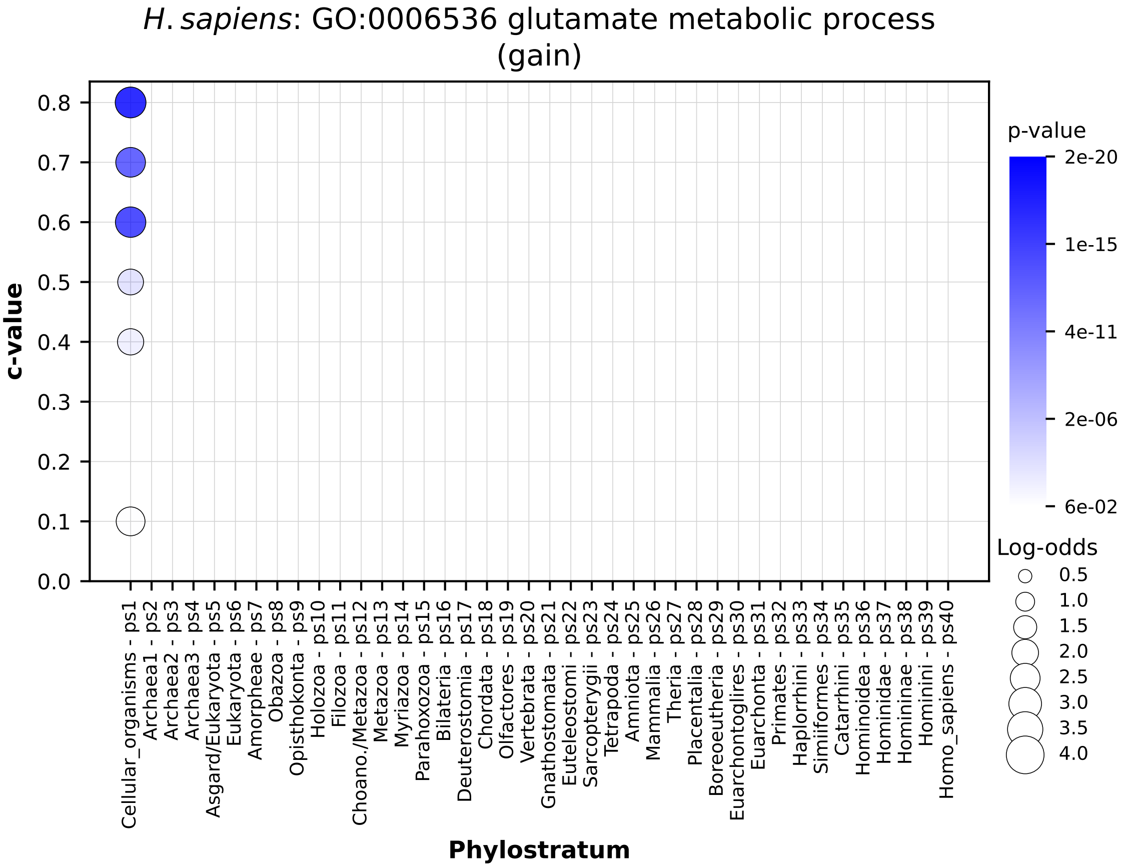

H. sapiens: GO:0006536 glutamate metabolic process
(gain)
c-value
0.0
0.1
0.2
0.3
0.4
0.5
0.6
0.7
0.8
Phylostratum
Cellular_organisms - ps1
Archaea1 - ps2
Archaea2 - ps3
Archaea3 - ps4
Asgard/Eukaryota - ps5
Eukaryota - ps6
Amorpheae - ps7
Obazoa - ps8
Opisthokonta - ps9
Holozoa - ps10
Filozoa - ps11
Choano./Metazoa - ps12
Metazoa - ps13
Myriazoa - ps14
Parahoxozoa - ps15
Bilateria - ps16
Deuterostomia - ps17
Chordata - ps18
Olfactores - ps19
Vertebrata - ps20
Gnathostomata - ps21
Euteleostomi - ps22
Sarcopterygii - ps23
Tetrapoda - ps24
Amniota - ps25
Mammalia - ps26
Theria - ps27
Placentalia - ps28
Boreoeutheria - ps29
Euarchontoglires - ps30
Euarchonta - ps31
Primates - ps32
Haplorrhini - ps33
Simiiformes - ps34
Catarrhini - ps35
Hominoidea - ps36
Hominidae - ps37
Homininae - ps38
Hominini - ps39
Homo_sapiens - ps40
p-value
2e-20
1e-15
4e-11
2e-06
6e-02
Log-odds
0.5
1.0
1.5
2.0
2.5
3.0
3.5
4.0

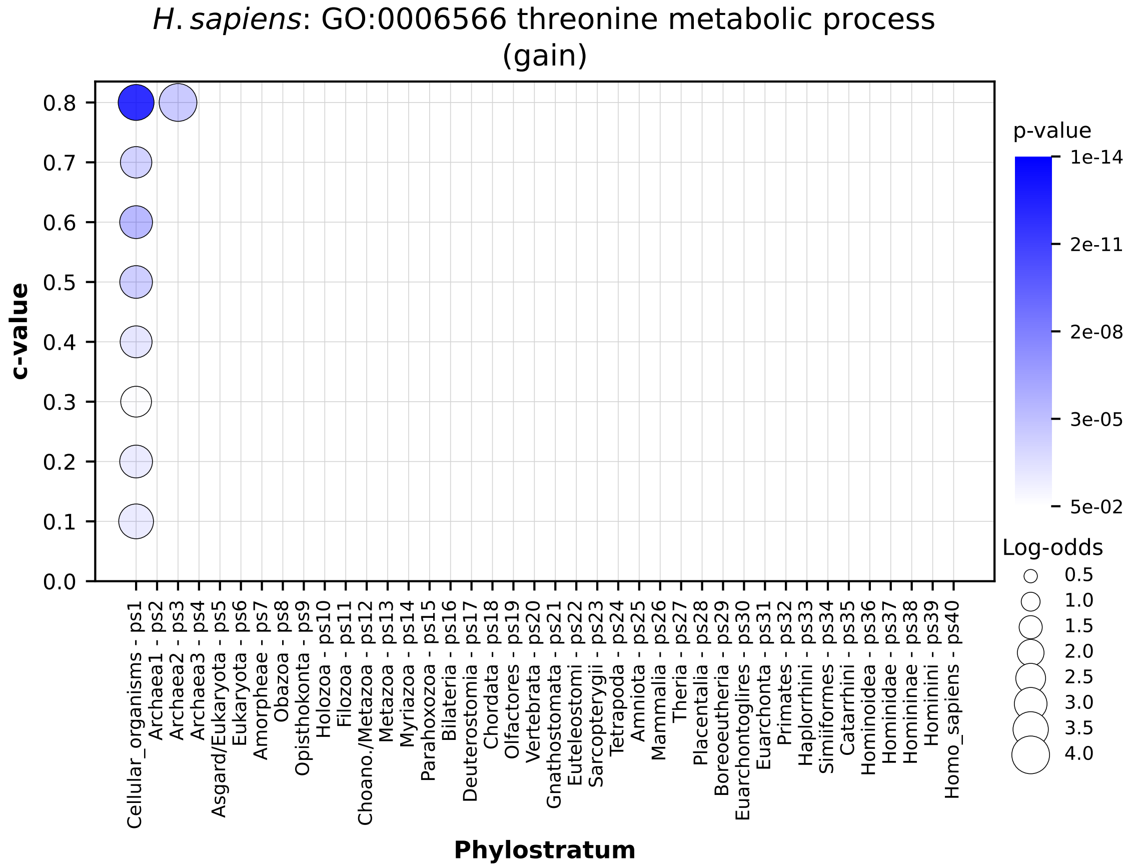
H. sapiens: GO:0006566 threonine metabolic process
(gain)
c-value
0.0
0.1
0.2
0.3
0.4
0.5
0.6
0.7
0.8
p-value
1e-14
2e-11
2e-08
3e-05
5e-02
Log-odds
0.5
1.0
1.5
2.0
2.5
3.0
3.5
4.0
Cellular_organisms - ps1
Archaea1 - ps2
Archaea2 - ps3
Archaea3 - ps4
Asgard/Eukaryota - ps5
Eukaryota - ps6
Amorpheae - ps7
Obazoa - ps8
Opisthokonta - ps9
Holozoa - ps10
Filozoa - ps11
Choano./Metazoa - ps12
Metazoa - ps13
Myriazoa - ps14
Parahoxozoa - ps15
Bilateria - ps16
Deuterostomia - ps17
Chordata - ps18
Olfactores - ps19
Vertebrata - ps20
Gnathostomata - ps21
Euteleostomi - ps22
Sarcopterygii - ps23
Tetrapoda - ps24
Amniota - ps25
Mammalia - ps26
Theria - ps27
Placentalia - ps28
Boreoeutheria - ps29
Euarchontoglires - ps30
Euarchonta - ps31
Primates - ps32
Haplorrhini - ps33
Simiiformes - ps34
Catarrhini - ps35
Hominoidea - ps36
Hominidae - ps37
Homininae - ps38
Hominini - ps39
Homo_sapiens - ps40
Phylostratum

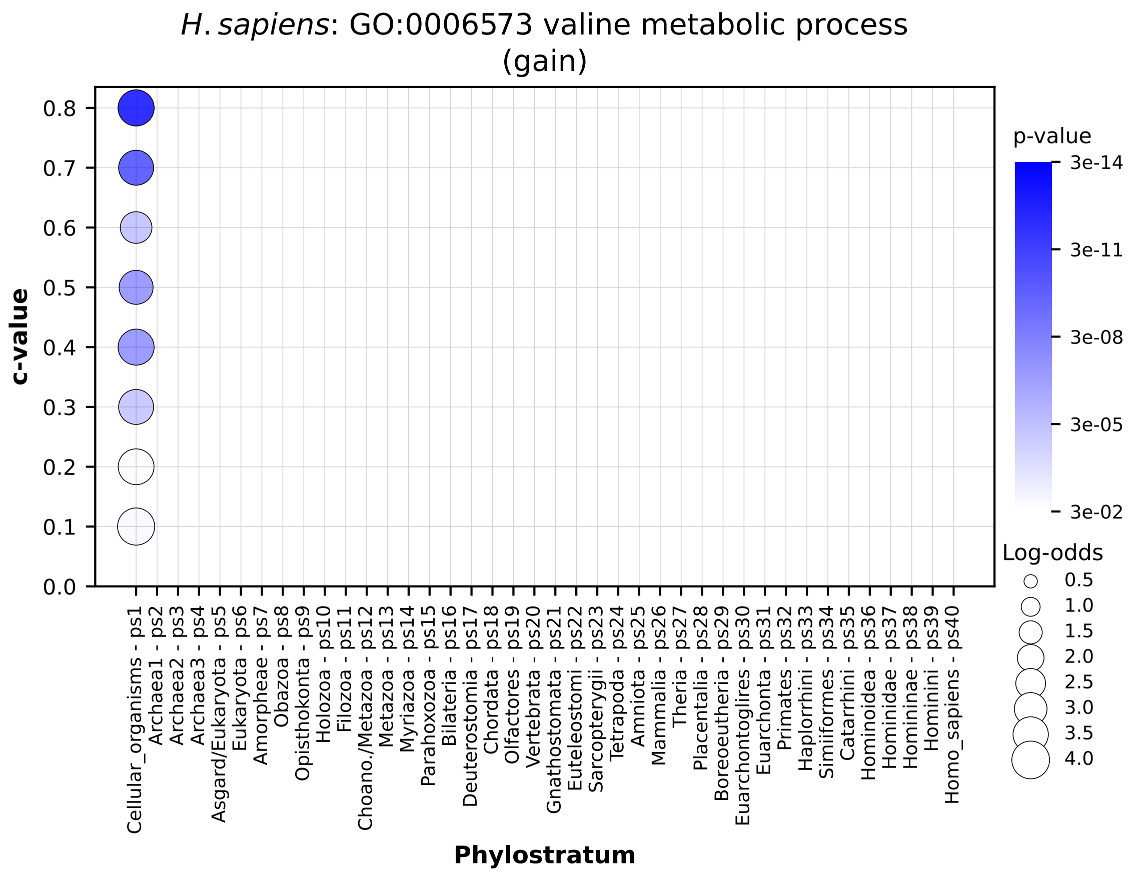

H. sapiens: GO:0006573 valine metabolic process
(gain)
c-value
0.0
0.1
0.2
0.3
0.4
0.5
0.6
0.7
0.8
p-value
3e-14
3e-11
3e-08
3e-05
3e-02
Log-odds
0.5
1.0
1.5
2.0
2.5
3.0
3.5
4.0
Cellular_organisms - ps1
Archaea1 - ps2
Archaea2 - ps3
Archaea3 - ps4
Asgard/Eukaryota - ps5
Eukaryota - ps6
Amorpheae - ps7
Obazoa - ps8
Opisthokonta - ps9
Holozoa - ps10
Filozoa - ps11
Choano./Metazoa - ps12
Metazoa - ps13
Myriazoa - ps14
Parahoxozoa - ps15
Bilateria - ps16
Deuterostomia - ps17
Chordata - ps18
Olfactores - ps19
Vertebrata - ps20
Gnathostomata - ps21
Euteleostomi - ps22
Sarcopterygii - ps23
Tetrapoda - ps24
Amniota - ps25
Mammalia - ps26
Theria - ps27
Placentalia - ps28
Boreoeutheria - ps29
Euarchontoglires - ps30
Euarchonta - ps31
Primates - ps32
Haplorrhini - ps33
Simiiformes - ps34
Catarrhini - ps35
Hominoidea - ps36
Hominidae - ps37
Homininae - ps38
Hominini - ps39
Homo_sapiens - ps40
Phylostratum

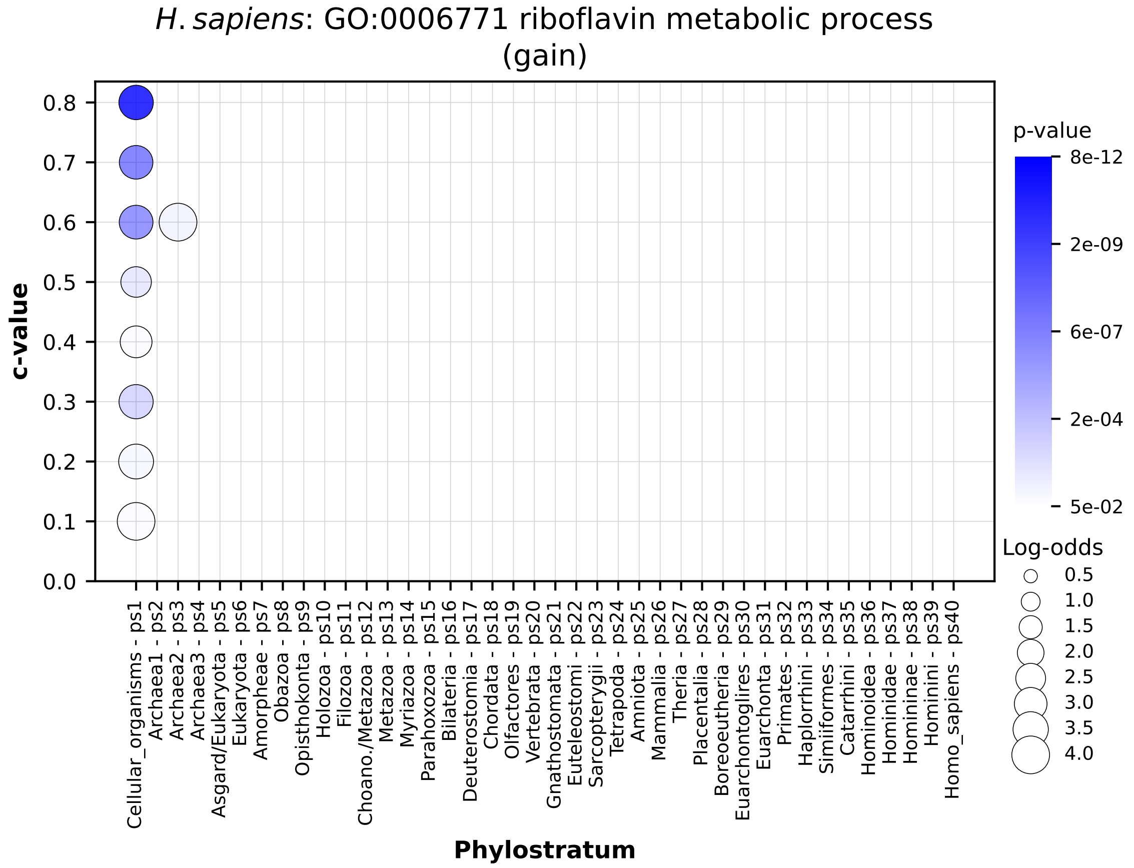

H. sapiens: GO:0006771 riboflavin metabolic process
(gain)
c-value
0.0
0.1
0.2
0.3
0.4
0.5
0.6
0.7
0.8
p-value
8e-12
2e-09
6e-07
2e-04
5e-02
Log-odds
0.5
1.0
1.5
2.0
2.5
3.0
3.5
4.0
Cellular_organisms - ps1
Archaea1 - ps2
Archaea2 - ps3
Archaea3 - ps4
Asgard/Eukaryota - ps5
Eukaryota - ps6
Amorpheae - ps7
Obazoa - ps8
Opisthokonta - ps9
Holozoa - ps10
Filozoa - ps11
Choano./Metazoa - ps12
Metazoa - ps13
Myriazoa - ps14
Parahoxozoa - ps15
Bilateria - ps16
Deuterostomia - ps17
Chordata - ps18
Olfactores - ps19
Vertebrata - ps20
Gnathostomata - ps21
Euteleostomi - ps22
Sarcopterygii - ps23
Tetrapoda - ps24
Amniota - ps25
Mammalia - ps26
Theria - ps27
Placentalia - ps28
Boreoeutheria - ps29
Euarchontoglires - ps30
Euarchonta - ps31
Primates - ps32
Haplorrhini - ps33
Simiiformes - ps34
Catarrhini - ps35
Hominoidea - ps36
Hominidae - ps37
Homininae - ps38
Hominini - ps39
Homo_sapiens - ps40
Phylostratum

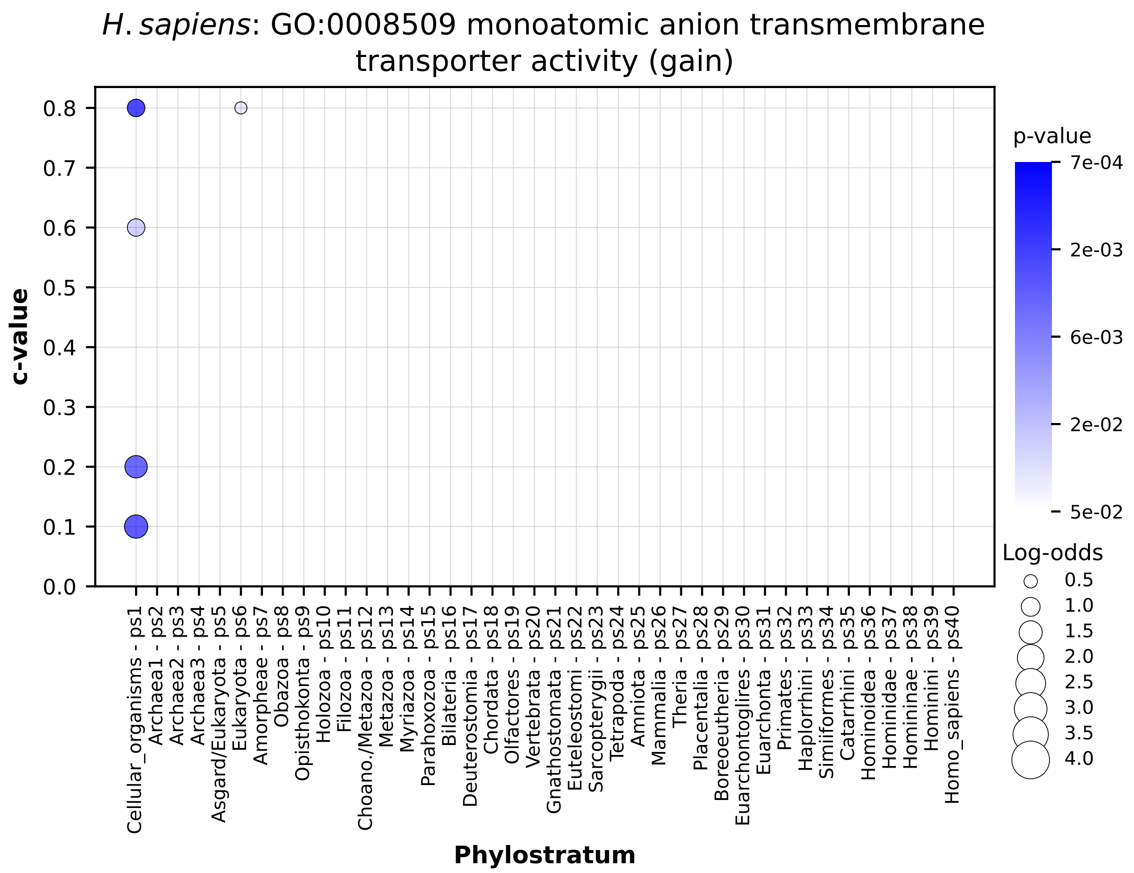
H. sapiens: GO:0008509 monoatomic anion transmembrane transporter activity (gain)
c-value
0.0
0.1
0.2
0.3
0.4
0.5
0.6
0.7
0.8
p-value
7e-04
2e-03
6e-03
2e-02
5e-02
Log-odds
0.5
1.0
1.5
2.0
2.5
3.0
3.5
4.0
Cellular_organisms - ps1
Archaea1 - ps2
Archaea2 - ps3
Archaea3 - ps4
Asgard/Eukaryota - ps5
Eukaryota - ps6
Amorpheae - ps7
Obazoa - ps8
Opisthokonta - ps9
Holozoa - ps10
Filozoa - ps11
Choano./Metazoa - ps12
Metazoa - ps13
Myriazoa - ps14
Parahoxozoa - ps15
Bilateria - ps16
Deuterostomia - ps17
Chordata - ps18
Olfactores - ps19
Vertebrata - ps20
Gnathostomata - ps21
Euteleostomi - ps22
Sarcopterygii - ps23
Tetrapoda - ps24
Amniota - ps25
Mammalia - ps26
Theria - ps27
Placentalia - ps28
Boreoeutheria - ps29
Euarchontoglires - ps30
Euarchonta - ps31
Primates - ps32
Haplorrhini - ps33
Simiiformes - ps34
Catarrhini - ps35
Hominoidea - ps36
Hominidae - ps37
Homininae - ps38
Hominini - ps39
Homo_sapiens - ps40
Phylostratum

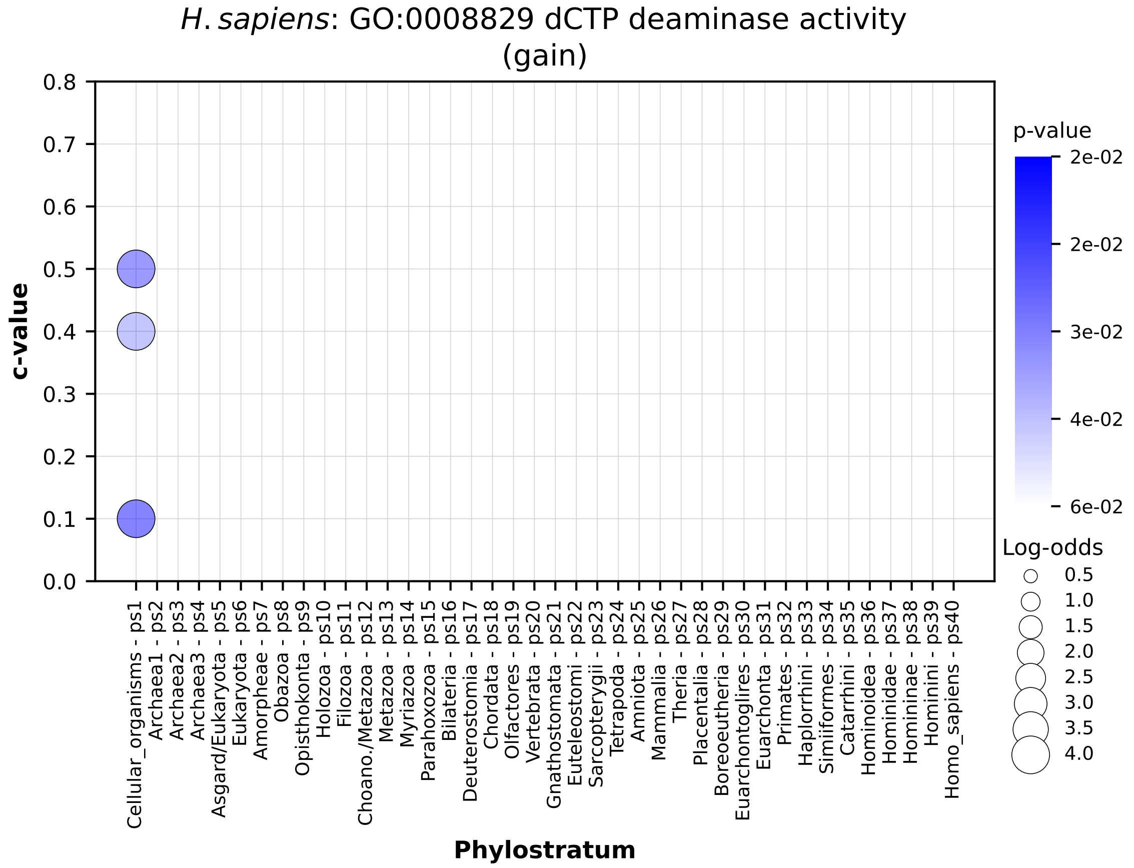

H. sapiens: GO:0008829 dCTP deaminase activity
(gain)
c-value
0.0
0.1
0.2
0.3
0.4
0.5
0.6
0.7
0.8
p-value
2e-02
2e-02
3e-02
4e-02
6e-02
Log-odds
0.5
1.0
1.5
2.0
2.5
3.0
3.5
4.0
Cellular_organisms - ps1
Archaea1 - ps2
Archaea2 - ps3
Archaea3 - ps4
Asgard/Eukaryota - ps5
Eukaryota - ps6
Amorpheae - ps7
Obazoa - ps8
Opisthokonta - ps9
Holozoa - ps10
Filozoa - ps11
Choano./Metazoa - ps12
Metazoa - ps13
Myriazoa - ps14
Parahoxozoa - ps15
Bilateria - ps16
Deuterostomia - ps17
Chordata - ps18
Olfactores - ps19
Vertebrata - ps20
Gnathostomata - ps21
Euteleostomi - ps22
Sarcopterygii - ps23
Tetrapoda - ps24
Amniota - ps25
Mammalia - ps26
Theria - ps27
Placentalia - ps28
Boreoeutheria - ps29
Euarchontoglires - ps30
Euarchonta - ps31
Primates - ps32
Haplorrhini - ps33
Simiiformes - ps34
Catarrhini - ps35
Hominoidea - ps36
Hominidae - ps37
Homininae - ps38
Hominini - ps39
Homo_sapiens - ps40
Phylostratum

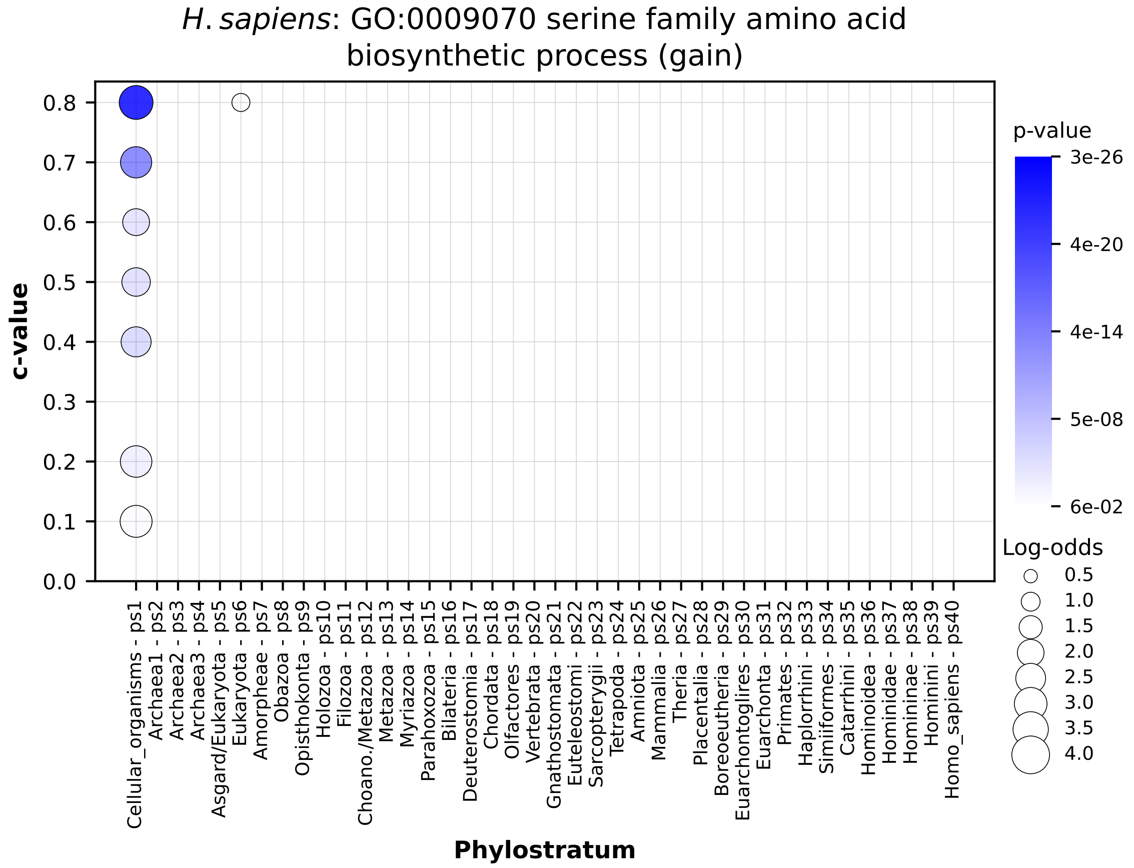

H. sapiens: GO:0009070 serine family amino acid biosynthetic process (gain)
c-value
0.0
0.1
0.2
0.3
0.4
0.5
0.6
0.7
0.8
Phylostratum
Cellular_organisms - ps1
Archaea1 - ps2
Archaea2 - ps3
Archaea3 - ps4
Asgard/Eukaryota - ps5
Eukaryota - ps6
Amorpheae - ps7
Obazoa - ps8
Opisthokonta - ps9
Holozoa - ps10
Filozoa - ps11
Choano./Metazoa - ps12
Metazoa - ps13
Myriazoa - ps14
Parahoxozoa - ps15
Bilateria - ps16
Deuterostomia - ps17
Chordata - ps18
Olfactores - ps19
Vertebrata - ps20
Gnathostomata - ps21
Euteleostomi - ps22
Sarcopterygii - ps23
Tetrapoda - ps24
Amniota - ps25
Mammalia - ps26
Theria - ps27
Placentalia - ps28
Boreoeutheria - ps29
Euarchontoglires - ps30
Euarchonta - ps31
Primates - ps32
Haplorrhini - ps33
Simiiformes - ps34
Catarrhini - ps35
Hominoidea - ps36
Hominidae - ps37
Homininae - ps38
Hominini - ps39
Homo_sapiens - ps40
p-value
3e-26
4e-20
4e-14
5e-08
6e-02
Log-odds
0.5
1.0
1.5
2.0
2.5
3.0
3.5
4.0

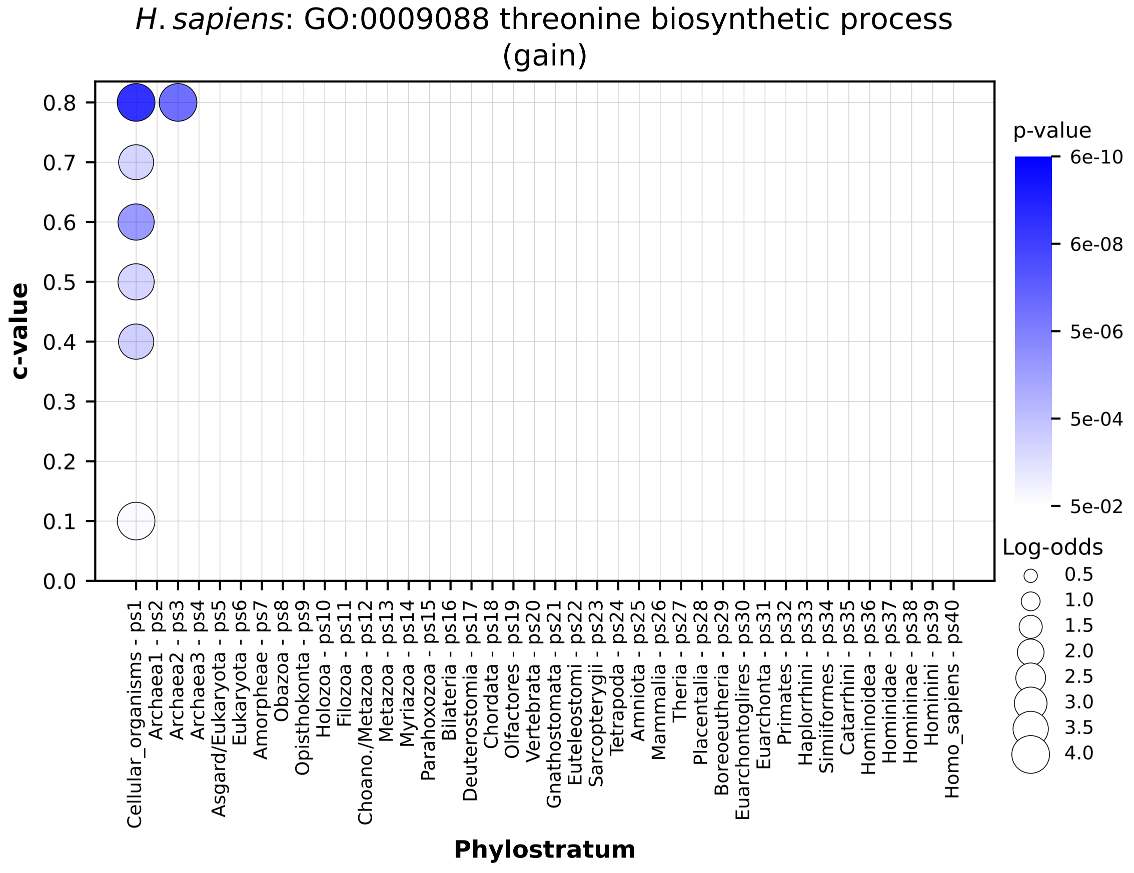

H. sapiens: GO:0009088 threonine biosynthetic process
(gain)
c-value
0.0
0.1
0.2
0.3
0.4
0.5
0.6
0.7
0.8
Phylostratum
Cellular_organisms - ps1
Archaea1 - ps2
Archaea2 - ps3
Archaea3 - ps4
Asgard/Eukaryota - ps5
Eukaryota - ps6
Amorpheae - ps7
Obazoa - ps8
Opisthokonta - ps9
Holozoa - ps10
Filozoa - ps11
Choano./Metazoa - ps12
Metazoa - ps13
Myriazoa - ps14
Parahoxozoa - ps15
Bilateria - ps16
Deuterostomia - ps17
Chordata - ps18
Olfactores - ps19
Vertebrata - ps20
Gnathostomata - ps21
Euteleostomi - ps22
Sarcopterygii - ps23
Tetrapoda - ps24
Amniota - ps25
Mammalia - ps26
Theria - ps27
Placentalia - ps28
Boreoeutheria - ps29
Euarchontoglires - ps30
Euarchonta - ps31
Primates - ps32
Haplorrhini - ps33
Simiiformes - ps34
Catarrhini - ps35
Hominoidea - ps36
Hominidae - ps37
Homininae - ps38
Hominini - ps39
Homo_sapiens - ps40
p-value
6e-10
6e-08
5e-06
5e-04
5e-02
Log-odds
0.5
1.0
1.5
2.0
2.5
3.0
3.5
4.0

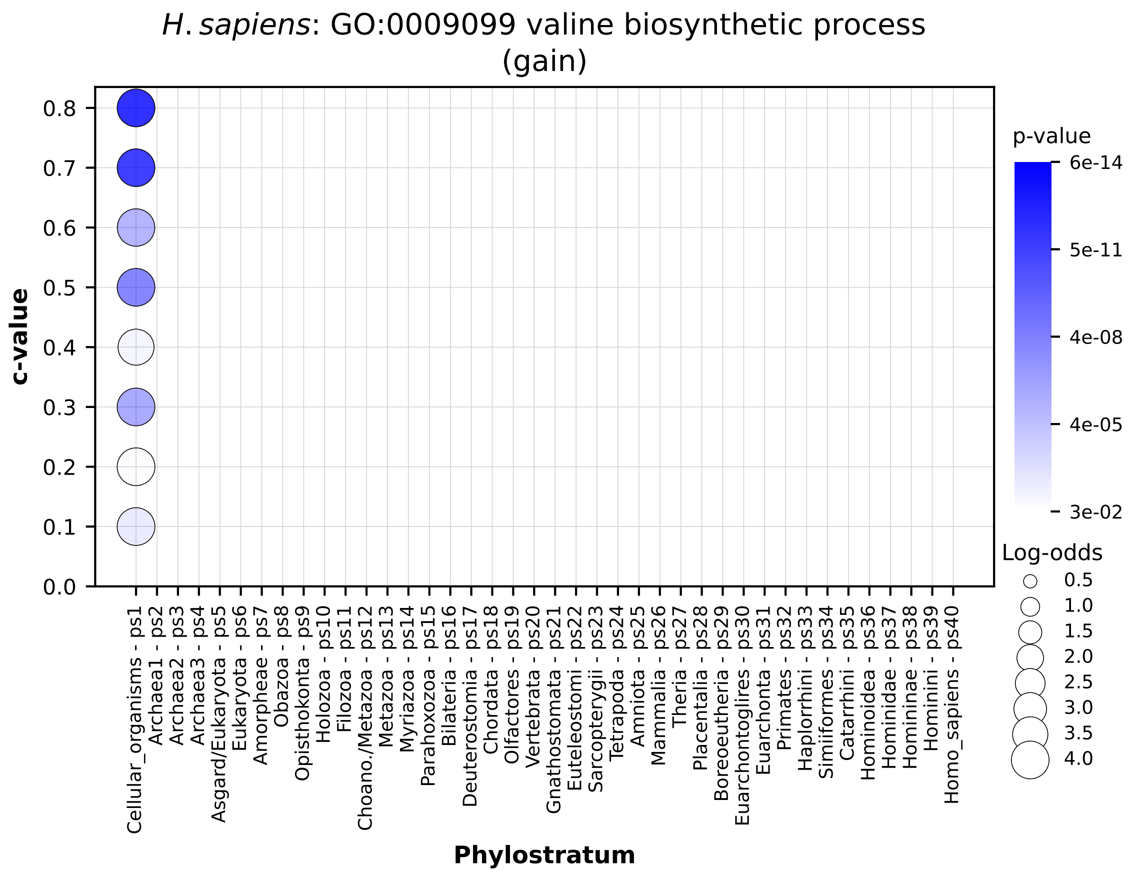

H. sapiens: GO:0009099 valine biosynthetic process
(gain)
c-value
0.0
0.1
0.2
0.3
0.4
0.5
0.6
0.7
0.8
p-value
6e-14
5e-11
4e-08
4e-05
3e-02
Log-odds
0.5
1.0
1.5
2.0
2.5
3.0
3.5
4.0
Cellular_organisms - ps1
Archaea1 - ps2
Archaea2 - ps3
Archaea3 - ps4
Asgard/Eukaryota - ps5
Eukaryota - ps6
Amorpheae - ps7
Obazoa - ps8
Opisthokonta - ps9
Holozoa - ps10
Filozoa - ps11
Choano./Metazoa - ps12
Metazoa - ps13
Myriazoa - ps14
Parahoxozoa - ps15
Bilateria - ps16
Deuterostomia - ps17
Chordata - ps18
Olfactores - ps19
Vertebrata - ps20
Gnathostomata - ps21
Euteleostomi - ps22
Sarcopterygii - ps23
Tetrapoda - ps24
Amniota - ps25
Mammalia - ps26
Theria - ps27
Placentalia - ps28
Boreoeutheria - ps29
Euarchontoglires - ps30
Euarchonta - ps31
Primates - ps32
Haplorrhini - ps33
Simiiformes - ps34
Catarrhini - ps35
Hominoidea - ps36
Hominidae - ps37
Homininae - ps38
Hominini - ps39
Homo_sapiens - ps40
Phylostratum

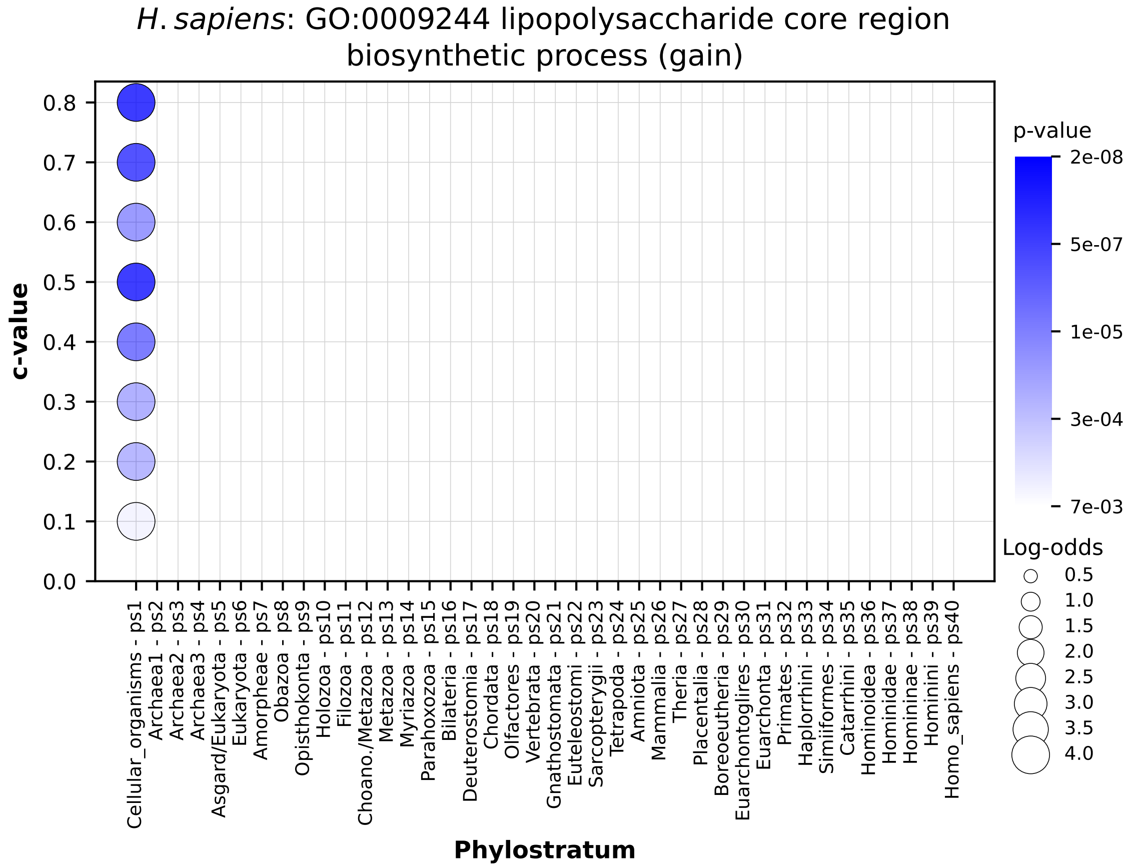

H. sapiens: GO:0009244 lipopolysaccharide core region biosynthetic process (gain)
c-value
0.0
0.1
0.2
0.3
0.4
0.5
0.6
0.7
0.8
Phylostratum
Cellular_organisms - ps1
Archaea1 - ps2
Archaea2 - ps3
Archaea3 - ps4
Asgard/Eukaryota - ps5
Eukaryota - ps6
Amorpheae - ps7
Obazoa - ps8
Opisthokonta - ps9
Holozoa - ps10
Filozoa - ps11
Choano./Metazoa - ps12
Metazoa - ps13
Myriazoa - ps14
Parahoxozoa - ps15
Bilateria - ps16
Deuterostomia - ps17
Chordata - ps18
Olfactores - ps19
Vertebrata - ps20
Gnathostomata - ps21
Euteleostomi - ps22
Sarcopterygii - ps23
Tetrapoda - ps24
Amniota - ps25
Mammalia - ps26
Theria - ps27
Placentalia - ps28
Boreoeutheria - ps29
Euarchontoglires - ps30
Euarchonta - ps31
Primates - ps32
Haplorrhini - ps33
Simiiformes - ps34
Catarrhini - ps35
Hominoidea - ps36
Hominidae - ps37
Homininae - ps38
Hominini - ps39
Homo_sapiens - ps40
p-value
2e-08
5e-07
1e-05
3e-04
7e-03
Log-odds
0.5
1.0
1.5
2.0
2.5
3.0
3.5
4.0

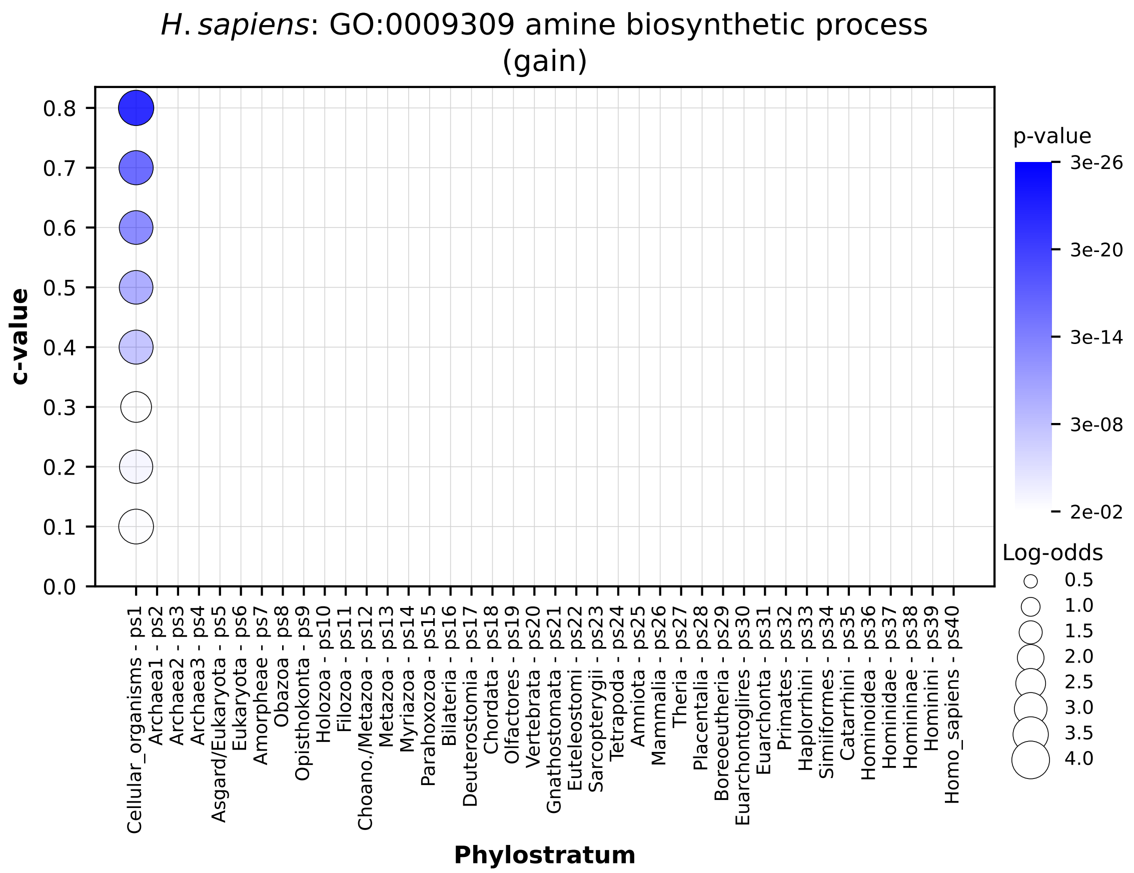

H. sapiens: GO:0009309 amine biosynthetic process
(gain)
c-value
0.0
0.1
0.2
0.3
0.4
0.5
0.6
0.7
0.8
p-value
3e-26
3e-20
3e-14
3e-08
2e-02
Log-odds
0.5
1.0
1.5
2.0
2.5
3.0
3.5
4.0
Cellular_organisms - ps1
Archaea1 - ps2
Archaea2 - ps3
Archaea3 - ps4
Asgard/Eukaryota - ps5
Eukaryota - ps6
Amorpheae - ps7
Obazoa - ps8
Opisthokonta - ps9
Holozoa - ps10
Filozoa - ps11
Choano./Metazoa - ps12
Metazoa - ps13
Myriazoa - ps14
Parahoxozoa - ps15
Bilateria - ps16
Deuterostomia - ps17
Chordata - ps18
Olfactores - ps19
Vertebrata - ps20
Gnathostomata - ps21
Euteleostomi - ps22
Sarcopterygii - ps23
Tetrapoda - ps24
Amniota - ps25
Mammalia - ps26
Theria - ps27
Placentalia - ps28
Boreoeutheria - ps29
Euarchontoglires - ps30
Euarchonta - ps31
Primates - ps32
Haplorrhini - ps33
Simiiformes - ps34
Catarrhini - ps35
Hominoidea - ps36
Hominidae - ps37
Homininae - ps38
Hominini - ps39
Homo_sapiens - ps40
Phylostratum

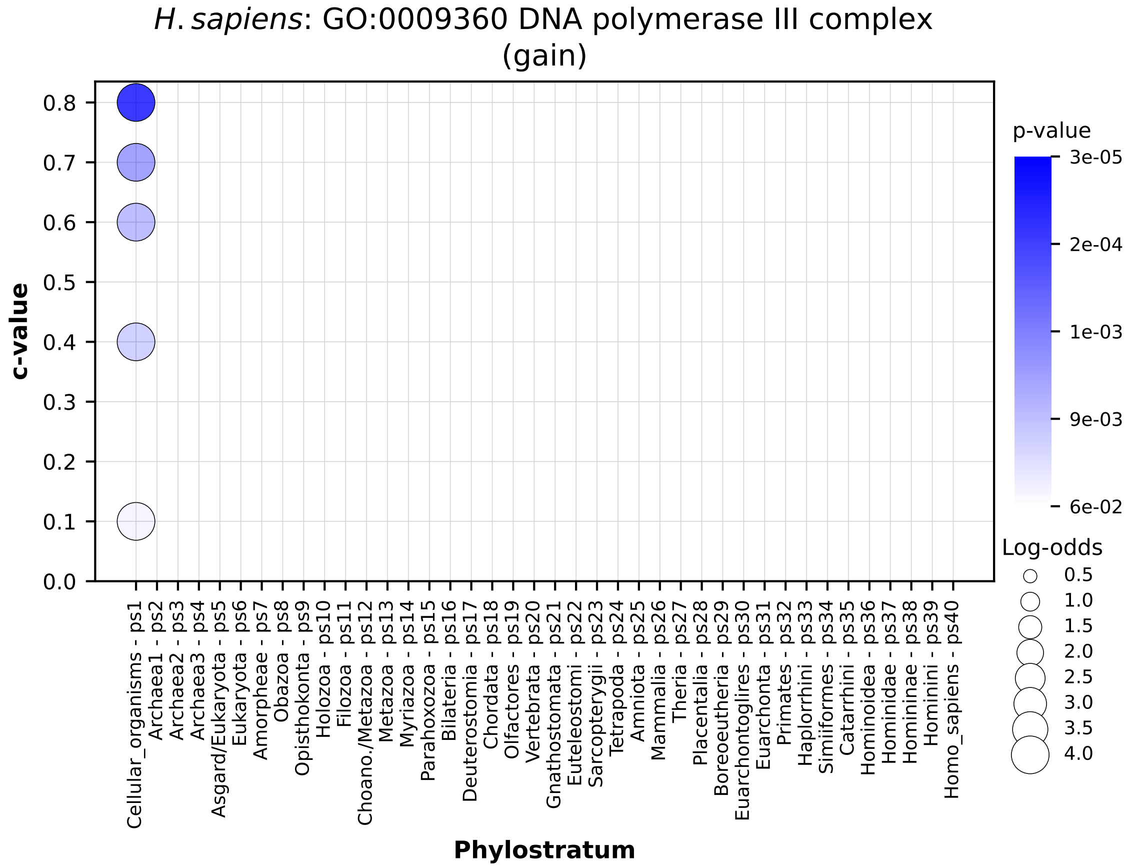

H. sapiens: GO:0009360 DNA polymerase III complex
(gain)
c-value
0.0
0.1
0.2
0.3
0.4
0.5
0.6
0.7
0.8
Phylostratum
Cellular_organisms - ps1
Archaea1 - ps2
Archaea2 - ps3
Archaea3 - ps4
Asgard/Eukaryota - ps5
Eukaryota - ps6
Amorpheae - ps7
Obazoa - ps8
Opisthokonta - ps9
Holozoa - ps10
Filozoa - ps11
Choano./Metazoa - ps12
Metazoa - ps13
Myriazoa - ps14
Parahoxozoa - ps15
Bilateria - ps16
Deuterostomia - ps17
Chordata - ps18
Olfactores - ps19
Vertebrata - ps20
Gnathostomata - ps21
Euteleostomi - ps22
Sarcopterygii - ps23
Tetrapoda - ps24
Amniota - ps25
Mammalia - ps26
Theria - ps27
Placentalia - ps28
Boreoeutheria - ps29
Euarchontoglires - ps30
Euarchonta - ps31
Primates - ps32
Haplorrhini - ps33
Simiiformes - ps34
Catarrhini - ps35
Hominoidea - ps36
Hominidae - ps37
Homininae - ps38
Hominini - ps39
Homo_sapiens - ps40
p-value
3e-05
2e-04
1e-03
9e-03
6e-02
Log-odds
0.5
1.0
1.5
2.0
2.5
3.0
3.5
4.0

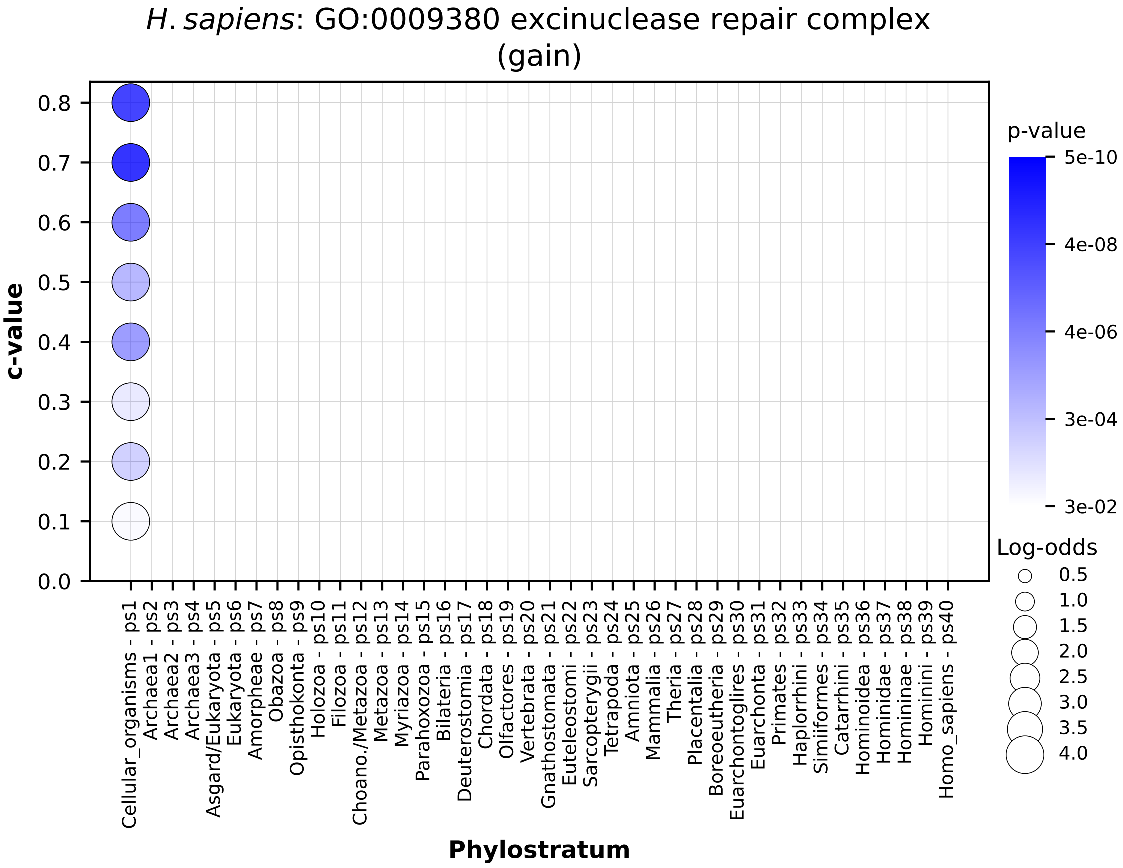

*H. sapiens*: GO:0009380 excinuclease repair complex
(gain)
c-value
0.0
0.1
0.2
0.3
0.4
0.5
0.6
0.7
0.8
Phylostratum
Cellular_organisms - ps1
Archaea1 - ps2
Archaea2 - ps3
Archaea3 - ps4
Asgard/Eukaryota - ps5
Eukaryota - ps6
Amorpheae - ps7
Obazoa - ps8
Opisthokonta - ps9
Holozoa - ps10
Filozoa - ps11
Choano./Metazoa - ps12
Metazoa - ps13
Myriazoa - ps14
Parahoxozoa - ps15
Bilateria - ps16
Deuterostomia - ps17
Chordata - ps18
Olfactores - ps19
Vertebrata - ps20
Gnathostomata - ps21
Euteleostomi - ps22
Sarcopterygii - ps23
Tetrapoda - ps24
Amniota - ps25
Mammalia - ps26
Theria - ps27
Placentalia - ps28
Boreoeutheria - ps29
Euarchontoglires - ps30
Euarchonta - ps31
Primates - ps32
Haplorrhini - ps33
Simiiformes - ps34
Catarrhini - ps35
Hominoidea - ps36
Hominidae - ps37
Homininae - ps38
Hominini - ps39
Homo_sapiens - ps40
p-value
5e-10
4e-08
4e-06
3e-04
3e-02
Log-odds
0.5
1.0
1.5
2.0
2.5
3.0
3.5
4.0

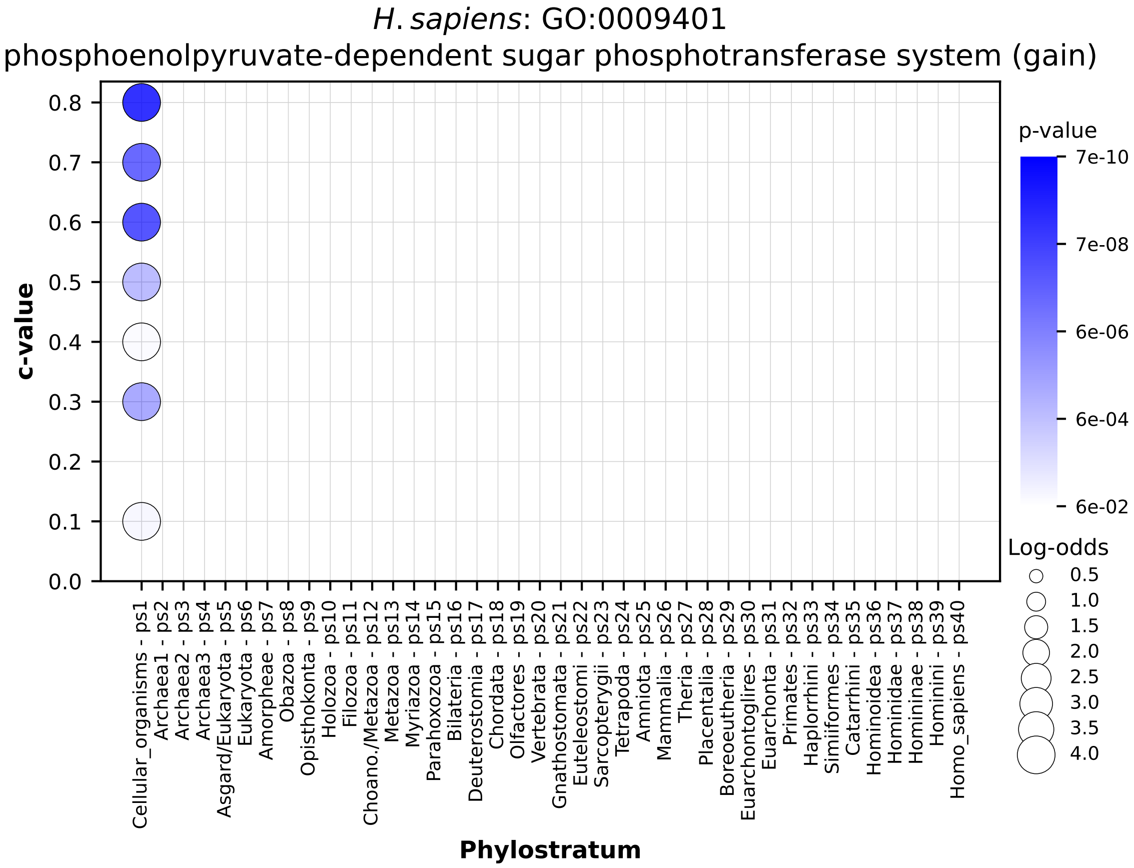

H. sapiens: GO:0009401
phosphoenolpyruvate-dependent sugar phosphotransferase system (gain)
c-value
0.0
0.1
0.2
0.3
0.4
0.5
0.6
0.7
0.8
Phylostratum
Cellular_organisms - ps1
Archaea1 - ps2
Archaea2 - ps3
Archaea3 - ps4
Asgard/Eukaryota - ps5
Eukaryota - ps6
Amorpheae - ps7
Obazoa - ps8
Opisthokonta - ps9
Holozoa - ps10
Filozoa - ps11
Choano./Metazoa - ps12
Metazoa - ps13
Myriazoa - ps14
Parahoxozoa - ps15
Bilateria - ps16
Deuterostomia - ps17
Chordata - ps18
Olfactores - ps19
Vertebrata - ps20
Gnathostomata - ps21
Euteleostomi - ps22
Sarcopterygii - ps23
Tetrapoda - ps24
Amniota - ps25
Mammalia - ps26
Theria - ps27
Placentalia - ps28
Boreoeutheria - ps29
Euarchontoglires - ps30
Euarchonta - ps31
Primates - ps32
Haplorrhini - ps33
Simiiformes - ps34
Catarrhini - ps35
Hominoidea - ps36
Hominidae - ps37
Homininae - ps38
Hominini - ps39
Homo_sapiens - ps40
p-value
7e-10
7e-08
6e-06
6e-04
6e-02
Log-odds
0.5
1.0
1.5
2.0
2.5
3.0
3.5
4.0

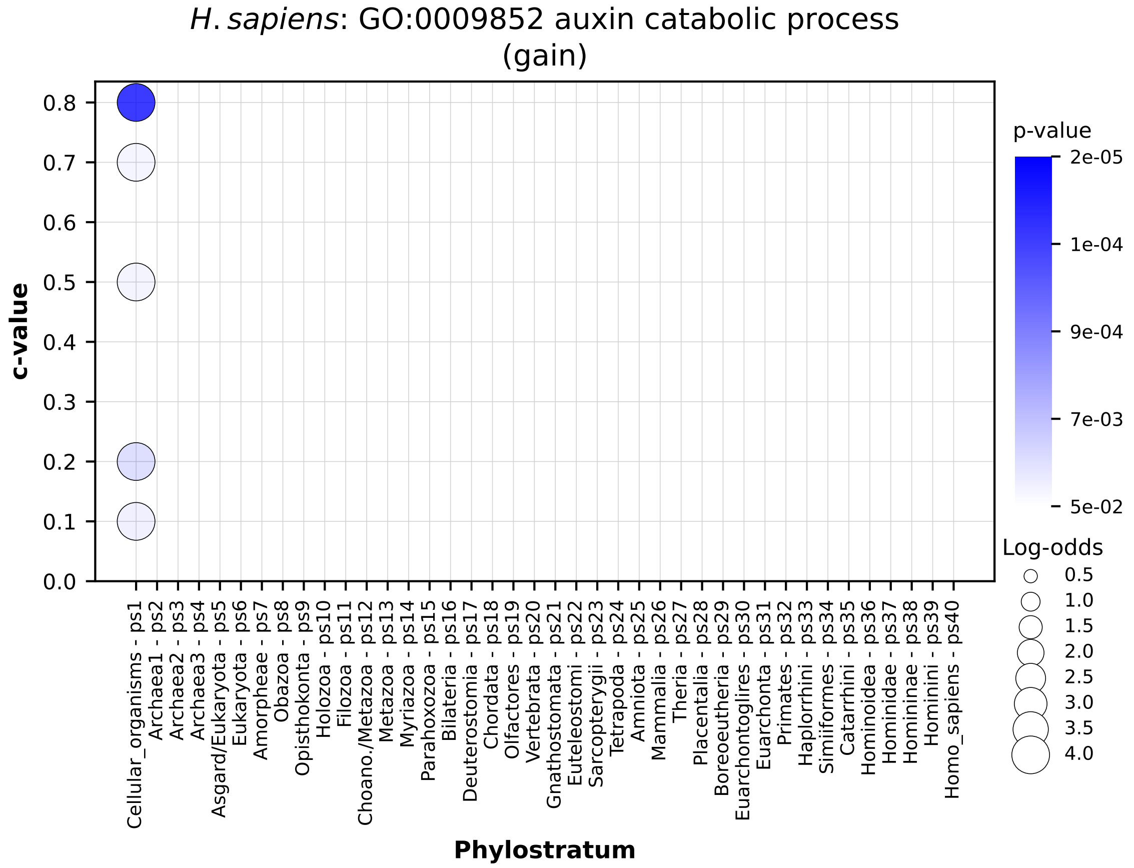

*H. sapiens*: GO:0009852 auxin catabolic process
(gain)
c-value
0.8
0.7
0.6
0.5
0.4
0.3
0.2
0.1
0.0
Phylostratum
Cellular_organisms - ps1
Archaea1 - ps2
Archaea2 - ps3
Archaea3 - ps4
Asgard/Eukaryota - ps5
Eukaryota - ps6
Amorpheae - ps7
Obazoa - ps8
Opisthokonta - ps9
Holozoa - ps10
Filozoa - ps11
Choano./Metazoa - ps12
Metazoa - ps13
Myriazoa - ps14
Parahoxozoa - ps15
Bilateria - ps16
Deuterostomia - ps17
Chordata - ps18
Olfactores - ps19
Vertebrata - ps20
Gnathostomata - ps21
Euteleostomi - ps22
Sarcopterygii - ps23
Tetrapoda - ps24
Amniota - ps25
Mammalia - ps26
Theria - ps27
Placentalia - ps28
Boreoeutheria - ps29
Euarchontoglires - ps30
Euarchonta - ps31
Primates - ps32
Haplorrhini - ps33
Simiiformes - ps34
Catarrhini - ps35
Hominoidea - ps36
Hominidae - ps37
Homininae - ps38
Hominini - ps39
Homo_sapiens - ps40
p-value
2e-05
1e-04
9e-04
7e-03
5e-02
Log-odds
0.5
1.0
1.5
2.0
2.5
3.0
3.5
4.0

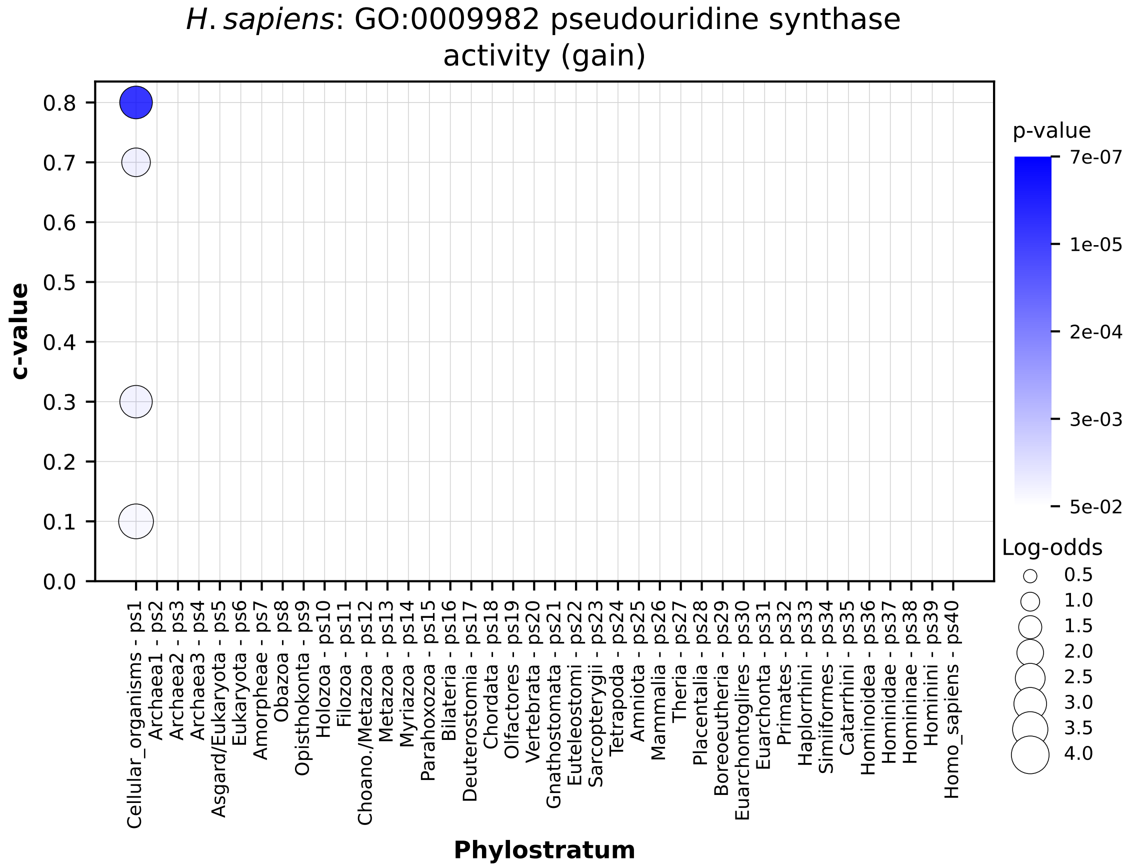

H. sapiens: GO:0009982 pseudouridine synthase activity (gain)
c-value
0.0
0.1
0.2
0.3
0.4
0.5
0.6
0.7
0.8
Phylostratum
Cellular_organisms - ps1
Archaea1 - ps2
Archaea2 - ps3
Archaea3 - ps4
Asgard/Eukaryota - ps5
Eukaryota - ps6
Amorpheae - ps7
Obazoa - ps8
Opisthokonta - ps9
Holozoa - ps10
Filozoa - ps11
Choano./Metazoa - ps12
Metazoa - ps13
Myriazoa - ps14
Parahoxozoa - ps15
Bilateria - ps16
Deuterostomia - ps17
Chordata - ps18
Olfactores - ps19
Vertebrata - ps20
Gnathostomata - ps21
Euteleostomi - ps22
Sarcopterygii - ps23
Tetrapoda - ps24
Amniota - ps25
Mammalia - ps26
Theria - ps27
Placentalia - ps28
Boreoeutheria - ps29
Euarchontoglires - ps30
Euarchonta - ps31
Primates - ps32
Haplorrhini - ps33
Simiiformes - ps34
Catarrhini - ps35
Hominoidea - ps36
Hominidae - ps37
Homininae - ps38
Hominini - ps39
Homo_sapiens - ps40
p-value
7e-07
1e-05
2e-04
3e-03
5e-02
Log-odds
0.5
1.0
1.5
2.0
2.5
3.0
3.5
4.0

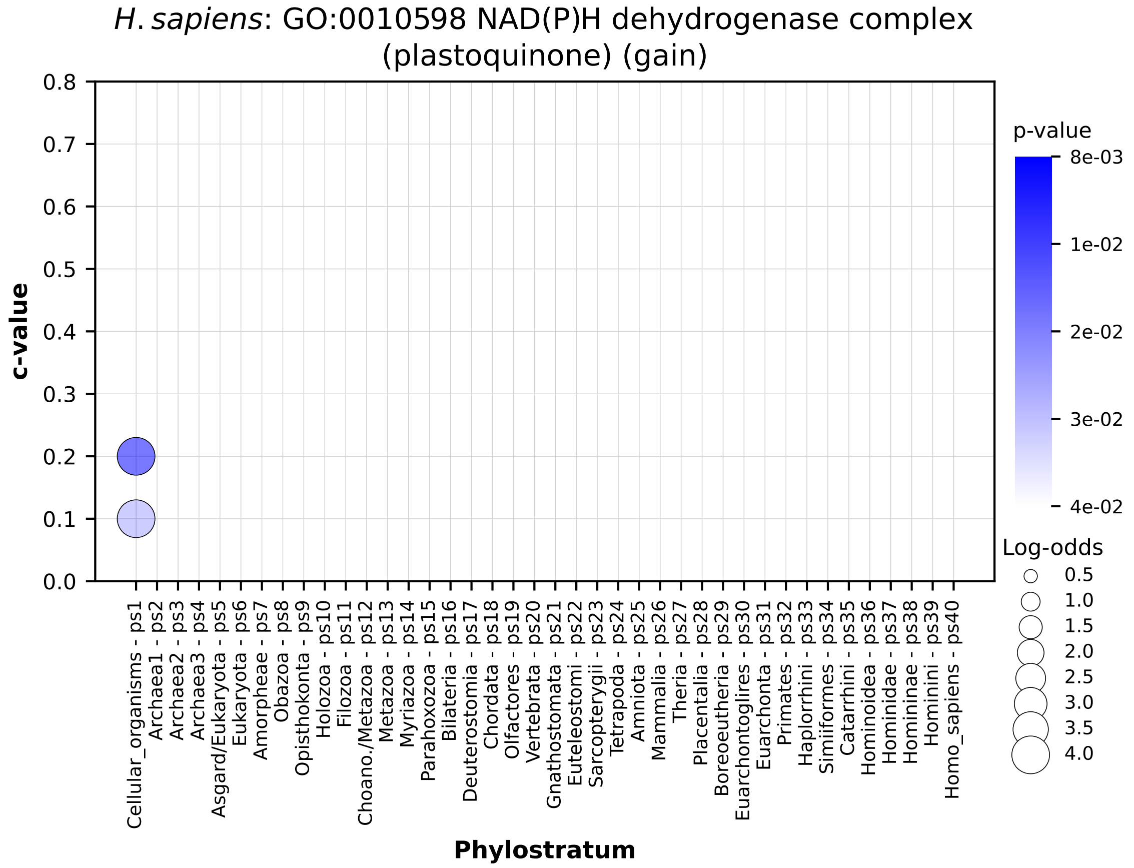
H. sapiens: GO:0010598 NAD(P)H dehydrogenase complex (plastoquinone) (gain)
c-value
0.0
0.1
0.2
0.3
0.4
0.5
0.6
0.7
0.8
Phylostratum
Cellular_organisms - ps1
Archaea1 - ps2
Archaea2 - ps3
Archaea3 - ps4
Asgard/Eukaryota - ps5
Eukaryota - ps6
Amorpheae - ps7
Obazoa - ps8
Opisthokonta - ps9
Holozoa - ps10
Filozoa - ps11
Choano./Metazoa - ps12
Metazoa - ps13
Myriazoa - ps14
Parahoxozoa - ps15
Bilateria - ps16
Deuterostomia - ps17
Chordata - ps18
Olfactores - ps19
Vertebrata - ps20
Gnathostomata - ps21
Euteleostomi - ps22
Sarcopterygii - ps23
Tetrapoda - ps24
Amniota - ps25
Mammalia - ps26
Theria - ps27
Placentalia - ps28
Boreoeutheria - ps29
Euarchontoglires - ps30
Euarchonta - ps31
Primates - ps32
Haplorrhini - ps33
Simiiformes - ps34
Catarrhini - ps35
Hominoidea - ps36
Hominidae - ps37
Homininae - ps38
Hominini - ps39
Homo_sapiens - ps40
p-value
8e-03
1e-02
2e-02
3e-02
4e-02
Log-odds
0.5
1.0
1.5
2.0
2.5
3.0
3.5
4.0

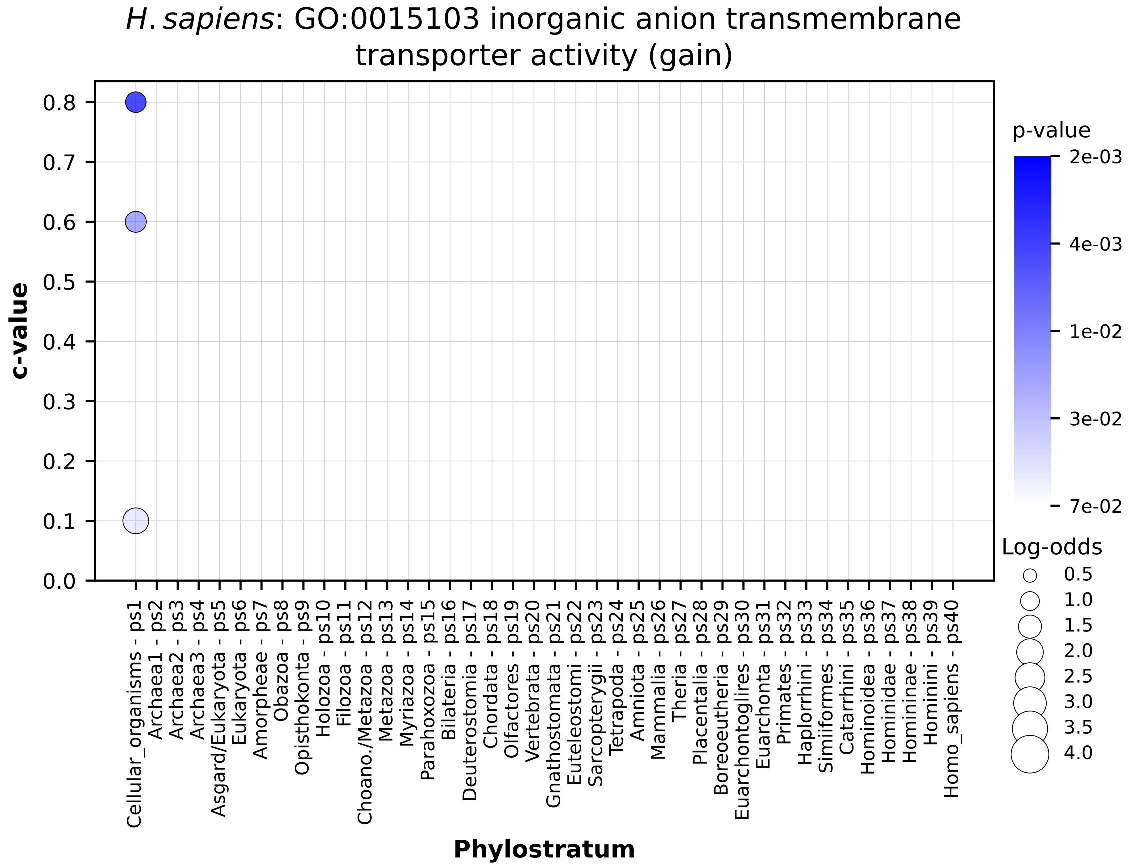

H. sapiens: GO:0015103 inorganic anion transmembrane transporter activity (gain)
c-value
0.0
0.1
0.2
0.3
0.4
0.5
0.6
0.7
0.8
Phylostratum
Cellular_organisms - ps1
Archaea1 - ps2
Archaea2 - ps3
Archaea3 - ps4
Asgard/Eukaryota - ps5
Eukaryota - ps6
Amorpheae - ps7
Obazoa - ps8
Opisthokonta - ps9
Holozoa - ps10
Filozoa - ps11
Choano./Metazoa - ps12
Metazoa - ps13
Myriazoa - ps14
Parahoxozoa - ps15
Bilateria - ps16
Deuterostomia - ps17
Chordata - ps18
Olfactores - ps19
Vertebrata - ps20
Gnathostomata - ps21
Euteleostomi - ps22
Sarcopterygii - ps23
Tetrapoda - ps24
Amniota - ps25
Mammalia - ps26
Theria - ps27
Placentalia - ps28
Boreoeutheria - ps29
Euarchontoglires - ps30
Euarchonta - ps31
Primates - ps32
Haplorrhini - ps33
Simiiformes - ps34
Catarrhini - ps35
Hominoidea - ps36
Hominidae - ps37
Homininae - ps38
Hominini - ps39
Homo_sapiens - ps40
p-value
2e-03
4e-03
1e-02
3e-02
7e-02
Log-odds
0.5
1.0
1.5
2.0
2.5
3.0
3.5
4.0

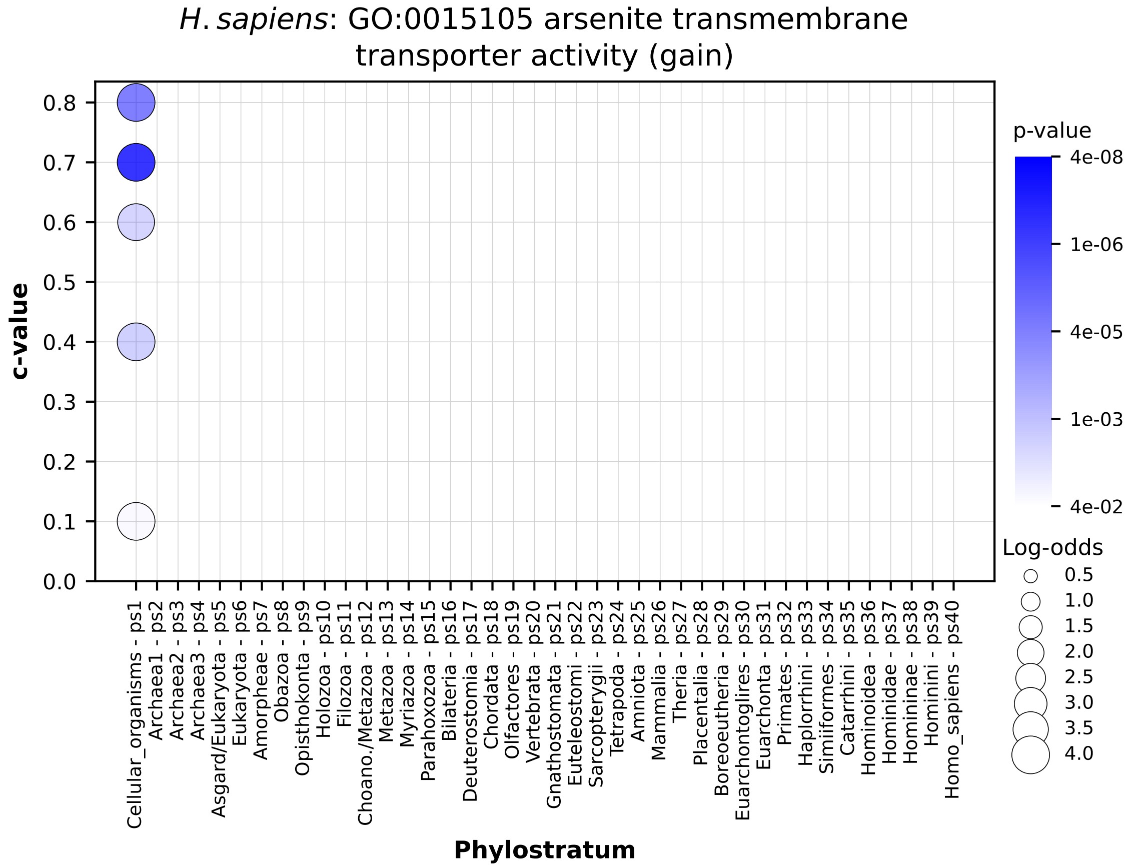

H. sapiens: GO:0015105 arsenite transmembrane transporter activity (gain)
c-value
0.0
0.1
0.2
0.3
0.4
0.5
0.6
0.7
0.8
Phylostratum
Cellular_organisms - ps1
Archaea1 - ps2
Archaea2 - ps3
Archaea3 - ps4
Asgard/Eukaryota - ps5
Eukaryota - ps6
Amorpheae - ps7
Obazoa - ps8
Opisthokonta - ps9
Holozoa - ps10
Filozoa - ps11
Choano./Metazoa - ps12
Metazoa - ps13
Myriazoa - ps14
Parahoxozoa - ps15
Bilateria - ps16
Deuterostomia - ps17
Chordata - ps18
Olfactores - ps19
Vertebrata - ps20
Gnathostomata - ps21
Euteleostomi - ps22
Sarcopterygii - ps23
Tetrapoda - ps24
Amniota - ps25
Mammalia - ps26
Theria - ps27
Placentalia - ps28
Boreoeutheria - ps29
Euarchontoglires - ps30
Euarchonta - ps31
Primates - ps32
Haplorrhini - ps33
Simiiformes - ps34
Catarrhini - ps35
Hominoidea - ps36
Hominidae - ps37
Homininae - ps38
Hominini - ps39
Homo_sapiens - ps40
p-value
4e-08
1e-06
4e-05
1e-03
4e-02
Log-odds
0.5
1.0
1.5
2.0
2.5
3.0
3.5
4.0

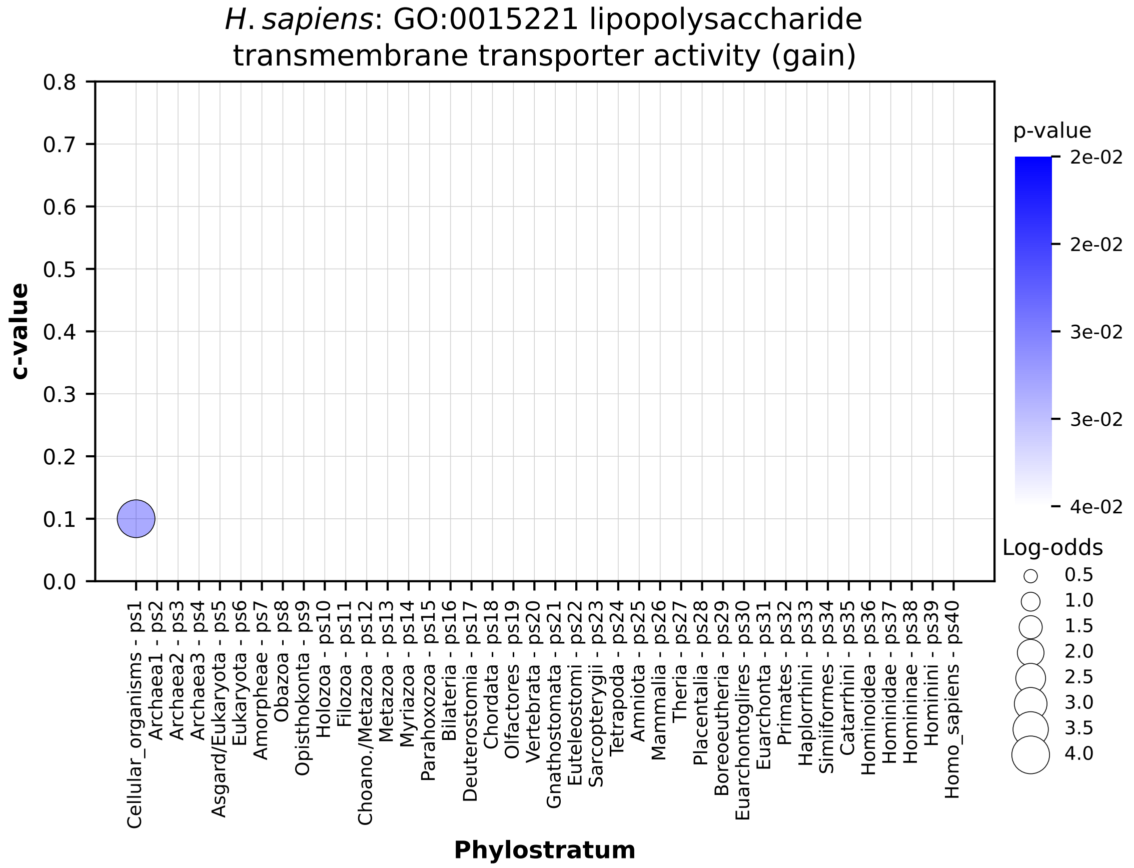
H. sapiens: GO:0015221 lipopolysaccharide transmembrane transporter activity (gain)
c-value
0.0
0.1
0.2
0.3
0.4
0.5
0.6
0.7
0.8
Phylostratum
Cellular_organisms - ps1
Archaea1 - ps2
Archaea2 - ps3
Archaea3 - ps4
Asgard/Eukaryota - ps5
Eukaryota - ps6
Amorpheae - ps7
Obazoa - ps8
Opisthokonta - ps9
Holozoa - ps10
Filozoa - ps11
Choano./Metazoa - ps12
Metazoa - ps13
Myriazoa - ps14
Parahoxozoa - ps15
Bilateria - ps16
Deuterostomia - ps17
Chordata - ps18
Olfactores - ps19
Vertebrata - ps20
Gnathostomata - ps21
Euteleostomi - ps22
Sarcopterygii - ps23
Tetrapoda - ps24
Amniota - ps25
Mammalia - ps26
Theria - ps27
Placentalia - ps28
Boreoeutheria - ps29
Euarchontoglires - ps30
Euarchonta - ps31
Primates - ps32
Haplorrhini - ps33
Simiiformes - ps34
Catarrhini - ps35
Hominoidea - ps36
Hominidae - ps37
Homininae - ps38
Hominini - ps39
Homo_sapiens - ps40
p-value
2e-02
2e-02
3e-02
3e-02
4e-02
Log-odds
0.5
1.0
1.5
2.0
2.5
3.0
3.5
4.0

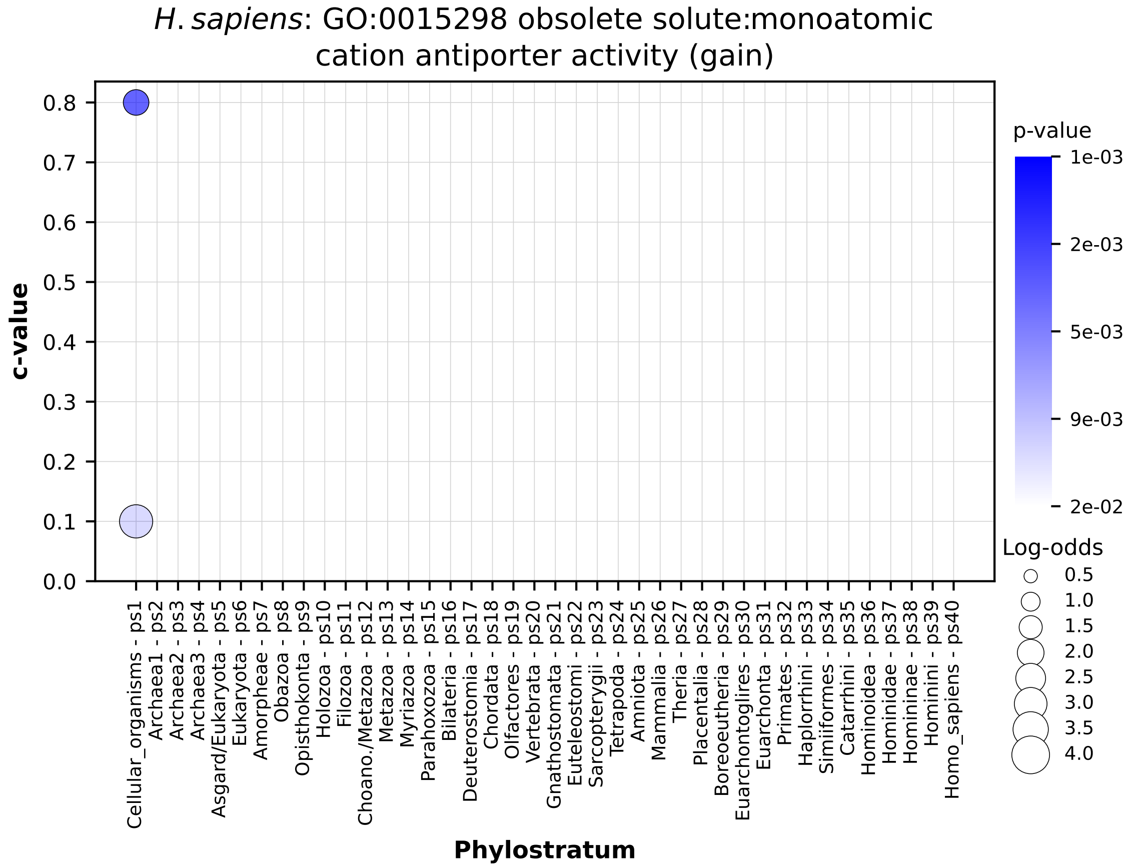

H. sapiens: GO:0015298 obsolete solute:monoatomic cation antiporter activity (gain)
c-value
0.0
0.1
0.2
0.3
0.4
0.5
0.6
0.7
0.8
Phylostratum
Cellular_organisms - ps1
Archaea1 - ps2
Archaea2 - ps3
Archaea3 - ps4
Asgard/Eukaryota - ps5
Eukaryota - ps6
Amorpheae - ps7
Obazoa - ps8
Opisthokonta - ps9
Holozoa - ps10
Filozoa - ps11
Choano./Metazoa - ps12
Metazoa - ps13
Myriazoa - ps14
Parahoxozoa - ps15
Bilateria - ps16
Deuterostomia - ps17
Chordata - ps18
Olfactores - ps19
Vertebrata - ps20
Gnathostomata - ps21
Euteleostomi - ps22
Sarcopterygii - ps23
Tetrapoda - ps24
Amniota - ps25
Mammalia - ps26
Theria - ps27
Placentalia - ps28
Boreoeutheria - ps29
Euarchontoglires - ps30
Euarchonta - ps31
Primates - ps32
Haplorrhini - ps33
Simiiformes - ps34
Catarrhini - ps35
Hominoidea - ps36
Hominidae - ps37
Homininae - ps38
Hominini - ps39
Homo_sapiens - ps40
p-value
1e-03
2e-03
5e-03
9e-03
2e-02
Log-odds
0.5
1.0
1.5
2.0
2.5
3.0
3.5
4.0

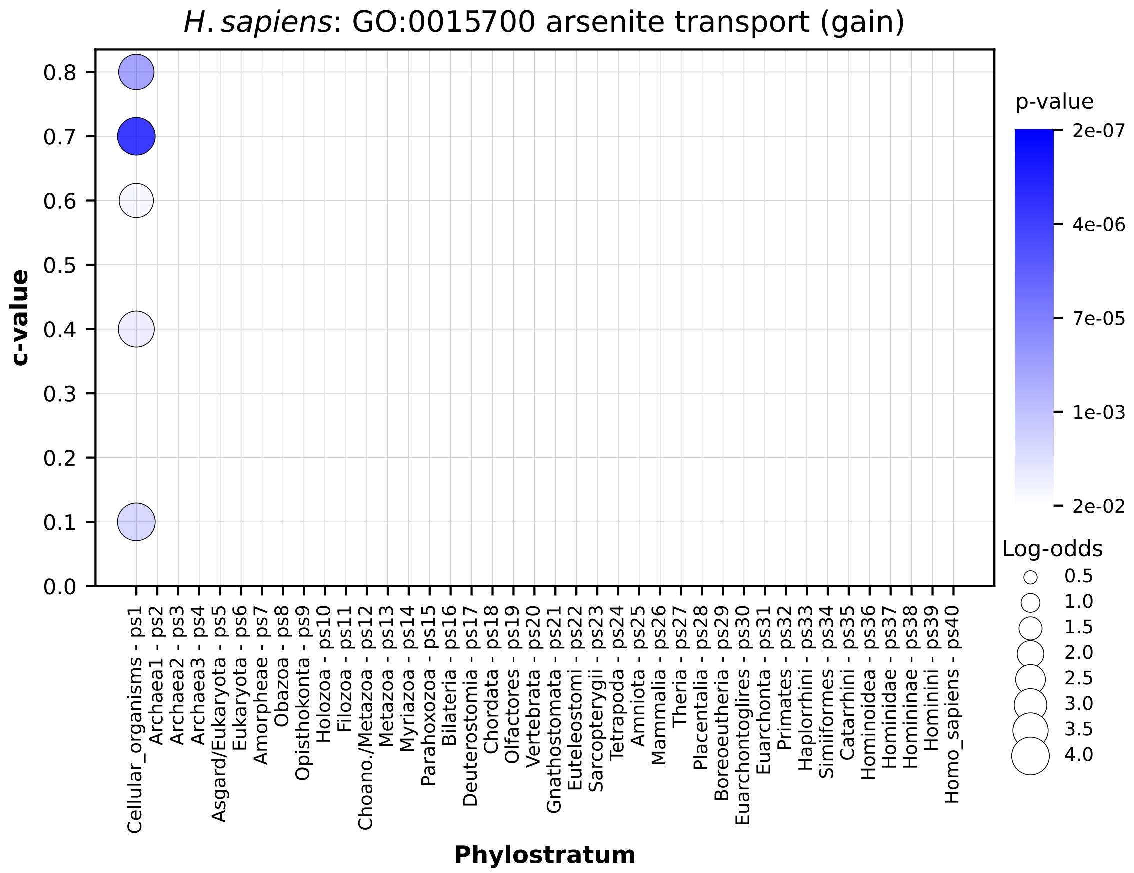

H. sapiens: GO:0015700 arsenite transport (gain)
c-value
0.0
0.1
0.2
0.3
0.4
0.5
0.6
0.7
0.8
Phylostratum
Cellular_organisms - ps1
Archaea1 - ps2
Archaea2 - ps3
Archaea3 - ps4
Asgard/Eukaryota - ps5
Eukaryota - ps6
Amorpheae - ps7
Obazoa - ps8
Opisthokonta - ps9
Holozoa - ps10
Filozoa - ps11
Choano./Metazoa - ps12
Metazoa - ps13
Myriazoa - ps14
Parahoxozoa - ps15
Bilateria - ps16
Deuterostomia - ps17
Chordata - ps18
Olfactores - ps19
Vertebrata - ps20
Gnathostomata - ps21
Euteleostomi - ps22
Sarcopterygii - ps23
Tetrapoda - ps24
Amniota - ps25
Mammalia - ps26
Theria - ps27
Placentalia - ps28
Boreoeutheria - ps29
Euarchontoglires - ps30
Euarchonta - ps31
Primates - ps32
Haplorrhini - ps33
Simiiformes - ps34
Catarrhini - ps35
Hominoidea - ps36
Hominidae - ps37
Homininae - ps38
Hominini - ps39
Homo_sapiens - ps40
p-value
2e-07
4e-06
7e-05
1e-03
2e-02
Log-odds
0.5
1.0
1.5
2.0
2.5
3.0
3.5
4.0

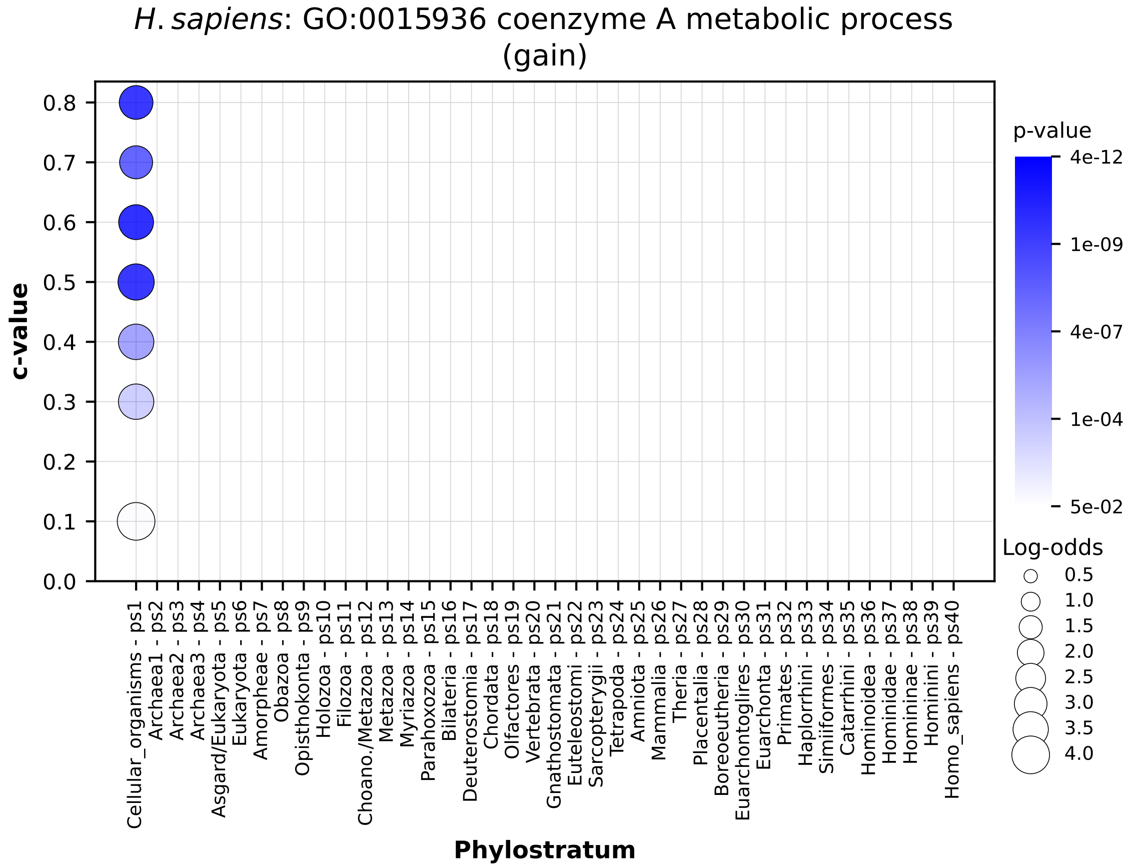

*H. sapiens*: GO:0015936 coenzyme A metabolic process
(gain)
c-value
0.0
0.1
0.2
0.3
0.4
0.5
0.6
0.7
0.8
Phylostratum
Cellular_organisms - ps1
Archaea1 - ps2
Archaea2 - ps3
Archaea3 - ps4
Asgard/Eukaryota - ps5
Eukaryota - ps6
Amorpheae - ps7
Obazoa - ps8
Opisthokonta - ps9
Holozoa - ps10
Filozoa - ps11
Choano./Metazoa - ps12
Metazoa - ps13
Myriazoa - ps14
Parahoxozoa - ps15
Bilateria - ps16
Deuterostomia - ps17
Chordata - ps18
Olfactores - ps19
Vertebrata - ps20
Gnathostomata - ps21
Euteleostomi - ps22
Sarcopterygii - ps23
Tetrapoda - ps24
Amniota - ps25
Mammalia - ps26
Theria - ps27
Placentalia - ps28
Boreoeutheria - ps29
Euarchontoglires - ps30
Euarchonta - ps31
Primates - ps32
Haplorrhini - ps33
Simiiformes - ps34
Catarrhini - ps35
Hominoidea - ps36
Hominidae - ps37
Homininae - ps38
Hominini - ps39
Homo_sapiens - ps40
p-value
4e-12
1e-09
4e-07
1e-04
5e-02
Log-odds
0.5
1.0
1.5
2.0
2.5
3.0
3.5
4.0

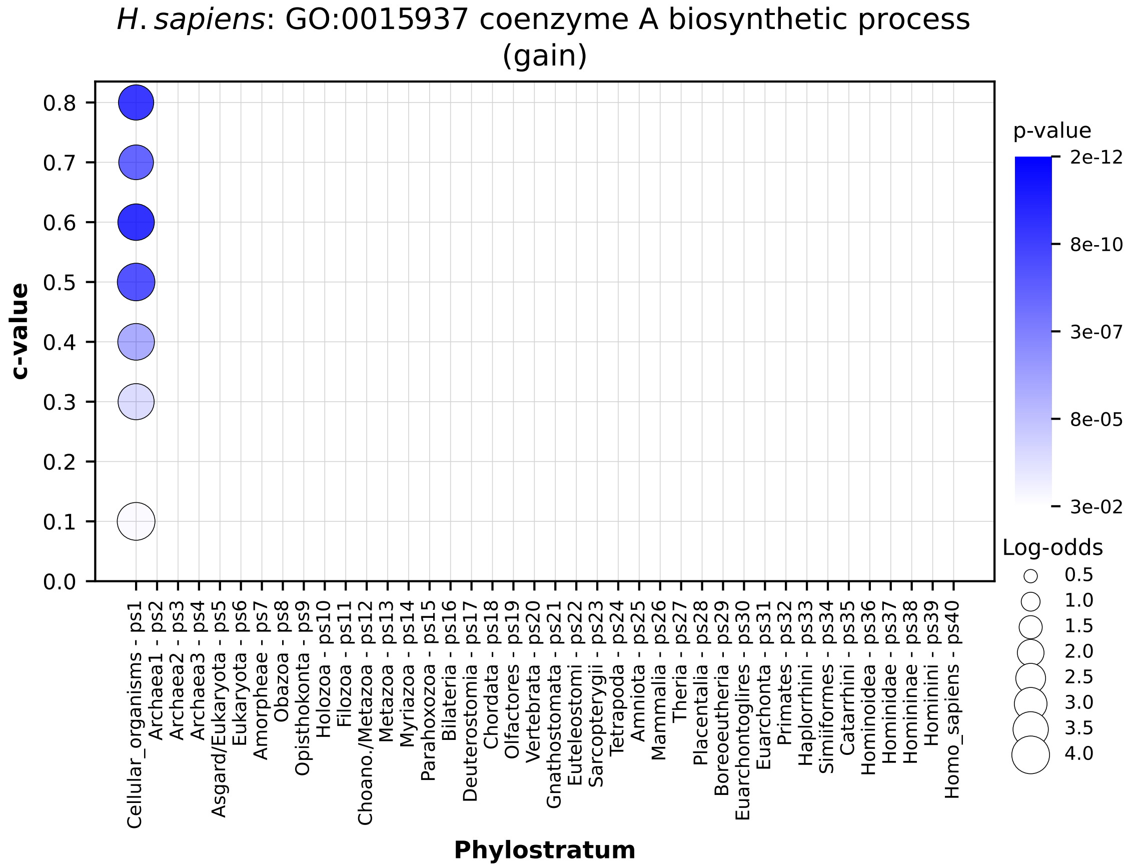

H. sapiens: GO:0015937 coenzyme A biosynthetic process
(gain)
c-value
0.0
0.1
0.2
0.3
0.4
0.5
0.6
0.7
0.8
p-value
2e-12
8e-10
3e-07
8e-05
3e-02
Log-odds
0.5
1.0
1.5
2.0
2.5
3.0
3.5
4.0
Cellular_organisms - ps1
Archaea1 - ps2
Archaea2 - ps3
Archaea3 - ps4
Asgard/Eukaryota - ps5
Eukaryota - ps6
Amorpheae - ps7
Obazoa - ps8
Opisthokonta - ps9
Holozoa - ps10
Filozoa - ps11
Choano./Metazoa - ps12
Metazoa - ps13
Myriazoa - ps14
Parahoxozoa - ps15
Bilateria - ps16
Deuterostomia - ps17
Chordata - ps18
Olfactores - ps19
Vertebrata - ps20
Gnathostomata - ps21
Euteleostomi - ps22
Sarcopterygii - ps23
Tetrapoda - ps24
Amniota - ps25
Mammalia - ps26
Theria - ps27
Placentalia - ps28
Boreoeutheria - ps29
Euarchontoglires - ps30
Euarchonta - ps31
Primates - ps32
Haplorrhini - ps33
Simiiformes - ps34
Catarrhini - ps35
Hominoidea - ps36
Hominidae - ps37
Homininae - ps38
Hominini - ps39
Homo_sapiens - ps40
Phylostratum

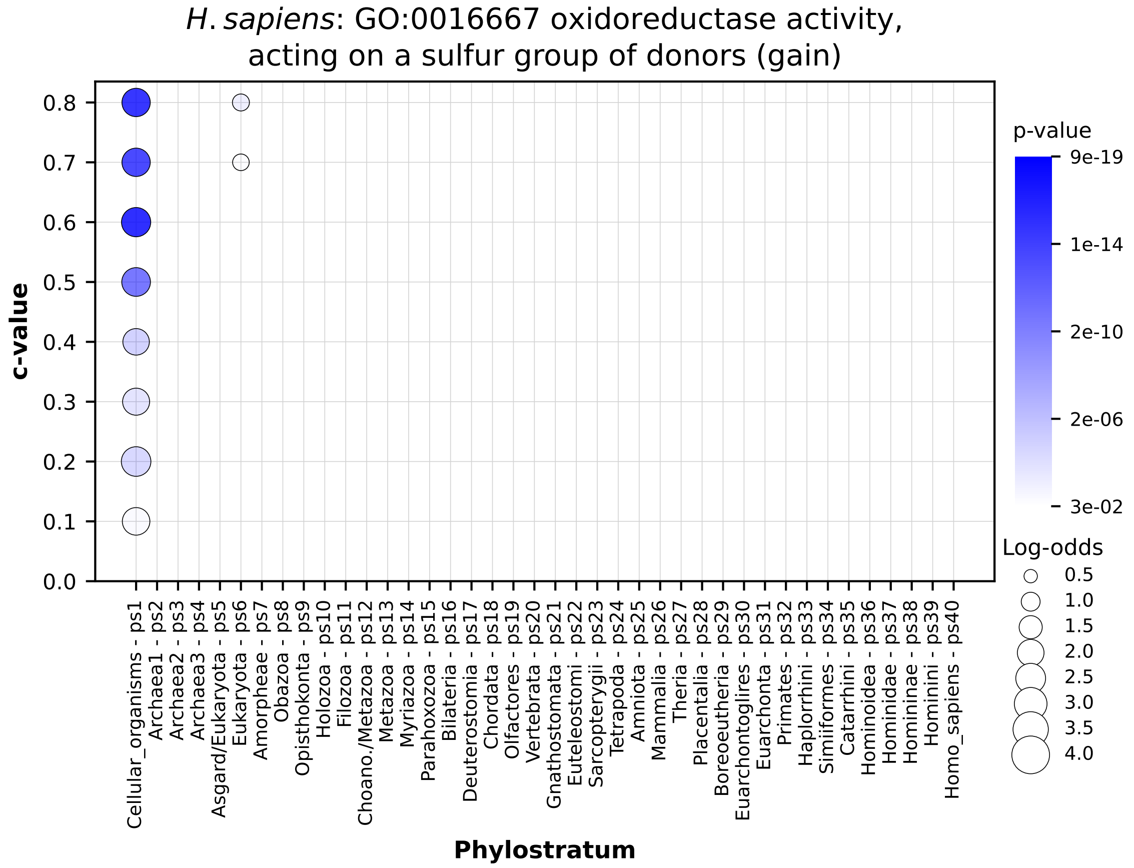
H. sapiens: GO:0016667 oxidoreductase activity, acting on a sulfur group of donors (gain)
c-value
0.0
0.1
0.2
0.3
0.4
0.5
0.6
0.7
0.8
Phylostratum
Cellular_organisms - ps1
Archaea1 - ps2
Archaea2 - ps3
Archaea3 - ps4
Asgard/Eukaryota - ps5
Eukaryota - ps6
Amorpheae - ps7
Obazoa - ps8
Opisthokonta - ps9
Holozoa - ps10
Filozoa - ps11
Choano./Metazoa - ps12
Metazoa - ps13
Myriazoa - ps14
Parahoxozoa - ps15
Bilateria - ps16
Deuterostomia - ps17
Chordata - ps18
Olfactores - ps19
Vertebrata - ps20
Gnathostomata - ps21
Euteleostomi - ps22
Sarcopterygii - ps23
Tetrapoda - ps24
Amniota - ps25
Mammalia - ps26
Theria - ps27
Placentalia - ps28
Boreoeutheria - ps29
Euarchontoglires - ps30
Euarchonta - ps31
Primates - ps32
Haplorrhini - ps33
Simiiformes - ps34
Catarrhini - ps35
Hominoidea - ps36
Hominidae - ps37
Homininae - ps38
Hominini - ps39
Homo_sapiens - ps40
p-value
9e-19
1e-14
2e-10
2e-06
3e-02
Log-odds
0.5
1.0
1.5
2.0
2.5
3.0
3.5
4.0

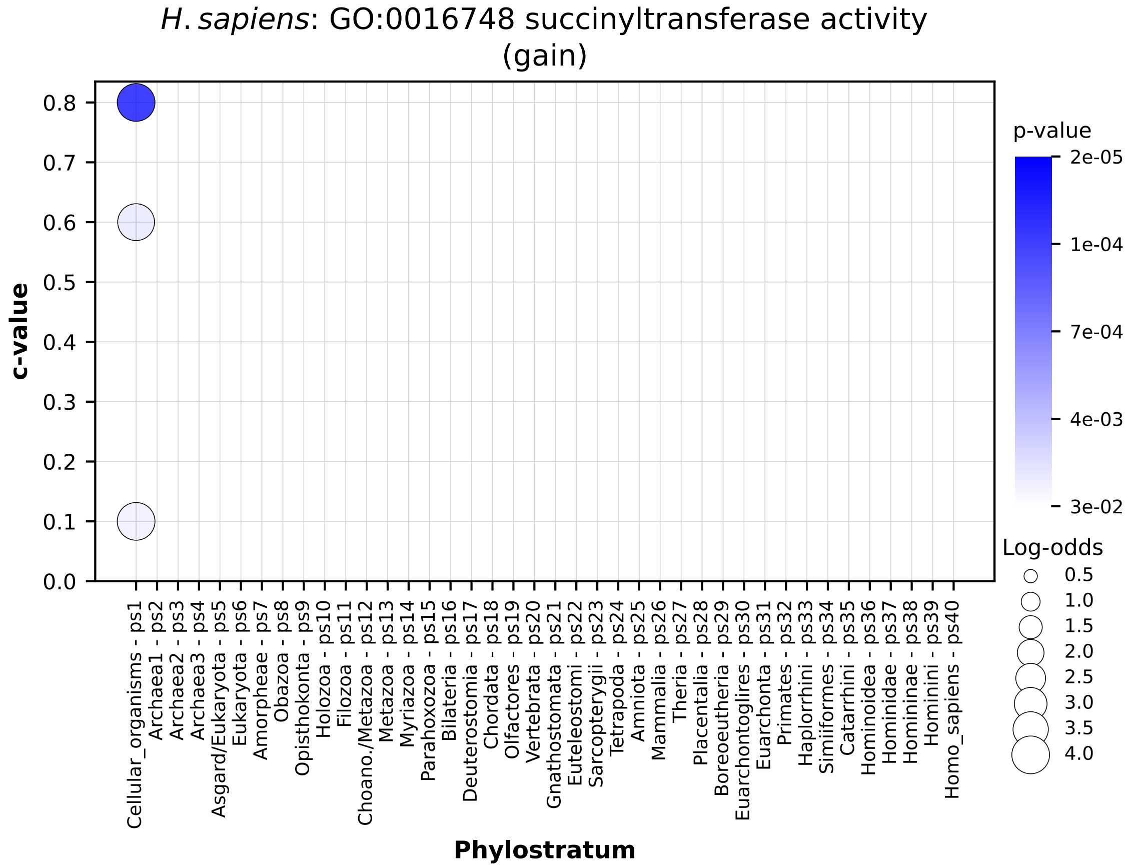

H. sapiens: GO:0016748 succinyltransferase activity
(gain)
c-value
0.0
0.1
0.2
0.3
0.4
0.5
0.6
0.7
0.8
p-value
2e-05
1e-04
7e-04
4e-03
3e-02
Log-odds
0.5
1.0
1.5
2.0
2.5
3.0
3.5
4.0
Cellular_organisms - ps1
Archaea1 - ps2
Archaea2 - ps3
Archaea3 - ps4
Asgard/Eukaryota - ps5
Eukaryota - ps6
Amorpheae - ps7
Obazoa - ps8
Opisthokonta - ps9
Holozoa - ps10
Filozoa - ps11
Choano./Metazoa - ps12
Metazoa - ps13
Myriazoa - ps14
Parahoxozoa - ps15
Bilateria - ps16
Deuterostomia - ps17
Chordata - ps18
Olfactores - ps19
Vertebrata - ps20
Gnathostomata - ps21
Euteleostomi - ps22
Sarcopterygii - ps23
Tetrapoda - ps24
Amniota - ps25
Mammalia - ps26
Theria - ps27
Placentalia - ps28
Boreoeutheria - ps29
Euarchontoglires - ps30
Euarchonta - ps31
Primates - ps32
Haplorrhini - ps33
Simiiformes - ps34
Catarrhini - ps35
Hominoidea - ps36
Hominidae - ps37
Homininae - ps38
Hominini - ps39
Homo_sapiens - ps40
Phylostratum

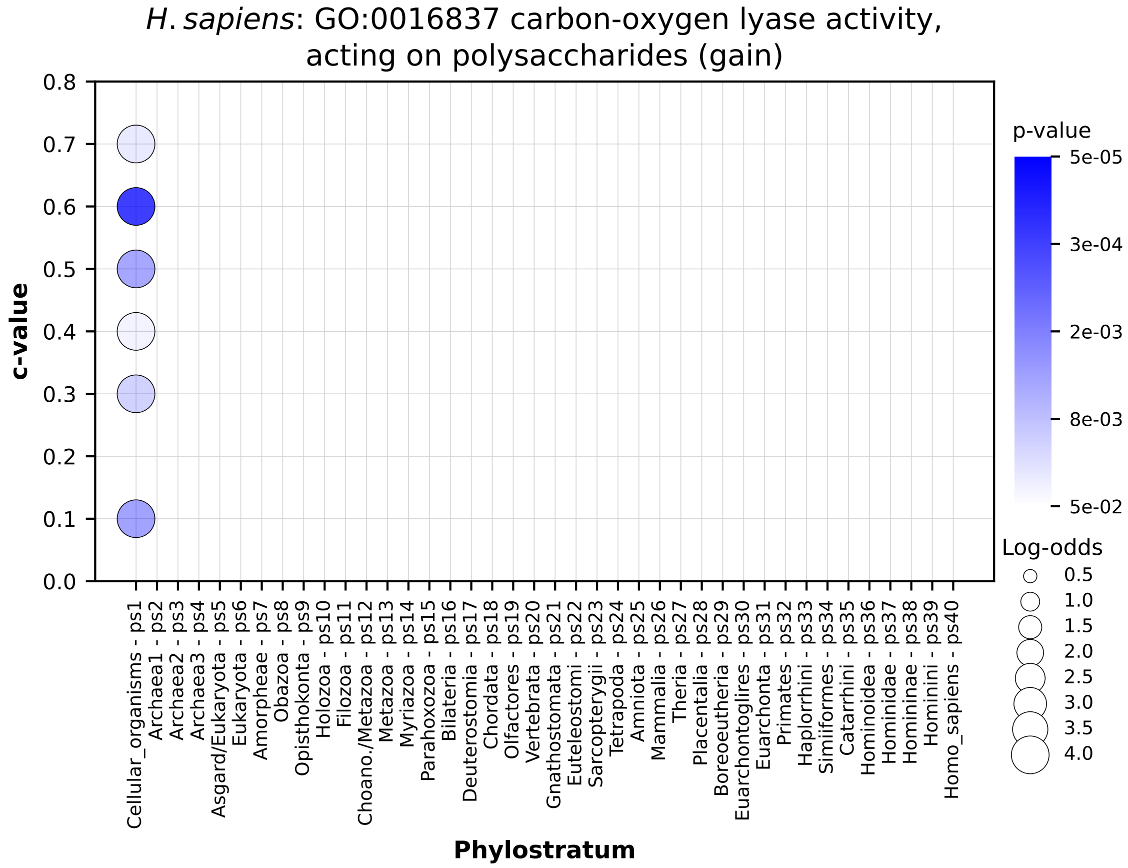

H. sapiens: GO:0016837 carbon-oxygen lyase activity, acting on polysaccharides (gain)
c-value
0.0
0.1
0.2
0.3
0.4
0.5
0.6
0.7
0.8
Phylostratum
Cellular_organisms - ps1
Archaea1 - ps2
Archaea2 - ps3
Archaea3 - ps4
Asgard/Eukaryota - ps5
Eukaryota - ps6
Amorpheae - ps7
Obazoa - ps8
Opisthokonta - ps9
Holozoa - ps10
Filozoa - ps11
Choano./Metazoa - ps12
Metazoa - ps13
Myriazoa - ps14
Parahoxozoa - ps15
Bilateria - ps16
Deuterostomia - ps17
Chordata - ps18
Olfactores - ps19
Vertebrata - ps20
Gnathostomata - ps21
Euteleostomi - ps22
Sarcopterygii - ps23
Tetrapoda - ps24
Amniota - ps25
Mammalia - ps26
Theria - ps27
Placentalia - ps28
Boreoeutheria - ps29
Euarchontoglires - ps30
Euarchonta - ps31
Primates - ps32
Haplorrhini - ps33
Simiiformes - ps34
Catarrhini - ps35
Hominoidea - ps36
Hominidae - ps37
Homininae - ps38
Hominini - ps39
Homo_sapiens - ps40
p-value
5e-05
3e-04
2e-03
8e-03
5e-02
Log-odds
0.5
1.0
1.5
2.0
2.5
3.0
3.5
4.0

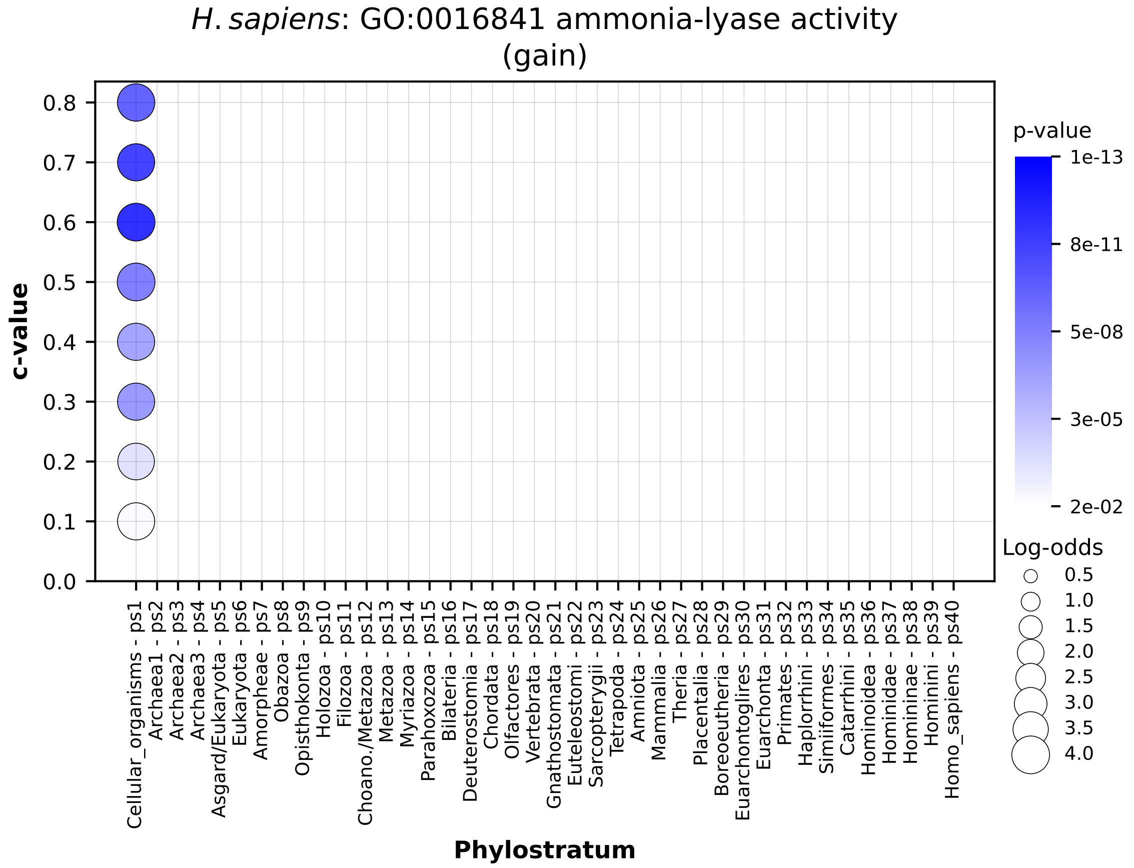

H. sapiens: GO:0016841 ammonia-lyase activity
(gain)
c-value
0.0
0.1
0.2
0.3
0.4
0.5
0.6
0.7
0.8
p-value
1e-13
8e-11
5e-08
3e-05
2e-02
Log-odds
0.5
1.0
1.5
2.0
2.5
3.0
3.5
4.0
Cellular_organisms - ps1
Archaea1 - ps2
Archaea2 - ps3
Archaea3 - ps4
Asgard/Eukaryota - ps5
Eukaryota - ps6
Amorpheae - ps7
Obazoa - ps8
Opisthokonta - ps9
Holozoa - ps10
Filozoa - ps11
Choano./Metazoa - ps12
Metazoa - ps13
Myriazoa - ps14
Parahoxozoa - ps15
Bilateria - ps16
Deuterostomia - ps17
Chordata - ps18
Olfactores - ps19
Vertebrata - ps20
Gnathostomata - ps21
Euteleostomi - ps22
Sarcopterygii - ps23
Tetrapoda - ps24
Amniota - ps25
Mammalia - ps26
Theria - ps27
Placentalia - ps28
Boreoeutheria - ps29
Euarchontoglires - ps30
Euarchonta - ps31
Primates - ps32
Haplorrhini - ps33
Simiiformes - ps34
Catarrhini - ps35
Hominoidea - ps36
Hominidae - ps37
Homininae - ps38
Hominini - ps39
Homo_sapiens - ps40
Phylostratum

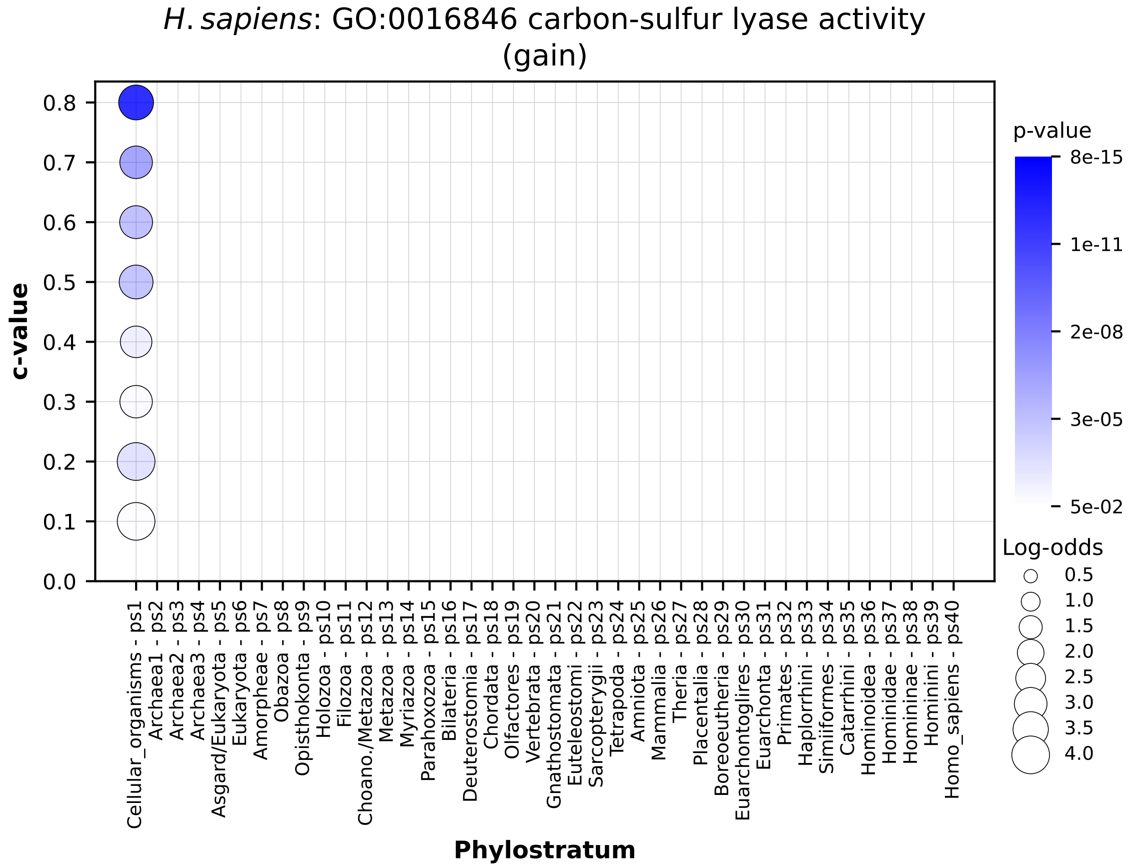

H. sapiens: GO:0016846 carbon-sulfur lyase activity
(gain)
c-value
0.0
0.1
0.2
0.3
0.4
0.5
0.6
0.7
0.8
p-value
8e-15
1e-11
2e-08
3e-05
5e-02
Log-odds
0.5
1.0
1.5
2.0
2.5
3.0
3.5
4.0
Cellular_organisms - ps1
Archaea1 - ps2
Archaea2 - ps3
Archaea3 - ps4
Asgard/Eukaryota - ps5
Eukaryota - ps6
Amorpheae - ps7
Obazoa - ps8
Opisthokonta - ps9
Holozoa - ps10
Filozoa - ps11
Choano./Metazoa - ps12
Metazoa - ps13
Myriazoa - ps14
Parahoxozoa - ps15
Bilateria - ps16
Deuterostomia - ps17
Chordata - ps18
Olfactores - ps19
Vertebrata - ps20
Gnathostomata - ps21
Euteleostomi - ps22
Sarcopterygii - ps23
Tetrapoda - ps24
Amniota - ps25
Mammalia - ps26
Theria - ps27
Placentalia - ps28
Boreoeutheria - ps29
Euarchontoglires - ps30
Euarchonta - ps31
Primates - ps32
Haplorrhini - ps33
Simiiformes - ps34
Catarrhini - ps35
Hominoidea - ps36
Hominidae - ps37
Homininae - ps38
Hominini - ps39
Homo_sapiens - ps40
Phylostratum

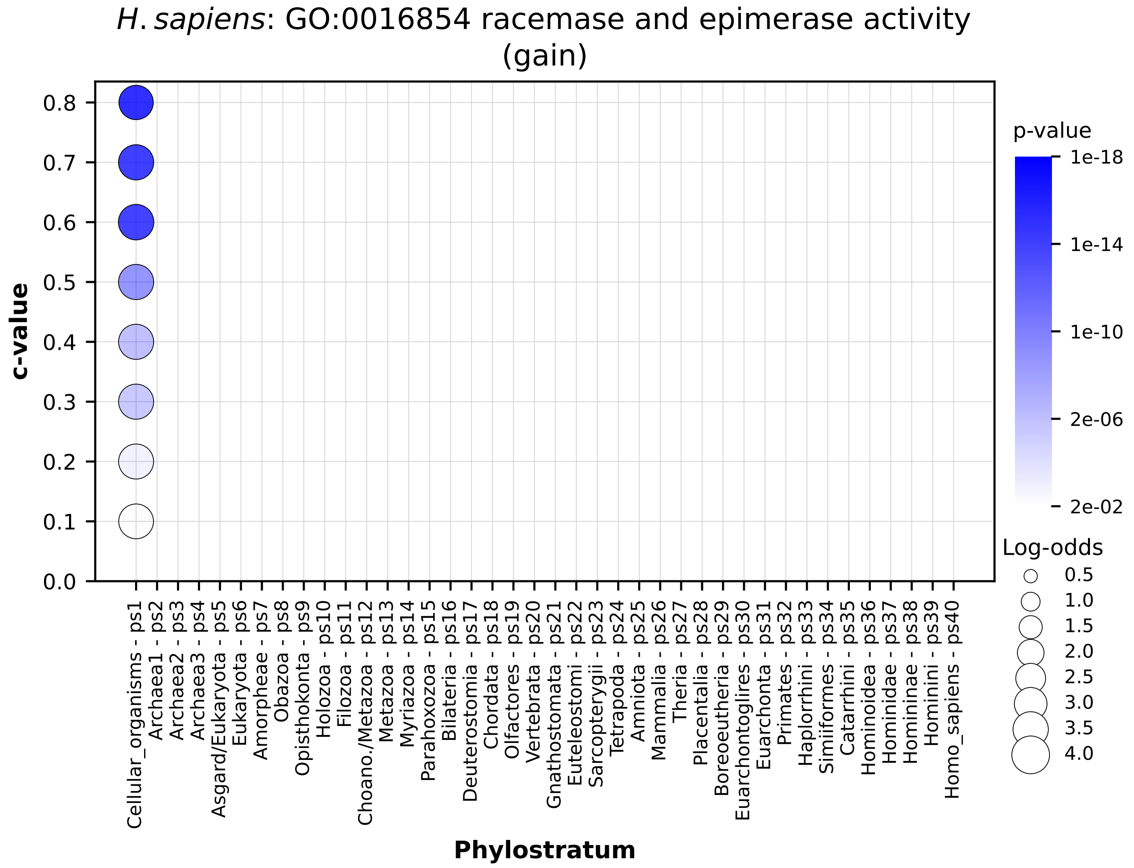

H. sapiens: GO:0016854 racemase and epimerase activity (gain)
c-value
0.0
0.1
0.2
0.3
0.4
0.5
0.6
0.7
0.8
Phylostratum
Cellular_organisms - ps1
Archaea1 - ps2
Archaea2 - ps3
Archaea3 - ps4
Asgard/Eukaryota - ps5
Eukaryota - ps6
Amorpheae - ps7
Obazoa - ps8
Opisthokonta - ps9
Holozoa - ps10
Filozoa - ps11
Choano./Metazoa - ps12
Metazoa - ps13
Myriazoa - ps14
Parahoxozoa - ps15
Bilateria - ps16
Deuterostomia - ps17
Chordata - ps18
Olfactores - ps19
Vertebrata - ps20
Gnathostomata - ps21
Euteleostomi - ps22
Sarcopterygii - ps23
Tetrapoda - ps24
Amniota - ps25
Mammalia - ps26
Theria - ps27
Placentalia - ps28
Boreoeutheria - ps29
Euarchontoglires - ps30
Euarchonta - ps31
Primates - ps32
Haplorrhini - ps33
Simiiformes - ps34
Catarrhini - ps35
Hominoidea - ps36
Hominidae - ps37
Homininae - ps38
Hominini - ps39
Homo_sapiens - ps40
p-value
1e-18
1e-14
1e-10
2e-06
2e-02
Log-odds
0.5
1.0
1.5
2.0
2.5
3.0
3.5
4.0

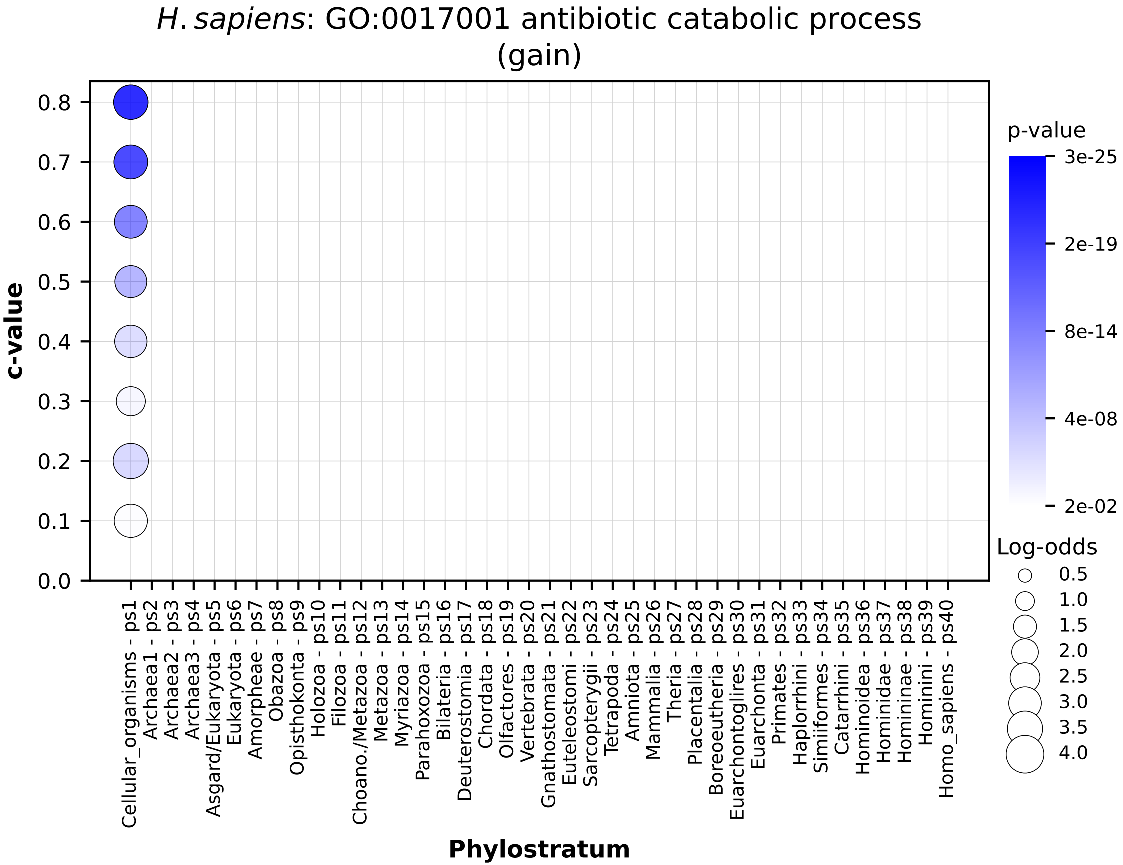

*H. sapiens*: GO:0017001 antibiotic catabolic process
(gain)
c-value
0.0
0.1
0.2
0.3
0.4
0.5
0.6
0.7
0.8
p-value
3e-25
2e-19
8e-14
4e-08
2e-02
Log-odds
0.5
1.0
1.5
2.0
2.5
3.0
3.5
4.0
Cellular_organisms - ps1
Archaea1 - ps2
Archaea2 - ps3
Archaea3 - ps4
Asgard/Eukaryota - ps5
Eukaryota - ps6
Amorpheae - ps7
Obazoa - ps8
Opisthokonta - ps9
Holozoa - ps10
Filozoa - ps11
Choano./Metazoa - ps12
Metazoa - ps13
Myriazoa - ps14
Parahoxozoa - ps15
Bilateria - ps16
Deuterostomia - ps17
Chordata - ps18
Olfactores - ps19
Vertebrata - ps20
Gnathostomata - ps21
Euteleostomi - ps22
Sarcopterygii - ps23
Tetrapoda - ps24
Amniota - ps25
Mammalia - ps26
Theria - ps27
Placentalia - ps28
Boreoeutheria - ps29
Euarchontoglires - ps30
Euarchonta - ps31
Primates - ps32
Haplorrhini - ps33
Simiiformes - ps34
Catarrhini - ps35
Hominoidea - ps36
Hominidae - ps37
Homininae - ps38
Hominini - ps39
Homo_sapiens - ps40
Phylostratum

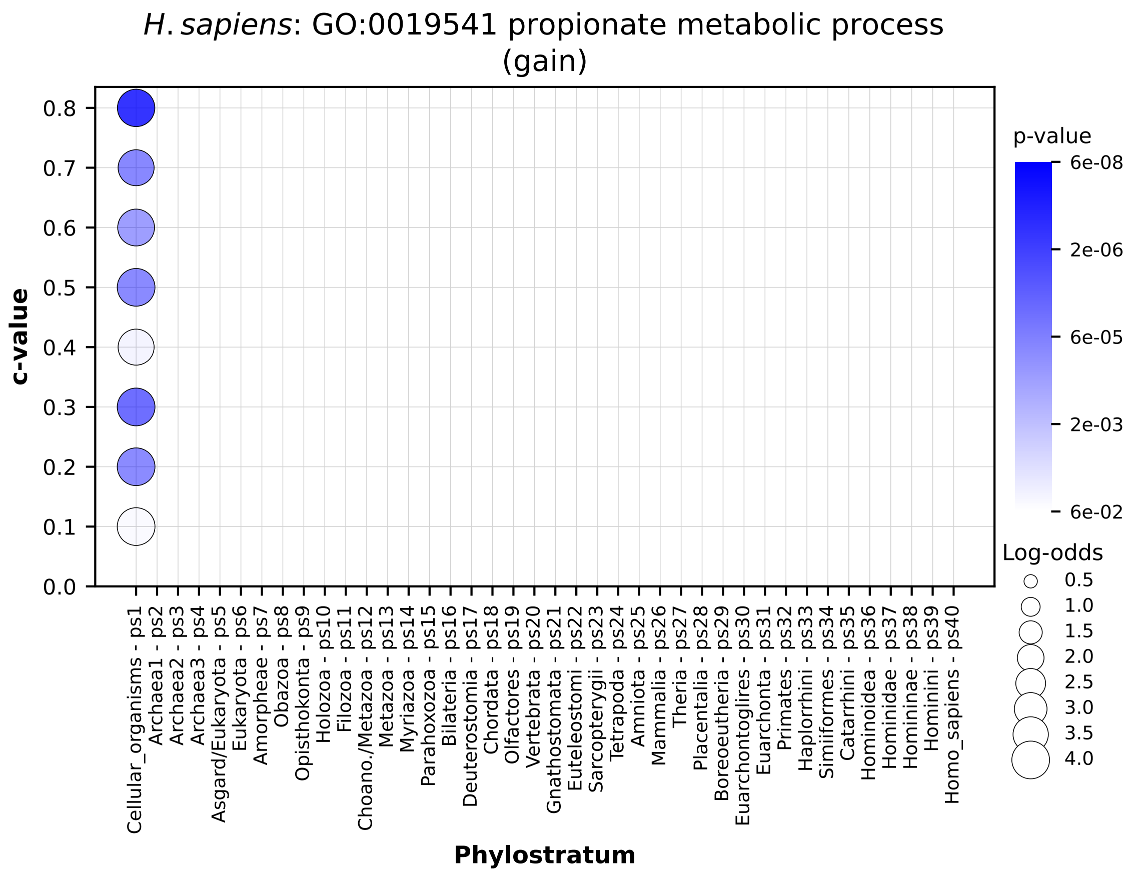
H. sapiens: GO:0019541 propionate metabolic process
(gain)
c-value
0.0
0.1
0.2
0.3
0.4
0.5
0.6
0.7
0.8
Phylostratum
Cellular_organisms - ps1
Archaea1 - ps2
Archaea2 - ps3
Archaea3 - ps4
Asgard/Eukaryota - ps5
Eukaryota - ps6
Amorpheae - ps7
Obazoa - ps8
Opisthokonta - ps9
Holozoa - ps10
Filozoa - ps11
Choano./Metazoa - ps12
Metazoa - ps13
Myriazoa - ps14
Parahoxozoa - ps15
Bilateria - ps16
Deuterostomia - ps17
Chordata - ps18
Olfactores - ps19
Vertebrata - ps20
Gnathostomata - ps21
Euteleostomi - ps22
Sarcopterygii - ps23
Tetrapoda - ps24
Amniota - ps25
Mammalia - ps26
Theria - ps27
Placentalia - ps28
Boreoeutheria - ps29
Euarchontoglires - ps30
Euarchonta - ps31
Primates - ps32
Haplorrhini - ps33
Simiiformes - ps34
Catarrhini - ps35
Hominoidea - ps36
Hominidae - ps37
Homininae - ps38
Hominini - ps39
Homo_sapiens - ps40
p-value
6e-08
2e-06
6e-05
2e-03
6e-02
Log-odds
0.5
1.0
1.5
2.0
2.5
3.0
3.5
4.0

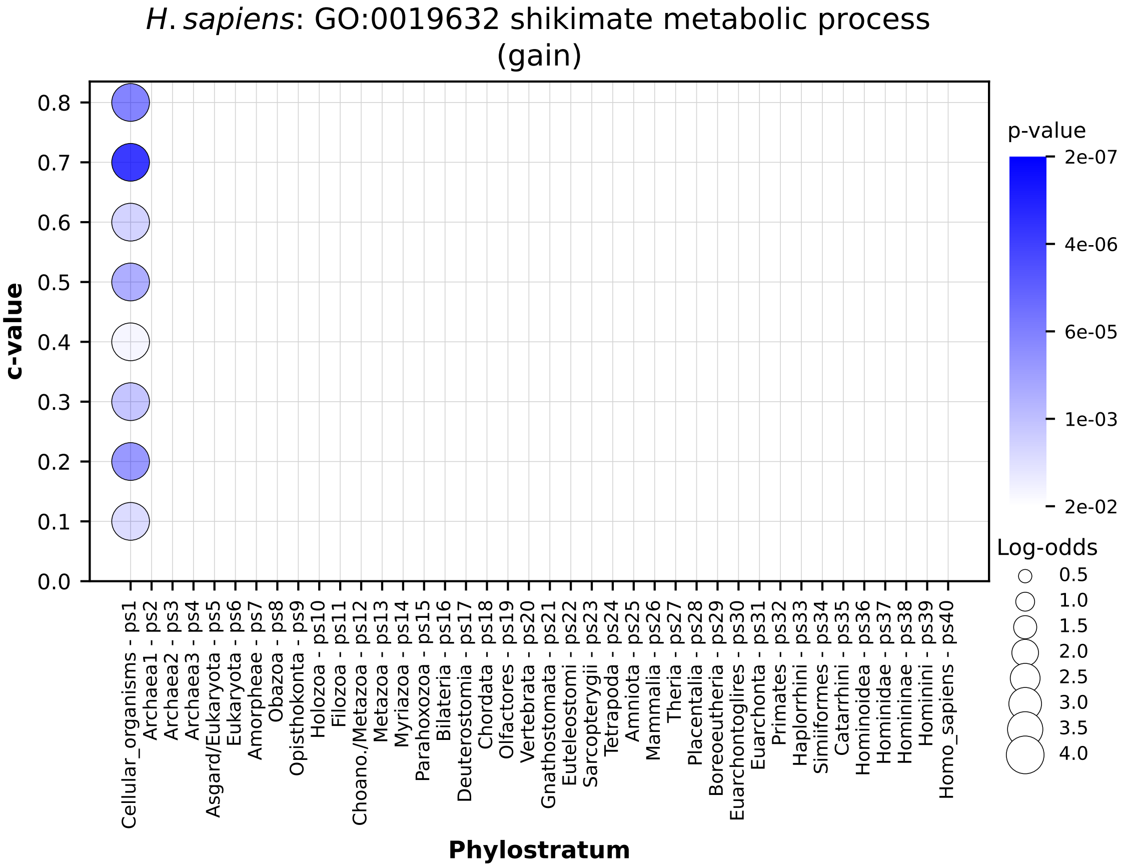

*H. sapiens*: GO:0019632 shikimate metabolic process (gain)
c-value
0.0
0.1
0.2
0.3
0.4
0.5
0.6
0.7
0.8
Phylostratum
Cellular_organisms - ps1
Archaea1 - ps2
Archaea2 - ps3
Archaea3 - ps4
Asgard/Eukaryota - ps5
Eukaryota - ps6
Amorpheae - ps7
Obazoa - ps8
Opisthokonta - ps9
Holozoa - ps10
Filozoa - ps11
Choano./Metazoa - ps12
Metazoa - ps13
Myriazoa - ps14
Parahoxozoa - ps15
Bilateria - ps16
Deuterostomia - ps17
Chordata - ps18
Olfactores - ps19
Vertebrata - ps20
Gnathostomata - ps21
Euteleostomi - ps22
Sarcopterygii - ps23
Tetrapoda - ps24
Amniota - ps25
Mammalia - ps26
Theria - ps27
Placentalia - ps28
Boreoeutheria - ps29
Euarchontoglires - ps30
Euarchonta - ps31
Primates - ps32
Haplorrhini - ps33
Simiiformes - ps34
Catarrhini - ps35
Hominoidea - ps36
Hominidae - ps37
Homininae - ps38
Hominini - ps39
Homo_sapiens - ps40
p-value
2e-07
4e-06
6e-05
1e-03
2e-02
Log-odds
0.5
1.0
1.5
2.0
2.5
3.0
3.5
4.0

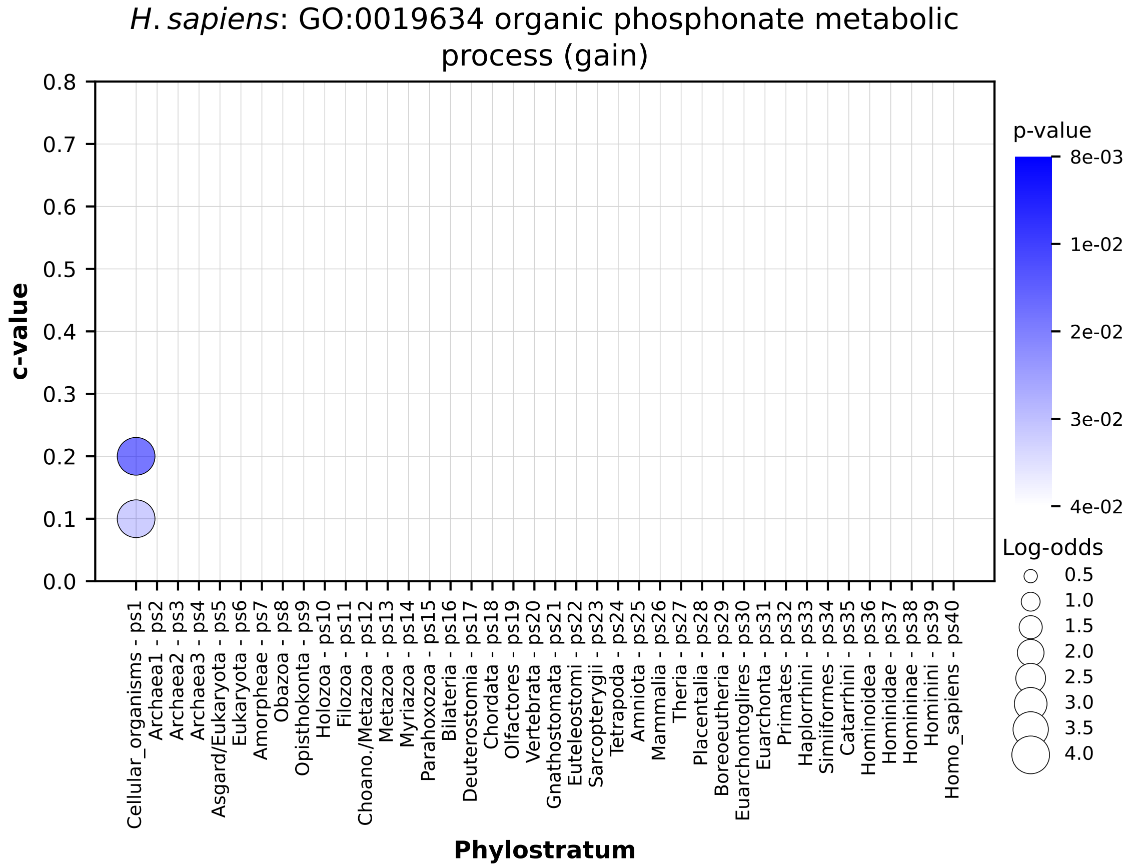

*H. sapiens*: GO:0019634 organic phosphonate metabolic process (gain)
c-value
0.8
0.7
0.6
0.5
0.4
0.3
0.2
0.1
0.0
p-value
8e-03
1e-02
2e-02
3e-02
4e-02
Log-odds
0.5
1.0
1.5
2.0
2.5
3.0
3.5
4.0
Cellular_organisms - ps1
Archaea1 - ps2
Archaea2 - ps3
Archaea3 - ps4
Asgard/Eukaryota - ps5
Eukaryota - ps6
Amorpheae - ps7
Obazoa - ps8
Opisthokonta - ps9
Holozoa - ps10
Filozoa - ps11
Choano./Metazoa - ps12
Metazoa - ps13
Myriazoa - ps14
Parahoxozoa - ps15
Bilateria - ps16
Deuterostomia - ps17
Chordata - ps18
Olfactores - ps19
Vertebrata - ps20
Gnathostomata - ps21
Euteleostomi - ps22
Sarcopterygii - ps23
Tetrapoda - ps24
Amniota - ps25
Mammalia - ps26
Theria - ps27
Placentalia - ps28
Boreoeutheria - ps29
Euarchontoglires - ps30
Euarchonta - ps31
Primates - ps32
Haplorrhini - ps33
Simiiformes - ps34
Catarrhini - ps35
Hominoidea - ps36
Hominidae - ps37
Homininae - ps38
Hominini - ps39
Homo_sapiens - ps40
Phylostratum

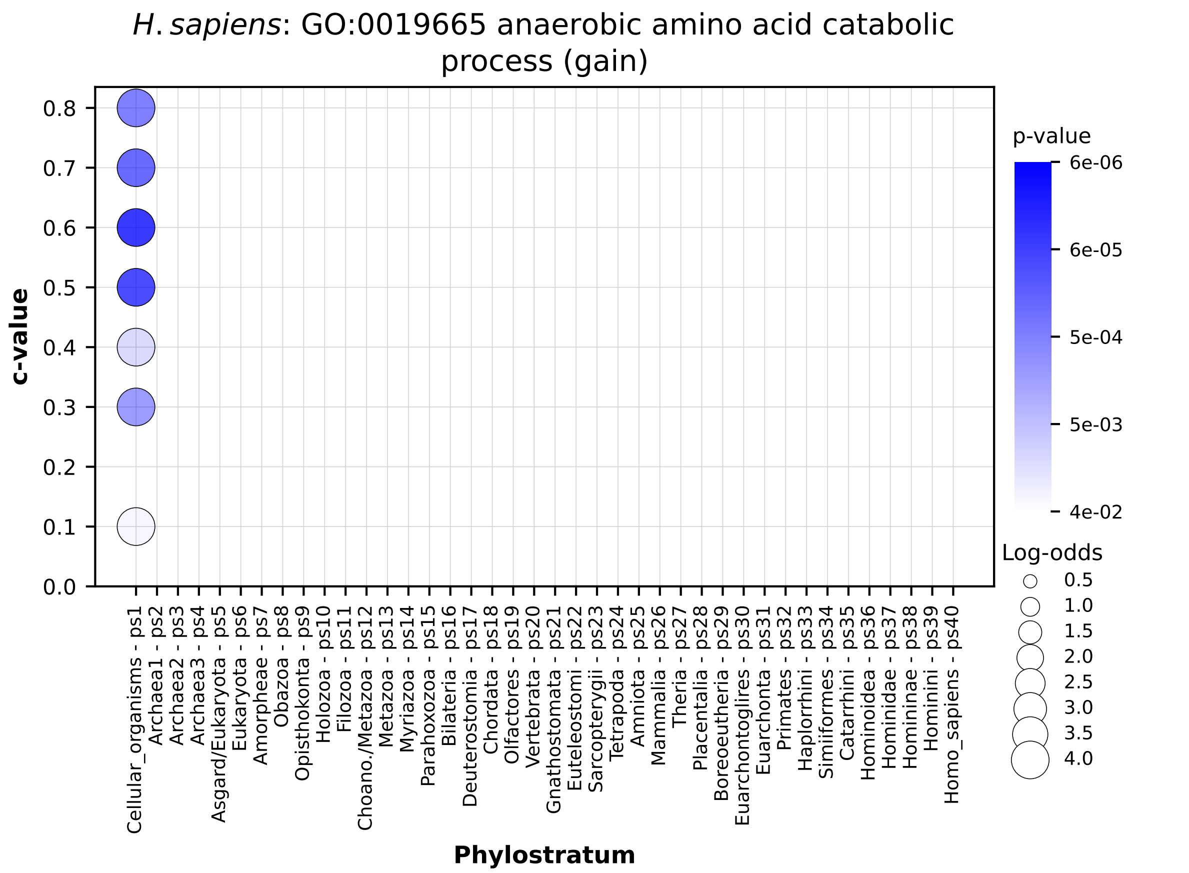

H. sapiens: GO:0019665 anaerobic amino acid catabolic process (gain)
c-value
0.0
0.1
0.2
0.3
0.4
0.5
0.6
0.7
0.8
Phylostratum
Cellular_organisms - ps1
Archaea1 - ps2
Archaea2 - ps3
Archaea3 - ps4
Asgard/Eukaryota - ps5
Eukaryota - ps6
Amorpheae - ps7
Obazoa - ps8
Opisthokonta - ps9
Holozoa - ps10
Filozoa - ps11
Choano./Metazoa - ps12
Metazoa - ps13
Myriazoa - ps14
Parahoxozoa - ps15
Bilateria - ps16
Deuterostomia - ps17
Chordata - ps18
Olfactores - ps19
Vertebrata - ps20
Gnathostomata - ps21
Euteleostomi - ps22
Sarcopterygii - ps23
Tetrapoda - ps24
Amniota - ps25
Mammalia - ps26
Theria - ps27
Placentalia - ps28
Boreoeutheria - ps29
Euarchontoglires - ps30
Euarchonta - ps31
Primates - ps32
Haplorrhini - ps33
Simiiformes - ps34
Catarrhini - ps35
Hominoidea - ps36
Hominidae - ps37
Homininae - ps38
Hominini - ps39
Homo_sapiens - ps40
p-value
6e-06
6e-05
5e-04
5e-03
4e-02
Log-odds
0.5
1.0
1.5
2.0
2.5
3.0
3.5
4.0

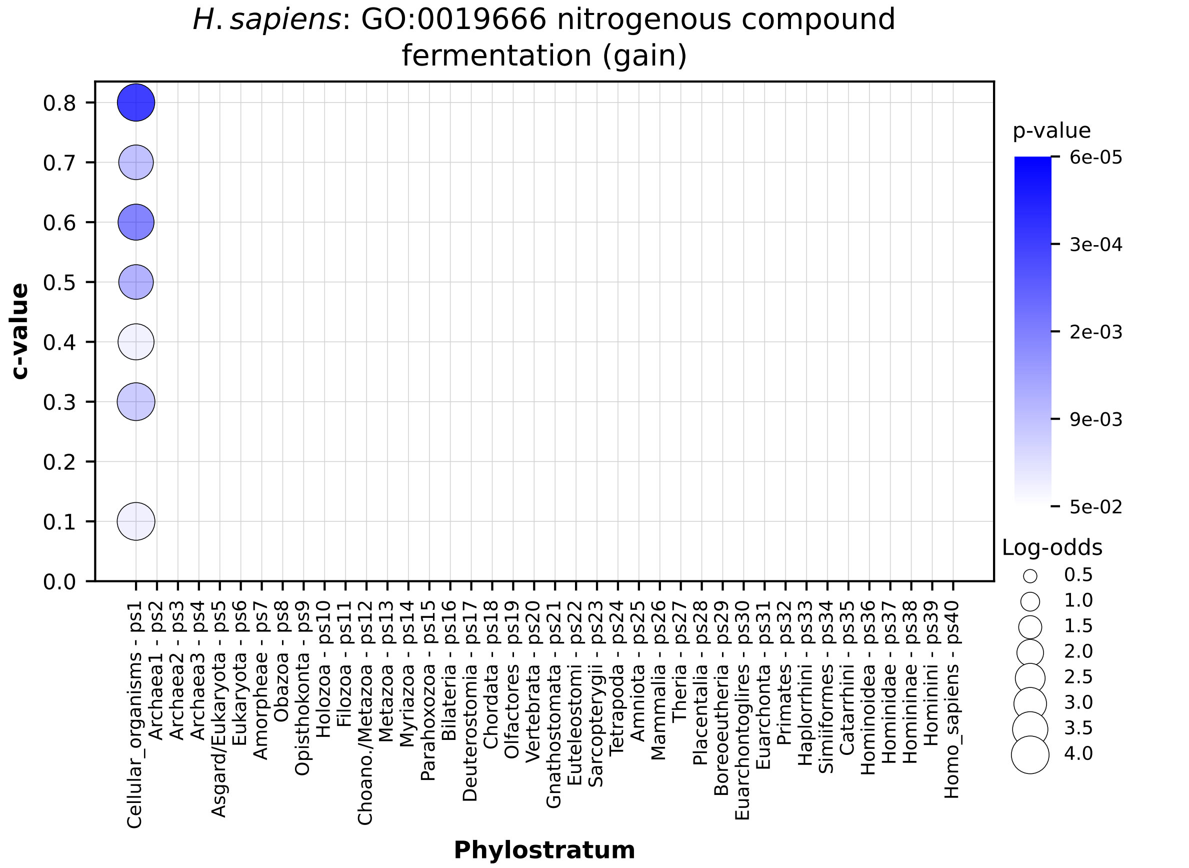

H. sapiens: GO:0019666 nitrogenous compound fermentation (gain)
c-value
0.0
0.1
0.2
0.3
0.4
0.5
0.6
0.7
0.8
p-value
6e-05
3e-04
2e-03
9e-03
5e-02
Log-odds
0.5
1.0
1.5
2.0
2.5
3.0
3.5
4.0
Cellular_organisms - ps1
Archaea1 - ps2
Archaea2 - ps3
Archaea3 - ps4
Asgard/Eukaryota - ps5
Eukaryota - ps6
Amorpheae - ps7
Obazoa - ps8
Opisthokonta - ps9
Holozoa - ps10
Filozoa - ps11
Choano./Metazoa - ps12
Metazoa - ps13
Myriazoa - ps14
Parahoxozoa - ps15
Bilateria - ps16
Deuterostomia - ps17
Chordata - ps18
Olfactores - ps19
Vertebrata - ps20
Gnathostomata - ps21
Euteleostomi - ps22
Sarcopterygii - ps23
Tetrapoda - ps24
Amniota - ps25
Mammalia - ps26
Theria - ps27
Placentalia - ps28
Boreoeutheria - ps29
Euarchontoglires - ps30
Euarchonta - ps31
Primates - ps32
Haplorrhini - ps33
Simiiformes - ps34
Catarrhini - ps35
Hominoidea - ps36
Hominidae - ps37
Homininae - ps38
Hominini - ps39
Homo_sapiens - ps40
Phylostratum

# *H. sapiens*: GO:0019700 organic phosphonate catabolic process (gain)

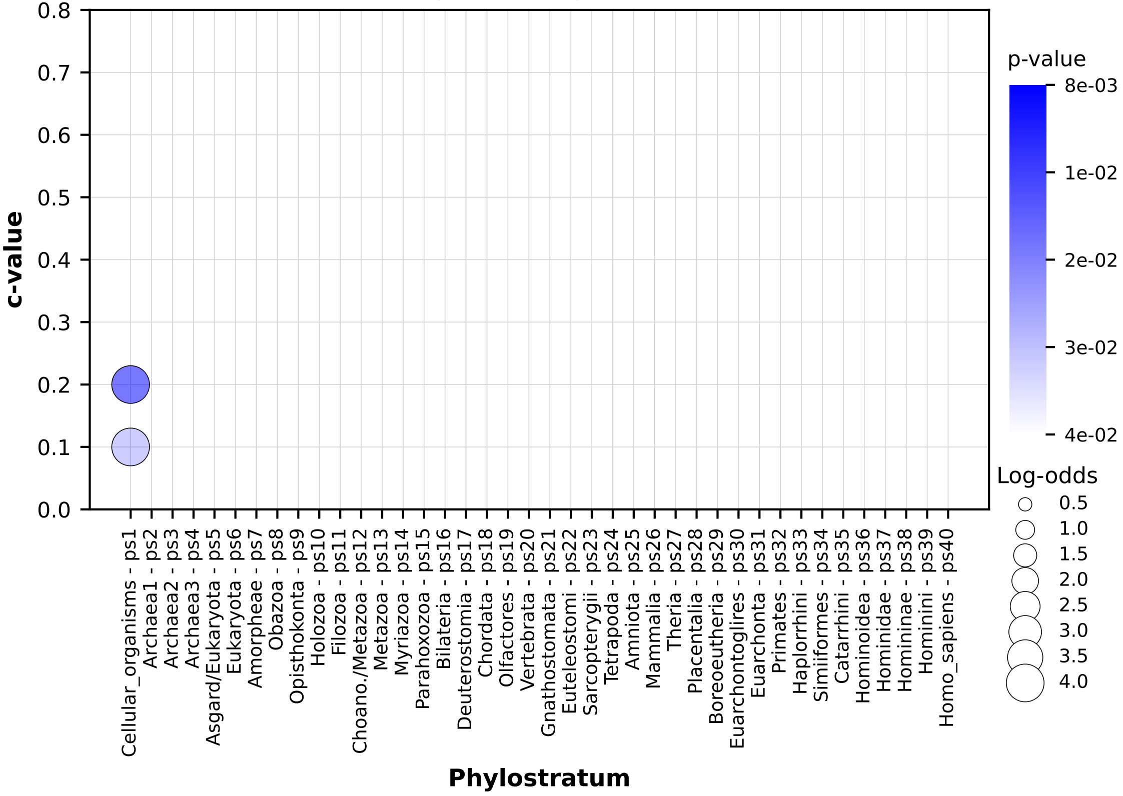

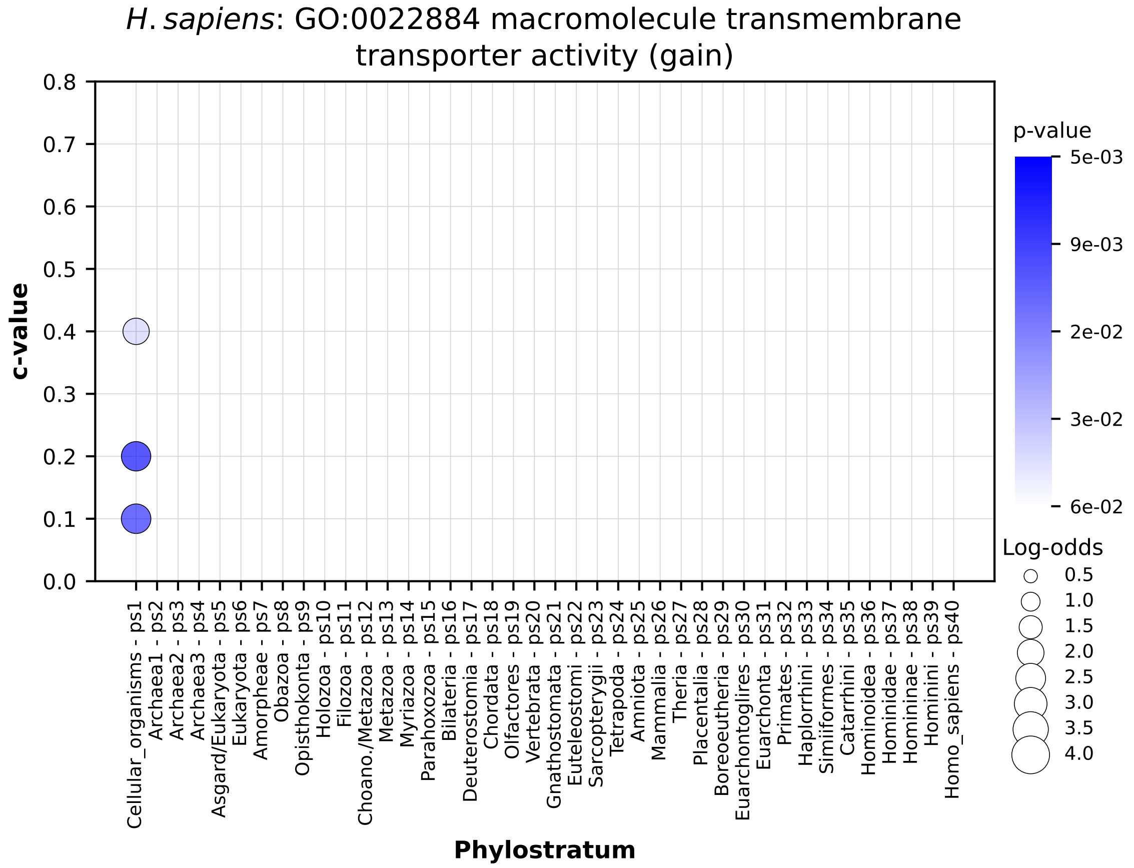
H. sapiens: GO:0022884 macromolecule transmembrane transporter activity (gain)
c-value
0.0
0.1
0.2
0.3
0.4
0.5
0.6
0.7
0.8
Phylostratum
Cellular_organisms - ps1
Archaea1 - ps2
Archaea2 - ps3
Archaea3 - ps4
Asgard/Eukaryota - ps5
Eukaryota - ps6
Amorpheae - ps7
Obazoa - ps8
Opisthokonta - ps9
Holozoa - ps10
Filozoa - ps11
Choano./Metazoa - ps12
Metazoa - ps13
Myriazoa - ps14
Parahoxozoa - ps15
Bilateria - ps16
Deuterostomia - ps17
Chordata - ps18
Olfactores - ps19
Vertebrata - ps20
Gnathostomata - ps21
Euteleostomi - ps22
Sarcopterygii - ps23
Tetrapoda - ps24
Amniota - ps25
Mammalia - ps26
Theria - ps27
Placentalia - ps28
Boreoeutheria - ps29
Euarchontoglires - ps30
Euarchonta - ps31
Primates - ps32
Haplorrhini - ps33
Simiiformes - ps34
Catarrhini - ps35
Hominoidea - ps36
Hominidae - ps37
Homininae - ps38
Hominini - ps39
Homo_sapiens - ps40
p-value
5e-03
9e-03
2e-02
3e-02
6e-02
Log-odds
0.5
1.0
1.5
2.0
2.5
3.0
3.5
4.0

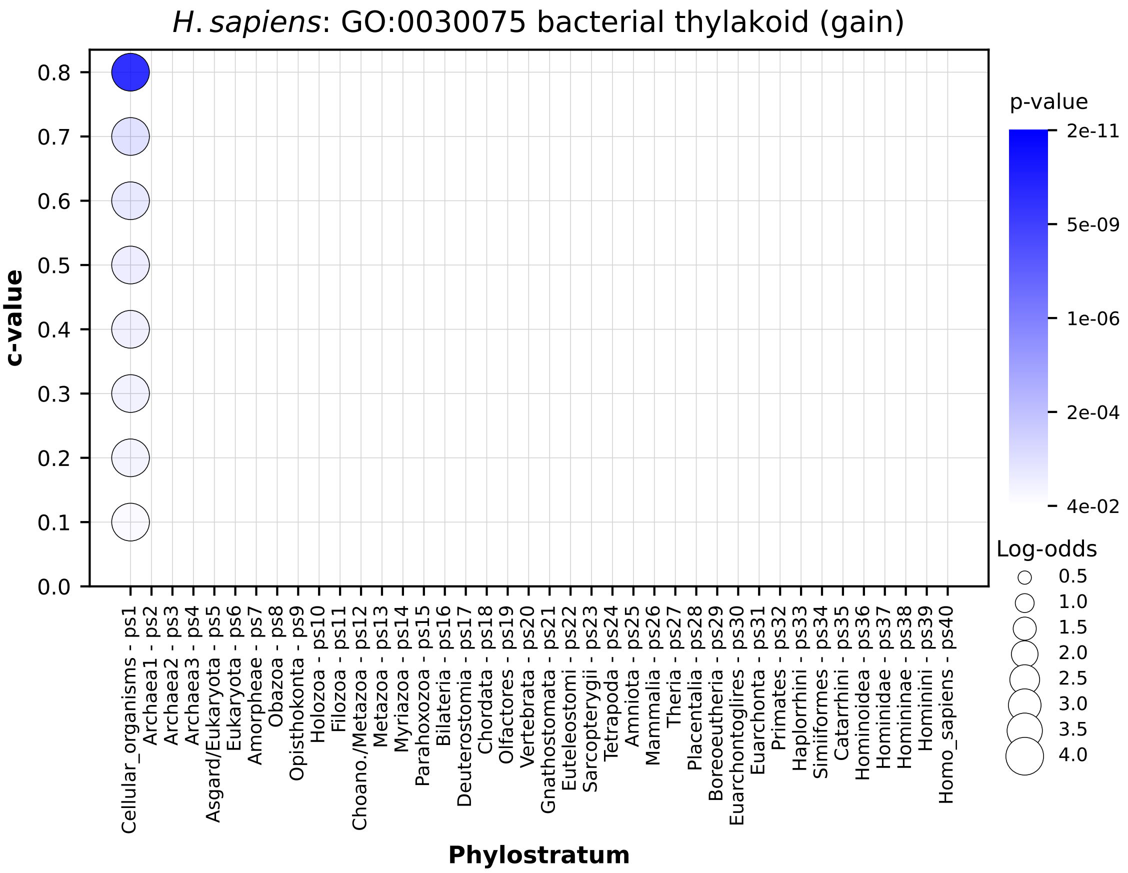

H. sapiens: GO:0030075 bacterial thylakoid (gain)
c-value
0.0
0.1
0.2
0.3
0.4
0.5
0.6
0.7
0.8
Phylostratum
Cellular_organisms - ps1
Archaea1 - ps2
Archaea2 - ps3
Archaea3 - ps4
Asgard/Eukaryota - ps5
Eukaryota - ps6
Amorpheae - ps7
Obazoa - ps8
Opisthokonta - ps9
Holozoa - ps10
Filozoa - ps11
Choano./Metazoa - ps12
Metazoa - ps13
Myriazoa - ps14
Parahoxozoa - ps15
Bilateria - ps16
Deuterostomia - ps17
Chordata - ps18
Olfactores - ps19
Vertebrata - ps20
Gnathostomata - ps21
Euteleostomi - ps22
Sarcopterygii - ps23
Tetrapoda - ps24
Amniota - ps25
Mammalia - ps26
Theria - ps27
Placentalia - ps28
Boreoeutheria - ps29
Euarchontoglires - ps30
Euarchonta - ps31
Primates - ps32
Haplorrhini - ps33
Simiiformes - ps34
Catarrhini - ps35
Hominoidea - ps36
Hominidae - ps37
Homininae - ps38
Hominini - ps39
Homo_sapiens - ps40
p-value
2e-11
5e-09
1e-06
2e-04
4e-02
Log-odds
0.5
1.0
1.5
2.0
2.5
3.0
3.5
4.0

*H. sapiens*: GO:0030076 light-harvesting complex (gain)

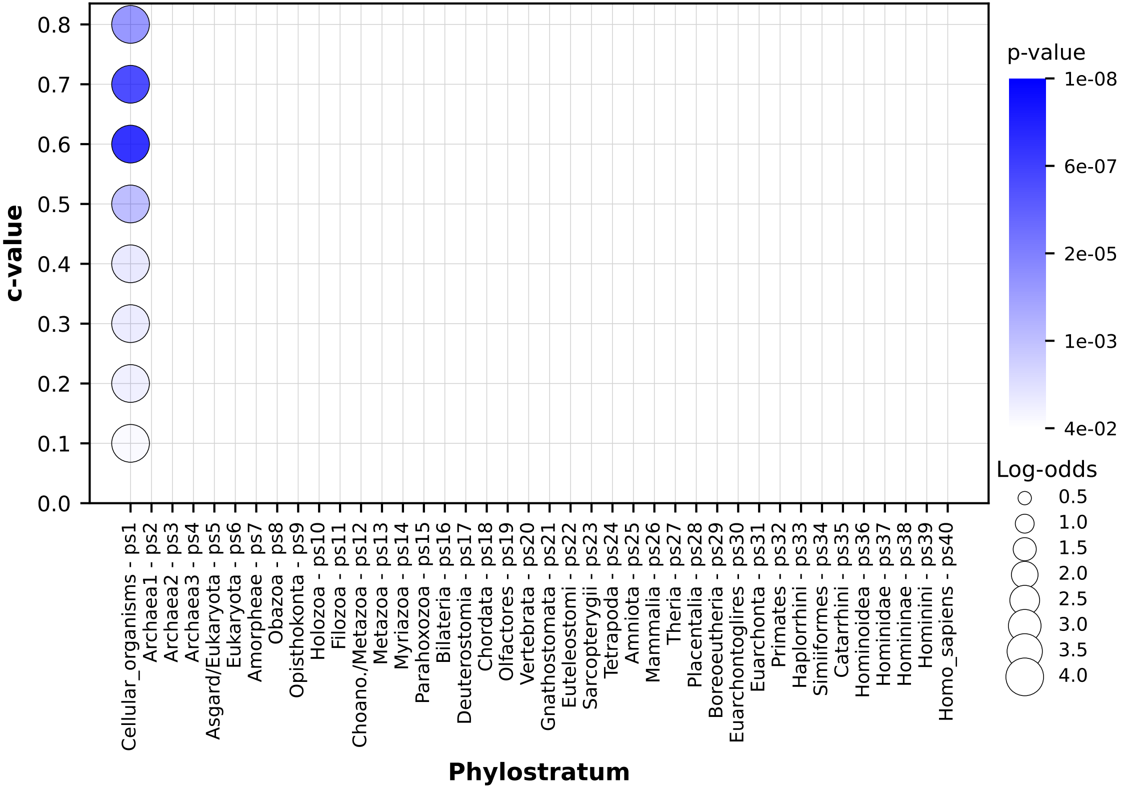

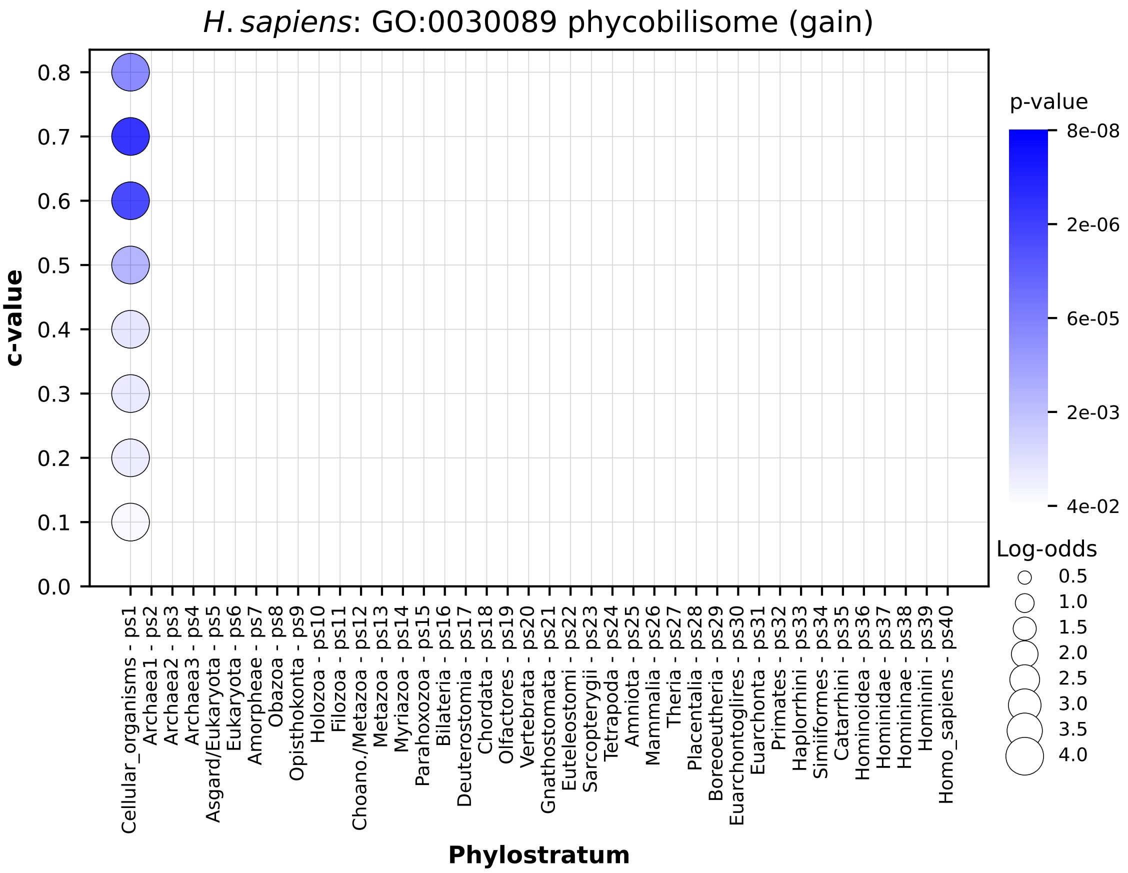

H. sapiens: GO:0030089 phycobilisome (gain)
c-value
0.8
0.7
0.6
0.5
0.4
0.3
0.2
0.1
0.0
p-value
8e-08
2e-06
6e-05
2e-03
4e-02
Log-odds
0.5
1.0
1.5
2.0
2.5
3.0
3.5
4.0
Cellular_organisms - ps1
Archaea1 - ps2
Archaea2 - ps3
Archaea3 - ps4
Asgard/Eukaryota - ps5
Eukaryota - ps6
Amorpheae - ps7
Obazoa - ps8
Opisthokonta - ps9
Holozoa - ps10
Filozoa - ps11
Choano./Metazoa - ps12
Metazoa - ps13
Myriazoa - ps14
Parahoxozoa - ps15
Bilateria - ps16
Deuterostomia - ps17
Chordata - ps18
Olfactores - ps19
Vertebrata - ps20
Gnathostomata - ps21
Euteleostomi - ps22
Sarcopterygii - ps23
Tetrapoda - ps24
Amniota - ps25
Mammalia - ps26
Theria - ps27
Placentalia - ps28
Boreoeutheria - ps29
Euarchontoglires - ps30
Euarchonta - ps31
Primates - ps32
Haplorrhini - ps33
Simiiformes - ps34
Catarrhini - ps35
Hominoidea - ps36
Hominidae - ps37
Homininae - ps38
Hominini - ps39
Homo_sapiens - ps40
Phylostratum

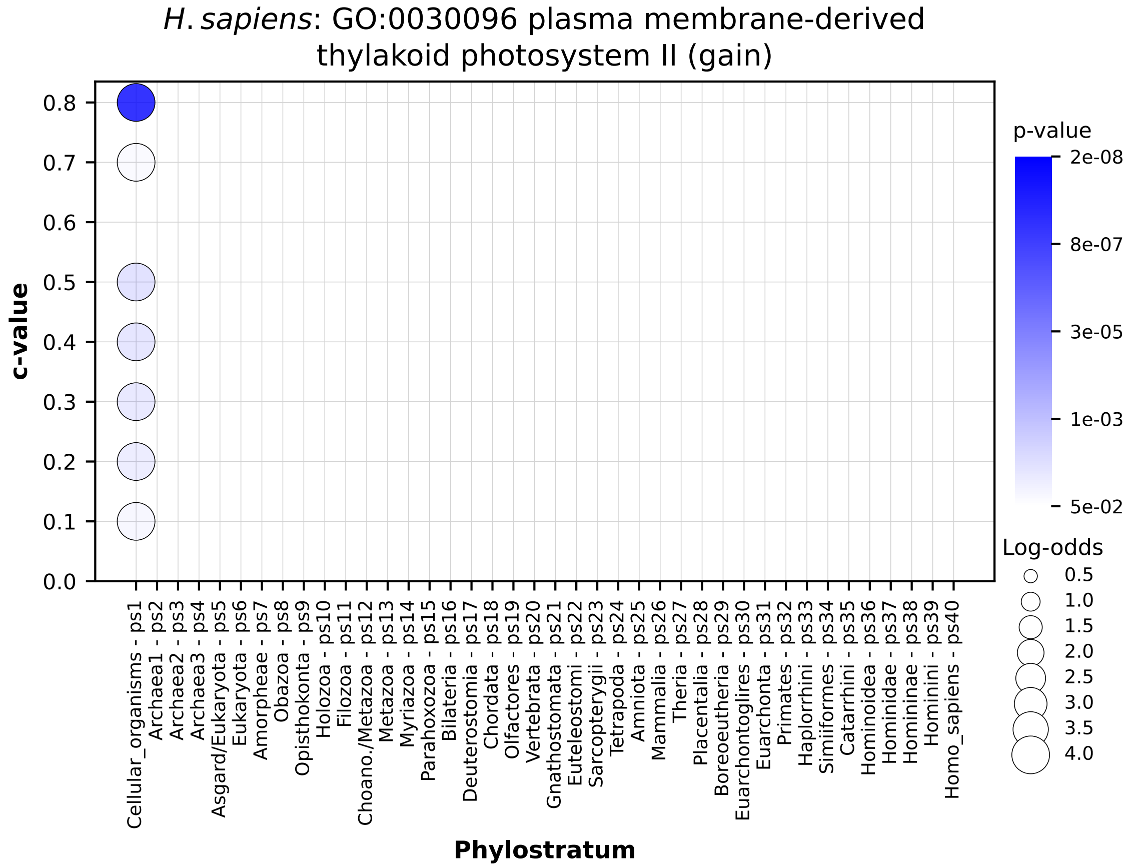

H. sapiens: GO:0030096 plasma membrane-derived thylakoid photosystem II (gain)
c-value
0.0
0.1
0.2
0.3
0.4
0.5
0.6
0.7
0.8
p-value
2e-08
8e-07
3e-05
1e-03
5e-02
Log-odds
0.5
1.0
1.5
2.0
2.5
3.0
3.5
4.0
Cellular_organisms - ps1
Archaea1 - ps2
Archaea2 - ps3
Archaea3 - ps4
Asgard/Eukaryota - ps5
Eukaryota - ps6
Amorpheae - ps7
Obazoa - ps8
Opisthokonta - ps9
Holozoa - ps10
Filozoa - ps11
Choano./Metazoa - ps12
Metazoa - ps13
Myriazoa - ps14
Parahoxozoa - ps15
Bilateria - ps16
Deuterostomia - ps17
Chordata - ps18
Olfactores - ps19
Vertebrata - ps20
Gnathostomata - ps21
Euteleostomi - ps22
Sarcopterygii - ps23
Tetrapoda - ps24
Amniota - ps25
Mammalia - ps26
Theria - ps27
Placentalia - ps28
Boreoeutheria - ps29
Euarchontoglires - ps30
Euarchonta - ps31
Primates - ps32
Haplorrhini - ps33
Simiiformes - ps34
Catarrhini - ps35
Hominoidea - ps36
Hominidae - ps37
Homininae - ps38
Hominini - ps39
Homo_sapiens - ps40
Phylostratum

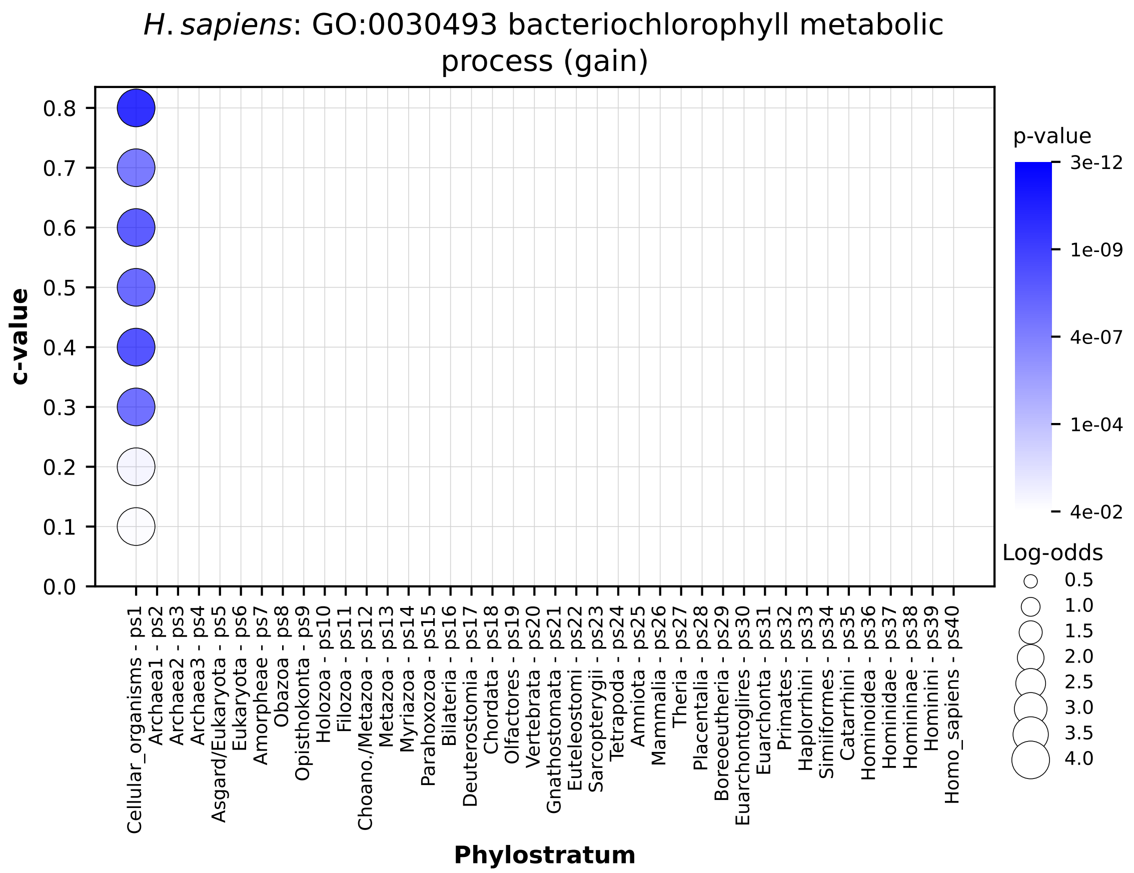

*H. sapiens*: GO:0030493 bacteriochlorophyll metabolic process (gain)
c-value
0.0
0.1
0.2
0.3
0.4
0.5
0.6
0.7
0.8
Phylostratum
Cellular_organisms - ps1
Archaea1 - ps2
Archaea2 - ps3
Archaea3 - ps4
Asgard/Eukaryota - ps5
Eukaryota - ps6
Amorpheae - ps7
Obazoa - ps8
Opisthokonta - ps9
Holozoa - ps10
Filozoa - ps11
Choano./Metazoa - ps12
Metazoa - ps13
Myriazoa - ps14
Parahoxozoa - ps15
Bilateria - ps16
Deuterostomia - ps17
Chordata - ps18
Olfactores - ps19
Vertebrata - ps20
Gnathostomata - ps21
Euteleostomi - ps22
Sarcopterygii - ps23
Tetrapoda - ps24
Amniota - ps25
Mammalia - ps26
Theria - ps27
Placentalia - ps28
Boreoeutheria - ps29
Euarchontoglires - ps30
Euarchonta - ps31
Primates - ps32
Haplorrhini - ps33
Simiiformes - ps34
Catarrhini - ps35
Hominoidea - ps36
Hominidae - ps37
Homininae - ps38
Hominini - ps39
Homo_sapiens - ps40
p-value
3e-12
1e-09
4e-07
1e-04
4e-02
Log-odds
0.5
1.0
1.5
2.0
2.5
3.0
3.5
4.0

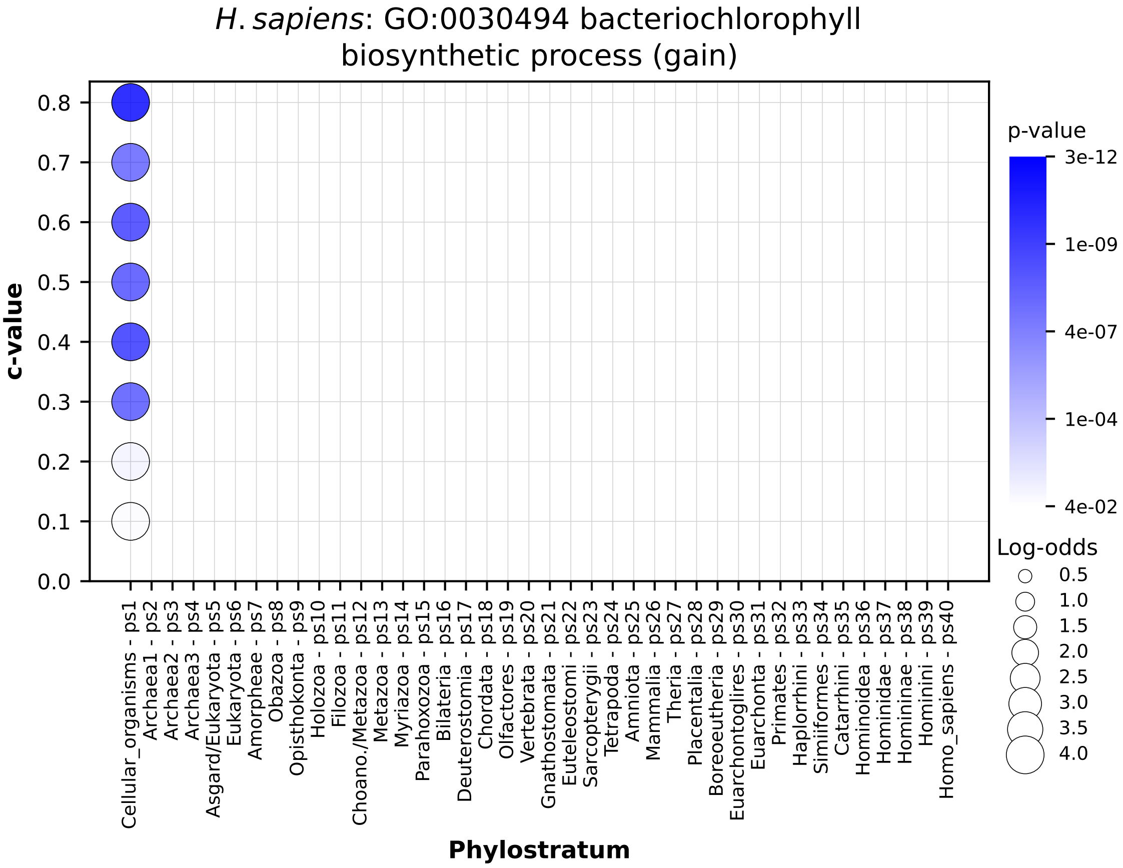

*H. sapiens*: GO:0030494 bacteriochlorophyll biosynthetic process (gain)
c-value
0.0
0.1
0.2
0.3
0.4
0.5
0.6
0.7
0.8
Phylostratum
Cellular_organisms - ps1
Archaea1 - ps2
Archaea2 - ps3
Archaea3 - ps4
Asgard/Eukaryota - ps5
Eukaryota - ps6
Amorpheae - ps7
Obazoa - ps8
Opisthokonta - ps9
Holozoa - ps10
Filozoa - ps11
Choano./Metazoa - ps12
Metazoa - ps13
Myriazoa - ps14
Parahoxozoa - ps15
Bilateria - ps16
Deuterostomia - ps17
Chordata - ps18
Olfactores - ps19
Vertebrata - ps20
Gnathostomata - ps21
Euteleostomi - ps22
Sarcopterygii - ps23
Tetrapoda - ps24
Amniota - ps25
Mammalia - ps26
Theria - ps27
Placentalia - ps28
Boreoeutheria - ps29
Euarchontoglires - ps30
Euarchonta - ps31
Primates - ps32
Haplorrhini - ps33
Simiiformes - ps34
Catarrhini - ps35
Hominoidea - ps36
Hominidae - ps37
Homininae - ps38
Hominini - ps39
Homo_sapiens - ps40
p-value
3e-12
1e-09
4e-07
1e-04
4e-02
Log-odds
0.5
1.0
1.5
2.0
2.5
3.0
3.5
4.0

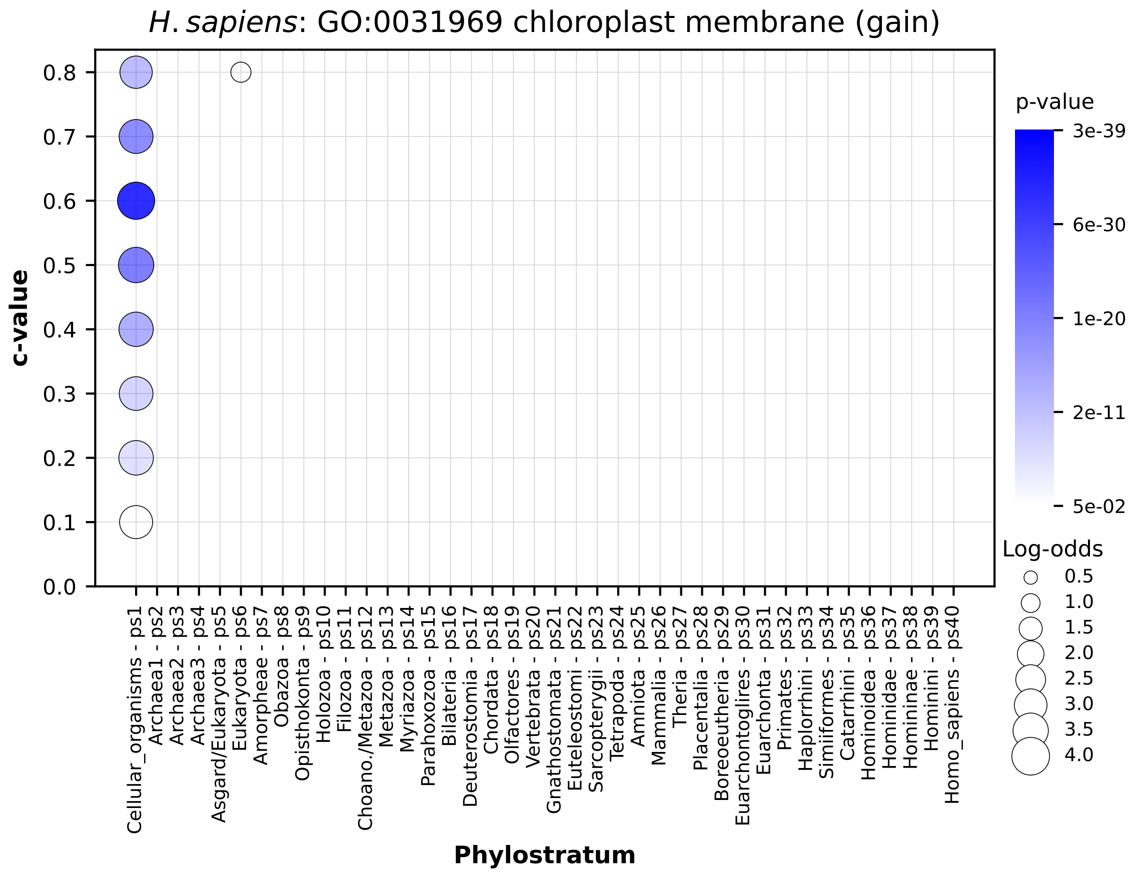

*H. sapiens*: GO:0031969 chloroplast membrane (gain)
c-value
0.0
0.1
0.2
0.3
0.4
0.5
0.6
0.7
0.8
p-value
3e-39
6e-30
1e-20
2e-11
5e-02
Log-odds
0.5
1.0
1.5
2.0
2.5
3.0
3.5
4.0
Cellular_organisms - ps1
Archaea1 - ps2
Archaea2 - ps3
Archaea3 - ps4
Asgard/Eukaryota - ps5
Eukaryota - ps6
Amorpheae - ps7
Obazoa - ps8
Opisthokonta - ps9
Holozoa - ps10
Filozoa - ps11
Choano./Metazoa - ps12
Metazoa - ps13
Myriazoa - ps14
Parahoxozoa - ps15
Bilateria - ps16
Deuterostomia - ps17
Chordata - ps18
Olfactores - ps19
Vertebrata - ps20
Gnathostomata - ps21
Euteleostomi - ps22
Sarcopterygii - ps23
Tetrapoda - ps24
Amniota - ps25
Mammalia - ps26
Theria - ps27
Placentalia - ps28
Boreoeutheria - ps29
Euarchontoglires - ps30
Euarchonta - ps31
Primates - ps32
Haplorrhini - ps33
Simiiformes - ps34
Catarrhini - ps35
Hominoidea - ps36
Hominidae - ps37
Homininae - ps38
Hominini - ps39
Homo_sapiens - ps40
Phylostratum

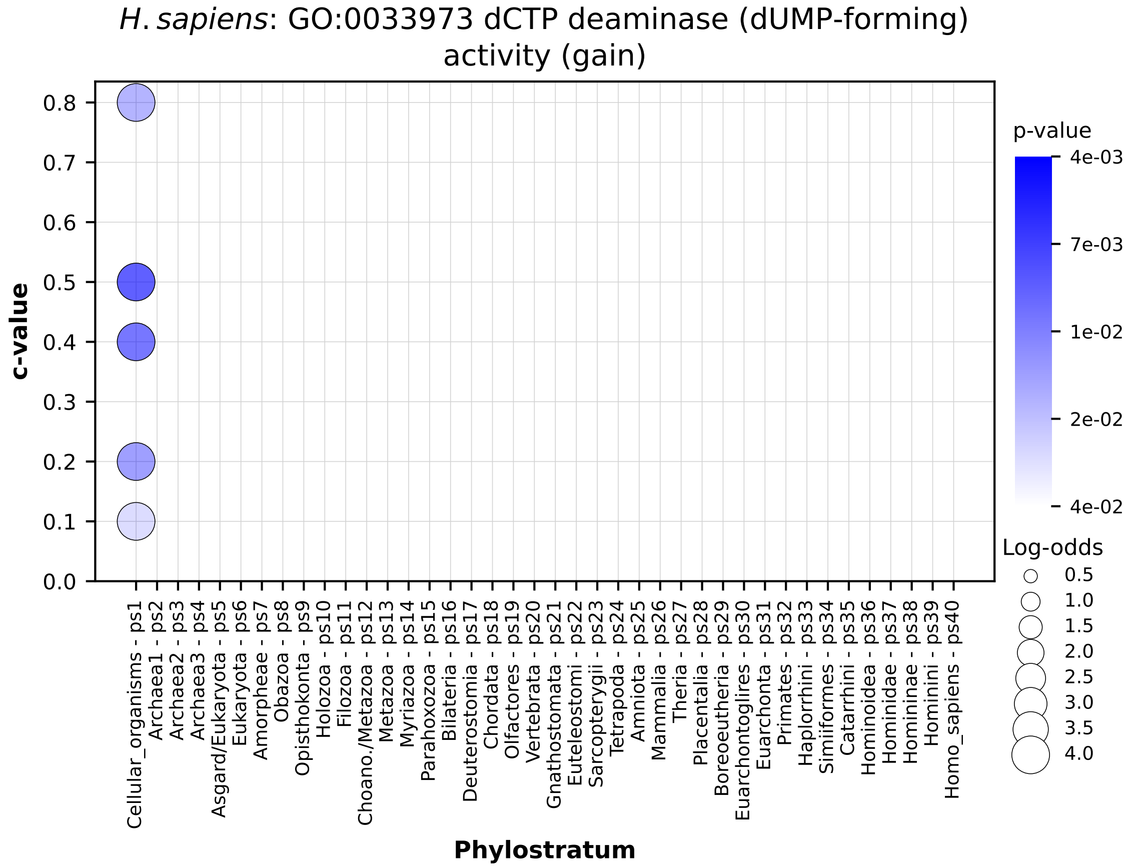

*H. sapiens*: GO:0033973 dCTP deaminase (dUMP-forming) activity (gain)
c-value
0.0
0.1
0.2
0.3
0.4
0.5
0.6
0.7
0.8
p-value
4e-03
7e-03
1e-02
2e-02
4e-02
Log-odds
0.5
1.0
1.5
2.0
2.5
3.0
3.5
4.0
Cellular_organisms - ps1
Archaea1 - ps2
Archaea2 - ps3
Archaea3 - ps4
Asgard/Eukaryota - ps5
Eukaryota - ps6
Amorpheae - ps7
Obazoa - ps8
Opisthokonta - ps9
Holozoa - ps10
Filozoa - ps11
Choano./Metazoa - ps12
Metazoa - ps13
Myriazoa - ps14
Parahoxozoa - ps15
Bilateria - ps16
Deuterostomia - ps17
Chordata - ps18
Olfactores - ps19
Vertebrata - ps20
Gnathostomata - ps21
Euteleostomi - ps22
Sarcopterygii - ps23
Tetrapoda - ps24
Amniota - ps25
Mammalia - ps26
Theria - ps27
Placentalia - ps28
Boreoeutheria - ps29
Euarchontoglires - ps30
Euarchonta - ps31
Primates - ps32
Haplorrhini - ps33
Simiiformes - ps34
Catarrhini - ps35
Hominoidea - ps36
Hominidae - ps37
Homininae - ps38
Hominini - ps39
Homo_sapiens - ps40
Phylostratum

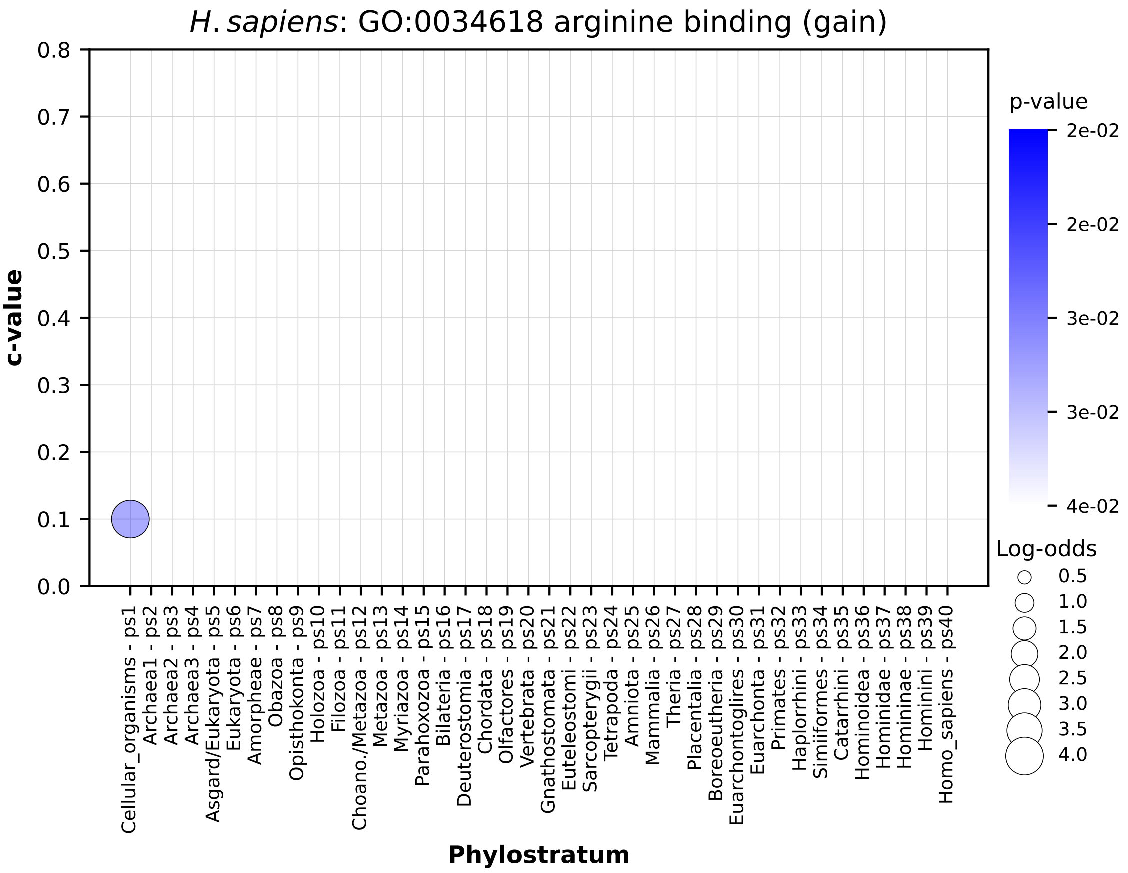
H. sapiens: GO:0034618 arginine binding (gain)
c-value
0.0
0.1
0.2
0.3
0.4
0.5
0.6
0.7
0.8
Phylostratum
Cellular_organisms - ps1
Archaea1 - ps2
Archaea2 - ps3
Archaea3 - ps4
Asgard/Eukaryota - ps5
Eukaryota - ps6
Amorpheae - ps7
Obazoa - ps8
Opisthokonta - ps9
Holozoa - ps10
Filozoa - ps11
Choano./Metazoa - ps12
Metazoa - ps13
Myriazoa - ps14
Parahoxozoa - ps15
Bilateria - ps16
Deuterostomia - ps17
Chordata - ps18
Olfactores - ps19
Vertebrata - ps20
Gnathostomata - ps21
Euteleostomi - ps22
Sarcopterygii - ps23
Tetrapoda - ps24
Amniota - ps25
Mammalia - ps26
Theria - ps27
Placentalia - ps28
Boreoeutheria - ps29
Euarchontoglires - ps30
Euarchonta - ps31
Primates - ps32
Haplorrhini - ps33
Simiiformes - ps34
Catarrhini - ps35
Hominoidea - ps36
Hominidae - ps37
Homininae - ps38
Hominini - ps39
Homo_sapiens - ps40
p-value
2e-02
2e-02
3e-02
3e-02
4e-02
Log-odds
0.5
1.0
1.5
2.0
2.5
3.0
3.5
4.0

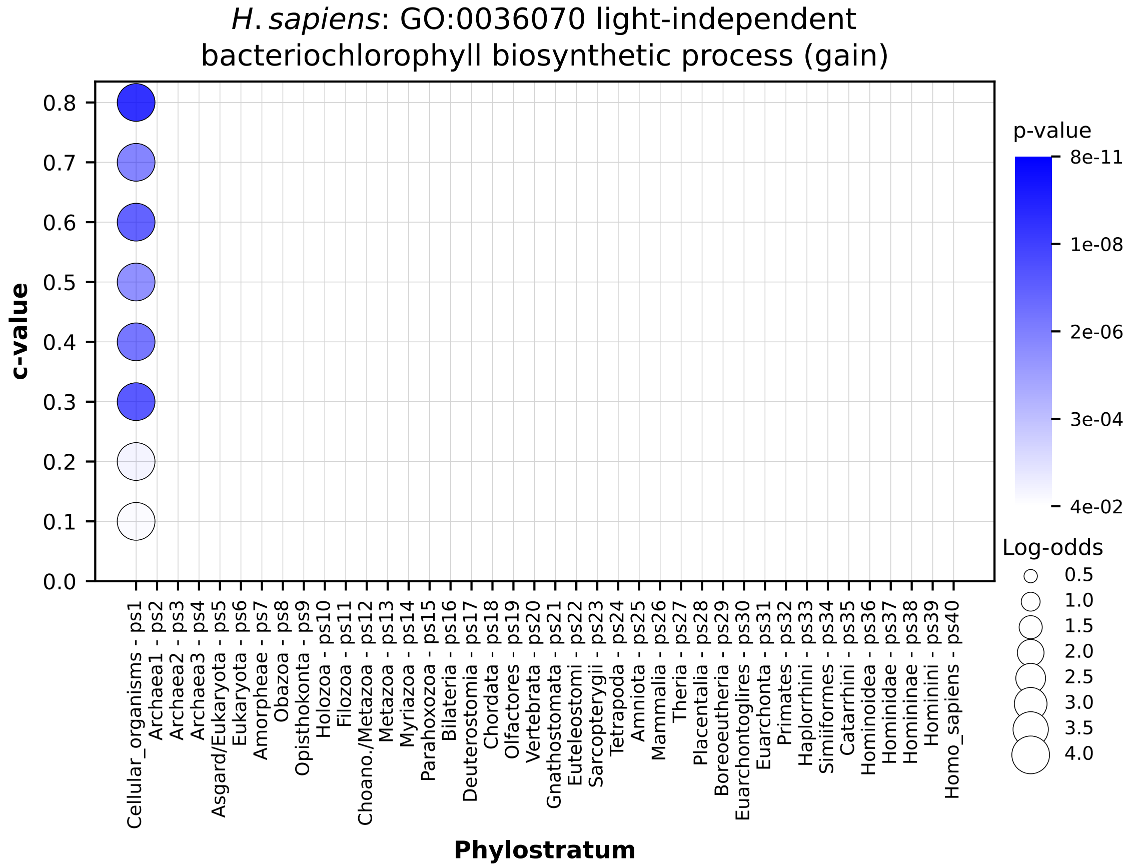

H. sapiens: GO:0036070 light-independent bacteriochlorophyll biosynthetic process (gain)
c-value
0.0
0.1
0.2
0.3
0.4
0.5
0.6
0.7
0.8
Phylostratum
Cellular_organisms - ps1
Archaea1 - ps2
Archaea2 - ps3
Archaea3 - ps4
Asgard/Eukaryota - ps5
Eukaryota - ps6
Amorpheae - ps7
Obazoa - ps8
Opisthokonta - ps9
Holozoa - ps10
Filozoa - ps11
Choano./Metazoa - ps12
Metazoa - ps13
Myriazoa - ps14
Parahoxozoa - ps15
Bilateria - ps16
Deuterostomia - ps17
Chordata - ps18
Olfactores - ps19
Vertebrata - ps20
Gnathostomata - ps21
Euteleostomi - ps22
Sarcopterygii - ps23
Tetrapoda - ps24
Amniota - ps25
Mammalia - ps26
Theria - ps27
Placentalia - ps28
Boreoeutheria - ps29
Euarchontoglires - ps30
Euarchonta - ps31
Primates - ps32
Haplorrhini - ps33
Simiiformes - ps34
Catarrhini - ps35
Hominoidea - ps36
Hominidae - ps37
Homininae - ps38
Hominini - ps39
Homo_sapiens - ps40
p-value
8e-11
1e-08
2e-06
3e-04
4e-02
Log-odds
0.5
1.0
1.5
2.0
2.5
3.0
3.5
4.0

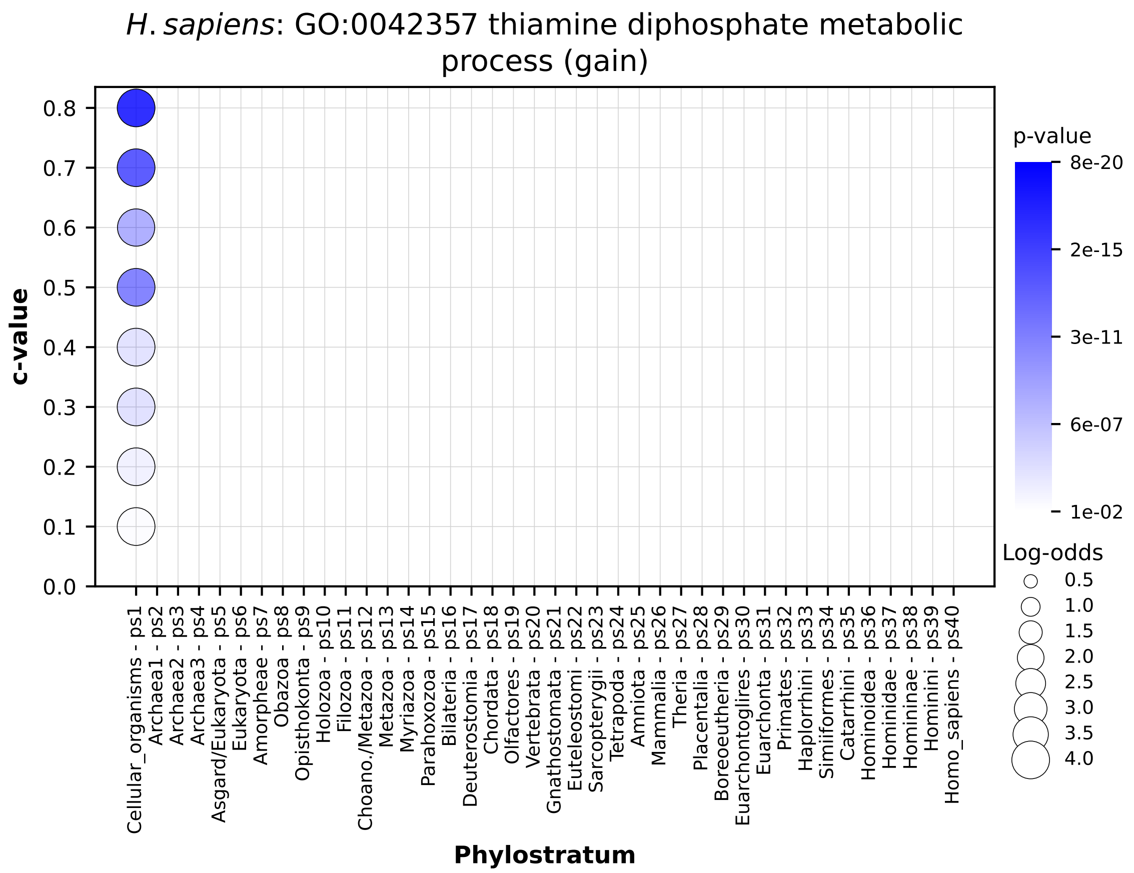
*H. sapiens*: GO:0042357 thiamine diphosphate metabolic process (gain)
c-value
0.0
0.1
0.2
0.3
0.4
0.5
0.6
0.7
0.8
Phylostratum
Cellular_organisms - ps1
Archaea1 - ps2
Archaea2 - ps3
Archaea3 - ps4
Asgard/Eukaryota - ps5
Eukaryota - ps6
Amorpheae - ps7
Obazoa - ps8
Opisthokonta - ps9
Holozoa - ps10
Filozoa - ps11
Choano./Metazoa - ps12
Metazoa - ps13
Myriazoa - ps14
Parahoxozoa - ps15
Bilateria - ps16
Deuterostomia - ps17
Chordata - ps18
Olfactores - ps19
Vertebrata - ps20
Gnathostomata - ps21
Euteleostomi - ps22
Sarcopterygii - ps23
Tetrapoda - ps24
Amniota - ps25
Mammalia - ps26
Theria - ps27
Placentalia - ps28
Boreoeutheria - ps29
Euarchontoglires - ps30
Euarchonta - ps31
Primates - ps32
Haplorrhini - ps33
Simiiformes - ps34
Catarrhini - ps35
Hominoidea - ps36
Hominidae - ps37
Homininae - ps38
Hominini - ps39
Homo_sapiens - ps40
p-value
8e-20
2e-15
3e-11
6e-07
1e-02
Log-odds
0.5
1.0
1.5
2.0
2.5
3.0
3.5
4.0

# *H. sapiens*: GO:0042401 biogenic amine biosynthetic process (gain)

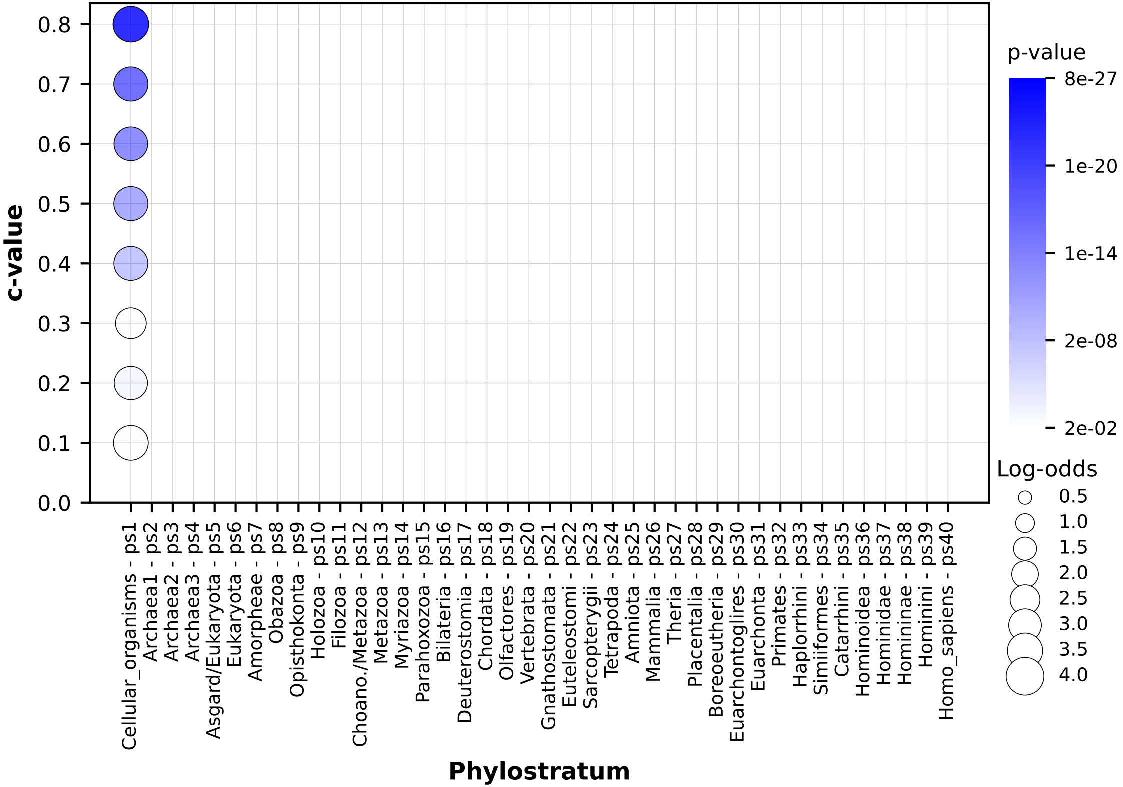

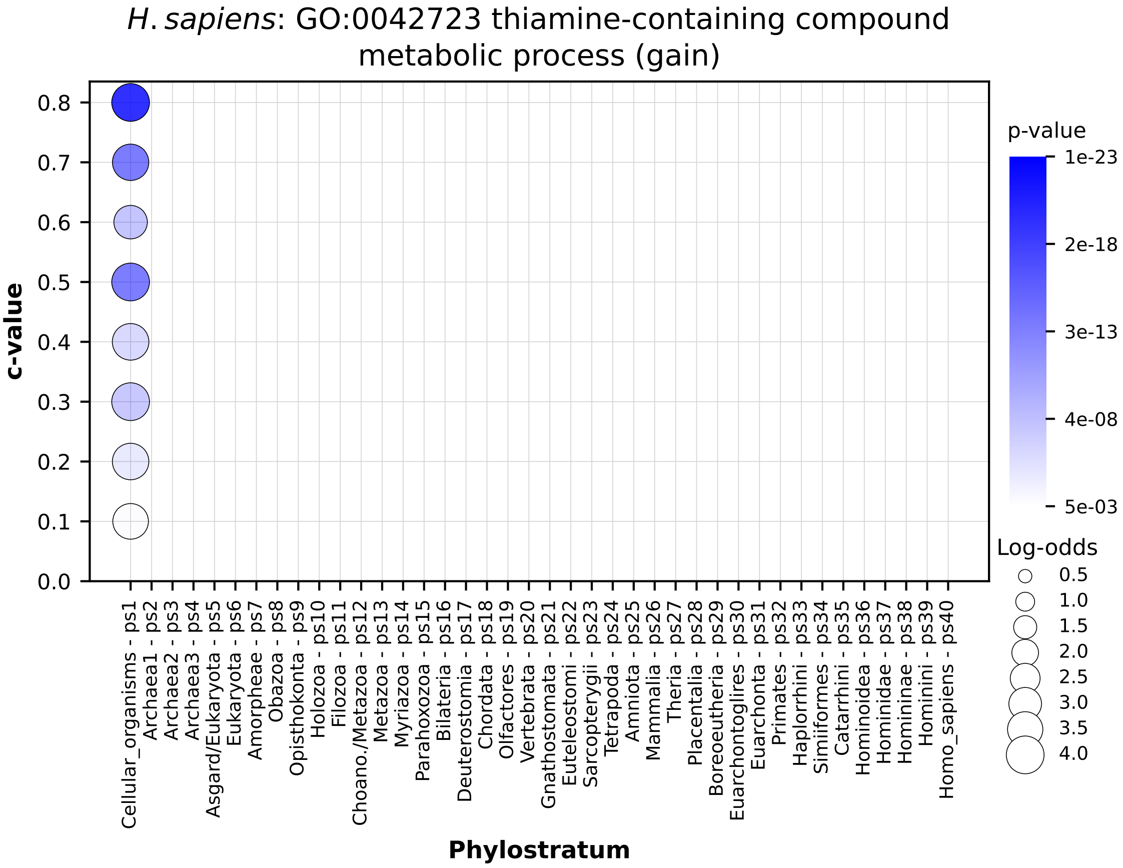

*H. sapiens*: GO:0042723 thiamine-containing compound metabolic process (gain)
c-value
0.0
0.1
0.2
0.3
0.4
0.5
0.6
0.7
0.8
Phylostratum
Cellular_organisms - ps1
Archaea1 - ps2
Archaea2 - ps3
Archaea3 - ps4
Asgard/Eukaryota - ps5
Eukaryota - ps6
Amorpheae - ps7
Obazoa - ps8
Opisthokonta - ps9
Holozoa - ps10
Filozoa - ps11
Choano./Metazoa - ps12
Metazoa - ps13
Myriazoa - ps14
Parahoxozoa - ps15
Bilateria - ps16
Deuterostomia - ps17
Chordata - ps18
Olfactores - ps19
Vertebrata - ps20
Gnathostomata - ps21
Euteleostomi - ps22
Sarcopterygii - ps23
Tetrapoda - ps24
Amniota - ps25
Mammalia - ps26
Theria - ps27
Placentalia - ps28
Boreoeutheria - ps29
Euarchontoglires - ps30
Euarchonta - ps31
Primates - ps32
Haplorrhini - ps33
Simiiformes - ps34
Catarrhini - ps35
Hominoidea - ps36
Hominidae - ps37
Homininae - ps38
Hominini - ps39
Homo_sapiens - ps40
p-value
1e-23
2e-18
3e-13
4e-08
5e-03
Log-odds
0.5
1.0
1.5
2.0
2.5
3.0
3.5
4.0

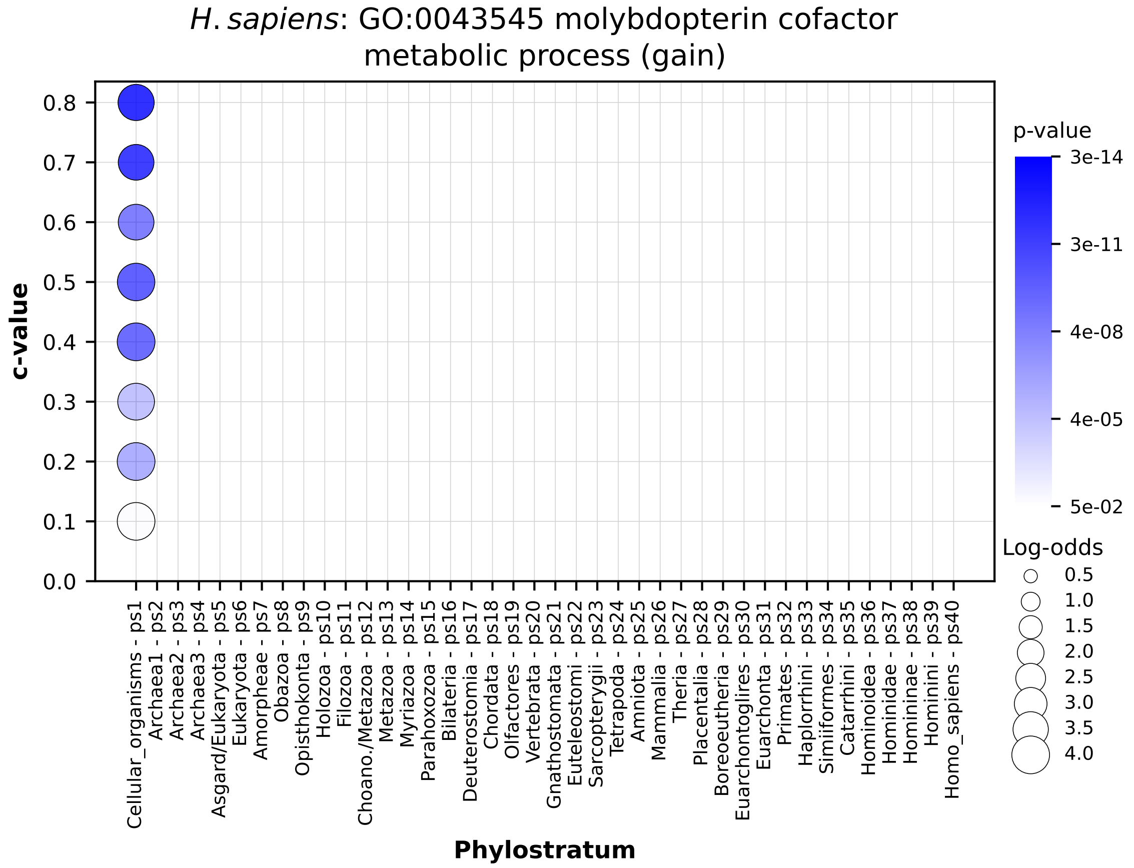

*H. sapiens*: GO:0043545 molybdopterin cofactor metabolic process (gain)
c-value
0.0
0.1
0.2
0.3
0.4
0.5
0.6
0.7
0.8
Phylostratum
Cellular_organisms - ps1
Archaea1 - ps2
Archaea2 - ps3
Archaea3 - ps4
Asgard/Eukaryota - ps5
Eukaryota - ps6
Amorpheae - ps7
Obazoa - ps8
Opisthokonta - ps9
Holozoa - ps10
Filozoa - ps11
Choano./Metazoa - ps12
Metazoa - ps13
Myriazoa - ps14
Parahoxozoa - ps15
Bilateria - ps16
Deuterostomia - ps17
Chordata - ps18
Olfactores - ps19
Vertebrata - ps20
Gnathostomata - ps21
Euteleostomi - ps22
Sarcopterygii - ps23
Tetrapoda - ps24
Amniota - ps25
Mammalia - ps26
Theria - ps27
Placentalia - ps28
Boreoeutheria - ps29
Euarchontoglires - ps30
Euarchonta - ps31
Primates - ps32
Haplorrhini - ps33
Simiiformes - ps34
Catarrhini - ps35
Hominoidea - ps36
Hominidae - ps37
Homininae - ps38
Hominini - ps39
Homo_sapiens - ps40
p-value
3e-14
3e-11
4e-08
4e-05
5e-02
Log-odds
0.5
1.0
1.5
2.0
2.5
3.0
3.5
4.0

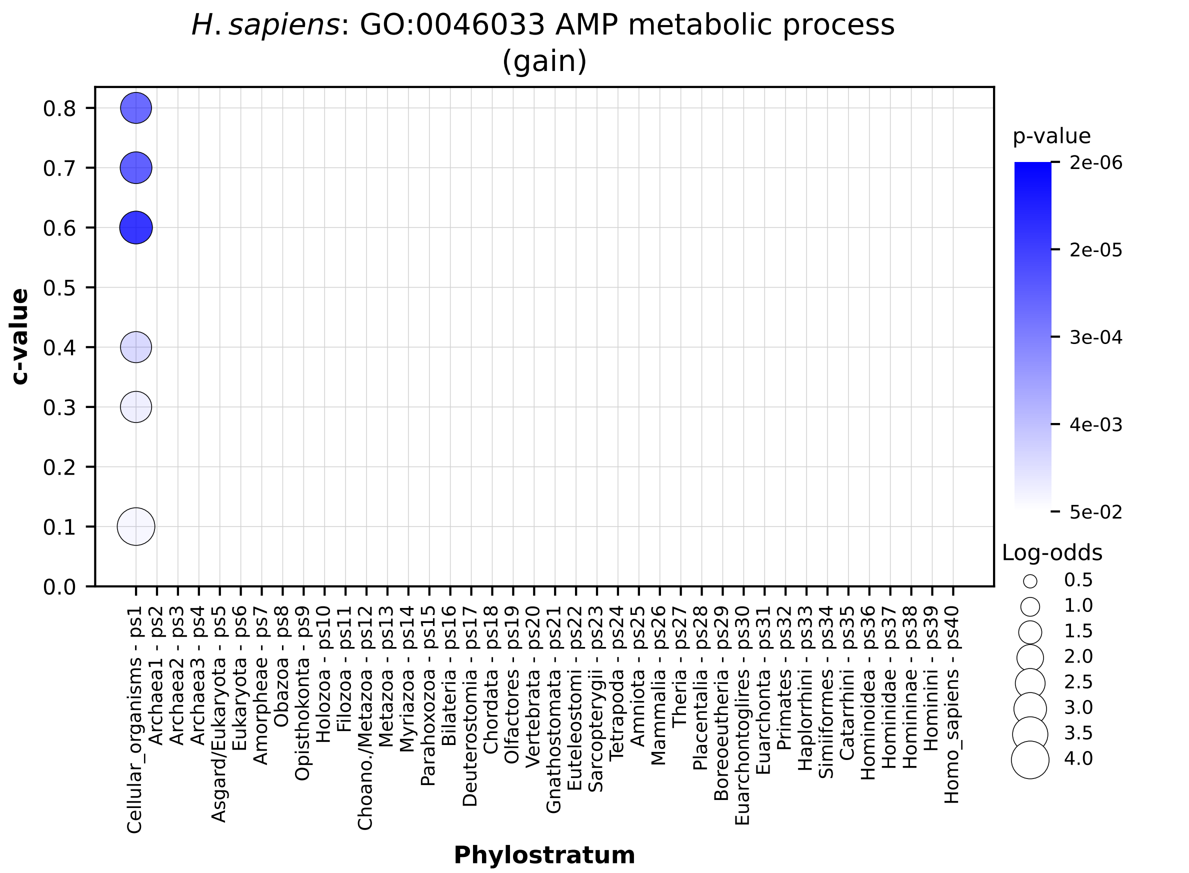

*H. sapiens*: GO:0046033 AMP metabolic process
(gain)
c-value
0.0
0.1
0.2
0.3
0.4
0.5
0.6
0.7
0.8
Phylostratum
Cellular_organisms - ps1
Archaea1 - ps2
Archaea2 - ps3
Archaea3 - ps4
Asgard/Eukaryota - ps5
Eukaryota - ps6
Amorpheae - ps7
Obazoa - ps8
Opisthokonta - ps9
Holozoa - ps10
Filozoa - ps11
Choano./Metazoa - ps12
Metazoa - ps13
Myriazoa - ps14
Parahoxozoa - ps15
Bilateria - ps16
Deuterostomia - ps17
Chordata - ps18
Olfactores - ps19
Vertebrata - ps20
Gnathostomata - ps21
Euteleostomi - ps22
Sarcopterygii - ps23
Tetrapoda - ps24
Amniota - ps25
Mammalia - ps26
Theria - ps27
Placentalia - ps28
Boreoeutheria - ps29
Euarchontoglires - ps30
Euarchonta - ps31
Primates - ps32
Haplorrhini - ps33
Simiiformes - ps34
Catarrhini - ps35
Hominoidea - ps36
Hominidae - ps37
Homininae - ps38
Hominini - ps39
Homo_sapiens - ps40
p-value
2e-06
2e-05
3e-04
4e-03
5e-02
Log-odds
0.5
1.0
1.5
2.0
2.5
3.0
3.5
4.0

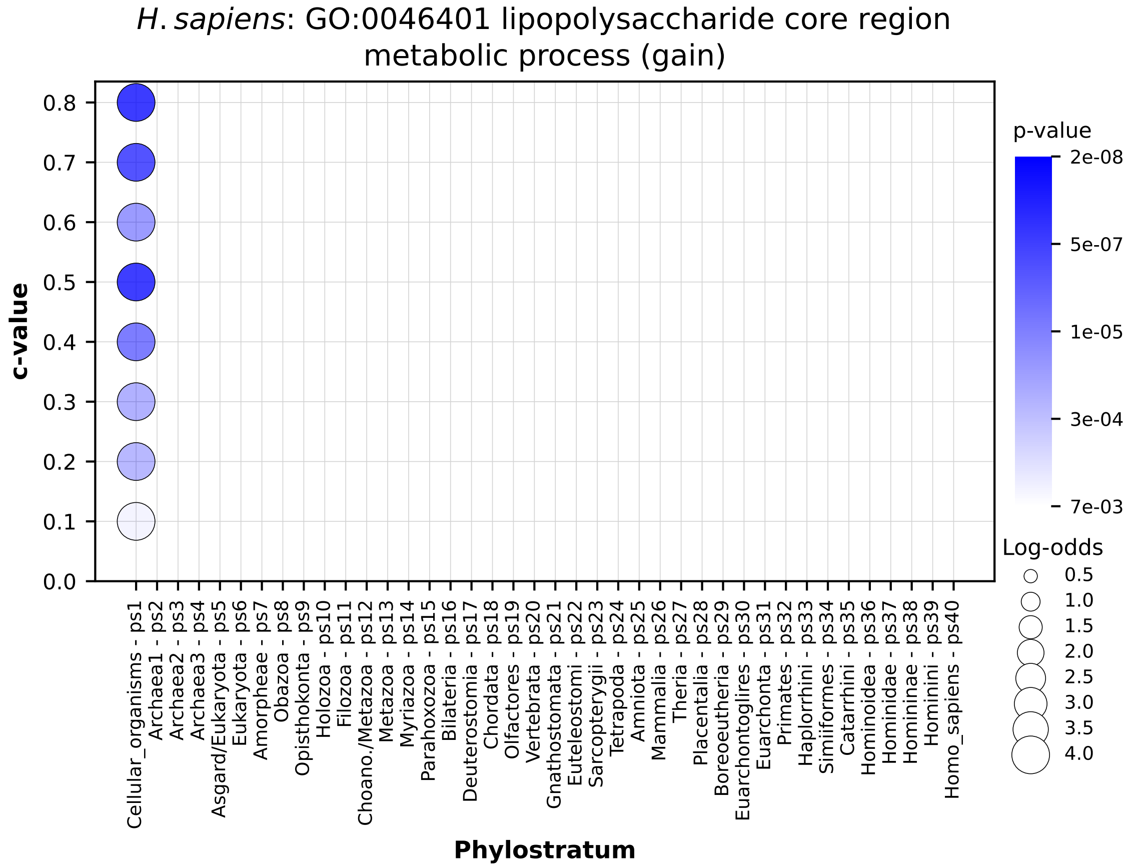

H. sapiens: GO:0046401 lipopolysaccharide core region metabolic process (gain)
c-value
0.0
0.1
0.2
0.3
0.4
0.5
0.6
0.7
0.8
p-value
2e-08
5e-07
1e-05
3e-04
7e-03
Log-odds
0.5
1.0
1.5
2.0
2.5
3.0
3.5
4.0
Cellular_organisms - ps1
Archaea1 - ps2
Archaea2 - ps3
Archaea3 - ps4
Asgard/Eukaryota - ps5
Eukaryota - ps6
Amorpheae - ps7
Obazoa - ps8
Opisthokonta - ps9
Holozoa - ps10
Filozoa - ps11
Choano./Metazoa - ps12
Metazoa - ps13
Myriazoa - ps14
Parahoxozoa - ps15
Bilateria - ps16
Deuterostomia - ps17
Chordata - ps18
Olfactores - ps19
Vertebrata - ps20
Gnathostomata - ps21
Euteleostomi - ps22
Sarcopterygii - ps23
Tetrapoda - ps24
Amniota - ps25
Mammalia - ps26
Theria - ps27
Placentalia - ps28
Boreoeutheria - ps29
Euarchontoglires - ps30
Euarchonta - ps31
Primates - ps32
Haplorrhini - ps33
Simiiformes - ps34
Catarrhini - ps35
Hominoidea - ps36
Hominidae - ps37
Homininae - ps38
Hominini - ps39
Homo_sapiens - ps40
Phylostratum

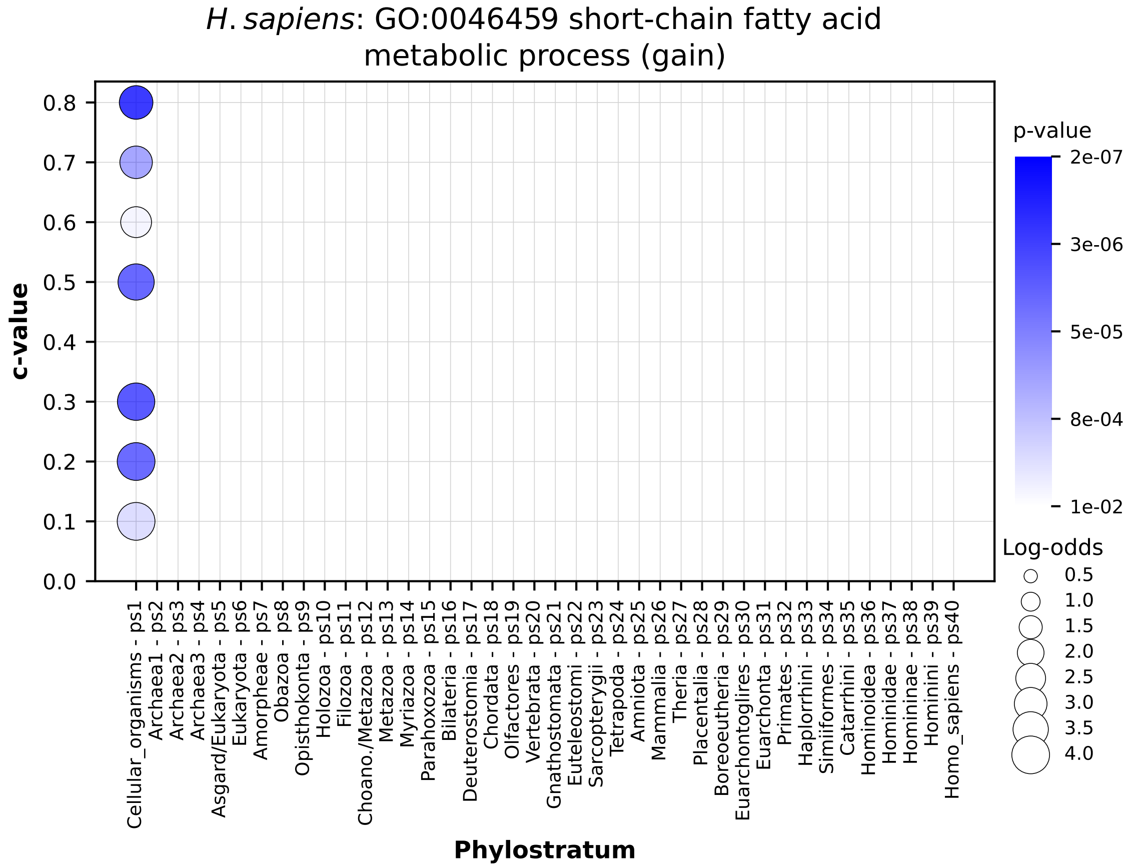

H. sapiens: GO:0046459 short-chain fatty acid metabolic process (gain)
c-value
0.0
0.1
0.2
0.3
0.4
0.5
0.6
0.7
0.8
Phylostratum
Cellular_organisms - ps1
Archaea1 - ps2
Archaea2 - ps3
Archaea3 - ps4
Asgard/Eukaryota - ps5
Eukaryota - ps6
Amorpheae - ps7
Obazoa - ps8
Opisthokonta - ps9
Holozoa - ps10
Filozoa - ps11
Choano./Metazoa - ps12
Metazoa - ps13
Myriazoa - ps14
Parahoxozoa - ps15
Bilateria - ps16
Deuterostomia - ps17
Chordata - ps18
Olfactores - ps19
Vertebrata - ps20
Gnathostomata - ps21
Euteleostomi - ps22
Sarcopterygii - ps23
Tetrapoda - ps24
Amniota - ps25
Mammalia - ps26
Theria - ps27
Placentalia - ps28
Boreoeutheria - ps29
Euarchontoglires - ps30
Euarchonta - ps31
Primates - ps32
Haplorrhini - ps33
Simiiformes - ps34
Catarrhini - ps35
Hominoidea - ps36
Hominidae - ps37
Homininae - ps38
Hominini - ps39
Homo_sapiens - ps40
p-value
2e-07
3e-06
5e-05
8e-04
1e-02
Log-odds
0.5
1.0
1.5
2.0
2.5
3.0
3.5
4.0

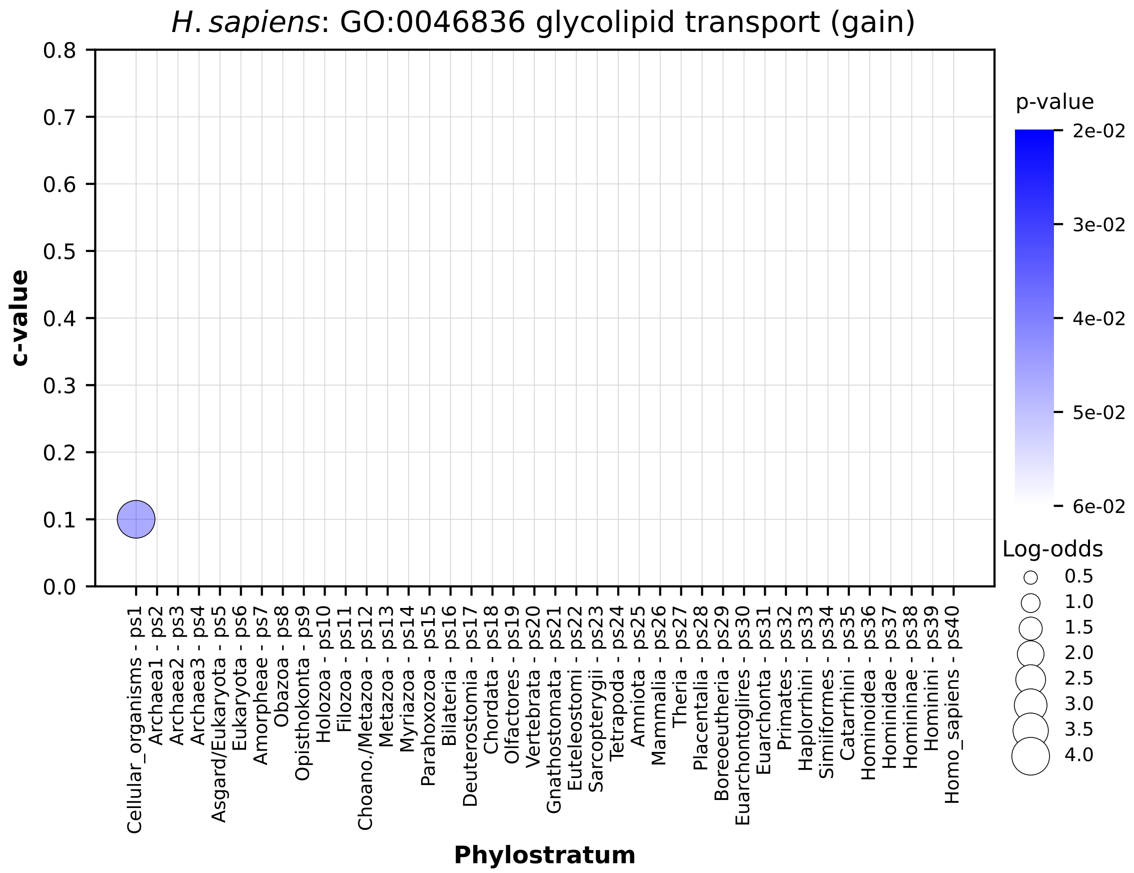

*H. sapiens*: GO:0046836 glycolipid transport (gain)
c-value
0.8
0.7
0.6
0.5
0.4
0.3
0.2
0.1
0.0
Phylostratum
Cellular_organisms - ps1
Archaea1 - ps2
Archaea2 - ps3
Archaea3 - ps4
Asgard/Eukaryota - ps5
Eukaryota - ps6
Amorpheae - ps7
Obazoa - ps8
Opisthokonta - ps9
Holozoa - ps10
Filozoa - ps11
Choano./Metazoa - ps12
Metazoa - ps13
Myriazoa - ps14
Parahoxozoa - ps15
Bilateria - ps16
Deuterostomia - ps17
Chordata - ps18
Olfactores - ps19
Vertebrata - ps20
Gnathostomata - ps21
Euteleostomi - ps22
Sarcopterygii - ps23
Tetrapoda - ps24
Amniota - ps25
Mammalia - ps26
Theria - ps27
Placentalia - ps28
Boreoeutheria - ps29
Euarchontoglires - ps30
Euarchonta - ps31
Primates - ps32
Haplorrhini - ps33
Simiiformes - ps34
Catarrhini - ps35
Hominoidea - ps36
Hominidae - ps37
Homininae - ps38
Hominini - ps39
Homo_sapiens - ps40
p-value
2e-02
3e-02
4e-02
5e-02
6e-02
Log-odds
0.5
1.0
1.5
2.0
2.5
3.0
3.5
4.0

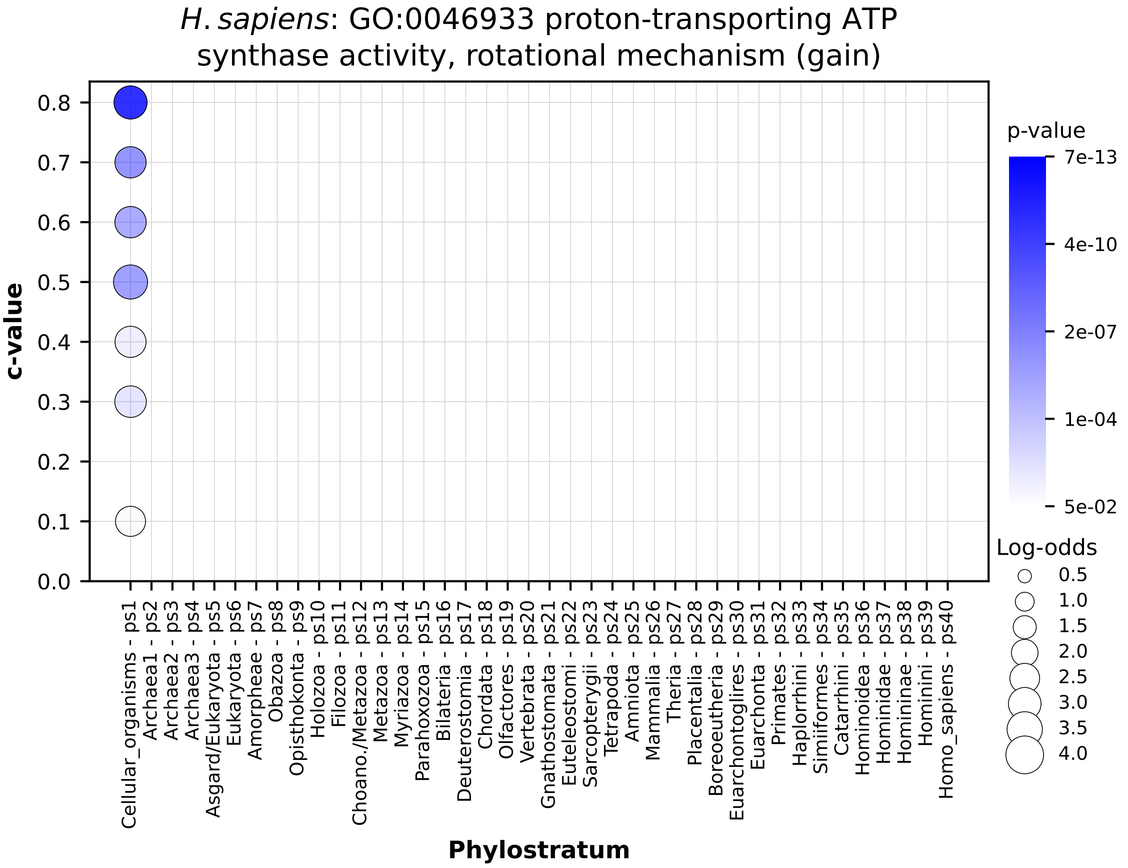

H. sapiens: GO:0046933 proton-transporting ATP synthase activity, rotational mechanism (gain)
c-value
0.0
0.1
0.2
0.3
0.4
0.5
0.6
0.7
0.8
Phylostratum
Cellular_organisms - ps1
Archaea1 - ps2
Archaea2 - ps3
Archaea3 - ps4
Asgard/Eukaryota - ps5
Eukaryota - ps6
Amorpheae - ps7
Obazoa - ps8
Opisthokonta - ps9
Holozoa - ps10
Filozoa - ps11
Choano./Metazoa - ps12
Metazoa - ps13
Myriazoa - ps14
Parahoxozoa - ps15
Bilateria - ps16
Deuterostomia - ps17
Chordata - ps18
Olfactores - ps19
Vertebrata - ps20
Gnathostomata - ps21
Euteleostomi - ps22
Sarcopterygii - ps23
Tetrapoda - ps24
Amniota - ps25
Mammalia - ps26
Theria - ps27
Placentalia - ps28
Boreoeutheria - ps29
Euarchontoglires - ps30
Euarchonta - ps31
Primates - ps32
Haplorrhini - ps33
Simiiformes - ps34
Catarrhini - ps35
Hominoidea - ps36
Hominidae - ps37
Homininae - ps38
Hominini - ps39
Homo_sapiens - ps40
p-value
7e-13
4e-10
2e-07
1e-04
5e-02
Log-odds
0.5
1.0
1.5
2.0
2.5
3.0
3.5
4.0

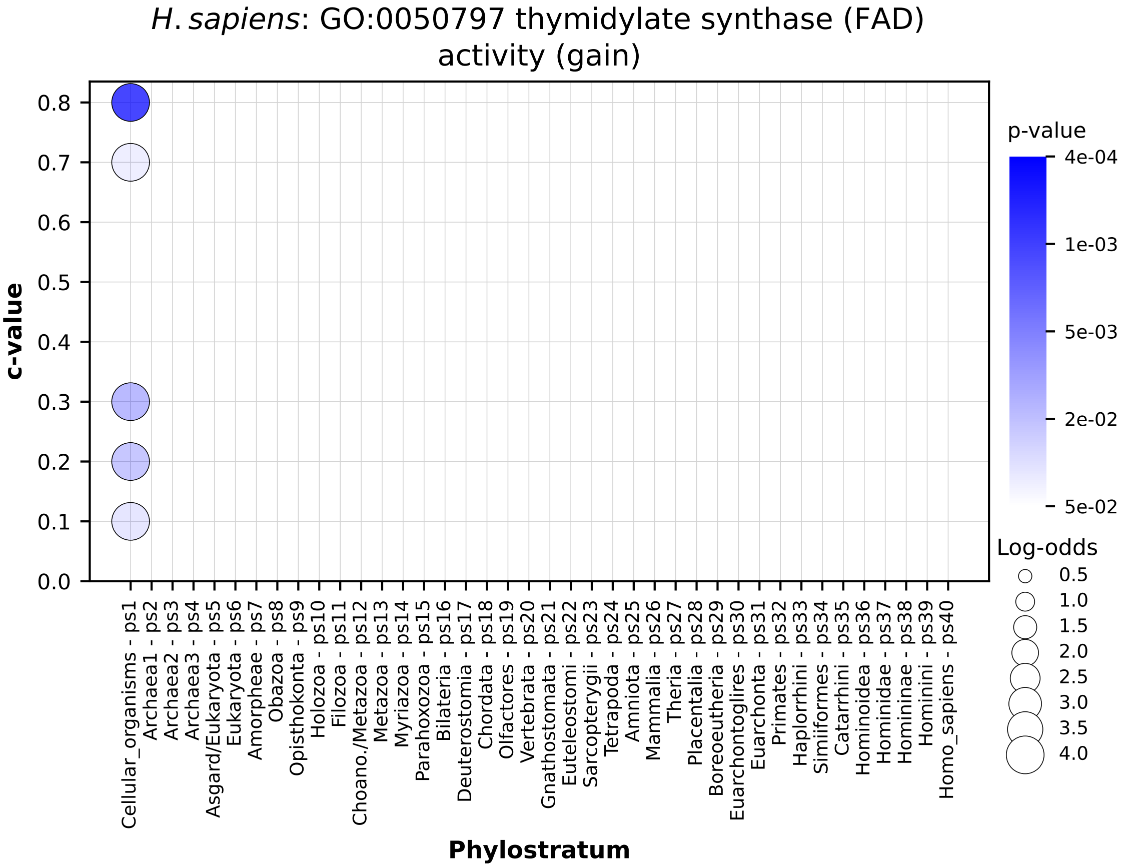

*H. sapiens*: GO:0050797 thymidylate synthase (FAD) activity (gain)
c-value
0.0
0.1
0.2
0.3
0.4
0.5
0.6
0.7
0.8
p-value
4e-04
1e-03
5e-03
2e-02
5e-02
Log-odds
0.5
1.0
1.5
2.0
2.5
3.0
3.5
4.0
Cellular_organisms - ps1
Archaea1 - ps2
Archaea2 - ps3
Archaea3 - ps4
Asgard/Eukaryota - ps5
Eukaryota - ps6
Amorpheae - ps7
Obazoa - ps8
Opisthokonta - ps9
Holozoa - ps10
Filozoa - ps11
Choano./Metazoa - ps12
Metazoa - ps13
Myriazoa - ps14
Parahoxozoa - ps15
Bilateria - ps16
Deuterostomia - ps17
Chordata - ps18
Olfactores - ps19
Vertebrata - ps20
Gnathostomata - ps21
Euteleostomi - ps22
Sarcopterygii - ps23
Tetrapoda - ps24
Amniota - ps25
Mammalia - ps26
Theria - ps27
Placentalia - ps28
Boreoeutheria - ps29
Euarchontoglires - ps30
Euarchonta - ps31
Primates - ps32
Haplorrhini - ps33
Simiiformes - ps34
Catarrhini - ps35
Hominoidea - ps36
Hominidae - ps37
Homininae - ps38
Hominini - ps39
Homo_sapiens - ps40
Phylostratum

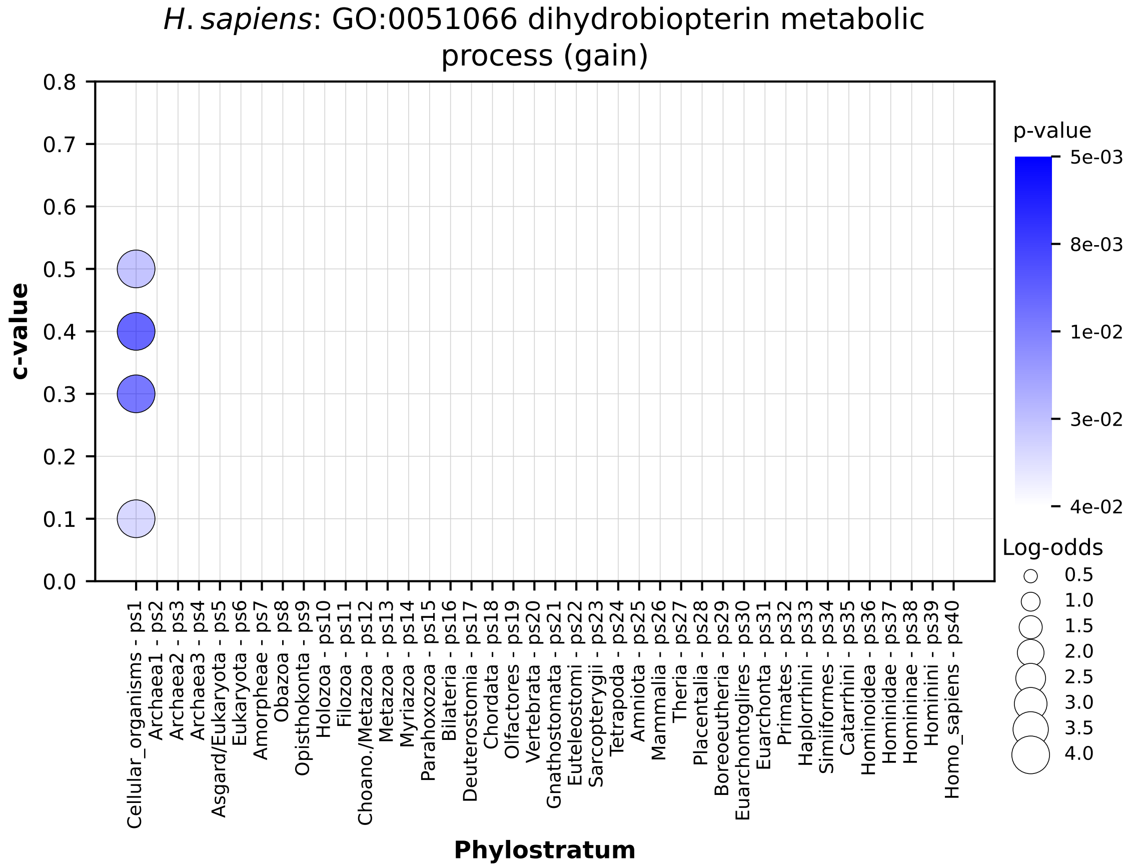

H. sapiens: GO:0051066 dihydrobiopterin metabolic process (gain)
c-value
0.0
0.1
0.2
0.3
0.4
0.5
0.6
0.7
0.8
Phylostratum
Cellular_organisms - ps1
Archaea1 - ps2
Archaea2 - ps3
Archaea3 - ps4
Asgard/Eukaryota - ps5
Eukaryota - ps6
Amorpheae - ps7
Obazoa - ps8
Opisthokonta - ps9
Holozoa - ps10
Filozoa - ps11
Choano./Metazoa - ps12
Metazoa - ps13
Myriazoa - ps14
Parahoxozoa - ps15
Bilateria - ps16
Deuterostomia - ps17
Chordata - ps18
Olfactores - ps19
Vertebrata - ps20
Gnathostomata - ps21
Euteleostomi - ps22
Sarcopterygii - ps23
Tetrapoda - ps24
Amniota - ps25
Mammalia - ps26
Theria - ps27
Placentalia - ps28
Boreoeutheria - ps29
Euarchontoglires - ps30
Euarchonta - ps31
Primates - ps32
Haplorrhini - ps33
Simiiformes - ps34
Catarrhini - ps35
Hominoidea - ps36
Hominidae - ps37
Homininae - ps38
Hominini - ps39
Homo_sapiens - ps40
p-value
5e-03
8e-03
1e-02
3e-02
4e-02
Log-odds
0.5
1.0
1.5
2.0
2.5
3.0
3.5
4.0

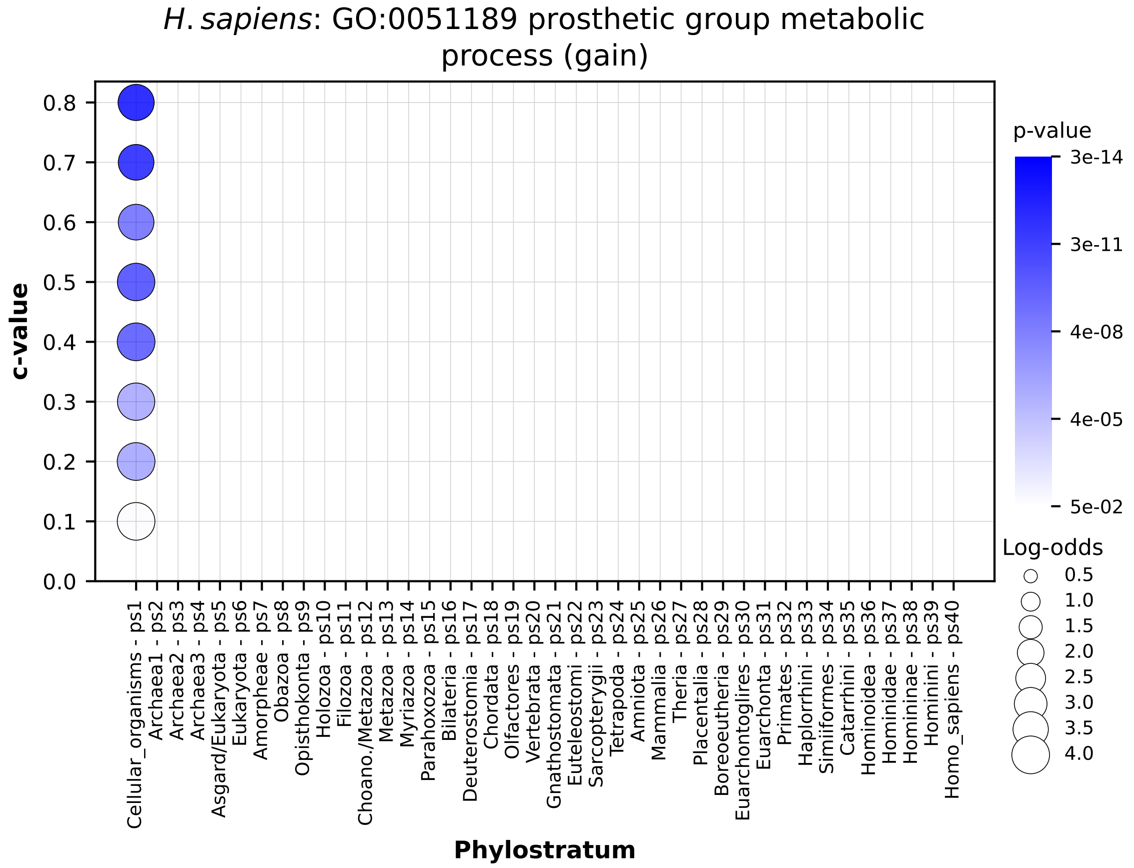
H. sapiens: GO:0051189 prosthetic group metabolic process (gain)
c-value
0.0
0.1
0.2
0.3
0.4
0.5
0.6
0.7
0.8
p-value
3e-14
3e-11
4e-08
4e-05
5e-02
Log-odds
0.5
1.0
1.5
2.0
2.5
3.0
3.5
4.0
Cellular_organisms - ps1
Archaea1 - ps2
Archaea2 - ps3
Archaea3 - ps4
Asgard/Eukaryota - ps5
Eukaryota - ps6
Amorpheae - ps7
Obazoa - ps8
Opisthokonta - ps9
Holozoa - ps10
Filozoa - ps11
Choano./Metazoa - ps12
Metazoa - ps13
Myriazoa - ps14
Parahoxozoa - ps15
Bilateria - ps16
Deuterostomia - ps17
Chordata - ps18
Olfactores - ps19
Vertebrata - ps20
Gnathostomata - ps21
Euteleostomi - ps22
Sarcopterygii - ps23
Tetrapoda - ps24
Amniota - ps25
Mammalia - ps26
Theria - ps27
Placentalia - ps28
Boreoeutheria - ps29
Euarchontoglires - ps30
Euarchonta - ps31
Primates - ps32
Haplorrhini - ps33
Simiiformes - ps34
Catarrhini - ps35
Hominoidea - ps36
Hominidae - ps37
Homininae - ps38
Hominini - ps39
Homo_sapiens - ps40
Phylostratum

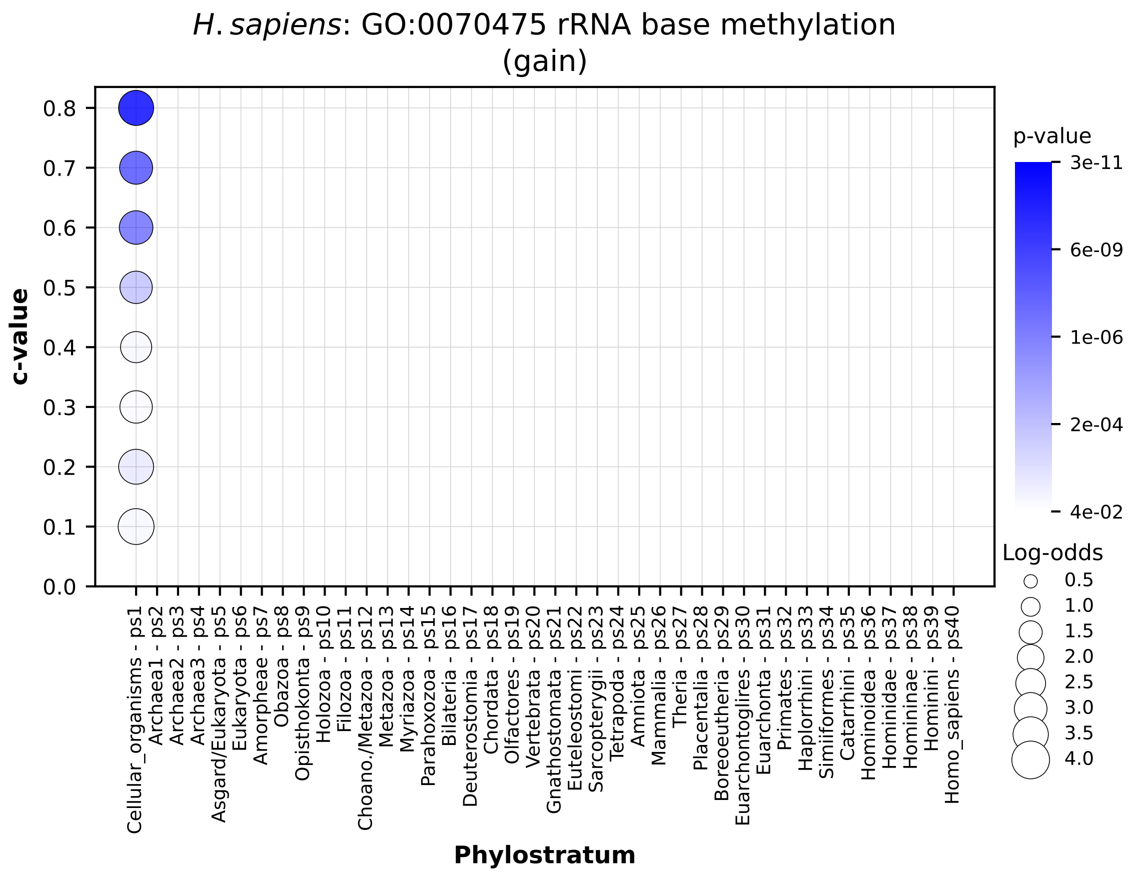

*H. sapiens*: GO:0070475 rRNA base methylation
(gain)
c-value
0.0
0.1
0.2
0.3
0.4
0.5
0.6
0.7
0.8
Phylostratum
Cellular_organisms - ps1
Archaea1 - ps2
Archaea2 - ps3
Archaea3 - ps4
Asgard/Eukaryota - ps5
Eukaryota - ps6
Amorpheae - ps7
Obazoa - ps8
Opisthokonta - ps9
Holozoa - ps10
Filozoa - ps11
Choano./Metazoa - ps12
Metazoa - ps13
Myriazoa - ps14
Parahoxozoa - ps15
Bilateria - ps16
Deuterostomia - ps17
Chordata - ps18
Olfactores - ps19
Vertebrata - ps20
Gnathostomata - ps21
Euteleostomi - ps22
Sarcopterygii - ps23
Tetrapoda - ps24
Amniota - ps25
Mammalia - ps26
Theria - ps27
Placentalia - ps28
Boreoeutheria - ps29
Euarchontoglires - ps30
Euarchonta - ps31
Primates - ps32
Haplorrhini - ps33
Simiiformes - ps34
Catarrhini - ps35
Hominoidea - ps36
Hominidae - ps37
Homininae - ps38
Hominini - ps39
Homo_sapiens - ps40
p-value
3e-11
6e-09
1e-06
2e-04
4e-02
Log-odds
0.5
1.0
1.5
2.0
2.5
3.0
3.5
4.0

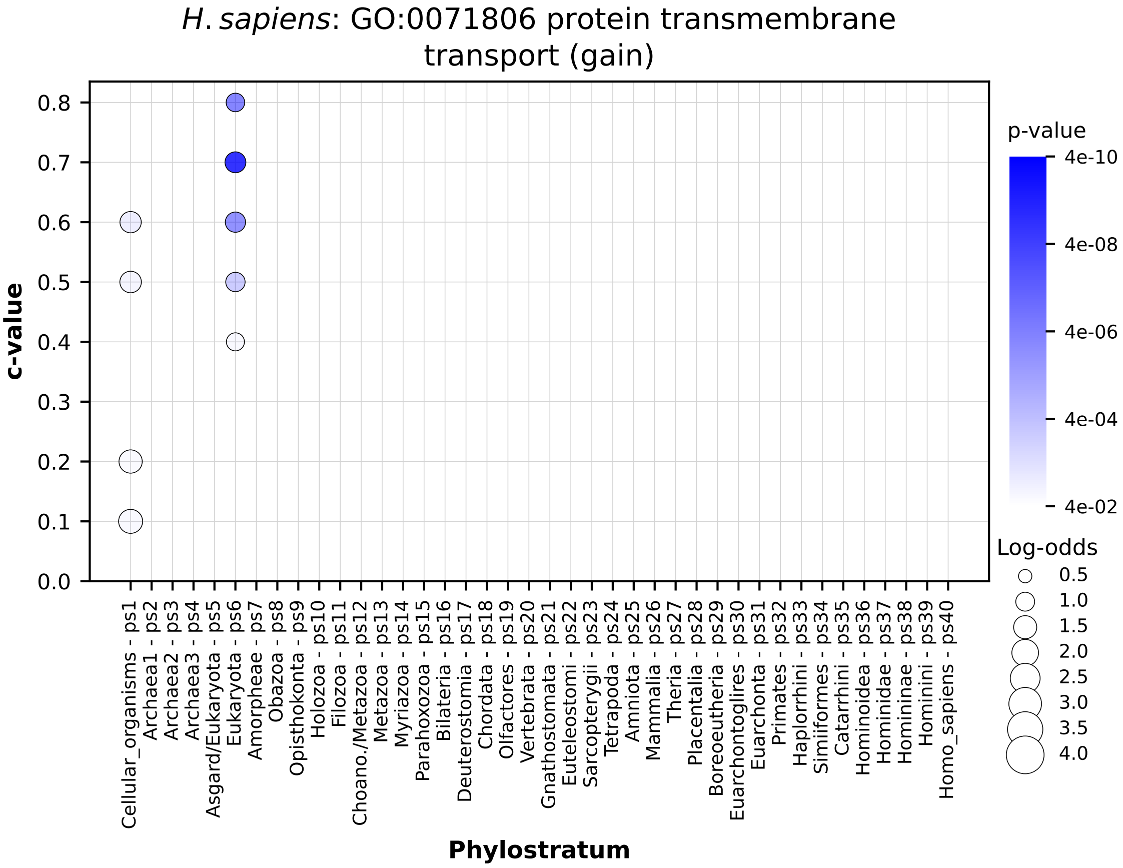

*H. sapiens*: GO:0071806 protein transmembrane transport (gain)
c-value
0.0
0.1
0.2
0.3
0.4
0.5
0.6
0.7
0.8
Phylostratum
Cellular_organisms - ps1
Archaea1 - ps2
Archaea2 - ps3
Archaea3 - ps4
Asgard/Eukaryota - ps5
Eukaryota - ps6
Amorpheae - ps7
Obazoa - ps8
Opisthokonta - ps9
Holozoa - ps10
Filozoa - ps11
Choano./Metazoa - ps12
Metazoa - ps13
Myriazoa - ps14
Parahoxozoa - ps15
Bilateria - ps16
Deuterostomia - ps17
Chordata - ps18
Olfactores - ps19
Vertebrata - ps20
Gnathostomata - ps21
Euteleostomi - ps22
Sarcopterygii - ps23
Tetrapoda - ps24
Amniota - ps25
Mammalia - ps26
Theria - ps27
Placentalia - ps28
Boreoeutheria - ps29
Euarchontoglires - ps30
Euarchonta - ps31
Primates - ps32
Haplorrhini - ps33
Simiiformes - ps34
Catarrhini - ps35
Hominoidea - ps36
Hominidae - ps37
Homininae - ps38
Hominini - ps39
Homo_sapiens - ps40
p-value
4e-10
4e-08
4e-06
4e-04
4e-02
Log-odds
0.5
1.0
1.5
2.0
2.5
3.0
3.5
4.0

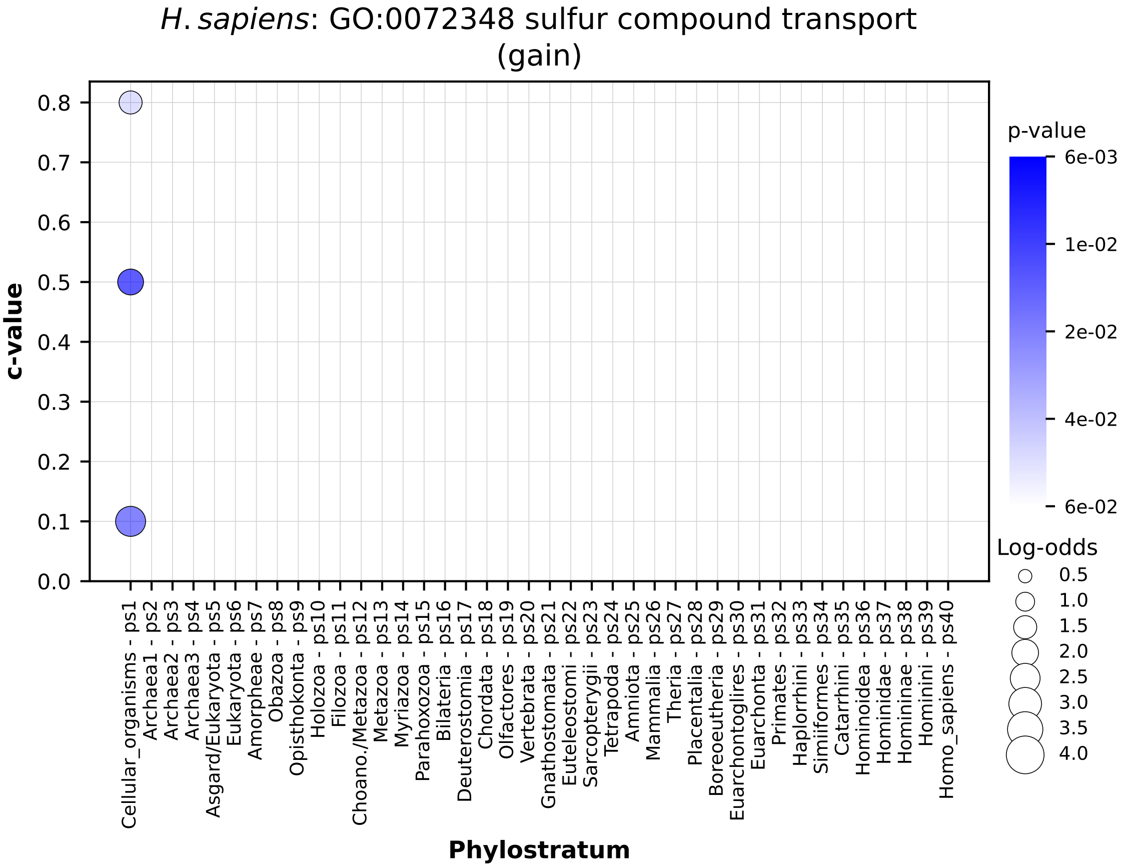

H. sapiens: GO:0072348 sulfur compound transport
(gain)
c-value
0.0
0.1
0.2
0.3
0.4
0.5
0.6
0.7
0.8
Phylostratum
Cellular_organisms - ps1
Archaea1 - ps2
Archaea2 - ps3
Archaea3 - ps4
Asgard/Eukaryota - ps5
Eukaryota - ps6
Amorpheae - ps7
Obazoa - ps8
Opisthokonta - ps9
Holozoa - ps10
Filozoa - ps11
Choano./Metazoa - ps12
Metazoa - ps13
Myriazoa - ps14
Parahoxozoa - ps15
Bilateria - ps16
Deuterostomia - ps17
Chordata - ps18
Olfactores - ps19
Vertebrata - ps20
Gnathostomata - ps21
Euteleostomi - ps22
Sarcopterygii - ps23
Tetrapoda - ps24
Amniota - ps25
Mammalia - ps26
Theria - ps27
Placentalia - ps28
Boreoeutheria - ps29
Euarchontoglires - ps30
Euarchonta - ps31
Primates - ps32
Haplorrhini - ps33
Simiiformes - ps34
Catarrhini - ps35
Hominoidea - ps36
Hominidae - ps37
Homininae - ps38
Hominini - ps39
Homo_sapiens - ps40
p-value
6e-03
1e-02
2e-02
4e-02
6e-02
Log-odds
0.5
1.0
1.5
2.0
2.5
3.0
3.5
4.0

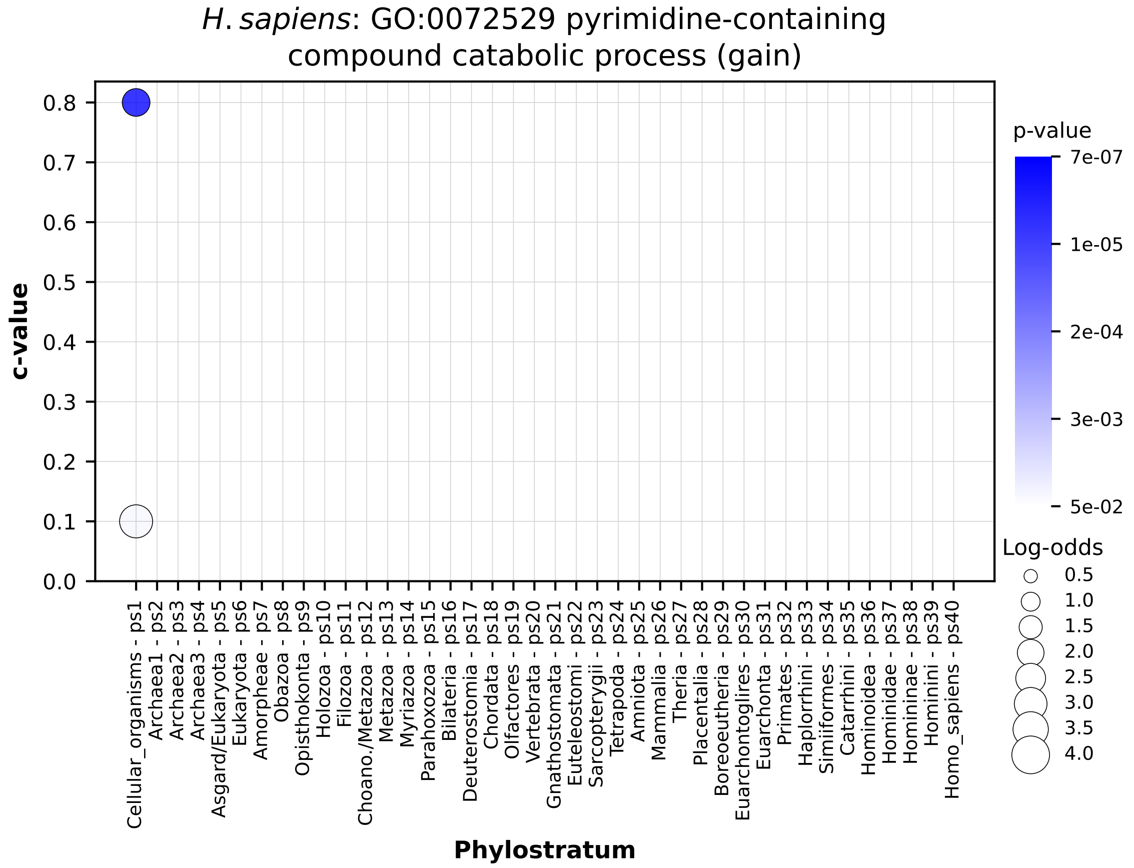

H. sapiens: GO:0072529 pyrimidine-containing compound catabolic process (gain)
c-value
0.0
0.1
0.2
0.3
0.4
0.5
0.6
0.7
0.8
Phylostratum
Cellular_organisms - ps1
Archaea1 - ps2
Archaea2 - ps3
Archaea3 - ps4
Asgard/Eukaryota - ps5
Eukaryota - ps6
Amorpheae - ps7
Obazoa - ps8
Opisthokonta - ps9
Holozoa - ps10
Filozoa - ps11
Choano./Metazoa - ps12
Metazoa - ps13
Myriazoa - ps14
Parahoxozoa - ps15
Bilateria - ps16
Deuterostomia - ps17
Chordata - ps18
Olfactores - ps19
Vertebrata - ps20
Gnathostomata - ps21
Euteleostomi - ps22
Sarcopterygii - ps23
Tetrapoda - ps24
Amniota - ps25
Mammalia - ps26
Theria - ps27
Placentalia - ps28
Boreoeutheria - ps29
Euarchontoglires - ps30
Euarchonta - ps31
Primates - ps32
Haplorrhini - ps33
Simiiformes - ps34
Catarrhini - ps35
Hominoidea - ps36
Hominidae - ps37
Homininae - ps38
Hominini - ps39
Homo_sapiens - ps40
p-value
7e-07
1e-05
2e-04
3e-03
5e-02
Log-odds
0.5
1.0
1.5
2.0
2.5
3.0
3.5
4.0

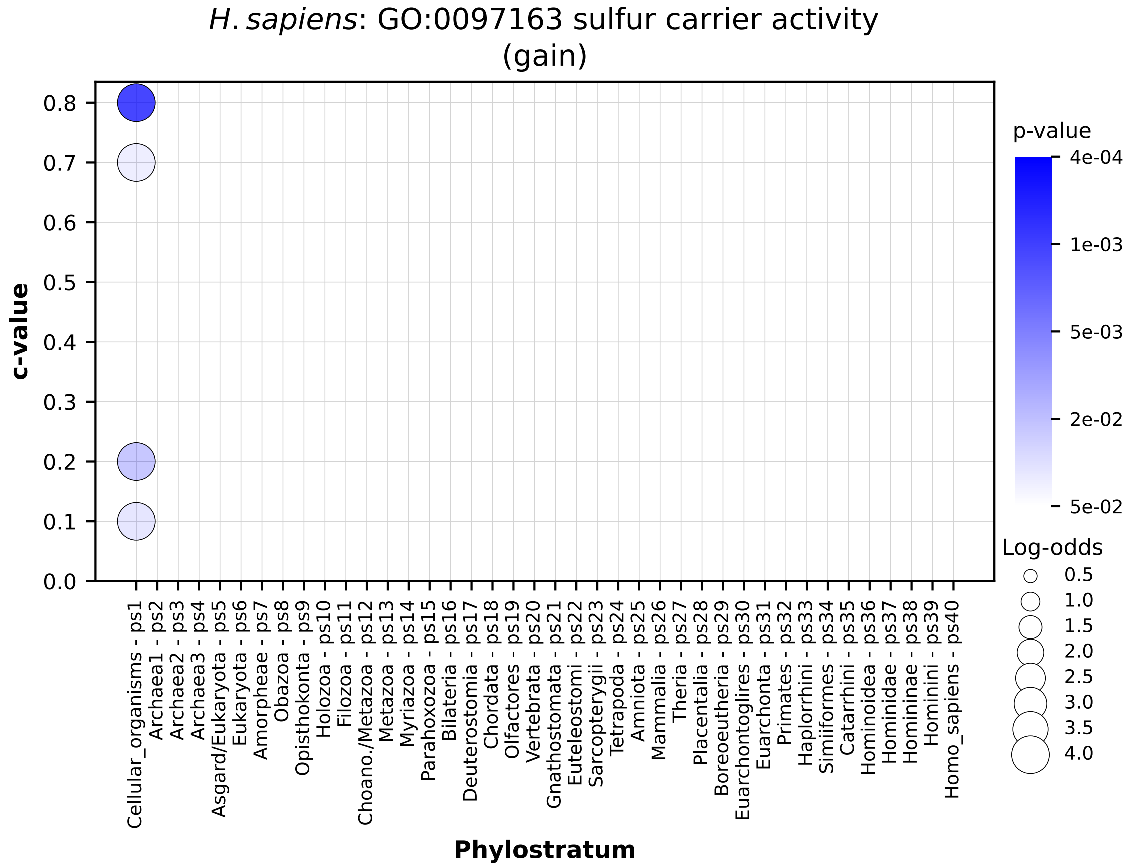

H. sapiens: GO:0097163 sulfur carrier activity
(gain)
c-value
0.0
0.1
0.2
0.3
0.4
0.5
0.6
0.7
0.8
Phylostratum
Cellular_organisms - ps1
Archaea1 - ps2
Archaea2 - ps3
Archaea3 - ps4
Asgard/Eukaryota - ps5
Eukaryota - ps6
Amorpheae - ps7
Obazoa - ps8
Opisthokonta - ps9
Holozoa - ps10
Filozoa - ps11
Choano./Metazoa - ps12
Metazoa - ps13
Myriazoa - ps14
Parahoxozoa - ps15
Bilateria - ps16
Deuterostomia - ps17
Chordata - ps18
Olfactores - ps19
Vertebrata - ps20
Gnathostomata - ps21
Euteleostomi - ps22
Sarcopterygii - ps23
Tetrapoda - ps24
Amniota - ps25
Mammalia - ps26
Theria - ps27
Placentalia - ps28
Boreoeutheria - ps29
Euarchontoglires - ps30
Euarchonta - ps31
Primates - ps32
Haplorrhini - ps33
Simiiformes - ps34
Catarrhini - ps35
Hominoidea - ps36
Hominidae - ps37
Homininae - ps38
Hominini - ps39
Homo_sapiens - ps40
p-value
4e-04
1e-03
5e-03
2e-02
5e-02
Log-odds
0.5
1.0
1.5
2.0
2.5
3.0
3.5
4.0

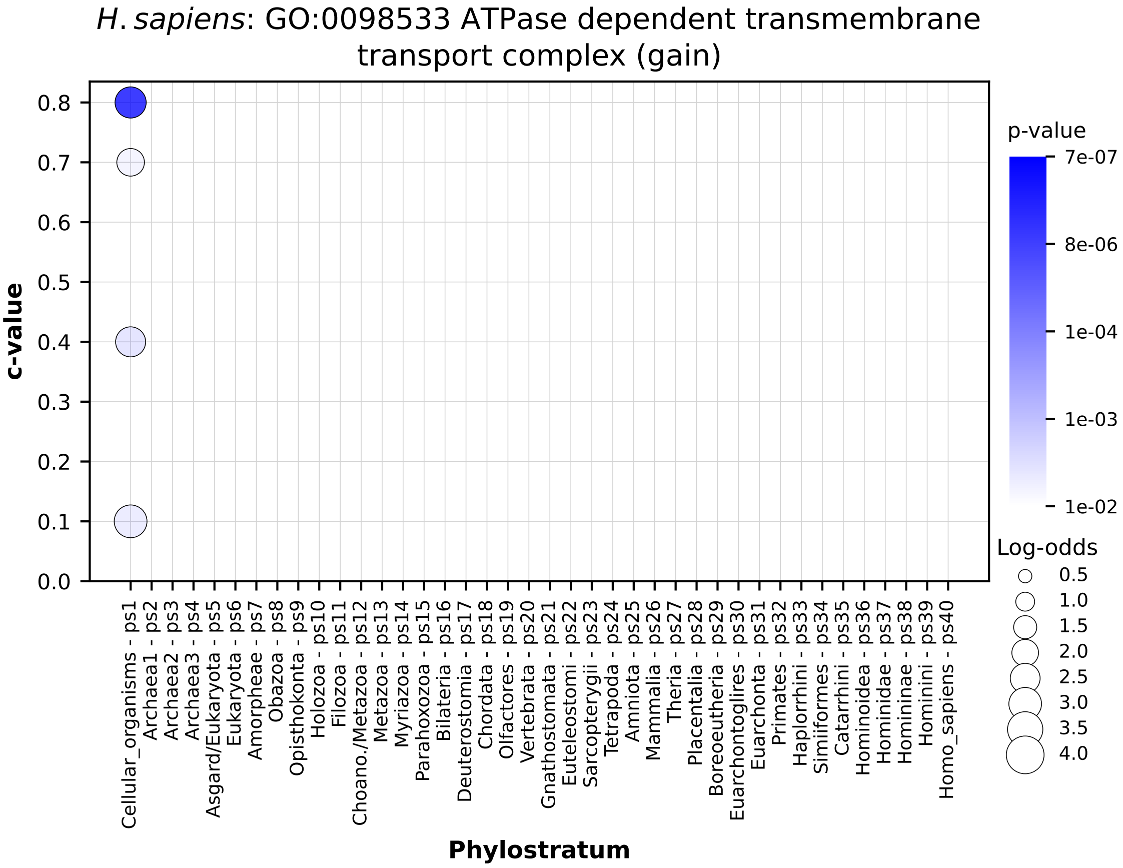

H. sapiens: GO:0098533 ATPase dependent transmembrane transport complex (gain)
c-value
0.0
0.1
0.2
0.3
0.4
0.5
0.6
0.7
0.8
Phylostratum
Cellular_organisms - ps1
Archaea1 - ps2
Archaea2 - ps3
Archaea3 - ps4
Asgard/Eukaryota - ps5
Eukaryota - ps6
Amorpheae - ps7
Obazoa - ps8
Opisthokonta - ps9
Holozoa - ps10
Filozoa - ps11
Choano./Metazoa - ps12
Metazoa - ps13
Myriazoa - ps14
Parahoxozoa - ps15
Bilateria - ps16
Deuterostomia - ps17
Chordata - ps18
Olfactores - ps19
Vertebrata - ps20
Gnathostomata - ps21
Euteleostomi - ps22
Sarcopterygii - ps23
Tetrapoda - ps24
Amniota - ps25
Mammalia - ps26
Theria - ps27
Placentalia - ps28
Boreoeutheria - ps29
Euarchontoglires - ps30
Euarchonta - ps31
Primates - ps32
Haplorrhini - ps33
Simiiformes - ps34
Catarrhini - ps35
Hominoidea - ps36
Hominidae - ps37
Homininae - ps38
Hominini - ps39
Homo_sapiens - ps40
p-value
7e-07
8e-06
1e-04
1e-03
1e-02
Log-odds
0.5
1.0
1.5
2.0
2.5
3.0
3.5
4.0

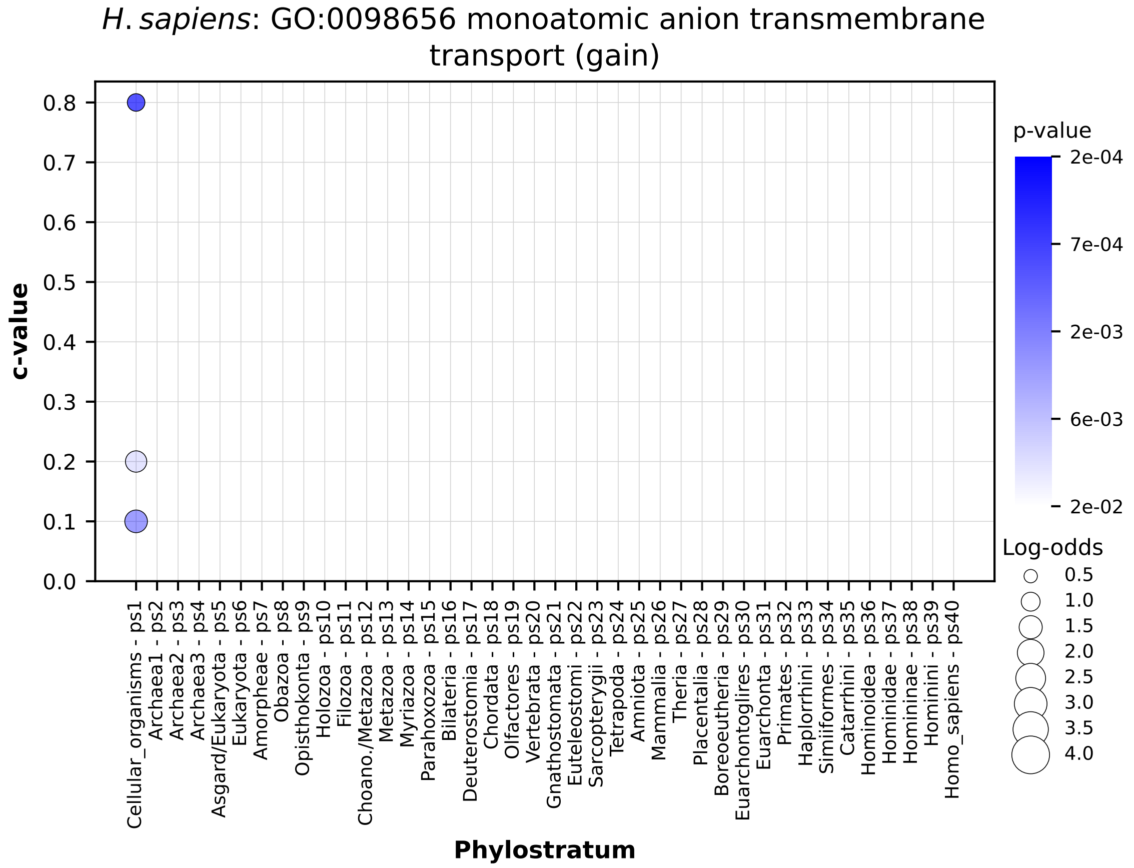
H. sapiens: GO:0098656 monoatomic anion transmembrane transport (gain)
c-value
0.0
0.1
0.2
0.3
0.4
0.5
0.6
0.7
0.8
Phylostratum
Cellular_organisms - ps1
Archaea1 - ps2
Archaea2 - ps3
Archaea3 - ps4
Asgard/Eukaryota - ps5
Eukaryota - ps6
Amorpheae - ps7
Obazoa - ps8
Opisthokonta - ps9
Holozoa - ps10
Filozoa - ps11
Choano./Metazoa - ps12
Metazoa - ps13
Myriazoa - ps14
Parahoxozoa - ps15
Bilateria - ps16
Deuterostomia - ps17
Chordata - ps18
Olfactores - ps19
Vertebrata - ps20
Gnathostomata - ps21
Euteleostomi - ps22
Sarcopterygii - ps23
Tetrapoda - ps24
Amniota - ps25
Mammalia - ps26
Theria - ps27
Placentalia - ps28
Boreoeutheria - ps29
Euarchontoglires - ps30
Euarchonta - ps31
Primates - ps32
Haplorrhini - ps33
Simiiformes - ps34
Catarrhini - ps35
Hominoidea - ps36
Hominidae - ps37
Homininae - ps38
Hominini - ps39
Homo_sapiens - ps40
p-value
2e-04
7e-04
2e-03
6e-03
2e-02
Log-odds
0.5
1.0
1.5
2.0
2.5
3.0
3.5
4.0

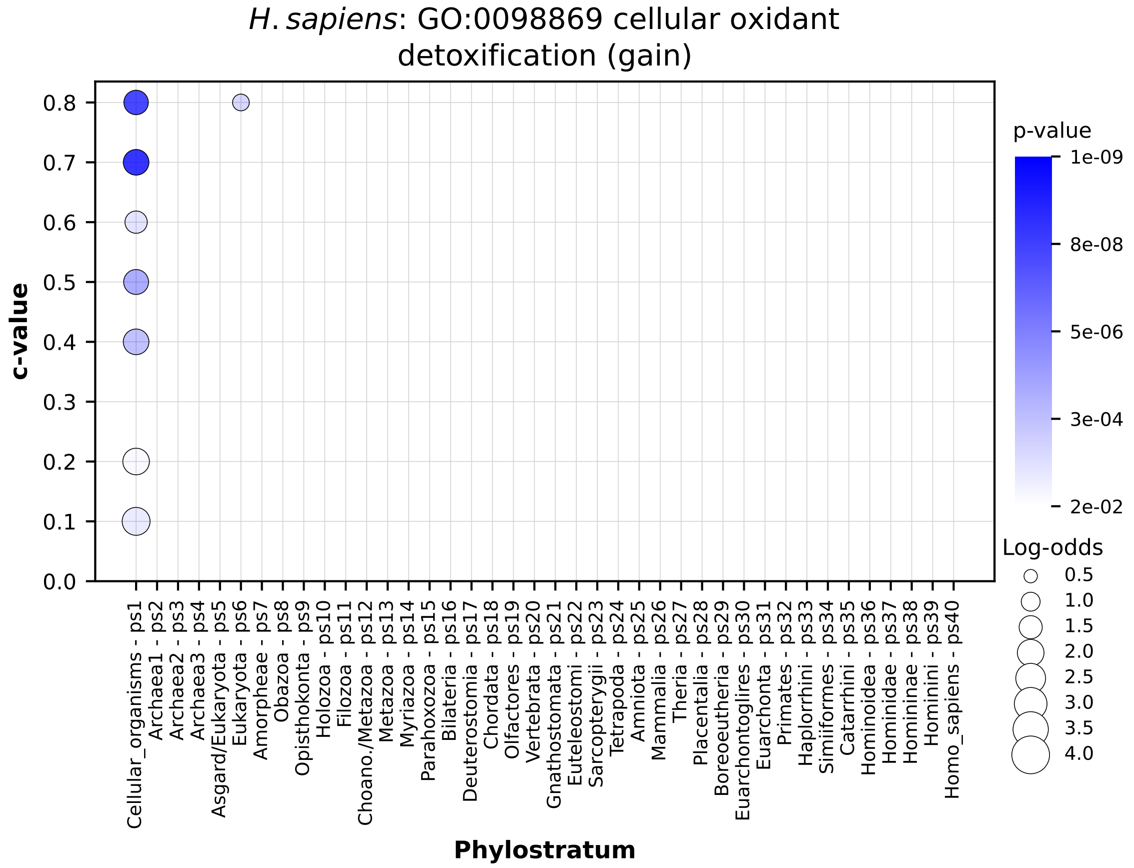

H. sapiens: GO:0098869 cellular oxidant detoxification (gain)
c-value
0.0
0.1
0.2
0.3
0.4
0.5
0.6
0.7
0.8
Phylostratum
Cellular_organisms - ps1
Archaea1 - ps2
Archaea2 - ps3
Archaea3 - ps4
Asgard/Eukaryota - ps5
Eukaryota - ps6
Amorpheae - ps7
Obazoa - ps8
Opisthokonta - ps9
Holozoa - ps10
Filozoa - ps11
Choano./Metazoa - ps12
Metazoa - ps13
Myriazoa - ps14
Parahoxozoa - ps15
Bilateria - ps16
Deuterostomia - ps17
Chordata - ps18
Olfactores - ps19
Vertebrata - ps20
Gnathostomata - ps21
Euteleostomi - ps22
Sarcopterygii - ps23
Tetrapoda - ps24
Amniota - ps25
Mammalia - ps26
Theria - ps27
Placentalia - ps28
Boreoeutheria - ps29
Euarchontoglires - ps30
Euarchonta - ps31
Primates - ps32
Haplorrhini - ps33
Simiiformes - ps34
Catarrhini - ps35
Hominoidea - ps36
Hominidae - ps37
Homininae - ps38
Hominini - ps39
Homo_sapiens - ps40
p-value
1e-09
8e-08
5e-06
3e-04
2e-02
Log-odds
0.5
1.0
1.5
2.0
2.5
3.0
3.5
4.0

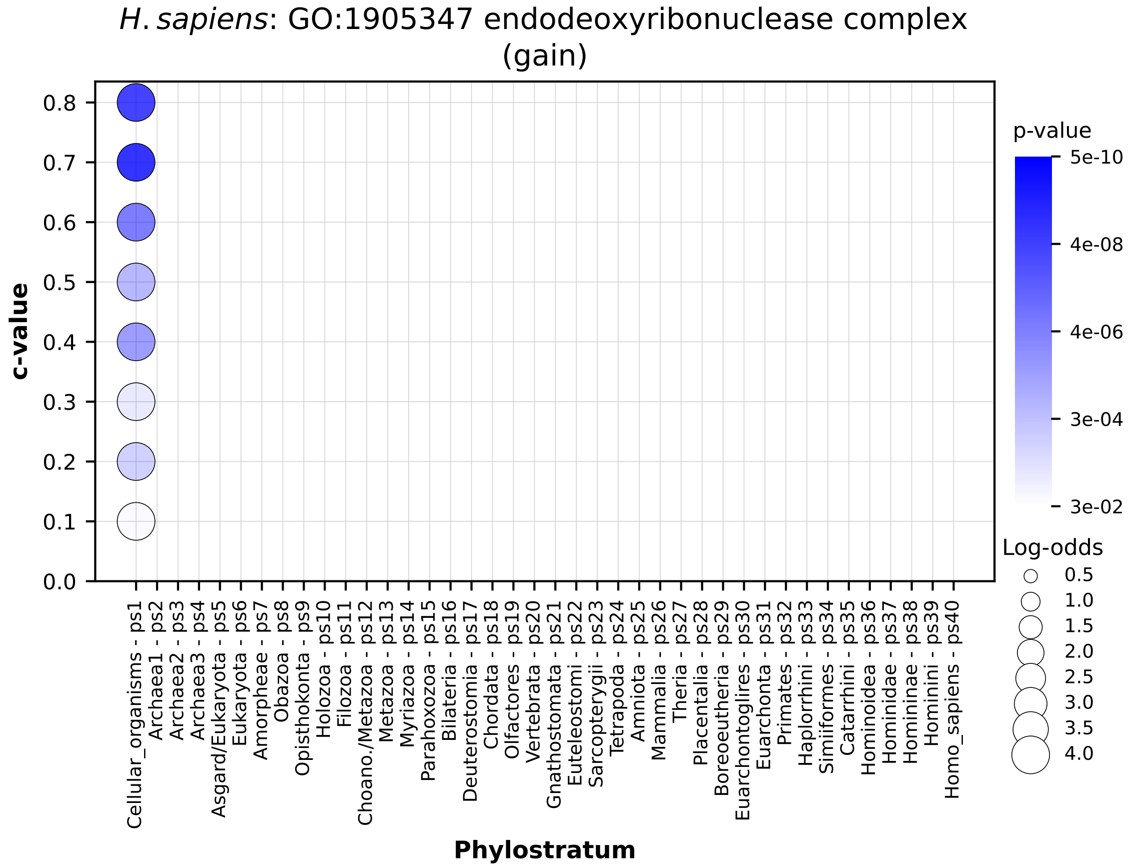
*H. sapiens*: GO:1905347 endodeoxyribonuclease complex
(gain)
c-value
0.0
0.1
0.2
0.3
0.4
0.5
0.6
0.7
0.8
Phylostratum
Cellular_organisms - ps1
Archaea1 - ps2
Archaea2 - ps3
Archaea3 - ps4
Asgard/Eukaryota - ps5
Eukaryota - ps6
Amorpheae - ps7
Obazoa - ps8
Opisthokonta - ps9
Holozoa - ps10
Filozoa - ps11
Choano./Metazoa - ps12
Metazoa - ps13
Myriazoa - ps14
Parahoxozoa - ps15
Bilateria - ps16
Deuterostomia - ps17
Chordata - ps18
Olfactores - ps19
Vertebrata - ps20
Gnathostomata - ps21
Euteleostomi - ps22
Sarcopterygii - ps23
Tetrapoda - ps24
Amniota - ps25
Mammalia - ps26
Theria - ps27
Placentalia - ps28
Boreoeutheria - ps29
Euarchontoglires - ps30
Euarchonta - ps31
Primates - ps32
Haplorrhini - ps33
Simiiformes - ps34
Catarrhini - ps35
Hominoidea - ps36
Hominidae - ps37
Homininae - ps38
Hominini - ps39
Homo_sapiens - ps40
p-value
5e-10
4e-08
4e-06
3e-04
3e-02
Log-odds
0.5
1.0
1.5
2.0
2.5
3.0
3.5
4.0

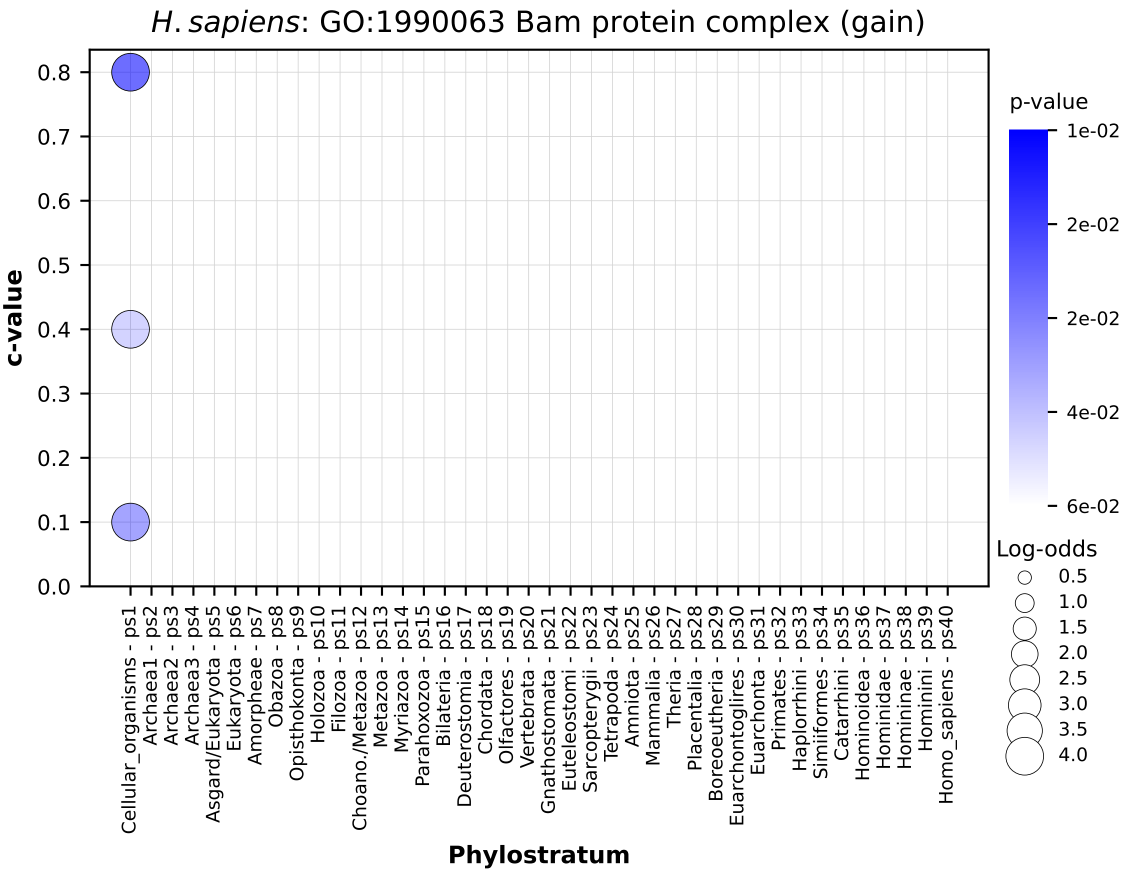

H. sapiens: GO:1990063 Bam protein complex (gain)
c-value
0.0
0.1
0.2
0.3
0.4
0.5
0.6
0.7
0.8
p-value
1e-02
2e-02
2e-02
4e-02
6e-02
Log-odds
0.5
1.0
1.5
2.0
2.5
3.0
3.5
4.0
Cellular_organisms - ps1
Archaea1 - ps2
Archaea2 - ps3
Archaea3 - ps4
Asgard/Eukaryota - ps5
Eukaryota - ps6
Amorpheae - ps7
Obazoa - ps8
Opisthokonta - ps9
Holozoa - ps10
Filozoa - ps11
Choano./Metazoa - ps12
Metazoa - ps13
Myriazoa - ps14
Parahoxozoa - ps15
Bilateria - ps16
Deuterostomia - ps17
Chordata - ps18
Olfactores - ps19
Vertebrata - ps20
Gnathostomata - ps21
Euteleostomi - ps22
Sarcopterygii - ps23
Tetrapoda - ps24
Amniota - ps25
Mammalia - ps26
Theria - ps27
Placentalia - ps28
Boreoeutheria - ps29
Euarchontoglires - ps30
Euarchonta - ps31
Primates - ps32
Haplorrhini - ps33
Simiiformes - ps34
Catarrhini - ps35
Hominoidea - ps36
Hominidae - ps37
Homininae - ps38
Hominini - ps39
Homo_sapiens - ps40
Phylostratum

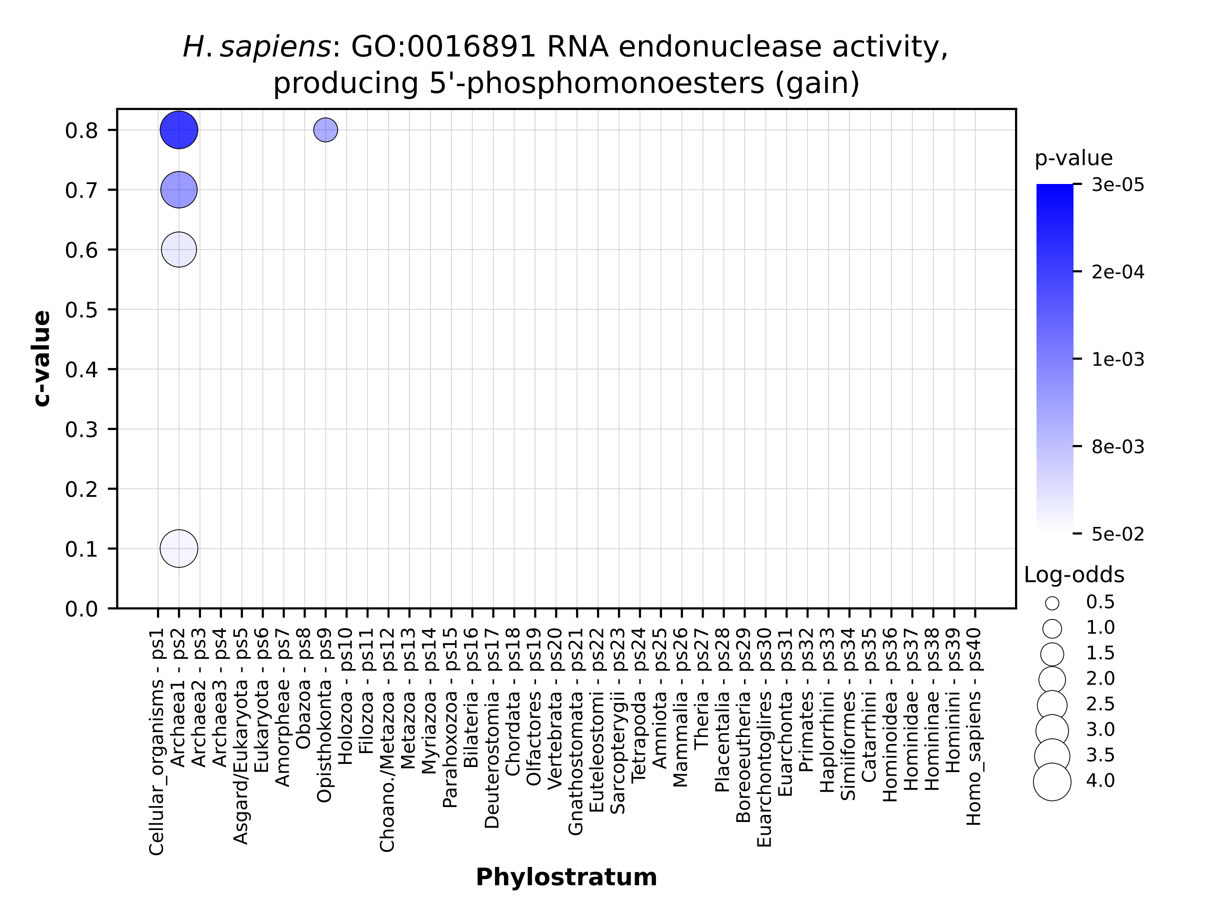

*H. sapiens*: GO:0016891 RNA endonuclease activity, producing 5'-phosphomonoesters (gain)
c-value
0.8
0.7
0.6
0.5
0.4
0.3
0.2
0.1
0.0
Phylostratum
Cellular_organisms - ps1
Archaea1 - ps2
Archaea2 - ps3
Archaea3 - ps4
Asgard/Eukaryota - ps5
Eukaryota - ps6
Amorpheae - ps7
Obazoa - ps8
Opisthokonta - ps9
Holozoa - ps10
Filozoa - ps11
Choano./Metazoa - ps12
Metazoa - ps13
Myriazoa - ps14
Parahoxozoa - ps15
Bilateria - ps16
Deuterostomia - ps17
Chordata - ps18
Olfactores - ps19
Vertebrata - ps20
Gnathostomata - ps21
Euteleostomi - ps22
Sarcopterygii - ps23
Tetrapoda - ps24
Amniota - ps25
Mammalia - ps26
Theria - ps27
Placentalia - ps28
Boreoeutheria - ps29
Euarchontoglires - ps30
Euarchonta - ps31
Primates - ps32
Haplorrhini - ps33
Simiiformes - ps34
Catarrhini - ps35
Hominoidea - ps36
Hominidae - ps37
Homininae - ps38
Hominini - ps39
Homo_sapiens - ps40
p-value
3e-05
2e-04
1e-03
8e-03
5e-02
Log-odds
0.5
1.0
1.5
2.0
2.5
3.0
3.5
4.0

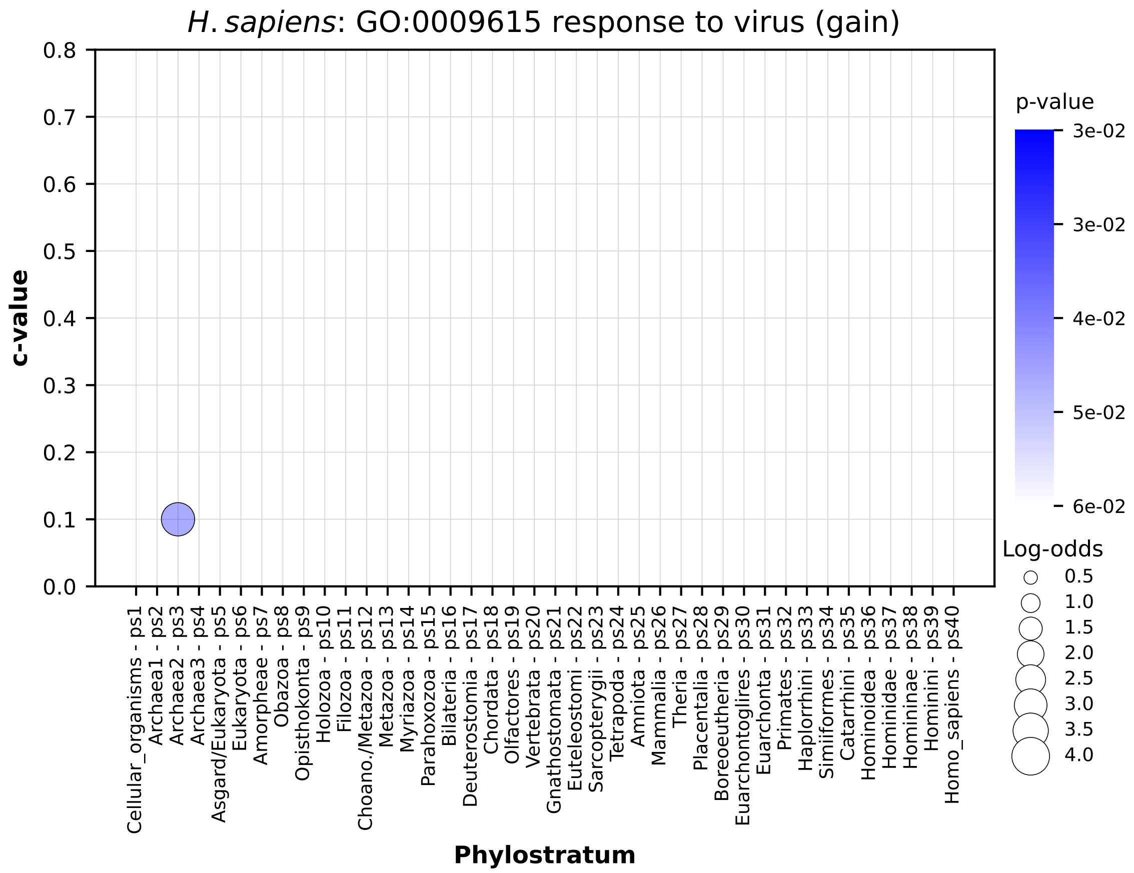

H. sapiens: GO:0009615 response to virus (gain)
c-value
0.0
0.1
0.2
0.3
0.4
0.5
0.6
0.7
0.8
Phylostratum
Cellular_organisms - ps1
Archaea1 - ps2
Archaea2 - ps3
Archaea3 - ps4
Asgard/Eukaryota - ps5
Eukaryota - ps6
Amorpheae - ps7
Obazoa - ps8
Opisthokonta - ps9
Holozoa - ps10
Filozoa - ps11
Choano./Metazoa - ps12
Metazoa - ps13
Myriazoa - ps14
Parahoxozoa - ps15
Bilateria - ps16
Deuterostomia - ps17
Chordata - ps18
Olfactores - ps19
Vertebrata - ps20
Gnathostomata - ps21
Euteleostomi - ps22
Sarcopterygii - ps23
Tetrapoda - ps24
Amniota - ps25
Mammalia - ps26
Theria - ps27
Placentalia - ps28
Boreoeutheria - ps29
Euarchontoglires - ps30
Euarchonta - ps31
Primates - ps32
Haplorrhini - ps33
Simiiformes - ps34
Catarrhini - ps35
Hominoidea - ps36
Hominidae - ps37
Homininae - ps38
Hominini - ps39
Homo_sapiens - ps40
p-value
3e-02
3e-02
4e-02
5e-02
6e-02
Log-odds
0.5
1.0
1.5
2.0
2.5
3.0
3.5
4.0

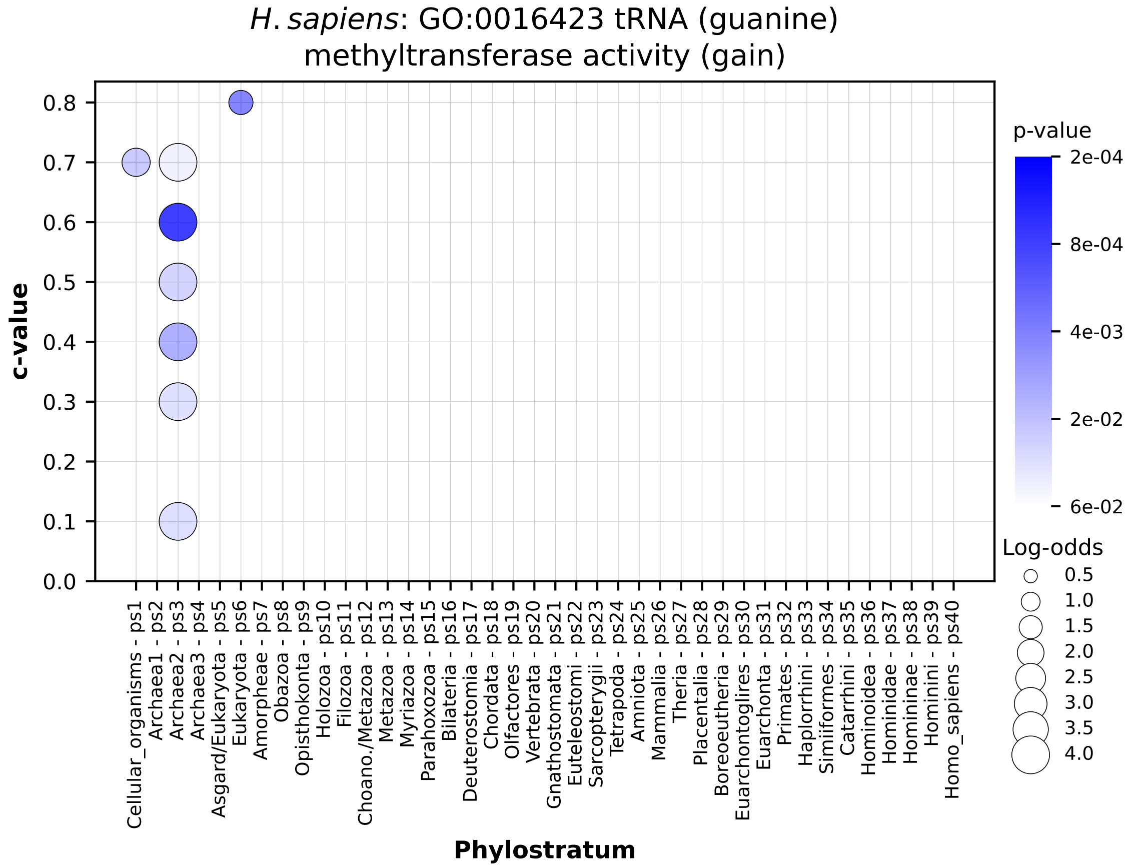

H. sapiens: GO:0016423 tRNA (guanine) methyltransferase activity (gain)
c-value
0.0
0.1
0.2
0.3
0.4
0.5
0.6
0.7
0.8
Phylostratum
Cellular_organisms - ps1
Archaea1 - ps2
Archaea2 - ps3
Archaea3 - ps4
Asgard/Eukaryota - ps5
Eukaryota - ps6
Amorpheae - ps7
Obazoa - ps8
Opisthokonta - ps9
Holozoa - ps10
Filozoa - ps11
Choano./Metazoa - ps12
Metazoa - ps13
Myriazoa - ps14
Parahoxozoa - ps15
Bilateria - ps16
Deuterostomia - ps17
Chordata - ps18
Olfactores - ps19
Vertebrata - ps20
Gnathostomata - ps21
Euteleostomi - ps22
Sarcopterygii - ps23
Tetrapoda - ps24
Amniota - ps25
Mammalia - ps26
Theria - ps27
Placentalia - ps28
Boreoeutheria - ps29
Euarchontoglires - ps30
Euarchonta - ps31
Primates - ps32
Haplorrhini - ps33
Simiiformes - ps34
Catarrhini - ps35
Hominoidea - ps36
Hominidae - ps37
Homininae - ps38
Hominini - ps39
Homo_sapiens - ps40
p-value
2e-04
8e-04
4e-03
2e-02
6e-02
Log-odds
0.5
1.0
1.5
2.0
2.5
3.0
3.5
4.0

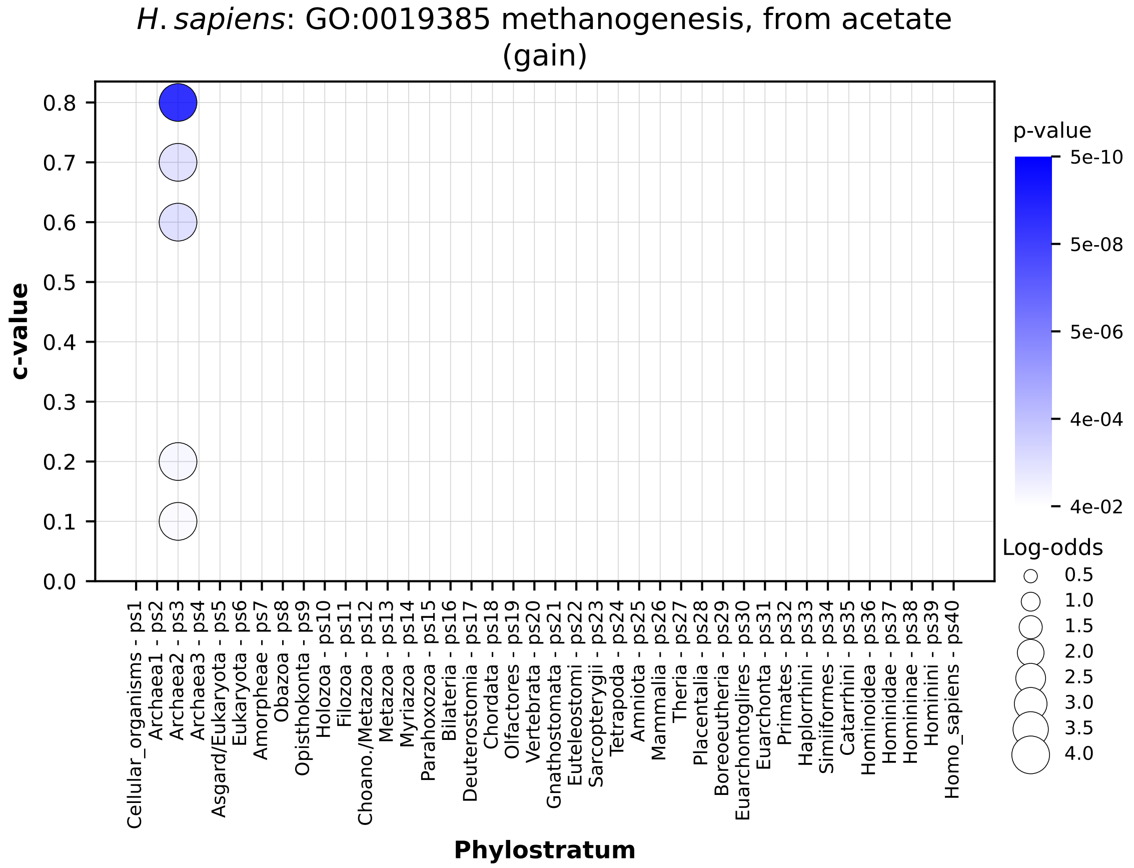
H. sapiens: GO:0019385 methanogenesis, from acetate
(gain)
c-value
0.0
0.1
0.2
0.3
0.4
0.5
0.6
0.7
0.8
p-value
5e-10
5e-08
5e-06
4e-04
4e-02
Log-odds
0.5
1.0
1.5
2.0
2.5
3.0
3.5
4.0
Cellular_organisms - ps1
Archaea1 - ps2
Archaea2 - ps3
Archaea3 - ps4
Asgard/Eukaryota - ps5
Eukaryota - ps6
Amorpheae - ps7
Obazoa - ps8
Opisthokonta - ps9
Holozoa - ps10
Filozoa - ps11
Choano./Metazoa - ps12
Metazoa - ps13
Myriazoa - ps14
Parahoxozoa - ps15
Bilateria - ps16
Deuterostomia - ps17
Chordata - ps18
Olfactores - ps19
Vertebrata - ps20
Gnathostomata - ps21
Euteleostomi - ps22
Sarcopterygii - ps23
Tetrapoda - ps24
Amniota - ps25
Mammalia - ps26
Theria - ps27
Placentalia - ps28
Boreoeutheria - ps29
Euarchontoglires - ps30
Euarchonta - ps31
Primates - ps32
Haplorrhini - ps33
Simiiformes - ps34
Catarrhini - ps35
Hominoidea - ps36
Hominidae - ps37
Homininae - ps38
Hominini - ps39
Homo_sapiens - ps40
Phylostratum

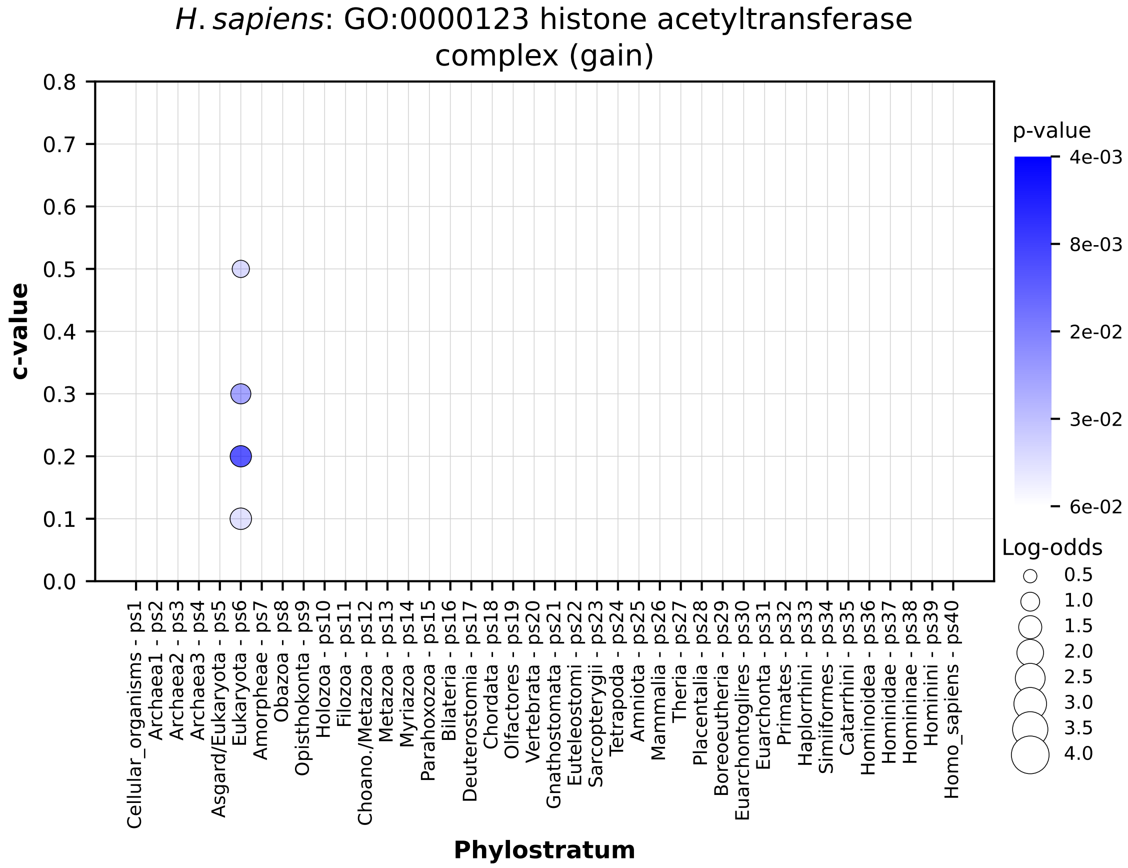

*H. sapiens*: GO:0000123 histone acetyltransferase complex (gain)
c-value
0.0
0.1
0.2
0.3
0.4
0.5
0.6
0.7
0.8
Phylostratum
Cellular_organisms - ps1
Archaea1 - ps2
Archaea2 - ps3
Archaea3 - ps4
Asgard/Eukaryota - ps5
Eukaryota - ps6
Amorpheae - ps7
Obazoa - ps8
Opisthokonta - ps9
Holozoa - ps10
Filozoa - ps11
Choano./Metazoa - ps12
Metazoa - ps13
Myriazoa - ps14
Parahoxozoa - ps15
Bilateria - ps16
Deuterostomia - ps17
Chordata - ps18
Olfactores - ps19
Vertebrata - ps20
Gnathostomata - ps21
Euteleostomi - ps22
Sarcopterygii - ps23
Tetrapoda - ps24
Amniota - ps25
Mammalia - ps26
Theria - ps27
Placentalia - ps28
Boreoeutheria - ps29
Euarchontoglires - ps30
Euarchonta - ps31
Primates - ps32
Haplorrhini - ps33
Simiiformes - ps34
Catarrhini - ps35
Hominoidea - ps36
Hominidae - ps37
Homininae - ps38
Hominini - ps39
Homo_sapiens - ps40
p-value
4e-03
8e-03
2e-02
3e-02
6e-02
Log-odds
0.5
1.0
1.5
2.0
2.5
3.0
3.5
4.0

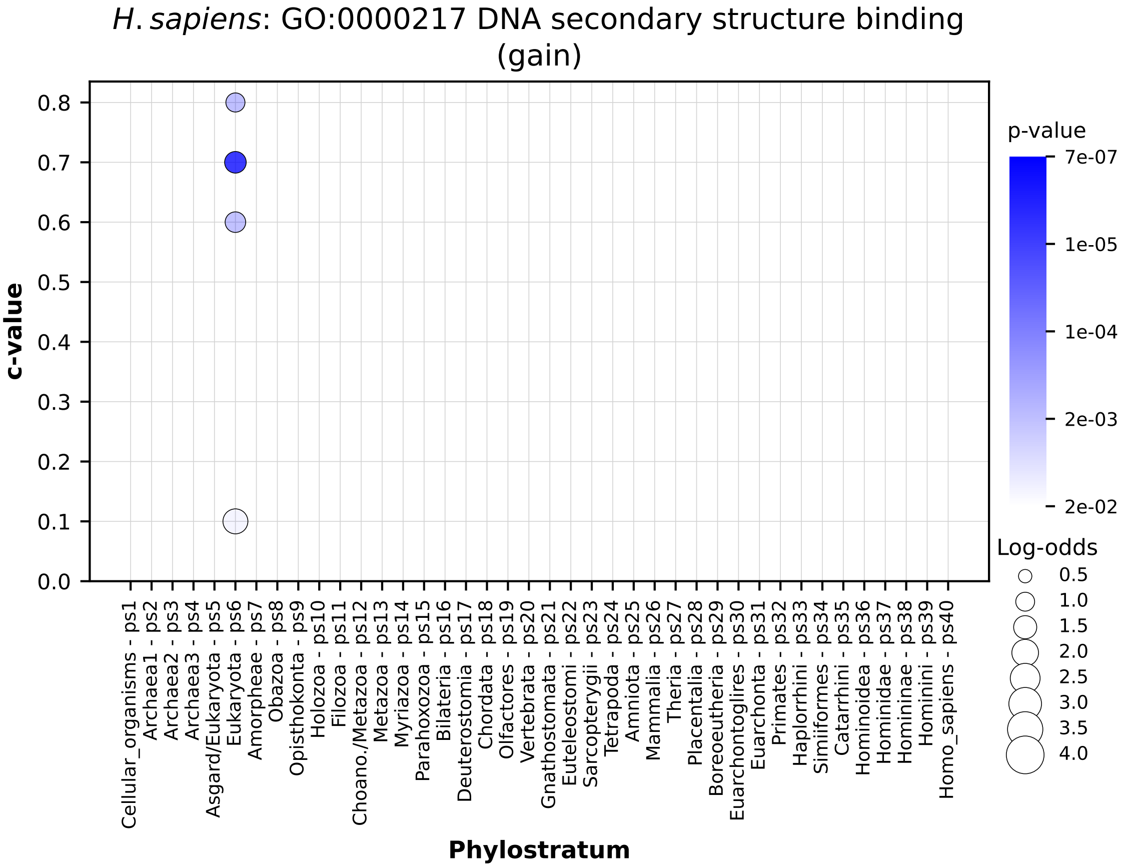
*H. sapiens*: GO:0000217 DNA secondary structure binding
(gain)
c-value
0.0
0.1
0.2
0.3
0.4
0.5
0.6
0.7
0.8
Phylostratum
Cellular_organisms - ps1
Archaea1 - ps2
Archaea2 - ps3
Archaea3 - ps4
Asgard/Eukaryota - ps5
Eukaryota - ps6
Amorpheae - ps7
Obazoa - ps8
Opisthokonta - ps9
Holozoa - ps10
Filozoa - ps11
Choano./Metazoa - ps12
Metazoa - ps13
Myriazoa - ps14
Parahoxozoa - ps15
Bilateria - ps16
Deuterostomia - ps17
Chordata - ps18
Olfactores - ps19
Vertebrata - ps20
Gnathostomata - ps21
Euteleostomi - ps22
Sarcopterygii - ps23
Tetrapoda - ps24
Amniota - ps25
Mammalia - ps26
Theria - ps27
Placentalia - ps28
Boreoeutheria - ps29
Euarchontoglires - ps30
Euarchonta - ps31
Primates - ps32
Haplorrhini - ps33
Simiiformes - ps34
Catarrhini - ps35
Hominoidea - ps36
Hominidae - ps37
Homininae - ps38
Hominini - ps39
Homo_sapiens - ps40
p-value
7e-07
1e-05
1e-04
2e-03
2e-02
Log-odds
0.5
1.0
1.5
2.0
2.5
3.0
3.5
4.0

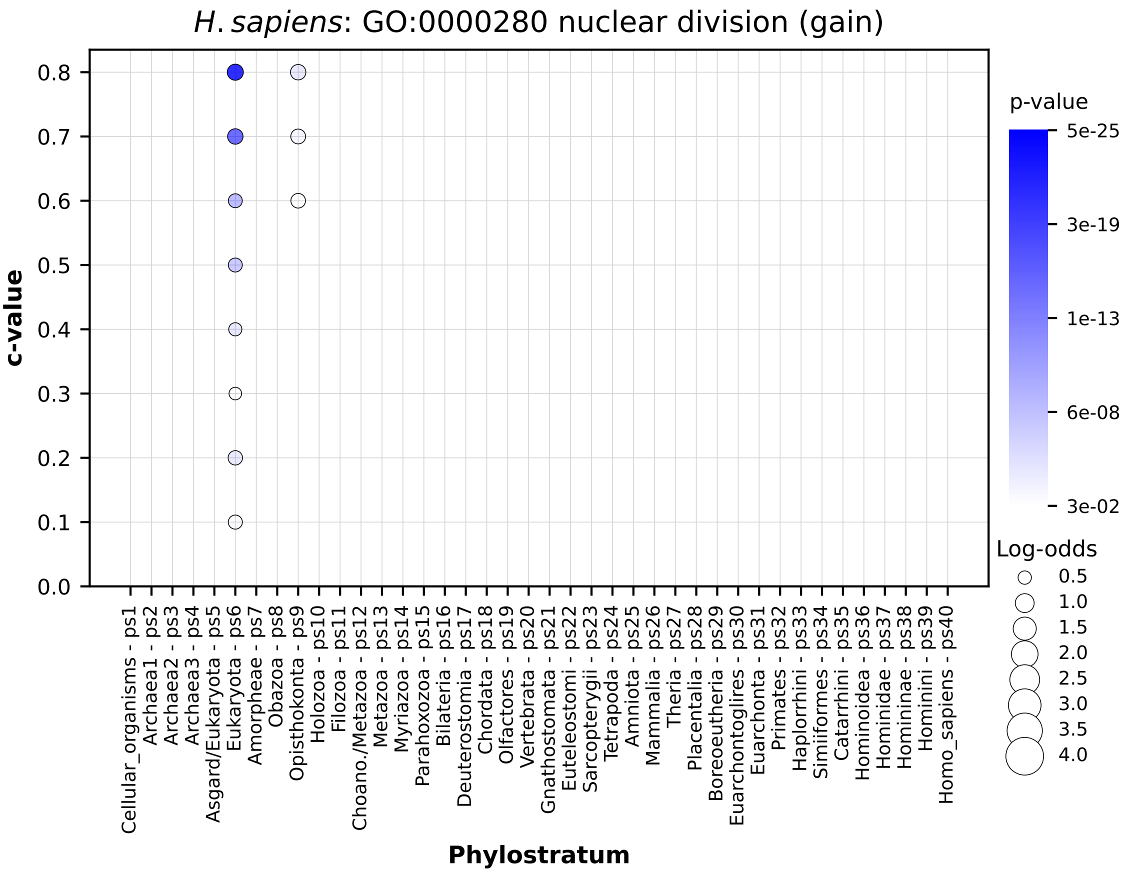

H. sapiens: GO:0000280 nuclear division (gain)
c-value
0.0
0.1
0.2
0.3
0.4
0.5
0.6
0.7
0.8
Phylostratum
Cellular_organisms - ps1
Archaea1 - ps2
Archaea2 - ps3
Archaea3 - ps4
Asgard/Eukaryota - ps5
Eukaryota - ps6
Amorpheae - ps7
Obazoa - ps8
Opisthokonta - ps9
Holozoa - ps10
Filozoa - ps11
Choano./Metazoa - ps12
Metazoa - ps13
Myriazoa - ps14
Parahoxozoa - ps15
Bilateria - ps16
Deuterostomia - ps17
Chordata - ps18
Olfactores - ps19
Vertebrata - ps20
Gnathostomata - ps21
Euteleostomi - ps22
Sarcopterygii - ps23
Tetrapoda - ps24
Amniota - ps25
Mammalia - ps26
Theria - ps27
Placentalia - ps28
Boreoeutheria - ps29
Euarchontoglires - ps30
Euarchonta - ps31
Primates - ps32
Haplorrhini - ps33
Simiiformes - ps34
Catarrhini - ps35
Hominoidea - ps36
Hominidae - ps37
Homininae - ps38
Hominini - ps39
Homo_sapiens - ps40
p-value
5e-25
3e-19
1e-13
6e-08
3e-02
Log-odds
0.5
1.0
1.5
2.0
2.5
3.0
3.5
4.0

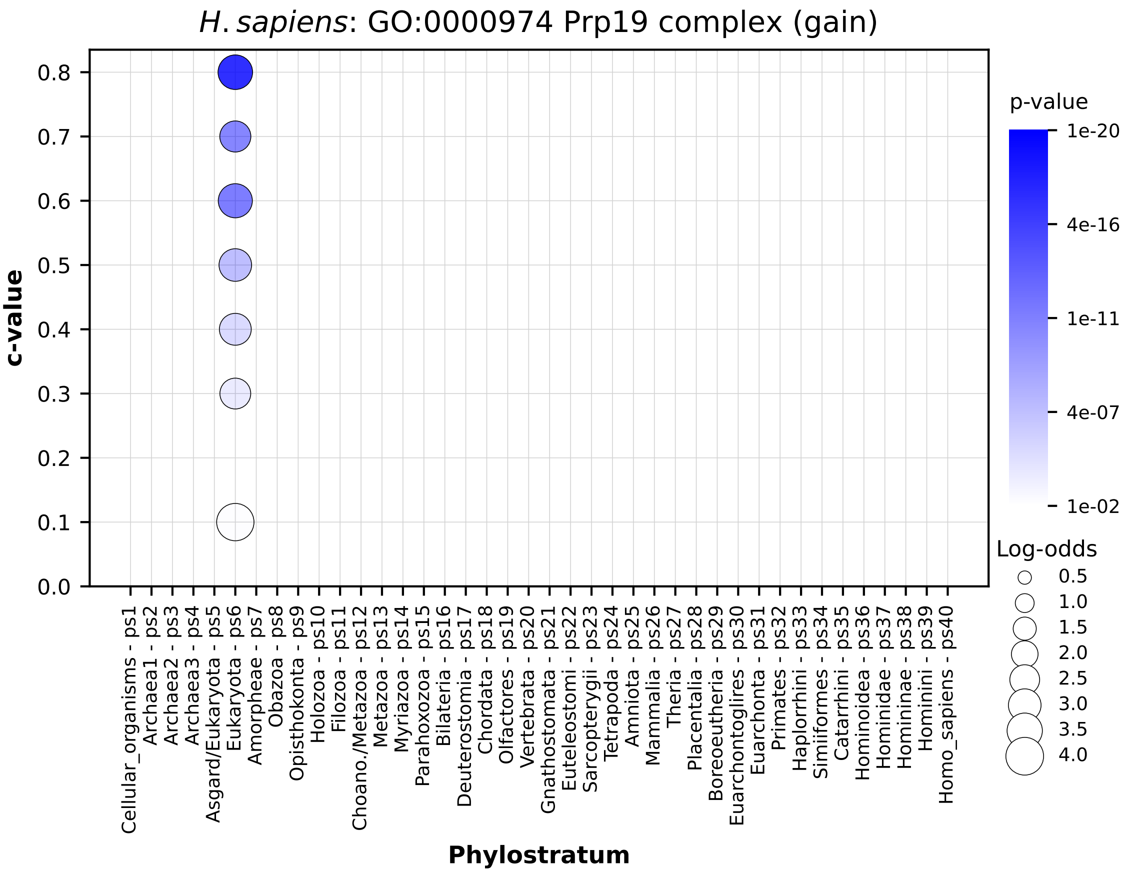
H. sapiens: GO:0000974 Prp19 complex (gain)
c-value
0.0
0.1
0.2
0.3
0.4
0.5
0.6
0.7
0.8
Phylostratum
Cellular_organisms - ps1
Archaea1 - ps2
Archaea2 - ps3
Archaea3 - ps4
Asgard/Eukaryota - ps5
Eukaryota - ps6
Amorpheae - ps7
Obazoa - ps8
Opisthokonta - ps9
Holozoa - ps10
Filozoa - ps11
Choano./Metazoa - ps12
Metazoa - ps13
Myriazoa - ps14
Parahoxozoa - ps15
Bilateria - ps16
Deuterostomia - ps17
Chordata - ps18
Olfactores - ps19
Vertebrata - ps20
Gnathostomata - ps21
Euteleostomi - ps22
Sarcopterygii - ps23
Tetrapoda - ps24
Amniota - ps25
Mammalia - ps26
Theria - ps27
Placentalia - ps28
Boreoeutheria - ps29
Euarchontoglires - ps30
Euarchonta - ps31
Primates - ps32
Haplorrhini - ps33
Simiiformes - ps34
Catarrhini - ps35
Hominoidea - ps36
Hominidae - ps37
Homininae - ps38
Hominini - ps39
Homo_sapiens - ps40
p-value
1e-20
4e-16
1e-11
4e-07
1e-02
Log-odds
0.5
1.0
1.5
2.0
2.5
3.0
3.5
4.0

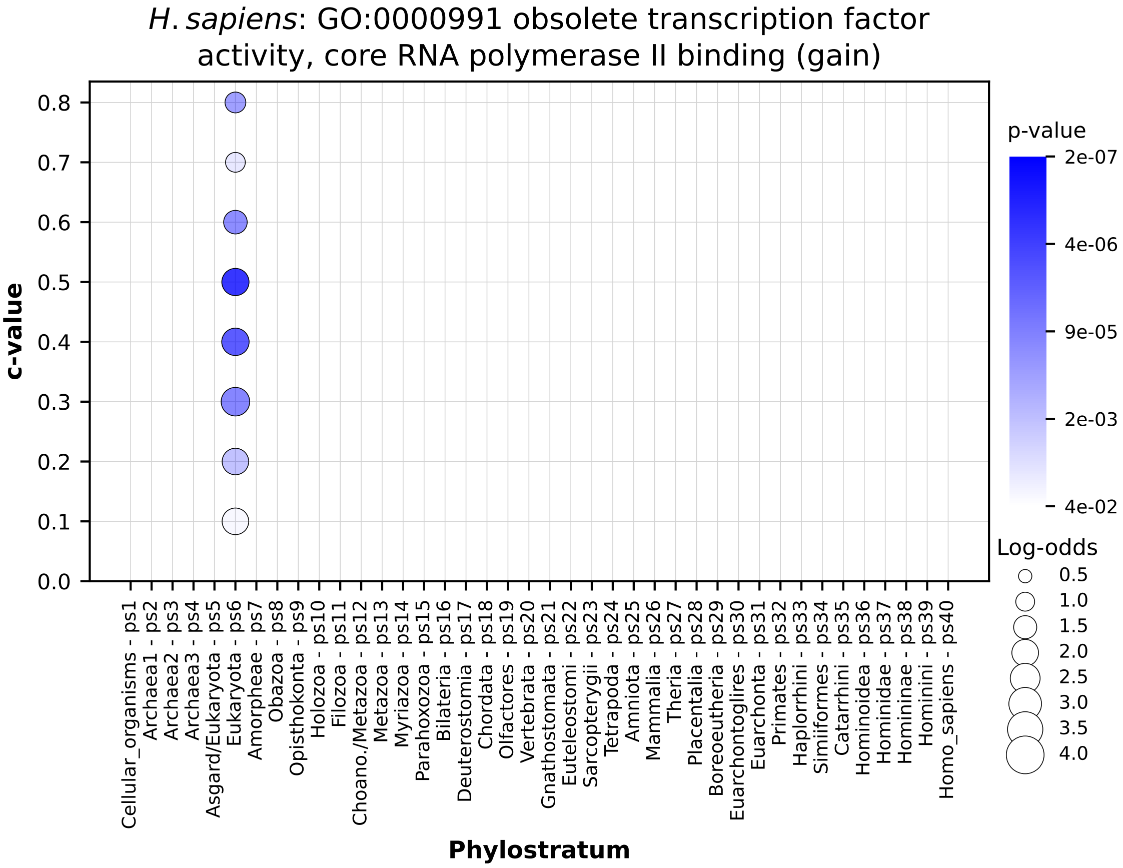
*H. sapiens*: GO:0000991 obsolete transcription factor activity, core RNA polymerase II binding (gain)
c-value
0.0
0.1
0.2
0.3
0.4
0.5
0.6
0.7
0.8
Phylostratum
Cellular_organisms - ps1
Archaea1 - ps2
Archaea2 - ps3
Archaea3 - ps4
Asgard/Eukaryota - ps5
Eukaryota - ps6
Amorpheae - ps7
Obazoa - ps8
Opisthokonta - ps9
Holozoa - ps10
Filozoa - ps11
Choano./Metazoa - ps12
Metazoa - ps13
Myriazoa - ps14
Parahoxozoa - ps15
Bilateria - ps16
Deuterostomia - ps17
Chordata - ps18
Olfactores - ps19
Vertebrata - ps20
Gnathostomata - ps21
Euteleostomi - ps22
Sarcopterygii - ps23
Tetrapoda - ps24
Amniota - ps25
Mammalia - ps26
Theria - ps27
Placentalia - ps28
Boreoeutheria - ps29
Euarchontoglires - ps30
Euarchonta - ps31
Primates - ps32
Haplorrhini - ps33
Simiiformes - ps34
Catarrhini - ps35
Hominoidea - ps36
Hominidae - ps37
Homininae - ps38
Hominini - ps39
Homo_sapiens - ps40
p-value
2e-07
4e-06
9e-05
2e-03
4e-02
Log-odds
0.5
1.0
1.5
2.0
2.5
3.0
3.5
4.0

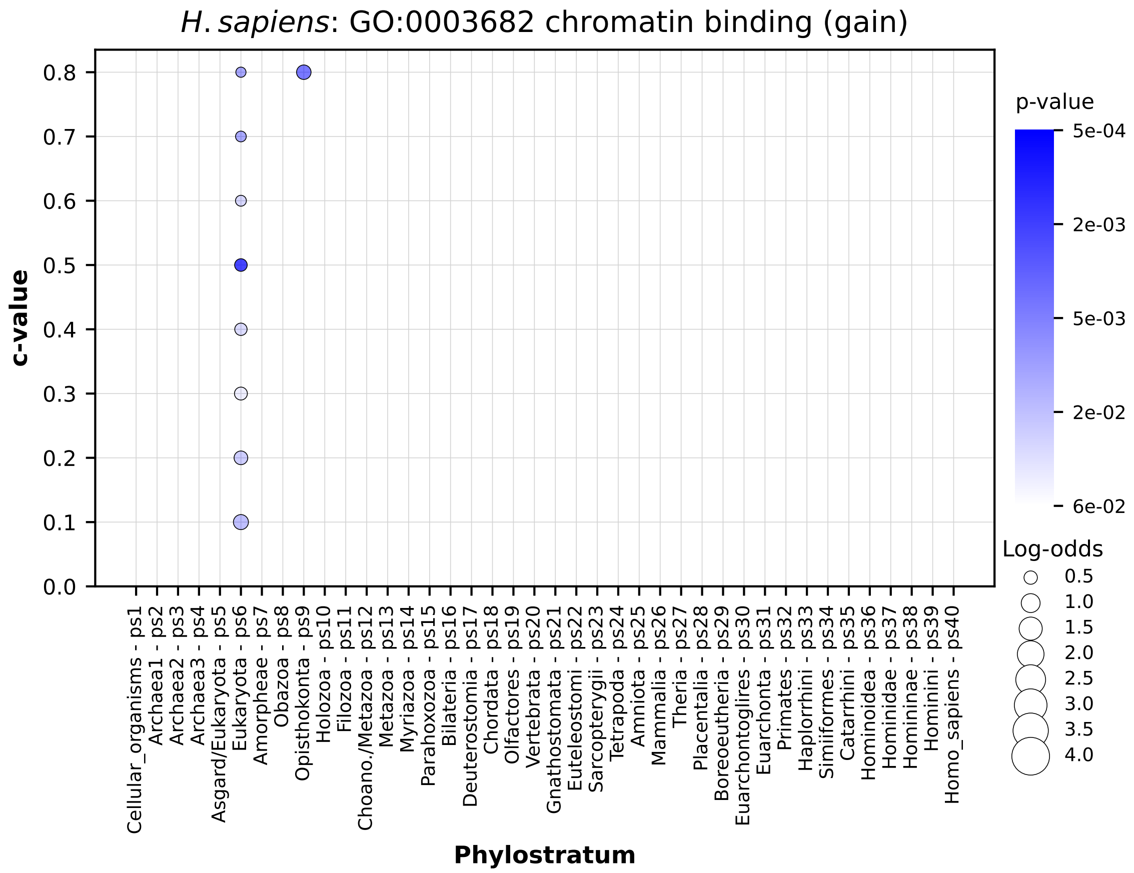

$H. sapiens$: GO:0003682 chromatin binding (gain)
c-value
0.0
0.1
0.2
0.3
0.4
0.5
0.6
0.7
0.8
Phylostratum
Cellular_organisms - ps1
Archaea1 - ps2
Archaea2 - ps3
Archaea3 - ps4
Asgard/Eukaryota - ps5
Eukaryota - ps6
Amorpheae - ps7
Obazoa - ps8
Opisthokonta - ps9
Holozoa - ps10
Filozoa - ps11
Choano./Metazoa - ps12
Metazoa - ps13
Myriazoa - ps14
Parahoxozoa - ps15
Bilateria - ps16
Deuterostomia - ps17
Chordata - ps18
Olfactores - ps19
Vertebrata - ps20
Gnathostomata - ps21
Euteleostomi - ps22
Sarcopterygii - ps23
Tetrapoda - ps24
Amniota - ps25
Mammalia - ps26
Theria - ps27
Placentalia - ps28
Boreoeutheria - ps29
Euarchontoglires - ps30
Euarchonta - ps31
Primates - ps32
Haplorrhini - ps33
Simiiformes - ps34
Catarrhini - ps35
Hominoidea - ps36
Hominidae - ps37
Homininae - ps38
Hominini - ps39
Homo_sapiens - ps40
p-value
5e-04
2e-03
5e-03
2e-02
6e-02
Log-odds
0.5
1.0
1.5
2.0
2.5
3.0
3.5
4.0

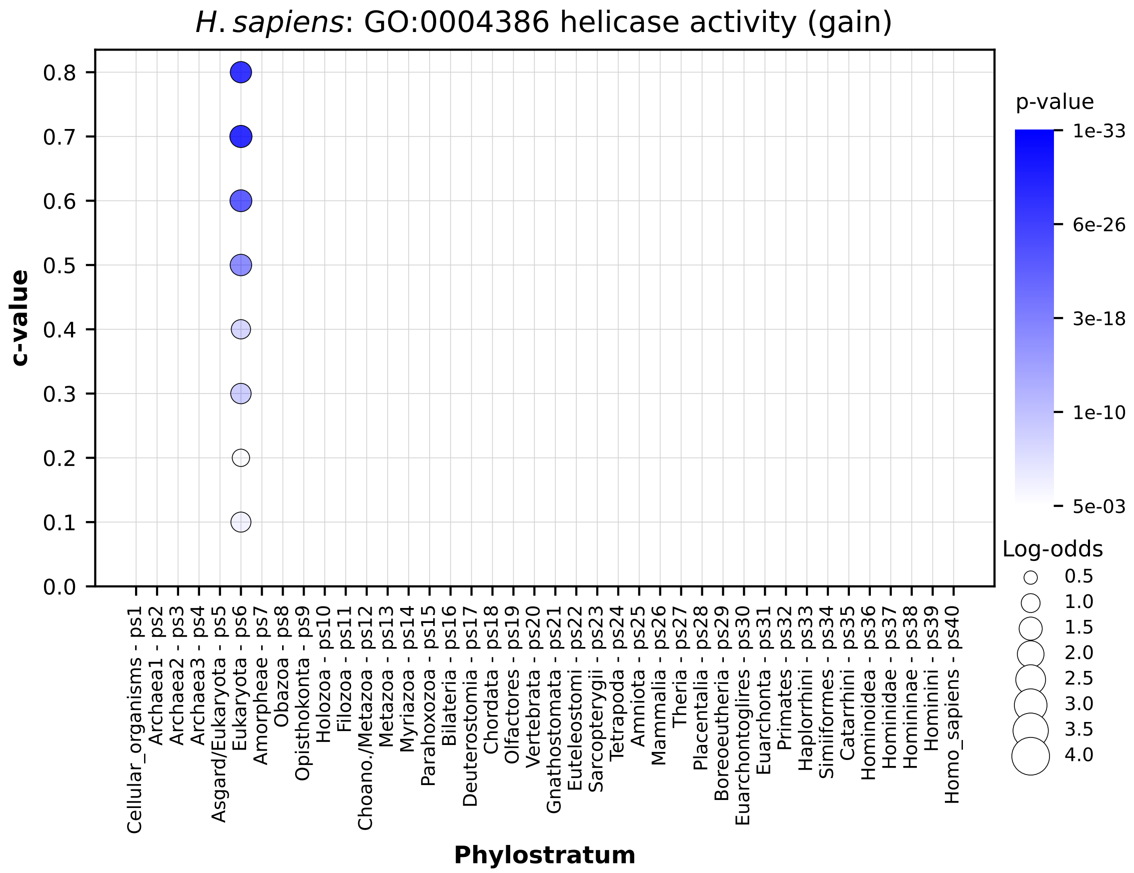

H. sapiens: GO:0004386 helicase activity (gain)
c-value
0.0
0.1
0.2
0.3
0.4
0.5
0.6
0.7
0.8
p-value
1e-33
6e-26
3e-18
1e-10
5e-03
Log-odds
0.5
1.0
1.5
2.0
2.5
3.0
3.5
4.0
Cellular_organisms - ps1
Archaea1 - ps2
Archaea2 - ps3
Archaea3 - ps4
Asgard/Eukaryota - ps5
Eukaryota - ps6
Amorpheae - ps7
Obazoa - ps8
Opisthokonta - ps9
Holozoa - ps10
Filozoa - ps11
Choano./Metazoa - ps12
Metazoa - ps13
Myriazoa - ps14
Parahoxozoa - ps15
Bilateria - ps16
Deuterostomia - ps17
Chordata - ps18
Olfactores - ps19
Vertebrata - ps20
Gnathostomata - ps21
Euteleostomi - ps22
Sarcopterygii - ps23
Tetrapoda - ps24
Amniota - ps25
Mammalia - ps26
Theria - ps27
Placentalia - ps28
Boreoeutheria - ps29
Euarchontoglires - ps30
Euarchonta - ps31
Primates - ps32
Haplorrhini - ps33
Simiiformes - ps34
Catarrhini - ps35
Hominoidea - ps36
Hominidae - ps37
Homininae - ps38
Hominini - ps39
Homo_sapiens - ps40
Phylostratum

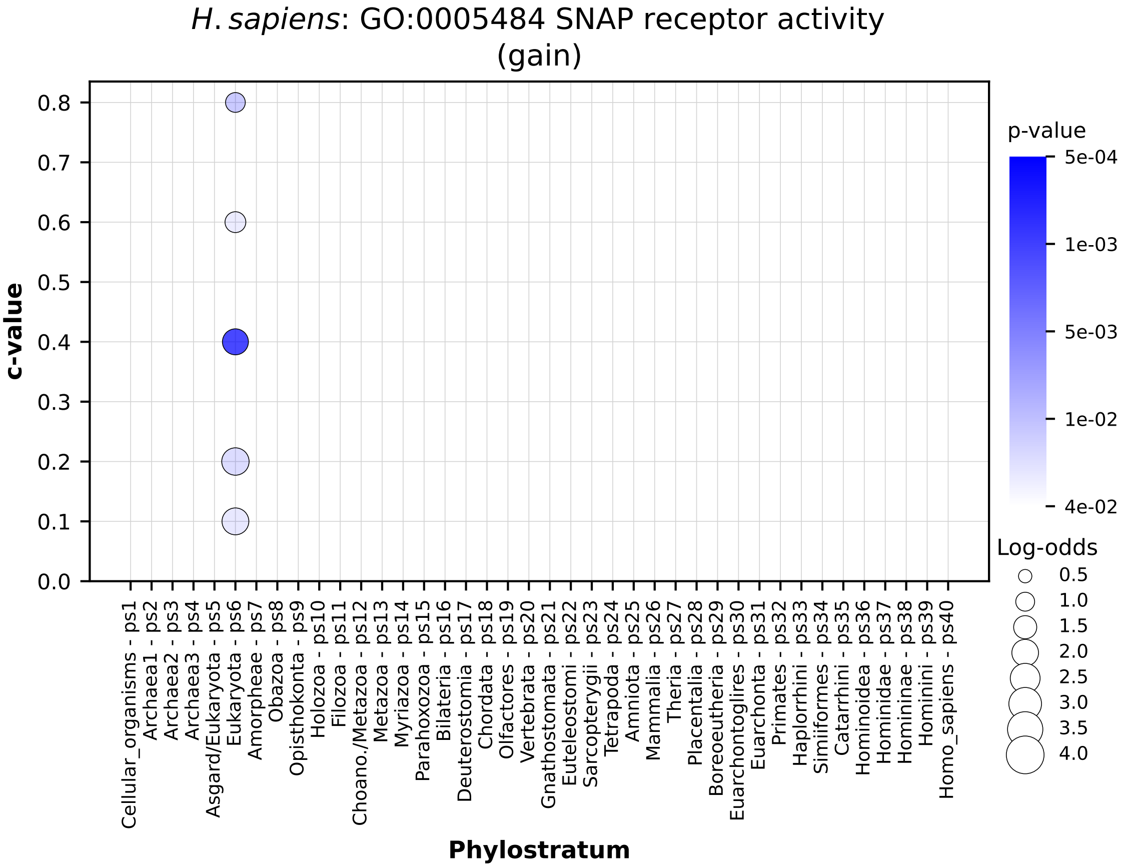

H. sapiens: GO:0005484 SNAP receptor activity
(gain)
c-value
0.0
0.1
0.2
0.3
0.4
0.5
0.6
0.7
0.8
Phylostratum
Cellular_organisms - ps1
Archaea1 - ps2
Archaea2 - ps3
Archaea3 - ps4
Asgard/Eukaryota - ps5
Eukaryota - ps6
Amorpheae - ps7
Obazoa - ps8
Opisthokonta - ps9
Holozoa - ps10
Filozoa - ps11
Choano./Metazoa - ps12
Metazoa - ps13
Myriazoa - ps14
Parahoxozoa - ps15
Bilateria - ps16
Deuterostomia - ps17
Chordata - ps18
Olfactores - ps19
Vertebrata - ps20
Gnathostomata - ps21
Euteleostomi - ps22
Sarcopterygii - ps23
Tetrapoda - ps24
Amniota - ps25
Mammalia - ps26
Theria - ps27
Placentalia - ps28
Boreoeutheria - ps29
Euarchontoglires - ps30
Euarchonta - ps31
Primates - ps32
Haplorrhini - ps33
Simiiformes - ps34
Catarrhini - ps35
Hominoidea - ps36
Hominidae - ps37
Homininae - ps38
Hominini - ps39
Homo_sapiens - ps40
p-value
5e-04
1e-03
5e-03
1e-02
4e-02
Log-odds
0.5
1.0
1.5
2.0
2.5
3.0
3.5
4.0

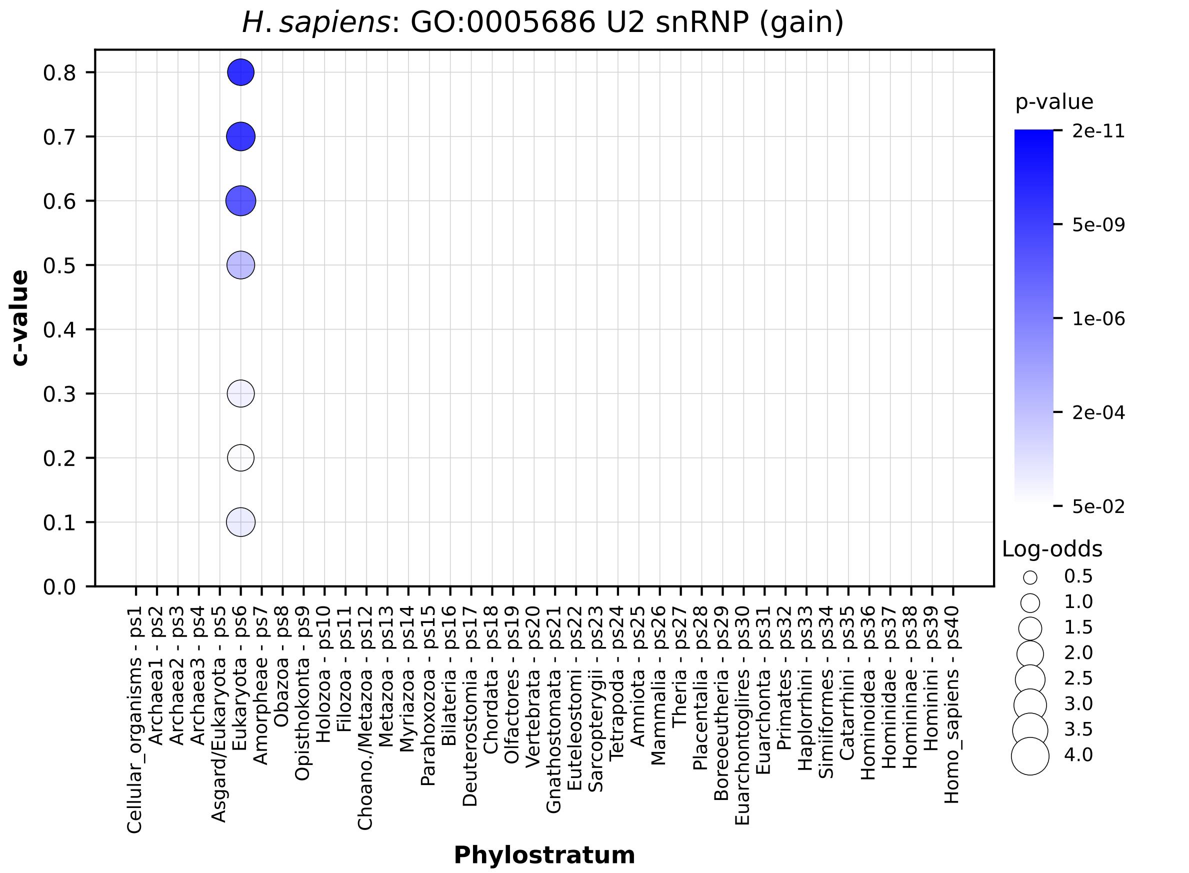
H. sapiens: GO:0005686 U2 snRNP (gain)
c-value
0.0
0.1
0.2
0.3
0.4
0.5
0.6
0.7
0.8
Phylostratum
Cellular_organisms - ps1
Archaea1 - ps2
Archaea2 - ps3
Archaea3 - ps4
Asgard/Eukaryota - ps5
Eukaryota - ps6
Amorpheae - ps7
Obazoa - ps8
Opisthokonta - ps9
Holozoa - ps10
Filozoa - ps11
Choano./Metazoa - ps12
Metazoa - ps13
Myriazoa - ps14
Parahoxozoa - ps15
Bilateria - ps16
Deuterostomia - ps17
Chordata - ps18
Olfactores - ps19
Vertebrata - ps20
Gnathostomata - ps21
Euteleostomi - ps22
Sarcopterygii - ps23
Tetrapoda - ps24
Amniota - ps25
Mammalia - ps26
Theria - ps27
Placentalia - ps28
Boreoeutheria - ps29
Euarchontoglires - ps30
Euarchonta - ps31
Primates - ps32
Haplorrhini - ps33
Simiiformes - ps34
Catarrhini - ps35
Hominoidea - ps36
Hominidae - ps37
Homininae - ps38
Hominini - ps39
Homo_sapiens - ps40
p-value
2e-11
5e-09
1e-06
2e-04
5e-02
Log-odds
0.5
1.0
1.5
2.0
2.5
3.0
3.5
4.0

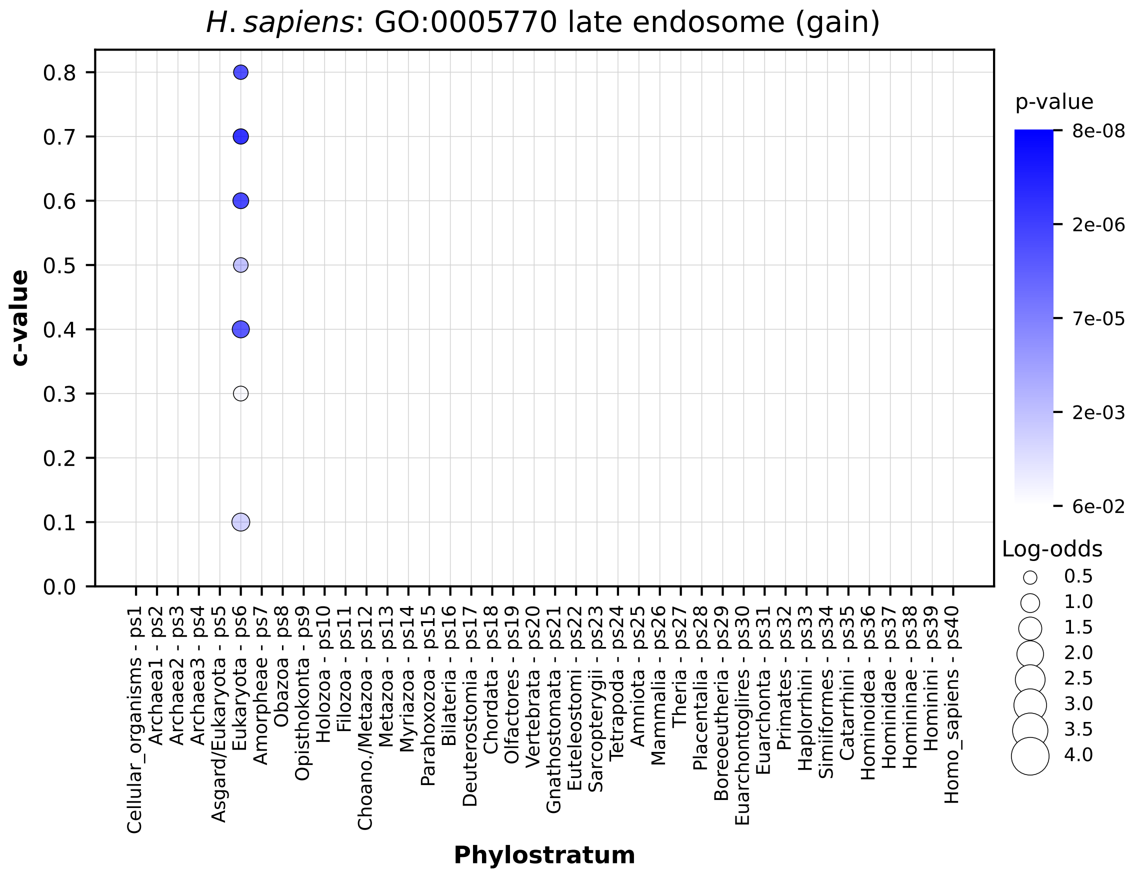

H. sapiens: GO:0005770 late endosome (gain)
c-value
0.0
0.1
0.2
0.3
0.4
0.5
0.6
0.7
0.8
Phylostratum
Cellular_organisms - ps1
Archaea1 - ps2
Archaea2 - ps3
Archaea3 - ps4
Asgard/Eukaryota - ps5
Eukaryota - ps6
Amorpheae - ps7
Obazoa - ps8
Opisthokonta - ps9
Holozoa - ps10
Filozoa - ps11
Choano./Metazoa - ps12
Metazoa - ps13
Myriazoa - ps14
Parahoxozoa - ps15
Bilateria - ps16
Deuterostomia - ps17
Chordata - ps18
Olfactores - ps19
Vertebrata - ps20
Gnathostomata - ps21
Euteleostomi - ps22
Sarcopterygii - ps23
Tetrapoda - ps24
Amniota - ps25
Mammalia - ps26
Theria - ps27
Placentalia - ps28
Boreoeutheria - ps29
Euarchontoglires - ps30
Euarchonta - ps31
Primates - ps32
Haplorrhini - ps33
Simiiformes - ps34
Catarrhini - ps35
Hominoidea - ps36
Hominidae - ps37
Homininae - ps38
Hominini - ps39
Homo_sapiens - ps40
p-value
8e-08
2e-06
7e-05
2e-03
6e-02
Log-odds
0.5
1.0
1.5
2.0
2.5
3.0
3.5
4.0

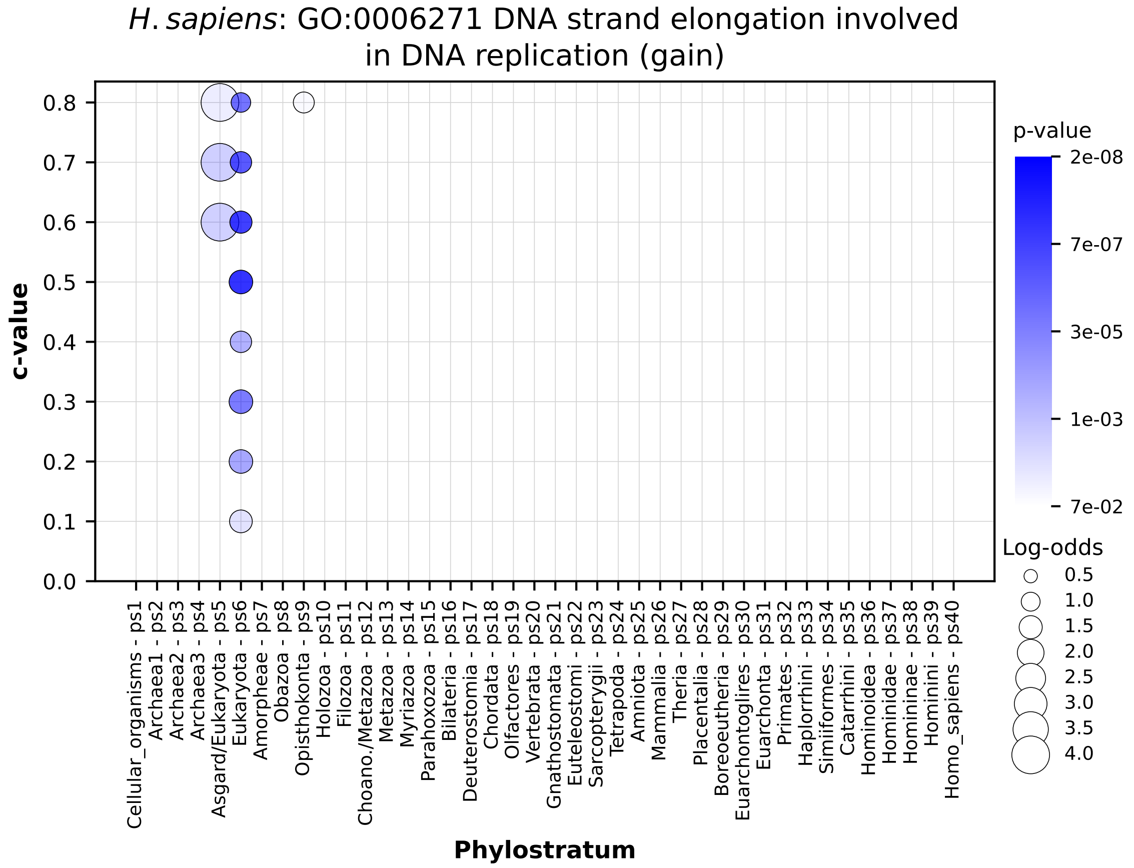

H. sapiens: GO:0006271 DNA strand elongation involved in DNA replication (gain)
c-value
0.0
0.1
0.2
0.3
0.4
0.5
0.6
0.7
0.8
Phylostratum
Cellular_organisms - ps1
Archaea1 - ps2
Archaea2 - ps3
Archaea3 - ps4
Asgard/Eukaryota - ps5
Eukaryota - ps6
Amorpheae - ps7
Obazoa - ps8
Opisthokonta - ps9
Holozoa - ps10
Filozoa - ps11
Choano./Metazoa - ps12
Metazoa - ps13
Myriazoa - ps14
Parahoxozoa - ps15
Bilateria - ps16
Deuterostomia - ps17
Chordata - ps18
Olfactores - ps19
Vertebrata - ps20
Gnathostomata - ps21
Euteleostomi - ps22
Sarcopterygii - ps23
Tetrapoda - ps24
Amniota - ps25
Mammalia - ps26
Theria - ps27
Placentalia - ps28
Boreoeutheria - ps29
Euarchontoglires - ps30
Euarchonta - ps31
Primates - ps32
Haplorrhini - ps33
Simiiformes - ps34
Catarrhini - ps35
Hominoidea - ps36
Hominidae - ps37
Homininae - ps38
Hominini - ps39
Homo_sapiens - ps40
p-value
2e-08
7e-07
3e-05
1e-03
7e-02
Log-odds
0.5
1.0
1.5
2.0
2.5
3.0
3.5
4.0

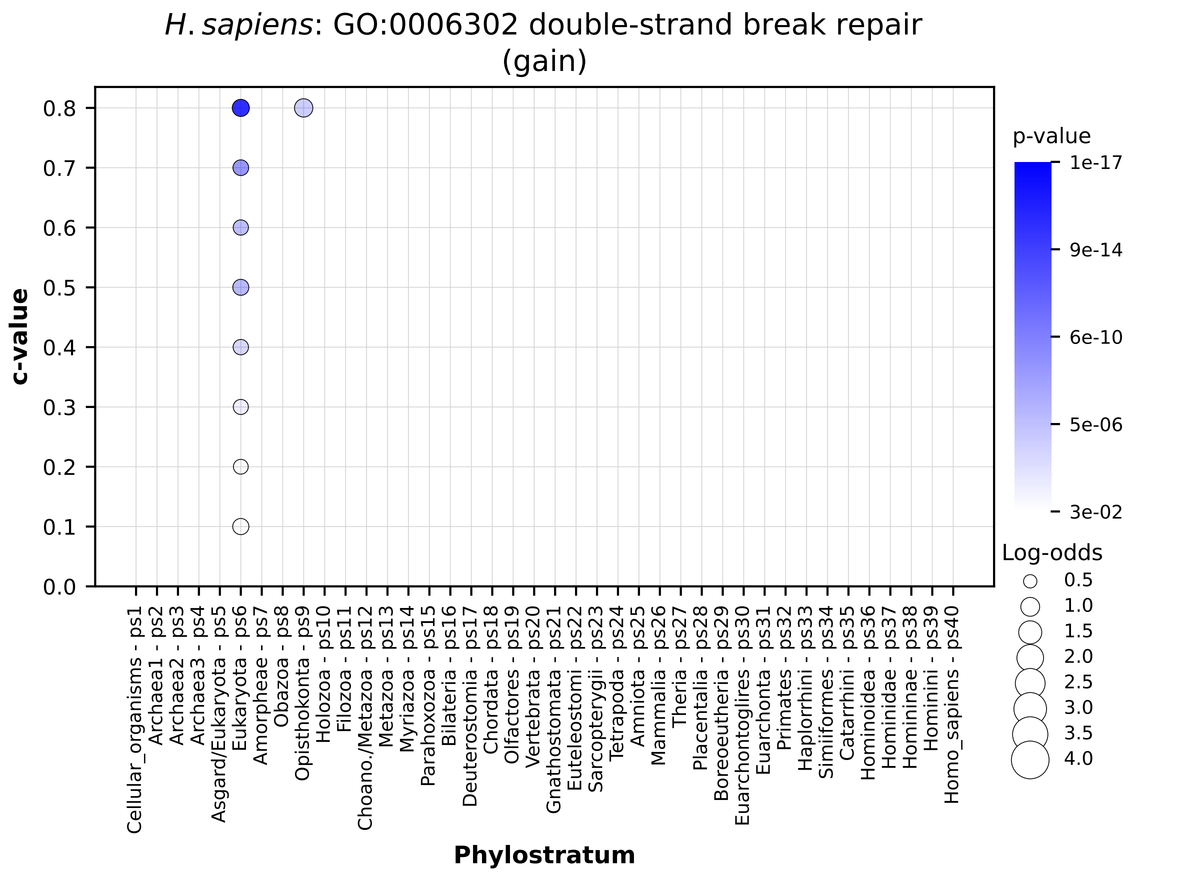
H. sapiens: GO:0006302 double-strand break repair
(gain)
c-value
0.0
0.1
0.2
0.3
0.4
0.5
0.6
0.7
0.8
Phylostratum
Cellular_organisms - ps1
Archaea1 - ps2
Archaea2 - ps3
Archaea3 - ps4
Asgard/Eukaryota - ps5
Eukaryota - ps6
Amorpheae - ps7
Obazoa - ps8
Opisthokonta - ps9
Holozoa - ps10
Filozoa - ps11
Choano./Metazoa - ps12
Metazoa - ps13
Myriazoa - ps14
Parahoxozoa - ps15
Bilateria - ps16
Deuterostomia - ps17
Chordata - ps18
Olfactores - ps19
Vertebrata - ps20
Gnathostomata - ps21
Euteleostomi - ps22
Sarcopterygii - ps23
Tetrapoda - ps24
Amniota - ps25
Mammalia - ps26
Theria - ps27
Placentalia - ps28
Boreoeutheria - ps29
Euarchontoglires - ps30
Euarchonta - ps31
Primates - ps32
Haplorrhini - ps33
Simiiformes - ps34
Catarrhini - ps35
Hominoidea - ps36
Hominidae - ps37
Homininae - ps38
Hominini - ps39
Homo_sapiens - ps40
p-value
1e-17
9e-14
6e-10
5e-06
3e-02
Log-odds
0.5
1.0
1.5
2.0
2.5
3.0
3.5
4.0

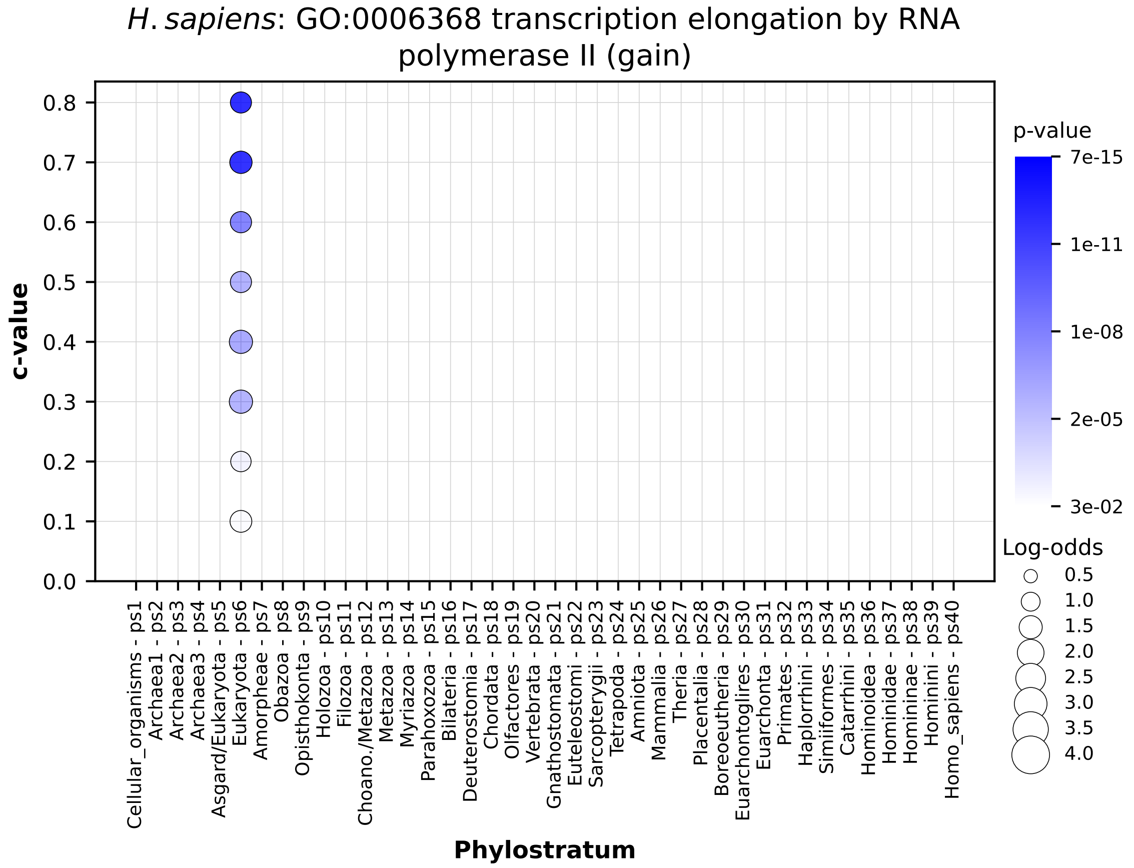

*H. sapiens*: GO:0006368 transcription elongation by RNA polymerase II (gain)
c-value
0.8
0.7
0.6
0.5
0.4
0.3
0.2
0.1
0.0
p-value
7e-15
1e-11
1e-08
2e-05
3e-02
Log-odds
0.5
1.0
1.5
2.0
2.5
3.0
3.5
4.0
Cellular_organisms - ps1
Archaea1 - ps2
Archaea2 - ps3
Archaea3 - ps4
Asgard/Eukaryota - ps5
Eukaryota - ps6
Amorpheae - ps7
Obazoa - ps8
Opisthokonta - ps9
Holozoa - ps10
Filozoa - ps11
Choano./Metazoa - ps12
Metazoa - ps13
Myriazoa - ps14
Parahoxozoa - ps15
Bilateria - ps16
Deuterostomia - ps17
Chordata - ps18
Olfactores - ps19
Vertebrata - ps20
Gnathostomata - ps21
Euteleostomi - ps22
Sarcopterygii - ps23
Tetrapoda - ps24
Amniota - ps25
Mammalia - ps26
Theria - ps27
Placentalia - ps28
Boreoeutheria - ps29
Euarchontoglires - ps30
Euarchonta - ps31
Primates - ps32
Haplorrhini - ps33
Simiiformes - ps34
Catarrhini - ps35
Hominoidea - ps36
Hominidae - ps37
Homininae - ps38
Hominini - ps39
Homo_sapiens - ps40
Phylostratum

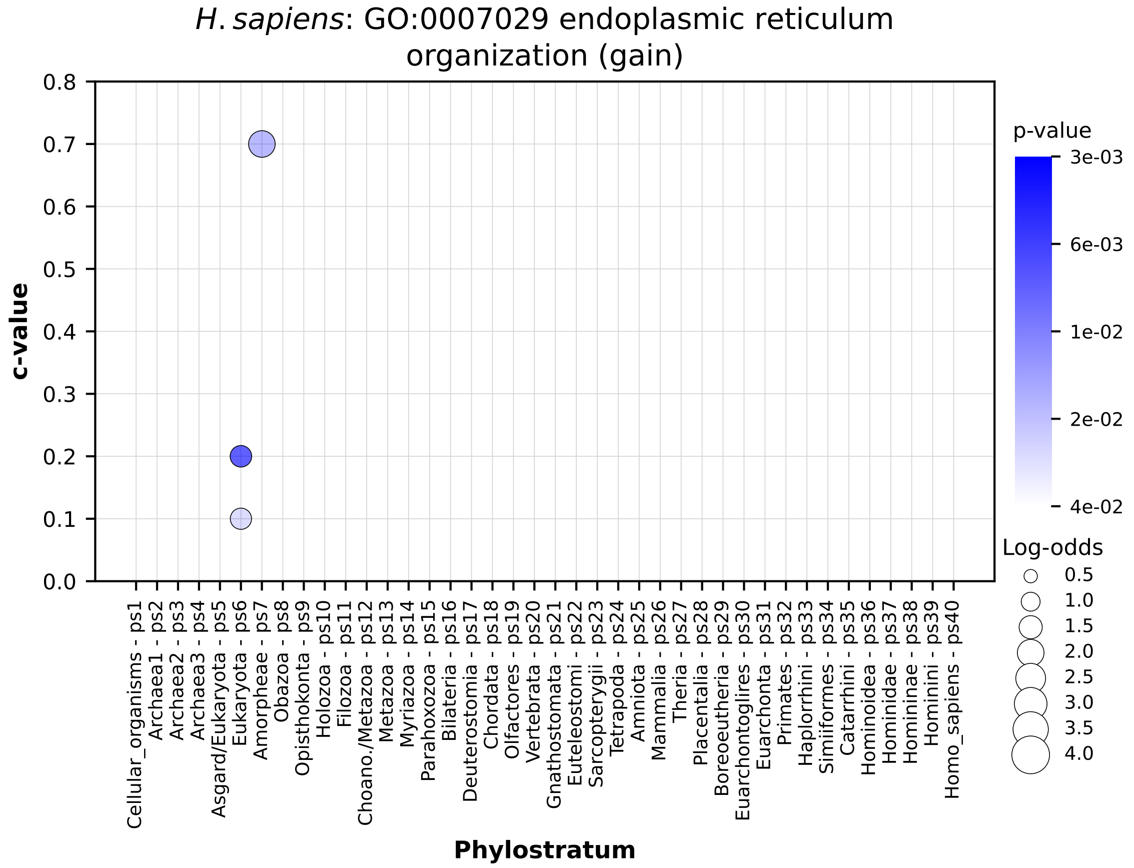

*H. sapiens*: GO:0007029 endoplasmic reticulum organization (gain)
c-value
0.8
0.7
0.6
0.5
0.4
0.3
0.2
0.1
0.0
Phylostratum
Cellular_organisms - ps1
Archaea1 - ps2
Archaea2 - ps3
Archaea3 - ps4
Asgard/Eukaryota - ps5
Eukaryota - ps6
Amorpheae - ps7
Obazoa - ps8
Opisthokonta - ps9
Holozoa - ps10
Filozoa - ps11
Choano./Metazoa - ps12
Metazoa - ps13
Myriazoa - ps14
Parahoxozoa - ps15
Bilateria - ps16
Deuterostomia - ps17
Chordata - ps18
Olfactores - ps19
Vertebrata - ps20
Gnathostomata - ps21
Euteleostomi - ps22
Sarcopterygii - ps23
Tetrapoda - ps24
Amniota - ps25
Mammalia - ps26
Theria - ps27
Placentalia - ps28
Boreoeutheria - ps29
Euarchontoglires - ps30
Euarchonta - ps31
Primates - ps32
Haplorrhini - ps33
Simiiformes - ps34
Catarrhini - ps35
Hominoidea - ps36
Hominidae - ps37
Homininae - ps38
Hominini - ps39
Homo_sapiens - ps40
p-value
3e-03
6e-03
1e-02
2e-02
4e-02
Log-odds
0.5
1.0
1.5
2.0
2.5
3.0
3.5
4.0

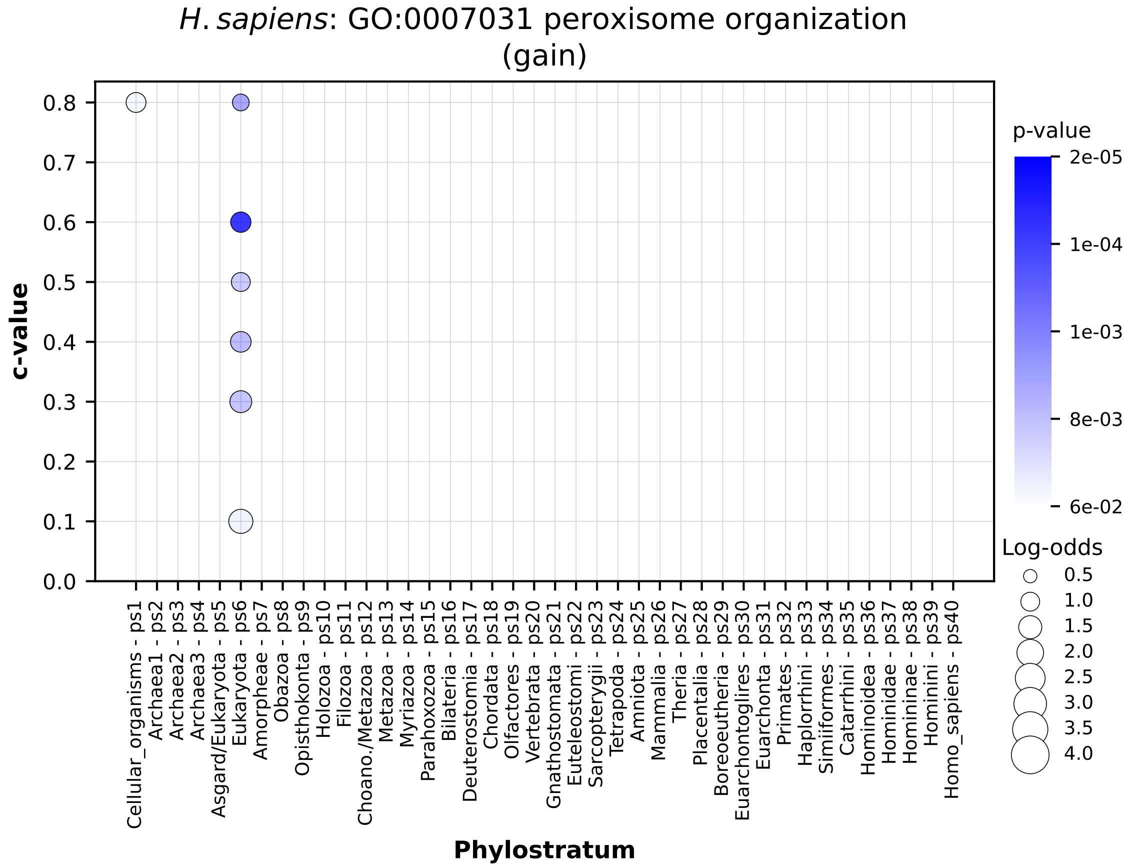

H. sapiens: GO:0007031 peroxisome organization (gain)
c-value
0.0
0.1
0.2
0.3
0.4
0.5
0.6
0.7
0.8
Phylostratum
Cellular_organisms - ps1
Archaea1 - ps2
Archaea2 - ps3
Archaea3 - ps4
Asgard/Eukaryota - ps5
Eukaryota - ps6
Amorpheae - ps7
Obazoa - ps8
Opisthokonta - ps9
Holozoa - ps10
Filozoa - ps11
Choano./Metazoa - ps12
Metazoa - ps13
Myriazoa - ps14
Parahoxozoa - ps15
Bilateria - ps16
Deuterostomia - ps17
Chordata - ps18
Olfactores - ps19
Vertebrata - ps20
Gnathostomata - ps21
Euteleostomi - ps22
Sarcopterygii - ps23
Tetrapoda - ps24
Amniota - ps25
Mammalia - ps26
Theria - ps27
Placentalia - ps28
Boreoeutheria - ps29
Euarchontoglires - ps30
Euarchonta - ps31
Primates - ps32
Haplorrhini - ps33
Simiiformes - ps34
Catarrhini - ps35
Hominoidea - ps36
Hominidae - ps37
Homininae - ps38
Hominini - ps39
Homo_sapiens - ps40
p-value
2e-05
1e-04
1e-03
8e-03
6e-02
Log-odds
0.5
1.0
1.5
2.0
2.5
3.0
3.5
4.0

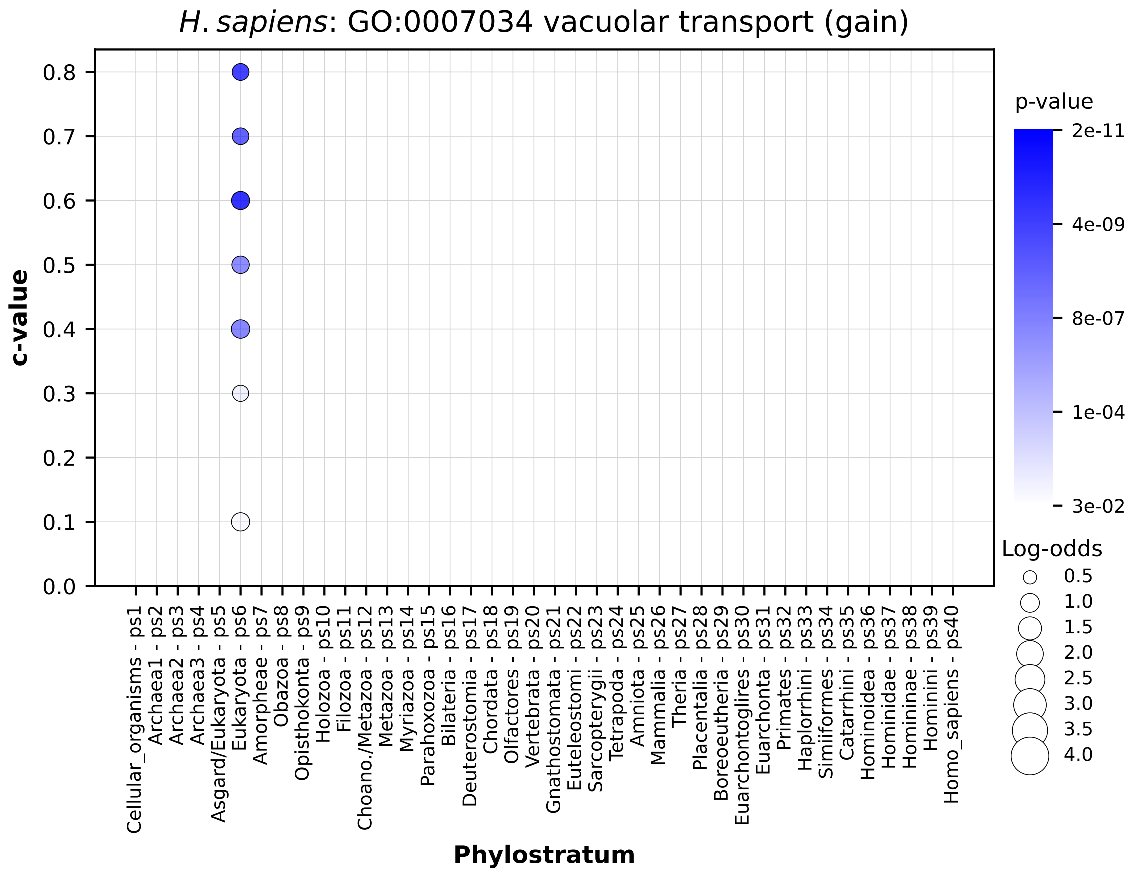

H. sapiens: GO:0007034 vacuolar transport (gain)
c-value
0.0
0.1
0.2
0.3
0.4
0.5
0.6
0.7
0.8
Phylostratum
Cellular_organisms - ps1
Archaea1 - ps2
Archaea2 - ps3
Archaea3 - ps4
Asgard/Eukaryota - ps5
Eukaryota - ps6
Amorpheae - ps7
Obazoa - ps8
Opisthokonta - ps9
Holozoa - ps10
Filozoa - ps11
Choano./Metazoa - ps12
Metazoa - ps13
Myriazoa - ps14
Parahoxozoa - ps15
Bilateria - ps16
Deuterostomia - ps17
Chordata - ps18
Olfactores - ps19
Vertebrata - ps20
Gnathostomata - ps21
Euteleostomi - ps22
Sarcopterygii - ps23
Tetrapoda - ps24
Amniota - ps25
Mammalia - ps26
Theria - ps27
Placentalia - ps28
Boreoeutheria - ps29
Euarchontoglires - ps30
Euarchonta - ps31
Primates - ps32
Haplorrhini - ps33
Simiiformes - ps34
Catarrhini - ps35
Hominoidea - ps36
Hominidae - ps37
Homininae - ps38
Hominini - ps39
Homo_sapiens - ps40
p-value
2e-11
4e-09
8e-07
1e-04
3e-02
Log-odds
0.5
1.0
1.5
2.0
2.5
3.0
3.5
4.0

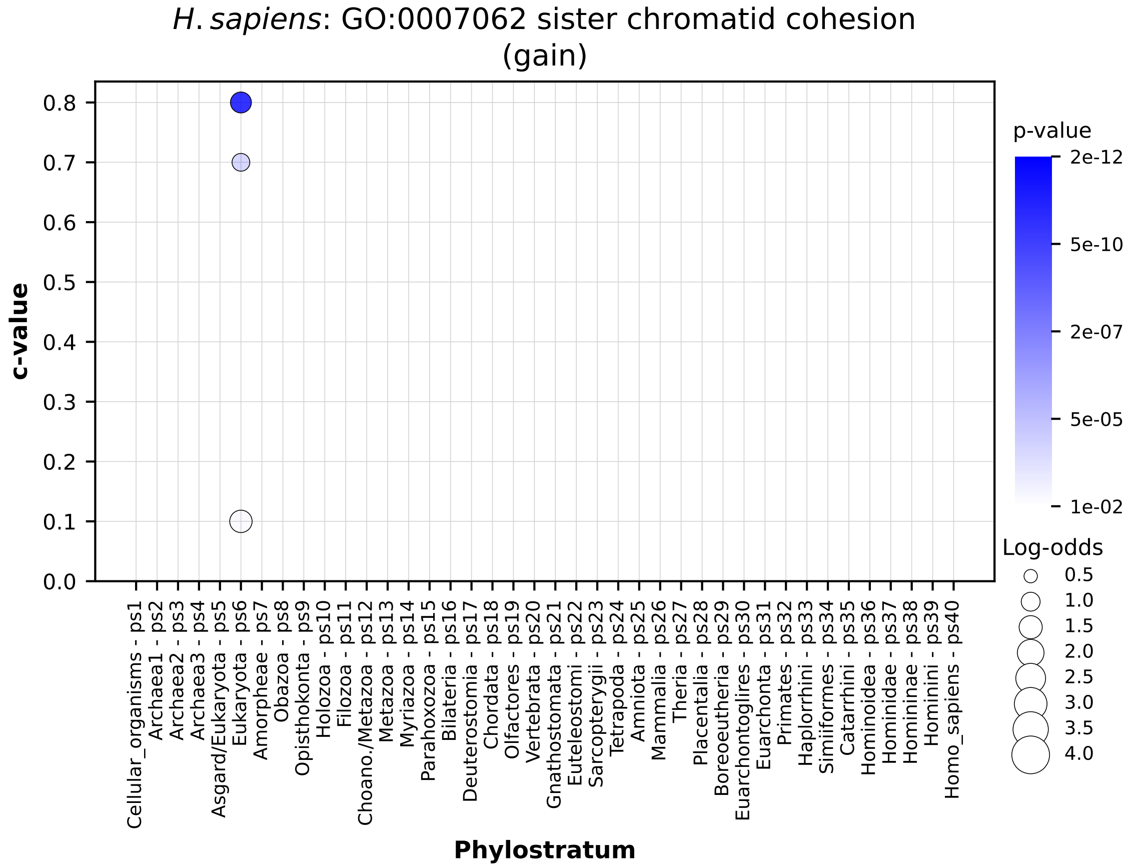

*H. sapiens*: GO:0007062 sister chromatid cohesion
(gain)
c-value
0.0
0.1
0.2
0.3
0.4
0.5
0.6
0.7
0.8
Phylostratum
Cellular_organisms - ps1
Archaea1 - ps2
Archaea2 - ps3
Archaea3 - ps4
Asgard/Eukaryota - ps5
Eukaryota - ps6
Amorpheae - ps7
Obazoa - ps8
Opisthokonta - ps9
Holozoa - ps10
Filozoa - ps11
Choano./Metazoa - ps12
Metazoa - ps13
Myriazoa - ps14
Parahoxozoa - ps15
Bilateria - ps16
Deuterostomia - ps17
Chordata - ps18
Olfactores - ps19
Vertebrata - ps20
Gnathostomata - ps21
Euteleostomi - ps22
Sarcopterygii - ps23
Tetrapoda - ps24
Amniota - ps25
Mammalia - ps26
Theria - ps27
Placentalia - ps28
Boreoeutheria - ps29
Euarchontoglires - ps30
Euarchonta - ps31
Primates - ps32
Haplorrhini - ps33
Simiiformes - ps34
Catarrhini - ps35
Hominoidea - ps36
Hominidae - ps37
Homininae - ps38
Hominini - ps39
Homo_sapiens - ps40
p-value
2e-12
5e-10
2e-07
5e-05
1e-02
Log-odds
0.5
1.0
1.5
2.0
2.5
3.0
3.5
4.0

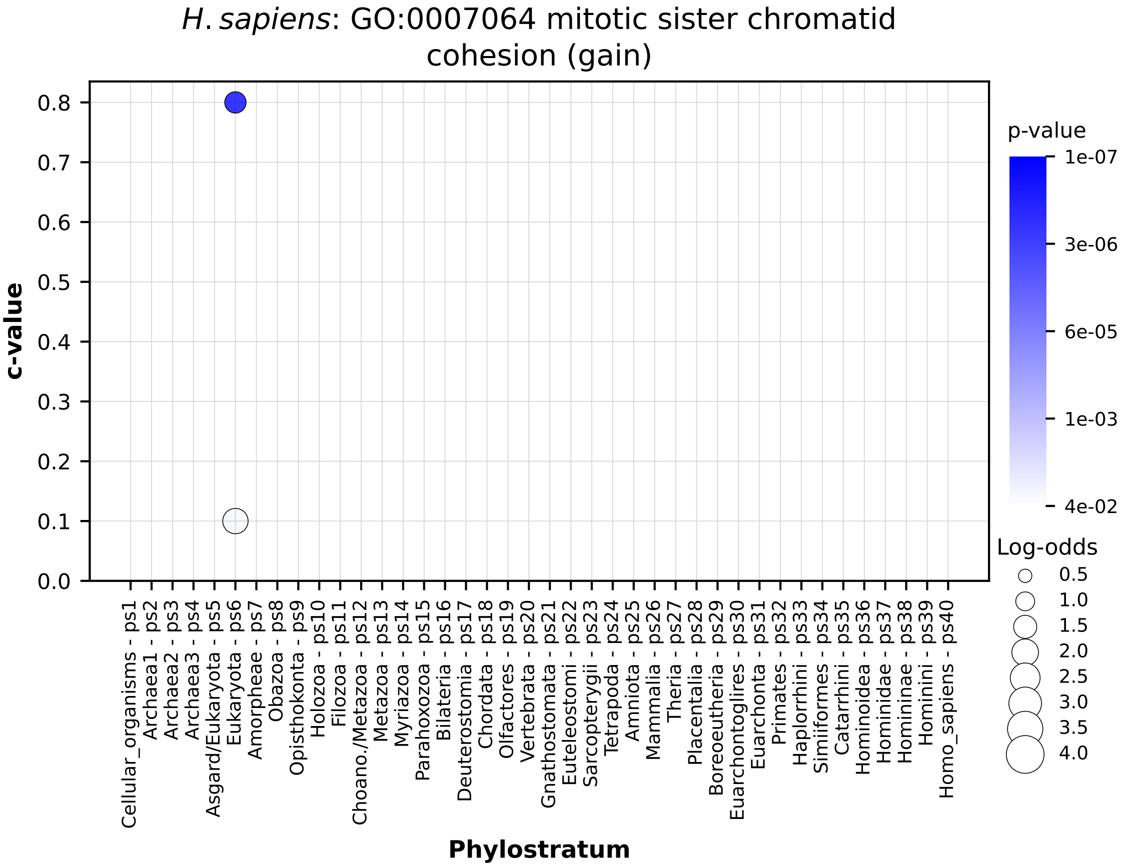

*H. sapiens*: GO:0007064 mitotic sister chromatid cohesion (gain)
c-value
0.8
0.7
0.6
0.5
0.4
0.3
0.2
0.1
0.0
p-value
1e-07
3e-06
6e-05
1e-03
4e-02
Log-odds
0.5
1.0
1.5
2.0
2.5
3.0
3.5
4.0
Cellular_organisms - ps1
Archaea1 - ps2
Archaea2 - ps3
Archaea3 - ps4
Asgard/Eukaryota - ps5
Eukaryota - ps6
Amorpheae - ps7
Obazoa - ps8
Opisthokonta - ps9
Holozoa - ps10
Filozoa - ps11
Choano./Metazoa - ps12
Metazoa - ps13
Myriazoa - ps14
Parahoxozoa - ps15
Bilateria - ps16
Deuterostomia - ps17
Chordata - ps18
Olfactores - ps19
Vertebrata - ps20
Gnathostomata - ps21
Euteleostomi - ps22
Sarcopterygii - ps23
Tetrapoda - ps24
Amniota - ps25
Mammalia - ps26
Theria - ps27
Placentalia - ps28
Boreoeutheria - ps29
Euarchontoglires - ps30
Euarchonta - ps31
Primates - ps32
Haplorrhini - ps33
Simiiformes - ps34
Catarrhini - ps35
Hominoidea - ps36
Hominidae - ps37
Homininae - ps38
Hominini - ps39
Homo_sapiens - ps40
Phylostratum

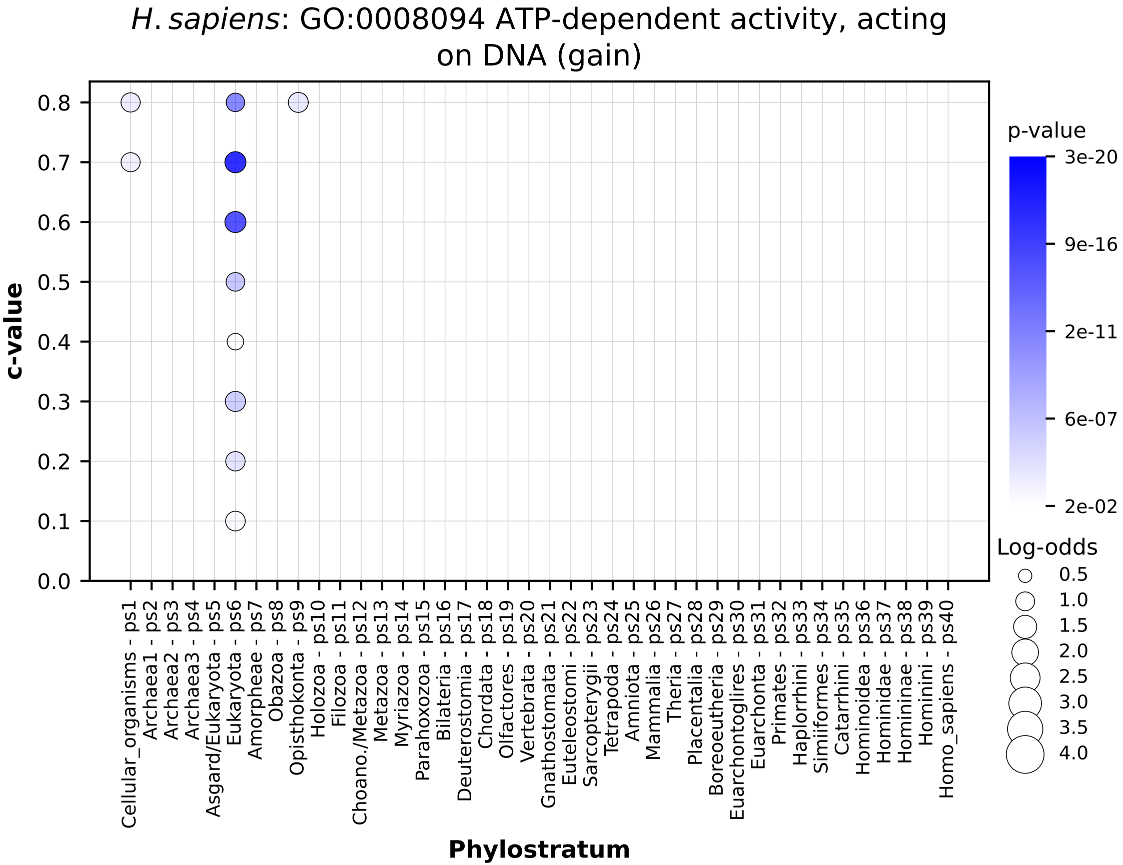
H. sapiens: GO:0008094 ATP-dependent activity, acting on DNA (gain)
c-value
0.0
0.1
0.2
0.3
0.4
0.5
0.6
0.7
0.8
Phylostratum
Cellular_organisms - ps1
Archaea1 - ps2
Archaea2 - ps3
Archaea3 - ps4
Asgard/Eukaryota - ps5
Eukaryota - ps6
Amorpheae - ps7
Obazoa - ps8
Opisthokonta - ps9
Holozoa - ps10
Filozoa - ps11
Choano./Metazoa - ps12
Metazoa - ps13
Myriazoa - ps14
Parahoxozoa - ps15
Bilateria - ps16
Deuterostomia - ps17
Chordata - ps18
Olfactores - ps19
Vertebrata - ps20
Gnathostomata - ps21
Euteleostomi - ps22
Sarcopterygii - ps23
Tetrapoda - ps24
Amniota - ps25
Mammalia - ps26
Theria - ps27
Placentalia - ps28
Boreoeutheria - ps29
Euarchontoglires - ps30
Euarchonta - ps31
Primates - ps32
Haplorrhini - ps33
Simiiformes - ps34
Catarrhini - ps35
Hominoidea - ps36
Hominidae - ps37
Homininae - ps38
Hominini - ps39
Homo_sapiens - ps40
p-value
3e-20
9e-16
2e-11
6e-07
2e-02
Log-odds
0.5
1.0
1.5
2.0
2.5
3.0
3.5
4.0

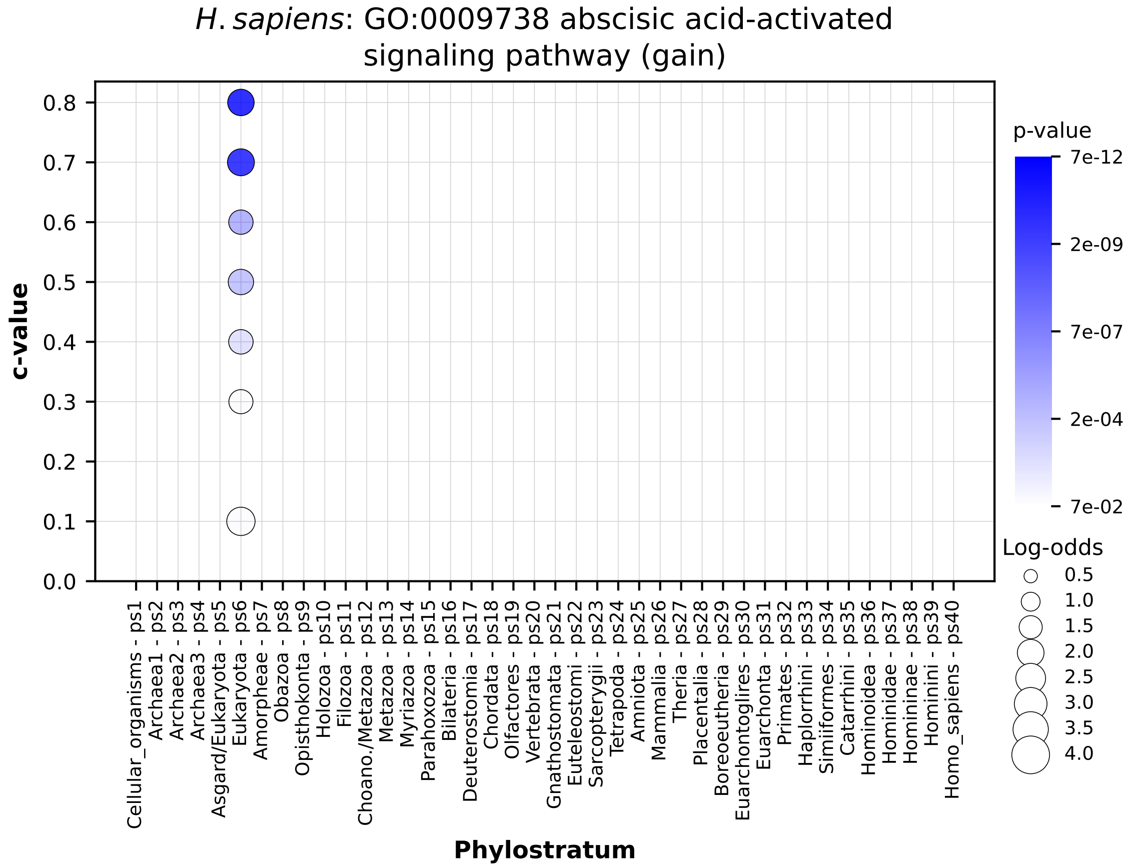

*H. sapiens*: GO:0009738 abscisic acid-activated signaling pathway (gain)
c-value
0.8
0.7
0.6
0.5
0.4
0.3
0.2
0.1
0.0
p-value
7e-12
2e-09
7e-07
2e-04
7e-02
Log-odds
0.5
1.0
1.5
2.0
2.5
3.0
3.5
4.0
Cellular_organisms - ps1
Archaea1 - ps2
Archaea2 - ps3
Archaea3 - ps4
Asgard/Eukaryota - ps5
Eukaryota - ps6
Amorpheae - ps7
Obazoa - ps8
Opisthokonta - ps9
Holozoa - ps10
Filozoa - ps11
Choano./Metazoa - ps12
Metazoa - ps13
Myriazoa - ps14
Parahoxozoa - ps15
Bilateria - ps16
Deuterostomia - ps17
Chordata - ps18
Olfactores - ps19
Vertebrata - ps20
Gnathostomata - ps21
Euteleostomi - ps22
Sarcopterygii - ps23
Tetrapoda - ps24
Amniota - ps25
Mammalia - ps26
Theria - ps27
Placentalia - ps28
Boreoeutheria - ps29
Euarchontoglires - ps30
Euarchonta - ps31
Primates - ps32
Haplorrhini - ps33
Simiiformes - ps34
Catarrhini - ps35
Hominoidea - ps36
Hominidae - ps37
Homininae - ps38
Hominini - ps39
Homo_sapiens - ps40
Phylostratum

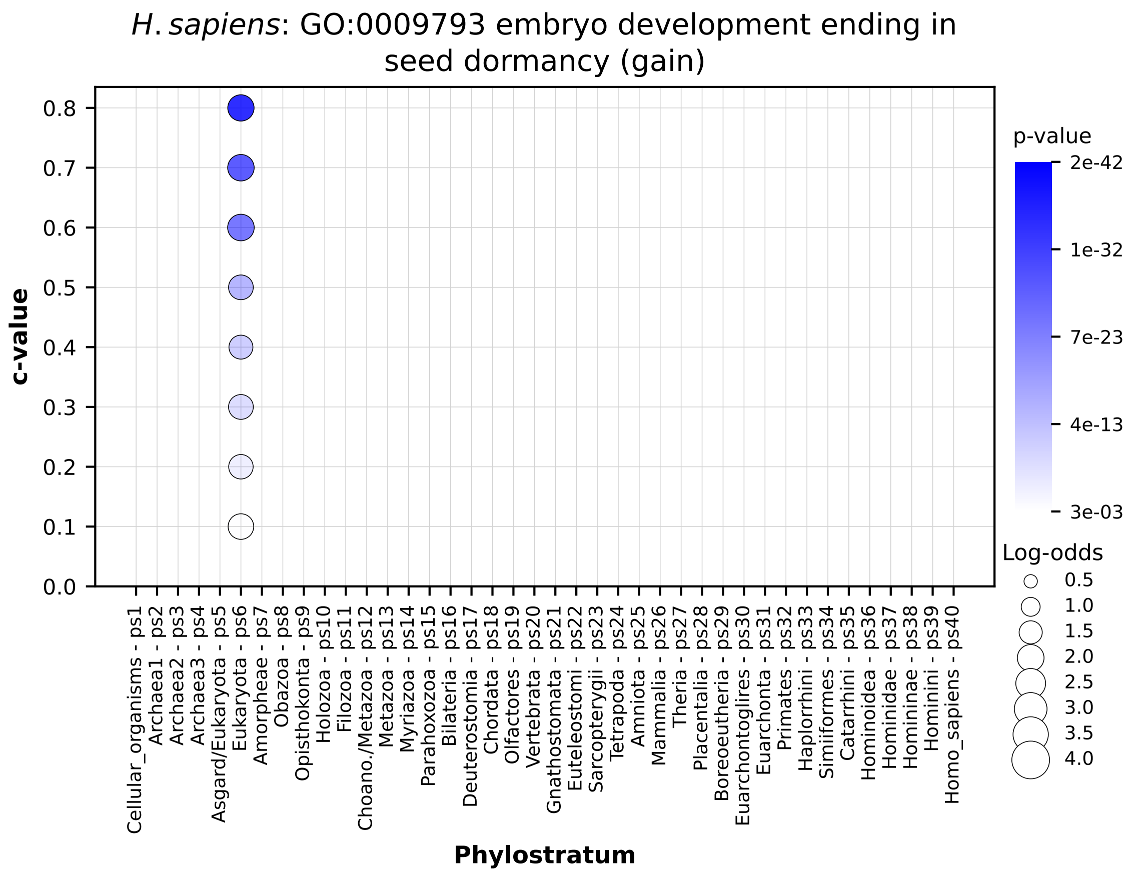

H. sapiens: GO:0009793 embryo development ending in seed dormancy (gain)
c-value
0.0
0.1
0.2
0.3
0.4
0.5
0.6
0.7
0.8
Phylostratum
Cellular_organisms - ps1
Archaea1 - ps2
Archaea2 - ps3
Archaea3 - ps4
Asgard/Eukaryota - ps5
Eukaryota - ps6
Amorpheae - ps7
Obazoa - ps8
Opisthokonta - ps9
Holozoa - ps10
Filozoa - ps11
Choano./Metazoa - ps12
Metazoa - ps13
Myriazoa - ps14
Parahoxozoa - ps15
Bilateria - ps16
Deuterostomia - ps17
Chordata - ps18
Olfactores - ps19
Vertebrata - ps20
Gnathostomata - ps21
Euteleostomi - ps22
Sarcopterygii - ps23
Tetrapoda - ps24
Amniota - ps25
Mammalia - ps26
Theria - ps27
Placentalia - ps28
Boreoeutheria - ps29
Euarchontoglires - ps30
Euarchonta - ps31
Primates - ps32
Haplorrhini - ps33
Simiiformes - ps34
Catarrhini - ps35
Hominoidea - ps36
Hominidae - ps37
Homininae - ps38
Hominini - ps39
Homo_sapiens - ps40
p-value
2e-42
1e-32
7e-23
4e-13
3e-03
Log-odds
0.5
1.0
1.5
2.0
2.5
3.0
3.5
4.0

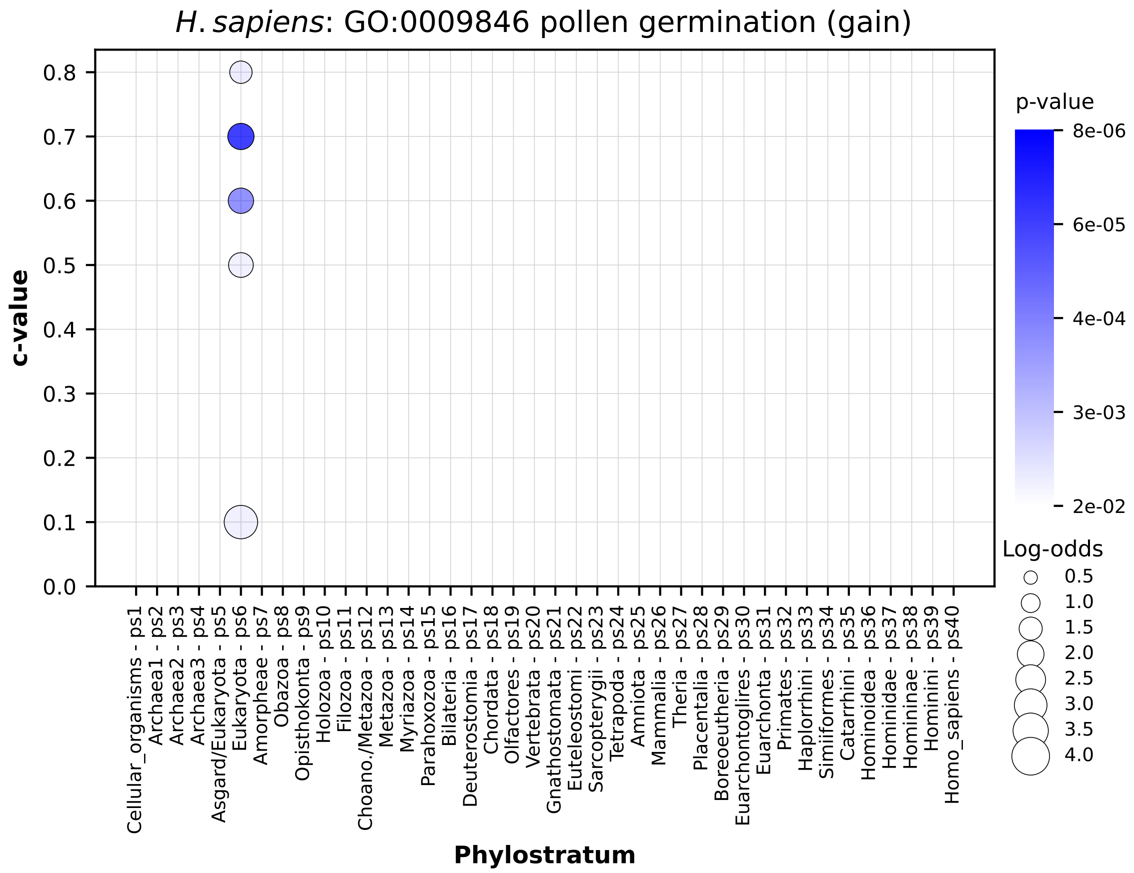

H. sapiens: GO:0009846 pollen germination (gain)
c-value
0.0
0.1
0.2
0.3
0.4
0.5
0.6
0.7
0.8
p-value
8e-06
6e-05
4e-04
3e-03
2e-02
Log-odds
0.5
1.0
1.5
2.0
2.5
3.0
3.5
4.0
Cellular_organisms - ps1
Archaea1 - ps2
Archaea2 - ps3
Archaea3 - ps4
Asgard/Eukaryota - ps5
Eukaryota - ps6
Amorpheae - ps7
Obazoa - ps8
Opisthokonta - ps9
Holozoa - ps10
Filozoa - ps11
Choano./Metazoa - ps12
Metazoa - ps13
Myriazoa - ps14
Parahoxozoa - ps15
Bilateria - ps16
Deuterostomia - ps17
Chordata - ps18
Olfactores - ps19
Vertebrata - ps20
Gnathostomata - ps21
Euteleostomi - ps22
Sarcopterygii - ps23
Tetrapoda - ps24
Amniota - ps25
Mammalia - ps26
Theria - ps27
Placentalia - ps28
Boreoeutheria - ps29
Euarchontoglires - ps30
Euarchonta - ps31
Primates - ps32
Haplorrhini - ps33
Simiiformes - ps34
Catarrhini - ps35
Hominoidea - ps36
Hominidae - ps37
Homininae - ps38
Hominini - ps39
Homo_sapiens - ps40
Phylostratum

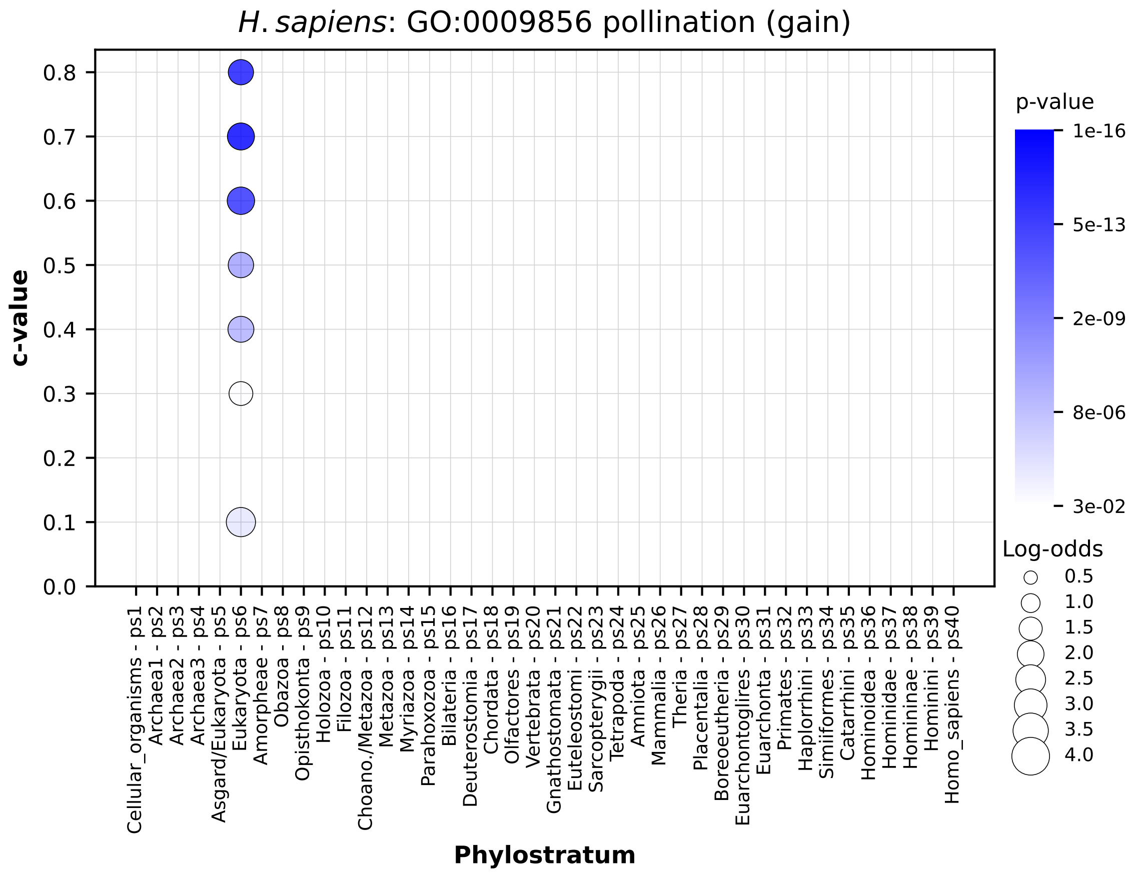

*H. sapiens*: GO:0009856 pollination (gain)
c-value
0.0
0.1
0.2
0.3
0.4
0.5
0.6
0.7
0.8
Phylostratum
Cellular_organisms - ps1
Archaea1 - ps2
Archaea2 - ps3
Archaea3 - ps4
Asgard/Eukaryota - ps5
Eukaryota - ps6
Amorpheae - ps7
Obazoa - ps8
Opisthokonta - ps9
Holozoa - ps10
Filozoa - ps11
Choano./Metazoa - ps12
Metazoa - ps13
Myriazoa - ps14
Parahoxozoa - ps15
Bilateria - ps16
Deuterostomia - ps17
Chordata - ps18
Olfactores - ps19
Vertebrata - ps20
Gnathostomata - ps21
Euteleostomi - ps22
Sarcopterygii - ps23
Tetrapoda - ps24
Amniota - ps25
Mammalia - ps26
Theria - ps27
Placentalia - ps28
Boreoeutheria - ps29
Euarchontoglires - ps30
Euarchonta - ps31
Primates - ps32
Haplorrhini - ps33
Simiiformes - ps34
Catarrhini - ps35
Hominoidea - ps36
Hominidae - ps37
Homininae - ps38
Hominini - ps39
Homo_sapiens - ps40
p-value
1e-16
5e-13
2e-09
8e-06
3e-02
Log-odds
0.5
1.0
1.5
2.0
2.5
3.0
3.5
4.0

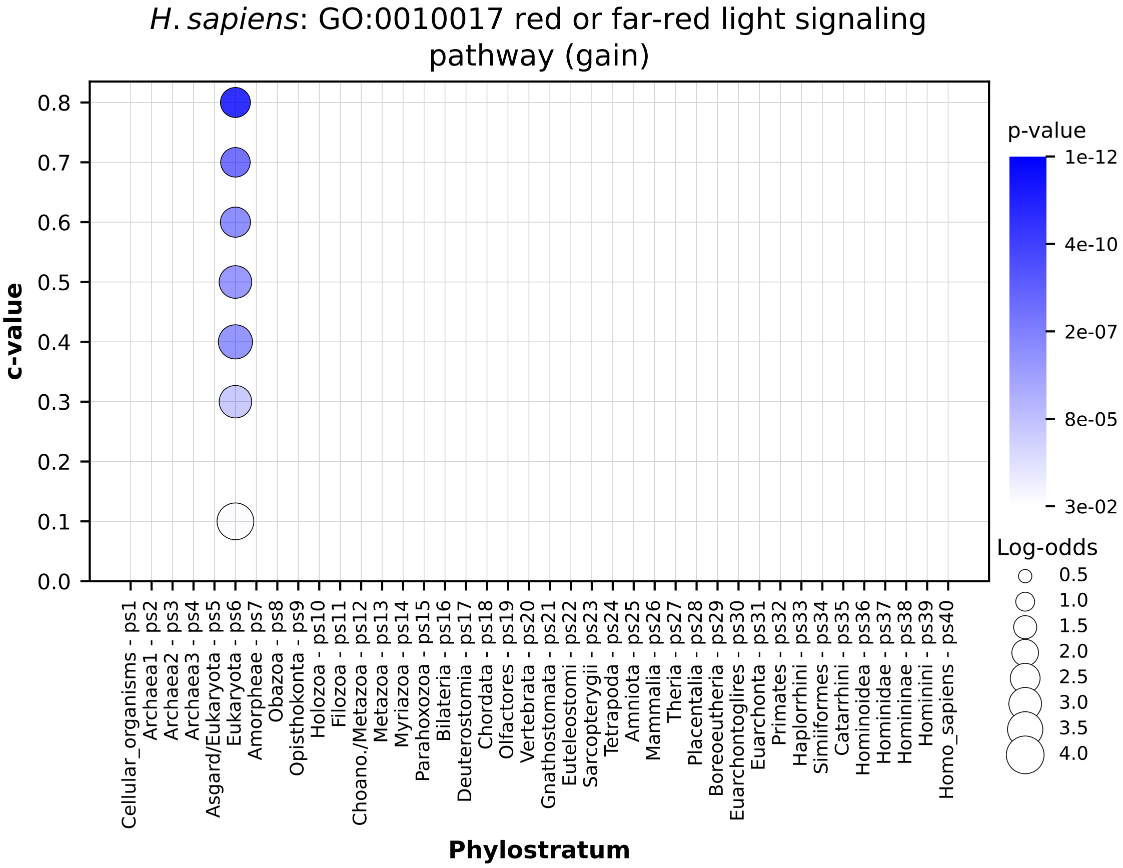
H. sapiens: GO:0010017 red or far-red light signaling pathway (gain)
c-value
0.0
0.1
0.2
0.3
0.4
0.5
0.6
0.7
0.8
Phylostratum
Cellular_organisms - ps1
Archaea1 - ps2
Archaea2 - ps3
Archaea3 - ps4
Asgard/Eukaryota - ps5
Eukaryota - ps6
Amorpheae - ps7
Obazoa - ps8
Opisthokonta - ps9
Holozoa - ps10
Filozoa - ps11
Choano./Metazoa - ps12
Metazoa - ps13
Myriazoa - ps14
Parahoxozoa - ps15
Bilateria - ps16
Deuterostomia - ps17
Chordata - ps18
Olfactores - ps19
Vertebrata - ps20
Gnathostomata - ps21
Euteleostomi - ps22
Sarcopterygii - ps23
Tetrapoda - ps24
Amniota - ps25
Mammalia - ps26
Theria - ps27
Placentalia - ps28
Boreoeutheria - ps29
Euarchontoglires - ps30
Euarchonta - ps31
Primates - ps32
Haplorrhini - ps33
Simiiformes - ps34
Catarrhini - ps35
Hominoidea - ps36
Hominidae - ps37
Homininae - ps38
Hominini - ps39
Homo_sapiens - ps40
p-value
1e-12
4e-10
2e-07
8e-05
3e-02
Log-odds
0.5
1.0
1.5
2.0
2.5
3.0
3.5
4.0

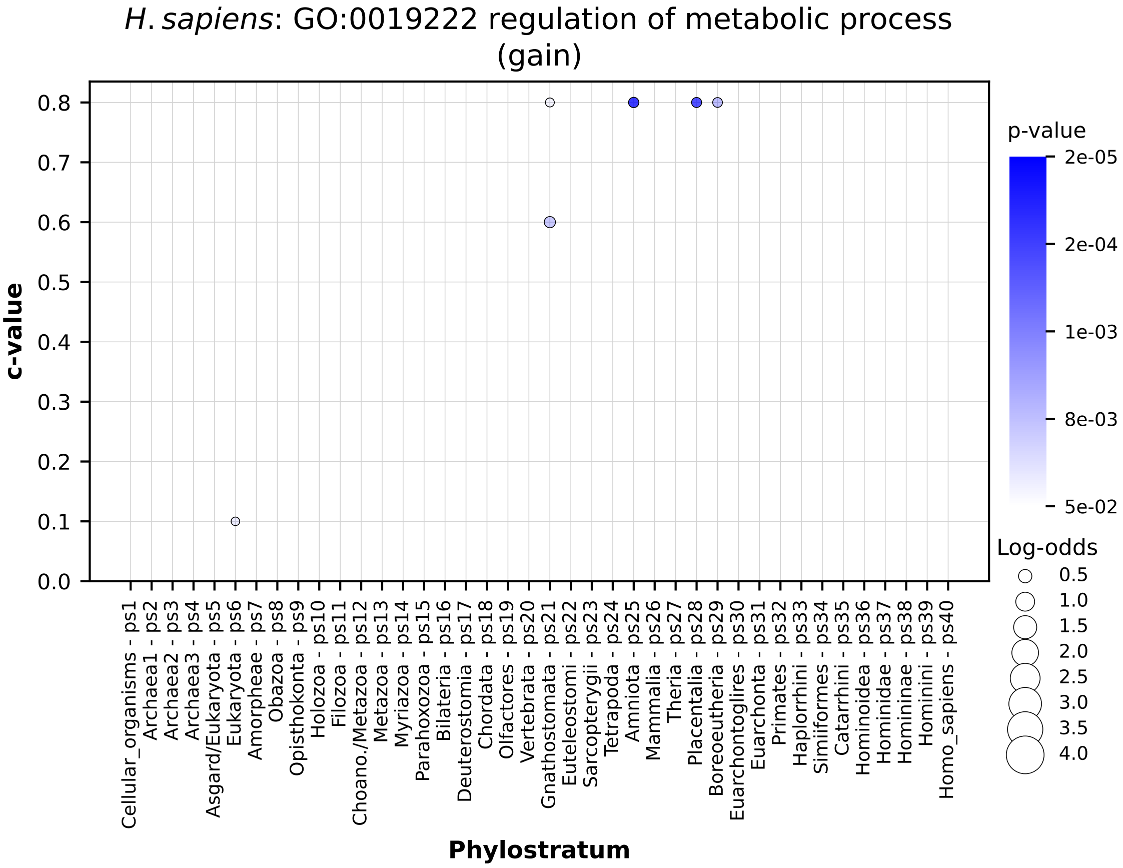

H. sapiens: GO:0019222 regulation of metabolic process
(gain)
c-value
0.0
0.1
0.2
0.3
0.4
0.5
0.6
0.7
0.8
Phylostratum
Cellular_organisms - ps1
Archaea1 - ps2
Archaea2 - ps3
Archaea3 - ps4
Asgard/Eukaryota - ps5
Eukaryota - ps6
Amorpheae - ps7
Obazoa - ps8
Opisthokonta - ps9
Holozoa - ps10
Filozoa - ps11
Choano./Metazoa - ps12
Metazoa - ps13
Myriazoa - ps14
Parahoxozoa - ps15
Bilateria - ps16
Deuterostomia - ps17
Chordata - ps18
Olfactores - ps19
Vertebrata - ps20
Gnathostomata - ps21
Euteleostomi - ps22
Sarcopterygii - ps23
Tetrapoda - ps24
Amniota - ps25
Mammalia - ps26
Theria - ps27
Placentalia - ps28
Boreoeutheria - ps29
Euarchontoglires - ps30
Euarchonta - ps31
Primates - ps32
Haplorrhini - ps33
Simiiformes - ps34
Catarrhini - ps35
Hominoidea - ps36
Hominidae - ps37
Homininae - ps38
Hominini - ps39
Homo_sapiens - ps40
p-value
2e-05
2e-04
1e-03
8e-03
5e-02
Log-odds
0.5
1.0
1.5
2.0
2.5
3.0
3.5
4.0

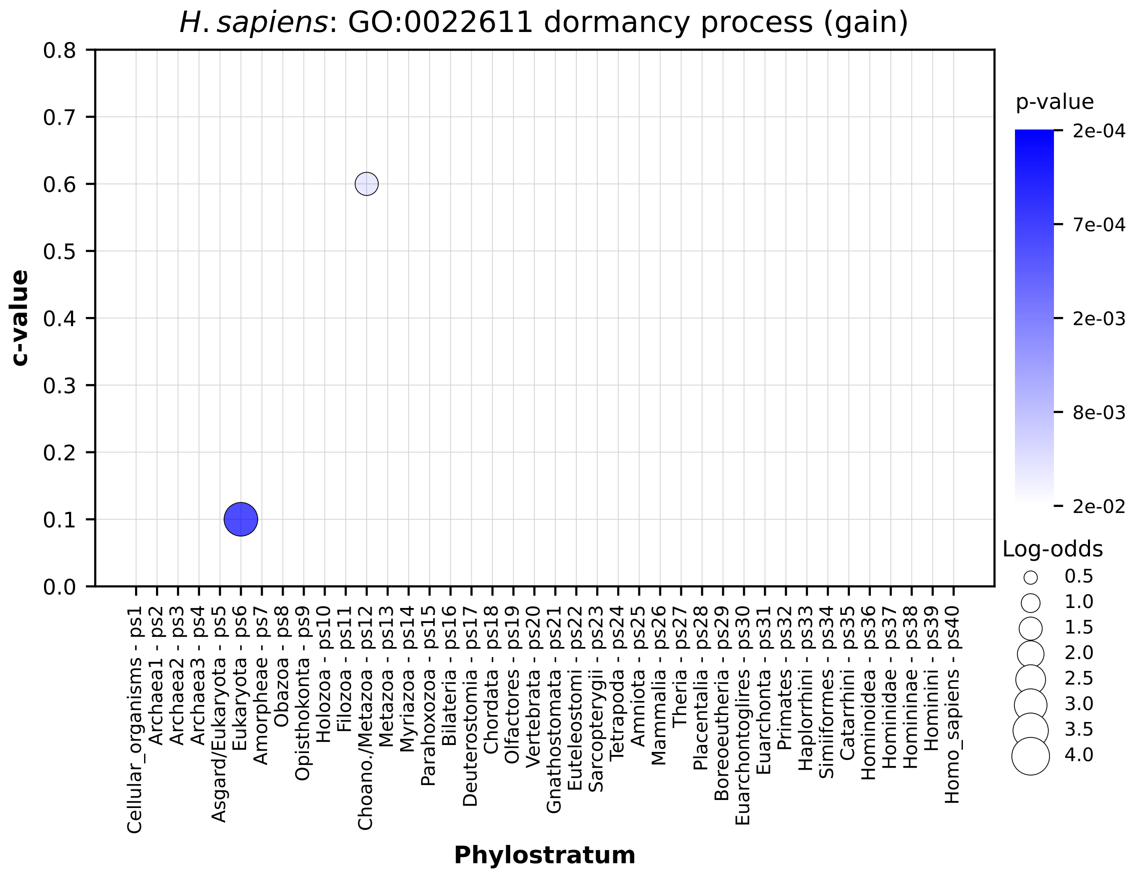

H. sapiens: GO:0022611 dormancy process (gain)
c-value
0.8
0.7
0.6
0.5
0.4
0.3
0.2
0.1
0.0
Phylostratum
Cellular_organisms - ps1
Archaea1 - ps2
Archaea2 - ps3
Archaea3 - ps4
Asgard/Eukaryota - ps5
Eukaryota - ps6
Amorpheae - ps7
Obazoa - ps8
Opisthokonta - ps9
Holozoa - ps10
Filozoa - ps11
Choano./Metazoa - ps12
Metazoa - ps13
Myriazoa - ps14
Parahoxozoa - ps15
Bilateria - ps16
Deuterostomia - ps17
Chordata - ps18
Olfactores - ps19
Vertebrata - ps20
Gnathostomata - ps21
Euteleostomi - ps22
Sarcopterygii - ps23
Tetrapoda - ps24
Amniota - ps25
Mammalia - ps26
Theria - ps27
Placentalia - ps28
Boreoeutheria - ps29
Euarchontoglires - ps30
Euarchonta - ps31
Primates - ps32
Haplorrhini - ps33
Simiiformes - ps34
Catarrhini - ps35
Hominoidea - ps36
Hominidae - ps37
Homininae - ps38
Hominini - ps39
Homo_sapiens - ps40
p-value
2e-04
7e-04
2e-03
8e-03
2e-02
Log-odds
0.5
1.0
1.5
2.0
2.5
3.0
3.5
4.0

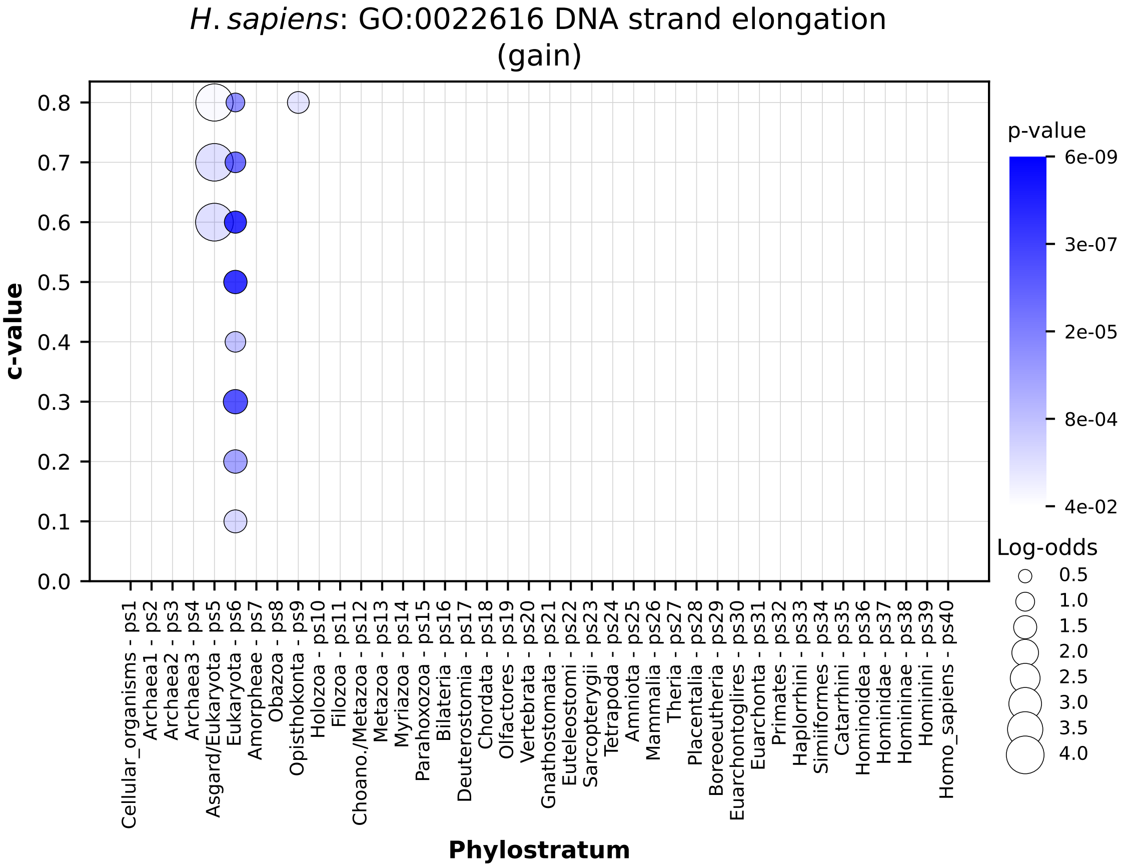

*H. sapiens*: GO:0022616 DNA strand elongation
(gain)
c-value
0.0
0.1
0.2
0.3
0.4
0.5
0.6
0.7
0.8
Phylostratum
Cellular_organisms - ps1
Archaea1 - ps2
Archaea2 - ps3
Archaea3 - ps4
Asgard/Eukaryota - ps5
Eukaryota - ps6
Amorpheae - ps7
Obazoa - ps8
Opisthokonta - ps9
Holozoa - ps10
Filozoa - ps11
Choano./Metazoa - ps12
Metazoa - ps13
Myriazoa - ps14
Parahoxozoa - ps15
Bilateria - ps16
Deuterostomia - ps17
Chordata - ps18
Olfactores - ps19
Vertebrata - ps20
Gnathostomata - ps21
Euteleostomi - ps22
Sarcopterygii - ps23
Tetrapoda - ps24
Amniota - ps25
Mammalia - ps26
Theria - ps27
Placentalia - ps28
Boreoeutheria - ps29
Euarchontoglires - ps30
Euarchonta - ps31
Primates - ps32
Haplorrhini - ps33
Simiiformes - ps34
Catarrhini - ps35
Hominoidea - ps36
Hominidae - ps37
Homininae - ps38
Hominini - ps39
Homo_sapiens - ps40
p-value
6e-09
3e-07
2e-05
8e-04
4e-02
Log-odds
0.5
1.0
1.5
2.0
2.5
3.0
3.5
4.0

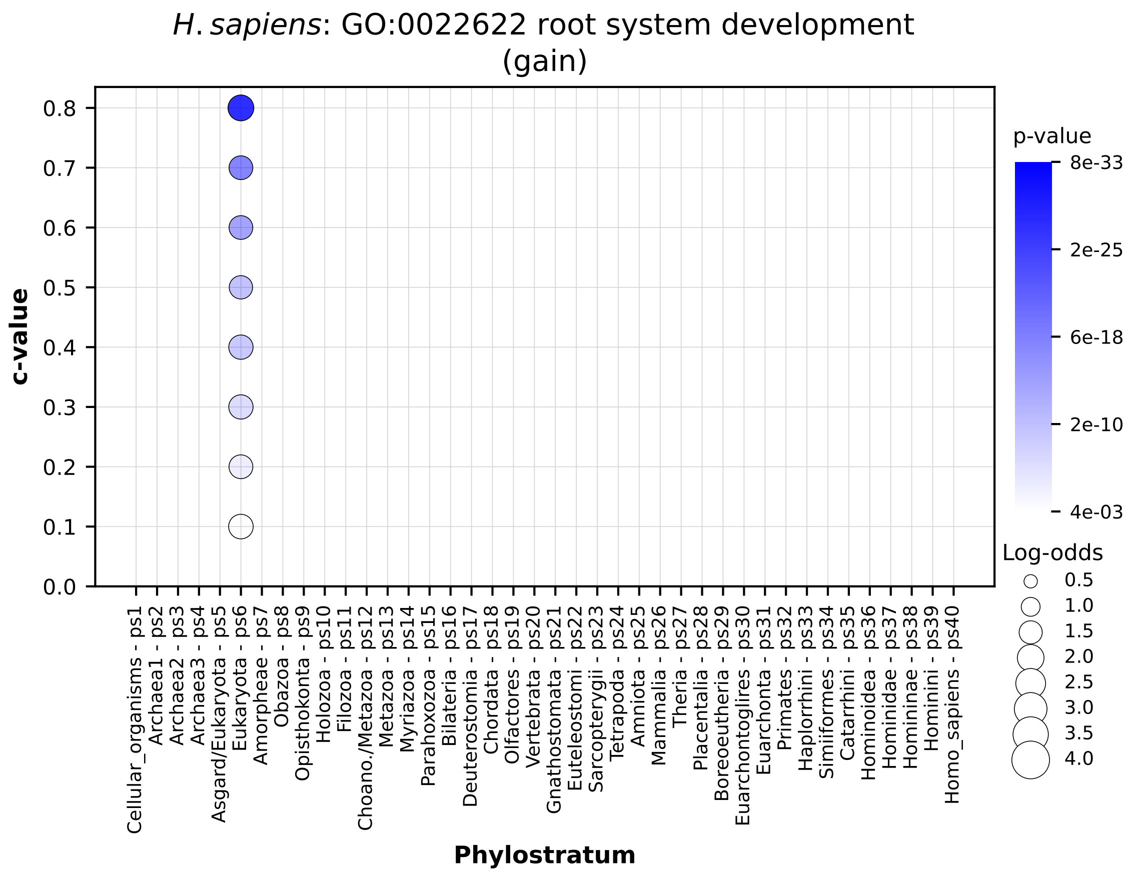

H. sapiens: GO:0022622 root system development
(gain)
c-value
0.0
0.1
0.2
0.3
0.4
0.5
0.6
0.7
0.8
p-value
8e-33
2e-25
6e-18
2e-10
4e-03
Log-odds
0.5
1.0
1.5
2.0
2.5
3.0
3.5
4.0
Cellular_organisms - ps1
Archaea1 - ps2
Archaea2 - ps3
Archaea3 - ps4
Asgard/Eukaryota - ps5
Eukaryota - ps6
Amorpheae - ps7
Obazoa - ps8
Opisthokonta - ps9
Holozoa - ps10
Filozoa - ps11
Choano./Metazoa - ps12
Metazoa - ps13
Myriazoa - ps14
Parahoxozoa - ps15
Bilateria - ps16
Deuterostomia - ps17
Chordata - ps18
Olfactores - ps19
Vertebrata - ps20
Gnathostomata - ps21
Euteleostomi - ps22
Sarcopterygii - ps23
Tetrapoda - ps24
Amniota - ps25
Mammalia - ps26
Theria - ps27
Placentalia - ps28
Boreoeutheria - ps29
Euarchontoglires - ps30
Euarchonta - ps31
Primates - ps32
Haplorrhini - ps33
Simiiformes - ps34
Catarrhini - ps35
Hominoidea - ps36
Hominidae - ps37
Homininae - ps38
Hominini - ps39
Homo_sapiens - ps40
Phylostratum

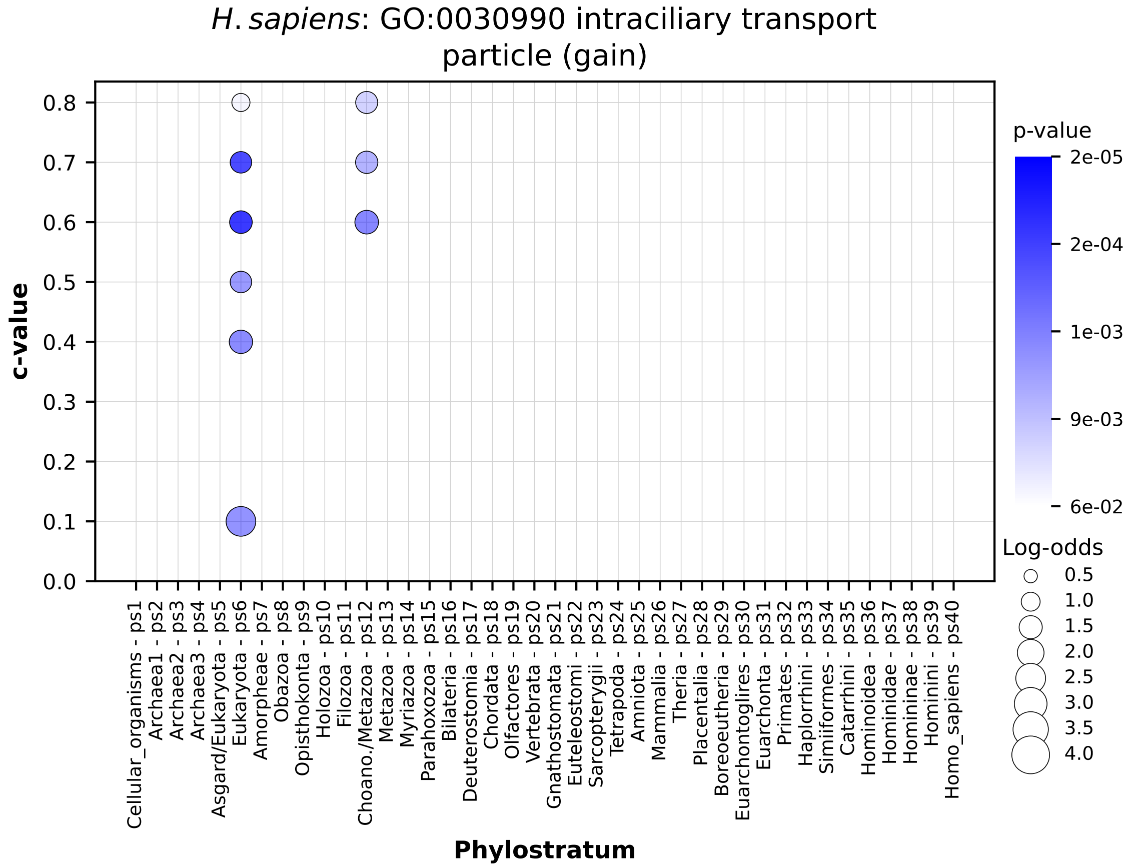

H. sapiens: GO:0030990 intraciliary transport particle (gain)
c-value
0.0
0.1
0.2
0.3
0.4
0.5
0.6
0.7
0.8
p-value
2e-05
2e-04
1e-03
9e-03
6e-02
Log-odds
0.5
1.0
1.5
2.0
2.5
3.0
3.5
4.0
Cellular_organisms - ps1
Archaea1 - ps2
Archaea2 - ps3
Archaea3 - ps4
Asgard/Eukaryota - ps5
Eukaryota - ps6
Amorpheae - ps7
Obazoa - ps8
Opisthokonta - ps9
Holozoa - ps10
Filozoa - ps11
Choano./Metazoa - ps12
Metazoa - ps13
Myriazoa - ps14
Parahoxozoa - ps15
Bilateria - ps16
Deuterostomia - ps17
Chordata - ps18
Olfactores - ps19
Vertebrata - ps20
Gnathostomata - ps21
Euteleostomi - ps22
Sarcopterygii - ps23
Tetrapoda - ps24
Amniota - ps25
Mammalia - ps26
Theria - ps27
Placentalia - ps28
Boreoeutheria - ps29
Euarchontoglires - ps30
Euarchonta - ps31
Primates - ps32
Haplorrhini - ps33
Simiiformes - ps34
Catarrhini - ps35
Hominoidea - ps36
Hominidae - ps37
Homininae - ps38
Hominini - ps39
Homo_sapiens - ps40
Phylostratum

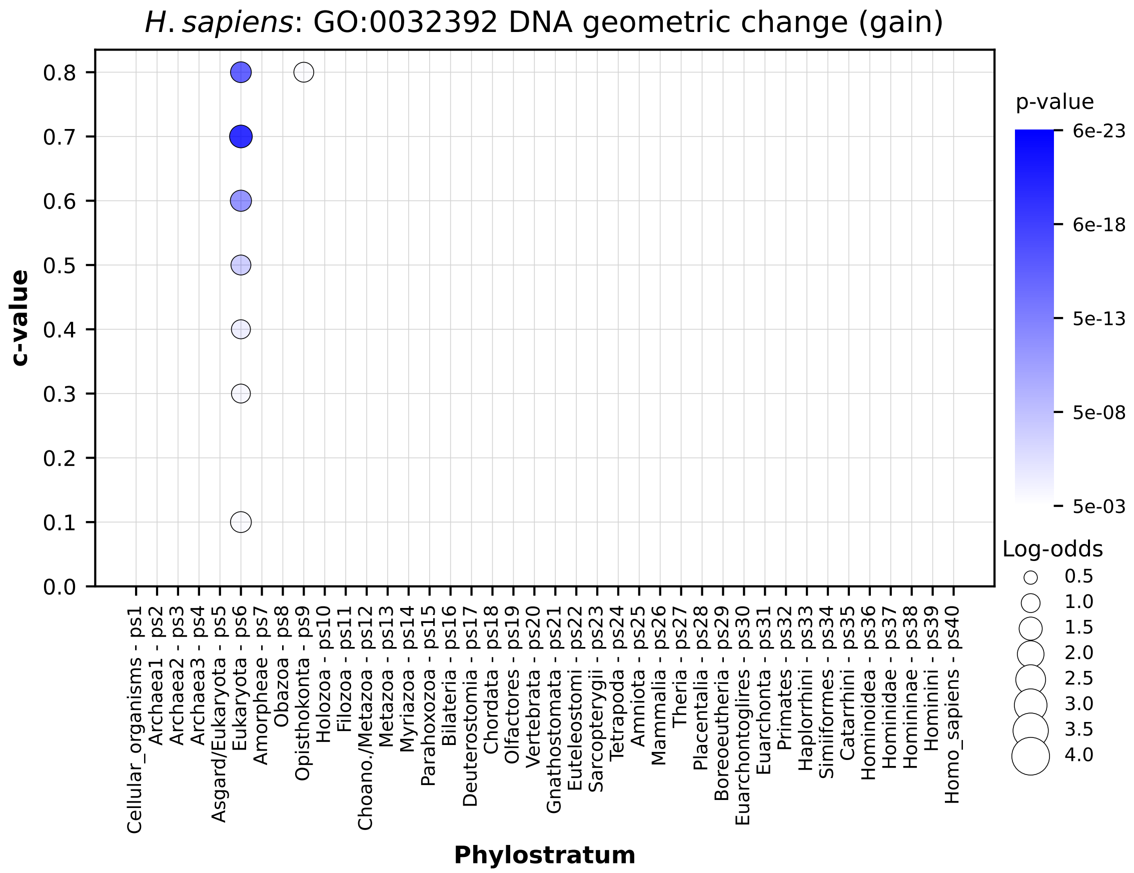

*H. sapiens*: GO:0032392 DNA geometric change (gain)
c-value
0.0
0.1
0.2
0.3
0.4
0.5
0.6
0.7
0.8
Phylostratum
Cellular_organisms - ps1
Archaea1 - ps2
Archaea2 - ps3
Archaea3 - ps4
Asgard/Eukaryota - ps5
Eukaryota - ps6
Amorpheae - ps7
Obazoa - ps8
Opisthokonta - ps9
Holozoa - ps10
Filozoa - ps11
Choano./Metazoa - ps12
Metazoa - ps13
Myriazoa - ps14
Parahoxozoa - ps15
Bilateria - ps16
Deuterostomia - ps17
Chordata - ps18
Olfactores - ps19
Vertebrata - ps20
Gnathostomata - ps21
Euteleostomi - ps22
Sarcopterygii - ps23
Tetrapoda - ps24
Amniota - ps25
Mammalia - ps26
Theria - ps27
Placentalia - ps28
Boreoeutheria - ps29
Euarchontoglires - ps30
Euarchonta - ps31
Primates - ps32
Haplorrhini - ps33
Simiiformes - ps34
Catarrhini - ps35
Hominoidea - ps36
Hominidae - ps37
Homininae - ps38
Hominini - ps39
Homo_sapiens - ps40
p-value
6e-23
6e-18
5e-13
5e-08
5e-03
Log-odds
0.5
1.0
1.5
2.0
2.5
3.0
3.5
4.0

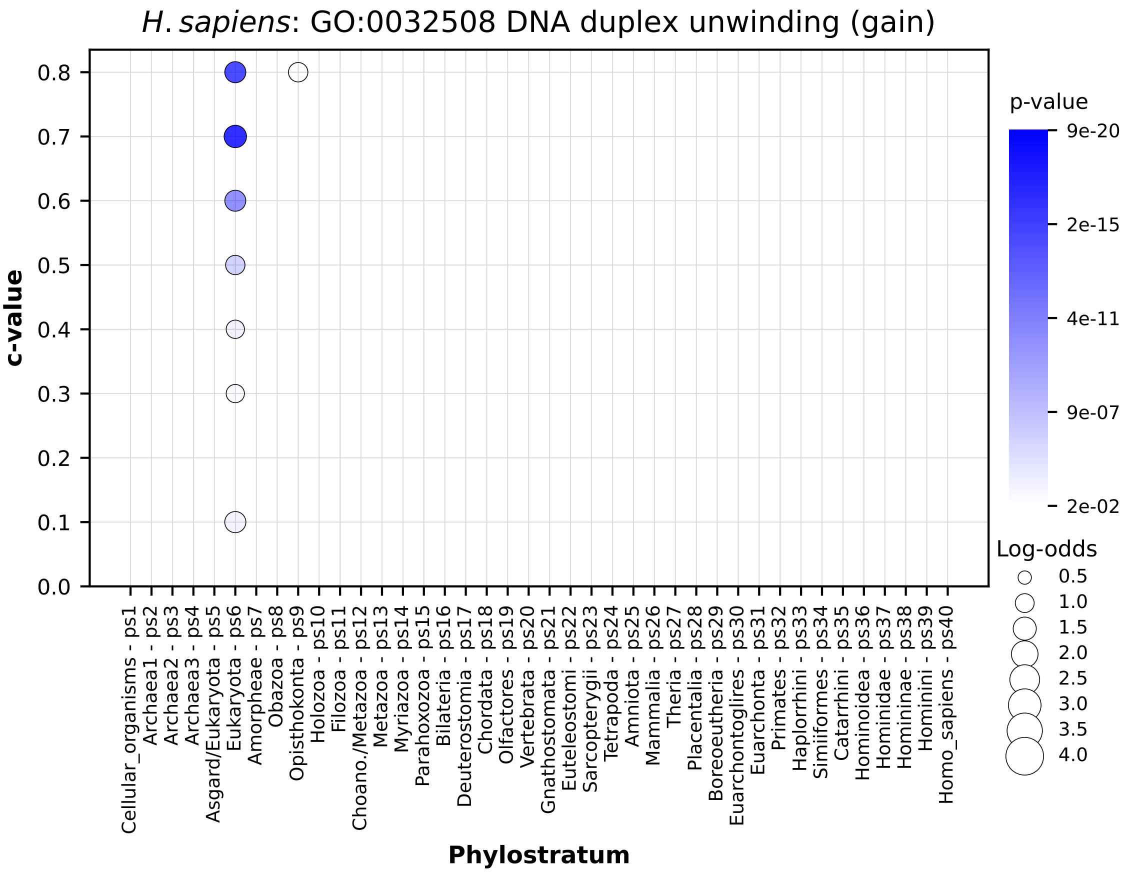
H. sapiens: GO:0032508 DNA duplex unwinding (gain)
c-value
0.0
0.1
0.2
0.3
0.4
0.5
0.6
0.7
0.8
Phylostratum
Cellular_organisms - ps1
Archaea1 - ps2
Archaea2 - ps3
Archaea3 - ps4
Asgard/Eukaryota - ps5
Eukaryota - ps6
Amorpheae - ps7
Obazoa - ps8
Opisthokonta - ps9
Holozoa - ps10
Filozoa - ps11
Choano./Metazoa - ps12
Metazoa - ps13
Myriazoa - ps14
Parahoxozoa - ps15
Bilateria - ps16
Deuterostomia - ps17
Chordata - ps18
Olfactores - ps19
Vertebrata - ps20
Gnathostomata - ps21
Euteleostomi - ps22
Sarcopterygii - ps23
Tetrapoda - ps24
Amniota - ps25
Mammalia - ps26
Theria - ps27
Placentalia - ps28
Boreoeutheria - ps29
Euarchontoglires - ps30
Euarchonta - ps31
Primates - ps32
Haplorrhini - ps33
Simiiformes - ps34
Catarrhini - ps35
Hominoidea - ps36
Hominidae - ps37
Homininae - ps38
Hominini - ps39
Homo_sapiens - ps40
p-value
9e-20
2e-15
4e-11
9e-07
2e-02
Log-odds
0.5
1.0
1.5
2.0
2.5
3.0
3.5
4.0

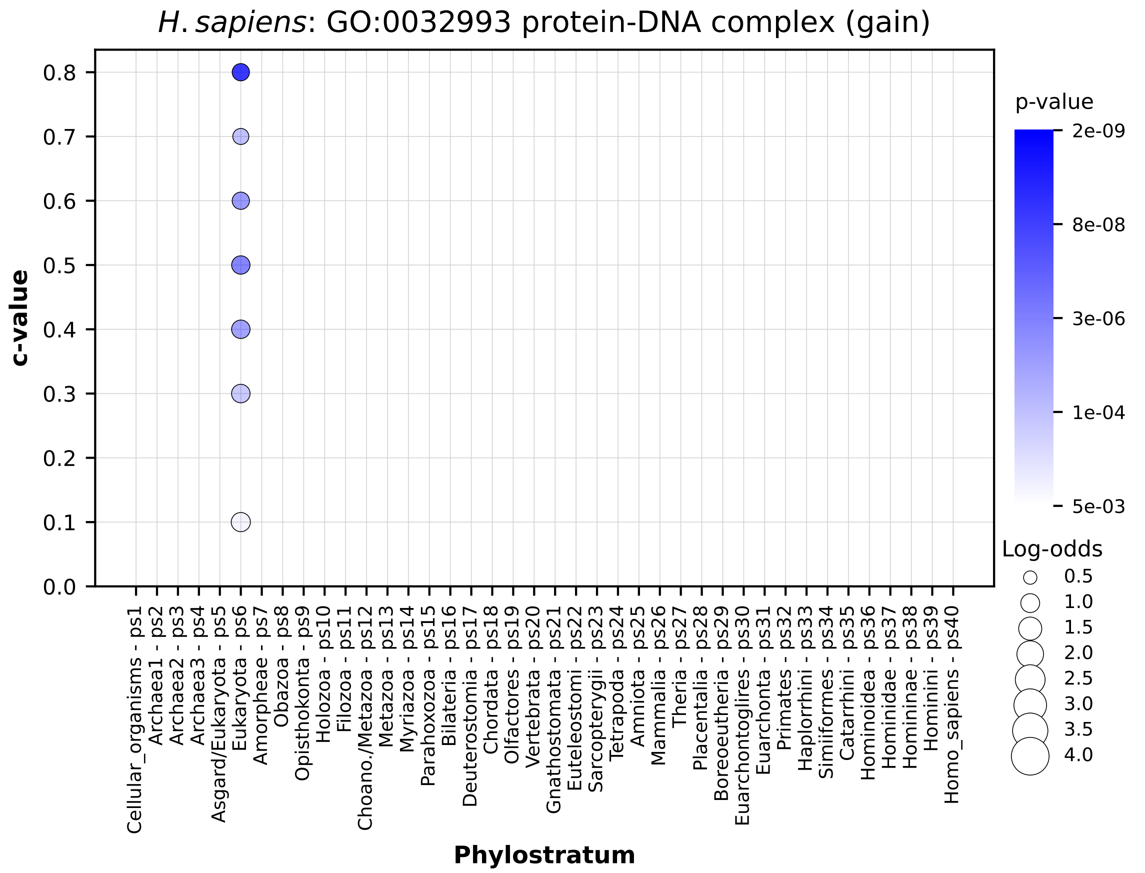
H. sapiens: GO:0032993 protein-DNA complex (gain)
c-value
0.0
0.1
0.2
0.3
0.4
0.5
0.6
0.7
0.8
Phylostratum
Cellular_organisms - ps1
Archaea1 - ps2
Archaea2 - ps3
Archaea3 - ps4
Asgard/Eukaryota - ps5
Eukaryota - ps6
Amorpheae - ps7
Obazoa - ps8
Opisthokonta - ps9
Holozoa - ps10
Filozoa - ps11
Choano./Metazoa - ps12
Metazoa - ps13
Myriazoa - ps14
Parahoxozoa - ps15
Bilateria - ps16
Deuterostomia - ps17
Chordata - ps18
Olfactores - ps19
Vertebrata - ps20
Gnathostomata - ps21
Euteleostomi - ps22
Sarcopterygii - ps23
Tetrapoda - ps24
Amniota - ps25
Mammalia - ps26
Theria - ps27
Placentalia - ps28
Boreoeutheria - ps29
Euarchontoglires - ps30
Euarchonta - ps31
Primates - ps32
Haplorrhini - ps33
Simiiformes - ps34
Catarrhini - ps35
Hominoidea - ps36
Hominidae - ps37
Homininae - ps38
Hominini - ps39
Homo_sapiens - ps40
p-value
2e-09
8e-08
3e-06
1e-04
5e-03
Log-odds
0.5
1.0
1.5
2.0
2.5
3.0
3.5
4.0

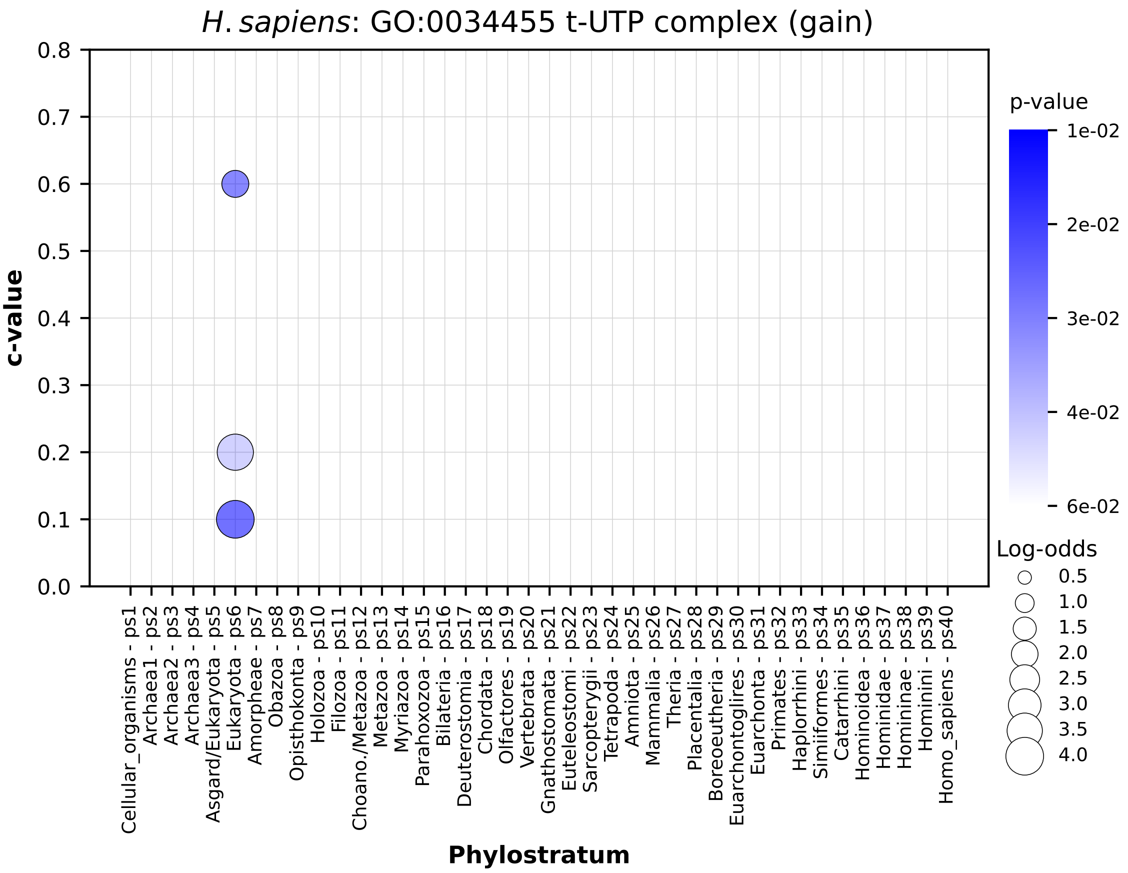

H. sapiens: GO:0034455 t-UTP complex (gain)
c-value
0.0
0.1
0.2
0.3
0.4
0.5
0.6
0.7
0.8
Phylostratum
Cellular_organisms - ps1
Archaea1 - ps2
Archaea2 - ps3
Archaea3 - ps4
Asgard/Eukaryota - ps5
Eukaryota - ps6
Amorpheae - ps7
Obazoa - ps8
Opisthokonta - ps9
Holozoa - ps10
Filozoa - ps11
Choano./Metazoa - ps12
Metazoa - ps13
Myriazoa - ps14
Parahoxozoa - ps15
Bilateria - ps16
Deuterostomia - ps17
Chordata - ps18
Olfactores - ps19
Vertebrata - ps20
Gnathostomata - ps21
Euteleostomi - ps22
Sarcopterygii - ps23
Tetrapoda - ps24
Amniota - ps25
Mammalia - ps26
Theria - ps27
Placentalia - ps28
Boreoeutheria - ps29
Euarchontoglires - ps30
Euarchonta - ps31
Primates - ps32
Haplorrhini - ps33
Simiiformes - ps34
Catarrhini - ps35
Hominoidea - ps36
Hominidae - ps37
Homininae - ps38
Hominini - ps39
Homo_sapiens - ps40
p-value
1e-02
2e-02
3e-02
4e-02
6e-02
Log-odds
0.5
1.0
1.5
2.0
2.5
3.0
3.5
4.0

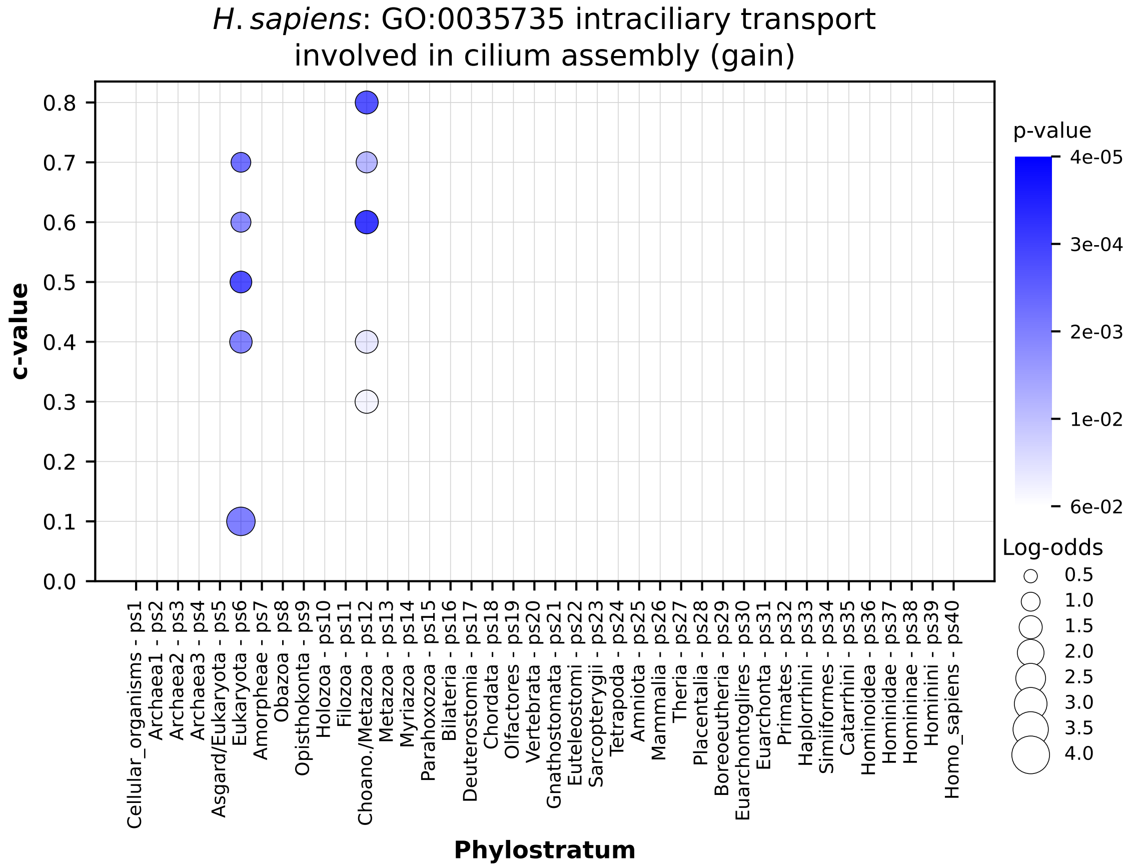

H. sapiens: GO:0035735 intraciliary transport involved in cilium assembly (gain)
c-value
0.0
0.1
0.2
0.3
0.4
0.5
0.6
0.7
0.8
Phylostratum
Cellular_organisms - ps1
Archaea1 - ps2
Archaea2 - ps3
Archaea3 - ps4
Asgard/Eukaryota - ps5
Eukaryota - ps6
Amorpheae - ps7
Obazoa - ps8
Opisthokonta - ps9
Holozoa - ps10
Filozoa - ps11
Choano./Metazoa - ps12
Metazoa - ps13
Myriazoa - ps14
Parahoxozoa - ps15
Bilateria - ps16
Deuterostomia - ps17
Chordata - ps18
Olfactores - ps19
Vertebrata - ps20
Gnathostomata - ps21
Euteleostomi - ps22
Sarcopterygii - ps23
Tetrapoda - ps24
Amniota - ps25
Mammalia - ps26
Theria - ps27
Placentalia - ps28
Boreoeutheria - ps29
Euarchontoglires - ps30
Euarchonta - ps31
Primates - ps32
Haplorrhini - ps33
Simiiformes - ps34
Catarrhini - ps35
Hominoidea - ps36
Hominidae - ps37
Homininae - ps38
Hominini - ps39
Homo_sapiens - ps40
p-value
4e-05
3e-04
2e-03
1e-02
6e-02
Log-odds
0.5
1.0
1.5
2.0
2.5
3.0
3.5
4.0

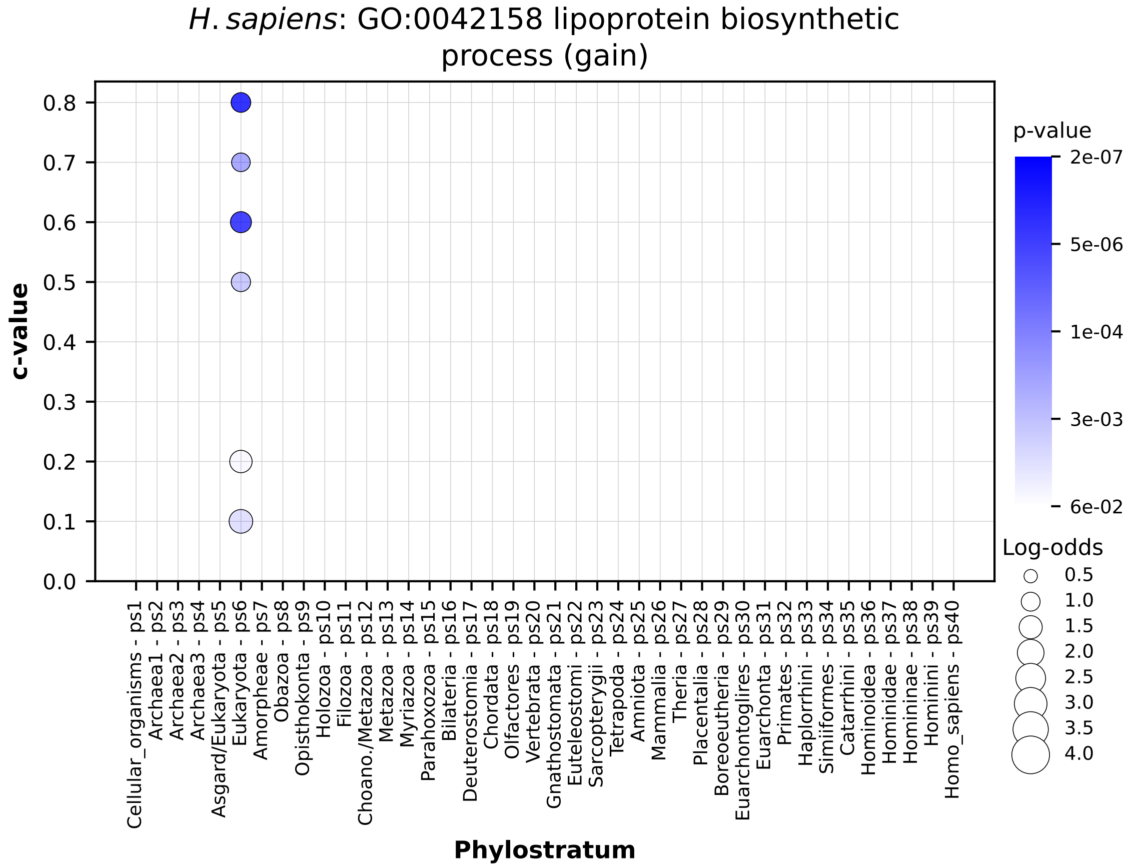

H. sapiens: GO:0042158 lipoprotein biosynthetic process (gain)
c-value
0.0
0.1
0.2
0.3
0.4
0.5
0.6
0.7
0.8
Phylostratum
Cellular_organisms - ps1
Archaea1 - ps2
Archaea2 - ps3
Archaea3 - ps4
Asgard/Eukaryota - ps5
Eukaryota - ps6
Amorpheae - ps7
Obazoa - ps8
Opisthokonta - ps9
Holozoa - ps10
Filozoa - ps11
Choano./Metazoa - ps12
Metazoa - ps13
Myriazoa - ps14
Parahoxozoa - ps15
Bilateria - ps16
Deuterostomia - ps17
Chordata - ps18
Olfactores - ps19
Vertebrata - ps20
Gnathostomata - ps21
Euteleostomi - ps22
Sarcopterygii - ps23
Tetrapoda - ps24
Amniota - ps25
Mammalia - ps26
Theria - ps27
Placentalia - ps28
Boreoeutheria - ps29
Euarchontoglires - ps30
Euarchonta - ps31
Primates - ps32
Haplorrhini - ps33
Simiiformes - ps34
Catarrhini - ps35
Hominoidea - ps36
Hominidae - ps37
Homininae - ps38
Hominini - ps39
Homo_sapiens - ps40
p-value
2e-07
5e-06
1e-04
3e-03
6e-02
Log-odds
0.5
1.0
1.5
2.0
2.5
3.0
3.5
4.0

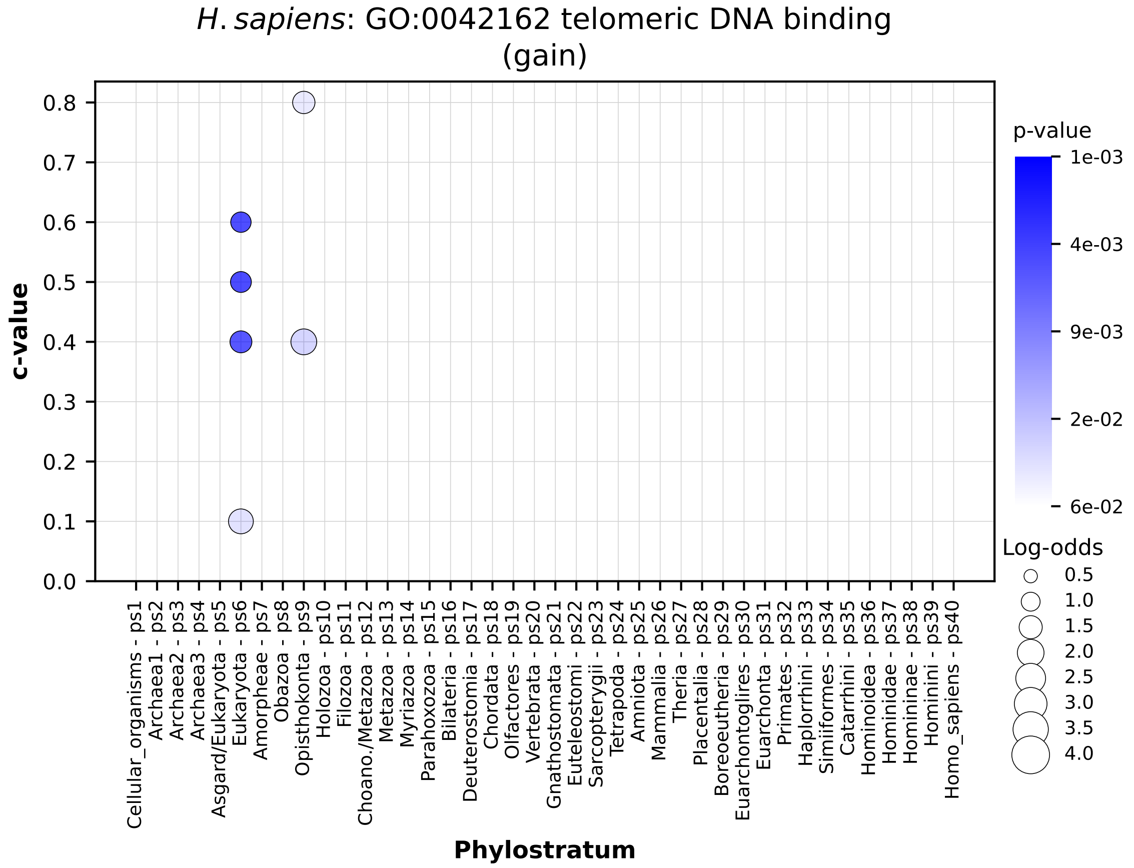

H. sapiens: GO:0042162 telomeric DNA binding
(gain)
c-value
0.0
0.1
0.2
0.3
0.4
0.5
0.6
0.7
0.8
Phylostratum
Cellular_organisms - ps1
Archaea1 - ps2
Archaea2 - ps3
Archaea3 - ps4
Asgard/Eukaryota - ps5
Eukaryota - ps6
Amorpheae - ps7
Obazoa - ps8
Opisthokonta - ps9
Holozoa - ps10
Filozoa - ps11
Choano./Metazoa - ps12
Metazoa - ps13
Myriazoa - ps14
Parahoxozoa - ps15
Bilateria - ps16
Deuterostomia - ps17
Chordata - ps18
Olfactores - ps19
Vertebrata - ps20
Gnathostomata - ps21
Euteleostomi - ps22
Sarcopterygii - ps23
Tetrapoda - ps24
Amniota - ps25
Mammalia - ps26
Theria - ps27
Placentalia - ps28
Boreoeutheria - ps29
Euarchontoglires - ps30
Euarchonta - ps31
Primates - ps32
Haplorrhini - ps33
Simiiformes - ps34
Catarrhini - ps35
Hominoidea - ps36
Hominidae - ps37
Homininae - ps38
Hominini - ps39
Homo_sapiens - ps40
p-value
1e-03
4e-03
9e-03
2e-02
6e-02
Log-odds
0.5
1.0
1.5
2.0
2.5
3.0
3.5
4.0

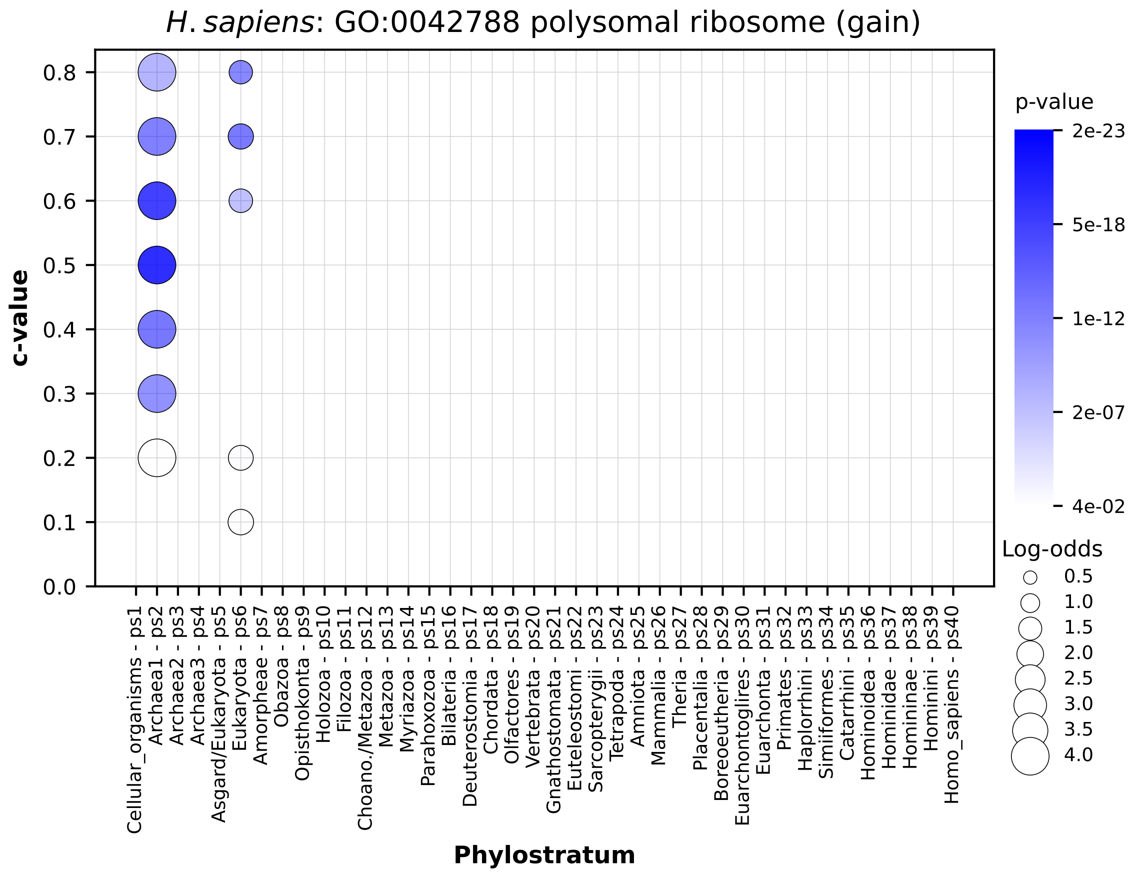

H. sapiens: GO:0042788 polysomal ribosome (gain)
c-value
0.0
0.1
0.2
0.3
0.4
0.5
0.6
0.7
0.8
Phylostratum
Cellular_organisms - ps1
Archaea1 - ps2
Archaea2 - ps3
Archaea3 - ps4
Asgard/Eukaryota - ps5
Eukaryota - ps6
Amorpheae - ps7
Obazoa - ps8
Opisthokonta - ps9
Holozoa - ps10
Filozoa - ps11
Choano./Metazoa - ps12
Metazoa - ps13
Myriazoa - ps14
Parahoxozoa - ps15
Bilateria - ps16
Deuterostomia - ps17
Chordata - ps18
Olfactores - ps19
Vertebrata - ps20
Gnathostomata - ps21
Euteleostomi - ps22
Sarcopterygii - ps23
Tetrapoda - ps24
Amniota - ps25
Mammalia - ps26
Theria - ps27
Placentalia - ps28
Boreoeutheria - ps29
Euarchontoglires - ps30
Euarchonta - ps31
Primates - ps32
Haplorrhini - ps33
Simiiformes - ps34
Catarrhini - ps35
Hominoidea - ps36
Hominidae - ps37
Homininae - ps38
Hominini - ps39
Homo_sapiens - ps40
p-value
2e-23
5e-18
1e-12
2e-07
4e-02
Log-odds
0.5
1.0
1.5
2.0
2.5
3.0
3.5
4.0

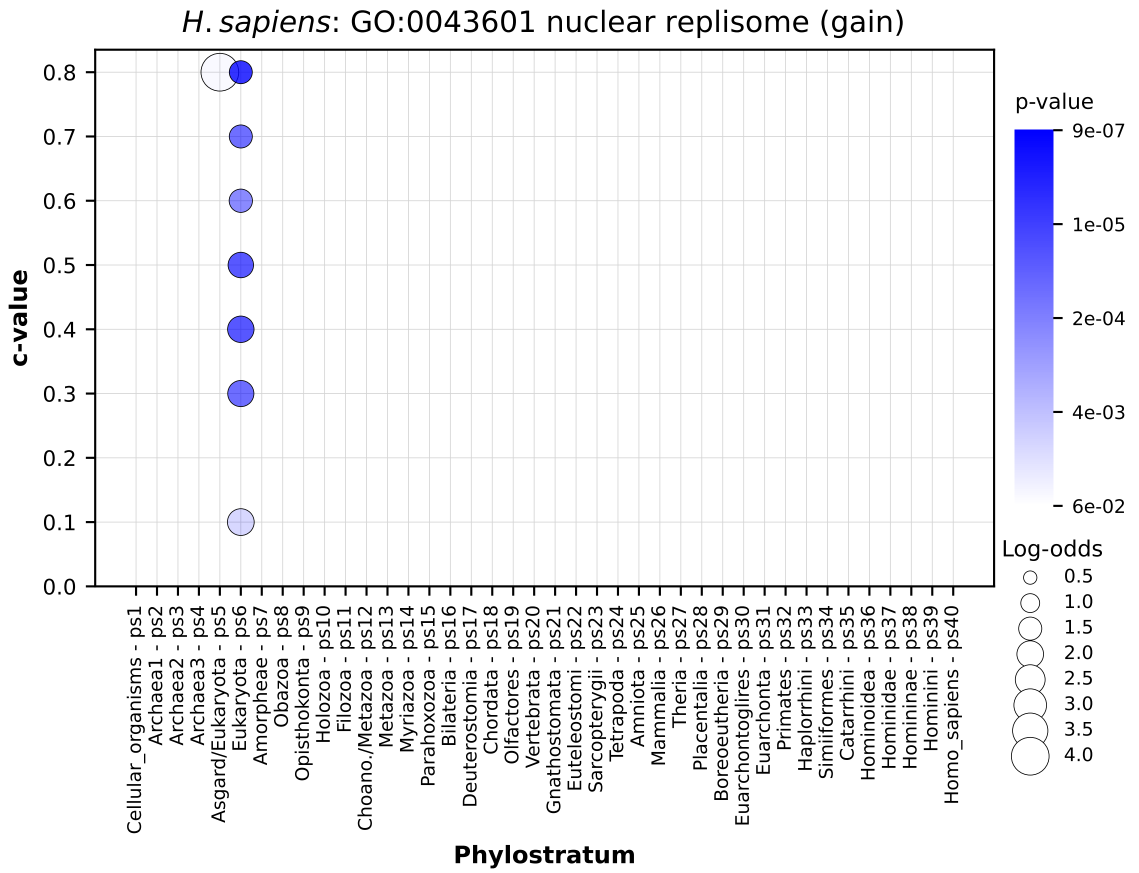
H. sapiens: GO:0043601 nuclear replisome (gain)
c-value
0.0
0.1
0.2
0.3
0.4
0.5
0.6
0.7
0.8
Phylostratum
Cellular_organisms - ps1
Archaea1 - ps2
Archaea2 - ps3
Archaea3 - ps4
Asgard/Eukaryota - ps5
Eukaryota - ps6
Amorpheae - ps7
Obazoa - ps8
Opisthokonta - ps9
Holozoa - ps10
Filozoa - ps11
Choano./Metazoa - ps12
Metazoa - ps13
Myriazoa - ps14
Parahoxozoa - ps15
Bilateria - ps16
Deuterostomia - ps17
Chordata - ps18
Olfactores - ps19
Vertebrata - ps20
Gnathostomata - ps21
Euteleostomi - ps22
Sarcopterygii - ps23
Tetrapoda - ps24
Amniota - ps25
Mammalia - ps26
Theria - ps27
Placentalia - ps28
Boreoeutheria - ps29
Euarchontoglires - ps30
Euarchonta - ps31
Primates - ps32
Haplorrhini - ps33
Simiiformes - ps34
Catarrhini - ps35
Hominoidea - ps36
Hominidae - ps37
Homininae - ps38
Hominini - ps39
Homo_sapiens - ps40
p-value
9e-07
1e-05
2e-04
4e-03
6e-02
Log-odds
0.5
1.0
1.5
2.0
2.5
3.0
3.5
4.0

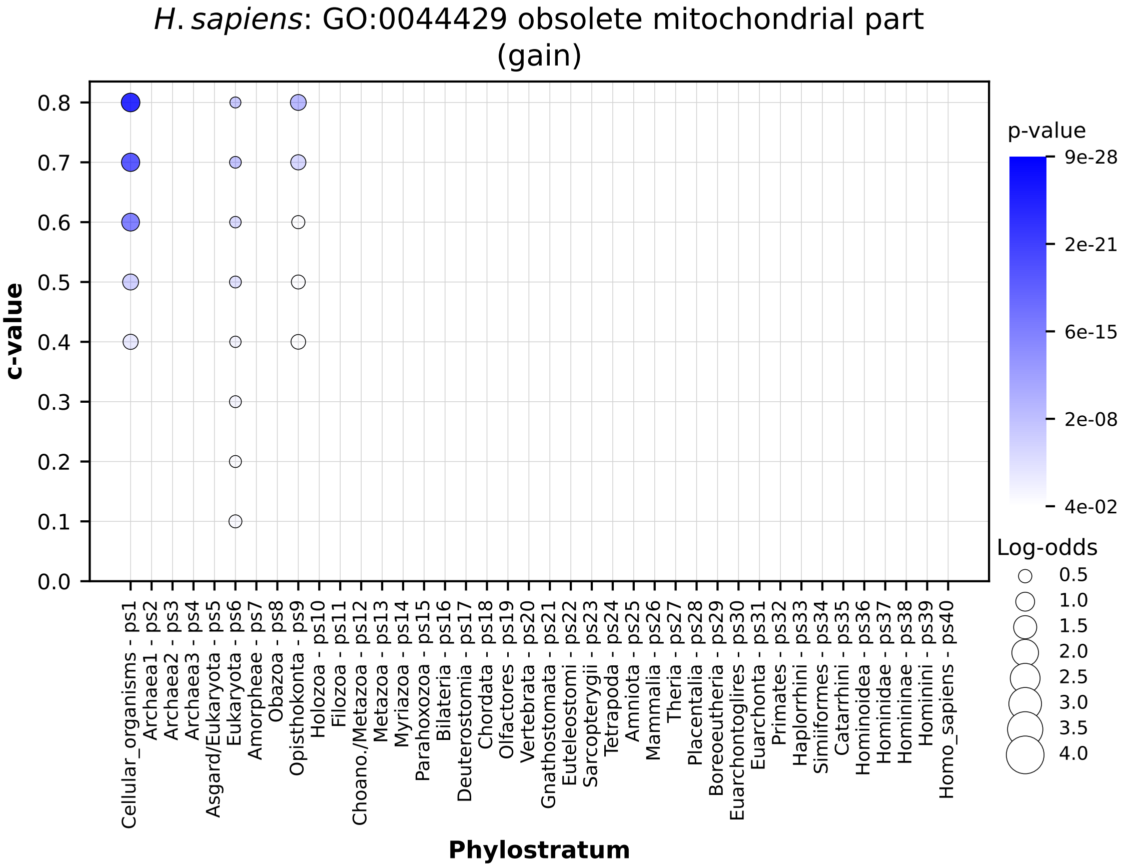

*H. sapiens*: GO:0044429 obsolete mitochondrial part (gain)
c-value
0.0
0.1
0.2
0.3
0.4
0.5
0.6
0.7
0.8
Phylostratum
Cellular_organisms - ps1
Archaea1 - ps2
Archaea2 - ps3
Archaea3 - ps4
Asgard/Eukaryota - ps5
Eukaryota - ps6
Amorpheae - ps7
Obazoa - ps8
Opisthokonta - ps9
Holozoa - ps10
Filozoa - ps11
Choano./Metazoa - ps12
Metazoa - ps13
Myriazoa - ps14
Parahoxozoa - ps15
Bilateria - ps16
Deuterostomia - ps17
Chordata - ps18
Olfactores - ps19
Vertebrata - ps20
Gnathostomata - ps21
Euteleostomi - ps22
Sarcopterygii - ps23
Tetrapoda - ps24
Amniota - ps25
Mammalia - ps26
Theria - ps27
Placentalia - ps28
Boreoeutheria - ps29
Euarchontoglires - ps30
Euarchonta - ps31
Primates - ps32
Haplorrhini - ps33
Simiiformes - ps34
Catarrhini - ps35
Hominoidea - ps36
Hominidae - ps37
Homininae - ps38
Hominini - ps39
Homo_sapiens - ps40
p-value
9e-28
2e-21
6e-15
2e-08
4e-02
Log-odds
0.5
1.0
1.5
2.0
2.5
3.0
3.5
4.0

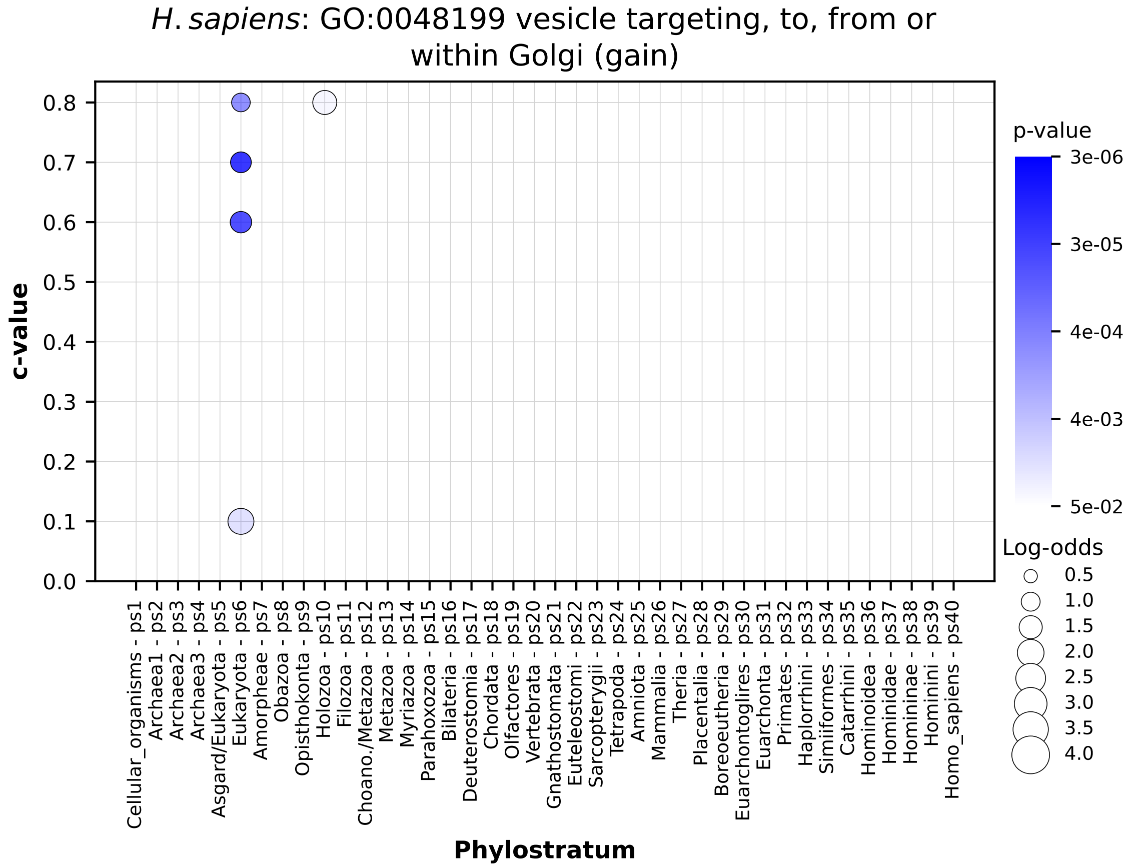
H. sapiens: GO:0048199 vesicle targeting, to, from or within Golgi (gain)
c-value
0.0
0.1
0.2
0.3
0.4
0.5
0.6
0.7
0.8
p-value
3e-06
3e-05
4e-04
4e-03
5e-02
Log-odds
0.5
1.0
1.5
2.0
2.5
3.0
3.5
4.0
Cellular_organisms - ps1
Archaea1 - ps2
Archaea2 - ps3
Archaea3 - ps4
Asgard/Eukaryota - ps5
Eukaryota - ps6
Amorpheae - ps7
Obazoa - ps8
Opisthokonta - ps9
Holozoa - ps10
Filozoa - ps11
Choano./Metazoa - ps12
Metazoa - ps13
Myriazoa - ps14
Parahoxozoa - ps15
Bilateria - ps16
Deuterostomia - ps17
Chordata - ps18
Olfactores - ps19
Vertebrata - ps20
Gnathostomata - ps21
Euteleostomi - ps22
Sarcopterygii - ps23
Tetrapoda - ps24
Amniota - ps25
Mammalia - ps26
Theria - ps27
Placentalia - ps28
Boreoeutheria - ps29
Euarchontoglires - ps30
Euarchonta - ps31
Primates - ps32
Haplorrhini - ps33
Simiiformes - ps34
Catarrhini - ps35
Hominoidea - ps36
Hominidae - ps37
Homininae - ps38
Hominini - ps39
Homo_sapiens - ps40
Phylostratum

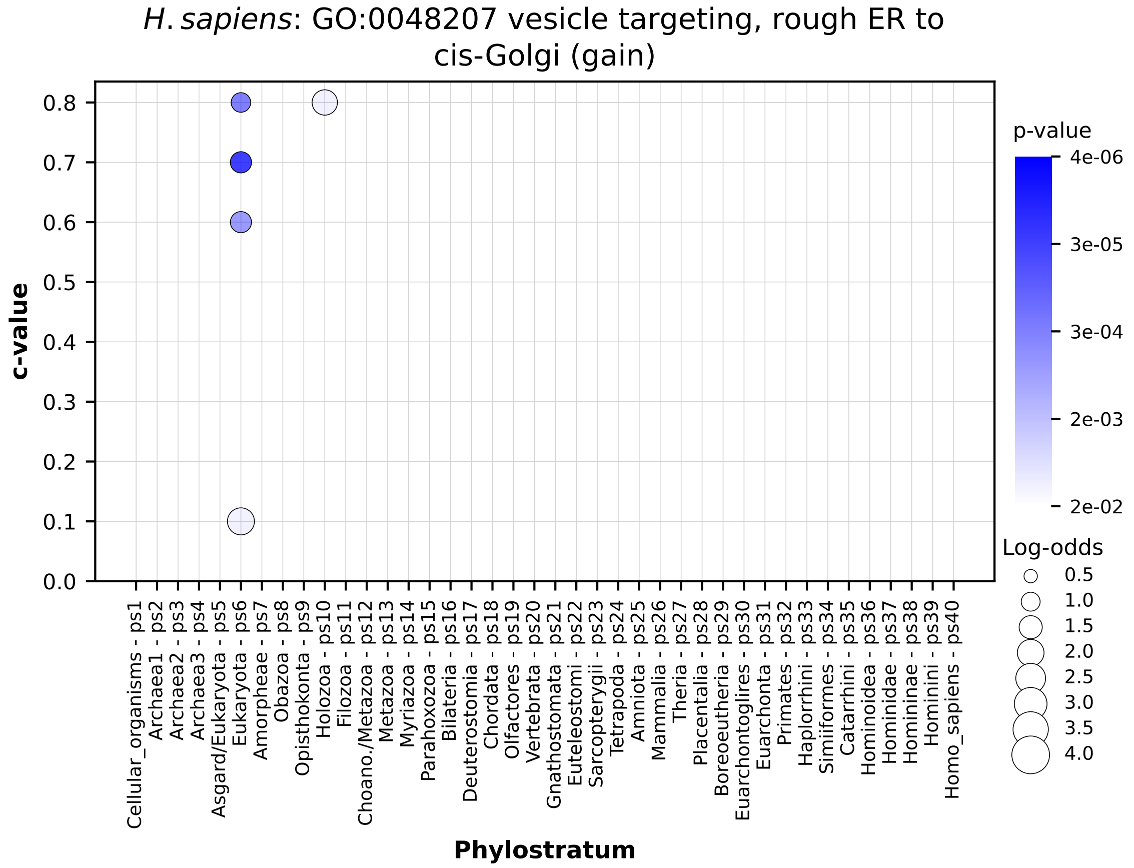
*H. sapiens*: GO:0048207 vesicle targeting, rough ER to cis-Golgi (gain)
c-value
0.0
0.1
0.2
0.3
0.4
0.5
0.6
0.7
0.8
p-value
4e-06
3e-05
3e-04
2e-03
2e-02
Log-odds
0.5
1.0
1.5
2.0
2.5
3.0
3.5
4.0
Cellular_organisms - ps1
Archaea1 - ps2
Archaea2 - ps3
Archaea3 - ps4
Asgard/Eukaryota - ps5
Eukaryota - ps6
Amorpheae - ps7
Obazoa - ps8
Opisthokonta - ps9
Holozoa - ps10
Filozoa - ps11
Choano./Metazoa - ps12
Metazoa - ps13
Myriazoa - ps14
Parahoxozoa - ps15
Bilateria - ps16
Deuterostomia - ps17
Chordata - ps18
Olfactores - ps19
Vertebrata - ps20
Gnathostomata - ps21
Euteleostomi - ps22
Sarcopterygii - ps23
Tetrapoda - ps24
Amniota - ps25
Mammalia - ps26
Theria - ps27
Placentalia - ps28
Boreoeutheria - ps29
Euarchontoglires - ps30
Euarchonta - ps31
Primates - ps32
Haplorrhini - ps33
Simiiformes - ps34
Catarrhini - ps35
Hominoidea - ps36
Hominidae - ps37
Homininae - ps38
Hominini - ps39
Homo_sapiens - ps40
Phylostratum

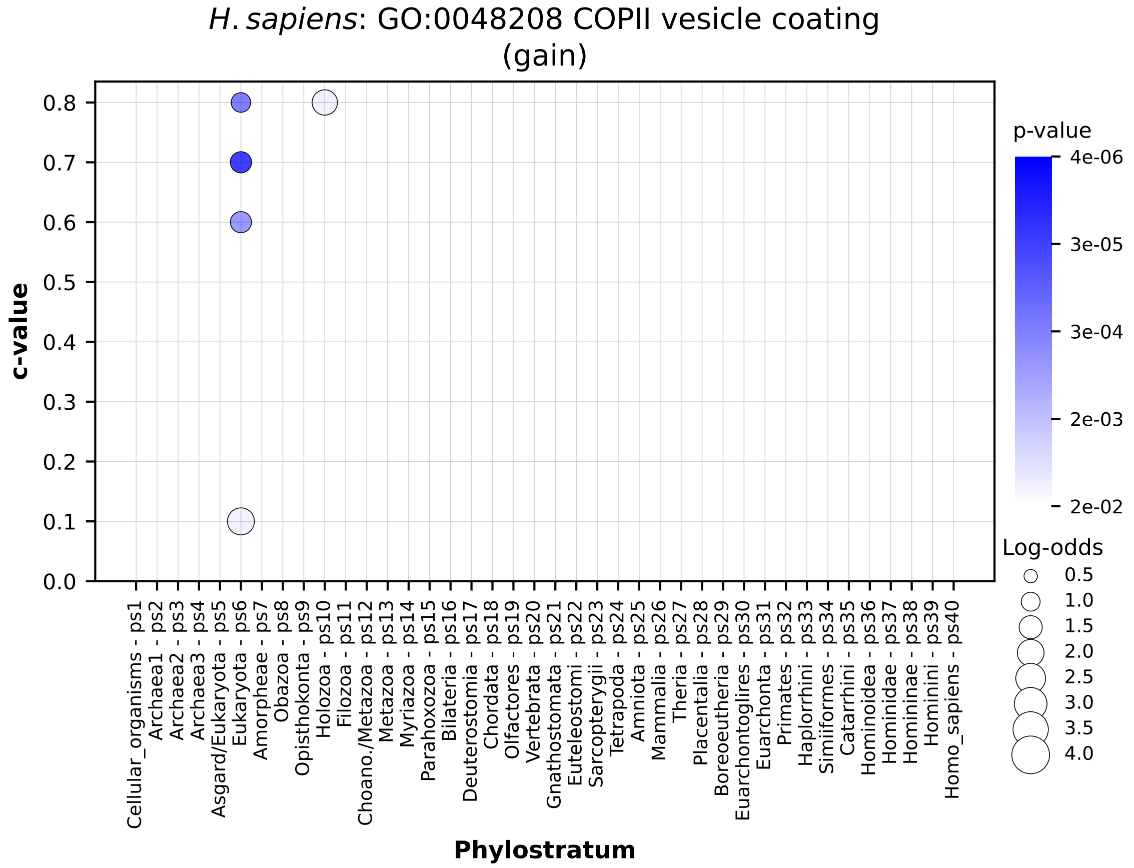
H. sapiens: GO:0048208 COPII vesicle coating
(gain)
c-value
0.0
0.1
0.2
0.3
0.4
0.5
0.6
0.7
0.8
p-value
4e-06
3e-05
3e-04
2e-03
2e-02
Log-odds
0.5
1.0
1.5
2.0
2.5
3.0
3.5
4.0
Cellular_organisms - ps1
Archaea1 - ps2
Archaea2 - ps3
Archaea3 - ps4
Asgard/Eukaryota - ps5
Eukaryota - ps6
Amorpheae - ps7
Obazoa - ps8
Opisthokonta - ps9
Holozoa - ps10
Filozoa - ps11
Choano./Metazoa - ps12
Metazoa - ps13
Myriazoa - ps14
Parahoxozoa - ps15
Bilateria - ps16
Deuterostomia - ps17
Chordata - ps18
Olfactores - ps19
Vertebrata - ps20
Gnathostomata - ps21
Euteleostomi - ps22
Sarcopterygii - ps23
Tetrapoda - ps24
Amniota - ps25
Mammalia - ps26
Theria - ps27
Placentalia - ps28
Boreoeutheria - ps29
Euarchontoglires - ps30
Euarchonta - ps31
Primates - ps32
Haplorrhini - ps33
Simiiformes - ps34
Catarrhini - ps35
Hominoidea - ps36
Hominidae - ps37
Homininae - ps38
Hominini - ps39
Homo_sapiens - ps40
Phylostratum

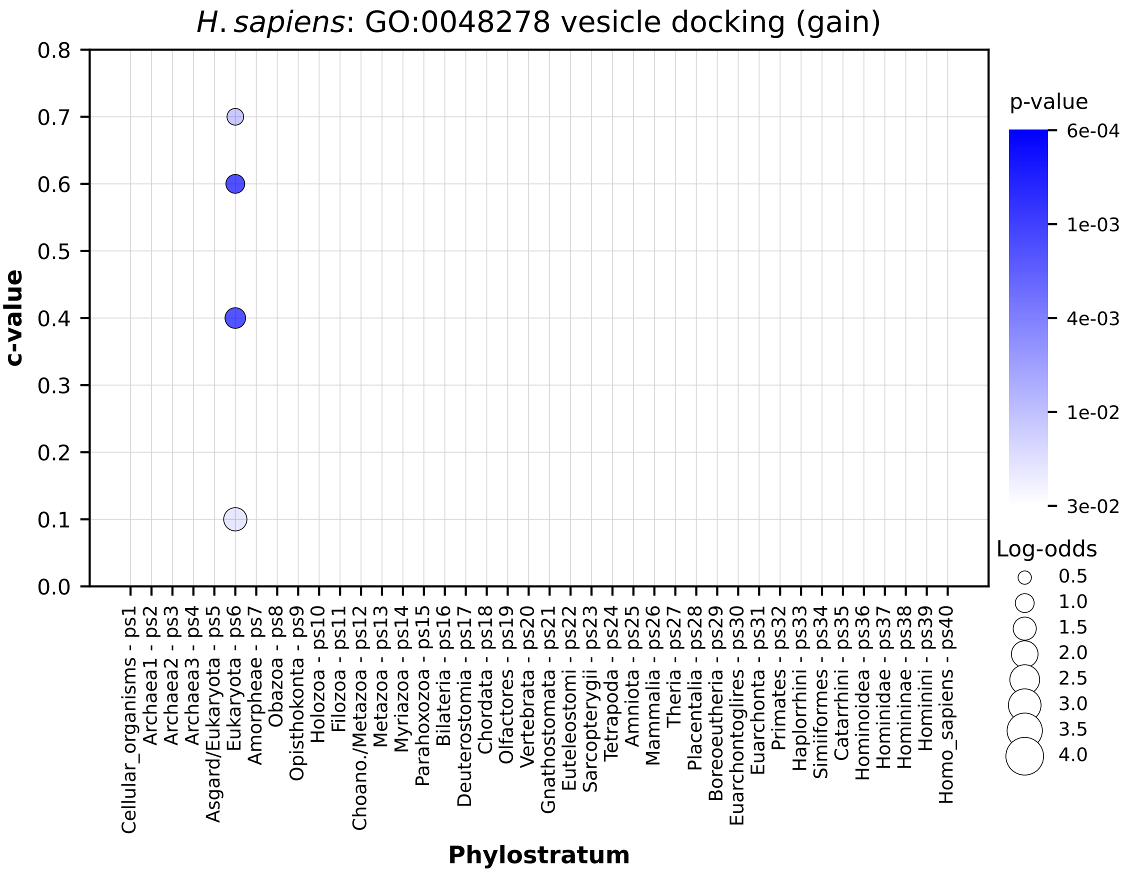

H. sapiens: GO:0048278 vesicle docking (gain)
c-value
0.0
0.1
0.2
0.3
0.4
0.5
0.6
0.7
0.8
Phylostratum
Cellular_organisms - ps1
Archaea1 - ps2
Archaea2 - ps3
Archaea3 - ps4
Asgard/Eukaryota - ps5
Eukaryota - ps6
Amorpheae - ps7
Obazoa - ps8
Opisthokonta - ps9
Holozoa - ps10
Filozoa - ps11
Choano./Metazoa - ps12
Metazoa - ps13
Myriazoa - ps14
Parahoxozoa - ps15
Bilateria - ps16
Deuterostomia - ps17
Chordata - ps18
Olfactores - ps19
Vertebrata - ps20
Gnathostomata - ps21
Euteleostomi - ps22
Sarcopterygii - ps23
Tetrapoda - ps24
Amniota - ps25
Mammalia - ps26
Theria - ps27
Placentalia - ps28
Boreoeutheria - ps29
Euarchontoglires - ps30
Euarchonta - ps31
Primates - ps32
Haplorrhini - ps33
Simiiformes - ps34
Catarrhini - ps35
Hominoidea - ps36
Hominidae - ps37
Homininae - ps38
Hominini - ps39
Homo_sapiens - ps40
p-value
6e-04
1e-03
4e-03
1e-02
3e-02
Log-odds
0.5
1.0
1.5
2.0
2.5
3.0
3.5
4.0

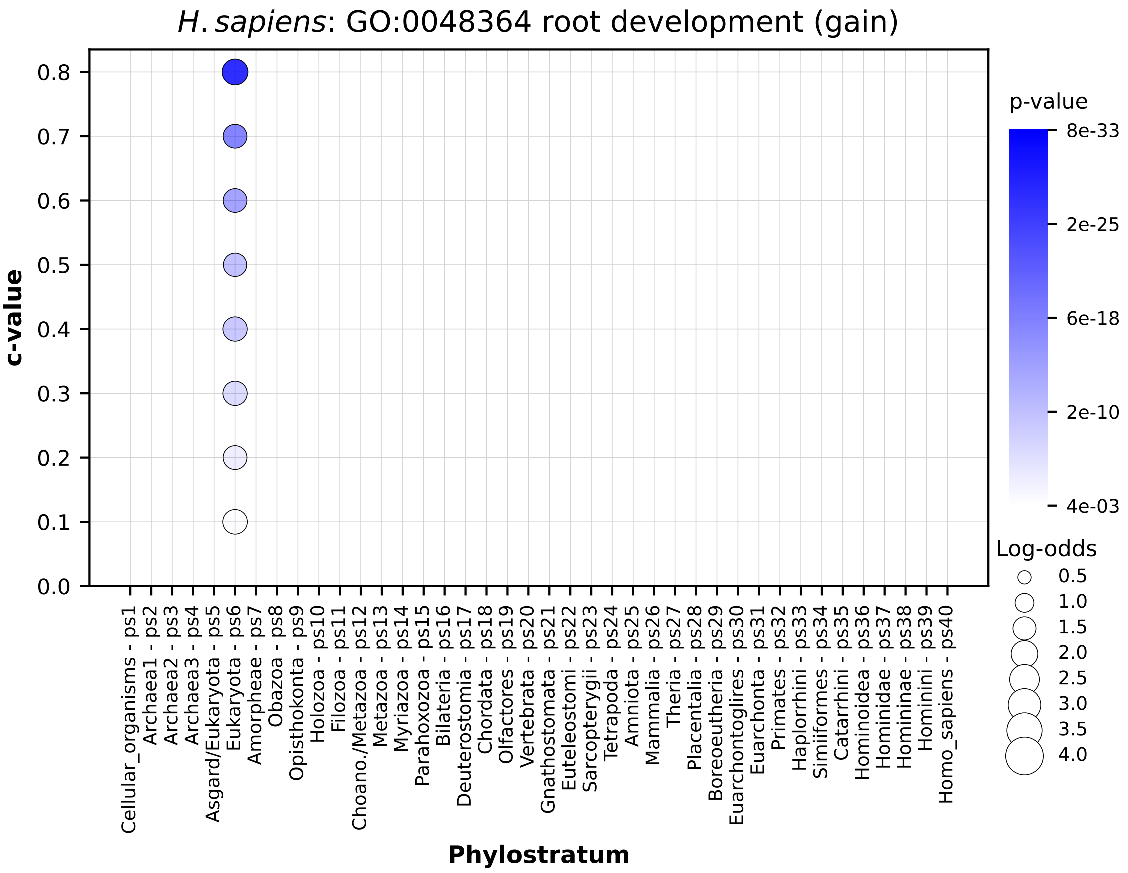

*H. sapiens*: GO:0048364 root development (gain)
c-value
0.0
0.1
0.2
0.3
0.4
0.5
0.6
0.7
0.8
Phylostratum
Cellular_organisms - ps1
Archaea1 - ps2
Archaea2 - ps3
Archaea3 - ps4
Asgard/Eukaryota - ps5
Eukaryota - ps6
Amorpheae - ps7
Obazoa - ps8
Opisthokonta - ps9
Holozoa - ps10
Filozoa - ps11
Choano./Metazoa - ps12
Metazoa - ps13
Myriazoa - ps14
Parahoxozoa - ps15
Bilateria - ps16
Deuterostomia - ps17
Chordata - ps18
Olfactores - ps19
Vertebrata - ps20
Gnathostomata - ps21
Euteleostomi - ps22
Sarcopterygii - ps23
Tetrapoda - ps24
Amniota - ps25
Mammalia - ps26
Theria - ps27
Placentalia - ps28
Boreoeutheria - ps29
Euarchontoglires - ps30
Euarchonta - ps31
Primates - ps32
Haplorrhini - ps33
Simiiformes - ps34
Catarrhini - ps35
Hominoidea - ps36
Hominidae - ps37
Homininae - ps38
Hominini - ps39
Homo_sapiens - ps40
p-value
8e-33
2e-25
6e-18
2e-10
4e-03
Log-odds
0.5
1.0
1.5
2.0
2.5
3.0
3.5
4.0

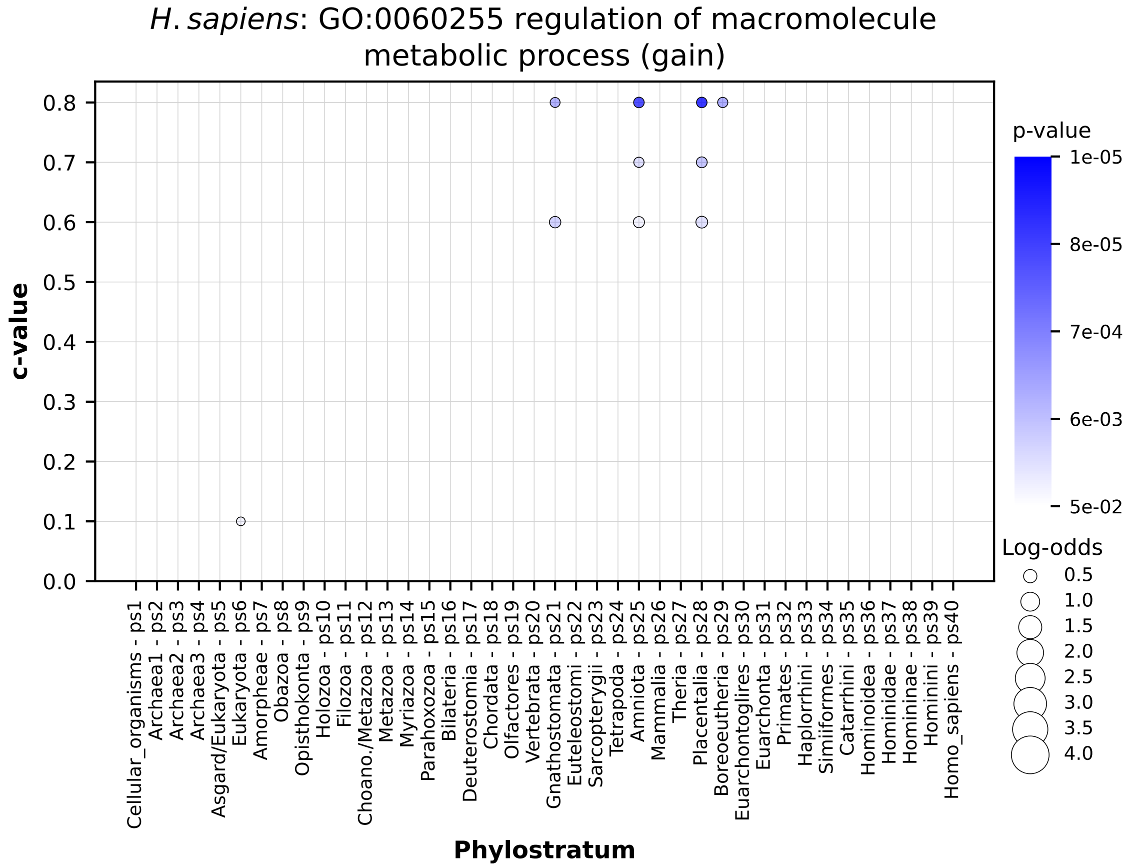

H. sapiens: GO:0060255 regulation of macromolecule metabolic process (gain)
c-value
0.0
0.1
0.2
0.3
0.4
0.5
0.6
0.7
0.8
Phylostratum
Cellular_organisms - ps1
Archaea1 - ps2
Archaea2 - ps3
Archaea3 - ps4
Asgard/Eukaryota - ps5
Eukaryota - ps6
Amorpheae - ps7
Obazoa - ps8
Opisthokonta - ps9
Holozoa - ps10
Filozoa - ps11
Choano./Metazoa - ps12
Metazoa - ps13
Myriazoa - ps14
Parahoxozoa - ps15
Bilateria - ps16
Deuterostomia - ps17
Chordata - ps18
Olfactores - ps19
Vertebrata - ps20
Gnathostomata - ps21
Euteleostomi - ps22
Sarcopterygii - ps23
Tetrapoda - ps24
Amniota - ps25
Mammalia - ps26
Theria - ps27
Placentalia - ps28
Boreoeutheria - ps29
Euarchontoglires - ps30
Euarchonta - ps31
Primates - ps32
Haplorrhini - ps33
Simiiformes - ps34
Catarrhini - ps35
Hominoidea - ps36
Hominidae - ps37
Homininae - ps38
Hominini - ps39
Homo_sapiens - ps40
p-value
1e-05
8e-05
7e-04
6e-03
5e-02
Log-odds
0.5
1.0
1.5
2.0
2.5
3.0
3.5
4.0

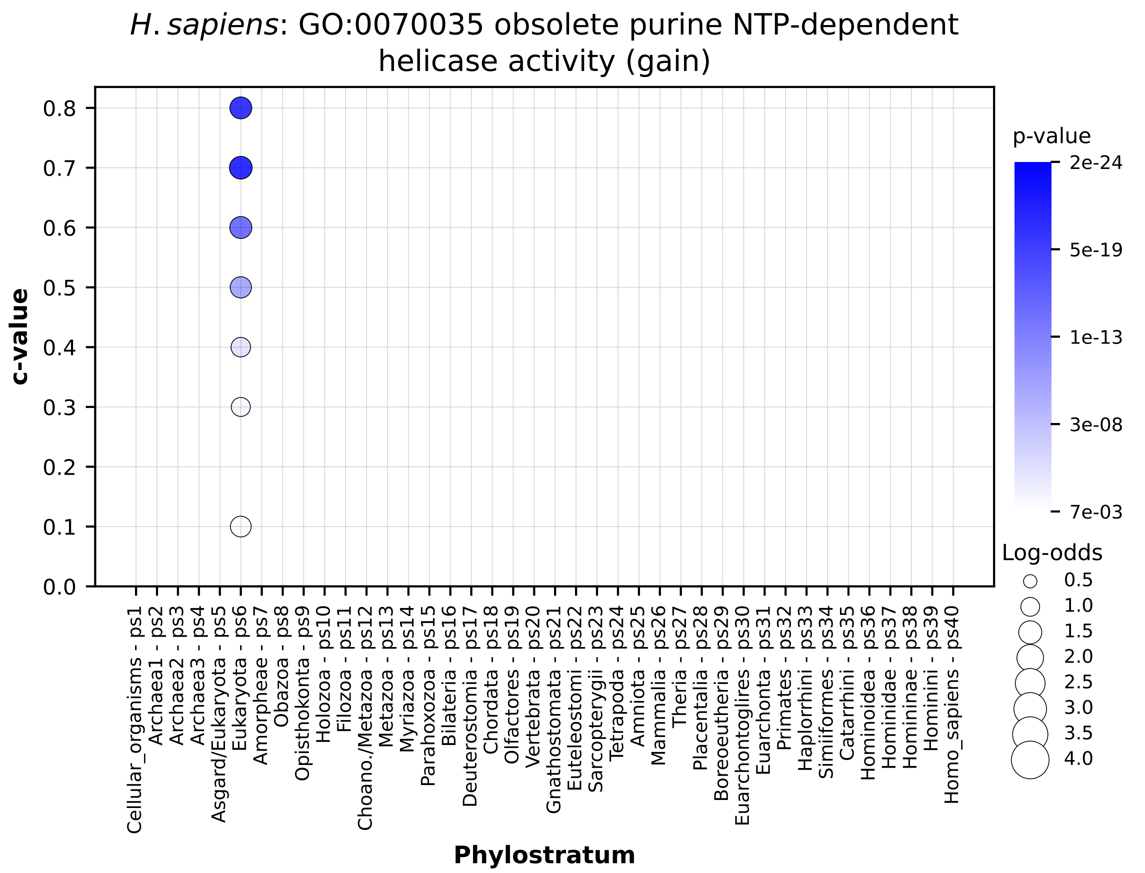

H. sapiens: GO:0070035 obsolete purine NTP-dependent helicase activity (gain)
c-value
0.0
0.1
0.2
0.3
0.4
0.5
0.6
0.7
0.8
Phylostratum
Cellular_organisms - ps1
Archaea1 - ps2
Archaea2 - ps3
Archaea3 - ps4
Asgard/Eukaryota - ps5
Eukaryota - ps6
Amorpheae - ps7
Obazoa - ps8
Opisthokonta - ps9
Holozoa - ps10
Filozoa - ps11
Choano./Metazoa - ps12
Metazoa - ps13
Myriazoa - ps14
Parahoxozoa - ps15
Bilateria - ps16
Deuterostomia - ps17
Chordata - ps18
Olfactores - ps19
Vertebrata - ps20
Gnathostomata - ps21
Euteleostomi - ps22
Sarcopterygii - ps23
Tetrapoda - ps24
Amniota - ps25
Mammalia - ps26
Theria - ps27
Placentalia - ps28
Boreoeutheria - ps29
Euarchontoglires - ps30
Euarchonta - ps31
Primates - ps32
Haplorrhini - ps33
Simiiformes - ps34
Catarrhini - ps35
Hominoidea - ps36
Hominidae - ps37
Homininae - ps38
Hominini - ps39
Homo_sapiens - ps40
p-value
2e-24
5e-19
1e-13
3e-08
7e-03
Log-odds
0.5
1.0
1.5
2.0
2.5
3.0
3.5
4.0

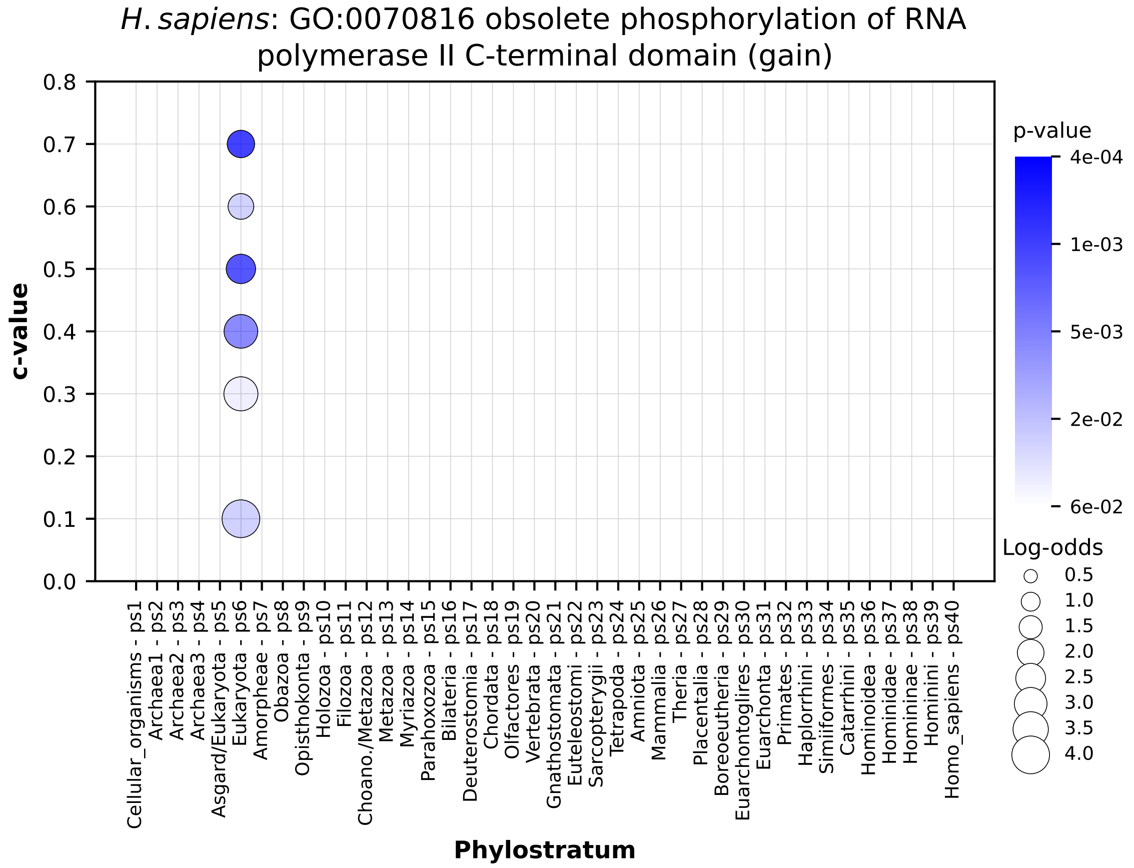
H. sapiens: GO:0070816 obsolete phosphorylation of RNA polymerase II C-terminal domain (gain)
c-value
0.0
0.1
0.2
0.3
0.4
0.5
0.6
0.7
0.8
Phylostratum
Cellular_organisms - ps1
Archaea1 - ps2
Archaea2 - ps3
Archaea3 - ps4
Asgard/Eukaryota - ps5
Eukaryota - ps6
Amorpheae - ps7
Obazoa - ps8
Opisthokonta - ps9
Holozoa - ps10
Filozoa - ps11
Choano./Metazoa - ps12
Metazoa - ps13
Myriazoa - ps14
Parahoxozoa - ps15
Bilateria - ps16
Deuterostomia - ps17
Chordata - ps18
Olfactores - ps19
Vertebrata - ps20
Gnathostomata - ps21
Euteleostomi - ps22
Sarcopterygii - ps23
Tetrapoda - ps24
Amniota - ps25
Mammalia - ps26
Theria - ps27
Placentalia - ps28
Boreoeutheria - ps29
Euarchontoglires - ps30
Euarchonta - ps31
Primates - ps32
Haplorrhini - ps33
Simiiformes - ps34
Catarrhini - ps35
Hominoidea - ps36
Hominidae - ps37
Homininae - ps38
Hominini - ps39
Homo_sapiens - ps40
p-value
4e-04
1e-03
5e-03
2e-02
6e-02
Log-odds
0.5
1.0
1.5
2.0
2.5
3.0
3.5
4.0

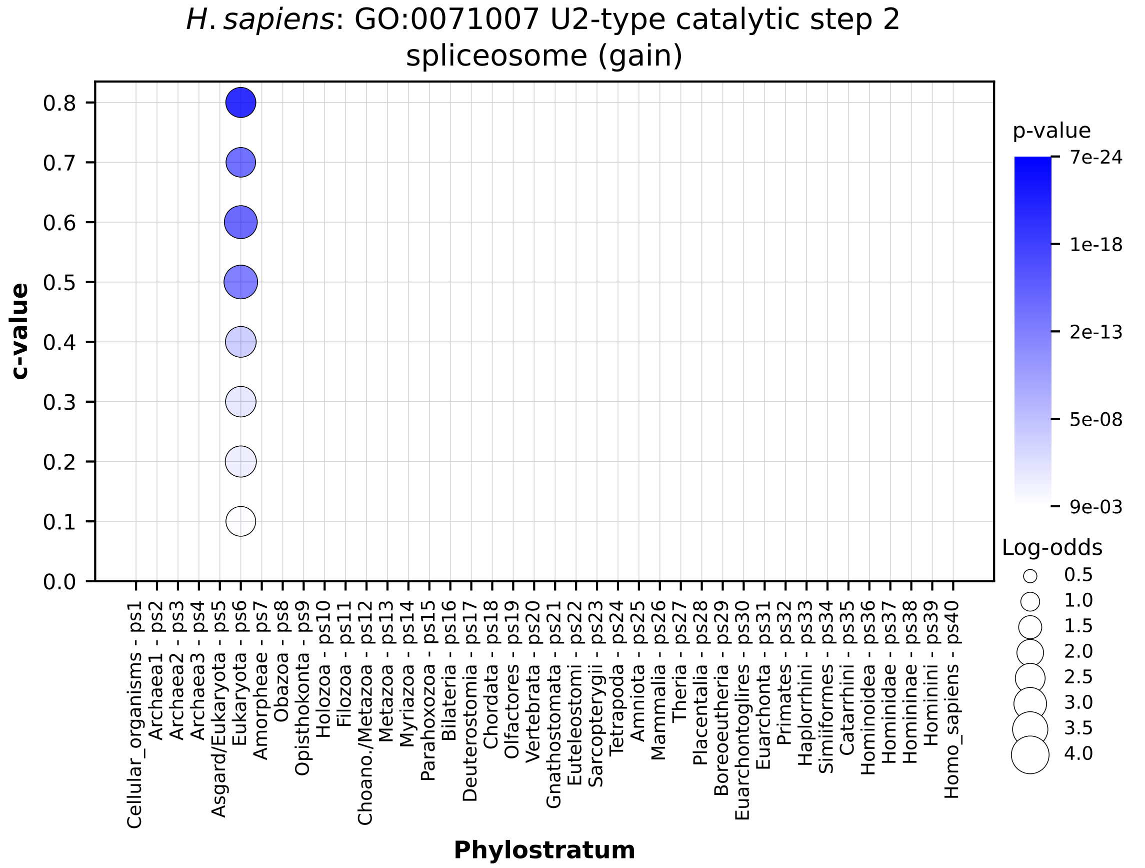
H. sapiens: GO:0071007 U2-type catalytic step 2 spliceosome (gain)
c-value
0.0
0.1
0.2
0.3
0.4
0.5
0.6
0.7
0.8
Phylostratum
Cellular_organisms - ps1
Archaea1 - ps2
Archaea2 - ps3
Archaea3 - ps4
Asgard/Eukaryota - ps5
Eukaryota - ps6
Amorpheae - ps7
Obazoa - ps8
Opisthokonta - ps9
Holozoa - ps10
Filozoa - ps11
Choano./Metazoa - ps12
Metazoa - ps13
Myriazoa - ps14
Parahoxozoa - ps15
Bilateria - ps16
Deuterostomia - ps17
Chordata - ps18
Olfactores - ps19
Vertebrata - ps20
Gnathostomata - ps21
Euteleostomi - ps22
Sarcopterygii - ps23
Tetrapoda - ps24
Amniota - ps25
Mammalia - ps26
Theria - ps27
Placentalia - ps28
Boreoeutheria - ps29
Euarchontoglires - ps30
Euarchonta - ps31
Primates - ps32
Haplorrhini - ps33
Simiiformes - ps34
Catarrhini - ps35
Hominoidea - ps36
Hominidae - ps37
Homininae - ps38
Hominini - ps39
Homo_sapiens - ps40
p-value
7e-24
1e-18
2e-13
5e-08
9e-03
Log-odds
0.5
1.0
1.5
2.0
2.5
3.0
3.5
4.0

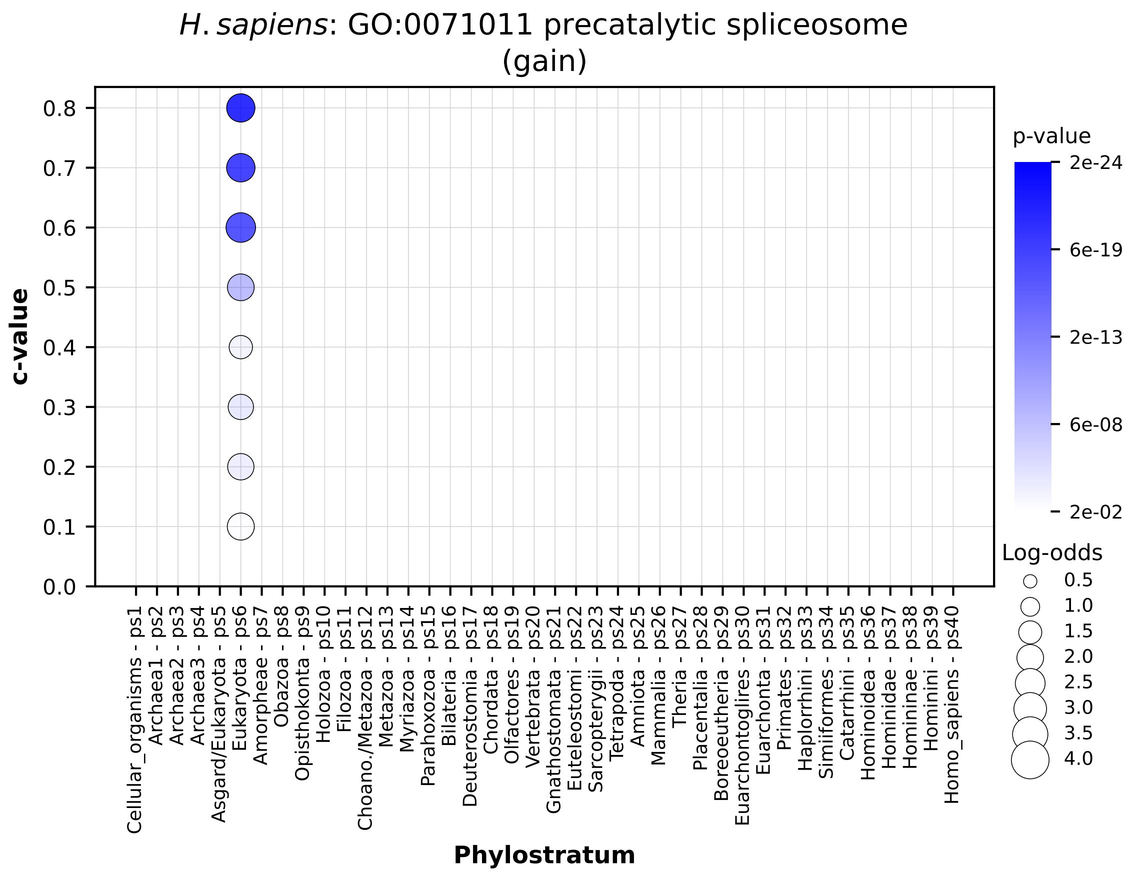

H. sapiens: GO:0071011 precatalytic spliceosome
(gain)
c-value
0.0
0.1
0.2
0.3
0.4
0.5
0.6
0.7
0.8
p-value
2e-24
6e-19
2e-13
6e-08
2e-02
Log-odds
0.5
1.0
1.5
2.0
2.5
3.0
3.5
4.0
Cellular_organisms - ps1
Archaea1 - ps2
Archaea2 - ps3
Archaea3 - ps4
Asgard/Eukaryota - ps5
Eukaryota - ps6
Amorpheae - ps7
Obazoa - ps8
Opisthokonta - ps9
Holozoa - ps10
Filozoa - ps11
Choano./Metazoa - ps12
Metazoa - ps13
Myriazoa - ps14
Parahoxozoa - ps15
Bilateria - ps16
Deuterostomia - ps17
Chordata - ps18
Olfactores - ps19
Vertebrata - ps20
Gnathostomata - ps21
Euteleostomi - ps22
Sarcopterygii - ps23
Tetrapoda - ps24
Amniota - ps25
Mammalia - ps26
Theria - ps27
Placentalia - ps28
Boreoeutheria - ps29
Euarchontoglires - ps30
Euarchonta - ps31
Primates - ps32
Haplorrhini - ps33
Simiiformes - ps34
Catarrhini - ps35
Hominoidea - ps36
Hominidae - ps37
Homininae - ps38
Hominini - ps39
Homo_sapiens - ps40
Phylostratum

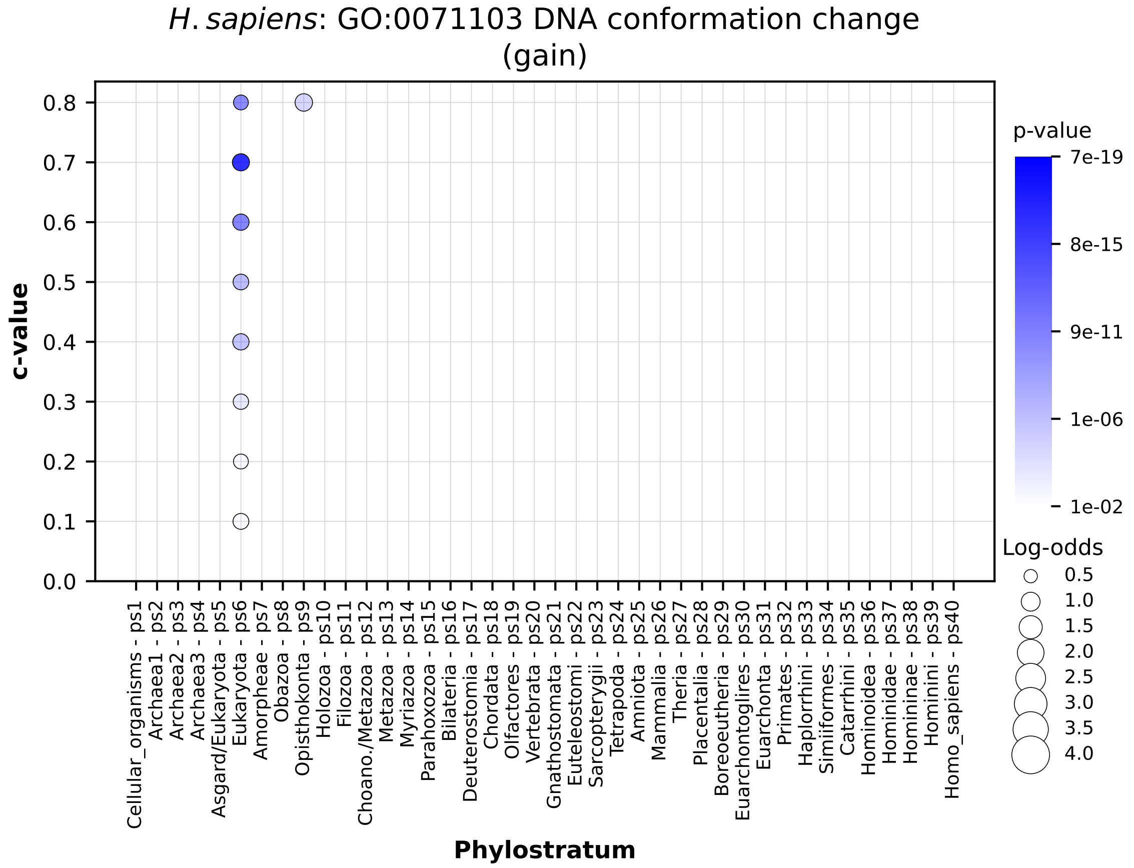

$H. sapiens$: GO:0071103 DNA conformation change
(gain)
c-value
0.0
0.1
0.2
0.3
0.4
0.5
0.6
0.7
0.8
p-value
7e-19
8e-15
9e-11
1e-06
1e-02
Log-odds
0.5
1.0
1.5
2.0
2.5
3.0
3.5
4.0
Cellular_organisms - ps1
Archaea1 - ps2
Archaea2 - ps3
Archaea3 - ps4
Asgard/Eukaryota - ps5
Eukaryota - ps6
Amorpheae - ps7
Obazoa - ps8
Opisthokonta - ps9
Holozoa - ps10
Filozoa - ps11
Choano./Metazoa - ps12
Metazoa - ps13
Myriazoa - ps14
Parahoxozoa - ps15
Bilateria - ps16
Deuterostomia - ps17
Chordata - ps18
Olfactores - ps19
Vertebrata - ps20
Gnathostomata - ps21
Euteleostomi - ps22
Sarcopterygii - ps23
Tetrapoda - ps24
Amniota - ps25
Mammalia - ps26
Theria - ps27
Placentalia - ps28
Boreoeutheria - ps29
Euarchontoglires - ps30
Euarchonta - ps31
Primates - ps32
Haplorrhini - ps33
Simiiformes - ps34
Catarrhini - ps35
Hominoidea - ps36
Hominidae - ps37
Homininae - ps38
Hominini - ps39
Homo_sapiens - ps40
Phylostratum

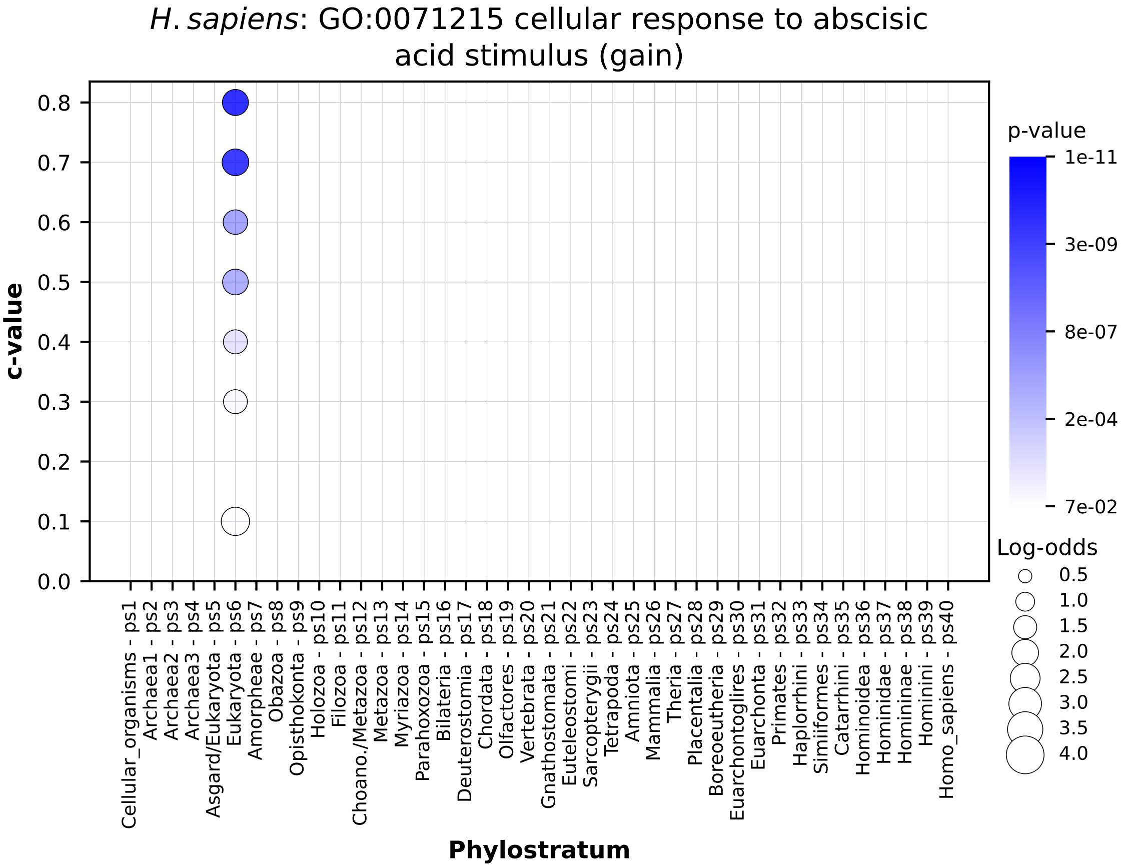

H. sapiens: GO:0071215 cellular response to abscisic acid stimulus (gain)
c-value
0.0
0.1
0.2
0.3
0.4
0.5
0.6
0.7
0.8
Phylostratum
Cellular_organisms - ps1
Archaea1 - ps2
Archaea2 - ps3
Archaea3 - ps4
Asgard/Eukaryota - ps5
Eukaryota - ps6
Amorpheae - ps7
Obazoa - ps8
Opisthokonta - ps9
Holozoa - ps10
Filozoa - ps11
Choano./Metazoa - ps12
Metazoa - ps13
Myriazoa - ps14
Parahoxozoa - ps15
Bilateria - ps16
Deuterostomia - ps17
Chordata - ps18
Olfactores - ps19
Vertebrata - ps20
Gnathostomata - ps21
Euteleostomi - ps22
Sarcopterygii - ps23
Tetrapoda - ps24
Amniota - ps25
Mammalia - ps26
Theria - ps27
Placentalia - ps28
Boreoeutheria - ps29
Euarchontoglires - ps30
Euarchonta - ps31
Primates - ps32
Haplorrhini - ps33
Simiiformes - ps34
Catarrhini - ps35
Hominoidea - ps36
Hominidae - ps37
Homininae - ps38
Hominini - ps39
Homo_sapiens - ps40
p-value
1e-11
3e-09
8e-07
2e-04
7e-02
Log-odds
0.5
1.0
1.5
2.0
2.5
3.0
3.5
4.0

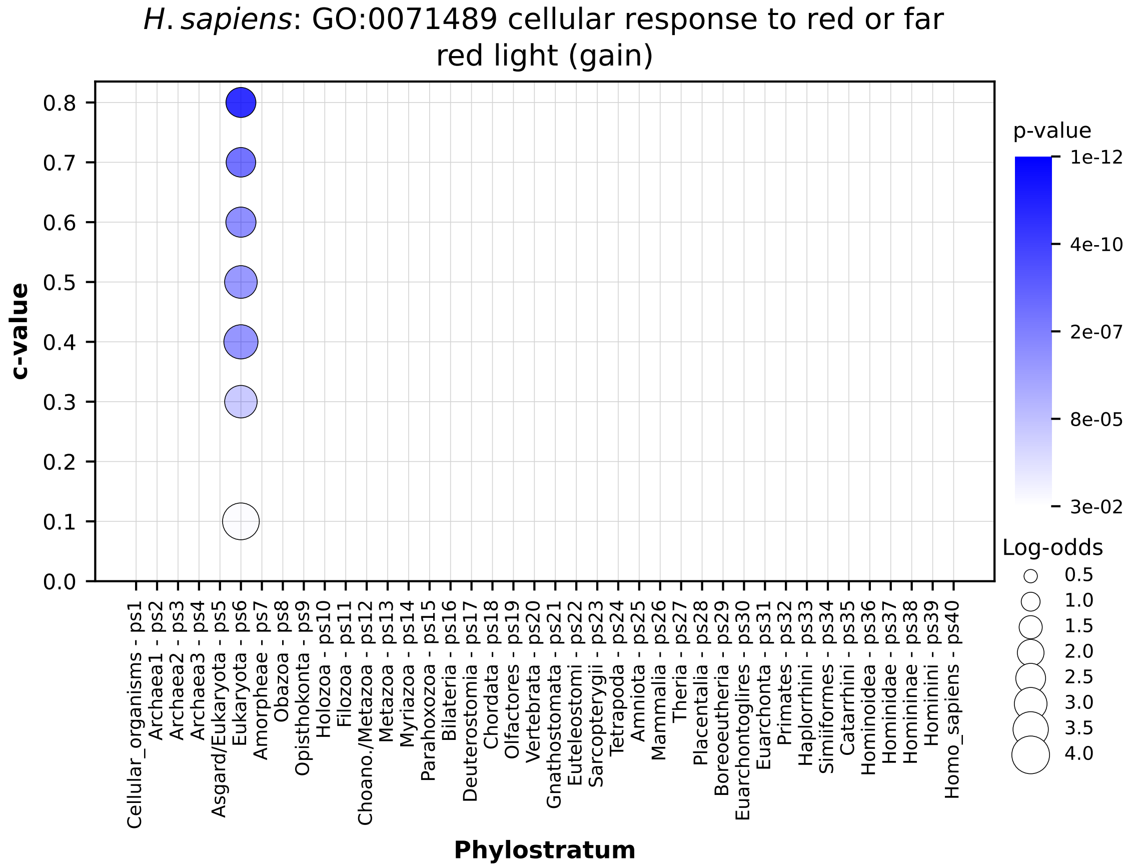

H. sapiens: GO:0071489 cellular response to red or far red light (gain)
c-value
0.0
0.1
0.2
0.3
0.4
0.5
0.6
0.7
0.8
Phylostratum
Cellular_organisms - ps1
Archaea1 - ps2
Archaea2 - ps3
Archaea3 - ps4
Asgard/Eukaryota - ps5
Eukaryota - ps6
Amorpheae - ps7
Obazoa - ps8
Opisthokonta - ps9
Holozoa - ps10
Filozoa - ps11
Choano./Metazoa - ps12
Metazoa - ps13
Myriazoa - ps14
Parahoxozoa - ps15
Bilateria - ps16
Deuterostomia - ps17
Chordata - ps18
Olfactores - ps19
Vertebrata - ps20
Gnathostomata - ps21
Euteleostomi - ps22
Sarcopterygii - ps23
Tetrapoda - ps24
Amniota - ps25
Mammalia - ps26
Theria - ps27
Placentalia - ps28
Boreoeutheria - ps29
Euarchontoglires - ps30
Euarchonta - ps31
Primates - ps32
Haplorrhini - ps33
Simiiformes - ps34
Catarrhini - ps35
Hominoidea - ps36
Hominidae - ps37
Homininae - ps38
Hominini - ps39
Homo_sapiens - ps40
p-value
1e-12
4e-10
2e-07
8e-05
3e-02
Log-odds
0.5
1.0
1.5
2.0
2.5
3.0
3.5
4.0

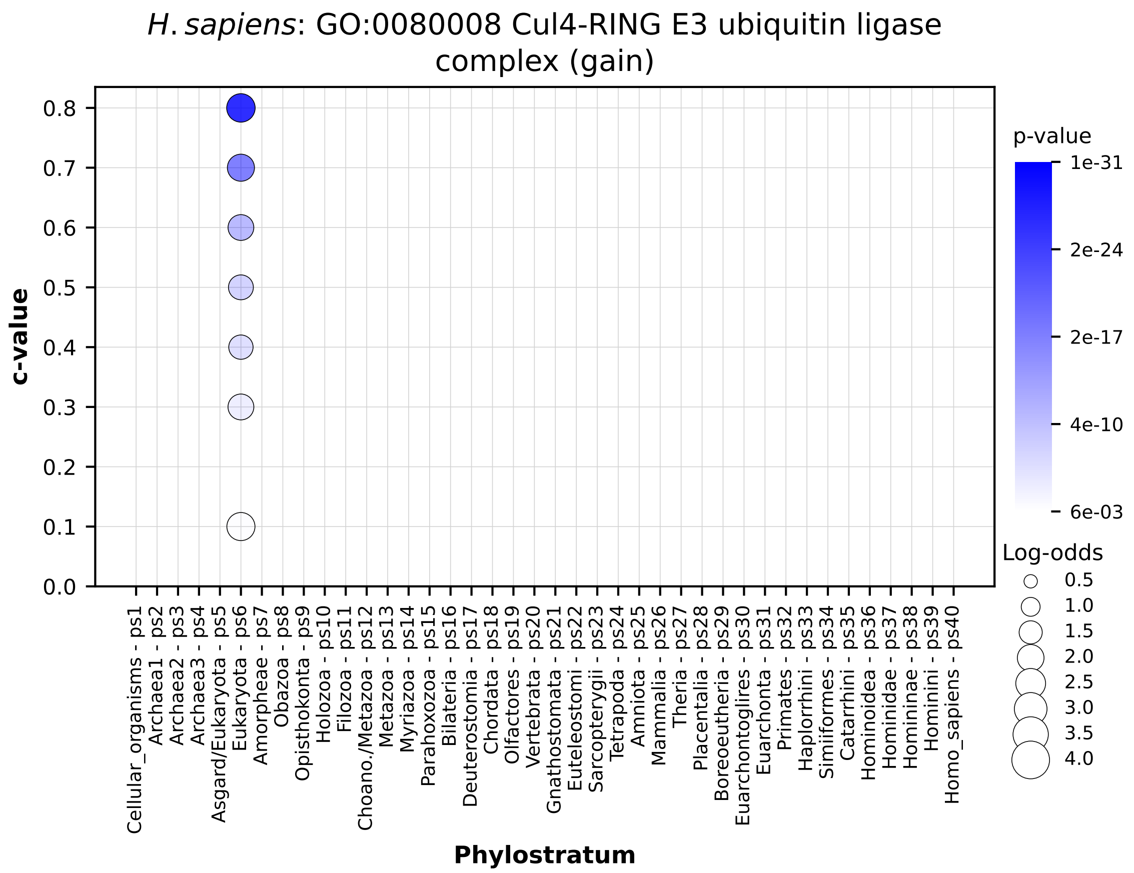

H. sapiens: GO:0080008 Cul4-RING E3 ubiquitin ligase complex (gain)
c-value
0.0
0.1
0.2
0.3
0.4
0.5
0.6
0.7
0.8
Phylostratum
Cellular_organisms - ps1
Archaea1 - ps2
Archaea2 - ps3
Archaea3 - ps4
Asgard/Eukaryota - ps5
Eukaryota - ps6
Amorpheae - ps7
Obazoa - ps8
Opisthokonta - ps9
Holozoa - ps10
Filozoa - ps11
Choano./Metazoa - ps12
Metazoa - ps13
Myriazoa - ps14
Parahoxozoa - ps15
Bilateria - ps16
Deuterostomia - ps17
Chordata - ps18
Olfactores - ps19
Vertebrata - ps20
Gnathostomata - ps21
Euteleostomi - ps22
Sarcopterygii - ps23
Tetrapoda - ps24
Amniota - ps25
Mammalia - ps26
Theria - ps27
Placentalia - ps28
Boreoeutheria - ps29
Euarchontoglires - ps30
Euarchonta - ps31
Primates - ps32
Haplorrhini - ps33
Simiiformes - ps34
Catarrhini - ps35
Hominoidea - ps36
Hominidae - ps37
Homininae - ps38
Hominini - ps39
Homo_sapiens - ps40
p-value
1e-31
2e-24
2e-17
4e-10
6e-03
Log-odds
0.5
1.0
1.5
2.0
2.5
3.0
3.5
4.0

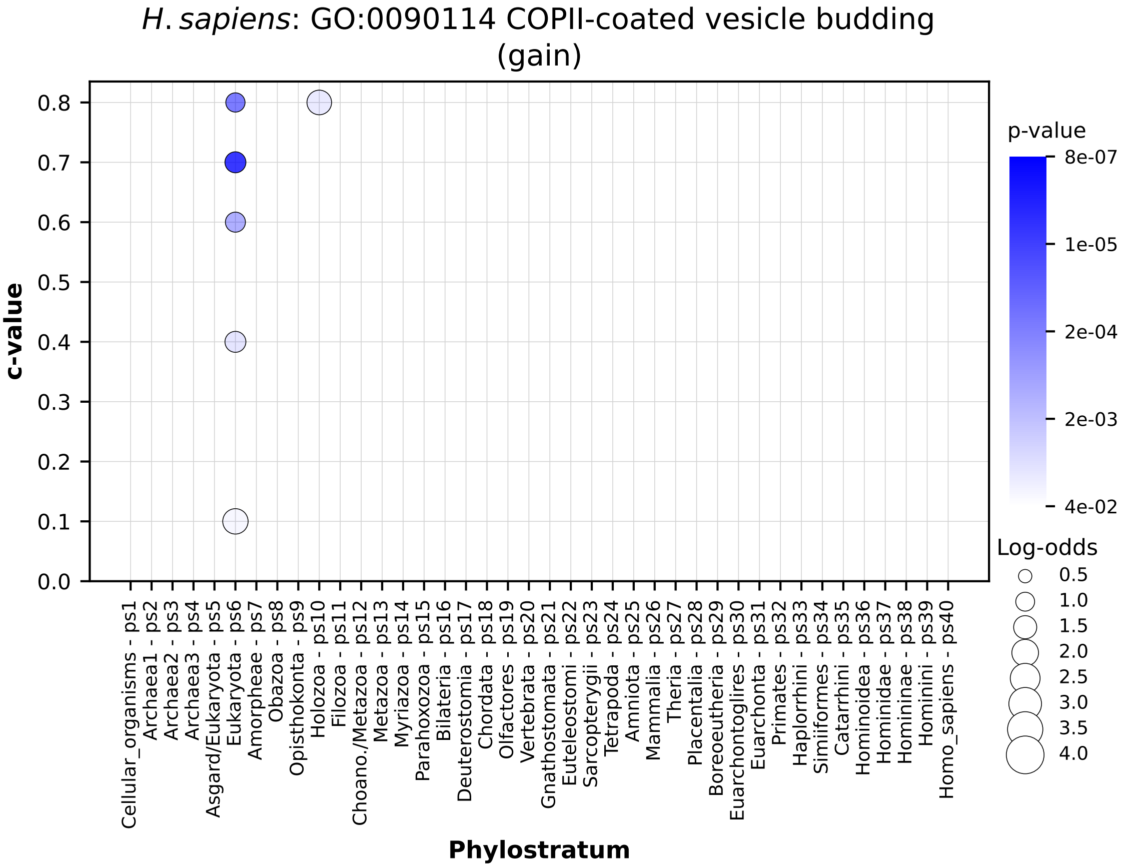

H. sapiens: GO:0090114 COPII-coated vesicle budding (gain)
c-value
0.0
0.1
0.2
0.3
0.4
0.5
0.6
0.7
0.8
p-value
8e-07
1e-05
2e-04
2e-03
4e-02
Log-odds
0.5
1.0
1.5
2.0
2.5
3.0
3.5
4.0
Cellular_organisms - ps1
Archaea1 - ps2
Archaea2 - ps3
Archaea3 - ps4
Asgard/Eukaryota - ps5
Eukaryota - ps6
Amorpheae - ps7
Obazoa - ps8
Opisthokonta - ps9
Holozoa - ps10
Filozoa - ps11
Choano./Metazoa - ps12
Metazoa - ps13
Myriazoa - ps14
Parahoxozoa - ps15
Bilateria - ps16
Deuterostomia - ps17
Chordata - ps18
Olfactores - ps19
Vertebrata - ps20
Gnathostomata - ps21
Euteleostomi - ps22
Sarcopterygii - ps23
Tetrapoda - ps24
Amniota - ps25
Mammalia - ps26
Theria - ps27
Placentalia - ps28
Boreoeutheria - ps29
Euarchontoglires - ps30
Euarchonta - ps31
Primates - ps32
Haplorrhini - ps33
Simiiformes - ps34
Catarrhini - ps35
Hominoidea - ps36
Hominidae - ps37
Homininae - ps38
Hominini - ps39
Homo_sapiens - ps40
Phylostratum

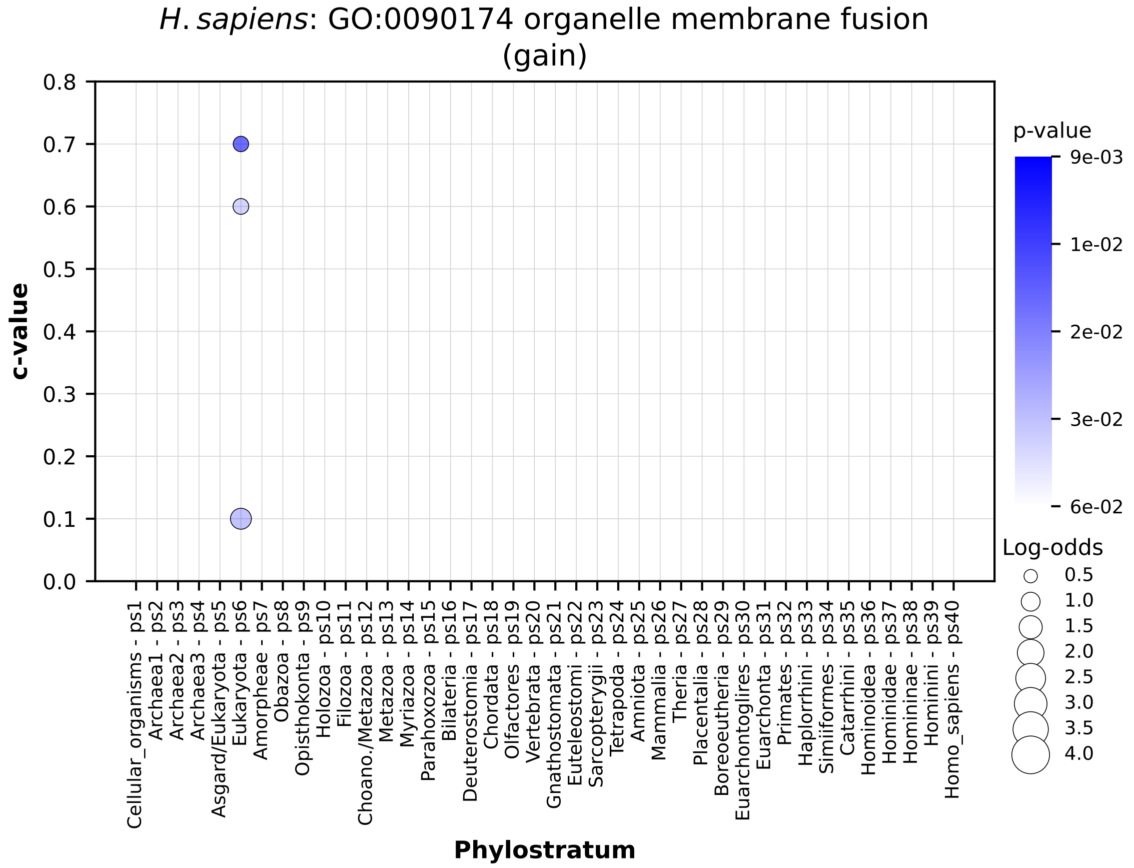
H. sapiens: GO:0090174 organelle membrane fusion
(gain)
c-value
0.0
0.1
0.2
0.3
0.4
0.5
0.6
0.7
0.8
Phylostratum
Cellular_organisms - ps1
Archaea1 - ps2
Archaea2 - ps3
Archaea3 - ps4
Asgard/Eukaryota - ps5
Eukaryota - ps6
Amorpheae - ps7
Obazoa - ps8
Opisthokonta - ps9
Holozoa - ps10
Filozoa - ps11
Choano./Metazoa - ps12
Metazoa - ps13
Myriazoa - ps14
Parahoxozoa - ps15
Bilateria - ps16
Deuterostomia - ps17
Chordata - ps18
Olfactores - ps19
Vertebrata - ps20
Gnathostomata - ps21
Euteleostomi - ps22
Sarcopterygii - ps23
Tetrapoda - ps24
Amniota - ps25
Mammalia - ps26
Theria - ps27
Placentalia - ps28
Boreoeutheria - ps29
Euarchontoglires - ps30
Euarchonta - ps31
Primates - ps32
Haplorrhini - ps33
Simiiformes - ps34
Catarrhini - ps35
Hominoidea - ps36
Hominidae - ps37
Homininae - ps38
Hominini - ps39
Homo_sapiens - ps40
p-value
9e-03
1e-02
2e-02
3e-02
6e-02
Log-odds
0.5
1.0
1.5
2.0
2.5
3.0
3.5
4.0

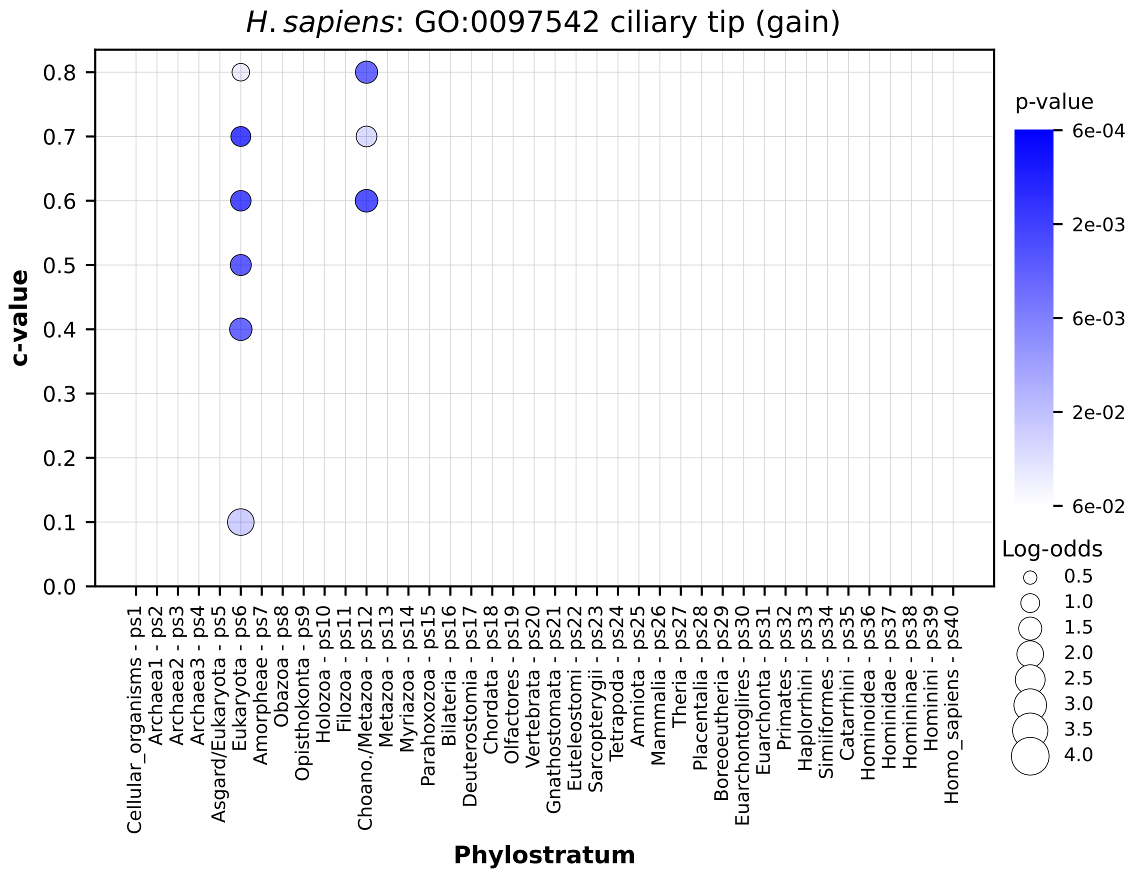
H. sapiens: GO:0097542 ciliary tip (gain)
c-value
0.8
0.7
0.6
0.5
0.4
0.3
0.2
0.1
0.0
p-value
6e-04
2e-03
6e-03
2e-02
6e-02
Log-odds
0.5
1.0
1.5
2.0
2.5
3.0
3.5
4.0
Cellular_organisms - ps1
Archaea1 - ps2
Archaea2 - ps3
Archaea3 - ps4
Asgard/Eukaryota - ps5
Eukaryota - ps6
Amorpheae - ps7
Obazoa - ps8
Opisthokonta - ps9
Holozoa - ps10
Filozoa - ps11
Choano./Metazoa - ps12
Metazoa - ps13
Myriazoa - ps14
Parahoxozoa - ps15
Bilateria - ps16
Deuterostomia - ps17
Chordata - ps18
Olfactores - ps19
Vertebrata - ps20
Gnathostomata - ps21
Euteleostomi - ps22
Sarcopterygii - ps23
Tetrapoda - ps24
Amniota - ps25
Mammalia - ps26
Theria - ps27
Placentalia - ps28
Boreoeutheria - ps29
Euarchontoglires - ps30
Euarchonta - ps31
Primates - ps32
Haplorrhini - ps33
Simiiformes - ps34
Catarrhini - ps35
Hominoidea - ps36
Hominidae - ps37
Homininae - ps38
Hominini - ps39
Homo_sapiens - ps40
Phylostratum

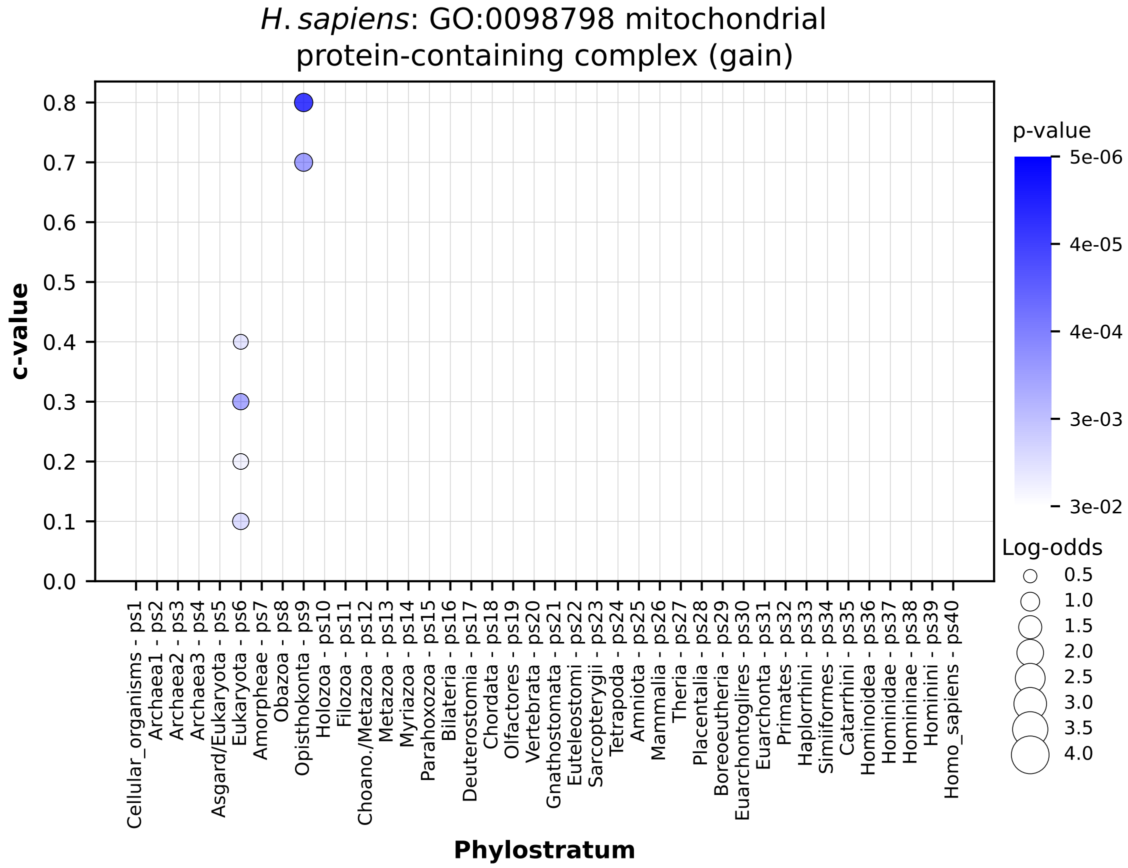

H. sapiens: GO:0098798 mitochondrial protein-containing complex (gain)
c-value
0.0
0.1
0.2
0.3
0.4
0.5
0.6
0.7
0.8
Phylostratum
Cellular_organisms - ps1
Archaea1 - ps2
Archaea2 - ps3
Archaea3 - ps4
Asgard/Eukaryota - ps5
Eukaryota - ps6
Amorpheae - ps7
Obazoa - ps8
Opisthokonta - ps9
Holozoa - ps10
Filozoa - ps11
Choano./Metazoa - ps12
Metazoa - ps13
Myriazoa - ps14
Parahoxozoa - ps15
Bilateria - ps16
Deuterostomia - ps17
Chordata - ps18
Olfactores - ps19
Vertebrata - ps20
Gnathostomata - ps21
Euteleostomi - ps22
Sarcopterygii - ps23
Tetrapoda - ps24
Amniota - ps25
Mammalia - ps26
Theria - ps27
Placentalia - ps28
Boreoeutheria - ps29
Euarchontoglires - ps30
Euarchonta - ps31
Primates - ps32
Haplorrhini - ps33
Simiiformes - ps34
Catarrhini - ps35
Hominoidea - ps36
Hominidae - ps37
Homininae - ps38
Hominini - ps39
Homo_sapiens - ps40
p-value
5e-06
4e-05
4e-04
3e-03
3e-02
Log-odds
0.5
1.0
1.5
2.0
2.5
3.0
3.5
4.0

# *H. sapiens*: GO:1903460 mitotic DNA replication leading strand elongation (gain)

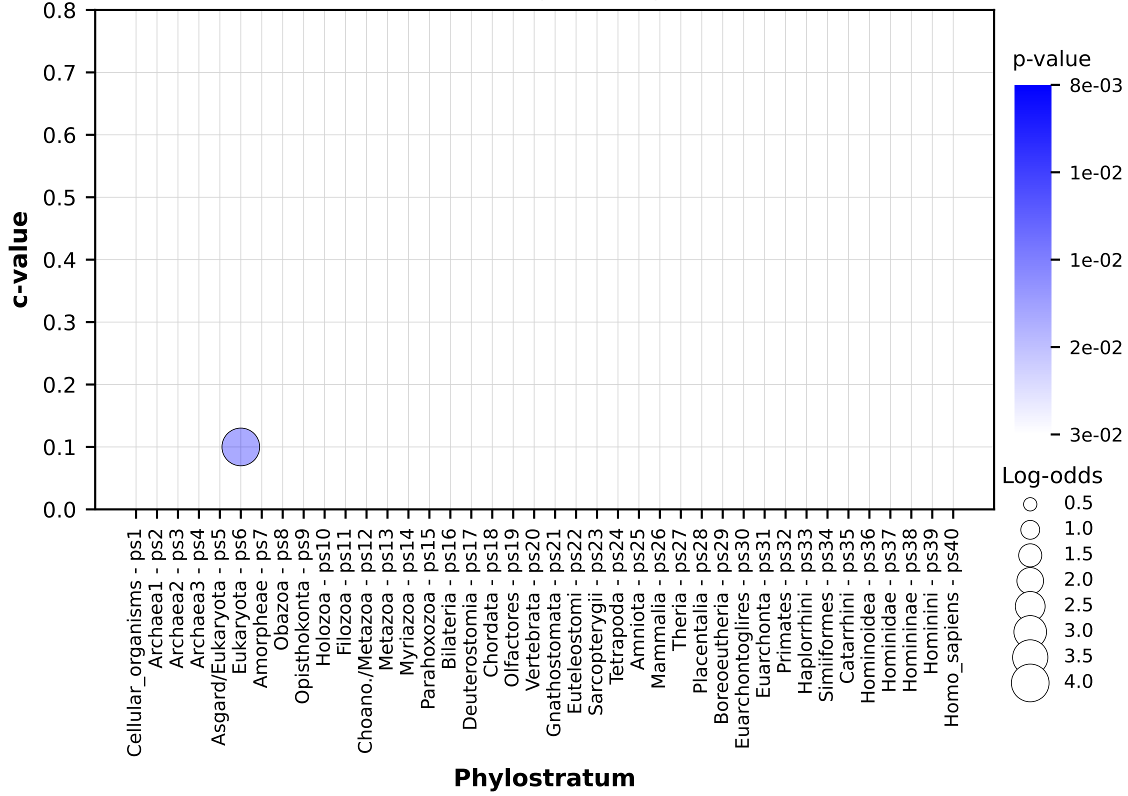

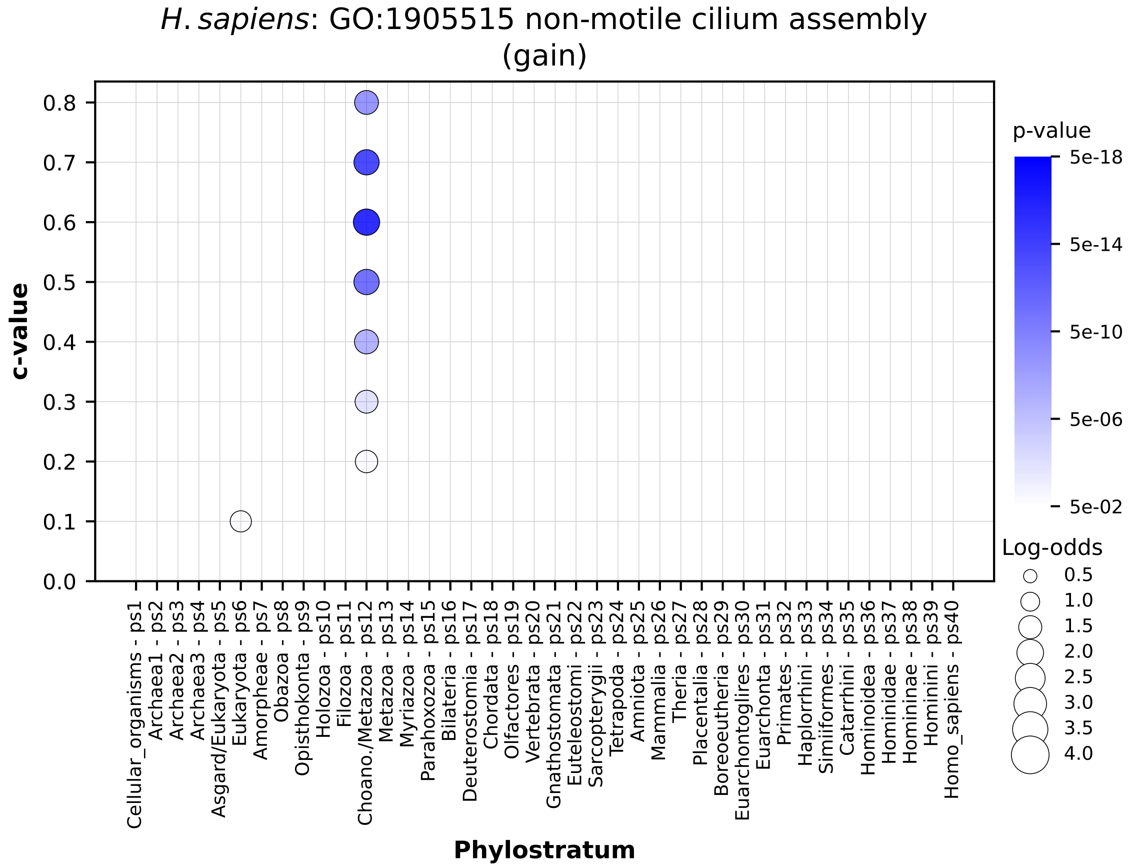

H. sapiens: GO:1905515 non-motile cilium assembly
(gain)
c-value
0.0
0.1
0.2
0.3
0.4
0.5
0.6
0.7
0.8
p-value
5e-18
5e-14
5e-10
5e-06
5e-02
Log-odds
0.5
1.0
1.5
2.0
2.5
3.0
3.5
4.0
Cellular_organisms - ps1
Archaea1 - ps2
Archaea2 - ps3
Archaea3 - ps4
Asgard/Eukaryota - ps5
Eukaryota - ps6
Amorpheae - ps7
Obazoa - ps8
Opisthokonta - ps9
Holozoa - ps10
Filozoa - ps11
Choano./Metazoa - ps12
Metazoa - ps13
Myriazoa - ps14
Parahoxozoa - ps15
Bilateria - ps16
Deuterostomia - ps17
Chordata - ps18
Olfactores - ps19
Vertebrata - ps20
Gnathostomata - ps21
Euteleostomi - ps22
Sarcopterygii - ps23
Tetrapoda - ps24
Amniota - ps25
Mammalia - ps26
Theria - ps27
Placentalia - ps28
Boreoeutheria - ps29
Euarchontoglires - ps30
Euarchonta - ps31
Primates - ps32
Haplorrhini - ps33
Simiiformes - ps34
Catarrhini - ps35
Hominoidea - ps36
Hominidae - ps37
Homininae - ps38
Hominini - ps39
Homo_sapiens - ps40
Phylostratum

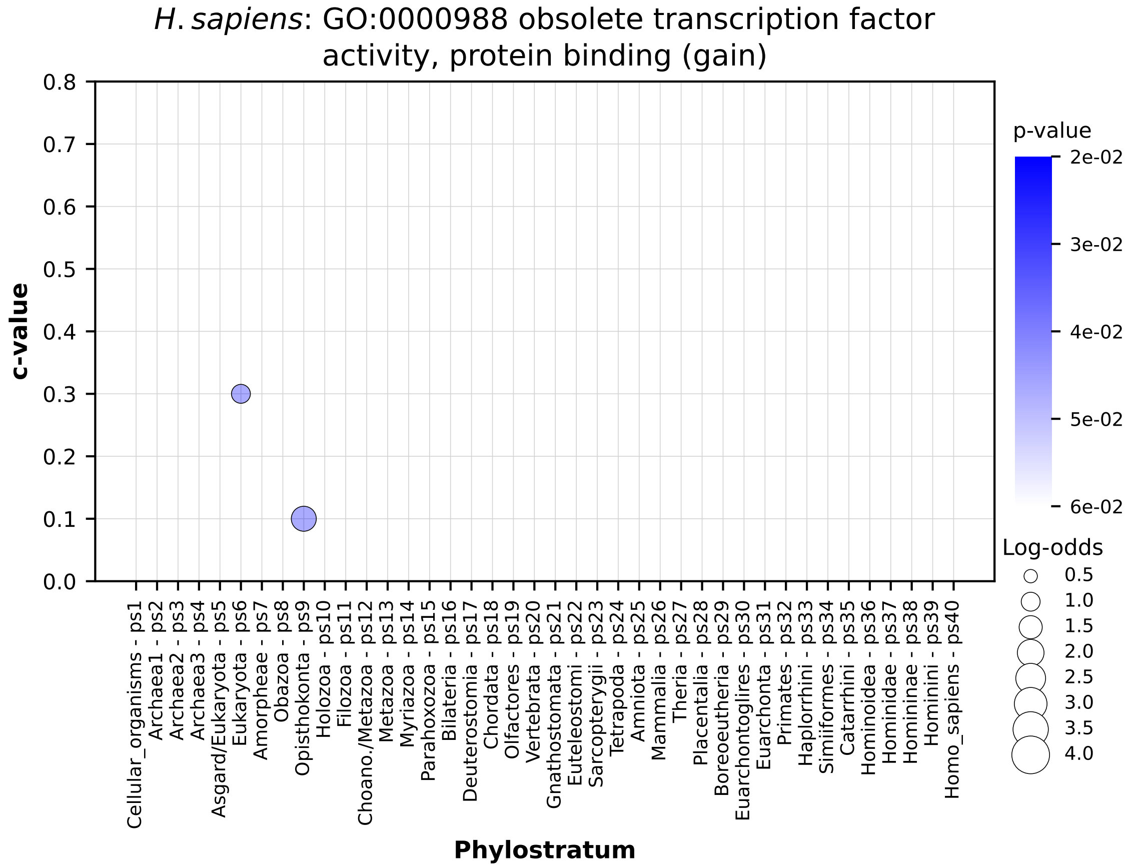

*H. sapiens*: GO:0000988 obsolete transcription factor activity, protein binding (gain)
c-value
0.0
0.1
0.2
0.3
0.4
0.5
0.6
0.7
0.8
Phylostratum
Cellular_organisms - ps1
Archaea1 - ps2
Archaea2 - ps3
Archaea3 - ps4
Asgard/Eukaryota - ps5
Eukaryota - ps6
Amorpheae - ps7
Obazoa - ps8
Opisthokonta - ps9
Holozoa - ps10
Filozoa - ps11
Choano./Metazoa - ps12
Metazoa - ps13
Myriazoa - ps14
Parahoxozoa - ps15
Bilateria - ps16
Deuterostomia - ps17
Chordata - ps18
Olfactores - ps19
Vertebrata - ps20
Gnathostomata - ps21
Euteleostomi - ps22
Sarcopterygii - ps23
Tetrapoda - ps24
Amniota - ps25
Mammalia - ps26
Theria - ps27
Placentalia - ps28
Boreoeutheria - ps29
Euarchontoglires - ps30
Euarchonta - ps31
Primates - ps32
Haplorrhini - ps33
Simiiformes - ps34
Catarrhini - ps35
Hominoidea - ps36
Hominidae - ps37
Homininae - ps38
Hominini - ps39
Homo_sapiens - ps40
p-value
2e-02
3e-02
4e-02
5e-02
6e-02
Log-odds
0.5
1.0
1.5
2.0
2.5
3.0
3.5
4.0

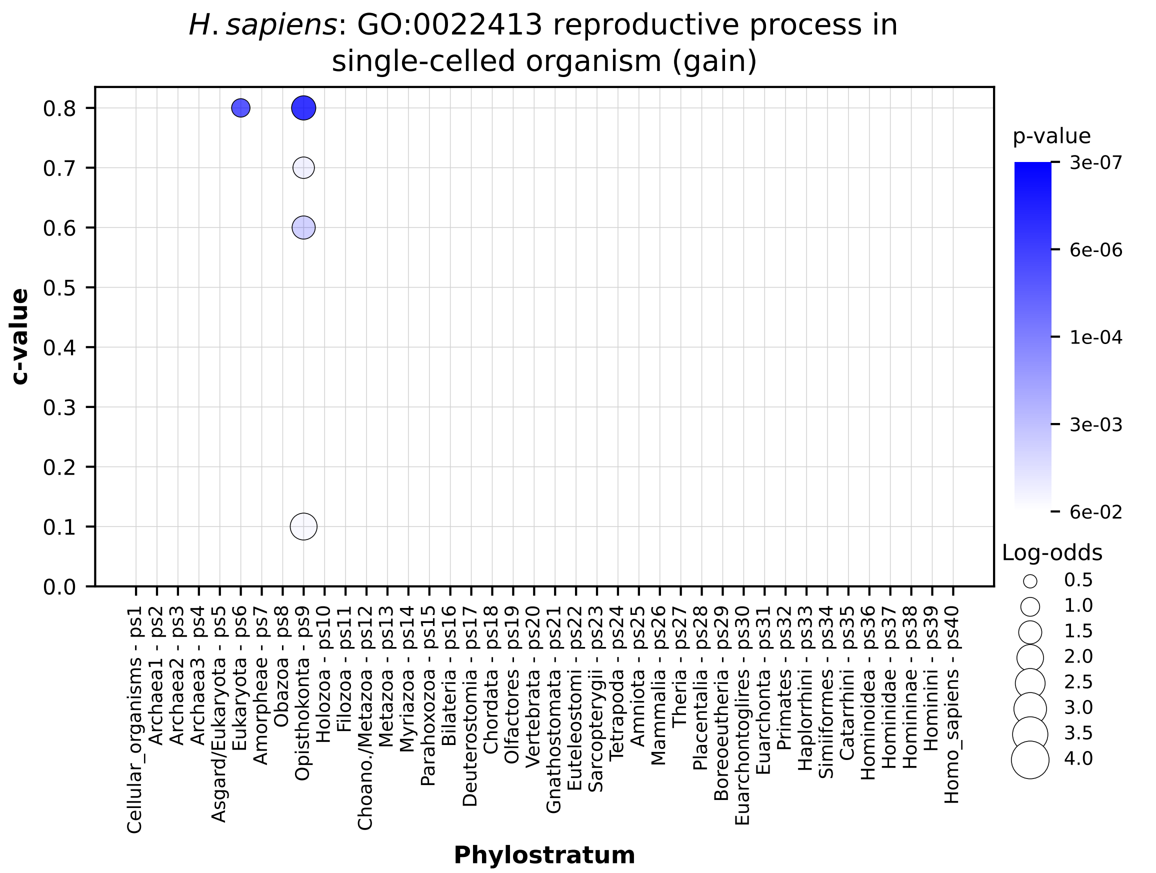

*H. sapiens*: GO:0022413 reproductive process in single-celled organism (gain)
c-value
0.0
0.1
0.2
0.3
0.4
0.5
0.6
0.7
0.8
Phylostratum
Cellular_organisms - ps1
Archaea1 - ps2
Archaea2 - ps3
Archaea3 - ps4
Asgard/Eukaryota - ps5
Eukaryota - ps6
Amorpheae - ps7
Obazoa - ps8
Opisthokonta - ps9
Holozoa - ps10
Filozoa - ps11
Choano./Metazoa - ps12
Metazoa - ps13
Myriazoa - ps14
Parahoxozoa - ps15
Bilateria - ps16
Deuterostomia - ps17
Chordata - ps18
Olfactores - ps19
Vertebrata - ps20
Gnathostomata - ps21
Euteleostomi - ps22
Sarcopterygii - ps23
Tetrapoda - ps24
Amniota - ps25
Mammalia - ps26
Theria - ps27
Placentalia - ps28
Boreoeutheria - ps29
Euarchontoglires - ps30
Euarchonta - ps31
Primates - ps32
Haplorrhini - ps33
Simiiformes - ps34
Catarrhini - ps35
Hominoidea - ps36
Hominidae - ps37
Homininae - ps38
Hominini - ps39
Homo_sapiens - ps40
p-value
3e-07
6e-06
1e-04
3e-03
6e-02
Log-odds
0.5
1.0
1.5
2.0
2.5
3.0
3.5
4.0

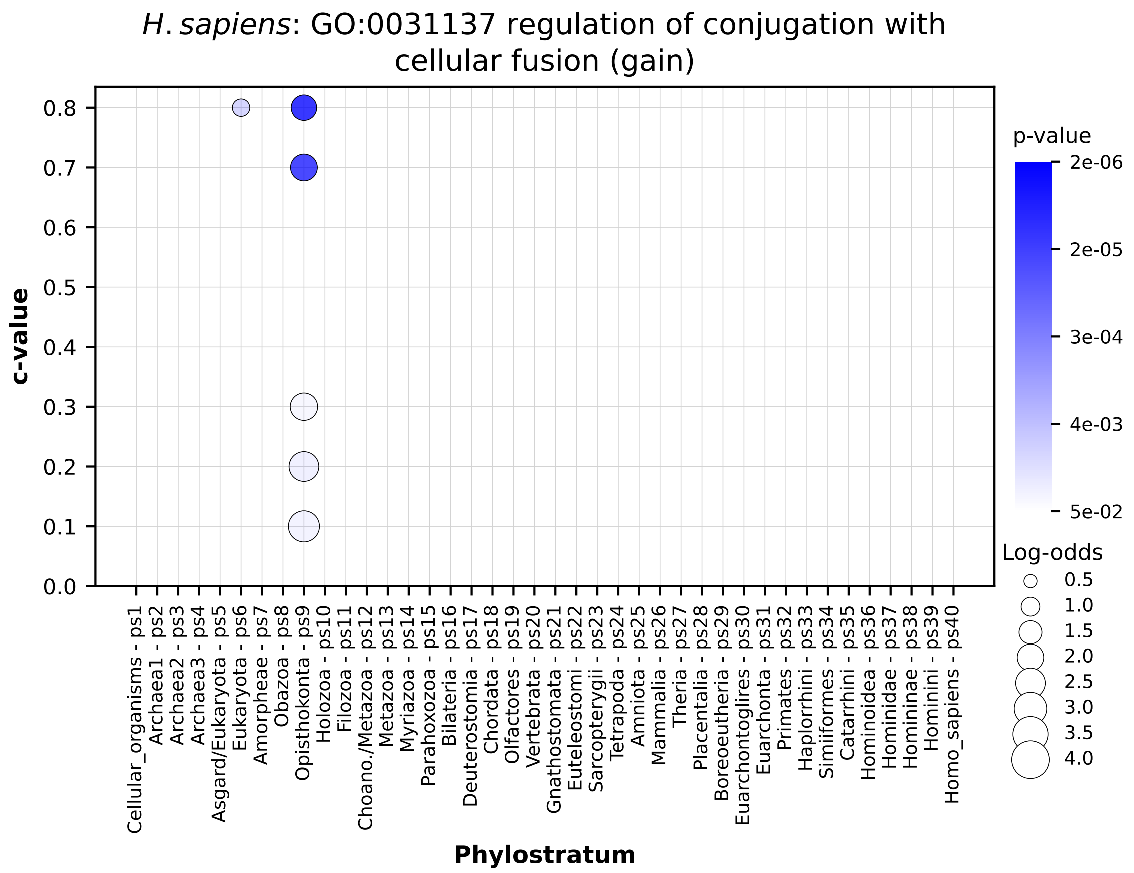

H. sapiens: GO:0031137 regulation of conjugation with cellular fusion (gain)
c-value
0.0
0.1
0.2
0.3
0.4
0.5
0.6
0.7
0.8
Phylostratum
Cellular_organisms - ps1
Archaea1 - ps2
Archaea2 - ps3
Archaea3 - ps4
Asgard/Eukaryota - ps5
Eukaryota - ps6
Amorpheae - ps7
Obazoa - ps8
Opisthokonta - ps9
Holozoa - ps10
Filozoa - ps11
Choano./Metazoa - ps12
Metazoa - ps13
Myriazoa - ps14
Parahoxozoa - ps15
Bilateria - ps16
Deuterostomia - ps17
Chordata - ps18
Olfactores - ps19
Vertebrata - ps20
Gnathostomata - ps21
Euteleostomi - ps22
Sarcopterygii - ps23
Tetrapoda - ps24
Amniota - ps25
Mammalia - ps26
Theria - ps27
Placentalia - ps28
Boreoeutheria - ps29
Euarchontoglires - ps30
Euarchonta - ps31
Primates - ps32
Haplorrhini - ps33
Simiiformes - ps34
Catarrhini - ps35
Hominoidea - ps36
Hominidae - ps37
Homininae - ps38
Hominini - ps39
Homo_sapiens - ps40
p-value
2e-06
2e-05
3e-04
4e-03
5e-02
Log-odds
0.5
1.0
1.5
2.0
2.5
3.0
3.5
4.0

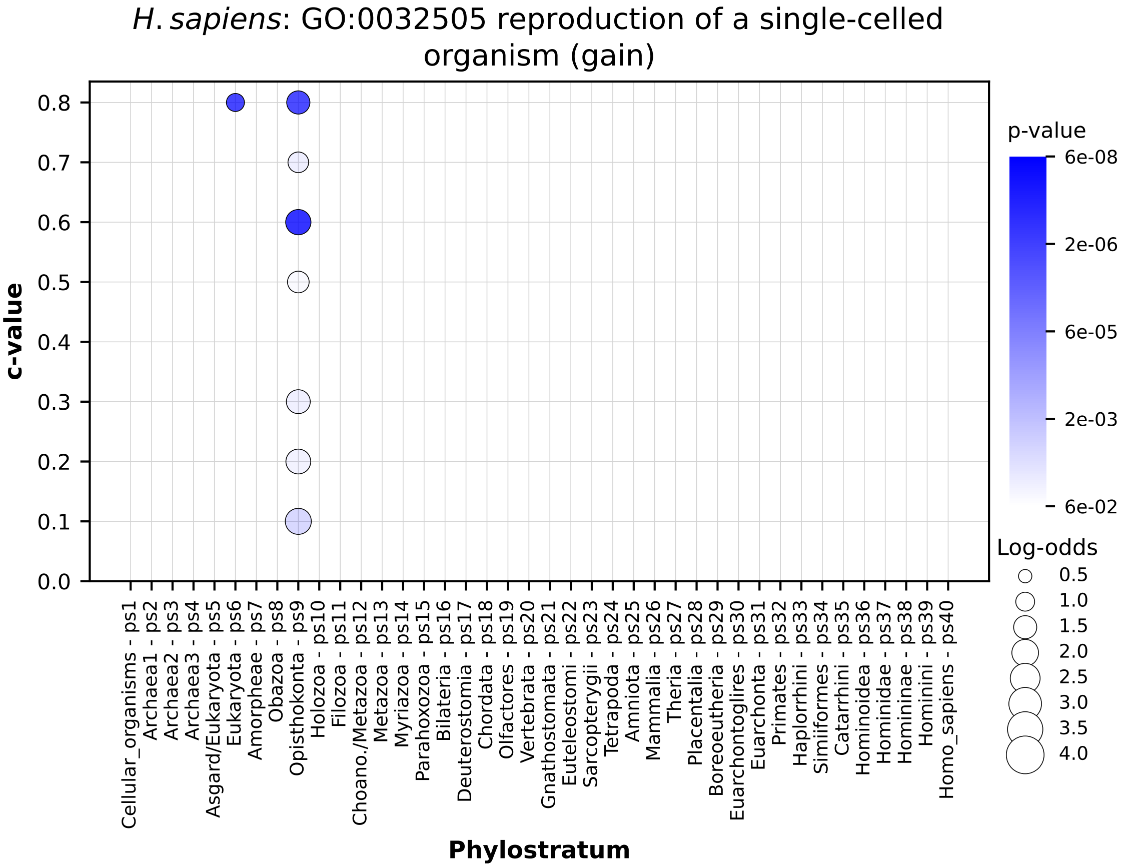

H. sapiens: GO:0032505 reproduction of a single-celled organism (gain)
c-value
0.8
0.7
0.6
0.5
0.4
0.3
0.2
0.1
0.0
Phylostratum
Cellular_organisms - ps1
Archaea1 - ps2
Archaea2 - ps3
Archaea3 - ps4
Asgard/Eukaryota - ps5
Eukaryota - ps6
Amorpheae - ps7
Obazoa - ps8
Opisthokonta - ps9
Holozoa - ps10
Filozoa - ps11
Choano./Metazoa - ps12
Metazoa - ps13
Myriazoa - ps14
Parahoxozoa - ps15
Bilateria - ps16
Deuterostomia - ps17
Chordata - ps18
Olfactores - ps19
Vertebrata - ps20
Gnathostomata - ps21
Euteleostomi - ps22
Sarcopterygii - ps23
Tetrapoda - ps24
Amniota - ps25
Mammalia - ps26
Theria - ps27
Placentalia - ps28
Boreoeutheria - ps29
Euarchontoglires - ps30
Euarchonta - ps31
Primates - ps32
Haplorrhini - ps33
Simiiformes - ps34
Catarrhini - ps35
Hominoidea - ps36
Hominidae - ps37
Homininae - ps38
Hominini - ps39
Homo_sapiens - ps40
p-value
6e-08
2e-06
6e-05
2e-03
6e-02
Log-odds
0.5
1.0
1.5
2.0
2.5
3.0
3.5
4.0

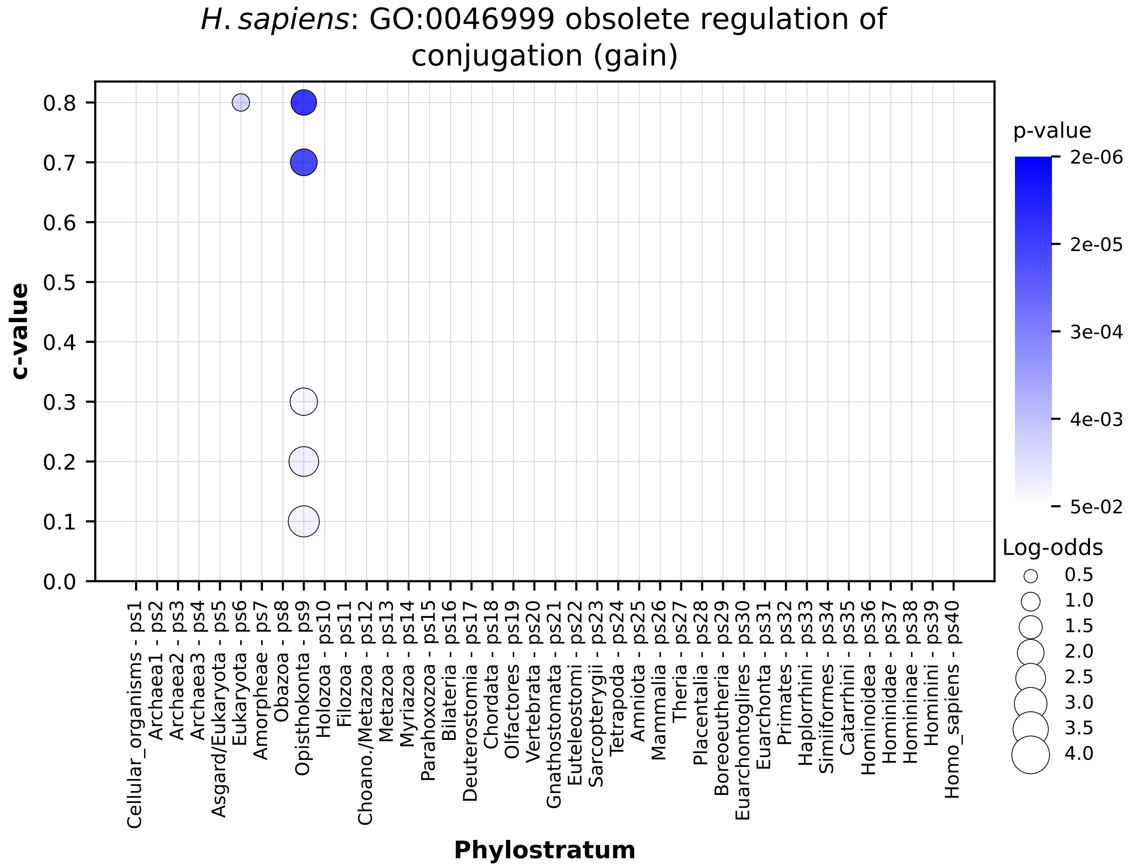
H. sapiens: GO:0046999 obsolete regulation of conjugation (gain)
c-value
0.0
0.1
0.2
0.3
0.4
0.5
0.6
0.7
0.8
Phylostratum
Cellular_organisms - ps1
Archaea1 - ps2
Archaea2 - ps3
Archaea3 - ps4
Asgard/Eukaryota - ps5
Eukaryota - ps6
Amorpheae - ps7
Obazoa - ps8
Opisthokonta - ps9
Holozoa - ps10
Filozoa - ps11
Choano./Metazoa - ps12
Metazoa - ps13
Myriazoa - ps14
Parahoxozoa - ps15
Bilateria - ps16
Deuterostomia - ps17
Chordata - ps18
Olfactores - ps19
Vertebrata - ps20
Gnathostomata - ps21
Euteleostomi - ps22
Sarcopterygii - ps23
Tetrapoda - ps24
Amniota - ps25
Mammalia - ps26
Theria - ps27
Placentalia - ps28
Boreoeutheria - ps29
Euarchontoglires - ps30
Euarchonta - ps31
Primates - ps32
Haplorrhini - ps33
Simiiformes - ps34
Catarrhini - ps35
Hominoidea - ps36
Hominidae - ps37
Homininae - ps38
Hominini - ps39
Homo_sapiens - ps40
p-value
2e-06
2e-05
3e-04
4e-03
5e-02
Log-odds
0.5
1.0
1.5
2.0
2.5
3.0
3.5
4.0

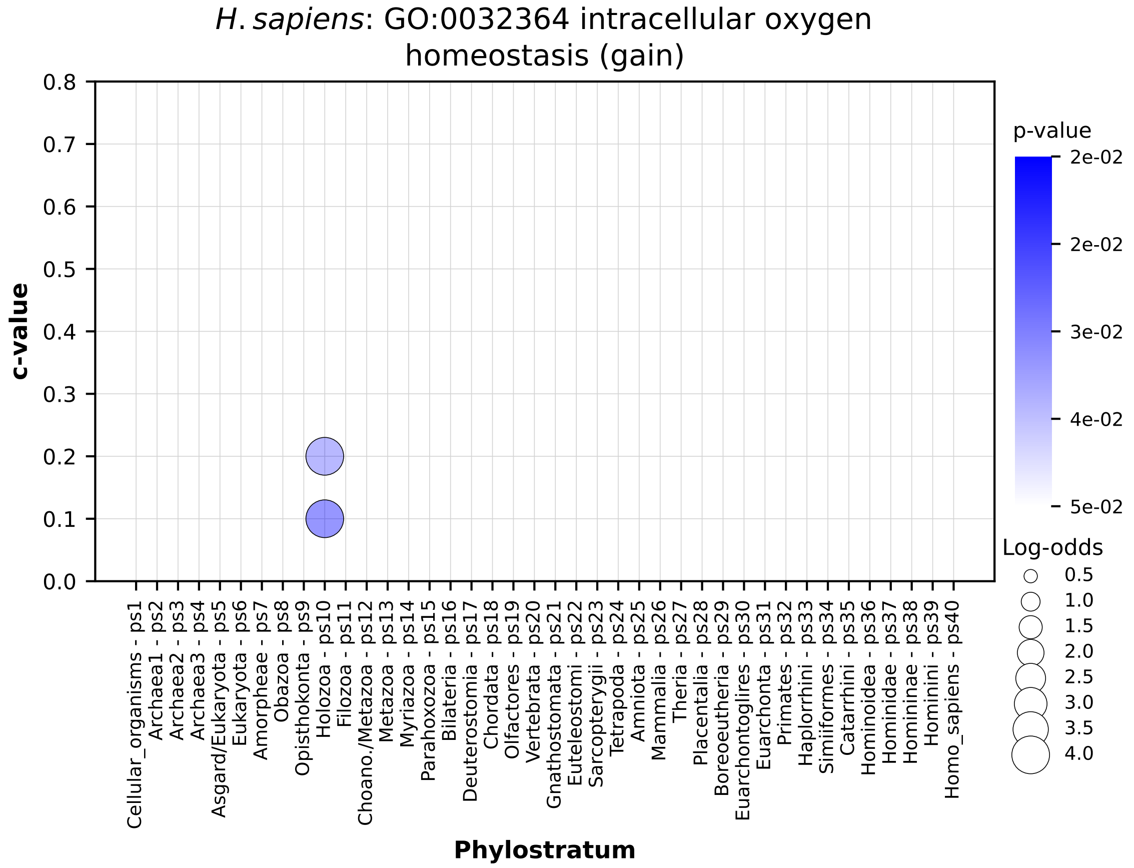

H. sapiens: GO:0032364 intracellular oxygen homeostasis (gain)
c-value
0.0
0.1
0.2
0.3
0.4
0.5
0.6
0.7
0.8
Phylostratum
Cellular_organisms - ps1
Archaea1 - ps2
Archaea2 - ps3
Archaea3 - ps4
Asgard/Eukaryota - ps5
Eukaryota - ps6
Amorpheae - ps7
Obazoa - ps8
Opisthokonta - ps9
Holozoa - ps10
Filozoa - ps11
Choano./Metazoa - ps12
Metazoa - ps13
Myriazoa - ps14
Parahoxozoa - ps15
Bilateria - ps16
Deuterostomia - ps17
Chordata - ps18
Olfactores - ps19
Vertebrata - ps20
Gnathostomata - ps21
Euteleostomi - ps22
Sarcopterygii - ps23
Tetrapoda - ps24
Amniota - ps25
Mammalia - ps26
Theria - ps27
Placentalia - ps28
Boreoeutheria - ps29
Euarchontoglires - ps30
Euarchonta - ps31
Primates - ps32
Haplorrhini - ps33
Simiiformes - ps34
Catarrhini - ps35
Hominoidea - ps36
Hominidae - ps37
Homininae - ps38
Hominini - ps39
Homo_sapiens - ps40
p-value
2e-02
2e-02
3e-02
4e-02
5e-02
Log-odds
0.5
1.0
1.5
2.0
2.5
3.0
3.5
4.0

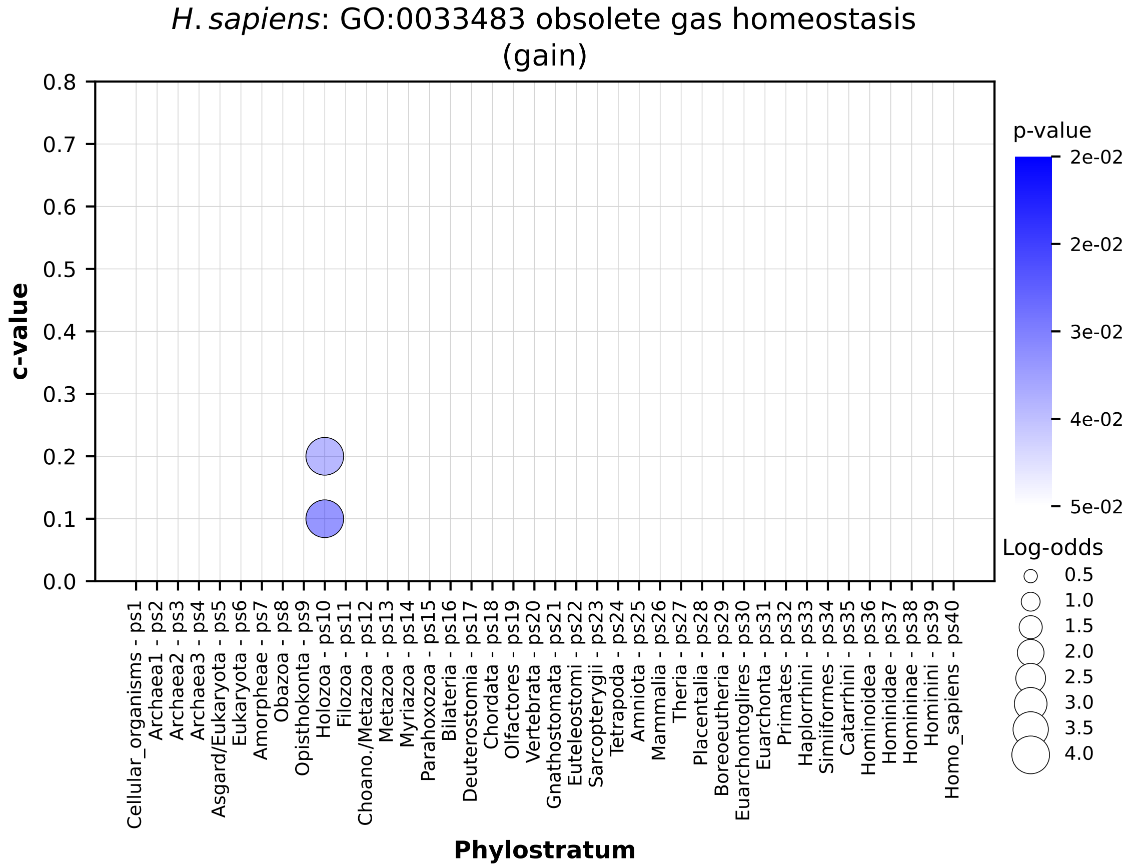
H. sapiens: GO:0033483 obsolete gas homeostasis
(gain)
c-value
0.8
0.7
0.6
0.5
0.4
0.3
0.2
0.1
0.0
p-value
2e-02
2e-02
3e-02
4e-02
5e-02
Log-odds
0.5
1.0
1.5
2.0
2.5
3.0
3.5
4.0
Cellular_organisms - ps1
Archaea1 - ps2
Archaea2 - ps3
Archaea3 - ps4
Asgard/Eukaryota - ps5
Eukaryota - ps6
Amorpheae - ps7
Obazoa - ps8
Opisthokonta - ps9
Holozoa - ps10
Filozoa - ps11
Choano./Metazoa - ps12
Metazoa - ps13
Myriazoa - ps14
Parahoxozoa - ps15
Bilateria - ps16
Deuterostomia - ps17
Chordata - ps18
Olfactores - ps19
Vertebrata - ps20
Gnathostomata - ps21
Euteleostomi - ps22
Sarcopterygii - ps23
Tetrapoda - ps24
Amniota - ps25
Mammalia - ps26
Theria - ps27
Placentalia - ps28
Boreoeutheria - ps29
Euarchontoglires - ps30
Euarchonta - ps31
Primates - ps32
Haplorrhini - ps33
Simiiformes - ps34
Catarrhini - ps35
Hominoidea - ps36
Hominidae - ps37
Homininae - ps38
Hominini - ps39
Homo_sapiens - ps40
Phylostratum

*H. sapiens*: GO:1902117 positive regulation of organelle assembly (gain)

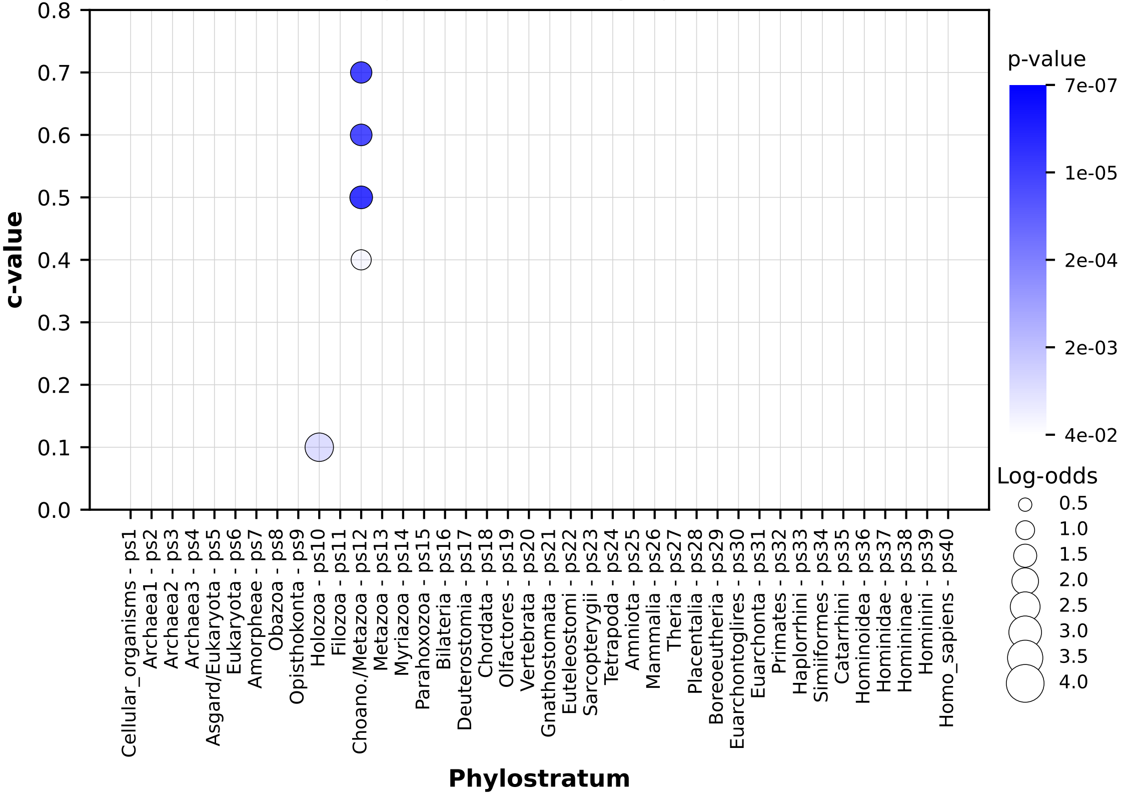

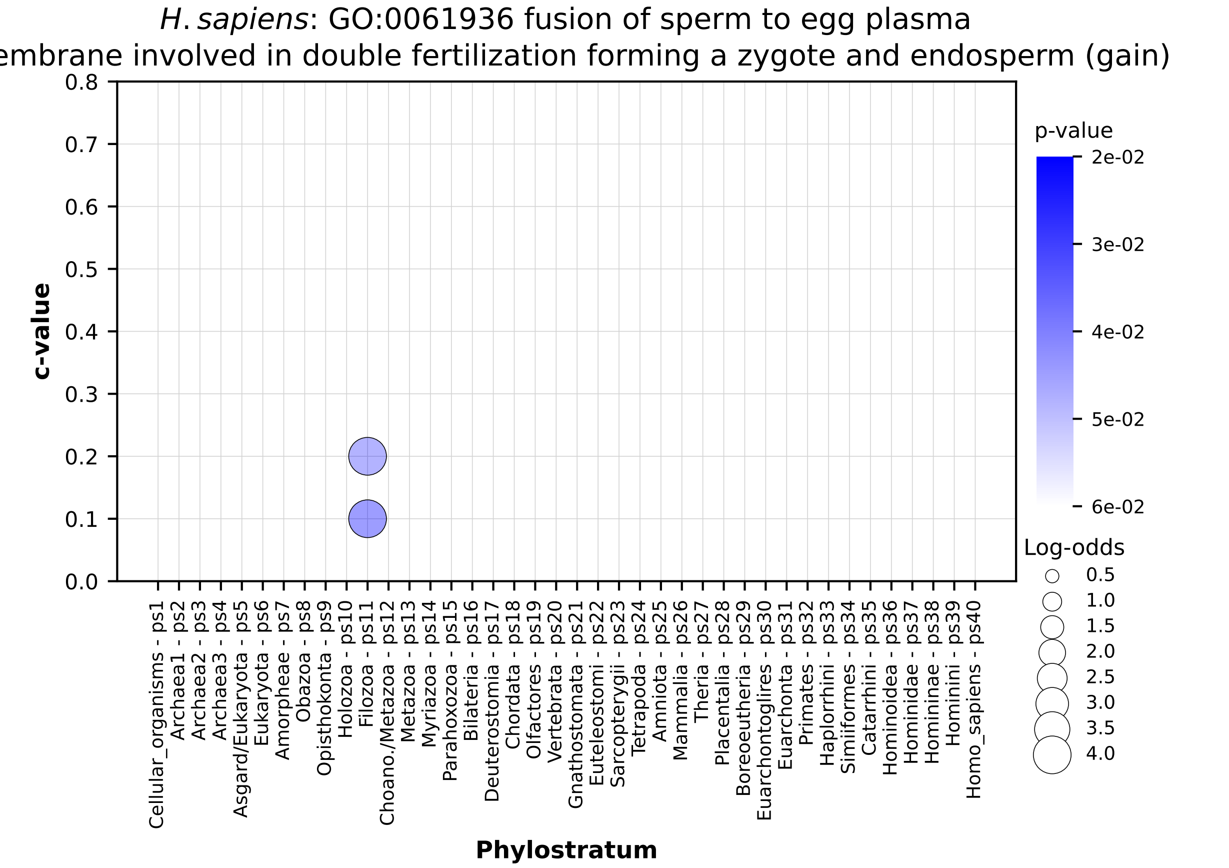

H. sapiens: GO:0061936 fusion of sperm to egg plasma
embrane involved in double fertilization forming a zygote and endosperm (gain)
c-value
0.0
0.1
0.2
0.3
0.4
0.5
0.6
0.7
0.8
Phylostratum
Cellular_organisms - ps1
Archaea1 - ps2
Archaea2 - ps3
Archaea3 - ps4
Asgard/Eukaryota - ps5
Eukaryota - ps6
Amorpheae - ps7
Obazoa - ps8
Opisthokonta - ps9
Holozoa - ps10
Filozoa - ps11
Choano./Metazoa - ps12
Metazoa - ps13
Myriazoa - ps14
Parahoxozoa - ps15
Bilateria - ps16
Deuterostomia - ps17
Chordata - ps18
Olfactores - ps19
Vertebrata - ps20
Gnathostomata - ps21
Euteleostomi - ps22
Sarcopterygii - ps23
Tetrapoda - ps24
Amniota - ps25
Mammalia - ps26
Theria - ps27
Placentalia - ps28
Boreoeutheria - ps29
Euarchontoglires - ps30
Euarchonta - ps31
Primates - ps32
Haplorrhini - ps33
Simiiformes - ps34
Catarrhini - ps35
Hominoidea - ps36
Hominidae - ps37
Homininae - ps38
Hominini - ps39
Homo_sapiens - ps40
p-value
2e-02
3e-02
4e-02
5e-02
6e-02
Log-odds
0.5
1.0
1.5
2.0
2.5
3.0
3.5
4.0

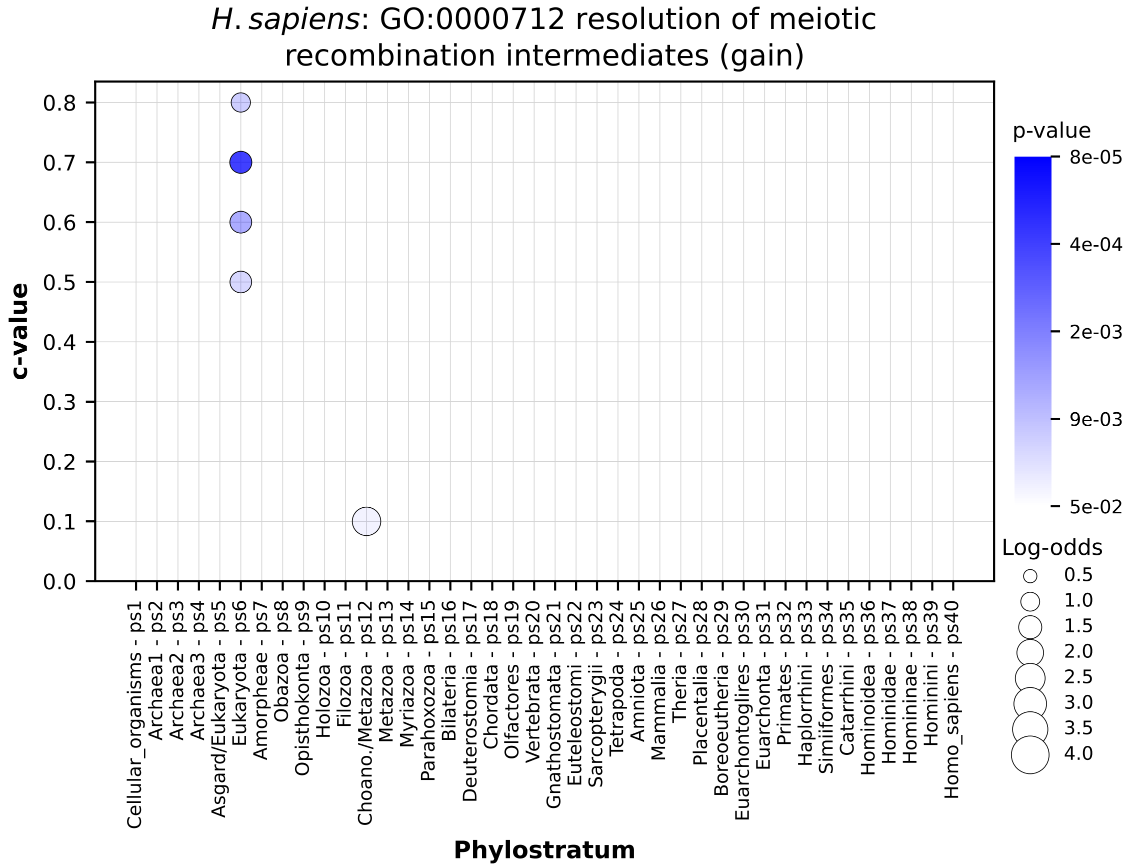

*H. sapiens*: GO:0000712 resolution of meiotic recombination intermediates (gain)
c-value
Phylostratum
p-value
8e-05
4e-04
2e-03
9e-03
5e-02
Log-odds
0.5
1.0
1.5
2.0
2.5
3.0
3.5
4.0
Cellular_organisms - ps1
Archaea1 - ps2
Archaea2 - ps3
Archaea3 - ps4
Asgard/Eukaryota - ps5
Eukaryota - ps6
Amorpheae - ps7
Obazoa - ps8
Opisthokonta - ps9
Holozoa - ps10
Filozoa - ps11
Choano./Metazoa - ps12
Metazoa - ps13
Myriazoa - ps14
Parahoxozoa - ps15
Bilateria - ps16
Deuterostomia - ps17
Chordata - ps18
Olfactores - ps19
Vertebrata - ps20
Gnathostomata - ps21
Euteleostomi - ps22
Sarcopterygii - ps23
Tetrapoda - ps24
Amniota - ps25
Mammalia - ps26
Theria - ps27
Placentalia - ps28
Boreoeutheria - ps29
Euarchontoglires - ps30
Euarchonta - ps31
Primates - ps32
Haplorrhini - ps33
Simiiformes - ps34
Catarrhini - ps35
Hominoidea - ps36
Hominidae - ps37
Homininae - ps38
Hominini - ps39
Homo_sapiens - ps40

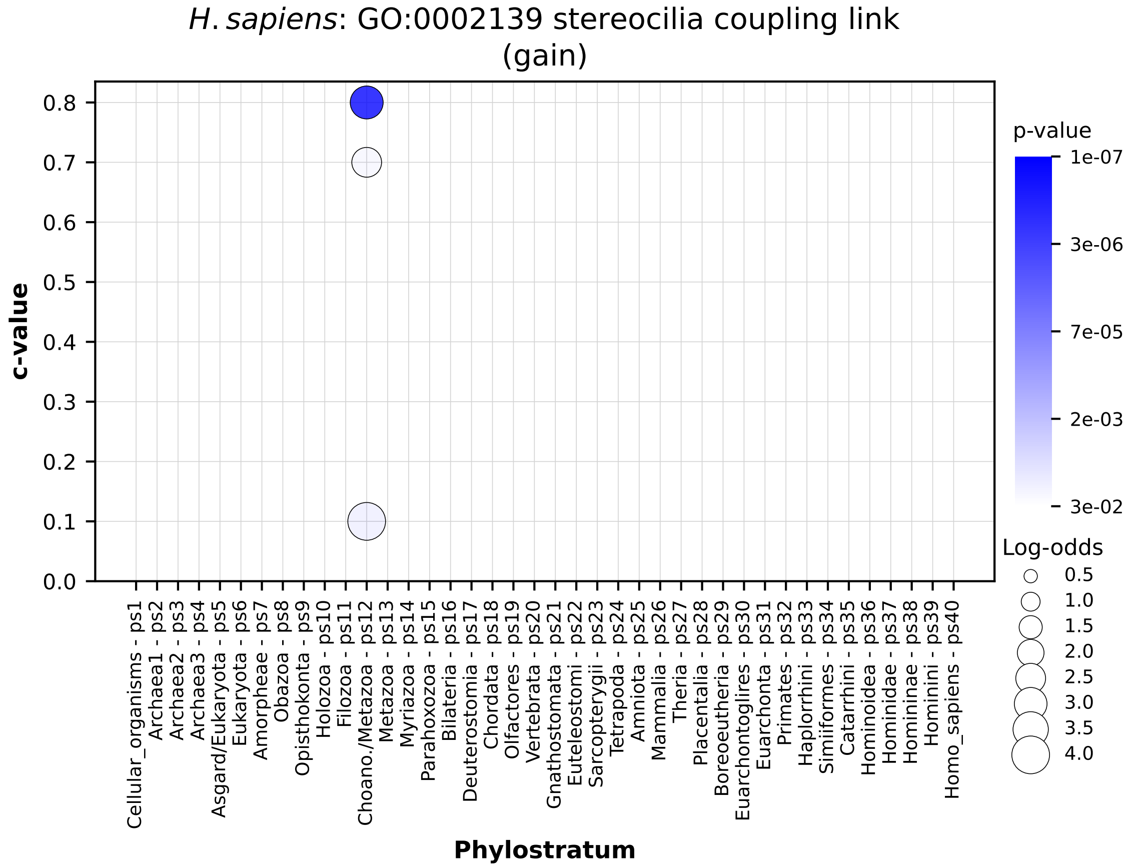

H. sapiens: GO:0002139 stereocilia coupling link
(gain)
c-value
0.0
0.1
0.2
0.3
0.4
0.5
0.6
0.7
0.8
Phylostratum
Cellular_organisms - ps1
Archaea1 - ps2
Archaea2 - ps3
Archaea3 - ps4
Asgard/Eukaryota - ps5
Eukaryota - ps6
Amorpheae - ps7
Obazoa - ps8
Opisthokonta - ps9
Holozoa - ps10
Filozoa - ps11
Choano./Metazoa - ps12
Metazoa - ps13
Myriazoa - ps14
Parahoxozoa - ps15
Bilateria - ps16
Deuterostomia - ps17
Chordata - ps18
Olfactores - ps19
Vertebrata - ps20
Gnathostomata - ps21
Euteleostomi - ps22
Sarcopterygii - ps23
Tetrapoda - ps24
Amniota - ps25
Mammalia - ps26
Theria - ps27
Placentalia - ps28
Boreoeutheria - ps29
Euarchontoglires - ps30
Euarchonta - ps31
Primates - ps32
Haplorrhini - ps33
Simiiformes - ps34
Catarrhini - ps35
Hominoidea - ps36
Hominidae - ps37
Homininae - ps38
Hominini - ps39
Homo_sapiens - ps40
p-value
1e-07
3e-06
7e-05
2e-03
3e-02
Log-odds
0.5
1.0
1.5
2.0
2.5
3.0
3.5
4.0

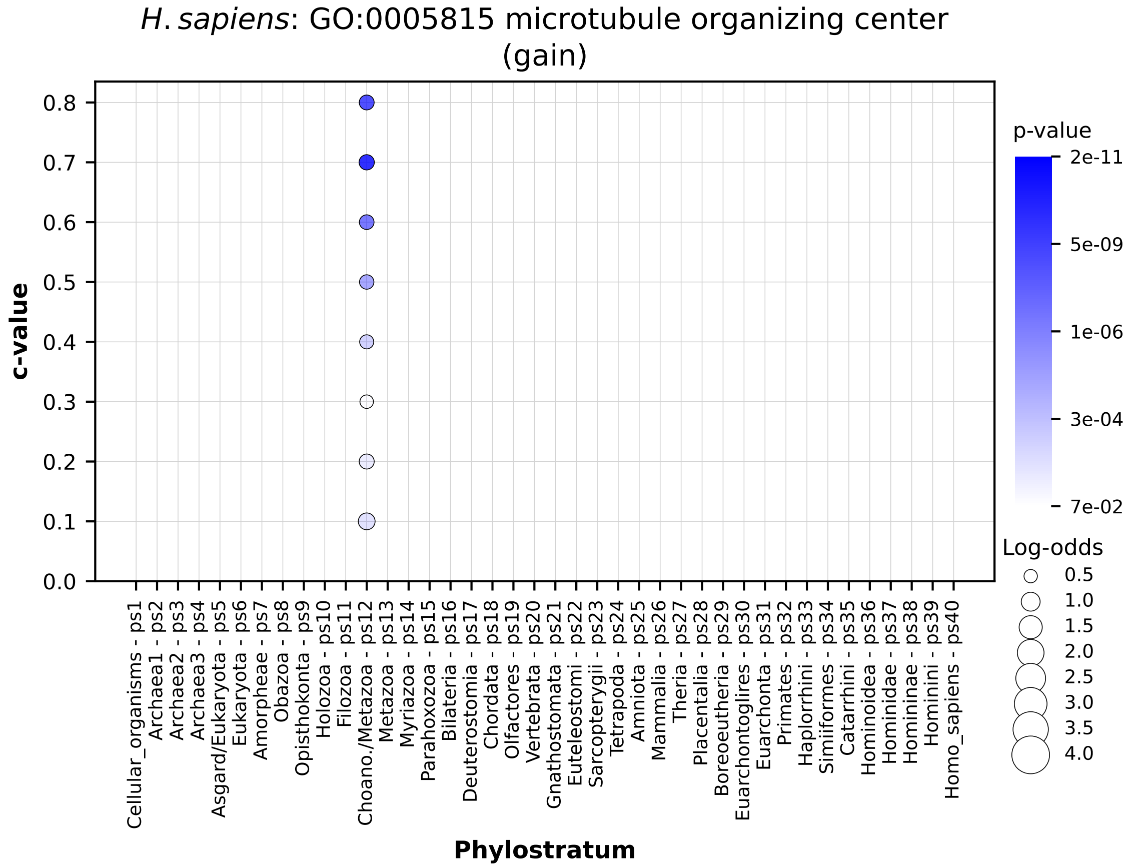

*H. sapiens*: GO:0005815 microtubule organizing center (gain)
c-value
0.0
0.1
0.2
0.3
0.4
0.5
0.6
0.7
0.8
p-value
2e-11
5e-09
1e-06
3e-04
7e-02
Log-odds
0.5
1.0
1.5
2.0
2.5
3.0
3.5
4.0
Cellular_organisms - ps1
Archaea1 - ps2
Archaea2 - ps3
Archaea3 - ps4
Asgard/Eukaryota - ps5
Eukaryota - ps6
Amorpheae - ps7
Obazoa - ps8
Opisthokonta - ps9
Holozoa - ps10
Filozoa - ps11
Choano./Metazoa - ps12
Metazoa - ps13
Myriazoa - ps14
Parahoxozoa - ps15
Bilateria - ps16
Deuterostomia - ps17
Chordata - ps18
Olfactores - ps19
Vertebrata - ps20
Gnathostomata - ps21
Euteleostomi - ps22
Sarcopterygii - ps23
Tetrapoda - ps24
Amniota - ps25
Mammalia - ps26
Theria - ps27
Placentalia - ps28
Boreoeutheria - ps29
Euarchontoglires - ps30
Euarchonta - ps31
Primates - ps32
Haplorrhini - ps33
Simiiformes - ps34
Catarrhini - ps35
Hominoidea - ps36
Hominidae - ps37
Homininae - ps38
Hominini - ps39
Homo_sapiens - ps40
Phylostratum

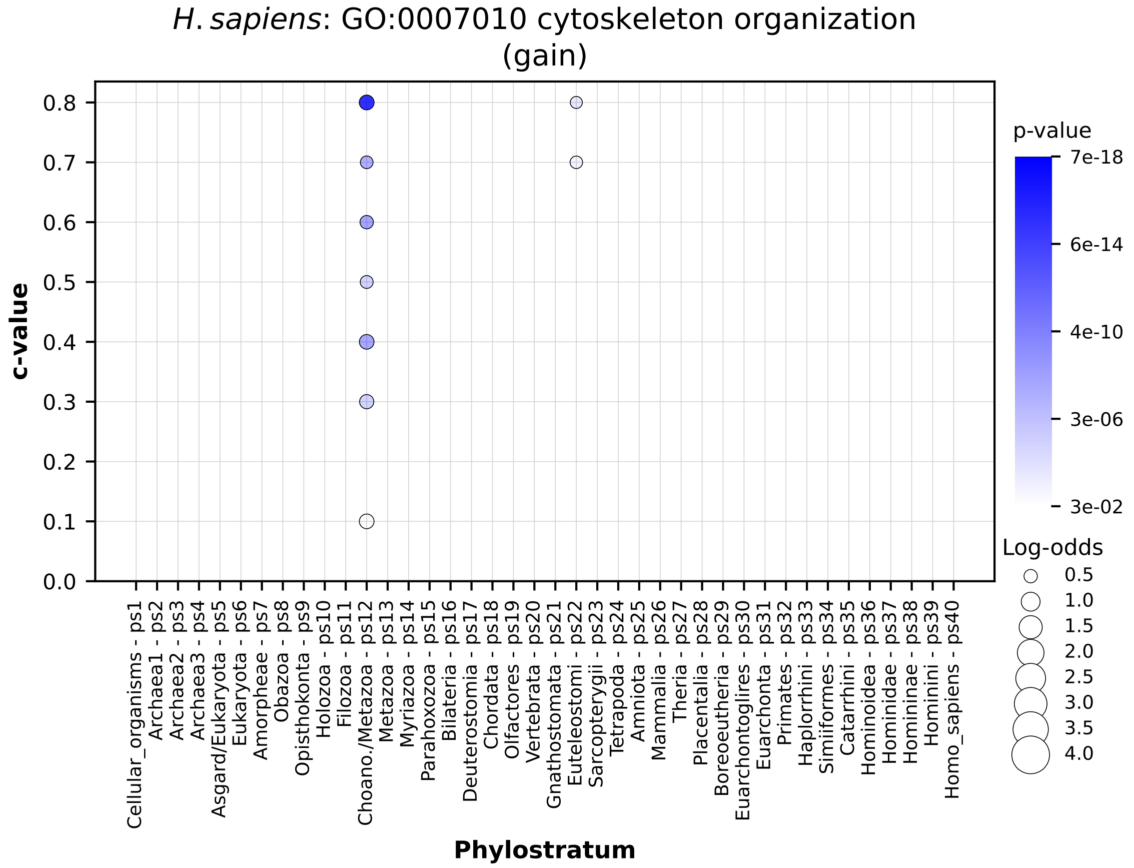

H. sapiens: GO:0007010 cytoskeleton organization
(gain)
c-value
0.0
0.1
0.2
0.3
0.4
0.5
0.6
0.7
0.8
p-value
7e-18
6e-14
4e-10
3e-06
3e-02
Log-odds
0.5
1.0
1.5
2.0
2.5
3.0
3.5
4.0
Cellular_organisms - ps1
Archaea1 - ps2
Archaea2 - ps3
Archaea3 - ps4
Asgard/Eukaryota - ps5
Eukaryota - ps6
Amorpheae - ps7
Obazoa - ps8
Opisthokonta - ps9
Holozoa - ps10
Filozoa - ps11
Choano./Metazoa - ps12
Metazoa - ps13
Myriazoa - ps14
Parahoxozoa - ps15
Bilateria - ps16
Deuterostomia - ps17
Chordata - ps18
Olfactores - ps19
Vertebrata - ps20
Gnathostomata - ps21
Euteleostomi - ps22
Sarcopterygii - ps23
Tetrapoda - ps24
Amniota - ps25
Mammalia - ps26
Theria - ps27
Placentalia - ps28
Boreoeutheria - ps29
Euarchontoglires - ps30
Euarchonta - ps31
Primates - ps32
Haplorrhini - ps33
Simiiformes - ps34
Catarrhini - ps35
Hominoidea - ps36
Hominidae - ps37
Homininae - ps38
Hominini - ps39
Homo_sapiens - ps40
Phylostratum

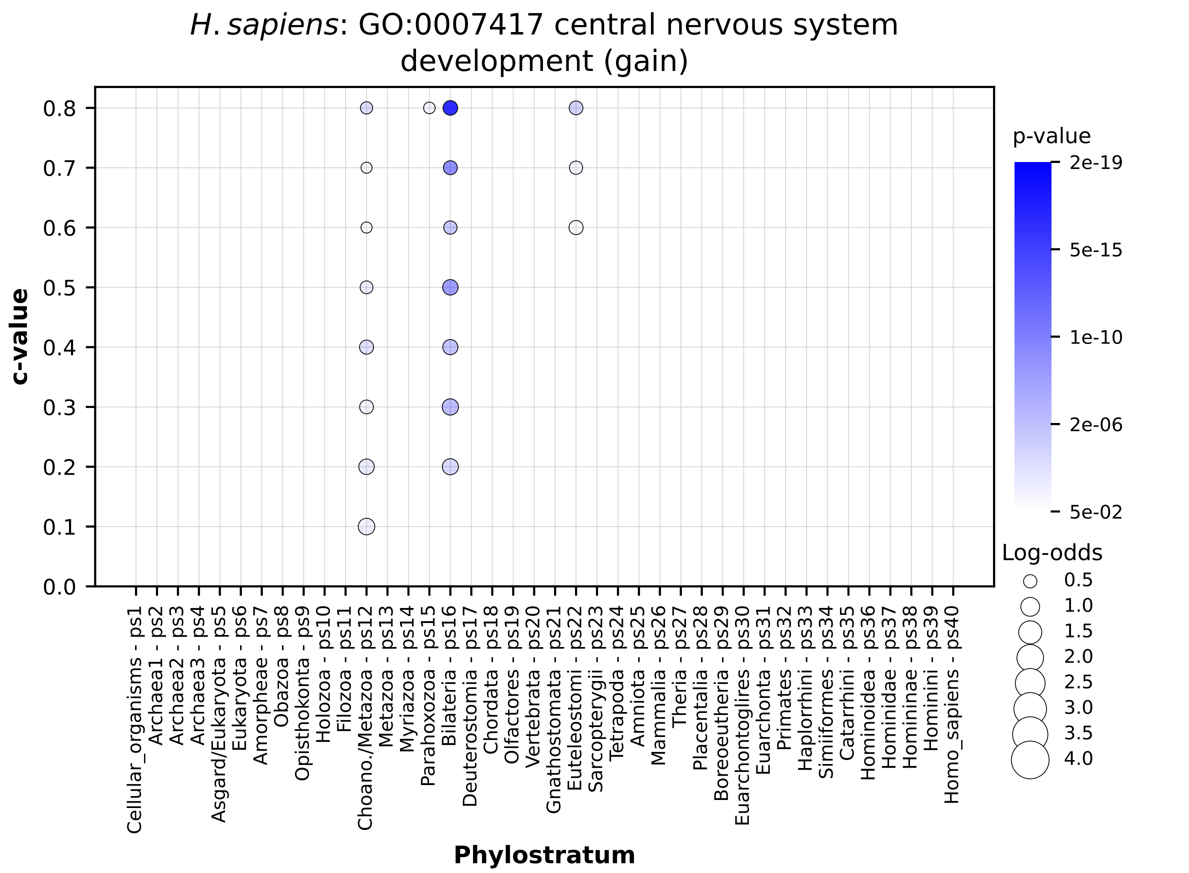

H. sapiens: GO:0007417 central nervous system development (gain)
c-value
0.0
0.1
0.2
0.3
0.4
0.5
0.6
0.7
0.8
Phylostratum
Cellular_organisms - ps1
Archaea1 - ps2
Archaea2 - ps3
Archaea3 - ps4
Asgard/Eukaryota - ps5
Eukaryota - ps6
Amorpheae - ps7
Obazoa - ps8
Opisthokonta - ps9
Holozoa - ps10
Filozoa - ps11
Choano./Metazoa - ps12
Metazoa - ps13
Myriazoa - ps14
Parahoxozoa - ps15
Bilateria - ps16
Deuterostomia - ps17
Chordata - ps18
Olfactores - ps19
Vertebrata - ps20
Gnathostomata - ps21
Euteleostomi - ps22
Sarcopterygii - ps23
Tetrapoda - ps24
Amniota - ps25
Mammalia - ps26
Theria - ps27
Placentalia - ps28
Boreoeutheria - ps29
Euarchontoglires - ps30
Euarchonta - ps31
Primates - ps32
Haplorrhini - ps33
Simiiformes - ps34
Catarrhini - ps35
Hominoidea - ps36
Hominidae - ps37
Homininae - ps38
Hominini - ps39
Homo_sapiens - ps40
p-value
2e-19
5e-15
1e-10
2e-06
5e-02
Log-odds
0.5
1.0
1.5
2.0
2.5
3.0
3.5
4.0

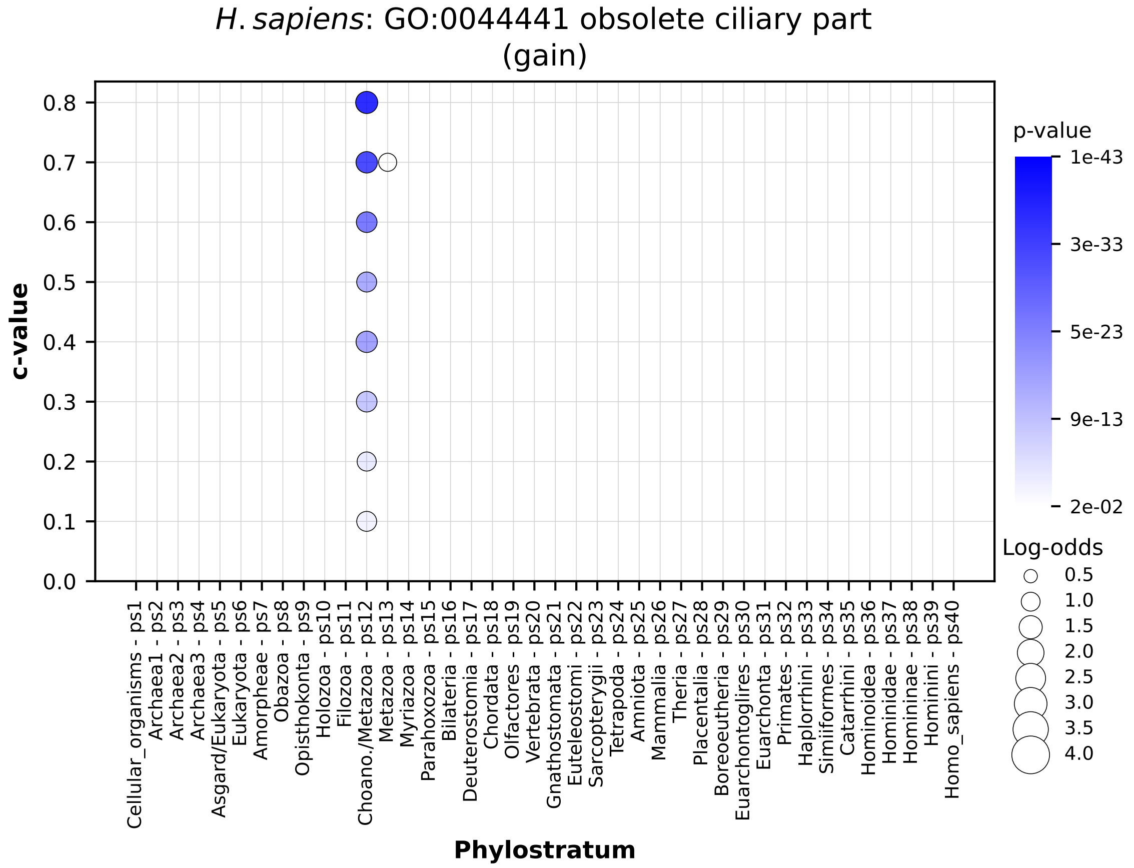

H. sapiens: GO:0044441 obsolete ciliary part
(gain)
c-value
0.0
0.1
0.2
0.3
0.4
0.5
0.6
0.7
0.8
p-value
1e-43
3e-33
5e-23
9e-13
2e-02
Log-odds
0.5
1.0
1.5
2.0
2.5
3.0
3.5
4.0
Cellular_organisms - ps1
Archaea1 - ps2
Archaea2 - ps3
Archaea3 - ps4
Asgard/Eukaryota - ps5
Eukaryota - ps6
Amorpheae - ps7
Obazoa - ps8
Opisthokonta - ps9
Holozoa - ps10
Filozoa - ps11
Choano./Metazoa - ps12
Metazoa - ps13
Myriazoa - ps14
Parahoxozoa - ps15
Bilateria - ps16
Deuterostomia - ps17
Chordata - ps18
Olfactores - ps19
Vertebrata - ps20
Gnathostomata - ps21
Euteleostomi - ps22
Sarcopterygii - ps23
Tetrapoda - ps24
Amniota - ps25
Mammalia - ps26
Theria - ps27
Placentalia - ps28
Boreoeutheria - ps29
Euarchontoglires - ps30
Euarchonta - ps31
Primates - ps32
Haplorrhini - ps33
Simiiformes - ps34
Catarrhini - ps35
Hominoidea - ps36
Hominidae - ps37
Homininae - ps38
Hominini - ps39
Homo_sapiens - ps40
Phylostratum

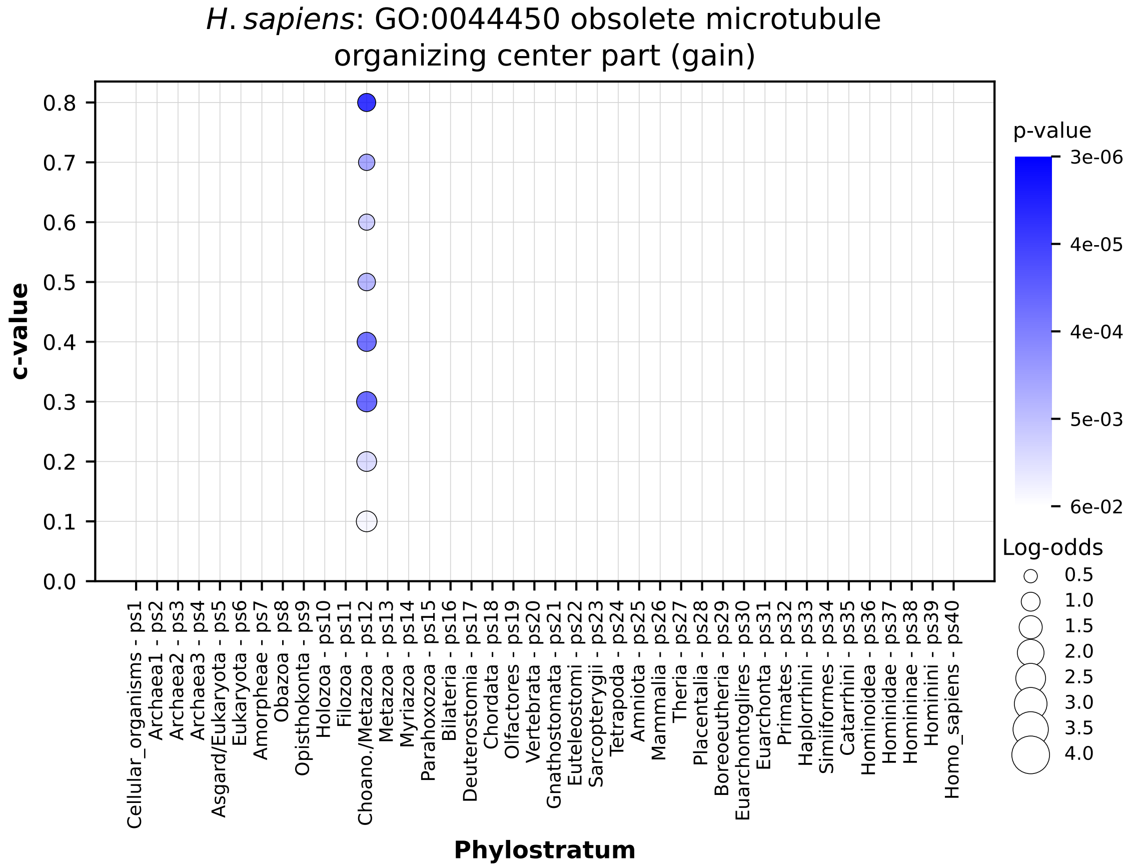

H. sapiens: GO:0044450 obsolete microtubule organizing center part (gain)
c-value
0.0
0.1
0.2
0.3
0.4
0.5
0.6
0.7
0.8
Phylostratum
Cellular_organisms - ps1
Archaea1 - ps2
Archaea2 - ps3
Archaea3 - ps4
Asgard/Eukaryota - ps5
Eukaryota - ps6
Amorpheae - ps7
Obazoa - ps8
Opisthokonta - ps9
Holozoa - ps10
Filozoa - ps11
Choano./Metazoa - ps12
Metazoa - ps13
Myriazoa - ps14
Parahoxozoa - ps15
Bilateria - ps16
Deuterostomia - ps17
Chordata - ps18
Olfactores - ps19
Vertebrata - ps20
Gnathostomata - ps21
Euteleostomi - ps22
Sarcopterygii - ps23
Tetrapoda - ps24
Amniota - ps25
Mammalia - ps26
Theria - ps27
Placentalia - ps28
Boreoeutheria - ps29
Euarchontoglires - ps30
Euarchonta - ps31
Primates - ps32
Haplorrhini - ps33
Simiiformes - ps34
Catarrhini - ps35
Hominoidea - ps36
Hominidae - ps37
Homininae - ps38
Hominini - ps39
Homo_sapiens - ps40
p-value
3e-06
4e-05
4e-04
5e-03
6e-02
Log-odds
0.5
1.0
1.5
2.0
2.5
3.0
3.5
4.0

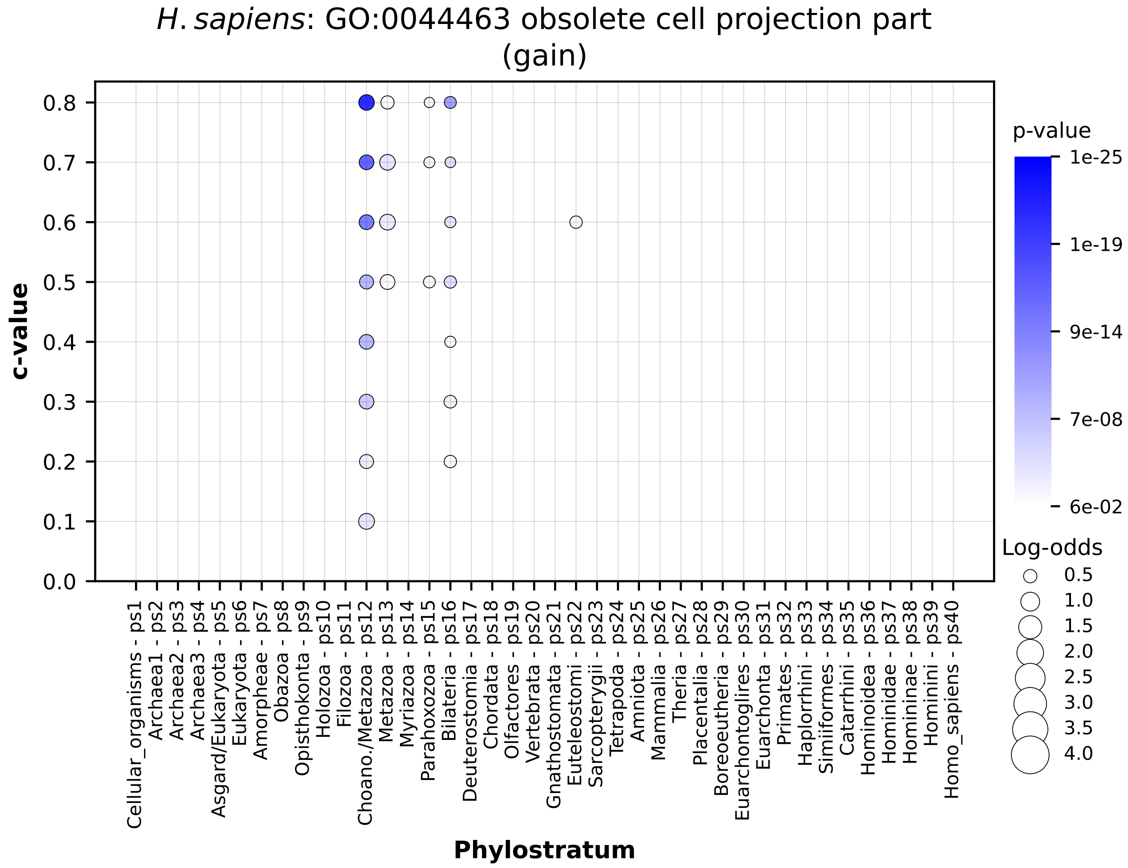

*H. sapiens*: GO:0044463 obsolete cell projection part
(gain)
c-value
0.8
0.7
0.6
0.5
0.4
0.3
0.2
0.1
0.0
Phylostratum
Cellular_organisms - ps1
Archaea1 - ps2
Archaea2 - ps3
Archaea3 - ps4
Asgard/Eukaryota - ps5
Eukaryota - ps6
Amorpheae - ps7
Obazoa - ps8
Opisthokonta - ps9
Holozoa - ps10
Filozoa - ps11
Choano./Metazoa - ps12
Metazoa - ps13
Myriazoa - ps14
Parahoxozoa - ps15
Bilateria - ps16
Deuterostomia - ps17
Chordata - ps18
Olfactores - ps19
Vertebrata - ps20
Gnathostomata - ps21
Euteleostomi - ps22
Sarcopterygii - ps23
Tetrapoda - ps24
Amniota - ps25
Mammalia - ps26
Theria - ps27
Placentalia - ps28
Boreoeutheria - ps29
Euarchontoglires - ps30
Euarchonta - ps31
Primates - ps32
Haplorrhini - ps33
Simiiformes - ps34
Catarrhini - ps35
Hominoidea - ps36
Hominidae - ps37
Homininae - ps38
Hominini - ps39
Homo_sapiens - ps40
p-value
1e-25
1e-19
9e-14
7e-08
6e-02
Log-odds
0.5
1.0
1.5
2.0
2.5
3.0
3.5
4.0

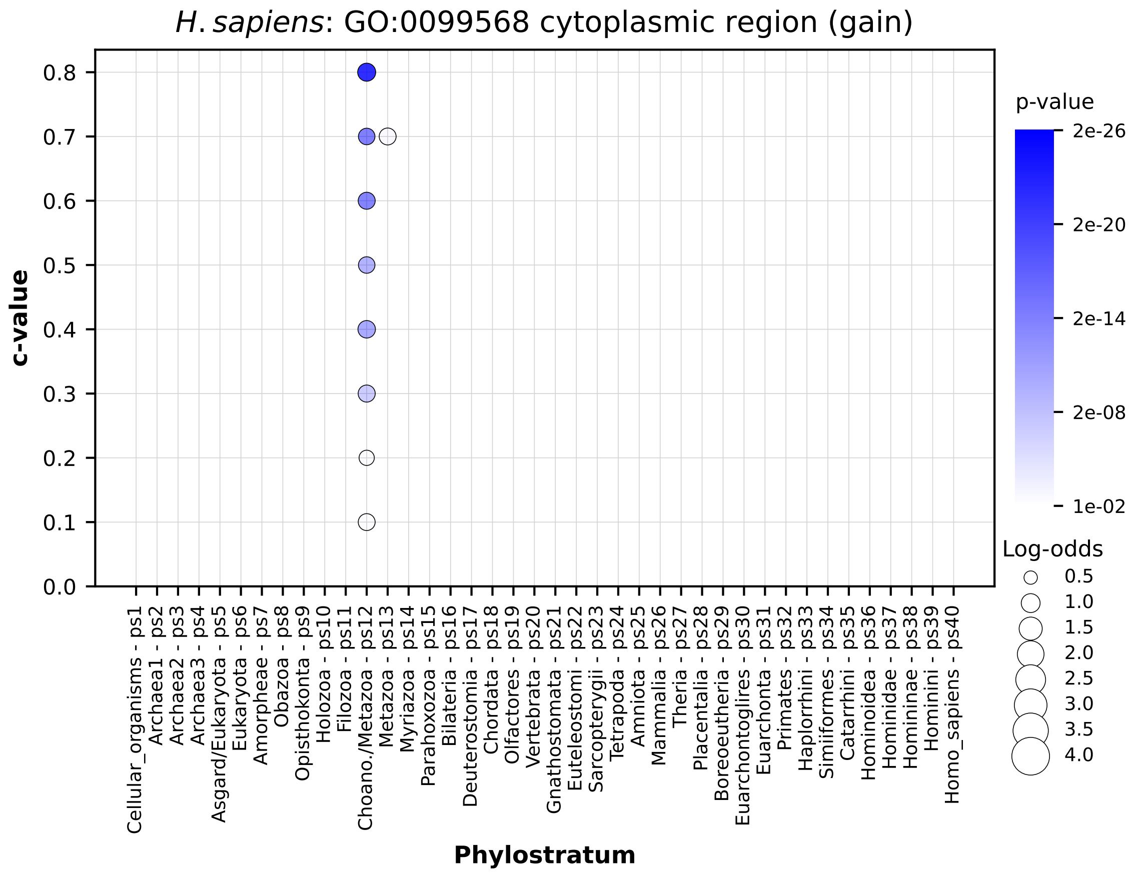

H. sapiens: GO:0099568 cytoplasmic region (gain)
c-value
0.0
0.1
0.2
0.3
0.4
0.5
0.6
0.7
0.8
p-value
2e-26
2e-20
2e-14
2e-08
1e-02
Log-odds
0.5
1.0
1.5
2.0
2.5
3.0
3.5
4.0
Cellular_organisms - ps1
Archaea1 - ps2
Archaea2 - ps3
Archaea3 - ps4
Asgard/Eukaryota - ps5
Eukaryota - ps6
Amorpheae - ps7
Obazoa - ps8
Opisthokonta - ps9
Holozoa - ps10
Filozoa - ps11
Choano./Metazoa - ps12
Metazoa - ps13
Myriazoa - ps14
Parahoxozoa - ps15
Bilateria - ps16
Deuterostomia - ps17
Chordata - ps18
Olfactores - ps19
Vertebrata - ps20
Gnathostomata - ps21
Euteleostomi - ps22
Sarcopterygii - ps23
Tetrapoda - ps24
Amniota - ps25
Mammalia - ps26
Theria - ps27
Placentalia - ps28
Boreoeutheria - ps29
Euarchontoglires - ps30
Euarchonta - ps31
Primates - ps32
Haplorrhini - ps33
Simiiformes - ps34
Catarrhini - ps35
Hominoidea - ps36
Hominidae - ps37
Homininae - ps38
Hominini - ps39
Homo_sapiens - ps40
Phylostratum

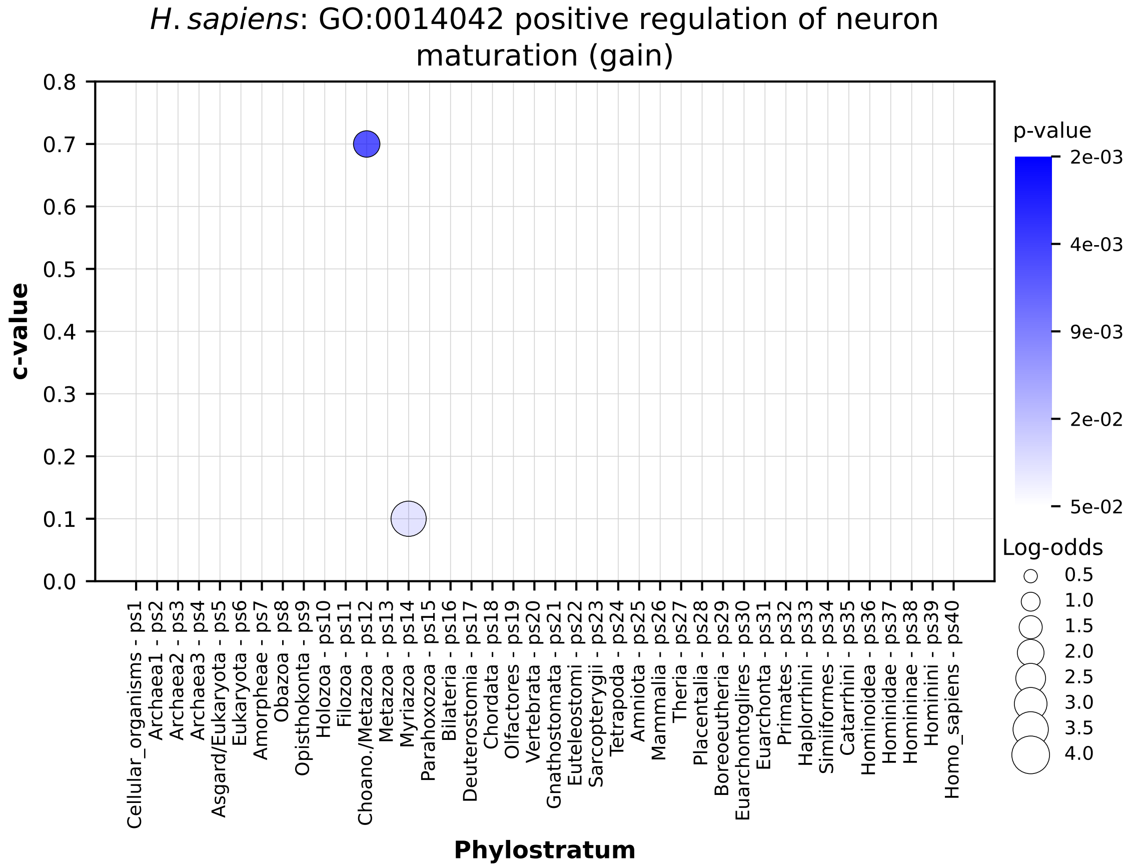

*H. sapiens*: GO:0014042 positive regulation of neuron maturation (gain)
c-value
0.8
0.7
0.6
0.5
0.4
0.3
0.2
0.1
0.0
Cellular_organisms - ps1
Archaea1 - ps2
Archaea2 - ps3
Archaea3 - ps4
Asgard/Eukaryota - ps5
Eukaryota - ps6
Amorpheae - ps7
Obazoa - ps8
Opisthokonta - ps9
Holozoa - ps10
Filozoa - ps11
Choano./Metazoa - ps12
Metazoa - ps13
Myriazoa - ps14
Parahoxozoa - ps15
Bilateria - ps16
Deuterostomia - ps17
Chordata - ps18
Olfactores - ps19
Vertebrata - ps20
Gnathostomata - ps21
Euteleostomi - ps22
Sarcopterygii - ps23
Tetrapoda - ps24
Amniota - ps25
Mammalia - ps26
Theria - ps27
Placentalia - ps28
Boreoeutheria - ps29
Euarchontoglires - ps30
Euarchonta - ps31
Primates - ps32
Haplorrhini - ps33
Simiiformes - ps34
Catarrhini - ps35
Hominoidea - ps36
Hominidae - ps37
Homininae - ps38
Hominini - ps39
Homo_sapiens - ps40
Phylostratum
p-value
2e-03
4e-03
9e-03
2e-02
5e-02
Log-odds
0.5
1.0
1.5
2.0
2.5
3.0
3.5
4.0

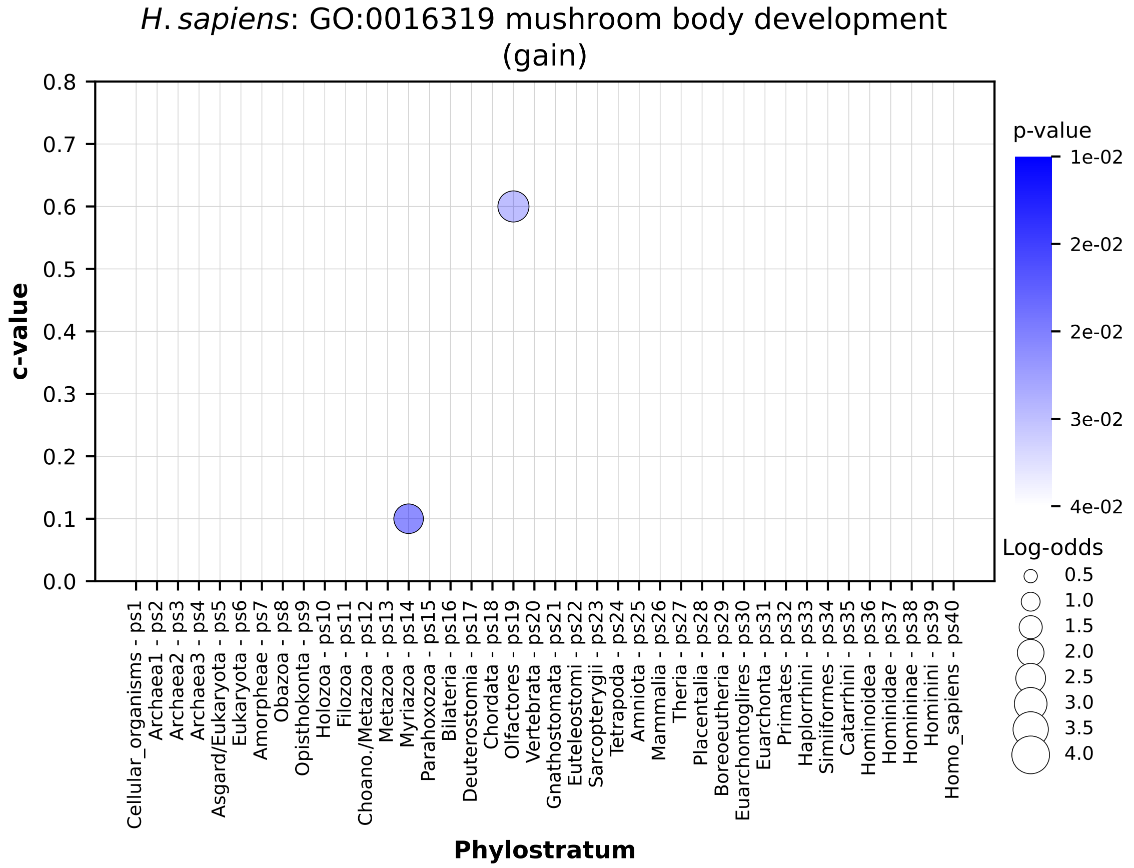
H. sapiens: GO:0016319 mushroom body development (gain)
c-value
0.0
0.1
0.2
0.3
0.4
0.5
0.6
0.7
0.8
Phylostratum
Cellular_organisms - ps1
Archaea1 - ps2
Archaea2 - ps3
Archaea3 - ps4
Asgard/Eukaryota - ps5
Eukaryota - ps6
Amorpheae - ps7
Obazoa - ps8
Opisthokonta - ps9
Holozoa - ps10
Filozoa - ps11
Choano./Metazoa - ps12
Metazoa - ps13
Myriazoa - ps14
Parahoxozoa - ps15
Bilateria - ps16
Deuterostomia - ps17
Chordata - ps18
Olfactores - ps19
Vertebrata - ps20
Gnathostomata - ps21
Euteleostomi - ps22
Sarcopterygii - ps23
Tetrapoda - ps24
Amniota - ps25
Mammalia - ps26
Theria - ps27
Placentalia - ps28
Boreoeutheria - ps29
Euarchontoglires - ps30
Euarchonta - ps31
Primates - ps32
Haplorrhini - ps33
Simiiformes - ps34
Catarrhini - ps35
Hominoidea - ps36
Hominidae - ps37
Homininae - ps38
Hominini - ps39
Homo_sapiens - ps40
p-value
1e-02
2e-02
2e-02
3e-02
4e-02
Log-odds
0.5
1.0
1.5
2.0
2.5
3.0
3.5
4.0

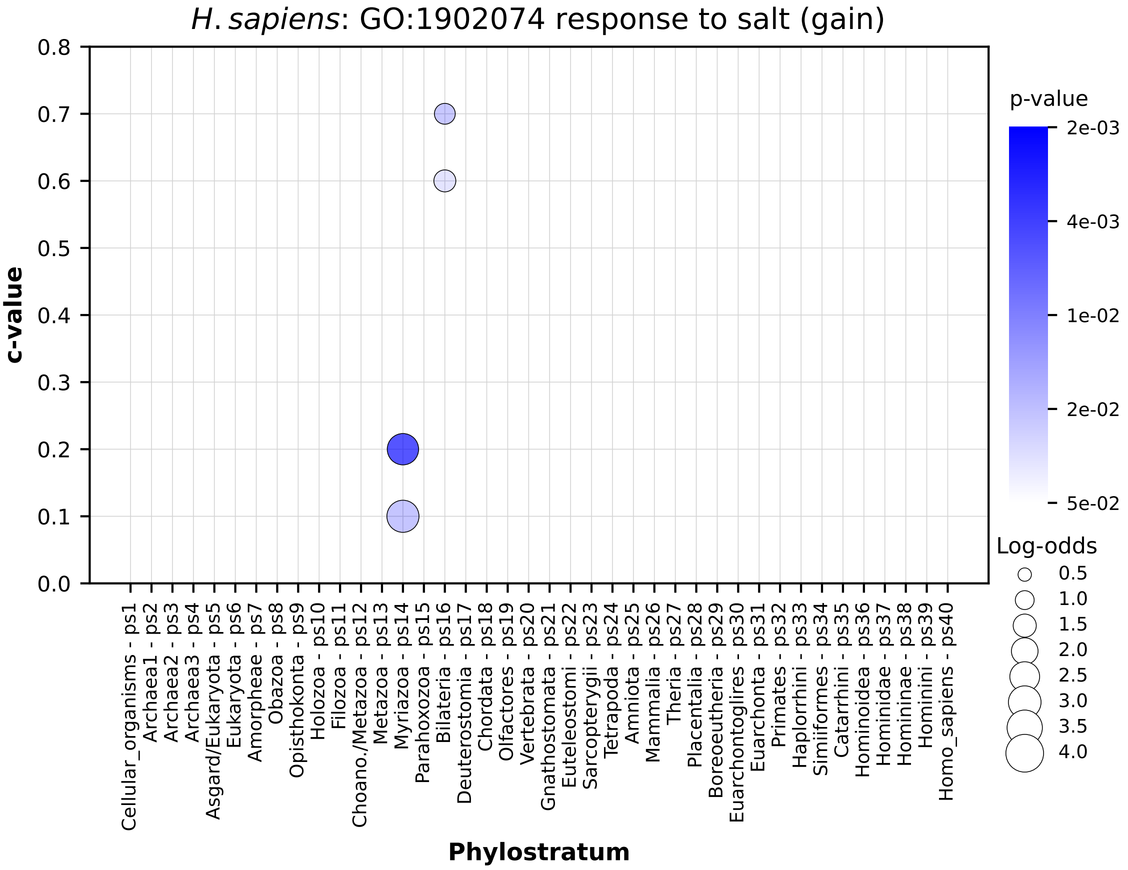

H. sapiens: GO:1902074 response to salt (gain)
c-value
0.0
0.1
0.2
0.3
0.4
0.5
0.6
0.7
0.8
Phylostratum
Cellular_organisms - ps1
Archaea1 - ps2
Archaea2 - ps3
Archaea3 - ps4
Asgard/Eukaryota - ps5
Eukaryota - ps6
Amorpheae - ps7
Obazoa - ps8
Opisthokonta - ps9
Holozoa - ps10
Filozoa - ps11
Choano./Metazoa - ps12
Metazoa - ps13
Myriazoa - ps14
Parahoxozoa - ps15
Bilateria - ps16
Deuterostomia - ps17
Chordata - ps18
Olfactores - ps19
Vertebrata - ps20
Gnathostomata - ps21
Euteleostomi - ps22
Sarcopterygii - ps23
Tetrapoda - ps24
Amniota - ps25
Mammalia - ps26
Theria - ps27
Placentalia - ps28
Boreoeutheria - ps29
Euarchontoglires - ps30
Euarchonta - ps31
Primates - ps32
Haplorrhini - ps33
Simiiformes - ps34
Catarrhini - ps35
Hominoidea - ps36
Hominidae - ps37
Homininae - ps38
Hominini - ps39
Homo_sapiens - ps40
p-value
2e-03
4e-03
1e-02
2e-02
5e-02
Log-odds
0.5
1.0
1.5
2.0
2.5
3.0
3.5
4.0

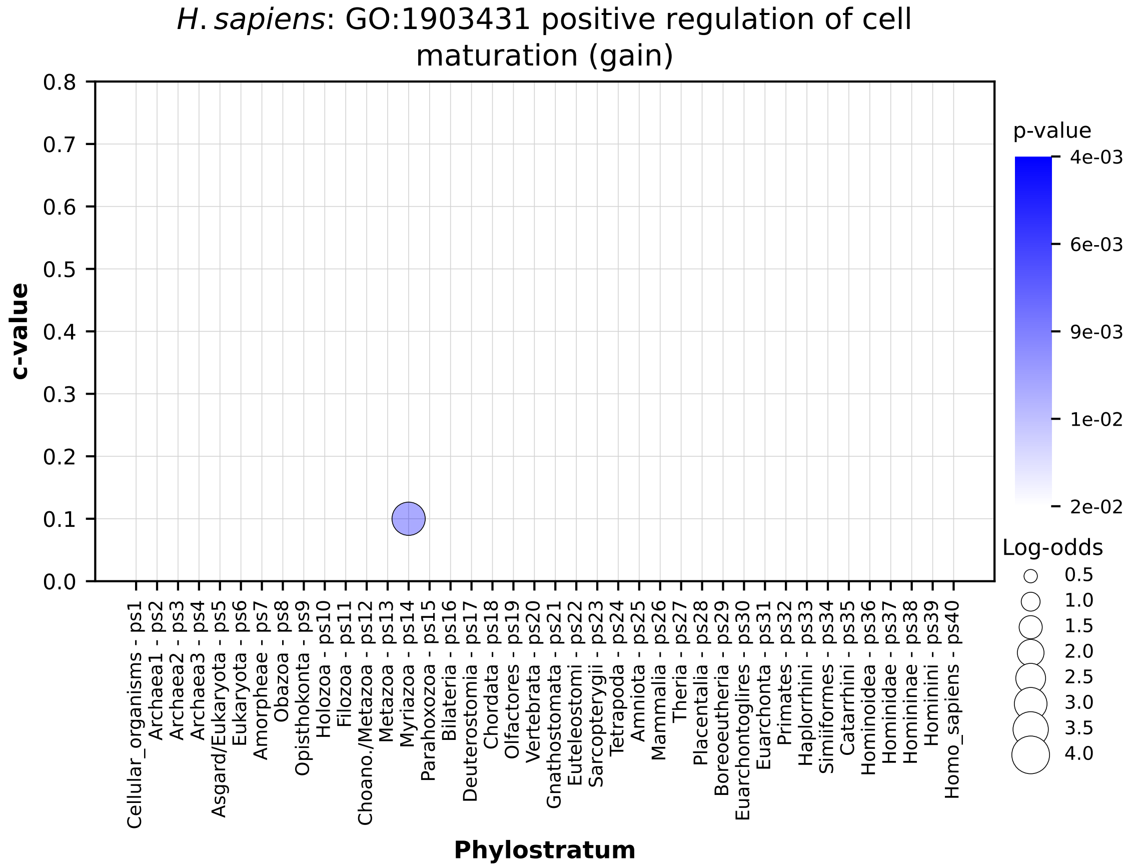

*H. sapiens*: GO:1903431 positive regulation of cell maturation (gain)
c-value
0.0
0.1
0.2
0.3
0.4
0.5
0.6
0.7
0.8
Phylostratum
Cellular_organisms - ps1
Archaea1 - ps2
Archaea2 - ps3
Archaea3 - ps4
Asgard/Eukaryota - ps5
Eukaryota - ps6
Amorpheae - ps7
Obazoa - ps8
Opisthokonta - ps9
Holozoa - ps10
Filozoa - ps11
Choano./Metazoa - ps12
Metazoa - ps13
Myriazoa - ps14
Parahoxozoa - ps15
Bilateria - ps16
Deuterostomia - ps17
Chordata - ps18
Olfactores - ps19
Vertebrata - ps20
Gnathostomata - ps21
Euteleostomi - ps22
Sarcopterygii - ps23
Tetrapoda - ps24
Amniota - ps25
Mammalia - ps26
Theria - ps27
Placentalia - ps28
Boreoeutheria - ps29
Euarchontoglires - ps30
Euarchonta - ps31
Primates - ps32
Haplorrhini - ps33
Simiiformes - ps34
Catarrhini - ps35
Hominoidea - ps36
Hominidae - ps37
Homininae - ps38
Hominini - ps39
Homo_sapiens - ps40
p-value
4e-03
6e-03
9e-03
1e-02
2e-02
Log-odds
0.5
1.0
1.5
2.0
2.5
3.0
3.5
4.0

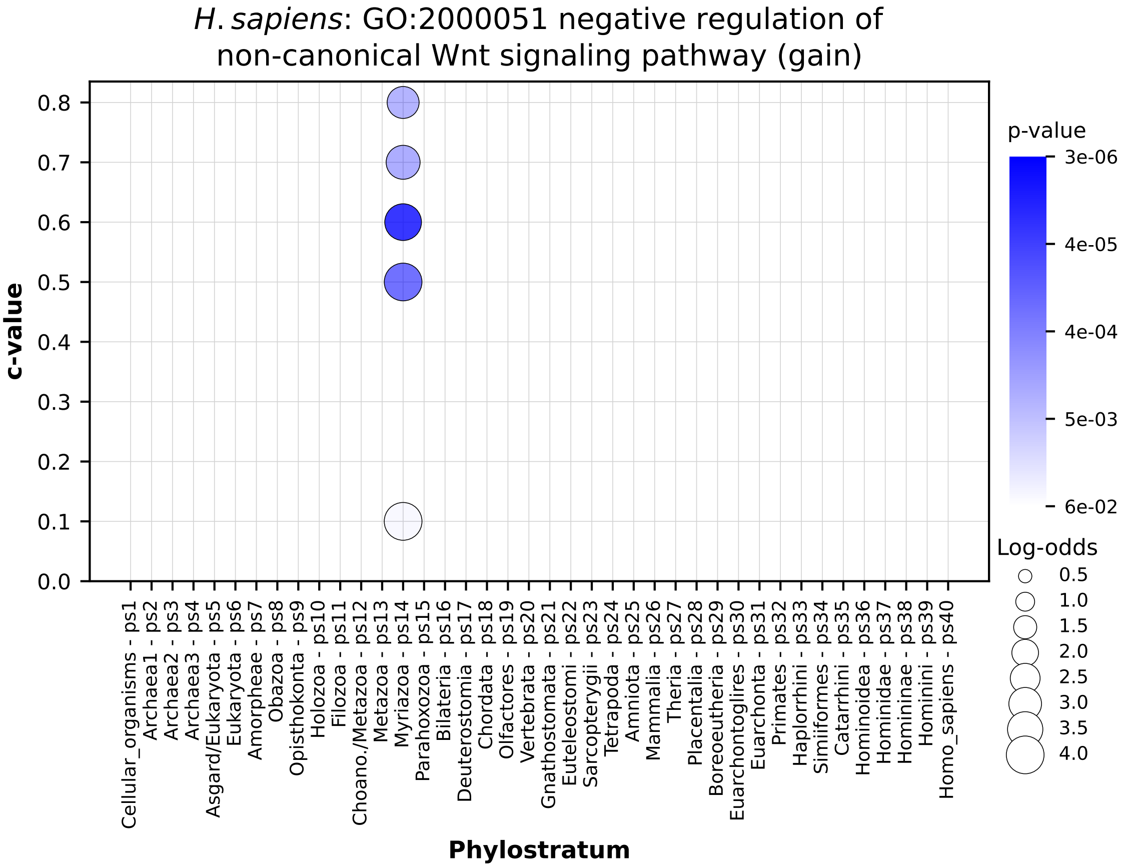

H. sapiens: GO:2000051 negative regulation of non-canonical Wnt signaling pathway (gain)
c-value
0.8
0.7
0.6
0.5
0.4
0.3
0.2
0.1
0.0
p-value
3e-06
4e-05
4e-04
5e-03
6e-02
Log-odds
0.5
1.0
1.5
2.0
2.5
3.0
3.5
4.0
Cellular_organisms - ps1
Archaea1 - ps2
Archaea2 - ps3
Archaea3 - ps4
Asgard/Eukaryota - ps5
Eukaryota - ps6
Amorpheae - ps7
Obazoa - ps8
Opisthokonta - ps9
Holozoa - ps10
Filozoa - ps11
Choano./Metazoa - ps12
Metazoa - ps13
Myriazoa - ps14
Parahoxozoa - ps15
Bilateria - ps16
Deuterostomia - ps17
Chordata - ps18
Olfactores - ps19
Vertebrata - ps20
Gnathostomata - ps21
Euteleostomi - ps22
Sarcopterygii - ps23
Tetrapoda - ps24
Amniota - ps25
Mammalia - ps26
Theria - ps27
Placentalia - ps28
Boreoeutheria - ps29
Euarchontoglires - ps30
Euarchonta - ps31
Primates - ps32
Haplorrhini - ps33
Simiiformes - ps34
Catarrhini - ps35
Hominoidea - ps36
Hominidae - ps37
Homininae - ps38
Hominini - ps39
Homo_sapiens - ps40
Phylostratum

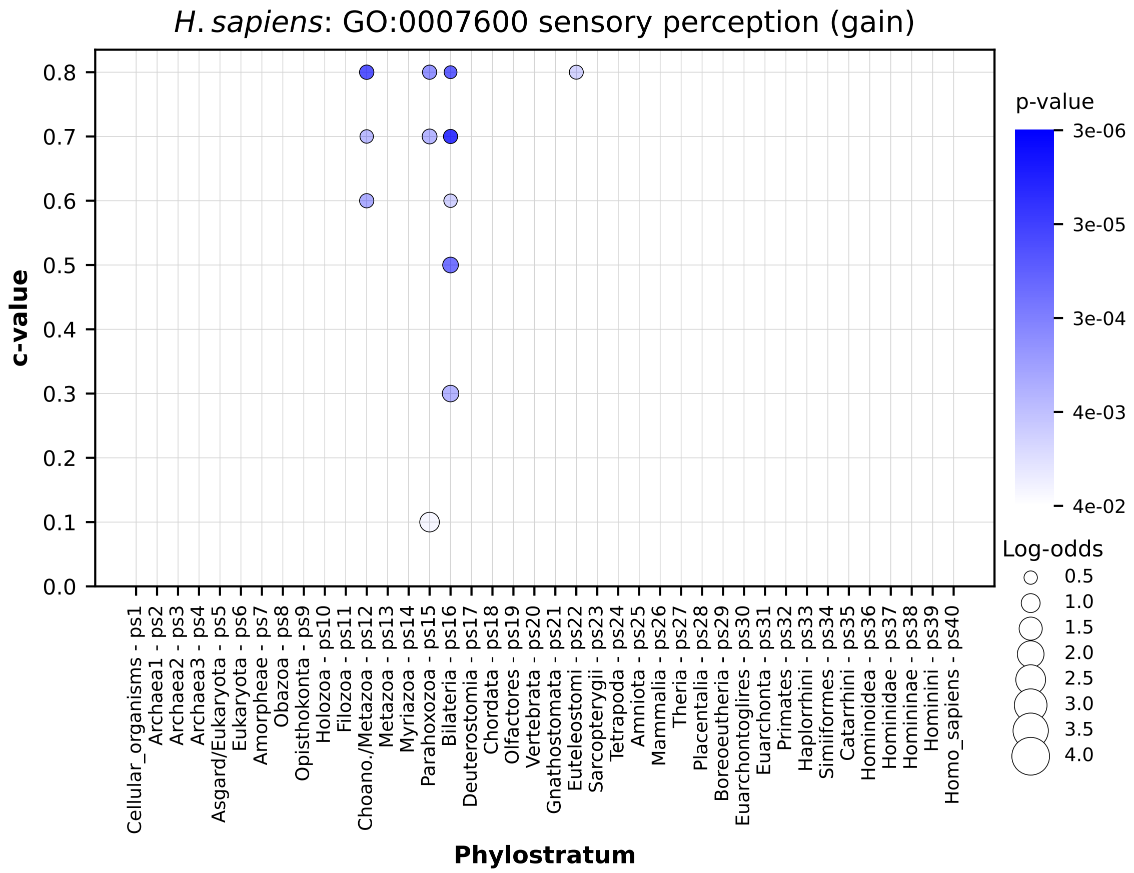

H. sapiens: GO:0007600 sensory perception (gain)
c-value
0.0
0.1
0.2
0.3
0.4
0.5
0.6
0.7
0.8
Phylostratum
Cellular_organisms - ps1
Archaea1 - ps2
Archaea2 - ps3
Archaea3 - ps4
Asgard/Eukaryota - ps5
Eukaryota - ps6
Amorpheae - ps7
Obazoa - ps8
Opisthokonta - ps9
Holozoa - ps10
Filozoa - ps11
Choano./Metazoa - ps12
Metazoa - ps13
Myriazoa - ps14
Parahoxozoa - ps15
Bilateria - ps16
Deuterostomia - ps17
Chordata - ps18
Olfactores - ps19
Vertebrata - ps20
Gnathostomata - ps21
Euteleostomi - ps22
Sarcopterygii - ps23
Tetrapoda - ps24
Amniota - ps25
Mammalia - ps26
Theria - ps27
Placentalia - ps28
Boreoeutheria - ps29
Euarchontoglires - ps30
Euarchonta - ps31
Primates - ps32
Haplorrhini - ps33
Simiiformes - ps34
Catarrhini - ps35
Hominoidea - ps36
Hominidae - ps37
Homininae - ps38
Hominini - ps39
Homo_sapiens - ps40
p-value
3e-06
3e-05
3e-04
4e-03
4e-02
Log-odds
0.5
1.0
1.5
2.0
2.5
3.0
3.5
4.0

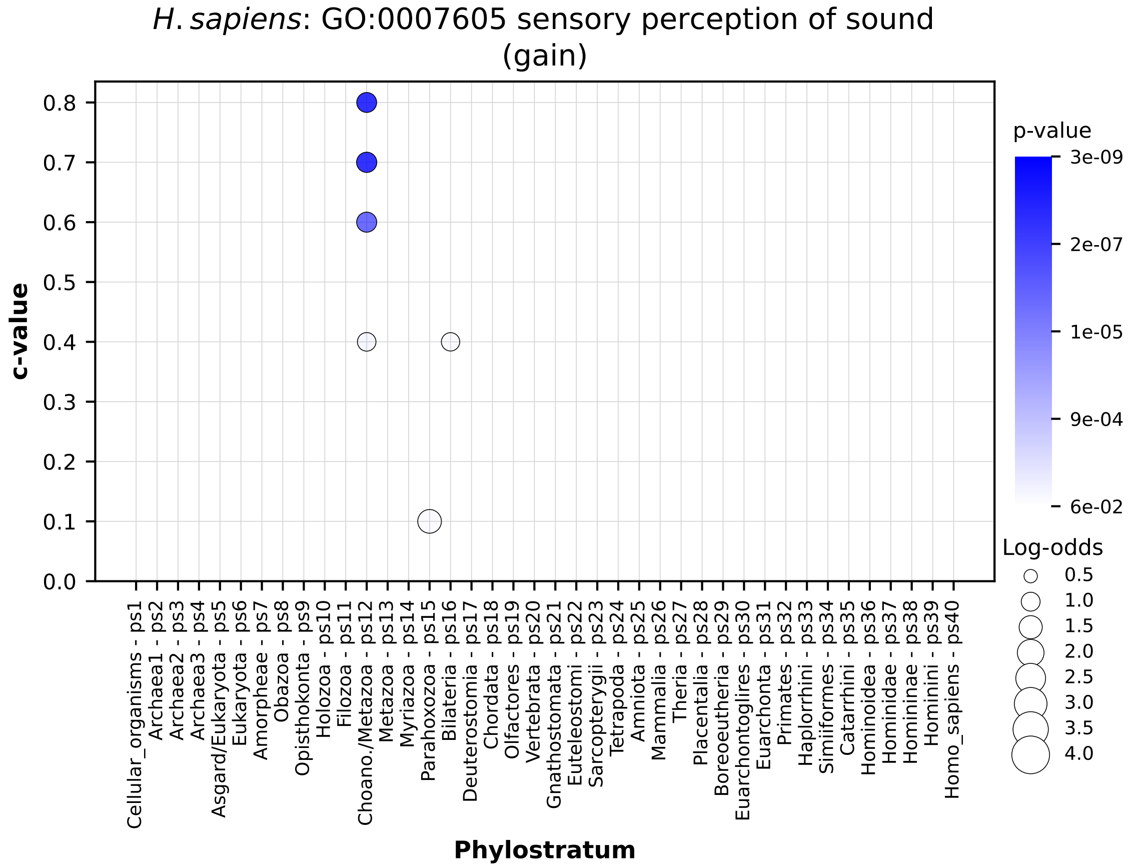

*H. sapiens*: GO:0007605 sensory perception of sound
(gain)
c-value
0.0
0.1
0.2
0.3
0.4
0.5
0.6
0.7
0.8
p-value
3e-09
2e-07
1e-05
9e-04
6e-02
Log-odds
0.5
1.0
1.5
2.0
2.5
3.0
3.5
4.0
Cellular_organisms - ps1
Archaea1 - ps2
Archaea2 - ps3
Archaea3 - ps4
Asgard/Eukaryota - ps5
Eukaryota - ps6
Amorpheae - ps7
Obazoa - ps8
Opisthokonta - ps9
Holozoa - ps10
Filozoa - ps11
Choano./Metazoa - ps12
Metazoa - ps13
Myriazoa - ps14
Parahoxozoa - ps15
Bilateria - ps16
Deuterostomia - ps17
Chordata - ps18
Olfactores - ps19
Vertebrata - ps20
Gnathostomata - ps21
Euteleostomi - ps22
Sarcopterygii - ps23
Tetrapoda - ps24
Amniota - ps25
Mammalia - ps26
Theria - ps27
Placentalia - ps28
Boreoeutheria - ps29
Euarchontoglires - ps30
Euarchonta - ps31
Primates - ps32
Haplorrhini - ps33
Simiiformes - ps34
Catarrhini - ps35
Hominoidea - ps36
Hominidae - ps37
Homininae - ps38
Hominini - ps39
Homo_sapiens - ps40
Phylostratum

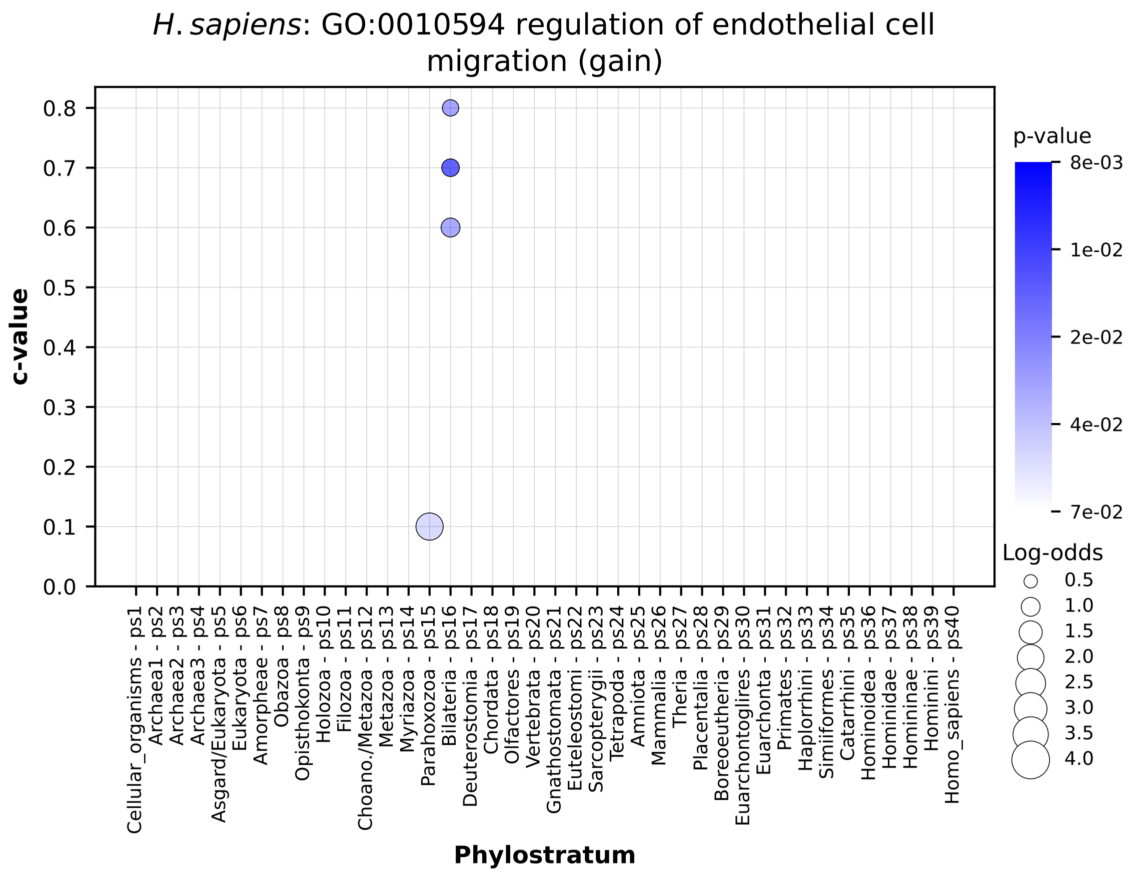

*H. sapiens*: GO:0010594 regulation of endothelial cell migration (gain)
c-value
0.8
0.7
0.6
0.5
0.4
0.3
0.2
0.1
0.0
p-value
8e-03
1e-02
2e-02
4e-02
7e-02
Log-odds
0.5
1.0
1.5
2.0
2.5
3.0
3.5
4.0
Cellular_organisms - ps1
Archaea1 - ps2
Archaea2 - ps3
Archaea3 - ps4
Asgard/Eukaryota - ps5
Eukaryota - ps6
Amorpheae - ps7
Obazoa - ps8
Opisthokonta - ps9
Holozoa - ps10
Filozoa - ps11
Choano./Metazoa - ps12
Metazoa - ps13
Myriazoa - ps14
Parahoxozoa - ps15
Bilateria - ps16
Deuterostomia - ps17
Chordata - ps18
Olfactores - ps19
Vertebrata - ps20
Gnathostomata - ps21
Euteleostomi - ps22
Sarcopterygii - ps23
Tetrapoda - ps24
Amniota - ps25
Mammalia - ps26
Theria - ps27
Placentalia - ps28
Boreoeutheria - ps29
Euarchontoglires - ps30
Euarchonta - ps31
Primates - ps32
Haplorrhini - ps33
Simiiformes - ps34
Catarrhini - ps35
Hominoidea - ps36
Hominidae - ps37
Homininae - ps38
Hominini - ps39
Homo_sapiens - ps40
Phylostratum

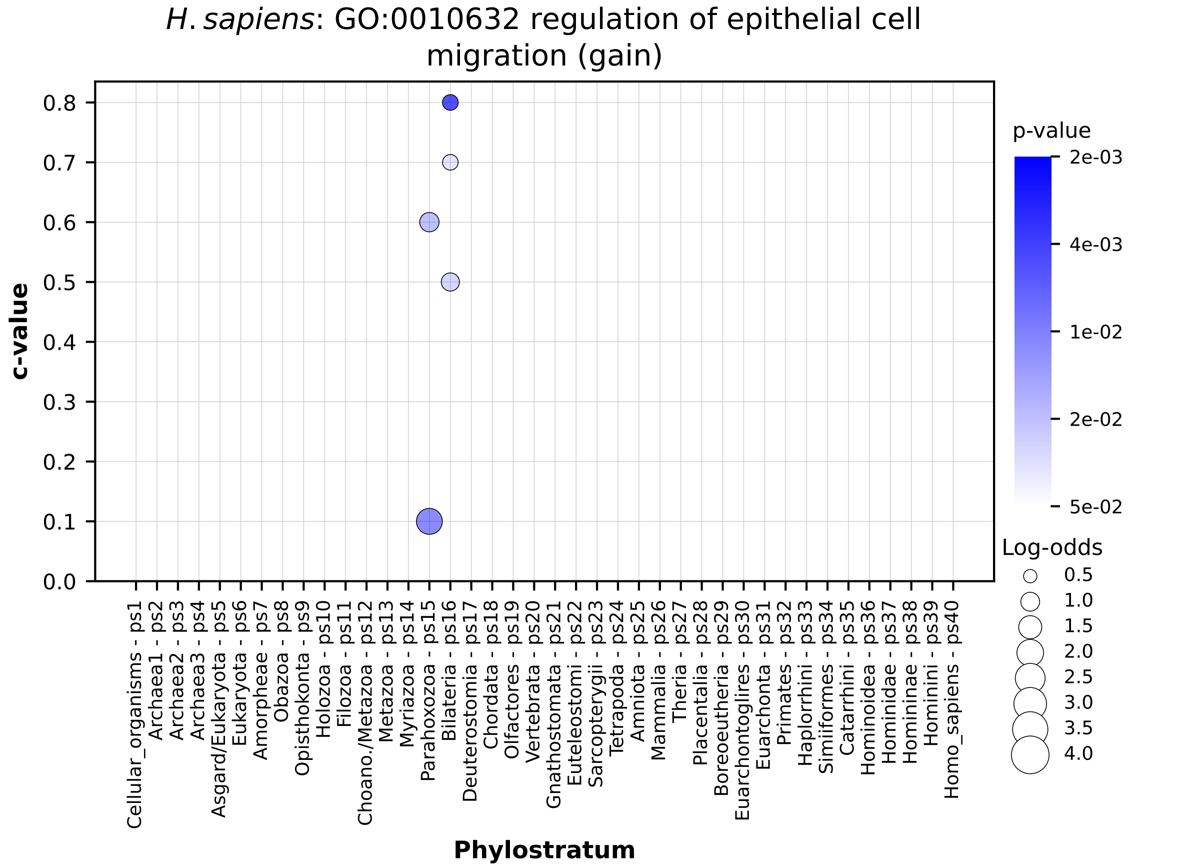
H. sapiens: GO:0010632 regulation of epithelial cell migration (gain)
c-value
0.0
0.1
0.2
0.3
0.4
0.5
0.6
0.7
0.8
Phylostratum
Cellular_organisms - ps1
Archaea1 - ps2
Archaea2 - ps3
Archaea3 - ps4
Asgard/Eukaryota - ps5
Eukaryota - ps6
Amorpheae - ps7
Obazoa - ps8
Opisthokonta - ps9
Holozoa - ps10
Filozoa - ps11
Choano./Metazoa - ps12
Metazoa - ps13
Myriazoa - ps14
Parahoxozoa - ps15
Bilateria - ps16
Deuterostomia - ps17
Chordata - ps18
Olfactores - ps19
Vertebrata - ps20
Gnathostomata - ps21
Euteleostomi - ps22
Sarcopterygii - ps23
Tetrapoda - ps24
Amniota - ps25
Mammalia - ps26
Theria - ps27
Placentalia - ps28
Boreoeutheria - ps29
Euarchontoglires - ps30
Euarchonta - ps31
Primates - ps32
Haplorrhini - ps33
Simiiformes - ps34
Catarrhini - ps35
Hominoidea - ps36
Hominidae - ps37
Homininae - ps38
Hominini - ps39
Homo_sapiens - ps40
p-value
2e-03
4e-03
1e-02
2e-02
5e-02
Log-odds
0.5
1.0
1.5
2.0
2.5
3.0
3.5
4.0

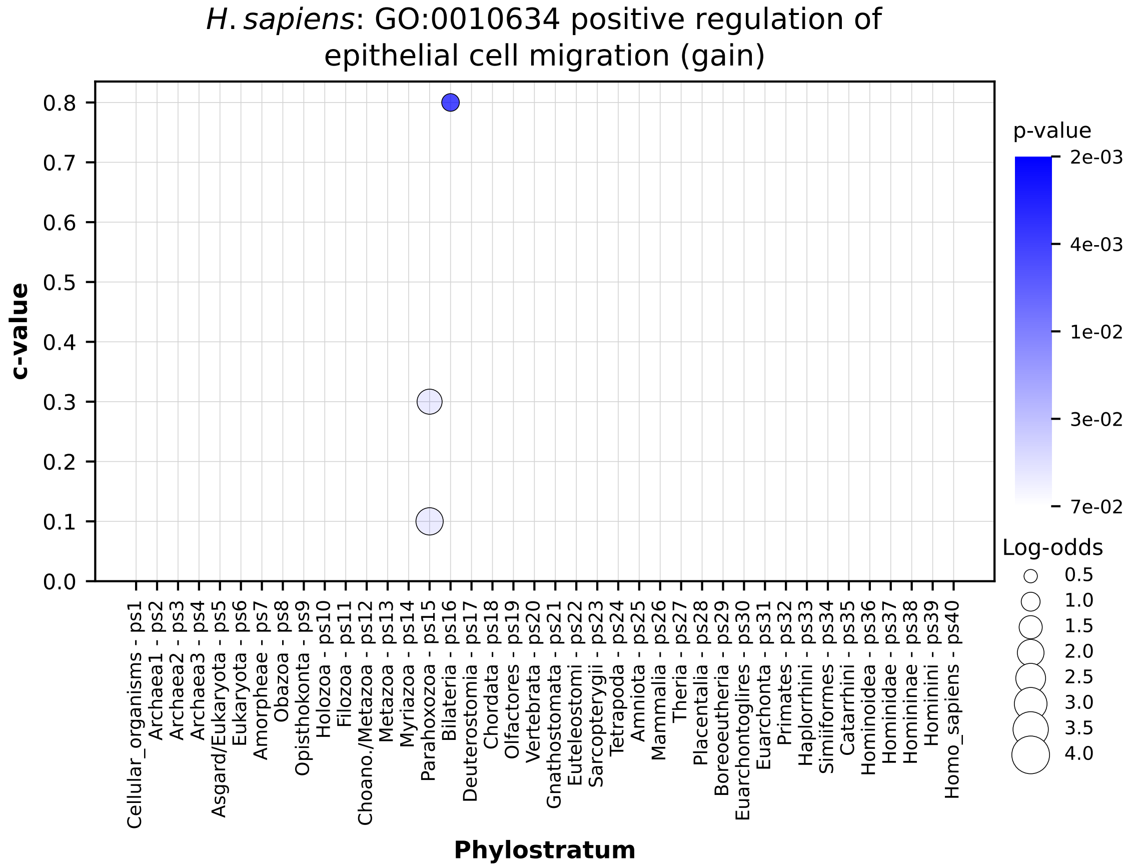

H. sapiens: GO:0010634 positive regulation of epithelial cell migration (gain)
c-value
0.0
0.1
0.2
0.3
0.4
0.5
0.6
0.7
0.8
Phylostratum
Cellular_organisms - ps1
Archaea1 - ps2
Archaea2 - ps3
Archaea3 - ps4
Asgard/Eukaryota - ps5
Eukaryota - ps6
Amorpheae - ps7
Obazoa - ps8
Opisthokonta - ps9
Holozoa - ps10
Filozoa - ps11
Choano./Metazoa - ps12
Metazoa - ps13
Myriazoa - ps14
Parahoxozoa - ps15
Bilateria - ps16
Deuterostomia - ps17
Chordata - ps18
Olfactores - ps19
Vertebrata - ps20
Gnathostomata - ps21
Euteleostomi - ps22
Sarcopterygii - ps23
Tetrapoda - ps24
Amniota - ps25
Mammalia - ps26
Theria - ps27
Placentalia - ps28
Boreoeutheria - ps29
Euarchontoglires - ps30
Euarchonta - ps31
Primates - ps32
Haplorrhini - ps33
Simiiformes - ps34
Catarrhini - ps35
Hominoidea - ps36
Hominidae - ps37
Homininae - ps38
Hominini - ps39
Homo_sapiens - ps40
p-value
2e-03
4e-03
1e-02
3e-02
7e-02
Log-odds
0.5
1.0
1.5
2.0
2.5
3.0
3.5
4.0

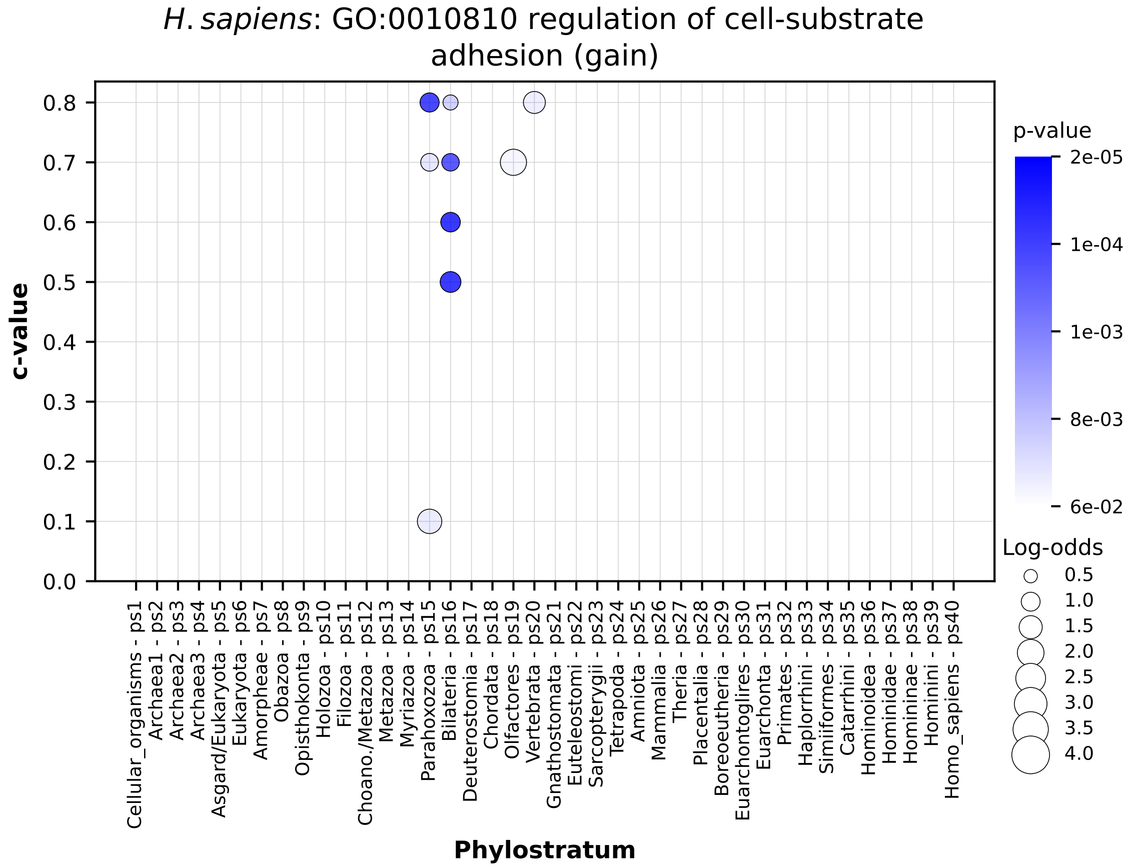

H. sapiens: GO:0010810 regulation of cell-substrate adhesion (gain)
c-value
0.0
0.1
0.2
0.3
0.4
0.5
0.6
0.7
0.8
Phylostratum
Cellular_organisms - ps1
Archaea1 - ps2
Archaea2 - ps3
Archaea3 - ps4
Asgard/Eukaryota - ps5
Eukaryota - ps6
Amorpheae - ps7
Obazoa - ps8
Opisthokonta - ps9
Holozoa - ps10
Filozoa - ps11
Choano./Metazoa - ps12
Metazoa - ps13
Myriazoa - ps14
Parahoxozoa - ps15
Bilateria - ps16
Deuterostomia - ps17
Chordata - ps18
Olfactores - ps19
Vertebrata - ps20
Gnathostomata - ps21
Euteleostomi - ps22
Sarcopterygii - ps23
Tetrapoda - ps24
Amniota - ps25
Mammalia - ps26
Theria - ps27
Placentalia - ps28
Boreoeutheria - ps29
Euarchontoglires - ps30
Euarchonta - ps31
Primates - ps32
Haplorrhini - ps33
Simiiformes - ps34
Catarrhini - ps35
Hominoidea - ps36
Hominidae - ps37
Homininae - ps38
Hominini - ps39
Homo_sapiens - ps40
p-value
2e-05
1e-04
1e-03
8e-03
6e-02
Log-odds
0.5
1.0
1.5
2.0
2.5
3.0
3.5
4.0

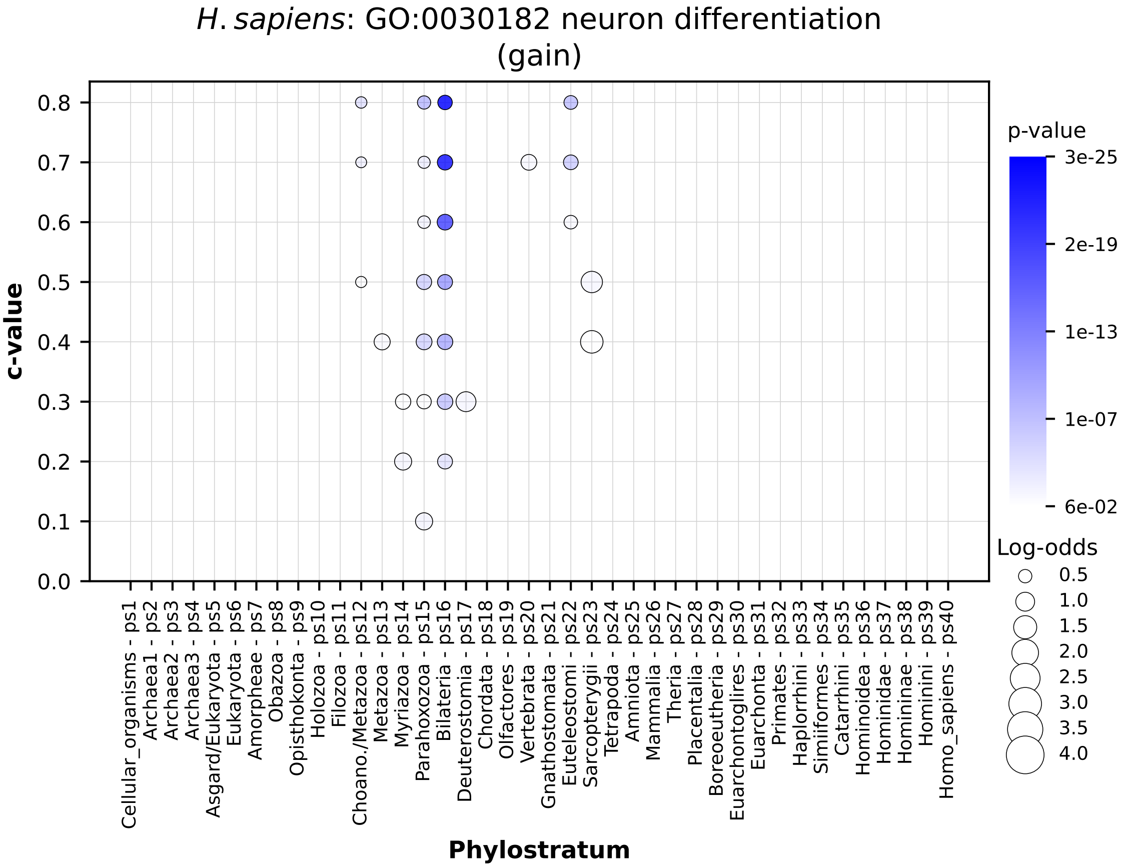

H. sapiens: GO:0030182 neuron differentiation
(gain)
c-value
0.8
0.7
0.6
0.5
0.4
0.3
0.2
0.1
0.0
Phylostratum
Cellular_organisms - ps1
Archaea1 - ps2
Archaea2 - ps3
Archaea3 - ps4
Asgard/Eukaryota - ps5
Eukaryota - ps6
Amorpheae - ps7
Obazoa - ps8
Opisthokonta - ps9
Holozoa - ps10
Filozoa - ps11
Choano./Metazoa - ps12
Metazoa - ps13
Myriazoa - ps14
Parahoxozoa - ps15
Bilateria - ps16
Deuterostomia - ps17
Chordata - ps18
Olfactores - ps19
Vertebrata - ps20
Gnathostomata - ps21
Euteleostomi - ps22
Sarcopterygii - ps23
Tetrapoda - ps24
Amniota - ps25
Mammalia - ps26
Theria - ps27
Placentalia - ps28
Boreoeutheria - ps29
Euarchontoglires - ps30
Euarchonta - ps31
Primates - ps32
Haplorrhini - ps33
Simiiformes - ps34
Catarrhini - ps35
Hominoidea - ps36
Hominidae - ps37
Homininae - ps38
Hominini - ps39
Homo_sapiens - ps40
p-value
3e-25
2e-19
1e-13
1e-07
6e-02
Log-odds
0.5
1.0
1.5
2.0
2.5
3.0
3.5
4.0

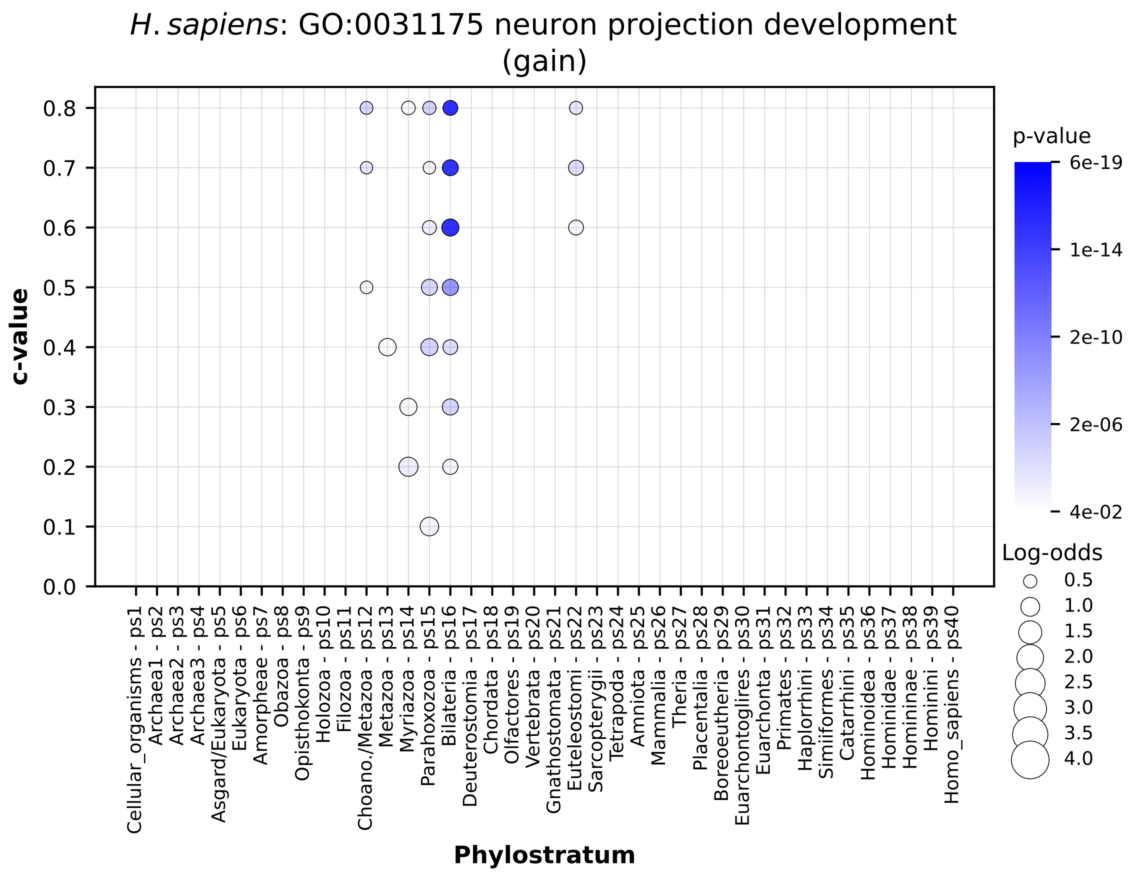

H. sapiens: GO:0031175 neuron projection development
(gain)
c-value
0.0
0.1
0.2
0.3
0.4
0.5
0.6
0.7
0.8
Phylostratum
Cellular_organisms - ps1
Archaea1 - ps2
Archaea2 - ps3
Archaea3 - ps4
Asgard/Eukaryota - ps5
Eukaryota - ps6
Amorpheae - ps7
Obazoa - ps8
Opisthokonta - ps9
Holozoa - ps10
Filozoa - ps11
Choano./Metazoa - ps12
Metazoa - ps13
Myriazoa - ps14
Parahoxozoa - ps15
Bilateria - ps16
Deuterostomia - ps17
Chordata - ps18
Olfactores - ps19
Vertebrata - ps20
Gnathostomata - ps21
Euteleostomi - ps22
Sarcopterygii - ps23
Tetrapoda - ps24
Amniota - ps25
Mammalia - ps26
Theria - ps27
Placentalia - ps28
Boreoeutheria - ps29
Euarchontoglires - ps30
Euarchonta - ps31
Primates - ps32
Haplorrhini - ps33
Simiiformes - ps34
Catarrhini - ps35
Hominoidea - ps36
Hominidae - ps37
Homininae - ps38
Hominini - ps39
Homo_sapiens - ps40
p-value
6e-19
1e-14
2e-10
2e-06
4e-02
Log-odds
0.5
1.0
1.5
2.0
2.5
3.0
3.5
4.0

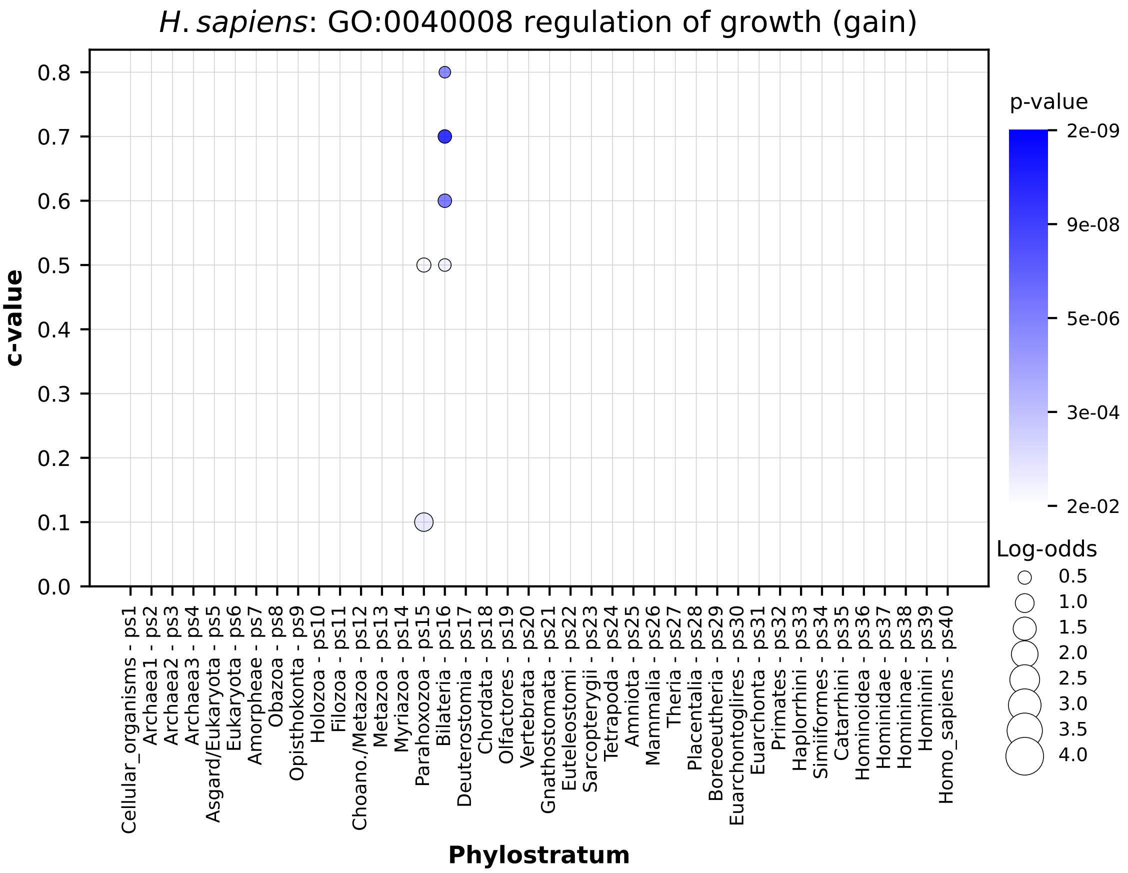

H. sapiens: GO:0040008 regulation of growth (gain)
c-value
0.0
0.1
0.2
0.3
0.4
0.5
0.6
0.7
0.8
Phylostratum
Cellular_organisms - ps1
Archaea1 - ps2
Archaea2 - ps3
Archaea3 - ps4
Asgard/Eukaryota - ps5
Eukaryota - ps6
Amorpheae - ps7
Obazoa - ps8
Opisthokonta - ps9
Holozoa - ps10
Filozoa - ps11
Choano./Metazoa - ps12
Metazoa - ps13
Myriazoa - ps14
Parahoxozoa - ps15
Bilateria - ps16
Deuterostomia - ps17
Chordata - ps18
Olfactores - ps19
Vertebrata - ps20
Gnathostomata - ps21
Euteleostomi - ps22
Sarcopterygii - ps23
Tetrapoda - ps24
Amniota - ps25
Mammalia - ps26
Theria - ps27
Placentalia - ps28
Boreoeutheria - ps29
Euarchontoglires - ps30
Euarchonta - ps31
Primates - ps32
Haplorrhini - ps33
Simiiformes - ps34
Catarrhini - ps35
Hominoidea - ps36
Hominidae - ps37
Homininae - ps38
Hominini - ps39
Homo_sapiens - ps40
p-value
2e-09
9e-08
5e-06
3e-04
2e-02
Log-odds
0.5
1.0
1.5
2.0
2.5
3.0
3.5
4.0

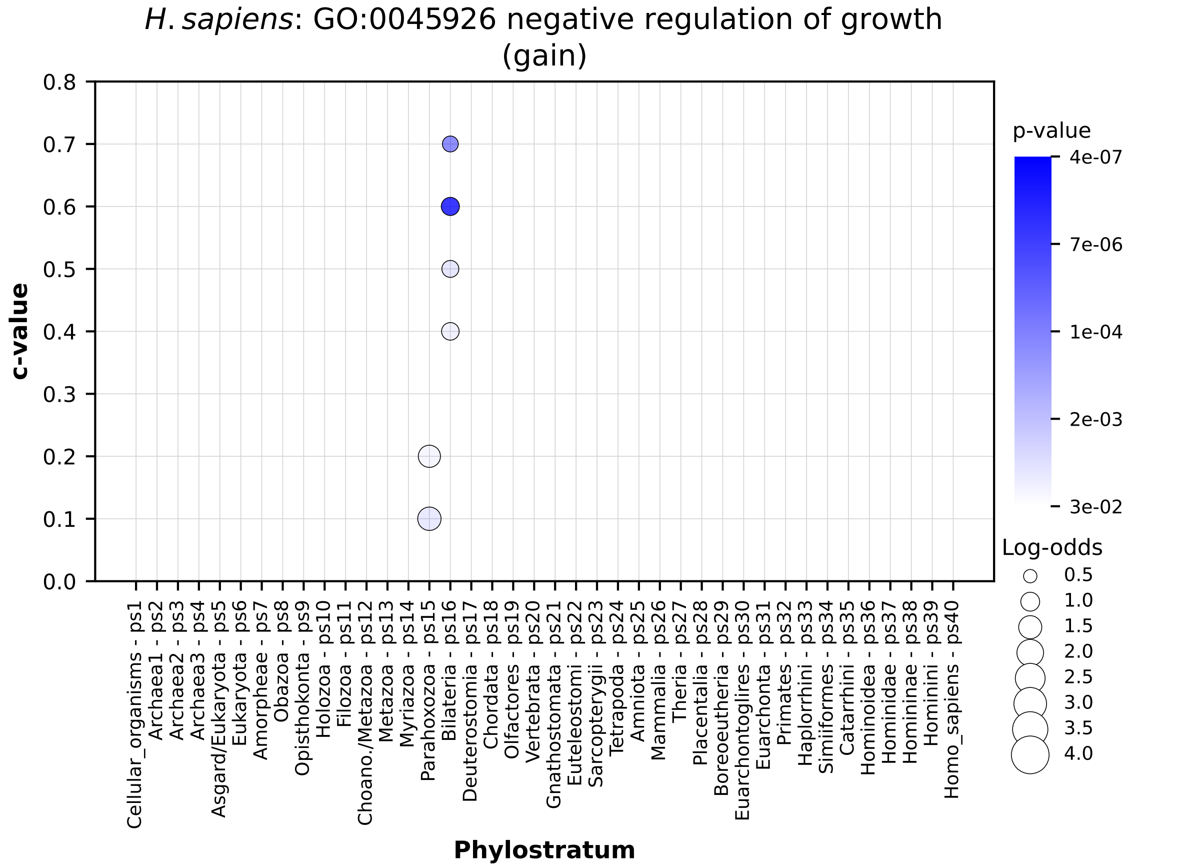

$H. sapiens$: GO:0045926 negative regulation of growth
(gain)
c-value
0.8
0.7
0.6
0.5
0.4
0.3
0.2
0.1
0.0
p-value
4e-07
7e-06
1e-04
2e-03
3e-02
Log-odds
0.5
1.0
1.5
2.0
2.5
3.0
3.5
4.0
Cellular_organisms - ps1
Archaea1 - ps2
Archaea2 - ps3
Archaea3 - ps4
Asgard/Eukaryota - ps5
Eukaryota - ps6
Amorpheae - ps7
Obazoa - ps8
Opisthokonta - ps9
Holozoa - ps10
Filozoa - ps11
Choano./Metazoa - ps12
Metazoa - ps13
Myriazoa - ps14
Parahoxozoa - ps15
Bilateria - ps16
Deuterostomia - ps17
Chordata - ps18
Olfactores - ps19
Vertebrata - ps20
Gnathostomata - ps21
Euteleostomi - ps22
Sarcopterygii - ps23
Tetrapoda - ps24
Amniota - ps25
Mammalia - ps26
Theria - ps27
Placentalia - ps28
Boreoeutheria - ps29
Euarchontoglires - ps30
Euarchonta - ps31
Primates - ps32
Haplorrhini - ps33
Simiiformes - ps34
Catarrhini - ps35
Hominoidea - ps36
Hominidae - ps37
Homininae - ps38
Hominini - ps39
Homo_sapiens - ps40
Phylostratum

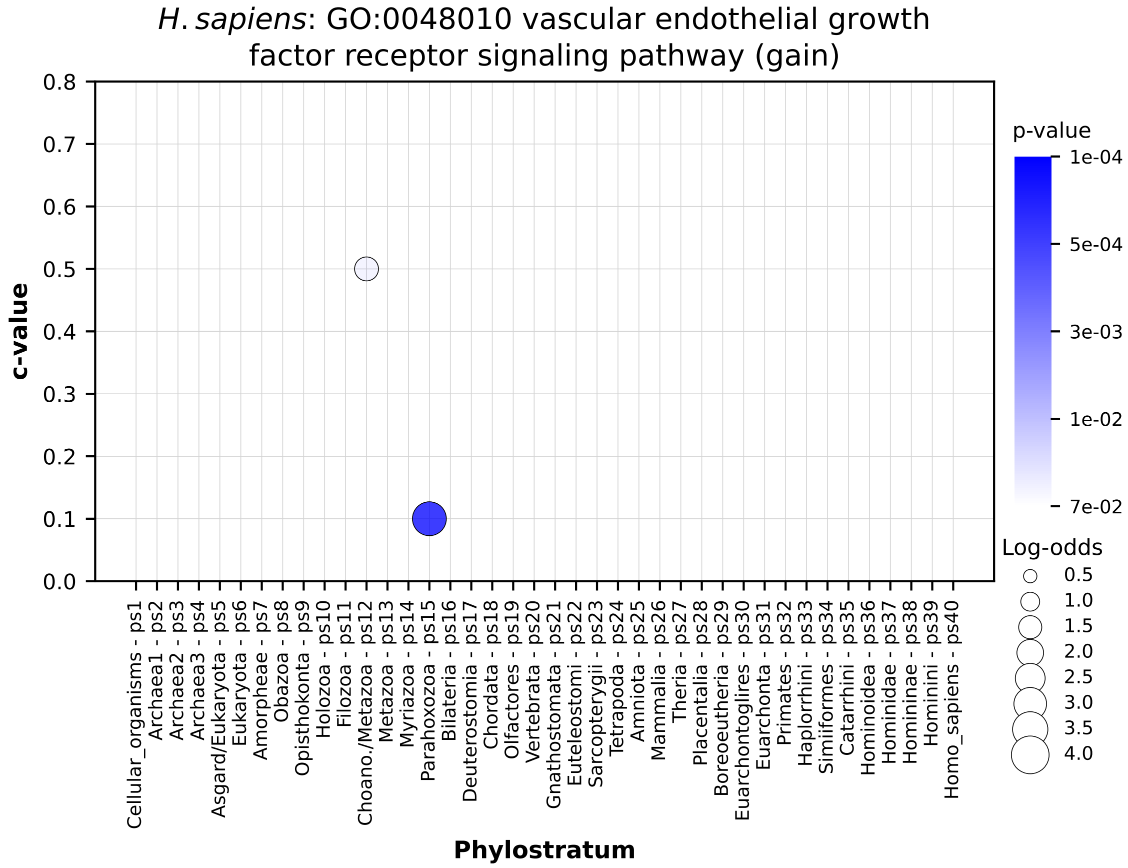

H. sapiens: GO:0048010 vascular endothelial growth factor receptor signaling pathway (gain)
c-value
0.8
0.7
0.6
0.5
0.4
0.3
0.2
0.1
0.0
Phylostratum
Cellular_organisms - ps1
Archaea1 - ps2
Archaea2 - ps3
Archaea3 - ps4
Asgard/Eukaryota - ps5
Eukaryota - ps6
Amorpheae - ps7
Obazoa - ps8
Opisthokonta - ps9
Holozoa - ps10
Filozoa - ps11
Choano./Metazoa - ps12
Metazoa - ps13
Myriazoa - ps14
Parahoxozoa - ps15
Bilateria - ps16
Deuterostomia - ps17
Chordata - ps18
Olfactores - ps19
Vertebrata - ps20
Gnathostomata - ps21
Euteleostomi - ps22
Sarcopterygii - ps23
Tetrapoda - ps24
Amniota - ps25
Mammalia - ps26
Theria - ps27
Placentalia - ps28
Boreoeutheria - ps29
Euarchontoglires - ps30
Euarchonta - ps31
Primates - ps32
Haplorrhini - ps33
Simiiformes - ps34
Catarrhini - ps35
Hominoidea - ps36
Hominidae - ps37
Homininae - ps38
Hominini - ps39
Homo_sapiens - ps40
p-value
1e-04
5e-04
3e-03
1e-02
7e-02
Log-odds
0.5
1.0
1.5
2.0
2.5
3.0
3.5
4.0

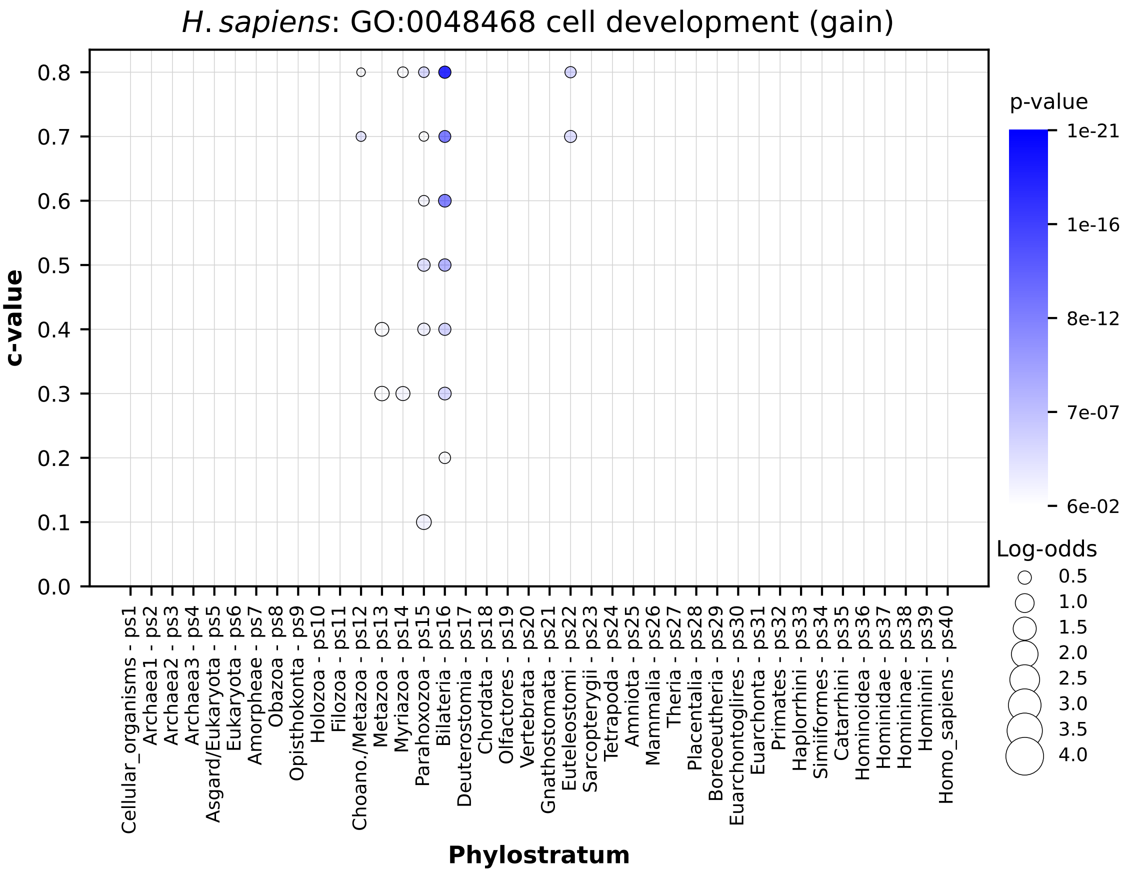

*H. sapiens*: GO:0048468 cell development (gain)
c-value
0.8
0.7
0.6
0.5
0.4
0.3
0.2
0.1
0.0
p-value
1e-21
1e-16
8e-12
7e-07
6e-02
Log-odds
0.5
1.0
1.5
2.0
2.5
3.0
3.5
4.0
Cellular_organisms - ps1
Archaea1 - ps2
Archaea2 - ps3
Archaea3 - ps4
Asgard/Eukaryota - ps5
Eukaryota - ps6
Amorpheae - ps7
Obazoa - ps8
Opisthokonta - ps9
Holozoa - ps10
Filozoa - ps11
Choano./Metazoa - ps12
Metazoa - ps13
Myriazoa - ps14
Parahoxozoa - ps15
Bilateria - ps16
Deuterostomia - ps17
Chordata - ps18
Olfactores - ps19
Vertebrata - ps20
Gnathostomata - ps21
Euteleostomi - ps22
Sarcopterygii - ps23
Tetrapoda - ps24
Amniota - ps25
Mammalia - ps26
Theria - ps27
Placentalia - ps28
Boreoeutheria - ps29
Euarchontoglires - ps30
Euarchonta - ps31
Primates - ps32
Haplorrhini - ps33
Simiiformes - ps34
Catarrhini - ps35
Hominoidea - ps36
Hominidae - ps37
Homininae - ps38
Hominini - ps39
Homo_sapiens - ps40
Phylostratum

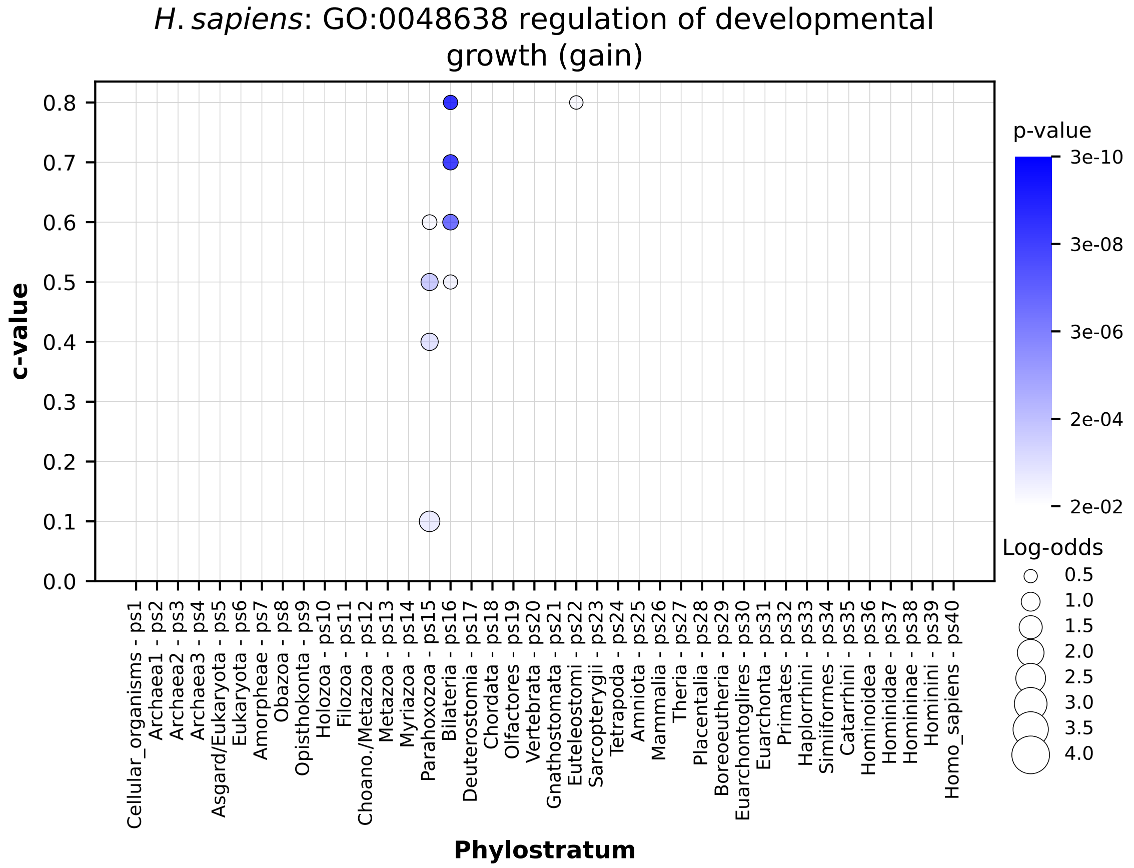

H. sapiens: GO:0048638 regulation of developmental growth (gain)
c-value
0.0
0.1
0.2
0.3
0.4
0.5
0.6
0.7
0.8
p-value
3e-10
3e-08
3e-06
2e-04
2e-02
Log-odds
0.5
1.0
1.5
2.0
2.5
3.0
3.5
4.0
Cellular_organisms - ps1
Archaea1 - ps2
Archaea2 - ps3
Archaea3 - ps4
Asgard/Eukaryota - ps5
Eukaryota - ps6
Amorpheae - ps7
Obazoa - ps8
Opisthokonta - ps9
Holozoa - ps10
Filozoa - ps11
Choano./Metazoa - ps12
Metazoa - ps13
Myriazoa - ps14
Parahoxozoa - ps15
Bilateria - ps16
Deuterostomia - ps17
Chordata - ps18
Olfactores - ps19
Vertebrata - ps20
Gnathostomata - ps21
Euteleostomi - ps22
Sarcopterygii - ps23
Tetrapoda - ps24
Amniota - ps25
Mammalia - ps26
Theria - ps27
Placentalia - ps28
Boreoeutheria - ps29
Euarchontoglires - ps30
Euarchonta - ps31
Primates - ps32
Haplorrhini - ps33
Simiiformes - ps34
Catarrhini - ps35
Hominoidea - ps36
Hominidae - ps37
Homininae - ps38
Hominini - ps39
Homo_sapiens - ps40
Phylostratum

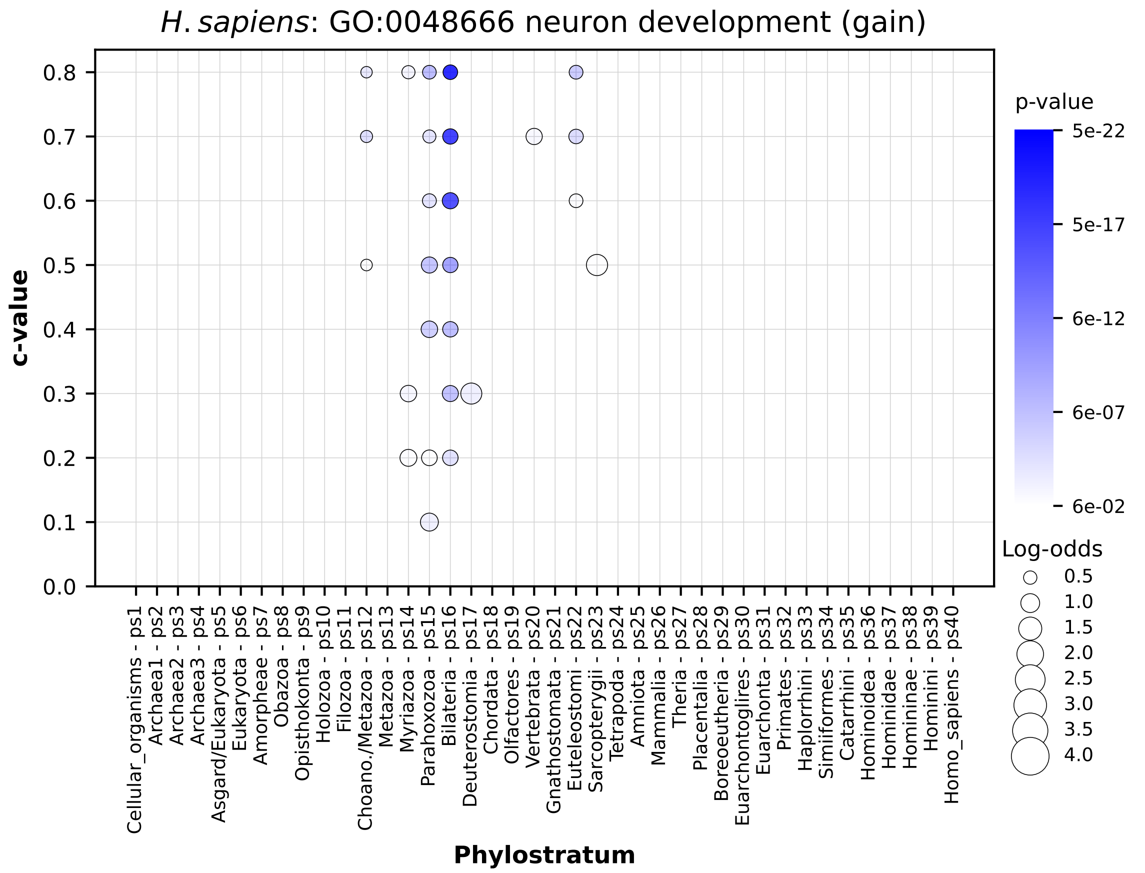

*H. sapiens*: GO:0048666 neuron development (gain)
c-value
0.8
0.7
0.6
0.5
0.4
0.3
0.2
0.1
0.0
Phylostratum
Cellular_organisms - ps1
Archaea1 - ps2
Archaea2 - ps3
Archaea3 - ps4
Asgard/Eukaryota - ps5
Eukaryota - ps6
Amorpheae - ps7
Obazoa - ps8
Opisthokonta - ps9
Holozoa - ps10
Filozoa - ps11
Choano./Metazoa - ps12
Metazoa - ps13
Myriazoa - ps14
Parahoxozoa - ps15
Bilateria - ps16
Deuterostomia - ps17
Chordata - ps18
Olfactores - ps19
Vertebrata - ps20
Gnathostomata - ps21
Euteleostomi - ps22
Sarcopterygii - ps23
Tetrapoda - ps24
Amniota - ps25
Mammalia - ps26
Theria - ps27
Placentalia - ps28
Boreoeutheria - ps29
Euarchontoglires - ps30
Euarchonta - ps31
Primates - ps32
Haplorrhini - ps33
Simiiformes - ps34
Catarrhini - ps35
Hominoidea - ps36
Hominidae - ps37
Homininae - ps38
Hominini - ps39
Homo_sapiens - ps40
p-value
5e-22
5e-17
6e-12
6e-07
6e-02
Log-odds
0.5
1.0
1.5
2.0
2.5
3.0
3.5
4.0

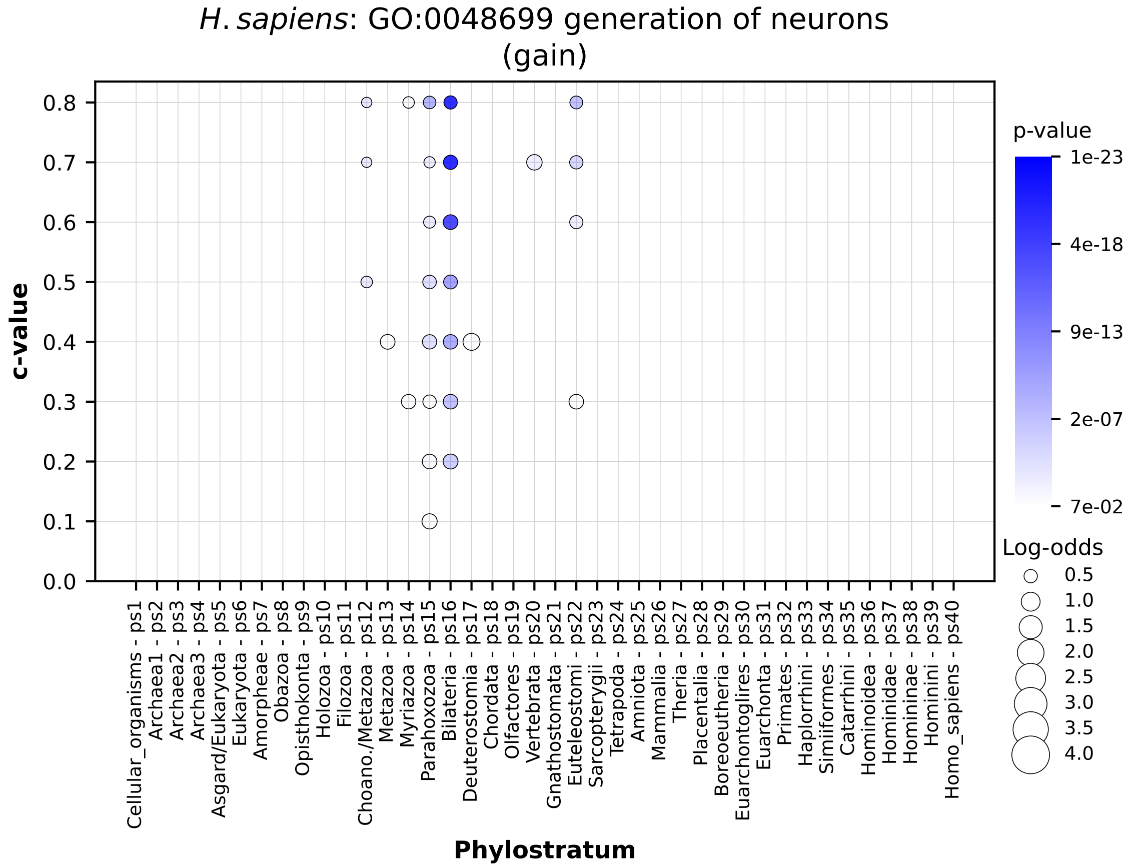

H. sapiens: GO:0048699 generation of neurons
(gain)
c-value
0.8
0.7
0.6
0.5
0.4
0.3
0.2
0.1
0.0
Phylostratum
Cellular_organisms - ps1
Archaea1 - ps2
Archaea2 - ps3
Archaea3 - ps4
Asgard/Eukaryota - ps5
Eukaryota - ps6
Amorpheae - ps7
Obazoa - ps8
Opisthokonta - ps9
Holozoa - ps10
Filozoa - ps11
Choano./Metazoa - ps12
Metazoa - ps13
Myriazoa - ps14
Parahoxozoa - ps15
Bilateria - ps16
Deuterostomia - ps17
Chordata - ps18
Olfactores - ps19
Vertebrata - ps20
Gnathostomata - ps21
Euteleostomi - ps22
Sarcopterygii - ps23
Tetrapoda - ps24
Amniota - ps25
Mammalia - ps26
Theria - ps27
Placentalia - ps28
Boreoeutheria - ps29
Euarchontoglires - ps30
Euarchonta - ps31
Primates - ps32
Haplorrhini - ps33
Simiiformes - ps34
Catarrhini - ps35
Hominoidea - ps36
Hominidae - ps37
Homininae - ps38
Hominini - ps39
Homo_sapiens - ps40
p-value
1e-23
4e-18
9e-13
2e-07
7e-02
Log-odds
0.5
1.0
1.5
2.0
2.5
3.0
3.5
4.0

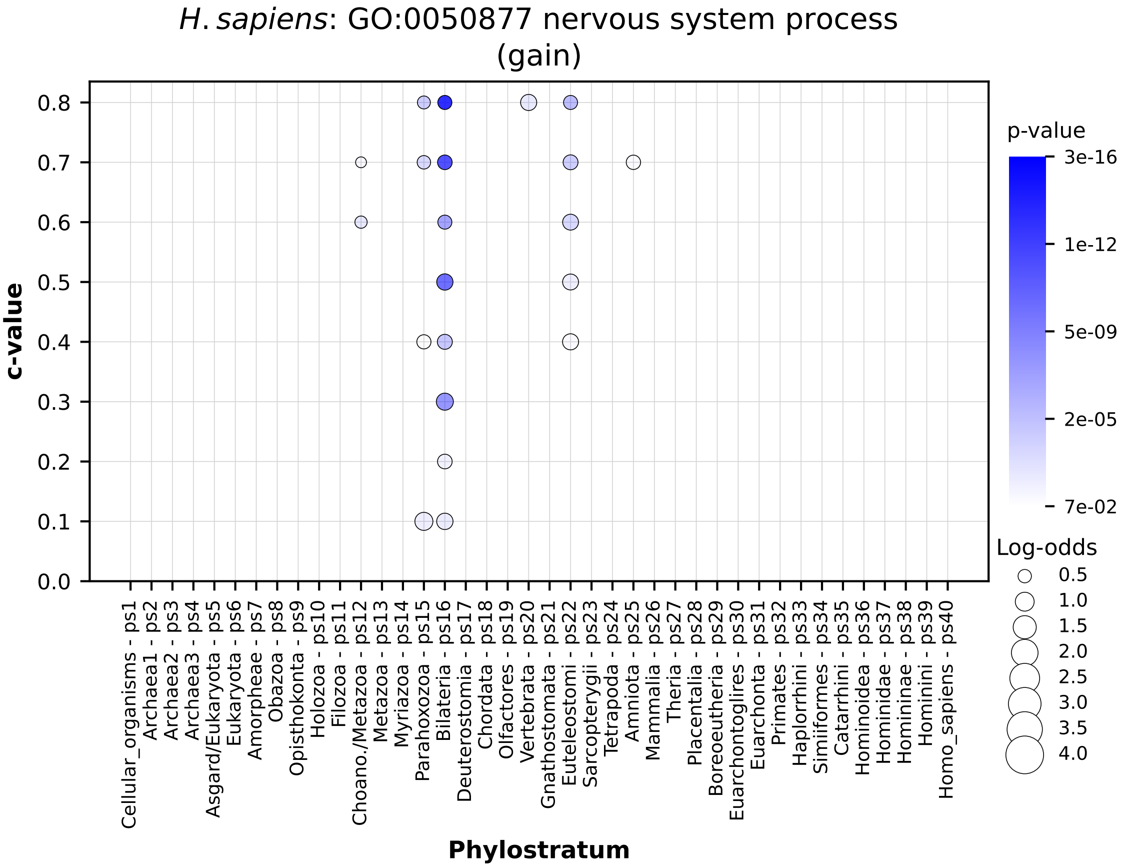

H. sapiens: GO:0050877 nervous system process
(gain)
c-value
0.0
0.1
0.2
0.3
0.4
0.5
0.6
0.7
0.8
Phylostratum
Cellular_organisms - ps1
Archaea1 - ps2
Archaea2 - ps3
Archaea3 - ps4
Asgard/Eukaryota - ps5
Eukaryota - ps6
Amorpheae - ps7
Obazoa - ps8
Opisthokonta - ps9
Holozoa - ps10
Filozoa - ps11
Choano./Metazoa - ps12
Metazoa - ps13
Myriazoa - ps14
Parahoxozoa - ps15
Bilateria - ps16
Deuterostomia - ps17
Chordata - ps18
Olfactores - ps19
Vertebrata - ps20
Gnathostomata - ps21
Euteleostomi - ps22
Sarcopterygii - ps23
Tetrapoda - ps24
Amniota - ps25
Mammalia - ps26
Theria - ps27
Placentalia - ps28
Boreoeutheria - ps29
Euarchontoglires - ps30
Euarchonta - ps31
Primates - ps32
Haplorrhini - ps33
Simiiformes - ps34
Catarrhini - ps35
Hominoidea - ps36
Hominidae - ps37
Homininae - ps38
Hominini - ps39
Homo_sapiens - ps40
p-value
3e-16
1e-12
5e-09
2e-05
7e-02
Log-odds
0.5
1.0
1.5
2.0
2.5
3.0
3.5
4.0

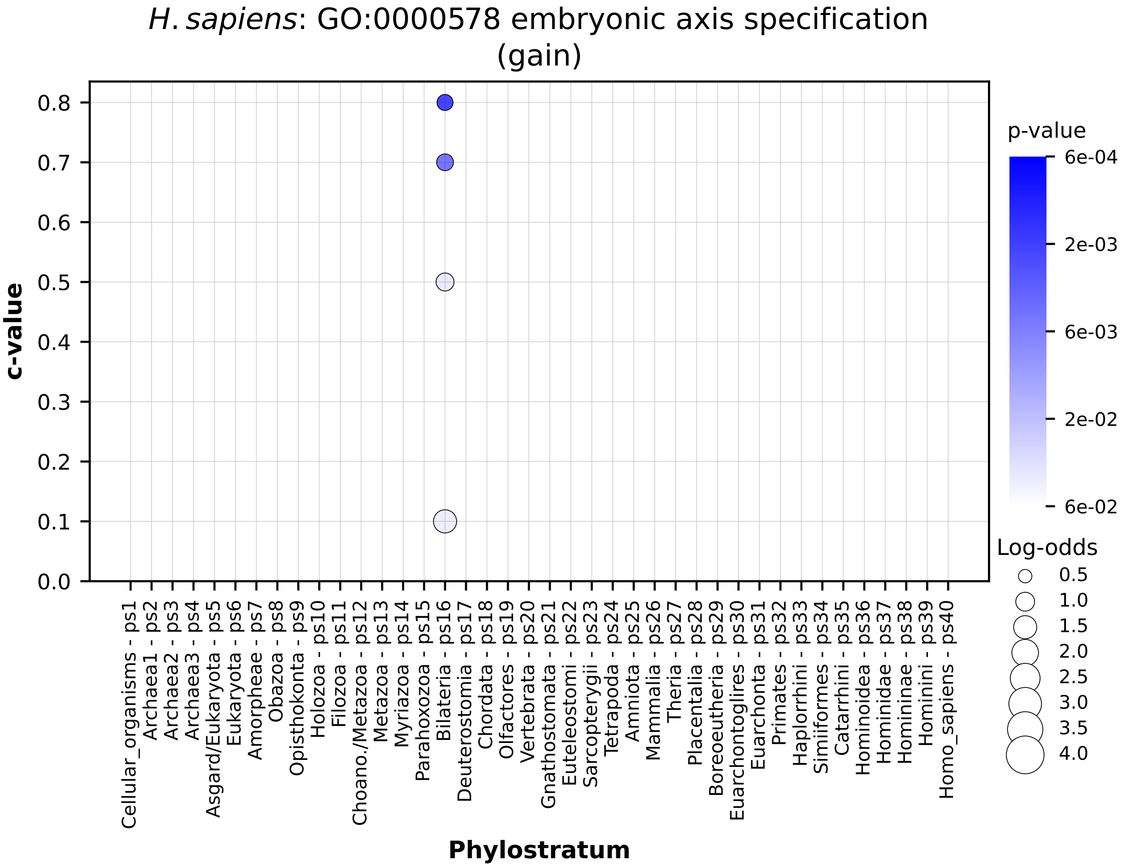

*H. sapiens*: GO:0000578 embryonic axis specification (gain)
c-value
0.0
0.1
0.2
0.3
0.4
0.5
0.6
0.7
0.8
Phylostratum
Cellular_organisms - ps1
Archaea1 - ps2
Archaea2 - ps3
Archaea3 - ps4
Asgard/Eukaryota - ps5
Eukaryota - ps6
Amorpheae - ps7
Obazoa - ps8
Opisthokonta - ps9
Holozoa - ps10
Filozoa - ps11
Choano./Metazoa - ps12
Metazoa - ps13
Myriazoa - ps14
Parahoxozoa - ps15
Bilateria - ps16
Deuterostomia - ps17
Chordata - ps18
Olfactores - ps19
Vertebrata - ps20
Gnathostomata - ps21
Euteleostomi - ps22
Sarcopterygii - ps23
Tetrapoda - ps24
Amniota - ps25
Mammalia - ps26
Theria - ps27
Placentalia - ps28
Boreoeutheria - ps29
Euarchontoglires - ps30
Euarchonta - ps31
Primates - ps32
Haplorrhini - ps33
Simiiformes - ps34
Catarrhini - ps35
Hominoidea - ps36
Hominidae - ps37
Homininae - ps38
Hominini - ps39
Homo_sapiens - ps40
p-value
6e-04
2e-03
6e-03
2e-02
6e-02
Log-odds
0.5
1.0
1.5
2.0
2.5
3.0
3.5
4.0

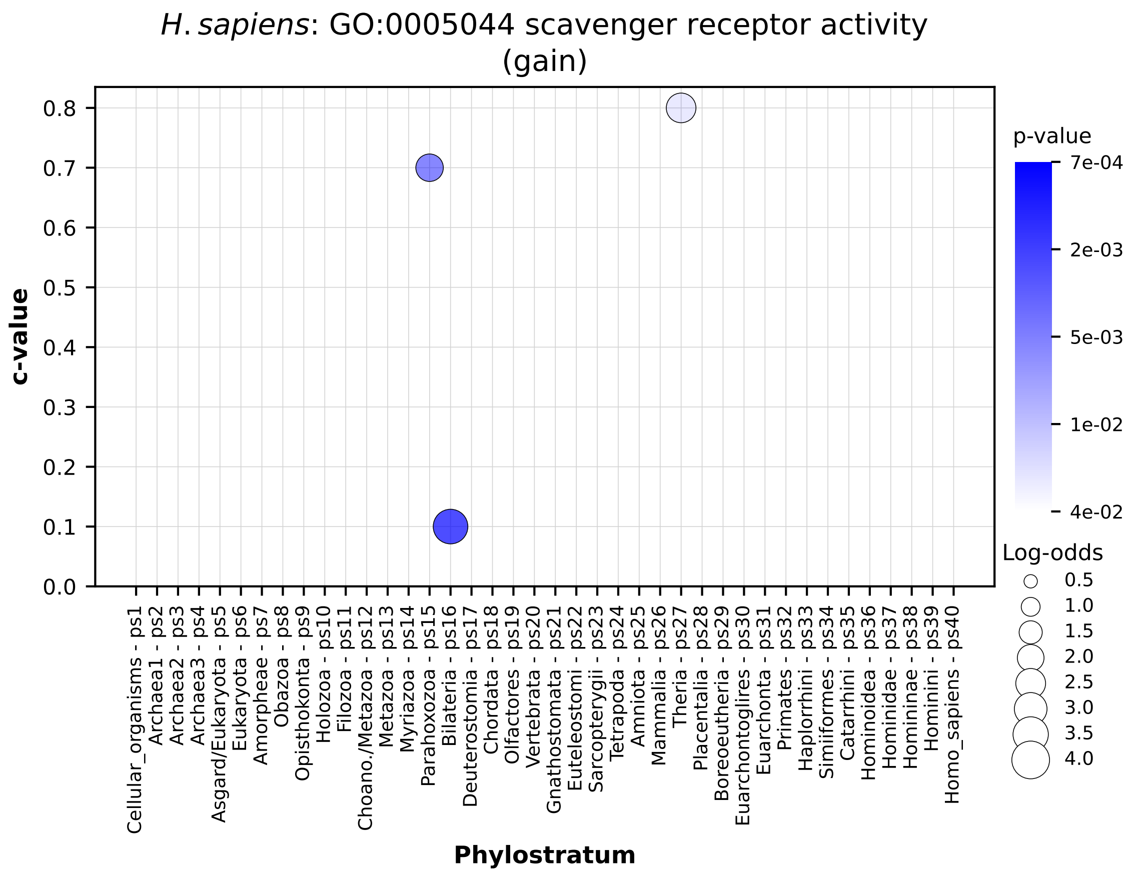

H. sapiens: GO:0005044 scavenger receptor activity
(gain)
c-value
0.0
0.1
0.2
0.3
0.4
0.5
0.6
0.7
0.8
Phylostratum
Cellular_organisms - ps1
Archaea1 - ps2
Archaea2 - ps3
Archaea3 - ps4
Asgard/Eukaryota - ps5
Eukaryota - ps6
Amorpheae - ps7
Obazoa - ps8
Opisthokonta - ps9
Holozoa - ps10
Filozoa - ps11
Choano./Metazoa - ps12
Metazoa - ps13
Myriazoa - ps14
Parahoxozoa - ps15
Bilateria - ps16
Deuterostomia - ps17
Chordata - ps18
Olfactores - ps19
Vertebrata - ps20
Gnathostomata - ps21
Euteleostomi - ps22
Sarcopterygii - ps23
Tetrapoda - ps24
Amniota - ps25
Mammalia - ps26
Theria - ps27
Placentalia - ps28
Boreoeutheria - ps29
Euarchontoglires - ps30
Euarchonta - ps31
Primates - ps32
Haplorrhini - ps33
Simiiformes - ps34
Catarrhini - ps35
Hominoidea - ps36
Hominidae - ps37
Homininae - ps38
Hominini - ps39
Homo_sapiens - ps40
p-value
7e-04
2e-03
5e-03
1e-02
4e-02
Log-odds
0.5
1.0
1.5
2.0
2.5
3.0
3.5
4.0

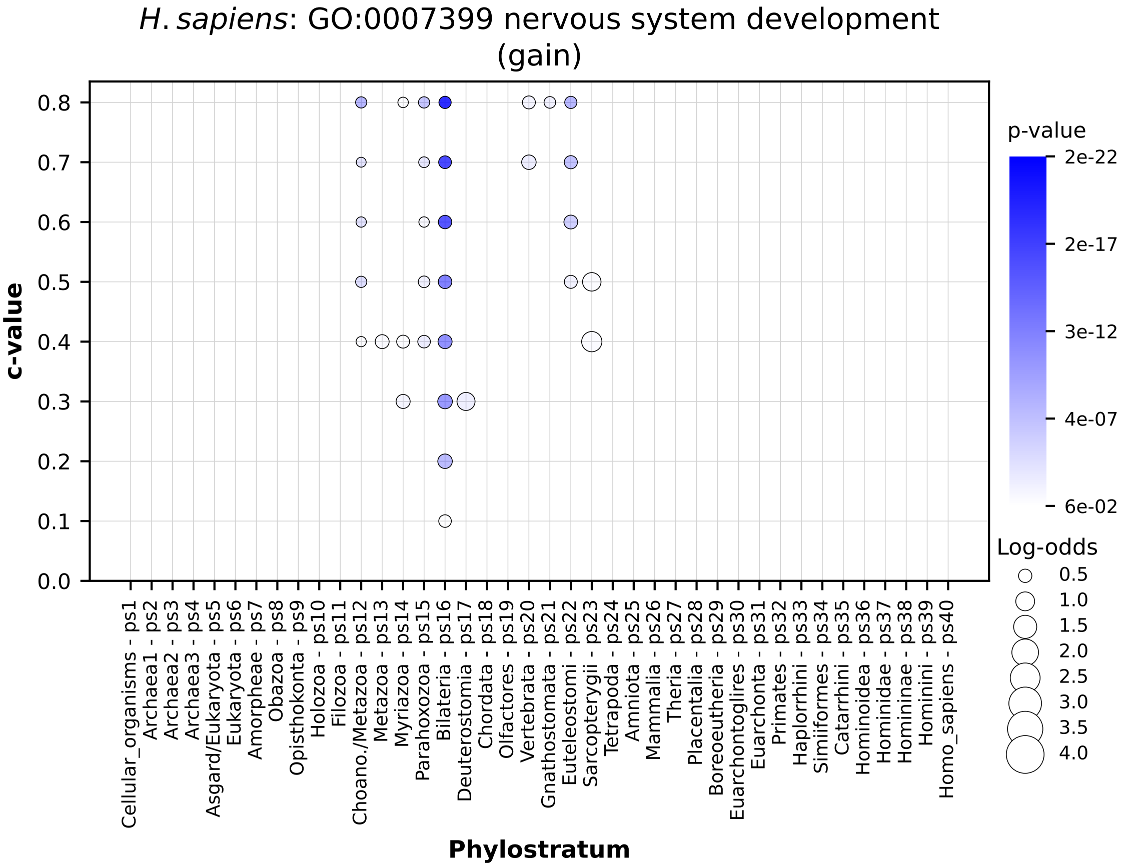

H. sapiens: GO:0007399 nervous system development (gain)
c-value
0.0
0.1
0.2
0.3
0.4
0.5
0.6
0.7
0.8
Phylostratum
Cellular_organisms - ps1
Archaea1 - ps2
Archaea2 - ps3
Archaea3 - ps4
Asgard/Eukaryota - ps5
Eukaryota - ps6
Amorpheae - ps7
Obazoa - ps8
Opisthokonta - ps9
Holozoa - ps10
Filozoa - ps11
Choano./Metazoa - ps12
Metazoa - ps13
Myriazoa - ps14
Parahoxozoa - ps15
Bilateria - ps16
Deuterostomia - ps17
Chordata - ps18
Olfactores - ps19
Vertebrata - ps20
Gnathostomata - ps21
Euteleostomi - ps22
Sarcopterygii - ps23
Tetrapoda - ps24
Amniota - ps25
Mammalia - ps26
Theria - ps27
Placentalia - ps28
Boreoeutheria - ps29
Euarchontoglires - ps30
Euarchonta - ps31
Primates - ps32
Haplorrhini - ps33
Simiiformes - ps34
Catarrhini - ps35
Hominoidea - ps36
Hominidae - ps37
Homininae - ps38
Hominini - ps39
Homo_sapiens - ps40
p-value
2e-22
2e-17
3e-12
4e-07
6e-02
Log-odds
0.5
1.0
1.5
2.0
2.5
3.0
3.5
4.0

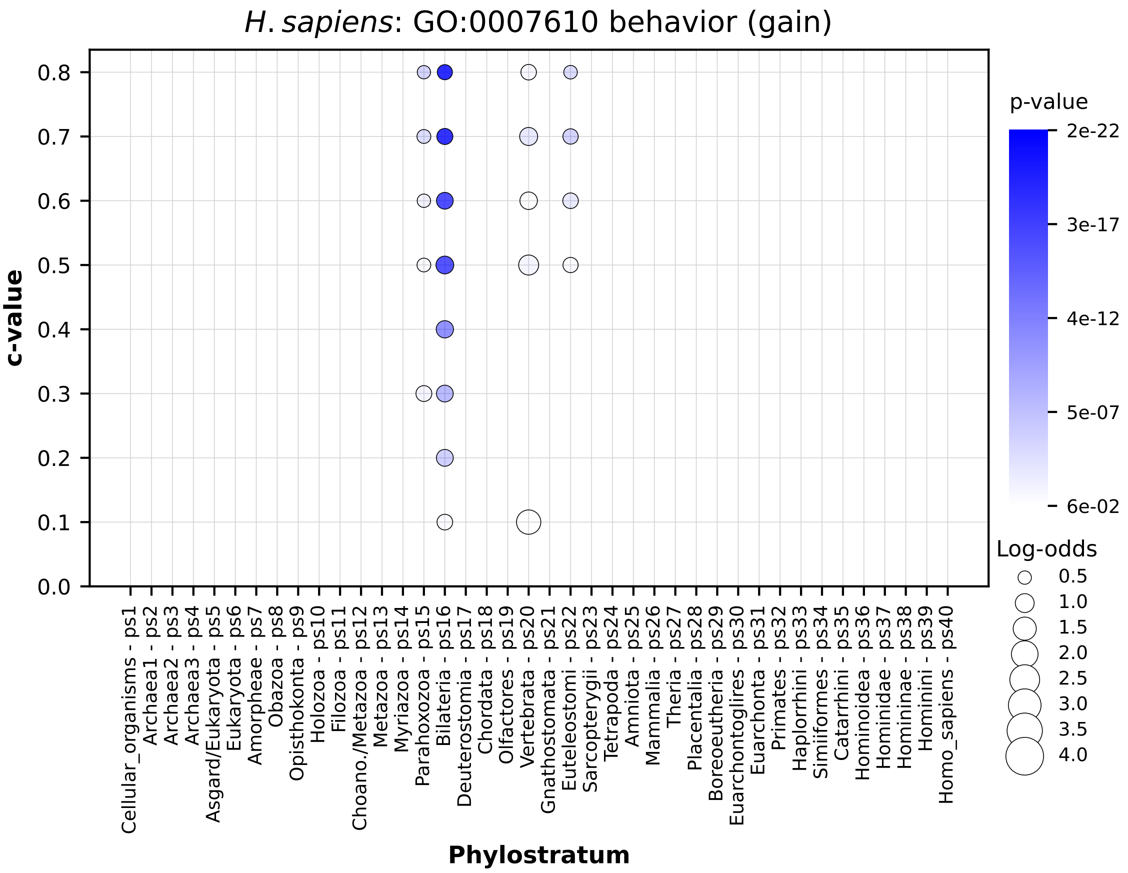

H. sapiens: GO:0007610 behavior (gain)
c-value
0.0
0.1
0.2
0.3
0.4
0.5
0.6
0.7
0.8
Phylostratum
Cellular_organisms - ps1
Archaea1 - ps2
Archaea2 - ps3
Archaea3 - ps4
Asgard/Eukaryota - ps5
Eukaryota - ps6
Amorpheae - ps7
Obazoa - ps8
Opisthokonta - ps9
Holozoa - ps10
Filozoa - ps11
Choano./Metazoa - ps12
Metazoa - ps13
Myriazoa - ps14
Parahoxozoa - ps15
Bilateria - ps16
Deuterostomia - ps17
Chordata - ps18
Olfactores - ps19
Vertebrata - ps20
Gnathostomata - ps21
Euteleostomi - ps22
Sarcopterygii - ps23
Tetrapoda - ps24
Amniota - ps25
Mammalia - ps26
Theria - ps27
Placentalia - ps28
Boreoeutheria - ps29
Euarchontoglires - ps30
Euarchonta - ps31
Primates - ps32
Haplorrhini - ps33
Simiiformes - ps34
Catarrhini - ps35
Hominoidea - ps36
Hominidae - ps37
Homininae - ps38
Hominini - ps39
Homo_sapiens - ps40
p-value
2e-22
3e-17
4e-12
5e-07
6e-02
Log-odds
0.5
1.0
1.5
2.0
2.5
3.0
3.5
4.0

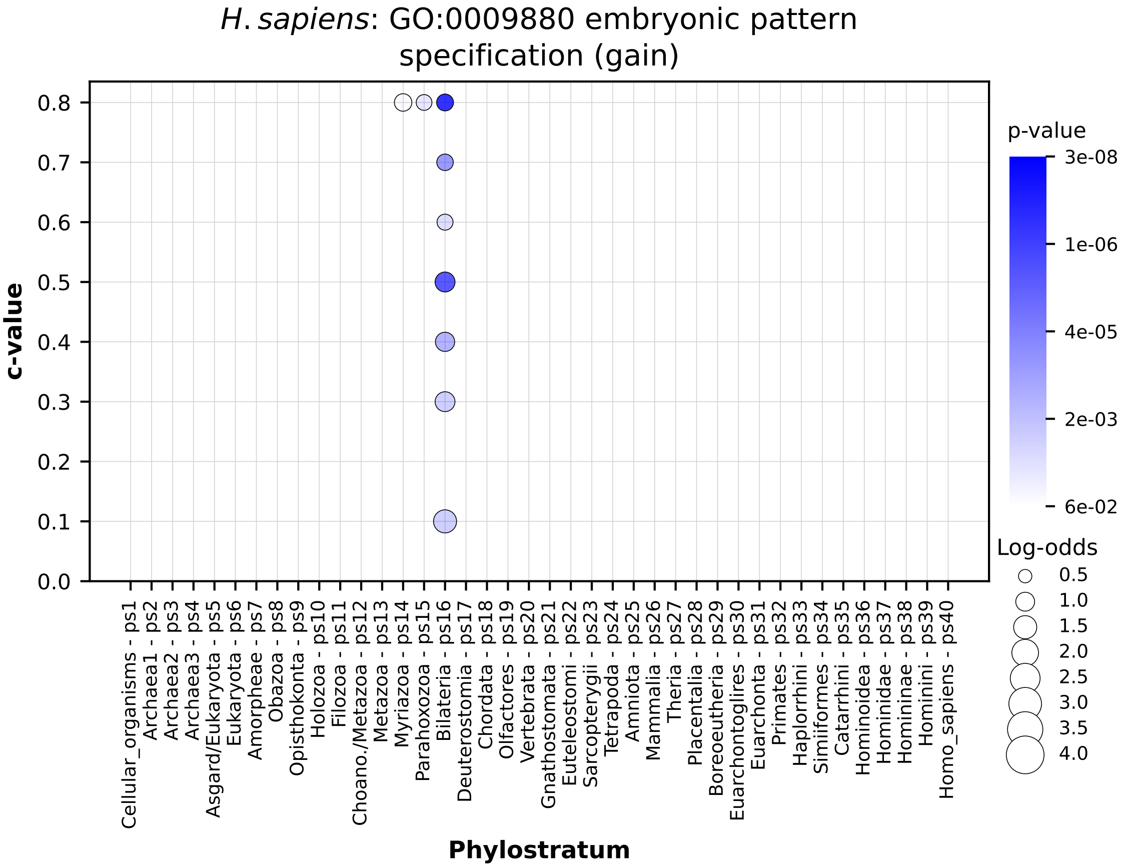

H. sapiens: GO:0009880 embryonic pattern specification (gain)
c-value
0.0
0.1
0.2
0.3
0.4
0.5
0.6
0.7
0.8
Phylostratum
Cellular_organisms - ps1
Archaea1 - ps2
Archaea2 - ps3
Archaea3 - ps4
Asgard/Eukaryota - ps5
Eukaryota - ps6
Amorpheae - ps7
Obazoa - ps8
Opisthokonta - ps9
Holozoa - ps10
Filozoa - ps11
Choano./Metazoa - ps12
Metazoa - ps13
Myriazoa - ps14
Parahoxozoa - ps15
Bilateria - ps16
Deuterostomia - ps17
Chordata - ps18
Olfactores - ps19
Vertebrata - ps20
Gnathostomata - ps21
Euteleostomi - ps22
Sarcopterygii - ps23
Tetrapoda - ps24
Amniota - ps25
Mammalia - ps26
Theria - ps27
Placentalia - ps28
Boreoeutheria - ps29
Euarchontoglires - ps30
Euarchonta - ps31
Primates - ps32
Haplorrhini - ps33
Simiiformes - ps34
Catarrhini - ps35
Hominoidea - ps36
Hominidae - ps37
Homininae - ps38
Hominini - ps39
Homo_sapiens - ps40
p-value
3e-08
1e-06
4e-05
2e-03
6e-02
Log-odds
0.5
1.0
1.5
2.0
2.5
3.0
3.5
4.0

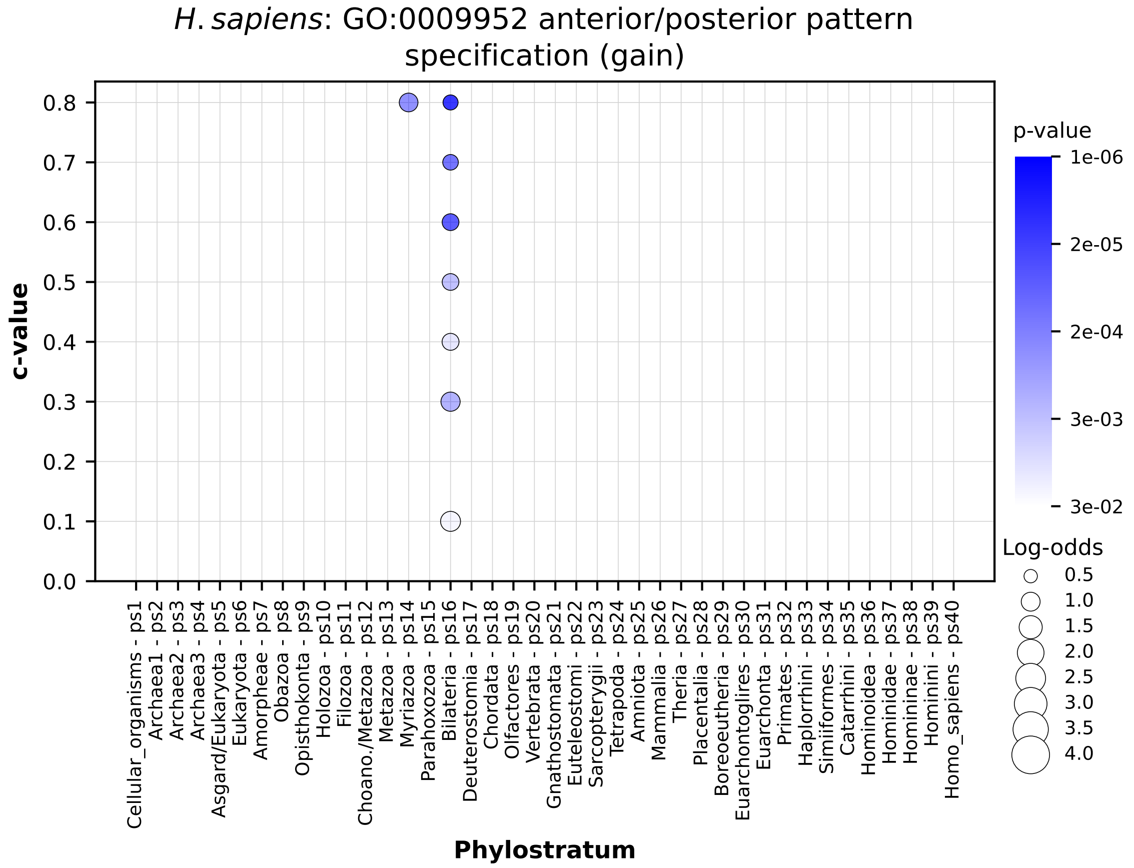
H. sapiens: GO:0009952 anterior/posterior pattern specification (gain)
c-value
0.0
0.1
0.2
0.3
0.4
0.5
0.6
0.7
0.8
Phylostratum
Cellular_organisms - ps1
Archaea1 - ps2
Archaea2 - ps3
Archaea3 - ps4
Asgard/Eukaryota - ps5
Eukaryota - ps6
Amorpheae - ps7
Obazoa - ps8
Opisthokonta - ps9
Holozoa - ps10
Filozoa - ps11
Choano./Metazoa - ps12
Metazoa - ps13
Myriazoa - ps14
Parahoxozoa - ps15
Bilateria - ps16
Deuterostomia - ps17
Chordata - ps18
Olfactores - ps19
Vertebrata - ps20
Gnathostomata - ps21
Euteleostomi - ps22
Sarcopterygii - ps23
Tetrapoda - ps24
Amniota - ps25
Mammalia - ps26
Theria - ps27
Placentalia - ps28
Boreoeutheria - ps29
Euarchontoglires - ps30
Euarchonta - ps31
Primates - ps32
Haplorrhini - ps33
Simiiformes - ps34
Catarrhini - ps35
Hominoidea - ps36
Hominidae - ps37
Homininae - ps38
Hominini - ps39
Homo_sapiens - ps40
p-value
1e-06
2e-05
2e-04
3e-03
3e-02
Log-odds
0.5
1.0
1.5
2.0
2.5
3.0
3.5
4.0

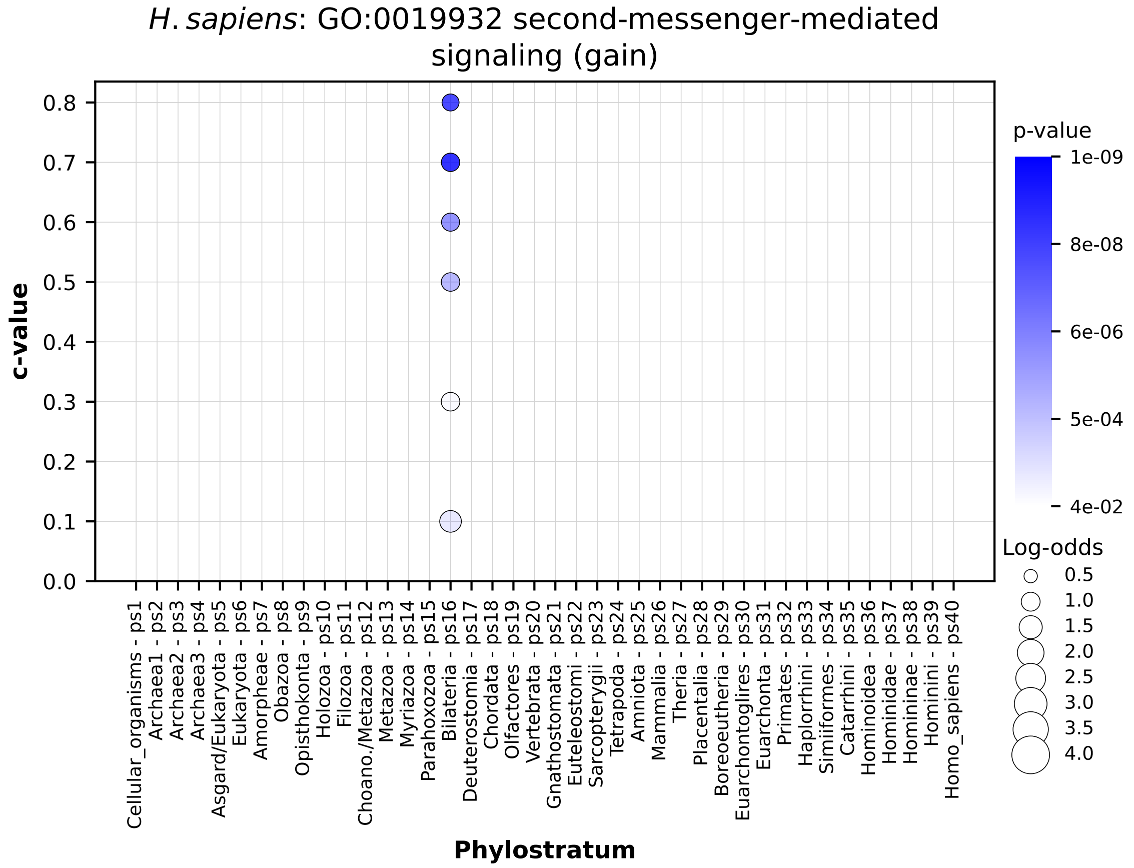

H. sapiens: GO:0019932 second-messenger-mediated signaling (gain)
c-value
0.0
0.1
0.2
0.3
0.4
0.5
0.6
0.7
0.8
Phylostratum
Cellular_organisms - ps1
Archaea1 - ps2
Archaea2 - ps3
Archaea3 - ps4
Asgard/Eukaryota - ps5
Eukaryota - ps6
Amorpheae - ps7
Obazoa - ps8
Opisthokonta - ps9
Holozoa - ps10
Filozoa - ps11
Choano./Metazoa - ps12
Metazoa - ps13
Myriazoa - ps14
Parahoxozoa - ps15
Bilateria - ps16
Deuterostomia - ps17
Chordata - ps18
Olfactores - ps19
Vertebrata - ps20
Gnathostomata - ps21
Euteleostomi - ps22
Sarcopterygii - ps23
Tetrapoda - ps24
Amniota - ps25
Mammalia - ps26
Theria - ps27
Placentalia - ps28
Boreoeutheria - ps29
Euarchontoglires - ps30
Euarchonta - ps31
Primates - ps32
Haplorrhini - ps33
Simiiformes - ps34
Catarrhini - ps35
Hominoidea - ps36
Hominidae - ps37
Homininae - ps38
Hominini - ps39
Homo_sapiens - ps40
p-value
1e-09
8e-08
6e-06
5e-04
4e-02
Log-odds
0.5
1.0
1.5
2.0
2.5
3.0
3.5
4.0

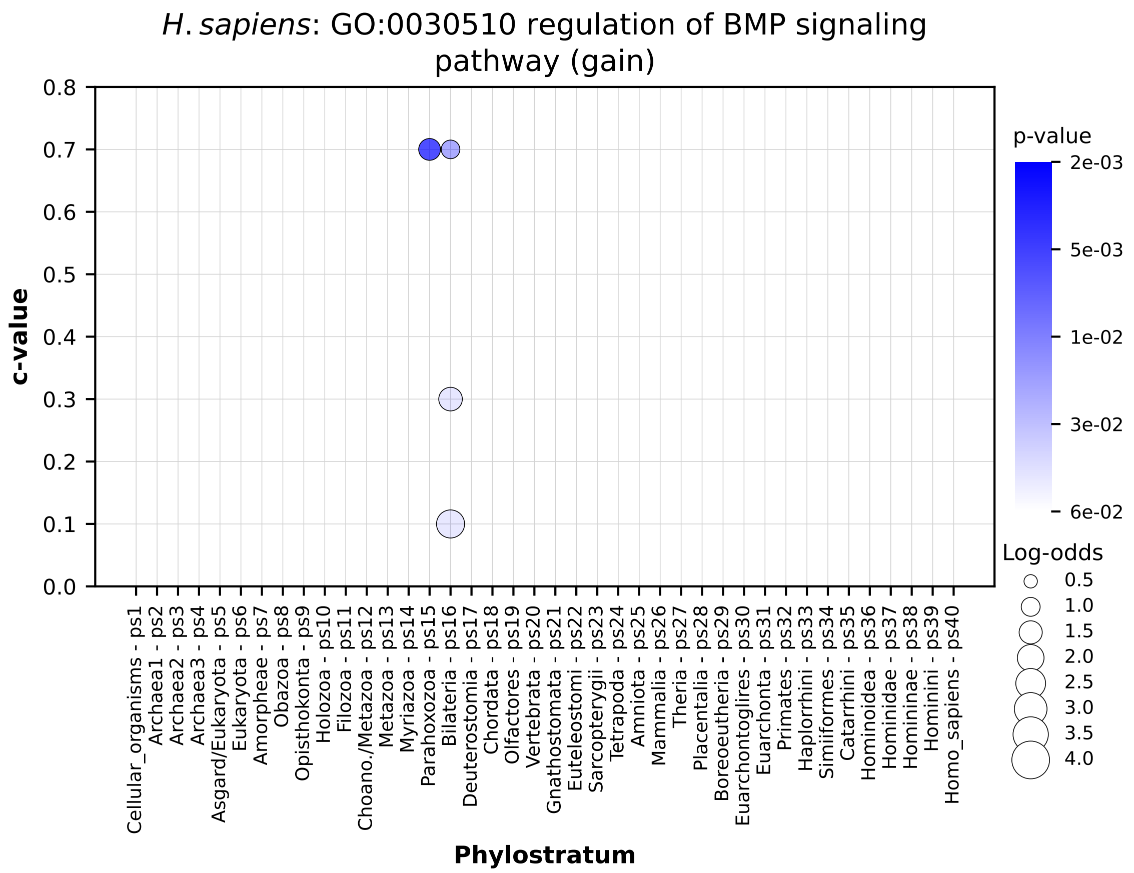

H. sapiens: GO:0030510 regulation of BMP signaling pathway (gain)
c-value
0.0
0.1
0.2
0.3
0.4
0.5
0.6
0.7
0.8
Phylostratum
Cellular_organisms - ps1
Archaea1 - ps2
Archaea2 - ps3
Archaea3 - ps4
Asgard/Eukaryota - ps5
Eukaryota - ps6
Amorpheae - ps7
Obazoa - ps8
Opisthokonta - ps9
Holozoa - ps10
Filozoa - ps11
Choano./Metazoa - ps12
Metazoa - ps13
Myriazoa - ps14
Parahoxozoa - ps15
Bilateria - ps16
Deuterostomia - ps17
Chordata - ps18
Olfactores - ps19
Vertebrata - ps20
Gnathostomata - ps21
Euteleostomi - ps22
Sarcopterygii - ps23
Tetrapoda - ps24
Amniota - ps25
Mammalia - ps26
Theria - ps27
Placentalia - ps28
Boreoeutheria - ps29
Euarchontoglires - ps30
Euarchonta - ps31
Primates - ps32
Haplorrhini - ps33
Simiiformes - ps34
Catarrhini - ps35
Hominoidea - ps36
Hominidae - ps37
Homininae - ps38
Hominini - ps39
Homo_sapiens - ps40
p-value
2e-03
5e-03
1e-02
3e-02
6e-02
Log-odds
0.5
1.0
1.5
2.0
2.5
3.0
3.5
4.0

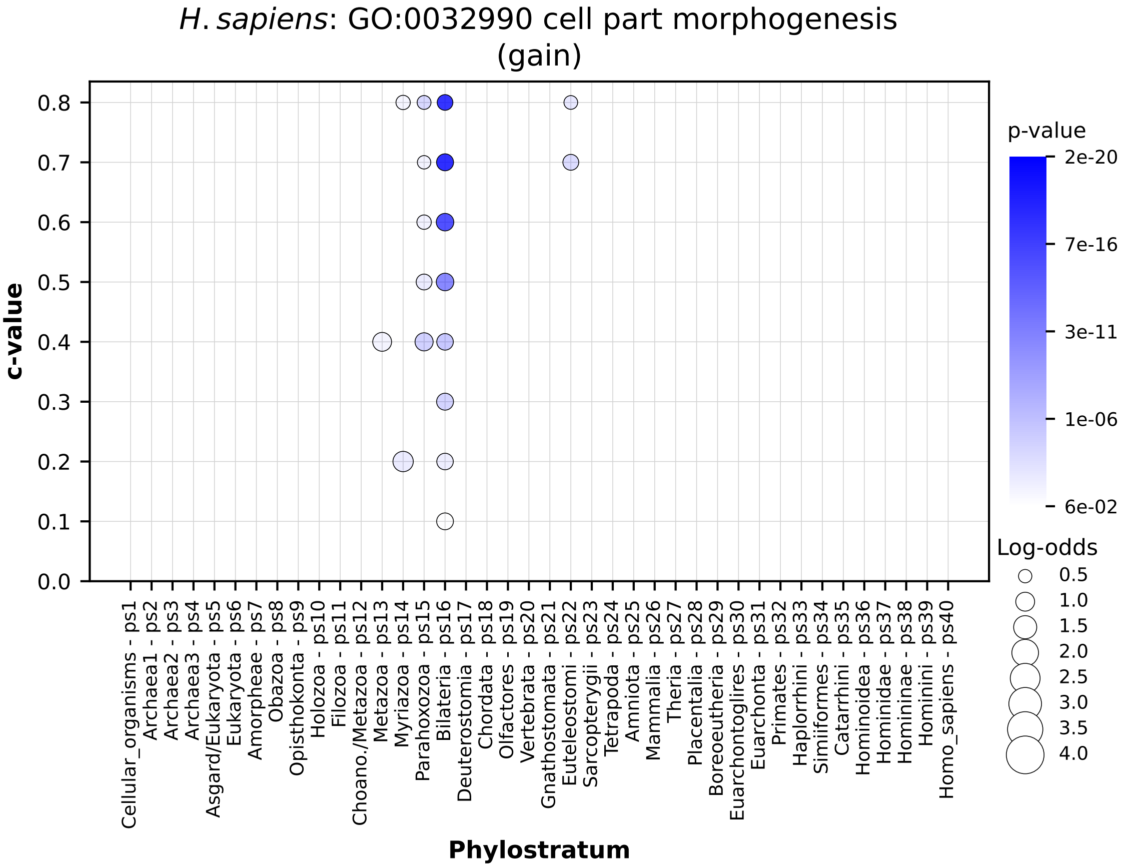

*H. sapiens*: GO:0032990 cell part morphogenesis
(gain)
c-value
0.0
0.1
0.2
0.3
0.4
0.5
0.6
0.7
0.8
Phylostratum
Cellular_organisms - ps1
Archaea1 - ps2
Archaea2 - ps3
Archaea3 - ps4
Asgard/Eukaryota - ps5
Eukaryota - ps6
Amorpheae - ps7
Obazoa - ps8
Opisthokonta - ps9
Holozoa - ps10
Filozoa - ps11
Choano./Metazoa - ps12
Metazoa - ps13
Myriazoa - ps14
Parahoxozoa - ps15
Bilateria - ps16
Deuterostomia - ps17
Chordata - ps18
Olfactores - ps19
Vertebrata - ps20
Gnathostomata - ps21
Euteleostomi - ps22
Sarcopterygii - ps23
Tetrapoda - ps24
Amniota - ps25
Mammalia - ps26
Theria - ps27
Placentalia - ps28
Boreoeutheria - ps29
Euarchontoglires - ps30
Euarchonta - ps31
Primates - ps32
Haplorrhini - ps33
Simiiformes - ps34
Catarrhini - ps35
Hominoidea - ps36
Hominidae - ps37
Homininae - ps38
Hominini - ps39
Homo_sapiens - ps40
p-value
2e-20
7e-16
3e-11
1e-06
6e-02
Log-odds
0.5
1.0
1.5
2.0
2.5
3.0
3.5
4.0

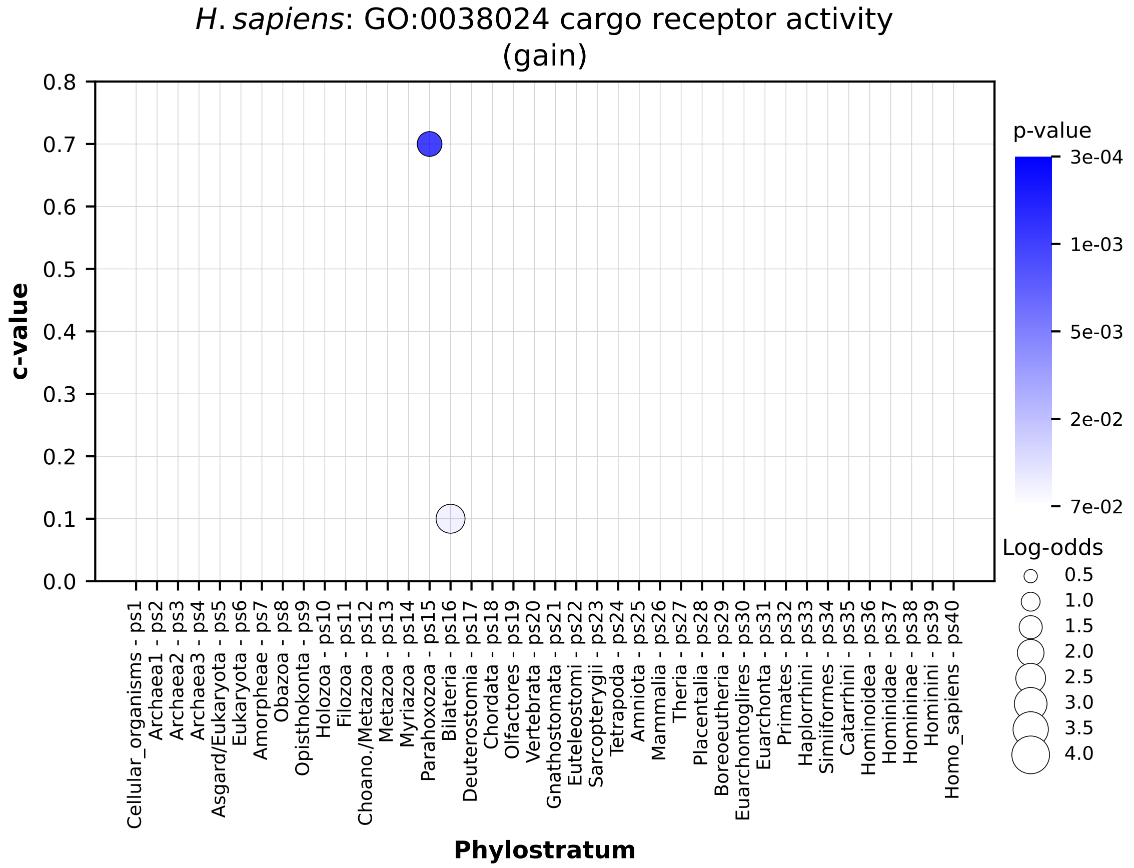

*H. sapiens*: GO:0038024 cargo receptor activity
(gain)
c-value
0.8
0.7
0.6
0.5
0.4
0.3
0.2
0.1
0.0
Phylostratum
Cellular_organisms - ps1
Archaea1 - ps2
Archaea2 - ps3
Archaea3 - ps4
Asgard/Eukaryota - ps5
Eukaryota - ps6
Amorpheae - ps7
Obazoa - ps8
Opisthokonta - ps9
Holozoa - ps10
Filozoa - ps11
Choano./Metazoa - ps12
Metazoa - ps13
Myriazoa - ps14
Parahoxozoa - ps15
Bilateria - ps16
Deuterostomia - ps17
Chordata - ps18
Olfactores - ps19
Vertebrata - ps20
Gnathostomata - ps21
Euteleostomi - ps22
Sarcopterygii - ps23
Tetrapoda - ps24
Amniota - ps25
Mammalia - ps26
Theria - ps27
Placentalia - ps28
Boreoeutheria - ps29
Euarchontoglires - ps30
Euarchonta - ps31
Primates - ps32
Haplorrhini - ps33
Simiiformes - ps34
Catarrhini - ps35
Hominoidea - ps36
Hominidae - ps37
Homininae - ps38
Hominini - ps39
Homo_sapiens - ps40
p-value
3e-04
1e-03
5e-03
2e-02
7e-02
Log-odds
0.5
1.0
1.5
2.0
2.5
3.0
3.5
4.0

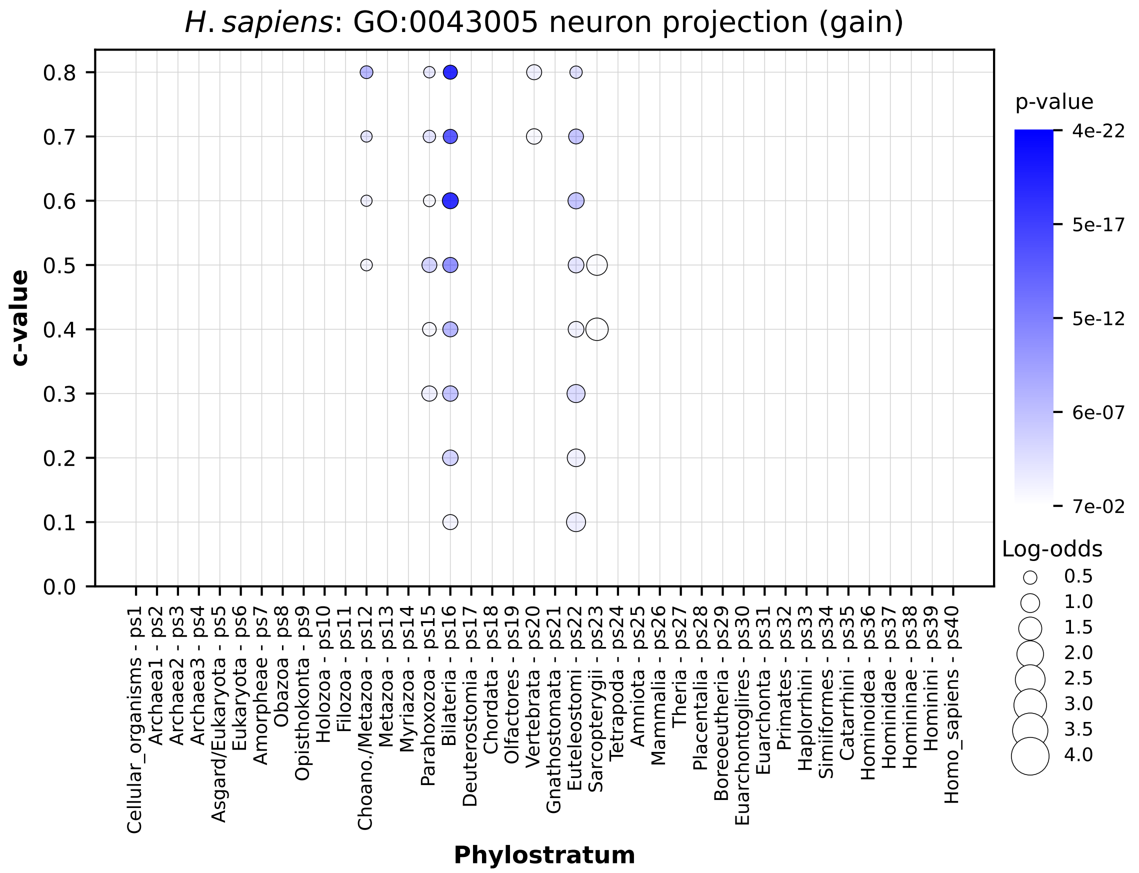
H. sapiens: GO:0043005 neuron projection (gain)
c-value
0.0
0.1
0.2
0.3
0.4
0.5
0.6
0.7
0.8
Phylostratum
Cellular_organisms - ps1
Archaea1 - ps2
Archaea2 - ps3
Archaea3 - ps4
Asgard/Eukaryota - ps5
Eukaryota - ps6
Amorpheae - ps7
Obazoa - ps8
Opisthokonta - ps9
Holozoa - ps10
Filozoa - ps11
Choano./Metazoa - ps12
Metazoa - ps13
Myriazoa - ps14
Parahoxozoa - ps15
Bilateria - ps16
Deuterostomia - ps17
Chordata - ps18
Olfactores - ps19
Vertebrata - ps20
Gnathostomata - ps21
Euteleostomi - ps22
Sarcopterygii - ps23
Tetrapoda - ps24
Amniota - ps25
Mammalia - ps26
Theria - ps27
Placentalia - ps28
Boreoeutheria - ps29
Euarchontoglires - ps30
Euarchonta - ps31
Primates - ps32
Haplorrhini - ps33
Simiiformes - ps34
Catarrhini - ps35
Hominoidea - ps36
Hominidae - ps37
Homininae - ps38
Hominini - ps39
Homo_sapiens - ps40
p-value
4e-22
5e-17
5e-12
6e-07
7e-02
Log-odds
0.5
1.0
1.5
2.0
2.5
3.0
3.5
4.0

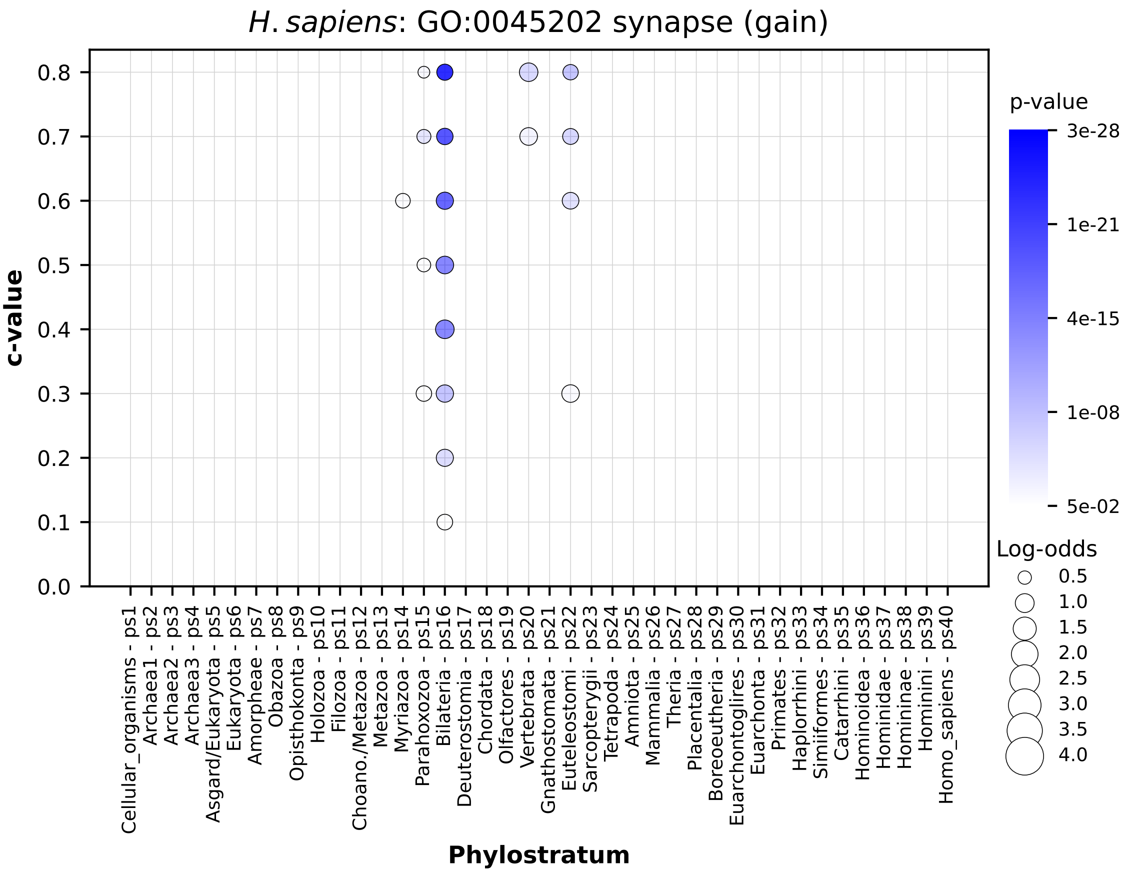

H. sapiens: GO:0045202 synapse (gain)
c-value
0.0
0.1
0.2
0.3
0.4
0.5
0.6
0.7
0.8
Phylostratum
Cellular_organisms - ps1
Archaea1 - ps2
Archaea2 - ps3
Archaea3 - ps4
Asgard/Eukaryota - ps5
Eukaryota - ps6
Amorpheae - ps7
Obazoa - ps8
Opisthokonta - ps9
Holozoa - ps10
Filozoa - ps11
Choano./Metazoa - ps12
Metazoa - ps13
Myriazoa - ps14
Parahoxozoa - ps15
Bilateria - ps16
Deuterostomia - ps17
Chordata - ps18
Olfactores - ps19
Vertebrata - ps20
Gnathostomata - ps21
Euteleostomi - ps22
Sarcopterygii - ps23
Tetrapoda - ps24
Amniota - ps25
Mammalia - ps26
Theria - ps27
Placentalia - ps28
Boreoeutheria - ps29
Euarchontoglires - ps30
Euarchonta - ps31
Primates - ps32
Haplorrhini - ps33
Simiiformes - ps34
Catarrhini - ps35
Hominoidea - ps36
Hominidae - ps37
Homininae - ps38
Hominini - ps39
Homo_sapiens - ps40
p-value
3e-28
1e-21
4e-15
1e-08
5e-02
Log-odds
0.5
1.0
1.5
2.0
2.5
3.0
3.5
4.0

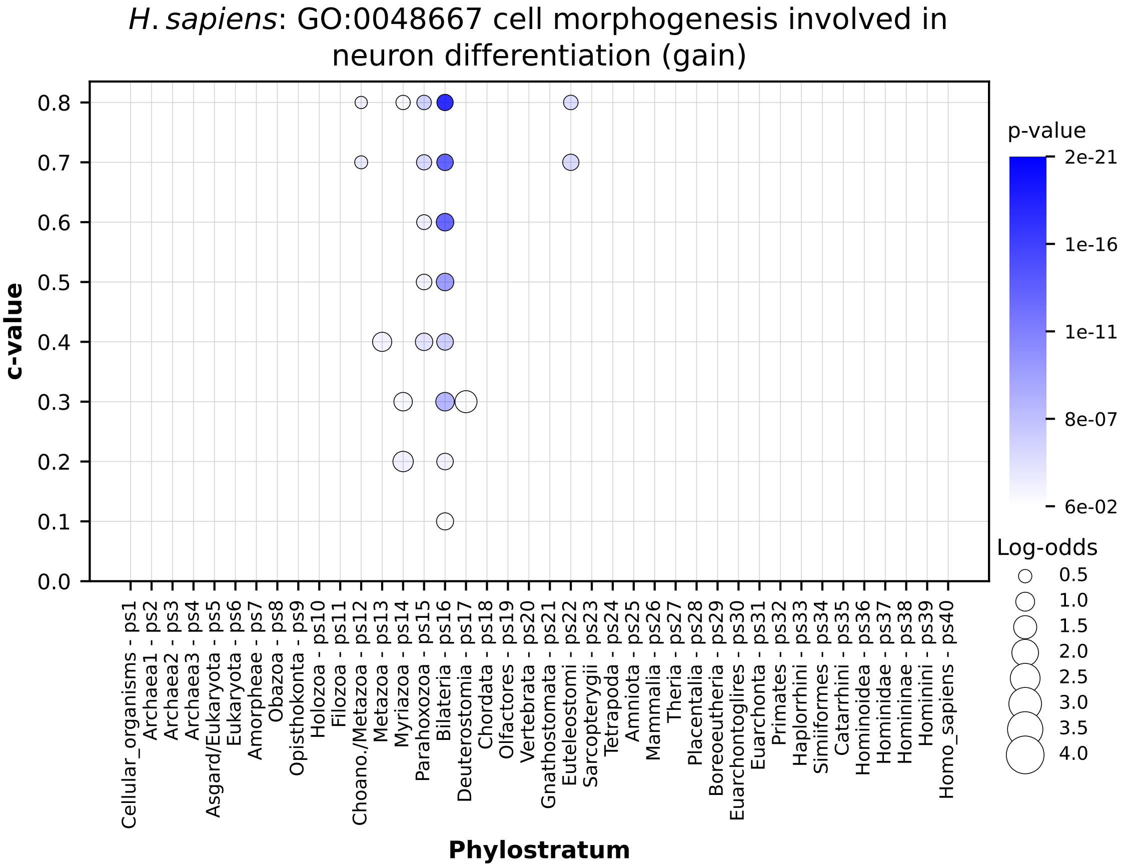

H. sapiens: GO:0048667 cell morphogenesis involved in neuron differentiation (gain)
c-value
0.0
0.1
0.2
0.3
0.4
0.5
0.6
0.7
0.8
Phylostratum
Cellular_organisms - ps1
Archaea1 - ps2
Archaea2 - ps3
Archaea3 - ps4
Asgard/Eukaryota - ps5
Eukaryota - ps6
Amorpheae - ps7
Obazoa - ps8
Opisthokonta - ps9
Holozoa - ps10
Filozoa - ps11
Choano./Metazoa - ps12
Metazoa - ps13
Myriazoa - ps14
Parahoxozoa - ps15
Bilateria - ps16
Deuterostomia - ps17
Chordata - ps18
Olfactores - ps19
Vertebrata - ps20
Gnathostomata - ps21
Euteleostomi - ps22
Sarcopterygii - ps23
Tetrapoda - ps24
Amniota - ps25
Mammalia - ps26
Theria - ps27
Placentalia - ps28
Boreoeutheria - ps29
Euarchontoglires - ps30
Euarchonta - ps31
Primates - ps32
Haplorrhini - ps33
Simiiformes - ps34
Catarrhini - ps35
Hominoidea - ps36
Hominidae - ps37
Homininae - ps38
Hominini - ps39
Homo_sapiens - ps40
p-value
2e-21
1e-16
1e-11
8e-07
6e-02
Log-odds
0.5
1.0
1.5
2.0
2.5
3.0
3.5
4.0

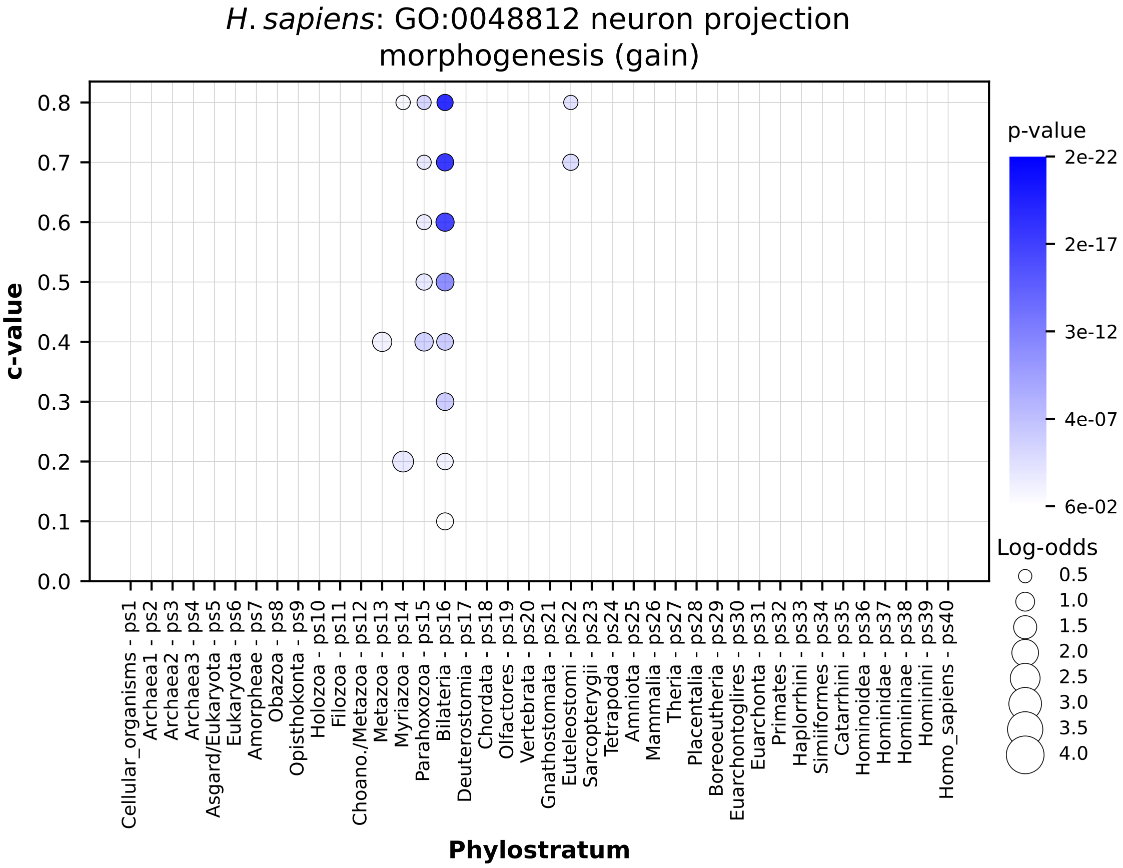

H. sapiens: GO:0048812 neuron projection morphogenesis (gain)
c-value
0.0
0.1
0.2
0.3
0.4
0.5
0.6
0.7
0.8
Phylostratum
Cellular_organisms - ps1
Archaea1 - ps2
Archaea2 - ps3
Archaea3 - ps4
Asgard/Eukaryota - ps5
Eukaryota - ps6
Amorpheae - ps7
Obazoa - ps8
Opisthokonta - ps9
Holozoa - ps10
Filozoa - ps11
Choano./Metazoa - ps12
Metazoa - ps13
Myriazoa - ps14
Parahoxozoa - ps15
Bilateria - ps16
Deuterostomia - ps17
Chordata - ps18
Olfactores - ps19
Vertebrata - ps20
Gnathostomata - ps21
Euteleostomi - ps22
Sarcopterygii - ps23
Tetrapoda - ps24
Amniota - ps25
Mammalia - ps26
Theria - ps27
Placentalia - ps28
Boreoeutheria - ps29
Euarchontoglires - ps30
Euarchonta - ps31
Primates - ps32
Haplorrhini - ps33
Simiiformes - ps34
Catarrhini - ps35
Hominoidea - ps36
Hominidae - ps37
Homininae - ps38
Hominini - ps39
Homo_sapiens - ps40
p-value
2e-22
2e-17
3e-12
4e-07
6e-02
Log-odds
0.5
1.0
1.5
2.0
2.5
3.0
3.5
4.0

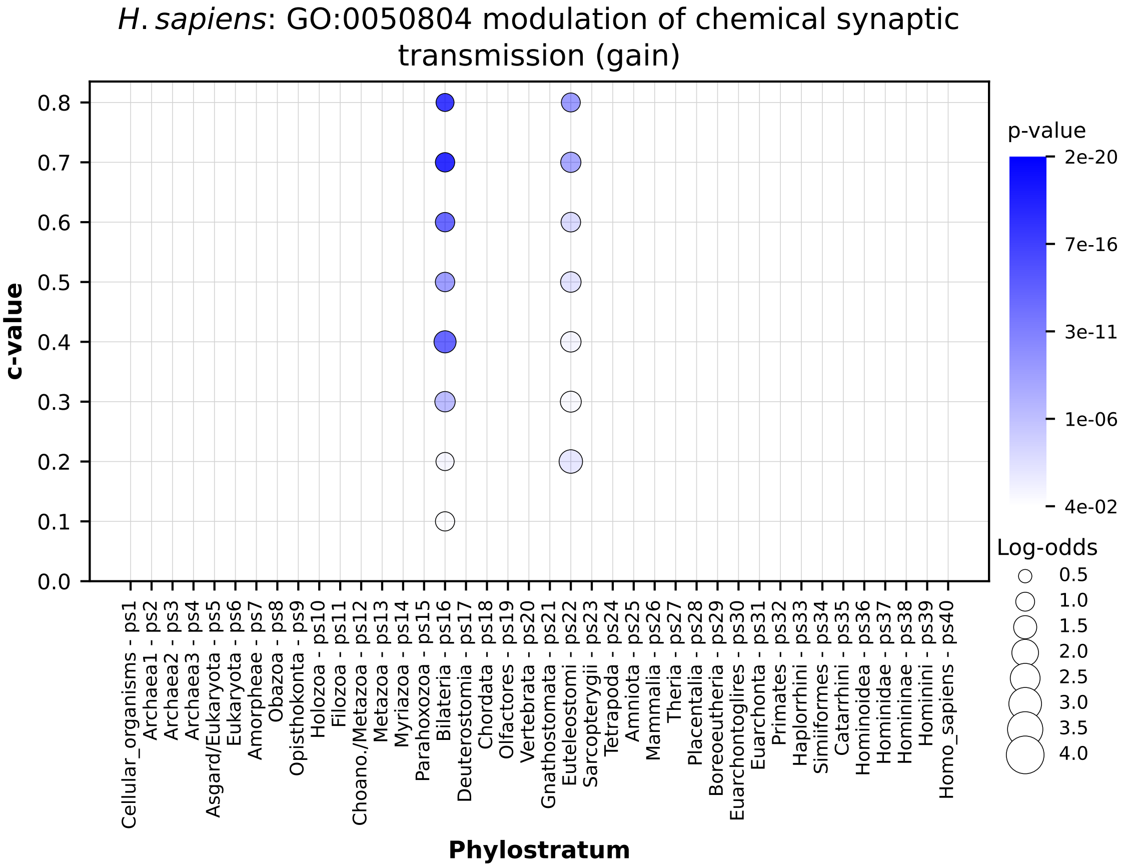
H. sapiens: GO:0050804 modulation of chemical synaptic transmission (gain)
c-value
0.0
0.1
0.2
0.3
0.4
0.5
0.6
0.7
0.8
Phylostratum
Cellular_organisms - ps1
Archaea1 - ps2
Archaea2 - ps3
Archaea3 - ps4
Asgard/Eukaryota - ps5
Eukaryota - ps6
Amorpheae - ps7
Obazoa - ps8
Opisthokonta - ps9
Holozoa - ps10
Filozoa - ps11
Choano./Metazoa - ps12
Metazoa - ps13
Myriazoa - ps14
Parahoxozoa - ps15
Bilateria - ps16
Deuterostomia - ps17
Chordata - ps18
Olfactores - ps19
Vertebrata - ps20
Gnathostomata - ps21
Euteleostomi - ps22
Sarcopterygii - ps23
Tetrapoda - ps24
Amniota - ps25
Mammalia - ps26
Theria - ps27
Placentalia - ps28
Boreoeutheria - ps29
Euarchontoglires - ps30
Euarchonta - ps31
Primates - ps32
Haplorrhini - ps33
Simiiformes - ps34
Catarrhini - ps35
Hominoidea - ps36
Hominidae - ps37
Homininae - ps38
Hominini - ps39
Homo_sapiens - ps40
p-value
2e-20
7e-16
3e-11
1e-06
4e-02
Log-odds
0.5
1.0
1.5
2.0
2.5
3.0
3.5
4.0

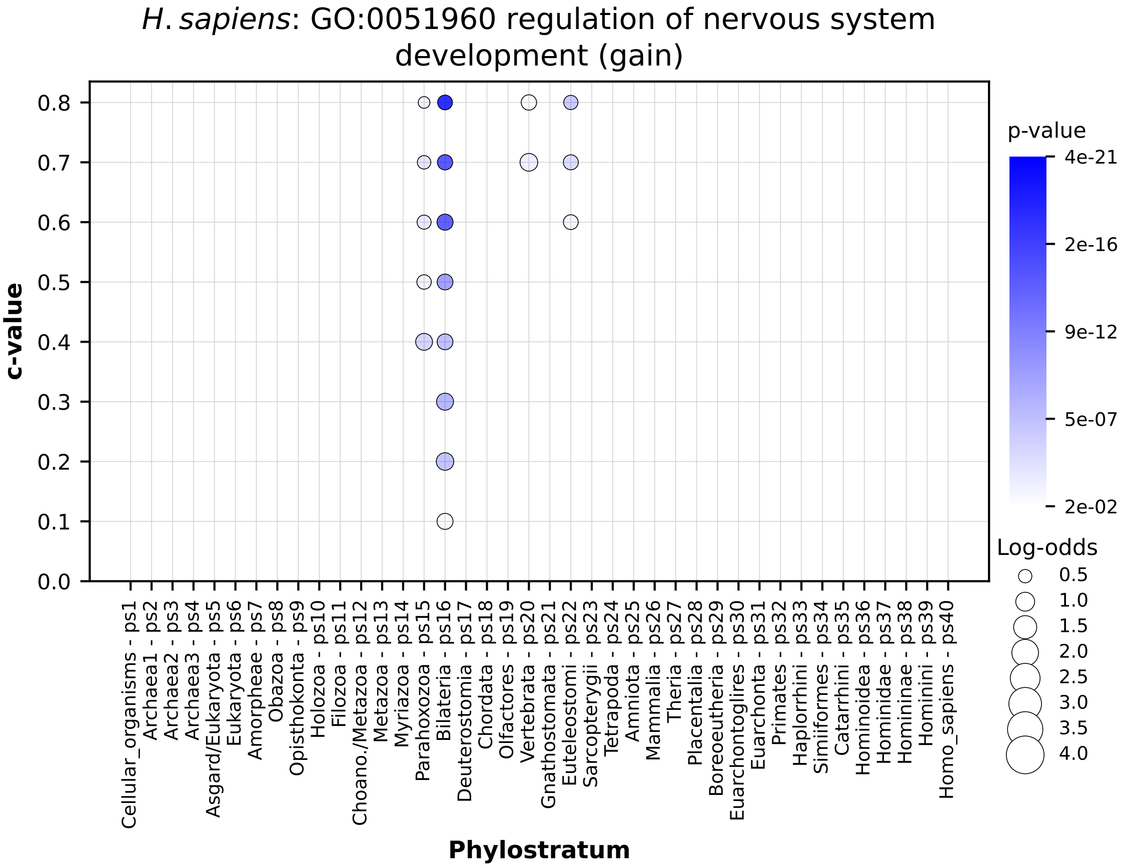
*H. sapiens*: GO:0051960 regulation of nervous system development (gain)
c-value
0.8
0.7
0.6
0.5
0.4
0.3
0.2
0.1
0.0
Phylostratum
Cellular_organisms - ps1
Archaea1 - ps2
Archaea2 - ps3
Archaea3 - ps4
Asgard/Eukaryota - ps5
Eukaryota - ps6
Amorpheae - ps7
Obazoa - ps8
Opisthokonta - ps9
Holozoa - ps10
Filozoa - ps11
Choano./Metazoa - ps12
Metazoa - ps13
Myriazoa - ps14
Parahoxozoa - ps15
Bilateria - ps16
Deuterostomia - ps17
Chordata - ps18
Olfactores - ps19
Vertebrata - ps20
Gnathostomata - ps21
Euteleostomi - ps22
Sarcopterygii - ps23
Tetrapoda - ps24
Amniota - ps25
Mammalia - ps26
Theria - ps27
Placentalia - ps28
Boreoeutheria - ps29
Euarchontoglires - ps30
Euarchonta - ps31
Primates - ps32
Haplorrhini - ps33
Simiiformes - ps34
Catarrhini - ps35
Hominoidea - ps36
Hominidae - ps37
Homininae - ps38
Hominini - ps39
Homo_sapiens - ps40
p-value
4e-21
2e-16
9e-12
5e-07
2e-02
Log-odds
0.5
1.0
1.5
2.0
2.5
3.0
3.5
4.0

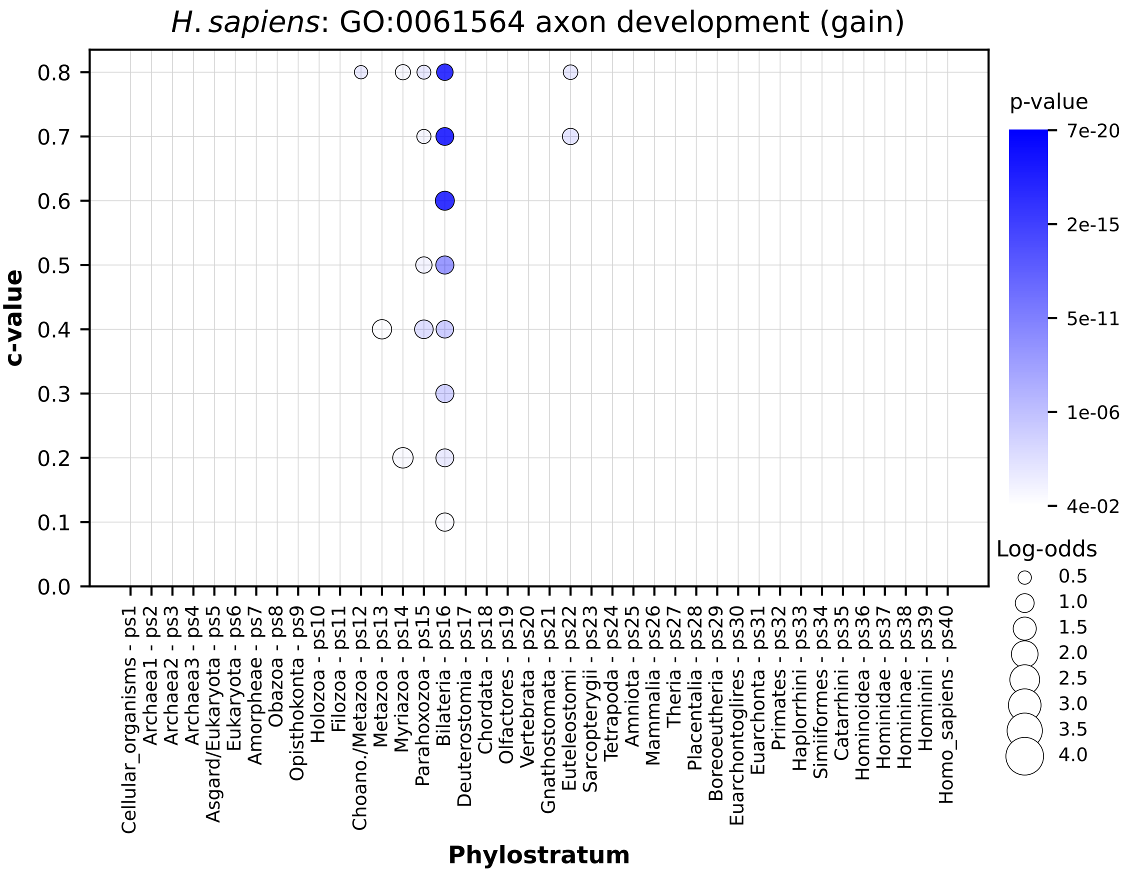

H. sapiens: GO:0061564 axon development (gain)
c-value
0.8
0.7
0.6
0.5
0.4
0.3
0.2
0.1
0.0
p-value
7e-20
2e-15
5e-11
1e-06
4e-02
Log-odds
0.5
1.0
1.5
2.0
2.5
3.0
3.5
4.0
Cellular_organisms - ps1
Archaea1 - ps2
Archaea2 - ps3
Archaea3 - ps4
Asgard/Eukaryota - ps5
Eukaryota - ps6
Amorpheae - ps7
Obazoa - ps8
Opisthokonta - ps9
Holozoa - ps10
Filozoa - ps11
Choano./Metazoa - ps12
Metazoa - ps13
Myriazoa - ps14
Parahoxozoa - ps15
Bilateria - ps16
Deuterostomia - ps17
Chordata - ps18
Olfactores - ps19
Vertebrata - ps20
Gnathostomata - ps21
Euteleostomi - ps22
Sarcopterygii - ps23
Tetrapoda - ps24
Amniota - ps25
Mammalia - ps26
Theria - ps27
Placentalia - ps28
Boreoeutheria - ps29
Euarchontoglires - ps30
Euarchonta - ps31
Primates - ps32
Haplorrhini - ps33
Simiiformes - ps34
Catarrhini - ps35
Hominoidea - ps36
Hominidae - ps37
Homininae - ps38
Hominini - ps39
Homo_sapiens - ps40
Phylostratum

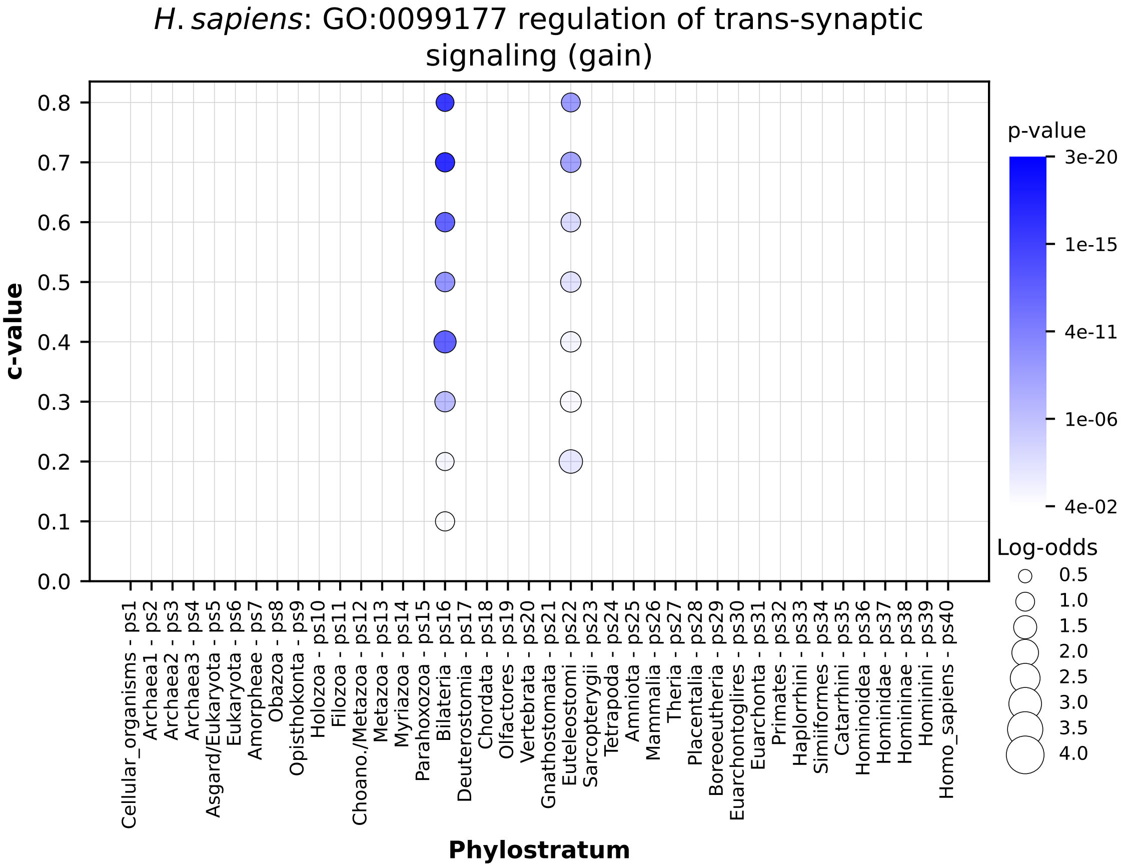
H. sapiens: GO:0099177 regulation of trans-synaptic signaling (gain)
c-value
Phylostratum
p-value
3e-20
1e-15
4e-11
1e-06
4e-02
Log-odds
0.5
1.0
1.5
2.0
2.5
3.0
3.5
4.0
Cellular_organisms - ps1
Archaea1 - ps2
Archaea2 - ps3
Archaea3 - ps4
Asgard/Eukaryota - ps5
Eukaryota - ps6
Amorpheae - ps7
Obazoa - ps8
Opisthokonta - ps9
Holozoa - ps10
Filozoa - ps11
Choano./Metazoa - ps12
Metazoa - ps13
Myriazoa - ps14
Parahoxozoa - ps15
Bilateria - ps16
Deuterostomia - ps17
Chordata - ps18
Olfactores - ps19
Vertebrata - ps20
Gnathostomata - ps21
Euteleostomi - ps22
Sarcopterygii - ps23
Tetrapoda - ps24
Amniota - ps25
Mammalia - ps26
Theria - ps27
Placentalia - ps28
Boreoeutheria - ps29
Euarchontoglires - ps30
Euarchonta - ps31
Primates - ps32
Haplorrhini - ps33
Simiiformes - ps34
Catarrhini - ps35
Hominoidea - ps36
Hominidae - ps37
Homininae - ps38
Hominini - ps39
Homo_sapiens - ps40

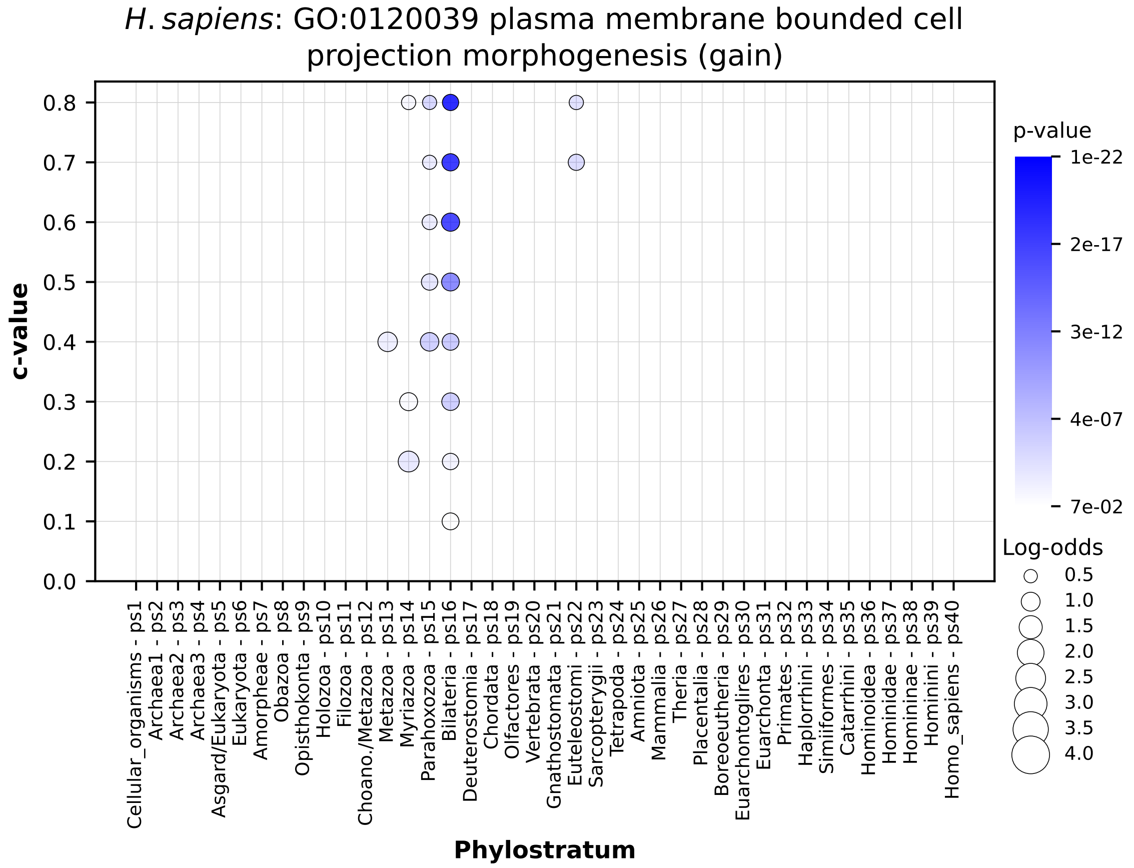
H. sapiens: GO:0120039 plasma membrane bounded cell projection morphogenesis (gain)
c-value
0.0
0.1
0.2
0.3
0.4
0.5
0.6
0.7
0.8
Phylostratum
Cellular_organisms - ps1
Archaea1 - ps2
Archaea2 - ps3
Archaea3 - ps4
Asgard/Eukaryota - ps5
Eukaryota - ps6
Amorpheae - ps7
Obazoa - ps8
Opisthokonta - ps9
Holozoa - ps10
Filozoa - ps11
Choano./Metazoa - ps12
Metazoa - ps13
Myriazoa - ps14
Parahoxozoa - ps15
Bilateria - ps16
Deuterostomia - ps17
Chordata - ps18
Olfactores - ps19
Vertebrata - ps20
Gnathostomata - ps21
Euteleostomi - ps22
Sarcopterygii - ps23
Tetrapoda - ps24
Amniota - ps25
Mammalia - ps26
Theria - ps27
Placentalia - ps28
Boreoeutheria - ps29
Euarchontoglires - ps30
Euarchonta - ps31
Primates - ps32
Haplorrhini - ps33
Simiiformes - ps34
Catarrhini - ps35
Hominoidea - ps36
Hominidae - ps37
Homininae - ps38
Hominini - ps39
Homo_sapiens - ps40
p-value
1e-22
2e-17
3e-12
4e-07
7e-02
Log-odds
0.5
1.0
1.5
2.0
2.5
3.0
3.5
4.0

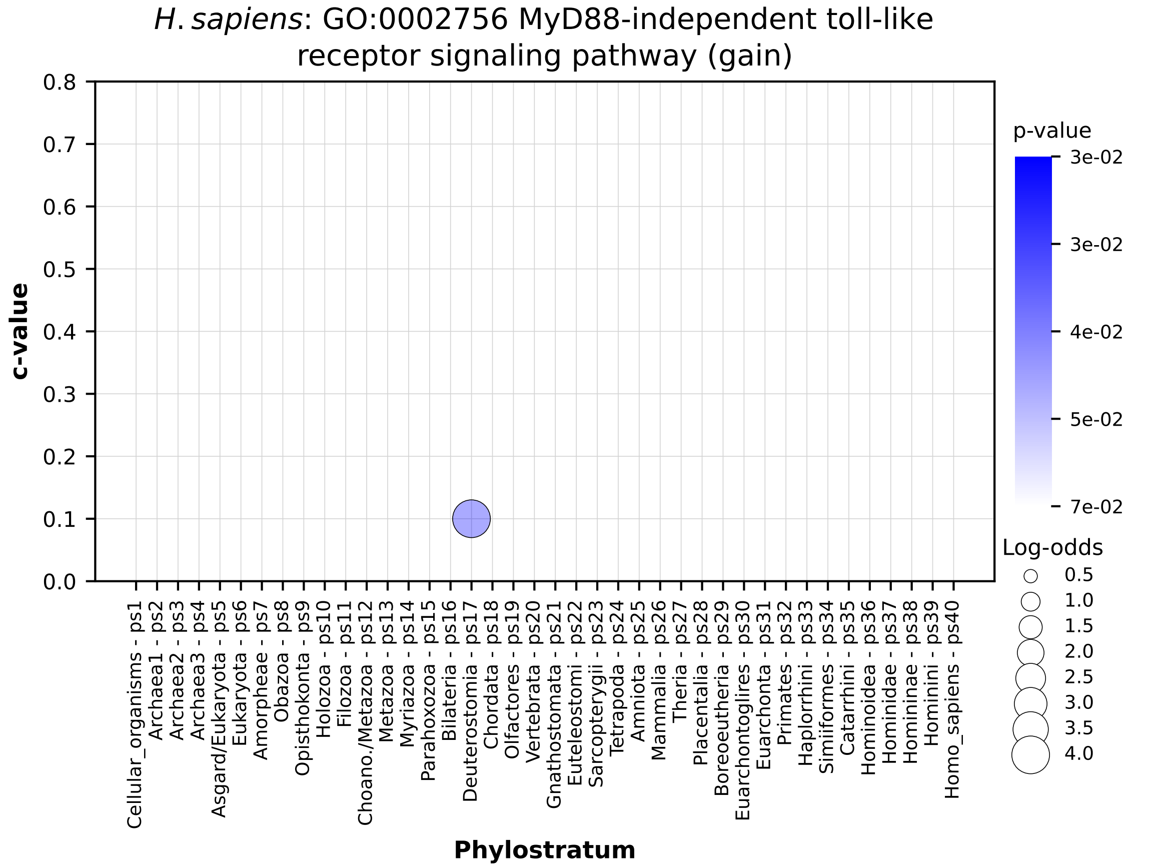

H. sapiens: GO:0002756 MyD88-independent toll-like receptor signaling pathway (gain)
c-value
0.8
0.7
0.6
0.5
0.4
0.3
0.2
0.1
0.0
p-value
3e-02
3e-02
4e-02
5e-02
7e-02
Log-odds
0.5
1.0
1.5
2.0
2.5
3.0
3.5
4.0
Cellular_organisms - ps1
Archaea1 - ps2
Archaea2 - ps3
Archaea3 - ps4
Asgard/Eukaryota - ps5
Eukaryota - ps6
Amorpheae - ps7
Obazoa - ps8
Opisthokonta - ps9
Holozoa - ps10
Filozoa - ps11
Choano./Metazoa - ps12
Metazoa - ps13
Myriazoa - ps14
Parahoxozoa - ps15
Bilateria - ps16
Deuterostomia - ps17
Chordata - ps18
Olfactores - ps19
Vertebrata - ps20
Gnathostomata - ps21
Euteleostomi - ps22
Sarcopterygii - ps23
Tetrapoda - ps24
Amniota - ps25
Mammalia - ps26
Theria - ps27
Placentalia - ps28
Boreoeutheria - ps29
Euarchontoglires - ps30
Euarchonta - ps31
Primates - ps32
Haplorrhini - ps33
Simiiformes - ps34
Catarrhini - ps35
Hominoidea - ps36
Hominidae - ps37
Homininae - ps38
Hominini - ps39
Homo_sapiens - ps40
Phylostratum

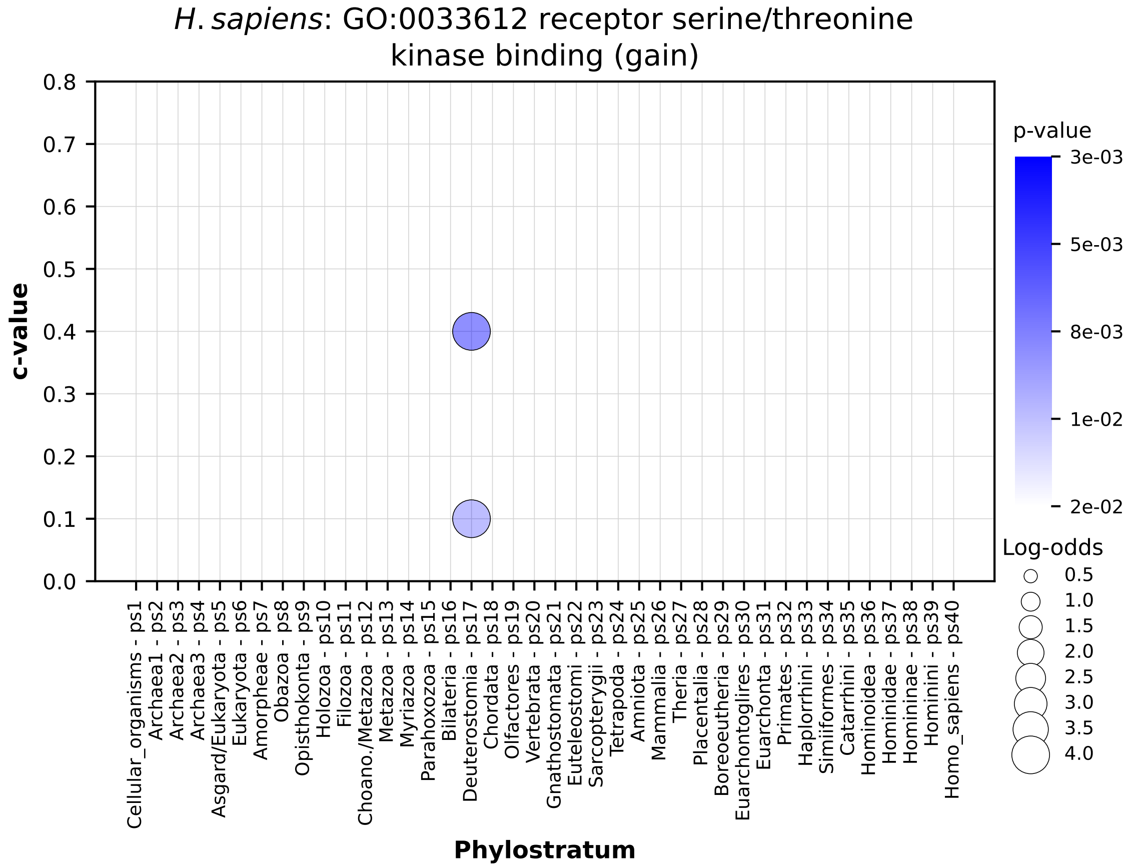

H. sapiens: GO:0033612 receptor serine/threonine kinase binding (gain)
c-value
0.8
0.7
0.6
0.5
0.4
0.3
0.2
0.1
0.0
p-value
3e-03
5e-03
8e-03
1e-02
2e-02
Log-odds
0.5
1.0
1.5
2.0
2.5
3.0
3.5
4.0
Cellular_organisms - ps1
Archaea1 - ps2
Archaea2 - ps3
Archaea3 - ps4
Asgard/Eukaryota - ps5
Eukaryota - ps6
Amorpheae - ps7
Obazoa - ps8
Opisthokonta - ps9
Holozoa - ps10
Filozoa - ps11
Choano./Metazoa - ps12
Metazoa - ps13
Myriazoa - ps14
Parahoxozoa - ps15
Bilateria - ps16
Deuterostomia - ps17
Chordata - ps18
Olfactores - ps19
Vertebrata - ps20
Gnathostomata - ps21
Euteleostomi - ps22
Sarcopterygii - ps23
Tetrapoda - ps24
Amniota - ps25
Mammalia - ps26
Theria - ps27
Placentalia - ps28
Boreoeutheria - ps29
Euarchontoglires - ps30
Euarchonta - ps31
Primates - ps32
Haplorrhini - ps33
Simiiformes - ps34
Catarrhini - ps35
Hominoidea - ps36
Hominidae - ps37
Homininae - ps38
Hominini - ps39
Homo_sapiens - ps40
Phylostratum

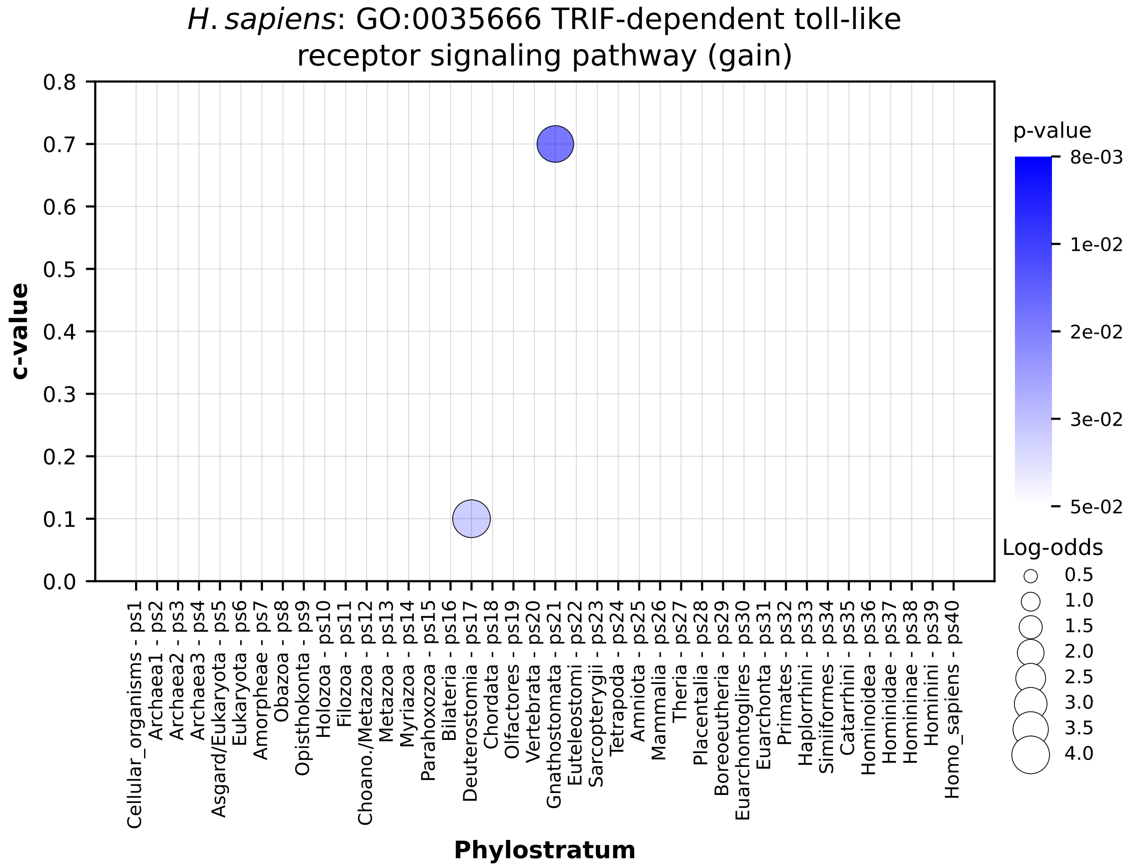

H. sapiens: GO:0035666 TRIF-dependent toll-like receptor signaling pathway (gain)
c-value
0.0
0.1
0.2
0.3
0.4
0.5
0.6
0.7
0.8
Phylostratum
Cellular_organisms - ps1
Archaea1 - ps2
Archaea2 - ps3
Archaea3 - ps4
Asgard/Eukaryota - ps5
Eukaryota - ps6
Amorpheae - ps7
Obazoa - ps8
Opisthokonta - ps9
Holozoa - ps10
Filozoa - ps11
Choano./Metazoa - ps12
Metazoa - ps13
Myriazoa - ps14
Parahoxozoa - ps15
Bilateria - ps16
Deuterostomia - ps17
Chordata - ps18
Olfactores - ps19
Vertebrata - ps20
Gnathostomata - ps21
Euteleostomi - ps22
Sarcopterygii - ps23
Tetrapoda - ps24
Amniota - ps25
Mammalia - ps26
Theria - ps27
Placentalia - ps28
Boreoeutheria - ps29
Euarchontoglires - ps30
Euarchonta - ps31
Primates - ps32
Haplorrhini - ps33
Simiiformes - ps34
Catarrhini - ps35
Hominoidea - ps36
Hominidae - ps37
Homininae - ps38
Hominini - ps39
Homo_sapiens - ps40
p-value
8e-03
1e-02
2e-02
3e-02
5e-02
Log-odds
0.5
1.0
1.5
2.0
2.5
3.0
3.5
4.0

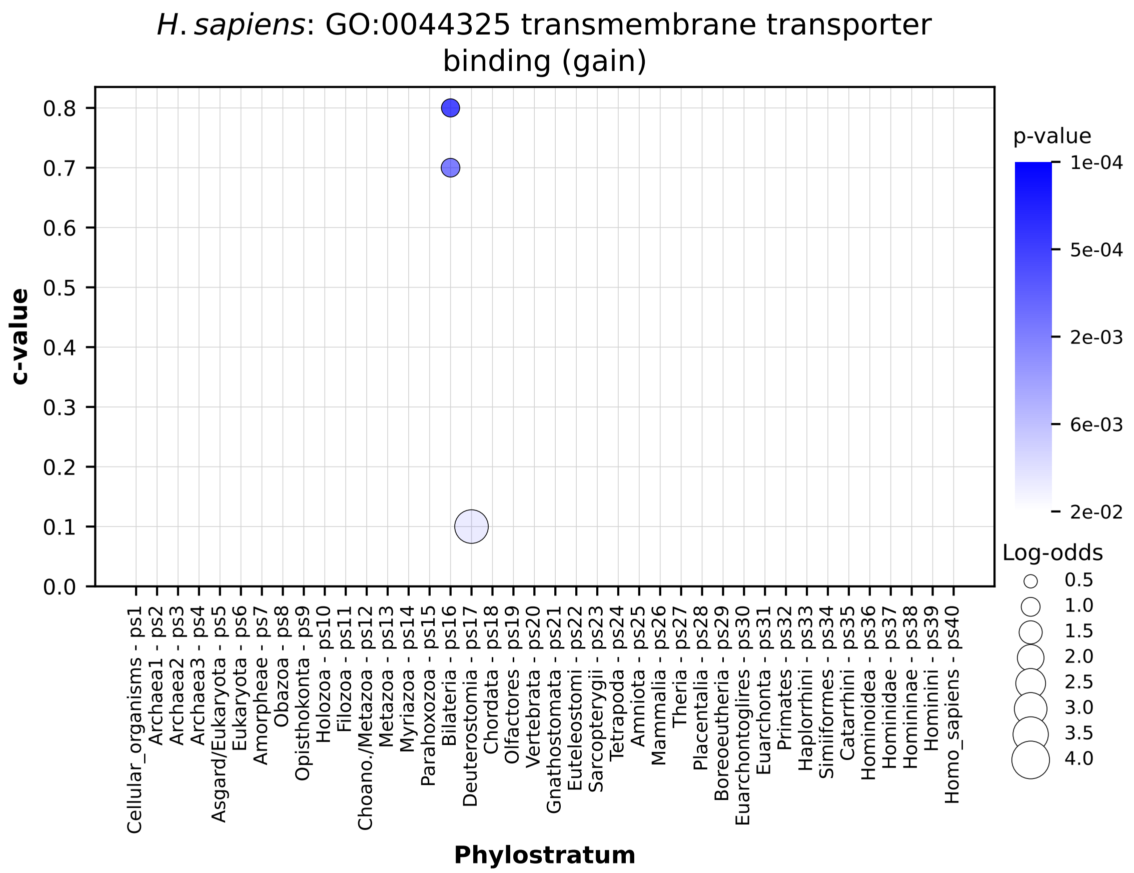

*H. sapiens*: GO:0044325 transmembrane transporter binding (gain)
c-value
0.8
0.7
0.6
0.5
0.4
0.3
0.2
0.1
0.0
p-value
1e-04
5e-04
2e-03
6e-03
2e-02
Log-odds
0.5
1.0
1.5
2.0
2.5
3.0
3.5
4.0
Cellular_organisms - ps1
Archaea1 - ps2
Archaea2 - ps3
Archaea3 - ps4
Asgard/Eukaryota - ps5
Eukaryota - ps6
Amorpheae - ps7
Obazoa - ps8
Opisthokonta - ps9
Holozoa - ps10
Filozoa - ps11
Choano./Metazoa - ps12
Metazoa - ps13
Myriazoa - ps14
Parahoxozoa - ps15
Bilateria - ps16
Deuterostomia - ps17
Chordata - ps18
Olfactores - ps19
Vertebrata - ps20
Gnathostomata - ps21
Euteleostomi - ps22
Sarcopterygii - ps23
Tetrapoda - ps24
Amniota - ps25
Mammalia - ps26
Theria - ps27
Placentalia - ps28
Boreoeutheria - ps29
Euarchontoglires - ps30
Euarchonta - ps31
Primates - ps32
Haplorrhini - ps33
Simiiformes - ps34
Catarrhini - ps35
Hominoidea - ps36
Hominidae - ps37
Homininae - ps38
Hominini - ps39
Homo_sapiens - ps40
Phylostratum

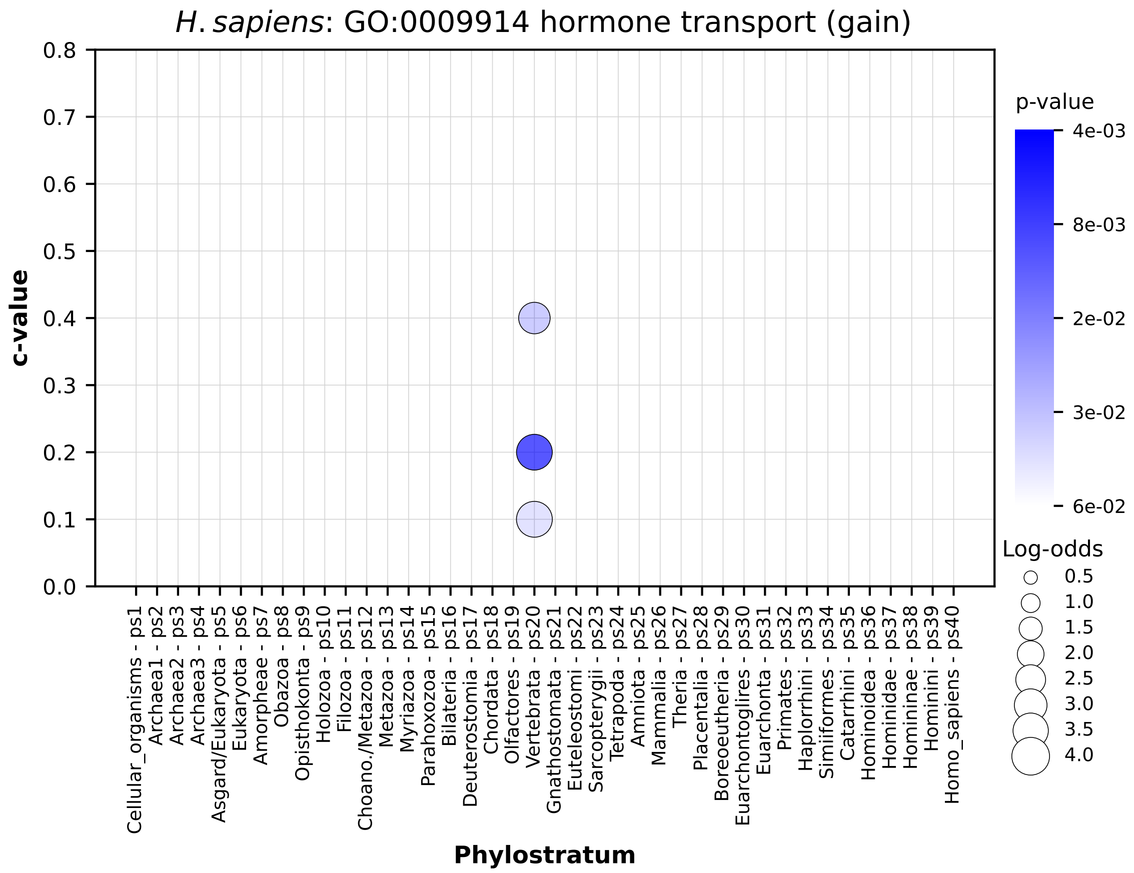
H. sapiens: GO:0009914 hormone transport (gain)
c-value
0.0
0.1
0.2
0.3
0.4
0.5
0.6
0.7
0.8
Phylostratum
Cellular_organisms - ps1
Archaea1 - ps2
Archaea2 - ps3
Archaea3 - ps4
Asgard/Eukaryota - ps5
Eukaryota - ps6
Amorpheae - ps7
Obazoa - ps8
Opisthokonta - ps9
Holozoa - ps10
Filozoa - ps11
Choano./Metazoa - ps12
Metazoa - ps13
Myriazoa - ps14
Parahoxozoa - ps15
Bilateria - ps16
Deuterostomia - ps17
Chordata - ps18
Olfactores - ps19
Vertebrata - ps20
Gnathostomata - ps21
Euteleostomi - ps22
Sarcopterygii - ps23
Tetrapoda - ps24
Amniota - ps25
Mammalia - ps26
Theria - ps27
Placentalia - ps28
Boreoeutheria - ps29
Euarchontoglires - ps30
Euarchonta - ps31
Primates - ps32
Haplorrhini - ps33
Simiiformes - ps34
Catarrhini - ps35
Hominoidea - ps36
Hominidae - ps37
Homininae - ps38
Hominini - ps39
Homo_sapiens - ps40
p-value
4e-03
8e-03
2e-02
3e-02
6e-02
Log-odds
0.5
1.0
1.5
2.0
2.5
3.0
3.5
4.0

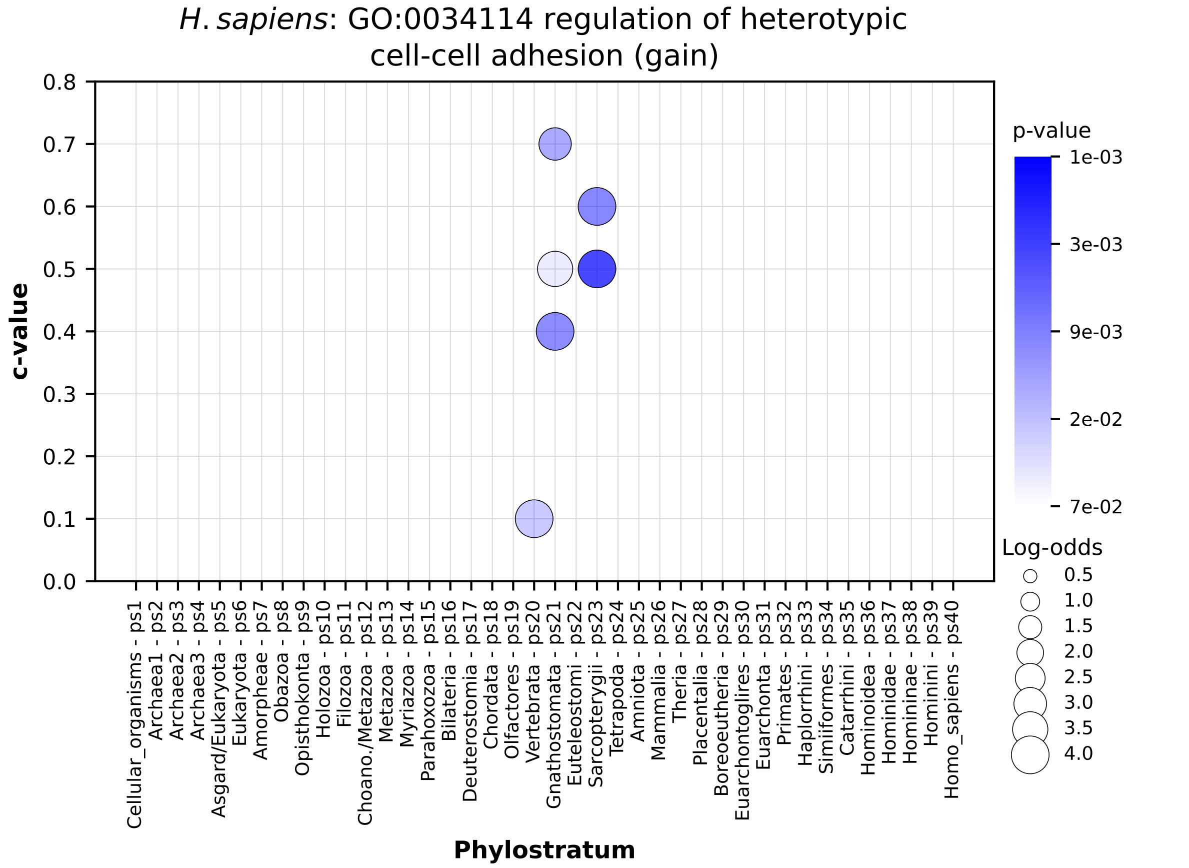

*H. sapiens*: GO:0034114 regulation of heterotypic cell-cell adhesion (gain)
c-value
0.0
0.1
0.2
0.3
0.4
0.5
0.6
0.7
0.8
Phylostratum
Cellular_organisms - ps1
Archaea1 - ps2
Archaea2 - ps3
Archaea3 - ps4
Asgard/Eukaryota - ps5
Eukaryota - ps6
Amorpheae - ps7
Obazoa - ps8
Opisthokonta - ps9
Holozoa - ps10
Filozoa - ps11
Choano./Metazoa - ps12
Metazoa - ps13
Myriazoa - ps14
Parahoxozoa - ps15
Bilateria - ps16
Deuterostomia - ps17
Chordata - ps18
Olfactores - ps19
Vertebrata - ps20
Gnathostomata - ps21
Euteleostomi - ps22
Sarcopterygii - ps23
Tetrapoda - ps24
Amniota - ps25
Mammalia - ps26
Theria - ps27
Placentalia - ps28
Boreoeutheria - ps29
Euarchontoglires - ps30
Euarchonta - ps31
Primates - ps32
Haplorrhini - ps33
Simiiformes - ps34
Catarrhini - ps35
Hominoidea - ps36
Hominidae - ps37
Homininae - ps38
Hominini - ps39
Homo_sapiens - ps40
p-value
1e-03
3e-03
9e-03
2e-02
7e-02
Log-odds
0.5
1.0
1.5
2.0
2.5
3.0
3.5
4.0

# *H. sapiens*: GO:0043410 positive regulation of MAPK cascade (gain)

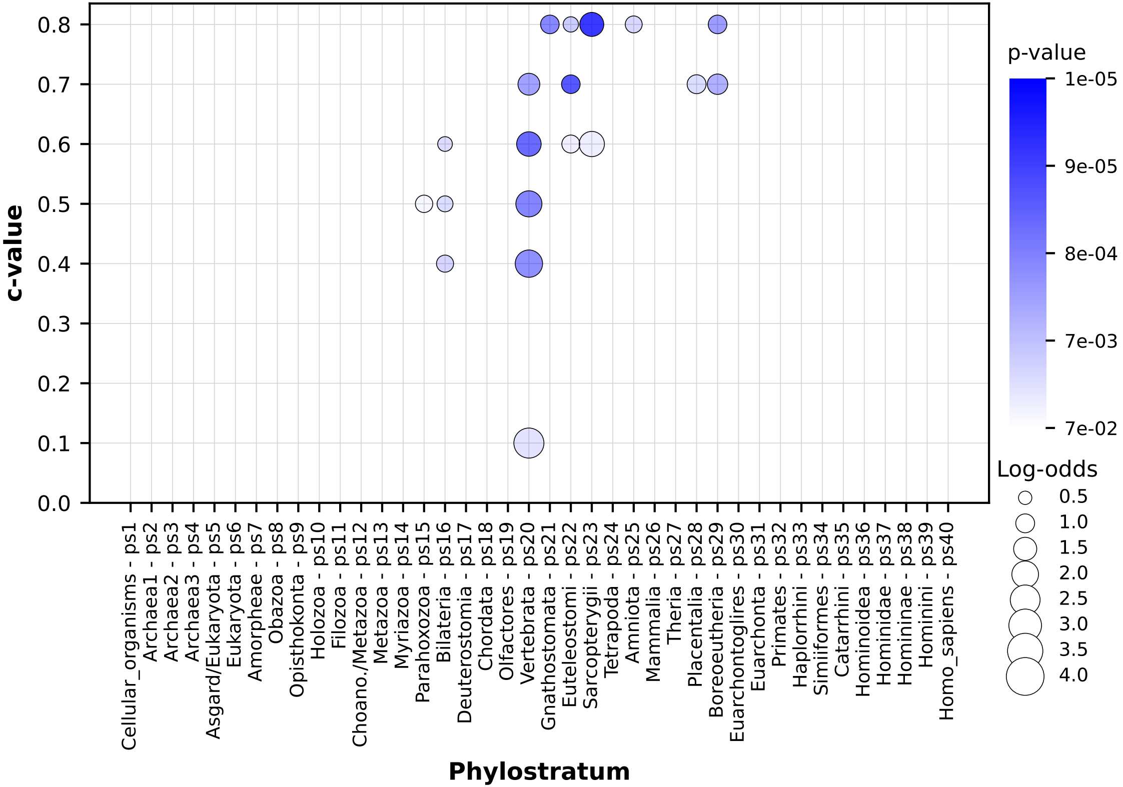

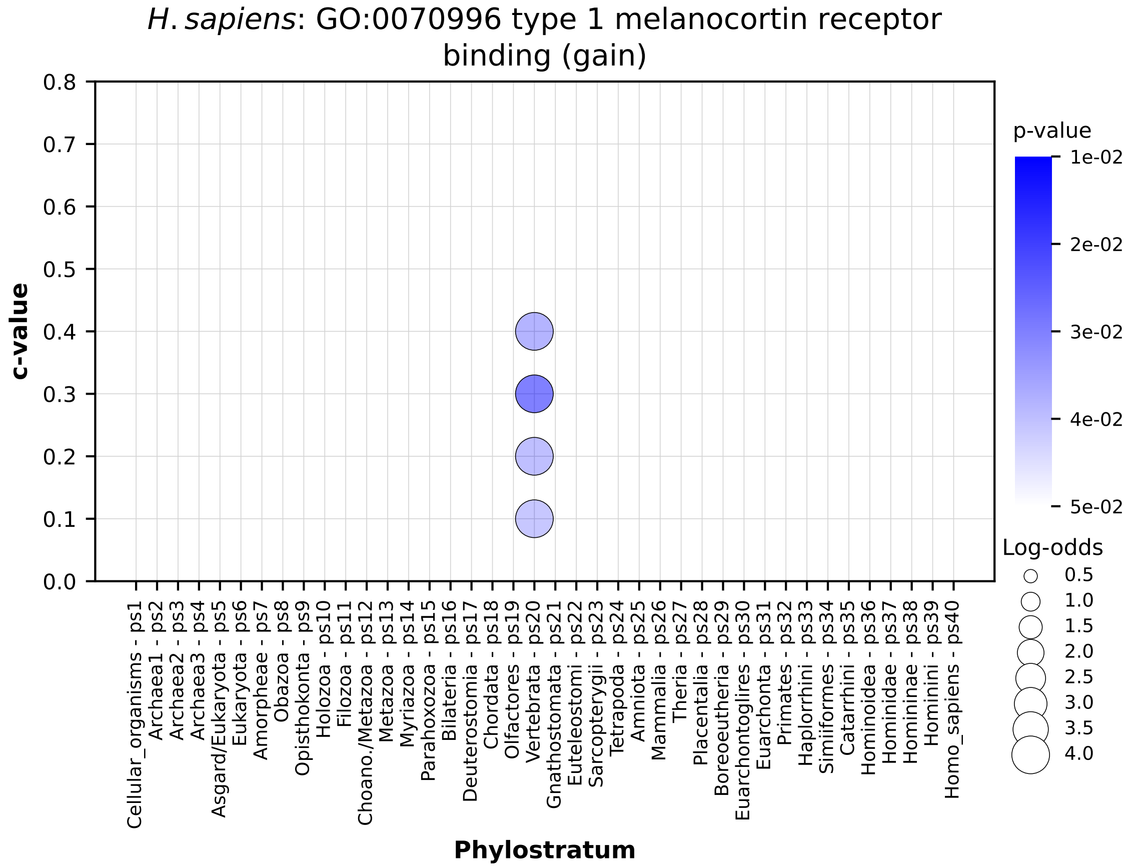

H. sapiens: GO:0070996 type 1 melanocortin receptor binding (gain)
c-value
0.0
0.1
0.2
0.3
0.4
0.5
0.6
0.7
0.8
Phylostratum
Cellular_organisms - ps1
Archaea1 - ps2
Archaea2 - ps3
Archaea3 - ps4
Asgard/Eukaryota - ps5
Eukaryota - ps6
Amorpheae - ps7
Obazoa - ps8
Opisthokonta - ps9
Holozoa - ps10
Filozoa - ps11
Choano./Metazoa - ps12
Metazoa - ps13
Myriazoa - ps14
Parahoxozoa - ps15
Bilateria - ps16
Deuterostomia - ps17
Chordata - ps18
Olfactores - ps19
Vertebrata - ps20
Gnathostomata - ps21
Euteleostomi - ps22
Sarcopterygii - ps23
Tetrapoda - ps24
Amniota - ps25
Mammalia - ps26
Theria - ps27
Placentalia - ps28
Boreoeutheria - ps29
Euarchontoglires - ps30
Euarchonta - ps31
Primates - ps32
Haplorrhini - ps33
Simiiformes - ps34
Catarrhini - ps35
Hominoidea - ps36
Hominidae - ps37
Homininae - ps38
Hominini - ps39
Homo_sapiens - ps40
p-value
1e-02
2e-02
3e-02
4e-02
5e-02
Log-odds
0.5
1.0
1.5
2.0
2.5
3.0
3.5
4.0

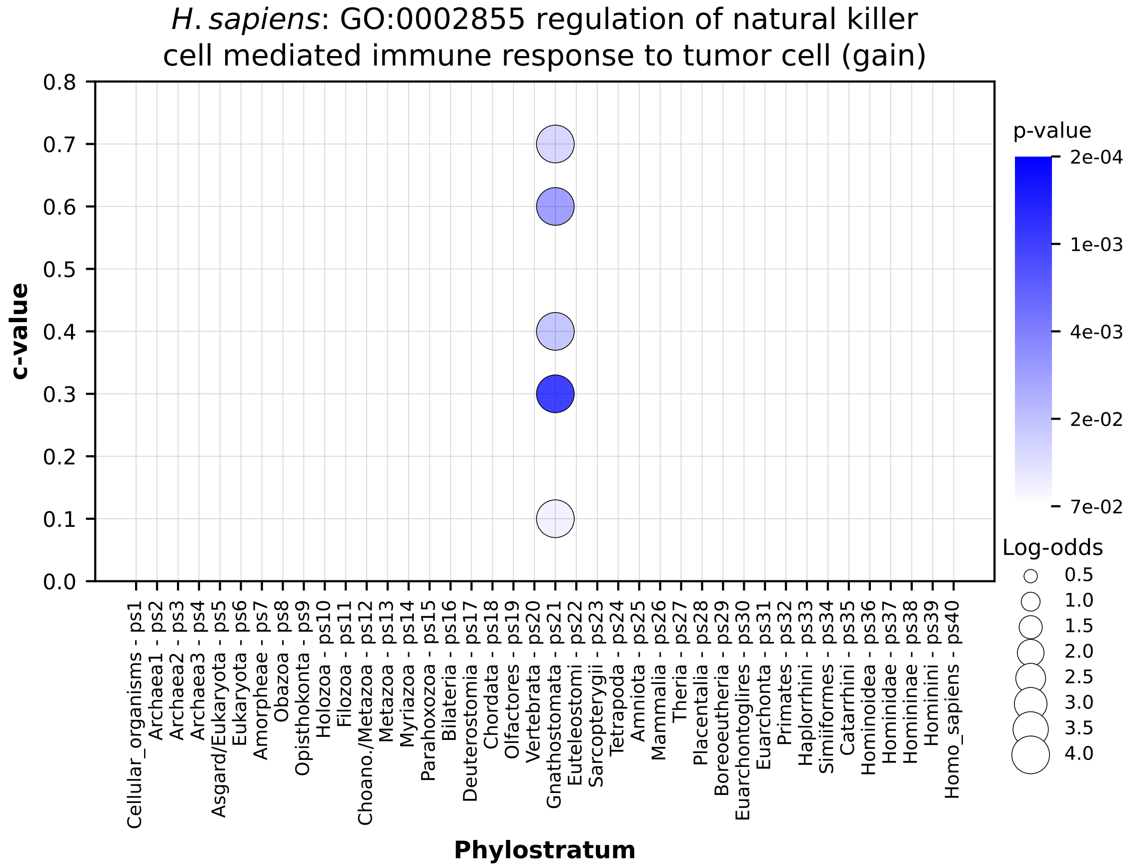

*H. sapiens*: GO:0002855 regulation of natural killer cell mediated immune response to tumor cell (gain)
c-value
0.0
0.1
0.2
0.3
0.4
0.5
0.6
0.7
0.8
Phylostratum
Cellular_organisms - ps1
Archaea1 - ps2
Archaea2 - ps3
Archaea3 - ps4
Asgard/Eukaryota - ps5
Eukaryota - ps6
Amorpheae - ps7
Obazoa - ps8
Opisthokonta - ps9
Holozoa - ps10
Filozoa - ps11
Choano./Metazoa - ps12
Metazoa - ps13
Myriazoa - ps14
Parahoxozoa - ps15
Bilateria - ps16
Deuterostomia - ps17
Chordata - ps18
Olfactores - ps19
Vertebrata - ps20
Gnathostomata - ps21
Euteleostomi - ps22
Sarcopterygii - ps23
Tetrapoda - ps24
Amniota - ps25
Mammalia - ps26
Theria - ps27
Placentalia - ps28
Boreoeutheria - ps29
Euarchontoglires - ps30
Euarchonta - ps31
Primates - ps32
Haplorrhini - ps33
Simiiformes - ps34
Catarrhini - ps35
Hominoidea - ps36
Hominidae - ps37
Homininae - ps38
Hominini - ps39
Homo_sapiens - ps40
p-value
2e-04
1e-03
4e-03
2e-02
7e-02
Log-odds
0.5
1.0
1.5
2.0
2.5
3.0
3.5
4.0

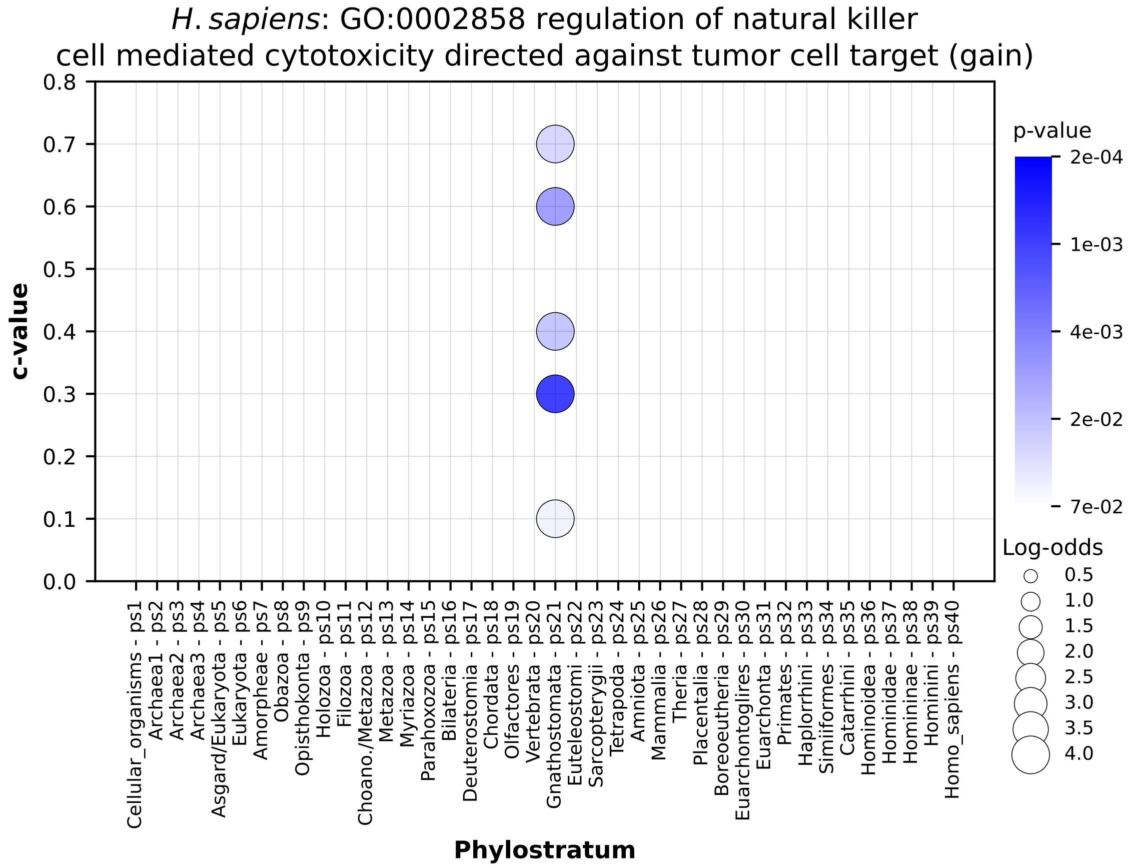

H. sapiens: GO:0002858 regulation of natural killer cell mediated cytotoxicity directed against tumor cell target (gain)
c-value
0.0
0.1
0.2
0.3
0.4
0.5
0.6
0.7
0.8
Phylostratum
Cellular_organisms - ps1
Archaea1 - ps2
Archaea2 - ps3
Archaea3 - ps4
Asgard/Eukaryota - ps5
Eukaryota - ps6
Amorpheae - ps7
Obazoa - ps8
Opisthokonta - ps9
Holozoa - ps10
Filozoa - ps11
Choano./Metazoa - ps12
Metazoa - ps13
Myriazoa - ps14
Parahoxozoa - ps15
Bilateria - ps16
Deuterostomia - ps17
Chordata - ps18
Olfactores - ps19
Vertebrata - ps20
Gnathostomata - ps21
Euteleostomi - ps22
Sarcopterygii - ps23
Tetrapoda - ps24
Amniota - ps25
Mammalia - ps26
Theria - ps27
Placentalia - ps28
Boreoeutheria - ps29
Euarchontoglires - ps30
Euarchonta - ps31
Primates - ps32
Haplorrhini - ps33
Simiiformes - ps34
Catarrhini - ps35
Hominoidea - ps36
Hominidae - ps37
Homininae - ps38
Hominini - ps39
Homo_sapiens - ps40
p-value
2e-04
1e-03
4e-03
2e-02
7e-02
Log-odds
0.5
1.0
1.5
2.0
2.5
3.0
3.5
4.0

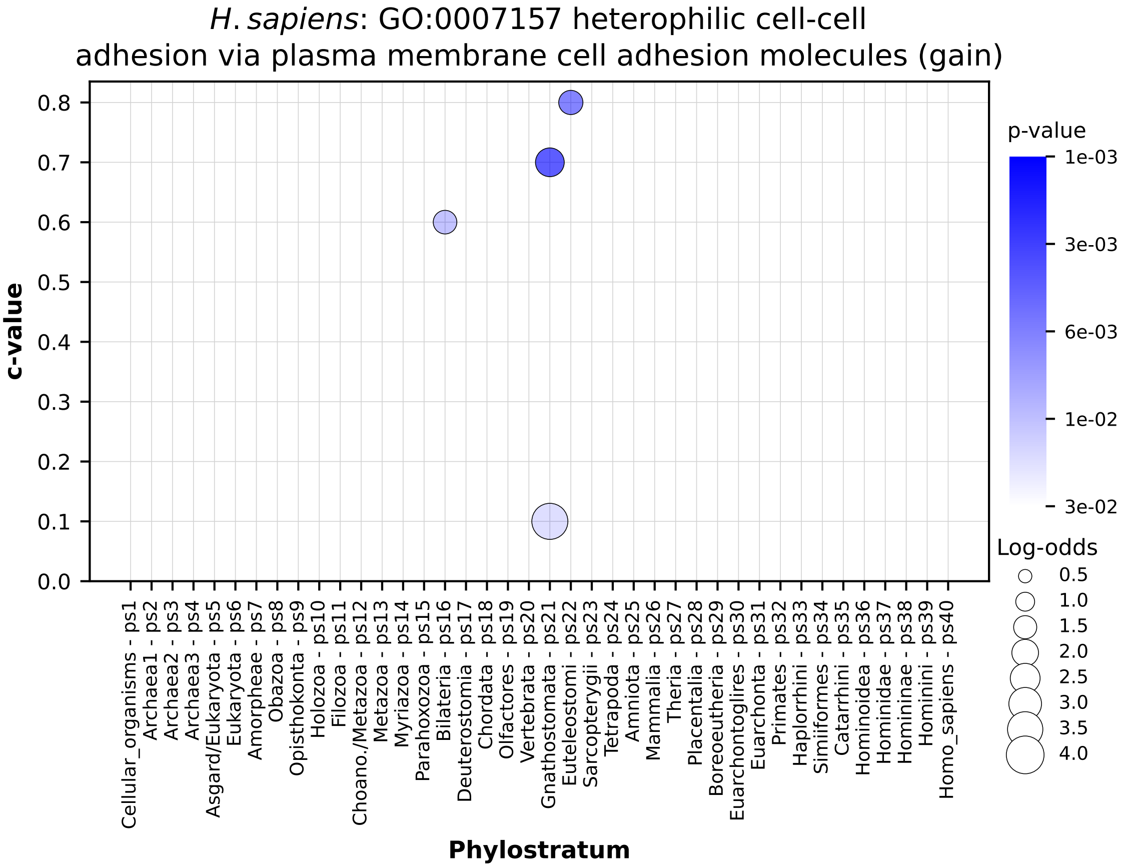

H. sapiens: GO:0007157 heterophilic cell-cell adhesion via plasma membrane cell adhesion molecules (gain)
c-value
0.0
0.1
0.2
0.3
0.4
0.5
0.6
0.7
0.8
p-value
1e-03
3e-03
6e-03
1e-02
3e-02
Log-odds
0.5
1.0
1.5
2.0
2.5
3.0
3.5
4.0
Cellular_organisms - ps1
Archaea1 - ps2
Archaea2 - ps3
Archaea3 - ps4
Asgard/Eukaryota - ps5
Eukaryota - ps6
Amorpheae - ps7
Obazoa - ps8
Opisthokonta - ps9
Holozoa - ps10
Filozoa - ps11
Choano./Metazoa - ps12
Metazoa - ps13
Myriazoa - ps14
Parahoxozoa - ps15
Bilateria - ps16
Deuterostomia - ps17
Chordata - ps18
Olfactores - ps19
Vertebrata - ps20
Gnathostomata - ps21
Euteleostomi - ps22
Sarcopterygii - ps23
Tetrapoda - ps24
Amniota - ps25
Mammalia - ps26
Theria - ps27
Placentalia - ps28
Boreoeutheria - ps29
Euarchontoglires - ps30
Euarchonta - ps31
Primates - ps32
Haplorrhini - ps33
Simiiformes - ps34
Catarrhini - ps35
Hominoidea - ps36
Hominidae - ps37
Homininae - ps38
Hominini - ps39
Homo_sapiens - ps40
Phylostratum

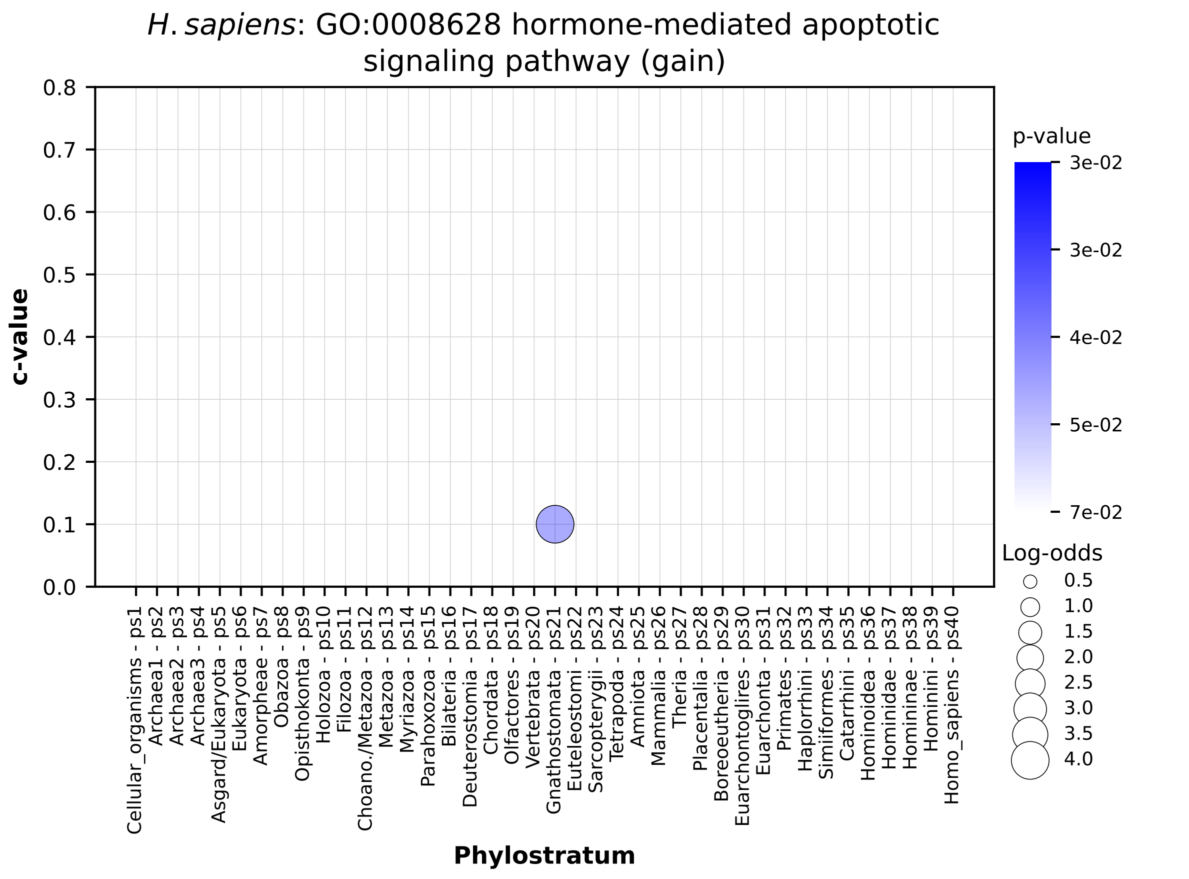

H. sapiens: GO:0008628 hormone-mediated apoptotic signaling pathway (gain)
c-value
0.0
0.1
0.2
0.3
0.4
0.5
0.6
0.7
0.8
Phylostratum
Cellular_organisms - ps1
Archaea1 - ps2
Archaea2 - ps3
Archaea3 - ps4
Asgard/Eukaryota - ps5
Eukaryota - ps6
Amorpheae - ps7
Obazoa - ps8
Opisthokonta - ps9
Holozoa - ps10
Filozoa - ps11
Choano./Metazoa - ps12
Metazoa - ps13
Myriazoa - ps14
Parahoxozoa - ps15
Bilateria - ps16
Deuterostomia - ps17
Chordata - ps18
Olfactores - ps19
Vertebrata - ps20
Gnathostomata - ps21
Euteleostomi - ps22
Sarcopterygii - ps23
Tetrapoda - ps24
Amniota - ps25
Mammalia - ps26
Theria - ps27
Placentalia - ps28
Boreoeutheria - ps29
Euarchontoglires - ps30
Euarchonta - ps31
Primates - ps32
Haplorrhini - ps33
Simiiformes - ps34
Catarrhini - ps35
Hominoidea - ps36
Hominidae - ps37
Homininae - ps38
Hominini - ps39
Homo_sapiens - ps40
p-value
3e-02
3e-02
4e-02
5e-02
7e-02
Log-odds
0.5
1.0
1.5
2.0
2.5
3.0
3.5
4.0

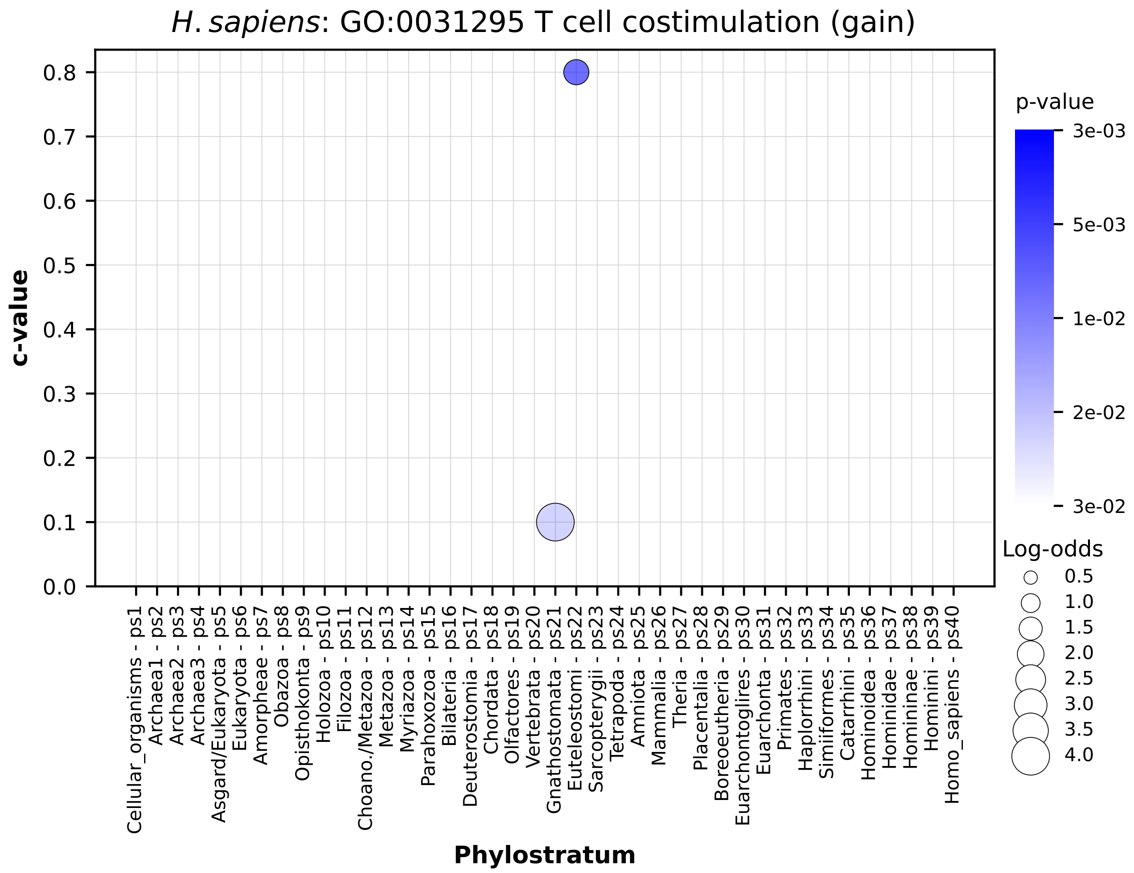
H. sapiens: GO:0031295 T cell costimulation (gain)
c-value
0.0
0.1
0.2
0.3
0.4
0.5
0.6
0.7
0.8
Phylostratum
Cellular_organisms - ps1
Archaea1 - ps2
Archaea2 - ps3
Archaea3 - ps4
Asgard/Eukaryota - ps5
Eukaryota - ps6
Amorpheae - ps7
Obazoa - ps8
Opisthokonta - ps9
Holozoa - ps10
Filozoa - ps11
Choano./Metazoa - ps12
Metazoa - ps13
Myriazoa - ps14
Parahoxozoa - ps15
Bilateria - ps16
Deuterostomia - ps17
Chordata - ps18
Olfactores - ps19
Vertebrata - ps20
Gnathostomata - ps21
Euteleostomi - ps22
Sarcopterygii - ps23
Tetrapoda - ps24
Amniota - ps25
Mammalia - ps26
Theria - ps27
Placentalia - ps28
Boreoeutheria - ps29
Euarchontoglires - ps30
Euarchonta - ps31
Primates - ps32
Haplorrhini - ps33
Simiiformes - ps34
Catarrhini - ps35
Hominoidea - ps36
Hominidae - ps37
Homininae - ps38
Hominini - ps39
Homo_sapiens - ps40
p-value
3e-03
5e-03
1e-02
2e-02
3e-02
Log-odds
0.5
1.0
1.5
2.0
2.5
3.0
3.5
4.0

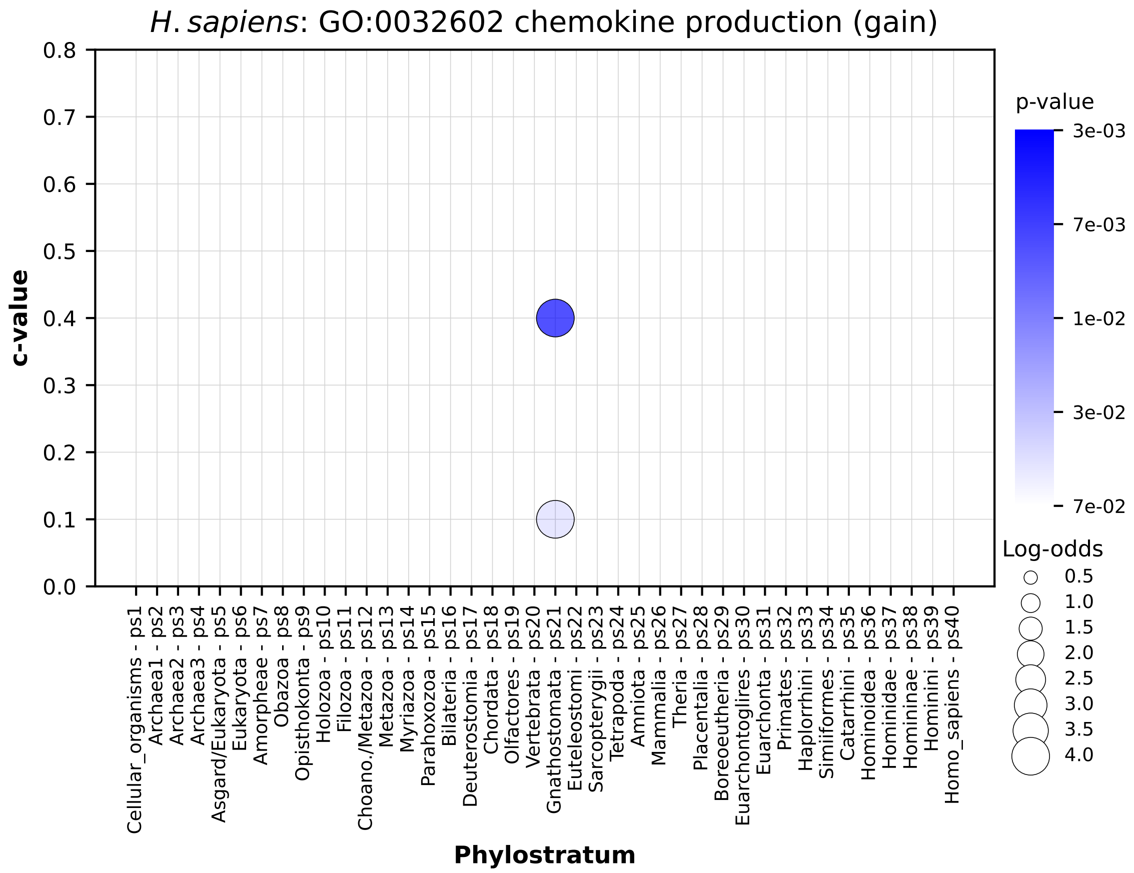

*H. sapiens*: GO:0032602 chemokine production (gain)
c-value
0.8
0.7
0.6
0.5
0.4
0.3
0.2
0.1
0.0
Phylostratum
Cellular_organisms - ps1
Archaea1 - ps2
Archaea2 - ps3
Archaea3 - ps4
Asgard/Eukaryota - ps5
Eukaryota - ps6
Amorpheae - ps7
Obazoa - ps8
Opisthokonta - ps9
Holozoa - ps10
Filozoa - ps11
Choano./Metazoa - ps12
Metazoa - ps13
Myriazoa - ps14
Parahoxozoa - ps15
Bilateria - ps16
Deuterostomia - ps17
Chordata - ps18
Olfactores - ps19
Vertebrata - ps20
Gnathostomata - ps21
Euteleostomi - ps22
Sarcopterygii - ps23
Tetrapoda - ps24
Amniota - ps25
Mammalia - ps26
Theria - ps27
Placentalia - ps28
Boreoeutheria - ps29
Euarchontoglires - ps30
Euarchonta - ps31
Primates - ps32
Haplorrhini - ps33
Simiiformes - ps34
Catarrhini - ps35
Hominoidea - ps36
Hominidae - ps37
Homininae - ps38
Hominini - ps39
Homo_sapiens - ps40
p-value
3e-03
7e-03
1e-02
3e-02
7e-02
Log-odds
0.5
1.0
1.5
2.0
2.5
3.0
3.5
4.0

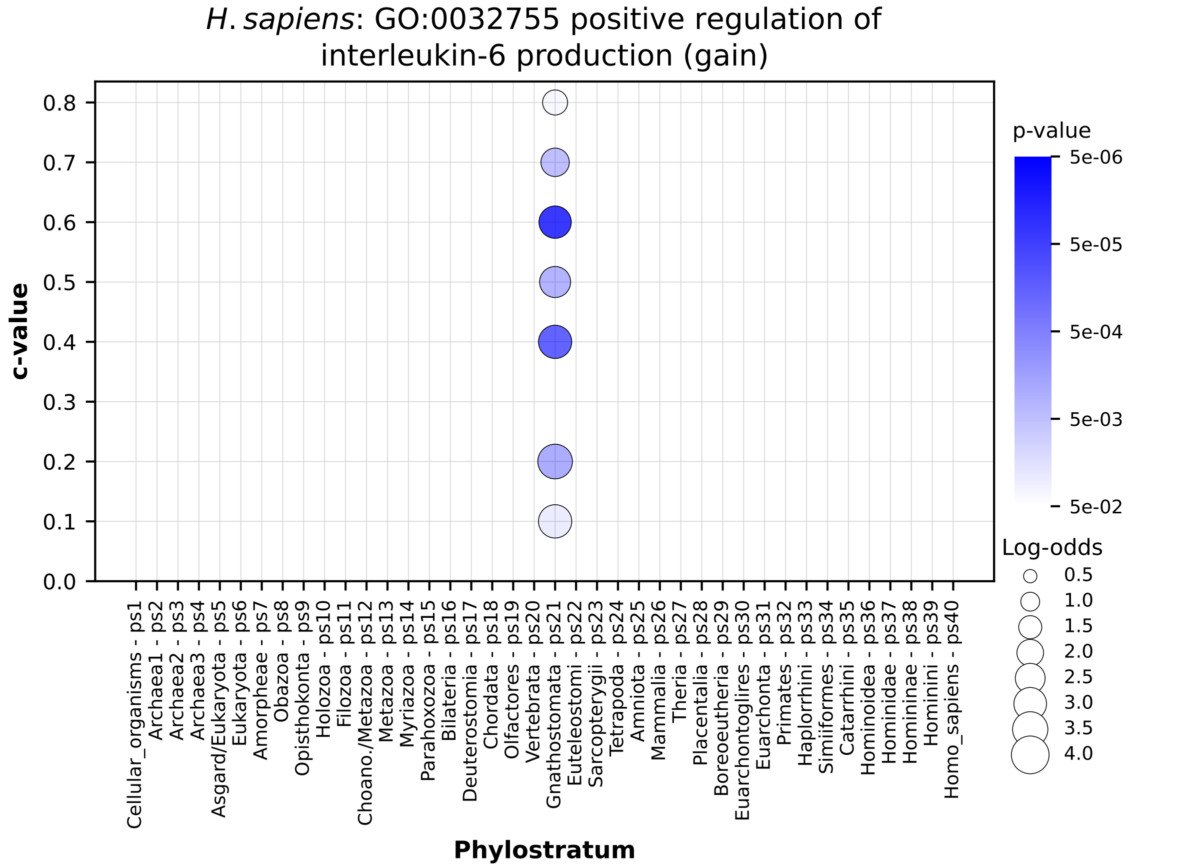

*H. sapiens*: GO:0032755 positive regulation of interleukin-6 production (gain)
c-value
0.0
0.1
0.2
0.3
0.4
0.5
0.6
0.7
0.8
Phylostratum
Cellular_organisms - ps1
Archaea1 - ps2
Archaea2 - ps3
Archaea3 - ps4
Asgard/Eukaryota - ps5
Eukaryota - ps6
Amorpheae - ps7
Obazoa - ps8
Opisthokonta - ps9
Holozoa - ps10
Filozoa - ps11
Choano./Metazoa - ps12
Metazoa - ps13
Myriazoa - ps14
Parahoxozoa - ps15
Bilateria - ps16
Deuterostomia - ps17
Chordata - ps18
Olfactores - ps19
Vertebrata - ps20
Gnathostomata - ps21
Euteleostomi - ps22
Sarcopterygii - ps23
Tetrapoda - ps24
Amniota - ps25
Mammalia - ps26
Theria - ps27
Placentalia - ps28
Boreoeutheria - ps29
Euarchontoglires - ps30
Euarchonta - ps31
Primates - ps32
Haplorrhini - ps33
Simiiformes - ps34
Catarrhini - ps35
Hominoidea - ps36
Hominidae - ps37
Homininae - ps38
Hominini - ps39
Homo_sapiens - ps40
p-value
5e-06
5e-05
5e-04
5e-03
5e-02
Log-odds
0.5
1.0
1.5
2.0
2.5
3.0
3.5
4.0

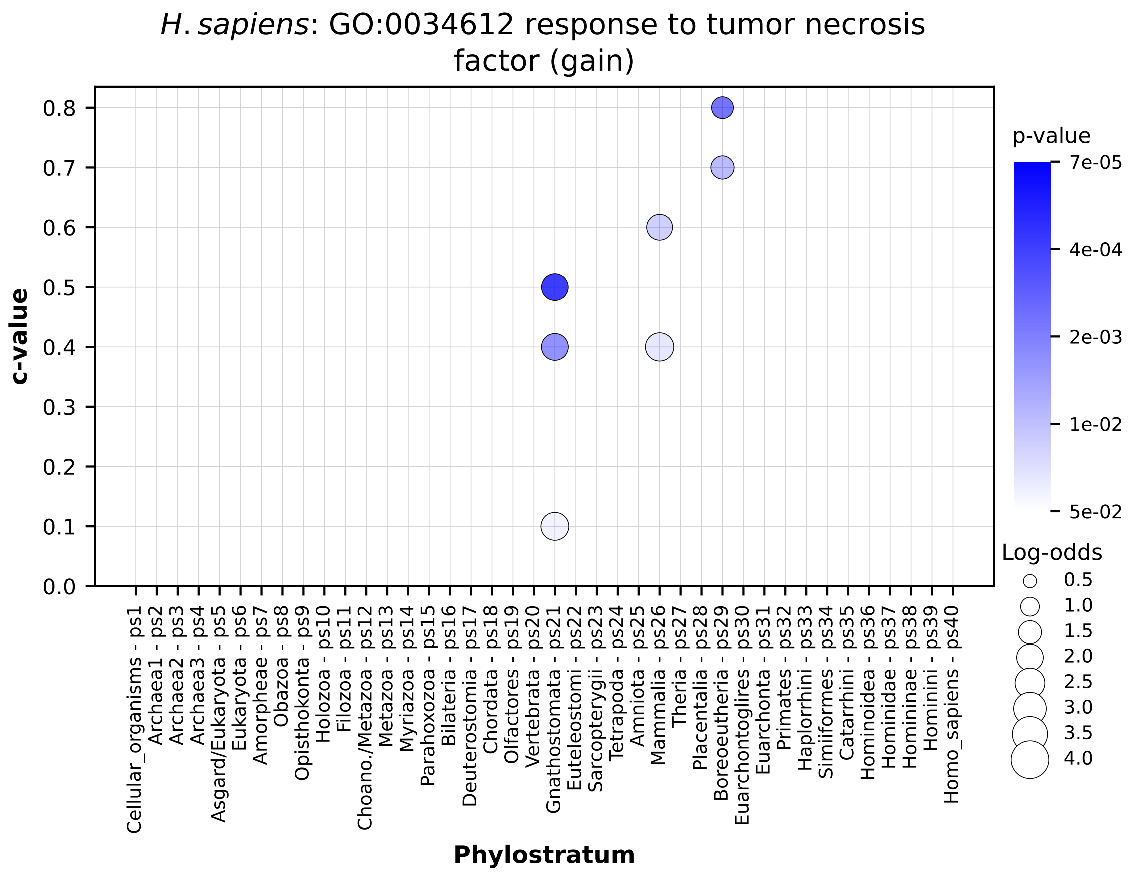

*H. sapiens*: GO:0034612 response to tumor necrosis factor (gain)
c-value
0.0
0.1
0.2
0.3
0.4
0.5
0.6
0.7
0.8
Phylostratum
Cellular_organisms - ps1
Archaea1 - ps2
Archaea2 - ps3
Archaea3 - ps4
Asgard/Eukaryota - ps5
Eukaryota - ps6
Amorpheae - ps7
Obazoa - ps8
Opisthokonta - ps9
Holozoa - ps10
Filozoa - ps11
Choano./Metazoa - ps12
Metazoa - ps13
Myriazoa - ps14
Parahoxozoa - ps15
Bilateria - ps16
Deuterostomia - ps17
Chordata - ps18
Olfactores - ps19
Vertebrata - ps20
Gnathostomata - ps21
Euteleostomi - ps22
Sarcopterygii - ps23
Tetrapoda - ps24
Amniota - ps25
Mammalia - ps26
Theria - ps27
Placentalia - ps28
Boreoeutheria - ps29
Euarchontoglires - ps30
Euarchonta - ps31
Primates - ps32
Haplorrhini - ps33
Simiiformes - ps34
Catarrhini - ps35
Hominoidea - ps36
Hominidae - ps37
Homininae - ps38
Hominini - ps39
Homo_sapiens - ps40
p-value
7e-05
4e-04
2e-03
1e-02
5e-02
Log-odds
0.5
1.0
1.5
2.0
2.5
3.0
3.5
4.0

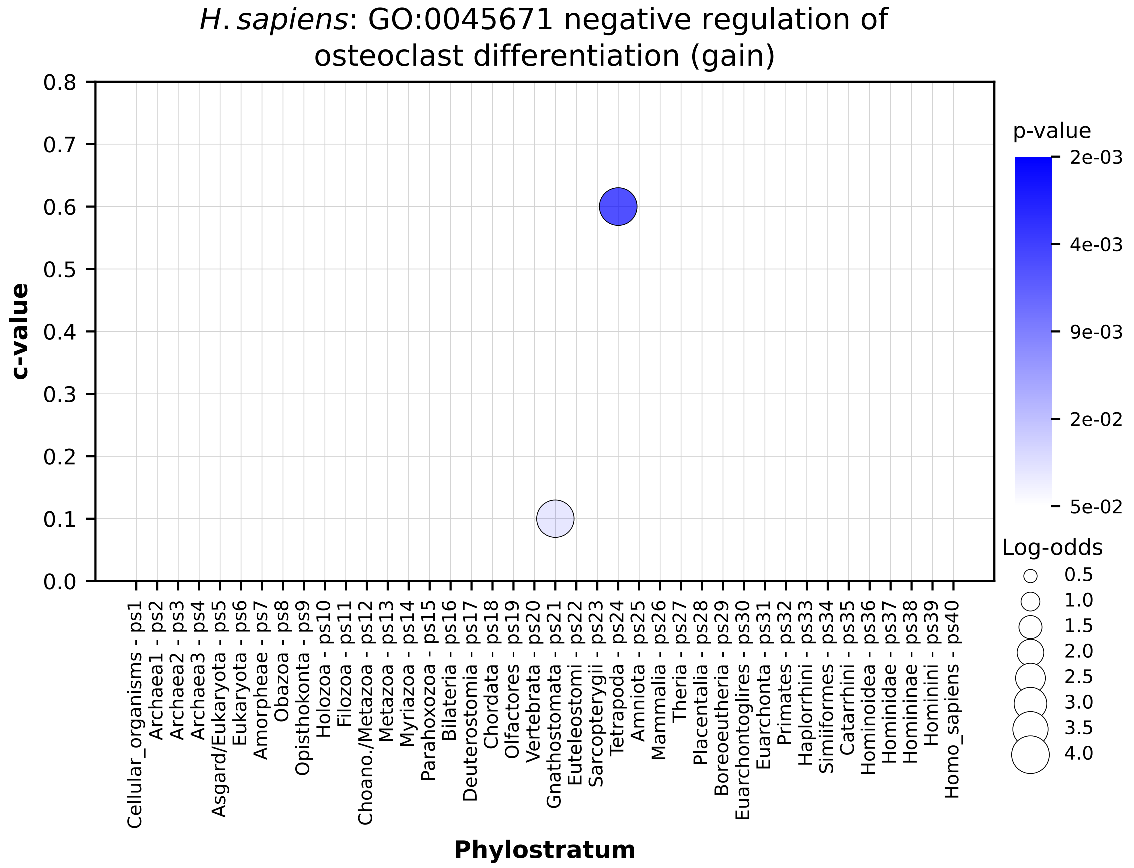

H. sapiens: GO:0045671 negative regulation of osteoclast differentiation (gain)
c-value
0.0
0.1
0.2
0.3
0.4
0.5
0.6
0.7
0.8
Phylostratum
Cellular_organisms - ps1
Archaea1 - ps2
Archaea2 - ps3
Archaea3 - ps4
Asgard/Eukaryota - ps5
Eukaryota - ps6
Amorpheae - ps7
Obazoa - ps8
Opisthokonta - ps9
Holozoa - ps10
Filozoa - ps11
Choano./Metazoa - ps12
Metazoa - ps13
Myriazoa - ps14
Parahoxozoa - ps15
Bilateria - ps16
Deuterostomia - ps17
Chordata - ps18
Olfactores - ps19
Vertebrata - ps20
Gnathostomata - ps21
Euteleostomi - ps22
Sarcopterygii - ps23
Tetrapoda - ps24
Amniota - ps25
Mammalia - ps26
Theria - ps27
Placentalia - ps28
Boreoeutheria - ps29
Euarchontoglires - ps30
Euarchonta - ps31
Primates - ps32
Haplorrhini - ps33
Simiiformes - ps34
Catarrhini - ps35
Hominoidea - ps36
Hominidae - ps37
Homininae - ps38
Hominini - ps39
Homo_sapiens - ps40
p-value
2e-03
4e-03
9e-03
2e-02
5e-02
Log-odds
0.5
1.0
1.5
2.0
2.5
3.0
3.5
4.0

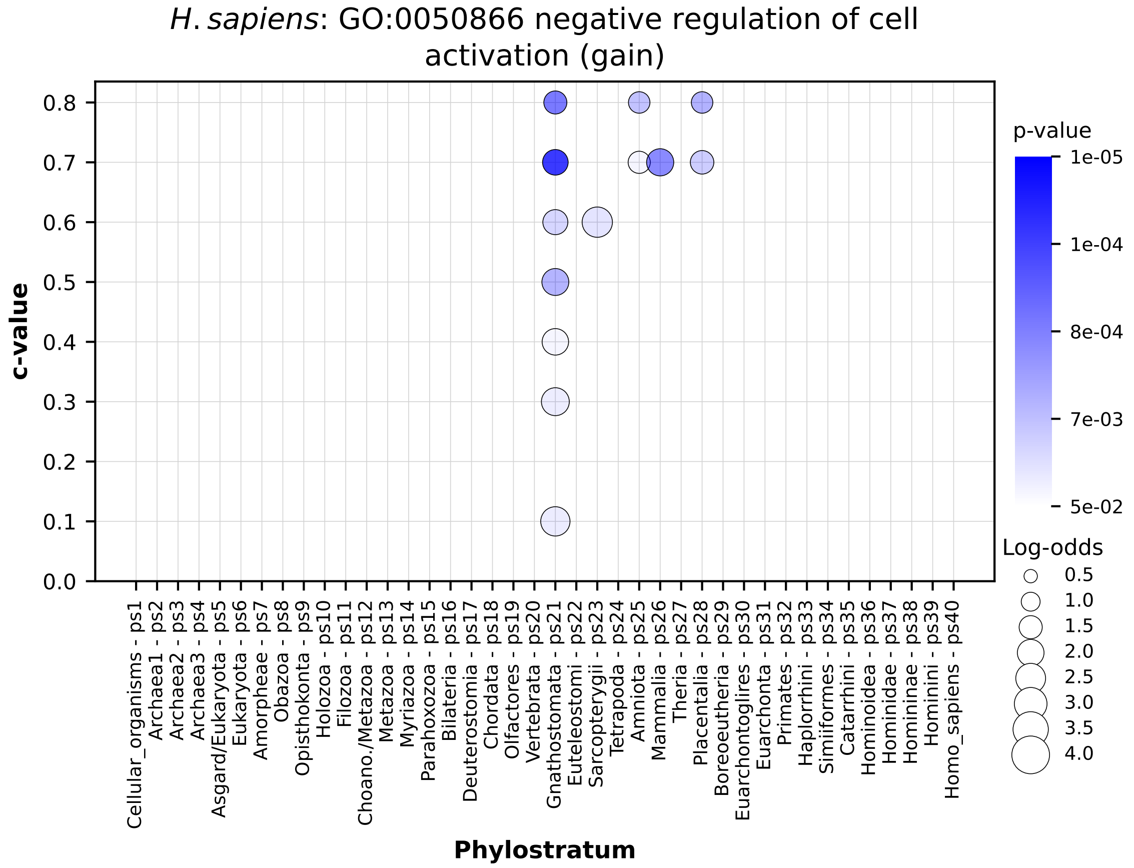

H. sapiens: GO:0050866 negative regulation of cell activation (gain)
c-value
0.0
0.1
0.2
0.3
0.4
0.5
0.6
0.7
0.8
Phylostratum
Cellular_organisms - ps1
Archaea1 - ps2
Archaea2 - ps3
Archaea3 - ps4
Asgard/Eukaryota - ps5
Eukaryota - ps6
Amorpheae - ps7
Obazoa - ps8
Opisthokonta - ps9
Holozoa - ps10
Filozoa - ps11
Choano./Metazoa - ps12
Metazoa - ps13
Myriazoa - ps14
Parahoxozoa - ps15
Bilateria - ps16
Deuterostomia - ps17
Chordata - ps18
Olfactores - ps19
Vertebrata - ps20
Gnathostomata - ps21
Euteleostomi - ps22
Sarcopterygii - ps23
Tetrapoda - ps24
Amniota - ps25
Mammalia - ps26
Theria - ps27
Placentalia - ps28
Boreoeutheria - ps29
Euarchontoglires - ps30
Euarchonta - ps31
Primates - ps32
Haplorrhini - ps33
Simiiformes - ps34
Catarrhini - ps35
Hominoidea - ps36
Hominidae - ps37
Homininae - ps38
Hominini - ps39
Homo_sapiens - ps40
p-value
1e-05
1e-04
8e-04
7e-03
5e-02
Log-odds
0.5
1.0
1.5
2.0
2.5
3.0
3.5
4.0

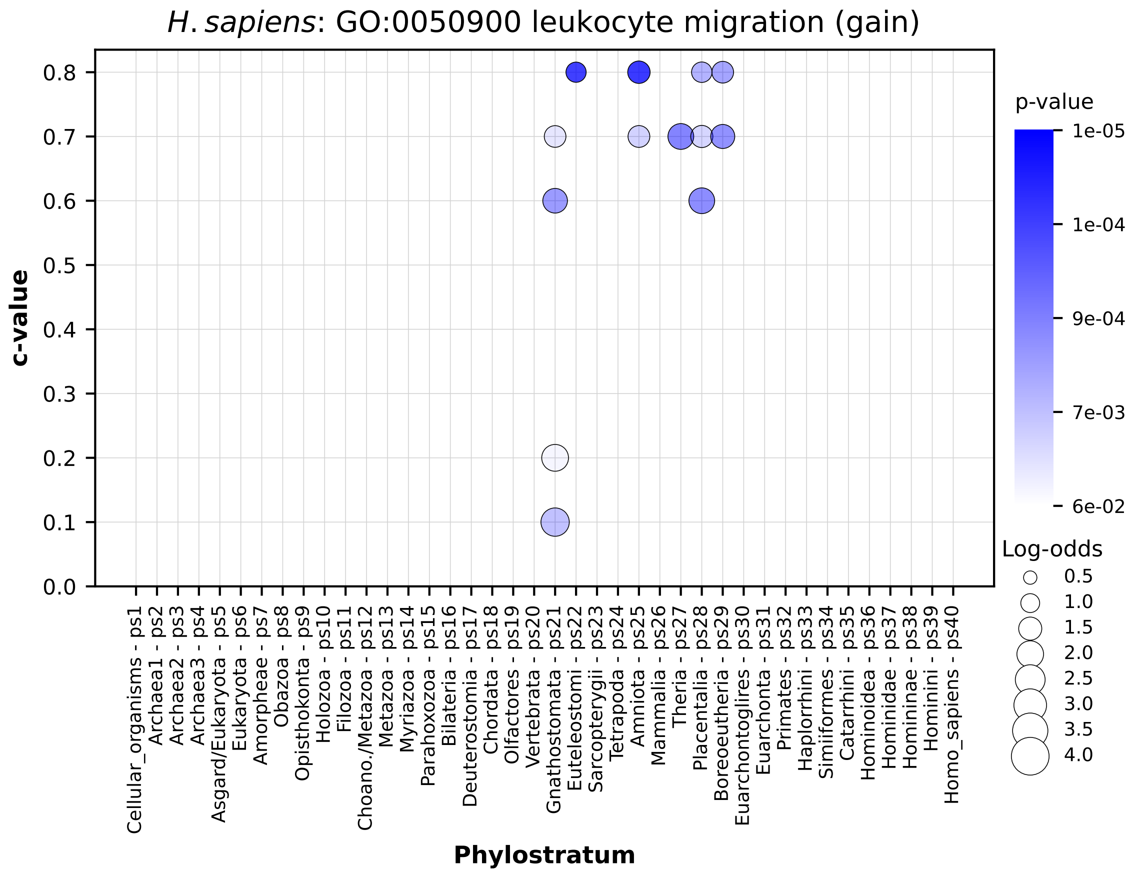

H. sapiens: GO:0050900 leukocyte migration (gain)
c-value
0.0
0.1
0.2
0.3
0.4
0.5
0.6
0.7
0.8
p-value
1e-05
1e-04
9e-04
7e-03
6e-02
Log-odds
0.5
1.0
1.5
2.0
2.5
3.0
3.5
4.0
Cellular_organisms - ps1
Archaea1 - ps2
Archaea2 - ps3
Archaea3 - ps4
Asgard/Eukaryota - ps5
Eukaryota - ps6
Amorpheae - ps7
Obazoa - ps8
Opisthokonta - ps9
Holozoa - ps10
Filozoa - ps11
Choano./Metazoa - ps12
Metazoa - ps13
Myriazoa - ps14
Parahoxozoa - ps15
Bilateria - ps16
Deuterostomia - ps17
Chordata - ps18
Olfactores - ps19
Vertebrata - ps20
Gnathostomata - ps21
Euteleostomi - ps22
Sarcopterygii - ps23
Tetrapoda - ps24
Amniota - ps25
Mammalia - ps26
Theria - ps27
Placentalia - ps28
Boreoeutheria - ps29
Euarchontoglires - ps30
Euarchonta - ps31
Primates - ps32
Haplorrhini - ps33
Simiiformes - ps34
Catarrhini - ps35
Hominoidea - ps36
Hominidae - ps37
Homininae - ps38
Hominini - ps39
Homo_sapiens - ps40
Phylostratum

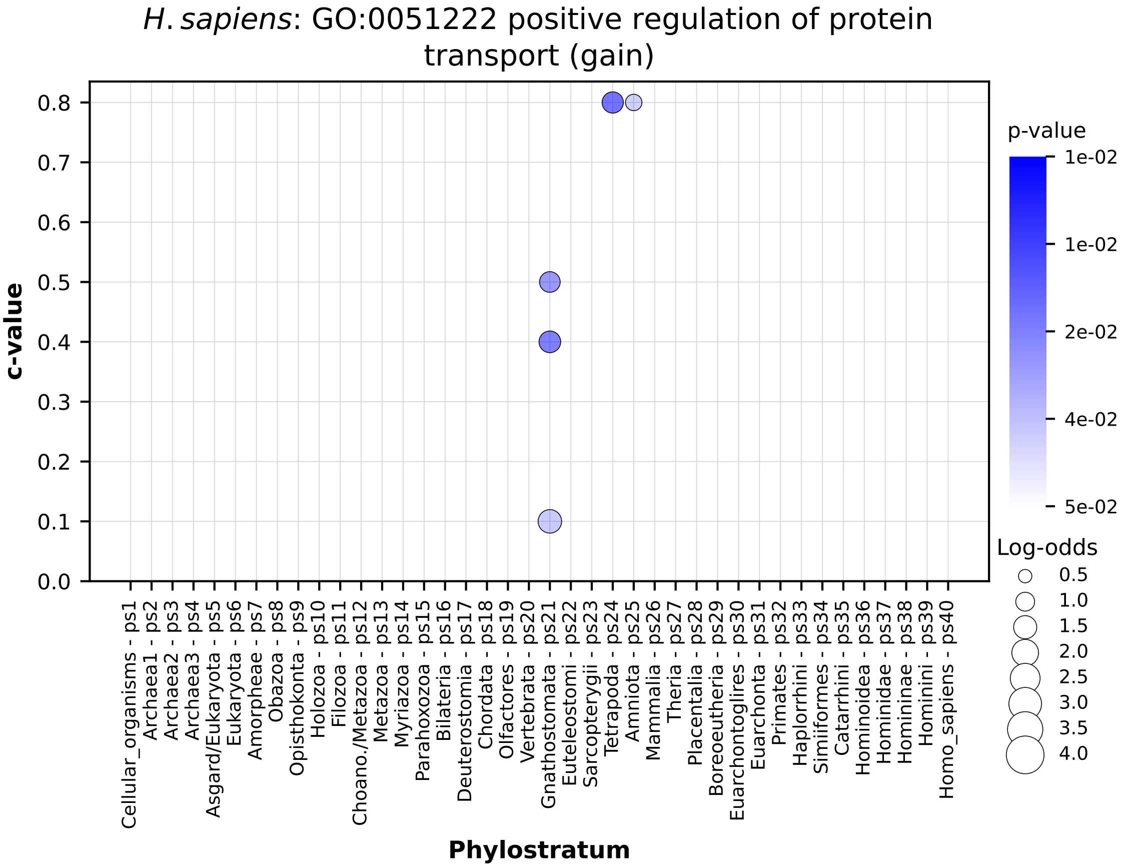
H. sapiens: GO:0051222 positive regulation of protein transport (gain)
c-value
0.0
0.1
0.2
0.3
0.4
0.5
0.6
0.7
0.8
Phylostratum
Cellular_organisms - ps1
Archaea1 - ps2
Archaea2 - ps3
Archaea3 - ps4
Asgard/Eukaryota - ps5
Eukaryota - ps6
Amorpheae - ps7
Obazoa - ps8
Opisthokonta - ps9
Holozoa - ps10
Filozoa - ps11
Choano./Metazoa - ps12
Metazoa - ps13
Myriazoa - ps14
Parahoxozoa - ps15
Bilateria - ps16
Deuterostomia - ps17
Chordata - ps18
Olfactores - ps19
Vertebrata - ps20
Gnathostomata - ps21
Euteleostomi - ps22
Sarcopterygii - ps23
Tetrapoda - ps24
Amniota - ps25
Mammalia - ps26
Theria - ps27
Placentalia - ps28
Boreoeutheria - ps29
Euarchontoglires - ps30
Euarchonta - ps31
Primates - ps32
Haplorrhini - ps33
Simiiformes - ps34
Catarrhini - ps35
Hominoidea - ps36
Hominidae - ps37
Homininae - ps38
Hominini - ps39
Homo_sapiens - ps40
p-value
1e-02
1e-02
2e-02
4e-02
5e-02
Log-odds
0.5
1.0
1.5
2.0
2.5
3.0
3.5
4.0

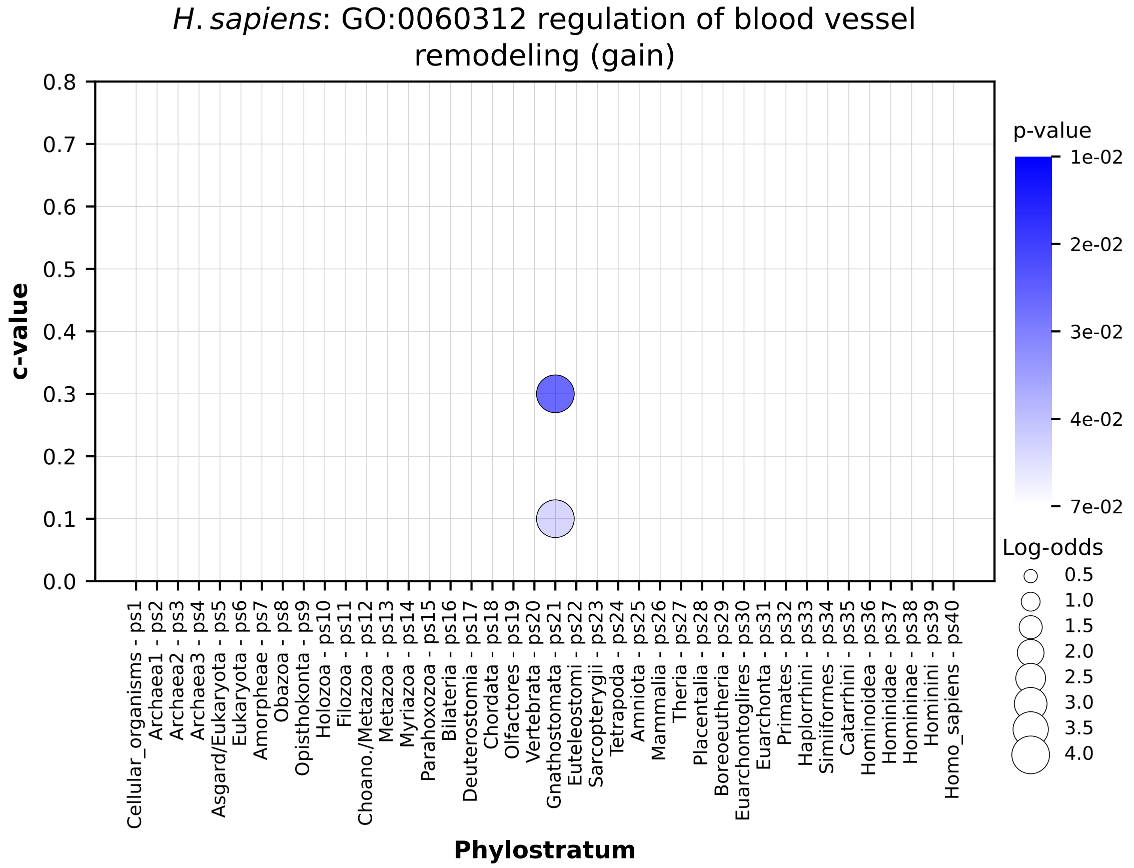
H. sapiens: GO:0060312 regulation of blood vessel remodeling (gain)
c-value
0.0
0.1
0.2
0.3
0.4
0.5
0.6
0.7
0.8
Phylostratum
Cellular_organisms - ps1
Archaea1 - ps2
Archaea2 - ps3
Archaea3 - ps4
Asgard/Eukaryota - ps5
Eukaryota - ps6
Amorpheae - ps7
Obazoa - ps8
Opisthokonta - ps9
Holozoa - ps10
Filozoa - ps11
Choano./Metazoa - ps12
Metazoa - ps13
Myriazoa - ps14
Parahoxozoa - ps15
Bilateria - ps16
Deuterostomia - ps17
Chordata - ps18
Olfactores - ps19
Vertebrata - ps20
Gnathostomata - ps21
Euteleostomi - ps22
Sarcopterygii - ps23
Tetrapoda - ps24
Amniota - ps25
Mammalia - ps26
Theria - ps27
Placentalia - ps28
Boreoeutheria - ps29
Euarchontoglires - ps30
Euarchonta - ps31
Primates - ps32
Haplorrhini - ps33
Simiiformes - ps34
Catarrhini - ps35
Hominoidea - ps36
Hominidae - ps37
Homininae - ps38
Hominini - ps39
Homo_sapiens - ps40
p-value
1e-02
2e-02
3e-02
4e-02
7e-02
Log-odds
0.5
1.0
1.5
2.0
2.5
3.0
3.5
4.0

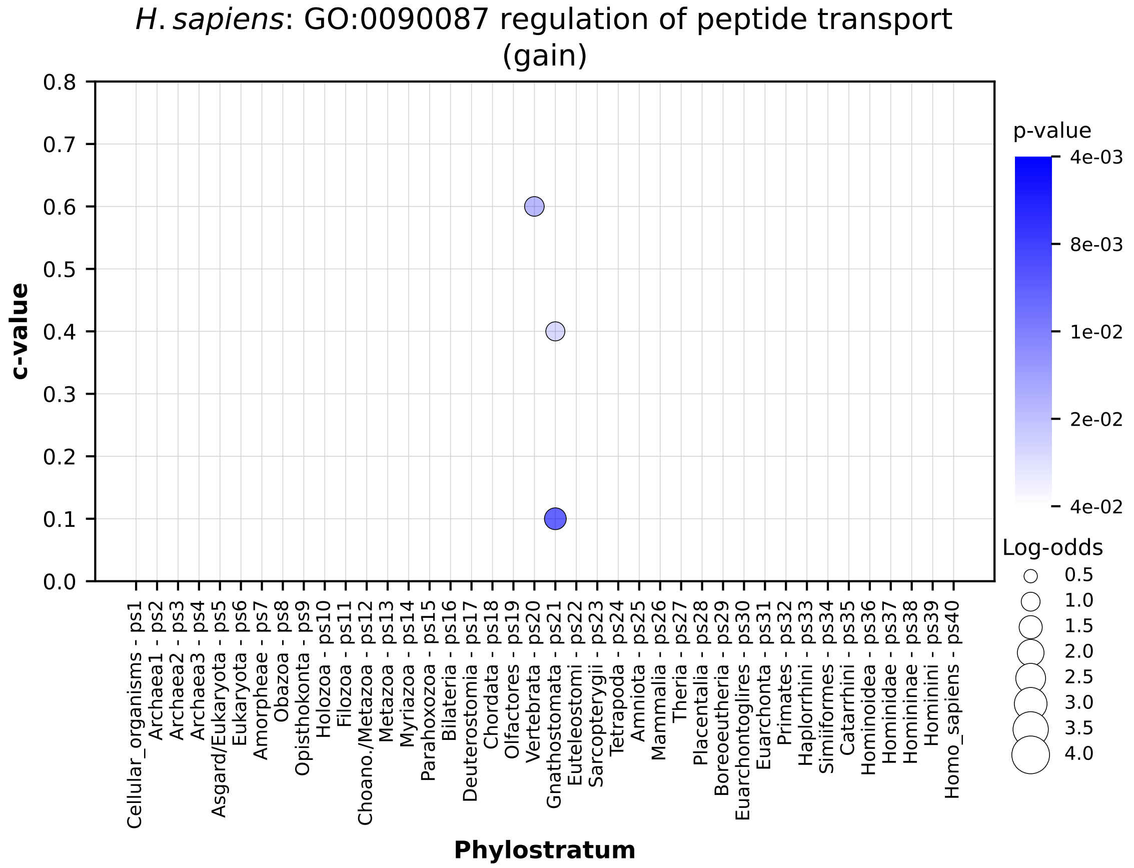

H. sapiens: GO:0090087 regulation of peptide transport
(gain)
c-value
0.0
0.1
0.2
0.3
0.4
0.5
0.6
0.7
0.8
Phylostratum
Cellular_organisms - ps1
Archaea1 - ps2
Archaea2 - ps3
Archaea3 - ps4
Asgard/Eukaryota - ps5
Eukaryota - ps6
Amorpheae - ps7
Obazoa - ps8
Opisthokonta - ps9
Holozoa - ps10
Filozoa - ps11
Choano./Metazoa - ps12
Metazoa - ps13
Myriazoa - ps14
Parahoxozoa - ps15
Bilateria - ps16
Deuterostomia - ps17
Chordata - ps18
Olfactores - ps19
Vertebrata - ps20
Gnathostomata - ps21
Euteleostomi - ps22
Sarcopterygii - ps23
Tetrapoda - ps24
Amniota - ps25
Mammalia - ps26
Theria - ps27
Placentalia - ps28
Boreoeutheria - ps29
Euarchontoglires - ps30
Euarchonta - ps31
Primates - ps32
Haplorrhini - ps33
Simiiformes - ps34
Catarrhini - ps35
Hominoidea - ps36
Hominidae - ps37
Homininae - ps38
Hominini - ps39
Homo_sapiens - ps40
p-value
4e-03
8e-03
1e-02
2e-02
4e-02
Log-odds
0.5
1.0
1.5
2.0
2.5
3.0
3.5
4.0

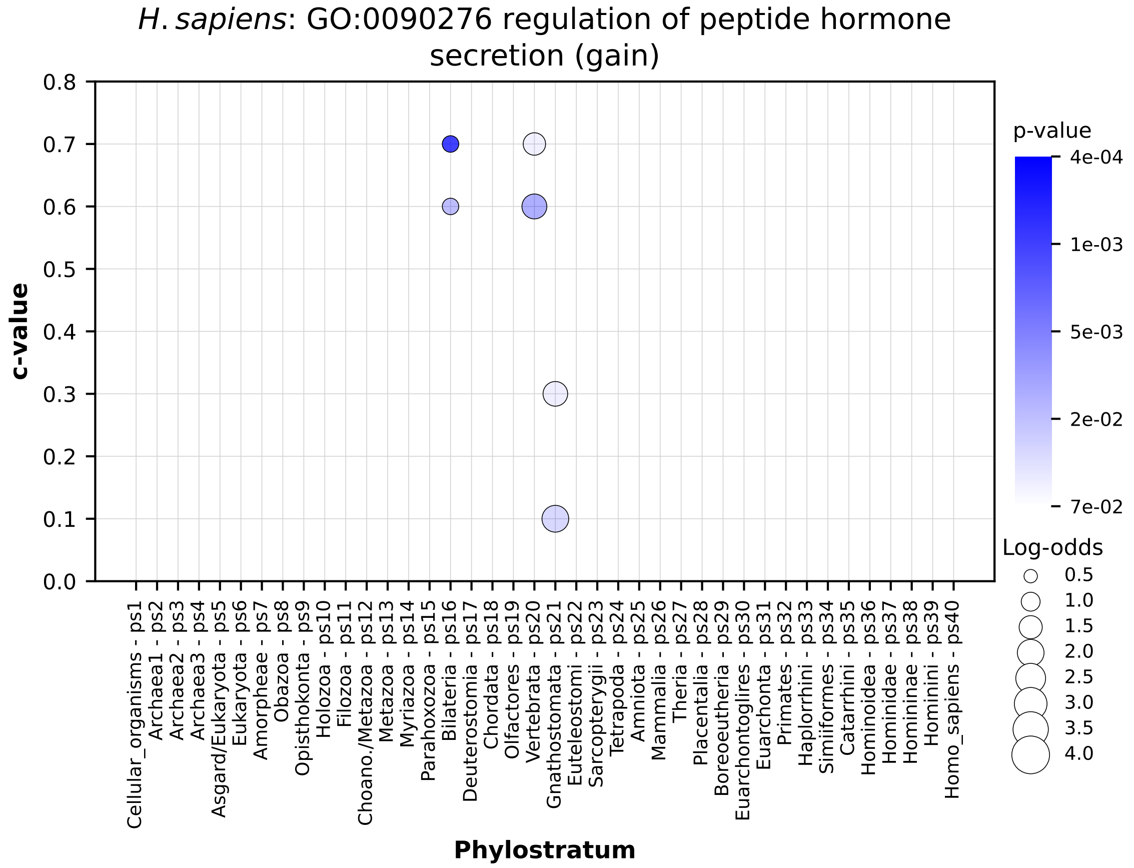

H. sapiens: GO:0090276 regulation of peptide hormone secretion (gain)
c-value
0.0
0.1
0.2
0.3
0.4
0.5
0.6
0.7
0.8
Phylostratum
Cellular_organisms - ps1
Archaea1 - ps2
Archaea2 - ps3
Archaea3 - ps4
Asgard/Eukaryota - ps5
Eukaryota - ps6
Amorpheae - ps7
Obazoa - ps8
Opisthokonta - ps9
Holozoa - ps10
Filozoa - ps11
Choano./Metazoa - ps12
Metazoa - ps13
Myriazoa - ps14
Parahoxozoa - ps15
Bilateria - ps16
Deuterostomia - ps17
Chordata - ps18
Olfactores - ps19
Vertebrata - ps20
Gnathostomata - ps21
Euteleostomi - ps22
Sarcopterygii - ps23
Tetrapoda - ps24
Amniota - ps25
Mammalia - ps26
Theria - ps27
Placentalia - ps28
Boreoeutheria - ps29
Euarchontoglires - ps30
Euarchonta - ps31
Primates - ps32
Haplorrhini - ps33
Simiiformes - ps34
Catarrhini - ps35
Hominoidea - ps36
Hominidae - ps37
Homininae - ps38
Hominini - ps39
Homo_sapiens - ps40
p-value
4e-04
1e-03
5e-03
2e-02
7e-02
Log-odds
0.5
1.0
1.5
2.0
2.5
3.0
3.5
4.0

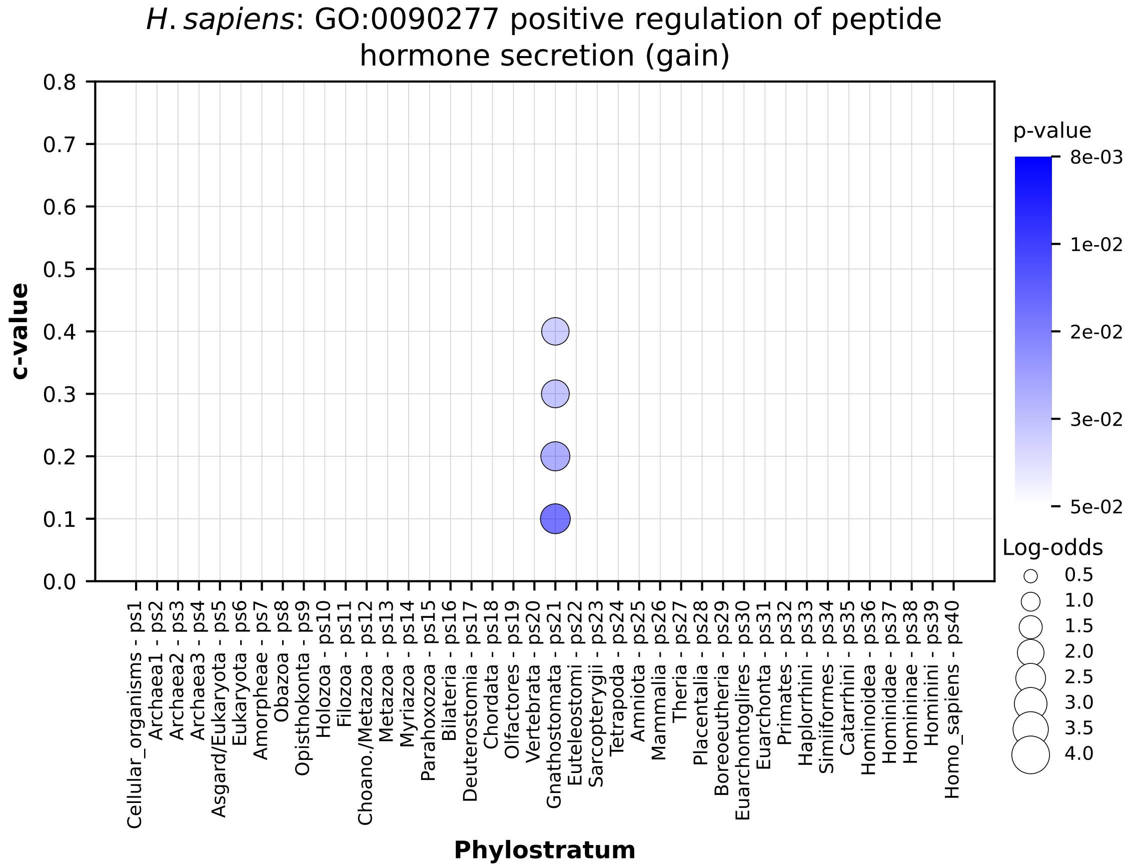

H. sapiens: GO:0090277 positive regulation of peptide hormone secretion (gain)
c-value
0.0
0.1
0.2
0.3
0.4
0.5
0.6
0.7
0.8
Phylostratum
Cellular_organisms - ps1
Archaea1 - ps2
Archaea2 - ps3
Archaea3 - ps4
Asgard/Eukaryota - ps5
Eukaryota - ps6
Amorpheae - ps7
Obazoa - ps8
Opisthokonta - ps9
Holozoa - ps10
Filozoa - ps11
Choano./Metazoa - ps12
Metazoa - ps13
Myriazoa - ps14
Parahoxozoa - ps15
Bilateria - ps16
Deuterostomia - ps17
Chordata - ps18
Olfactores - ps19
Vertebrata - ps20
Gnathostomata - ps21
Euteleostomi - ps22
Sarcopterygii - ps23
Tetrapoda - ps24
Amniota - ps25
Mammalia - ps26
Theria - ps27
Placentalia - ps28
Boreoeutheria - ps29
Euarchontoglires - ps30
Euarchonta - ps31
Primates - ps32
Haplorrhini - ps33
Simiiformes - ps34
Catarrhini - ps35
Hominoidea - ps36
Hominidae - ps37
Homininae - ps38
Hominini - ps39
Homo_sapiens - ps40
p-value
8e-03
1e-02
2e-02
3e-02
5e-02
Log-odds
0.5
1.0
1.5
2.0
2.5
3.0
3.5
4.0

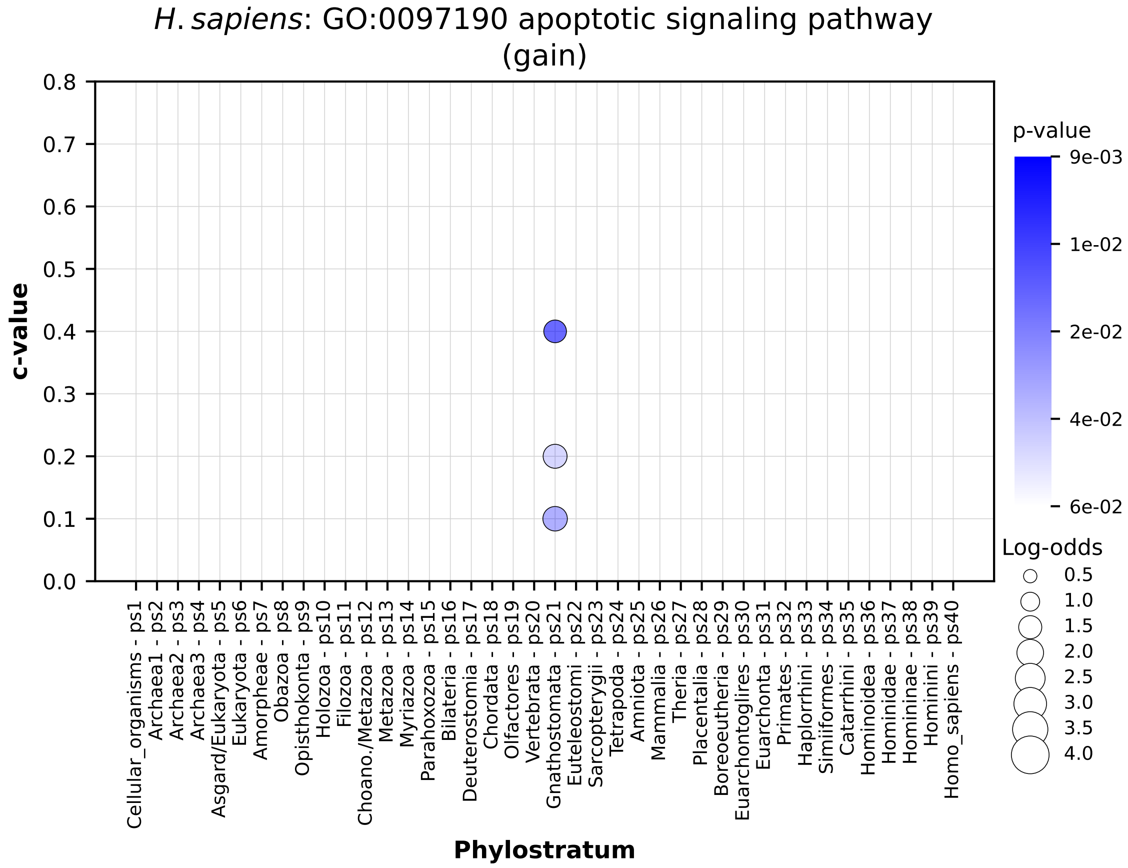

*H. sapiens*: GO:0097190 apoptotic signaling pathway (gain)
c-value
0.8
0.7
0.6
0.5
0.4
0.3
0.2
0.1
0.0
Phylostratum
Cellular_organisms - ps1
Archaea1 - ps2
Archaea2 - ps3
Archaea3 - ps4
Asgard/Eukaryota - ps5
Eukaryota - ps6
Amorpheae - ps7
Obazoa - ps8
Opisthokonta - ps9
Holozoa - ps10
Filozoa - ps11
Choano./Metazoa - ps12
Metazoa - ps13
Myriazoa - ps14
Parahoxozoa - ps15
Bilateria - ps16
Deuterostomia - ps17
Chordata - ps18
Olfactores - ps19
Vertebrata - ps20
Gnathostomata - ps21
Euteleostomi - ps22
Sarcopterygii - ps23
Tetrapoda - ps24
Amniota - ps25
Mammalia - ps26
Theria - ps27
Placentalia - ps28
Boreoeutheria - ps29
Euarchontoglires - ps30
Euarchonta - ps31
Primates - ps32
Haplorrhini - ps33
Simiiformes - ps34
Catarrhini - ps35
Hominoidea - ps36
Hominidae - ps37
Homininae - ps38
Hominini - ps39
Homo_sapiens - ps40
p-value
9e-03
1e-02
2e-02
4e-02
6e-02
Log-odds
0.5
1.0
1.5
2.0
2.5
3.0
3.5
4.0

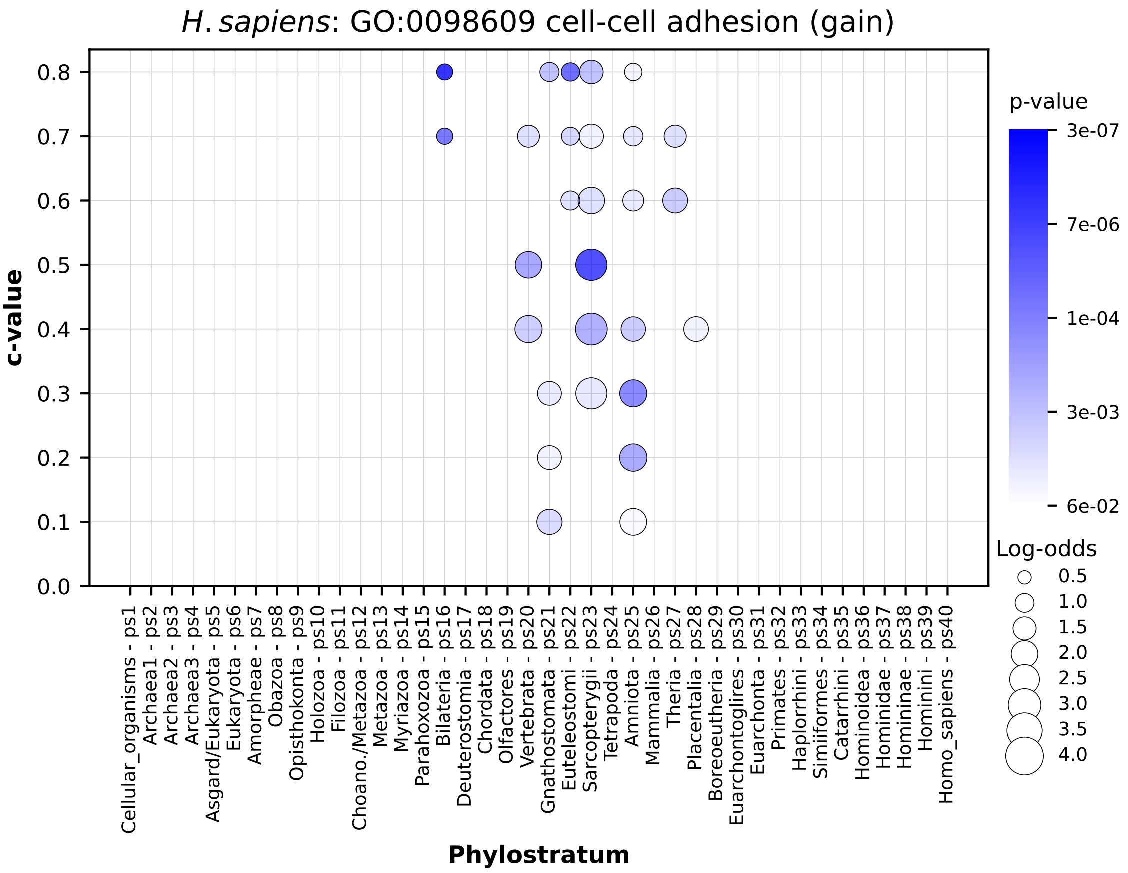

H. sapiens: GO:0098609 cell-cell adhesion (gain)
c-value
0.0
0.1
0.2
0.3
0.4
0.5
0.6
0.7
0.8
Phylostratum
Cellular_organisms - ps1
Archaea1 - ps2
Archaea2 - ps3
Archaea3 - ps4
Asgard/Eukaryota - ps5
Eukaryota - ps6
Amorpheae - ps7
Obazoa - ps8
Opisthokonta - ps9
Holozoa - ps10
Filozoa - ps11
Choano./Metazoa - ps12
Metazoa - ps13
Myriazoa - ps14
Parahoxozoa - ps15
Bilateria - ps16
Deuterostomia - ps17
Chordata - ps18
Olfactores - ps19
Vertebrata - ps20
Gnathostomata - ps21
Euteleostomi - ps22
Sarcopterygii - ps23
Tetrapoda - ps24
Amniota - ps25
Mammalia - ps26
Theria - ps27
Placentalia - ps28
Boreoeutheria - ps29
Euarchontoglires - ps30
Euarchonta - ps31
Primates - ps32
Haplorrhini - ps33
Simiiformes - ps34
Catarrhini - ps35
Hominoidea - ps36
Hominidae - ps37
Homininae - ps38
Hominini - ps39
Homo_sapiens - ps40
p-value
3e-07
7e-06
1e-04
3e-03
6e-02
Log-odds
0.5
1.0
1.5
2.0
2.5
3.0
3.5
4.0

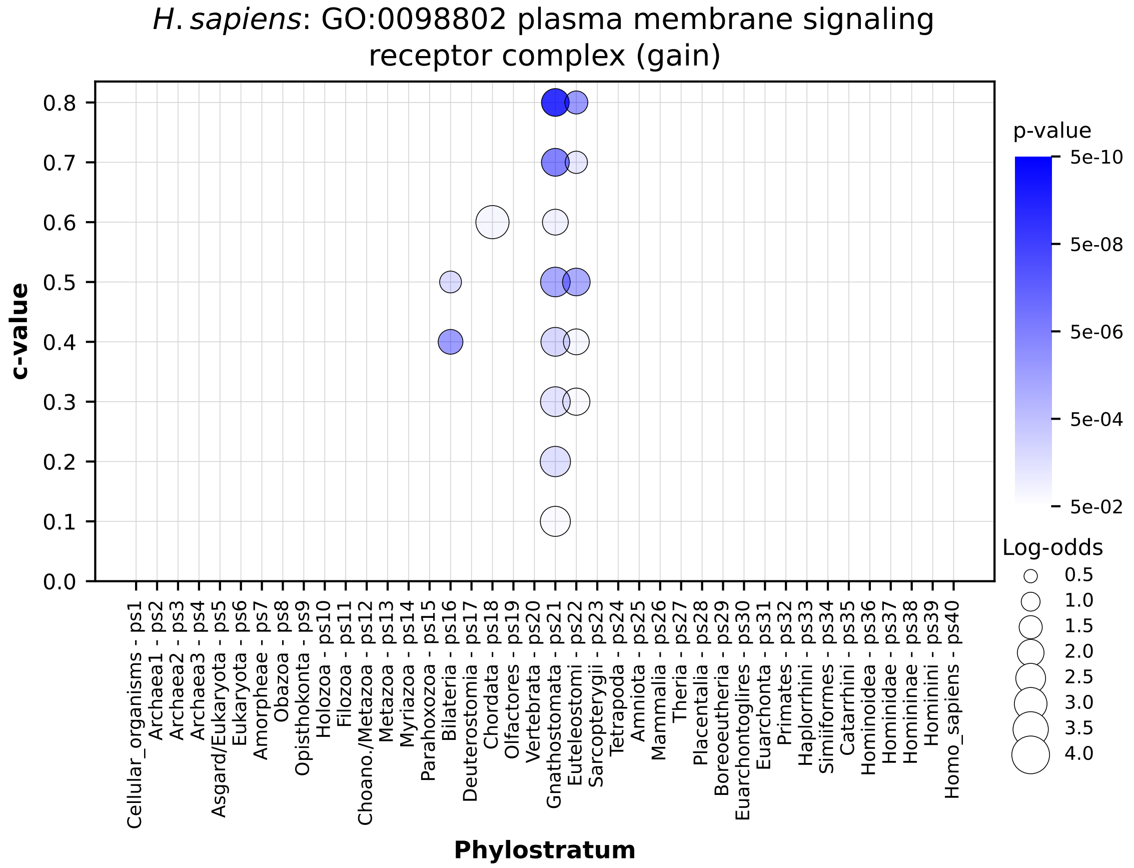

*H. sapiens*: GO:0098802 plasma membrane signaling receptor complex (gain)
c-value
0.0
0.1
0.2
0.3
0.4
0.5
0.6
0.7
0.8
p-value
5e-10
5e-08
5e-06
5e-04
5e-02
Log-odds
0.5
1.0
1.5
2.0
2.5
3.0
3.5
4.0
Cellular_organisms - ps1
Archaea1 - ps2
Archaea2 - ps3
Archaea3 - ps4
Asgard/Eukaryota - ps5
Eukaryota - ps6
Amorpheae - ps7
Obazoa - ps8
Opisthokonta - ps9
Holozoa - ps10
Filozoa - ps11
Choano./Metazoa - ps12
Metazoa - ps13
Myriazoa - ps14
Parahoxozoa - ps15
Bilateria - ps16
Deuterostomia - ps17
Chordata - ps18
Olfactores - ps19
Vertebrata - ps20
Gnathostomata - ps21
Euteleostomi - ps22
Sarcopterygii - ps23
Tetrapoda - ps24
Amniota - ps25
Mammalia - ps26
Theria - ps27
Placentalia - ps28
Boreoeutheria - ps29
Euarchontoglires - ps30
Euarchonta - ps31
Primates - ps32
Haplorrhini - ps33
Simiiformes - ps34
Catarrhini - ps35
Hominoidea - ps36
Hominidae - ps37
Homininae - ps38
Hominini - ps39
Homo_sapiens - ps40
Phylostratum

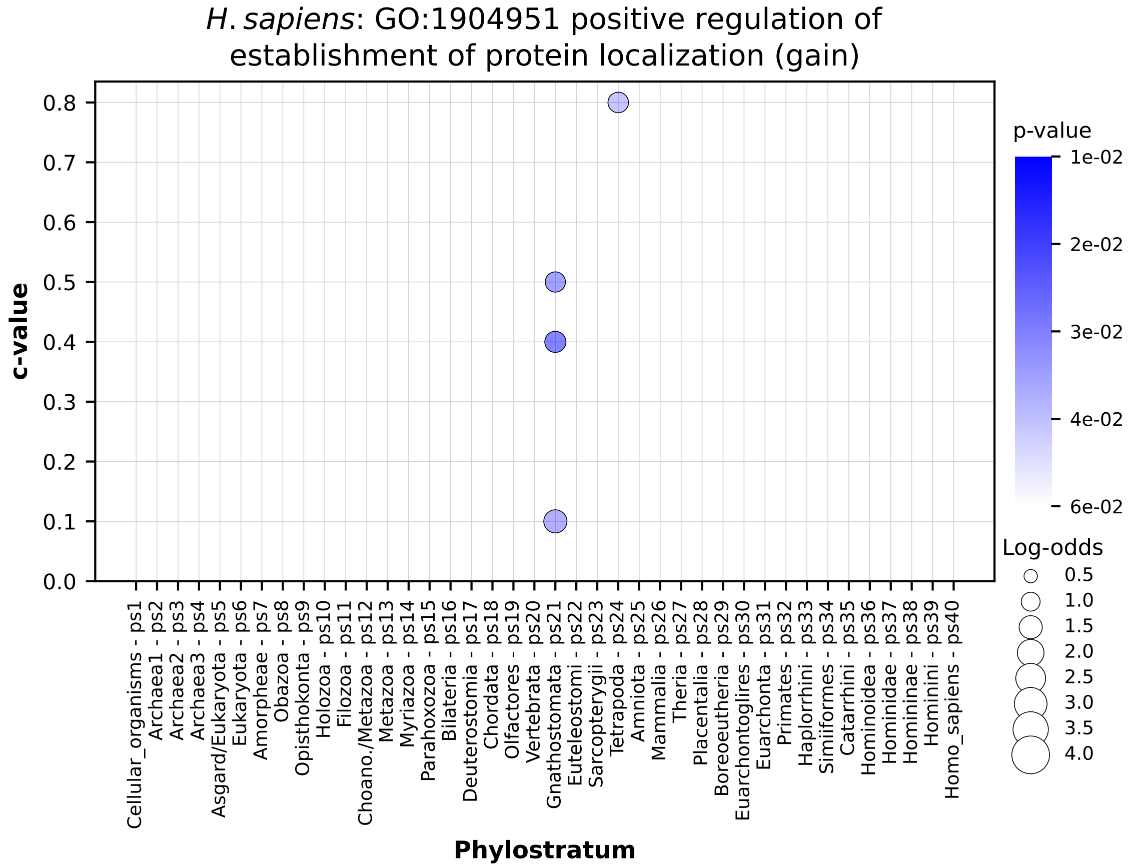
H. sapiens: GO:1904951 positive regulation of establishment of protein localization (gain)
c-value
0.0
0.1
0.2
0.3
0.4
0.5
0.6
0.7
0.8
Phylostratum
Cellular_organisms - ps1
Archaea1 - ps2
Archaea2 - ps3
Archaea3 - ps4
Asgard/Eukaryota - ps5
Eukaryota - ps6
Amorpheae - ps7
Obazoa - ps8
Opisthokonta - ps9
Holozoa - ps10
Filozoa - ps11
Choano./Metazoa - ps12
Metazoa - ps13
Myriazoa - ps14
Parahoxozoa - ps15
Bilateria - ps16
Deuterostomia - ps17
Chordata - ps18
Olfactores - ps19
Vertebrata - ps20
Gnathostomata - ps21
Euteleostomi - ps22
Sarcopterygii - ps23
Tetrapoda - ps24
Amniota - ps25
Mammalia - ps26
Theria - ps27
Placentalia - ps28
Boreoeutheria - ps29
Euarchontoglires - ps30
Euarchonta - ps31
Primates - ps32
Haplorrhini - ps33
Simiiformes - ps34
Catarrhini - ps35
Hominoidea - ps36
Hominidae - ps37
Homininae - ps38
Hominini - ps39
Homo_sapiens - ps40
p-value
1e-02
2e-02
3e-02
4e-02
6e-02
Log-odds
0.5
1.0
1.5
2.0
2.5
3.0
3.5
4.0

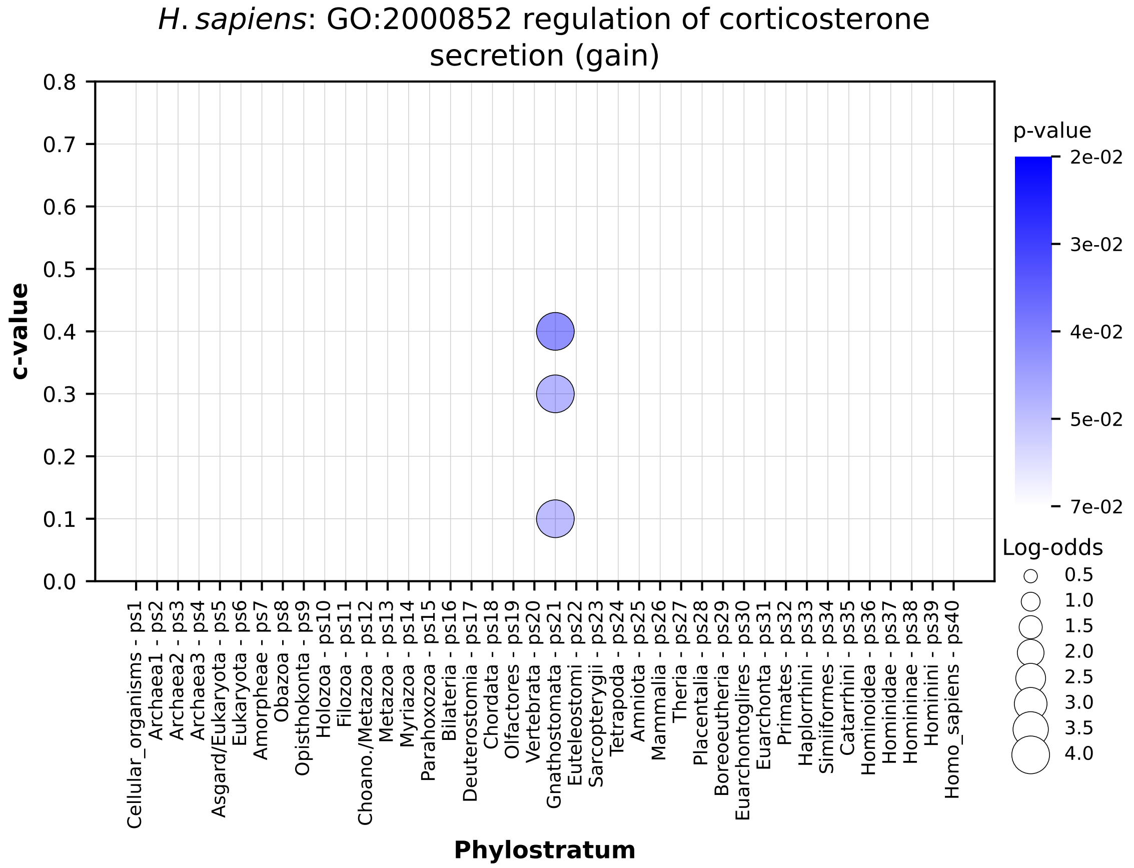

H. sapiens: GO:2000852 regulation of corticosterone secretion (gain)
c-value
0.0
0.1
0.2
0.3
0.4
0.5
0.6
0.7
0.8
Phylostratum
Cellular_organisms - ps1
Archaea1 - ps2
Archaea2 - ps3
Archaea3 - ps4
Asgard/Eukaryota - ps5
Eukaryota - ps6
Amorpheae - ps7
Obazoa - ps8
Opisthokonta - ps9
Holozoa - ps10
Filozoa - ps11
Choano./Metazoa - ps12
Metazoa - ps13
Myriazoa - ps14
Parahoxozoa - ps15
Bilateria - ps16
Deuterostomia - ps17
Chordata - ps18
Olfactores - ps19
Vertebrata - ps20
Gnathostomata - ps21
Euteleostomi - ps22
Sarcopterygii - ps23
Tetrapoda - ps24
Amniota - ps25
Mammalia - ps26
Theria - ps27
Placentalia - ps28
Boreoeutheria - ps29
Euarchontoglires - ps30
Euarchonta - ps31
Primates - ps32
Haplorrhini - ps33
Simiiformes - ps34
Catarrhini - ps35
Hominoidea - ps36
Hominidae - ps37
Homininae - ps38
Hominini - ps39
Homo_sapiens - ps40
p-value
2e-02
3e-02
4e-02
5e-02
7e-02
Log-odds
0.5
1.0
1.5
2.0
2.5
3.0
3.5
4.0

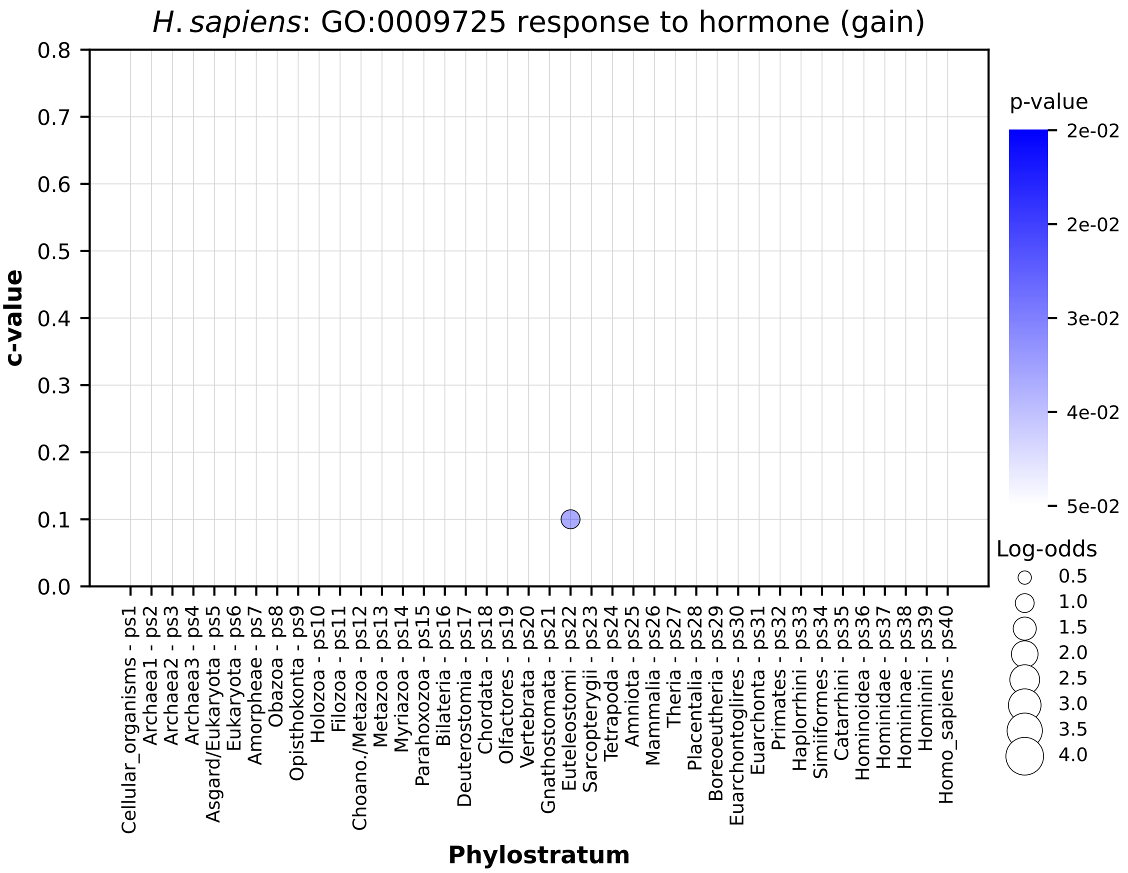
H. sapiens: GO:0009725 response to hormone (gain)
c-value
0.8
0.7
0.6
0.5
0.4
0.3
0.2
0.1
0.0
p-value
2e-02
2e-02
3e-02
4e-02
5e-02
Log-odds
0.5
1.0
1.5
2.0
2.5
3.0
3.5
4.0
Cellular_organisms - ps1
Archaea1 - ps2
Archaea2 - ps3
Archaea3 - ps4
Asgard/Eukaryota - ps5
Eukaryota - ps6
Amorpheae - ps7
Obazoa - ps8
Opisthokonta - ps9
Holozoa - ps10
Filozoa - ps11
Choano./Metazoa - ps12
Metazoa - ps13
Myriazoa - ps14
Parahoxozoa - ps15
Bilateria - ps16
Deuterostomia - ps17
Chordata - ps18
Olfactores - ps19
Vertebrata - ps20
Gnathostomata - ps21
Euteleostomi - ps22
Sarcopterygii - ps23
Tetrapoda - ps24
Amniota - ps25
Mammalia - ps26
Theria - ps27
Placentalia - ps28
Boreoeutheria - ps29
Euarchontoglires - ps30
Euarchonta - ps31
Primates - ps32
Haplorrhini - ps33
Simiiformes - ps34
Catarrhini - ps35
Hominoidea - ps36
Hominidae - ps37
Homininae - ps38
Hominini - ps39
Homo_sapiens - ps40
Phylostratum

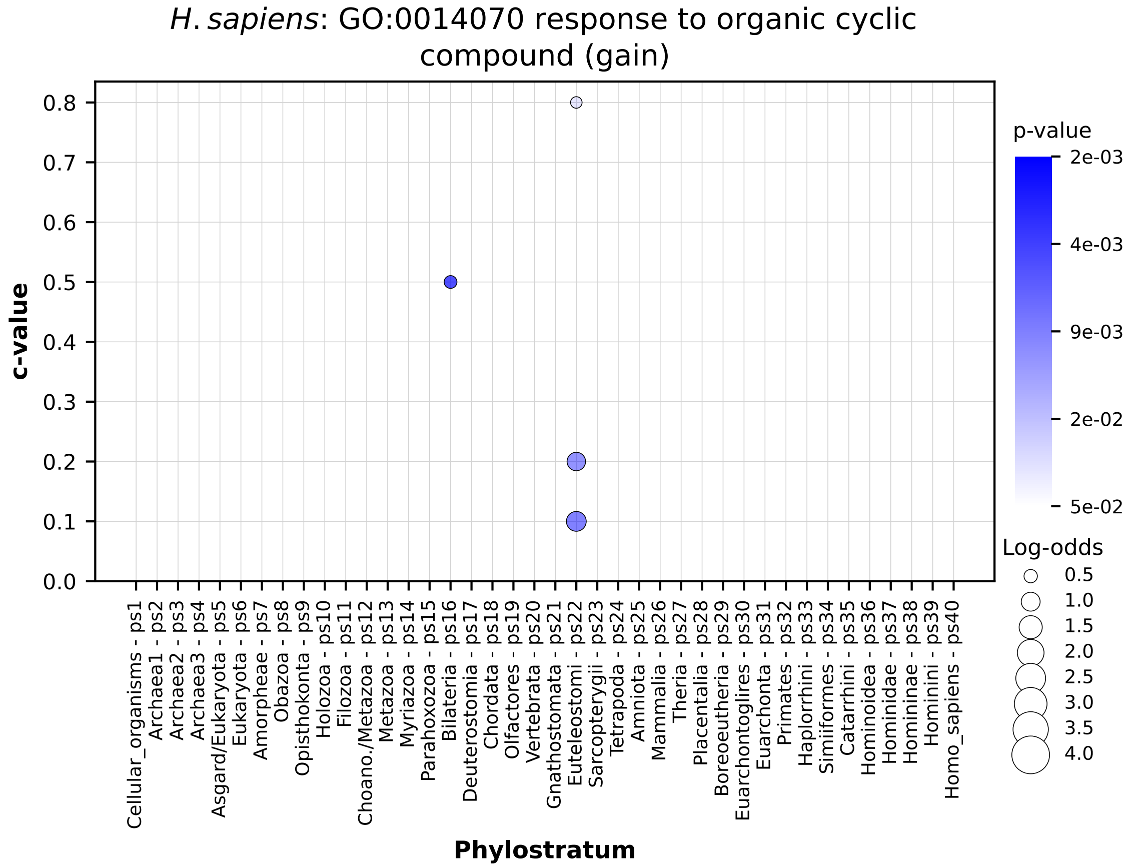

H. sapiens: GO:0014070 response to organic cyclic compound (gain)
c-value
0.8
0.7
0.6
0.5
0.4
0.3
0.2
0.1
0.0
Cellular_organisms - ps1
Archaea1 - ps2
Archaea2 - ps3
Archaea3 - ps4
Asgard/Eukaryota - ps5
Eukaryota - ps6
Amorpheae - ps7
Obazoa - ps8
Opisthokonta - ps9
Holozoa - ps10
Filozoa - ps11
Choano./Metazoa - ps12
Metazoa - ps13
Myriazoa - ps14
Parahoxozoa - ps15
Bilateria - ps16
Deuterostomia - ps17
Chordata - ps18
Olfactores - ps19
Vertebrata - ps20
Gnathostomata - ps21
Euteleostomi - ps22
Sarcopterygii - ps23
Tetrapoda - ps24
Amniota - ps25
Mammalia - ps26
Theria - ps27
Placentalia - ps28
Boreoeutheria - ps29
Euarchontoglires - ps30
Euarchonta - ps31
Primates - ps32
Haplorrhini - ps33
Simiiformes - ps34
Catarrhini - ps35
Hominoidea - ps36
Hominidae - ps37
Homininae - ps38
Hominini - ps39
Homo_sapiens - ps40
Phylostratum
p-value
2e-03
4e-03
9e-03
2e-02
5e-02
Log-odds
0.5
1.0
1.5
2.0
2.5
3.0
3.5
4.0

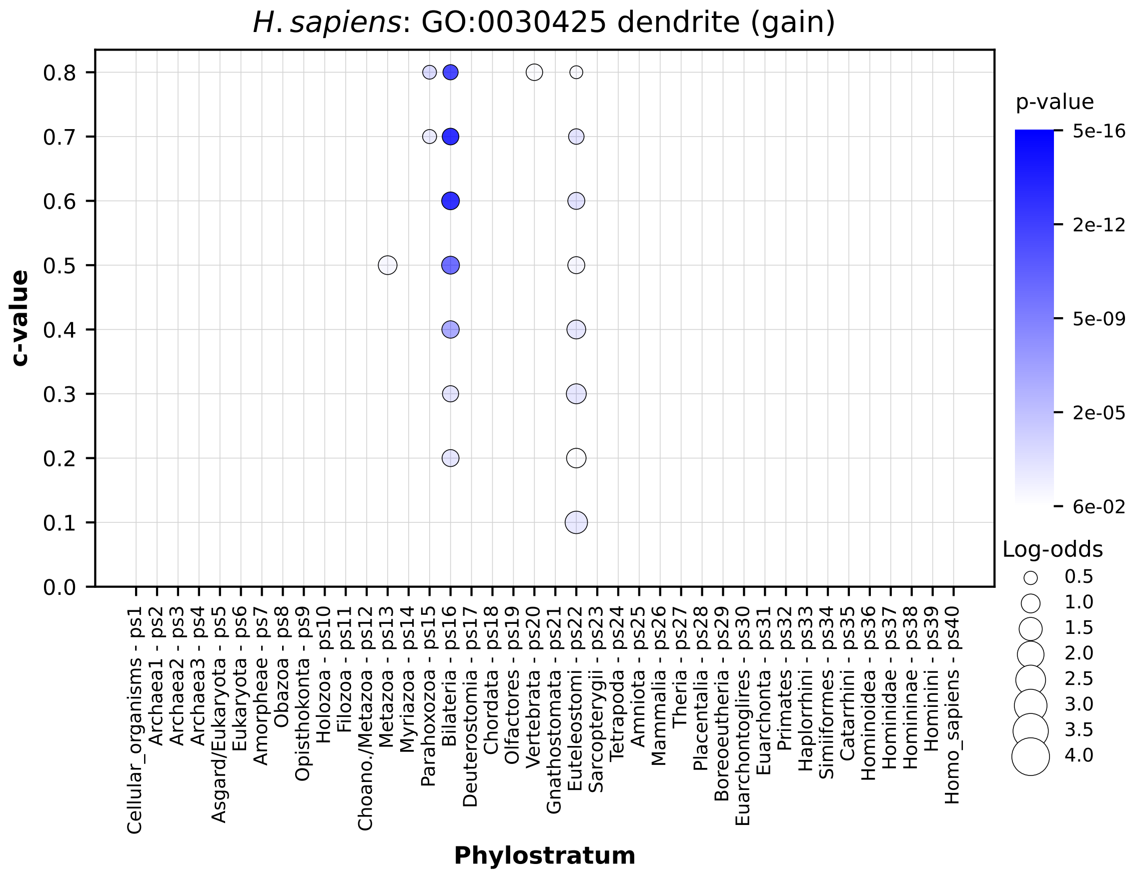

H. sapiens: GO:0030425 dendrite (gain)
c-value
0.8
0.7
0.6
0.5
0.4
0.3
0.2
0.1
0.0
Phylostratum
Cellular_organisms - ps1
Archaea1 - ps2
Archaea2 - ps3
Archaea3 - ps4
Asgard/Eukaryota - ps5
Eukaryota - ps6
Amorpheae - ps7
Obazoa - ps8
Opisthokonta - ps9
Holozoa - ps10
Filozoa - ps11
Choano./Metazoa - ps12
Metazoa - ps13
Myriazoa - ps14
Parahoxozoa - ps15
Bilateria - ps16
Deuterostomia - ps17
Chordata - ps18
Olfactores - ps19
Vertebrata - ps20
Gnathostomata - ps21
Euteleostomi - ps22
Sarcopterygii - ps23
Tetrapoda - ps24
Amniota - ps25
Mammalia - ps26
Theria - ps27
Placentalia - ps28
Boreoeutheria - ps29
Euarchontoglires - ps30
Euarchonta - ps31
Primates - ps32
Haplorrhini - ps33
Simiiformes - ps34
Catarrhini - ps35
Hominoidea - ps36
Hominidae - ps37
Homininae - ps38
Hominini - ps39
Homo_sapiens - ps40
p-value
5e-16
2e-12
5e-09
2e-05
6e-02
Log-odds
0.5
1.0
1.5
2.0
2.5
3.0
3.5
4.0

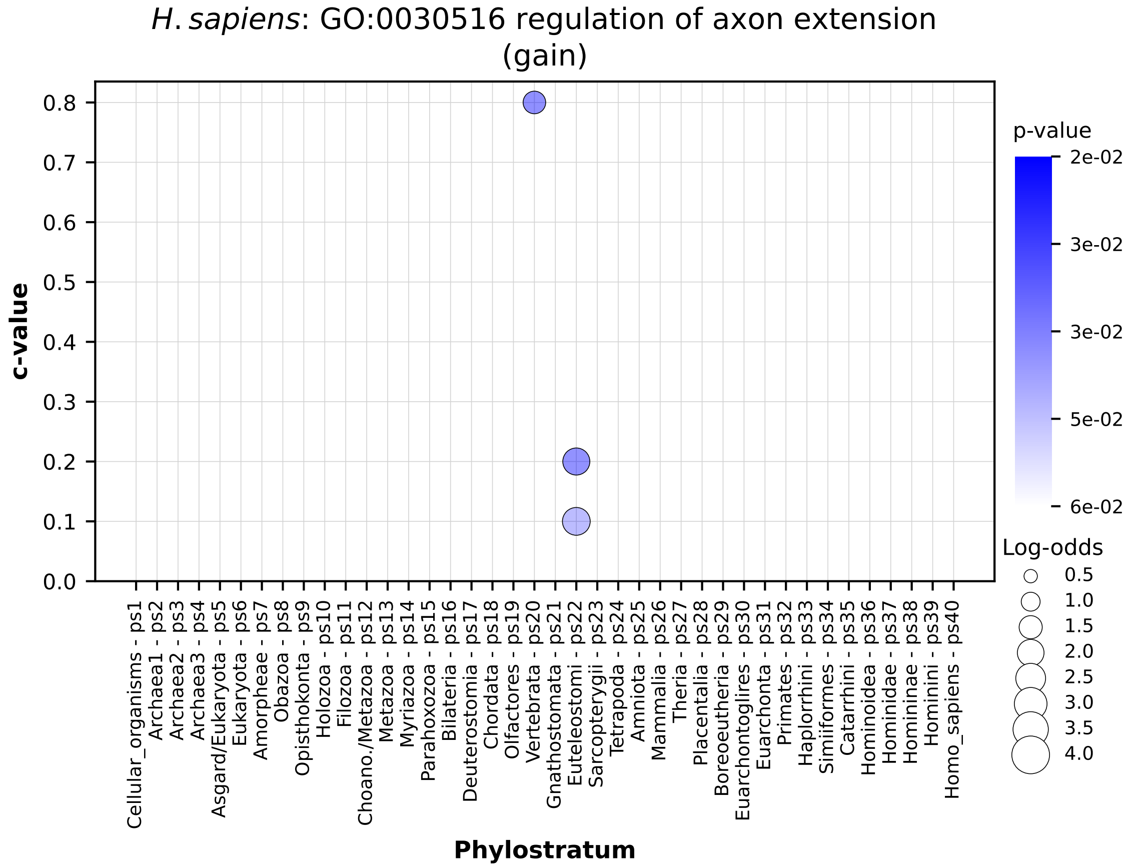

H. sapiens: GO:0030516 regulation of axon extension
(gain)
c-value
0.0
0.1
0.2
0.3
0.4
0.5
0.6
0.7
0.8
p-value
2e-02
3e-02
3e-02
5e-02
6e-02
Log-odds
0.5
1.0
1.5
2.0
2.5
3.0
3.5
4.0
Cellular_organisms - ps1
Archaea1 - ps2
Archaea2 - ps3
Archaea3 - ps4
Asgard/Eukaryota - ps5
Eukaryota - ps6
Amorpheae - ps7
Obazoa - ps8
Opisthokonta - ps9
Holozoa - ps10
Filozoa - ps11
Choano./Metazoa - ps12
Metazoa - ps13
Myriazoa - ps14
Parahoxozoa - ps15
Bilateria - ps16
Deuterostomia - ps17
Chordata - ps18
Olfactores - ps19
Vertebrata - ps20
Gnathostomata - ps21
Euteleostomi - ps22
Sarcopterygii - ps23
Tetrapoda - ps24
Amniota - ps25
Mammalia - ps26
Theria - ps27
Placentalia - ps28
Boreoeutheria - ps29
Euarchontoglires - ps30
Euarchonta - ps31
Primates - ps32
Haplorrhini - ps33
Simiiformes - ps34
Catarrhini - ps35
Hominoidea - ps36
Hominidae - ps37
Homininae - ps38
Hominini - ps39
Homo_sapiens - ps40
Phylostratum

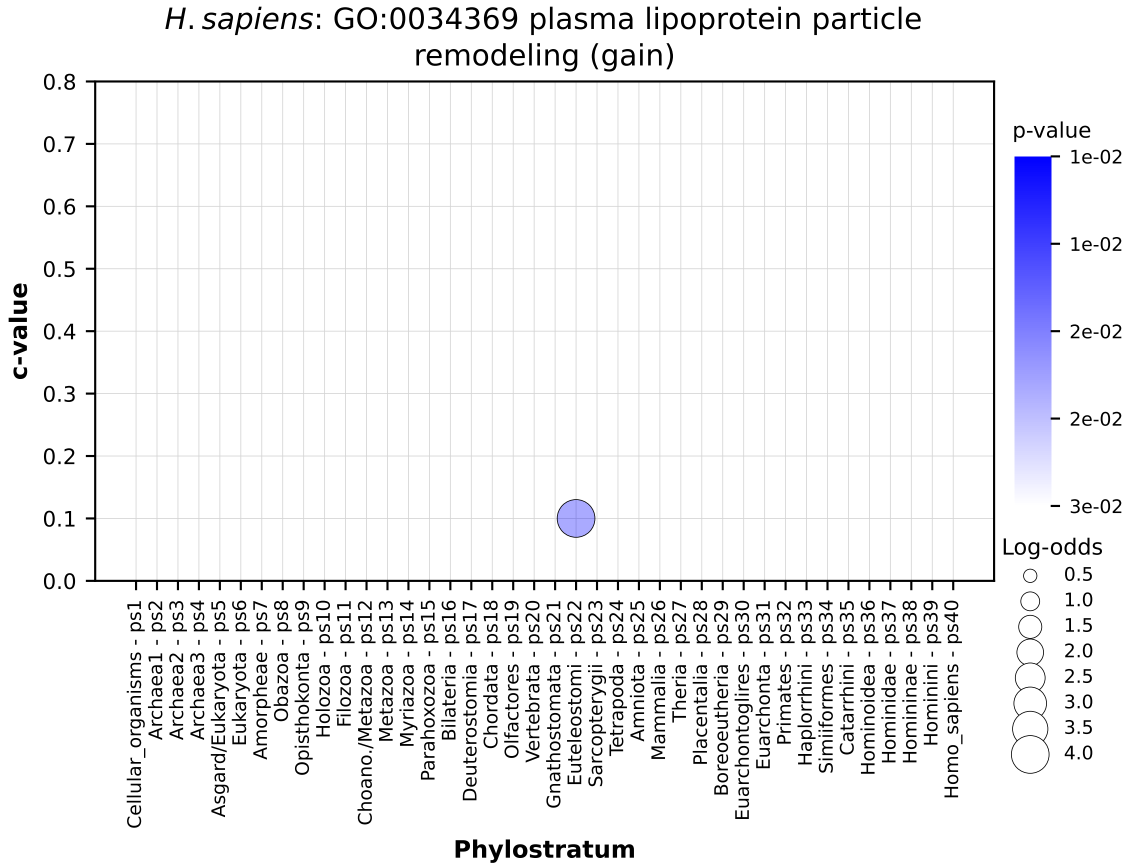

H. sapiens: GO:0034369 plasma lipoprotein particle remodeling (gain)
c-value
0.0
0.1
0.2
0.3
0.4
0.5
0.6
0.7
0.8
p-value
1e-02
1e-02
2e-02
2e-02
3e-02
Log-odds
0.5
1.0
1.5
2.0
2.5
3.0
3.5
4.0
Cellular_organisms - ps1
Archaea1 - ps2
Archaea2 - ps3
Archaea3 - ps4
Asgard/Eukaryota - ps5
Eukaryota - ps6
Amorpheae - ps7
Obazoa - ps8
Opisthokonta - ps9
Holozoa - ps10
Filozoa - ps11
Choano./Metazoa - ps12
Metazoa - ps13
Myriazoa - ps14
Parahoxozoa - ps15
Bilateria - ps16
Deuterostomia - ps17
Chordata - ps18
Olfactores - ps19
Vertebrata - ps20
Gnathostomata - ps21
Euteleostomi - ps22
Sarcopterygii - ps23
Tetrapoda - ps24
Amniota - ps25
Mammalia - ps26
Theria - ps27
Placentalia - ps28
Boreoeutheria - ps29
Euarchontoglires - ps30
Euarchonta - ps31
Primates - ps32
Haplorrhini - ps33
Simiiformes - ps34
Catarrhini - ps35
Hominoidea - ps36
Hominidae - ps37
Homininae - ps38
Hominini - ps39
Homo_sapiens - ps40
Phylostratum

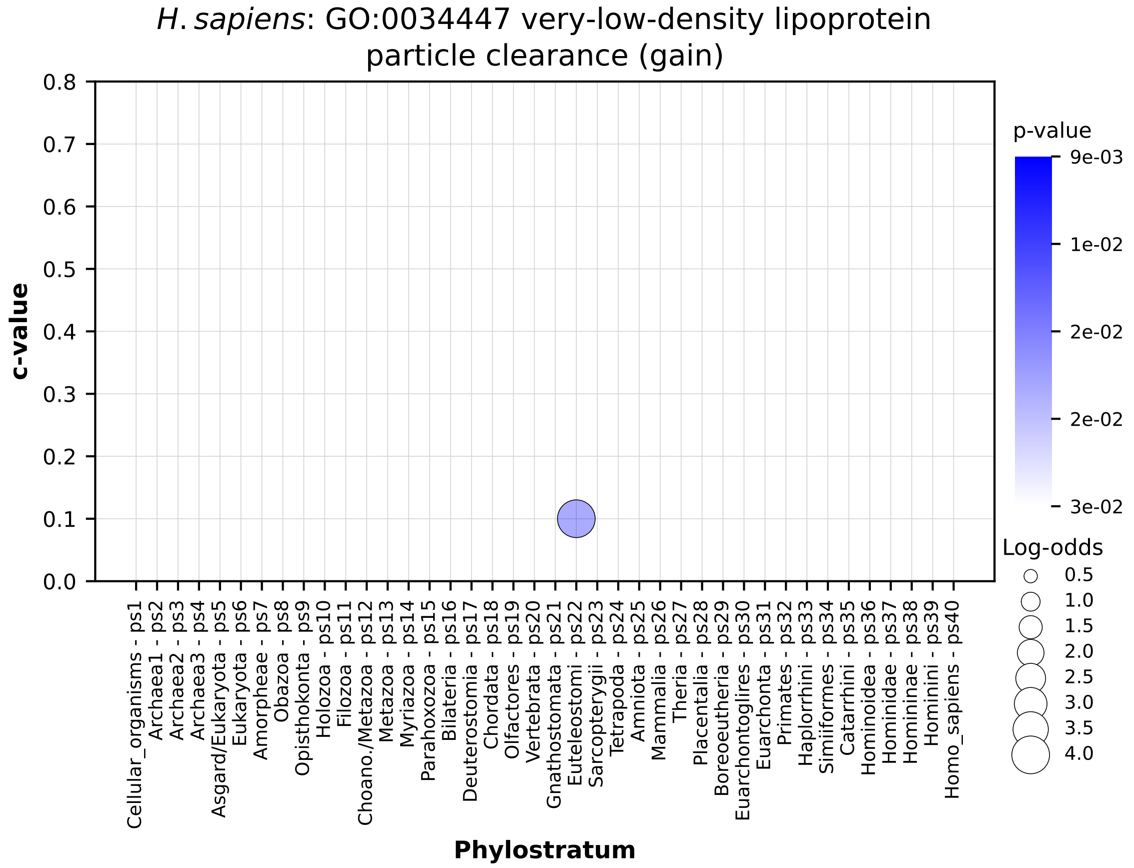

*H. sapiens*: GO:0034447 very-low-density lipoprotein particle clearance (gain)
c-value
0.8
0.7
0.6
0.5
0.4
0.3
0.2
0.1
0.0
Cellular_organisms - ps1
Archaea1 - ps2
Archaea2 - ps3
Archaea3 - ps4
Asgard/Eukaryota - ps5
Eukaryota - ps6
Amorpheae - ps7
Obazoa - ps8
Opisthokonta - ps9
Holozoa - ps10
Filozoa - ps11
Choano./Metazoa - ps12
Metazoa - ps13
Myriazoa - ps14
Parahoxozoa - ps15
Bilateria - ps16
Deuterostomia - ps17
Chordata - ps18
Olfactores - ps19
Vertebrata - ps20
Gnathostomata - ps21
Euteleostomi - ps22
Sarcopterygii - ps23
Tetrapoda - ps24
Amniota - ps25
Mammalia - ps26
Theria - ps27
Placentalia - ps28
Boreoeutheria - ps29
Euarchontoglires - ps30
Euarchonta - ps31
Primates - ps32
Haplorrhini - ps33
Simiiformes - ps34
Catarrhini - ps35
Hominoidea - ps36
Hominidae - ps37
Homininae - ps38
Hominini - ps39
Homo_sapiens - ps40
Phylostratum
p-value
9e-03
1e-02
2e-02
2e-02
3e-02
Log-odds
0.5
1.0
1.5
2.0
2.5
3.0
3.5
4.0

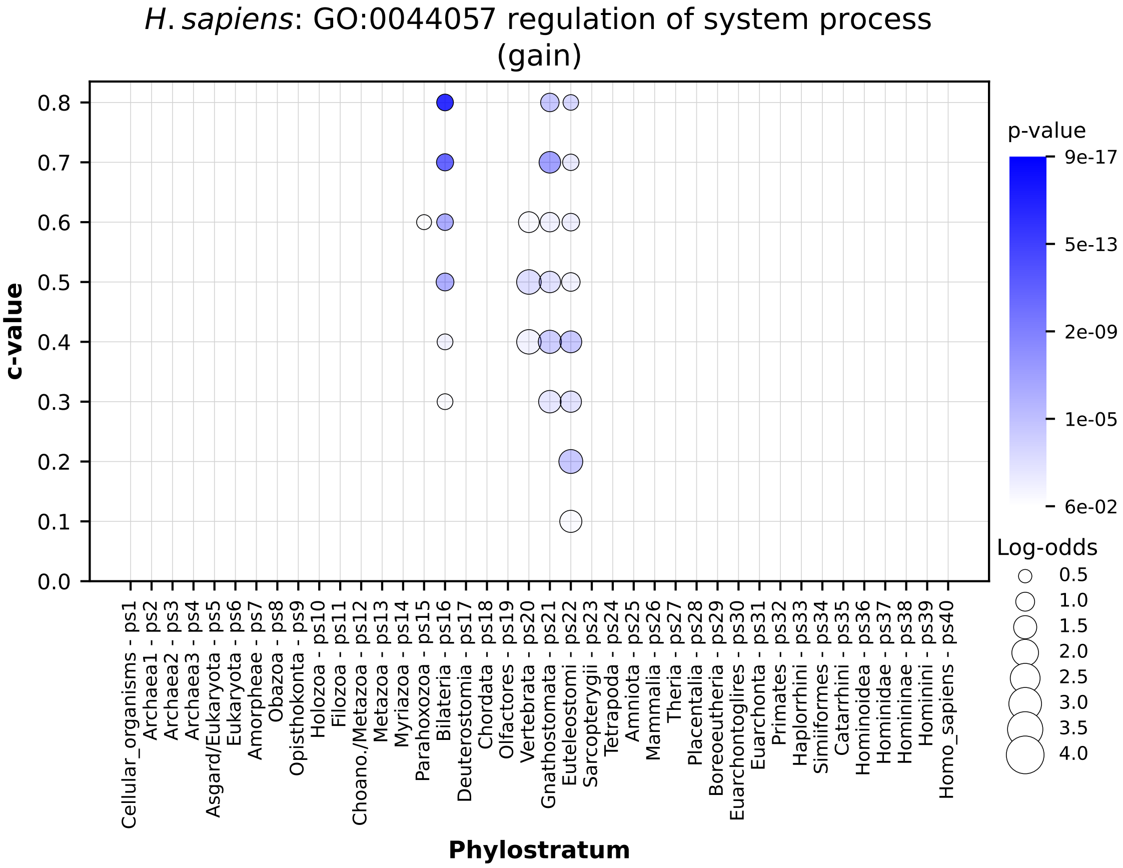
H. sapiens: GO:0044057 regulation of system process
(gain)
c-value
0.0
0.1
0.2
0.3
0.4
0.5
0.6
0.7
0.8
Phylostratum
Cellular_organisms - ps1
Archaea1 - ps2
Archaea2 - ps3
Archaea3 - ps4
Asgard/Eukaryota - ps5
Eukaryota - ps6
Amorpheae - ps7
Obazoa - ps8
Opisthokonta - ps9
Holozoa - ps10
Filozoa - ps11
Choano./Metazoa - ps12
Metazoa - ps13
Myriazoa - ps14
Parahoxozoa - ps15
Bilateria - ps16
Deuterostomia - ps17
Chordata - ps18
Olfactores - ps19
Vertebrata - ps20
Gnathostomata - ps21
Euteleostomi - ps22
Sarcopterygii - ps23
Tetrapoda - ps24
Amniota - ps25
Mammalia - ps26
Theria - ps27
Placentalia - ps28
Boreoeutheria - ps29
Euarchontoglires - ps30
Euarchonta - ps31
Primates - ps32
Haplorrhini - ps33
Simiiformes - ps34
Catarrhini - ps35
Hominoidea - ps36
Hominidae - ps37
Homininae - ps38
Hominini - ps39
Homo_sapiens - ps40
p-value
9e-17
5e-13
2e-09
1e-05
6e-02
Log-odds
0.5
1.0
1.5
2.0
2.5
3.0
3.5
4.0

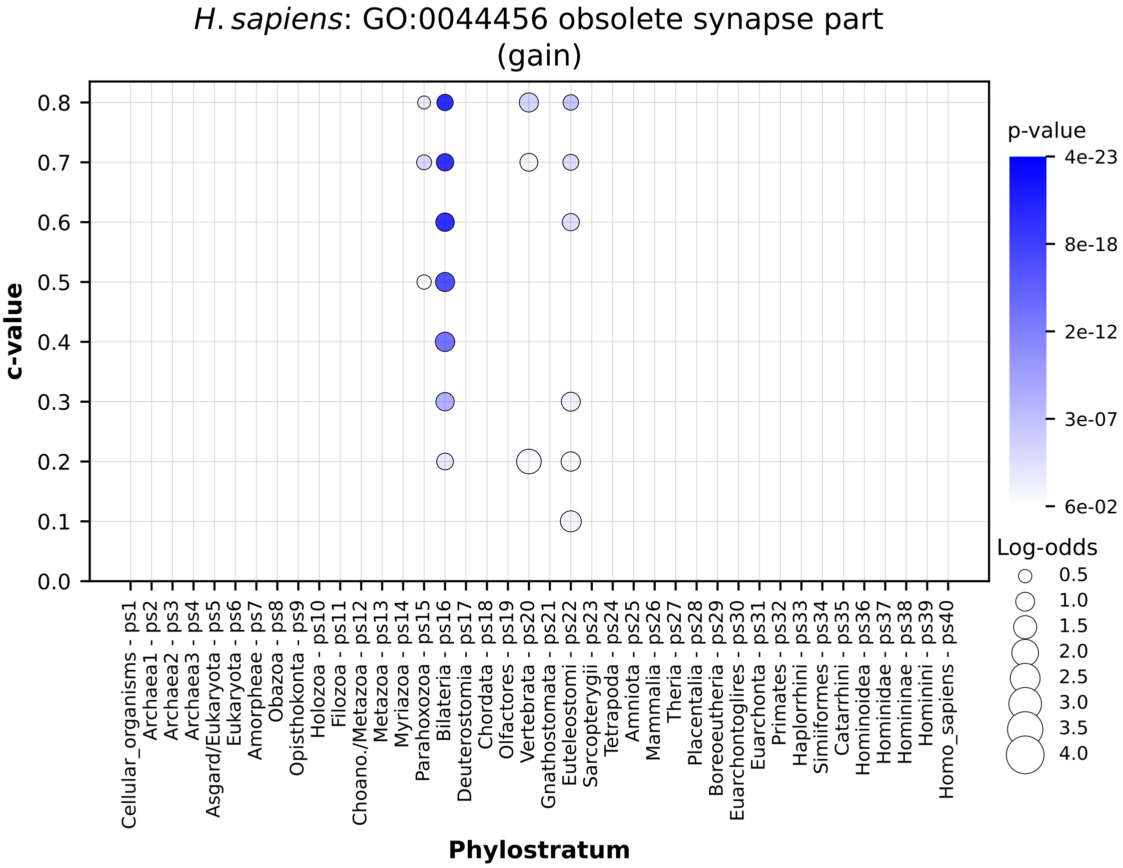
H. sapiens: GO:0044456 obsolete synapse part
(gain)
c-value
0.8
0.7
0.6
0.5
0.4
0.3
0.2
0.1
0.0
p-value
4e-23
8e-18
2e-12
3e-07
6e-02
Log-odds
0.5
1.0
1.5
2.0
2.5
3.0
3.5
4.0
Cellular_organisms - ps1
Archaea1 - ps2
Archaea2 - ps3
Archaea3 - ps4
Asgard/Eukaryota - ps5
Eukaryota - ps6
Amorpheae - ps7
Obazoa - ps8
Opisthokonta - ps9
Holozoa - ps10
Filozoa - ps11
Choano./Metazoa - ps12
Metazoa - ps13
Myriazoa - ps14
Parahoxozoa - ps15
Bilateria - ps16
Deuterostomia - ps17
Chordata - ps18
Olfactores - ps19
Vertebrata - ps20
Gnathostomata - ps21
Euteleostomi - ps22
Sarcopterygii - ps23
Tetrapoda - ps24
Amniota - ps25
Mammalia - ps26
Theria - ps27
Placentalia - ps28
Boreoeutheria - ps29
Euarchontoglires - ps30
Euarchonta - ps31
Primates - ps32
Haplorrhini - ps33
Simiiformes - ps34
Catarrhini - ps35
Hominoidea - ps36
Hominidae - ps37
Homininae - ps38
Hominini - ps39
Homo_sapiens - ps40
Phylostratum

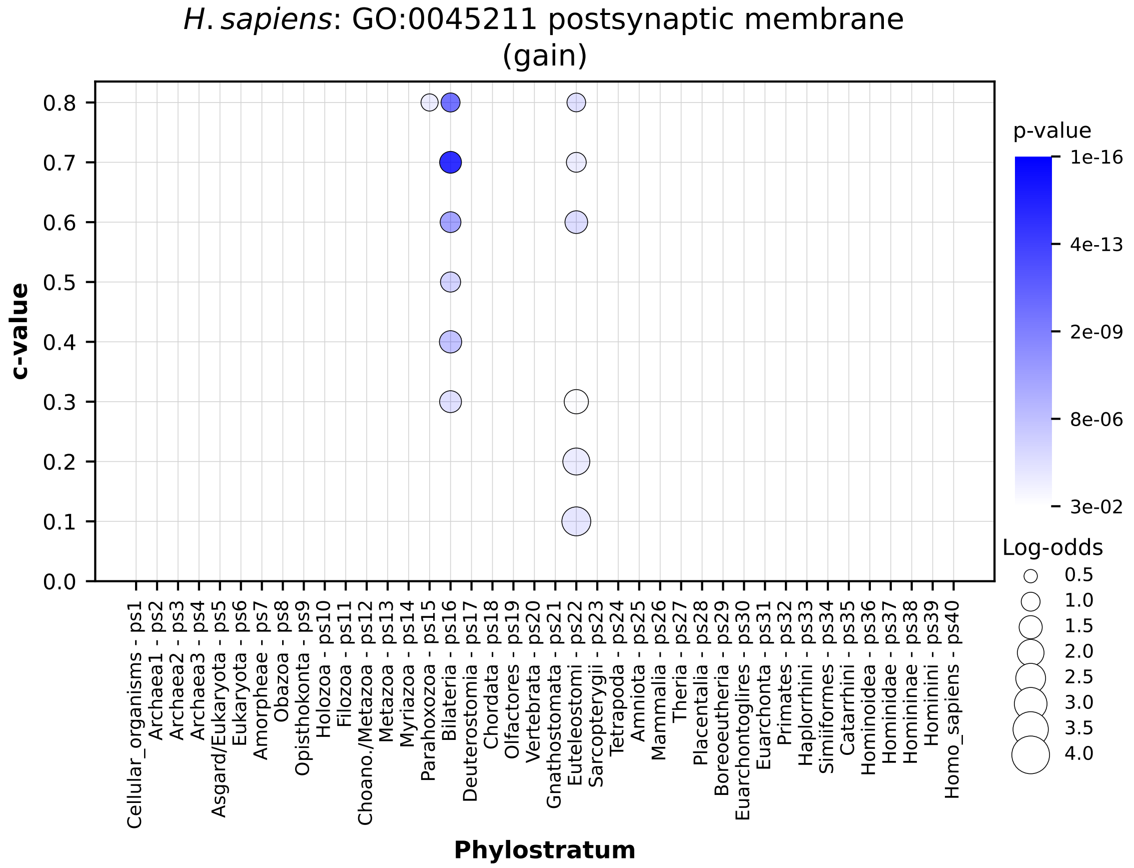

H. sapiens: GO:0045211 postsynaptic membrane
(gain)
c-value
0.0
0.1
0.2
0.3
0.4
0.5
0.6
0.7
0.8
Phylostratum
Cellular_organisms - ps1
Archaea1 - ps2
Archaea2 - ps3
Archaea3 - ps4
Asgard/Eukaryota - ps5
Eukaryota - ps6
Amorpheae - ps7
Obazoa - ps8
Opisthokonta - ps9
Holozoa - ps10
Filozoa - ps11
Choano./Metazoa - ps12
Metazoa - ps13
Myriazoa - ps14
Parahoxozoa - ps15
Bilateria - ps16
Deuterostomia - ps17
Chordata - ps18
Olfactores - ps19
Vertebrata - ps20
Gnathostomata - ps21
Euteleostomi - ps22
Sarcopterygii - ps23
Tetrapoda - ps24
Amniota - ps25
Mammalia - ps26
Theria - ps27
Placentalia - ps28
Boreoeutheria - ps29
Euarchontoglires - ps30
Euarchonta - ps31
Primates - ps32
Haplorrhini - ps33
Simiiformes - ps34
Catarrhini - ps35
Hominoidea - ps36
Hominidae - ps37
Homininae - ps38
Hominini - ps39
Homo_sapiens - ps40
p-value
1e-16
4e-13
2e-09
8e-06
3e-02
Log-odds
0.5
1.0
1.5
2.0
2.5
3.0
3.5
4.0

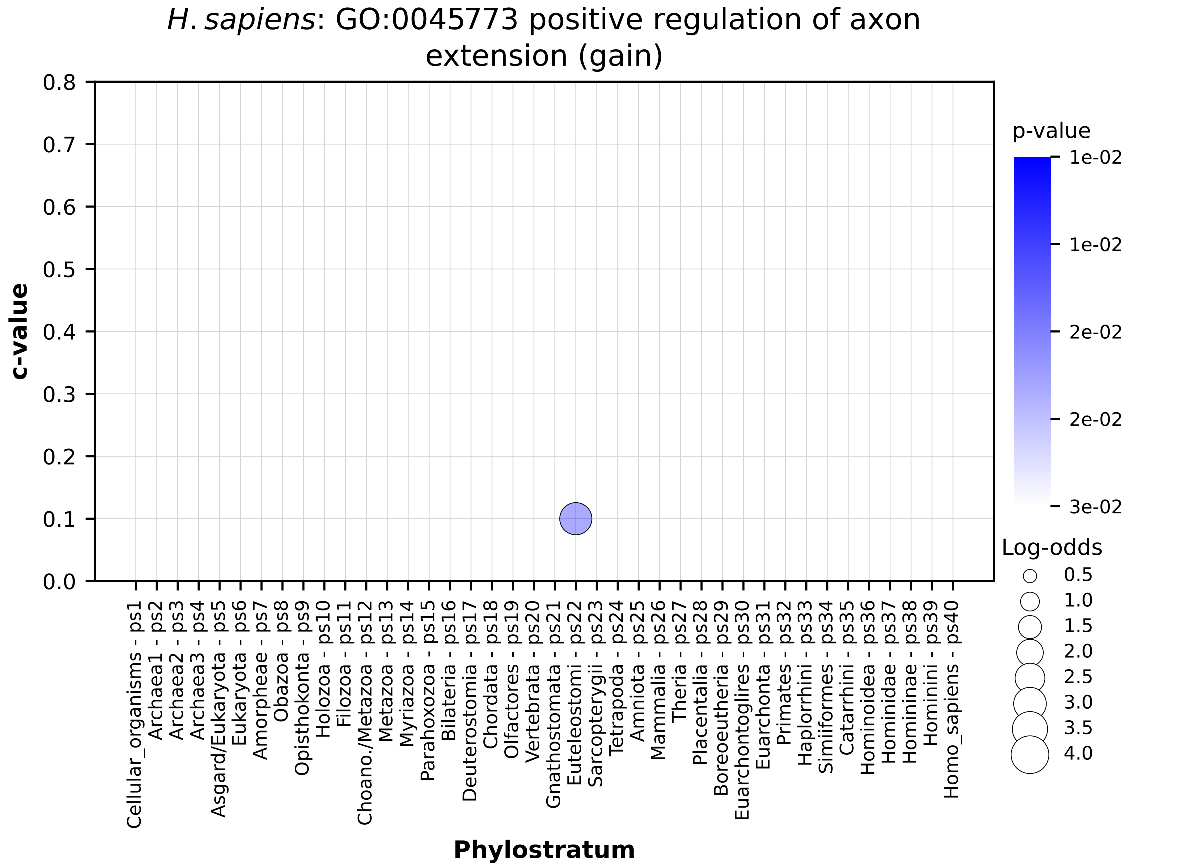

H. sapiens: GO:0045773 positive regulation of axon extension (gain)
c-value
0.0
0.1
0.2
0.3
0.4
0.5
0.6
0.7
0.8
p-value
1e-02
1e-02
2e-02
2e-02
3e-02
Log-odds
0.5
1.0
1.5
2.0
2.5
3.0
3.5
4.0
Cellular_organisms - ps1
Archaea1 - ps2
Archaea2 - ps3
Archaea3 - ps4
Asgard/Eukaryota - ps5
Eukaryota - ps6
Amorpheae - ps7
Obazoa - ps8
Opisthokonta - ps9
Holozoa - ps10
Filozoa - ps11
Choano./Metazoa - ps12
Metazoa - ps13
Myriazoa - ps14
Parahoxozoa - ps15
Bilateria - ps16
Deuterostomia - ps17
Chordata - ps18
Olfactores - ps19
Vertebrata - ps20
Gnathostomata - ps21
Euteleostomi - ps22
Sarcopterygii - ps23
Tetrapoda - ps24
Amniota - ps25
Mammalia - ps26
Theria - ps27
Placentalia - ps28
Boreoeutheria - ps29
Euarchontoglires - ps30
Euarchonta - ps31
Primates - ps32
Haplorrhini - ps33
Simiiformes - ps34
Catarrhini - ps35
Hominoidea - ps36
Hominidae - ps37
Homininae - ps38
Hominini - ps39
Homo_sapiens - ps40
Phylostratum

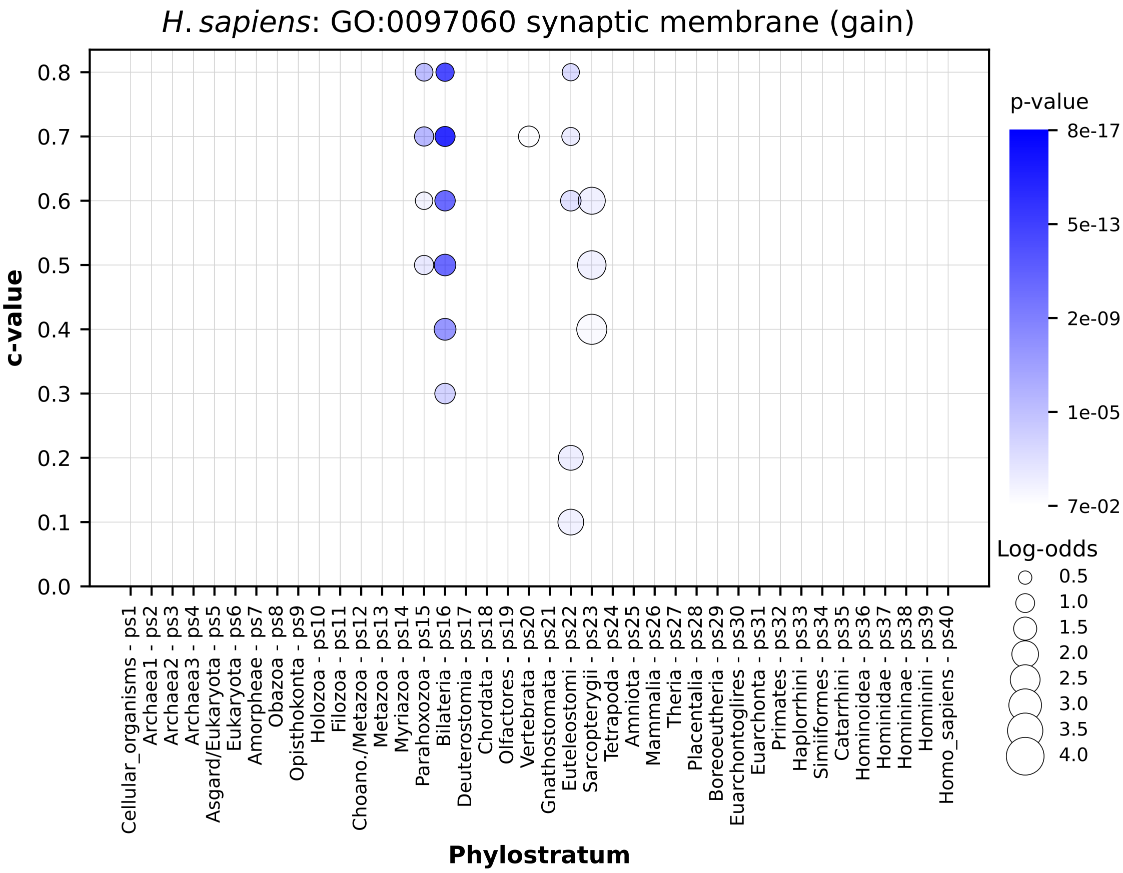
H. sapiens: GO:0097060 synaptic membrane (gain)
c-value
0.0
0.1
0.2
0.3
0.4
0.5
0.6
0.7
0.8
Phylostratum
Cellular_organisms - ps1
Archaea1 - ps2
Archaea2 - ps3
Archaea3 - ps4
Asgard/Eukaryota - ps5
Eukaryota - ps6
Amorpheae - ps7
Obazoa - ps8
Opisthokonta - ps9
Holozoa - ps10
Filozoa - ps11
Choano./Metazoa - ps12
Metazoa - ps13
Myriazoa - ps14
Parahoxozoa - ps15
Bilateria - ps16
Deuterostomia - ps17
Chordata - ps18
Olfactores - ps19
Vertebrata - ps20
Gnathostomata - ps21
Euteleostomi - ps22
Sarcopterygii - ps23
Tetrapoda - ps24
Amniota - ps25
Mammalia - ps26
Theria - ps27
Placentalia - ps28
Boreoeutheria - ps29
Euarchontoglires - ps30
Euarchonta - ps31
Primates - ps32
Haplorrhini - ps33
Simiiformes - ps34
Catarrhini - ps35
Hominoidea - ps36
Hominidae - ps37
Homininae - ps38
Hominini - ps39
Homo_sapiens - ps40
p-value
8e-17
5e-13
2e-09
1e-05
7e-02
Log-odds
0.5
1.0
1.5
2.0
2.5
3.0
3.5
4.0

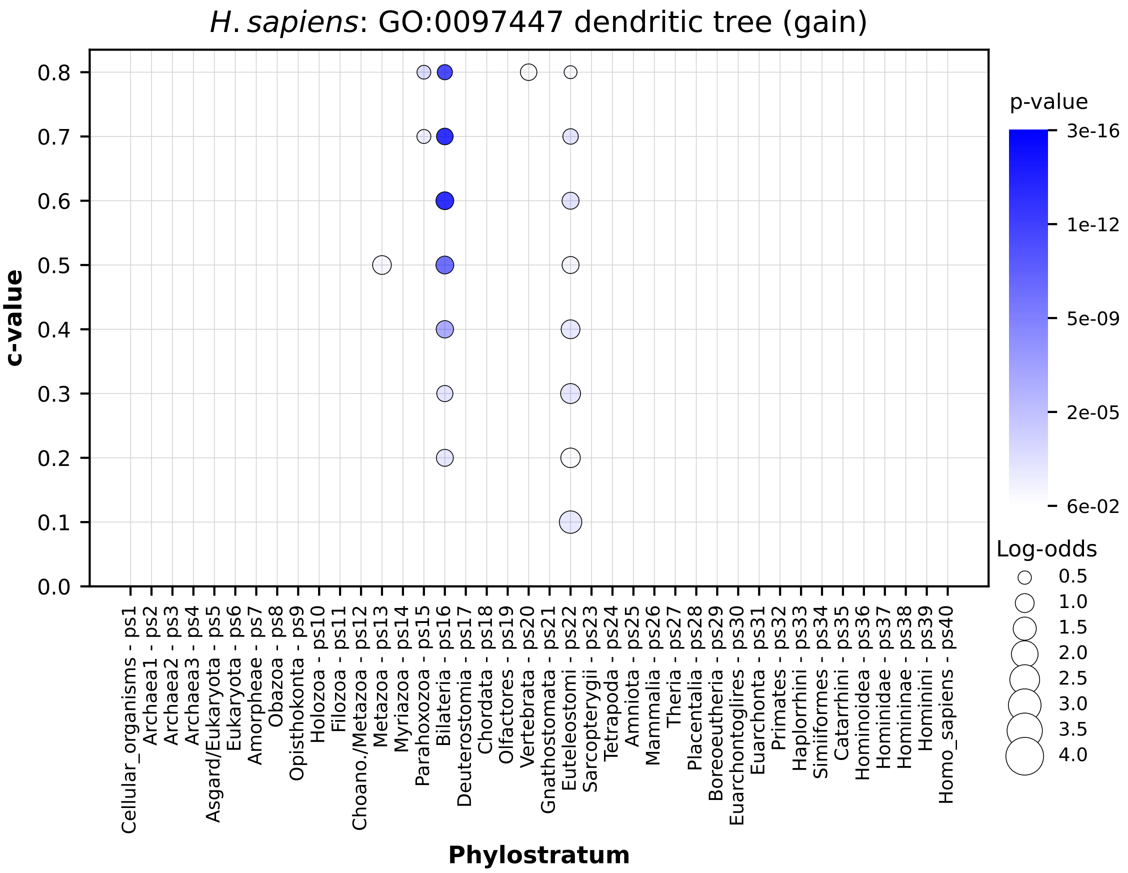

H. sapiens: GO:0097447 dendritic tree (gain)
c-value
0.0
0.1
0.2
0.3
0.4
0.5
0.6
0.7
0.8
Phylostratum
Cellular_organisms - ps1
Archaea1 - ps2
Archaea2 - ps3
Archaea3 - ps4
Asgard/Eukaryota - ps5
Eukaryota - ps6
Amorpheae - ps7
Obazoa - ps8
Opisthokonta - ps9
Holozoa - ps10
Filozoa - ps11
Choano./Metazoa - ps12
Metazoa - ps13
Myriazoa - ps14
Parahoxozoa - ps15
Bilateria - ps16
Deuterostomia - ps17
Chordata - ps18
Olfactores - ps19
Vertebrata - ps20
Gnathostomata - ps21
Euteleostomi - ps22
Sarcopterygii - ps23
Tetrapoda - ps24
Amniota - ps25
Mammalia - ps26
Theria - ps27
Placentalia - ps28
Boreoeutheria - ps29
Euarchontoglires - ps30
Euarchonta - ps31
Primates - ps32
Haplorrhini - ps33
Simiiformes - ps34
Catarrhini - ps35
Hominoidea - ps36
Hominidae - ps37
Homininae - ps38
Hominini - ps39
Homo_sapiens - ps40
p-value
3e-16
1e-12
5e-09
2e-05
6e-02
Log-odds
0.5
1.0
1.5
2.0
2.5
3.0
3.5
4.0

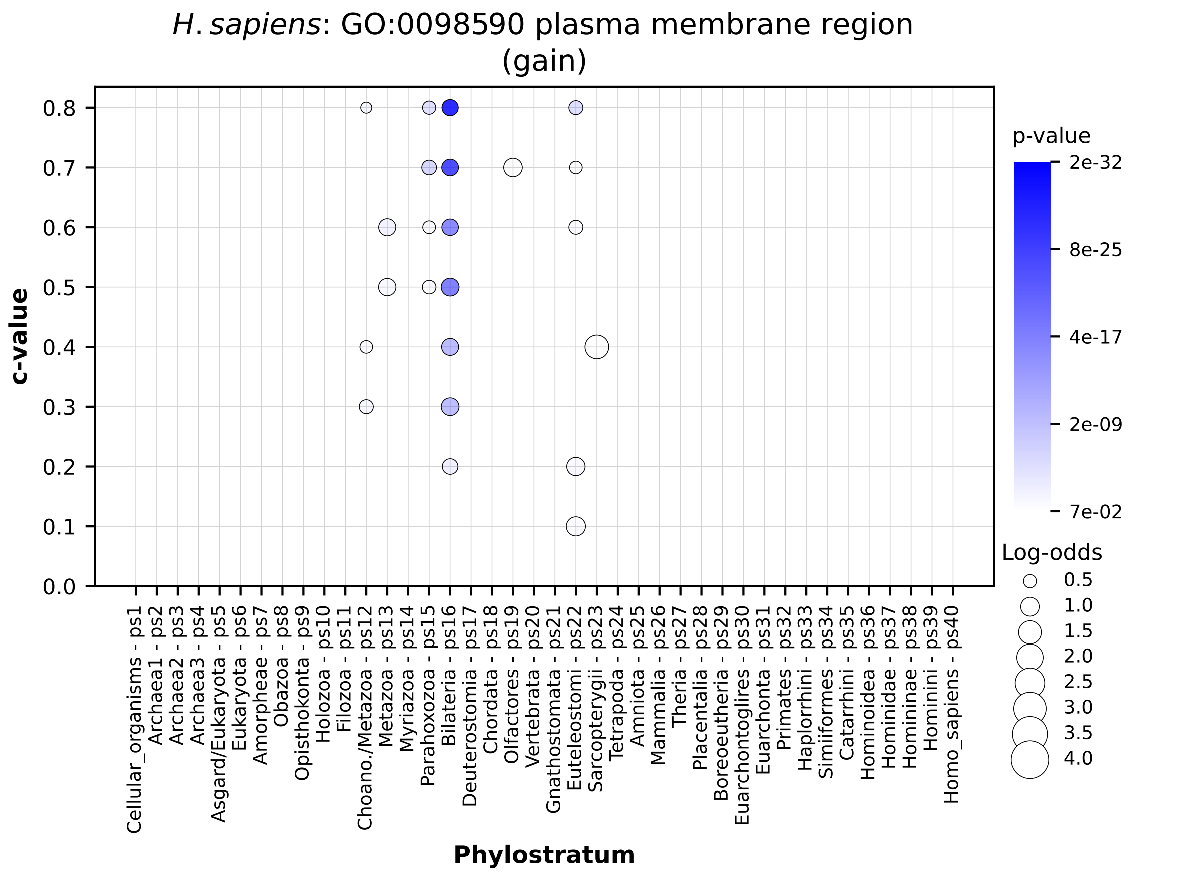

H. sapiens: GO:0098590 plasma membrane region
(gain)
c-value
0.0
0.1
0.2
0.3
0.4
0.5
0.6
0.7
0.8
Phylostratum
Cellular_organisms - ps1
Archaea1 - ps2
Archaea2 - ps3
Archaea3 - ps4
Asgard/Eukaryota - ps5
Eukaryota - ps6
Amorpheae - ps7
Obazoa - ps8
Opisthokonta - ps9
Holozoa - ps10
Filozoa - ps11
Choano./Metazoa - ps12
Metazoa - ps13
Myriazoa - ps14
Parahoxozoa - ps15
Bilateria - ps16
Deuterostomia - ps17
Chordata - ps18
Olfactores - ps19
Vertebrata - ps20
Gnathostomata - ps21
Euteleostomi - ps22
Sarcopterygii - ps23
Tetrapoda - ps24
Amniota - ps25
Mammalia - ps26
Theria - ps27
Placentalia - ps28
Boreoeutheria - ps29
Euarchontoglires - ps30
Euarchonta - ps31
Primates - ps32
Haplorrhini - ps33
Simiiformes - ps34
Catarrhini - ps35
Hominoidea - ps36
Hominidae - ps37
Homininae - ps38
Hominini - ps39
Homo_sapiens - ps40
p-value
2e-32
8e-25
4e-17
2e-09
7e-02
Log-odds
0.5
1.0
1.5
2.0
2.5
3.0
3.5
4.0

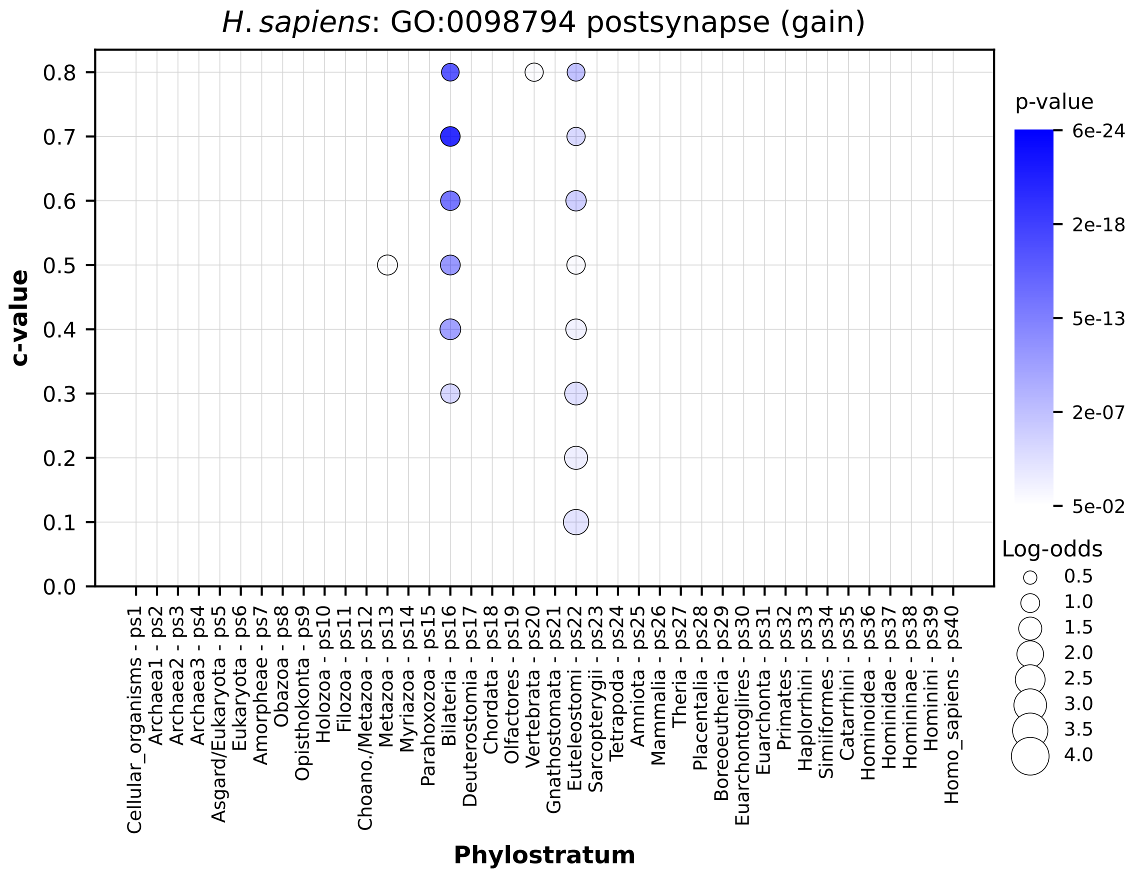

H. sapiens: GO:0098794 postsynapse (gain)
c-value
0.0
0.1
0.2
0.3
0.4
0.5
0.6
0.7
0.8
Phylostratum
Cellular_organisms - ps1
Archaea1 - ps2
Archaea2 - ps3
Archaea3 - ps4
Asgard/Eukaryota - ps5
Eukaryota - ps6
Amorpheae - ps7
Obazoa - ps8
Opisthokonta - ps9
Holozoa - ps10
Filozoa - ps11
Choano./Metazoa - ps12
Metazoa - ps13
Myriazoa - ps14
Parahoxozoa - ps15
Bilateria - ps16
Deuterostomia - ps17
Chordata - ps18
Olfactores - ps19
Vertebrata - ps20
Gnathostomata - ps21
Euteleostomi - ps22
Sarcopterygii - ps23
Tetrapoda - ps24
Amniota - ps25
Mammalia - ps26
Theria - ps27
Placentalia - ps28
Boreoeutheria - ps29
Euarchontoglires - ps30
Euarchonta - ps31
Primates - ps32
Haplorrhini - ps33
Simiiformes - ps34
Catarrhini - ps35
Hominoidea - ps36
Hominidae - ps37
Homininae - ps38
Hominini - ps39
Homo_sapiens - ps40
p-value
6e-24
2e-18
5e-13
2e-07
5e-02
Log-odds
0.5
1.0
1.5
2.0
2.5
3.0
3.5
4.0

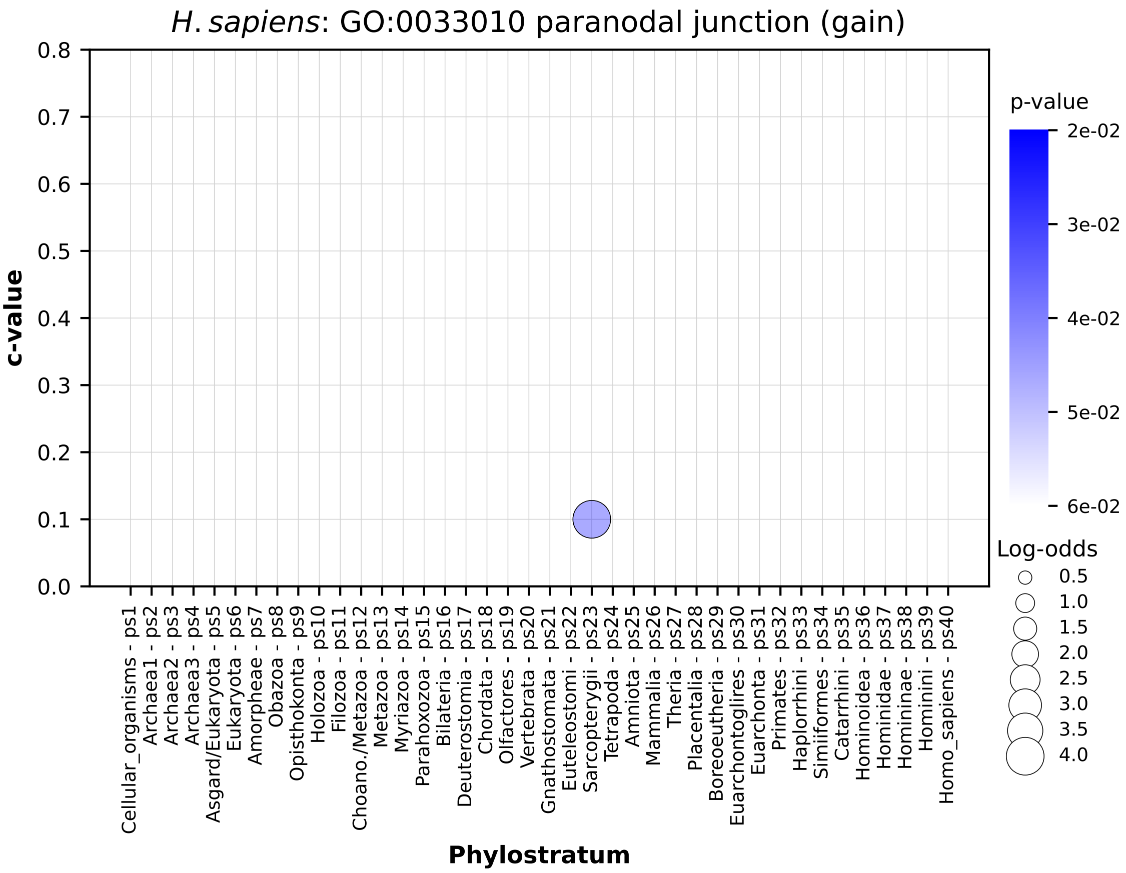

H. sapiens: GO:0033010 paranodal junction (gain)
c-value
0.8
0.7
0.6
0.5
0.4
0.3
0.2
0.1
0.0
Phylostratum
Cellular_organisms - ps1
Archaea1 - ps2
Archaea2 - ps3
Archaea3 - ps4
Asgard/Eukaryota - ps5
Eukaryota - ps6
Amorpheae - ps7
Obazoa - ps8
Opisthokonta - ps9
Holozoa - ps10
Filozoa - ps11
Choano./Metazoa - ps12
Metazoa - ps13
Myriazoa - ps14
Parahoxozoa - ps15
Bilateria - ps16
Deuterostomia - ps17
Chordata - ps18
Olfactores - ps19
Vertebrata - ps20
Gnathostomata - ps21
Euteleostomi - ps22
Sarcopterygii - ps23
Tetrapoda - ps24
Amniota - ps25
Mammalia - ps26
Theria - ps27
Placentalia - ps28
Boreoeutheria - ps29
Euarchontoglires - ps30
Euarchonta - ps31
Primates - ps32
Haplorrhini - ps33
Simiiformes - ps34
Catarrhini - ps35
Hominoidea - ps36
Hominidae - ps37
Homininae - ps38
Hominini - ps39
Homo_sapiens - ps40
p-value
2e-02
3e-02
4e-02
5e-02
6e-02
Log-odds
0.5
1.0
1.5
2.0
2.5
3.0
3.5
4.0

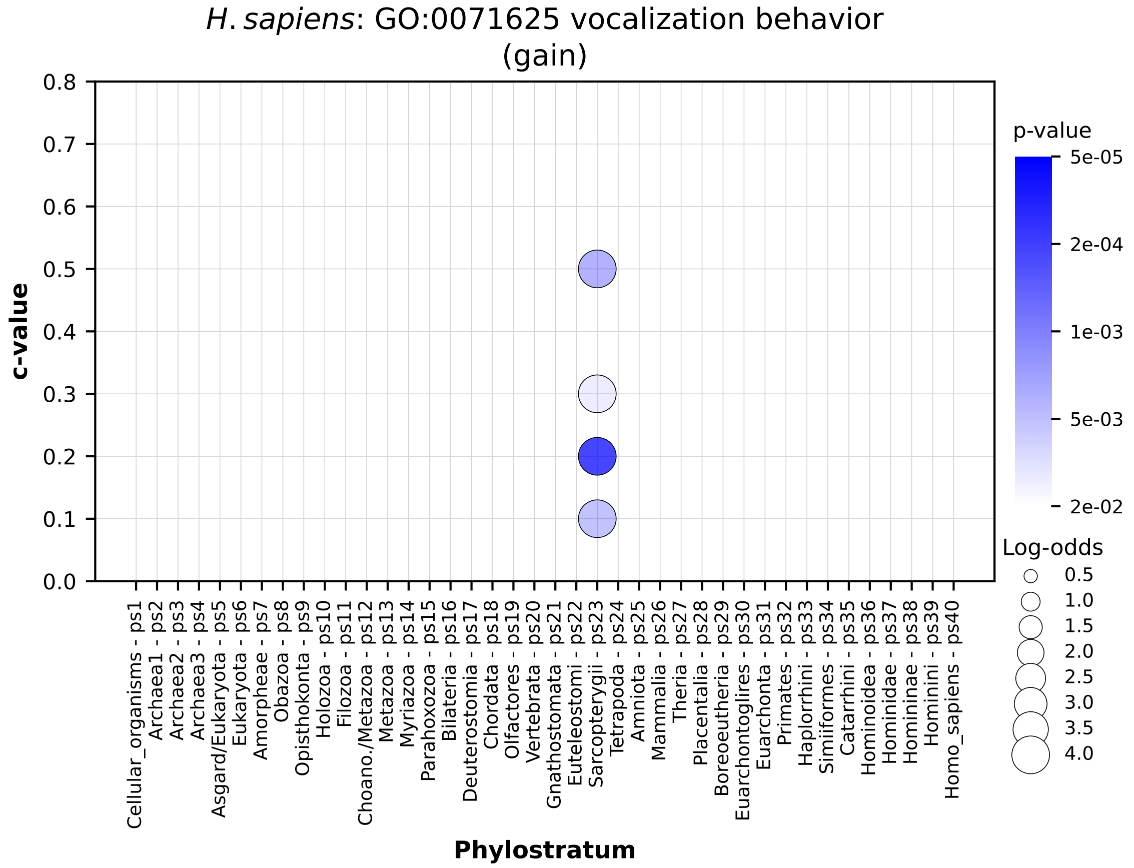

H. sapiens: GO:0071625 vocalization behavior
(gain)
c-value
0.0
0.1
0.2
0.3
0.4
0.5
0.6
0.7
0.8
Phylostratum
Cellular_organisms - ps1
Archaea1 - ps2
Archaea2 - ps3
Archaea3 - ps4
Asgard/Eukaryota - ps5
Eukaryota - ps6
Amorpheae - ps7
Obazoa - ps8
Opisthokonta - ps9
Holozoa - ps10
Filozoa - ps11
Choano./Metazoa - ps12
Metazoa - ps13
Myriazoa - ps14
Parahoxozoa - ps15
Bilateria - ps16
Deuterostomia - ps17
Chordata - ps18
Olfactores - ps19
Vertebrata - ps20
Gnathostomata - ps21
Euteleostomi - ps22
Sarcopterygii - ps23
Tetrapoda - ps24
Amniota - ps25
Mammalia - ps26
Theria - ps27
Placentalia - ps28
Boreoeutheria - ps29
Euarchontoglires - ps30
Euarchonta - ps31
Primates - ps32
Haplorrhini - ps33
Simiiformes - ps34
Catarrhini - ps35
Hominoidea - ps36
Hominidae - ps37
Homininae - ps38
Hominini - ps39
Homo_sapiens - ps40
p-value
5e-05
2e-04
1e-03
5e-03
2e-02
Log-odds
0.5
1.0
1.5
2.0
2.5
3.0
3.5
4.0

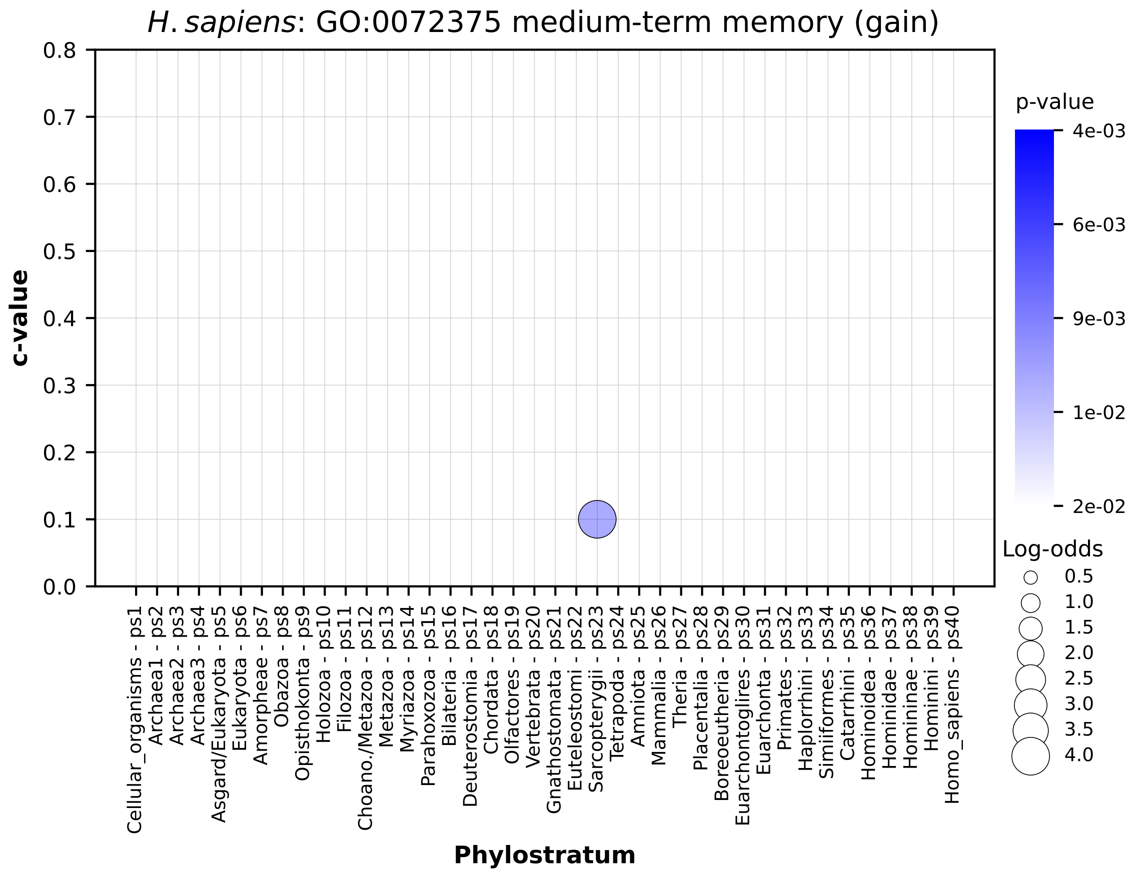

H. sapiens: GO:0072375 medium-term memory (gain)
c-value
0.8
0.7
0.6
0.5
0.4
0.3
0.2
0.1
0.0
Phylostratum
Cellular_organisms - ps1
Archaea1 - ps2
Archaea2 - ps3
Archaea3 - ps4
Asgard/Eukaryota - ps5
Eukaryota - ps6
Amorpheae - ps7
Obazoa - ps8
Opisthokonta - ps9
Holozoa - ps10
Filozoa - ps11
Choano./Metazoa - ps12
Metazoa - ps13
Myriazoa - ps14
Parahoxozoa - ps15
Bilateria - ps16
Deuterostomia - ps17
Chordata - ps18
Olfactores - ps19
Vertebrata - ps20
Gnathostomata - ps21
Euteleostomi - ps22
Sarcopterygii - ps23
Tetrapoda - ps24
Amniota - ps25
Mammalia - ps26
Theria - ps27
Placentalia - ps28
Boreoeutheria - ps29
Euarchontoglires - ps30
Euarchonta - ps31
Primates - ps32
Haplorrhini - ps33
Simiiformes - ps34
Catarrhini - ps35
Hominoidea - ps36
Hominidae - ps37
Homininae - ps38
Hominini - ps39
Homo_sapiens - ps40
p-value
4e-03
6e-03
9e-03
1e-02
2e-02
Log-odds
0.5
1.0
1.5
2.0
2.5
3.0
3.5
4.0

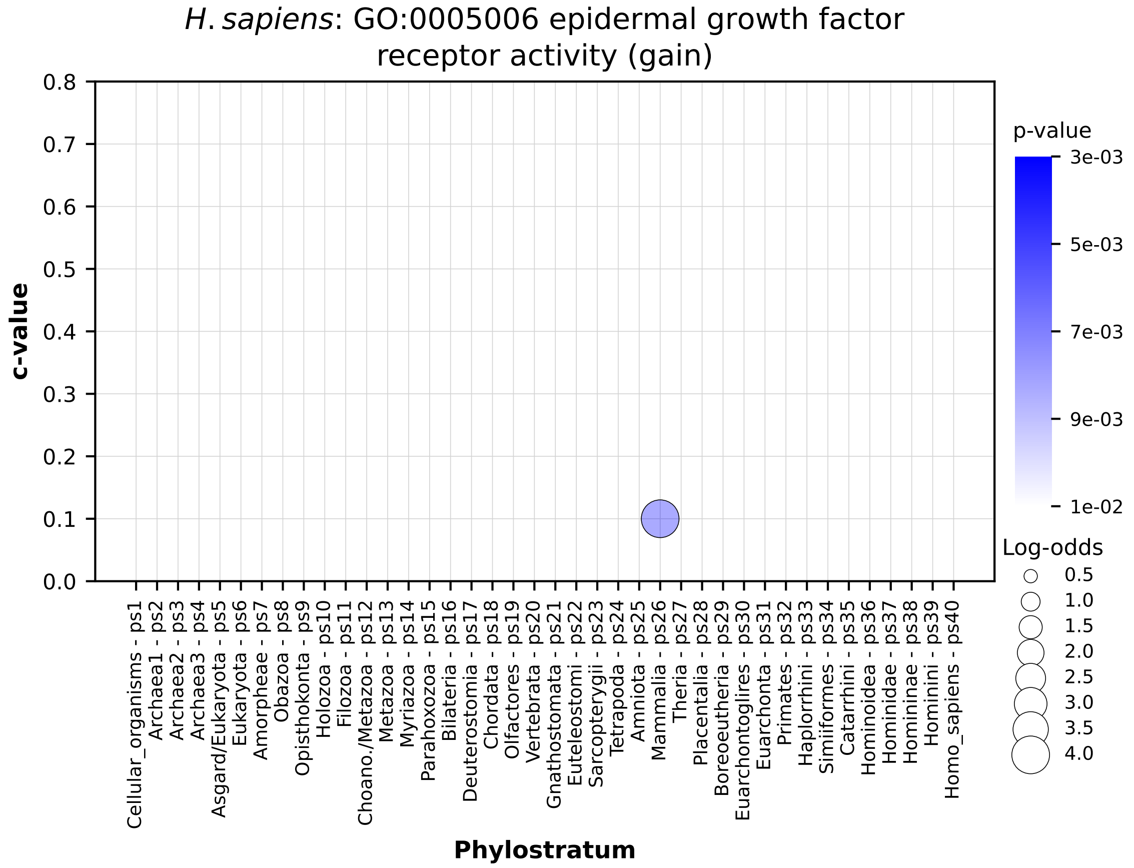

H. sapiens: GO:0005006 epidermal growth factor receptor activity (gain)
c-value
0.0
0.1
0.2
0.3
0.4
0.5
0.6
0.7
0.8
Phylostratum
Cellular_organisms - ps1
Archaea1 - ps2
Archaea2 - ps3
Archaea3 - ps4
Asgard/Eukaryota - ps5
Eukaryota - ps6
Amorpheae - ps7
Obazoa - ps8
Opisthokonta - ps9
Holozoa - ps10
Filozoa - ps11
Choano./Metazoa - ps12
Metazoa - ps13
Myriazoa - ps14
Parahoxozoa - ps15
Bilateria - ps16
Deuterostomia - ps17
Chordata - ps18
Olfactores - ps19
Vertebrata - ps20
Gnathostomata - ps21
Euteleostomi - ps22
Sarcopterygii - ps23
Tetrapoda - ps24
Amniota - ps25
Mammalia - ps26
Theria - ps27
Placentalia - ps28
Boreoeutheria - ps29
Euarchontoglires - ps30
Euarchonta - ps31
Primates - ps32
Haplorrhini - ps33
Simiiformes - ps34
Catarrhini - ps35
Hominoidea - ps36
Hominidae - ps37
Homininae - ps38
Hominini - ps39
Homo_sapiens - ps40
p-value
3e-03
5e-03
7e-03
9e-03
1e-02
Log-odds
0.5
1.0
1.5
2.0
2.5
3.0
3.5
4.0

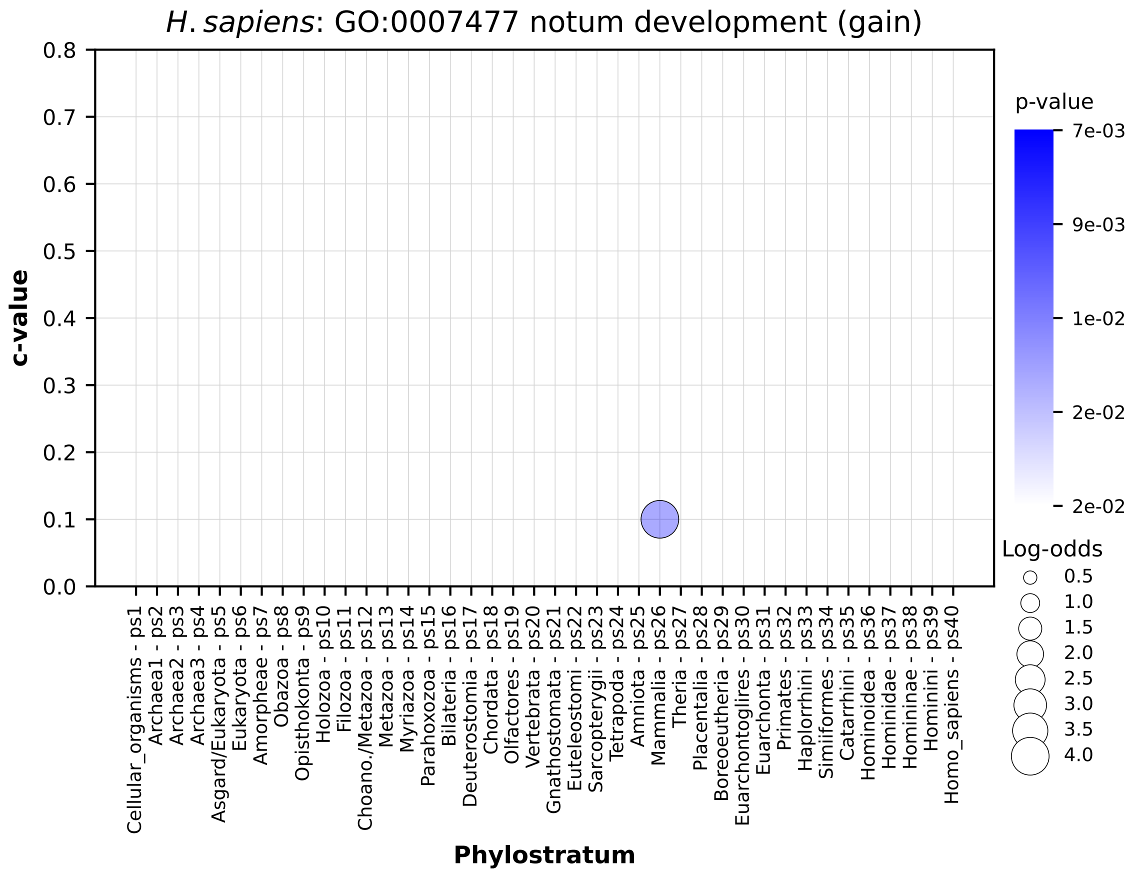

*H. sapiens*: GO:0007477 notum development (gain)
c-value
0.8
0.7
0.6
0.5
0.4
0.3
0.2
0.1
0.0
Phylostratum
Cellular_organisms - ps1
Archaea1 - ps2
Archaea2 - ps3
Archaea3 - ps4
Asgard/Eukaryota - ps5
Eukaryota - ps6
Amorpheae - ps7
Obazoa - ps8
Opisthokonta - ps9
Holozoa - ps10
Filozoa - ps11
Choano./Metazoa - ps12
Metazoa - ps13
Myriazoa - ps14
Parahoxozoa - ps15
Bilateria - ps16
Deuterostomia - ps17
Chordata - ps18
Olfactores - ps19
Vertebrata - ps20
Gnathostomata - ps21
Euteleostomi - ps22
Sarcopterygii - ps23
Tetrapoda - ps24
Amniota - ps25
Mammalia - ps26
Theria - ps27
Placentalia - ps28
Boreoeutheria - ps29
Euarchontoglires - ps30
Euarchonta - ps31
Primates - ps32
Haplorrhini - ps33
Simiiformes - ps34
Catarrhini - ps35
Hominoidea - ps36
Hominidae - ps37
Homininae - ps38
Hominini - ps39
Homo_sapiens - ps40
p-value
7e-03
9e-03
1e-02
2e-02
2e-02
Log-odds
0.5
1.0
1.5
2.0
2.5
3.0
3.5
4.0

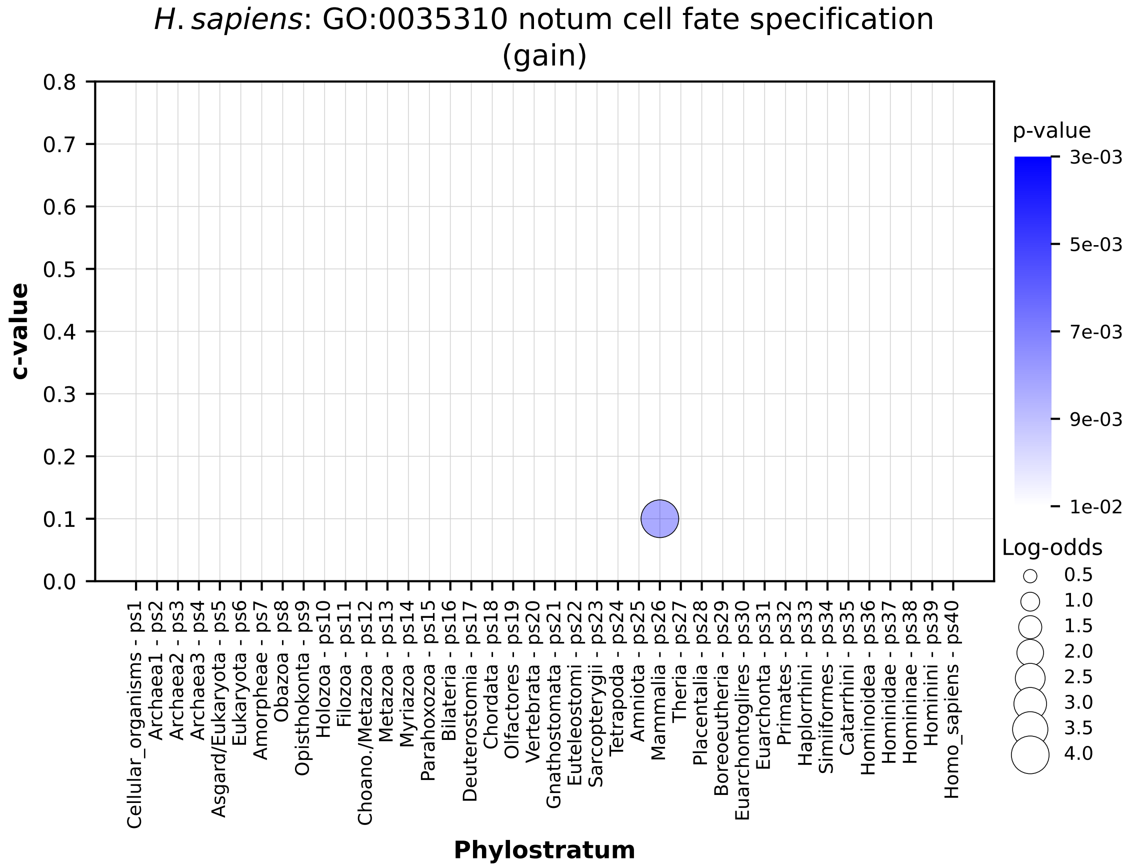

*H. sapiens*: GO:0035310 notum cell fate specification
(gain)
c-value
0.8
0.7
0.6
0.5
0.4
0.3
0.2
0.1
0.0
p-value
3e-03
5e-03
7e-03
9e-03
1e-02
Log-odds
0.5
1.0
1.5
2.0
2.5
3.0
3.5
4.0
Cellular_organisms - ps1
Archaea1 - ps2
Archaea2 - ps3
Archaea3 - ps4
Asgard/Eukaryota - ps5
Eukaryota - ps6
Amorpheae - ps7
Obazoa - ps8
Opisthokonta - ps9
Holozoa - ps10
Filozoa - ps11
Choano./Metazoa - ps12
Metazoa - ps13
Myriazoa - ps14
Parahoxozoa - ps15
Bilateria - ps16
Deuterostomia - ps17
Chordata - ps18
Olfactores - ps19
Vertebrata - ps20
Gnathostomata - ps21
Euteleostomi - ps22
Sarcopterygii - ps23
Tetrapoda - ps24
Amniota - ps25
Mammalia - ps26
Theria - ps27
Placentalia - ps28
Boreoeutheria - ps29
Euarchontoglires - ps30
Euarchonta - ps31
Primates - ps32
Haplorrhini - ps33
Simiiformes - ps34
Catarrhini - ps35
Hominoidea - ps36
Hominidae - ps37
Homininae - ps38
Hominini - ps39
Homo_sapiens - ps40
Phylostratum

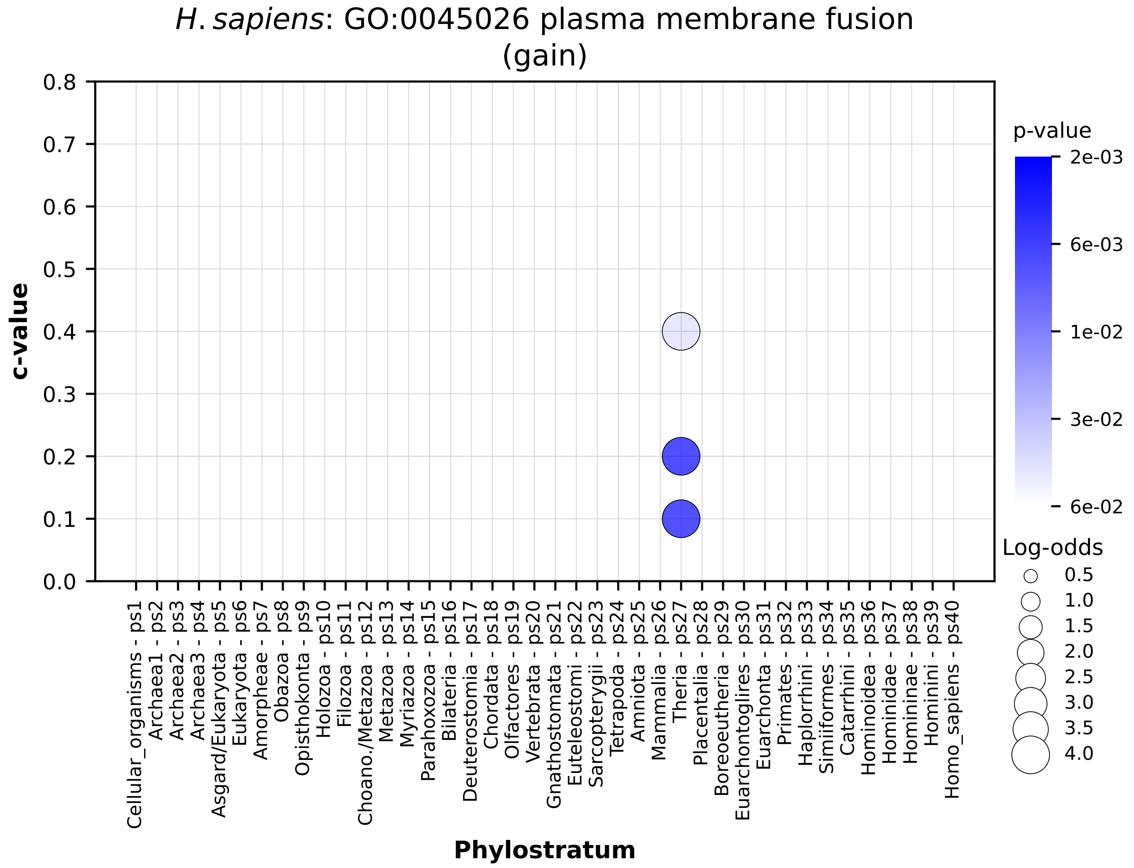
H. sapiens: GO:0045026 plasma membrane fusion
(gain)
c-value
0.0
0.1
0.2
0.3
0.4
0.5
0.6
0.7
0.8
Cellular_organisms - ps1
Archaea1 - ps2
Archaea2 - ps3
Archaea3 - ps4
Asgard/Eukaryota - ps5
Eukaryota - ps6
Amorpheae - ps7
Obazoa - ps8
Opisthokonta - ps9
Holozoa - ps10
Filozoa - ps11
Choano./Metazoa - ps12
Metazoa - ps13
Myriazoa - ps14
Parahoxozoa - ps15
Bilateria - ps16
Deuterostomia - ps17
Chordata - ps18
Olfactores - ps19
Vertebrata - ps20
Gnathostomata - ps21
Euteleostomi - ps22
Sarcopterygii - ps23
Tetrapoda - ps24
Amniota - ps25
Mammalia - ps26
Theria - ps27
Placentalia - ps28
Boreoeutheria - ps29
Euarchontoglires - ps30
Euarchonta - ps31
Primates - ps32
Haplorrhini - ps33
Simiiformes - ps34
Catarrhini - ps35
Hominoidea - ps36
Hominidae - ps37
Homininae - ps38
Hominini - ps39
Homo_sapiens - ps40
Phylostratum
p-value
2e-03
6e-03
1e-02
3e-02
6e-02
Log-odds
0.5
1.0
1.5
2.0
2.5
3.0
3.5
4.0

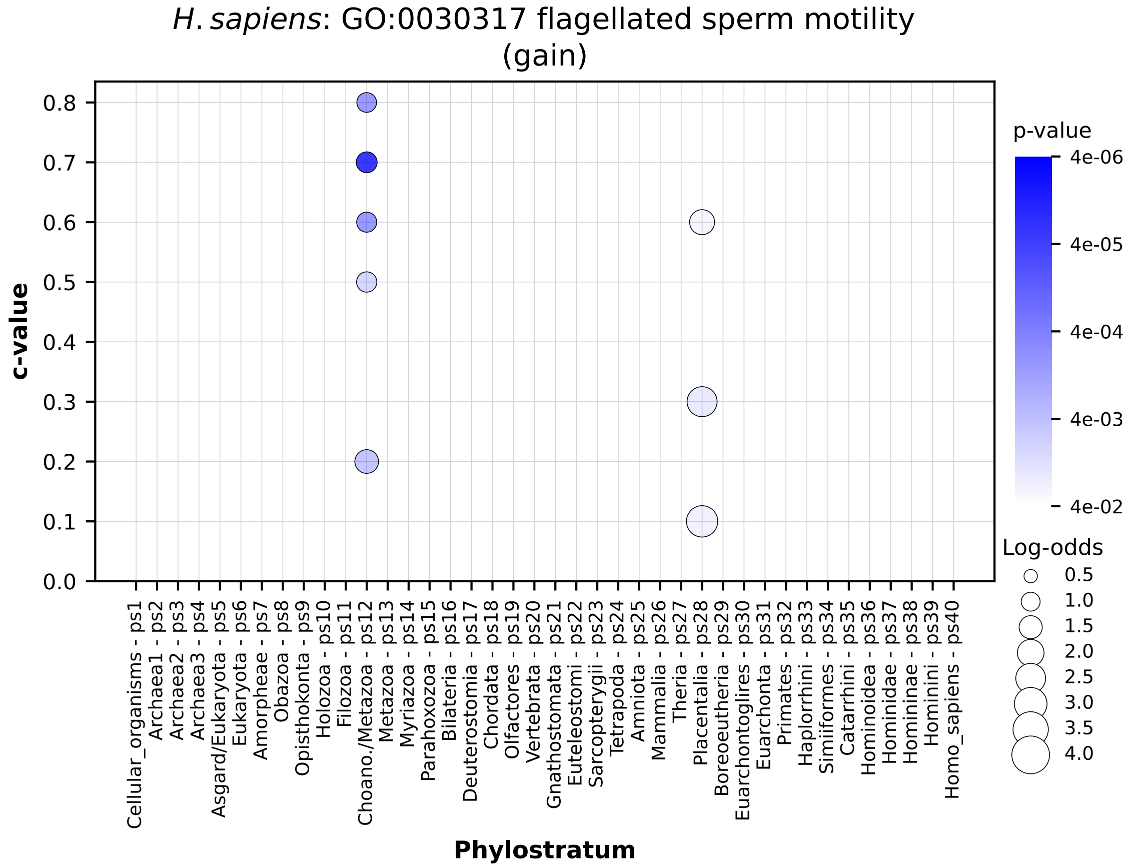

H. sapiens: GO:0030317 flagellated sperm motility
(gain)
c-value
0.0
0.1
0.2
0.3
0.4
0.5
0.6
0.7
0.8
Phylostratum
Cellular_organisms - ps1
Archaea1 - ps2
Archaea2 - ps3
Archaea3 - ps4
Asgard/Eukaryota - ps5
Eukaryota - ps6
Amorpheae - ps7
Obazoa - ps8
Opisthokonta - ps9
Holozoa - ps10
Filozoa - ps11
Choano./Metazoa - ps12
Metazoa - ps13
Myriazoa - ps14
Parahoxozoa - ps15
Bilateria - ps16
Deuterostomia - ps17
Chordata - ps18
Olfactores - ps19
Vertebrata - ps20
Gnathostomata - ps21
Euteleostomi - ps22
Sarcopterygii - ps23
Tetrapoda - ps24
Amniota - ps25
Mammalia - ps26
Theria - ps27
Placentalia - ps28
Boreoeutheria - ps29
Euarchontoglires - ps30
Euarchonta - ps31
Primates - ps32
Haplorrhini - ps33
Simiiformes - ps34
Catarrhini - ps35
Hominoidea - ps36
Hominidae - ps37
Homininae - ps38
Hominini - ps39
Homo_sapiens - ps40
p-value
4e-06
4e-05
4e-04
4e-03
4e-02
Log-odds
0.5
1.0
1.5
2.0
2.5
3.0
3.5
4.0

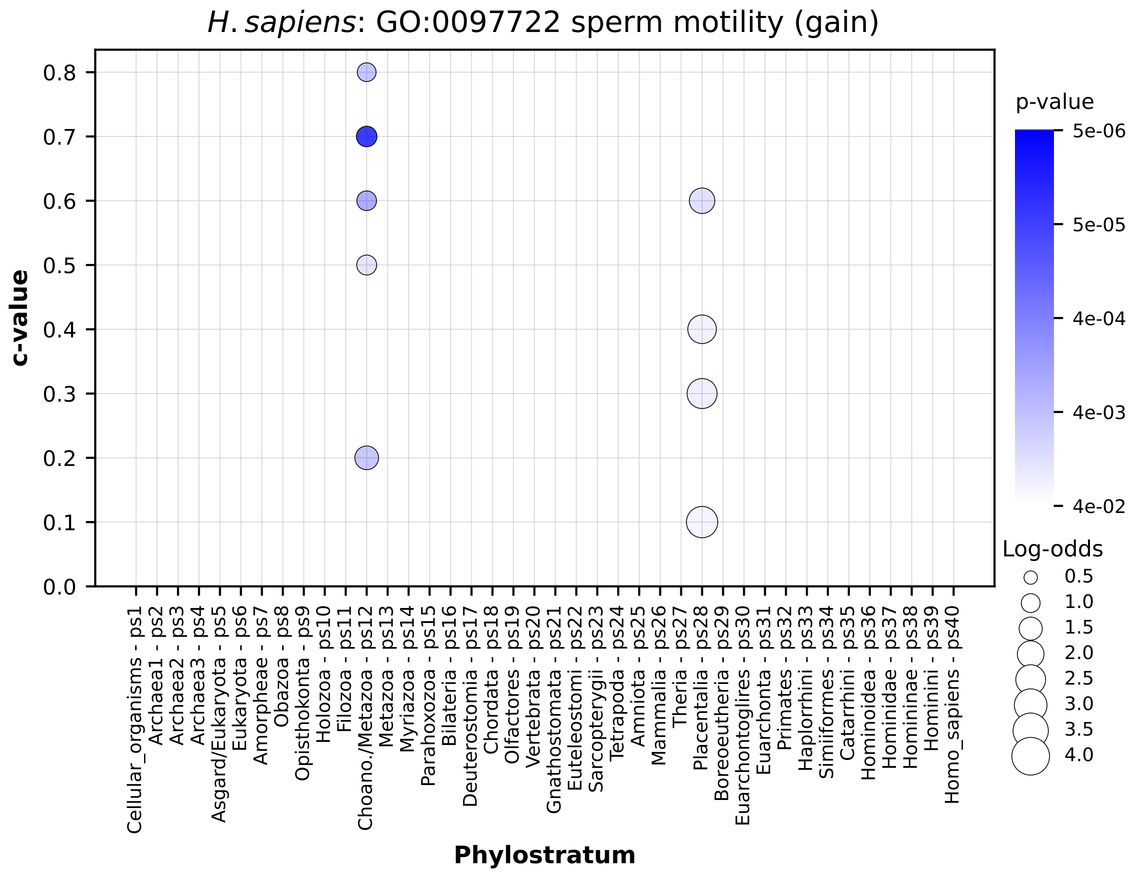

H. sapiens: GO:0097722 sperm motility (gain)
c-value
0.0
0.1
0.2
0.3
0.4
0.5
0.6
0.7
0.8
Phylostratum
Cellular_organisms - ps1
Archaea1 - ps2
Archaea2 - ps3
Archaea3 - ps4
Asgard/Eukaryota - ps5
Eukaryota - ps6
Amorpheae - ps7
Obazoa - ps8
Opisthokonta - ps9
Holozoa - ps10
Filozoa - ps11
Choano./Metazoa - ps12
Metazoa - ps13
Myriazoa - ps14
Parahoxozoa - ps15
Bilateria - ps16
Deuterostomia - ps17
Chordata - ps18
Olfactores - ps19
Vertebrata - ps20
Gnathostomata - ps21
Euteleostomi - ps22
Sarcopterygii - ps23
Tetrapoda - ps24
Amniota - ps25
Mammalia - ps26
Theria - ps27
Placentalia - ps28
Boreoeutheria - ps29
Euarchontoglires - ps30
Euarchonta - ps31
Primates - ps32
Haplorrhini - ps33
Simiiformes - ps34
Catarrhini - ps35
Hominoidea - ps36
Hominidae - ps37
Homininae - ps38
Hominini - ps39
Homo_sapiens - ps40
p-value
5e-06
5e-05
4e-04
4e-03
4e-02
Log-odds
0.5
1.0
1.5
2.0
2.5
3.0
3.5
4.0

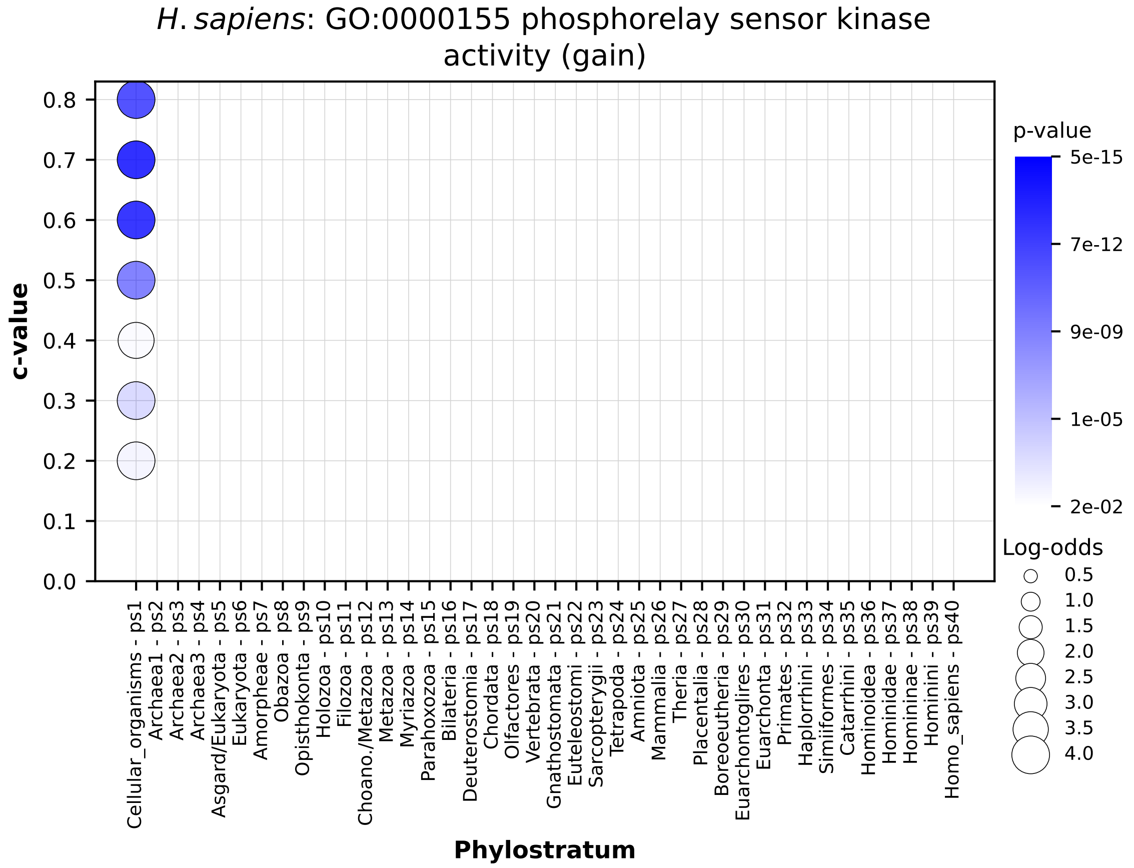

H. sapiens: GO:0000155 phosphorelay sensor kinase activity (gain)
c-value
0.0
0.1
0.2
0.3
0.4
0.5
0.6
0.7
0.8
Phylostratum
Cellular_organisms - ps1
Archaea1 - ps2
Archaea2 - ps3
Archaea3 - ps4
Asgard/Eukaryota - ps5
Eukaryota - ps6
Amorpheae - ps7
Obazoa - ps8
Opisthokonta - ps9
Holozoa - ps10
Filozoa - ps11
Choano./Metazoa - ps12
Metazoa - ps13
Myriazoa - ps14
Parahoxozoa - ps15
Bilateria - ps16
Deuterostomia - ps17
Chordata - ps18
Olfactores - ps19
Vertebrata - ps20
Gnathostomata - ps21
Euteleostomi - ps22
Sarcopterygii - ps23
Tetrapoda - ps24
Amniota - ps25
Mammalia - ps26
Theria - ps27
Placentalia - ps28
Boreoeutheria - ps29
Euarchontoglires - ps30
Euarchonta - ps31
Primates - ps32
Haplorrhini - ps33
Simiiformes - ps34
Catarrhini - ps35
Hominoidea - ps36
Hominidae - ps37
Homininae - ps38
Hominini - ps39
Homo_sapiens - ps40
p-value
5e-15
7e-12
9e-09
1e-05
2e-02
Log-odds
0.5
1.0
1.5
2.0
2.5
3.0
3.5
4.0

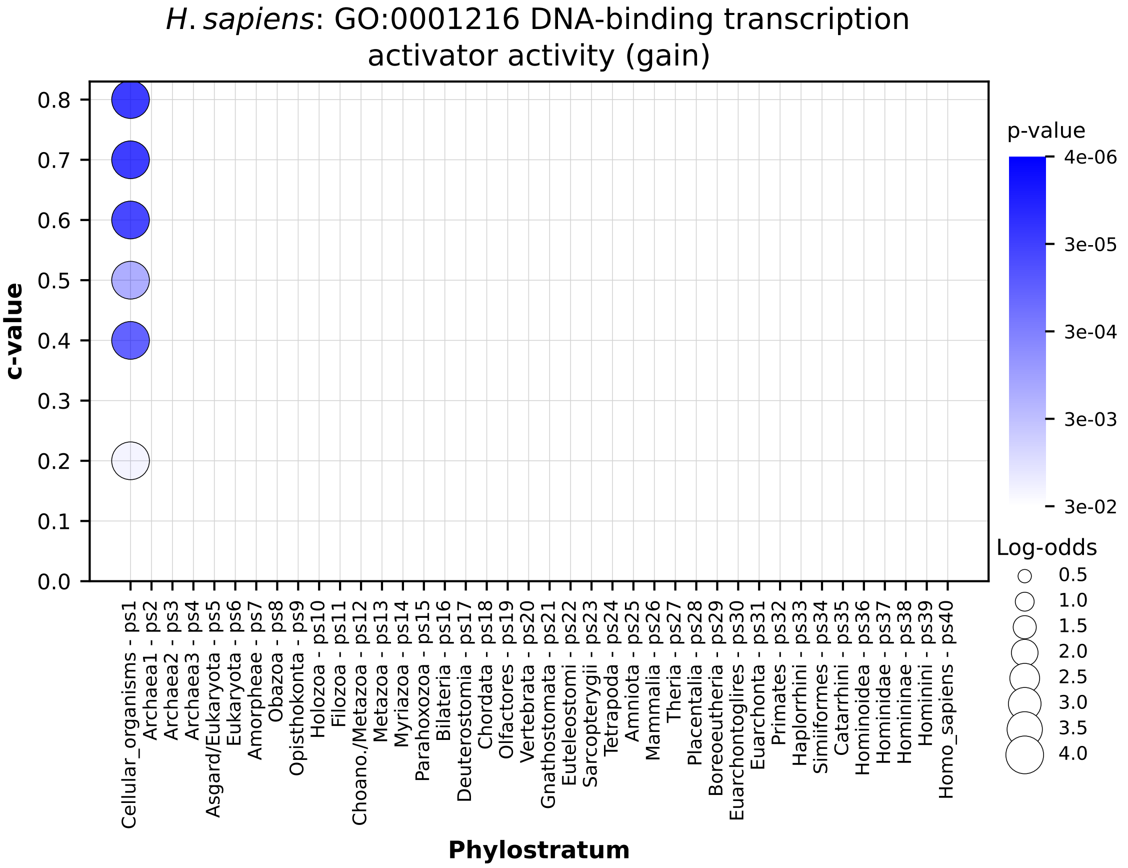

H. sapiens: GO:0001216 DNA-binding transcription activator activity (gain)
c-value
0.0
0.1
0.2
0.3
0.4
0.5
0.6
0.7
0.8
Phylostratum
Cellular_organisms - ps1
Archaea1 - ps2
Archaea2 - ps3
Archaea3 - ps4
Asgard/Eukaryota - ps5
Eukaryota - ps6
Amorpheae - ps7
Obazoa - ps8
Opisthokonta - ps9
Holozoa - ps10
Filozoa - ps11
Choano./Metazoa - ps12
Metazoa - ps13
Myriazoa - ps14
Parahoxozoa - ps15
Bilateria - ps16
Deuterostomia - ps17
Chordata - ps18
Olfactores - ps19
Vertebrata - ps20
Gnathostomata - ps21
Euteleostomi - ps22
Sarcopterygii - ps23
Tetrapoda - ps24
Amniota - ps25
Mammalia - ps26
Theria - ps27
Placentalia - ps28
Boreoeutheria - ps29
Euarchontoglires - ps30
Euarchonta - ps31
Primates - ps32
Haplorrhini - ps33
Simiiformes - ps34
Catarrhini - ps35
Hominoidea - ps36
Hominidae - ps37
Homininae - ps38
Hominini - ps39
Homo_sapiens - ps40
p-value
4e-06
3e-05
3e-04
3e-03
3e-02
Log-odds
0.5
1.0
1.5
2.0
2.5
3.0
3.5
4.0

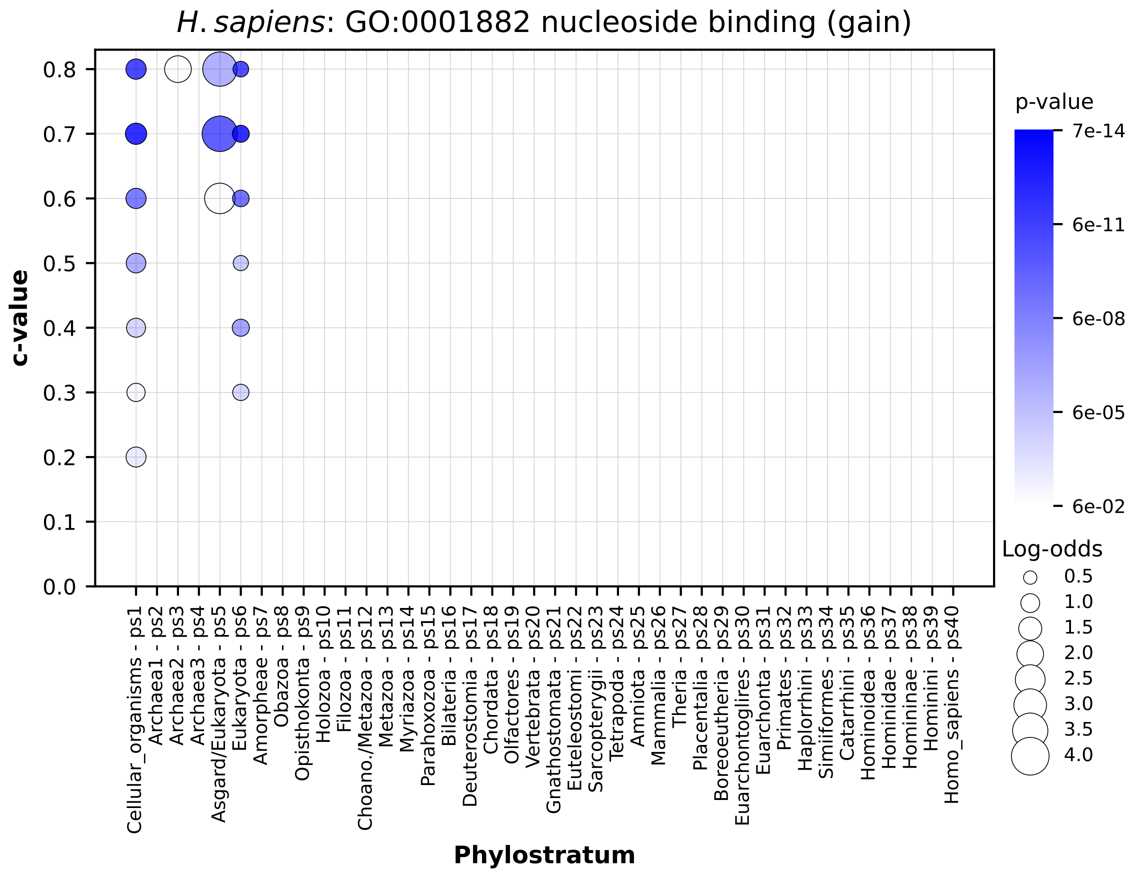

H. sapiens: GO:0001882 nucleoside binding (gain)
c-value
0.8
0.7
0.6
0.5
0.4
0.3
0.2
0.1
0.0
Phylostratum
Cellular_organisms - ps1
Archaea1 - ps2
Archaea2 - ps3
Archaea3 - ps4
Asgard/Eukaryota - ps5
Eukaryota - ps6
Amorpheae - ps7
Obazoa - ps8
Opisthokonta - ps9
Holozoa - ps10
Filozoa - ps11
Choano./Metazoa - ps12
Metazoa - ps13
Myriazoa - ps14
Parahoxozoa - ps15
Bilateria - ps16
Deuterostomia - ps17
Chordata - ps18
Olfactores - ps19
Vertebrata - ps20
Gnathostomata - ps21
Euteleostomi - ps22
Sarcopterygii - ps23
Tetrapoda - ps24
Amniota - ps25
Mammalia - ps26
Theria - ps27
Placentalia - ps28
Boreoeutheria - ps29
Euarchontoglires - ps30
Euarchonta - ps31
Primates - ps32
Haplorrhini - ps33
Simiiformes - ps34
Catarrhini - ps35
Hominoidea - ps36
Hominidae - ps37
Homininae - ps38
Hominini - ps39
Homo_sapiens - ps40
p-value
7e-14
6e-11
6e-08
6e-05
6e-02
Log-odds
0.5
1.0
1.5
2.0
2.5
3.0
3.5
4.0

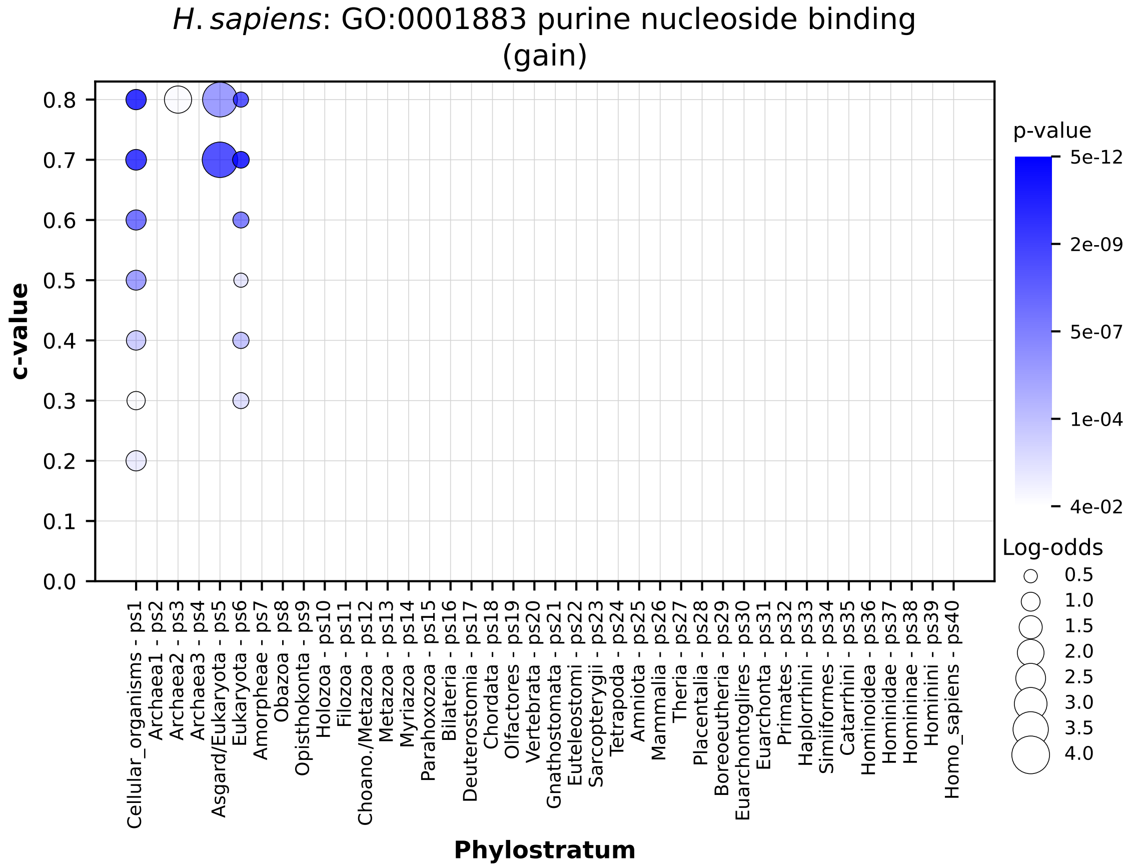

H. sapiens: GO:0001883 purine nucleoside binding
(gain)
c-value
0.8
0.7
0.6
0.5
0.4
0.3
0.2
0.1
0.0
Phylostratum
Cellular_organisms - ps1
Archaea1 - ps2
Archaea2 - ps3
Archaea3 - ps4
Asgard/Eukaryota - ps5
Eukaryota - ps6
Amorpheae - ps7
Obazoa - ps8
Opisthokonta - ps9
Holozoa - ps10
Filozoa - ps11
Choano./Metazoa - ps12
Metazoa - ps13
Myriazoa - ps14
Parahoxozoa - ps15
Bilateria - ps16
Deuterostomia - ps17
Chordata - ps18
Olfactores - ps19
Vertebrata - ps20
Gnathostomata - ps21
Euteleostomi - ps22
Sarcopterygii - ps23
Tetrapoda - ps24
Amniota - ps25
Mammalia - ps26
Theria - ps27
Placentalia - ps28
Boreoeutheria - ps29
Euarchontoglires - ps30
Euarchonta - ps31
Primates - ps32
Haplorrhini - ps33
Simiiformes - ps34
Catarrhini - ps35
Hominoidea - ps36
Hominidae - ps37
Homininae - ps38
Hominini - ps39
Homo_sapiens - ps40
p-value
5e-12
2e-09
5e-07
1e-04
4e-02
Log-odds
0.5
1.0
1.5
2.0
2.5
3.0
3.5
4.0

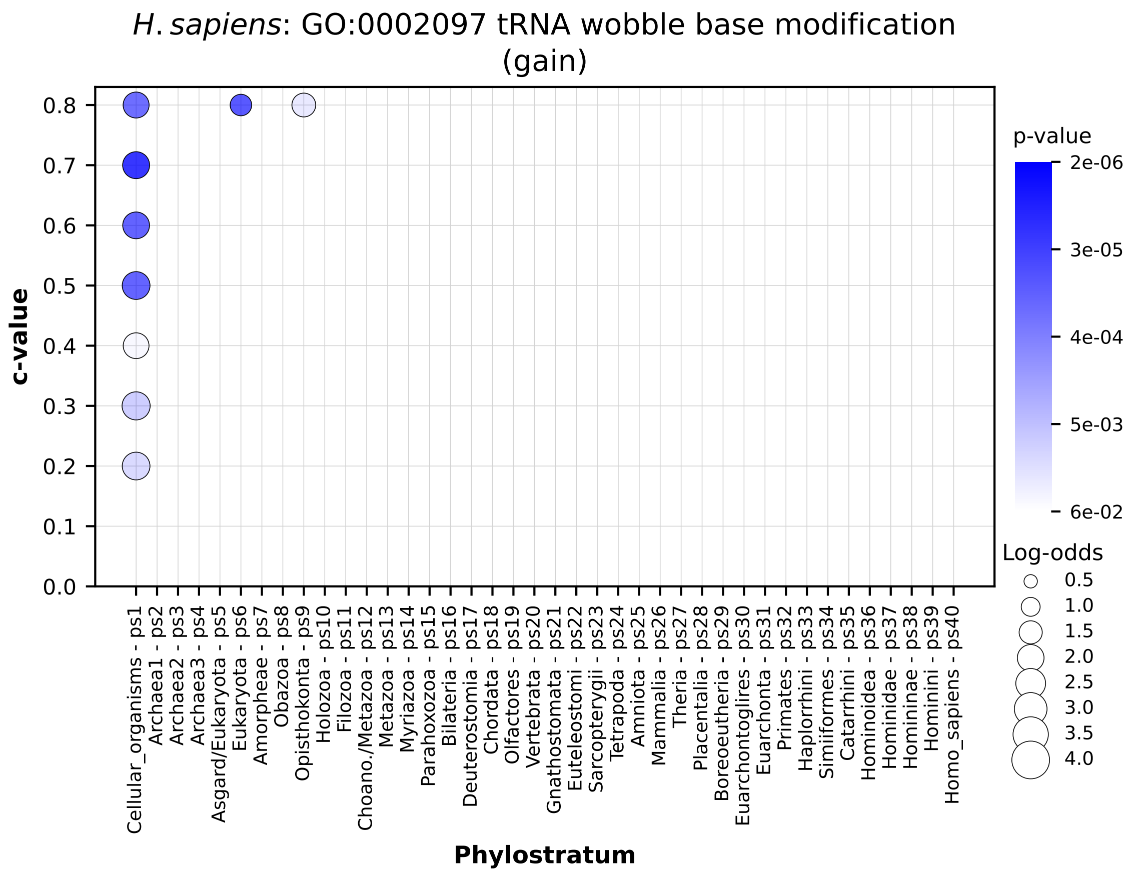

H. sapiens: GO:0002097 tRNA wobble base modification (gain)
c-value
0.0
0.1
0.2
0.3
0.4
0.5
0.6
0.7
0.8
Phylostratum
Cellular_organisms - ps1
Archaea1 - ps2
Archaea2 - ps3
Archaea3 - ps4
Asgard/Eukaryota - ps5
Eukaryota - ps6
Amorpheae - ps7
Obazoa - ps8
Opisthokonta - ps9
Holozoa - ps10
Filozoa - ps11
Choano./Metazoa - ps12
Metazoa - ps13
Myriazoa - ps14
Parahoxozoa - ps15
Bilateria - ps16
Deuterostomia - ps17
Chordata - ps18
Olfactores - ps19
Vertebrata - ps20
Gnathostomata - ps21
Euteleostomi - ps22
Sarcopterygii - ps23
Tetrapoda - ps24
Amniota - ps25
Mammalia - ps26
Theria - ps27
Placentalia - ps28
Boreoeutheria - ps29
Euarchontoglires - ps30
Euarchonta - ps31
Primates - ps32
Haplorrhini - ps33
Simiiformes - ps34
Catarrhini - ps35
Hominoidea - ps36
Hominidae - ps37
Homininae - ps38
Hominini - ps39
Homo_sapiens - ps40
p-value
2e-06
3e-05
4e-04
5e-03
6e-02
Log-odds
0.5
1.0
1.5
2.0
2.5
3.0
3.5
4.0

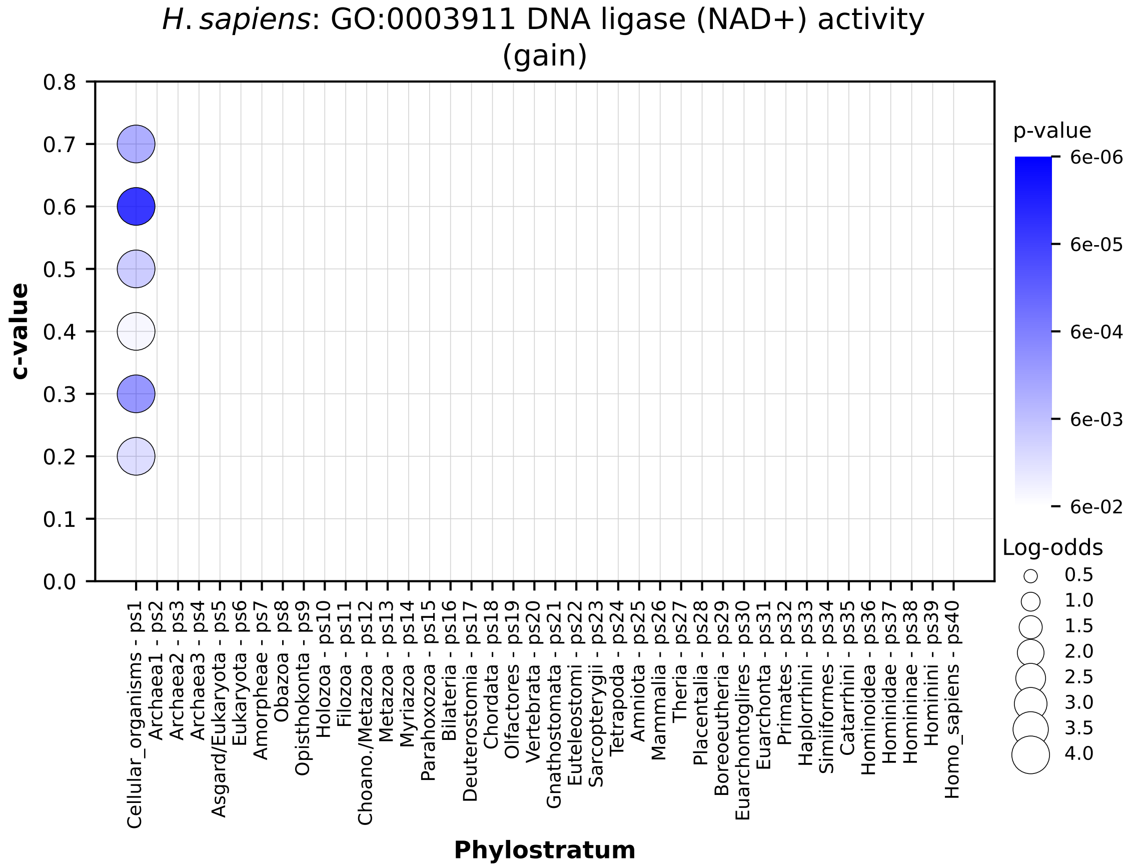

*H. sapiens*: GO:0003911 DNA ligase (NAD+) activity (gain)
c-value
0.0
0.1
0.2
0.3
0.4
0.5
0.6
0.7
0.8
Phylostratum
Cellular_organisms - ps1
Archaea1 - ps2
Archaea2 - ps3
Archaea3 - ps4
Asgard/Eukaryota - ps5
Eukaryota - ps6
Amorpheae - ps7
Obazoa - ps8
Opisthokonta - ps9
Holozoa - ps10
Filozoa - ps11
Choano./Metazoa - ps12
Metazoa - ps13
Myriazoa - ps14
Parahoxozoa - ps15
Bilateria - ps16
Deuterostomia - ps17
Chordata - ps18
Olfactores - ps19
Vertebrata - ps20
Gnathostomata - ps21
Euteleostomi - ps22
Sarcopterygii - ps23
Tetrapoda - ps24
Amniota - ps25
Mammalia - ps26
Theria - ps27
Placentalia - ps28
Boreoeutheria - ps29
Euarchontoglires - ps30
Euarchonta - ps31
Primates - ps32
Haplorrhini - ps33
Simiiformes - ps34
Catarrhini - ps35
Hominoidea - ps36
Hominidae - ps37
Homininae - ps38
Hominini - ps39
Homo_sapiens - ps40
p-value
6e-06
6e-05
6e-04
6e-03
6e-02
Log-odds
0.5
1.0
1.5
2.0
2.5
3.0
3.5
4.0

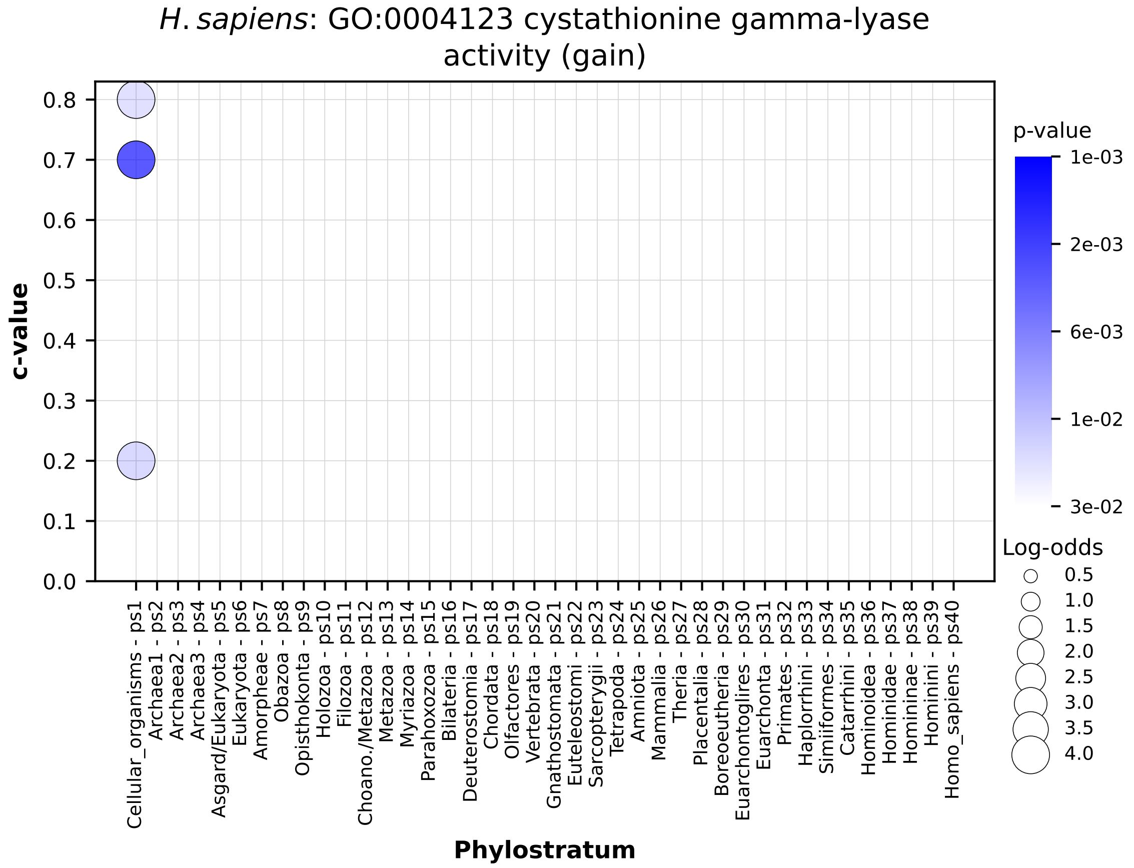
H. sapiens: GO:0004123 cystathionine gamma-lyase activity (gain)
c-value
0.0
0.1
0.2
0.3
0.4
0.5
0.6
0.7
0.8
Phylostratum
Cellular_organisms - ps1
Archaea1 - ps2
Archaea2 - ps3
Archaea3 - ps4
Asgard/Eukaryota - ps5
Eukaryota - ps6
Amorpheae - ps7
Obazoa - ps8
Opisthokonta - ps9
Holozoa - ps10
Filozoa - ps11
Choano./Metazoa - ps12
Metazoa - ps13
Myriazoa - ps14
Parahoxozoa - ps15
Bilateria - ps16
Deuterostomia - ps17
Chordata - ps18
Olfactores - ps19
Vertebrata - ps20
Gnathostomata - ps21
Euteleostomi - ps22
Sarcopterygii - ps23
Tetrapoda - ps24
Amniota - ps25
Mammalia - ps26
Theria - ps27
Placentalia - ps28
Boreoeutheria - ps29
Euarchontoglires - ps30
Euarchonta - ps31
Primates - ps32
Haplorrhini - ps33
Simiiformes - ps34
Catarrhini - ps35
Hominoidea - ps36
Hominidae - ps37
Homininae - ps38
Hominini - ps39
Homo_sapiens - ps40
p-value
1e-03
2e-03
6e-03
1e-02
3e-02
Log-odds
0.5
1.0
1.5
2.0
2.5
3.0
3.5
4.0

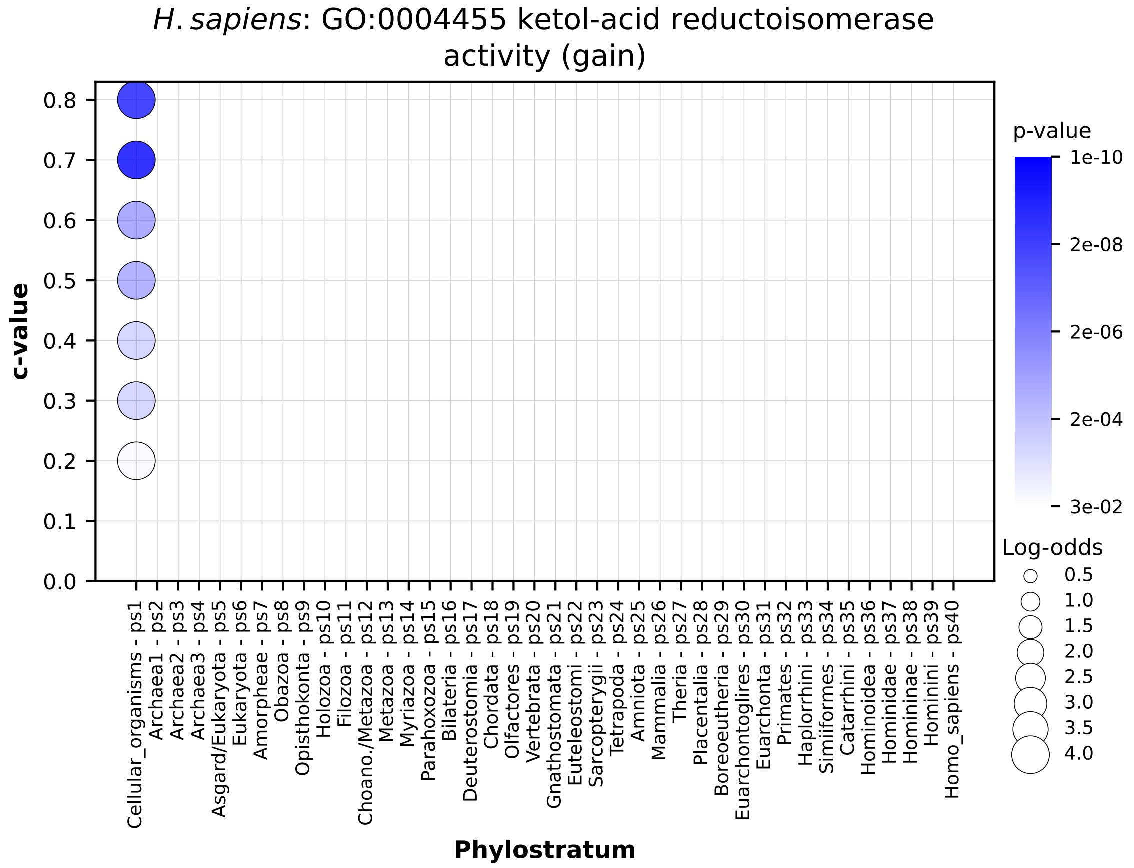

H. sapiens: GO:0004455 ketol-acid reductoisomerase activity (gain)
c-value
0.0
0.1
0.2
0.3
0.4
0.5
0.6
0.7
0.8
Phylostratum
Cellular_organisms - ps1
Archaea1 - ps2
Archaea2 - ps3
Archaea3 - ps4
Asgard/Eukaryota - ps5
Eukaryota - ps6
Amorpheae - ps7
Obazoa - ps8
Opisthokonta - ps9
Holozoa - ps10
Filozoa - ps11
Choano./Metazoa - ps12
Metazoa - ps13
Myriazoa - ps14
Parahoxozoa - ps15
Bilateria - ps16
Deuterostomia - ps17
Chordata - ps18
Olfactores - ps19
Vertebrata - ps20
Gnathostomata - ps21
Euteleostomi - ps22
Sarcopterygii - ps23
Tetrapoda - ps24
Amniota - ps25
Mammalia - ps26
Theria - ps27
Placentalia - ps28
Boreoeutheria - ps29
Euarchontoglires - ps30
Euarchonta - ps31
Primates - ps32
Haplorrhini - ps33
Simiiformes - ps34
Catarrhini - ps35
Hominoidea - ps36
Hominidae - ps37
Homininae - ps38
Hominini - ps39
Homo_sapiens - ps40
p-value
1e-10
2e-08
2e-06
2e-04
3e-02
Log-odds
0.5
1.0
1.5
2.0
2.5
3.0
3.5
4.0

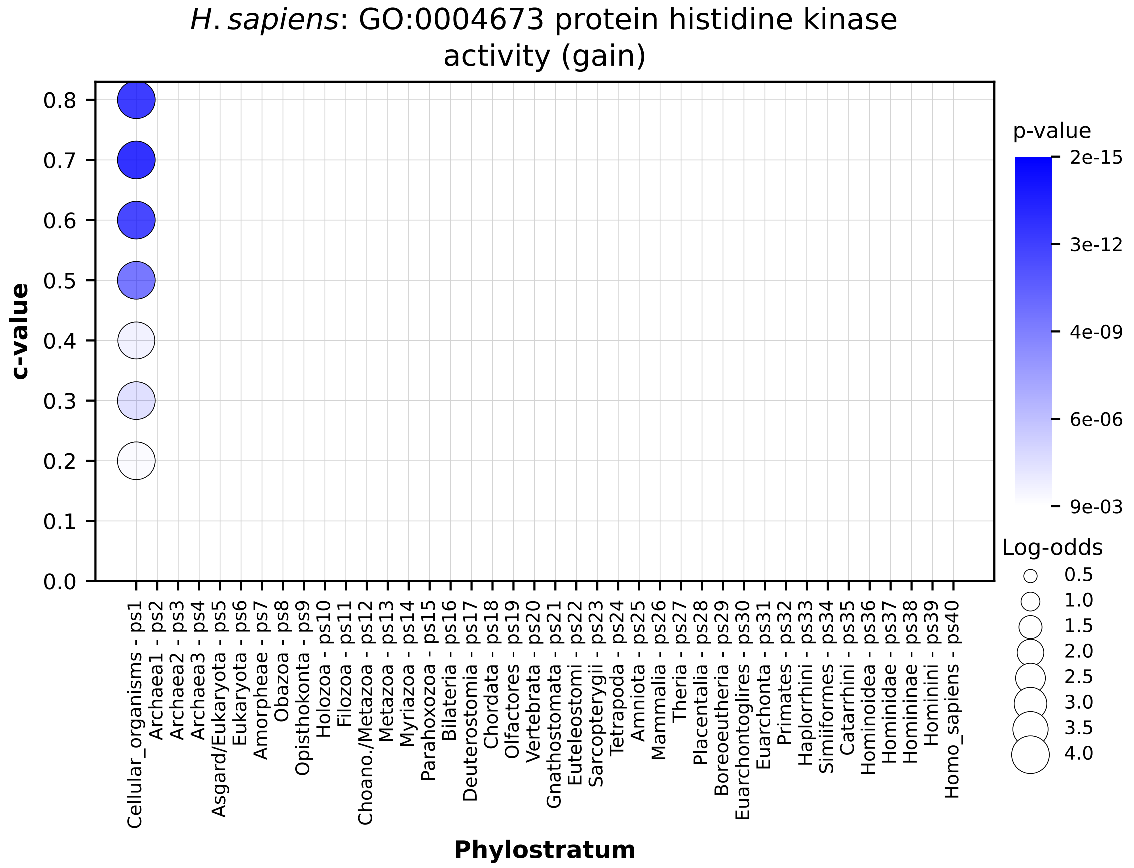

H. sapiens: GO:0004673 protein histidine kinase activity (gain)
c-value
0.0
0.1
0.2
0.3
0.4
0.5
0.6
0.7
0.8
Phylostratum
Cellular_organisms - ps1
Archaea1 - ps2
Archaea2 - ps3
Archaea3 - ps4
Asgard/Eukaryota - ps5
Eukaryota - ps6
Amorpheae - ps7
Obazoa - ps8
Opisthokonta - ps9
Holozoa - ps10
Filozoa - ps11
Choano./Metazoa - ps12
Metazoa - ps13
Myriazoa - ps14
Parahoxozoa - ps15
Bilateria - ps16
Deuterostomia - ps17
Chordata - ps18
Olfactores - ps19
Vertebrata - ps20
Gnathostomata - ps21
Euteleostomi - ps22
Sarcopterygii - ps23
Tetrapoda - ps24
Amniota - ps25
Mammalia - ps26
Theria - ps27
Placentalia - ps28
Boreoeutheria - ps29
Euarchontoglires - ps30
Euarchonta - ps31
Primates - ps32
Haplorrhini - ps33
Simiiformes - ps34
Catarrhini - ps35
Hominoidea - ps36
Hominidae - ps37
Homininae - ps38
Hominini - ps39
Homo_sapiens - ps40
p-value
2e-15
3e-12
4e-09
6e-06
9e-03
Log-odds
0.5
1.0
1.5
2.0
2.5
3.0
3.5
4.0

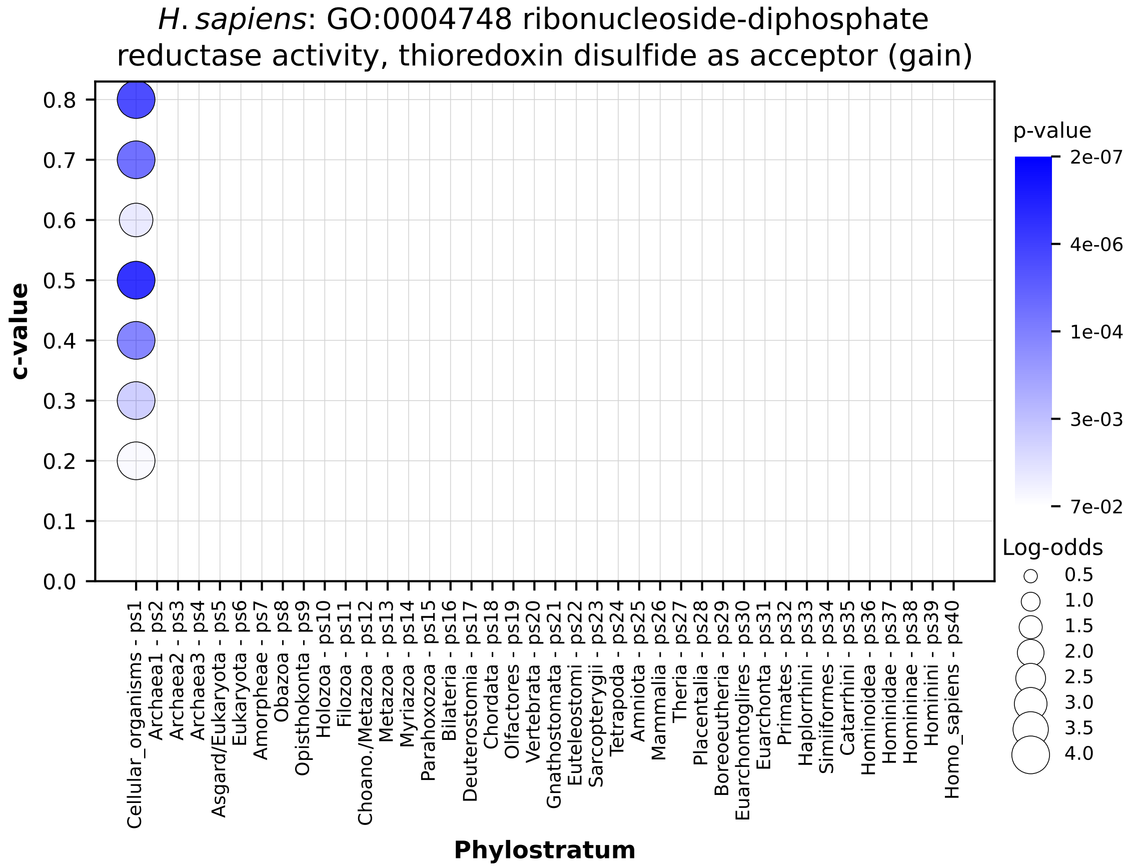

H. sapiens: GO:0004748 ribonucleoside-diphosphate reductase activity, thioredoxin disulfide as acceptor (gain)
c-value
0.0
0.1
0.2
0.3
0.4
0.5
0.6
0.7
0.8
Phylostratum
Cellular_organisms - ps1
Archaea1 - ps2
Archaea2 - ps3
Archaea3 - ps4
Asgard/Eukaryota - ps5
Eukaryota - ps6
Amorpheae - ps7
Obazoa - ps8
Opisthokonta - ps9
Holozoa - ps10
Filozoa - ps11
Choano./Metazoa - ps12
Metazoa - ps13
Myriazoa - ps14
Parahoxozoa - ps15
Bilateria - ps16
Deuterostomia - ps17
Chordata - ps18
Olfactores - ps19
Vertebrata - ps20
Gnathostomata - ps21
Euteleostomi - ps22
Sarcopterygii - ps23
Tetrapoda - ps24
Amniota - ps25
Mammalia - ps26
Theria - ps27
Placentalia - ps28
Boreoeutheria - ps29
Euarchontoglires - ps30
Euarchonta - ps31
Primates - ps32
Haplorrhini - ps33
Simiiformes - ps34
Catarrhini - ps35
Hominoidea - ps36
Hominidae - ps37
Homininae - ps38
Hominini - ps39
Homo_sapiens - ps40
p-value
2e-07
4e-06
1e-04
3e-03
7e-02
Log-odds
0.5
1.0
1.5
2.0
2.5
3.0
3.5
4.0

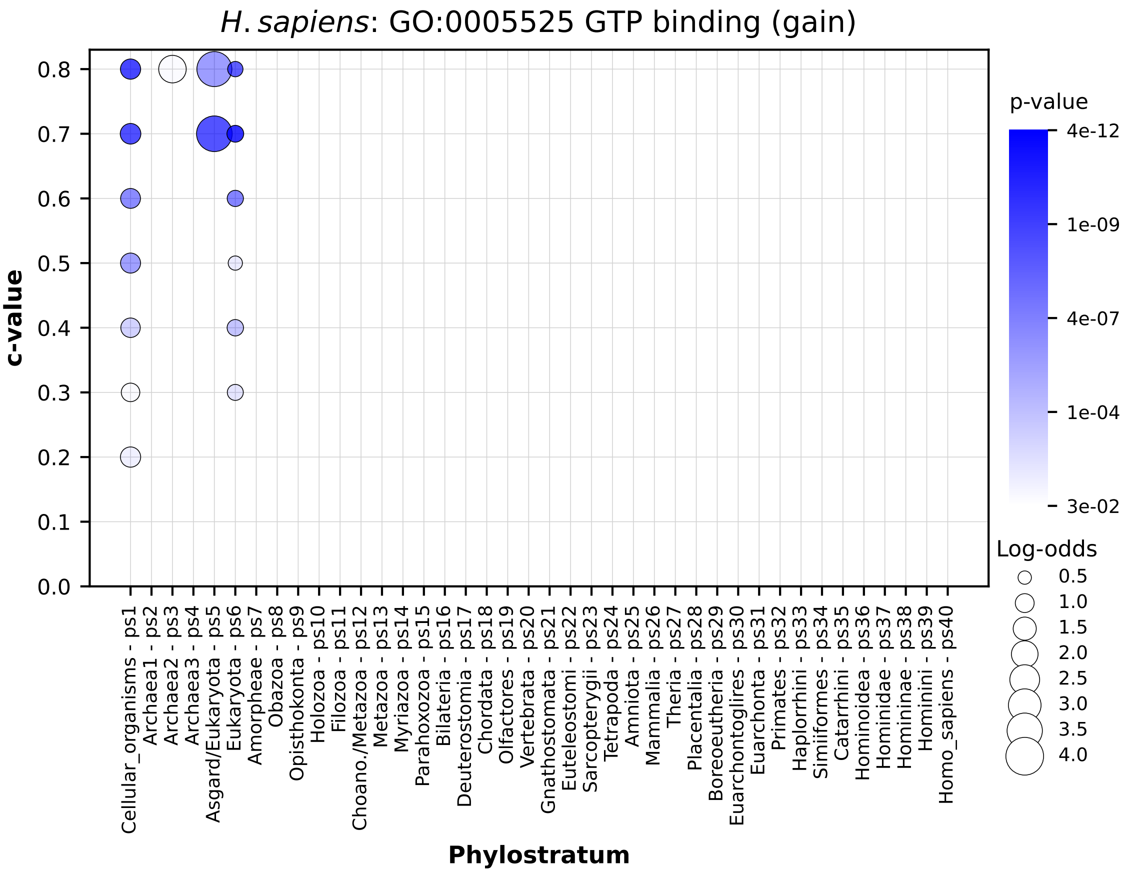

H. sapiens: GO:0005525 GTP binding (gain)
c-value
0.0
0.1
0.2
0.3
0.4
0.5
0.6
0.7
0.8
Phylostratum
Cellular_organisms - ps1
Archaea1 - ps2
Archaea2 - ps3
Archaea3 - ps4
Asgard/Eukaryota - ps5
Eukaryota - ps6
Amorpheae - ps7
Obazoa - ps8
Opisthokonta - ps9
Holozoa - ps10
Filozoa - ps11
Choano./Metazoa - ps12
Metazoa - ps13
Myriazoa - ps14
Parahoxozoa - ps15
Bilateria - ps16
Deuterostomia - ps17
Chordata - ps18
Olfactores - ps19
Vertebrata - ps20
Gnathostomata - ps21
Euteleostomi - ps22
Sarcopterygii - ps23
Tetrapoda - ps24
Amniota - ps25
Mammalia - ps26
Theria - ps27
Placentalia - ps28
Boreoeutheria - ps29
Euarchontoglires - ps30
Euarchonta - ps31
Primates - ps32
Haplorrhini - ps33
Simiiformes - ps34
Catarrhini - ps35
Hominoidea - ps36
Hominidae - ps37
Homininae - ps38
Hominini - ps39
Homo_sapiens - ps40
p-value
4e-12
1e-09
4e-07
1e-04
3e-02
Log-odds
0.5
1.0
1.5
2.0
2.5
3.0
3.5
4.0

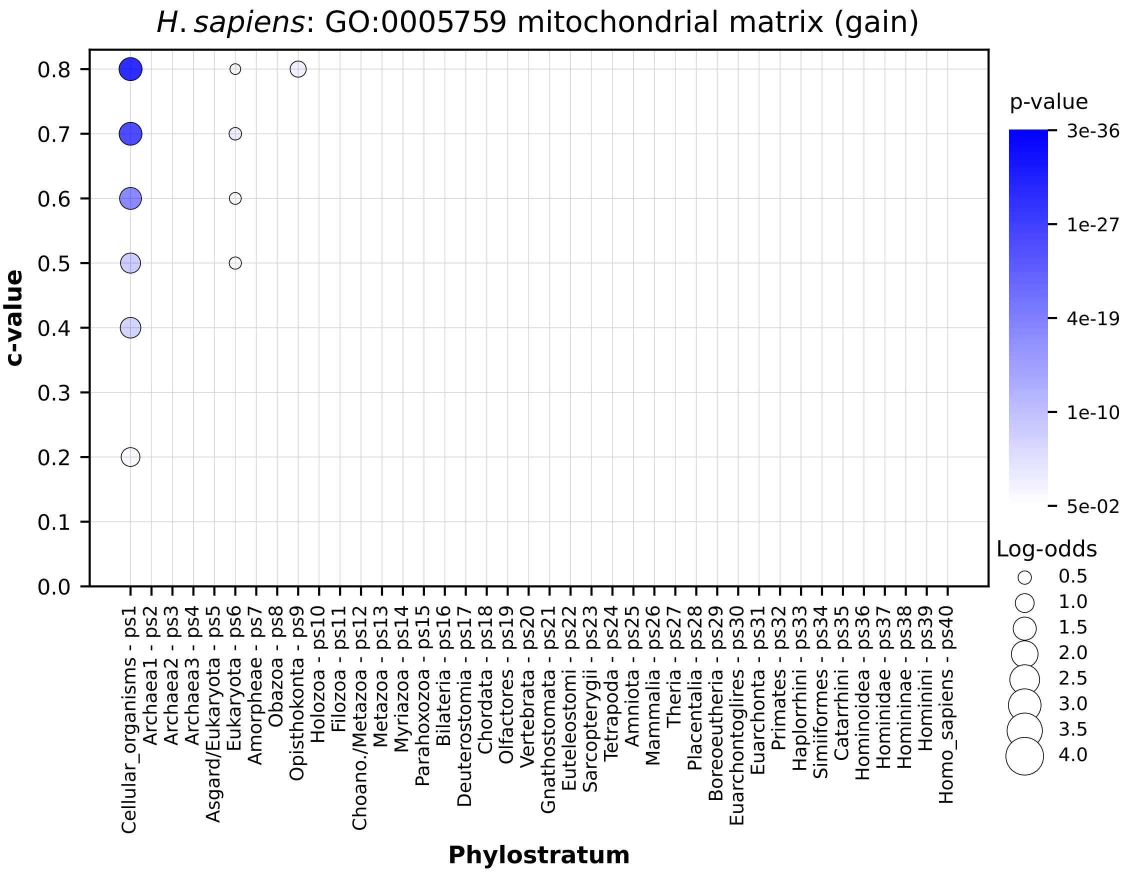
H. sapiens: GO:0005759 mitochondrial matrix (gain)
c-value
0.8
0.7
0.6
0.5
0.4
0.3
0.2
0.1
0.0
Phylostratum
Cellular_organisms - ps1
Archaea1 - ps2
Archaea2 - ps3
Archaea3 - ps4
Asgard/Eukaryota - ps5
Eukaryota - ps6
Amorpheae - ps7
Obazoa - ps8
Opisthokonta - ps9
Holozoa - ps10
Filozoa - ps11
Choano./Metazoa - ps12
Metazoa - ps13
Myriazoa - ps14
Parahoxozoa - ps15
Bilateria - ps16
Deuterostomia - ps17
Chordata - ps18
Olfactores - ps19
Vertebrata - ps20
Gnathostomata - ps21
Euteleostomi - ps22
Sarcopterygii - ps23
Tetrapoda - ps24
Amniota - ps25
Mammalia - ps26
Theria - ps27
Placentalia - ps28
Boreoeutheria - ps29
Euarchontoglires - ps30
Euarchonta - ps31
Primates - ps32
Haplorrhini - ps33
Simiiformes - ps34
Catarrhini - ps35
Hominoidea - ps36
Hominidae - ps37
Homininae - ps38
Hominini - ps39
Homo_sapiens - ps40
p-value
3e-36
1e-27
4e-19
1e-10
5e-02
Log-odds
0.5
1.0
1.5
2.0
2.5
3.0
3.5
4.0

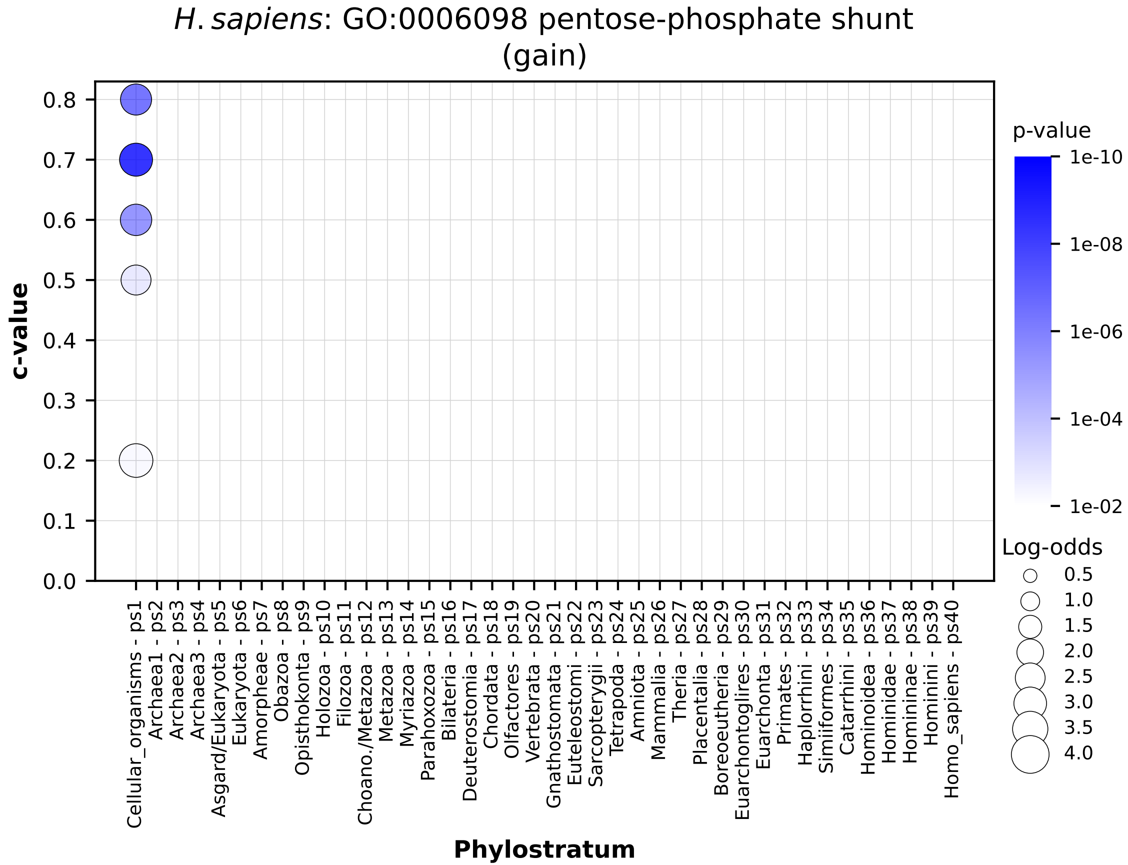
H. sapiens: GO:0006098 pentose-phosphate shunt
(gain)
c-value
0.0
0.1
0.2
0.3
0.4
0.5
0.6
0.7
0.8
p-value
1e-10
1e-08
1e-06
1e-04
1e-02
Log-odds
0.5
1.0
1.5
2.0
2.5
3.0
3.5
4.0
Cellular_organisms - ps1
Archaea1 - ps2
Archaea2 - ps3
Archaea3 - ps4
Asgard/Eukaryota - ps5
Eukaryota - ps6
Amorpheae - ps7
Obazoa - ps8
Opisthokonta - ps9
Holozoa - ps10
Filozoa - ps11
Choano./Metazoa - ps12
Metazoa - ps13
Myriazoa - ps14
Parahoxozoa - ps15
Bilateria - ps16
Deuterostomia - ps17
Chordata - ps18
Olfactores - ps19
Vertebrata - ps20
Gnathostomata - ps21
Euteleostomi - ps22
Sarcopterygii - ps23
Tetrapoda - ps24
Amniota - ps25
Mammalia - ps26
Theria - ps27
Placentalia - ps28
Boreoeutheria - ps29
Euarchontoglires - ps30
Euarchonta - ps31
Primates - ps32
Haplorrhini - ps33
Simiiformes - ps34
Catarrhini - ps35
Hominoidea - ps36
Hominidae - ps37
Homininae - ps38
Hominini - ps39
Homo_sapiens - ps40
Phylostratum

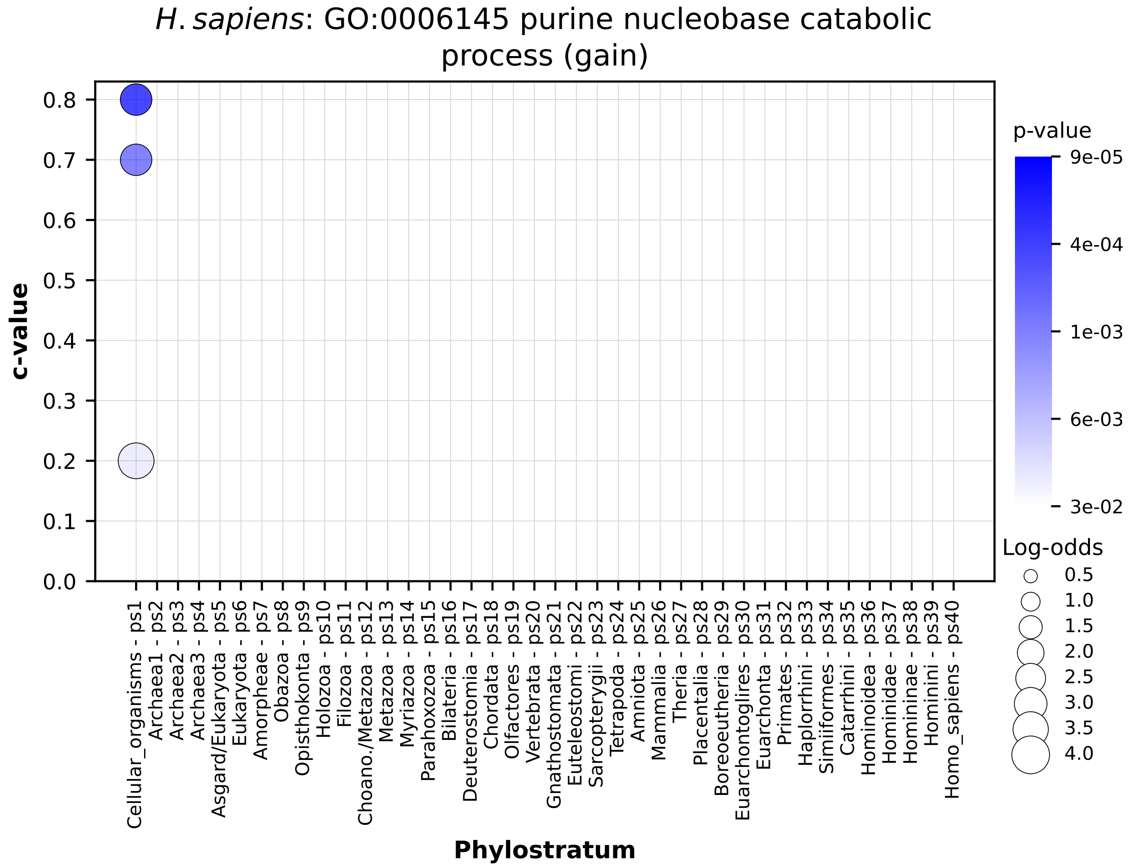
H. sapiens: GO:0006145 purine nucleobase catabolic process (gain)
c-value
0.8
0.7
0.6
0.5
0.4
0.3
0.2
0.1
0.0
p-value
9e-05
4e-04
1e-03
6e-03
3e-02
Log-odds
0.5
1.0
1.5
2.0
2.5
3.0
3.5
4.0
Cellular_organisms - ps1
Archaea1 - ps2
Archaea2 - ps3
Archaea3 - ps4
Asgard/Eukaryota - ps5
Eukaryota - ps6
Amorpheae - ps7
Obazoa - ps8
Opisthokonta - ps9
Holozoa - ps10
Filozoa - ps11
Choano./Metazoa - ps12
Metazoa - ps13
Myriazoa - ps14
Parahoxozoa - ps15
Bilateria - ps16
Deuterostomia - ps17
Chordata - ps18
Olfactores - ps19
Vertebrata - ps20
Gnathostomata - ps21
Euteleostomi - ps22
Sarcopterygii - ps23
Tetrapoda - ps24
Amniota - ps25
Mammalia - ps26
Theria - ps27
Placentalia - ps28
Boreoeutheria - ps29
Euarchontoglires - ps30
Euarchonta - ps31
Primates - ps32
Haplorrhini - ps33
Simiiformes - ps34
Catarrhini - ps35
Hominoidea - ps36
Hominidae - ps37
Homininae - ps38
Hominini - ps39
Homo_sapiens - ps40
Phylostratum

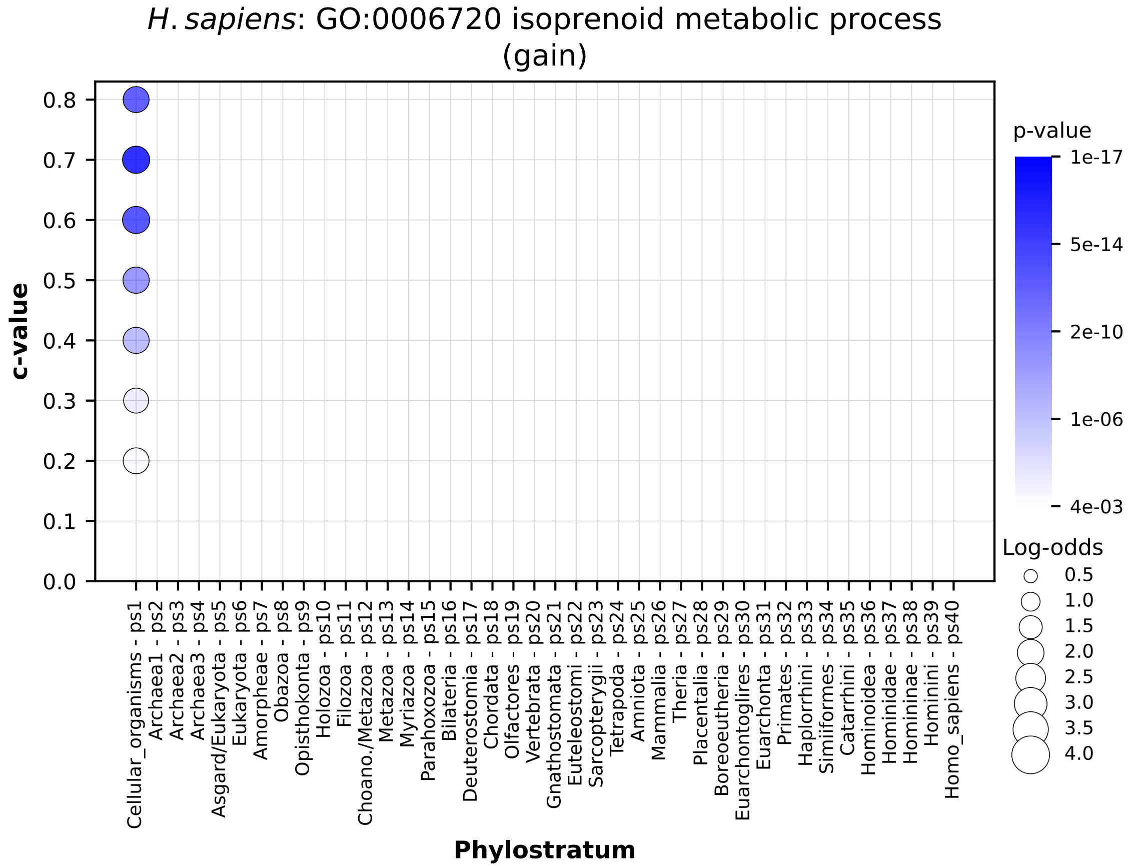

H. sapiens: GO:0006720 isoprenoid metabolic process
(gain)
c-value
0.0
0.1
0.2
0.3
0.4
0.5
0.6
0.7
0.8
p-value
1e-17
5e-14
2e-10
1e-06
4e-03
Log-odds
0.5
1.0
1.5
2.0
2.5
3.0
3.5
4.0
Cellular_organisms - ps1
Archaea1 - ps2
Archaea2 - ps3
Archaea3 - ps4
Asgard/Eukaryota - ps5
Eukaryota - ps6
Amorpheae - ps7
Obazoa - ps8
Opisthokonta - ps9
Holozoa - ps10
Filozoa - ps11
Choano./Metazoa - ps12
Metazoa - ps13
Myriazoa - ps14
Parahoxozoa - ps15
Bilateria - ps16
Deuterostomia - ps17
Chordata - ps18
Olfactores - ps19
Vertebrata - ps20
Gnathostomata - ps21
Euteleostomi - ps22
Sarcopterygii - ps23
Tetrapoda - ps24
Amniota - ps25
Mammalia - ps26
Theria - ps27
Placentalia - ps28
Boreoeutheria - ps29
Euarchontoglires - ps30
Euarchonta - ps31
Primates - ps32
Haplorrhini - ps33
Simiiformes - ps34
Catarrhini - ps35
Hominoidea - ps36
Hominidae - ps37
Homininae - ps38
Hominini - ps39
Homo_sapiens - ps40
Phylostratum

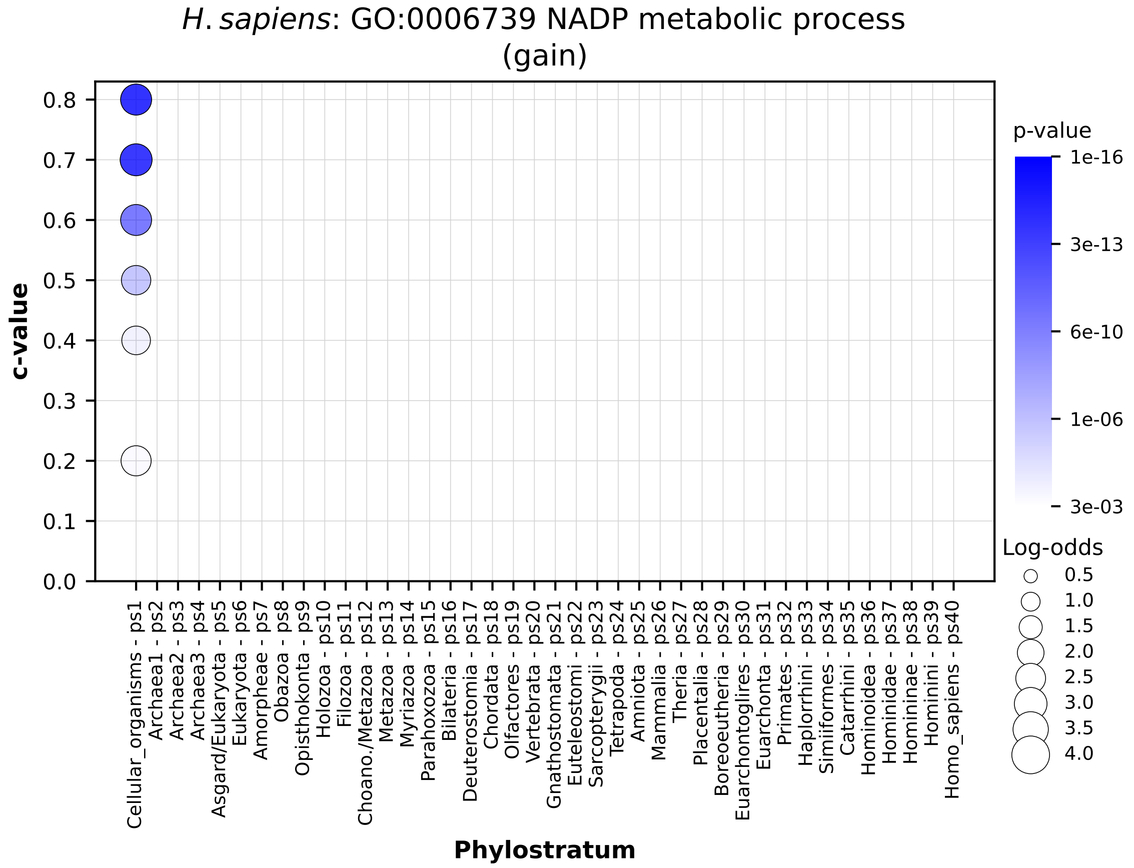

*H. sapiens*: GO:0006739 NADP metabolic process
(gain)
c-value
0.0
0.1
0.2
0.3
0.4
0.5
0.6
0.7
0.8
Phylostratum
Cellular_organisms - ps1
Archaea1 - ps2
Archaea2 - ps3
Archaea3 - ps4
Asgard/Eukaryota - ps5
Eukaryota - ps6
Amorpheae - ps7
Obazoa - ps8
Opisthokonta - ps9
Holozoa - ps10
Filozoa - ps11
Choano./Metazoa - ps12
Metazoa - ps13
Myriazoa - ps14
Parahoxozoa - ps15
Bilateria - ps16
Deuterostomia - ps17
Chordata - ps18
Olfactores - ps19
Vertebrata - ps20
Gnathostomata - ps21
Euteleostomi - ps22
Sarcopterygii - ps23
Tetrapoda - ps24
Amniota - ps25
Mammalia - ps26
Theria - ps27
Placentalia - ps28
Boreoeutheria - ps29
Euarchontoglires - ps30
Euarchonta - ps31
Primates - ps32
Haplorrhini - ps33
Simiiformes - ps34
Catarrhini - ps35
Hominoidea - ps36
Hominidae - ps37
Homininae - ps38
Hominini - ps39
Homo_sapiens - ps40
p-value
1e-16
3e-13
6e-10
1e-06
3e-03
Log-odds
0.5
1.0
1.5
2.0
2.5
3.0
3.5
4.0

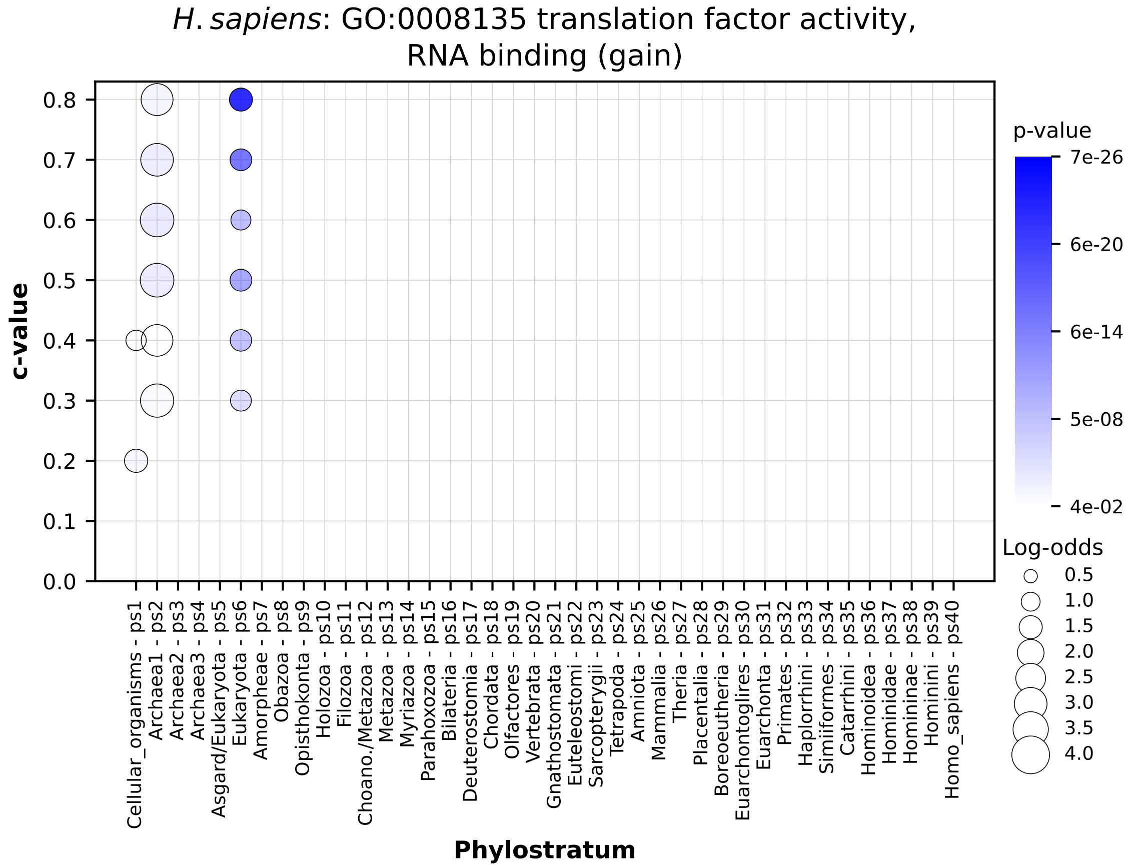

H. sapiens: GO:0008135 translation factor activity, RNA binding (gain)
c-value
Phylostratum
p-value
7e-26
6e-20
6e-14
5e-08
4e-02
Log-odds
0.5
1.0
1.5
2.0
2.5
3.0
3.5
4.0
Cellular_organisms - ps1
Archaea1 - ps2
Archaea2 - ps3
Archaea3 - ps4
Asgard/Eukaryota - ps5
Eukaryota - ps6
Amorpheae - ps7
Obazoa - ps8
Opisthokonta - ps9
Holozoa - ps10
Filozoa - ps11
Choano./Metazoa - ps12
Metazoa - ps13
Myriazoa - ps14
Parahoxozoa - ps15
Bilateria - ps16
Deuterostomia - ps17
Chordata - ps18
Olfactores - ps19
Vertebrata - ps20
Gnathostomata - ps21
Euteleostomi - ps22
Sarcopterygii - ps23
Tetrapoda - ps24
Amniota - ps25
Mammalia - ps26
Theria - ps27
Placentalia - ps28
Boreoeutheria - ps29
Euarchontoglires - ps30
Euarchonta - ps31
Primates - ps32
Haplorrhini - ps33
Simiiformes - ps34
Catarrhini - ps35
Hominoidea - ps36
Hominidae - ps37
Homininae - ps38
Hominini - ps39
Homo_sapiens - ps40

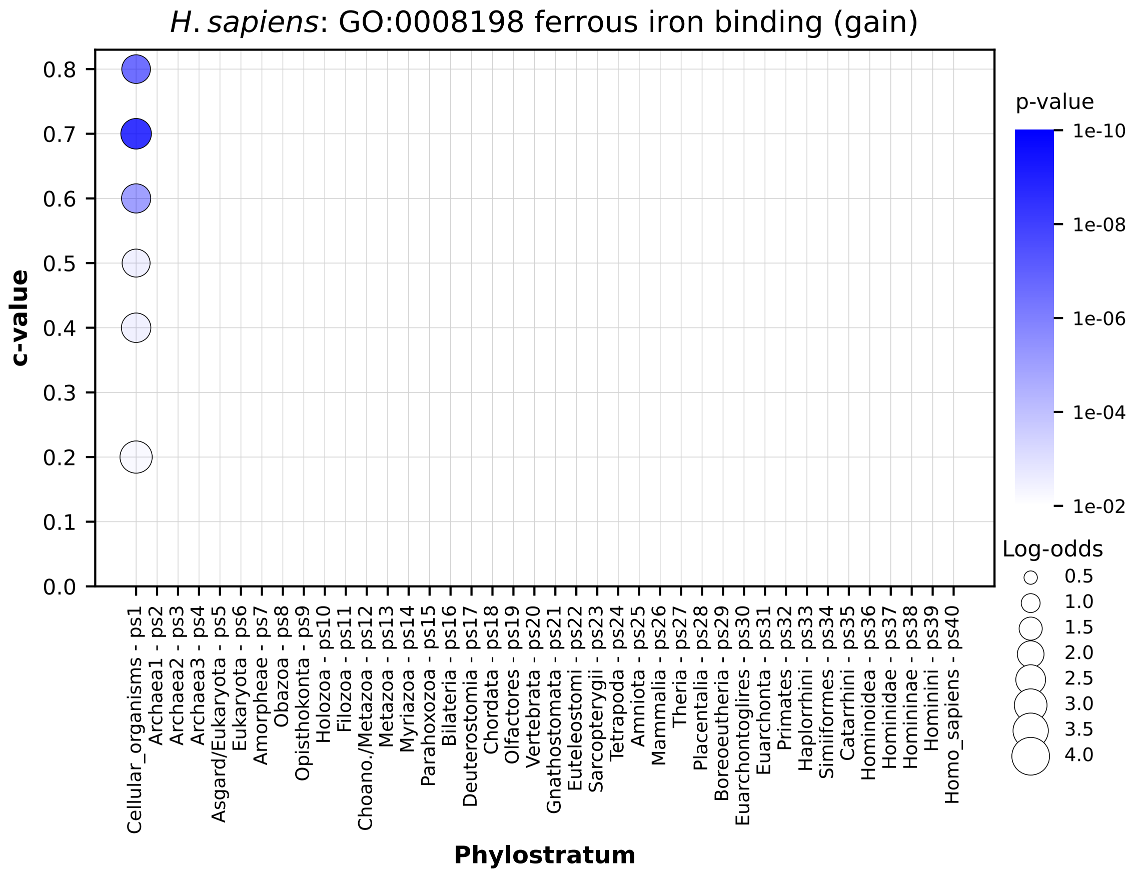

H. sapiens: GO:0008198 ferrous iron binding (gain)
c-value
0.0
0.1
0.2
0.3
0.4
0.5
0.6
0.7
0.8
Phylostratum
Cellular_organisms - ps1
Archaea1 - ps2
Archaea2 - ps3
Archaea3 - ps4
Asgard/Eukaryota - ps5
Eukaryota - ps6
Amorpheae - ps7
Obazoa - ps8
Opisthokonta - ps9
Holozoa - ps10
Filozoa - ps11
Choano./Metazoa - ps12
Metazoa - ps13
Myriazoa - ps14
Parahoxozoa - ps15
Bilateria - ps16
Deuterostomia - ps17
Chordata - ps18
Olfactores - ps19
Vertebrata - ps20
Gnathostomata - ps21
Euteleostomi - ps22
Sarcopterygii - ps23
Tetrapoda - ps24
Amniota - ps25
Mammalia - ps26
Theria - ps27
Placentalia - ps28
Boreoeutheria - ps29
Euarchontoglires - ps30
Euarchonta - ps31
Primates - ps32
Haplorrhini - ps33
Simiiformes - ps34
Catarrhini - ps35
Hominoidea - ps36
Hominidae - ps37
Homininae - ps38
Hominini - ps39
Homo_sapiens - ps40
p-value
1e-10
1e-08
1e-06
1e-04
1e-02
Log-odds
0.5
1.0
1.5
2.0
2.5
3.0
3.5
4.0

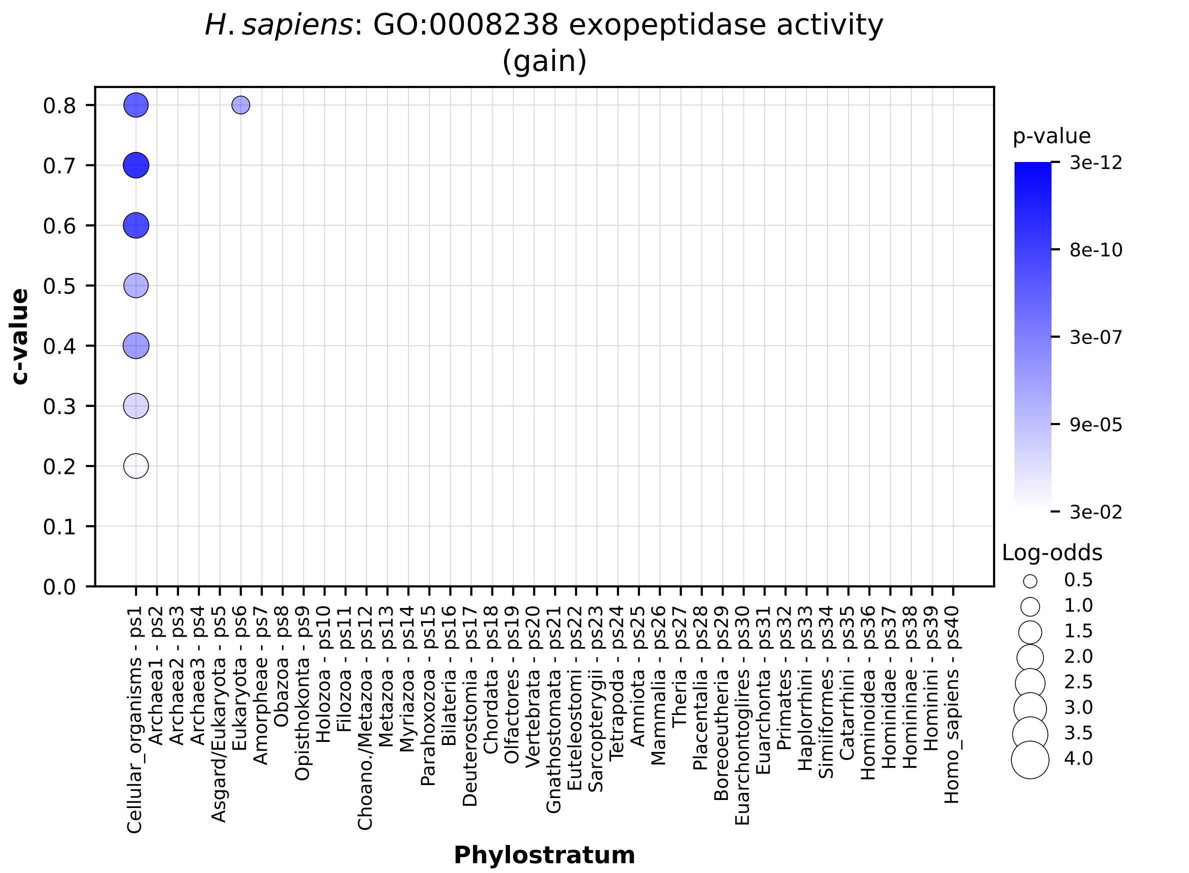

H. sapiens: GO:0008238 exopeptidase activity
(gain)
c-value
0.0
0.1
0.2
0.3
0.4
0.5
0.6
0.7
0.8
Phylostratum
Cellular_organisms - ps1
Archaea1 - ps2
Archaea2 - ps3
Archaea3 - ps4
Asgard/Eukaryota - ps5
Eukaryota - ps6
Amorpheae - ps7
Obazoa - ps8
Opisthokonta - ps9
Holozoa - ps10
Filozoa - ps11
Choano./Metazoa - ps12
Metazoa - ps13
Myriazoa - ps14
Parahoxozoa - ps15
Bilateria - ps16
Deuterostomia - ps17
Chordata - ps18
Olfactores - ps19
Vertebrata - ps20
Gnathostomata - ps21
Euteleostomi - ps22
Sarcopterygii - ps23
Tetrapoda - ps24
Amniota - ps25
Mammalia - ps26
Theria - ps27
Placentalia - ps28
Boreoeutheria - ps29
Euarchontoglires - ps30
Euarchonta - ps31
Primates - ps32
Haplorrhini - ps33
Simiiformes - ps34
Catarrhini - ps35
Hominoidea - ps36
Hominidae - ps37
Homininae - ps38
Hominini - ps39
Homo_sapiens - ps40
p-value
3e-12
8e-10
3e-07
9e-05
3e-02
Log-odds
0.5
1.0
1.5
2.0
2.5
3.0
3.5
4.0

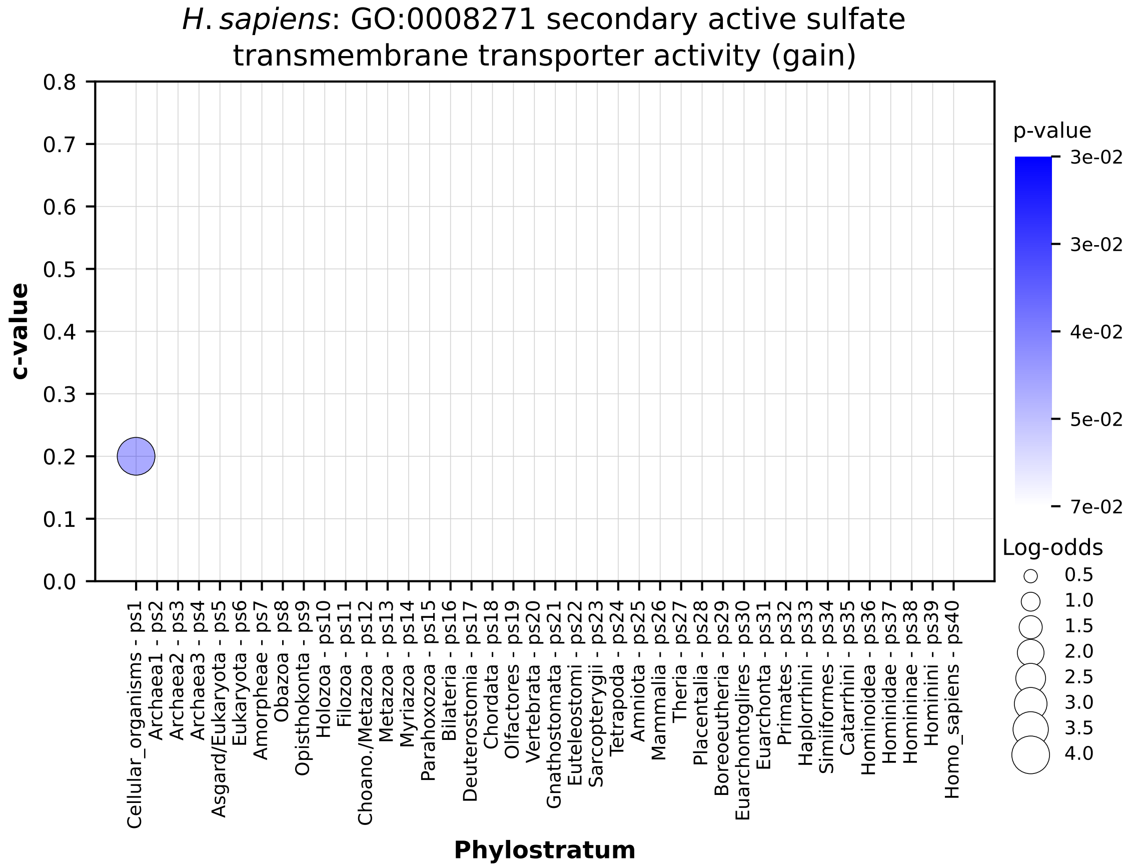

*H. sapiens*: GO:0008271 secondary active sulfate transmembrane transporter activity (gain)
c-value
0.0
0.1
0.2
0.3
0.4
0.5
0.6
0.7
0.8
Phylostratum
Cellular_organisms - ps1
Archaea1 - ps2
Archaea2 - ps3
Archaea3 - ps4
Asgard/Eukaryota - ps5
Eukaryota - ps6
Amorpheae - ps7
Obazoa - ps8
Opisthokonta - ps9
Holozoa - ps10
Filozoa - ps11
Choano./Metazoa - ps12
Metazoa - ps13
Myriazoa - ps14
Parahoxozoa - ps15
Bilateria - ps16
Deuterostomia - ps17
Chordata - ps18
Olfactores - ps19
Vertebrata - ps20
Gnathostomata - ps21
Euteleostomi - ps22
Sarcopterygii - ps23
Tetrapoda - ps24
Amniota - ps25
Mammalia - ps26
Theria - ps27
Placentalia - ps28
Boreoeutheria - ps29
Euarchontoglires - ps30
Euarchonta - ps31
Primates - ps32
Haplorrhini - ps33
Simiiformes - ps34
Catarrhini - ps35
Hominoidea - ps36
Hominidae - ps37
Homininae - ps38
Hominini - ps39
Homo_sapiens - ps40
p-value
3e-02
3e-02
4e-02
5e-02
7e-02
Log-odds
0.5
1.0
1.5
2.0
2.5
3.0
3.5
4.0

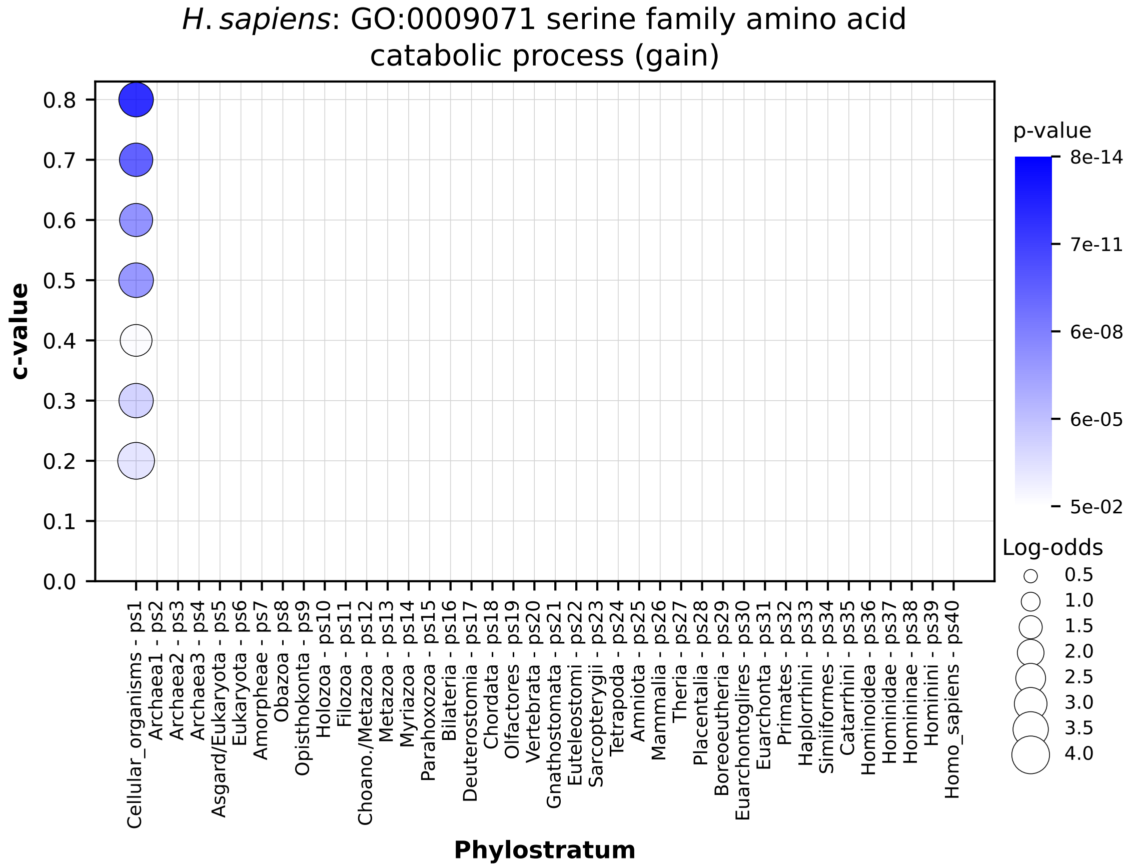

H. sapiens: GO:0009071 serine family amino acid catabolic process (gain)
c-value
0.0
0.1
0.2
0.3
0.4
0.5
0.6
0.7
0.8
Phylostratum
Cellular_organisms - ps1
Archaea1 - ps2
Archaea2 - ps3
Archaea3 - ps4
Asgard/Eukaryota - ps5
Eukaryota - ps6
Amorpheae - ps7
Obazoa - ps8
Opisthokonta - ps9
Holozoa - ps10
Filozoa - ps11
Choano./Metazoa - ps12
Metazoa - ps13
Myriazoa - ps14
Parahoxozoa - ps15
Bilateria - ps16
Deuterostomia - ps17
Chordata - ps18
Olfactores - ps19
Vertebrata - ps20
Gnathostomata - ps21
Euteleostomi - ps22
Sarcopterygii - ps23
Tetrapoda - ps24
Amniota - ps25
Mammalia - ps26
Theria - ps27
Placentalia - ps28
Boreoeutheria - ps29
Euarchontoglires - ps30
Euarchonta - ps31
Primates - ps32
Haplorrhini - ps33
Simiiformes - ps34
Catarrhini - ps35
Hominoidea - ps36
Hominidae - ps37
Homininae - ps38
Hominini - ps39
Homo_sapiens - ps40
p-value
8e-14
7e-11
6e-08
6e-05
5e-02
Log-odds
0.5
1.0
1.5
2.0
2.5
3.0
3.5
4.0

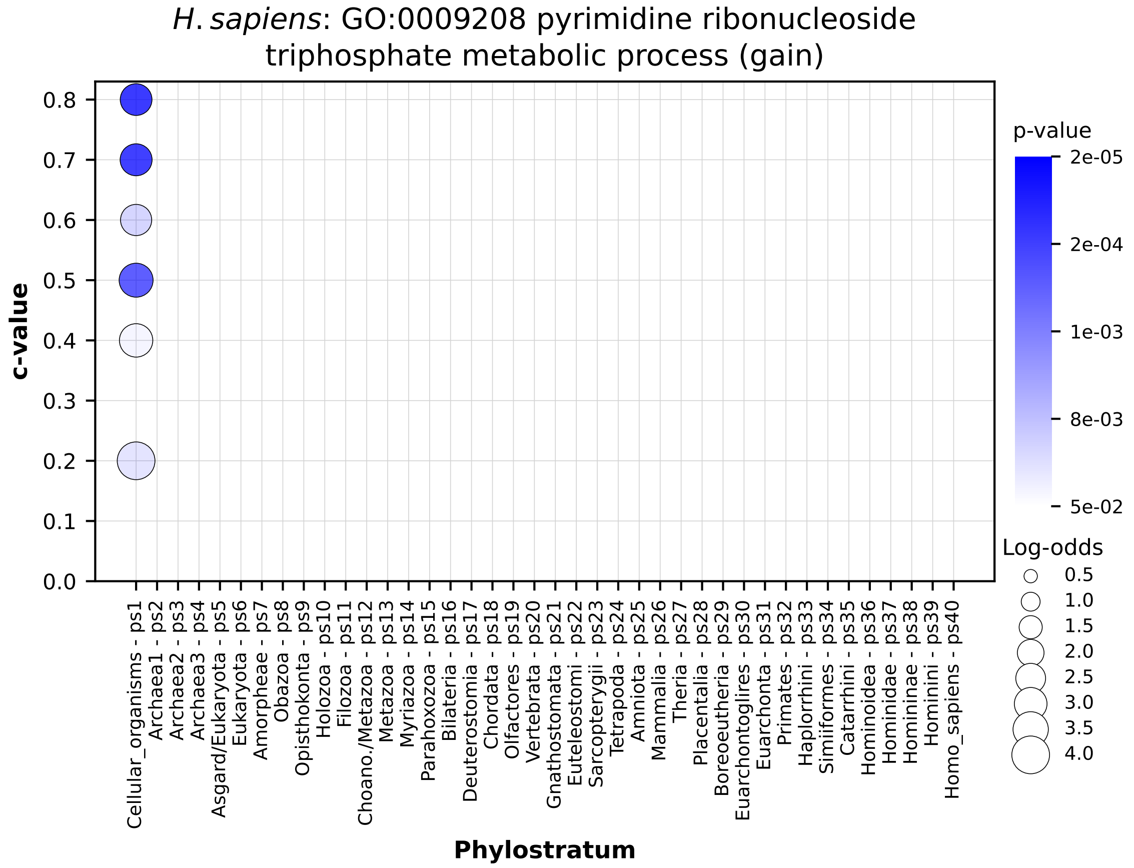

H. sapiens: GO:0009208 pyrimidine ribonucleoside triphosphate metabolic process (gain)
c-value
0.0
0.1
0.2
0.3
0.4
0.5
0.6
0.7
0.8
Phylostratum
Cellular_organisms - ps1
Archaea1 - ps2
Archaea2 - ps3
Archaea3 - ps4
Asgard/Eukaryota - ps5
Eukaryota - ps6
Amorpheae - ps7
Obazoa - ps8
Opisthokonta - ps9
Holozoa - ps10
Filozoa - ps11
Choano./Metazoa - ps12
Metazoa - ps13
Myriazoa - ps14
Parahoxozoa - ps15
Bilateria - ps16
Deuterostomia - ps17
Chordata - ps18
Olfactores - ps19
Vertebrata - ps20
Gnathostomata - ps21
Euteleostomi - ps22
Sarcopterygii - ps23
Tetrapoda - ps24
Amniota - ps25
Mammalia - ps26
Theria - ps27
Placentalia - ps28
Boreoeutheria - ps29
Euarchontoglires - ps30
Euarchonta - ps31
Primates - ps32
Haplorrhini - ps33
Simiiformes - ps34
Catarrhini - ps35
Hominoidea - ps36
Hominidae - ps37
Homininae - ps38
Hominini - ps39
Homo_sapiens - ps40
p-value
2e-05
2e-04
1e-03
8e-03
5e-02
Log-odds
0.5
1.0
1.5
2.0
2.5
3.0
3.5
4.0

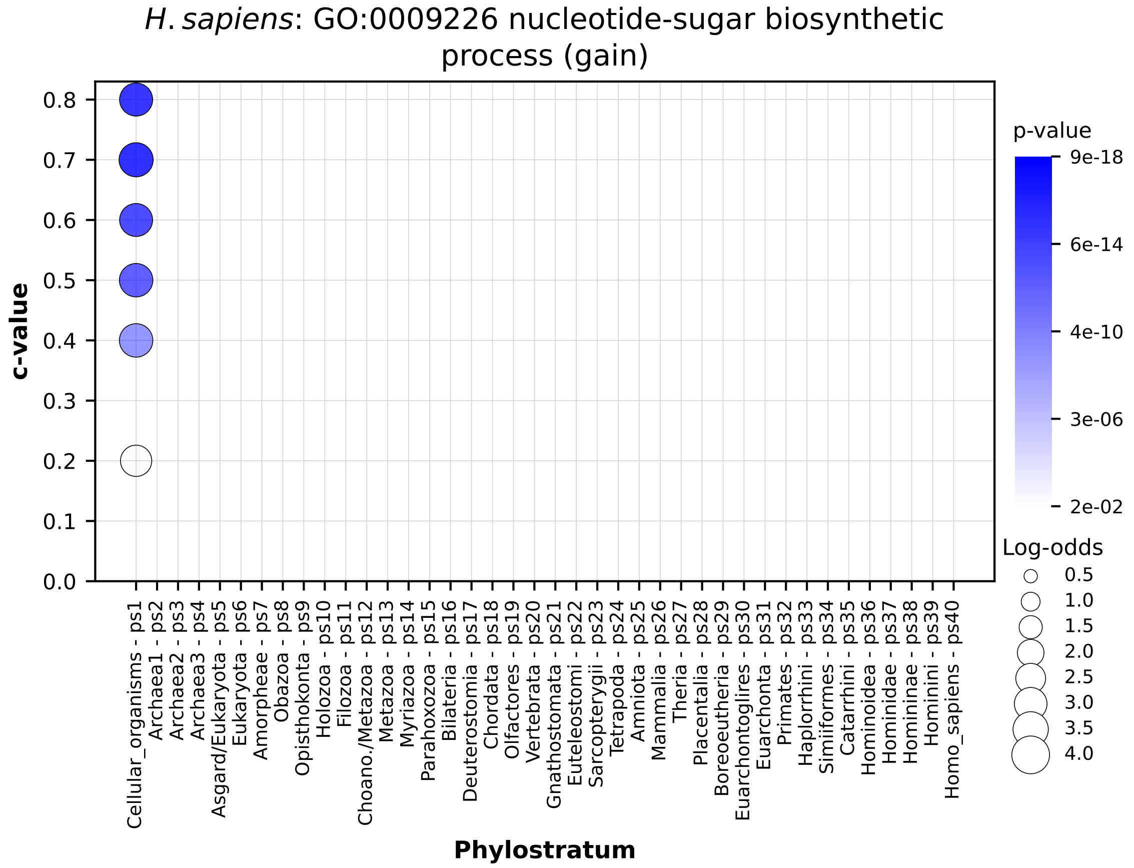

*H. sapiens*: GO:0009226 nucleotide-sugar biosynthetic process (gain)
c-value
0.0
0.1
0.2
0.3
0.4
0.5
0.6
0.7
0.8
Phylostratum
Cellular_organisms - ps1
Archaea1 - ps2
Archaea2 - ps3
Archaea3 - ps4
Asgard/Eukaryota - ps5
Eukaryota - ps6
Amorpheae - ps7
Obazoa - ps8
Opisthokonta - ps9
Holozoa - ps10
Filozoa - ps11
Choano./Metazoa - ps12
Metazoa - ps13
Myriazoa - ps14
Parahoxozoa - ps15
Bilateria - ps16
Deuterostomia - ps17
Chordata - ps18
Olfactores - ps19
Vertebrata - ps20
Gnathostomata - ps21
Euteleostomi - ps22
Sarcopterygii - ps23
Tetrapoda - ps24
Amniota - ps25
Mammalia - ps26
Theria - ps27
Placentalia - ps28
Boreoeutheria - ps29
Euarchontoglires - ps30
Euarchonta - ps31
Primates - ps32
Haplorrhini - ps33
Simiiformes - ps34
Catarrhini - ps35
Hominoidea - ps36
Hominidae - ps37
Homininae - ps38
Hominini - ps39
Homo_sapiens - ps40
p-value
9e-18
6e-14
4e-10
3e-06
2e-02
Log-odds
0.5
1.0
1.5
2.0
2.5
3.0
3.5
4.0

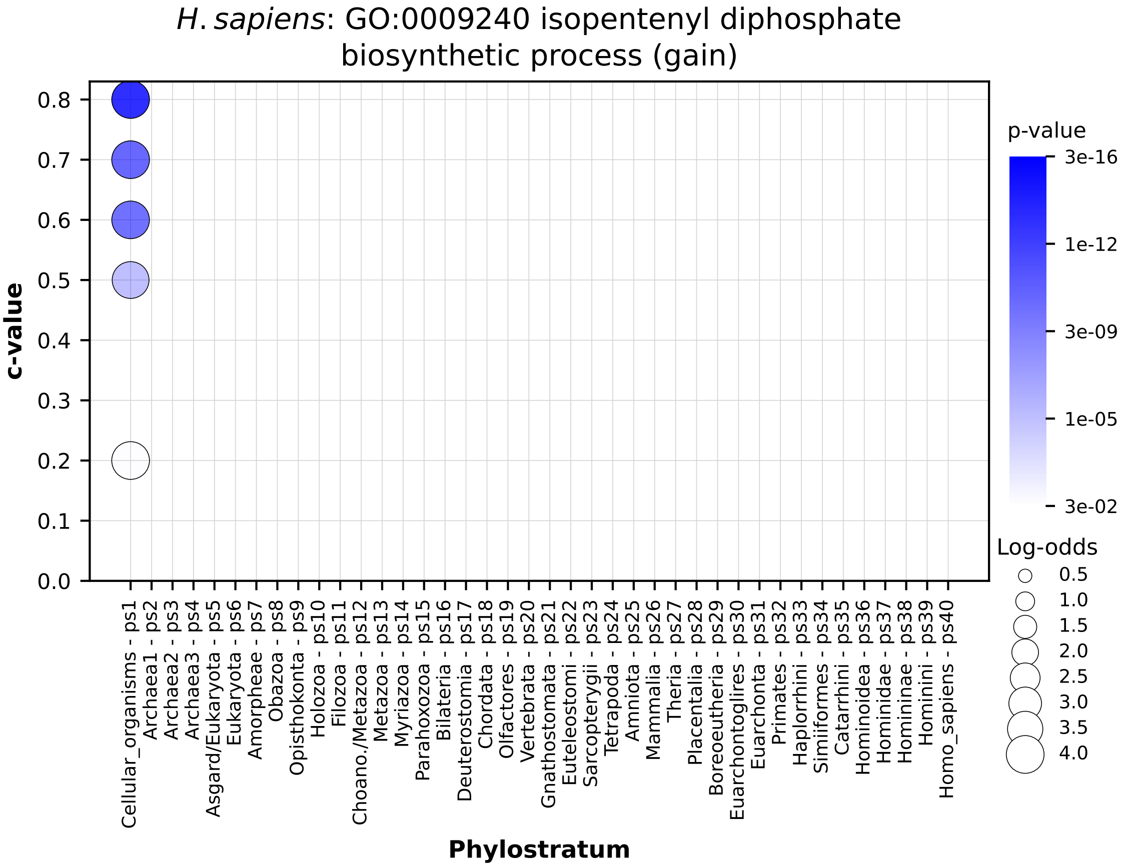

*H. sapiens*: GO:0009240 isopentenyl diphosphate biosynthetic process (gain)
c-value
0.8
0.7
0.6
0.5
0.4
0.3
0.2
0.1
0.0
Phylostratum
Cellular_organisms - ps1
Archaea1 - ps2
Archaea2 - ps3
Archaea3 - ps4
Asgard/Eukaryota - ps5
Eukaryota - ps6
Amorpheae - ps7
Obazoa - ps8
Opisthokonta - ps9
Holozoa - ps10
Filozoa - ps11
Choano./Metazoa - ps12
Metazoa - ps13
Myriazoa - ps14
Parahoxozoa - ps15
Bilateria - ps16
Deuterostomia - ps17
Chordata - ps18
Olfactores - ps19
Vertebrata - ps20
Gnathostomata - ps21
Euteleostomi - ps22
Sarcopterygii - ps23
Tetrapoda - ps24
Amniota - ps25
Mammalia - ps26
Theria - ps27
Placentalia - ps28
Boreoeutheria - ps29
Euarchontoglires - ps30
Euarchonta - ps31
Primates - ps32
Haplorrhini - ps33
Simiiformes - ps34
Catarrhini - ps35
Hominoidea - ps36
Hominidae - ps37
Homininae - ps38
Hominini - ps39
Homo_sapiens - ps40
p-value
3e-16
1e-12
3e-09
1e-05
3e-02
Log-odds
0.5
1.0
1.5
2.0
2.5
3.0
3.5
4.0

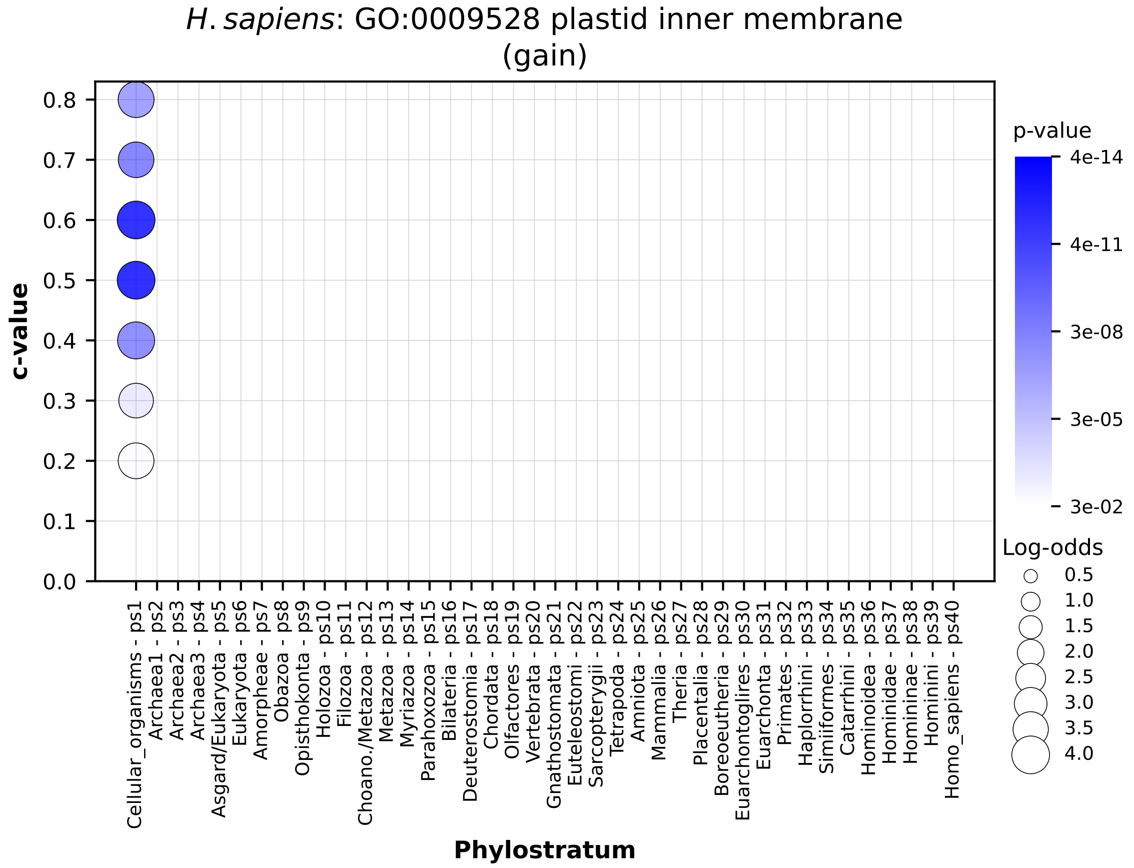

H. sapiens: GO:0009528 plastid inner membrane
(gain)
c-value
0.0
0.1
0.2
0.3
0.4
0.5
0.6
0.7
0.8
Phylostratum
Cellular_organisms - ps1
Archaea1 - ps2
Archaea2 - ps3
Archaea3 - ps4
Asgard/Eukaryota - ps5
Eukaryota - ps6
Amorpheae - ps7
Obazoa - ps8
Opisthokonta - ps9
Holozoa - ps10
Filozoa - ps11
Choano./Metazoa - ps12
Metazoa - ps13
Myriazoa - ps14
Parahoxozoa - ps15
Bilateria - ps16
Deuterostomia - ps17
Chordata - ps18
Olfactores - ps19
Vertebrata - ps20
Gnathostomata - ps21
Euteleostomi - ps22
Sarcopterygii - ps23
Tetrapoda - ps24
Amniota - ps25
Mammalia - ps26
Theria - ps27
Placentalia - ps28
Boreoeutheria - ps29
Euarchontoglires - ps30
Euarchonta - ps31
Primates - ps32
Haplorrhini - ps33
Simiiformes - ps34
Catarrhini - ps35
Hominoidea - ps36
Hominidae - ps37
Homininae - ps38
Hominini - ps39
Homo_sapiens - ps40
p-value
4e-14
4e-11
3e-08
3e-05
3e-02
Log-odds
0.5
1.0
1.5
2.0
2.5
3.0
3.5
4.0

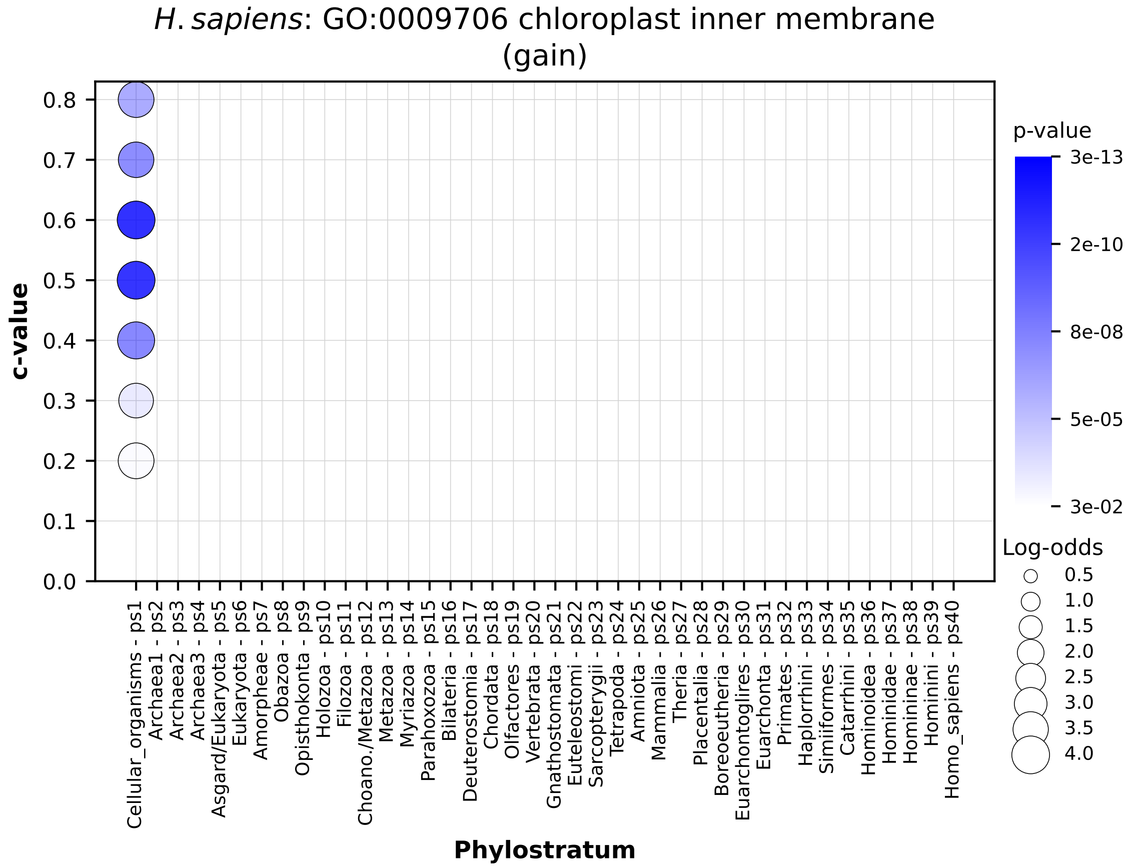

H. sapiens: GO:0009706 chloroplast inner membrane
(gain)
c-value
0.0
0.1
0.2
0.3
0.4
0.5
0.6
0.7
0.8
Phylostratum
Cellular_organisms - ps1
Archaea1 - ps2
Archaea2 - ps3
Archaea3 - ps4
Asgard/Eukaryota - ps5
Eukaryota - ps6
Amorpheae - ps7
Obazoa - ps8
Opisthokonta - ps9
Holozoa - ps10
Filozoa - ps11
Choano./Metazoa - ps12
Metazoa - ps13
Myriazoa - ps14
Parahoxozoa - ps15
Bilateria - ps16
Deuterostomia - ps17
Chordata - ps18
Olfactores - ps19
Vertebrata - ps20
Gnathostomata - ps21
Euteleostomi - ps22
Sarcopterygii - ps23
Tetrapoda - ps24
Amniota - ps25
Mammalia - ps26
Theria - ps27
Placentalia - ps28
Boreoeutheria - ps29
Euarchontoglires - ps30
Euarchonta - ps31
Primates - ps32
Haplorrhini - ps33
Simiiformes - ps34
Catarrhini - ps35
Hominoidea - ps36
Hominidae - ps37
Homininae - ps38
Hominini - ps39
Homo_sapiens - ps40
p-value
3e-13
2e-10
8e-08
5e-05
3e-02
Log-odds
0.5
1.0
1.5
2.0
2.5
3.0
3.5
4.0

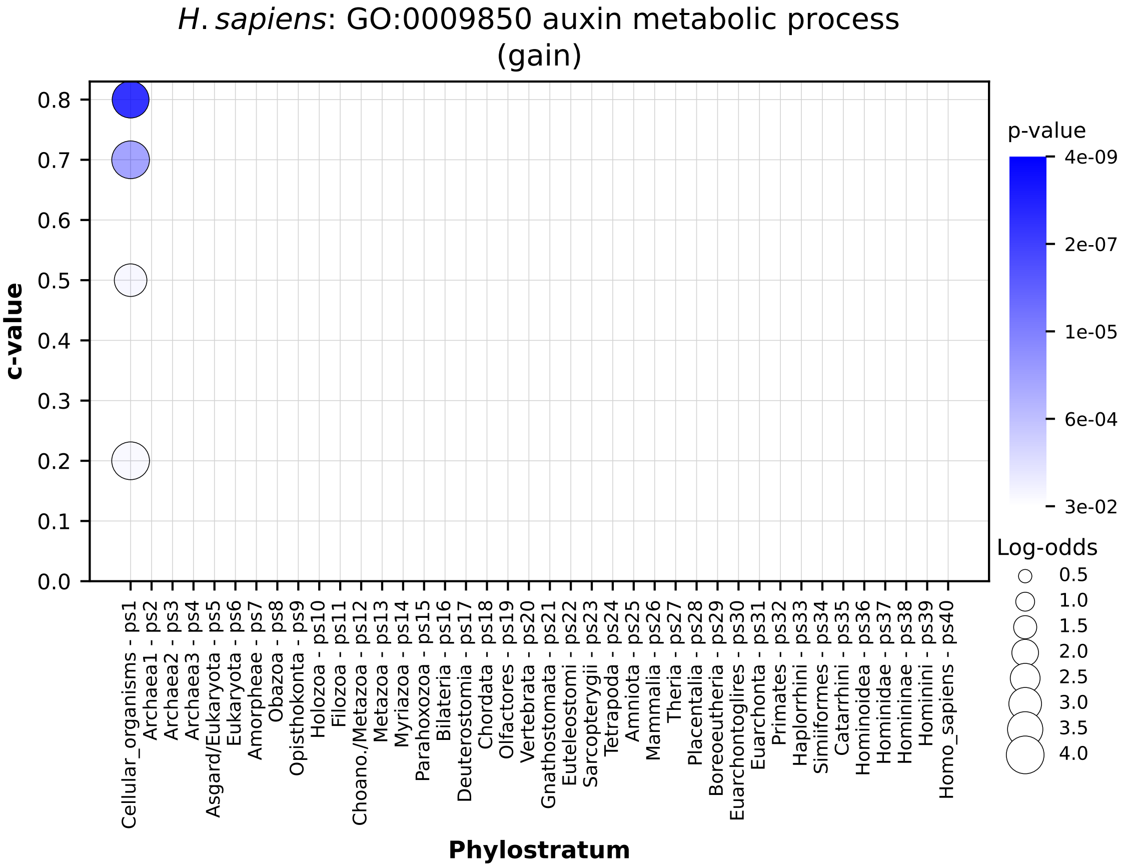

H. sapiens: GO:0009850 auxin metabolic process
(gain)
c-value
0.0
0.1
0.2
0.3
0.4
0.5
0.6
0.7
0.8
p-value
4e-09
2e-07
1e-05
6e-04
3e-02
Log-odds
0.5
1.0
1.5
2.0
2.5
3.0
3.5
4.0
Cellular_organisms - ps1
Archaea1 - ps2
Archaea2 - ps3
Archaea3 - ps4
Asgard/Eukaryota - ps5
Eukaryota - ps6
Amorpheae - ps7
Obazoa - ps8
Opisthokonta - ps9
Holozoa - ps10
Filozoa - ps11
Choano./Metazoa - ps12
Metazoa - ps13
Myriazoa - ps14
Parahoxozoa - ps15
Bilateria - ps16
Deuterostomia - ps17
Chordata - ps18
Olfactores - ps19
Vertebrata - ps20
Gnathostomata - ps21
Euteleostomi - ps22
Sarcopterygii - ps23
Tetrapoda - ps24
Amniota - ps25
Mammalia - ps26
Theria - ps27
Placentalia - ps28
Boreoeutheria - ps29
Euarchontoglires - ps30
Euarchonta - ps31
Primates - ps32
Haplorrhini - ps33
Simiiformes - ps34
Catarrhini - ps35
Hominoidea - ps36
Hominidae - ps37
Homininae - ps38
Hominini - ps39
Homo_sapiens - ps40
Phylostratum

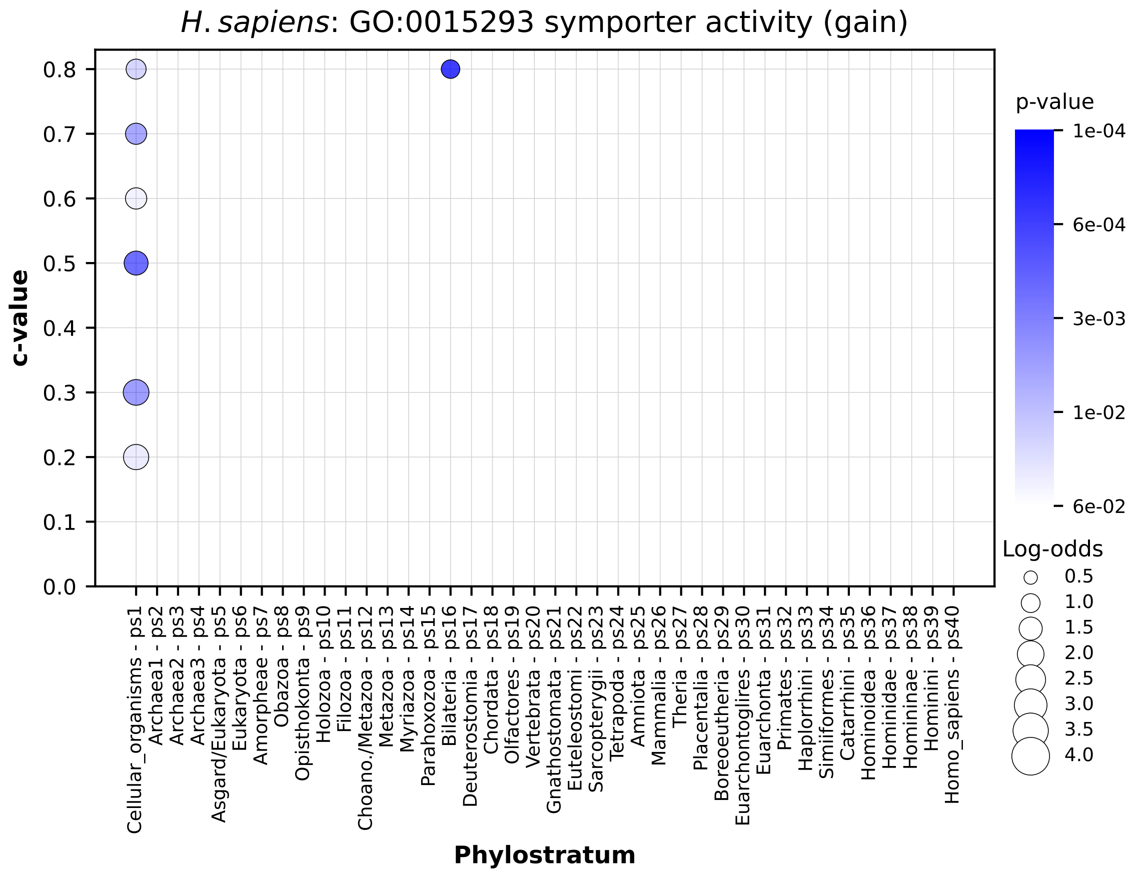

H. sapiens: GO:0015293 symporter activity (gain)
c-value
0.0
0.1
0.2
0.3
0.4
0.5
0.6
0.7
0.8
Phylostratum
Cellular_organisms - ps1
Archaea1 - ps2
Archaea2 - ps3
Archaea3 - ps4
Asgard/Eukaryota - ps5
Eukaryota - ps6
Amorpheae - ps7
Obazoa - ps8
Opisthokonta - ps9
Holozoa - ps10
Filozoa - ps11
Choano./Metazoa - ps12
Metazoa - ps13
Myriazoa - ps14
Parahoxozoa - ps15
Bilateria - ps16
Deuterostomia - ps17
Chordata - ps18
Olfactores - ps19
Vertebrata - ps20
Gnathostomata - ps21
Euteleostomi - ps22
Sarcopterygii - ps23
Tetrapoda - ps24
Amniota - ps25
Mammalia - ps26
Theria - ps27
Placentalia - ps28
Boreoeutheria - ps29
Euarchontoglires - ps30
Euarchonta - ps31
Primates - ps32
Haplorrhini - ps33
Simiiformes - ps34
Catarrhini - ps35
Hominoidea - ps36
Hominidae - ps37
Homininae - ps38
Hominini - ps39
Homo_sapiens - ps40
p-value
1e-04
6e-04
3e-03
1e-02
6e-02
Log-odds
0.5
1.0
1.5
2.0
2.5
3.0
3.5
4.0

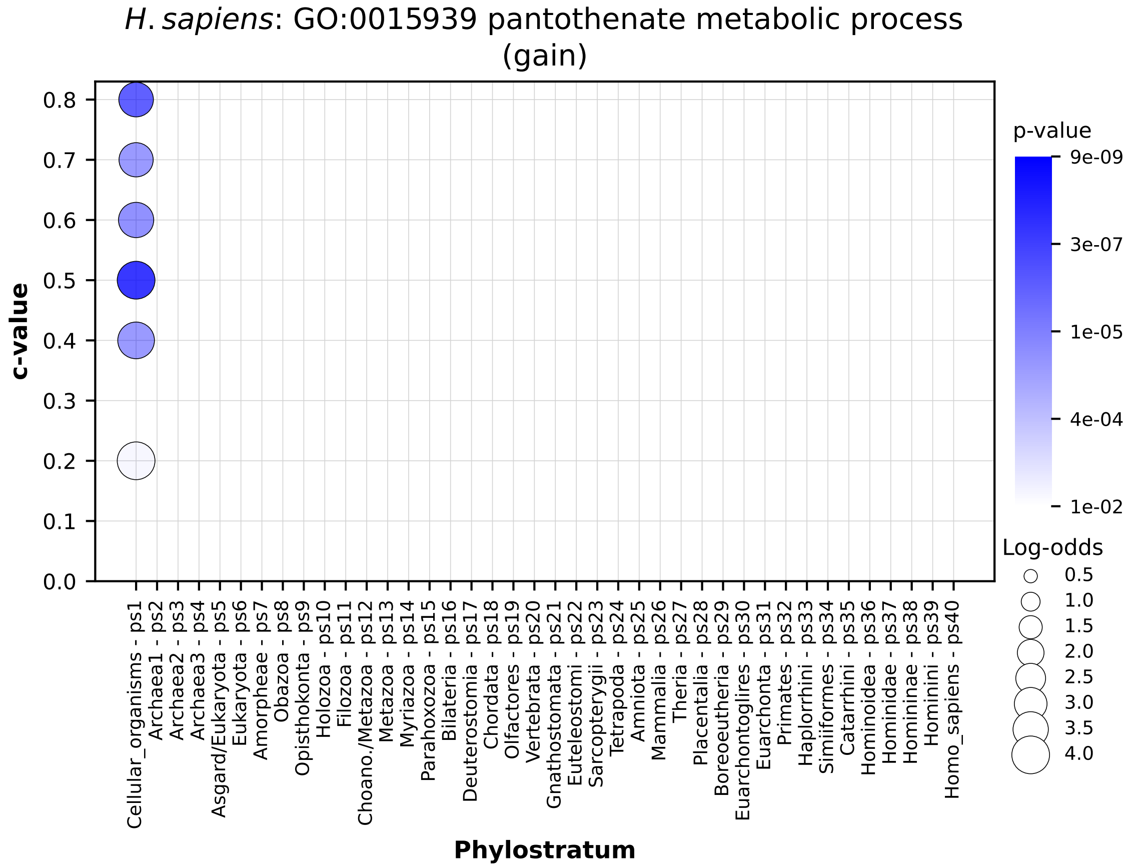
H. sapiens: GO:0015939 pantothenate metabolic process
(gain)
c-value
0.0
0.1
0.2
0.3
0.4
0.5
0.6
0.7
0.8
Phylostratum
Cellular_organisms - ps1
Archaea1 - ps2
Archaea2 - ps3
Archaea3 - ps4
Asgard/Eukaryota - ps5
Eukaryota - ps6
Amorpheae - ps7
Obazoa - ps8
Opisthokonta - ps9
Holozoa - ps10
Filozoa - ps11
Choano./Metazoa - ps12
Metazoa - ps13
Myriazoa - ps14
Parahoxozoa - ps15
Bilateria - ps16
Deuterostomia - ps17
Chordata - ps18
Olfactores - ps19
Vertebrata - ps20
Gnathostomata - ps21
Euteleostomi - ps22
Sarcopterygii - ps23
Tetrapoda - ps24
Amniota - ps25
Mammalia - ps26
Theria - ps27
Placentalia - ps28
Boreoeutheria - ps29
Euarchontoglires - ps30
Euarchonta - ps31
Primates - ps32
Haplorrhini - ps33
Simiiformes - ps34
Catarrhini - ps35
Hominoidea - ps36
Hominidae - ps37
Homininae - ps38
Hominini - ps39
Homo_sapiens - ps40
p-value
9e-09
3e-07
1e-05
4e-04
1e-02
Log-odds
0.5
1.0
1.5
2.0
2.5
3.0
3.5
4.0

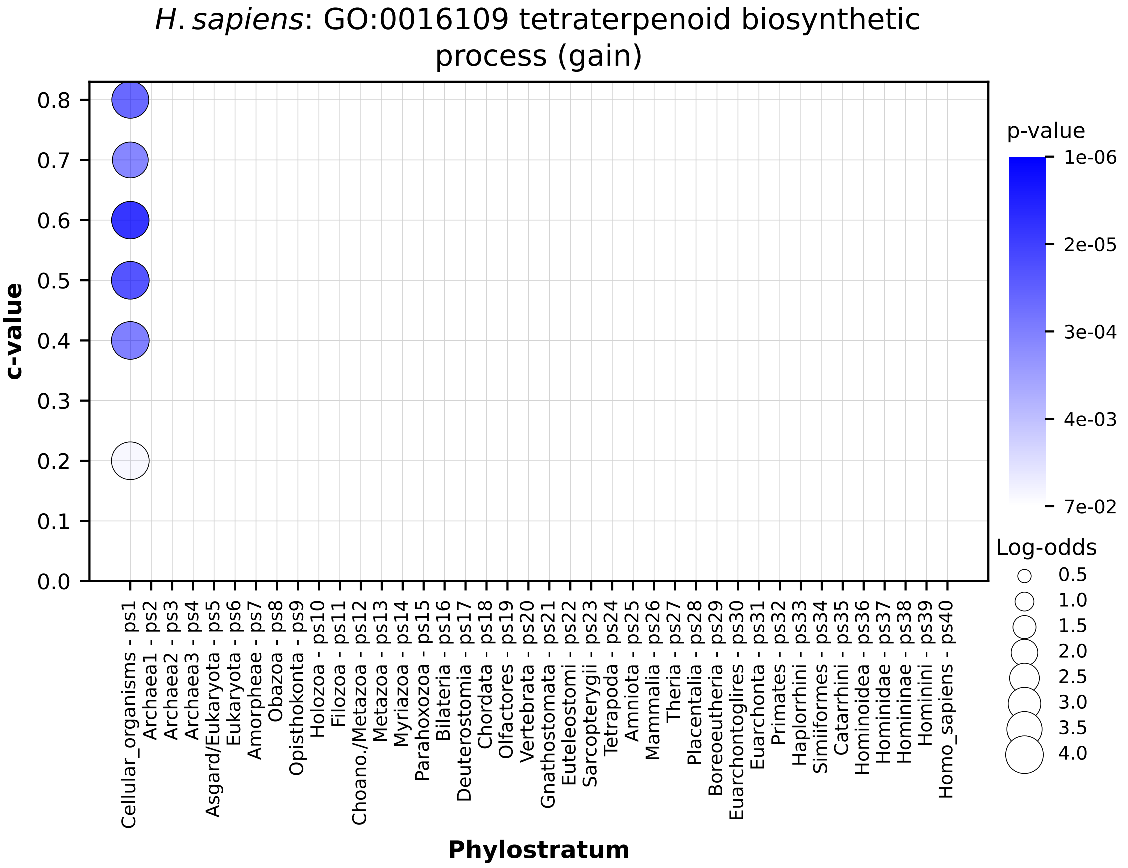

*H. sapiens*: GO:0016109 tetraterpenoid biosynthetic process (gain)
c-value
0.8
0.7
0.6
0.5
0.4
0.3
0.2
0.1
0.0
Phylostratum
Cellular_organisms - ps1
Archaea1 - ps2
Archaea2 - ps3
Archaea3 - ps4
Asgard/Eukaryota - ps5
Eukaryota - ps6
Amorpheae - ps7
Obazoa - ps8
Opisthokonta - ps9
Holozoa - ps10
Filozoa - ps11
Choano./Metazoa - ps12
Metazoa - ps13
Myriazoa - ps14
Parahoxozoa - ps15
Bilateria - ps16
Deuterostomia - ps17
Chordata - ps18
Olfactores - ps19
Vertebrata - ps20
Gnathostomata - ps21
Euteleostomi - ps22
Sarcopterygii - ps23
Tetrapoda - ps24
Amniota - ps25
Mammalia - ps26
Theria - ps27
Placentalia - ps28
Boreoeutheria - ps29
Euarchontoglires - ps30
Euarchonta - ps31
Primates - ps32
Haplorrhini - ps33
Simiiformes - ps34
Catarrhini - ps35
Hominoidea - ps36
Hominidae - ps37
Homininae - ps38
Hominini - ps39
Homo_sapiens - ps40
p-value
1e-06
2e-05
3e-04
4e-03
7e-02
Log-odds
0.5
1.0
1.5
2.0
2.5
3.0
3.5
4.0

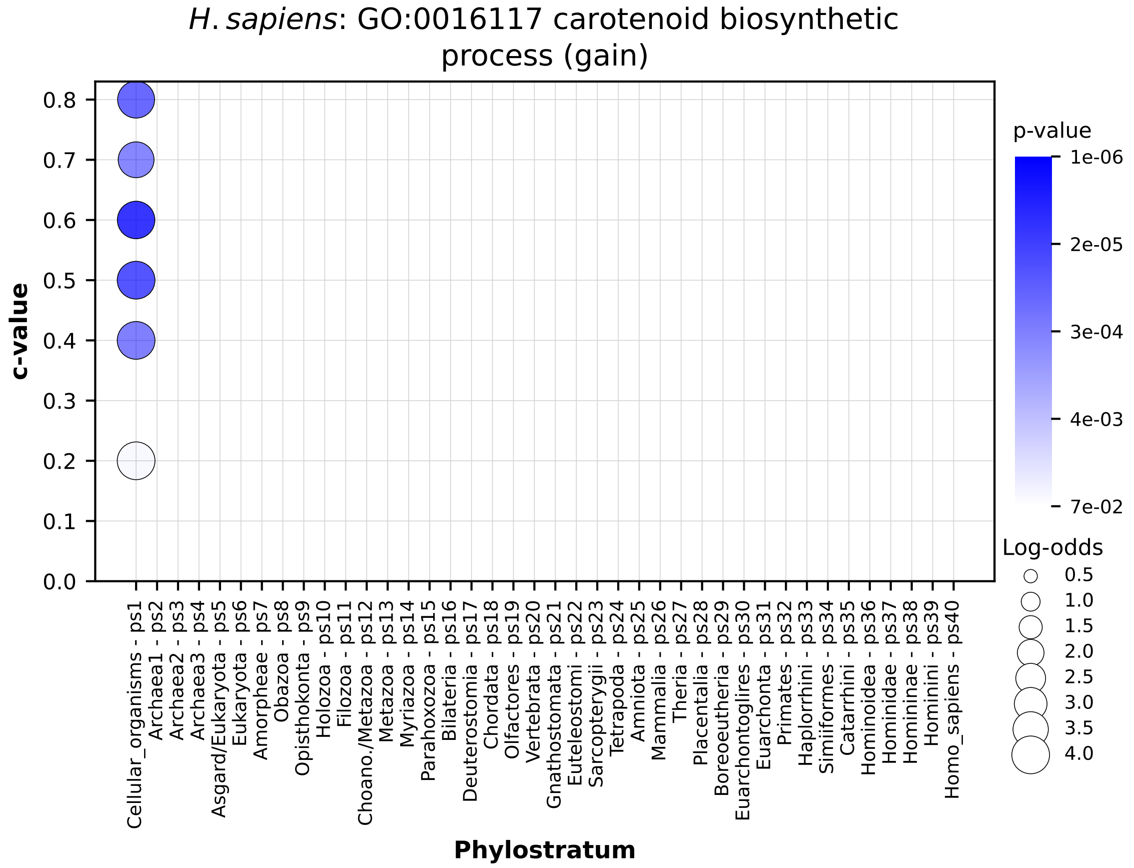

H. sapiens: GO:0016117 carotenoid biosynthetic process (gain)
c-value
0.8
0.7
0.6
0.5
0.4
0.3
0.2
0.1
0.0
p-value
1e-06
2e-05
3e-04
4e-03
7e-02
Log-odds
0.5
1.0
1.5
2.0
2.5
3.0
3.5
4.0
Cellular_organisms - ps1
Archaea1 - ps2
Archaea2 - ps3
Archaea3 - ps4
Asgard/Eukaryota - ps5
Eukaryota - ps6
Amorpheae - ps7
Obazoa - ps8
Opisthokonta - ps9
Holozoa - ps10
Filozoa - ps11
Choano./Metazoa - ps12
Metazoa - ps13
Myriazoa - ps14
Parahoxozoa - ps15
Bilateria - ps16
Deuterostomia - ps17
Chordata - ps18
Olfactores - ps19
Vertebrata - ps20
Gnathostomata - ps21
Euteleostomi - ps22
Sarcopterygii - ps23
Tetrapoda - ps24
Amniota - ps25
Mammalia - ps26
Theria - ps27
Placentalia - ps28
Boreoeutheria - ps29
Euarchontoglires - ps30
Euarchonta - ps31
Primates - ps32
Haplorrhini - ps33
Simiiformes - ps34
Catarrhini - ps35
Hominoidea - ps36
Hominidae - ps37
Homininae - ps38
Hominini - ps39
Homo_sapiens - ps40
Phylostratum

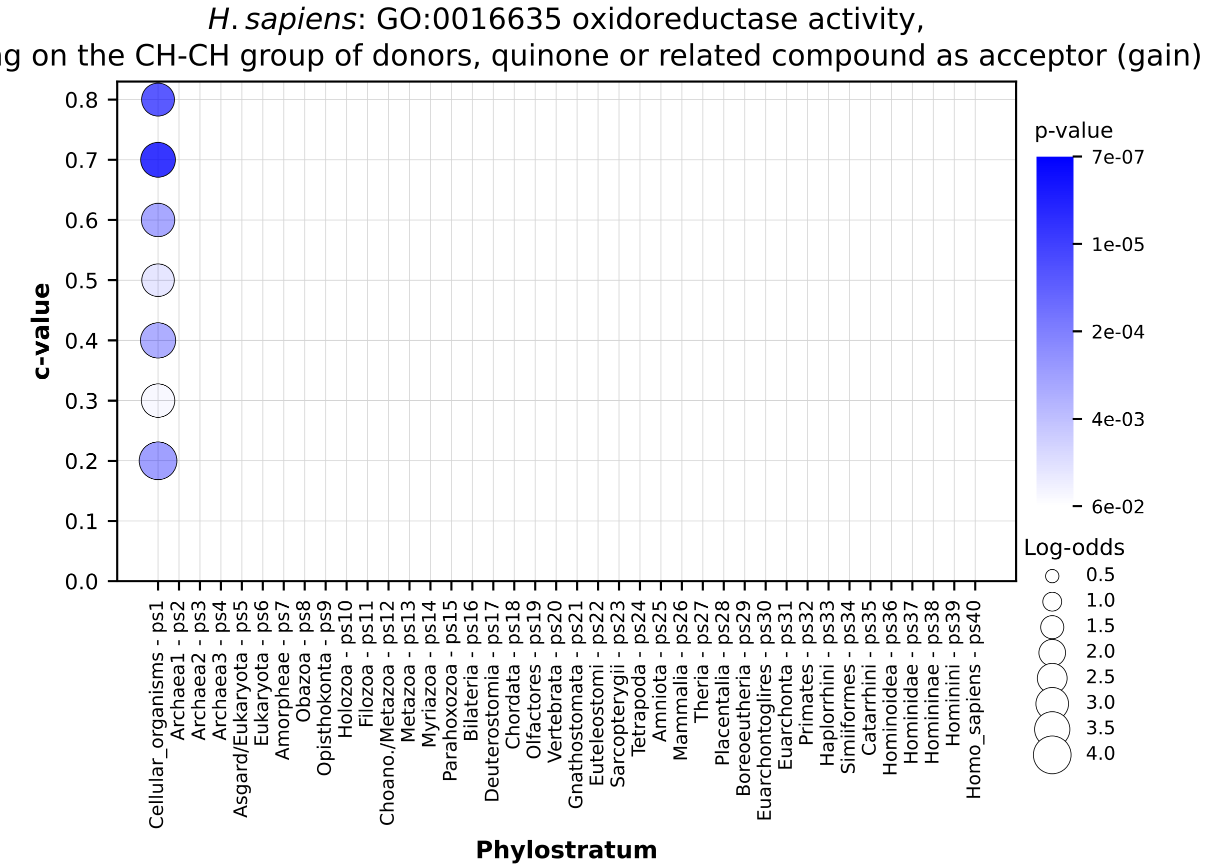
H. sapiens: GO:0016635 oxidoreductase activity,
g on the CH-CH group of donors, quinone or related compound as acceptor (gain)
c-value
0.0
0.1
0.2
0.3
0.4
0.5
0.6
0.7
0.8
Phylostratum
Cellular_organisms - ps1
Archaea1 - ps2
Archaea2 - ps3
Archaea3 - ps4
Asgard/Eukaryota - ps5
Eukaryota - ps6
Amorpheae - ps7
Obazoa - ps8
Opisthokonta - ps9
Holozoa - ps10
Filozoa - ps11
Choano./Metazoa - ps12
Metazoa - ps13
Myriazoa - ps14
Parahoxozoa - ps15
Bilateria - ps16
Deuterostomia - ps17
Chordata - ps18
Olfactores - ps19
Vertebrata - ps20
Gnathostomata - ps21
Euteleostomi - ps22
Sarcopterygii - ps23
Tetrapoda - ps24
Amniota - ps25
Mammalia - ps26
Theria - ps27
Placentalia - ps28
Boreoeutheria - ps29
Euarchontoglires - ps30
Euarchonta - ps31
Primates - ps32
Haplorrhini - ps33
Simiiformes - ps34
Catarrhini - ps35
Hominoidea - ps36
Hominidae - ps37
Homininae - ps38
Hominini - ps39
Homo_sapiens - ps40
p-value
7e-07
1e-05
2e-04
4e-03
6e-02
Log-odds
0.5
1.0
1.5
2.0
2.5
3.0
3.5
4.0

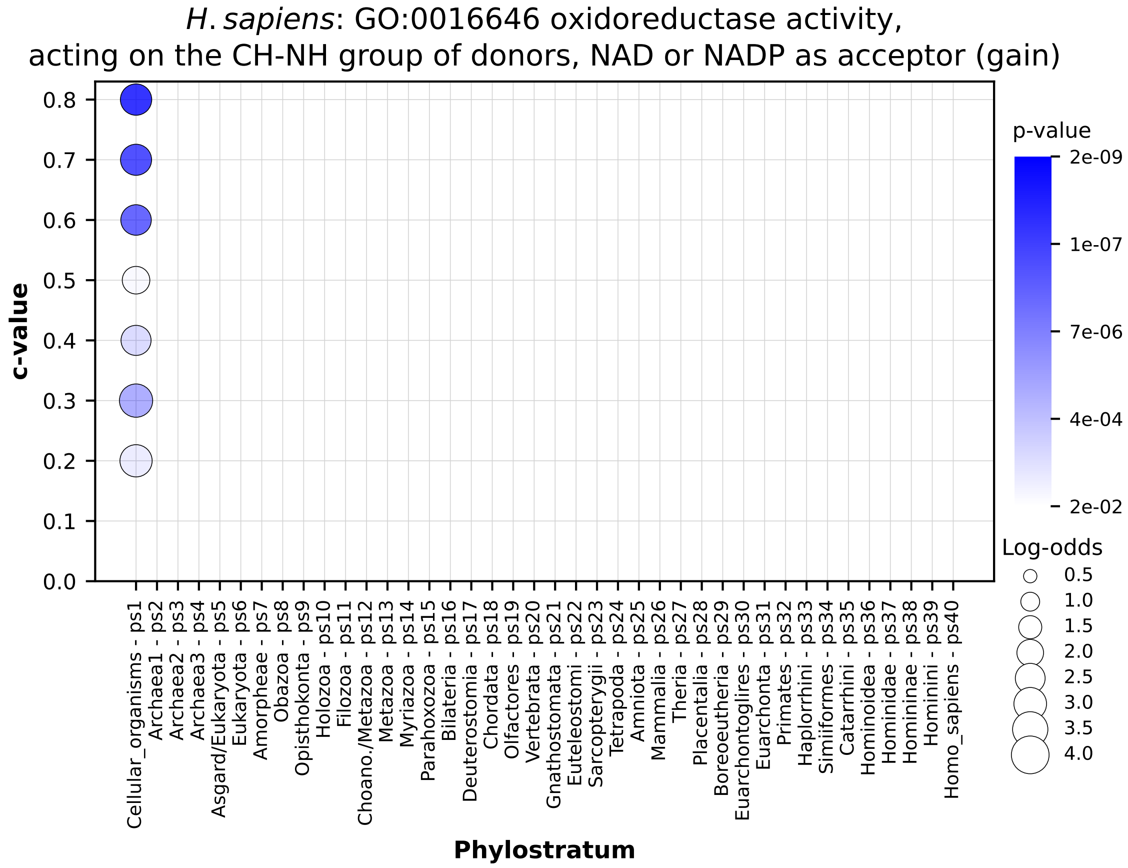

*H. sapiens*: GO:0016646 oxidoreductase activity,
acting on the CH-NH group of donors, NAD or NADP as acceptor (gain)
c-value
0.0
0.1
0.2
0.3
0.4
0.5
0.6
0.7
0.8
Phylostratum
Cellular_organisms - ps1
Archaea1 - ps2
Archaea2 - ps3
Archaea3 - ps4
Asgard/Eukaryota - ps5
Eukaryota - ps6
Amorpheae - ps7
Obazoa - ps8
Opisthokonta - ps9
Holozoa - ps10
Filozoa - ps11
Choano./Metazoa - ps12
Metazoa - ps13
Myriazoa - ps14
Parahoxozoa - ps15
Bilateria - ps16
Deuterostomia - ps17
Chordata - ps18
Olfactores - ps19
Vertebrata - ps20
Gnathostomata - ps21
Euteleostomi - ps22
Sarcopterygii - ps23
Tetrapoda - ps24
Amniota - ps25
Mammalia - ps26
Theria - ps27
Placentalia - ps28
Boreoeutheria - ps29
Euarchontoglires - ps30
Euarchonta - ps31
Primates - ps32
Haplorrhini - ps33
Simiiformes - ps34
Catarrhini - ps35
Hominoidea - ps36
Hominidae - ps37
Homininae - ps38
Hominini - ps39
Homo_sapiens - ps40
p-value
2e-09
1e-07
7e-06
4e-04
2e-02
Log-odds
0.5
1.0
1.5
2.0
2.5
3.0
3.5
4.0

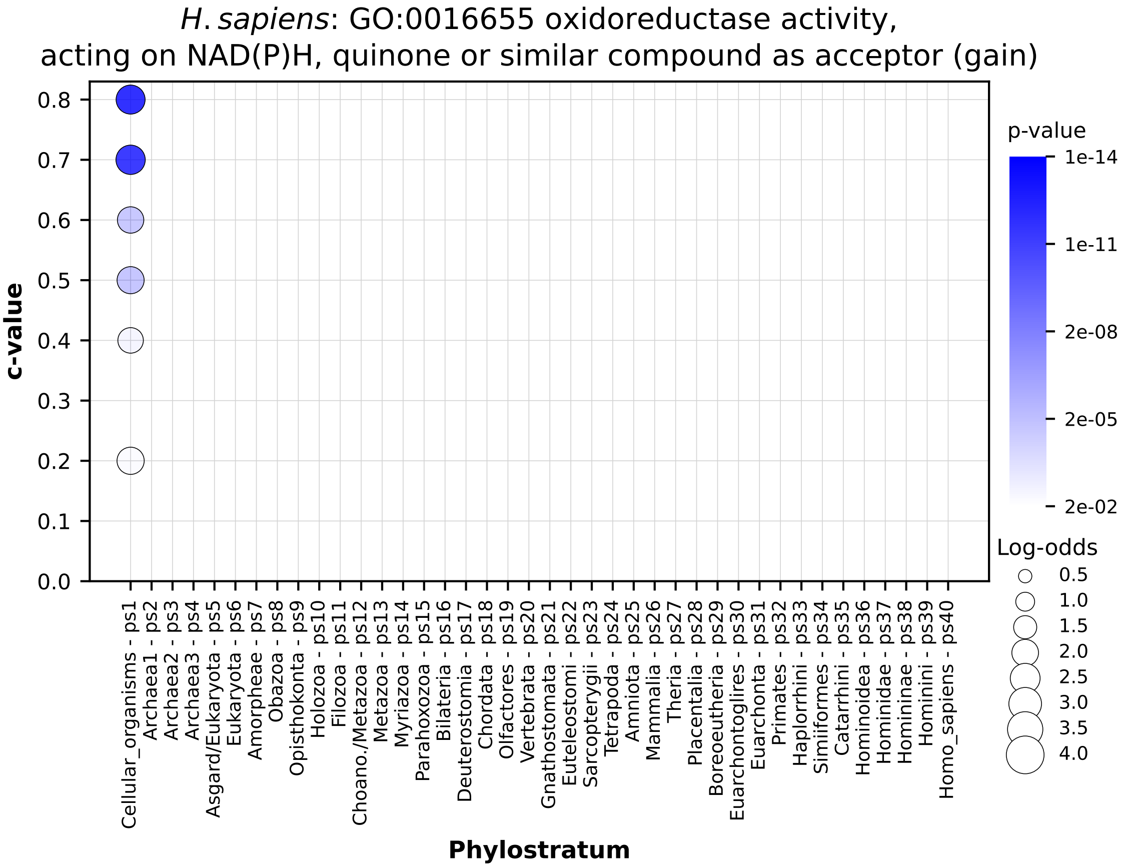

*H. sapiens*: GO:0016655 oxidoreductase activity,
acting on NAD(P)H, quinone or similar compound as acceptor (gain)
c-value
0.0
0.1
0.2
0.3
0.4
0.5
0.6
0.7
0.8
Phylostratum
Cellular_organisms - ps1
Archaea1 - ps2
Archaea2 - ps3
Archaea3 - ps4
Asgard/Eukaryota - ps5
Eukaryota - ps6
Amorpheae - ps7
Obazoa - ps8
Opisthokonta - ps9
Holozoa - ps10
Filozoa - ps11
Choano./Metazoa - ps12
Metazoa - ps13
Myriazoa - ps14
Parahoxozoa - ps15
Bilateria - ps16
Deuterostomia - ps17
Chordata - ps18
Olfactores - ps19
Vertebrata - ps20
Gnathostomata - ps21
Euteleostomi - ps22
Sarcopterygii - ps23
Tetrapoda - ps24
Amniota - ps25
Mammalia - ps26
Theria - ps27
Placentalia - ps28
Boreoeutheria - ps29
Euarchontoglires - ps30
Euarchonta - ps31
Primates - ps32
Haplorrhini - ps33
Simiiformes - ps34
Catarrhini - ps35
Hominoidea - ps36
Hominidae - ps37
Homininae - ps38
Hominini - ps39
Homo_sapiens - ps40
p-value
1e-14
1e-11
2e-08
2e-05
2e-02
Log-odds
0.5
1.0
1.5
2.0
2.5
3.0
3.5
4.0

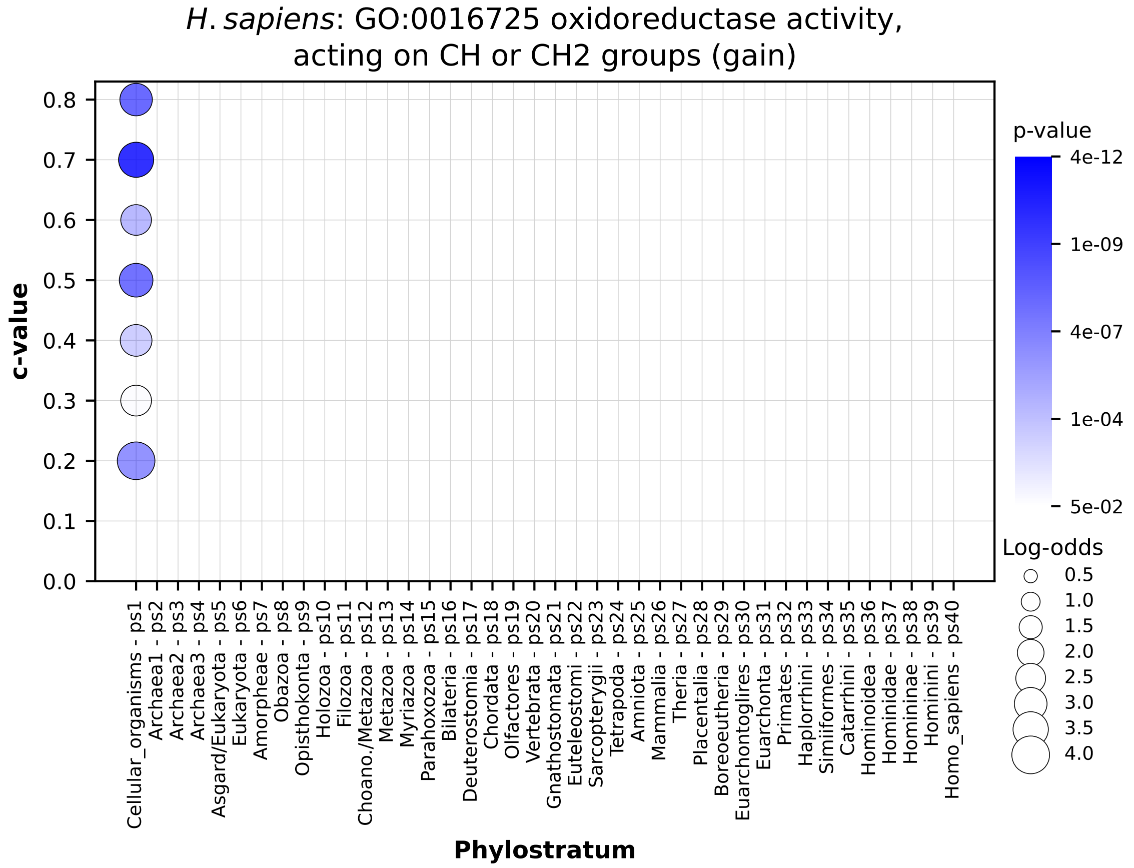

H. sapiens: GO:0016725 oxidoreductase activity, acting on CH or CH2 groups (gain)
c-value
0.0
0.1
0.2
0.3
0.4
0.5
0.6
0.7
0.8
Phylostratum
Cellular_organisms - ps1
Archaea1 - ps2
Archaea2 - ps3
Archaea3 - ps4
Asgard/Eukaryota - ps5
Eukaryota - ps6
Amorpheae - ps7
Obazoa - ps8
Opisthokonta - ps9
Holozoa - ps10
Filozoa - ps11
Choano./Metazoa - ps12
Metazoa - ps13
Myriazoa - ps14
Parahoxozoa - ps15
Bilateria - ps16
Deuterostomia - ps17
Chordata - ps18
Olfactores - ps19
Vertebrata - ps20
Gnathostomata - ps21
Euteleostomi - ps22
Sarcopterygii - ps23
Tetrapoda - ps24
Amniota - ps25
Mammalia - ps26
Theria - ps27
Placentalia - ps28
Boreoeutheria - ps29
Euarchontoglires - ps30
Euarchonta - ps31
Primates - ps32
Haplorrhini - ps33
Simiiformes - ps34
Catarrhini - ps35
Hominoidea - ps36
Hominidae - ps37
Homininae - ps38
Hominini - ps39
Homo_sapiens - ps40
p-value
4e-12
1e-09
4e-07
1e-04
5e-02
Log-odds
0.5
1.0
1.5
2.0
2.5
3.0
3.5
4.0

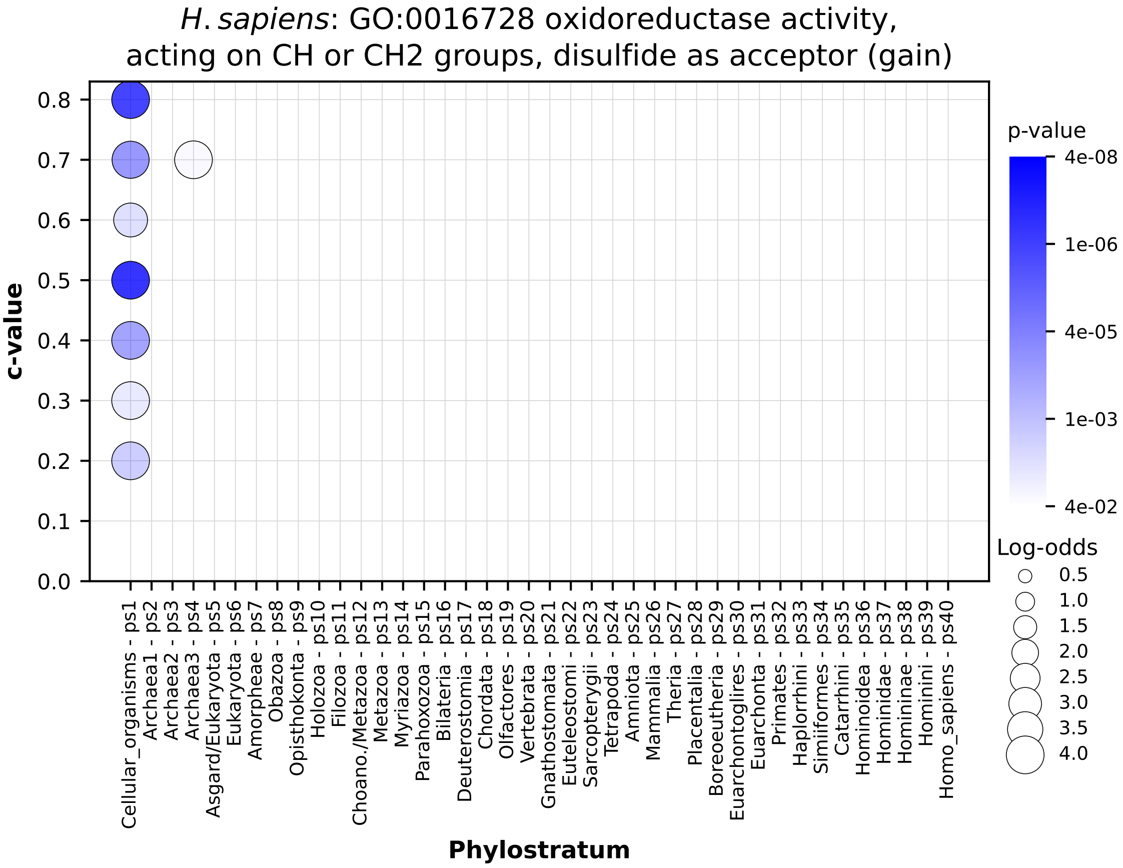
*H. sapiens*: GO:0016728 oxidoreductase activity,
acting on CH or CH2 groups, disulfide as acceptor (gain)
c-value
0.0
0.1
0.2
0.3
0.4
0.5
0.6
0.7
0.8
Phylostratum
Cellular_organisms - ps1
Archaea1 - ps2
Archaea2 - ps3
Archaea3 - ps4
Asgard/Eukaryota - ps5
Eukaryota - ps6
Amorpheae - ps7
Obazoa - ps8
Opisthokonta - ps9
Holozoa - ps10
Filozoa - ps11
Choano./Metazoa - ps12
Metazoa - ps13
Myriazoa - ps14
Parahoxozoa - ps15
Bilateria - ps16
Deuterostomia - ps17
Chordata - ps18
Olfactores - ps19
Vertebrata - ps20
Gnathostomata - ps21
Euteleostomi - ps22
Sarcopterygii - ps23
Tetrapoda - ps24
Amniota - ps25
Mammalia - ps26
Theria - ps27
Placentalia - ps28
Boreoeutheria - ps29
Euarchontoglires - ps30
Euarchonta - ps31
Primates - ps32
Haplorrhini - ps33
Simiiformes - ps34
Catarrhini - ps35
Hominoidea - ps36
Hominidae - ps37
Homininae - ps38
Hominini - ps39
Homo_sapiens - ps40
p-value
4e-08
1e-06
4e-05
1e-03
4e-02
Log-odds
0.5
1.0
1.5
2.0
2.5
3.0
3.5
4.0

*H. sapiens*: GO:0016775 phosphotransferase activity, nitrogenous group as acceptor (gain)

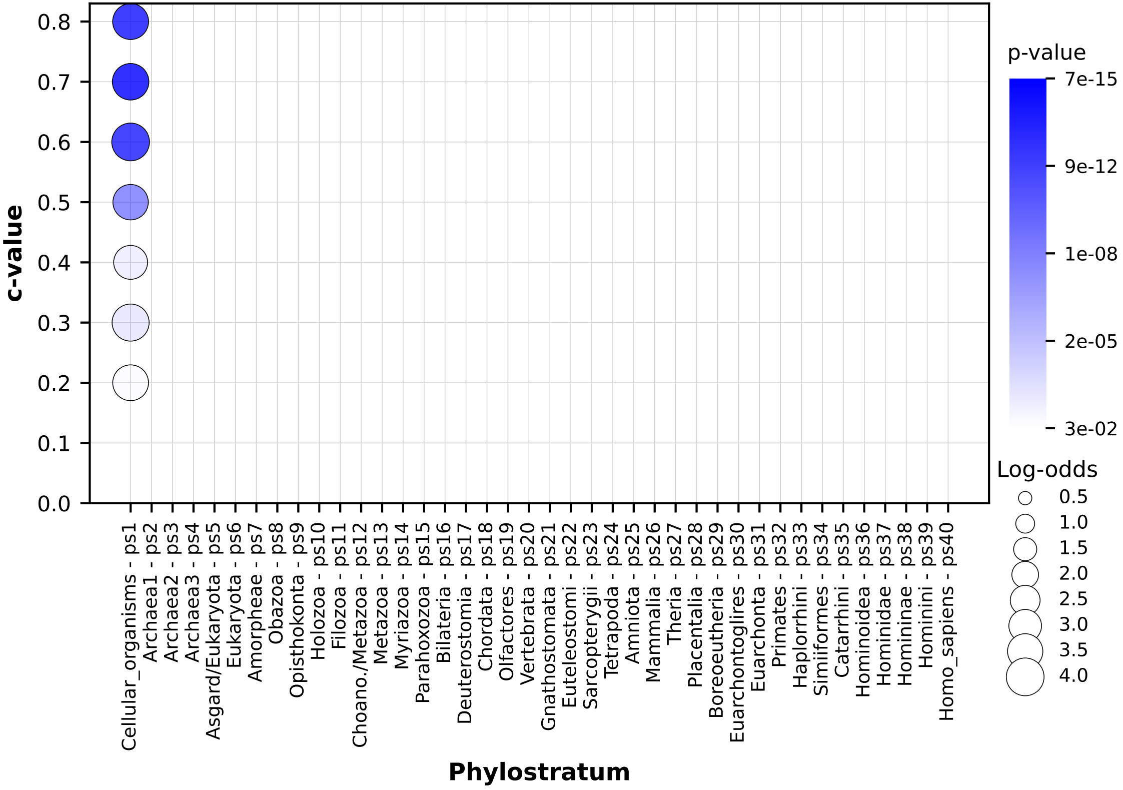

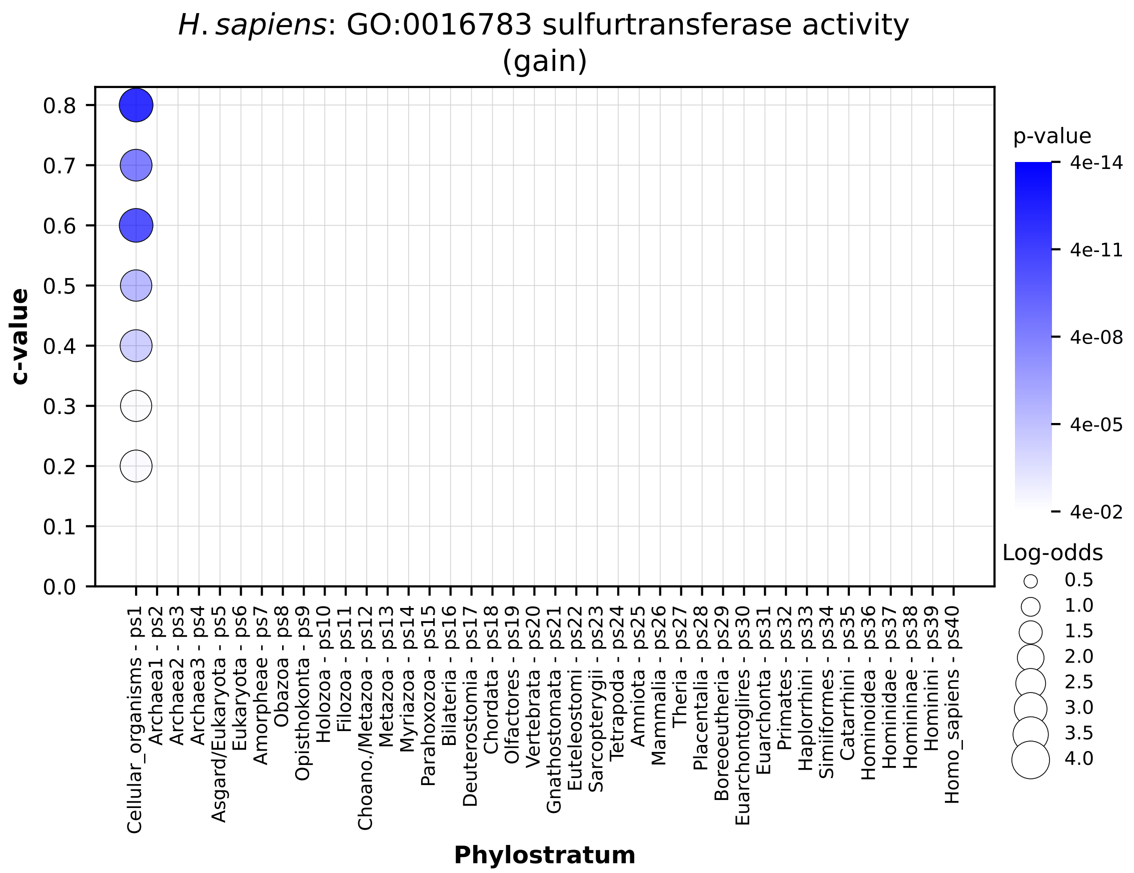

*H. sapiens*: GO:0016783 sulfurtransferase activity
(gain)
c-value
0.0
0.1
0.2
0.3
0.4
0.5
0.6
0.7
0.8
Phylostratum
Cellular_organisms - ps1
Archaea1 - ps2
Archaea2 - ps3
Archaea3 - ps4
Asgard/Eukaryota - ps5
Eukaryota - ps6
Amorpheae - ps7
Obazoa - ps8
Opisthokonta - ps9
Holozoa - ps10
Filozoa - ps11
Choano./Metazoa - ps12
Metazoa - ps13
Myriazoa - ps14
Parahoxozoa - ps15
Bilateria - ps16
Deuterostomia - ps17
Chordata - ps18
Olfactores - ps19
Vertebrata - ps20
Gnathostomata - ps21
Euteleostomi - ps22
Sarcopterygii - ps23
Tetrapoda - ps24
Amniota - ps25
Mammalia - ps26
Theria - ps27
Placentalia - ps28
Boreoeutheria - ps29
Euarchontoglires - ps30
Euarchonta - ps31
Primates - ps32
Haplorrhini - ps33
Simiiformes - ps34
Catarrhini - ps35
Hominoidea - ps36
Hominidae - ps37
Homininae - ps38
Hominini - ps39
Homo_sapiens - ps40
p-value
4e-14
4e-11
4e-08
4e-05
4e-02
Log-odds
0.5
1.0
1.5
2.0
2.5
3.0
3.5
4.0

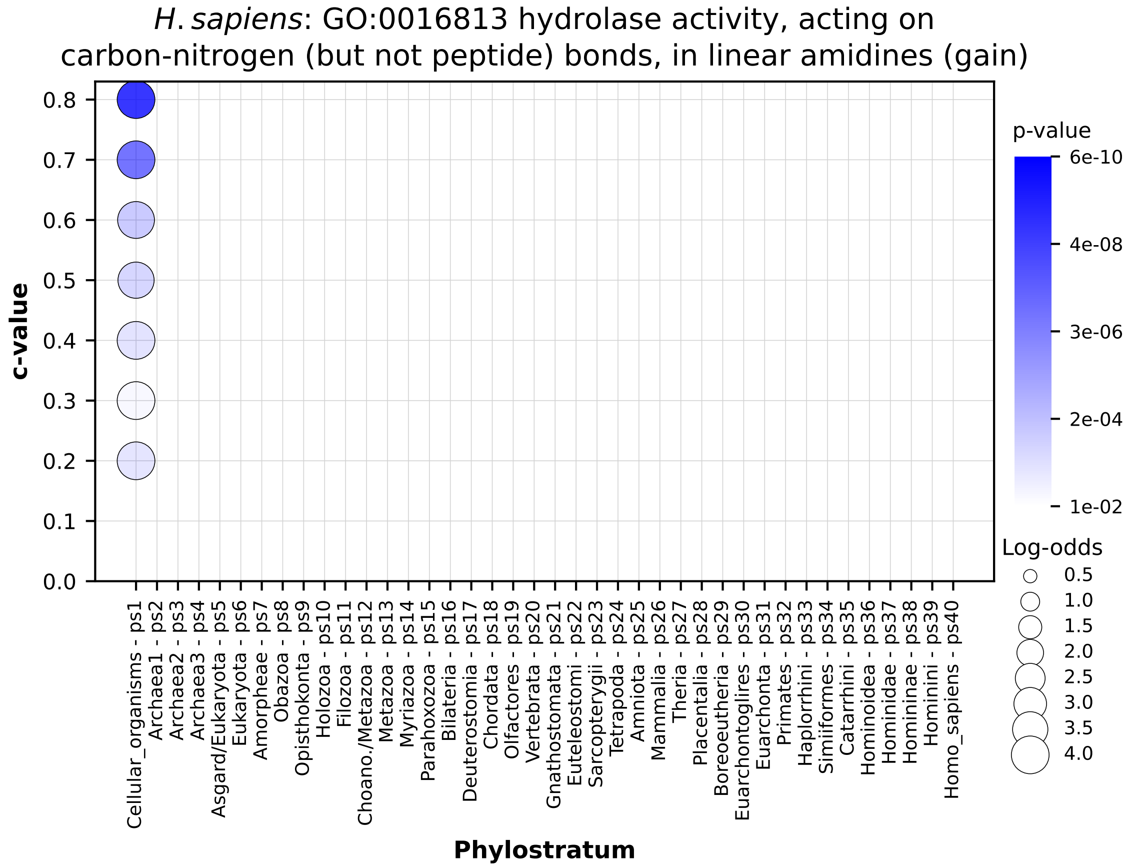
*H. sapiens*: GO:0016813 hydrolase activity, acting on carbon-nitrogen (but not peptide) bonds, in linear amidines (gain)
c-value
0.0
0.1
0.2
0.3
0.4
0.5
0.6
0.7
0.8
p-value
6e-10
4e-08
3e-06
2e-04
1e-02
Log-odds
0.5
1.0
1.5
2.0
2.5
3.0
3.5
4.0
Cellular_organisms - ps1
Archaea1 - ps2
Archaea2 - ps3
Archaea3 - ps4
Asgard/Eukaryota - ps5
Eukaryota - ps6
Amorpheae - ps7
Obazoa - ps8
Opisthokonta - ps9
Holozoa - ps10
Filozoa - ps11
Choano./Metazoa - ps12
Metazoa - ps13
Myriazoa - ps14
Parahoxozoa - ps15
Bilateria - ps16
Deuterostomia - ps17
Chordata - ps18
Olfactores - ps19
Vertebrata - ps20
Gnathostomata - ps21
Euteleostomi - ps22
Sarcopterygii - ps23
Tetrapoda - ps24
Amniota - ps25
Mammalia - ps26
Theria - ps27
Placentalia - ps28
Boreoeutheria - ps29
Euarchontoglires - ps30
Euarchonta - ps31
Primates - ps32
Haplorrhini - ps33
Simiiformes - ps34
Catarrhini - ps35
Hominoidea - ps36
Hominidae - ps37
Homininae - ps38
Hominini - ps39
Homo_sapiens - ps40
Phylostratum

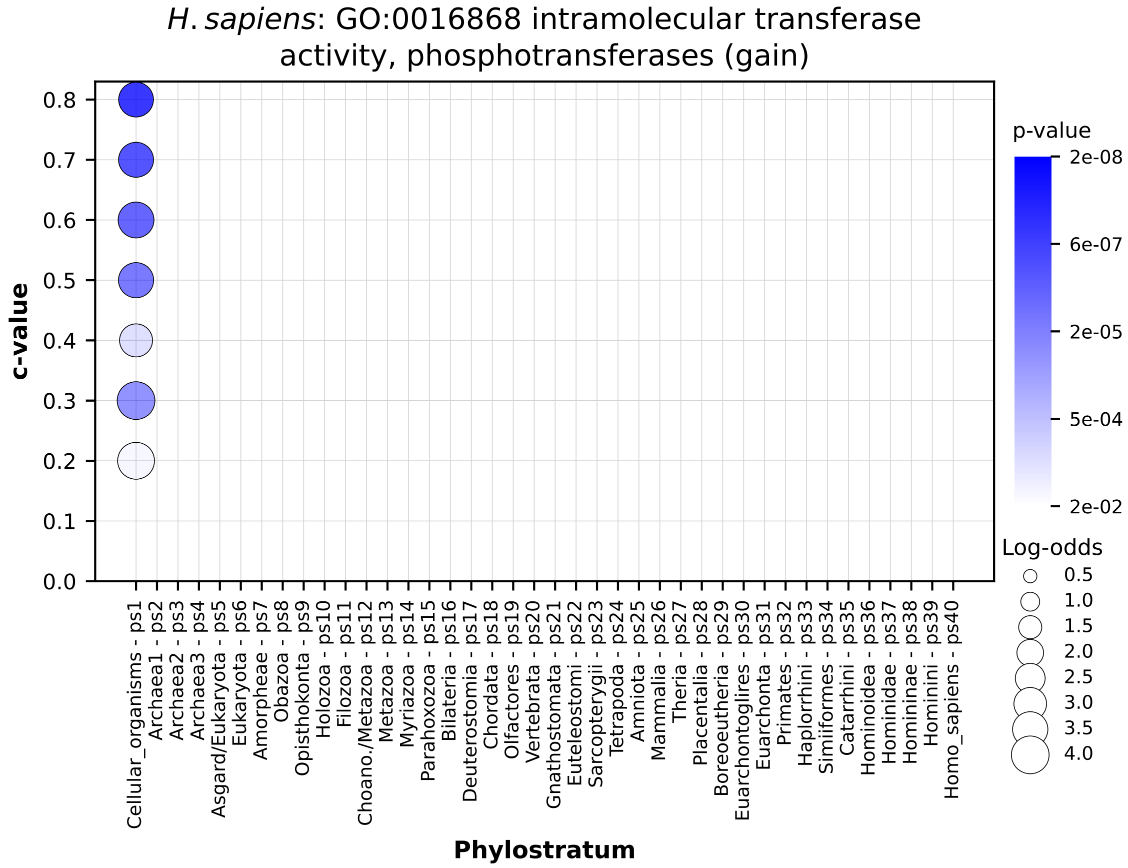

H. sapiens: GO:0016868 intramolecular transferase activity, phosphotransferases (gain)
c-value
0.0
0.1
0.2
0.3
0.4
0.5
0.6
0.7
0.8
Phylostratum
Cellular_organisms - ps1
Archaea1 - ps2
Archaea2 - ps3
Archaea3 - ps4
Asgard/Eukaryota - ps5
Eukaryota - ps6
Amorpheae - ps7
Obazoa - ps8
Opisthokonta - ps9
Holozoa - ps10
Filozoa - ps11
Choano./Metazoa - ps12
Metazoa - ps13
Myriazoa - ps14
Parahoxozoa - ps15
Bilateria - ps16
Deuterostomia - ps17
Chordata - ps18
Olfactores - ps19
Vertebrata - ps20
Gnathostomata - ps21
Euteleostomi - ps22
Sarcopterygii - ps23
Tetrapoda - ps24
Amniota - ps25
Mammalia - ps26
Theria - ps27
Placentalia - ps28
Boreoeutheria - ps29
Euarchontoglires - ps30
Euarchonta - ps31
Primates - ps32
Haplorrhini - ps33
Simiiformes - ps34
Catarrhini - ps35
Hominoidea - ps36
Hominidae - ps37
Homininae - ps38
Hominini - ps39
Homo_sapiens - ps40
p-value
2e-08
6e-07
2e-05
5e-04
2e-02
Log-odds
0.5
1.0
1.5
2.0
2.5
3.0
3.5
4.0

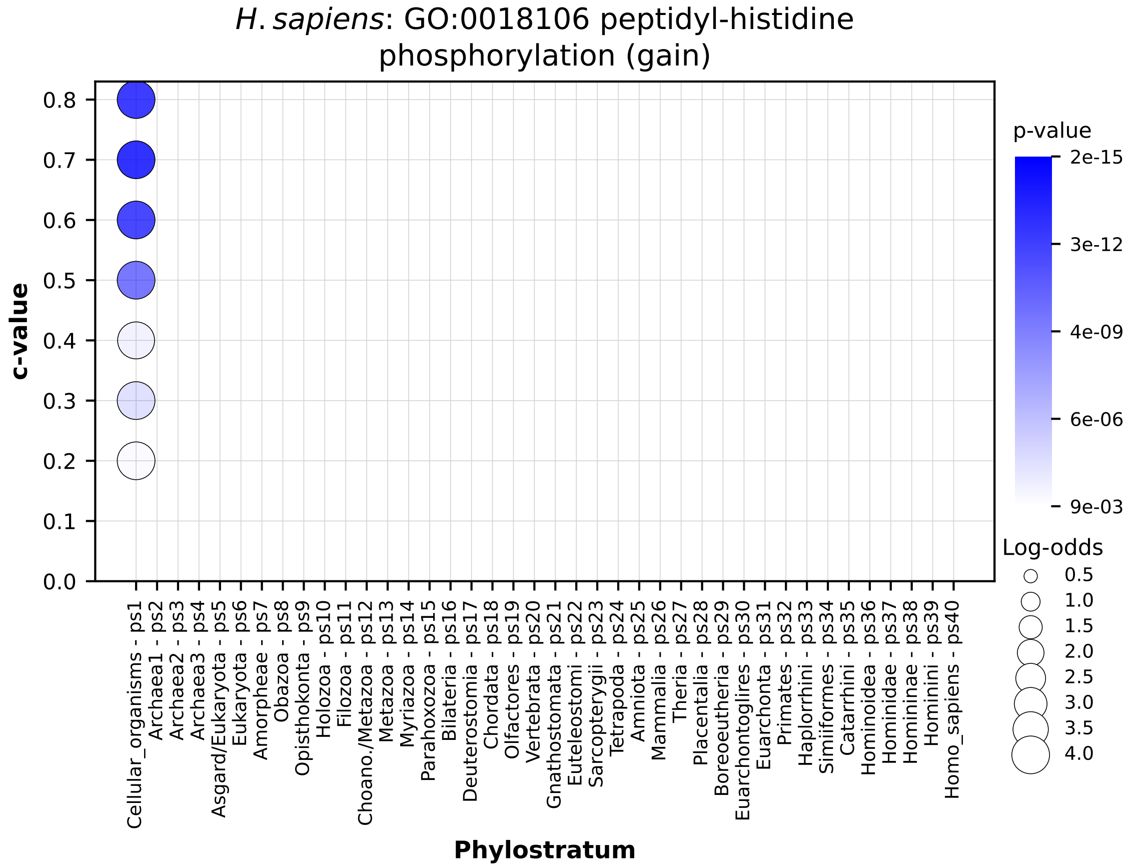

H. sapiens: GO:0018106 peptidyl-histidine phosphorylation (gain)
c-value
0.8
0.7
0.6
0.5
0.4
0.3
0.2
0.1
0.0
Phylostratum
Cellular_organisms - ps1
Archaea1 - ps2
Archaea2 - ps3
Archaea3 - ps4
Asgard/Eukaryota - ps5
Eukaryota - ps6
Amorpheae - ps7
Obazoa - ps8
Opisthokonta - ps9
Holozoa - ps10
Filozoa - ps11
Choano./Metazoa - ps12
Metazoa - ps13
Myriazoa - ps14
Parahoxozoa - ps15
Bilateria - ps16
Deuterostomia - ps17
Chordata - ps18
Olfactores - ps19
Vertebrata - ps20
Gnathostomata - ps21
Euteleostomi - ps22
Sarcopterygii - ps23
Tetrapoda - ps24
Amniota - ps25
Mammalia - ps26
Theria - ps27
Placentalia - ps28
Boreoeutheria - ps29
Euarchontoglires - ps30
Euarchonta - ps31
Primates - ps32
Haplorrhini - ps33
Simiiformes - ps34
Catarrhini - ps35
Hominoidea - ps36
Hominidae - ps37
Homininae - ps38
Hominini - ps39
Homo_sapiens - ps40
p-value
2e-15
3e-12
4e-09
6e-06
9e-03
Log-odds
0.5
1.0
1.5
2.0
2.5
3.0
3.5
4.0

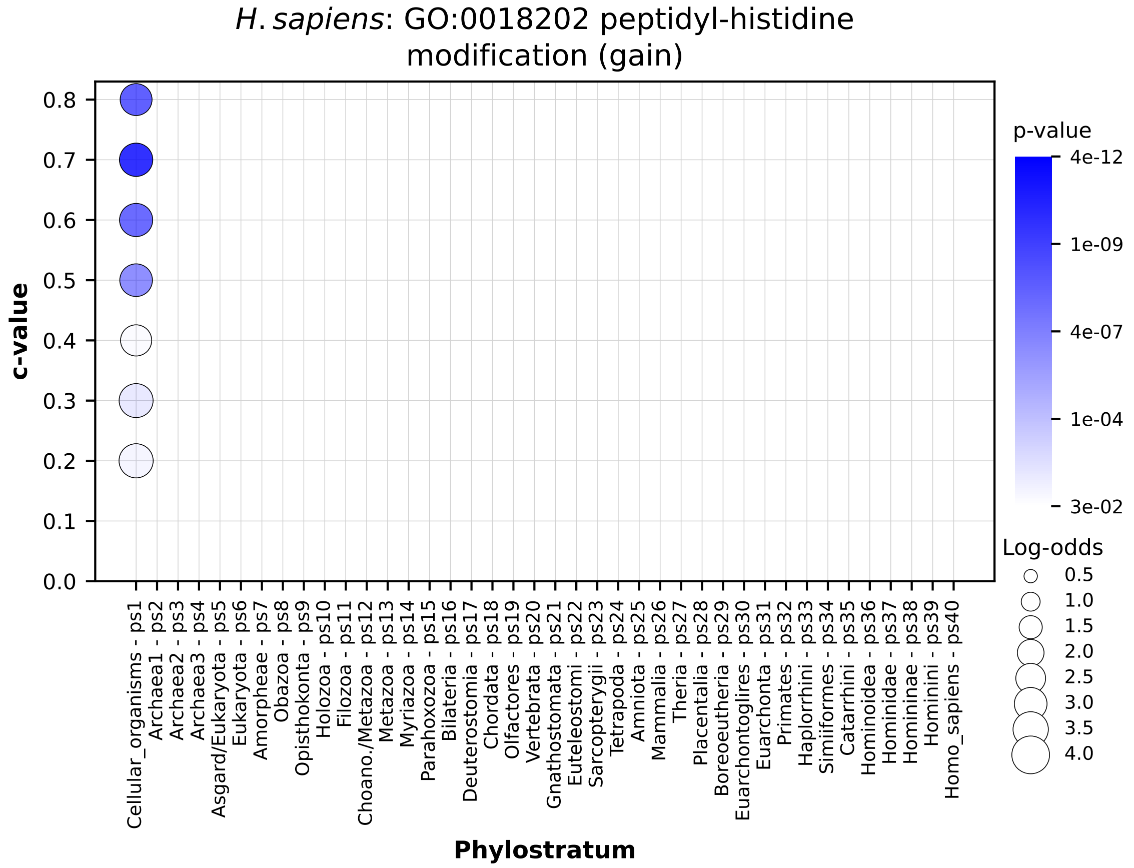

H. sapiens: GO:0018202 peptidyl-histidine modification (gain)
c-value
0.0
0.1
0.2
0.3
0.4
0.5
0.6
0.7
0.8
Phylostratum
Cellular_organisms - ps1
Archaea1 - ps2
Archaea2 - ps3
Archaea3 - ps4
Asgard/Eukaryota - ps5
Eukaryota - ps6
Amorpheae - ps7
Obazoa - ps8
Opisthokonta - ps9
Holozoa - ps10
Filozoa - ps11
Choano./Metazoa - ps12
Metazoa - ps13
Myriazoa - ps14
Parahoxozoa - ps15
Bilateria - ps16
Deuterostomia - ps17
Chordata - ps18
Olfactores - ps19
Vertebrata - ps20
Gnathostomata - ps21
Euteleostomi - ps22
Sarcopterygii - ps23
Tetrapoda - ps24
Amniota - ps25
Mammalia - ps26
Theria - ps27
Placentalia - ps28
Boreoeutheria - ps29
Euarchontoglires - ps30
Euarchonta - ps31
Primates - ps32
Haplorrhini - ps33
Simiiformes - ps34
Catarrhini - ps35
Hominoidea - ps36
Hominidae - ps37
Homininae - ps38
Hominini - ps39
Homo_sapiens - ps40
p-value
4e-12
1e-09
4e-07
1e-04
3e-02
Log-odds
0.5
1.0
1.5
2.0
2.5
3.0
3.5
4.0

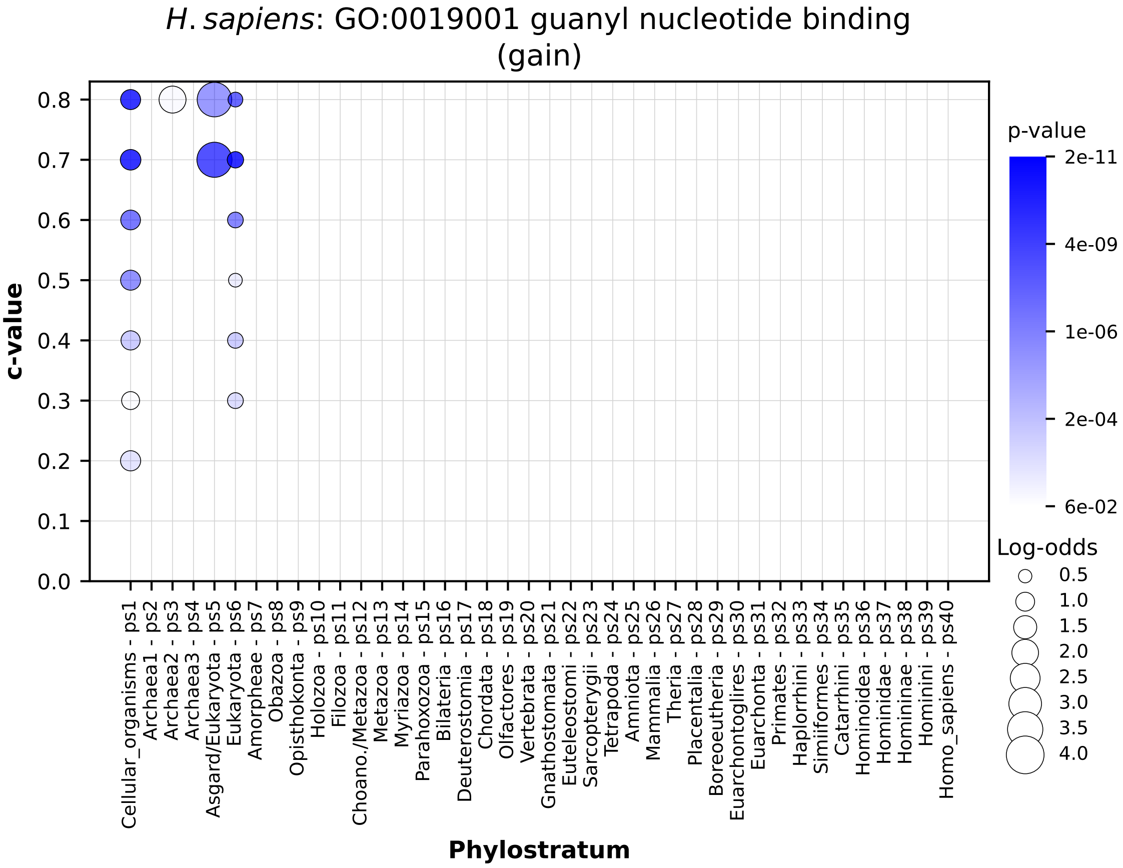

H. sapiens: GO:0019001 guanyl nucleotide binding
(gain)
c-value
0.0
0.1
0.2
0.3
0.4
0.5
0.6
0.7
0.8
Phylostratum
Cellular_organisms - ps1
Archaea1 - ps2
Archaea2 - ps3
Archaea3 - ps4
Asgard/Eukaryota - ps5
Eukaryota - ps6
Amorpheae - ps7
Obazoa - ps8
Opisthokonta - ps9
Holozoa - ps10
Filozoa - ps11
Choano./Metazoa - ps12
Metazoa - ps13
Myriazoa - ps14
Parahoxozoa - ps15
Bilateria - ps16
Deuterostomia - ps17
Chordata - ps18
Olfactores - ps19
Vertebrata - ps20
Gnathostomata - ps21
Euteleostomi - ps22
Sarcopterygii - ps23
Tetrapoda - ps24
Amniota - ps25
Mammalia - ps26
Theria - ps27
Placentalia - ps28
Boreoeutheria - ps29
Euarchontoglires - ps30
Euarchonta - ps31
Primates - ps32
Haplorrhini - ps33
Simiiformes - ps34
Catarrhini - ps35
Hominoidea - ps36
Hominidae - ps37
Homininae - ps38
Hominini - ps39
Homo_sapiens - ps40
p-value
2e-11
4e-09
1e-06
2e-04
6e-02
Log-odds
0.5
1.0
1.5
2.0
2.5
3.0
3.5
4.0

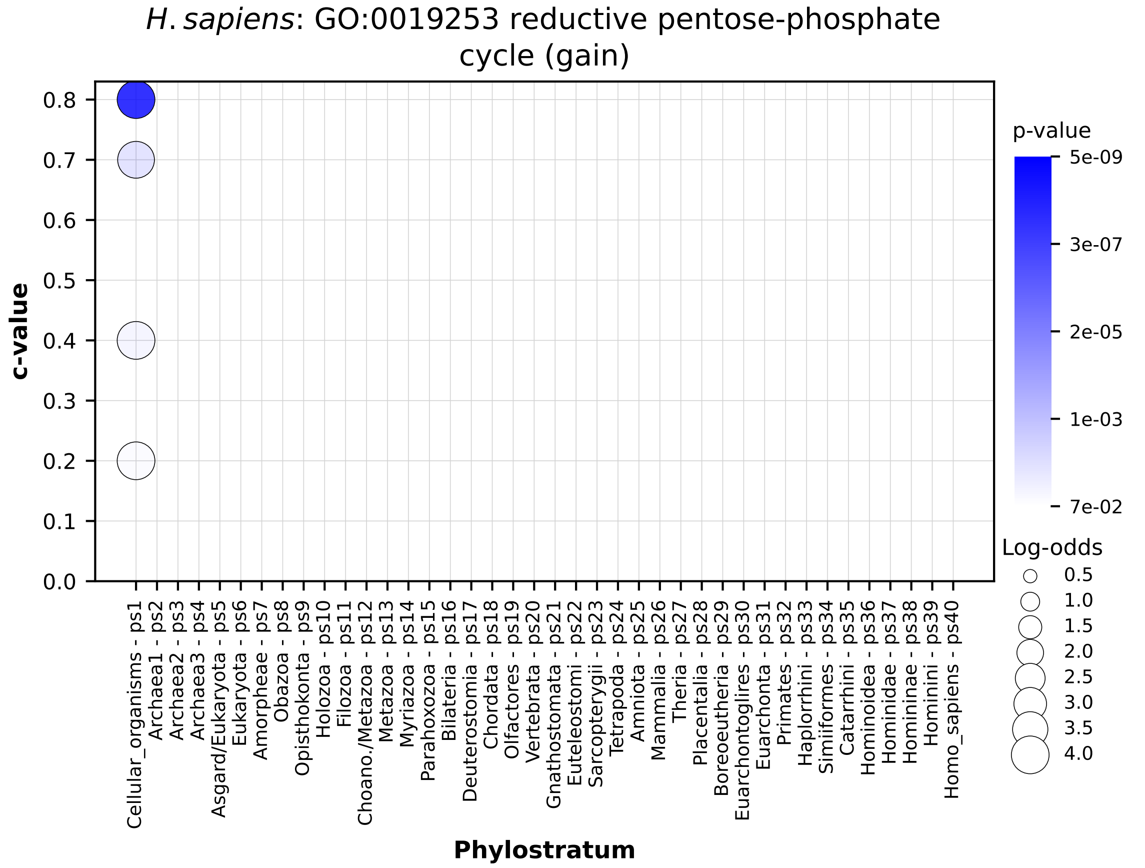
H. sapiens: GO:0019253 reductive pentose-phosphate cycle (gain)
c-value
Phylostratum
p-value
5e-09
3e-07
2e-05
1e-03
7e-02
Log-odds
0.5
1.0
1.5
2.0
2.5
3.0
3.5
4.0
0.0
0.1
0.2
0.3
0.4
0.5
0.6
0.7
0.8
Cellular_organisms - ps1
Archaea1 - ps2
Archaea2 - ps3
Archaea3 - ps4
Asgard/Eukaryota - ps5
Eukaryota - ps6
Amorpheae - ps7
Obazoa - ps8
Opisthokonta - ps9
Holozoa - ps10
Filozoa - ps11
Choano./Metazoa - ps12
Metazoa - ps13
Myriazoa - ps14
Parahoxozoa - ps15
Bilateria - ps16
Deuterostomia - ps17
Chordata - ps18
Olfactores - ps19
Vertebrata - ps20
Gnathostomata - ps21
Euteleostomi - ps22
Sarcopterygii - ps23
Tetrapoda - ps24
Amniota - ps25
Mammalia - ps26
Theria - ps27
Placentalia - ps28
Boreoeutheria - ps29
Euarchontoglires - ps30
Euarchonta - ps31
Primates - ps32
Haplorrhini - ps33
Simiiformes - ps34
Catarrhini - ps35
Hominoidea - ps36
Hominidae - ps37
Homininae - ps38
Hominini - ps39
Homo_sapiens - ps40

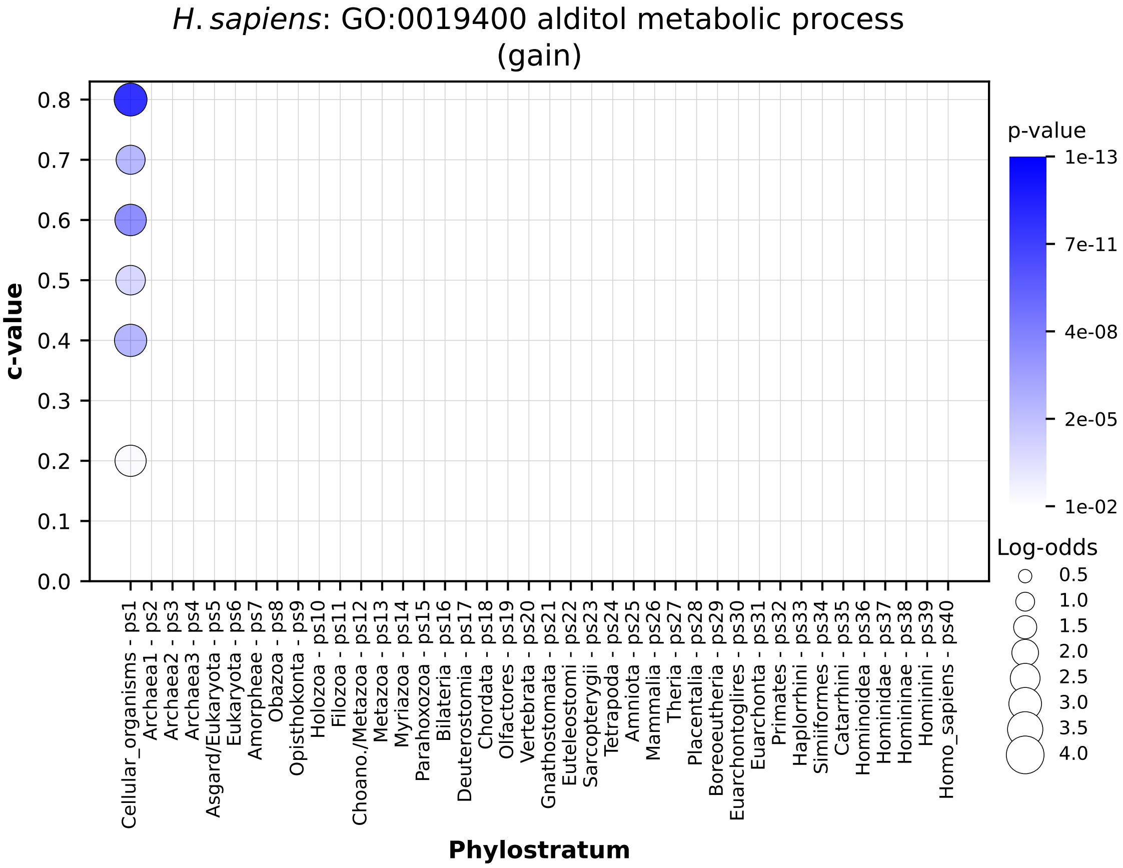

H. sapiens: GO:0019400 alditol metabolic process
(gain)
c-value
0.0
0.1
0.2
0.3
0.4
0.5
0.6
0.7
0.8
Phylostratum
Cellular_organisms - ps1
Archaea1 - ps2
Archaea2 - ps3
Archaea3 - ps4
Asgard/Eukaryota - ps5
Eukaryota - ps6
Amorpheae - ps7
Obazoa - ps8
Opisthokonta - ps9
Holozoa - ps10
Filozoa - ps11
Choano./Metazoa - ps12
Metazoa - ps13
Myriazoa - ps14
Parahoxozoa - ps15
Bilateria - ps16
Deuterostomia - ps17
Chordata - ps18
Olfactores - ps19
Vertebrata - ps20
Gnathostomata - ps21
Euteleostomi - ps22
Sarcopterygii - ps23
Tetrapoda - ps24
Amniota - ps25
Mammalia - ps26
Theria - ps27
Placentalia - ps28
Boreoeutheria - ps29
Euarchontoglires - ps30
Euarchonta - ps31
Primates - ps32
Haplorrhini - ps33
Simiiformes - ps34
Catarrhini - ps35
Hominoidea - ps36
Hominidae - ps37
Homininae - ps38
Hominini - ps39
Homo_sapiens - ps40
p-value
1e-13
7e-11
4e-08
2e-05
1e-02
Log-odds
0.5
1.0
1.5
2.0
2.5
3.0
3.5
4.0

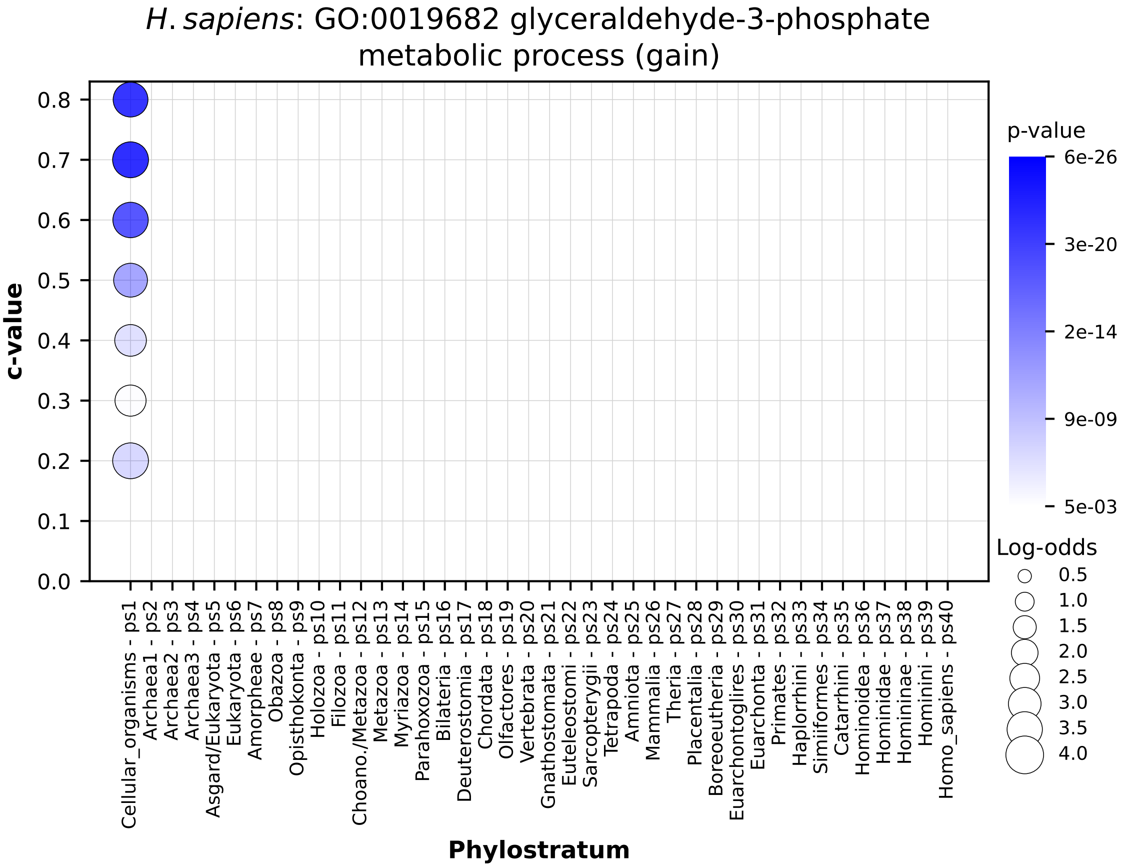

*H. sapiens*: GO:0019682 glyceraldehyde-3-phosphate metabolic process (gain)
c-value
0.0
0.1
0.2
0.3
0.4
0.5
0.6
0.7
0.8
Phylostratum
Cellular_organisms - ps1
Archaea1 - ps2
Archaea2 - ps3
Archaea3 - ps4
Asgard/Eukaryota - ps5
Eukaryota - ps6
Amorpheae - ps7
Obazoa - ps8
Opisthokonta - ps9
Holozoa - ps10
Filozoa - ps11
Choano./Metazoa - ps12
Metazoa - ps13
Myriazoa - ps14
Parahoxozoa - ps15
Bilateria - ps16
Deuterostomia - ps17
Chordata - ps18
Olfactores - ps19
Vertebrata - ps20
Gnathostomata - ps21
Euteleostomi - ps22
Sarcopterygii - ps23
Tetrapoda - ps24
Amniota - ps25
Mammalia - ps26
Theria - ps27
Placentalia - ps28
Boreoeutheria - ps29
Euarchontoglires - ps30
Euarchonta - ps31
Primates - ps32
Haplorrhini - ps33
Simiiformes - ps34
Catarrhini - ps35
Hominoidea - ps36
Hominidae - ps37
Homininae - ps38
Hominini - ps39
Homo_sapiens - ps40
p-value
6e-26
3e-20
2e-14
9e-09
5e-03
Log-odds
0.5
1.0
1.5
2.0
2.5
3.0
3.5
4.0

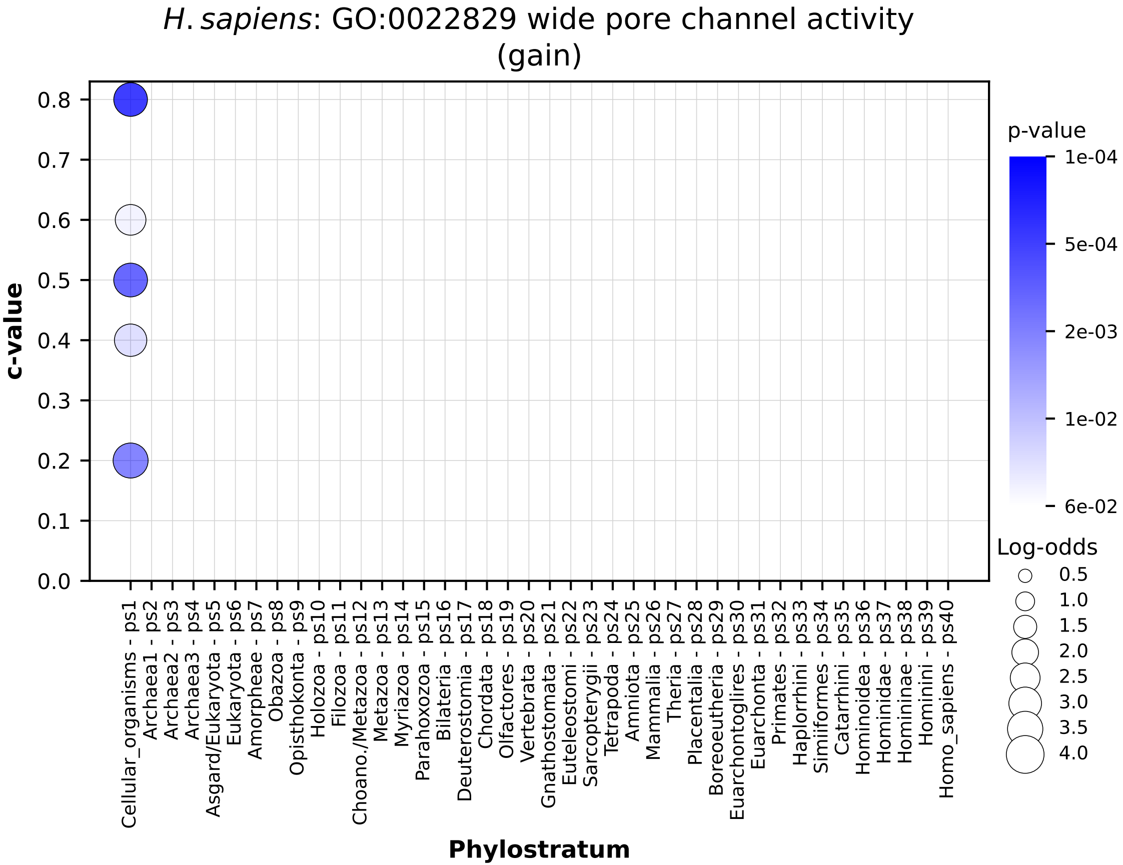

H. sapiens: GO:0022829 wide pore channel activity
(gain)
c-value
0.0
0.1
0.2
0.3
0.4
0.5
0.6
0.7
0.8
Phylostratum
Cellular_organisms - ps1
Archaea1 - ps2
Archaea2 - ps3
Archaea3 - ps4
Asgard/Eukaryota - ps5
Eukaryota - ps6
Amorpheae - ps7
Obazoa - ps8
Opisthokonta - ps9
Holozoa - ps10
Filozoa - ps11
Choano./Metazoa - ps12
Metazoa - ps13
Myriazoa - ps14
Parahoxozoa - ps15
Bilateria - ps16
Deuterostomia - ps17
Chordata - ps18
Olfactores - ps19
Vertebrata - ps20
Gnathostomata - ps21
Euteleostomi - ps22
Sarcopterygii - ps23
Tetrapoda - ps24
Amniota - ps25
Mammalia - ps26
Theria - ps27
Placentalia - ps28
Boreoeutheria - ps29
Euarchontoglires - ps30
Euarchonta - ps31
Primates - ps32
Haplorrhini - ps33
Simiiformes - ps34
Catarrhini - ps35
Hominoidea - ps36
Hominidae - ps37
Homininae - ps38
Hominini - ps39
Homo_sapiens - ps40
p-value
1e-04
5e-04
2e-03
1e-02
6e-02
Log-odds
0.5
1.0
1.5
2.0
2.5
3.0
3.5
4.0

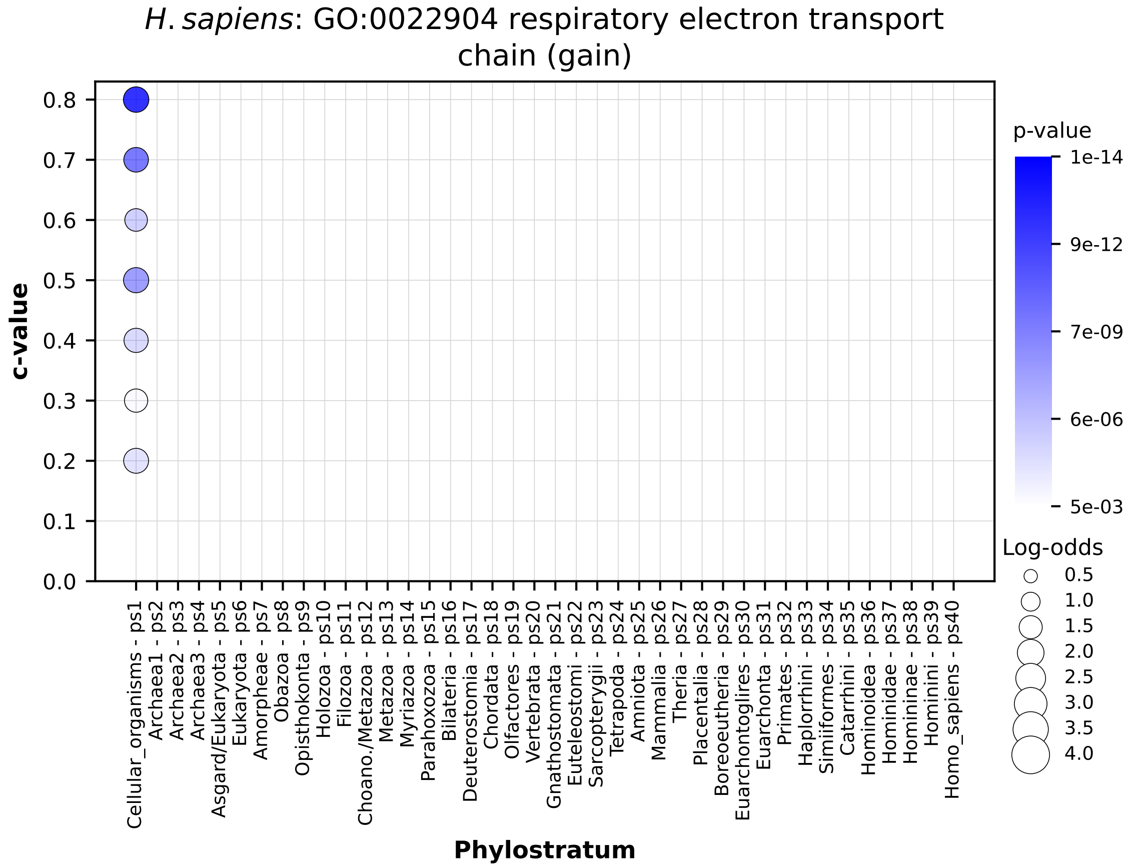

*H. sapiens*: GO:0022904 respiratory electron transport chain (gain)
c-value
0.0
0.1
0.2
0.3
0.4
0.5
0.6
0.7
0.8
Phylostratum
Cellular_organisms - ps1
Archaea1 - ps2
Archaea2 - ps3
Archaea3 - ps4
Asgard/Eukaryota - ps5
Eukaryota - ps6
Amorpheae - ps7
Obazoa - ps8
Opisthokonta - ps9
Holozoa - ps10
Filozoa - ps11
Choano./Metazoa - ps12
Metazoa - ps13
Myriazoa - ps14
Parahoxozoa - ps15
Bilateria - ps16
Deuterostomia - ps17
Chordata - ps18
Olfactores - ps19
Vertebrata - ps20
Gnathostomata - ps21
Euteleostomi - ps22
Sarcopterygii - ps23
Tetrapoda - ps24
Amniota - ps25
Mammalia - ps26
Theria - ps27
Placentalia - ps28
Boreoeutheria - ps29
Euarchontoglires - ps30
Euarchonta - ps31
Primates - ps32
Haplorrhini - ps33
Simiiformes - ps34
Catarrhini - ps35
Hominoidea - ps36
Hominidae - ps37
Homininae - ps38
Hominini - ps39
Homo_sapiens - ps40
p-value
1e-14
9e-12
7e-09
6e-06
5e-03
Log-odds
0.5
1.0
1.5
2.0
2.5
3.0
3.5
4.0

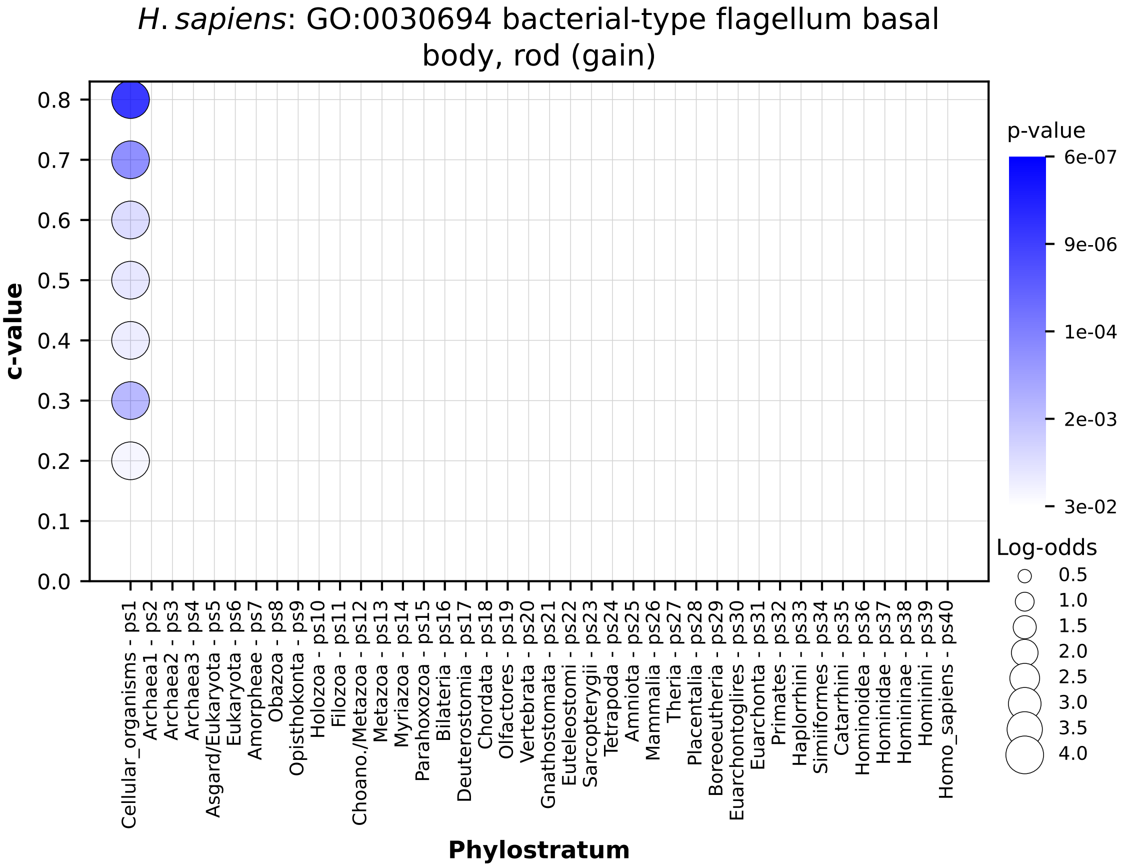
H. sapiens: GO:0030694 bacterial-type flagellum basal body, rod (gain)
c-value
0.8
0.7
0.6
0.5
0.4
0.3
0.2
0.1
0.0
Phylostratum
Cellular_organisms - ps1
Archaea1 - ps2
Archaea2 - ps3
Archaea3 - ps4
Asgard/Eukaryota - ps5
Eukaryota - ps6
Amorpheae - ps7
Obazoa - ps8
Opisthokonta - ps9
Holozoa - ps10
Filozoa - ps11
Choano./Metazoa - ps12
Metazoa - ps13
Myriazoa - ps14
Parahoxozoa - ps15
Bilateria - ps16
Deuterostomia - ps17
Chordata - ps18
Olfactores - ps19
Vertebrata - ps20
Gnathostomata - ps21
Euteleostomi - ps22
Sarcopterygii - ps23
Tetrapoda - ps24
Amniota - ps25
Mammalia - ps26
Theria - ps27
Placentalia - ps28
Boreoeutheria - ps29
Euarchontoglires - ps30
Euarchonta - ps31
Primates - ps32
Haplorrhini - ps33
Simiiformes - ps34
Catarrhini - ps35
Hominoidea - ps36
Hominidae - ps37
Homininae - ps38
Hominini - ps39
Homo_sapiens - ps40
p-value
6e-07
9e-06
1e-04
2e-03
3e-02
Log-odds
0.5
1.0
1.5
2.0
2.5
3.0
3.5
4.0

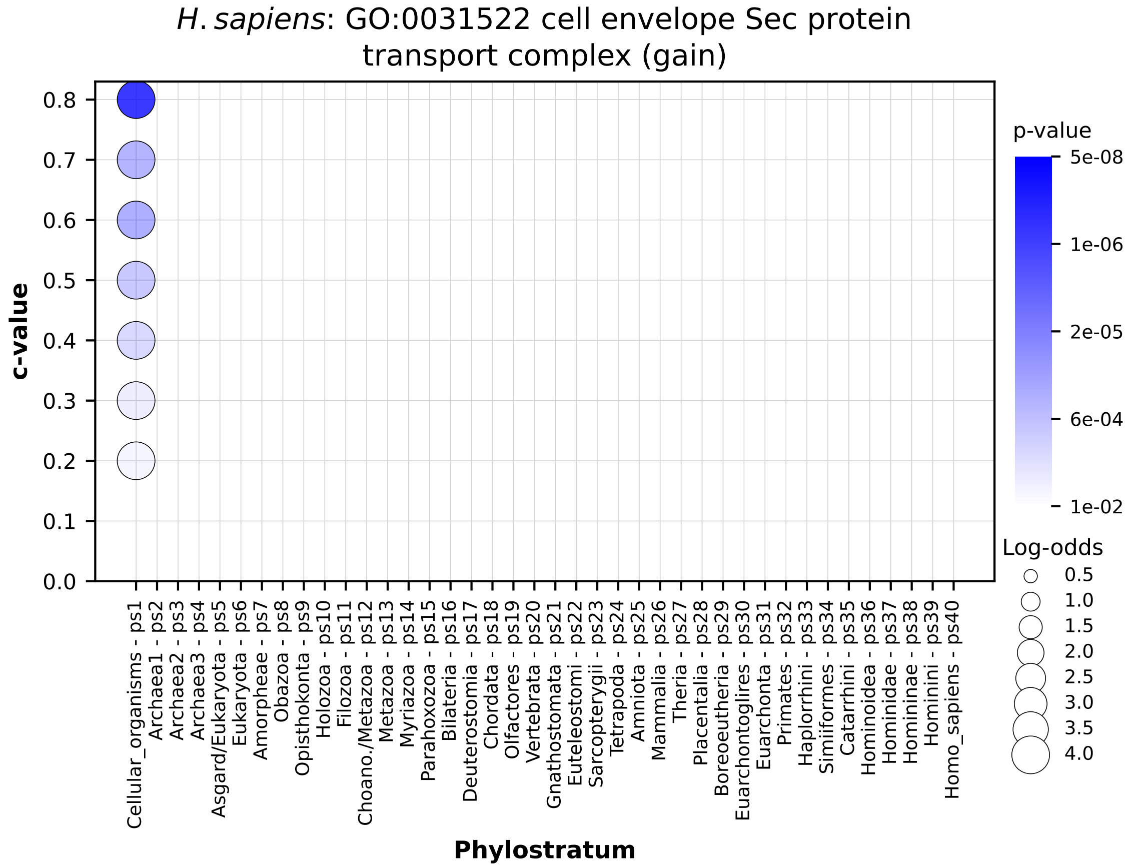
*H. sapiens*: GO:0031522 cell envelope Sec protein transport complex (gain)
c-value
0.0
0.1
0.2
0.3
0.4
0.5
0.6
0.7
0.8
p-value
5e-08
1e-06
2e-05
6e-04
1e-02
Log-odds
0.5
1.0
1.5
2.0
2.5
3.0
3.5
4.0
Cellular_organisms - ps1
Archaea1 - ps2
Archaea2 - ps3
Archaea3 - ps4
Asgard/Eukaryota - ps5
Eukaryota - ps6
Amorpheae - ps7
Obazoa - ps8
Opisthokonta - ps9
Holozoa - ps10
Filozoa - ps11
Choano./Metazoa - ps12
Metazoa - ps13
Myriazoa - ps14
Parahoxozoa - ps15
Bilateria - ps16
Deuterostomia - ps17
Chordata - ps18
Olfactores - ps19
Vertebrata - ps20
Gnathostomata - ps21
Euteleostomi - ps22
Sarcopterygii - ps23
Tetrapoda - ps24
Amniota - ps25
Mammalia - ps26
Theria - ps27
Placentalia - ps28
Boreoeutheria - ps29
Euarchontoglires - ps30
Euarchonta - ps31
Primates - ps32
Haplorrhini - ps33
Simiiformes - ps34
Catarrhini - ps35
Hominoidea - ps36
Hominidae - ps37
Homininae - ps38
Hominini - ps39
Homo_sapiens - ps40
Phylostratum

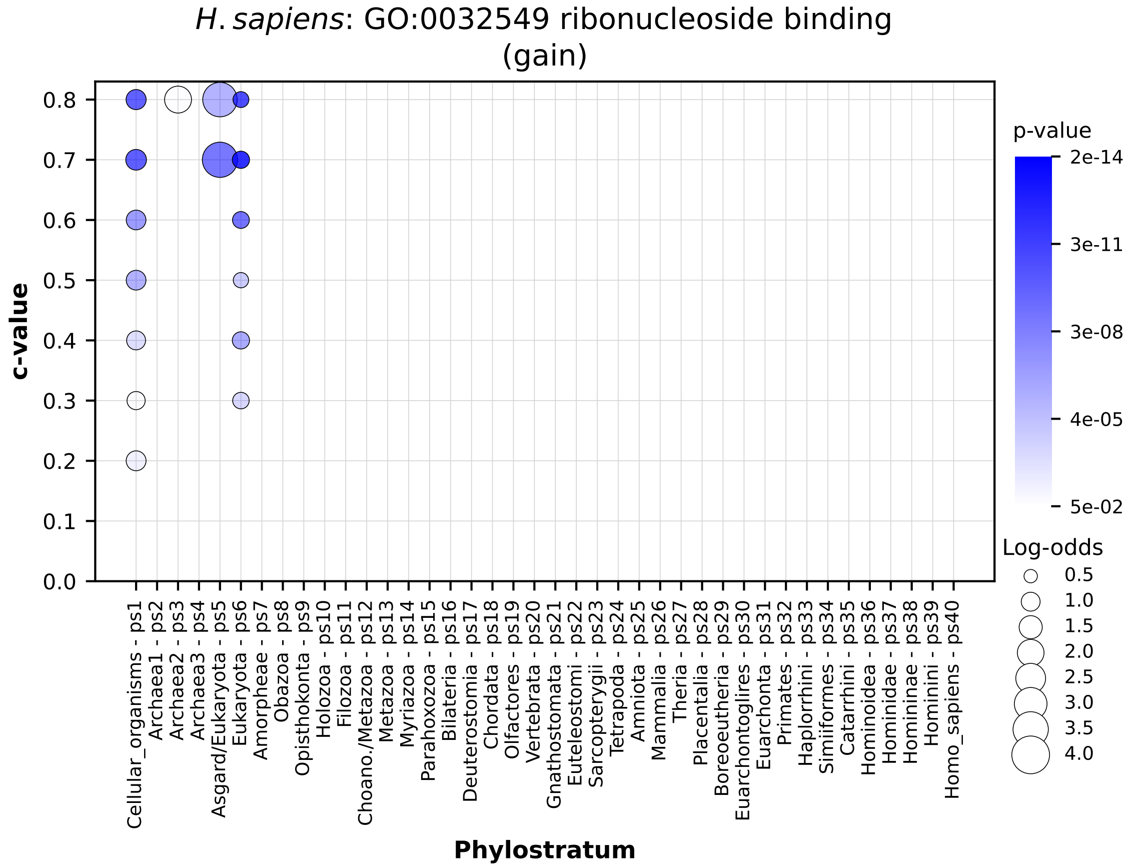

H. sapiens: GO:0032549 ribonucleoside binding
(gain)
c-value
0.8
0.7
0.6
0.5
0.4
0.3
0.2
0.1
0.0
p-value
2e-14
3e-11
3e-08
4e-05
5e-02
Log-odds
0.5
1.0
1.5
2.0
2.5
3.0
3.5
4.0
Cellular_organisms - ps1
Archaea1 - ps2
Archaea2 - ps3
Archaea3 - ps4
Asgard/Eukaryota - ps5
Eukaryota - ps6
Amorpheae - ps7
Obazoa - ps8
Opisthokonta - ps9
Holozoa - ps10
Filozoa - ps11
Choano./Metazoa - ps12
Metazoa - ps13
Myriazoa - ps14
Parahoxozoa - ps15
Bilateria - ps16
Deuterostomia - ps17
Chordata - ps18
Olfactores - ps19
Vertebrata - ps20
Gnathostomata - ps21
Euteleostomi - ps22
Sarcopterygii - ps23
Tetrapoda - ps24
Amniota - ps25
Mammalia - ps26
Theria - ps27
Placentalia - ps28
Boreoeutheria - ps29
Euarchontoglires - ps30
Euarchonta - ps31
Primates - ps32
Haplorrhini - ps33
Simiiformes - ps34
Catarrhini - ps35
Hominoidea - ps36
Hominidae - ps37
Homininae - ps38
Hominini - ps39
Homo_sapiens - ps40
Phylostratum

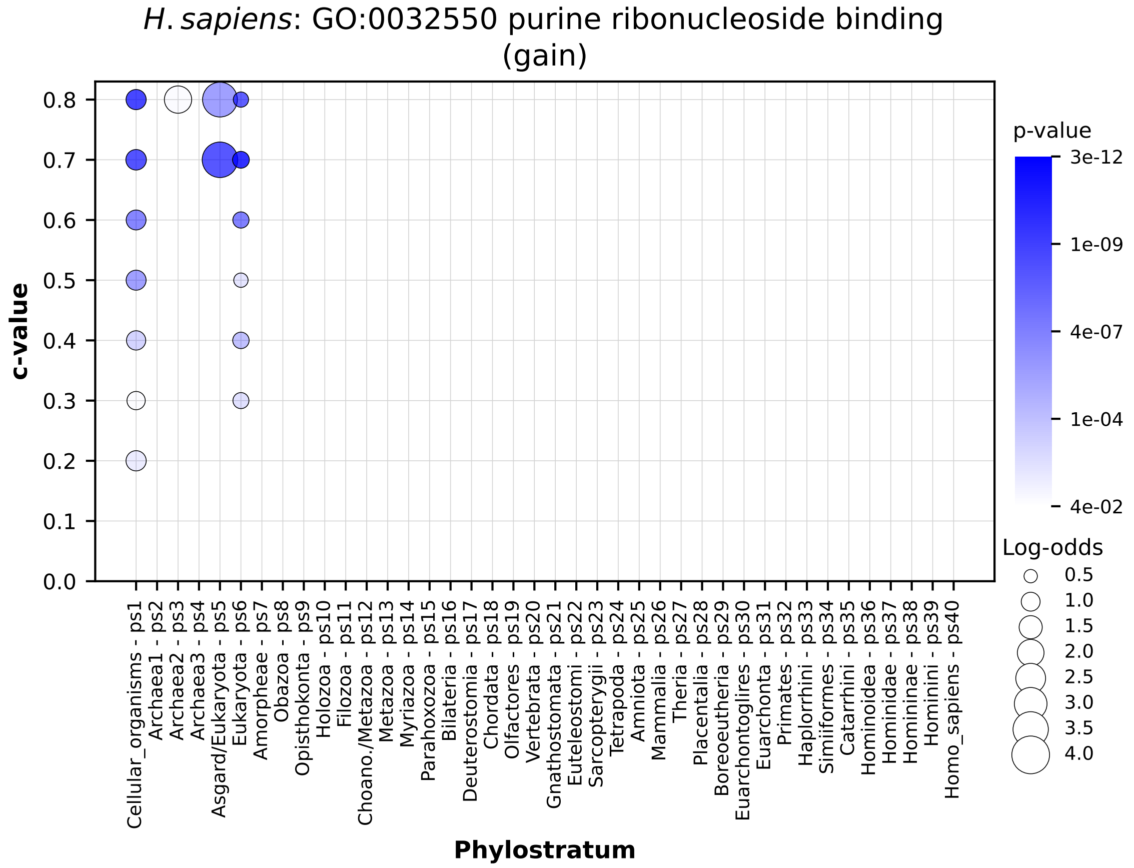

*H. sapiens*: GO:0032550 purine ribonucleoside binding (gain)
c-value
0.8
0.7
0.6
0.5
0.4
0.3
0.2
0.1
0.0
Phylostratum
Cellular_organisms - ps1
Archaea1 - ps2
Archaea2 - ps3
Archaea3 - ps4
Asgard/Eukaryota - ps5
Eukaryota - ps6
Amorpheae - ps7
Obazoa - ps8
Opisthokonta - ps9
Holozoa - ps10
Filozoa - ps11
Choano./Metazoa - ps12
Metazoa - ps13
Myriazoa - ps14
Parahoxozoa - ps15
Bilateria - ps16
Deuterostomia - ps17
Chordata - ps18
Olfactores - ps19
Vertebrata - ps20
Gnathostomata - ps21
Euteleostomi - ps22
Sarcopterygii - ps23
Tetrapoda - ps24
Amniota - ps25
Mammalia - ps26
Theria - ps27
Placentalia - ps28
Boreoeutheria - ps29
Euarchontoglires - ps30
Euarchonta - ps31
Primates - ps32
Haplorrhini - ps33
Simiiformes - ps34
Catarrhini - ps35
Hominoidea - ps36
Hominidae - ps37
Homininae - ps38
Hominini - ps39
Homo_sapiens - ps40
p-value
3e-12
1e-09
4e-07
1e-04
4e-02
Log-odds
0.5
1.0
1.5
2.0
2.5
3.0
3.5
4.0

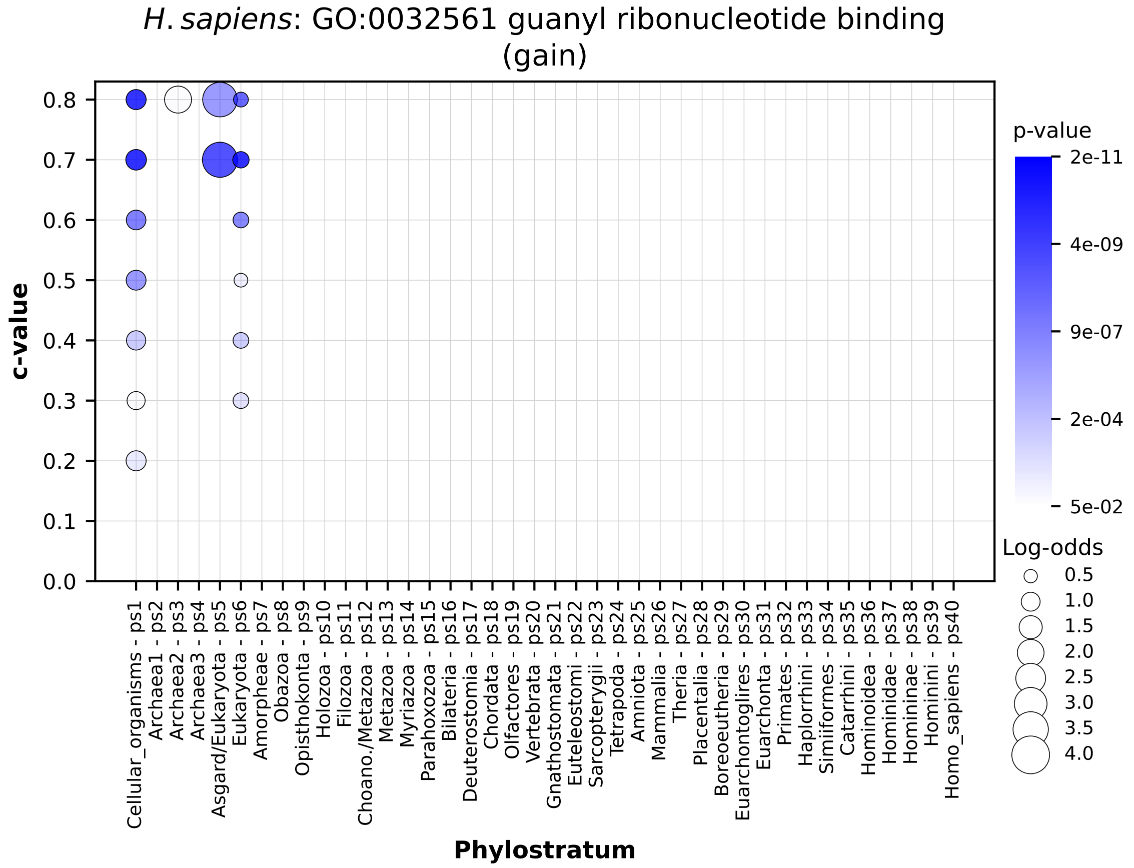

H. sapiens: GO:0032561 guanyl ribonucleotide binding (gain)
c-value
0.0
0.1
0.2
0.3
0.4
0.5
0.6
0.7
0.8
Phylostratum
Cellular_organisms - ps1
Archaea1 - ps2
Archaea2 - ps3
Archaea3 - ps4
Asgard/Eukaryota - ps5
Eukaryota - ps6
Amorpheae - ps7
Obazoa - ps8
Opisthokonta - ps9
Holozoa - ps10
Filozoa - ps11
Choano./Metazoa - ps12
Metazoa - ps13
Myriazoa - ps14
Parahoxozoa - ps15
Bilateria - ps16
Deuterostomia - ps17
Chordata - ps18
Olfactores - ps19
Vertebrata - ps20
Gnathostomata - ps21
Euteleostomi - ps22
Sarcopterygii - ps23
Tetrapoda - ps24
Amniota - ps25
Mammalia - ps26
Theria - ps27
Placentalia - ps28
Boreoeutheria - ps29
Euarchontoglires - ps30
Euarchonta - ps31
Primates - ps32
Haplorrhini - ps33
Simiiformes - ps34
Catarrhini - ps35
Hominoidea - ps36
Hominidae - ps37
Homininae - ps38
Hominini - ps39
Homo_sapiens - ps40
p-value
2e-11
4e-09
9e-07
2e-04
5e-02
Log-odds
0.5
1.0
1.5
2.0
2.5
3.0
3.5
4.0

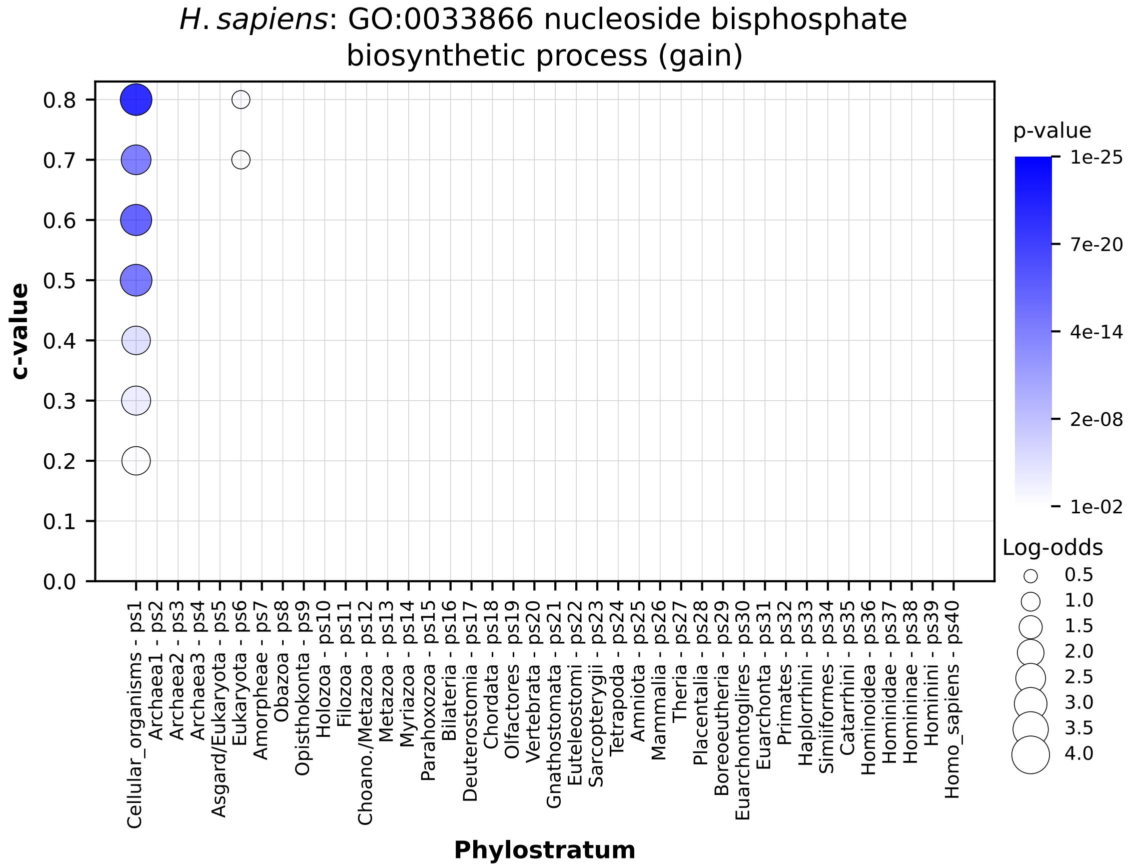

H. sapiens: GO:0033866 nucleoside bisphosphate biosynthetic process (gain)
c-value
0.0
0.1
0.2
0.3
0.4
0.5
0.6
0.7
0.8
Phylostratum
Cellular_organisms - ps1
Archaea1 - ps2
Archaea2 - ps3
Archaea3 - ps4
Asgard/Eukaryota - ps5
Eukaryota - ps6
Amorpheae - ps7
Obazoa - ps8
Opisthokonta - ps9
Holozoa - ps10
Filozoa - ps11
Choano./Metazoa - ps12
Metazoa - ps13
Myriazoa - ps14
Parahoxozoa - ps15
Bilateria - ps16
Deuterostomia - ps17
Chordata - ps18
Olfactores - ps19
Vertebrata - ps20
Gnathostomata - ps21
Euteleostomi - ps22
Sarcopterygii - ps23
Tetrapoda - ps24
Amniota - ps25
Mammalia - ps26
Theria - ps27
Placentalia - ps28
Boreoeutheria - ps29
Euarchontoglires - ps30
Euarchonta - ps31
Primates - ps32
Haplorrhini - ps33
Simiiformes - ps34
Catarrhini - ps35
Hominoidea - ps36
Hominidae - ps37
Homininae - ps38
Hominini - ps39
Homo_sapiens - ps40
p-value
1e-25
7e-20
4e-14
2e-08
1e-02
Log-odds
0.5
1.0
1.5
2.0
2.5
3.0
3.5
4.0

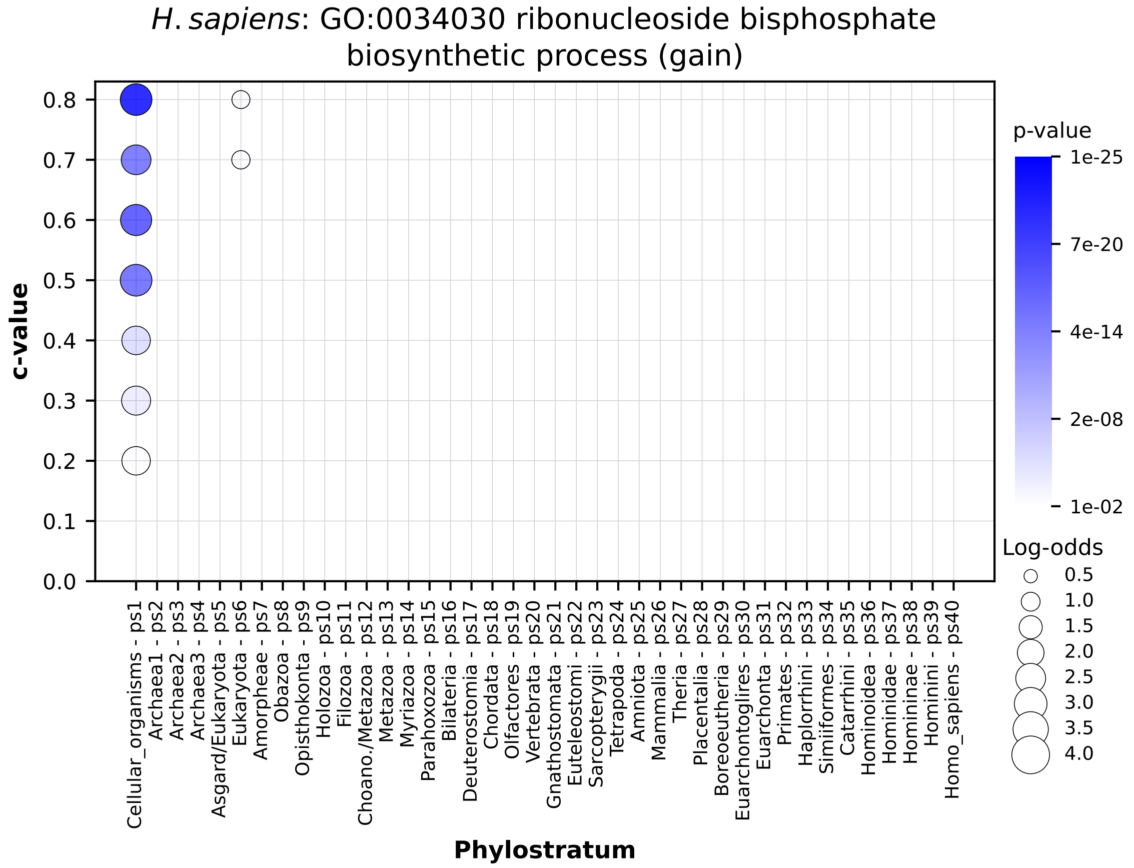
H. sapiens: GO:0034030 ribonucleoside bisphosphate biosynthetic process (gain)
c-value
0.0
0.1
0.2
0.3
0.4
0.5
0.6
0.7
0.8
Phylostratum
Cellular_organisms - ps1
Archaea1 - ps2
Archaea2 - ps3
Archaea3 - ps4
Asgard/Eukaryota - ps5
Eukaryota - ps6
Amorpheae - ps7
Obazoa - ps8
Opisthokonta - ps9
Holozoa - ps10
Filozoa - ps11
Choano./Metazoa - ps12
Metazoa - ps13
Myriazoa - ps14
Parahoxozoa - ps15
Bilateria - ps16
Deuterostomia - ps17
Chordata - ps18
Olfactores - ps19
Vertebrata - ps20
Gnathostomata - ps21
Euteleostomi - ps22
Sarcopterygii - ps23
Tetrapoda - ps24
Amniota - ps25
Mammalia - ps26
Theria - ps27
Placentalia - ps28
Boreoeutheria - ps29
Euarchontoglires - ps30
Euarchonta - ps31
Primates - ps32
Haplorrhini - ps33
Simiiformes - ps34
Catarrhini - ps35
Hominoidea - ps36
Hominidae - ps37
Homininae - ps38
Hominini - ps39
Homo_sapiens - ps40
p-value
1e-25
7e-20
4e-14
2e-08
1e-02
Log-odds
0.5
1.0
1.5
2.0
2.5
3.0
3.5
4.0

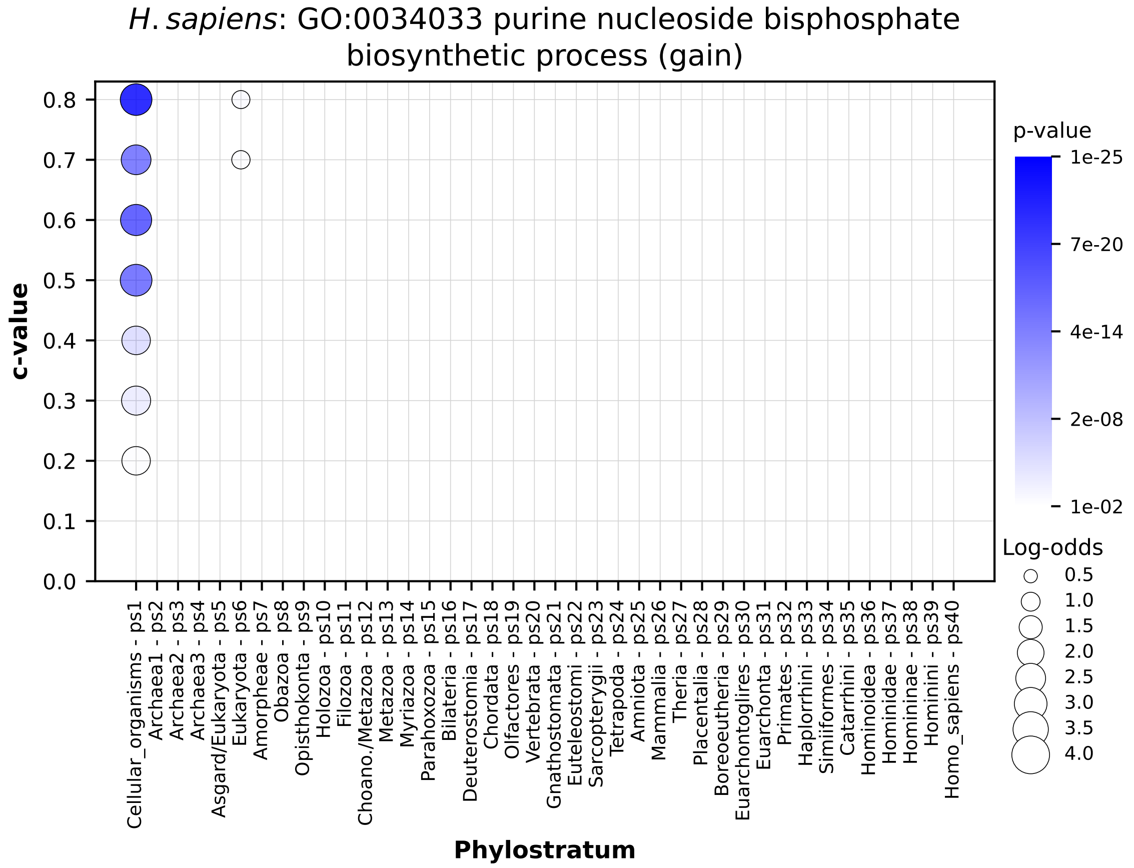

*H. sapiens*: GO:0034033 purine nucleoside bisphosphate biosynthetic process (gain)
c-value
0.0
0.1
0.2
0.3
0.4
0.5
0.6
0.7
0.8
Phylostratum
Cellular_organisms - ps1
Archaea1 - ps2
Archaea2 - ps3
Archaea3 - ps4
Asgard/Eukaryota - ps5
Eukaryota - ps6
Amorpheae - ps7
Obazoa - ps8
Opisthokonta - ps9
Holozoa - ps10
Filozoa - ps11
Choano./Metazoa - ps12
Metazoa - ps13
Myriazoa - ps14
Parahoxozoa - ps15
Bilateria - ps16
Deuterostomia - ps17
Chordata - ps18
Olfactores - ps19
Vertebrata - ps20
Gnathostomata - ps21
Euteleostomi - ps22
Sarcopterygii - ps23
Tetrapoda - ps24
Amniota - ps25
Mammalia - ps26
Theria - ps27
Placentalia - ps28
Boreoeutheria - ps29
Euarchontoglires - ps30
Euarchonta - ps31
Primates - ps32
Haplorrhini - ps33
Simiiformes - ps34
Catarrhini - ps35
Hominoidea - ps36
Hominidae - ps37
Homininae - ps38
Hominini - ps39
Homo_sapiens - ps40
p-value
1e-25
7e-20
4e-14
2e-08
1e-02
Log-odds
0.5
1.0
1.5
2.0
2.5
3.0
3.5
4.0

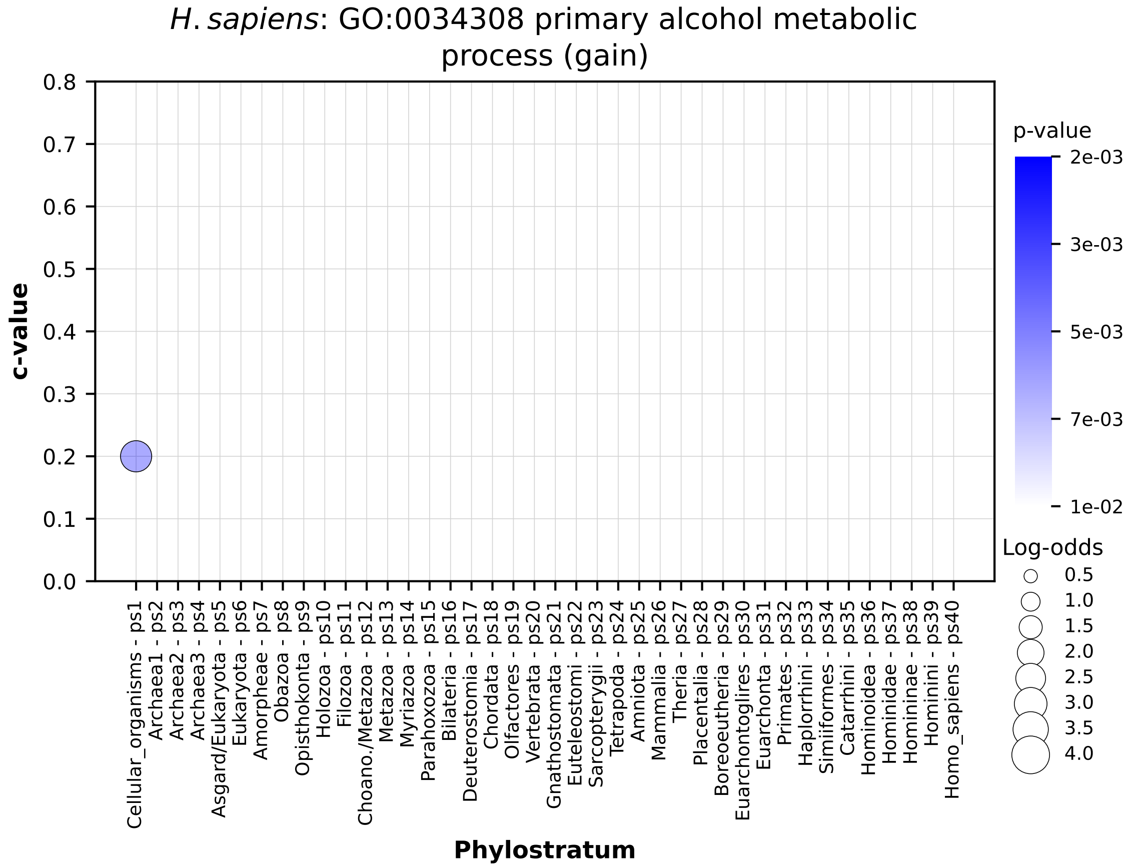

H. sapiens: GO:0034308 primary alcohol metabolic process (gain)
c-value
0.0
0.1
0.2
0.3
0.4
0.5
0.6
0.7
0.8
Phylostratum
Cellular_organisms - ps1
Archaea1 - ps2
Archaea2 - ps3
Archaea3 - ps4
Asgard/Eukaryota - ps5
Eukaryota - ps6
Amorpheae - ps7
Obazoa - ps8
Opisthokonta - ps9
Holozoa - ps10
Filozoa - ps11
Choano./Metazoa - ps12
Metazoa - ps13
Myriazoa - ps14
Parahoxozoa - ps15
Bilateria - ps16
Deuterostomia - ps17
Chordata - ps18
Olfactores - ps19
Vertebrata - ps20
Gnathostomata - ps21
Euteleostomi - ps22
Sarcopterygii - ps23
Tetrapoda - ps24
Amniota - ps25
Mammalia - ps26
Theria - ps27
Placentalia - ps28
Boreoeutheria - ps29
Euarchontoglires - ps30
Euarchonta - ps31
Primates - ps32
Haplorrhini - ps33
Simiiformes - ps34
Catarrhini - ps35
Hominoidea - ps36
Hominidae - ps37
Homininae - ps38
Hominini - ps39
Homo_sapiens - ps40
p-value
2e-03
3e-03
5e-03
7e-03
1e-02
Log-odds
0.5
1.0
1.5
2.0
2.5
3.0
3.5
4.0

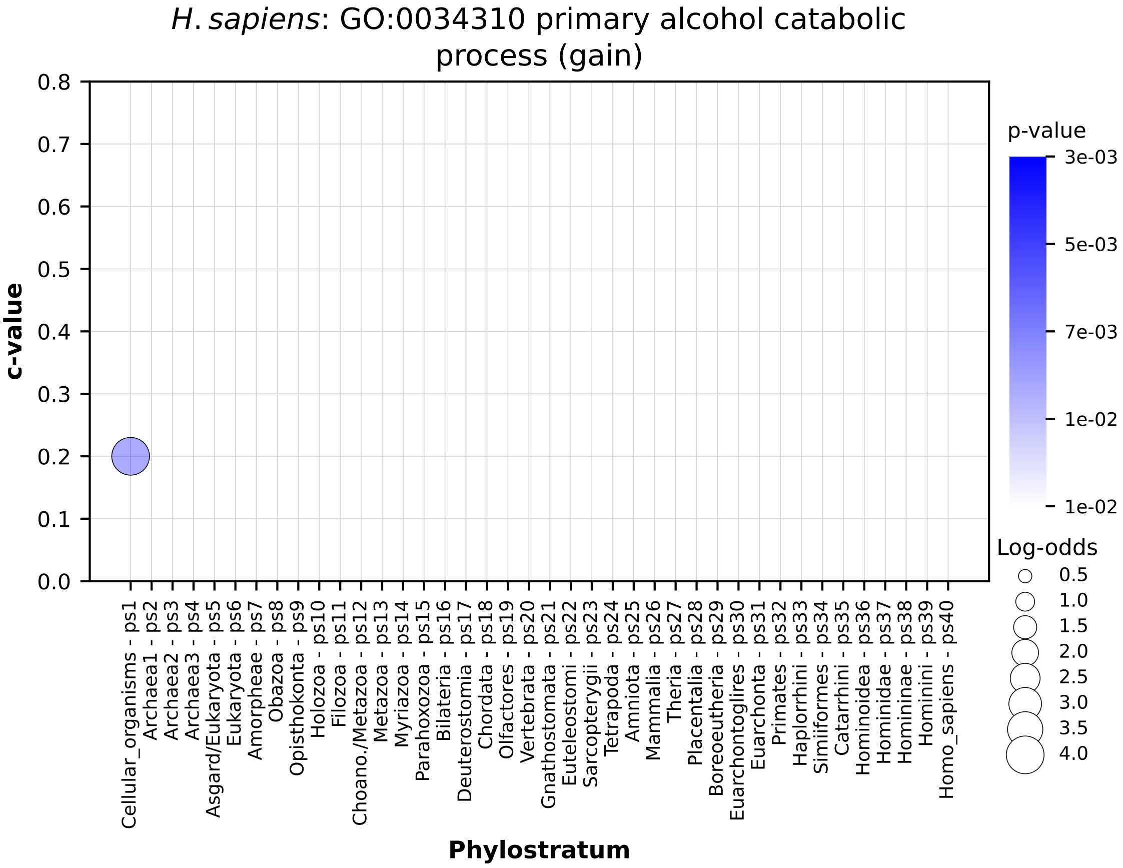

*H. sapiens*: GO:0034310 primary alcohol catabolic process (gain)
c-value
0.8
0.7
0.6
0.5
0.4
0.3
0.2
0.1
0.0
Phylostratum
Cellular_organisms - ps1
Archaea1 - ps2
Archaea2 - ps3
Archaea3 - ps4
Asgard/Eukaryota - ps5
Eukaryota - ps6
Amorpheae - ps7
Obazoa - ps8
Opisthokonta - ps9
Holozoa - ps10
Filozoa - ps11
Choano./Metazoa - ps12
Metazoa - ps13
Myriazoa - ps14
Parahoxozoa - ps15
Bilateria - ps16
Deuterostomia - ps17
Chordata - ps18
Olfactores - ps19
Vertebrata - ps20
Gnathostomata - ps21
Euteleostomi - ps22
Sarcopterygii - ps23
Tetrapoda - ps24
Amniota - ps25
Mammalia - ps26
Theria - ps27
Placentalia - ps28
Boreoeutheria - ps29
Euarchontoglires - ps30
Euarchonta - ps31
Primates - ps32
Haplorrhini - ps33
Simiiformes - ps34
Catarrhini - ps35
Hominoidea - ps36
Hominidae - ps37
Homininae - ps38
Hominini - ps39
Homo_sapiens - ps40
p-value
3e-03
5e-03
7e-03
1e-02
1e-02
Log-odds
0.5
1.0
1.5
2.0
2.5
3.0
3.5
4.0

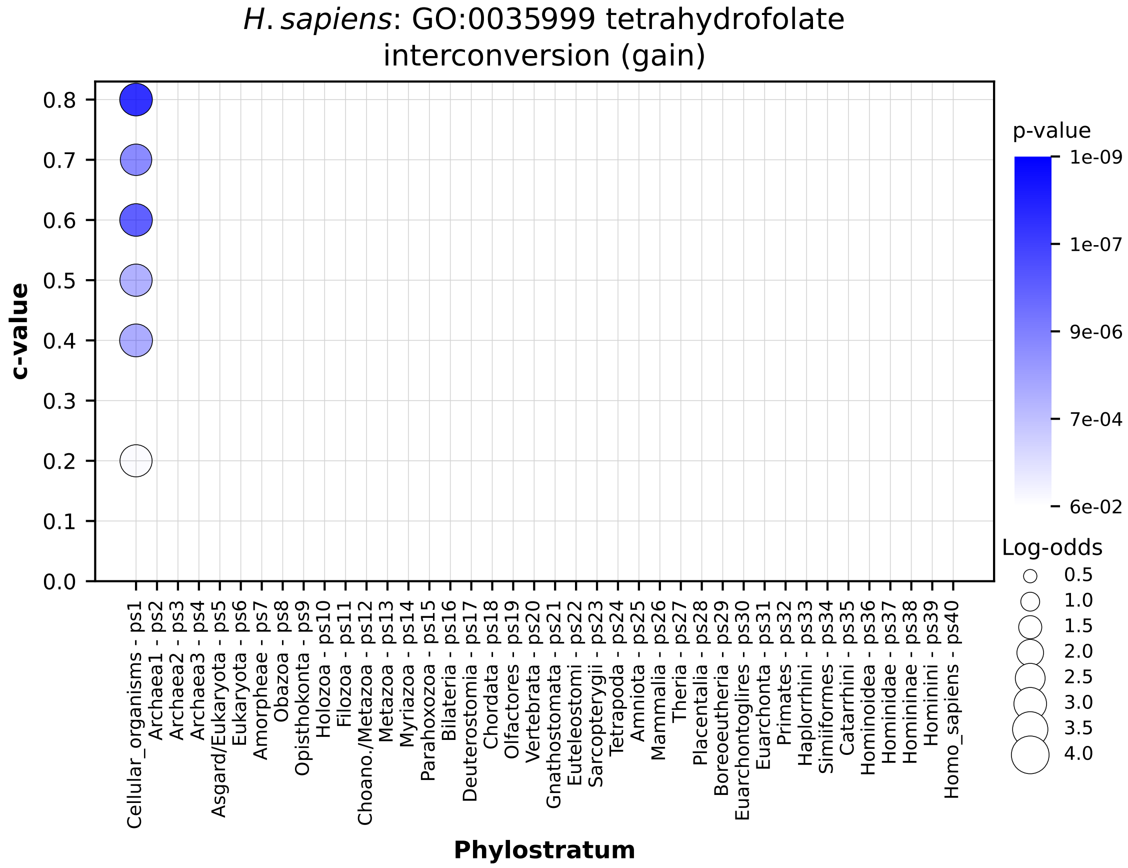

H. sapiens: GO:0035999 tetrahydrofolate interconversion (gain)
c-value
0.8
0.7
0.6
0.5
0.4
0.3
0.2
0.1
0.0
p-value
1e-09
1e-07
9e-06
7e-04
6e-02
Log-odds
0.5
1.0
1.5
2.0
2.5
3.0
3.5
4.0
Cellular_organisms - ps1
Archaea1 - ps2
Archaea2 - ps3
Archaea3 - ps4
Asgard/Eukaryota - ps5
Eukaryota - ps6
Amorpheae - ps7
Obazoa - ps8
Opisthokonta - ps9
Holozoa - ps10
Filozoa - ps11
Choano./Metazoa - ps12
Metazoa - ps13
Myriazoa - ps14
Parahoxozoa - ps15
Bilateria - ps16
Deuterostomia - ps17
Chordata - ps18
Olfactores - ps19
Vertebrata - ps20
Gnathostomata - ps21
Euteleostomi - ps22
Sarcopterygii - ps23
Tetrapoda - ps24
Amniota - ps25
Mammalia - ps26
Theria - ps27
Placentalia - ps28
Boreoeutheria - ps29
Euarchontoglires - ps30
Euarchonta - ps31
Primates - ps32
Haplorrhini - ps33
Simiiformes - ps34
Catarrhini - ps35
Hominoidea - ps36
Hominidae - ps37
Homininae - ps38
Hominini - ps39
Homo_sapiens - ps40
Phylostratum

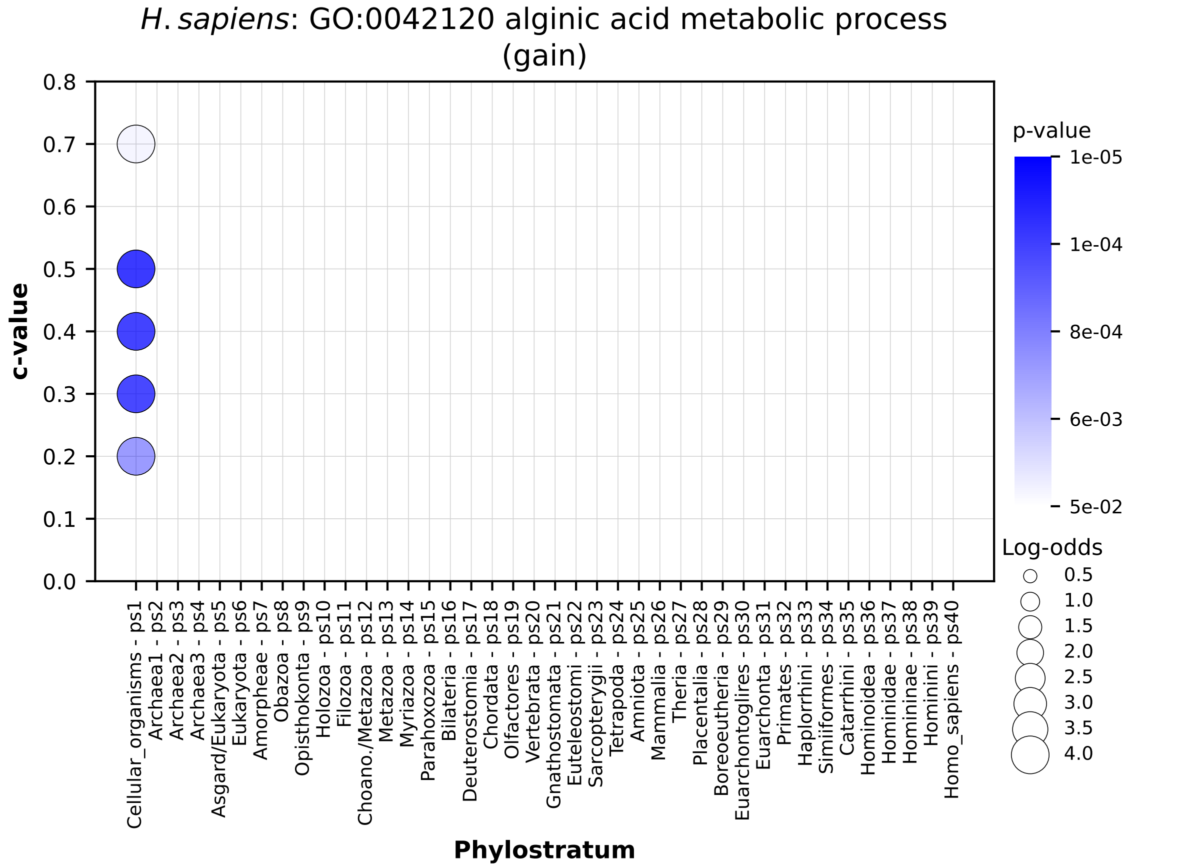
H. sapiens: GO:0042120 alginic acid metabolic process
(gain)
c-value
0.0
0.1
0.2
0.3
0.4
0.5
0.6
0.7
0.8
p-value
1e-05
1e-04
8e-04
6e-03
5e-02
Log-odds
0.5
1.0
1.5
2.0
2.5
3.0
3.5
4.0
Cellular_organisms - ps1
Archaea1 - ps2
Archaea2 - ps3
Archaea3 - ps4
Asgard/Eukaryota - ps5
Eukaryota - ps6
Amorpheae - ps7
Obazoa - ps8
Opisthokonta - ps9
Holozoa - ps10
Filozoa - ps11
Choano./Metazoa - ps12
Metazoa - ps13
Myriazoa - ps14
Parahoxozoa - ps15
Bilateria - ps16
Deuterostomia - ps17
Chordata - ps18
Olfactores - ps19
Vertebrata - ps20
Gnathostomata - ps21
Euteleostomi - ps22
Sarcopterygii - ps23
Tetrapoda - ps24
Amniota - ps25
Mammalia - ps26
Theria - ps27
Placentalia - ps28
Boreoeutheria - ps29
Euarchontoglires - ps30
Euarchonta - ps31
Primates - ps32
Haplorrhini - ps33
Simiiformes - ps34
Catarrhini - ps35
Hominoidea - ps36
Hominidae - ps37
Homininae - ps38
Hominini - ps39
Homo_sapiens - ps40
Phylostratum

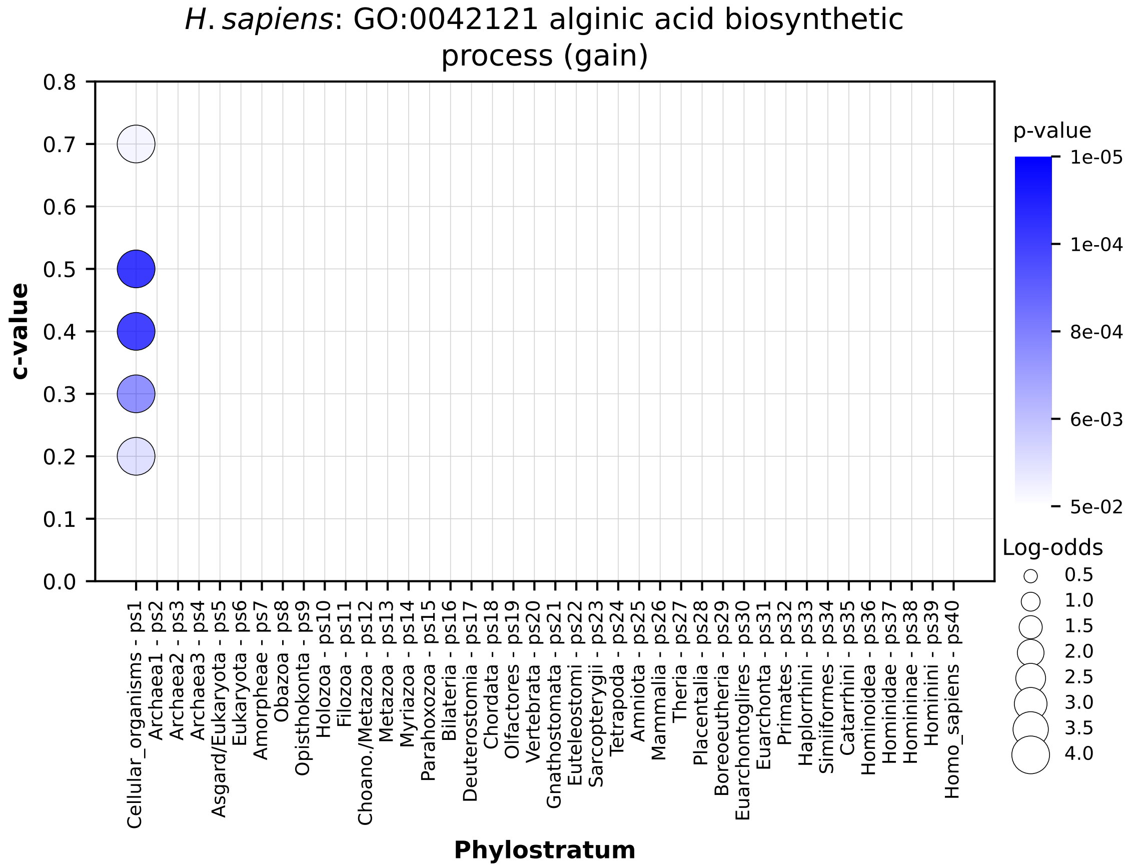

H. sapiens: GO:0042121 alginic acid biosynthetic process (gain)
c-value
0.0
0.1
0.2
0.3
0.4
0.5
0.6
0.7
0.8
Phylostratum
Cellular_organisms - ps1
Archaea1 - ps2
Archaea2 - ps3
Archaea3 - ps4
Asgard/Eukaryota - ps5
Eukaryota - ps6
Amorpheae - ps7
Obazoa - ps8
Opisthokonta - ps9
Holozoa - ps10
Filozoa - ps11
Choano./Metazoa - ps12
Metazoa - ps13
Myriazoa - ps14
Parahoxozoa - ps15
Bilateria - ps16
Deuterostomia - ps17
Chordata - ps18
Olfactores - ps19
Vertebrata - ps20
Gnathostomata - ps21
Euteleostomi - ps22
Sarcopterygii - ps23
Tetrapoda - ps24
Amniota - ps25
Mammalia - ps26
Theria - ps27
Placentalia - ps28
Boreoeutheria - ps29
Euarchontoglires - ps30
Euarchonta - ps31
Primates - ps32
Haplorrhini - ps33
Simiiformes - ps34
Catarrhini - ps35
Hominoidea - ps36
Hominidae - ps37
Homininae - ps38
Hominini - ps39
Homo_sapiens - ps40
p-value
1e-05
1e-04
8e-04
6e-03
5e-02
Log-odds
0.5
1.0
1.5
2.0
2.5
3.0
3.5
4.0

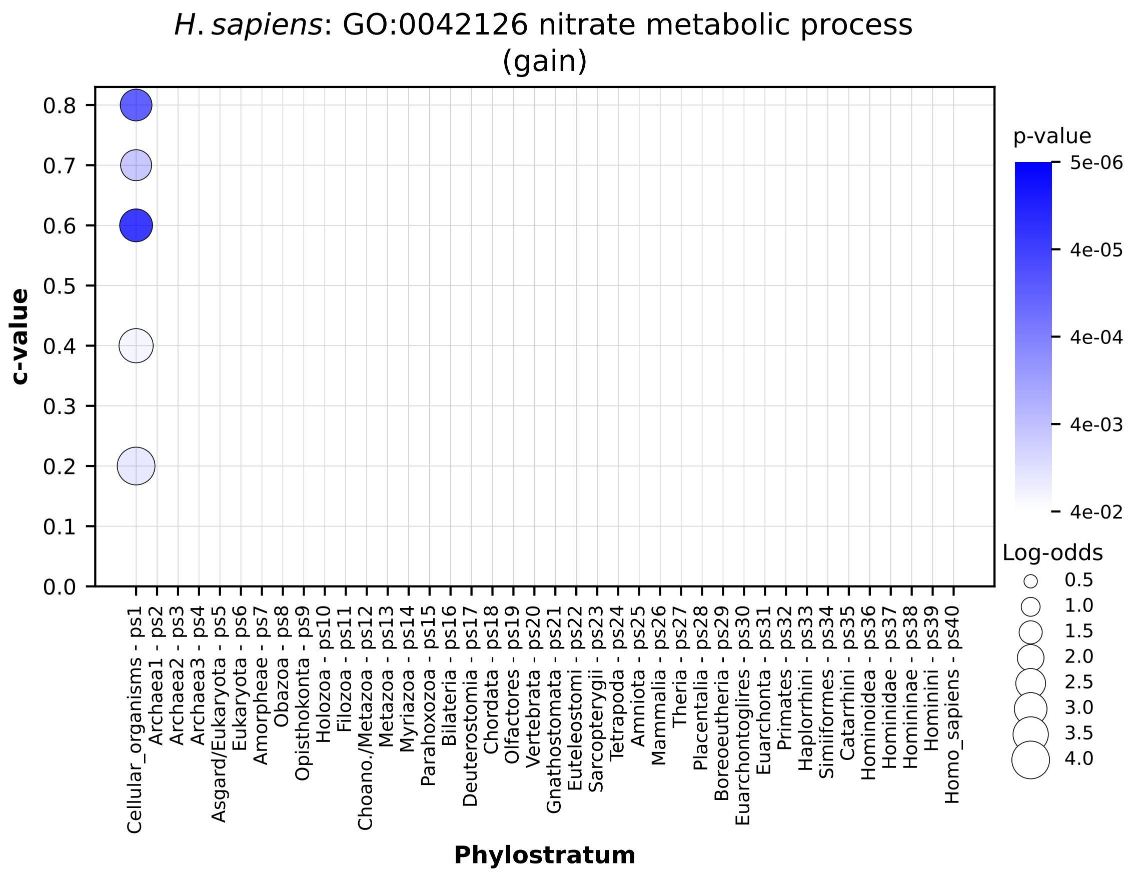

H. sapiens: GO:0042126 nitrate metabolic process
(gain)
c-value
0.0
0.1
0.2
0.3
0.4
0.5
0.6
0.7
0.8
Phylostratum
Cellular_organisms - ps1
Archaea1 - ps2
Archaea2 - ps3
Archaea3 - ps4
Asgard/Eukaryota - ps5
Eukaryota - ps6
Amorpheae - ps7
Obazoa - ps8
Opisthokonta - ps9
Holozoa - ps10
Filozoa - ps11
Choano./Metazoa - ps12
Metazoa - ps13
Myriazoa - ps14
Parahoxozoa - ps15
Bilateria - ps16
Deuterostomia - ps17
Chordata - ps18
Olfactores - ps19
Vertebrata - ps20
Gnathostomata - ps21
Euteleostomi - ps22
Sarcopterygii - ps23
Tetrapoda - ps24
Amniota - ps25
Mammalia - ps26
Theria - ps27
Placentalia - ps28
Boreoeutheria - ps29
Euarchontoglires - ps30
Euarchonta - ps31
Primates - ps32
Haplorrhini - ps33
Simiiformes - ps34
Catarrhini - ps35
Hominoidea - ps36
Hominidae - ps37
Homininae - ps38
Hominini - ps39
Homo_sapiens - ps40
p-value
5e-06
4e-05
4e-04
4e-03
4e-02
Log-odds
0.5
1.0
1.5
2.0
2.5
3.0
3.5
4.0

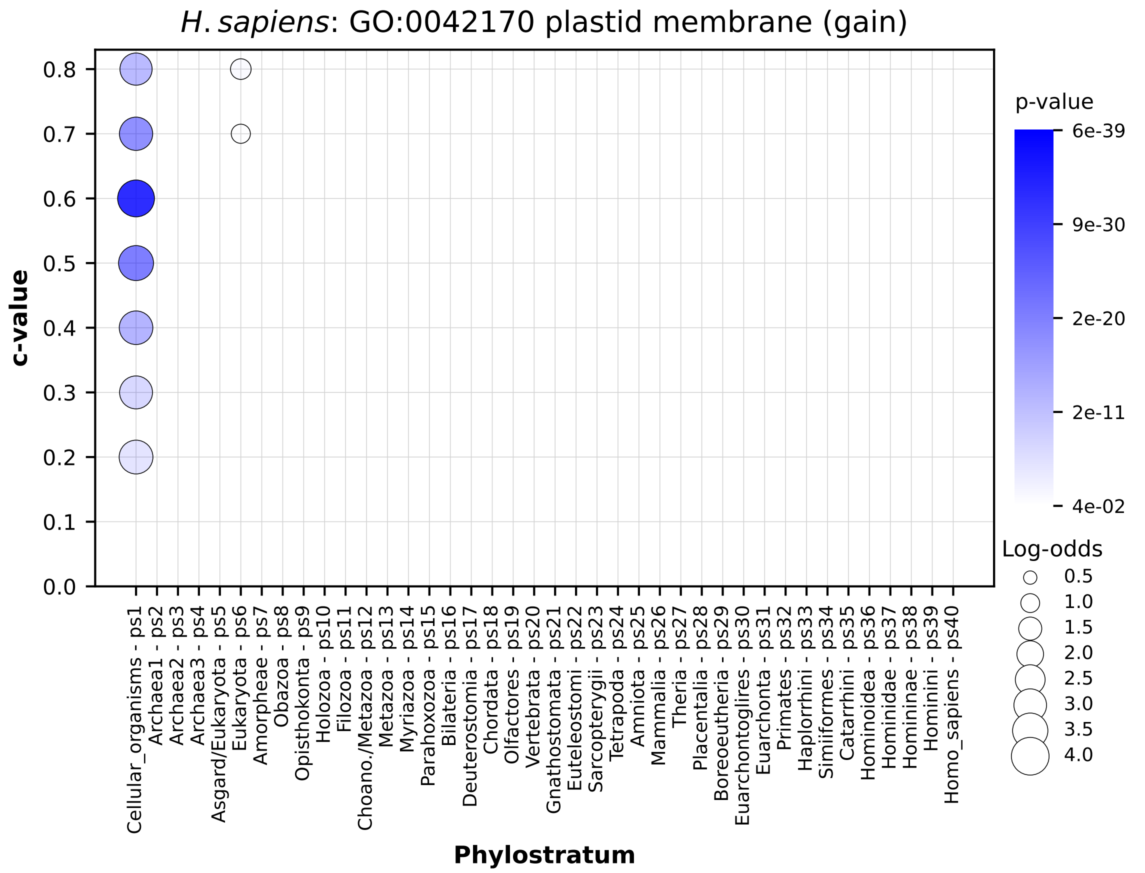

*H. sapiens*: GO:0042170 plastid membrane (gain)
c-value
0.8
0.7
0.6
0.5
0.4
0.3
0.2
0.1
0.0
Phylostratum
Cellular_organisms - ps1
Archaea1 - ps2
Archaea2 - ps3
Archaea3 - ps4
Asgard/Eukaryota - ps5
Eukaryota - ps6
Amorpheae - ps7
Obazoa - ps8
Opisthokonta - ps9
Holozoa - ps10
Filozoa - ps11
Choano./Metazoa - ps12
Metazoa - ps13
Myriazoa - ps14
Parahoxozoa - ps15
Bilateria - ps16
Deuterostomia - ps17
Chordata - ps18
Olfactores - ps19
Vertebrata - ps20
Gnathostomata - ps21
Euteleostomi - ps22
Sarcopterygii - ps23
Tetrapoda - ps24
Amniota - ps25
Mammalia - ps26
Theria - ps27
Placentalia - ps28
Boreoeutheria - ps29
Euarchontoglires - ps30
Euarchonta - ps31
Primates - ps32
Haplorrhini - ps33
Simiiformes - ps34
Catarrhini - ps35
Hominoidea - ps36
Hominidae - ps37
Homininae - ps38
Hominini - ps39
Homo_sapiens - ps40
p-value
6e-39
9e-30
2e-20
2e-11
4e-02
Log-odds
0.5
1.0
1.5
2.0
2.5
3.0
3.5
4.0

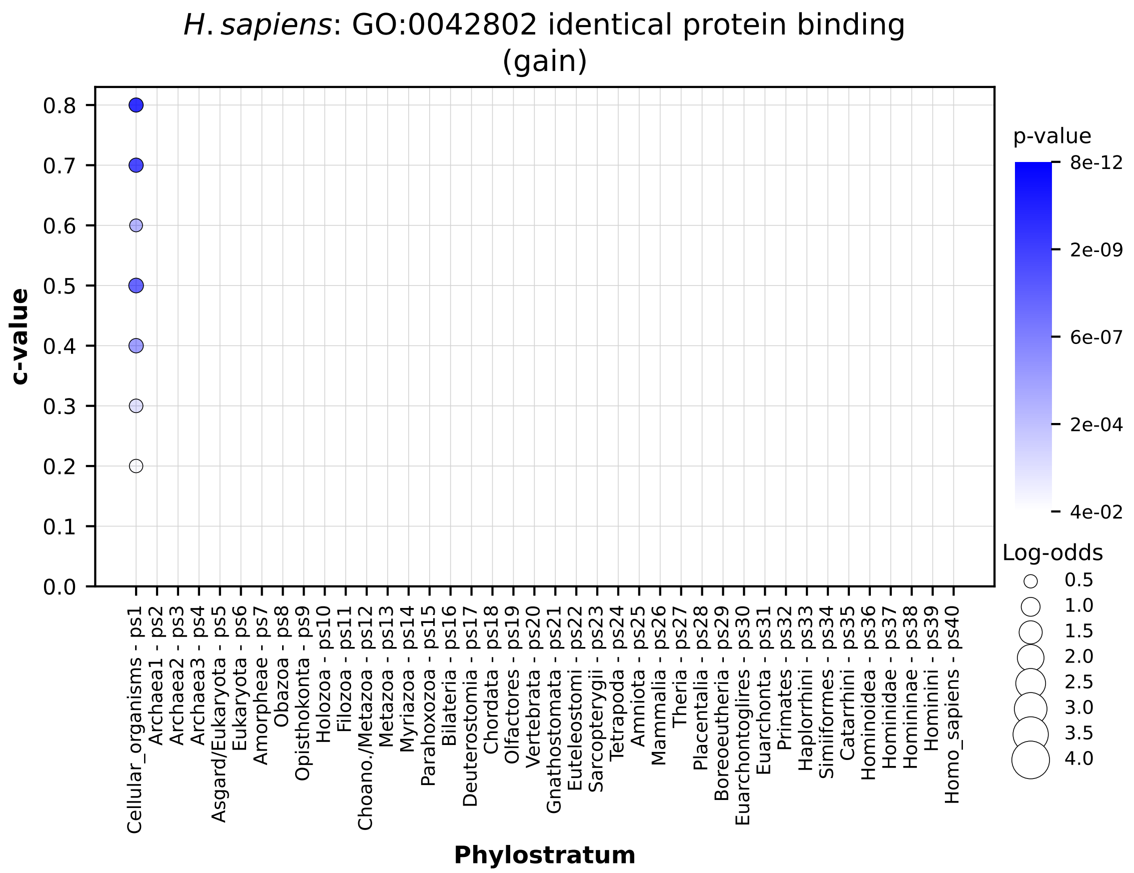

H. sapiens: GO:0042802 identical protein binding
(gain)
c-value
0.0
0.1
0.2
0.3
0.4
0.5
0.6
0.7
0.8
Phylostratum
Cellular_organisms - ps1
Archaea1 - ps2
Archaea2 - ps3
Archaea3 - ps4
Asgard/Eukaryota - ps5
Eukaryota - ps6
Amorpheae - ps7
Obazoa - ps8
Opisthokonta - ps9
Holozoa - ps10
Filozoa - ps11
Choano./Metazoa - ps12
Metazoa - ps13
Myriazoa - ps14
Parahoxozoa - ps15
Bilateria - ps16
Deuterostomia - ps17
Chordata - ps18
Olfactores - ps19
Vertebrata - ps20
Gnathostomata - ps21
Euteleostomi - ps22
Sarcopterygii - ps23
Tetrapoda - ps24
Amniota - ps25
Mammalia - ps26
Theria - ps27
Placentalia - ps28
Boreoeutheria - ps29
Euarchontoglires - ps30
Euarchonta - ps31
Primates - ps32
Haplorrhini - ps33
Simiiformes - ps34
Catarrhini - ps35
Hominoidea - ps36
Hominidae - ps37
Homininae - ps38
Hominini - ps39
Homo_sapiens - ps40
p-value
8e-12
2e-09
6e-07
2e-04
4e-02
Log-odds
0.5
1.0
1.5
2.0
2.5
3.0
3.5
4.0

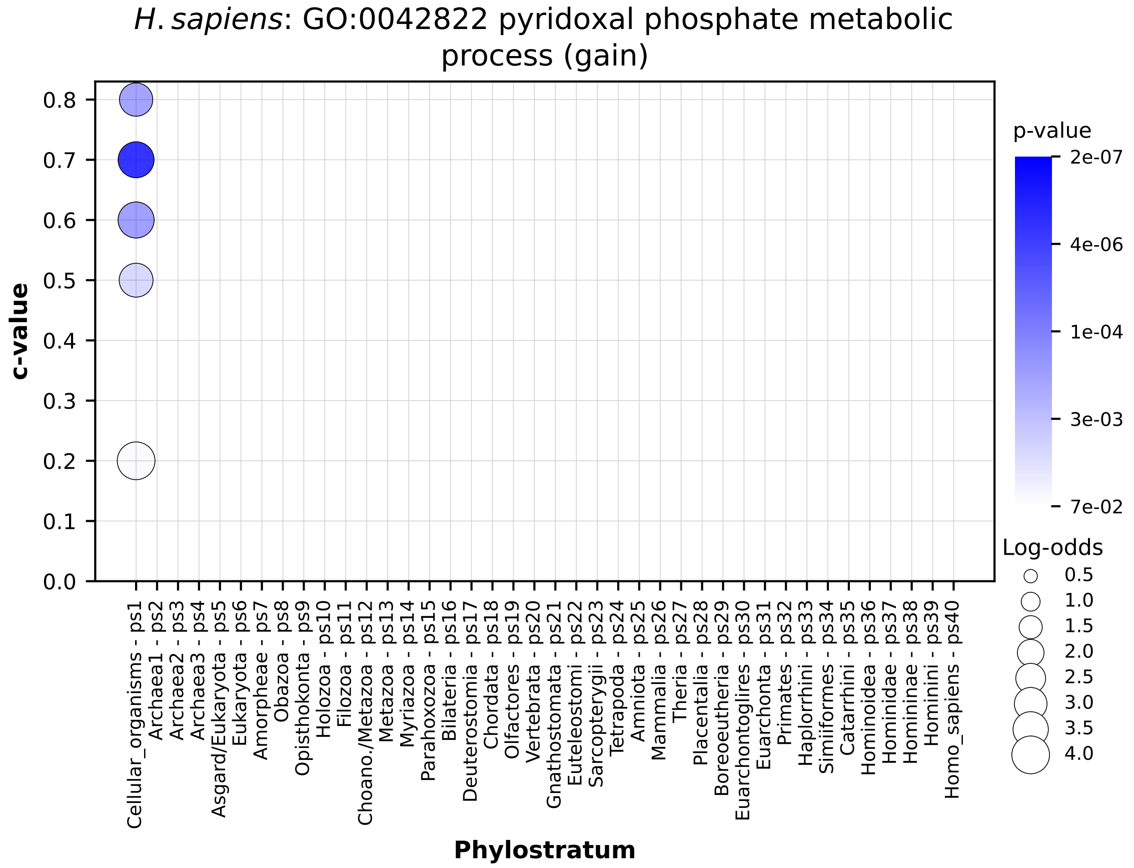

H. sapiens: GO:0042822 pyridoxal phosphate metabolic process (gain)
c-value
0.0
0.1
0.2
0.3
0.4
0.5
0.6
0.7
0.8
Phylostratum
Cellular_organisms - ps1
Archaea1 - ps2
Archaea2 - ps3
Archaea3 - ps4
Asgard/Eukaryota - ps5
Eukaryota - ps6
Amorpheae - ps7
Obazoa - ps8
Opisthokonta - ps9
Holozoa - ps10
Filozoa - ps11
Choano./Metazoa - ps12
Metazoa - ps13
Myriazoa - ps14
Parahoxozoa - ps15
Bilateria - ps16
Deuterostomia - ps17
Chordata - ps18
Olfactores - ps19
Vertebrata - ps20
Gnathostomata - ps21
Euteleostomi - ps22
Sarcopterygii - ps23
Tetrapoda - ps24
Amniota - ps25
Mammalia - ps26
Theria - ps27
Placentalia - ps28
Boreoeutheria - ps29
Euarchontoglires - ps30
Euarchonta - ps31
Primates - ps32
Haplorrhini - ps33
Simiiformes - ps34
Catarrhini - ps35
Hominoidea - ps36
Hominidae - ps37
Homininae - ps38
Hominini - ps39
Homo_sapiens - ps40
p-value
2e-07
4e-06
1e-04
3e-03
7e-02
Log-odds
0.5
1.0
1.5
2.0
2.5
3.0
3.5
4.0

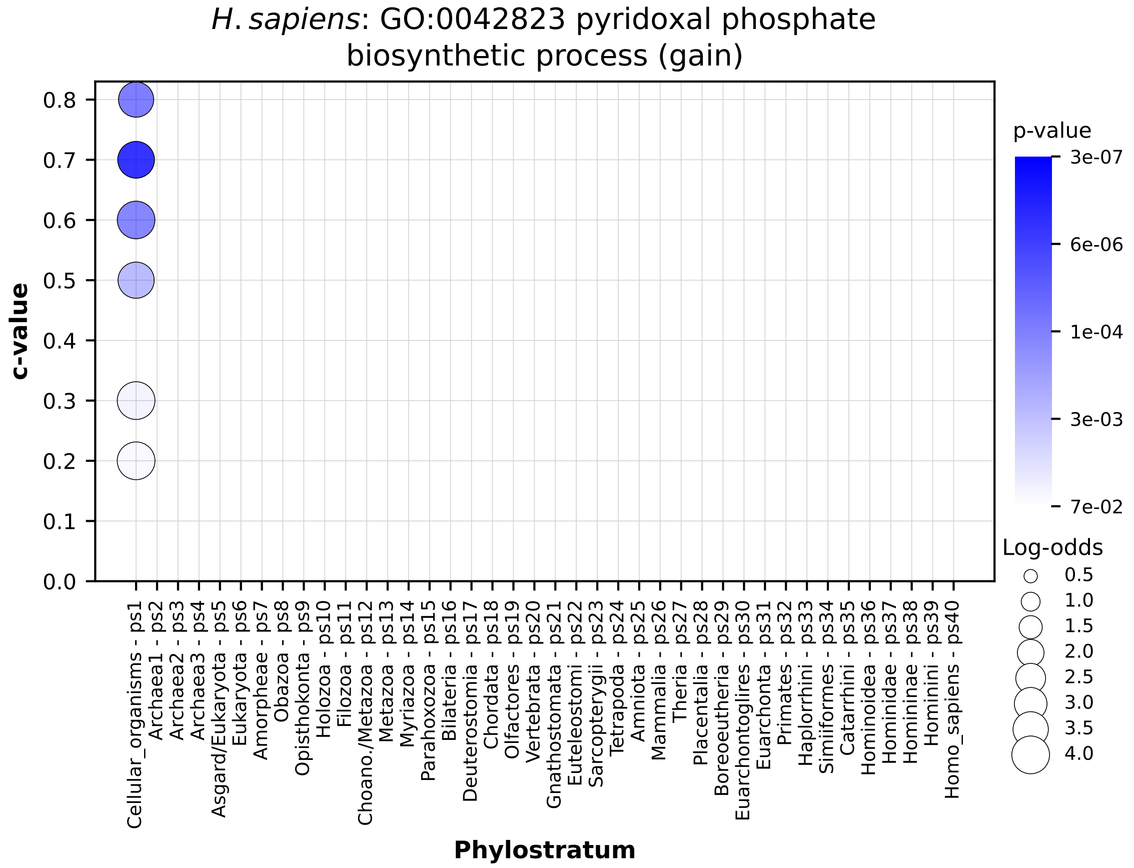

H. sapiens: GO:0042823 pyridoxal phosphate biosynthetic process (gain)
c-value
0.0
0.1
0.2
0.3
0.4
0.5
0.6
0.7
0.8
Phylostratum
Cellular_organisms - ps1
Archaea1 - ps2
Archaea2 - ps3
Archaea3 - ps4
Asgard/Eukaryota - ps5
Eukaryota - ps6
Amorpheae - ps7
Obazoa - ps8
Opisthokonta - ps9
Holozoa - ps10
Filozoa - ps11
Choano./Metazoa - ps12
Metazoa - ps13
Myriazoa - ps14
Parahoxozoa - ps15
Bilateria - ps16
Deuterostomia - ps17
Chordata - ps18
Olfactores - ps19
Vertebrata - ps20
Gnathostomata - ps21
Euteleostomi - ps22
Sarcopterygii - ps23
Tetrapoda - ps24
Amniota - ps25
Mammalia - ps26
Theria - ps27
Placentalia - ps28
Boreoeutheria - ps29
Euarchontoglires - ps30
Euarchonta - ps31
Primates - ps32
Haplorrhini - ps33
Simiiformes - ps34
Catarrhini - ps35
Hominoidea - ps36
Hominidae - ps37
Homininae - ps38
Hominini - ps39
Homo_sapiens - ps40
p-value
3e-07
6e-06
1e-04
3e-03
7e-02
Log-odds
0.5
1.0
1.5
2.0
2.5
3.0
3.5
4.0

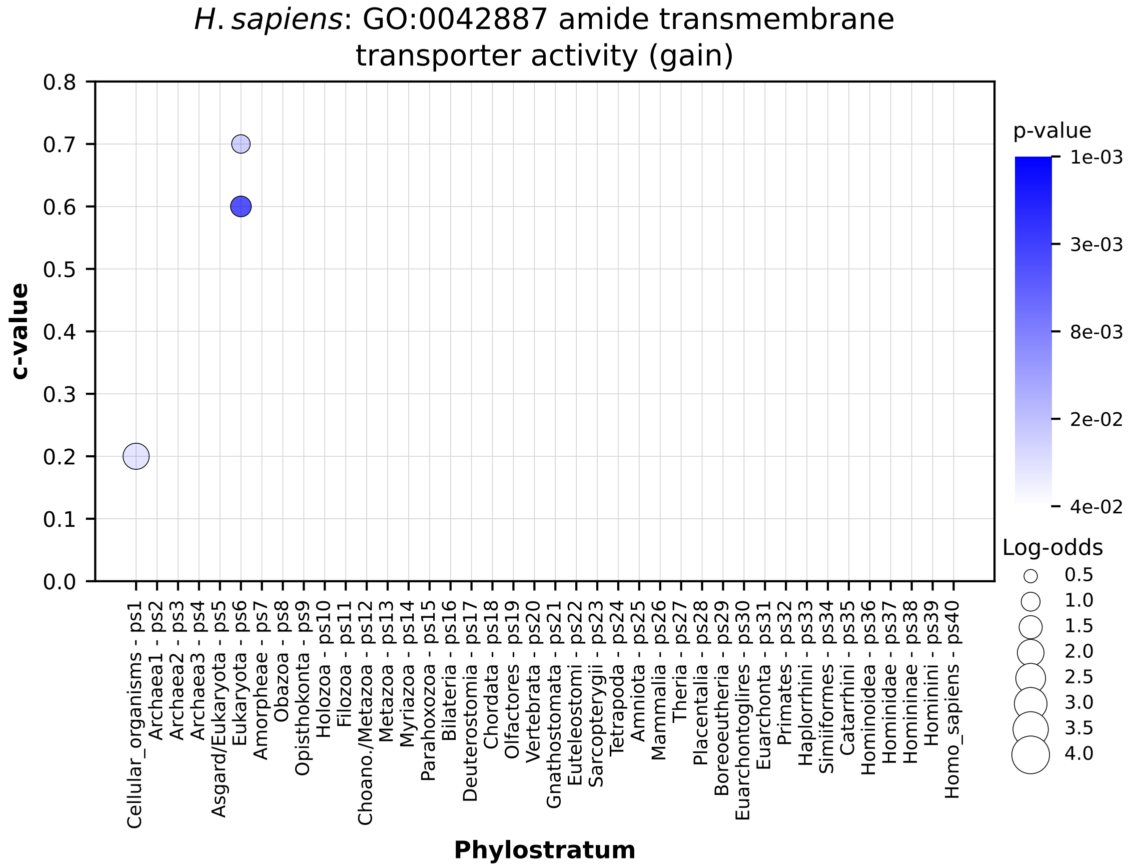

H. sapiens: GO:0042887 amide transmembrane transporter activity (gain)
c-value
0.0
0.1
0.2
0.3
0.4
0.5
0.6
0.7
0.8
Phylostratum
Cellular_organisms - ps1
Archaea1 - ps2
Archaea2 - ps3
Archaea3 - ps4
Asgard/Eukaryota - ps5
Eukaryota - ps6
Amorpheae - ps7
Obazoa - ps8
Opisthokonta - ps9
Holozoa - ps10
Filozoa - ps11
Choano./Metazoa - ps12
Metazoa - ps13
Myriazoa - ps14
Parahoxozoa - ps15
Bilateria - ps16
Deuterostomia - ps17
Chordata - ps18
Olfactores - ps19
Vertebrata - ps20
Gnathostomata - ps21
Euteleostomi - ps22
Sarcopterygii - ps23
Tetrapoda - ps24
Amniota - ps25
Mammalia - ps26
Theria - ps27
Placentalia - ps28
Boreoeutheria - ps29
Euarchontoglires - ps30
Euarchonta - ps31
Primates - ps32
Haplorrhini - ps33
Simiiformes - ps34
Catarrhini - ps35
Hominoidea - ps36
Hominidae - ps37
Homininae - ps38
Hominini - ps39
Homo_sapiens - ps40
p-value
1e-03
3e-03
8e-03
2e-02
4e-02
Log-odds
0.5
1.0
1.5
2.0
2.5
3.0
3.5
4.0

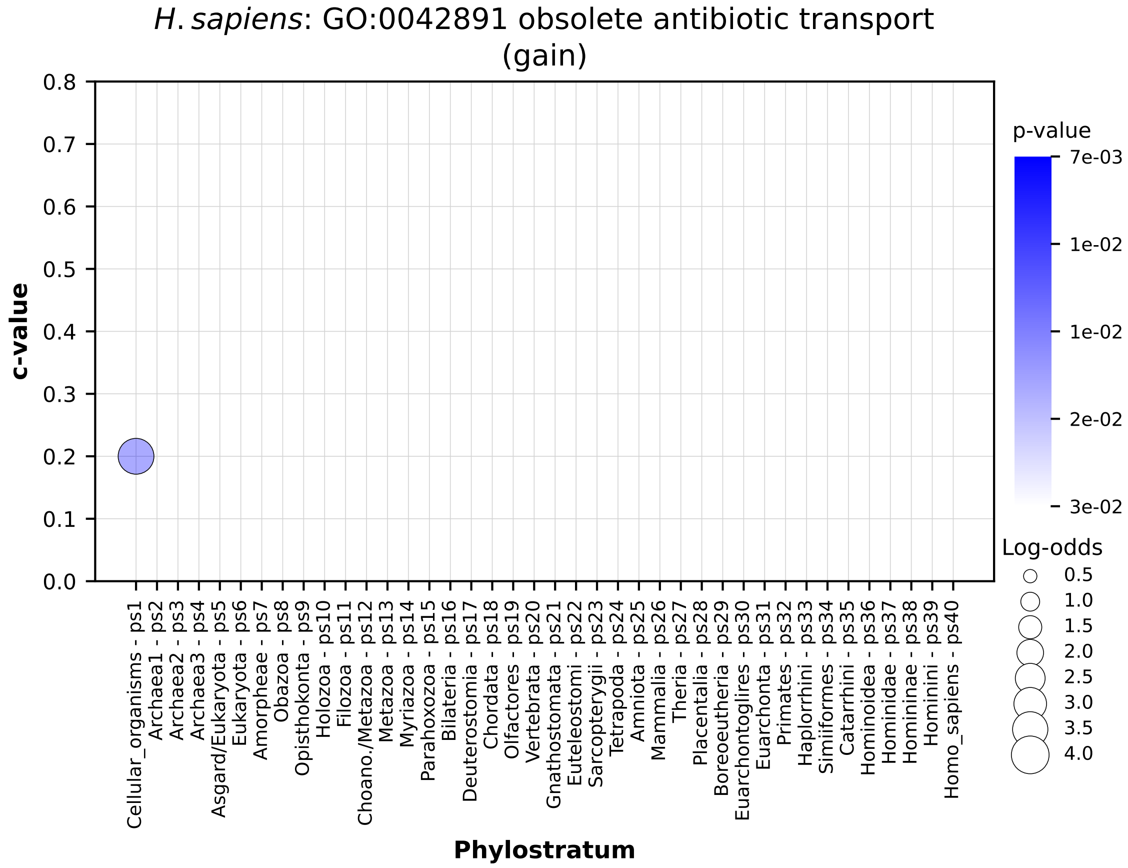

*H. sapiens*: GO:0042891 obsolete antibiotic transport (gain)
c-value
0.8
0.7
0.6
0.5
0.4
0.3
0.2
0.1
0.0
p-value
7e-03
1e-02
1e-02
2e-02
3e-02
Log-odds
0.5
1.0
1.5
2.0
2.5
3.0
3.5
4.0
Cellular_organisms - ps1
Archaea1 - ps2
Archaea2 - ps3
Archaea3 - ps4
Asgard/Eukaryota - ps5
Eukaryota - ps6
Amorpheae - ps7
Obazoa - ps8
Opisthokonta - ps9
Holozoa - ps10
Filozoa - ps11
Choano./Metazoa - ps12
Metazoa - ps13
Myriazoa - ps14
Parahoxozoa - ps15
Bilateria - ps16
Deuterostomia - ps17
Chordata - ps18
Olfactores - ps19
Vertebrata - ps20
Gnathostomata - ps21
Euteleostomi - ps22
Sarcopterygii - ps23
Tetrapoda - ps24
Amniota - ps25
Mammalia - ps26
Theria - ps27
Placentalia - ps28
Boreoeutheria - ps29
Euarchontoglires - ps30
Euarchonta - ps31
Primates - ps32
Haplorrhini - ps33
Simiiformes - ps34
Catarrhini - ps35
Hominoidea - ps36
Hominidae - ps37
Homininae - ps38
Hominini - ps39
Homo_sapiens - ps40
Phylostratum

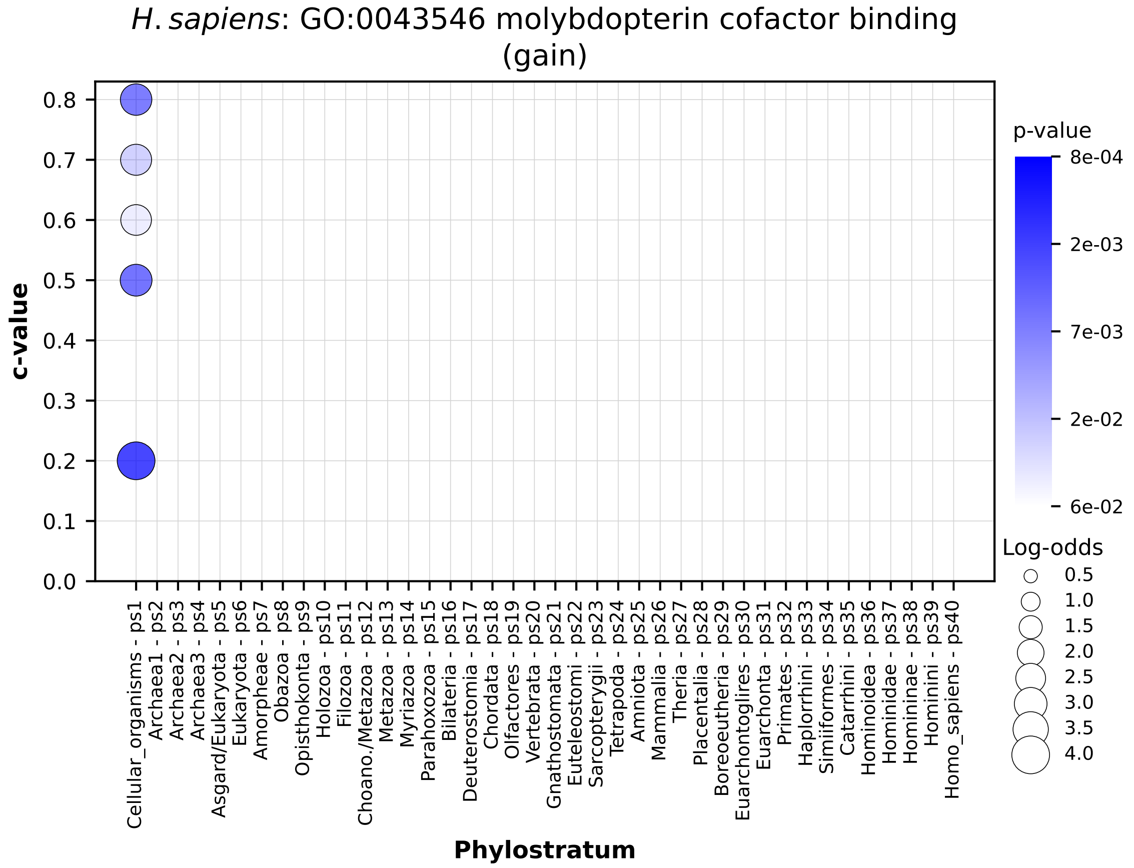

H. sapiens: GO:0043546 molybdopterin cofactor binding
(gain)
c-value
0.0
0.1
0.2
0.3
0.4
0.5
0.6
0.7
0.8
Phylostratum
Cellular_organisms - ps1
Archaea1 - ps2
Archaea2 - ps3
Archaea3 - ps4
Asgard/Eukaryota - ps5
Eukaryota - ps6
Amorpheae - ps7
Obazoa - ps8
Opisthokonta - ps9
Holozoa - ps10
Filozoa - ps11
Choano./Metazoa - ps12
Metazoa - ps13
Myriazoa - ps14
Parahoxozoa - ps15
Bilateria - ps16
Deuterostomia - ps17
Chordata - ps18
Olfactores - ps19
Vertebrata - ps20
Gnathostomata - ps21
Euteleostomi - ps22
Sarcopterygii - ps23
Tetrapoda - ps24
Amniota - ps25
Mammalia - ps26
Theria - ps27
Placentalia - ps28
Boreoeutheria - ps29
Euarchontoglires - ps30
Euarchonta - ps31
Primates - ps32
Haplorrhini - ps33
Simiiformes - ps34
Catarrhini - ps35
Hominoidea - ps36
Hominidae - ps37
Homininae - ps38
Hominini - ps39
Homo_sapiens - ps40
p-value
8e-04
2e-03
7e-03
2e-02
6e-02
Log-odds
0.5
1.0
1.5
2.0
2.5
3.0
3.5
4.0

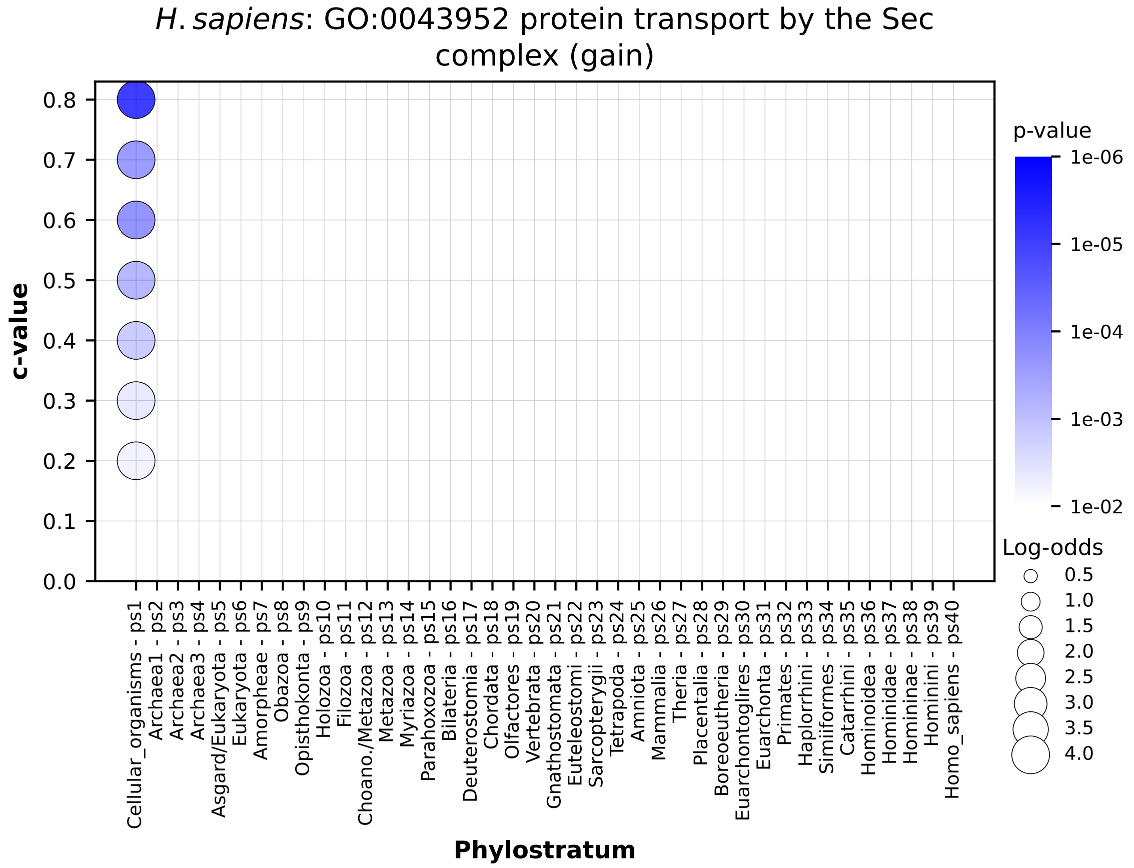

H. sapiens: GO:0043952 protein transport by the Sec complex (gain)
c-value
0.0
0.1
0.2
0.3
0.4
0.5
0.6
0.7
0.8
Phylostratum
Cellular_organisms - ps1
Archaea1 - ps2
Archaea2 - ps3
Archaea3 - ps4
Asgard/Eukaryota - ps5
Eukaryota - ps6
Amorpheae - ps7
Obazoa - ps8
Opisthokonta - ps9
Holozoa - ps10
Filozoa - ps11
Choano./Metazoa - ps12
Metazoa - ps13
Myriazoa - ps14
Parahoxozoa - ps15
Bilateria - ps16
Deuterostomia - ps17
Chordata - ps18
Olfactores - ps19
Vertebrata - ps20
Gnathostomata - ps21
Euteleostomi - ps22
Sarcopterygii - ps23
Tetrapoda - ps24
Amniota - ps25
Mammalia - ps26
Theria - ps27
Placentalia - ps28
Boreoeutheria - ps29
Euarchontoglires - ps30
Euarchonta - ps31
Primates - ps32
Haplorrhini - ps33
Simiiformes - ps34
Catarrhini - ps35
Hominoidea - ps36
Hominidae - ps37
Homininae - ps38
Hominini - ps39
Homo_sapiens - ps40
p-value
1e-06
1e-05
1e-04
1e-03
1e-02
Log-odds
0.5
1.0
1.5
2.0
2.5
3.0
3.5
4.0

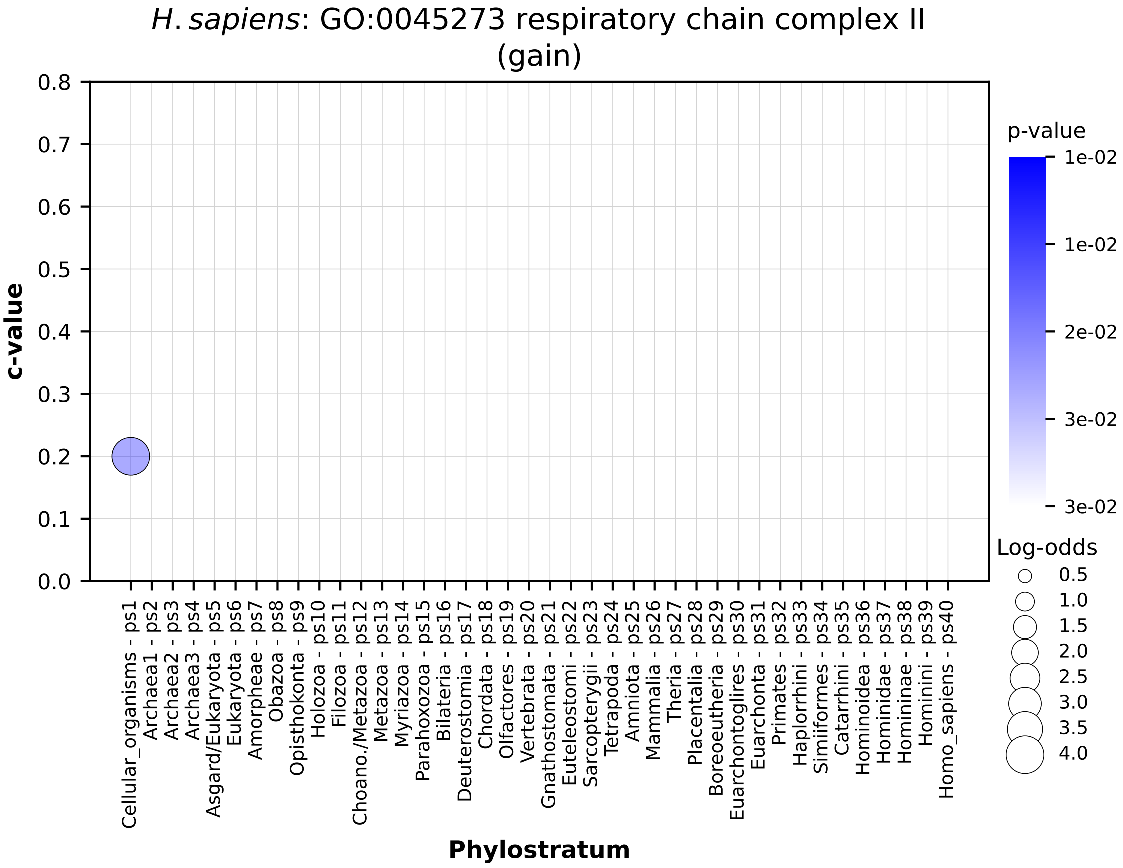

H. sapiens: GO:0045273 respiratory chain complex II (gain)
c-value
0.0
0.1
0.2
0.3
0.4
0.5
0.6
0.7
0.8
Phylostratum
Cellular_organisms - ps1
Archaea1 - ps2
Archaea2 - ps3
Archaea3 - ps4
Asgard/Eukaryota - ps5
Eukaryota - ps6
Amorpheae - ps7
Obazoa - ps8
Opisthokonta - ps9
Holozoa - ps10
Filozoa - ps11
Choano./Metazoa - ps12
Metazoa - ps13
Myriazoa - ps14
Parahoxozoa - ps15
Bilateria - ps16
Deuterostomia - ps17
Chordata - ps18
Olfactores - ps19
Vertebrata - ps20
Gnathostomata - ps21
Euteleostomi - ps22
Sarcopterygii - ps23
Tetrapoda - ps24
Amniota - ps25
Mammalia - ps26
Theria - ps27
Placentalia - ps28
Boreoeutheria - ps29
Euarchontoglires - ps30
Euarchonta - ps31
Primates - ps32
Haplorrhini - ps33
Simiiformes - ps34
Catarrhini - ps35
Hominoidea - ps36
Hominidae - ps37
Homininae - ps38
Hominini - ps39
Homo_sapiens - ps40
p-value
1e-02
1e-02
2e-02
3e-02
3e-02
Log-odds
0.5
1.0
1.5
2.0
2.5
3.0
3.5
4.0

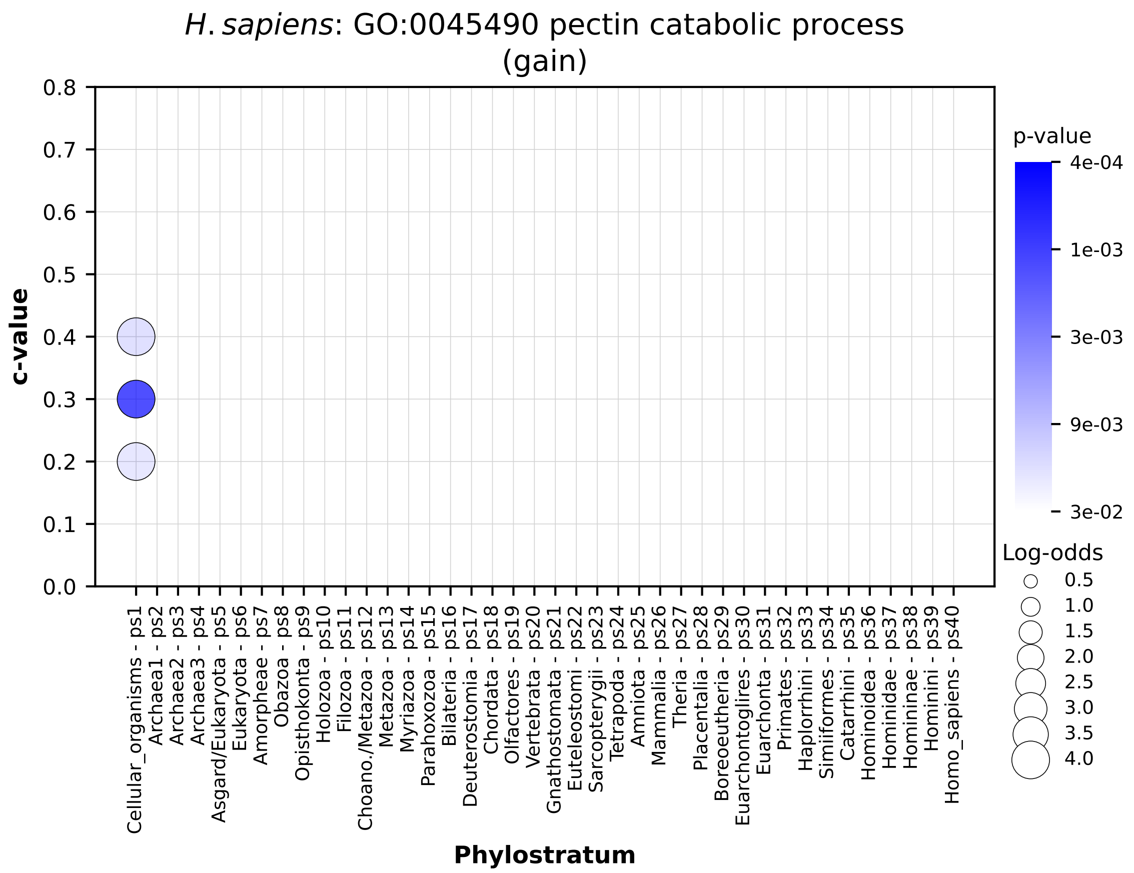

H. sapiens: GO:0045490 pectin catabolic process
(gain)
c-value
0.0
0.1
0.2
0.3
0.4
0.5
0.6
0.7
0.8
Phylostratum
Cellular_organisms - ps1
Archaea1 - ps2
Archaea2 - ps3
Archaea3 - ps4
Asgard/Eukaryota - ps5
Eukaryota - ps6
Amorpheae - ps7
Obazoa - ps8
Opisthokonta - ps9
Holozoa - ps10
Filozoa - ps11
Choano./Metazoa - ps12
Metazoa - ps13
Myriazoa - ps14
Parahoxozoa - ps15
Bilateria - ps16
Deuterostomia - ps17
Chordata - ps18
Olfactores - ps19
Vertebrata - ps20
Gnathostomata - ps21
Euteleostomi - ps22
Sarcopterygii - ps23
Tetrapoda - ps24
Amniota - ps25
Mammalia - ps26
Theria - ps27
Placentalia - ps28
Boreoeutheria - ps29
Euarchontoglires - ps30
Euarchonta - ps31
Primates - ps32
Haplorrhini - ps33
Simiiformes - ps34
Catarrhini - ps35
Hominoidea - ps36
Hominidae - ps37
Homininae - ps38
Hominini - ps39
Homo_sapiens - ps40
p-value
4e-04
1e-03
3e-03
9e-03
3e-02
Log-odds
0.5
1.0
1.5
2.0
2.5
3.0
3.5
4.0

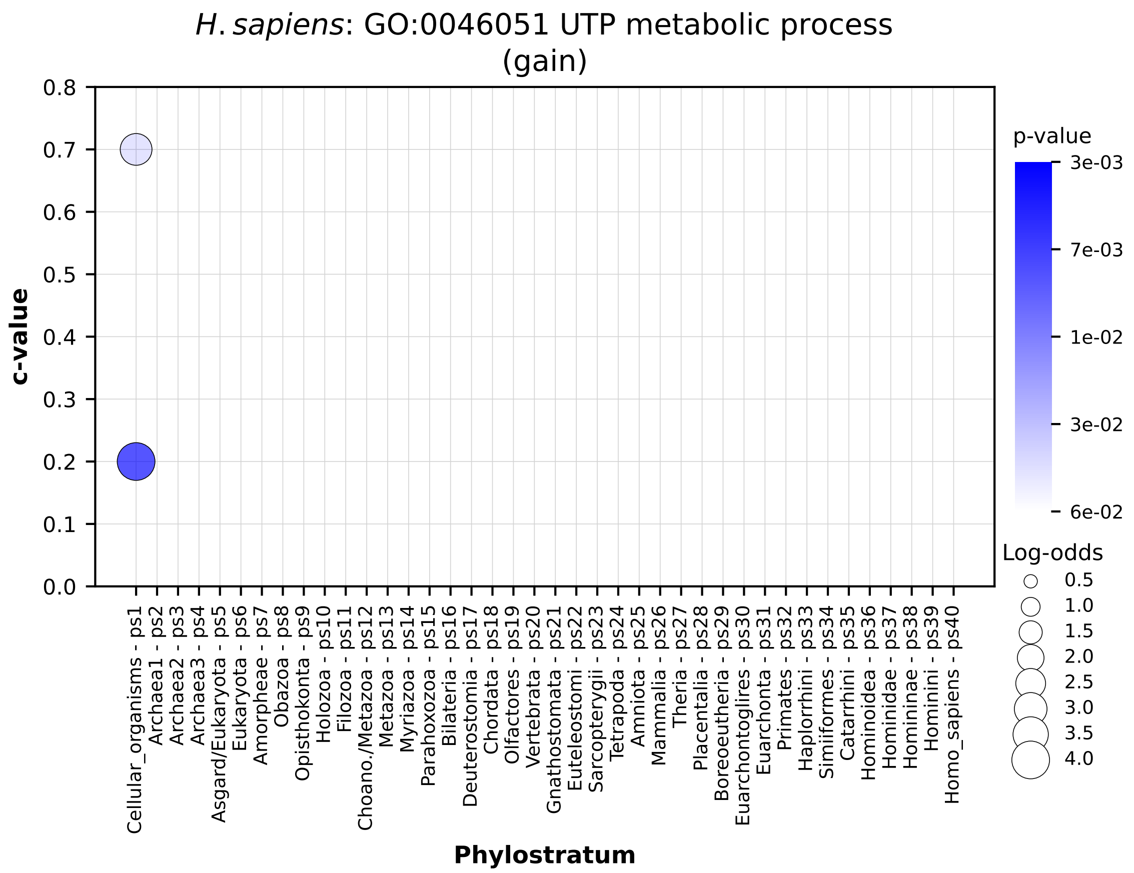

*H. sapiens*: GO:0046051 UTP metabolic process
(gain)
c-value
0.0
0.1
0.2
0.3
0.4
0.5
0.6
0.7
0.8
Phylostratum
Cellular_organisms - ps1
Archaea1 - ps2
Archaea2 - ps3
Archaea3 - ps4
Asgard/Eukaryota - ps5
Eukaryota - ps6
Amorpheae - ps7
Obazoa - ps8
Opisthokonta - ps9
Holozoa - ps10
Filozoa - ps11
Choano./Metazoa - ps12
Metazoa - ps13
Myriazoa - ps14
Parahoxozoa - ps15
Bilateria - ps16
Deuterostomia - ps17
Chordata - ps18
Olfactores - ps19
Vertebrata - ps20
Gnathostomata - ps21
Euteleostomi - ps22
Sarcopterygii - ps23
Tetrapoda - ps24
Amniota - ps25
Mammalia - ps26
Theria - ps27
Placentalia - ps28
Boreoeutheria - ps29
Euarchontoglires - ps30
Euarchonta - ps31
Primates - ps32
Haplorrhini - ps33
Simiiformes - ps34
Catarrhini - ps35
Hominoidea - ps36
Hominidae - ps37
Homininae - ps38
Hominini - ps39
Homo_sapiens - ps40
p-value
3e-03
7e-03
1e-02
3e-02
6e-02
Log-odds
0.5
1.0
1.5
2.0
2.5
3.0
3.5
4.0

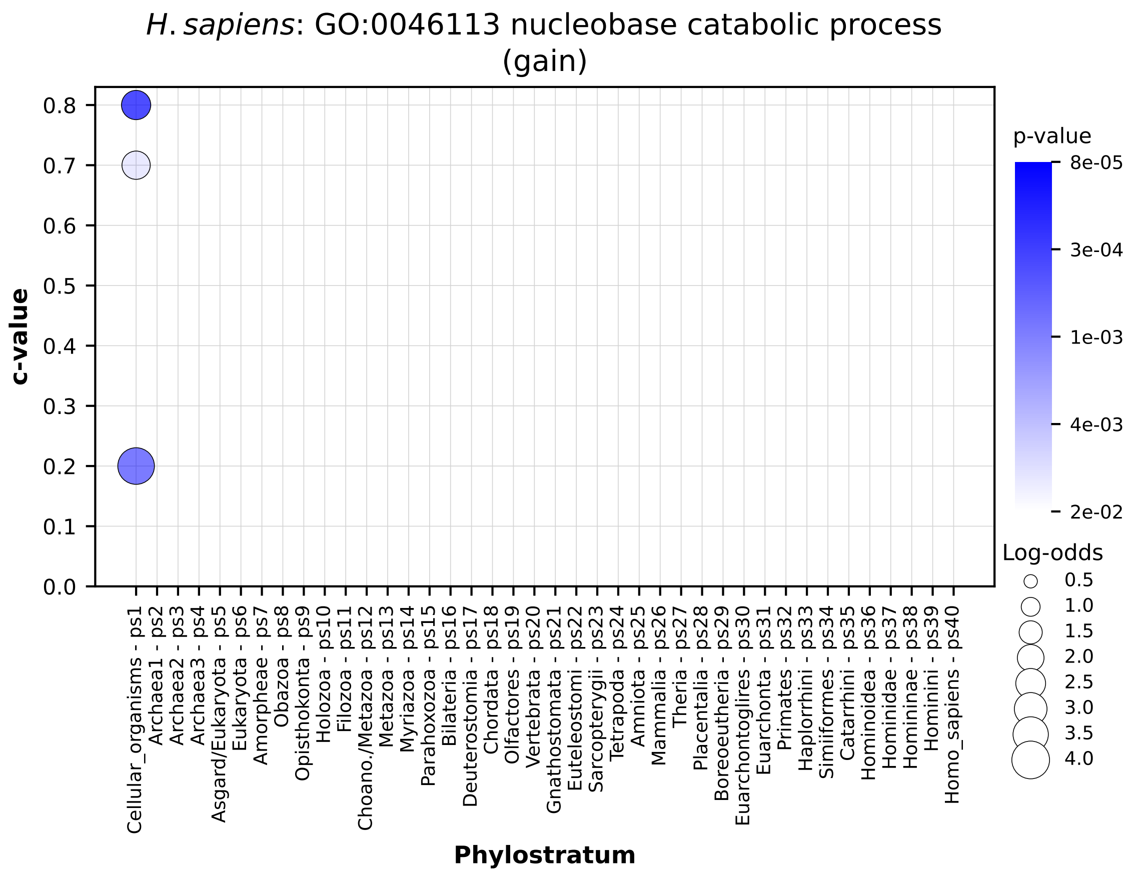
H. sapiens: GO:0046113 nucleobase catabolic process
(gain)
c-value
0.0
0.1
0.2
0.3
0.4
0.5
0.6
0.7
0.8
Phylostratum
Cellular_organisms - ps1
Archaea1 - ps2
Archaea2 - ps3
Archaea3 - ps4
Asgard/Eukaryota - ps5
Eukaryota - ps6
Amorpheae - ps7
Obazoa - ps8
Opisthokonta - ps9
Holozoa - ps10
Filozoa - ps11
Choano./Metazoa - ps12
Metazoa - ps13
Myriazoa - ps14
Parahoxozoa - ps15
Bilateria - ps16
Deuterostomia - ps17
Chordata - ps18
Olfactores - ps19
Vertebrata - ps20
Gnathostomata - ps21
Euteleostomi - ps22
Sarcopterygii - ps23
Tetrapoda - ps24
Amniota - ps25
Mammalia - ps26
Theria - ps27
Placentalia - ps28
Boreoeutheria - ps29
Euarchontoglires - ps30
Euarchonta - ps31
Primates - ps32
Haplorrhini - ps33
Simiiformes - ps34
Catarrhini - ps35
Hominoidea - ps36
Hominidae - ps37
Homininae - ps38
Hominini - ps39
Homo_sapiens - ps40
p-value
8e-05
3e-04
1e-03
4e-03
2e-02
Log-odds
0.5
1.0
1.5
2.0
2.5
3.0
3.5
4.0

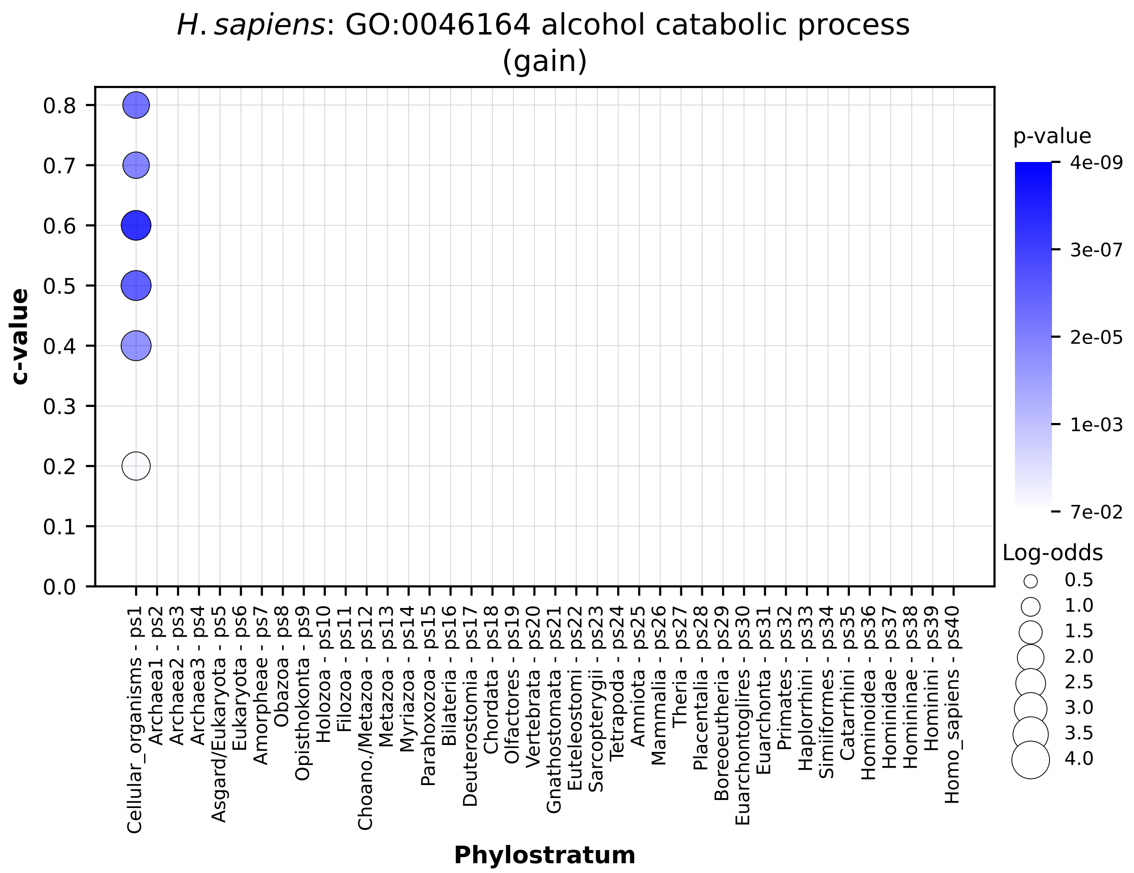

*H. sapiens*: GO:0046164 alcohol catabolic process
(gain)
c-value
0.0
0.1
0.2
0.3
0.4
0.5
0.6
0.7
0.8
Phylostratum
Cellular_organisms - ps1
Archaea1 - ps2
Archaea2 - ps3
Archaea3 - ps4
Asgard/Eukaryota - ps5
Eukaryota - ps6
Amorpheae - ps7
Obazoa - ps8
Opisthokonta - ps9
Holozoa - ps10
Filozoa - ps11
Choano./Metazoa - ps12
Metazoa - ps13
Myriazoa - ps14
Parahoxozoa - ps15
Bilateria - ps16
Deuterostomia - ps17
Chordata - ps18
Olfactores - ps19
Vertebrata - ps20
Gnathostomata - ps21
Euteleostomi - ps22
Sarcopterygii - ps23
Tetrapoda - ps24
Amniota - ps25
Mammalia - ps26
Theria - ps27
Placentalia - ps28
Boreoeutheria - ps29
Euarchontoglires - ps30
Euarchonta - ps31
Primates - ps32
Haplorrhini - ps33
Simiiformes - ps34
Catarrhini - ps35
Hominoidea - ps36
Hominidae - ps37
Homininae - ps38
Hominini - ps39
Homo_sapiens - ps40
p-value
4e-09
3e-07
2e-05
1e-03
7e-02
Log-odds
0.5
1.0
1.5
2.0
2.5
3.0
3.5
4.0

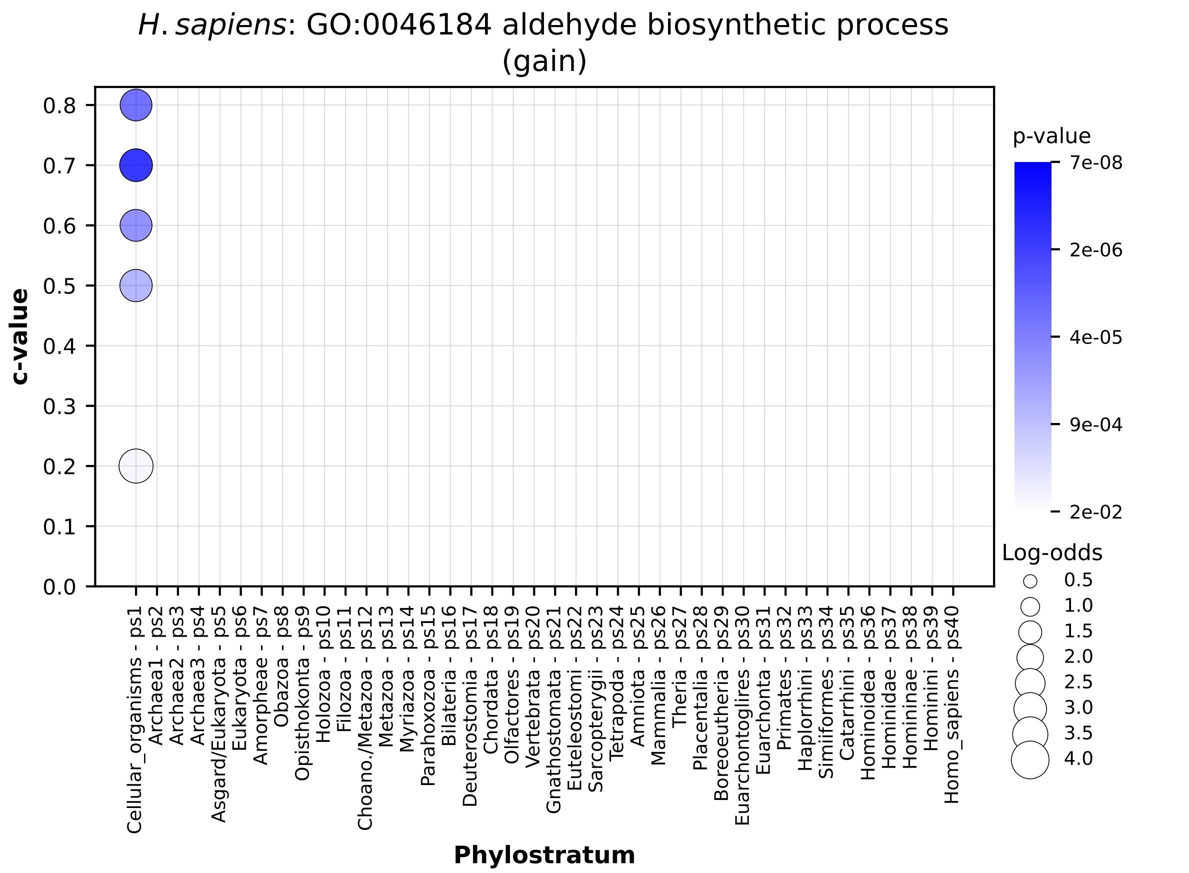
H. sapiens: GO:0046184 aldehyde biosynthetic process
(gain)
c-value
0.0
0.1
0.2
0.3
0.4
0.5
0.6
0.7
0.8
Phylostratum
Cellular_organisms - ps1
Archaea1 - ps2
Archaea2 - ps3
Archaea3 - ps4
Asgard/Eukaryota - ps5
Eukaryota - ps6
Amorpheae - ps7
Obazoa - ps8
Opisthokonta - ps9
Holozoa - ps10
Filozoa - ps11
Choano./Metazoa - ps12
Metazoa - ps13
Myriazoa - ps14
Parahoxozoa - ps15
Bilateria - ps16
Deuterostomia - ps17
Chordata - ps18
Olfactores - ps19
Vertebrata - ps20
Gnathostomata - ps21
Euteleostomi - ps22
Sarcopterygii - ps23
Tetrapoda - ps24
Amniota - ps25
Mammalia - ps26
Theria - ps27
Placentalia - ps28
Boreoeutheria - ps29
Euarchontoglires - ps30
Euarchonta - ps31
Primates - ps32
Haplorrhini - ps33
Simiiformes - ps34
Catarrhini - ps35
Hominoidea - ps36
Hominidae - ps37
Homininae - ps38
Hominini - ps39
Homo_sapiens - ps40
p-value
7e-08
2e-06
4e-05
9e-04
2e-02
Log-odds
0.5
1.0
1.5
2.0
2.5
3.0
3.5
4.0

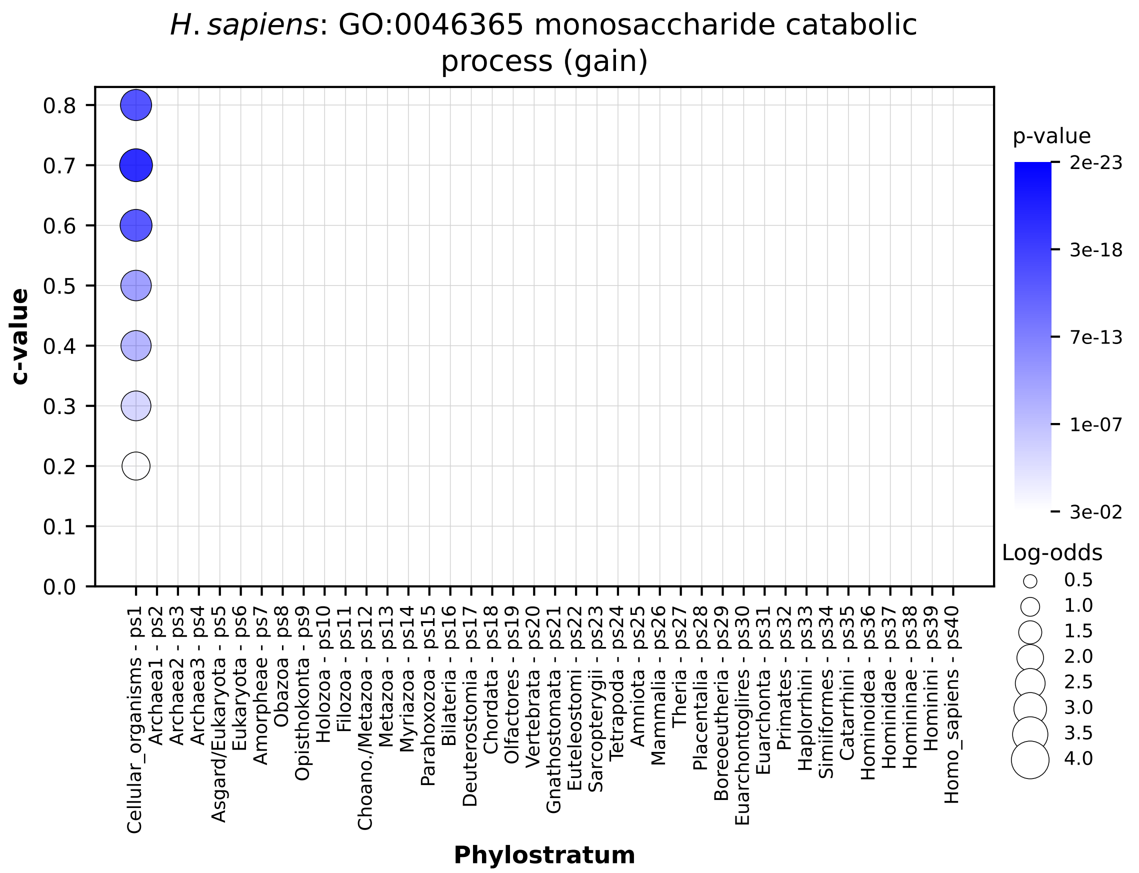
H. sapiens: GO:0046365 monosaccharide catabolic process (gain)
c-value
0.0
0.1
0.2
0.3
0.4
0.5
0.6
0.7
0.8
Phylostratum
Cellular_organisms - ps1
Archaea1 - ps2
Archaea2 - ps3
Archaea3 - ps4
Asgard/Eukaryota - ps5
Eukaryota - ps6
Amorpheae - ps7
Obazoa - ps8
Opisthokonta - ps9
Holozoa - ps10
Filozoa - ps11
Choano./Metazoa - ps12
Metazoa - ps13
Myriazoa - ps14
Parahoxozoa - ps15
Bilateria - ps16
Deuterostomia - ps17
Chordata - ps18
Olfactores - ps19
Vertebrata - ps20
Gnathostomata - ps21
Euteleostomi - ps22
Sarcopterygii - ps23
Tetrapoda - ps24
Amniota - ps25
Mammalia - ps26
Theria - ps27
Placentalia - ps28
Boreoeutheria - ps29
Euarchontoglires - ps30
Euarchonta - ps31
Primates - ps32
Haplorrhini - ps33
Simiiformes - ps34
Catarrhini - ps35
Hominoidea - ps36
Hominidae - ps37
Homininae - ps38
Hominini - ps39
Homo_sapiens - ps40
p-value
2e-23
3e-18
7e-13
1e-07
3e-02
Log-odds
0.5
1.0
1.5
2.0
2.5
3.0
3.5
4.0

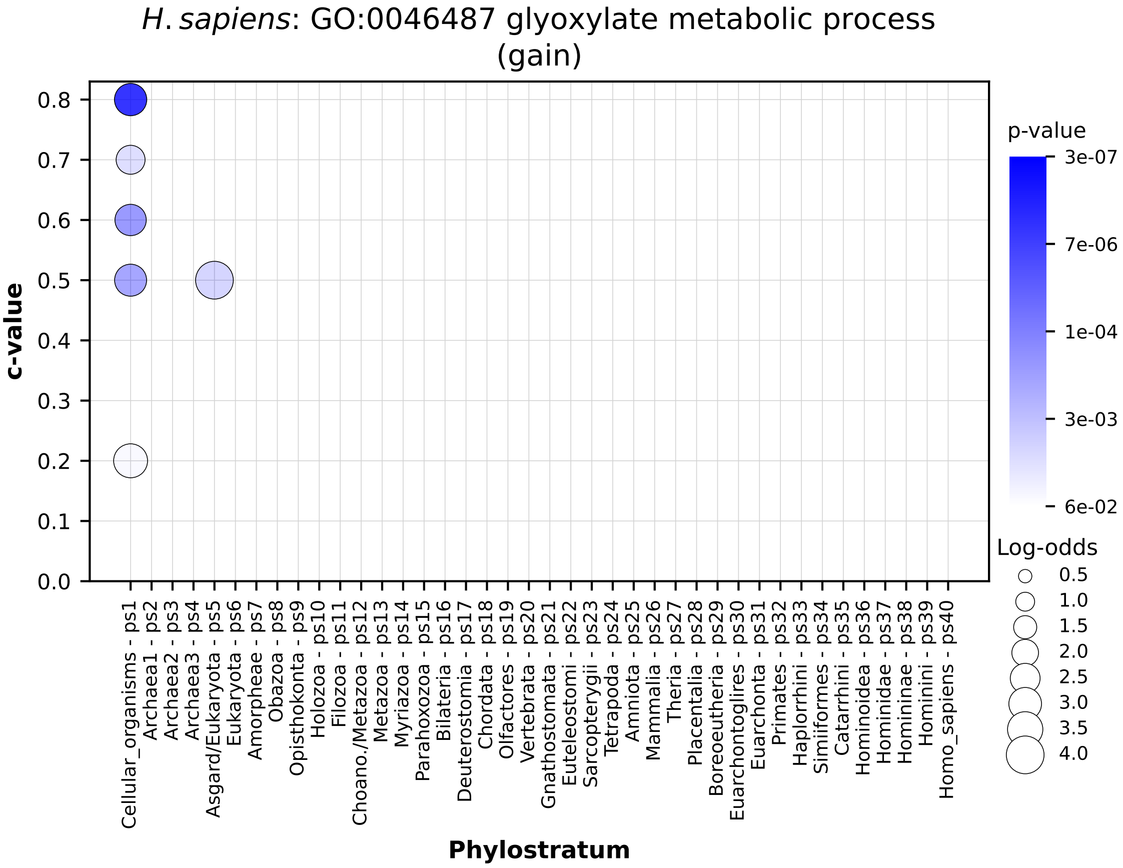
H. sapiens: GO:0046487 glyoxylate metabolic process
(gain)
c-value
0.0
0.1
0.2
0.3
0.4
0.5
0.6
0.7
0.8
Cellular_organisms - ps1
Archaea1 - ps2
Archaea2 - ps3
Archaea3 - ps4
Asgard/Eukaryota - ps5
Eukaryota - ps6
Amorpheae - ps7
Obazoa - ps8
Opisthokonta - ps9
Holozoa - ps10
Filozoa - ps11
Choano./Metazoa - ps12
Metazoa - ps13
Myriazoa - ps14
Parahoxozoa - ps15
Bilateria - ps16
Deuterostomia - ps17
Chordata - ps18
Olfactores - ps19
Vertebrata - ps20
Gnathostomata - ps21
Euteleostomi - ps22
Sarcopterygii - ps23
Tetrapoda - ps24
Amniota - ps25
Mammalia - ps26
Theria - ps27
Placentalia - ps28
Boreoeutheria - ps29
Euarchontoglires - ps30
Euarchonta - ps31
Primates - ps32
Haplorrhini - ps33
Simiiformes - ps34
Catarrhini - ps35
Hominoidea - ps36
Hominidae - ps37
Homininae - ps38
Hominini - ps39
Homo_sapiens - ps40
Phylostratum
p-value
3e-07
7e-06
1e-04
3e-03
6e-02
Log-odds
0.5
1.0
1.5
2.0
2.5
3.0
3.5
4.0

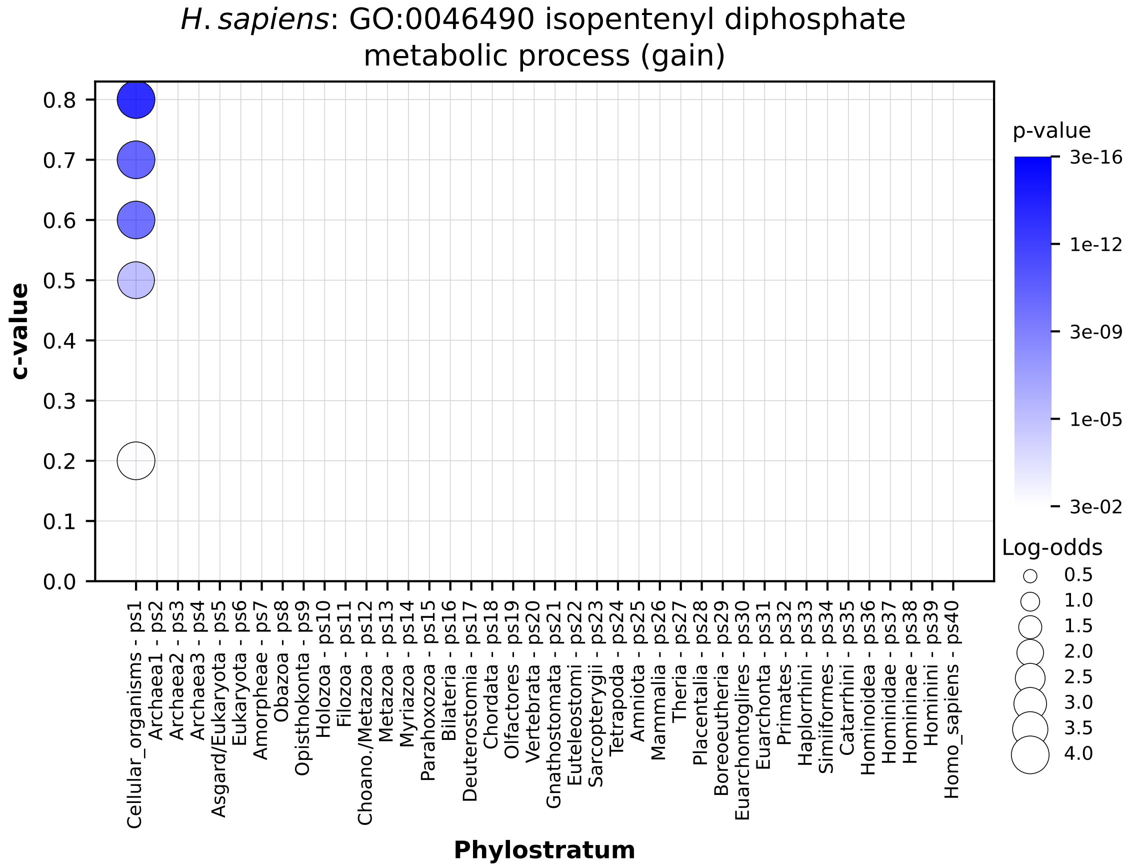

H. sapiens: GO:0046490 isopentenyl diphosphate metabolic process (gain)
c-value
0.0
0.1
0.2
0.3
0.4
0.5
0.6
0.7
0.8
Phylostratum
Cellular_organisms - ps1
Archaea1 - ps2
Archaea2 - ps3
Archaea3 - ps4
Asgard/Eukaryota - ps5
Eukaryota - ps6
Amorpheae - ps7
Obazoa - ps8
Opisthokonta - ps9
Holozoa - ps10
Filozoa - ps11
Choano./Metazoa - ps12
Metazoa - ps13
Myriazoa - ps14
Parahoxozoa - ps15
Bilateria - ps16
Deuterostomia - ps17
Chordata - ps18
Olfactores - ps19
Vertebrata - ps20
Gnathostomata - ps21
Euteleostomi - ps22
Sarcopterygii - ps23
Tetrapoda - ps24
Amniota - ps25
Mammalia - ps26
Theria - ps27
Placentalia - ps28
Boreoeutheria - ps29
Euarchontoglires - ps30
Euarchonta - ps31
Primates - ps32
Haplorrhini - ps33
Simiiformes - ps34
Catarrhini - ps35
Hominoidea - ps36
Hominidae - ps37
Homininae - ps38
Hominini - ps39
Homo_sapiens - ps40
p-value
3e-16
1e-12
3e-09
1e-05
3e-02
Log-odds
0.5
1.0
1.5
2.0
2.5
3.0
3.5
4.0

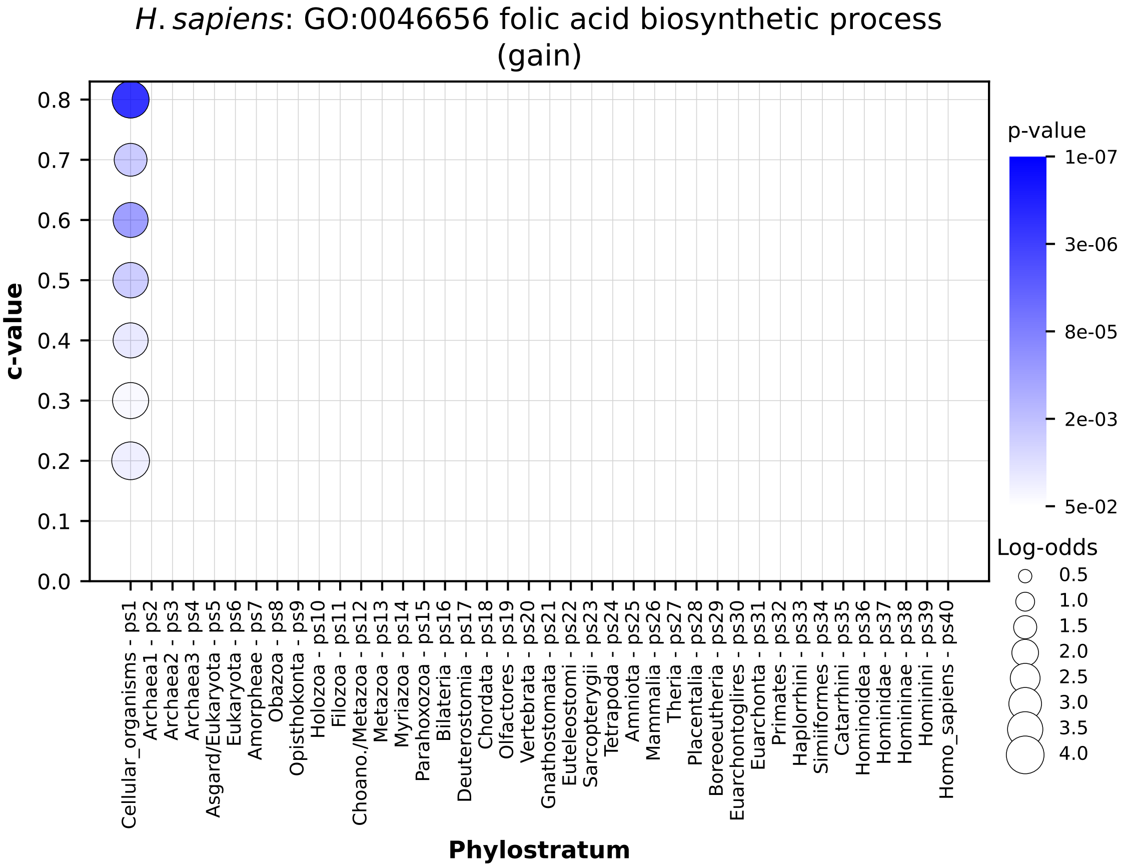

*H. sapiens*: GO:0046656 folic acid biosynthetic process
(gain)
c-value
0.0
0.1
0.2
0.3
0.4
0.5
0.6
0.7
0.8
p-value
1e-07
3e-06
8e-05
2e-03
5e-02
Log-odds
0.5
1.0
1.5
2.0
2.5
3.0
3.5
4.0
Cellular_organisms - ps1
Archaea1 - ps2
Archaea2 - ps3
Archaea3 - ps4
Asgard/Eukaryota - ps5
Eukaryota - ps6
Amorpheae - ps7
Obazoa - ps8
Opisthokonta - ps9
Holozoa - ps10
Filozoa - ps11
Choano./Metazoa - ps12
Metazoa - ps13
Myriazoa - ps14
Parahoxozoa - ps15
Bilateria - ps16
Deuterostomia - ps17
Chordata - ps18
Olfactores - ps19
Vertebrata - ps20
Gnathostomata - ps21
Euteleostomi - ps22
Sarcopterygii - ps23
Tetrapoda - ps24
Amniota - ps25
Mammalia - ps26
Theria - ps27
Placentalia - ps28
Boreoeutheria - ps29
Euarchontoglires - ps30
Euarchonta - ps31
Primates - ps32
Haplorrhini - ps33
Simiiformes - ps34
Catarrhini - ps35
Hominoidea - ps36
Hominidae - ps37
Homininae - ps38
Hominini - ps39
Homo_sapiens - ps40
Phylostratum

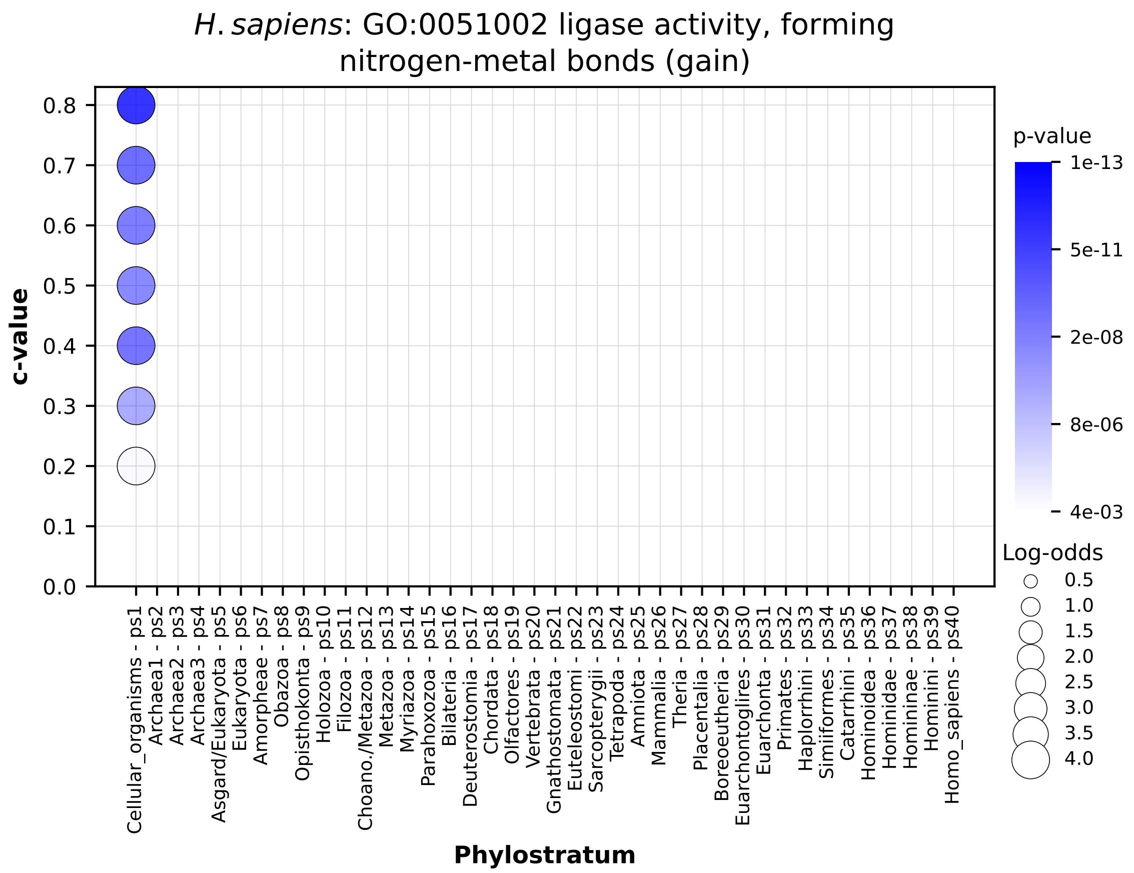
H. sapiens: GO:0051002 ligase activity, forming nitrogen-metal bonds (gain)
c-value
0.8
0.7
0.6
0.5
0.4
0.3
0.2
0.1
0.0
p-value
1e-13
5e-11
2e-08
8e-06
4e-03
Log-odds
0.5
1.0
1.5
2.0
2.5
3.0
3.5
4.0
Cellular_organisms - ps1
Archaea1 - ps2
Archaea2 - ps3
Archaea3 - ps4
Asgard/Eukaryota - ps5
Eukaryota - ps6
Amorpheae - ps7
Obazoa - ps8
Opisthokonta - ps9
Holozoa - ps10
Filozoa - ps11
Choano./Metazoa - ps12
Metazoa - ps13
Myriazoa - ps14
Parahoxozoa - ps15
Bilateria - ps16
Deuterostomia - ps17
Chordata - ps18
Olfactores - ps19
Vertebrata - ps20
Gnathostomata - ps21
Euteleostomi - ps22
Sarcopterygii - ps23
Tetrapoda - ps24
Amniota - ps25
Mammalia - ps26
Theria - ps27
Placentalia - ps28
Boreoeutheria - ps29
Euarchontoglires - ps30
Euarchonta - ps31
Primates - ps32
Haplorrhini - ps33
Simiiformes - ps34
Catarrhini - ps35
Hominoidea - ps36
Hominidae - ps37
Homininae - ps38
Hominini - ps39
Homo_sapiens - ps40
Phylostratum

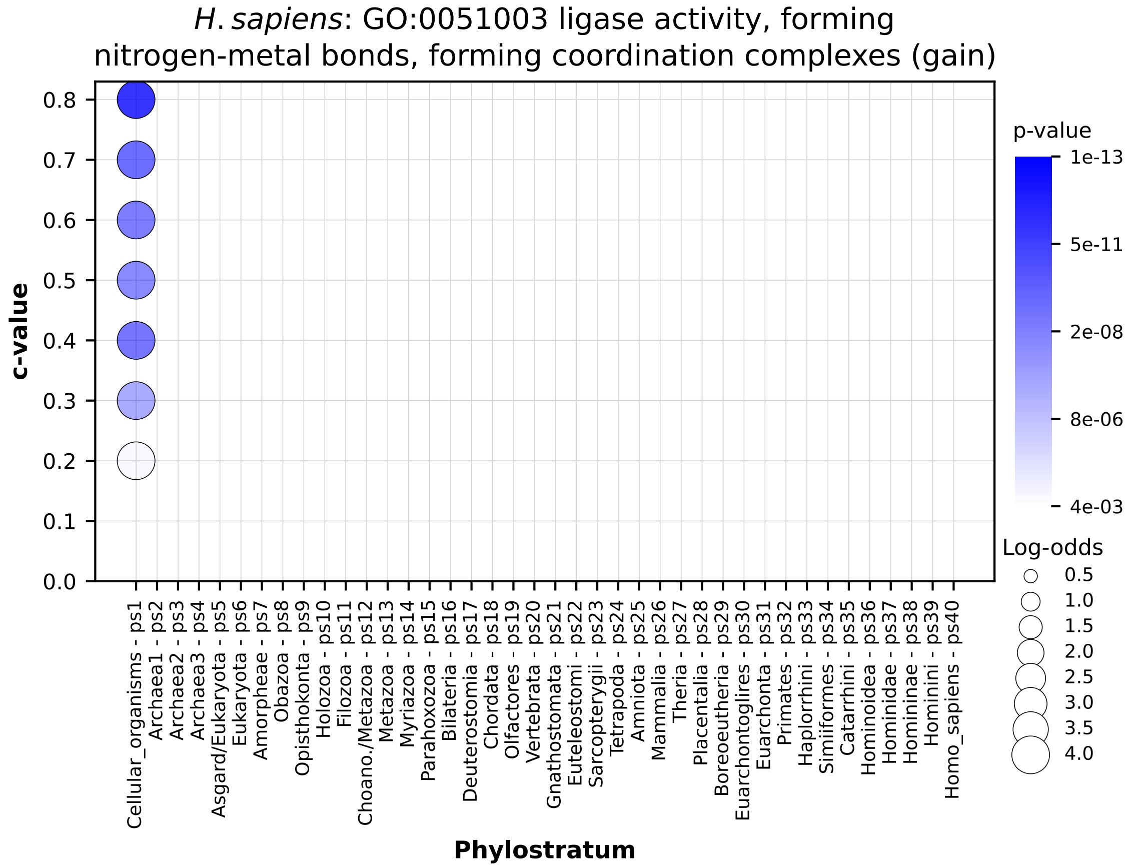

H. sapiens: GO:0051003 ligase activity, forming nitrogen-metal bonds, forming coordination complexes (gain)
c-value
0.0
0.1
0.2
0.3
0.4
0.5
0.6
0.7
0.8
Phylostratum
Cellular_organisms - ps1
Archaea1 - ps2
Archaea2 - ps3
Archaea3 - ps4
Asgard/Eukaryota - ps5
Eukaryota - ps6
Amorpheae - ps7
Obazoa - ps8
Opisthokonta - ps9
Holozoa - ps10
Filozoa - ps11
Choano./Metazoa - ps12
Metazoa - ps13
Myriazoa - ps14
Parahoxozoa - ps15
Bilateria - ps16
Deuterostomia - ps17
Chordata - ps18
Olfactores - ps19
Vertebrata - ps20
Gnathostomata - ps21
Euteleostomi - ps22
Sarcopterygii - ps23
Tetrapoda - ps24
Amniota - ps25
Mammalia - ps26
Theria - ps27
Placentalia - ps28
Boreoeutheria - ps29
Euarchontoglires - ps30
Euarchonta - ps31
Primates - ps32
Haplorrhini - ps33
Simiiformes - ps34
Catarrhini - ps35
Hominoidea - ps36
Hominidae - ps37
Homininae - ps38
Hominini - ps39
Homo_sapiens - ps40
p-value
1e-13
5e-11
2e-08
8e-06
4e-03
Log-odds
0.5
1.0
1.5
2.0
2.5
3.0
3.5
4.0

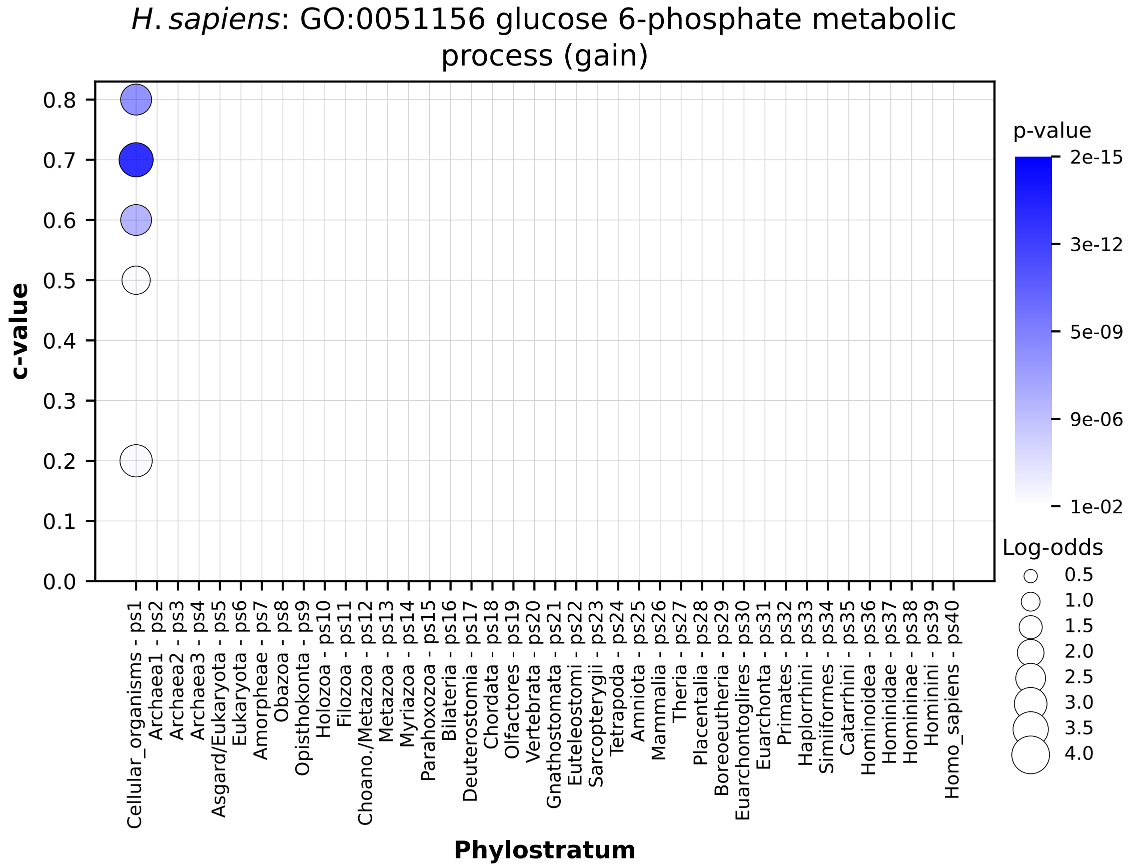

H. sapiens: GO:0051156 glucose 6-phosphate metabolic process (gain)
c-value
0.0
0.1
0.2
0.3
0.4
0.5
0.6
0.7
0.8
Cellular_organisms - ps1
Archaea1 - ps2
Archaea2 - ps3
Archaea3 - ps4
Asgard/Eukaryota - ps5
Eukaryota - ps6
Amorpheae - ps7
Obazoa - ps8
Opisthokonta - ps9
Holozoa - ps10
Filozoa - ps11
Choano./Metazoa - ps12
Metazoa - ps13
Myriazoa - ps14
Parahoxozoa - ps15
Bilateria - ps16
Deuterostomia - ps17
Chordata - ps18
Olfactores - ps19
Vertebrata - ps20
Gnathostomata - ps21
Euteleostomi - ps22
Sarcopterygii - ps23
Tetrapoda - ps24
Amniota - ps25
Mammalia - ps26
Theria - ps27
Placentalia - ps28
Boreoeutheria - ps29
Euarchontoglires - ps30
Euarchonta - ps31
Primates - ps32
Haplorrhini - ps33
Simiiformes - ps34
Catarrhini - ps35
Hominoidea - ps36
Hominidae - ps37
Homininae - ps38
Hominini - ps39
Homo_sapiens - ps40
Phylostratum
p-value
2e-15
3e-12
5e-09
9e-06
1e-02
Log-odds
0.5
1.0
1.5
2.0
2.5
3.0
3.5
4.0

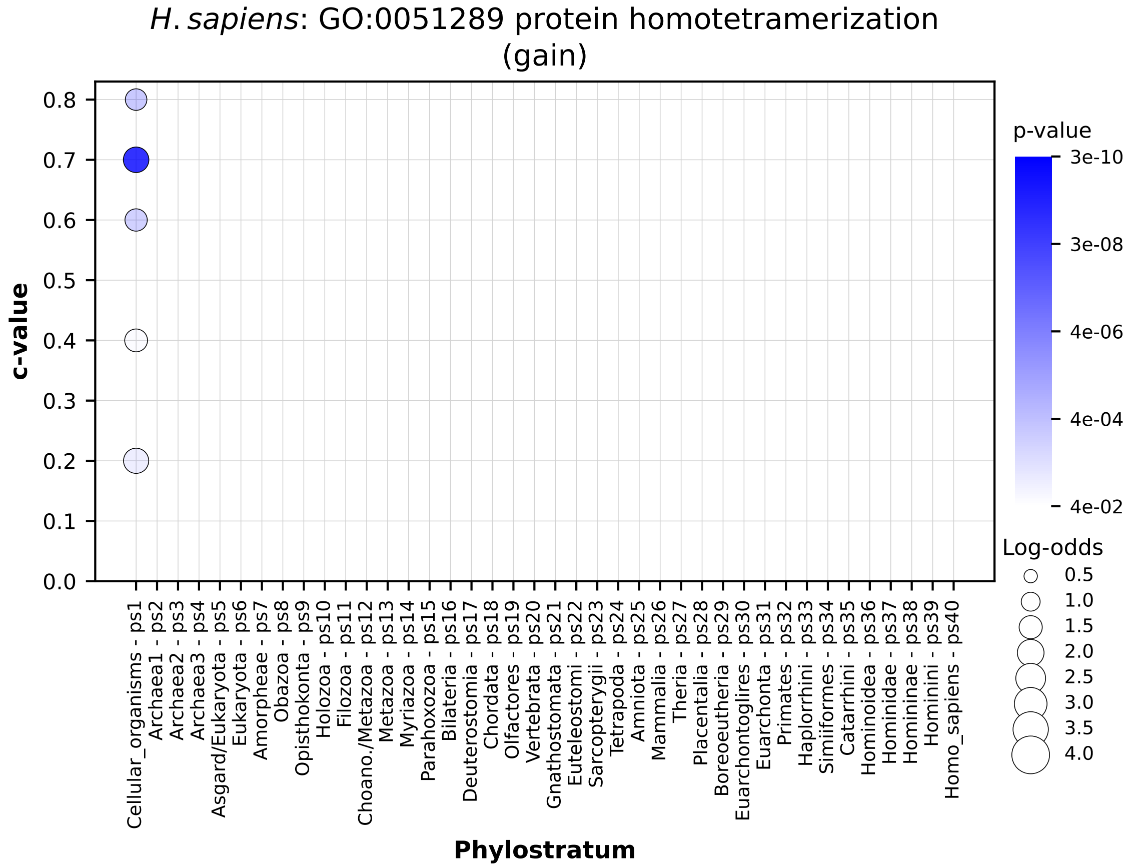

H. sapiens: GO:0051289 protein homotetramerization
(gain)
c-value
0.0
0.1
0.2
0.3
0.4
0.5
0.6
0.7
0.8
Phylostratum
Cellular_organisms - ps1
Archaea1 - ps2
Archaea2 - ps3
Archaea3 - ps4
Asgard/Eukaryota - ps5
Eukaryota - ps6
Amorpheae - ps7
Obazoa - ps8
Opisthokonta - ps9
Holozoa - ps10
Filozoa - ps11
Choano./Metazoa - ps12
Metazoa - ps13
Myriazoa - ps14
Parahoxozoa - ps15
Bilateria - ps16
Deuterostomia - ps17
Chordata - ps18
Olfactores - ps19
Vertebrata - ps20
Gnathostomata - ps21
Euteleostomi - ps22
Sarcopterygii - ps23
Tetrapoda - ps24
Amniota - ps25
Mammalia - ps26
Theria - ps27
Placentalia - ps28
Boreoeutheria - ps29
Euarchontoglires - ps30
Euarchonta - ps31
Primates - ps32
Haplorrhini - ps33
Simiiformes - ps34
Catarrhini - ps35
Hominoidea - ps36
Hominidae - ps37
Homininae - ps38
Hominini - ps39
Homo_sapiens - ps40
p-value
3e-10
3e-08
4e-06
4e-04
4e-02
Log-odds
0.5
1.0
1.5
2.0
2.5
3.0
3.5
4.0

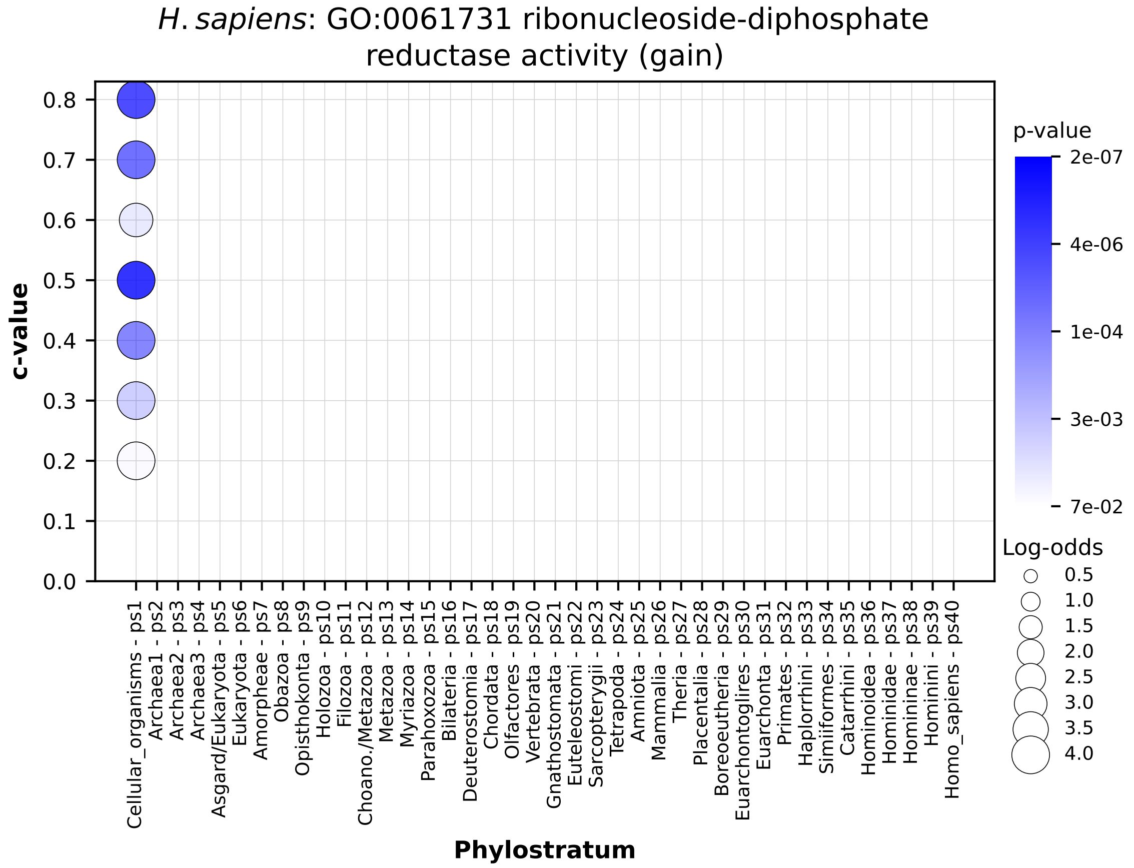

H. sapiens: GO:0061731 ribonucleoside-diphosphate reductase activity (gain)
c-value
0.0
0.1
0.2
0.3
0.4
0.5
0.6
0.7
0.8
Phylostratum
Cellular_organisms - ps1
Archaea1 - ps2
Archaea2 - ps3
Archaea3 - ps4
Asgard/Eukaryota - ps5
Eukaryota - ps6
Amorpheae - ps7
Obazoa - ps8
Opisthokonta - ps9
Holozoa - ps10
Filozoa - ps11
Choano./Metazoa - ps12
Metazoa - ps13
Myriazoa - ps14
Parahoxozoa - ps15
Bilateria - ps16
Deuterostomia - ps17
Chordata - ps18
Olfactores - ps19
Vertebrata - ps20
Gnathostomata - ps21
Euteleostomi - ps22
Sarcopterygii - ps23
Tetrapoda - ps24
Amniota - ps25
Mammalia - ps26
Theria - ps27
Placentalia - ps28
Boreoeutheria - ps29
Euarchontoglires - ps30
Euarchonta - ps31
Primates - ps32
Haplorrhini - ps33
Simiiformes - ps34
Catarrhini - ps35
Hominoidea - ps36
Hominidae - ps37
Homininae - ps38
Hominini - ps39
Homo_sapiens - ps40
p-value
2e-07
4e-06
1e-04
3e-03
7e-02
Log-odds
0.5
1.0
1.5
2.0
2.5
3.0
3.5
4.0

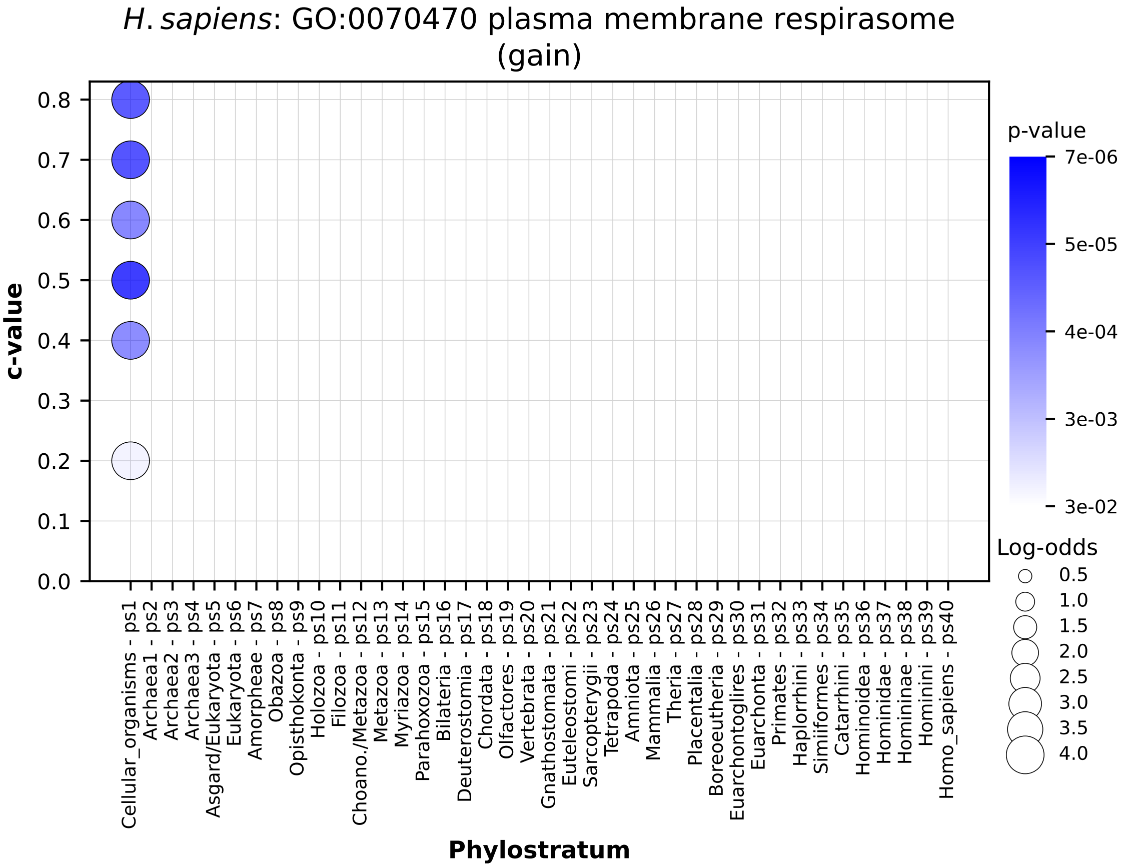

*H. sapiens*: GO:0070470 plasma membrane respirasome
(gain)
c-value
0.0
0.1
0.2
0.3
0.4
0.5
0.6
0.7
0.8
Phylostratum
Cellular_organisms - ps1
Archaea1 - ps2
Archaea2 - ps3
Archaea3 - ps4
Asgard/Eukaryota - ps5
Eukaryota - ps6
Amorpheae - ps7
Obazoa - ps8
Opisthokonta - ps9
Holozoa - ps10
Filozoa - ps11
Choano./Metazoa - ps12
Metazoa - ps13
Myriazoa - ps14
Parahoxozoa - ps15
Bilateria - ps16
Deuterostomia - ps17
Chordata - ps18
Olfactores - ps19
Vertebrata - ps20
Gnathostomata - ps21
Euteleostomi - ps22
Sarcopterygii - ps23
Tetrapoda - ps24
Amniota - ps25
Mammalia - ps26
Theria - ps27
Placentalia - ps28
Boreoeutheria - ps29
Euarchontoglires - ps30
Euarchonta - ps31
Primates - ps32
Haplorrhini - ps33
Simiiformes - ps34
Catarrhini - ps35
Hominoidea - ps36
Hominidae - ps37
Homininae - ps38
Hominini - ps39
Homo_sapiens - ps40
p-value
7e-06
5e-05
4e-04
3e-03
3e-02
Log-odds
0.5
1.0
1.5
2.0
2.5
3.0
3.5
4.0

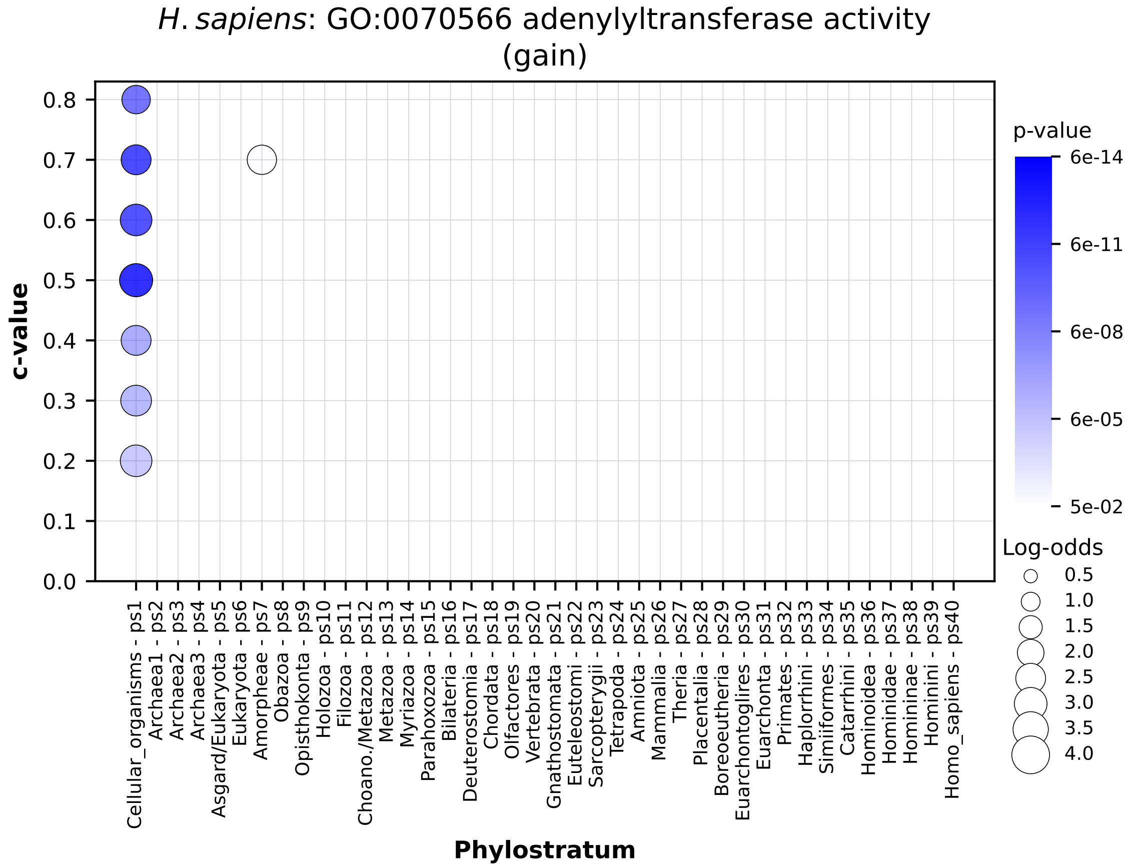

H. sapiens: GO:0070566 adenylyltransferase activity
(gain)
c-value
0.0
0.1
0.2
0.3
0.4
0.5
0.6
0.7
0.8
Phylostratum
Cellular_organisms - ps1
Archaea1 - ps2
Archaea2 - ps3
Archaea3 - ps4
Asgard/Eukaryota - ps5
Eukaryota - ps6
Amorpheae - ps7
Obazoa - ps8
Opisthokonta - ps9
Holozoa - ps10
Filozoa - ps11
Choano./Metazoa - ps12
Metazoa - ps13
Myriazoa - ps14
Parahoxozoa - ps15
Bilateria - ps16
Deuterostomia - ps17
Chordata - ps18
Olfactores - ps19
Vertebrata - ps20
Gnathostomata - ps21
Euteleostomi - ps22
Sarcopterygii - ps23
Tetrapoda - ps24
Amniota - ps25
Mammalia - ps26
Theria - ps27
Placentalia - ps28
Boreoeutheria - ps29
Euarchontoglires - ps30
Euarchonta - ps31
Primates - ps32
Haplorrhini - ps33
Simiiformes - ps34
Catarrhini - ps35
Hominoidea - ps36
Hominidae - ps37
Homininae - ps38
Hominini - ps39
Homo_sapiens - ps40
p-value
6e-14
6e-11
6e-08
6e-05
5e-02
Log-odds
0.5
1.0
1.5
2.0
2.5
3.0
3.5
4.0

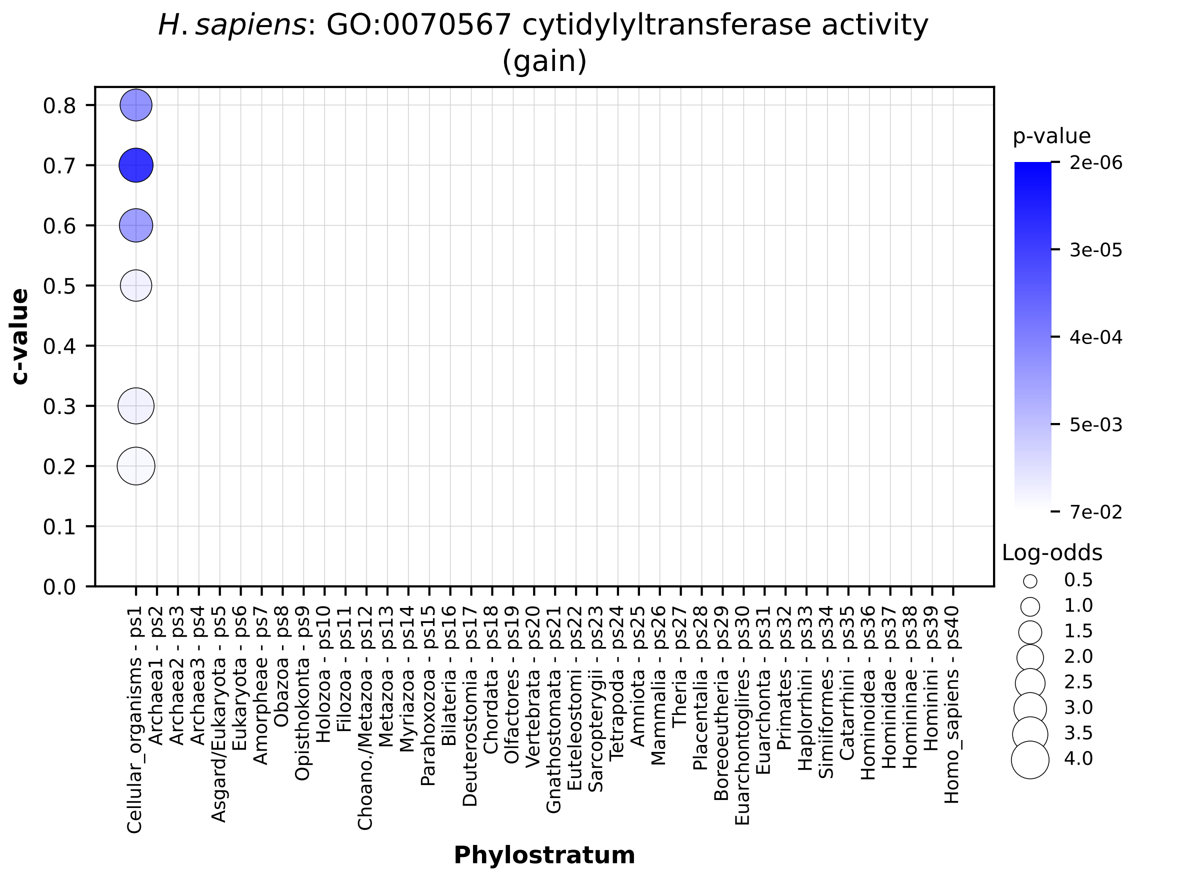

H. sapiens: GO:0070567 cytidylyltransferase activity
(gain)
c-value
0.0
0.1
0.2
0.3
0.4
0.5
0.6
0.7
0.8
p-value
2e-06
3e-05
4e-04
5e-03
7e-02
Log-odds
0.5
1.0
1.5
2.0
2.5
3.0
3.5
4.0
Cellular_organisms - ps1
Archaea1 - ps2
Archaea2 - ps3
Archaea3 - ps4
Asgard/Eukaryota - ps5
Eukaryota - ps6
Amorpheae - ps7
Obazoa - ps8
Opisthokonta - ps9
Holozoa - ps10
Filozoa - ps11
Choano./Metazoa - ps12
Metazoa - ps13
Myriazoa - ps14
Parahoxozoa - ps15
Bilateria - ps16
Deuterostomia - ps17
Chordata - ps18
Olfactores - ps19
Vertebrata - ps20
Gnathostomata - ps21
Euteleostomi - ps22
Sarcopterygii - ps23
Tetrapoda - ps24
Amniota - ps25
Mammalia - ps26
Theria - ps27
Placentalia - ps28
Boreoeutheria - ps29
Euarchontoglires - ps30
Euarchonta - ps31
Primates - ps32
Haplorrhini - ps33
Simiiformes - ps34
Catarrhini - ps35
Hominoidea - ps36
Hominidae - ps37
Homininae - ps38
Hominini - ps39
Homo_sapiens - ps40
Phylostratum

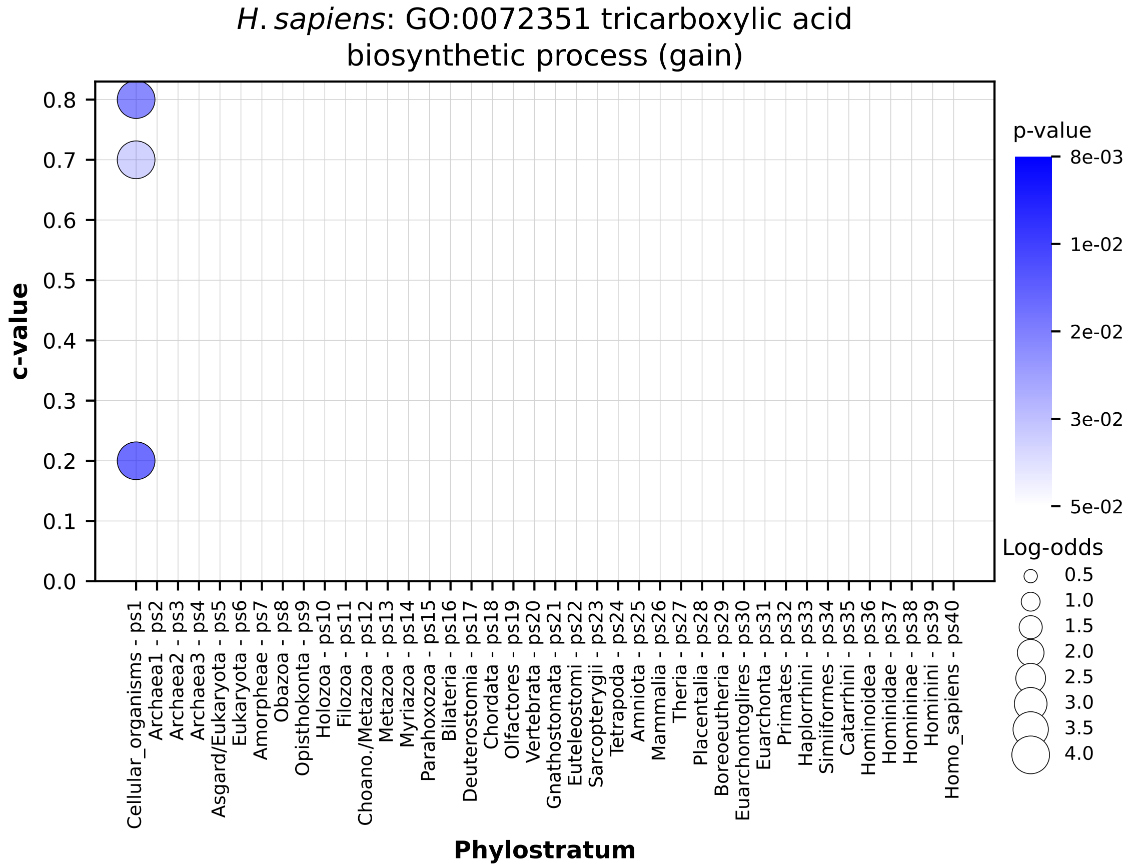

*H. sapiens*: GO:0072351 tricarboxylic acid biosynthetic process (gain)
c-value
0.0
0.1
0.2
0.3
0.4
0.5
0.6
0.7
0.8
Cellular_organisms - ps1
Archaea1 - ps2
Archaea2 - ps3
Archaea3 - ps4
Asgard/Eukaryota - ps5
Eukaryota - ps6
Amorpheae - ps7
Obazoa - ps8
Opisthokonta - ps9
Holozoa - ps10
Filozoa - ps11
Choano./Metazoa - ps12
Metazoa - ps13
Myriazoa - ps14
Parahoxozoa - ps15
Bilateria - ps16
Deuterostomia - ps17
Chordata - ps18
Olfactores - ps19
Vertebrata - ps20
Gnathostomata - ps21
Euteleostomi - ps22
Sarcopterygii - ps23
Tetrapoda - ps24
Amniota - ps25
Mammalia - ps26
Theria - ps27
Placentalia - ps28
Boreoeutheria - ps29
Euarchontoglires - ps30
Euarchonta - ps31
Primates - ps32
Haplorrhini - ps33
Simiiformes - ps34
Catarrhini - ps35
Hominoidea - ps36
Hominidae - ps37
Homininae - ps38
Hominini - ps39
Homo_sapiens - ps40
Phylostratum
p-value
8e-03
1e-02
2e-02
3e-02
5e-02
Log-odds
0.5
1.0
1.5
2.0
2.5
3.0
3.5
4.0

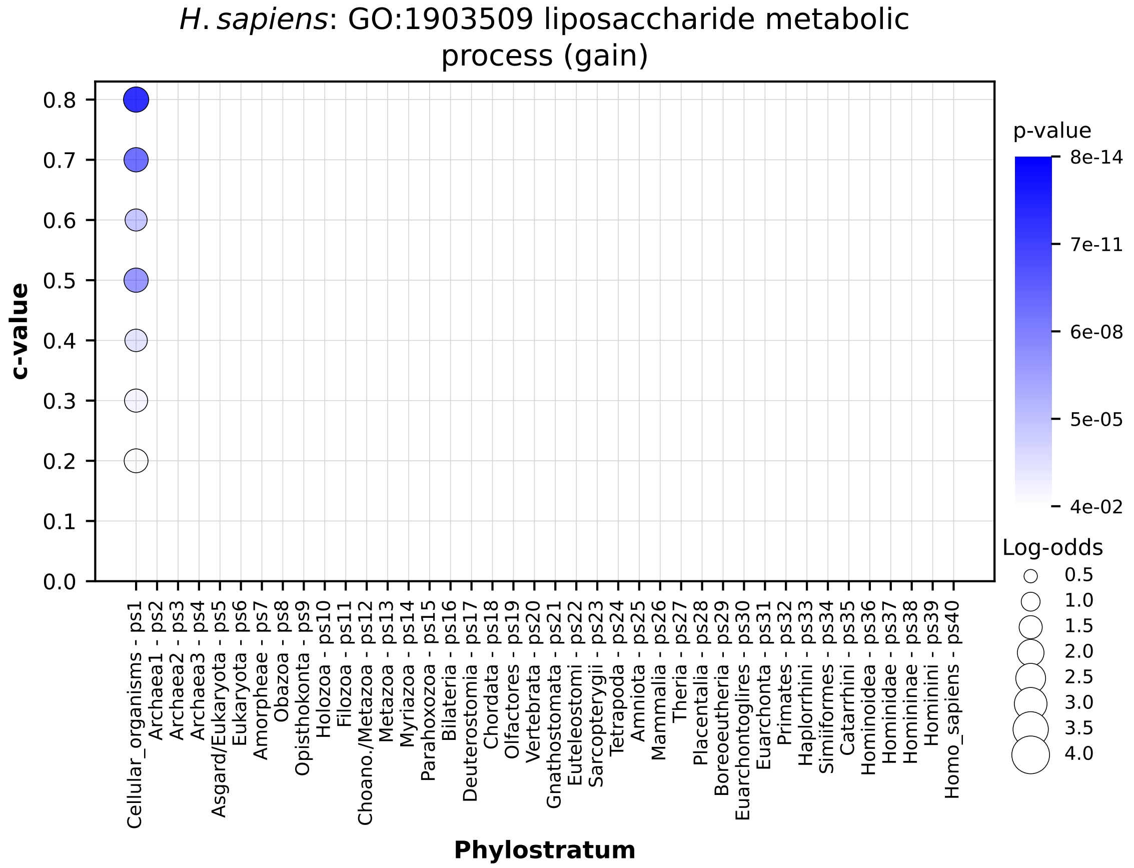

H. sapiens: GO:1903509 liposaccharide metabolic process (gain)
c-value
0.0
0.1
0.2
0.3
0.4
0.5
0.6
0.7
0.8
p-value
8e-14
7e-11
6e-08
5e-05
4e-02
Log-odds
0.5
1.0
1.5
2.0
2.5
3.0
3.5
4.0
Cellular_organisms - ps1
Archaea1 - ps2
Archaea2 - ps3
Archaea3 - ps4
Asgard/Eukaryota - ps5
Eukaryota - ps6
Amorpheae - ps7
Obazoa - ps8
Opisthokonta - ps9
Holozoa - ps10
Filozoa - ps11
Choano./Metazoa - ps12
Metazoa - ps13
Myriazoa - ps14
Parahoxozoa - ps15
Bilateria - ps16
Deuterostomia - ps17
Chordata - ps18
Olfactores - ps19
Vertebrata - ps20
Gnathostomata - ps21
Euteleostomi - ps22
Sarcopterygii - ps23
Tetrapoda - ps24
Amniota - ps25
Mammalia - ps26
Theria - ps27
Placentalia - ps28
Boreoeutheria - ps29
Euarchontoglires - ps30
Euarchonta - ps31
Primates - ps32
Haplorrhini - ps33
Simiiformes - ps34
Catarrhini - ps35
Hominoidea - ps36
Hominidae - ps37
Homininae - ps38
Hominini - ps39
Homo_sapiens - ps40
Phylostratum

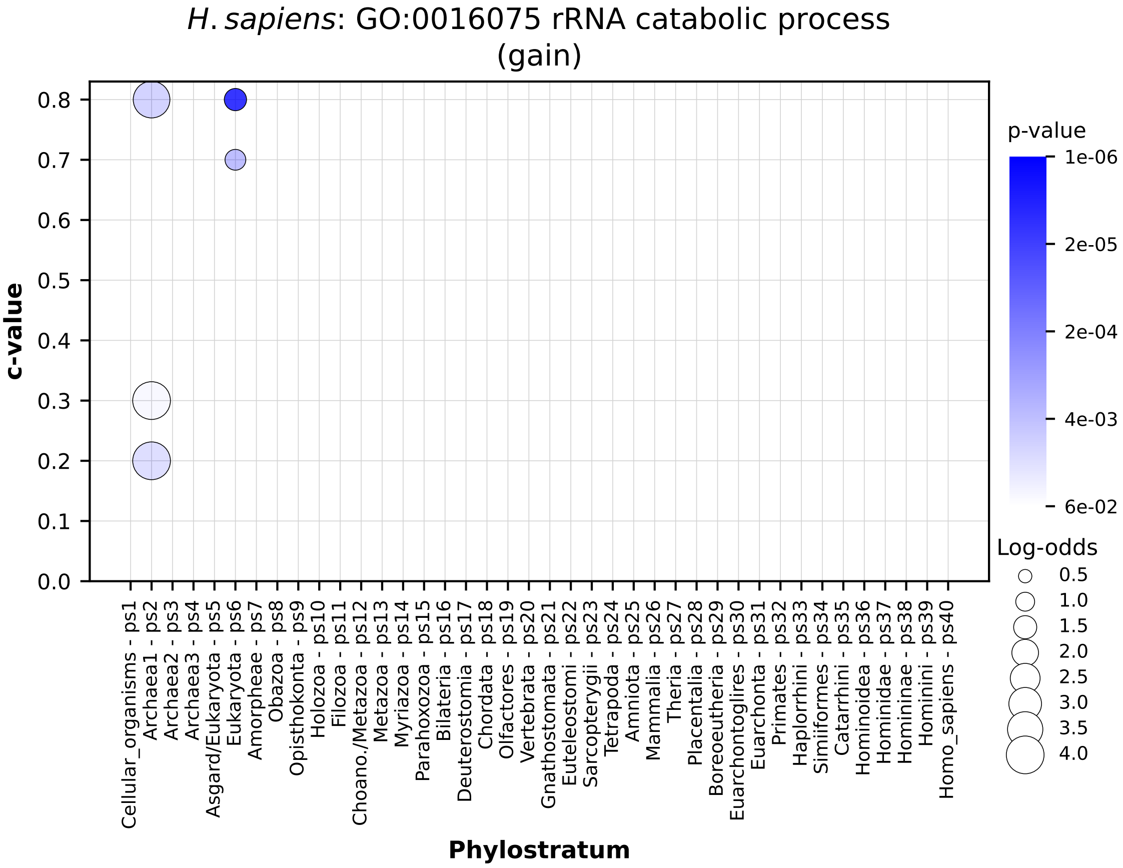
H. sapiens: GO:0016075 rRNA catabolic process
(gain)
c-value
0.0
0.1
0.2
0.3
0.4
0.5
0.6
0.7
0.8
Phylostratum
Cellular_organisms - ps1
Archaea1 - ps2
Archaea2 - ps3
Archaea3 - ps4
Asgard/Eukaryota - ps5
Eukaryota - ps6
Amorpheae - ps7
Obazoa - ps8
Opisthokonta - ps9
Holozoa - ps10
Filozoa - ps11
Choano./Metazoa - ps12
Metazoa - ps13
Myriazoa - ps14
Parahoxozoa - ps15
Bilateria - ps16
Deuterostomia - ps17
Chordata - ps18
Olfactores - ps19
Vertebrata - ps20
Gnathostomata - ps21
Euteleostomi - ps22
Sarcopterygii - ps23
Tetrapoda - ps24
Amniota - ps25
Mammalia - ps26
Theria - ps27
Placentalia - ps28
Boreoeutheria - ps29
Euarchontoglires - ps30
Euarchonta - ps31
Primates - ps32
Haplorrhini - ps33
Simiiformes - ps34
Catarrhini - ps35
Hominoidea - ps36
Hominidae - ps37
Homininae - ps38
Hominini - ps39
Homo_sapiens - ps40
p-value
1e-06
2e-05
2e-04
4e-03
6e-02
Log-odds
0.5
1.0
1.5
2.0
2.5
3.0
3.5
4.0

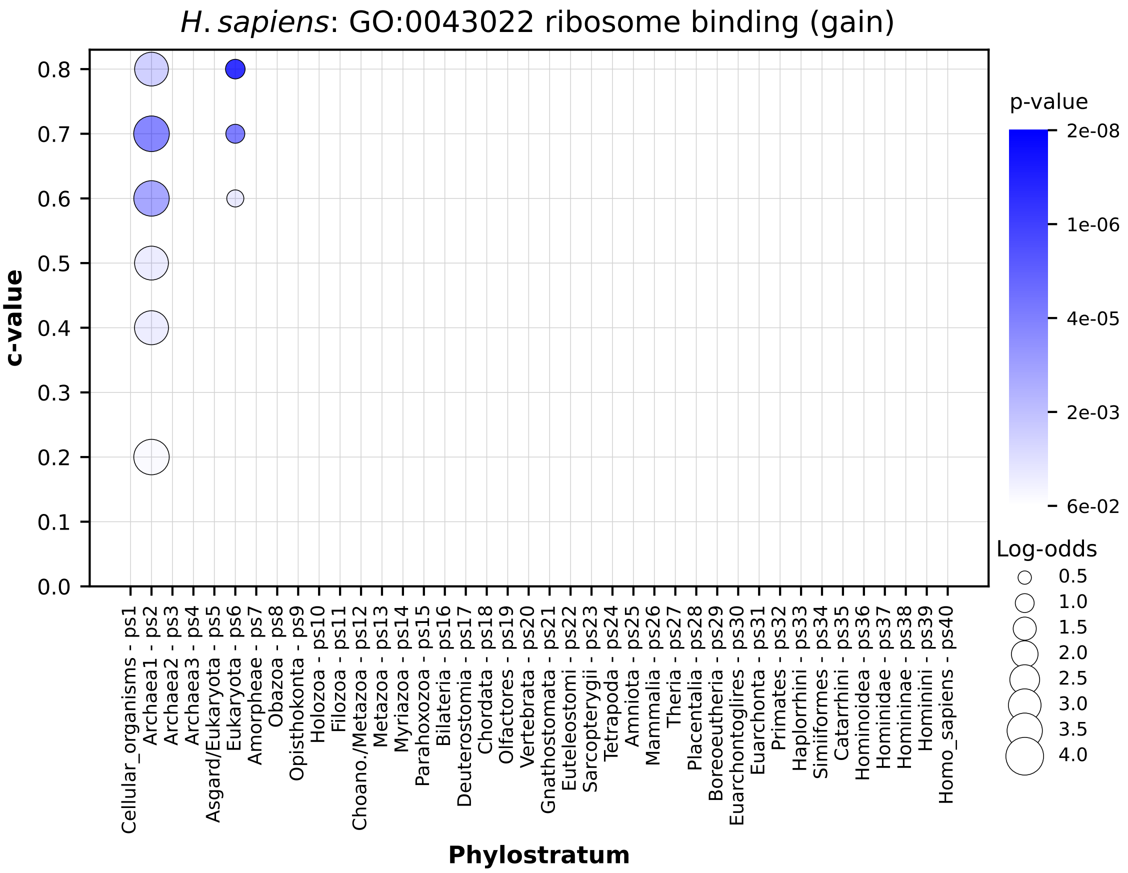
H. sapiens: GO:0043022 ribosome binding (gain)
c-value
0.0
0.1
0.2
0.3
0.4
0.5
0.6
0.7
0.8
Phylostratum
Cellular_organisms - ps1
Archaea1 - ps2
Archaea2 - ps3
Archaea3 - ps4
Asgard/Eukaryota - ps5
Eukaryota - ps6
Amorpheae - ps7
Obazoa - ps8
Opisthokonta - ps9
Holozoa - ps10
Filozoa - ps11
Choano./Metazoa - ps12
Metazoa - ps13
Myriazoa - ps14
Parahoxozoa - ps15
Bilateria - ps16
Deuterostomia - ps17
Chordata - ps18
Olfactores - ps19
Vertebrata - ps20
Gnathostomata - ps21
Euteleostomi - ps22
Sarcopterygii - ps23
Tetrapoda - ps24
Amniota - ps25
Mammalia - ps26
Theria - ps27
Placentalia - ps28
Boreoeutheria - ps29
Euarchontoglires - ps30
Euarchonta - ps31
Primates - ps32
Haplorrhini - ps33
Simiiformes - ps34
Catarrhini - ps35
Hominoidea - ps36
Hominidae - ps37
Homininae - ps38
Hominini - ps39
Homo_sapiens - ps40
p-value
2e-08
1e-06
4e-05
2e-03
6e-02
Log-odds
0.5
1.0
1.5
2.0
2.5
3.0
3.5
4.0

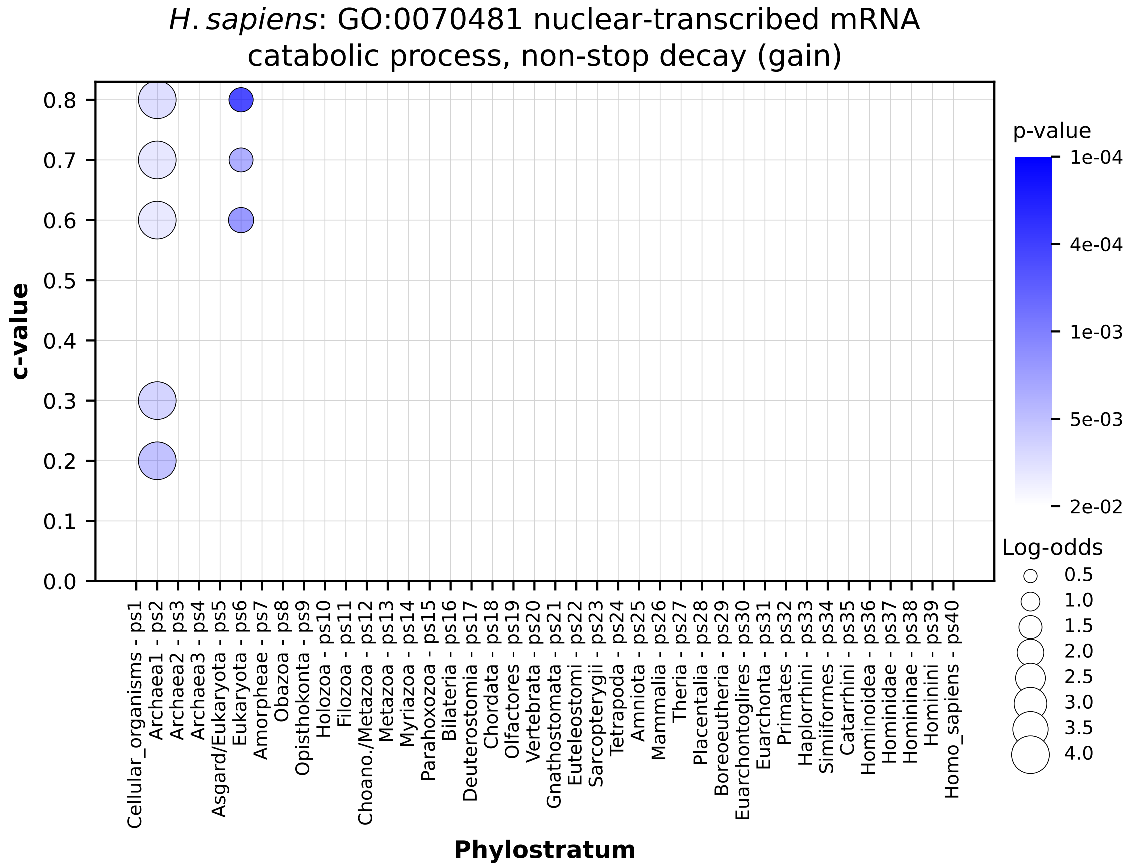

H. sapiens: GO:0070481 nuclear-transcribed mRNA catabolic process, non-stop decay (gain)
c-value
0.0
0.1
0.2
0.3
0.4
0.5
0.6
0.7
0.8
Phylostratum
Cellular_organisms - ps1
Archaea1 - ps2
Archaea2 - ps3
Archaea3 - ps4
Asgard/Eukaryota - ps5
Eukaryota - ps6
Amorpheae - ps7
Obazoa - ps8
Opisthokonta - ps9
Holozoa - ps10
Filozoa - ps11
Choano./Metazoa - ps12
Metazoa - ps13
Myriazoa - ps14
Parahoxozoa - ps15
Bilateria - ps16
Deuterostomia - ps17
Chordata - ps18
Olfactores - ps19
Vertebrata - ps20
Gnathostomata - ps21
Euteleostomi - ps22
Sarcopterygii - ps23
Tetrapoda - ps24
Amniota - ps25
Mammalia - ps26
Theria - ps27
Placentalia - ps28
Boreoeutheria - ps29
Euarchontoglires - ps30
Euarchonta - ps31
Primates - ps32
Haplorrhini - ps33
Simiiformes - ps34
Catarrhini - ps35
Hominoidea - ps36
Hominidae - ps37
Homininae - ps38
Hominini - ps39
Homo_sapiens - ps40
p-value
1e-04
4e-04
1e-03
5e-03
2e-02
Log-odds
0.5
1.0
1.5
2.0
2.5
3.0
3.5
4.0

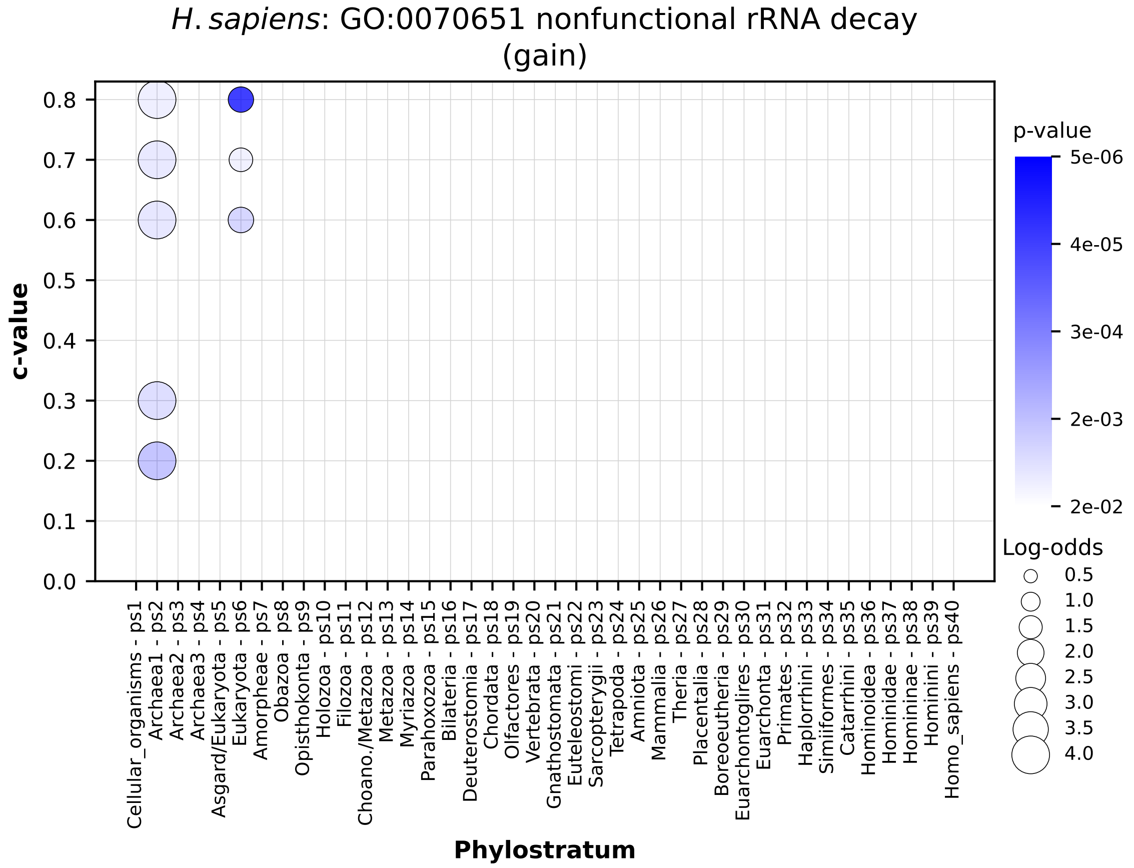

H. sapiens: GO:0070651 nonfunctional rRNA decay
(gain)
c-value
0.0
0.1
0.2
0.3
0.4
0.5
0.6
0.7
0.8
Phylostratum
Cellular_organisms - ps1
Archaea1 - ps2
Archaea2 - ps3
Archaea3 - ps4
Asgard/Eukaryota - ps5
Eukaryota - ps6
Amorpheae - ps7
Obazoa - ps8
Opisthokonta - ps9
Holozoa - ps10
Filozoa - ps11
Choano./Metazoa - ps12
Metazoa - ps13
Myriazoa - ps14
Parahoxozoa - ps15
Bilateria - ps16
Deuterostomia - ps17
Chordata - ps18
Olfactores - ps19
Vertebrata - ps20
Gnathostomata - ps21
Euteleostomi - ps22
Sarcopterygii - ps23
Tetrapoda - ps24
Amniota - ps25
Mammalia - ps26
Theria - ps27
Placentalia - ps28
Boreoeutheria - ps29
Euarchontoglires - ps30
Euarchonta - ps31
Primates - ps32
Haplorrhini - ps33
Simiiformes - ps34
Catarrhini - ps35
Hominoidea - ps36
Hominidae - ps37
Homininae - ps38
Hominini - ps39
Homo_sapiens - ps40
p-value
5e-06
4e-05
3e-04
2e-03
2e-02
Log-odds
0.5
1.0
1.5
2.0
2.5
3.0
3.5
4.0

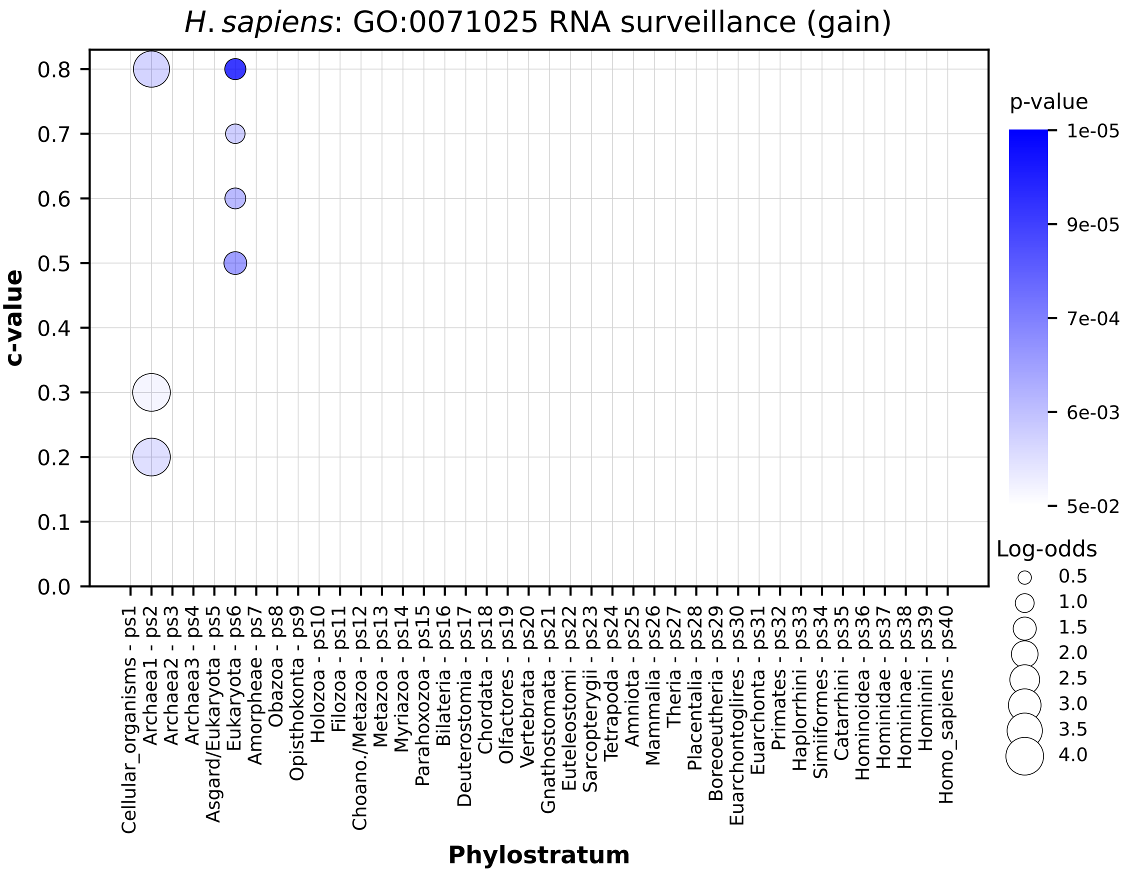
*H. sapiens*: GO:0071025 RNA surveillance (gain)
c-value
0.0
0.1
0.2
0.3
0.4
0.5
0.6
0.7
0.8
Phylostratum
Cellular_organisms - ps1
Archaea1 - ps2
Archaea2 - ps3
Archaea3 - ps4
Asgard/Eukaryota - ps5
Eukaryota - ps6
Amorpheae - ps7
Obazoa - ps8
Opisthokonta - ps9
Holozoa - ps10
Filozoa - ps11
Choano./Metazoa - ps12
Metazoa - ps13
Myriazoa - ps14
Parahoxozoa - ps15
Bilateria - ps16
Deuterostomia - ps17
Chordata - ps18
Olfactores - ps19
Vertebrata - ps20
Gnathostomata - ps21
Euteleostomi - ps22
Sarcopterygii - ps23
Tetrapoda - ps24
Amniota - ps25
Mammalia - ps26
Theria - ps27
Placentalia - ps28
Boreoeutheria - ps29
Euarchontoglires - ps30
Euarchonta - ps31
Primates - ps32
Haplorrhini - ps33
Simiiformes - ps34
Catarrhini - ps35
Hominoidea - ps36
Hominidae - ps37
Homininae - ps38
Hominini - ps39
Homo_sapiens - ps40
p-value
1e-05
9e-05
7e-04
6e-03
5e-02
Log-odds
0.5
1.0
1.5
2.0
2.5
3.0
3.5
4.0

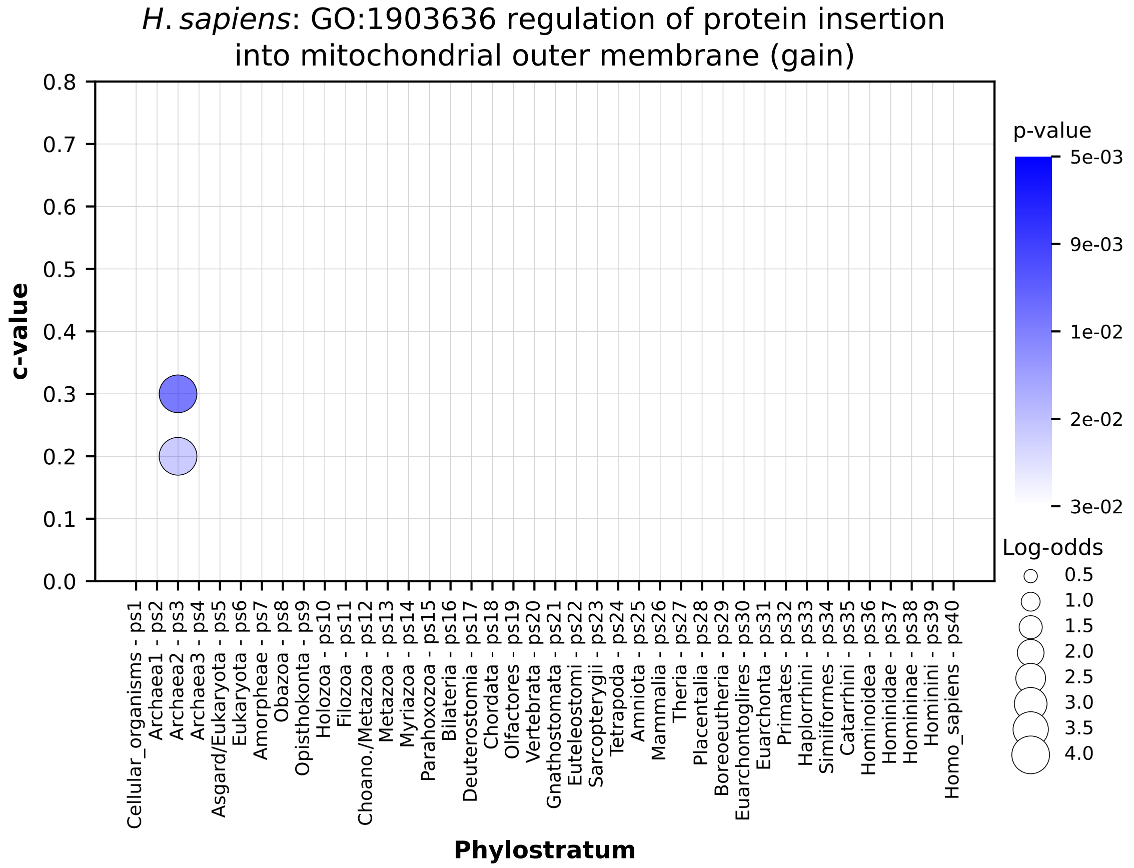

*H. sapiens*: GO:1903636 regulation of protein insertion into mitochondrial outer membrane (gain)
c-value
0.0
0.1
0.2
0.3
0.4
0.5
0.6
0.7
0.8
Phylostratum
Cellular_organisms - ps1
Archaea1 - ps2
Archaea2 - ps3
Archaea3 - ps4
Asgard/Eukaryota - ps5
Eukaryota - ps6
Amorpheae - ps7
Obazoa - ps8
Opisthokonta - ps9
Holozoa - ps10
Filozoa - ps11
Choano./Metazoa - ps12
Metazoa - ps13
Myriazoa - ps14
Parahoxozoa - ps15
Bilateria - ps16
Deuterostomia - ps17
Chordata - ps18
Olfactores - ps19
Vertebrata - ps20
Gnathostomata - ps21
Euteleostomi - ps22
Sarcopterygii - ps23
Tetrapoda - ps24
Amniota - ps25
Mammalia - ps26
Theria - ps27
Placentalia - ps28
Boreoeutheria - ps29
Euarchontoglires - ps30
Euarchonta - ps31
Primates - ps32
Haplorrhini - ps33
Simiiformes - ps34
Catarrhini - ps35
Hominoidea - ps36
Hominidae - ps37
Homininae - ps38
Hominini - ps39
Homo_sapiens - ps40
p-value
5e-03
9e-03
1e-02
2e-02
3e-02
Log-odds
0.5
1.0
1.5
2.0
2.5
3.0
3.5
4.0

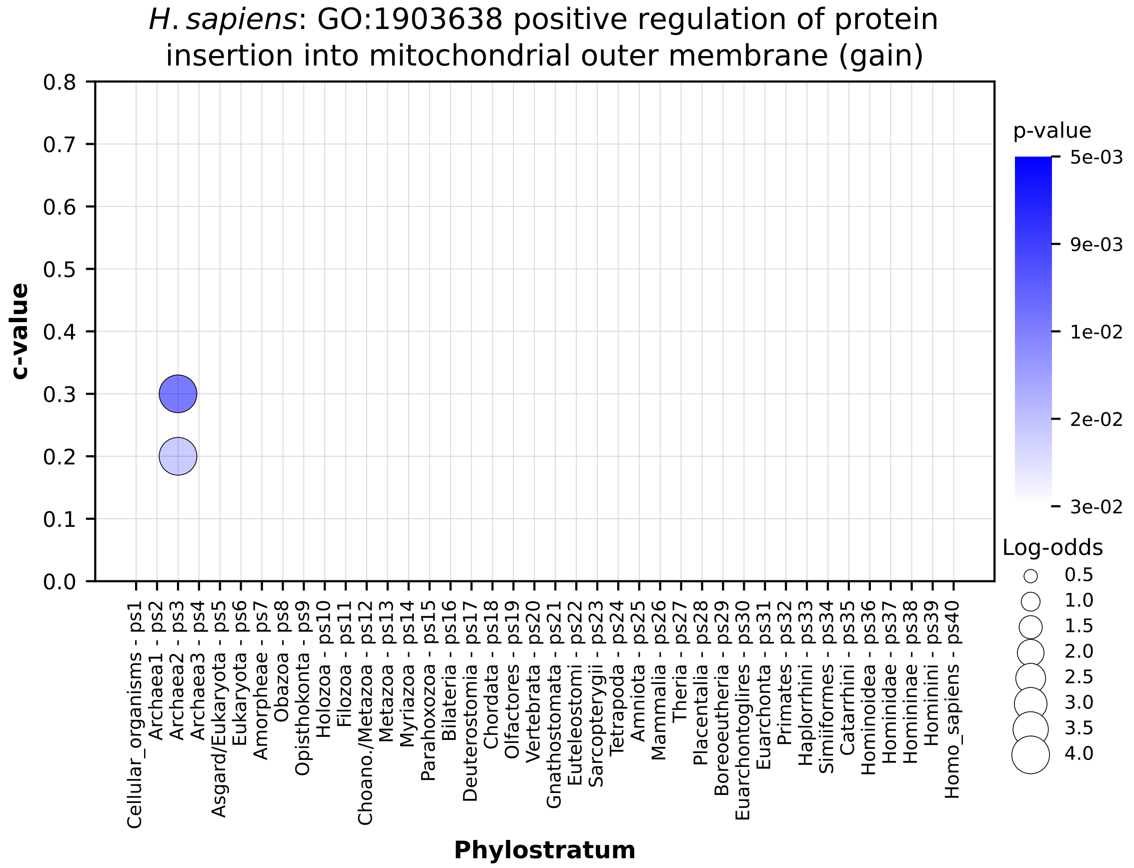

H. sapiens: GO:1903638 positive regulation of protein insertion into mitochondrial outer membrane (gain)
c-value
0.0
0.1
0.2
0.3
0.4
0.5
0.6
0.7
0.8
Phylostratum
Cellular_organisms - ps1
Archaea1 - ps2
Archaea2 - ps3
Archaea3 - ps4
Asgard/Eukaryota - ps5
Eukaryota - ps6
Amorpheae - ps7
Obazoa - ps8
Opisthokonta - ps9
Holozoa - ps10
Filozoa - ps11
Choano./Metazoa - ps12
Metazoa - ps13
Myriazoa - ps14
Parahoxozoa - ps15
Bilateria - ps16
Deuterostomia - ps17
Chordata - ps18
Olfactores - ps19
Vertebrata - ps20
Gnathostomata - ps21
Euteleostomi - ps22
Sarcopterygii - ps23
Tetrapoda - ps24
Amniota - ps25
Mammalia - ps26
Theria - ps27
Placentalia - ps28
Boreoeutheria - ps29
Euarchontoglires - ps30
Euarchonta - ps31
Primates - ps32
Haplorrhini - ps33
Simiiformes - ps34
Catarrhini - ps35
Hominoidea - ps36
Hominidae - ps37
Homininae - ps38
Hominini - ps39
Homo_sapiens - ps40
p-value
5e-03
9e-03
1e-02
2e-02
3e-02
Log-odds
0.5
1.0
1.5
2.0
2.5
3.0
3.5
4.0

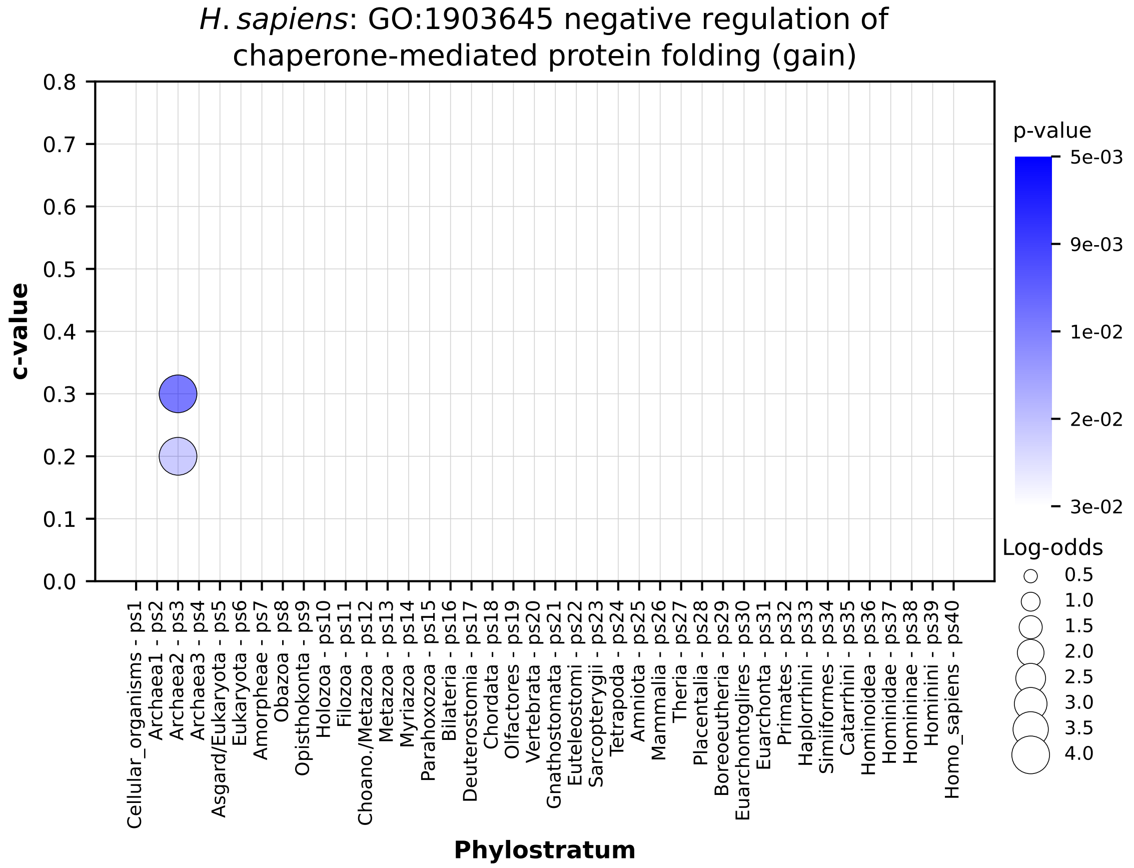
H. sapiens: GO:1903645 negative regulation of chaperone-mediated protein folding (gain)
c-value
0.8
0.7
0.6
0.5
0.4
0.3
0.2
0.1
0.0
p-value
5e-03
9e-03
1e-02
2e-02
3e-02
Log-odds
0.5
1.0
1.5
2.0
2.5
3.0
3.5
4.0
Cellular_organisms - ps1
Archaea1 - ps2
Archaea2 - ps3
Archaea3 - ps4
Asgard/Eukaryota - ps5
Eukaryota - ps6
Amorpheae - ps7
Obazoa - ps8
Opisthokonta - ps9
Holozoa - ps10
Filozoa - ps11
Choano./Metazoa - ps12
Metazoa - ps13
Myriazoa - ps14
Parahoxozoa - ps15
Bilateria - ps16
Deuterostomia - ps17
Chordata - ps18
Olfactores - ps19
Vertebrata - ps20
Gnathostomata - ps21
Euteleostomi - ps22
Sarcopterygii - ps23
Tetrapoda - ps24
Amniota - ps25
Mammalia - ps26
Theria - ps27
Placentalia - ps28
Boreoeutheria - ps29
Euarchontoglires - ps30
Euarchonta - ps31
Primates - ps32
Haplorrhini - ps33
Simiiformes - ps34
Catarrhini - ps35
Hominoidea - ps36
Hominidae - ps37
Homininae - ps38
Hominini - ps39
Homo_sapiens - ps40
Phylostratum

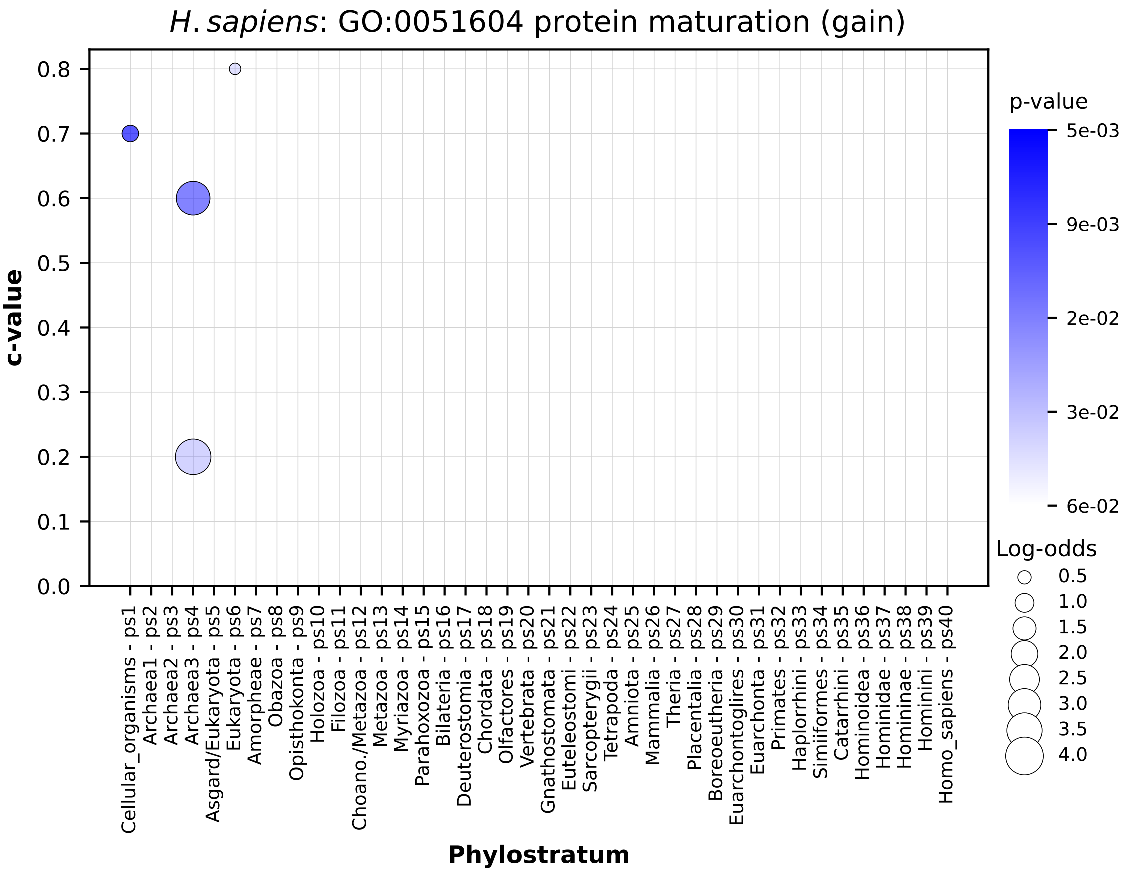

H. sapiens: GO:0051604 protein maturation (gain)
c-value
0.0
0.1
0.2
0.3
0.4
0.5
0.6
0.7
0.8
Phylostratum
Cellular_organisms - ps1
Archaea1 - ps2
Archaea2 - ps3
Archaea3 - ps4
Asgard/Eukaryota - ps5
Eukaryota - ps6
Amorpheae - ps7
Obazoa - ps8
Opisthokonta - ps9
Holozoa - ps10
Filozoa - ps11
Choano./Metazoa - ps12
Metazoa - ps13
Myriazoa - ps14
Parahoxozoa - ps15
Bilateria - ps16
Deuterostomia - ps17
Chordata - ps18
Olfactores - ps19
Vertebrata - ps20
Gnathostomata - ps21
Euteleostomi - ps22
Sarcopterygii - ps23
Tetrapoda - ps24
Amniota - ps25
Mammalia - ps26
Theria - ps27
Placentalia - ps28
Boreoeutheria - ps29
Euarchontoglires - ps30
Euarchonta - ps31
Primates - ps32
Haplorrhini - ps33
Simiiformes - ps34
Catarrhini - ps35
Hominoidea - ps36
Hominidae - ps37
Homininae - ps38
Hominini - ps39
Homo_sapiens - ps40
p-value
5e-03
9e-03
2e-02
3e-02
6e-02
Log-odds
0.5
1.0
1.5
2.0
2.5
3.0
3.5
4.0

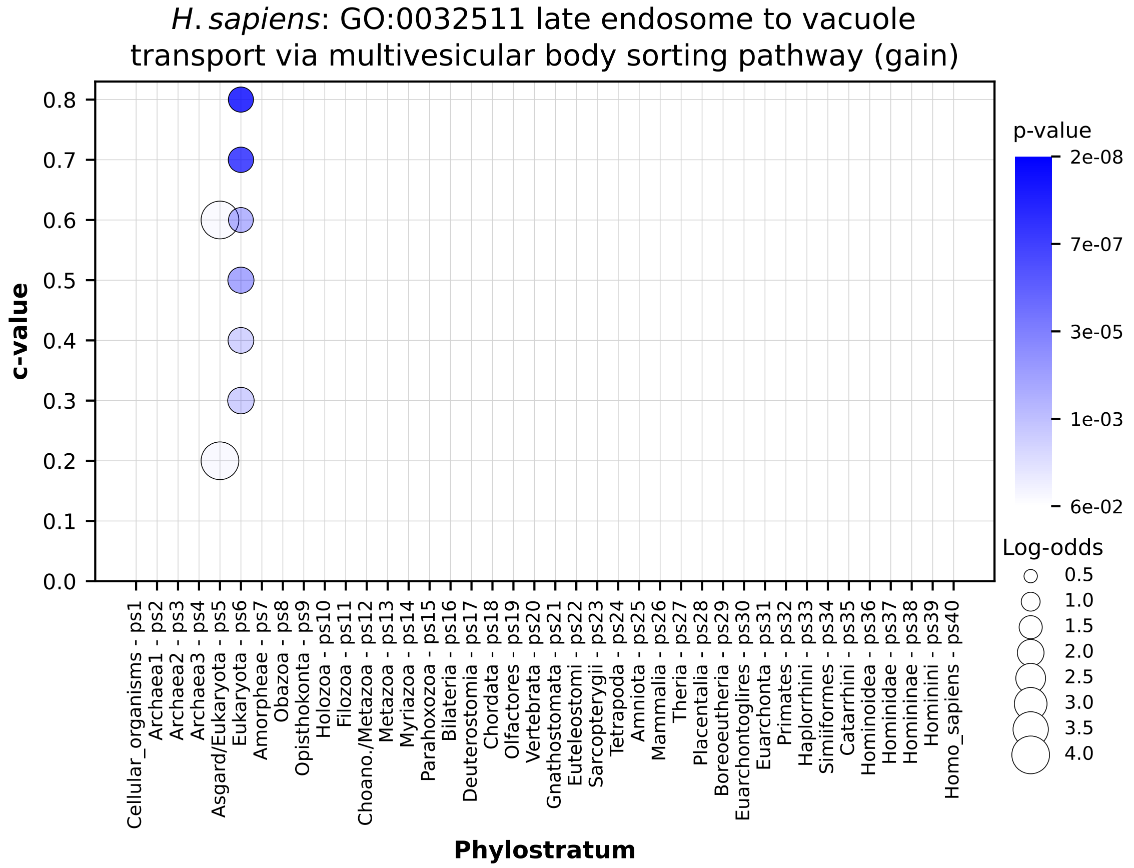

*H. sapiens*: GO:0032511 late endosome to vacuole transport via multivesicular body sorting pathway (gain)
c-value
0.8
0.7
0.6
0.5
0.4
0.3
0.2
0.1
0.0
Cellular_organisms - ps1
Archaea1 - ps2
Archaea2 - ps3
Archaea3 - ps4
Asgard/Eukaryota - ps5
Eukaryota - ps6
Amorpheae - ps7
Obazoa - ps8
Opisthokonta - ps9
Holozoa - ps10
Filozoa - ps11
Choano./Metazoa - ps12
Metazoa - ps13
Myriazoa - ps14
Parahoxozoa - ps15
Bilateria - ps16
Deuterostomia - ps17
Chordata - ps18
Olfactores - ps19
Vertebrata - ps20
Gnathostomata - ps21
Euteleostomi - ps22
Sarcopterygii - ps23
Tetrapoda - ps24
Amniota - ps25
Mammalia - ps26
Theria - ps27
Placentalia - ps28
Boreoeutheria - ps29
Euarchontoglires - ps30
Euarchonta - ps31
Primates - ps32
Haplorrhini - ps33
Simiiformes - ps34
Catarrhini - ps35
Hominoidea - ps36
Hominidae - ps37
Homininae - ps38
Hominini - ps39
Homo_sapiens - ps40
Phylostratum
p-value
2e-08
7e-07
3e-05
1e-03
6e-02
Log-odds
0.5
1.0
1.5
2.0
2.5
3.0
3.5
4.0

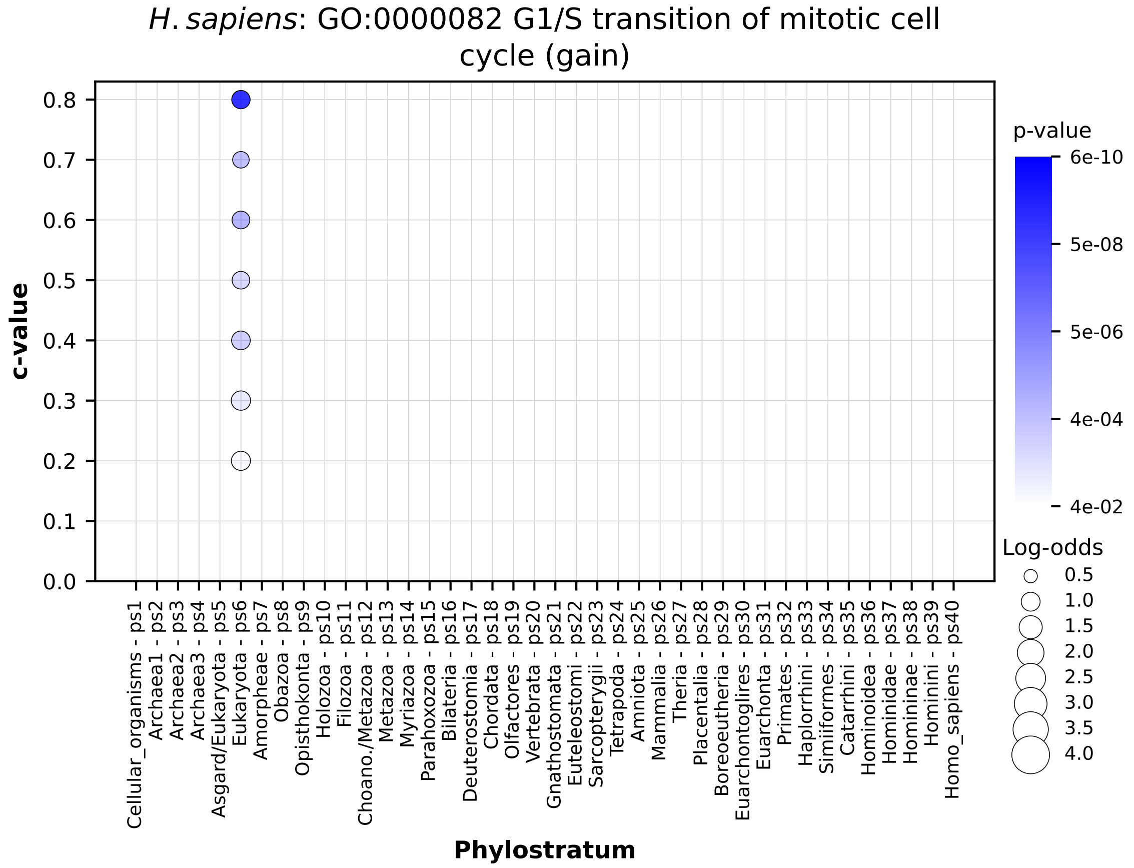
*H. sapiens*: GO:0000082 G1/S transition of mitotic cell cycle (gain)
c-value
0.0
0.1
0.2
0.3
0.4
0.5
0.6
0.7
0.8
p-value
6e-10
5e-08
5e-06
4e-04
4e-02
Log-odds
0.5
1.0
1.5
2.0
2.5
3.0
3.5
4.0
Cellular_organisms - ps1
Archaea1 - ps2
Archaea2 - ps3
Archaea3 - ps4
Asgard/Eukaryota - ps5
Eukaryota - ps6
Amorpheae - ps7
Obazoa - ps8
Opisthokonta - ps9
Holozoa - ps10
Filozoa - ps11
Choano./Metazoa - ps12
Metazoa - ps13
Myriazoa - ps14
Parahoxozoa - ps15
Bilateria - ps16
Deuterostomia - ps17
Chordata - ps18
Olfactores - ps19
Vertebrata - ps20
Gnathostomata - ps21
Euteleostomi - ps22
Sarcopterygii - ps23
Tetrapoda - ps24
Amniota - ps25
Mammalia - ps26
Theria - ps27
Placentalia - ps28
Boreoeutheria - ps29
Euarchontoglires - ps30
Euarchonta - ps31
Primates - ps32
Haplorrhini - ps33
Simiiformes - ps34
Catarrhini - ps35
Hominoidea - ps36
Hominidae - ps37
Homininae - ps38
Hominini - ps39
Homo_sapiens - ps40
Phylostratum

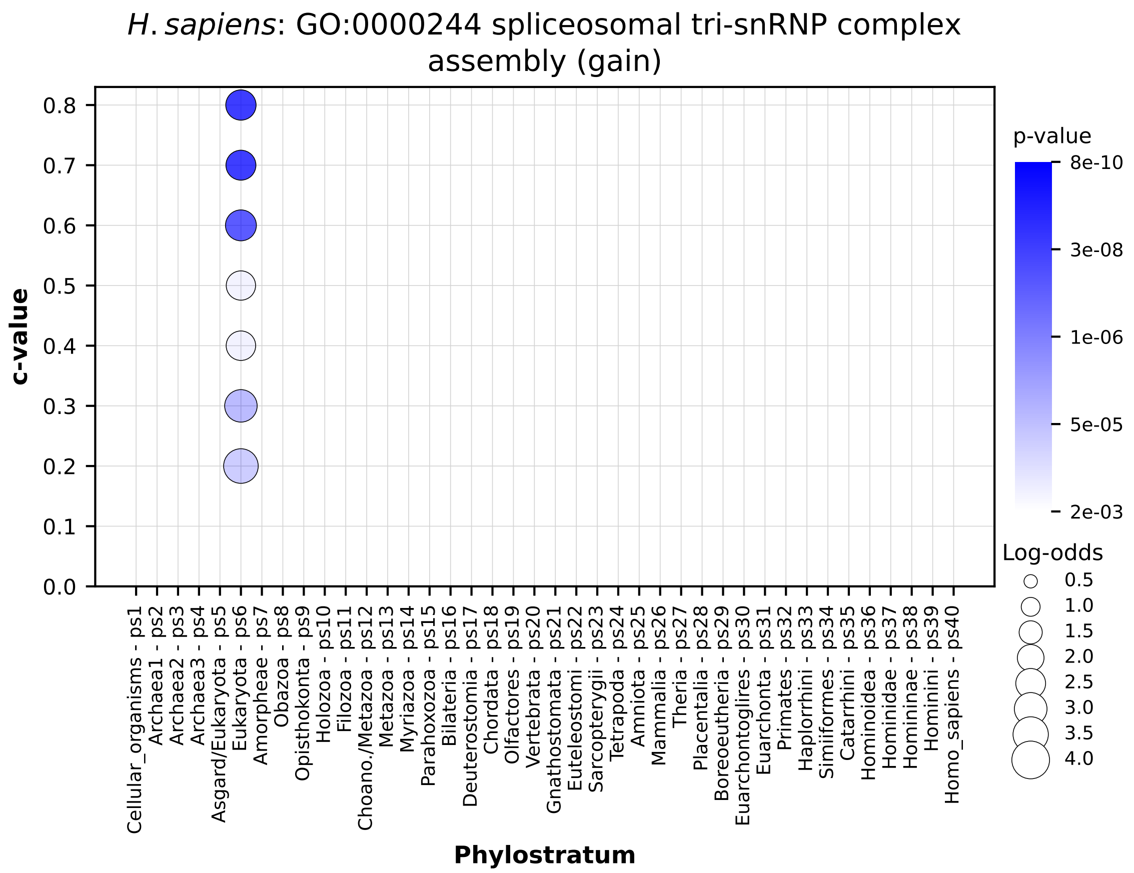

*H. sapiens*: GO:0000244 spliceosomal tri-snRNP complex assembly (gain)
c-value
0.0
0.1
0.2
0.3
0.4
0.5
0.6
0.7
0.8
Phylostratum
Cellular_organisms - ps1
Archaea1 - ps2
Archaea2 - ps3
Archaea3 - ps4
Asgard/Eukaryota - ps5
Eukaryota - ps6
Amorpheae - ps7
Obazoa - ps8
Opisthokonta - ps9
Holozoa - ps10
Filozoa - ps11
Choano./Metazoa - ps12
Metazoa - ps13
Myriazoa - ps14
Parahoxozoa - ps15
Bilateria - ps16
Deuterostomia - ps17
Chordata - ps18
Olfactores - ps19
Vertebrata - ps20
Gnathostomata - ps21
Euteleostomi - ps22
Sarcopterygii - ps23
Tetrapoda - ps24
Amniota - ps25
Mammalia - ps26
Theria - ps27
Placentalia - ps28
Boreoeutheria - ps29
Euarchontoglires - ps30
Euarchonta - ps31
Primates - ps32
Haplorrhini - ps33
Simiiformes - ps34
Catarrhini - ps35
Hominoidea - ps36
Hominidae - ps37
Homininae - ps38
Hominini - ps39
Homo_sapiens - ps40
p-value
8e-10
3e-08
1e-06
5e-05
2e-03
Log-odds
0.5
1.0
1.5
2.0
2.5
3.0
3.5
4.0

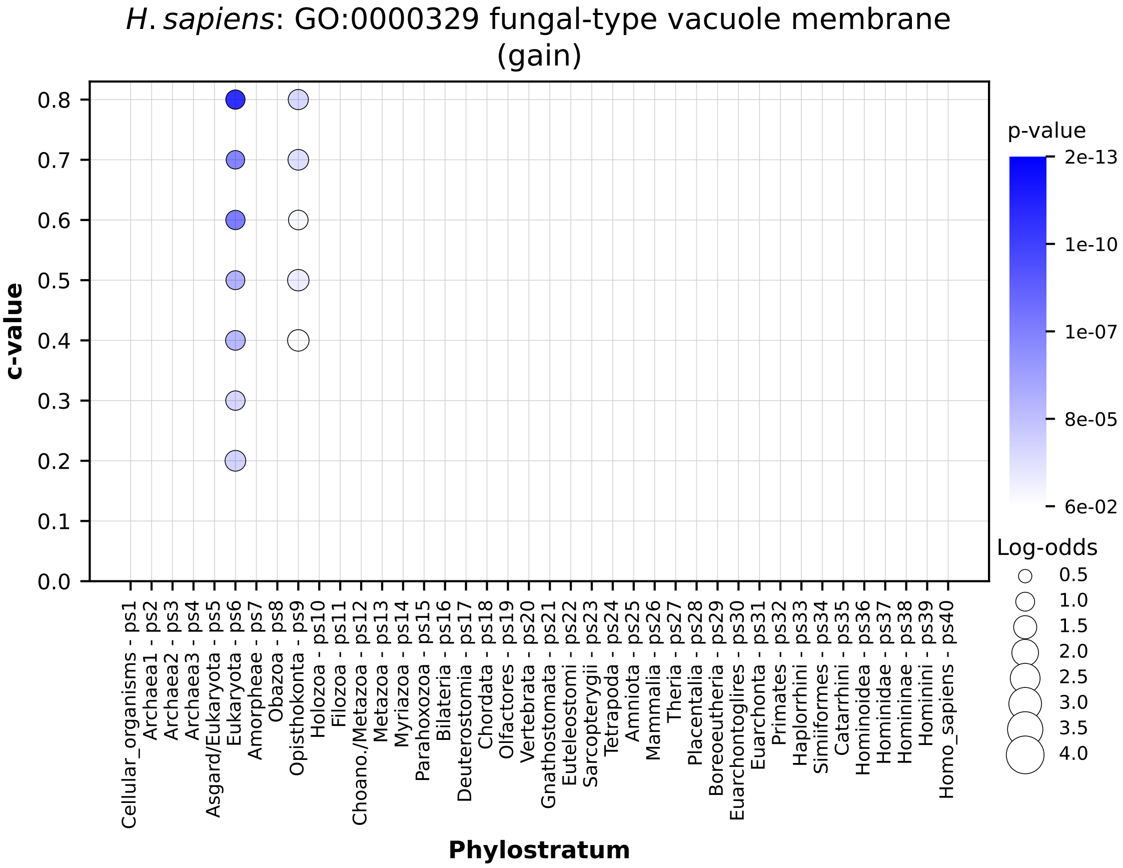

*H. sapiens*: GO:0000329 fungal-type vacuole membrane
(gain)
c-value
0.0
0.1
0.2
0.3
0.4
0.5
0.6
0.7
0.8
Phylostratum
Cellular_organisms - ps1
Archaea1 - ps2
Archaea2 - ps3
Archaea3 - ps4
Asgard/Eukaryota - ps5
Eukaryota - ps6
Amorpheae - ps7
Obazoa - ps8
Opisthokonta - ps9
Holozoa - ps10
Filozoa - ps11
Choano./Metazoa - ps12
Metazoa - ps13
Myriazoa - ps14
Parahoxozoa - ps15
Bilateria - ps16
Deuterostomia - ps17
Chordata - ps18
Olfactores - ps19
Vertebrata - ps20
Gnathostomata - ps21
Euteleostomi - ps22
Sarcopterygii - ps23
Tetrapoda - ps24
Amniota - ps25
Mammalia - ps26
Theria - ps27
Placentalia - ps28
Boreoeutheria - ps29
Euarchontoglires - ps30
Euarchonta - ps31
Primates - ps32
Haplorrhini - ps33
Simiiformes - ps34
Catarrhini - ps35
Hominoidea - ps36
Hominidae - ps37
Homininae - ps38
Hominini - ps39
Homo_sapiens - ps40
p-value
2e-13
1e-10
1e-07
8e-05
6e-02
Log-odds
0.5
1.0
1.5
2.0
2.5
3.0
3.5
4.0

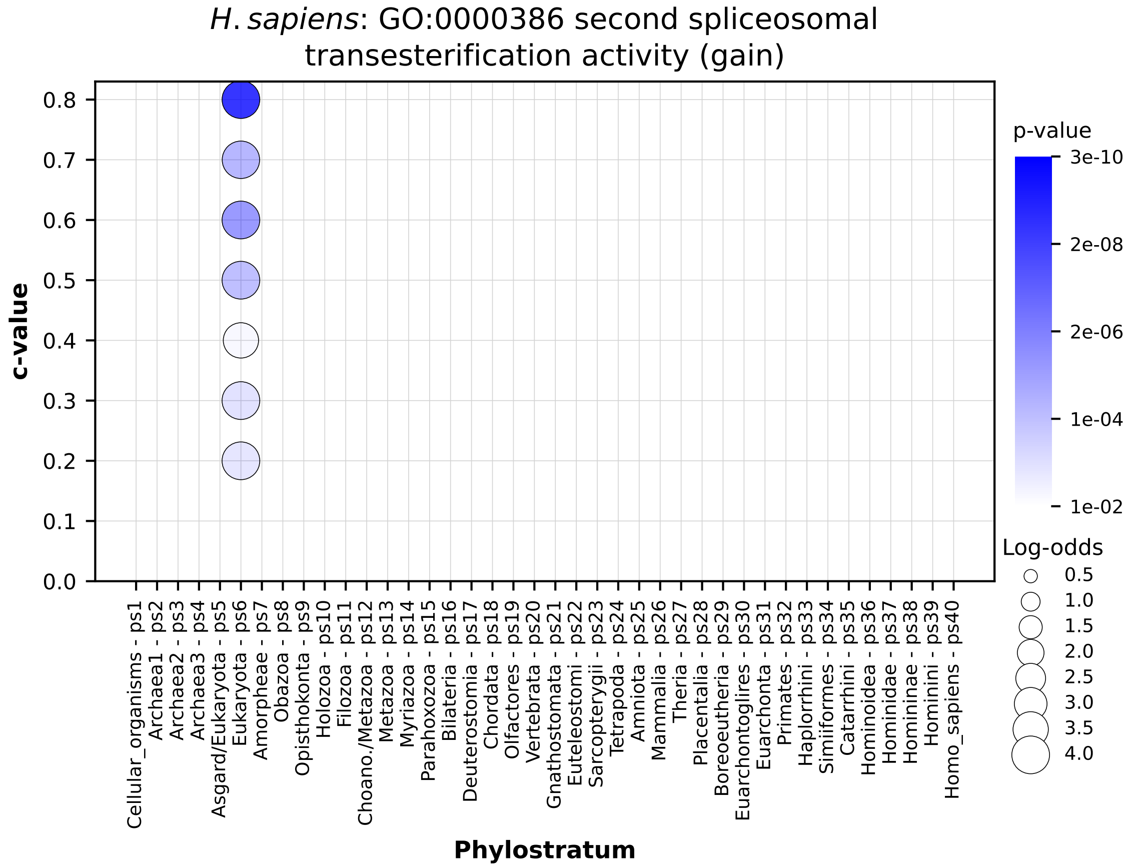

H. sapiens: GO:0000386 second spliceosomal transesterification activity (gain)
c-value
0.0
0.1
0.2
0.3
0.4
0.5
0.6
0.7
0.8
Phylostratum
Cellular_organisms - ps1
Archaea1 - ps2
Archaea2 - ps3
Archaea3 - ps4
Asgard/Eukaryota - ps5
Eukaryota - ps6
Amorpheae - ps7
Obazoa - ps8
Opisthokonta - ps9
Holozoa - ps10
Filozoa - ps11
Choano./Metazoa - ps12
Metazoa - ps13
Myriazoa - ps14
Parahoxozoa - ps15
Bilateria - ps16
Deuterostomia - ps17
Chordata - ps18
Olfactores - ps19
Vertebrata - ps20
Gnathostomata - ps21
Euteleostomi - ps22
Sarcopterygii - ps23
Tetrapoda - ps24
Amniota - ps25
Mammalia - ps26
Theria - ps27
Placentalia - ps28
Boreoeutheria - ps29
Euarchontoglires - ps30
Euarchonta - ps31
Primates - ps32
Haplorrhini - ps33
Simiiformes - ps34
Catarrhini - ps35
Hominoidea - ps36
Hominidae - ps37
Homininae - ps38
Hominini - ps39
Homo_sapiens - ps40
p-value
3e-10
2e-08
2e-06
1e-04
1e-02
Log-odds
0.5
1.0
1.5
2.0
2.5
3.0
3.5
4.0

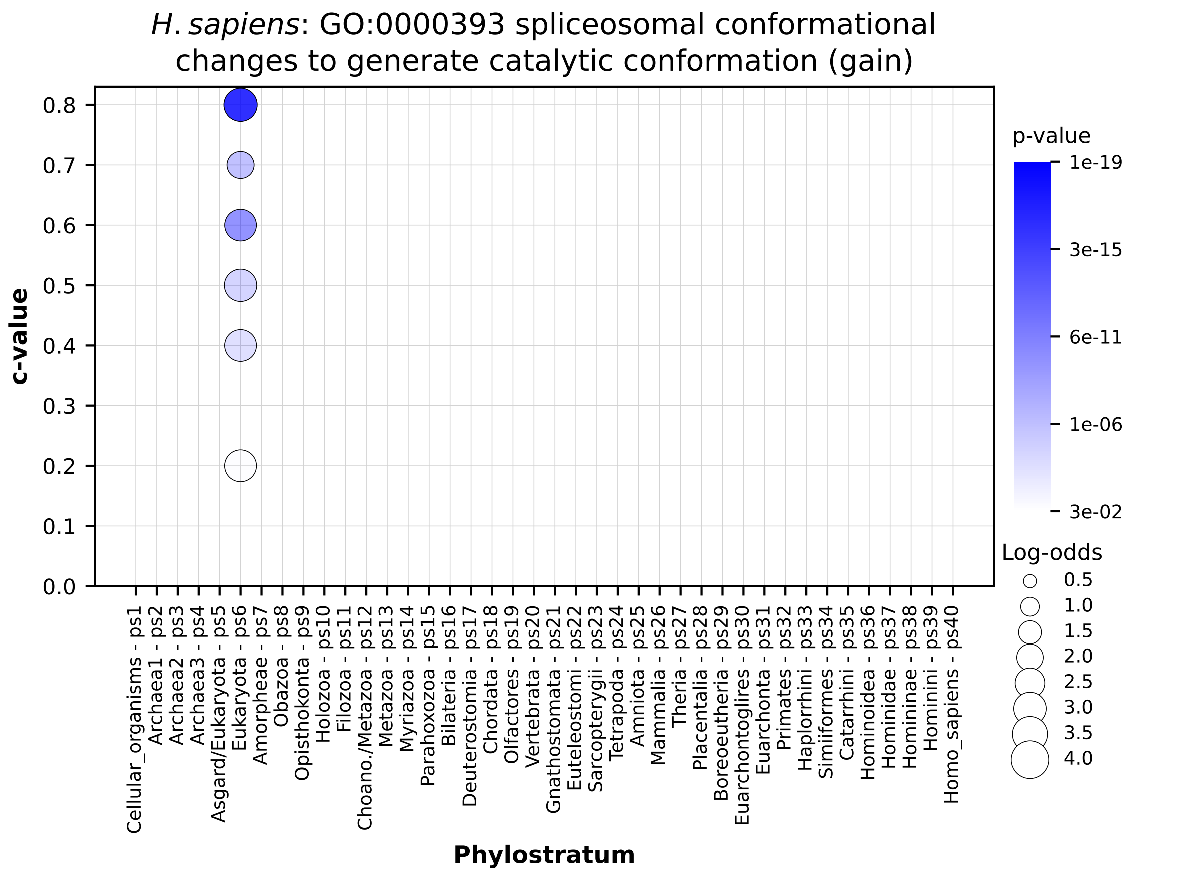

H. sapiens: GO:0000393 spliceosomal conformational changes to generate catalytic conformation (gain)
c-value
0.0
0.1
0.2
0.3
0.4
0.5
0.6
0.7
0.8
Phylostratum
Cellular_organisms - ps1
Archaea1 - ps2
Archaea2 - ps3
Archaea3 - ps4
Asgard/Eukaryota - ps5
Eukaryota - ps6
Amorpheae - ps7
Obazoa - ps8
Opisthokonta - ps9
Holozoa - ps10
Filozoa - ps11
Choano./Metazoa - ps12
Metazoa - ps13
Myriazoa - ps14
Parahoxozoa - ps15
Bilateria - ps16
Deuterostomia - ps17
Chordata - ps18
Olfactores - ps19
Vertebrata - ps20
Gnathostomata - ps21
Euteleostomi - ps22
Sarcopterygii - ps23
Tetrapoda - ps24
Amniota - ps25
Mammalia - ps26
Theria - ps27
Placentalia - ps28
Boreoeutheria - ps29
Euarchontoglires - ps30
Euarchonta - ps31
Primates - ps32
Haplorrhini - ps33
Simiiformes - ps34
Catarrhini - ps35
Hominoidea - ps36
Hominidae - ps37
Homininae - ps38
Hominini - ps39
Homo_sapiens - ps40
p-value
1e-19
3e-15
6e-11
1e-06
3e-02
Log-odds
0.5
1.0
1.5
2.0
2.5
3.0
3.5
4.0

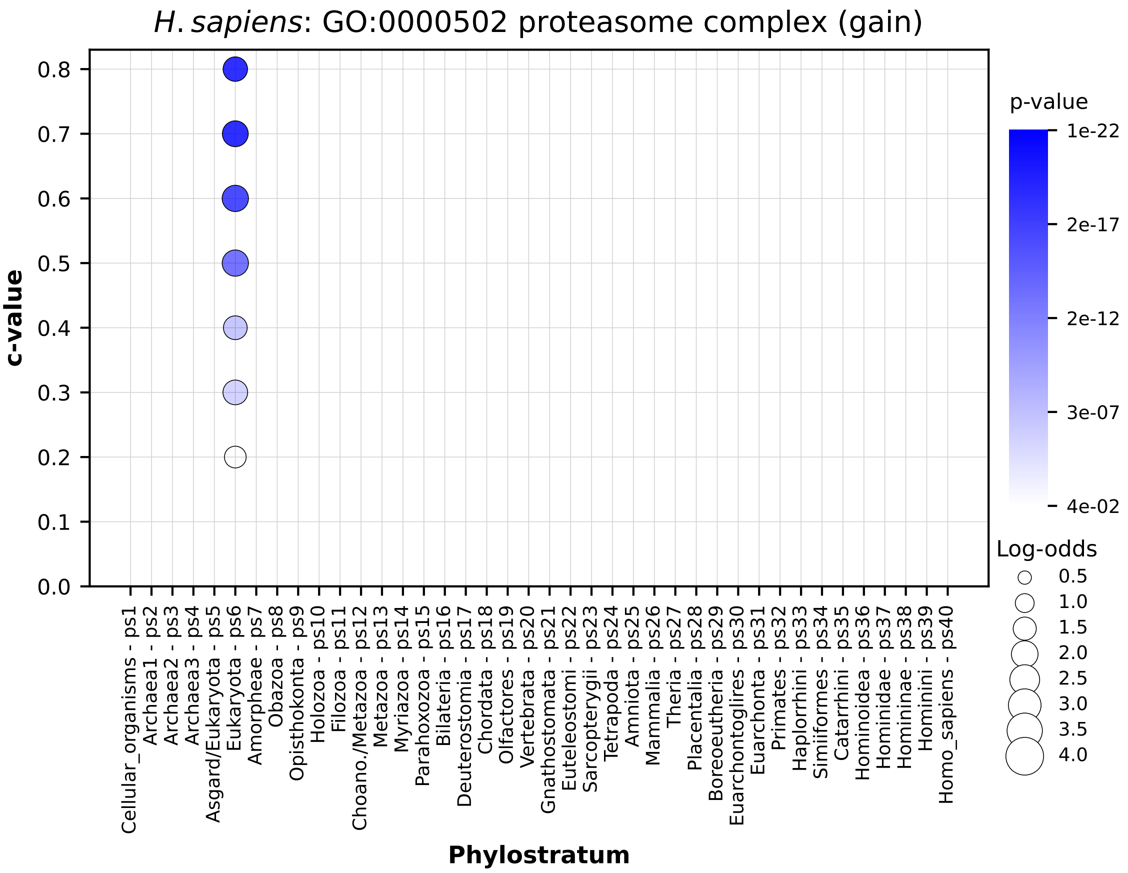

H. sapiens: GO:0000502 proteasome complex (gain)
c-value
0.0
0.1
0.2
0.3
0.4
0.5
0.6
0.7
0.8
Phylostratum
Cellular_organisms - ps1
Archaea1 - ps2
Archaea2 - ps3
Archaea3 - ps4
Asgard/Eukaryota - ps5
Eukaryota - ps6
Amorpheae - ps7
Obazoa - ps8
Opisthokonta - ps9
Holozoa - ps10
Filozoa - ps11
Choano./Metazoa - ps12
Metazoa - ps13
Myriazoa - ps14
Parahoxozoa - ps15
Bilateria - ps16
Deuterostomia - ps17
Chordata - ps18
Olfactores - ps19
Vertebrata - ps20
Gnathostomata - ps21
Euteleostomi - ps22
Sarcopterygii - ps23
Tetrapoda - ps24
Amniota - ps25
Mammalia - ps26
Theria - ps27
Placentalia - ps28
Boreoeutheria - ps29
Euarchontoglires - ps30
Euarchonta - ps31
Primates - ps32
Haplorrhini - ps33
Simiiformes - ps34
Catarrhini - ps35
Hominoidea - ps36
Hominidae - ps37
Homininae - ps38
Hominini - ps39
Homo_sapiens - ps40
p-value
1e-22
2e-17
2e-12
3e-07
4e-02
Log-odds
0.5
1.0
1.5
2.0
2.5
3.0
3.5
4.0

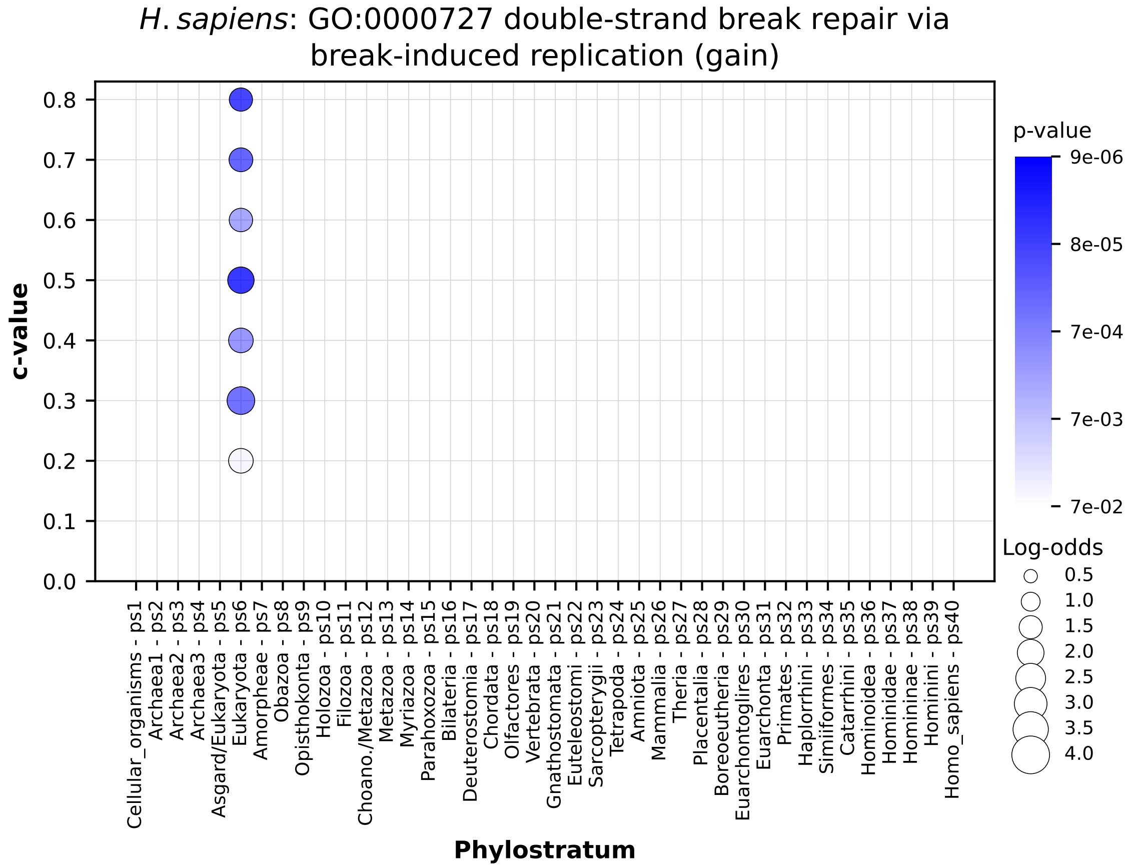

H. sapiens: GO:0000727 double-strand break repair via break-induced replication (gain)
c-value
0.0
0.1
0.2
0.3
0.4
0.5
0.6
0.7
0.8
Phylostratum
Cellular_organisms - ps1
Archaea1 - ps2
Archaea2 - ps3
Archaea3 - ps4
Asgard/Eukaryota - ps5
Eukaryota - ps6
Amorpheae - ps7
Obazoa - ps8
Opisthokonta - ps9
Holozoa - ps10
Filozoa - ps11
Choano./Metazoa - ps12
Metazoa - ps13
Myriazoa - ps14
Parahoxozoa - ps15
Bilateria - ps16
Deuterostomia - ps17
Chordata - ps18
Olfactores - ps19
Vertebrata - ps20
Gnathostomata - ps21
Euteleostomi - ps22
Sarcopterygii - ps23
Tetrapoda - ps24
Amniota - ps25
Mammalia - ps26
Theria - ps27
Placentalia - ps28
Boreoeutheria - ps29
Euarchontoglires - ps30
Euarchonta - ps31
Primates - ps32
Haplorrhini - ps33
Simiiformes - ps34
Catarrhini - ps35
Hominoidea - ps36
Hominidae - ps37
Homininae - ps38
Hominini - ps39
Homo_sapiens - ps40
p-value
9e-06
8e-05
7e-04
7e-03
7e-02
Log-odds
0.5
1.0
1.5
2.0
2.5
3.0
3.5
4.0

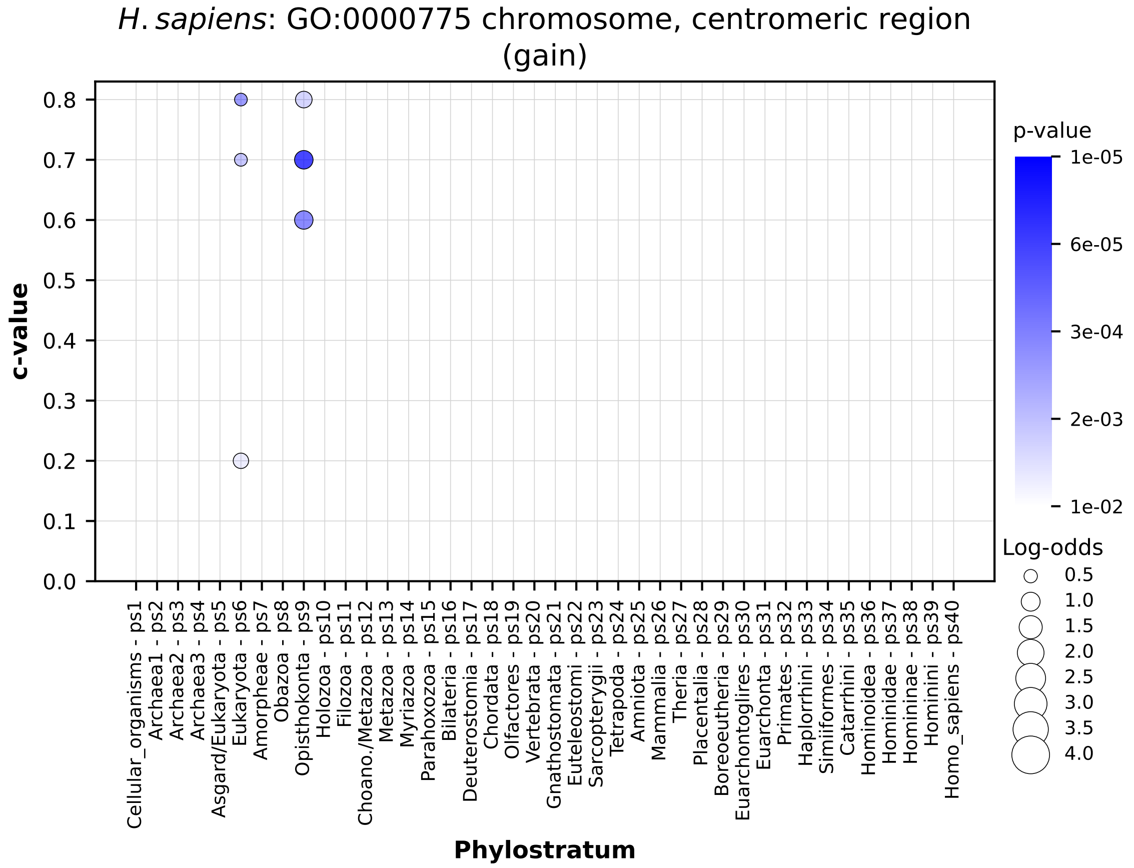

H. sapiens: GO:0000775 chromosome, centromeric region (gain)
c-value
0.0
0.1
0.2
0.3
0.4
0.5
0.6
0.7
0.8
Phylostratum
Cellular_organisms - ps1
Archaea1 - ps2
Archaea2 - ps3
Archaea3 - ps4
Asgard/Eukaryota - ps5
Eukaryota - ps6
Amorpheae - ps7
Obazoa - ps8
Opisthokonta - ps9
Holozoa - ps10
Filozoa - ps11
Choano./Metazoa - ps12
Metazoa - ps13
Myriazoa - ps14
Parahoxozoa - ps15
Bilateria - ps16
Deuterostomia - ps17
Chordata - ps18
Olfactores - ps19
Vertebrata - ps20
Gnathostomata - ps21
Euteleostomi - ps22
Sarcopterygii - ps23
Tetrapoda - ps24
Amniota - ps25
Mammalia - ps26
Theria - ps27
Placentalia - ps28
Boreoeutheria - ps29
Euarchontoglires - ps30
Euarchonta - ps31
Primates - ps32
Haplorrhini - ps33
Simiiformes - ps34
Catarrhini - ps35
Hominoidea - ps36
Hominidae - ps37
Homininae - ps38
Hominini - ps39
Homo_sapiens - ps40
p-value
1e-05
6e-05
3e-04
2e-03
1e-02
Log-odds
0.5
1.0
1.5
2.0
2.5
3.0
3.5
4.0

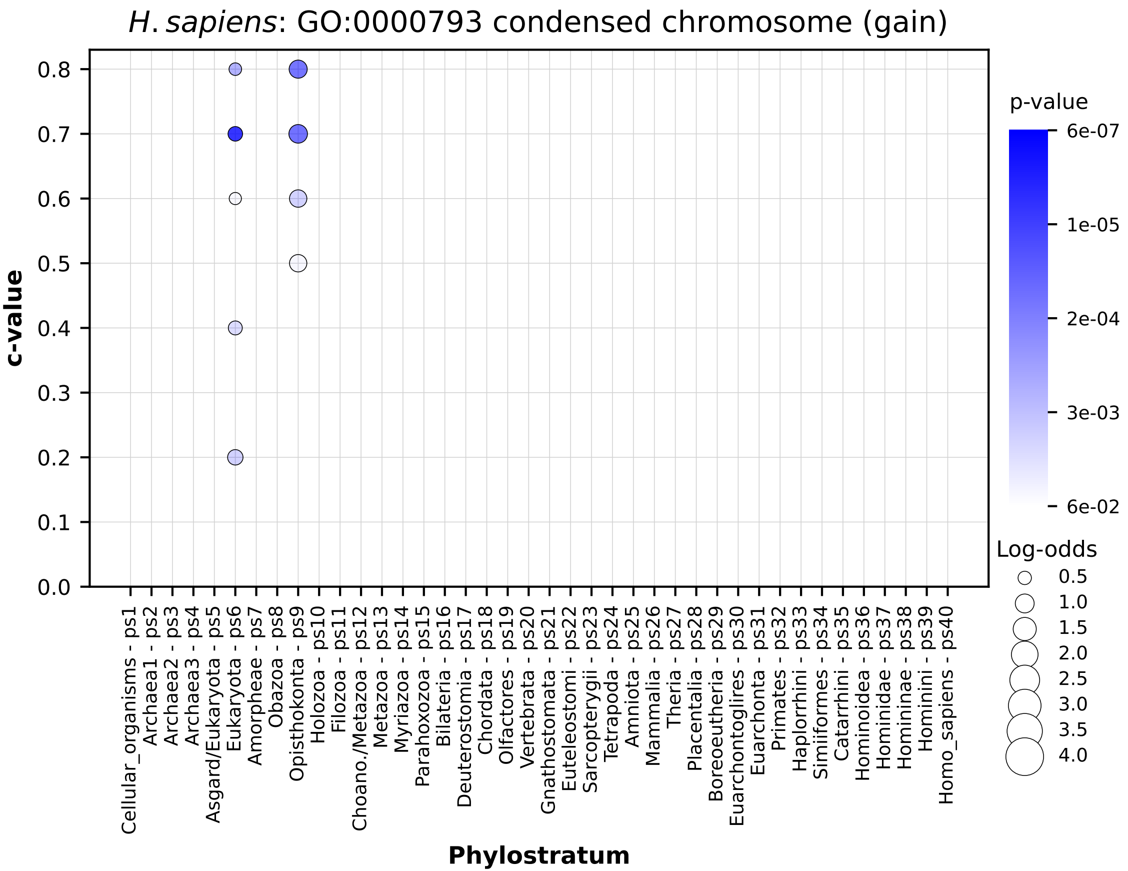

H. sapiens: GO:0000793 condensed chromosome (gain)
c-value
0.8
0.7
0.6
0.5
0.4
0.3
0.2
0.1
0.0
p-value
6e-07
1e-05
2e-04
3e-03
6e-02
Log-odds
0.5
1.0
1.5
2.0
2.5
3.0
3.5
4.0
Cellular_organisms - ps1
Archaea1 - ps2
Archaea2 - ps3
Archaea3 - ps4
Asgard/Eukaryota - ps5
Eukaryota - ps6
Amorpheae - ps7
Obazoa - ps8
Opisthokonta - ps9
Holozoa - ps10
Filozoa - ps11
Choano./Metazoa - ps12
Metazoa - ps13
Myriazoa - ps14
Parahoxozoa - ps15
Bilateria - ps16
Deuterostomia - ps17
Chordata - ps18
Olfactores - ps19
Vertebrata - ps20
Gnathostomata - ps21
Euteleostomi - ps22
Sarcopterygii - ps23
Tetrapoda - ps24
Amniota - ps25
Mammalia - ps26
Theria - ps27
Placentalia - ps28
Boreoeutheria - ps29
Euarchontoglires - ps30
Euarchonta - ps31
Primates - ps32
Haplorrhini - ps33
Simiiformes - ps34
Catarrhini - ps35
Hominoidea - ps36
Hominidae - ps37
Homininae - ps38
Hominini - ps39
Homo_sapiens - ps40
Phylostratum

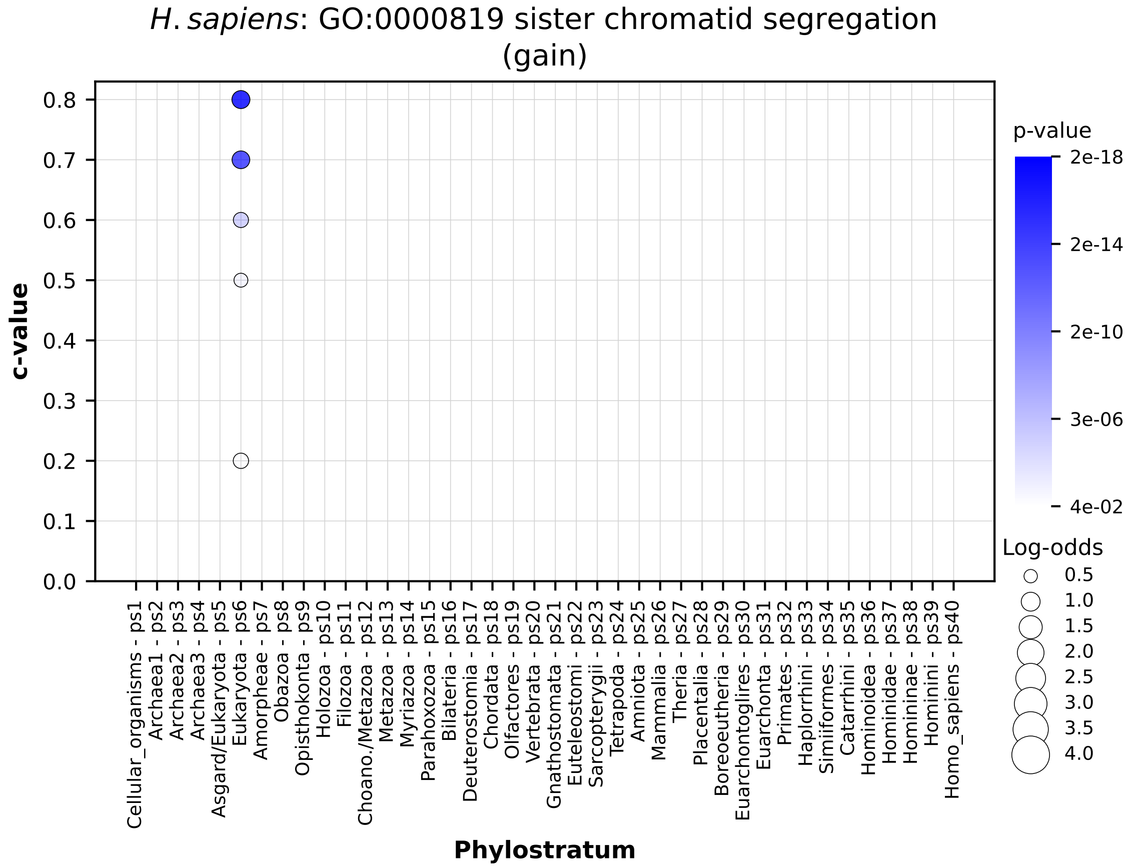

H. sapiens: GO:0000819 sister chromatid segregation
(gain)
c-value
0.0
0.1
0.2
0.3
0.4
0.5
0.6
0.7
0.8
Phylostratum
Cellular_organisms - ps1
Archaea1 - ps2
Archaea2 - ps3
Archaea3 - ps4
Asgard/Eukaryota - ps5
Eukaryota - ps6
Amorpheae - ps7
Obazoa - ps8
Opisthokonta - ps9
Holozoa - ps10
Filozoa - ps11
Choano./Metazoa - ps12
Metazoa - ps13
Myriazoa - ps14
Parahoxozoa - ps15
Bilateria - ps16
Deuterostomia - ps17
Chordata - ps18
Olfactores - ps19
Vertebrata - ps20
Gnathostomata - ps21
Euteleostomi - ps22
Sarcopterygii - ps23
Tetrapoda - ps24
Amniota - ps25
Mammalia - ps26
Theria - ps27
Placentalia - ps28
Boreoeutheria - ps29
Euarchontoglires - ps30
Euarchonta - ps31
Primates - ps32
Haplorrhini - ps33
Simiiformes - ps34
Catarrhini - ps35
Hominoidea - ps36
Hominidae - ps37
Homininae - ps38
Hominini - ps39
Homo_sapiens - ps40
p-value
2e-18
2e-14
2e-10
3e-06
4e-02
Log-odds
0.5
1.0
1.5
2.0
2.5
3.0
3.5
4.0

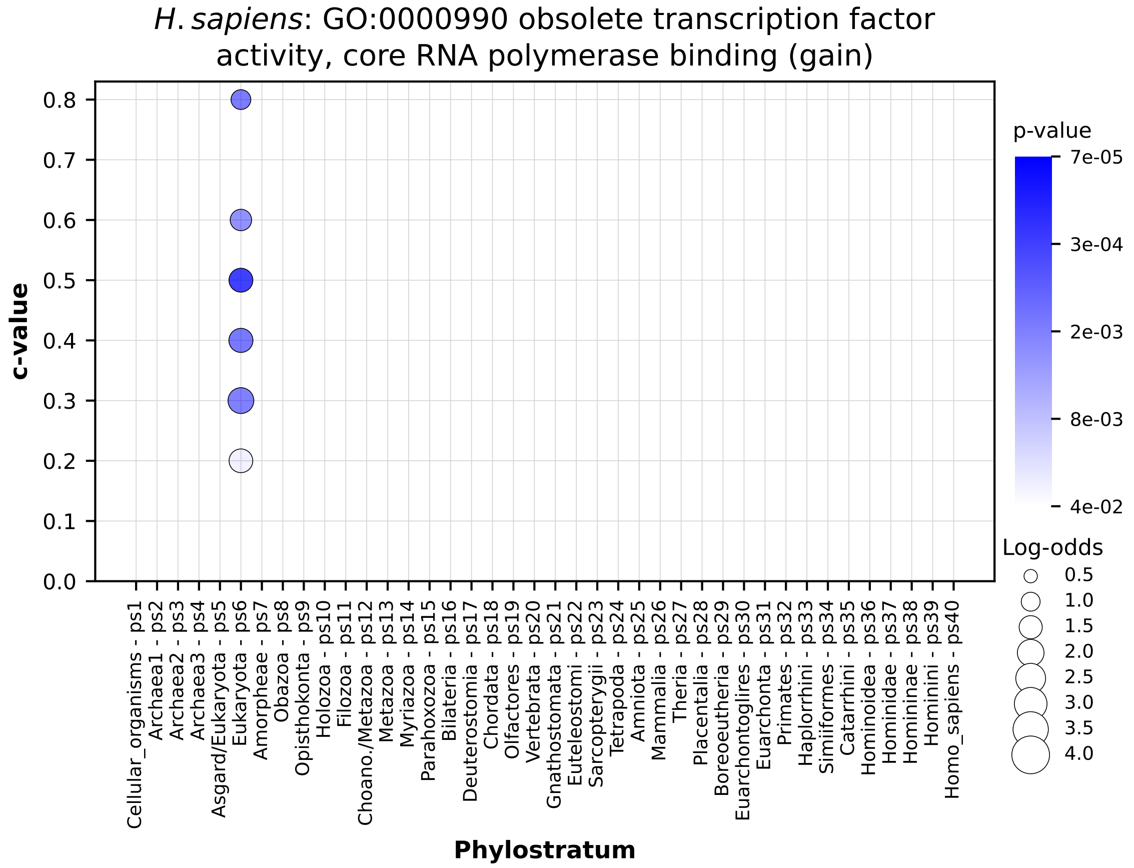

*H. sapiens*: GO:0000990 obsolete transcription factor activity, core RNA polymerase binding (gain)
c-value
0.0
0.1
0.2
0.3
0.4
0.5
0.6
0.7
0.8
p-value
7e-05
3e-04
2e-03
8e-03
4e-02
Log-odds
0.5
1.0
1.5
2.0
2.5
3.0
3.5
4.0
Cellular_organisms - ps1
Archaea1 - ps2
Archaea2 - ps3
Archaea3 - ps4
Asgard/Eukaryota - ps5
Eukaryota - ps6
Amorpheae - ps7
Obazoa - ps8
Opisthokonta - ps9
Holozoa - ps10
Filozoa - ps11
Choano./Metazoa - ps12
Metazoa - ps13
Myriazoa - ps14
Parahoxozoa - ps15
Bilateria - ps16
Deuterostomia - ps17
Chordata - ps18
Olfactores - ps19
Vertebrata - ps20
Gnathostomata - ps21
Euteleostomi - ps22
Sarcopterygii - ps23
Tetrapoda - ps24
Amniota - ps25
Mammalia - ps26
Theria - ps27
Placentalia - ps28
Boreoeutheria - ps29
Euarchontoglires - ps30
Euarchonta - ps31
Primates - ps32
Haplorrhini - ps33
Simiiformes - ps34
Catarrhini - ps35
Hominoidea - ps36
Hominidae - ps37
Homininae - ps38
Hominini - ps39
Homo_sapiens - ps40
Phylostratum

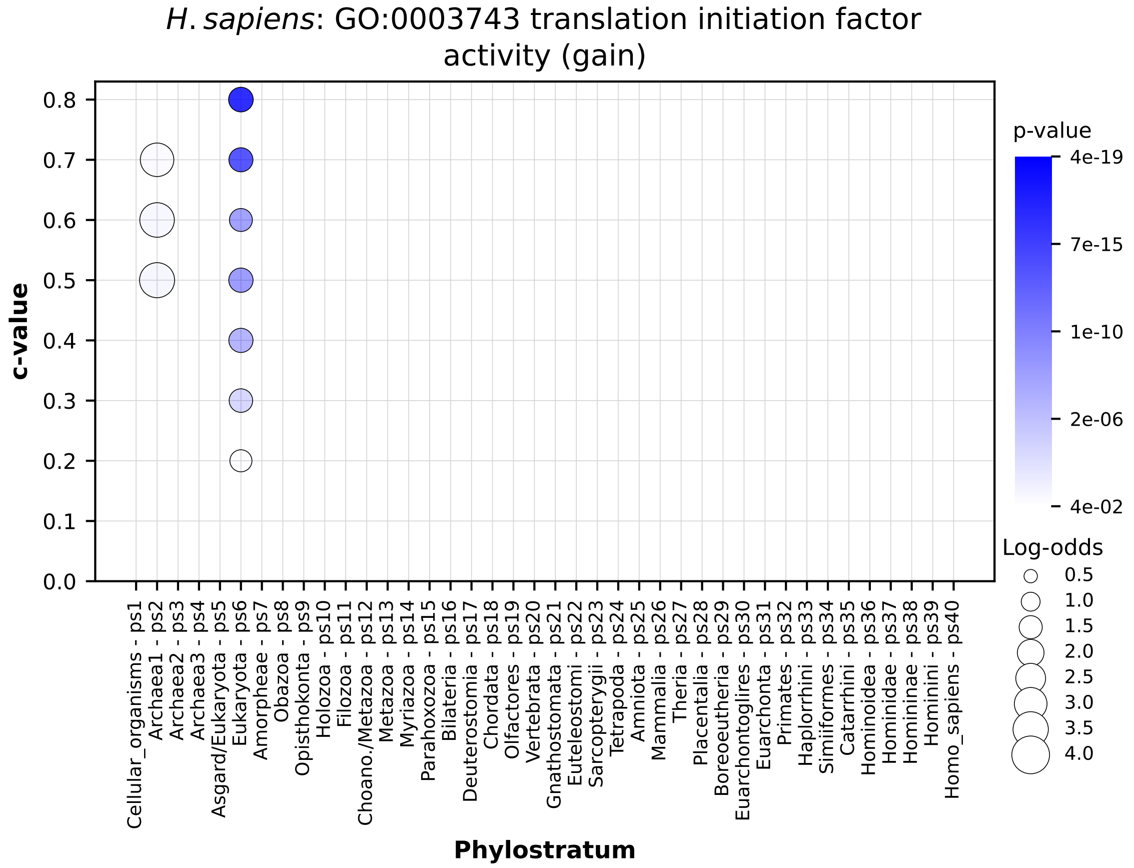

H. sapiens: GO:0003743 translation initiation factor activity (gain)
c-value
0.0
0.1
0.2
0.3
0.4
0.5
0.6
0.7
0.8
Phylostratum
Cellular_organisms - ps1
Archaea1 - ps2
Archaea2 - ps3
Archaea3 - ps4
Asgard/Eukaryota - ps5
Eukaryota - ps6
Amorpheae - ps7
Obazoa - ps8
Opisthokonta - ps9
Holozoa - ps10
Filozoa - ps11
Choano./Metazoa - ps12
Metazoa - ps13
Myriazoa - ps14
Parahoxozoa - ps15
Bilateria - ps16
Deuterostomia - ps17
Chordata - ps18
Olfactores - ps19
Vertebrata - ps20
Gnathostomata - ps21
Euteleostomi - ps22
Sarcopterygii - ps23
Tetrapoda - ps24
Amniota - ps25
Mammalia - ps26
Theria - ps27
Placentalia - ps28
Boreoeutheria - ps29
Euarchontoglires - ps30
Euarchonta - ps31
Primates - ps32
Haplorrhini - ps33
Simiiformes - ps34
Catarrhini - ps35
Hominoidea - ps36
Hominidae - ps37
Homininae - ps38
Hominini - ps39
Homo_sapiens - ps40
p-value
4e-19
7e-15
1e-10
2e-06
4e-02
Log-odds
0.5
1.0
1.5
2.0
2.5
3.0
3.5
4.0

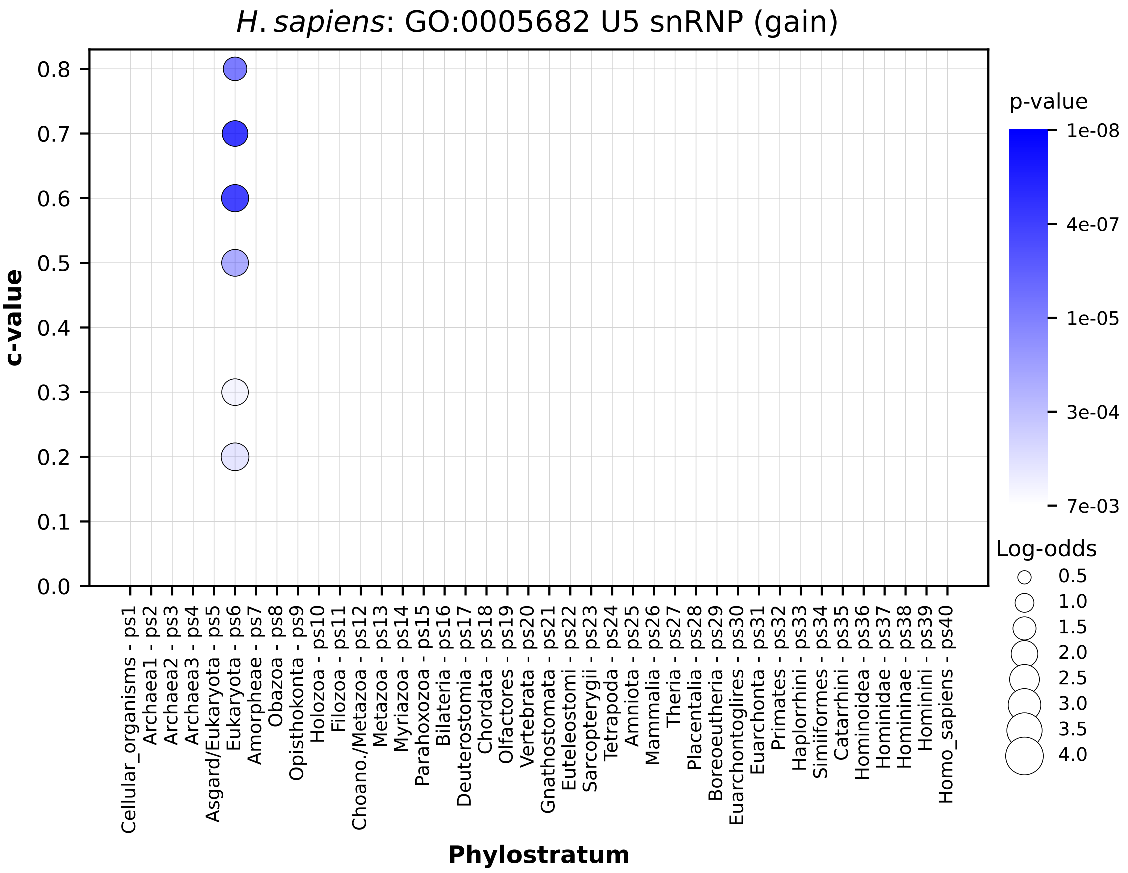

H. sapiens: GO:0005682 U5 snRNP (gain)
c-value
0.0
0.1
0.2
0.3
0.4
0.5
0.6
0.7
0.8
Phylostratum
Cellular_organisms - ps1
Archaea1 - ps2
Archaea2 - ps3
Archaea3 - ps4
Asgard/Eukaryota - ps5
Eukaryota - ps6
Amorpheae - ps7
Obazoa - ps8
Opisthokonta - ps9
Holozoa - ps10
Filozoa - ps11
Choano./Metazoa - ps12
Metazoa - ps13
Myriazoa - ps14
Parahoxozoa - ps15
Bilateria - ps16
Deuterostomia - ps17
Chordata - ps18
Olfactores - ps19
Vertebrata - ps20
Gnathostomata - ps21
Euteleostomi - ps22
Sarcopterygii - ps23
Tetrapoda - ps24
Amniota - ps25
Mammalia - ps26
Theria - ps27
Placentalia - ps28
Boreoeutheria - ps29
Euarchontoglires - ps30
Euarchonta - ps31
Primates - ps32
Haplorrhini - ps33
Simiiformes - ps34
Catarrhini - ps35
Hominoidea - ps36
Hominidae - ps37
Homininae - ps38
Hominini - ps39
Homo_sapiens - ps40
p-value
1e-08
4e-07
1e-05
3e-04
7e-03
Log-odds
0.5
1.0
1.5
2.0
2.5
3.0
3.5
4.0

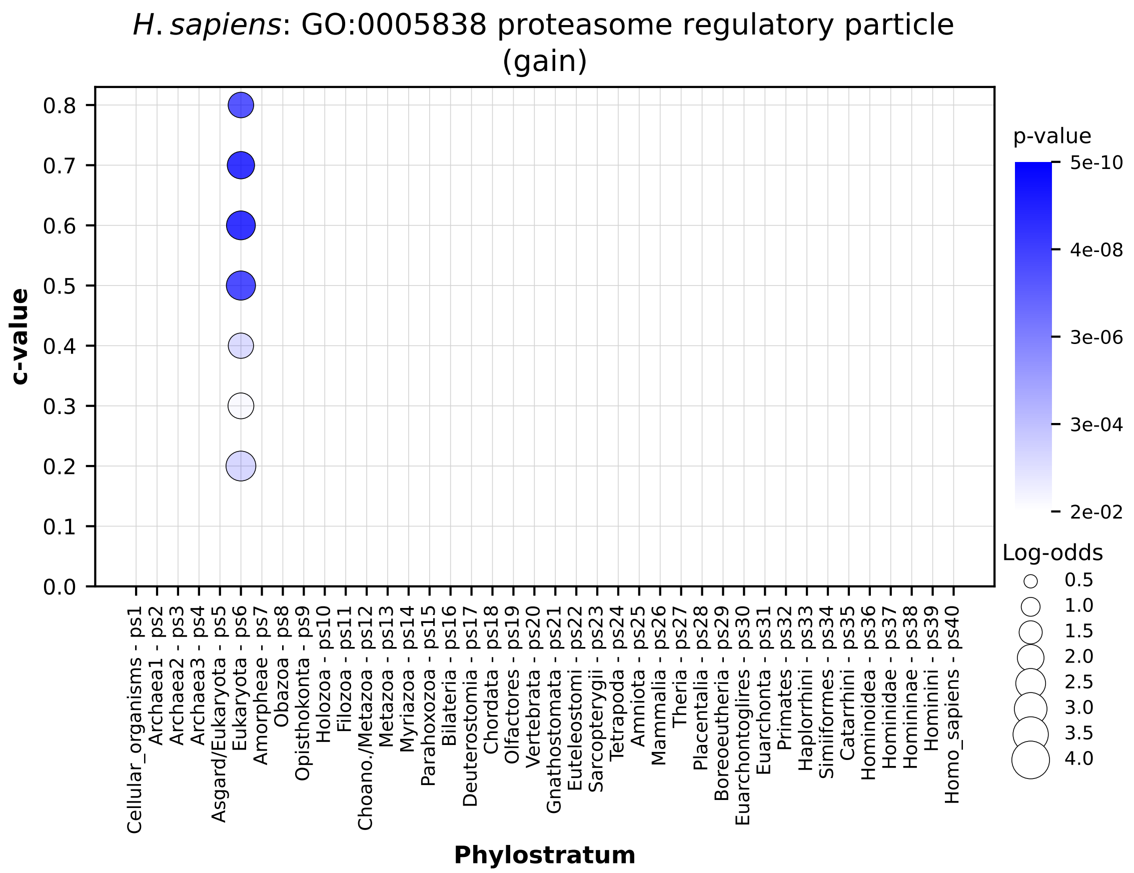

H. sapiens: GO:0005838 proteasome regulatory particle
(gain)
c-value
0.0
0.1
0.2
0.3
0.4
0.5
0.6
0.7
0.8
Phylostratum
Cellular_organisms - ps1
Archaea1 - ps2
Archaea2 - ps3
Archaea3 - ps4
Asgard/Eukaryota - ps5
Eukaryota - ps6
Amorpheae - ps7
Obazoa - ps8
Opisthokonta - ps9
Holozoa - ps10
Filozoa - ps11
Choano./Metazoa - ps12
Metazoa - ps13
Myriazoa - ps14
Parahoxozoa - ps15
Bilateria - ps16
Deuterostomia - ps17
Chordata - ps18
Olfactores - ps19
Vertebrata - ps20
Gnathostomata - ps21
Euteleostomi - ps22
Sarcopterygii - ps23
Tetrapoda - ps24
Amniota - ps25
Mammalia - ps26
Theria - ps27
Placentalia - ps28
Boreoeutheria - ps29
Euarchontoglires - ps30
Euarchonta - ps31
Primates - ps32
Haplorrhini - ps33
Simiiformes - ps34
Catarrhini - ps35
Hominoidea - ps36
Hominidae - ps37
Homininae - ps38
Hominini - ps39
Homo_sapiens - ps40
p-value
5e-10
4e-08
3e-06
3e-04
2e-02
Log-odds
0.5
1.0
1.5
2.0
2.5
3.0
3.5
4.0

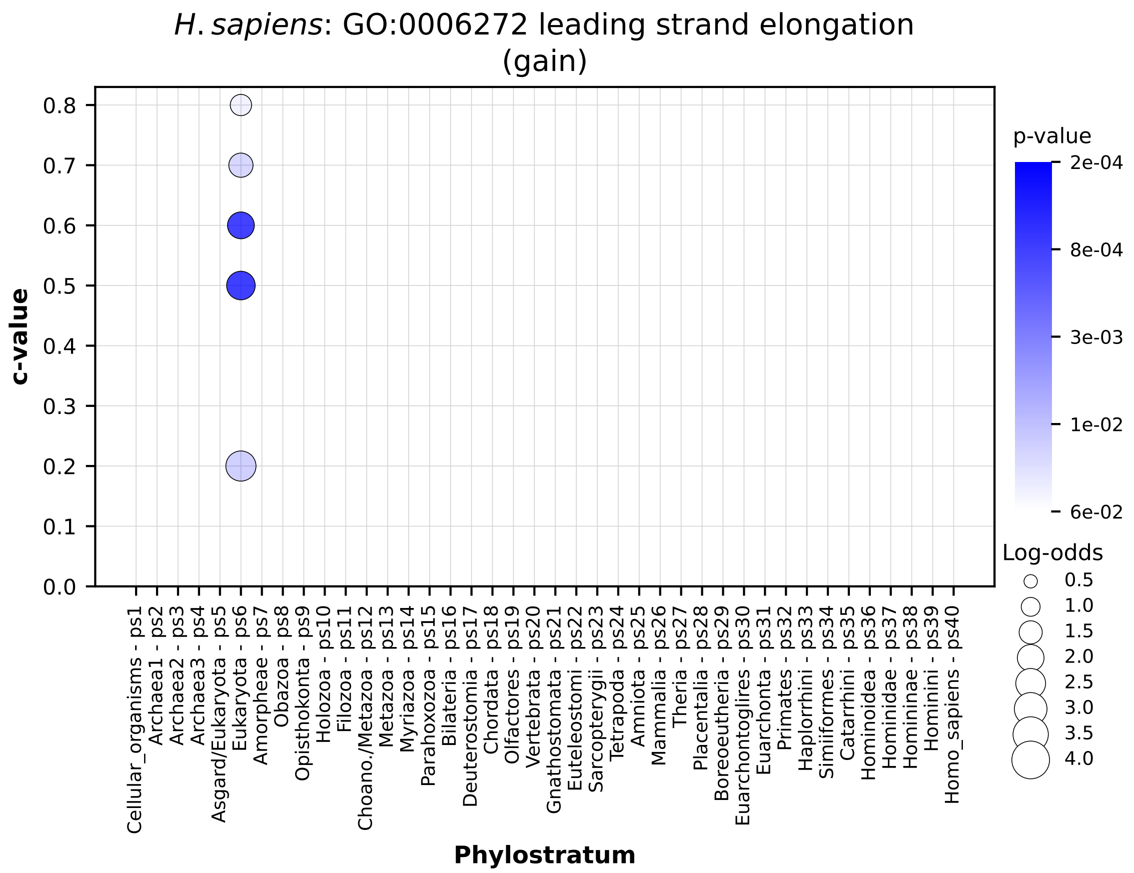
*H. sapiens*: GO:0006272 leading strand elongation
(gain)
c-value
0.8
0.7
0.6
0.5
0.4
0.3
0.2
0.1
0.0
Phylostratum
Cellular_organisms - ps1
Archaea1 - ps2
Archaea2 - ps3
Archaea3 - ps4
Asgard/Eukaryota - ps5
Eukaryota - ps6
Amorpheae - ps7
Obazoa - ps8
Opisthokonta - ps9
Holozoa - ps10
Filozoa - ps11
Choano./Metazoa - ps12
Metazoa - ps13
Myriazoa - ps14
Parahoxozoa - ps15
Bilateria - ps16
Deuterostomia - ps17
Chordata - ps18
Olfactores - ps19
Vertebrata - ps20
Gnathostomata - ps21
Euteleostomi - ps22
Sarcopterygii - ps23
Tetrapoda - ps24
Amniota - ps25
Mammalia - ps26
Theria - ps27
Placentalia - ps28
Boreoeutheria - ps29
Euarchontoglires - ps30
Euarchonta - ps31
Primates - ps32
Haplorrhini - ps33
Simiiformes - ps34
Catarrhini - ps35
Hominoidea - ps36
Hominidae - ps37
Homininae - ps38
Hominini - ps39
Homo_sapiens - ps40
p-value
2e-04
8e-04
3e-03
1e-02
6e-02
Log-odds
0.5
1.0
1.5
2.0
2.5
3.0
3.5
4.0

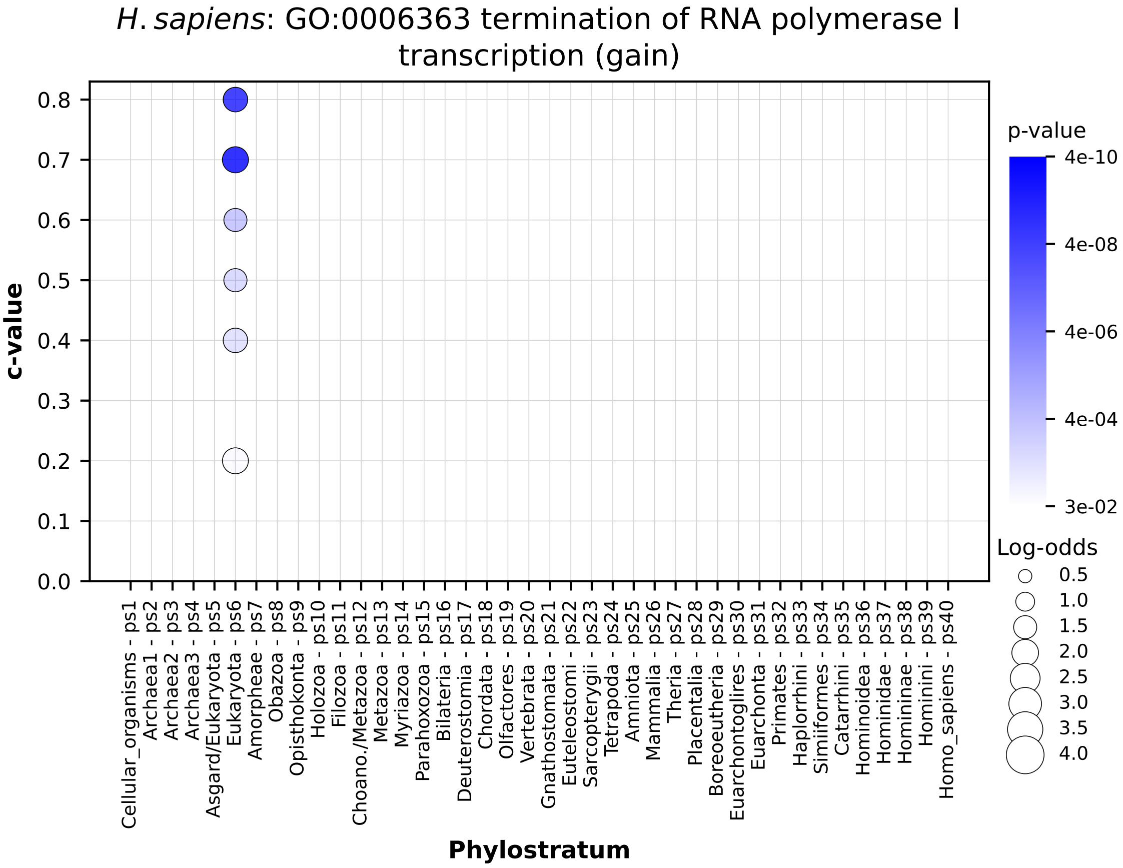

*H. sapiens*: GO:0006363 termination of RNA polymerase I transcription (gain)
c-value
0.0
0.1
0.2
0.3
0.4
0.5
0.6
0.7
0.8
Phylostratum
Cellular_organisms - ps1
Archaea1 - ps2
Archaea2 - ps3
Archaea3 - ps4
Asgard/Eukaryota - ps5
Eukaryota - ps6
Amorpheae - ps7
Obazoa - ps8
Opisthokonta - ps9
Holozoa - ps10
Filozoa - ps11
Choano./Metazoa - ps12
Metazoa - ps13
Myriazoa - ps14
Parahoxozoa - ps15
Bilateria - ps16
Deuterostomia - ps17
Chordata - ps18
Olfactores - ps19
Vertebrata - ps20
Gnathostomata - ps21
Euteleostomi - ps22
Sarcopterygii - ps23
Tetrapoda - ps24
Amniota - ps25
Mammalia - ps26
Theria - ps27
Placentalia - ps28
Boreoeutheria - ps29
Euarchontoglires - ps30
Euarchonta - ps31
Primates - ps32
Haplorrhini - ps33
Simiiformes - ps34
Catarrhini - ps35
Hominoidea - ps36
Hominidae - ps37
Homininae - ps38
Hominini - ps39
Homo_sapiens - ps40
p-value
4e-10
4e-08
4e-06
4e-04
3e-02
Log-odds
0.5
1.0
1.5
2.0
2.5
3.0
3.5
4.0

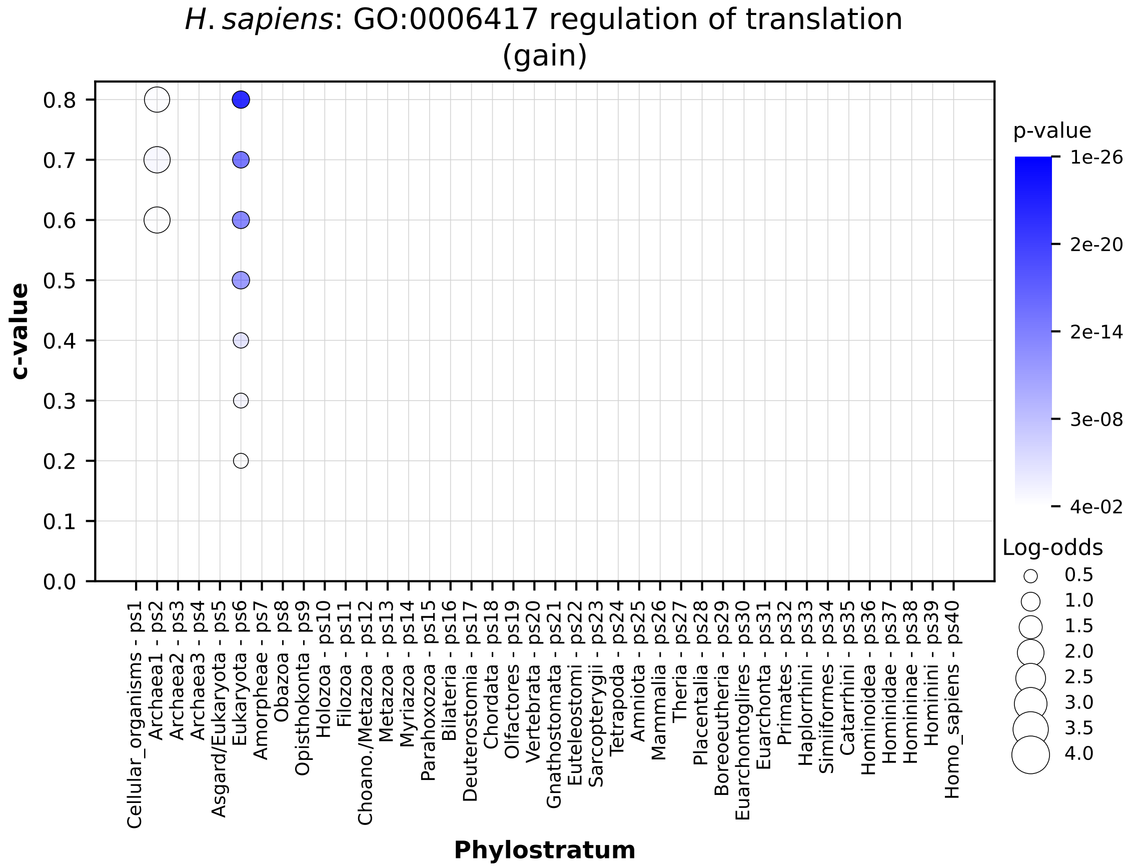

*H. sapiens*: GO:0006417 regulation of translation
(gain)
c-value
0.8
0.7
0.6
0.5
0.4
0.3
0.2
0.1
0.0
p-value
1e-26
2e-20
2e-14
3e-08
4e-02
Log-odds
0.5
1.0
1.5
2.0
2.5
3.0
3.5
4.0
Cellular_organisms - ps1
Archaea1 - ps2
Archaea2 - ps3
Archaea3 - ps4
Asgard/Eukaryota - ps5
Eukaryota - ps6
Amorpheae - ps7
Obazoa - ps8
Opisthokonta - ps9
Holozoa - ps10
Filozoa - ps11
Choano./Metazoa - ps12
Metazoa - ps13
Myriazoa - ps14
Parahoxozoa - ps15
Bilateria - ps16
Deuterostomia - ps17
Chordata - ps18
Olfactores - ps19
Vertebrata - ps20
Gnathostomata - ps21
Euteleostomi - ps22
Sarcopterygii - ps23
Tetrapoda - ps24
Amniota - ps25
Mammalia - ps26
Theria - ps27
Placentalia - ps28
Boreoeutheria - ps29
Euarchontoglires - ps30
Euarchonta - ps31
Primates - ps32
Haplorrhini - ps33
Simiiformes - ps34
Catarrhini - ps35
Hominoidea - ps36
Hominidae - ps37
Homininae - ps38
Hominini - ps39
Homo_sapiens - ps40
Phylostratum

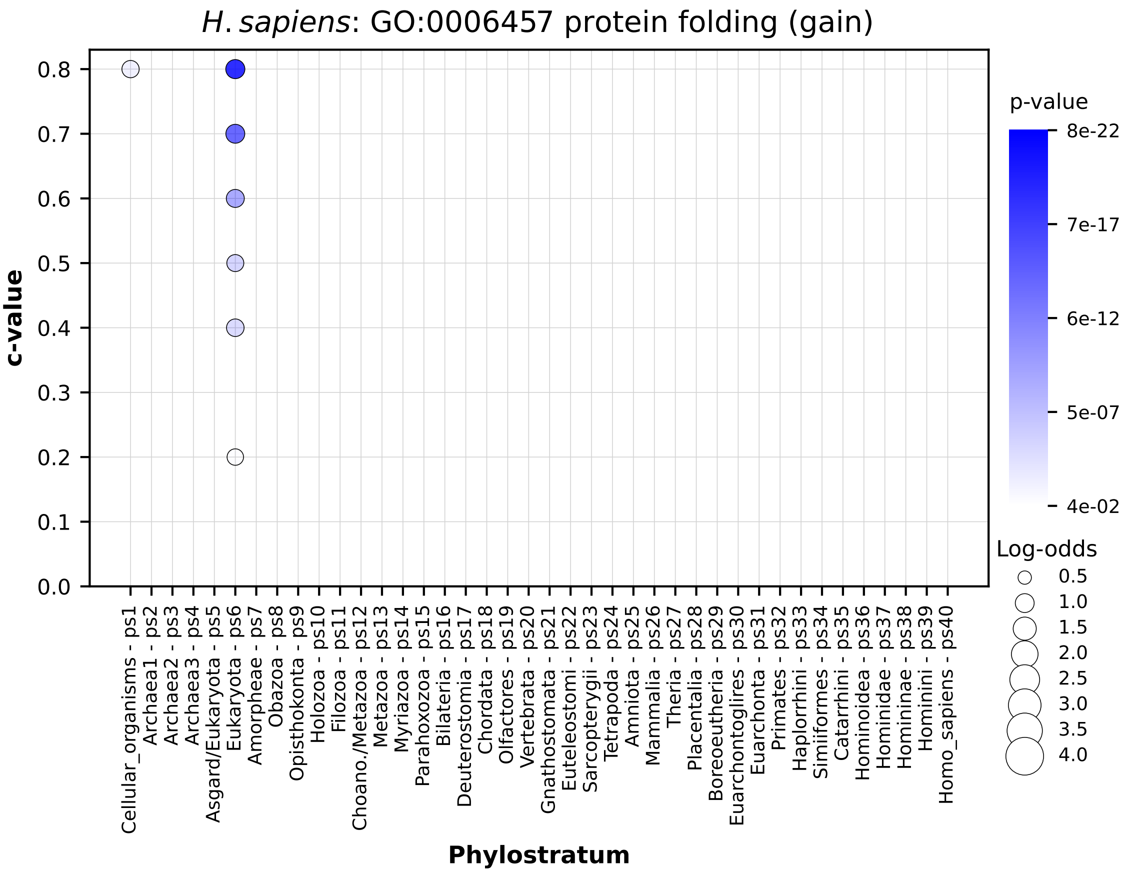

H. sapiens: GO:0006457 protein folding (gain)
c-value
0.0
0.1
0.2
0.3
0.4
0.5
0.6
0.7
0.8
Phylostratum
Cellular_organisms - ps1
Archaea1 - ps2
Archaea2 - ps3
Archaea3 - ps4
Asgard/Eukaryota - ps5
Eukaryota - ps6
Amorpheae - ps7
Obazoa - ps8
Opisthokonta - ps9
Holozoa - ps10
Filozoa - ps11
Choano./Metazoa - ps12
Metazoa - ps13
Myriazoa - ps14
Parahoxozoa - ps15
Bilateria - ps16
Deuterostomia - ps17
Chordata - ps18
Olfactores - ps19
Vertebrata - ps20
Gnathostomata - ps21
Euteleostomi - ps22
Sarcopterygii - ps23
Tetrapoda - ps24
Amniota - ps25
Mammalia - ps26
Theria - ps27
Placentalia - ps28
Boreoeutheria - ps29
Euarchontoglires - ps30
Euarchonta - ps31
Primates - ps32
Haplorrhini - ps33
Simiiformes - ps34
Catarrhini - ps35
Hominoidea - ps36
Hominidae - ps37
Homininae - ps38
Hominini - ps39
Homo_sapiens - ps40
p-value
8e-22
7e-17
6e-12
5e-07
4e-02
Log-odds
0.5
1.0
1.5
2.0
2.5
3.0
3.5
4.0

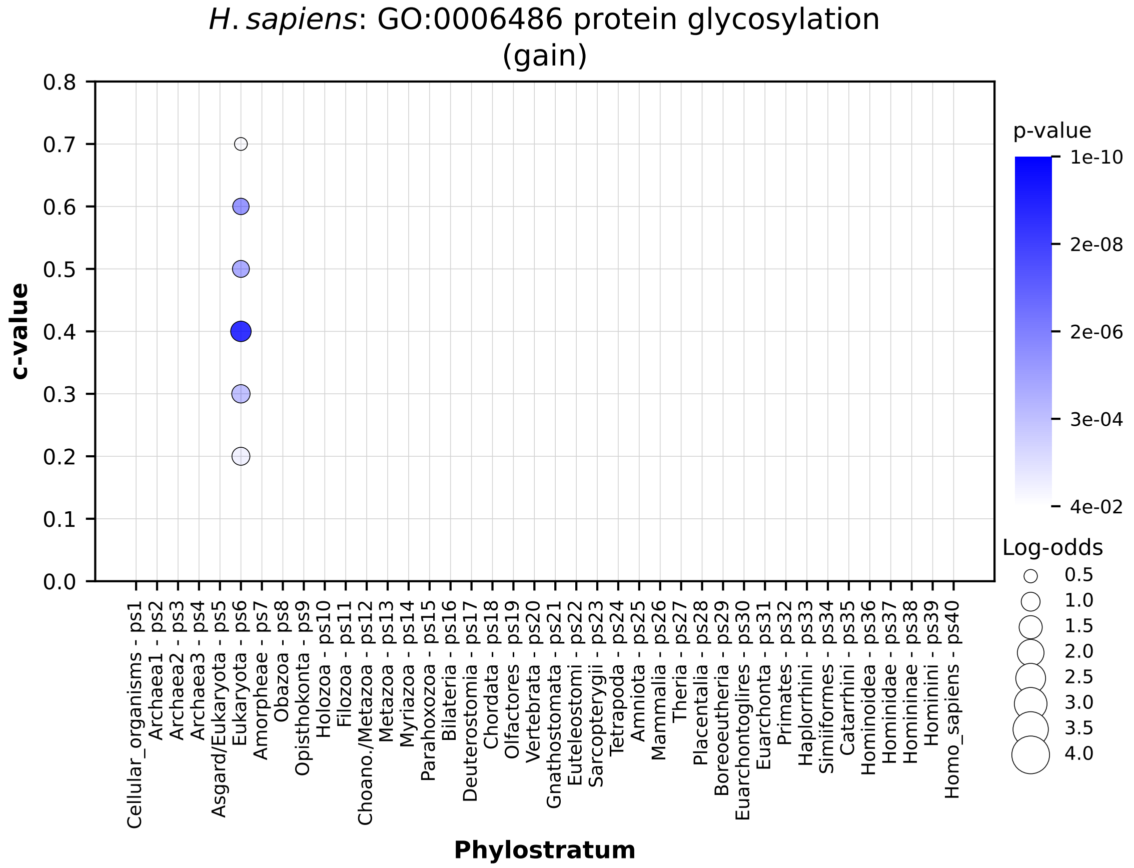

H. sapiens: GO:0006486 protein glycosylation
(gain)
c-value
0.8
0.7
0.6
0.5
0.4
0.3
0.2
0.1
0.0
Phylostratum
Cellular_organisms - ps1
Archaea1 - ps2
Archaea2 - ps3
Archaea3 - ps4
Asgard/Eukaryota - ps5
Eukaryota - ps6
Amorpheae - ps7
Obazoa - ps8
Opisthokonta - ps9
Holozoa - ps10
Filozoa - ps11
Choano./Metazoa - ps12
Metazoa - ps13
Myriazoa - ps14
Parahoxozoa - ps15
Bilateria - ps16
Deuterostomia - ps17
Chordata - ps18
Olfactores - ps19
Vertebrata - ps20
Gnathostomata - ps21
Euteleostomi - ps22
Sarcopterygii - ps23
Tetrapoda - ps24
Amniota - ps25
Mammalia - ps26
Theria - ps27
Placentalia - ps28
Boreoeutheria - ps29
Euarchontoglires - ps30
Euarchonta - ps31
Primates - ps32
Haplorrhini - ps33
Simiiformes - ps34
Catarrhini - ps35
Hominoidea - ps36
Hominidae - ps37
Homininae - ps38
Hominini - ps39
Homo_sapiens - ps40
p-value
1e-10
2e-08
2e-06
3e-04
4e-02
Log-odds
0.5
1.0
1.5
2.0
2.5
3.0
3.5
4.0

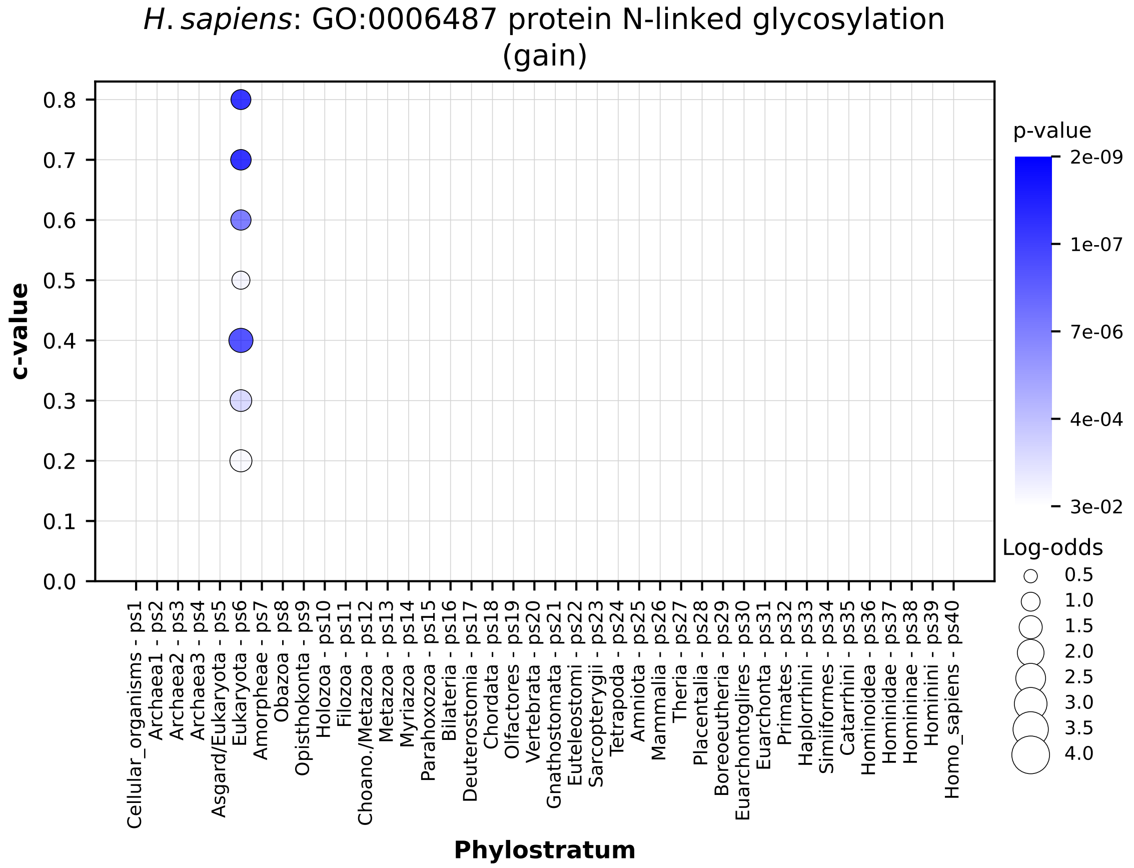

H. sapiens: GO:0006487 protein N-linked glycosylation
(gain)
c-value
0.0
0.1
0.2
0.3
0.4
0.5
0.6
0.7
0.8
p-value
2e-09
1e-07
7e-06
4e-04
3e-02
Log-odds
0.5
1.0
1.5
2.0
2.5
3.0
3.5
4.0
Cellular_organisms - ps1
Archaea1 - ps2
Archaea2 - ps3
Archaea3 - ps4
Asgard/Eukaryota - ps5
Eukaryota - ps6
Amorpheae - ps7
Obazoa - ps8
Opisthokonta - ps9
Holozoa - ps10
Filozoa - ps11
Choano./Metazoa - ps12
Metazoa - ps13
Myriazoa - ps14
Parahoxozoa - ps15
Bilateria - ps16
Deuterostomia - ps17
Chordata - ps18
Olfactores - ps19
Vertebrata - ps20
Gnathostomata - ps21
Euteleostomi - ps22
Sarcopterygii - ps23
Tetrapoda - ps24
Amniota - ps25
Mammalia - ps26
Theria - ps27
Placentalia - ps28
Boreoeutheria - ps29
Euarchontoglires - ps30
Euarchonta - ps31
Primates - ps32
Haplorrhini - ps33
Simiiformes - ps34
Catarrhini - ps35
Hominoidea - ps36
Hominidae - ps37
Homininae - ps38
Hominini - ps39
Homo_sapiens - ps40
Phylostratum

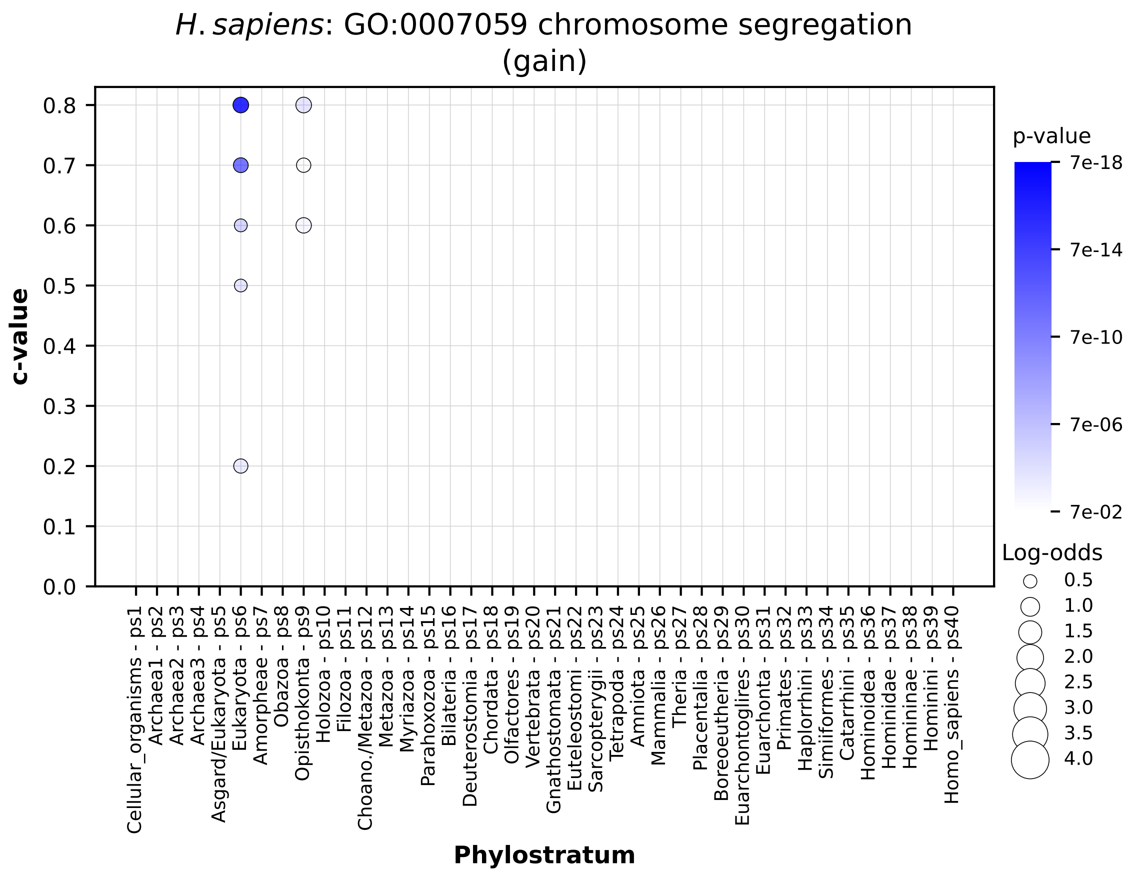

H. sapiens: GO:0007059 chromosome segregation
(gain)
c-value
0.0
0.1
0.2
0.3
0.4
0.5
0.6
0.7
0.8
Phylostratum
Cellular_organisms - ps1
Archaea1 - ps2
Archaea2 - ps3
Archaea3 - ps4
Asgard/Eukaryota - ps5
Eukaryota - ps6
Amorpheae - ps7
Obazoa - ps8
Opisthokonta - ps9
Holozoa - ps10
Filozoa - ps11
Choano./Metazoa - ps12
Metazoa - ps13
Myriazoa - ps14
Parahoxozoa - ps15
Bilateria - ps16
Deuterostomia - ps17
Chordata - ps18
Olfactores - ps19
Vertebrata - ps20
Gnathostomata - ps21
Euteleostomi - ps22
Sarcopterygii - ps23
Tetrapoda - ps24
Amniota - ps25
Mammalia - ps26
Theria - ps27
Placentalia - ps28
Boreoeutheria - ps29
Euarchontoglires - ps30
Euarchonta - ps31
Primates - ps32
Haplorrhini - ps33
Simiiformes - ps34
Catarrhini - ps35
Hominoidea - ps36
Hominidae - ps37
Homininae - ps38
Hominini - ps39
Homo_sapiens - ps40
p-value
7e-18
7e-14
7e-10
7e-06
7e-02
Log-odds
0.5
1.0
1.5
2.0
2.5
3.0
3.5
4.0

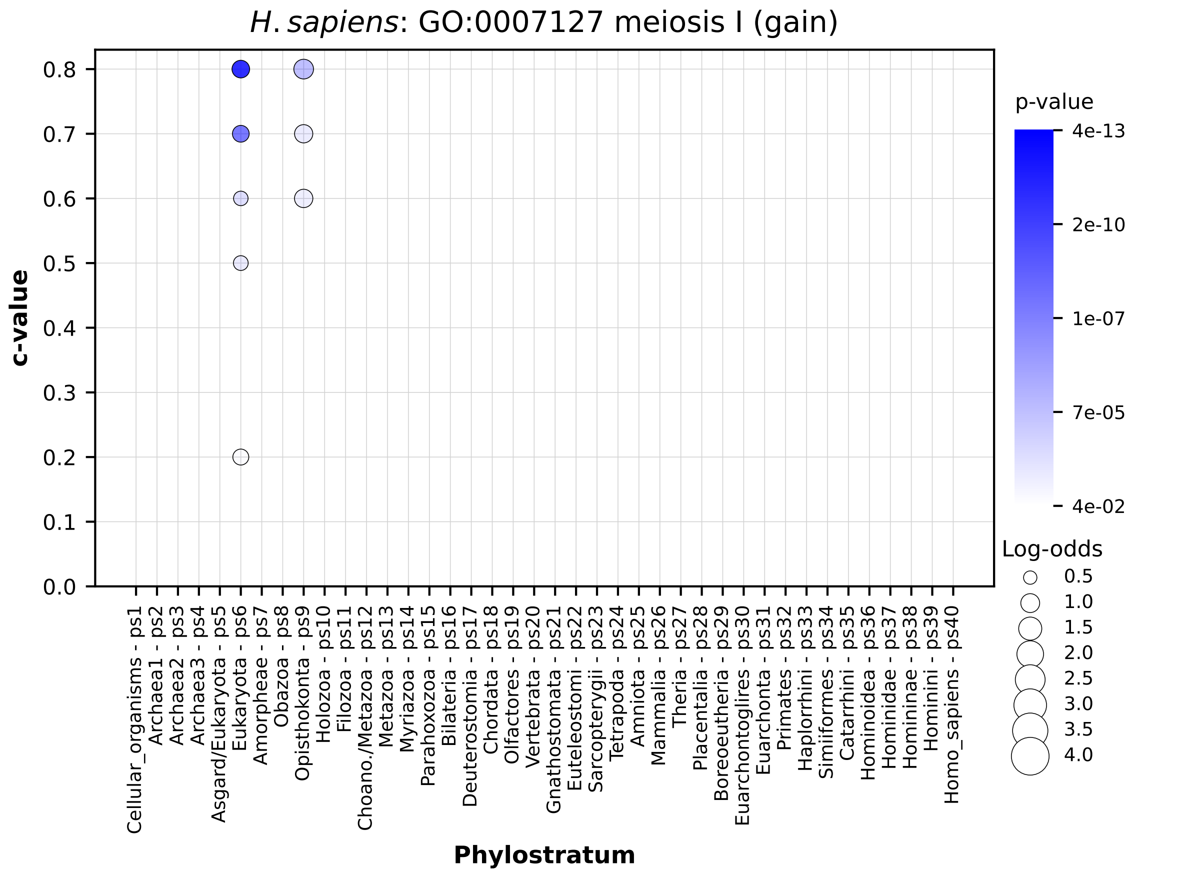

$H. sapiens$: GO:0007127 meiosis I (gain)
c-value
0.0
0.1
0.2
0.3
0.4
0.5
0.6
0.7
0.8
Phylostratum
Cellular_organisms - ps1
Archaea1 - ps2
Archaea2 - ps3
Archaea3 - ps4
Asgard/Eukaryota - ps5
Eukaryota - ps6
Amorpheae - ps7
Obazoa - ps8
Opisthokonta - ps9
Holozoa - ps10
Filozoa - ps11
Choano./Metazoa - ps12
Metazoa - ps13
Myriazoa - ps14
Parahoxozoa - ps15
Bilateria - ps16
Deuterostomia - ps17
Chordata - ps18
Olfactores - ps19
Vertebrata - ps20
Gnathostomata - ps21
Euteleostomi - ps22
Sarcopterygii - ps23
Tetrapoda - ps24
Amniota - ps25
Mammalia - ps26
Theria - ps27
Placentalia - ps28
Boreoeutheria - ps29
Euarchontoglires - ps30
Euarchonta - ps31
Primates - ps32
Haplorrhini - ps33
Simiiformes - ps34
Catarrhini - ps35
Hominoidea - ps36
Hominidae - ps37
Homininae - ps38
Hominini - ps39
Homo_sapiens - ps40
p-value
4e-13
2e-10
1e-07
7e-05
4e-02
Log-odds
0.5
1.0
1.5
2.0
2.5
3.0
3.5
4.0

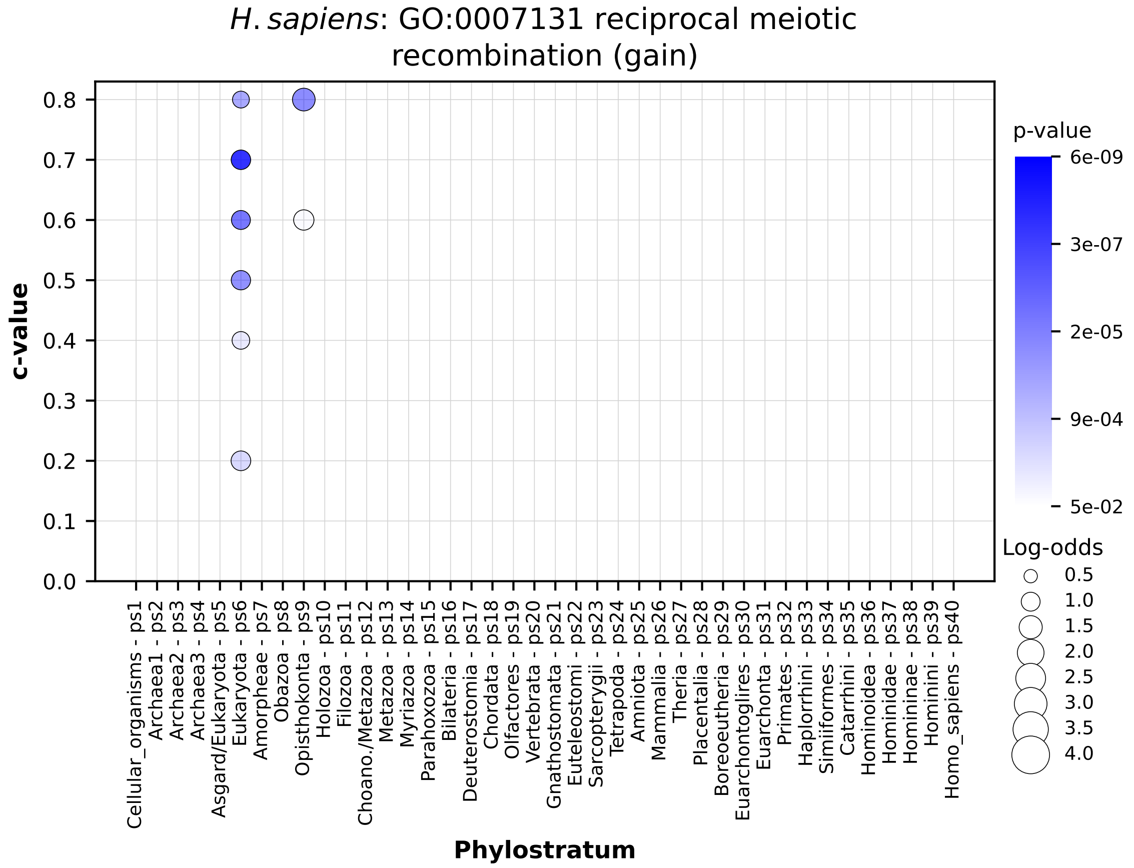
H. sapiens: GO:0007131 reciprocal meiotic recombination (gain)
c-value
0.0
0.1
0.2
0.3
0.4
0.5
0.6
0.7
0.8
Phylostratum
Cellular_organisms - ps1
Archaea1 - ps2
Archaea2 - ps3
Archaea3 - ps4
Asgard/Eukaryota - ps5
Eukaryota - ps6
Amorpheae - ps7
Obazoa - ps8
Opisthokonta - ps9
Holozoa - ps10
Filozoa - ps11
Choano./Metazoa - ps12
Metazoa - ps13
Myriazoa - ps14
Parahoxozoa - ps15
Bilateria - ps16
Deuterostomia - ps17
Chordata - ps18
Olfactores - ps19
Vertebrata - ps20
Gnathostomata - ps21
Euteleostomi - ps22
Sarcopterygii - ps23
Tetrapoda - ps24
Amniota - ps25
Mammalia - ps26
Theria - ps27
Placentalia - ps28
Boreoeutheria - ps29
Euarchontoglires - ps30
Euarchonta - ps31
Primates - ps32
Haplorrhini - ps33
Simiiformes - ps34
Catarrhini - ps35
Hominoidea - ps36
Hominidae - ps37
Homininae - ps38
Hominini - ps39
Homo_sapiens - ps40
p-value
6e-09
3e-07
2e-05
9e-04
5e-02
Log-odds
0.5
1.0
1.5
2.0
2.5
3.0
3.5
4.0

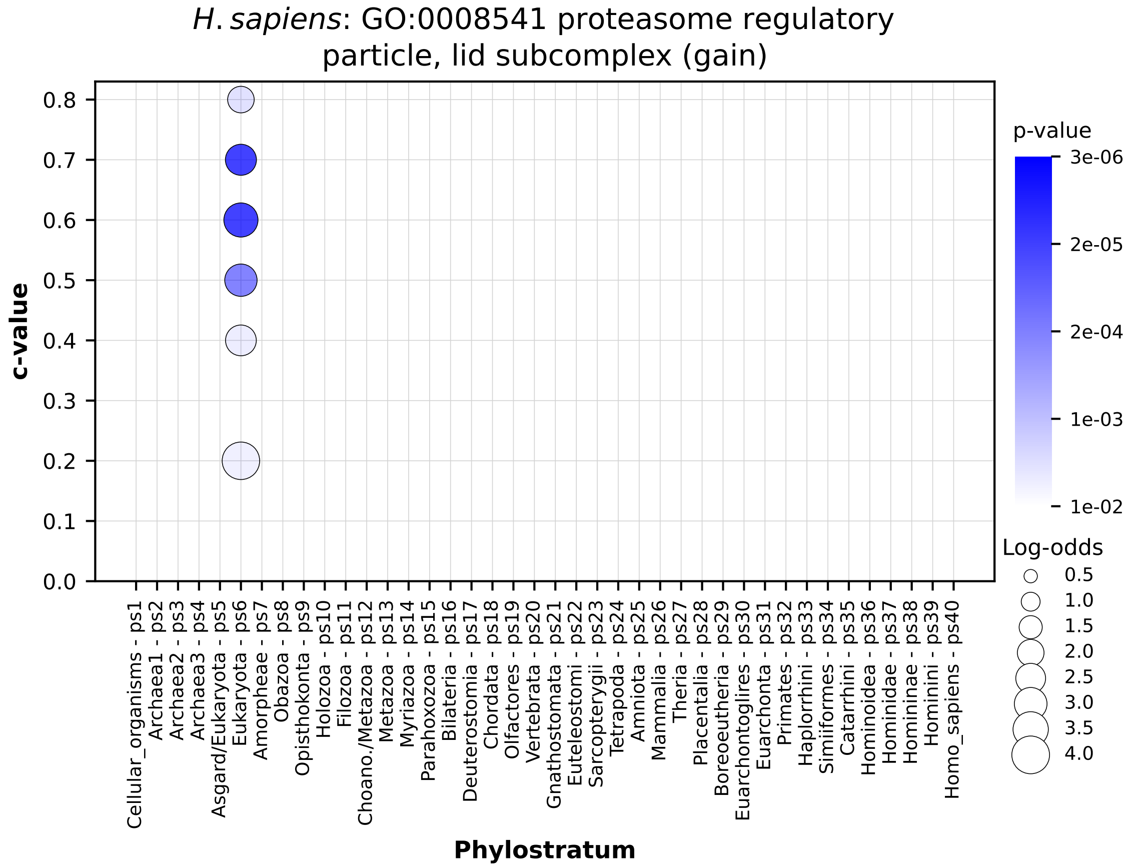

H. sapiens: GO:0008541 proteasome regulatory particle, lid subcomplex (gain)
c-value
0.0
0.1
0.2
0.3
0.4
0.5
0.6
0.7
0.8
Phylostratum
Cellular_organisms - ps1
Archaea1 - ps2
Archaea2 - ps3
Archaea3 - ps4
Asgard/Eukaryota - ps5
Eukaryota - ps6
Amorpheae - ps7
Obazoa - ps8
Opisthokonta - ps9
Holozoa - ps10
Filozoa - ps11
Choano./Metazoa - ps12
Metazoa - ps13
Myriazoa - ps14
Parahoxozoa - ps15
Bilateria - ps16
Deuterostomia - ps17
Chordata - ps18
Olfactores - ps19
Vertebrata - ps20
Gnathostomata - ps21
Euteleostomi - ps22
Sarcopterygii - ps23
Tetrapoda - ps24
Amniota - ps25
Mammalia - ps26
Theria - ps27
Placentalia - ps28
Boreoeutheria - ps29
Euarchontoglires - ps30
Euarchonta - ps31
Primates - ps32
Haplorrhini - ps33
Simiiformes - ps34
Catarrhini - ps35
Hominoidea - ps36
Hominidae - ps37
Homininae - ps38
Hominini - ps39
Homo_sapiens - ps40
p-value
3e-06
2e-05
2e-04
1e-03
1e-02
Log-odds
0.5
1.0
1.5
2.0
2.5
3.0
3.5
4.0

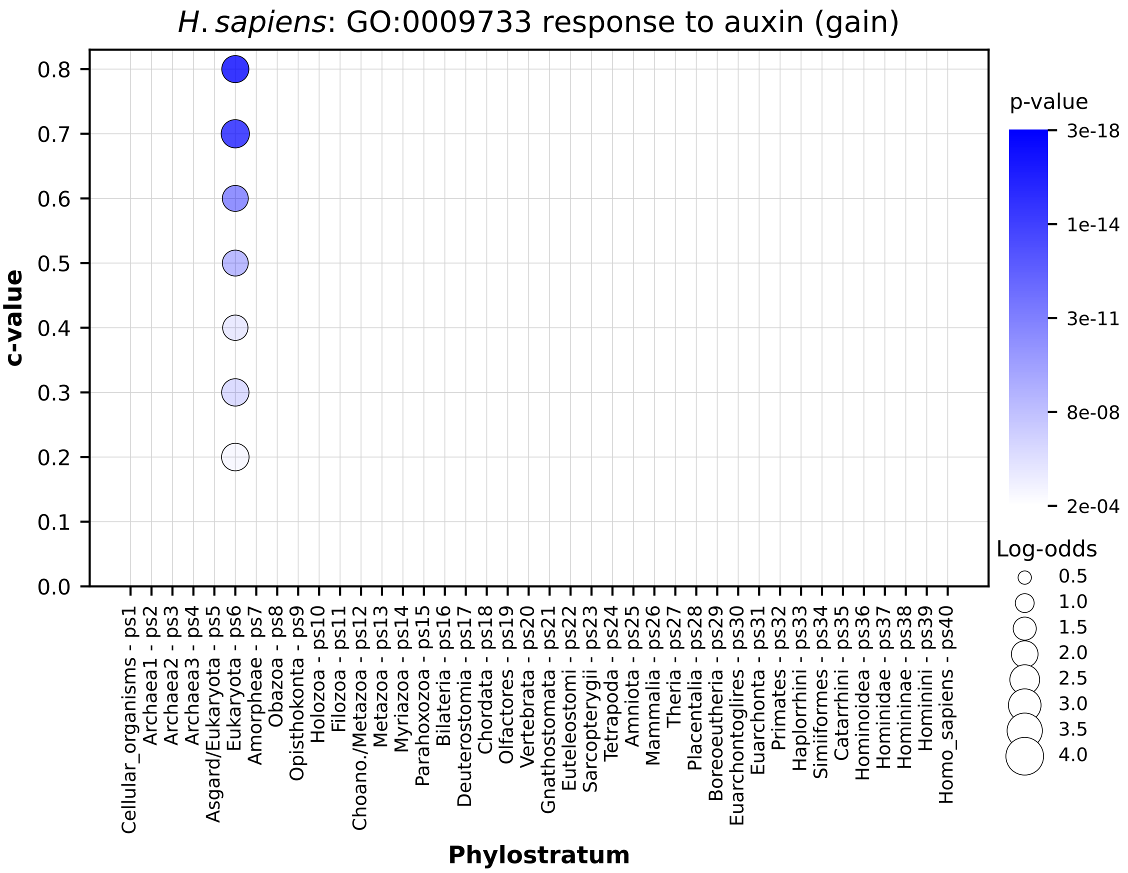

H. sapiens: GO:0009733 response to auxin (gain)
c-value
0.0
0.1
0.2
0.3
0.4
0.5
0.6
0.7
0.8
Phylostratum
Cellular_organisms - ps1
Archaea1 - ps2
Archaea2 - ps3
Archaea3 - ps4
Asgard/Eukaryota - ps5
Eukaryota - ps6
Amorpheae - ps7
Obazoa - ps8
Opisthokonta - ps9
Holozoa - ps10
Filozoa - ps11
Choano./Metazoa - ps12
Metazoa - ps13
Myriazoa - ps14
Parahoxozoa - ps15
Bilateria - ps16
Deuterostomia - ps17
Chordata - ps18
Olfactores - ps19
Vertebrata - ps20
Gnathostomata - ps21
Euteleostomi - ps22
Sarcopterygii - ps23
Tetrapoda - ps24
Amniota - ps25
Mammalia - ps26
Theria - ps27
Placentalia - ps28
Boreoeutheria - ps29
Euarchontoglires - ps30
Euarchonta - ps31
Primates - ps32
Haplorrhini - ps33
Simiiformes - ps34
Catarrhini - ps35
Hominoidea - ps36
Hominidae - ps37
Homininae - ps38
Hominini - ps39
Homo_sapiens - ps40
p-value
3e-18
1e-14
3e-11
8e-08
2e-04
Log-odds
0.5
1.0
1.5
2.0
2.5
3.0
3.5
4.0

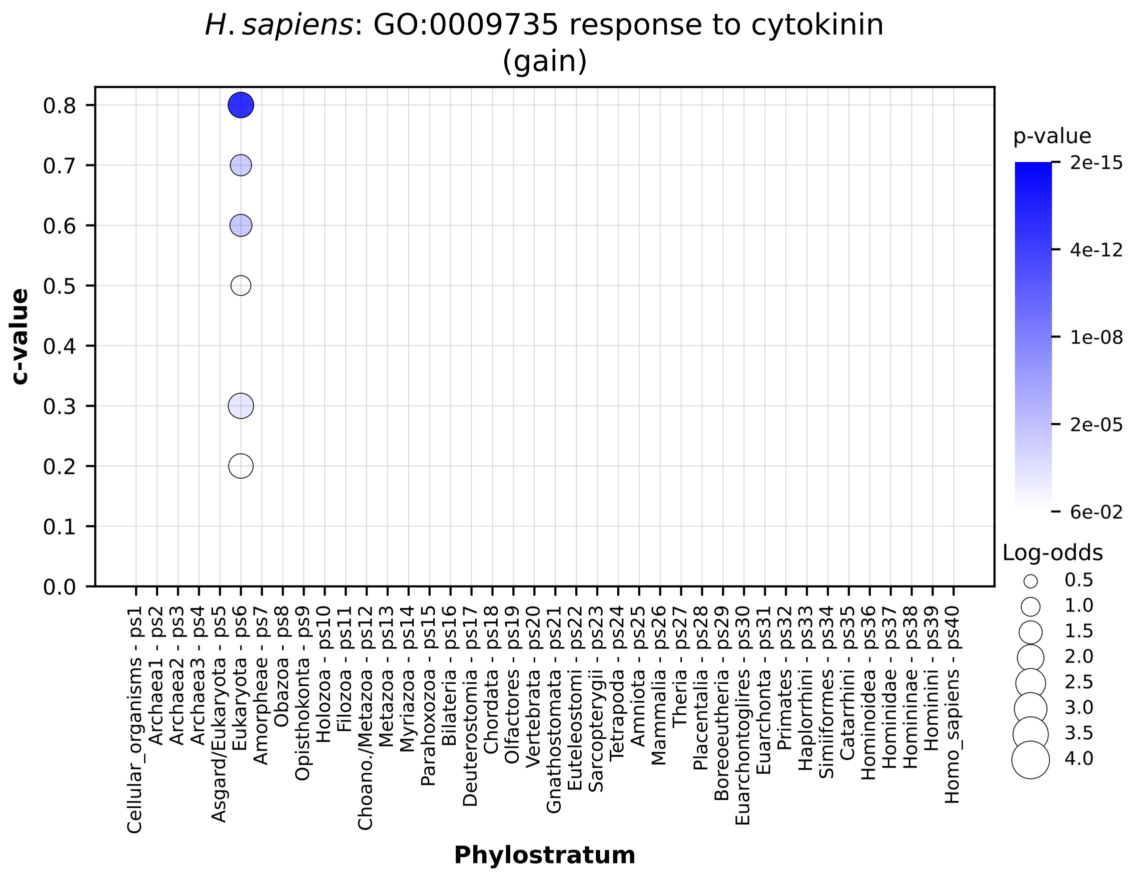

H. sapiens: GO:0009735 response to cytokinin
(gain)
c-value
0.0
0.1
0.2
0.3
0.4
0.5
0.6
0.7
0.8
Phylostratum
Cellular_organisms - ps1
Archaea1 - ps2
Archaea2 - ps3
Archaea3 - ps4
Asgard/Eukaryota - ps5
Eukaryota - ps6
Amorpheae - ps7
Obazoa - ps8
Opisthokonta - ps9
Holozoa - ps10
Filozoa - ps11
Choano./Metazoa - ps12
Metazoa - ps13
Myriazoa - ps14
Parahoxozoa - ps15
Bilateria - ps16
Deuterostomia - ps17
Chordata - ps18
Olfactores - ps19
Vertebrata - ps20
Gnathostomata - ps21
Euteleostomi - ps22
Sarcopterygii - ps23
Tetrapoda - ps24
Amniota - ps25
Mammalia - ps26
Theria - ps27
Placentalia - ps28
Boreoeutheria - ps29
Euarchontoglires - ps30
Euarchonta - ps31
Primates - ps32
Haplorrhini - ps33
Simiiformes - ps34
Catarrhini - ps35
Hominoidea - ps36
Hominidae - ps37
Homininae - ps38
Hominini - ps39
Homo_sapiens - ps40
p-value
2e-15
4e-12
1e-08
2e-05
6e-02
Log-odds
0.5
1.0
1.5
2.0
2.5
3.0
3.5
4.0

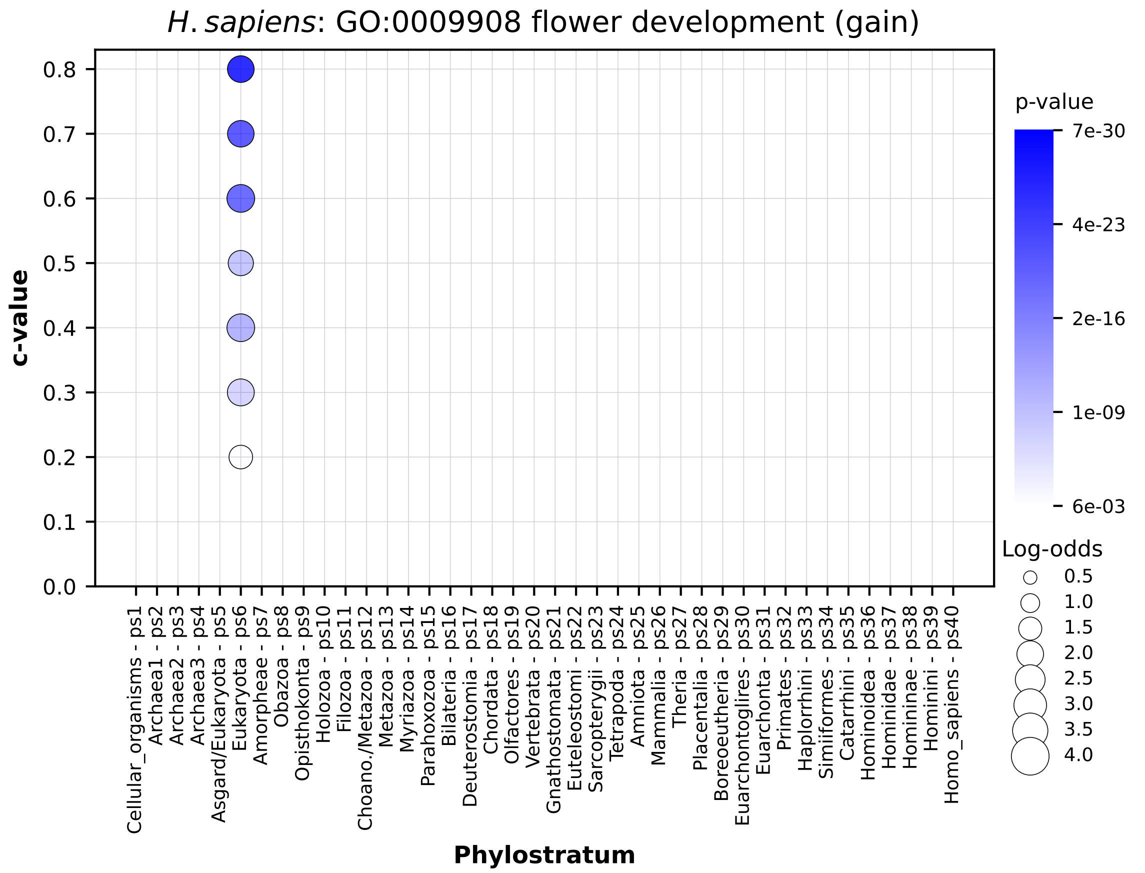

H. sapiens: GO:0009908 flower development (gain)
c-value
0.8
0.7
0.6
0.5
0.4
0.3
0.2
0.1
0.0
p-value
7e-30
4e-23
2e-16
1e-09
6e-03
Log-odds
0.5
1.0
1.5
2.0
2.5
3.0
3.5
4.0
Cellular_organisms - ps1
Archaea1 - ps2
Archaea2 - ps3
Archaea3 - ps4
Asgard/Eukaryota - ps5
Eukaryota - ps6
Amorpheae - ps7
Obazoa - ps8
Opisthokonta - ps9
Holozoa - ps10
Filozoa - ps11
Choano./Metazoa - ps12
Metazoa - ps13
Myriazoa - ps14
Parahoxozoa - ps15
Bilateria - ps16
Deuterostomia - ps17
Chordata - ps18
Olfactores - ps19
Vertebrata - ps20
Gnathostomata - ps21
Euteleostomi - ps22
Sarcopterygii - ps23
Tetrapoda - ps24
Amniota - ps25
Mammalia - ps26
Theria - ps27
Placentalia - ps28
Boreoeutheria - ps29
Euarchontoglires - ps30
Euarchonta - ps31
Primates - ps32
Haplorrhini - ps33
Simiiformes - ps34
Catarrhini - ps35
Hominoidea - ps36
Hominidae - ps37
Homininae - ps38
Hominini - ps39
Homo_sapiens - ps40
Phylostratum

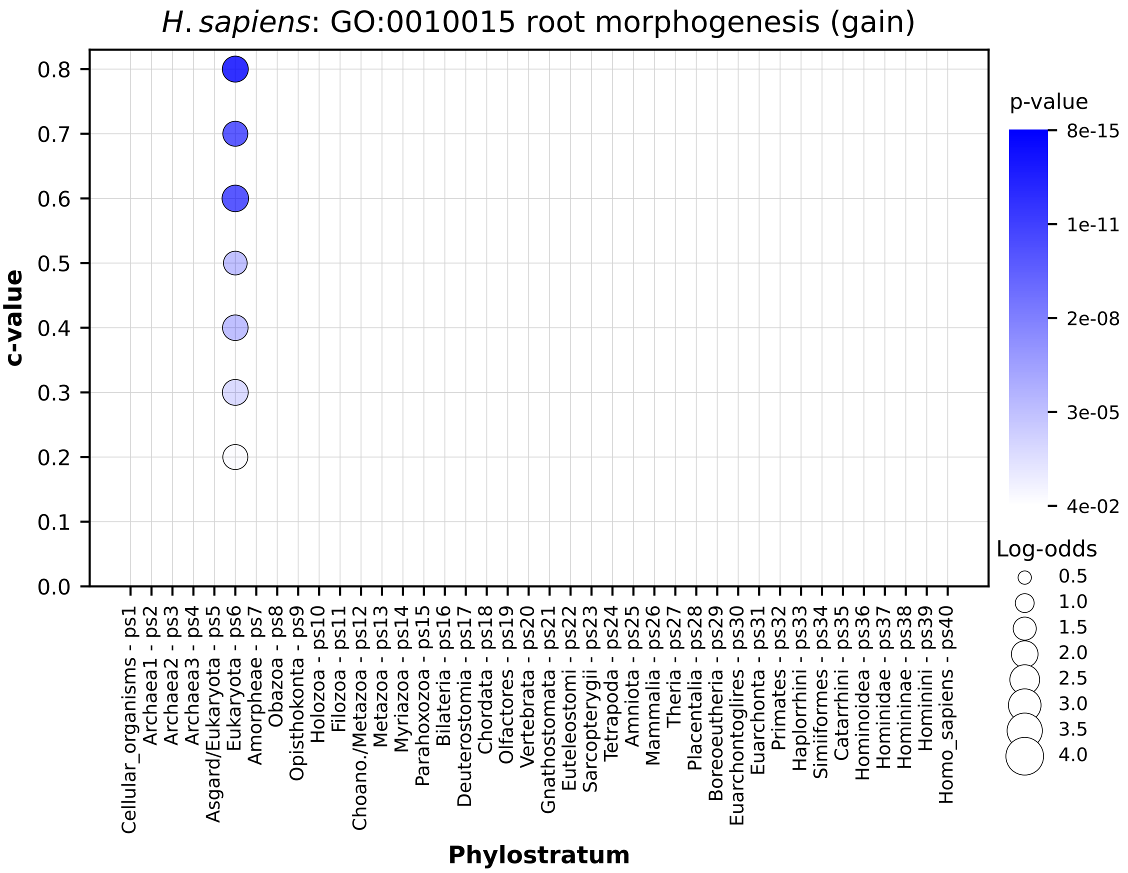

*H. sapiens*: GO:0010015 root morphogenesis (gain)
c-value
0.8
0.7
0.6
0.5
0.4
0.3
0.2
0.1
0.0
Phylostratum
Cellular_organisms - ps1
Archaea1 - ps2
Archaea2 - ps3
Archaea3 - ps4
Asgard/Eukaryota - ps5
Eukaryota - ps6
Amorpheae - ps7
Obazoa - ps8
Opisthokonta - ps9
Holozoa - ps10
Filozoa - ps11
Choano./Metazoa - ps12
Metazoa - ps13
Myriazoa - ps14
Parahoxozoa - ps15
Bilateria - ps16
Deuterostomia - ps17
Chordata - ps18
Olfactores - ps19
Vertebrata - ps20
Gnathostomata - ps21
Euteleostomi - ps22
Sarcopterygii - ps23
Tetrapoda - ps24
Amniota - ps25
Mammalia - ps26
Theria - ps27
Placentalia - ps28
Boreoeutheria - ps29
Euarchontoglires - ps30
Euarchonta - ps31
Primates - ps32
Haplorrhini - ps33
Simiiformes - ps34
Catarrhini - ps35
Hominoidea - ps36
Hominidae - ps37
Homininae - ps38
Hominini - ps39
Homo_sapiens - ps40
p-value
8e-15
1e-11
2e-08
3e-05
4e-02
Log-odds
0.5
1.0
1.5
2.0
2.5
3.0
3.5
4.0

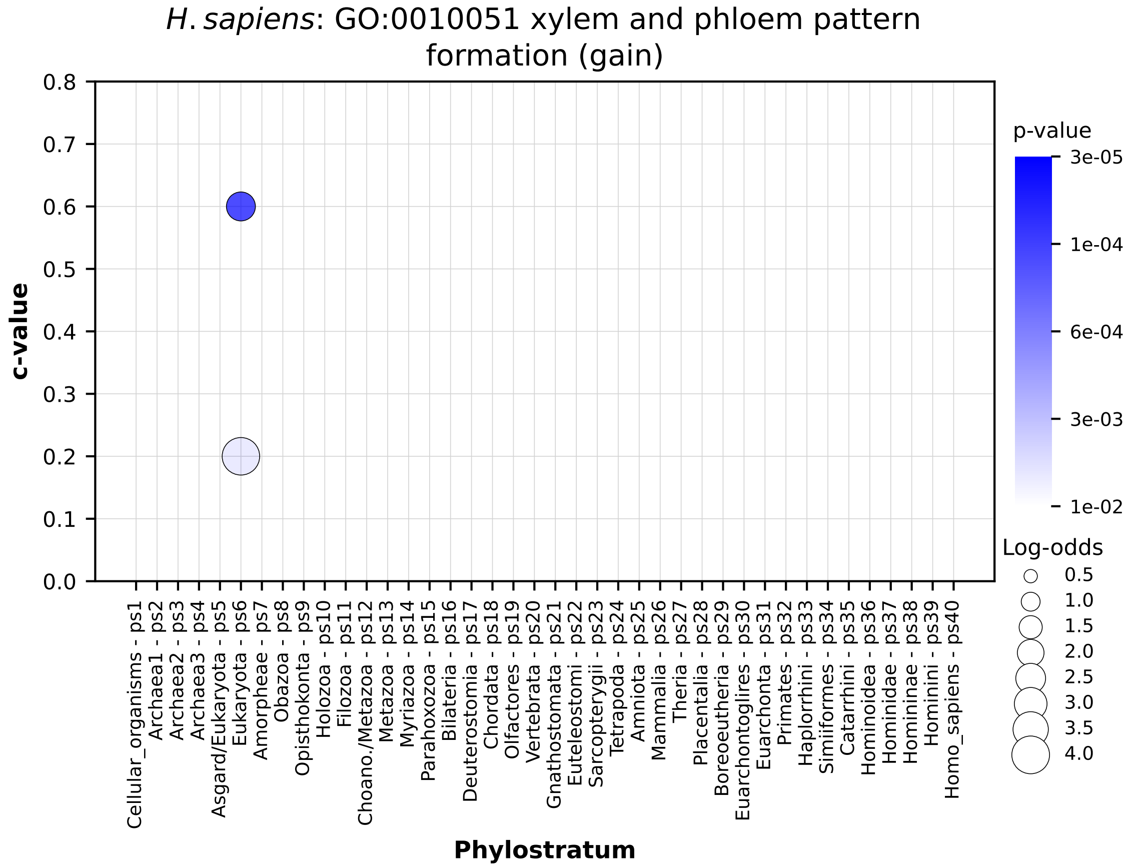
H. sapiens: GO:0010051 xylem and phloem pattern formation (gain)
c-value
0.0
0.1
0.2
0.3
0.4
0.5
0.6
0.7
0.8
Phylostratum
Cellular_organisms - ps1
Archaea1 - ps2
Archaea2 - ps3
Archaea3 - ps4
Asgard/Eukaryota - ps5
Eukaryota - ps6
Amorpheae - ps7
Obazoa - ps8
Opisthokonta - ps9
Holozoa - ps10
Filozoa - ps11
Choano./Metazoa - ps12
Metazoa - ps13
Myriazoa - ps14
Parahoxozoa - ps15
Bilateria - ps16
Deuterostomia - ps17
Chordata - ps18
Olfactores - ps19
Vertebrata - ps20
Gnathostomata - ps21
Euteleostomi - ps22
Sarcopterygii - ps23
Tetrapoda - ps24
Amniota - ps25
Mammalia - ps26
Theria - ps27
Placentalia - ps28
Boreoeutheria - ps29
Euarchontoglires - ps30
Euarchonta - ps31
Primates - ps32
Haplorrhini - ps33
Simiiformes - ps34
Catarrhini - ps35
Hominoidea - ps36
Hominidae - ps37
Homininae - ps38
Hominini - ps39
Homo_sapiens - ps40
p-value
3e-05
1e-04
6e-04
3e-03
1e-02
Log-odds
0.5
1.0
1.5
2.0
2.5
3.0
3.5
4.0

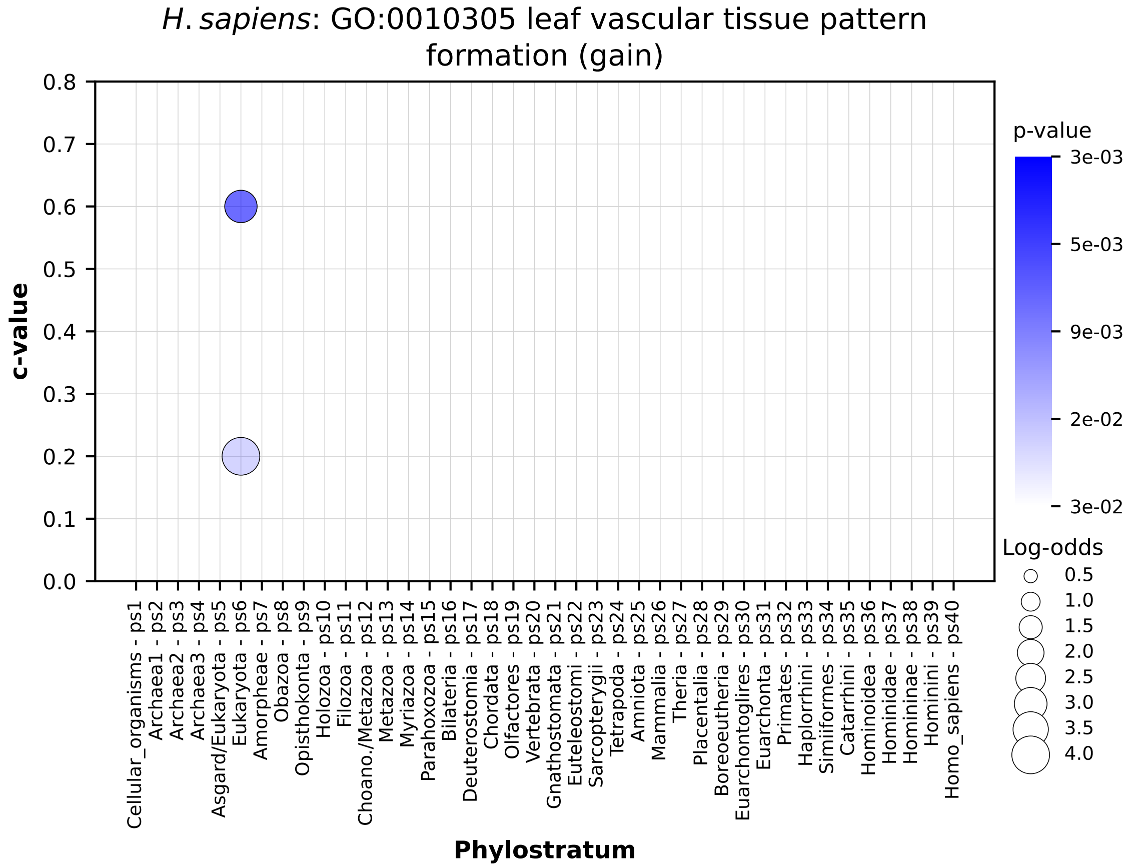

*H. sapiens*: GO:0010305 leaf vascular tissue pattern formation (gain)
c-value
0.0
0.1
0.2
0.3
0.4
0.5
0.6
0.7
0.8
Phylostratum
Cellular_organisms - ps1
Archaea1 - ps2
Archaea2 - ps3
Archaea3 - ps4
Asgard/Eukaryota - ps5
Eukaryota - ps6
Amorpheae - ps7
Obazoa - ps8
Opisthokonta - ps9
Holozoa - ps10
Filozoa - ps11
Choano./Metazoa - ps12
Metazoa - ps13
Myriazoa - ps14
Parahoxozoa - ps15
Bilateria - ps16
Deuterostomia - ps17
Chordata - ps18
Olfactores - ps19
Vertebrata - ps20
Gnathostomata - ps21
Euteleostomi - ps22
Sarcopterygii - ps23
Tetrapoda - ps24
Amniota - ps25
Mammalia - ps26
Theria - ps27
Placentalia - ps28
Boreoeutheria - ps29
Euarchontoglires - ps30
Euarchonta - ps31
Primates - ps32
Haplorrhini - ps33
Simiiformes - ps34
Catarrhini - ps35
Hominoidea - ps36
Hominidae - ps37
Homininae - ps38
Hominini - ps39
Homo_sapiens - ps40
p-value
3e-03
5e-03
9e-03
2e-02
3e-02
Log-odds
0.5
1.0
1.5
2.0
2.5
3.0
3.5
4.0

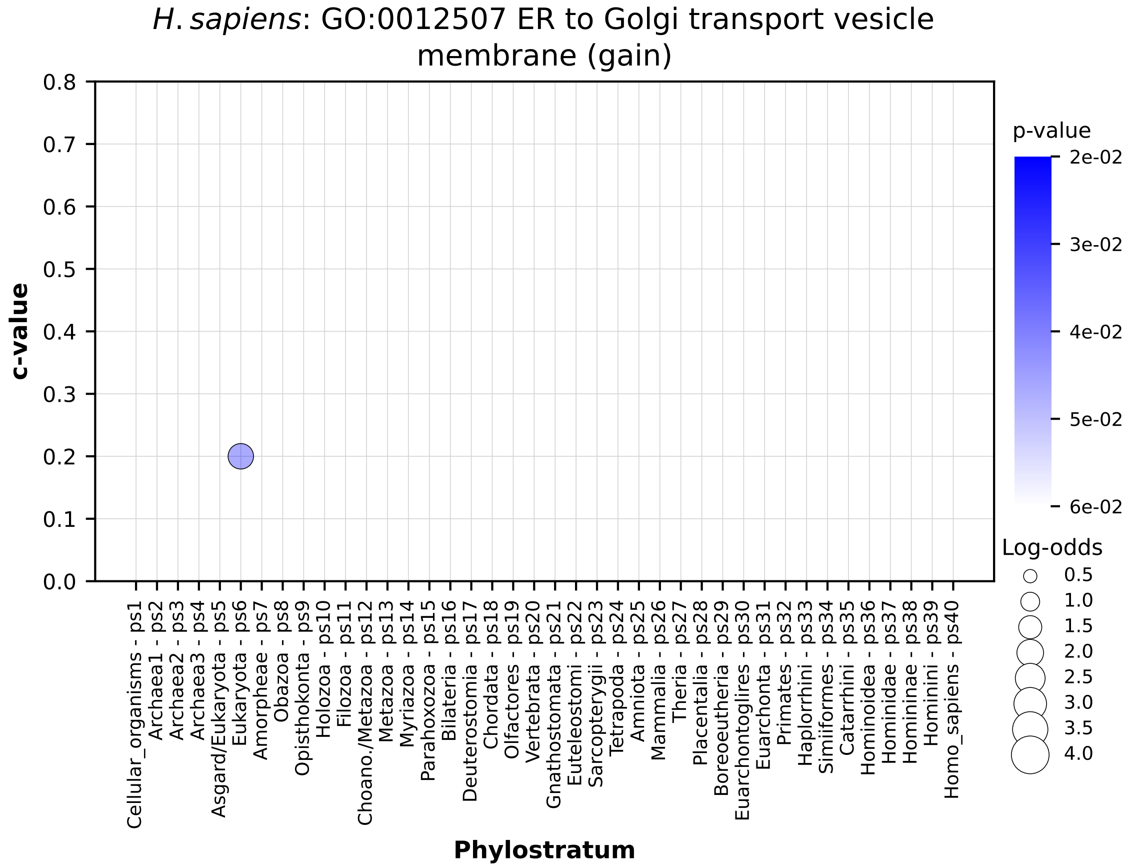

*H. sapiens*: GO:0012507 ER to Golgi transport vesicle membrane (gain)
c-value
0.8
0.7
0.6
0.5
0.4
0.3
0.2
0.1
0.0
Cellular_organisms - ps1
Archaea1 - ps2
Archaea2 - ps3
Archaea3 - ps4
Asgard/Eukaryota - ps5
Eukaryota - ps6
Amorpheae - ps7
Obazoa - ps8
Opisthokonta - ps9
Holozoa - ps10
Filozoa - ps11
Choano./Metazoa - ps12
Metazoa - ps13
Myriazoa - ps14
Parahoxozoa - ps15
Bilateria - ps16
Deuterostomia - ps17
Chordata - ps18
Olfactores - ps19
Vertebrata - ps20
Gnathostomata - ps21
Euteleostomi - ps22
Sarcopterygii - ps23
Tetrapoda - ps24
Amniota - ps25
Mammalia - ps26
Theria - ps27
Placentalia - ps28
Boreoeutheria - ps29
Euarchontoglires - ps30
Euarchonta - ps31
Primates - ps32
Haplorrhini - ps33
Simiiformes - ps34
Catarrhini - ps35
Hominoidea - ps36
Hominidae - ps37
Homininae - ps38
Hominini - ps39
Homo_sapiens - ps40
Phylostratum
p-value
2e-02
3e-02
4e-02
5e-02
6e-02
Log-odds
0.5
1.0
1.5
2.0
2.5
3.0
3.5
4.0

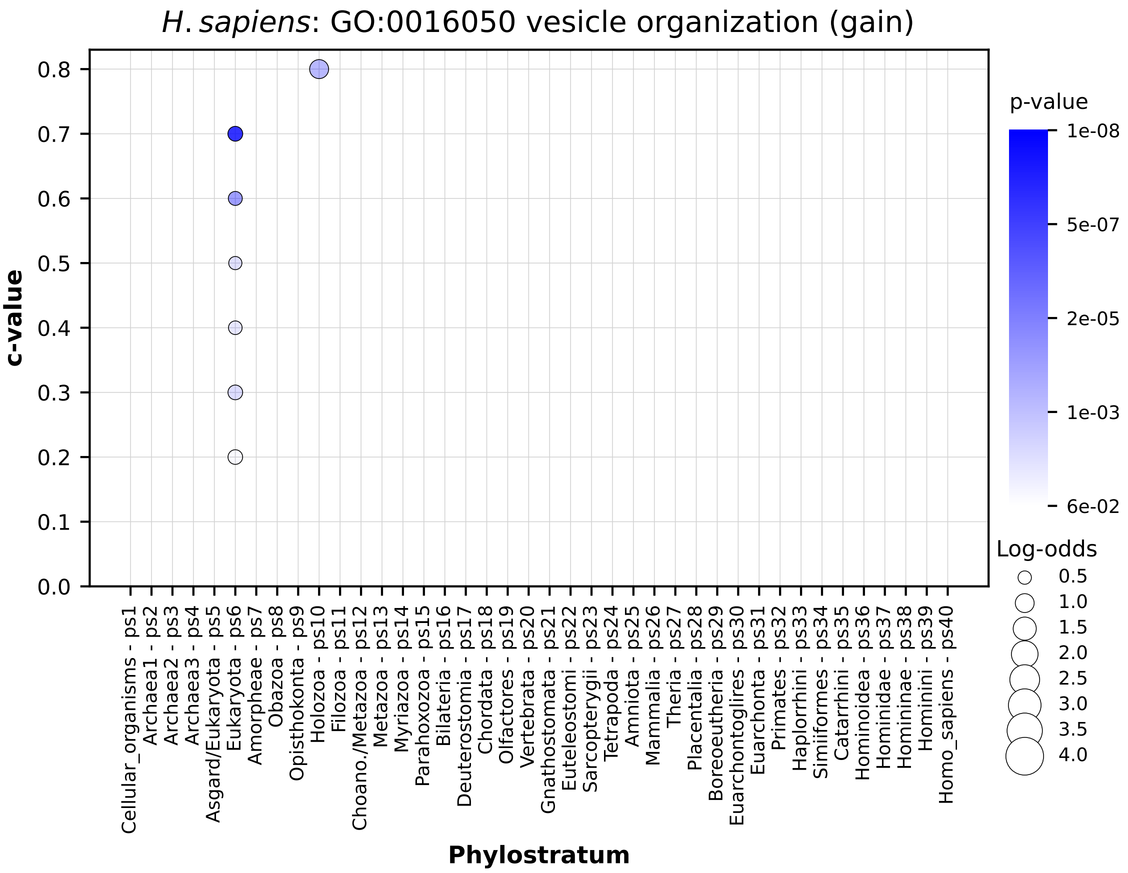
H. sapiens: GO:0016050 vesicle organization (gain)
c-value
0.0
0.1
0.2
0.3
0.4
0.5
0.6
0.7
0.8
Phylostratum
Cellular_organisms - ps1
Archaea1 - ps2
Archaea2 - ps3
Archaea3 - ps4
Asgard/Eukaryota - ps5
Eukaryota - ps6
Amorpheae - ps7
Obazoa - ps8
Opisthokonta - ps9
Holozoa - ps10
Filozoa - ps11
Choano./Metazoa - ps12
Metazoa - ps13
Myriazoa - ps14
Parahoxozoa - ps15
Bilateria - ps16
Deuterostomia - ps17
Chordata - ps18
Olfactores - ps19
Vertebrata - ps20
Gnathostomata - ps21
Euteleostomi - ps22
Sarcopterygii - ps23
Tetrapoda - ps24
Amniota - ps25
Mammalia - ps26
Theria - ps27
Placentalia - ps28
Boreoeutheria - ps29
Euarchontoglires - ps30
Euarchonta - ps31
Primates - ps32
Haplorrhini - ps33
Simiiformes - ps34
Catarrhini - ps35
Hominoidea - ps36
Hominidae - ps37
Homininae - ps38
Hominini - ps39
Homo_sapiens - ps40
p-value
1e-08
5e-07
2e-05
1e-03
6e-02
Log-odds
0.5
1.0
1.5
2.0
2.5
3.0
3.5
4.0

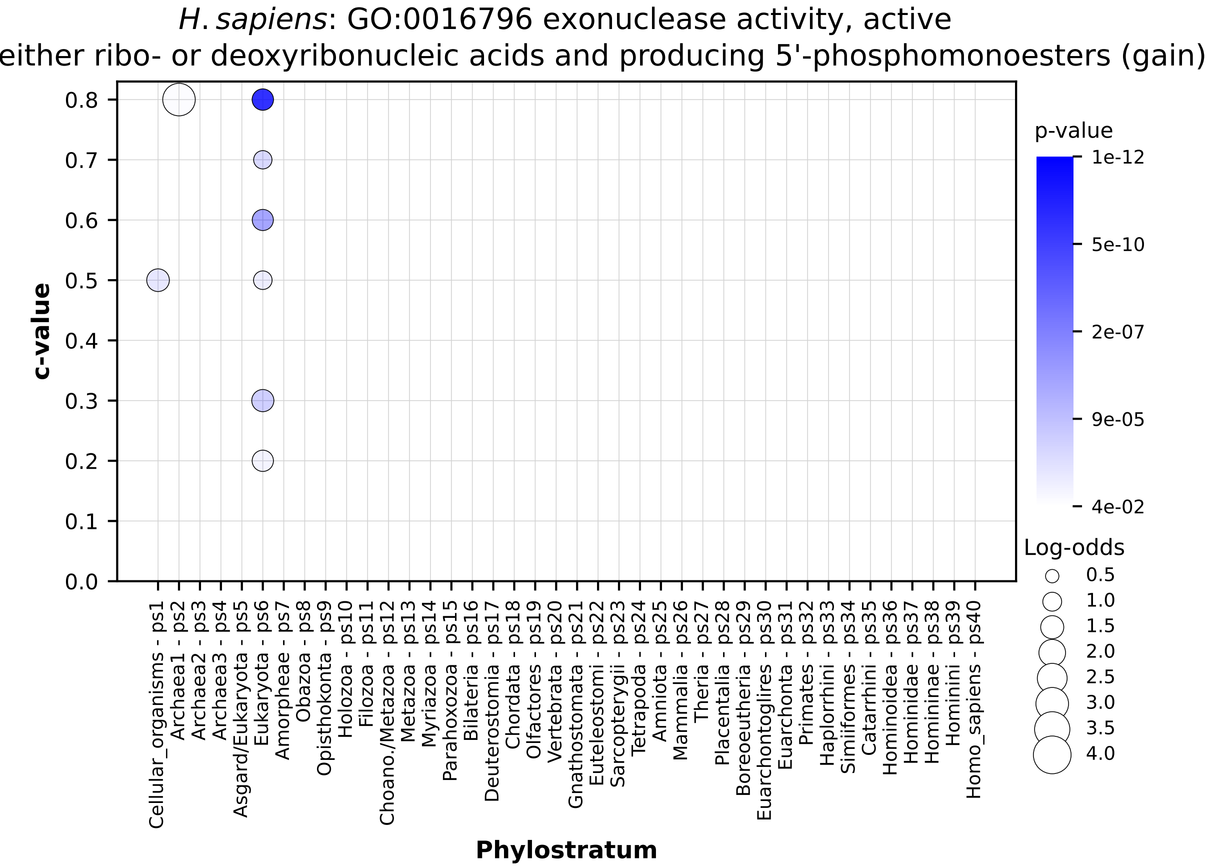

H. sapiens: GO:0016796 exonuclease activity, active
either ribo- or deoxyribonucleic acids and producing 5'-phosphomonoesters (gain)
c-value
Phylostratum
p-value
1e-12
5e-10
2e-07
9e-05
4e-02
Log-odds
0.5
1.0
1.5
2.0
2.5
3.0
3.5
4.0
Cellular_organisms - ps1
Archaea1 - ps2
Archaea2 - ps3
Archaea3 - ps4
Asgard/Eukaryota - ps5
Eukaryota - ps6
Amorpheae - ps7
Obazoa - ps8
Opisthokonta - ps9
Holozoa - ps10
Filozoa - ps11
Choano./Metazoa - ps12
Metazoa - ps13
Myriazoa - ps14
Parahoxozoa - ps15
Bilateria - ps16
Deuterostomia - ps17
Chordata - ps18
Olfactores - ps19
Vertebrata - ps20
Gnathostomata - ps21
Euteleostomi - ps22
Sarcopterygii - ps23
Tetrapoda - ps24
Amniota - ps25
Mammalia - ps26
Theria - ps27
Placentalia - ps28
Boreoeutheria - ps29
Euarchontoglires - ps30
Euarchonta - ps31
Primates - ps32
Haplorrhini - ps33
Simiiformes - ps34
Catarrhini - ps35
Hominoidea - ps36
Hominidae - ps37
Homininae - ps38
Hominini - ps39
Homo_sapiens - ps40

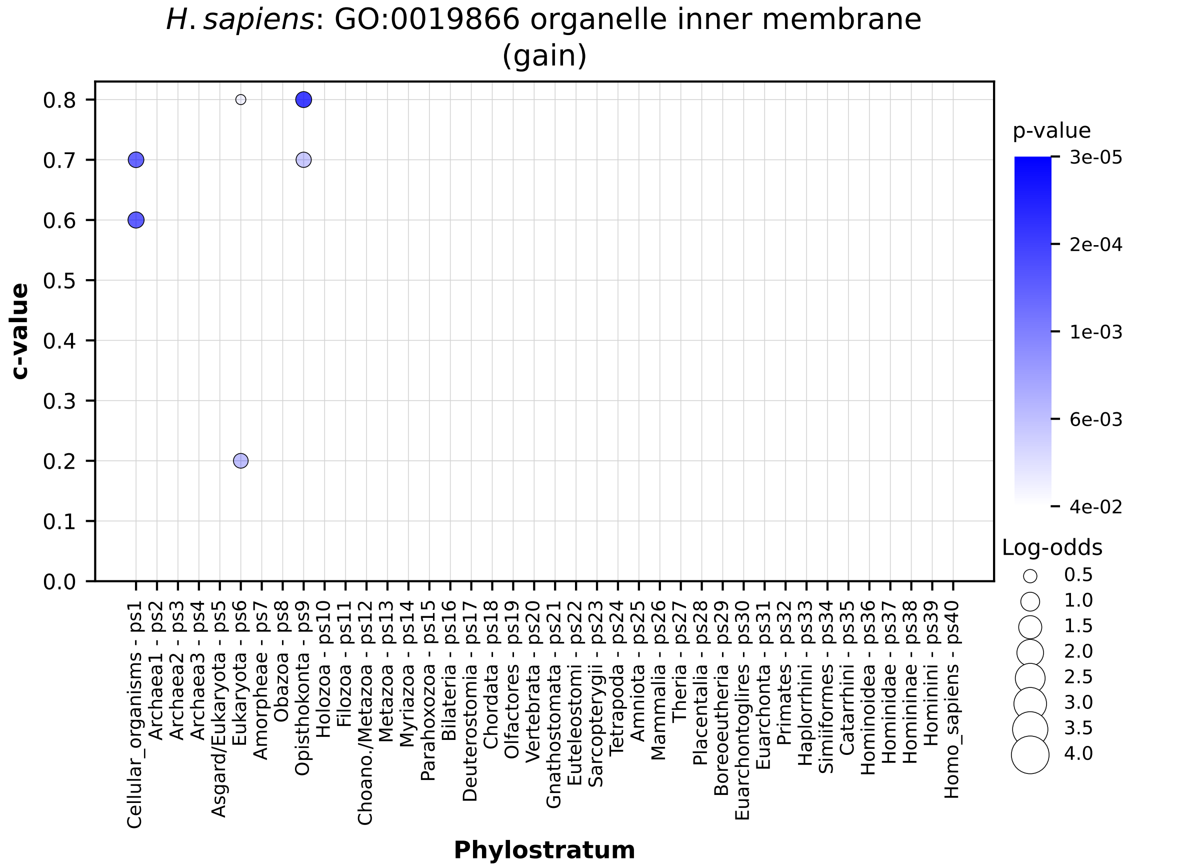

H. sapiens: GO:0019866 organelle inner membrane
(gain)
c-value
0.8
0.7
0.6
0.5
0.4
0.3
0.2
0.1
0.0
p-value
3e-05
2e-04
1e-03
6e-03
4e-02
Log-odds
0.5
1.0
1.5
2.0
2.5
3.0
3.5
4.0
Cellular_organisms - ps1
Archaea1 - ps2
Archaea2 - ps3
Archaea3 - ps4
Asgard/Eukaryota - ps5
Eukaryota - ps6
Amorpheae - ps7
Obazoa - ps8
Opisthokonta - ps9
Holozoa - ps10
Filozoa - ps11
Choano./Metazoa - ps12
Metazoa - ps13
Myriazoa - ps14
Parahoxozoa - ps15
Bilateria - ps16
Deuterostomia - ps17
Chordata - ps18
Olfactores - ps19
Vertebrata - ps20
Gnathostomata - ps21
Euteleostomi - ps22
Sarcopterygii - ps23
Tetrapoda - ps24
Amniota - ps25
Mammalia - ps26
Theria - ps27
Placentalia - ps28
Boreoeutheria - ps29
Euarchontoglires - ps30
Euarchonta - ps31
Primates - ps32
Haplorrhini - ps33
Simiiformes - ps34
Catarrhini - ps35
Hominoidea - ps36
Hominidae - ps37
Homininae - ps38
Hominini - ps39
Homo_sapiens - ps40
Phylostratum

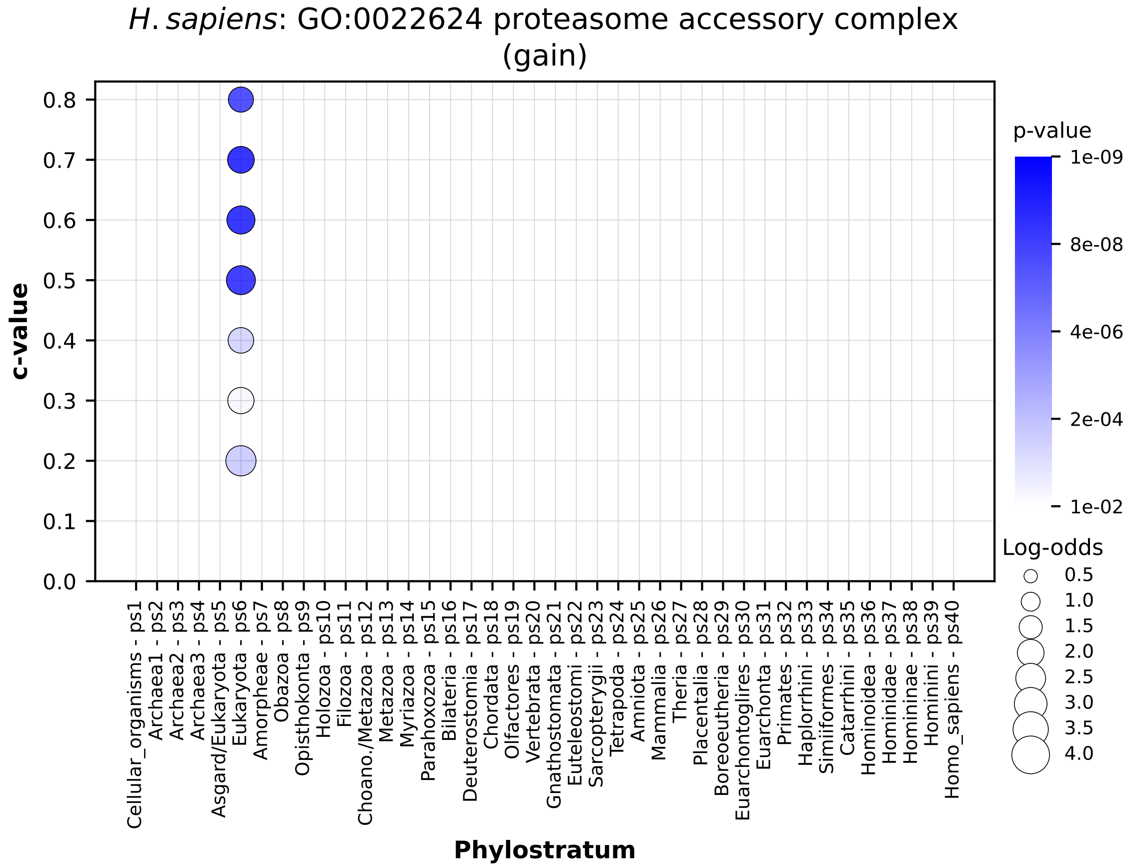

H. sapiens: GO:0022624 proteasome accessory complex
(gain)
c-value
0.0
0.1
0.2
0.3
0.4
0.5
0.6
0.7
0.8
Phylostratum
Cellular_organisms - ps1
Archaea1 - ps2
Archaea2 - ps3
Archaea3 - ps4
Asgard/Eukaryota - ps5
Eukaryota - ps6
Amorpheae - ps7
Obazoa - ps8
Opisthokonta - ps9
Holozoa - ps10
Filozoa - ps11
Choano./Metazoa - ps12
Metazoa - ps13
Myriazoa - ps14
Parahoxozoa - ps15
Bilateria - ps16
Deuterostomia - ps17
Chordata - ps18
Olfactores - ps19
Vertebrata - ps20
Gnathostomata - ps21
Euteleostomi - ps22
Sarcopterygii - ps23
Tetrapoda - ps24
Amniota - ps25
Mammalia - ps26
Theria - ps27
Placentalia - ps28
Boreoeutheria - ps29
Euarchontoglires - ps30
Euarchonta - ps31
Primates - ps32
Haplorrhini - ps33
Simiiformes - ps34
Catarrhini - ps35
Hominoidea - ps36
Hominidae - ps37
Homininae - ps38
Hominini - ps39
Homo_sapiens - ps40
p-value
1e-09
8e-08
4e-06
2e-04
1e-02
Log-odds
0.5
1.0
1.5
2.0
2.5
3.0
3.5
4.0

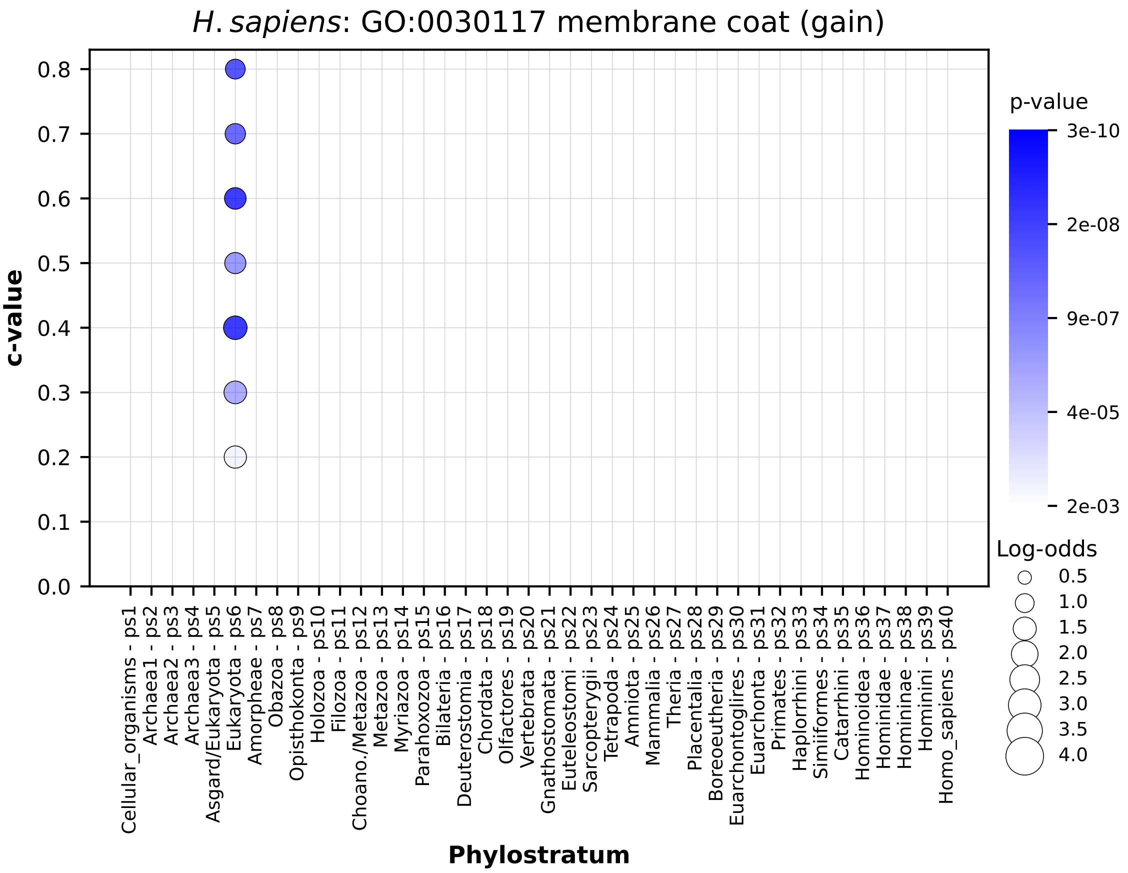

H. sapiens: GO:0030117 membrane coat (gain)
c-value
0.0
0.1
0.2
0.3
0.4
0.5
0.6
0.7
0.8
Phylostratum
Cellular_organisms - ps1
Archaea1 - ps2
Archaea2 - ps3
Archaea3 - ps4
Asgard/Eukaryota - ps5
Eukaryota - ps6
Amorpheae - ps7
Obazoa - ps8
Opisthokonta - ps9
Holozoa - ps10
Filozoa - ps11
Choano./Metazoa - ps12
Metazoa - ps13
Myriazoa - ps14
Parahoxozoa - ps15
Bilateria - ps16
Deuterostomia - ps17
Chordata - ps18
Olfactores - ps19
Vertebrata - ps20
Gnathostomata - ps21
Euteleostomi - ps22
Sarcopterygii - ps23
Tetrapoda - ps24
Amniota - ps25
Mammalia - ps26
Theria - ps27
Placentalia - ps28
Boreoeutheria - ps29
Euarchontoglires - ps30
Euarchonta - ps31
Primates - ps32
Haplorrhini - ps33
Simiiformes - ps34
Catarrhini - ps35
Hominoidea - ps36
Hominidae - ps37
Homininae - ps38
Hominini - ps39
Homo_sapiens - ps40
p-value
3e-10
2e-08
9e-07
4e-05
2e-03
Log-odds
0.5
1.0
1.5
2.0
2.5
3.0
3.5
4.0

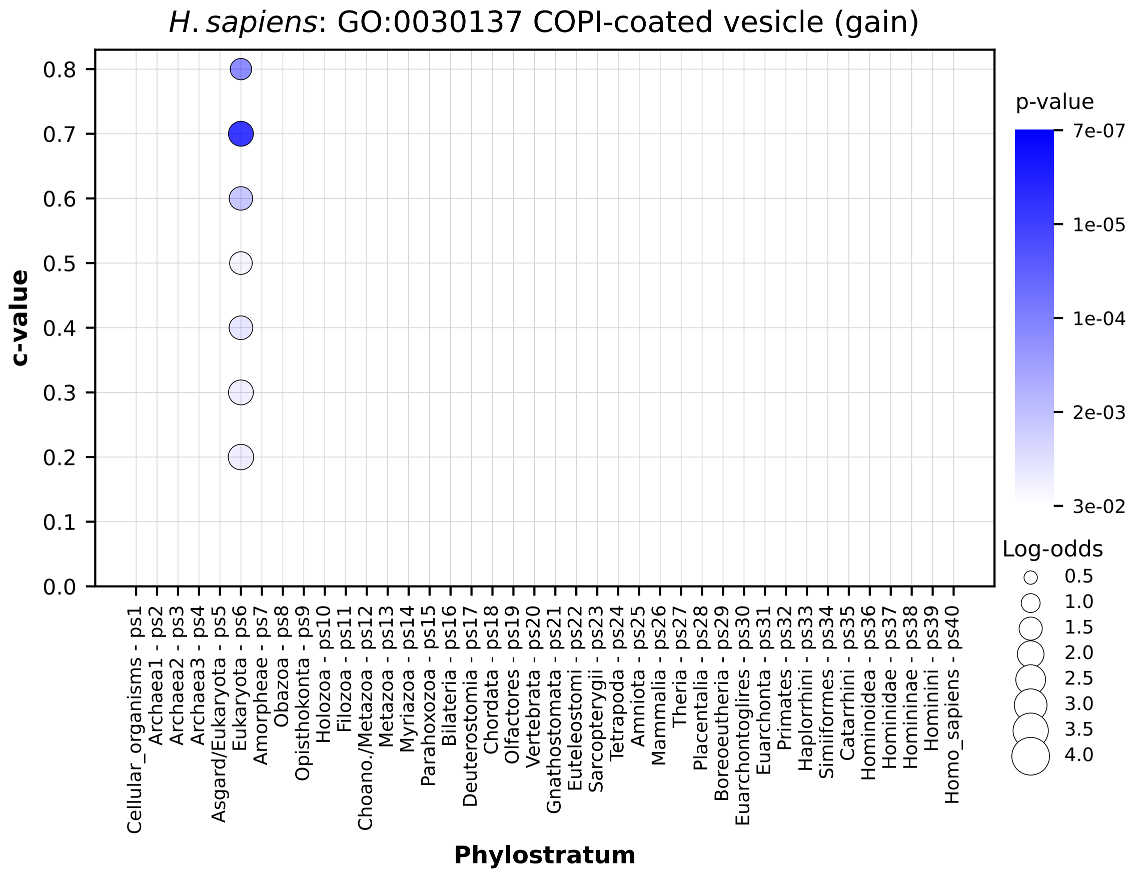

H. sapiens: GO:0030137 COPI-coated vesicle (gain)
c-value
0.0
0.1
0.2
0.3
0.4
0.5
0.6
0.7
0.8
Phylostratum
Cellular_organisms - ps1
Archaea1 - ps2
Archaea2 - ps3
Archaea3 - ps4
Asgard/Eukaryota - ps5
Eukaryota - ps6
Amorpheae - ps7
Obazoa - ps8
Opisthokonta - ps9
Holozoa - ps10
Filozoa - ps11
Choano./Metazoa - ps12
Metazoa - ps13
Myriazoa - ps14
Parahoxozoa - ps15
Bilateria - ps16
Deuterostomia - ps17
Chordata - ps18
Olfactores - ps19
Vertebrata - ps20
Gnathostomata - ps21
Euteleostomi - ps22
Sarcopterygii - ps23
Tetrapoda - ps24
Amniota - ps25
Mammalia - ps26
Theria - ps27
Placentalia - ps28
Boreoeutheria - ps29
Euarchontoglires - ps30
Euarchonta - ps31
Primates - ps32
Haplorrhini - ps33
Simiiformes - ps34
Catarrhini - ps35
Hominoidea - ps36
Hominidae - ps37
Homininae - ps38
Hominini - ps39
Homo_sapiens - ps40
p-value
7e-07
1e-05
1e-04
2e-03
3e-02
Log-odds
0.5
1.0
1.5
2.0
2.5
3.0
3.5
4.0

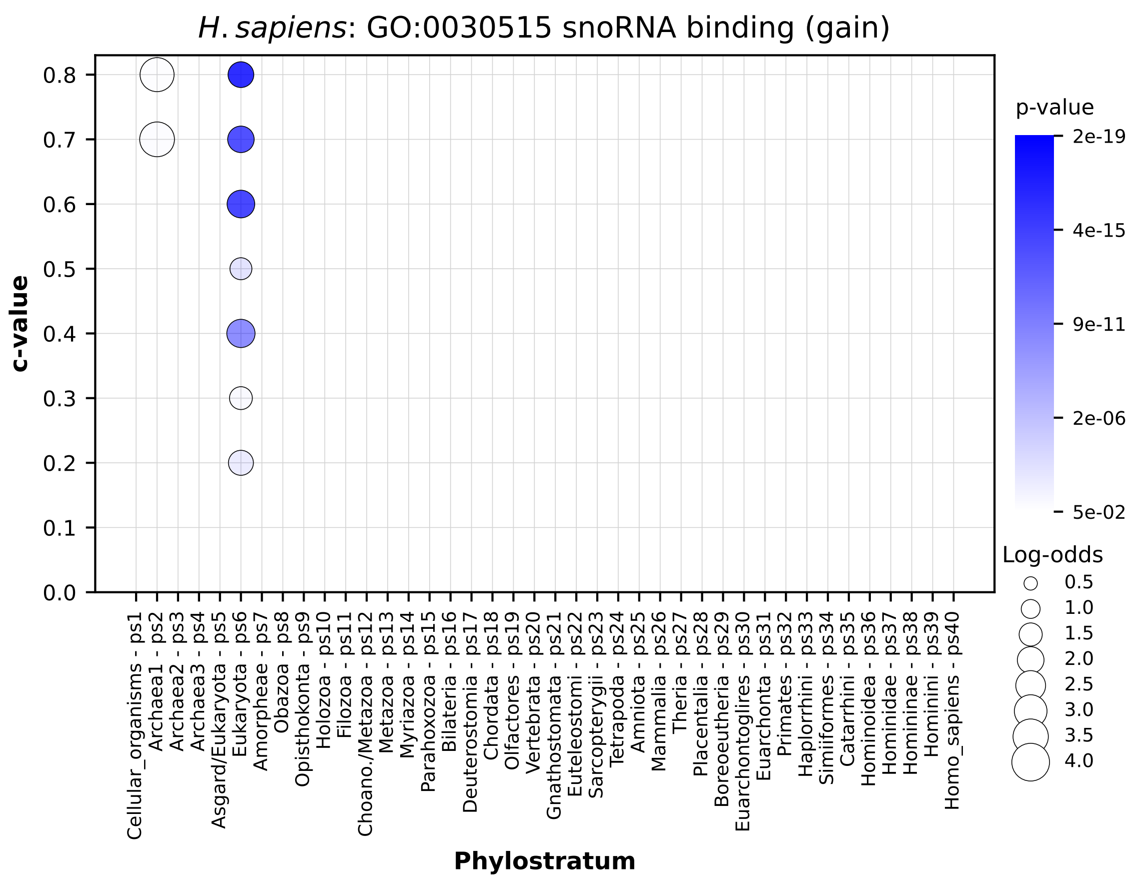
H. sapiens: GO:0030515 snoRNA binding (gain)
c-value
0.0
0.1
0.2
0.3
0.4
0.5
0.6
0.7
0.8
Phylostratum
Cellular_organisms - ps1
Archaea1 - ps2
Archaea2 - ps3
Archaea3 - ps4
Asgard/Eukaryota - ps5
Eukaryota - ps6
Amorpheae - ps7
Obazoa - ps8
Opisthokonta - ps9
Holozoa - ps10
Filozoa - ps11
Choano./Metazoa - ps12
Metazoa - ps13
Myriazoa - ps14
Parahoxozoa - ps15
Bilateria - ps16
Deuterostomia - ps17
Chordata - ps18
Olfactores - ps19
Vertebrata - ps20
Gnathostomata - ps21
Euteleostomi - ps22
Sarcopterygii - ps23
Tetrapoda - ps24
Amniota - ps25
Mammalia - ps26
Theria - ps27
Placentalia - ps28
Boreoeutheria - ps29
Euarchontoglires - ps30
Euarchonta - ps31
Primates - ps32
Haplorrhini - ps33
Simiiformes - ps34
Catarrhini - ps35
Hominoidea - ps36
Hominidae - ps37
Homininae - ps38
Hominini - ps39
Homo_sapiens - ps40
p-value
2e-19
4e-15
9e-11
2e-06
5e-02
Log-odds
0.5
1.0
1.5
2.0
2.5
3.0
3.5
4.0

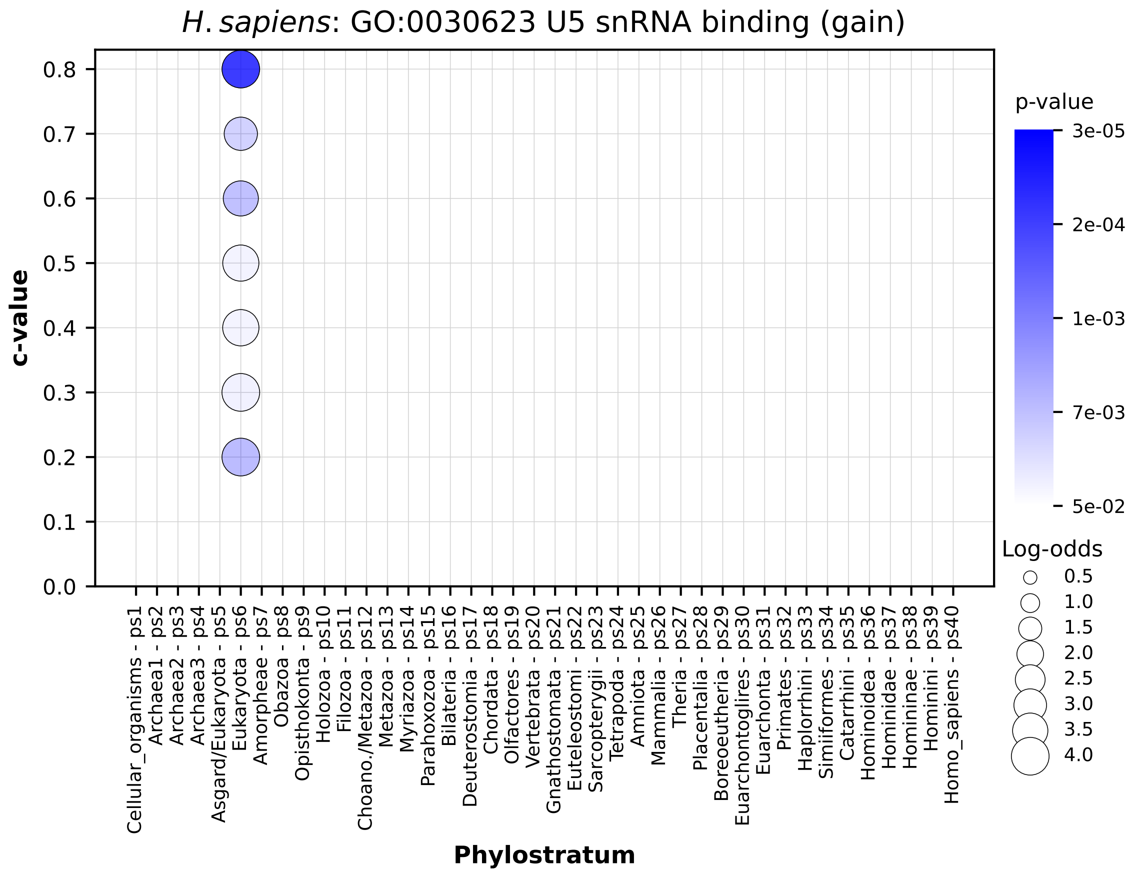
*H. sapiens*: GO:0030623 U5 snRNA binding (gain)
c-value
0.0
0.1
0.2
0.3
0.4
0.5
0.6
0.7
0.8
Phylostratum
Cellular_organisms - ps1
Archaea1 - ps2
Archaea2 - ps3
Archaea3 - ps4
Asgard/Eukaryota - ps5
Eukaryota - ps6
Amorpheae - ps7
Obazoa - ps8
Opisthokonta - ps9
Holozoa - ps10
Filozoa - ps11
Choano./Metazoa - ps12
Metazoa - ps13
Myriazoa - ps14
Parahoxozoa - ps15
Bilateria - ps16
Deuterostomia - ps17
Chordata - ps18
Olfactores - ps19
Vertebrata - ps20
Gnathostomata - ps21
Euteleostomi - ps22
Sarcopterygii - ps23
Tetrapoda - ps24
Amniota - ps25
Mammalia - ps26
Theria - ps27
Placentalia - ps28
Boreoeutheria - ps29
Euarchontoglires - ps30
Euarchonta - ps31
Primates - ps32
Haplorrhini - ps33
Simiiformes - ps34
Catarrhini - ps35
Hominoidea - ps36
Hominidae - ps37
Homininae - ps38
Hominini - ps39
Homo_sapiens - ps40
p-value
3e-05
2e-04
1e-03
7e-03
5e-02
Log-odds
0.5
1.0
1.5
2.0
2.5
3.0
3.5
4.0

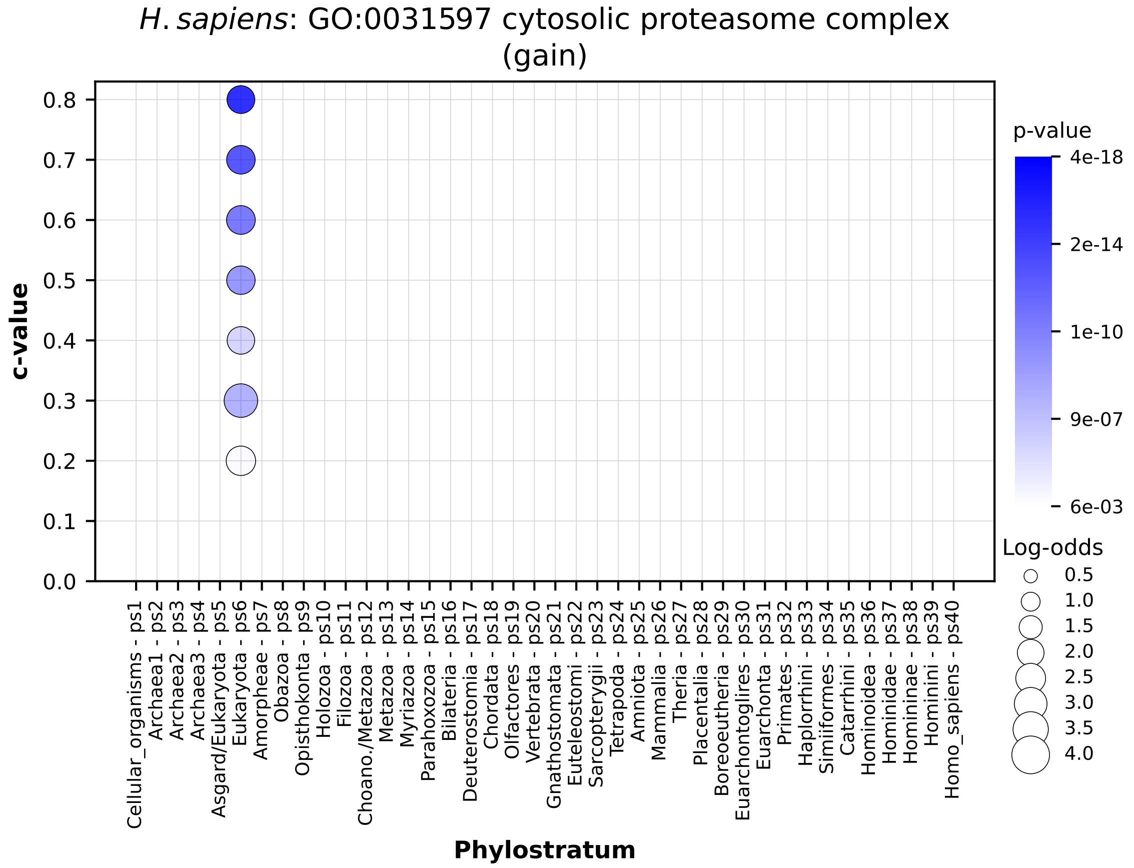

*H. sapiens*: GO:0031597 cytosolic proteasome complex (gain)
c-value
0.0
0.1
0.2
0.3
0.4
0.5
0.6
0.7
0.8
p-value
4e-18
2e-14
1e-10
9e-07
6e-03
Log-odds
0.5
1.0
1.5
2.0
2.5
3.0
3.5
4.0
Cellular_organisms - ps1
Archaea1 - ps2
Archaea2 - ps3
Archaea3 - ps4
Asgard/Eukaryota - ps5
Eukaryota - ps6
Amorpheae - ps7
Obazoa - ps8
Opisthokonta - ps9
Holozoa - ps10
Filozoa - ps11
Choano./Metazoa - ps12
Metazoa - ps13
Myriazoa - ps14
Parahoxozoa - ps15
Bilateria - ps16
Deuterostomia - ps17
Chordata - ps18
Olfactores - ps19
Vertebrata - ps20
Gnathostomata - ps21
Euteleostomi - ps22
Sarcopterygii - ps23
Tetrapoda - ps24
Amniota - ps25
Mammalia - ps26
Theria - ps27
Placentalia - ps28
Boreoeutheria - ps29
Euarchontoglires - ps30
Euarchonta - ps31
Primates - ps32
Haplorrhini - ps33
Simiiformes - ps34
Catarrhini - ps35
Hominoidea - ps36
Hominidae - ps37
Homininae - ps38
Hominini - ps39
Homo_sapiens - ps40
Phylostratum

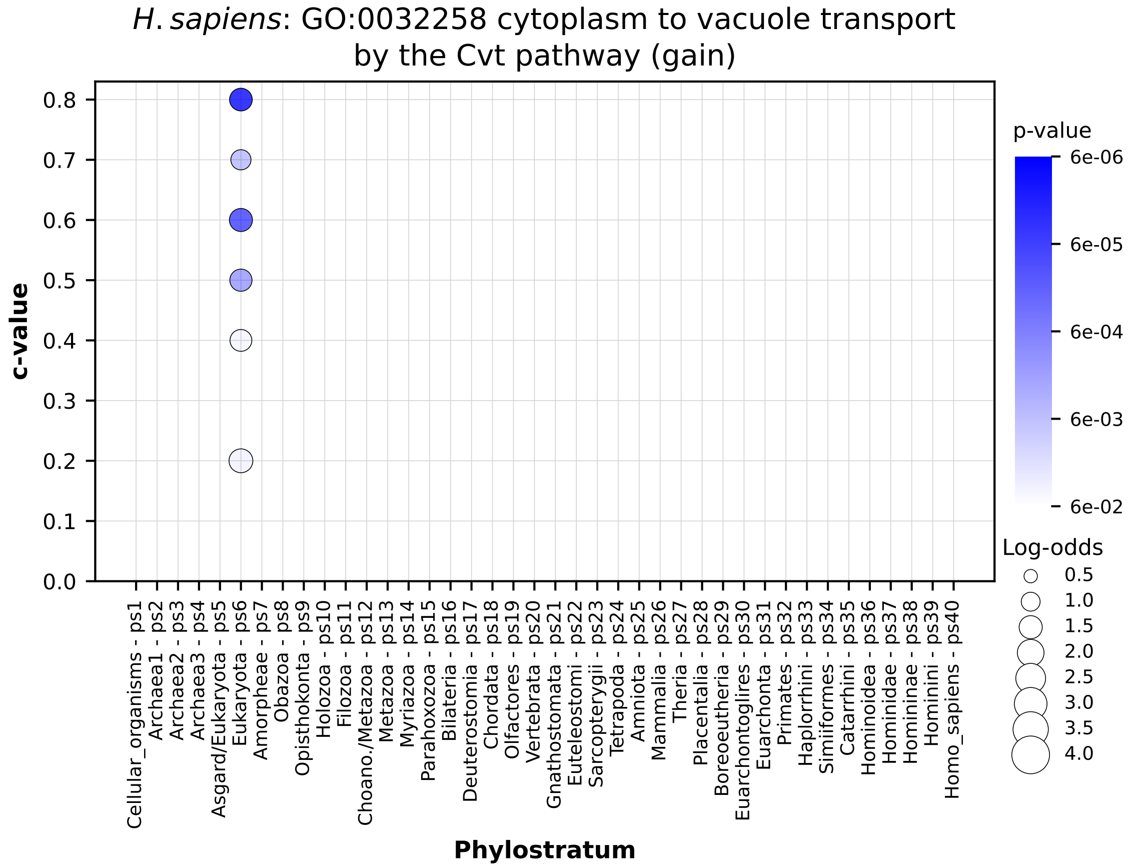

H. sapiens: GO:0032258 cytoplasm to vacuole transport by the Cvt pathway (gain)
c-value
0.0
0.1
0.2
0.3
0.4
0.5
0.6
0.7
0.8
Phylostratum
Cellular_organisms - ps1
Archaea1 - ps2
Archaea2 - ps3
Archaea3 - ps4
Asgard/Eukaryota - ps5
Eukaryota - ps6
Amorpheae - ps7
Obazoa - ps8
Opisthokonta - ps9
Holozoa - ps10
Filozoa - ps11
Choano./Metazoa - ps12
Metazoa - ps13
Myriazoa - ps14
Parahoxozoa - ps15
Bilateria - ps16
Deuterostomia - ps17
Chordata - ps18
Olfactores - ps19
Vertebrata - ps20
Gnathostomata - ps21
Euteleostomi - ps22
Sarcopterygii - ps23
Tetrapoda - ps24
Amniota - ps25
Mammalia - ps26
Theria - ps27
Placentalia - ps28
Boreoeutheria - ps29
Euarchontoglires - ps30
Euarchonta - ps31
Primates - ps32
Haplorrhini - ps33
Simiiformes - ps34
Catarrhini - ps35
Hominoidea - ps36
Hominidae - ps37
Homininae - ps38
Hominini - ps39
Homo_sapiens - ps40
p-value
6e-06
6e-05
6e-04
6e-03
6e-02
Log-odds
0.5
1.0
1.5
2.0
2.5
3.0
3.5
4.0

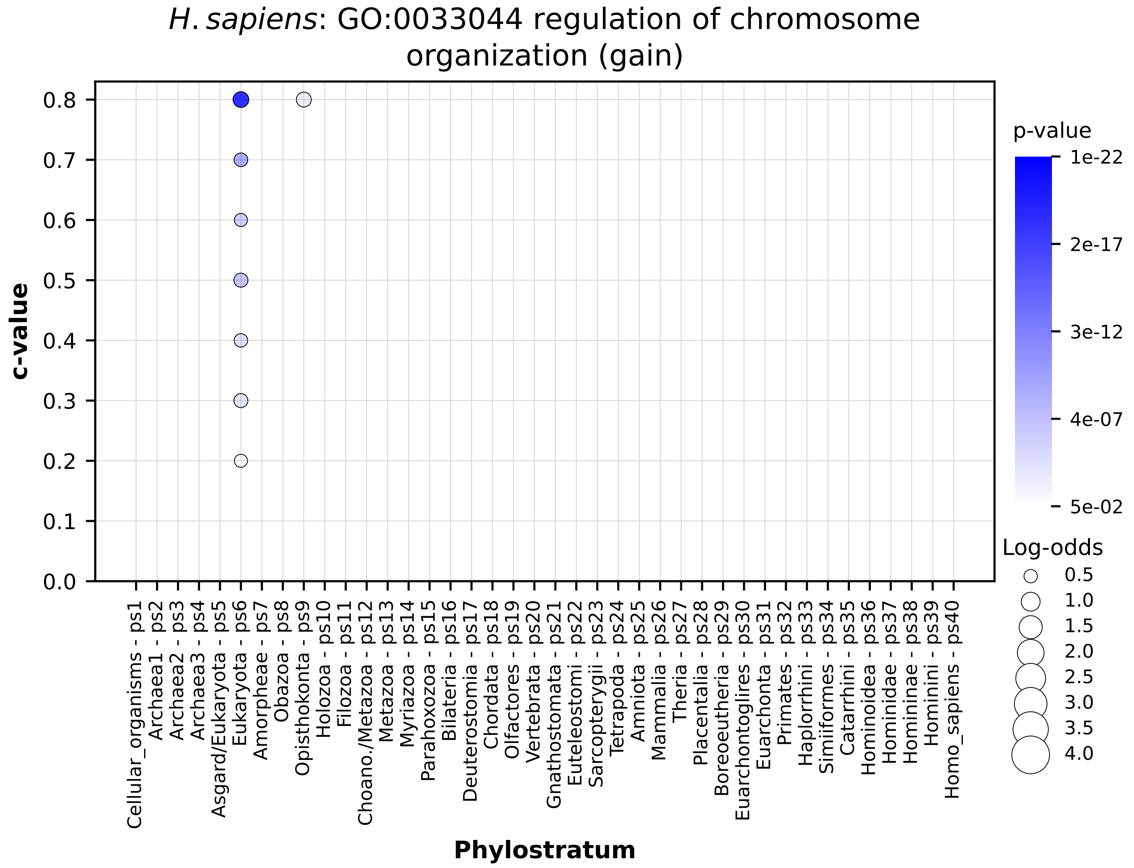

H. sapiens: GO:0033044 regulation of chromosome organization (gain)
c-value
0.0
0.1
0.2
0.3
0.4
0.5
0.6
0.7
0.8
Phylostratum
Cellular_organisms - ps1
Archaea1 - ps2
Archaea2 - ps3
Archaea3 - ps4
Asgard/Eukaryota - ps5
Eukaryota - ps6
Amorpheae - ps7
Obazoa - ps8
Opisthokonta - ps9
Holozoa - ps10
Filozoa - ps11
Choano./Metazoa - ps12
Metazoa - ps13
Myriazoa - ps14
Parahoxozoa - ps15
Bilateria - ps16
Deuterostomia - ps17
Chordata - ps18
Olfactores - ps19
Vertebrata - ps20
Gnathostomata - ps21
Euteleostomi - ps22
Sarcopterygii - ps23
Tetrapoda - ps24
Amniota - ps25
Mammalia - ps26
Theria - ps27
Placentalia - ps28
Boreoeutheria - ps29
Euarchontoglires - ps30
Euarchonta - ps31
Primates - ps32
Haplorrhini - ps33
Simiiformes - ps34
Catarrhini - ps35
Hominoidea - ps36
Hominidae - ps37
Homininae - ps38
Hominini - ps39
Homo_sapiens - ps40
p-value
1e-22
2e-17
3e-12
4e-07
5e-02
Log-odds
0.5
1.0
1.5
2.0
2.5
3.0
3.5
4.0

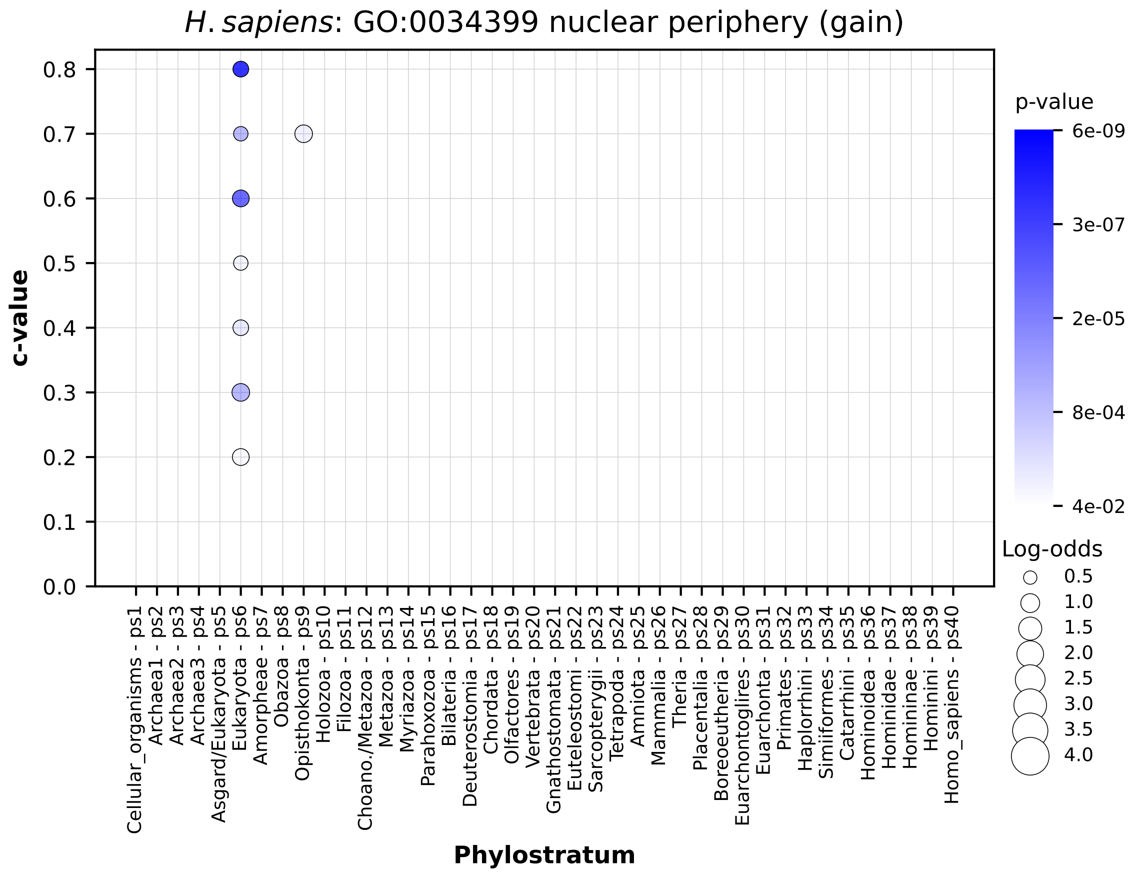

H. sapiens: GO:0034399 nuclear periphery (gain)
c-value
0.0
0.1
0.2
0.3
0.4
0.5
0.6
0.7
0.8
Phylostratum
Cellular_organisms - ps1
Archaea1 - ps2
Archaea2 - ps3
Archaea3 - ps4
Asgard/Eukaryota - ps5
Eukaryota - ps6
Amorpheae - ps7
Obazoa - ps8
Opisthokonta - ps9
Holozoa - ps10
Filozoa - ps11
Choano./Metazoa - ps12
Metazoa - ps13
Myriazoa - ps14
Parahoxozoa - ps15
Bilateria - ps16
Deuterostomia - ps17
Chordata - ps18
Olfactores - ps19
Vertebrata - ps20
Gnathostomata - ps21
Euteleostomi - ps22
Sarcopterygii - ps23
Tetrapoda - ps24
Amniota - ps25
Mammalia - ps26
Theria - ps27
Placentalia - ps28
Boreoeutheria - ps29
Euarchontoglires - ps30
Euarchonta - ps31
Primates - ps32
Haplorrhini - ps33
Simiiformes - ps34
Catarrhini - ps35
Hominoidea - ps36
Hominidae - ps37
Homininae - ps38
Hominini - ps39
Homo_sapiens - ps40
p-value
6e-09
3e-07
2e-05
8e-04
4e-02
Log-odds
0.5
1.0
1.5
2.0
2.5
3.0
3.5
4.0

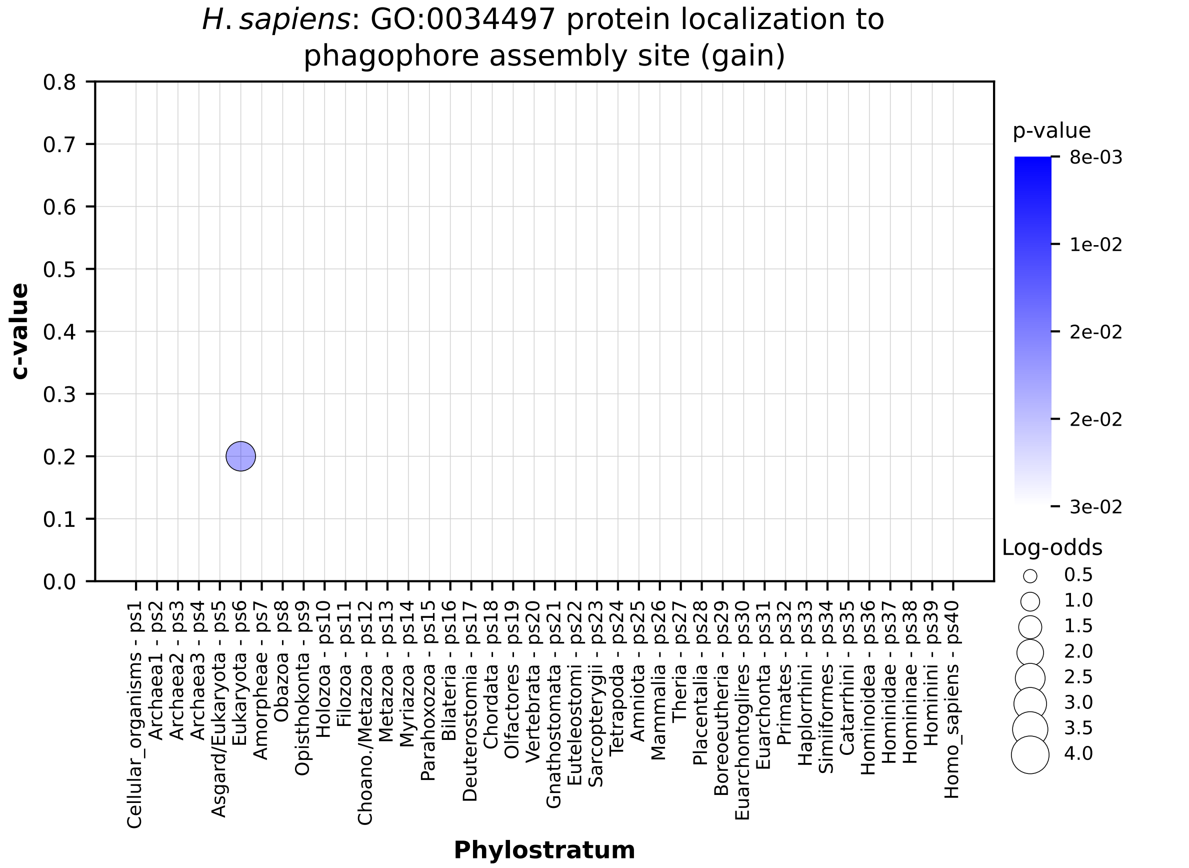

*H. sapiens*: GO:0034497 protein localization to phagophore assembly site (gain)
c-value
0.8
0.7
0.6
0.5
0.4
0.3
0.2
0.1
0.0
Cellular_organisms - ps1
Archaea1 - ps2
Archaea2 - ps3
Archaea3 - ps4
Asgard/Eukaryota - ps5
Eukaryota - ps6
Amorpheae - ps7
Obazoa - ps8
Opisthokonta - ps9
Holozoa - ps10
Filozoa - ps11
Choano./Metazoa - ps12
Metazoa - ps13
Myriazoa - ps14
Parahoxozoa - ps15
Bilateria - ps16
Deuterostomia - ps17
Chordata - ps18
Olfactores - ps19
Vertebrata - ps20
Gnathostomata - ps21
Euteleostomi - ps22
Sarcopterygii - ps23
Tetrapoda - ps24
Amniota - ps25
Mammalia - ps26
Theria - ps27
Placentalia - ps28
Boreoeutheria - ps29
Euarchontoglires - ps30
Euarchonta - ps31
Primates - ps32
Haplorrhini - ps33
Simiiformes - ps34
Catarrhini - ps35
Hominoidea - ps36
Hominidae - ps37
Homininae - ps38
Hominini - ps39
Homo_sapiens - ps40
Phylostratum
p-value
8e-03
1e-02
2e-02
2e-02
3e-02
Log-odds
0.5
1.0
1.5
2.0
2.5
3.0
3.5
4.0

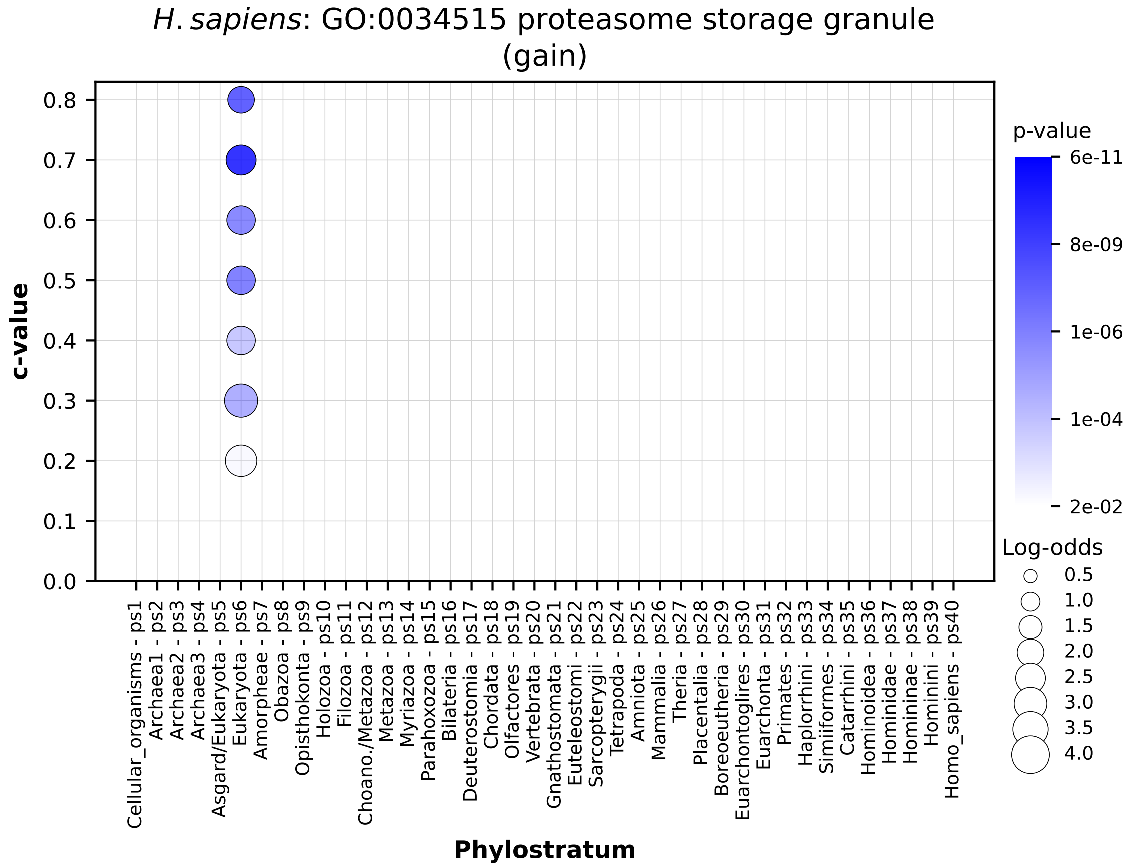

H. sapiens: GO:0034515 proteasome storage granule
(gain)
c-value
0.0
0.1
0.2
0.3
0.4
0.5
0.6
0.7
0.8
Phylostratum
Cellular_organisms - ps1
Archaea1 - ps2
Archaea2 - ps3
Archaea3 - ps4
Asgard/Eukaryota - ps5
Eukaryota - ps6
Amorpheae - ps7
Obazoa - ps8
Opisthokonta - ps9
Holozoa - ps10
Filozoa - ps11
Choano./Metazoa - ps12
Metazoa - ps13
Myriazoa - ps14
Parahoxozoa - ps15
Bilateria - ps16
Deuterostomia - ps17
Chordata - ps18
Olfactores - ps19
Vertebrata - ps20
Gnathostomata - ps21
Euteleostomi - ps22
Sarcopterygii - ps23
Tetrapoda - ps24
Amniota - ps25
Mammalia - ps26
Theria - ps27
Placentalia - ps28
Boreoeutheria - ps29
Euarchontoglires - ps30
Euarchonta - ps31
Primates - ps32
Haplorrhini - ps33
Simiiformes - ps34
Catarrhini - ps35
Hominoidea - ps36
Hominidae - ps37
Homininae - ps38
Hominini - ps39
Homo_sapiens - ps40
p-value
6e-11
8e-09
1e-06
1e-04
2e-02
Log-odds
0.5
1.0
1.5
2.0
2.5
3.0
3.5
4.0

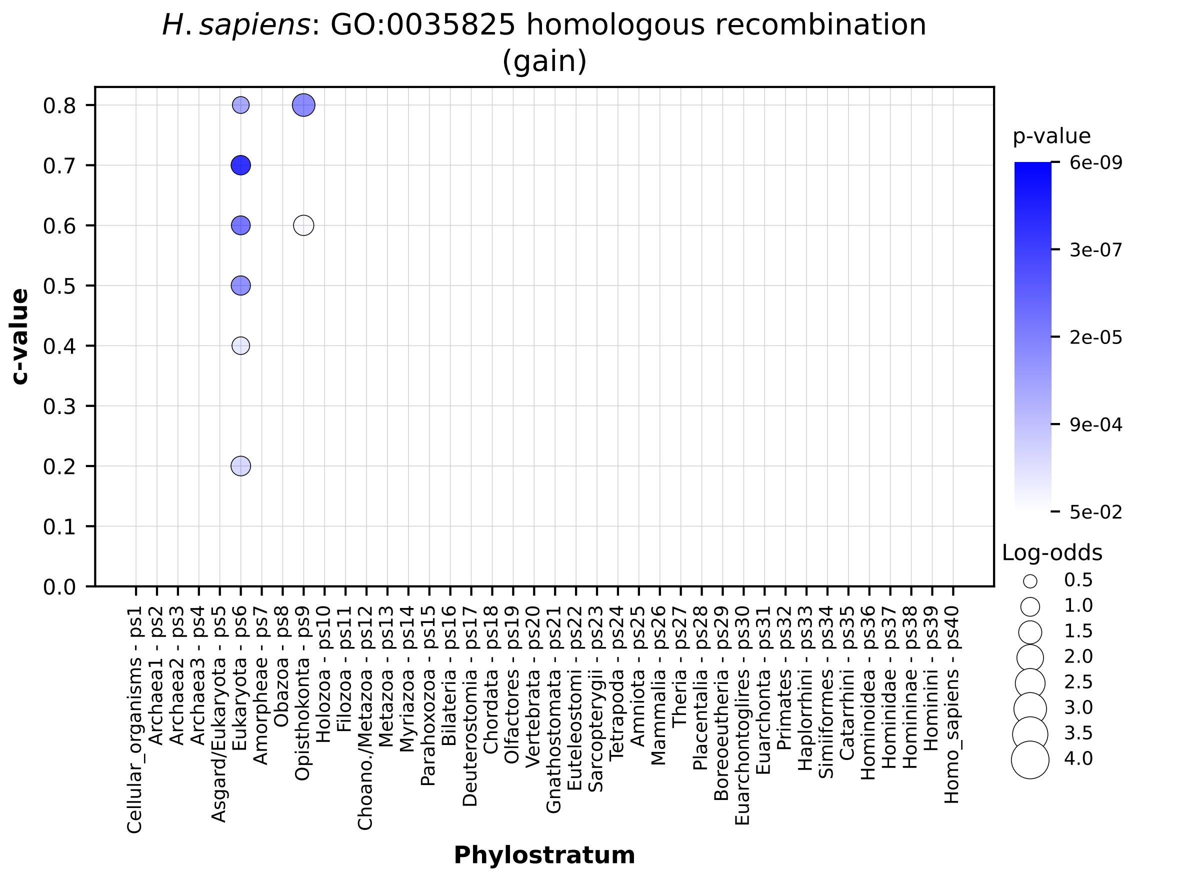

H. sapiens: GO:0035825 homologous recombination
(gain)
c-value
0.0
0.1
0.2
0.3
0.4
0.5
0.6
0.7
0.8
Phylostratum
Cellular_organisms - ps1
Archaea1 - ps2
Archaea2 - ps3
Archaea3 - ps4
Asgard/Eukaryota - ps5
Eukaryota - ps6
Amorpheae - ps7
Obazoa - ps8
Opisthokonta - ps9
Holozoa - ps10
Filozoa - ps11
Choano./Metazoa - ps12
Metazoa - ps13
Myriazoa - ps14
Parahoxozoa - ps15
Bilateria - ps16
Deuterostomia - ps17
Chordata - ps18
Olfactores - ps19
Vertebrata - ps20
Gnathostomata - ps21
Euteleostomi - ps22
Sarcopterygii - ps23
Tetrapoda - ps24
Amniota - ps25
Mammalia - ps26
Theria - ps27
Placentalia - ps28
Boreoeutheria - ps29
Euarchontoglires - ps30
Euarchonta - ps31
Primates - ps32
Haplorrhini - ps33
Simiiformes - ps34
Catarrhini - ps35
Hominoidea - ps36
Hominidae - ps37
Homininae - ps38
Hominini - ps39
Homo_sapiens - ps40
p-value
6e-09
3e-07
2e-05
9e-04
5e-02
Log-odds
0.5
1.0
1.5
2.0
2.5
3.0
3.5
4.0

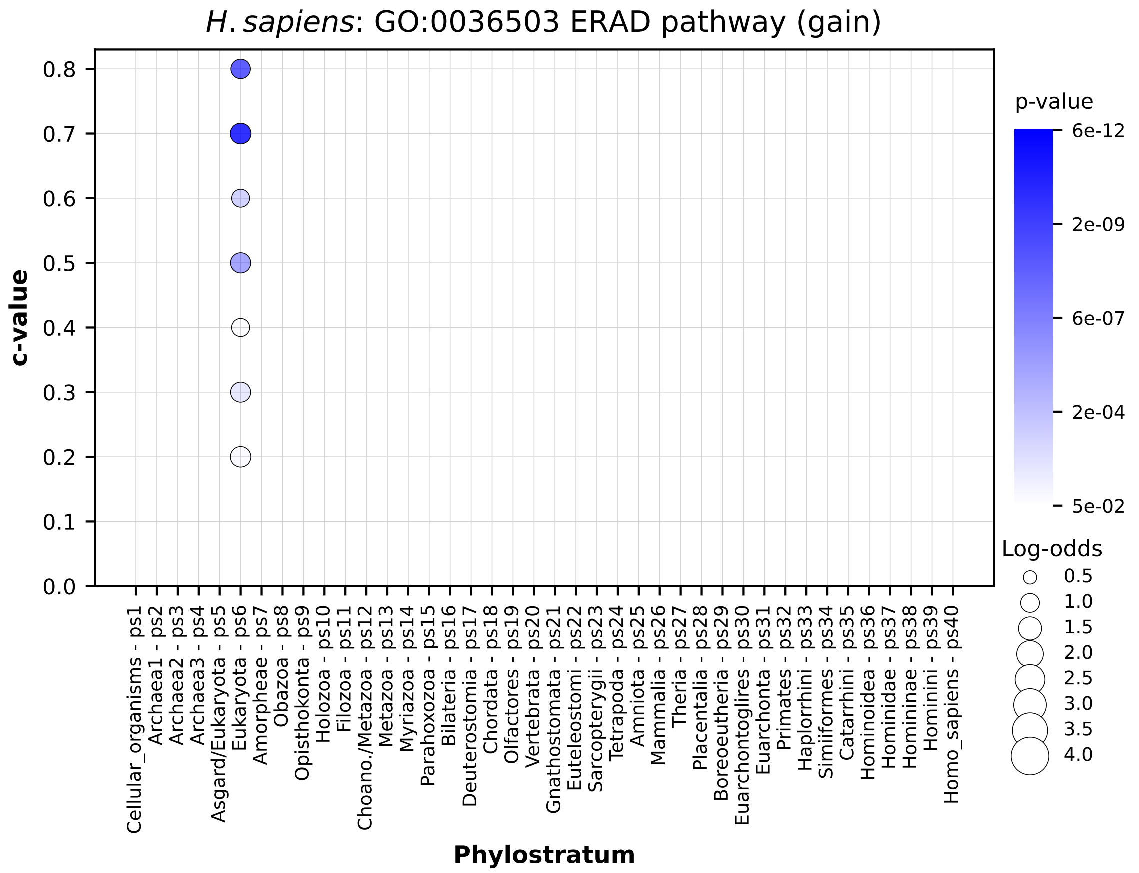

H. sapiens: GO:0036503 ERAD pathway (gain)
c-value
0.8
0.7
0.6
0.5
0.4
0.3
0.2
0.1
0.0
p-value
6e-12
2e-09
6e-07
2e-04
5e-02
Log-odds
0.5
1.0
1.5
2.0
2.5
3.0
3.5
4.0
Cellular_organisms - ps1
Archaea1 - ps2
Archaea2 - ps3
Archaea3 - ps4
Asgard/Eukaryota - ps5
Eukaryota - ps6
Amorpheae - ps7
Obazoa - ps8
Opisthokonta - ps9
Holozoa - ps10
Filozoa - ps11
Choano./Metazoa - ps12
Metazoa - ps13
Myriazoa - ps14
Parahoxozoa - ps15
Bilateria - ps16
Deuterostomia - ps17
Chordata - ps18
Olfactores - ps19
Vertebrata - ps20
Gnathostomata - ps21
Euteleostomi - ps22
Sarcopterygii - ps23
Tetrapoda - ps24
Amniota - ps25
Mammalia - ps26
Theria - ps27
Placentalia - ps28
Boreoeutheria - ps29
Euarchontoglires - ps30
Euarchonta - ps31
Primates - ps32
Haplorrhini - ps33
Simiiformes - ps34
Catarrhini - ps35
Hominoidea - ps36
Hominidae - ps37
Homininae - ps38
Hominini - ps39
Homo_sapiens - ps40
Phylostratum

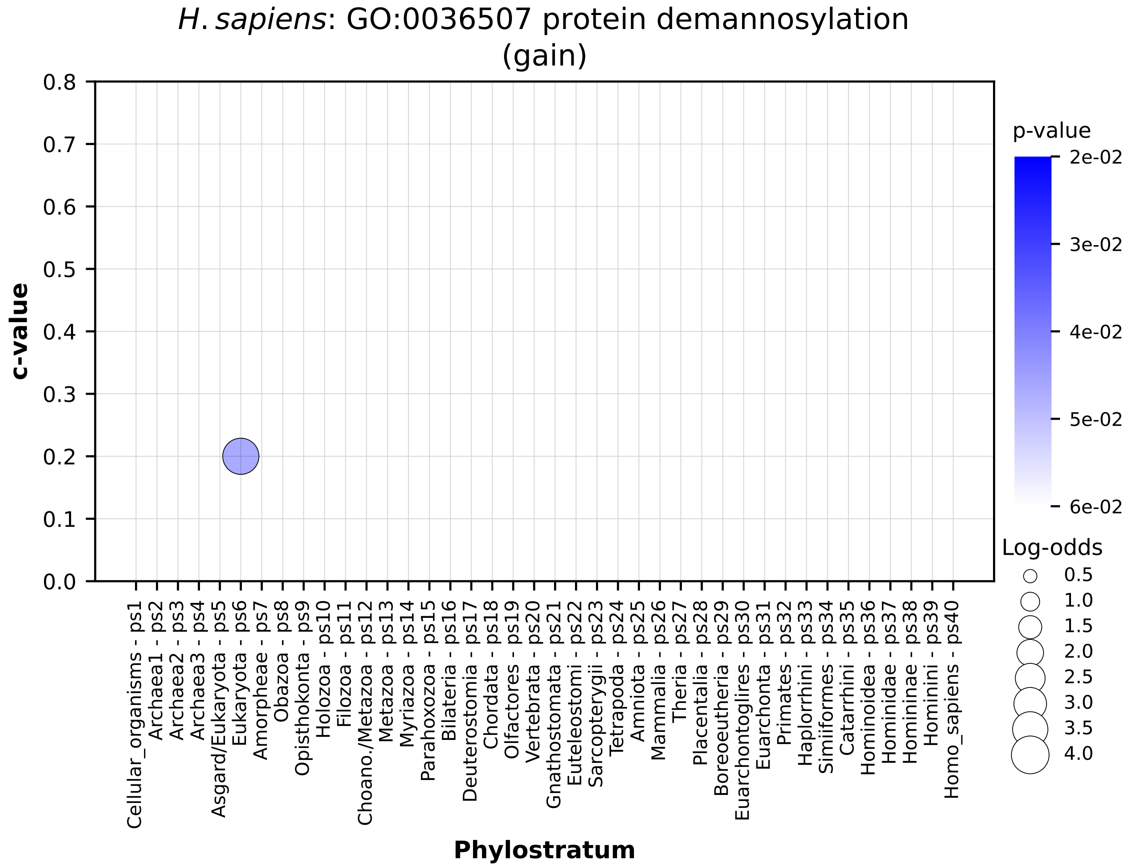

H. sapiens: GO:0036507 protein demannosylation (gain)
c-value
0.8
0.7
0.6
0.5
0.4
0.3
0.2
0.1
0.0
Cellular_organisms - ps1
Archaea1 - ps2
Archaea2 - ps3
Archaea3 - ps4
Asgard/Eukaryota - ps5
Eukaryota - ps6
Amorpheae - ps7
Obazoa - ps8
Opisthokonta - ps9
Holozoa - ps10
Filozoa - ps11
Choano./Metazoa - ps12
Metazoa - ps13
Myriazoa - ps14
Parahoxozoa - ps15
Bilateria - ps16
Deuterostomia - ps17
Chordata - ps18
Olfactores - ps19
Vertebrata - ps20
Gnathostomata - ps21
Euteleostomi - ps22
Sarcopterygii - ps23
Tetrapoda - ps24
Amniota - ps25
Mammalia - ps26
Theria - ps27
Placentalia - ps28
Boreoeutheria - ps29
Euarchontoglires - ps30
Euarchonta - ps31
Primates - ps32
Haplorrhini - ps33
Simiiformes - ps34
Catarrhini - ps35
Hominoidea - ps36
Hominidae - ps37
Homininae - ps38
Hominini - ps39
Homo_sapiens - ps40
Phylostratum
p-value
2e-02
3e-02
4e-02
5e-02
6e-02
Log-odds
0.5
1.0
1.5
2.0
2.5
3.0
3.5
4.0

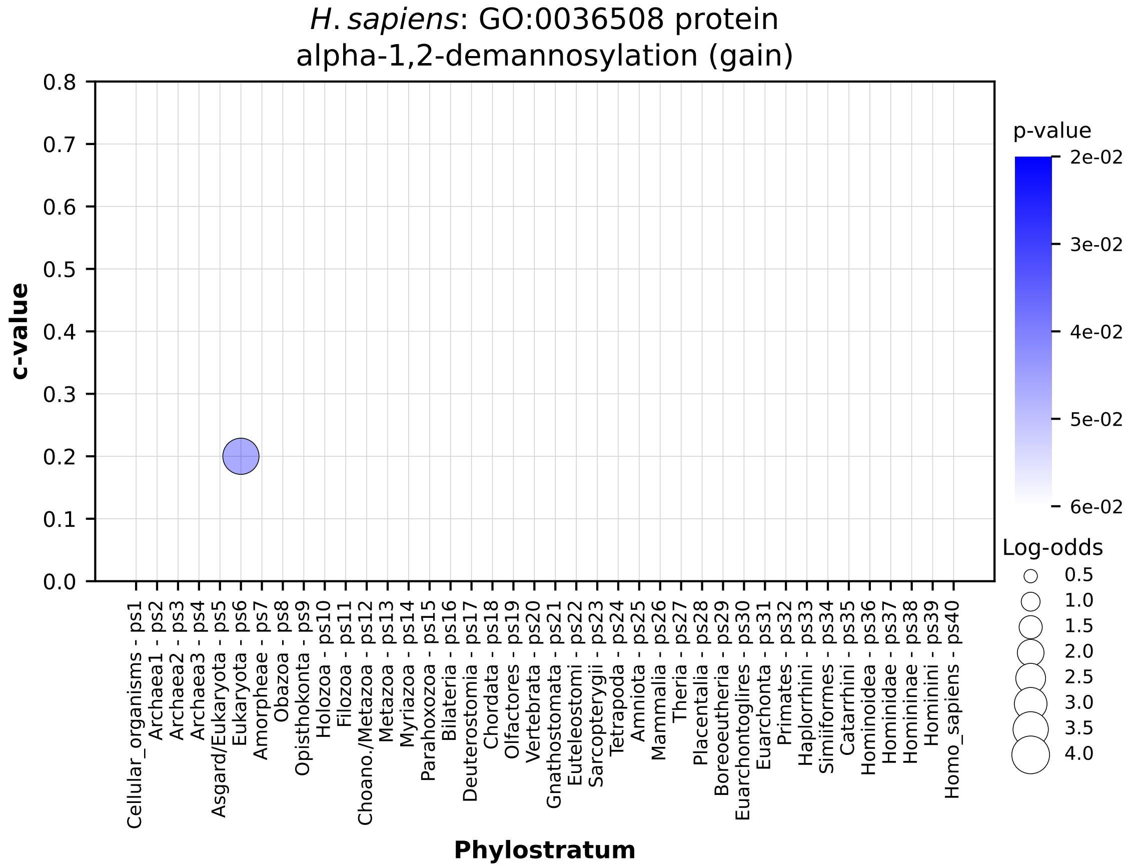

H. sapiens: GO:0036508 protein alpha-1,2-demannosylation (gain)
c-value
0.0
0.1
0.2
0.3
0.4
0.5
0.6
0.7
0.8
Phylostratum
Cellular_organisms - ps1
Archaea1 - ps2
Archaea2 - ps3
Archaea3 - ps4
Asgard/Eukaryota - ps5
Eukaryota - ps6
Amorpheae - ps7
Obazoa - ps8
Opisthokonta - ps9
Holozoa - ps10
Filozoa - ps11
Choano./Metazoa - ps12
Metazoa - ps13
Myriazoa - ps14
Parahoxozoa - ps15
Bilateria - ps16
Deuterostomia - ps17
Chordata - ps18
Olfactores - ps19
Vertebrata - ps20
Gnathostomata - ps21
Euteleostomi - ps22
Sarcopterygii - ps23
Tetrapoda - ps24
Amniota - ps25
Mammalia - ps26
Theria - ps27
Placentalia - ps28
Boreoeutheria - ps29
Euarchontoglires - ps30
Euarchonta - ps31
Primates - ps32
Haplorrhini - ps33
Simiiformes - ps34
Catarrhini - ps35
Hominoidea - ps36
Hominidae - ps37
Homininae - ps38
Hominini - ps39
Homo_sapiens - ps40
p-value
2e-02
3e-02
4e-02
5e-02
6e-02
Log-odds
0.5
1.0
1.5
2.0
2.5
3.0
3.5
4.0

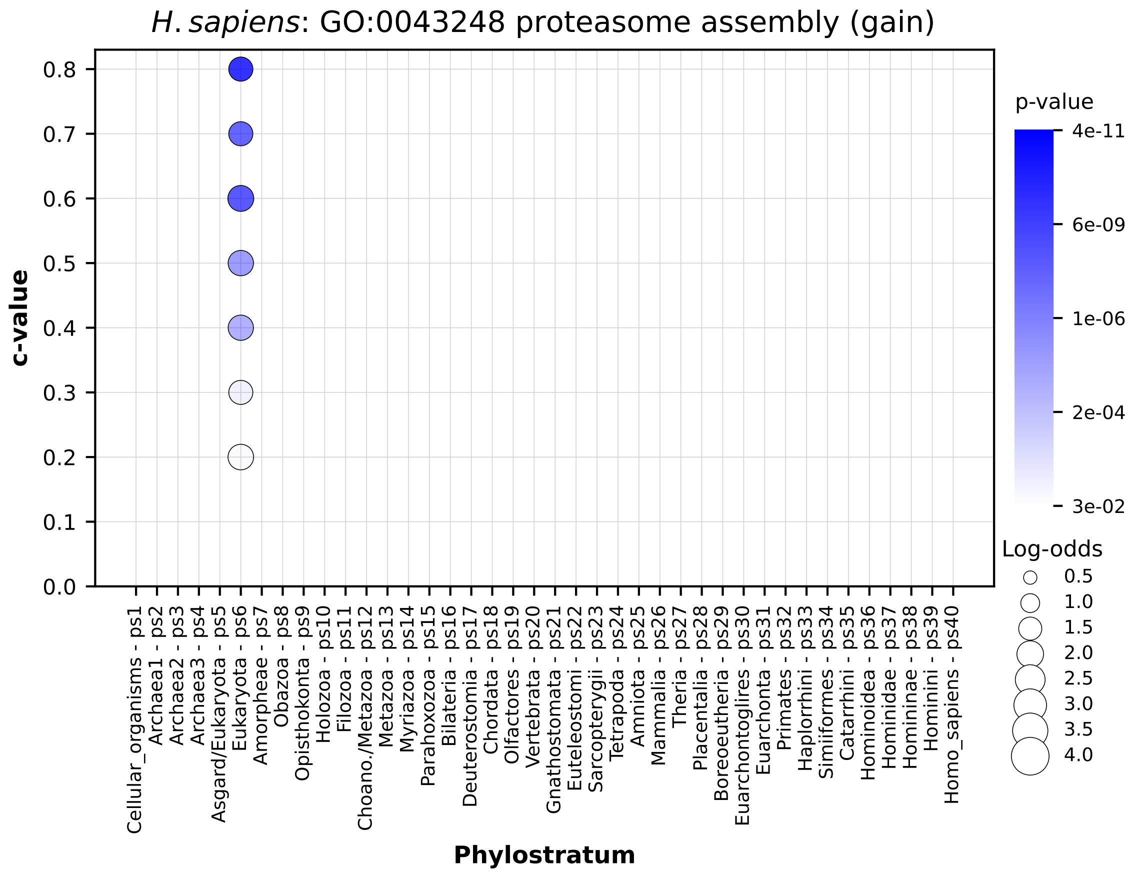
H. sapiens: GO:0043248 proteasome assembly (gain)
c-value
0.0
0.1
0.2
0.3
0.4
0.5
0.6
0.7
0.8
Phylostratum
Cellular_organisms - ps1
Archaea1 - ps2
Archaea2 - ps3
Archaea3 - ps4
Asgard/Eukaryota - ps5
Eukaryota - ps6
Amorpheae - ps7
Obazoa - ps8
Opisthokonta - ps9
Holozoa - ps10
Filozoa - ps11
Choano./Metazoa - ps12
Metazoa - ps13
Myriazoa - ps14
Parahoxozoa - ps15
Bilateria - ps16
Deuterostomia - ps17
Chordata - ps18
Olfactores - ps19
Vertebrata - ps20
Gnathostomata - ps21
Euteleostomi - ps22
Sarcopterygii - ps23
Tetrapoda - ps24
Amniota - ps25
Mammalia - ps26
Theria - ps27
Placentalia - ps28
Boreoeutheria - ps29
Euarchontoglires - ps30
Euarchonta - ps31
Primates - ps32
Haplorrhini - ps33
Simiiformes - ps34
Catarrhini - ps35
Hominoidea - ps36
Hominidae - ps37
Homininae - ps38
Hominini - ps39
Homo_sapiens - ps40
p-value
4e-11
6e-09
1e-06
2e-04
3e-02
Log-odds
0.5
1.0
1.5
2.0
2.5
3.0
3.5
4.0

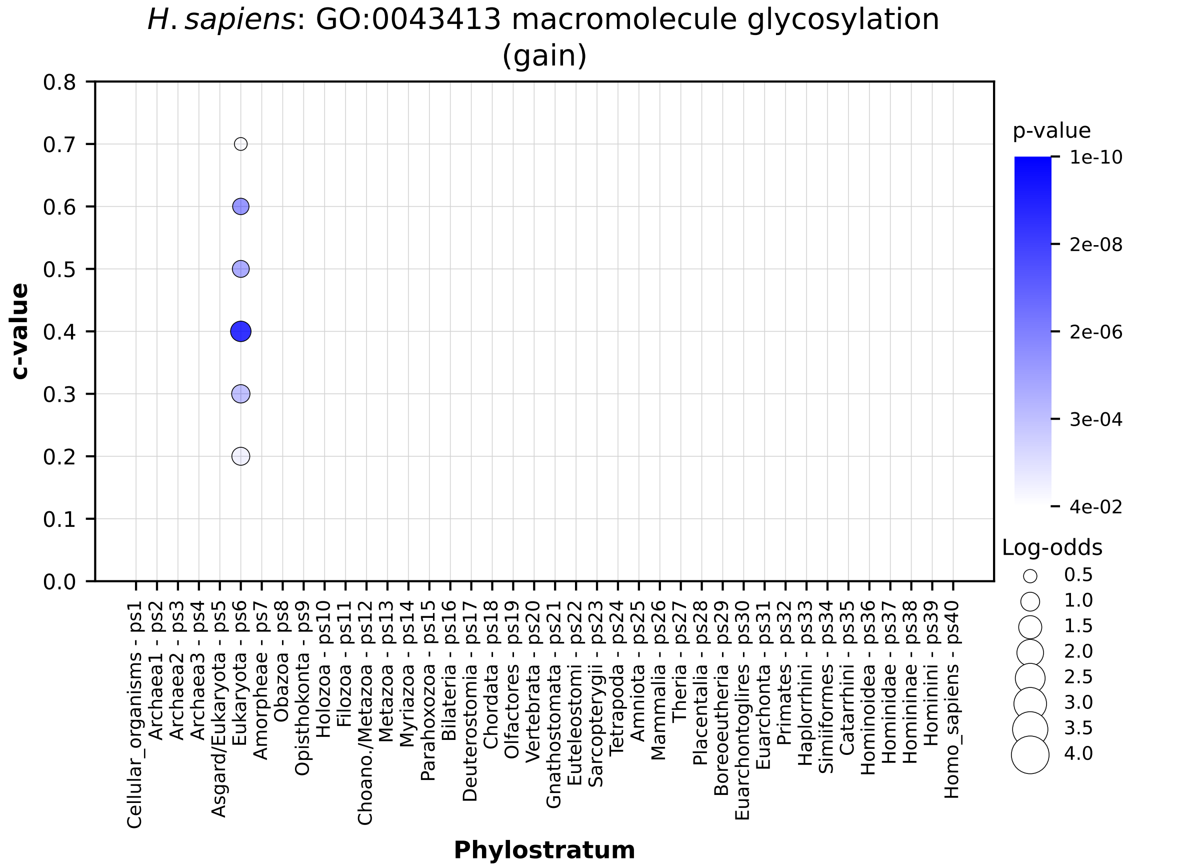

H. sapiens: GO:0043413 macromolecule glycosylation
(gain)
c-value
0.8
0.7
0.6
0.5
0.4
0.3
0.2
0.1
0.0
p-value
1e-10
2e-08
2e-06
3e-04
4e-02
Log-odds
0.5
1.0
1.5
2.0
2.5
3.0
3.5
4.0
Cellular_organisms - ps1
Archaea1 - ps2
Archaea2 - ps3
Archaea3 - ps4
Asgard/Eukaryota - ps5
Eukaryota - ps6
Amorpheae - ps7
Obazoa - ps8
Opisthokonta - ps9
Holozoa - ps10
Filozoa - ps11
Choano./Metazoa - ps12
Metazoa - ps13
Myriazoa - ps14
Parahoxozoa - ps15
Bilateria - ps16
Deuterostomia - ps17
Chordata - ps18
Olfactores - ps19
Vertebrata - ps20
Gnathostomata - ps21
Euteleostomi - ps22
Sarcopterygii - ps23
Tetrapoda - ps24
Amniota - ps25
Mammalia - ps26
Theria - ps27
Placentalia - ps28
Boreoeutheria - ps29
Euarchontoglires - ps30
Euarchonta - ps31
Primates - ps32
Haplorrhini - ps33
Simiiformes - ps34
Catarrhini - ps35
Hominoidea - ps36
Hominidae - ps37
Homininae - ps38
Hominini - ps39
Homo_sapiens - ps40
Phylostratum

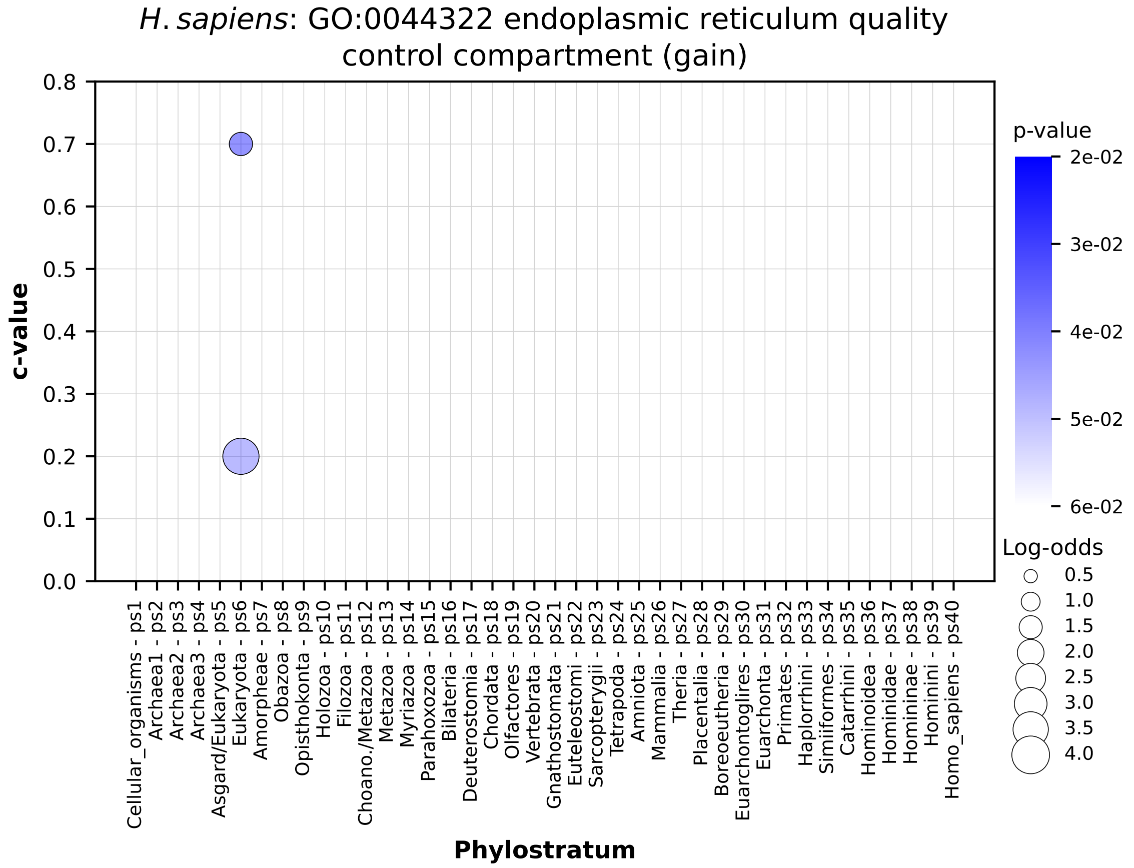

H. sapiens: GO:0044322 endoplasmic reticulum quality control compartment (gain)
c-value
0.0
0.1
0.2
0.3
0.4
0.5
0.6
0.7
0.8
Phylostratum
Cellular_organisms - ps1
Archaea1 - ps2
Archaea2 - ps3
Archaea3 - ps4
Asgard/Eukaryota - ps5
Eukaryota - ps6
Amorpheae - ps7
Obazoa - ps8
Opisthokonta - ps9
Holozoa - ps10
Filozoa - ps11
Choano./Metazoa - ps12
Metazoa - ps13
Myriazoa - ps14
Parahoxozoa - ps15
Bilateria - ps16
Deuterostomia - ps17
Chordata - ps18
Olfactores - ps19
Vertebrata - ps20
Gnathostomata - ps21
Euteleostomi - ps22
Sarcopterygii - ps23
Tetrapoda - ps24
Amniota - ps25
Mammalia - ps26
Theria - ps27
Placentalia - ps28
Boreoeutheria - ps29
Euarchontoglires - ps30
Euarchonta - ps31
Primates - ps32
Haplorrhini - ps33
Simiiformes - ps34
Catarrhini - ps35
Hominoidea - ps36
Hominidae - ps37
Homininae - ps38
Hominini - ps39
Homo_sapiens - ps40
p-value
2e-02
3e-02
4e-02
5e-02
6e-02
Log-odds
0.5
1.0
1.5
2.0
2.5
3.0
3.5
4.0

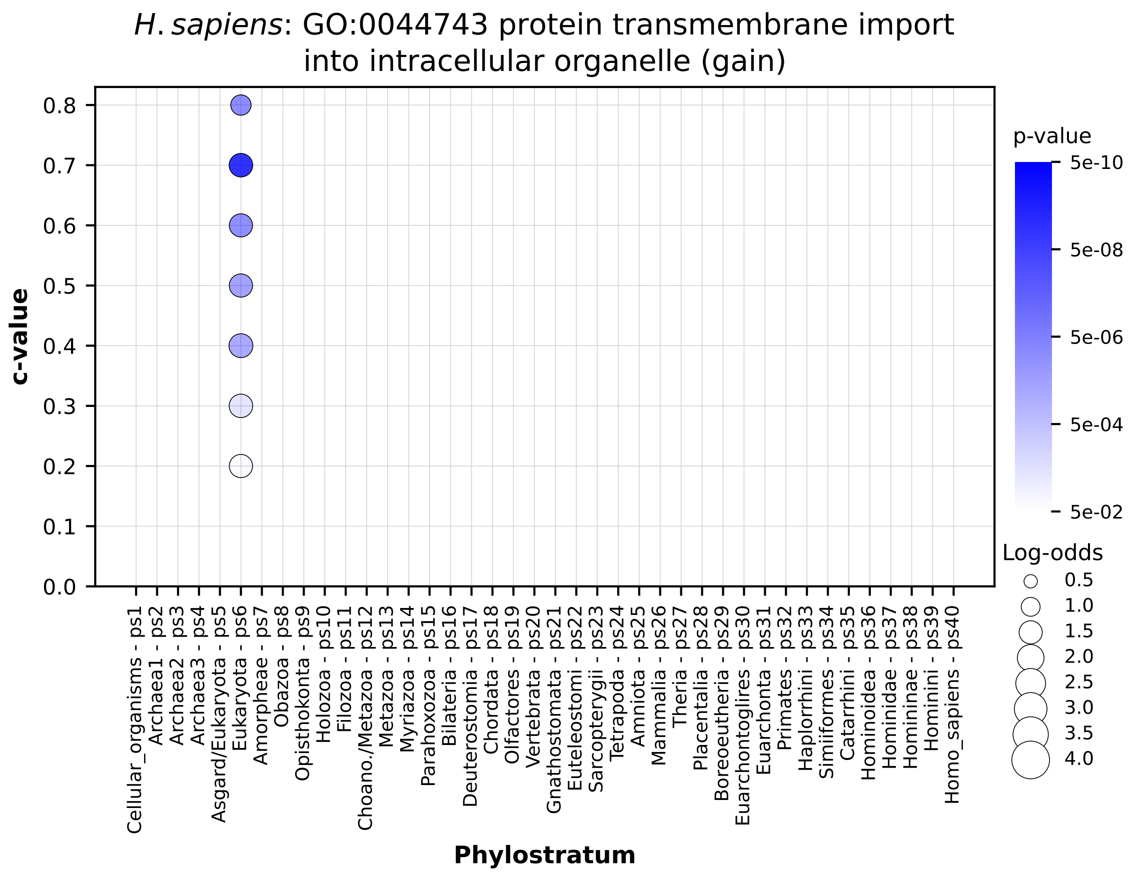

H. sapiens: GO:0044743 protein transmembrane import into intracellular organelle (gain)
c-value
0.0
0.1
0.2
0.3
0.4
0.5
0.6
0.7
0.8
Phylostratum
Cellular_organisms - ps1
Archaea1 - ps2
Archaea2 - ps3
Archaea3 - ps4
Asgard/Eukaryota - ps5
Eukaryota - ps6
Amorpheae - ps7
Obazoa - ps8
Opisthokonta - ps9
Holozoa - ps10
Filozoa - ps11
Choano./Metazoa - ps12
Metazoa - ps13
Myriazoa - ps14
Parahoxozoa - ps15
Bilateria - ps16
Deuterostomia - ps17
Chordata - ps18
Olfactores - ps19
Vertebrata - ps20
Gnathostomata - ps21
Euteleostomi - ps22
Sarcopterygii - ps23
Tetrapoda - ps24
Amniota - ps25
Mammalia - ps26
Theria - ps27
Placentalia - ps28
Boreoeutheria - ps29
Euarchontoglires - ps30
Euarchonta - ps31
Primates - ps32
Haplorrhini - ps33
Simiiformes - ps34
Catarrhini - ps35
Hominoidea - ps36
Hominidae - ps37
Homininae - ps38
Hominini - ps39
Homo_sapiens - ps40
p-value
5e-10
5e-08
5e-06
5e-04
5e-02
Log-odds
0.5
1.0
1.5
2.0
2.5
3.0
3.5
4.0

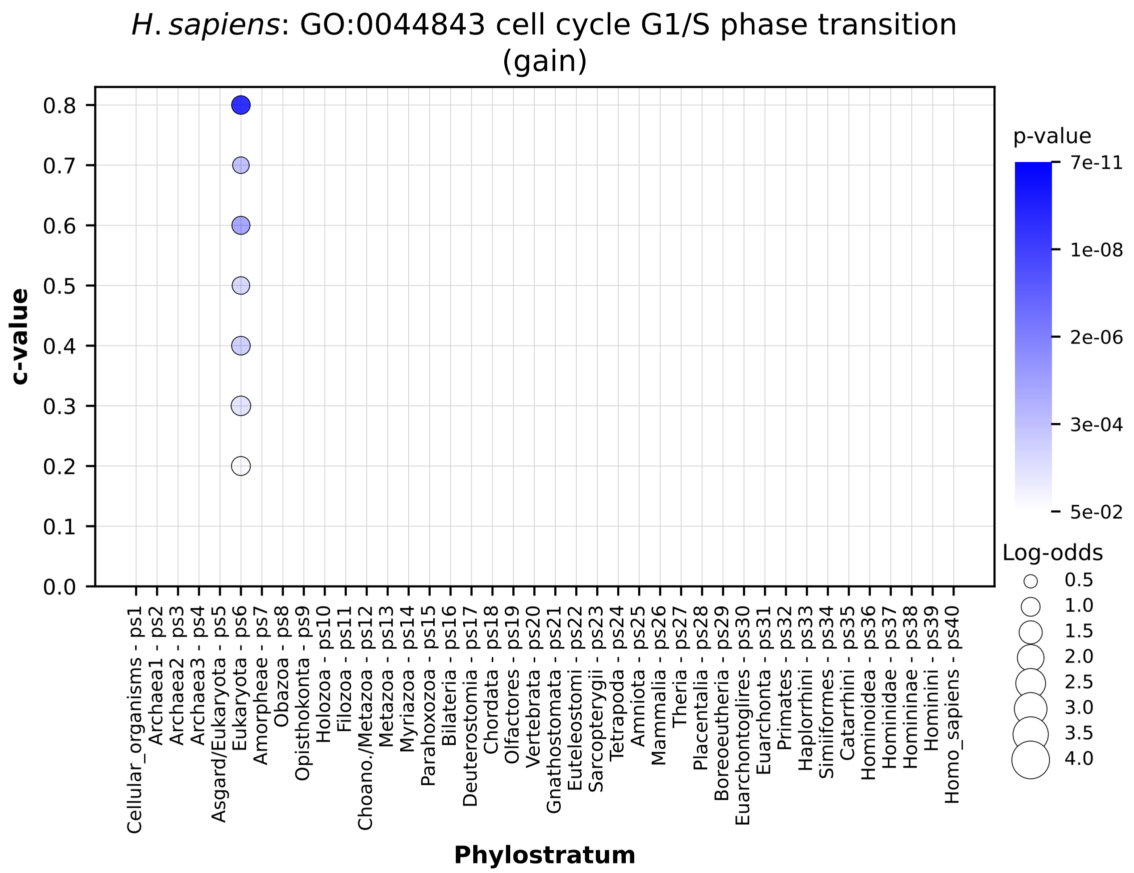

H. sapiens: GO:0044843 cell cycle G1/S phase transition (gain)
c-value
0.0
0.1
0.2
0.3
0.4
0.5
0.6
0.7
0.8
Phylostratum
Cellular_organisms - ps1
Archaea1 - ps2
Archaea2 - ps3
Archaea3 - ps4
Asgard/Eukaryota - ps5
Eukaryota - ps6
Amorpheae - ps7
Obazoa - ps8
Opisthokonta - ps9
Holozoa - ps10
Filozoa - ps11
Choano./Metazoa - ps12
Metazoa - ps13
Myriazoa - ps14
Parahoxozoa - ps15
Bilateria - ps16
Deuterostomia - ps17
Chordata - ps18
Olfactores - ps19
Vertebrata - ps20
Gnathostomata - ps21
Euteleostomi - ps22
Sarcopterygii - ps23
Tetrapoda - ps24
Amniota - ps25
Mammalia - ps26
Theria - ps27
Placentalia - ps28
Boreoeutheria - ps29
Euarchontoglires - ps30
Euarchonta - ps31
Primates - ps32
Haplorrhini - ps33
Simiiformes - ps34
Catarrhini - ps35
Hominoidea - ps36
Hominidae - ps37
Homininae - ps38
Hominini - ps39
Homo_sapiens - ps40
p-value
7e-11
1e-08
2e-06
3e-04
5e-02
Log-odds
0.5
1.0
1.5
2.0
2.5
3.0
3.5
4.0

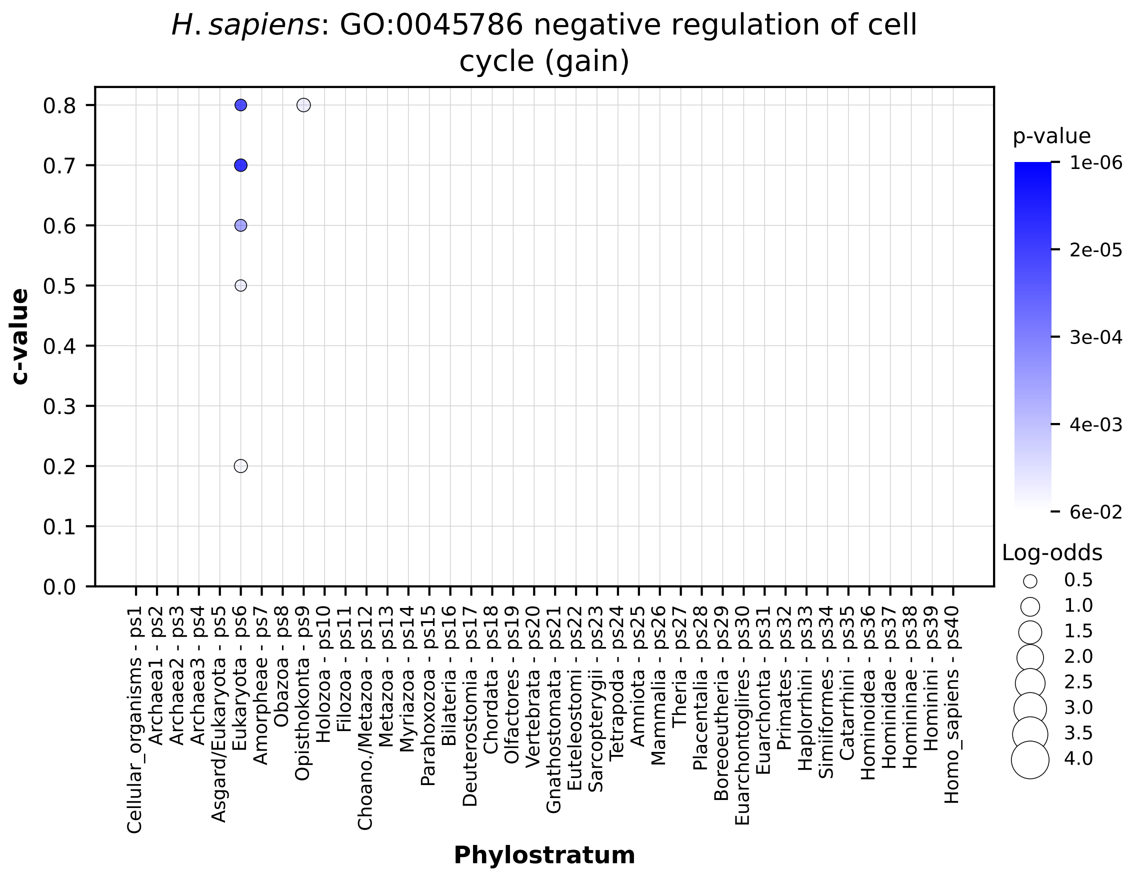

H. sapiens: GO:0045786 negative regulation of cell cycle (gain)
c-value
0.0
0.1
0.2
0.3
0.4
0.5
0.6
0.7
0.8
Phylostratum
Cellular_organisms - ps1
Archaea1 - ps2
Archaea2 - ps3
Archaea3 - ps4
Asgard/Eukaryota - ps5
Eukaryota - ps6
Amorpheae - ps7
Obazoa - ps8
Opisthokonta - ps9
Holozoa - ps10
Filozoa - ps11
Choano./Metazoa - ps12
Metazoa - ps13
Myriazoa - ps14
Parahoxozoa - ps15
Bilateria - ps16
Deuterostomia - ps17
Chordata - ps18
Olfactores - ps19
Vertebrata - ps20
Gnathostomata - ps21
Euteleostomi - ps22
Sarcopterygii - ps23
Tetrapoda - ps24
Amniota - ps25
Mammalia - ps26
Theria - ps27
Placentalia - ps28
Boreoeutheria - ps29
Euarchontoglires - ps30
Euarchonta - ps31
Primates - ps32
Haplorrhini - ps33
Simiiformes - ps34
Catarrhini - ps35
Hominoidea - ps36
Hominidae - ps37
Homininae - ps38
Hominini - ps39
Homo_sapiens - ps40
p-value
1e-06
2e-05
3e-04
4e-03
6e-02
Log-odds
0.5
1.0
1.5
2.0
2.5
3.0
3.5
4.0

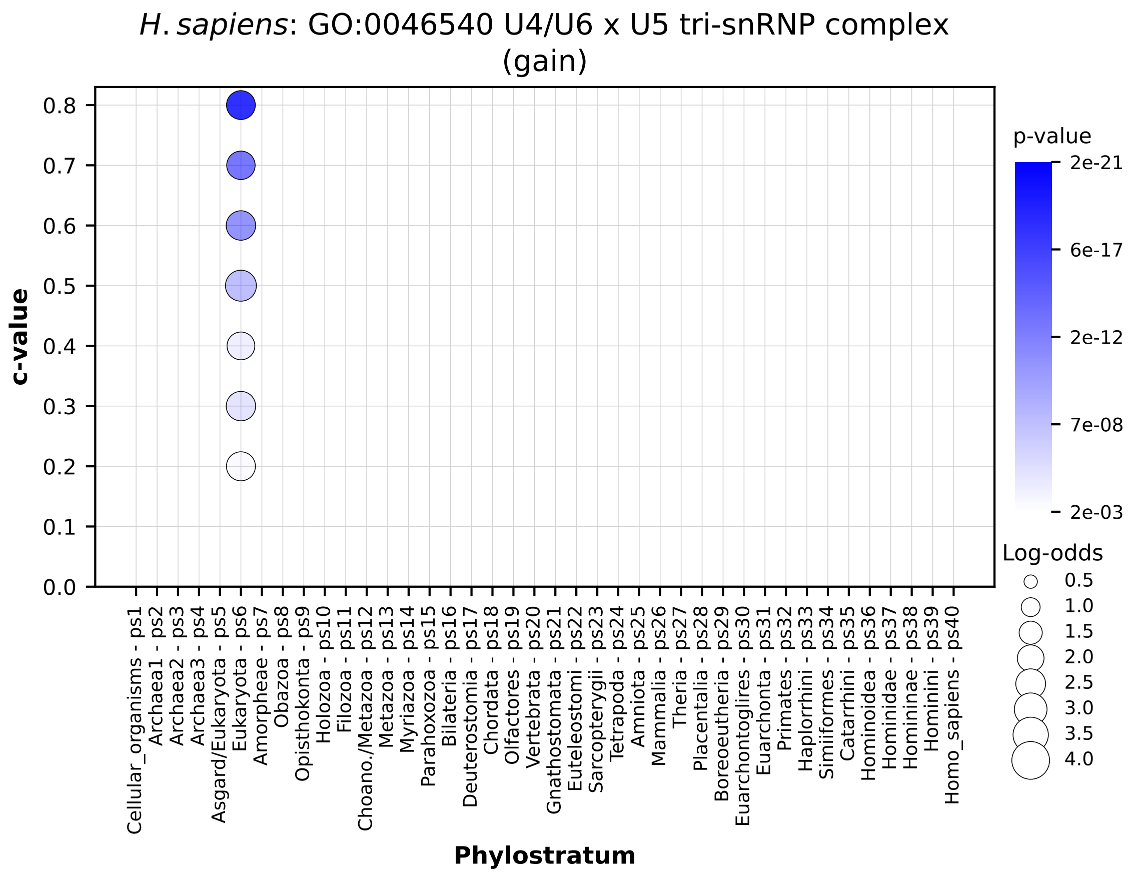
H. sapiens: GO:0046540 U4/U6 x U5 tri-snRNP complex
(gain)
c-value
0.0
0.1
0.2
0.3
0.4
0.5
0.6
0.7
0.8
p-value
2e-21
6e-17
2e-12
7e-08
2e-03
Log-odds
0.5
1.0
1.5
2.0
2.5
3.0
3.5
4.0
Cellular_organisms - ps1
Archaea1 - ps2
Archaea2 - ps3
Archaea3 - ps4
Asgard/Eukaryota - ps5
Eukaryota - ps6
Amorpheae - ps7
Obazoa - ps8
Opisthokonta - ps9
Holozoa - ps10
Filozoa - ps11
Choano./Metazoa - ps12
Metazoa - ps13
Myriazoa - ps14
Parahoxozoa - ps15
Bilateria - ps16
Deuterostomia - ps17
Chordata - ps18
Olfactores - ps19
Vertebrata - ps20
Gnathostomata - ps21
Euteleostomi - ps22
Sarcopterygii - ps23
Tetrapoda - ps24
Amniota - ps25
Mammalia - ps26
Theria - ps27
Placentalia - ps28
Boreoeutheria - ps29
Euarchontoglires - ps30
Euarchonta - ps31
Primates - ps32
Haplorrhini - ps33
Simiiformes - ps34
Catarrhini - ps35
Hominoidea - ps36
Hominidae - ps37
Homininae - ps38
Hominini - ps39
Homo_sapiens - ps40
Phylostratum

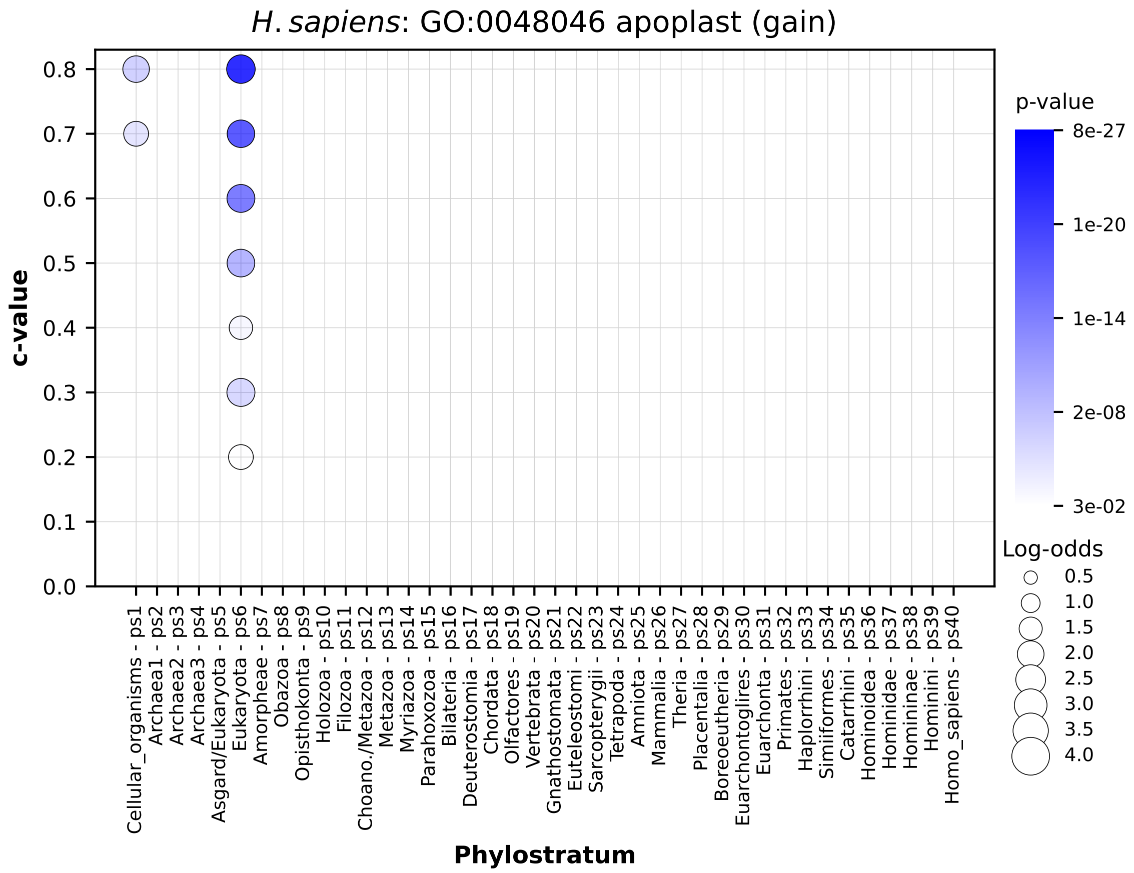

H. sapiens: GO:0048046 apoplast (gain)
c-value
0.0
0.1
0.2
0.3
0.4
0.5
0.6
0.7
0.8
Phylostratum
Cellular_organisms - ps1
Archaea1 - ps2
Archaea2 - ps3
Archaea3 - ps4
Asgard/Eukaryota - ps5
Eukaryota - ps6
Amorpheae - ps7
Obazoa - ps8
Opisthokonta - ps9
Holozoa - ps10
Filozoa - ps11
Choano./Metazoa - ps12
Metazoa - ps13
Myriazoa - ps14
Parahoxozoa - ps15
Bilateria - ps16
Deuterostomia - ps17
Chordata - ps18
Olfactores - ps19
Vertebrata - ps20
Gnathostomata - ps21
Euteleostomi - ps22
Sarcopterygii - ps23
Tetrapoda - ps24
Amniota - ps25
Mammalia - ps26
Theria - ps27
Placentalia - ps28
Boreoeutheria - ps29
Euarchontoglires - ps30
Euarchonta - ps31
Primates - ps32
Haplorrhini - ps33
Simiiformes - ps34
Catarrhini - ps35
Hominoidea - ps36
Hominidae - ps37
Homininae - ps38
Hominini - ps39
Homo_sapiens - ps40
p-value
8e-27
1e-20
1e-14
2e-08
3e-02
Log-odds
0.5
1.0
1.5
2.0
2.5
3.0
3.5
4.0

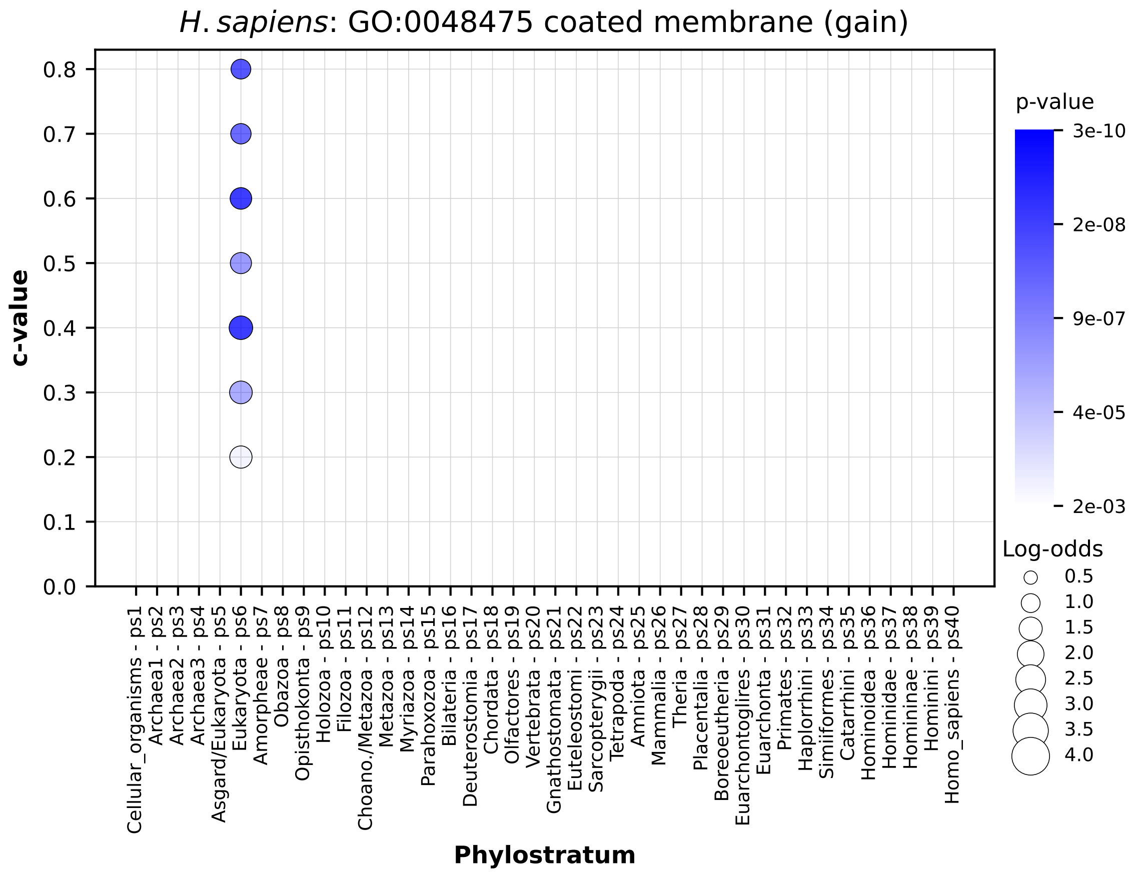
H. sapiens: GO:0048475 coated membrane (gain)
c-value
0.8
0.7
0.6
0.5
0.4
0.3
0.2
0.1
0.0
Phylostratum
Cellular_organisms - ps1
Archaea1 - ps2
Archaea2 - ps3
Archaea3 - ps4
Asgard/Eukaryota - ps5
Eukaryota - ps6
Amorpheae - ps7
Obazoa - ps8
Opisthokonta - ps9
Holozoa - ps10
Filozoa - ps11
Choano./Metazoa - ps12
Metazoa - ps13
Myriazoa - ps14
Parahoxozoa - ps15
Bilateria - ps16
Deuterostomia - ps17
Chordata - ps18
Olfactores - ps19
Vertebrata - ps20
Gnathostomata - ps21
Euteleostomi - ps22
Sarcopterygii - ps23
Tetrapoda - ps24
Amniota - ps25
Mammalia - ps26
Theria - ps27
Placentalia - ps28
Boreoeutheria - ps29
Euarchontoglires - ps30
Euarchonta - ps31
Primates - ps32
Haplorrhini - ps33
Simiiformes - ps34
Catarrhini - ps35
Hominoidea - ps36
Hominidae - ps37
Homininae - ps38
Hominini - ps39
Homo_sapiens - ps40
p-value
3e-10
2e-08
9e-07
4e-05
2e-03
Log-odds
0.5
1.0
1.5
2.0
2.5
3.0
3.5
4.0

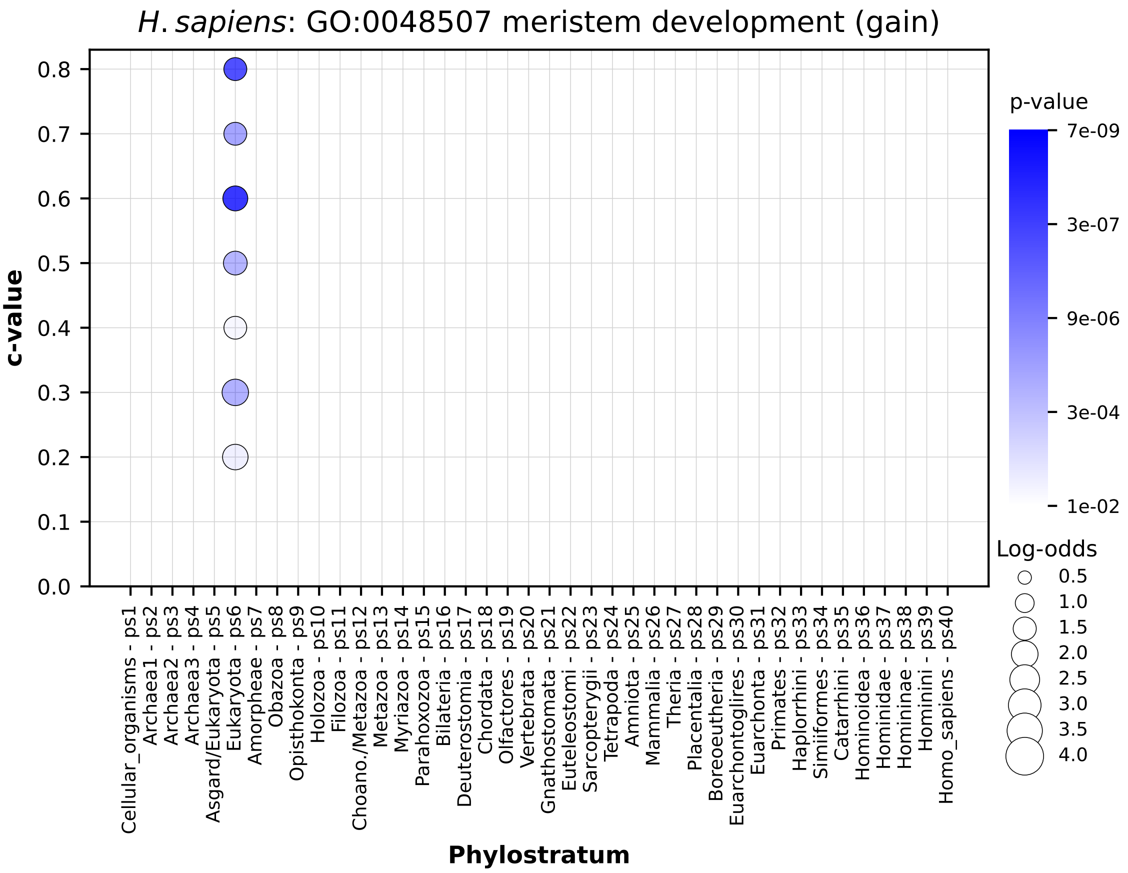

H. sapiens: GO:0048507 meristem development (gain)
c-value
0.0
0.1
0.2
0.3
0.4
0.5
0.6
0.7
0.8
Phylostratum
Cellular_organisms - ps1
Archaea1 - ps2
Archaea2 - ps3
Archaea3 - ps4
Asgard/Eukaryota - ps5
Eukaryota - ps6
Amorpheae - ps7
Obazoa - ps8
Opisthokonta - ps9
Holozoa - ps10
Filozoa - ps11
Choano./Metazoa - ps12
Metazoa - ps13
Myriazoa - ps14
Parahoxozoa - ps15
Bilateria - ps16
Deuterostomia - ps17
Chordata - ps18
Olfactores - ps19
Vertebrata - ps20
Gnathostomata - ps21
Euteleostomi - ps22
Sarcopterygii - ps23
Tetrapoda - ps24
Amniota - ps25
Mammalia - ps26
Theria - ps27
Placentalia - ps28
Boreoeutheria - ps29
Euarchontoglires - ps30
Euarchonta - ps31
Primates - ps32
Haplorrhini - ps33
Simiiformes - ps34
Catarrhini - ps35
Hominoidea - ps36
Hominidae - ps37
Homininae - ps38
Hominini - ps39
Homo_sapiens - ps40
p-value
7e-09
3e-07
9e-06
3e-04
1e-02
Log-odds
0.5
1.0
1.5
2.0
2.5
3.0
3.5
4.0

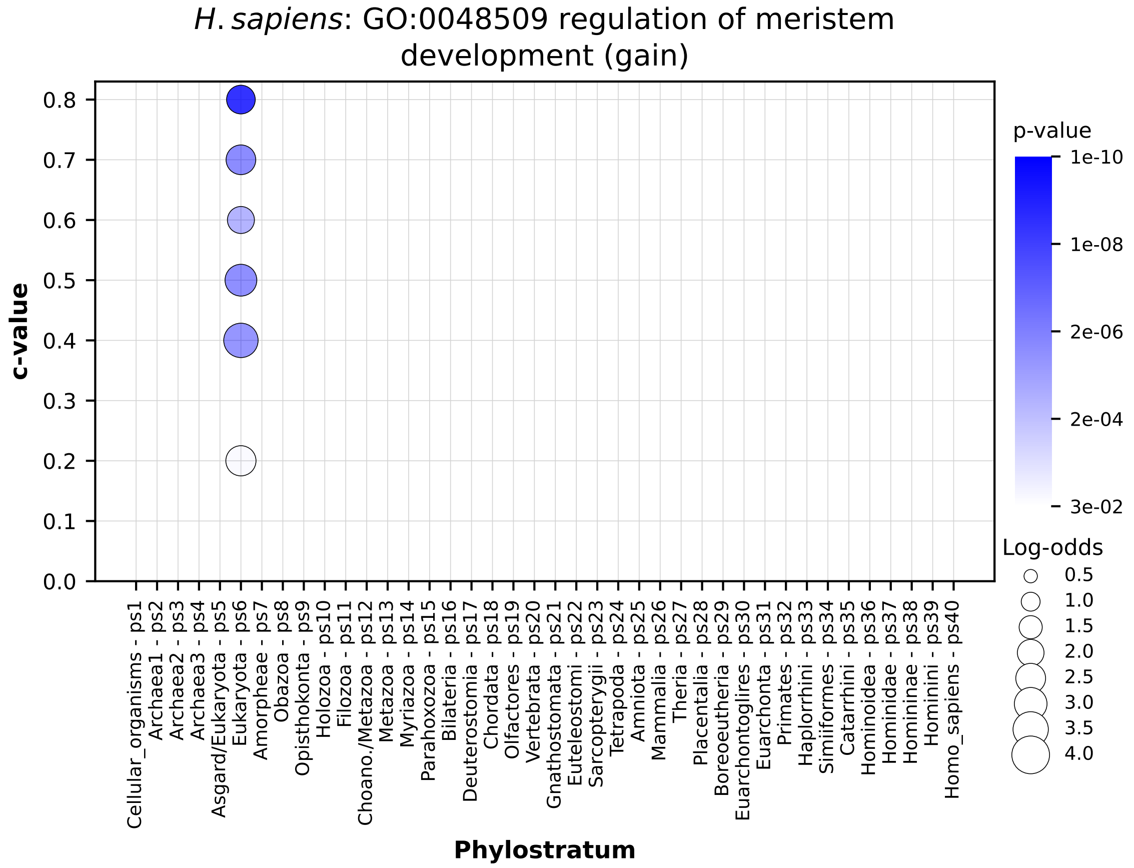

*H. sapiens*: GO:0048509 regulation of meristem development (gain)
c-value
0.8
0.7
0.6
0.5
0.4
0.3
0.2
0.1
0.0
Phylostratum
Cellular_organisms - ps1
Archaea1 - ps2
Archaea2 - ps3
Archaea3 - ps4
Asgard/Eukaryota - ps5
Eukaryota - ps6
Amorpheae - ps7
Obazoa - ps8
Opisthokonta - ps9
Holozoa - ps10
Filozoa - ps11
Choano./Metazoa - ps12
Metazoa - ps13
Myriazoa - ps14
Parahoxozoa - ps15
Bilateria - ps16
Deuterostomia - ps17
Chordata - ps18
Olfactores - ps19
Vertebrata - ps20
Gnathostomata - ps21
Euteleostomi - ps22
Sarcopterygii - ps23
Tetrapoda - ps24
Amniota - ps25
Mammalia - ps26
Theria - ps27
Placentalia - ps28
Boreoeutheria - ps29
Euarchontoglires - ps30
Euarchonta - ps31
Primates - ps32
Haplorrhini - ps33
Simiiformes - ps34
Catarrhini - ps35
Hominoidea - ps36
Hominidae - ps37
Homininae - ps38
Hominini - ps39
Homo_sapiens - ps40
p-value
1e-10
1e-08
2e-06
2e-04
3e-02
Log-odds
0.5
1.0
1.5
2.0
2.5
3.0
3.5
4.0

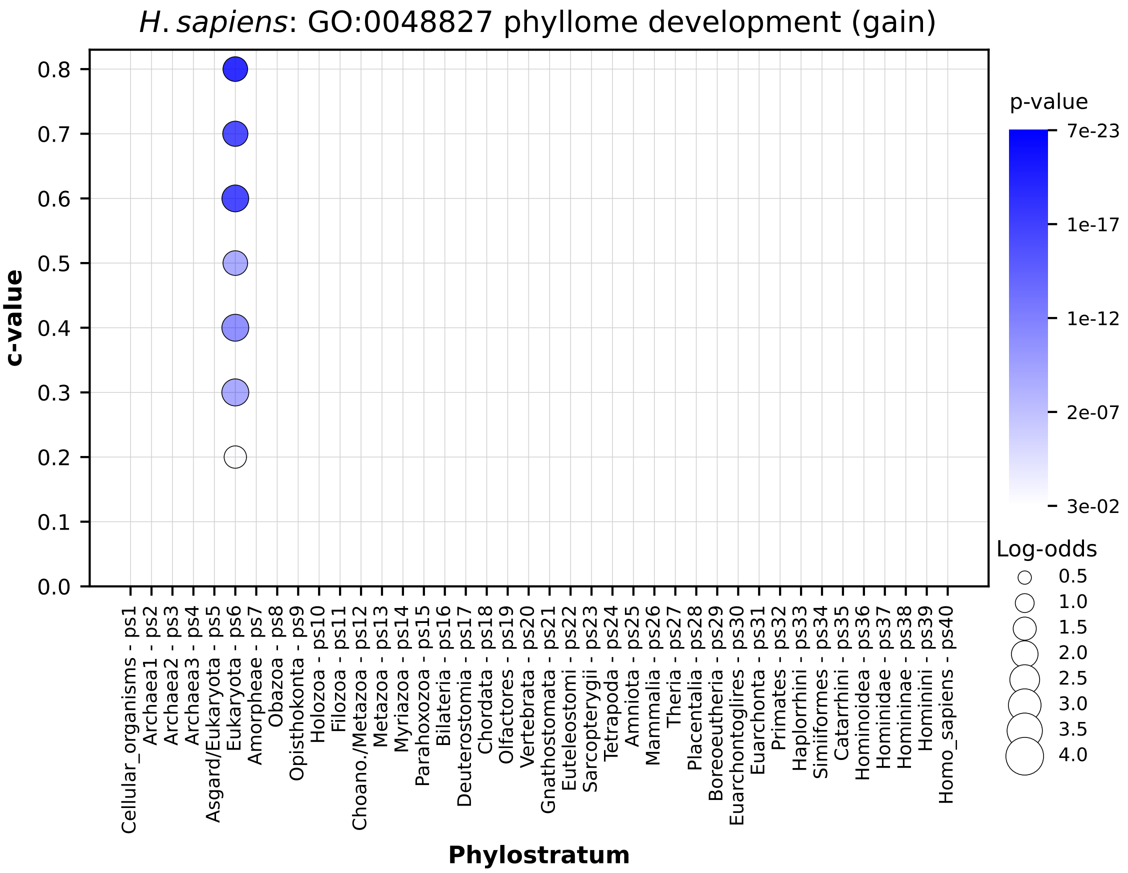

H. sapiens: GO:0048827 phyllome development (gain)
c-value
0.0
0.1
0.2
0.3
0.4
0.5
0.6
0.7
0.8
Phylostratum
Cellular_organisms - ps1
Archaea1 - ps2
Archaea2 - ps3
Archaea3 - ps4
Asgard/Eukaryota - ps5
Eukaryota - ps6
Amorpheae - ps7
Obazoa - ps8
Opisthokonta - ps9
Holozoa - ps10
Filozoa - ps11
Choano./Metazoa - ps12
Metazoa - ps13
Myriazoa - ps14
Parahoxozoa - ps15
Bilateria - ps16
Deuterostomia - ps17
Chordata - ps18
Olfactores - ps19
Vertebrata - ps20
Gnathostomata - ps21
Euteleostomi - ps22
Sarcopterygii - ps23
Tetrapoda - ps24
Amniota - ps25
Mammalia - ps26
Theria - ps27
Placentalia - ps28
Boreoeutheria - ps29
Euarchontoglires - ps30
Euarchonta - ps31
Primates - ps32
Haplorrhini - ps33
Simiiformes - ps34
Catarrhini - ps35
Hominoidea - ps36
Hominidae - ps37
Homininae - ps38
Hominini - ps39
Homo_sapiens - ps40
p-value
7e-23
1e-17
1e-12
2e-07
3e-02
Log-odds
0.5
1.0
1.5
2.0
2.5
3.0
3.5
4.0

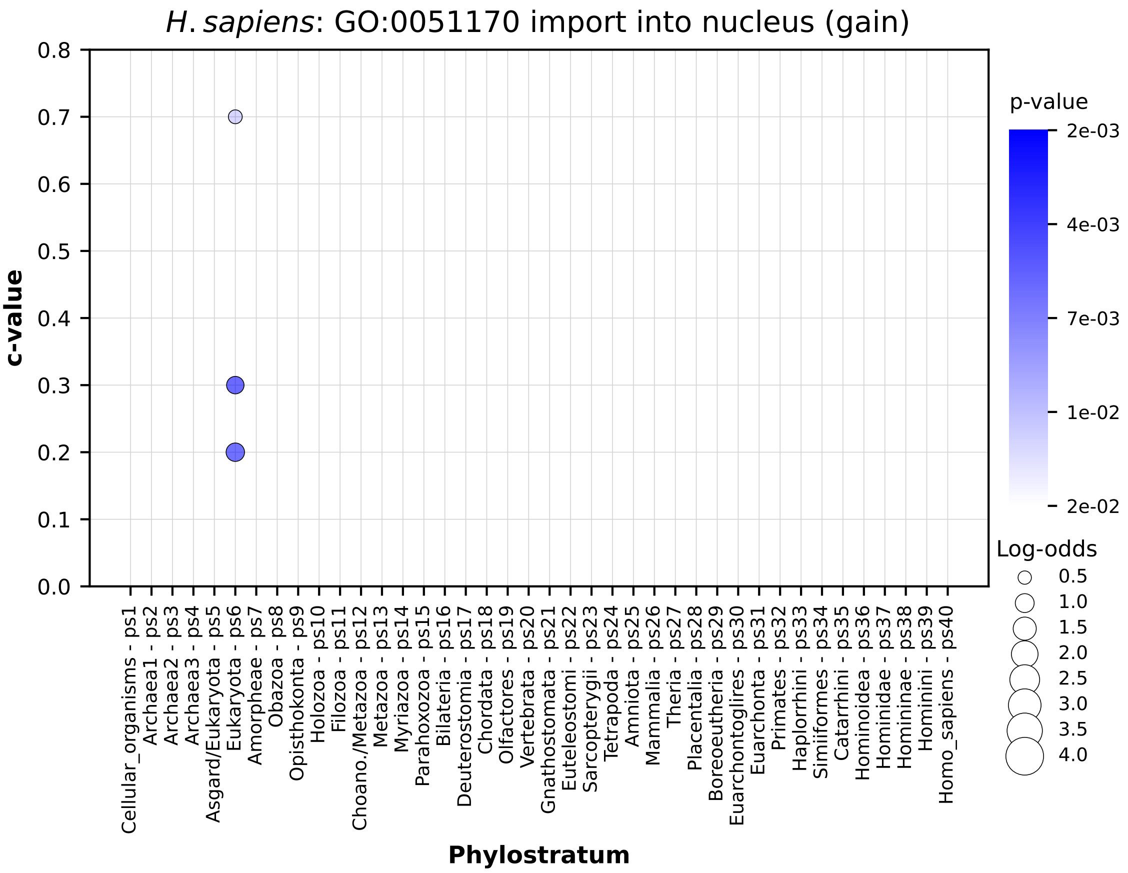

*H. sapiens*: GO:0051170 import into nucleus (gain)
c-value
0.8
0.7
0.6
0.5
0.4
0.3
0.2
0.1
0.0
p-value
2e-03
4e-03
7e-03
1e-02
2e-02
Log-odds
0.5
1.0
1.5
2.0
2.5
3.0
3.5
4.0
Cellular_organisms - ps1
Archaea1 - ps2
Archaea2 - ps3
Archaea3 - ps4
Asgard/Eukaryota - ps5
Eukaryota - ps6
Amorpheae - ps7
Obazoa - ps8
Opisthokonta - ps9
Holozoa - ps10
Filozoa - ps11
Choano./Metazoa - ps12
Metazoa - ps13
Myriazoa - ps14
Parahoxozoa - ps15
Bilateria - ps16
Deuterostomia - ps17
Chordata - ps18
Olfactores - ps19
Vertebrata - ps20
Gnathostomata - ps21
Euteleostomi - ps22
Sarcopterygii - ps23
Tetrapoda - ps24
Amniota - ps25
Mammalia - ps26
Theria - ps27
Placentalia - ps28
Boreoeutheria - ps29
Euarchontoglires - ps30
Euarchonta - ps31
Primates - ps32
Haplorrhini - ps33
Simiiformes - ps34
Catarrhini - ps35
Hominoidea - ps36
Hominidae - ps37
Homininae - ps38
Hominini - ps39
Homo_sapiens - ps40
Phylostratum

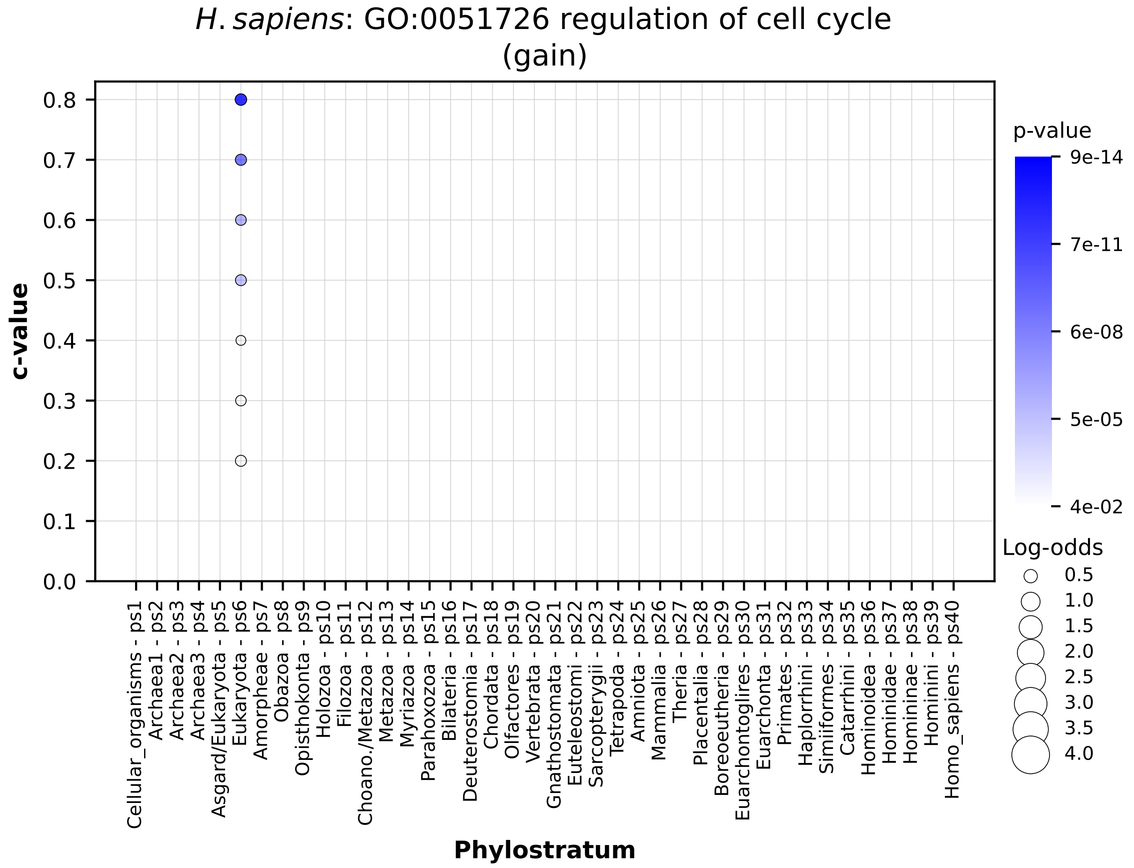

H. sapiens: GO:0051726 regulation of cell cycle
(gain)
c-value
0.0
0.1
0.2
0.3
0.4
0.5
0.6
0.7
0.8
Phylostratum
Cellular_organisms - ps1
Archaea1 - ps2
Archaea2 - ps3
Archaea3 - ps4
Asgard/Eukaryota - ps5
Eukaryota - ps6
Amorpheae - ps7
Obazoa - ps8
Opisthokonta - ps9
Holozoa - ps10
Filozoa - ps11
Choano./Metazoa - ps12
Metazoa - ps13
Myriazoa - ps14
Parahoxozoa - ps15
Bilateria - ps16
Deuterostomia - ps17
Chordata - ps18
Olfactores - ps19
Vertebrata - ps20
Gnathostomata - ps21
Euteleostomi - ps22
Sarcopterygii - ps23
Tetrapoda - ps24
Amniota - ps25
Mammalia - ps26
Theria - ps27
Placentalia - ps28
Boreoeutheria - ps29
Euarchontoglires - ps30
Euarchonta - ps31
Primates - ps32
Haplorrhini - ps33
Simiiformes - ps34
Catarrhini - ps35
Hominoidea - ps36
Hominidae - ps37
Homininae - ps38
Hominini - ps39
Homo_sapiens - ps40
p-value
9e-14
7e-11
6e-08
5e-05
4e-02
Log-odds
0.5
1.0
1.5
2.0
2.5
3.0
3.5
4.0

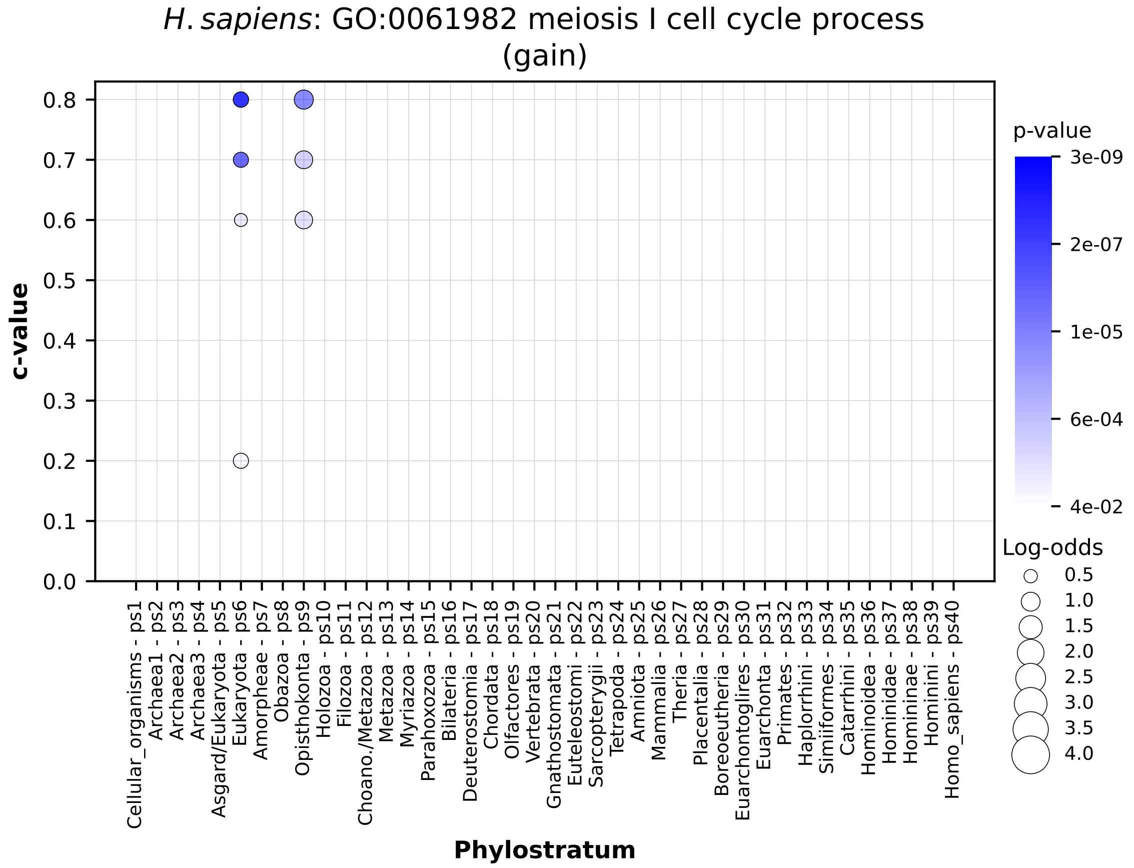
H. sapiens: GO:0061982 meiosis I cell cycle process
(gain)
c-value
0.0
0.1
0.2
0.3
0.4
0.5
0.6
0.7
0.8
p-value
3e-09
2e-07
1e-05
6e-04
4e-02
Log-odds
0.5
1.0
1.5
2.0
2.5
3.0
3.5
4.0
Cellular_organisms - ps1
Archaea1 - ps2
Archaea2 - ps3
Archaea3 - ps4
Asgard/Eukaryota - ps5
Eukaryota - ps6
Amorpheae - ps7
Obazoa - ps8
Opisthokonta - ps9
Holozoa - ps10
Filozoa - ps11
Choano./Metazoa - ps12
Metazoa - ps13
Myriazoa - ps14
Parahoxozoa - ps15
Bilateria - ps16
Deuterostomia - ps17
Chordata - ps18
Olfactores - ps19
Vertebrata - ps20
Gnathostomata - ps21
Euteleostomi - ps22
Sarcopterygii - ps23
Tetrapoda - ps24
Amniota - ps25
Mammalia - ps26
Theria - ps27
Placentalia - ps28
Boreoeutheria - ps29
Euarchontoglires - ps30
Euarchonta - ps31
Primates - ps32
Haplorrhini - ps33
Simiiformes - ps34
Catarrhini - ps35
Hominoidea - ps36
Hominidae - ps37
Homininae - ps38
Hominini - ps39
Homo_sapiens - ps40
Phylostratum

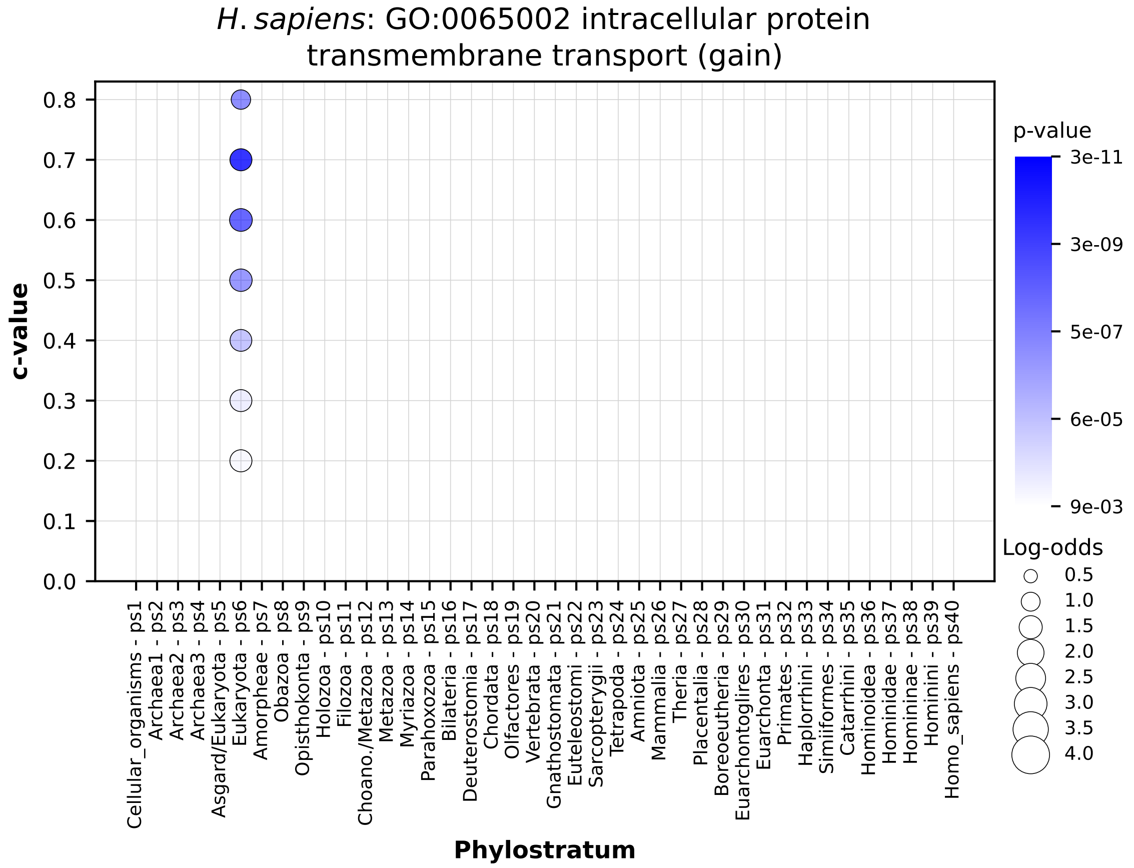
H. sapiens: GO:0065002 intracellular protein transmembrane transport (gain)
c-value
0.0
0.1
0.2
0.3
0.4
0.5
0.6
0.7
0.8
Phylostratum
Cellular_organisms - ps1
Archaea1 - ps2
Archaea2 - ps3
Archaea3 - ps4
Asgard/Eukaryota - ps5
Eukaryota - ps6
Amorpheae - ps7
Obazoa - ps8
Opisthokonta - ps9
Holozoa - ps10
Filozoa - ps11
Choano./Metazoa - ps12
Metazoa - ps13
Myriazoa - ps14
Parahoxozoa - ps15
Bilateria - ps16
Deuterostomia - ps17
Chordata - ps18
Olfactores - ps19
Vertebrata - ps20
Gnathostomata - ps21
Euteleostomi - ps22
Sarcopterygii - ps23
Tetrapoda - ps24
Amniota - ps25
Mammalia - ps26
Theria - ps27
Placentalia - ps28
Boreoeutheria - ps29
Euarchontoglires - ps30
Euarchonta - ps31
Primates - ps32
Haplorrhini - ps33
Simiiformes - ps34
Catarrhini - ps35
Hominoidea - ps36
Hominidae - ps37
Homininae - ps38
Hominini - ps39
Homo_sapiens - ps40
p-value
3e-11
3e-09
5e-07
6e-05
9e-03
Log-odds
0.5
1.0
1.5
2.0
2.5
3.0
3.5
4.0

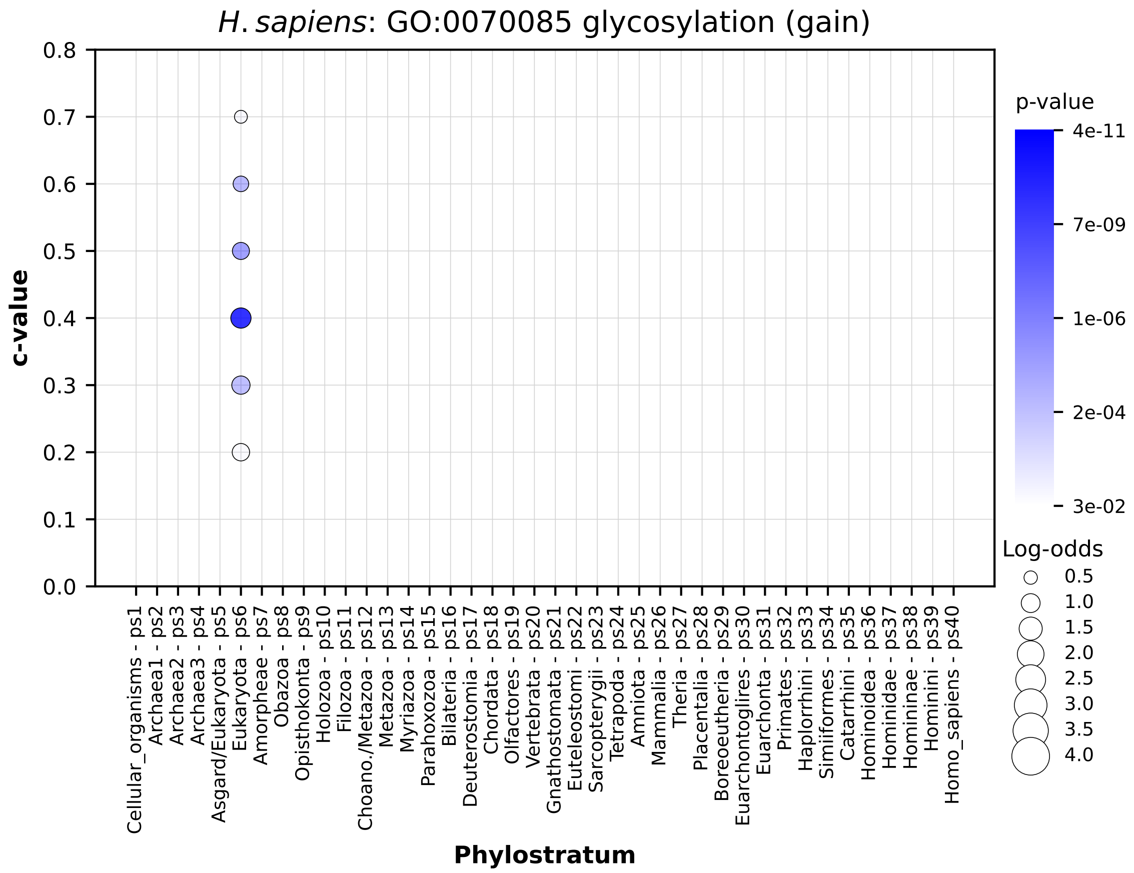

H. sapiens: GO:0070085 glycosylation (gain)
c-value
Phylostratum
p-value
4e-11
7e-09
1e-06
2e-04
3e-02
Log-odds
0.5
1.0
1.5
2.0
2.5
3.0
3.5
4.0
Cellular_organisms - ps1
Archaea1 - ps2
Archaea2 - ps3
Archaea3 - ps4
Asgard/Eukaryota - ps5
Eukaryota - ps6
Amorpheae - ps7
Obazoa - ps8
Opisthokonta - ps9
Holozoa - ps10
Filozoa - ps11
Choano./Metazoa - ps12
Metazoa - ps13
Myriazoa - ps14
Parahoxozoa - ps15
Bilateria - ps16
Deuterostomia - ps17
Chordata - ps18
Olfactores - ps19
Vertebrata - ps20
Gnathostomata - ps21
Euteleostomi - ps22
Sarcopterygii - ps23
Tetrapoda - ps24
Amniota - ps25
Mammalia - ps26
Theria - ps27
Placentalia - ps28
Boreoeutheria - ps29
Euarchontoglires - ps30
Euarchonta - ps31
Primates - ps32
Haplorrhini - ps33
Simiiformes - ps34
Catarrhini - ps35
Hominoidea - ps36
Hominidae - ps37
Homininae - ps38
Hominini - ps39
Homo_sapiens - ps40

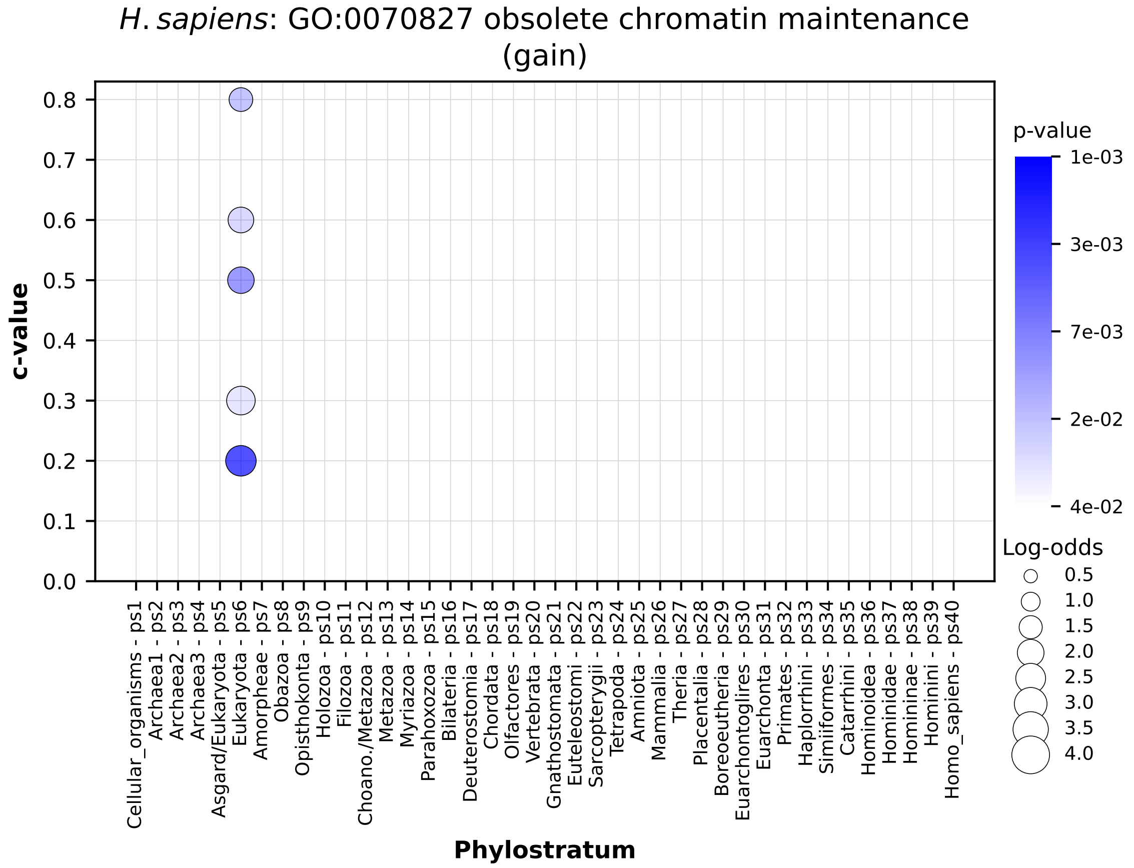

H. sapiens: GO:0070827 obsolete chromatin maintenance (gain)
c-value
0.0
0.1
0.2
0.3
0.4
0.5
0.6
0.7
0.8
Phylostratum
Cellular_organisms - ps1
Archaea1 - ps2
Archaea2 - ps3
Archaea3 - ps4
Asgard/Eukaryota - ps5
Eukaryota - ps6
Amorpheae - ps7
Obazoa - ps8
Opisthokonta - ps9
Holozoa - ps10
Filozoa - ps11
Choano./Metazoa - ps12
Metazoa - ps13
Myriazoa - ps14
Parahoxozoa - ps15
Bilateria - ps16
Deuterostomia - ps17
Chordata - ps18
Olfactores - ps19
Vertebrata - ps20
Gnathostomata - ps21
Euteleostomi - ps22
Sarcopterygii - ps23
Tetrapoda - ps24
Amniota - ps25
Mammalia - ps26
Theria - ps27
Placentalia - ps28
Boreoeutheria - ps29
Euarchontoglires - ps30
Euarchonta - ps31
Primates - ps32
Haplorrhini - ps33
Simiiformes - ps34
Catarrhini - ps35
Hominoidea - ps36
Hominidae - ps37
Homininae - ps38
Hominini - ps39
Homo_sapiens - ps40
p-value
1e-03
3e-03
7e-03
2e-02
4e-02
Log-odds
0.5
1.0
1.5
2.0
2.5
3.0
3.5
4.0

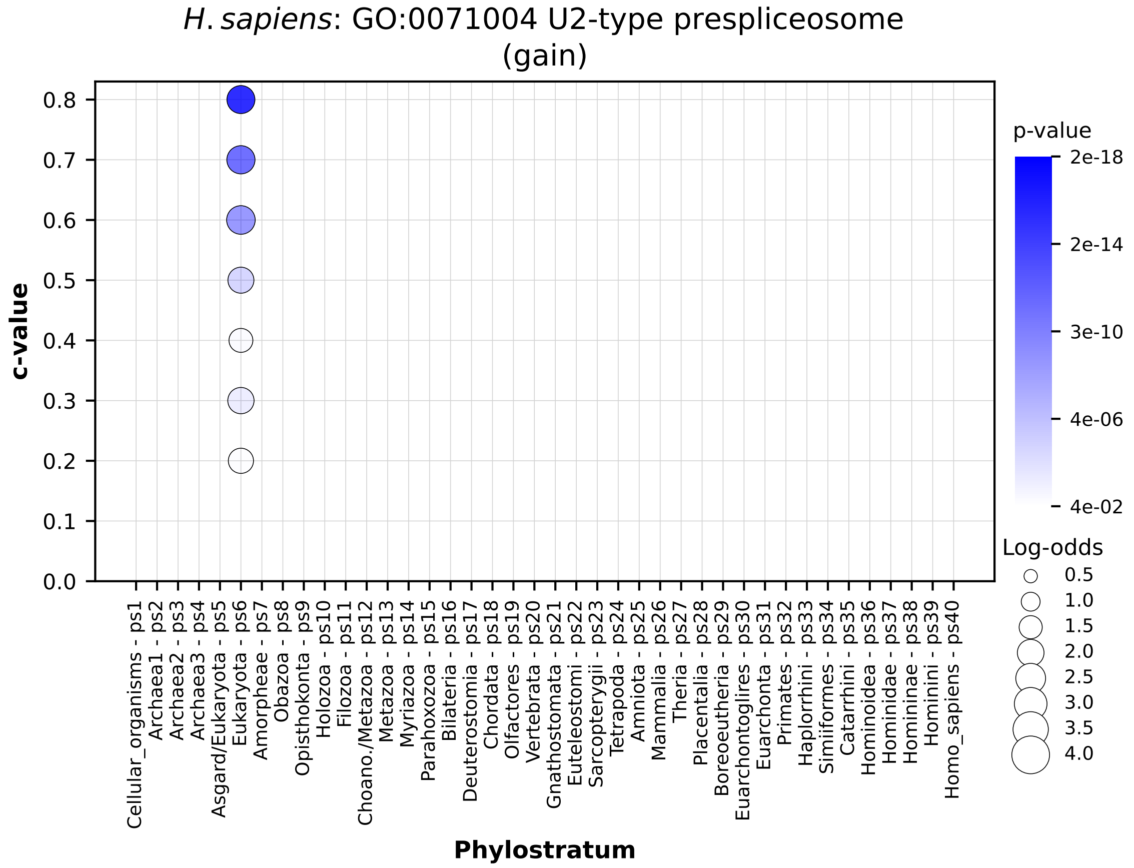

$H. sapiens$: GO:0071004 U2-type prespliceosome
(gain)
c-value
0.0
0.1
0.2
0.3
0.4
0.5
0.6
0.7
0.8
Phylostratum
Cellular_organisms - ps1
Archaea1 - ps2
Archaea2 - ps3
Archaea3 - ps4
Asgard/Eukaryota - ps5
Eukaryota - ps6
Amorpheae - ps7
Obazoa - ps8
Opisthokonta - ps9
Holozoa - ps10
Filozoa - ps11
Choano./Metazoa - ps12
Metazoa - ps13
Myriazoa - ps14
Parahoxozoa - ps15
Bilateria - ps16
Deuterostomia - ps17
Chordata - ps18
Olfactores - ps19
Vertebrata - ps20
Gnathostomata - ps21
Euteleostomi - ps22
Sarcopterygii - ps23
Tetrapoda - ps24
Amniota - ps25
Mammalia - ps26
Theria - ps27
Placentalia - ps28
Boreoeutheria - ps29
Euarchontoglires - ps30
Euarchonta - ps31
Primates - ps32
Haplorrhini - ps33
Simiiformes - ps34
Catarrhini - ps35
Hominoidea - ps36
Hominidae - ps37
Homininae - ps38
Hominini - ps39
Homo_sapiens - ps40
p-value
2e-18
2e-14
3e-10
4e-06
4e-02
Log-odds
0.5
1.0
1.5
2.0
2.5
3.0
3.5
4.0

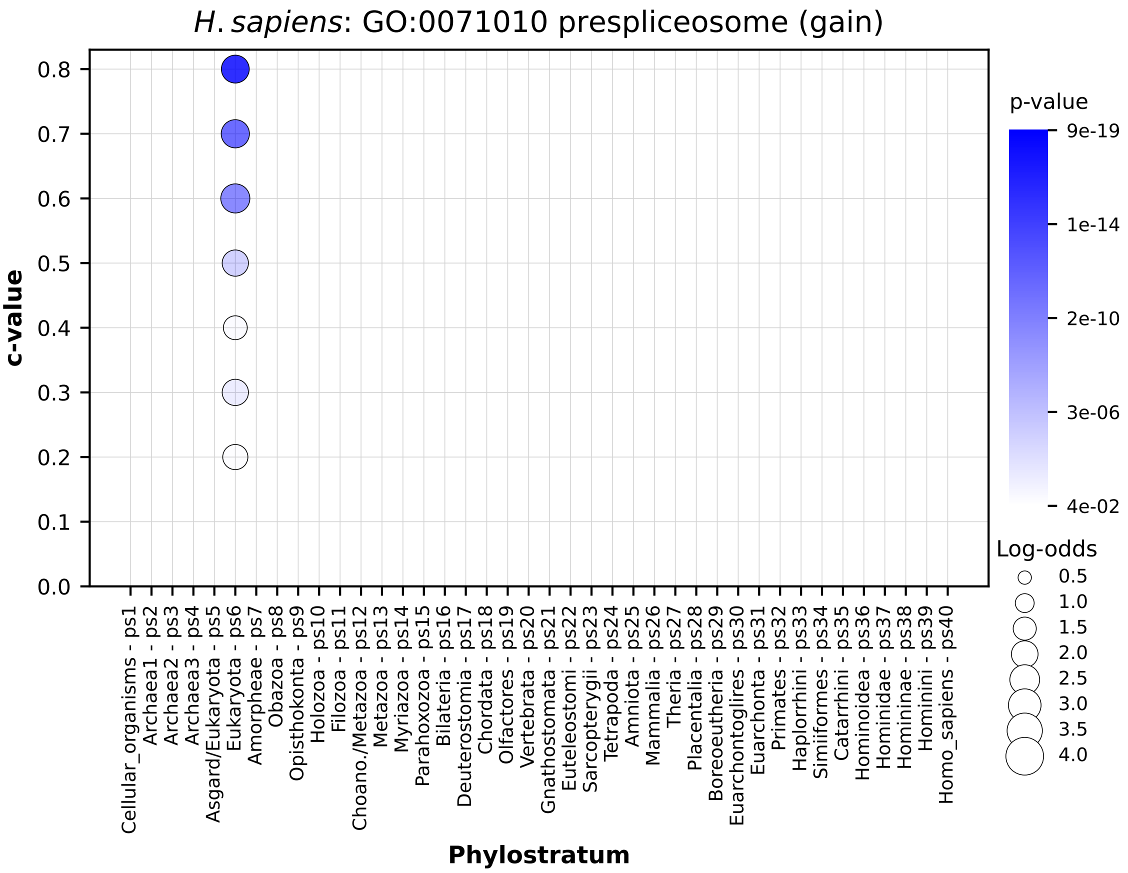

H. sapiens: GO:0071010 prespliceosome (gain)
c-value
0.0
0.1
0.2
0.3
0.4
0.5
0.6
0.7
0.8
Phylostratum
Cellular_organisms - ps1
Archaea1 - ps2
Archaea2 - ps3
Archaea3 - ps4
Asgard/Eukaryota - ps5
Eukaryota - ps6
Amorpheae - ps7
Obazoa - ps8
Opisthokonta - ps9
Holozoa - ps10
Filozoa - ps11
Choano./Metazoa - ps12
Metazoa - ps13
Myriazoa - ps14
Parahoxozoa - ps15
Bilateria - ps16
Deuterostomia - ps17
Chordata - ps18
Olfactores - ps19
Vertebrata - ps20
Gnathostomata - ps21
Euteleostomi - ps22
Sarcopterygii - ps23
Tetrapoda - ps24
Amniota - ps25
Mammalia - ps26
Theria - ps27
Placentalia - ps28
Boreoeutheria - ps29
Euarchontoglires - ps30
Euarchonta - ps31
Primates - ps32
Haplorrhini - ps33
Simiiformes - ps34
Catarrhini - ps35
Hominoidea - ps36
Hominidae - ps37
Homininae - ps38
Hominini - ps39
Homo_sapiens - ps40
p-value
9e-19
1e-14
2e-10
3e-06
4e-02
Log-odds
0.5
1.0
1.5
2.0
2.5
3.0
3.5
4.0

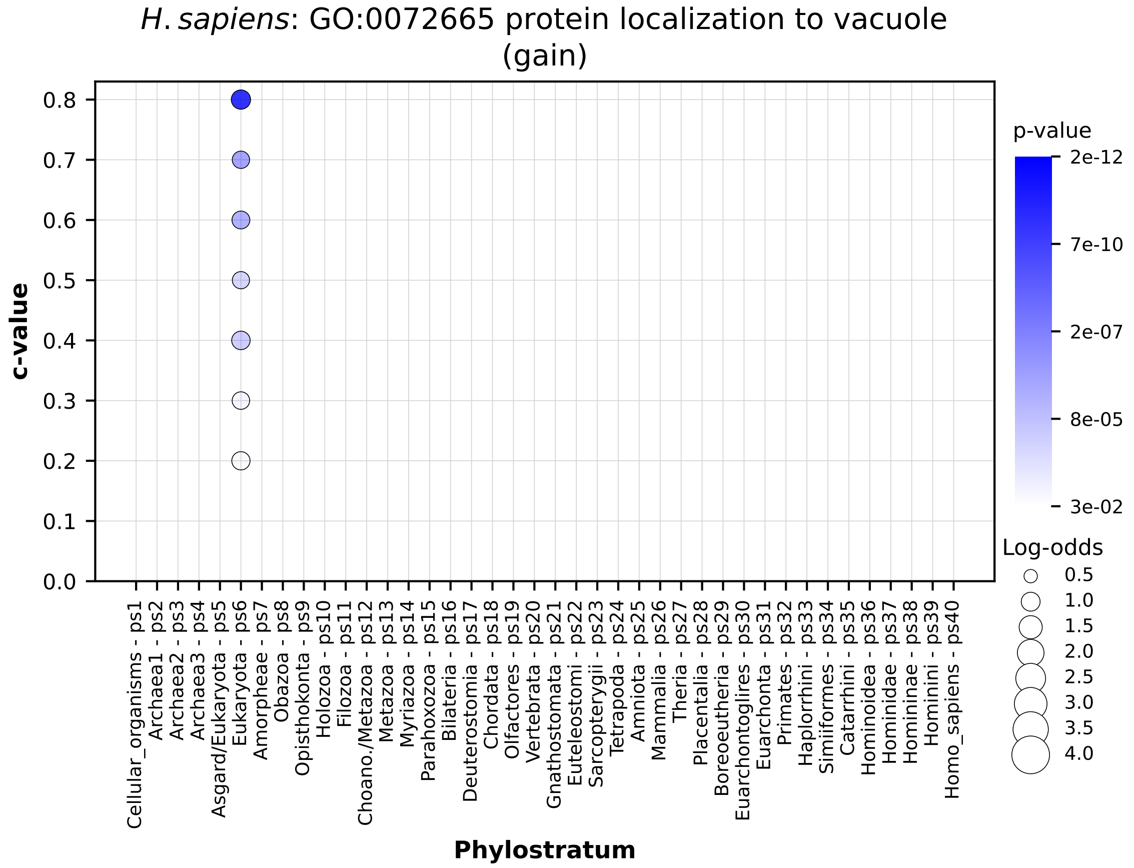

H. sapiens: GO:0072665 protein localization to vacuole (gain)
c-value
0.0
0.1
0.2
0.3
0.4
0.5
0.6
0.7
0.8
Phylostratum
Cellular_organisms - ps1
Archaea1 - ps2
Archaea2 - ps3
Archaea3 - ps4
Asgard/Eukaryota - ps5
Eukaryota - ps6
Amorpheae - ps7
Obazoa - ps8
Opisthokonta - ps9
Holozoa - ps10
Filozoa - ps11
Choano./Metazoa - ps12
Metazoa - ps13
Myriazoa - ps14
Parahoxozoa - ps15
Bilateria - ps16
Deuterostomia - ps17
Chordata - ps18
Olfactores - ps19
Vertebrata - ps20
Gnathostomata - ps21
Euteleostomi - ps22
Sarcopterygii - ps23
Tetrapoda - ps24
Amniota - ps25
Mammalia - ps26
Theria - ps27
Placentalia - ps28
Boreoeutheria - ps29
Euarchontoglires - ps30
Euarchonta - ps31
Primates - ps32
Haplorrhini - ps33
Simiiformes - ps34
Catarrhini - ps35
Hominoidea - ps36
Hominidae - ps37
Homininae - ps38
Hominini - ps39
Homo_sapiens - ps40
p-value
2e-12
7e-10
2e-07
8e-05
3e-02
Log-odds
0.5
1.0
1.5
2.0
2.5
3.0
3.5
4.0

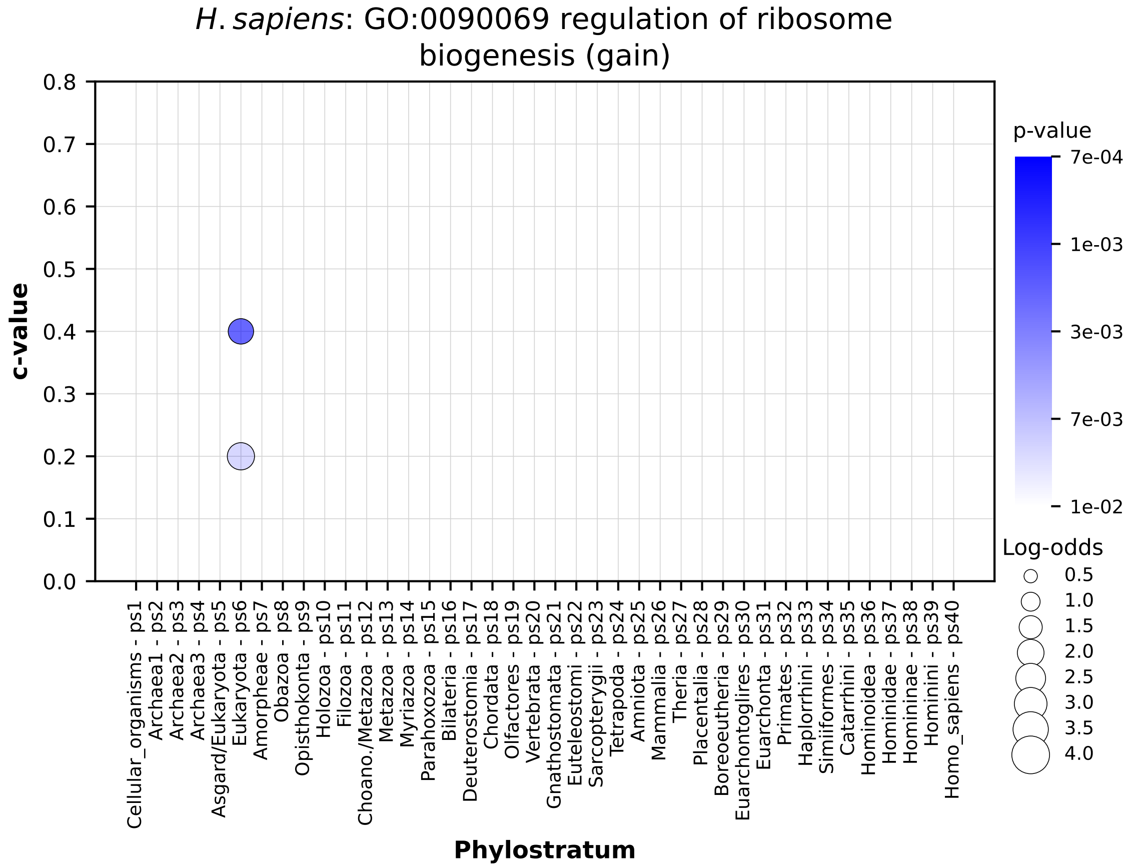

*H. sapiens*: GO:0090069 regulation of ribosome biogenesis (gain)
c-value
0.0
0.1
0.2
0.3
0.4
0.5
0.6
0.7
0.8
Phylostratum
Cellular_organisms - ps1
Archaea1 - ps2
Archaea2 - ps3
Archaea3 - ps4
Asgard/Eukaryota - ps5
Eukaryota - ps6
Amorpheae - ps7
Obazoa - ps8
Opisthokonta - ps9
Holozoa - ps10
Filozoa - ps11
Choano./Metazoa - ps12
Metazoa - ps13
Myriazoa - ps14
Parahoxozoa - ps15
Bilateria - ps16
Deuterostomia - ps17
Chordata - ps18
Olfactores - ps19
Vertebrata - ps20
Gnathostomata - ps21
Euteleostomi - ps22
Sarcopterygii - ps23
Tetrapoda - ps24
Amniota - ps25
Mammalia - ps26
Theria - ps27
Placentalia - ps28
Boreoeutheria - ps29
Euarchontoglires - ps30
Euarchonta - ps31
Primates - ps32
Haplorrhini - ps33
Simiiformes - ps34
Catarrhini - ps35
Hominoidea - ps36
Hominidae - ps37
Homininae - ps38
Hominini - ps39
Homo_sapiens - ps40
p-value
7e-04
1e-03
3e-03
7e-03
1e-02
Log-odds
0.5
1.0
1.5
2.0
2.5
3.0
3.5
4.0

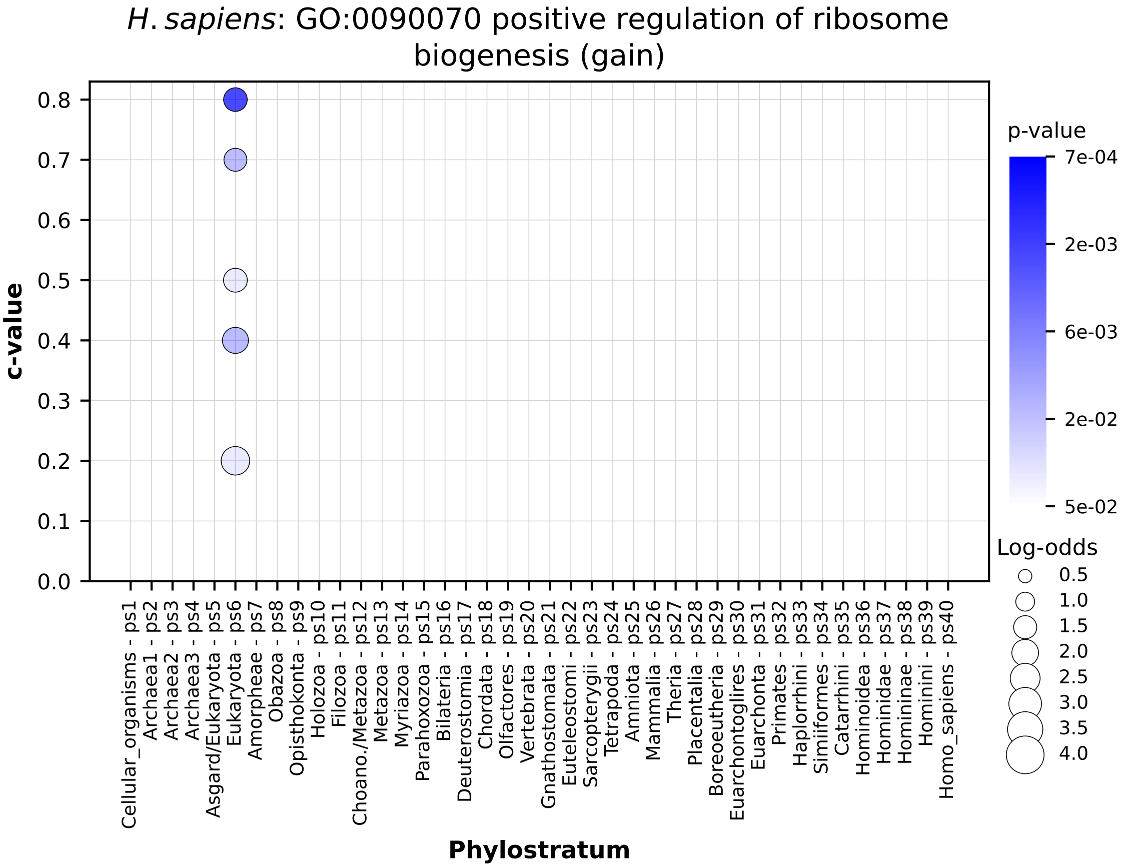

H. sapiens: GO:0090070 positive regulation of ribosome biogenesis (gain)
c-value
0.0
0.1
0.2
0.3
0.4
0.5
0.6
0.7
0.8
Cellular_organisms - ps1
Archaea1 - ps2
Archaea2 - ps3
Archaea3 - ps4
Asgard/Eukaryota - ps5
Eukaryota - ps6
Amorpheae - ps7
Obazoa - ps8
Opisthokonta - ps9
Holozoa - ps10
Filozoa - ps11
Choano./Metazoa - ps12
Metazoa - ps13
Myriazoa - ps14
Parahoxozoa - ps15
Bilateria - ps16
Deuterostomia - ps17
Chordata - ps18
Olfactores - ps19
Vertebrata - ps20
Gnathostomata - ps21
Euteleostomi - ps22
Sarcopterygii - ps23
Tetrapoda - ps24
Amniota - ps25
Mammalia - ps26
Theria - ps27
Placentalia - ps28
Boreoeutheria - ps29
Euarchontoglires - ps30
Euarchonta - ps31
Primates - ps32
Haplorrhini - ps33
Simiiformes - ps34
Catarrhini - ps35
Hominoidea - ps36
Hominidae - ps37
Homininae - ps38
Hominini - ps39
Homo_sapiens - ps40
Phylostratum
p-value
7e-04
2e-03
6e-03
2e-02
5e-02
Log-odds
0.5
1.0
1.5
2.0
2.5
3.0
3.5
4.0

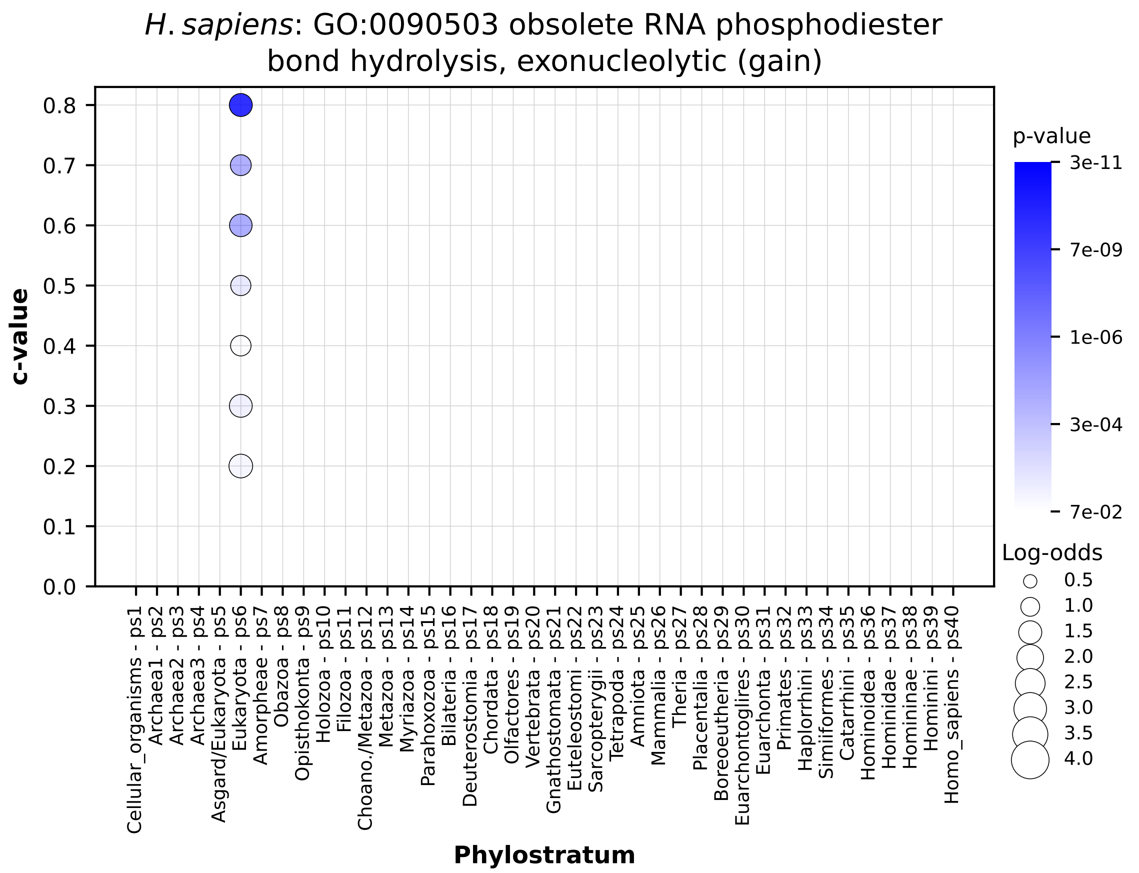

H. sapiens: GO:0090503 obsolete RNA phosphodiester bond hydrolysis, exonucleolytic (gain)
c-value
0.0
0.1
0.2
0.3
0.4
0.5
0.6
0.7
0.8
Phylostratum
Cellular_organisms - ps1
Archaea1 - ps2
Archaea2 - ps3
Archaea3 - ps4
Asgard/Eukaryota - ps5
Eukaryota - ps6
Amorpheae - ps7
Obazoa - ps8
Opisthokonta - ps9
Holozoa - ps10
Filozoa - ps11
Choano./Metazoa - ps12
Metazoa - ps13
Myriazoa - ps14
Parahoxozoa - ps15
Bilateria - ps16
Deuterostomia - ps17
Chordata - ps18
Olfactores - ps19
Vertebrata - ps20
Gnathostomata - ps21
Euteleostomi - ps22
Sarcopterygii - ps23
Tetrapoda - ps24
Amniota - ps25
Mammalia - ps26
Theria - ps27
Placentalia - ps28
Boreoeutheria - ps29
Euarchontoglires - ps30
Euarchonta - ps31
Primates - ps32
Haplorrhini - ps33
Simiiformes - ps34
Catarrhini - ps35
Hominoidea - ps36
Hominidae - ps37
Homininae - ps38
Hominini - ps39
Homo_sapiens - ps40
p-value
3e-11
7e-09
1e-06
3e-04
7e-02
Log-odds
0.5
1.0
1.5
2.0
2.5
3.0
3.5
4.0

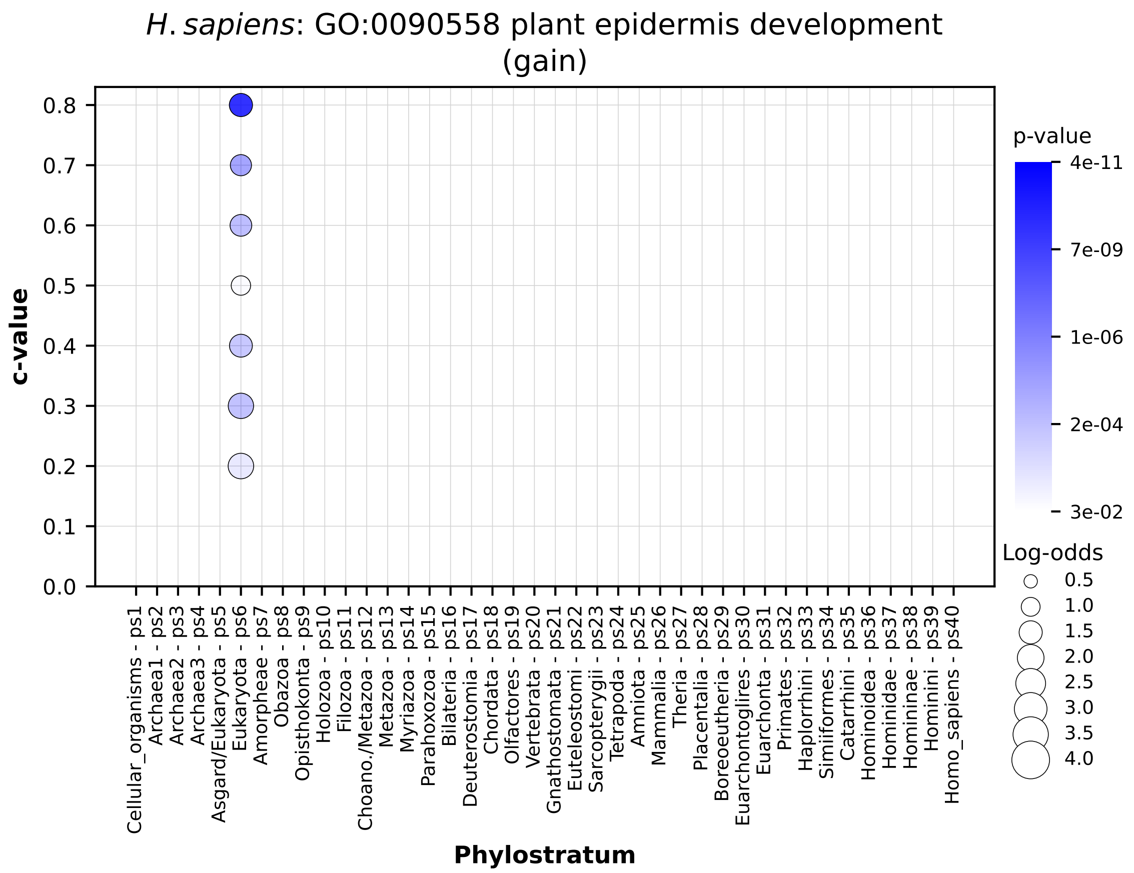
H. sapiens: GO:0090558 plant epidermis development
(gain)
c-value
0.8
0.7
0.6
0.5
0.4
0.3
0.2
0.1
0.0
Phylostratum
Cellular_organisms - ps1
Archaea1 - ps2
Archaea2 - ps3
Archaea3 - ps4
Asgard/Eukaryota - ps5
Eukaryota - ps6
Amorpheae - ps7
Obazoa - ps8
Opisthokonta - ps9
Holozoa - ps10
Filozoa - ps11
Choano./Metazoa - ps12
Metazoa - ps13
Myriazoa - ps14
Parahoxozoa - ps15
Bilateria - ps16
Deuterostomia - ps17
Chordata - ps18
Olfactores - ps19
Vertebrata - ps20
Gnathostomata - ps21
Euteleostomi - ps22
Sarcopterygii - ps23
Tetrapoda - ps24
Amniota - ps25
Mammalia - ps26
Theria - ps27
Placentalia - ps28
Boreoeutheria - ps29
Euarchontoglires - ps30
Euarchonta - ps31
Primates - ps32
Haplorrhini - ps33
Simiiformes - ps34
Catarrhini - ps35
Hominoidea - ps36
Hominidae - ps37
Homininae - ps38
Hominini - ps39
Homo_sapiens - ps40
p-value
4e-11
7e-09
1e-06
2e-04
3e-02
Log-odds
0.5
1.0
1.5
2.0
2.5
3.0
3.5
4.0

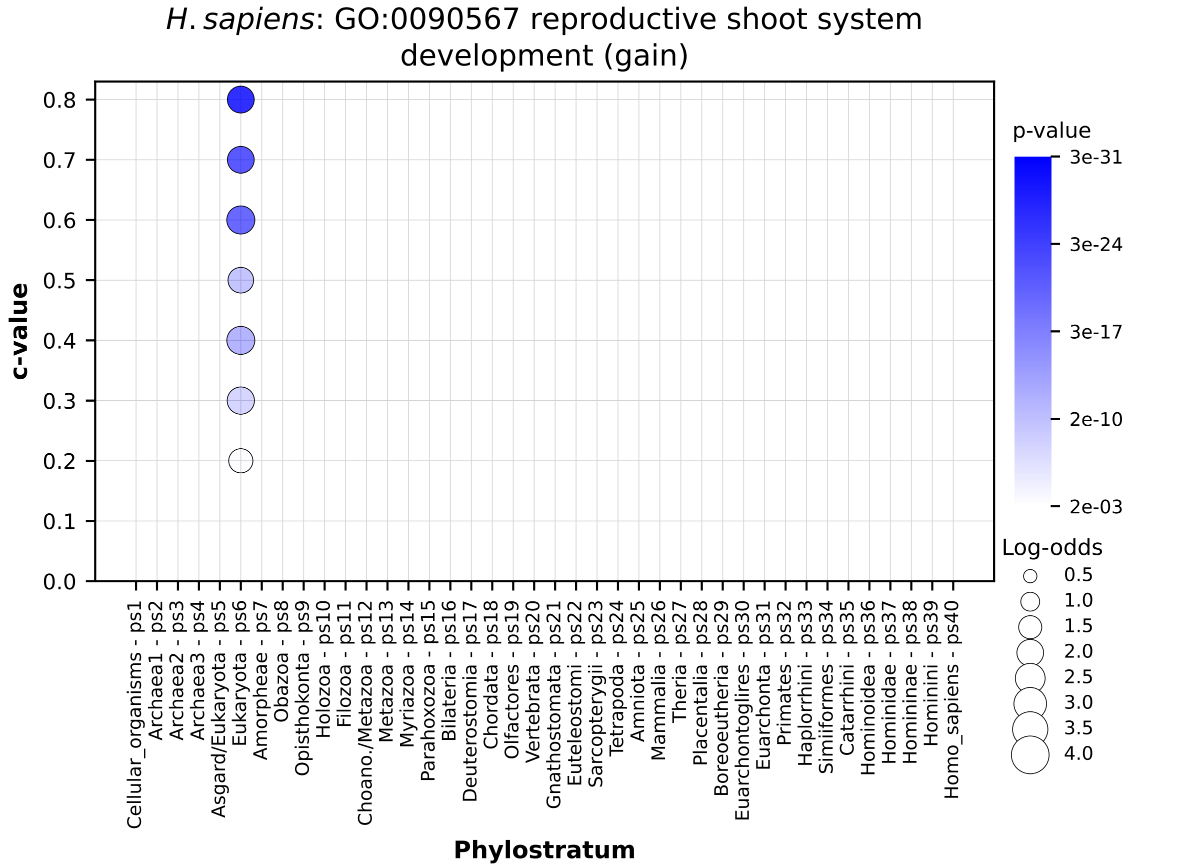
H. sapiens: GO:0090567 reproductive shoot system development (gain)
c-value
0.0
0.1
0.2
0.3
0.4
0.5
0.6
0.7
0.8
Phylostratum
Cellular_organisms - ps1
Archaea1 - ps2
Archaea2 - ps3
Archaea3 - ps4
Asgard/Eukaryota - ps5
Eukaryota - ps6
Amorpheae - ps7
Obazoa - ps8
Opisthokonta - ps9
Holozoa - ps10
Filozoa - ps11
Choano./Metazoa - ps12
Metazoa - ps13
Myriazoa - ps14
Parahoxozoa - ps15
Bilateria - ps16
Deuterostomia - ps17
Chordata - ps18
Olfactores - ps19
Vertebrata - ps20
Gnathostomata - ps21
Euteleostomi - ps22
Sarcopterygii - ps23
Tetrapoda - ps24
Amniota - ps25
Mammalia - ps26
Theria - ps27
Placentalia - ps28
Boreoeutheria - ps29
Euarchontoglires - ps30
Euarchonta - ps31
Primates - ps32
Haplorrhini - ps33
Simiiformes - ps34
Catarrhini - ps35
Hominoidea - ps36
Hominidae - ps37
Homininae - ps38
Hominini - ps39
Homo_sapiens - ps40
p-value
3e-31
3e-24
3e-17
2e-10
2e-03
Log-odds
0.5
1.0
1.5
2.0
2.5
3.0
3.5
4.0

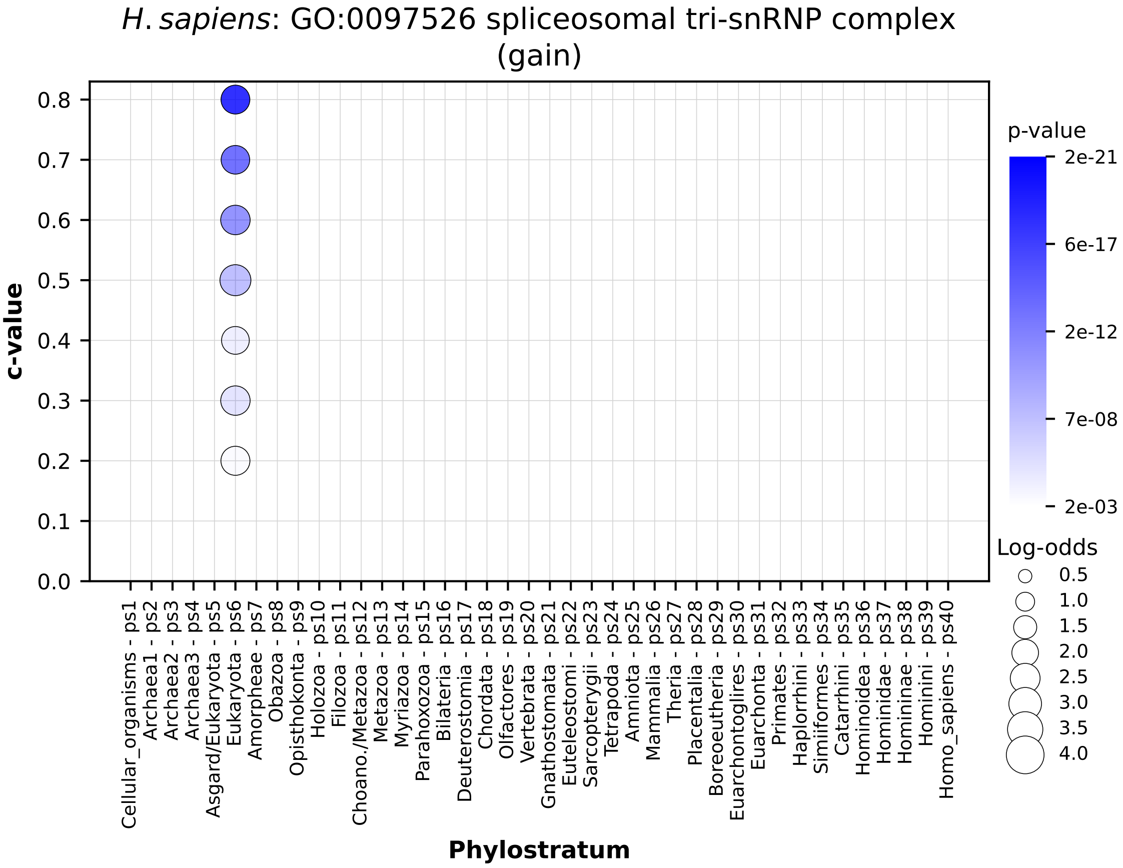

*H. sapiens*: GO:0097526 spliceosomal tri-snRNP complex (gain)
c-value
0.0
0.1
0.2
0.3
0.4
0.5
0.6
0.7
0.8
p-value
2e-21
6e-17
2e-12
7e-08
2e-03
Log-odds
0.5
1.0
1.5
2.0
2.5
3.0
3.5
4.0
Cellular_organisms - ps1
Archaea1 - ps2
Archaea2 - ps3
Archaea3 - ps4
Asgard/Eukaryota - ps5
Eukaryota - ps6
Amorpheae - ps7
Obazoa - ps8
Opisthokonta - ps9
Holozoa - ps10
Filozoa - ps11
Choano./Metazoa - ps12
Metazoa - ps13
Myriazoa - ps14
Parahoxozoa - ps15
Bilateria - ps16
Deuterostomia - ps17
Chordata - ps18
Olfactores - ps19
Vertebrata - ps20
Gnathostomata - ps21
Euteleostomi - ps22
Sarcopterygii - ps23
Tetrapoda - ps24
Amniota - ps25
Mammalia - ps26
Theria - ps27
Placentalia - ps28
Boreoeutheria - ps29
Euarchontoglires - ps30
Euarchonta - ps31
Primates - ps32
Haplorrhini - ps33
Simiiformes - ps34
Catarrhini - ps35
Hominoidea - ps36
Hominidae - ps37
Homininae - ps38
Hominini - ps39
Homo_sapiens - ps40
Phylostratum

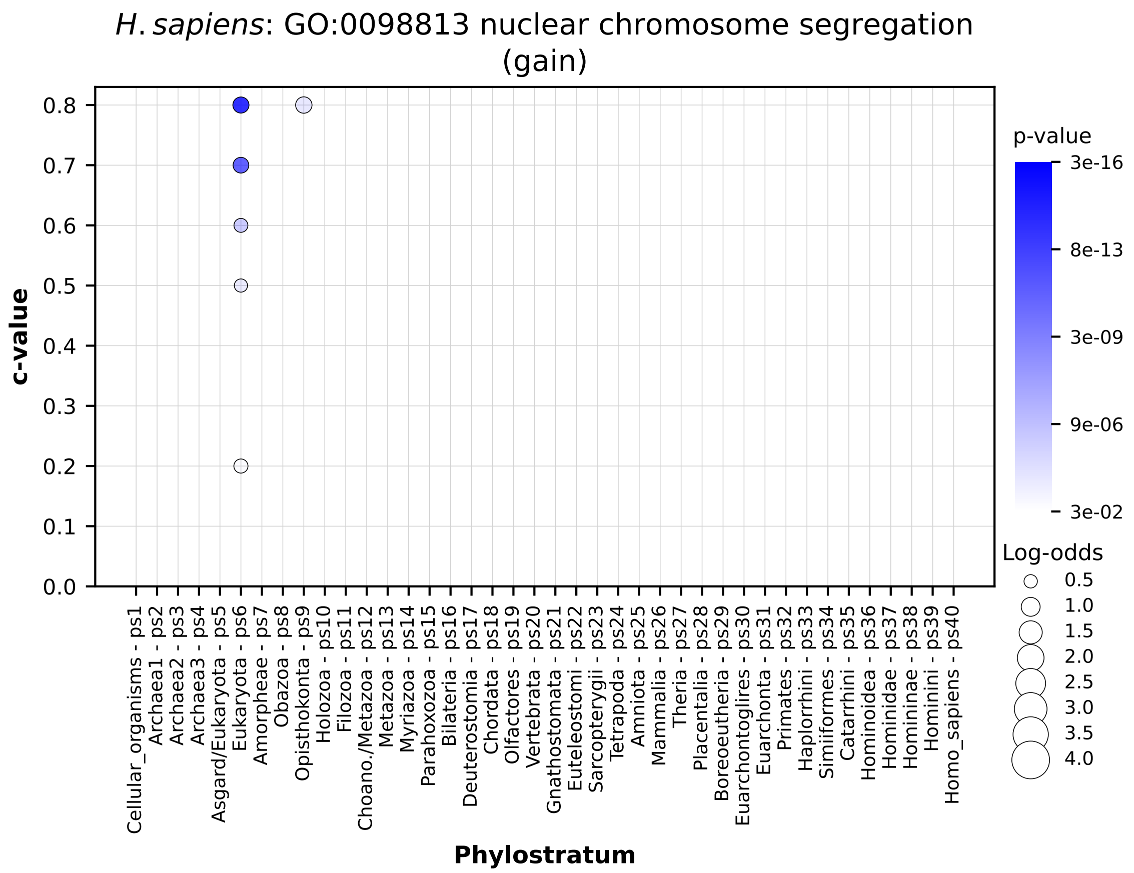
H. sapiens: GO:0098813 nuclear chromosome segregation
(gain)
c-value
0.0
0.1
0.2
0.3
0.4
0.5
0.6
0.7
0.8
Phylostratum
Cellular_organisms - ps1
Archaea1 - ps2
Archaea2 - ps3
Archaea3 - ps4
Asgard/Eukaryota - ps5
Eukaryota - ps6
Amorpheae - ps7
Obazoa - ps8
Opisthokonta - ps9
Holozoa - ps10
Filozoa - ps11
Choano./Metazoa - ps12
Metazoa - ps13
Myriazoa - ps14
Parahoxozoa - ps15
Bilateria - ps16
Deuterostomia - ps17
Chordata - ps18
Olfactores - ps19
Vertebrata - ps20
Gnathostomata - ps21
Euteleostomi - ps22
Sarcopterygii - ps23
Tetrapoda - ps24
Amniota - ps25
Mammalia - ps26
Theria - ps27
Placentalia - ps28
Boreoeutheria - ps29
Euarchontoglires - ps30
Euarchonta - ps31
Primates - ps32
Haplorrhini - ps33
Simiiformes - ps34
Catarrhini - ps35
Hominoidea - ps36
Hominidae - ps37
Homininae - ps38
Hominini - ps39
Homo_sapiens - ps40
p-value
3e-16
8e-13
3e-09
9e-06
3e-02
Log-odds
0.5
1.0
1.5
2.0
2.5
3.0
3.5
4.0

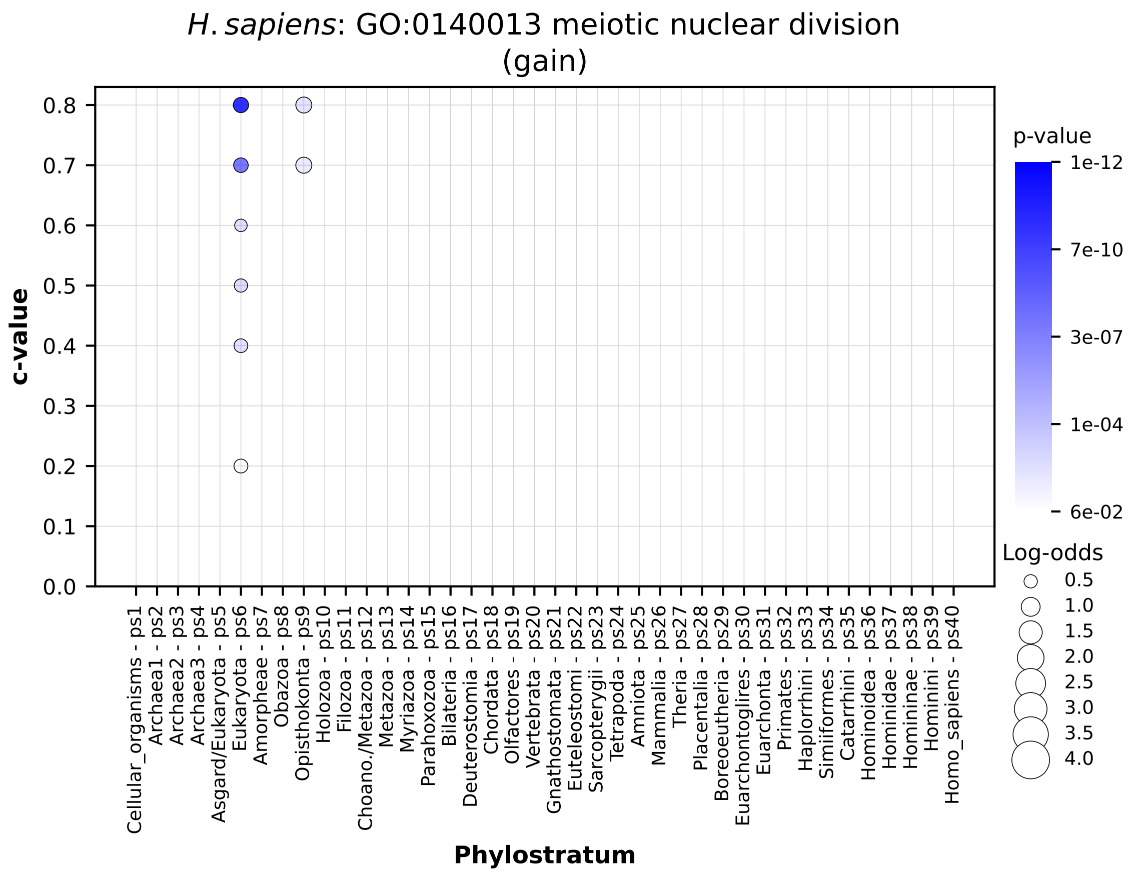

H. sapiens: GO:0140013 meiotic nuclear division
(gain)
c-value
0.0
0.1
0.2
0.3
0.4
0.5
0.6
0.7
0.8
Phylostratum
Cellular_organisms - ps1
Archaea1 - ps2
Archaea2 - ps3
Archaea3 - ps4
Asgard/Eukaryota - ps5
Eukaryota - ps6
Amorpheae - ps7
Obazoa - ps8
Opisthokonta - ps9
Holozoa - ps10
Filozoa - ps11
Choano./Metazoa - ps12
Metazoa - ps13
Myriazoa - ps14
Parahoxozoa - ps15
Bilateria - ps16
Deuterostomia - ps17
Chordata - ps18
Olfactores - ps19
Vertebrata - ps20
Gnathostomata - ps21
Euteleostomi - ps22
Sarcopterygii - ps23
Tetrapoda - ps24
Amniota - ps25
Mammalia - ps26
Theria - ps27
Placentalia - ps28
Boreoeutheria - ps29
Euarchontoglires - ps30
Euarchonta - ps31
Primates - ps32
Haplorrhini - ps33
Simiiformes - ps34
Catarrhini - ps35
Hominoidea - ps36
Hominidae - ps37
Homininae - ps38
Hominini - ps39
Homo_sapiens - ps40
p-value
1e-12
7e-10
3e-07
1e-04
6e-02
Log-odds
0.5
1.0
1.5
2.0
2.5
3.0
3.5
4.0

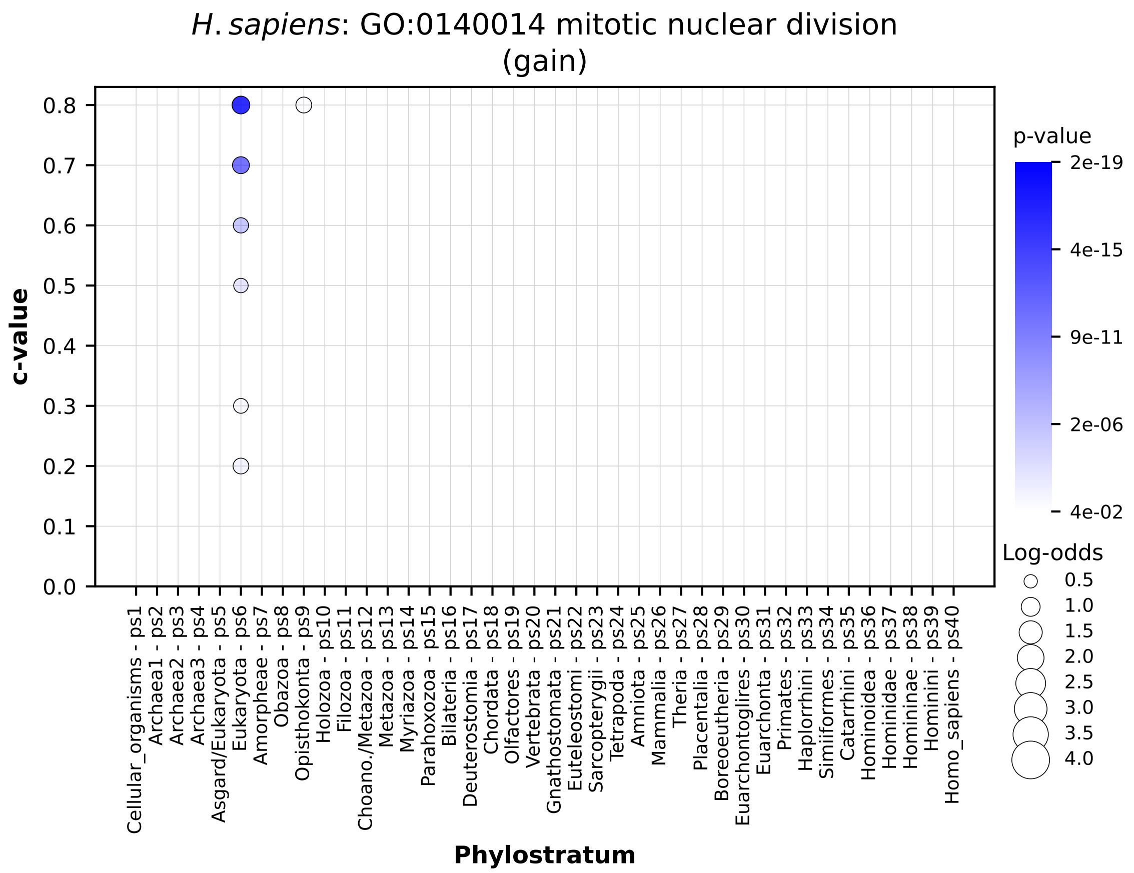

H. sapiens: GO:0140014 mitotic nuclear division
(gain)
c-value
0.0
0.1
0.2
0.3
0.4
0.5
0.6
0.7
0.8
Phylostratum
Cellular_organisms - ps1
Archaea1 - ps2
Archaea2 - ps3
Archaea3 - ps4
Asgard/Eukaryota - ps5
Eukaryota - ps6
Amorpheae - ps7
Obazoa - ps8
Opisthokonta - ps9
Holozoa - ps10
Filozoa - ps11
Choano./Metazoa - ps12
Metazoa - ps13
Myriazoa - ps14
Parahoxozoa - ps15
Bilateria - ps16
Deuterostomia - ps17
Chordata - ps18
Olfactores - ps19
Vertebrata - ps20
Gnathostomata - ps21
Euteleostomi - ps22
Sarcopterygii - ps23
Tetrapoda - ps24
Amniota - ps25
Mammalia - ps26
Theria - ps27
Placentalia - ps28
Boreoeutheria - ps29
Euarchontoglires - ps30
Euarchonta - ps31
Primates - ps32
Haplorrhini - ps33
Simiiformes - ps34
Catarrhini - ps35
Hominoidea - ps36
Hominidae - ps37
Homininae - ps38
Hominini - ps39
Homo_sapiens - ps40
p-value
2e-19
4e-15
9e-11
2e-06
4e-02
Log-odds
0.5
1.0
1.5
2.0
2.5
3.0
3.5
4.0

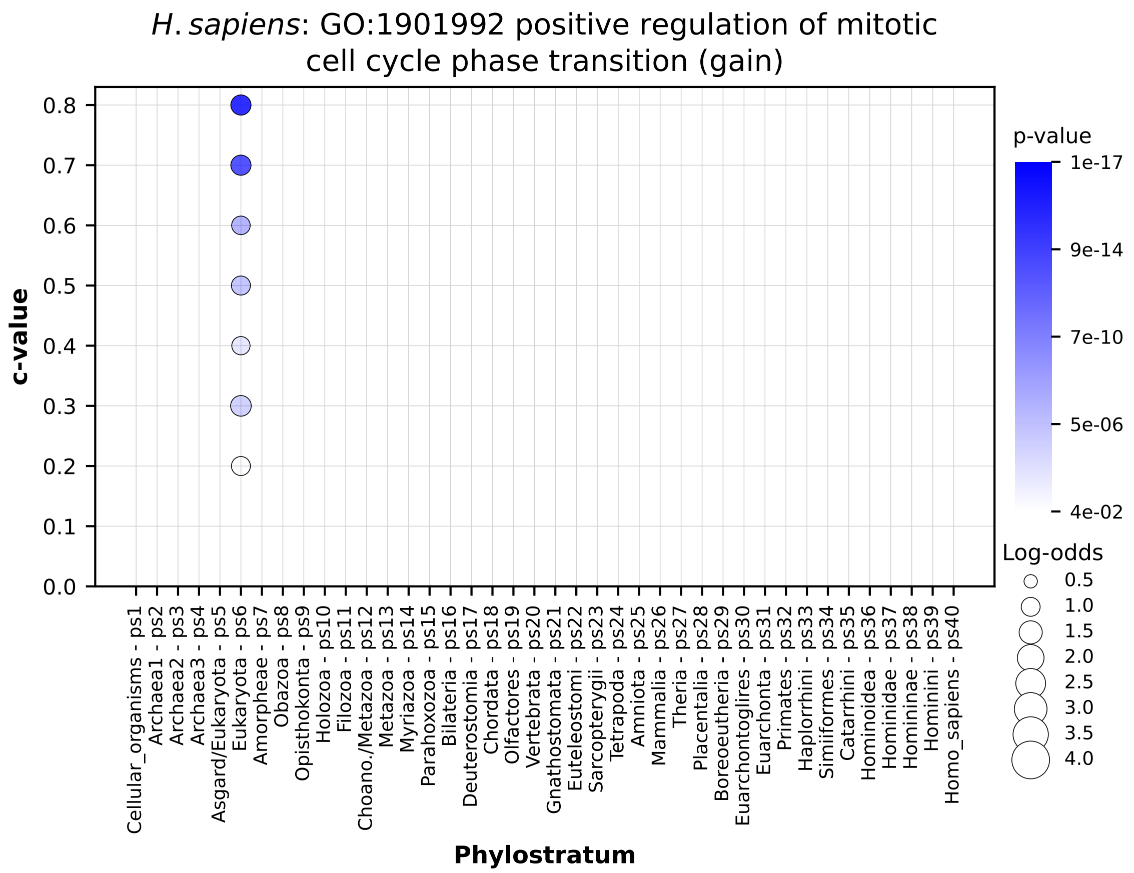

H. sapiens: GO:1901992 positive regulation of mitotic cell cycle phase transition (gain)
c-value
0.0
0.1
0.2
0.3
0.4
0.5
0.6
0.7
0.8
Phylostratum
Cellular_organisms - ps1
Archaea1 - ps2
Archaea2 - ps3
Archaea3 - ps4
Asgard/Eukaryota - ps5
Eukaryota - ps6
Amorpheae - ps7
Obazoa - ps8
Opisthokonta - ps9
Holozoa - ps10
Filozoa - ps11
Choano./Metazoa - ps12
Metazoa - ps13
Myriazoa - ps14
Parahoxozoa - ps15
Bilateria - ps16
Deuterostomia - ps17
Chordata - ps18
Olfactores - ps19
Vertebrata - ps20
Gnathostomata - ps21
Euteleostomi - ps22
Sarcopterygii - ps23
Tetrapoda - ps24
Amniota - ps25
Mammalia - ps26
Theria - ps27
Placentalia - ps28
Boreoeutheria - ps29
Euarchontoglires - ps30
Euarchonta - ps31
Primates - ps32
Haplorrhini - ps33
Simiiformes - ps34
Catarrhini - ps35
Hominoidea - ps36
Hominidae - ps37
Homininae - ps38
Hominini - ps39
Homo_sapiens - ps40
p-value
1e-17
9e-14
7e-10
5e-06
4e-02
Log-odds
0.5
1.0
1.5
2.0
2.5
3.0
3.5
4.0

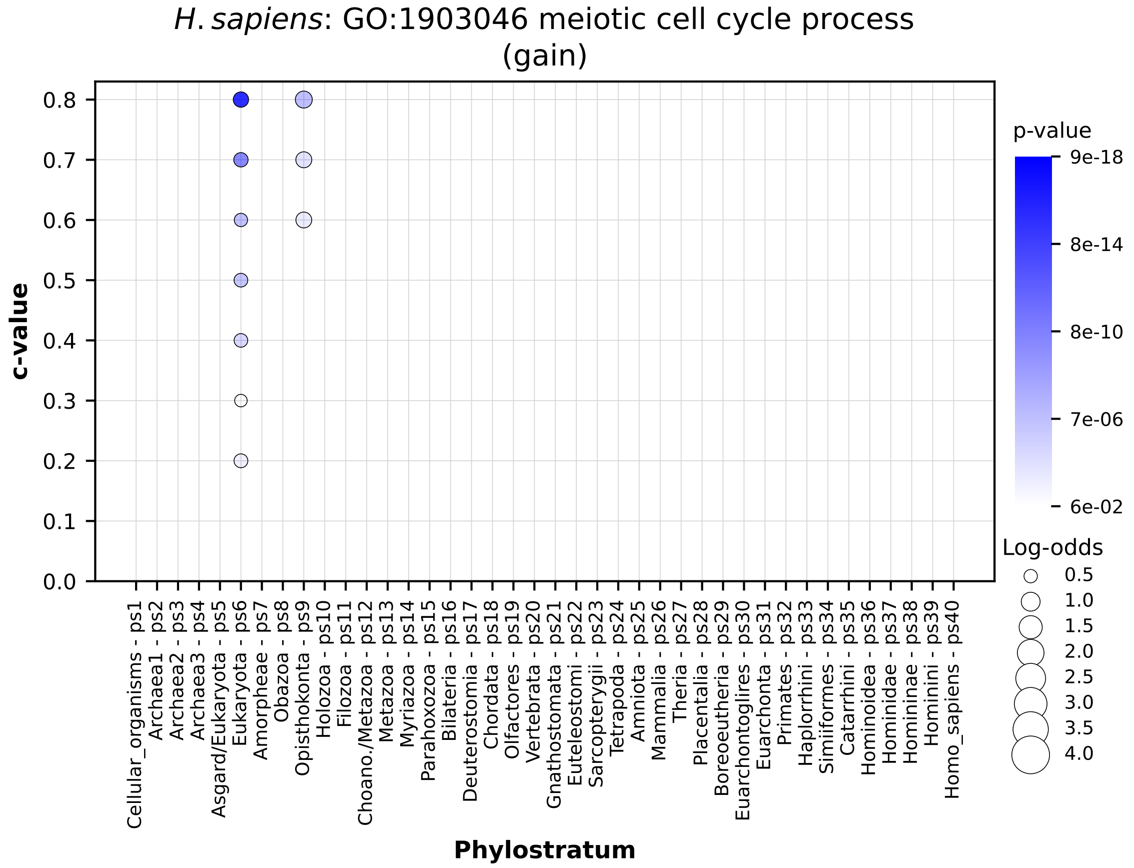

H. sapiens: GO:1903046 meiotic cell cycle process
(gain)
c-value
0.0
0.1
0.2
0.3
0.4
0.5
0.6
0.7
0.8
Phylostratum
Cellular_organisms - ps1
Archaea1 - ps2
Archaea2 - ps3
Archaea3 - ps4
Asgard/Eukaryota - ps5
Eukaryota - ps6
Amorpheae - ps7
Obazoa - ps8
Opisthokonta - ps9
Holozoa - ps10
Filozoa - ps11
Choano./Metazoa - ps12
Metazoa - ps13
Myriazoa - ps14
Parahoxozoa - ps15
Bilateria - ps16
Deuterostomia - ps17
Chordata - ps18
Olfactores - ps19
Vertebrata - ps20
Gnathostomata - ps21
Euteleostomi - ps22
Sarcopterygii - ps23
Tetrapoda - ps24
Amniota - ps25
Mammalia - ps26
Theria - ps27
Placentalia - ps28
Boreoeutheria - ps29
Euarchontoglires - ps30
Euarchonta - ps31
Primates - ps32
Haplorrhini - ps33
Simiiformes - ps34
Catarrhini - ps35
Hominoidea - ps36
Hominidae - ps37
Homininae - ps38
Hominini - ps39
Homo_sapiens - ps40
p-value
9e-18
8e-14
8e-10
7e-06
6e-02
Log-odds
0.5
1.0
1.5
2.0
2.5
3.0
3.5
4.0

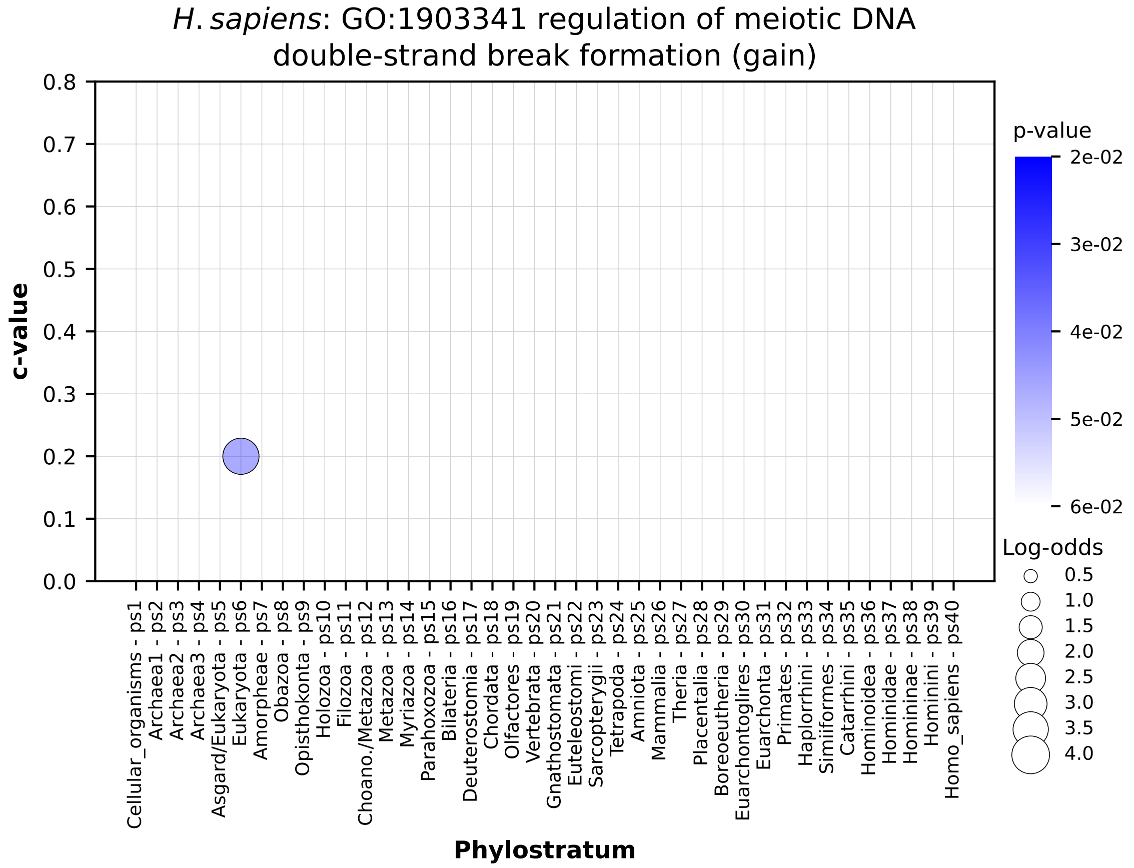
H. sapiens: GO:1903341 regulation of meiotic DNA double-strand break formation (gain)
c-value
0.0
0.1
0.2
0.3
0.4
0.5
0.6
0.7
0.8
Phylostratum
Cellular_organisms - ps1
Archaea1 - ps2
Archaea2 - ps3
Archaea3 - ps4
Asgard/Eukaryota - ps5
Eukaryota - ps6
Amorpheae - ps7
Obazoa - ps8
Opisthokonta - ps9
Holozoa - ps10
Filozoa - ps11
Choano./Metazoa - ps12
Metazoa - ps13
Myriazoa - ps14
Parahoxozoa - ps15
Bilateria - ps16
Deuterostomia - ps17
Chordata - ps18
Olfactores - ps19
Vertebrata - ps20
Gnathostomata - ps21
Euteleostomi - ps22
Sarcopterygii - ps23
Tetrapoda - ps24
Amniota - ps25
Mammalia - ps26
Theria - ps27
Placentalia - ps28
Boreoeutheria - ps29
Euarchontoglires - ps30
Euarchonta - ps31
Primates - ps32
Haplorrhini - ps33
Simiiformes - ps34
Catarrhini - ps35
Hominoidea - ps36
Hominidae - ps37
Homininae - ps38
Hominini - ps39
Homo_sapiens - ps40
p-value
2e-02
3e-02
4e-02
5e-02
6e-02
Log-odds
0.5
1.0
1.5
2.0
2.5
3.0
3.5
4.0

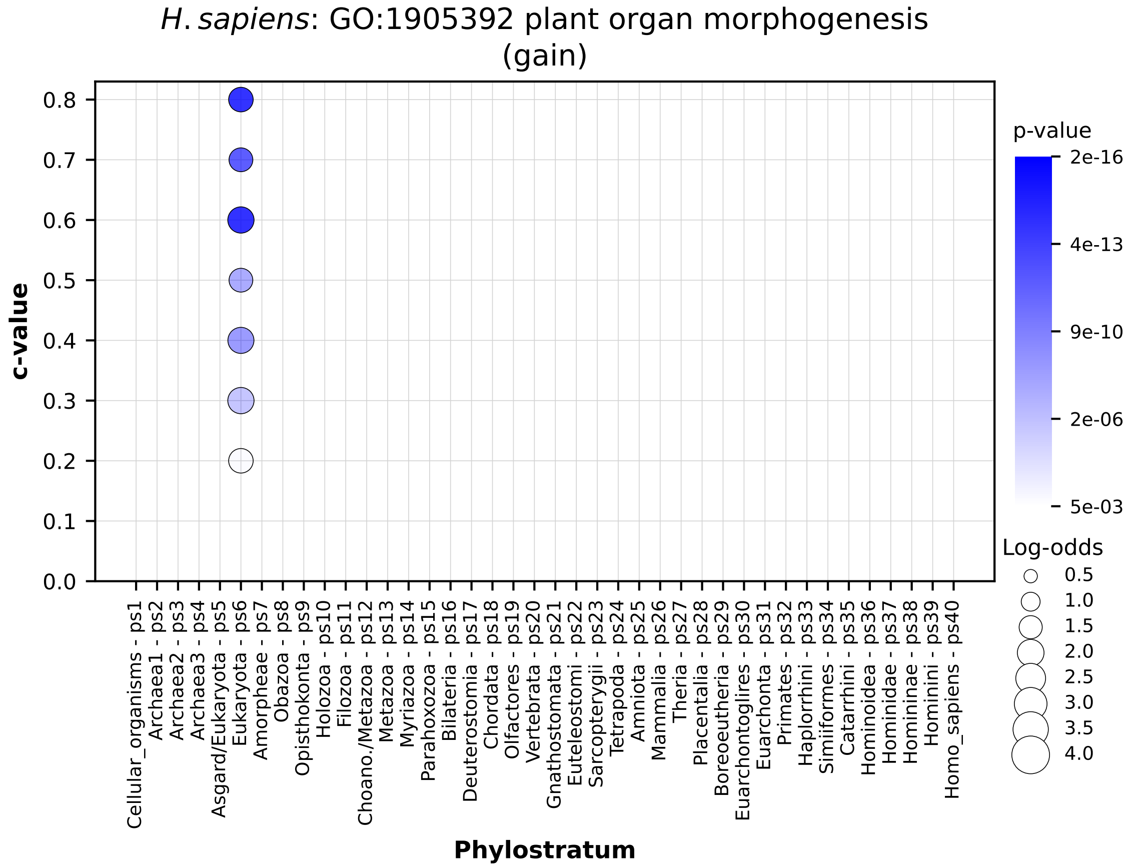

H. sapiens: GO:1905392 plant organ morphogenesis
(gain)
c-value
0.0
0.1
0.2
0.3
0.4
0.5
0.6
0.7
0.8
Phylostratum
Cellular_organisms - ps1
Archaea1 - ps2
Archaea2 - ps3
Archaea3 - ps4
Asgard/Eukaryota - ps5
Eukaryota - ps6
Amorpheae - ps7
Obazoa - ps8
Opisthokonta - ps9
Holozoa - ps10
Filozoa - ps11
Choano./Metazoa - ps12
Metazoa - ps13
Myriazoa - ps14
Parahoxozoa - ps15
Bilateria - ps16
Deuterostomia - ps17
Chordata - ps18
Olfactores - ps19
Vertebrata - ps20
Gnathostomata - ps21
Euteleostomi - ps22
Sarcopterygii - ps23
Tetrapoda - ps24
Amniota - ps25
Mammalia - ps26
Theria - ps27
Placentalia - ps28
Boreoeutheria - ps29
Euarchontoglires - ps30
Euarchonta - ps31
Primates - ps32
Haplorrhini - ps33
Simiiformes - ps34
Catarrhini - ps35
Hominoidea - ps36
Hominidae - ps37
Homininae - ps38
Hominini - ps39
Homo_sapiens - ps40
p-value
2e-16
4e-13
9e-10
2e-06
5e-03
Log-odds
0.5
1.0
1.5
2.0
2.5
3.0
3.5
4.0

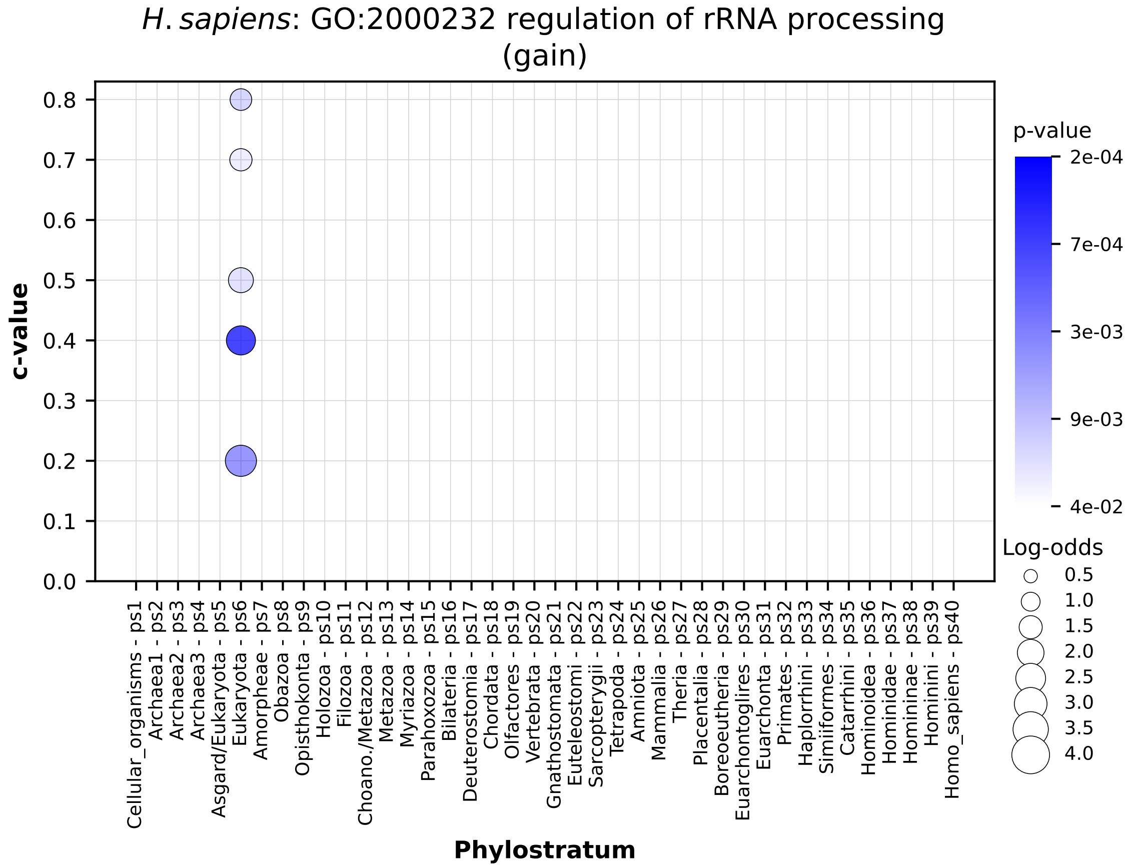

H. sapiens: GO:2000232 regulation of rRNA processing (gain)
c-value
0.0
0.1
0.2
0.3
0.4
0.5
0.6
0.7
0.8
Phylostratum
Cellular_organisms - ps1
Archaea1 - ps2
Archaea2 - ps3
Archaea3 - ps4
Asgard/Eukaryota - ps5
Eukaryota - ps6
Amorpheae - ps7
Obazoa - ps8
Opisthokonta - ps9
Holozoa - ps10
Filozoa - ps11
Choano./Metazoa - ps12
Metazoa - ps13
Myriazoa - ps14
Parahoxozoa - ps15
Bilateria - ps16
Deuterostomia - ps17
Chordata - ps18
Olfactores - ps19
Vertebrata - ps20
Gnathostomata - ps21
Euteleostomi - ps22
Sarcopterygii - ps23
Tetrapoda - ps24
Amniota - ps25
Mammalia - ps26
Theria - ps27
Placentalia - ps28
Boreoeutheria - ps29
Euarchontoglires - ps30
Euarchonta - ps31
Primates - ps32
Haplorrhini - ps33
Simiiformes - ps34
Catarrhini - ps35
Hominoidea - ps36
Hominidae - ps37
Homininae - ps38
Hominini - ps39
Homo_sapiens - ps40
p-value
2e-04
7e-04
3e-03
9e-03
4e-02
Log-odds
0.5
1.0
1.5
2.0
2.5
3.0
3.5
4.0

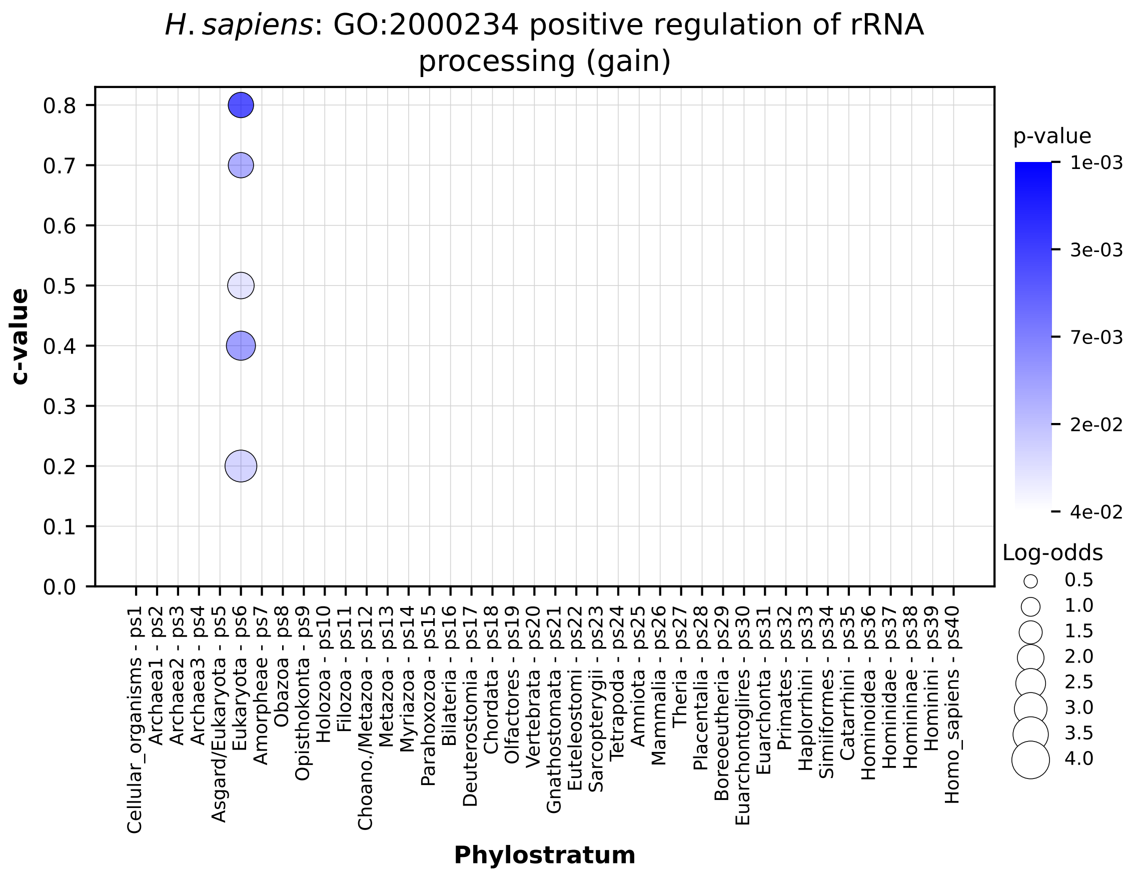

H. sapiens: GO:2000234 positive regulation of rRNA processing (gain)
c-value
0.0
0.1
0.2
0.3
0.4
0.5
0.6
0.7
0.8
Phylostratum
Cellular_organisms - ps1
Archaea1 - ps2
Archaea2 - ps3
Archaea3 - ps4
Asgard/Eukaryota - ps5
Eukaryota - ps6
Amorpheae - ps7
Obazoa - ps8
Opisthokonta - ps9
Holozoa - ps10
Filozoa - ps11
Choano./Metazoa - ps12
Metazoa - ps13
Myriazoa - ps14
Parahoxozoa - ps15
Bilateria - ps16
Deuterostomia - ps17
Chordata - ps18
Olfactores - ps19
Vertebrata - ps20
Gnathostomata - ps21
Euteleostomi - ps22
Sarcopterygii - ps23
Tetrapoda - ps24
Amniota - ps25
Mammalia - ps26
Theria - ps27
Placentalia - ps28
Boreoeutheria - ps29
Euarchontoglires - ps30
Euarchonta - ps31
Primates - ps32
Haplorrhini - ps33
Simiiformes - ps34
Catarrhini - ps35
Hominoidea - ps36
Hominidae - ps37
Homininae - ps38
Hominini - ps39
Homo_sapiens - ps40
p-value
1e-03
3e-03
7e-03
2e-02
4e-02
Log-odds
0.5
1.0
1.5
2.0
2.5
3.0
3.5
4.0

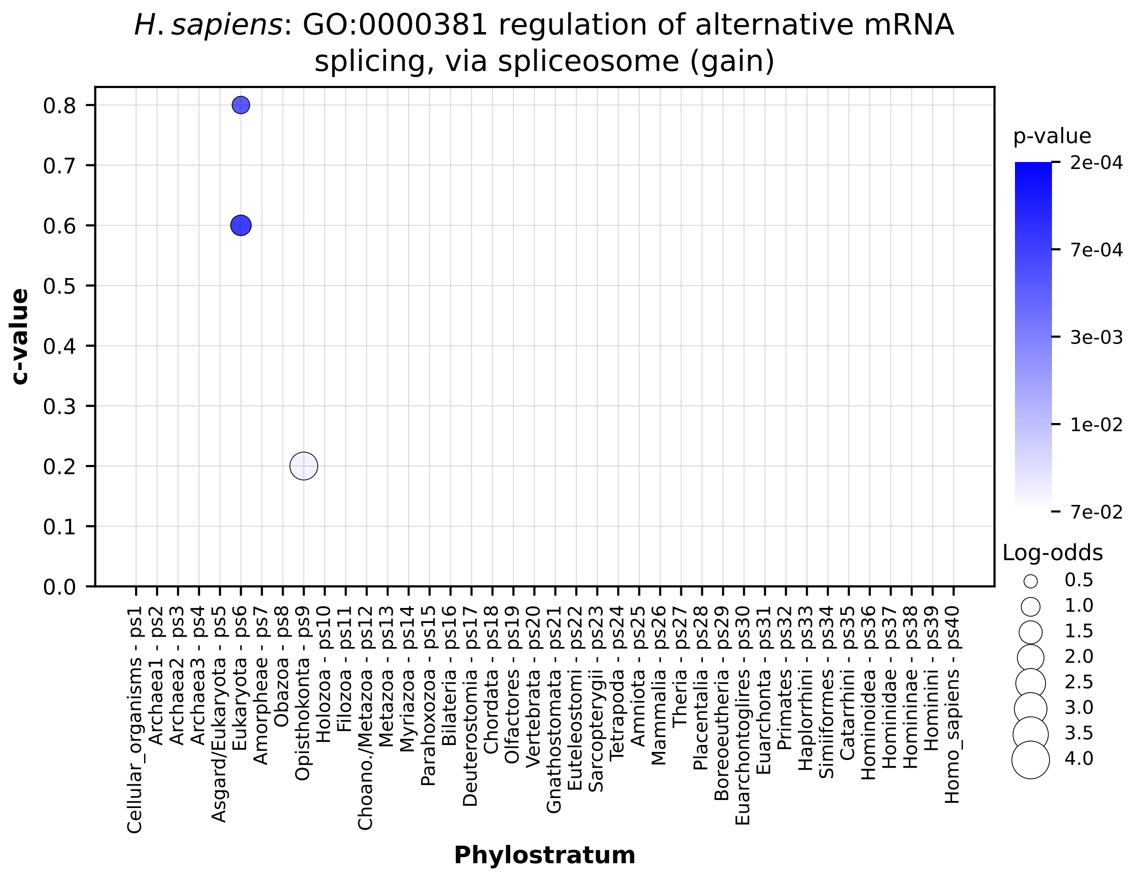

*H. sapiens*: GO:0000381 regulation of alternative mRNA splicing, via spliceosome (gain)
c-value
0.0
0.1
0.2
0.3
0.4
0.5
0.6
0.7
0.8
Phylostratum
Cellular_organisms - ps1
Archaea1 - ps2
Archaea2 - ps3
Archaea3 - ps4
Asgard/Eukaryota - ps5
Eukaryota - ps6
Amorpheae - ps7
Obazoa - ps8
Opisthokonta - ps9
Holozoa - ps10
Filozoa - ps11
Choano./Metazoa - ps12
Metazoa - ps13
Myriazoa - ps14
Parahoxozoa - ps15
Bilateria - ps16
Deuterostomia - ps17
Chordata - ps18
Olfactores - ps19
Vertebrata - ps20
Gnathostomata - ps21
Euteleostomi - ps22
Sarcopterygii - ps23
Tetrapoda - ps24
Amniota - ps25
Mammalia - ps26
Theria - ps27
Placentalia - ps28
Boreoeutheria - ps29
Euarchontoglires - ps30
Euarchonta - ps31
Primates - ps32
Haplorrhini - ps33
Simiiformes - ps34
Catarrhini - ps35
Hominoidea - ps36
Hominidae - ps37
Homininae - ps38
Hominini - ps39
Homo_sapiens - ps40
p-value
2e-04
7e-04
3e-03
1e-02
7e-02
Log-odds
0.5
1.0
1.5
2.0
2.5
3.0
3.5
4.0

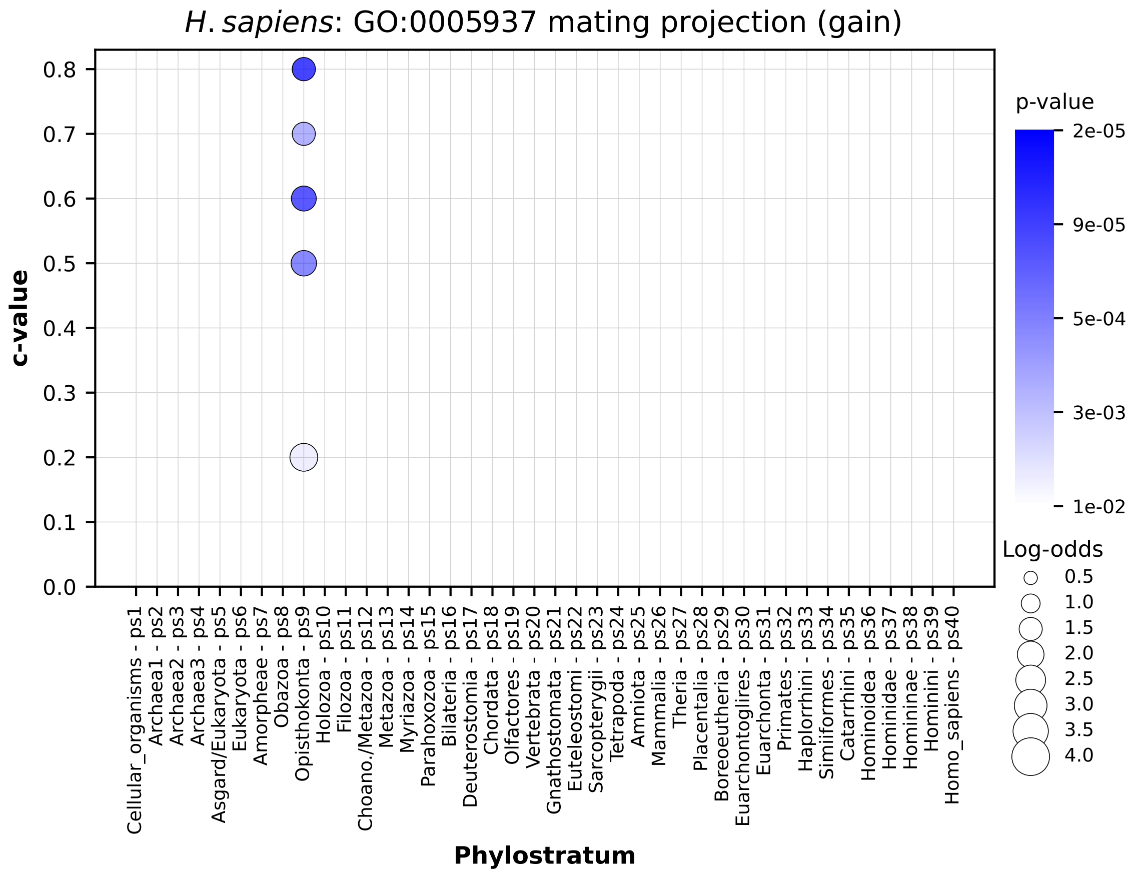

*H. sapiens*: GO:0005937 mating projection (gain)
c-value
0.8
0.7
0.6
0.5
0.4
0.3
0.2
0.1
0.0
Cellular_organisms - ps1
Archaea1 - ps2
Archaea2 - ps3
Archaea3 - ps4
Asgard/Eukaryota - ps5
Eukaryota - ps6
Amorpheae - ps7
Obazoa - ps8
Opisthokonta - ps9
Holozoa - ps10
Filozoa - ps11
Choano./Metazoa - ps12
Metazoa - ps13
Myriazoa - ps14
Parahoxozoa - ps15
Bilateria - ps16
Deuterostomia - ps17
Chordata - ps18
Olfactores - ps19
Vertebrata - ps20
Gnathostomata - ps21
Euteleostomi - ps22
Sarcopterygii - ps23
Tetrapoda - ps24
Amniota - ps25
Mammalia - ps26
Theria - ps27
Placentalia - ps28
Boreoeutheria - ps29
Euarchontoglires - ps30
Euarchonta - ps31
Primates - ps32
Haplorrhini - ps33
Simiiformes - ps34
Catarrhini - ps35
Hominoidea - ps36
Hominidae - ps37
Homininae - ps38
Hominini - ps39
Homo_sapiens - ps40
Phylostratum
p-value
2e-05
9e-05
5e-04
3e-03
1e-02
Log-odds
0.5
1.0
1.5
2.0
2.5
3.0
3.5
4.0

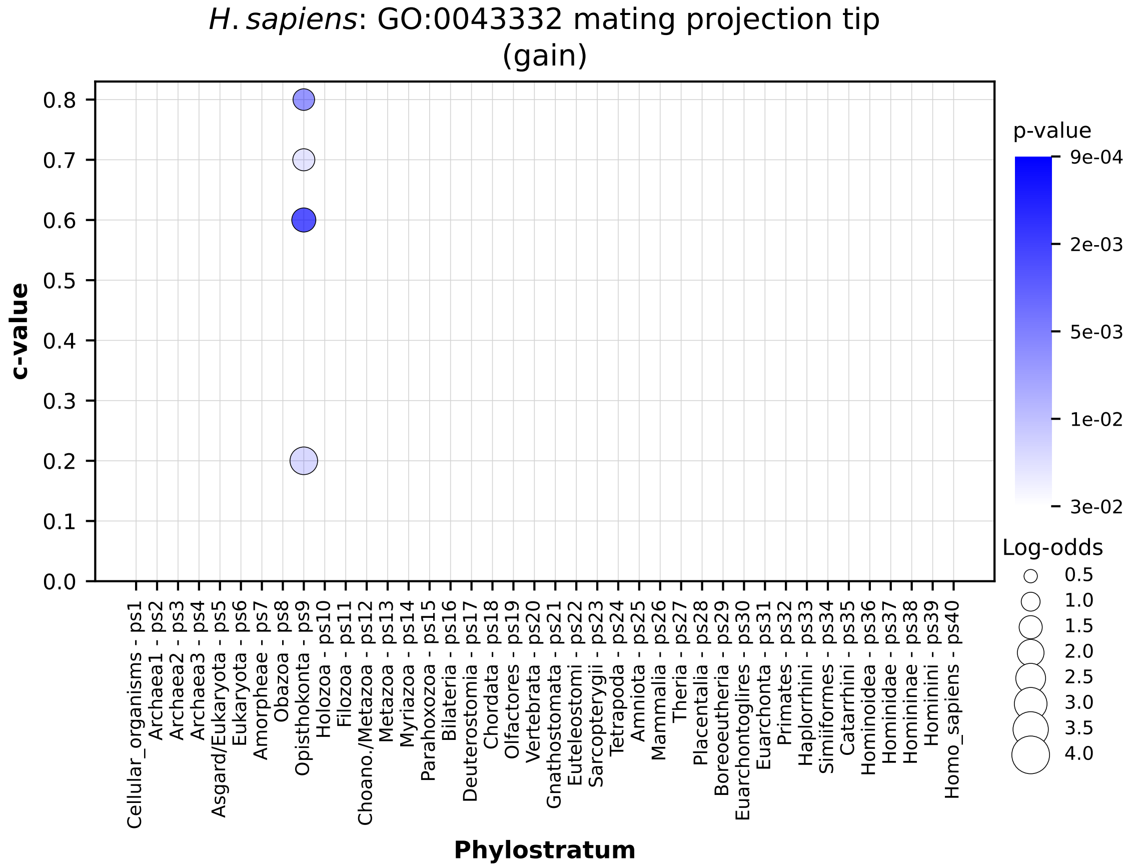

H. sapiens: GO:0043332 mating projection tip
(gain)
c-value
0.0
0.1
0.2
0.3
0.4
0.5
0.6
0.7
0.8
Phylostratum
Cellular_organisms - ps1
Archaea1 - ps2
Archaea2 - ps3
Archaea3 - ps4
Asgard/Eukaryota - ps5
Eukaryota - ps6
Amorpheae - ps7
Obazoa - ps8
Opisthokonta - ps9
Holozoa - ps10
Filozoa - ps11
Choano./Metazoa - ps12
Metazoa - ps13
Myriazoa - ps14
Parahoxozoa - ps15
Bilateria - ps16
Deuterostomia - ps17
Chordata - ps18
Olfactores - ps19
Vertebrata - ps20
Gnathostomata - ps21
Euteleostomi - ps22
Sarcopterygii - ps23
Tetrapoda - ps24
Amniota - ps25
Mammalia - ps26
Theria - ps27
Placentalia - ps28
Boreoeutheria - ps29
Euarchontoglires - ps30
Euarchonta - ps31
Primates - ps32
Haplorrhini - ps33
Simiiformes - ps34
Catarrhini - ps35
Hominoidea - ps36
Hominidae - ps37
Homininae - ps38
Hominini - ps39
Homo_sapiens - ps40
p-value
9e-04
2e-03
5e-03
1e-02
3e-02
Log-odds
0.5
1.0
1.5
2.0
2.5
3.0
3.5
4.0

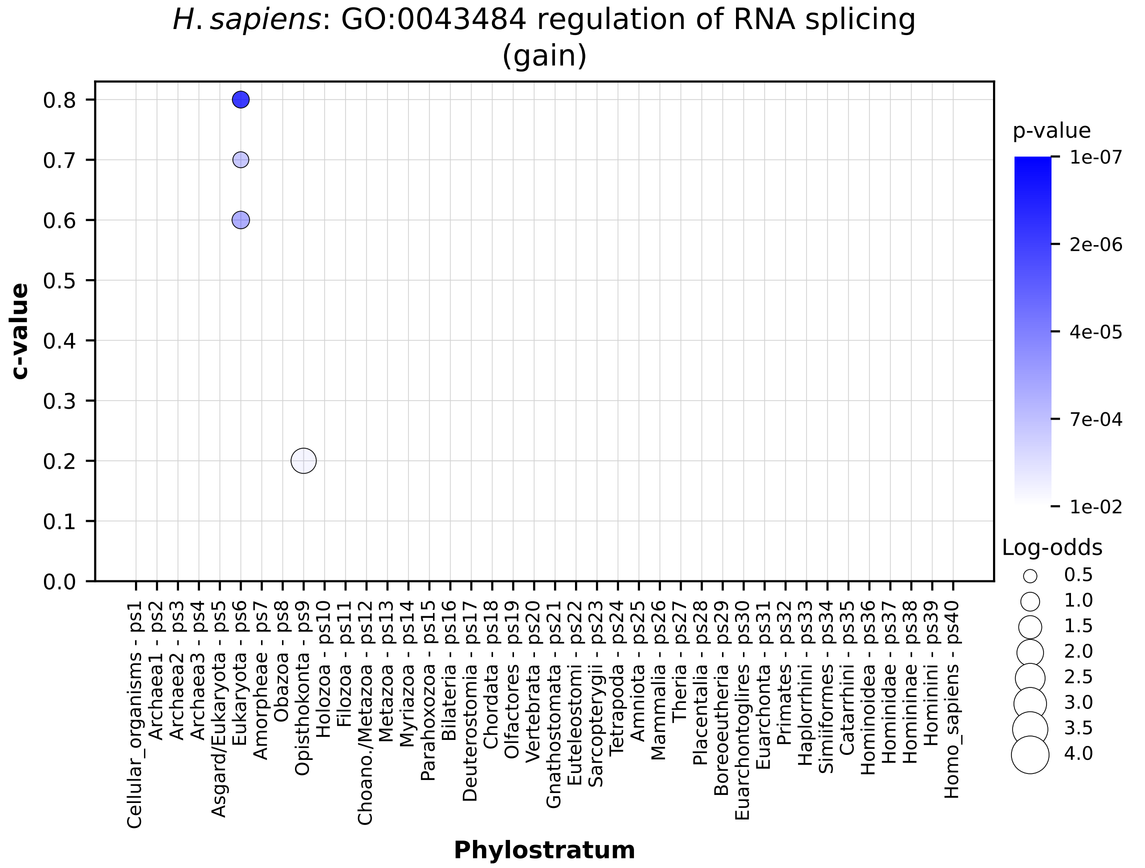

H. sapiens: GO:0043484 regulation of RNA splicing (gain)
c-value
0.0
0.1
0.2
0.3
0.4
0.5
0.6
0.7
0.8
Phylostratum
Cellular_organisms - ps1
Archaea1 - ps2
Archaea2 - ps3
Archaea3 - ps4
Asgard/Eukaryota - ps5
Eukaryota - ps6
Amorpheae - ps7
Obazoa - ps8
Opisthokonta - ps9
Holozoa - ps10
Filozoa - ps11
Choano./Metazoa - ps12
Metazoa - ps13
Myriazoa - ps14
Parahoxozoa - ps15
Bilateria - ps16
Deuterostomia - ps17
Chordata - ps18
Olfactores - ps19
Vertebrata - ps20
Gnathostomata - ps21
Euteleostomi - ps22
Sarcopterygii - ps23
Tetrapoda - ps24
Amniota - ps25
Mammalia - ps26
Theria - ps27
Placentalia - ps28
Boreoeutheria - ps29
Euarchontoglires - ps30
Euarchonta - ps31
Primates - ps32
Haplorrhini - ps33
Simiiformes - ps34
Catarrhini - ps35
Hominoidea - ps36
Hominidae - ps37
Homininae - ps38
Hominini - ps39
Homo_sapiens - ps40
p-value
1e-07
2e-06
4e-05
7e-04
1e-02
Log-odds
0.5
1.0
1.5
2.0
2.5
3.0
3.5
4.0

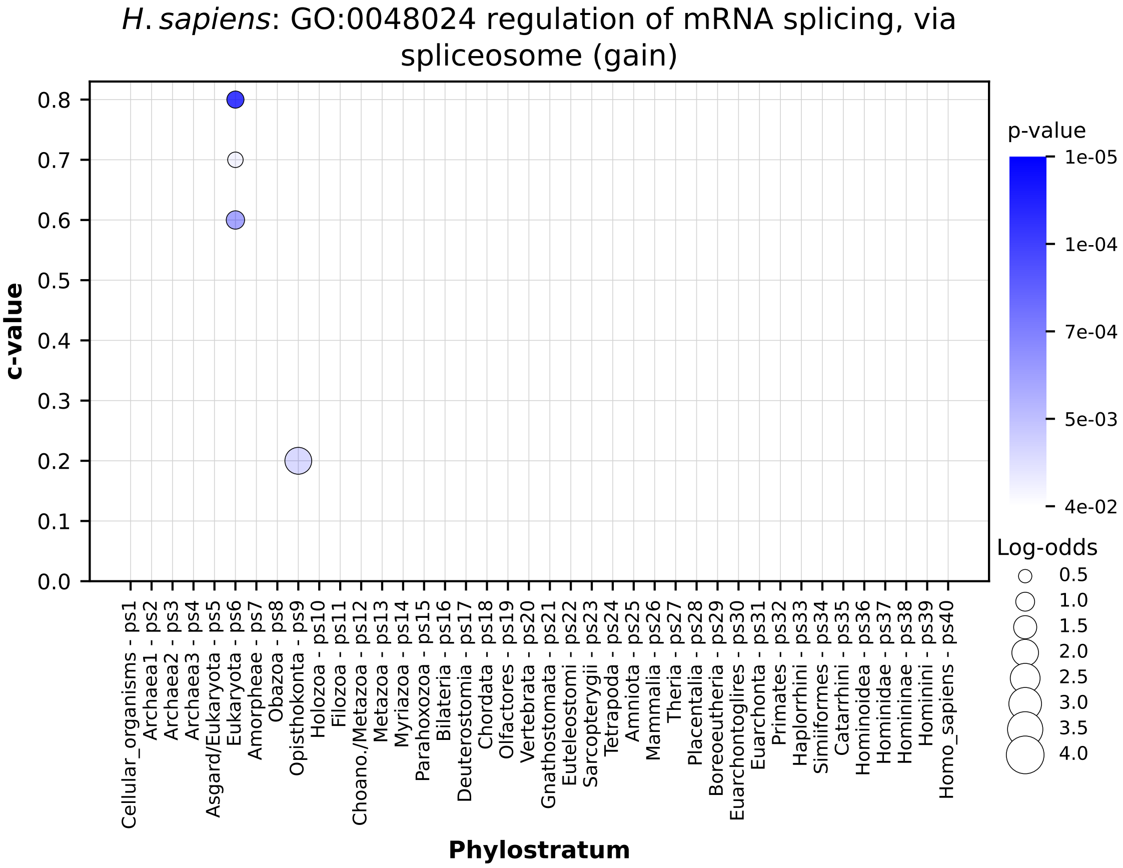

H. sapiens: GO:0048024 regulation of mRNA splicing, via spliceosome (gain)
c-value
0.0
0.1
0.2
0.3
0.4
0.5
0.6
0.7
0.8
Phylostratum
Cellular_organisms - ps1
Archaea1 - ps2
Archaea2 - ps3
Archaea3 - ps4
Asgard/Eukaryota - ps5
Eukaryota - ps6
Amorpheae - ps7
Obazoa - ps8
Opisthokonta - ps9
Holozoa - ps10
Filozoa - ps11
Choano./Metazoa - ps12
Metazoa - ps13
Myriazoa - ps14
Parahoxozoa - ps15
Bilateria - ps16
Deuterostomia - ps17
Chordata - ps18
Olfactores - ps19
Vertebrata - ps20
Gnathostomata - ps21
Euteleostomi - ps22
Sarcopterygii - ps23
Tetrapoda - ps24
Amniota - ps25
Mammalia - ps26
Theria - ps27
Placentalia - ps28
Boreoeutheria - ps29
Euarchontoglires - ps30
Euarchonta - ps31
Primates - ps32
Haplorrhini - ps33
Simiiformes - ps34
Catarrhini - ps35
Hominoidea - ps36
Hominidae - ps37
Homininae - ps38
Hominini - ps39
Homo_sapiens - ps40
p-value
1e-05
1e-04
7e-04
5e-03
4e-02
Log-odds
0.5
1.0
1.5
2.0
2.5
3.0
3.5
4.0

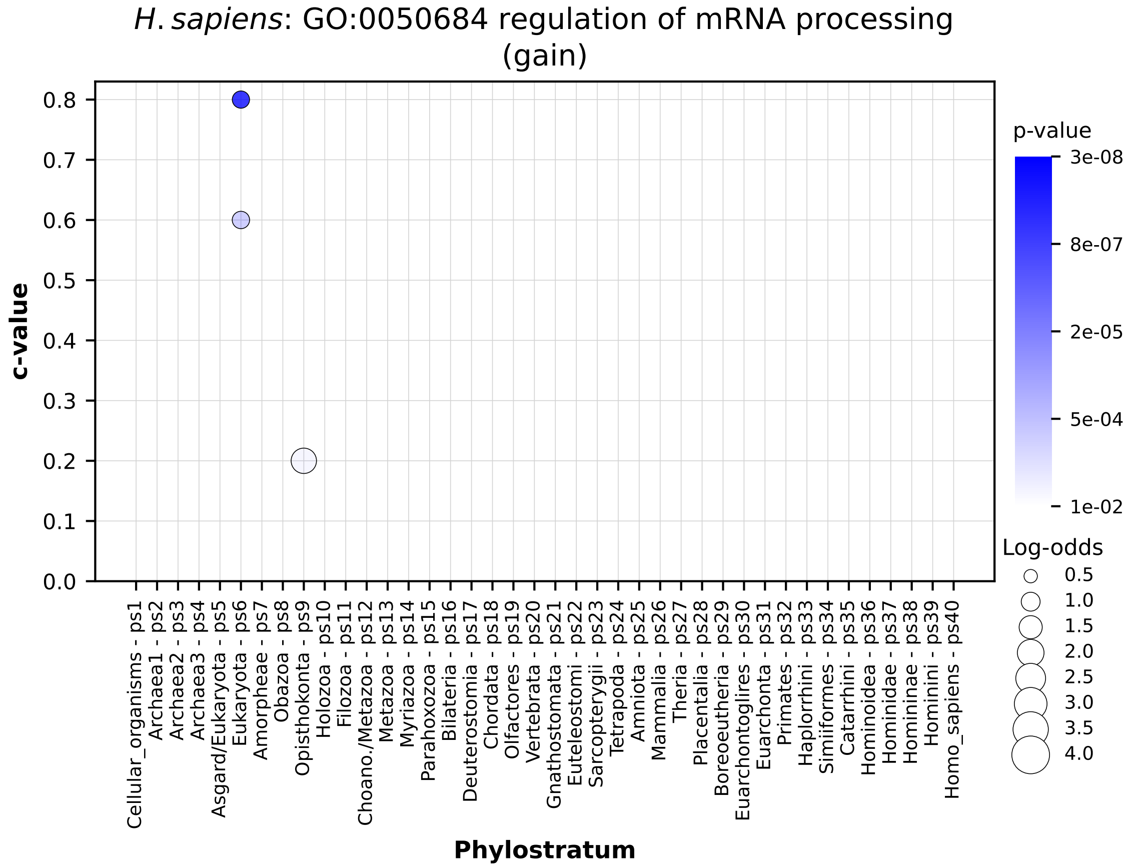

H. sapiens: GO:0050684 regulation of mRNA processing (gain)
c-value
0.0
0.1
0.2
0.3
0.4
0.5
0.6
0.7
0.8
Phylostratum
Cellular_organisms - ps1
Archaea1 - ps2
Archaea2 - ps3
Archaea3 - ps4
Asgard/Eukaryota - ps5
Eukaryota - ps6
Amorpheae - ps7
Obazoa - ps8
Opisthokonta - ps9
Holozoa - ps10
Filozoa - ps11
Choano./Metazoa - ps12
Metazoa - ps13
Myriazoa - ps14
Parahoxozoa - ps15
Bilateria - ps16
Deuterostomia - ps17
Chordata - ps18
Olfactores - ps19
Vertebrata - ps20
Gnathostomata - ps21
Euteleostomi - ps22
Sarcopterygii - ps23
Tetrapoda - ps24
Amniota - ps25
Mammalia - ps26
Theria - ps27
Placentalia - ps28
Boreoeutheria - ps29
Euarchontoglires - ps30
Euarchonta - ps31
Primates - ps32
Haplorrhini - ps33
Simiiformes - ps34
Catarrhini - ps35
Hominoidea - ps36
Hominidae - ps37
Homininae - ps38
Hominini - ps39
Homo_sapiens - ps40
p-value
3e-08
8e-07
2e-05
5e-04
1e-02
Log-odds
0.5
1.0
1.5
2.0
2.5
3.0
3.5
4.0

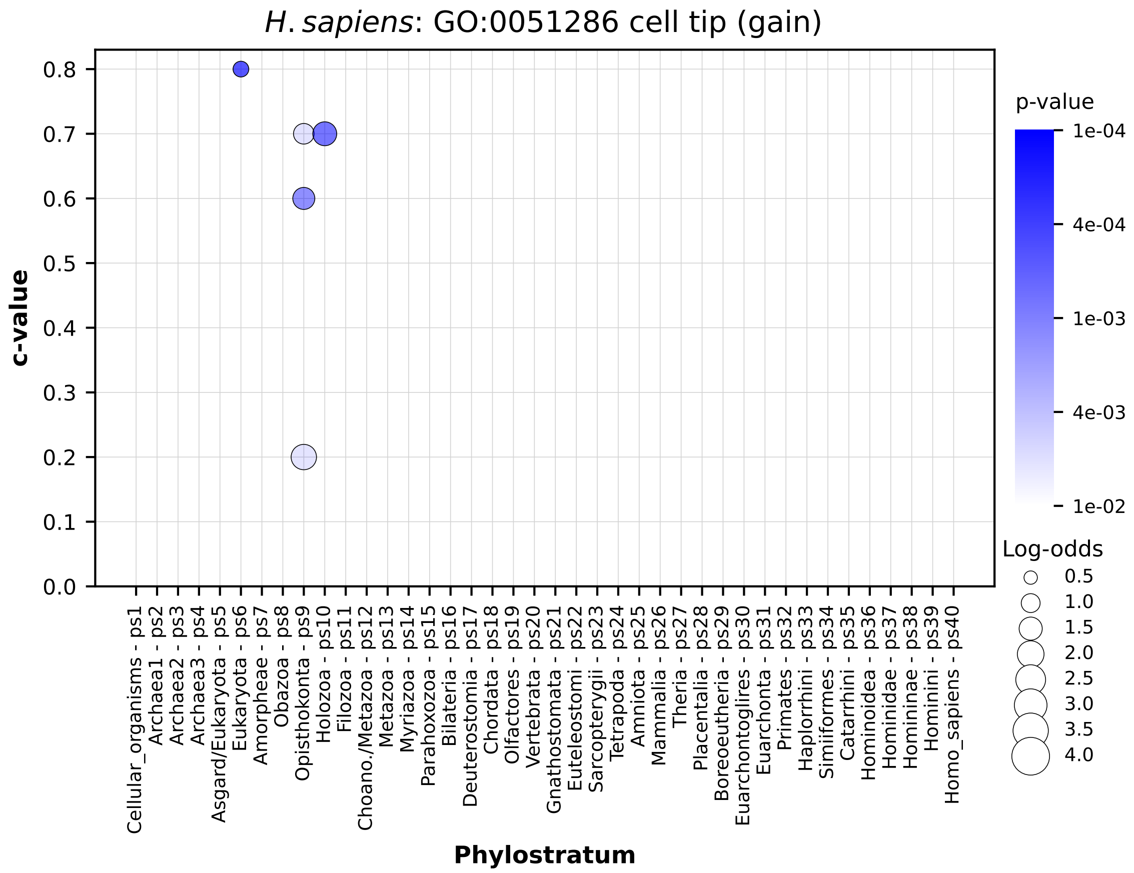

H. sapiens: GO:0051286 cell tip (gain)
c-value
0.0
0.1
0.2
0.3
0.4
0.5
0.6
0.7
0.8
Phylostratum
Cellular_organisms - ps1
Archaea1 - ps2
Archaea2 - ps3
Archaea3 - ps4
Asgard/Eukaryota - ps5
Eukaryota - ps6
Amorpheae - ps7
Obazoa - ps8
Opisthokonta - ps9
Holozoa - ps10
Filozoa - ps11
Choano./Metazoa - ps12
Metazoa - ps13
Myriazoa - ps14
Parahoxozoa - ps15
Bilateria - ps16
Deuterostomia - ps17
Chordata - ps18
Olfactores - ps19
Vertebrata - ps20
Gnathostomata - ps21
Euteleostomi - ps22
Sarcopterygii - ps23
Tetrapoda - ps24
Amniota - ps25
Mammalia - ps26
Theria - ps27
Placentalia - ps28
Boreoeutheria - ps29
Euarchontoglires - ps30
Euarchonta - ps31
Primates - ps32
Haplorrhini - ps33
Simiiformes - ps34
Catarrhini - ps35
Hominoidea - ps36
Hominidae - ps37
Homininae - ps38
Hominini - ps39
Homo_sapiens - ps40
p-value
1e-04
4e-04
1e-03
4e-03
1e-02
Log-odds
0.5
1.0
1.5
2.0
2.5
3.0
3.5
4.0

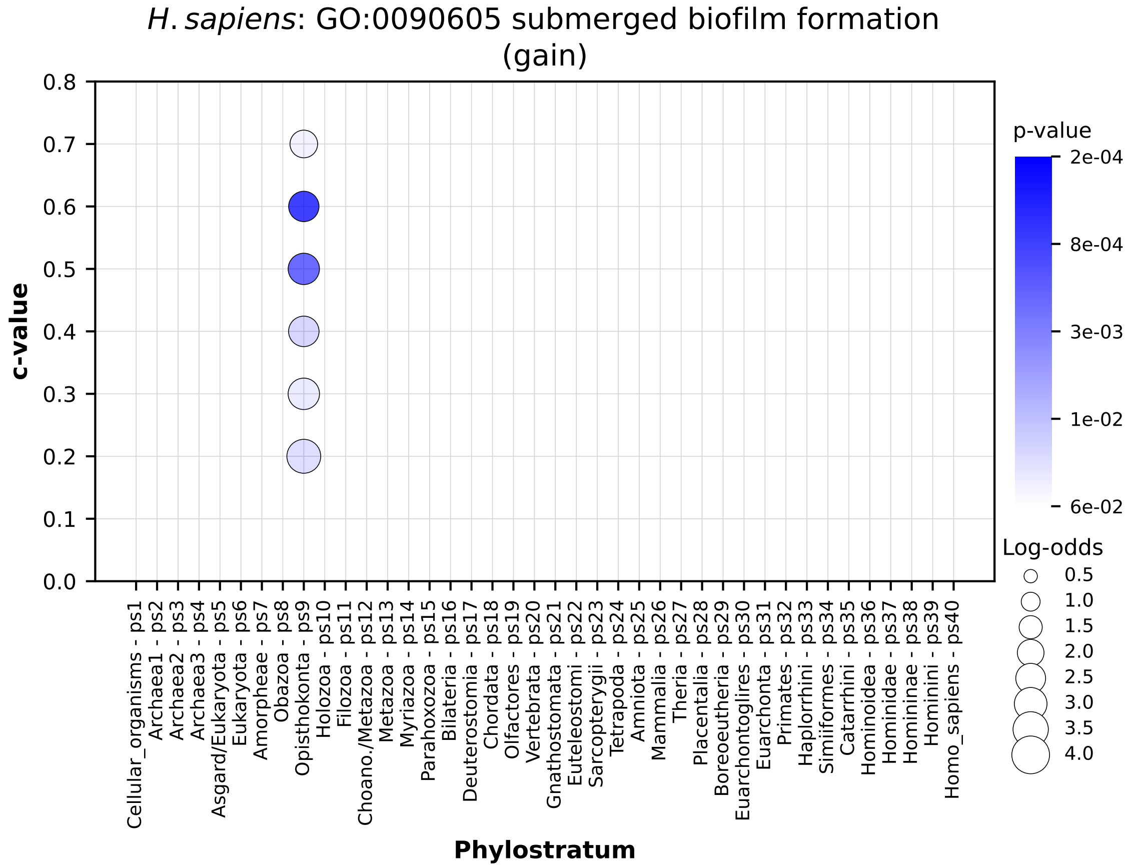
H. sapiens: GO:0090605 submerged biofilm formation
(gain)
c-value
0.8
0.7
0.6
0.5
0.4
0.3
0.2
0.1
0.0
p-value
2e-04
8e-04
3e-03
1e-02
6e-02
Log-odds
0.5
1.0
1.5
2.0
2.5
3.0
3.5
4.0
Cellular_organisms - ps1
Archaea1 - ps2
Archaea2 - ps3
Archaea3 - ps4
Asgard/Eukaryota - ps5
Eukaryota - ps6
Amorpheae - ps7
Obazoa - ps8
Opisthokonta - ps9
Holozoa - ps10
Filozoa - ps11
Choano./Metazoa - ps12
Metazoa - ps13
Myriazoa - ps14
Parahoxozoa - ps15
Bilateria - ps16
Deuterostomia - ps17
Chordata - ps18
Olfactores - ps19
Vertebrata - ps20
Gnathostomata - ps21
Euteleostomi - ps22
Sarcopterygii - ps23
Tetrapoda - ps24
Amniota - ps25
Mammalia - ps26
Theria - ps27
Placentalia - ps28
Boreoeutheria - ps29
Euarchontoglires - ps30
Euarchonta - ps31
Primates - ps32
Haplorrhini - ps33
Simiiformes - ps34
Catarrhini - ps35
Hominoidea - ps36
Hominidae - ps37
Homininae - ps38
Hominini - ps39
Homo_sapiens - ps40
Phylostratum

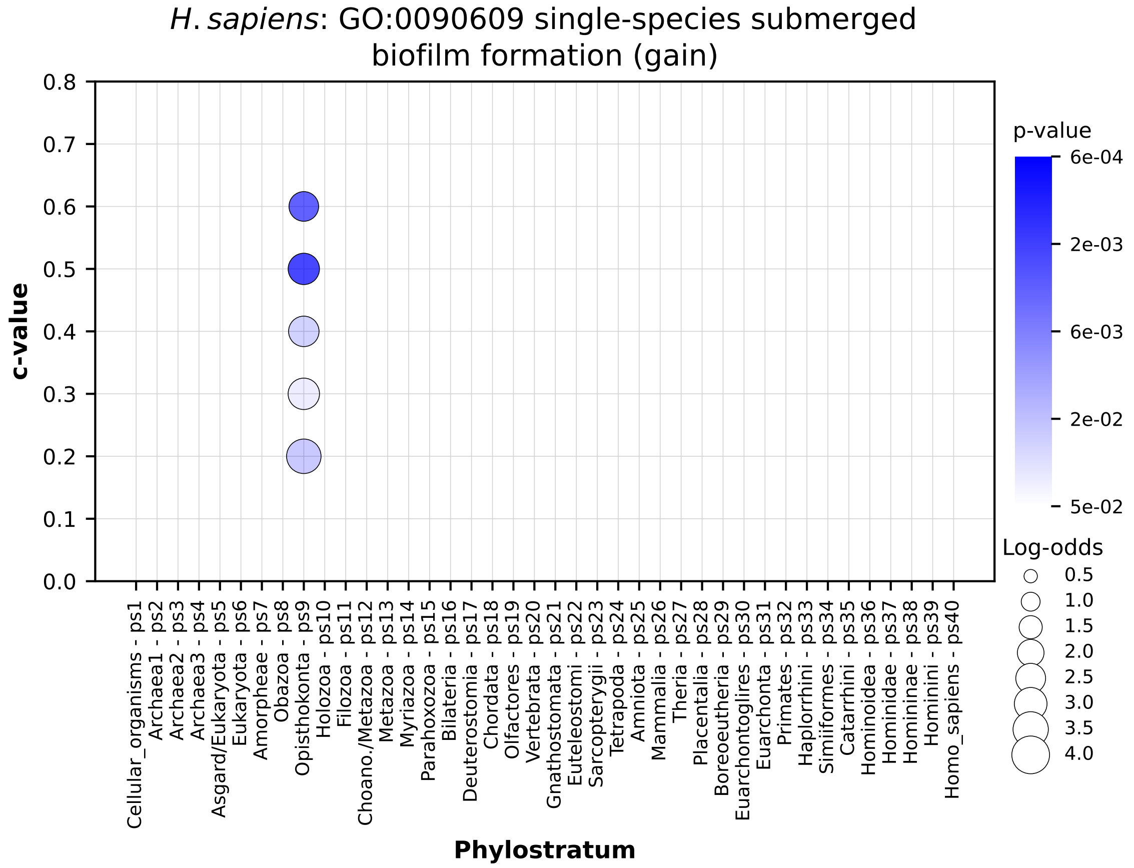
H. sapiens: GO:0090609 single-species submerged biofilm formation (gain)
c-value
0.0
0.1
0.2
0.3
0.4
0.5
0.6
0.7
0.8
Phylostratum
Cellular_organisms - ps1
Archaea1 - ps2
Archaea2 - ps3
Archaea3 - ps4
Asgard/Eukaryota - ps5
Eukaryota - ps6
Amorpheae - ps7
Obazoa - ps8
Opisthokonta - ps9
Holozoa - ps10
Filozoa - ps11
Choano./Metazoa - ps12
Metazoa - ps13
Myriazoa - ps14
Parahoxozoa - ps15
Bilateria - ps16
Deuterostomia - ps17
Chordata - ps18
Olfactores - ps19
Vertebrata - ps20
Gnathostomata - ps21
Euteleostomi - ps22
Sarcopterygii - ps23
Tetrapoda - ps24
Amniota - ps25
Mammalia - ps26
Theria - ps27
Placentalia - ps28
Boreoeutheria - ps29
Euarchontoglires - ps30
Euarchonta - ps31
Primates - ps32
Haplorrhini - ps33
Simiiformes - ps34
Catarrhini - ps35
Hominoidea - ps36
Hominidae - ps37
Homininae - ps38
Hominini - ps39
Homo_sapiens - ps40
p-value
6e-04
2e-03
6e-03
2e-02
5e-02
Log-odds
0.5
1.0
1.5
2.0
2.5
3.0
3.5
4.0

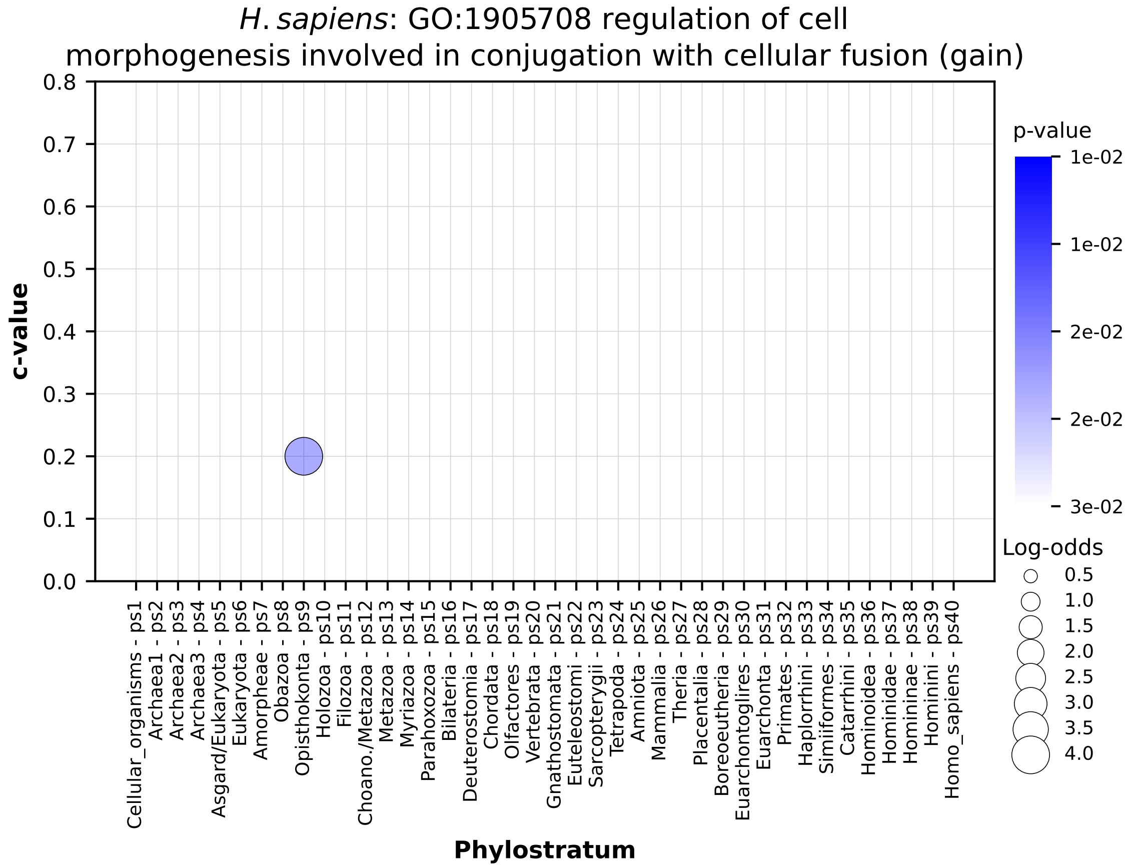

H. sapiens: GO:1905708 regulation of cell morphogenesis involved in conjugation with cellular fusion (gain)
c-value
0.0
0.1
0.2
0.3
0.4
0.5
0.6
0.7
0.8
Phylostratum
Cellular_organisms - ps1
Archaea1 - ps2
Archaea2 - ps3
Archaea3 - ps4
Asgard/Eukaryota - ps5
Eukaryota - ps6
Amorpheae - ps7
Obazoa - ps8
Opisthokonta - ps9
Holozoa - ps10
Filozoa - ps11
Choano./Metazoa - ps12
Metazoa - ps13
Myriazoa - ps14
Parahoxozoa - ps15
Bilateria - ps16
Deuterostomia - ps17
Chordata - ps18
Olfactores - ps19
Vertebrata - ps20
Gnathostomata - ps21
Euteleostomi - ps22
Sarcopterygii - ps23
Tetrapoda - ps24
Amniota - ps25
Mammalia - ps26
Theria - ps27
Placentalia - ps28
Boreoeutheria - ps29
Euarchontoglires - ps30
Euarchonta - ps31
Primates - ps32
Haplorrhini - ps33
Simiiformes - ps34
Catarrhini - ps35
Hominoidea - ps36
Hominidae - ps37
Homininae - ps38
Hominini - ps39
Homo_sapiens - ps40
p-value
1e-02
1e-02
2e-02
2e-02
3e-02
Log-odds
0.5
1.0
1.5
2.0
2.5
3.0
3.5
4.0

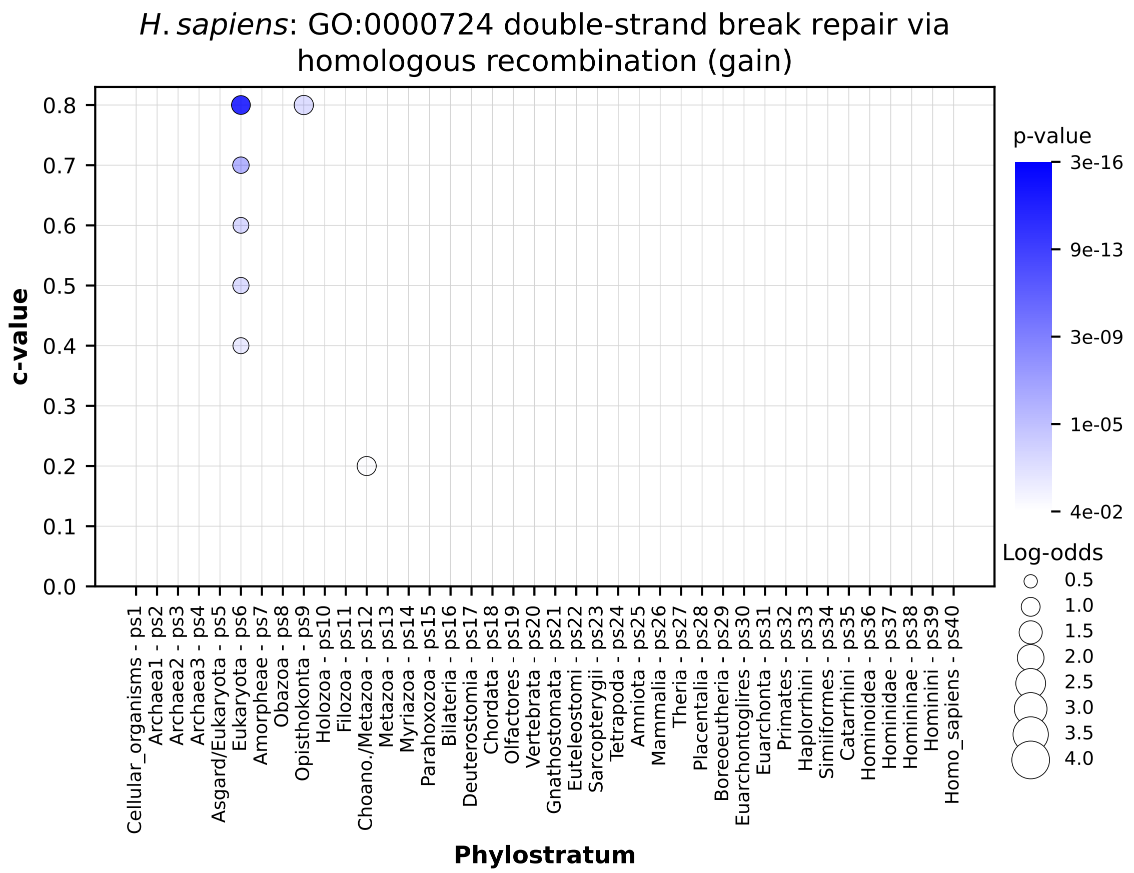

H. sapiens: GO:0000724 double-strand break repair via homologous recombination (gain)
c-value
0.0
0.1
0.2
0.3
0.4
0.5
0.6
0.7
0.8
Phylostratum
Cellular_organisms - ps1
Archaea1 - ps2
Archaea2 - ps3
Archaea3 - ps4
Asgard/Eukaryota - ps5
Eukaryota - ps6
Amorpheae - ps7
Obazoa - ps8
Opisthokonta - ps9
Holozoa - ps10
Filozoa - ps11
Choano./Metazoa - ps12
Metazoa - ps13
Myriazoa - ps14
Parahoxozoa - ps15
Bilateria - ps16
Deuterostomia - ps17
Chordata - ps18
Olfactores - ps19
Vertebrata - ps20
Gnathostomata - ps21
Euteleostomi - ps22
Sarcopterygii - ps23
Tetrapoda - ps24
Amniota - ps25
Mammalia - ps26
Theria - ps27
Placentalia - ps28
Boreoeutheria - ps29
Euarchontoglires - ps30
Euarchonta - ps31
Primates - ps32
Haplorrhini - ps33
Simiiformes - ps34
Catarrhini - ps35
Hominoidea - ps36
Hominidae - ps37
Homininae - ps38
Hominini - ps39
Homo_sapiens - ps40
p-value
3e-16
9e-13
3e-09
1e-05
4e-02
Log-odds
0.5
1.0
1.5
2.0
2.5
3.0
3.5
4.0

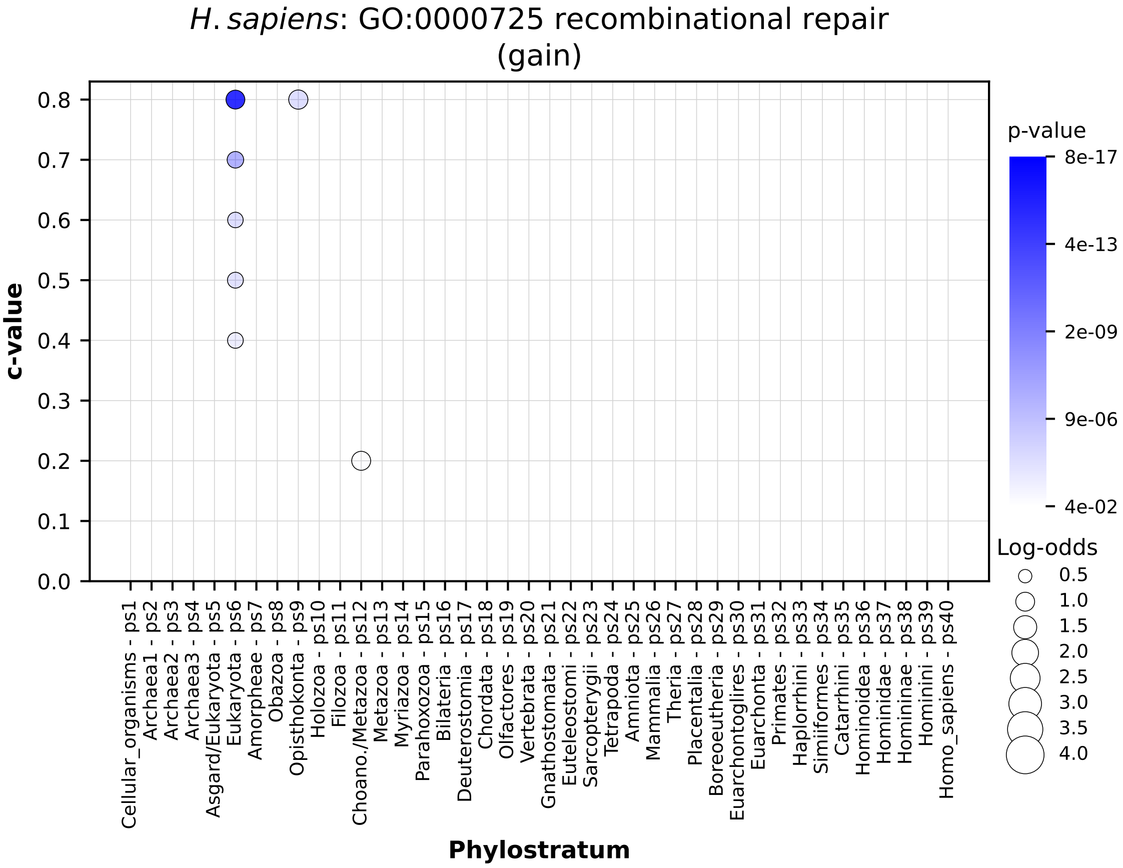

H. sapiens: GO:0000725 recombinational repair
(gain)
c-value
0.0
0.1
0.2
0.3
0.4
0.5
0.6
0.7
0.8
Phylostratum
Cellular_organisms - ps1
Archaea1 - ps2
Archaea2 - ps3
Archaea3 - ps4
Asgard/Eukaryota - ps5
Eukaryota - ps6
Amorpheae - ps7
Obazoa - ps8
Opisthokonta - ps9
Holozoa - ps10
Filozoa - ps11
Choano./Metazoa - ps12
Metazoa - ps13
Myriazoa - ps14
Parahoxozoa - ps15
Bilateria - ps16
Deuterostomia - ps17
Chordata - ps18
Olfactores - ps19
Vertebrata - ps20
Gnathostomata - ps21
Euteleostomi - ps22
Sarcopterygii - ps23
Tetrapoda - ps24
Amniota - ps25
Mammalia - ps26
Theria - ps27
Placentalia - ps28
Boreoeutheria - ps29
Euarchontoglires - ps30
Euarchonta - ps31
Primates - ps32
Haplorrhini - ps33
Simiiformes - ps34
Catarrhini - ps35
Hominoidea - ps36
Hominidae - ps37
Homininae - ps38
Hominini - ps39
Homo_sapiens - ps40
p-value
8e-17
4e-13
2e-09
9e-06
4e-02
Log-odds
0.5
1.0
1.5
2.0
2.5
3.0
3.5
4.0

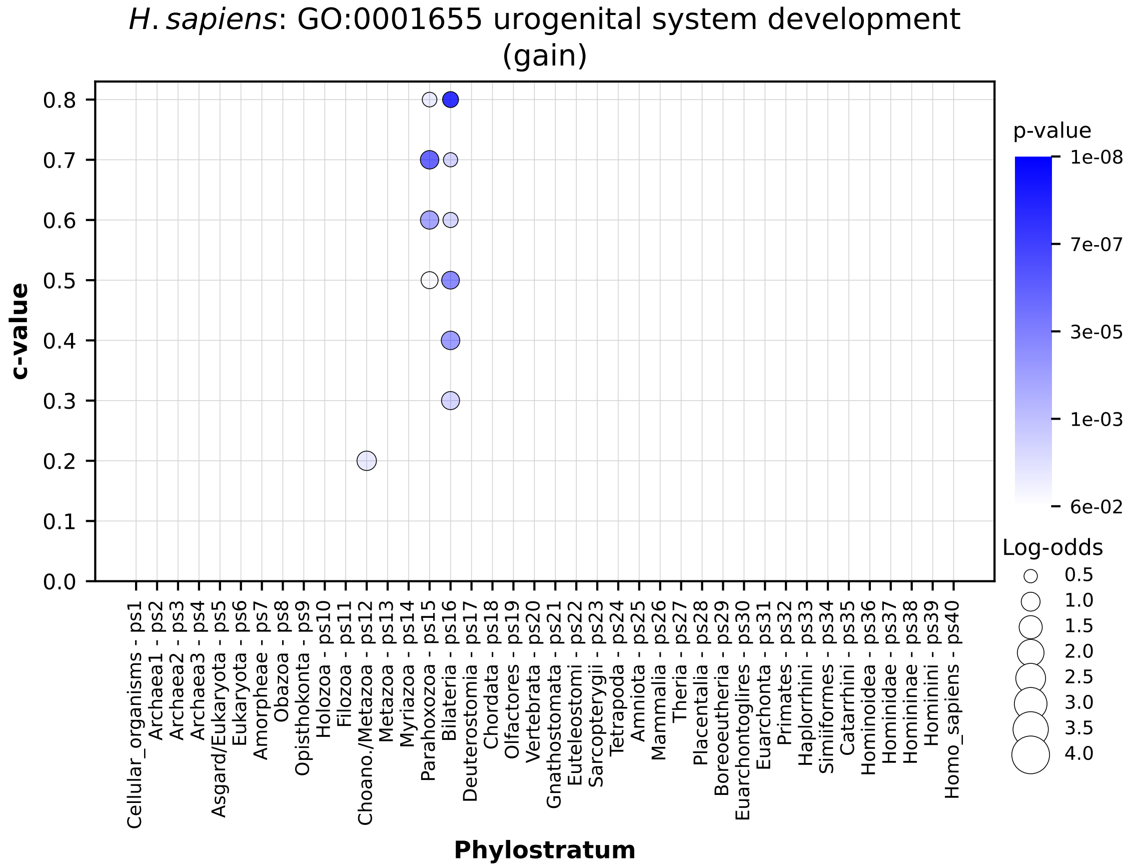
H. sapiens: GO:0001655 urogenital system development
(gain)
c-value
0.0
0.1
0.2
0.3
0.4
0.5
0.6
0.7
0.8
Phylostratum
Cellular_organisms - ps1
Archaea1 - ps2
Archaea2 - ps3
Archaea3 - ps4
Asgard/Eukaryota - ps5
Eukaryota - ps6
Amorpheae - ps7
Obazoa - ps8
Opisthokonta - ps9
Holozoa - ps10
Filozoa - ps11
Choano./Metazoa - ps12
Metazoa - ps13
Myriazoa - ps14
Parahoxozoa - ps15
Bilateria - ps16
Deuterostomia - ps17
Chordata - ps18
Olfactores - ps19
Vertebrata - ps20
Gnathostomata - ps21
Euteleostomi - ps22
Sarcopterygii - ps23
Tetrapoda - ps24
Amniota - ps25
Mammalia - ps26
Theria - ps27
Placentalia - ps28
Boreoeutheria - ps29
Euarchontoglires - ps30
Euarchonta - ps31
Primates - ps32
Haplorrhini - ps33
Simiiformes - ps34
Catarrhini - ps35
Hominoidea - ps36
Hominidae - ps37
Homininae - ps38
Hominini - ps39
Homo_sapiens - ps40
p-value
1e-08
7e-07
3e-05
1e-03
6e-02
Log-odds
0.5
1.0
1.5
2.0
2.5
3.0
3.5
4.0

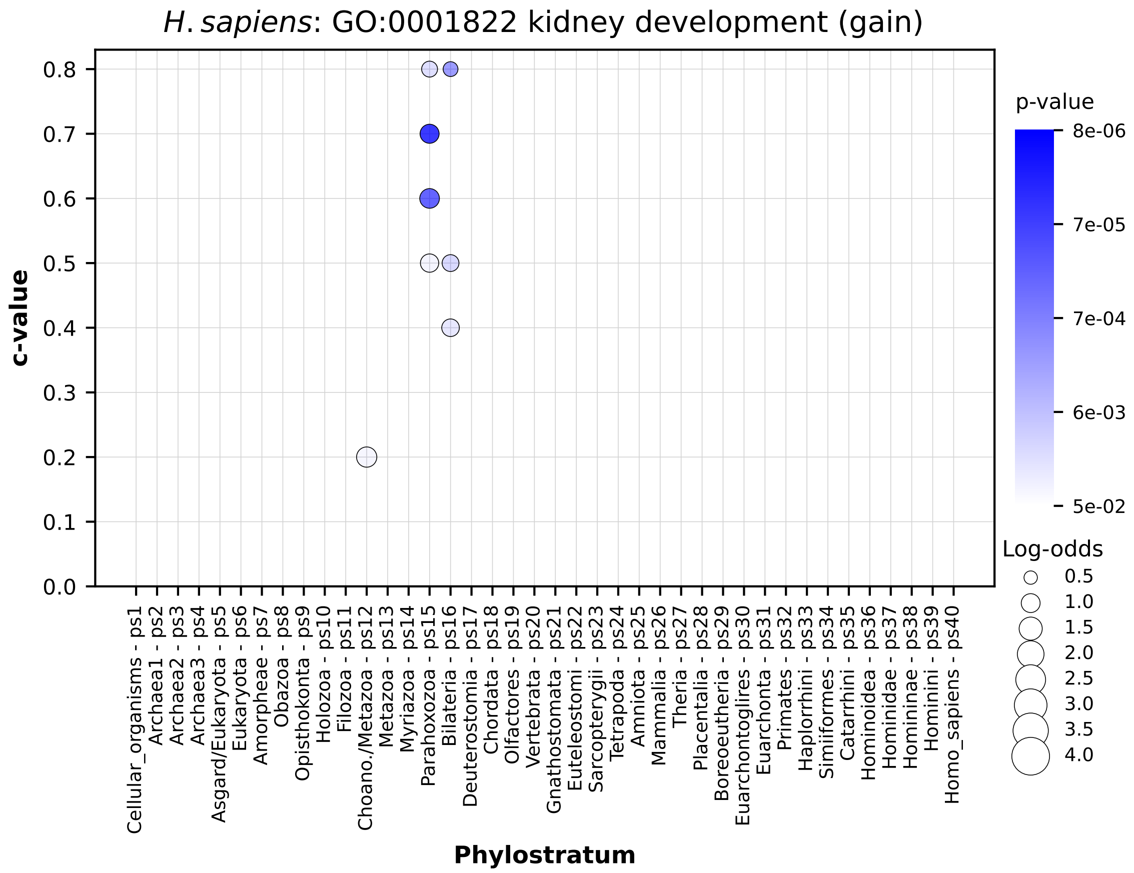
H. sapiens: GO:0001822 kidney development (gain)
c-value
0.0
0.1
0.2
0.3
0.4
0.5
0.6
0.7
0.8
p-value
8e-06
7e-05
7e-04
6e-03
5e-02
Log-odds
0.5
1.0
1.5
2.0
2.5
3.0
3.5
4.0
Cellular_organisms - ps1
Archaea1 - ps2
Archaea2 - ps3
Archaea3 - ps4
Asgard/Eukaryota - ps5
Eukaryota - ps6
Amorpheae - ps7
Obazoa - ps8
Opisthokonta - ps9
Holozoa - ps10
Filozoa - ps11
Choano./Metazoa - ps12
Metazoa - ps13
Myriazoa - ps14
Parahoxozoa - ps15
Bilateria - ps16
Deuterostomia - ps17
Chordata - ps18
Olfactores - ps19
Vertebrata - ps20
Gnathostomata - ps21
Euteleostomi - ps22
Sarcopterygii - ps23
Tetrapoda - ps24
Amniota - ps25
Mammalia - ps26
Theria - ps27
Placentalia - ps28
Boreoeutheria - ps29
Euarchontoglires - ps30
Euarchonta - ps31
Primates - ps32
Haplorrhini - ps33
Simiiformes - ps34
Catarrhini - ps35
Hominoidea - ps36
Hominidae - ps37
Homininae - ps38
Hominini - ps39
Homo_sapiens - ps40
Phylostratum

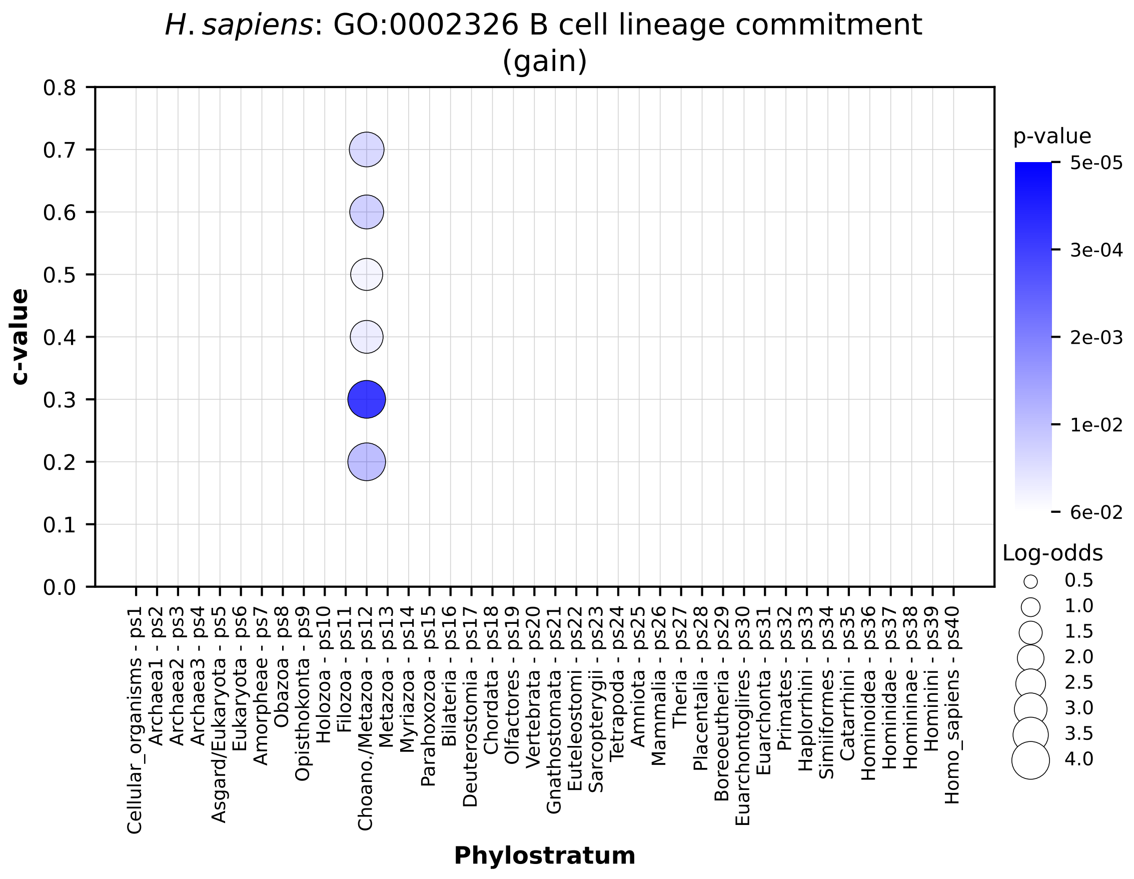

H. sapiens: GO:0002326 B cell lineage commitment
(gain)
c-value
0.0
0.1
0.2
0.3
0.4
0.5
0.6
0.7
0.8
p-value
5e-05
3e-04
2e-03
1e-02
6e-02
Log-odds
0.5
1.0
1.5
2.0
2.5
3.0
3.5
4.0
Cellular_organisms - ps1
Archaea1 - ps2
Archaea2 - ps3
Archaea3 - ps4
Asgard/Eukaryota - ps5
Eukaryota - ps6
Amorpheae - ps7
Obazoa - ps8
Opisthokonta - ps9
Holozoa - ps10
Filozoa - ps11
Choano./Metazoa - ps12
Metazoa - ps13
Myriazoa - ps14
Parahoxozoa - ps15
Bilateria - ps16
Deuterostomia - ps17
Chordata - ps18
Olfactores - ps19
Vertebrata - ps20
Gnathostomata - ps21
Euteleostomi - ps22
Sarcopterygii - ps23
Tetrapoda - ps24
Amniota - ps25
Mammalia - ps26
Theria - ps27
Placentalia - ps28
Boreoeutheria - ps29
Euarchontoglires - ps30
Euarchonta - ps31
Primates - ps32
Haplorrhini - ps33
Simiiformes - ps34
Catarrhini - ps35
Hominoidea - ps36
Hominidae - ps37
Homininae - ps38
Hominini - ps39
Homo_sapiens - ps40
Phylostratum

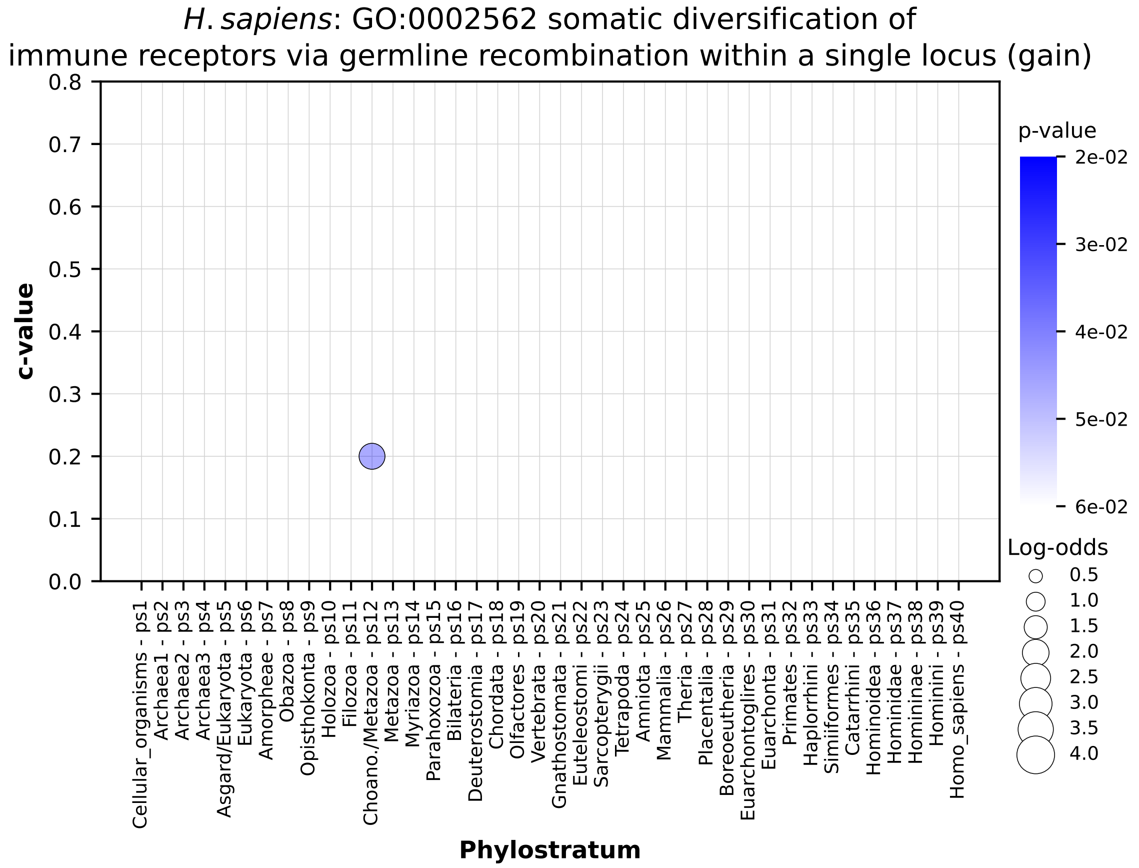
*H. sapiens*: GO:0002562 somatic diversification of
immune receptors via germline recombination within a single locus (gain)
c-value
0.0
0.1
0.2
0.3
0.4
0.5
0.6
0.7
0.8
Phylostratum
Cellular_organisms - ps1
Archaea1 - ps2
Archaea2 - ps3
Archaea3 - ps4
Asgard/Eukaryota - ps5
Eukaryota - ps6
Amorpheae - ps7
Obazoa - ps8
Opisthokonta - ps9
Holozoa - ps10
Filozoa - ps11
Choano./Metazoa - ps12
Metazoa - ps13
Myriazoa - ps14
Parahoxozoa - ps15
Bilateria - ps16
Deuterostomia - ps17
Chordata - ps18
Olfactores - ps19
Vertebrata - ps20
Gnathostomata - ps21
Euteleostomi - ps22
Sarcopterygii - ps23
Tetrapoda - ps24
Amniota - ps25
Mammalia - ps26
Theria - ps27
Placentalia - ps28
Boreoeutheria - ps29
Euarchontoglires - ps30
Euarchonta - ps31
Primates - ps32
Haplorrhini - ps33
Simiiformes - ps34
Catarrhini - ps35
Hominoidea - ps36
Hominidae - ps37
Homininae - ps38
Hominini - ps39
Homo_sapiens - ps40
p-value
2e-02
3e-02
4e-02
5e-02
6e-02
Log-odds
0.5
1.0
1.5
2.0
2.5
3.0
3.5
4.0

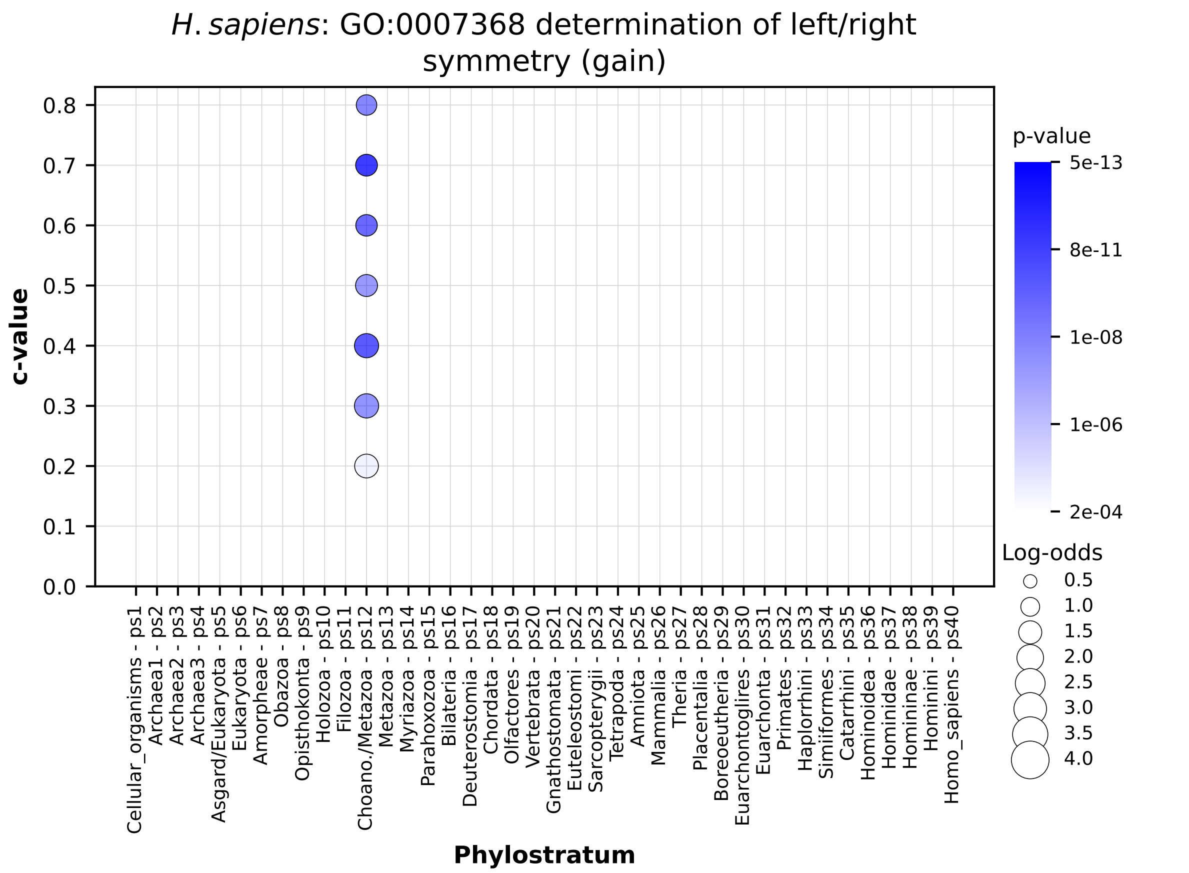

*H. sapiens*: GO:0007368 determination of left/right symmetry (gain)
c-value
0.0
0.1
0.2
0.3
0.4
0.5
0.6
0.7
0.8
Phylostratum
Cellular_organisms - ps1
Archaea1 - ps2
Archaea2 - ps3
Archaea3 - ps4
Asgard/Eukaryota - ps5
Eukaryota - ps6
Amorpheae - ps7
Obazoa - ps8
Opisthokonta - ps9
Holozoa - ps10
Filozoa - ps11
Choano./Metazoa - ps12
Metazoa - ps13
Myriazoa - ps14
Parahoxozoa - ps15
Bilateria - ps16
Deuterostomia - ps17
Chordata - ps18
Olfactores - ps19
Vertebrata - ps20
Gnathostomata - ps21
Euteleostomi - ps22
Sarcopterygii - ps23
Tetrapoda - ps24
Amniota - ps25
Mammalia - ps26
Theria - ps27
Placentalia - ps28
Boreoeutheria - ps29
Euarchontoglires - ps30
Euarchonta - ps31
Primates - ps32
Haplorrhini - ps33
Simiiformes - ps34
Catarrhini - ps35
Hominoidea - ps36
Hominidae - ps37
Homininae - ps38
Hominini - ps39
Homo_sapiens - ps40
p-value
5e-13
8e-11
1e-08
1e-06
2e-04
Log-odds
0.5
1.0
1.5
2.0
2.5
3.0
3.5
4.0

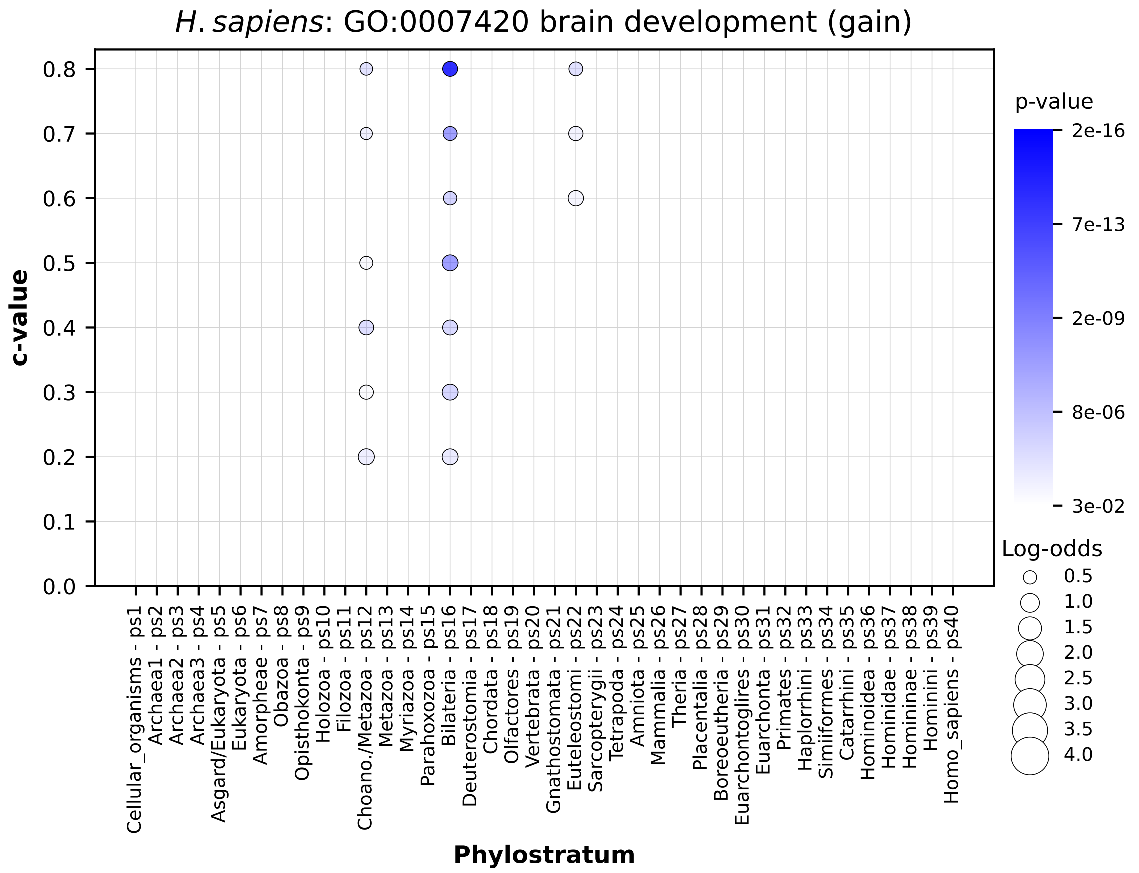

H. sapiens: GO:0007420 brain development (gain)
c-value
0.0
0.1
0.2
0.3
0.4
0.5
0.6
0.7
0.8
Phylostratum
Cellular_organisms - ps1
Archaea1 - ps2
Archaea2 - ps3
Archaea3 - ps4
Asgard/Eukaryota - ps5
Eukaryota - ps6
Amorpheae - ps7
Obazoa - ps8
Opisthokonta - ps9
Holozoa - ps10
Filozoa - ps11
Choano./Metazoa - ps12
Metazoa - ps13
Myriazoa - ps14
Parahoxozoa - ps15
Bilateria - ps16
Deuterostomia - ps17
Chordata - ps18
Olfactores - ps19
Vertebrata - ps20
Gnathostomata - ps21
Euteleostomi - ps22
Sarcopterygii - ps23
Tetrapoda - ps24
Amniota - ps25
Mammalia - ps26
Theria - ps27
Placentalia - ps28
Boreoeutheria - ps29
Euarchontoglires - ps30
Euarchonta - ps31
Primates - ps32
Haplorrhini - ps33
Simiiformes - ps34
Catarrhini - ps35
Hominoidea - ps36
Hominidae - ps37
Homininae - ps38
Hominini - ps39
Homo_sapiens - ps40
p-value
2e-16
7e-13
2e-09
8e-06
3e-02
Log-odds
0.5
1.0
1.5
2.0
2.5
3.0
3.5
4.0

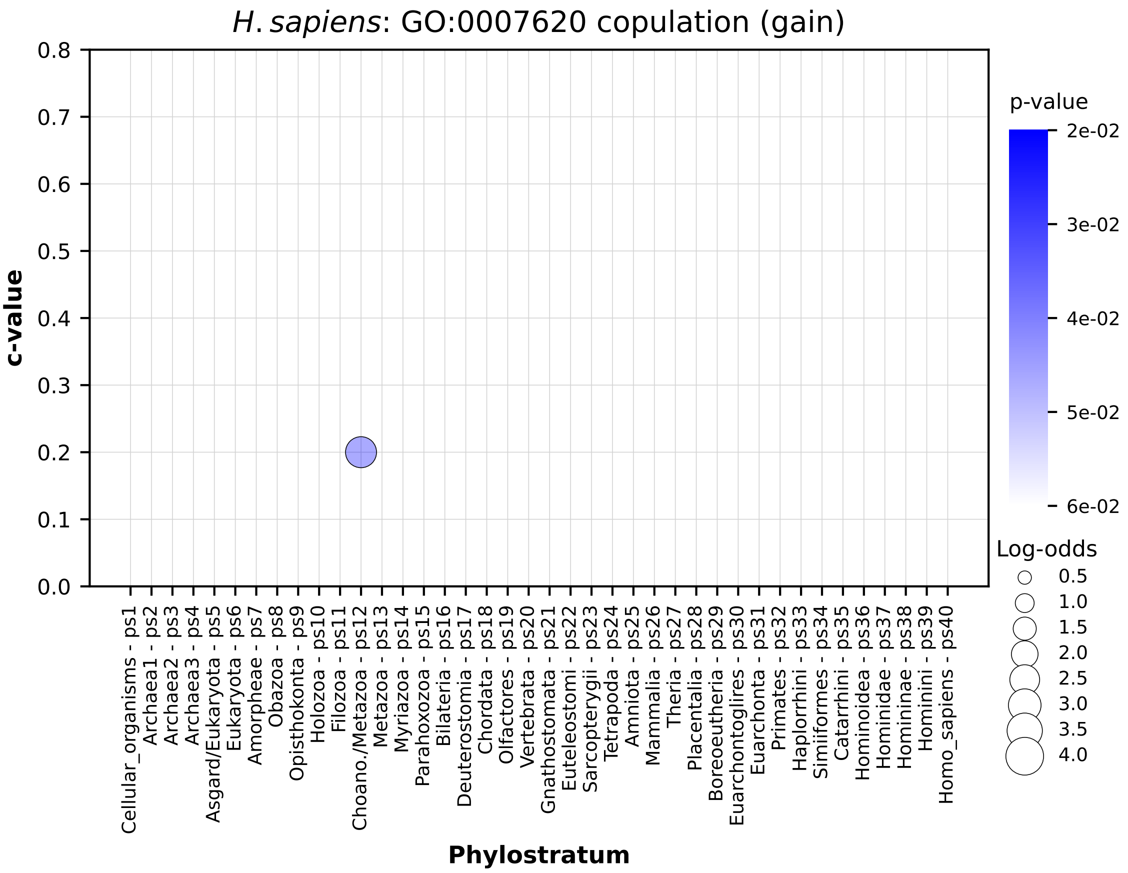
H. sapiens: GO:0007620 copulation (gain)
c-value
0.8
0.7
0.6
0.5
0.4
0.3
0.2
0.1
0.0
Phylostratum
Cellular_organisms - ps1
Archaea1 - ps2
Archaea2 - ps3
Archaea3 - ps4
Asgard/Eukaryota - ps5
Eukaryota - ps6
Amorpheae - ps7
Obazoa - ps8
Opisthokonta - ps9
Holozoa - ps10
Filozoa - ps11
Choano./Metazoa - ps12
Metazoa - ps13
Myriazoa - ps14
Parahoxozoa - ps15
Bilateria - ps16
Deuterostomia - ps17
Chordata - ps18
Olfactores - ps19
Vertebrata - ps20
Gnathostomata - ps21
Euteleostomi - ps22
Sarcopterygii - ps23
Tetrapoda - ps24
Amniota - ps25
Mammalia - ps26
Theria - ps27
Placentalia - ps28
Boreoeutheria - ps29
Euarchontoglires - ps30
Euarchonta - ps31
Primates - ps32
Haplorrhini - ps33
Simiiformes - ps34
Catarrhini - ps35
Hominoidea - ps36
Hominidae - ps37
Homininae - ps38
Hominini - ps39
Homo_sapiens - ps40
p-value
2e-02
3e-02
4e-02
5e-02
6e-02
Log-odds
0.5
1.0
1.5
2.0
2.5
3.0
3.5
4.0

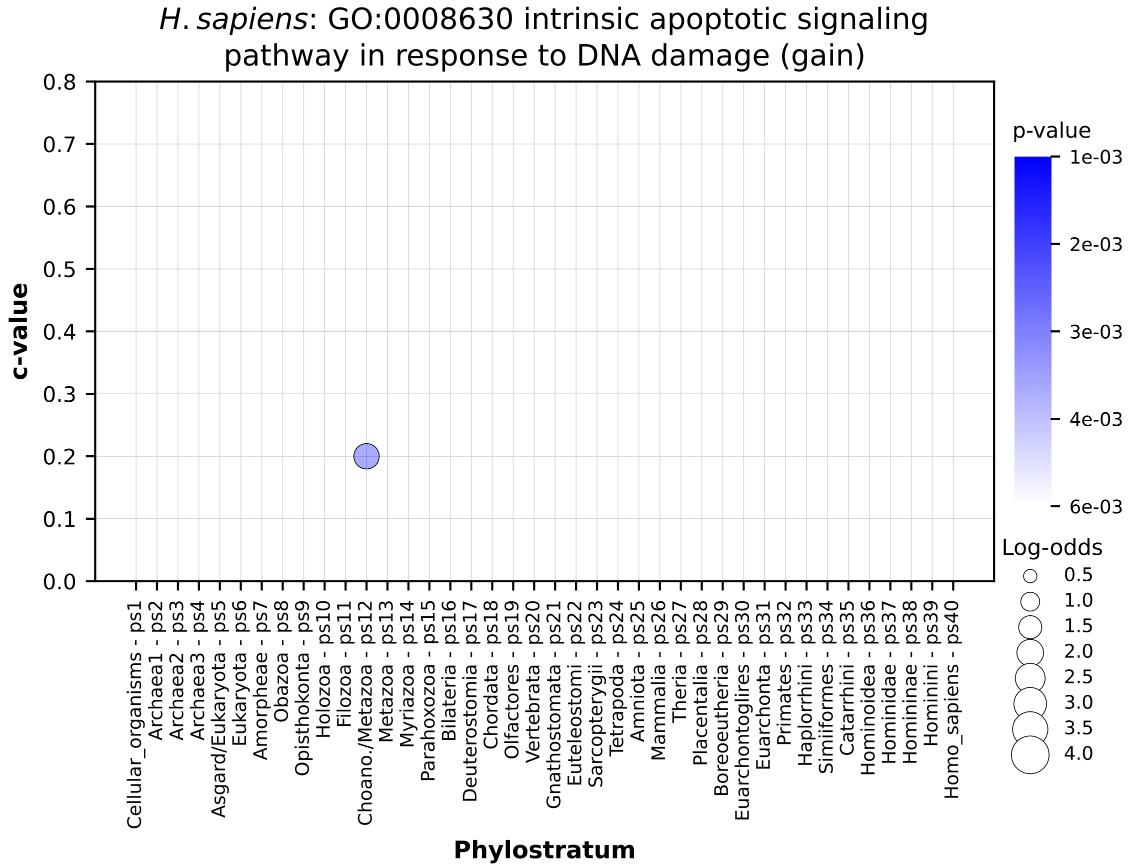
H. sapiens: GO:0008630 intrinsic apoptotic signaling pathway in response to DNA damage (gain)
c-value
0.0
0.1
0.2
0.3
0.4
0.5
0.6
0.7
0.8
Phylostratum
Cellular_organisms - ps1
Archaea1 - ps2
Archaea2 - ps3
Archaea3 - ps4
Asgard/Eukaryota - ps5
Eukaryota - ps6
Amorpheae - ps7
Obazoa - ps8
Opisthokonta - ps9
Holozoa - ps10
Filozoa - ps11
Choano./Metazoa - ps12
Metazoa - ps13
Myriazoa - ps14
Parahoxozoa - ps15
Bilateria - ps16
Deuterostomia - ps17
Chordata - ps18
Olfactores - ps19
Vertebrata - ps20
Gnathostomata - ps21
Euteleostomi - ps22
Sarcopterygii - ps23
Tetrapoda - ps24
Amniota - ps25
Mammalia - ps26
Theria - ps27
Placentalia - ps28
Boreoeutheria - ps29
Euarchontoglires - ps30
Euarchonta - ps31
Primates - ps32
Haplorrhini - ps33
Simiiformes - ps34
Catarrhini - ps35
Hominoidea - ps36
Hominidae - ps37
Homininae - ps38
Hominini - ps39
Homo_sapiens - ps40
p-value
1e-03
2e-03
3e-03
4e-03
6e-03
Log-odds
0.5
1.0
1.5
2.0
2.5
3.0
3.5
4.0

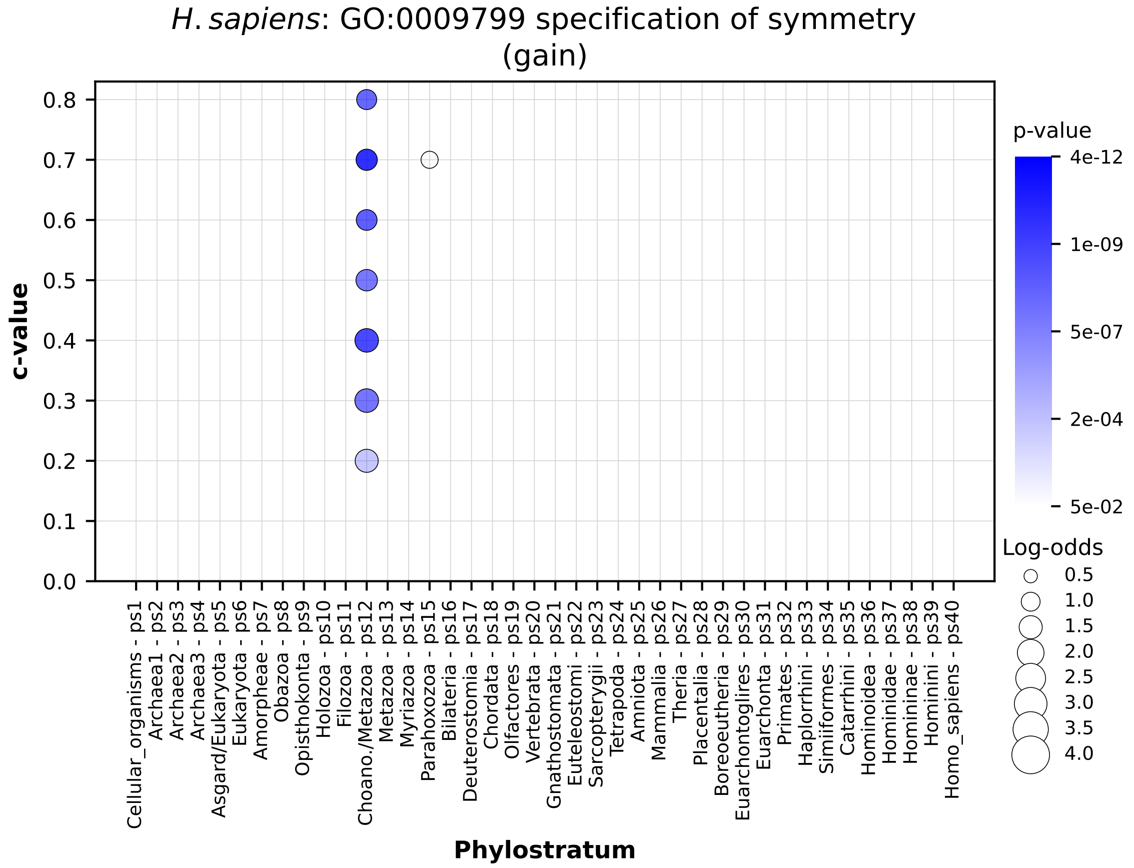

*H. sapiens*: GO:0009799 specification of symmetry
(gain)
c-value
0.0
0.1
0.2
0.3
0.4
0.5
0.6
0.7
0.8
Phylostratum
Cellular_organisms - ps1
Archaea1 - ps2
Archaea2 - ps3
Archaea3 - ps4
Asgard/Eukaryota - ps5
Eukaryota - ps6
Amorpheae - ps7
Obazoa - ps8
Opisthokonta - ps9
Holozoa - ps10
Filozoa - ps11
Choano./Metazoa - ps12
Metazoa - ps13
Myriazoa - ps14
Parahoxozoa - ps15
Bilateria - ps16
Deuterostomia - ps17
Chordata - ps18
Olfactores - ps19
Vertebrata - ps20
Gnathostomata - ps21
Euteleostomi - ps22
Sarcopterygii - ps23
Tetrapoda - ps24
Amniota - ps25
Mammalia - ps26
Theria - ps27
Placentalia - ps28
Boreoeutheria - ps29
Euarchontoglires - ps30
Euarchonta - ps31
Primates - ps32
Haplorrhini - ps33
Simiiformes - ps34
Catarrhini - ps35
Hominoidea - ps36
Hominidae - ps37
Homininae - ps38
Hominini - ps39
Homo_sapiens - ps40
p-value
4e-12
1e-09
5e-07
2e-04
5e-02
Log-odds
0.5
1.0
1.5
2.0
2.5
3.0
3.5
4.0

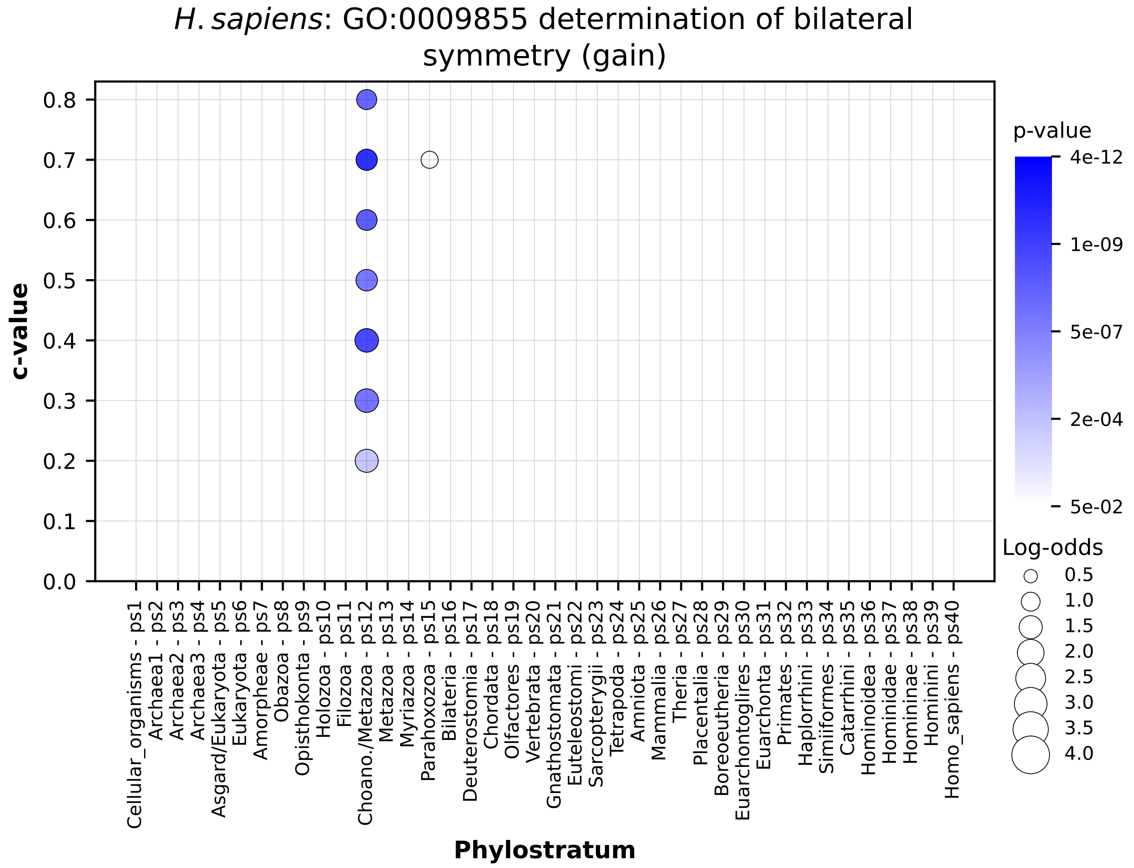
H. sapiens: GO:0009855 determination of bilateral symmetry (gain)
c-value
0.0
0.1
0.2
0.3
0.4
0.5
0.6
0.7
0.8
Phylostratum
Cellular_organisms - ps1
Archaea1 - ps2
Archaea2 - ps3
Archaea3 - ps4
Asgard/Eukaryota - ps5
Eukaryota - ps6
Amorpheae - ps7
Obazoa - ps8
Opisthokonta - ps9
Holozoa - ps10
Filozoa - ps11
Choano./Metazoa - ps12
Metazoa - ps13
Myriazoa - ps14
Parahoxozoa - ps15
Bilateria - ps16
Deuterostomia - ps17
Chordata - ps18
Olfactores - ps19
Vertebrata - ps20
Gnathostomata - ps21
Euteleostomi - ps22
Sarcopterygii - ps23
Tetrapoda - ps24
Amniota - ps25
Mammalia - ps26
Theria - ps27
Placentalia - ps28
Boreoeutheria - ps29
Euarchontoglires - ps30
Euarchonta - ps31
Primates - ps32
Haplorrhini - ps33
Simiiformes - ps34
Catarrhini - ps35
Hominoidea - ps36
Hominidae - ps37
Homininae - ps38
Hominini - ps39
Homo_sapiens - ps40
p-value
4e-12
1e-09
5e-07
2e-04
5e-02
Log-odds
0.5
1.0
1.5
2.0
2.5
3.0
3.5
4.0

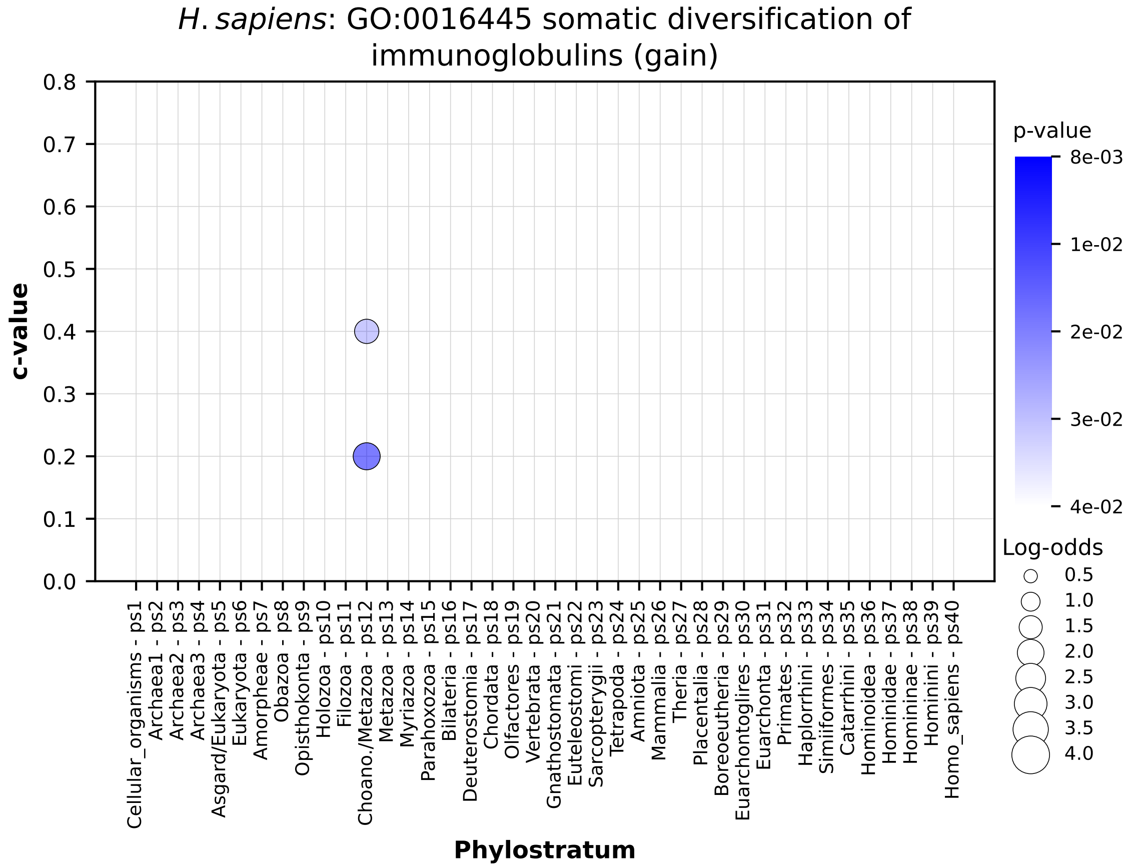

H. sapiens: GO:0016445 somatic diversification of immunoglobulins (gain)
c-value
0.0
0.1
0.2
0.3
0.4
0.5
0.6
0.7
0.8
Phylostratum
Cellular_organisms - ps1
Archaea1 - ps2
Archaea2 - ps3
Archaea3 - ps4
Asgard/Eukaryota - ps5
Eukaryota - ps6
Amorpheae - ps7
Obazoa - ps8
Opisthokonta - ps9
Holozoa - ps10
Filozoa - ps11
Choano./Metazoa - ps12
Metazoa - ps13
Myriazoa - ps14
Parahoxozoa - ps15
Bilateria - ps16
Deuterostomia - ps17
Chordata - ps18
Olfactores - ps19
Vertebrata - ps20
Gnathostomata - ps21
Euteleostomi - ps22
Sarcopterygii - ps23
Tetrapoda - ps24
Amniota - ps25
Mammalia - ps26
Theria - ps27
Placentalia - ps28
Boreoeutheria - ps29
Euarchontoglires - ps30
Euarchonta - ps31
Primates - ps32
Haplorrhini - ps33
Simiiformes - ps34
Catarrhini - ps35
Hominoidea - ps36
Hominidae - ps37
Homininae - ps38
Hominini - ps39
Homo_sapiens - ps40
p-value
8e-03
1e-02
2e-02
3e-02
4e-02
Log-odds
0.5
1.0
1.5
2.0
2.5
3.0
3.5
4.0

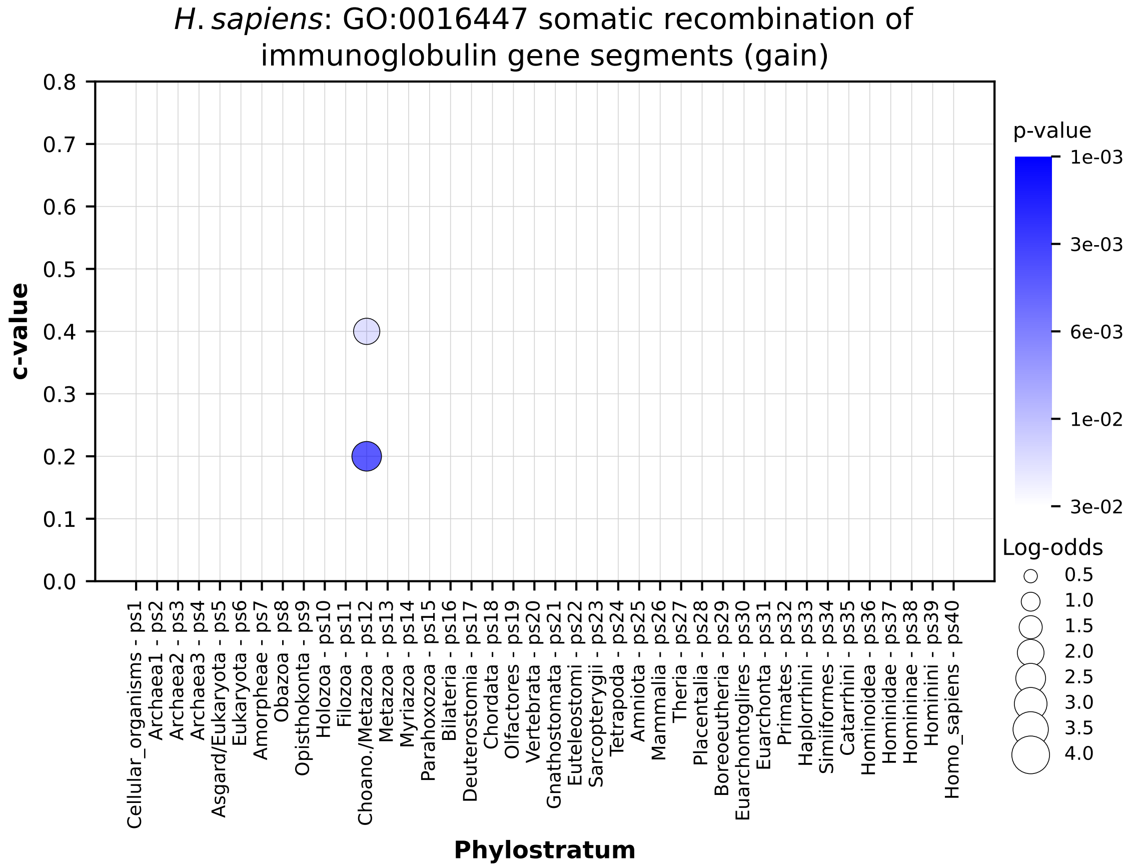

*H. sapiens*: GO:0016447 somatic recombination of immunoglobulin gene segments (gain)
c-value
0.8
0.7
0.6
0.5
0.4
0.3
0.2
0.1
0.0
Phylostratum
Cellular_organisms - ps1
Archaea1 - ps2
Archaea2 - ps3
Archaea3 - ps4
Asgard/Eukaryota - ps5
Eukaryota - ps6
Amorpheae - ps7
Obazoa - ps8
Opisthokonta - ps9
Holozoa - ps10
Filozoa - ps11
Choano./Metazoa - ps12
Metazoa - ps13
Myriazoa - ps14
Parahoxozoa - ps15
Bilateria - ps16
Deuterostomia - ps17
Chordata - ps18
Olfactores - ps19
Vertebrata - ps20
Gnathostomata - ps21
Euteleostomi - ps22
Sarcopterygii - ps23
Tetrapoda - ps24
Amniota - ps25
Mammalia - ps26
Theria - ps27
Placentalia - ps28
Boreoeutheria - ps29
Euarchontoglires - ps30
Euarchonta - ps31
Primates - ps32
Haplorrhini - ps33
Simiiformes - ps34
Catarrhini - ps35
Hominoidea - ps36
Hominidae - ps37
Homininae - ps38
Hominini - ps39
Homo_sapiens - ps40
p-value
1e-03
3e-03
6e-03
1e-02
3e-02
Log-odds
0.5
1.0
1.5
2.0
2.5
3.0
3.5
4.0

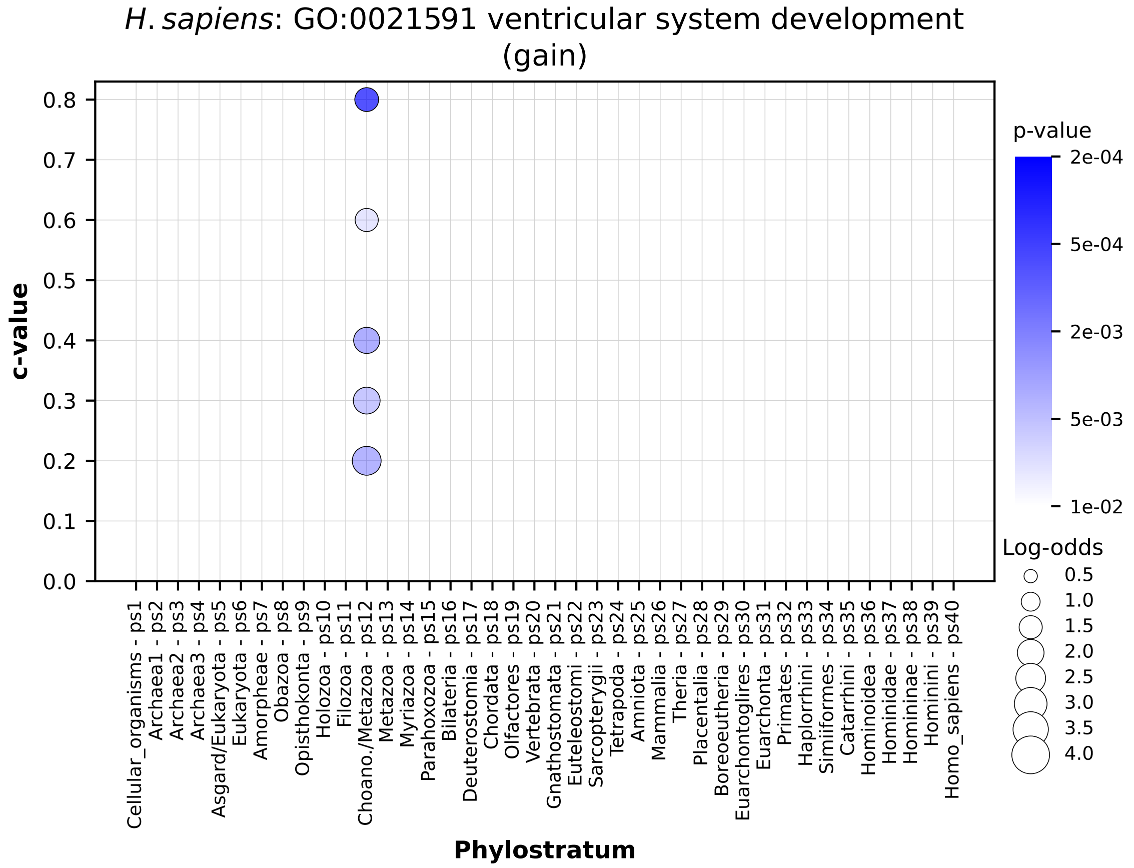
H. sapiens: GO:0021591 ventricular system development
(gain)
c-value
0.0
0.1
0.2
0.3
0.4
0.5
0.6
0.7
0.8
p-value
2e-04
5e-04
2e-03
5e-03
1e-02
Log-odds
0.5
1.0
1.5
2.0
2.5
3.0
3.5
4.0
Cellular_organisms - ps1
Archaea1 - ps2
Archaea2 - ps3
Archaea3 - ps4
Asgard/Eukaryota - ps5
Eukaryota - ps6
Amorpheae - ps7
Obazoa - ps8
Opisthokonta - ps9
Holozoa - ps10
Filozoa - ps11
Choano./Metazoa - ps12
Metazoa - ps13
Myriazoa - ps14
Parahoxozoa - ps15
Bilateria - ps16
Deuterostomia - ps17
Chordata - ps18
Olfactores - ps19
Vertebrata - ps20
Gnathostomata - ps21
Euteleostomi - ps22
Sarcopterygii - ps23
Tetrapoda - ps24
Amniota - ps25
Mammalia - ps26
Theria - ps27
Placentalia - ps28
Boreoeutheria - ps29
Euarchontoglires - ps30
Euarchonta - ps31
Primates - ps32
Haplorrhini - ps33
Simiiformes - ps34
Catarrhini - ps35
Hominoidea - ps36
Hominidae - ps37
Homininae - ps38
Hominini - ps39
Homo_sapiens - ps40
Phylostratum

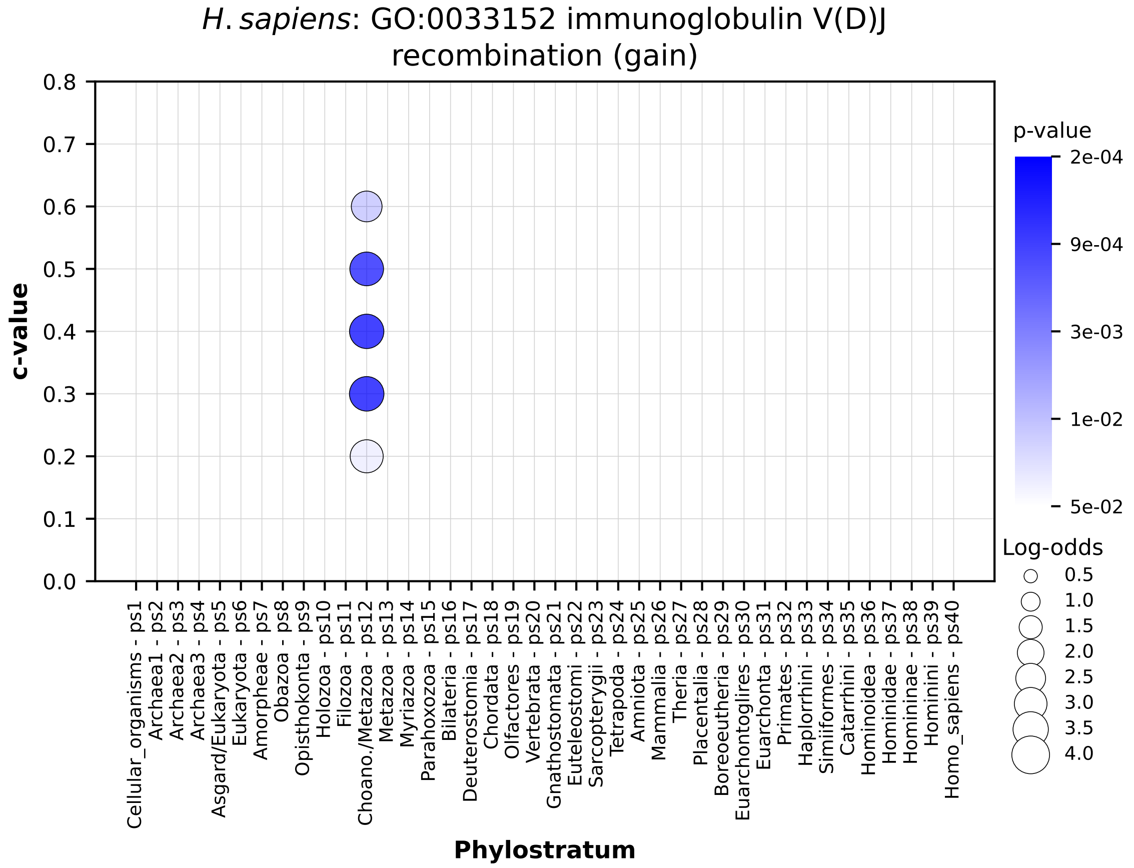
H. sapiens: GO:0033152 immunoglobulin V(D)J recombination (gain)
c-value
0.0
0.1
0.2
0.3
0.4
0.5
0.6
0.7
0.8
Phylostratum
Cellular_organisms - ps1
Archaea1 - ps2
Archaea2 - ps3
Archaea3 - ps4
Asgard/Eukaryota - ps5
Eukaryota - ps6
Amorpheae - ps7
Obazoa - ps8
Opisthokonta - ps9
Holozoa - ps10
Filozoa - ps11
Choano./Metazoa - ps12
Metazoa - ps13
Myriazoa - ps14
Parahoxozoa - ps15
Bilateria - ps16
Deuterostomia - ps17
Chordata - ps18
Olfactores - ps19
Vertebrata - ps20
Gnathostomata - ps21
Euteleostomi - ps22
Sarcopterygii - ps23
Tetrapoda - ps24
Amniota - ps25
Mammalia - ps26
Theria - ps27
Placentalia - ps28
Boreoeutheria - ps29
Euarchontoglires - ps30
Euarchonta - ps31
Primates - ps32
Haplorrhini - ps33
Simiiformes - ps34
Catarrhini - ps35
Hominoidea - ps36
Hominidae - ps37
Homininae - ps38
Hominini - ps39
Homo_sapiens - ps40
p-value
2e-04
9e-04
3e-03
1e-02
5e-02
Log-odds
0.5
1.0
1.5
2.0
2.5
3.0
3.5
4.0

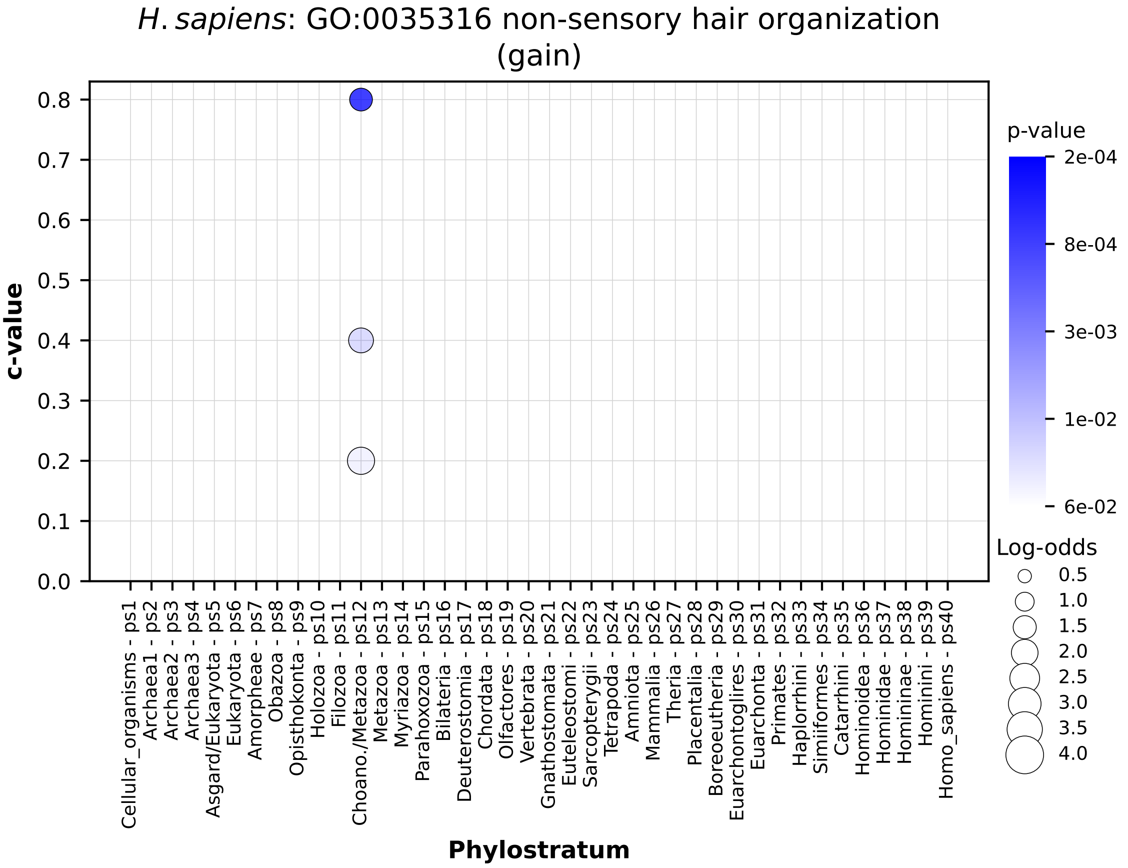

H. sapiens: GO:0035316 non-sensory hair organization
(gain)
c-value
0.0
0.1
0.2
0.3
0.4
0.5
0.6
0.7
0.8
Cellular_organisms - ps1
Archaea1 - ps2
Archaea2 - ps3
Archaea3 - ps4
Asgard/Eukaryota - ps5
Eukaryota - ps6
Amorpheae - ps7
Obazoa - ps8
Opisthokonta - ps9
Holozoa - ps10
Filozoa - ps11
Choano./Metazoa - ps12
Metazoa - ps13
Myriazoa - ps14
Parahoxozoa - ps15
Bilateria - ps16
Deuterostomia - ps17
Chordata - ps18
Olfactores - ps19
Vertebrata - ps20
Gnathostomata - ps21
Euteleostomi - ps22
Sarcopterygii - ps23
Tetrapoda - ps24
Amniota - ps25
Mammalia - ps26
Theria - ps27
Placentalia - ps28
Boreoeutheria - ps29
Euarchontoglires - ps30
Euarchonta - ps31
Primates - ps32
Haplorrhini - ps33
Simiiformes - ps34
Catarrhini - ps35
Hominoidea - ps36
Hominidae - ps37
Homininae - ps38
Hominini - ps39
Homo_sapiens - ps40
Phylostratum
p-value
2e-04
8e-04
3e-03
1e-02
6e-02
Log-odds
0.5
1.0
1.5
2.0
2.5
3.0
3.5
4.0

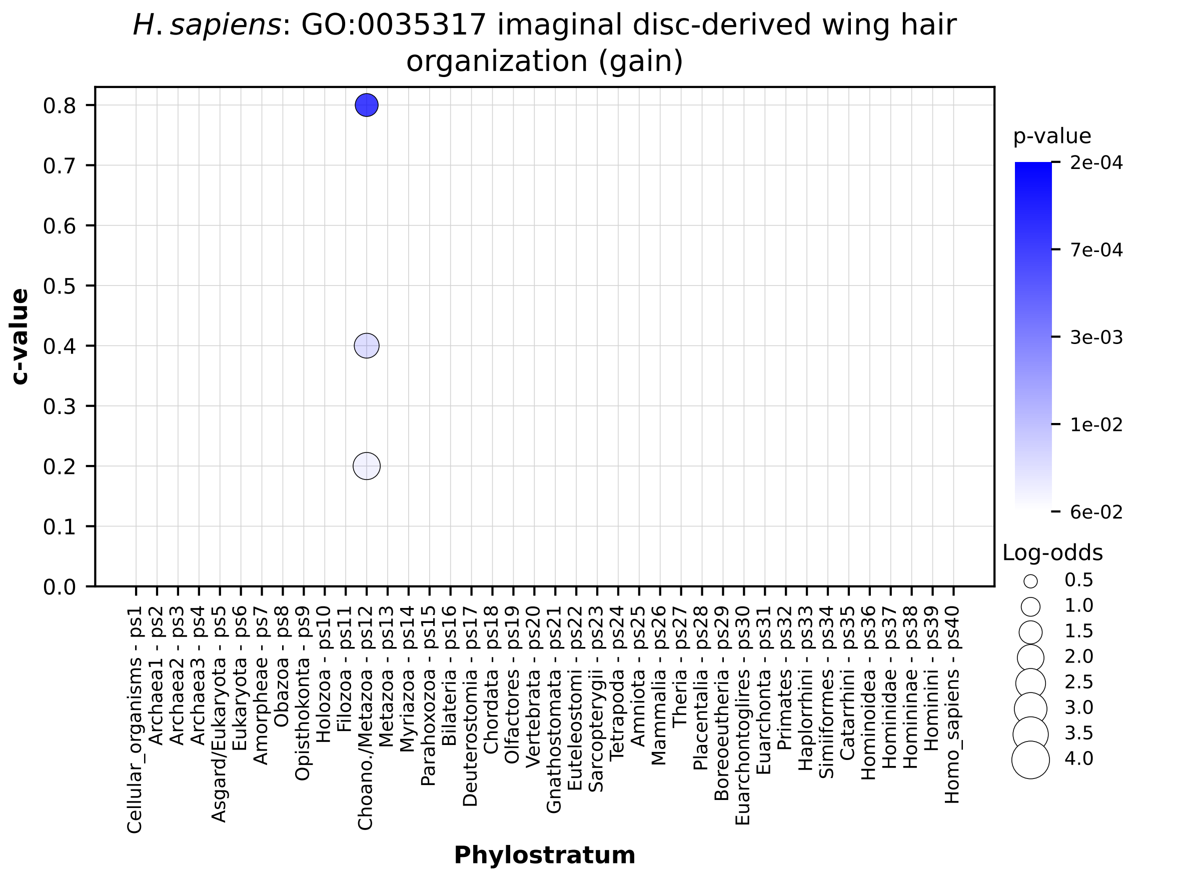

H. sapiens: GO:0035317 imaginal disc-derived wing hair organization (gain)
c-value
0.0
0.1
0.2
0.3
0.4
0.5
0.6
0.7
0.8
Phylostratum
Cellular_organisms - ps1
Archaea1 - ps2
Archaea2 - ps3
Archaea3 - ps4
Asgard/Eukaryota - ps5
Eukaryota - ps6
Amorpheae - ps7
Obazoa - ps8
Opisthokonta - ps9
Holozoa - ps10
Filozoa - ps11
Choano./Metazoa - ps12
Metazoa - ps13
Myriazoa - ps14
Parahoxozoa - ps15
Bilateria - ps16
Deuterostomia - ps17
Chordata - ps18
Olfactores - ps19
Vertebrata - ps20
Gnathostomata - ps21
Euteleostomi - ps22
Sarcopterygii - ps23
Tetrapoda - ps24
Amniota - ps25
Mammalia - ps26
Theria - ps27
Placentalia - ps28
Boreoeutheria - ps29
Euarchontoglires - ps30
Euarchonta - ps31
Primates - ps32
Haplorrhini - ps33
Simiiformes - ps34
Catarrhini - ps35
Hominoidea - ps36
Hominidae - ps37
Homininae - ps38
Hominini - ps39
Homo_sapiens - ps40
p-value
2e-04
7e-04
3e-03
1e-02
6e-02
Log-odds
0.5
1.0
1.5
2.0
2.5
3.0
3.5
4.0

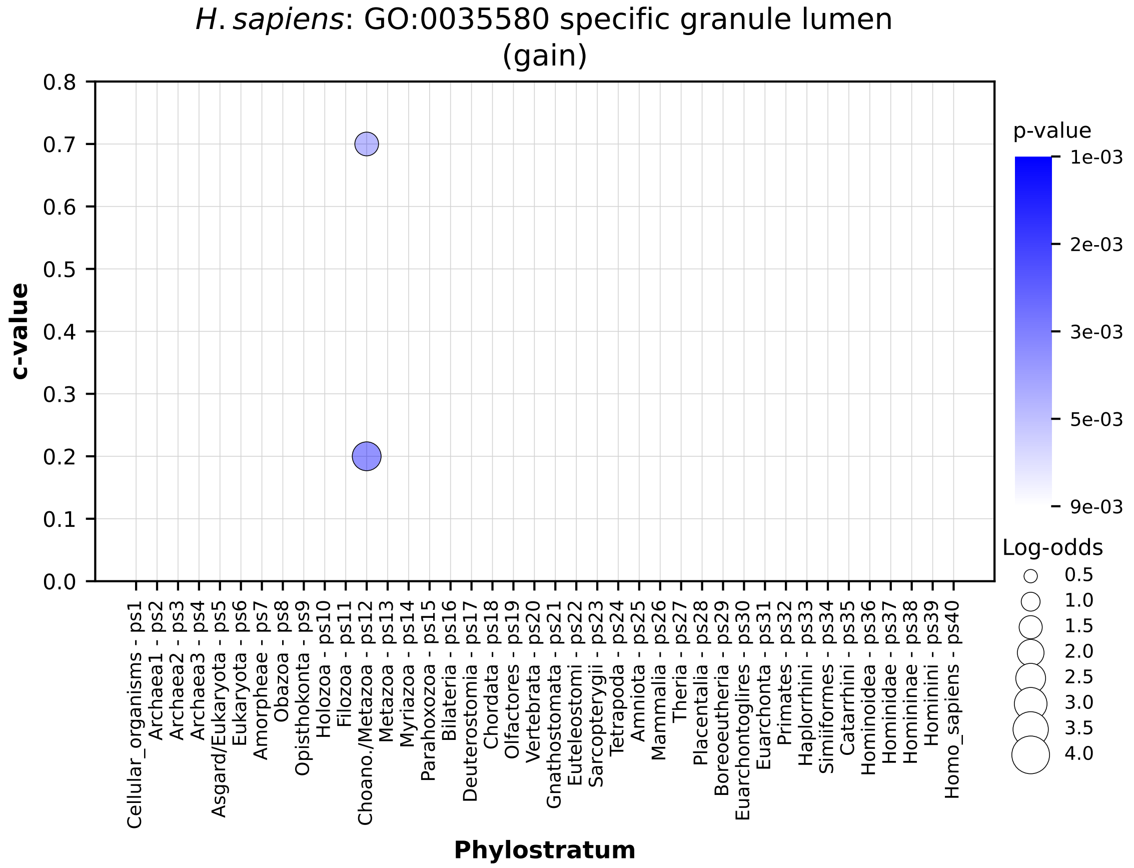

H. sapiens: GO:0035580 specific granule lumen
(gain)
c-value
0.0
0.1
0.2
0.3
0.4
0.5
0.6
0.7
0.8
Phylostratum
Cellular_organisms - ps1
Archaea1 - ps2
Archaea2 - ps3
Archaea3 - ps4
Asgard/Eukaryota - ps5
Eukaryota - ps6
Amorpheae - ps7
Obazoa - ps8
Opisthokonta - ps9
Holozoa - ps10
Filozoa - ps11
Choano./Metazoa - ps12
Metazoa - ps13
Myriazoa - ps14
Parahoxozoa - ps15
Bilateria - ps16
Deuterostomia - ps17
Chordata - ps18
Olfactores - ps19
Vertebrata - ps20
Gnathostomata - ps21
Euteleostomi - ps22
Sarcopterygii - ps23
Tetrapoda - ps24
Amniota - ps25
Mammalia - ps26
Theria - ps27
Placentalia - ps28
Boreoeutheria - ps29
Euarchontoglires - ps30
Euarchonta - ps31
Primates - ps32
Haplorrhini - ps33
Simiiformes - ps34
Catarrhini - ps35
Hominoidea - ps36
Hominidae - ps37
Homininae - ps38
Hominini - ps39
Homo_sapiens - ps40
p-value
1e-03
2e-03
3e-03
5e-03
9e-03
Log-odds
0.5
1.0
1.5
2.0
2.5
3.0
3.5
4.0

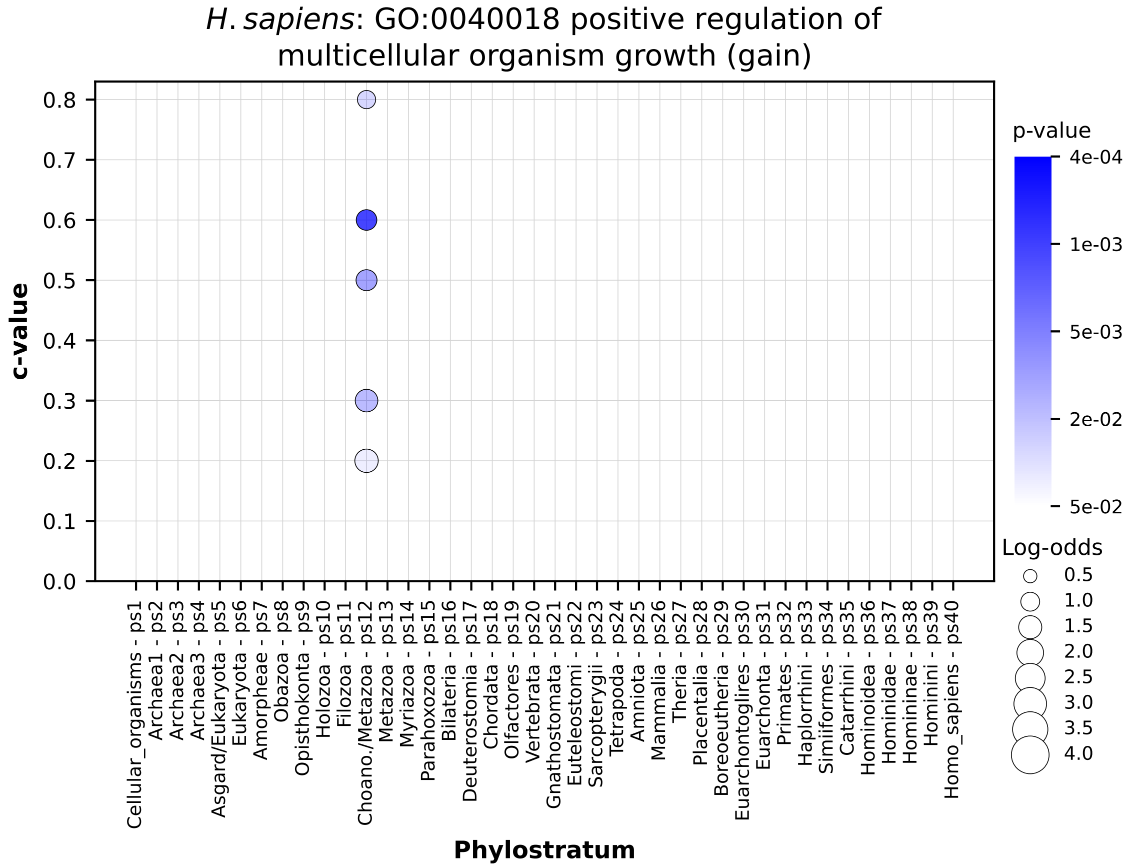

H. sapiens: GO:0040018 positive regulation of multicellular organism growth (gain)
c-value
0.0
0.1
0.2
0.3
0.4
0.5
0.6
0.7
0.8
p-value
4e-04
1e-03
5e-03
2e-02
5e-02
Log-odds
0.5
1.0
1.5
2.0
2.5
3.0
3.5
4.0
Cellular_organisms - ps1
Archaea1 - ps2
Archaea2 - ps3
Archaea3 - ps4
Asgard/Eukaryota - ps5
Eukaryota - ps6
Amorpheae - ps7
Obazoa - ps8
Opisthokonta - ps9
Holozoa - ps10
Filozoa - ps11
Choano./Metazoa - ps12
Metazoa - ps13
Myriazoa - ps14
Parahoxozoa - ps15
Bilateria - ps16
Deuterostomia - ps17
Chordata - ps18
Olfactores - ps19
Vertebrata - ps20
Gnathostomata - ps21
Euteleostomi - ps22
Sarcopterygii - ps23
Tetrapoda - ps24
Amniota - ps25
Mammalia - ps26
Theria - ps27
Placentalia - ps28
Boreoeutheria - ps29
Euarchontoglires - ps30
Euarchonta - ps31
Primates - ps32
Haplorrhini - ps33
Simiiformes - ps34
Catarrhini - ps35
Hominoidea - ps36
Hominidae - ps37
Homininae - ps38
Hominini - ps39
Homo_sapiens - ps40
Phylostratum

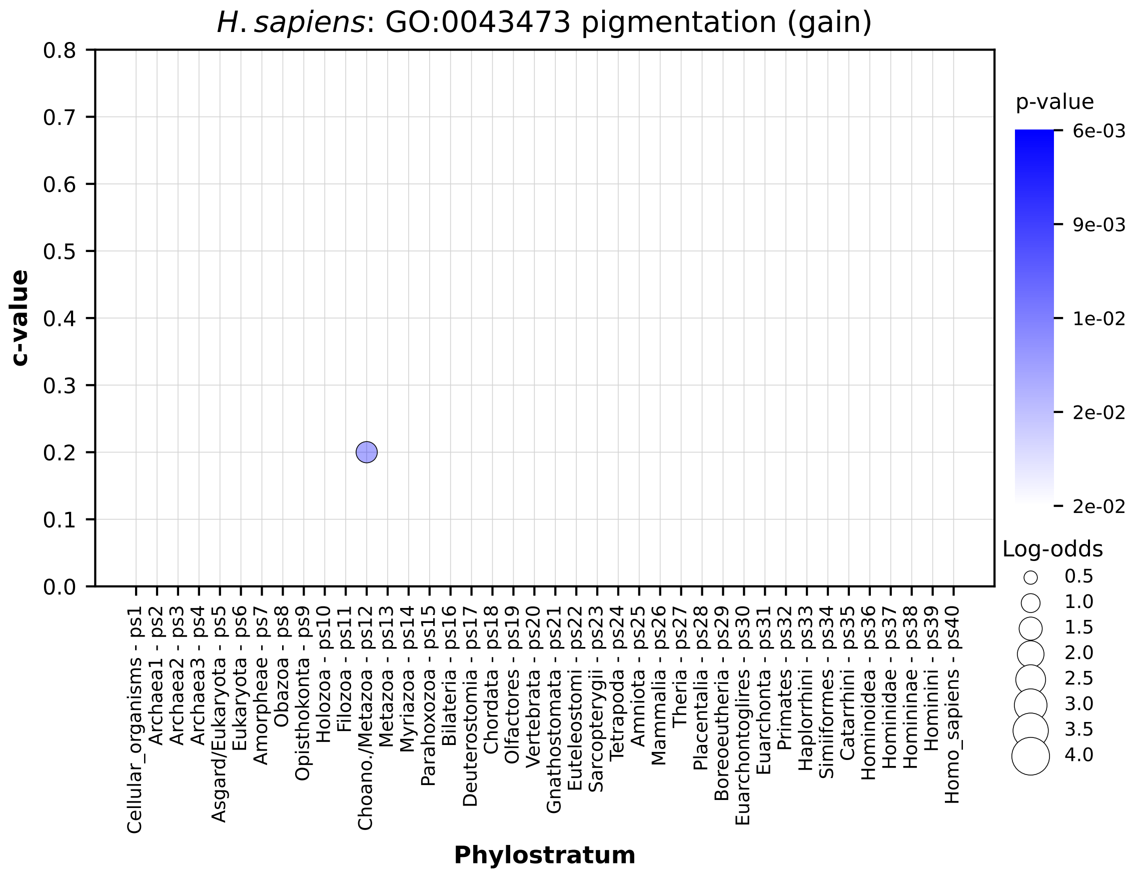

H. sapiens: GO:0043473 pigmentation (gain)
c-value
0.8
0.7
0.6
0.5
0.4
0.3
0.2
0.1
0.0
Cellular_organisms - ps1
Archaea1 - ps2
Archaea2 - ps3
Archaea3 - ps4
Asgard/Eukaryota - ps5
Eukaryota - ps6
Amorpheae - ps7
Obazoa - ps8
Opisthokonta - ps9
Holozoa - ps10
Filozoa - ps11
Choano./Metazoa - ps12
Metazoa - ps13
Myriazoa - ps14
Parahoxozoa - ps15
Bilateria - ps16
Deuterostomia - ps17
Chordata - ps18
Olfactores - ps19
Vertebrata - ps20
Gnathostomata - ps21
Euteleostomi - ps22
Sarcopterygii - ps23
Tetrapoda - ps24
Amniota - ps25
Mammalia - ps26
Theria - ps27
Placentalia - ps28
Boreoeutheria - ps29
Euarchontoglires - ps30
Euarchonta - ps31
Primates - ps32
Haplorrhini - ps33
Simiiformes - ps34
Catarrhini - ps35
Hominoidea - ps36
Hominidae - ps37
Homininae - ps38
Hominini - ps39
Homo_sapiens - ps40
Phylostratum
p-value
6e-03
9e-03
1e-02
2e-02
2e-02
Log-odds
0.5
1.0
1.5
2.0
2.5
3.0
3.5
4.0

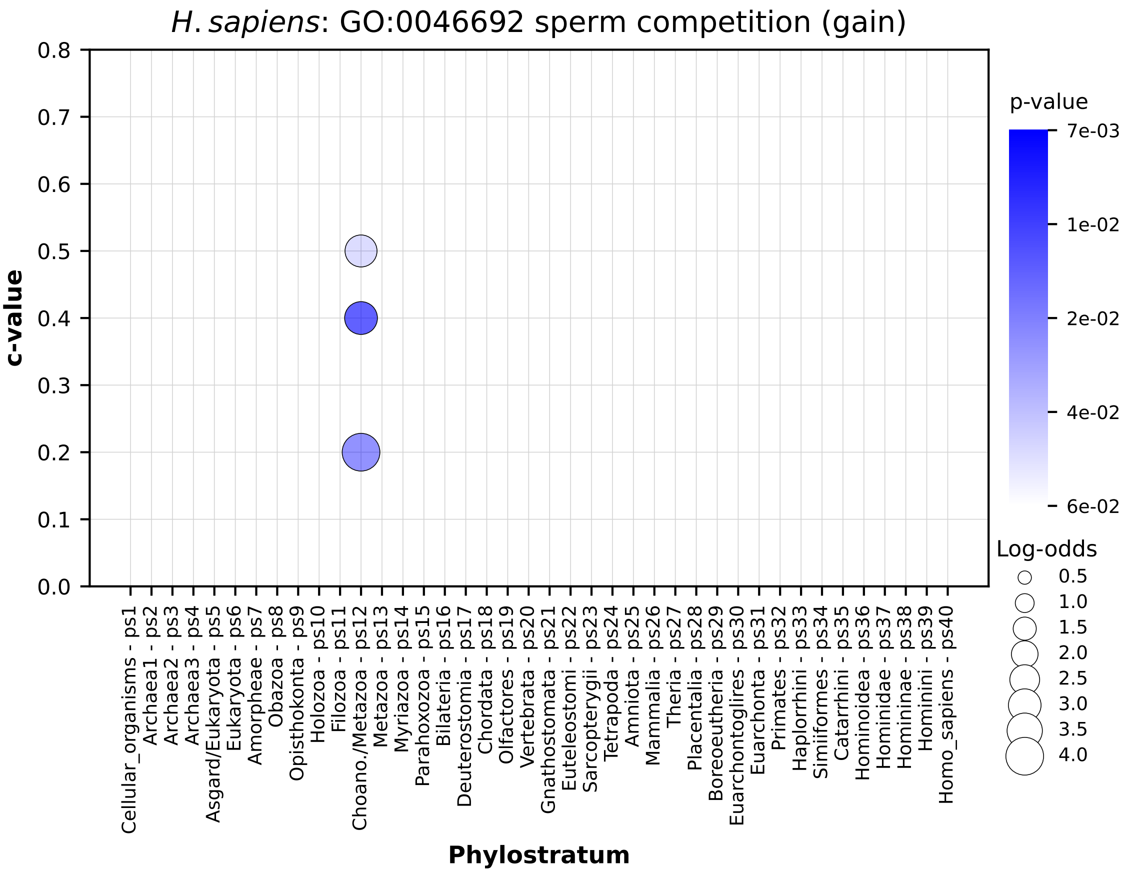

*H. sapiens*: GO:0046692 sperm competition (gain)
c-value
0.8
0.7
0.6
0.5
0.4
0.3
0.2
0.1
0.0
Cellular_organisms - ps1
Archaea1 - ps2
Archaea2 - ps3
Archaea3 - ps4
Asgard/Eukaryota - ps5
Eukaryota - ps6
Amorpheae - ps7
Obazoa - ps8
Opisthokonta - ps9
Holozoa - ps10
Filozoa - ps11
Choano./Metazoa - ps12
Metazoa - ps13
Myriazoa - ps14
Parahoxozoa - ps15
Bilateria - ps16
Deuterostomia - ps17
Chordata - ps18
Olfactores - ps19
Vertebrata - ps20
Gnathostomata - ps21
Euteleostomi - ps22
Sarcopterygii - ps23
Tetrapoda - ps24
Amniota - ps25
Mammalia - ps26
Theria - ps27
Placentalia - ps28
Boreoeutheria - ps29
Euarchontoglires - ps30
Euarchonta - ps31
Primates - ps32
Haplorrhini - ps33
Simiiformes - ps34
Catarrhini - ps35
Hominoidea - ps36
Hominidae - ps37
Homininae - ps38
Hominini - ps39
Homo_sapiens - ps40
Phylostratum
p-value
7e-03
1e-02
2e-02
4e-02
6e-02
Log-odds
0.5
1.0
1.5
2.0
2.5
3.0
3.5
4.0

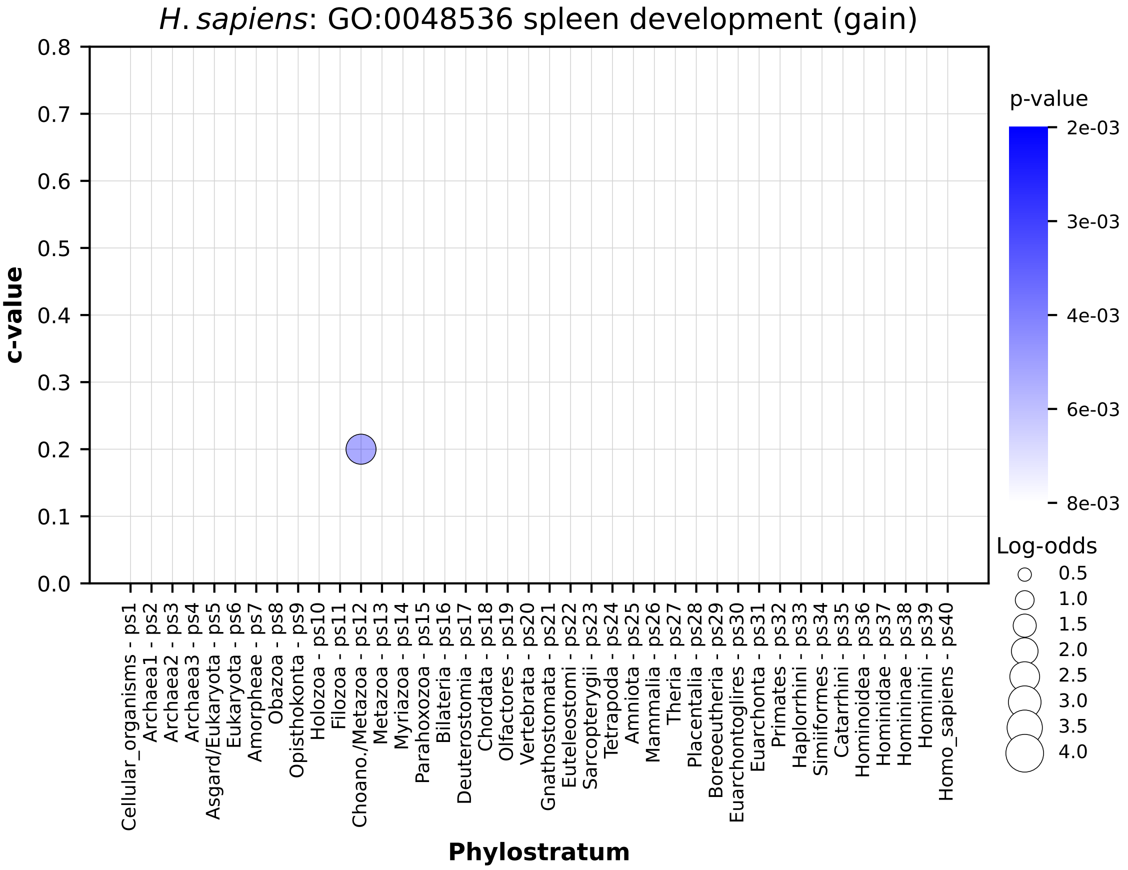

*H. sapiens*: GO:0048536 spleen development (gain)
c-value
0.8
0.7
0.6
0.5
0.4
0.3
0.2
0.1
0.0
p-value
2e-03
3e-03
4e-03
6e-03
8e-03
Log-odds
0.5
1.0
1.5
2.0
2.5
3.0
3.5
4.0
Cellular_organisms - ps1
Archaea1 - ps2
Archaea2 - ps3
Archaea3 - ps4
Asgard/Eukaryota - ps5
Eukaryota - ps6
Amorpheae - ps7
Obazoa - ps8
Opisthokonta - ps9
Holozoa - ps10
Filozoa - ps11
Choano./Metazoa - ps12
Metazoa - ps13
Myriazoa - ps14
Parahoxozoa - ps15
Bilateria - ps16
Deuterostomia - ps17
Chordata - ps18
Olfactores - ps19
Vertebrata - ps20
Gnathostomata - ps21
Euteleostomi - ps22
Sarcopterygii - ps23
Tetrapoda - ps24
Amniota - ps25
Mammalia - ps26
Theria - ps27
Placentalia - ps28
Boreoeutheria - ps29
Euarchontoglires - ps30
Euarchonta - ps31
Primates - ps32
Haplorrhini - ps33
Simiiformes - ps34
Catarrhini - ps35
Hominoidea - ps36
Hominidae - ps37
Homininae - ps38
Hominini - ps39
Homo_sapiens - ps40
Phylostratum

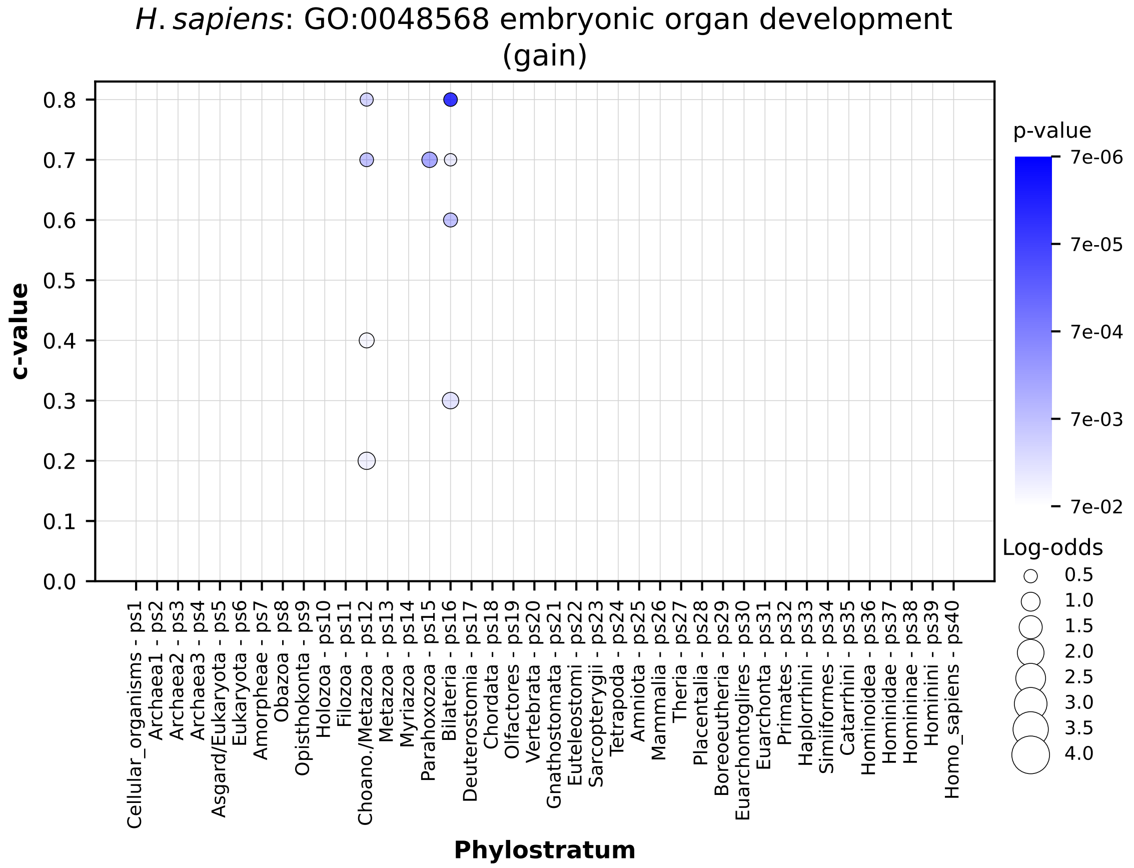
H. sapiens: GO:0048568 embryonic organ development
(gain)
c-value
0.0
0.1
0.2
0.3
0.4
0.5
0.6
0.7
0.8
Phylostratum
Cellular_organisms - ps1
Archaea1 - ps2
Archaea2 - ps3
Archaea3 - ps4
Asgard/Eukaryota - ps5
Eukaryota - ps6
Amorpheae - ps7
Obazoa - ps8
Opisthokonta - ps9
Holozoa - ps10
Filozoa - ps11
Choano./Metazoa - ps12
Metazoa - ps13
Myriazoa - ps14
Parahoxozoa - ps15
Bilateria - ps16
Deuterostomia - ps17
Chordata - ps18
Olfactores - ps19
Vertebrata - ps20
Gnathostomata - ps21
Euteleostomi - ps22
Sarcopterygii - ps23
Tetrapoda - ps24
Amniota - ps25
Mammalia - ps26
Theria - ps27
Placentalia - ps28
Boreoeutheria - ps29
Euarchontoglires - ps30
Euarchonta - ps31
Primates - ps32
Haplorrhini - ps33
Simiiformes - ps34
Catarrhini - ps35
Hominoidea - ps36
Hominidae - ps37
Homininae - ps38
Hominini - ps39
Homo_sapiens - ps40
p-value
7e-06
7e-05
7e-04
7e-03
7e-02
Log-odds
0.5
1.0
1.5
2.0
2.5
3.0
3.5
4.0

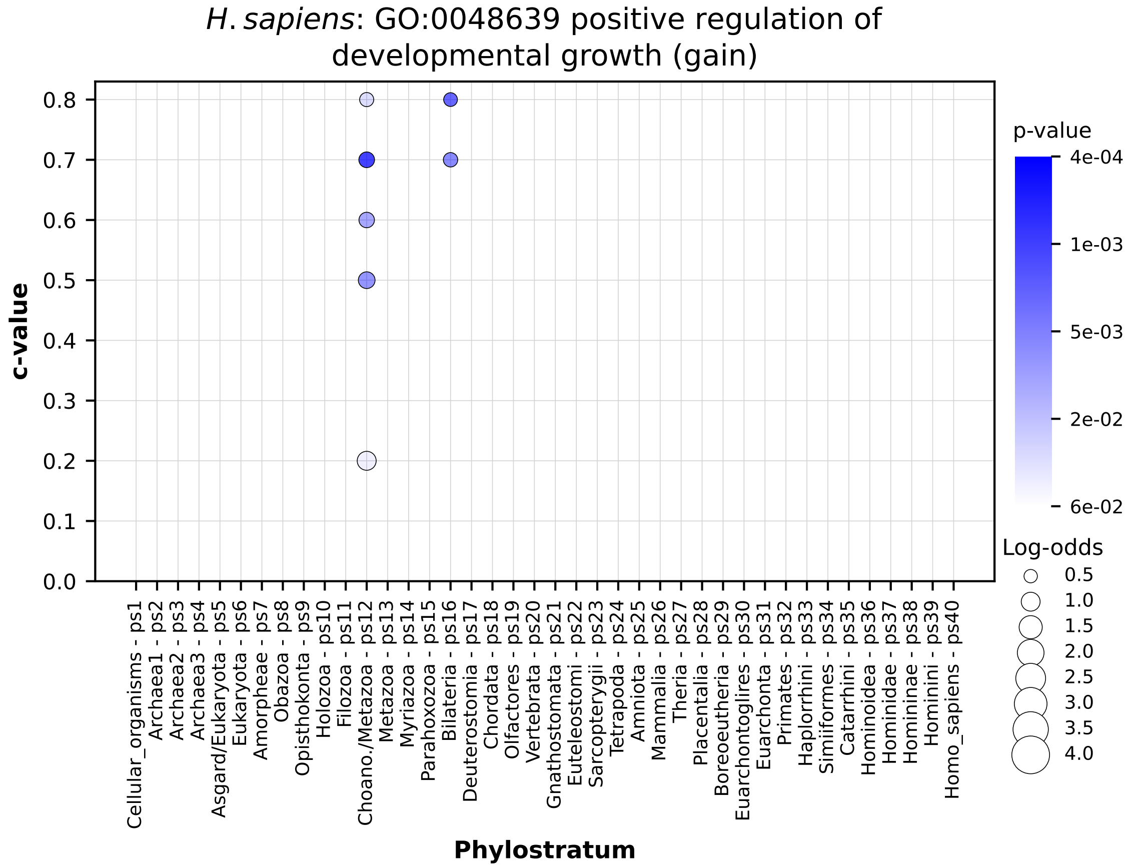

*H. sapiens*: GO:0048639 positive regulation of developmental growth (gain)
c-value
0.0
0.1
0.2
0.3
0.4
0.5
0.6
0.7
0.8
Phylostratum
Cellular_organisms - ps1
Archaea1 - ps2
Archaea2 - ps3
Archaea3 - ps4
Asgard/Eukaryota - ps5
Eukaryota - ps6
Amorpheae - ps7
Obazoa - ps8
Opisthokonta - ps9
Holozoa - ps10
Filozoa - ps11
Choano./Metazoa - ps12
Metazoa - ps13
Myriazoa - ps14
Parahoxozoa - ps15
Bilateria - ps16
Deuterostomia - ps17
Chordata - ps18
Olfactores - ps19
Vertebrata - ps20
Gnathostomata - ps21
Euteleostomi - ps22
Sarcopterygii - ps23
Tetrapoda - ps24
Amniota - ps25
Mammalia - ps26
Theria - ps27
Placentalia - ps28
Boreoeutheria - ps29
Euarchontoglires - ps30
Euarchonta - ps31
Primates - ps32
Haplorrhini - ps33
Simiiformes - ps34
Catarrhini - ps35
Hominoidea - ps36
Hominidae - ps37
Homininae - ps38
Hominini - ps39
Homo_sapiens - ps40
p-value
4e-04
1e-03
5e-03
2e-02
6e-02
Log-odds
0.5
1.0
1.5
2.0
2.5
3.0
3.5
4.0

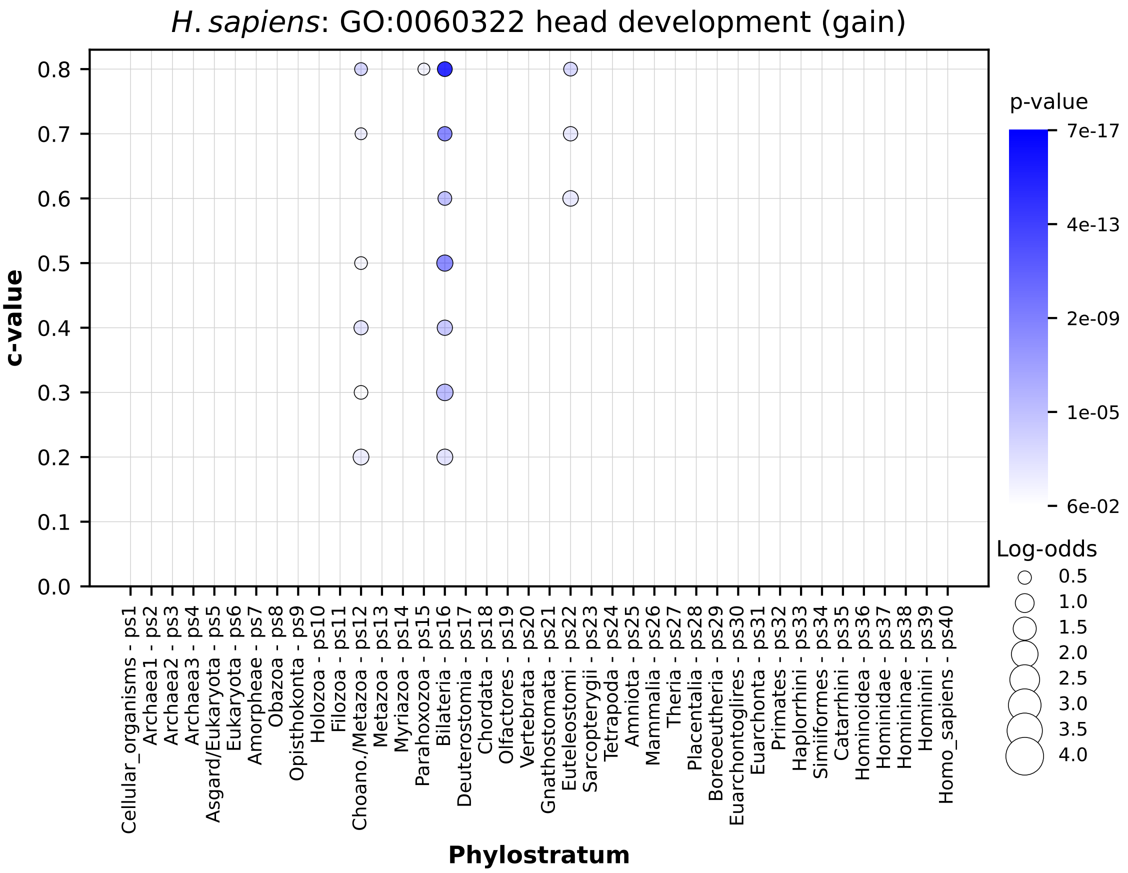

*H. sapiens*: GO:0060322 head development (gain)
c-value
0.0
0.1
0.2
0.3
0.4
0.5
0.6
0.7
0.8
Phylostratum
Cellular_organisms - ps1
Archaea1 - ps2
Archaea2 - ps3
Archaea3 - ps4
Asgard/Eukaryota - ps5
Eukaryota - ps6
Amorpheae - ps7
Obazoa - ps8
Opisthokonta - ps9
Holozoa - ps10
Filozoa - ps11
Choano./Metazoa - ps12
Metazoa - ps13
Myriazoa - ps14
Parahoxozoa - ps15
Bilateria - ps16
Deuterostomia - ps17
Chordata - ps18
Olfactores - ps19
Vertebrata - ps20
Gnathostomata - ps21
Euteleostomi - ps22
Sarcopterygii - ps23
Tetrapoda - ps24
Amniota - ps25
Mammalia - ps26
Theria - ps27
Placentalia - ps28
Boreoeutheria - ps29
Euarchontoglires - ps30
Euarchonta - ps31
Primates - ps32
Haplorrhini - ps33
Simiiformes - ps34
Catarrhini - ps35
Hominoidea - ps36
Hominidae - ps37
Homininae - ps38
Hominini - ps39
Homo_sapiens - ps40
p-value
7e-17
4e-13
2e-09
1e-05
6e-02
Log-odds
0.5
1.0
1.5
2.0
2.5
3.0
3.5
4.0

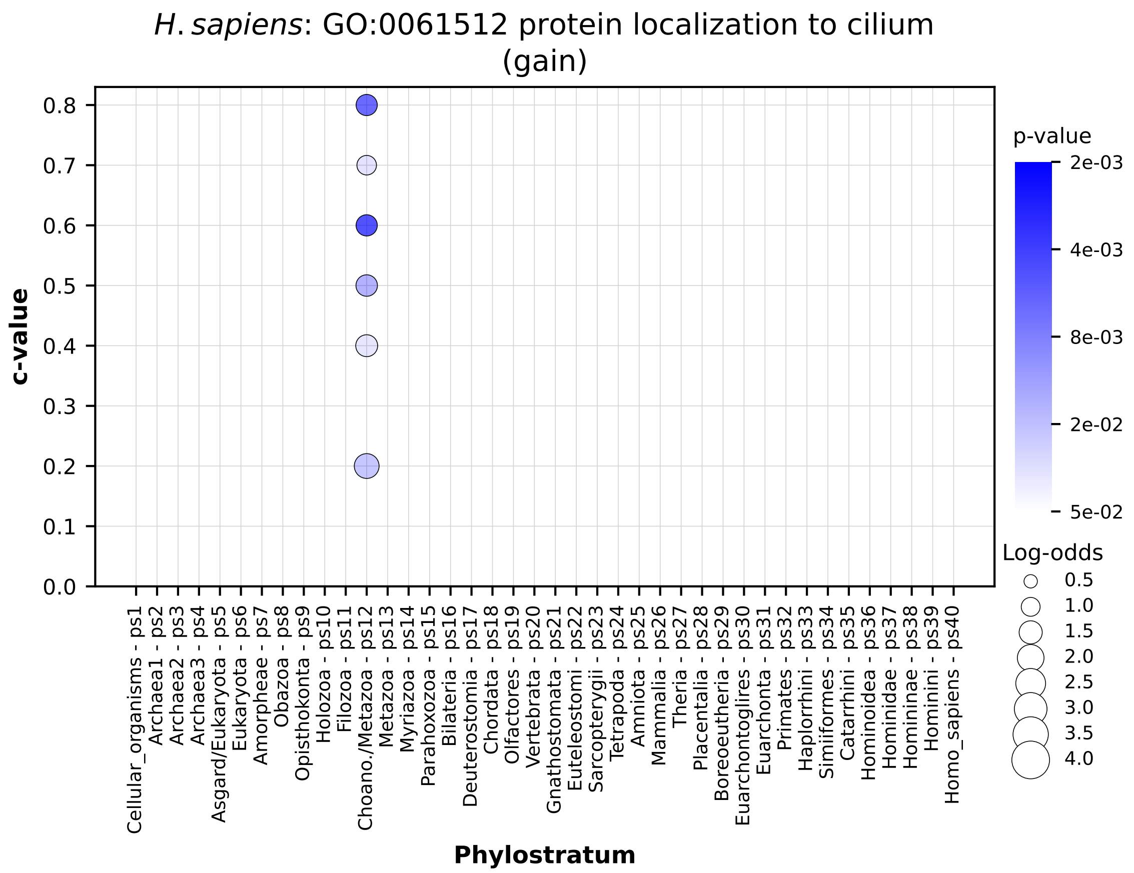

*H. sapiens*: GO:0061512 protein localization to cilium (gain)
c-value
0.8
0.7
0.6
0.5
0.4
0.3
0.2
0.1
0.0
Phylostratum
Cellular_organisms - ps1
Archaea1 - ps2
Archaea2 - ps3
Archaea3 - ps4
Asgard/Eukaryota - ps5
Eukaryota - ps6
Amorpheae - ps7
Obazoa - ps8
Opisthokonta - ps9
Holozoa - ps10
Filozoa - ps11
Choano./Metazoa - ps12
Metazoa - ps13
Myriazoa - ps14
Parahoxozoa - ps15
Bilateria - ps16
Deuterostomia - ps17
Chordata - ps18
Olfactores - ps19
Vertebrata - ps20
Gnathostomata - ps21
Euteleostomi - ps22
Sarcopterygii - ps23
Tetrapoda - ps24
Amniota - ps25
Mammalia - ps26
Theria - ps27
Placentalia - ps28
Boreoeutheria - ps29
Euarchontoglires - ps30
Euarchonta - ps31
Primates - ps32
Haplorrhini - ps33
Simiiformes - ps34
Catarrhini - ps35
Hominoidea - ps36
Hominidae - ps37
Homininae - ps38
Hominini - ps39
Homo_sapiens - ps40
p-value
2e-03
4e-03
8e-03
2e-02
5e-02
Log-odds
0.5
1.0
1.5
2.0
2.5
3.0
3.5
4.0

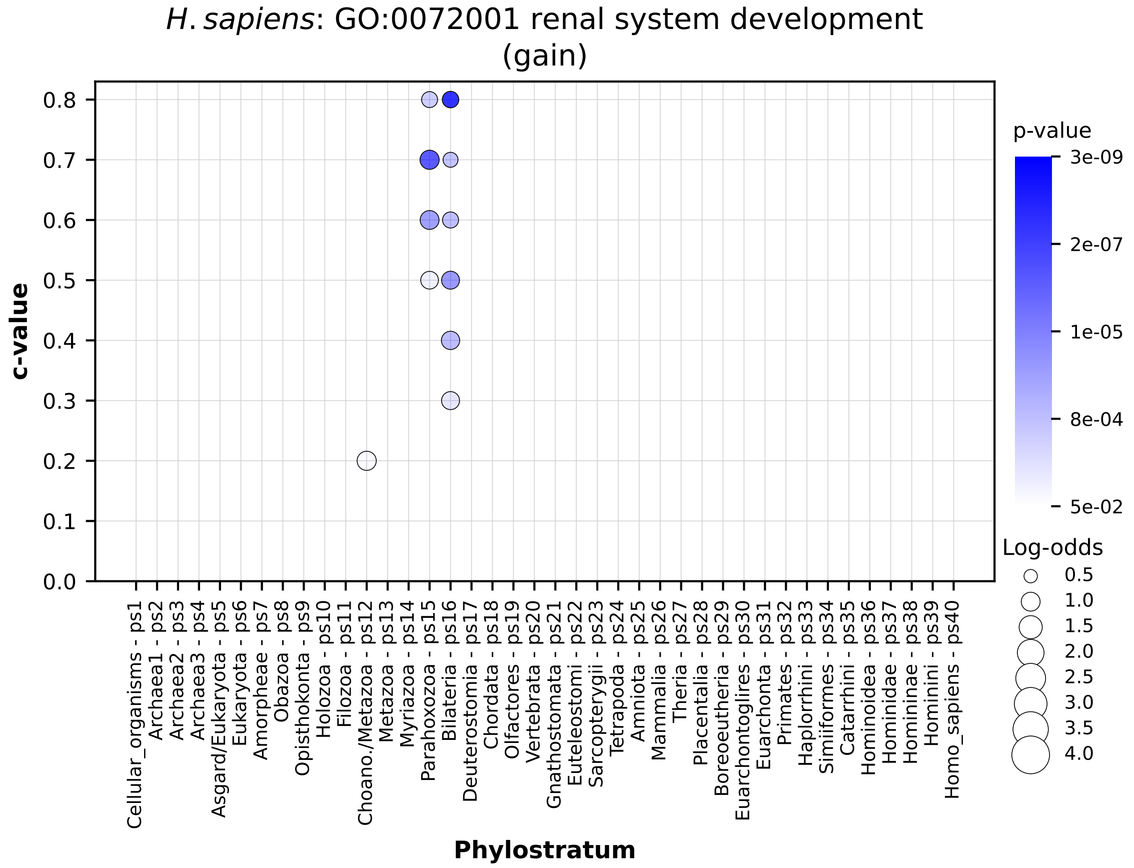

H. sapiens: GO:0072001 renal system development
(gain)
c-value
0.0
0.1
0.2
0.3
0.4
0.5
0.6
0.7
0.8
Phylostratum
Cellular_organisms - ps1
Archaea1 - ps2
Archaea2 - ps3
Archaea3 - ps4
Asgard/Eukaryota - ps5
Eukaryota - ps6
Amorpheae - ps7
Obazoa - ps8
Opisthokonta - ps9
Holozoa - ps10
Filozoa - ps11
Choano./Metazoa - ps12
Metazoa - ps13
Myriazoa - ps14
Parahoxozoa - ps15
Bilateria - ps16
Deuterostomia - ps17
Chordata - ps18
Olfactores - ps19
Vertebrata - ps20
Gnathostomata - ps21
Euteleostomi - ps22
Sarcopterygii - ps23
Tetrapoda - ps24
Amniota - ps25
Mammalia - ps26
Theria - ps27
Placentalia - ps28
Boreoeutheria - ps29
Euarchontoglires - ps30
Euarchonta - ps31
Primates - ps32
Haplorrhini - ps33
Simiiformes - ps34
Catarrhini - ps35
Hominoidea - ps36
Hominidae - ps37
Homininae - ps38
Hominini - ps39
Homo_sapiens - ps40
p-value
3e-09
2e-07
1e-05
8e-04
5e-02
Log-odds
0.5
1.0
1.5
2.0
2.5
3.0
3.5
4.0

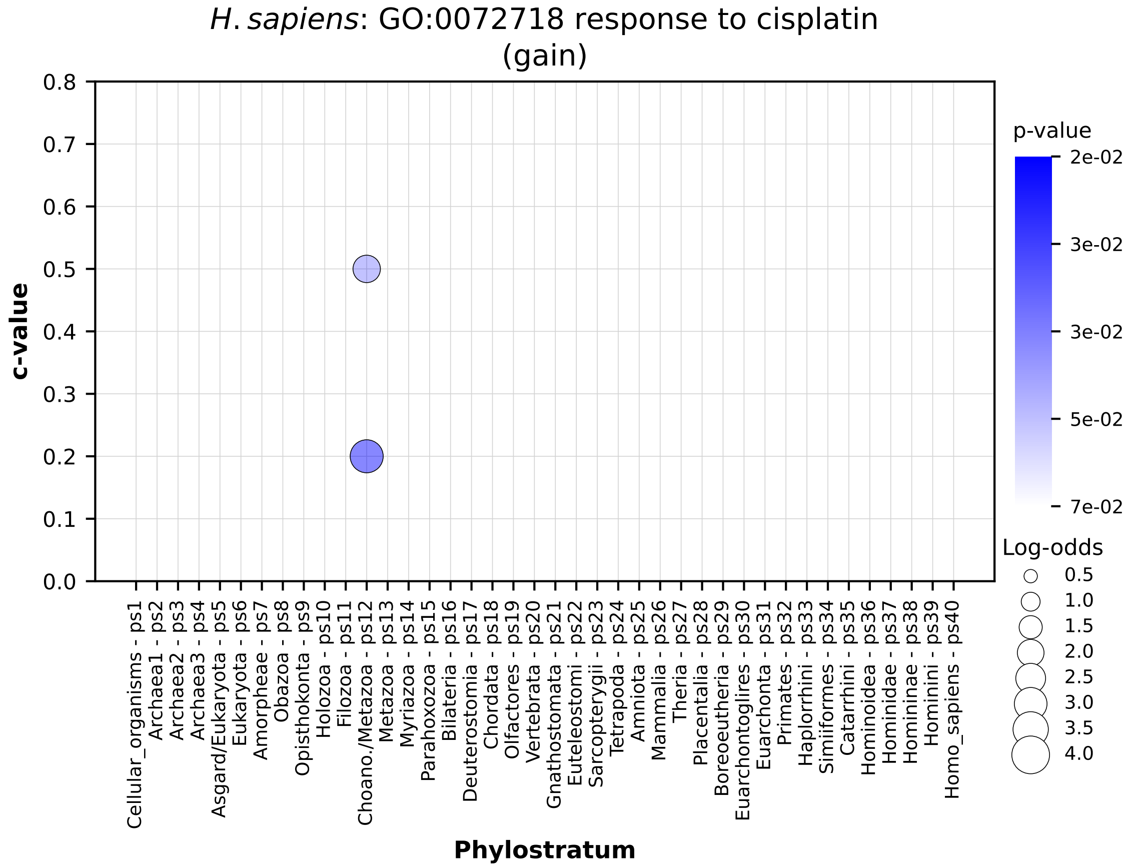

*H. sapiens*: GO:0072718 response to cisplatin
(gain)
c-value
0.0
0.1
0.2
0.3
0.4
0.5
0.6
0.7
0.8
p-value
2e-02
3e-02
3e-02
5e-02
7e-02
Log-odds
0.5
1.0
1.5
2.0
2.5
3.0
3.5
4.0
Cellular_organisms - ps1
Archaea1 - ps2
Archaea2 - ps3
Archaea3 - ps4
Asgard/Eukaryota - ps5
Eukaryota - ps6
Amorpheae - ps7
Obazoa - ps8
Opisthokonta - ps9
Holozoa - ps10
Filozoa - ps11
Choano./Metazoa - ps12
Metazoa - ps13
Myriazoa - ps14
Parahoxozoa - ps15
Bilateria - ps16
Deuterostomia - ps17
Chordata - ps18
Olfactores - ps19
Vertebrata - ps20
Gnathostomata - ps21
Euteleostomi - ps22
Sarcopterygii - ps23
Tetrapoda - ps24
Amniota - ps25
Mammalia - ps26
Theria - ps27
Placentalia - ps28
Boreoeutheria - ps29
Euarchontoglires - ps30
Euarchonta - ps31
Primates - ps32
Haplorrhini - ps33
Simiiformes - ps34
Catarrhini - ps35
Hominoidea - ps36
Hominidae - ps37
Homininae - ps38
Hominini - ps39
Homo_sapiens - ps40
Phylostratum

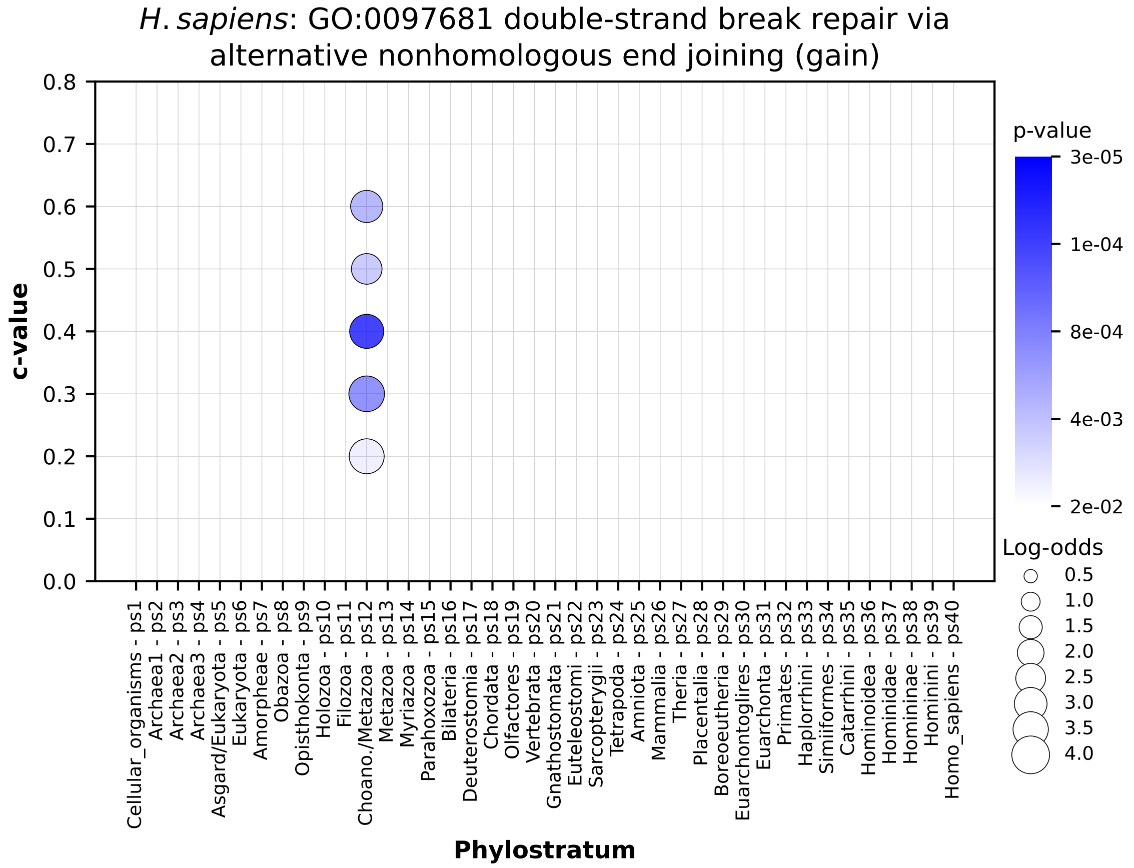

H. sapiens: GO:0097681 double-strand break repair via alternative nonhomologous end joining (gain)
c-value
0.0
0.1
0.2
0.3
0.4
0.5
0.6
0.7
0.8
Phylostratum
Cellular_organisms - ps1
Archaea1 - ps2
Archaea2 - ps3
Archaea3 - ps4
Asgard/Eukaryota - ps5
Eukaryota - ps6
Amorpheae - ps7
Obazoa - ps8
Opisthokonta - ps9
Holozoa - ps10
Filozoa - ps11
Choano./Metazoa - ps12
Metazoa - ps13
Myriazoa - ps14
Parahoxozoa - ps15
Bilateria - ps16
Deuterostomia - ps17
Chordata - ps18
Olfactores - ps19
Vertebrata - ps20
Gnathostomata - ps21
Euteleostomi - ps22
Sarcopterygii - ps23
Tetrapoda - ps24
Amniota - ps25
Mammalia - ps26
Theria - ps27
Placentalia - ps28
Boreoeutheria - ps29
Euarchontoglires - ps30
Euarchonta - ps31
Primates - ps32
Haplorrhini - ps33
Simiiformes - ps34
Catarrhini - ps35
Hominoidea - ps36
Hominidae - ps37
Homininae - ps38
Hominini - ps39
Homo_sapiens - ps40
p-value
3e-05
1e-04
8e-04
4e-03
2e-02
Log-odds
0.5
1.0
1.5
2.0
2.5
3.0
3.5
4.0

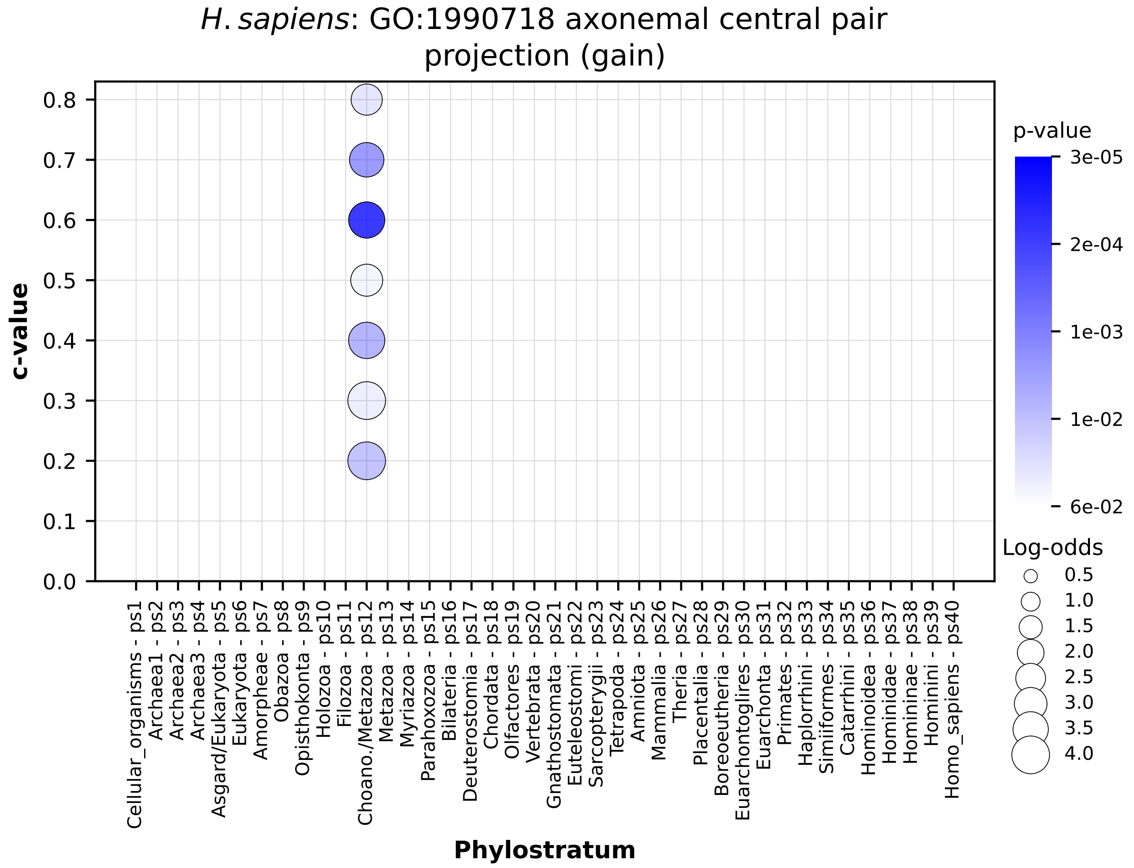

H. sapiens: GO:1990718 axonemal central pair projection (gain)
c-value
0.0
0.1
0.2
0.3
0.4
0.5
0.6
0.7
0.8
Phylostratum
Cellular_organisms - ps1
Archaea1 - ps2
Archaea2 - ps3
Archaea3 - ps4
Asgard/Eukaryota - ps5
Eukaryota - ps6
Amorpheae - ps7
Obazoa - ps8
Opisthokonta - ps9
Holozoa - ps10
Filozoa - ps11
Choano./Metazoa - ps12
Metazoa - ps13
Myriazoa - ps14
Parahoxozoa - ps15
Bilateria - ps16
Deuterostomia - ps17
Chordata - ps18
Olfactores - ps19
Vertebrata - ps20
Gnathostomata - ps21
Euteleostomi - ps22
Sarcopterygii - ps23
Tetrapoda - ps24
Amniota - ps25
Mammalia - ps26
Theria - ps27
Placentalia - ps28
Boreoeutheria - ps29
Euarchontoglires - ps30
Euarchonta - ps31
Primates - ps32
Haplorrhini - ps33
Simiiformes - ps34
Catarrhini - ps35
Hominoidea - ps36
Hominidae - ps37
Homininae - ps38
Hominini - ps39
Homo_sapiens - ps40
p-value
3e-05
2e-04
1e-03
1e-02
6e-02
Log-odds
0.5
1.0
1.5
2.0
2.5
3.0
3.5
4.0

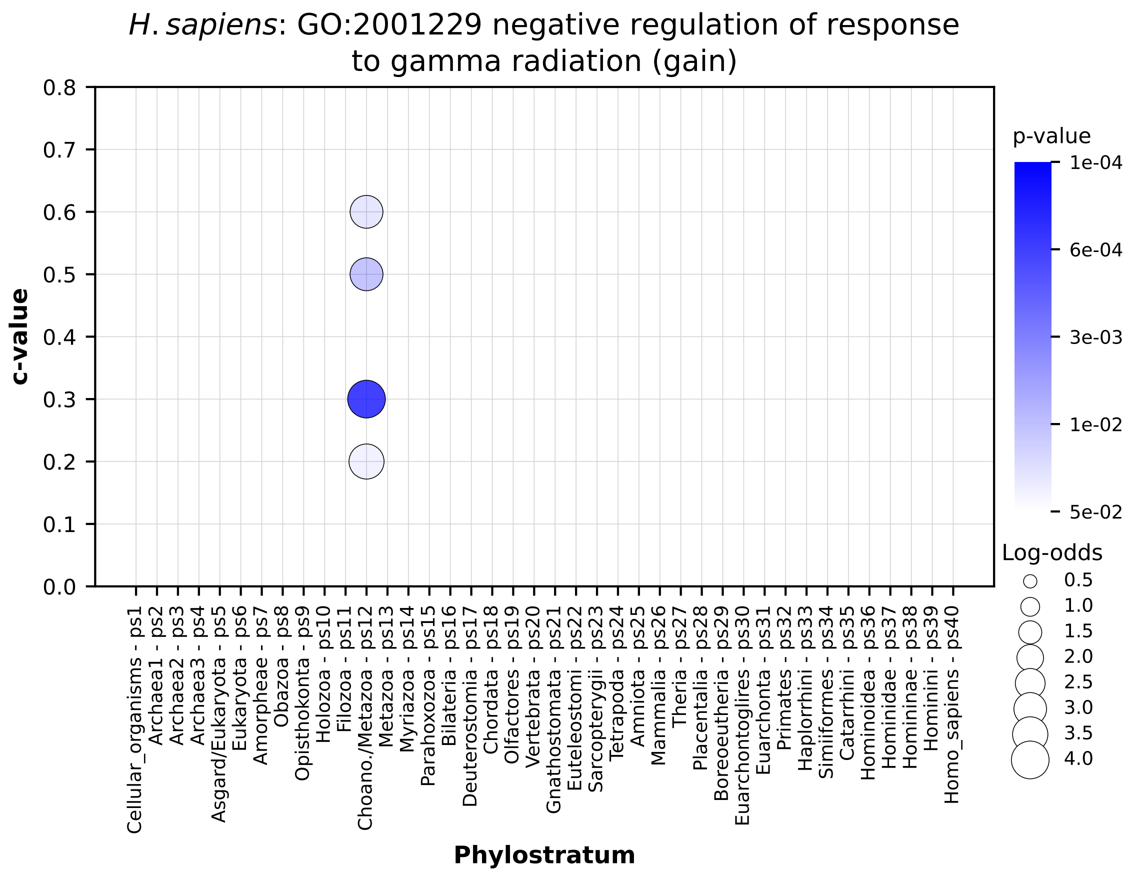

H. sapiens: GO:2001229 negative regulation of response to gamma radiation (gain)
c-value
0.0
0.1
0.2
0.3
0.4
0.5
0.6
0.7
0.8
Phylostratum
Cellular_organisms - ps1
Archaea1 - ps2
Archaea2 - ps3
Archaea3 - ps4
Asgard/Eukaryota - ps5
Eukaryota - ps6
Amorpheae - ps7
Obazoa - ps8
Opisthokonta - ps9
Holozoa - ps10
Filozoa - ps11
Choano./Metazoa - ps12
Metazoa - ps13
Myriazoa - ps14
Parahoxozoa - ps15
Bilateria - ps16
Deuterostomia - ps17
Chordata - ps18
Olfactores - ps19
Vertebrata - ps20
Gnathostomata - ps21
Euteleostomi - ps22
Sarcopterygii - ps23
Tetrapoda - ps24
Amniota - ps25
Mammalia - ps26
Theria - ps27
Placentalia - ps28
Boreoeutheria - ps29
Euarchontoglires - ps30
Euarchonta - ps31
Primates - ps32
Haplorrhini - ps33
Simiiformes - ps34
Catarrhini - ps35
Hominoidea - ps36
Hominidae - ps37
Homininae - ps38
Hominini - ps39
Homo_sapiens - ps40
p-value
1e-04
6e-04
3e-03
1e-02
5e-02
Log-odds
0.5
1.0
1.5
2.0
2.5
3.0
3.5
4.0

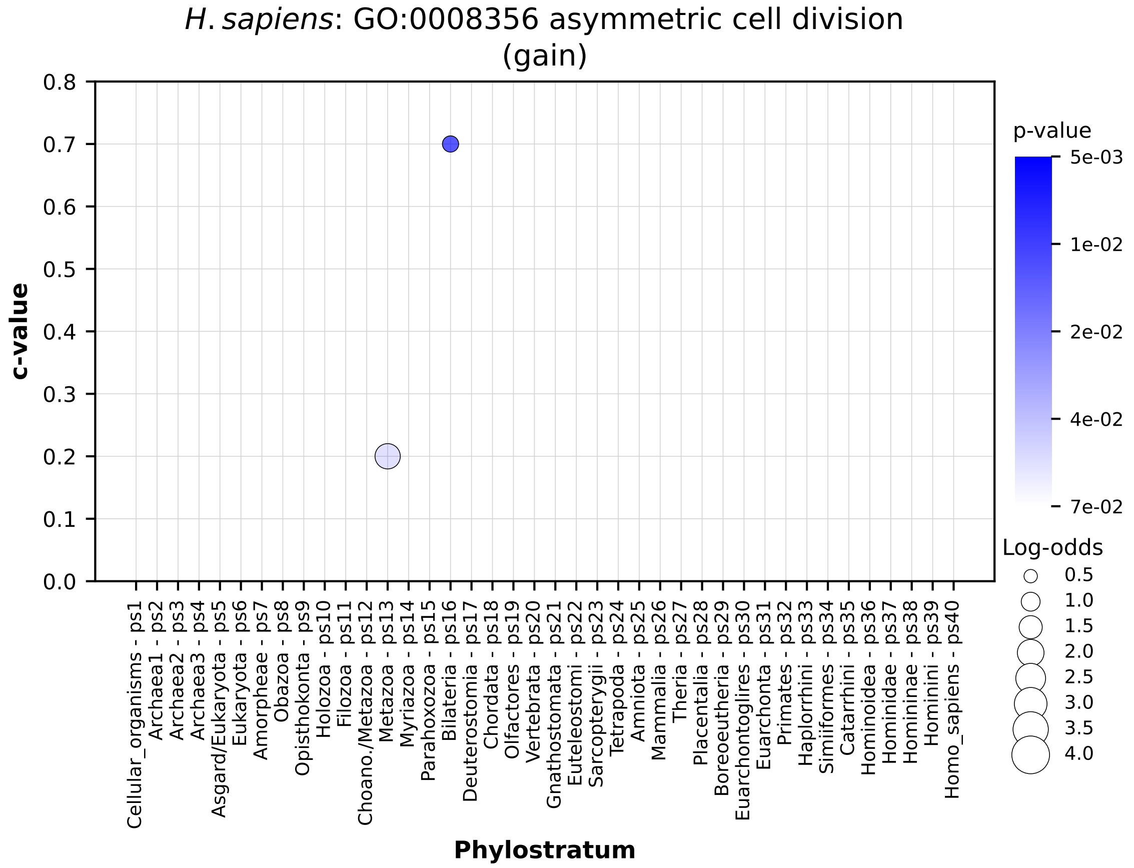
H. sapiens: GO:0008356 asymmetric cell division
(gain)
c-value
0.0
0.1
0.2
0.3
0.4
0.5
0.6
0.7
0.8
Phylostratum
Cellular_organisms - ps1
Archaea1 - ps2
Archaea2 - ps3
Archaea3 - ps4
Asgard/Eukaryota - ps5
Eukaryota - ps6
Amorpheae - ps7
Obazoa - ps8
Opisthokonta - ps9
Holozoa - ps10
Filozoa - ps11
Choano./Metazoa - ps12
Metazoa - ps13
Myriazoa - ps14
Parahoxozoa - ps15
Bilateria - ps16
Deuterostomia - ps17
Chordata - ps18
Olfactores - ps19
Vertebrata - ps20
Gnathostomata - ps21
Euteleostomi - ps22
Sarcopterygii - ps23
Tetrapoda - ps24
Amniota - ps25
Mammalia - ps26
Theria - ps27
Placentalia - ps28
Boreoeutheria - ps29
Euarchontoglires - ps30
Euarchonta - ps31
Primates - ps32
Haplorrhini - ps33
Simiiformes - ps34
Catarrhini - ps35
Hominoidea - ps36
Hominidae - ps37
Homininae - ps38
Hominini - ps39
Homo_sapiens - ps40
p-value
5e-03
1e-02
2e-02
4e-02
7e-02
Log-odds
0.5
1.0
1.5
2.0
2.5
3.0
3.5
4.0

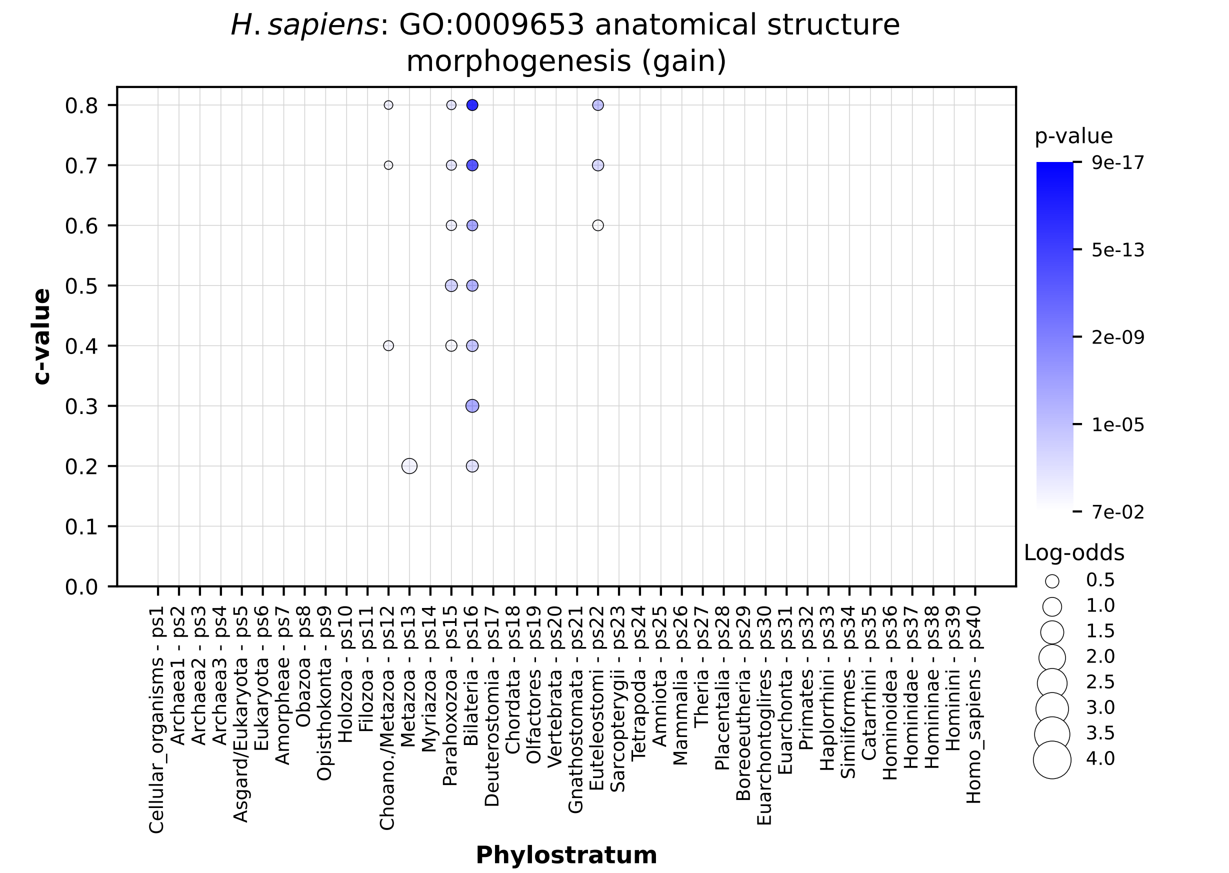

*H. sapiens*: GO:0009653 anatomical structure morphogenesis (gain)
c-value
0.8
0.7
0.6
0.5
0.4
0.3
0.2
0.1
0.0
Phylostratum
Cellular_organisms - ps1
Archaea1 - ps2
Archaea2 - ps3
Archaea3 - ps4
Asgard/Eukaryota - ps5
Eukaryota - ps6
Amorpheae - ps7
Obazoa - ps8
Opisthokonta - ps9
Holozoa - ps10
Filozoa - ps11
Choano./Metazoa - ps12
Metazoa - ps13
Myriazoa - ps14
Parahoxozoa - ps15
Bilateria - ps16
Deuterostomia - ps17
Chordata - ps18
Olfactores - ps19
Vertebrata - ps20
Gnathostomata - ps21
Euteleostomi - ps22
Sarcopterygii - ps23
Tetrapoda - ps24
Amniota - ps25
Mammalia - ps26
Theria - ps27
Placentalia - ps28
Boreoeutheria - ps29
Euarchontoglires - ps30
Euarchonta - ps31
Primates - ps32
Haplorrhini - ps33
Simiiformes - ps34
Catarrhini - ps35
Hominoidea - ps36
Hominidae - ps37
Homininae - ps38
Hominini - ps39
Homo_sapiens - ps40
p-value
9e-17
5e-13
2e-09
1e-05
7e-02
Log-odds
0.5
1.0
1.5
2.0
2.5
3.0
3.5
4.0

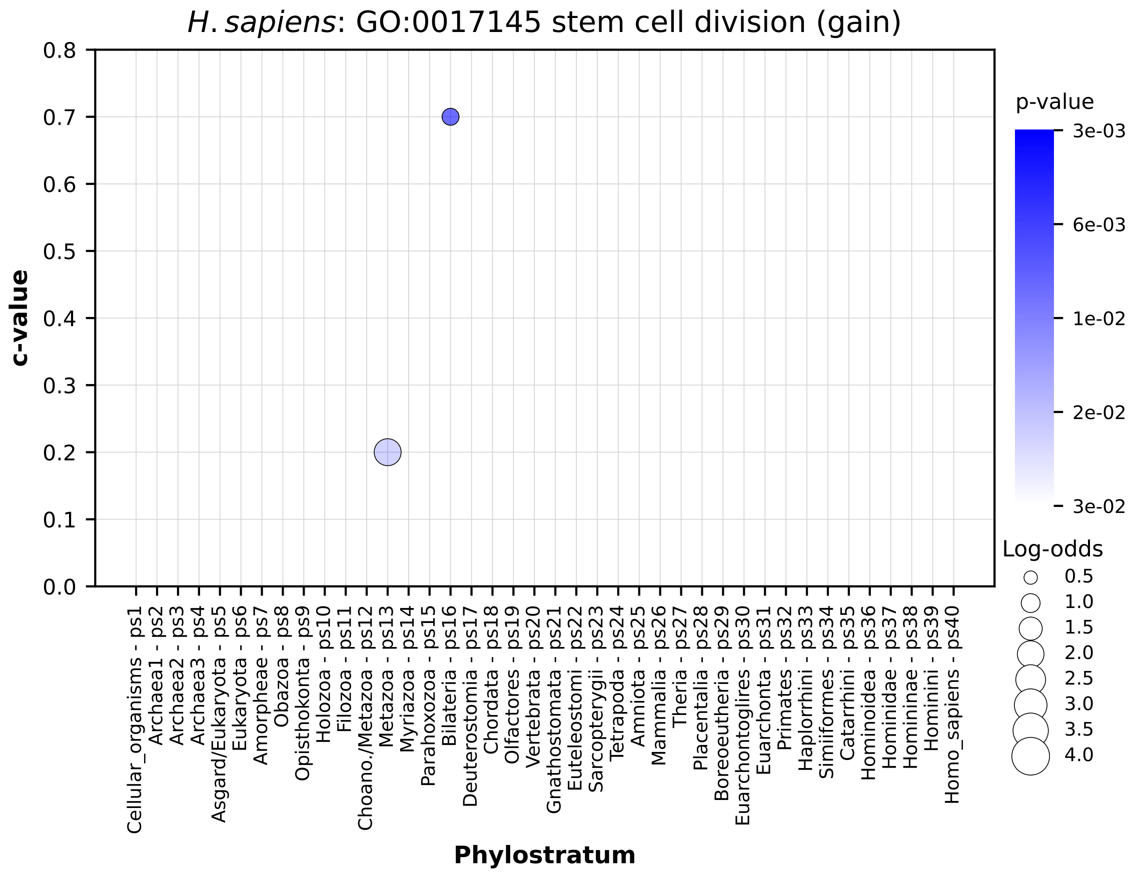
H. sapiens: GO:0017145 stem cell division (gain)
c-value
0.0
0.1
0.2
0.3
0.4
0.5
0.6
0.7
0.8
Phylostratum
Cellular_organisms - ps1
Archaea1 - ps2
Archaea2 - ps3
Archaea3 - ps4
Asgard/Eukaryota - ps5
Eukaryota - ps6
Amorpheae - ps7
Obazoa - ps8
Opisthokonta - ps9
Holozoa - ps10
Filozoa - ps11
Choano./Metazoa - ps12
Metazoa - ps13
Myriazoa - ps14
Parahoxozoa - ps15
Bilateria - ps16
Deuterostomia - ps17
Chordata - ps18
Olfactores - ps19
Vertebrata - ps20
Gnathostomata - ps21
Euteleostomi - ps22
Sarcopterygii - ps23
Tetrapoda - ps24
Amniota - ps25
Mammalia - ps26
Theria - ps27
Placentalia - ps28
Boreoeutheria - ps29
Euarchontoglires - ps30
Euarchonta - ps31
Primates - ps32
Haplorrhini - ps33
Simiiformes - ps34
Catarrhini - ps35
Hominoidea - ps36
Hominidae - ps37
Homininae - ps38
Hominini - ps39
Homo_sapiens - ps40
p-value
3e-03
6e-03
1e-02
2e-02
3e-02
Log-odds
0.5
1.0
1.5
2.0
2.5
3.0
3.5
4.0

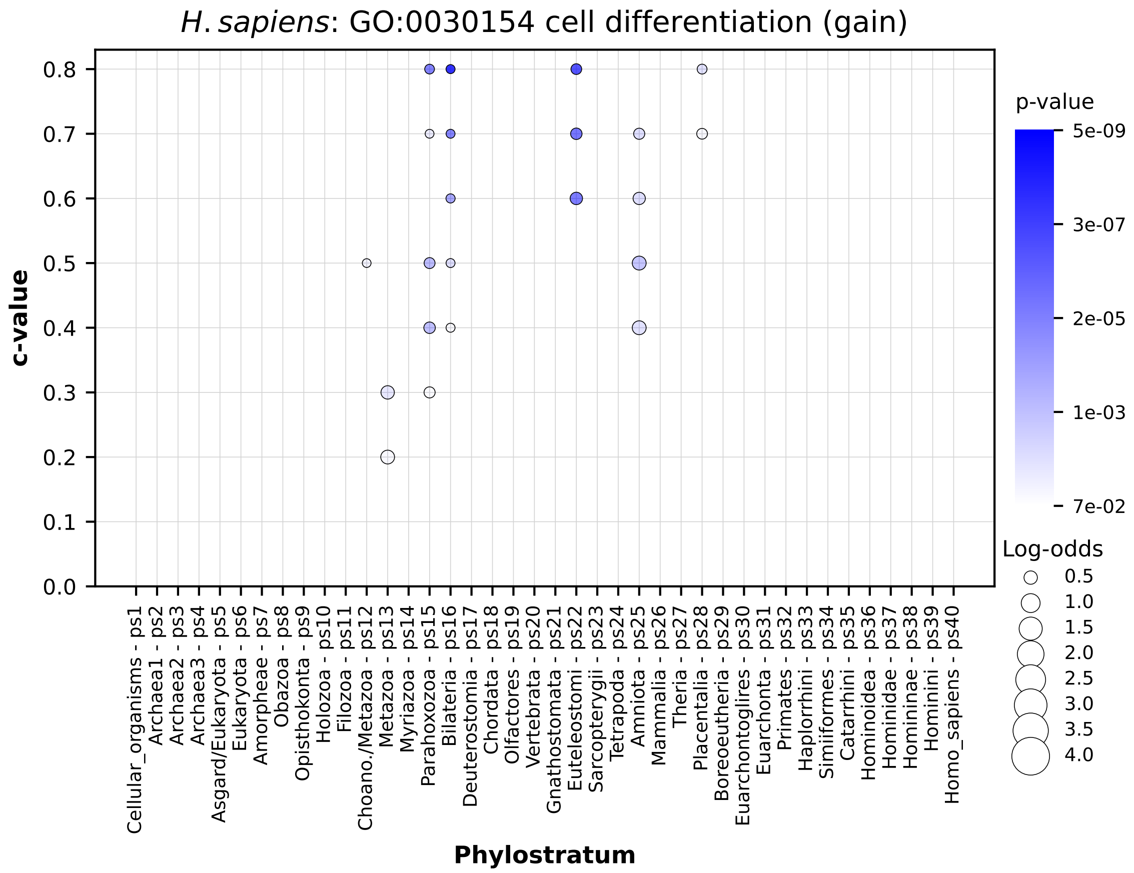

H. sapiens: GO:0030154 cell differentiation (gain)
c-value
0.0
0.1
0.2
0.3
0.4
0.5
0.6
0.7
0.8
Phylostratum
Cellular_organisms - ps1
Archaea1 - ps2
Archaea2 - ps3
Archaea3 - ps4
Asgard/Eukaryota - ps5
Eukaryota - ps6
Amorpheae - ps7
Obazoa - ps8
Opisthokonta - ps9
Holozoa - ps10
Filozoa - ps11
Choano./Metazoa - ps12
Metazoa - ps13
Myriazoa - ps14
Parahoxozoa - ps15
Bilateria - ps16
Deuterostomia - ps17
Chordata - ps18
Olfactores - ps19
Vertebrata - ps20
Gnathostomata - ps21
Euteleostomi - ps22
Sarcopterygii - ps23
Tetrapoda - ps24
Amniota - ps25
Mammalia - ps26
Theria - ps27
Placentalia - ps28
Boreoeutheria - ps29
Euarchontoglires - ps30
Euarchonta - ps31
Primates - ps32
Haplorrhini - ps33
Simiiformes - ps34
Catarrhini - ps35
Hominoidea - ps36
Hominidae - ps37
Homininae - ps38
Hominini - ps39
Homo_sapiens - ps40
p-value
5e-09
3e-07
2e-05
1e-03
7e-02
Log-odds
0.5
1.0
1.5
2.0
2.5
3.0
3.5
4.0

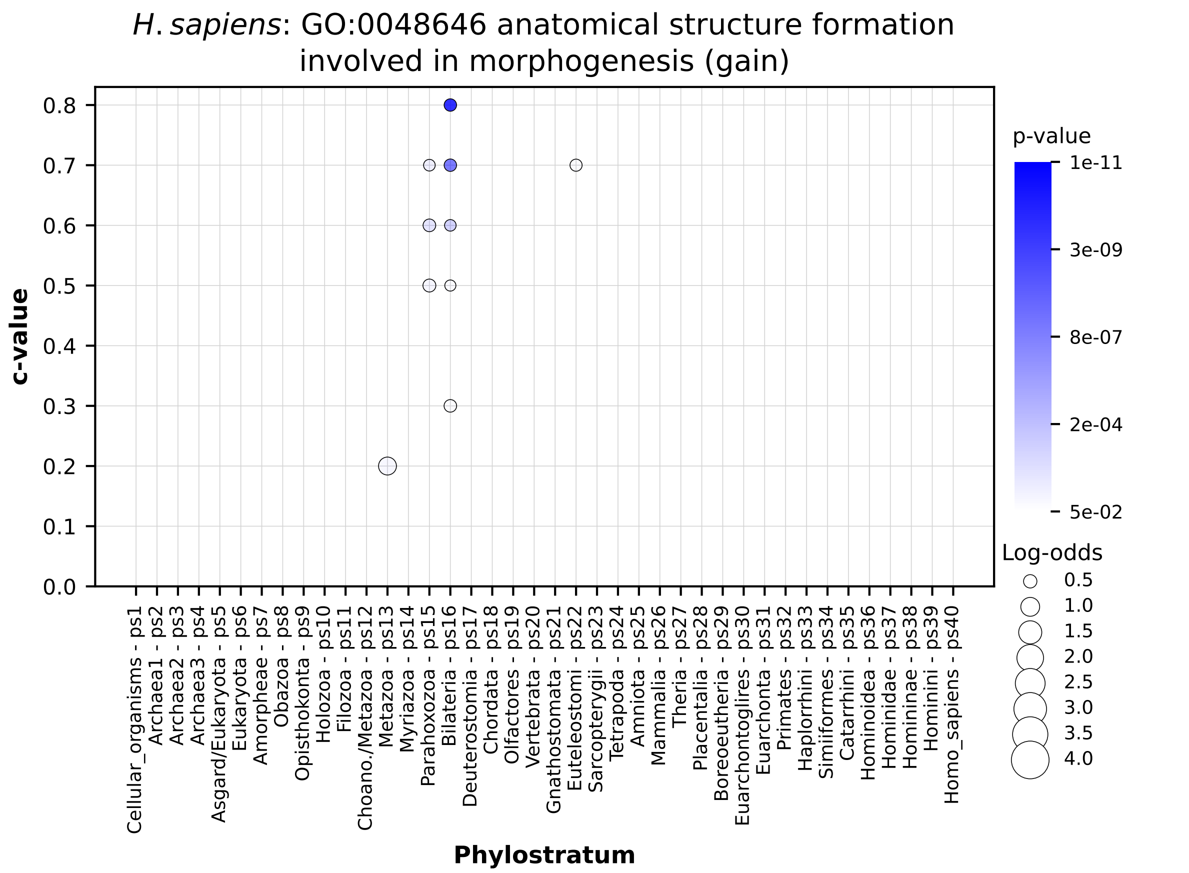

H. sapiens: GO:0048646 anatomical structure formation involved in morphogenesis (gain)
c-value
0.0
0.1
0.2
0.3
0.4
0.5
0.6
0.7
0.8
Phylostratum
Cellular_organisms - ps1
Archaea1 - ps2
Archaea2 - ps3
Archaea3 - ps4
Asgard/Eukaryota - ps5
Eukaryota - ps6
Amorpheae - ps7
Obazoa - ps8
Opisthokonta - ps9
Holozoa - ps10
Filozoa - ps11
Choano./Metazoa - ps12
Metazoa - ps13
Myriazoa - ps14
Parahoxozoa - ps15
Bilateria - ps16
Deuterostomia - ps17
Chordata - ps18
Olfactores - ps19
Vertebrata - ps20
Gnathostomata - ps21
Euteleostomi - ps22
Sarcopterygii - ps23
Tetrapoda - ps24
Amniota - ps25
Mammalia - ps26
Theria - ps27
Placentalia - ps28
Boreoeutheria - ps29
Euarchontoglires - ps30
Euarchonta - ps31
Primates - ps32
Haplorrhini - ps33
Simiiformes - ps34
Catarrhini - ps35
Hominoidea - ps36
Hominidae - ps37
Homininae - ps38
Hominini - ps39
Homo_sapiens - ps40
p-value
1e-11
3e-09
8e-07
2e-04
5e-02
Log-odds
0.5
1.0
1.5
2.0
2.5
3.0
3.5
4.0

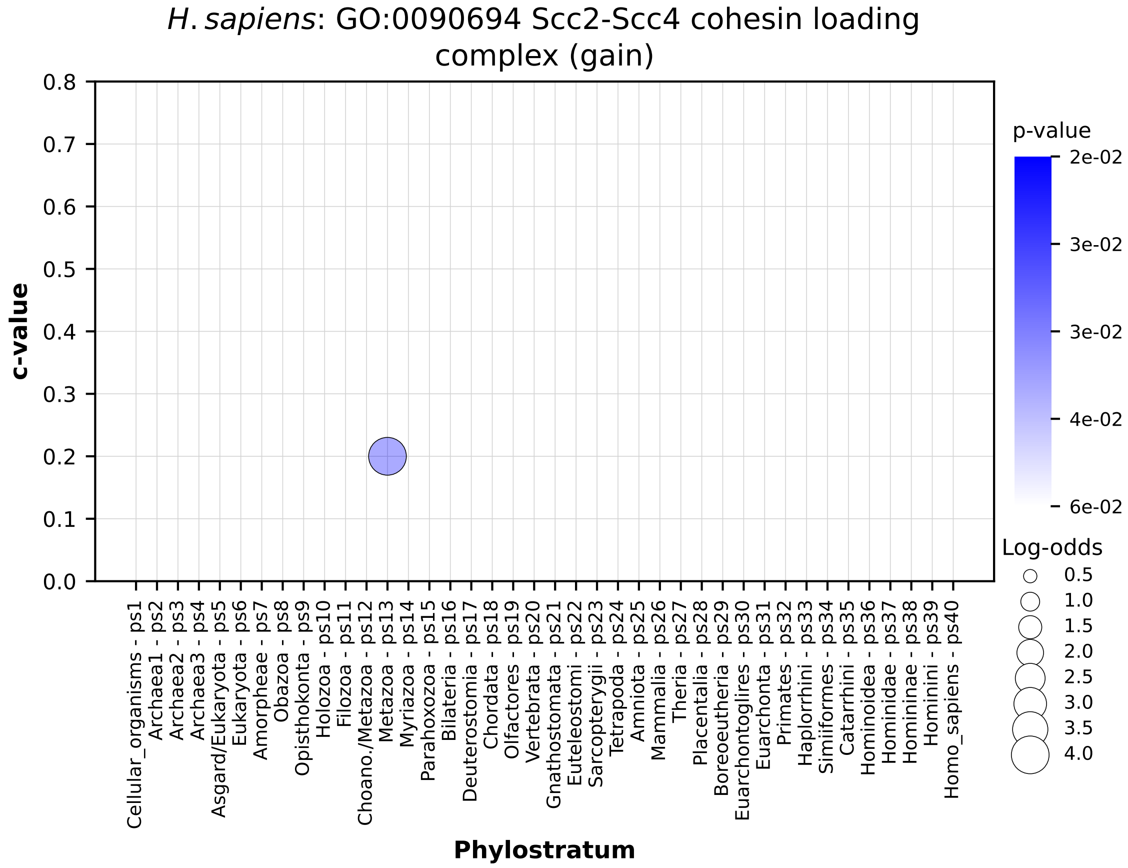

H. sapiens: GO:0090694 Scc2-Scc4 cohesin loading complex (gain)
c-value
0.0
0.1
0.2
0.3
0.4
0.5
0.6
0.7
0.8
Phylostratum
Cellular_organisms - ps1
Archaea1 - ps2
Archaea2 - ps3
Archaea3 - ps4
Asgard/Eukaryota - ps5
Eukaryota - ps6
Amorpheae - ps7
Obazoa - ps8
Opisthokonta - ps9
Holozoa - ps10
Filozoa - ps11
Choano./Metazoa - ps12
Metazoa - ps13
Myriazoa - ps14
Parahoxozoa - ps15
Bilateria - ps16
Deuterostomia - ps17
Chordata - ps18
Olfactores - ps19
Vertebrata - ps20
Gnathostomata - ps21
Euteleostomi - ps22
Sarcopterygii - ps23
Tetrapoda - ps24
Amniota - ps25
Mammalia - ps26
Theria - ps27
Placentalia - ps28
Boreoeutheria - ps29
Euarchontoglires - ps30
Euarchonta - ps31
Primates - ps32
Haplorrhini - ps33
Simiiformes - ps34
Catarrhini - ps35
Hominoidea - ps36
Hominidae - ps37
Homininae - ps38
Hominini - ps39
Homo_sapiens - ps40
p-value
2e-02
3e-02
3e-02
4e-02
6e-02
Log-odds
0.5
1.0
1.5
2.0
2.5
3.0
3.5
4.0

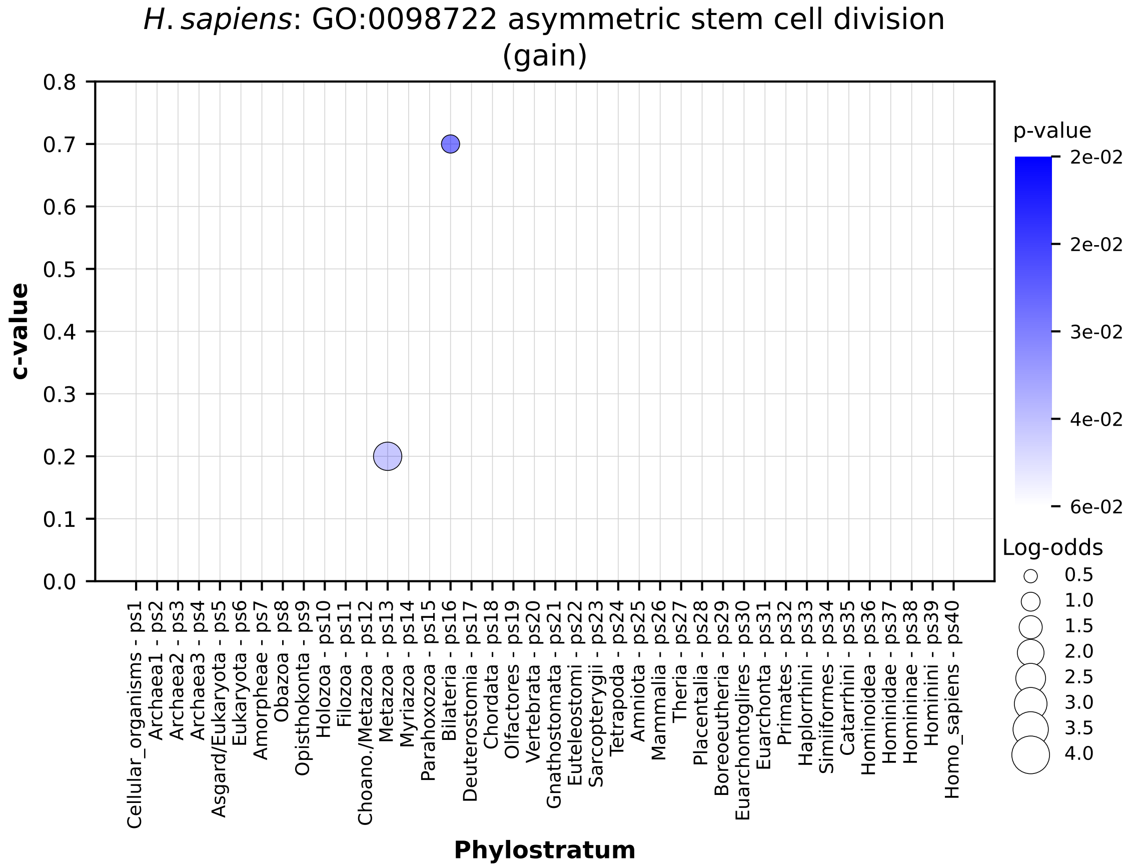

H. sapiens: GO:0098722 asymmetric stem cell division
(gain)
c-value
0.8
0.7
0.6
0.5
0.4
0.3
0.2
0.1
0.0
p-value
2e-02
2e-02
3e-02
4e-02
6e-02
Log-odds
0.5
1.0
1.5
2.0
2.5
3.0
3.5
4.0
Cellular_organisms - ps1
Archaea1 - ps2
Archaea2 - ps3
Archaea3 - ps4
Asgard/Eukaryota - ps5
Eukaryota - ps6
Amorpheae - ps7
Obazoa - ps8
Opisthokonta - ps9
Holozoa - ps10
Filozoa - ps11
Choano./Metazoa - ps12
Metazoa - ps13
Myriazoa - ps14
Parahoxozoa - ps15
Bilateria - ps16
Deuterostomia - ps17
Chordata - ps18
Olfactores - ps19
Vertebrata - ps20
Gnathostomata - ps21
Euteleostomi - ps22
Sarcopterygii - ps23
Tetrapoda - ps24
Amniota - ps25
Mammalia - ps26
Theria - ps27
Placentalia - ps28
Boreoeutheria - ps29
Euarchontoglires - ps30
Euarchonta - ps31
Primates - ps32
Haplorrhini - ps33
Simiiformes - ps34
Catarrhini - ps35
Hominoidea - ps36
Hominidae - ps37
Homininae - ps38
Hominini - ps39
Homo_sapiens - ps40
Phylostratum

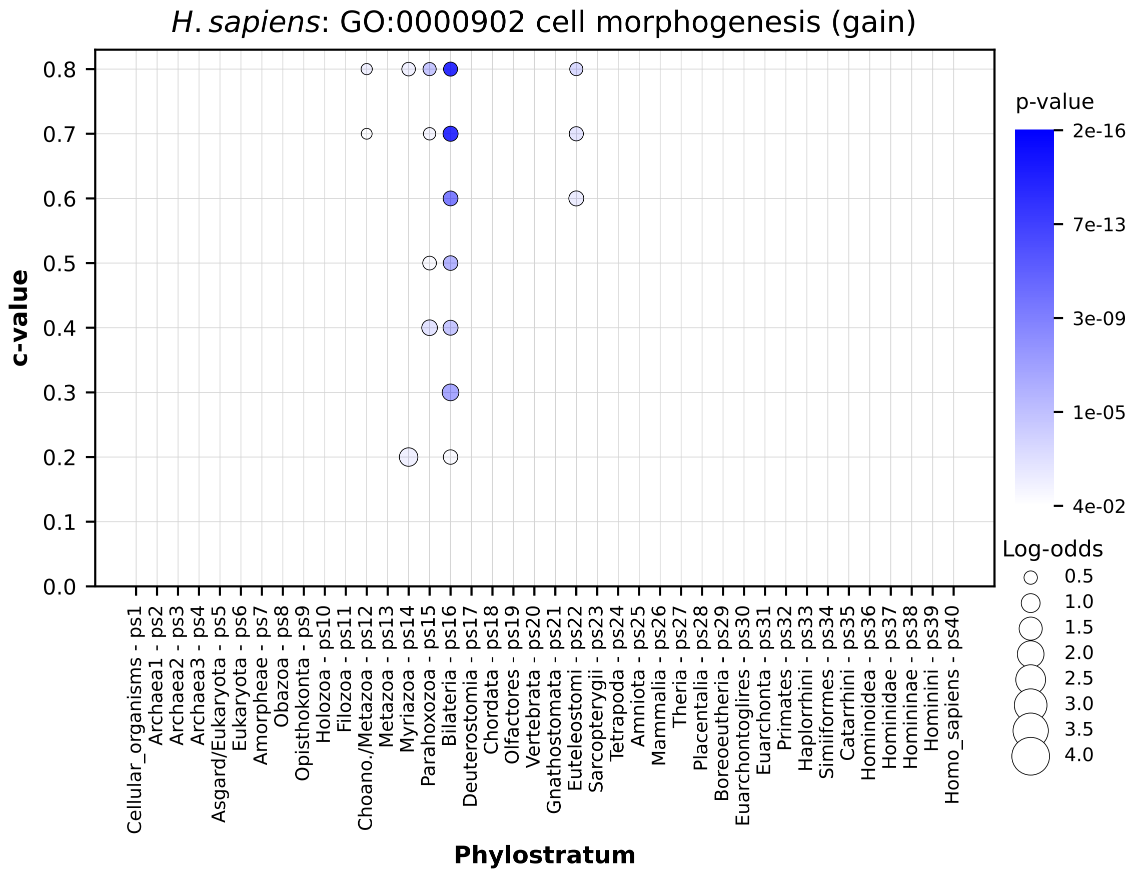

H. sapiens: GO:0000902 cell morphogenesis (gain)
c-value
0.0
0.1
0.2
0.3
0.4
0.5
0.6
0.7
0.8
Phylostratum
Cellular_organisms - ps1
Archaea1 - ps2
Archaea2 - ps3
Archaea3 - ps4
Asgard/Eukaryota - ps5
Eukaryota - ps6
Amorpheae - ps7
Obazoa - ps8
Opisthokonta - ps9
Holozoa - ps10
Filozoa - ps11
Choano./Metazoa - ps12
Metazoa - ps13
Myriazoa - ps14
Parahoxozoa - ps15
Bilateria - ps16
Deuterostomia - ps17
Chordata - ps18
Olfactores - ps19
Vertebrata - ps20
Gnathostomata - ps21
Euteleostomi - ps22
Sarcopterygii - ps23
Tetrapoda - ps24
Amniota - ps25
Mammalia - ps26
Theria - ps27
Placentalia - ps28
Boreoeutheria - ps29
Euarchontoglires - ps30
Euarchonta - ps31
Primates - ps32
Haplorrhini - ps33
Simiiformes - ps34
Catarrhini - ps35
Hominoidea - ps36
Hominidae - ps37
Homininae - ps38
Hominini - ps39
Homo_sapiens - ps40
p-value
2e-16
7e-13
3e-09
1e-05
4e-02
Log-odds
0.5
1.0
1.5
2.0
2.5
3.0
3.5
4.0

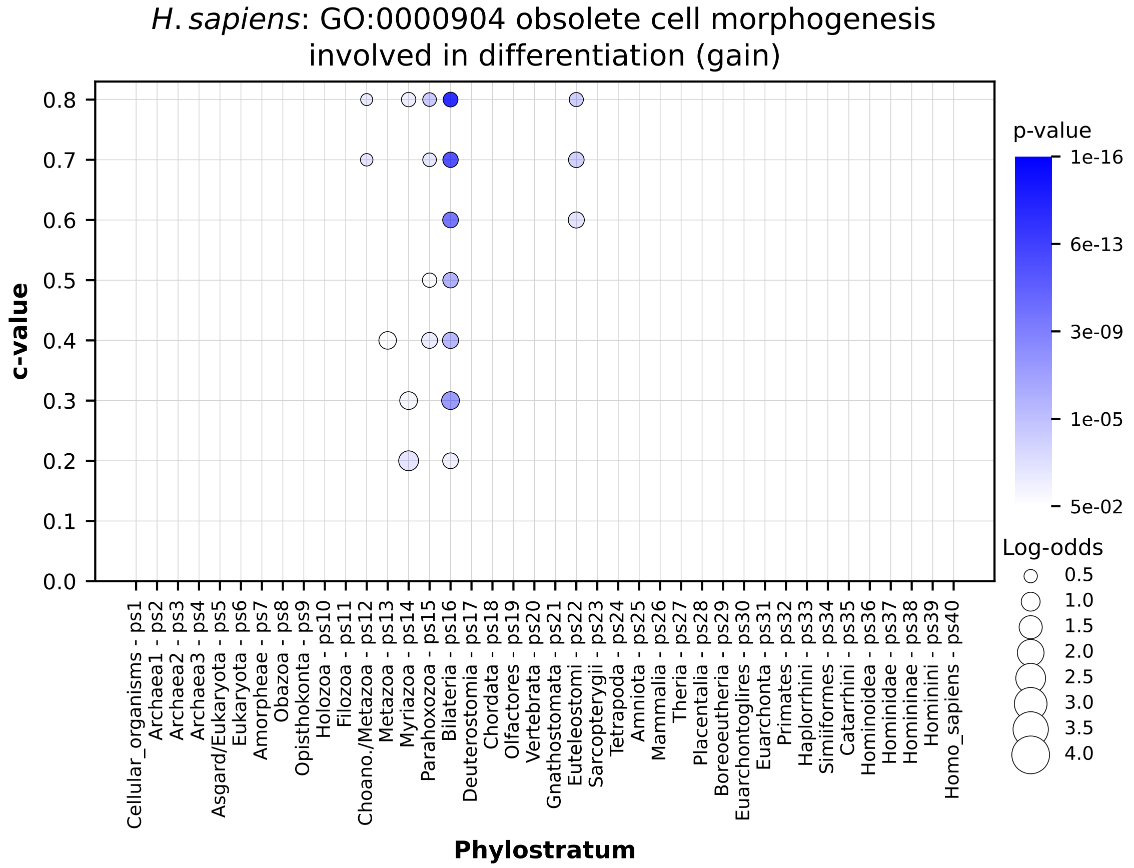
H. sapiens: GO:0000904 obsolete cell morphogenesis involved in differentiation (gain)
c-value
0.0
0.1
0.2
0.3
0.4
0.5
0.6
0.7
0.8
Phylostratum
Cellular_organisms - ps1
Archaea1 - ps2
Archaea2 - ps3
Archaea3 - ps4
Asgard/Eukaryota - ps5
Eukaryota - ps6
Amorpheae - ps7
Obazoa - ps8
Opisthokonta - ps9
Holozoa - ps10
Filozoa - ps11
Choano./Metazoa - ps12
Metazoa - ps13
Myriazoa - ps14
Parahoxozoa - ps15
Bilateria - ps16
Deuterostomia - ps17
Chordata - ps18
Olfactores - ps19
Vertebrata - ps20
Gnathostomata - ps21
Euteleostomi - ps22
Sarcopterygii - ps23
Tetrapoda - ps24
Amniota - ps25
Mammalia - ps26
Theria - ps27
Placentalia - ps28
Boreoeutheria - ps29
Euarchontoglires - ps30
Euarchonta - ps31
Primates - ps32
Haplorrhini - ps33
Simiiformes - ps34
Catarrhini - ps35
Hominoidea - ps36
Hominidae - ps37
Homininae - ps38
Hominini - ps39
Homo_sapiens - ps40
p-value
1e-16
6e-13
3e-09
1e-05
5e-02
Log-odds
0.5
1.0
1.5
2.0
2.5
3.0
3.5
4.0

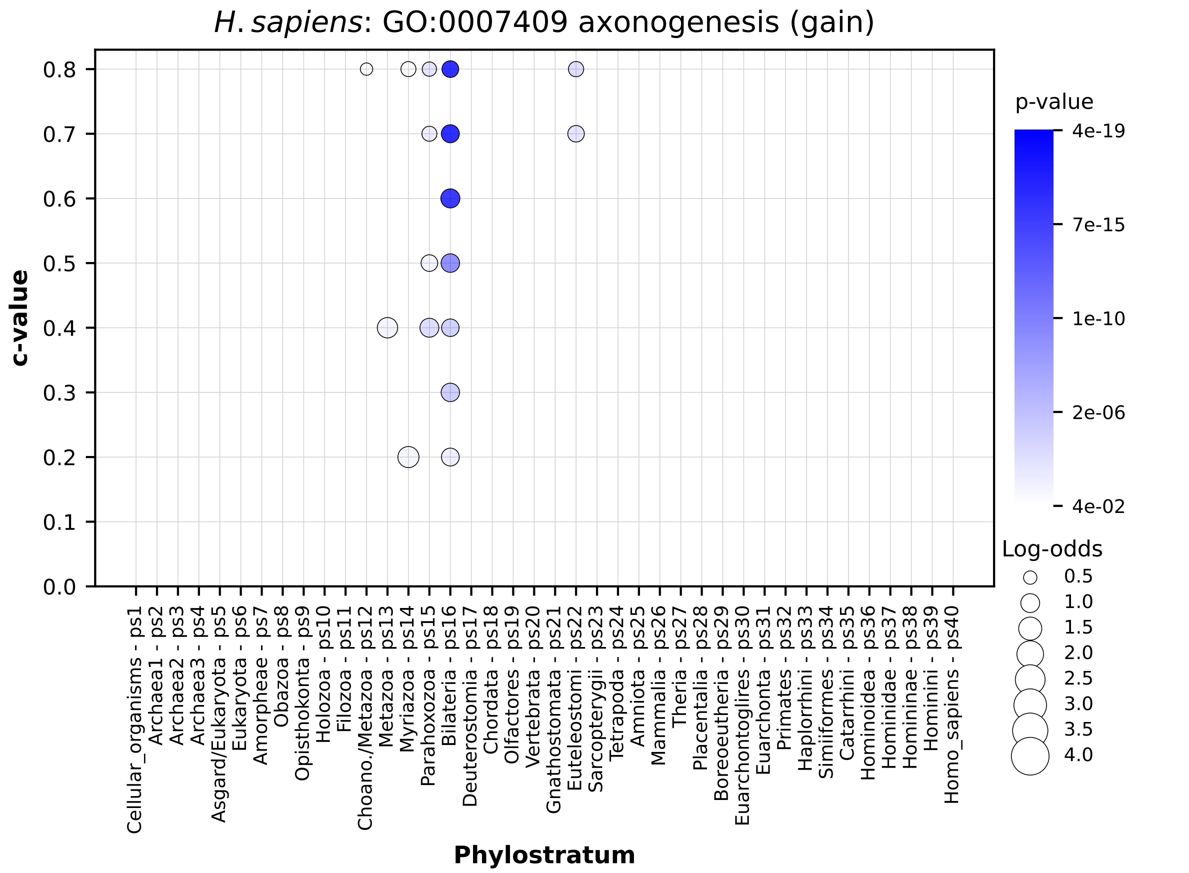

H. sapiens: GO:0007409 axonogenesis (gain)
c-value
0.8
0.7
0.6
0.5
0.4
0.3
0.2
0.1
0.0
p-value
4e-19
7e-15
1e-10
2e-06
4e-02
Log-odds
0.5
1.0
1.5
2.0
2.5
3.0
3.5
4.0
Cellular_organisms - ps1
Archaea1 - ps2
Archaea2 - ps3
Archaea3 - ps4
Asgard/Eukaryota - ps5
Eukaryota - ps6
Amorpheae - ps7
Obazoa - ps8
Opisthokonta - ps9
Holozoa - ps10
Filozoa - ps11
Choano./Metazoa - ps12
Metazoa - ps13
Myriazoa - ps14
Parahoxozoa - ps15
Bilateria - ps16
Deuterostomia - ps17
Chordata - ps18
Olfactores - ps19
Vertebrata - ps20
Gnathostomata - ps21
Euteleostomi - ps22
Sarcopterygii - ps23
Tetrapoda - ps24
Amniota - ps25
Mammalia - ps26
Theria - ps27
Placentalia - ps28
Boreoeutheria - ps29
Euarchontoglires - ps30
Euarchonta - ps31
Primates - ps32
Haplorrhini - ps33
Simiiformes - ps34
Catarrhini - ps35
Hominoidea - ps36
Hominidae - ps37
Homininae - ps38
Hominini - ps39
Homo_sapiens - ps40
Phylostratum

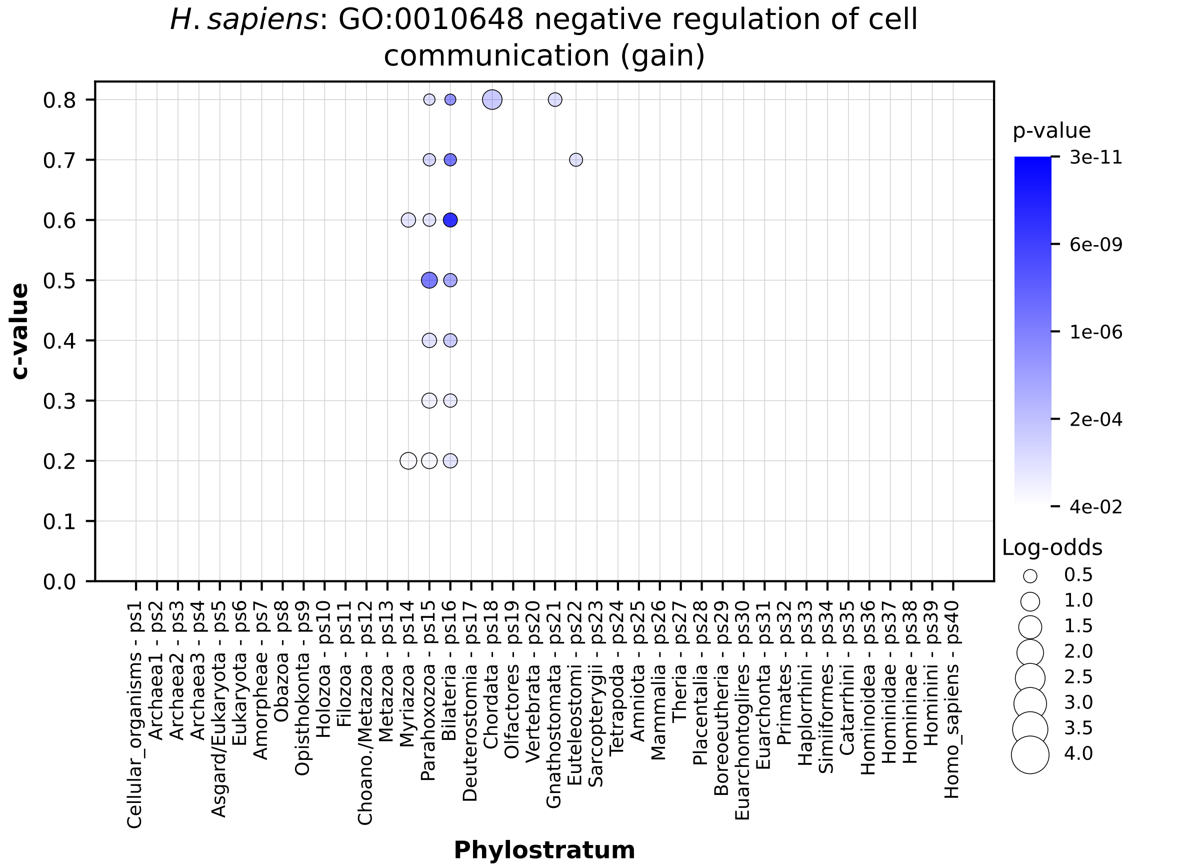
H. sapiens: GO:0010648 negative regulation of cell communication (gain)
c-value
Phylostratum
p-value
3e-11
6e-09
1e-06
2e-04
4e-02
Log-odds
0.5
1.0
1.5
2.0
2.5
3.0
3.5
4.0
Cellular_organisms - ps1
Archaea1 - ps2
Archaea2 - ps3
Archaea3 - ps4
Asgard/Eukaryota - ps5
Eukaryota - ps6
Amorpheae - ps7
Obazoa - ps8
Opisthokonta - ps9
Holozoa - ps10
Filozoa - ps11
Choano./Metazoa - ps12
Metazoa - ps13
Myriazoa - ps14
Parahoxozoa - ps15
Bilateria - ps16
Deuterostomia - ps17
Chordata - ps18
Olfactores - ps19
Vertebrata - ps20
Gnathostomata - ps21
Euteleostomi - ps22
Sarcopterygii - ps23
Tetrapoda - ps24
Amniota - ps25
Mammalia - ps26
Theria - ps27
Placentalia - ps28
Boreoeutheria - ps29
Euarchontoglires - ps30
Euarchonta - ps31
Primates - ps32
Haplorrhini - ps33
Simiiformes - ps34
Catarrhini - ps35
Hominoidea - ps36
Hominidae - ps37
Homininae - ps38
Hominini - ps39
Homo_sapiens - ps40

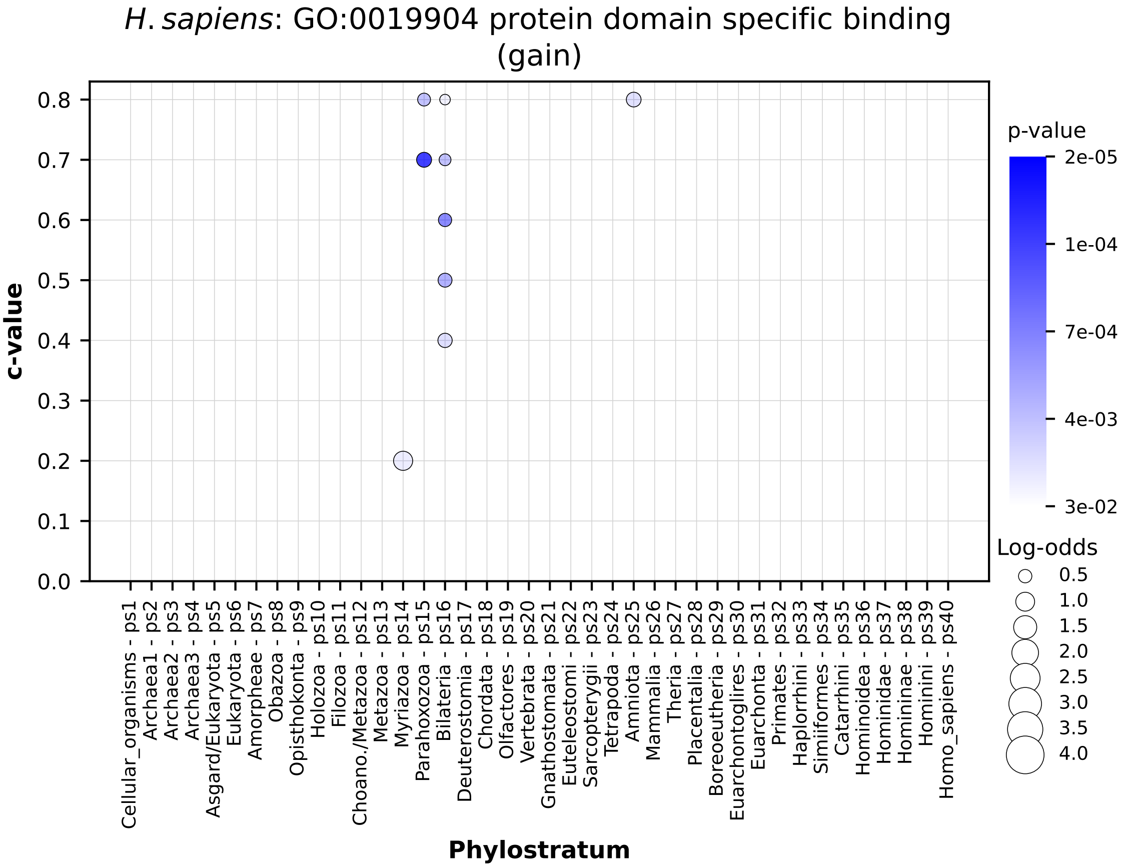

H. sapiens: GO:0019904 protein domain specific binding (gain)
c-value
0.0
0.1
0.2
0.3
0.4
0.5
0.6
0.7
0.8
Phylostratum
Cellular_organisms - ps1
Archaea1 - ps2
Archaea2 - ps3
Archaea3 - ps4
Asgard/Eukaryota - ps5
Eukaryota - ps6
Amorpheae - ps7
Obazoa - ps8
Opisthokonta - ps9
Holozoa - ps10
Filozoa - ps11
Choano./Metazoa - ps12
Metazoa - ps13
Myriazoa - ps14
Parahoxozoa - ps15
Bilateria - ps16
Deuterostomia - ps17
Chordata - ps18
Olfactores - ps19
Vertebrata - ps20
Gnathostomata - ps21
Euteleostomi - ps22
Sarcopterygii - ps23
Tetrapoda - ps24
Amniota - ps25
Mammalia - ps26
Theria - ps27
Placentalia - ps28
Boreoeutheria - ps29
Euarchontoglires - ps30
Euarchonta - ps31
Primates - ps32
Haplorrhini - ps33
Simiiformes - ps34
Catarrhini - ps35
Hominoidea - ps36
Hominidae - ps37
Homininae - ps38
Hominini - ps39
Homo_sapiens - ps40
p-value
2e-05
1e-04
7e-04
4e-03
3e-02
Log-odds
0.5
1.0
1.5
2.0
2.5
3.0
3.5
4.0

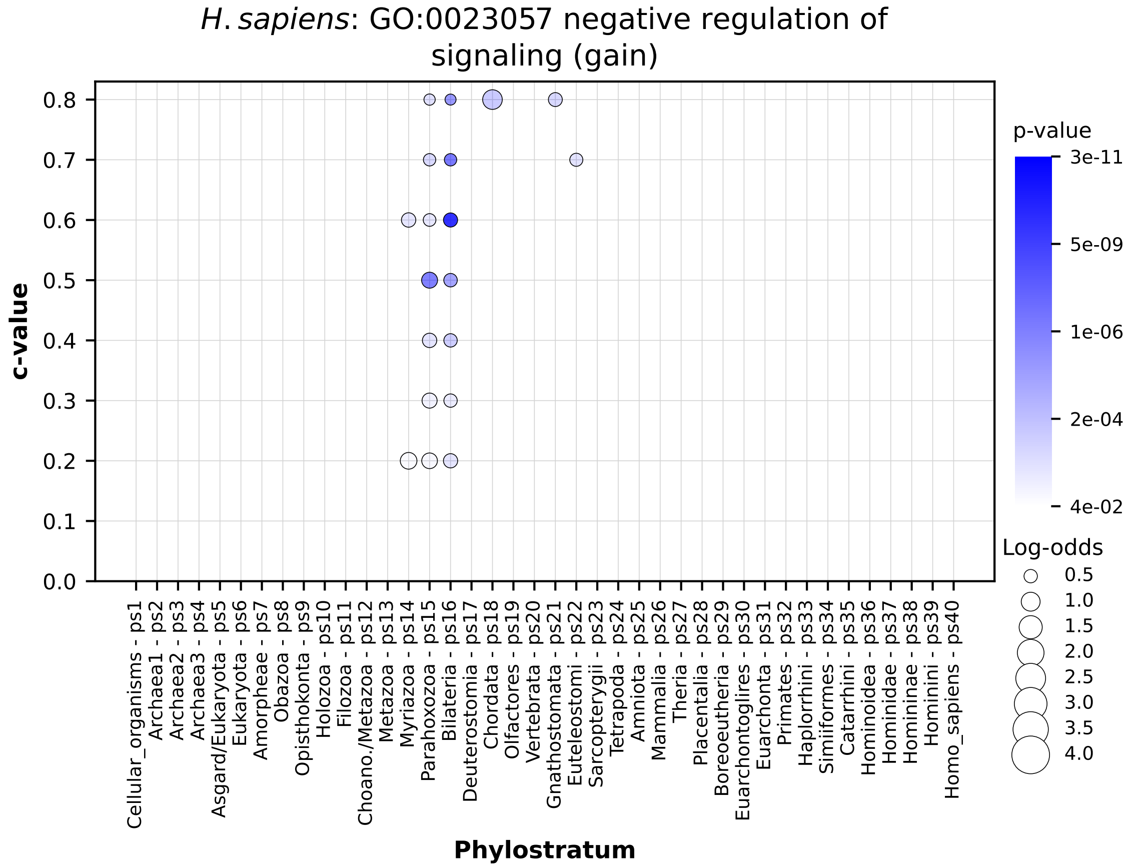

H. sapiens: GO:0023057 negative regulation of signaling (gain)
c-value
0.0
0.1
0.2
0.3
0.4
0.5
0.6
0.7
0.8
Phylostratum
Cellular_organisms - ps1
Archaea1 - ps2
Archaea2 - ps3
Archaea3 - ps4
Asgard/Eukaryota - ps5
Eukaryota - ps6
Amorpheae - ps7
Obazoa - ps8
Opisthokonta - ps9
Holozoa - ps10
Filozoa - ps11
Choano./Metazoa - ps12
Metazoa - ps13
Myriazoa - ps14
Parahoxozoa - ps15
Bilateria - ps16
Deuterostomia - ps17
Chordata - ps18
Olfactores - ps19
Vertebrata - ps20
Gnathostomata - ps21
Euteleostomi - ps22
Sarcopterygii - ps23
Tetrapoda - ps24
Amniota - ps25
Mammalia - ps26
Theria - ps27
Placentalia - ps28
Boreoeutheria - ps29
Euarchontoglires - ps30
Euarchonta - ps31
Primates - ps32
Haplorrhini - ps33
Simiiformes - ps34
Catarrhini - ps35
Hominoidea - ps36
Hominidae - ps37
Homininae - ps38
Hominini - ps39
Homo_sapiens - ps40
p-value
3e-11
5e-09
1e-06
2e-04
4e-02
Log-odds
0.5
1.0
1.5
2.0
2.5
3.0
3.5
4.0

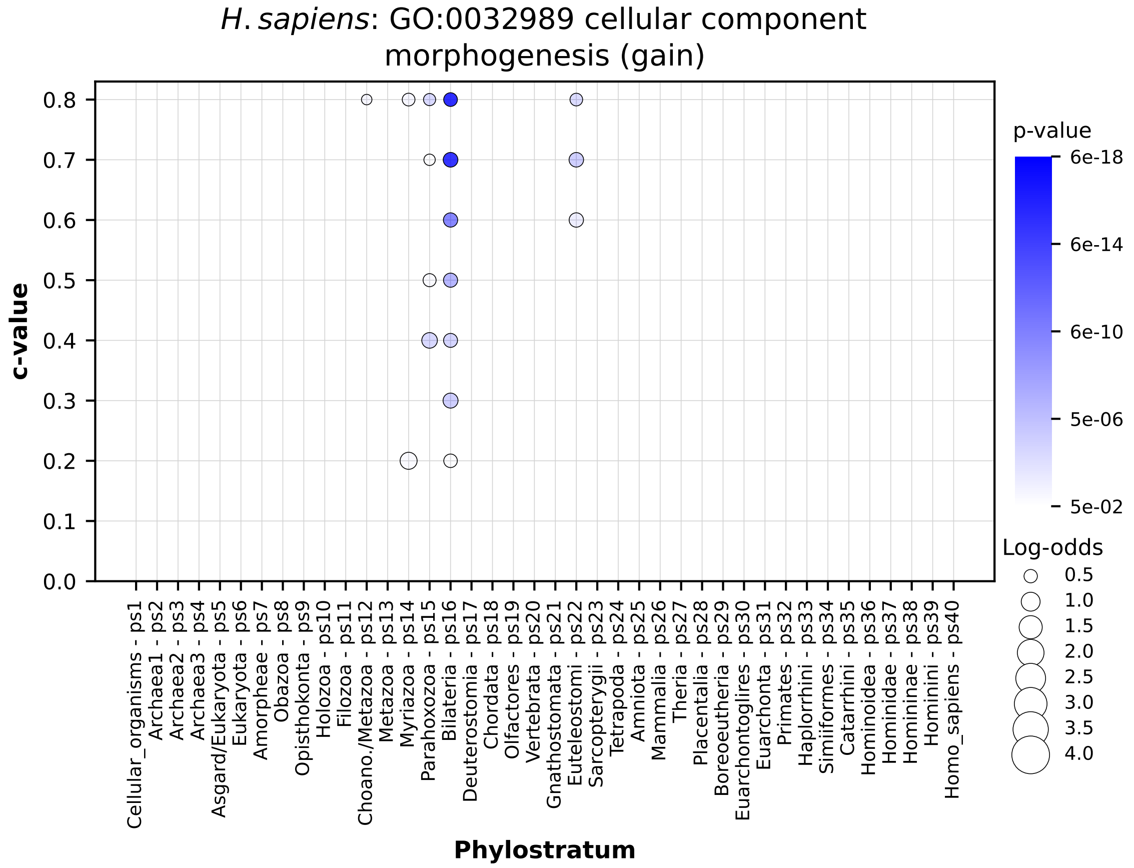

H. sapiens: GO:0032989 cellular component morphogenesis (gain)
c-value
0.0
0.1
0.2
0.3
0.4
0.5
0.6
0.7
0.8
Phylostratum
Cellular_organisms - ps1
Archaea1 - ps2
Archaea2 - ps3
Archaea3 - ps4
Asgard/Eukaryota - ps5
Eukaryota - ps6
Amorpheae - ps7
Obazoa - ps8
Opisthokonta - ps9
Holozoa - ps10
Filozoa - ps11
Choano./Metazoa - ps12
Metazoa - ps13
Myriazoa - ps14
Parahoxozoa - ps15
Bilateria - ps16
Deuterostomia - ps17
Chordata - ps18
Olfactores - ps19
Vertebrata - ps20
Gnathostomata - ps21
Euteleostomi - ps22
Sarcopterygii - ps23
Tetrapoda - ps24
Amniota - ps25
Mammalia - ps26
Theria - ps27
Placentalia - ps28
Boreoeutheria - ps29
Euarchontoglires - ps30
Euarchonta - ps31
Primates - ps32
Haplorrhini - ps33
Simiiformes - ps34
Catarrhini - ps35
Hominoidea - ps36
Hominidae - ps37
Homininae - ps38
Hominini - ps39
Homo_sapiens - ps40
p-value
6e-18
6e-14
6e-10
5e-06
5e-02
Log-odds
0.5
1.0
1.5
2.0
2.5
3.0
3.5
4.0

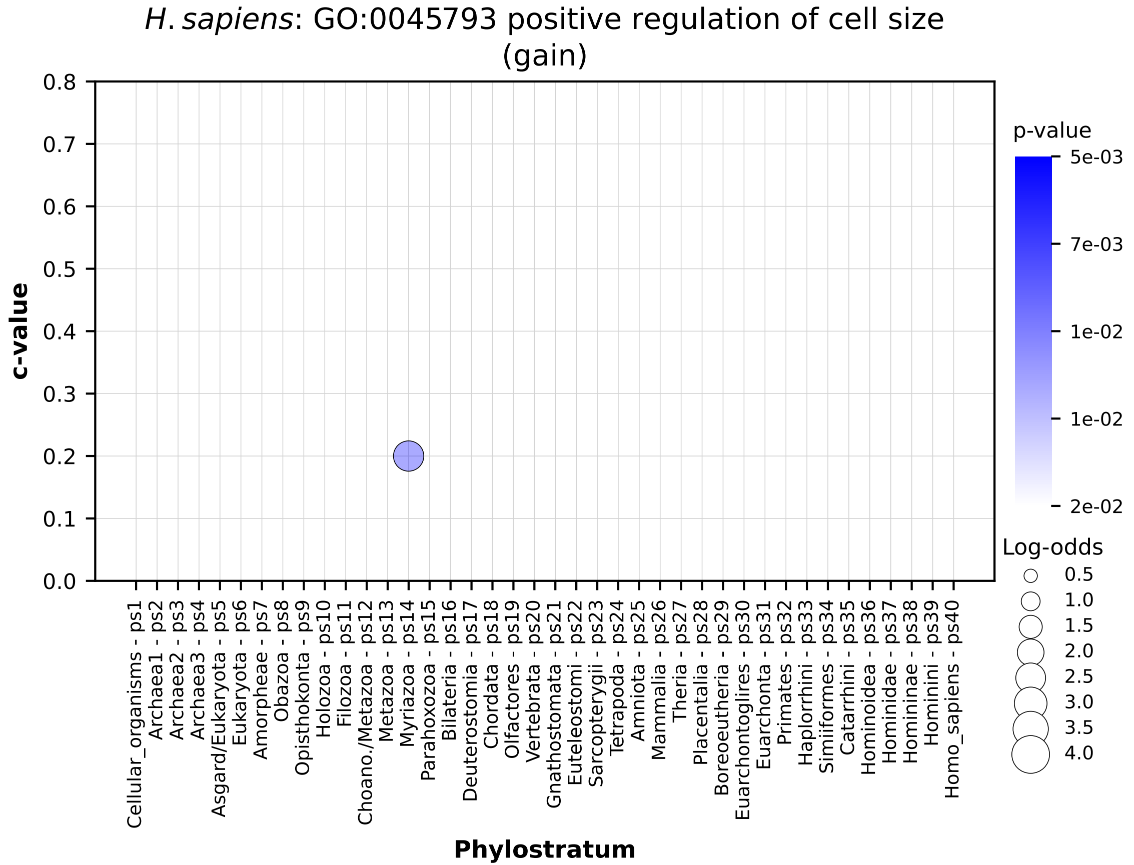

*H. sapiens*: GO:0045793 positive regulation of cell size
(gain)
c-value
0.8
0.7
0.6
0.5
0.4
0.3
0.2
0.1
0.0
p-value
5e-03
7e-03
1e-02
1e-02
2e-02
Log-odds
0.5
1.0
1.5
2.0
2.5
3.0
3.5
4.0
Cellular_organisms - ps1
Archaea1 - ps2
Archaea2 - ps3
Archaea3 - ps4
Asgard/Eukaryota - ps5
Eukaryota - ps6
Amorpheae - ps7
Obazoa - ps8
Opisthokonta - ps9
Holozoa - ps10
Filozoa - ps11
Choano./Metazoa - ps12
Metazoa - ps13
Myriazoa - ps14
Parahoxozoa - ps15
Bilateria - ps16
Deuterostomia - ps17
Chordata - ps18
Olfactores - ps19
Vertebrata - ps20
Gnathostomata - ps21
Euteleostomi - ps22
Sarcopterygii - ps23
Tetrapoda - ps24
Amniota - ps25
Mammalia - ps26
Theria - ps27
Placentalia - ps28
Boreoeutheria - ps29
Euarchontoglires - ps30
Euarchonta - ps31
Primates - ps32
Haplorrhini - ps33
Simiiformes - ps34
Catarrhini - ps35
Hominoidea - ps36
Hominidae - ps37
Homininae - ps38
Hominini - ps39
Homo_sapiens - ps40
Phylostratum

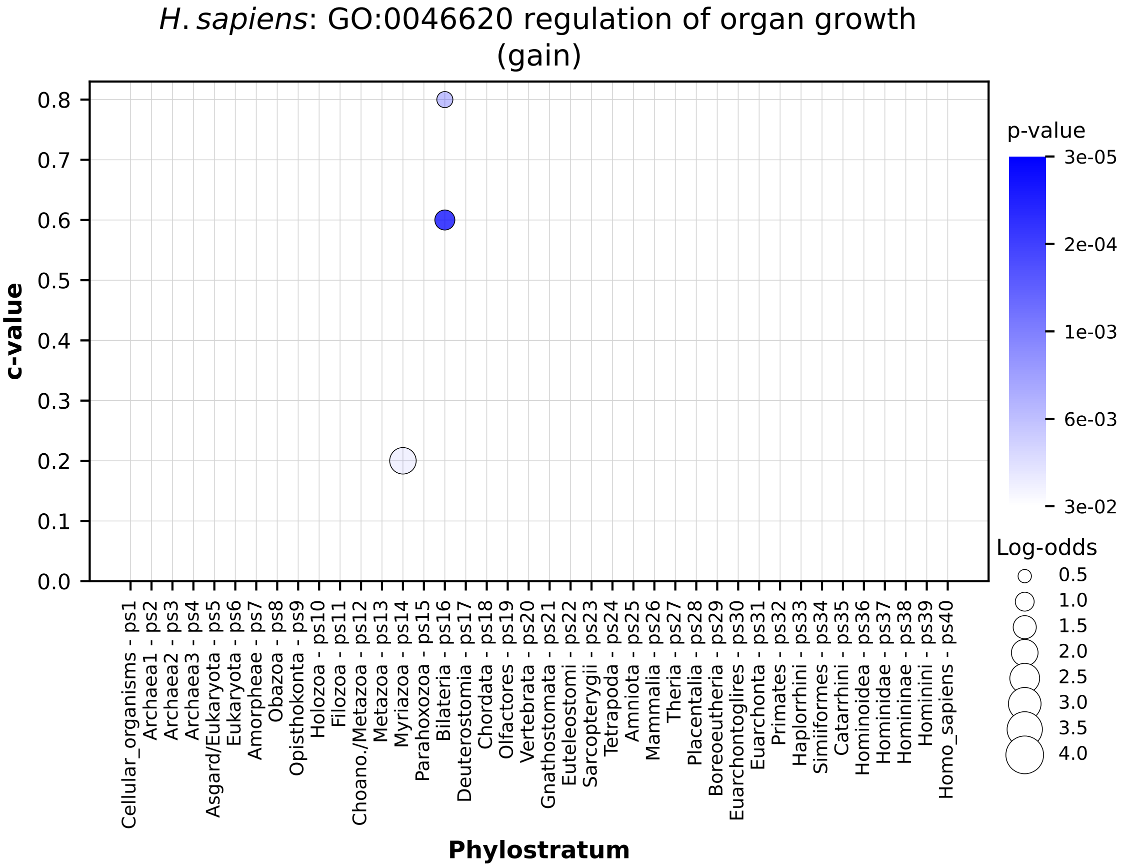
*H. sapiens*: GO:0046620 regulation of organ growth
(gain)
c-value
0.0
0.1
0.2
0.3
0.4
0.5
0.6
0.7
0.8
Phylostratum
Cellular_organisms - ps1
Archaea1 - ps2
Archaea2 - ps3
Archaea3 - ps4
Asgard/Eukaryota - ps5
Eukaryota - ps6
Amorpheae - ps7
Obazoa - ps8
Opisthokonta - ps9
Holozoa - ps10
Filozoa - ps11
Choano./Metazoa - ps12
Metazoa - ps13
Myriazoa - ps14
Parahoxozoa - ps15
Bilateria - ps16
Deuterostomia - ps17
Chordata - ps18
Olfactores - ps19
Vertebrata - ps20
Gnathostomata - ps21
Euteleostomi - ps22
Sarcopterygii - ps23
Tetrapoda - ps24
Amniota - ps25
Mammalia - ps26
Theria - ps27
Placentalia - ps28
Boreoeutheria - ps29
Euarchontoglires - ps30
Euarchonta - ps31
Primates - ps32
Haplorrhini - ps33
Simiiformes - ps34
Catarrhini - ps35
Hominoidea - ps36
Hominidae - ps37
Homininae - ps38
Hominini - ps39
Homo_sapiens - ps40
p-value
3e-05
2e-04
1e-03
6e-03
3e-02
Log-odds
0.5
1.0
1.5
2.0
2.5
3.0
3.5
4.0

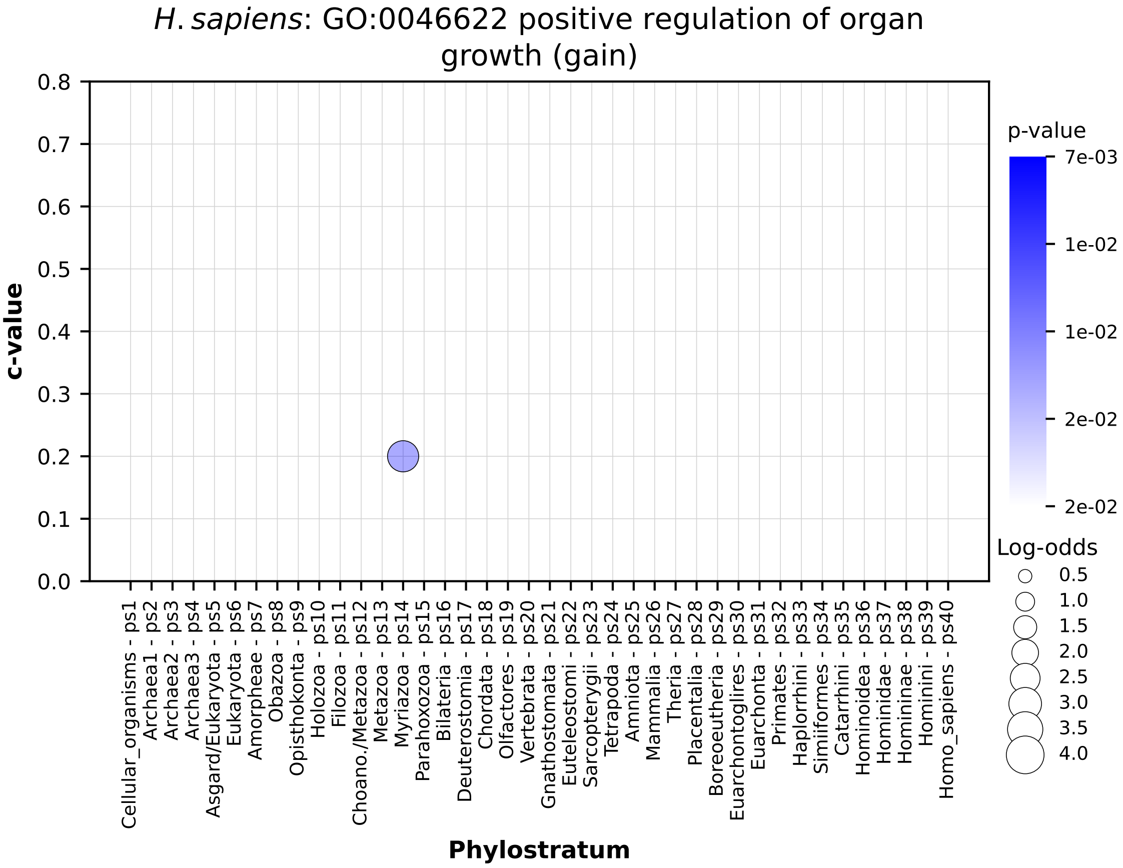

*H. sapiens*: GO:0046622 positive regulation of organ growth (gain)
c-value
0.8
0.7
0.6
0.5
0.4
0.3
0.2
0.1
0.0
Cellular_organisms - ps1
Archaea1 - ps2
Archaea2 - ps3
Archaea3 - ps4
Asgard/Eukaryota - ps5
Eukaryota - ps6
Amorpheae - ps7
Obazoa - ps8
Opisthokonta - ps9
Holozoa - ps10
Filozoa - ps11
Choano./Metazoa - ps12
Metazoa - ps13
Myriazoa - ps14
Parahoxozoa - ps15
Bilateria - ps16
Deuterostomia - ps17
Chordata - ps18
Olfactores - ps19
Vertebrata - ps20
Gnathostomata - ps21
Euteleostomi - ps22
Sarcopterygii - ps23
Tetrapoda - ps24
Amniota - ps25
Mammalia - ps26
Theria - ps27
Placentalia - ps28
Boreoeutheria - ps29
Euarchontoglires - ps30
Euarchonta - ps31
Primates - ps32
Haplorrhini - ps33
Simiiformes - ps34
Catarrhini - ps35
Hominoidea - ps36
Hominidae - ps37
Homininae - ps38
Hominini - ps39
Homo_sapiens - ps40
Phylostratum
p-value
7e-03
1e-02
1e-02
2e-02
2e-02
Log-odds
0.5
1.0
1.5
2.0
2.5
3.0
3.5
4.0

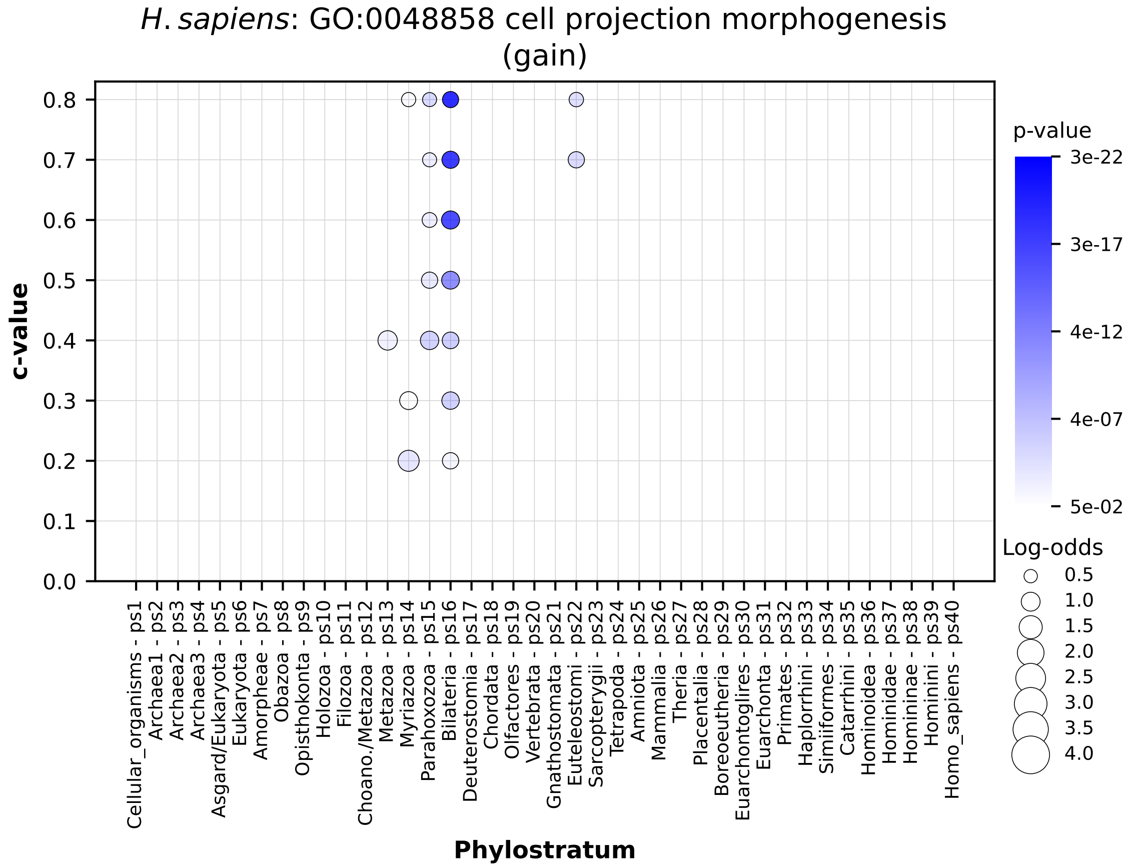

*H. sapiens*: GO:0048858 cell projection morphogenesis
(gain)
c-value
0.8
0.7
0.6
0.5
0.4
0.3
0.2
0.1
0.0
Phylostratum
Cellular_organisms - ps1
Archaea1 - ps2
Archaea2 - ps3
Archaea3 - ps4
Asgard/Eukaryota - ps5
Eukaryota - ps6
Amorpheae - ps7
Obazoa - ps8
Opisthokonta - ps9
Holozoa - ps10
Filozoa - ps11
Choano./Metazoa - ps12
Metazoa - ps13
Myriazoa - ps14
Parahoxozoa - ps15
Bilateria - ps16
Deuterostomia - ps17
Chordata - ps18
Olfactores - ps19
Vertebrata - ps20
Gnathostomata - ps21
Euteleostomi - ps22
Sarcopterygii - ps23
Tetrapoda - ps24
Amniota - ps25
Mammalia - ps26
Theria - ps27
Placentalia - ps28
Boreoeutheria - ps29
Euarchontoglires - ps30
Euarchonta - ps31
Primates - ps32
Haplorrhini - ps33
Simiiformes - ps34
Catarrhini - ps35
Hominoidea - ps36
Hominidae - ps37
Homininae - ps38
Hominini - ps39
Homo_sapiens - ps40
p-value
3e-22
3e-17
4e-12
4e-07
5e-02
Log-odds
0.5
1.0
1.5
2.0
2.5
3.0
3.5
4.0

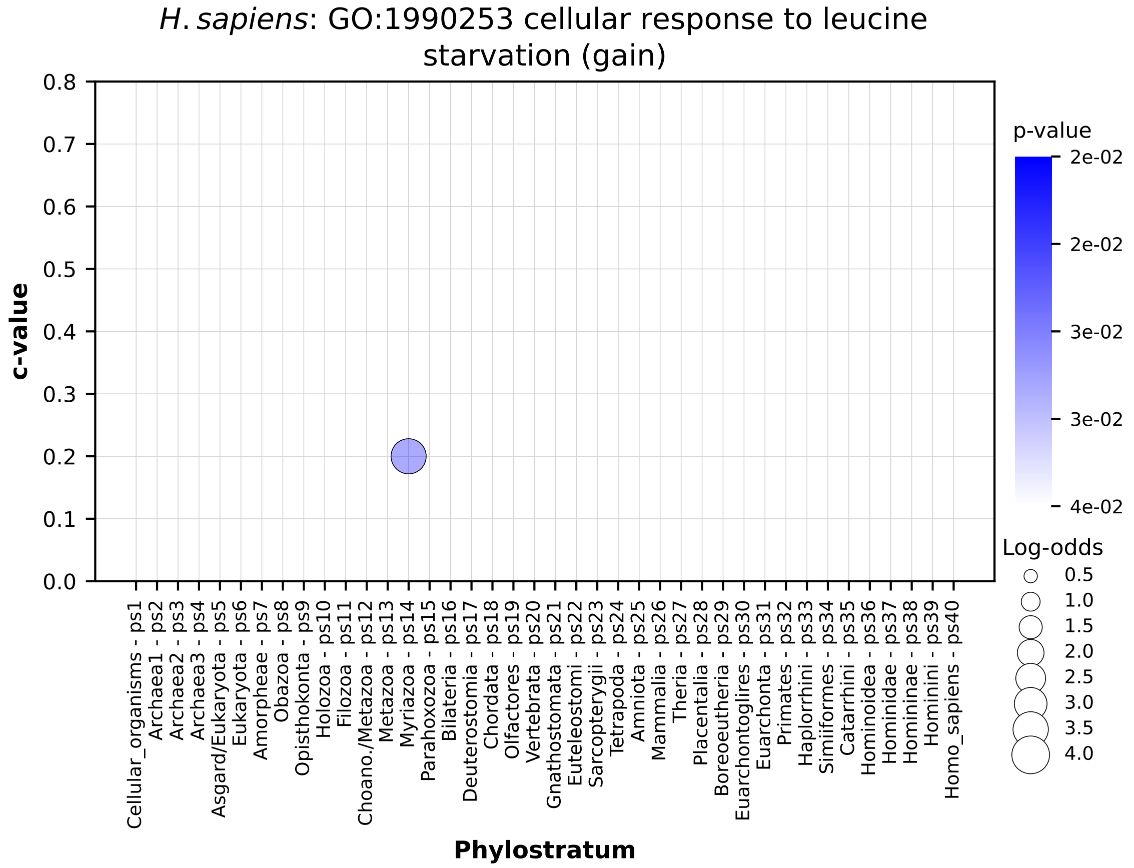

H. sapiens: GO:1990253 cellular response to leucine starvation (gain)
c-value
0.0
0.1
0.2
0.3
0.4
0.5
0.6
0.7
0.8
Phylostratum
Cellular_organisms - ps1
Archaea1 - ps2
Archaea2 - ps3
Archaea3 - ps4
Asgard/Eukaryota - ps5
Eukaryota - ps6
Amorpheae - ps7
Obazoa - ps8
Opisthokonta - ps9
Holozoa - ps10
Filozoa - ps11
Choano./Metazoa - ps12
Metazoa - ps13
Myriazoa - ps14
Parahoxozoa - ps15
Bilateria - ps16
Deuterostomia - ps17
Chordata - ps18
Olfactores - ps19
Vertebrata - ps20
Gnathostomata - ps21
Euteleostomi - ps22
Sarcopterygii - ps23
Tetrapoda - ps24
Amniota - ps25
Mammalia - ps26
Theria - ps27
Placentalia - ps28
Boreoeutheria - ps29
Euarchontoglires - ps30
Euarchonta - ps31
Primates - ps32
Haplorrhini - ps33
Simiiformes - ps34
Catarrhini - ps35
Hominoidea - ps36
Hominidae - ps37
Homininae - ps38
Hominini - ps39
Homo_sapiens - ps40
p-value
2e-02
2e-02
3e-02
3e-02
4e-02
Log-odds
0.5
1.0
1.5
2.0
2.5
3.0
3.5
4.0

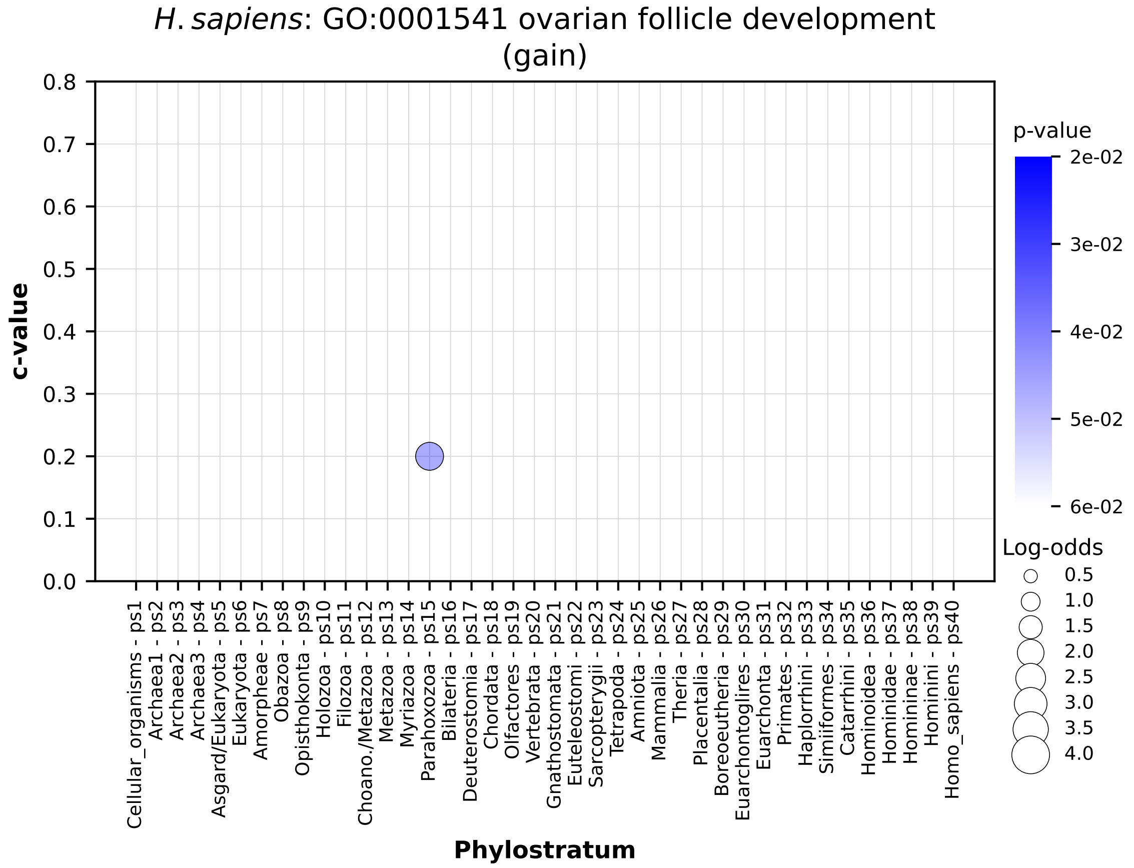

H. sapiens: GO:0001541 ovarian follicle development
(gain)
c-value
0.8
0.7
0.6
0.5
0.4
0.3
0.2
0.1
0.0
Phylostratum
Cellular_organisms - ps1
Archaea1 - ps2
Archaea2 - ps3
Archaea3 - ps4
Asgard/Eukaryota - ps5
Eukaryota - ps6
Amorpheae - ps7
Obazoa - ps8
Opisthokonta - ps9
Holozoa - ps10
Filozoa - ps11
Choano./Metazoa - ps12
Metazoa - ps13
Myriazoa - ps14
Parahoxozoa - ps15
Bilateria - ps16
Deuterostomia - ps17
Chordata - ps18
Olfactores - ps19
Vertebrata - ps20
Gnathostomata - ps21
Euteleostomi - ps22
Sarcopterygii - ps23
Tetrapoda - ps24
Amniota - ps25
Mammalia - ps26
Theria - ps27
Placentalia - ps28
Boreoeutheria - ps29
Euarchontoglires - ps30
Euarchonta - ps31
Primates - ps32
Haplorrhini - ps33
Simiiformes - ps34
Catarrhini - ps35
Hominoidea - ps36
Hominidae - ps37
Homininae - ps38
Hominini - ps39
Homo_sapiens - ps40
p-value
2e-02
3e-02
4e-02
5e-02
6e-02
Log-odds
0.5
1.0
1.5
2.0
2.5
3.0
3.5
4.0

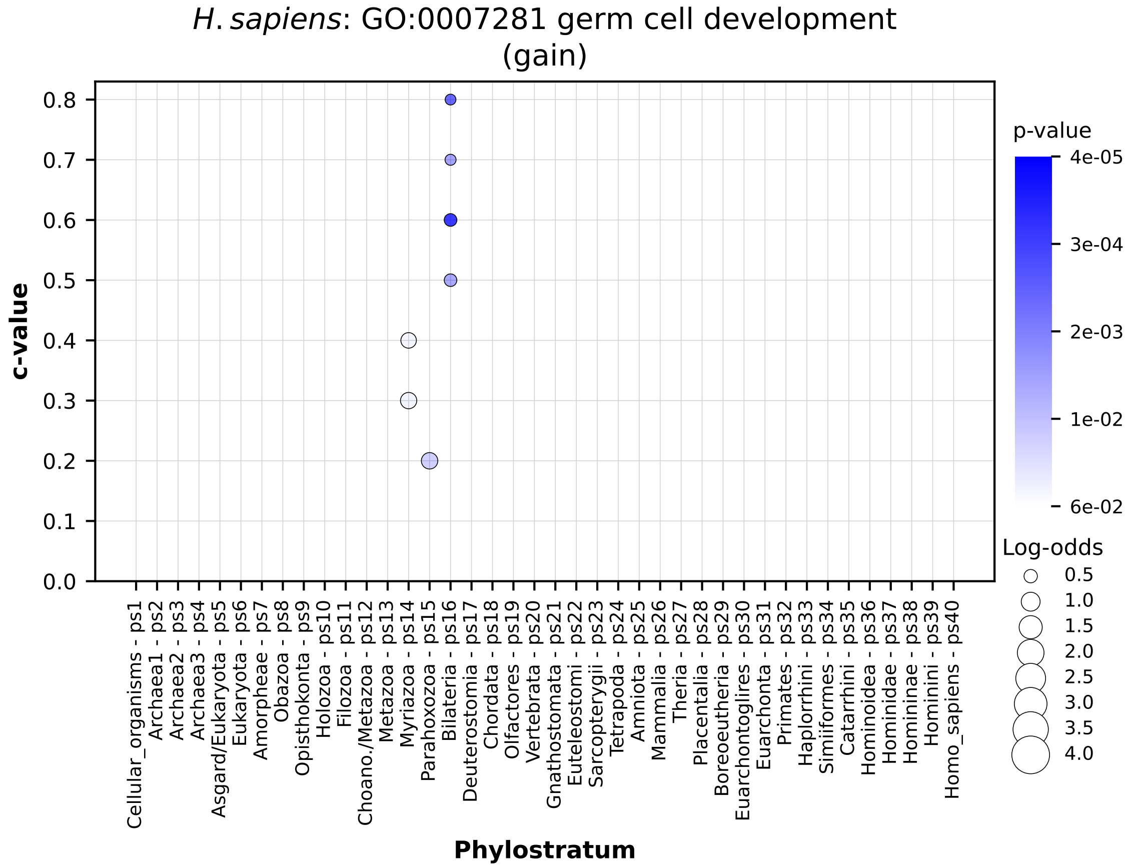

H. sapiens: GO:0007281 germ cell development
(gain)
c-value
0.0
0.1
0.2
0.3
0.4
0.5
0.6
0.7
0.8
Phylostratum
Cellular_organisms - ps1
Archaea1 - ps2
Archaea2 - ps3
Archaea3 - ps4
Asgard/Eukaryota - ps5
Eukaryota - ps6
Amorpheae - ps7
Obazoa - ps8
Opisthokonta - ps9
Holozoa - ps10
Filozoa - ps11
Choano./Metazoa - ps12
Metazoa - ps13
Myriazoa - ps14
Parahoxozoa - ps15
Bilateria - ps16
Deuterostomia - ps17
Chordata - ps18
Olfactores - ps19
Vertebrata - ps20
Gnathostomata - ps21
Euteleostomi - ps22
Sarcopterygii - ps23
Tetrapoda - ps24
Amniota - ps25
Mammalia - ps26
Theria - ps27
Placentalia - ps28
Boreoeutheria - ps29
Euarchontoglires - ps30
Euarchonta - ps31
Primates - ps32
Haplorrhini - ps33
Simiiformes - ps34
Catarrhini - ps35
Hominoidea - ps36
Hominidae - ps37
Homininae - ps38
Hominini - ps39
Homo_sapiens - ps40
p-value
4e-05
3e-04
2e-03
1e-02
6e-02
Log-odds
0.5
1.0
1.5
2.0
2.5
3.0
3.5
4.0

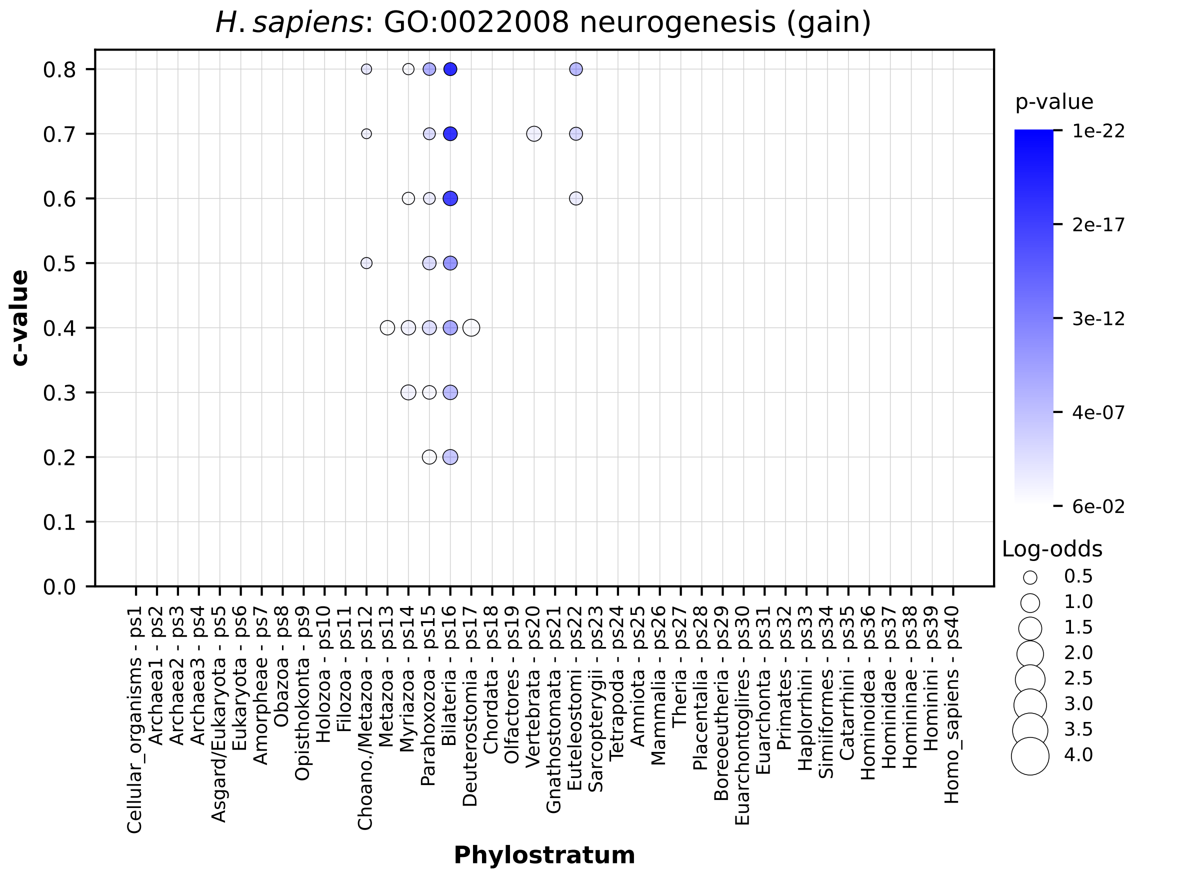

H. sapiens: GO:0022008 neurogenesis (gain)
c-value
0.0
0.1
0.2
0.3
0.4
0.5
0.6
0.7
0.8
Phylostratum
Cellular_organisms - ps1
Archaea1 - ps2
Archaea2 - ps3
Archaea3 - ps4
Asgard/Eukaryota - ps5
Eukaryota - ps6
Amorpheae - ps7
Obazoa - ps8
Opisthokonta - ps9
Holozoa - ps10
Filozoa - ps11
Choano./Metazoa - ps12
Metazoa - ps13
Myriazoa - ps14
Parahoxozoa - ps15
Bilateria - ps16
Deuterostomia - ps17
Chordata - ps18
Olfactores - ps19
Vertebrata - ps20
Gnathostomata - ps21
Euteleostomi - ps22
Sarcopterygii - ps23
Tetrapoda - ps24
Amniota - ps25
Mammalia - ps26
Theria - ps27
Placentalia - ps28
Boreoeutheria - ps29
Euarchontoglires - ps30
Euarchonta - ps31
Primates - ps32
Haplorrhini - ps33
Simiiformes - ps34
Catarrhini - ps35
Hominoidea - ps36
Hominidae - ps37
Homininae - ps38
Hominini - ps39
Homo_sapiens - ps40
p-value
1e-22
2e-17
3e-12
4e-07
6e-02
Log-odds
0.5
1.0
1.5
2.0
2.5
3.0
3.5
4.0

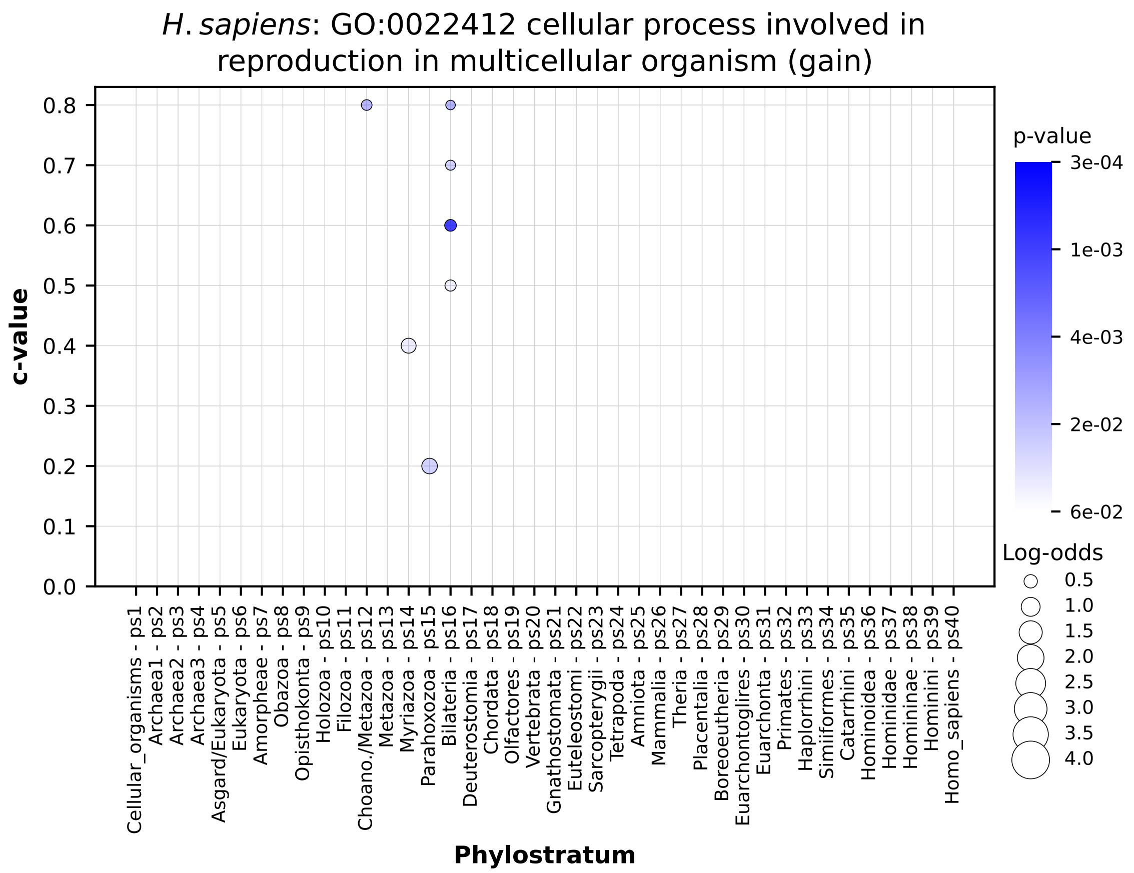

H. sapiens: GO:0022412 cellular process involved in reproduction in multicellular organism (gain)
c-value
0.0
0.1
0.2
0.3
0.4
0.5
0.6
0.7
0.8
Phylostratum
Cellular_organisms - ps1
Archaea1 - ps2
Archaea2 - ps3
Archaea3 - ps4
Asgard/Eukaryota - ps5
Eukaryota - ps6
Amorpheae - ps7
Obazoa - ps8
Opisthokonta - ps9
Holozoa - ps10
Filozoa - ps11
Choano./Metazoa - ps12
Metazoa - ps13
Myriazoa - ps14
Parahoxozoa - ps15
Bilateria - ps16
Deuterostomia - ps17
Chordata - ps18
Olfactores - ps19
Vertebrata - ps20
Gnathostomata - ps21
Euteleostomi - ps22
Sarcopterygii - ps23
Tetrapoda - ps24
Amniota - ps25
Mammalia - ps26
Theria - ps27
Placentalia - ps28
Boreoeutheria - ps29
Euarchontoglires - ps30
Euarchonta - ps31
Primates - ps32
Haplorrhini - ps33
Simiiformes - ps34
Catarrhini - ps35
Hominoidea - ps36
Hominidae - ps37
Homininae - ps38
Hominini - ps39
Homo_sapiens - ps40
p-value
3e-04
1e-03
4e-03
2e-02
6e-02
Log-odds
0.5
1.0
1.5
2.0
2.5
3.0
3.5
4.0

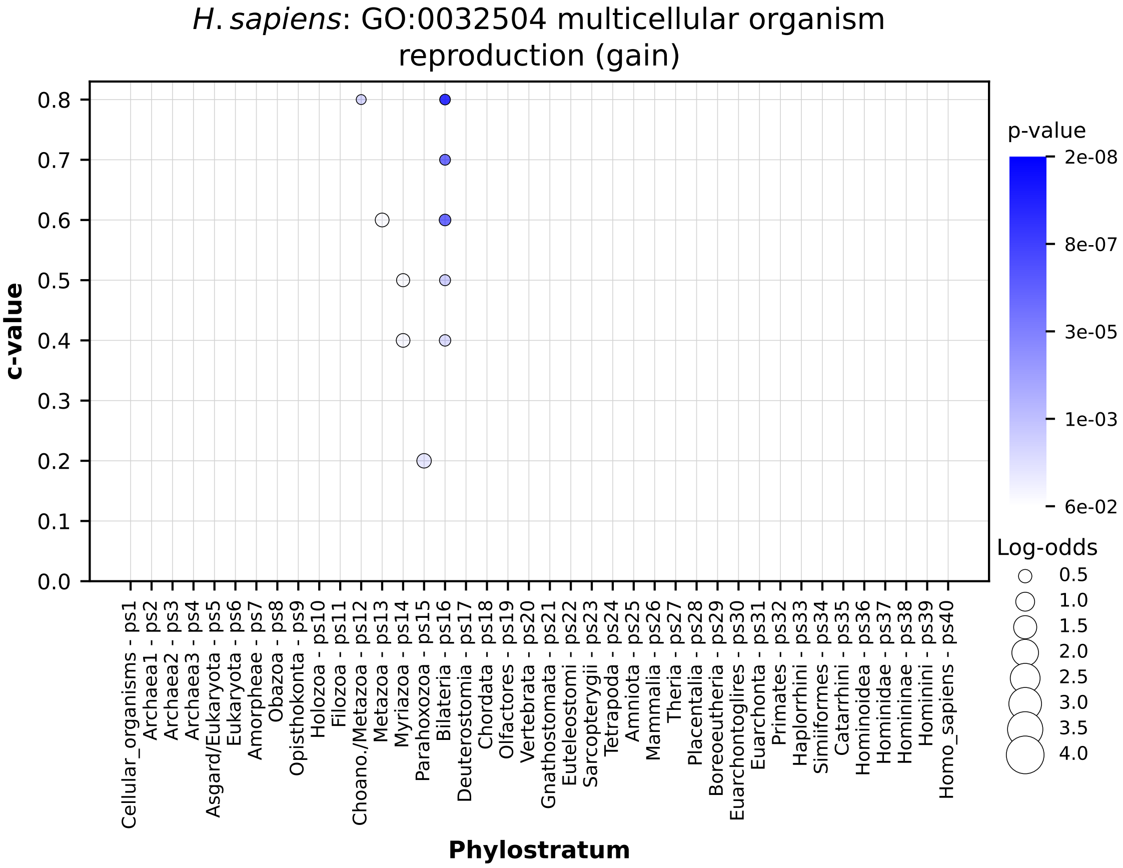

H. sapiens: GO:0032504 multicellular organism reproduction (gain)
c-value
0.0
0.1
0.2
0.3
0.4
0.5
0.6
0.7
0.8
Phylostratum
Cellular_organisms - ps1
Archaea1 - ps2
Archaea2 - ps3
Archaea3 - ps4
Asgard/Eukaryota - ps5
Eukaryota - ps6
Amorpheae - ps7
Obazoa - ps8
Opisthokonta - ps9
Holozoa - ps10
Filozoa - ps11
Choano./Metazoa - ps12
Metazoa - ps13
Myriazoa - ps14
Parahoxozoa - ps15
Bilateria - ps16
Deuterostomia - ps17
Chordata - ps18
Olfactores - ps19
Vertebrata - ps20
Gnathostomata - ps21
Euteleostomi - ps22
Sarcopterygii - ps23
Tetrapoda - ps24
Amniota - ps25
Mammalia - ps26
Theria - ps27
Placentalia - ps28
Boreoeutheria - ps29
Euarchontoglires - ps30
Euarchonta - ps31
Primates - ps32
Haplorrhini - ps33
Simiiformes - ps34
Catarrhini - ps35
Hominoidea - ps36
Hominidae - ps37
Homininae - ps38
Hominini - ps39
Homo_sapiens - ps40
p-value
2e-08
8e-07
3e-05
1e-03
6e-02
Log-odds
0.5
1.0
1.5
2.0
2.5
3.0
3.5
4.0

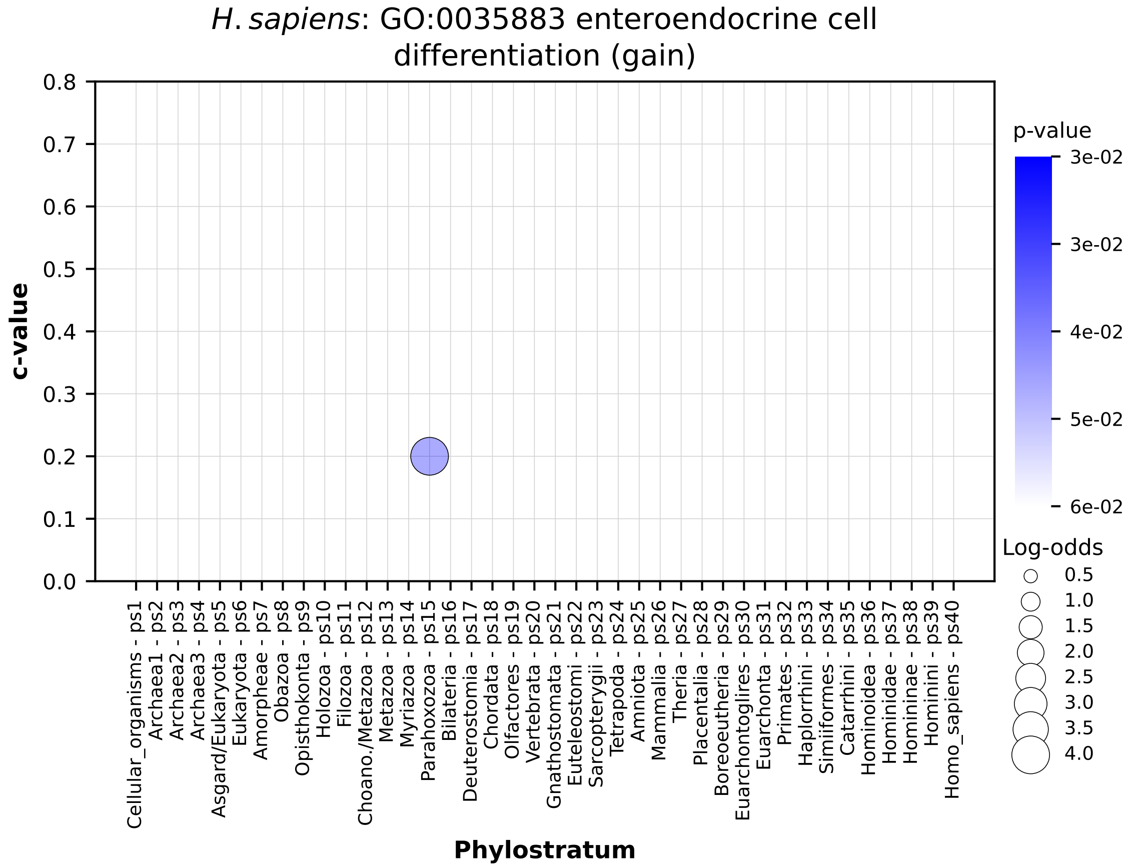

*H. sapiens*: GO:0035883 enteroendocrine cell differentiation (gain)
c-value
0.8
0.7
0.6
0.5
0.4
0.3
0.2
0.1
0.0
Phylostratum
Cellular_organisms - ps1
Archaea1 - ps2
Archaea2 - ps3
Archaea3 - ps4
Asgard/Eukaryota - ps5
Eukaryota - ps6
Amorpheae - ps7
Obazoa - ps8
Opisthokonta - ps9
Holozoa - ps10
Filozoa - ps11
Choano./Metazoa - ps12
Metazoa - ps13
Myriazoa - ps14
Parahoxozoa - ps15
Bilateria - ps16
Deuterostomia - ps17
Chordata - ps18
Olfactores - ps19
Vertebrata - ps20
Gnathostomata - ps21
Euteleostomi - ps22
Sarcopterygii - ps23
Tetrapoda - ps24
Amniota - ps25
Mammalia - ps26
Theria - ps27
Placentalia - ps28
Boreoeutheria - ps29
Euarchontoglires - ps30
Euarchonta - ps31
Primates - ps32
Haplorrhini - ps33
Simiiformes - ps34
Catarrhini - ps35
Hominoidea - ps36
Hominidae - ps37
Homininae - ps38
Hominini - ps39
Homo_sapiens - ps40
p-value
3e-02
3e-02
4e-02
5e-02
6e-02
Log-odds
0.5
1.0
1.5
2.0
2.5
3.0
3.5
4.0

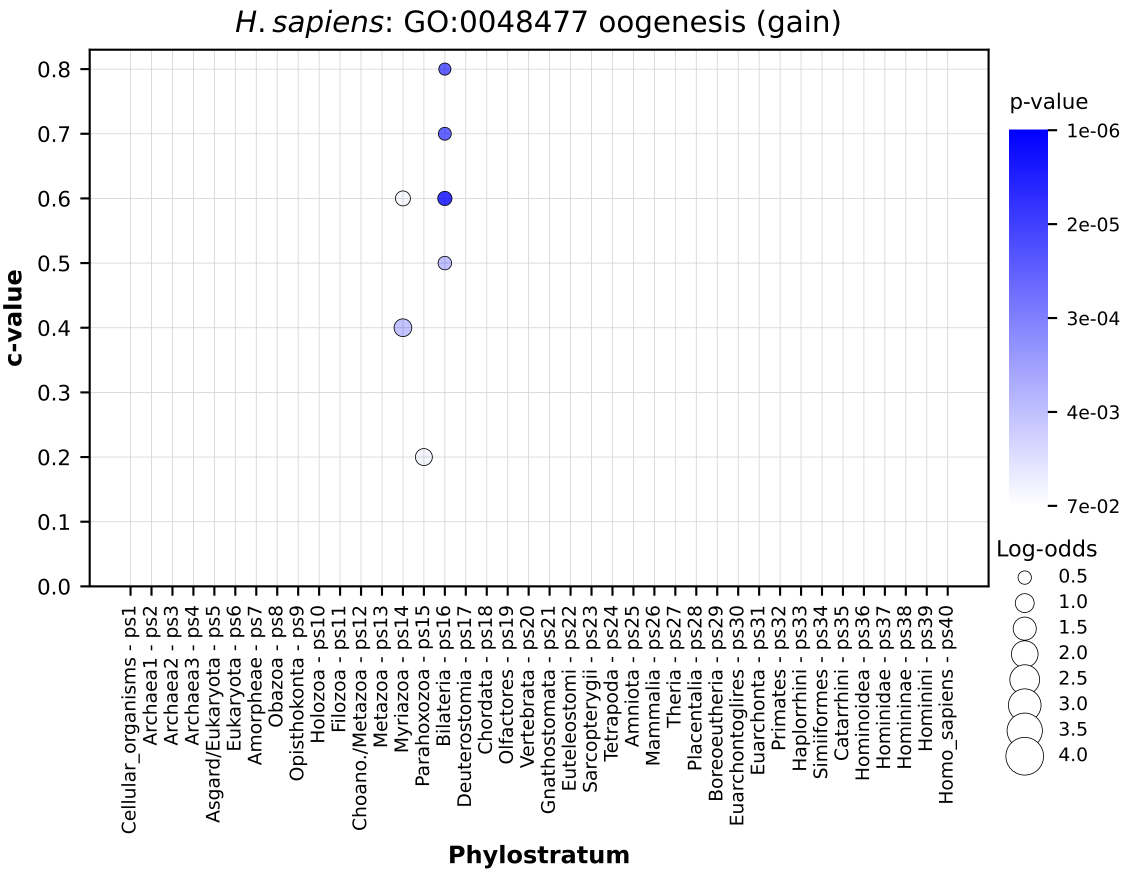

*H. sapiens*: GO:0048477 oogenesis (gain)
c-value
0.8
0.7
0.6
0.5
0.4
0.3
0.2
0.1
0.0
Phylostratum
Cellular_organisms - ps1
Archaea1 - ps2
Archaea2 - ps3
Archaea3 - ps4
Asgard/Eukaryota - ps5
Eukaryota - ps6
Amorpheae - ps7
Obazoa - ps8
Opisthokonta - ps9
Holozoa - ps10
Filozoa - ps11
Choano./Metazoa - ps12
Metazoa - ps13
Myriazoa - ps14
Parahoxozoa - ps15
Bilateria - ps16
Deuterostomia - ps17
Chordata - ps18
Olfactores - ps19
Vertebrata - ps20
Gnathostomata - ps21
Euteleostomi - ps22
Sarcopterygii - ps23
Tetrapoda - ps24
Amniota - ps25
Mammalia - ps26
Theria - ps27
Placentalia - ps28
Boreoeutheria - ps29
Euarchontoglires - ps30
Euarchonta - ps31
Primates - ps32
Haplorrhini - ps33
Simiiformes - ps34
Catarrhini - ps35
Hominoidea - ps36
Hominidae - ps37
Homininae - ps38
Hominini - ps39
Homo_sapiens - ps40
p-value
1e-06
2e-05
3e-04
4e-03
7e-02
Log-odds
0.5
1.0
1.5
2.0
2.5
3.0
3.5
4.0

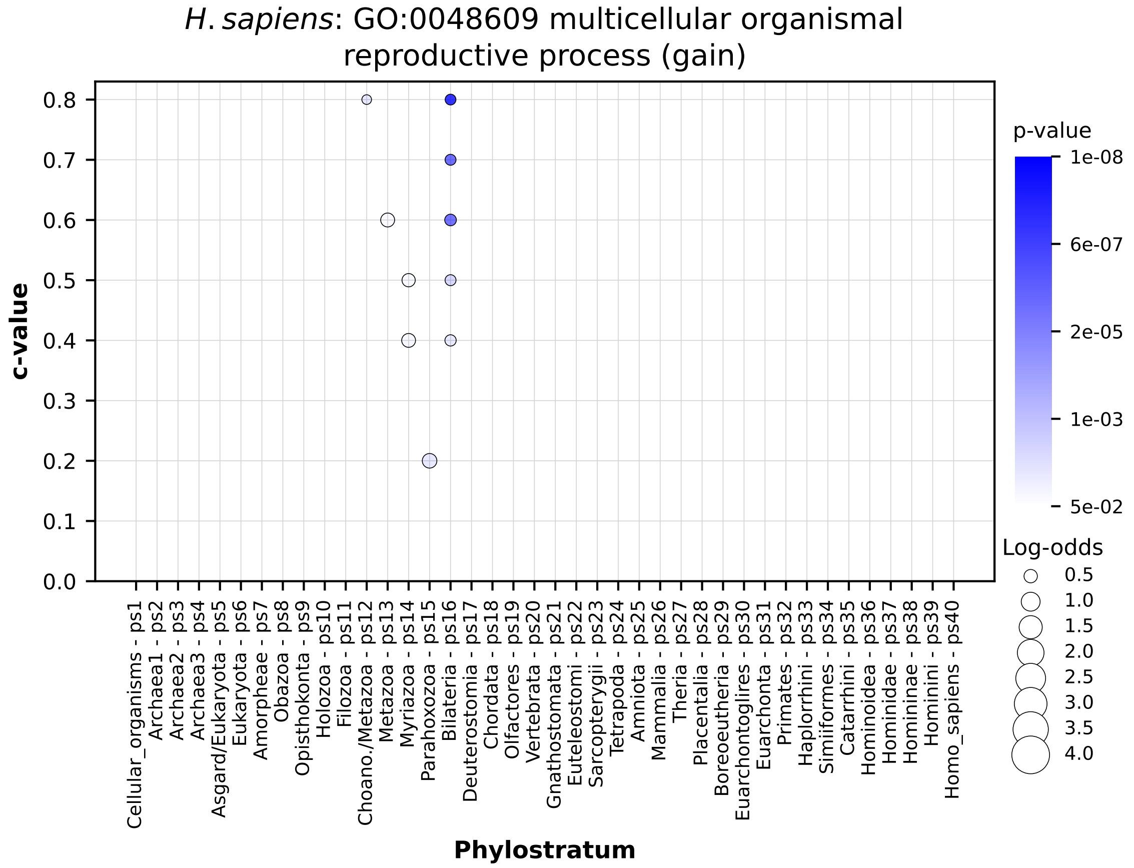
*H. sapiens*: GO:0048609 multicellular organismal reproductive process (gain)
c-value
0.0
0.1
0.2
0.3
0.4
0.5
0.6
0.7
0.8
Phylostratum
Cellular_organisms - ps1
Archaea1 - ps2
Archaea2 - ps3
Archaea3 - ps4
Asgard/Eukaryota - ps5
Eukaryota - ps6
Amorpheae - ps7
Obazoa - ps8
Opisthokonta - ps9
Holozoa - ps10
Filozoa - ps11
Choano./Metazoa - ps12
Metazoa - ps13
Myriazoa - ps14
Parahoxozoa - ps15
Bilateria - ps16
Deuterostomia - ps17
Chordata - ps18
Olfactores - ps19
Vertebrata - ps20
Gnathostomata - ps21
Euteleostomi - ps22
Sarcopterygii - ps23
Tetrapoda - ps24
Amniota - ps25
Mammalia - ps26
Theria - ps27
Placentalia - ps28
Boreoeutheria - ps29
Euarchontoglires - ps30
Euarchonta - ps31
Primates - ps32
Haplorrhini - ps33
Simiiformes - ps34
Catarrhini - ps35
Hominoidea - ps36
Hominidae - ps37
Homininae - ps38
Hominini - ps39
Homo_sapiens - ps40
p-value
1e-08
6e-07
2e-05
1e-03
5e-02
Log-odds
0.5
1.0
1.5
2.0
2.5
3.0
3.5
4.0

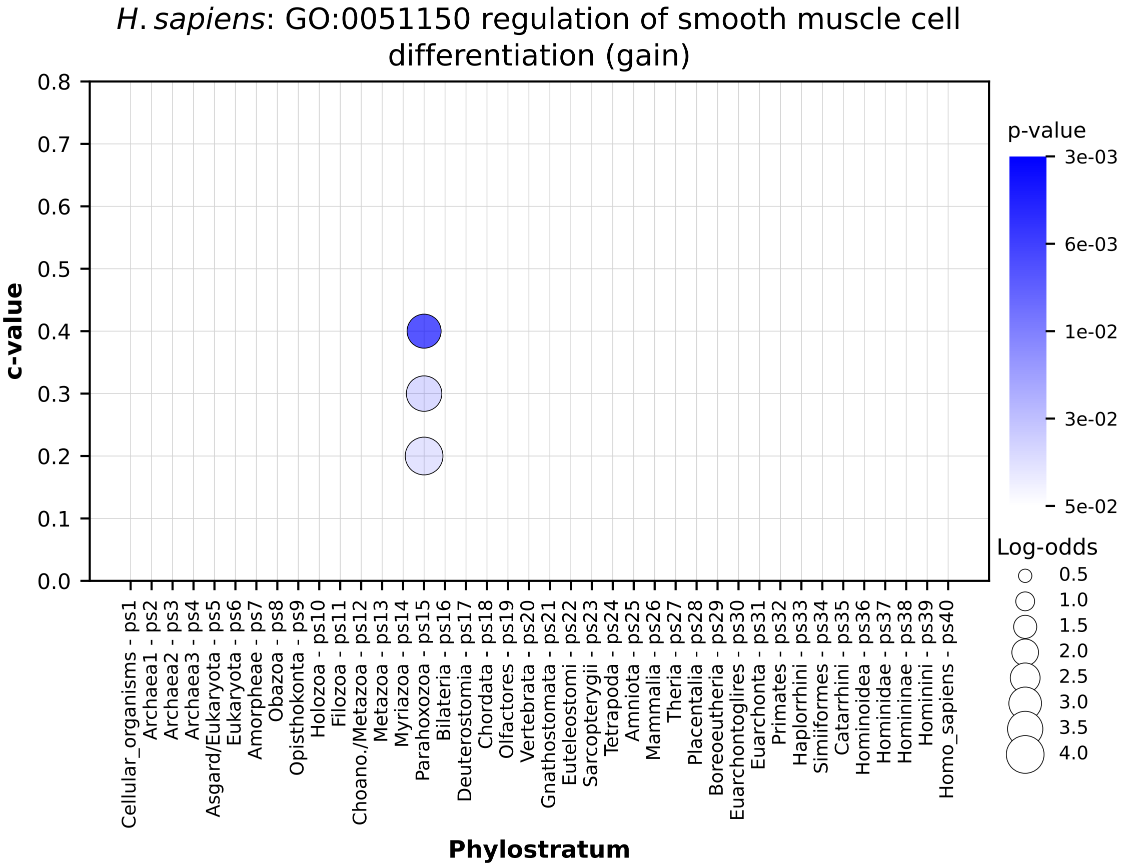
*H. sapiens*: GO:0051150 regulation of smooth muscle cell differentiation (gain)
c-value
0.8
0.7
0.6
0.5
0.4
0.3
0.2
0.1
0.0
p-value
3e-03
6e-03
1e-02
3e-02
5e-02
Log-odds
0.5
1.0
1.5
2.0
2.5
3.0
3.5
4.0
Cellular_organisms - ps1
Archaea1 - ps2
Archaea2 - ps3
Archaea3 - ps4
Asgard/Eukaryota - ps5
Eukaryota - ps6
Amorpheae - ps7
Obazoa - ps8
Opisthokonta - ps9
Holozoa - ps10
Filozoa - ps11
Choano./Metazoa - ps12
Metazoa - ps13
Myriazoa - ps14
Parahoxozoa - ps15
Bilateria - ps16
Deuterostomia - ps17
Chordata - ps18
Olfactores - ps19
Vertebrata - ps20
Gnathostomata - ps21
Euteleostomi - ps22
Sarcopterygii - ps23
Tetrapoda - ps24
Amniota - ps25
Mammalia - ps26
Theria - ps27
Placentalia - ps28
Boreoeutheria - ps29
Euarchontoglires - ps30
Euarchonta - ps31
Primates - ps32
Haplorrhini - ps33
Simiiformes - ps34
Catarrhini - ps35
Hominoidea - ps36
Hominidae - ps37
Homininae - ps38
Hominini - ps39
Homo_sapiens - ps40
Phylostratum

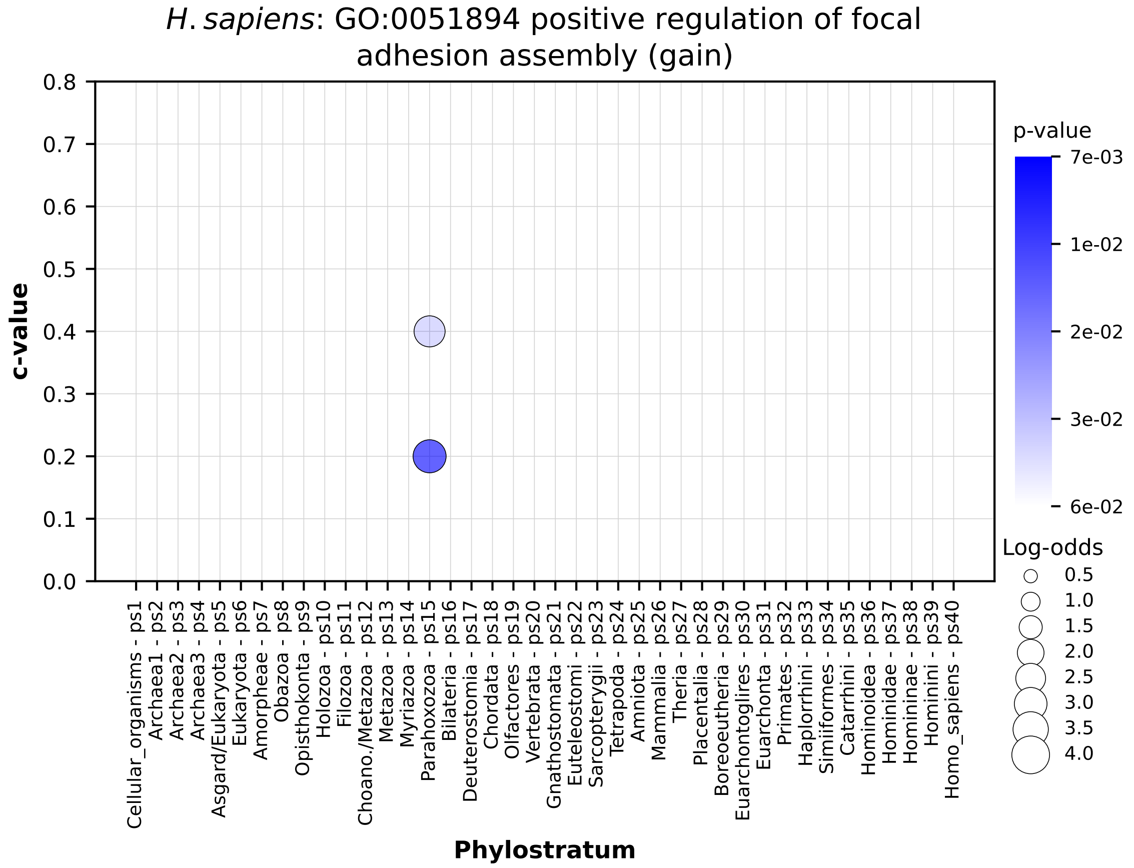

H. sapiens: GO:0051894 positive regulation of focal adhesion assembly (gain)
c-value
0.8
0.7
0.6
0.5
0.4
0.3
0.2
0.1
0.0
p-value
7e-03
1e-02
2e-02
3e-02
6e-02
Log-odds
0.5
1.0
1.5
2.0
2.5
3.0
3.5
4.0
Cellular_organisms - ps1
Archaea1 - ps2
Archaea2 - ps3
Archaea3 - ps4
Asgard/Eukaryota - ps5
Eukaryota - ps6
Amorpheae - ps7
Obazoa - ps8
Opisthokonta - ps9
Holozoa - ps10
Filozoa - ps11
Choano./Metazoa - ps12
Metazoa - ps13
Myriazoa - ps14
Parahoxozoa - ps15
Bilateria - ps16
Deuterostomia - ps17
Chordata - ps18
Olfactores - ps19
Vertebrata - ps20
Gnathostomata - ps21
Euteleostomi - ps22
Sarcopterygii - ps23
Tetrapoda - ps24
Amniota - ps25
Mammalia - ps26
Theria - ps27
Placentalia - ps28
Boreoeutheria - ps29
Euarchontoglires - ps30
Euarchonta - ps31
Primates - ps32
Haplorrhini - ps33
Simiiformes - ps34
Catarrhini - ps35
Hominoidea - ps36
Hominidae - ps37
Homininae - ps38
Hominini - ps39
Homo_sapiens - ps40
Phylostratum

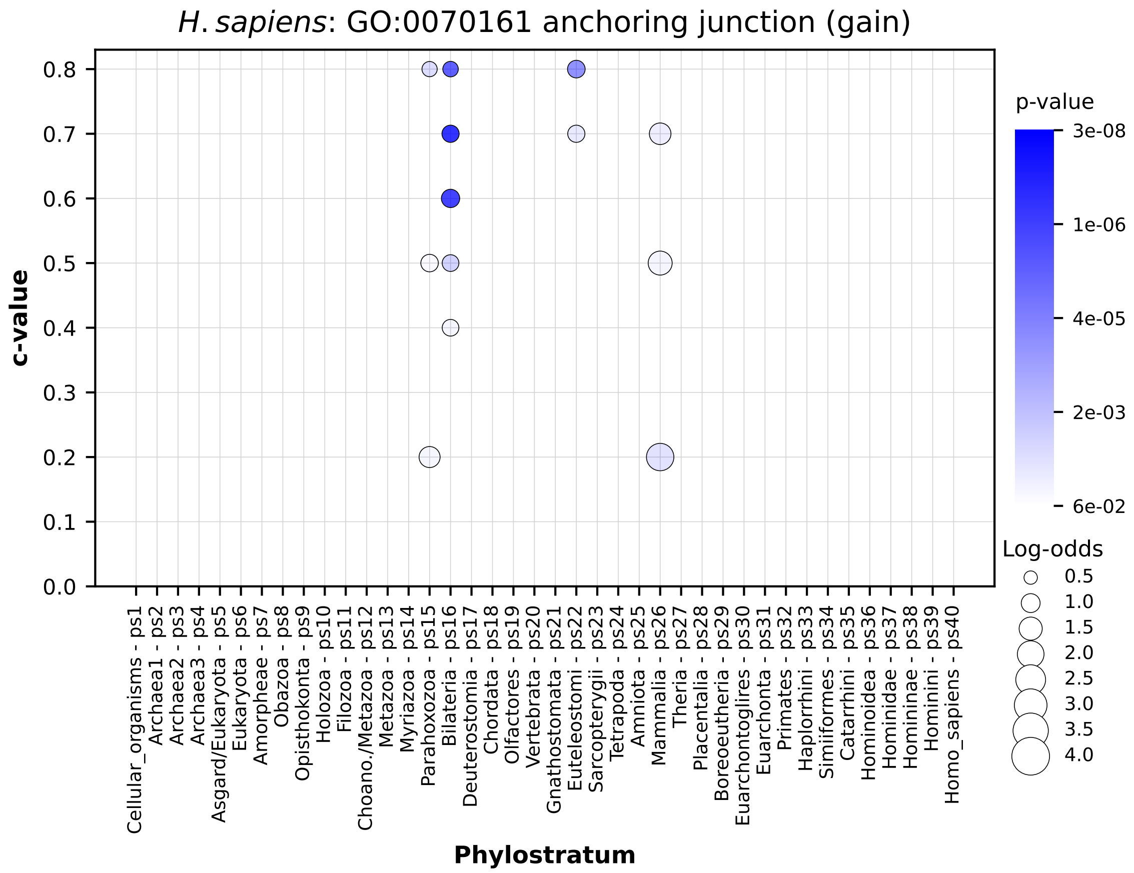

H. sapiens: GO:0070161 anchoring junction (gain)
c-value
0.0
0.1
0.2
0.3
0.4
0.5
0.6
0.7
0.8
Phylostratum
Cellular_organisms - ps1
Archaea1 - ps2
Archaea2 - ps3
Archaea3 - ps4
Asgard/Eukaryota - ps5
Eukaryota - ps6
Amorpheae - ps7
Obazoa - ps8
Opisthokonta - ps9
Holozoa - ps10
Filozoa - ps11
Choano./Metazoa - ps12
Metazoa - ps13
Myriazoa - ps14
Parahoxozoa - ps15
Bilateria - ps16
Deuterostomia - ps17
Chordata - ps18
Olfactores - ps19
Vertebrata - ps20
Gnathostomata - ps21
Euteleostomi - ps22
Sarcopterygii - ps23
Tetrapoda - ps24
Amniota - ps25
Mammalia - ps26
Theria - ps27
Placentalia - ps28
Boreoeutheria - ps29
Euarchontoglires - ps30
Euarchonta - ps31
Primates - ps32
Haplorrhini - ps33
Simiiformes - ps34
Catarrhini - ps35
Hominoidea - ps36
Hominidae - ps37
Homininae - ps38
Hominini - ps39
Homo_sapiens - ps40
p-value
3e-08
1e-06
4e-05
2e-03
6e-02
Log-odds
0.5
1.0
1.5
2.0
2.5
3.0
3.5
4.0

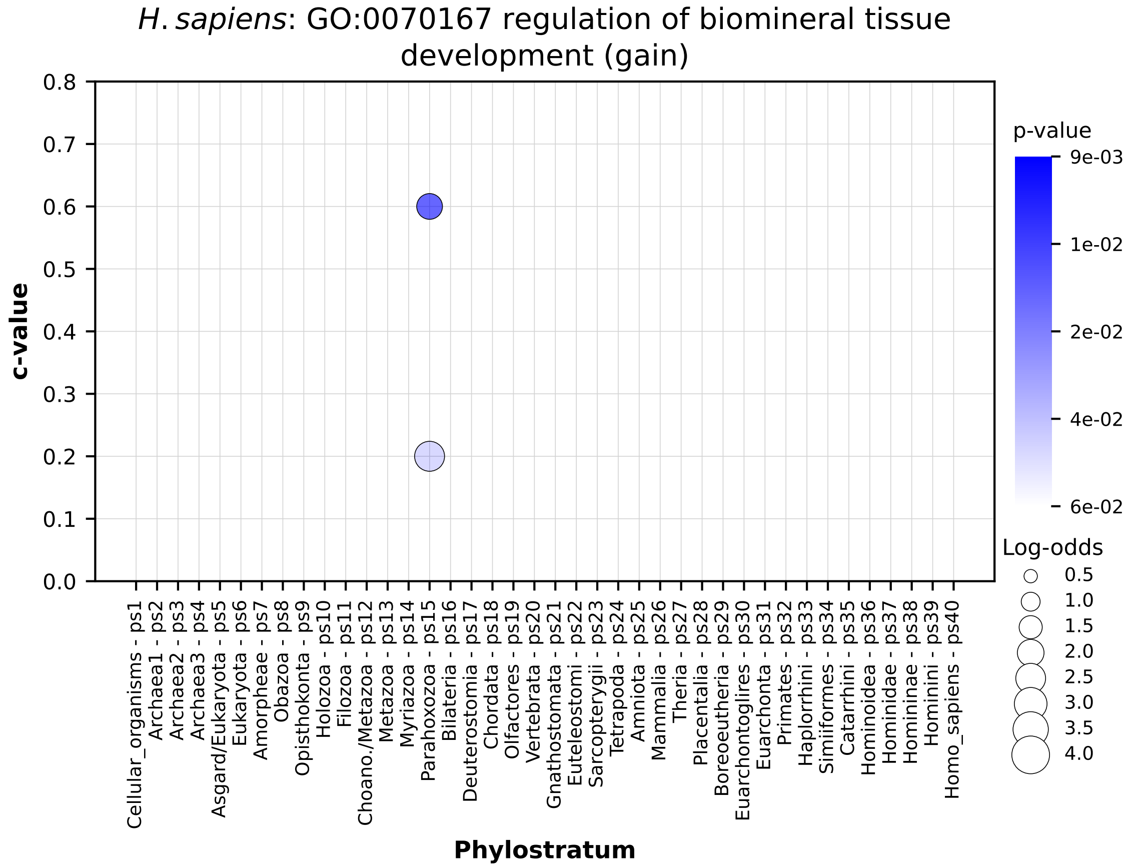

H. sapiens: GO:0070167 regulation of biomineral tissue development (gain)
c-value
0.0
0.1
0.2
0.3
0.4
0.5
0.6
0.7
0.8
Phylostratum
Cellular_organisms - ps1
Archaea1 - ps2
Archaea2 - ps3
Archaea3 - ps4
Asgard/Eukaryota - ps5
Eukaryota - ps6
Amorpheae - ps7
Obazoa - ps8
Opisthokonta - ps9
Holozoa - ps10
Filozoa - ps11
Choano./Metazoa - ps12
Metazoa - ps13
Myriazoa - ps14
Parahoxozoa - ps15
Bilateria - ps16
Deuterostomia - ps17
Chordata - ps18
Olfactores - ps19
Vertebrata - ps20
Gnathostomata - ps21
Euteleostomi - ps22
Sarcopterygii - ps23
Tetrapoda - ps24
Amniota - ps25
Mammalia - ps26
Theria - ps27
Placentalia - ps28
Boreoeutheria - ps29
Euarchontoglires - ps30
Euarchonta - ps31
Primates - ps32
Haplorrhini - ps33
Simiiformes - ps34
Catarrhini - ps35
Hominoidea - ps36
Hominidae - ps37
Homininae - ps38
Hominini - ps39
Homo_sapiens - ps40
p-value
9e-03
1e-02
2e-02
4e-02
6e-02
Log-odds
0.5
1.0
1.5
2.0
2.5
3.0
3.5
4.0

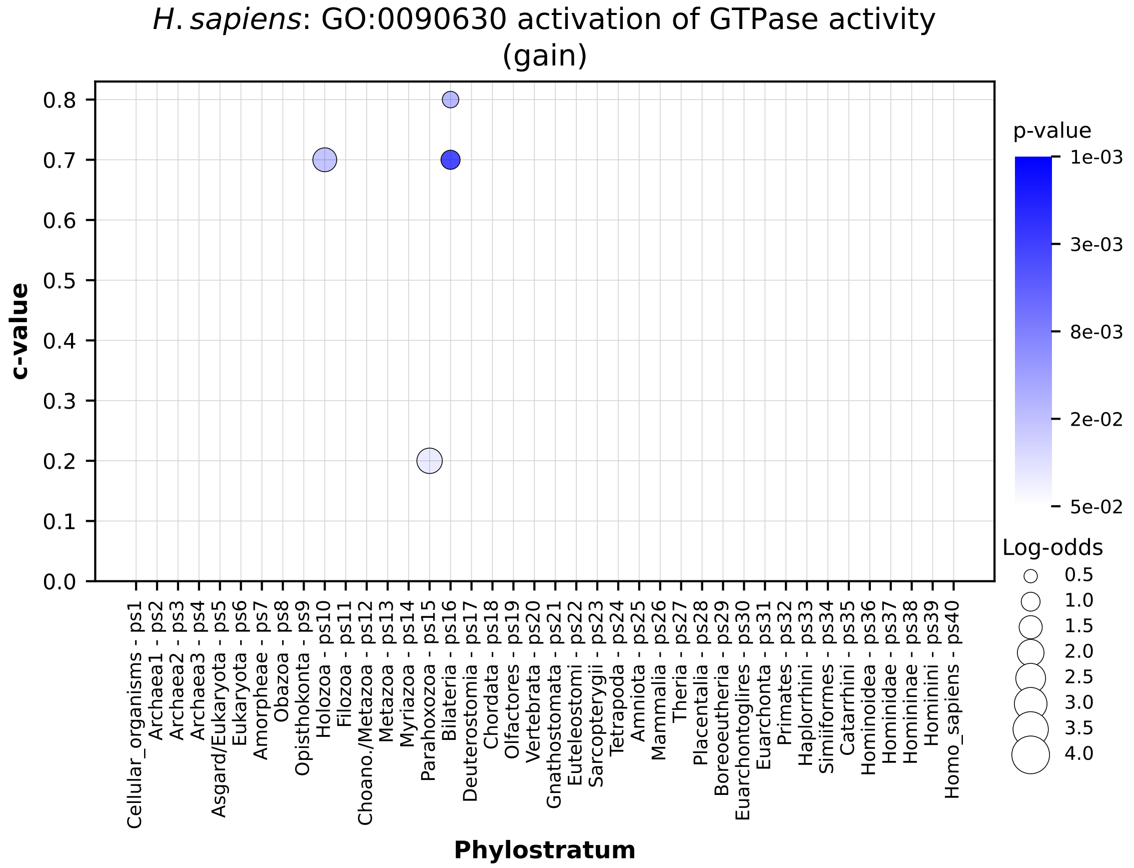
H. sapiens: GO:0090630 activation of GTPase activity
(gain)
c-value
0.0
0.1
0.2
0.3
0.4
0.5
0.6
0.7
0.8
Phylostratum
Cellular_organisms - ps1
Archaea1 - ps2
Archaea2 - ps3
Archaea3 - ps4
Asgard/Eukaryota - ps5
Eukaryota - ps6
Amorpheae - ps7
Obazoa - ps8
Opisthokonta - ps9
Holozoa - ps10
Filozoa - ps11
Choano./Metazoa - ps12
Metazoa - ps13
Myriazoa - ps14
Parahoxozoa - ps15
Bilateria - ps16
Deuterostomia - ps17
Chordata - ps18
Olfactores - ps19
Vertebrata - ps20
Gnathostomata - ps21
Euteleostomi - ps22
Sarcopterygii - ps23
Tetrapoda - ps24
Amniota - ps25
Mammalia - ps26
Theria - ps27
Placentalia - ps28
Boreoeutheria - ps29
Euarchontoglires - ps30
Euarchonta - ps31
Primates - ps32
Haplorrhini - ps33
Simiiformes - ps34
Catarrhini - ps35
Hominoidea - ps36
Hominidae - ps37
Homininae - ps38
Hominini - ps39
Homo_sapiens - ps40
p-value
1e-03
3e-03
8e-03
2e-02
5e-02
Log-odds
0.5
1.0
1.5
2.0
2.5
3.0
3.5
4.0

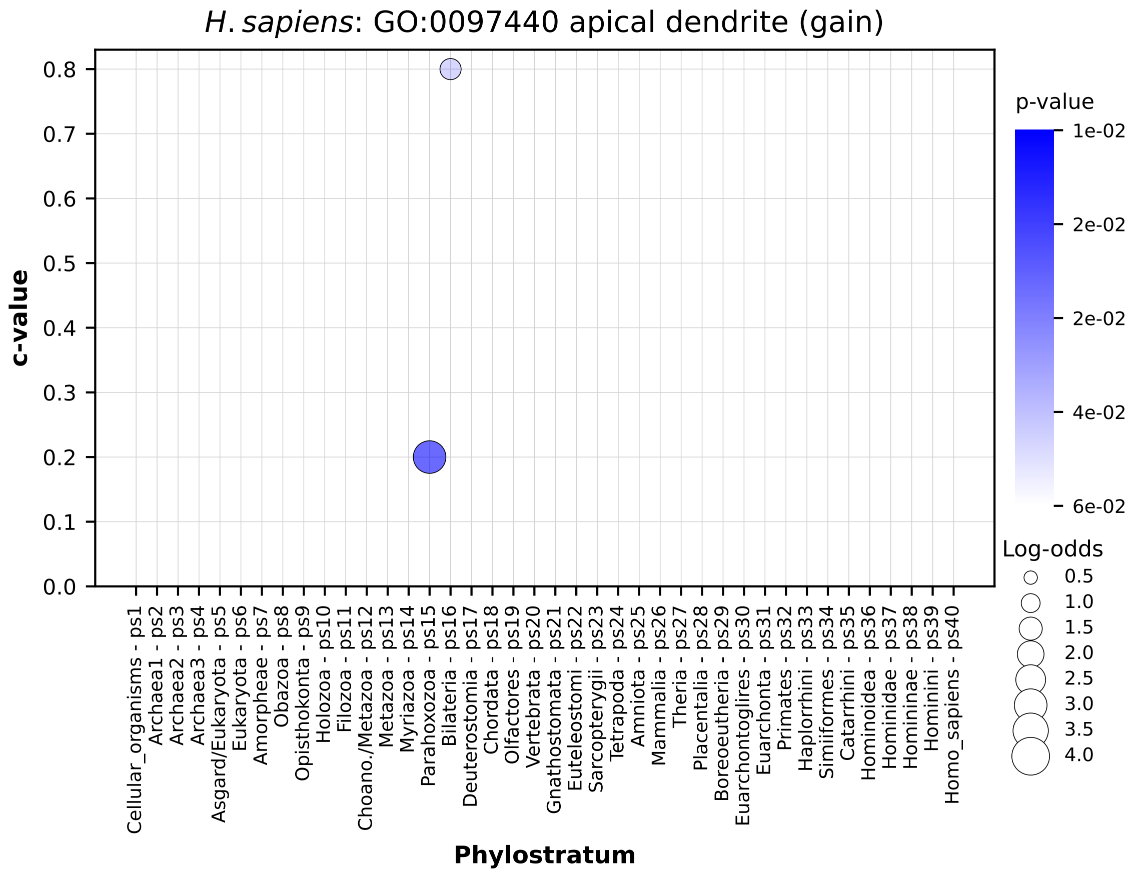

H. sapiens: GO:0097440 apical dendrite (gain)
c-value
0.0
0.1
0.2
0.3
0.4
0.5
0.6
0.7
0.8
p-value
1e-02
2e-02
2e-02
4e-02
6e-02
Log-odds
0.5
1.0
1.5
2.0
2.5
3.0
3.5
4.0
Cellular_organisms - ps1
Archaea1 - ps2
Archaea2 - ps3
Archaea3 - ps4
Asgard/Eukaryota - ps5
Eukaryota - ps6
Amorpheae - ps7
Obazoa - ps8
Opisthokonta - ps9
Holozoa - ps10
Filozoa - ps11
Choano./Metazoa - ps12
Metazoa - ps13
Myriazoa - ps14
Parahoxozoa - ps15
Bilateria - ps16
Deuterostomia - ps17
Chordata - ps18
Olfactores - ps19
Vertebrata - ps20
Gnathostomata - ps21
Euteleostomi - ps22
Sarcopterygii - ps23
Tetrapoda - ps24
Amniota - ps25
Mammalia - ps26
Theria - ps27
Placentalia - ps28
Boreoeutheria - ps29
Euarchontoglires - ps30
Euarchonta - ps31
Primates - ps32
Haplorrhini - ps33
Simiiformes - ps34
Catarrhini - ps35
Hominoidea - ps36
Hominidae - ps37
Homininae - ps38
Hominini - ps39
Homo_sapiens - ps40
Phylostratum

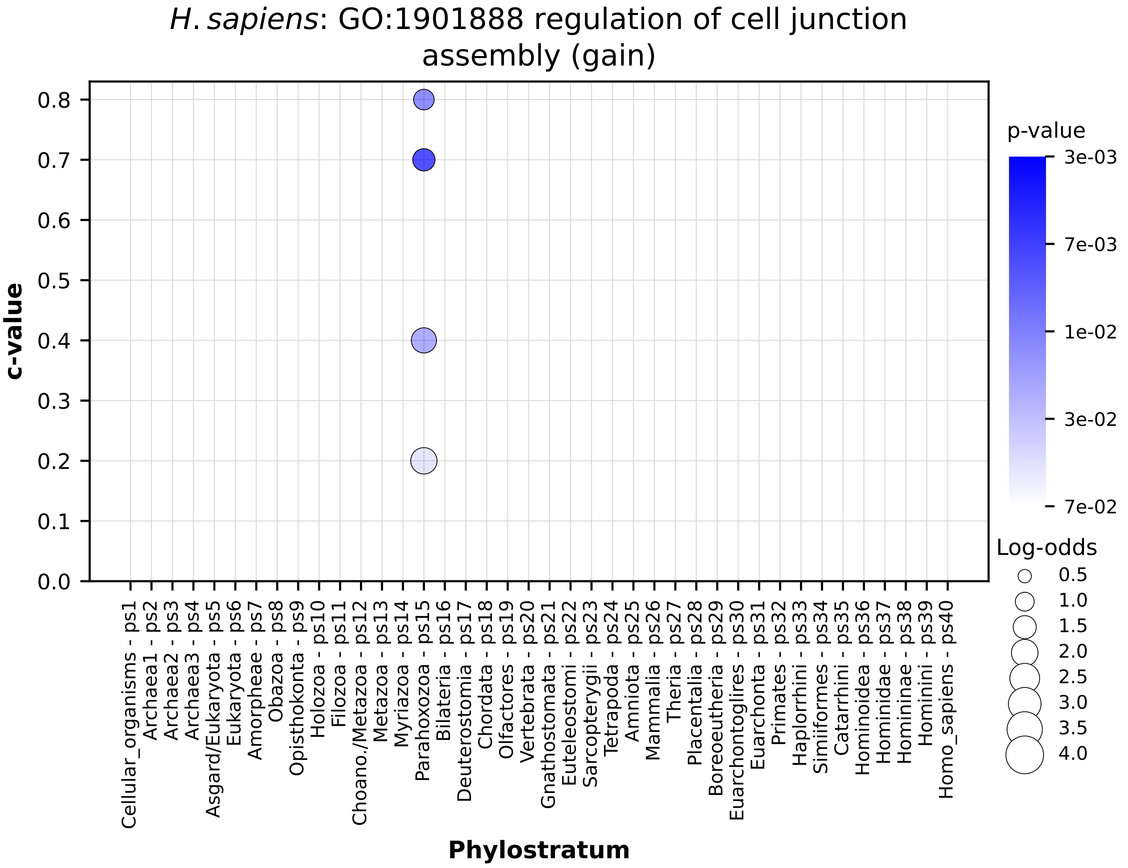

H. sapiens: GO:1901888 regulation of cell junction assembly (gain)
c-value
0.0
0.1
0.2
0.3
0.4
0.5
0.6
0.7
0.8
Phylostratum
Cellular_organisms - ps1
Archaea1 - ps2
Archaea2 - ps3
Archaea3 - ps4
Asgard/Eukaryota - ps5
Eukaryota - ps6
Amorpheae - ps7
Obazoa - ps8
Opisthokonta - ps9
Holozoa - ps10
Filozoa - ps11
Choano./Metazoa - ps12
Metazoa - ps13
Myriazoa - ps14
Parahoxozoa - ps15
Bilateria - ps16
Deuterostomia - ps17
Chordata - ps18
Olfactores - ps19
Vertebrata - ps20
Gnathostomata - ps21
Euteleostomi - ps22
Sarcopterygii - ps23
Tetrapoda - ps24
Amniota - ps25
Mammalia - ps26
Theria - ps27
Placentalia - ps28
Boreoeutheria - ps29
Euarchontoglires - ps30
Euarchonta - ps31
Primates - ps32
Haplorrhini - ps33
Simiiformes - ps34
Catarrhini - ps35
Hominoidea - ps36
Hominidae - ps37
Homininae - ps38
Hominini - ps39
Homo_sapiens - ps40
p-value
3e-03
7e-03
1e-02
3e-02
7e-02
Log-odds
0.5
1.0
1.5
2.0
2.5
3.0
3.5
4.0

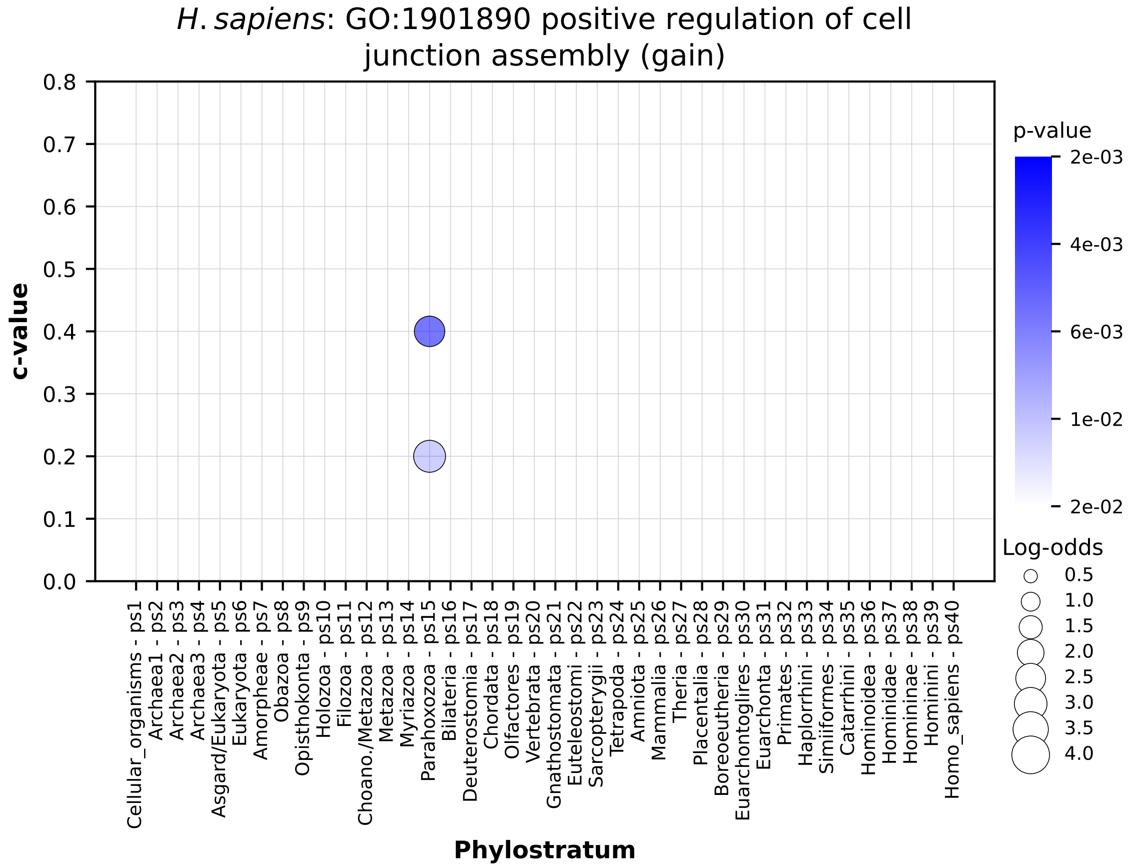

H. sapiens: GO:1901890 positive regulation of cell junction assembly (gain)
c-value
0.0
0.1
0.2
0.3
0.4
0.5
0.6
0.7
0.8
Phylostratum
Cellular_organisms - ps1
Archaea1 - ps2
Archaea2 - ps3
Archaea3 - ps4
Asgard/Eukaryota - ps5
Eukaryota - ps6
Amorpheae - ps7
Obazoa - ps8
Opisthokonta - ps9
Holozoa - ps10
Filozoa - ps11
Choano./Metazoa - ps12
Metazoa - ps13
Myriazoa - ps14
Parahoxozoa - ps15
Bilateria - ps16
Deuterostomia - ps17
Chordata - ps18
Olfactores - ps19
Vertebrata - ps20
Gnathostomata - ps21
Euteleostomi - ps22
Sarcopterygii - ps23
Tetrapoda - ps24
Amniota - ps25
Mammalia - ps26
Theria - ps27
Placentalia - ps28
Boreoeutheria - ps29
Euarchontoglires - ps30
Euarchonta - ps31
Primates - ps32
Haplorrhini - ps33
Simiiformes - ps34
Catarrhini - ps35
Hominoidea - ps36
Hominidae - ps37
Homininae - ps38
Hominini - ps39
Homo_sapiens - ps40
p-value
2e-03
4e-03
6e-03
1e-02
2e-02
Log-odds
0.5
1.0
1.5
2.0
2.5
3.0
3.5
4.0

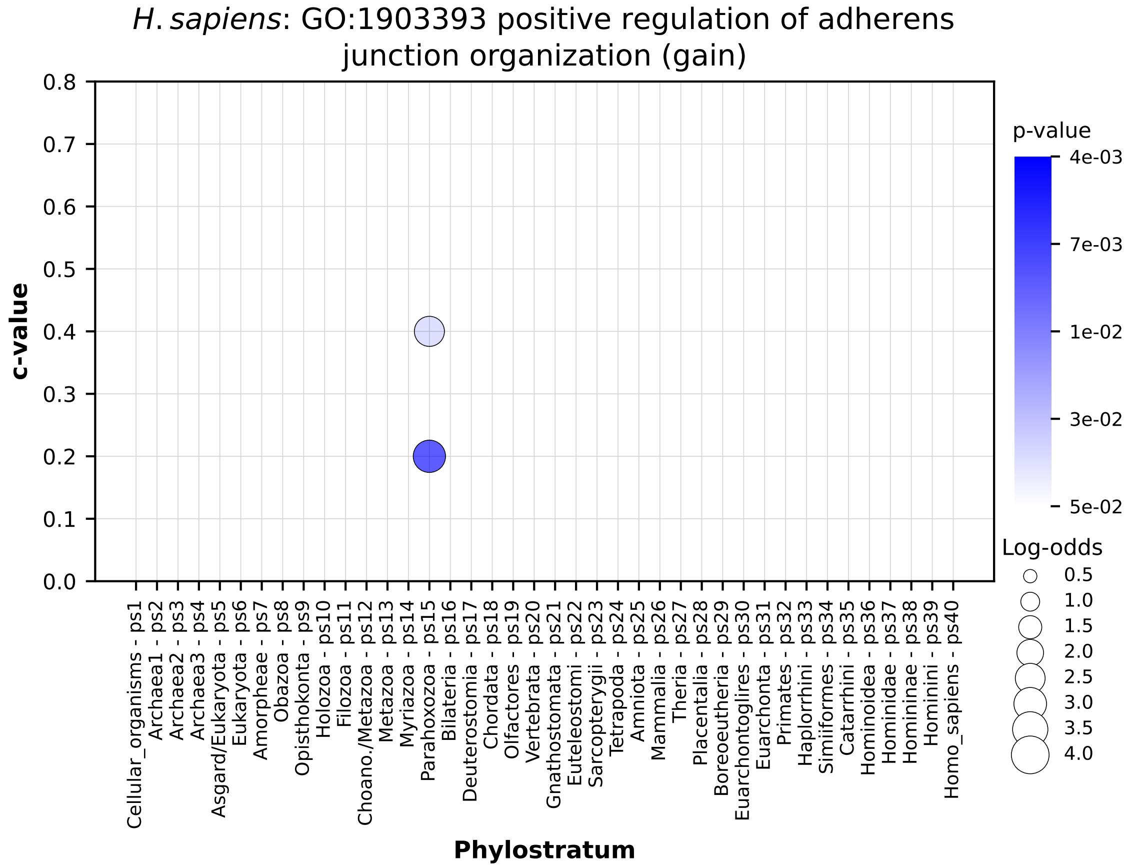

H. sapiens: GO:1903393 positive regulation of adherens junction organization (gain)
c-value
0.8
0.7
0.6
0.5
0.4
0.3
0.2
0.1
0.0
p-value
4e-03
7e-03
1e-02
3e-02
5e-02
Log-odds
0.5
1.0
1.5
2.0
2.5
3.0
3.5
4.0
Cellular_organisms - ps1
Archaea1 - ps2
Archaea2 - ps3
Archaea3 - ps4
Asgard/Eukaryota - ps5
Eukaryota - ps6
Amorpheae - ps7
Obazoa - ps8
Opisthokonta - ps9
Holozoa - ps10
Filozoa - ps11
Choano./Metazoa - ps12
Metazoa - ps13
Myriazoa - ps14
Parahoxozoa - ps15
Bilateria - ps16
Deuterostomia - ps17
Chordata - ps18
Olfactores - ps19
Vertebrata - ps20
Gnathostomata - ps21
Euteleostomi - ps22
Sarcopterygii - ps23
Tetrapoda - ps24
Amniota - ps25
Mammalia - ps26
Theria - ps27
Placentalia - ps28
Boreoeutheria - ps29
Euarchontoglires - ps30
Euarchonta - ps31
Primates - ps32
Haplorrhini - ps33
Simiiformes - ps34
Catarrhini - ps35
Hominoidea - ps36
Hominidae - ps37
Homininae - ps38
Hominini - ps39
Homo_sapiens - ps40
Phylostratum

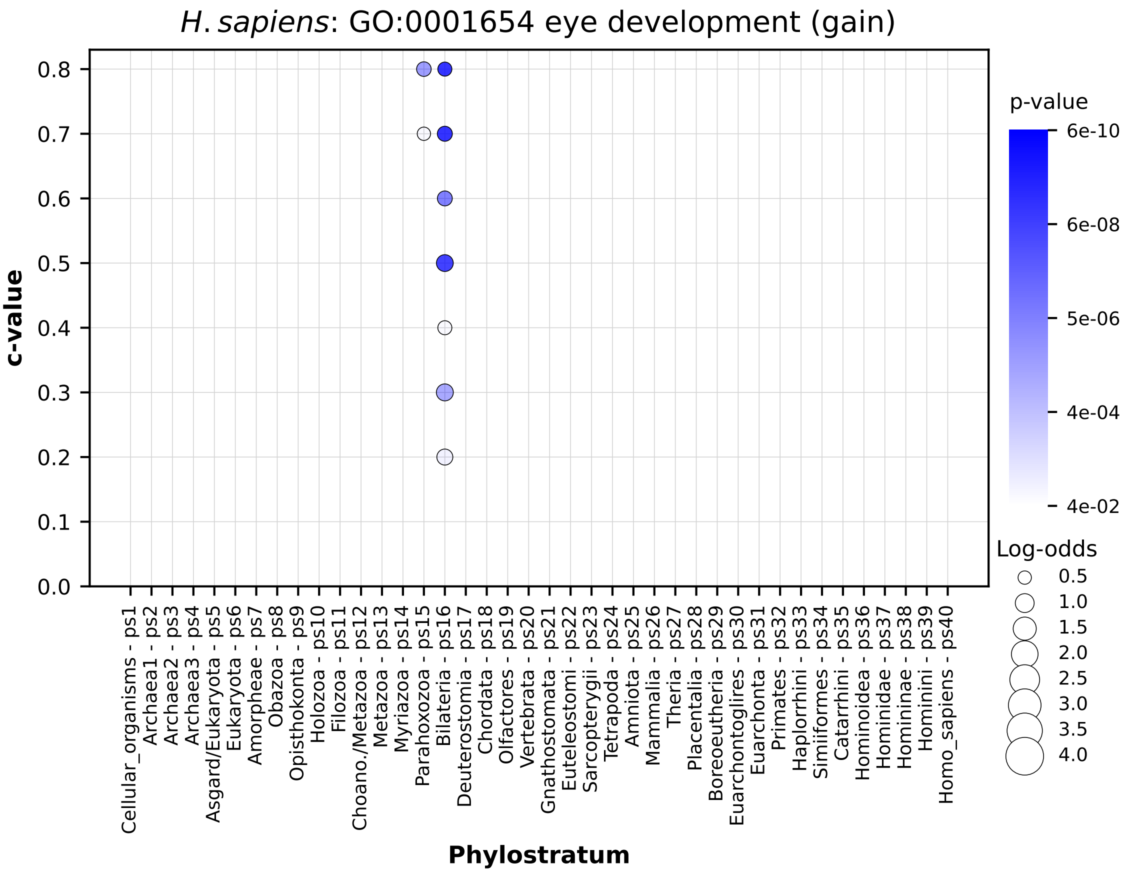

*H. sapiens*: GO:0001654 eye development (gain)
c-value
0.0
0.1
0.2
0.3
0.4
0.5
0.6
0.7
0.8
Phylostratum
Cellular_organisms - ps1
Archaea1 - ps2
Archaea2 - ps3
Archaea3 - ps4
Asgard/Eukaryota - ps5
Eukaryota - ps6
Amorpheae - ps7
Obazoa - ps8
Opisthokonta - ps9
Holozoa - ps10
Filozoa - ps11
Choano./Metazoa - ps12
Metazoa - ps13
Myriazoa - ps14
Parahoxozoa - ps15
Bilateria - ps16
Deuterostomia - ps17
Chordata - ps18
Olfactores - ps19
Vertebrata - ps20
Gnathostomata - ps21
Euteleostomi - ps22
Sarcopterygii - ps23
Tetrapoda - ps24
Amniota - ps25
Mammalia - ps26
Theria - ps27
Placentalia - ps28
Boreoeutheria - ps29
Euarchontoglires - ps30
Euarchonta - ps31
Primates - ps32
Haplorrhini - ps33
Simiiformes - ps34
Catarrhini - ps35
Hominoidea - ps36
Hominidae - ps37
Homininae - ps38
Hominini - ps39
Homo_sapiens - ps40
p-value
6e-10
6e-08
5e-06
4e-04
4e-02
Log-odds
0.5
1.0
1.5
2.0
2.5
3.0
3.5
4.0

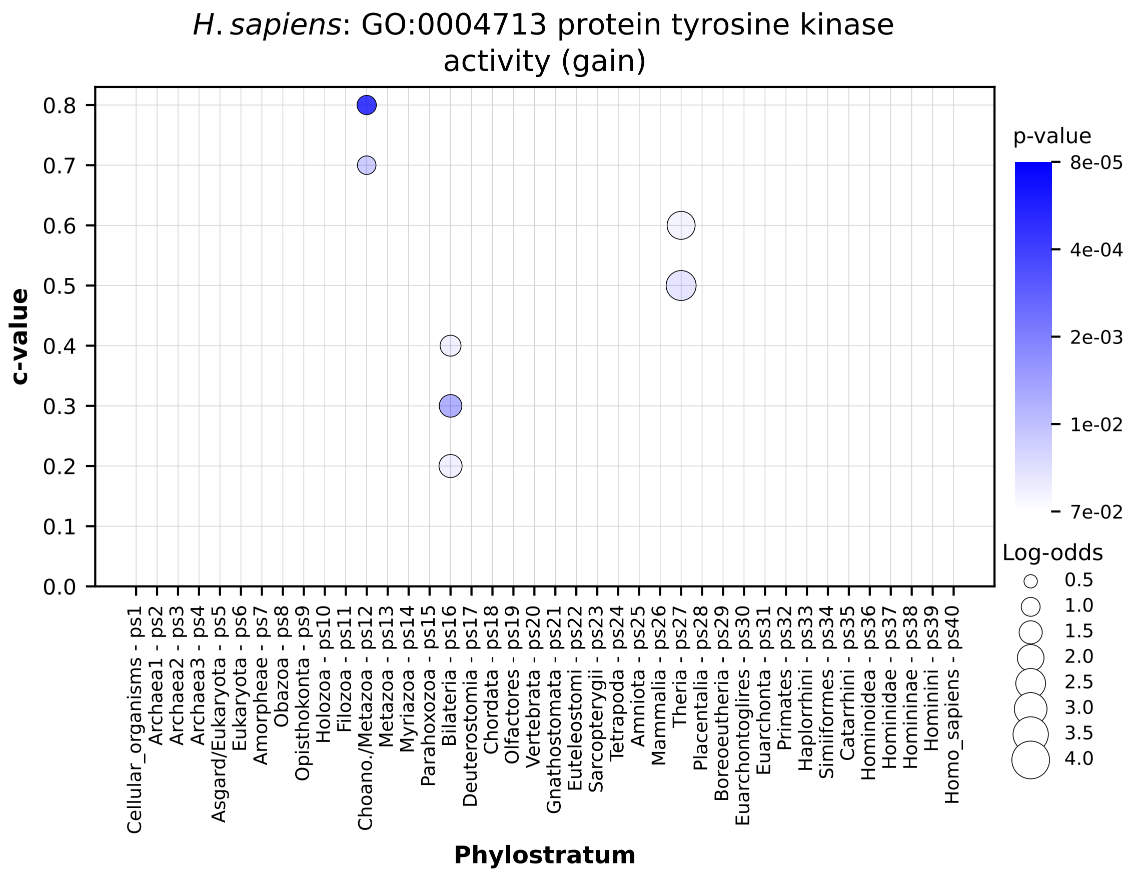

*H. sapiens*: GO:0004713 protein tyrosine kinase activity (gain)
c-value
0.0
0.1
0.2
0.3
0.4
0.5
0.6
0.7
0.8
Phylostratum
Cellular_organisms - ps1
Archaea1 - ps2
Archaea2 - ps3
Archaea3 - ps4
Asgard/Eukaryota - ps5
Eukaryota - ps6
Amorpheae - ps7
Obazoa - ps8
Opisthokonta - ps9
Holozoa - ps10
Filozoa - ps11
Choano./Metazoa - ps12
Metazoa - ps13
Myriazoa - ps14
Parahoxozoa - ps15
Bilateria - ps16
Deuterostomia - ps17
Chordata - ps18
Olfactores - ps19
Vertebrata - ps20
Gnathostomata - ps21
Euteleostomi - ps22
Sarcopterygii - ps23
Tetrapoda - ps24
Amniota - ps25
Mammalia - ps26
Theria - ps27
Placentalia - ps28
Boreoeutheria - ps29
Euarchontoglires - ps30
Euarchonta - ps31
Primates - ps32
Haplorrhini - ps33
Simiiformes - ps34
Catarrhini - ps35
Hominoidea - ps36
Hominidae - ps37
Homininae - ps38
Hominini - ps39
Homo_sapiens - ps40
p-value
8e-05
4e-04
2e-03
1e-02
7e-02
Log-odds
0.5
1.0
1.5
2.0
2.5
3.0
3.5
4.0

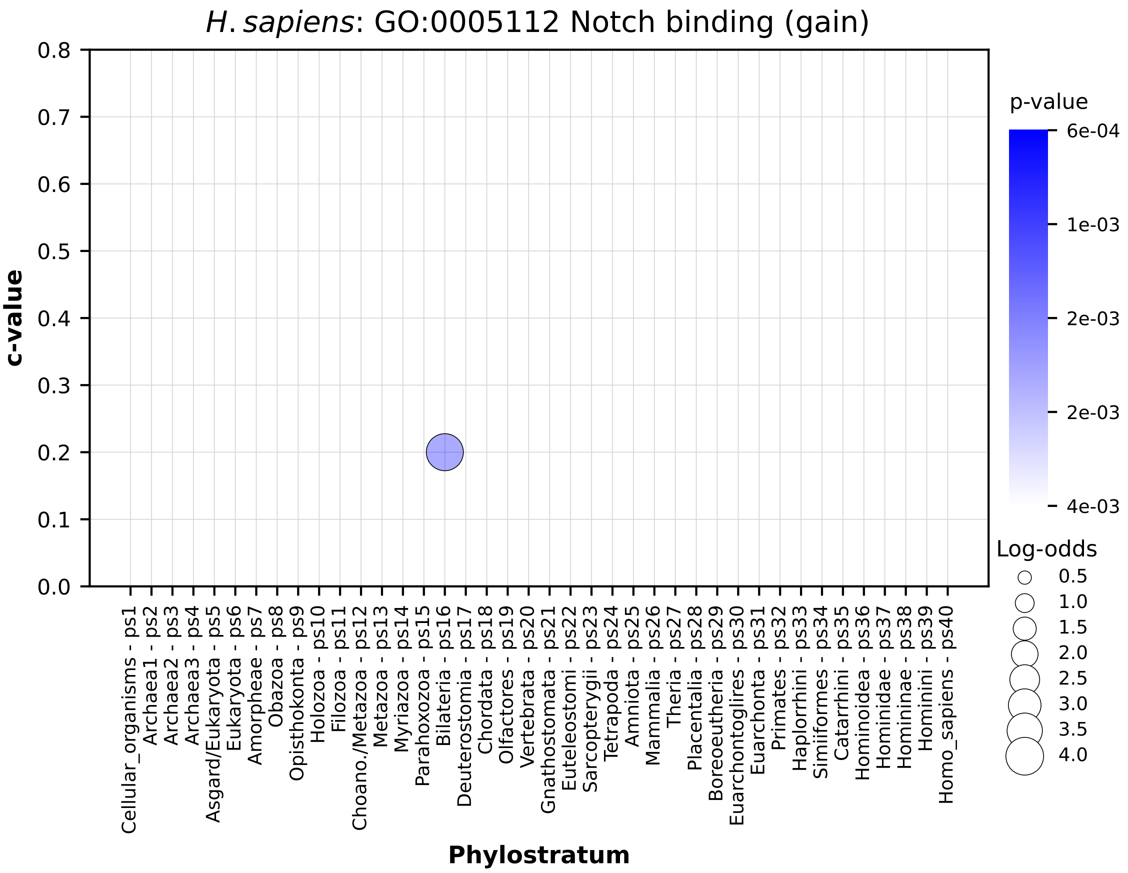

*H. sapiens*: GO:0005112 Notch binding (gain)
c-value
0.8
0.7
0.6
0.5
0.4
0.3
0.2
0.1
0.0
Phylostratum
Cellular_organisms - ps1
Archaea1 - ps2
Archaea2 - ps3
Archaea3 - ps4
Asgard/Eukaryota - ps5
Eukaryota - ps6
Amorpheae - ps7
Obazoa - ps8
Opisthokonta - ps9
Holozoa - ps10
Filozoa - ps11
Choano./Metazoa - ps12
Metazoa - ps13
Myriazoa - ps14
Parahoxozoa - ps15
Bilateria - ps16
Deuterostomia - ps17
Chordata - ps18
Olfactores - ps19
Vertebrata - ps20
Gnathostomata - ps21
Euteleostomi - ps22
Sarcopterygii - ps23
Tetrapoda - ps24
Amniota - ps25
Mammalia - ps26
Theria - ps27
Placentalia - ps28
Boreoeutheria - ps29
Euarchontoglires - ps30
Euarchonta - ps31
Primates - ps32
Haplorrhini - ps33
Simiiformes - ps34
Catarrhini - ps35
Hominoidea - ps36
Hominidae - ps37
Homininae - ps38
Hominini - ps39
Homo_sapiens - ps40
p-value
6e-04
1e-03
2e-03
2e-03
4e-03
Log-odds
0.5
1.0
1.5
2.0
2.5
3.0
3.5
4.0

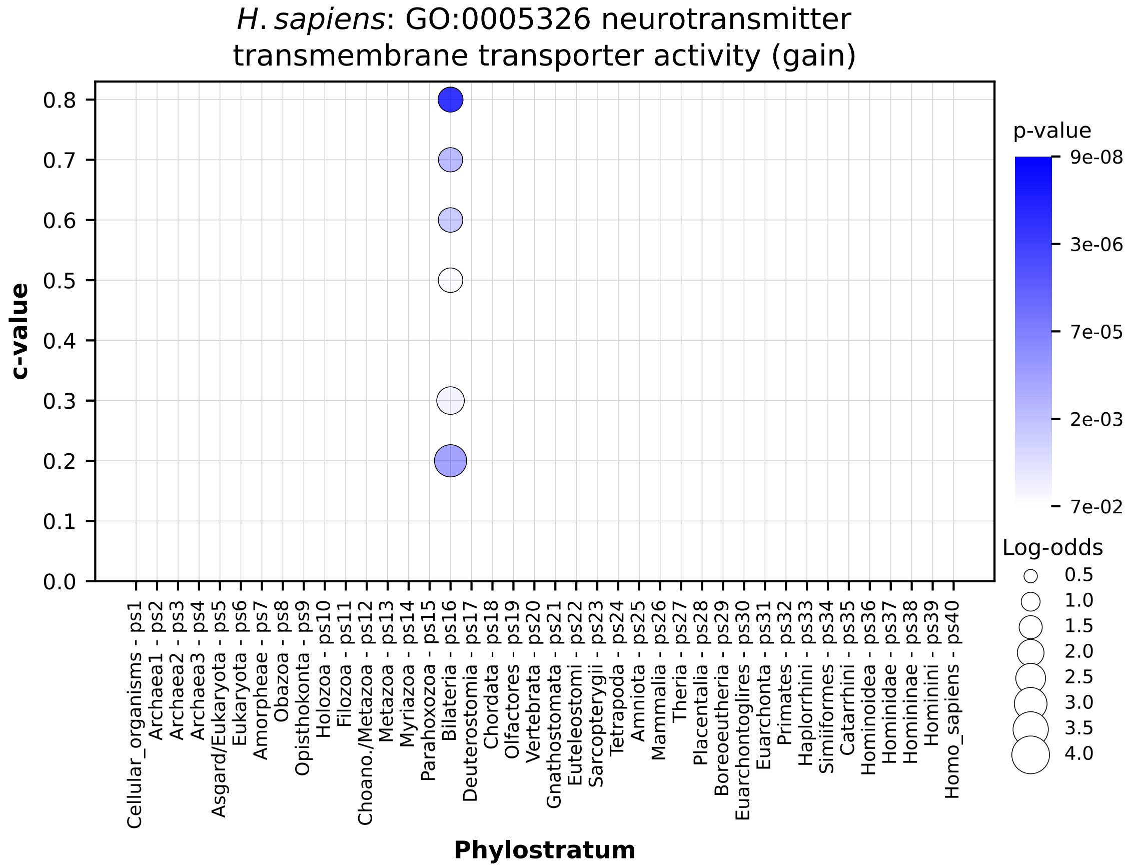

H. sapiens: GO:0005326 neurotransmitter transmembrane transporter activity (gain)
c-value
0.0
0.1
0.2
0.3
0.4
0.5
0.6
0.7
0.8
Phylostratum
Cellular_organisms - ps1
Archaea1 - ps2
Archaea2 - ps3
Archaea3 - ps4
Asgard/Eukaryota - ps5
Eukaryota - ps6
Amorpheae - ps7
Obazoa - ps8
Opisthokonta - ps9
Holozoa - ps10
Filozoa - ps11
Choano./Metazoa - ps12
Metazoa - ps13
Myriazoa - ps14
Parahoxozoa - ps15
Bilateria - ps16
Deuterostomia - ps17
Chordata - ps18
Olfactores - ps19
Vertebrata - ps20
Gnathostomata - ps21
Euteleostomi - ps22
Sarcopterygii - ps23
Tetrapoda - ps24
Amniota - ps25
Mammalia - ps26
Theria - ps27
Placentalia - ps28
Boreoeutheria - ps29
Euarchontoglires - ps30
Euarchonta - ps31
Primates - ps32
Haplorrhini - ps33
Simiiformes - ps34
Catarrhini - ps35
Hominoidea - ps36
Hominidae - ps37
Homininae - ps38
Hominini - ps39
Homo_sapiens - ps40
p-value
9e-08
3e-06
7e-05
2e-03
7e-02
Log-odds
0.5
1.0
1.5
2.0
2.5
3.0
3.5
4.0

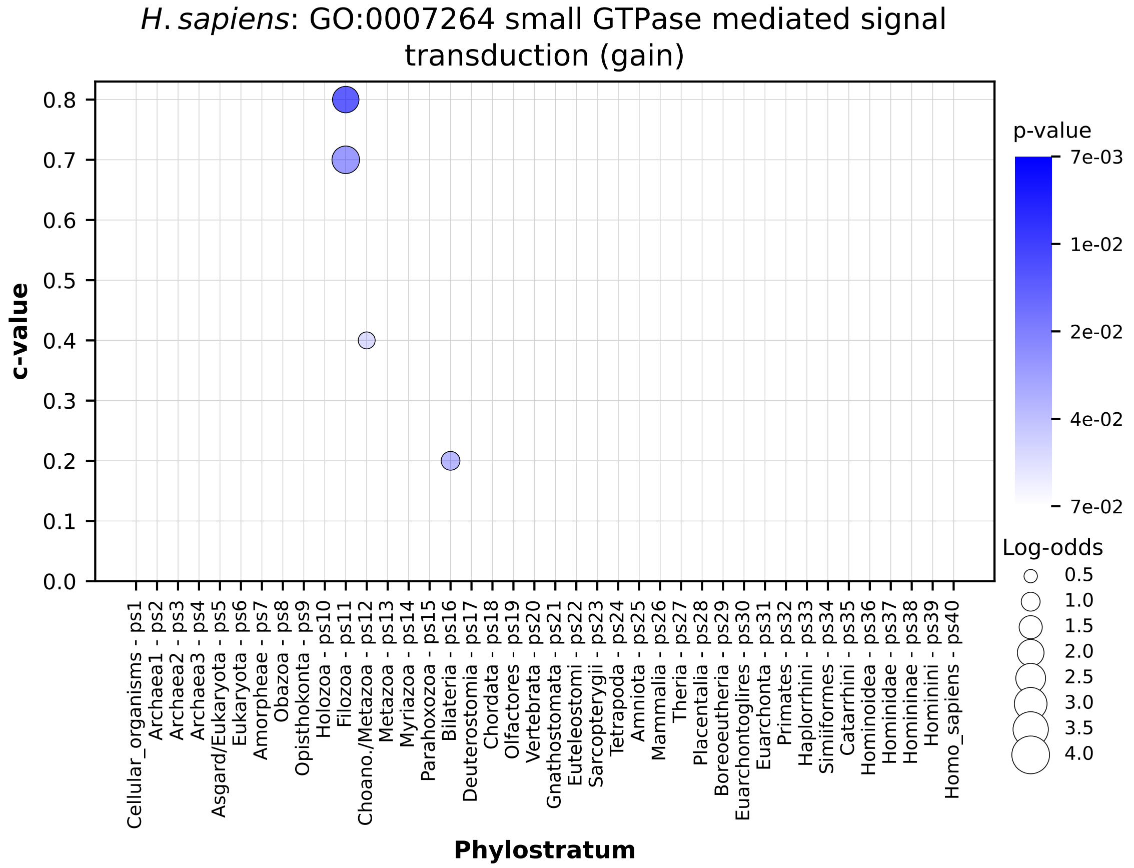
H. sapiens: GO:0007264 small GTPase mediated signal transduction (gain)
c-value
0.0
0.1
0.2
0.3
0.4
0.5
0.6
0.7
0.8
Phylostratum
Cellular_organisms - ps1
Archaea1 - ps2
Archaea2 - ps3
Archaea3 - ps4
Asgard/Eukaryota - ps5
Eukaryota - ps6
Amorpheae - ps7
Obazoa - ps8
Opisthokonta - ps9
Holozoa - ps10
Filozoa - ps11
Choano./Metazoa - ps12
Metazoa - ps13
Myriazoa - ps14
Parahoxozoa - ps15
Bilateria - ps16
Deuterostomia - ps17
Chordata - ps18
Olfactores - ps19
Vertebrata - ps20
Gnathostomata - ps21
Euteleostomi - ps22
Sarcopterygii - ps23
Tetrapoda - ps24
Amniota - ps25
Mammalia - ps26
Theria - ps27
Placentalia - ps28
Boreoeutheria - ps29
Euarchontoglires - ps30
Euarchonta - ps31
Primates - ps32
Haplorrhini - ps33
Simiiformes - ps34
Catarrhini - ps35
Hominoidea - ps36
Hominidae - ps37
Homininae - ps38
Hominini - ps39
Homo_sapiens - ps40
p-value
7e-03
1e-02
2e-02
4e-02
7e-02
Log-odds
0.5
1.0
1.5
2.0
2.5
3.0
3.5
4.0

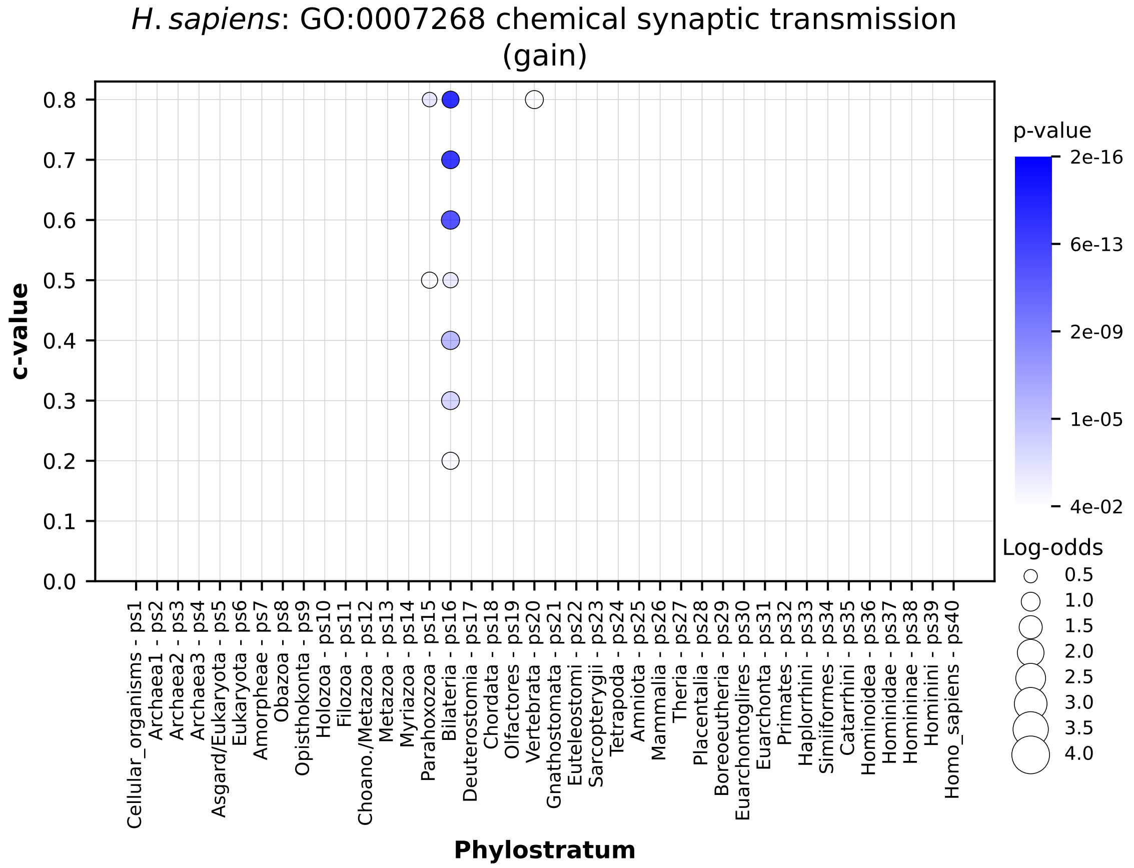

H. sapiens: GO:0007268 chemical synaptic transmission
(gain)
c-value
0.8
0.7
0.6
0.5
0.4
0.3
0.2
0.1
0.0
Phylostratum
Cellular_organisms - ps1
Archaea1 - ps2
Archaea2 - ps3
Archaea3 - ps4
Asgard/Eukaryota - ps5
Eukaryota - ps6
Amorpheae - ps7
Obazoa - ps8
Opisthokonta - ps9
Holozoa - ps10
Filozoa - ps11
Choano./Metazoa - ps12
Metazoa - ps13
Myriazoa - ps14
Parahoxozoa - ps15
Bilateria - ps16
Deuterostomia - ps17
Chordata - ps18
Olfactores - ps19
Vertebrata - ps20
Gnathostomata - ps21
Euteleostomi - ps22
Sarcopterygii - ps23
Tetrapoda - ps24
Amniota - ps25
Mammalia - ps26
Theria - ps27
Placentalia - ps28
Boreoeutheria - ps29
Euarchontoglires - ps30
Euarchonta - ps31
Primates - ps32
Haplorrhini - ps33
Simiiformes - ps34
Catarrhini - ps35
Hominoidea - ps36
Hominidae - ps37
Homininae - ps38
Hominini - ps39
Homo_sapiens - ps40
p-value
2e-16
6e-13
2e-09
1e-05
4e-02
Log-odds
0.5
1.0
1.5
2.0
2.5
3.0
3.5
4.0

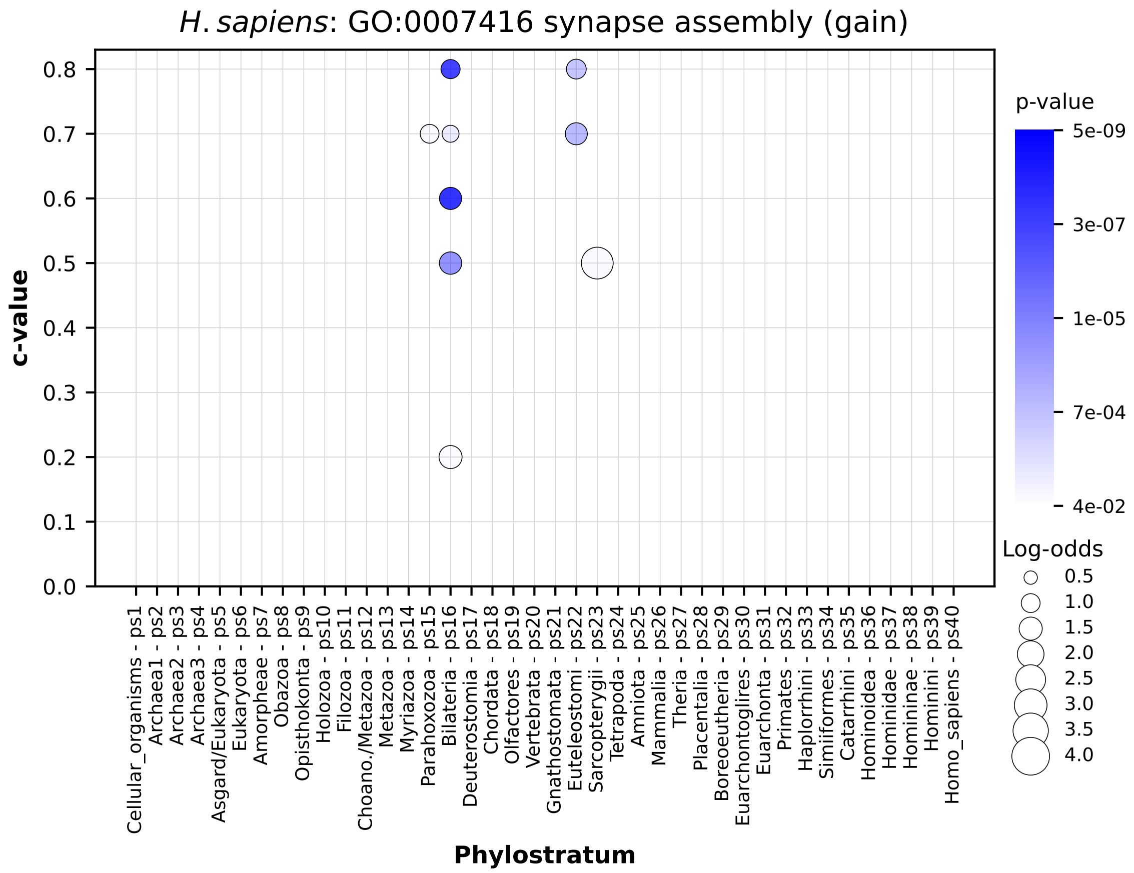
*H. sapiens*: GO:0007416 synapse assembly (gain)
c-value
Phylostratum
p-value
5e-09
3e-07
1e-05
7e-04
4e-02
Log-odds
0.5
1.0
1.5
2.0
2.5
3.0
3.5
4.0
Cellular_organisms - ps1
Archaea1 - ps2
Archaea2 - ps3
Archaea3 - ps4
Asgard/Eukaryota - ps5
Eukaryota - ps6
Amorpheae - ps7
Obazoa - ps8
Opisthokonta - ps9
Holozoa - ps10
Filozoa - ps11
Choano./Metazoa - ps12
Metazoa - ps13
Myriazoa - ps14
Parahoxozoa - ps15
Bilateria - ps16
Deuterostomia - ps17
Chordata - ps18
Olfactores - ps19
Vertebrata - ps20
Gnathostomata - ps21
Euteleostomi - ps22
Sarcopterygii - ps23
Tetrapoda - ps24
Amniota - ps25
Mammalia - ps26
Theria - ps27
Placentalia - ps28
Boreoeutheria - ps29
Euarchontoglires - ps30
Euarchonta - ps31
Primates - ps32
Haplorrhini - ps33
Simiiformes - ps34
Catarrhini - ps35
Hominoidea - ps36
Hominidae - ps37
Homininae - ps38
Hominini - ps39
Homo_sapiens - ps40

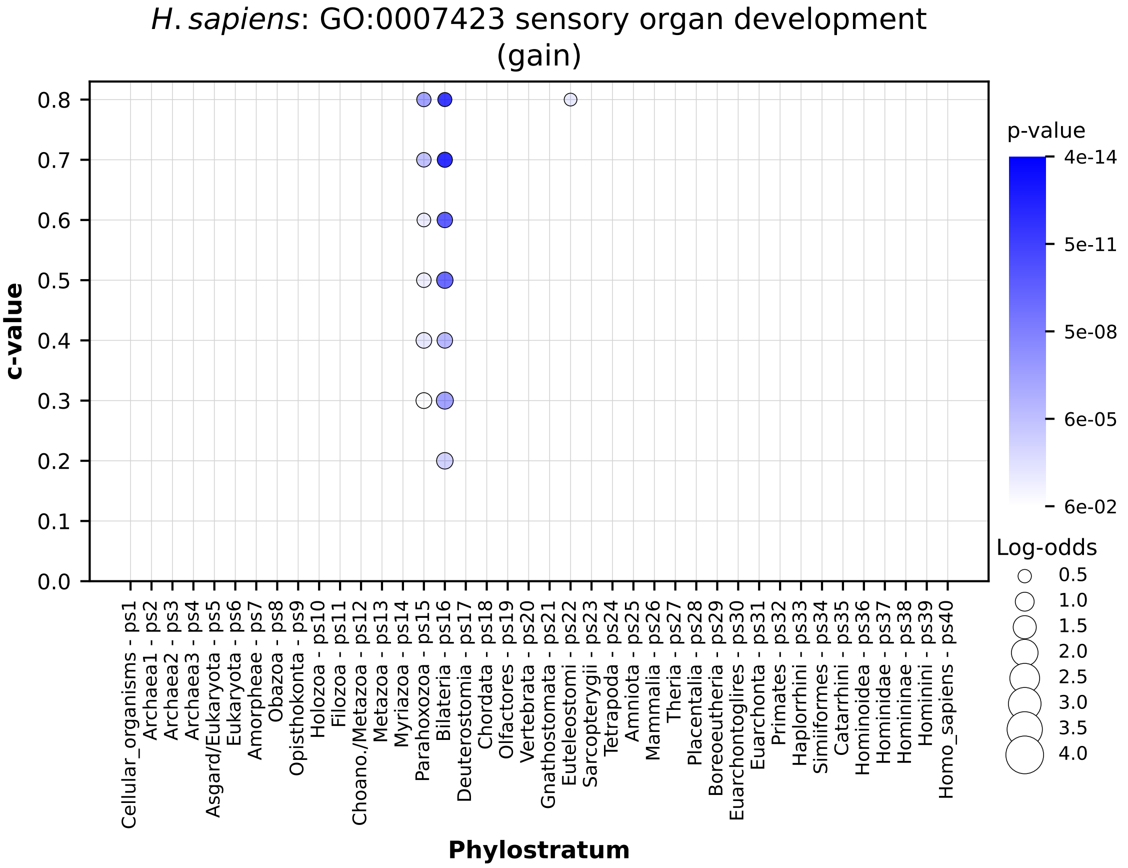

H. sapiens: GO:0007423 sensory organ development
(gain)
c-value
0.0
0.1
0.2
0.3
0.4
0.5
0.6
0.7
0.8
Phylostratum
Cellular_organisms - ps1
Archaea1 - ps2
Archaea2 - ps3
Archaea3 - ps4
Asgard/Eukaryota - ps5
Eukaryota - ps6
Amorpheae - ps7
Obazoa - ps8
Opisthokonta - ps9
Holozoa - ps10
Filozoa - ps11
Choano./Metazoa - ps12
Metazoa - ps13
Myriazoa - ps14
Parahoxozoa - ps15
Bilateria - ps16
Deuterostomia - ps17
Chordata - ps18
Olfactores - ps19
Vertebrata - ps20
Gnathostomata - ps21
Euteleostomi - ps22
Sarcopterygii - ps23
Tetrapoda - ps24
Amniota - ps25
Mammalia - ps26
Theria - ps27
Placentalia - ps28
Boreoeutheria - ps29
Euarchontoglires - ps30
Euarchonta - ps31
Primates - ps32
Haplorrhini - ps33
Simiiformes - ps34
Catarrhini - ps35
Hominoidea - ps36
Hominidae - ps37
Homininae - ps38
Hominini - ps39
Homo_sapiens - ps40
p-value
4e-14
5e-11
5e-08
6e-05
6e-02
Log-odds
0.5
1.0
1.5
2.0
2.5
3.0
3.5
4.0

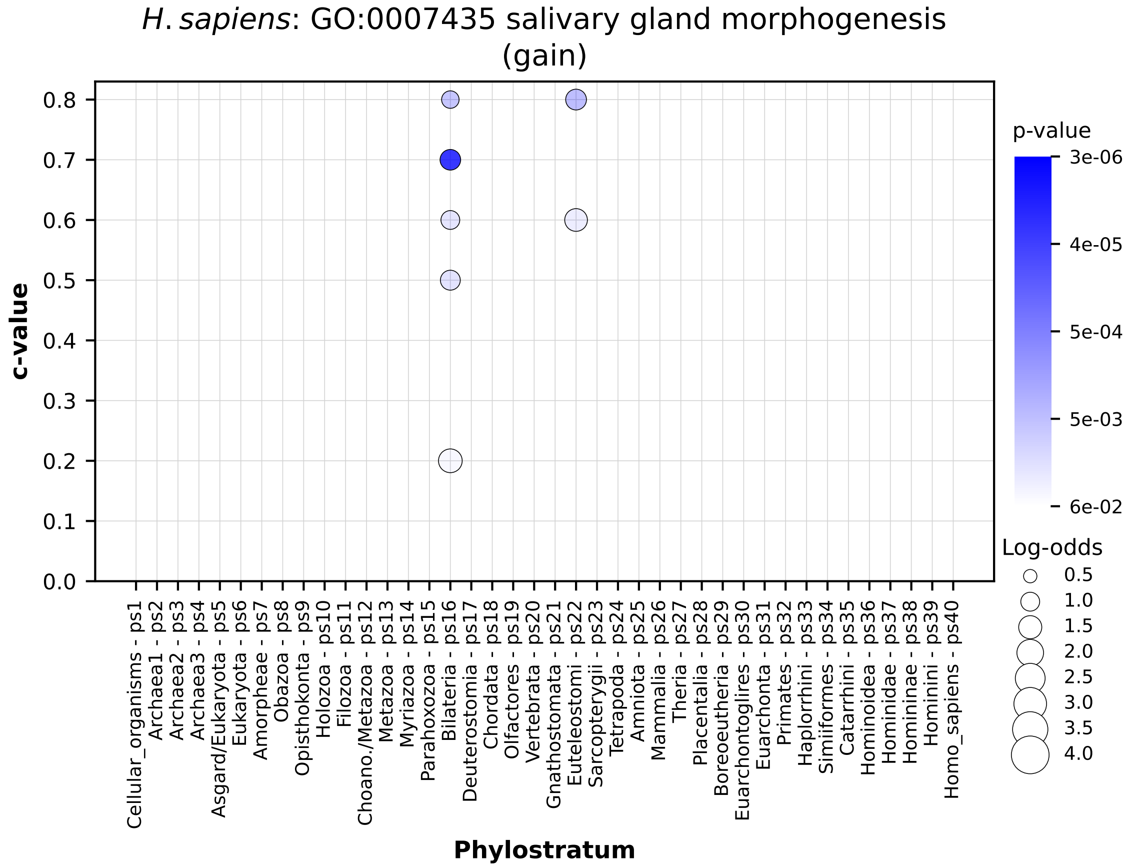

*H. sapiens*: GO:0007435 salivary gland morphogenesis
(gain)
c-value
0.8
0.7
0.6
0.5
0.4
0.3
0.2
0.1
0.0
p-value
3e-06
4e-05
5e-04
5e-03
6e-02
Log-odds
0.5
1.0
1.5
2.0
2.5
3.0
3.5
4.0
Cellular_organisms - ps1
Archaea1 - ps2
Archaea2 - ps3
Archaea3 - ps4
Asgard/Eukaryota - ps5
Eukaryota - ps6
Amorpheae - ps7
Obazoa - ps8
Opisthokonta - ps9
Holozoa - ps10
Filozoa - ps11
Choano./Metazoa - ps12
Metazoa - ps13
Myriazoa - ps14
Parahoxozoa - ps15
Bilateria - ps16
Deuterostomia - ps17
Chordata - ps18
Olfactores - ps19
Vertebrata - ps20
Gnathostomata - ps21
Euteleostomi - ps22
Sarcopterygii - ps23
Tetrapoda - ps24
Amniota - ps25
Mammalia - ps26
Theria - ps27
Placentalia - ps28
Boreoeutheria - ps29
Euarchontoglires - ps30
Euarchonta - ps31
Primates - ps32
Haplorrhini - ps33
Simiiformes - ps34
Catarrhini - ps35
Hominoidea - ps36
Hominidae - ps37
Homininae - ps38
Hominini - ps39
Homo_sapiens - ps40
Phylostratum

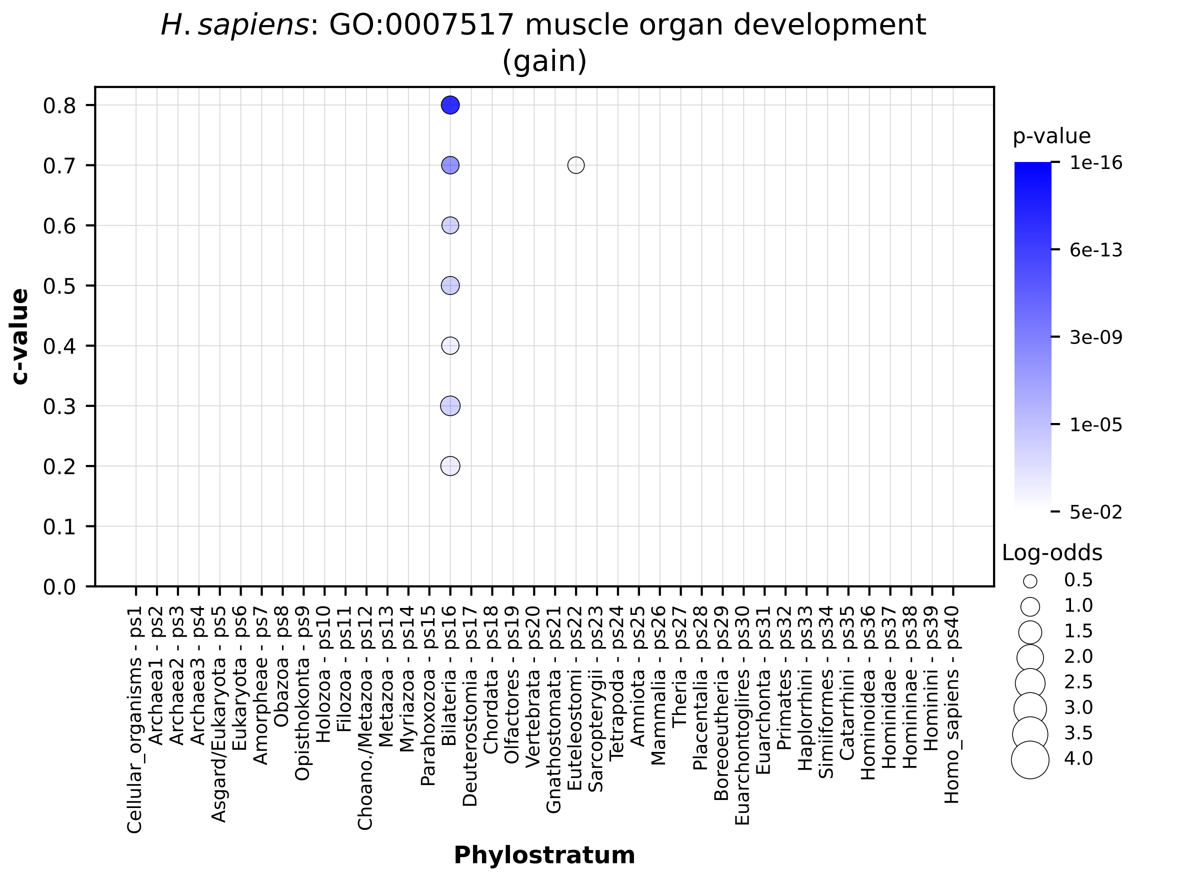
H. sapiens: GO:0007517 muscle organ development
(gain)
c-value
0.0
0.1
0.2
0.3
0.4
0.5
0.6
0.7
0.8
Phylostratum
Cellular_organisms - ps1
Archaea1 - ps2
Archaea2 - ps3
Archaea3 - ps4
Asgard/Eukaryota - ps5
Eukaryota - ps6
Amorpheae - ps7
Obazoa - ps8
Opisthokonta - ps9
Holozoa - ps10
Filozoa - ps11
Choano./Metazoa - ps12
Metazoa - ps13
Myriazoa - ps14
Parahoxozoa - ps15
Bilateria - ps16
Deuterostomia - ps17
Chordata - ps18
Olfactores - ps19
Vertebrata - ps20
Gnathostomata - ps21
Euteleostomi - ps22
Sarcopterygii - ps23
Tetrapoda - ps24
Amniota - ps25
Mammalia - ps26
Theria - ps27
Placentalia - ps28
Boreoeutheria - ps29
Euarchontoglires - ps30
Euarchonta - ps31
Primates - ps32
Haplorrhini - ps33
Simiiformes - ps34
Catarrhini - ps35
Hominoidea - ps36
Hominidae - ps37
Homininae - ps38
Hominini - ps39
Homo_sapiens - ps40
p-value
1e-16
6e-13
3e-09
1e-05
5e-02
Log-odds
0.5
1.0
1.5
2.0
2.5
3.0
3.5
4.0

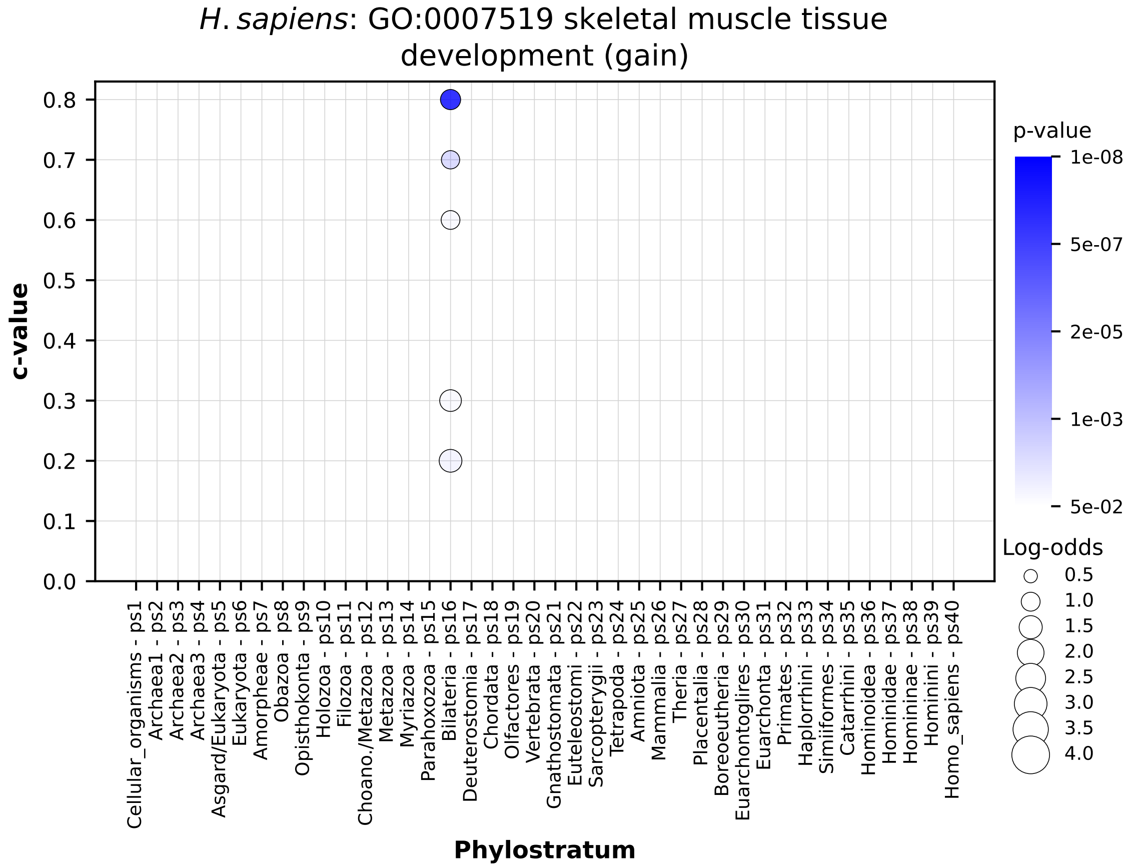
H. sapiens: GO:0007519 skeletal muscle tissue development (gain)
c-value
0.0
0.1
0.2
0.3
0.4
0.5
0.6
0.7
0.8
Phylostratum
Cellular_organisms - ps1
Archaea1 - ps2
Archaea2 - ps3
Archaea3 - ps4
Asgard/Eukaryota - ps5
Eukaryota - ps6
Amorpheae - ps7
Obazoa - ps8
Opisthokonta - ps9
Holozoa - ps10
Filozoa - ps11
Choano./Metazoa - ps12
Metazoa - ps13
Myriazoa - ps14
Parahoxozoa - ps15
Bilateria - ps16
Deuterostomia - ps17
Chordata - ps18
Olfactores - ps19
Vertebrata - ps20
Gnathostomata - ps21
Euteleostomi - ps22
Sarcopterygii - ps23
Tetrapoda - ps24
Amniota - ps25
Mammalia - ps26
Theria - ps27
Placentalia - ps28
Boreoeutheria - ps29
Euarchontoglires - ps30
Euarchonta - ps31
Primates - ps32
Haplorrhini - ps33
Simiiformes - ps34
Catarrhini - ps35
Hominoidea - ps36
Hominidae - ps37
Homininae - ps38
Hominini - ps39
Homo_sapiens - ps40
p-value
1e-08
5e-07
2e-05
1e-03
5e-02
Log-odds
0.5
1.0
1.5
2.0
2.5
3.0
3.5
4.0

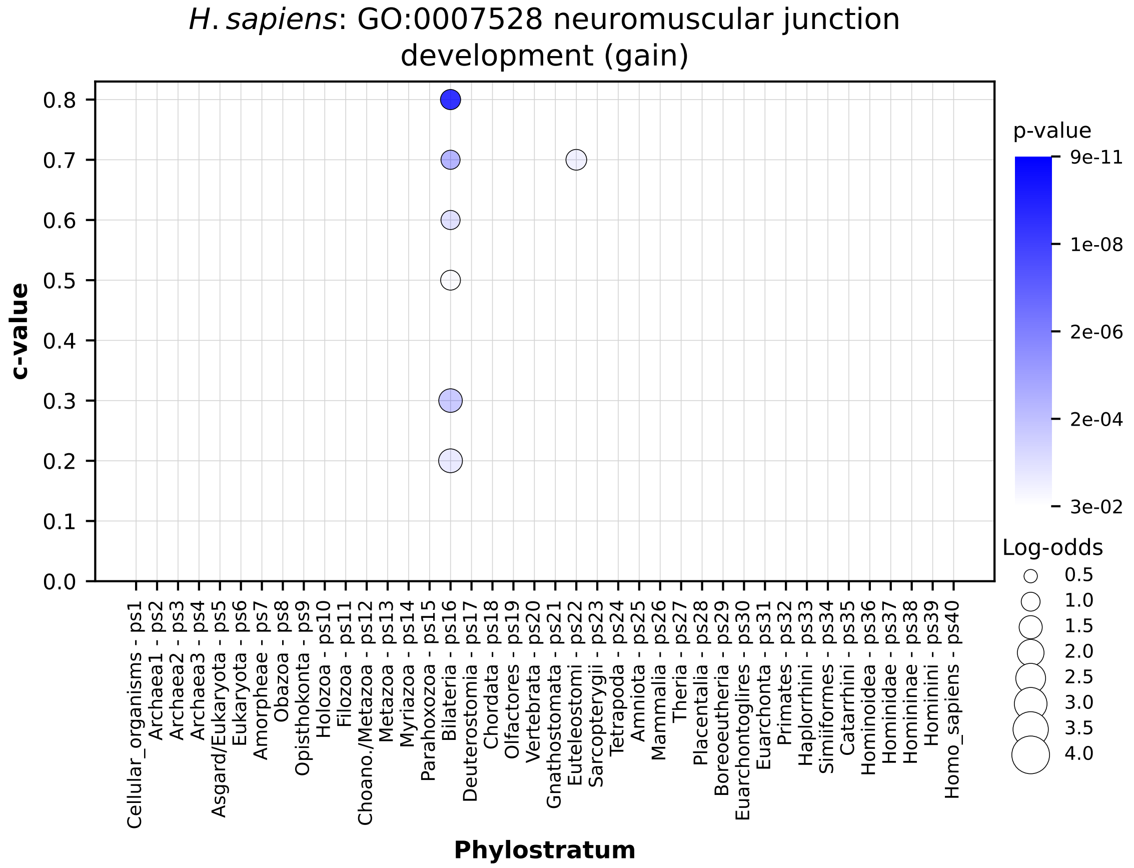

H. sapiens: GO:0007528 neuromuscular junction development (gain)
c-value
0.0
0.1
0.2
0.3
0.4
0.5
0.6
0.7
0.8
Phylostratum
Cellular_organisms - ps1
Archaea1 - ps2
Archaea2 - ps3
Archaea3 - ps4
Asgard/Eukaryota - ps5
Eukaryota - ps6
Amorpheae - ps7
Obazoa - ps8
Opisthokonta - ps9
Holozoa - ps10
Filozoa - ps11
Choano./Metazoa - ps12
Metazoa - ps13
Myriazoa - ps14
Parahoxozoa - ps15
Bilateria - ps16
Deuterostomia - ps17
Chordata - ps18
Olfactores - ps19
Vertebrata - ps20
Gnathostomata - ps21
Euteleostomi - ps22
Sarcopterygii - ps23
Tetrapoda - ps24
Amniota - ps25
Mammalia - ps26
Theria - ps27
Placentalia - ps28
Boreoeutheria - ps29
Euarchontoglires - ps30
Euarchonta - ps31
Primates - ps32
Haplorrhini - ps33
Simiiformes - ps34
Catarrhini - ps35
Hominoidea - ps36
Hominidae - ps37
Homininae - ps38
Hominini - ps39
Homo_sapiens - ps40
p-value
9e-11
1e-08
2e-06
2e-04
3e-02
Log-odds
0.5
1.0
1.5
2.0
2.5
3.0
3.5
4.0

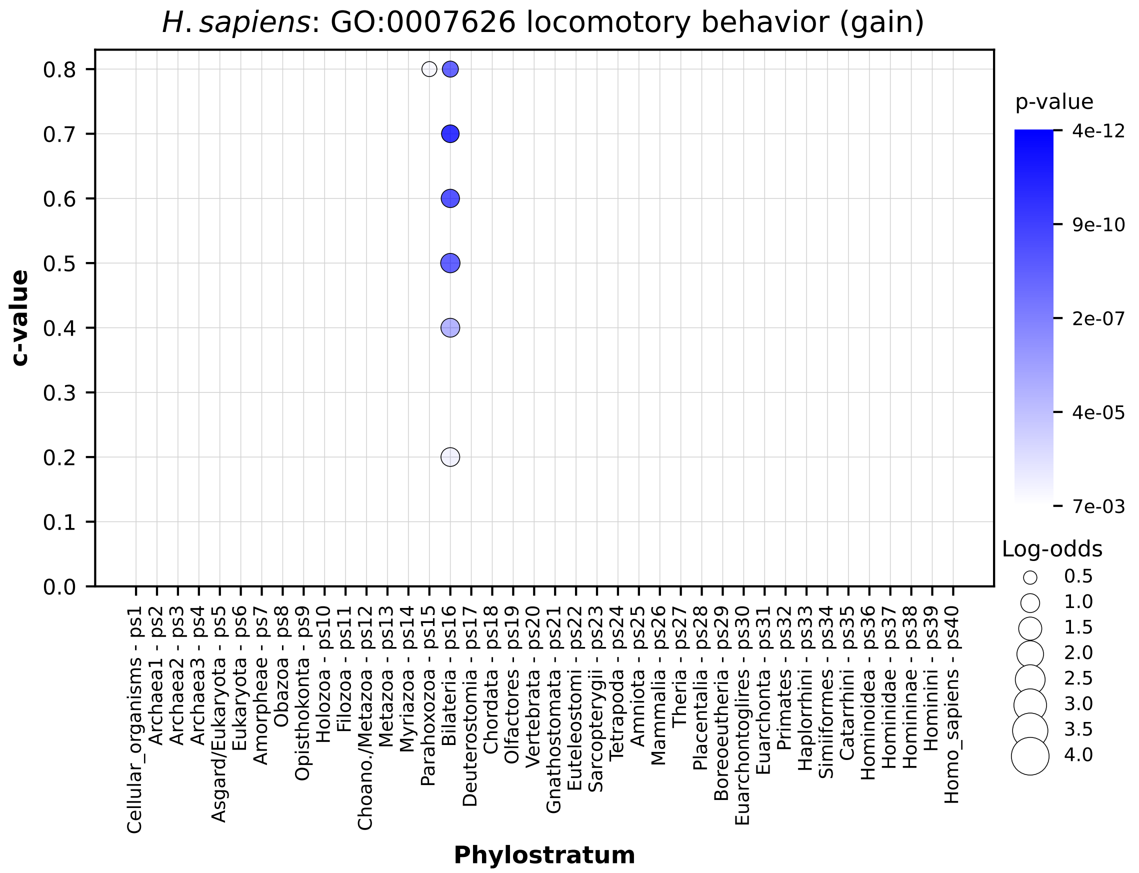
H. sapiens: GO:0007626 locomotory behavior (gain)
c-value
0.0
0.1
0.2
0.3
0.4
0.5
0.6
0.7
0.8
Phylostratum
Cellular_organisms - ps1
Archaea1 - ps2
Archaea2 - ps3
Archaea3 - ps4
Asgard/Eukaryota - ps5
Eukaryota - ps6
Amorpheae - ps7
Obazoa - ps8
Opisthokonta - ps9
Holozoa - ps10
Filozoa - ps11
Choano./Metazoa - ps12
Metazoa - ps13
Myriazoa - ps14
Parahoxozoa - ps15
Bilateria - ps16
Deuterostomia - ps17
Chordata - ps18
Olfactores - ps19
Vertebrata - ps20
Gnathostomata - ps21
Euteleostomi - ps22
Sarcopterygii - ps23
Tetrapoda - ps24
Amniota - ps25
Mammalia - ps26
Theria - ps27
Placentalia - ps28
Boreoeutheria - ps29
Euarchontoglires - ps30
Euarchonta - ps31
Primates - ps32
Haplorrhini - ps33
Simiiformes - ps34
Catarrhini - ps35
Hominoidea - ps36
Hominidae - ps37
Homininae - ps38
Hominini - ps39
Homo_sapiens - ps40
p-value
4e-12
9e-10
2e-07
4e-05
7e-03
Log-odds
0.5
1.0
1.5
2.0
2.5
3.0
3.5
4.0

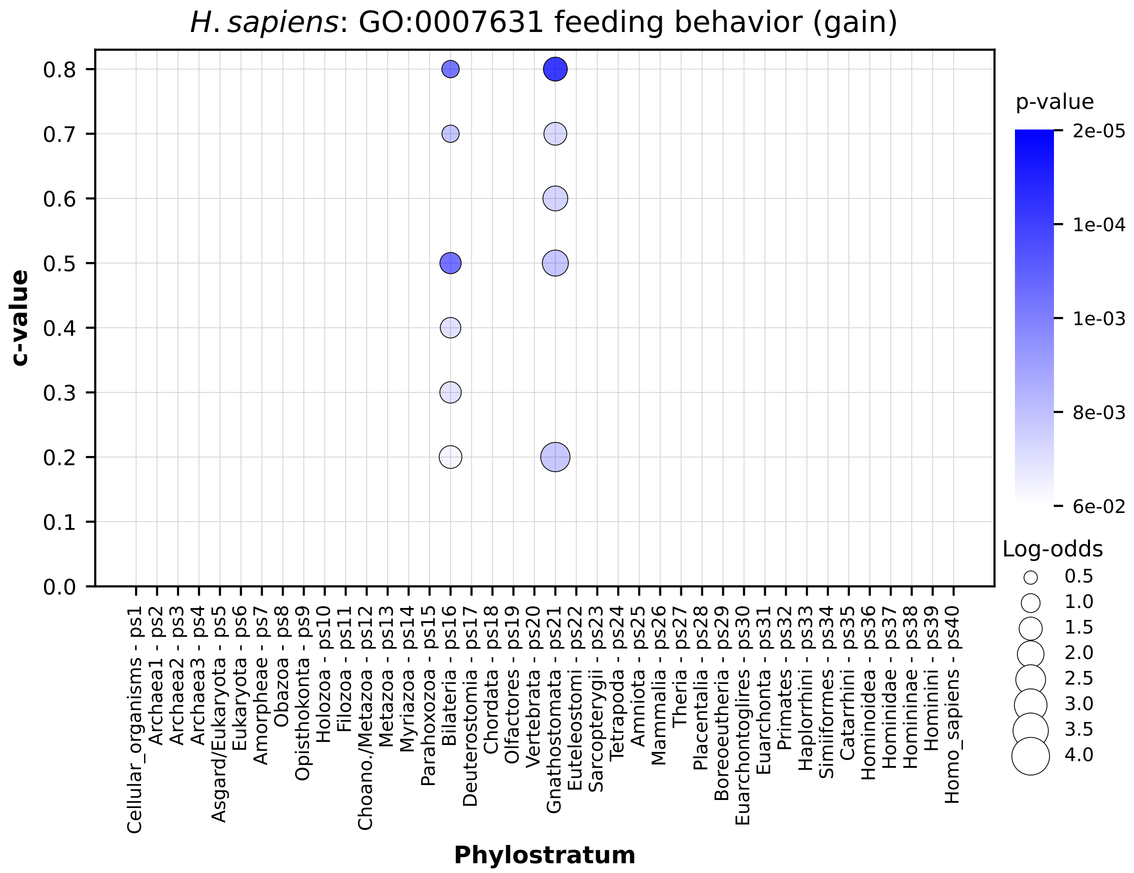

H. sapiens: GO:0007631 feeding behavior (gain)
c-value
0.0
0.1
0.2
0.3
0.4
0.5
0.6
0.7
0.8
Phylostratum
Cellular_organisms - ps1
Archaea1 - ps2
Archaea2 - ps3
Archaea3 - ps4
Asgard/Eukaryota - ps5
Eukaryota - ps6
Amorpheae - ps7
Obazoa - ps8
Opisthokonta - ps9
Holozoa - ps10
Filozoa - ps11
Choano./Metazoa - ps12
Metazoa - ps13
Myriazoa - ps14
Parahoxozoa - ps15
Bilateria - ps16
Deuterostomia - ps17
Chordata - ps18
Olfactores - ps19
Vertebrata - ps20
Gnathostomata - ps21
Euteleostomi - ps22
Sarcopterygii - ps23
Tetrapoda - ps24
Amniota - ps25
Mammalia - ps26
Theria - ps27
Placentalia - ps28
Boreoeutheria - ps29
Euarchontoglires - ps30
Euarchonta - ps31
Primates - ps32
Haplorrhini - ps33
Simiiformes - ps34
Catarrhini - ps35
Hominoidea - ps36
Hominidae - ps37
Homininae - ps38
Hominini - ps39
Homo_sapiens - ps40
p-value
2e-05
1e-04
1e-03
8e-03
6e-02
Log-odds
0.5
1.0
1.5
2.0
2.5
3.0
3.5
4.0

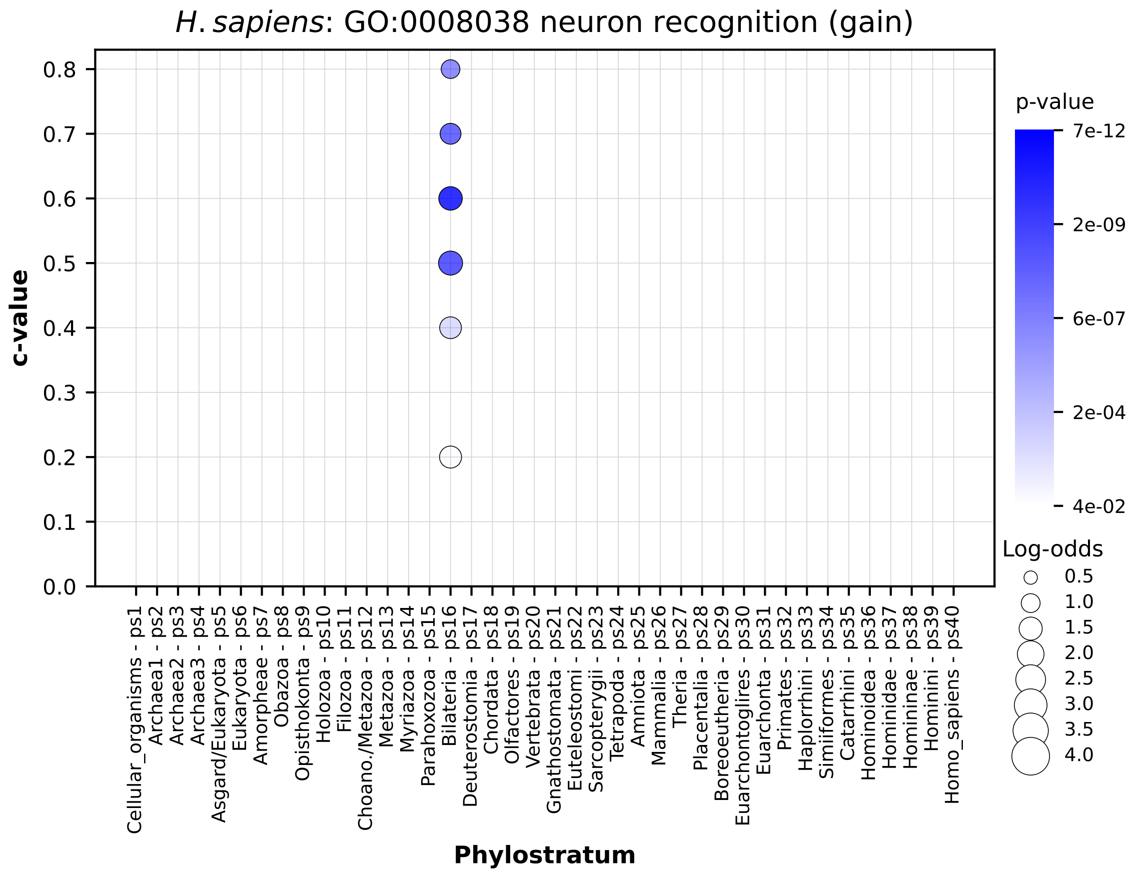

H. sapiens: GO:0008038 neuron recognition (gain)
c-value
0.0
0.1
0.2
0.3
0.4
0.5
0.6
0.7
0.8
Phylostratum
Cellular_organisms - ps1
Archaea1 - ps2
Archaea2 - ps3
Archaea3 - ps4
Asgard/Eukaryota - ps5
Eukaryota - ps6
Amorpheae - ps7
Obazoa - ps8
Opisthokonta - ps9
Holozoa - ps10
Filozoa - ps11
Choano./Metazoa - ps12
Metazoa - ps13
Myriazoa - ps14
Parahoxozoa - ps15
Bilateria - ps16
Deuterostomia - ps17
Chordata - ps18
Olfactores - ps19
Vertebrata - ps20
Gnathostomata - ps21
Euteleostomi - ps22
Sarcopterygii - ps23
Tetrapoda - ps24
Amniota - ps25
Mammalia - ps26
Theria - ps27
Placentalia - ps28
Boreoeutheria - ps29
Euarchontoglires - ps30
Euarchonta - ps31
Primates - ps32
Haplorrhini - ps33
Simiiformes - ps34
Catarrhini - ps35
Hominoidea - ps36
Hominidae - ps37
Homininae - ps38
Hominini - ps39
Homo_sapiens - ps40
p-value
7e-12
2e-09
6e-07
2e-04
4e-02
Log-odds
0.5
1.0
1.5
2.0
2.5
3.0
3.5
4.0

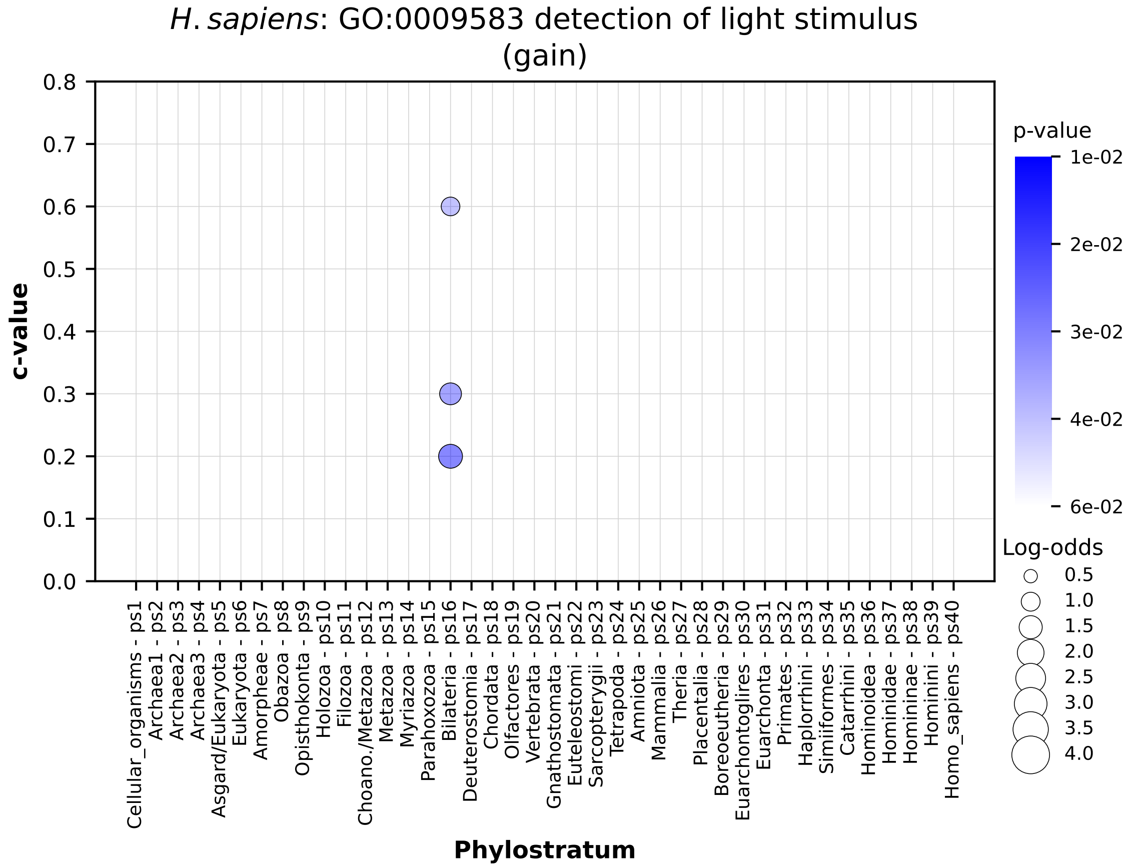
H. sapiens: GO:0009583 detection of light stimulus (gain)
c-value
0.0
0.1
0.2
0.3
0.4
0.5
0.6
0.7
0.8
Phylostratum
Cellular_organisms - ps1
Archaea1 - ps2
Archaea2 - ps3
Archaea3 - ps4
Asgard/Eukaryota - ps5
Eukaryota - ps6
Amorpheae - ps7
Obazoa - ps8
Opisthokonta - ps9
Holozoa - ps10
Filozoa - ps11
Choano./Metazoa - ps12
Metazoa - ps13
Myriazoa - ps14
Parahoxozoa - ps15
Bilateria - ps16
Deuterostomia - ps17
Chordata - ps18
Olfactores - ps19
Vertebrata - ps20
Gnathostomata - ps21
Euteleostomi - ps22
Sarcopterygii - ps23
Tetrapoda - ps24
Amniota - ps25
Mammalia - ps26
Theria - ps27
Placentalia - ps28
Boreoeutheria - ps29
Euarchontoglires - ps30
Euarchonta - ps31
Primates - ps32
Haplorrhini - ps33
Simiiformes - ps34
Catarrhini - ps35
Hominoidea - ps36
Hominidae - ps37
Homininae - ps38
Hominini - ps39
Homo_sapiens - ps40
p-value
1e-02
2e-02
3e-02
4e-02
6e-02
Log-odds
0.5
1.0
1.5
2.0
2.5
3.0
3.5
4.0

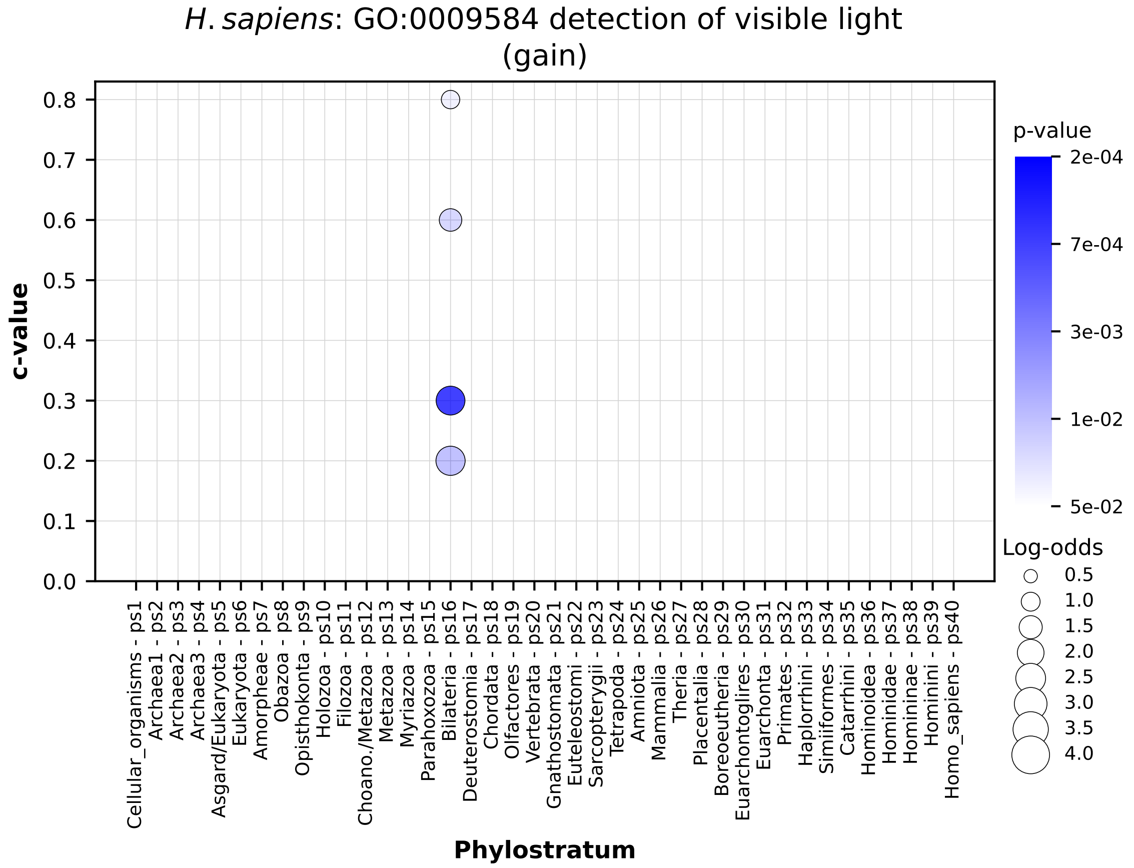

H. sapiens: GO:0009584 detection of visible light
(gain)
c-value
0.0
0.1
0.2
0.3
0.4
0.5
0.6
0.7
0.8
Phylostratum
Cellular_organisms - ps1
Archaea1 - ps2
Archaea2 - ps3
Archaea3 - ps4
Asgard/Eukaryota - ps5
Eukaryota - ps6
Amorpheae - ps7
Obazoa - ps8
Opisthokonta - ps9
Holozoa - ps10
Filozoa - ps11
Choano./Metazoa - ps12
Metazoa - ps13
Myriazoa - ps14
Parahoxozoa - ps15
Bilateria - ps16
Deuterostomia - ps17
Chordata - ps18
Olfactores - ps19
Vertebrata - ps20
Gnathostomata - ps21
Euteleostomi - ps22
Sarcopterygii - ps23
Tetrapoda - ps24
Amniota - ps25
Mammalia - ps26
Theria - ps27
Placentalia - ps28
Boreoeutheria - ps29
Euarchontoglires - ps30
Euarchonta - ps31
Primates - ps32
Haplorrhini - ps33
Simiiformes - ps34
Catarrhini - ps35
Hominoidea - ps36
Hominidae - ps37
Homininae - ps38
Hominini - ps39
Homo_sapiens - ps40
p-value
2e-04
7e-04
3e-03
1e-02
5e-02
Log-odds
0.5
1.0
1.5
2.0
2.5
3.0
3.5
4.0

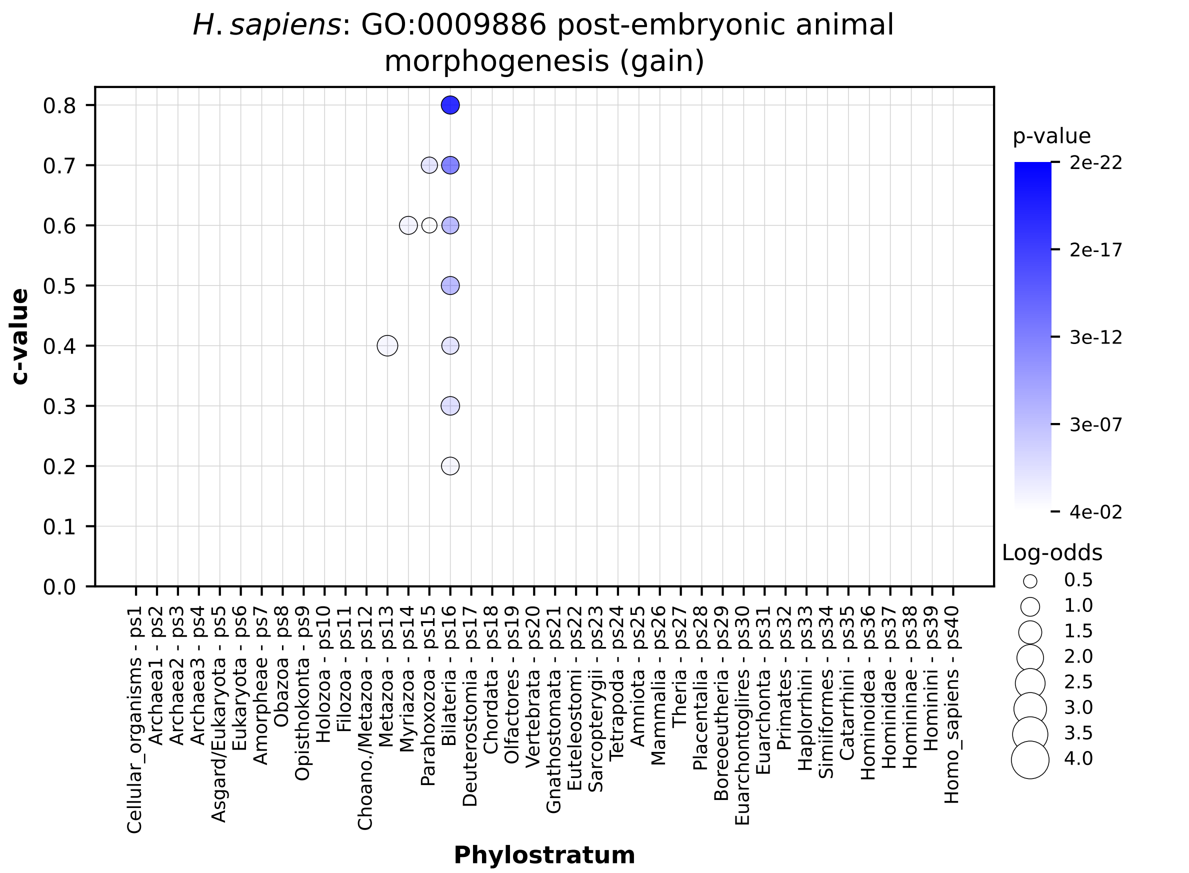
*H. sapiens*: GO:0009886 post-embryonic animal morphogenesis (gain)
c-value
0.8
0.7
0.6
0.5
0.4
0.3
0.2
0.1
0.0
Phylostratum
Cellular_organisms - ps1
Archaea1 - ps2
Archaea2 - ps3
Archaea3 - ps4
Asgard/Eukaryota - ps5
Eukaryota - ps6
Amorpheae - ps7
Obazoa - ps8
Opisthokonta - ps9
Holozoa - ps10
Filozoa - ps11
Choano./Metazoa - ps12
Metazoa - ps13
Myriazoa - ps14
Parahoxozoa - ps15
Bilateria - ps16
Deuterostomia - ps17
Chordata - ps18
Olfactores - ps19
Vertebrata - ps20
Gnathostomata - ps21
Euteleostomi - ps22
Sarcopterygii - ps23
Tetrapoda - ps24
Amniota - ps25
Mammalia - ps26
Theria - ps27
Placentalia - ps28
Boreoeutheria - ps29
Euarchontoglires - ps30
Euarchonta - ps31
Primates - ps32
Haplorrhini - ps33
Simiiformes - ps34
Catarrhini - ps35
Hominoidea - ps36
Hominidae - ps37
Homininae - ps38
Hominini - ps39
Homo_sapiens - ps40
p-value
2e-22
2e-17
3e-12
3e-07
4e-02
Log-odds
0.5
1.0
1.5
2.0
2.5
3.0
3.5
4.0

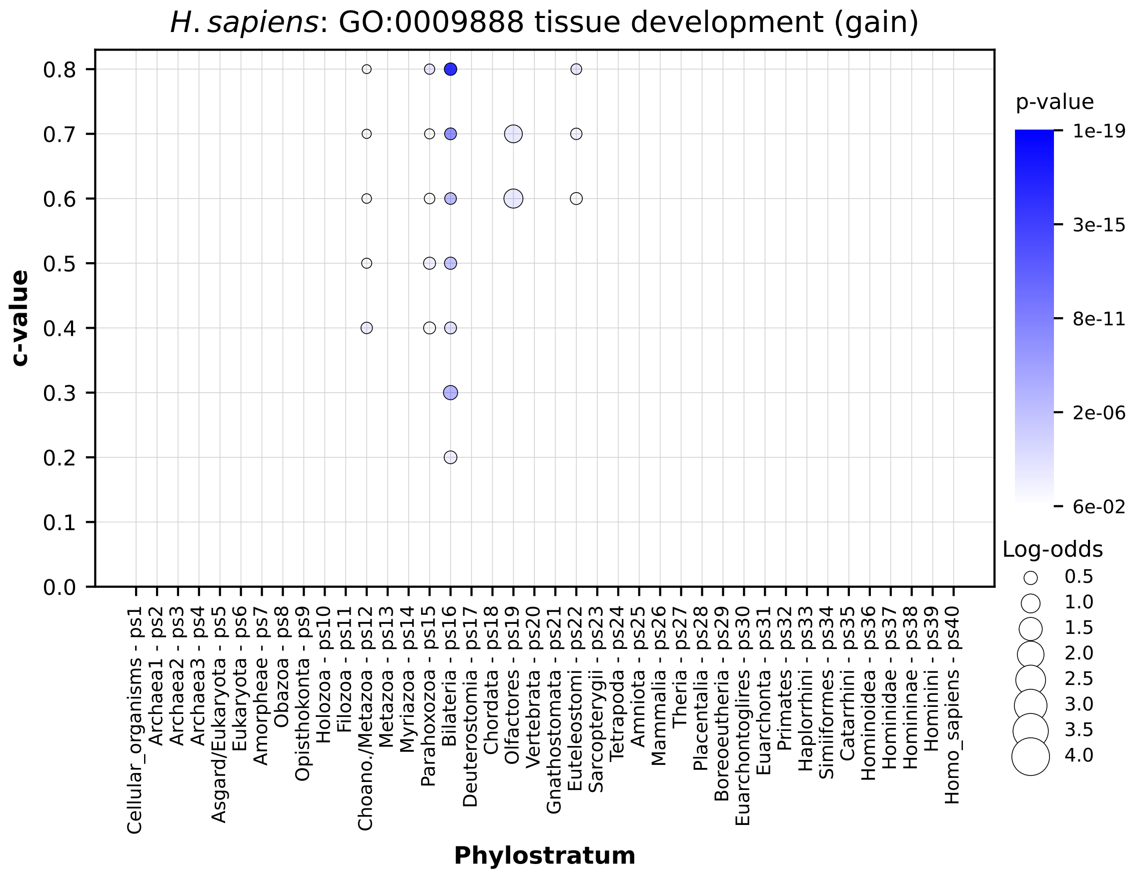
*H. sapiens*: GO:0009888 tissue development (gain)
c-value
0.0
0.1
0.2
0.3
0.4
0.5
0.6
0.7
0.8
Phylostratum
Cellular_organisms - ps1
Archaea1 - ps2
Archaea2 - ps3
Archaea3 - ps4
Asgard/Eukaryota - ps5
Eukaryota - ps6
Amorpheae - ps7
Obazoa - ps8
Opisthokonta - ps9
Holozoa - ps10
Filozoa - ps11
Choano./Metazoa - ps12
Metazoa - ps13
Myriazoa - ps14
Parahoxozoa - ps15
Bilateria - ps16
Deuterostomia - ps17
Chordata - ps18
Olfactores - ps19
Vertebrata - ps20
Gnathostomata - ps21
Euteleostomi - ps22
Sarcopterygii - ps23
Tetrapoda - ps24
Amniota - ps25
Mammalia - ps26
Theria - ps27
Placentalia - ps28
Boreoeutheria - ps29
Euarchontoglires - ps30
Euarchonta - ps31
Primates - ps32
Haplorrhini - ps33
Simiiformes - ps34
Catarrhini - ps35
Hominoidea - ps36
Hominidae - ps37
Homininae - ps38
Hominini - ps39
Homo_sapiens - ps40
p-value
1e-19
3e-15
8e-11
2e-06
6e-02
Log-odds
0.5
1.0
1.5
2.0
2.5
3.0
3.5
4.0

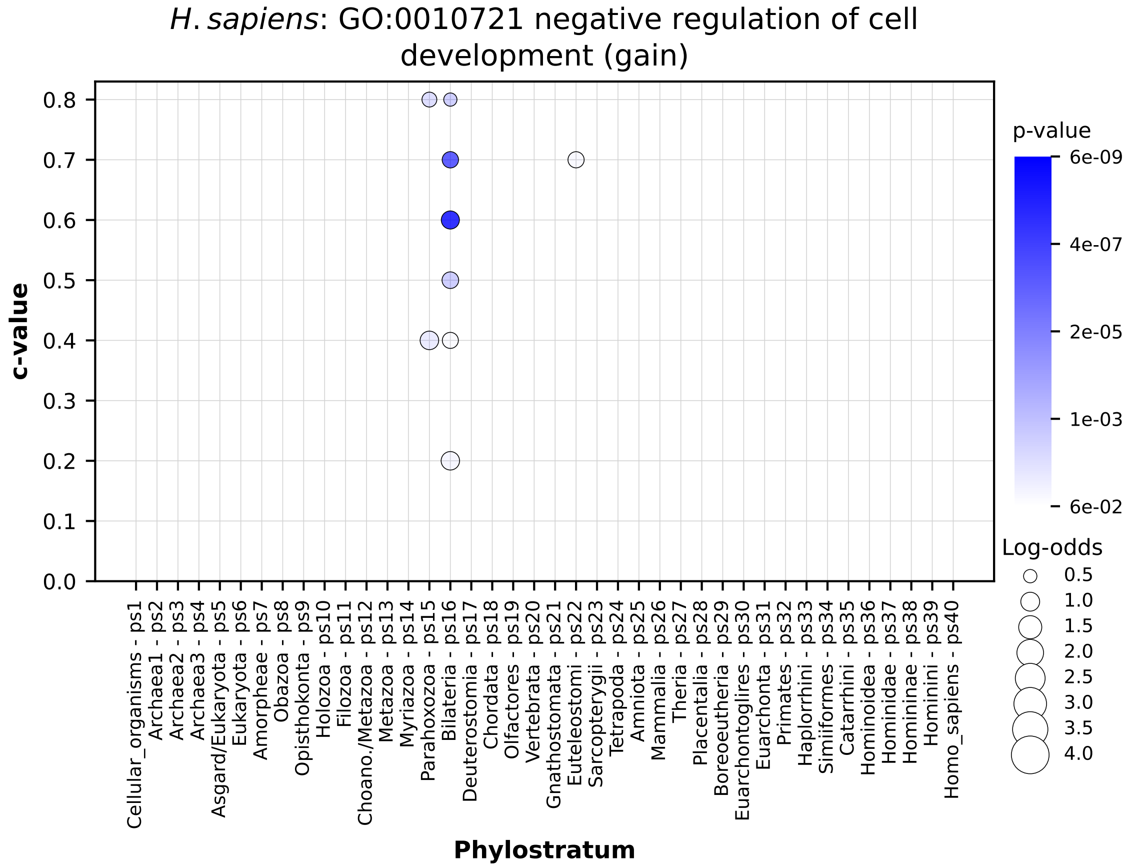
H. sapiens: GO:0010721 negative regulation of cell development (gain)
c-value
0.0
0.1
0.2
0.3
0.4
0.5
0.6
0.7
0.8
Phylostratum
Cellular_organisms - ps1
Archaea1 - ps2
Archaea2 - ps3
Archaea3 - ps4
Asgard/Eukaryota - ps5
Eukaryota - ps6
Amorpheae - ps7
Obazoa - ps8
Opisthokonta - ps9
Holozoa - ps10
Filozoa - ps11
Choano./Metazoa - ps12
Metazoa - ps13
Myriazoa - ps14
Parahoxozoa - ps15
Bilateria - ps16
Deuterostomia - ps17
Chordata - ps18
Olfactores - ps19
Vertebrata - ps20
Gnathostomata - ps21
Euteleostomi - ps22
Sarcopterygii - ps23
Tetrapoda - ps24
Amniota - ps25
Mammalia - ps26
Theria - ps27
Placentalia - ps28
Boreoeutheria - ps29
Euarchontoglires - ps30
Euarchonta - ps31
Primates - ps32
Haplorrhini - ps33
Simiiformes - ps34
Catarrhini - ps35
Hominoidea - ps36
Hominidae - ps37
Homininae - ps38
Hominini - ps39
Homo_sapiens - ps40
p-value
6e-09
4e-07
2e-05
1e-03
6e-02
Log-odds
0.5
1.0
1.5
2.0
2.5
3.0
3.5
4.0

# *H. sapiens*: GO:0010975 regulation of neuron projection development (gain)

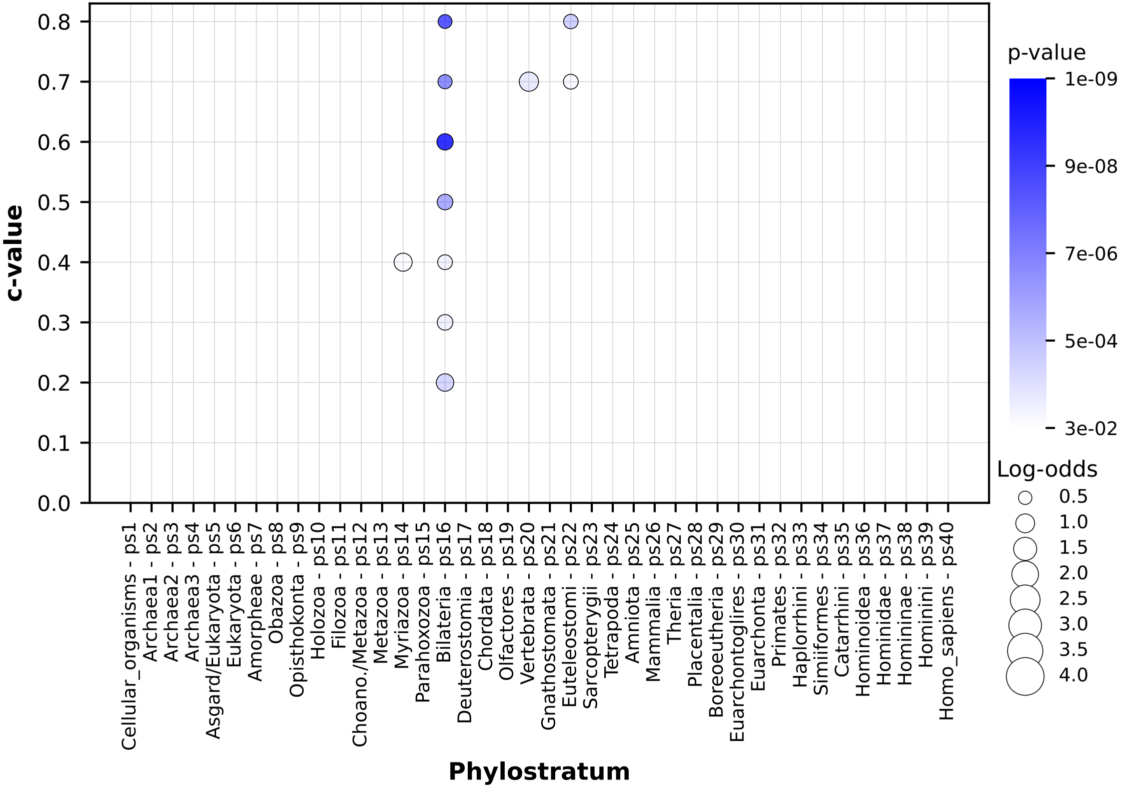

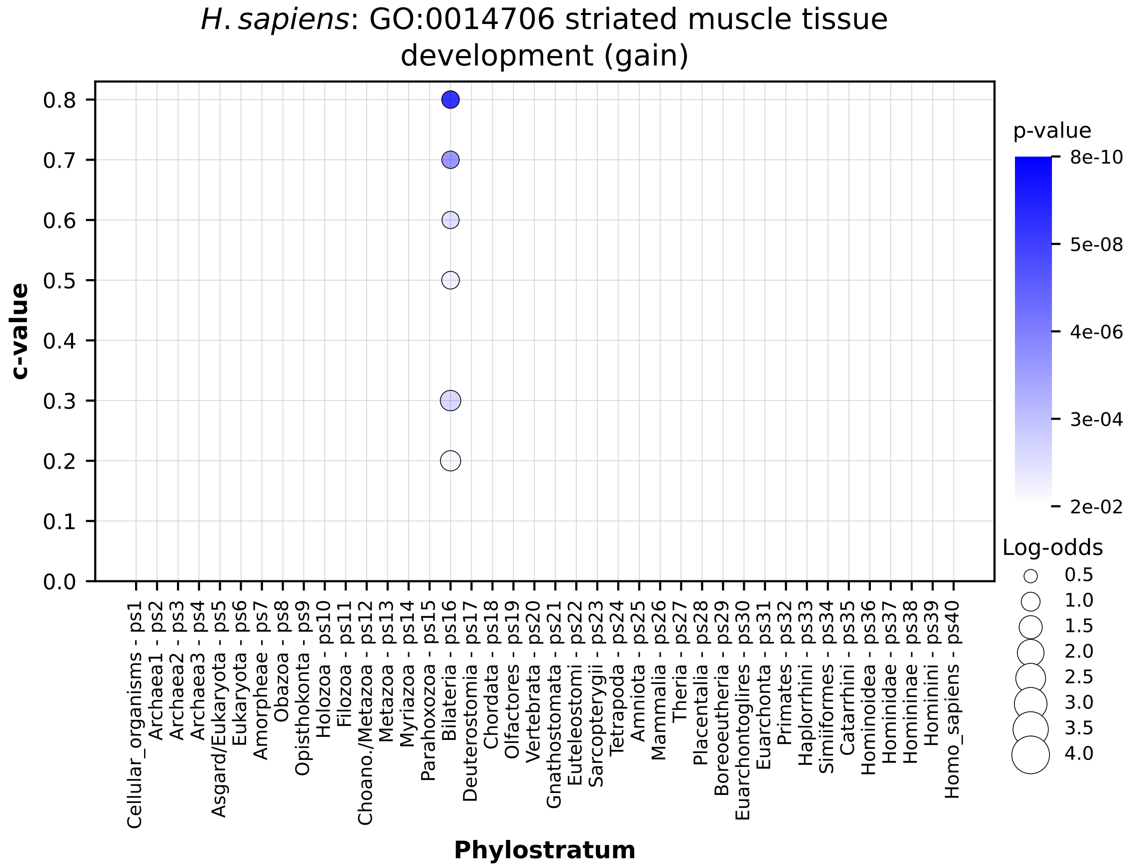

H. sapiens: GO:0014706 striated muscle tissue development (gain)
c-value
0.0
0.1
0.2
0.3
0.4
0.5
0.6
0.7
0.8
Phylostratum
Cellular_organisms - ps1
Archaea1 - ps2
Archaea2 - ps3
Archaea3 - ps4
Asgard/Eukaryota - ps5
Eukaryota - ps6
Amorpheae - ps7
Obazoa - ps8
Opisthokonta - ps9
Holozoa - ps10
Filozoa - ps11
Choano./Metazoa - ps12
Metazoa - ps13
Myriazoa - ps14
Parahoxozoa - ps15
Bilateria - ps16
Deuterostomia - ps17
Chordata - ps18
Olfactores - ps19
Vertebrata - ps20
Gnathostomata - ps21
Euteleostomi - ps22
Sarcopterygii - ps23
Tetrapoda - ps24
Amniota - ps25
Mammalia - ps26
Theria - ps27
Placentalia - ps28
Boreoeutheria - ps29
Euarchontoglires - ps30
Euarchonta - ps31
Primates - ps32
Haplorrhini - ps33
Simiiformes - ps34
Catarrhini - ps35
Hominoidea - ps36
Hominidae - ps37
Homininae - ps38
Hominini - ps39
Homo_sapiens - ps40
p-value
8e-10
5e-08
4e-06
3e-04
2e-02
Log-odds
0.5
1.0
1.5
2.0
2.5
3.0
3.5
4.0

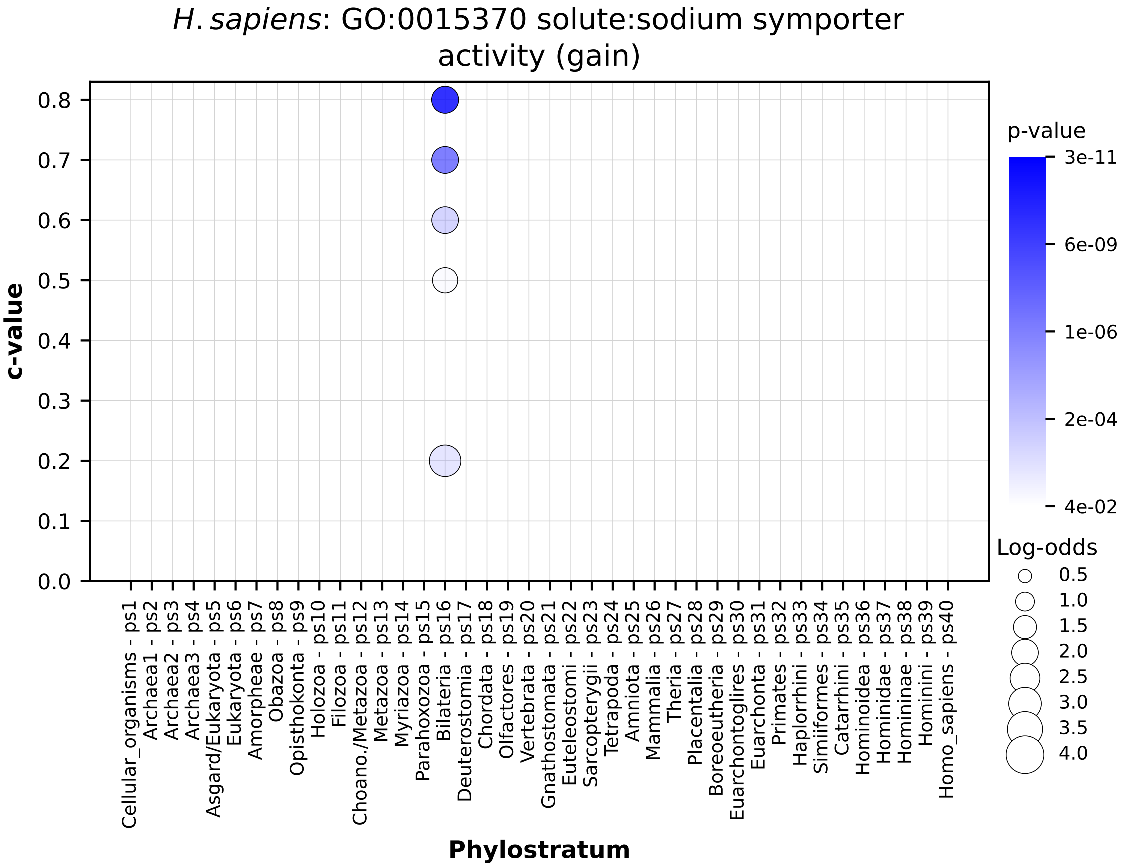

H. sapiens: GO:0015370 solute:sodium symporter activity (gain)
c-value
0.0
0.1
0.2
0.3
0.4
0.5
0.6
0.7
0.8
p-value
3e-11
6e-09
1e-06
2e-04
4e-02
Log-odds
0.5
1.0
1.5
2.0
2.5
3.0
3.5
4.0
Cellular_organisms - ps1
Archaea1 - ps2
Archaea2 - ps3
Archaea3 - ps4
Asgard/Eukaryota - ps5
Eukaryota - ps6
Amorpheae - ps7
Obazoa - ps8
Opisthokonta - ps9
Holozoa - ps10
Filozoa - ps11
Choano./Metazoa - ps12
Metazoa - ps13
Myriazoa - ps14
Parahoxozoa - ps15
Bilateria - ps16
Deuterostomia - ps17
Chordata - ps18
Olfactores - ps19
Vertebrata - ps20
Gnathostomata - ps21
Euteleostomi - ps22
Sarcopterygii - ps23
Tetrapoda - ps24
Amniota - ps25
Mammalia - ps26
Theria - ps27
Placentalia - ps28
Boreoeutheria - ps29
Euarchontoglires - ps30
Euarchonta - ps31
Primates - ps32
Haplorrhini - ps33
Simiiformes - ps34
Catarrhini - ps35
Hominoidea - ps36
Hominidae - ps37
Homininae - ps38
Hominini - ps39
Homo_sapiens - ps40
Phylostratum

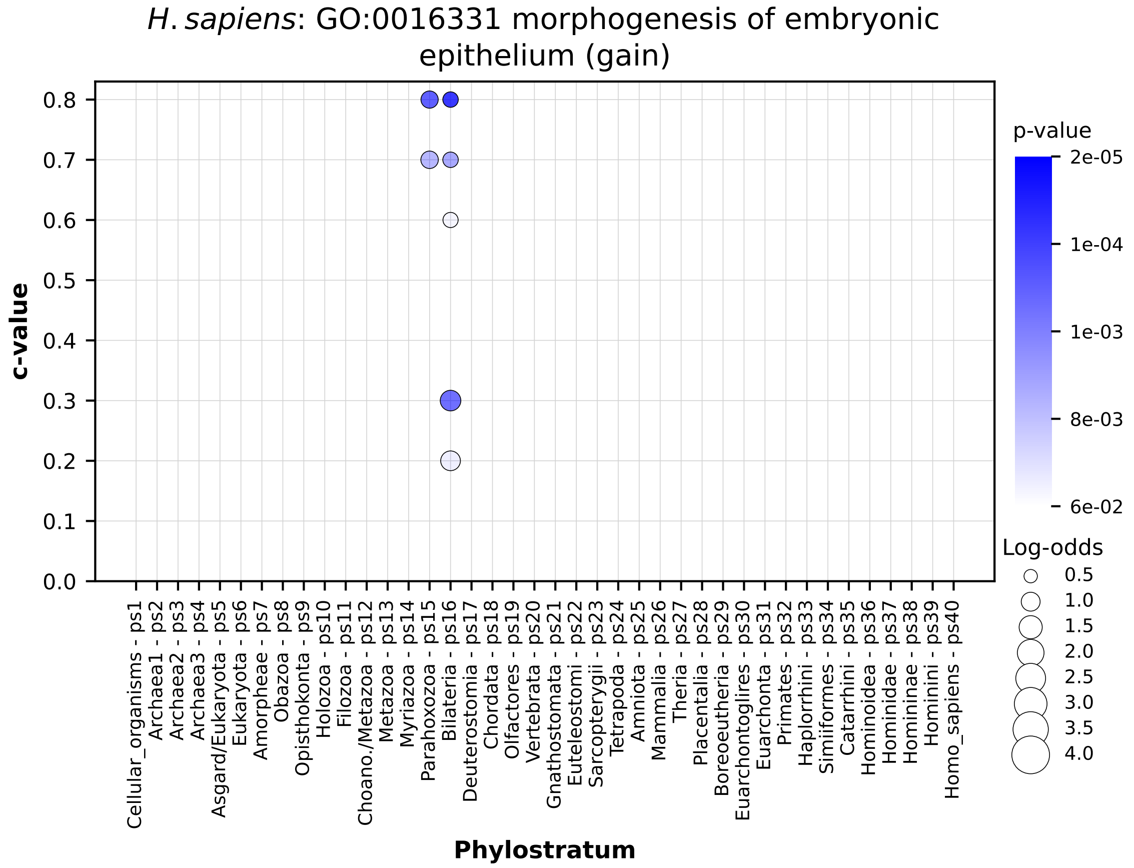
*H. sapiens*: GO:0016331 morphogenesis of embryonic epithelium (gain)
c-value
0.0
0.1
0.2
0.3
0.4
0.5
0.6
0.7
0.8
Phylostratum
Cellular_organisms - ps1
Archaea1 - ps2
Archaea2 - ps3
Archaea3 - ps4
Asgard/Eukaryota - ps5
Eukaryota - ps6
Amorpheae - ps7
Obazoa - ps8
Opisthokonta - ps9
Holozoa - ps10
Filozoa - ps11
Choano./Metazoa - ps12
Metazoa - ps13
Myriazoa - ps14
Parahoxozoa - ps15
Bilateria - ps16
Deuterostomia - ps17
Chordata - ps18
Olfactores - ps19
Vertebrata - ps20
Gnathostomata - ps21
Euteleostomi - ps22
Sarcopterygii - ps23
Tetrapoda - ps24
Amniota - ps25
Mammalia - ps26
Theria - ps27
Placentalia - ps28
Boreoeutheria - ps29
Euarchontoglires - ps30
Euarchonta - ps31
Primates - ps32
Haplorrhini - ps33
Simiiformes - ps34
Catarrhini - ps35
Hominoidea - ps36
Hominidae - ps37
Homininae - ps38
Hominini - ps39
Homo_sapiens - ps40
p-value
2e-05
1e-04
1e-03
8e-03
6e-02
Log-odds
0.5
1.0
1.5
2.0
2.5
3.0
3.5
4.0

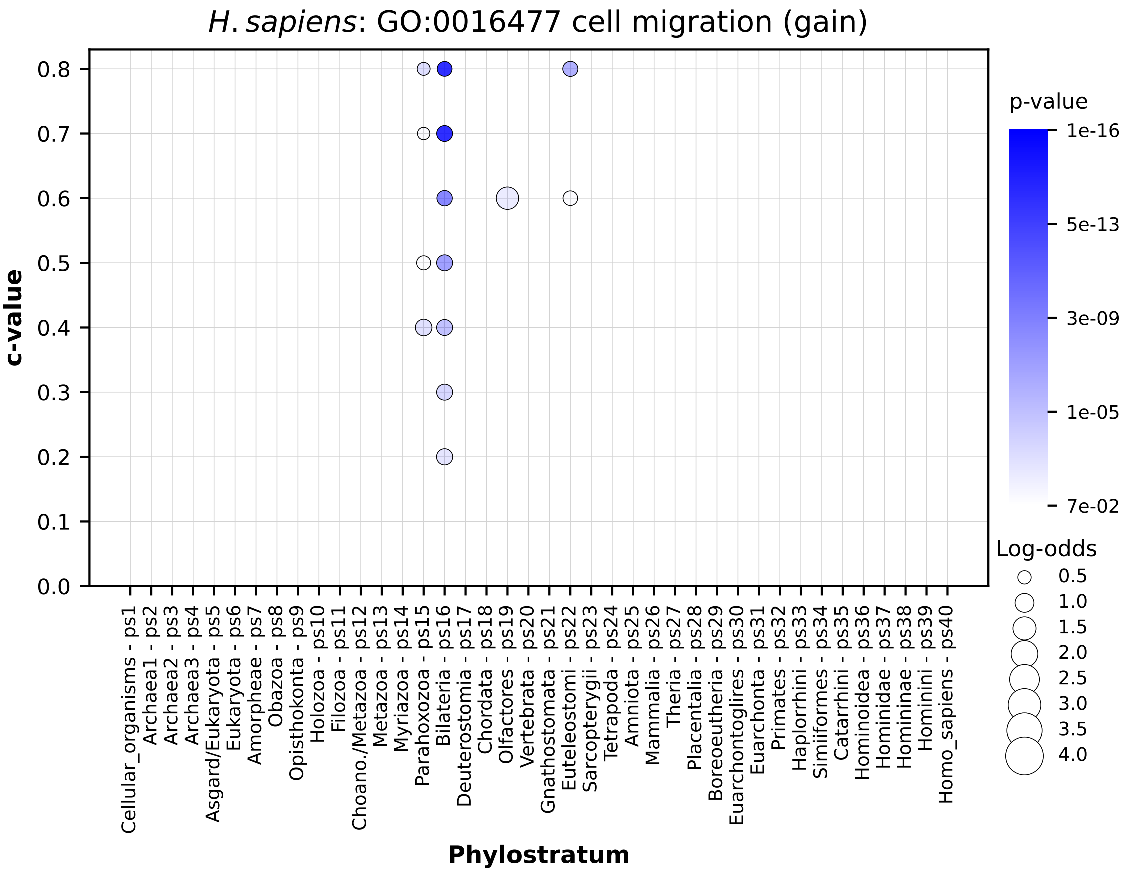

H. sapiens: GO:0016477 cell migration (gain)
c-value
0.0
0.1
0.2
0.3
0.4
0.5
0.6
0.7
0.8
Phylostratum
Cellular_organisms - ps1
Archaea1 - ps2
Archaea2 - ps3
Archaea3 - ps4
Asgard/Eukaryota - ps5
Eukaryota - ps6
Amorpheae - ps7
Obazoa - ps8
Opisthokonta - ps9
Holozoa - ps10
Filozoa - ps11
Choano./Metazoa - ps12
Metazoa - ps13
Myriazoa - ps14
Parahoxozoa - ps15
Bilateria - ps16
Deuterostomia - ps17
Chordata - ps18
Olfactores - ps19
Vertebrata - ps20
Gnathostomata - ps21
Euteleostomi - ps22
Sarcopterygii - ps23
Tetrapoda - ps24
Amniota - ps25
Mammalia - ps26
Theria - ps27
Placentalia - ps28
Boreoeutheria - ps29
Euarchontoglires - ps30
Euarchonta - ps31
Primates - ps32
Haplorrhini - ps33
Simiiformes - ps34
Catarrhini - ps35
Hominoidea - ps36
Hominidae - ps37
Homininae - ps38
Hominini - ps39
Homo_sapiens - ps40
p-value
1e-16
5e-13
3e-09
1e-05
7e-02
Log-odds
0.5
1.0
1.5
2.0
2.5
3.0
3.5
4.0

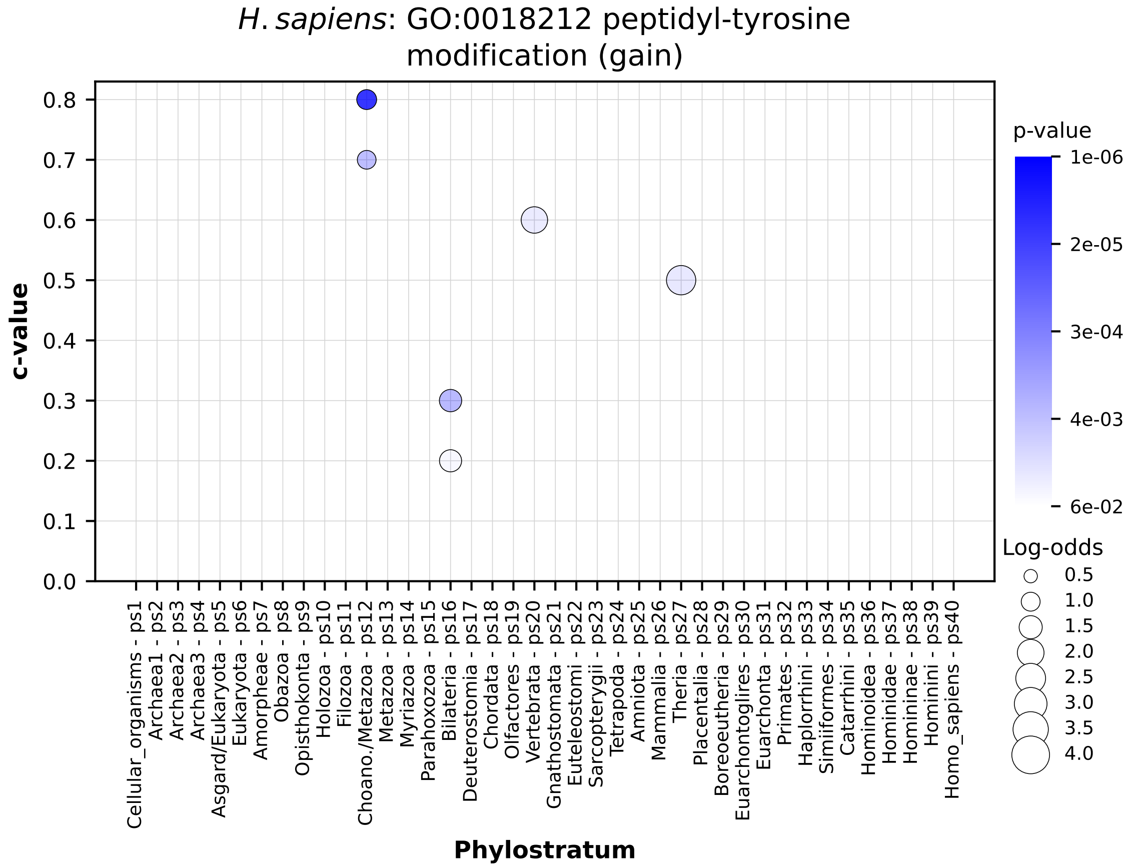

H. sapiens: GO:0018212 peptidyl-tyrosine modification (gain)
c-value
0.0
0.1
0.2
0.3
0.4
0.5
0.6
0.7
0.8
Phylostratum
Cellular_organisms - ps1
Archaea1 - ps2
Archaea2 - ps3
Archaea3 - ps4
Asgard/Eukaryota - ps5
Eukaryota - ps6
Amorpheae - ps7
Obazoa - ps8
Opisthokonta - ps9
Holozoa - ps10
Filozoa - ps11
Choano./Metazoa - ps12
Metazoa - ps13
Myriazoa - ps14
Parahoxozoa - ps15
Bilateria - ps16
Deuterostomia - ps17
Chordata - ps18
Olfactores - ps19
Vertebrata - ps20
Gnathostomata - ps21
Euteleostomi - ps22
Sarcopterygii - ps23
Tetrapoda - ps24
Amniota - ps25
Mammalia - ps26
Theria - ps27
Placentalia - ps28
Boreoeutheria - ps29
Euarchontoglires - ps30
Euarchonta - ps31
Primates - ps32
Haplorrhini - ps33
Simiiformes - ps34
Catarrhini - ps35
Hominoidea - ps36
Hominidae - ps37
Homininae - ps38
Hominini - ps39
Homo_sapiens - ps40
p-value
1e-06
2e-05
3e-04
4e-03
6e-02
Log-odds
0.5
1.0
1.5
2.0
2.5
3.0
3.5
4.0

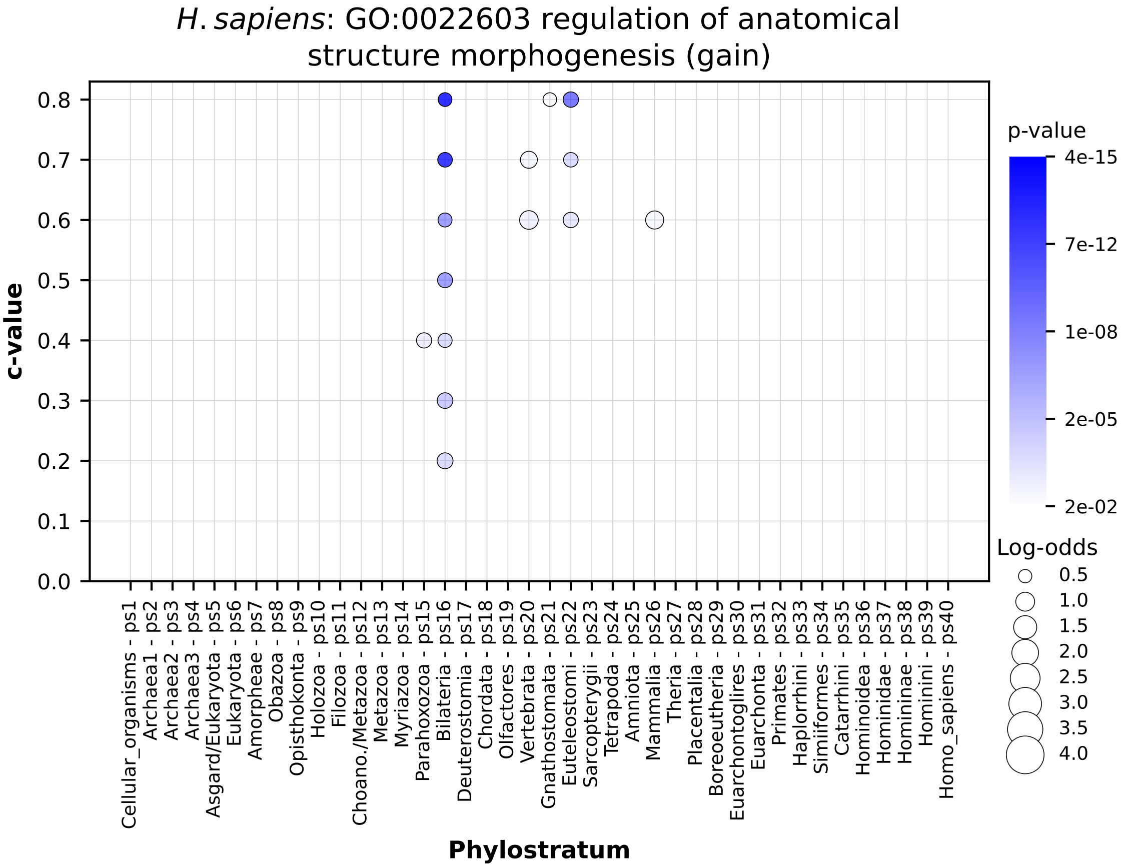

*H. sapiens*: GO:0022603 regulation of anatomical structure morphogenesis (gain)
c-value
0.0
0.1
0.2
0.3
0.4
0.5
0.6
0.7
0.8
Phylostratum
Cellular_organisms - ps1
Archaea1 - ps2
Archaea2 - ps3
Archaea3 - ps4
Asgard/Eukaryota - ps5
Eukaryota - ps6
Amorpheae - ps7
Obazoa - ps8
Opisthokonta - ps9
Holozoa - ps10
Filozoa - ps11
Choano./Metazoa - ps12
Metazoa - ps13
Myriazoa - ps14
Parahoxozoa - ps15
Bilateria - ps16
Deuterostomia - ps17
Chordata - ps18
Olfactores - ps19
Vertebrata - ps20
Gnathostomata - ps21
Euteleostomi - ps22
Sarcopterygii - ps23
Tetrapoda - ps24
Amniota - ps25
Mammalia - ps26
Theria - ps27
Placentalia - ps28
Boreoeutheria - ps29
Euarchontoglires - ps30
Euarchonta - ps31
Primates - ps32
Haplorrhini - ps33
Simiiformes - ps34
Catarrhini - ps35
Hominoidea - ps36
Hominidae - ps37
Homininae - ps38
Hominini - ps39
Homo_sapiens - ps40
p-value
4e-15
7e-12
1e-08
2e-05
2e-02
Log-odds
0.5
1.0
1.5
2.0
2.5
3.0
3.5
4.0

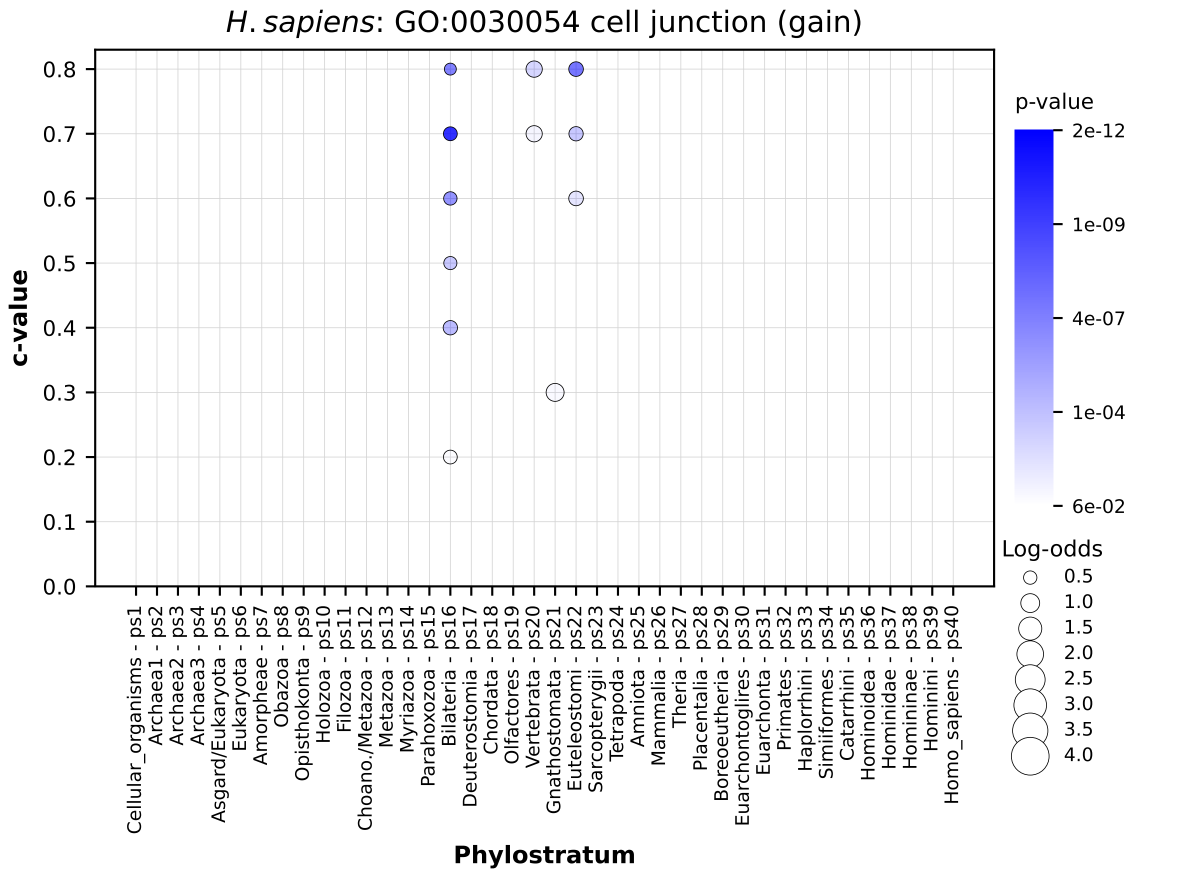

H. sapiens: GO:0030054 cell junction (gain)
c-value
0.8
0.7
0.6
0.5
0.4
0.3
0.2
0.1
0.0
p-value
2e-12
1e-09
4e-07
1e-04
6e-02
Log-odds
0.5
1.0
1.5
2.0
2.5
3.0
3.5
4.0
Cellular_organisms - ps1
Archaea1 - ps2
Archaea2 - ps3
Archaea3 - ps4
Asgard/Eukaryota - ps5
Eukaryota - ps6
Amorpheae - ps7
Obazoa - ps8
Opisthokonta - ps9
Holozoa - ps10
Filozoa - ps11
Choano./Metazoa - ps12
Metazoa - ps13
Myriazoa - ps14
Parahoxozoa - ps15
Bilateria - ps16
Deuterostomia - ps17
Chordata - ps18
Olfactores - ps19
Vertebrata - ps20
Gnathostomata - ps21
Euteleostomi - ps22
Sarcopterygii - ps23
Tetrapoda - ps24
Amniota - ps25
Mammalia - ps26
Theria - ps27
Placentalia - ps28
Boreoeutheria - ps29
Euarchontoglires - ps30
Euarchonta - ps31
Primates - ps32
Haplorrhini - ps33
Simiiformes - ps34
Catarrhini - ps35
Hominoidea - ps36
Hominidae - ps37
Homininae - ps38
Hominini - ps39
Homo_sapiens - ps40
Phylostratum

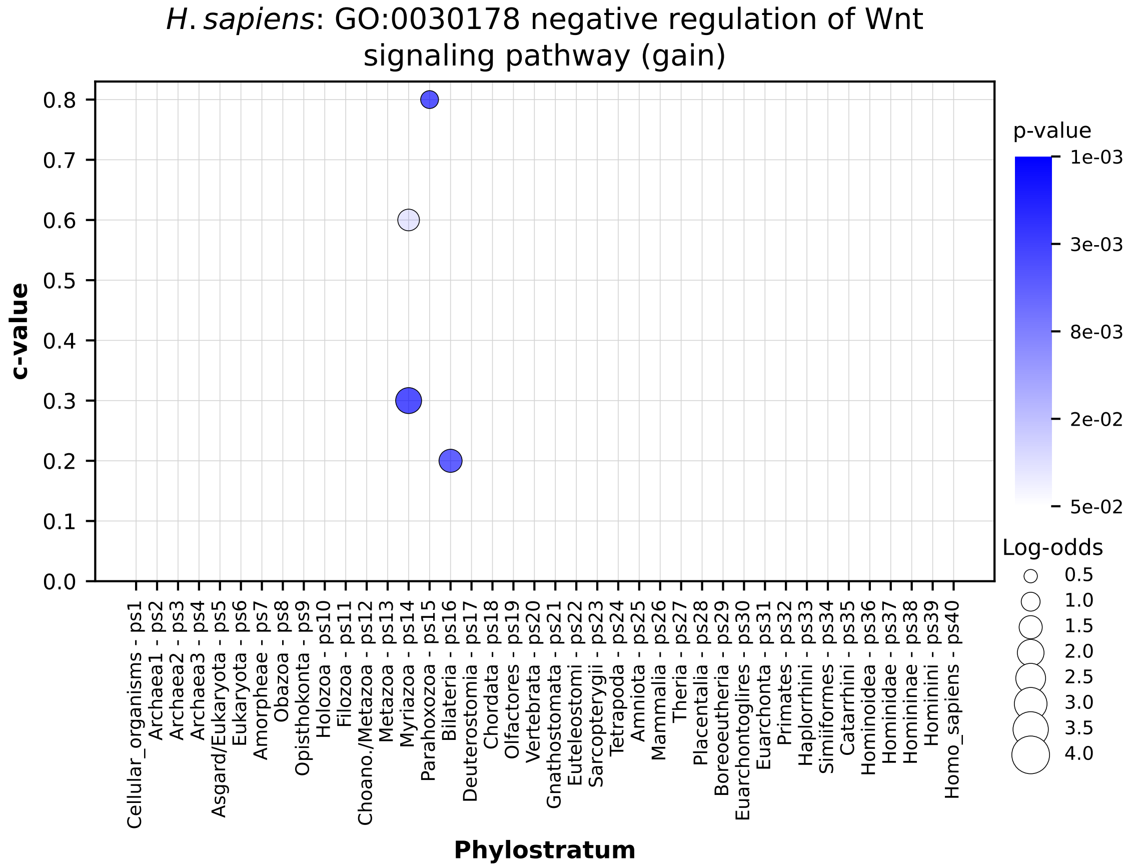

H. sapiens: GO:0030178 negative regulation of Wnt signaling pathway (gain)
c-value
0.0
0.1
0.2
0.3
0.4
0.5
0.6
0.7
0.8
Phylostratum
Cellular_organisms - ps1
Archaea1 - ps2
Archaea2 - ps3
Archaea3 - ps4
Asgard/Eukaryota - ps5
Eukaryota - ps6
Amorpheae - ps7
Obazoa - ps8
Opisthokonta - ps9
Holozoa - ps10
Filozoa - ps11
Choano./Metazoa - ps12
Metazoa - ps13
Myriazoa - ps14
Parahoxozoa - ps15
Bilateria - ps16
Deuterostomia - ps17
Chordata - ps18
Olfactores - ps19
Vertebrata - ps20
Gnathostomata - ps21
Euteleostomi - ps22
Sarcopterygii - ps23
Tetrapoda - ps24
Amniota - ps25
Mammalia - ps26
Theria - ps27
Placentalia - ps28
Boreoeutheria - ps29
Euarchontoglires - ps30
Euarchonta - ps31
Primates - ps32
Haplorrhini - ps33
Simiiformes - ps34
Catarrhini - ps35
Hominoidea - ps36
Hominidae - ps37
Homininae - ps38
Hominini - ps39
Homo_sapiens - ps40
p-value
1e-03
3e-03
8e-03
2e-02
5e-02
Log-odds
0.5
1.0
1.5
2.0
2.5
3.0
3.5
4.0

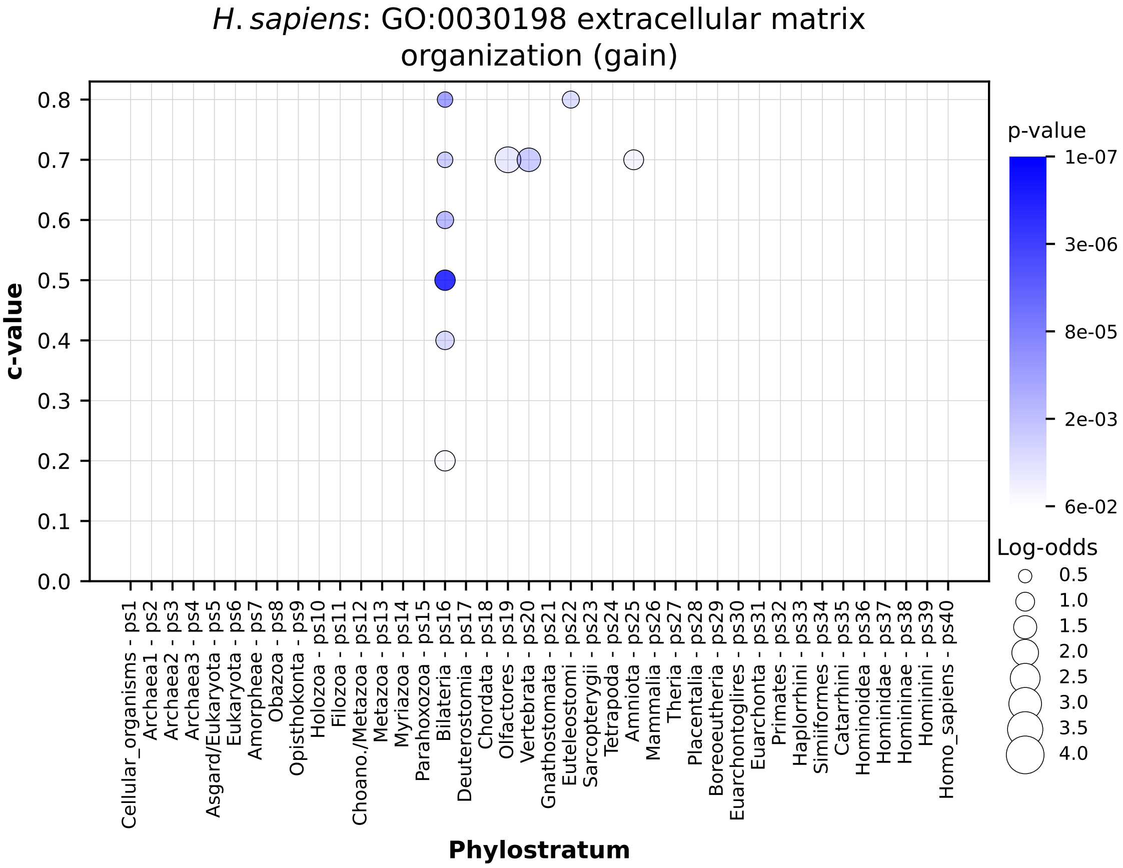

H. sapiens: GO:0030198 extracellular matrix organization (gain)
c-value
0.0
0.1
0.2
0.3
0.4
0.5
0.6
0.7
0.8
Phylostratum
Cellular_organisms - ps1
Archaea1 - ps2
Archaea2 - ps3
Archaea3 - ps4
Asgard/Eukaryota - ps5
Eukaryota - ps6
Amorpheae - ps7
Obazoa - ps8
Opisthokonta - ps9
Holozoa - ps10
Filozoa - ps11
Choano./Metazoa - ps12
Metazoa - ps13
Myriazoa - ps14
Parahoxozoa - ps15
Bilateria - ps16
Deuterostomia - ps17
Chordata - ps18
Olfactores - ps19
Vertebrata - ps20
Gnathostomata - ps21
Euteleostomi - ps22
Sarcopterygii - ps23
Tetrapoda - ps24
Amniota - ps25
Mammalia - ps26
Theria - ps27
Placentalia - ps28
Boreoeutheria - ps29
Euarchontoglires - ps30
Euarchonta - ps31
Primates - ps32
Haplorrhini - ps33
Simiiformes - ps34
Catarrhini - ps35
Hominoidea - ps36
Hominidae - ps37
Homininae - ps38
Hominini - ps39
Homo_sapiens - ps40
p-value
1e-07
3e-06
8e-05
2e-03
6e-02
Log-odds
0.5
1.0
1.5
2.0
2.5
3.0
3.5
4.0

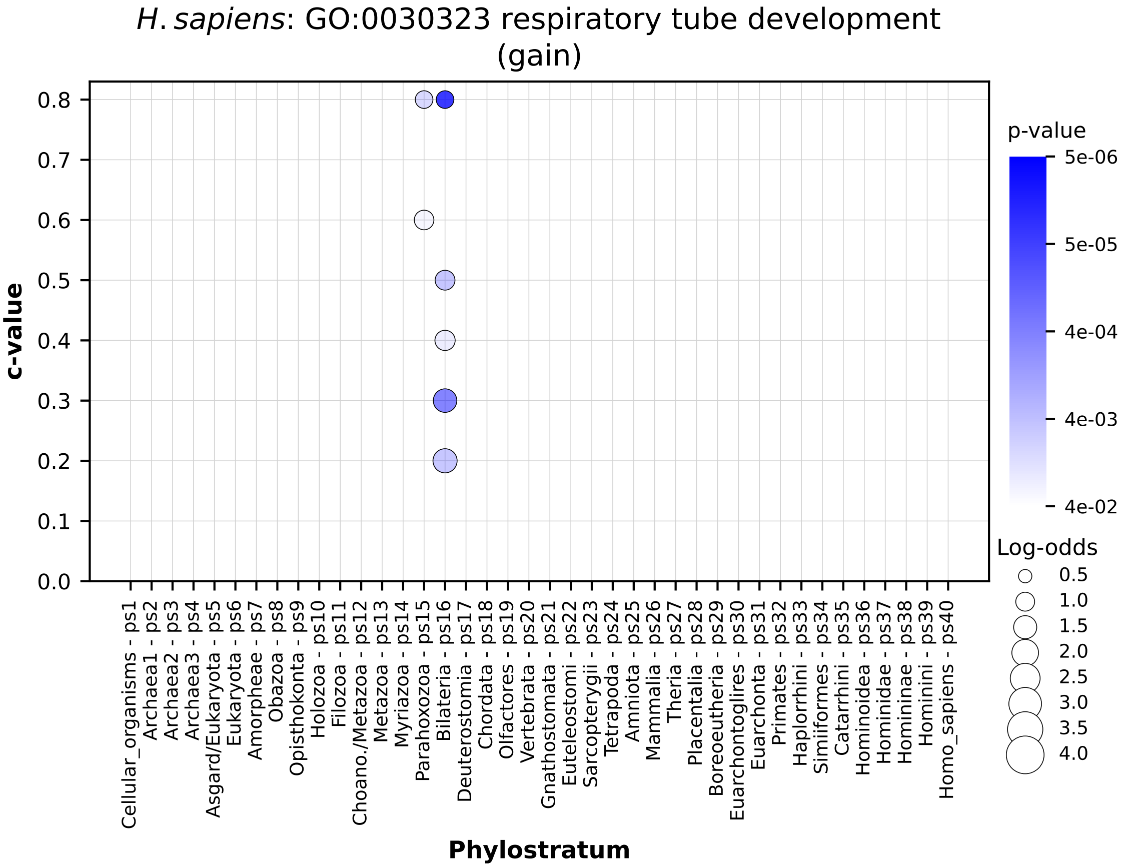

H. sapiens: GO:0030323 respiratory tube development
(gain)
c-value
0.0
0.1
0.2
0.3
0.4
0.5
0.6
0.7
0.8
Phylostratum
Cellular_organisms - ps1
Archaea1 - ps2
Archaea2 - ps3
Archaea3 - ps4
Asgard/Eukaryota - ps5
Eukaryota - ps6
Amorpheae - ps7
Obazoa - ps8
Opisthokonta - ps9
Holozoa - ps10
Filozoa - ps11
Choano./Metazoa - ps12
Metazoa - ps13
Myriazoa - ps14
Parahoxozoa - ps15
Bilateria - ps16
Deuterostomia - ps17
Chordata - ps18
Olfactores - ps19
Vertebrata - ps20
Gnathostomata - ps21
Euteleostomi - ps22
Sarcopterygii - ps23
Tetrapoda - ps24
Amniota - ps25
Mammalia - ps26
Theria - ps27
Placentalia - ps28
Boreoeutheria - ps29
Euarchontoglires - ps30
Euarchonta - ps31
Primates - ps32
Haplorrhini - ps33
Simiiformes - ps34
Catarrhini - ps35
Hominoidea - ps36
Hominidae - ps37
Homininae - ps38
Hominini - ps39
Homo_sapiens - ps40
p-value
5e-06
5e-05
4e-04
4e-03
4e-02
Log-odds
0.5
1.0
1.5
2.0
2.5
3.0
3.5
4.0

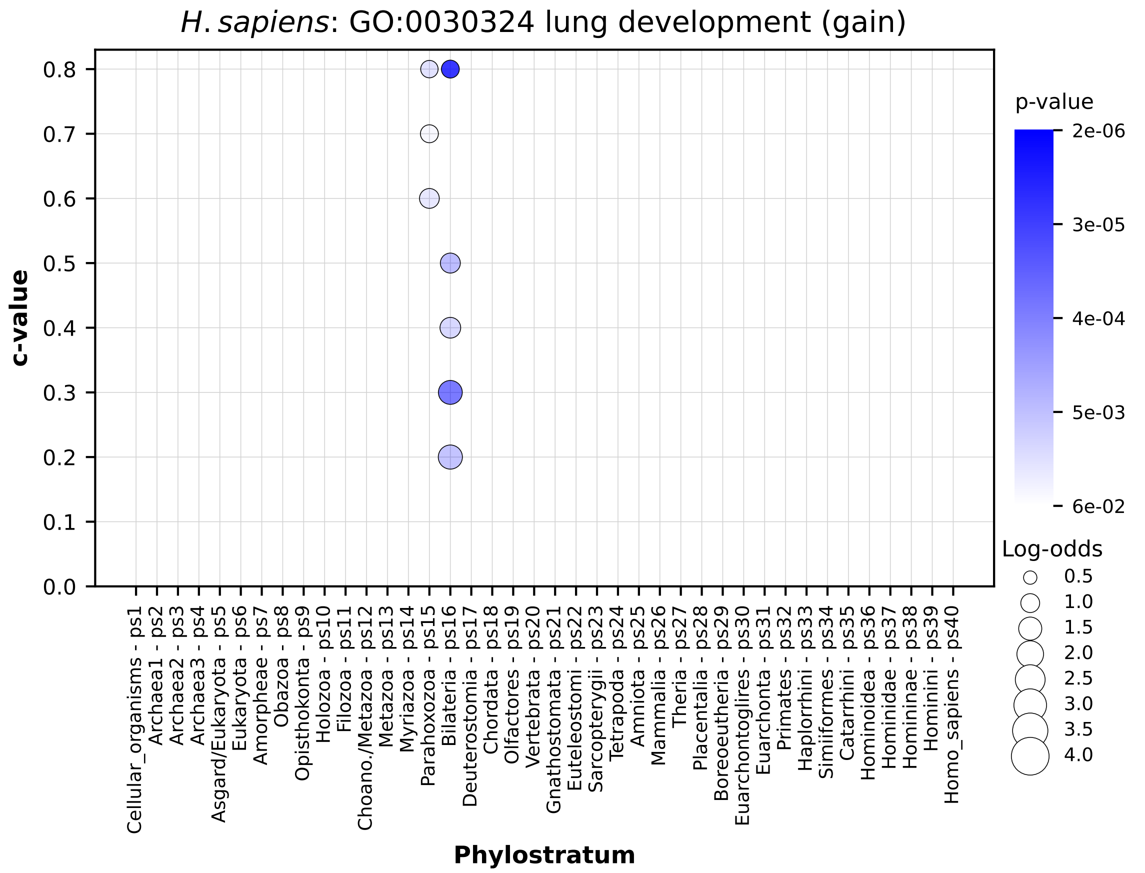

H. sapiens: GO:0030324 lung development (gain)
c-value
0.0
0.1
0.2
0.3
0.4
0.5
0.6
0.7
0.8
p-value
2e-06
3e-05
4e-04
5e-03
6e-02
Log-odds
0.5
1.0
1.5
2.0
2.5
3.0
3.5
4.0
Cellular_organisms - ps1
Archaea1 - ps2
Archaea2 - ps3
Archaea3 - ps4
Asgard/Eukaryota - ps5
Eukaryota - ps6
Amorpheae - ps7
Obazoa - ps8
Opisthokonta - ps9
Holozoa - ps10
Filozoa - ps11
Choano./Metazoa - ps12
Metazoa - ps13
Myriazoa - ps14
Parahoxozoa - ps15
Bilateria - ps16
Deuterostomia - ps17
Chordata - ps18
Olfactores - ps19
Vertebrata - ps20
Gnathostomata - ps21
Euteleostomi - ps22
Sarcopterygii - ps23
Tetrapoda - ps24
Amniota - ps25
Mammalia - ps26
Theria - ps27
Placentalia - ps28
Boreoeutheria - ps29
Euarchontoglires - ps30
Euarchonta - ps31
Primates - ps32
Haplorrhini - ps33
Simiiformes - ps34
Catarrhini - ps35
Hominoidea - ps36
Hominidae - ps37
Homininae - ps38
Hominini - ps39
Homo_sapiens - ps40
Phylostratum

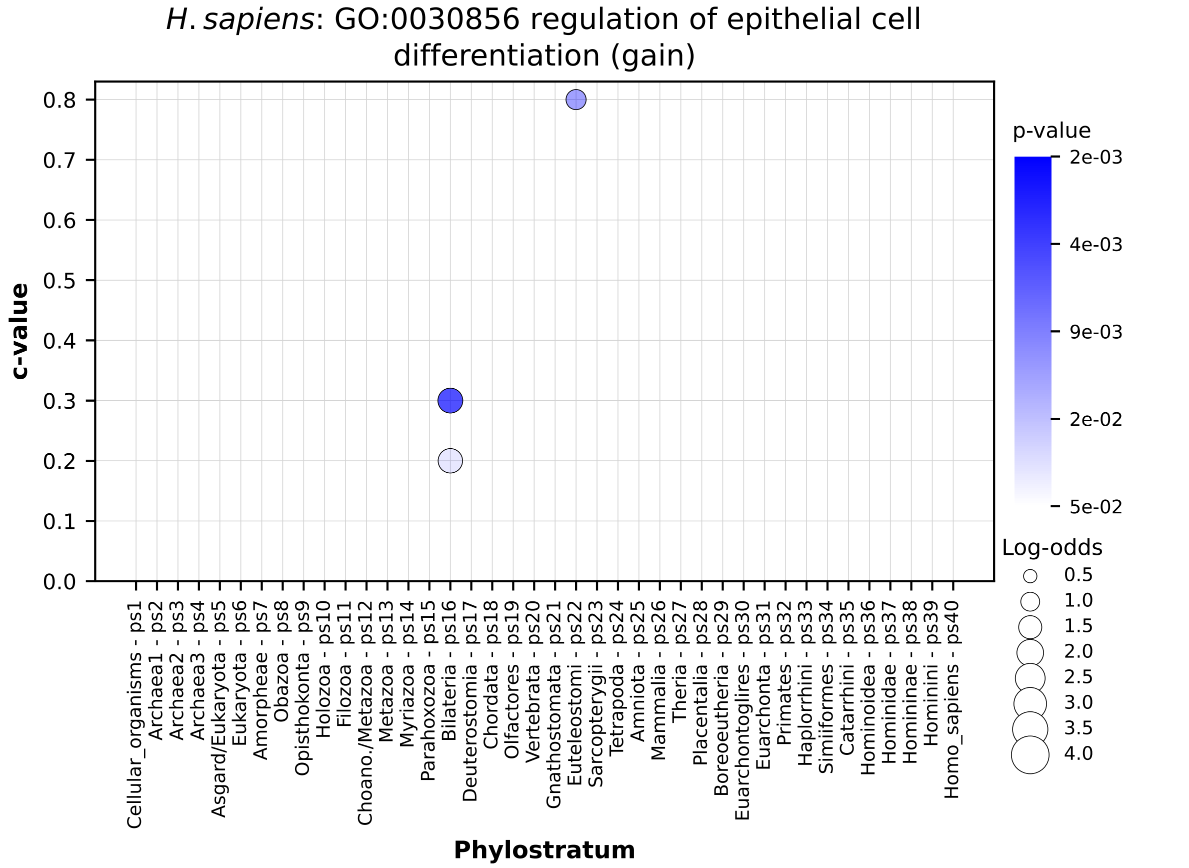

H. sapiens: GO:0030856 regulation of epithelial cell differentiation (gain)
c-value
0.0
0.1
0.2
0.3
0.4
0.5
0.6
0.7
0.8
Phylostratum
Cellular_organisms - ps1
Archaea1 - ps2
Archaea2 - ps3
Archaea3 - ps4
Asgard/Eukaryota - ps5
Eukaryota - ps6
Amorpheae - ps7
Obazoa - ps8
Opisthokonta - ps9
Holozoa - ps10
Filozoa - ps11
Choano./Metazoa - ps12
Metazoa - ps13
Myriazoa - ps14
Parahoxozoa - ps15
Bilateria - ps16
Deuterostomia - ps17
Chordata - ps18
Olfactores - ps19
Vertebrata - ps20
Gnathostomata - ps21
Euteleostomi - ps22
Sarcopterygii - ps23
Tetrapoda - ps24
Amniota - ps25
Mammalia - ps26
Theria - ps27
Placentalia - ps28
Boreoeutheria - ps29
Euarchontoglires - ps30
Euarchonta - ps31
Primates - ps32
Haplorrhini - ps33
Simiiformes - ps34
Catarrhini - ps35
Hominoidea - ps36
Hominidae - ps37
Homininae - ps38
Hominini - ps39
Homo_sapiens - ps40
p-value
2e-03
4e-03
9e-03
2e-02
5e-02
Log-odds
0.5
1.0
1.5
2.0
2.5
3.0
3.5
4.0

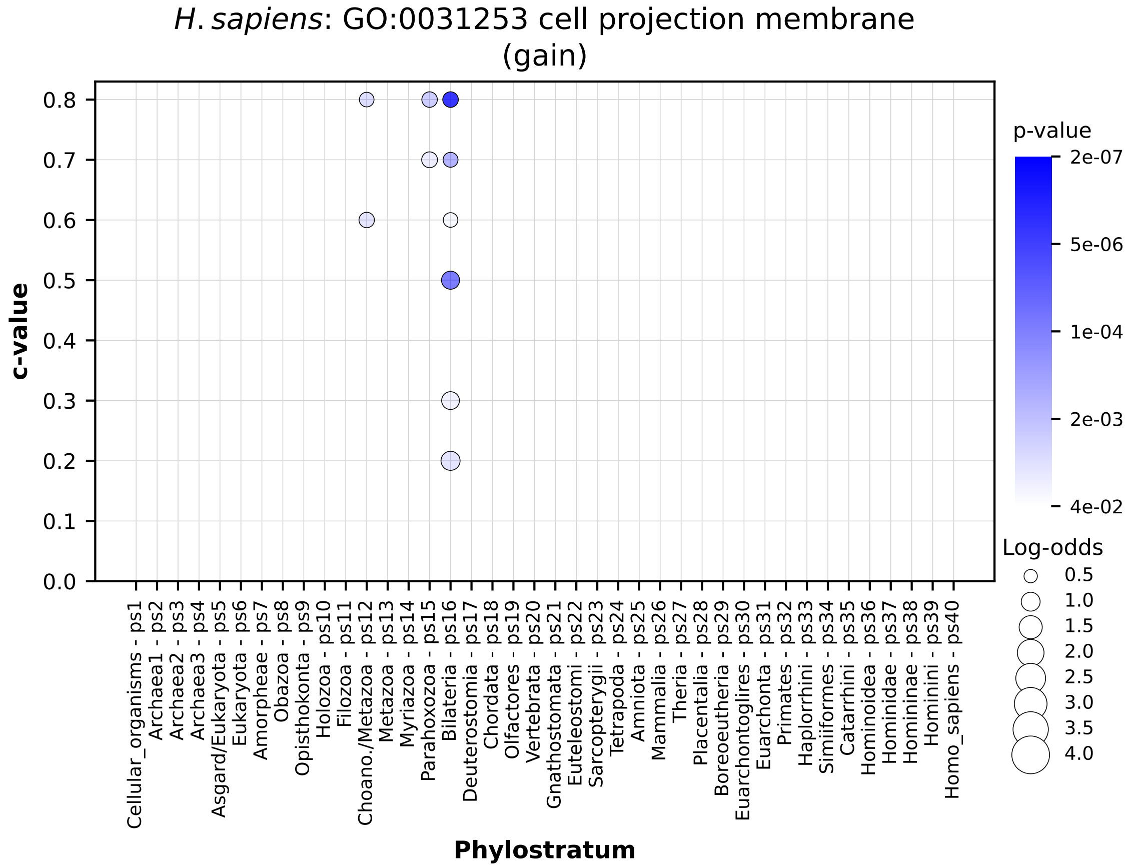
H. sapiens: GO:0031253 cell projection membrane
(gain)
c-value
0.8
0.7
0.6
0.5
0.4
0.3
0.2
0.1
0.0
Phylostratum
Cellular_organisms - ps1
Archaea1 - ps2
Archaea2 - ps3
Archaea3 - ps4
Asgard/Eukaryota - ps5
Eukaryota - ps6
Amorpheae - ps7
Obazoa - ps8
Opisthokonta - ps9
Holozoa - ps10
Filozoa - ps11
Choano./Metazoa - ps12
Metazoa - ps13
Myriazoa - ps14
Parahoxozoa - ps15
Bilateria - ps16
Deuterostomia - ps17
Chordata - ps18
Olfactores - ps19
Vertebrata - ps20
Gnathostomata - ps21
Euteleostomi - ps22
Sarcopterygii - ps23
Tetrapoda - ps24
Amniota - ps25
Mammalia - ps26
Theria - ps27
Placentalia - ps28
Boreoeutheria - ps29
Euarchontoglires - ps30
Euarchonta - ps31
Primates - ps32
Haplorrhini - ps33
Simiiformes - ps34
Catarrhini - ps35
Hominoidea - ps36
Hominidae - ps37
Homininae - ps38
Hominini - ps39
Homo_sapiens - ps40
p-value
2e-07
5e-06
1e-04
2e-03
4e-02
Log-odds
0.5
1.0
1.5
2.0
2.5
3.0
3.5
4.0

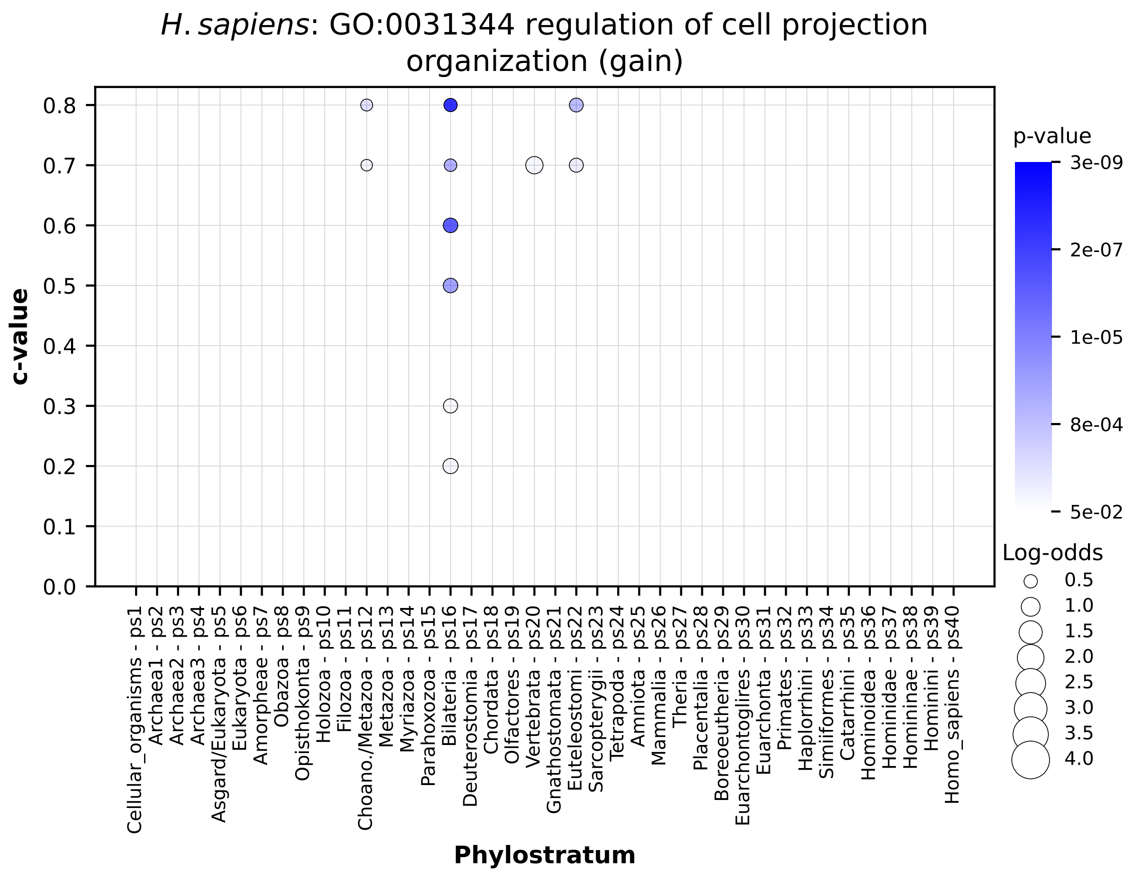

H. sapiens: GO:0031344 regulation of cell projection organization (gain)
c-value
Phylostratum
p-value
3e-09
2e-07
1e-05
8e-04
5e-02
Log-odds
0.5
1.0
1.5
2.0
2.5
3.0
3.5
4.0
Cellular_organisms - ps1
Archaea1 - ps2
Archaea2 - ps3
Archaea3 - ps4
Asgard/Eukaryota - ps5
Eukaryota - ps6
Amorpheae - ps7
Obazoa - ps8
Opisthokonta - ps9
Holozoa - ps10
Filozoa - ps11
Choano./Metazoa - ps12
Metazoa - ps13
Myriazoa - ps14
Parahoxozoa - ps15
Bilateria - ps16
Deuterostomia - ps17
Chordata - ps18
Olfactores - ps19
Vertebrata - ps20
Gnathostomata - ps21
Euteleostomi - ps22
Sarcopterygii - ps23
Tetrapoda - ps24
Amniota - ps25
Mammalia - ps26
Theria - ps27
Placentalia - ps28
Boreoeutheria - ps29
Euarchontoglires - ps30
Euarchonta - ps31
Primates - ps32
Haplorrhini - ps33
Simiiformes - ps34
Catarrhini - ps35
Hominoidea - ps36
Hominidae - ps37
Homininae - ps38
Hominini - ps39
Homo_sapiens - ps40

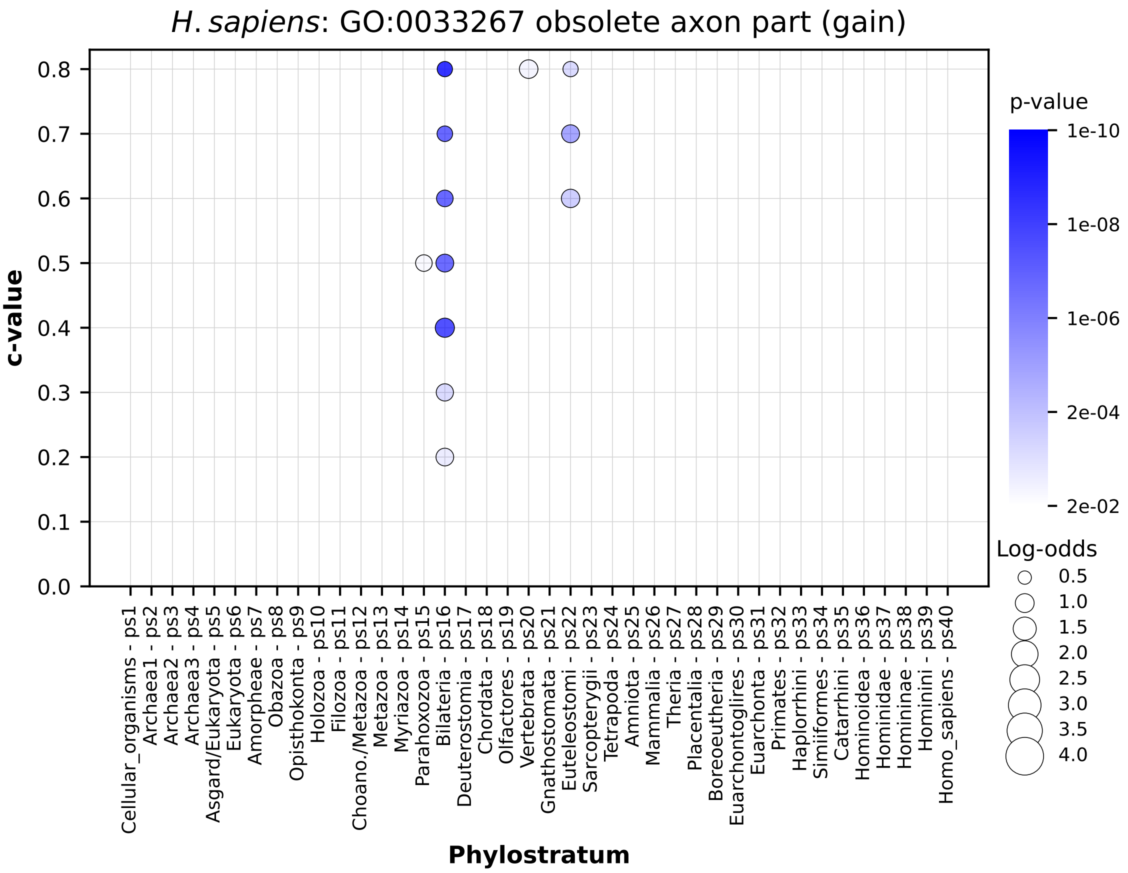
H. sapiens: GO:0033267 obsolete axon part (gain)
c-value
0.0
0.1
0.2
0.3
0.4
0.5
0.6
0.7
0.8
Phylostratum
Cellular_organisms - ps1
Archaea1 - ps2
Archaea2 - ps3
Archaea3 - ps4
Asgard/Eukaryota - ps5
Eukaryota - ps6
Amorpheae - ps7
Obazoa - ps8
Opisthokonta - ps9
Holozoa - ps10
Filozoa - ps11
Choano./Metazoa - ps12
Metazoa - ps13
Myriazoa - ps14
Parahoxozoa - ps15
Bilateria - ps16
Deuterostomia - ps17
Chordata - ps18
Olfactores - ps19
Vertebrata - ps20
Gnathostomata - ps21
Euteleostomi - ps22
Sarcopterygii - ps23
Tetrapoda - ps24
Amniota - ps25
Mammalia - ps26
Theria - ps27
Placentalia - ps28
Boreoeutheria - ps29
Euarchontoglires - ps30
Euarchonta - ps31
Primates - ps32
Haplorrhini - ps33
Simiiformes - ps34
Catarrhini - ps35
Hominoidea - ps36
Hominidae - ps37
Homininae - ps38
Hominini - ps39
Homo_sapiens - ps40
p-value
1e-10
1e-08
1e-06
2e-04
2e-02
Log-odds
0.5
1.0
1.5
2.0
2.5
3.0
3.5
4.0

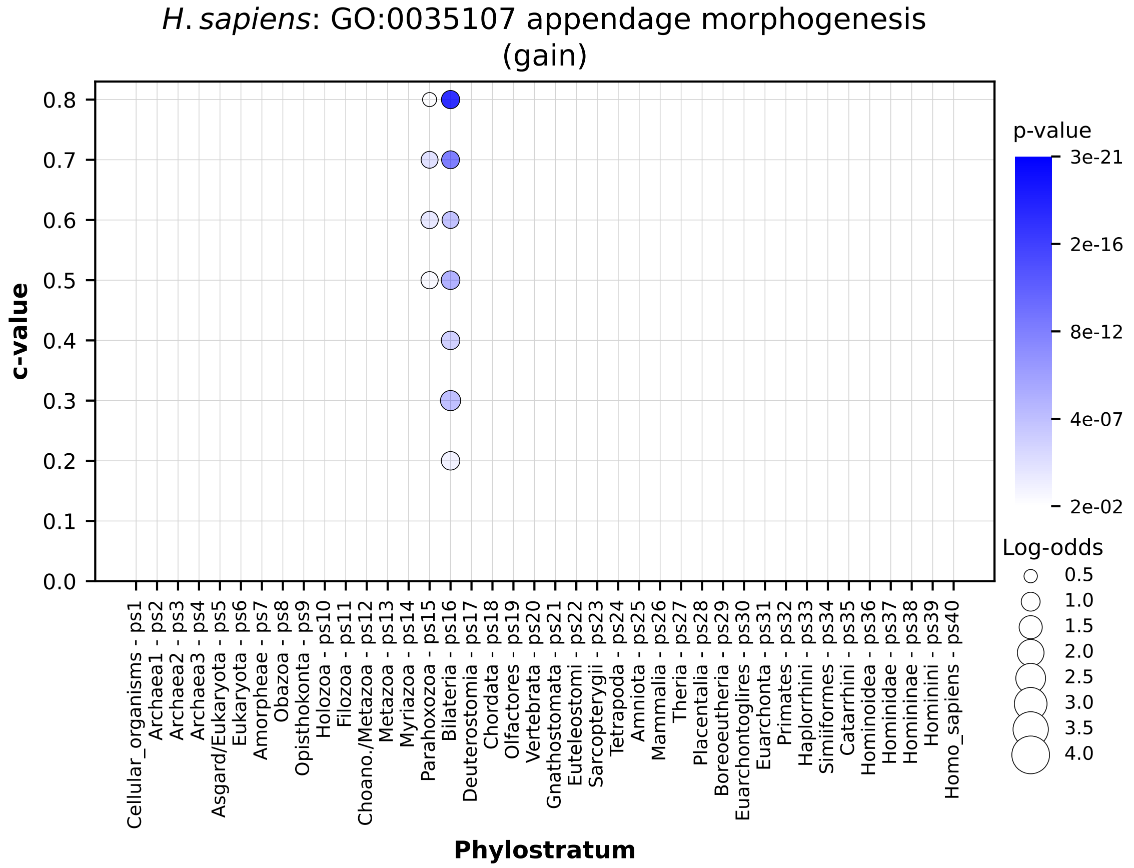

H. sapiens: GO:0035107 appendage morphogenesis
(gain)
c-value
0.0
0.1
0.2
0.3
0.4
0.5
0.6
0.7
0.8
Phylostratum
Cellular_organisms - ps1
Archaea1 - ps2
Archaea2 - ps3
Archaea3 - ps4
Asgard/Eukaryota - ps5
Eukaryota - ps6
Amorpheae - ps7
Obazoa - ps8
Opisthokonta - ps9
Holozoa - ps10
Filozoa - ps11
Choano./Metazoa - ps12
Metazoa - ps13
Myriazoa - ps14
Parahoxozoa - ps15
Bilateria - ps16
Deuterostomia - ps17
Chordata - ps18
Olfactores - ps19
Vertebrata - ps20
Gnathostomata - ps21
Euteleostomi - ps22
Sarcopterygii - ps23
Tetrapoda - ps24
Amniota - ps25
Mammalia - ps26
Theria - ps27
Placentalia - ps28
Boreoeutheria - ps29
Euarchontoglires - ps30
Euarchonta - ps31
Primates - ps32
Haplorrhini - ps33
Simiiformes - ps34
Catarrhini - ps35
Hominoidea - ps36
Hominidae - ps37
Homininae - ps38
Hominini - ps39
Homo_sapiens - ps40
p-value
3e-21
2e-16
8e-12
4e-07
2e-02
Log-odds
0.5
1.0
1.5
2.0
2.5
3.0
3.5
4.0

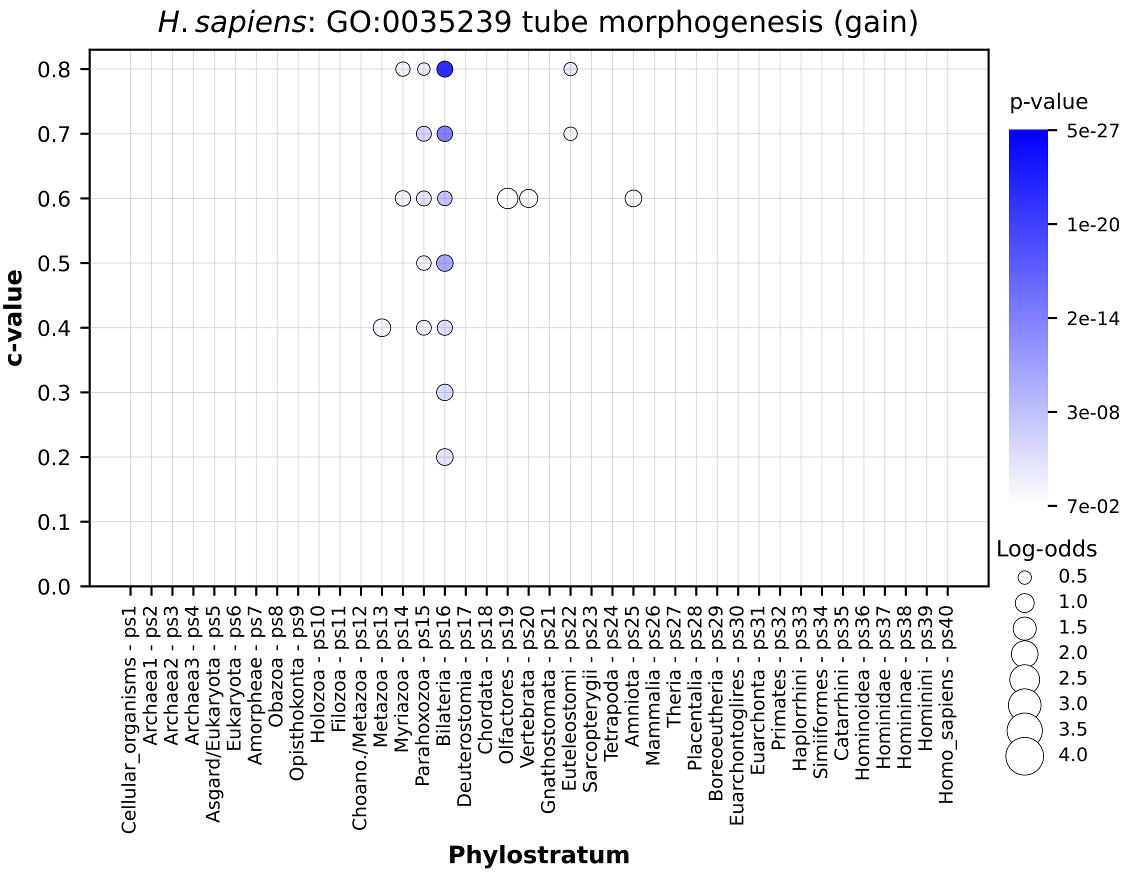

H. sapiens: GO:0035239 tube morphogenesis (gain)
c-value
0.8
0.7
0.6
0.5
0.4
0.3
0.2
0.1
0.0
Phylostratum
Cellular_organisms - ps1
Archaea1 - ps2
Archaea2 - ps3
Archaea3 - ps4
Asgard/Eukaryota - ps5
Eukaryota - ps6
Amorpheae - ps7
Obazoa - ps8
Opisthokonta - ps9
Holozoa - ps10
Filozoa - ps11
Choano./Metazoa - ps12
Metazoa - ps13
Myriazoa - ps14
Parahoxozoa - ps15
Bilateria - ps16
Deuterostomia - ps17
Chordata - ps18
Olfactores - ps19
Vertebrata - ps20
Gnathostomata - ps21
Euteleostomi - ps22
Sarcopterygii - ps23
Tetrapoda - ps24
Amniota - ps25
Mammalia - ps26
Theria - ps27
Placentalia - ps28
Boreoeutheria - ps29
Euarchontoglires - ps30
Euarchonta - ps31
Primates - ps32
Haplorrhini - ps33
Simiiformes - ps34
Catarrhini - ps35
Hominoidea - ps36
Hominidae - ps37
Homininae - ps38
Hominini - ps39
Homo_sapiens - ps40
p-value
5e-27
1e-20
2e-14
3e-08
7e-02
Log-odds
0.5
1.0
1.5
2.0
2.5
3.0
3.5
4.0

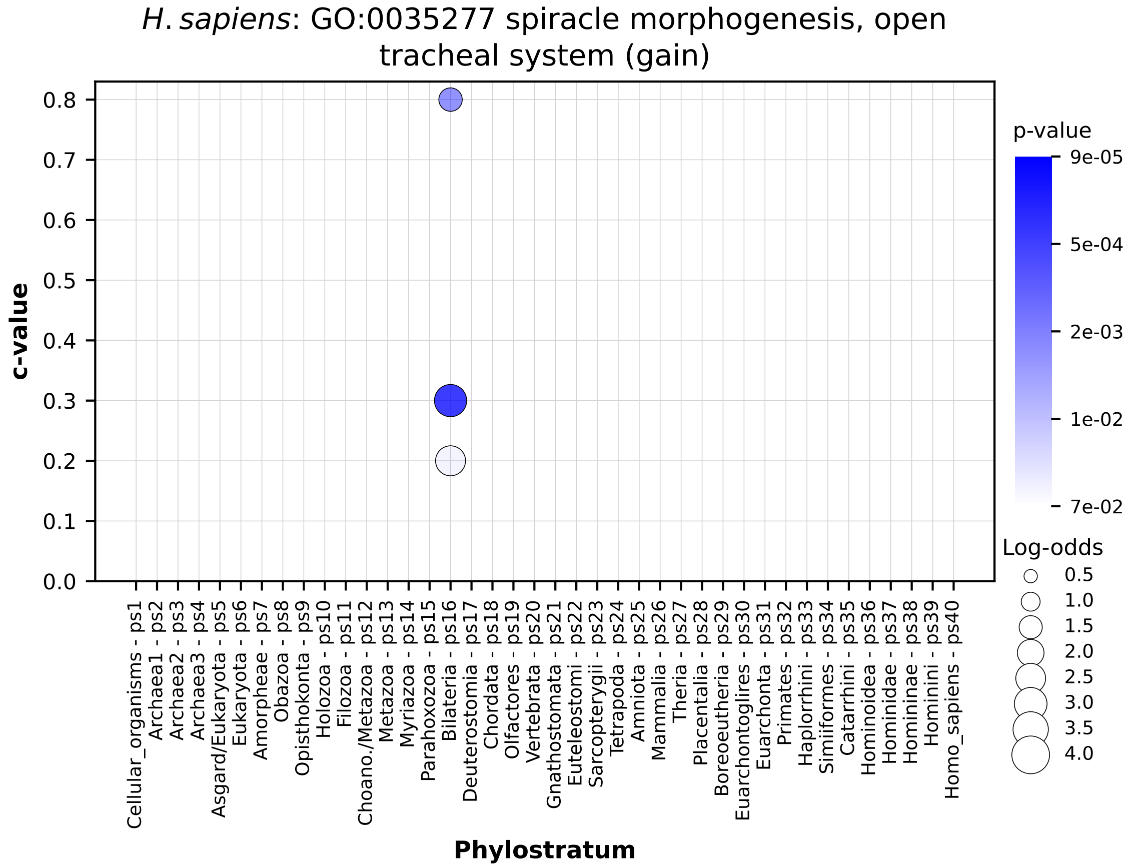

H. sapiens: GO:0035277 spiracle morphogenesis, open tracheal system (gain)
c-value
0.8
0.7
0.6
0.5
0.4
0.3
0.2
0.1
0.0
Phylostratum
Cellular_organisms - ps1
Archaea1 - ps2
Archaea2 - ps3
Archaea3 - ps4
Asgard/Eukaryota - ps5
Eukaryota - ps6
Amorpheae - ps7
Obazoa - ps8
Opisthokonta - ps9
Holozoa - ps10
Filozoa - ps11
Choano./Metazoa - ps12
Metazoa - ps13
Myriazoa - ps14
Parahoxozoa - ps15
Bilateria - ps16
Deuterostomia - ps17
Chordata - ps18
Olfactores - ps19
Vertebrata - ps20
Gnathostomata - ps21
Euteleostomi - ps22
Sarcopterygii - ps23
Tetrapoda - ps24
Amniota - ps25
Mammalia - ps26
Theria - ps27
Placentalia - ps28
Boreoeutheria - ps29
Euarchontoglires - ps30
Euarchonta - ps31
Primates - ps32
Haplorrhini - ps33
Simiiformes - ps34
Catarrhini - ps35
Hominoidea - ps36
Hominidae - ps37
Homininae - ps38
Hominini - ps39
Homo_sapiens - ps40
p-value
9e-05
5e-04
2e-03
1e-02
7e-02
Log-odds
0.5
1.0
1.5
2.0
2.5
3.0
3.5
4.0

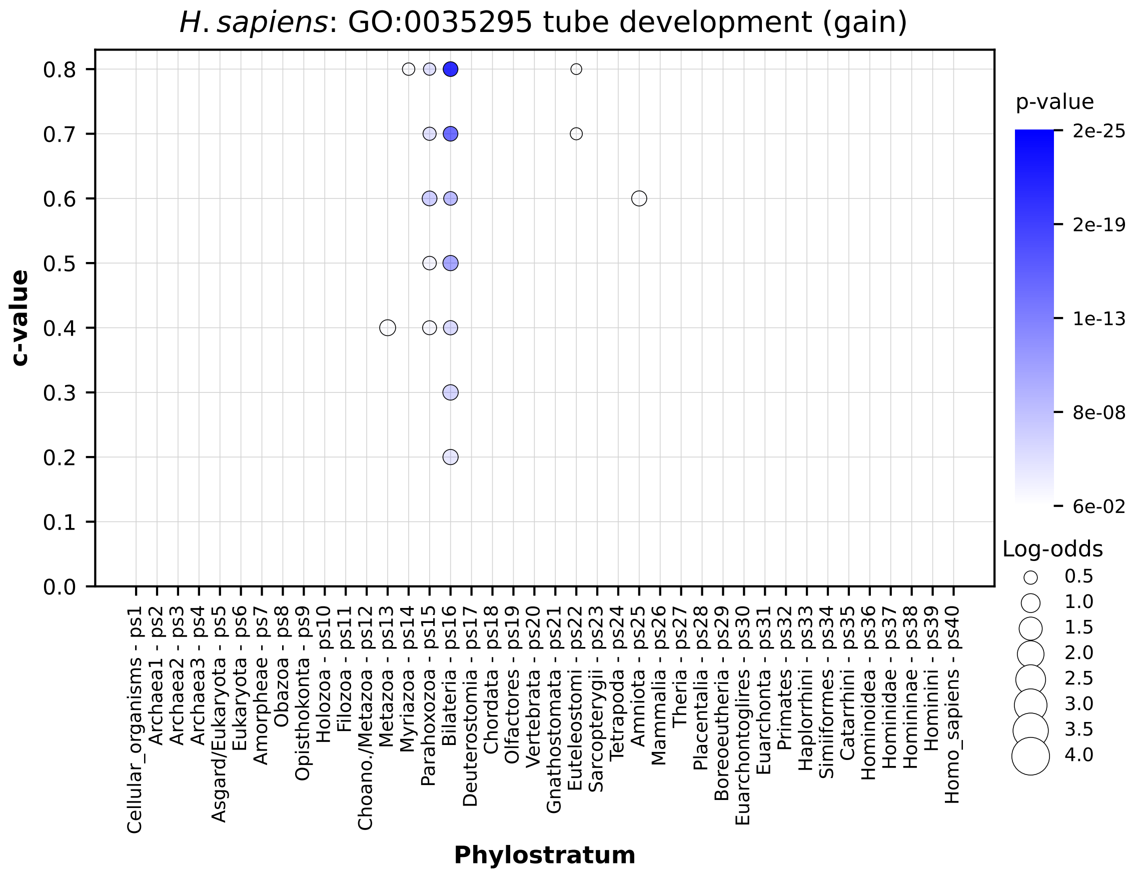

*H. sapiens*: GO:0035295 tube development (gain)
c-value
Phylostratum
p-value
2e-25
2e-19
1e-13
8e-08
6e-02
Log-odds
0.5
1.0
1.5
2.0
2.5
3.0
3.5
4.0
Cellular_organisms - ps1
Archaea1 - ps2
Archaea2 - ps3
Archaea3 - ps4
Asgard/Eukaryota - ps5
Eukaryota - ps6
Amorpheae - ps7
Obazoa - ps8
Opisthokonta - ps9
Holozoa - ps10
Filozoa - ps11
Choano./Metazoa - ps12
Metazoa - ps13
Myriazoa - ps14
Parahoxozoa - ps15
Bilateria - ps16
Deuterostomia - ps17
Chordata - ps18
Olfactores - ps19
Vertebrata - ps20
Gnathostomata - ps21
Euteleostomi - ps22
Sarcopterygii - ps23
Tetrapoda - ps24
Amniota - ps25
Mammalia - ps26
Theria - ps27
Placentalia - ps28
Boreoeutheria - ps29
Euarchontoglires - ps30
Euarchonta - ps31
Primates - ps32
Haplorrhini - ps33
Simiiformes - ps34
Catarrhini - ps35
Hominoidea - ps36
Hominidae - ps37
Homininae - ps38
Hominini - ps39
Homo_sapiens - ps40

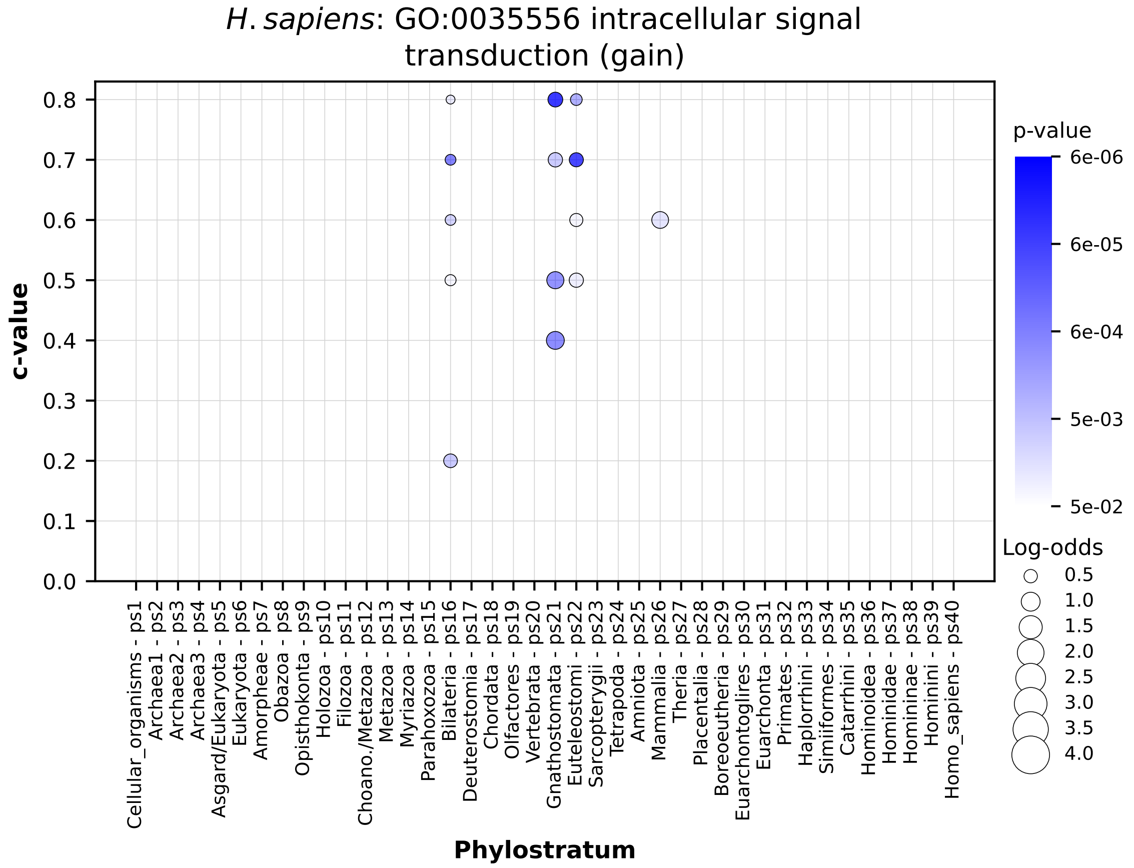

H. sapiens: GO:0035556 intracellular signal transduction (gain)
c-value
0.0
0.1
0.2
0.3
0.4
0.5
0.6
0.7
0.8
Phylostratum
Cellular_organisms - ps1
Archaea1 - ps2
Archaea2 - ps3
Archaea3 - ps4
Asgard/Eukaryota - ps5
Eukaryota - ps6
Amorpheae - ps7
Obazoa - ps8
Opisthokonta - ps9
Holozoa - ps10
Filozoa - ps11
Choano./Metazoa - ps12
Metazoa - ps13
Myriazoa - ps14
Parahoxozoa - ps15
Bilateria - ps16
Deuterostomia - ps17
Chordata - ps18
Olfactores - ps19
Vertebrata - ps20
Gnathostomata - ps21
Euteleostomi - ps22
Sarcopterygii - ps23
Tetrapoda - ps24
Amniota - ps25
Mammalia - ps26
Theria - ps27
Placentalia - ps28
Boreoeutheria - ps29
Euarchontoglires - ps30
Euarchonta - ps31
Primates - ps32
Haplorrhini - ps33
Simiiformes - ps34
Catarrhini - ps35
Hominoidea - ps36
Hominidae - ps37
Homininae - ps38
Hominini - ps39
Homo_sapiens - ps40
p-value
6e-06
6e-05
6e-04
5e-03
5e-02
Log-odds
0.5
1.0
1.5
2.0
2.5
3.0
3.5
4.0

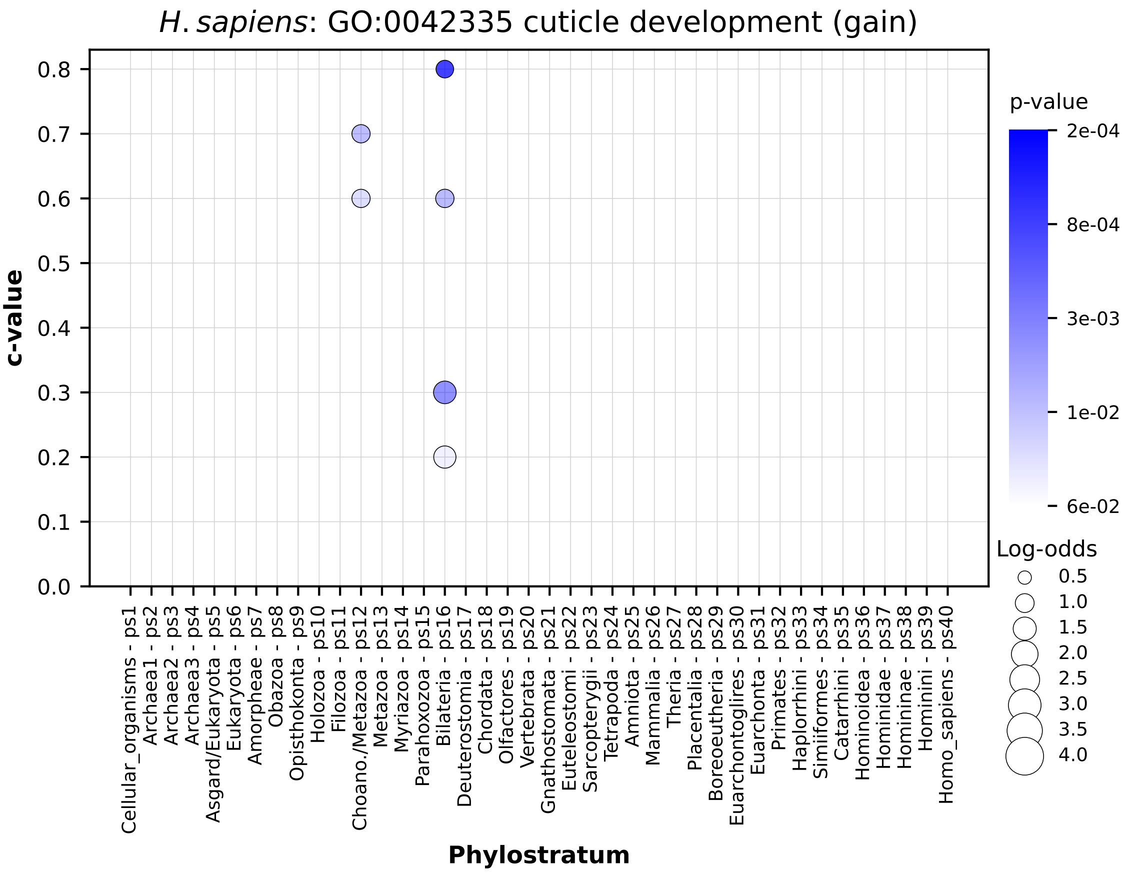

H. sapiens: GO:0042335 cuticle development (gain)
c-value
0.0
0.1
0.2
0.3
0.4
0.5
0.6
0.7
0.8
Phylostratum
Cellular_organisms - ps1
Archaea1 - ps2
Archaea2 - ps3
Archaea3 - ps4
Asgard/Eukaryota - ps5
Eukaryota - ps6
Amorpheae - ps7
Obazoa - ps8
Opisthokonta - ps9
Holozoa - ps10
Filozoa - ps11
Choano./Metazoa - ps12
Metazoa - ps13
Myriazoa - ps14
Parahoxozoa - ps15
Bilateria - ps16
Deuterostomia - ps17
Chordata - ps18
Olfactores - ps19
Vertebrata - ps20
Gnathostomata - ps21
Euteleostomi - ps22
Sarcopterygii - ps23
Tetrapoda - ps24
Amniota - ps25
Mammalia - ps26
Theria - ps27
Placentalia - ps28
Boreoeutheria - ps29
Euarchontoglires - ps30
Euarchonta - ps31
Primates - ps32
Haplorrhini - ps33
Simiiformes - ps34
Catarrhini - ps35
Hominoidea - ps36
Hominidae - ps37
Homininae - ps38
Hominini - ps39
Homo_sapiens - ps40
p-value
2e-04
8e-04
3e-03
1e-02
6e-02
Log-odds
0.5
1.0
1.5
2.0
2.5
3.0
3.5
4.0

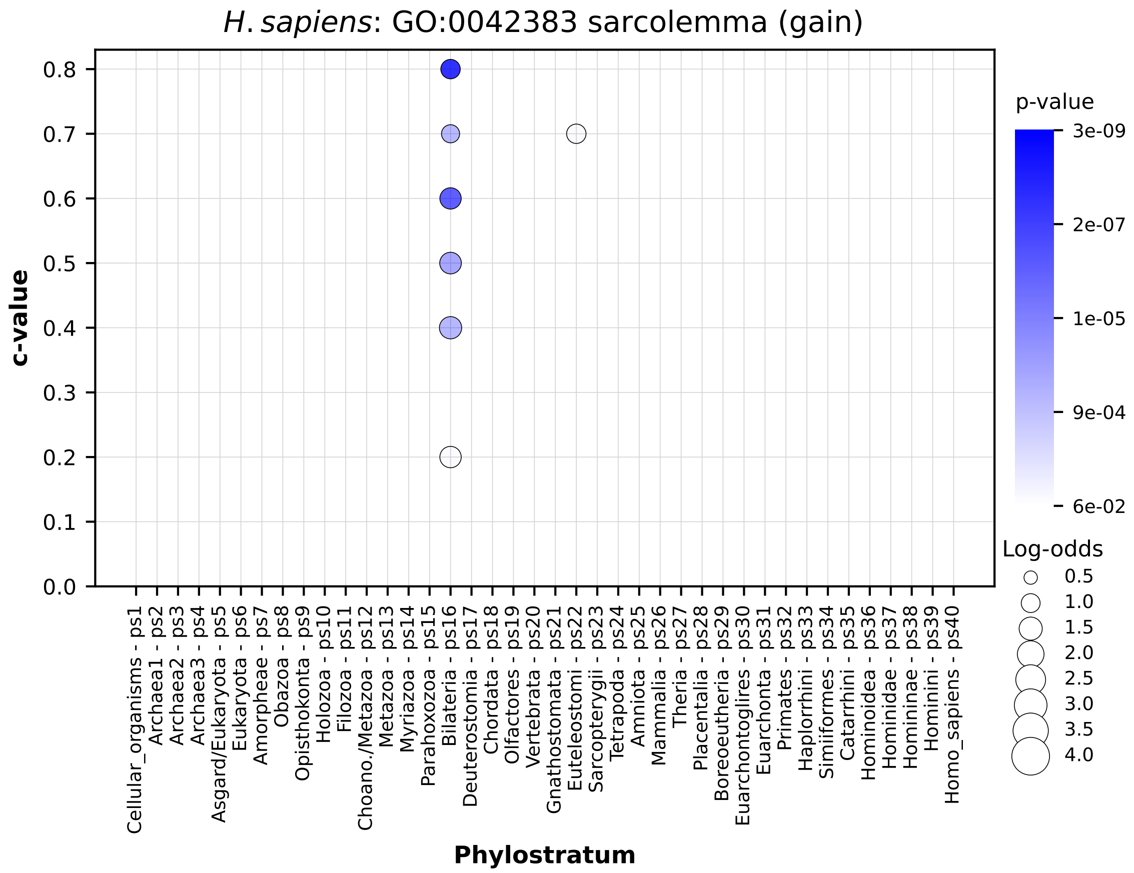

H. sapiens: GO:0042383 sarcolemma (gain)
c-value
0.0
0.1
0.2
0.3
0.4
0.5
0.6
0.7
0.8
Phylostratum
Cellular_organisms - ps1
Archaea1 - ps2
Archaea2 - ps3
Archaea3 - ps4
Asgard/Eukaryota - ps5
Eukaryota - ps6
Amorpheae - ps7
Obazoa - ps8
Opisthokonta - ps9
Holozoa - ps10
Filozoa - ps11
Choano./Metazoa - ps12
Metazoa - ps13
Myriazoa - ps14
Parahoxozoa - ps15
Bilateria - ps16
Deuterostomia - ps17
Chordata - ps18
Olfactores - ps19
Vertebrata - ps20
Gnathostomata - ps21
Euteleostomi - ps22
Sarcopterygii - ps23
Tetrapoda - ps24
Amniota - ps25
Mammalia - ps26
Theria - ps27
Placentalia - ps28
Boreoeutheria - ps29
Euarchontoglires - ps30
Euarchonta - ps31
Primates - ps32
Haplorrhini - ps33
Simiiformes - ps34
Catarrhini - ps35
Hominoidea - ps36
Hominidae - ps37
Homininae - ps38
Hominini - ps39
Homo_sapiens - ps40
p-value
3e-09
2e-07
1e-05
9e-04
6e-02
Log-odds
0.5
1.0
1.5
2.0
2.5
3.0
3.5
4.0

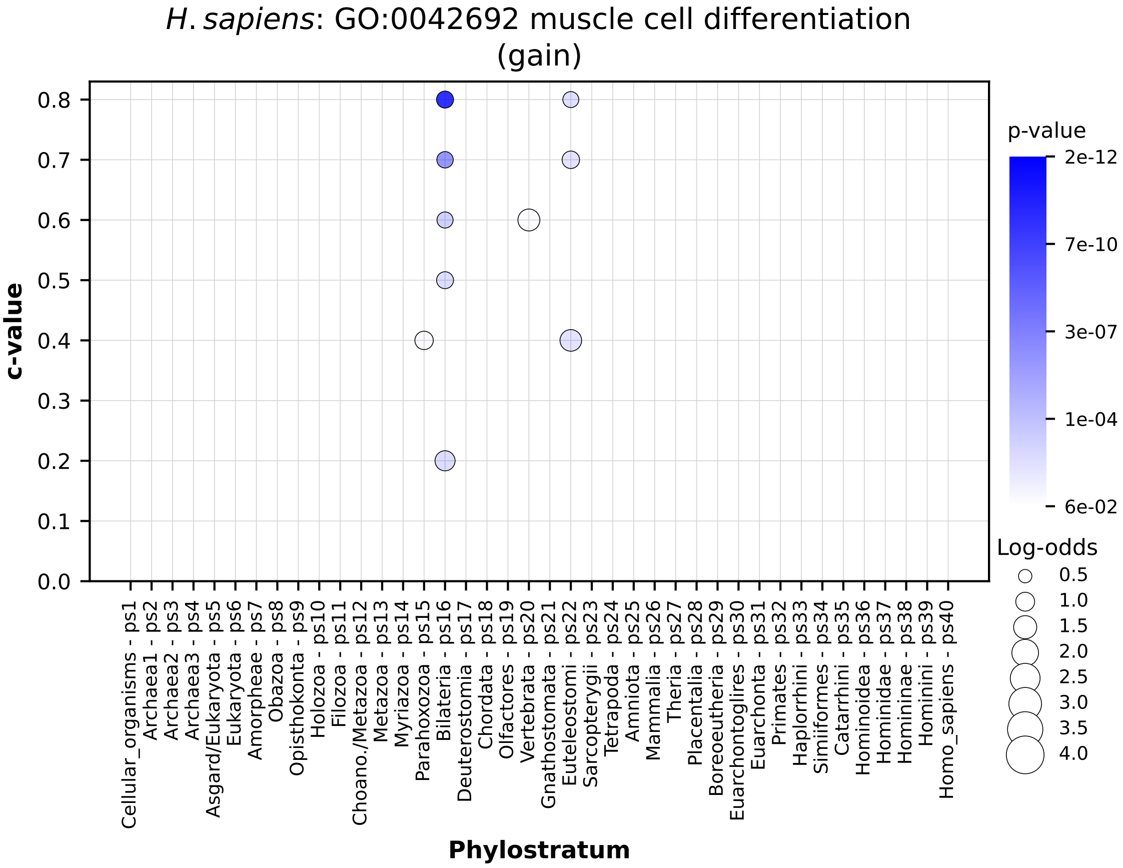
H. sapiens: GO:0042692 muscle cell differentiation
(gain)
c-value
0.0
0.1
0.2
0.3
0.4
0.5
0.6
0.7
0.8
Phylostratum
Cellular_organisms - ps1
Archaea1 - ps2
Archaea2 - ps3
Archaea3 - ps4
Asgard/Eukaryota - ps5
Eukaryota - ps6
Amorpheae - ps7
Obazoa - ps8
Opisthokonta - ps9
Holozoa - ps10
Filozoa - ps11
Choano./Metazoa - ps12
Metazoa - ps13
Myriazoa - ps14
Parahoxozoa - ps15
Bilateria - ps16
Deuterostomia - ps17
Chordata - ps18
Olfactores - ps19
Vertebrata - ps20
Gnathostomata - ps21
Euteleostomi - ps22
Sarcopterygii - ps23
Tetrapoda - ps24
Amniota - ps25
Mammalia - ps26
Theria - ps27
Placentalia - ps28
Boreoeutheria - ps29
Euarchontoglires - ps30
Euarchonta - ps31
Primates - ps32
Haplorrhini - ps33
Simiiformes - ps34
Catarrhini - ps35
Hominoidea - ps36
Hominidae - ps37
Homininae - ps38
Hominini - ps39
Homo_sapiens - ps40
p-value
2e-12
7e-10
3e-07
1e-04
6e-02
Log-odds
0.5
1.0
1.5
2.0
2.5
3.0
3.5
4.0

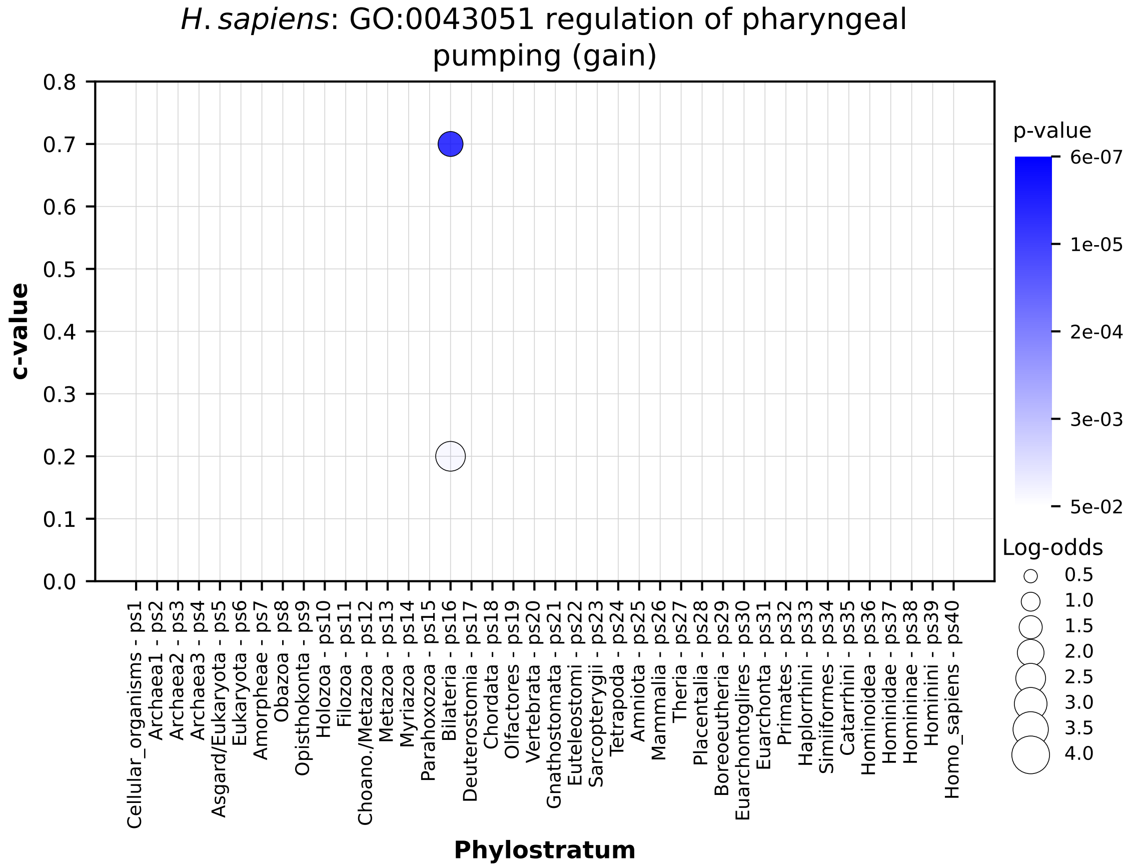

H. sapiens: GO:0043051 regulation of pharyngeal pumping (gain)
c-value
Phylostratum
p-value
6e-07
1e-05
2e-04
3e-03
5e-02
Log-odds
0.5
1.0
1.5
2.0
2.5
3.0
3.5
4.0
0.0
0.1
0.2
0.3
0.4
0.5
0.6
0.7
0.8
Cellular_organisms - ps1
Archaea1 - ps2
Archaea2 - ps3
Archaea3 - ps4
Asgard/Eukaryota - ps5
Eukaryota - ps6
Amorpheae - ps7
Obazoa - ps8
Opisthokonta - ps9
Holozoa - ps10
Filozoa - ps11
Choano./Metazoa - ps12
Metazoa - ps13
Myriazoa - ps14
Parahoxozoa - ps15
Bilateria - ps16
Deuterostomia - ps17
Chordata - ps18
Olfactores - ps19
Vertebrata - ps20
Gnathostomata - ps21
Euteleostomi - ps22
Sarcopterygii - ps23
Tetrapoda - ps24
Amniota - ps25
Mammalia - ps26
Theria - ps27
Placentalia - ps28
Boreoeutheria - ps29
Euarchontoglires - ps30
Euarchonta - ps31
Primates - ps32
Haplorrhini - ps33
Simiiformes - ps34
Catarrhini - ps35
Hominoidea - ps36
Hominidae - ps37
Homininae - ps38
Hominini - ps39
Homo_sapiens - ps40

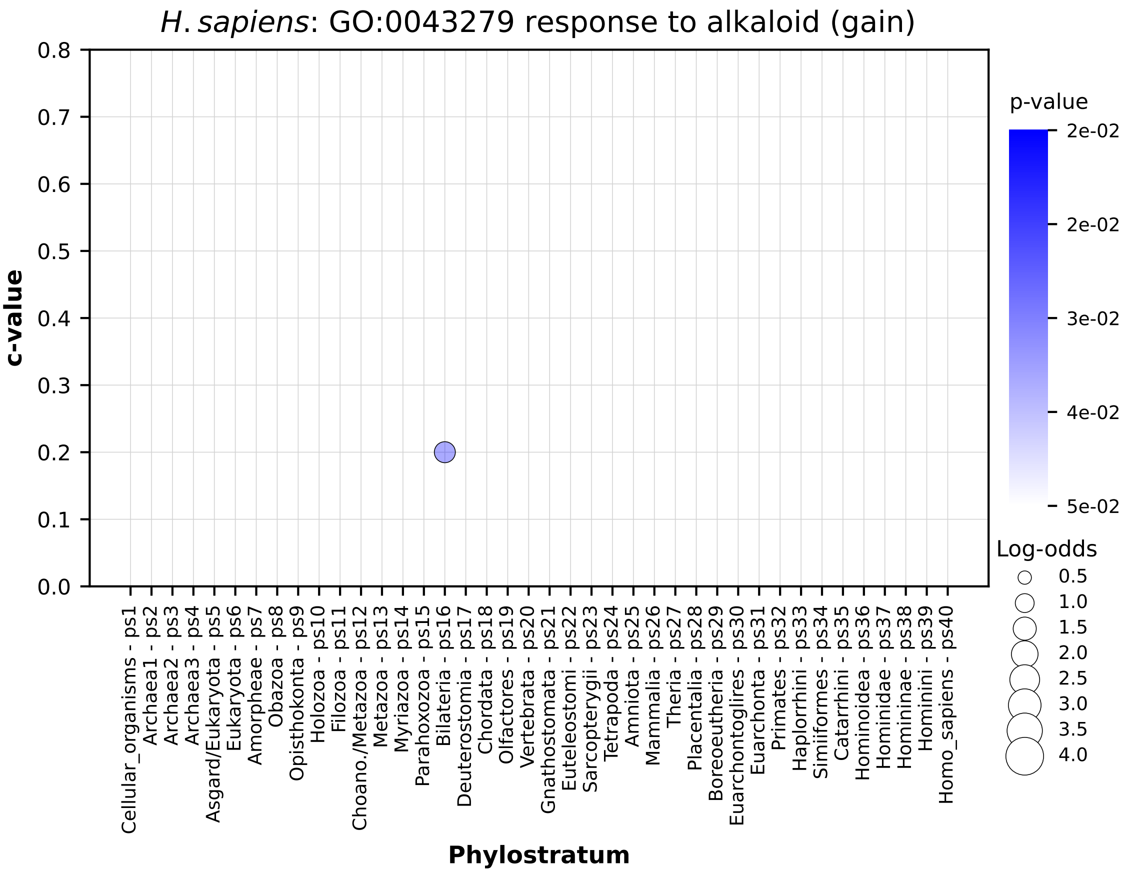

*H. sapiens*: GO:0043279 response to alkaloid (gain)
c-value
0.0
0.1
0.2
0.3
0.4
0.5
0.6
0.7
0.8
Cellular_organisms - ps1
Archaea1 - ps2
Archaea2 - ps3
Archaea3 - ps4
Asgard/Eukaryota - ps5
Eukaryota - ps6
Amorpheae - ps7
Obazoa - ps8
Opisthokonta - ps9
Holozoa - ps10
Filozoa - ps11
Choano./Metazoa - ps12
Metazoa - ps13
Myriazoa - ps14
Parahoxozoa - ps15
Bilateria - ps16
Deuterostomia - ps17
Chordata - ps18
Olfactores - ps19
Vertebrata - ps20
Gnathostomata - ps21
Euteleostomi - ps22
Sarcopterygii - ps23
Tetrapoda - ps24
Amniota - ps25
Mammalia - ps26
Theria - ps27
Placentalia - ps28
Boreoeutheria - ps29
Euarchontoglires - ps30
Euarchonta - ps31
Primates - ps32
Haplorrhini - ps33
Simiiformes - ps34
Catarrhini - ps35
Hominoidea - ps36
Hominidae - ps37
Homininae - ps38
Hominini - ps39
Homo_sapiens - ps40
Phylostratum
p-value
2e-02
2e-02
3e-02
4e-02
5e-02
Log-odds
0.5
1.0
1.5
2.0
2.5
3.0
3.5
4.0

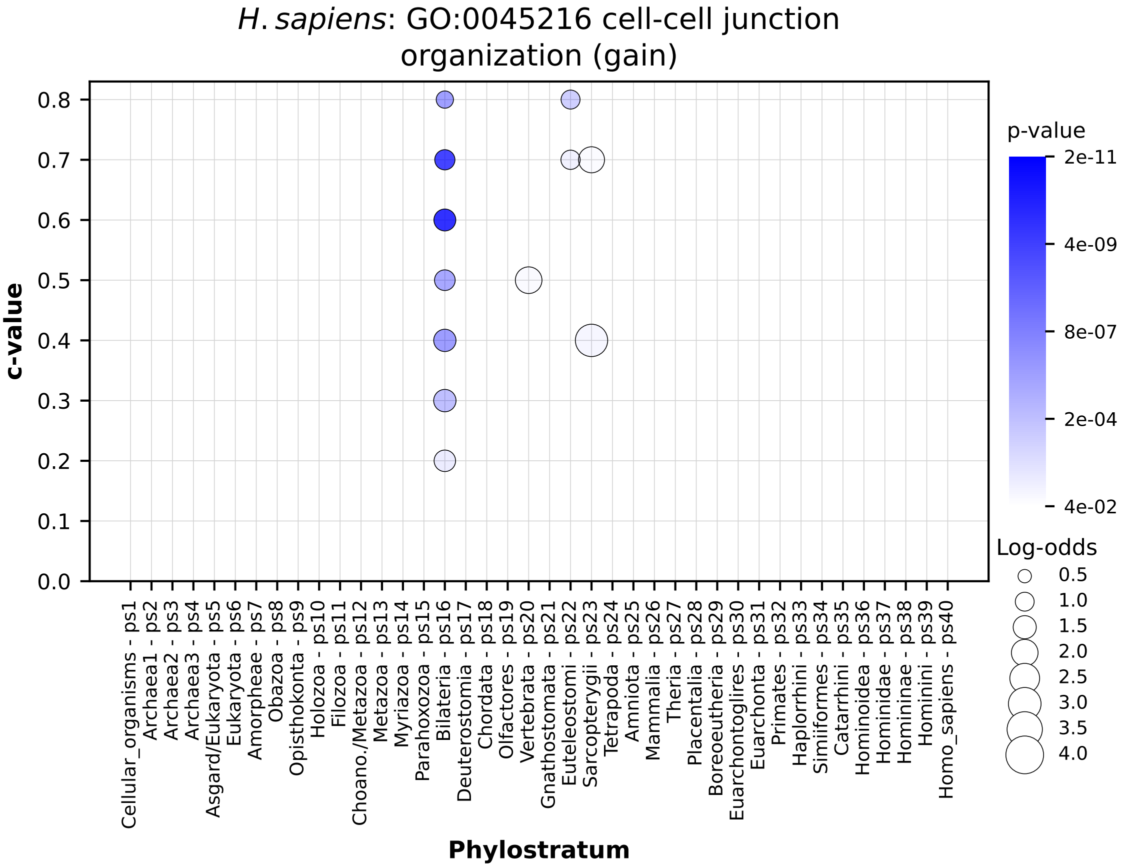

H. sapiens: GO:0045216 cell-cell junction organization (gain)
c-value
0.8
0.7
0.6
0.5
0.4
0.3
0.2
0.1
0.0
Phylostratum
Cellular_organisms - ps1
Archaea1 - ps2
Archaea2 - ps3
Archaea3 - ps4
Asgard/Eukaryota - ps5
Eukaryota - ps6
Amorpheae - ps7
Obazoa - ps8
Opisthokonta - ps9
Holozoa - ps10
Filozoa - ps11
Choano./Metazoa - ps12
Metazoa - ps13
Myriazoa - ps14
Parahoxozoa - ps15
Bilateria - ps16
Deuterostomia - ps17
Chordata - ps18
Olfactores - ps19
Vertebrata - ps20
Gnathostomata - ps21
Euteleostomi - ps22
Sarcopterygii - ps23
Tetrapoda - ps24
Amniota - ps25
Mammalia - ps26
Theria - ps27
Placentalia - ps28
Boreoeutheria - ps29
Euarchontoglires - ps30
Euarchonta - ps31
Primates - ps32
Haplorrhini - ps33
Simiiformes - ps34
Catarrhini - ps35
Hominoidea - ps36
Hominidae - ps37
Homininae - ps38
Hominini - ps39
Homo_sapiens - ps40
p-value
2e-11
4e-09
8e-07
2e-04
4e-02
Log-odds
0.5
1.0
1.5
2.0
2.5
3.0
3.5
4.0

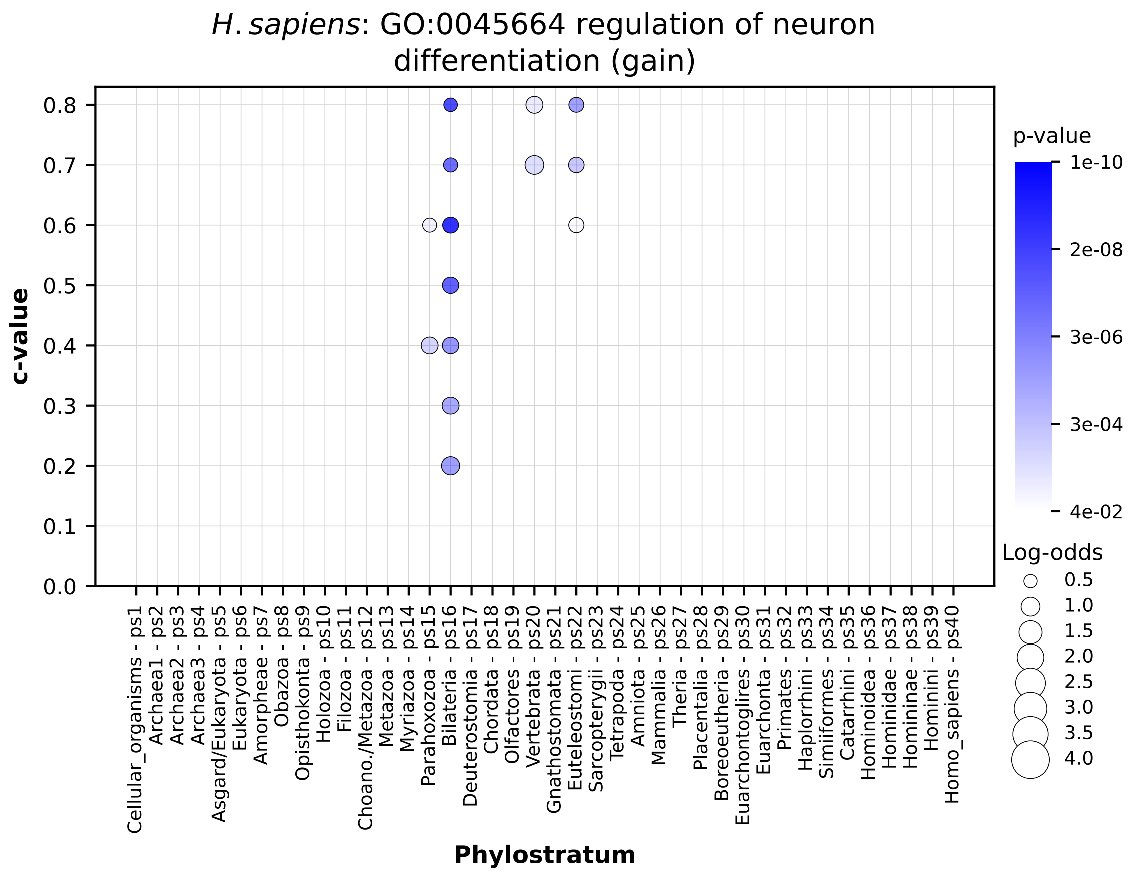

H. sapiens: GO:0045664 regulation of neuron differentiation (gain)
c-value
0.0
0.1
0.2
0.3
0.4
0.5
0.6
0.7
0.8
Phylostratum
Cellular_organisms - ps1
Archaea1 - ps2
Archaea2 - ps3
Archaea3 - ps4
Asgard/Eukaryota - ps5
Eukaryota - ps6
Amorpheae - ps7
Obazoa - ps8
Opisthokonta - ps9
Holozoa - ps10
Filozoa - ps11
Choano./Metazoa - ps12
Metazoa - ps13
Myriazoa - ps14
Parahoxozoa - ps15
Bilateria - ps16
Deuterostomia - ps17
Chordata - ps18
Olfactores - ps19
Vertebrata - ps20
Gnathostomata - ps21
Euteleostomi - ps22
Sarcopterygii - ps23
Tetrapoda - ps24
Amniota - ps25
Mammalia - ps26
Theria - ps27
Placentalia - ps28
Boreoeutheria - ps29
Euarchontoglires - ps30
Euarchonta - ps31
Primates - ps32
Haplorrhini - ps33
Simiiformes - ps34
Catarrhini - ps35
Hominoidea - ps36
Hominidae - ps37
Homininae - ps38
Hominini - ps39
Homo_sapiens - ps40
p-value
1e-10
2e-08
3e-06
3e-04
4e-02
Log-odds
0.5
1.0
1.5
2.0
2.5
3.0
3.5
4.0

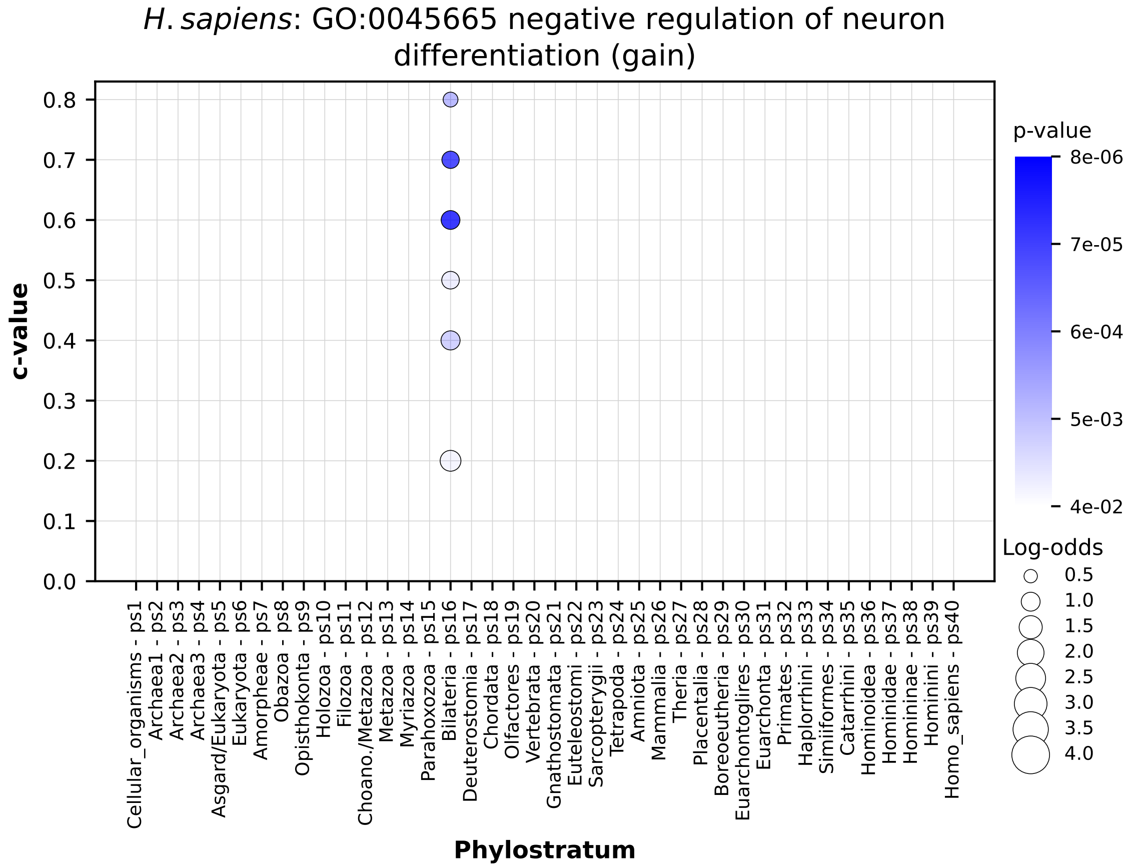

H. sapiens: GO:0045665 negative regulation of neuron differentiation (gain)
c-value
0.0
0.1
0.2
0.3
0.4
0.5
0.6
0.7
0.8
Phylostratum
Cellular_organisms - ps1
Archaea1 - ps2
Archaea2 - ps3
Archaea3 - ps4
Asgard/Eukaryota - ps5
Eukaryota - ps6
Amorpheae - ps7
Obazoa - ps8
Opisthokonta - ps9
Holozoa - ps10
Filozoa - ps11
Choano./Metazoa - ps12
Metazoa - ps13
Myriazoa - ps14
Parahoxozoa - ps15
Bilateria - ps16
Deuterostomia - ps17
Chordata - ps18
Olfactores - ps19
Vertebrata - ps20
Gnathostomata - ps21
Euteleostomi - ps22
Sarcopterygii - ps23
Tetrapoda - ps24
Amniota - ps25
Mammalia - ps26
Theria - ps27
Placentalia - ps28
Boreoeutheria - ps29
Euarchontoglires - ps30
Euarchonta - ps31
Primates - ps32
Haplorrhini - ps33
Simiiformes - ps34
Catarrhini - ps35
Hominoidea - ps36
Hominidae - ps37
Homininae - ps38
Hominini - ps39
Homo_sapiens - ps40
p-value
8e-06
7e-05
6e-04
5e-03
4e-02
Log-odds
0.5
1.0
1.5
2.0
2.5
3.0
3.5
4.0

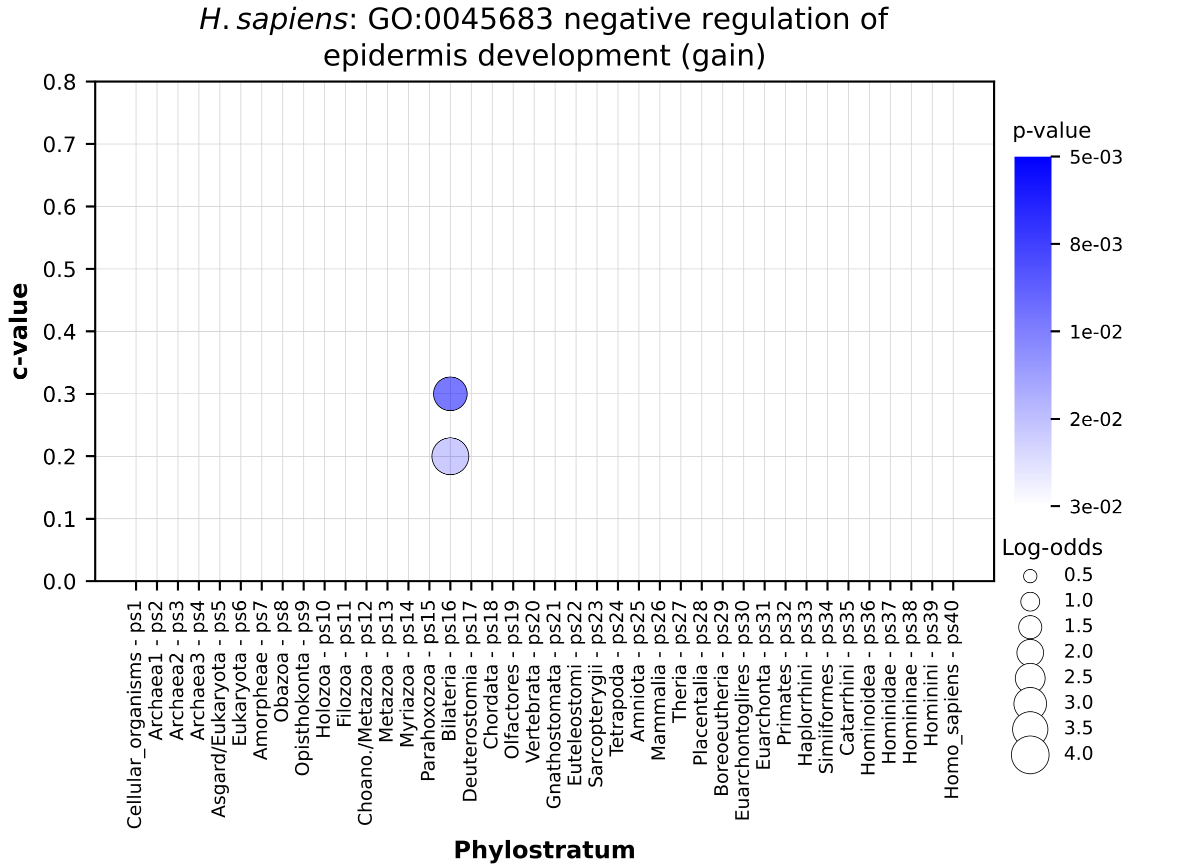

*H. sapiens*: GO:0045683 negative regulation of epidermis development (gain)
c-value
0.0
0.1
0.2
0.3
0.4
0.5
0.6
0.7
0.8
p-value
5e-03
8e-03
1e-02
2e-02
3e-02
Log-odds
0.5
1.0
1.5
2.0
2.5
3.0
3.5
4.0
Cellular_organisms - ps1
Archaea1 - ps2
Archaea2 - ps3
Archaea3 - ps4
Asgard/Eukaryota - ps5
Eukaryota - ps6
Amorpheae - ps7
Obazoa - ps8
Opisthokonta - ps9
Holozoa - ps10
Filozoa - ps11
Choano./Metazoa - ps12
Metazoa - ps13
Myriazoa - ps14
Parahoxozoa - ps15
Bilateria - ps16
Deuterostomia - ps17
Chordata - ps18
Olfactores - ps19
Vertebrata - ps20
Gnathostomata - ps21
Euteleostomi - ps22
Sarcopterygii - ps23
Tetrapoda - ps24
Amniota - ps25
Mammalia - ps26
Theria - ps27
Placentalia - ps28
Boreoeutheria - ps29
Euarchontoglires - ps30
Euarchonta - ps31
Primates - ps32
Haplorrhini - ps33
Simiiformes - ps34
Catarrhini - ps35
Hominoidea - ps36
Hominidae - ps37
Homininae - ps38
Hominini - ps39
Homo_sapiens - ps40
Phylostratum

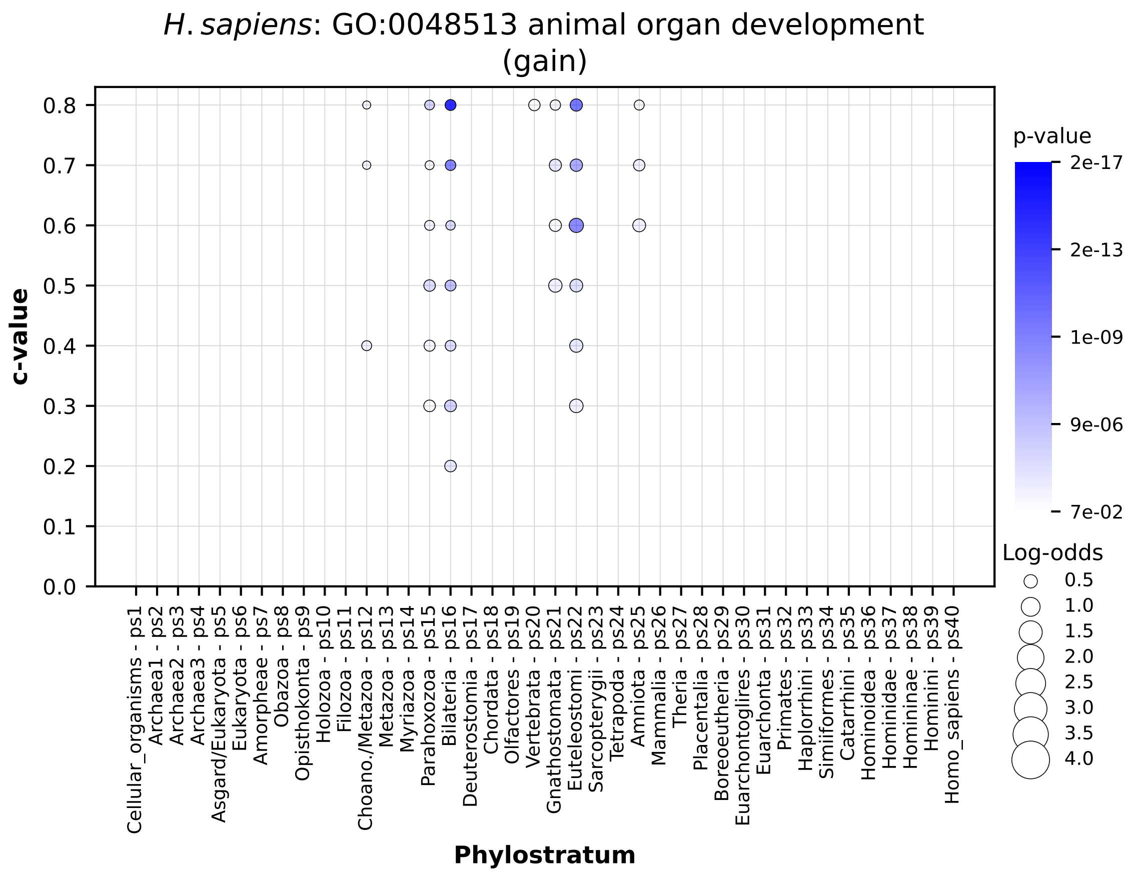

*H. sapiens*: GO:0048513 animal organ development
(gain)
c-value
0.0
0.1
0.2
0.3
0.4
0.5
0.6
0.7
0.8
Phylostratum
Cellular_organisms - ps1
Archaea1 - ps2
Archaea2 - ps3
Archaea3 - ps4
Asgard/Eukaryota - ps5
Eukaryota - ps6
Amorpheae - ps7
Obazoa - ps8
Opisthokonta - ps9
Holozoa - ps10
Filozoa - ps11
Choano./Metazoa - ps12
Metazoa - ps13
Myriazoa - ps14
Parahoxozoa - ps15
Bilateria - ps16
Deuterostomia - ps17
Chordata - ps18
Olfactores - ps19
Vertebrata - ps20
Gnathostomata - ps21
Euteleostomi - ps22
Sarcopterygii - ps23
Tetrapoda - ps24
Amniota - ps25
Mammalia - ps26
Theria - ps27
Placentalia - ps28
Boreoeutheria - ps29
Euarchontoglires - ps30
Euarchonta - ps31
Primates - ps32
Haplorrhini - ps33
Simiiformes - ps34
Catarrhini - ps35
Hominoidea - ps36
Hominidae - ps37
Homininae - ps38
Hominini - ps39
Homo_sapiens - ps40
p-value
2e-17
2e-13
1e-09
9e-06
7e-02
Log-odds
0.5
1.0
1.5
2.0
2.5
3.0
3.5
4.0

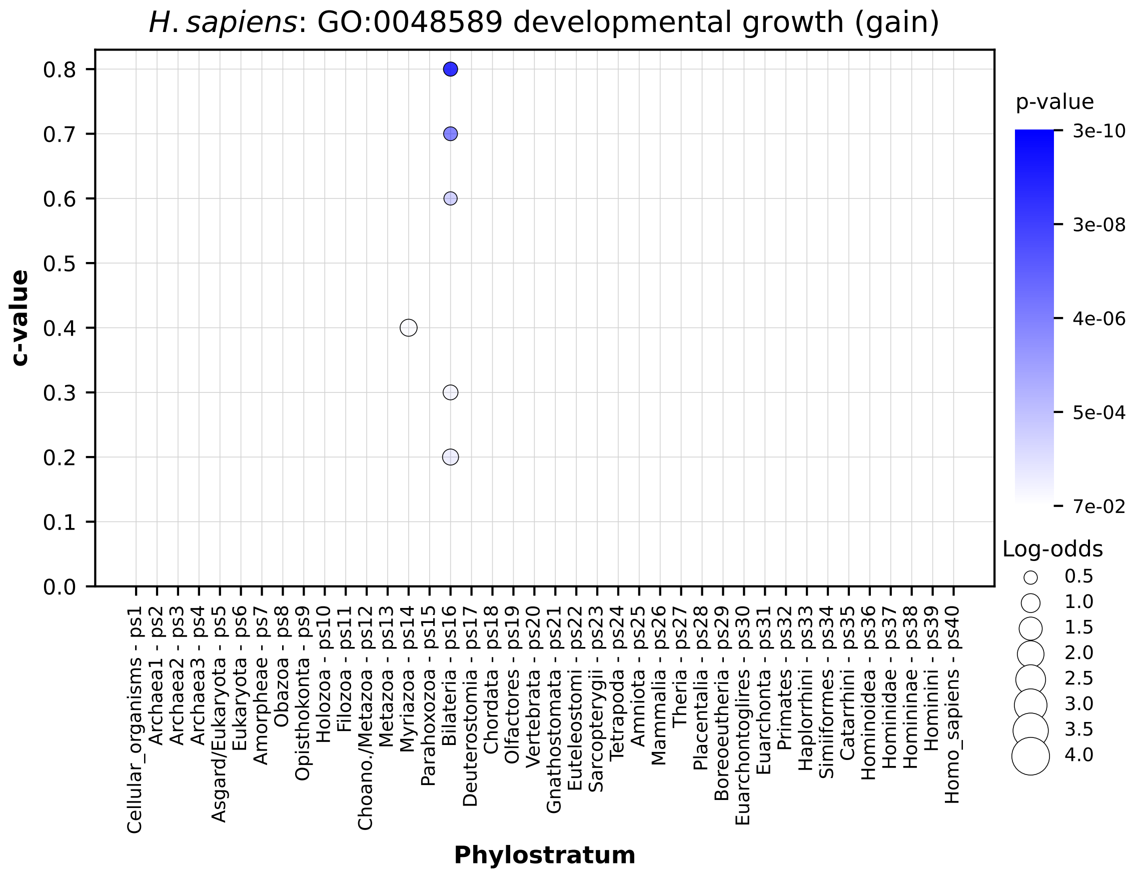

H. sapiens: GO:0048589 developmental growth (gain)
c-value
0.0
0.1
0.2
0.3
0.4
0.5
0.6
0.7
0.8
Phylostratum
Cellular_organisms - ps1
Archaea1 - ps2
Archaea2 - ps3
Archaea3 - ps4
Asgard/Eukaryota - ps5
Eukaryota - ps6
Amorpheae - ps7
Obazoa - ps8
Opisthokonta - ps9
Holozoa - ps10
Filozoa - ps11
Choano./Metazoa - ps12
Metazoa - ps13
Myriazoa - ps14
Parahoxozoa - ps15
Bilateria - ps16
Deuterostomia - ps17
Chordata - ps18
Olfactores - ps19
Vertebrata - ps20
Gnathostomata - ps21
Euteleostomi - ps22
Sarcopterygii - ps23
Tetrapoda - ps24
Amniota - ps25
Mammalia - ps26
Theria - ps27
Placentalia - ps28
Boreoeutheria - ps29
Euarchontoglires - ps30
Euarchonta - ps31
Primates - ps32
Haplorrhini - ps33
Simiiformes - ps34
Catarrhini - ps35
Hominoidea - ps36
Hominidae - ps37
Homininae - ps38
Hominini - ps39
Homo_sapiens - ps40
p-value
3e-10
3e-08
4e-06
5e-04
7e-02
Log-odds
0.5
1.0
1.5
2.0
2.5
3.0
3.5
4.0

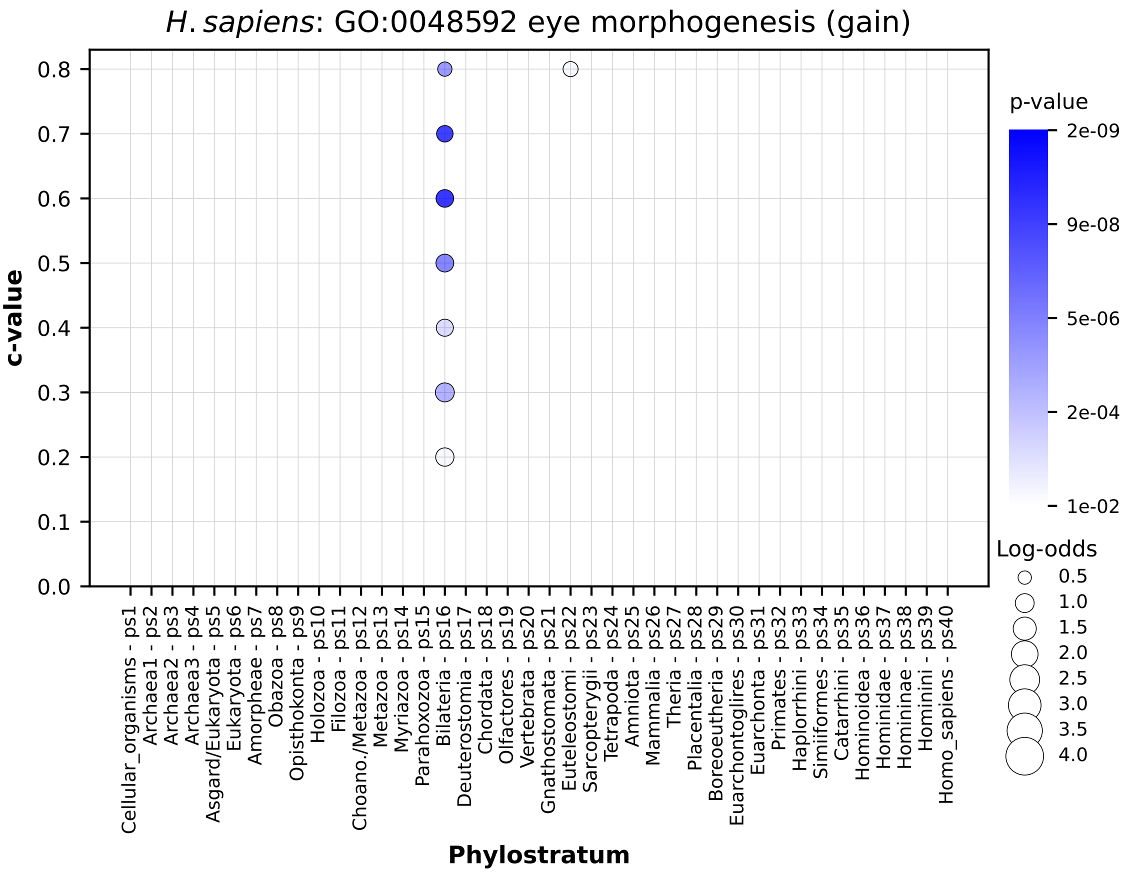

*H. sapiens*: GO:0048592 eye morphogenesis (gain)
c-value
0.0
0.1
0.2
0.3
0.4
0.5
0.6
0.7
0.8
Phylostratum
Cellular_organisms - ps1
Archaea1 - ps2
Archaea2 - ps3
Archaea3 - ps4
Asgard/Eukaryota - ps5
Eukaryota - ps6
Amorpheae - ps7
Obazoa - ps8
Opisthokonta - ps9
Holozoa - ps10
Filozoa - ps11
Choano./Metazoa - ps12
Metazoa - ps13
Myriazoa - ps14
Parahoxozoa - ps15
Bilateria - ps16
Deuterostomia - ps17
Chordata - ps18
Olfactores - ps19
Vertebrata - ps20
Gnathostomata - ps21
Euteleostomi - ps22
Sarcopterygii - ps23
Tetrapoda - ps24
Amniota - ps25
Mammalia - ps26
Theria - ps27
Placentalia - ps28
Boreoeutheria - ps29
Euarchontoglires - ps30
Euarchonta - ps31
Primates - ps32
Haplorrhini - ps33
Simiiformes - ps34
Catarrhini - ps35
Hominoidea - ps36
Hominidae - ps37
Homininae - ps38
Hominini - ps39
Homo_sapiens - ps40
p-value
2e-09
9e-08
5e-06
2e-04
1e-02
Log-odds
0.5
1.0
1.5
2.0
2.5
3.0
3.5
4.0

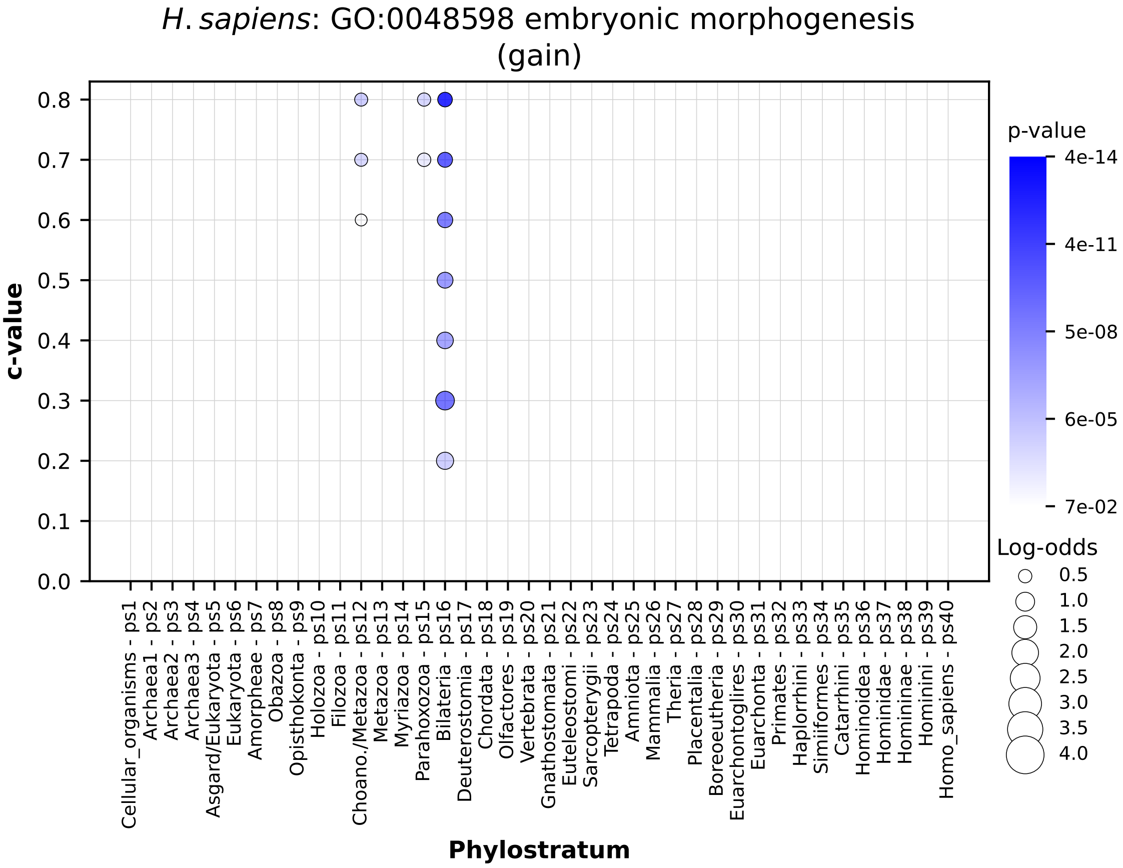

*H. sapiens*: GO:0048598 embryonic morphogenesis
(gain)
c-value
0.0
0.1
0.2
0.3
0.4
0.5
0.6
0.7
0.8
p-value
4e-14
4e-11
5e-08
6e-05
7e-02
Log-odds
0.5
1.0
1.5
2.0
2.5
3.0
3.5
4.0
Cellular_organisms - ps1
Archaea1 - ps2
Archaea2 - ps3
Archaea3 - ps4
Asgard/Eukaryota - ps5
Eukaryota - ps6
Amorpheae - ps7
Obazoa - ps8
Opisthokonta - ps9
Holozoa - ps10
Filozoa - ps11
Choano./Metazoa - ps12
Metazoa - ps13
Myriazoa - ps14
Parahoxozoa - ps15
Bilateria - ps16
Deuterostomia - ps17
Chordata - ps18
Olfactores - ps19
Vertebrata - ps20
Gnathostomata - ps21
Euteleostomi - ps22
Sarcopterygii - ps23
Tetrapoda - ps24
Amniota - ps25
Mammalia - ps26
Theria - ps27
Placentalia - ps28
Boreoeutheria - ps29
Euarchontoglires - ps30
Euarchonta - ps31
Primates - ps32
Haplorrhini - ps33
Simiiformes - ps34
Catarrhini - ps35
Hominoidea - ps36
Hominidae - ps37
Homininae - ps38
Hominini - ps39
Homo_sapiens - ps40
Phylostratum

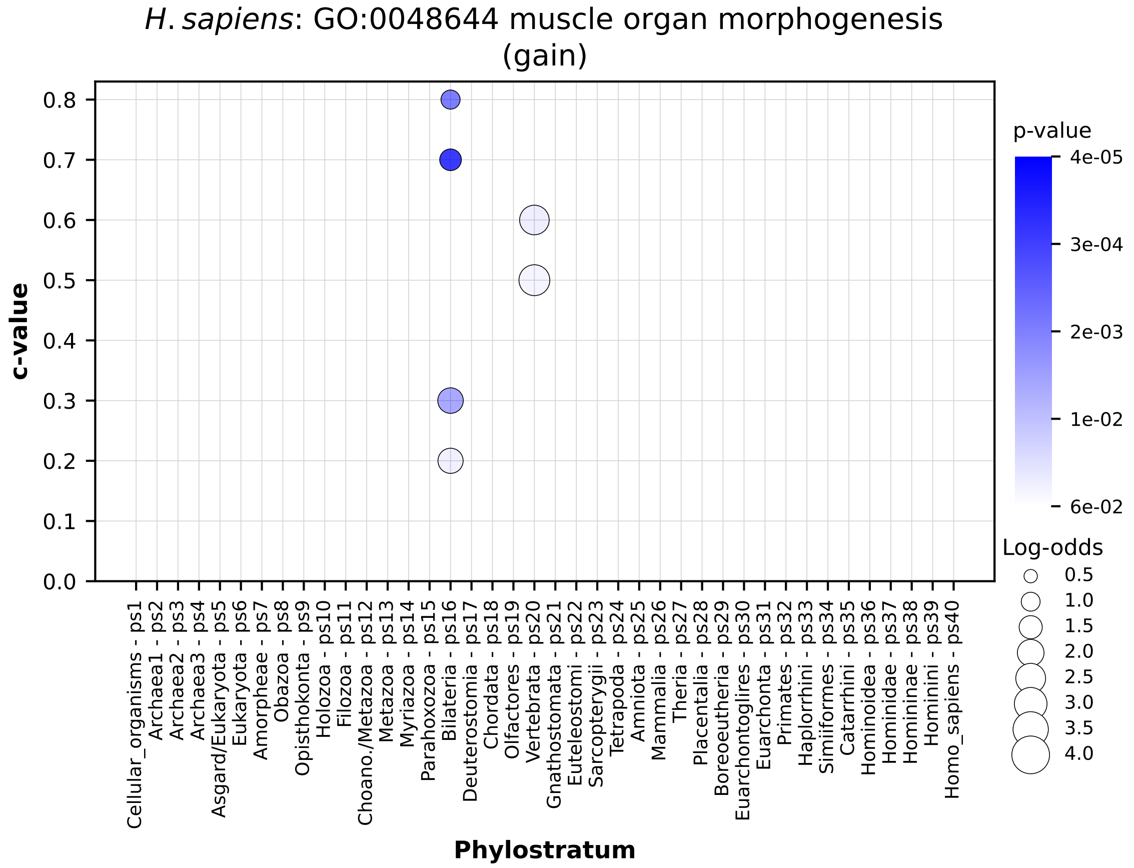

*H. sapiens*: GO:0048644 muscle organ morphogenesis
(gain)
c-value
0.8
0.7
0.6
0.5
0.4
0.3
0.2
0.1
0.0
Cellular_organisms - ps1
Archaea1 - ps2
Archaea2 - ps3
Archaea3 - ps4
Asgard/Eukaryota - ps5
Eukaryota - ps6
Amorpheae - ps7
Obazoa - ps8
Opisthokonta - ps9
Holozoa - ps10
Filozoa - ps11
Choano./Metazoa - ps12
Metazoa - ps13
Myriazoa - ps14
Parahoxozoa - ps15
Bilateria - ps16
Deuterostomia - ps17
Chordata - ps18
Olfactores - ps19
Vertebrata - ps20
Gnathostomata - ps21
Euteleostomi - ps22
Sarcopterygii - ps23
Tetrapoda - ps24
Amniota - ps25
Mammalia - ps26
Theria - ps27
Placentalia - ps28
Boreoeutheria - ps29
Euarchontoglires - ps30
Euarchonta - ps31
Primates - ps32
Haplorrhini - ps33
Simiiformes - ps34
Catarrhini - ps35
Hominoidea - ps36
Hominidae - ps37
Homininae - ps38
Hominini - ps39
Homo_sapiens - ps40
Phylostratum
p-value
4e-05
3e-04
2e-03
1e-02
6e-02
Log-odds
0.5
1.0
1.5
2.0
2.5
3.0
3.5
4.0

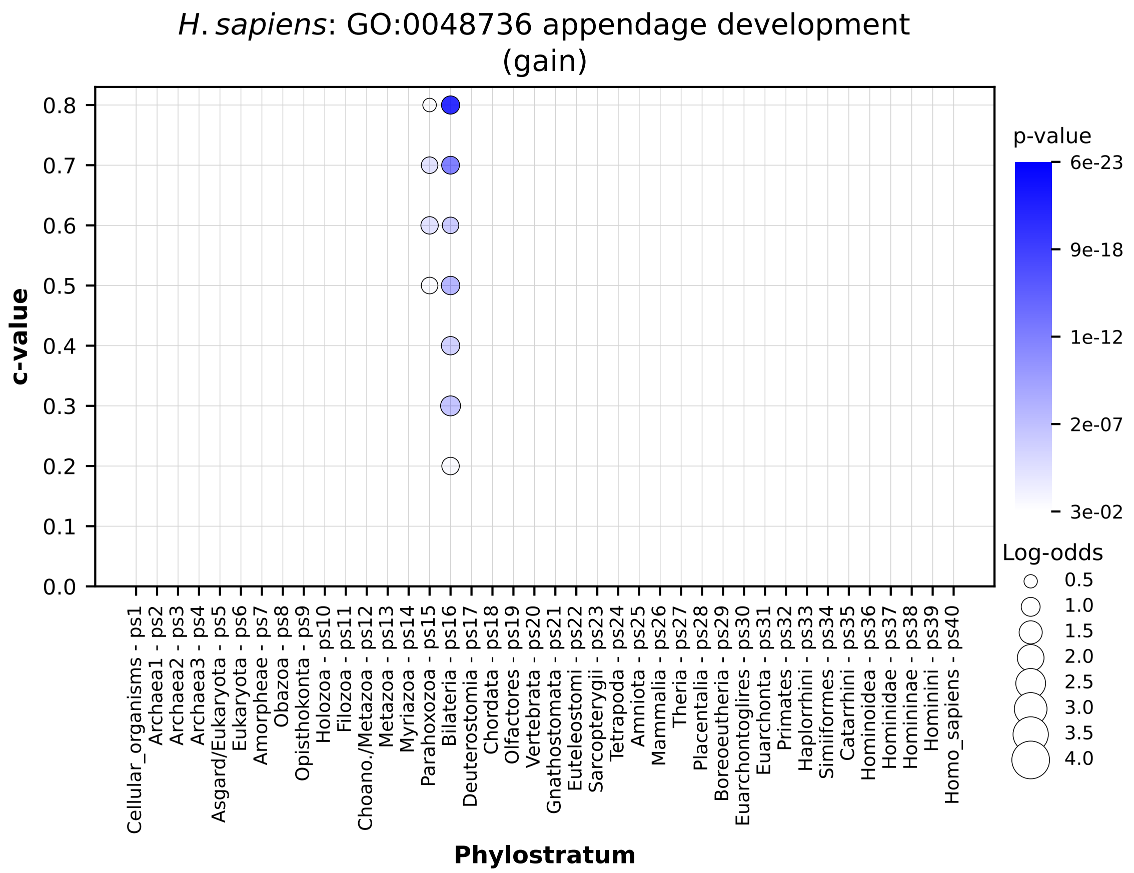

H. sapiens: GO:0048736 appendage development
(gain)
c-value
Phylostratum
p-value
6e-23
9e-18
1e-12
2e-07
3e-02
Log-odds
0.5
1.0
1.5
2.0
2.5
3.0
3.5
4.0
Cellular_organisms - ps1
Archaea1 - ps2
Archaea2 - ps3
Archaea3 - ps4
Asgard/Eukaryota - ps5
Eukaryota - ps6
Amorpheae - ps7
Obazoa - ps8
Opisthokonta - ps9
Holozoa - ps10
Filozoa - ps11
Choano./Metazoa - ps12
Metazoa - ps13
Myriazoa - ps14
Parahoxozoa - ps15
Bilateria - ps16
Deuterostomia - ps17
Chordata - ps18
Olfactores - ps19
Vertebrata - ps20
Gnathostomata - ps21
Euteleostomi - ps22
Sarcopterygii - ps23
Tetrapoda - ps24
Amniota - ps25
Mammalia - ps26
Theria - ps27
Placentalia - ps28
Boreoeutheria - ps29
Euarchontoglires - ps30
Euarchonta - ps31
Primates - ps32
Haplorrhini - ps33
Simiiformes - ps34
Catarrhini - ps35
Hominoidea - ps36
Hominidae - ps37
Homininae - ps38
Hominini - ps39
Homo_sapiens - ps40

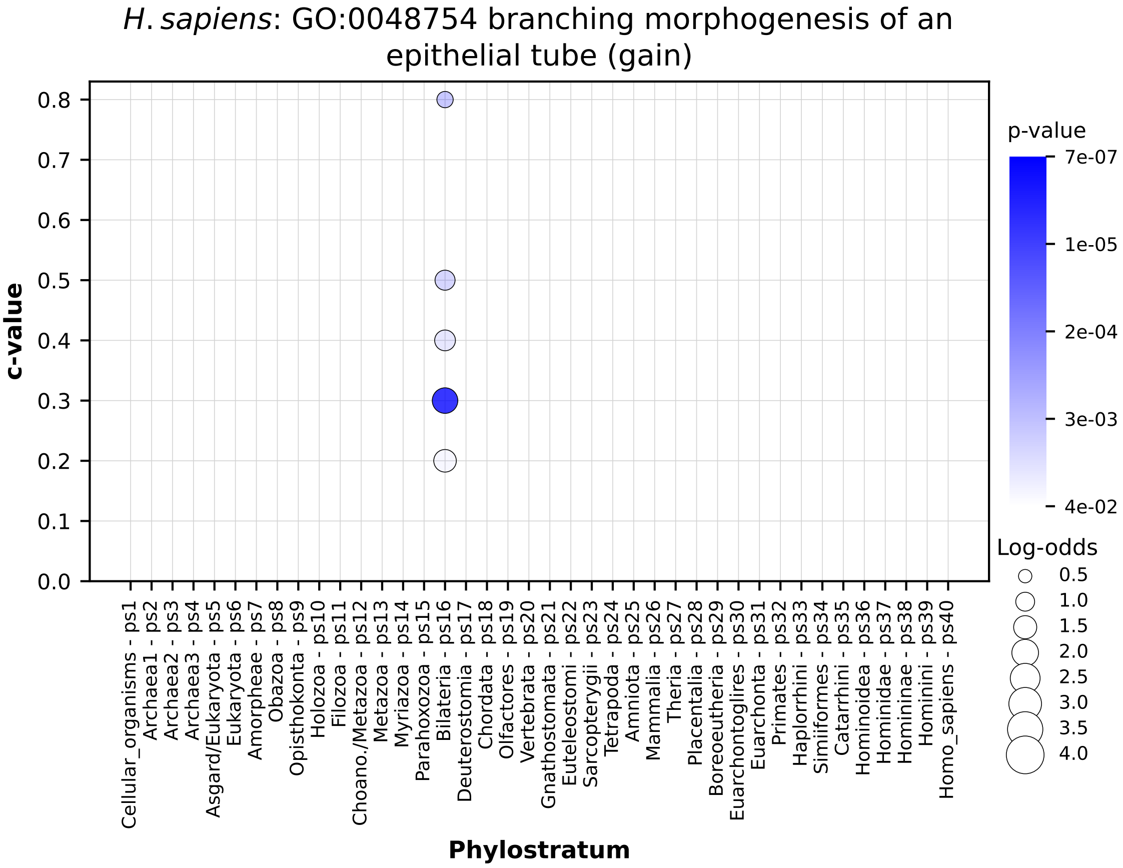

H. sapiens: GO:0048754 branching morphogenesis of an epithelial tube (gain)
c-value
0.0
0.1
0.2
0.3
0.4
0.5
0.6
0.7
0.8
Phylostratum
Cellular_organisms - ps1
Archaea1 - ps2
Archaea2 - ps3
Archaea3 - ps4
Asgard/Eukaryota - ps5
Eukaryota - ps6
Amorpheae - ps7
Obazoa - ps8
Opisthokonta - ps9
Holozoa - ps10
Filozoa - ps11
Choano./Metazoa - ps12
Metazoa - ps13
Myriazoa - ps14
Parahoxozoa - ps15
Bilateria - ps16
Deuterostomia - ps17
Chordata - ps18
Olfactores - ps19
Vertebrata - ps20
Gnathostomata - ps21
Euteleostomi - ps22
Sarcopterygii - ps23
Tetrapoda - ps24
Amniota - ps25
Mammalia - ps26
Theria - ps27
Placentalia - ps28
Boreoeutheria - ps29
Euarchontoglires - ps30
Euarchonta - ps31
Primates - ps32
Haplorrhini - ps33
Simiiformes - ps34
Catarrhini - ps35
Hominoidea - ps36
Hominidae - ps37
Homininae - ps38
Hominini - ps39
Homo_sapiens - ps40
p-value
7e-07
1e-05
2e-04
3e-03
4e-02
Log-odds
0.5
1.0
1.5
2.0
2.5
3.0
3.5
4.0

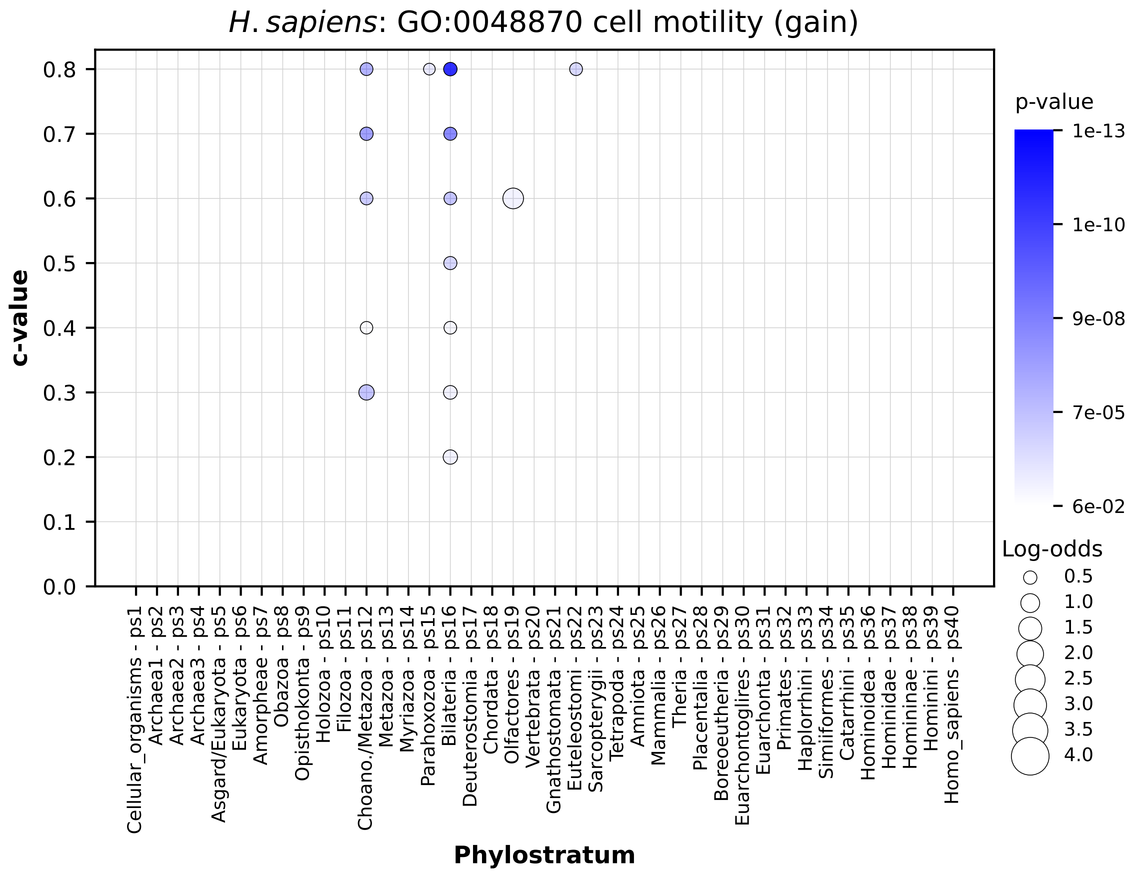

H. sapiens: GO:0048870 cell motility (gain)
c-value
0.0
0.1
0.2
0.3
0.4
0.5
0.6
0.7
0.8
Phylostratum
Cellular_organisms - ps1
Archaea1 - ps2
Archaea2 - ps3
Archaea3 - ps4
Asgard/Eukaryota - ps5
Eukaryota - ps6
Amorpheae - ps7
Obazoa - ps8
Opisthokonta - ps9
Holozoa - ps10
Filozoa - ps11
Choano./Metazoa - ps12
Metazoa - ps13
Myriazoa - ps14
Parahoxozoa - ps15
Bilateria - ps16
Deuterostomia - ps17
Chordata - ps18
Olfactores - ps19
Vertebrata - ps20
Gnathostomata - ps21
Euteleostomi - ps22
Sarcopterygii - ps23
Tetrapoda - ps24
Amniota - ps25
Mammalia - ps26
Theria - ps27
Placentalia - ps28
Boreoeutheria - ps29
Euarchontoglires - ps30
Euarchonta - ps31
Primates - ps32
Haplorrhini - ps33
Simiiformes - ps34
Catarrhini - ps35
Hominoidea - ps36
Hominidae - ps37
Homininae - ps38
Hominini - ps39
Homo_sapiens - ps40
p-value
1e-13
1e-10
9e-08
7e-05
6e-02
Log-odds
0.5
1.0
1.5
2.0
2.5
3.0
3.5
4.0

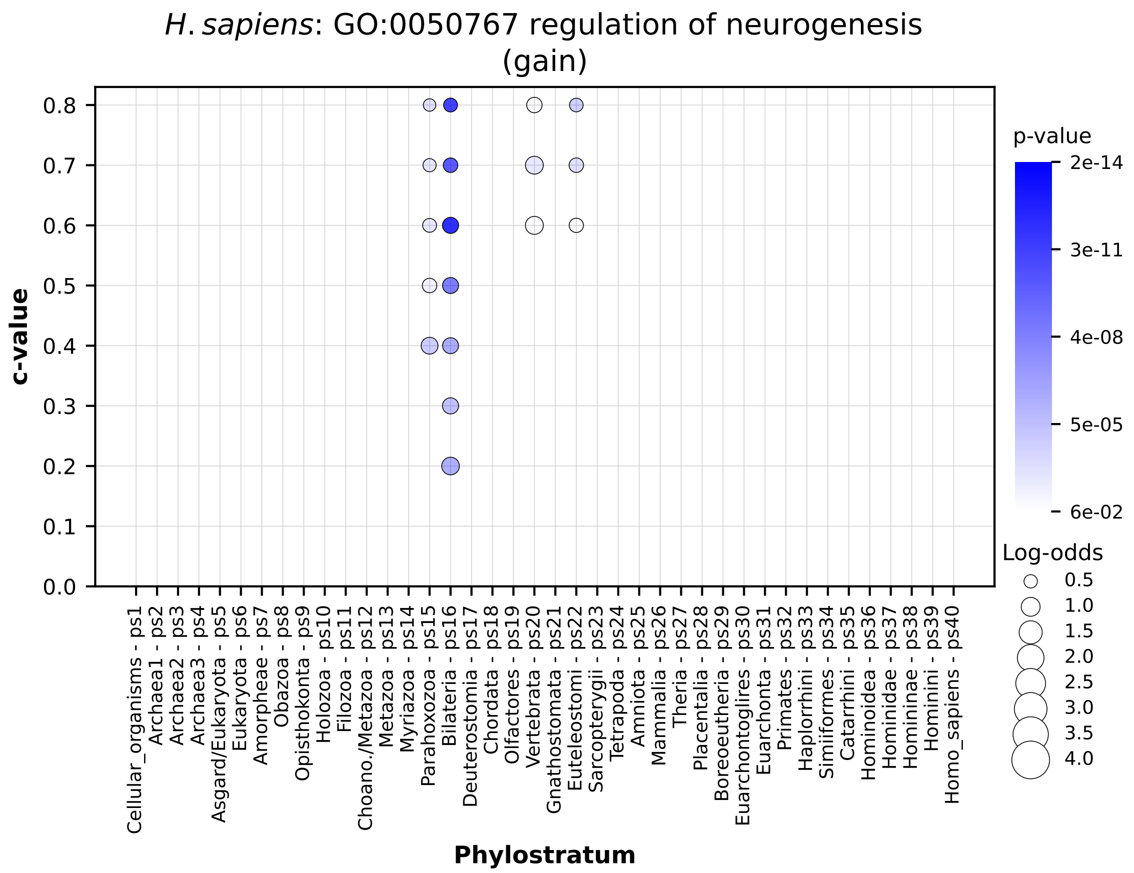

H. sapiens: GO:0050767 regulation of neurogenesis
(gain)
c-value
0.0
0.1
0.2
0.3
0.4
0.5
0.6
0.7
0.8
Phylostratum
Cellular_organisms - ps1
Archaea1 - ps2
Archaea2 - ps3
Archaea3 - ps4
Asgard/Eukaryota - ps5
Eukaryota - ps6
Amorpheae - ps7
Obazoa - ps8
Opisthokonta - ps9
Holozoa - ps10
Filozoa - ps11
Choano./Metazoa - ps12
Metazoa - ps13
Myriazoa - ps14
Parahoxozoa - ps15
Bilateria - ps16
Deuterostomia - ps17
Chordata - ps18
Olfactores - ps19
Vertebrata - ps20
Gnathostomata - ps21
Euteleostomi - ps22
Sarcopterygii - ps23
Tetrapoda - ps24
Amniota - ps25
Mammalia - ps26
Theria - ps27
Placentalia - ps28
Boreoeutheria - ps29
Euarchontoglires - ps30
Euarchonta - ps31
Primates - ps32
Haplorrhini - ps33
Simiiformes - ps34
Catarrhini - ps35
Hominoidea - ps36
Hominidae - ps37
Homininae - ps38
Hominini - ps39
Homo_sapiens - ps40
p-value
2e-14
3e-11
4e-08
5e-05
6e-02
Log-odds
0.5
1.0
1.5
2.0
2.5
3.0
3.5
4.0

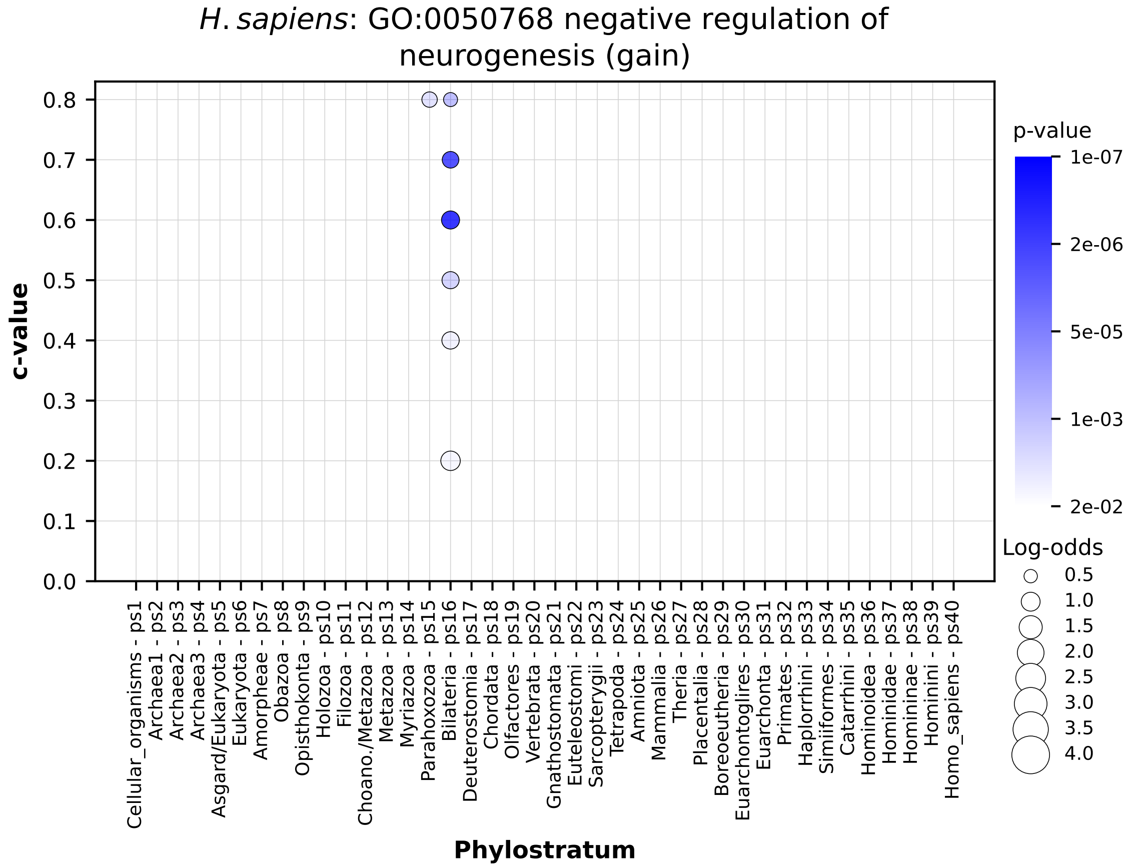
H. sapiens: GO:0050768 negative regulation of neurogenesis (gain)
c-value
0.8
0.7
0.6
0.5
0.4
0.3
0.2
0.1
0.0
Phylostratum
Cellular_organisms - ps1
Archaea1 - ps2
Archaea2 - ps3
Archaea3 - ps4
Asgard/Eukaryota - ps5
Eukaryota - ps6
Amorpheae - ps7
Obazoa - ps8
Opisthokonta - ps9
Holozoa - ps10
Filozoa - ps11
Choano./Metazoa - ps12
Metazoa - ps13
Myriazoa - ps14
Parahoxozoa - ps15
Bilateria - ps16
Deuterostomia - ps17
Chordata - ps18
Olfactores - ps19
Vertebrata - ps20
Gnathostomata - ps21
Euteleostomi - ps22
Sarcopterygii - ps23
Tetrapoda - ps24
Amniota - ps25
Mammalia - ps26
Theria - ps27
Placentalia - ps28
Boreoeutheria - ps29
Euarchontoglires - ps30
Euarchonta - ps31
Primates - ps32
Haplorrhini - ps33
Simiiformes - ps34
Catarrhini - ps35
Hominoidea - ps36
Hominidae - ps37
Homininae - ps38
Hominini - ps39
Homo_sapiens - ps40
p-value
1e-07
2e-06
5e-05
1e-03
2e-02
Log-odds
0.5
1.0
1.5
2.0
2.5
3.0
3.5
4.0

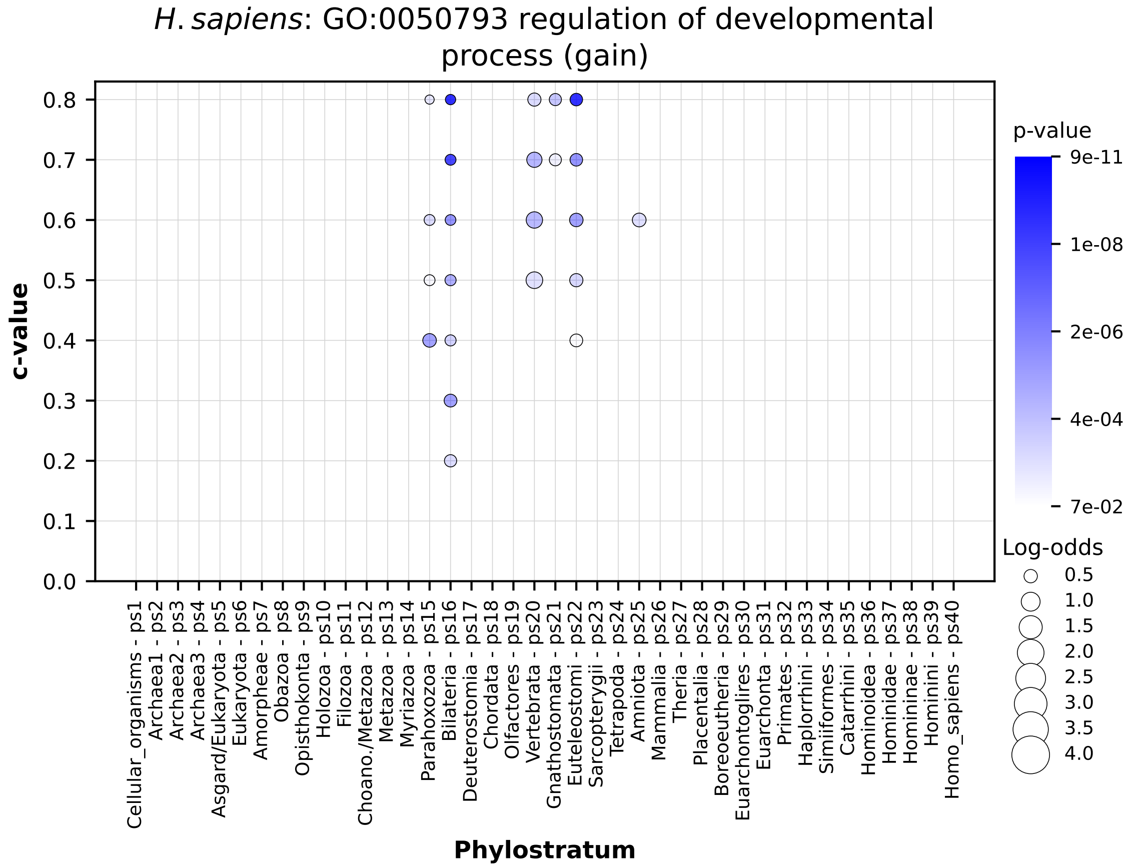

*H. sapiens*: GO:0050793 regulation of developmental process (gain)
c-value
Phylostratum
p-value
9e-11
1e-08
2e-06
4e-04
7e-02
Log-odds
0.5
1.0
1.5
2.0
2.5
3.0
3.5
4.0
Cellular_organisms - ps1
Archaea1 - ps2
Archaea2 - ps3
Archaea3 - ps4
Asgard/Eukaryota - ps5
Eukaryota - ps6
Amorpheae - ps7
Obazoa - ps8
Opisthokonta - ps9
Holozoa - ps10
Filozoa - ps11
Choano./Metazoa - ps12
Metazoa - ps13
Myriazoa - ps14
Parahoxozoa - ps15
Bilateria - ps16
Deuterostomia - ps17
Chordata - ps18
Olfactores - ps19
Vertebrata - ps20
Gnathostomata - ps21
Euteleostomi - ps22
Sarcopterygii - ps23
Tetrapoda - ps24
Amniota - ps25
Mammalia - ps26
Theria - ps27
Placentalia - ps28
Boreoeutheria - ps29
Euarchontoglires - ps30
Euarchonta - ps31
Primates - ps32
Haplorrhini - ps33
Simiiformes - ps34
Catarrhini - ps35
Hominoidea - ps36
Hominidae - ps37
Homininae - ps38
Hominini - ps39
Homo_sapiens - ps40

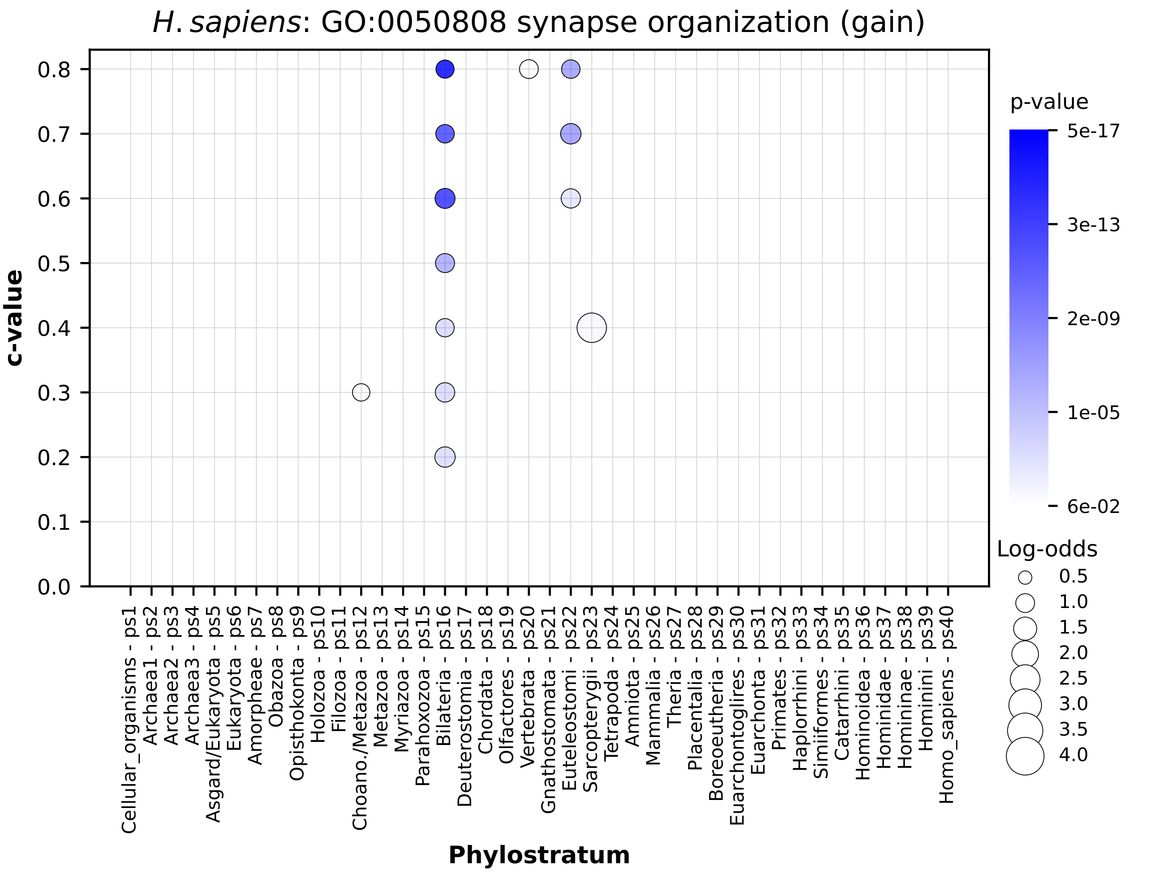

H. sapiens: GO:0050808 synapse organization (gain)
c-value
0.0
0.1
0.2
0.3
0.4
0.5
0.6
0.7
0.8
Phylostratum
Cellular_organisms - ps1
Archaea1 - ps2
Archaea2 - ps3
Archaea3 - ps4
Asgard/Eukaryota - ps5
Eukaryota - ps6
Amorpheae - ps7
Obazoa - ps8
Opisthokonta - ps9
Holozoa - ps10
Filozoa - ps11
Choano./Metazoa - ps12
Metazoa - ps13
Myriazoa - ps14
Parahoxozoa - ps15
Bilateria - ps16
Deuterostomia - ps17
Chordata - ps18
Olfactores - ps19
Vertebrata - ps20
Gnathostomata - ps21
Euteleostomi - ps22
Sarcopterygii - ps23
Tetrapoda - ps24
Amniota - ps25
Mammalia - ps26
Theria - ps27
Placentalia - ps28
Boreoeutheria - ps29
Euarchontoglires - ps30
Euarchonta - ps31
Primates - ps32
Haplorrhini - ps33
Simiiformes - ps34
Catarrhini - ps35
Hominoidea - ps36
Hominidae - ps37
Homininae - ps38
Hominini - ps39
Homo_sapiens - ps40
p-value
5e-17
3e-13
2e-09
1e-05
6e-02
Log-odds
0.5
1.0
1.5
2.0
2.5
3.0
3.5
4.0

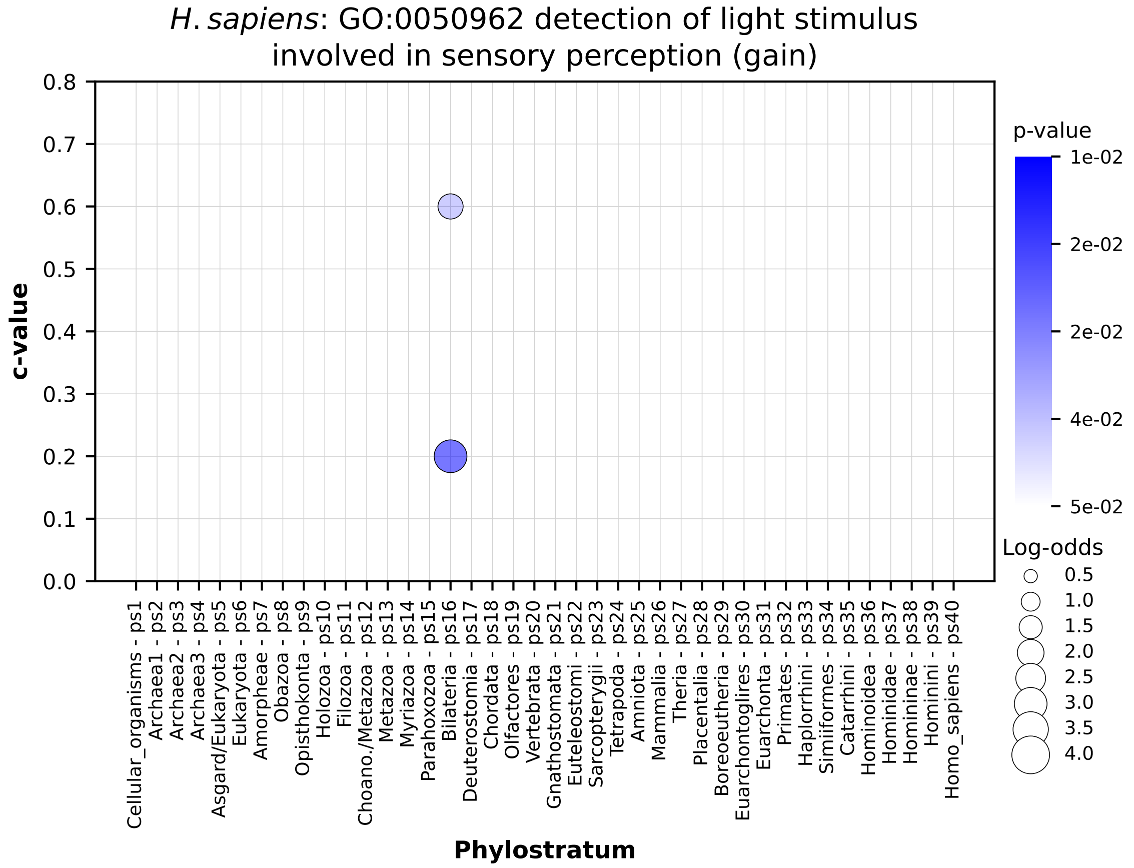

H. sapiens: GO:0050962 detection of light stimulus involved in sensory perception (gain)
c-value
0.0
0.1
0.2
0.3
0.4
0.5
0.6
0.7
0.8
Phylostratum
Cellular_organisms - ps1
Archaea1 - ps2
Archaea2 - ps3
Archaea3 - ps4
Asgard/Eukaryota - ps5
Eukaryota - ps6
Amorpheae - ps7
Obazoa - ps8
Opisthokonta - ps9
Holozoa - ps10
Filozoa - ps11
Choano./Metazoa - ps12
Metazoa - ps13
Myriazoa - ps14
Parahoxozoa - ps15
Bilateria - ps16
Deuterostomia - ps17
Chordata - ps18
Olfactores - ps19
Vertebrata - ps20
Gnathostomata - ps21
Euteleostomi - ps22
Sarcopterygii - ps23
Tetrapoda - ps24
Amniota - ps25
Mammalia - ps26
Theria - ps27
Placentalia - ps28
Boreoeutheria - ps29
Euarchontoglires - ps30
Euarchonta - ps31
Primates - ps32
Haplorrhini - ps33
Simiiformes - ps34
Catarrhini - ps35
Hominoidea - ps36
Hominidae - ps37
Homininae - ps38
Hominini - ps39
Homo_sapiens - ps40
p-value
1e-02
2e-02
2e-02
4e-02
5e-02
Log-odds
0.5
1.0
1.5
2.0
2.5
3.0
3.5
4.0

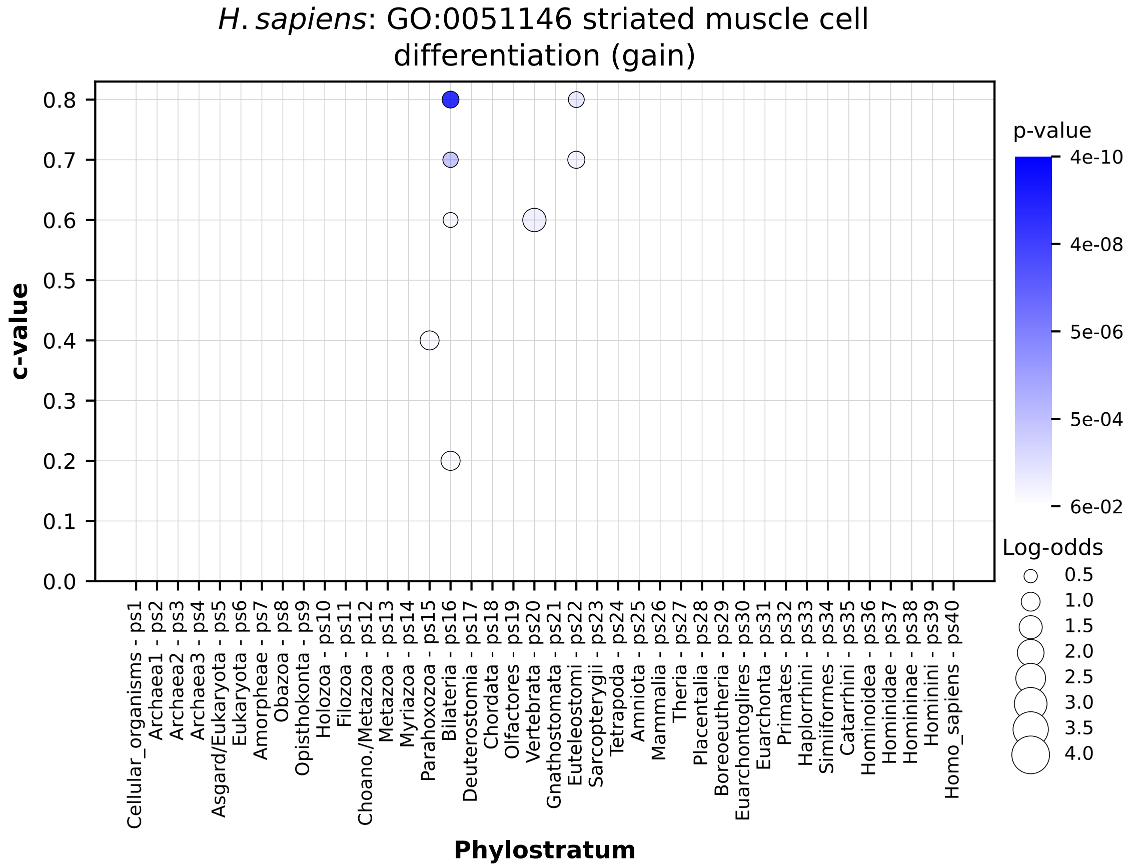
H. sapiens: GO:0051146 striated muscle cell differentiation (gain)
c-value
0.0
0.1
0.2
0.3
0.4
0.5
0.6
0.7
0.8
Phylostratum
Cellular_organisms - ps1
Archaea1 - ps2
Archaea2 - ps3
Archaea3 - ps4
Asgard/Eukaryota - ps5
Eukaryota - ps6
Amorpheae - ps7
Obazoa - ps8
Opisthokonta - ps9
Holozoa - ps10
Filozoa - ps11
Choano./Metazoa - ps12
Metazoa - ps13
Myriazoa - ps14
Parahoxozoa - ps15
Bilateria - ps16
Deuterostomia - ps17
Chordata - ps18
Olfactores - ps19
Vertebrata - ps20
Gnathostomata - ps21
Euteleostomi - ps22
Sarcopterygii - ps23
Tetrapoda - ps24
Amniota - ps25
Mammalia - ps26
Theria - ps27
Placentalia - ps28
Boreoeutheria - ps29
Euarchontoglires - ps30
Euarchonta - ps31
Primates - ps32
Haplorrhini - ps33
Simiiformes - ps34
Catarrhini - ps35
Hominoidea - ps36
Hominidae - ps37
Homininae - ps38
Hominini - ps39
Homo_sapiens - ps40
p-value
4e-10
4e-08
5e-06
5e-04
6e-02
Log-odds
0.5
1.0
1.5
2.0
2.5
3.0
3.5
4.0

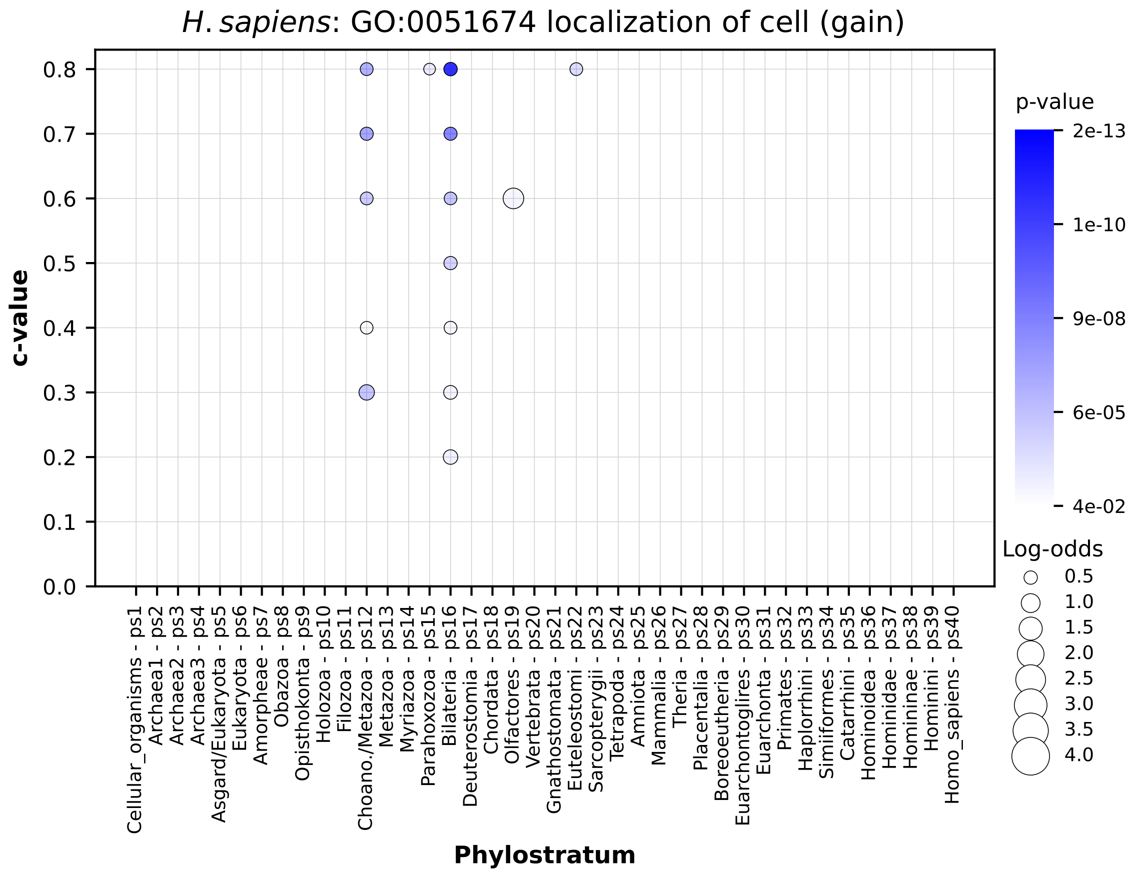

H. sapiens: GO:0051674 localization of cell (gain)
c-value
0.0
0.1
0.2
0.3
0.4
0.5
0.6
0.7
0.8
p-value
2e-13
1e-10
9e-08
6e-05
4e-02
Log-odds
0.5
1.0
1.5
2.0
2.5
3.0
3.5
4.0
Cellular_organisms - ps1
Archaea1 - ps2
Archaea2 - ps3
Archaea3 - ps4
Asgard/Eukaryota - ps5
Eukaryota - ps6
Amorpheae - ps7
Obazoa - ps8
Opisthokonta - ps9
Holozoa - ps10
Filozoa - ps11
Choano./Metazoa - ps12
Metazoa - ps13
Myriazoa - ps14
Parahoxozoa - ps15
Bilateria - ps16
Deuterostomia - ps17
Chordata - ps18
Olfactores - ps19
Vertebrata - ps20
Gnathostomata - ps21
Euteleostomi - ps22
Sarcopterygii - ps23
Tetrapoda - ps24
Amniota - ps25
Mammalia - ps26
Theria - ps27
Placentalia - ps28
Boreoeutheria - ps29
Euarchontoglires - ps30
Euarchonta - ps31
Primates - ps32
Haplorrhini - ps33
Simiiformes - ps34
Catarrhini - ps35
Hominoidea - ps36
Hominidae - ps37
Homininae - ps38
Hominini - ps39
Homo_sapiens - ps40
Phylostratum

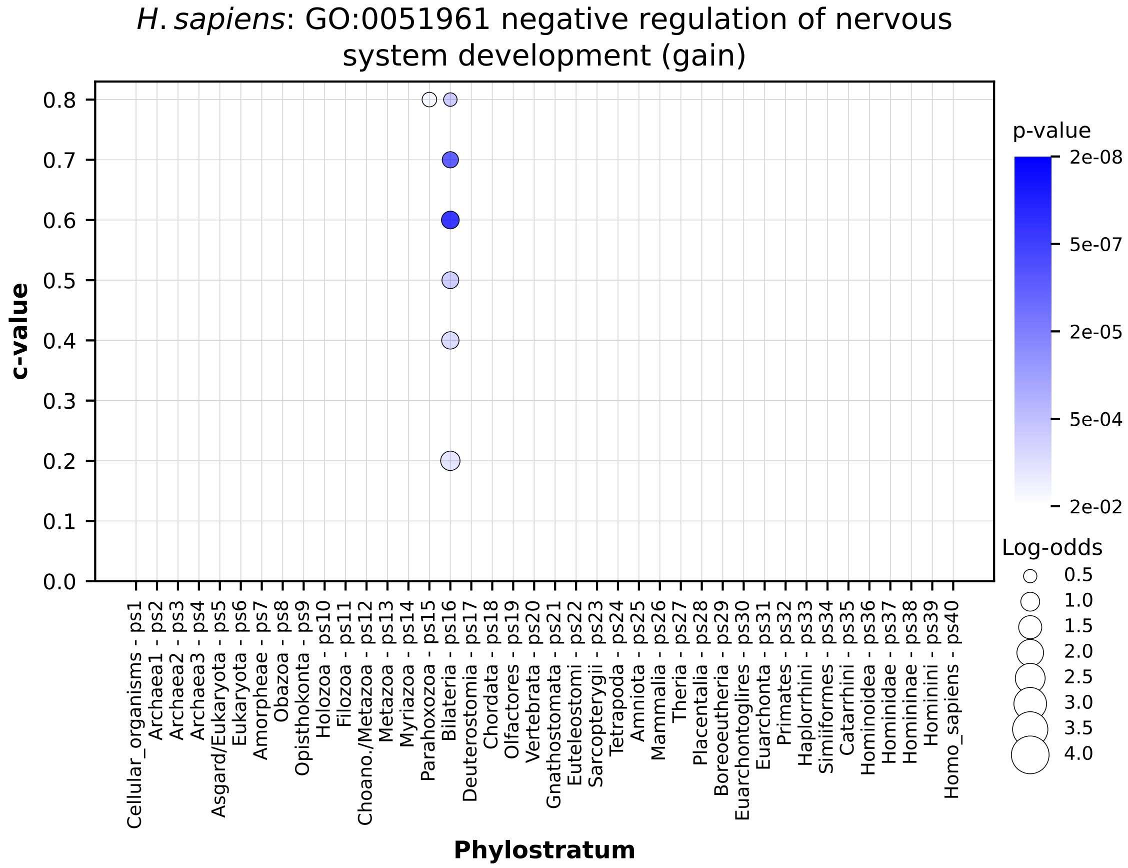

H. sapiens: GO:0051961 negative regulation of nervous system development (gain)
c-value
0.0
0.1
0.2
0.3
0.4
0.5
0.6
0.7
0.8
Phylostratum
Cellular_organisms - ps1
Archaea1 - ps2
Archaea2 - ps3
Archaea3 - ps4
Asgard/Eukaryota - ps5
Eukaryota - ps6
Amorpheae - ps7
Obazoa - ps8
Opisthokonta - ps9
Holozoa - ps10
Filozoa - ps11
Choano./Metazoa - ps12
Metazoa - ps13
Myriazoa - ps14
Parahoxozoa - ps15
Bilateria - ps16
Deuterostomia - ps17
Chordata - ps18
Olfactores - ps19
Vertebrata - ps20
Gnathostomata - ps21
Euteleostomi - ps22
Sarcopterygii - ps23
Tetrapoda - ps24
Amniota - ps25
Mammalia - ps26
Theria - ps27
Placentalia - ps28
Boreoeutheria - ps29
Euarchontoglires - ps30
Euarchonta - ps31
Primates - ps32
Haplorrhini - ps33
Simiiformes - ps34
Catarrhini - ps35
Hominoidea - ps36
Hominidae - ps37
Homininae - ps38
Hominini - ps39
Homo_sapiens - ps40
p-value
2e-08
5e-07
2e-05
5e-04
2e-02
Log-odds
0.5
1.0
1.5
2.0
2.5
3.0
3.5
4.0

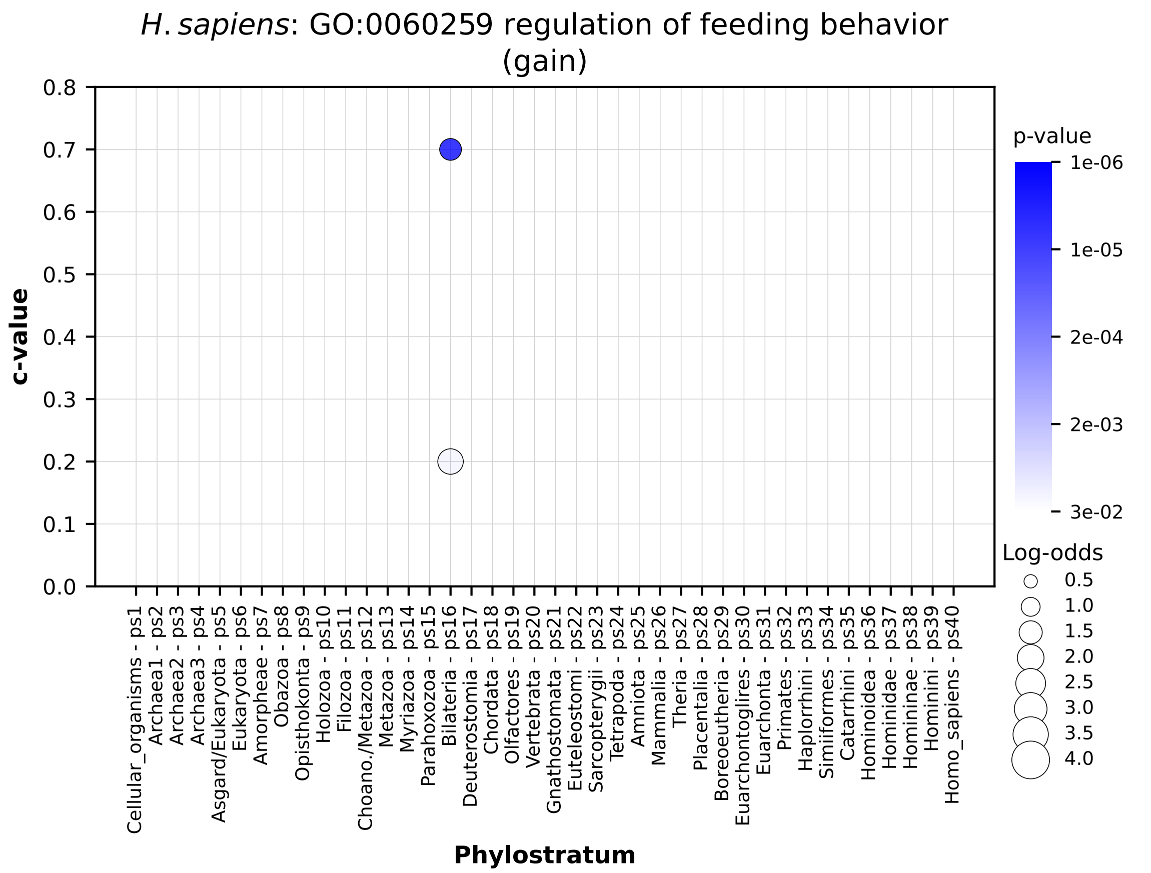
*H. sapiens*: GO:0060259 regulation of feeding behavior
(gain)
c-value
0.0
0.1
0.2
0.3
0.4
0.5
0.6
0.7
0.8
Phylostratum
Cellular_organisms - ps1
Archaea1 - ps2
Archaea2 - ps3
Archaea3 - ps4
Asgard/Eukaryota - ps5
Eukaryota - ps6
Amorpheae - ps7
Obazoa - ps8
Opisthokonta - ps9
Holozoa - ps10
Filozoa - ps11
Choano./Metazoa - ps12
Metazoa - ps13
Myriazoa - ps14
Parahoxozoa - ps15
Bilateria - ps16
Deuterostomia - ps17
Chordata - ps18
Olfactores - ps19
Vertebrata - ps20
Gnathostomata - ps21
Euteleostomi - ps22
Sarcopterygii - ps23
Tetrapoda - ps24
Amniota - ps25
Mammalia - ps26
Theria - ps27
Placentalia - ps28
Boreoeutheria - ps29
Euarchontoglires - ps30
Euarchonta - ps31
Primates - ps32
Haplorrhini - ps33
Simiiformes - ps34
Catarrhini - ps35
Hominoidea - ps36
Hominidae - ps37
Homininae - ps38
Hominini - ps39
Homo_sapiens - ps40
p-value
1e-06
1e-05
2e-04
2e-03
3e-02
Log-odds
0.5
1.0
1.5
2.0
2.5
3.0
3.5
4.0

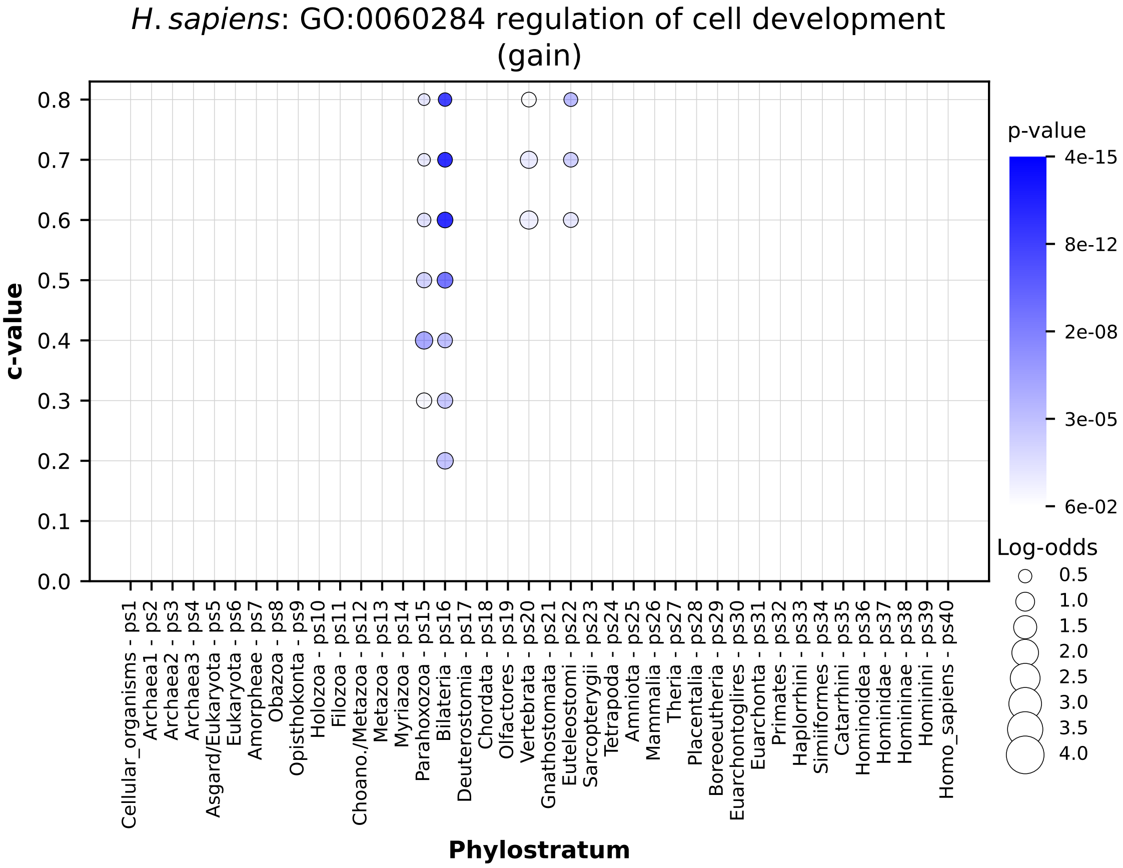
H. sapiens: GO:0060284 regulation of cell development
(gain)
c-value
0.0
0.1
0.2
0.3
0.4
0.5
0.6
0.7
0.8
Phylostratum
Cellular_organisms - ps1
Archaea1 - ps2
Archaea2 - ps3
Archaea3 - ps4
Asgard/Eukaryota - ps5
Eukaryota - ps6
Amorpheae - ps7
Obazoa - ps8
Opisthokonta - ps9
Holozoa - ps10
Filozoa - ps11
Choano./Metazoa - ps12
Metazoa - ps13
Myriazoa - ps14
Parahoxozoa - ps15
Bilateria - ps16
Deuterostomia - ps17
Chordata - ps18
Olfactores - ps19
Vertebrata - ps20
Gnathostomata - ps21
Euteleostomi - ps22
Sarcopterygii - ps23
Tetrapoda - ps24
Amniota - ps25
Mammalia - ps26
Theria - ps27
Placentalia - ps28
Boreoeutheria - ps29
Euarchontoglires - ps30
Euarchonta - ps31
Primates - ps32
Haplorrhini - ps33
Simiiformes - ps34
Catarrhini - ps35
Hominoidea - ps36
Hominidae - ps37
Homininae - ps38
Hominini - ps39
Homo_sapiens - ps40
p-value
4e-15
8e-12
2e-08
3e-05
6e-02
Log-odds
0.5
1.0
1.5
2.0
2.5
3.0
3.5
4.0

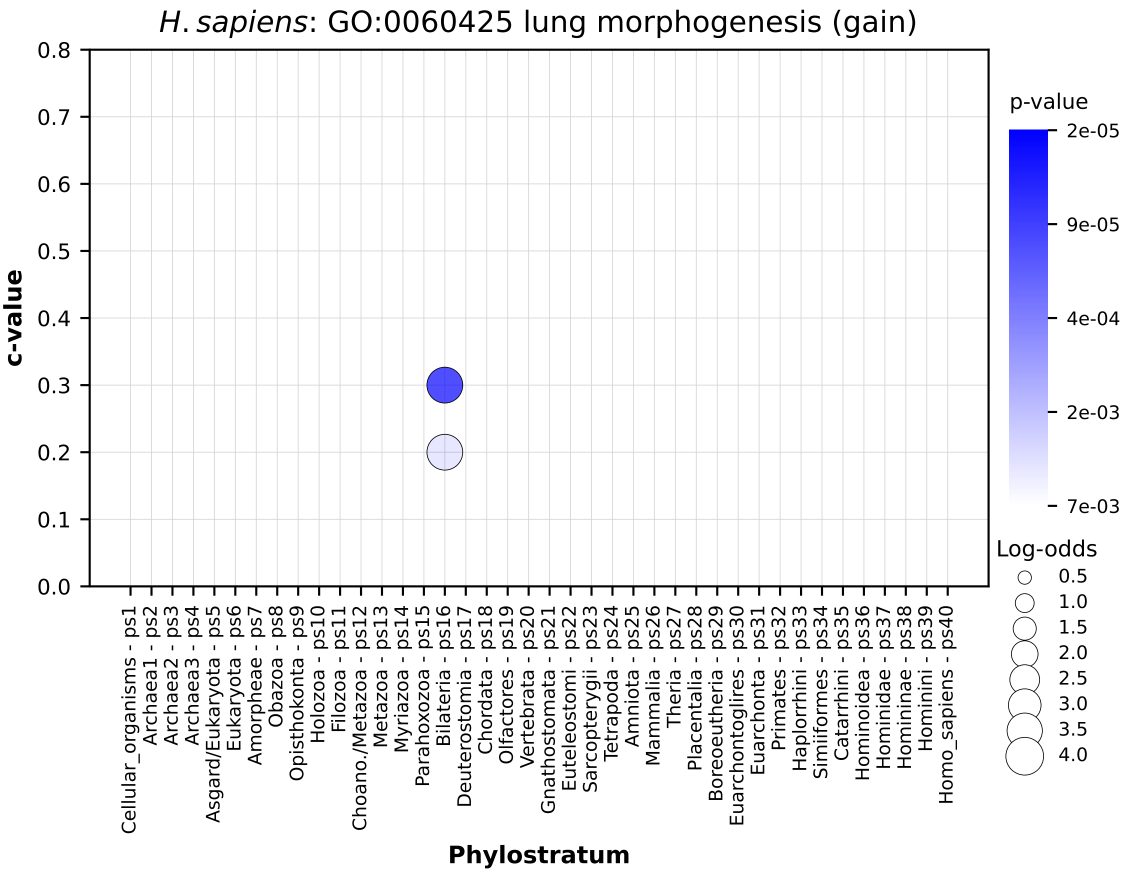

H. sapiens: GO:0060425 lung morphogenesis (gain)
c-value
0.0
0.1
0.2
0.3
0.4
0.5
0.6
0.7
0.8
Phylostratum
Cellular_organisms - ps1
Archaea1 - ps2
Archaea2 - ps3
Archaea3 - ps4
Asgard/Eukaryota - ps5
Eukaryota - ps6
Amorpheae - ps7
Obazoa - ps8
Opisthokonta - ps9
Holozoa - ps10
Filozoa - ps11
Choano./Metazoa - ps12
Metazoa - ps13
Myriazoa - ps14
Parahoxozoa - ps15
Bilateria - ps16
Deuterostomia - ps17
Chordata - ps18
Olfactores - ps19
Vertebrata - ps20
Gnathostomata - ps21
Euteleostomi - ps22
Sarcopterygii - ps23
Tetrapoda - ps24
Amniota - ps25
Mammalia - ps26
Theria - ps27
Placentalia - ps28
Boreoeutheria - ps29
Euarchontoglires - ps30
Euarchonta - ps31
Primates - ps32
Haplorrhini - ps33
Simiiformes - ps34
Catarrhini - ps35
Hominoidea - ps36
Hominidae - ps37
Homininae - ps38
Hominini - ps39
Homo_sapiens - ps40
p-value
2e-05
9e-05
4e-04
2e-03
7e-03
Log-odds
0.5
1.0
1.5
2.0
2.5
3.0
3.5
4.0

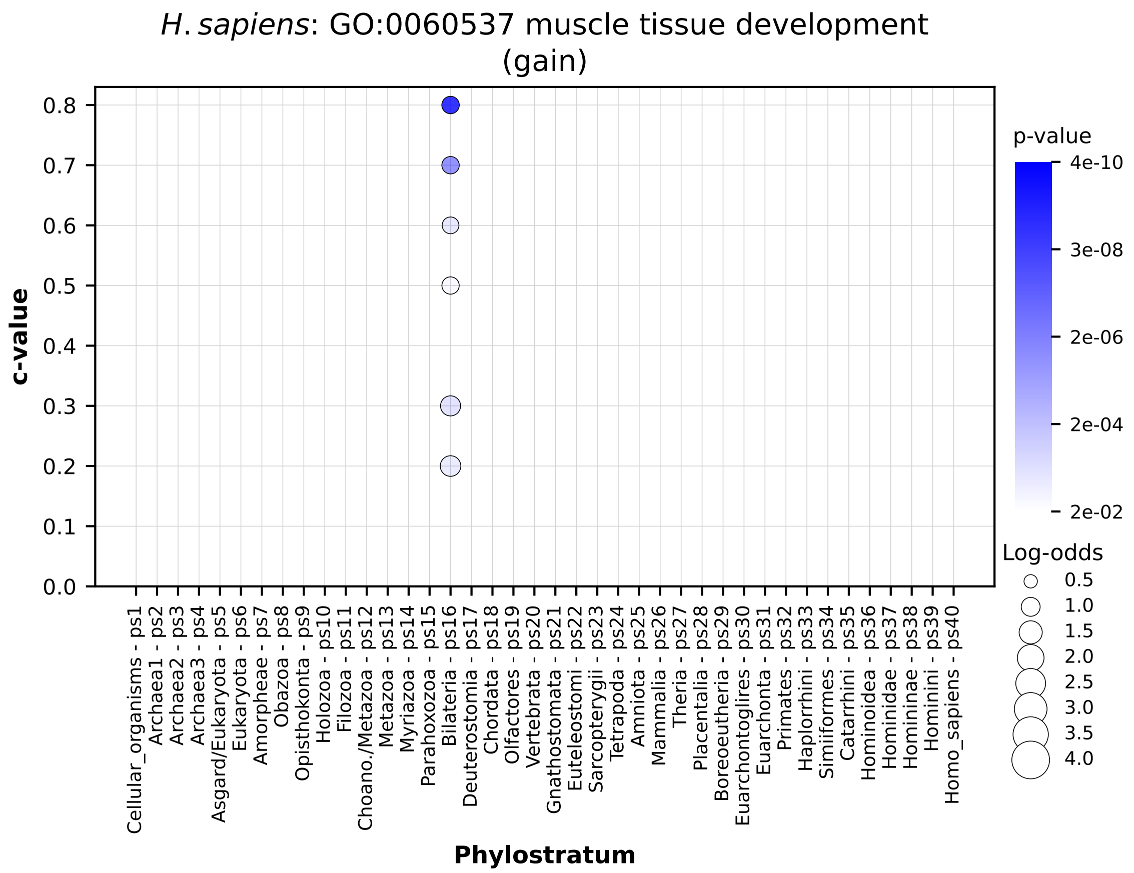

H. sapiens: GO:0060537 muscle tissue development
(gain)
c-value
0.0
0.1
0.2
0.3
0.4
0.5
0.6
0.7
0.8
Phylostratum
Cellular_organisms - ps1
Archaea1 - ps2
Archaea2 - ps3
Archaea3 - ps4
Asgard/Eukaryota - ps5
Eukaryota - ps6
Amorpheae - ps7
Obazoa - ps8
Opisthokonta - ps9
Holozoa - ps10
Filozoa - ps11
Choano./Metazoa - ps12
Metazoa - ps13
Myriazoa - ps14
Parahoxozoa - ps15
Bilateria - ps16
Deuterostomia - ps17
Chordata - ps18
Olfactores - ps19
Vertebrata - ps20
Gnathostomata - ps21
Euteleostomi - ps22
Sarcopterygii - ps23
Tetrapoda - ps24
Amniota - ps25
Mammalia - ps26
Theria - ps27
Placentalia - ps28
Boreoeutheria - ps29
Euarchontoglires - ps30
Euarchonta - ps31
Primates - ps32
Haplorrhini - ps33
Simiiformes - ps34
Catarrhini - ps35
Hominoidea - ps36
Hominidae - ps37
Homininae - ps38
Hominini - ps39
Homo_sapiens - ps40
p-value
4e-10
3e-08
2e-06
2e-04
2e-02
Log-odds
0.5
1.0
1.5
2.0
2.5
3.0
3.5
4.0

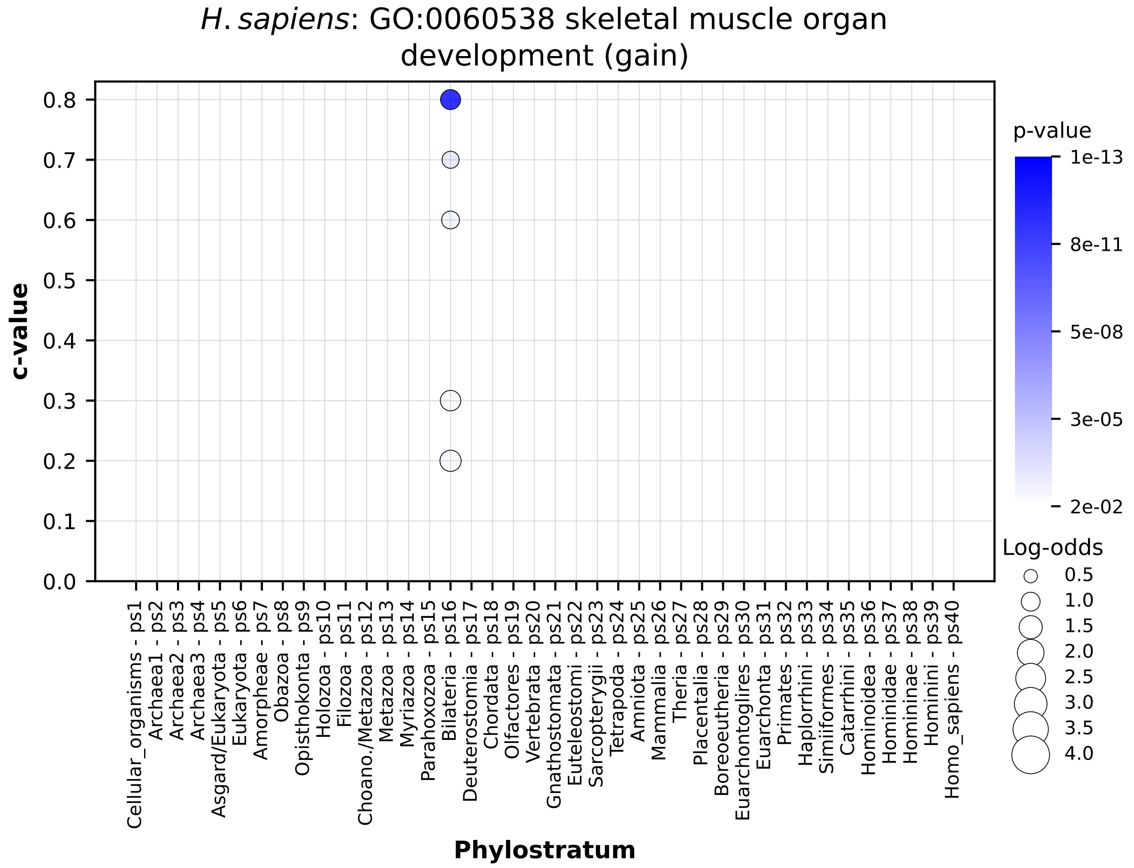

H. sapiens: GO:0060538 skeletal muscle organ development (gain)
c-value
0.0
0.1
0.2
0.3
0.4
0.5
0.6
0.7
0.8
Phylostratum
Cellular_organisms - ps1
Archaea1 - ps2
Archaea2 - ps3
Archaea3 - ps4
Asgard/Eukaryota - ps5
Eukaryota - ps6
Amorpheae - ps7
Obazoa - ps8
Opisthokonta - ps9
Holozoa - ps10
Filozoa - ps11
Choano./Metazoa - ps12
Metazoa - ps13
Myriazoa - ps14
Parahoxozoa - ps15
Bilateria - ps16
Deuterostomia - ps17
Chordata - ps18
Olfactores - ps19
Vertebrata - ps20
Gnathostomata - ps21
Euteleostomi - ps22
Sarcopterygii - ps23
Tetrapoda - ps24
Amniota - ps25
Mammalia - ps26
Theria - ps27
Placentalia - ps28
Boreoeutheria - ps29
Euarchontoglires - ps30
Euarchonta - ps31
Primates - ps32
Haplorrhini - ps33
Simiiformes - ps34
Catarrhini - ps35
Hominoidea - ps36
Hominidae - ps37
Homininae - ps38
Hominini - ps39
Homo_sapiens - ps40
p-value
1e-13
8e-11
5e-08
3e-05
2e-02
Log-odds
0.5
1.0
1.5
2.0
2.5
3.0
3.5
4.0

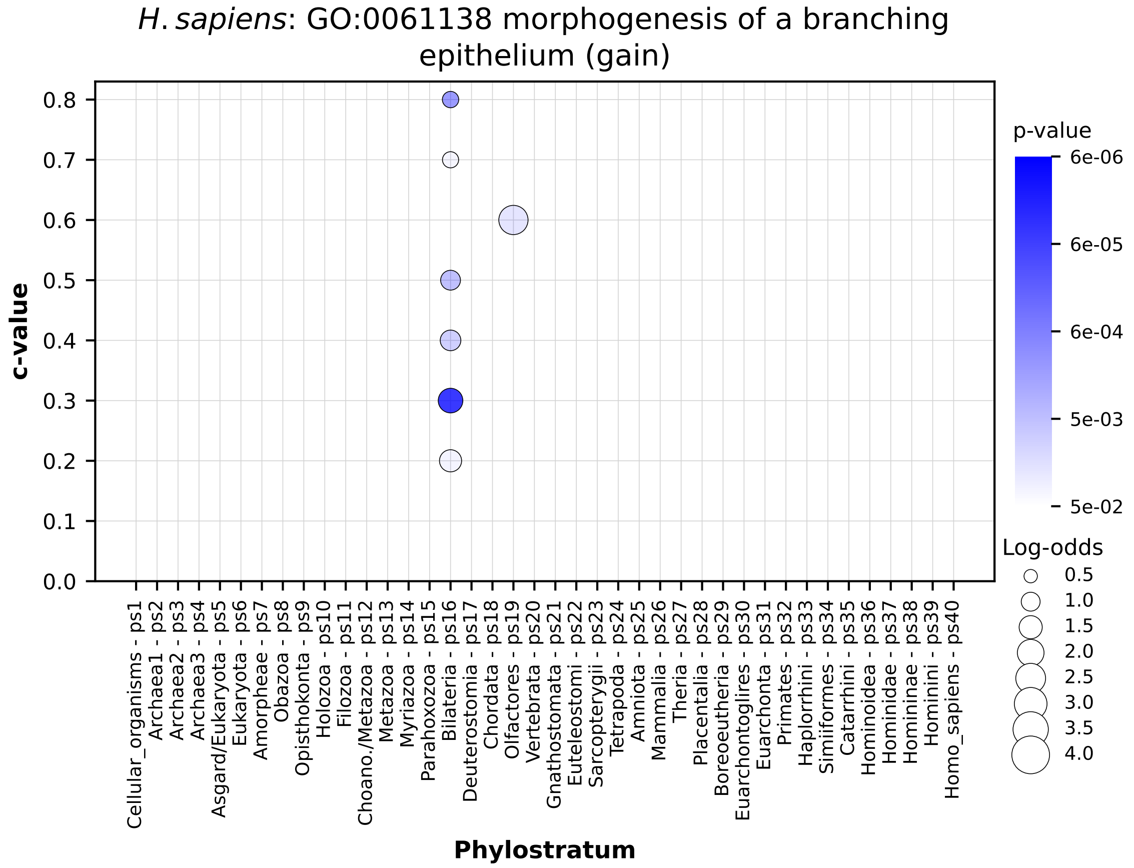

H. sapiens: GO:0061138 morphogenesis of a branching epithelium (gain)
c-value
0.8
0.7
0.6
0.5
0.4
0.3
0.2
0.1
0.0
Phylostratum
Cellular_organisms - ps1
Archaea1 - ps2
Archaea2 - ps3
Archaea3 - ps4
Asgard/Eukaryota - ps5
Eukaryota - ps6
Amorpheae - ps7
Obazoa - ps8
Opisthokonta - ps9
Holozoa - ps10
Filozoa - ps11
Choano./Metazoa - ps12
Metazoa - ps13
Myriazoa - ps14
Parahoxozoa - ps15
Bilateria - ps16
Deuterostomia - ps17
Chordata - ps18
Olfactores - ps19
Vertebrata - ps20
Gnathostomata - ps21
Euteleostomi - ps22
Sarcopterygii - ps23
Tetrapoda - ps24
Amniota - ps25
Mammalia - ps26
Theria - ps27
Placentalia - ps28
Boreoeutheria - ps29
Euarchontoglires - ps30
Euarchonta - ps31
Primates - ps32
Haplorrhini - ps33
Simiiformes - ps34
Catarrhini - ps35
Hominoidea - ps36
Hominidae - ps37
Homininae - ps38
Hominini - ps39
Homo_sapiens - ps40
p-value
6e-06
6e-05
6e-04
5e-03
5e-02
Log-odds
0.5
1.0
1.5
2.0
2.5
3.0
3.5
4.0

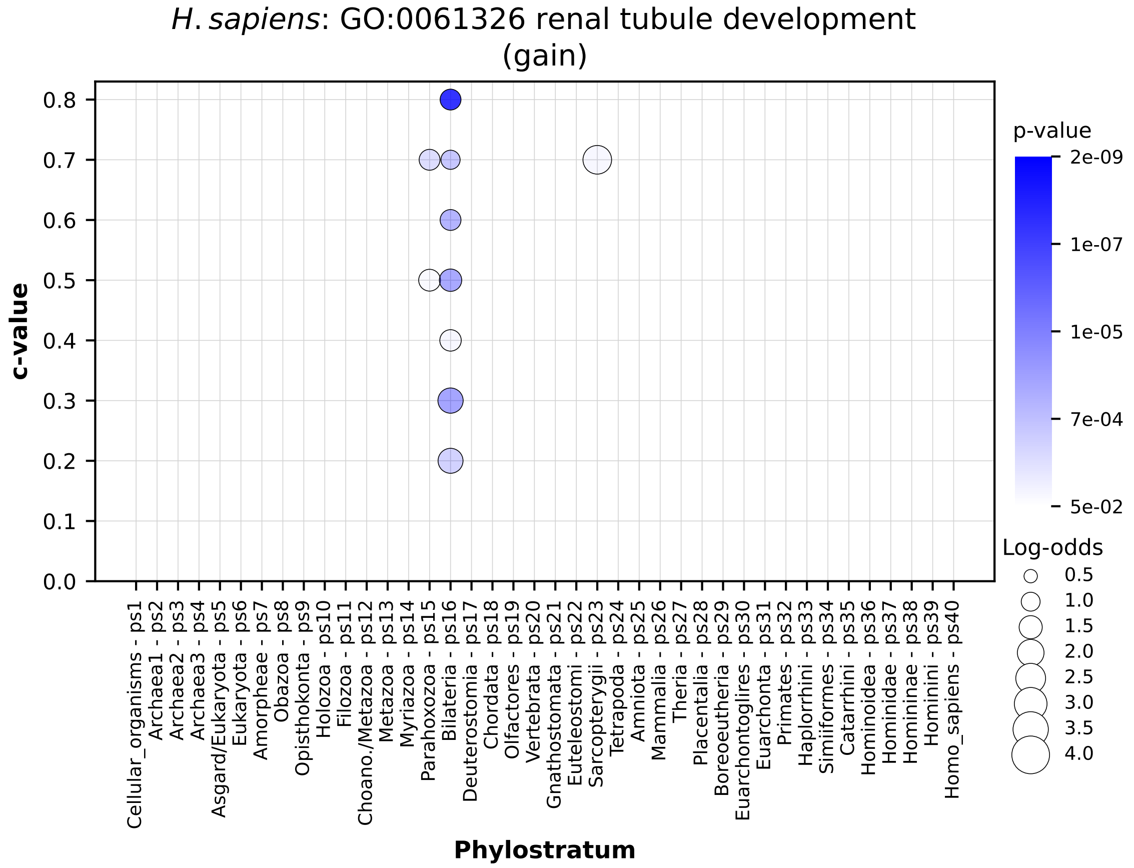

H. sapiens: GO:0061326 renal tubule development
(gain)
c-value
0.0
0.1
0.2
0.3
0.4
0.5
0.6
0.7
0.8
Phylostratum
Cellular_organisms - ps1
Archaea1 - ps2
Archaea2 - ps3
Archaea3 - ps4
Asgard/Eukaryota - ps5
Eukaryota - ps6
Amorpheae - ps7
Obazoa - ps8
Opisthokonta - ps9
Holozoa - ps10
Filozoa - ps11
Choano./Metazoa - ps12
Metazoa - ps13
Myriazoa - ps14
Parahoxozoa - ps15
Bilateria - ps16
Deuterostomia - ps17
Chordata - ps18
Olfactores - ps19
Vertebrata - ps20
Gnathostomata - ps21
Euteleostomi - ps22
Sarcopterygii - ps23
Tetrapoda - ps24
Amniota - ps25
Mammalia - ps26
Theria - ps27
Placentalia - ps28
Boreoeutheria - ps29
Euarchontoglires - ps30
Euarchonta - ps31
Primates - ps32
Haplorrhini - ps33
Simiiformes - ps34
Catarrhini - ps35
Hominoidea - ps36
Hominidae - ps37
Homininae - ps38
Hominini - ps39
Homo_sapiens - ps40
p-value
2e-09
1e-07
1e-05
7e-04
5e-02
Log-odds
0.5
1.0
1.5
2.0
2.5
3.0
3.5
4.0

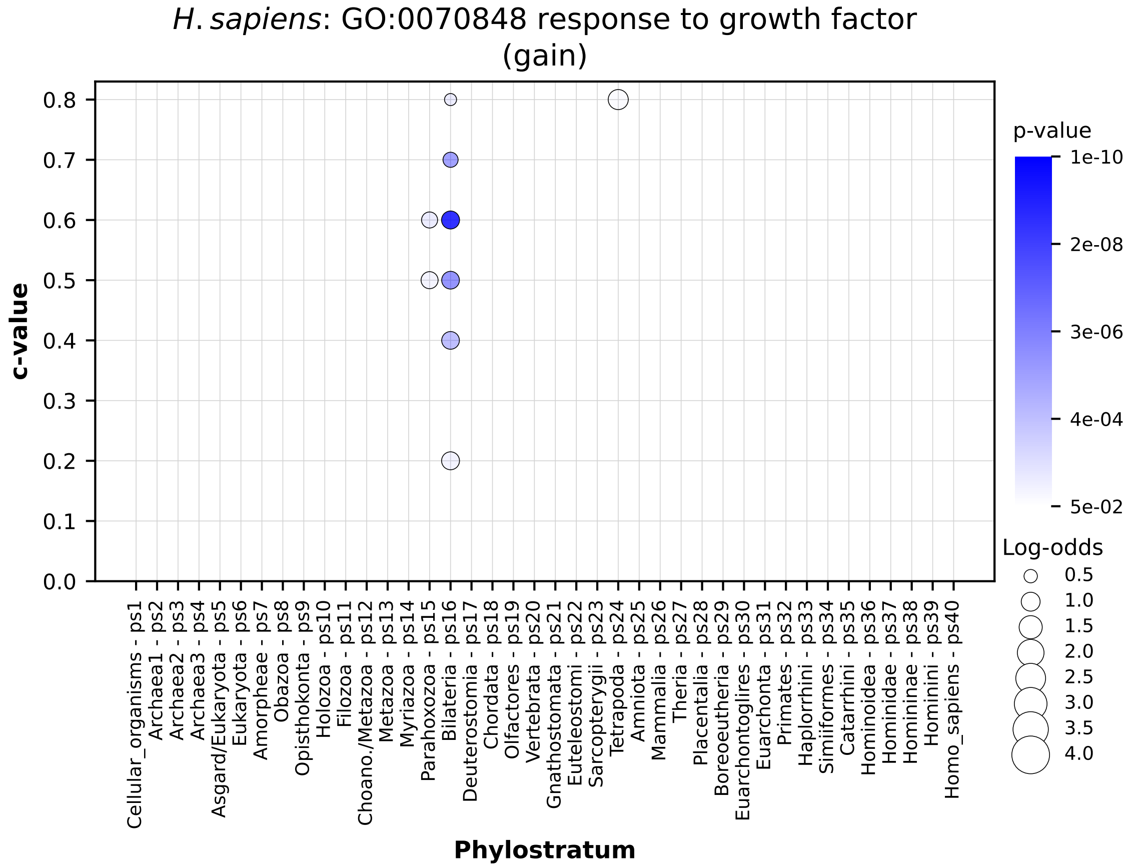
H. sapiens: GO:0070848 response to growth factor
(gain)
c-value
0.0
0.1
0.2
0.3
0.4
0.5
0.6
0.7
0.8
Phylostratum
Cellular_organisms - ps1
Archaea1 - ps2
Archaea2 - ps3
Archaea3 - ps4
Asgard/Eukaryota - ps5
Eukaryota - ps6
Amorpheae - ps7
Obazoa - ps8
Opisthokonta - ps9
Holozoa - ps10
Filozoa - ps11
Choano./Metazoa - ps12
Metazoa - ps13
Myriazoa - ps14
Parahoxozoa - ps15
Bilateria - ps16
Deuterostomia - ps17
Chordata - ps18
Olfactores - ps19
Vertebrata - ps20
Gnathostomata - ps21
Euteleostomi - ps22
Sarcopterygii - ps23
Tetrapoda - ps24
Amniota - ps25
Mammalia - ps26
Theria - ps27
Placentalia - ps28
Boreoeutheria - ps29
Euarchontoglires - ps30
Euarchonta - ps31
Primates - ps32
Haplorrhini - ps33
Simiiformes - ps34
Catarrhini - ps35
Hominoidea - ps36
Hominidae - ps37
Homininae - ps38
Hominini - ps39
Homo_sapiens - ps40
p-value
1e-10
2e-08
3e-06
4e-04
5e-02
Log-odds
0.5
1.0
1.5
2.0
2.5
3.0
3.5
4.0

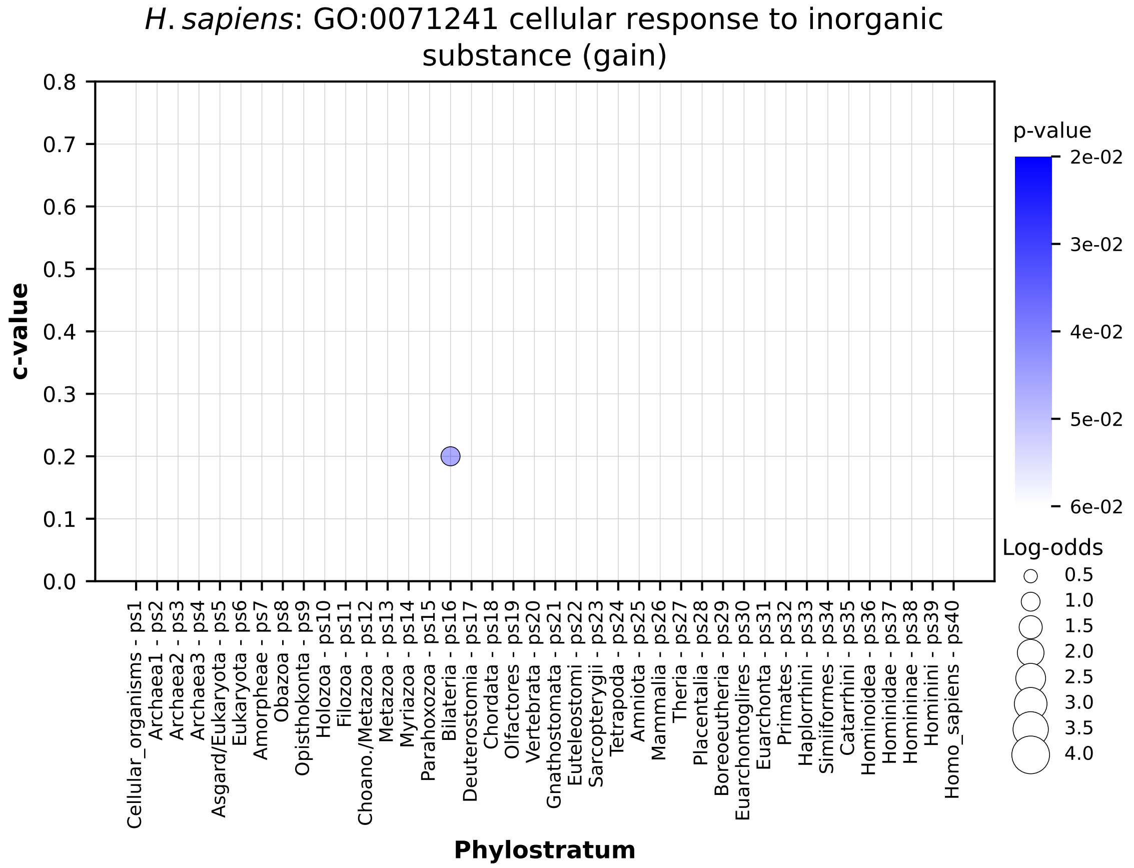
H. sapiens: GO:0071241 cellular response to inorganic substance (gain)
c-value
0.0
0.1
0.2
0.3
0.4
0.5
0.6
0.7
0.8
Phylostratum
Cellular_organisms - ps1
Archaea1 - ps2
Archaea2 - ps3
Archaea3 - ps4
Asgard/Eukaryota - ps5
Eukaryota - ps6
Amorpheae - ps7
Obazoa - ps8
Opisthokonta - ps9
Holozoa - ps10
Filozoa - ps11
Choano./Metazoa - ps12
Metazoa - ps13
Myriazoa - ps14
Parahoxozoa - ps15
Bilateria - ps16
Deuterostomia - ps17
Chordata - ps18
Olfactores - ps19
Vertebrata - ps20
Gnathostomata - ps21
Euteleostomi - ps22
Sarcopterygii - ps23
Tetrapoda - ps24
Amniota - ps25
Mammalia - ps26
Theria - ps27
Placentalia - ps28
Boreoeutheria - ps29
Euarchontoglires - ps30
Euarchonta - ps31
Primates - ps32
Haplorrhini - ps33
Simiiformes - ps34
Catarrhini - ps35
Hominoidea - ps36
Hominidae - ps37
Homininae - ps38
Hominini - ps39
Homo_sapiens - ps40
p-value
2e-02
3e-02
4e-02
5e-02
6e-02
Log-odds
0.5
1.0
1.5
2.0
2.5
3.0
3.5
4.0

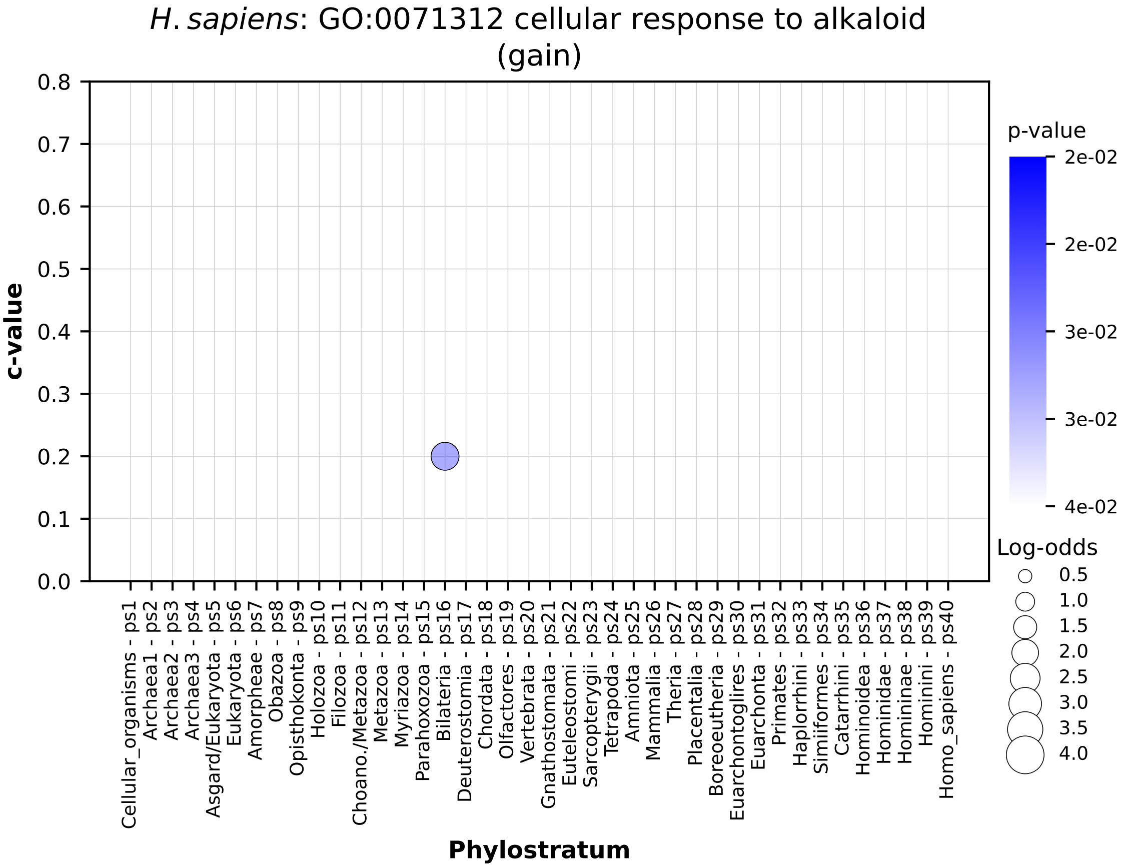

H. sapiens: GO:0071312 cellular response to alkaloid (gain)
c-value
0.0
0.1
0.2
0.3
0.4
0.5
0.6
0.7
0.8
Phylostratum
Cellular_organisms - ps1
Archaea1 - ps2
Archaea2 - ps3
Archaea3 - ps4
Asgard/Eukaryota - ps5
Eukaryota - ps6
Amorpheae - ps7
Obazoa - ps8
Opisthokonta - ps9
Holozoa - ps10
Filozoa - ps11
Choano./Metazoa - ps12
Metazoa - ps13
Myriazoa - ps14
Parahoxozoa - ps15
Bilateria - ps16
Deuterostomia - ps17
Chordata - ps18
Olfactores - ps19
Vertebrata - ps20
Gnathostomata - ps21
Euteleostomi - ps22
Sarcopterygii - ps23
Tetrapoda - ps24
Amniota - ps25
Mammalia - ps26
Theria - ps27
Placentalia - ps28
Boreoeutheria - ps29
Euarchontoglires - ps30
Euarchonta - ps31
Primates - ps32
Haplorrhini - ps33
Simiiformes - ps34
Catarrhini - ps35
Hominoidea - ps36
Hominidae - ps37
Homininae - ps38
Hominini - ps39
Homo_sapiens - ps40
p-value
2e-02
2e-02
3e-02
3e-02
4e-02
Log-odds
0.5
1.0
1.5
2.0
2.5
3.0
3.5
4.0

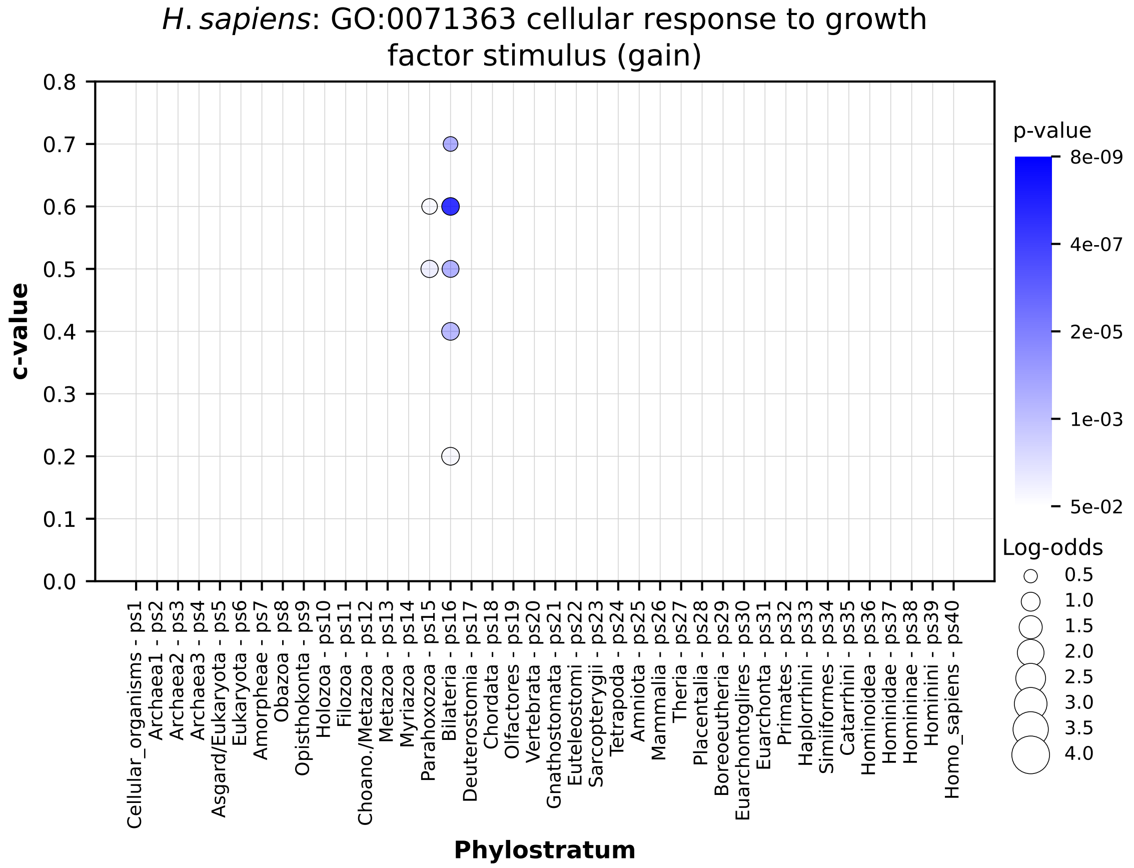

H. sapiens: GO:0071363 cellular response to growth factor stimulus (gain)
c-value
0.0
0.1
0.2
0.3
0.4
0.5
0.6
0.7
0.8
Phylostratum
Cellular_organisms - ps1
Archaea1 - ps2
Archaea2 - ps3
Archaea3 - ps4
Asgard/Eukaryota - ps5
Eukaryota - ps6
Amorpheae - ps7
Obazoa - ps8
Opisthokonta - ps9
Holozoa - ps10
Filozoa - ps11
Choano./Metazoa - ps12
Metazoa - ps13
Myriazoa - ps14
Parahoxozoa - ps15
Bilateria - ps16
Deuterostomia - ps17
Chordata - ps18
Olfactores - ps19
Vertebrata - ps20
Gnathostomata - ps21
Euteleostomi - ps22
Sarcopterygii - ps23
Tetrapoda - ps24
Amniota - ps25
Mammalia - ps26
Theria - ps27
Placentalia - ps28
Boreoeutheria - ps29
Euarchontoglires - ps30
Euarchonta - ps31
Primates - ps32
Haplorrhini - ps33
Simiiformes - ps34
Catarrhini - ps35
Hominoidea - ps36
Hominidae - ps37
Homininae - ps38
Hominini - ps39
Homo_sapiens - ps40
p-value
8e-09
4e-07
2e-05
1e-03
5e-02
Log-odds
0.5
1.0
1.5
2.0
2.5
3.0
3.5
4.0

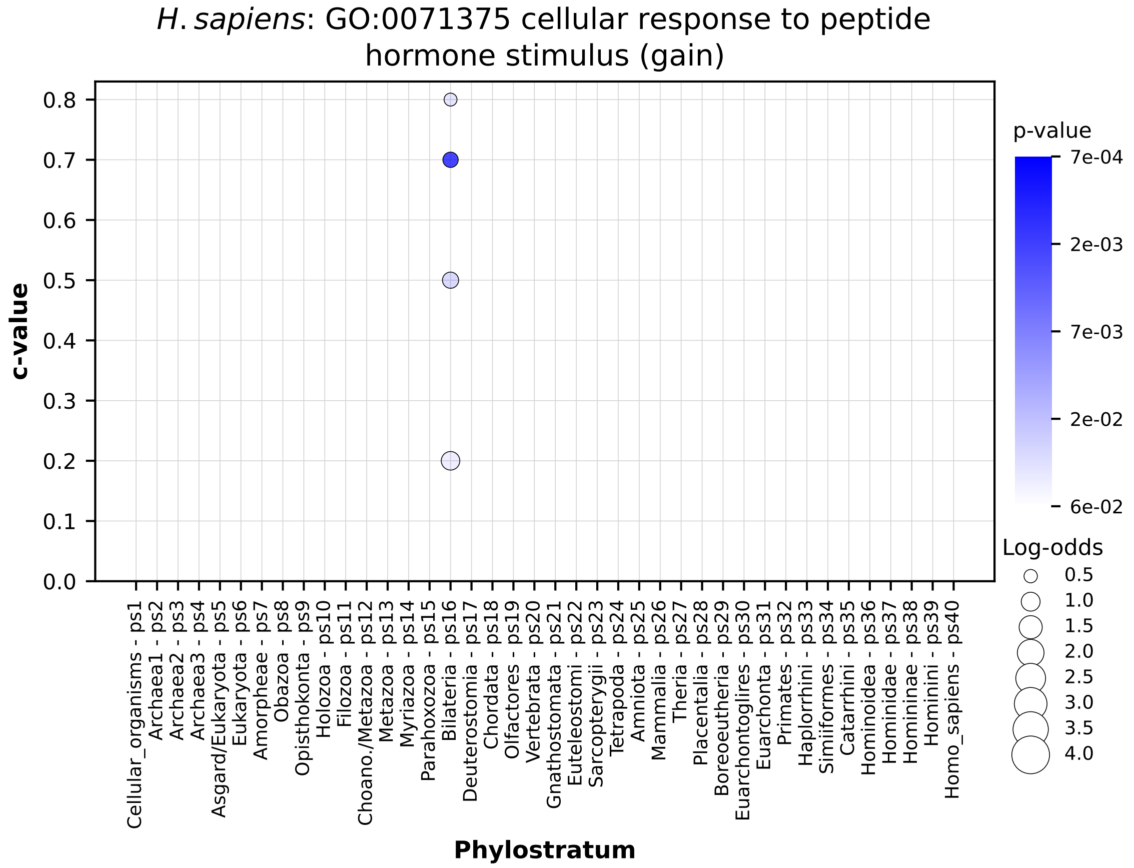
H. sapiens: GO:0071375 cellular response to peptide hormone stimulus (gain)
c-value
0.0
0.1
0.2
0.3
0.4
0.5
0.6
0.7
0.8
Phylostratum
Cellular_organisms - ps1
Archaea1 - ps2
Archaea2 - ps3
Archaea3 - ps4
Asgard/Eukaryota - ps5
Eukaryota - ps6
Amorpheae - ps7
Obazoa - ps8
Opisthokonta - ps9
Holozoa - ps10
Filozoa - ps11
Choano./Metazoa - ps12
Metazoa - ps13
Myriazoa - ps14
Parahoxozoa - ps15
Bilateria - ps16
Deuterostomia - ps17
Chordata - ps18
Olfactores - ps19
Vertebrata - ps20
Gnathostomata - ps21
Euteleostomi - ps22
Sarcopterygii - ps23
Tetrapoda - ps24
Amniota - ps25
Mammalia - ps26
Theria - ps27
Placentalia - ps28
Boreoeutheria - ps29
Euarchontoglires - ps30
Euarchonta - ps31
Primates - ps32
Haplorrhini - ps33
Simiiformes - ps34
Catarrhini - ps35
Hominoidea - ps36
Hominidae - ps37
Homininae - ps38
Hominini - ps39
Homo_sapiens - ps40
p-value
7e-04
2e-03
7e-03
2e-02
6e-02
Log-odds
0.5
1.0
1.5
2.0
2.5
3.0
3.5
4.0

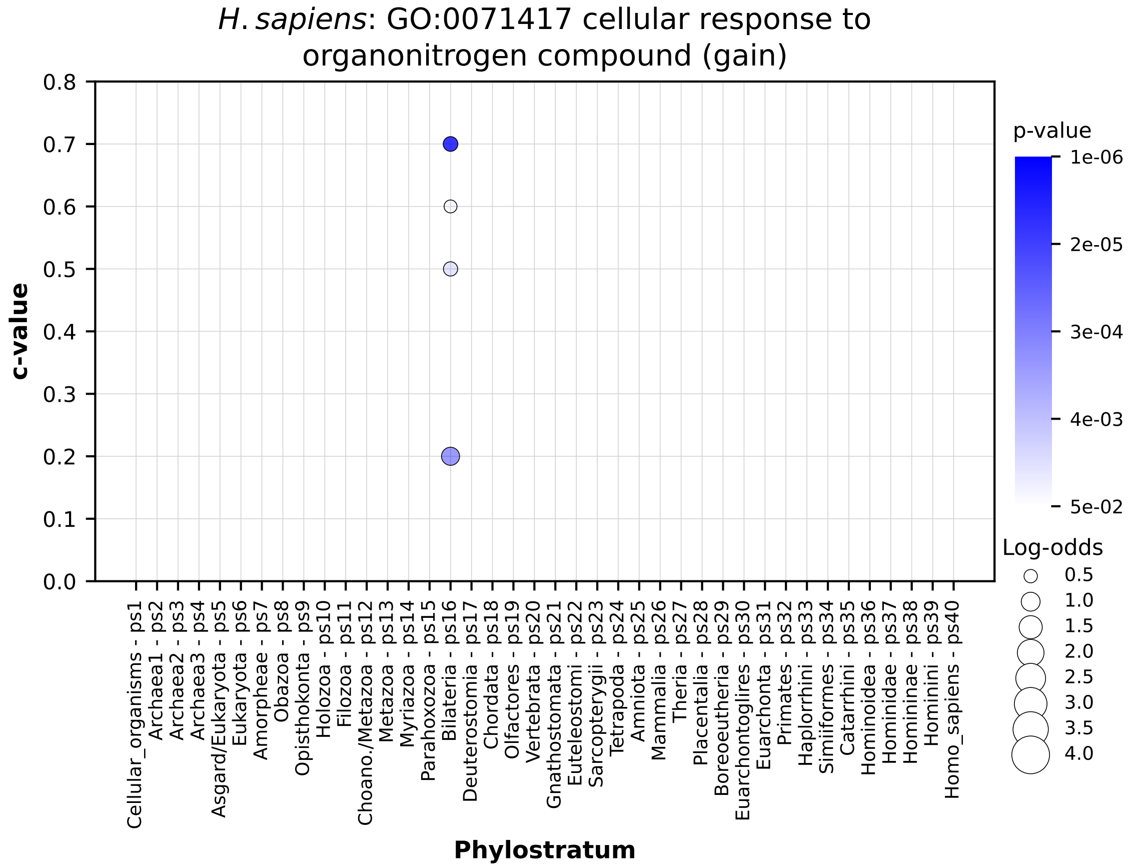
H. sapiens: GO:0071417 cellular response to organonitrogen compound (gain)
c-value
Phylostratum
p-value
1e-06
2e-05
3e-04
4e-03
5e-02
Log-odds
0.5
1.0
1.5
2.0
2.5
3.0
3.5
4.0
0.0
0.1
0.2
0.3
0.4
0.5
0.6
0.7
0.8
Cellular_organisms - ps1
Archaea1 - ps2
Archaea2 - ps3
Archaea3 - ps4
Asgard/Eukaryota - ps5
Eukaryota - ps6
Amorpheae - ps7
Obazoa - ps8
Opisthokonta - ps9
Holozoa - ps10
Filozoa - ps11
Choano./Metazoa - ps12
Metazoa - ps13
Myriazoa - ps14
Parahoxozoa - ps15
Bilateria - ps16
Deuterostomia - ps17
Chordata - ps18
Olfactores - ps19
Vertebrata - ps20
Gnathostomata - ps21
Euteleostomi - ps22
Sarcopterygii - ps23
Tetrapoda - ps24
Amniota - ps25
Mammalia - ps26
Theria - ps27
Placentalia - ps28
Boreoeutheria - ps29
Euarchontoglires - ps30
Euarchonta - ps31
Primates - ps32
Haplorrhini - ps33
Simiiformes - ps34
Catarrhini - ps35
Hominoidea - ps36
Hominidae - ps37
Homininae - ps38
Hominini - ps39
Homo_sapiens - ps40

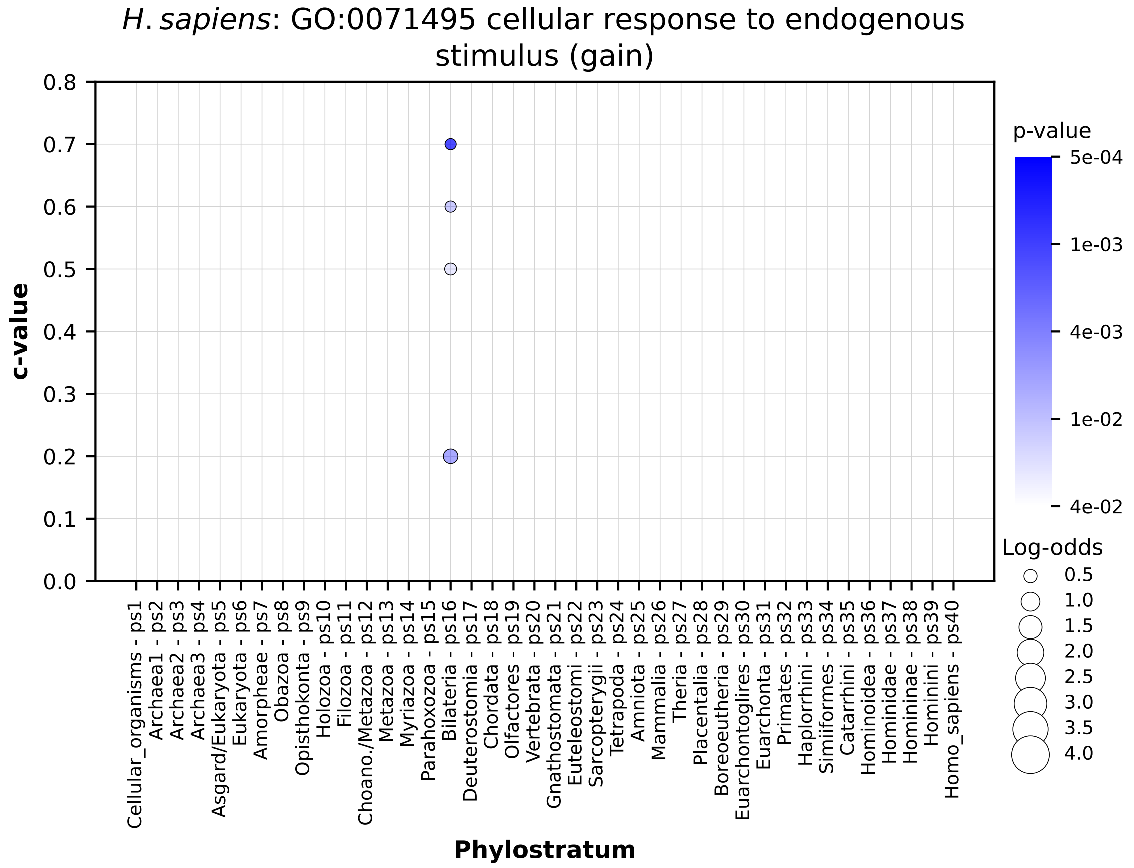

H. sapiens: GO:0071495 cellular response to endogenous stimulus (gain)
c-value
0.0
0.1
0.2
0.3
0.4
0.5
0.6
0.7
0.8
Phylostratum
Cellular_organisms - ps1
Archaea1 - ps2
Archaea2 - ps3
Archaea3 - ps4
Asgard/Eukaryota - ps5
Eukaryota - ps6
Amorpheae - ps7
Obazoa - ps8
Opisthokonta - ps9
Holozoa - ps10
Filozoa - ps11
Choano./Metazoa - ps12
Metazoa - ps13
Myriazoa - ps14
Parahoxozoa - ps15
Bilateria - ps16
Deuterostomia - ps17
Chordata - ps18
Olfactores - ps19
Vertebrata - ps20
Gnathostomata - ps21
Euteleostomi - ps22
Sarcopterygii - ps23
Tetrapoda - ps24
Amniota - ps25
Mammalia - ps26
Theria - ps27
Placentalia - ps28
Boreoeutheria - ps29
Euarchontoglires - ps30
Euarchonta - ps31
Primates - ps32
Haplorrhini - ps33
Simiiformes - ps34
Catarrhini - ps35
Hominoidea - ps36
Hominidae - ps37
Homininae - ps38
Hominini - ps39
Homo_sapiens - ps40
p-value
5e-04
1e-03
4e-03
1e-02
4e-02
Log-odds
0.5
1.0
1.5
2.0
2.5
3.0
3.5
4.0

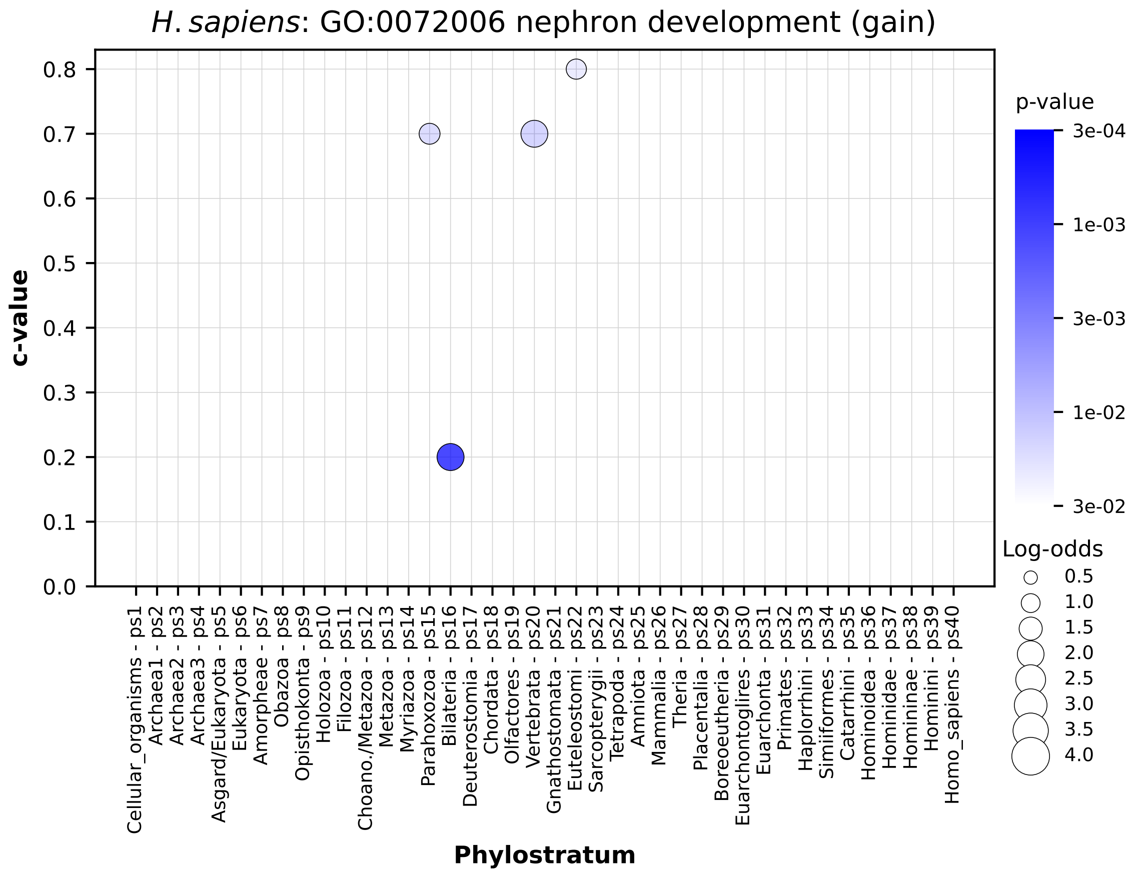

H. sapiens: GO:0072006 nephron development (gain)
c-value
0.0
0.1
0.2
0.3
0.4
0.5
0.6
0.7
0.8
Phylostratum
Cellular_organisms - ps1
Archaea1 - ps2
Archaea2 - ps3
Archaea3 - ps4
Asgard/Eukaryota - ps5
Eukaryota - ps6
Amorpheae - ps7
Obazoa - ps8
Opisthokonta - ps9
Holozoa - ps10
Filozoa - ps11
Choano./Metazoa - ps12
Metazoa - ps13
Myriazoa - ps14
Parahoxozoa - ps15
Bilateria - ps16
Deuterostomia - ps17
Chordata - ps18
Olfactores - ps19
Vertebrata - ps20
Gnathostomata - ps21
Euteleostomi - ps22
Sarcopterygii - ps23
Tetrapoda - ps24
Amniota - ps25
Mammalia - ps26
Theria - ps27
Placentalia - ps28
Boreoeutheria - ps29
Euarchontoglires - ps30
Euarchonta - ps31
Primates - ps32
Haplorrhini - ps33
Simiiformes - ps34
Catarrhini - ps35
Hominoidea - ps36
Hominidae - ps37
Homininae - ps38
Hominini - ps39
Homo_sapiens - ps40
p-value
3e-04
1e-03
3e-03
1e-02
3e-02
Log-odds
0.5
1.0
1.5
2.0
2.5
3.0
3.5
4.0

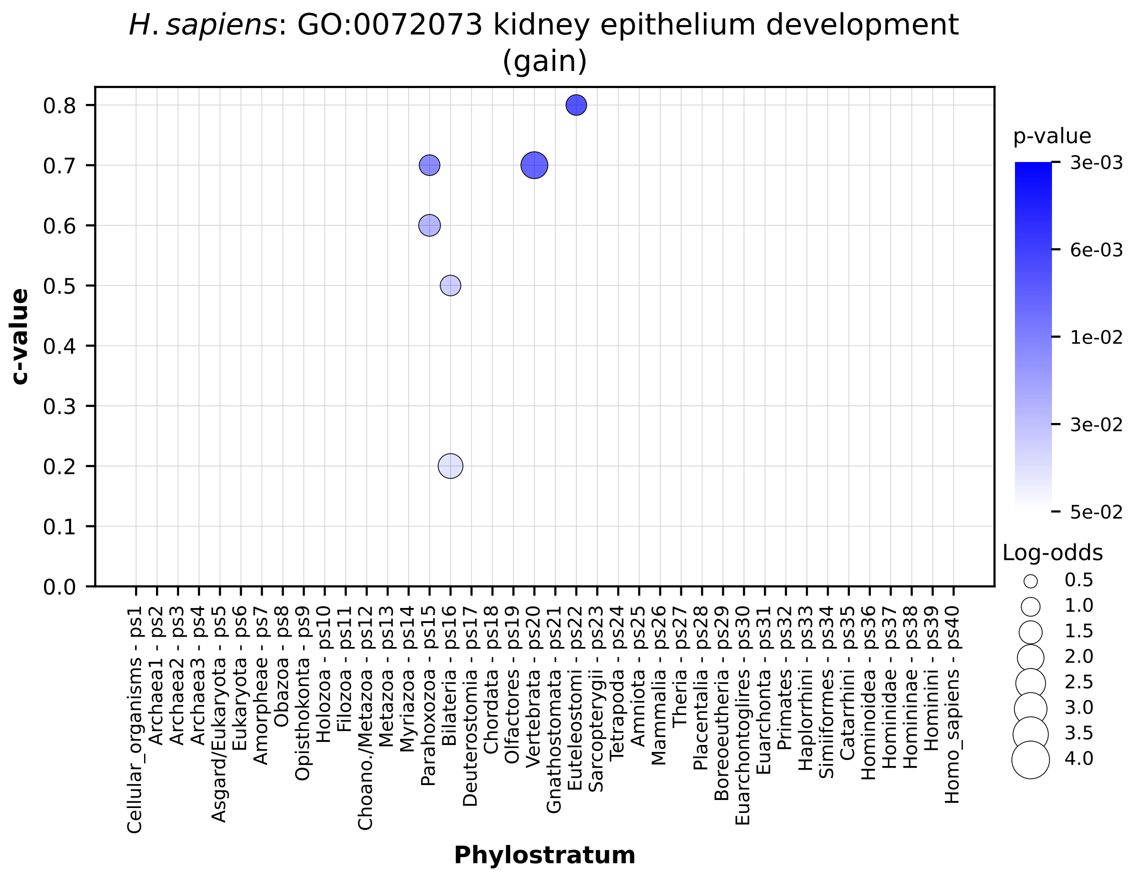

H. sapiens: GO:0072073 kidney epithelium development (gain)
c-value
0.0
0.1
0.2
0.3
0.4
0.5
0.6
0.7
0.8
Phylostratum
Cellular_organisms - ps1
Archaea1 - ps2
Archaea2 - ps3
Archaea3 - ps4
Asgard/Eukaryota - ps5
Eukaryota - ps6
Amorpheae - ps7
Obazoa - ps8
Opisthokonta - ps9
Holozoa - ps10
Filozoa - ps11
Choano./Metazoa - ps12
Metazoa - ps13
Myriazoa - ps14
Parahoxozoa - ps15
Bilateria - ps16
Deuterostomia - ps17
Chordata - ps18
Olfactores - ps19
Vertebrata - ps20
Gnathostomata - ps21
Euteleostomi - ps22
Sarcopterygii - ps23
Tetrapoda - ps24
Amniota - ps25
Mammalia - ps26
Theria - ps27
Placentalia - ps28
Boreoeutheria - ps29
Euarchontoglires - ps30
Euarchonta - ps31
Primates - ps32
Haplorrhini - ps33
Simiiformes - ps34
Catarrhini - ps35
Hominoidea - ps36
Hominidae - ps37
Homininae - ps38
Hominini - ps39
Homo_sapiens - ps40
p-value
3e-03
6e-03
1e-02
3e-02
5e-02
Log-odds
0.5
1.0
1.5
2.0
2.5
3.0
3.5
4.0

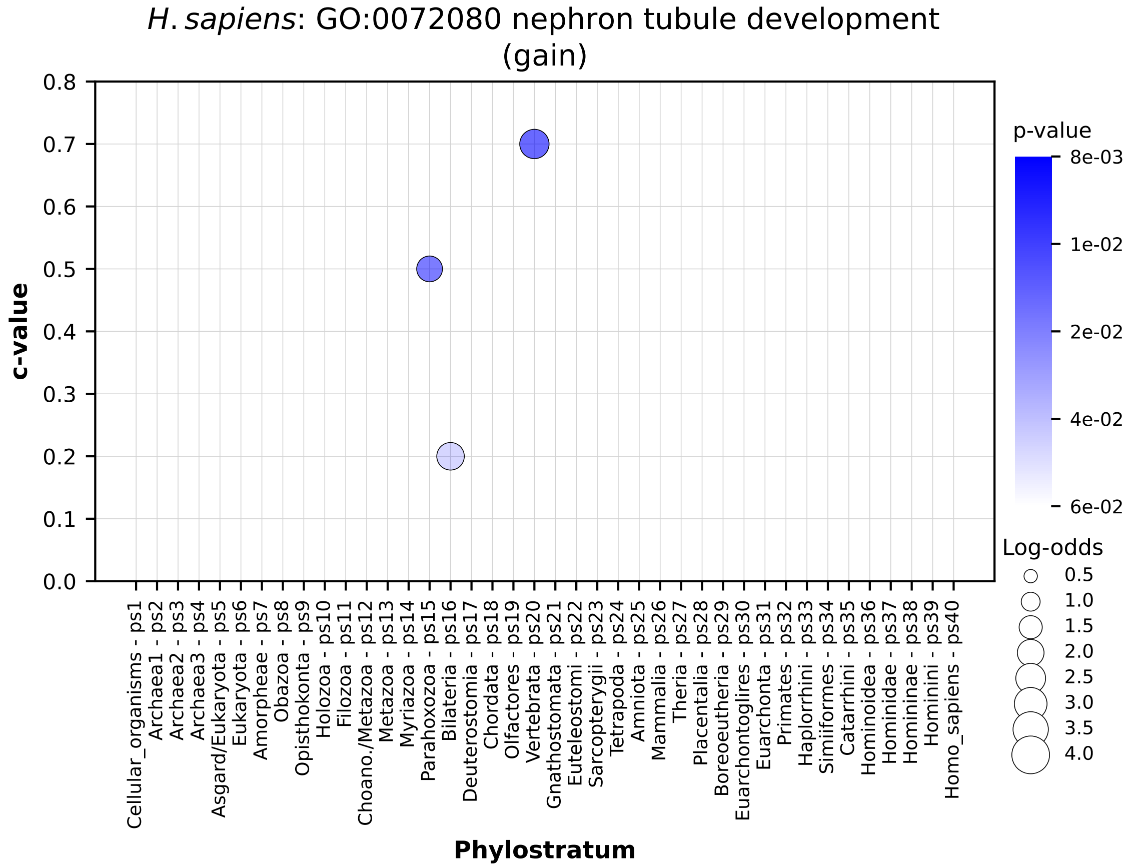
H. sapiens: GO:0072080 nephron tubule development
(gain)
c-value
0.0
0.1
0.2
0.3
0.4
0.5
0.6
0.7
0.8
p-value
8e-03
1e-02
2e-02
4e-02
6e-02
Log-odds
0.5
1.0
1.5
2.0
2.5
3.0
3.5
4.0
Cellular_organisms - ps1
Archaea1 - ps2
Archaea2 - ps3
Archaea3 - ps4
Asgard/Eukaryota - ps5
Eukaryota - ps6
Amorpheae - ps7
Obazoa - ps8
Opisthokonta - ps9
Holozoa - ps10
Filozoa - ps11
Choano./Metazoa - ps12
Metazoa - ps13
Myriazoa - ps14
Parahoxozoa - ps15
Bilateria - ps16
Deuterostomia - ps17
Chordata - ps18
Olfactores - ps19
Vertebrata - ps20
Gnathostomata - ps21
Euteleostomi - ps22
Sarcopterygii - ps23
Tetrapoda - ps24
Amniota - ps25
Mammalia - ps26
Theria - ps27
Placentalia - ps28
Boreoeutheria - ps29
Euarchontoglires - ps30
Euarchonta - ps31
Primates - ps32
Haplorrhini - ps33
Simiiformes - ps34
Catarrhini - ps35
Hominoidea - ps36
Hominidae - ps37
Homininae - ps38
Hominini - ps39
Homo_sapiens - ps40
Phylostratum

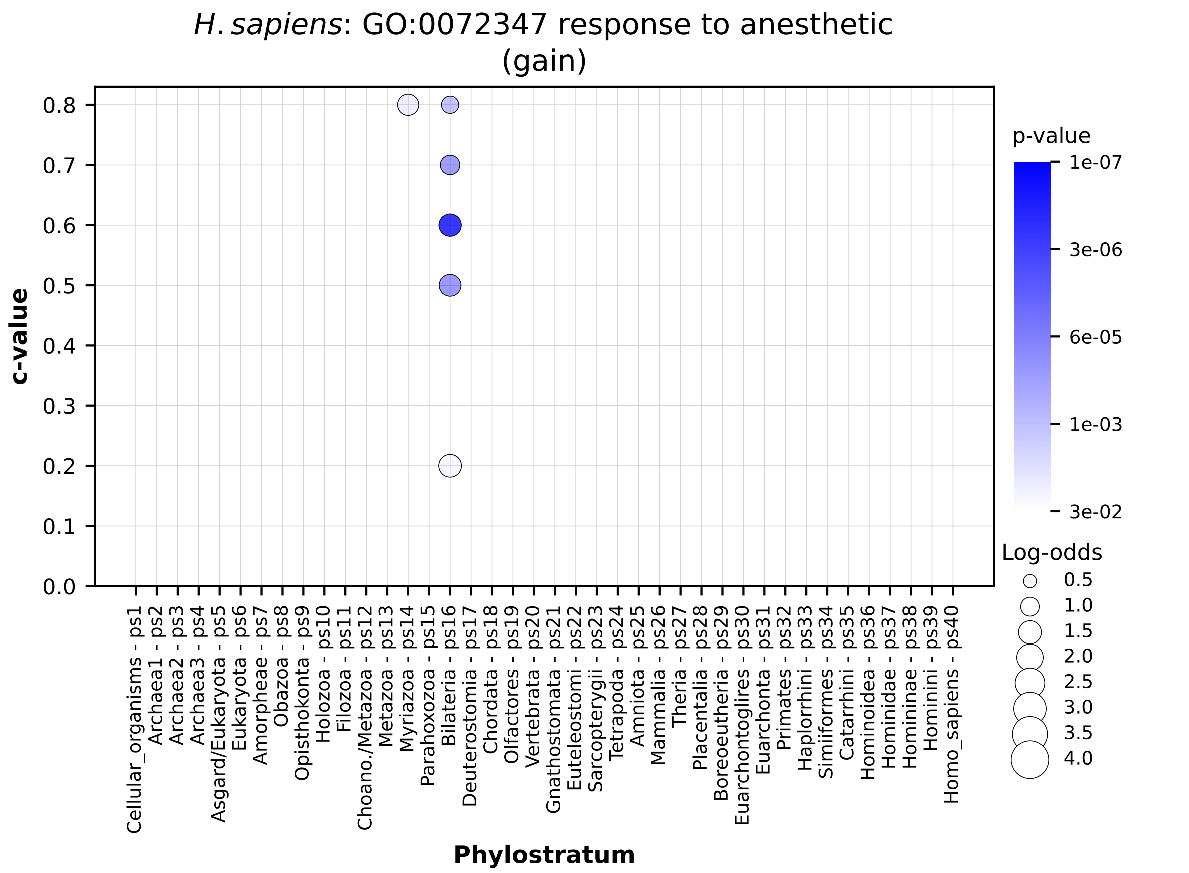
H. sapiens: GO:0072347 response to anesthetic
(gain)
c-value
0.8
0.7
0.6
0.5
0.4
0.3
0.2
0.1
0.0
p-value
1e-07
3e-06
6e-05
1e-03
3e-02
Log-odds
0.5
1.0
1.5
2.0
2.5
3.0
3.5
4.0
Cellular_organisms - ps1
Archaea1 - ps2
Archaea2 - ps3
Archaea3 - ps4
Asgard/Eukaryota - ps5
Eukaryota - ps6
Amorpheae - ps7
Obazoa - ps8
Opisthokonta - ps9
Holozoa - ps10
Filozoa - ps11
Choano./Metazoa - ps12
Metazoa - ps13
Myriazoa - ps14
Parahoxozoa - ps15
Bilateria - ps16
Deuterostomia - ps17
Chordata - ps18
Olfactores - ps19
Vertebrata - ps20
Gnathostomata - ps21
Euteleostomi - ps22
Sarcopterygii - ps23
Tetrapoda - ps24
Amniota - ps25
Mammalia - ps26
Theria - ps27
Placentalia - ps28
Boreoeutheria - ps29
Euarchontoglires - ps30
Euarchonta - ps31
Primates - ps32
Haplorrhini - ps33
Simiiformes - ps34
Catarrhini - ps35
Hominoidea - ps36
Hominidae - ps37
Homininae - ps38
Hominini - ps39
Homo_sapiens - ps40
Phylostratum

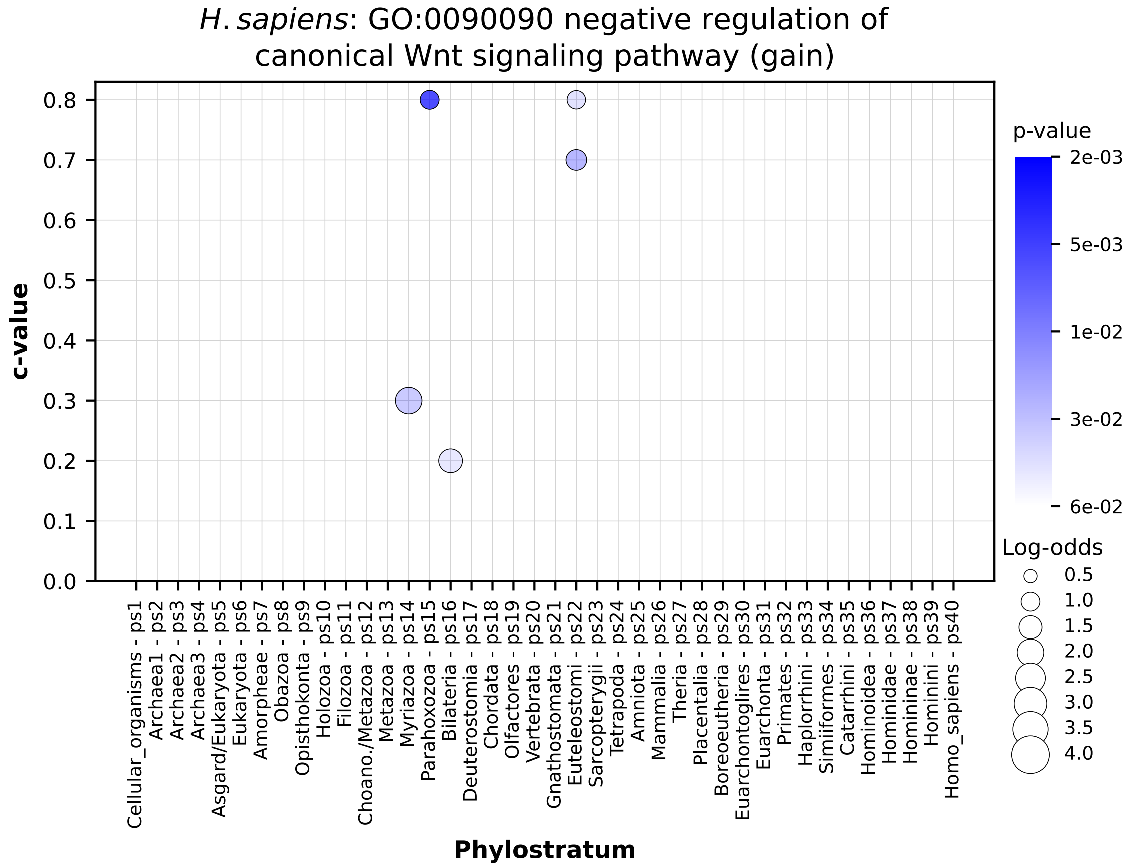

H. sapiens: GO:0090090 negative regulation of canonical Wnt signaling pathway (gain)
c-value
0.8
0.7
0.6
0.5
0.4
0.3
0.2
0.1
0.0
Phylostratum
Cellular_organisms - ps1
Archaea1 - ps2
Archaea2 - ps3
Archaea3 - ps4
Asgard/Eukaryota - ps5
Eukaryota - ps6
Amorpheae - ps7
Obazoa - ps8
Opisthokonta - ps9
Holozoa - ps10
Filozoa - ps11
Choano./Metazoa - ps12
Metazoa - ps13
Myriazoa - ps14
Parahoxozoa - ps15
Bilateria - ps16
Deuterostomia - ps17
Chordata - ps18
Olfactores - ps19
Vertebrata - ps20
Gnathostomata - ps21
Euteleostomi - ps22
Sarcopterygii - ps23
Tetrapoda - ps24
Amniota - ps25
Mammalia - ps26
Theria - ps27
Placentalia - ps28
Boreoeutheria - ps29
Euarchontoglires - ps30
Euarchonta - ps31
Primates - ps32
Haplorrhini - ps33
Simiiformes - ps34
Catarrhini - ps35
Hominoidea - ps36
Hominidae - ps37
Homininae - ps38
Hominini - ps39
Homo_sapiens - ps40
p-value
2e-03
5e-03
1e-02
3e-02
6e-02
Log-odds
0.5
1.0
1.5
2.0
2.5
3.0
3.5
4.0

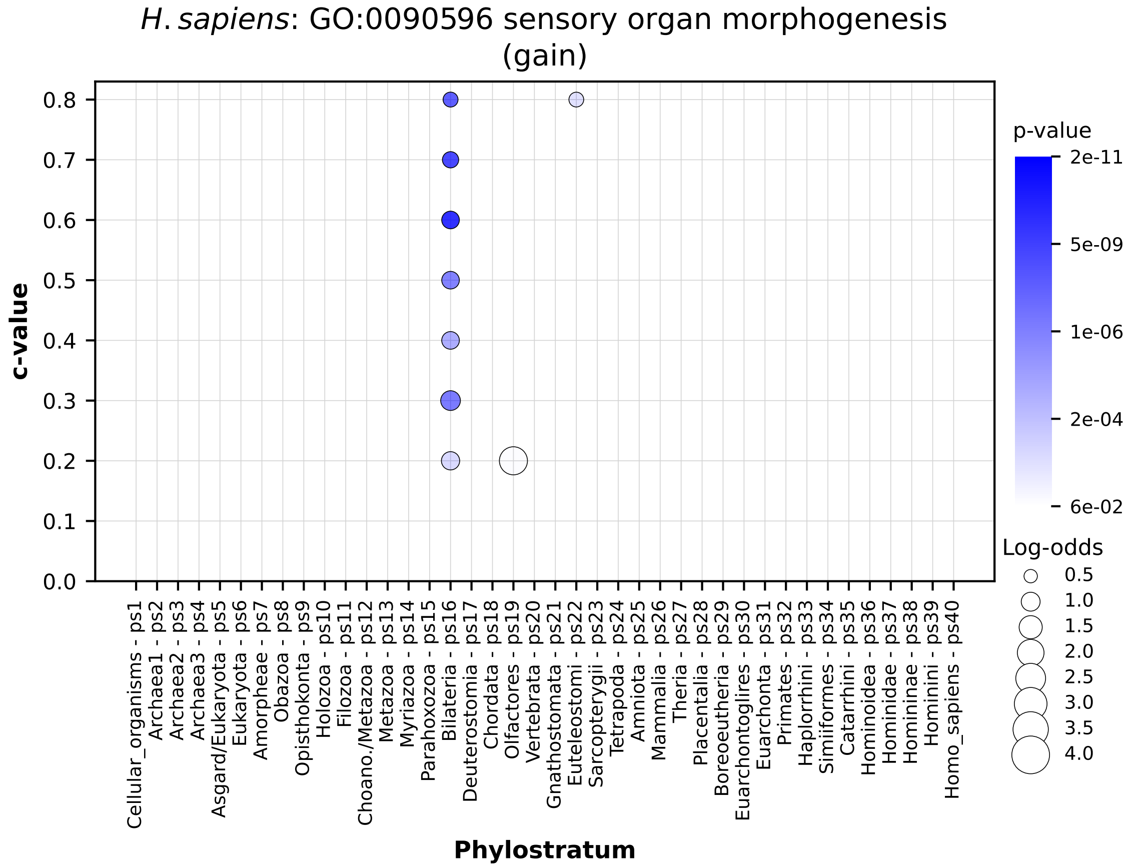
H. sapiens: GO:0090596 sensory organ morphogenesis (gain)
c-value
0.0
0.1
0.2
0.3
0.4
0.5
0.6
0.7
0.8
Phylostratum
Cellular_organisms - ps1
Archaea1 - ps2
Archaea2 - ps3
Archaea3 - ps4
Asgard/Eukaryota - ps5
Eukaryota - ps6
Amorpheae - ps7
Obazoa - ps8
Opisthokonta - ps9
Holozoa - ps10
Filozoa - ps11
Choano./Metazoa - ps12
Metazoa - ps13
Myriazoa - ps14
Parahoxozoa - ps15
Bilateria - ps16
Deuterostomia - ps17
Chordata - ps18
Olfactores - ps19
Vertebrata - ps20
Gnathostomata - ps21
Euteleostomi - ps22
Sarcopterygii - ps23
Tetrapoda - ps24
Amniota - ps25
Mammalia - ps26
Theria - ps27
Placentalia - ps28
Boreoeutheria - ps29
Euarchontoglires - ps30
Euarchonta - ps31
Primates - ps32
Haplorrhini - ps33
Simiiformes - ps34
Catarrhini - ps35
Hominoidea - ps36
Hominidae - ps37
Homininae - ps38
Hominini - ps39
Homo_sapiens - ps40
p-value
2e-11
5e-09
1e-06
2e-04
6e-02
Log-odds
0.5
1.0
1.5
2.0
2.5
3.0
3.5
4.0

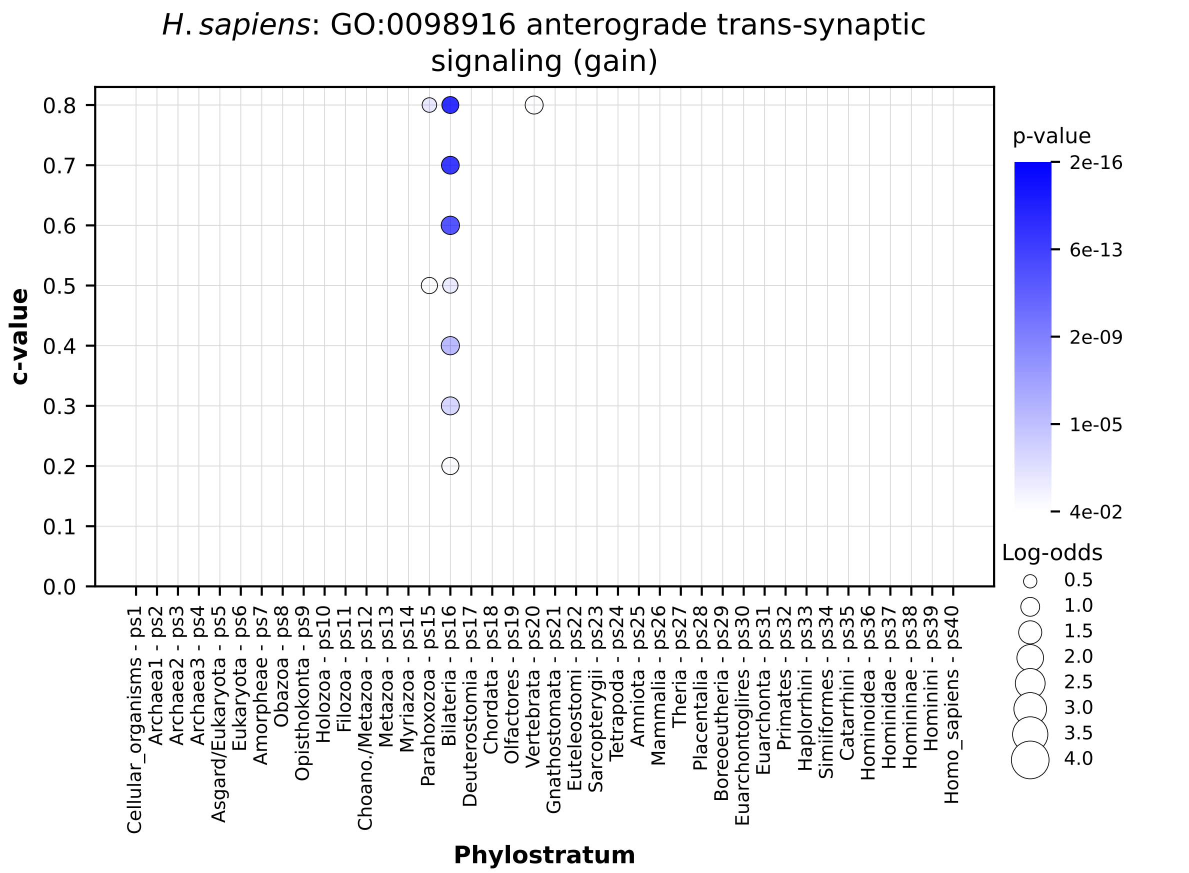

H. sapiens: GO:0098916 anterograde trans-synaptic signaling (gain)
c-value
0.0
0.1
0.2
0.3
0.4
0.5
0.6
0.7
0.8
Phylostratum
Cellular_organisms - ps1
Archaea1 - ps2
Archaea2 - ps3
Archaea3 - ps4
Asgard/Eukaryota - ps5
Eukaryota - ps6
Amorpheae - ps7
Obazoa - ps8
Opisthokonta - ps9
Holozoa - ps10
Filozoa - ps11
Choano./Metazoa - ps12
Metazoa - ps13
Myriazoa - ps14
Parahoxozoa - ps15
Bilateria - ps16
Deuterostomia - ps17
Chordata - ps18
Olfactores - ps19
Vertebrata - ps20
Gnathostomata - ps21
Euteleostomi - ps22
Sarcopterygii - ps23
Tetrapoda - ps24
Amniota - ps25
Mammalia - ps26
Theria - ps27
Placentalia - ps28
Boreoeutheria - ps29
Euarchontoglires - ps30
Euarchonta - ps31
Primates - ps32
Haplorrhini - ps33
Simiiformes - ps34
Catarrhini - ps35
Hominoidea - ps36
Hominidae - ps37
Homininae - ps38
Hominini - ps39
Homo_sapiens - ps40
p-value
2e-16
6e-13
2e-09
1e-05
4e-02
Log-odds
0.5
1.0
1.5
2.0
2.5
3.0
3.5
4.0

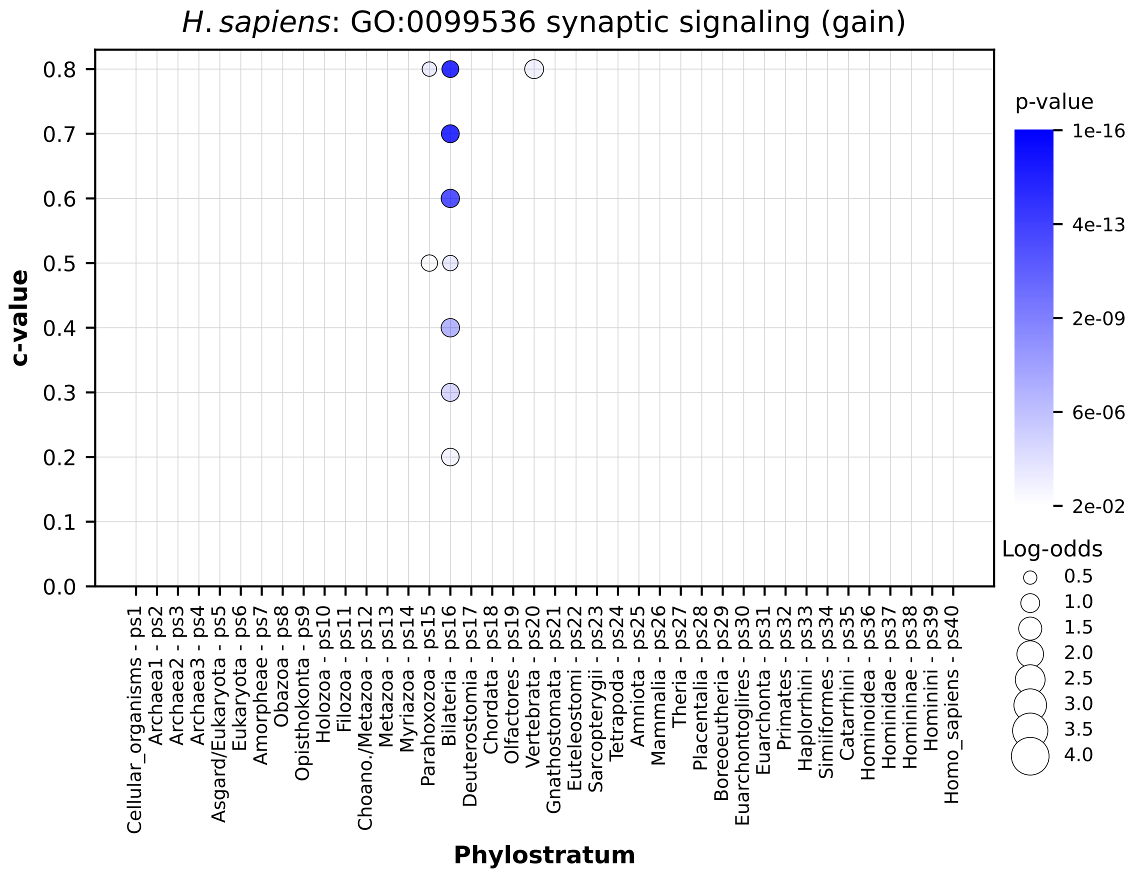

H. sapiens: GO:0099536 synaptic signaling (gain)
c-value
0.0
0.1
0.2
0.3
0.4
0.5
0.6
0.7
0.8
p-value
1e-16
4e-13
2e-09
6e-06
2e-02
Log-odds
0.5
1.0
1.5
2.0
2.5
3.0
3.5
4.0
Cellular_organisms - ps1
Archaea1 - ps2
Archaea2 - ps3
Archaea3 - ps4
Asgard/Eukaryota - ps5
Eukaryota - ps6
Amorpheae - ps7
Obazoa - ps8
Opisthokonta - ps9
Holozoa - ps10
Filozoa - ps11
Choano./Metazoa - ps12
Metazoa - ps13
Myriazoa - ps14
Parahoxozoa - ps15
Bilateria - ps16
Deuterostomia - ps17
Chordata - ps18
Olfactores - ps19
Vertebrata - ps20
Gnathostomata - ps21
Euteleostomi - ps22
Sarcopterygii - ps23
Tetrapoda - ps24
Amniota - ps25
Mammalia - ps26
Theria - ps27
Placentalia - ps28
Boreoeutheria - ps29
Euarchontoglires - ps30
Euarchonta - ps31
Primates - ps32
Haplorrhini - ps33
Simiiformes - ps34
Catarrhini - ps35
Hominoidea - ps36
Hominidae - ps37
Homininae - ps38
Hominini - ps39
Homo_sapiens - ps40
Phylostratum

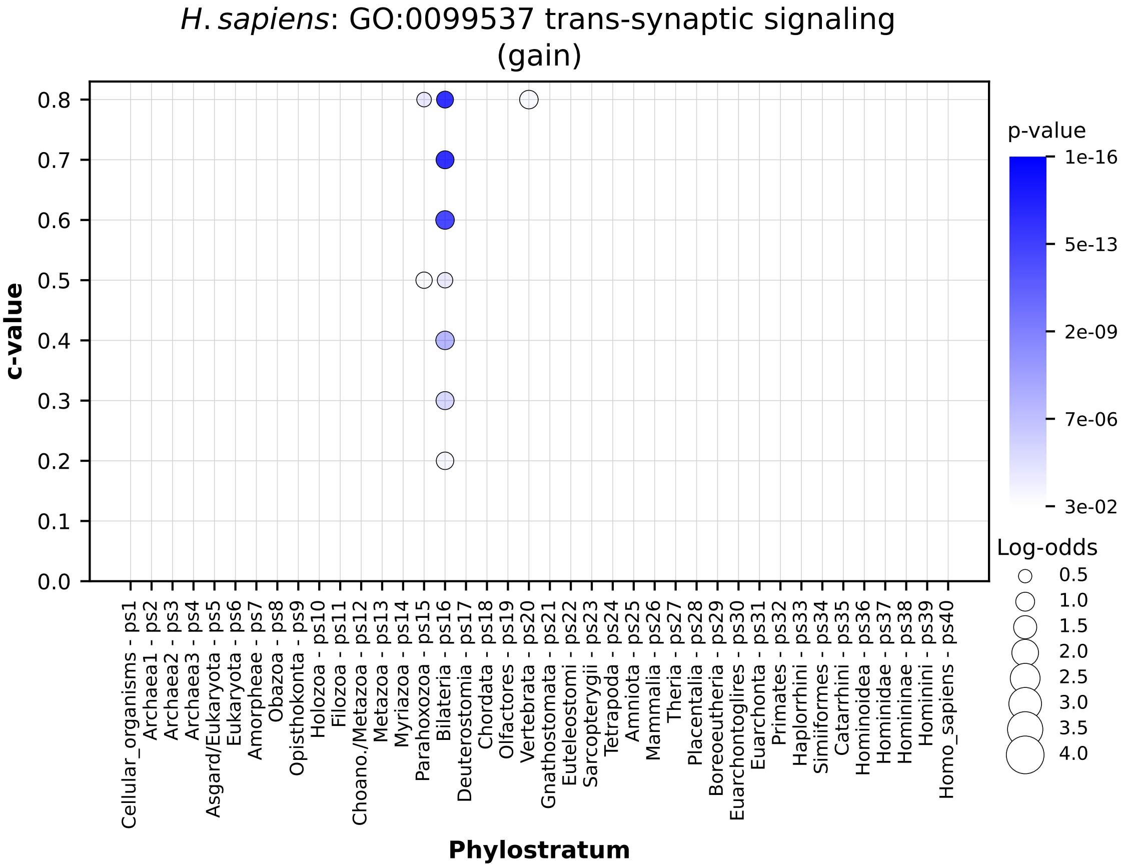

H. sapiens: GO:0099537 trans-synaptic signaling
(gain)
c-value
0.0
0.1
0.2
0.3
0.4
0.5
0.6
0.7
0.8
Phylostratum
Cellular_organisms - ps1
Archaea1 - ps2
Archaea2 - ps3
Archaea3 - ps4
Asgard/Eukaryota - ps5
Eukaryota - ps6
Amorpheae - ps7
Obazoa - ps8
Opisthokonta - ps9
Holozoa - ps10
Filozoa - ps11
Choano./Metazoa - ps12
Metazoa - ps13
Myriazoa - ps14
Parahoxozoa - ps15
Bilateria - ps16
Deuterostomia - ps17
Chordata - ps18
Olfactores - ps19
Vertebrata - ps20
Gnathostomata - ps21
Euteleostomi - ps22
Sarcopterygii - ps23
Tetrapoda - ps24
Amniota - ps25
Mammalia - ps26
Theria - ps27
Placentalia - ps28
Boreoeutheria - ps29
Euarchontoglires - ps30
Euarchonta - ps31
Primates - ps32
Haplorrhini - ps33
Simiiformes - ps34
Catarrhini - ps35
Hominoidea - ps36
Hominidae - ps37
Homininae - ps38
Hominini - ps39
Homo_sapiens - ps40
p-value
1e-16
5e-13
2e-09
7e-06
3e-02
Log-odds
0.5
1.0
1.5
2.0
2.5
3.0
3.5
4.0

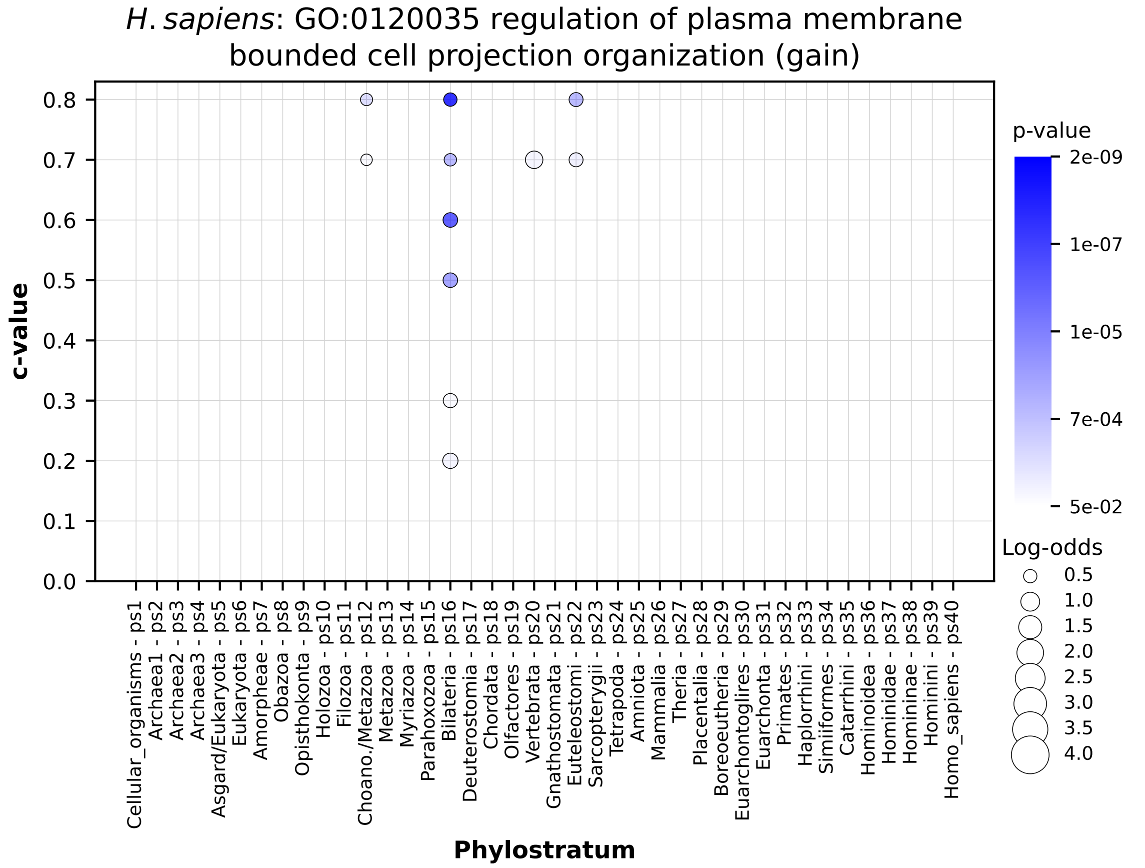

H. sapiens: GO:0120035 regulation of plasma membrane bounded cell projection organization (gain)
c-value
0.8
0.7
0.6
0.5
0.4
0.3
0.2
0.1
0.0
p-value
2e-09
1e-07
1e-05
7e-04
5e-02
Log-odds
0.5
1.0
1.5
2.0
2.5
3.0
3.5
4.0
Cellular_organisms - ps1
Archaea1 - ps2
Archaea2 - ps3
Archaea3 - ps4
Asgard/Eukaryota - ps5
Eukaryota - ps6
Amorpheae - ps7
Obazoa - ps8
Opisthokonta - ps9
Holozoa - ps10
Filozoa - ps11
Choano./Metazoa - ps12
Metazoa - ps13
Myriazoa - ps14
Parahoxozoa - ps15
Bilateria - ps16
Deuterostomia - ps17
Chordata - ps18
Olfactores - ps19
Vertebrata - ps20
Gnathostomata - ps21
Euteleostomi - ps22
Sarcopterygii - ps23
Tetrapoda - ps24
Amniota - ps25
Mammalia - ps26
Theria - ps27
Placentalia - ps28
Boreoeutheria - ps29
Euarchontoglires - ps30
Euarchonta - ps31
Primates - ps32
Haplorrhini - ps33
Simiiformes - ps34
Catarrhini - ps35
Hominoidea - ps36
Hominidae - ps37
Homininae - ps38
Hominini - ps39
Homo_sapiens - ps40
Phylostratum

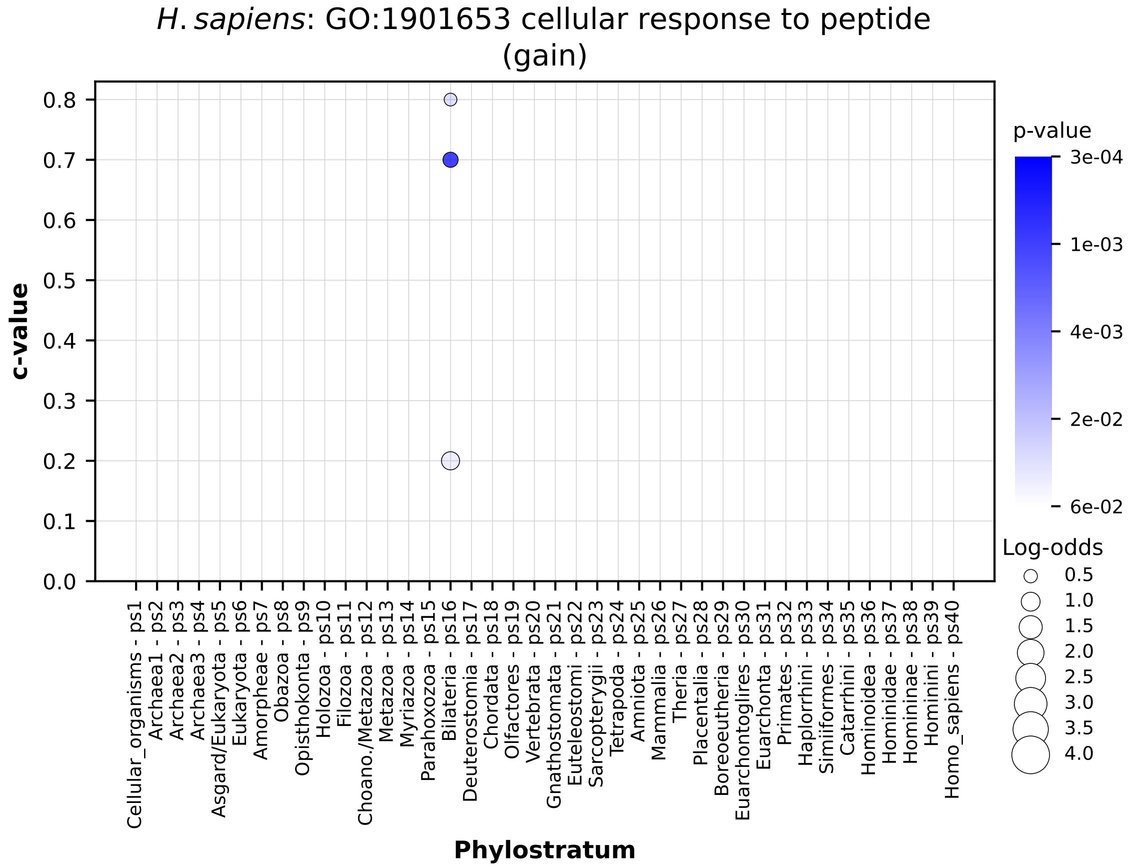
H. sapiens: GO:1901653 cellular response to peptide (gain)
c-value
0.0
0.1
0.2
0.3
0.4
0.5
0.6
0.7
0.8
Phylostratum
Cellular_organisms - ps1
Archaea1 - ps2
Archaea2 - ps3
Archaea3 - ps4
Asgard/Eukaryota - ps5
Eukaryota - ps6
Amorpheae - ps7
Obazoa - ps8
Opisthokonta - ps9
Holozoa - ps10
Filozoa - ps11
Choano./Metazoa - ps12
Metazoa - ps13
Myriazoa - ps14
Parahoxozoa - ps15
Bilateria - ps16
Deuterostomia - ps17
Chordata - ps18
Olfactores - ps19
Vertebrata - ps20
Gnathostomata - ps21
Euteleostomi - ps22
Sarcopterygii - ps23
Tetrapoda - ps24
Amniota - ps25
Mammalia - ps26
Theria - ps27
Placentalia - ps28
Boreoeutheria - ps29
Euarchontoglires - ps30
Euarchonta - ps31
Primates - ps32
Haplorrhini - ps33
Simiiformes - ps34
Catarrhini - ps35
Hominoidea - ps36
Hominidae - ps37
Homininae - ps38
Hominini - ps39
Homo_sapiens - ps40
p-value
3e-04
1e-03
4e-03
2e-02
6e-02
Log-odds
0.5
1.0
1.5
2.0
2.5
3.0
3.5
4.0

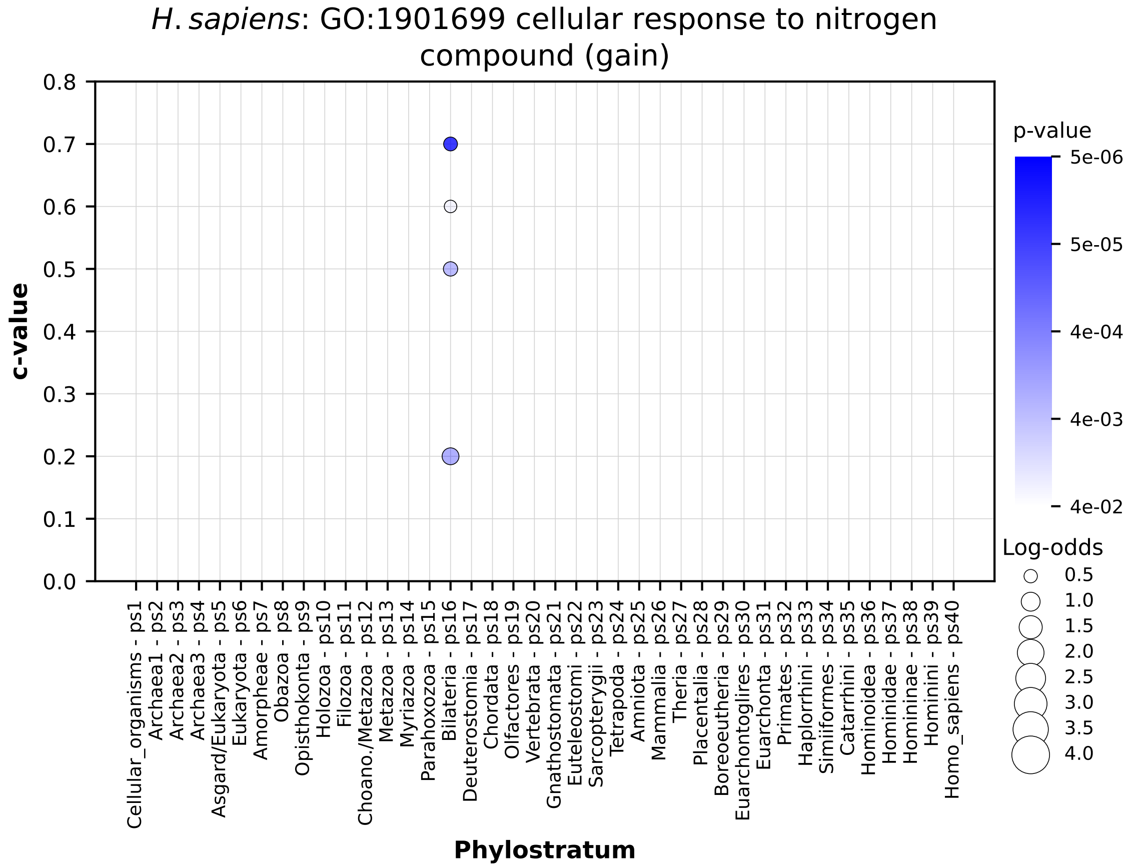

*H. sapiens*: GO:1901699 cellular response to nitrogen compound (gain)
c-value
0.8
0.7
0.6
0.5
0.4
0.3
0.2
0.1
0.0
Phylostratum
Cellular_organisms - ps1
Archaea1 - ps2
Archaea2 - ps3
Archaea3 - ps4
Asgard/Eukaryota - ps5
Eukaryota - ps6
Amorpheae - ps7
Obazoa - ps8
Opisthokonta - ps9
Holozoa - ps10
Filozoa - ps11
Choano./Metazoa - ps12
Metazoa - ps13
Myriazoa - ps14
Parahoxozoa - ps15
Bilateria - ps16
Deuterostomia - ps17
Chordata - ps18
Olfactores - ps19
Vertebrata - ps20
Gnathostomata - ps21
Euteleostomi - ps22
Sarcopterygii - ps23
Tetrapoda - ps24
Amniota - ps25
Mammalia - ps26
Theria - ps27
Placentalia - ps28
Boreoeutheria - ps29
Euarchontoglires - ps30
Euarchonta - ps31
Primates - ps32
Haplorrhini - ps33
Simiiformes - ps34
Catarrhini - ps35
Hominoidea - ps36
Hominidae - ps37
Homininae - ps38
Hominini - ps39
Homo_sapiens - ps40
p-value
5e-06
5e-05
4e-04
4e-03
4e-02
Log-odds
0.5
1.0
1.5
2.0
2.5
3.0
3.5
4.0

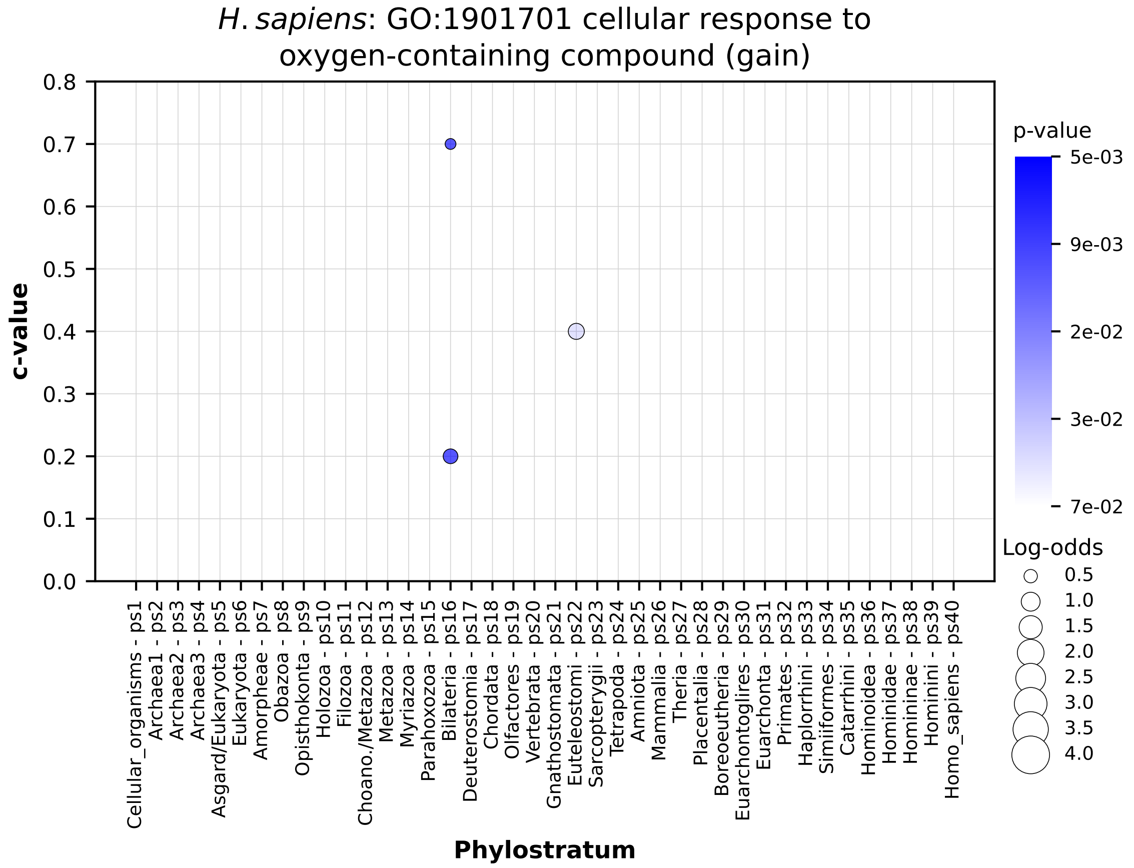
H. sapiens: GO:1901701 cellular response to oxygen-containing compound (gain)
c-value
0.0
0.1
0.2
0.3
0.4
0.5
0.6
0.7
0.8
Phylostratum
Cellular_organisms - ps1
Archaea1 - ps2
Archaea2 - ps3
Archaea3 - ps4
Asgard/Eukaryota - ps5
Eukaryota - ps6
Amorpheae - ps7
Obazoa - ps8
Opisthokonta - ps9
Holozoa - ps10
Filozoa - ps11
Choano./Metazoa - ps12
Metazoa - ps13
Myriazoa - ps14
Parahoxozoa - ps15
Bilateria - ps16
Deuterostomia - ps17
Chordata - ps18
Olfactores - ps19
Vertebrata - ps20
Gnathostomata - ps21
Euteleostomi - ps22
Sarcopterygii - ps23
Tetrapoda - ps24
Amniota - ps25
Mammalia - ps26
Theria - ps27
Placentalia - ps28
Boreoeutheria - ps29
Euarchontoglires - ps30
Euarchonta - ps31
Primates - ps32
Haplorrhini - ps33
Simiiformes - ps34
Catarrhini - ps35
Hominoidea - ps36
Hominidae - ps37
Homininae - ps38
Hominini - ps39
Homo_sapiens - ps40
p-value
5e-03
9e-03
2e-02
3e-02
7e-02
Log-odds
0.5
1.0
1.5
2.0
2.5
3.0
3.5
4.0

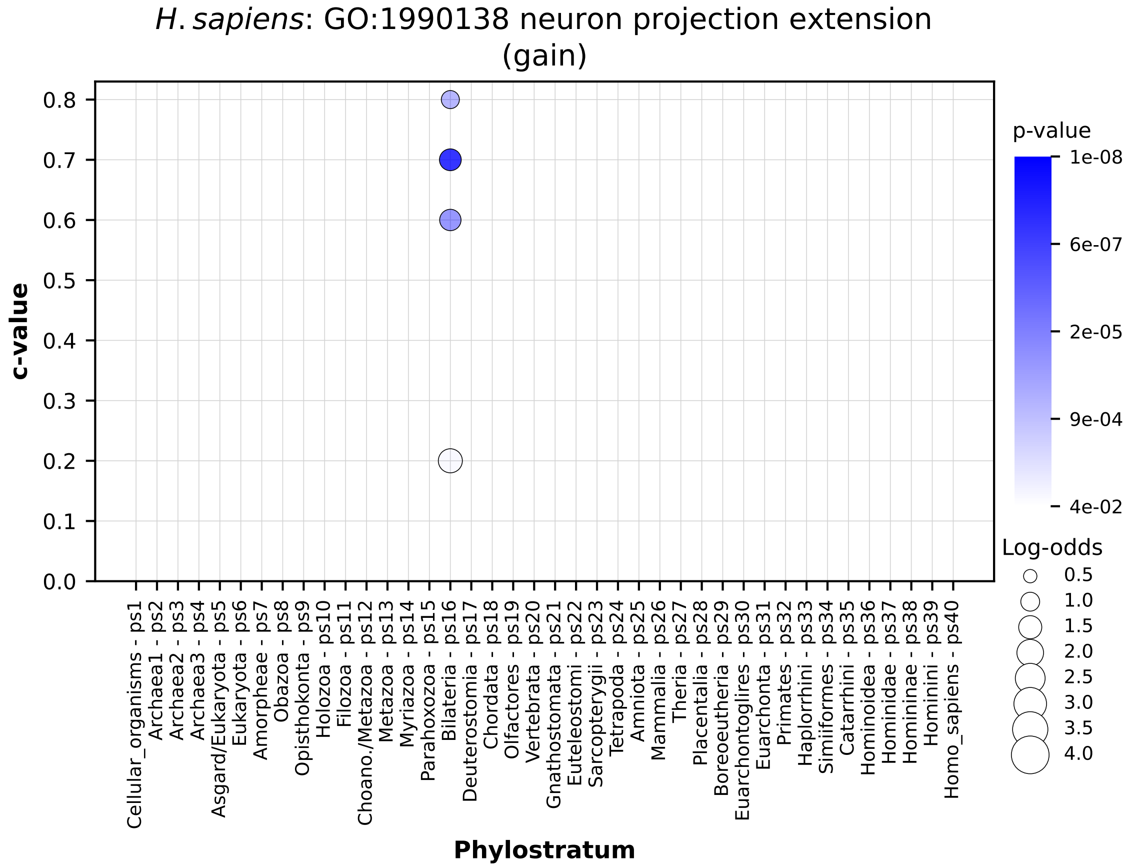

H. sapiens: GO:1990138 neuron projection extension (gain)
c-value
0.8
0.7
0.6
0.5
0.4
0.3
0.2
0.1
0.0
p-value
1e-08
6e-07
2e-05
9e-04
4e-02
Log-odds
0.5
1.0
1.5
2.0
2.5
3.0
3.5
4.0
Cellular_organisms - ps1
Archaea1 - ps2
Archaea2 - ps3
Archaea3 - ps4
Asgard/Eukaryota - ps5
Eukaryota - ps6
Amorpheae - ps7
Obazoa - ps8
Opisthokonta - ps9
Holozoa - ps10
Filozoa - ps11
Choano./Metazoa - ps12
Metazoa - ps13
Myriazoa - ps14
Parahoxozoa - ps15
Bilateria - ps16
Deuterostomia - ps17
Chordata - ps18
Olfactores - ps19
Vertebrata - ps20
Gnathostomata - ps21
Euteleostomi - ps22
Sarcopterygii - ps23
Tetrapoda - ps24
Amniota - ps25
Mammalia - ps26
Theria - ps27
Placentalia - ps28
Boreoeutheria - ps29
Euarchontoglires - ps30
Euarchonta - ps31
Primates - ps32
Haplorrhini - ps33
Simiiformes - ps34
Catarrhini - ps35
Hominoidea - ps36
Hominidae - ps37
Homininae - ps38
Hominini - ps39
Homo_sapiens - ps40
Phylostratum

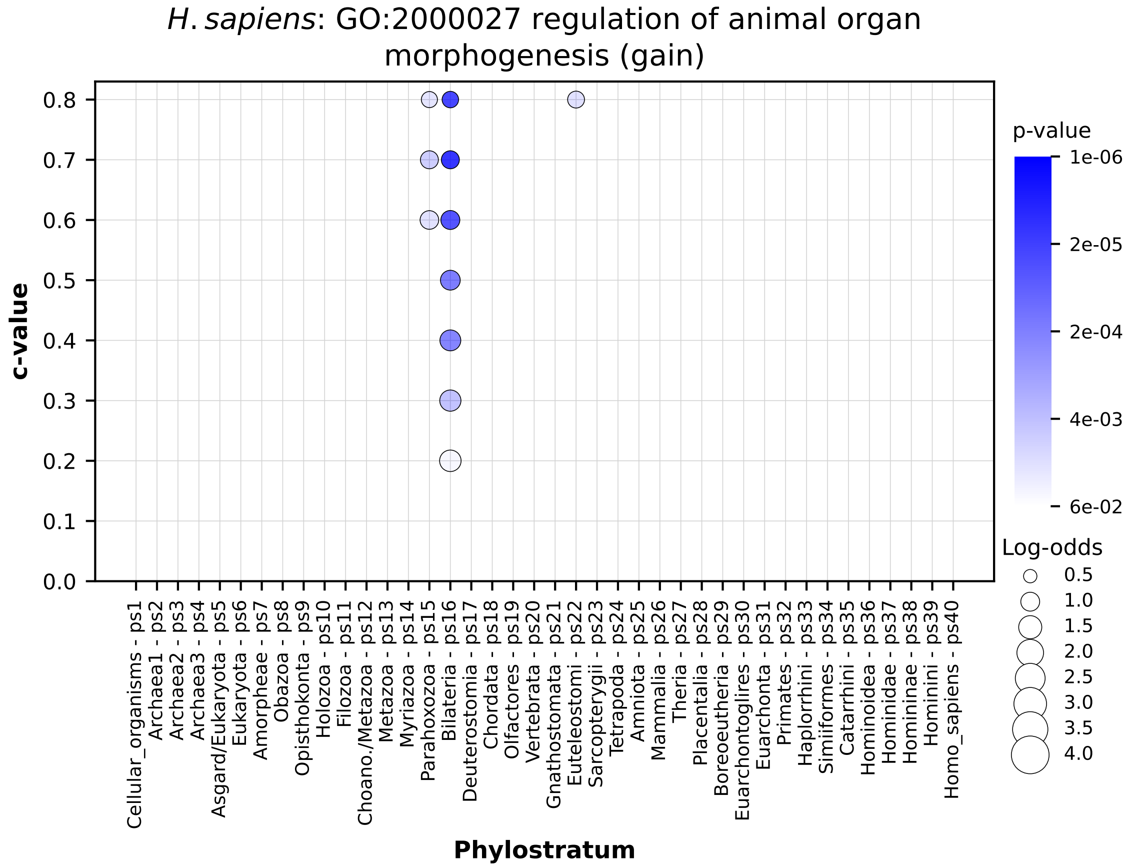

H. sapiens: GO:2000027 regulation of animal organ morphogenesis (gain)
c-value
0.0
0.1
0.2
0.3
0.4
0.5
0.6
0.7
0.8
p-value
1e-06
2e-05
2e-04
4e-03
6e-02
Log-odds
0.5
1.0
1.5
2.0
2.5
3.0
3.5
4.0
Cellular_organisms - ps1
Archaea1 - ps2
Archaea2 - ps3
Archaea3 - ps4
Asgard/Eukaryota - ps5
Eukaryota - ps6
Amorpheae - ps7
Obazoa - ps8
Opisthokonta - ps9
Holozoa - ps10
Filozoa - ps11
Choano./Metazoa - ps12
Metazoa - ps13
Myriazoa - ps14
Parahoxozoa - ps15
Bilateria - ps16
Deuterostomia - ps17
Chordata - ps18
Olfactores - ps19
Vertebrata - ps20
Gnathostomata - ps21
Euteleostomi - ps22
Sarcopterygii - ps23
Tetrapoda - ps24
Amniota - ps25
Mammalia - ps26
Theria - ps27
Placentalia - ps28
Boreoeutheria - ps29
Euarchontoglires - ps30
Euarchonta - ps31
Primates - ps32
Haplorrhini - ps33
Simiiformes - ps34
Catarrhini - ps35
Hominoidea - ps36
Hominidae - ps37
Homininae - ps38
Hominini - ps39
Homo_sapiens - ps40
Phylostratum

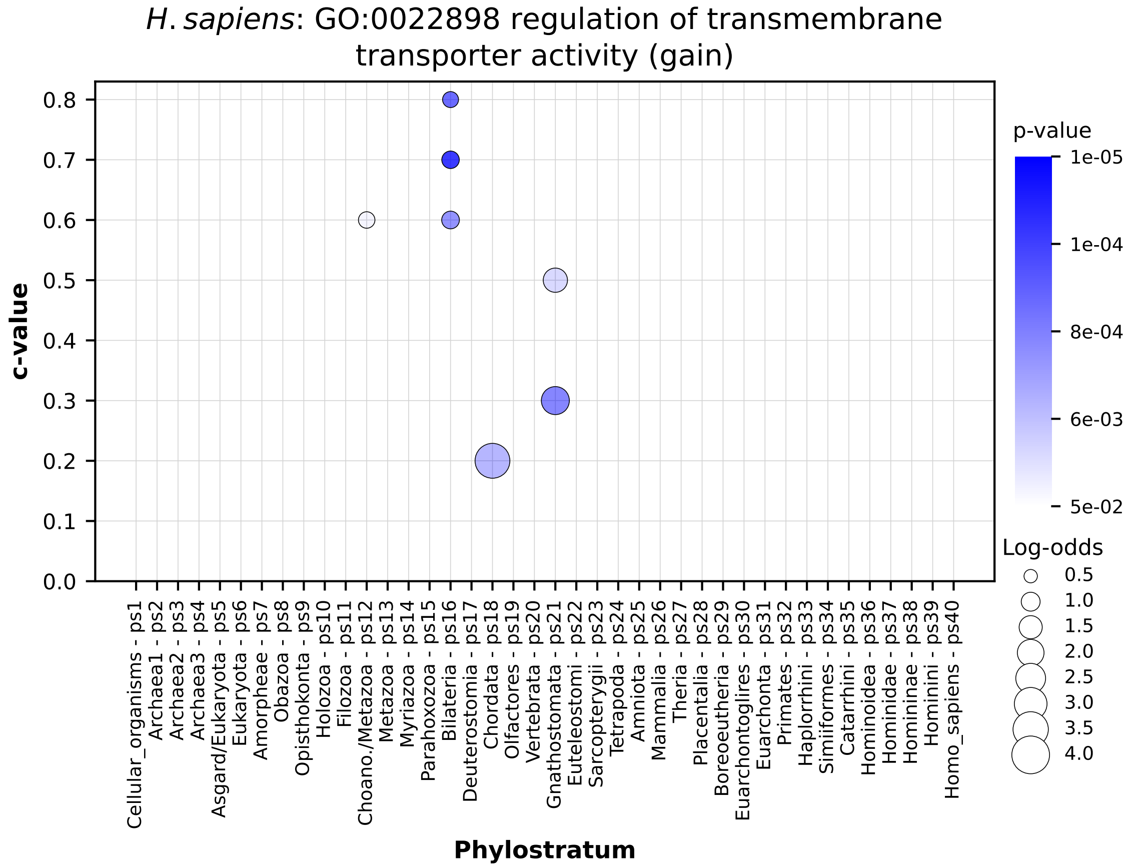

H. sapiens: GO:0022898 regulation of transmembrane transporter activity (gain)
c-value
0.0
0.1
0.2
0.3
0.4
0.5
0.6
0.7
0.8
Phylostratum
Cellular_organisms - ps1
Archaea1 - ps2
Archaea2 - ps3
Archaea3 - ps4
Asgard/Eukaryota - ps5
Eukaryota - ps6
Amorpheae - ps7
Obazoa - ps8
Opisthokonta - ps9
Holozoa - ps10
Filozoa - ps11
Choano./Metazoa - ps12
Metazoa - ps13
Myriazoa - ps14
Parahoxozoa - ps15
Bilateria - ps16
Deuterostomia - ps17
Chordata - ps18
Olfactores - ps19
Vertebrata - ps20
Gnathostomata - ps21
Euteleostomi - ps22
Sarcopterygii - ps23
Tetrapoda - ps24
Amniota - ps25
Mammalia - ps26
Theria - ps27
Placentalia - ps28
Boreoeutheria - ps29
Euarchontoglires - ps30
Euarchonta - ps31
Primates - ps32
Haplorrhini - ps33
Simiiformes - ps34
Catarrhini - ps35
Hominoidea - ps36
Hominidae - ps37
Homininae - ps38
Hominini - ps39
Homo_sapiens - ps40
p-value
1e-05
1e-04
8e-04
6e-03
5e-02
Log-odds
0.5
1.0
1.5
2.0
2.5
3.0
3.5
4.0

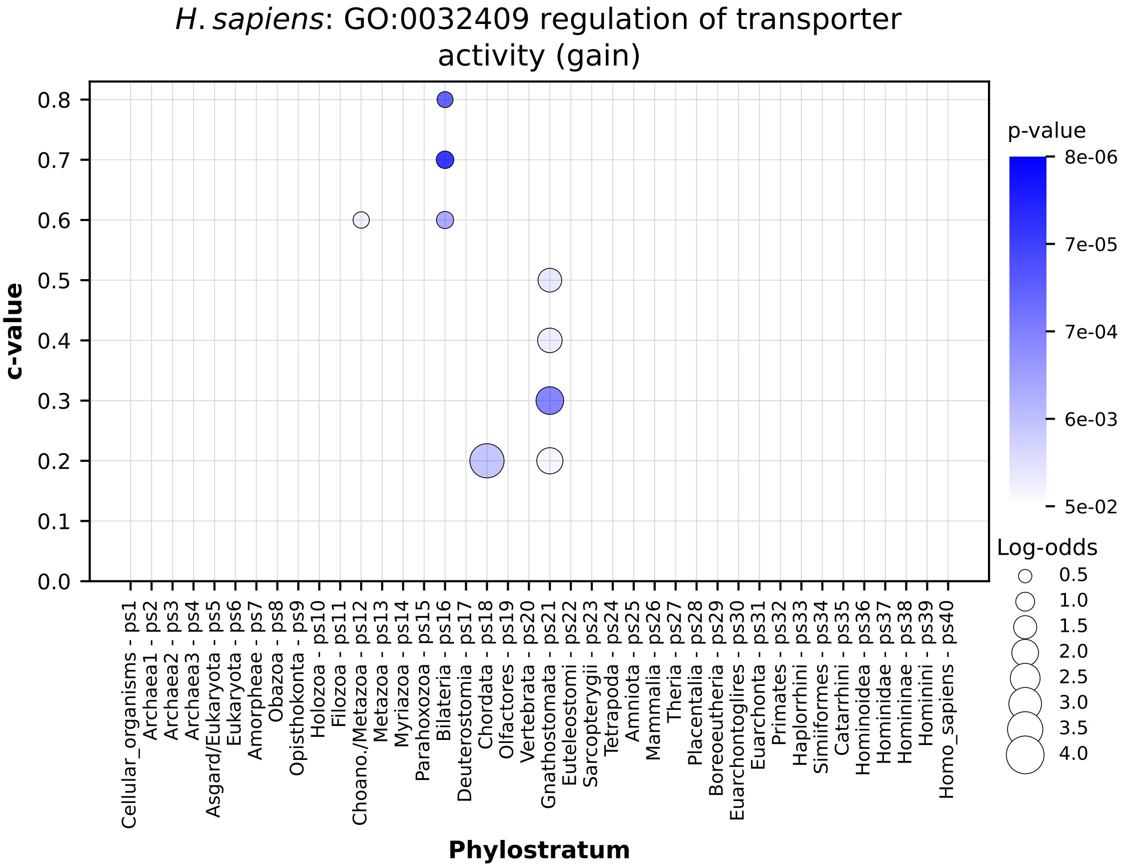

H. sapiens: GO:0032409 regulation of transporter activity (gain)
c-value
0.0
0.1
0.2
0.3
0.4
0.5
0.6
0.7
0.8
Phylostratum
Cellular_organisms - ps1
Archaea1 - ps2
Archaea2 - ps3
Archaea3 - ps4
Asgard/Eukaryota - ps5
Eukaryota - ps6
Amorpheae - ps7
Obazoa - ps8
Opisthokonta - ps9
Holozoa - ps10
Filozoa - ps11
Choano./Metazoa - ps12
Metazoa - ps13
Myriazoa - ps14
Parahoxozoa - ps15
Bilateria - ps16
Deuterostomia - ps17
Chordata - ps18
Olfactores - ps19
Vertebrata - ps20
Gnathostomata - ps21
Euteleostomi - ps22
Sarcopterygii - ps23
Tetrapoda - ps24
Amniota - ps25
Mammalia - ps26
Theria - ps27
Placentalia - ps28
Boreoeutheria - ps29
Euarchontoglires - ps30
Euarchonta - ps31
Primates - ps32
Haplorrhini - ps33
Simiiformes - ps34
Catarrhini - ps35
Hominoidea - ps36
Hominidae - ps37
Homininae - ps38
Hominini - ps39
Homo_sapiens - ps40
p-value
8e-06
7e-05
7e-04
6e-03
5e-02
Log-odds
0.5
1.0
1.5
2.0
2.5
3.0
3.5
4.0

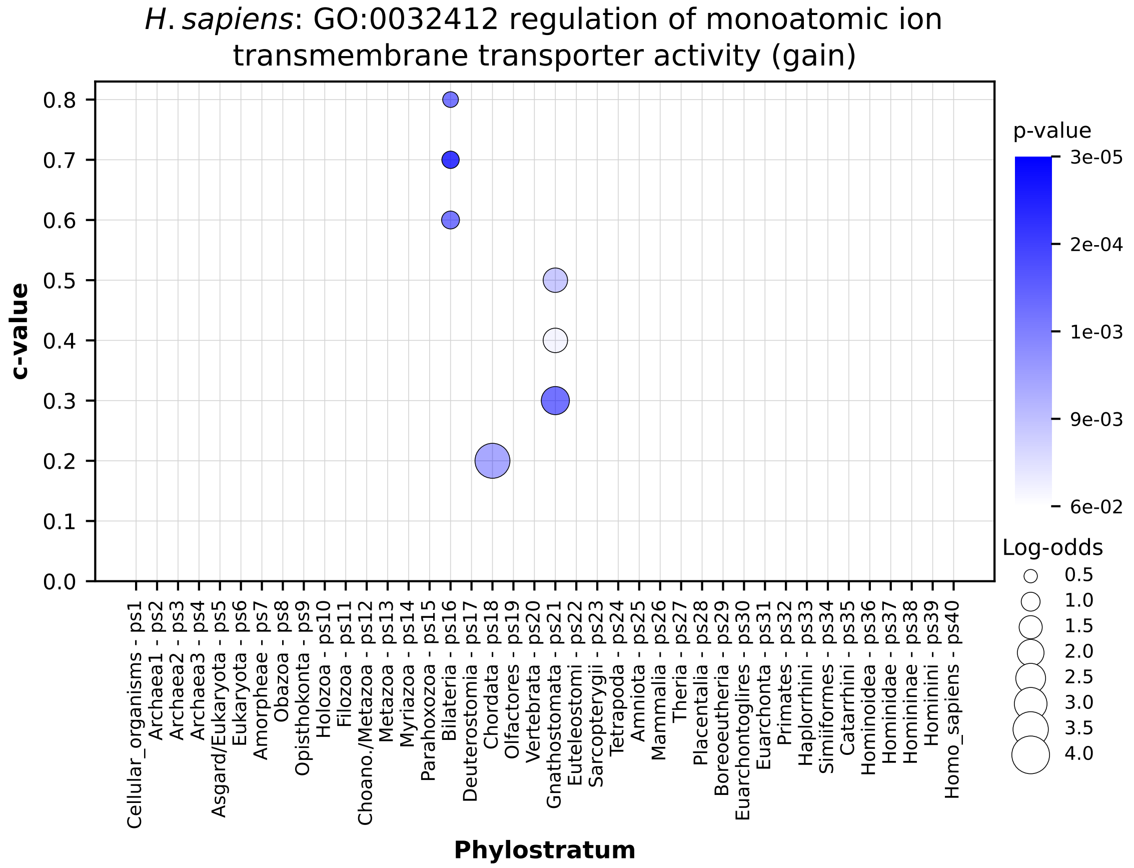

*H. sapiens*: GO:0032412 regulation of monoatomic ion transmembrane transporter activity (gain)
c-value
0.0
0.1
0.2
0.3
0.4
0.5
0.6
0.7
0.8
Phylostratum
Cellular_organisms - ps1
Archaea1 - ps2
Archaea2 - ps3
Archaea3 - ps4
Asgard/Eukaryota - ps5
Eukaryota - ps6
Amorpheae - ps7
Obazoa - ps8
Opisthokonta - ps9
Holozoa - ps10
Filozoa - ps11
Choano./Metazoa - ps12
Metazoa - ps13
Myriazoa - ps14
Parahoxozoa - ps15
Bilateria - ps16
Deuterostomia - ps17
Chordata - ps18
Olfactores - ps19
Vertebrata - ps20
Gnathostomata - ps21
Euteleostomi - ps22
Sarcopterygii - ps23
Tetrapoda - ps24
Amniota - ps25
Mammalia - ps26
Theria - ps27
Placentalia - ps28
Boreoeutheria - ps29
Euarchontoglires - ps30
Euarchonta - ps31
Primates - ps32
Haplorrhini - ps33
Simiiformes - ps34
Catarrhini - ps35
Hominoidea - ps36
Hominidae - ps37
Homininae - ps38
Hominini - ps39
Homo_sapiens - ps40
p-value
3e-05
2e-04
1e-03
9e-03
6e-02
Log-odds
0.5
1.0
1.5
2.0
2.5
3.0
3.5
4.0

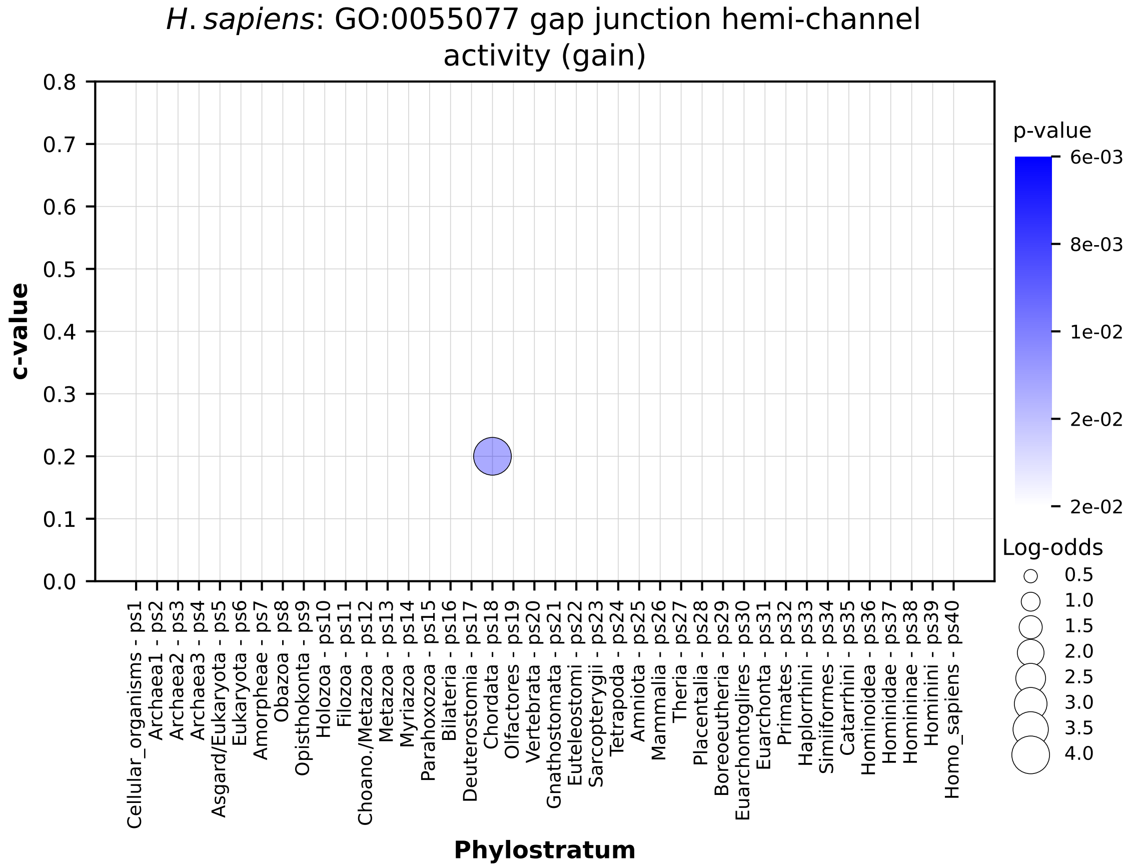

H. sapiens: GO:0055077 gap junction hemi-channel activity (gain)
c-value
0.8
0.7
0.6
0.5
0.4
0.3
0.2
0.1
0.0
p-value
6e-03
8e-03
1e-02
2e-02
2e-02
Log-odds
0.5
1.0
1.5
2.0
2.5
3.0
3.5
4.0
Cellular_organisms - ps1
Archaea1 - ps2
Archaea2 - ps3
Archaea3 - ps4
Asgard/Eukaryota - ps5
Eukaryota - ps6
Amorpheae - ps7
Obazoa - ps8
Opisthokonta - ps9
Holozoa - ps10
Filozoa - ps11
Choano./Metazoa - ps12
Metazoa - ps13
Myriazoa - ps14
Parahoxozoa - ps15
Bilateria - ps16
Deuterostomia - ps17
Chordata - ps18
Olfactores - ps19
Vertebrata - ps20
Gnathostomata - ps21
Euteleostomi - ps22
Sarcopterygii - ps23
Tetrapoda - ps24
Amniota - ps25
Mammalia - ps26
Theria - ps27
Placentalia - ps28
Boreoeutheria - ps29
Euarchontoglires - ps30
Euarchonta - ps31
Primates - ps32
Haplorrhini - ps33
Simiiformes - ps34
Catarrhini - ps35
Hominoidea - ps36
Hominidae - ps37
Homininae - ps38
Hominini - ps39
Homo_sapiens - ps40
Phylostratum

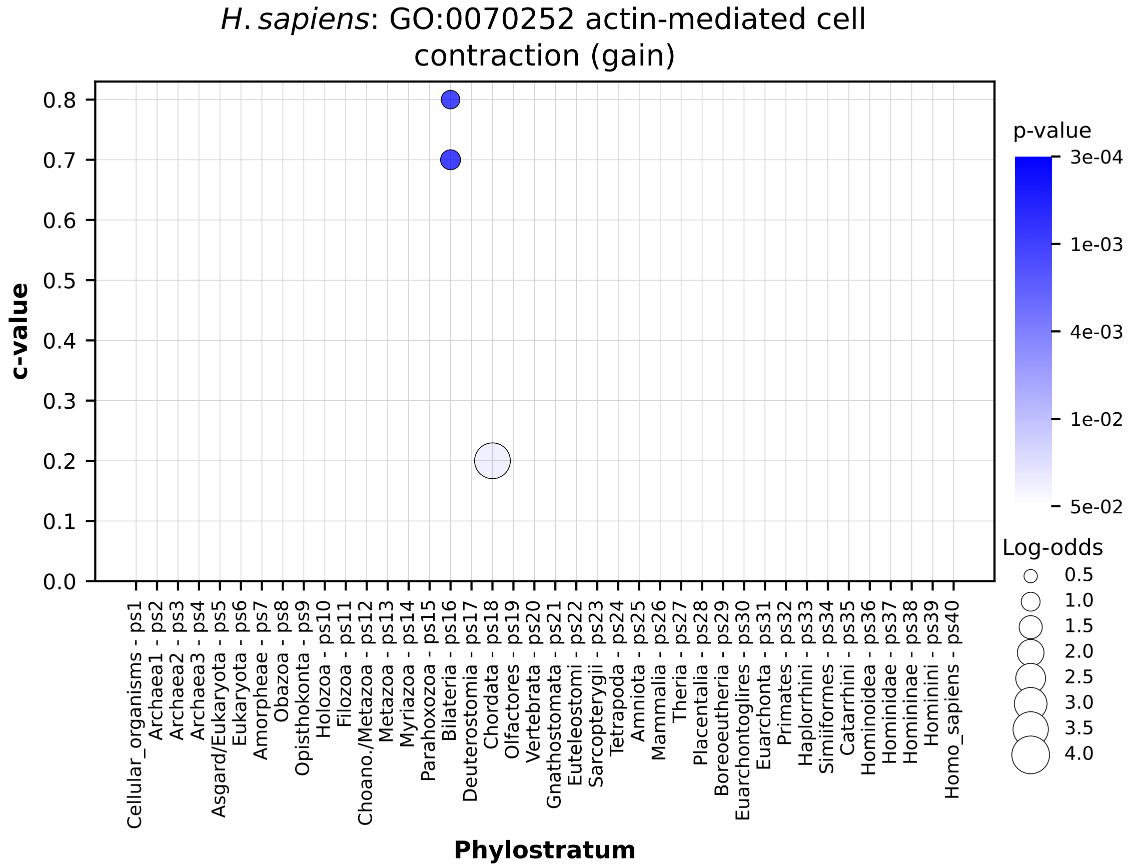

*H. sapiens*: GO:0070252 actin-mediated cell contraction (gain)
c-value
0.0
0.1
0.2
0.3
0.4
0.5
0.6
0.7
0.8
p-value
3e-04
1e-03
4e-03
1e-02
5e-02
Log-odds
0.5
1.0
1.5
2.0
2.5
3.0
3.5
4.0
Cellular_organisms - ps1
Archaea1 - ps2
Archaea2 - ps3
Archaea3 - ps4
Asgard/Eukaryota - ps5
Eukaryota - ps6
Amorpheae - ps7
Obazoa - ps8
Opisthokonta - ps9
Holozoa - ps10
Filozoa - ps11
Choano./Metazoa - ps12
Metazoa - ps13
Myriazoa - ps14
Parahoxozoa - ps15
Bilateria - ps16
Deuterostomia - ps17
Chordata - ps18
Olfactores - ps19
Vertebrata - ps20
Gnathostomata - ps21
Euteleostomi - ps22
Sarcopterygii - ps23
Tetrapoda - ps24
Amniota - ps25
Mammalia - ps26
Theria - ps27
Placentalia - ps28
Boreoeutheria - ps29
Euarchontoglires - ps30
Euarchonta - ps31
Primates - ps32
Haplorrhini - ps33
Simiiformes - ps34
Catarrhini - ps35
Hominoidea - ps36
Hominidae - ps37
Homininae - ps38
Hominini - ps39
Homo_sapiens - ps40
Phylostratum

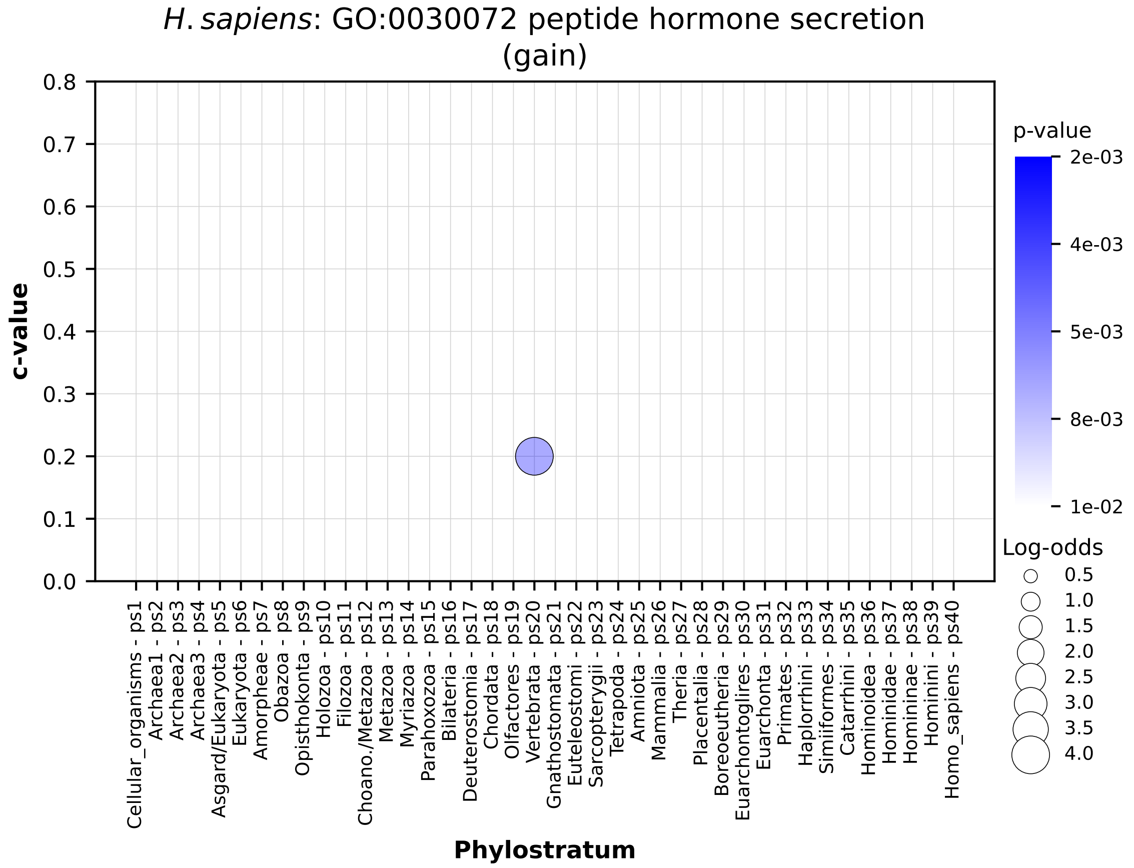
H. sapiens: GO:0030072 peptide hormone secretion
(gain)
c-value
0.0
0.1
0.2
0.3
0.4
0.5
0.6
0.7
0.8
Phylostratum
Cellular_organisms - ps1
Archaea1 - ps2
Archaea2 - ps3
Archaea3 - ps4
Asgard/Eukaryota - ps5
Eukaryota - ps6
Amorpheae - ps7
Obazoa - ps8
Opisthokonta - ps9
Holozoa - ps10
Filozoa - ps11
Choano./Metazoa - ps12
Metazoa - ps13
Myriazoa - ps14
Parahoxozoa - ps15
Bilateria - ps16
Deuterostomia - ps17
Chordata - ps18
Olfactores - ps19
Vertebrata - ps20
Gnathostomata - ps21
Euteleostomi - ps22
Sarcopterygii - ps23
Tetrapoda - ps24
Amniota - ps25
Mammalia - ps26
Theria - ps27
Placentalia - ps28
Boreoeutheria - ps29
Euarchontoglires - ps30
Euarchonta - ps31
Primates - ps32
Haplorrhini - ps33
Simiiformes - ps34
Catarrhini - ps35
Hominoidea - ps36
Hominidae - ps37
Homininae - ps38
Hominini - ps39
Homo_sapiens - ps40
p-value
2e-03
4e-03
5e-03
8e-03
1e-02
Log-odds
0.5
1.0
1.5
2.0
2.5
3.0
3.5
4.0

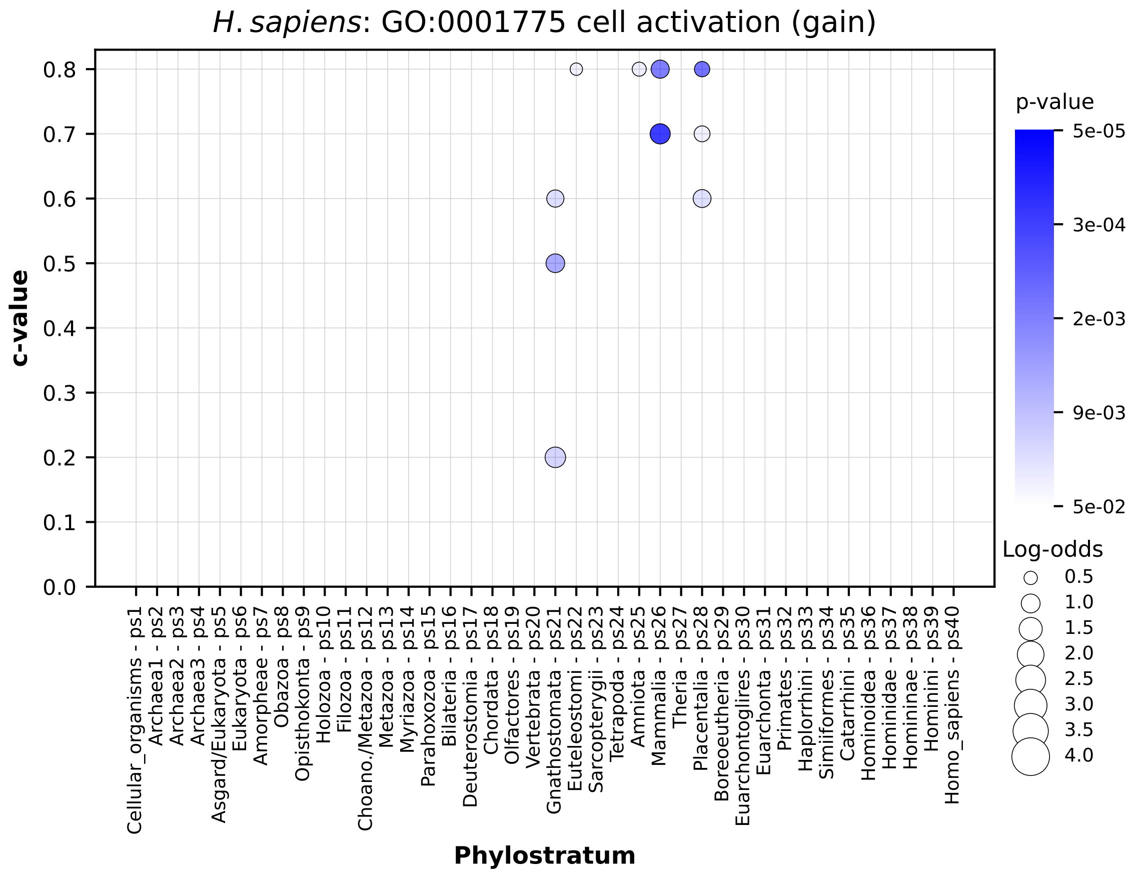

*H. sapiens*: GO:0001775 cell activation (gain)
c-value
0.0
0.1
0.2
0.3
0.4
0.5
0.6
0.7
0.8
Phylostratum
Cellular_organisms - ps1
Archaea1 - ps2
Archaea2 - ps3
Archaea3 - ps4
Asgard/Eukaryota - ps5
Eukaryota - ps6
Amorpheae - ps7
Obazoa - ps8
Opisthokonta - ps9
Holozoa - ps10
Filozoa - ps11
Choano./Metazoa - ps12
Metazoa - ps13
Myriazoa - ps14
Parahoxozoa - ps15
Bilateria - ps16
Deuterostomia - ps17
Chordata - ps18
Olfactores - ps19
Vertebrata - ps20
Gnathostomata - ps21
Euteleostomi - ps22
Sarcopterygii - ps23
Tetrapoda - ps24
Amniota - ps25
Mammalia - ps26
Theria - ps27
Placentalia - ps28
Boreoeutheria - ps29
Euarchontoglires - ps30
Euarchonta - ps31
Primates - ps32
Haplorrhini - ps33
Simiiformes - ps34
Catarrhini - ps35
Hominoidea - ps36
Hominidae - ps37
Homininae - ps38
Hominini - ps39
Homo_sapiens - ps40
p-value
5e-05
3e-04
2e-03
9e-03
5e-02
Log-odds
0.5
1.0
1.5
2.0
2.5
3.0
3.5
4.0

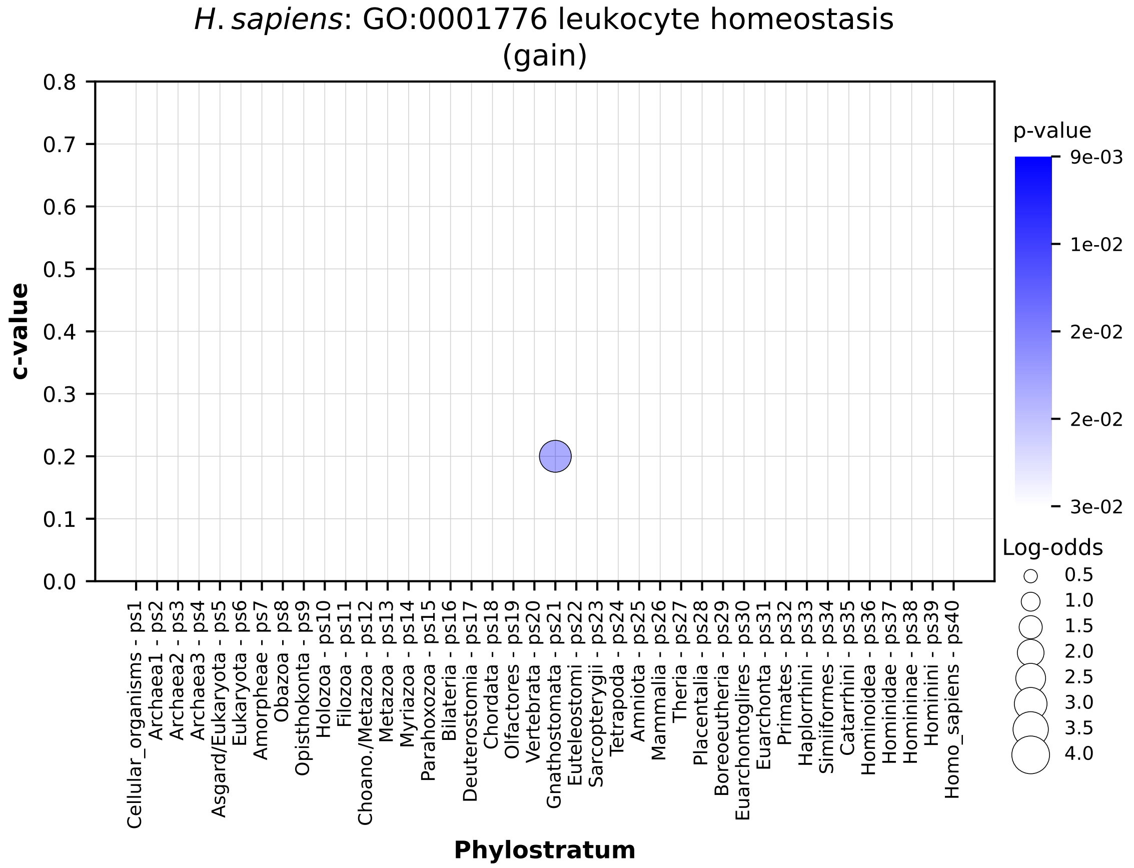

H. sapiens: GO:0001776 leukocyte homeostasis
(gain)
c-value
0.0
0.1
0.2
0.3
0.4
0.5
0.6
0.7
0.8
Phylostratum
Cellular_organisms - ps1
Archaea1 - ps2
Archaea2 - ps3
Archaea3 - ps4
Asgard/Eukaryota - ps5
Eukaryota - ps6
Amorpheae - ps7
Obazoa - ps8
Opisthokonta - ps9
Holozoa - ps10
Filozoa - ps11
Choano./Metazoa - ps12
Metazoa - ps13
Myriazoa - ps14
Parahoxozoa - ps15
Bilateria - ps16
Deuterostomia - ps17
Chordata - ps18
Olfactores - ps19
Vertebrata - ps20
Gnathostomata - ps21
Euteleostomi - ps22
Sarcopterygii - ps23
Tetrapoda - ps24
Amniota - ps25
Mammalia - ps26
Theria - ps27
Placentalia - ps28
Boreoeutheria - ps29
Euarchontoglires - ps30
Euarchonta - ps31
Primates - ps32
Haplorrhini - ps33
Simiiformes - ps34
Catarrhini - ps35
Hominoidea - ps36
Hominidae - ps37
Homininae - ps38
Hominini - ps39
Homo_sapiens - ps40
p-value
9e-03
1e-02
2e-02
2e-02
3e-02
Log-odds
0.5
1.0
1.5
2.0
2.5
3.0
3.5
4.0

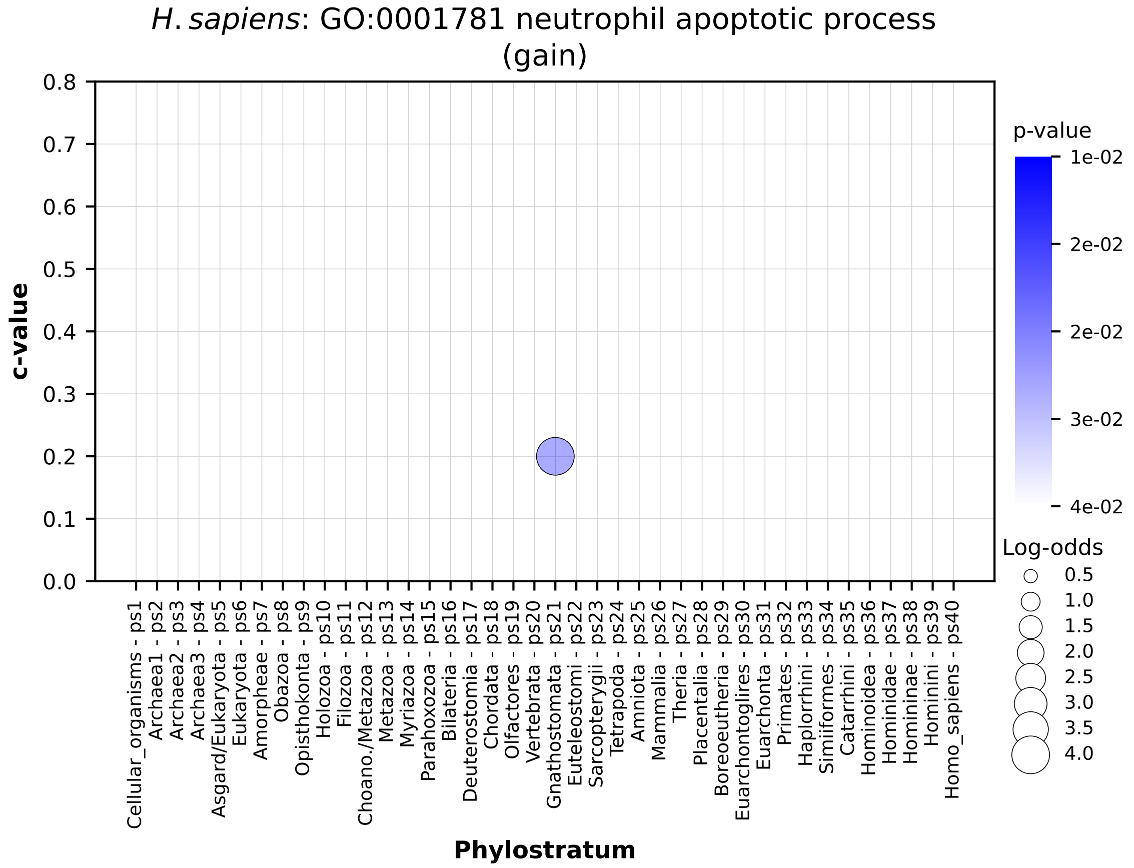
H. sapiens: GO:0001781 neutrophil apoptotic process (gain)
c-value
0.0
0.1
0.2
0.3
0.4
0.5
0.6
0.7
0.8
Phylostratum
Cellular_organisms - ps1
Archaea1 - ps2
Archaea2 - ps3
Archaea3 - ps4
Asgard/Eukaryota - ps5
Eukaryota - ps6
Amorpheae - ps7
Obazoa - ps8
Opisthokonta - ps9
Holozoa - ps10
Filozoa - ps11
Choano./Metazoa - ps12
Metazoa - ps13
Myriazoa - ps14
Parahoxozoa - ps15
Bilateria - ps16
Deuterostomia - ps17
Chordata - ps18
Olfactores - ps19
Vertebrata - ps20
Gnathostomata - ps21
Euteleostomi - ps22
Sarcopterygii - ps23
Tetrapoda - ps24
Amniota - ps25
Mammalia - ps26
Theria - ps27
Placentalia - ps28
Boreoeutheria - ps29
Euarchontoglires - ps30
Euarchonta - ps31
Primates - ps32
Haplorrhini - ps33
Simiiformes - ps34
Catarrhini - ps35
Hominoidea - ps36
Hominidae - ps37
Homininae - ps38
Hominini - ps39
Homo_sapiens - ps40
p-value
1e-02
2e-02
2e-02
3e-02
4e-02
Log-odds
0.5
1.0
1.5
2.0
2.5
3.0
3.5
4.0

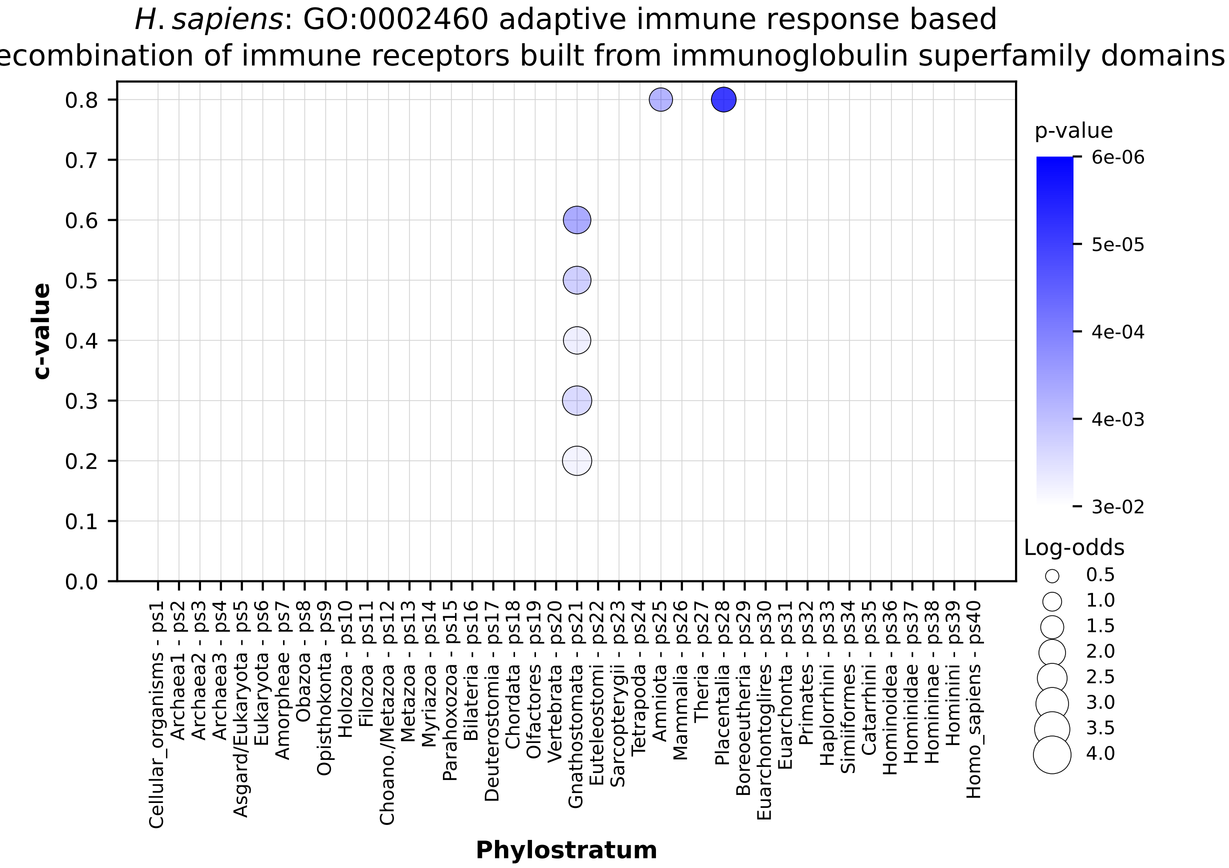

H. sapiens: GO:0002460 adaptive immune response based
ecombination of immune receptors built from immunoglobulin superfamily domains
c-value
0.0
0.1
0.2
0.3
0.4
0.5
0.6
0.7
0.8
Phylostratum
Cellular_organisms - ps1
Archaea1 - ps2
Archaea2 - ps3
Archaea3 - ps4
Asgard/Eukaryota - ps5
Eukaryota - ps6
Amorpheae - ps7
Obazoa - ps8
Opisthokonta - ps9
Holozoa - ps10
Filozoa - ps11
Choano./Metazoa - ps12
Metazoa - ps13
Myriazoa - ps14
Parahoxozoa - ps15
Bilateria - ps16
Deuterostomia - ps17
Chordata - ps18
Olfactores - ps19
Vertebrata - ps20
Gnathostomata - ps21
Euteleostomi - ps22
Sarcopterygii - ps23
Tetrapoda - ps24
Amniota - ps25
Mammalia - ps26
Theria - ps27
Placentalia - ps28
Boreoeutheria - ps29
Euarchontoglires - ps30
Euarchonta - ps31
Primates - ps32
Haplorrhini - ps33
Simiiformes - ps34
Catarrhini - ps35
Hominoidea - ps36
Hominidae - ps37
Homininae - ps38
Hominini - ps39
Homo_sapiens - ps40
p-value
6e-06
5e-05
4e-04
4e-03
3e-02
Log-odds
0.5
1.0
1.5
2.0
2.5
3.0
3.5
4.0

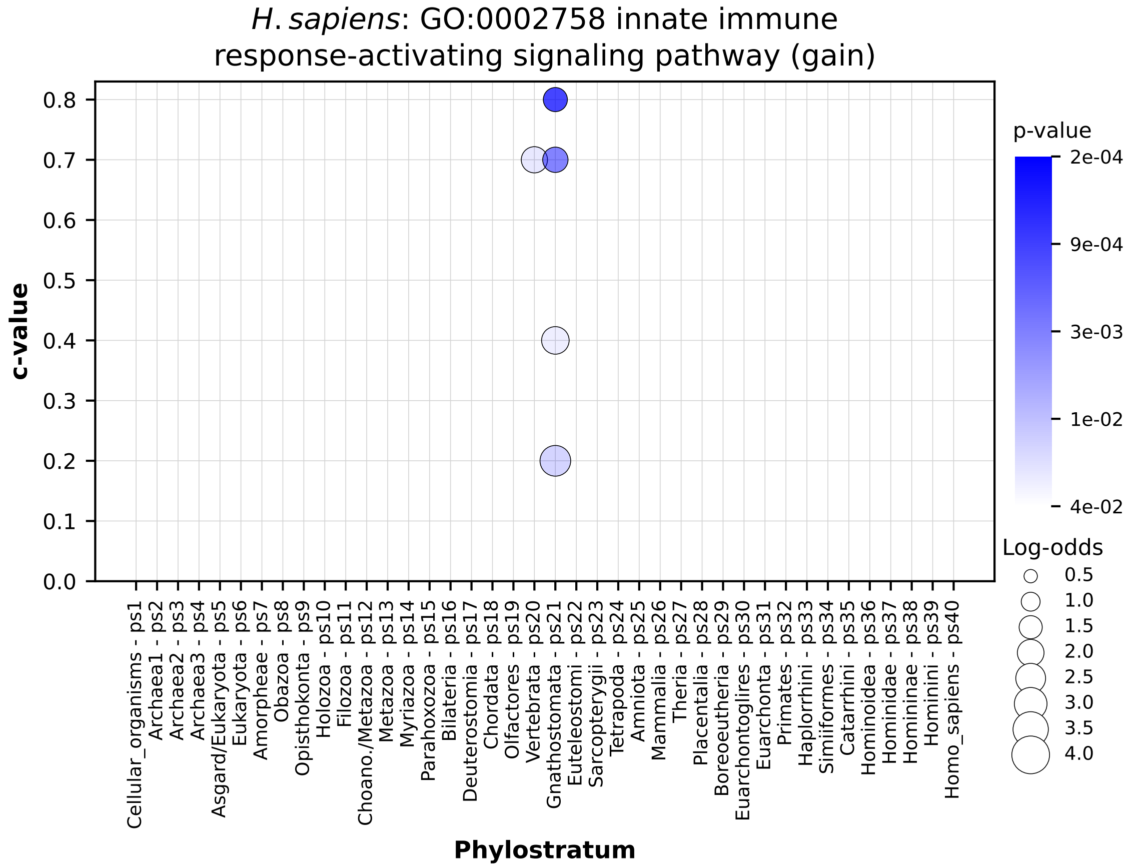

H. sapiens: GO:0002758 innate immune response-activating signaling pathway (gain)
c-value
0.0
0.1
0.2
0.3
0.4
0.5
0.6
0.7
0.8
Phylostratum
Cellular_organisms - ps1
Archaea1 - ps2
Archaea2 - ps3
Archaea3 - ps4
Asgard/Eukaryota - ps5
Eukaryota - ps6
Amorpheae - ps7
Obazoa - ps8
Opisthokonta - ps9
Holozoa - ps10
Filozoa - ps11
Choano./Metazoa - ps12
Metazoa - ps13
Myriazoa - ps14
Parahoxozoa - ps15
Bilateria - ps16
Deuterostomia - ps17
Chordata - ps18
Olfactores - ps19
Vertebrata - ps20
Gnathostomata - ps21
Euteleostomi - ps22
Sarcopterygii - ps23
Tetrapoda - ps24
Amniota - ps25
Mammalia - ps26
Theria - ps27
Placentalia - ps28
Boreoeutheria - ps29
Euarchontoglires - ps30
Euarchonta - ps31
Primates - ps32
Haplorrhini - ps33
Simiiformes - ps34
Catarrhini - ps35
Hominoidea - ps36
Hominidae - ps37
Homininae - ps38
Hominini - ps39
Homo_sapiens - ps40
p-value
2e-04
9e-04
3e-03
1e-02
4e-02
Log-odds
0.5
1.0
1.5
2.0
2.5
3.0
3.5
4.0

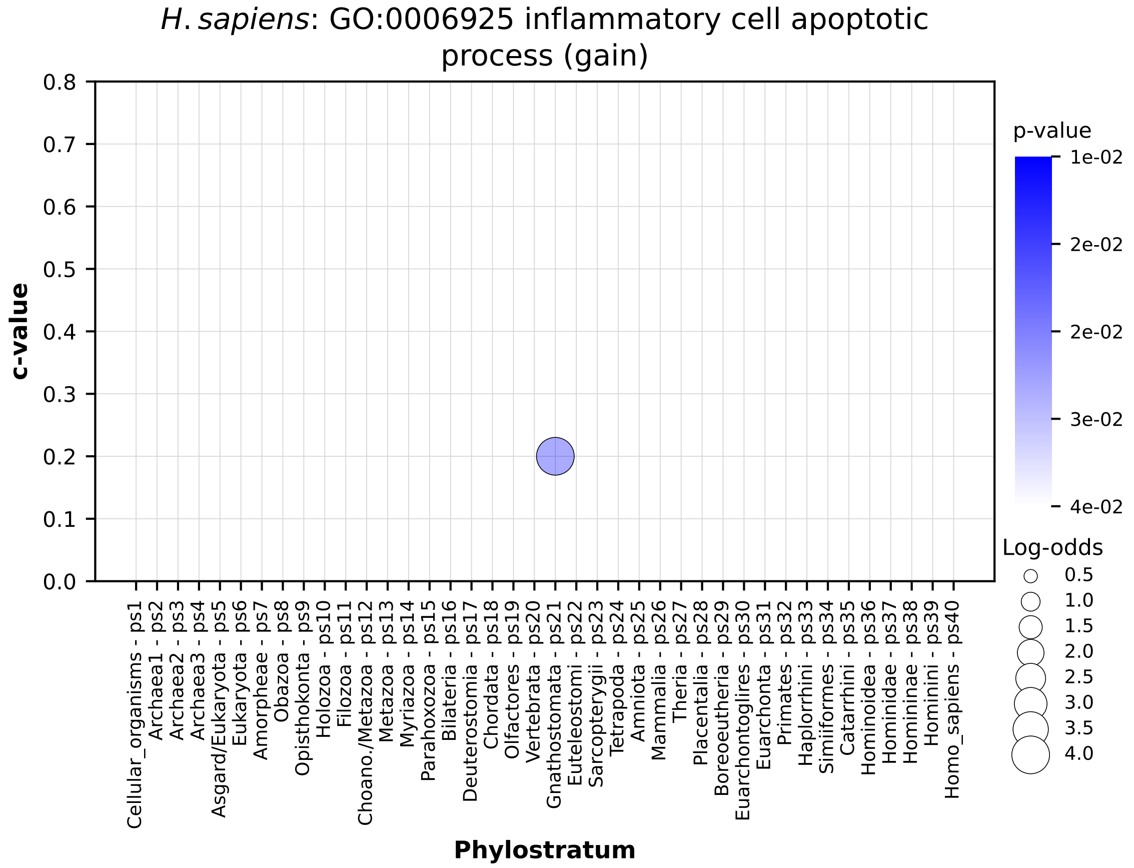

H. sapiens: GO:0006925 inflammatory cell apoptotic process (gain)
c-value
0.8
0.7
0.6
0.5
0.4
0.3
0.2
0.1
0.0
Phylostratum
Cellular_organisms - ps1
Archaea1 - ps2
Archaea2 - ps3
Archaea3 - ps4
Asgard/Eukaryota - ps5
Eukaryota - ps6
Amorpheae - ps7
Obazoa - ps8
Opisthokonta - ps9
Holozoa - ps10
Filozoa - ps11
Choano./Metazoa - ps12
Metazoa - ps13
Myriazoa - ps14
Parahoxozoa - ps15
Bilateria - ps16
Deuterostomia - ps17
Chordata - ps18
Olfactores - ps19
Vertebrata - ps20
Gnathostomata - ps21
Euteleostomi - ps22
Sarcopterygii - ps23
Tetrapoda - ps24
Amniota - ps25
Mammalia - ps26
Theria - ps27
Placentalia - ps28
Boreoeutheria - ps29
Euarchontoglires - ps30
Euarchonta - ps31
Primates - ps32
Haplorrhini - ps33
Simiiformes - ps34
Catarrhini - ps35
Hominoidea - ps36
Hominidae - ps37
Homininae - ps38
Hominini - ps39
Homo_sapiens - ps40
p-value
1e-02
2e-02
2e-02
3e-02
4e-02
Log-odds
0.5
1.0
1.5
2.0
2.5
3.0
3.5
4.0

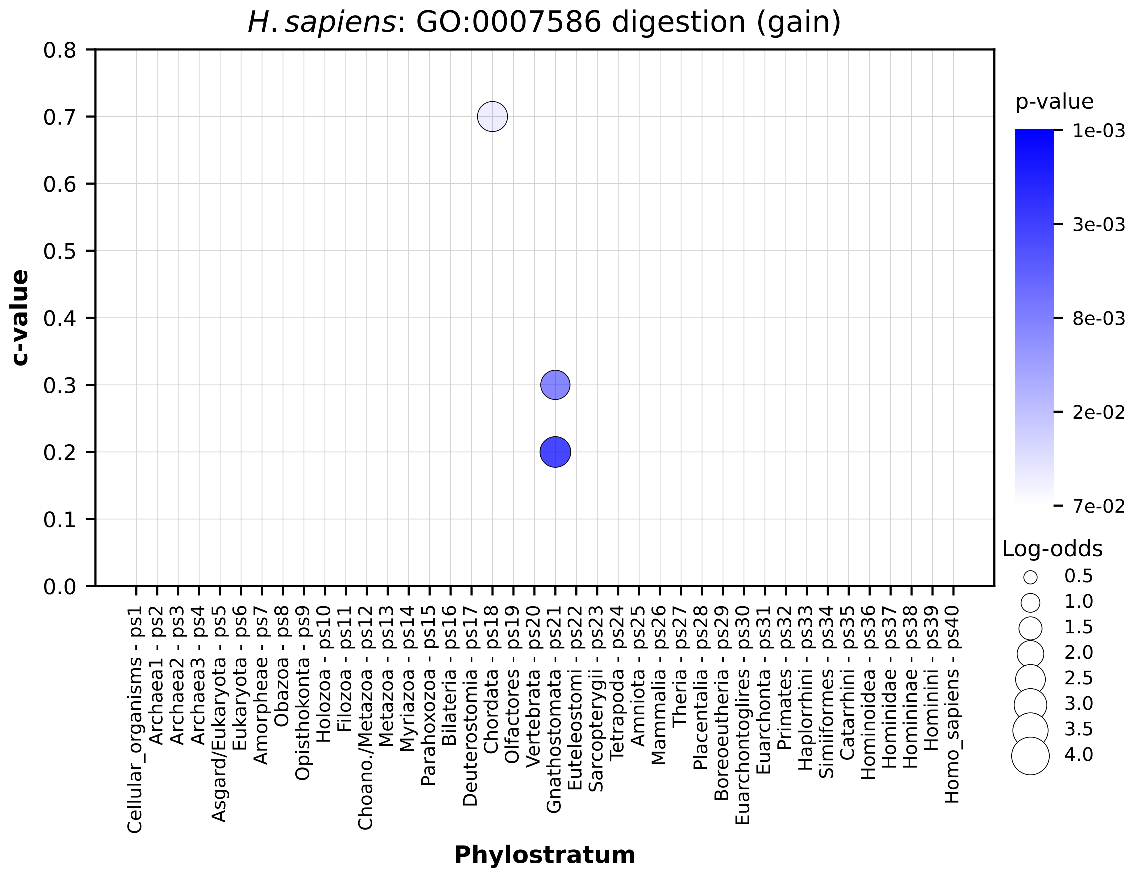

H. sapiens: GO:0007586 digestion (gain)
c-value
0.0
0.1
0.2
0.3
0.4
0.5
0.6
0.7
0.8
Phylostratum
Cellular_organisms - ps1
Archaea1 - ps2
Archaea2 - ps3
Archaea3 - ps4
Asgard/Eukaryota - ps5
Eukaryota - ps6
Amorpheae - ps7
Obazoa - ps8
Opisthokonta - ps9
Holozoa - ps10
Filozoa - ps11
Choano./Metazoa - ps12
Metazoa - ps13
Myriazoa - ps14
Parahoxozoa - ps15
Bilateria - ps16
Deuterostomia - ps17
Chordata - ps18
Olfactores - ps19
Vertebrata - ps20
Gnathostomata - ps21
Euteleostomi - ps22
Sarcopterygii - ps23
Tetrapoda - ps24
Amniota - ps25
Mammalia - ps26
Theria - ps27
Placentalia - ps28
Boreoeutheria - ps29
Euarchontoglires - ps30
Euarchonta - ps31
Primates - ps32
Haplorrhini - ps33
Simiiformes - ps34
Catarrhini - ps35
Hominoidea - ps36
Hominidae - ps37
Homininae - ps38
Hominini - ps39
Homo_sapiens - ps40
p-value
1e-03
3e-03
8e-03
2e-02
7e-02
Log-odds
0.5
1.0
1.5
2.0
2.5
3.0
3.5
4.0

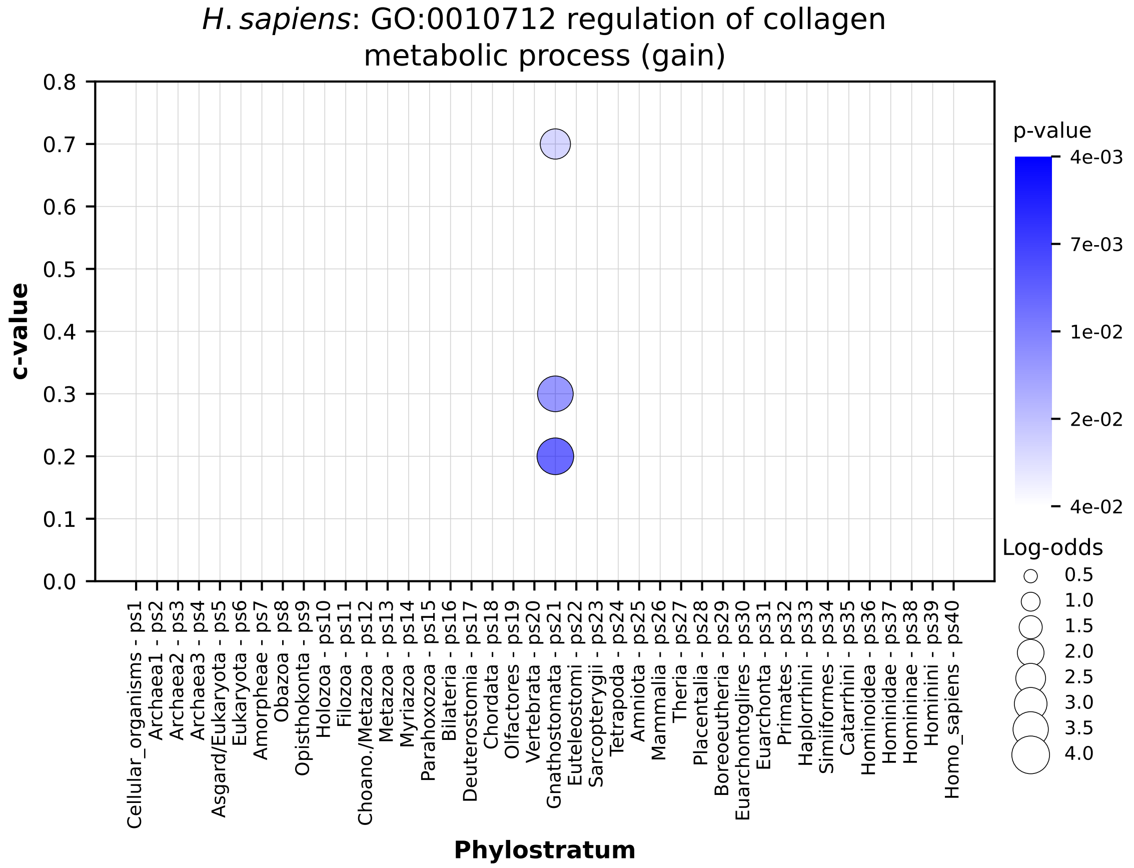
H. sapiens: GO:0010712 regulation of collagen metabolic process (gain)
c-value
0.8
0.7
0.6
0.5
0.4
0.3
0.2
0.1
0.0
p-value
4e-03
7e-03
1e-02
2e-02
4e-02
Log-odds
0.5
1.0
1.5
2.0
2.5
3.0
3.5
4.0
Cellular_organisms - ps1
Archaea1 - ps2
Archaea2 - ps3
Archaea3 - ps4
Asgard/Eukaryota - ps5
Eukaryota - ps6
Amorpheae - ps7
Obazoa - ps8
Opisthokonta - ps9
Holozoa - ps10
Filozoa - ps11
Choano./Metazoa - ps12
Metazoa - ps13
Myriazoa - ps14
Parahoxozoa - ps15
Bilateria - ps16
Deuterostomia - ps17
Chordata - ps18
Olfactores - ps19
Vertebrata - ps20
Gnathostomata - ps21
Euteleostomi - ps22
Sarcopterygii - ps23
Tetrapoda - ps24
Amniota - ps25
Mammalia - ps26
Theria - ps27
Placentalia - ps28
Boreoeutheria - ps29
Euarchontoglires - ps30
Euarchonta - ps31
Primates - ps32
Haplorrhini - ps33
Simiiformes - ps34
Catarrhini - ps35
Hominoidea - ps36
Hominidae - ps37
Homininae - ps38
Hominini - ps39
Homo_sapiens - ps40
Phylostratum

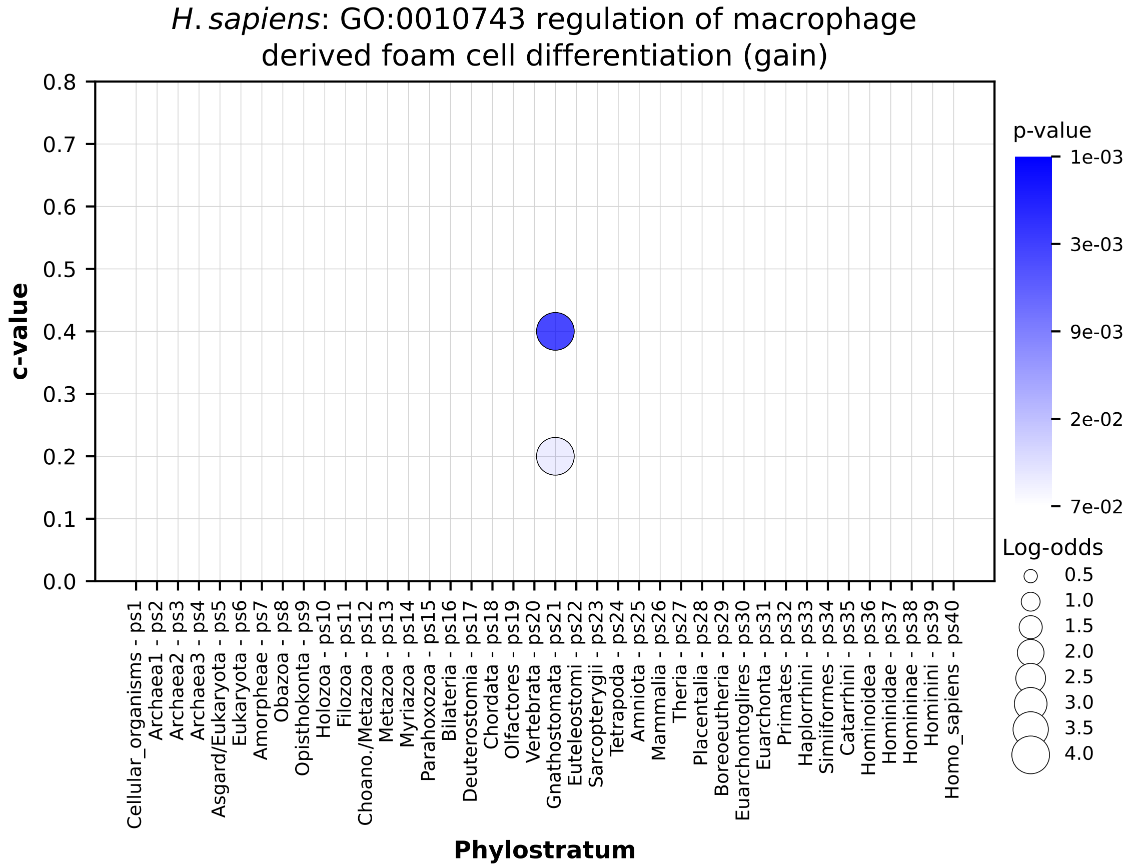
H. sapiens: GO:0010743 regulation of macrophage derived foam cell differentiation (gain)
c-value
0.0
0.1
0.2
0.3
0.4
0.5
0.6
0.7
0.8
Phylostratum
Cellular_organisms - ps1
Archaea1 - ps2
Archaea2 - ps3
Archaea3 - ps4
Asgard/Eukaryota - ps5
Eukaryota - ps6
Amorpheae - ps7
Obazoa - ps8
Opisthokonta - ps9
Holozoa - ps10
Filozoa - ps11
Choano./Metazoa - ps12
Metazoa - ps13
Myriazoa - ps14
Parahoxozoa - ps15
Bilateria - ps16
Deuterostomia - ps17
Chordata - ps18
Olfactores - ps19
Vertebrata - ps20
Gnathostomata - ps21
Euteleostomi - ps22
Sarcopterygii - ps23
Tetrapoda - ps24
Amniota - ps25
Mammalia - ps26
Theria - ps27
Placentalia - ps28
Boreoeutheria - ps29
Euarchontoglires - ps30
Euarchonta - ps31
Primates - ps32
Haplorrhini - ps33
Simiiformes - ps34
Catarrhini - ps35
Hominoidea - ps36
Hominidae - ps37
Homininae - ps38
Hominini - ps39
Homo_sapiens - ps40
p-value
1e-03
3e-03
9e-03
2e-02
7e-02
Log-odds
0.5
1.0
1.5
2.0
2.5
3.0
3.5
4.0

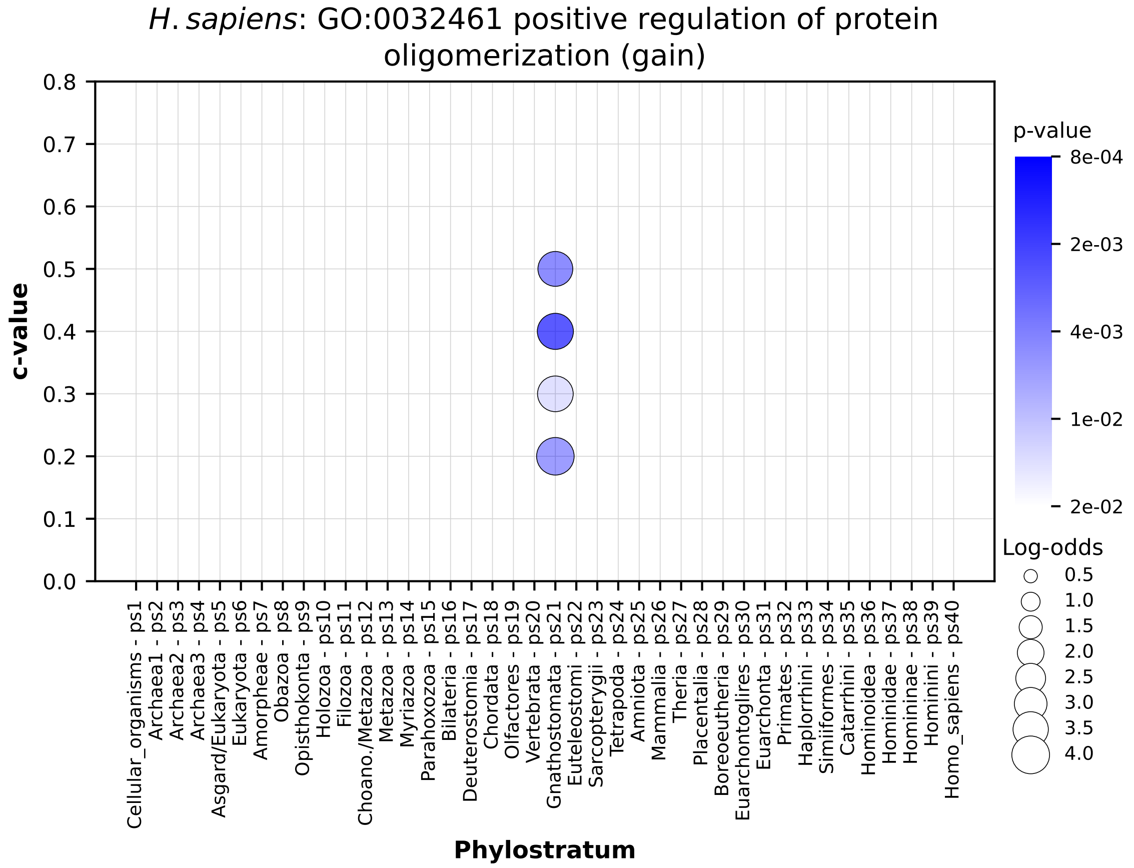

*H. sapiens*: GO:0032461 positive regulation of protein oligomerization (gain)
c-value
0.8
0.7
0.6
0.5
0.4
0.3
0.2
0.1
0.0
Cellular_organisms - ps1
Archaea1 - ps2
Archaea2 - ps3
Archaea3 - ps4
Asgard/Eukaryota - ps5
Eukaryota - ps6
Amorpheae - ps7
Obazoa - ps8
Opisthokonta - ps9
Holozoa - ps10
Filozoa - ps11
Choano./Metazoa - ps12
Metazoa - ps13
Myriazoa - ps14
Parahoxozoa - ps15
Bilateria - ps16
Deuterostomia - ps17
Chordata - ps18
Olfactores - ps19
Vertebrata - ps20
Gnathostomata - ps21
Euteleostomi - ps22
Sarcopterygii - ps23
Tetrapoda - ps24
Amniota - ps25
Mammalia - ps26
Theria - ps27
Placentalia - ps28
Boreoeutheria - ps29
Euarchontoglires - ps30
Euarchonta - ps31
Primates - ps32
Haplorrhini - ps33
Simiiformes - ps34
Catarrhini - ps35
Hominoidea - ps36
Hominidae - ps37
Homininae - ps38
Hominini - ps39
Homo_sapiens - ps40
Phylostratum
p-value
8e-04
2e-03
4e-03
1e-02
2e-02
Log-odds
0.5
1.0
1.5
2.0
2.5
3.0
3.5
4.0

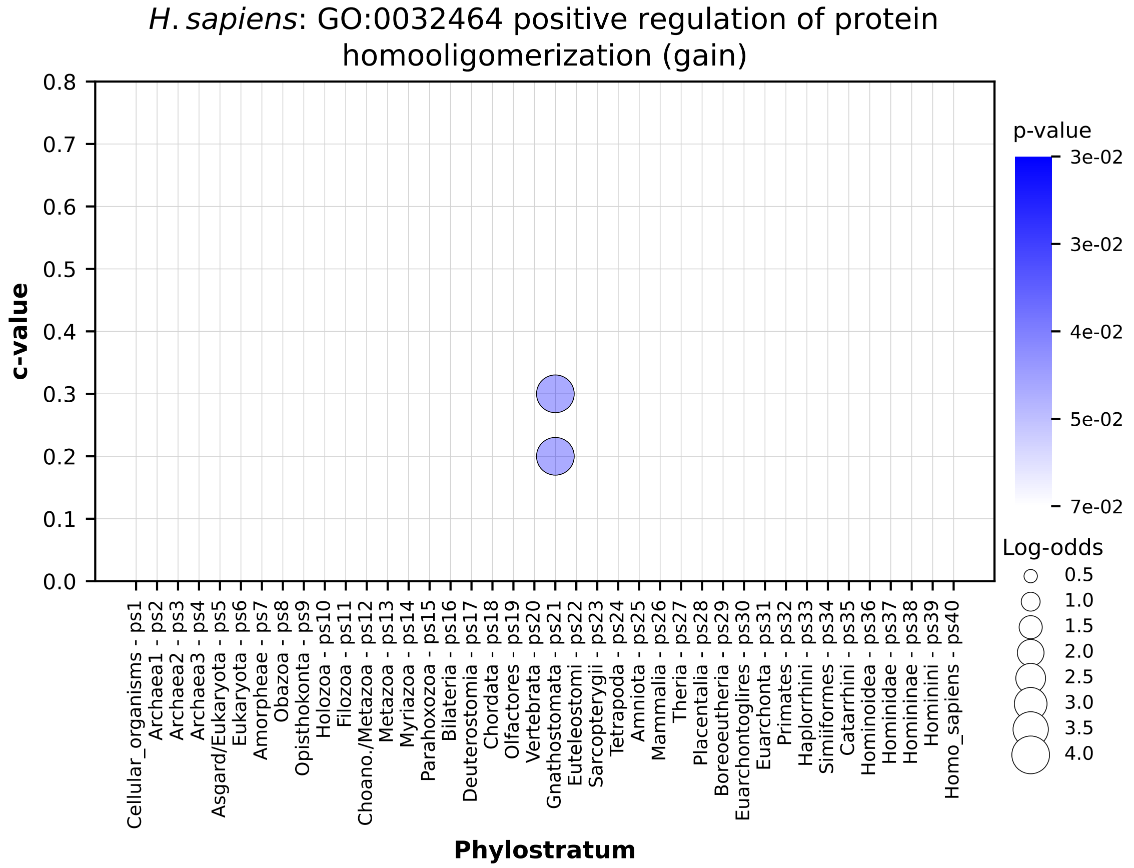
H. sapiens: GO:0032464 positive regulation of protein homooligomerization (gain)
c-value
0.0
0.1
0.2
0.3
0.4
0.5
0.6
0.7
0.8
Phylostratum
Cellular_organisms - ps1
Archaea1 - ps2
Archaea2 - ps3
Archaea3 - ps4
Asgard/Eukaryota - ps5
Eukaryota - ps6
Amorpheae - ps7
Obazoa - ps8
Opisthokonta - ps9
Holozoa - ps10
Filozoa - ps11
Choano./Metazoa - ps12
Metazoa - ps13
Myriazoa - ps14
Parahoxozoa - ps15
Bilateria - ps16
Deuterostomia - ps17
Chordata - ps18
Olfactores - ps19
Vertebrata - ps20
Gnathostomata - ps21
Euteleostomi - ps22
Sarcopterygii - ps23
Tetrapoda - ps24
Amniota - ps25
Mammalia - ps26
Theria - ps27
Placentalia - ps28
Boreoeutheria - ps29
Euarchontoglires - ps30
Euarchonta - ps31
Primates - ps32
Haplorrhini - ps33
Simiiformes - ps34
Catarrhini - ps35
Hominoidea - ps36
Hominidae - ps37
Homininae - ps38
Hominini - ps39
Homo_sapiens - ps40
p-value
3e-02
3e-02
4e-02
5e-02
7e-02
Log-odds
0.5
1.0
1.5
2.0
2.5
3.0
3.5
4.0

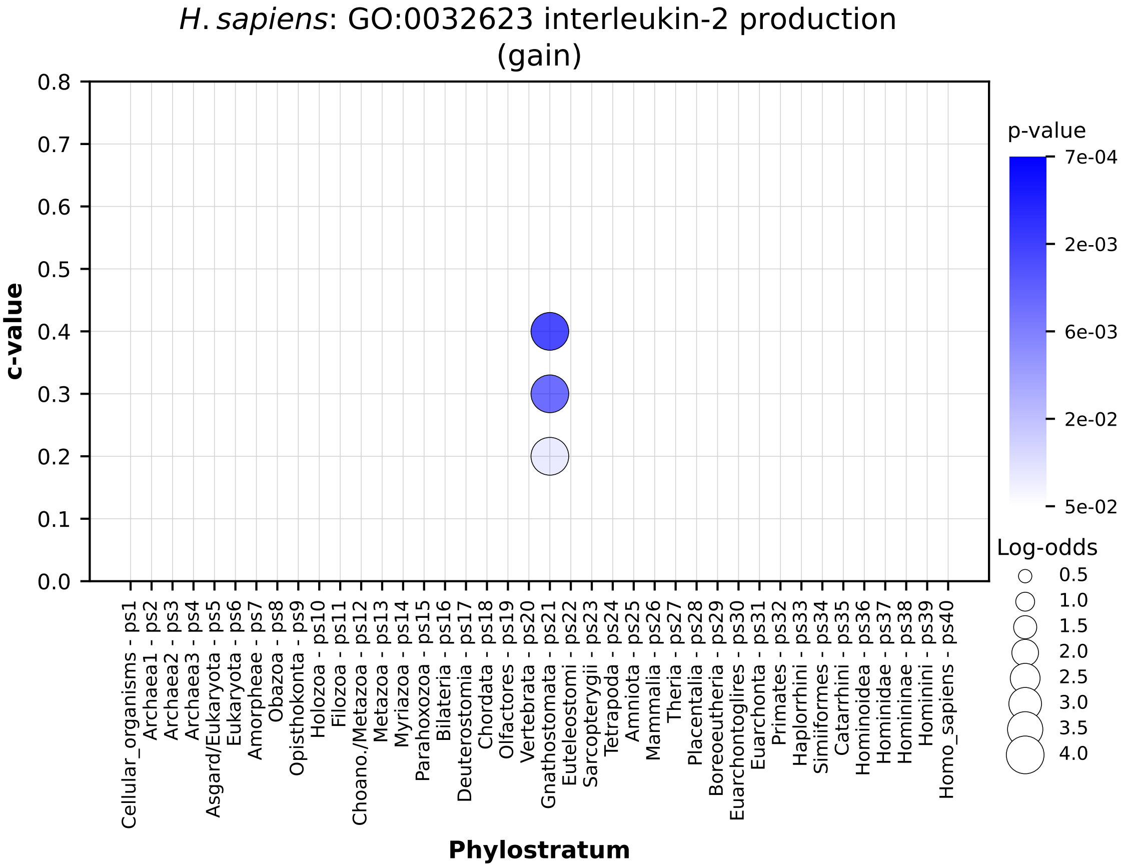

*H. sapiens*: GO:0032623 interleukin-2 production
(gain)
c-value
0.8
0.7
0.6
0.5
0.4
0.3
0.2
0.1
0.0
p-value
7e-04
2e-03
6e-03
2e-02
5e-02
Log-odds
0.5
1.0
1.5
2.0
2.5
3.0
3.5
4.0
Cellular_organisms - ps1
Archaea1 - ps2
Archaea2 - ps3
Archaea3 - ps4
Asgard/Eukaryota - ps5
Eukaryota - ps6
Amorpheae - ps7
Obazoa - ps8
Opisthokonta - ps9
Holozoa - ps10
Filozoa - ps11
Choano./Metazoa - ps12
Metazoa - ps13
Myriazoa - ps14
Parahoxozoa - ps15
Bilateria - ps16
Deuterostomia - ps17
Chordata - ps18
Olfactores - ps19
Vertebrata - ps20
Gnathostomata - ps21
Euteleostomi - ps22
Sarcopterygii - ps23
Tetrapoda - ps24
Amniota - ps25
Mammalia - ps26
Theria - ps27
Placentalia - ps28
Boreoeutheria - ps29
Euarchontoglires - ps30
Euarchonta - ps31
Primates - ps32
Haplorrhini - ps33
Simiiformes - ps34
Catarrhini - ps35
Hominoidea - ps36
Hominidae - ps37
Homininae - ps38
Hominini - ps39
Homo_sapiens - ps40
Phylostratum

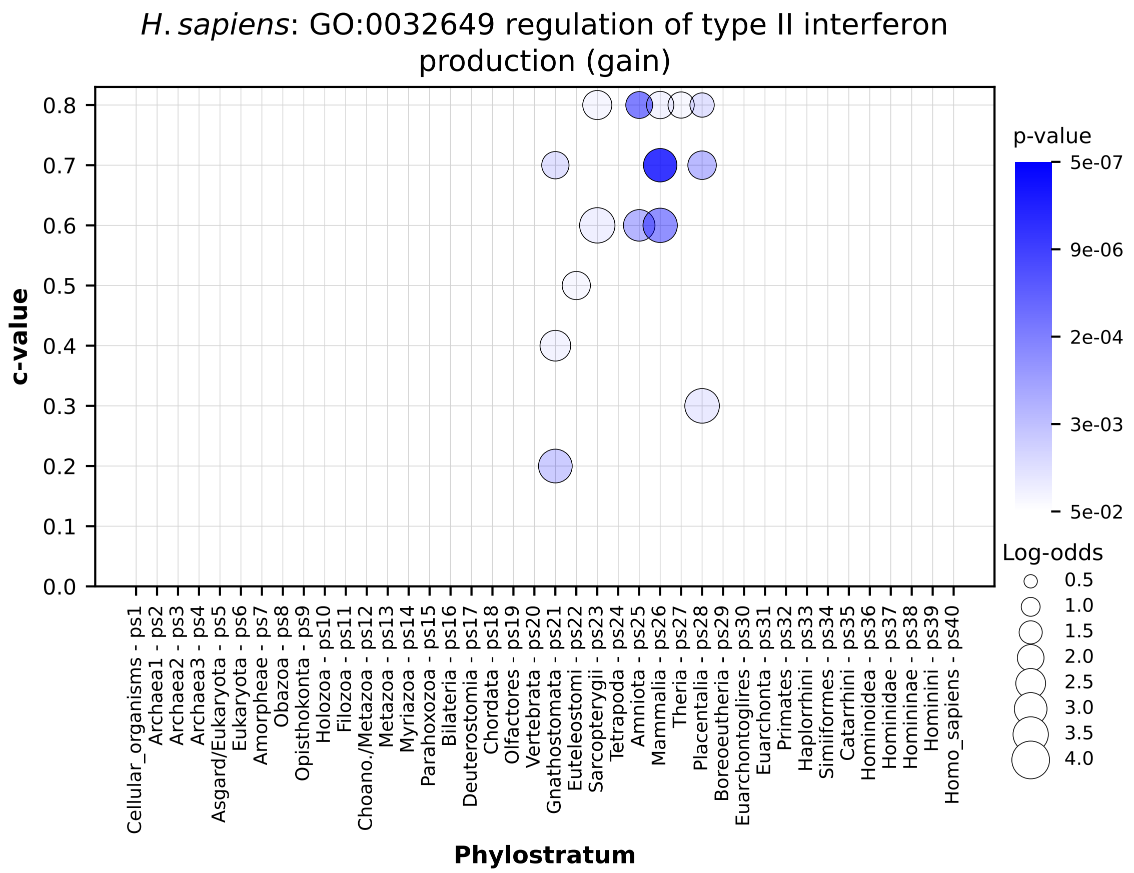

H. sapiens: GO:0032649 regulation of type II interferon production (gain)
c-value
0.0
0.1
0.2
0.3
0.4
0.5
0.6
0.7
0.8
Phylostratum
Cellular_organisms - ps1
Archaea1 - ps2
Archaea2 - ps3
Archaea3 - ps4
Asgard/Eukaryota - ps5
Eukaryota - ps6
Amorpheae - ps7
Obazoa - ps8
Opisthokonta - ps9
Holozoa - ps10
Filozoa - ps11
Choano./Metazoa - ps12
Metazoa - ps13
Myriazoa - ps14
Parahoxozoa - ps15
Bilateria - ps16
Deuterostomia - ps17
Chordata - ps18
Olfactores - ps19
Vertebrata - ps20
Gnathostomata - ps21
Euteleostomi - ps22
Sarcopterygii - ps23
Tetrapoda - ps24
Amniota - ps25
Mammalia - ps26
Theria - ps27
Placentalia - ps28
Boreoeutheria - ps29
Euarchontoglires - ps30
Euarchonta - ps31
Primates - ps32
Haplorrhini - ps33
Simiiformes - ps34
Catarrhini - ps35
Hominoidea - ps36
Hominidae - ps37
Homininae - ps38
Hominini - ps39
Homo_sapiens - ps40
p-value
5e-07
9e-06
2e-04
3e-03
5e-02
Log-odds
0.5
1.0
1.5
2.0
2.5
3.0
3.5
4.0

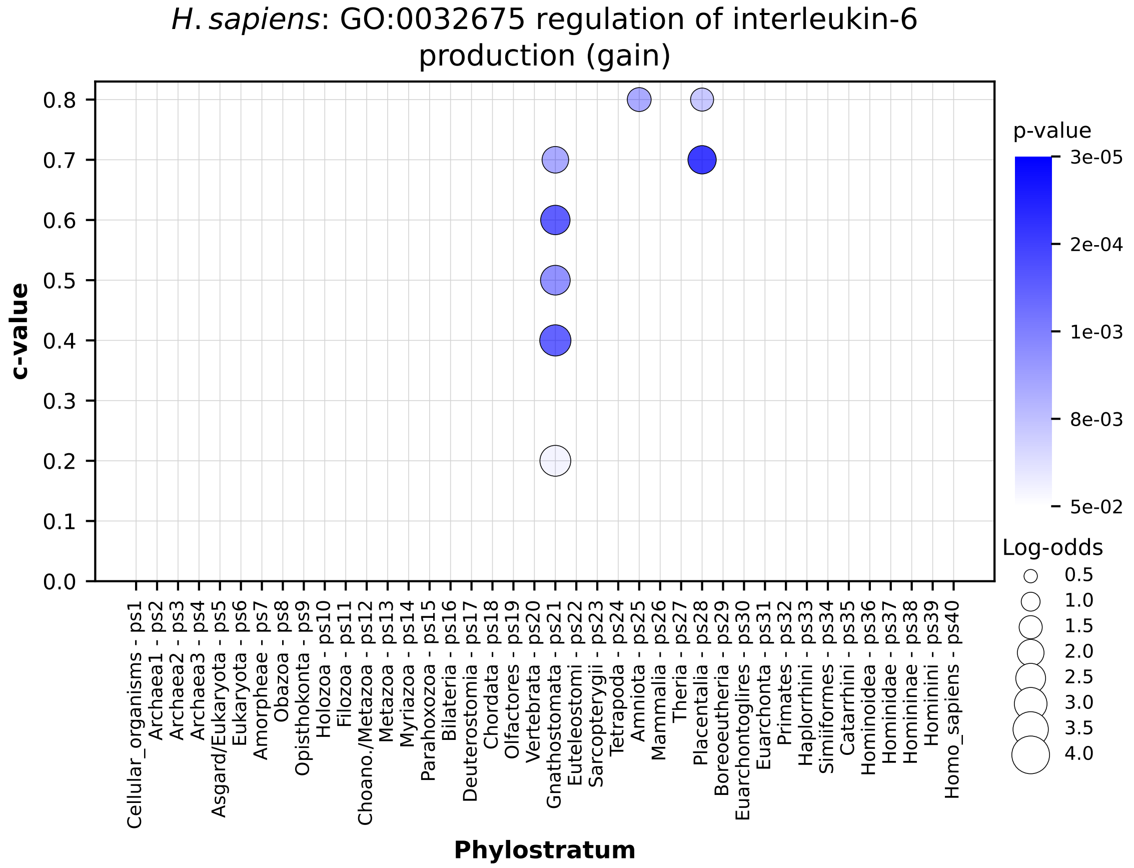

H. sapiens: GO:0032675 regulation of interleukin-6 production (gain)
c-value
0.0
0.1
0.2
0.3
0.4
0.5
0.6
0.7
0.8
Phylostratum
Cellular_organisms - ps1
Archaea1 - ps2
Archaea2 - ps3
Archaea3 - ps4
Asgard/Eukaryota - ps5
Eukaryota - ps6
Amorpheae - ps7
Obazoa - ps8
Opisthokonta - ps9
Holozoa - ps10
Filozoa - ps11
Choano./Metazoa - ps12
Metazoa - ps13
Myriazoa - ps14
Parahoxozoa - ps15
Bilateria - ps16
Deuterostomia - ps17
Chordata - ps18
Olfactores - ps19
Vertebrata - ps20
Gnathostomata - ps21
Euteleostomi - ps22
Sarcopterygii - ps23
Tetrapoda - ps24
Amniota - ps25
Mammalia - ps26
Theria - ps27
Placentalia - ps28
Boreoeutheria - ps29
Euarchontoglires - ps30
Euarchonta - ps31
Primates - ps32
Haplorrhini - ps33
Simiiformes - ps34
Catarrhini - ps35
Hominoidea - ps36
Hominidae - ps37
Homininae - ps38
Hominini - ps39
Homo_sapiens - ps40
p-value
3e-05
2e-04
1e-03
8e-03
5e-02
Log-odds
0.5
1.0
1.5
2.0
2.5
3.0
3.5
4.0

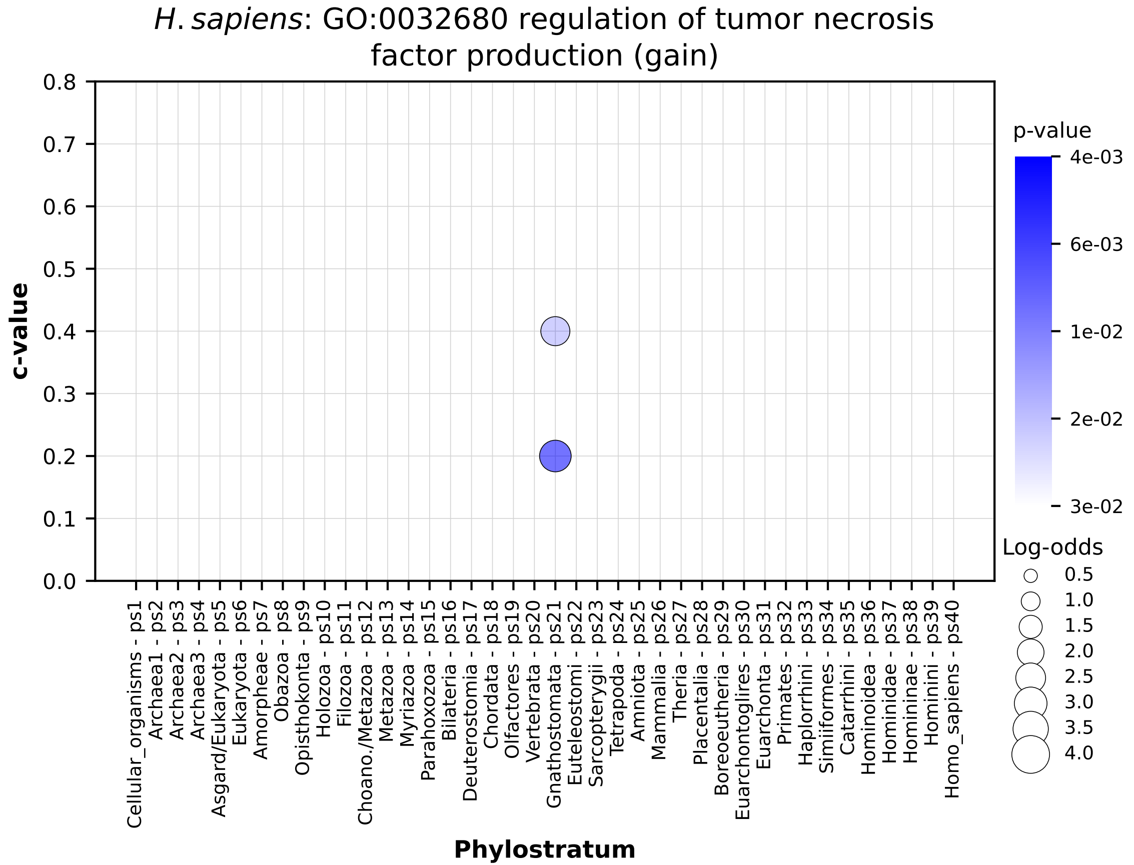

*H. sapiens*: GO:0032680 regulation of tumor necrosis factor production (gain)
c-value
0.8
0.7
0.6
0.5
0.4
0.3
0.2
0.1
0.0
Cellular_organisms - ps1
Archaea1 - ps2
Archaea2 - ps3
Archaea3 - ps4
Asgard/Eukaryota - ps5
Eukaryota - ps6
Amorpheae - ps7
Obazoa - ps8
Opisthokonta - ps9
Holozoa - ps10
Filozoa - ps11
Choano./Metazoa - ps12
Metazoa - ps13
Myriazoa - ps14
Parahoxozoa - ps15
Bilateria - ps16
Deuterostomia - ps17
Chordata - ps18
Olfactores - ps19
Vertebrata - ps20
Gnathostomata - ps21
Euteleostomi - ps22
Sarcopterygii - ps23
Tetrapoda - ps24
Amniota - ps25
Mammalia - ps26
Theria - ps27
Placentalia - ps28
Boreoeutheria - ps29
Euarchontoglires - ps30
Euarchonta - ps31
Primates - ps32
Haplorrhini - ps33
Simiiformes - ps34
Catarrhini - ps35
Hominoidea - ps36
Hominidae - ps37
Homininae - ps38
Hominini - ps39
Homo_sapiens - ps40
Phylostratum
p-value
4e-03
6e-03
1e-02
2e-02
3e-02
Log-odds
0.5
1.0
1.5
2.0
2.5
3.0
3.5
4.0

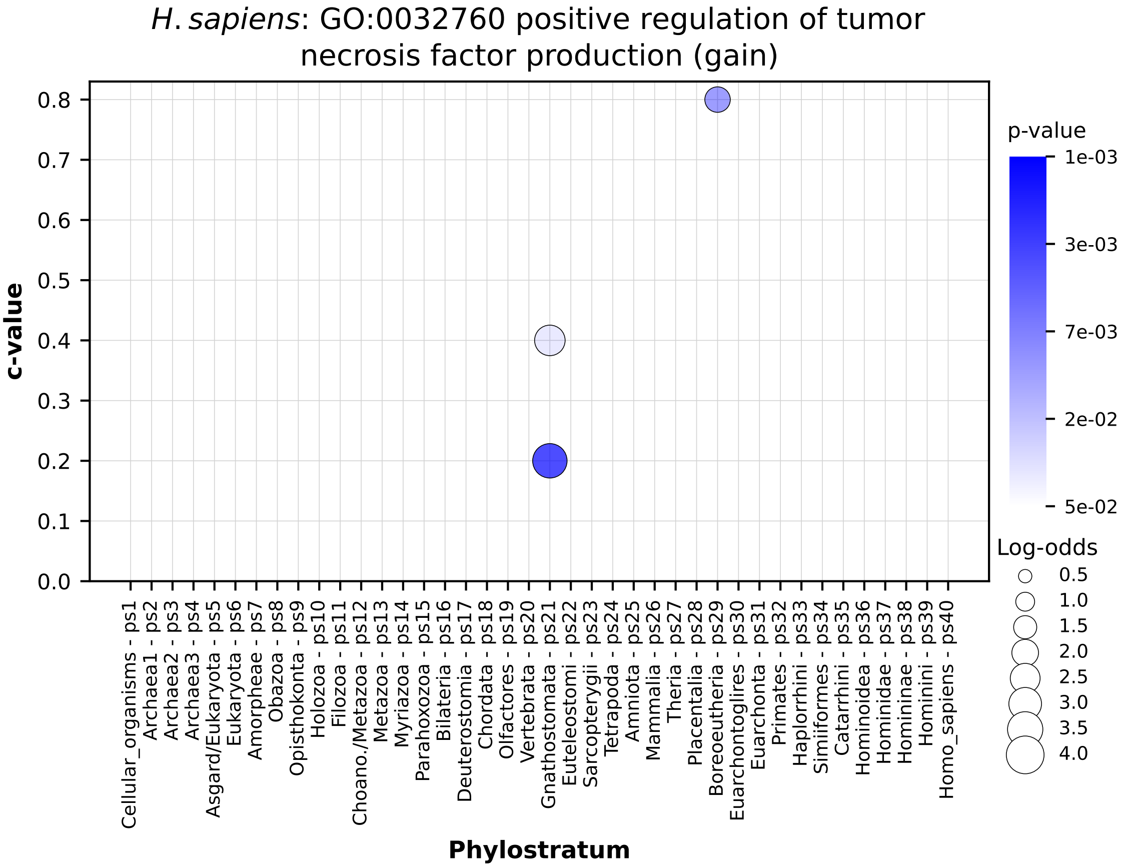
H. sapiens: GO:0032760 positive regulation of tumor necrosis factor production (gain)
c-value
0.8
0.7
0.6
0.5
0.4
0.3
0.2
0.1
0.0
p-value
1e-03
3e-03
7e-03
2e-02
5e-02
Log-odds
0.5
1.0
1.5
2.0
2.5
3.0
3.5
4.0
Cellular_organisms - ps1
Archaea1 - ps2
Archaea2 - ps3
Archaea3 - ps4
Asgard/Eukaryota - ps5
Eukaryota - ps6
Amorpheae - ps7
Obazoa - ps8
Opisthokonta - ps9
Holozoa - ps10
Filozoa - ps11
Choano./Metazoa - ps12
Metazoa - ps13
Myriazoa - ps14
Parahoxozoa - ps15
Bilateria - ps16
Deuterostomia - ps17
Chordata - ps18
Olfactores - ps19
Vertebrata - ps20
Gnathostomata - ps21
Euteleostomi - ps22
Sarcopterygii - ps23
Tetrapoda - ps24
Amniota - ps25
Mammalia - ps26
Theria - ps27
Placentalia - ps28
Boreoeutheria - ps29
Euarchontoglires - ps30
Euarchonta - ps31
Primates - ps32
Haplorrhini - ps33
Simiiformes - ps34
Catarrhini - ps35
Hominoidea - ps36
Hominidae - ps37
Homininae - ps38
Hominini - ps39
Homo_sapiens - ps40
Phylostratum

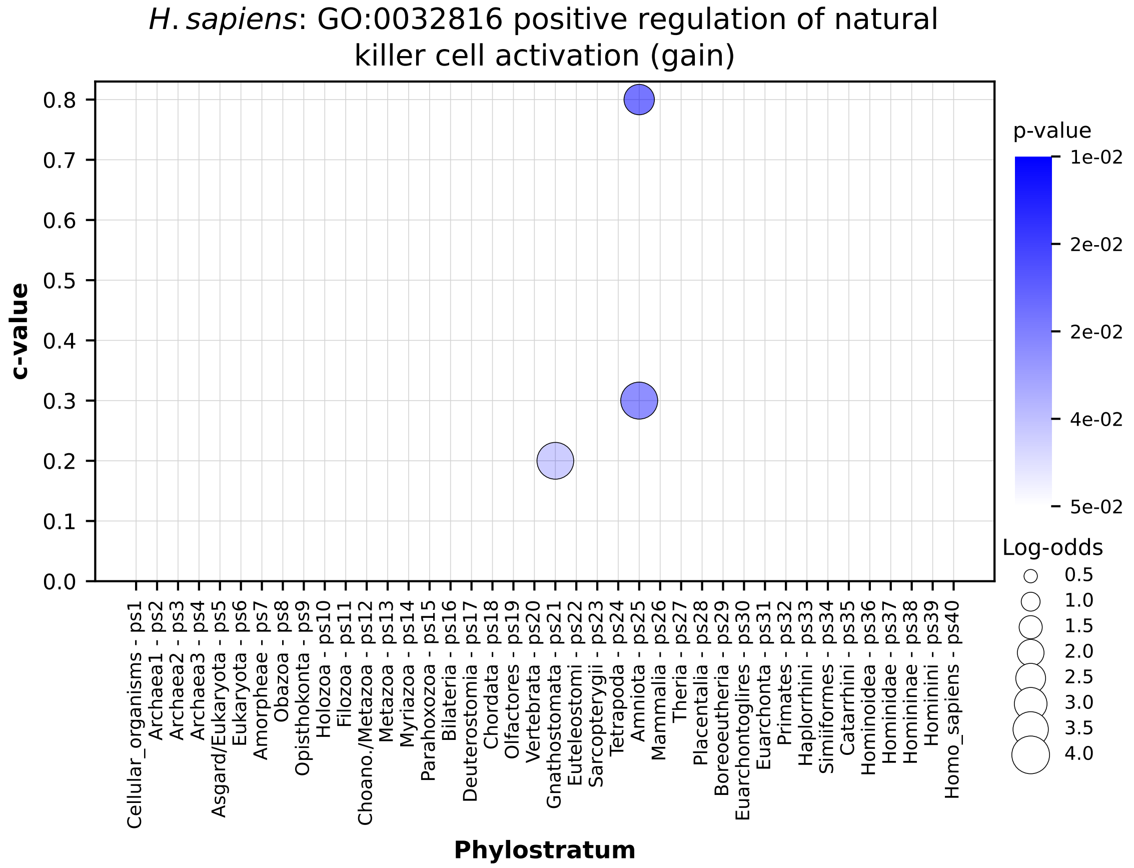

H. sapiens: GO:0032816 positive regulation of natural killer cell activation (gain)
c-value
0.0
0.1
0.2
0.3
0.4
0.5
0.6
0.7
0.8
Phylostratum
Cellular_organisms - ps1
Archaea1 - ps2
Archaea2 - ps3
Archaea3 - ps4
Asgard/Eukaryota - ps5
Eukaryota - ps6
Amorpheae - ps7
Obazoa - ps8
Opisthokonta - ps9
Holozoa - ps10
Filozoa - ps11
Choano./Metazoa - ps12
Metazoa - ps13
Myriazoa - ps14
Parahoxozoa - ps15
Bilateria - ps16
Deuterostomia - ps17
Chordata - ps18
Olfactores - ps19
Vertebrata - ps20
Gnathostomata - ps21
Euteleostomi - ps22
Sarcopterygii - ps23
Tetrapoda - ps24
Amniota - ps25
Mammalia - ps26
Theria - ps27
Placentalia - ps28
Boreoeutheria - ps29
Euarchontoglires - ps30
Euarchonta - ps31
Primates - ps32
Haplorrhini - ps33
Simiiformes - ps34
Catarrhini - ps35
Hominoidea - ps36
Hominidae - ps37
Homininae - ps38
Hominini - ps39
Homo_sapiens - ps40
p-value
1e-02
2e-02
2e-02
4e-02
5e-02
Log-odds
0.5
1.0
1.5
2.0
2.5
3.0
3.5
4.0

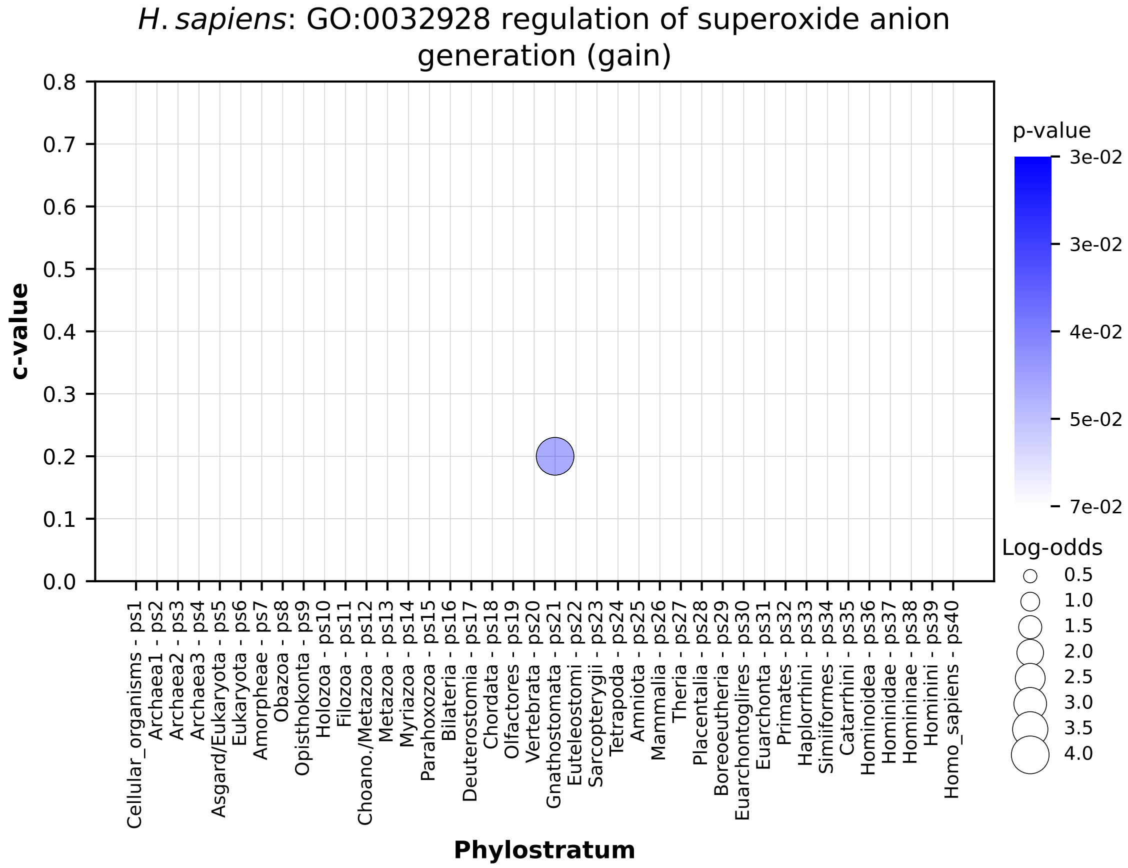

H. sapiens: GO:0032928 regulation of superoxide anion generation (gain)
c-value
0.0
0.1
0.2
0.3
0.4
0.5
0.6
0.7
0.8
Phylostratum
Cellular_organisms - ps1
Archaea1 - ps2
Archaea2 - ps3
Archaea3 - ps4
Asgard/Eukaryota - ps5
Eukaryota - ps6
Amorpheae - ps7
Obazoa - ps8
Opisthokonta - ps9
Holozoa - ps10
Filozoa - ps11
Choano./Metazoa - ps12
Metazoa - ps13
Myriazoa - ps14
Parahoxozoa - ps15
Bilateria - ps16
Deuterostomia - ps17
Chordata - ps18
Olfactores - ps19
Vertebrata - ps20
Gnathostomata - ps21
Euteleostomi - ps22
Sarcopterygii - ps23
Tetrapoda - ps24
Amniota - ps25
Mammalia - ps26
Theria - ps27
Placentalia - ps28
Boreoeutheria - ps29
Euarchontoglires - ps30
Euarchonta - ps31
Primates - ps32
Haplorrhini - ps33
Simiiformes - ps34
Catarrhini - ps35
Hominoidea - ps36
Hominidae - ps37
Homininae - ps38
Hominini - ps39
Homo_sapiens - ps40
p-value
3e-02
3e-02
4e-02
5e-02
7e-02
Log-odds
0.5
1.0
1.5
2.0
2.5
3.0
3.5
4.0

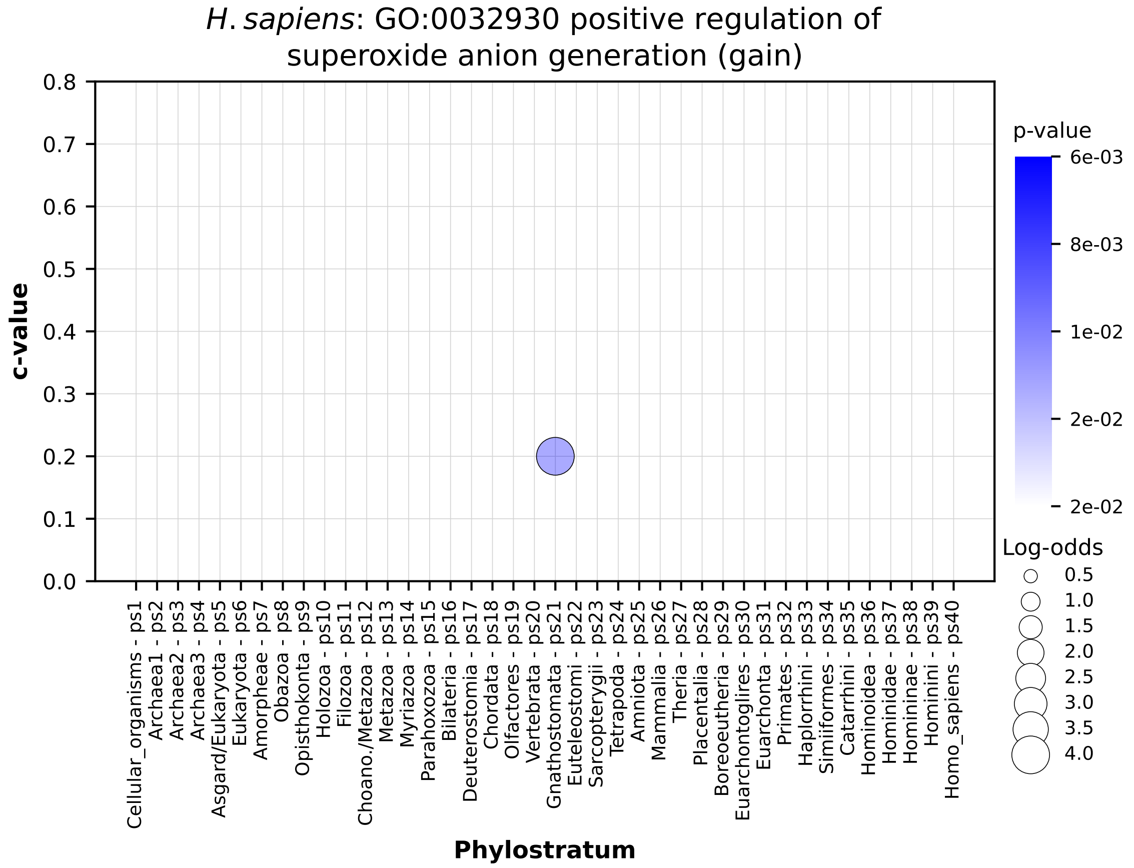

H. sapiens: GO:0032930 positive regulation of superoxide anion generation (gain)
c-value
0.0
0.1
0.2
0.3
0.4
0.5
0.6
0.7
0.8
Phylostratum
Cellular_organisms - ps1
Archaea1 - ps2
Archaea2 - ps3
Archaea3 - ps4
Asgard/Eukaryota - ps5
Eukaryota - ps6
Amorpheae - ps7
Obazoa - ps8
Opisthokonta - ps9
Holozoa - ps10
Filozoa - ps11
Choano./Metazoa - ps12
Metazoa - ps13
Myriazoa - ps14
Parahoxozoa - ps15
Bilateria - ps16
Deuterostomia - ps17
Chordata - ps18
Olfactores - ps19
Vertebrata - ps20
Gnathostomata - ps21
Euteleostomi - ps22
Sarcopterygii - ps23
Tetrapoda - ps24
Amniota - ps25
Mammalia - ps26
Theria - ps27
Placentalia - ps28
Boreoeutheria - ps29
Euarchontoglires - ps30
Euarchonta - ps31
Primates - ps32
Haplorrhini - ps33
Simiiformes - ps34
Catarrhini - ps35
Hominoidea - ps36
Hominidae - ps37
Homininae - ps38
Hominini - ps39
Homo_sapiens - ps40
p-value
6e-03
8e-03
1e-02
2e-02
2e-02
Log-odds
0.5
1.0
1.5
2.0
2.5
3.0
3.5
4.0

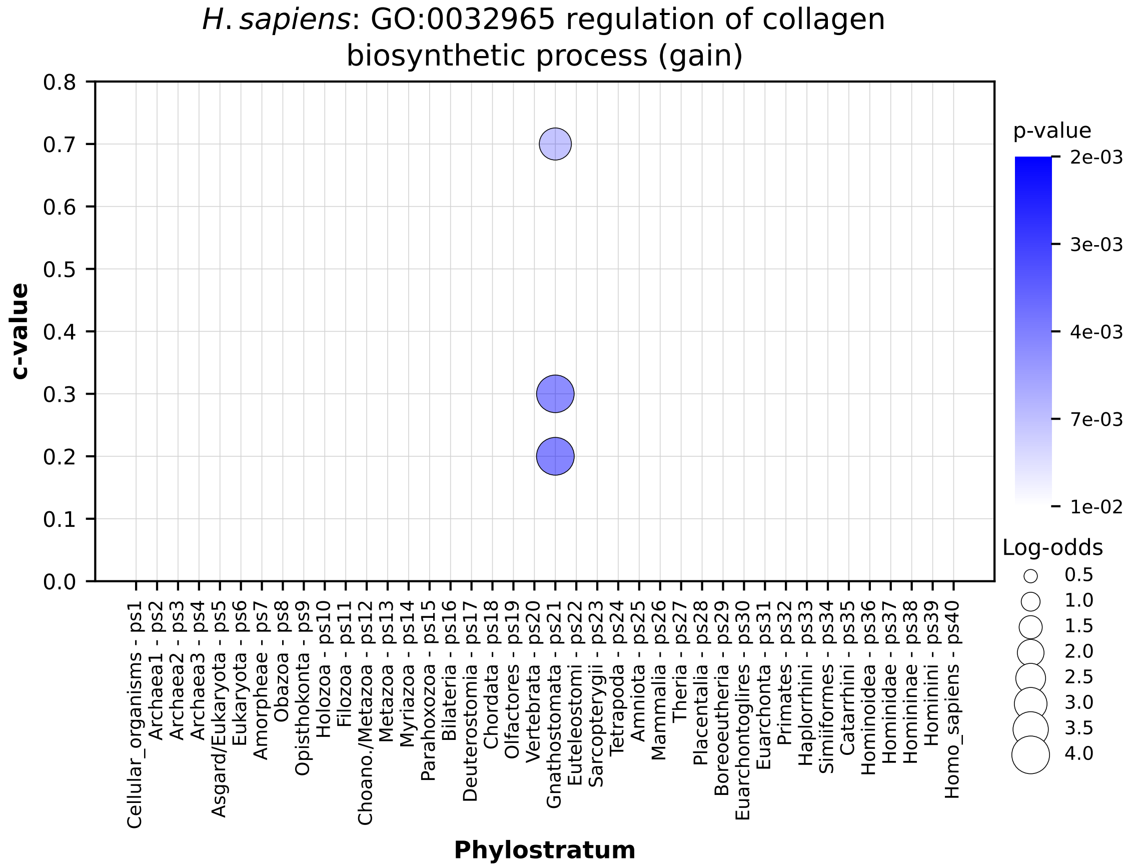
H. sapiens: GO:0032965 regulation of collagen biosynthetic process (gain)
c-value
0.8
0.7
0.6
0.5
0.4
0.3
0.2
0.1
0.0
Phylostratum
Cellular_organisms - ps1
Archaea1 - ps2
Archaea2 - ps3
Archaea3 - ps4
Asgard/Eukaryota - ps5
Eukaryota - ps6
Amorpheae - ps7
Obazoa - ps8
Opisthokonta - ps9
Holozoa - ps10
Filozoa - ps11
Choano./Metazoa - ps12
Metazoa - ps13
Myriazoa - ps14
Parahoxozoa - ps15
Bilateria - ps16
Deuterostomia - ps17
Chordata - ps18
Olfactores - ps19
Vertebrata - ps20
Gnathostomata - ps21
Euteleostomi - ps22
Sarcopterygii - ps23
Tetrapoda - ps24
Amniota - ps25
Mammalia - ps26
Theria - ps27
Placentalia - ps28
Boreoeutheria - ps29
Euarchontoglires - ps30
Euarchonta - ps31
Primates - ps32
Haplorrhini - ps33
Simiiformes - ps34
Catarrhini - ps35
Hominoidea - ps36
Hominidae - ps37
Homininae - ps38
Hominini - ps39
Homo_sapiens - ps40
p-value
2e-03
3e-03
4e-03
7e-03
1e-02
Log-odds
0.5
1.0
1.5
2.0
2.5
3.0
3.5
4.0

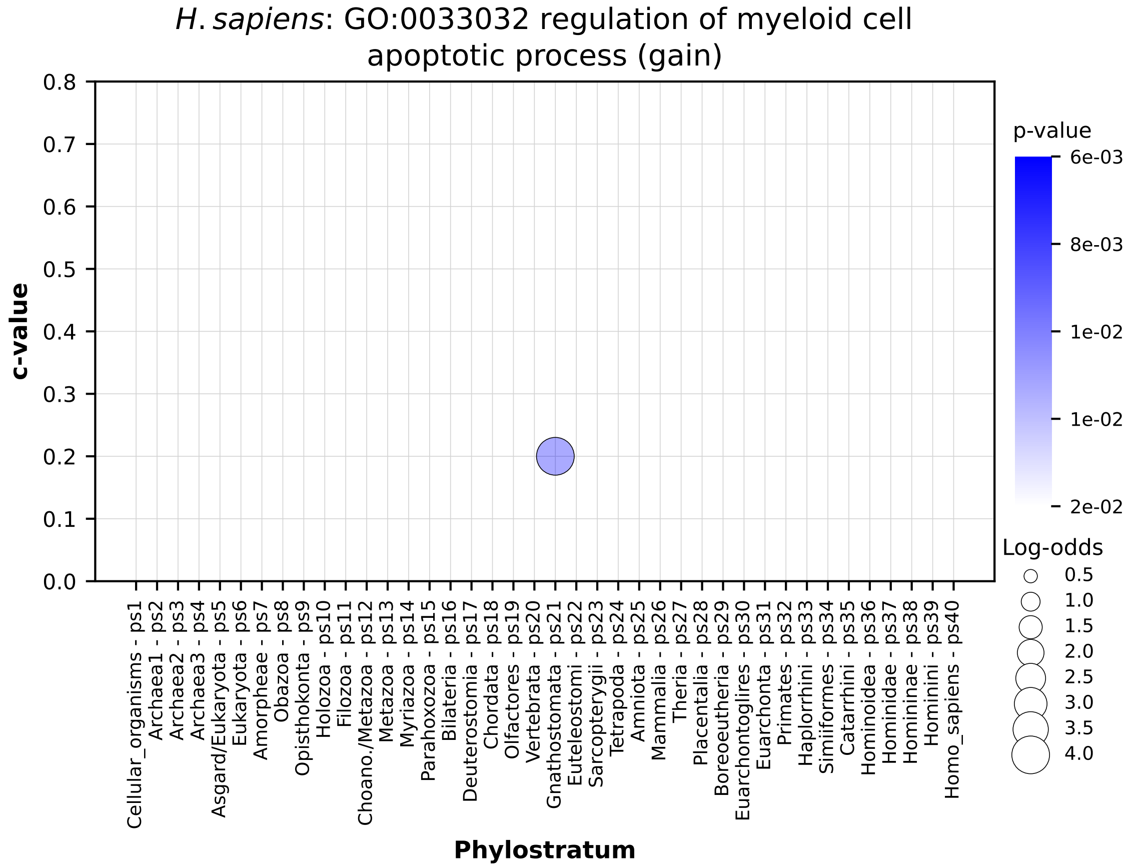

$H. sapiens$: GO:0033032 regulation of myeloid cell apoptotic process (gain)
c-value
0.8
0.7
0.6
0.5
0.4
0.3
0.2
0.1
0.0
p-value
6e-03
8e-03
1e-02
1e-02
2e-02
Log-odds
0.5
1.0
1.5
2.0
2.5
3.0
3.5
4.0
Cellular_organisms - ps1
Archaea1 - ps2
Archaea2 - ps3
Archaea3 - ps4
Asgard/Eukaryota - ps5
Eukaryota - ps6
Amorpheae - ps7
Obazoa - ps8
Opisthokonta - ps9
Holozoa - ps10
Filozoa - ps11
Choano./Metazoa - ps12
Metazoa - ps13
Myriazoa - ps14
Parahoxozoa - ps15
Bilateria - ps16
Deuterostomia - ps17
Chordata - ps18
Olfactores - ps19
Vertebrata - ps20
Gnathostomata - ps21
Euteleostomi - ps22
Sarcopterygii - ps23
Tetrapoda - ps24
Amniota - ps25
Mammalia - ps26
Theria - ps27
Placentalia - ps28
Boreoeutheria - ps29
Euarchontoglires - ps30
Euarchonta - ps31
Primates - ps32
Haplorrhini - ps33
Simiiformes - ps34
Catarrhini - ps35
Hominoidea - ps36
Hominidae - ps37
Homininae - ps38
Hominini - ps39
Homo_sapiens - ps40
Phylostratum

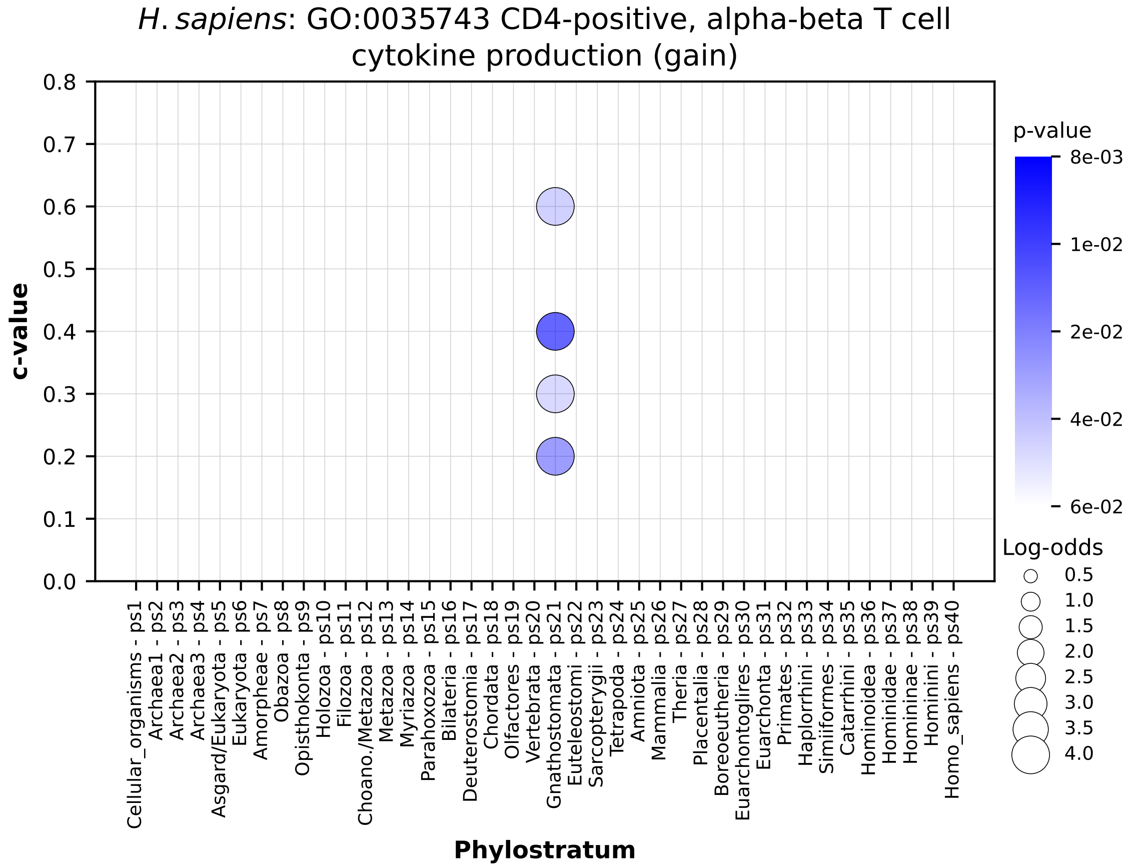

*H. sapiens*: GO:0035743 CD4-positive, alpha-beta T cell cytokine production (gain)
c-value
0.0
0.1
0.2
0.3
0.4
0.5
0.6
0.7
0.8
Phylostratum
Cellular_organisms - ps1
Archaea1 - ps2
Archaea2 - ps3
Archaea3 - ps4
Asgard/Eukaryota - ps5
Eukaryota - ps6
Amorpheae - ps7
Obazoa - ps8
Opisthokonta - ps9
Holozoa - ps10
Filozoa - ps11
Choano./Metazoa - ps12
Metazoa - ps13
Myriazoa - ps14
Parahoxozoa - ps15
Bilateria - ps16
Deuterostomia - ps17
Chordata - ps18
Olfactores - ps19
Vertebrata - ps20
Gnathostomata - ps21
Euteleostomi - ps22
Sarcopterygii - ps23
Tetrapoda - ps24
Amniota - ps25
Mammalia - ps26
Theria - ps27
Placentalia - ps28
Boreoeutheria - ps29
Euarchontoglires - ps30
Euarchonta - ps31
Primates - ps32
Haplorrhini - ps33
Simiiformes - ps34
Catarrhini - ps35
Hominoidea - ps36
Hominidae - ps37
Homininae - ps38
Hominini - ps39
Homo_sapiens - ps40
p-value
8e-03
1e-02
2e-02
4e-02
6e-02
Log-odds
0.5
1.0
1.5
2.0
2.5
3.0
3.5
4.0

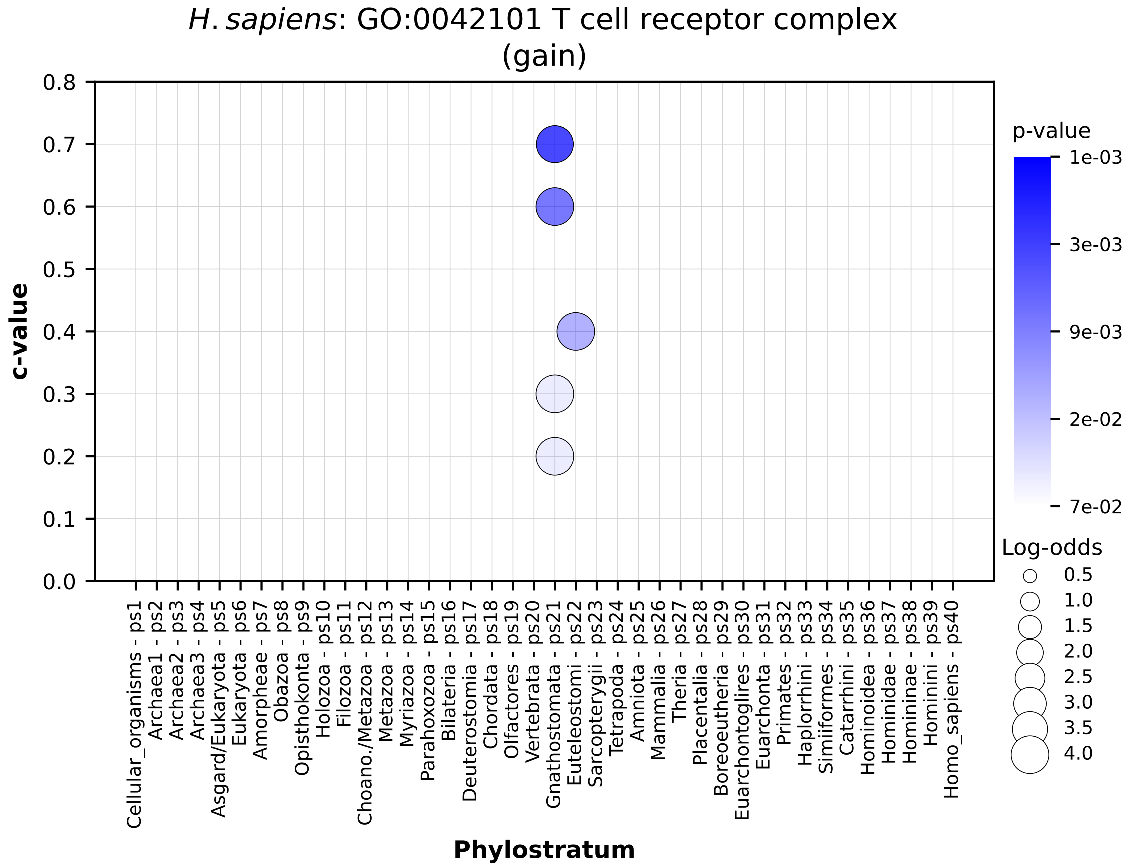

H. sapiens: GO:0042101 T cell receptor complex
(gain)
c-value
0.0
0.1
0.2
0.3
0.4
0.5
0.6
0.7
0.8
Phylostratum
Cellular_organisms - ps1
Archaea1 - ps2
Archaea2 - ps3
Archaea3 - ps4
Asgard/Eukaryota - ps5
Eukaryota - ps6
Amorpheae - ps7
Obazoa - ps8
Opisthokonta - ps9
Holozoa - ps10
Filozoa - ps11
Choano./Metazoa - ps12
Metazoa - ps13
Myriazoa - ps14
Parahoxozoa - ps15
Bilateria - ps16
Deuterostomia - ps17
Chordata - ps18
Olfactores - ps19
Vertebrata - ps20
Gnathostomata - ps21
Euteleostomi - ps22
Sarcopterygii - ps23
Tetrapoda - ps24
Amniota - ps25
Mammalia - ps26
Theria - ps27
Placentalia - ps28
Boreoeutheria - ps29
Euarchontoglires - ps30
Euarchonta - ps31
Primates - ps32
Haplorrhini - ps33
Simiiformes - ps34
Catarrhini - ps35
Hominoidea - ps36
Hominidae - ps37
Homininae - ps38
Hominini - ps39
Homo_sapiens - ps40
p-value
1e-03
3e-03
9e-03
2e-02
7e-02
Log-odds
0.5
1.0
1.5
2.0
2.5
3.0
3.5
4.0

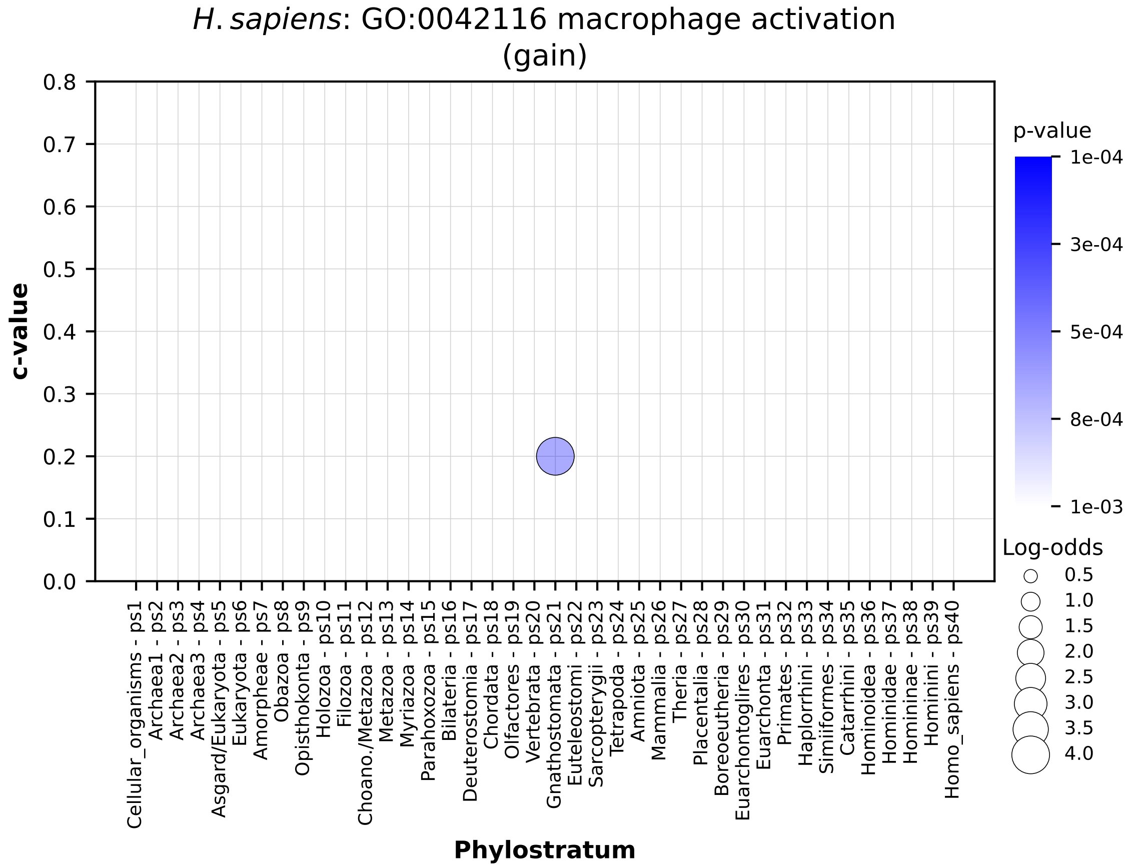
H. sapiens: GO:0042116 macrophage activation
(gain)
c-value
0.0
0.1
0.2
0.3
0.4
0.5
0.6
0.7
0.8
Phylostratum
Cellular_organisms - ps1
Archaea1 - ps2
Archaea2 - ps3
Archaea3 - ps4
Asgard/Eukaryota - ps5
Eukaryota - ps6
Amorpheae - ps7
Obazoa - ps8
Opisthokonta - ps9
Holozoa - ps10
Filozoa - ps11
Choano./Metazoa - ps12
Metazoa - ps13
Myriazoa - ps14
Parahoxozoa - ps15
Bilateria - ps16
Deuterostomia - ps17
Chordata - ps18
Olfactores - ps19
Vertebrata - ps20
Gnathostomata - ps21
Euteleostomi - ps22
Sarcopterygii - ps23
Tetrapoda - ps24
Amniota - ps25
Mammalia - ps26
Theria - ps27
Placentalia - ps28
Boreoeutheria - ps29
Euarchontoglires - ps30
Euarchonta - ps31
Primates - ps32
Haplorrhini - ps33
Simiiformes - ps34
Catarrhini - ps35
Hominoidea - ps36
Hominidae - ps37
Homininae - ps38
Hominini - ps39
Homo_sapiens - ps40
p-value
1e-04
3e-04
5e-04
8e-04
1e-03
Log-odds
0.5
1.0
1.5
2.0
2.5
3.0
3.5
4.0

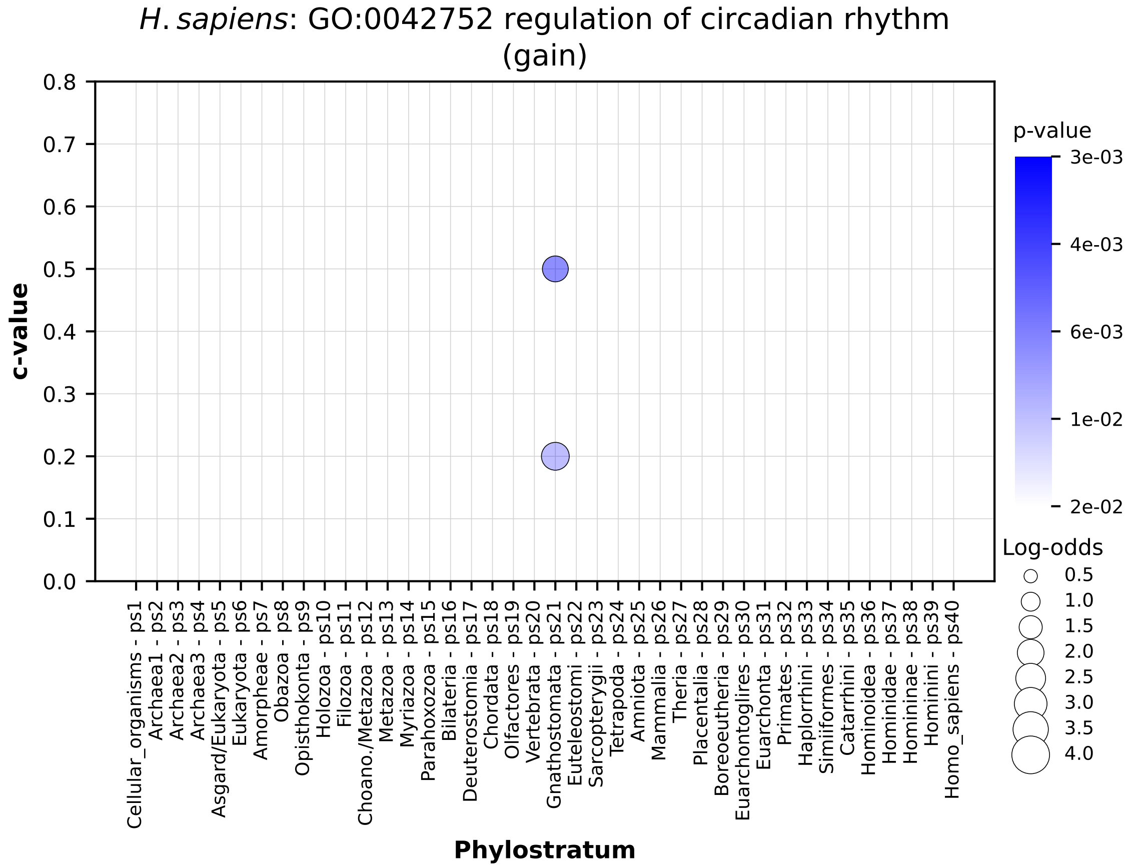
H. sapiens: GO:0042752 regulation of circadian rhythm
(gain)
c-value
0.8
0.7
0.6
0.5
0.4
0.3
0.2
0.1
0.0
Phylostratum
Cellular_organisms - ps1
Archaea1 - ps2
Archaea2 - ps3
Archaea3 - ps4
Asgard/Eukaryota - ps5
Eukaryota - ps6
Amorpheae - ps7
Obazoa - ps8
Opisthokonta - ps9
Holozoa - ps10
Filozoa - ps11
Choano./Metazoa - ps12
Metazoa - ps13
Myriazoa - ps14
Parahoxozoa - ps15
Bilateria - ps16
Deuterostomia - ps17
Chordata - ps18
Olfactores - ps19
Vertebrata - ps20
Gnathostomata - ps21
Euteleostomi - ps22
Sarcopterygii - ps23
Tetrapoda - ps24
Amniota - ps25
Mammalia - ps26
Theria - ps27
Placentalia - ps28
Boreoeutheria - ps29
Euarchontoglires - ps30
Euarchonta - ps31
Primates - ps32
Haplorrhini - ps33
Simiiformes - ps34
Catarrhini - ps35
Hominoidea - ps36
Hominidae - ps37
Homininae - ps38
Hominini - ps39
Homo_sapiens - ps40
p-value
3e-03
4e-03
6e-03
1e-02
2e-02
Log-odds
0.5
1.0
1.5
2.0
2.5
3.0
3.5
4.0

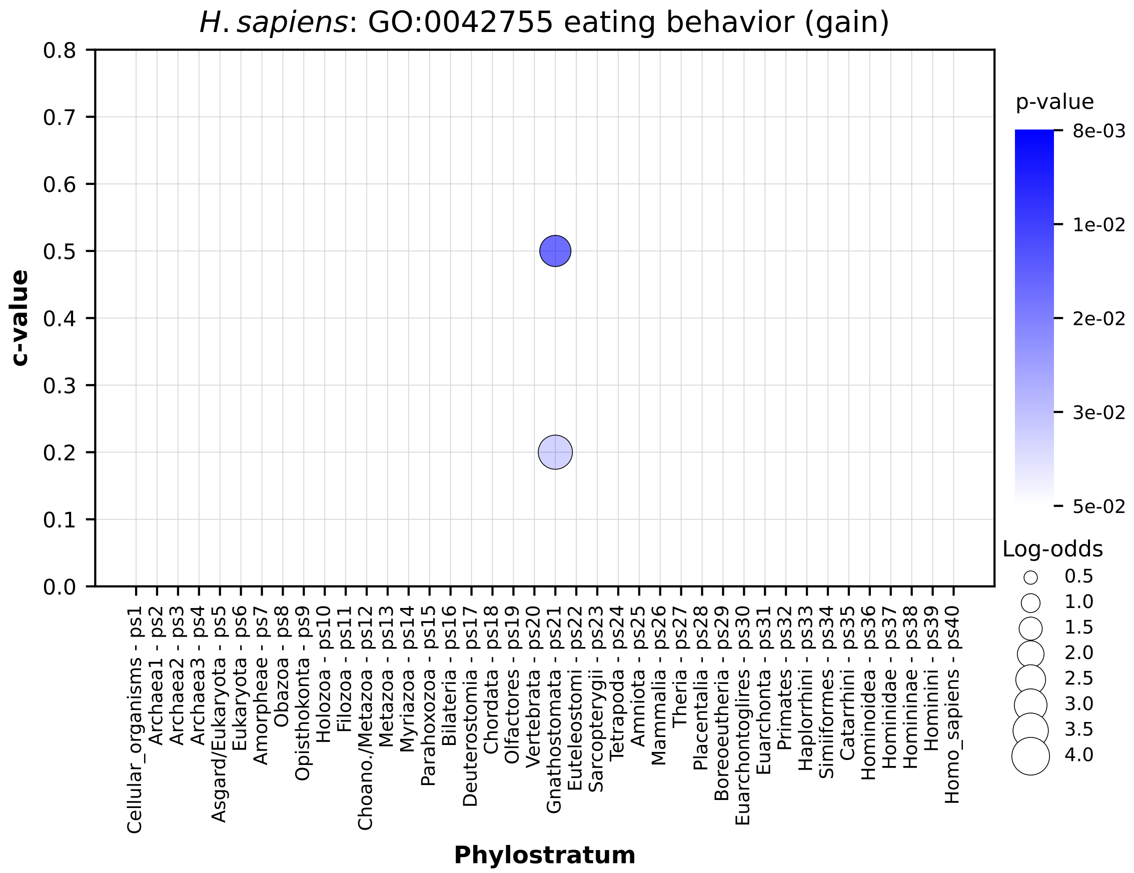
H. sapiens: GO:0042755 eating behavior (gain)
c-value
0.8
0.7
0.6
0.5
0.4
0.3
0.2
0.1
0.0
p-value
8e-03
1e-02
2e-02
3e-02
5e-02
Log-odds
0.5
1.0
1.5
2.0
2.5
3.0
3.5
4.0
Cellular_organisms - ps1
Archaea1 - ps2
Archaea2 - ps3
Archaea3 - ps4
Asgard/Eukaryota - ps5
Eukaryota - ps6
Amorpheae - ps7
Obazoa - ps8
Opisthokonta - ps9
Holozoa - ps10
Filozoa - ps11
Choano./Metazoa - ps12
Metazoa - ps13
Myriazoa - ps14
Parahoxozoa - ps15
Bilateria - ps16
Deuterostomia - ps17
Chordata - ps18
Olfactores - ps19
Vertebrata - ps20
Gnathostomata - ps21
Euteleostomi - ps22
Sarcopterygii - ps23
Tetrapoda - ps24
Amniota - ps25
Mammalia - ps26
Theria - ps27
Placentalia - ps28
Boreoeutheria - ps29
Euarchontoglires - ps30
Euarchonta - ps31
Primates - ps32
Haplorrhini - ps33
Simiiformes - ps34
Catarrhini - ps35
Hominoidea - ps36
Hominidae - ps37
Homininae - ps38
Hominini - ps39
Homo_sapiens - ps40
Phylostratum

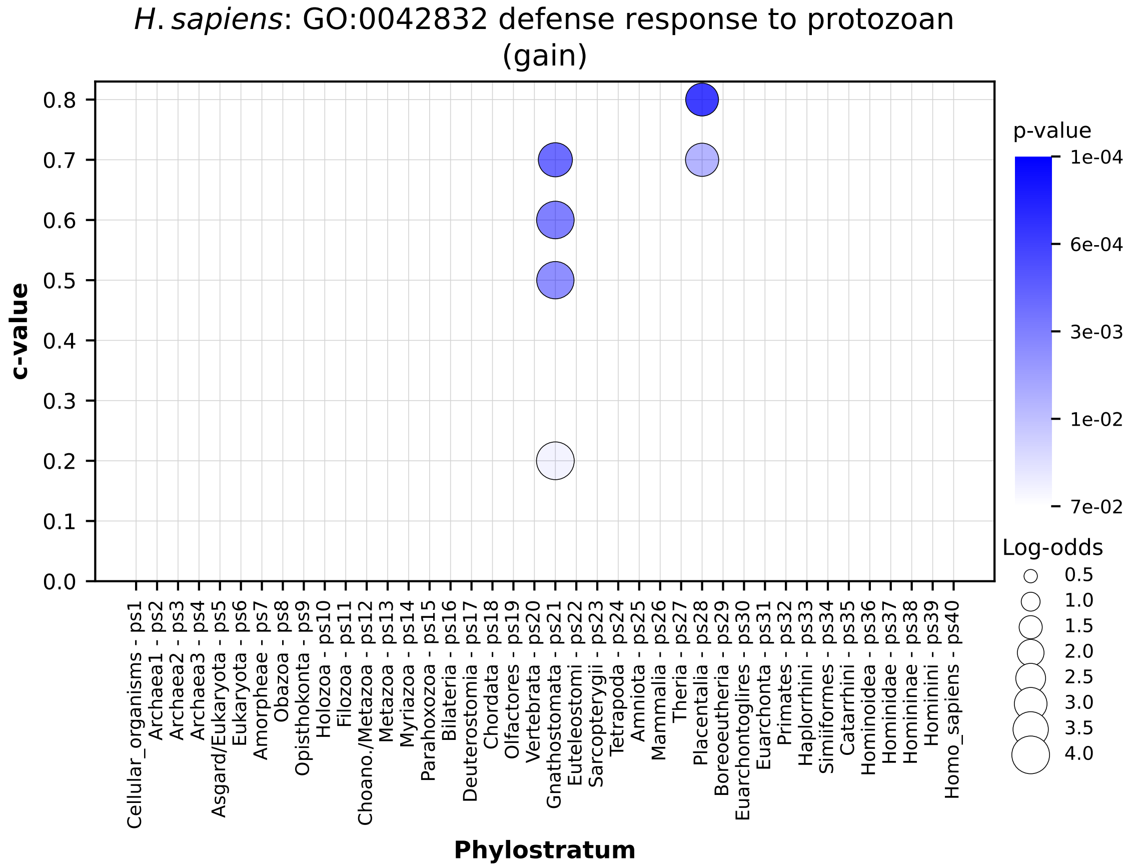

*H. sapiens*: GO:0042832 defense response to protozoan (gain)
c-value
0.0
0.1
0.2
0.3
0.4
0.5
0.6
0.7
0.8
p-value
1e-04
6e-04
3e-03
1e-02
7e-02
Log-odds
0.5
1.0
1.5
2.0
2.5
3.0
3.5
4.0
Cellular_organisms - ps1
Archaea1 - ps2
Archaea2 - ps3
Archaea3 - ps4
Asgard/Eukaryota - ps5
Eukaryota - ps6
Amorpheae - ps7
Obazoa - ps8
Opisthokonta - ps9
Holozoa - ps10
Filozoa - ps11
Choano./Metazoa - ps12
Metazoa - ps13
Myriazoa - ps14
Parahoxozoa - ps15
Bilateria - ps16
Deuterostomia - ps17
Chordata - ps18
Olfactores - ps19
Vertebrata - ps20
Gnathostomata - ps21
Euteleostomi - ps22
Sarcopterygii - ps23
Tetrapoda - ps24
Amniota - ps25
Mammalia - ps26
Theria - ps27
Placentalia - ps28
Boreoeutheria - ps29
Euarchontoglires - ps30
Euarchonta - ps31
Primates - ps32
Haplorrhini - ps33
Simiiformes - ps34
Catarrhini - ps35
Hominoidea - ps36
Hominidae - ps37
Homininae - ps38
Hominini - ps39
Homo_sapiens - ps40
Phylostratum

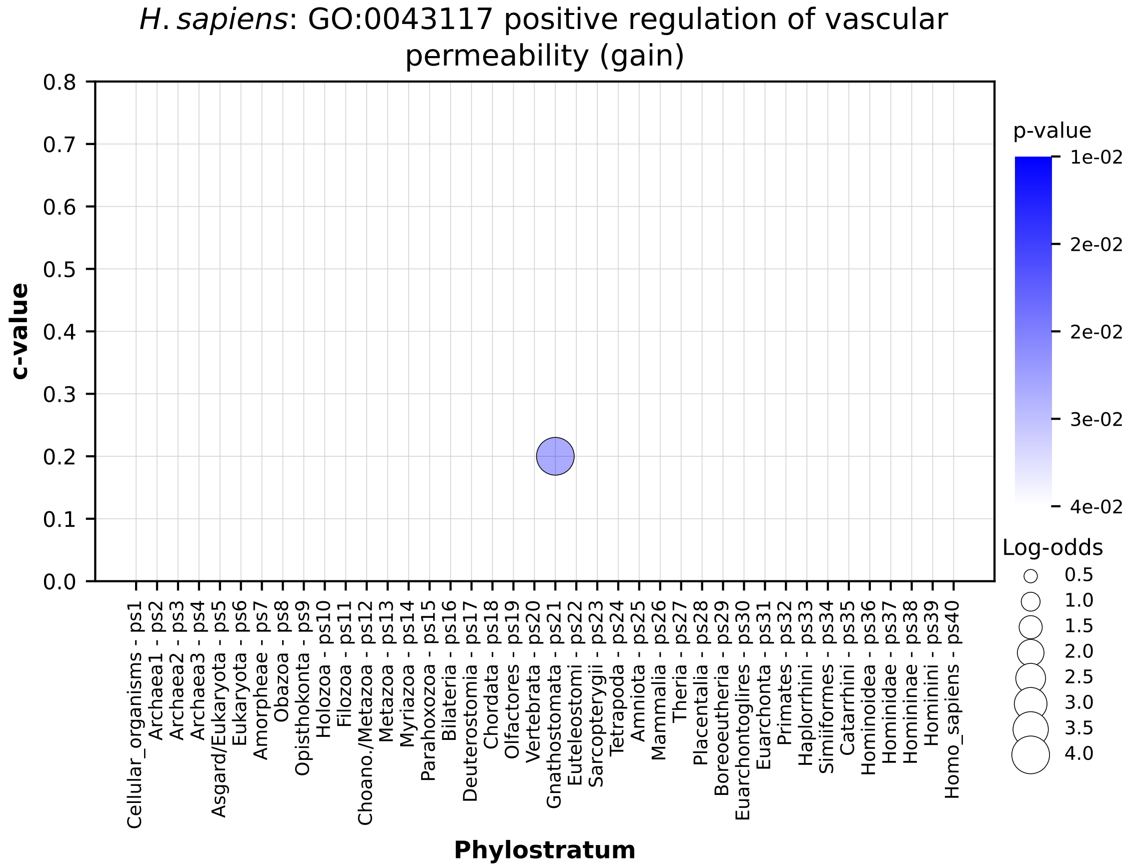

H. sapiens: GO:0043117 positive regulation of vascular permeability (gain)
c-value
0.0
0.1
0.2
0.3
0.4
0.5
0.6
0.7
0.8
Phylostratum
Cellular_organisms - ps1
Archaea1 - ps2
Archaea2 - ps3
Archaea3 - ps4
Asgard/Eukaryota - ps5
Eukaryota - ps6
Amorpheae - ps7
Obazoa - ps8
Opisthokonta - ps9
Holozoa - ps10
Filozoa - ps11
Choano./Metazoa - ps12
Metazoa - ps13
Myriazoa - ps14
Parahoxozoa - ps15
Bilateria - ps16
Deuterostomia - ps17
Chordata - ps18
Olfactores - ps19
Vertebrata - ps20
Gnathostomata - ps21
Euteleostomi - ps22
Sarcopterygii - ps23
Tetrapoda - ps24
Amniota - ps25
Mammalia - ps26
Theria - ps27
Placentalia - ps28
Boreoeutheria - ps29
Euarchontoglires - ps30
Euarchonta - ps31
Primates - ps32
Haplorrhini - ps33
Simiiformes - ps34
Catarrhini - ps35
Hominoidea - ps36
Hominidae - ps37
Homininae - ps38
Hominini - ps39
Homo_sapiens - ps40
p-value
1e-02
2e-02
2e-02
3e-02
4e-02
Log-odds
0.5
1.0
1.5
2.0
2.5
3.0
3.5
4.0

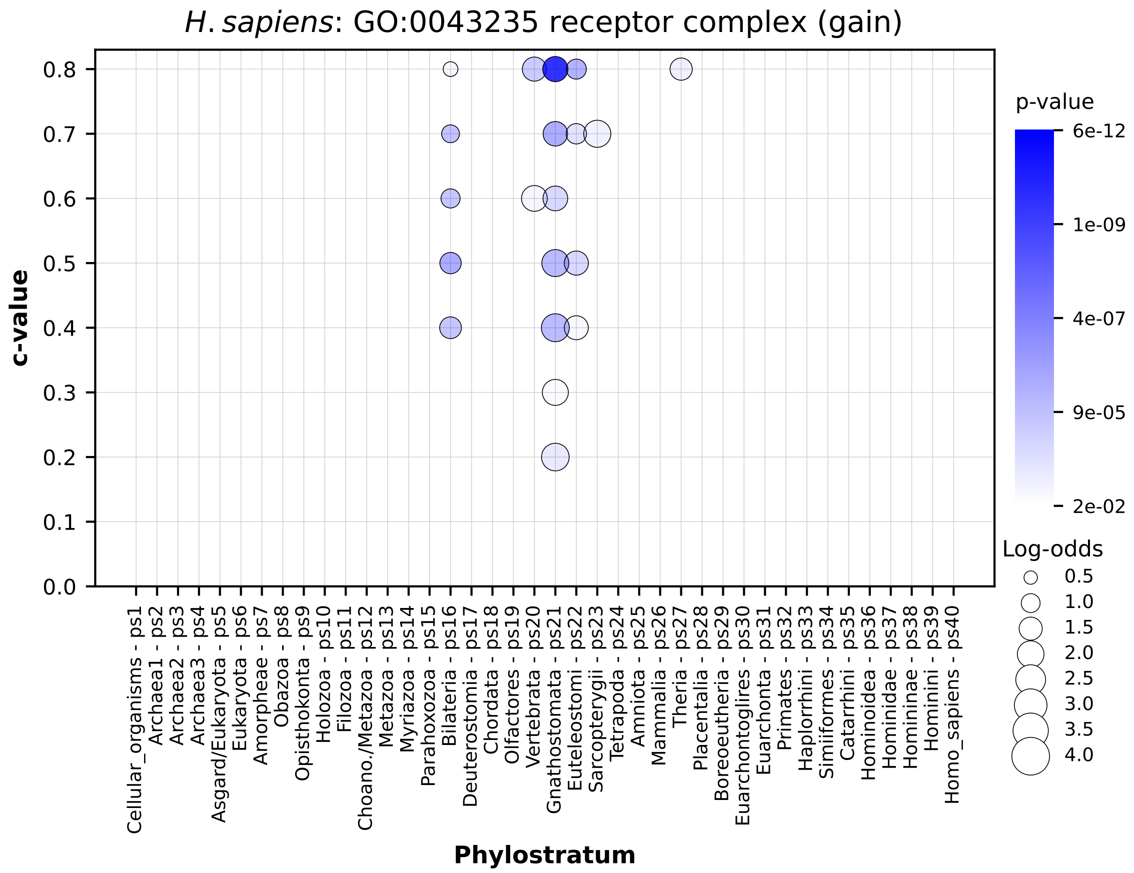

*H. sapiens*: GO:0043235 receptor complex (gain)
c-value
0.0
0.1
0.2
0.3
0.4
0.5
0.6
0.7
0.8
Phylostratum
Cellular_organisms - ps1
Archaea1 - ps2
Archaea2 - ps3
Archaea3 - ps4
Asgard/Eukaryota - ps5
Eukaryota - ps6
Amorpheae - ps7
Obazoa - ps8
Opisthokonta - ps9
Holozoa - ps10
Filozoa - ps11
Choano./Metazoa - ps12
Metazoa - ps13
Myriazoa - ps14
Parahoxozoa - ps15
Bilateria - ps16
Deuterostomia - ps17
Chordata - ps18
Olfactores - ps19
Vertebrata - ps20
Gnathostomata - ps21
Euteleostomi - ps22
Sarcopterygii - ps23
Tetrapoda - ps24
Amniota - ps25
Mammalia - ps26
Theria - ps27
Placentalia - ps28
Boreoeutheria - ps29
Euarchontoglires - ps30
Euarchonta - ps31
Primates - ps32
Haplorrhini - ps33
Simiiformes - ps34
Catarrhini - ps35
Hominoidea - ps36
Hominidae - ps37
Homininae - ps38
Hominini - ps39
Homo_sapiens - ps40
p-value
6e-12
1e-09
4e-07
9e-05
2e-02
Log-odds
0.5
1.0
1.5
2.0
2.5
3.0
3.5
4.0

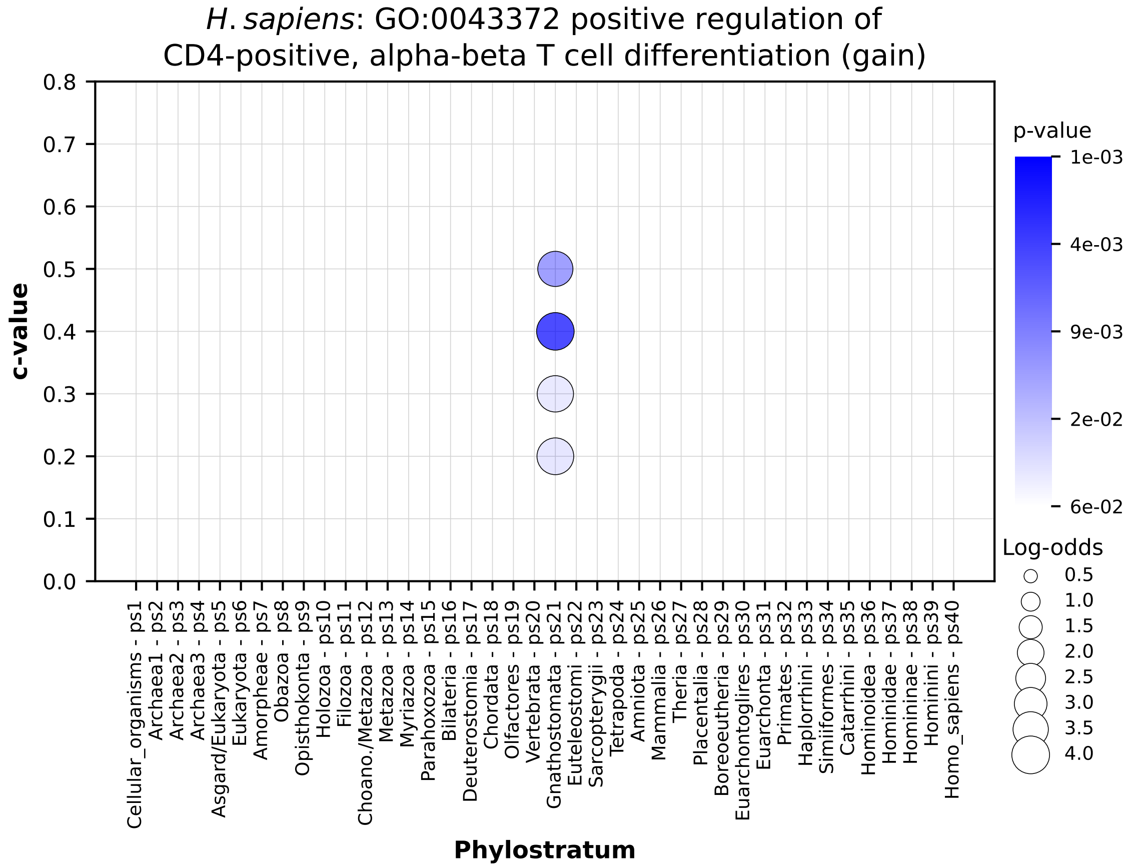

H. sapiens: GO:0043372 positive regulation of
CD4-positive, alpha-beta T cell differentiation (gain)
c-value
0.8
0.7
0.6
0.5
0.4
0.3
0.2
0.1
0.0
Phylostratum
Cellular_organisms - ps1
Archaea1 - ps2
Archaea2 - ps3
Archaea3 - ps4
Asgard/Eukaryota - ps5
Eukaryota - ps6
Amorpheae - ps7
Obazoa - ps8
Opisthokonta - ps9
Holozoa - ps10
Filozoa - ps11
Choano./Metazoa - ps12
Metazoa - ps13
Myriazoa - ps14
Parahoxozoa - ps15
Bilateria - ps16
Deuterostomia - ps17
Chordata - ps18
Olfactores - ps19
Vertebrata - ps20
Gnathostomata - ps21
Euteleostomi - ps22
Sarcopterygii - ps23
Tetrapoda - ps24
Amniota - ps25
Mammalia - ps26
Theria - ps27
Placentalia - ps28
Boreoeutheria - ps29
Euarchontoglires - ps30
Euarchonta - ps31
Primates - ps32
Haplorrhini - ps33
Simiiformes - ps34
Catarrhini - ps35
Hominoidea - ps36
Hominidae - ps37
Homininae - ps38
Hominini - ps39
Homo_sapiens - ps40
p-value
1e-03
4e-03
9e-03
2e-02
6e-02
Log-odds
0.5
1.0
1.5
2.0
2.5
3.0
3.5
4.0

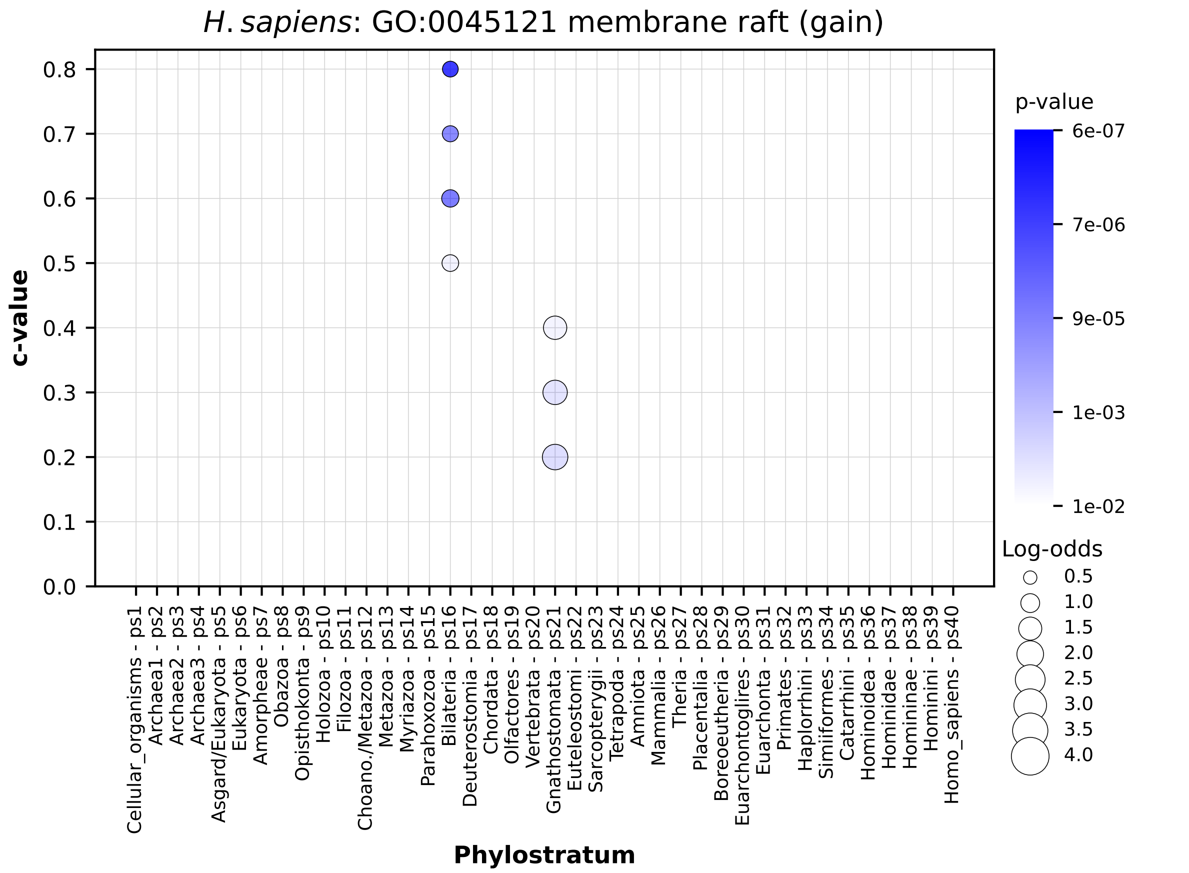

H. sapiens: GO:0045121 membrane raft (gain)
c-value
Phylostratum
p-value
6e-07
7e-06
9e-05
1e-03
1e-02
Log-odds
0.5
1.0
1.5
2.0
2.5
3.0
3.5
4.0
Cellular_organisms - ps1
Archaea1 - ps2
Archaea2 - ps3
Archaea3 - ps4
Asgard/Eukaryota - ps5
Eukaryota - ps6
Amorpheae - ps7
Obazoa - ps8
Opisthokonta - ps9
Holozoa - ps10
Filozoa - ps11
Choano./Metazoa - ps12
Metazoa - ps13
Myriazoa - ps14
Parahoxozoa - ps15
Bilateria - ps16
Deuterostomia - ps17
Chordata - ps18
Olfactores - ps19
Vertebrata - ps20
Gnathostomata - ps21
Euteleostomi - ps22
Sarcopterygii - ps23
Tetrapoda - ps24
Amniota - ps25
Mammalia - ps26
Theria - ps27
Placentalia - ps28
Boreoeutheria - ps29
Euarchontoglires - ps30
Euarchonta - ps31
Primates - ps32
Haplorrhini - ps33
Simiiformes - ps34
Catarrhini - ps35
Hominoidea - ps36
Hominidae - ps37
Homininae - ps38
Hominini - ps39
Homo_sapiens - ps40

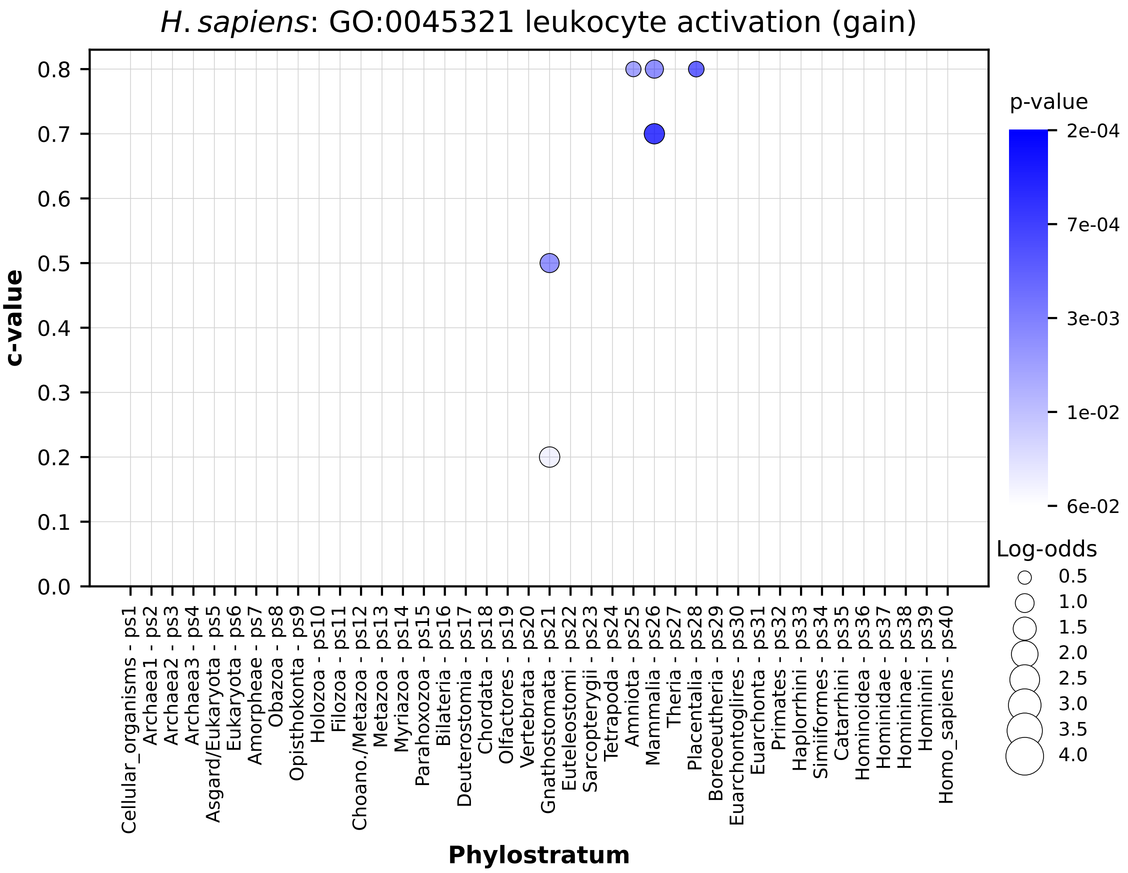

H. sapiens: GO:0045321 leukocyte activation (gain)
c-value
0.0
0.1
0.2
0.3
0.4
0.5
0.6
0.7
0.8
p-value
2e-04
7e-04
3e-03
1e-02
6e-02
Log-odds
0.5
1.0
1.5
2.0
2.5
3.0
3.5
4.0
Cellular_organisms - ps1
Archaea1 - ps2
Archaea2 - ps3
Archaea3 - ps4
Asgard/Eukaryota - ps5
Eukaryota - ps6
Amorpheae - ps7
Obazoa - ps8
Opisthokonta - ps9
Holozoa - ps10
Filozoa - ps11
Choano./Metazoa - ps12
Metazoa - ps13
Myriazoa - ps14
Parahoxozoa - ps15
Bilateria - ps16
Deuterostomia - ps17
Chordata - ps18
Olfactores - ps19
Vertebrata - ps20
Gnathostomata - ps21
Euteleostomi - ps22
Sarcopterygii - ps23
Tetrapoda - ps24
Amniota - ps25
Mammalia - ps26
Theria - ps27
Placentalia - ps28
Boreoeutheria - ps29
Euarchontoglires - ps30
Euarchonta - ps31
Primates - ps32
Haplorrhini - ps33
Simiiformes - ps34
Catarrhini - ps35
Hominoidea - ps36
Hominidae - ps37
Homininae - ps38
Hominini - ps39
Homo_sapiens - ps40
Phylostratum

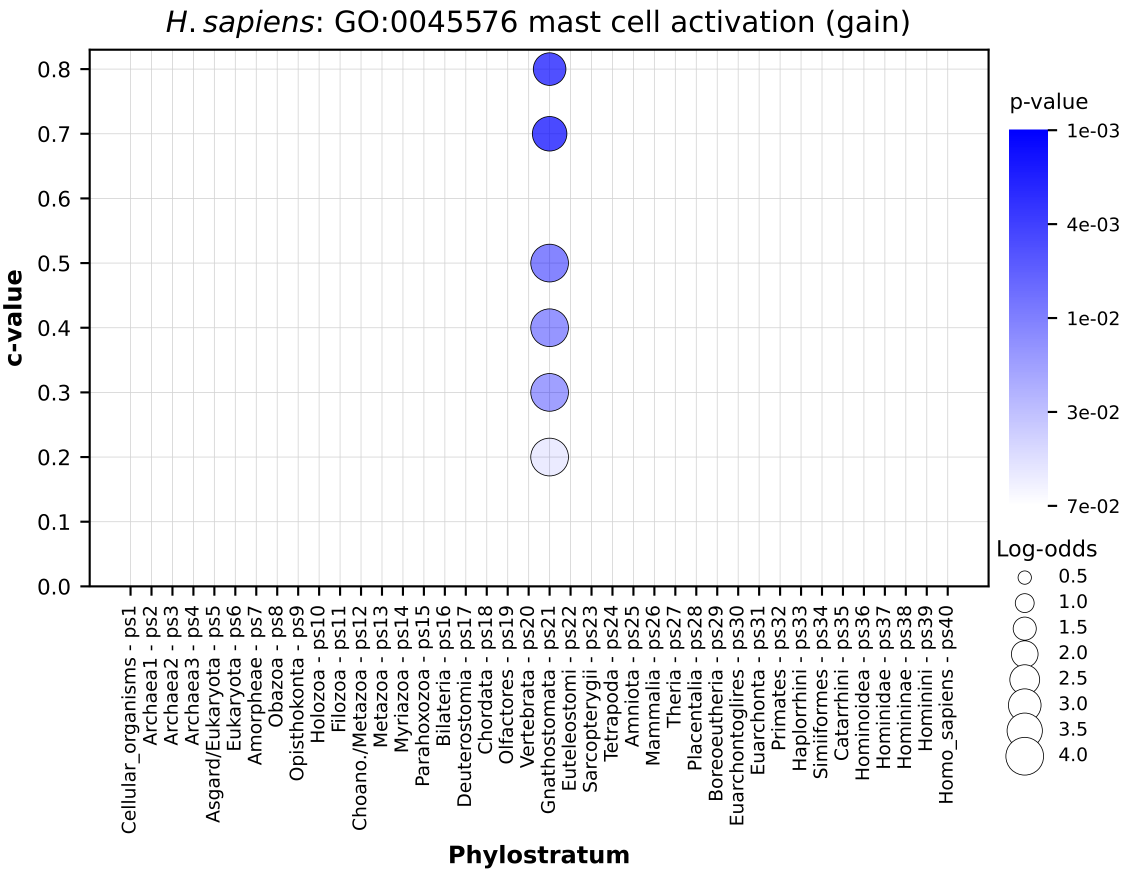

H. sapiens: GO:0045576 mast cell activation (gain)
c-value
0.0
0.1
0.2
0.3
0.4
0.5
0.6
0.7
0.8
Phylostratum
Cellular_organisms - ps1
Archaea1 - ps2
Archaea2 - ps3
Archaea3 - ps4
Asgard/Eukaryota - ps5
Eukaryota - ps6
Amorpheae - ps7
Obazoa - ps8
Opisthokonta - ps9
Holozoa - ps10
Filozoa - ps11
Choano./Metazoa - ps12
Metazoa - ps13
Myriazoa - ps14
Parahoxozoa - ps15
Bilateria - ps16
Deuterostomia - ps17
Chordata - ps18
Olfactores - ps19
Vertebrata - ps20
Gnathostomata - ps21
Euteleostomi - ps22
Sarcopterygii - ps23
Tetrapoda - ps24
Amniota - ps25
Mammalia - ps26
Theria - ps27
Placentalia - ps28
Boreoeutheria - ps29
Euarchontoglires - ps30
Euarchonta - ps31
Primates - ps32
Haplorrhini - ps33
Simiiformes - ps34
Catarrhini - ps35
Hominoidea - ps36
Hominidae - ps37
Homininae - ps38
Hominini - ps39
Homo_sapiens - ps40
p-value
1e-03
4e-03
1e-02
3e-02
7e-02
Log-odds
0.5
1.0
1.5
2.0
2.5
3.0
3.5
4.0

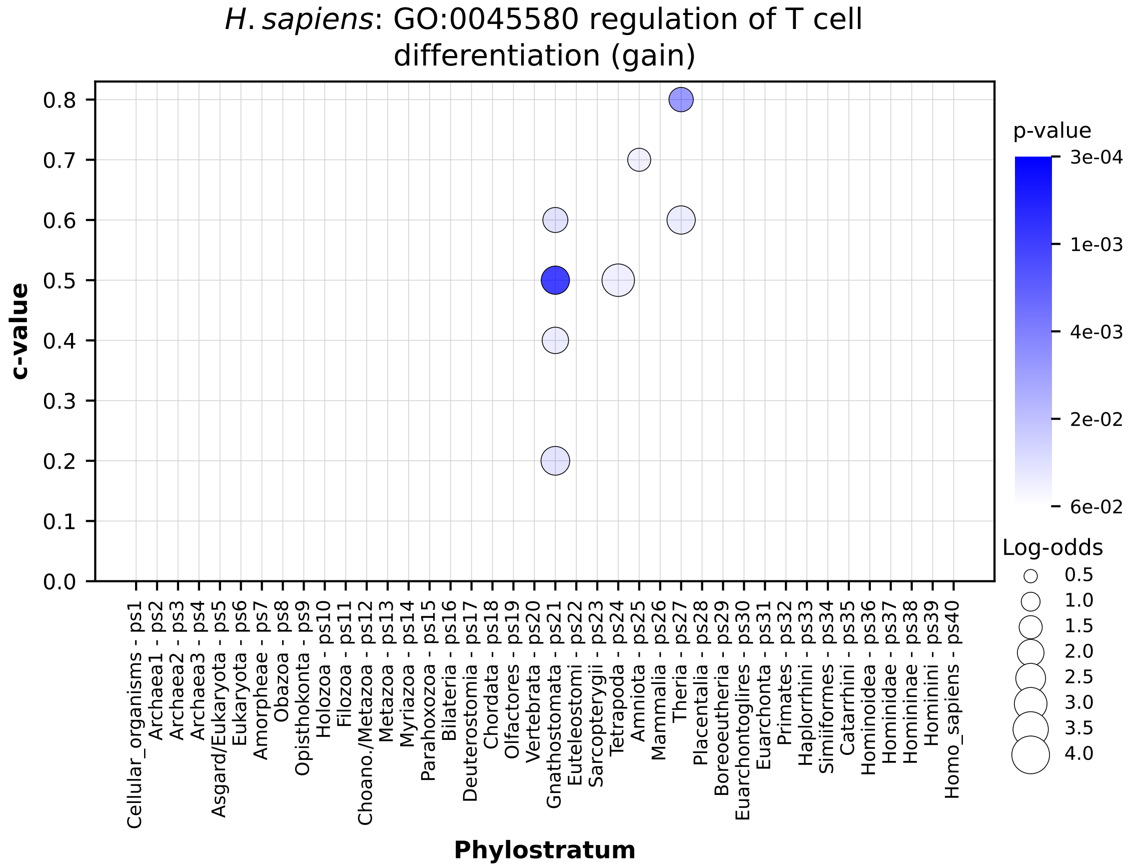

H. sapiens: GO:0045580 regulation of T cell differentiation (gain)
c-value
0.0
0.1
0.2
0.3
0.4
0.5
0.6
0.7
0.8
Phylostratum
Cellular_organisms - ps1
Archaea1 - ps2
Archaea2 - ps3
Archaea3 - ps4
Asgard/Eukaryota - ps5
Eukaryota - ps6
Amorpheae - ps7
Obazoa - ps8
Opisthokonta - ps9
Holozoa - ps10
Filozoa - ps11
Choano./Metazoa - ps12
Metazoa - ps13
Myriazoa - ps14
Parahoxozoa - ps15
Bilateria - ps16
Deuterostomia - ps17
Chordata - ps18
Olfactores - ps19
Vertebrata - ps20
Gnathostomata - ps21
Euteleostomi - ps22
Sarcopterygii - ps23
Tetrapoda - ps24
Amniota - ps25
Mammalia - ps26
Theria - ps27
Placentalia - ps28
Boreoeutheria - ps29
Euarchontoglires - ps30
Euarchonta - ps31
Primates - ps32
Haplorrhini - ps33
Simiiformes - ps34
Catarrhini - ps35
Hominoidea - ps36
Hominidae - ps37
Homininae - ps38
Hominini - ps39
Homo_sapiens - ps40
p-value
3e-04
1e-03
4e-03
2e-02
6e-02
Log-odds
0.5
1.0
1.5
2.0
2.5
3.0
3.5
4.0

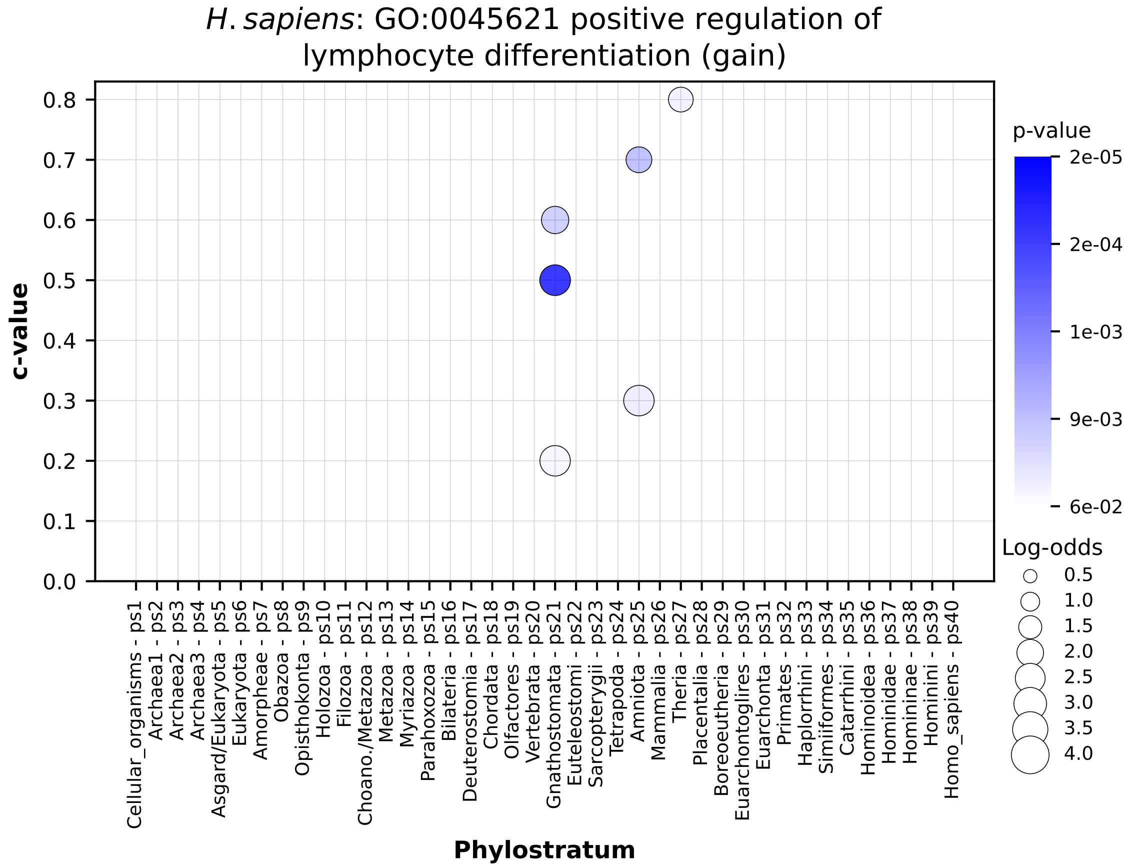

H. sapiens: GO:0045621 positive regulation of lymphocyte differentiation (gain)
c-value
0.0
0.1
0.2
0.3
0.4
0.5
0.6
0.7
0.8
Phylostratum
Cellular_organisms - ps1
Archaea1 - ps2
Archaea2 - ps3
Archaea3 - ps4
Asgard/Eukaryota - ps5
Eukaryota - ps6
Amorpheae - ps7
Obazoa - ps8
Opisthokonta - ps9
Holozoa - ps10
Filozoa - ps11
Choano./Metazoa - ps12
Metazoa - ps13
Myriazoa - ps14
Parahoxozoa - ps15
Bilateria - ps16
Deuterostomia - ps17
Chordata - ps18
Olfactores - ps19
Vertebrata - ps20
Gnathostomata - ps21
Euteleostomi - ps22
Sarcopterygii - ps23
Tetrapoda - ps24
Amniota - ps25
Mammalia - ps26
Theria - ps27
Placentalia - ps28
Boreoeutheria - ps29
Euarchontoglires - ps30
Euarchonta - ps31
Primates - ps32
Haplorrhini - ps33
Simiiformes - ps34
Catarrhini - ps35
Hominoidea - ps36
Hominidae - ps37
Homininae - ps38
Hominini - ps39
Homo_sapiens - ps40
p-value
2e-05
2e-04
1e-03
9e-03
6e-02
Log-odds
0.5
1.0
1.5
2.0
2.5
3.0
3.5
4.0

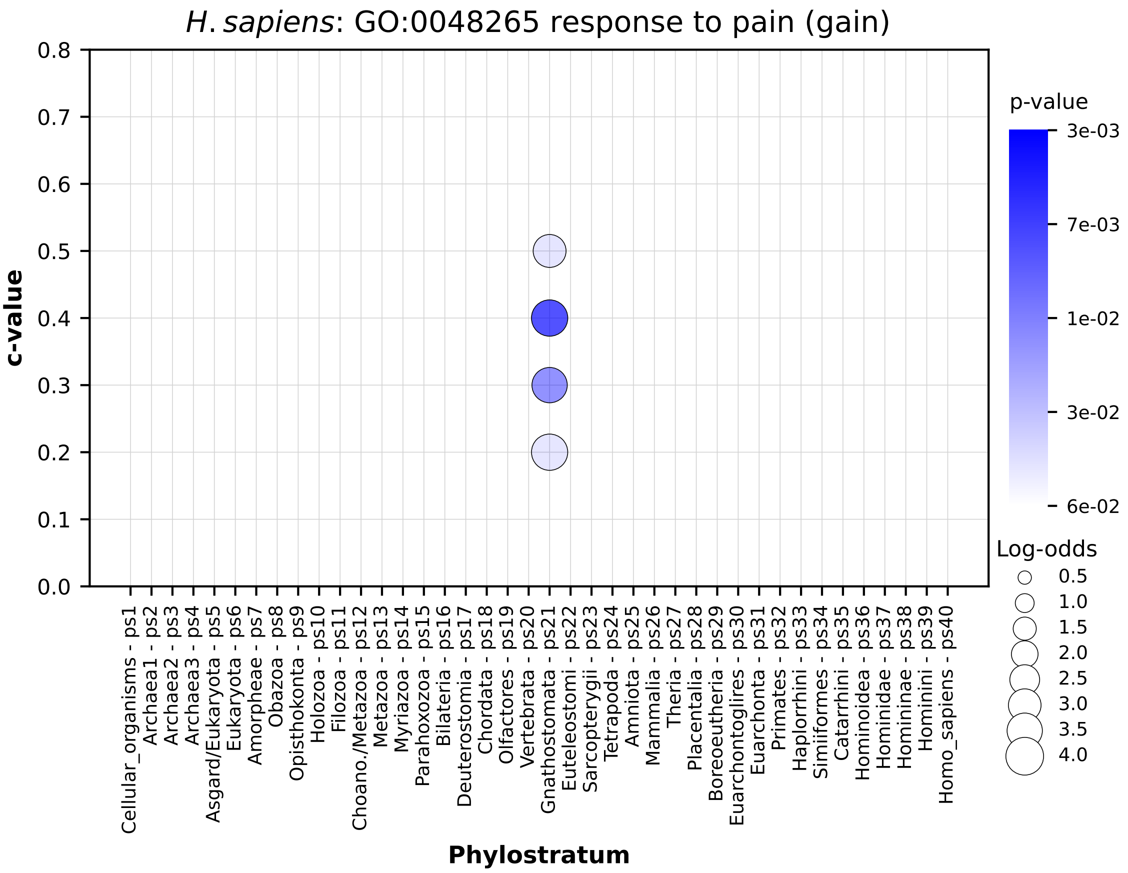

H. sapiens: GO:0048265 response to pain (gain)
c-value
0.0
0.1
0.2
0.3
0.4
0.5
0.6
0.7
0.8
Phylostratum
Cellular_organisms - ps1
Archaea1 - ps2
Archaea2 - ps3
Archaea3 - ps4
Asgard/Eukaryota - ps5
Eukaryota - ps6
Amorpheae - ps7
Obazoa - ps8
Opisthokonta - ps9
Holozoa - ps10
Filozoa - ps11
Choano./Metazoa - ps12
Metazoa - ps13
Myriazoa - ps14
Parahoxozoa - ps15
Bilateria - ps16
Deuterostomia - ps17
Chordata - ps18
Olfactores - ps19
Vertebrata - ps20
Gnathostomata - ps21
Euteleostomi - ps22
Sarcopterygii - ps23
Tetrapoda - ps24
Amniota - ps25
Mammalia - ps26
Theria - ps27
Placentalia - ps28
Boreoeutheria - ps29
Euarchontoglires - ps30
Euarchonta - ps31
Primates - ps32
Haplorrhini - ps33
Simiiformes - ps34
Catarrhini - ps35
Hominoidea - ps36
Hominidae - ps37
Homininae - ps38
Hominini - ps39
Homo_sapiens - ps40
p-value
3e-03
7e-03
1e-02
3e-02
6e-02
Log-odds
0.5
1.0
1.5
2.0
2.5
3.0
3.5
4.0

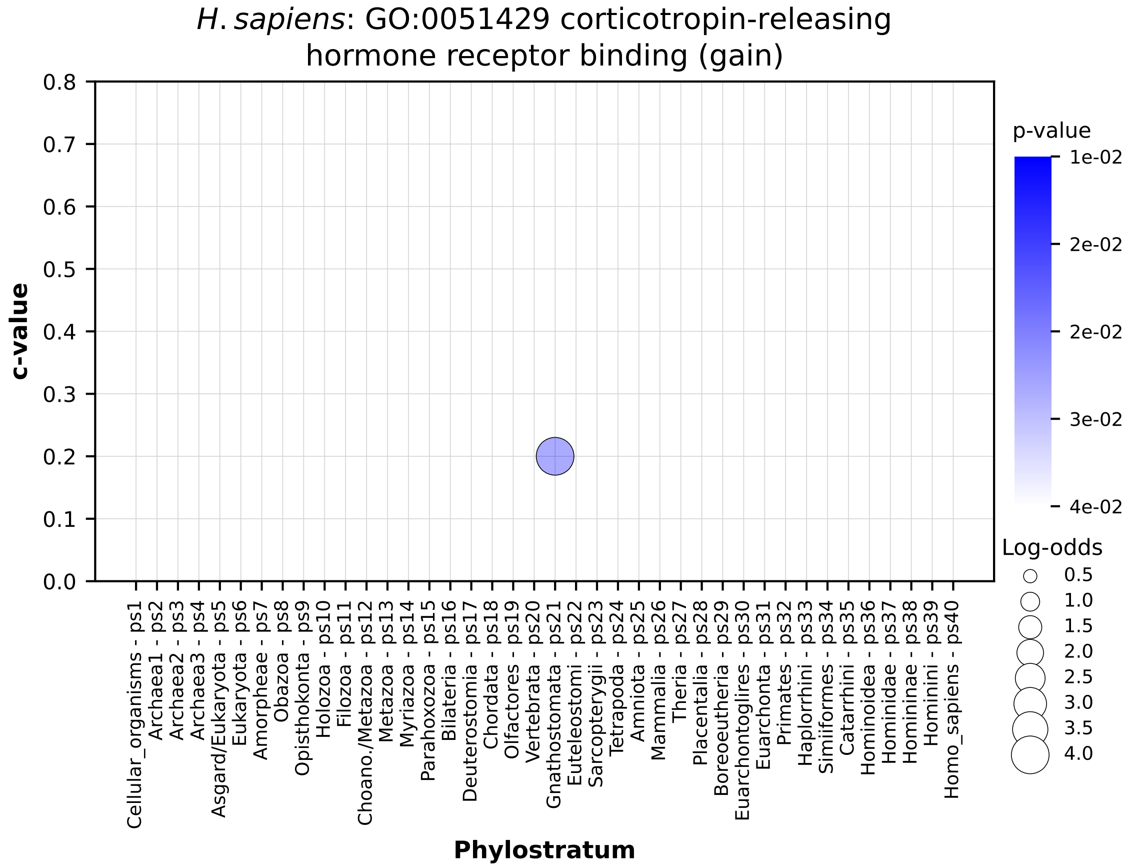

H. sapiens: GO:0051429 corticotropin-releasing hormone receptor binding (gain)
c-value
0.0
0.1
0.2
0.3
0.4
0.5
0.6
0.7
0.8
Phylostratum
Cellular_organisms - ps1
Archaea1 - ps2
Archaea2 - ps3
Archaea3 - ps4
Asgard/Eukaryota - ps5
Eukaryota - ps6
Amorpheae - ps7
Obazoa - ps8
Opisthokonta - ps9
Holozoa - ps10
Filozoa - ps11
Choano./Metazoa - ps12
Metazoa - ps13
Myriazoa - ps14
Parahoxozoa - ps15
Bilateria - ps16
Deuterostomia - ps17
Chordata - ps18
Olfactores - ps19
Vertebrata - ps20
Gnathostomata - ps21
Euteleostomi - ps22
Sarcopterygii - ps23
Tetrapoda - ps24
Amniota - ps25
Mammalia - ps26
Theria - ps27
Placentalia - ps28
Boreoeutheria - ps29
Euarchontoglires - ps30
Euarchonta - ps31
Primates - ps32
Haplorrhini - ps33
Simiiformes - ps34
Catarrhini - ps35
Hominoidea - ps36
Hominidae - ps37
Homininae - ps38
Hominini - ps39
Homo_sapiens - ps40
p-value
1e-02
2e-02
2e-02
3e-02
4e-02
Log-odds
0.5
1.0
1.5
2.0
2.5
3.0
3.5
4.0

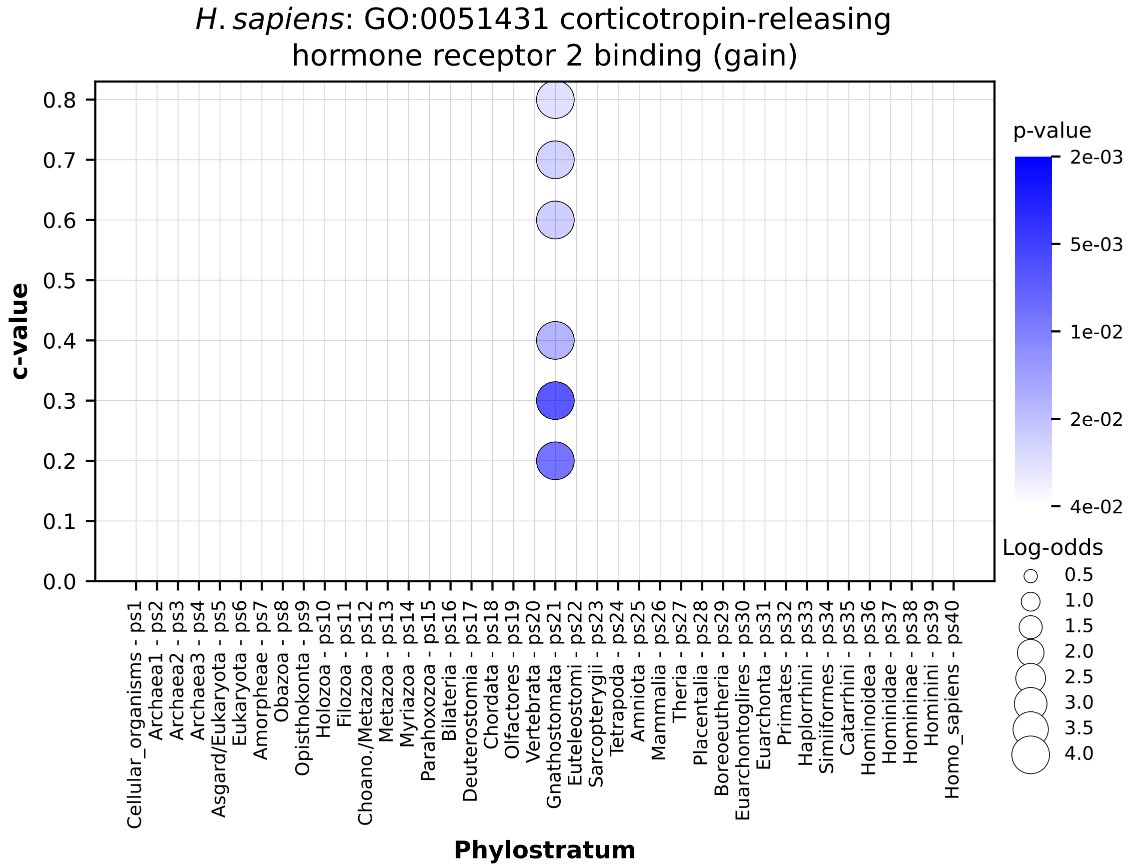

*H. sapiens*: GO:0051431 corticotropin-releasing hormone receptor 2 binding (gain)
c-value
0.8
0.7
0.6
0.5
0.4
0.3
0.2
0.1
0.0
p-value
2e-03
5e-03
1e-02
2e-02
4e-02
Log-odds
0.5
1.0
1.5
2.0
2.5
3.0
3.5
4.0
Cellular_organisms - ps1
Archaea1 - ps2
Archaea2 - ps3
Archaea3 - ps4
Asgard/Eukaryota - ps5
Eukaryota - ps6
Amorpheae - ps7
Obazoa - ps8
Opisthokonta - ps9
Holozoa - ps10
Filozoa - ps11
Choano./Metazoa - ps12
Metazoa - ps13
Myriazoa - ps14
Parahoxozoa - ps15
Bilateria - ps16
Deuterostomia - ps17
Chordata - ps18
Olfactores - ps19
Vertebrata - ps20
Gnathostomata - ps21
Euteleostomi - ps22
Sarcopterygii - ps23
Tetrapoda - ps24
Amniota - ps25
Mammalia - ps26
Theria - ps27
Placentalia - ps28
Boreoeutheria - ps29
Euarchontoglires - ps30
Euarchonta - ps31
Primates - ps32
Haplorrhini - ps33
Simiiformes - ps34
Catarrhini - ps35
Hominoidea - ps36
Hominidae - ps37
Homininae - ps38
Hominini - ps39
Homo_sapiens - ps40
Phylostratum

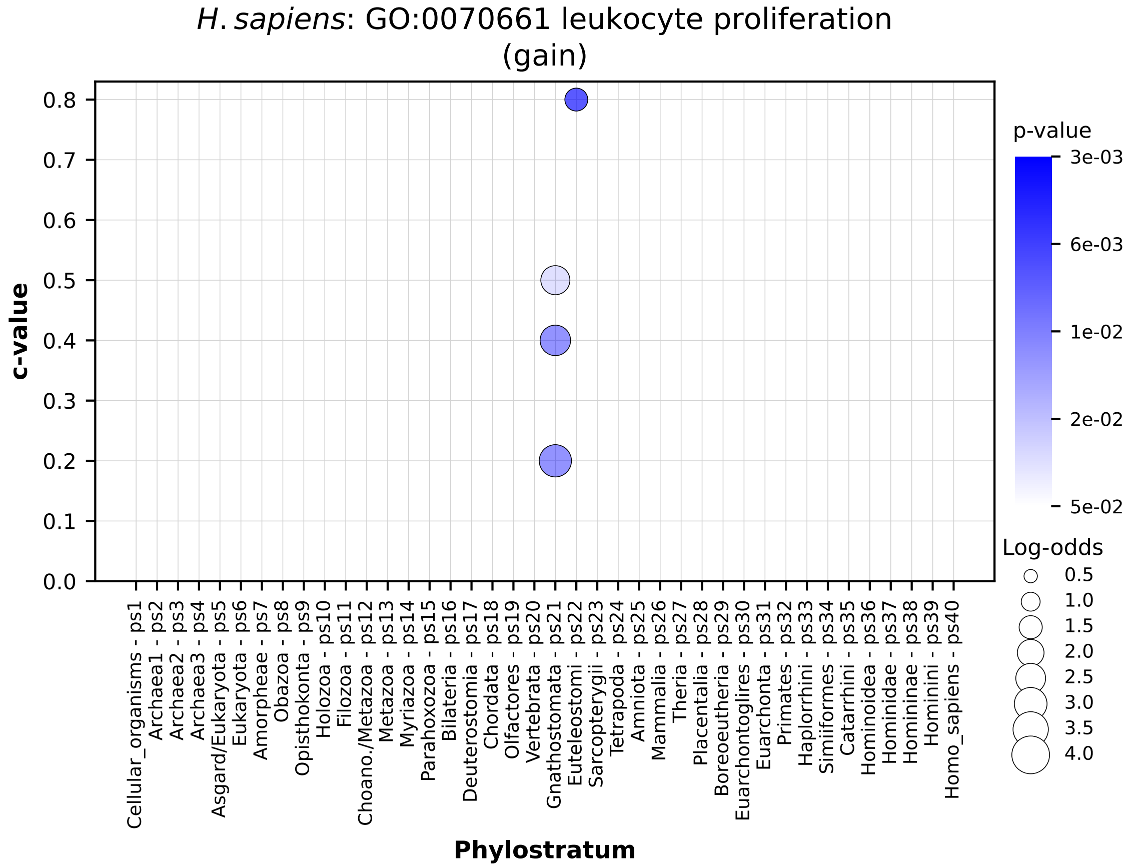

H. sapiens: GO:0070661 leukocyte proliferation
(gain)
c-value
0.0
0.1
0.2
0.3
0.4
0.5
0.6
0.7
0.8
Phylostratum
Cellular_organisms - ps1
Archaea1 - ps2
Archaea2 - ps3
Archaea3 - ps4
Asgard/Eukaryota - ps5
Eukaryota - ps6
Amorpheae - ps7
Obazoa - ps8
Opisthokonta - ps9
Holozoa - ps10
Filozoa - ps11
Choano./Metazoa - ps12
Metazoa - ps13
Myriazoa - ps14
Parahoxozoa - ps15
Bilateria - ps16
Deuterostomia - ps17
Chordata - ps18
Olfactores - ps19
Vertebrata - ps20
Gnathostomata - ps21
Euteleostomi - ps22
Sarcopterygii - ps23
Tetrapoda - ps24
Amniota - ps25
Mammalia - ps26
Theria - ps27
Placentalia - ps28
Boreoeutheria - ps29
Euarchontoglires - ps30
Euarchonta - ps31
Primates - ps32
Haplorrhini - ps33
Simiiformes - ps34
Catarrhini - ps35
Hominoidea - ps36
Hominidae - ps37
Homininae - ps38
Hominini - ps39
Homo_sapiens - ps40
p-value
3e-03
6e-03
1e-02
2e-02
5e-02
Log-odds
0.5
1.0
1.5
2.0
2.5
3.0
3.5
4.0

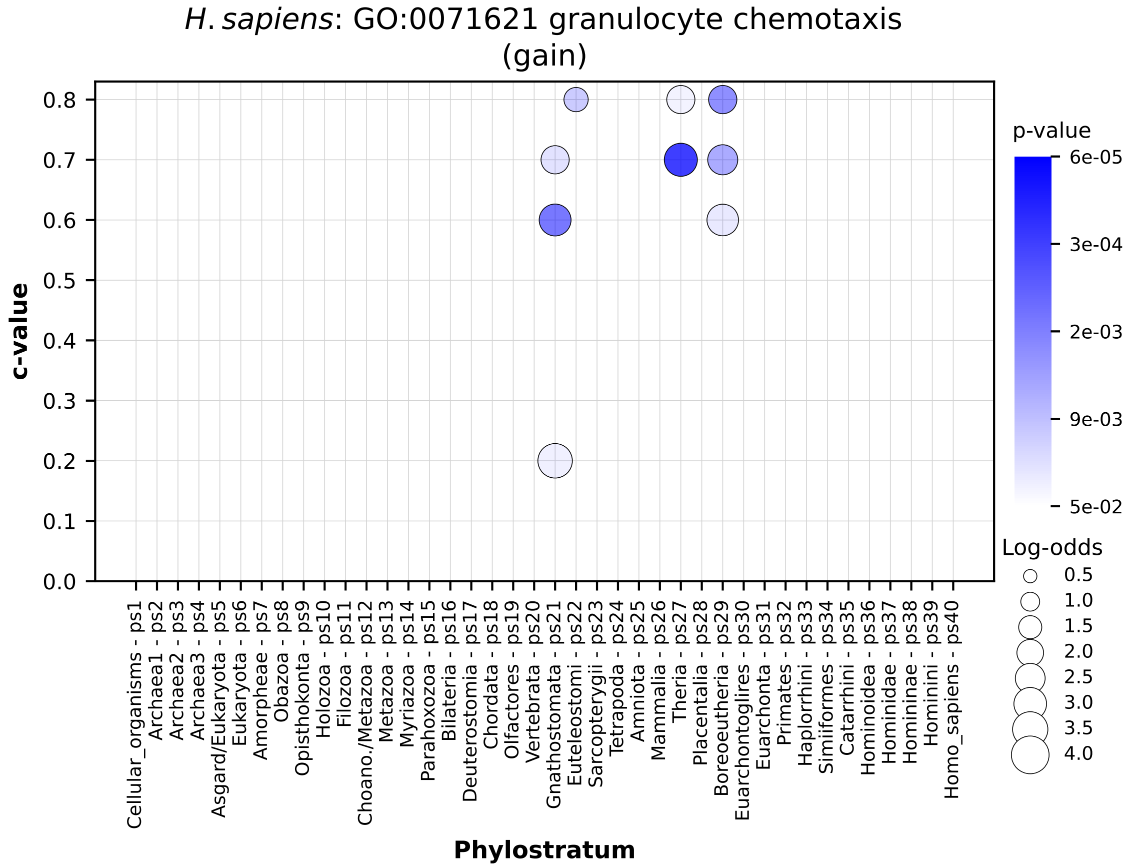

H. sapiens: GO:0071621 granulocyte chemotaxis
(gain)
c-value
0.0
0.1
0.2
0.3
0.4
0.5
0.6
0.7
0.8
Phylostratum
Cellular_organisms - ps1
Archaea1 - ps2
Archaea2 - ps3
Archaea3 - ps4
Asgard/Eukaryota - ps5
Eukaryota - ps6
Amorpheae - ps7
Obazoa - ps8
Opisthokonta - ps9
Holozoa - ps10
Filozoa - ps11
Choano./Metazoa - ps12
Metazoa - ps13
Myriazoa - ps14
Parahoxozoa - ps15
Bilateria - ps16
Deuterostomia - ps17
Chordata - ps18
Olfactores - ps19
Vertebrata - ps20
Gnathostomata - ps21
Euteleostomi - ps22
Sarcopterygii - ps23
Tetrapoda - ps24
Amniota - ps25
Mammalia - ps26
Theria - ps27
Placentalia - ps28
Boreoeutheria - ps29
Euarchontoglires - ps30
Euarchonta - ps31
Primates - ps32
Haplorrhini - ps33
Simiiformes - ps34
Catarrhini - ps35
Hominoidea - ps36
Hominidae - ps37
Homininae - ps38
Hominini - ps39
Homo_sapiens - ps40
p-value
6e-05
3e-04
2e-03
9e-03
5e-02
Log-odds
0.5
1.0
1.5
2.0
2.5
3.0
3.5
4.0

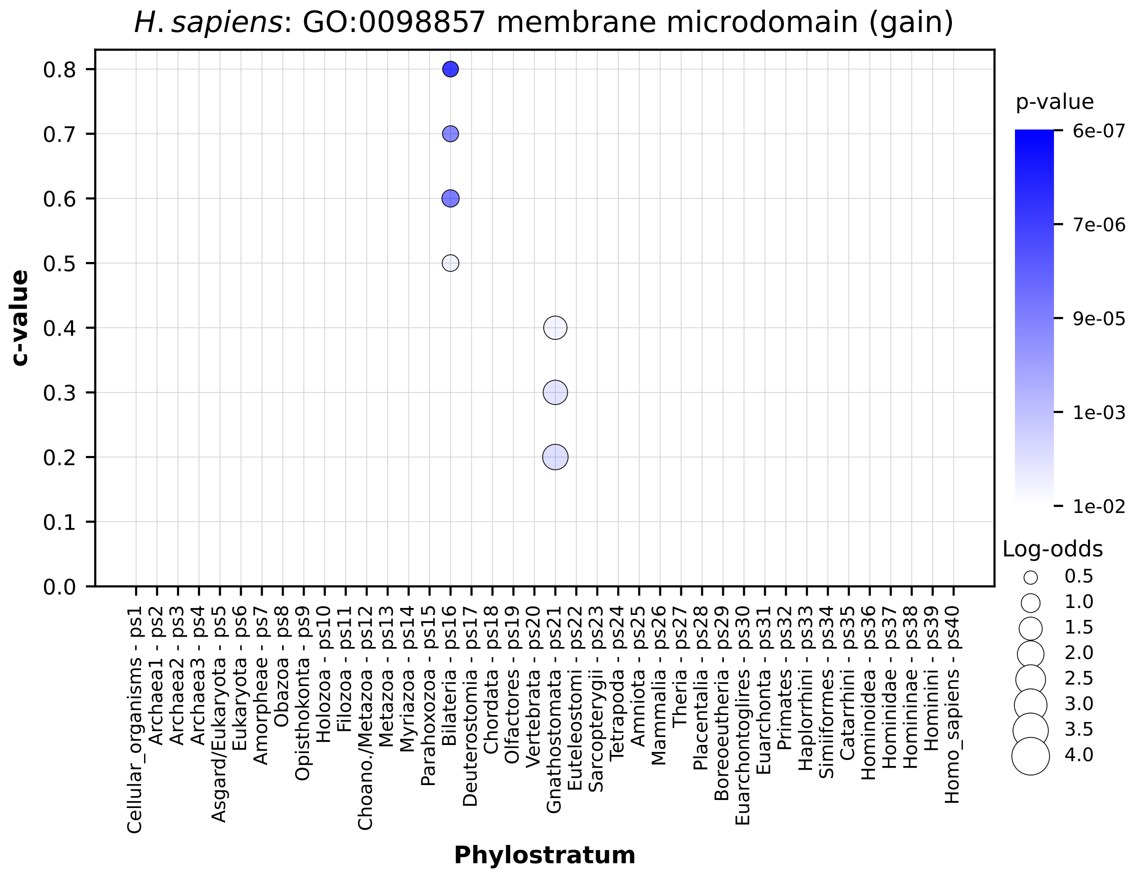

H. sapiens: GO:0098857 membrane microdomain (gain)
c-value
0.0
0.1
0.2
0.3
0.4
0.5
0.6
0.7
0.8
Phylostratum
Cellular_organisms - ps1
Archaea1 - ps2
Archaea2 - ps3
Archaea3 - ps4
Asgard/Eukaryota - ps5
Eukaryota - ps6
Amorpheae - ps7
Obazoa - ps8
Opisthokonta - ps9
Holozoa - ps10
Filozoa - ps11
Choano./Metazoa - ps12
Metazoa - ps13
Myriazoa - ps14
Parahoxozoa - ps15
Bilateria - ps16
Deuterostomia - ps17
Chordata - ps18
Olfactores - ps19
Vertebrata - ps20
Gnathostomata - ps21
Euteleostomi - ps22
Sarcopterygii - ps23
Tetrapoda - ps24
Amniota - ps25
Mammalia - ps26
Theria - ps27
Placentalia - ps28
Boreoeutheria - ps29
Euarchontoglires - ps30
Euarchonta - ps31
Primates - ps32
Haplorrhini - ps33
Simiiformes - ps34
Catarrhini - ps35
Hominoidea - ps36
Hominidae - ps37
Homininae - ps38
Hominini - ps39
Homo_sapiens - ps40
p-value
6e-07
7e-06
9e-05
1e-03
1e-02
Log-odds
0.5
1.0
1.5
2.0
2.5
3.0
3.5
4.0

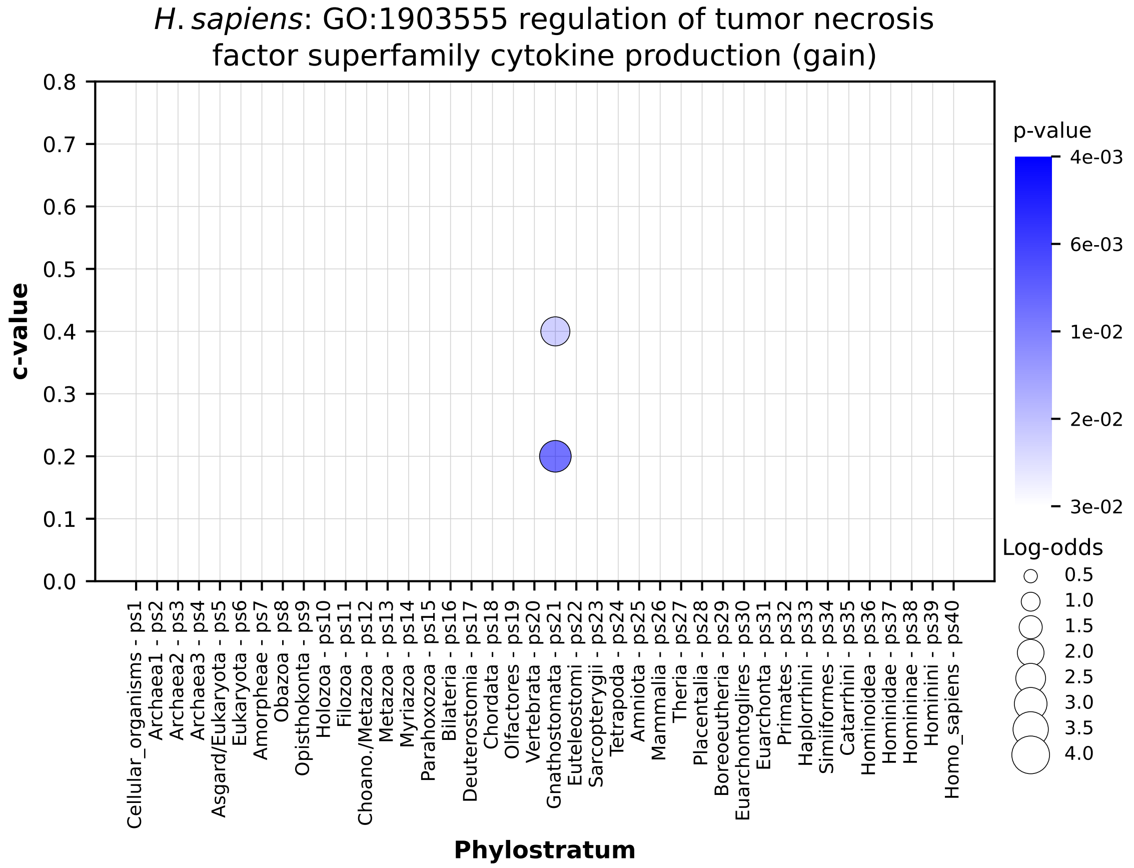

H. sapiens: GO:1903555 regulation of tumor necrosis factor superfamily cytokine production (gain)
c-value
0.8
0.7
0.6
0.5
0.4
0.3
0.2
0.1
0.0
Phylostratum
Cellular_organisms - ps1
Archaea1 - ps2
Archaea2 - ps3
Archaea3 - ps4
Asgard/Eukaryota - ps5
Eukaryota - ps6
Amorpheae - ps7
Obazoa - ps8
Opisthokonta - ps9
Holozoa - ps10
Filozoa - ps11
Choano./Metazoa - ps12
Metazoa - ps13
Myriazoa - ps14
Parahoxozoa - ps15
Bilateria - ps16
Deuterostomia - ps17
Chordata - ps18
Olfactores - ps19
Vertebrata - ps20
Gnathostomata - ps21
Euteleostomi - ps22
Sarcopterygii - ps23
Tetrapoda - ps24
Amniota - ps25
Mammalia - ps26
Theria - ps27
Placentalia - ps28
Boreoeutheria - ps29
Euarchontoglires - ps30
Euarchonta - ps31
Primates - ps32
Haplorrhini - ps33
Simiiformes - ps34
Catarrhini - ps35
Hominoidea - ps36
Hominidae - ps37
Homininae - ps38
Hominini - ps39
Homo_sapiens - ps40
p-value
4e-03
6e-03
1e-02
2e-02
3e-02
Log-odds
0.5
1.0
1.5
2.0
2.5
3.0
3.5
4.0

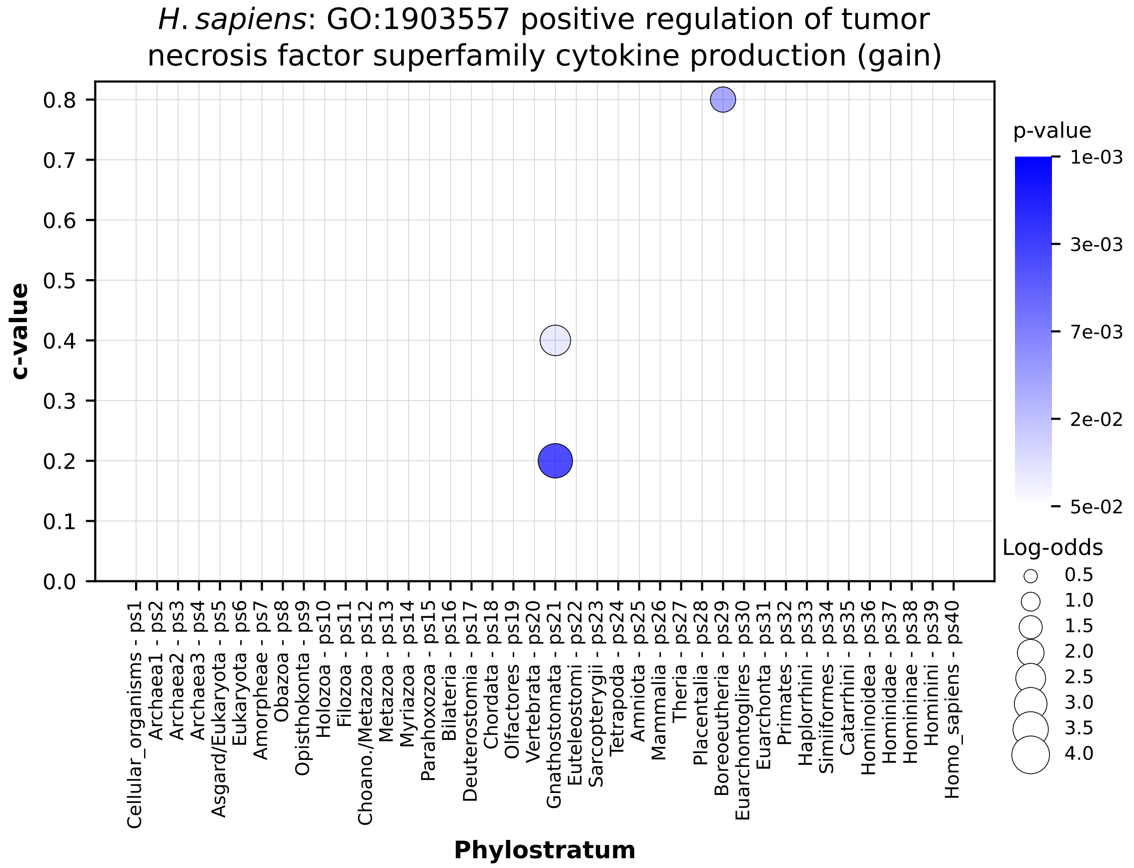

H. sapiens: GO:1903557 positive regulation of tumor necrosis factor superfamily cytokine production (gain)
c-value
0.0
0.1
0.2
0.3
0.4
0.5
0.6
0.7
0.8
Phylostratum
Cellular_organisms - ps1
Archaea1 - ps2
Archaea2 - ps3
Archaea3 - ps4
Asgard/Eukaryota - ps5
Eukaryota - ps6
Amorpheae - ps7
Obazoa - ps8
Opisthokonta - ps9
Holozoa - ps10
Filozoa - ps11
Choano./Metazoa - ps12
Metazoa - ps13
Myriazoa - ps14
Parahoxozoa - ps15
Bilateria - ps16
Deuterostomia - ps17
Chordata - ps18
Olfactores - ps19
Vertebrata - ps20
Gnathostomata - ps21
Euteleostomi - ps22
Sarcopterygii - ps23
Tetrapoda - ps24
Amniota - ps25
Mammalia - ps26
Theria - ps27
Placentalia - ps28
Boreoeutheria - ps29
Euarchontoglires - ps30
Euarchonta - ps31
Primates - ps32
Haplorrhini - ps33
Simiiformes - ps34
Catarrhini - ps35
Hominoidea - ps36
Hominidae - ps37
Homininae - ps38
Hominini - ps39
Homo_sapiens - ps40
p-value
1e-03
3e-03
7e-03
2e-02
5e-02
Log-odds
0.5
1.0
1.5
2.0
2.5
3.0
3.5
4.0

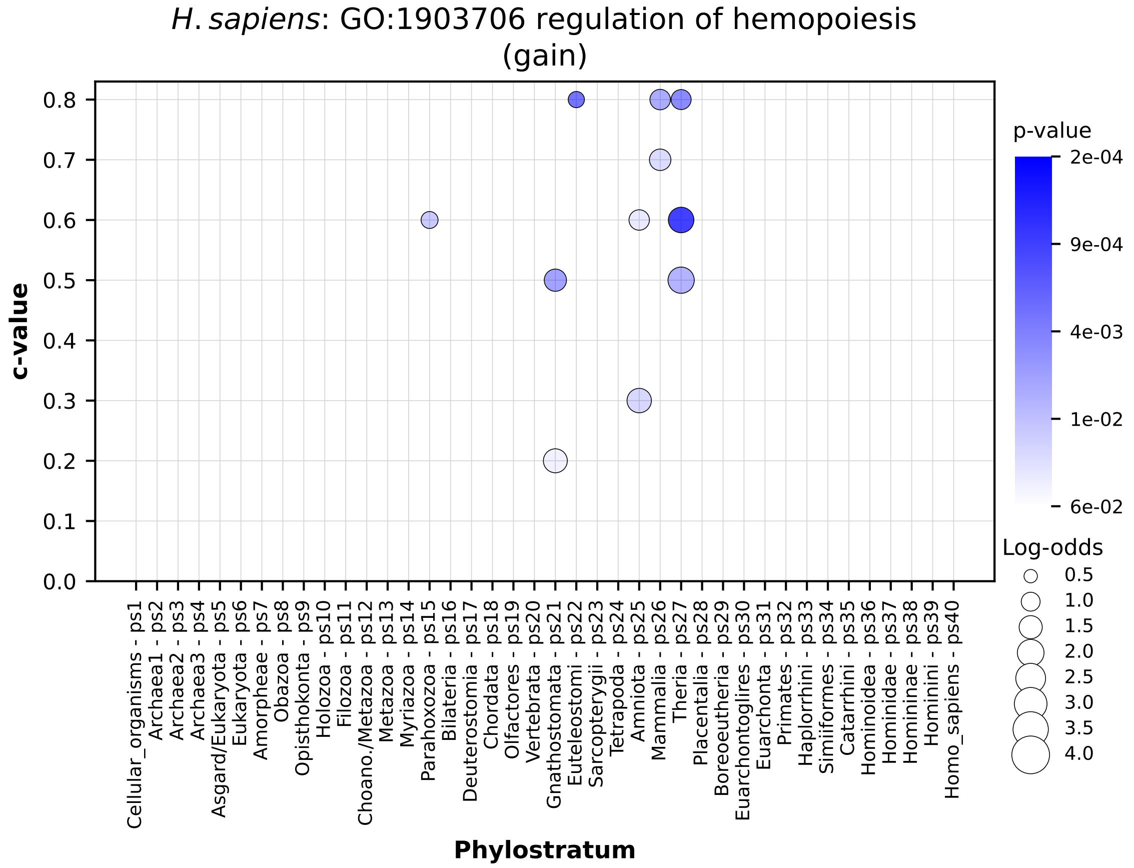

H. sapiens: GO:1903706 regulation of hemopoiesis (gain)
c-value
0.0
0.1
0.2
0.3
0.4
0.5
0.6
0.7
0.8
p-value
2e-04
9e-04
4e-03
1e-02
6e-02
Log-odds
0.5
1.0
1.5
2.0
2.5
3.0
3.5
4.0
Cellular_organisms - ps1
Archaea1 - ps2
Archaea2 - ps3
Archaea3 - ps4
Asgard/Eukaryota - ps5
Eukaryota - ps6
Amorpheae - ps7
Obazoa - ps8
Opisthokonta - ps9
Holozoa - ps10
Filozoa - ps11
Choano./Metazoa - ps12
Metazoa - ps13
Myriazoa - ps14
Parahoxozoa - ps15
Bilateria - ps16
Deuterostomia - ps17
Chordata - ps18
Olfactores - ps19
Vertebrata - ps20
Gnathostomata - ps21
Euteleostomi - ps22
Sarcopterygii - ps23
Tetrapoda - ps24
Amniota - ps25
Mammalia - ps26
Theria - ps27
Placentalia - ps28
Boreoeutheria - ps29
Euarchontoglires - ps30
Euarchonta - ps31
Primates - ps32
Haplorrhini - ps33
Simiiformes - ps34
Catarrhini - ps35
Hominoidea - ps36
Hominidae - ps37
Homininae - ps38
Hominini - ps39
Homo_sapiens - ps40
Phylostratum

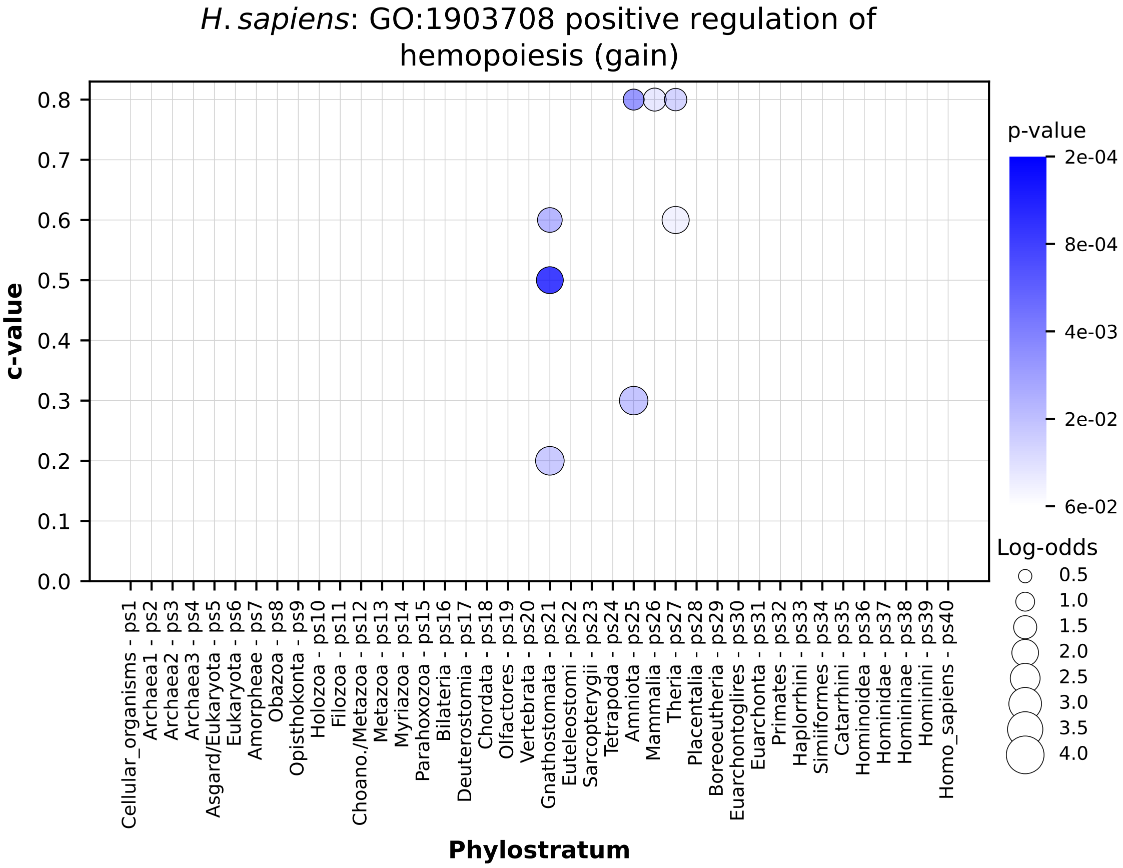

*H. sapiens*: GO:1903708 positive regulation of hemopoiesis (gain)
c-value
0.0
0.1
0.2
0.3
0.4
0.5
0.6
0.7
0.8
Phylostratum
Cellular_organisms - ps1
Archaea1 - ps2
Archaea2 - ps3
Archaea3 - ps4
Asgard/Eukaryota - ps5
Eukaryota - ps6
Amorpheae - ps7
Obazoa - ps8
Opisthokonta - ps9
Holozoa - ps10
Filozoa - ps11
Choano./Metazoa - ps12
Metazoa - ps13
Myriazoa - ps14
Parahoxozoa - ps15
Bilateria - ps16
Deuterostomia - ps17
Chordata - ps18
Olfactores - ps19
Vertebrata - ps20
Gnathostomata - ps21
Euteleostomi - ps22
Sarcopterygii - ps23
Tetrapoda - ps24
Amniota - ps25
Mammalia - ps26
Theria - ps27
Placentalia - ps28
Boreoeutheria - ps29
Euarchontoglires - ps30
Euarchonta - ps31
Primates - ps32
Haplorrhini - ps33
Simiiformes - ps34
Catarrhini - ps35
Hominoidea - ps36
Hominidae - ps37
Homininae - ps38
Hominini - ps39
Homo_sapiens - ps40
p-value
2e-04
8e-04
4e-03
2e-02
6e-02
Log-odds
0.5
1.0
1.5
2.0
2.5
3.0
3.5
4.0

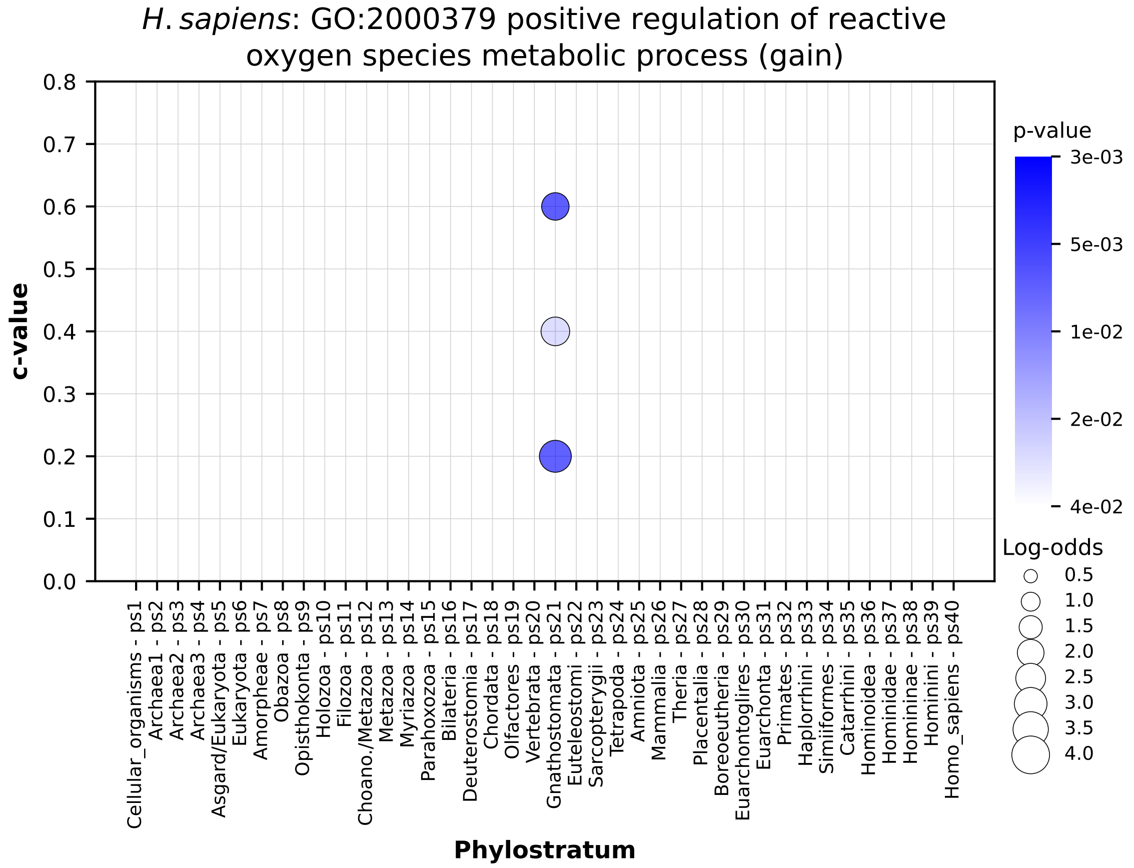

*H. sapiens*: GO:2000379 positive regulation of reactive oxygen species metabolic process (gain)
c-value
0.0
0.1
0.2
0.3
0.4
0.5
0.6
0.7
0.8
Phylostratum
Cellular_organisms - ps1
Archaea1 - ps2
Archaea2 - ps3
Archaea3 - ps4
Asgard/Eukaryota - ps5
Eukaryota - ps6
Amorpheae - ps7
Obazoa - ps8
Opisthokonta - ps9
Holozoa - ps10
Filozoa - ps11
Choano./Metazoa - ps12
Metazoa - ps13
Myriazoa - ps14
Parahoxozoa - ps15
Bilateria - ps16
Deuterostomia - ps17
Chordata - ps18
Olfactores - ps19
Vertebrata - ps20
Gnathostomata - ps21
Euteleostomi - ps22
Sarcopterygii - ps23
Tetrapoda - ps24
Amniota - ps25
Mammalia - ps26
Theria - ps27
Placentalia - ps28
Boreoeutheria - ps29
Euarchontoglires - ps30
Euarchonta - ps31
Primates - ps32
Haplorrhini - ps33
Simiiformes - ps34
Catarrhini - ps35
Hominoidea - ps36
Hominidae - ps37
Homininae - ps38
Hominini - ps39
Homo_sapiens - ps40
p-value
3e-03
5e-03
1e-02
2e-02
4e-02
Log-odds
0.5
1.0
1.5
2.0
2.5
3.0
3.5
4.0

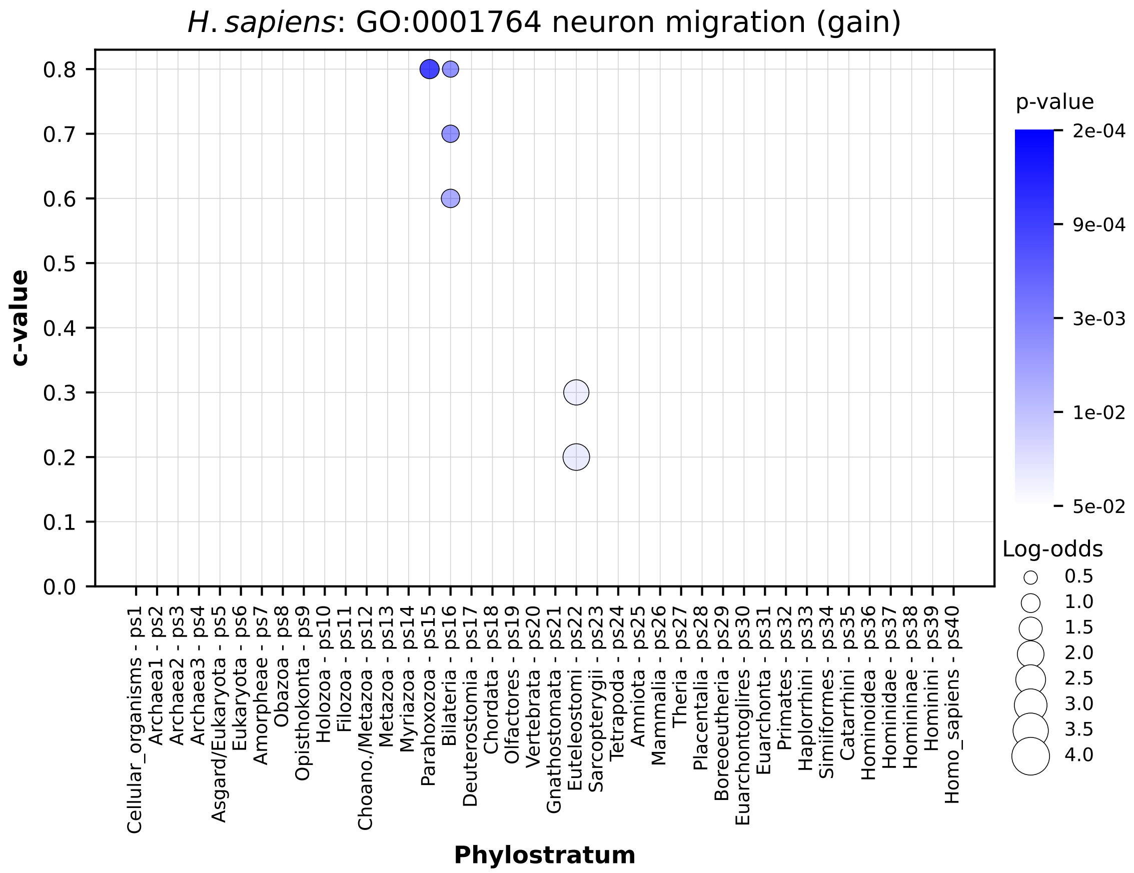

*H. sapiens*: GO:0001764 neuron migration (gain)
c-value
0.0
0.1
0.2
0.3
0.4
0.5
0.6
0.7
0.8
Phylostratum
Cellular_organisms - ps1
Archaea1 - ps2
Archaea2 - ps3
Archaea3 - ps4
Asgard/Eukaryota - ps5
Eukaryota - ps6
Amorpheae - ps7
Obazoa - ps8
Opisthokonta - ps9
Holozoa - ps10
Filozoa - ps11
Choano./Metazoa - ps12
Metazoa - ps13
Myriazoa - ps14
Parahoxozoa - ps15
Bilateria - ps16
Deuterostomia - ps17
Chordata - ps18
Olfactores - ps19
Vertebrata - ps20
Gnathostomata - ps21
Euteleostomi - ps22
Sarcopterygii - ps23
Tetrapoda - ps24
Amniota - ps25
Mammalia - ps26
Theria - ps27
Placentalia - ps28
Boreoeutheria - ps29
Euarchontoglires - ps30
Euarchonta - ps31
Primates - ps32
Haplorrhini - ps33
Simiiformes - ps34
Catarrhini - ps35
Hominoidea - ps36
Hominidae - ps37
Homininae - ps38
Hominini - ps39
Homo_sapiens - ps40
p-value
2e-04
9e-04
3e-03
1e-02
5e-02
Log-odds
0.5
1.0
1.5
2.0
2.5
3.0
3.5
4.0

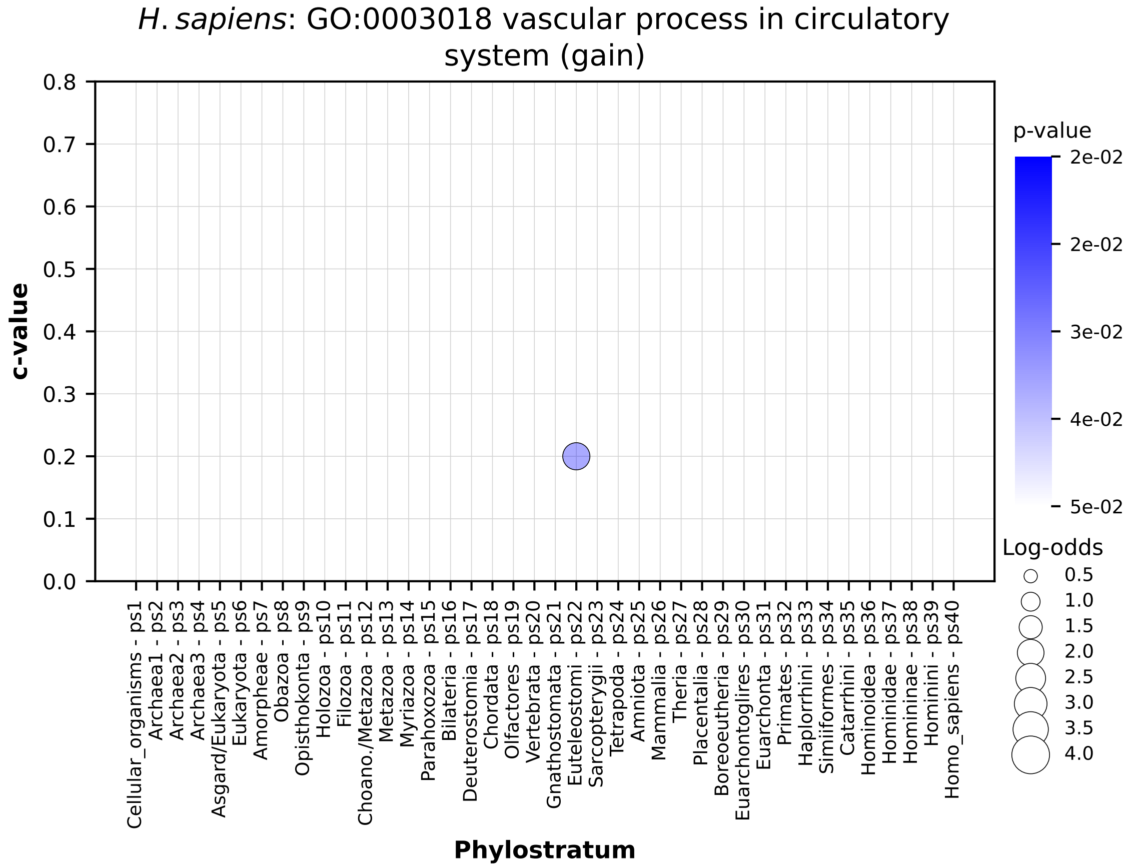
H. sapiens: GO:0003018 vascular process in circulatory system (gain)
c-value
0.0
0.1
0.2
0.3
0.4
0.5
0.6
0.7
0.8
Phylostratum
Cellular_organisms - ps1
Archaea1 - ps2
Archaea2 - ps3
Archaea3 - ps4
Asgard/Eukaryota - ps5
Eukaryota - ps6
Amorpheae - ps7
Obazoa - ps8
Opisthokonta - ps9
Holozoa - ps10
Filozoa - ps11
Choano./Metazoa - ps12
Metazoa - ps13
Myriazoa - ps14
Parahoxozoa - ps15
Bilateria - ps16
Deuterostomia - ps17
Chordata - ps18
Olfactores - ps19
Vertebrata - ps20
Gnathostomata - ps21
Euteleostomi - ps22
Sarcopterygii - ps23
Tetrapoda - ps24
Amniota - ps25
Mammalia - ps26
Theria - ps27
Placentalia - ps28
Boreoeutheria - ps29
Euarchontoglires - ps30
Euarchonta - ps31
Primates - ps32
Haplorrhini - ps33
Simiiformes - ps34
Catarrhini - ps35
Hominoidea - ps36
Hominidae - ps37
Homininae - ps38
Hominini - ps39
Homo_sapiens - ps40
p-value
2e-02
2e-02
3e-02
4e-02
5e-02
Log-odds
0.5
1.0
1.5
2.0
2.5
3.0
3.5
4.0

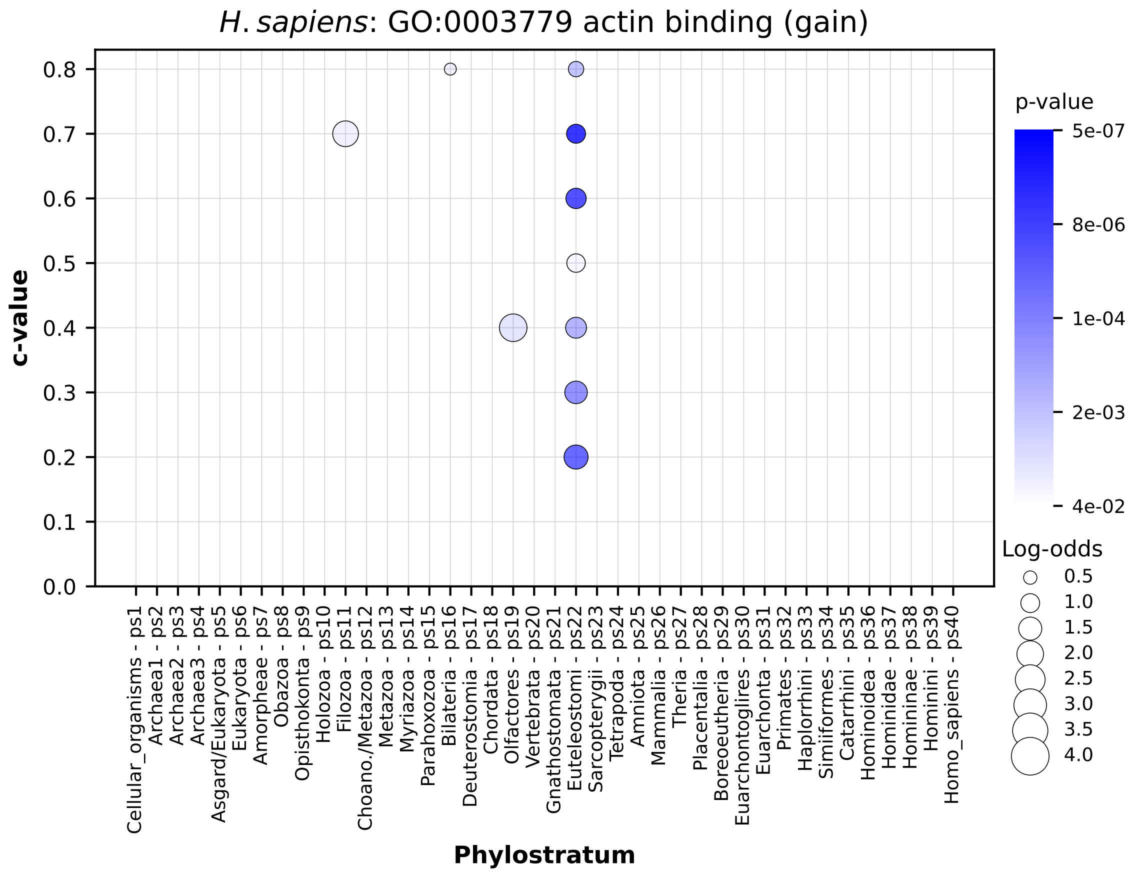

H. sapiens: GO:0003779 actin binding (gain)
c-value
0.0
0.1
0.2
0.3
0.4
0.5
0.6
0.7
0.8
Phylostratum
Cellular_organisms - ps1
Archaea1 - ps2
Archaea2 - ps3
Archaea3 - ps4
Asgard/Eukaryota - ps5
Eukaryota - ps6
Amorpheae - ps7
Obazoa - ps8
Opisthokonta - ps9
Holozoa - ps10
Filozoa - ps11
Choano./Metazoa - ps12
Metazoa - ps13
Myriazoa - ps14
Parahoxozoa - ps15
Bilateria - ps16
Deuterostomia - ps17
Chordata - ps18
Olfactores - ps19
Vertebrata - ps20
Gnathostomata - ps21
Euteleostomi - ps22
Sarcopterygii - ps23
Tetrapoda - ps24
Amniota - ps25
Mammalia - ps26
Theria - ps27
Placentalia - ps28
Boreoeutheria - ps29
Euarchontoglires - ps30
Euarchonta - ps31
Primates - ps32
Haplorrhini - ps33
Simiiformes - ps34
Catarrhini - ps35
Hominoidea - ps36
Hominidae - ps37
Homininae - ps38
Hominini - ps39
Homo_sapiens - ps40
p-value
5e-07
8e-06
1e-04
2e-03
4e-02
Log-odds
0.5
1.0
1.5
2.0
2.5
3.0
3.5
4.0

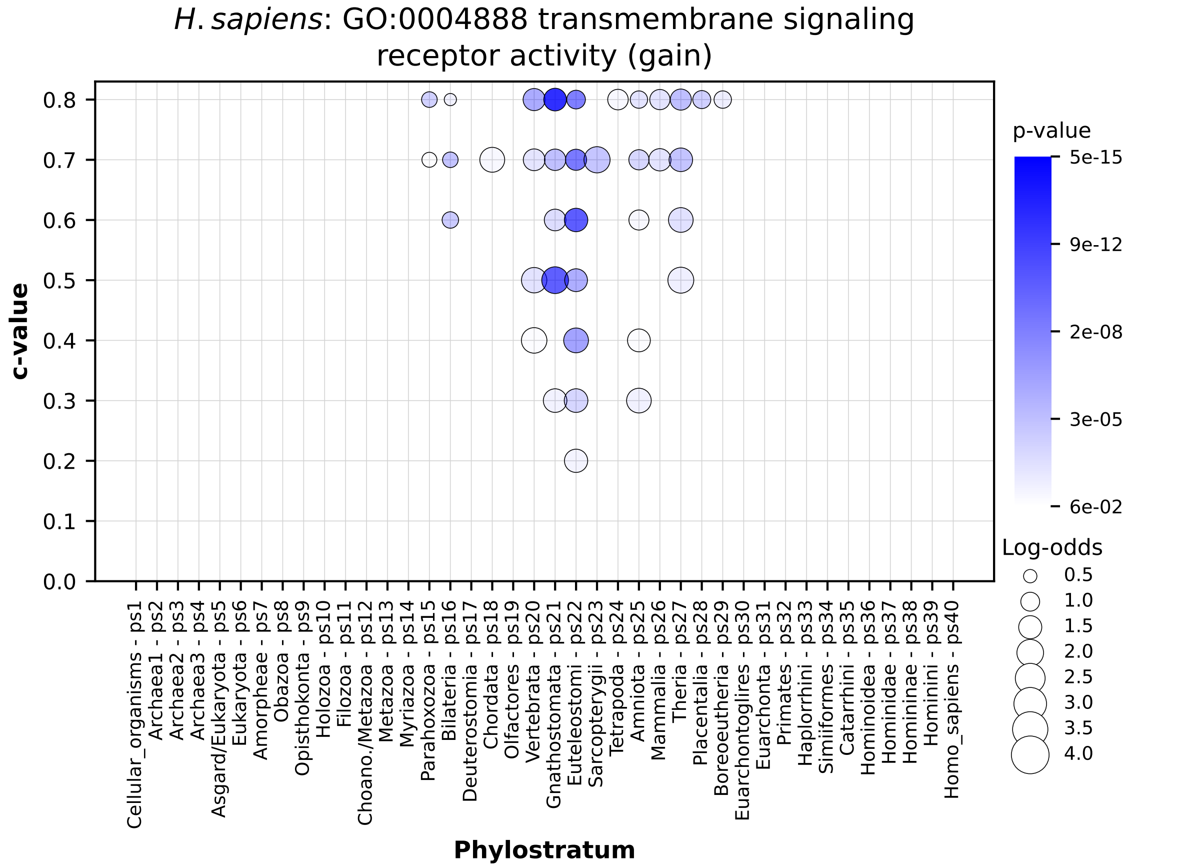

H. sapiens: GO:0004888 transmembrane signaling receptor activity (gain)
c-value
0.0
0.1
0.2
0.3
0.4
0.5
0.6
0.7
0.8
Phylostratum
Cellular_organisms - ps1
Archaea1 - ps2
Archaea2 - ps3
Archaea3 - ps4
Asgard/Eukaryota - ps5
Eukaryota - ps6
Amorpheae - ps7
Obazoa - ps8
Opisthokonta - ps9
Holozoa - ps10
Filozoa - ps11
Choano./Metazoa - ps12
Metazoa - ps13
Myriazoa - ps14
Parahoxozoa - ps15
Bilateria - ps16
Deuterostomia - ps17
Chordata - ps18
Olfactores - ps19
Vertebrata - ps20
Gnathostomata - ps21
Euteleostomi - ps22
Sarcopterygii - ps23
Tetrapoda - ps24
Amniota - ps25
Mammalia - ps26
Theria - ps27
Placentalia - ps28
Boreoeutheria - ps29
Euarchontoglires - ps30
Euarchonta - ps31
Primates - ps32
Haplorrhini - ps33
Simiiformes - ps34
Catarrhini - ps35
Hominoidea - ps36
Hominidae - ps37
Homininae - ps38
Hominini - ps39
Homo_sapiens - ps40
p-value
5e-15
9e-12
2e-08
3e-05
6e-02
Log-odds
0.5
1.0
1.5
2.0
2.5
3.0
3.5
4.0

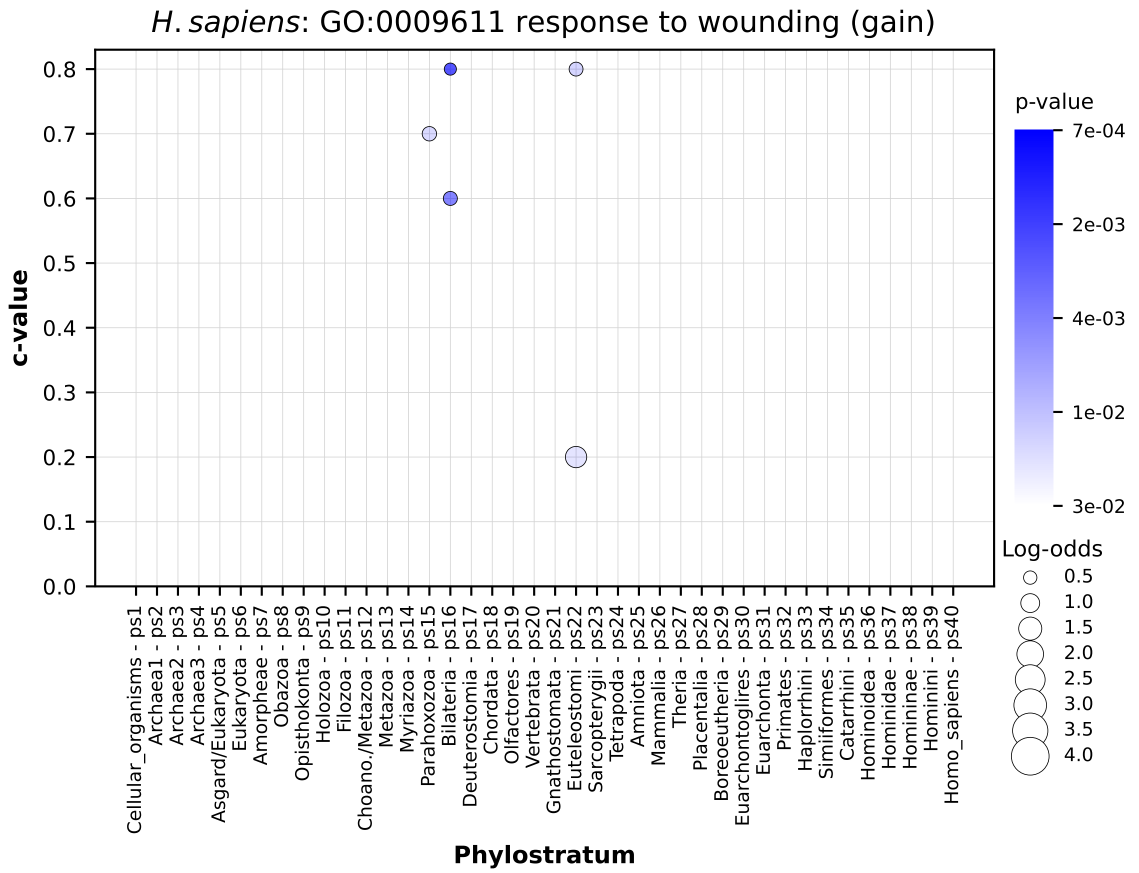

H. sapiens: GO:0009611 response to wounding (gain)
c-value
0.0
0.1
0.2
0.3
0.4
0.5
0.6
0.7
0.8
Phylostratum
Cellular_organisms - ps1
Archaea1 - ps2
Archaea2 - ps3
Archaea3 - ps4
Asgard/Eukaryota - ps5
Eukaryota - ps6
Amorpheae - ps7
Obazoa - ps8
Opisthokonta - ps9
Holozoa - ps10
Filozoa - ps11
Choano./Metazoa - ps12
Metazoa - ps13
Myriazoa - ps14
Parahoxozoa - ps15
Bilateria - ps16
Deuterostomia - ps17
Chordata - ps18
Olfactores - ps19
Vertebrata - ps20
Gnathostomata - ps21
Euteleostomi - ps22
Sarcopterygii - ps23
Tetrapoda - ps24
Amniota - ps25
Mammalia - ps26
Theria - ps27
Placentalia - ps28
Boreoeutheria - ps29
Euarchontoglires - ps30
Euarchonta - ps31
Primates - ps32
Haplorrhini - ps33
Simiiformes - ps34
Catarrhini - ps35
Hominoidea - ps36
Hominidae - ps37
Homininae - ps38
Hominini - ps39
Homo_sapiens - ps40
p-value
7e-04
2e-03
4e-03
1e-02
3e-02
Log-odds
0.5
1.0
1.5
2.0
2.5
3.0
3.5
4.0

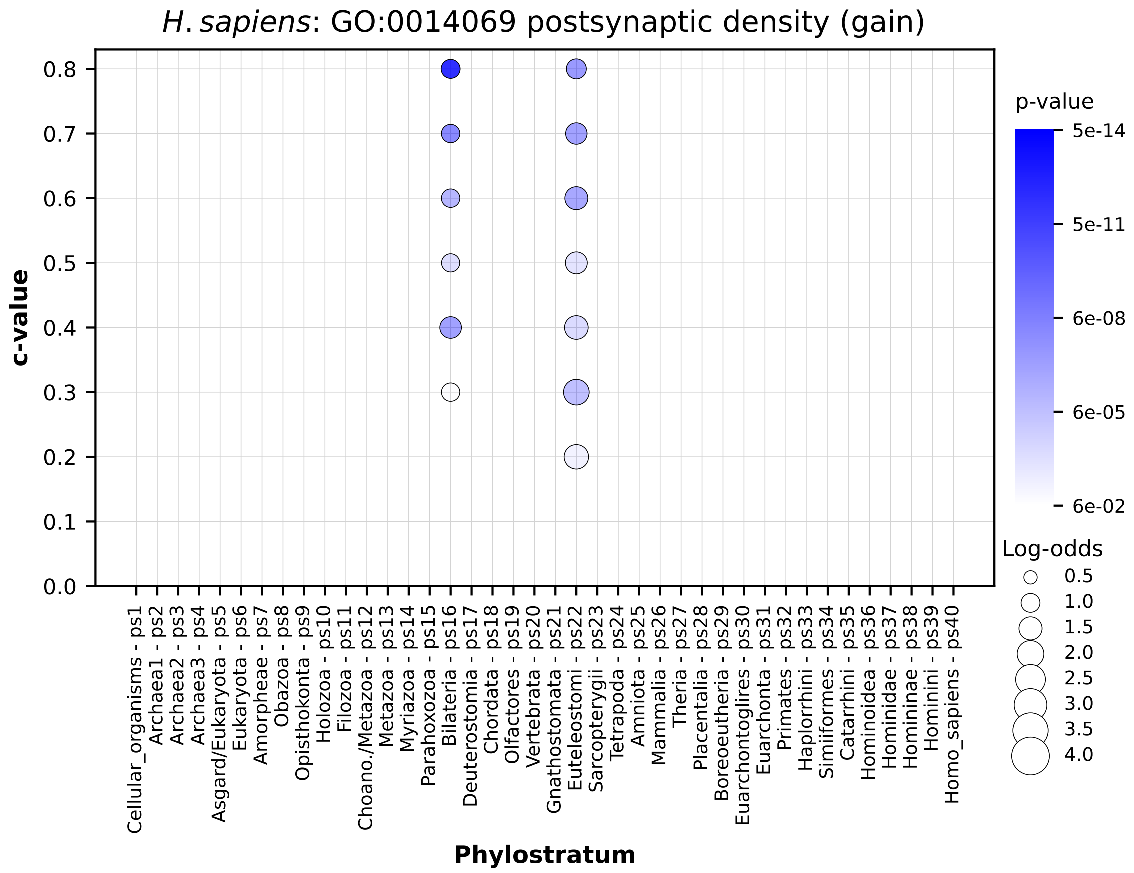
H. sapiens: GO:0014069 postsynaptic density (gain)
c-value
0.0
0.1
0.2
0.3
0.4
0.5
0.6
0.7
0.8
Phylostratum
Cellular_organisms - ps1
Archaea1 - ps2
Archaea2 - ps3
Archaea3 - ps4
Asgard/Eukaryota - ps5
Eukaryota - ps6
Amorpheae - ps7
Obazoa - ps8
Opisthokonta - ps9
Holozoa - ps10
Filozoa - ps11
Choano./Metazoa - ps12
Metazoa - ps13
Myriazoa - ps14
Parahoxozoa - ps15
Bilateria - ps16
Deuterostomia - ps17
Chordata - ps18
Olfactores - ps19
Vertebrata - ps20
Gnathostomata - ps21
Euteleostomi - ps22
Sarcopterygii - ps23
Tetrapoda - ps24
Amniota - ps25
Mammalia - ps26
Theria - ps27
Placentalia - ps28
Boreoeutheria - ps29
Euarchontoglires - ps30
Euarchonta - ps31
Primates - ps32
Haplorrhini - ps33
Simiiformes - ps34
Catarrhini - ps35
Hominoidea - ps36
Hominidae - ps37
Homininae - ps38
Hominini - ps39
Homo_sapiens - ps40
p-value
5e-14
5e-11
6e-08
6e-05
6e-02
Log-odds
0.5
1.0
1.5
2.0
2.5
3.0
3.5
4.0

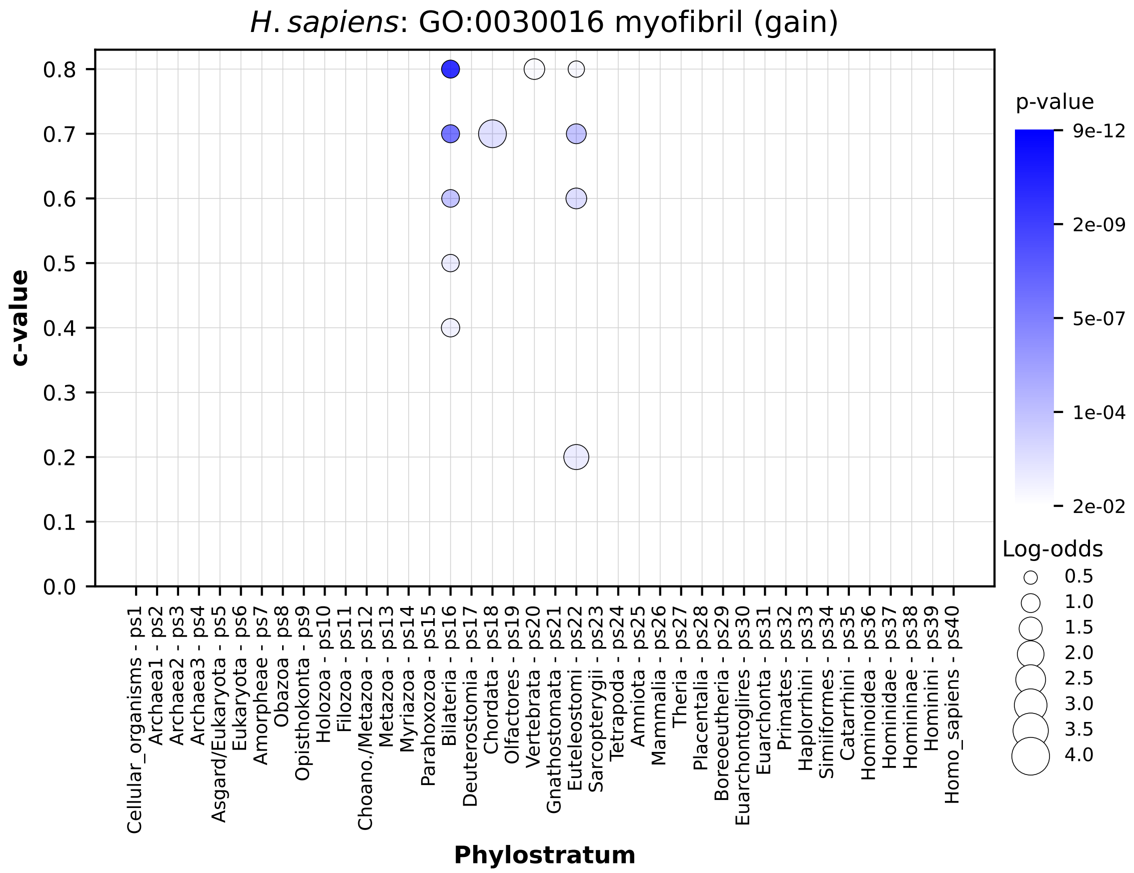

H. sapiens: GO:0030016 myofibril (gain)
c-value
0.0
0.1
0.2
0.3
0.4
0.5
0.6
0.7
0.8
p-value
9e-12
2e-09
5e-07
1e-04
2e-02
Log-odds
0.5
1.0
1.5
2.0
2.5
3.0
3.5
4.0
Cellular_organisms - ps1
Archaea1 - ps2
Archaea2 - ps3
Archaea3 - ps4
Asgard/Eukaryota - ps5
Eukaryota - ps6
Amorpheae - ps7
Obazoa - ps8
Opisthokonta - ps9
Holozoa - ps10
Filozoa - ps11
Choano./Metazoa - ps12
Metazoa - ps13
Myriazoa - ps14
Parahoxozoa - ps15
Bilateria - ps16
Deuterostomia - ps17
Chordata - ps18
Olfactores - ps19
Vertebrata - ps20
Gnathostomata - ps21
Euteleostomi - ps22
Sarcopterygii - ps23
Tetrapoda - ps24
Amniota - ps25
Mammalia - ps26
Theria - ps27
Placentalia - ps28
Boreoeutheria - ps29
Euarchontoglires - ps30
Euarchonta - ps31
Primates - ps32
Haplorrhini - ps33
Simiiformes - ps34
Catarrhini - ps35
Hominoidea - ps36
Hominidae - ps37
Homininae - ps38
Hominini - ps39
Homo_sapiens - ps40
Phylostratum

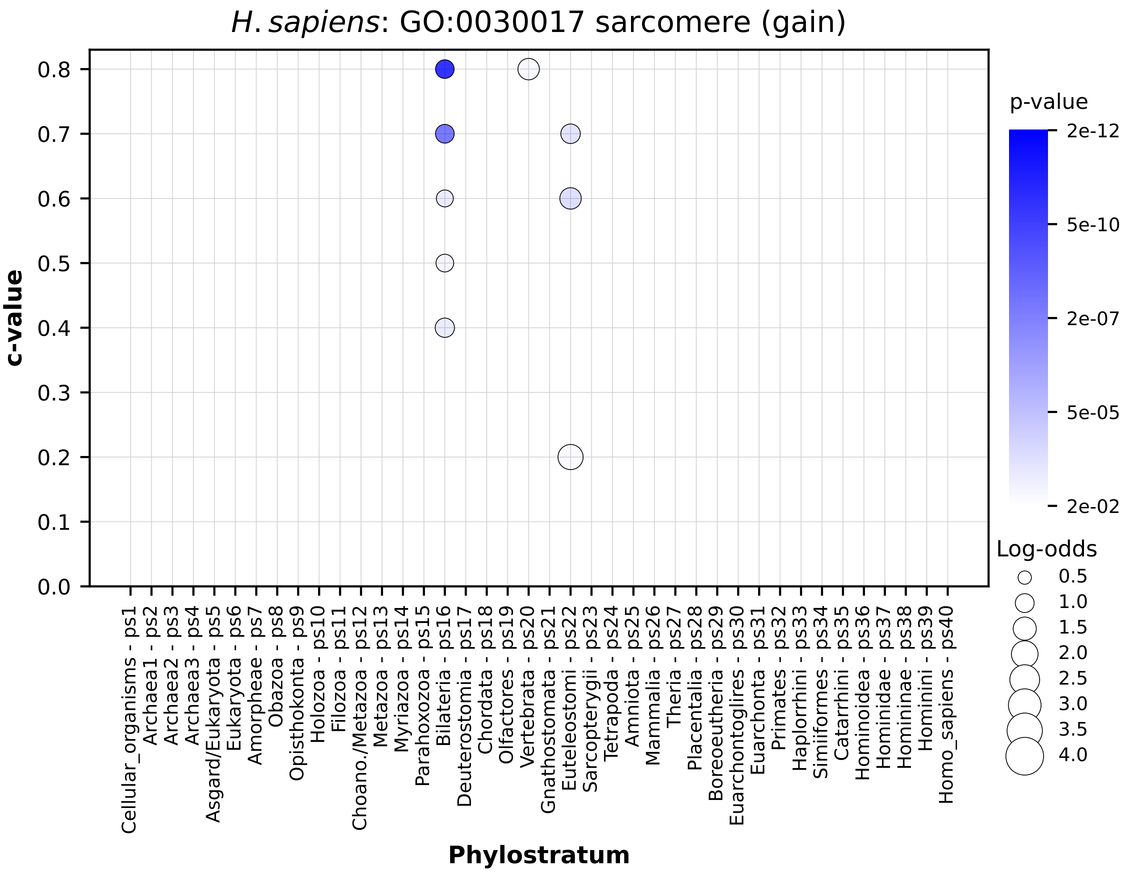

H. sapiens: GO:0030017 sarcomere (gain)
c-value
0.0
0.1
0.2
0.3
0.4
0.5
0.6
0.7
0.8
Phylostratum
Cellular_organisms - ps1
Archaea1 - ps2
Archaea2 - ps3
Archaea3 - ps4
Asgard/Eukaryota - ps5
Eukaryota - ps6
Amorpheae - ps7
Obazoa - ps8
Opisthokonta - ps9
Holozoa - ps10
Filozoa - ps11
Choano./Metazoa - ps12
Metazoa - ps13
Myriazoa - ps14
Parahoxozoa - ps15
Bilateria - ps16
Deuterostomia - ps17
Chordata - ps18
Olfactores - ps19
Vertebrata - ps20
Gnathostomata - ps21
Euteleostomi - ps22
Sarcopterygii - ps23
Tetrapoda - ps24
Amniota - ps25
Mammalia - ps26
Theria - ps27
Placentalia - ps28
Boreoeutheria - ps29
Euarchontoglires - ps30
Euarchonta - ps31
Primates - ps32
Haplorrhini - ps33
Simiiformes - ps34
Catarrhini - ps35
Hominoidea - ps36
Hominidae - ps37
Homininae - ps38
Hominini - ps39
Homo_sapiens - ps40
p-value
2e-12
5e-10
2e-07
5e-05
2e-02
Log-odds
0.5
1.0
1.5
2.0
2.5
3.0
3.5
4.0

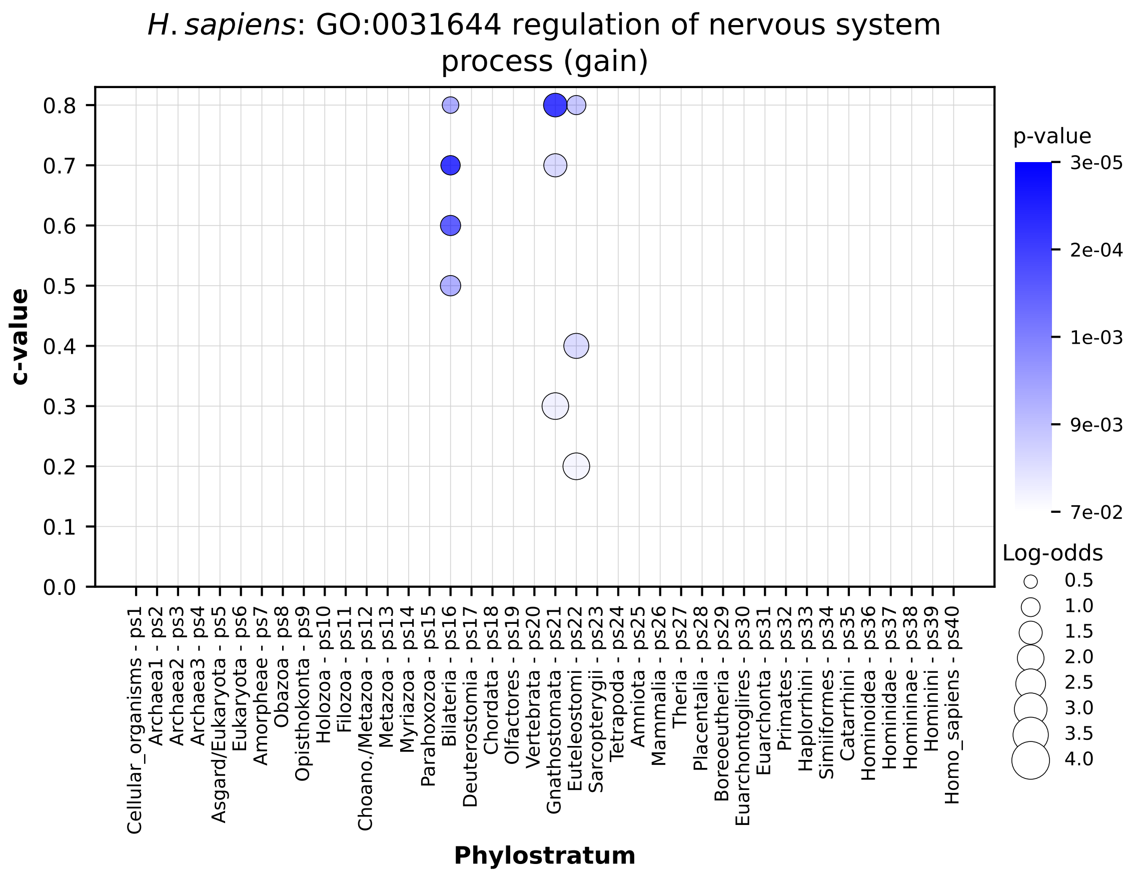

H. sapiens: GO:0031644 regulation of nervous system process (gain)
c-value
0.0
0.1
0.2
0.3
0.4
0.5
0.6
0.7
0.8
Phylostratum
Cellular_organisms - ps1
Archaea1 - ps2
Archaea2 - ps3
Archaea3 - ps4
Asgard/Eukaryota - ps5
Eukaryota - ps6
Amorpheae - ps7
Obazoa - ps8
Opisthokonta - ps9
Holozoa - ps10
Filozoa - ps11
Choano./Metazoa - ps12
Metazoa - ps13
Myriazoa - ps14
Parahoxozoa - ps15
Bilateria - ps16
Deuterostomia - ps17
Chordata - ps18
Olfactores - ps19
Vertebrata - ps20
Gnathostomata - ps21
Euteleostomi - ps22
Sarcopterygii - ps23
Tetrapoda - ps24
Amniota - ps25
Mammalia - ps26
Theria - ps27
Placentalia - ps28
Boreoeutheria - ps29
Euarchontoglires - ps30
Euarchonta - ps31
Primates - ps32
Haplorrhini - ps33
Simiiformes - ps34
Catarrhini - ps35
Hominoidea - ps36
Hominidae - ps37
Homininae - ps38
Hominini - ps39
Homo_sapiens - ps40
p-value
3e-05
2e-04
1e-03
9e-03
7e-02
Log-odds
0.5
1.0
1.5
2.0
2.5
3.0
3.5
4.0

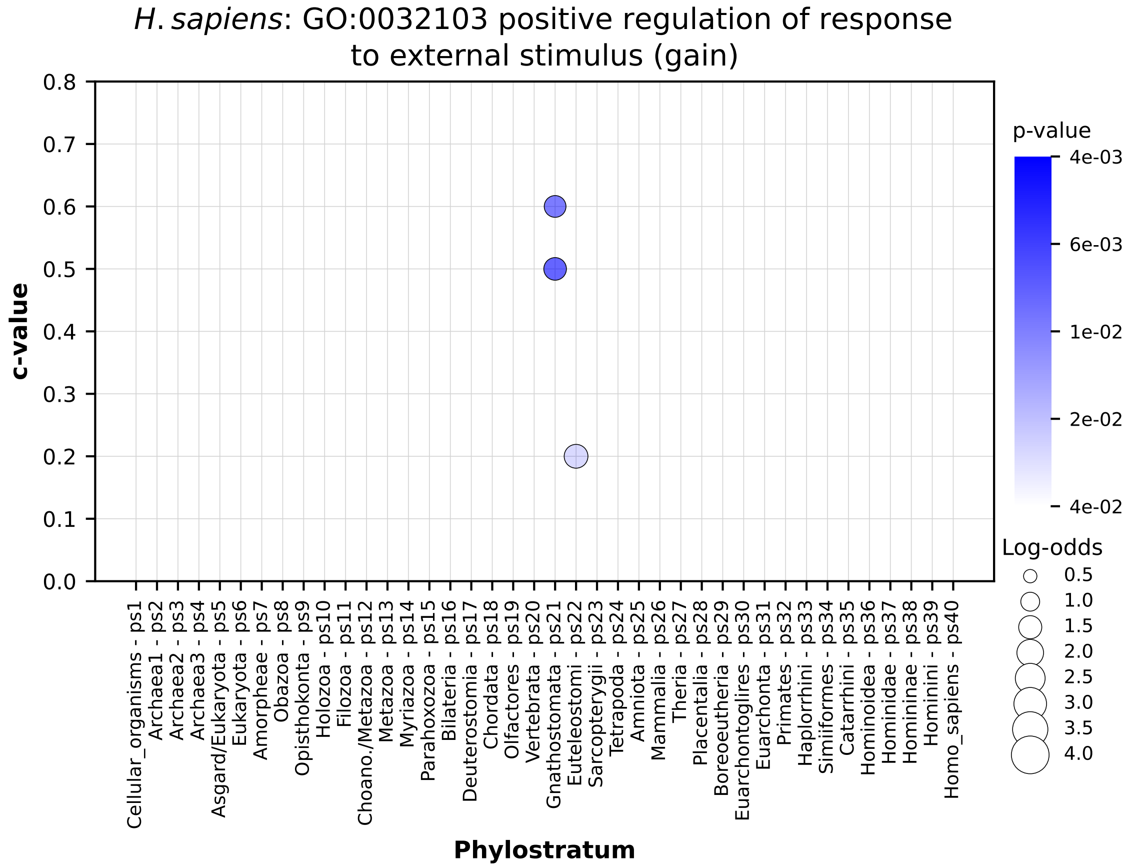

H. sapiens: GO:0032103 positive regulation of response to external stimulus (gain)
c-value
0.8
0.7
0.6
0.5
0.4
0.3
0.2
0.1
0.0
p-value
4e-03
6e-03
1e-02
2e-02
4e-02
Log-odds
0.5
1.0
1.5
2.0
2.5
3.0
3.5
4.0
Cellular_organisms - ps1
Archaea1 - ps2
Archaea2 - ps3
Archaea3 - ps4
Asgard/Eukaryota - ps5
Eukaryota - ps6
Amorpheae - ps7
Obazoa - ps8
Opisthokonta - ps9
Holozoa - ps10
Filozoa - ps11
Choano./Metazoa - ps12
Metazoa - ps13
Myriazoa - ps14
Parahoxozoa - ps15
Bilateria - ps16
Deuterostomia - ps17
Chordata - ps18
Olfactores - ps19
Vertebrata - ps20
Gnathostomata - ps21
Euteleostomi - ps22
Sarcopterygii - ps23
Tetrapoda - ps24
Amniota - ps25
Mammalia - ps26
Theria - ps27
Placentalia - ps28
Boreoeutheria - ps29
Euarchontoglires - ps30
Euarchonta - ps31
Primates - ps32
Haplorrhini - ps33
Simiiformes - ps34
Catarrhini - ps35
Hominoidea - ps36
Hominidae - ps37
Homininae - ps38
Hominini - ps39
Homo_sapiens - ps40
Phylostratum

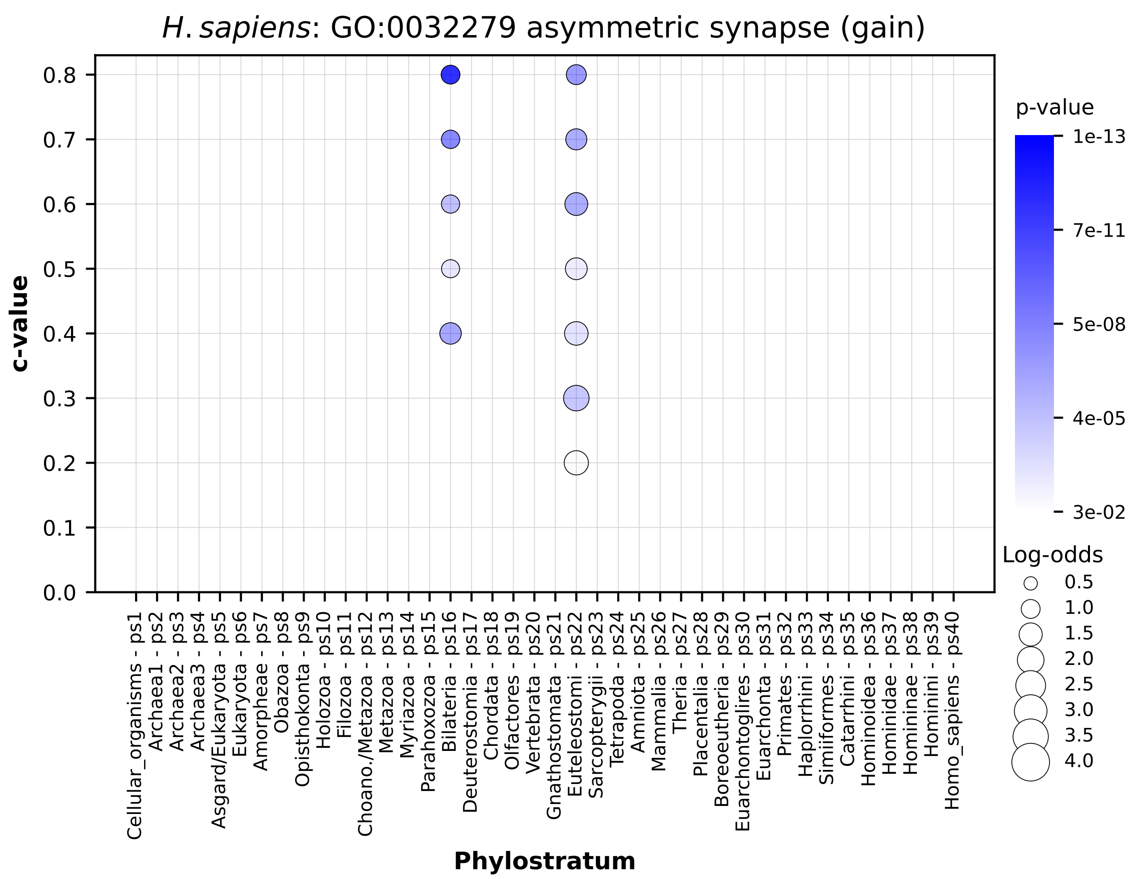

H. sapiens: GO:0032279 asymmetric synapse (gain)
c-value
0.0
0.1
0.2
0.3
0.4
0.5
0.6
0.7
0.8
p-value
1e-13
7e-11
5e-08
4e-05
3e-02
Log-odds
0.5
1.0
1.5
2.0
2.5
3.0
3.5
4.0
Cellular_organisms - ps1
Archaea1 - ps2
Archaea2 - ps3
Archaea3 - ps4
Asgard/Eukaryota - ps5
Eukaryota - ps6
Amorpheae - ps7
Obazoa - ps8
Opisthokonta - ps9
Holozoa - ps10
Filozoa - ps11
Choano./Metazoa - ps12
Metazoa - ps13
Myriazoa - ps14
Parahoxozoa - ps15
Bilateria - ps16
Deuterostomia - ps17
Chordata - ps18
Olfactores - ps19
Vertebrata - ps20
Gnathostomata - ps21
Euteleostomi - ps22
Sarcopterygii - ps23
Tetrapoda - ps24
Amniota - ps25
Mammalia - ps26
Theria - ps27
Placentalia - ps28
Boreoeutheria - ps29
Euarchontoglires - ps30
Euarchonta - ps31
Primates - ps32
Haplorrhini - ps33
Simiiformes - ps34
Catarrhini - ps35
Hominoidea - ps36
Hominidae - ps37
Homininae - ps38
Hominini - ps39
Homo_sapiens - ps40
Phylostratum

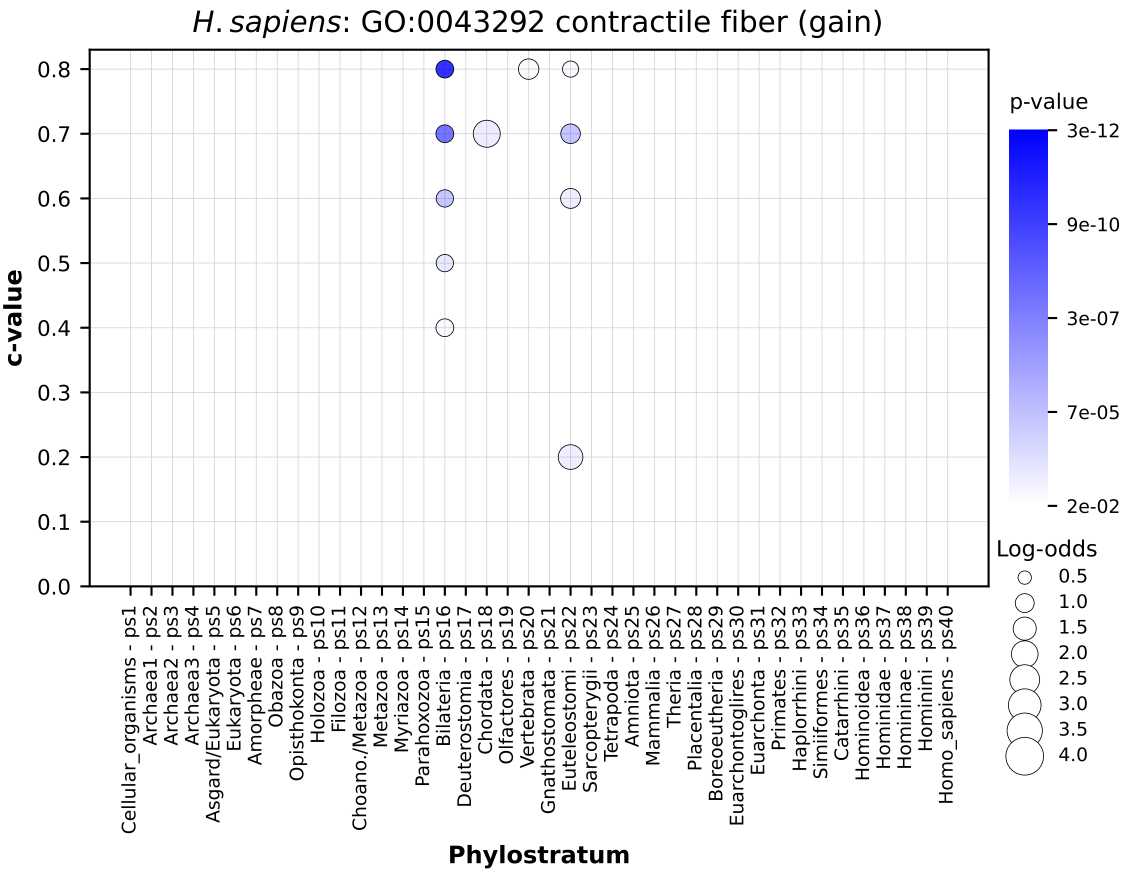

H. sapiens: GO:0043292 contractile fiber (gain)
c-value
0.0
0.1
0.2
0.3
0.4
0.5
0.6
0.7
0.8
Phylostratum
Cellular_organisms - ps1
Archaea1 - ps2
Archaea2 - ps3
Archaea3 - ps4
Asgard/Eukaryota - ps5
Eukaryota - ps6
Amorpheae - ps7
Obazoa - ps8
Opisthokonta - ps9
Holozoa - ps10
Filozoa - ps11
Choano./Metazoa - ps12
Metazoa - ps13
Myriazoa - ps14
Parahoxozoa - ps15
Bilateria - ps16
Deuterostomia - ps17
Chordata - ps18
Olfactores - ps19
Vertebrata - ps20
Gnathostomata - ps21
Euteleostomi - ps22
Sarcopterygii - ps23
Tetrapoda - ps24
Amniota - ps25
Mammalia - ps26
Theria - ps27
Placentalia - ps28
Boreoeutheria - ps29
Euarchontoglires - ps30
Euarchonta - ps31
Primates - ps32
Haplorrhini - ps33
Simiiformes - ps34
Catarrhini - ps35
Hominoidea - ps36
Hominidae - ps37
Homininae - ps38
Hominini - ps39
Homo_sapiens - ps40
p-value
3e-12
9e-10
3e-07
7e-05
2e-02
Log-odds
0.5
1.0
1.5
2.0
2.5
3.0
3.5
4.0

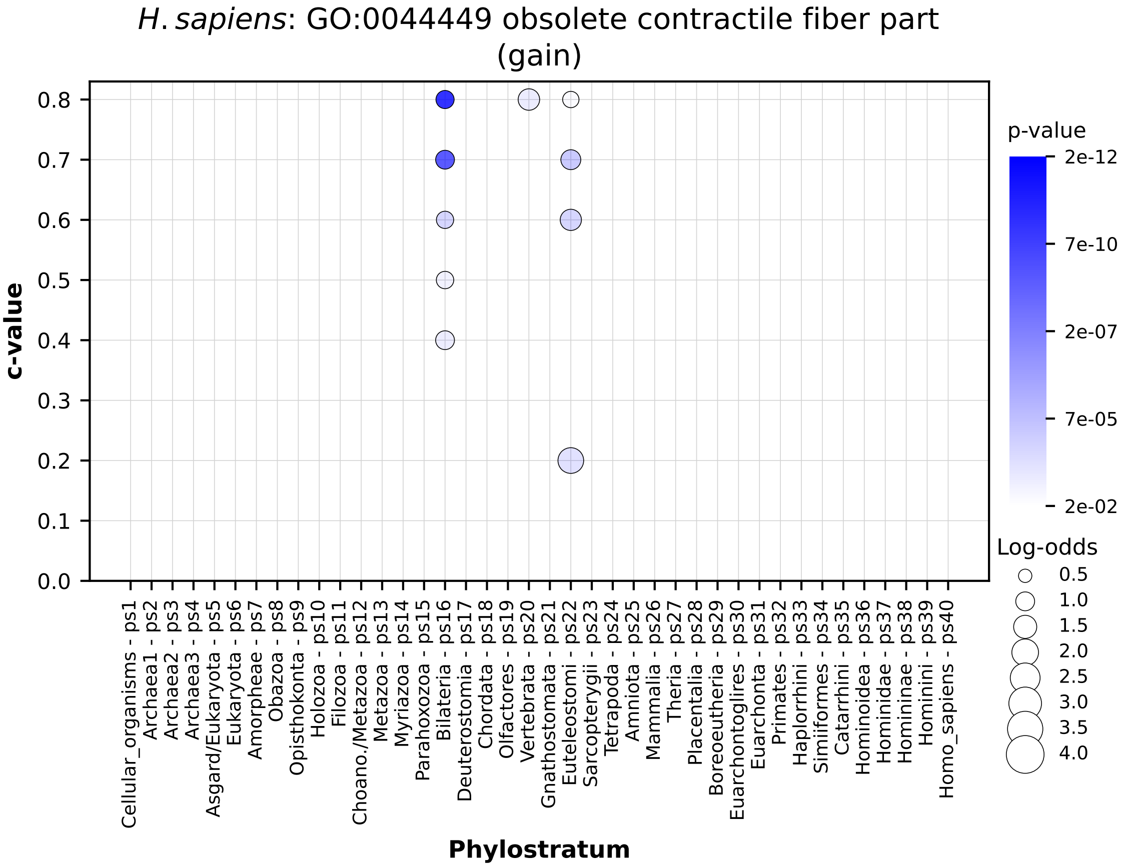

H. sapiens: GO:0044449 obsolete contractile fiber part
(gain)
c-value
0.0
0.1
0.2
0.3
0.4
0.5
0.6
0.7
0.8
Phylostratum
Cellular_organisms - ps1
Archaea1 - ps2
Archaea2 - ps3
Archaea3 - ps4
Asgard/Eukaryota - ps5
Eukaryota - ps6
Amorpheae - ps7
Obazoa - ps8
Opisthokonta - ps9
Holozoa - ps10
Filozoa - ps11
Choano./Metazoa - ps12
Metazoa - ps13
Myriazoa - ps14
Parahoxozoa - ps15
Bilateria - ps16
Deuterostomia - ps17
Chordata - ps18
Olfactores - ps19
Vertebrata - ps20
Gnathostomata - ps21
Euteleostomi - ps22
Sarcopterygii - ps23
Tetrapoda - ps24
Amniota - ps25
Mammalia - ps26
Theria - ps27
Placentalia - ps28
Boreoeutheria - ps29
Euarchontoglires - ps30
Euarchonta - ps31
Primates - ps32
Haplorrhini - ps33
Simiiformes - ps34
Catarrhini - ps35
Hominoidea - ps36
Hominidae - ps37
Homininae - ps38
Hominini - ps39
Homo_sapiens - ps40
p-value
2e-12
7e-10
2e-07
7e-05
2e-02
Log-odds
0.5
1.0
1.5
2.0
2.5
3.0
3.5
4.0

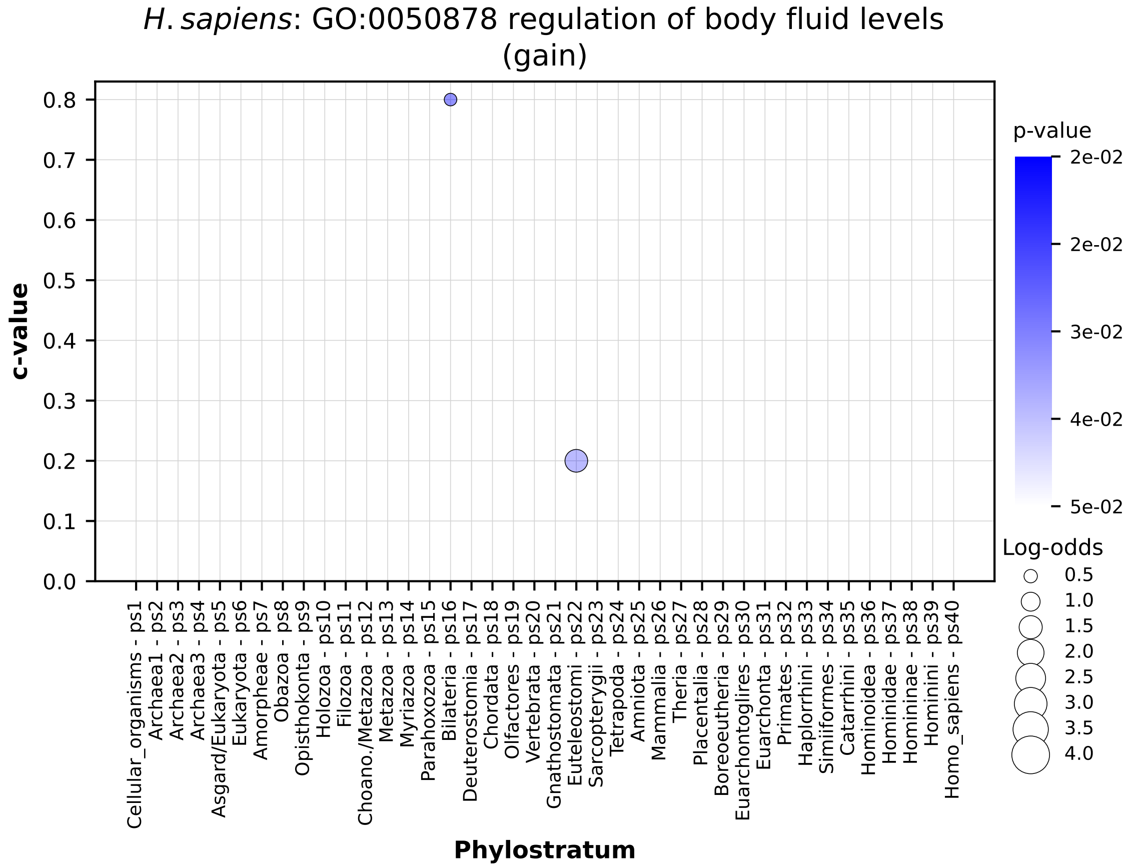
*H. sapiens*: GO:0050878 regulation of body fluid levels
(gain)
c-value
0.0
0.1
0.2
0.3
0.4
0.5
0.6
0.7
0.8
p-value
2e-02
2e-02
3e-02
4e-02
5e-02
Log-odds
0.5
1.0
1.5
2.0
2.5
3.0
3.5
4.0
Cellular_organisms - ps1
Archaea1 - ps2
Archaea2 - ps3
Archaea3 - ps4
Asgard/Eukaryota - ps5
Eukaryota - ps6
Amorpheae - ps7
Obazoa - ps8
Opisthokonta - ps9
Holozoa - ps10
Filozoa - ps11
Choano./Metazoa - ps12
Metazoa - ps13
Myriazoa - ps14
Parahoxozoa - ps15
Bilateria - ps16
Deuterostomia - ps17
Chordata - ps18
Olfactores - ps19
Vertebrata - ps20
Gnathostomata - ps21
Euteleostomi - ps22
Sarcopterygii - ps23
Tetrapoda - ps24
Amniota - ps25
Mammalia - ps26
Theria - ps27
Placentalia - ps28
Boreoeutheria - ps29
Euarchontoglires - ps30
Euarchonta - ps31
Primates - ps32
Haplorrhini - ps33
Simiiformes - ps34
Catarrhini - ps35
Hominoidea - ps36
Hominidae - ps37
Homininae - ps38
Hominini - ps39
Homo_sapiens - ps40
Phylostratum

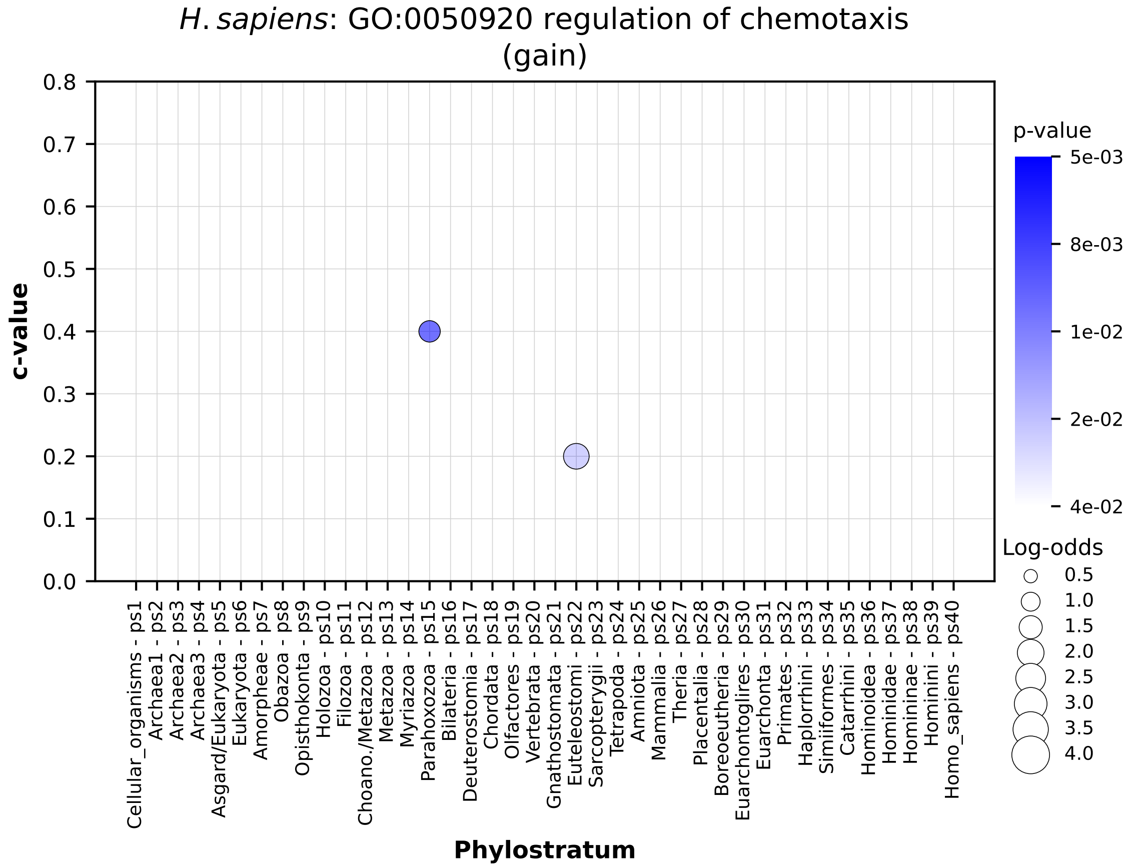
H. sapiens: GO:0050920 regulation of chemotaxis
(gain)
c-value
0.0
0.1
0.2
0.3
0.4
0.5
0.6
0.7
0.8
p-value
5e-03
8e-03
1e-02
2e-02
4e-02
Log-odds
0.5
1.0
1.5
2.0
2.5
3.0
3.5
4.0
Cellular_organisms - ps1
Archaea1 - ps2
Archaea2 - ps3
Archaea3 - ps4
Asgard/Eukaryota - ps5
Eukaryota - ps6
Amorpheae - ps7
Obazoa - ps8
Opisthokonta - ps9
Holozoa - ps10
Filozoa - ps11
Choano./Metazoa - ps12
Metazoa - ps13
Myriazoa - ps14
Parahoxozoa - ps15
Bilateria - ps16
Deuterostomia - ps17
Chordata - ps18
Olfactores - ps19
Vertebrata - ps20
Gnathostomata - ps21
Euteleostomi - ps22
Sarcopterygii - ps23
Tetrapoda - ps24
Amniota - ps25
Mammalia - ps26
Theria - ps27
Placentalia - ps28
Boreoeutheria - ps29
Euarchontoglires - ps30
Euarchonta - ps31
Primates - ps32
Haplorrhini - ps33
Simiiformes - ps34
Catarrhini - ps35
Hominoidea - ps36
Hominidae - ps37
Homininae - ps38
Hominini - ps39
Homo_sapiens - ps40
Phylostratum

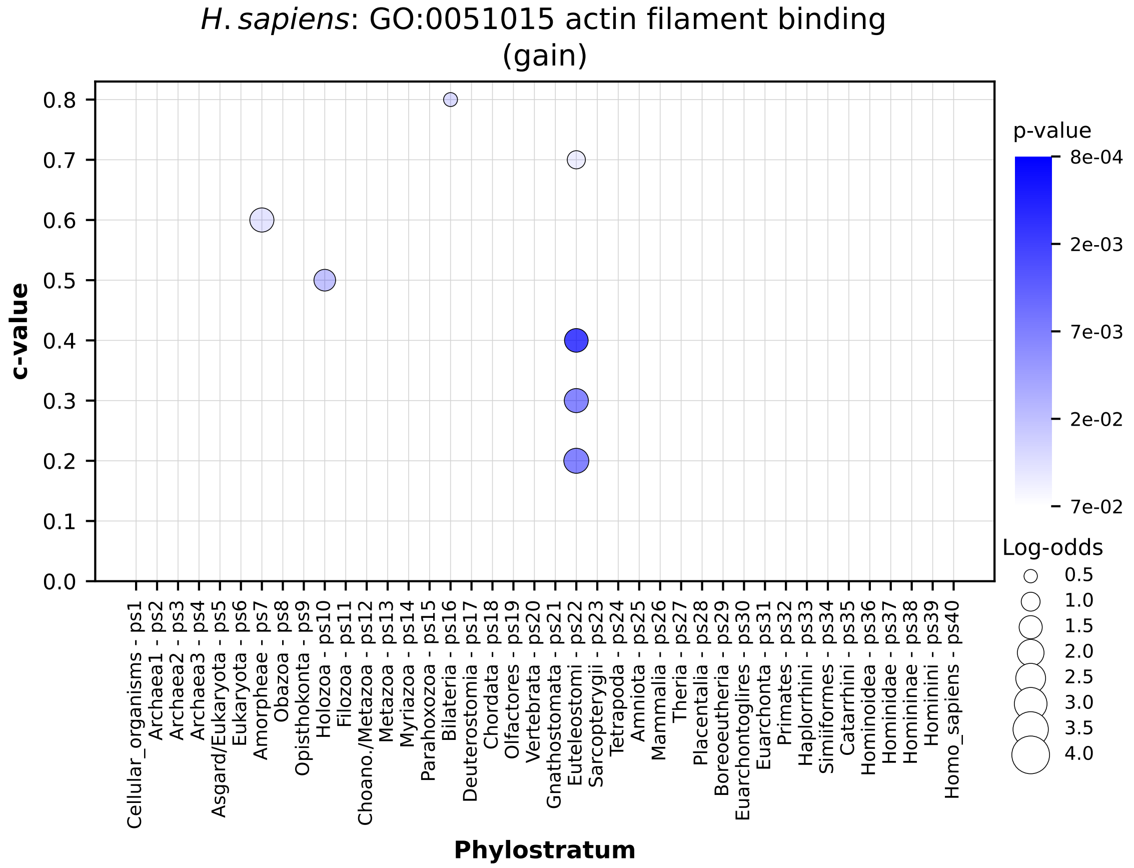

*H. sapiens*: GO:0051015 actin filament binding
(gain)
c-value
0.0
0.1
0.2
0.3
0.4
0.5
0.6
0.7
0.8
Phylostratum
Cellular_organisms - ps1
Archaea1 - ps2
Archaea2 - ps3
Archaea3 - ps4
Asgard/Eukaryota - ps5
Eukaryota - ps6
Amorpheae - ps7
Obazoa - ps8
Opisthokonta - ps9
Holozoa - ps10
Filozoa - ps11
Choano./Metazoa - ps12
Metazoa - ps13
Myriazoa - ps14
Parahoxozoa - ps15
Bilateria - ps16
Deuterostomia - ps17
Chordata - ps18
Olfactores - ps19
Vertebrata - ps20
Gnathostomata - ps21
Euteleostomi - ps22
Sarcopterygii - ps23
Tetrapoda - ps24
Amniota - ps25
Mammalia - ps26
Theria - ps27
Placentalia - ps28
Boreoeutheria - ps29
Euarchontoglires - ps30
Euarchonta - ps31
Primates - ps32
Haplorrhini - ps33
Simiiformes - ps34
Catarrhini - ps35
Hominoidea - ps36
Hominidae - ps37
Homininae - ps38
Hominini - ps39
Homo_sapiens - ps40
p-value
8e-04
2e-03
7e-03
2e-02
7e-02
Log-odds
0.5
1.0
1.5
2.0
2.5
3.0
3.5
4.0

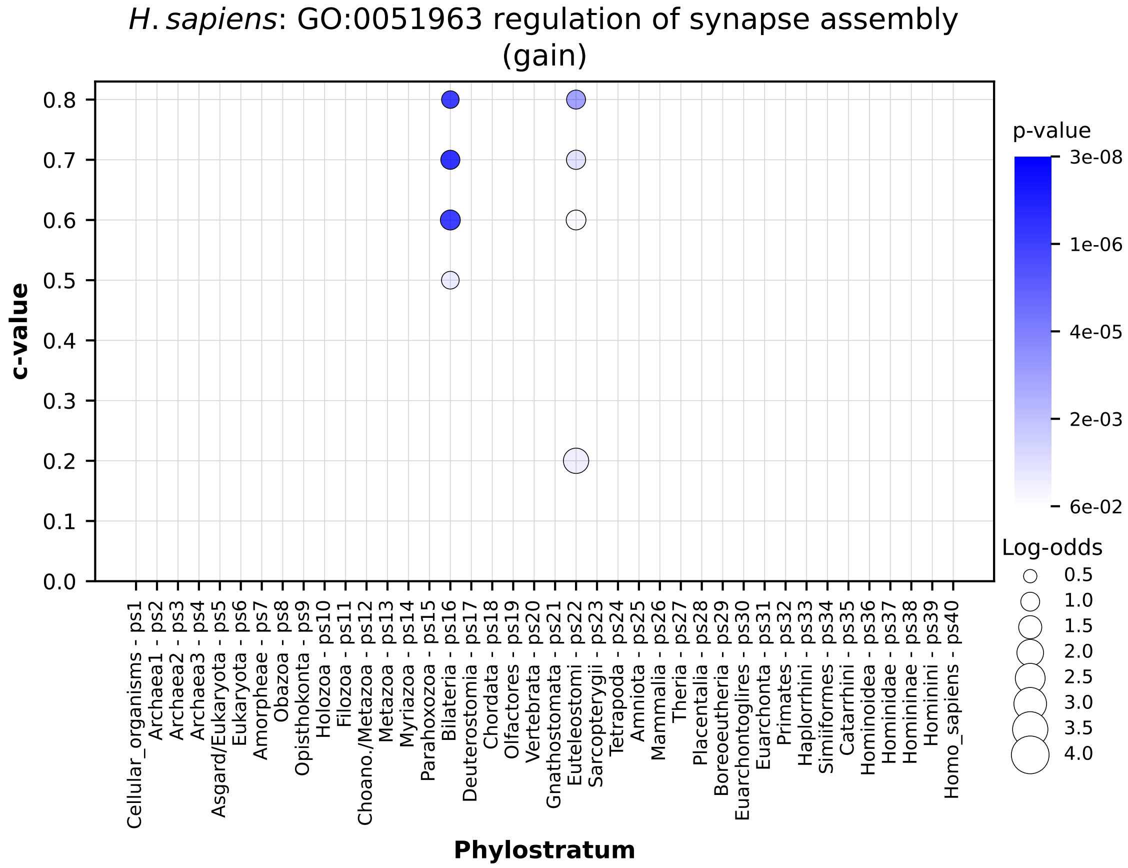

*H. sapiens*: GO:0051963 regulation of synapse assembly (gain)
c-value
0.0
0.1
0.2
0.3
0.4
0.5
0.6
0.7
0.8
Phylostratum
Cellular_organisms - ps1
Archaea1 - ps2
Archaea2 - ps3
Archaea3 - ps4
Asgard/Eukaryota - ps5
Eukaryota - ps6
Amorpheae - ps7
Obazoa - ps8
Opisthokonta - ps9
Holozoa - ps10
Filozoa - ps11
Choano./Metazoa - ps12
Metazoa - ps13
Myriazoa - ps14
Parahoxozoa - ps15
Bilateria - ps16
Deuterostomia - ps17
Chordata - ps18
Olfactores - ps19
Vertebrata - ps20
Gnathostomata - ps21
Euteleostomi - ps22
Sarcopterygii - ps23
Tetrapoda - ps24
Amniota - ps25
Mammalia - ps26
Theria - ps27
Placentalia - ps28
Boreoeutheria - ps29
Euarchontoglires - ps30
Euarchonta - ps31
Primates - ps32
Haplorrhini - ps33
Simiiformes - ps34
Catarrhini - ps35
Hominoidea - ps36
Hominidae - ps37
Homininae - ps38
Hominini - ps39
Homo_sapiens - ps40
p-value
3e-08
1e-06
4e-05
2e-03
6e-02
Log-odds
0.5
1.0
1.5
2.0
2.5
3.0
3.5
4.0

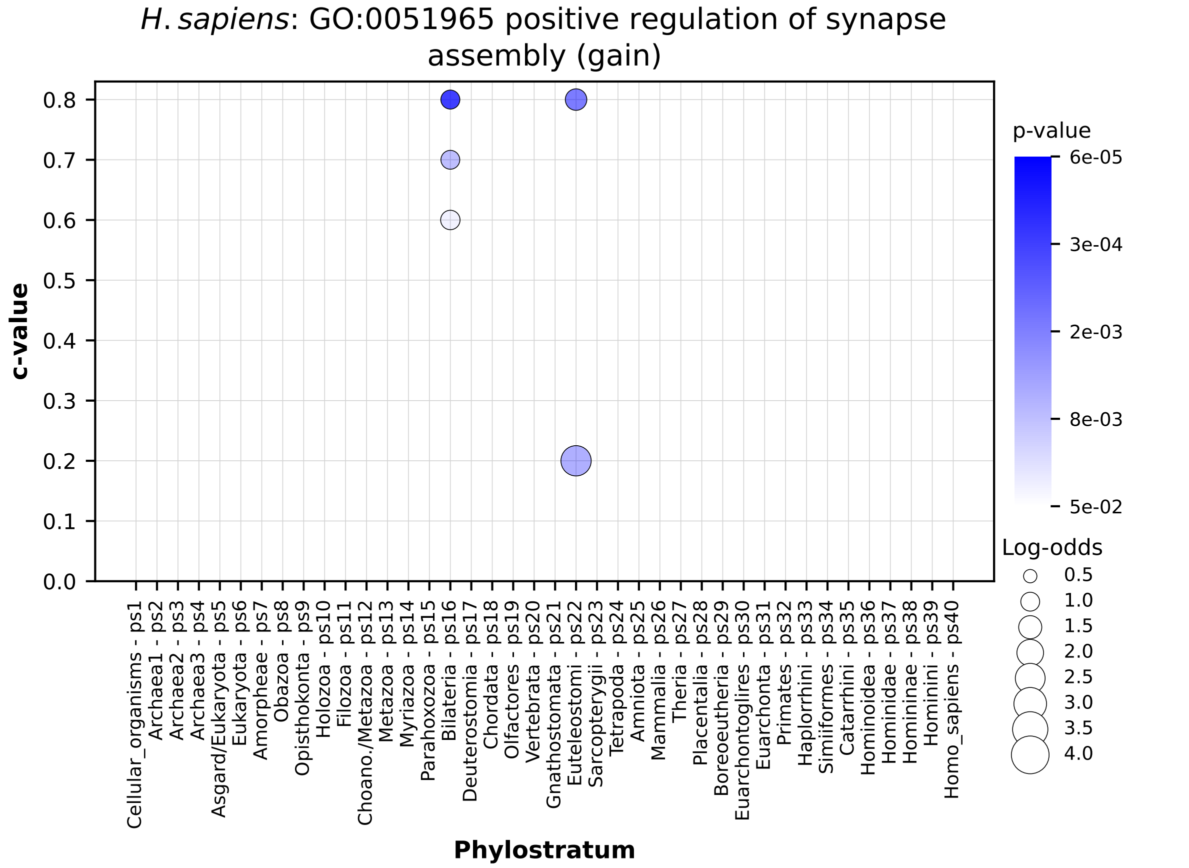

H. sapiens: GO:0051965 positive regulation of synapse assembly (gain)
c-value
0.8
0.7
0.6
0.5
0.4
0.3
0.2
0.1
0.0
p-value
6e-05
3e-04
2e-03
8e-03
5e-02
Log-odds
0.5
1.0
1.5
2.0
2.5
3.0
3.5
4.0
Cellular_organisms - ps1
Archaea1 - ps2
Archaea2 - ps3
Archaea3 - ps4
Asgard/Eukaryota - ps5
Eukaryota - ps6
Amorpheae - ps7
Obazoa - ps8
Opisthokonta - ps9
Holozoa - ps10
Filozoa - ps11
Choano./Metazoa - ps12
Metazoa - ps13
Myriazoa - ps14
Parahoxozoa - ps15
Bilateria - ps16
Deuterostomia - ps17
Chordata - ps18
Olfactores - ps19
Vertebrata - ps20
Gnathostomata - ps21
Euteleostomi - ps22
Sarcopterygii - ps23
Tetrapoda - ps24
Amniota - ps25
Mammalia - ps26
Theria - ps27
Placentalia - ps28
Boreoeutheria - ps29
Euarchontoglires - ps30
Euarchonta - ps31
Primates - ps32
Haplorrhini - ps33
Simiiformes - ps34
Catarrhini - ps35
Hominoidea - ps36
Hominidae - ps37
Homininae - ps38
Hominini - ps39
Homo_sapiens - ps40
Phylostratum

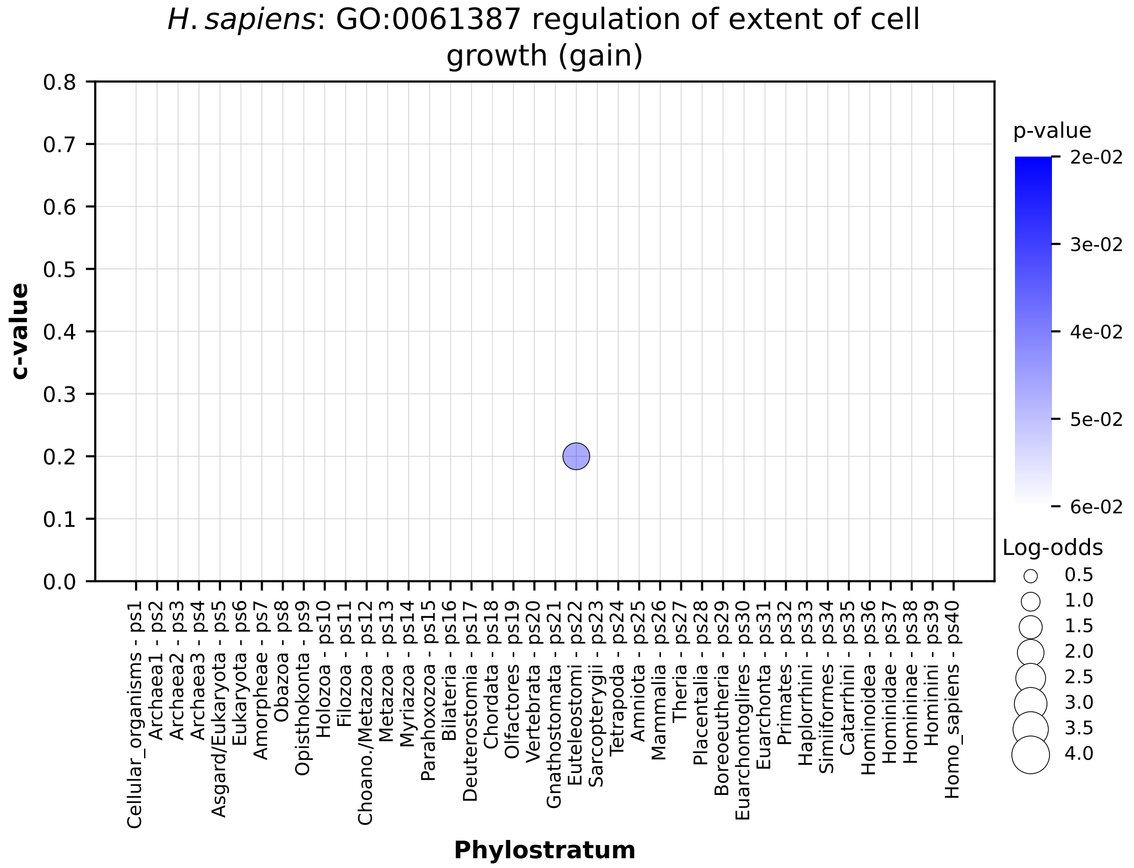

*H. sapiens*: GO:0061387 regulation of extent of cell growth (gain)
c-value
0.8
0.7
0.6
0.5
0.4
0.3
0.2
0.1
0.0
Phylostratum
Cellular_organisms - ps1
Archaea1 - ps2
Archaea2 - ps3
Archaea3 - ps4
Asgard/Eukaryota - ps5
Eukaryota - ps6
Amorpheae - ps7
Obazoa - ps8
Opisthokonta - ps9
Holozoa - ps10
Filozoa - ps11
Choano./Metazoa - ps12
Metazoa - ps13
Myriazoa - ps14
Parahoxozoa - ps15
Bilateria - ps16
Deuterostomia - ps17
Chordata - ps18
Olfactores - ps19
Vertebrata - ps20
Gnathostomata - ps21
Euteleostomi - ps22
Sarcopterygii - ps23
Tetrapoda - ps24
Amniota - ps25
Mammalia - ps26
Theria - ps27
Placentalia - ps28
Boreoeutheria - ps29
Euarchontoglires - ps30
Euarchonta - ps31
Primates - ps32
Haplorrhini - ps33
Simiiformes - ps34
Catarrhini - ps35
Hominoidea - ps36
Hominidae - ps37
Homininae - ps38
Hominini - ps39
Homo_sapiens - ps40
p-value
2e-02
3e-02
4e-02
5e-02
6e-02
Log-odds
0.5
1.0
1.5
2.0
2.5
3.0
3.5
4.0

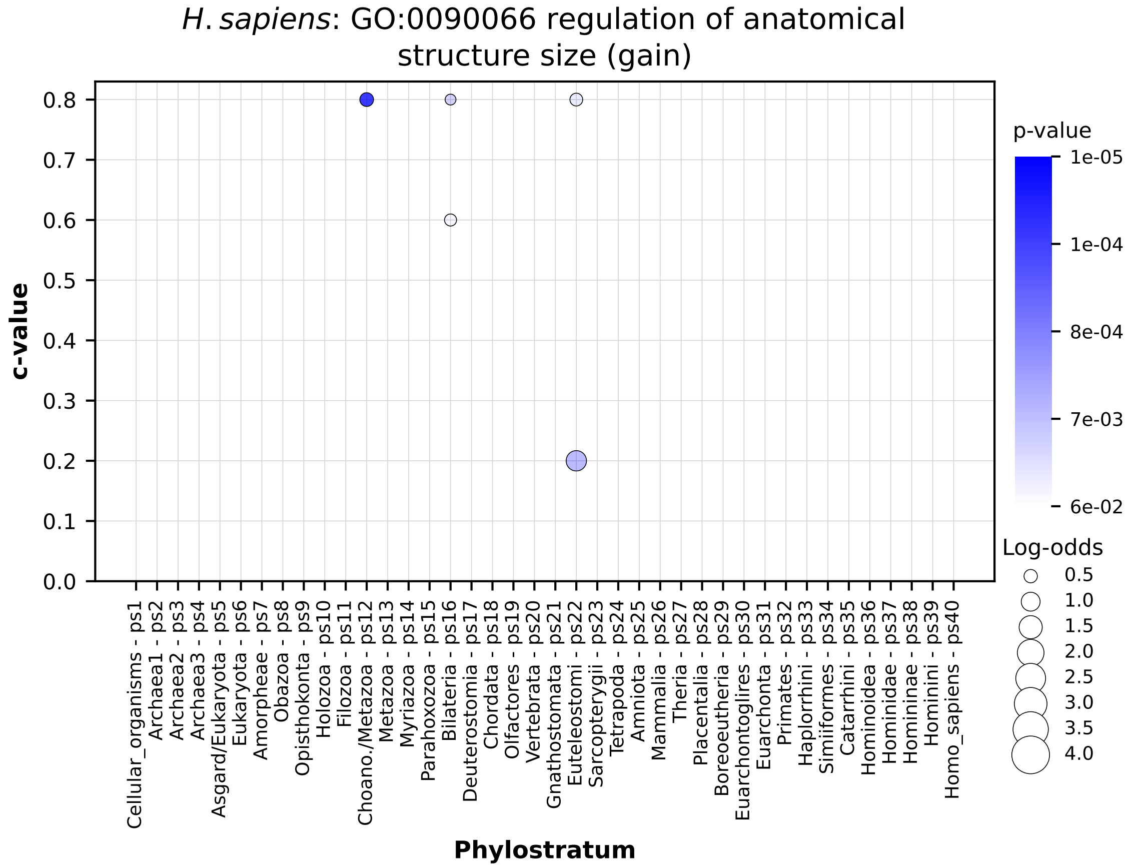
H. sapiens: GO:0090066 regulation of anatomical structure size (gain)
c-value
0.0
0.1
0.2
0.3
0.4
0.5
0.6
0.7
0.8
Phylostratum
Cellular_organisms - ps1
Archaea1 - ps2
Archaea2 - ps3
Archaea3 - ps4
Asgard/Eukaryota - ps5
Eukaryota - ps6
Amorpheae - ps7
Obazoa - ps8
Opisthokonta - ps9
Holozoa - ps10
Filozoa - ps11
Choano./Metazoa - ps12
Metazoa - ps13
Myriazoa - ps14
Parahoxozoa - ps15
Bilateria - ps16
Deuterostomia - ps17
Chordata - ps18
Olfactores - ps19
Vertebrata - ps20
Gnathostomata - ps21
Euteleostomi - ps22
Sarcopterygii - ps23
Tetrapoda - ps24
Amniota - ps25
Mammalia - ps26
Theria - ps27
Placentalia - ps28
Boreoeutheria - ps29
Euarchontoglires - ps30
Euarchonta - ps31
Primates - ps32
Haplorrhini - ps33
Simiiformes - ps34
Catarrhini - ps35
Hominoidea - ps36
Hominidae - ps37
Homininae - ps38
Hominini - ps39
Homo_sapiens - ps40
p-value
1e-05
1e-04
8e-04
7e-03
6e-02
Log-odds
0.5
1.0
1.5
2.0
2.5
3.0
3.5
4.0

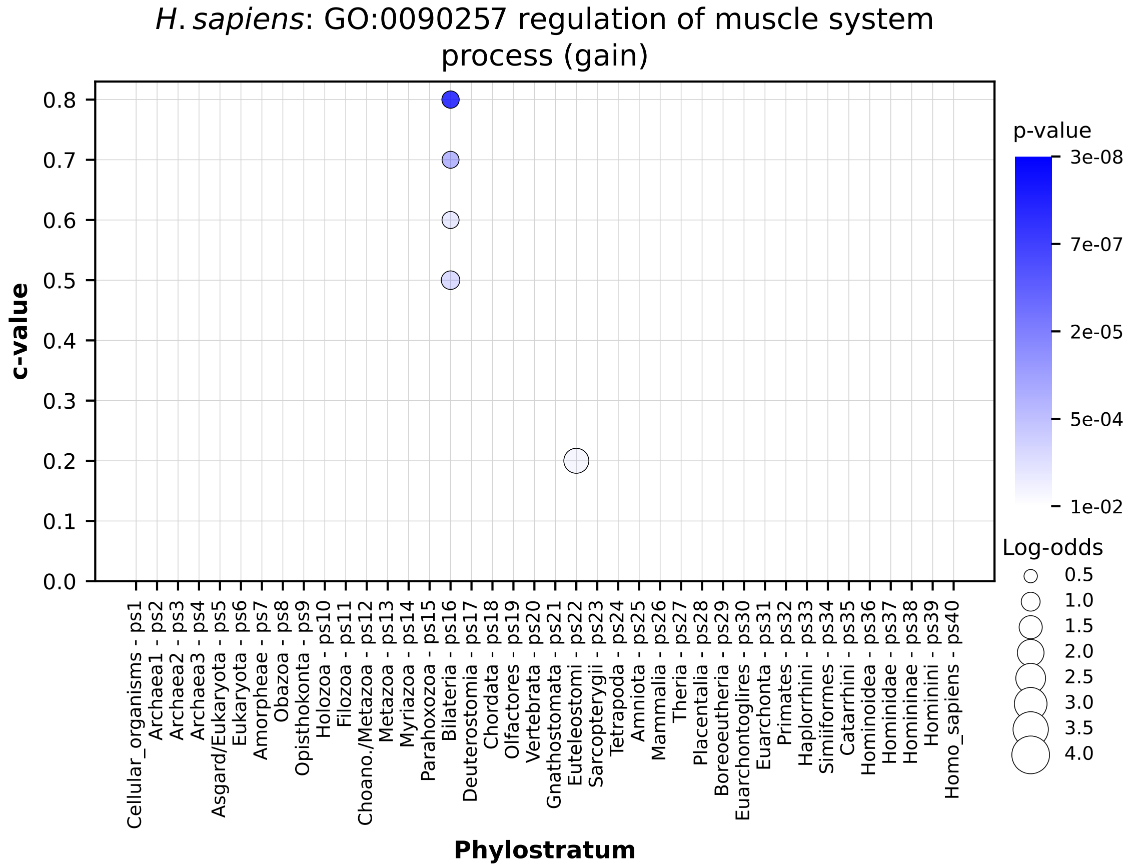
*H. sapiens*: GO:0090257 regulation of muscle system process (gain)
c-value
0.0
0.1
0.2
0.3
0.4
0.5
0.6
0.7
0.8
Phylostratum
Cellular_organisms - ps1
Archaea1 - ps2
Archaea2 - ps3
Archaea3 - ps4
Asgard/Eukaryota - ps5
Eukaryota - ps6
Amorpheae - ps7
Obazoa - ps8
Opisthokonta - ps9
Holozoa - ps10
Filozoa - ps11
Choano./Metazoa - ps12
Metazoa - ps13
Myriazoa - ps14
Parahoxozoa - ps15
Bilateria - ps16
Deuterostomia - ps17
Chordata - ps18
Olfactores - ps19
Vertebrata - ps20
Gnathostomata - ps21
Euteleostomi - ps22
Sarcopterygii - ps23
Tetrapoda - ps24
Amniota - ps25
Mammalia - ps26
Theria - ps27
Placentalia - ps28
Boreoeutheria - ps29
Euarchontoglires - ps30
Euarchonta - ps31
Primates - ps32
Haplorrhini - ps33
Simiiformes - ps34
Catarrhini - ps35
Hominoidea - ps36
Hominidae - ps37
Homininae - ps38
Hominini - ps39
Homo_sapiens - ps40
p-value
3e-08
7e-07
2e-05
5e-04
1e-02
Log-odds
0.5
1.0
1.5
2.0
2.5
3.0
3.5
4.0

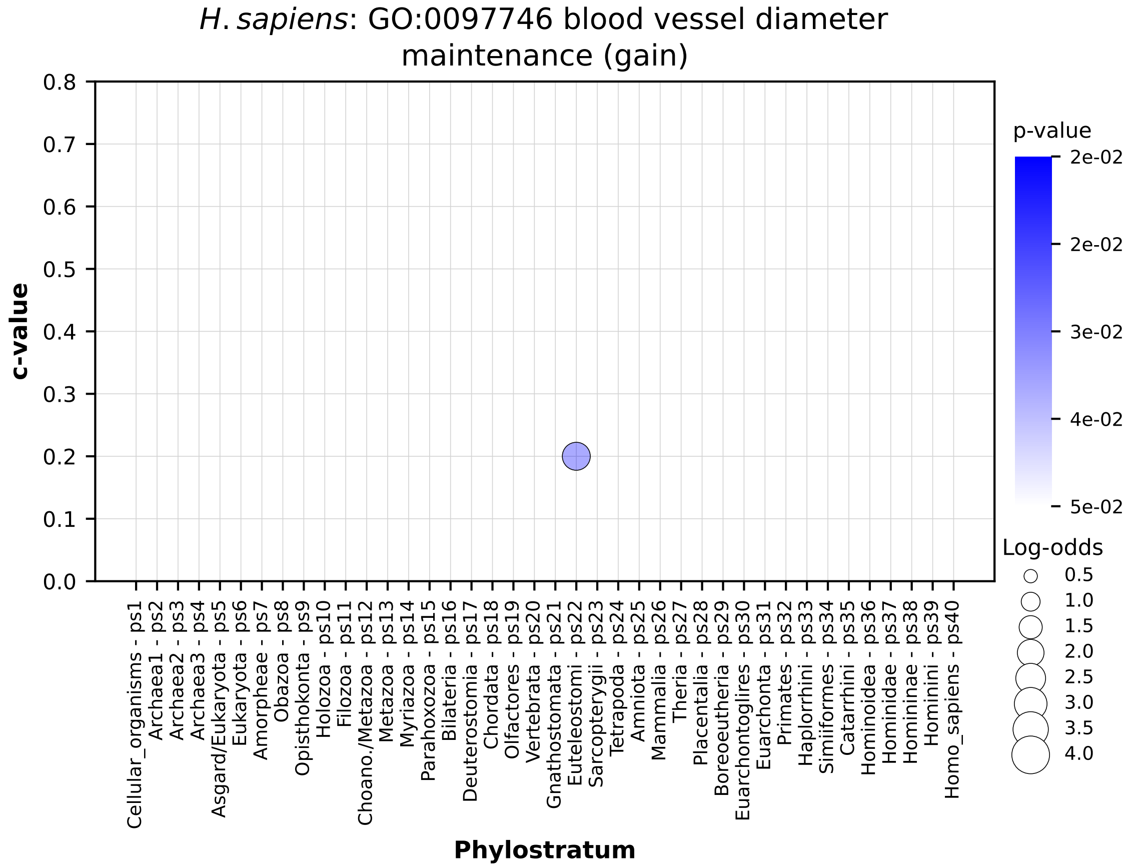

*H. sapiens*: GO:0097746 blood vessel diameter maintenance (gain)
c-value
0.0
0.1
0.2
0.3
0.4
0.5
0.6
0.7
0.8
Phylostratum
Cellular_organisms - ps1
Archaea1 - ps2
Archaea2 - ps3
Archaea3 - ps4
Asgard/Eukaryota - ps5
Eukaryota - ps6
Amorpheae - ps7
Obazoa - ps8
Opisthokonta - ps9
Holozoa - ps10
Filozoa - ps11
Choano./Metazoa - ps12
Metazoa - ps13
Myriazoa - ps14
Parahoxozoa - ps15
Bilateria - ps16
Deuterostomia - ps17
Chordata - ps18
Olfactores - ps19
Vertebrata - ps20
Gnathostomata - ps21
Euteleostomi - ps22
Sarcopterygii - ps23
Tetrapoda - ps24
Amniota - ps25
Mammalia - ps26
Theria - ps27
Placentalia - ps28
Boreoeutheria - ps29
Euarchontoglires - ps30
Euarchonta - ps31
Primates - ps32
Haplorrhini - ps33
Simiiformes - ps34
Catarrhini - ps35
Hominoidea - ps36
Hominidae - ps37
Homininae - ps38
Hominini - ps39
Homo_sapiens - ps40
p-value
2e-02
2e-02
3e-02
4e-02
5e-02
Log-odds
0.5
1.0
1.5
2.0
2.5
3.0
3.5
4.0

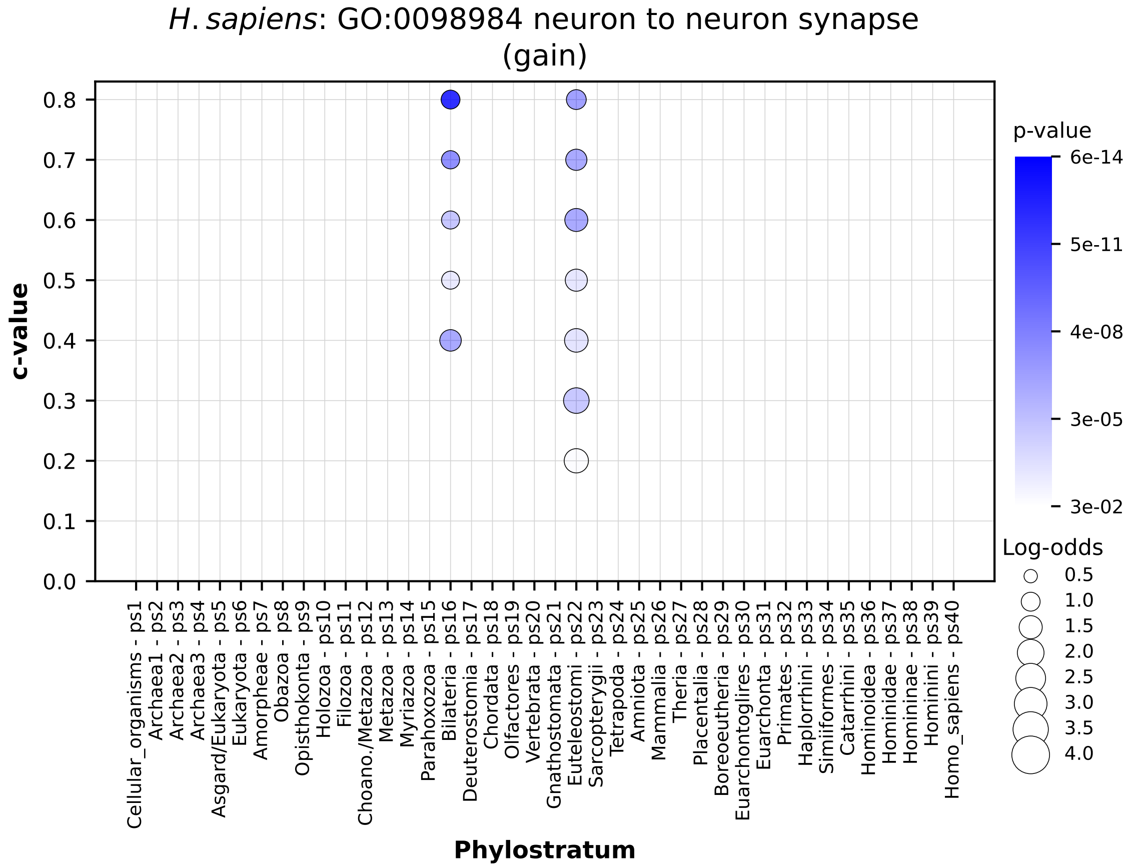

*H. sapiens*: GO:0098984 neuron to neuron synapse
(gain)
c-value
0.0
0.1
0.2
0.3
0.4
0.5
0.6
0.7
0.8
Phylostratum
Cellular_organisms - ps1
Archaea1 - ps2
Archaea2 - ps3
Archaea3 - ps4
Asgard/Eukaryota - ps5
Eukaryota - ps6
Amorpheae - ps7
Obazoa - ps8
Opisthokonta - ps9
Holozoa - ps10
Filozoa - ps11
Choano./Metazoa - ps12
Metazoa - ps13
Myriazoa - ps14
Parahoxozoa - ps15
Bilateria - ps16
Deuterostomia - ps17
Chordata - ps18
Olfactores - ps19
Vertebrata - ps20
Gnathostomata - ps21
Euteleostomi - ps22
Sarcopterygii - ps23
Tetrapoda - ps24
Amniota - ps25
Mammalia - ps26
Theria - ps27
Placentalia - ps28
Boreoeutheria - ps29
Euarchontoglires - ps30
Euarchonta - ps31
Primates - ps32
Haplorrhini - ps33
Simiiformes - ps34
Catarrhini - ps35
Hominoidea - ps36
Hominidae - ps37
Homininae - ps38
Hominini - ps39
Homo_sapiens - ps40
p-value
6e-14
5e-11
4e-08
3e-05
3e-02
Log-odds
0.5
1.0
1.5
2.0
2.5
3.0
3.5
4.0

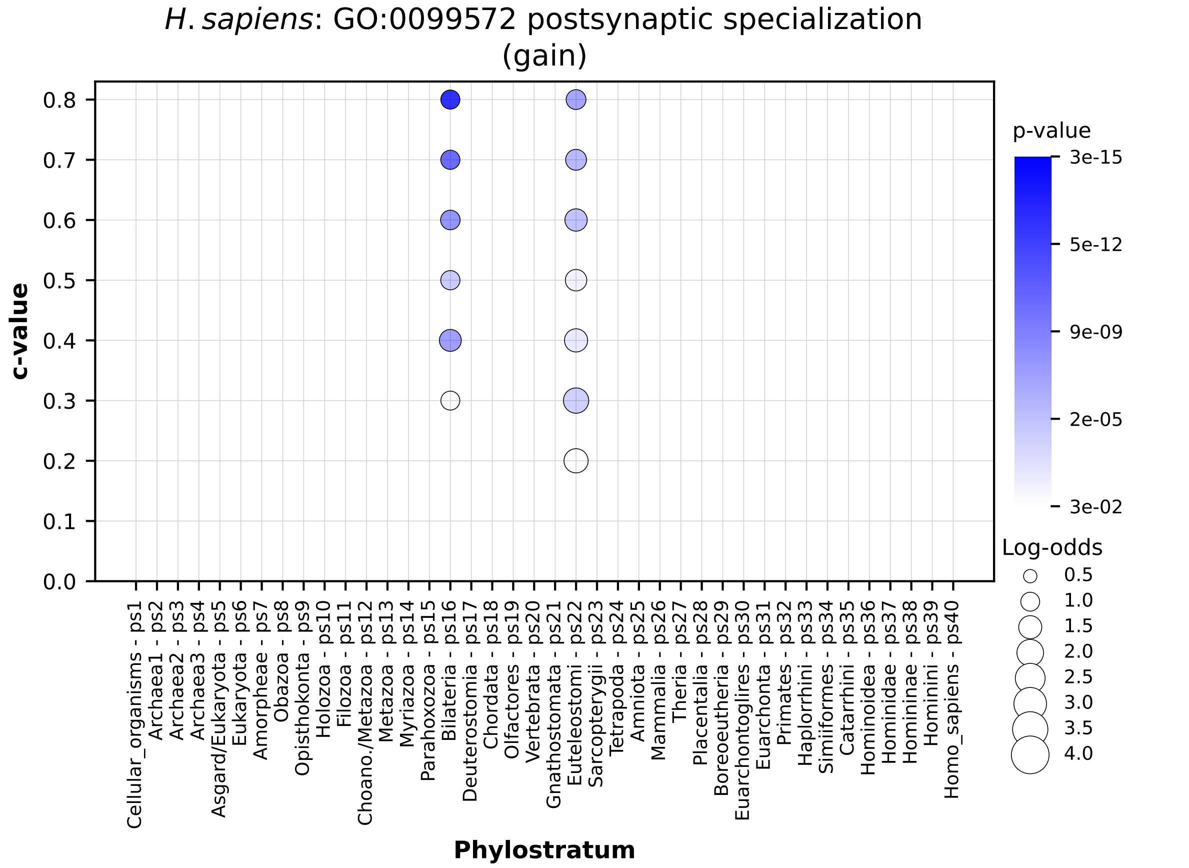

H. sapiens: GO:0099572 postsynaptic specialization
(gain)
c-value
0.8
0.7
0.6
0.5
0.4
0.3
0.2
0.1
0.0
p-value
3e-15
5e-12
9e-09
2e-05
3e-02
Log-odds
0.5
1.0
1.5
2.0
2.5
3.0
3.5
4.0
Cellular_organisms - ps1
Archaea1 - ps2
Archaea2 - ps3
Archaea3 - ps4
Asgard/Eukaryota - ps5
Eukaryota - ps6
Amorpheae - ps7
Obazoa - ps8
Opisthokonta - ps9
Holozoa - ps10
Filozoa - ps11
Choano./Metazoa - ps12
Metazoa - ps13
Myriazoa - ps14
Parahoxozoa - ps15
Bilateria - ps16
Deuterostomia - ps17
Chordata - ps18
Olfactores - ps19
Vertebrata - ps20
Gnathostomata - ps21
Euteleostomi - ps22
Sarcopterygii - ps23
Tetrapoda - ps24
Amniota - ps25
Mammalia - ps26
Theria - ps27
Placentalia - ps28
Boreoeutheria - ps29
Euarchontoglires - ps30
Euarchonta - ps31
Primates - ps32
Haplorrhini - ps33
Simiiformes - ps34
Catarrhini - ps35
Hominoidea - ps36
Hominidae - ps37
Homininae - ps38
Hominini - ps39
Homo_sapiens - ps40
Phylostratum

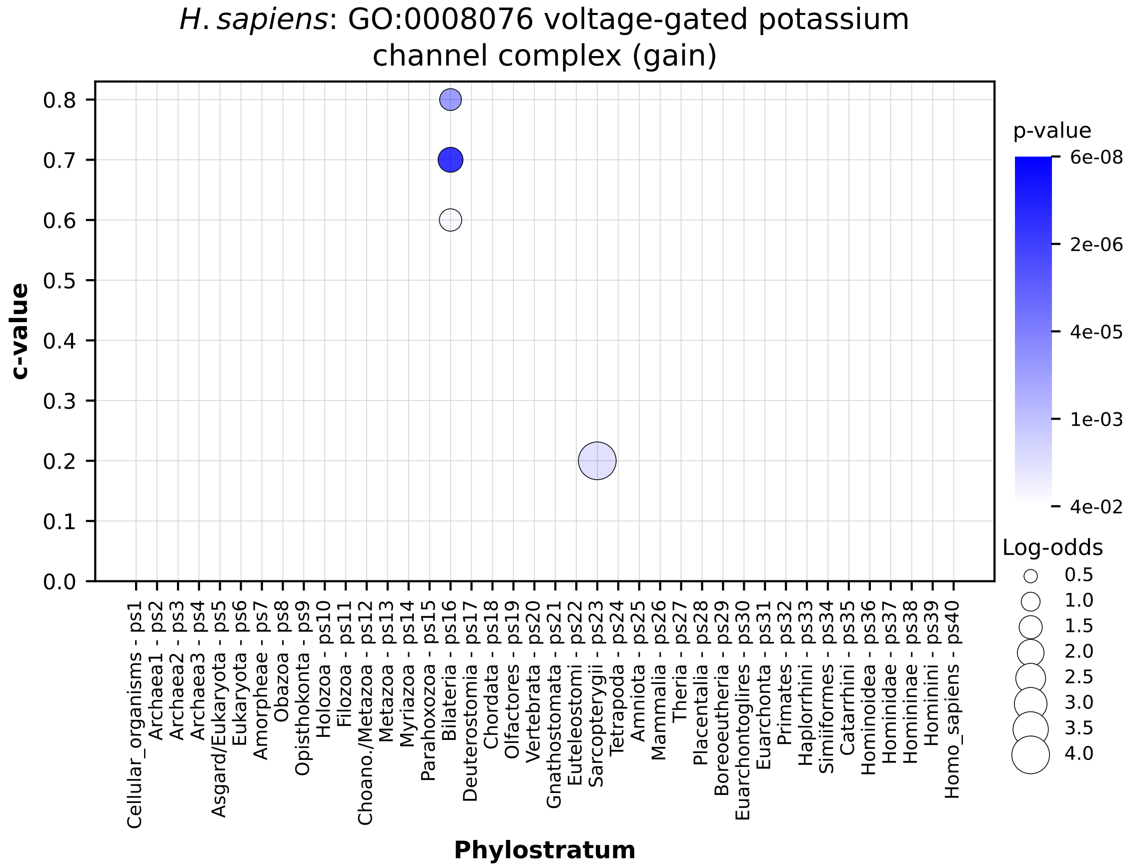

H. sapiens: GO:0008076 voltage-gated potassium channel complex (gain)
c-value
0.8
0.7
0.6
0.5
0.4
0.3
0.2
0.1
0.0
Phylostratum
Cellular_organisms - ps1
Archaea1 - ps2
Archaea2 - ps3
Archaea3 - ps4
Asgard/Eukaryota - ps5
Eukaryota - ps6
Amorpheae - ps7
Obazoa - ps8
Opisthokonta - ps9
Holozoa - ps10
Filozoa - ps11
Choano./Metazoa - ps12
Metazoa - ps13
Myriazoa - ps14
Parahoxozoa - ps15
Bilateria - ps16
Deuterostomia - ps17
Chordata - ps18
Olfactores - ps19
Vertebrata - ps20
Gnathostomata - ps21
Euteleostomi - ps22
Sarcopterygii - ps23
Tetrapoda - ps24
Amniota - ps25
Mammalia - ps26
Theria - ps27
Placentalia - ps28
Boreoeutheria - ps29
Euarchontoglires - ps30
Euarchonta - ps31
Primates - ps32
Haplorrhini - ps33
Simiiformes - ps34
Catarrhini - ps35
Hominoidea - ps36
Hominidae - ps37
Homininae - ps38
Hominini - ps39
Homo_sapiens - ps40
p-value
6e-08
2e-06
4e-05
1e-03
4e-02
Log-odds
0.5
1.0
1.5
2.0
2.5
3.0
3.5
4.0

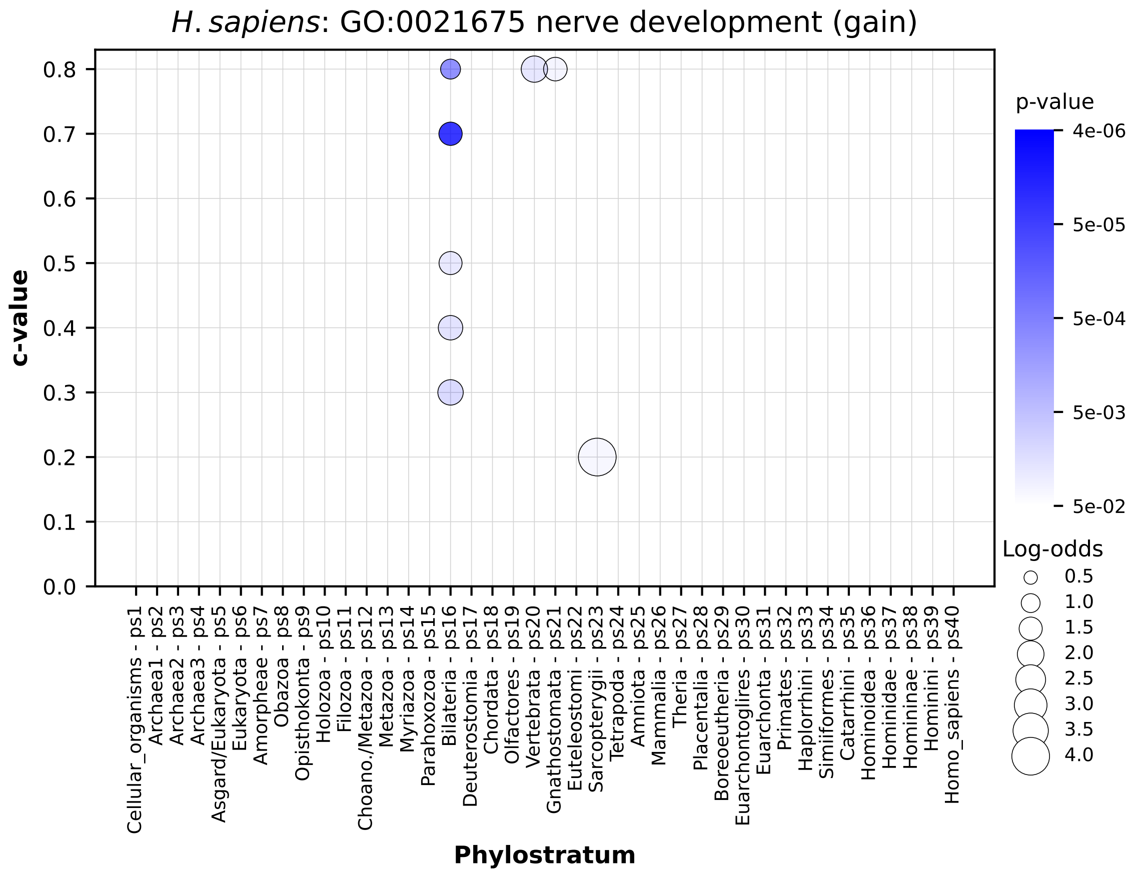

H. sapiens: GO:0021675 nerve development (gain)
c-value
0.0
0.1
0.2
0.3
0.4
0.5
0.6
0.7
0.8
Phylostratum
Cellular_organisms - ps1
Archaea1 - ps2
Archaea2 - ps3
Archaea3 - ps4
Asgard/Eukaryota - ps5
Eukaryota - ps6
Amorpheae - ps7
Obazoa - ps8
Opisthokonta - ps9
Holozoa - ps10
Filozoa - ps11
Choano./Metazoa - ps12
Metazoa - ps13
Myriazoa - ps14
Parahoxozoa - ps15
Bilateria - ps16
Deuterostomia - ps17
Chordata - ps18
Olfactores - ps19
Vertebrata - ps20
Gnathostomata - ps21
Euteleostomi - ps22
Sarcopterygii - ps23
Tetrapoda - ps24
Amniota - ps25
Mammalia - ps26
Theria - ps27
Placentalia - ps28
Boreoeutheria - ps29
Euarchontoglires - ps30
Euarchonta - ps31
Primates - ps32
Haplorrhini - ps33
Simiiformes - ps34
Catarrhini - ps35
Hominoidea - ps36
Hominidae - ps37
Homininae - ps38
Hominini - ps39
Homo_sapiens - ps40
p-value
4e-06
5e-05
5e-04
5e-03
5e-02
Log-odds
0.5
1.0
1.5
2.0
2.5
3.0
3.5
4.0

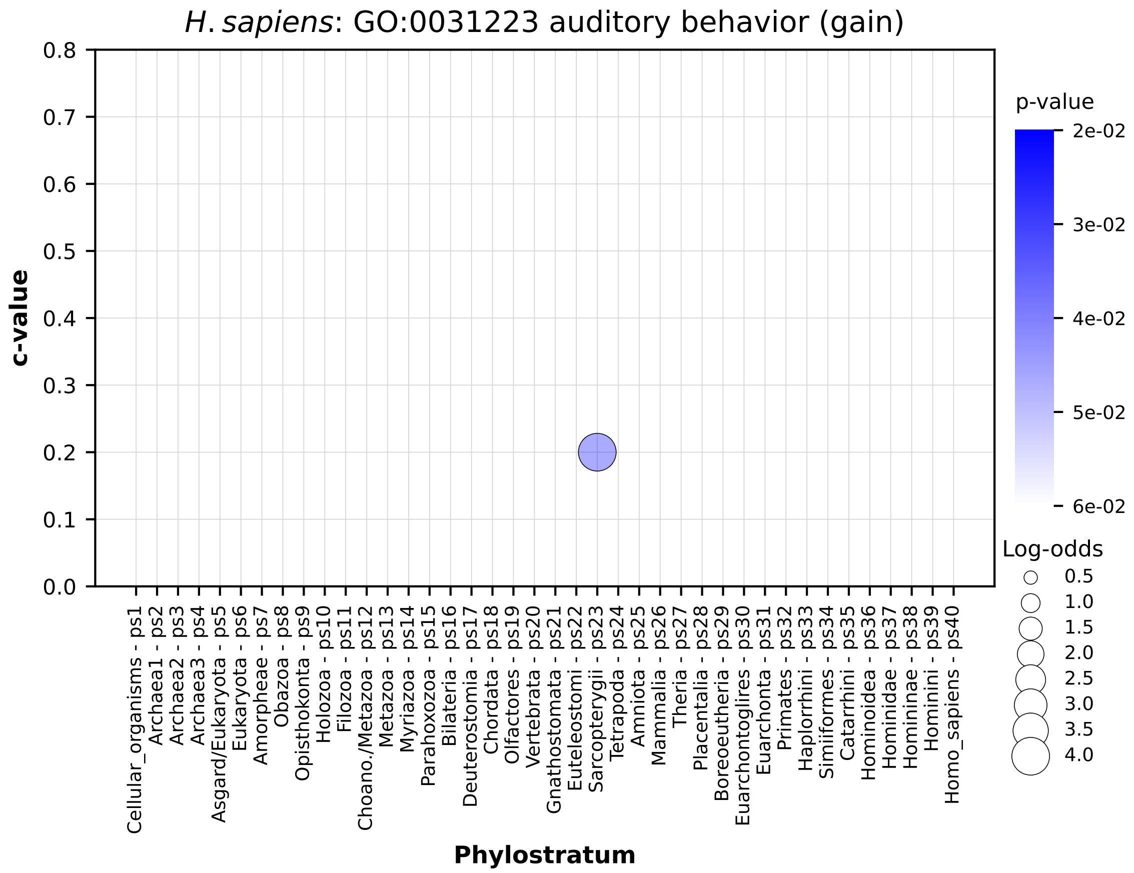

H. sapiens: GO:0031223 auditory behavior (gain)
c-value
0.0
0.1
0.2
0.3
0.4
0.5
0.6
0.7
0.8
Phylostratum
Cellular_organisms - ps1
Archaea1 - ps2
Archaea2 - ps3
Archaea3 - ps4
Asgard/Eukaryota - ps5
Eukaryota - ps6
Amorpheae - ps7
Obazoa - ps8
Opisthokonta - ps9
Holozoa - ps10
Filozoa - ps11
Choano./Metazoa - ps12
Metazoa - ps13
Myriazoa - ps14
Parahoxozoa - ps15
Bilateria - ps16
Deuterostomia - ps17
Chordata - ps18
Olfactores - ps19
Vertebrata - ps20
Gnathostomata - ps21
Euteleostomi - ps22
Sarcopterygii - ps23
Tetrapoda - ps24
Amniota - ps25
Mammalia - ps26
Theria - ps27
Placentalia - ps28
Boreoeutheria - ps29
Euarchontoglires - ps30
Euarchonta - ps31
Primates - ps32
Haplorrhini - ps33
Simiiformes - ps34
Catarrhini - ps35
Hominoidea - ps36
Hominidae - ps37
Homininae - ps38
Hominini - ps39
Homo_sapiens - ps40
p-value
2e-02
3e-02
4e-02
5e-02
6e-02
Log-odds
0.5
1.0
1.5
2.0
2.5
3.0
3.5
4.0

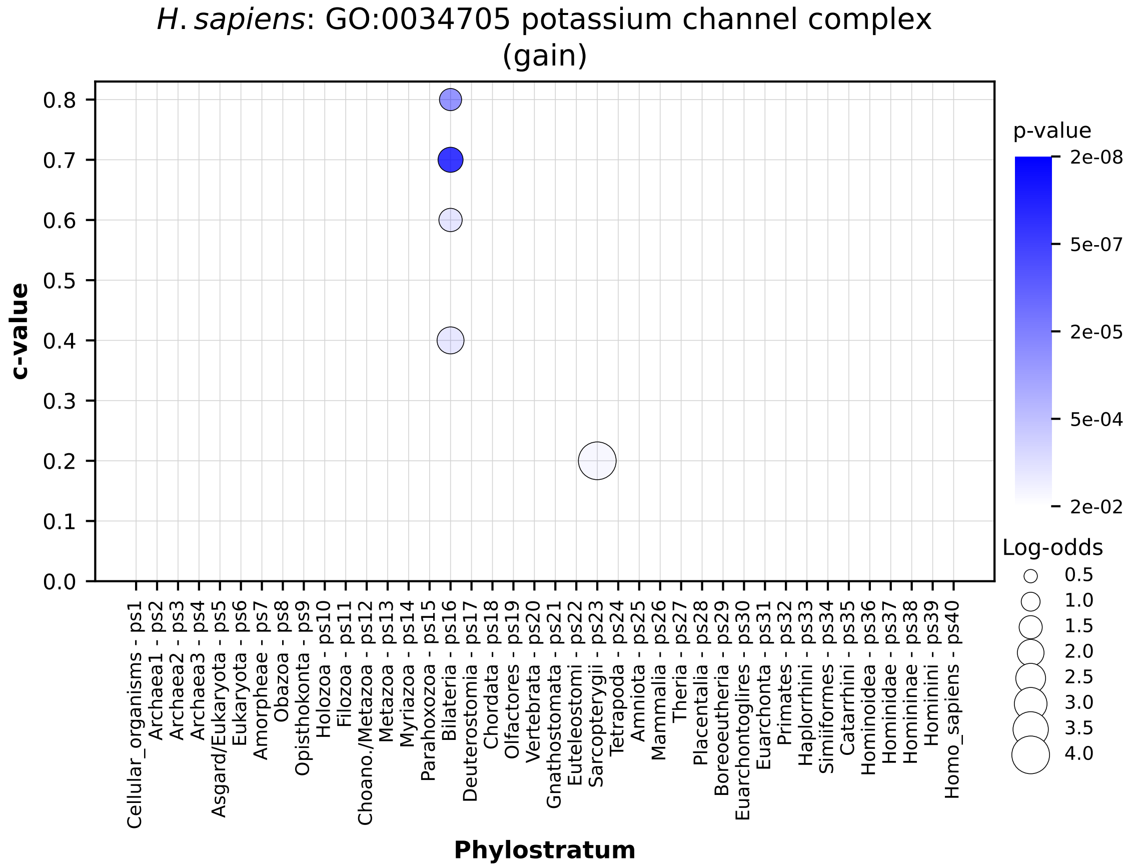
*H. sapiens*: GO:0034705 potassium channel complex
(gain)
c-value
0.0
0.1
0.2
0.3
0.4
0.5
0.6
0.7
0.8
Phylostratum
Cellular_organisms - ps1
Archaea1 - ps2
Archaea2 - ps3
Archaea3 - ps4
Asgard/Eukaryota - ps5
Eukaryota - ps6
Amorpheae - ps7
Obazoa - ps8
Opisthokonta - ps9
Holozoa - ps10
Filozoa - ps11
Choano./Metazoa - ps12
Metazoa - ps13
Myriazoa - ps14
Parahoxozoa - ps15
Bilateria - ps16
Deuterostomia - ps17
Chordata - ps18
Olfactores - ps19
Vertebrata - ps20
Gnathostomata - ps21
Euteleostomi - ps22
Sarcopterygii - ps23
Tetrapoda - ps24
Amniota - ps25
Mammalia - ps26
Theria - ps27
Placentalia - ps28
Boreoeutheria - ps29
Euarchontoglires - ps30
Euarchonta - ps31
Primates - ps32
Haplorrhini - ps33
Simiiformes - ps34
Catarrhini - ps35
Hominoidea - ps36
Hominidae - ps37
Homininae - ps38
Hominini - ps39
Homo_sapiens - ps40
p-value
2e-08
5e-07
2e-05
5e-04
2e-02
Log-odds
0.5
1.0
1.5
2.0
2.5
3.0
3.5
4.0

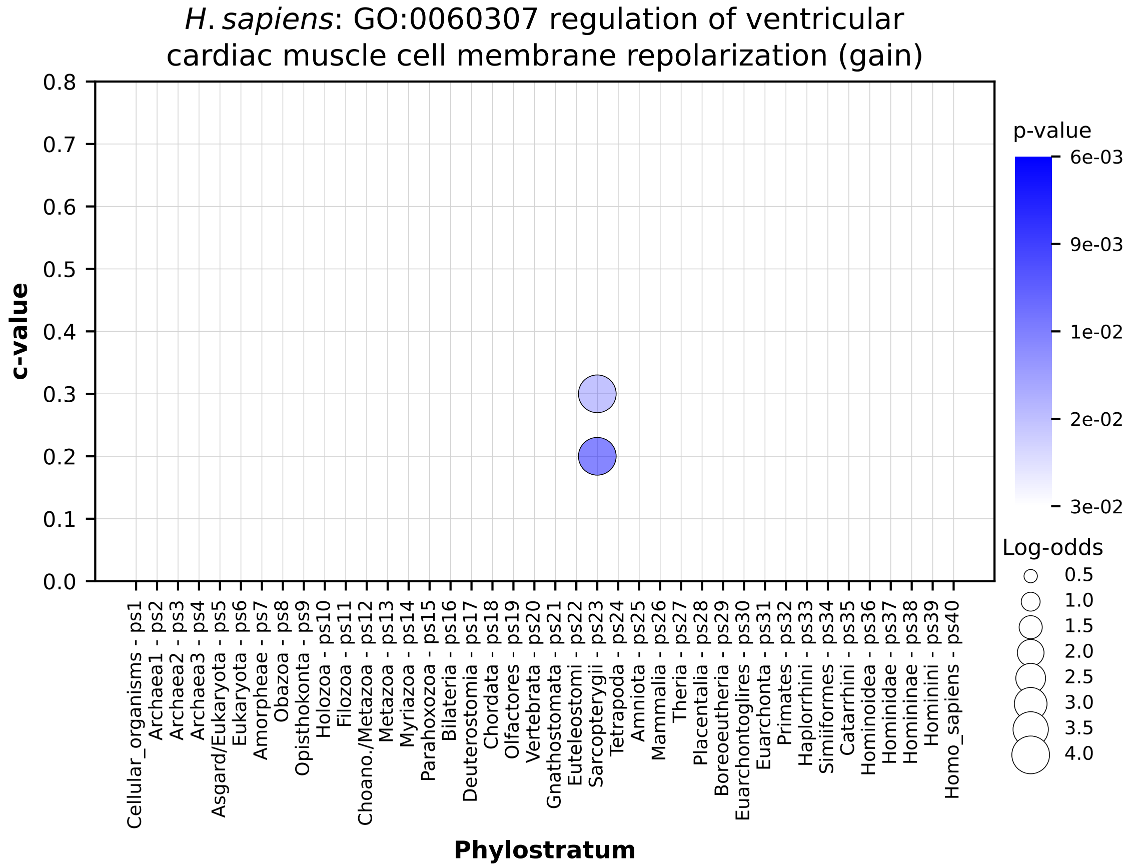

H. sapiens: GO:0060307 regulation of ventricular cardiac muscle cell membrane repolarization (gain)
c-value
0.8
0.7
0.6
0.5
0.4
0.3
0.2
0.1
0.0
p-value
6e-03
9e-03
1e-02
2e-02
3e-02
Log-odds
0.5
1.0
1.5
2.0
2.5
3.0
3.5
4.0
Cellular_organisms - ps1
Archaea1 - ps2
Archaea2 - ps3
Archaea3 - ps4
Asgard/Eukaryota - ps5
Eukaryota - ps6
Amorpheae - ps7
Obazoa - ps8
Opisthokonta - ps9
Holozoa - ps10
Filozoa - ps11
Choano./Metazoa - ps12
Metazoa - ps13
Myriazoa - ps14
Parahoxozoa - ps15
Bilateria - ps16
Deuterostomia - ps17
Chordata - ps18
Olfactores - ps19
Vertebrata - ps20
Gnathostomata - ps21
Euteleostomi - ps22
Sarcopterygii - ps23
Tetrapoda - ps24
Amniota - ps25
Mammalia - ps26
Theria - ps27
Placentalia - ps28
Boreoeutheria - ps29
Euarchontoglires - ps30
Euarchonta - ps31
Primates - ps32
Haplorrhini - ps33
Simiiformes - ps34
Catarrhini - ps35
Hominoidea - ps36
Hominidae - ps37
Homininae - ps38
Hominini - ps39
Homo_sapiens - ps40
Phylostratum

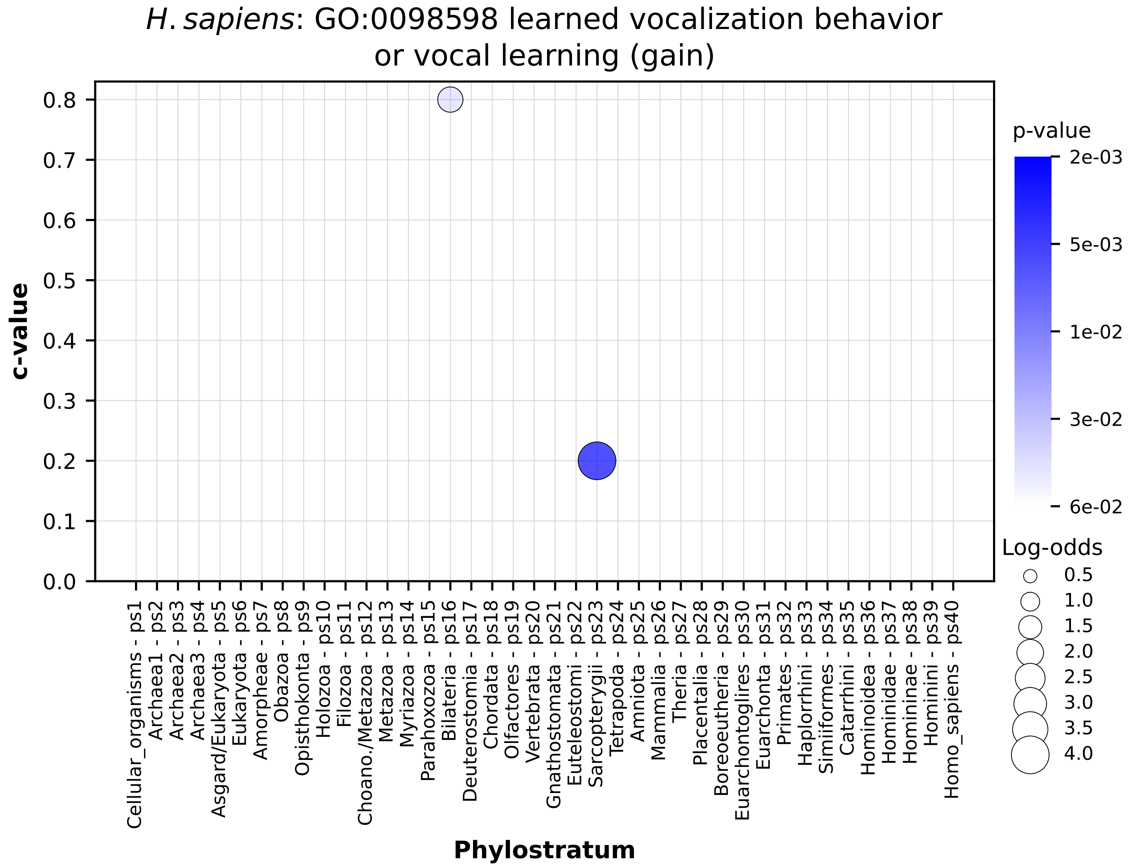

*H. sapiens*: GO:0098598 learned vocalization behavior or vocal learning (gain)
c-value
0.0
0.1
0.2
0.3
0.4
0.5
0.6
0.7
0.8
p-value
2e-03
5e-03
1e-02
3e-02
6e-02
Log-odds
0.5
1.0
1.5
2.0
2.5
3.0
3.5
4.0
Cellular_organisms - ps1
Archaea1 - ps2
Archaea2 - ps3
Archaea3 - ps4
Asgard/Eukaryota - ps5
Eukaryota - ps6
Amorpheae - ps7
Obazoa - ps8
Opisthokonta - ps9
Holozoa - ps10
Filozoa - ps11
Choano./Metazoa - ps12
Metazoa - ps13
Myriazoa - ps14
Parahoxozoa - ps15
Bilateria - ps16
Deuterostomia - ps17
Chordata - ps18
Olfactores - ps19
Vertebrata - ps20
Gnathostomata - ps21
Euteleostomi - ps22
Sarcopterygii - ps23
Tetrapoda - ps24
Amniota - ps25
Mammalia - ps26
Theria - ps27
Placentalia - ps28
Boreoeutheria - ps29
Euarchontoglires - ps30
Euarchonta - ps31
Primates - ps32
Haplorrhini - ps33
Simiiformes - ps34
Catarrhini - ps35
Hominoidea - ps36
Hominidae - ps37
Homininae - ps38
Hominini - ps39
Homo_sapiens - ps40
Phylostratum

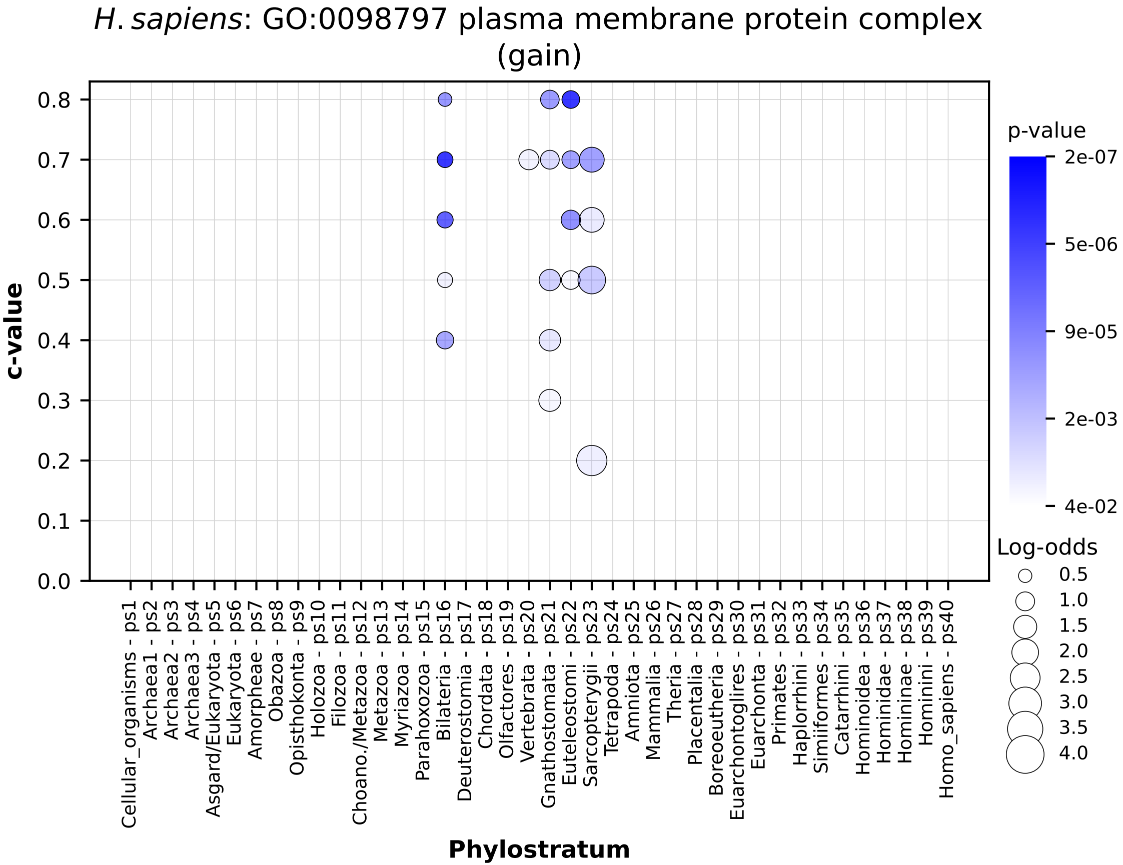

H. sapiens: GO:0098797 plasma membrane protein complex
(gain)
c-value
0.0
0.1
0.2
0.3
0.4
0.5
0.6
0.7
0.8
Phylostratum
Cellular_organisms - ps1
Archaea1 - ps2
Archaea2 - ps3
Archaea3 - ps4
Asgard/Eukaryota - ps5
Eukaryota - ps6
Amorpheae - ps7
Obazoa - ps8
Opisthokonta - ps9
Holozoa - ps10
Filozoa - ps11
Choano./Metazoa - ps12
Metazoa - ps13
Myriazoa - ps14
Parahoxozoa - ps15
Bilateria - ps16
Deuterostomia - ps17
Chordata - ps18
Olfactores - ps19
Vertebrata - ps20
Gnathostomata - ps21
Euteleostomi - ps22
Sarcopterygii - ps23
Tetrapoda - ps24
Amniota - ps25
Mammalia - ps26
Theria - ps27
Placentalia - ps28
Boreoeutheria - ps29
Euarchontoglires - ps30
Euarchonta - ps31
Primates - ps32
Haplorrhini - ps33
Simiiformes - ps34
Catarrhini - ps35
Hominoidea - ps36
Hominidae - ps37
Homininae - ps38
Hominini - ps39
Homo_sapiens - ps40
p-value
2e-07
5e-06
9e-05
2e-03
4e-02
Log-odds
0.5
1.0
1.5
2.0
2.5
3.0
3.5
4.0

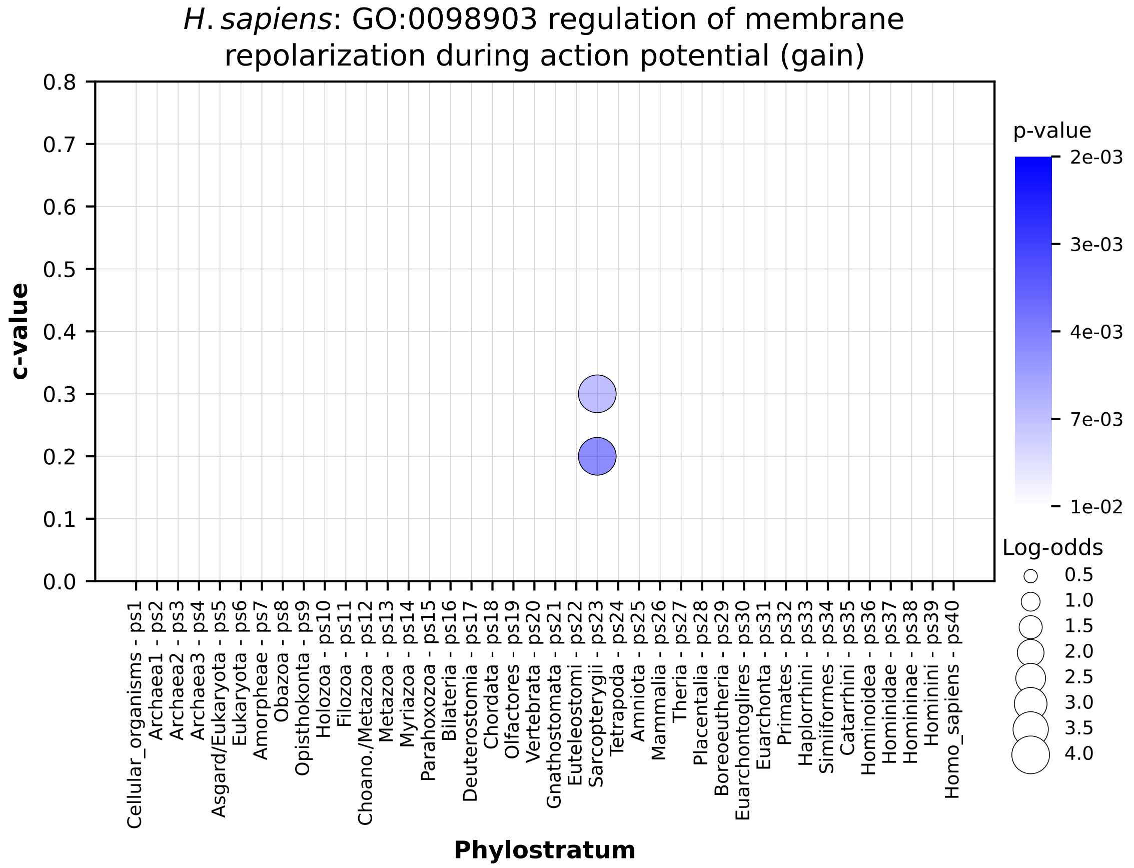

*H. sapiens*: GO:0098903 regulation of membrane repolarization during action potential (gain)
c-value
0.8
0.7
0.6
0.5
0.4
0.3
0.2
0.1
0.0
Phylostratum
Cellular_organisms - ps1
Archaea1 - ps2
Archaea2 - ps3
Archaea3 - ps4
Asgard/Eukaryota - ps5
Eukaryota - ps6
Amorpheae - ps7
Obazoa - ps8
Opisthokonta - ps9
Holozoa - ps10
Filozoa - ps11
Choano./Metazoa - ps12
Metazoa - ps13
Myriazoa - ps14
Parahoxozoa - ps15
Bilateria - ps16
Deuterostomia - ps17
Chordata - ps18
Olfactores - ps19
Vertebrata - ps20
Gnathostomata - ps21
Euteleostomi - ps22
Sarcopterygii - ps23
Tetrapoda - ps24
Amniota - ps25
Mammalia - ps26
Theria - ps27
Placentalia - ps28
Boreoeutheria - ps29
Euarchontoglires - ps30
Euarchonta - ps31
Primates - ps32
Haplorrhini - ps33
Simiiformes - ps34
Catarrhini - ps35
Hominoidea - ps36
Hominidae - ps37
Homininae - ps38
Hominini - ps39
Homo_sapiens - ps40
p-value
2e-03
3e-03
4e-03
7e-03
1e-02
Log-odds
0.5
1.0
1.5
2.0
2.5
3.0
3.5
4.0

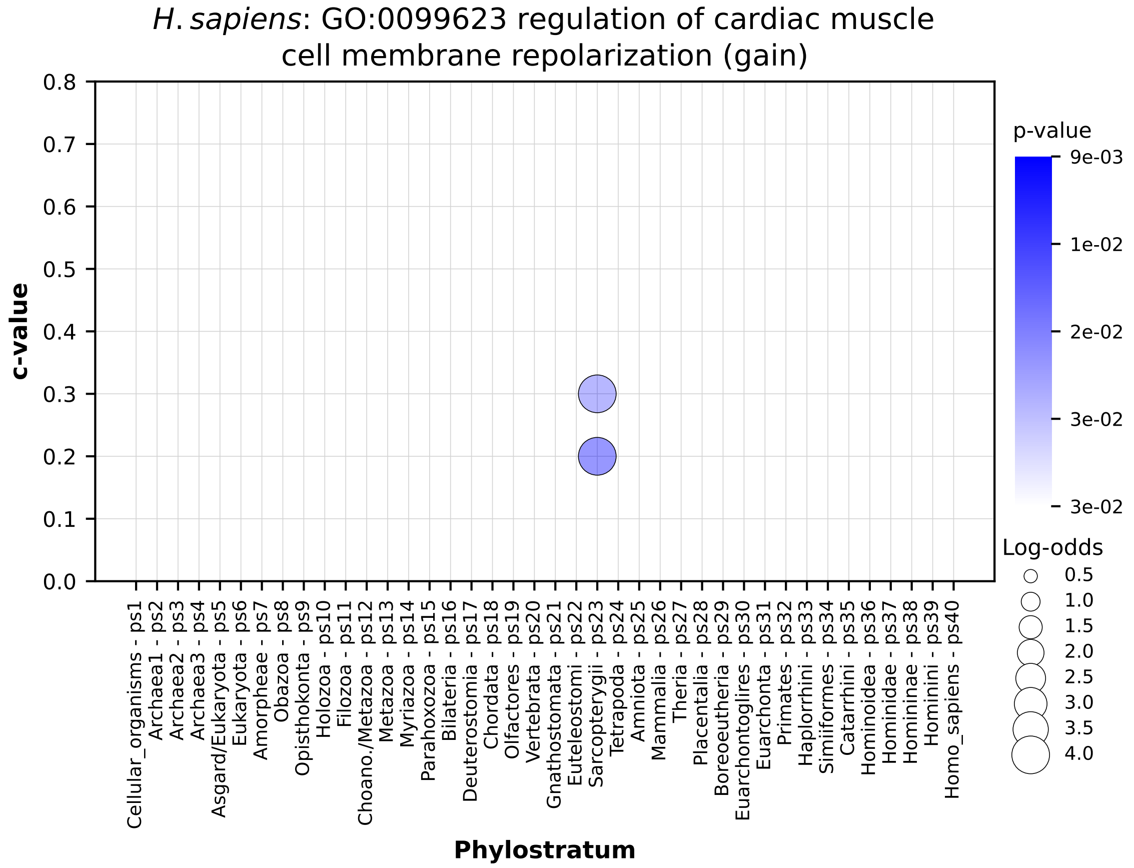

H. sapiens: GO:0099623 regulation of cardiac muscle cell membrane repolarization (gain)
c-value
0.0
0.1
0.2
0.3
0.4
0.5
0.6
0.7
0.8
Phylostratum
Cellular_organisms - ps1
Archaea1 - ps2
Archaea2 - ps3
Archaea3 - ps4
Asgard/Eukaryota - ps5
Eukaryota - ps6
Amorpheae - ps7
Obazoa - ps8
Opisthokonta - ps9
Holozoa - ps10
Filozoa - ps11
Choano./Metazoa - ps12
Metazoa - ps13
Myriazoa - ps14
Parahoxozoa - ps15
Bilateria - ps16
Deuterostomia - ps17
Chordata - ps18
Olfactores - ps19
Vertebrata - ps20
Gnathostomata - ps21
Euteleostomi - ps22
Sarcopterygii - ps23
Tetrapoda - ps24
Amniota - ps25
Mammalia - ps26
Theria - ps27
Placentalia - ps28
Boreoeutheria - ps29
Euarchontoglires - ps30
Euarchonta - ps31
Primates - ps32
Haplorrhini - ps33
Simiiformes - ps34
Catarrhini - ps35
Hominoidea - ps36
Hominidae - ps37
Homininae - ps38
Hominini - ps39
Homo_sapiens - ps40
p-value
9e-03
1e-02
2e-02
3e-02
3e-02
Log-odds
0.5
1.0
1.5
2.0
2.5
3.0
3.5
4.0

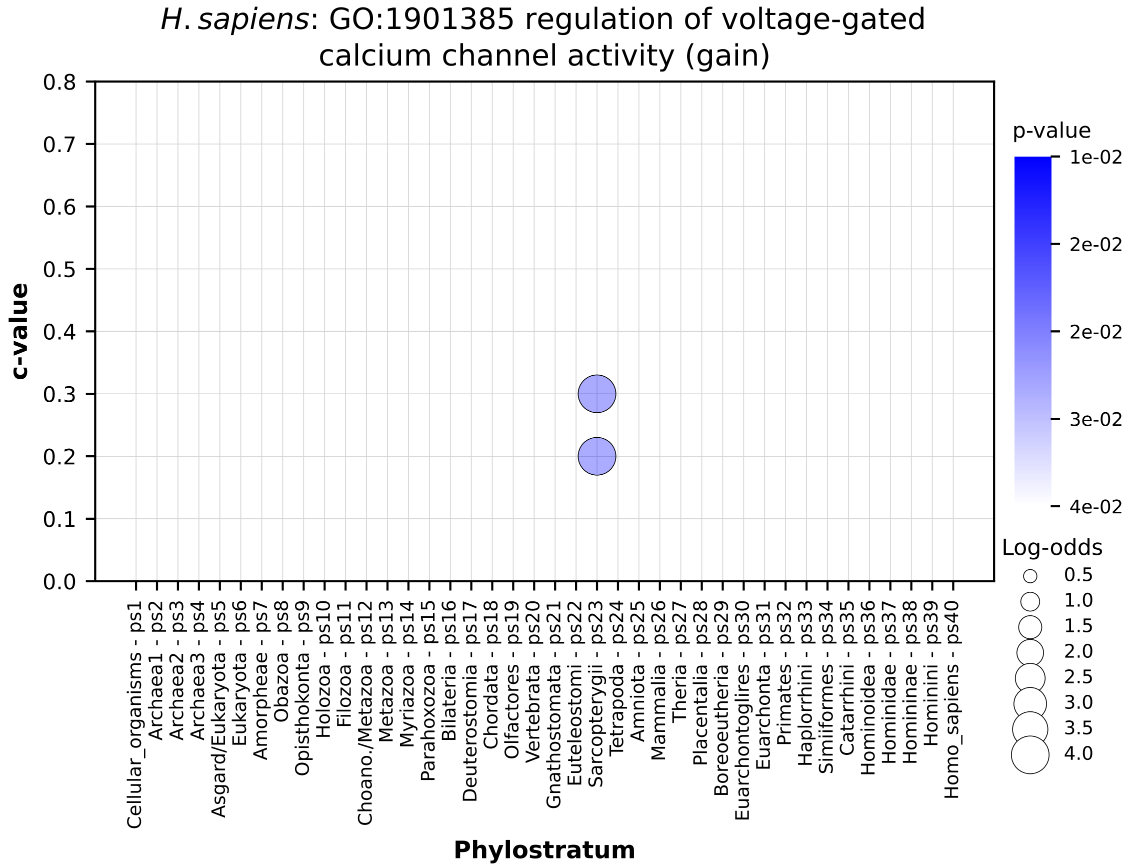
H. sapiens: GO:1901385 regulation of voltage-gated calcium channel activity (gain)
c-value
0.0
0.1
0.2
0.3
0.4
0.5
0.6
0.7
0.8
Phylostratum
Cellular_organisms - ps1
Archaea1 - ps2
Archaea2 - ps3
Archaea3 - ps4
Asgard/Eukaryota - ps5
Eukaryota - ps6
Amorpheae - ps7
Obazoa - ps8
Opisthokonta - ps9
Holozoa - ps10
Filozoa - ps11
Choano./Metazoa - ps12
Metazoa - ps13
Myriazoa - ps14
Parahoxozoa - ps15
Bilateria - ps16
Deuterostomia - ps17
Chordata - ps18
Olfactores - ps19
Vertebrata - ps20
Gnathostomata - ps21
Euteleostomi - ps22
Sarcopterygii - ps23
Tetrapoda - ps24
Amniota - ps25
Mammalia - ps26
Theria - ps27
Placentalia - ps28
Boreoeutheria - ps29
Euarchontoglires - ps30
Euarchonta - ps31
Primates - ps32
Haplorrhini - ps33
Simiiformes - ps34
Catarrhini - ps35
Hominoidea - ps36
Hominidae - ps37
Homininae - ps38
Hominini - ps39
Homo_sapiens - ps40
p-value
1e-02
2e-02
2e-02
3e-02
4e-02
Log-odds
0.5
1.0
1.5
2.0
2.5
3.0
3.5
4.0

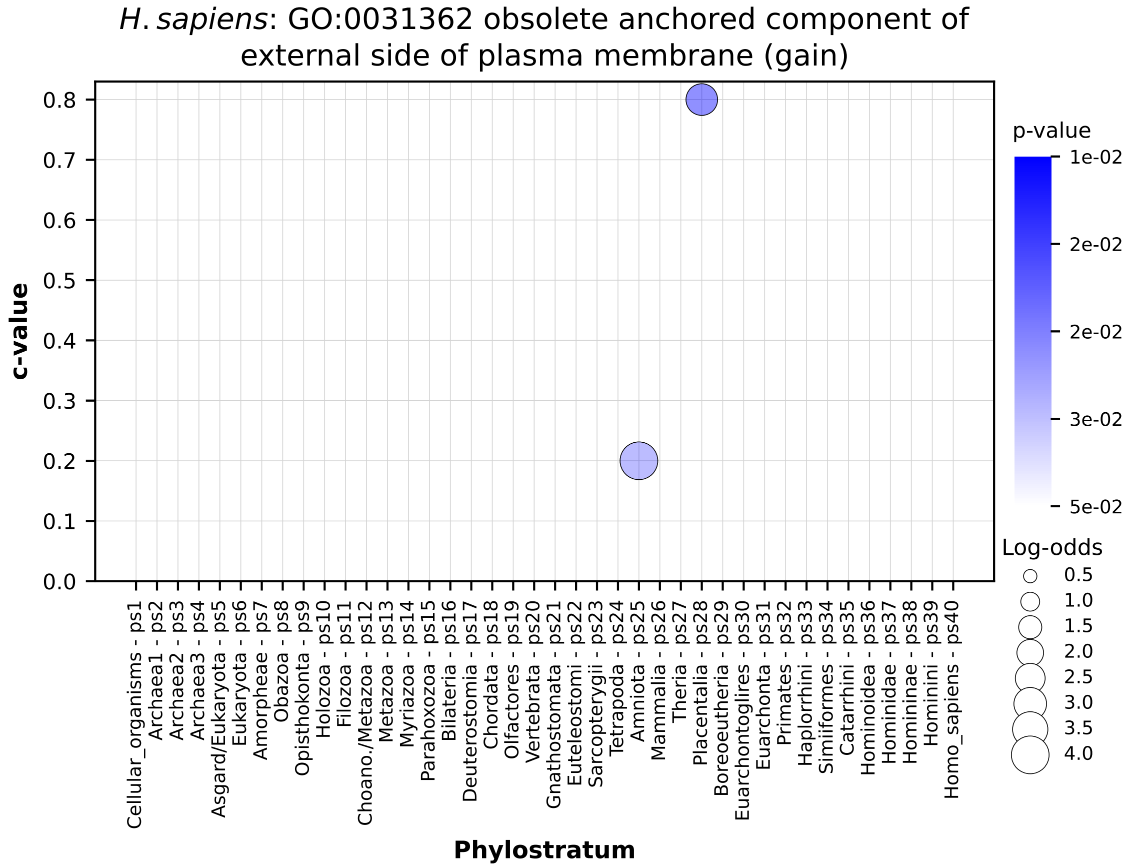

*H. sapiens*: GO:0031362 obsolete anchored component of external side of plasma membrane (gain)
c-value
0.8
0.7
0.6
0.5
0.4
0.3
0.2
0.1
0.0
p-value
1e-02
2e-02
2e-02
3e-02
5e-02
Log-odds
0.5
1.0
1.5
2.0
2.5
3.0
3.5
4.0
Cellular_organisms - ps1
Archaea1 - ps2
Archaea2 - ps3
Archaea3 - ps4
Asgard/Eukaryota - ps5
Eukaryota - ps6
Amorpheae - ps7
Obazoa - ps8
Opisthokonta - ps9
Holozoa - ps10
Filozoa - ps11
Choano./Metazoa - ps12
Metazoa - ps13
Myriazoa - ps14
Parahoxozoa - ps15
Bilateria - ps16
Deuterostomia - ps17
Chordata - ps18
Olfactores - ps19
Vertebrata - ps20
Gnathostomata - ps21
Euteleostomi - ps22
Sarcopterygii - ps23
Tetrapoda - ps24
Amniota - ps25
Mammalia - ps26
Theria - ps27
Placentalia - ps28
Boreoeutheria - ps29
Euarchontoglires - ps30
Euarchonta - ps31
Primates - ps32
Haplorrhini - ps33
Simiiformes - ps34
Catarrhini - ps35
Hominoidea - ps36
Hominidae - ps37
Homininae - ps38
Hominini - ps39
Homo_sapiens - ps40
Phylostratum

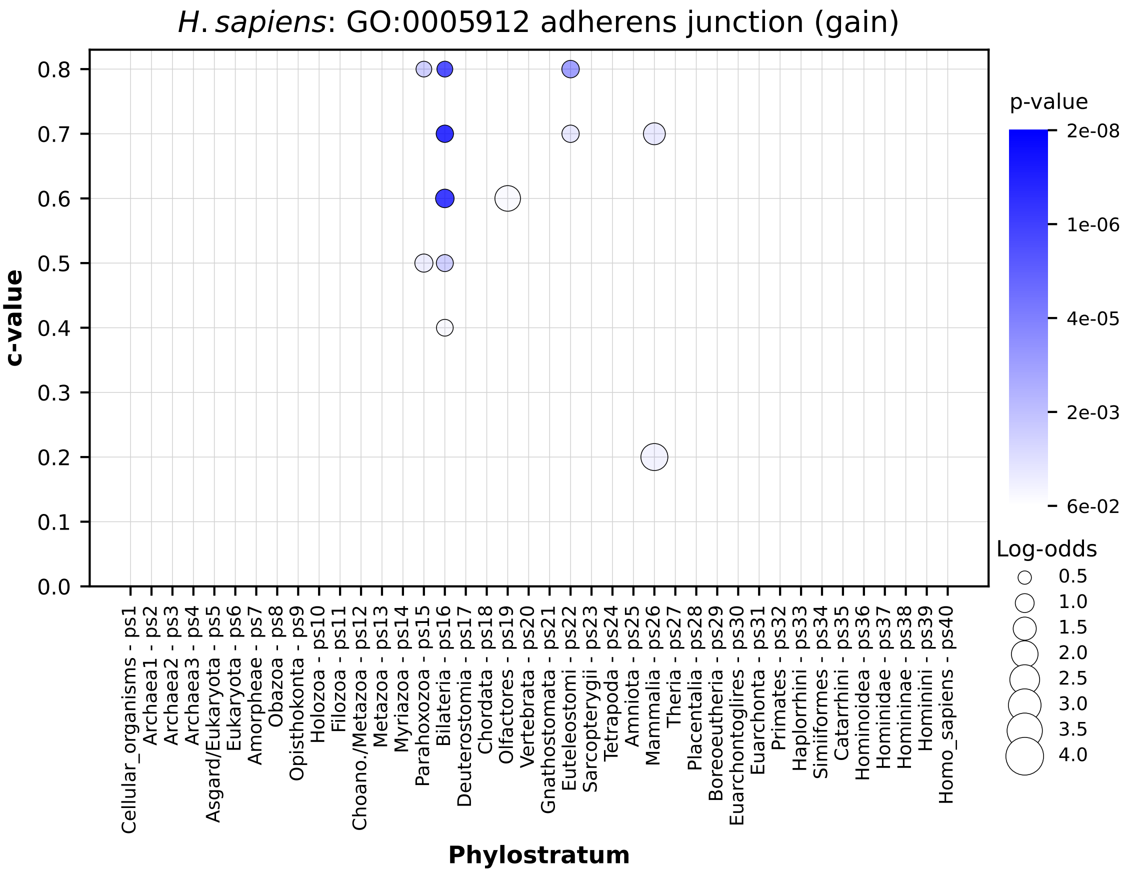

H. sapiens: GO:0005912 adherens junction (gain)
c-value
0.0
0.1
0.2
0.3
0.4
0.5
0.6
0.7
0.8
Phylostratum
Cellular_organisms - ps1
Archaea1 - ps2
Archaea2 - ps3
Archaea3 - ps4
Asgard/Eukaryota - ps5
Eukaryota - ps6
Amorpheae - ps7
Obazoa - ps8
Opisthokonta - ps9
Holozoa - ps10
Filozoa - ps11
Choano./Metazoa - ps12
Metazoa - ps13
Myriazoa - ps14
Parahoxozoa - ps15
Bilateria - ps16
Deuterostomia - ps17
Chordata - ps18
Olfactores - ps19
Vertebrata - ps20
Gnathostomata - ps21
Euteleostomi - ps22
Sarcopterygii - ps23
Tetrapoda - ps24
Amniota - ps25
Mammalia - ps26
Theria - ps27
Placentalia - ps28
Boreoeutheria - ps29
Euarchontoglires - ps30
Euarchonta - ps31
Primates - ps32
Haplorrhini - ps33
Simiiformes - ps34
Catarrhini - ps35
Hominoidea - ps36
Hominidae - ps37
Homininae - ps38
Hominini - ps39
Homo_sapiens - ps40
p-value
2e-08
1e-06
4e-05
2e-03
6e-02
Log-odds
0.5
1.0
1.5
2.0
2.5
3.0
3.5
4.0

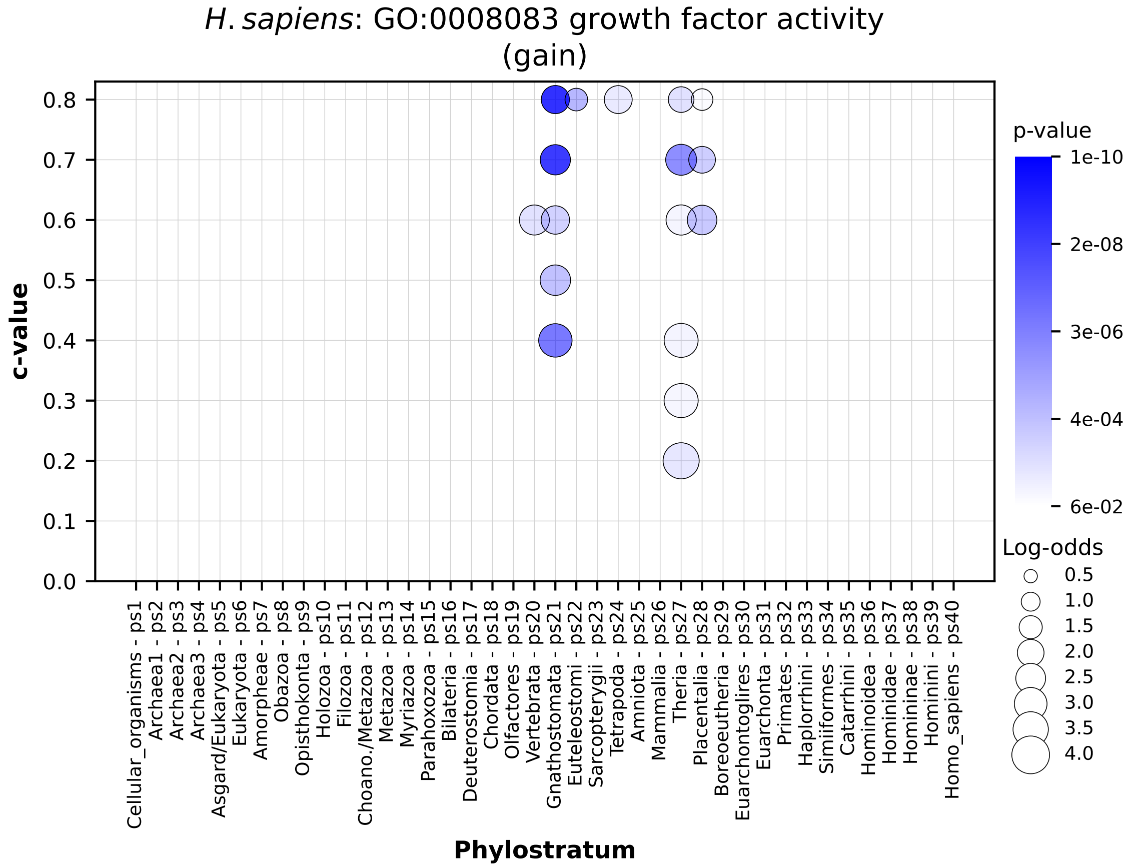

H. sapiens: GO:0008083 growth factor activity
(gain)
c-value
0.0
0.1
0.2
0.3
0.4
0.5
0.6
0.7
0.8
Phylostratum
Cellular_organisms - ps1
Archaea1 - ps2
Archaea2 - ps3
Archaea3 - ps4
Asgard/Eukaryota - ps5
Eukaryota - ps6
Amorpheae - ps7
Obazoa - ps8
Opisthokonta - ps9
Holozoa - ps10
Filozoa - ps11
Choano./Metazoa - ps12
Metazoa - ps13
Myriazoa - ps14
Parahoxozoa - ps15
Bilateria - ps16
Deuterostomia - ps17
Chordata - ps18
Olfactores - ps19
Vertebrata - ps20
Gnathostomata - ps21
Euteleostomi - ps22
Sarcopterygii - ps23
Tetrapoda - ps24
Amniota - ps25
Mammalia - ps26
Theria - ps27
Placentalia - ps28
Boreoeutheria - ps29
Euarchontoglires - ps30
Euarchonta - ps31
Primates - ps32
Haplorrhini - ps33
Simiiformes - ps34
Catarrhini - ps35
Hominoidea - ps36
Hominidae - ps37
Homininae - ps38
Hominini - ps39
Homo_sapiens - ps40
p-value
1e-10
2e-08
3e-06
4e-04
6e-02
Log-odds
0.5
1.0
1.5
2.0
2.5
3.0
3.5
4.0

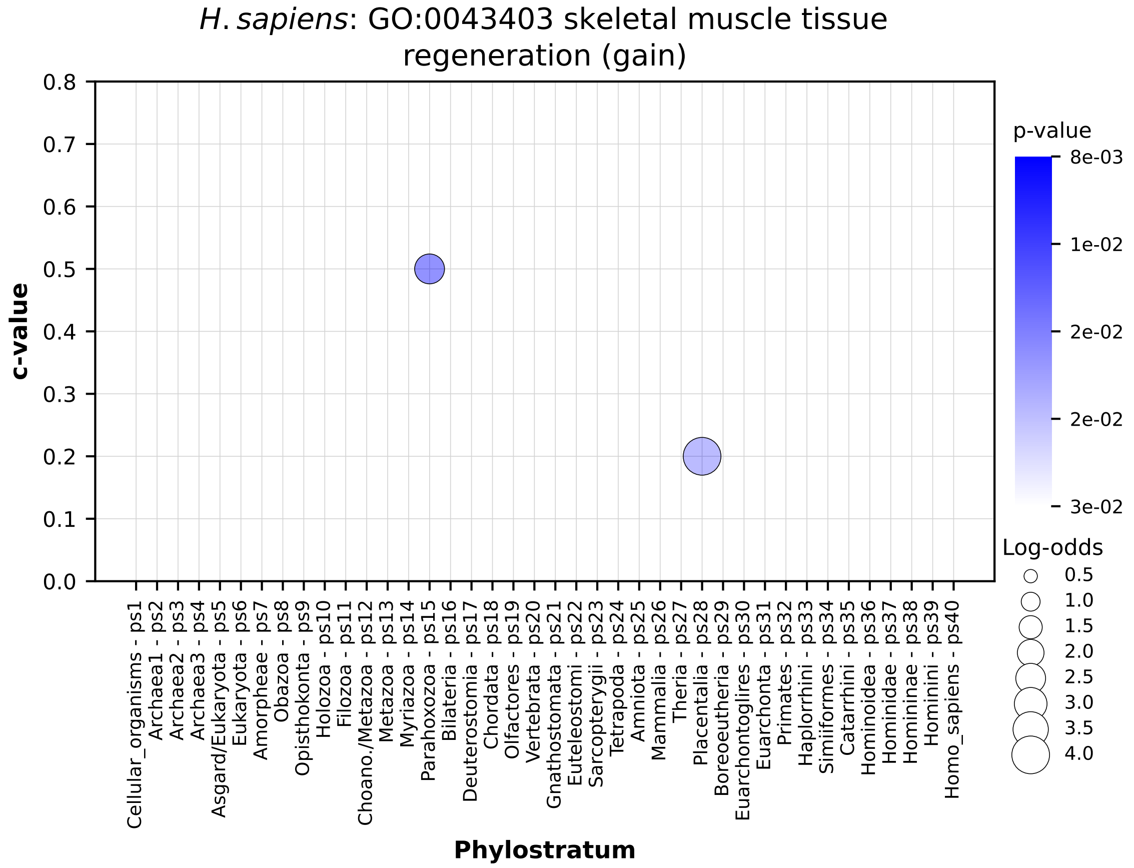

H. sapiens: GO:0043403 skeletal muscle tissue regeneration (gain)
c-value
0.0
0.1
0.2
0.3
0.4
0.5
0.6
0.7
0.8
Phylostratum
Cellular_organisms - ps1
Archaea1 - ps2
Archaea2 - ps3
Archaea3 - ps4
Asgard/Eukaryota - ps5
Eukaryota - ps6
Amorpheae - ps7
Obazoa - ps8
Opisthokonta - ps9
Holozoa - ps10
Filozoa - ps11
Choano./Metazoa - ps12
Metazoa - ps13
Myriazoa - ps14
Parahoxozoa - ps15
Bilateria - ps16
Deuterostomia - ps17
Chordata - ps18
Olfactores - ps19
Vertebrata - ps20
Gnathostomata - ps21
Euteleostomi - ps22
Sarcopterygii - ps23
Tetrapoda - ps24
Amniota - ps25
Mammalia - ps26
Theria - ps27
Placentalia - ps28
Boreoeutheria - ps29
Euarchontoglires - ps30
Euarchonta - ps31
Primates - ps32
Haplorrhini - ps33
Simiiformes - ps34
Catarrhini - ps35
Hominoidea - ps36
Hominidae - ps37
Homininae - ps38
Hominini - ps39
Homo_sapiens - ps40
p-value
8e-03
1e-02
2e-02
2e-02
3e-02
Log-odds
0.5
1.0
1.5
2.0
2.5
3.0
3.5
4.0

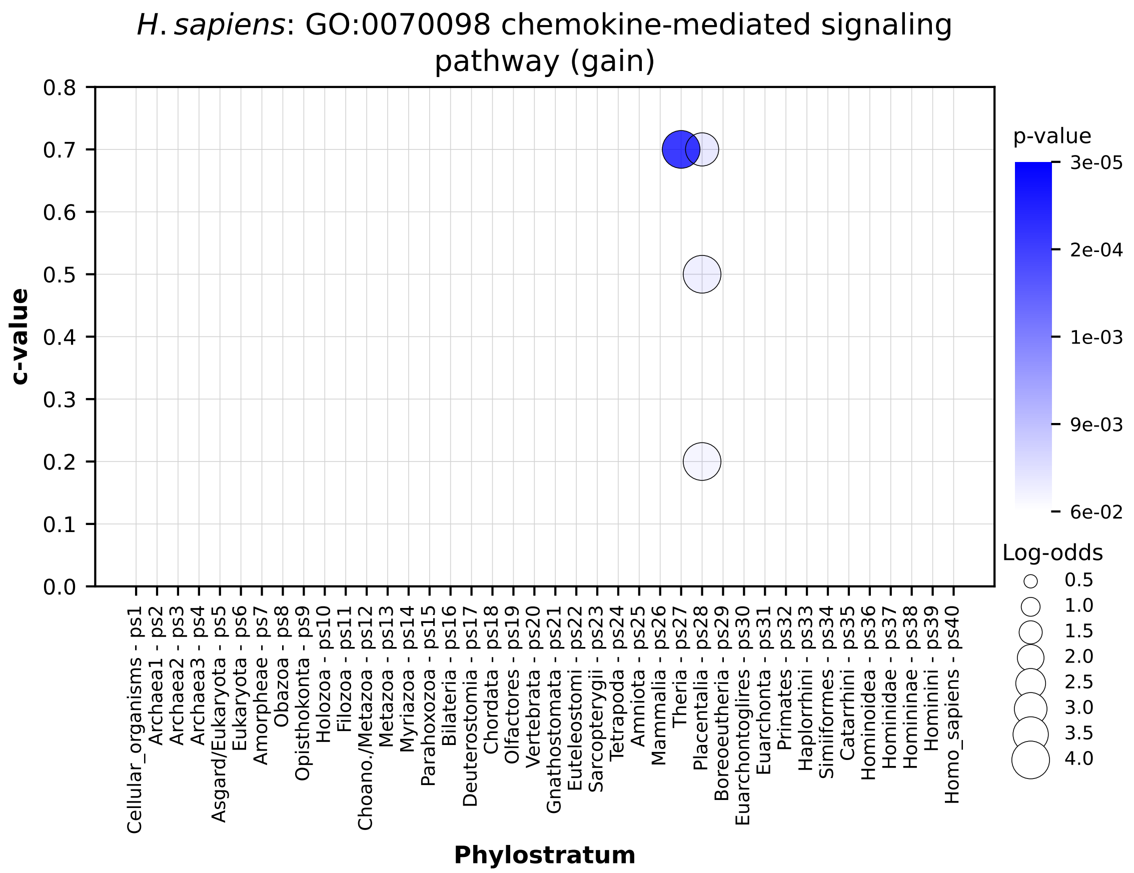

H. sapiens: GO:0070098 chemokine-mediated signaling pathway (gain)
c-value
0.0
0.1
0.2
0.3
0.4
0.5
0.6
0.7
0.8
Phylostratum
Cellular_organisms - ps1
Archaea1 - ps2
Archaea2 - ps3
Archaea3 - ps4
Asgard/Eukaryota - ps5
Eukaryota - ps6
Amorpheae - ps7
Obazoa - ps8
Opisthokonta - ps9
Holozoa - ps10
Filozoa - ps11
Choano./Metazoa - ps12
Metazoa - ps13
Myriazoa - ps14
Parahoxozoa - ps15
Bilateria - ps16
Deuterostomia - ps17
Chordata - ps18
Olfactores - ps19
Vertebrata - ps20
Gnathostomata - ps21
Euteleostomi - ps22
Sarcopterygii - ps23
Tetrapoda - ps24
Amniota - ps25
Mammalia - ps26
Theria - ps27
Placentalia - ps28
Boreoeutheria - ps29
Euarchontoglires - ps30
Euarchonta - ps31
Primates - ps32
Haplorrhini - ps33
Simiiformes - ps34
Catarrhini - ps35
Hominoidea - ps36
Hominidae - ps37
Homininae - ps38
Hominini - ps39
Homo_sapiens - ps40
p-value
3e-05
2e-04
1e-03
9e-03
6e-02
Log-odds
0.5
1.0
1.5
2.0
2.5
3.0
3.5
4.0

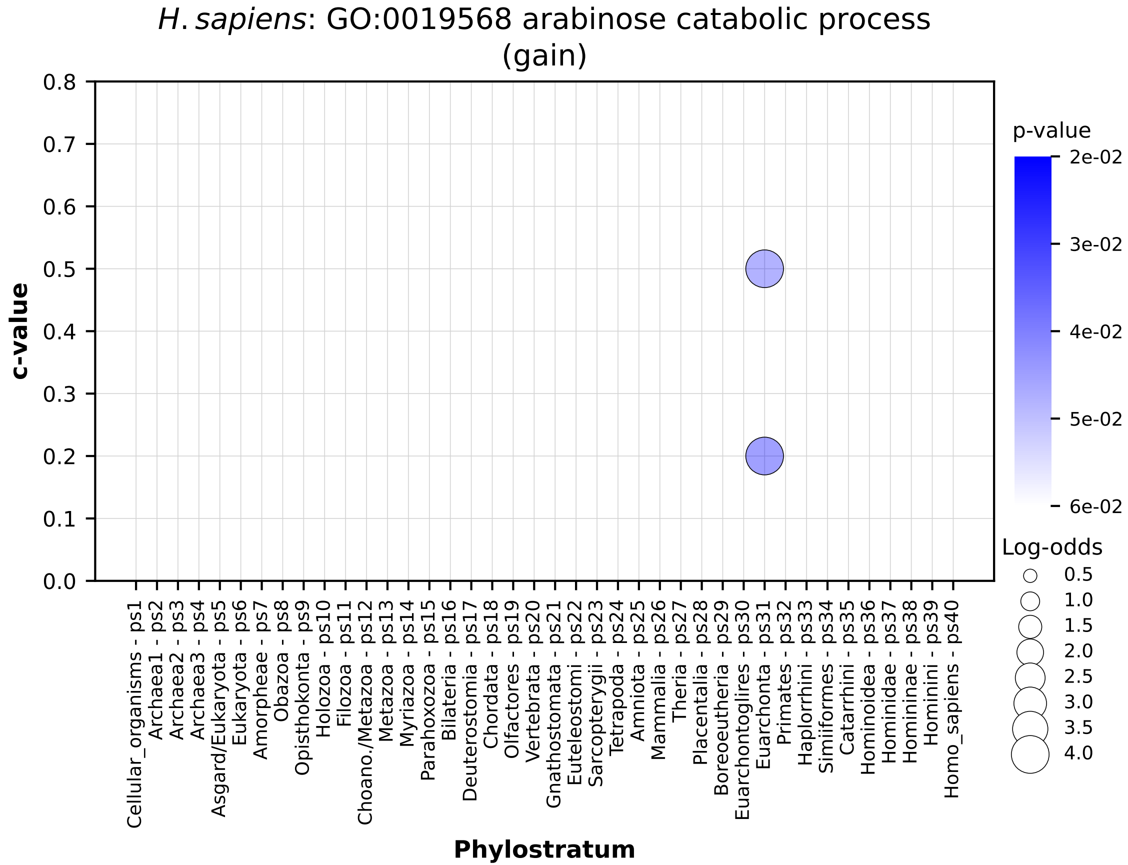

H. sapiens: GO:0019568 arabinose catabolic process (gain)
c-value
0.0
0.1
0.2
0.3
0.4
0.5
0.6
0.7
0.8
Phylostratum
Cellular_organisms - ps1
Archaea1 - ps2
Archaea2 - ps3
Archaea3 - ps4
Asgard/Eukaryota - ps5
Eukaryota - ps6
Amorpheae - ps7
Obazoa - ps8
Opisthokonta - ps9
Holozoa - ps10
Filozoa - ps11
Choano./Metazoa - ps12
Metazoa - ps13
Myriazoa - ps14
Parahoxozoa - ps15
Bilateria - ps16
Deuterostomia - ps17
Chordata - ps18
Olfactores - ps19
Vertebrata - ps20
Gnathostomata - ps21
Euteleostomi - ps22
Sarcopterygii - ps23
Tetrapoda - ps24
Amniota - ps25
Mammalia - ps26
Theria - ps27
Placentalia - ps28
Boreoeutheria - ps29
Euarchontoglires - ps30
Euarchonta - ps31
Primates - ps32
Haplorrhini - ps33
Simiiformes - ps34
Catarrhini - ps35
Hominoidea - ps36
Hominidae - ps37
Homininae - ps38
Hominini - ps39
Homo_sapiens - ps40
p-value
2e-02
3e-02
4e-02
5e-02
6e-02
Log-odds
0.5
1.0
1.5
2.0
2.5
3.0
3.5
4.0

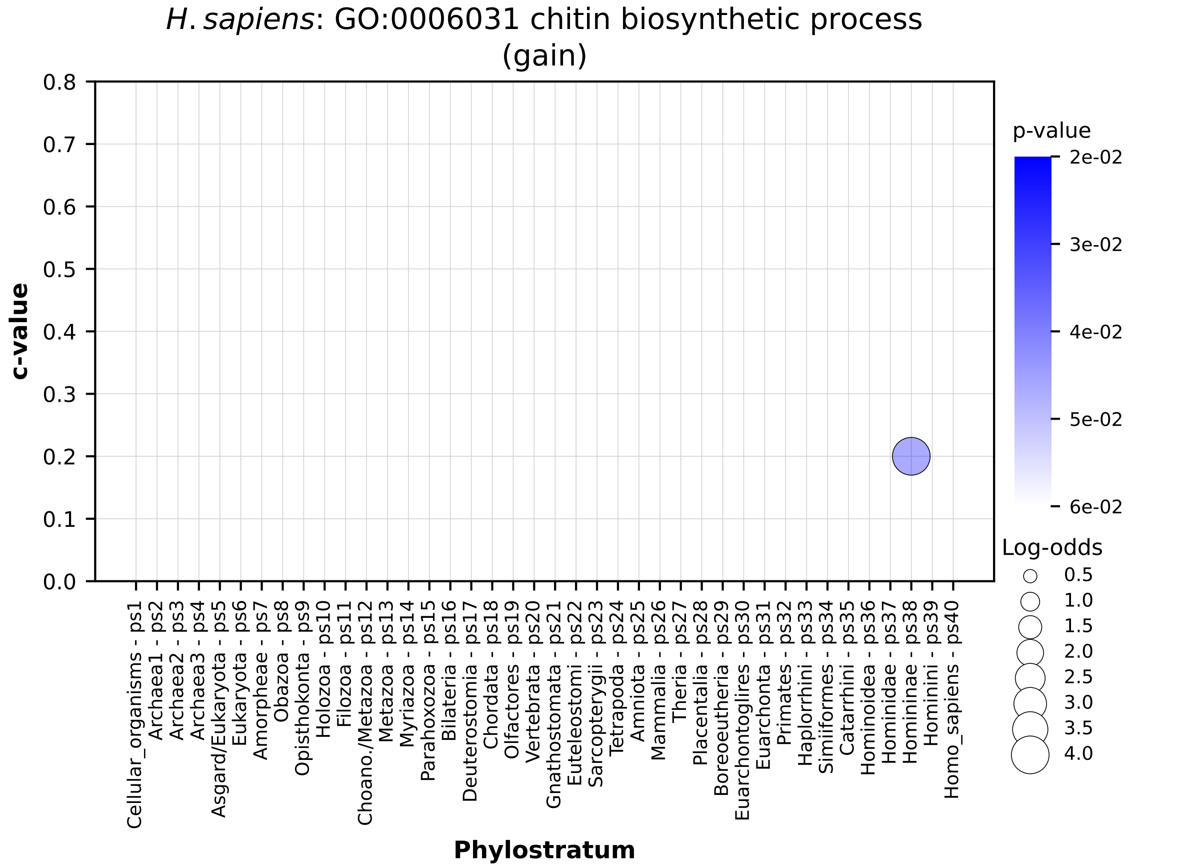

H. sapiens: GO:0006031 chitin biosynthetic process
(gain)
c-value
0.0
0.1
0.2
0.3
0.4
0.5
0.6
0.7
0.8
Phylostratum
Cellular_organisms - ps1
Archaea1 - ps2
Archaea2 - ps3
Archaea3 - ps4
Asgard/Eukaryota - ps5
Eukaryota - ps6
Amorpheae - ps7
Obazoa - ps8
Opisthokonta - ps9
Holozoa - ps10
Filozoa - ps11
Choano./Metazoa - ps12
Metazoa - ps13
Myriazoa - ps14
Parahoxozoa - ps15
Bilateria - ps16
Deuterostomia - ps17
Chordata - ps18
Olfactores - ps19
Vertebrata - ps20
Gnathostomata - ps21
Euteleostomi - ps22
Sarcopterygii - ps23
Tetrapoda - ps24
Amniota - ps25
Mammalia - ps26
Theria - ps27
Placentalia - ps28
Boreoeutheria - ps29
Euarchontoglires - ps30
Euarchonta - ps31
Primates - ps32
Haplorrhini - ps33
Simiiformes - ps34
Catarrhini - ps35
Hominoidea - ps36
Hominidae - ps37
Homininae - ps38
Hominini - ps39
Homo_sapiens - ps40
p-value
2e-02
3e-02
4e-02
5e-02
6e-02
Log-odds
0.5
1.0
1.5
2.0
2.5
3.0
3.5
4.0

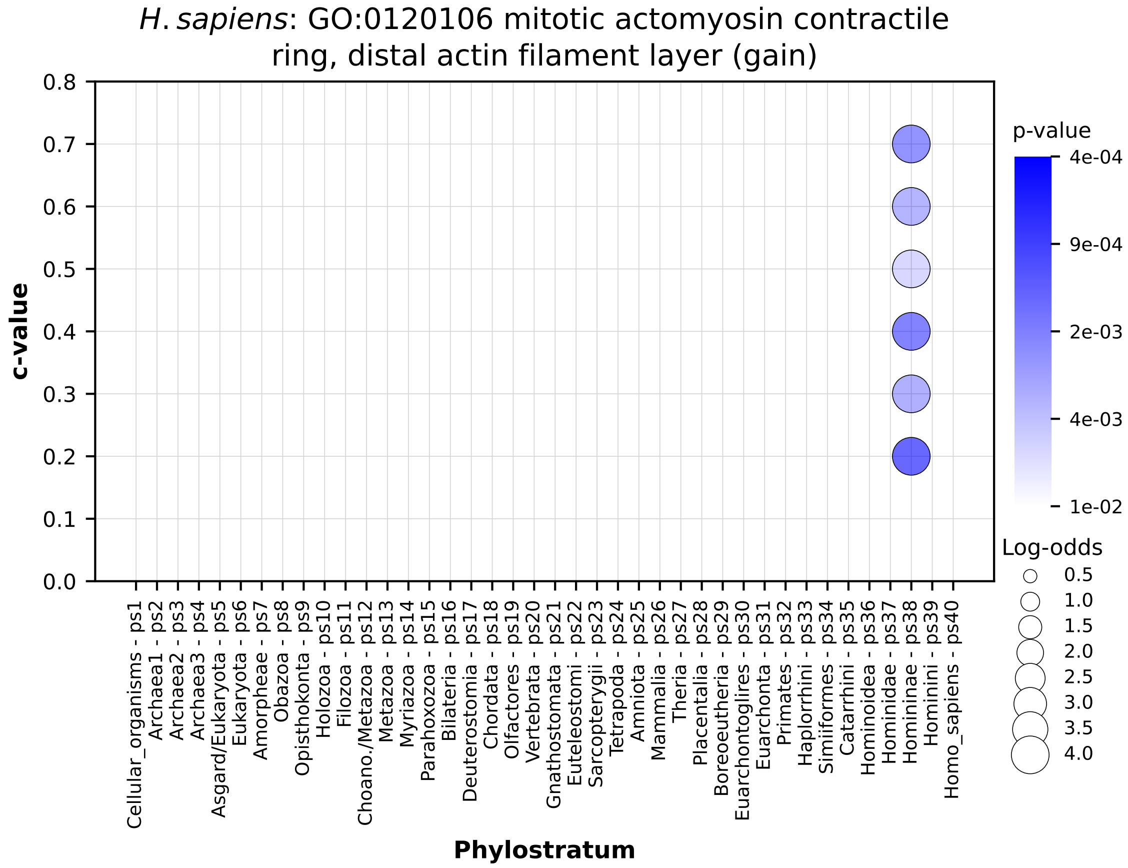
H. sapiens: GO:0120106 mitotic actomyosin contractile ring, distal actin filament layer (gain)
c-value
0.0
0.1
0.2
0.3
0.4
0.5
0.6
0.7
0.8
Phylostratum
Cellular_organisms - ps1
Archaea1 - ps2
Archaea2 - ps3
Archaea3 - ps4
Asgard/Eukaryota - ps5
Eukaryota - ps6
Amorpheae - ps7
Obazoa - ps8
Opisthokonta - ps9
Holozoa - ps10
Filozoa - ps11
Choano./Metazoa - ps12
Metazoa - ps13
Myriazoa - ps14
Parahoxozoa - ps15
Bilateria - ps16
Deuterostomia - ps17
Chordata - ps18
Olfactores - ps19
Vertebrata - ps20
Gnathostomata - ps21
Euteleostomi - ps22
Sarcopterygii - ps23
Tetrapoda - ps24
Amniota - ps25
Mammalia - ps26
Theria - ps27
Placentalia - ps28
Boreoeutheria - ps29
Euarchontoglires - ps30
Euarchonta - ps31
Primates - ps32
Haplorrhini - ps33
Simiiformes - ps34
Catarrhini - ps35
Hominoidea - ps36
Hominidae - ps37
Homininae - ps38
Hominini - ps39
Homo_sapiens - ps40
p-value
4e-04
9e-04
2e-03
4e-03
1e-02
Log-odds
0.5
1.0
1.5
2.0
2.5
3.0
3.5
4.0

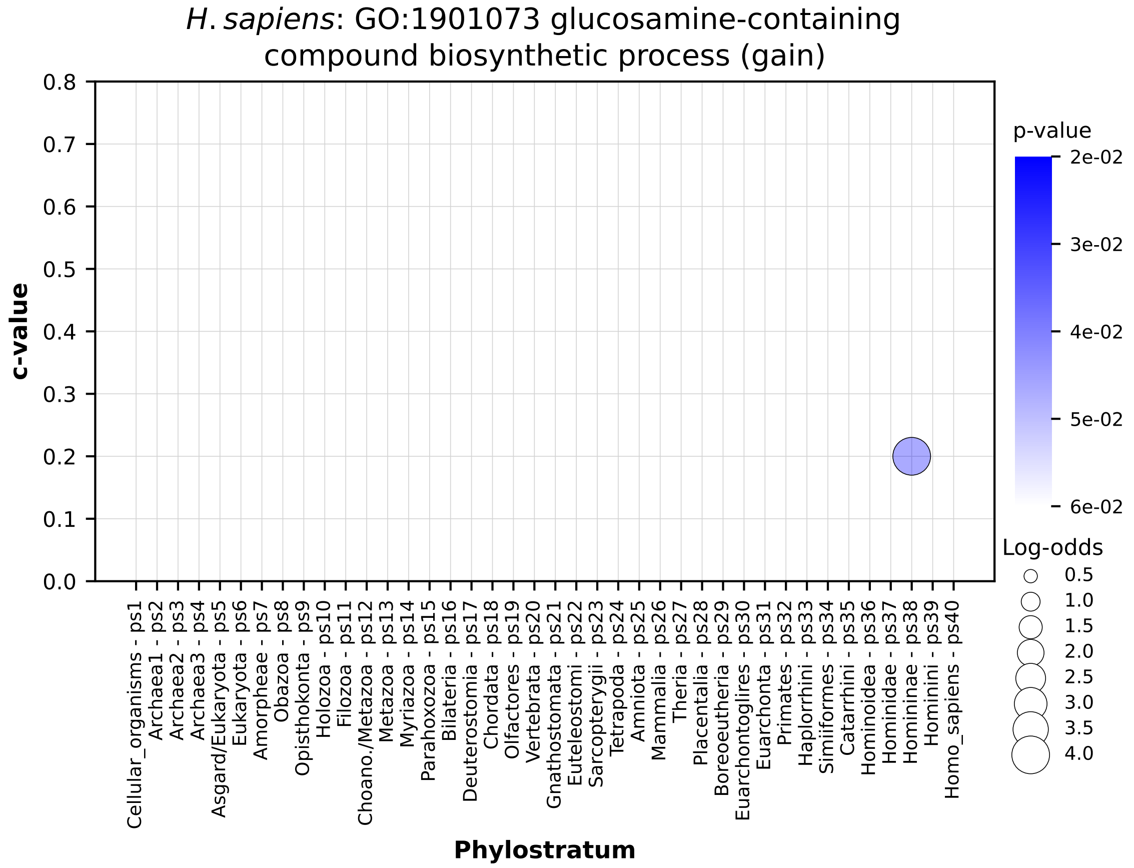
H. sapiens: GO:1901073 glucosamine-containing compound biosynthetic process (gain)
c-value
0.0
0.1
0.2
0.3
0.4
0.5
0.6
0.7
0.8
Phylostratum
Cellular_organisms - ps1
Archaea1 - ps2
Archaea2 - ps3
Archaea3 - ps4
Asgard/Eukaryota - ps5
Eukaryota - ps6
Amorpheae - ps7
Obazoa - ps8
Opisthokonta - ps9
Holozoa - ps10
Filozoa - ps11
Choano./Metazoa - ps12
Metazoa - ps13
Myriazoa - ps14
Parahoxozoa - ps15
Bilateria - ps16
Deuterostomia - ps17
Chordata - ps18
Olfactores - ps19
Vertebrata - ps20
Gnathostomata - ps21
Euteleostomi - ps22
Sarcopterygii - ps23
Tetrapoda - ps24
Amniota - ps25
Mammalia - ps26
Theria - ps27
Placentalia - ps28
Boreoeutheria - ps29
Euarchontoglires - ps30
Euarchonta - ps31
Primates - ps32
Haplorrhini - ps33
Simiiformes - ps34
Catarrhini - ps35
Hominoidea - ps36
Hominidae - ps37
Homininae - ps38
Hominini - ps39
Homo_sapiens - ps40
p-value
2e-02
3e-02
4e-02
5e-02
6e-02
Log-odds
0.5
1.0
1.5
2.0
2.5
3.0
3.5
4.0

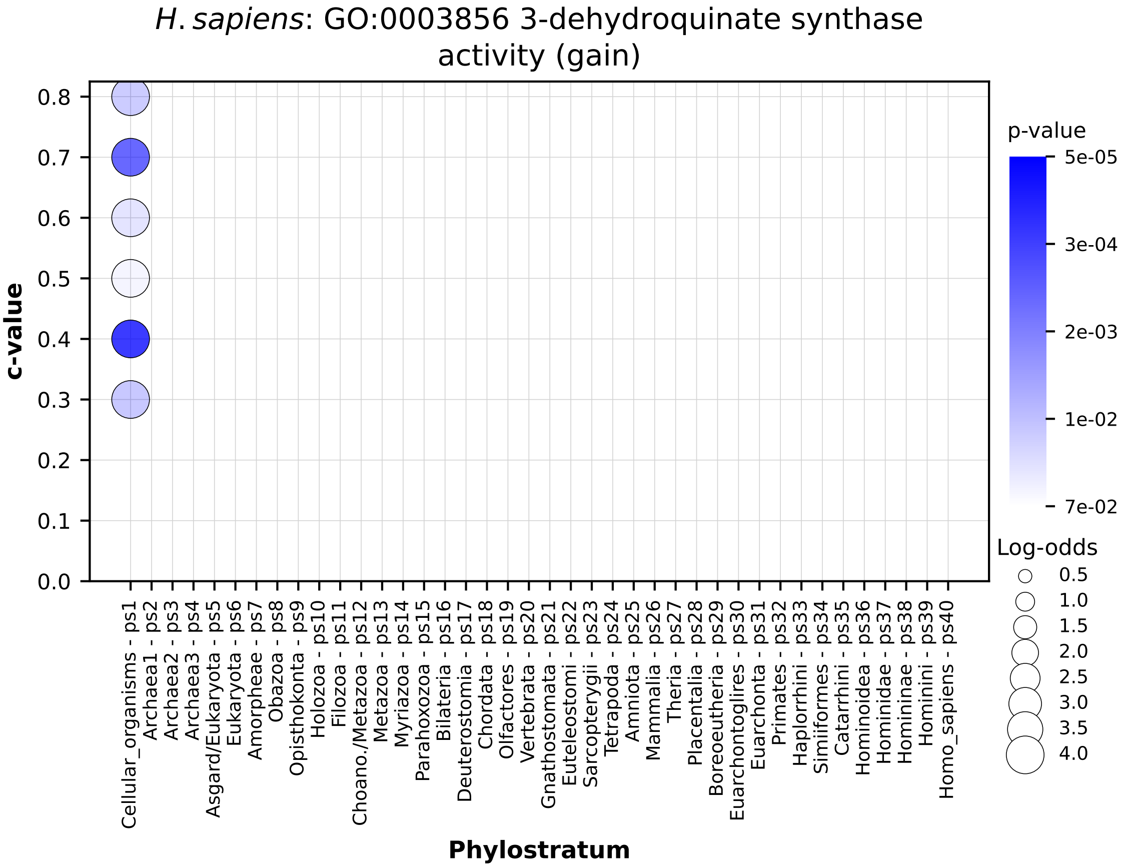

*H. sapiens*: GO:0003856 3-dehydroquinate synthase activity (gain)
c-value
0.8
0.7
0.6
0.5
0.4
0.3
0.2
0.1
0.0
Phylostratum
Cellular_organisms - ps1
Archaea1 - ps2
Archaea2 - ps3
Archaea3 - ps4
Asgard/Eukaryota - ps5
Eukaryota - ps6
Amorpheae - ps7
Obazoa - ps8
Opisthokonta - ps9
Holozoa - ps10
Filozoa - ps11
Choano./Metazoa - ps12
Metazoa - ps13
Myriazoa - ps14
Parahoxozoa - ps15
Bilateria - ps16
Deuterostomia - ps17
Chordata - ps18
Olfactores - ps19
Vertebrata - ps20
Gnathostomata - ps21
Euteleostomi - ps22
Sarcopterygii - ps23
Tetrapoda - ps24
Amniota - ps25
Mammalia - ps26
Theria - ps27
Placentalia - ps28
Boreoeutheria - ps29
Euarchontoglires - ps30
Euarchonta - ps31
Primates - ps32
Haplorrhini - ps33
Simiiformes - ps34
Catarrhini - ps35
Hominoidea - ps36
Hominidae - ps37
Homininae - ps38
Hominini - ps39
Homo_sapiens - ps40
p-value
5e-05
3e-04
2e-03
1e-02
7e-02
Log-odds
0.5
1.0
1.5
2.0
2.5
3.0
3.5
4.0

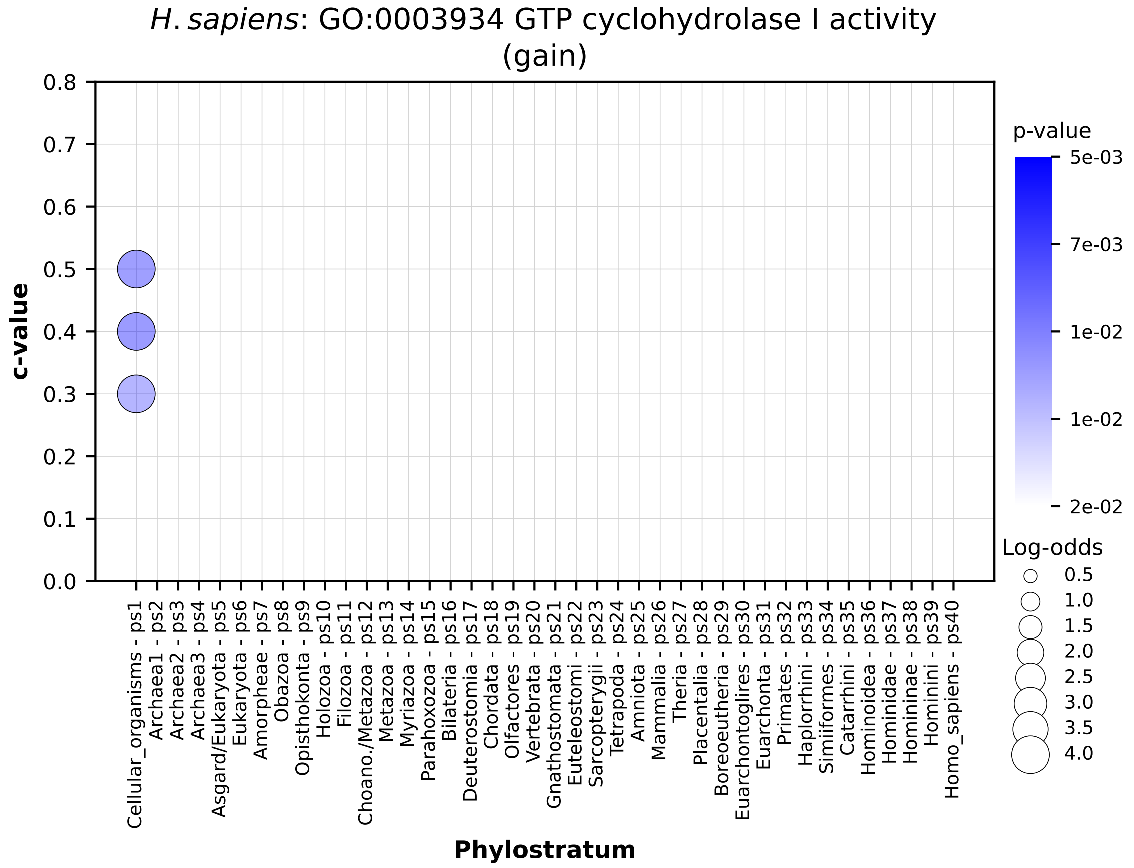

H. sapiens: GO:0003934 GTP cyclohydrolase I activity
(gain)
c-value
0.0
0.1
0.2
0.3
0.4
0.5
0.6
0.7
0.8
Phylostratum
Cellular_organisms - ps1
Archaea1 - ps2
Archaea2 - ps3
Archaea3 - ps4
Asgard/Eukaryota - ps5
Eukaryota - ps6
Amorpheae - ps7
Obazoa - ps8
Opisthokonta - ps9
Holozoa - ps10
Filozoa - ps11
Choano./Metazoa - ps12
Metazoa - ps13
Myriazoa - ps14
Parahoxozoa - ps15
Bilateria - ps16
Deuterostomia - ps17
Chordata - ps18
Olfactores - ps19
Vertebrata - ps20
Gnathostomata - ps21
Euteleostomi - ps22
Sarcopterygii - ps23
Tetrapoda - ps24
Amniota - ps25
Mammalia - ps26
Theria - ps27
Placentalia - ps28
Boreoeutheria - ps29
Euarchontoglires - ps30
Euarchonta - ps31
Primates - ps32
Haplorrhini - ps33
Simiiformes - ps34
Catarrhini - ps35
Hominoidea - ps36
Hominidae - ps37
Homininae - ps38
Hominini - ps39
Homo_sapiens - ps40
p-value
5e-03
7e-03
1e-02
1e-02
2e-02
Log-odds
0.5
1.0
1.5
2.0
2.5
3.0
3.5
4.0

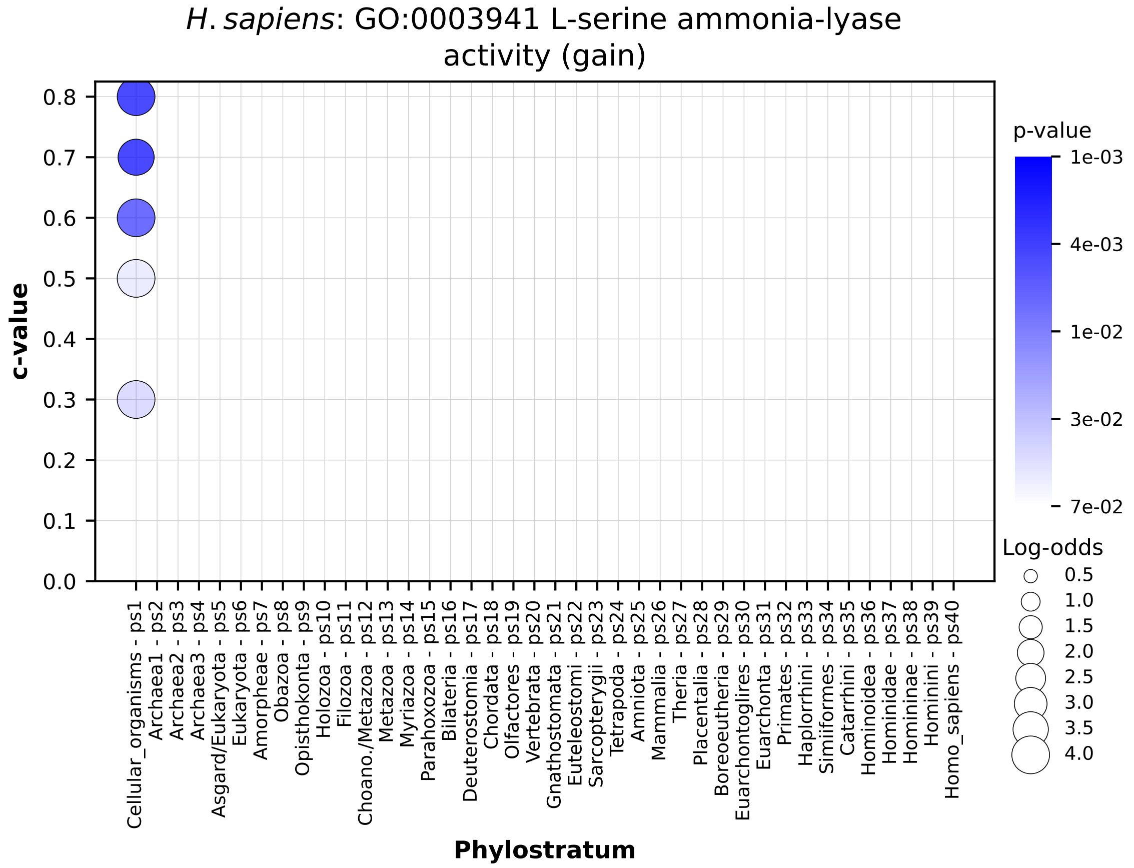

H. sapiens: GO:0003941 L-serine ammonia-lyase activity (gain)
c-value
0.0
0.1
0.2
0.3
0.4
0.5
0.6
0.7
0.8
Phylostratum
Cellular_organisms - ps1
Archaea1 - ps2
Archaea2 - ps3
Archaea3 - ps4
Asgard/Eukaryota - ps5
Eukaryota - ps6
Amorpheae - ps7
Obazoa - ps8
Opisthokonta - ps9
Holozoa - ps10
Filozoa - ps11
Choano./Metazoa - ps12
Metazoa - ps13
Myriazoa - ps14
Parahoxozoa - ps15
Bilateria - ps16
Deuterostomia - ps17
Chordata - ps18
Olfactores - ps19
Vertebrata - ps20
Gnathostomata - ps21
Euteleostomi - ps22
Sarcopterygii - ps23
Tetrapoda - ps24
Amniota - ps25
Mammalia - ps26
Theria - ps27
Placentalia - ps28
Boreoeutheria - ps29
Euarchontoglires - ps30
Euarchonta - ps31
Primates - ps32
Haplorrhini - ps33
Simiiformes - ps34
Catarrhini - ps35
Hominoidea - ps36
Hominidae - ps37
Homininae - ps38
Hominini - ps39
Homo_sapiens - ps40
p-value
1e-03
4e-03
1e-02
3e-02
7e-02
Log-odds
0.5
1.0
1.5
2.0
2.5
3.0
3.5
4.0

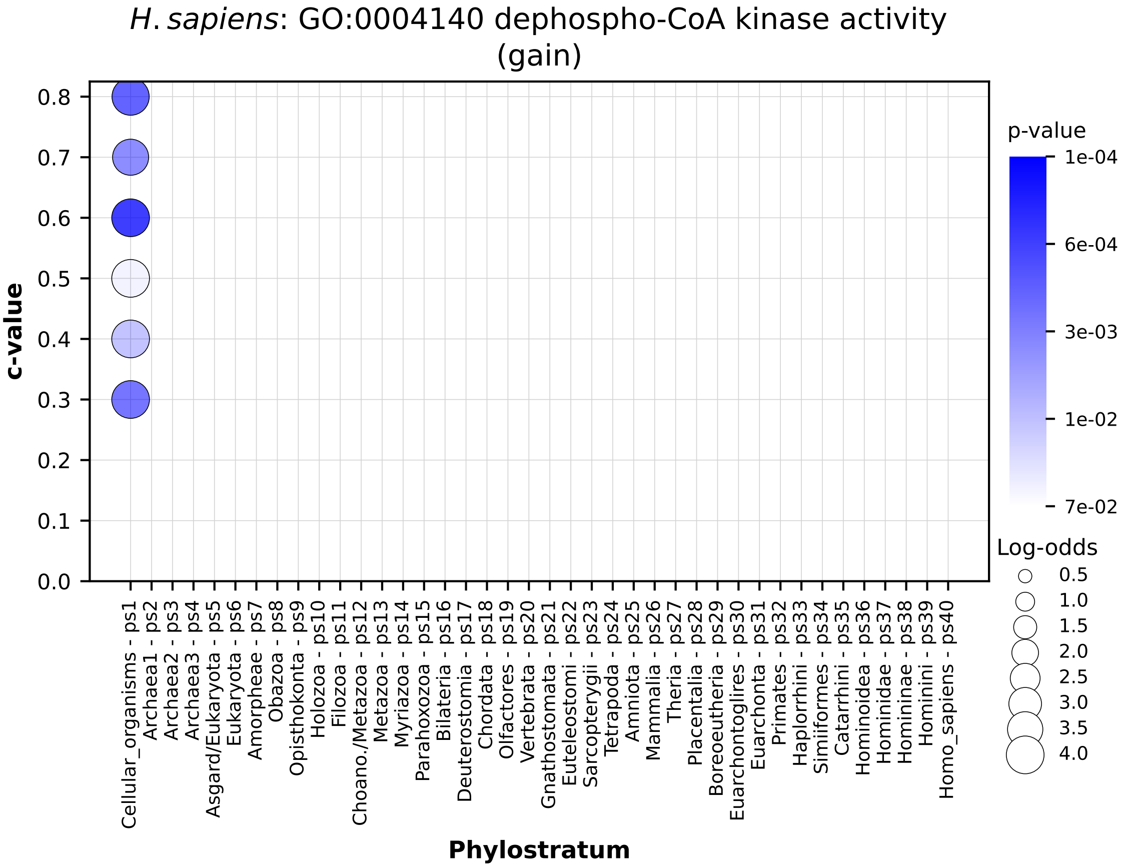

*H. sapiens*: GO:0004140 dephospho-CoA kinase activity (gain)
c-value
0.0
0.1
0.2
0.3
0.4
0.5
0.6
0.7
0.8
p-value
1e-04
6e-04
3e-03
1e-02
7e-02
Log-odds
0.5
1.0
1.5
2.0
2.5
3.0
3.5
4.0
Cellular_organisms - ps1
Archaea1 - ps2
Archaea2 - ps3
Archaea3 - ps4
Asgard/Eukaryota - ps5
Eukaryota - ps6
Amorpheae - ps7
Obazoa - ps8
Opisthokonta - ps9
Holozoa - ps10
Filozoa - ps11
Choano./Metazoa - ps12
Metazoa - ps13
Myriazoa - ps14
Parahoxozoa - ps15
Bilateria - ps16
Deuterostomia - ps17
Chordata - ps18
Olfactores - ps19
Vertebrata - ps20
Gnathostomata - ps21
Euteleostomi - ps22
Sarcopterygii - ps23
Tetrapoda - ps24
Amniota - ps25
Mammalia - ps26
Theria - ps27
Placentalia - ps28
Boreoeutheria - ps29
Euarchontoglires - ps30
Euarchonta - ps31
Primates - ps32
Haplorrhini - ps33
Simiiformes - ps34
Catarrhini - ps35
Hominoidea - ps36
Hominidae - ps37
Homininae - ps38
Hominini - ps39
Homo_sapiens - ps40
Phylostratum

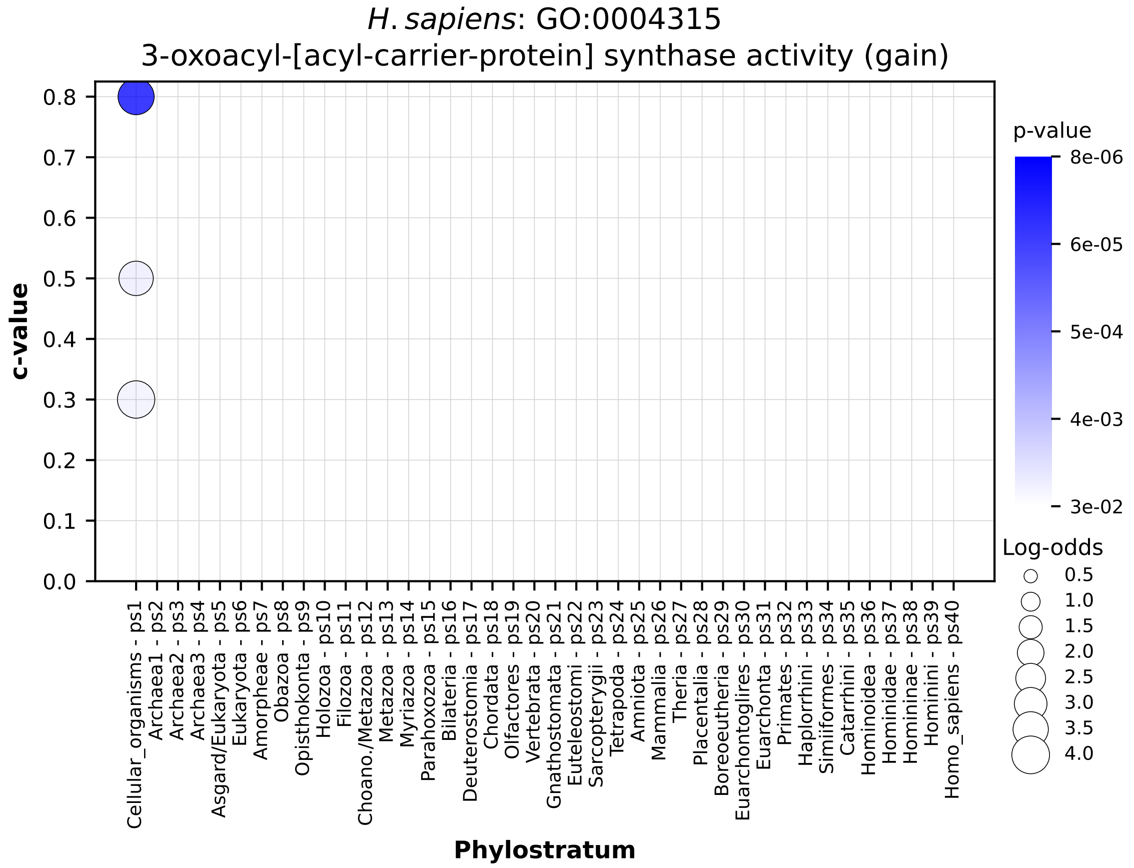

H. sapiens: GO:0004315
3-oxoacyl-[acyl-carrier-protein] synthase activity (gain)
c-value
0.0
0.1
0.2
0.3
0.4
0.5
0.6
0.7
0.8
Phylostratum
Cellular_organisms - ps1
Archaea1 - ps2
Archaea2 - ps3
Archaea3 - ps4
Asgard/Eukaryota - ps5
Eukaryota - ps6
Amorpheae - ps7
Obazoa - ps8
Opisthokonta - ps9
Holozoa - ps10
Filozoa - ps11
Choano./Metazoa - ps12
Metazoa - ps13
Myriazoa - ps14
Parahoxozoa - ps15
Bilateria - ps16
Deuterostomia - ps17
Chordata - ps18
Olfactores - ps19
Vertebrata - ps20
Gnathostomata - ps21
Euteleostomi - ps22
Sarcopterygii - ps23
Tetrapoda - ps24
Amniota - ps25
Mammalia - ps26
Theria - ps27
Placentalia - ps28
Boreoeutheria - ps29
Euarchontoglires - ps30
Euarchonta - ps31
Primates - ps32
Haplorrhini - ps33
Simiiformes - ps34
Catarrhini - ps35
Hominoidea - ps36
Hominidae - ps37
Homininae - ps38
Hominini - ps39
Homo_sapiens - ps40
p-value
8e-06
6e-05
5e-04
4e-03
3e-02
Log-odds
0.5
1.0
1.5
2.0
2.5
3.0
3.5
4.0

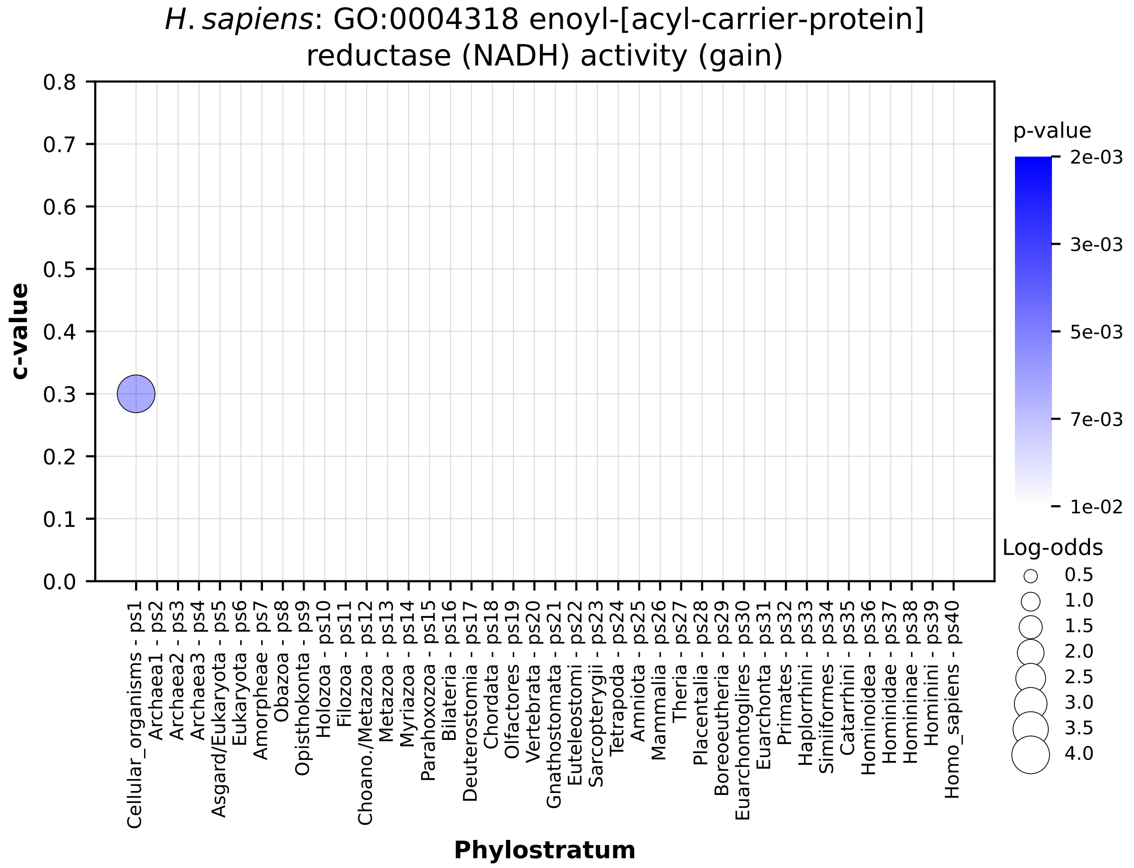

*H. sapiens*: GO:0004318 enoyl-[acyl-carrier-protein] reductase (NADH) activity (gain)
c-value
0.0
0.1
0.2
0.3
0.4
0.5
0.6
0.7
0.8
Phylostratum
Cellular_organisms - ps1
Archaea1 - ps2
Archaea2 - ps3
Archaea3 - ps4
Asgard/Eukaryota - ps5
Eukaryota - ps6
Amorpheae - ps7
Obazoa - ps8
Opisthokonta - ps9
Holozoa - ps10
Filozoa - ps11
Choano./Metazoa - ps12
Metazoa - ps13
Myriazoa - ps14
Parahoxozoa - ps15
Bilateria - ps16
Deuterostomia - ps17
Chordata - ps18
Olfactores - ps19
Vertebrata - ps20
Gnathostomata - ps21
Euteleostomi - ps22
Sarcopterygii - ps23
Tetrapoda - ps24
Amniota - ps25
Mammalia - ps26
Theria - ps27
Placentalia - ps28
Boreoeutheria - ps29
Euarchontoglires - ps30
Euarchonta - ps31
Primates - ps32
Haplorrhini - ps33
Simiiformes - ps34
Catarrhini - ps35
Hominoidea - ps36
Hominidae - ps37
Homininae - ps38
Hominini - ps39
Homo_sapiens - ps40
p-value
2e-03
3e-03
5e-03
7e-03
1e-02
Log-odds
0.5
1.0
1.5
2.0
2.5
3.0
3.5
4.0

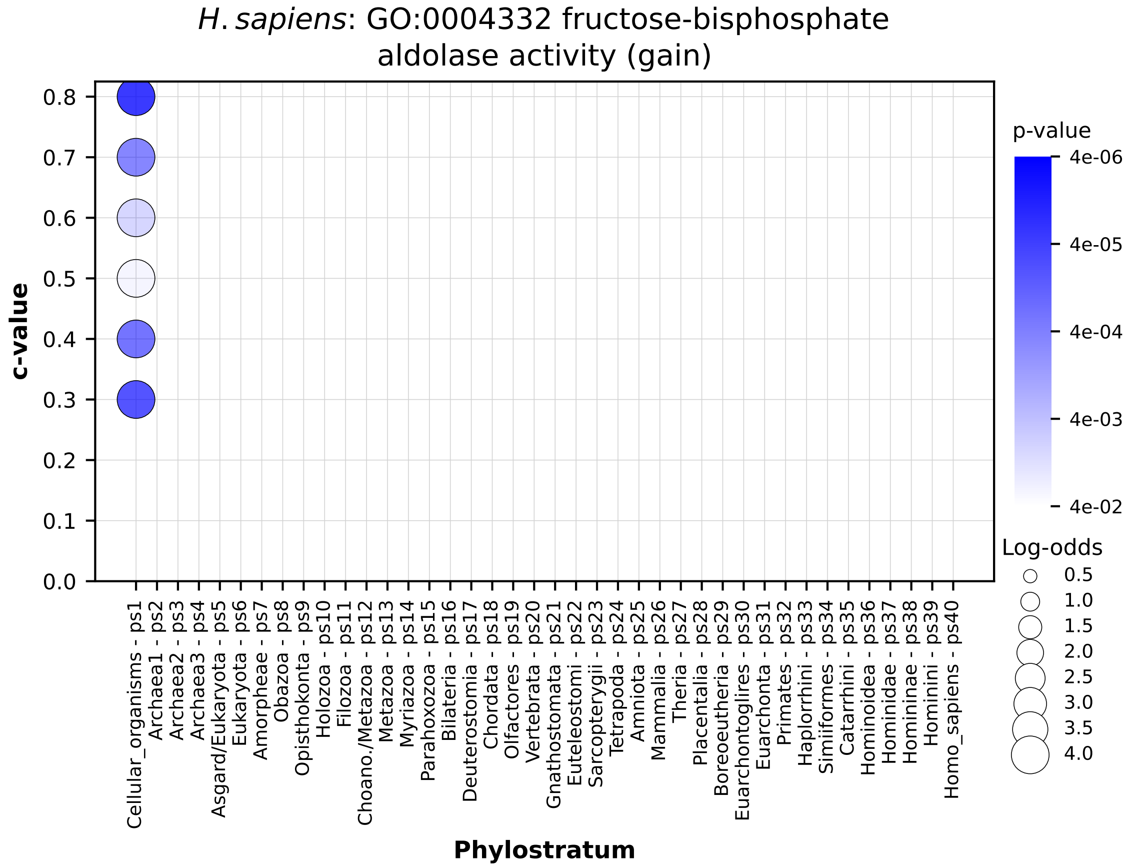
H. sapiens: GO:0004332 fructose-bisphosphate aldolase activity (gain)
c-value
0.0
0.1
0.2
0.3
0.4
0.5
0.6
0.7
0.8
Phylostratum
Cellular_organisms - ps1
Archaea1 - ps2
Archaea2 - ps3
Archaea3 - ps4
Asgard/Eukaryota - ps5
Eukaryota - ps6
Amorpheae - ps7
Obazoa - ps8
Opisthokonta - ps9
Holozoa - ps10
Filozoa - ps11
Choano./Metazoa - ps12
Metazoa - ps13
Myriazoa - ps14
Parahoxozoa - ps15
Bilateria - ps16
Deuterostomia - ps17
Chordata - ps18
Olfactores - ps19
Vertebrata - ps20
Gnathostomata - ps21
Euteleostomi - ps22
Sarcopterygii - ps23
Tetrapoda - ps24
Amniota - ps25
Mammalia - ps26
Theria - ps27
Placentalia - ps28
Boreoeutheria - ps29
Euarchontoglires - ps30
Euarchonta - ps31
Primates - ps32
Haplorrhini - ps33
Simiiformes - ps34
Catarrhini - ps35
Hominoidea - ps36
Hominidae - ps37
Homininae - ps38
Hominini - ps39
Homo_sapiens - ps40
p-value
4e-06
4e-05
4e-04
4e-03
4e-02
Log-odds
0.5
1.0
1.5
2.0
2.5
3.0
3.5
4.0

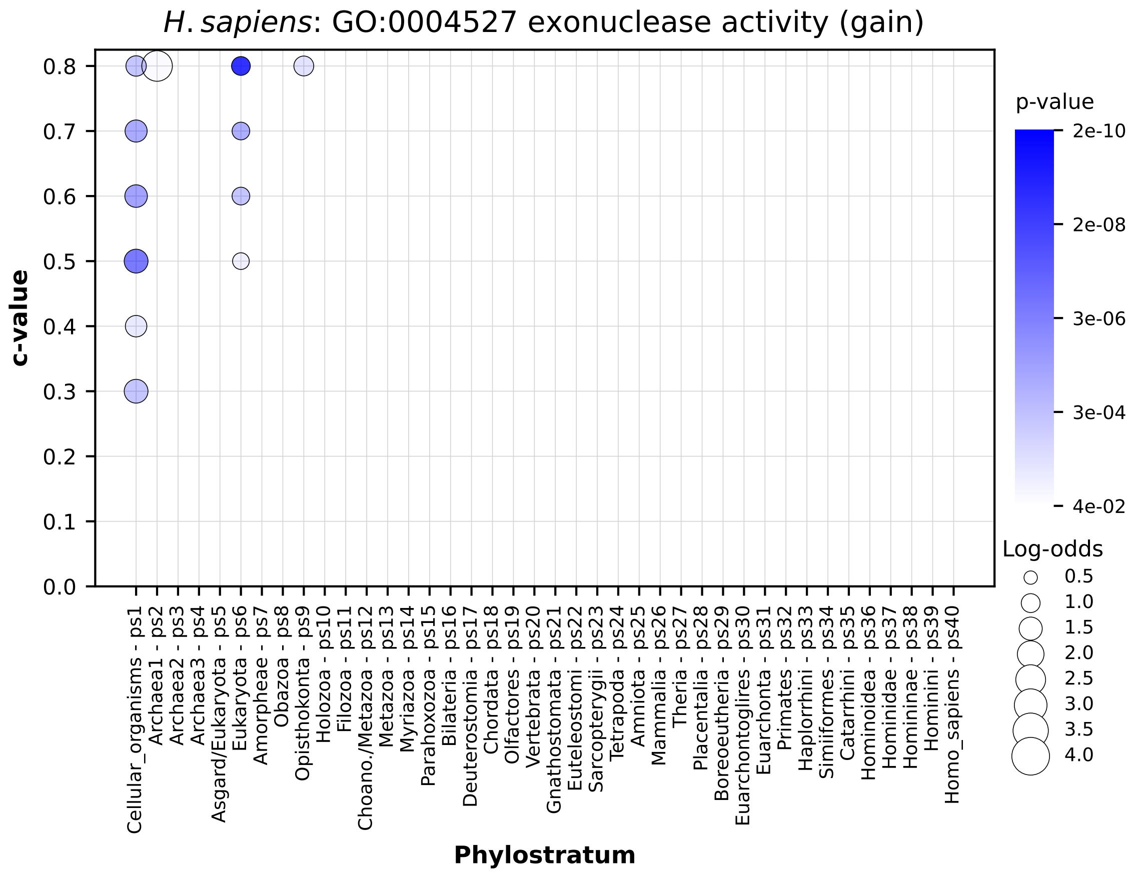

*H. sapiens*: GO:0004527 exonuclease activity (gain)
c-value
0.8
0.7
0.6
0.5
0.4
0.3
0.2
0.1
0.0
p-value
2e-10
2e-08
3e-06
3e-04
4e-02
Log-odds
0.5
1.0
1.5
2.0
2.5
3.0
3.5
4.0
Cellular_organisms - ps1
Archaea1 - ps2
Archaea2 - ps3
Archaea3 - ps4
Asgard/Eukaryota - ps5
Eukaryota - ps6
Amorpheae - ps7
Obazoa - ps8
Opisthokonta - ps9
Holozoa - ps10
Filozoa - ps11
Choano./Metazoa - ps12
Metazoa - ps13
Myriazoa - ps14
Parahoxozoa - ps15
Bilateria - ps16
Deuterostomia - ps17
Chordata - ps18
Olfactores - ps19
Vertebrata - ps20
Gnathostomata - ps21
Euteleostomi - ps22
Sarcopterygii - ps23
Tetrapoda - ps24
Amniota - ps25
Mammalia - ps26
Theria - ps27
Placentalia - ps28
Boreoeutheria - ps29
Euarchontoglires - ps30
Euarchonta - ps31
Primates - ps32
Haplorrhini - ps33
Simiiformes - ps34
Catarrhini - ps35
Hominoidea - ps36
Hominidae - ps37
Homininae - ps38
Hominini - ps39
Homo_sapiens - ps40
Phylostratum

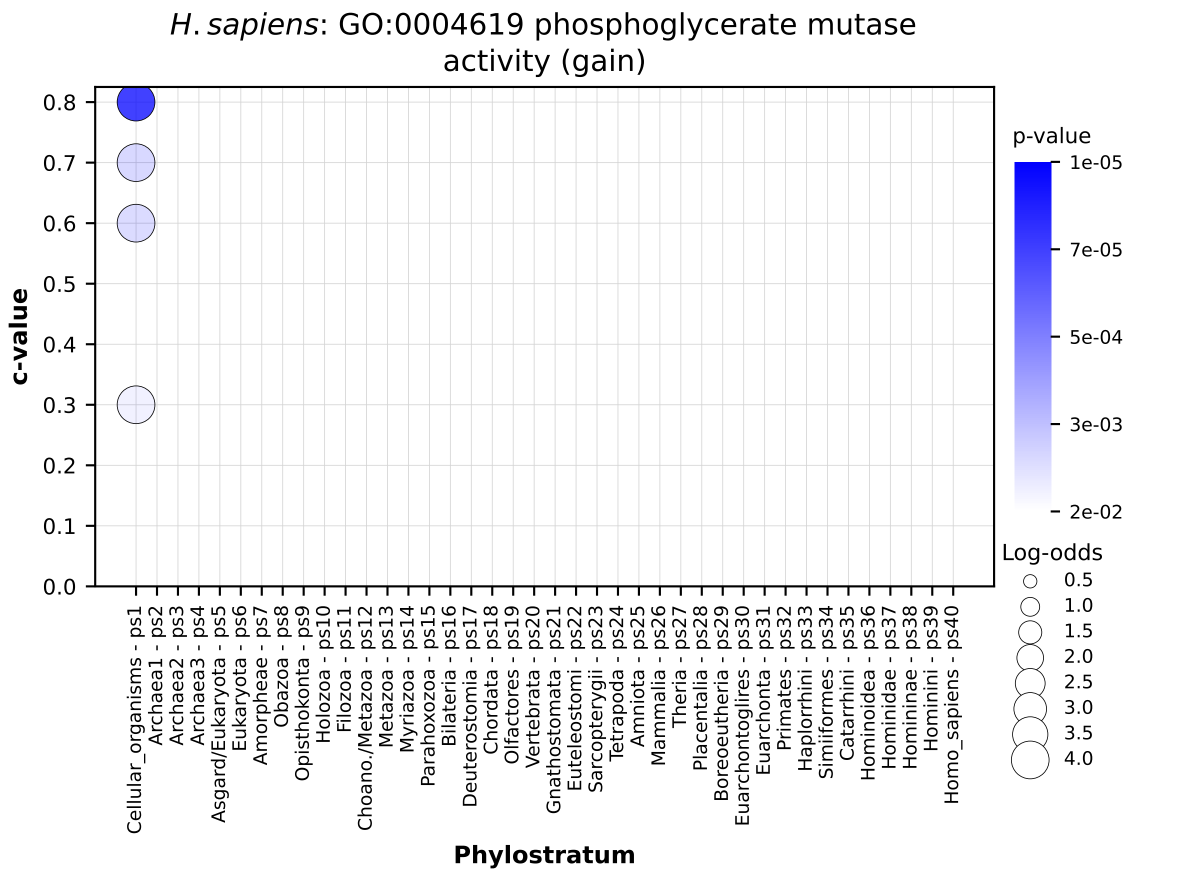
H. sapiens: GO:0004619 phosphoglycerate mutase activity (gain)
c-value
0.8
0.7
0.6
0.5
0.4
0.3
0.2
0.1
0.0
Phylostratum
Cellular_organisms - ps1
Archaea1 - ps2
Archaea2 - ps3
Archaea3 - ps4
Asgard/Eukaryota - ps5
Eukaryota - ps6
Amorpheae - ps7
Obazoa - ps8
Opisthokonta - ps9
Holozoa - ps10
Filozoa - ps11
Choano./Metazoa - ps12
Metazoa - ps13
Myriazoa - ps14
Parahoxozoa - ps15
Bilateria - ps16
Deuterostomia - ps17
Chordata - ps18
Olfactores - ps19
Vertebrata - ps20
Gnathostomata - ps21
Euteleostomi - ps22
Sarcopterygii - ps23
Tetrapoda - ps24
Amniota - ps25
Mammalia - ps26
Theria - ps27
Placentalia - ps28
Boreoeutheria - ps29
Euarchontoglires - ps30
Euarchonta - ps31
Primates - ps32
Haplorrhini - ps33
Simiiformes - ps34
Catarrhini - ps35
Hominoidea - ps36
Hominidae - ps37
Homininae - ps38
Hominini - ps39
Homo_sapiens - ps40
p-value
1e-05
7e-05
5e-04
3e-03
2e-02
Log-odds
0.5
1.0
1.5
2.0
2.5
3.0
3.5
4.0

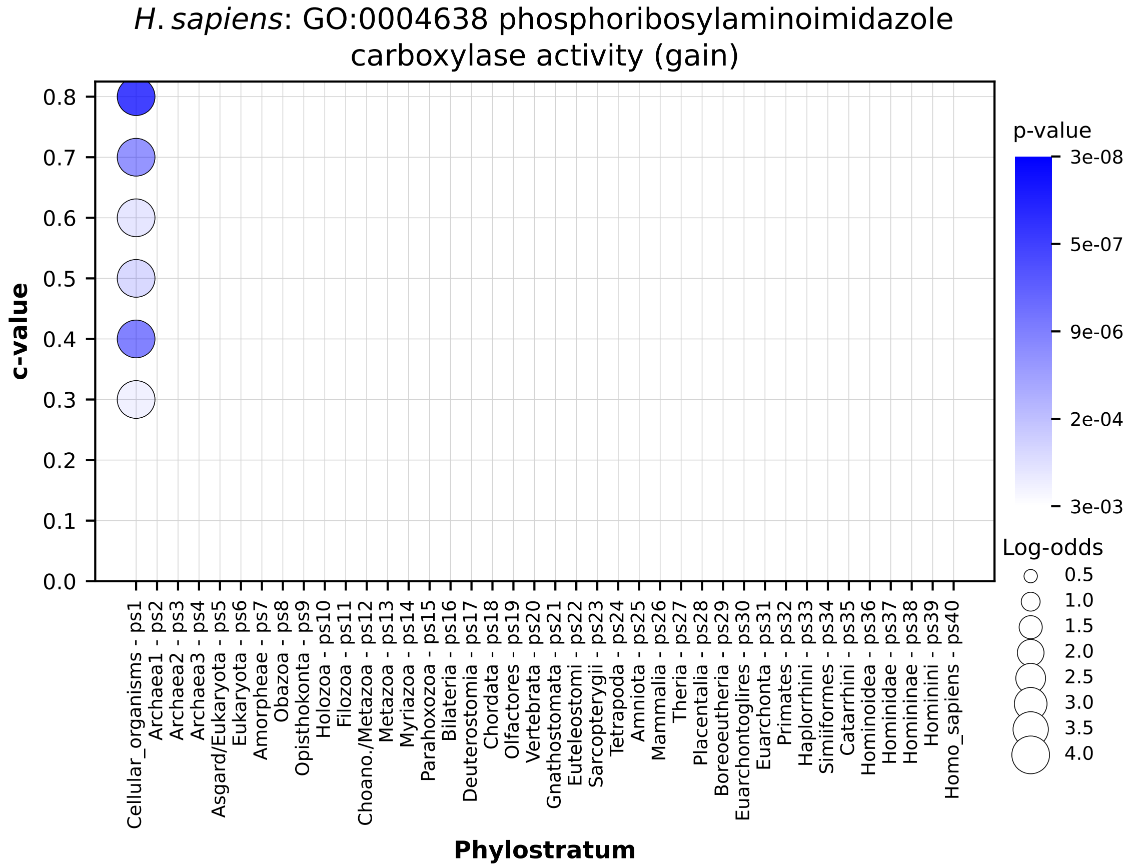

H. sapiens: GO:0004638 phosphoribosylaminoimidazole carboxylase activity (gain)
c-value
0.0
0.1
0.2
0.3
0.4
0.5
0.6
0.7
0.8
Phylostratum
Cellular_organisms - ps1
Archaea1 - ps2
Archaea2 - ps3
Archaea3 - ps4
Asgard/Eukaryota - ps5
Eukaryota - ps6
Amorpheae - ps7
Obazoa - ps8
Opisthokonta - ps9
Holozoa - ps10
Filozoa - ps11
Choano./Metazoa - ps12
Metazoa - ps13
Myriazoa - ps14
Parahoxozoa - ps15
Bilateria - ps16
Deuterostomia - ps17
Chordata - ps18
Olfactores - ps19
Vertebrata - ps20
Gnathostomata - ps21
Euteleostomi - ps22
Sarcopterygii - ps23
Tetrapoda - ps24
Amniota - ps25
Mammalia - ps26
Theria - ps27
Placentalia - ps28
Boreoeutheria - ps29
Euarchontoglires - ps30
Euarchonta - ps31
Primates - ps32
Haplorrhini - ps33
Simiiformes - ps34
Catarrhini - ps35
Hominoidea - ps36
Hominidae - ps37
Homininae - ps38
Hominini - ps39
Homo_sapiens - ps40
p-value
3e-08
5e-07
9e-06
2e-04
3e-03
Log-odds
0.5
1.0
1.5
2.0
2.5
3.0
3.5
4.0

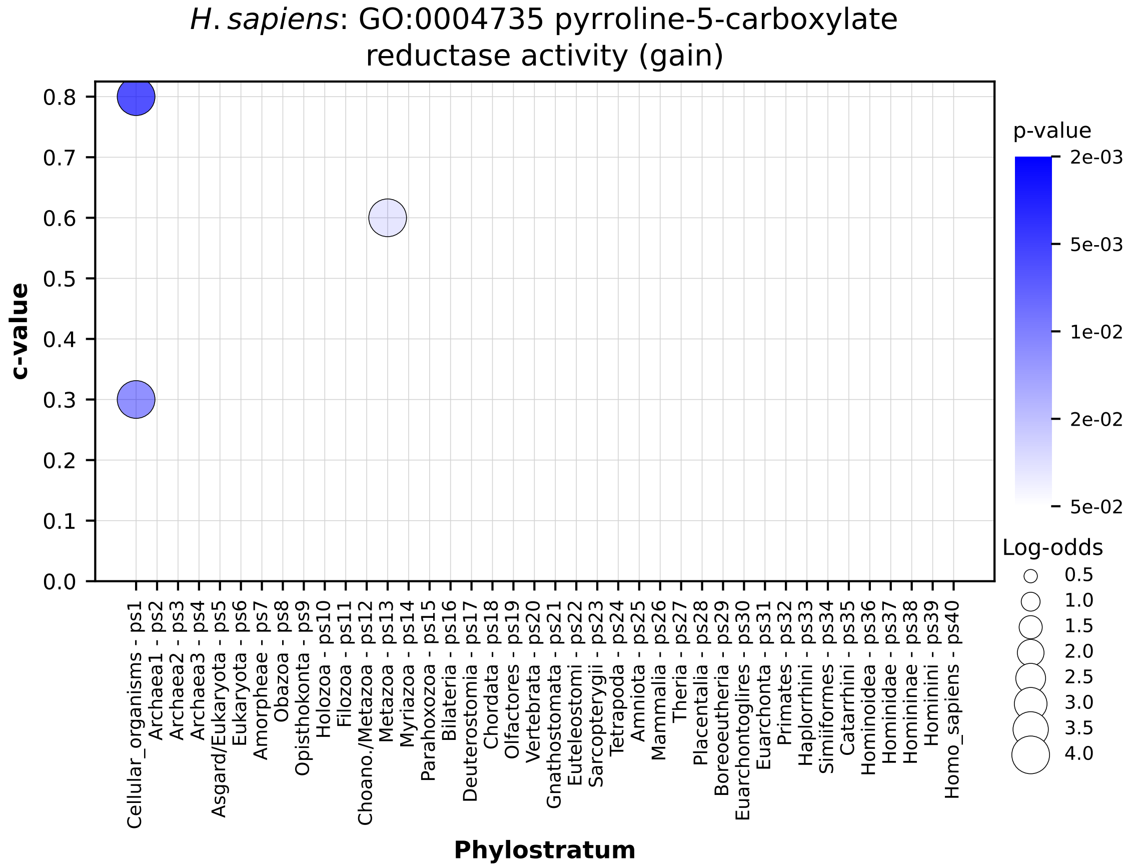

H. sapiens: GO:0004735 pyrroline-5-carboxylate reductase activity (gain)
c-value
0.0
0.1
0.2
0.3
0.4
0.5
0.6
0.7
0.8
Phylostratum
Cellular_organisms - ps1
Archaea1 - ps2
Archaea2 - ps3
Archaea3 - ps4
Asgard/Eukaryota - ps5
Eukaryota - ps6
Amorpheae - ps7
Obazoa - ps8
Opisthokonta - ps9
Holozoa - ps10
Filozoa - ps11
Choano./Metazoa - ps12
Metazoa - ps13
Myriazoa - ps14
Parahoxozoa - ps15
Bilateria - ps16
Deuterostomia - ps17
Chordata - ps18
Olfactores - ps19
Vertebrata - ps20
Gnathostomata - ps21
Euteleostomi - ps22
Sarcopterygii - ps23
Tetrapoda - ps24
Amniota - ps25
Mammalia - ps26
Theria - ps27
Placentalia - ps28
Boreoeutheria - ps29
Euarchontoglires - ps30
Euarchonta - ps31
Primates - ps32
Haplorrhini - ps33
Simiiformes - ps34
Catarrhini - ps35
Hominoidea - ps36
Hominidae - ps37
Homininae - ps38
Hominini - ps39
Homo_sapiens - ps40
p-value
2e-03
5e-03
1e-02
2e-02
5e-02
Log-odds
0.5
1.0
1.5
2.0
2.5
3.0
3.5
4.0

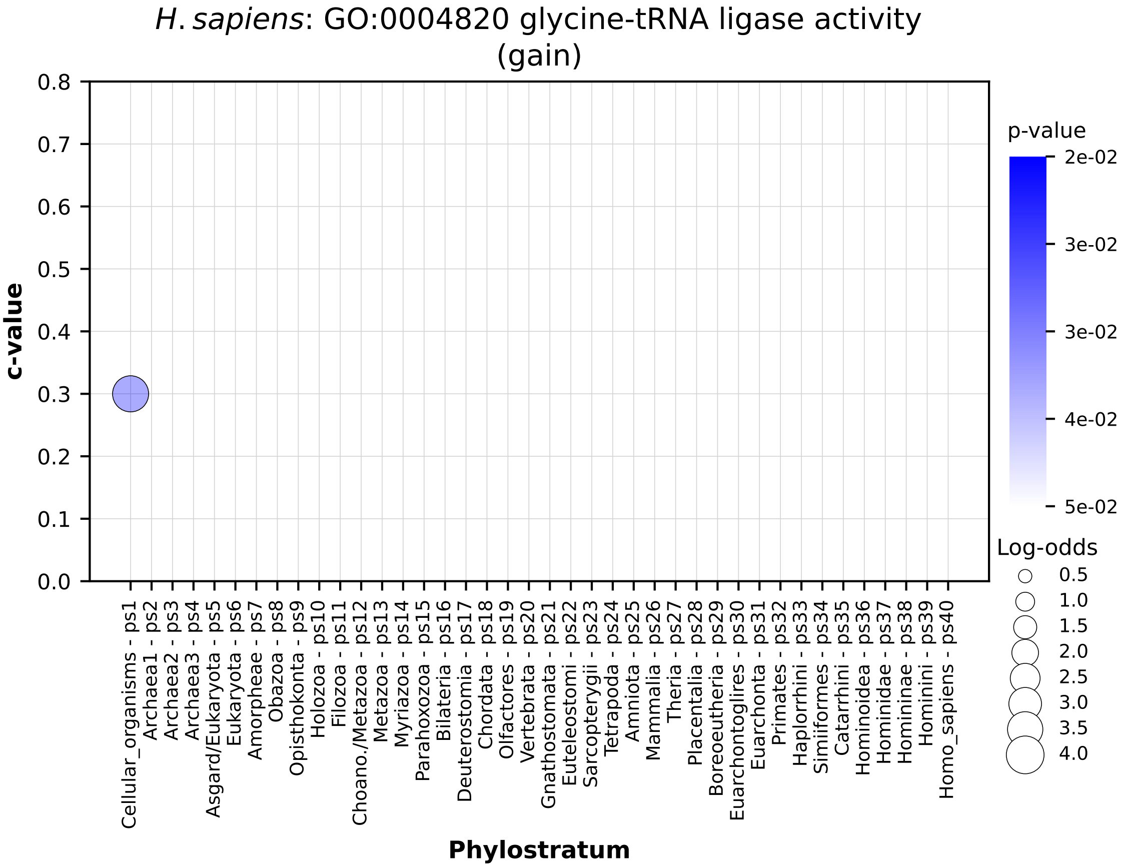

*H. sapiens*: GO:0004820 glycine-tRNA ligase activity
(gain)
c-value
0.8
0.7
0.6
0.5
0.4
0.3
0.2
0.1
0.0
Phylostratum
Cellular_organisms - ps1
Archaea1 - ps2
Archaea2 - ps3
Archaea3 - ps4
Asgard/Eukaryota - ps5
Eukaryota - ps6
Amorpheae - ps7
Obazoa - ps8
Opisthokonta - ps9
Holozoa - ps10
Filozoa - ps11
Choano./Metazoa - ps12
Metazoa - ps13
Myriazoa - ps14
Parahoxozoa - ps15
Bilateria - ps16
Deuterostomia - ps17
Chordata - ps18
Olfactores - ps19
Vertebrata - ps20
Gnathostomata - ps21
Euteleostomi - ps22
Sarcopterygii - ps23
Tetrapoda - ps24
Amniota - ps25
Mammalia - ps26
Theria - ps27
Placentalia - ps28
Boreoeutheria - ps29
Euarchontoglires - ps30
Euarchonta - ps31
Primates - ps32
Haplorrhini - ps33
Simiiformes - ps34
Catarrhini - ps35
Hominoidea - ps36
Hominidae - ps37
Homininae - ps38
Hominini - ps39
Homo_sapiens - ps40
p-value
2e-02
3e-02
3e-02
4e-02
5e-02
Log-odds
0.5
1.0
1.5
2.0
2.5
3.0
3.5
4.0

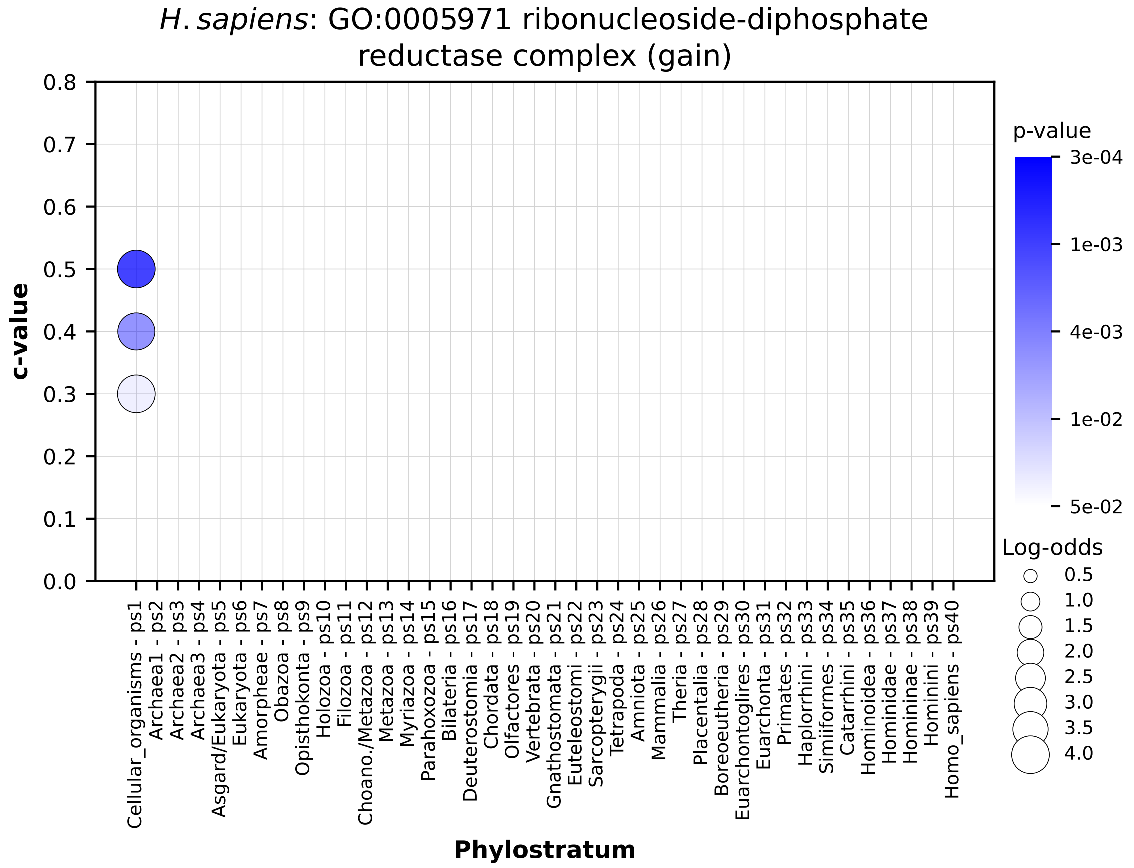
H. sapiens: GO:0005971 ribonucleoside-diphosphate reductase complex (gain)
c-value
0.8
0.7
0.6
0.5
0.4
0.3
0.2
0.1
0.0
p-value
3e-04
1e-03
4e-03
1e-02
5e-02
Log-odds
0.5
1.0
1.5
2.0
2.5
3.0
3.5
4.0
Cellular_organisms - ps1
Archaea1 - ps2
Archaea2 - ps3
Archaea3 - ps4
Asgard/Eukaryota - ps5
Eukaryota - ps6
Amorpheae - ps7
Obazoa - ps8
Opisthokonta - ps9
Holozoa - ps10
Filozoa - ps11
Choano./Metazoa - ps12
Metazoa - ps13
Myriazoa - ps14
Parahoxozoa - ps15
Bilateria - ps16
Deuterostomia - ps17
Chordata - ps18
Olfactores - ps19
Vertebrata - ps20
Gnathostomata - ps21
Euteleostomi - ps22
Sarcopterygii - ps23
Tetrapoda - ps24
Amniota - ps25
Mammalia - ps26
Theria - ps27
Placentalia - ps28
Boreoeutheria - ps29
Euarchontoglires - ps30
Euarchonta - ps31
Primates - ps32
Haplorrhini - ps33
Simiiformes - ps34
Catarrhini - ps35
Hominoidea - ps36
Hominidae - ps37
Homininae - ps38
Hominini - ps39
Homo_sapiens - ps40
Phylostratum

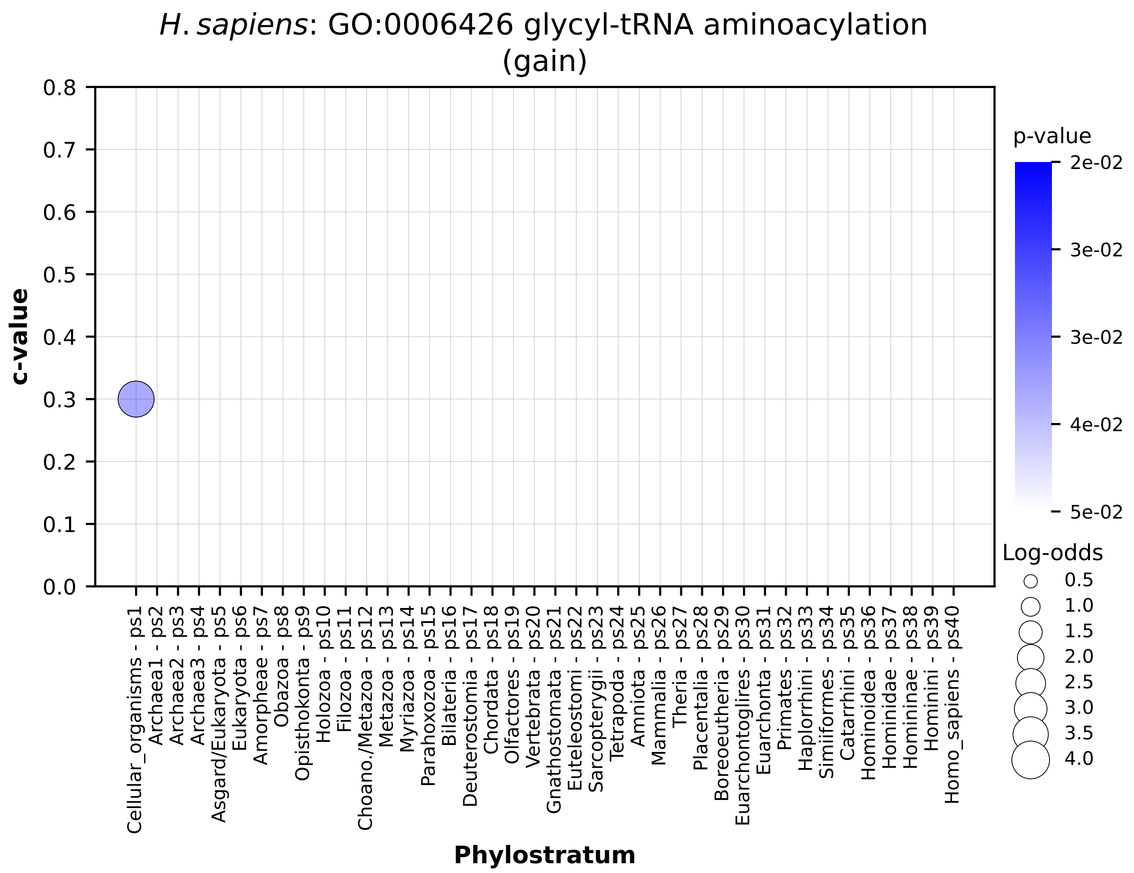

H. sapiens: GO:0006426 glycyl-tRNA aminoacylation
(gain)
c-value
0.0
0.1
0.2
0.3
0.4
0.5
0.6
0.7
0.8
Phylostratum
Cellular_organisms - ps1
Archaea1 - ps2
Archaea2 - ps3
Archaea3 - ps4
Asgard/Eukaryota - ps5
Eukaryota - ps6
Amorpheae - ps7
Obazoa - ps8
Opisthokonta - ps9
Holozoa - ps10
Filozoa - ps11
Choano./Metazoa - ps12
Metazoa - ps13
Myriazoa - ps14
Parahoxozoa - ps15
Bilateria - ps16
Deuterostomia - ps17
Chordata - ps18
Olfactores - ps19
Vertebrata - ps20
Gnathostomata - ps21
Euteleostomi - ps22
Sarcopterygii - ps23
Tetrapoda - ps24
Amniota - ps25
Mammalia - ps26
Theria - ps27
Placentalia - ps28
Boreoeutheria - ps29
Euarchontoglires - ps30
Euarchonta - ps31
Primates - ps32
Haplorrhini - ps33
Simiiformes - ps34
Catarrhini - ps35
Hominoidea - ps36
Hominidae - ps37
Homininae - ps38
Hominini - ps39
Homo_sapiens - ps40
p-value
2e-02
3e-02
3e-02
4e-02
5e-02
Log-odds
0.5
1.0
1.5
2.0
2.5
3.0
3.5
4.0

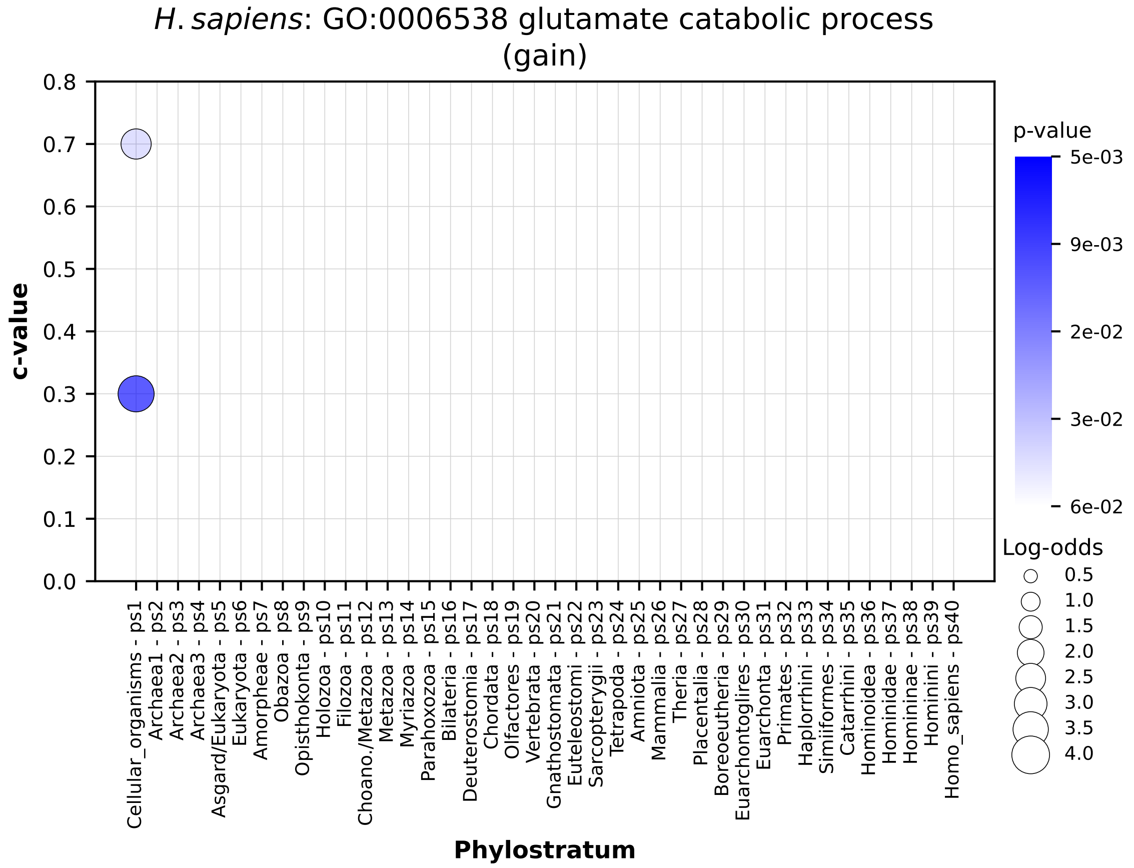

H. sapiens: GO:0006538 glutamate catabolic process (gain)
c-value
0.0
0.1
0.2
0.3
0.4
0.5
0.6
0.7
0.8
Phylostratum
Cellular_organisms - ps1
Archaea1 - ps2
Archaea2 - ps3
Archaea3 - ps4
Asgard/Eukaryota - ps5
Eukaryota - ps6
Amorpheae - ps7
Obazoa - ps8
Opisthokonta - ps9
Holozoa - ps10
Filozoa - ps11
Choano./Metazoa - ps12
Metazoa - ps13
Myriazoa - ps14
Parahoxozoa - ps15
Bilateria - ps16
Deuterostomia - ps17
Chordata - ps18
Olfactores - ps19
Vertebrata - ps20
Gnathostomata - ps21
Euteleostomi - ps22
Sarcopterygii - ps23
Tetrapoda - ps24
Amniota - ps25
Mammalia - ps26
Theria - ps27
Placentalia - ps28
Boreoeutheria - ps29
Euarchontoglires - ps30
Euarchonta - ps31
Primates - ps32
Haplorrhini - ps33
Simiiformes - ps34
Catarrhini - ps35
Hominoidea - ps36
Hominidae - ps37
Homininae - ps38
Hominini - ps39
Homo_sapiens - ps40
p-value
5e-03
9e-03
2e-02
3e-02
6e-02
Log-odds
0.5
1.0
1.5
2.0
2.5
3.0
3.5
4.0

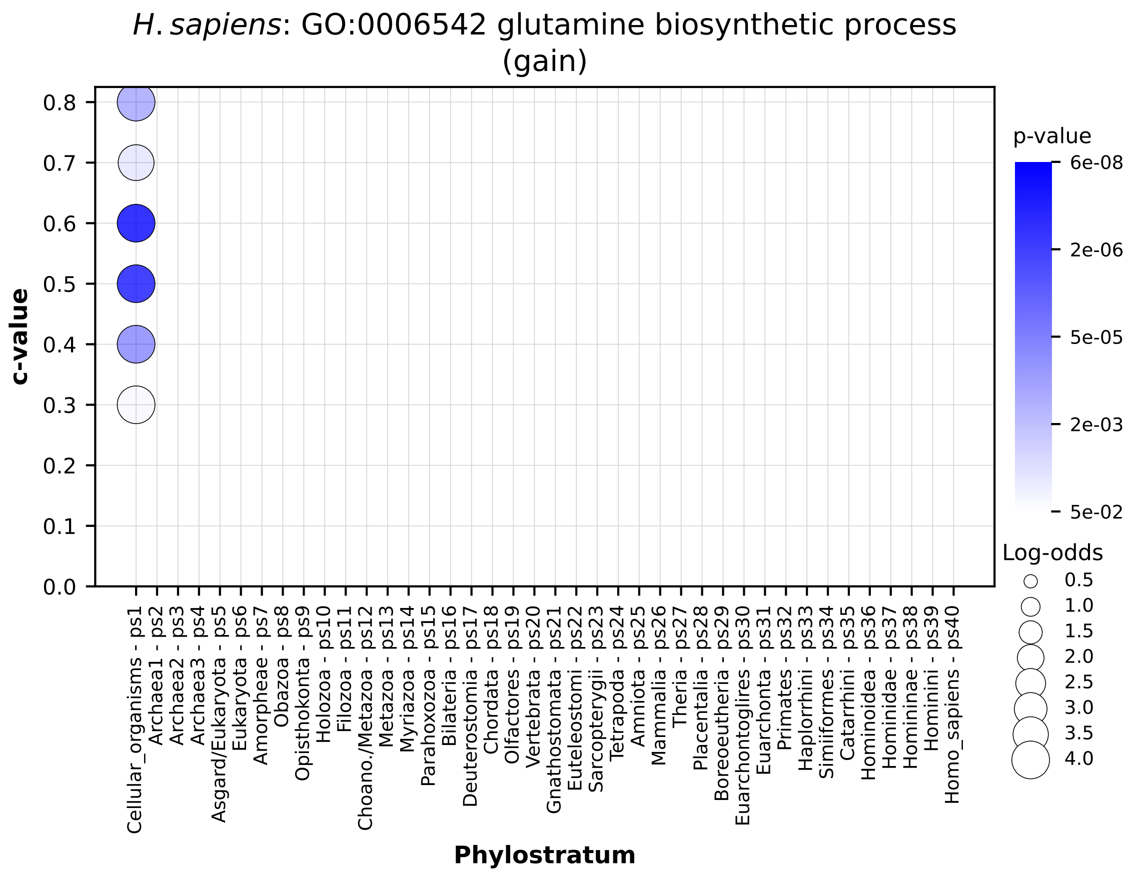

H. sapiens: GO:0006542 glutamine biosynthetic process
(gain)
c-value
0.0
0.1
0.2
0.3
0.4
0.5
0.6
0.7
0.8
p-value
6e-08
2e-06
5e-05
2e-03
5e-02
Log-odds
0.5
1.0
1.5
2.0
2.5
3.0
3.5
4.0
Cellular_organisms - ps1
Archaea1 - ps2
Archaea2 - ps3
Archaea3 - ps4
Asgard/Eukaryota - ps5
Eukaryota - ps6
Amorpheae - ps7
Obazoa - ps8
Opisthokonta - ps9
Holozoa - ps10
Filozoa - ps11
Choano./Metazoa - ps12
Metazoa - ps13
Myriazoa - ps14
Parahoxozoa - ps15
Bilateria - ps16
Deuterostomia - ps17
Chordata - ps18
Olfactores - ps19
Vertebrata - ps20
Gnathostomata - ps21
Euteleostomi - ps22
Sarcopterygii - ps23
Tetrapoda - ps24
Amniota - ps25
Mammalia - ps26
Theria - ps27
Placentalia - ps28
Boreoeutheria - ps29
Euarchontoglires - ps30
Euarchonta - ps31
Primates - ps32
Haplorrhini - ps33
Simiiformes - ps34
Catarrhini - ps35
Hominoidea - ps36
Hominidae - ps37
Homininae - ps38
Hominini - ps39
Homo_sapiens - ps40
Phylostratum

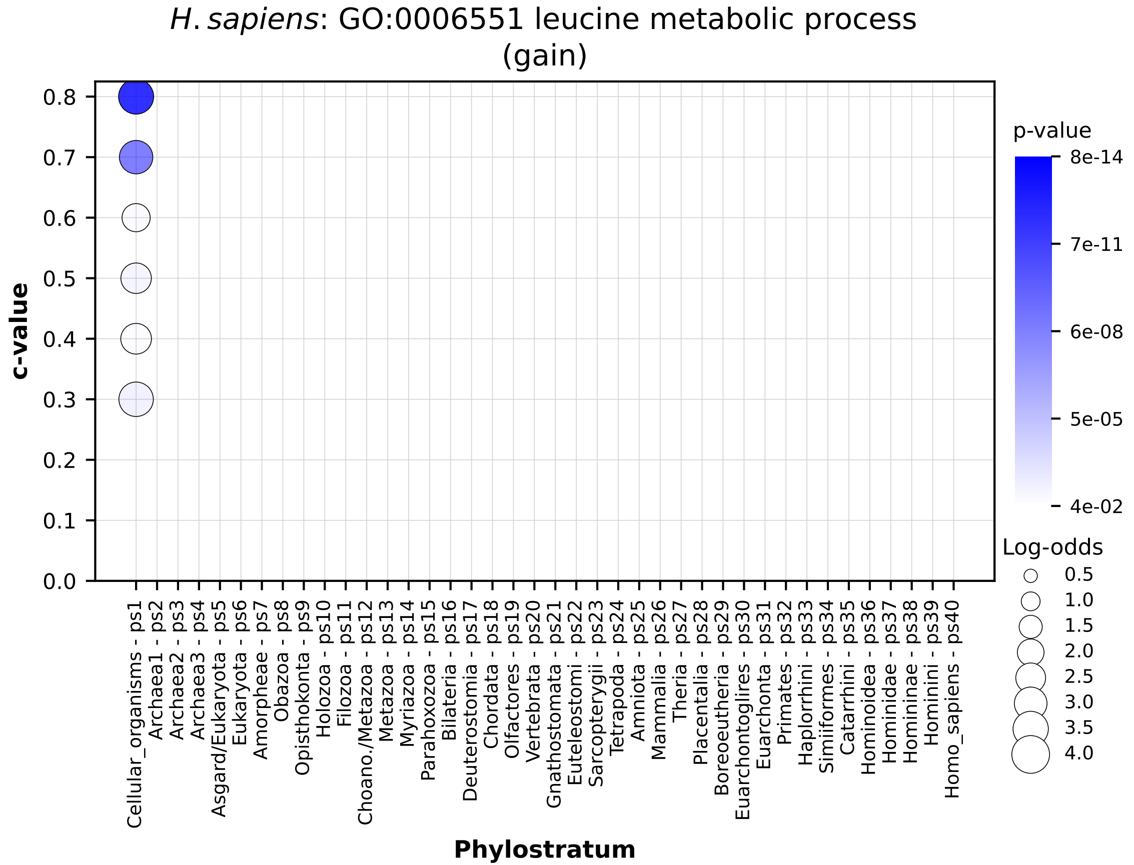

H. sapiens: GO:0006551 leucine metabolic process
(gain)
c-value
0.0
0.1
0.2
0.3
0.4
0.5
0.6
0.7
0.8
Phylostratum
Cellular_organisms - ps1
Archaea1 - ps2
Archaea2 - ps3
Archaea3 - ps4
Asgard/Eukaryota - ps5
Eukaryota - ps6
Amorpheae - ps7
Obazoa - ps8
Opisthokonta - ps9
Holozoa - ps10
Filozoa - ps11
Choano./Metazoa - ps12
Metazoa - ps13
Myriazoa - ps14
Parahoxozoa - ps15
Bilateria - ps16
Deuterostomia - ps17
Chordata - ps18
Olfactores - ps19
Vertebrata - ps20
Gnathostomata - ps21
Euteleostomi - ps22
Sarcopterygii - ps23
Tetrapoda - ps24
Amniota - ps25
Mammalia - ps26
Theria - ps27
Placentalia - ps28
Boreoeutheria - ps29
Euarchontoglires - ps30
Euarchonta - ps31
Primates - ps32
Haplorrhini - ps33
Simiiformes - ps34
Catarrhini - ps35
Hominoidea - ps36
Hominidae - ps37
Homininae - ps38
Hominini - ps39
Homo_sapiens - ps40
p-value
8e-14
7e-11
6e-08
5e-05
4e-02
Log-odds
0.5
1.0
1.5
2.0
2.5
3.0
3.5
4.0

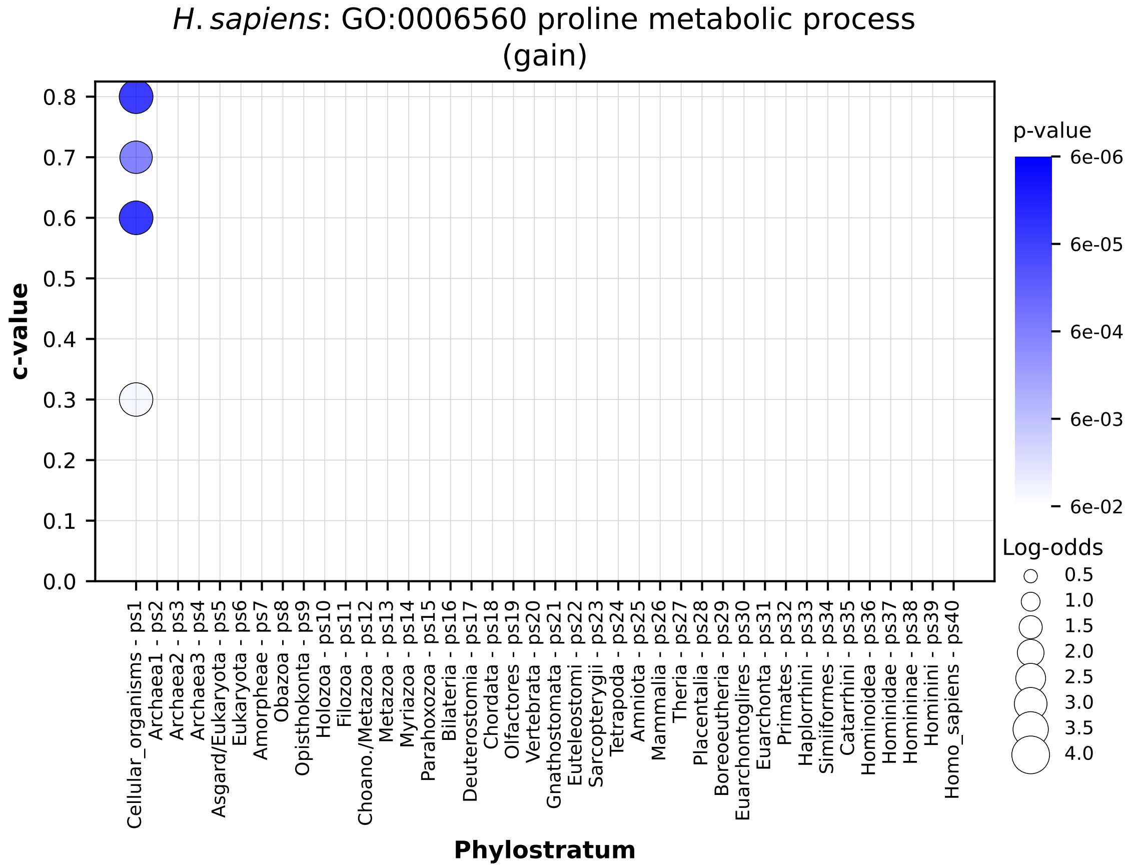

*H. sapiens*: GO:0006560 proline metabolic process
(gain)
c-value
0.8
0.7
0.6
0.5
0.4
0.3
0.2
0.1
0.0
Phylostratum
Cellular_organisms - ps1
Archaea1 - ps2
Archaea2 - ps3
Archaea3 - ps4
Asgard/Eukaryota - ps5
Eukaryota - ps6
Amorpheae - ps7
Obazoa - ps8
Opisthokonta - ps9
Holozoa - ps10
Filozoa - ps11
Choano./Metazoa - ps12
Metazoa - ps13
Myriazoa - ps14
Parahoxozoa - ps15
Bilateria - ps16
Deuterostomia - ps17
Chordata - ps18
Olfactores - ps19
Vertebrata - ps20
Gnathostomata - ps21
Euteleostomi - ps22
Sarcopterygii - ps23
Tetrapoda - ps24
Amniota - ps25
Mammalia - ps26
Theria - ps27
Placentalia - ps28
Boreoeutheria - ps29
Euarchontoglires - ps30
Euarchonta - ps31
Primates - ps32
Haplorrhini - ps33
Simiiformes - ps34
Catarrhini - ps35
Hominoidea - ps36
Hominidae - ps37
Homininae - ps38
Hominini - ps39
Homo_sapiens - ps40
p-value
6e-06
6e-05
6e-04
6e-03
6e-02
Log-odds
0.5
1.0
1.5
2.0
2.5
3.0
3.5
4.0

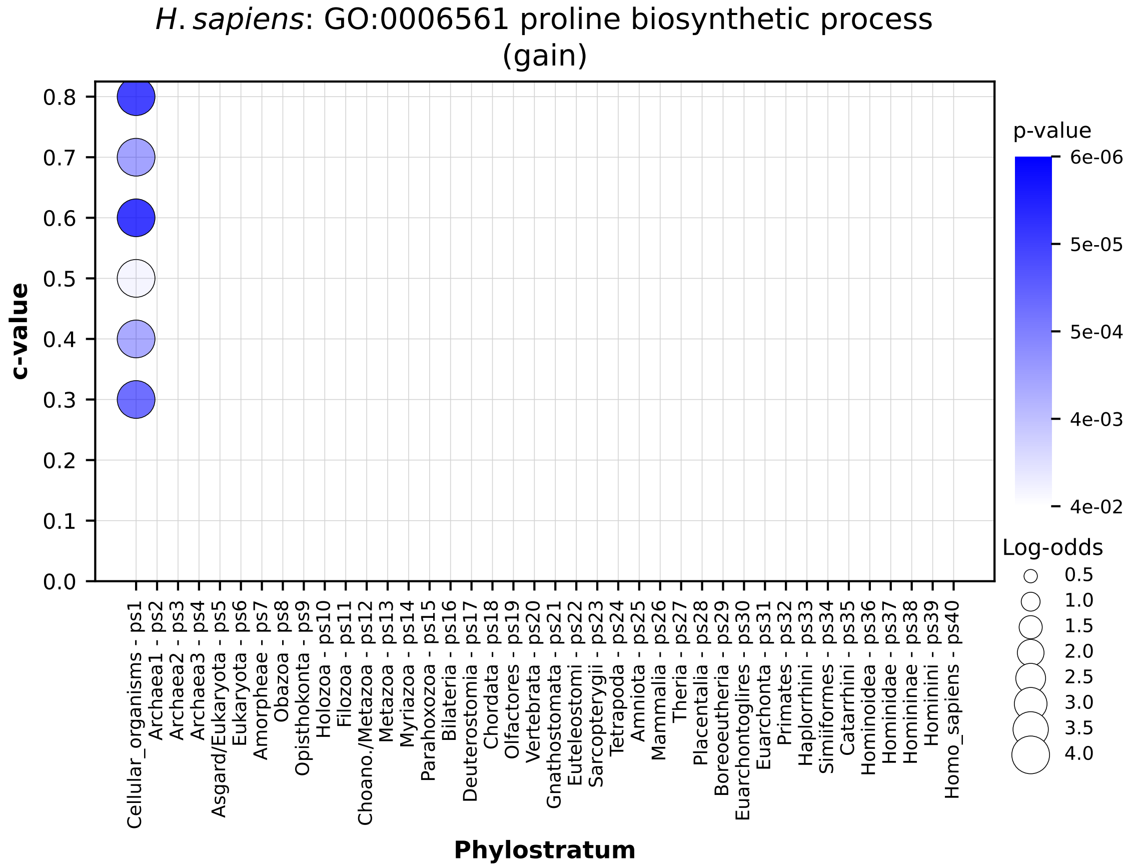

H. sapiens: GO:0006561 proline biosynthetic process
(gain)
c-value
0.0
0.1
0.2
0.3
0.4
0.5
0.6
0.7
0.8
Phylostratum
Cellular_organisms - ps1
Archaea1 - ps2
Archaea2 - ps3
Archaea3 - ps4
Asgard/Eukaryota - ps5
Eukaryota - ps6
Amorpheae - ps7
Obazoa - ps8
Opisthokonta - ps9
Holozoa - ps10
Filozoa - ps11
Choano./Metazoa - ps12
Metazoa - ps13
Myriazoa - ps14
Parahoxozoa - ps15
Bilateria - ps16
Deuterostomia - ps17
Chordata - ps18
Olfactores - ps19
Vertebrata - ps20
Gnathostomata - ps21
Euteleostomi - ps22
Sarcopterygii - ps23
Tetrapoda - ps24
Amniota - ps25
Mammalia - ps26
Theria - ps27
Placentalia - ps28
Boreoeutheria - ps29
Euarchontoglires - ps30
Euarchonta - ps31
Primates - ps32
Haplorrhini - ps33
Simiiformes - ps34
Catarrhini - ps35
Hominoidea - ps36
Hominidae - ps37
Homininae - ps38
Hominini - ps39
Homo_sapiens - ps40
p-value
6e-06
5e-05
5e-04
4e-03
4e-02
Log-odds
0.5
1.0
1.5
2.0
2.5
3.0
3.5
4.0

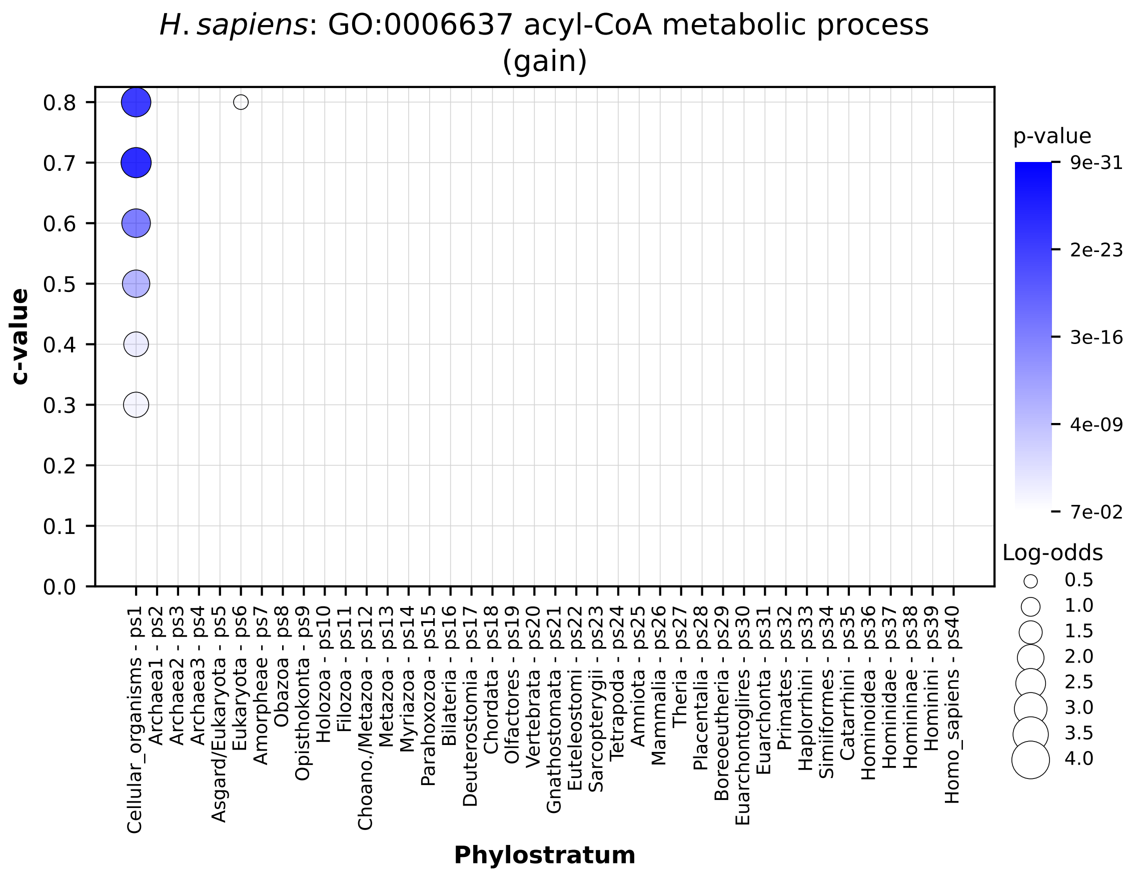
H. sapiens: GO:0006637 acyl-CoA metabolic process
(gain)
c-value
0.8
0.7
0.6
0.5
0.4
0.3
0.2
0.1
0.0
Phylostratum
Cellular_organisms - ps1
Archaea1 - ps2
Archaea2 - ps3
Archaea3 - ps4
Asgard/Eukaryota - ps5
Eukaryota - ps6
Amorpheae - ps7
Obazoa - ps8
Opisthokonta - ps9
Holozoa - ps10
Filozoa - ps11
Choano./Metazoa - ps12
Metazoa - ps13
Myriazoa - ps14
Parahoxozoa - ps15
Bilateria - ps16
Deuterostomia - ps17
Chordata - ps18
Olfactores - ps19
Vertebrata - ps20
Gnathostomata - ps21
Euteleostomi - ps22
Sarcopterygii - ps23
Tetrapoda - ps24
Amniota - ps25
Mammalia - ps26
Theria - ps27
Placentalia - ps28
Boreoeutheria - ps29
Euarchontoglires - ps30
Euarchonta - ps31
Primates - ps32
Haplorrhini - ps33
Simiiformes - ps34
Catarrhini - ps35
Hominoidea - ps36
Hominidae - ps37
Homininae - ps38
Hominini - ps39
Homo_sapiens - ps40
p-value
9e-31
2e-23
3e-16
4e-09
7e-02
Log-odds
0.5
1.0
1.5
2.0
2.5
3.0
3.5
4.0

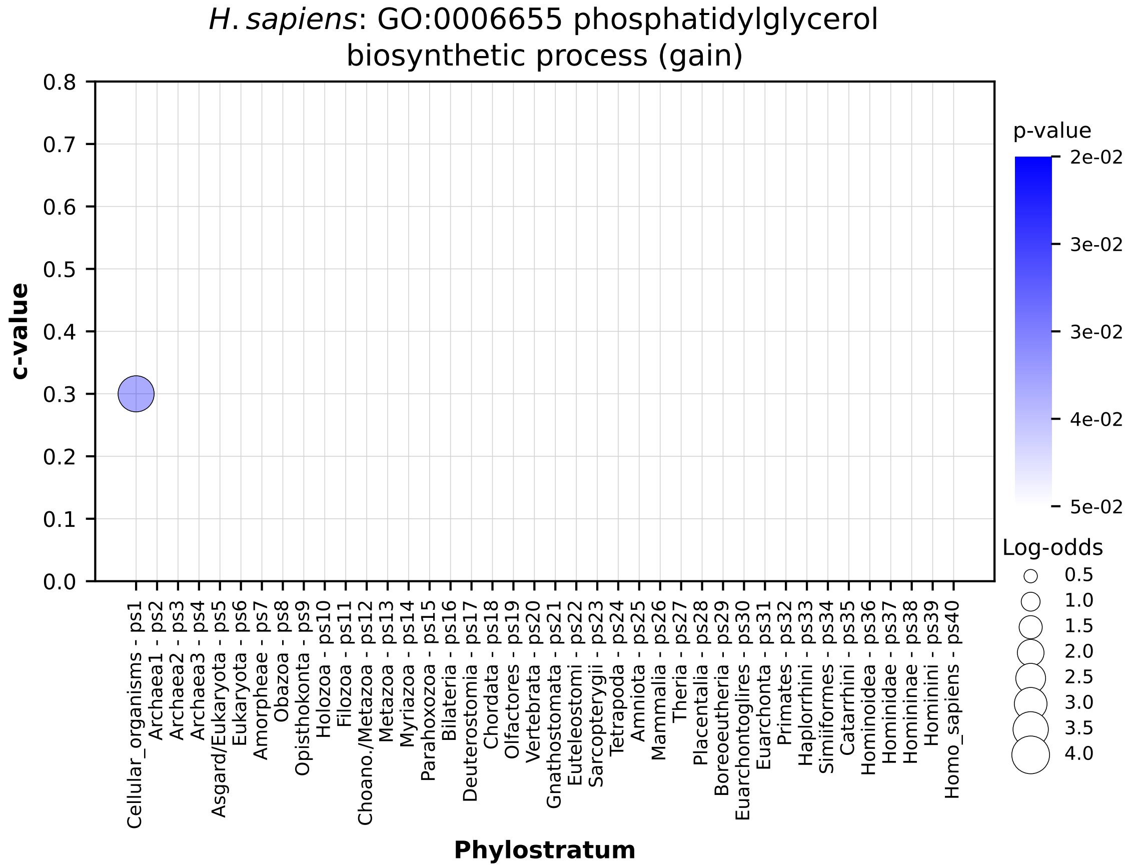

*H. sapiens*: GO:0006655 phosphatidylglycerol biosynthetic process (gain)
c-value
0.0
0.1
0.2
0.3
0.4
0.5
0.6
0.7
0.8
Phylostratum
Cellular_organisms - ps1
Archaea1 - ps2
Archaea2 - ps3
Archaea3 - ps4
Asgard/Eukaryota - ps5
Eukaryota - ps6
Amorpheae - ps7
Obazoa - ps8
Opisthokonta - ps9
Holozoa - ps10
Filozoa - ps11
Choano./Metazoa - ps12
Metazoa - ps13
Myriazoa - ps14
Parahoxozoa - ps15
Bilateria - ps16
Deuterostomia - ps17
Chordata - ps18
Olfactores - ps19
Vertebrata - ps20
Gnathostomata - ps21
Euteleostomi - ps22
Sarcopterygii - ps23
Tetrapoda - ps24
Amniota - ps25
Mammalia - ps26
Theria - ps27
Placentalia - ps28
Boreoeutheria - ps29
Euarchontoglires - ps30
Euarchonta - ps31
Primates - ps32
Haplorrhini - ps33
Simiiformes - ps34
Catarrhini - ps35
Hominoidea - ps36
Hominidae - ps37
Homininae - ps38
Hominini - ps39
Homo_sapiens - ps40
p-value
2e-02
3e-02
3e-02
4e-02
5e-02
Log-odds
0.5
1.0
1.5
2.0
2.5
3.0
3.5
4.0

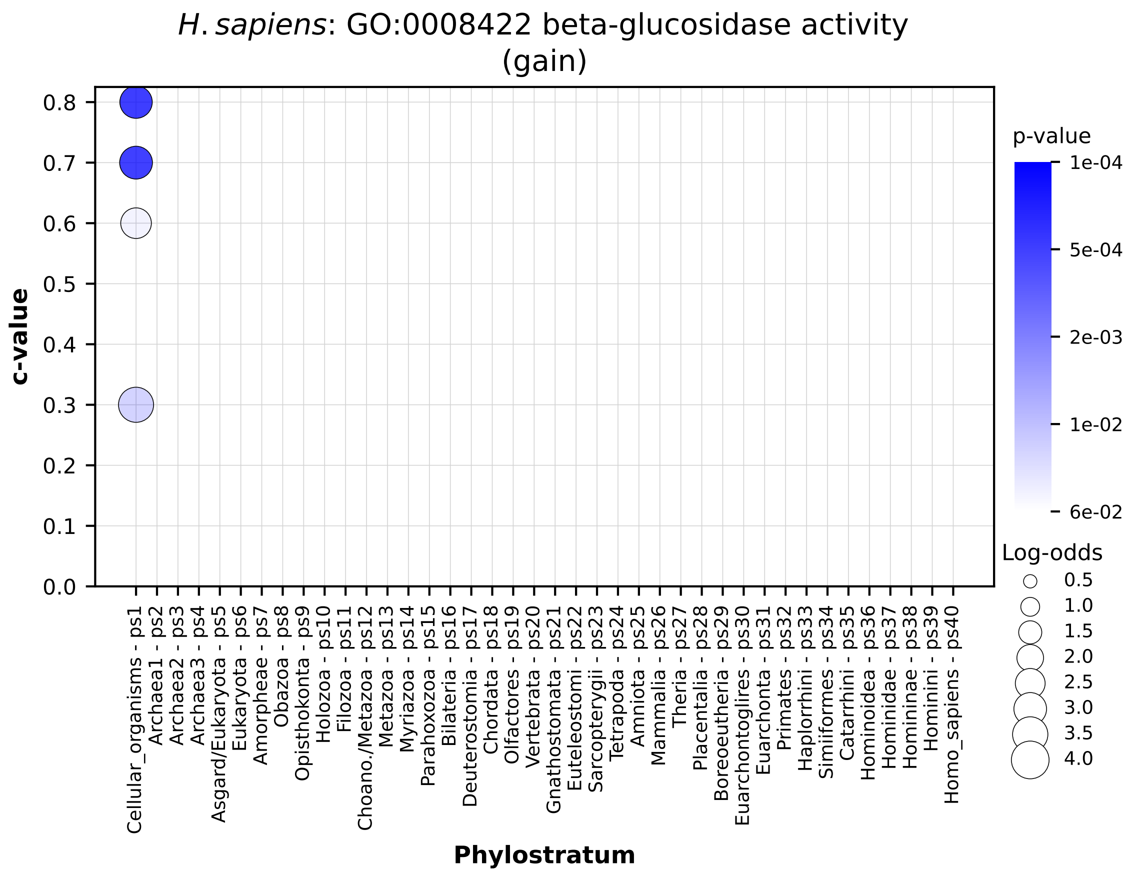
H. sapiens: GO:0008422 beta-glucosidase activity
(gain)
c-value
0.0
0.1
0.2
0.3
0.4
0.5
0.6
0.7
0.8
Phylostratum
Cellular_organisms - ps1
Archaea1 - ps2
Archaea2 - ps3
Archaea3 - ps4
Asgard/Eukaryota - ps5
Eukaryota - ps6
Amorpheae - ps7
Obazoa - ps8
Opisthokonta - ps9
Holozoa - ps10
Filozoa - ps11
Choano./Metazoa - ps12
Metazoa - ps13
Myriazoa - ps14
Parahoxozoa - ps15
Bilateria - ps16
Deuterostomia - ps17
Chordata - ps18
Olfactores - ps19
Vertebrata - ps20
Gnathostomata - ps21
Euteleostomi - ps22
Sarcopterygii - ps23
Tetrapoda - ps24
Amniota - ps25
Mammalia - ps26
Theria - ps27
Placentalia - ps28
Boreoeutheria - ps29
Euarchontoglires - ps30
Euarchonta - ps31
Primates - ps32
Haplorrhini - ps33
Simiiformes - ps34
Catarrhini - ps35
Hominoidea - ps36
Hominidae - ps37
Homininae - ps38
Hominini - ps39
Homo_sapiens - ps40
p-value
1e-04
5e-04
2e-03
1e-02
6e-02
Log-odds
0.5
1.0
1.5
2.0
2.5
3.0
3.5
4.0

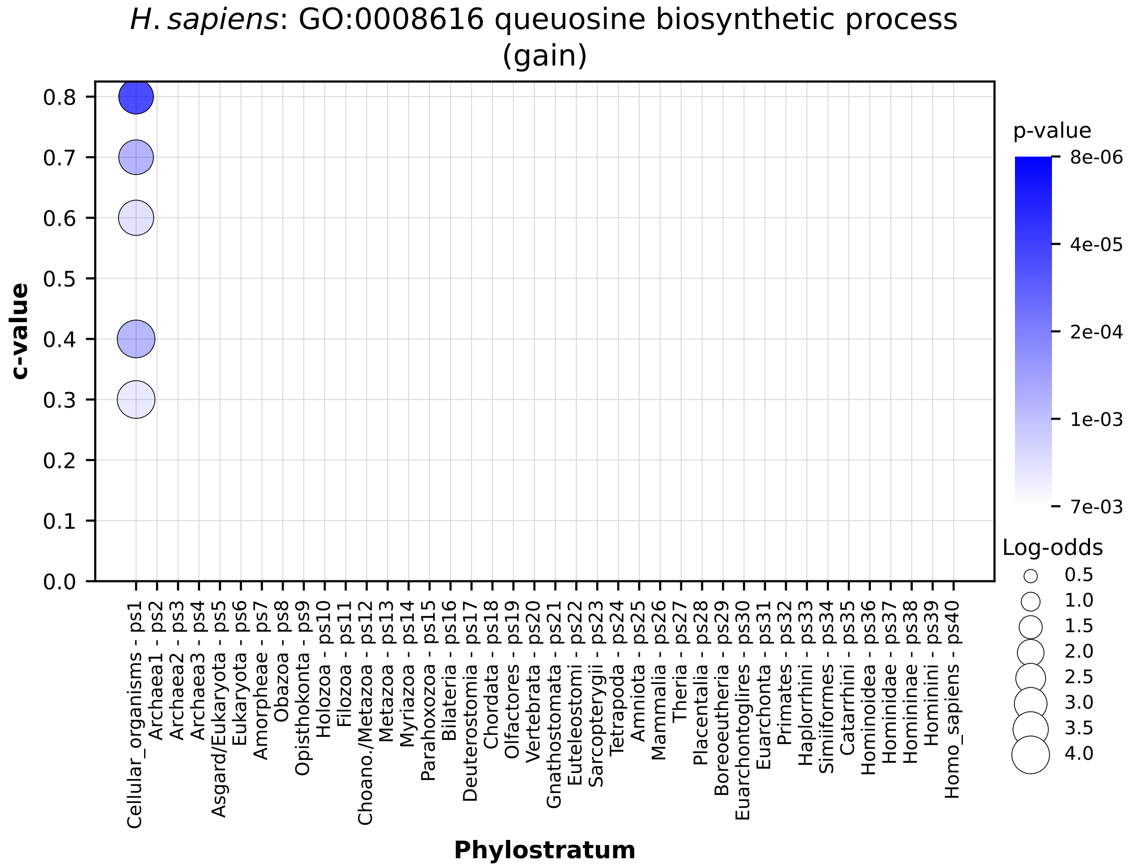
H. sapiens: GO:0008616 queuosine biosynthetic process
(gain)
c-value
0.0
0.1
0.2
0.3
0.4
0.5
0.6
0.7
0.8
Phylostratum
Cellular_organisms - ps1
Archaea1 - ps2
Archaea2 - ps3
Archaea3 - ps4
Asgard/Eukaryota - ps5
Eukaryota - ps6
Amorpheae - ps7
Obazoa - ps8
Opisthokonta - ps9
Holozoa - ps10
Filozoa - ps11
Choano./Metazoa - ps12
Metazoa - ps13
Myriazoa - ps14
Parahoxozoa - ps15
Bilateria - ps16
Deuterostomia - ps17
Chordata - ps18
Olfactores - ps19
Vertebrata - ps20
Gnathostomata - ps21
Euteleostomi - ps22
Sarcopterygii - ps23
Tetrapoda - ps24
Amniota - ps25
Mammalia - ps26
Theria - ps27
Placentalia - ps28
Boreoeutheria - ps29
Euarchontoglires - ps30
Euarchonta - ps31
Primates - ps32
Haplorrhini - ps33
Simiiformes - ps34
Catarrhini - ps35
Hominoidea - ps36
Hominidae - ps37
Homininae - ps38
Hominini - ps39
Homo_sapiens - ps40
p-value
8e-06
4e-05
2e-04
1e-03
7e-03
Log-odds
0.5
1.0
1.5
2.0
2.5
3.0
3.5
4.0

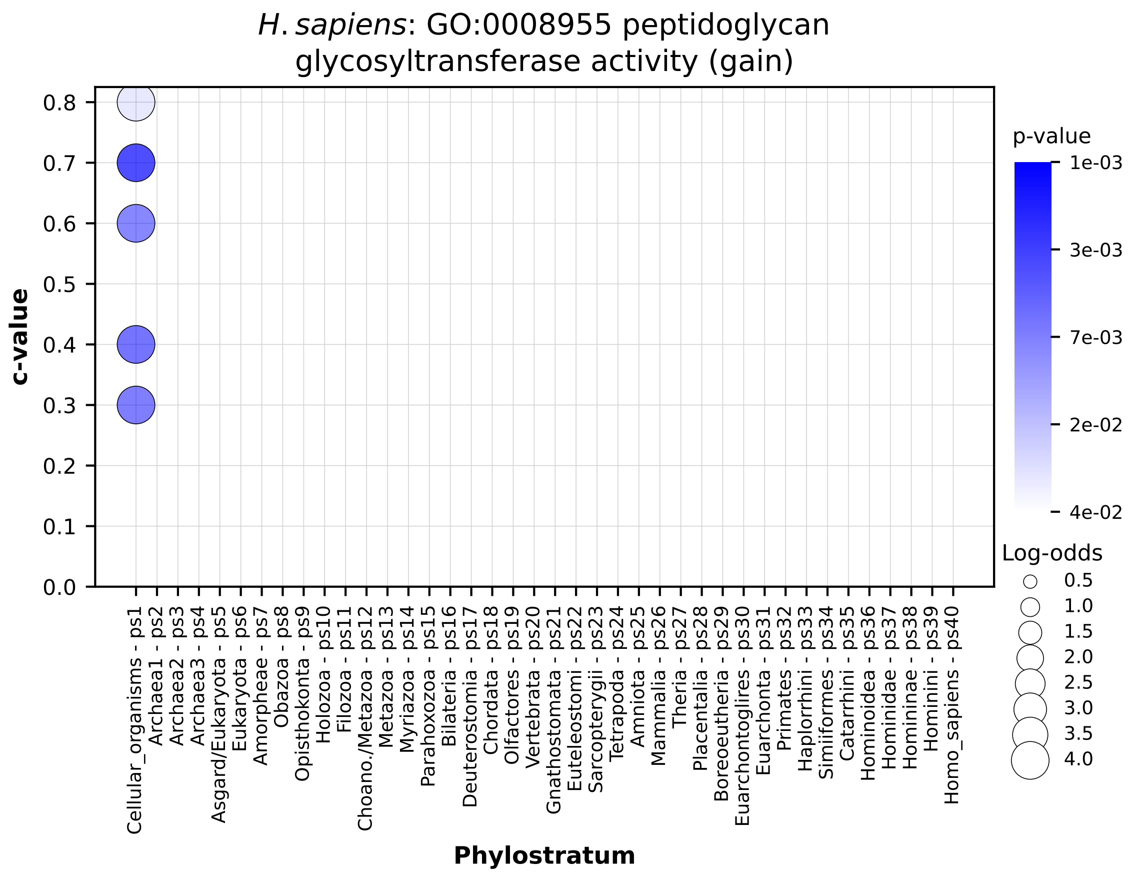

H. sapiens: GO:0008955 peptidoglycan glycosyltransferase activity (gain)
c-value
0.0
0.1
0.2
0.3
0.4
0.5
0.6
0.7
0.8
Phylostratum
Cellular_organisms - ps1
Archaea1 - ps2
Archaea2 - ps3
Archaea3 - ps4
Asgard/Eukaryota - ps5
Eukaryota - ps6
Amorpheae - ps7
Obazoa - ps8
Opisthokonta - ps9
Holozoa - ps10
Filozoa - ps11
Choano./Metazoa - ps12
Metazoa - ps13
Myriazoa - ps14
Parahoxozoa - ps15
Bilateria - ps16
Deuterostomia - ps17
Chordata - ps18
Olfactores - ps19
Vertebrata - ps20
Gnathostomata - ps21
Euteleostomi - ps22
Sarcopterygii - ps23
Tetrapoda - ps24
Amniota - ps25
Mammalia - ps26
Theria - ps27
Placentalia - ps28
Boreoeutheria - ps29
Euarchontoglires - ps30
Euarchonta - ps31
Primates - ps32
Haplorrhini - ps33
Simiiformes - ps34
Catarrhini - ps35
Hominoidea - ps36
Hominidae - ps37
Homininae - ps38
Hominini - ps39
Homo_sapiens - ps40
p-value
1e-03
3e-03
7e-03
2e-02
4e-02
Log-odds
0.5
1.0
1.5
2.0
2.5
3.0
3.5
4.0

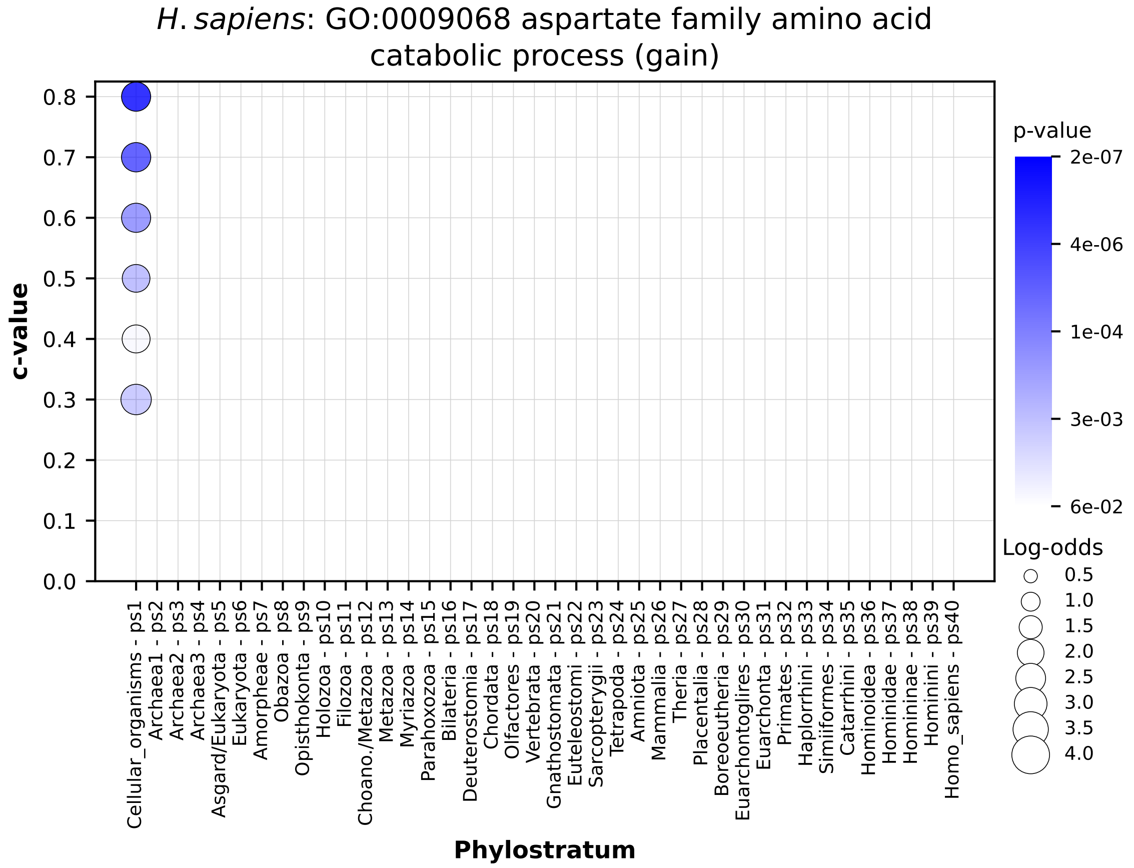
H. sapiens: GO:0009068 aspartate family amino acid catabolic process (gain)
c-value
0.0
0.1
0.2
0.3
0.4
0.5
0.6
0.7
0.8
Phylostratum
Cellular_organisms - ps1
Archaea1 - ps2
Archaea2 - ps3
Archaea3 - ps4
Asgard/Eukaryota - ps5
Eukaryota - ps6
Amorpheae - ps7
Obazoa - ps8
Opisthokonta - ps9
Holozoa - ps10
Filozoa - ps11
Choano./Metazoa - ps12
Metazoa - ps13
Myriazoa - ps14
Parahoxozoa - ps15
Bilateria - ps16
Deuterostomia - ps17
Chordata - ps18
Olfactores - ps19
Vertebrata - ps20
Gnathostomata - ps21
Euteleostomi - ps22
Sarcopterygii - ps23
Tetrapoda - ps24
Amniota - ps25
Mammalia - ps26
Theria - ps27
Placentalia - ps28
Boreoeutheria - ps29
Euarchontoglires - ps30
Euarchonta - ps31
Primates - ps32
Haplorrhini - ps33
Simiiformes - ps34
Catarrhini - ps35
Hominoidea - ps36
Hominidae - ps37
Homininae - ps38
Hominini - ps39
Homo_sapiens - ps40
p-value
2e-07
4e-06
1e-04
3e-03
6e-02
Log-odds
0.5
1.0
1.5
2.0
2.5
3.0
3.5
4.0

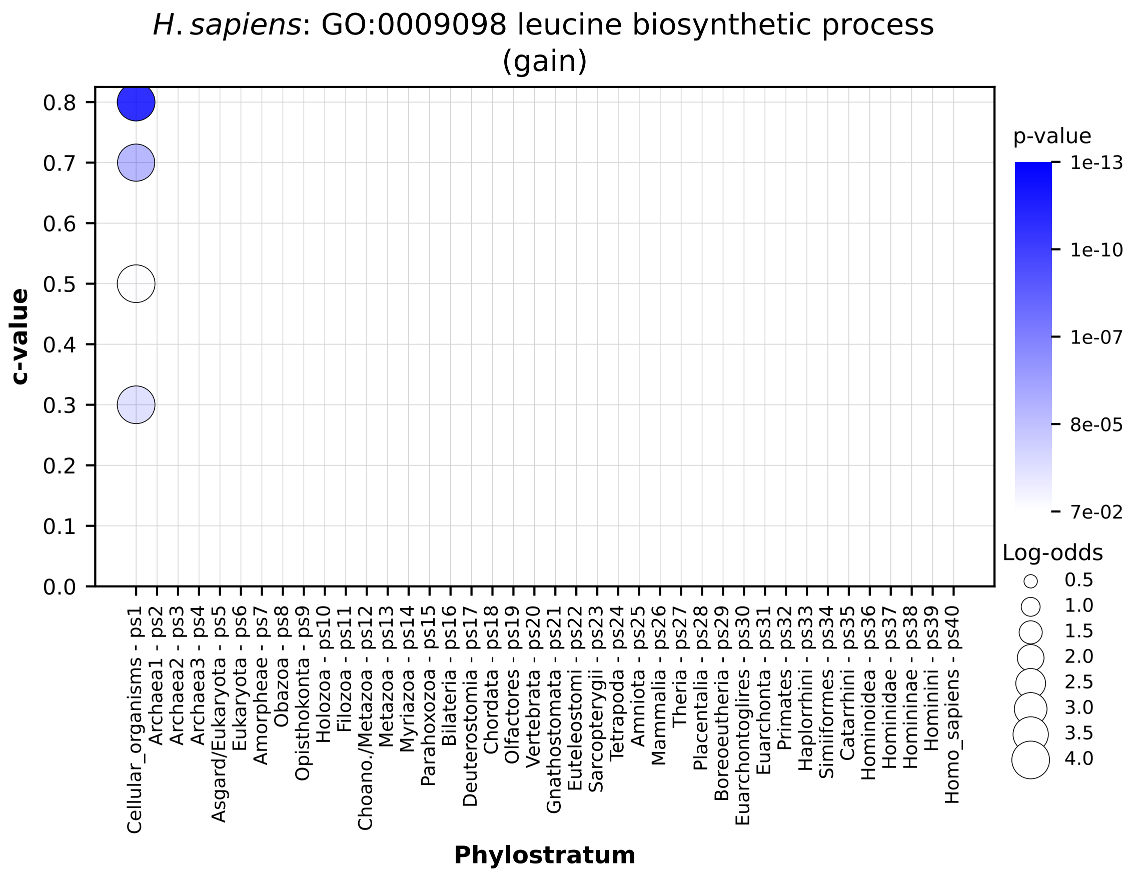
H. sapiens: GO:0009098 leucine biosynthetic process
(gain)
c-value
0.0
0.1
0.2
0.3
0.4
0.5
0.6
0.7
0.8
Phylostratum
Cellular_organisms - ps1
Archaea1 - ps2
Archaea2 - ps3
Archaea3 - ps4
Asgard/Eukaryota - ps5
Eukaryota - ps6
Amorpheae - ps7
Obazoa - ps8
Opisthokonta - ps9
Holozoa - ps10
Filozoa - ps11
Choano./Metazoa - ps12
Metazoa - ps13
Myriazoa - ps14
Parahoxozoa - ps15
Bilateria - ps16
Deuterostomia - ps17
Chordata - ps18
Olfactores - ps19
Vertebrata - ps20
Gnathostomata - ps21
Euteleostomi - ps22
Sarcopterygii - ps23
Tetrapoda - ps24
Amniota - ps25
Mammalia - ps26
Theria - ps27
Placentalia - ps28
Boreoeutheria - ps29
Euarchontoglires - ps30
Euarchonta - ps31
Primates - ps32
Haplorrhini - ps33
Simiiformes - ps34
Catarrhini - ps35
Hominoidea - ps36
Hominidae - ps37
Homininae - ps38
Hominini - ps39
Homo_sapiens - ps40
p-value
1e-13
1e-10
1e-07
8e-05
7e-02
Log-odds
0.5
1.0
1.5
2.0
2.5
3.0
3.5
4.0

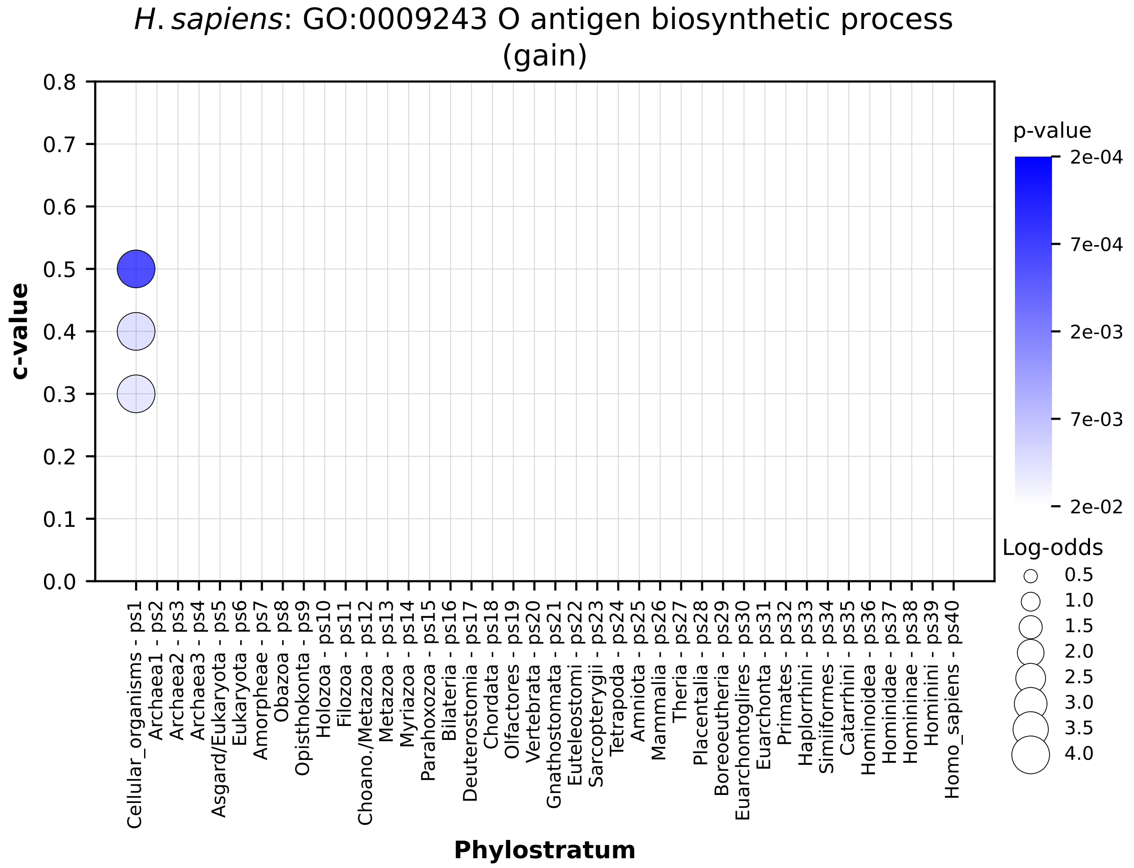

H. sapiens: GO:0009243 O antigen biosynthetic process (gain)
c-value
0.8
0.7
0.6
0.5
0.4
0.3
0.2
0.1
0.0
Phylostratum
Cellular_organisms - ps1
Archaea1 - ps2
Archaea2 - ps3
Archaea3 - ps4
Asgard/Eukaryota - ps5
Eukaryota - ps6
Amorpheae - ps7
Obazoa - ps8
Opisthokonta - ps9
Holozoa - ps10
Filozoa - ps11
Choano./Metazoa - ps12
Metazoa - ps13
Myriazoa - ps14
Parahoxozoa - ps15
Bilateria - ps16
Deuterostomia - ps17
Chordata - ps18
Olfactores - ps19
Vertebrata - ps20
Gnathostomata - ps21
Euteleostomi - ps22
Sarcopterygii - ps23
Tetrapoda - ps24
Amniota - ps25
Mammalia - ps26
Theria - ps27
Placentalia - ps28
Boreoeutheria - ps29
Euarchontoglires - ps30
Euarchonta - ps31
Primates - ps32
Haplorrhini - ps33
Simiiformes - ps34
Catarrhini - ps35
Hominoidea - ps36
Hominidae - ps37
Homininae - ps38
Hominini - ps39
Homo_sapiens - ps40
p-value
2e-04
7e-04
2e-03
7e-03
2e-02
Log-odds
0.5
1.0
1.5
2.0
2.5
3.0
3.5
4.0

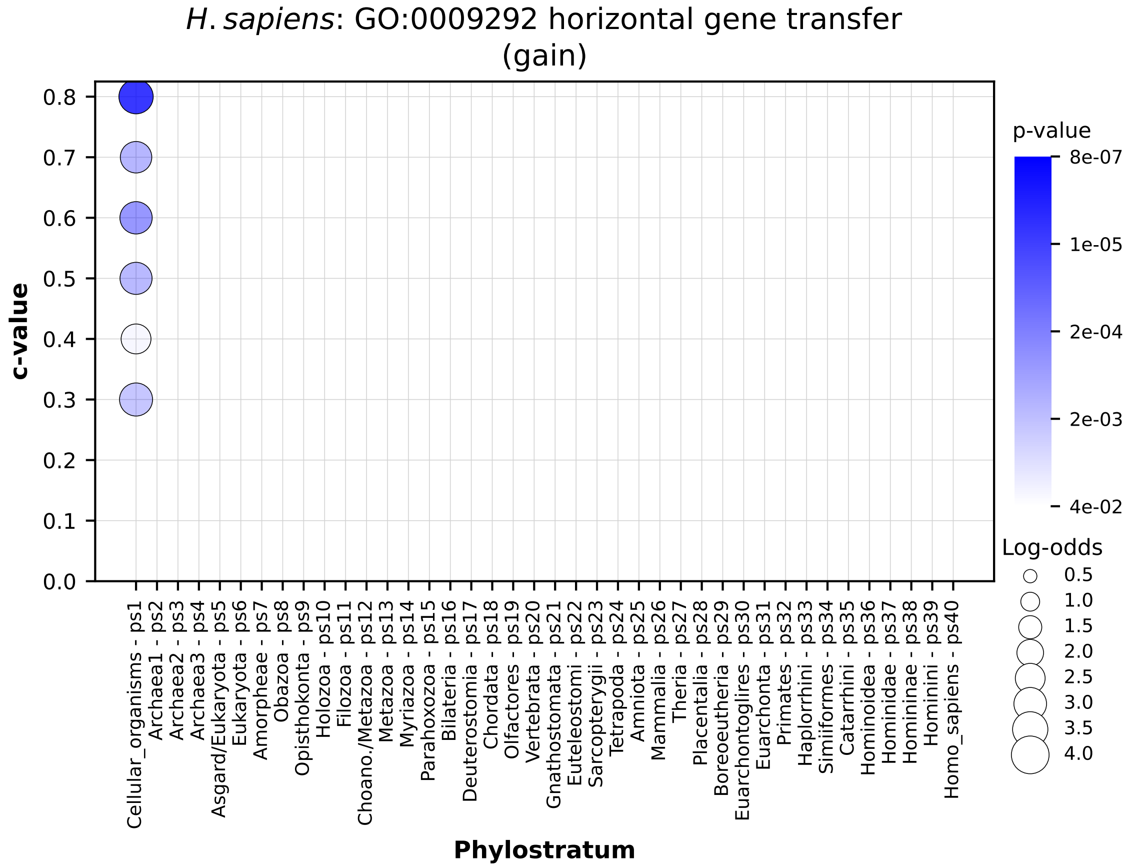

H. sapiens: GO:0009292 horizontal gene transfer
(gain)
c-value
0.0
0.1
0.2
0.3
0.4
0.5
0.6
0.7
0.8
Phylostratum
Cellular_organisms - ps1
Archaea1 - ps2
Archaea2 - ps3
Archaea3 - ps4
Asgard/Eukaryota - ps5
Eukaryota - ps6
Amorpheae - ps7
Obazoa - ps8
Opisthokonta - ps9
Holozoa - ps10
Filozoa - ps11
Choano./Metazoa - ps12
Metazoa - ps13
Myriazoa - ps14
Parahoxozoa - ps15
Bilateria - ps16
Deuterostomia - ps17
Chordata - ps18
Olfactores - ps19
Vertebrata - ps20
Gnathostomata - ps21
Euteleostomi - ps22
Sarcopterygii - ps23
Tetrapoda - ps24
Amniota - ps25
Mammalia - ps26
Theria - ps27
Placentalia - ps28
Boreoeutheria - ps29
Euarchontoglires - ps30
Euarchonta - ps31
Primates - ps32
Haplorrhini - ps33
Simiiformes - ps34
Catarrhini - ps35
Hominoidea - ps36
Hominidae - ps37
Homininae - ps38
Hominini - ps39
Homo_sapiens - ps40
p-value
8e-07
1e-05
2e-04
2e-03
4e-02
Log-odds
0.5
1.0
1.5
2.0
2.5
3.0
3.5
4.0

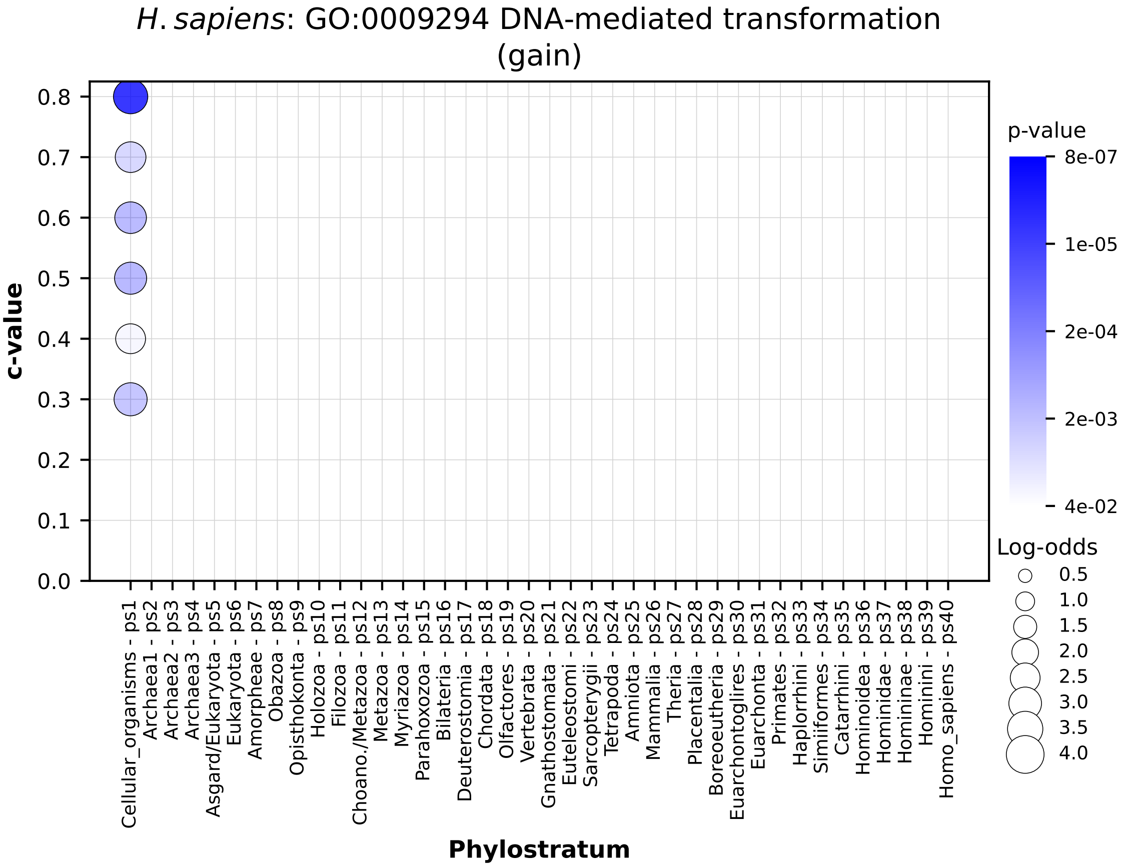

H. sapiens: GO:0009294 DNA-mediated transformation (gain)
c-value
0.8
0.7
0.6
0.5
0.4
0.3
0.2
0.1
0.0
Phylostratum
Cellular_organisms - ps1
Archaea1 - ps2
Archaea2 - ps3
Archaea3 - ps4
Asgard/Eukaryota - ps5
Eukaryota - ps6
Amorpheae - ps7
Obazoa - ps8
Opisthokonta - ps9
Holozoa - ps10
Filozoa - ps11
Choano./Metazoa - ps12
Metazoa - ps13
Myriazoa - ps14
Parahoxozoa - ps15
Bilateria - ps16
Deuterostomia - ps17
Chordata - ps18
Olfactores - ps19
Vertebrata - ps20
Gnathostomata - ps21
Euteleostomi - ps22
Sarcopterygii - ps23
Tetrapoda - ps24
Amniota - ps25
Mammalia - ps26
Theria - ps27
Placentalia - ps28
Boreoeutheria - ps29
Euarchontoglires - ps30
Euarchonta - ps31
Primates - ps32
Haplorrhini - ps33
Simiiformes - ps34
Catarrhini - ps35
Hominoidea - ps36
Hominidae - ps37
Homininae - ps38
Hominini - ps39
Homo_sapiens - ps40
p-value
8e-07
1e-05
2e-04
2e-03
4e-02
Log-odds
0.5
1.0
1.5
2.0
2.5
3.0
3.5
4.0

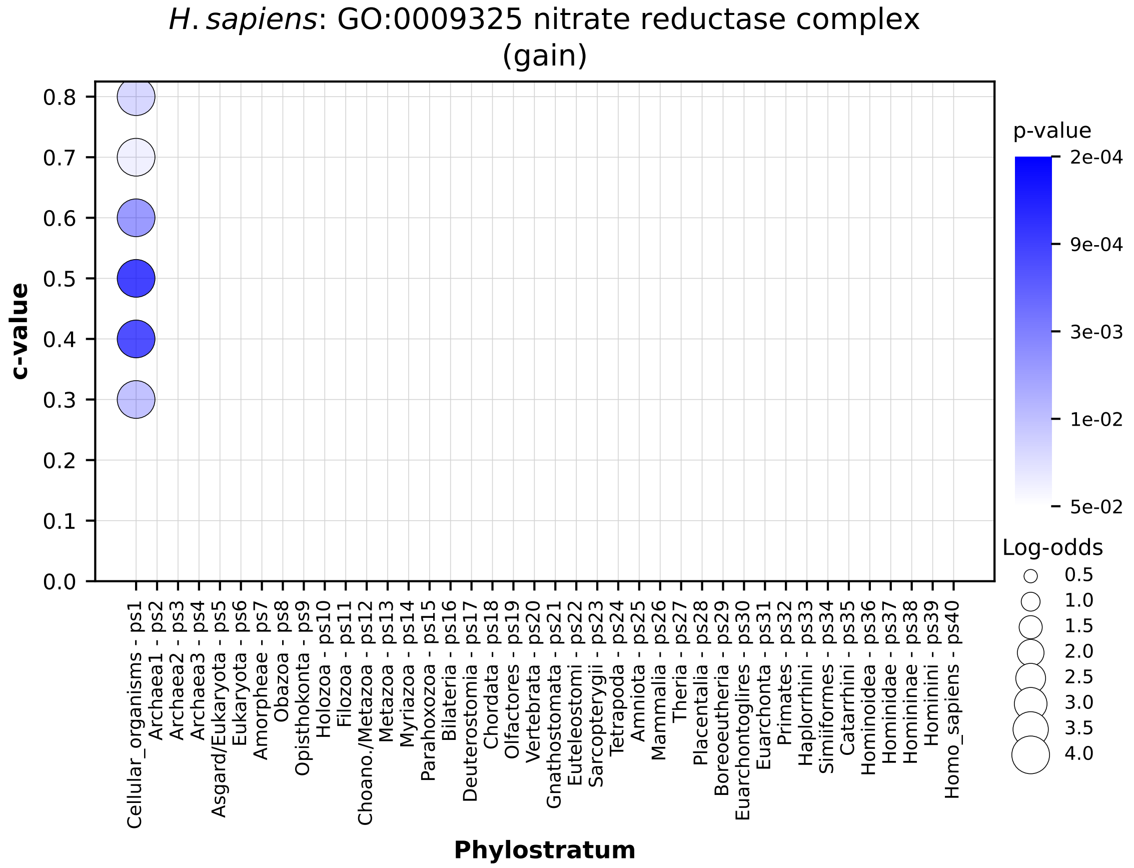

*H. sapiens*: GO:0009325 nitrate reductase complex
(gain)
c-value
0.8
0.7
0.6
0.5
0.4
0.3
0.2
0.1
0.0
Phylostratum
Cellular_organisms - ps1
Archaea1 - ps2
Archaea2 - ps3
Archaea3 - ps4
Asgard/Eukaryota - ps5
Eukaryota - ps6
Amorpheae - ps7
Obazoa - ps8
Opisthokonta - ps9
Holozoa - ps10
Filozoa - ps11
Choano./Metazoa - ps12
Metazoa - ps13
Myriazoa - ps14
Parahoxozoa - ps15
Bilateria - ps16
Deuterostomia - ps17
Chordata - ps18
Olfactores - ps19
Vertebrata - ps20
Gnathostomata - ps21
Euteleostomi - ps22
Sarcopterygii - ps23
Tetrapoda - ps24
Amniota - ps25
Mammalia - ps26
Theria - ps27
Placentalia - ps28
Boreoeutheria - ps29
Euarchontoglires - ps30
Euarchonta - ps31
Primates - ps32
Haplorrhini - ps33
Simiiformes - ps34
Catarrhini - ps35
Hominoidea - ps36
Hominidae - ps37
Homininae - ps38
Hominini - ps39
Homo_sapiens - ps40
p-value
2e-04
9e-04
3e-03
1e-02
5e-02
Log-odds
0.5
1.0
1.5
2.0
2.5
3.0
3.5
4.0

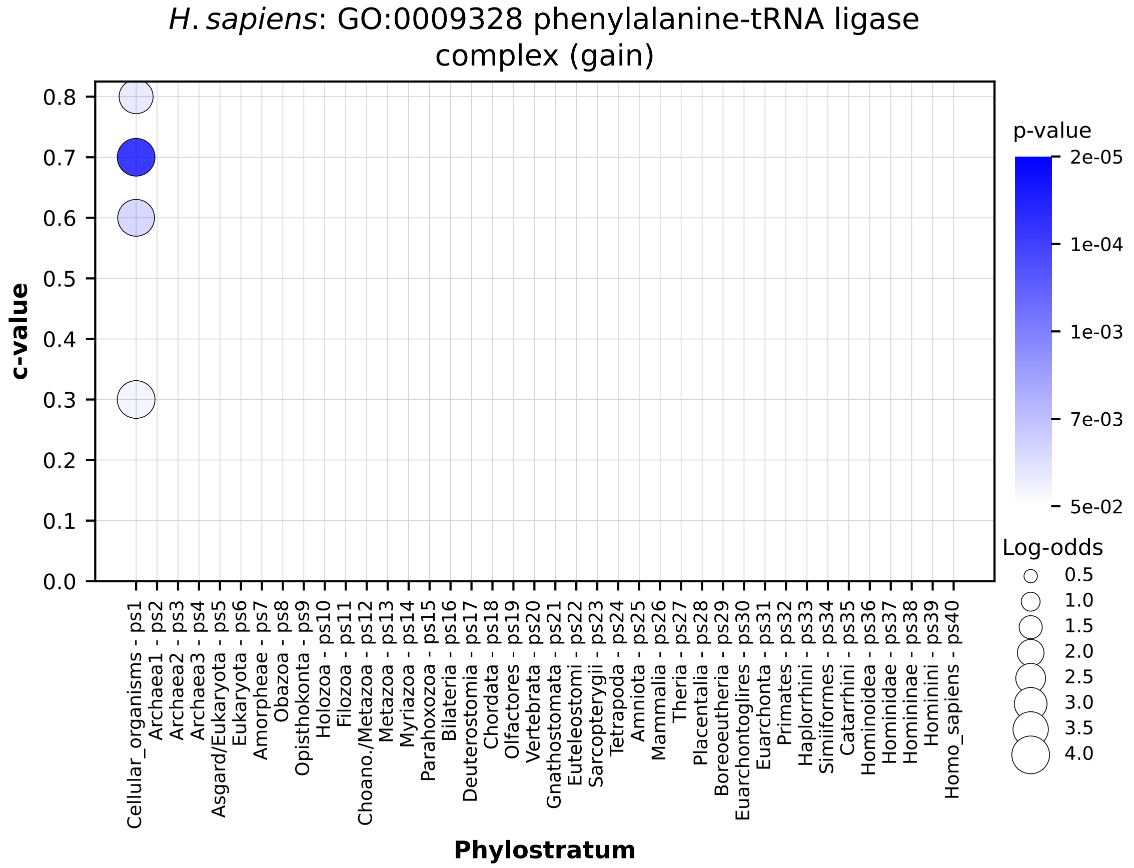

H. sapiens: GO:0009328 phenylalanine-tRNA ligase complex (gain)
c-value
0.0
0.1
0.2
0.3
0.4
0.5
0.6
0.7
0.8
Phylostratum
Cellular_organisms - ps1
Archaea1 - ps2
Archaea2 - ps3
Archaea3 - ps4
Asgard/Eukaryota - ps5
Eukaryota - ps6
Amorpheae - ps7
Obazoa - ps8
Opisthokonta - ps9
Holozoa - ps10
Filozoa - ps11
Choano./Metazoa - ps12
Metazoa - ps13
Myriazoa - ps14
Parahoxozoa - ps15
Bilateria - ps16
Deuterostomia - ps17
Chordata - ps18
Olfactores - ps19
Vertebrata - ps20
Gnathostomata - ps21
Euteleostomi - ps22
Sarcopterygii - ps23
Tetrapoda - ps24
Amniota - ps25
Mammalia - ps26
Theria - ps27
Placentalia - ps28
Boreoeutheria - ps29
Euarchontoglires - ps30
Euarchonta - ps31
Primates - ps32
Haplorrhini - ps33
Simiiformes - ps34
Catarrhini - ps35
Hominoidea - ps36
Hominidae - ps37
Homininae - ps38
Hominini - ps39
Homo_sapiens - ps40
p-value
2e-05
1e-04
1e-03
7e-03
5e-02
Log-odds
0.5
1.0
1.5
2.0
2.5
3.0
3.5
4.0

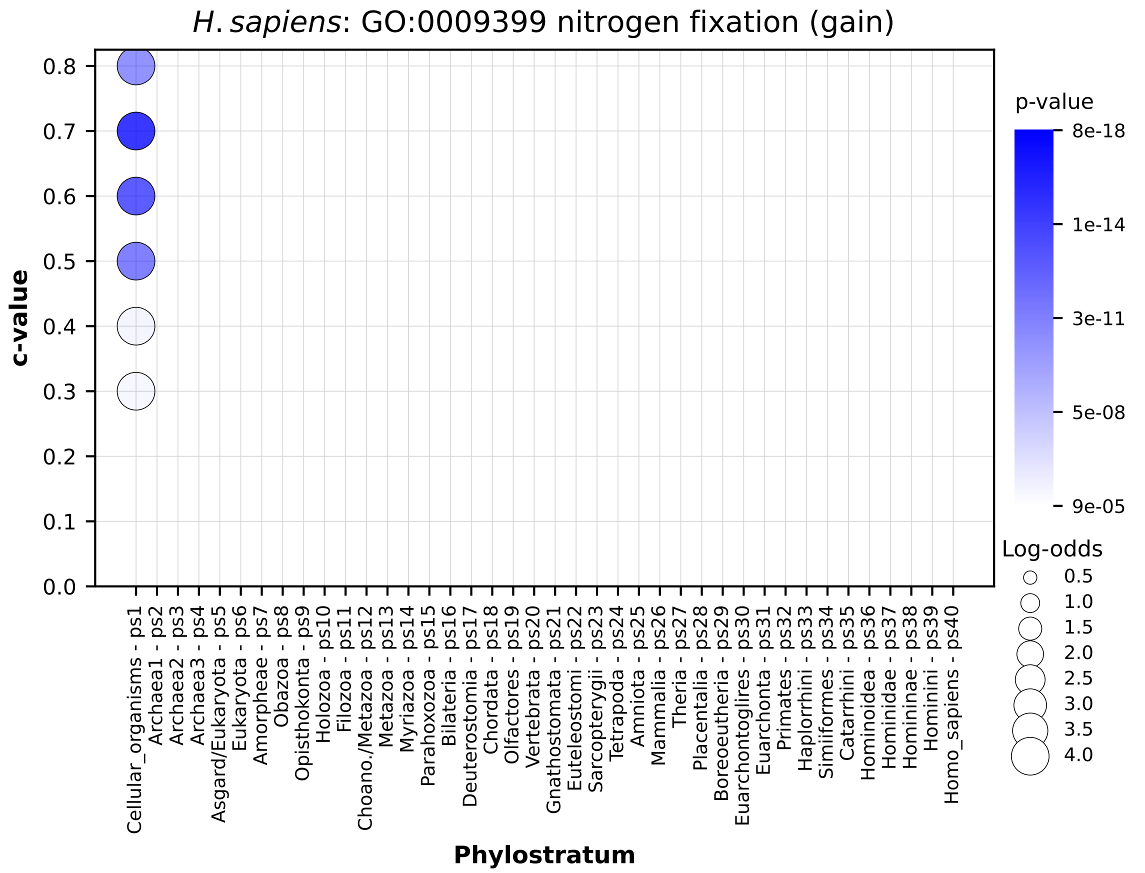

H. sapiens: GO:0009399 nitrogen fixation (gain)
c-value
0.0
0.1
0.2
0.3
0.4
0.5
0.6
0.7
0.8
Phylostratum
Cellular_organisms - ps1
Archaea1 - ps2
Archaea2 - ps3
Archaea3 - ps4
Asgard/Eukaryota - ps5
Eukaryota - ps6
Amorpheae - ps7
Obazoa - ps8
Opisthokonta - ps9
Holozoa - ps10
Filozoa - ps11
Choano./Metazoa - ps12
Metazoa - ps13
Myriazoa - ps14
Parahoxozoa - ps15
Bilateria - ps16
Deuterostomia - ps17
Chordata - ps18
Olfactores - ps19
Vertebrata - ps20
Gnathostomata - ps21
Euteleostomi - ps22
Sarcopterygii - ps23
Tetrapoda - ps24
Amniota - ps25
Mammalia - ps26
Theria - ps27
Placentalia - ps28
Boreoeutheria - ps29
Euarchontoglires - ps30
Euarchonta - ps31
Primates - ps32
Haplorrhini - ps33
Simiiformes - ps34
Catarrhini - ps35
Hominoidea - ps36
Hominidae - ps37
Homininae - ps38
Hominini - ps39
Homo_sapiens - ps40
p-value
8e-18
1e-14
3e-11
5e-08
9e-05
Log-odds
0.5
1.0
1.5
2.0
2.5
3.0
3.5
4.0

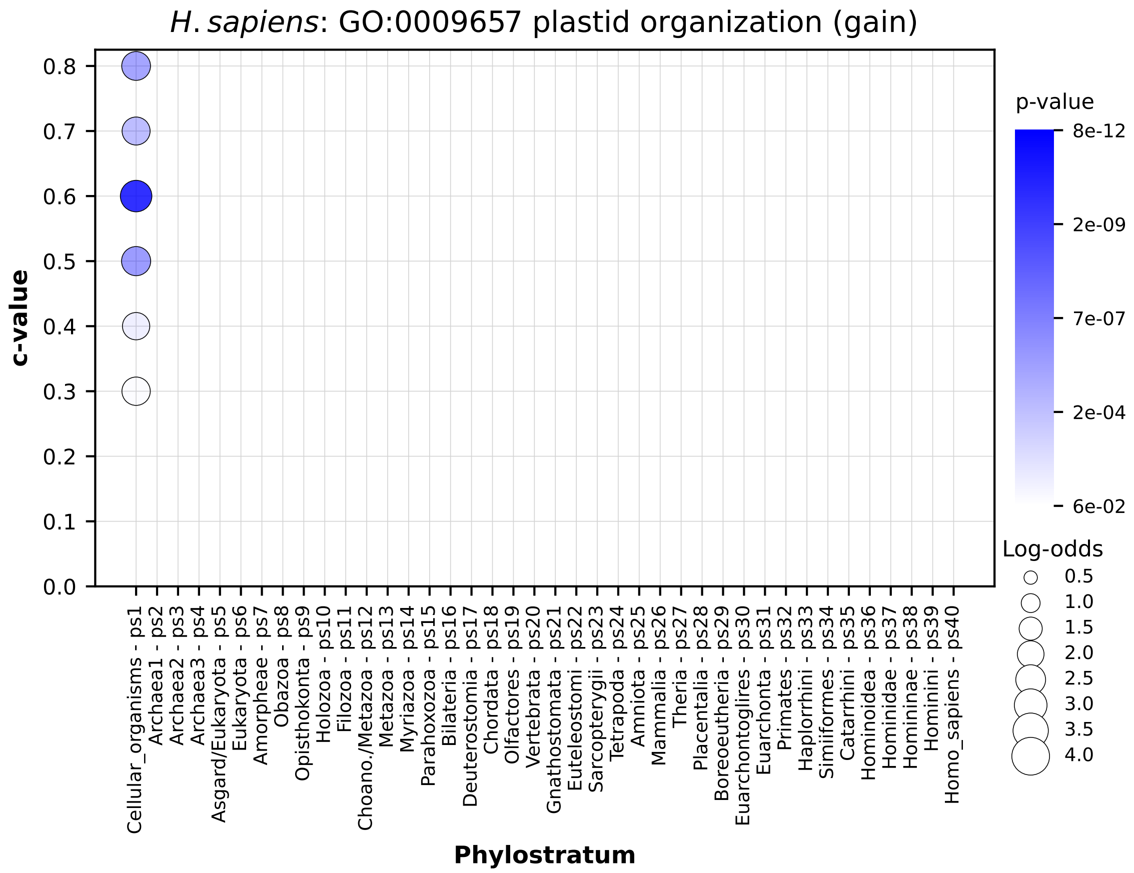
*H. sapiens*: GO:0009657 plastid organization (gain)
c-value
0.0
0.1
0.2
0.3
0.4
0.5
0.6
0.7
0.8
p-value
8e-12
2e-09
7e-07
2e-04
6e-02
Log-odds
0.5
1.0
1.5
2.0
2.5
3.0
3.5
4.0
Cellular_organisms - ps1
Archaea1 - ps2
Archaea2 - ps3
Archaea3 - ps4
Asgard/Eukaryota - ps5
Eukaryota - ps6
Amorpheae - ps7
Obazoa - ps8
Opisthokonta - ps9
Holozoa - ps10
Filozoa - ps11
Choano./Metazoa - ps12
Metazoa - ps13
Myriazoa - ps14
Parahoxozoa - ps15
Bilateria - ps16
Deuterostomia - ps17
Chordata - ps18
Olfactores - ps19
Vertebrata - ps20
Gnathostomata - ps21
Euteleostomi - ps22
Sarcopterygii - ps23
Tetrapoda - ps24
Amniota - ps25
Mammalia - ps26
Theria - ps27
Placentalia - ps28
Boreoeutheria - ps29
Euarchontoglires - ps30
Euarchonta - ps31
Primates - ps32
Haplorrhini - ps33
Simiiformes - ps34
Catarrhini - ps35
Hominoidea - ps36
Hominidae - ps37
Homininae - ps38
Hominini - ps39
Homo_sapiens - ps40
Phylostratum

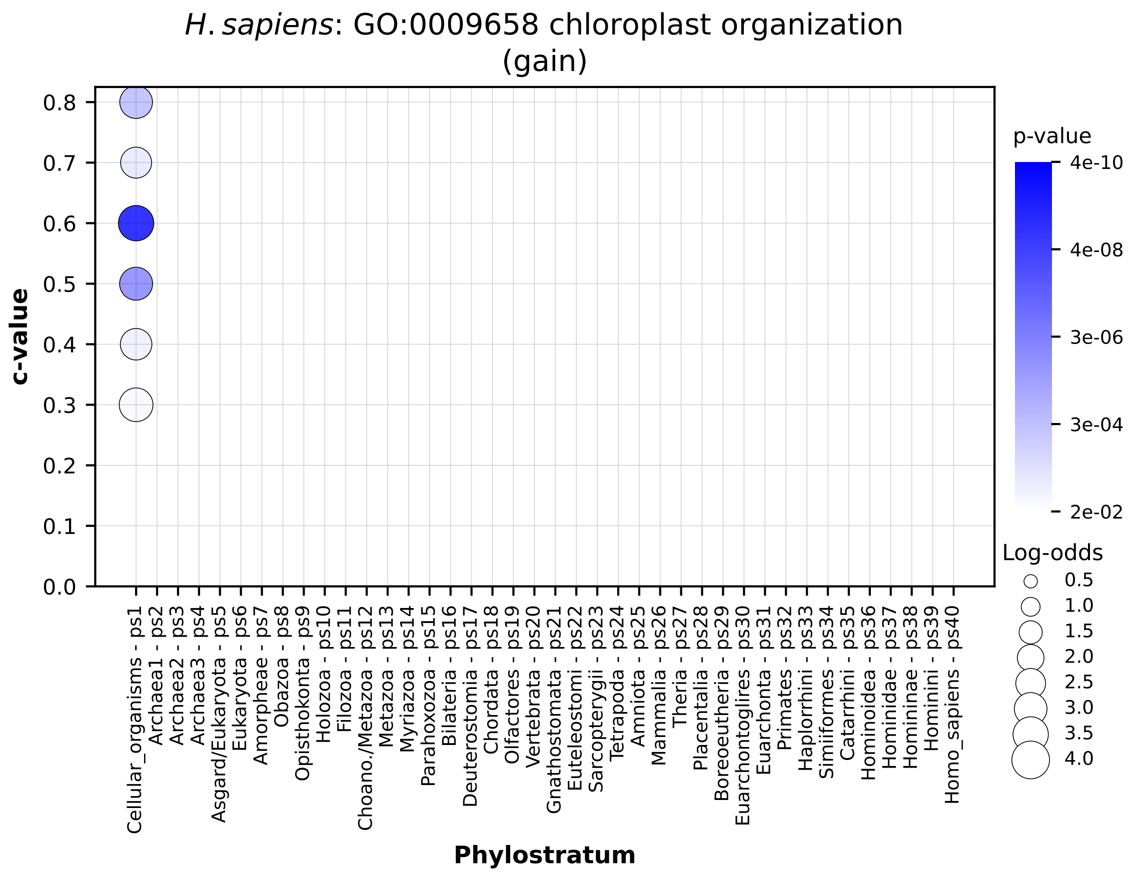

H. sapiens: GO:0009658 chloroplast organization
(gain)
c-value
0.0
0.1
0.2
0.3
0.4
0.5
0.6
0.7
0.8
Phylostratum
Cellular_organisms - ps1
Archaea1 - ps2
Archaea2 - ps3
Archaea3 - ps4
Asgard/Eukaryota - ps5
Eukaryota - ps6
Amorpheae - ps7
Obazoa - ps8
Opisthokonta - ps9
Holozoa - ps10
Filozoa - ps11
Choano./Metazoa - ps12
Metazoa - ps13
Myriazoa - ps14
Parahoxozoa - ps15
Bilateria - ps16
Deuterostomia - ps17
Chordata - ps18
Olfactores - ps19
Vertebrata - ps20
Gnathostomata - ps21
Euteleostomi - ps22
Sarcopterygii - ps23
Tetrapoda - ps24
Amniota - ps25
Mammalia - ps26
Theria - ps27
Placentalia - ps28
Boreoeutheria - ps29
Euarchontoglires - ps30
Euarchonta - ps31
Primates - ps32
Haplorrhini - ps33
Simiiformes - ps34
Catarrhini - ps35
Hominoidea - ps36
Hominidae - ps37
Homininae - ps38
Hominini - ps39
Homo_sapiens - ps40
p-value
4e-10
4e-08
3e-06
3e-04
2e-02
Log-odds
0.5
1.0
1.5
2.0
2.5
3.0
3.5
4.0

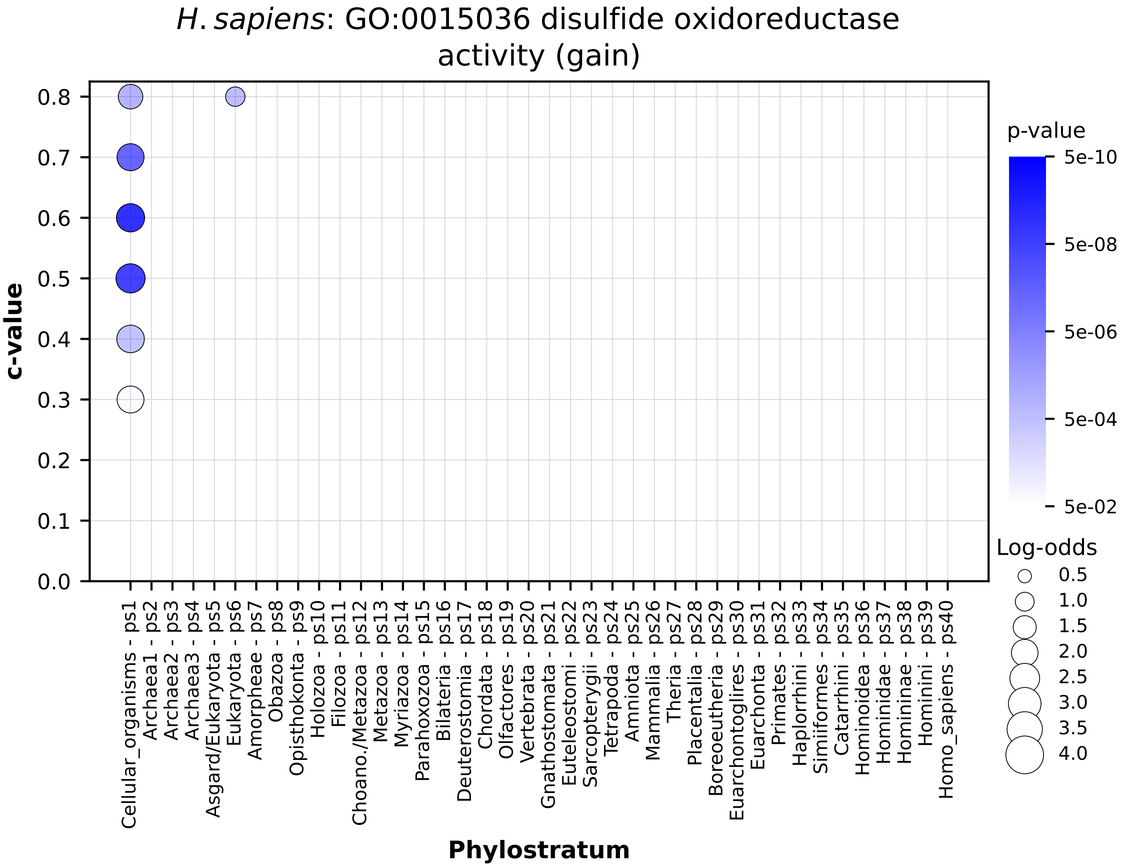

*H. sapiens*: GO:0015036 disulfide oxidoreductase activity (gain)
c-value
0.0
0.1
0.2
0.3
0.4
0.5
0.6
0.7
0.8
Phylostratum
Cellular_organisms - ps1
Archaea1 - ps2
Archaea2 - ps3
Archaea3 - ps4
Asgard/Eukaryota - ps5
Eukaryota - ps6
Amorpheae - ps7
Obazoa - ps8
Opisthokonta - ps9
Holozoa - ps10
Filozoa - ps11
Choano./Metazoa - ps12
Metazoa - ps13
Myriazoa - ps14
Parahoxozoa - ps15
Bilateria - ps16
Deuterostomia - ps17
Chordata - ps18
Olfactores - ps19
Vertebrata - ps20
Gnathostomata - ps21
Euteleostomi - ps22
Sarcopterygii - ps23
Tetrapoda - ps24
Amniota - ps25
Mammalia - ps26
Theria - ps27
Placentalia - ps28
Boreoeutheria - ps29
Euarchontoglires - ps30
Euarchonta - ps31
Primates - ps32
Haplorrhini - ps33
Simiiformes - ps34
Catarrhini - ps35
Hominoidea - ps36
Hominidae - ps37
Homininae - ps38
Hominini - ps39
Homo_sapiens - ps40
p-value
5e-10
5e-08
5e-06
5e-04
5e-02
Log-odds
0.5
1.0
1.5
2.0
2.5
3.0
3.5
4.0

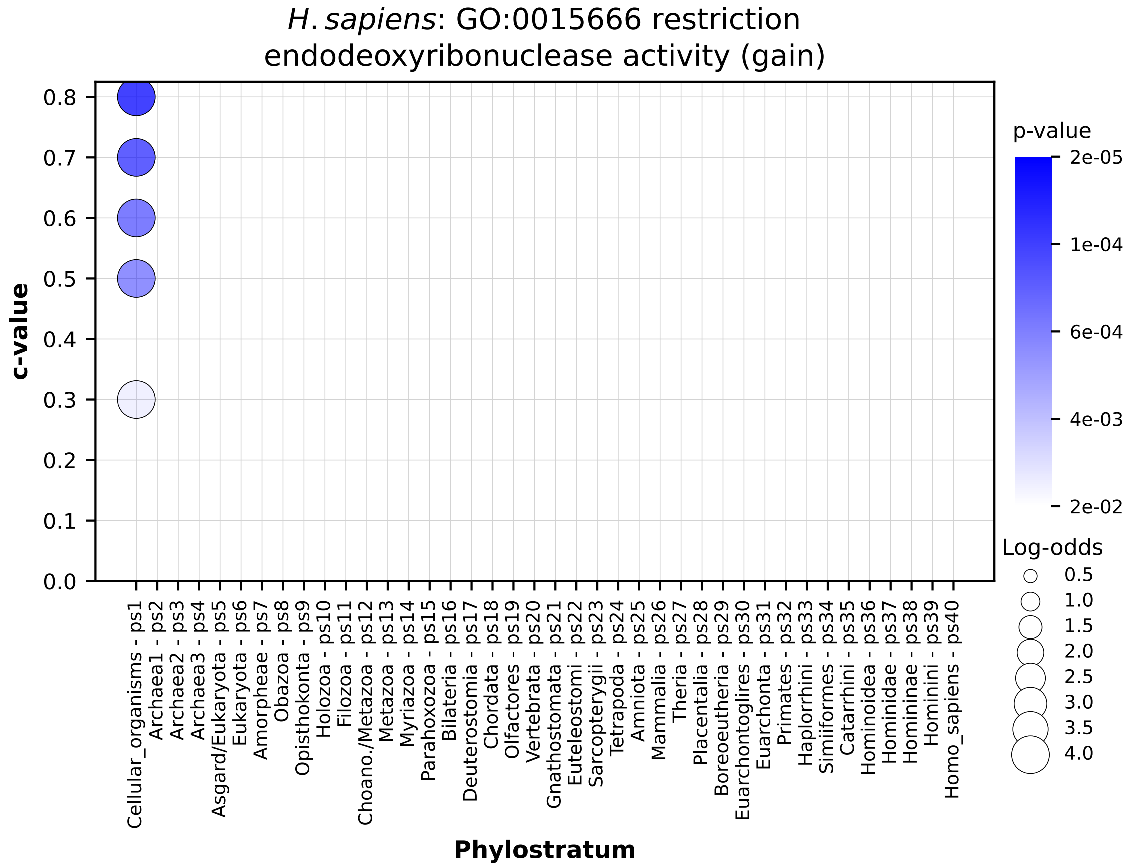
H. sapiens: GO:0015666 restriction endodeoxyribonuclease activity (gain)
c-value
0.0
0.1
0.2
0.3
0.4
0.5
0.6
0.7
0.8
Phylostratum
Cellular_organisms - ps1
Archaea1 - ps2
Archaea2 - ps3
Archaea3 - ps4
Asgard/Eukaryota - ps5
Eukaryota - ps6
Amorpheae - ps7
Obazoa - ps8
Opisthokonta - ps9
Holozoa - ps10
Filozoa - ps11
Choano./Metazoa - ps12
Metazoa - ps13
Myriazoa - ps14
Parahoxozoa - ps15
Bilateria - ps16
Deuterostomia - ps17
Chordata - ps18
Olfactores - ps19
Vertebrata - ps20
Gnathostomata - ps21
Euteleostomi - ps22
Sarcopterygii - ps23
Tetrapoda - ps24
Amniota - ps25
Mammalia - ps26
Theria - ps27
Placentalia - ps28
Boreoeutheria - ps29
Euarchontoglires - ps30
Euarchonta - ps31
Primates - ps32
Haplorrhini - ps33
Simiiformes - ps34
Catarrhini - ps35
Hominoidea - ps36
Hominidae - ps37
Homininae - ps38
Hominini - ps39
Homo_sapiens - ps40
p-value
2e-05
1e-04
6e-04
4e-03
2e-02
Log-odds
0.5
1.0
1.5
2.0
2.5
3.0
3.5
4.0

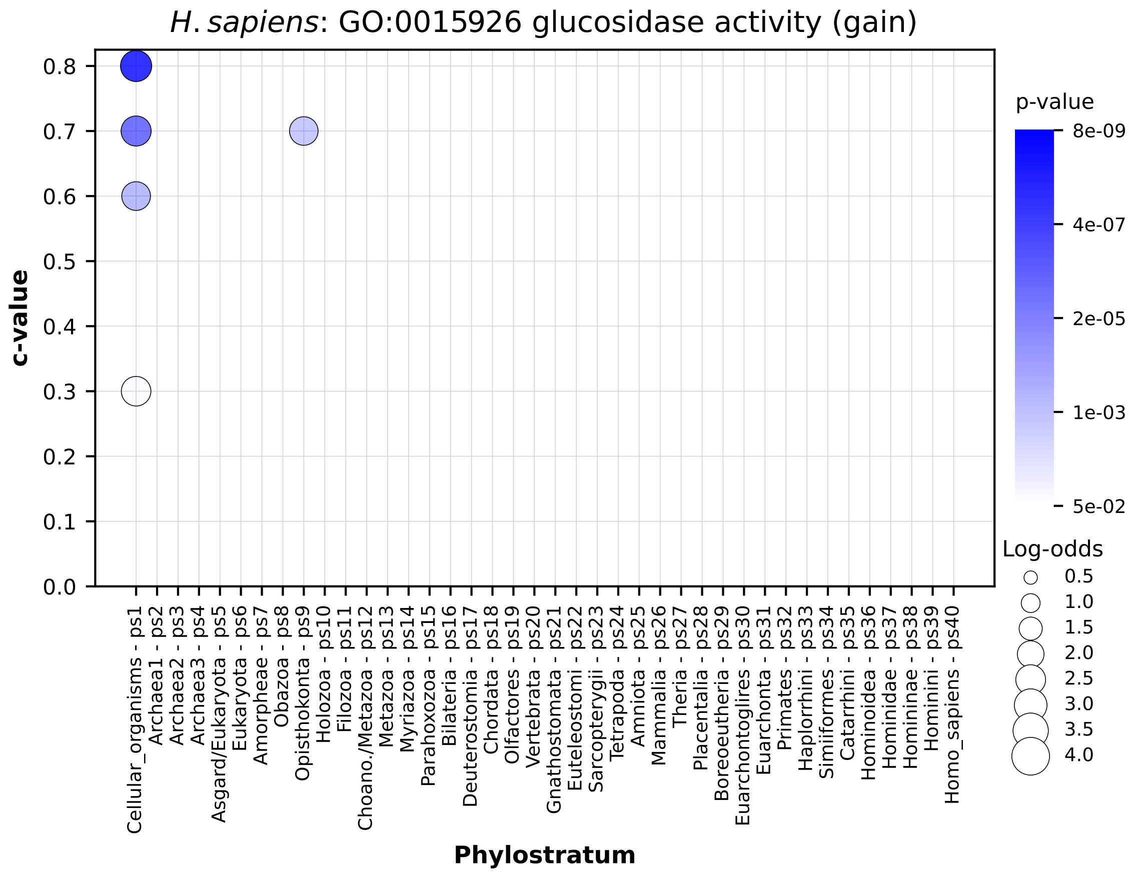

H. sapiens: GO:0015926 glucosidase activity (gain)
c-value
0.0
0.1
0.2
0.3
0.4
0.5
0.6
0.7
0.8
Phylostratum
Cellular_organisms - ps1
Archaea1 - ps2
Archaea2 - ps3
Archaea3 - ps4
Asgard/Eukaryota - ps5
Eukaryota - ps6
Amorpheae - ps7
Obazoa - ps8
Opisthokonta - ps9
Holozoa - ps10
Filozoa - ps11
Choano./Metazoa - ps12
Metazoa - ps13
Myriazoa - ps14
Parahoxozoa - ps15
Bilateria - ps16
Deuterostomia - ps17
Chordata - ps18
Olfactores - ps19
Vertebrata - ps20
Gnathostomata - ps21
Euteleostomi - ps22
Sarcopterygii - ps23
Tetrapoda - ps24
Amniota - ps25
Mammalia - ps26
Theria - ps27
Placentalia - ps28
Boreoeutheria - ps29
Euarchontoglires - ps30
Euarchonta - ps31
Primates - ps32
Haplorrhini - ps33
Simiiformes - ps34
Catarrhini - ps35
Hominoidea - ps36
Hominidae - ps37
Homininae - ps38
Hominini - ps39
Homo_sapiens - ps40
p-value
8e-09
4e-07
2e-05
1e-03
5e-02
Log-odds
0.5
1.0
1.5
2.0
2.5
3.0
3.5
4.0

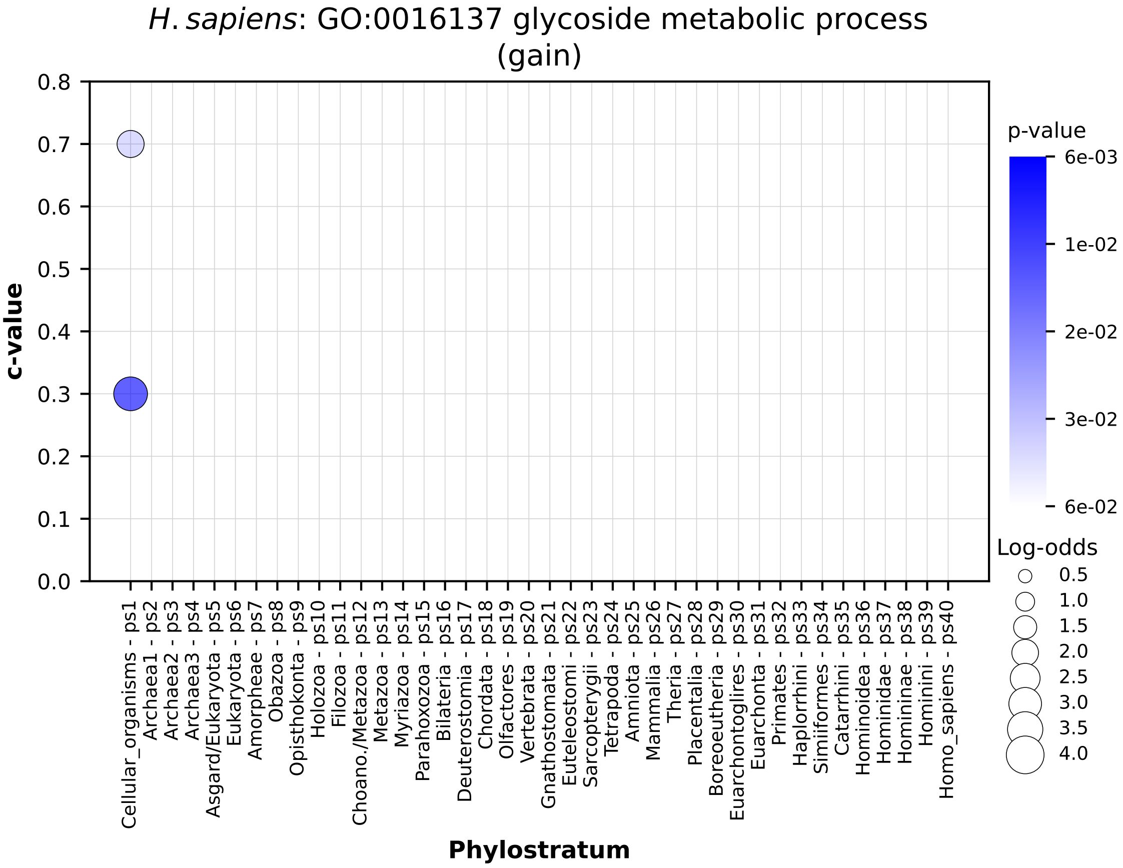

H. sapiens: GO:0016137 glycoside metabolic process (gain)
c-value
0.0
0.1
0.2
0.3
0.4
0.5
0.6
0.7
0.8
Phylostratum
Cellular_organisms - ps1
Archaea1 - ps2
Archaea2 - ps3
Archaea3 - ps4
Asgard/Eukaryota - ps5
Eukaryota - ps6
Amorpheae - ps7
Obazoa - ps8
Opisthokonta - ps9
Holozoa - ps10
Filozoa - ps11
Choano./Metazoa - ps12
Metazoa - ps13
Myriazoa - ps14
Parahoxozoa - ps15
Bilateria - ps16
Deuterostomia - ps17
Chordata - ps18
Olfactores - ps19
Vertebrata - ps20
Gnathostomata - ps21
Euteleostomi - ps22
Sarcopterygii - ps23
Tetrapoda - ps24
Amniota - ps25
Mammalia - ps26
Theria - ps27
Placentalia - ps28
Boreoeutheria - ps29
Euarchontoglires - ps30
Euarchonta - ps31
Primates - ps32
Haplorrhini - ps33
Simiiformes - ps34
Catarrhini - ps35
Hominoidea - ps36
Hominidae - ps37
Homininae - ps38
Hominini - ps39
Homo_sapiens - ps40
p-value
6e-03
1e-02
2e-02
3e-02
6e-02
Log-odds
0.5
1.0
1.5
2.0
2.5
3.0
3.5
4.0

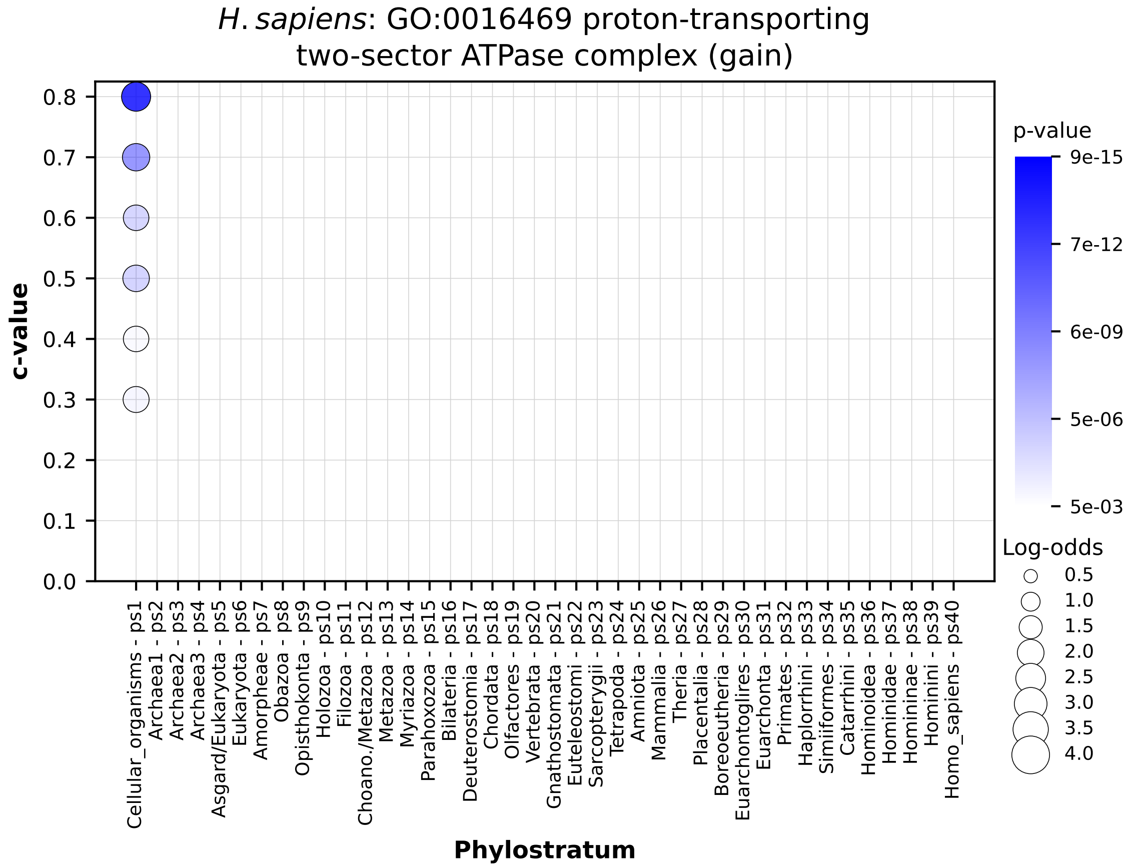
H. sapiens: GO:0016469 proton-transporting two-sector ATPase complex (gain)
c-value
0.0
0.1
0.2
0.3
0.4
0.5
0.6
0.7
0.8
Phylostratum
Cellular_organisms - ps1
Archaea1 - ps2
Archaea2 - ps3
Archaea3 - ps4
Asgard/Eukaryota - ps5
Eukaryota - ps6
Amorpheae - ps7
Obazoa - ps8
Opisthokonta - ps9
Holozoa - ps10
Filozoa - ps11
Choano./Metazoa - ps12
Metazoa - ps13
Myriazoa - ps14
Parahoxozoa - ps15
Bilateria - ps16
Deuterostomia - ps17
Chordata - ps18
Olfactores - ps19
Vertebrata - ps20
Gnathostomata - ps21
Euteleostomi - ps22
Sarcopterygii - ps23
Tetrapoda - ps24
Amniota - ps25
Mammalia - ps26
Theria - ps27
Placentalia - ps28
Boreoeutheria - ps29
Euarchontoglires - ps30
Euarchonta - ps31
Primates - ps32
Haplorrhini - ps33
Simiiformes - ps34
Catarrhini - ps35
Hominoidea - ps36
Hominidae - ps37
Homininae - ps38
Hominini - ps39
Homo_sapiens - ps40
p-value
9e-15
7e-12
6e-09
5e-06
5e-03
Log-odds
0.5
1.0
1.5
2.0
2.5
3.0
3.5
4.0

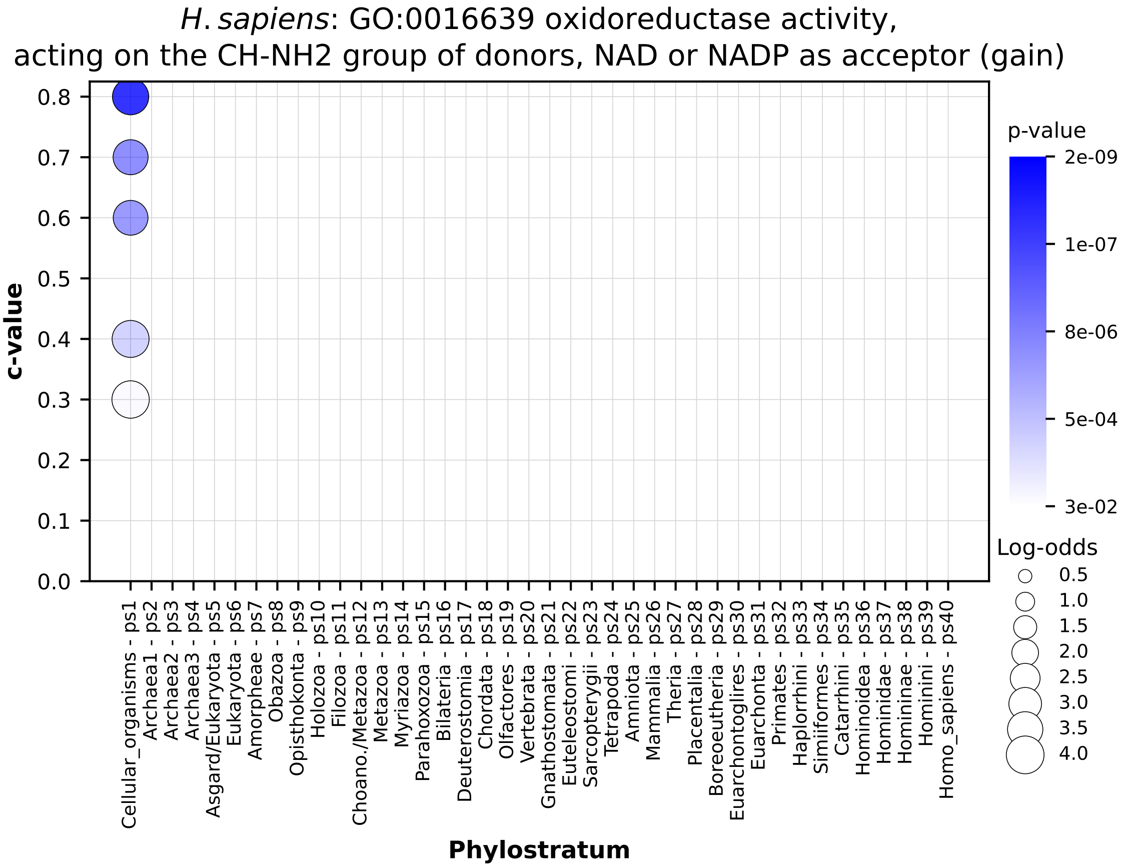

*H. sapiens*: GO:0016639 oxidoreductase activity, acting on the CH-NH2 group of donors, NAD or NADP as acceptor (gain)
c-value
0.0
0.1
0.2
0.3
0.4
0.5
0.6
0.7
0.8
Phylostratum
Cellular_organisms - ps1
Archaea1 - ps2
Archaea2 - ps3
Archaea3 - ps4
Asgard/Eukaryota - ps5
Eukaryota - ps6
Amorpheae - ps7
Obazoa - ps8
Opisthokonta - ps9
Holozoa - ps10
Filozoa - ps11
Choano./Metazoa - ps12
Metazoa - ps13
Myriazoa - ps14
Parahoxozoa - ps15
Bilateria - ps16
Deuterostomia - ps17
Chordata - ps18
Olfactores - ps19
Vertebrata - ps20
Gnathostomata - ps21
Euteleostomi - ps22
Sarcopterygii - ps23
Tetrapoda - ps24
Amniota - ps25
Mammalia - ps26
Theria - ps27
Placentalia - ps28
Boreoeutheria - ps29
Euarchontoglires - ps30
Euarchonta - ps31
Primates - ps32
Haplorrhini - ps33
Simiiformes - ps34
Catarrhini - ps35
Hominoidea - ps36
Hominidae - ps37
Homininae - ps38
Hominini - ps39
Homo_sapiens - ps40
p-value
2e-09
1e-07
8e-06
5e-04
3e-02
Log-odds
0.5
1.0
1.5
2.0
2.5
3.0
3.5
4.0

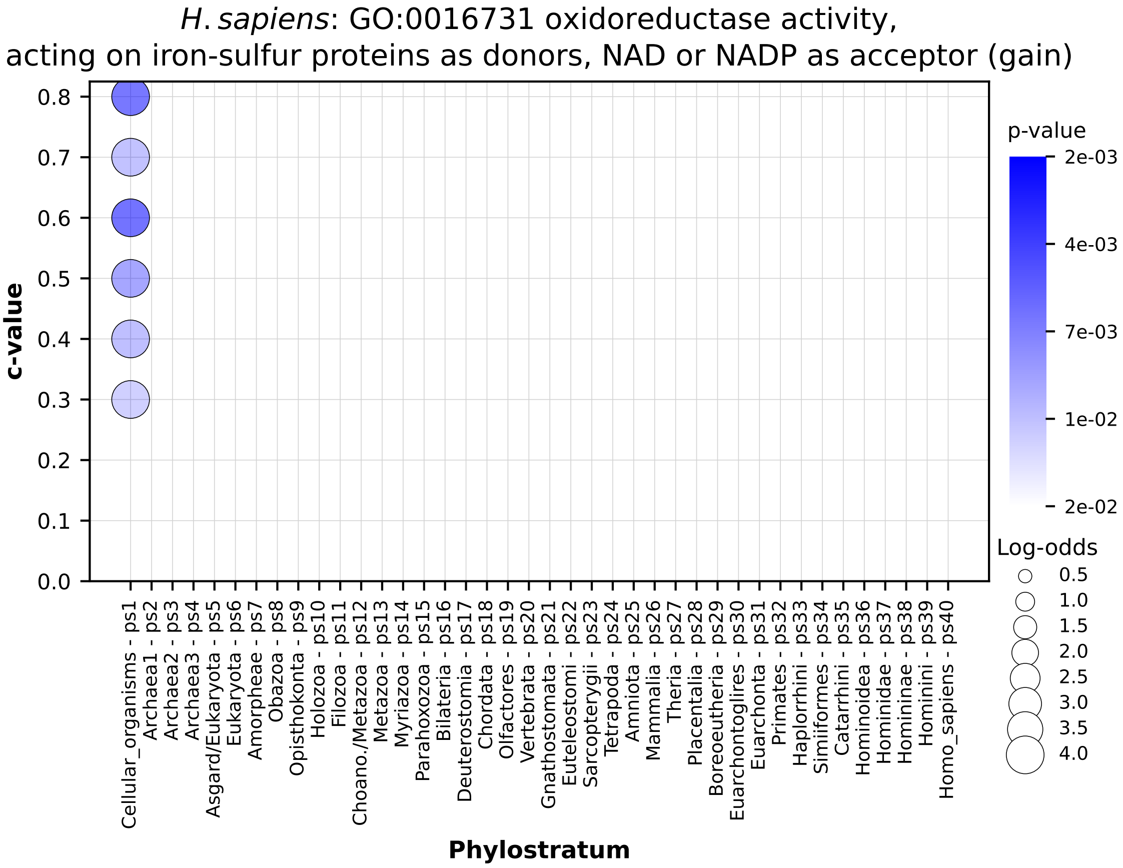

*H. sapiens*: GO:0016731 oxidoreductase activity,
acting on iron-sulfur proteins as donors, NAD or NADP as acceptor (gain)
c-value
0.8
0.7
0.6
0.5
0.4
0.3
0.2
0.1
0.0
p-value
2e-03
4e-03
7e-03
1e-02
2e-02
Log-odds
0.5
1.0
1.5
2.0
2.5
3.0
3.5
4.0
Cellular_organisms - ps1
Archaea1 - ps2
Archaea2 - ps3
Archaea3 - ps4
Asgard/Eukaryota - ps5
Eukaryota - ps6
Amorpheae - ps7
Obazoa - ps8
Opisthokonta - ps9
Holozoa - ps10
Filozoa - ps11
Choano./Metazoa - ps12
Metazoa - ps13
Myriazoa - ps14
Parahoxozoa - ps15
Bilateria - ps16
Deuterostomia - ps17
Chordata - ps18
Olfactores - ps19
Vertebrata - ps20
Gnathostomata - ps21
Euteleostomi - ps22
Sarcopterygii - ps23
Tetrapoda - ps24
Amniota - ps25
Mammalia - ps26
Theria - ps27
Placentalia - ps28
Boreoeutheria - ps29
Euarchontoglires - ps30
Euarchonta - ps31
Primates - ps32
Haplorrhini - ps33
Simiiformes - ps34
Catarrhini - ps35
Hominoidea - ps36
Hominidae - ps37
Homininae - ps38
Hominini - ps39
Homo_sapiens - ps40
Phylostratum

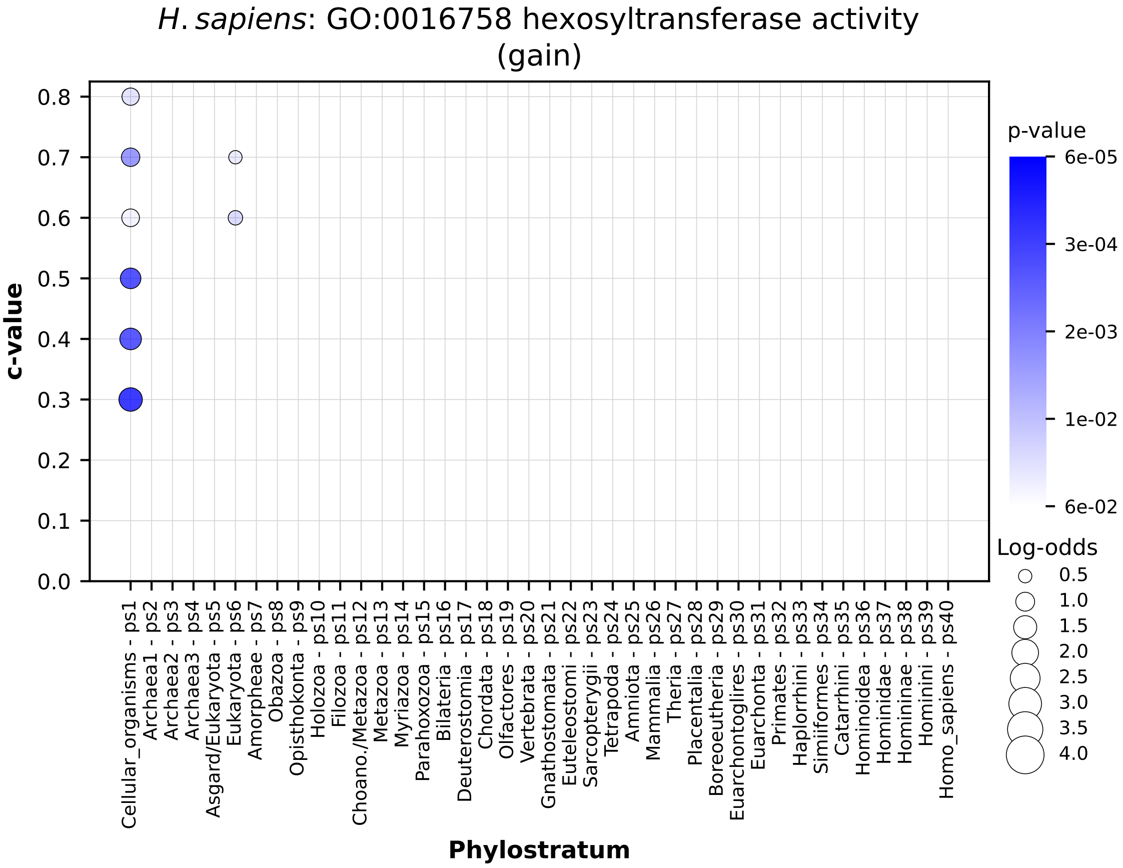

H. sapiens: GO:0016758 hexosyltransferase activity
(gain)
c-value
0.0
0.1
0.2
0.3
0.4
0.5
0.6
0.7
0.8
Phylostratum
Cellular_organisms - ps1
Archaea1 - ps2
Archaea2 - ps3
Archaea3 - ps4
Asgard/Eukaryota - ps5
Eukaryota - ps6
Amorpheae - ps7
Obazoa - ps8
Opisthokonta - ps9
Holozoa - ps10
Filozoa - ps11
Choano./Metazoa - ps12
Metazoa - ps13
Myriazoa - ps14
Parahoxozoa - ps15
Bilateria - ps16
Deuterostomia - ps17
Chordata - ps18
Olfactores - ps19
Vertebrata - ps20
Gnathostomata - ps21
Euteleostomi - ps22
Sarcopterygii - ps23
Tetrapoda - ps24
Amniota - ps25
Mammalia - ps26
Theria - ps27
Placentalia - ps28
Boreoeutheria - ps29
Euarchontoglires - ps30
Euarchonta - ps31
Primates - ps32
Haplorrhini - ps33
Simiiformes - ps34
Catarrhini - ps35
Hominoidea - ps36
Hominidae - ps37
Homininae - ps38
Hominini - ps39
Homo_sapiens - ps40
p-value
6e-05
3e-04
2e-03
1e-02
6e-02
Log-odds
0.5
1.0
1.5
2.0
2.5
3.0
3.5
4.0

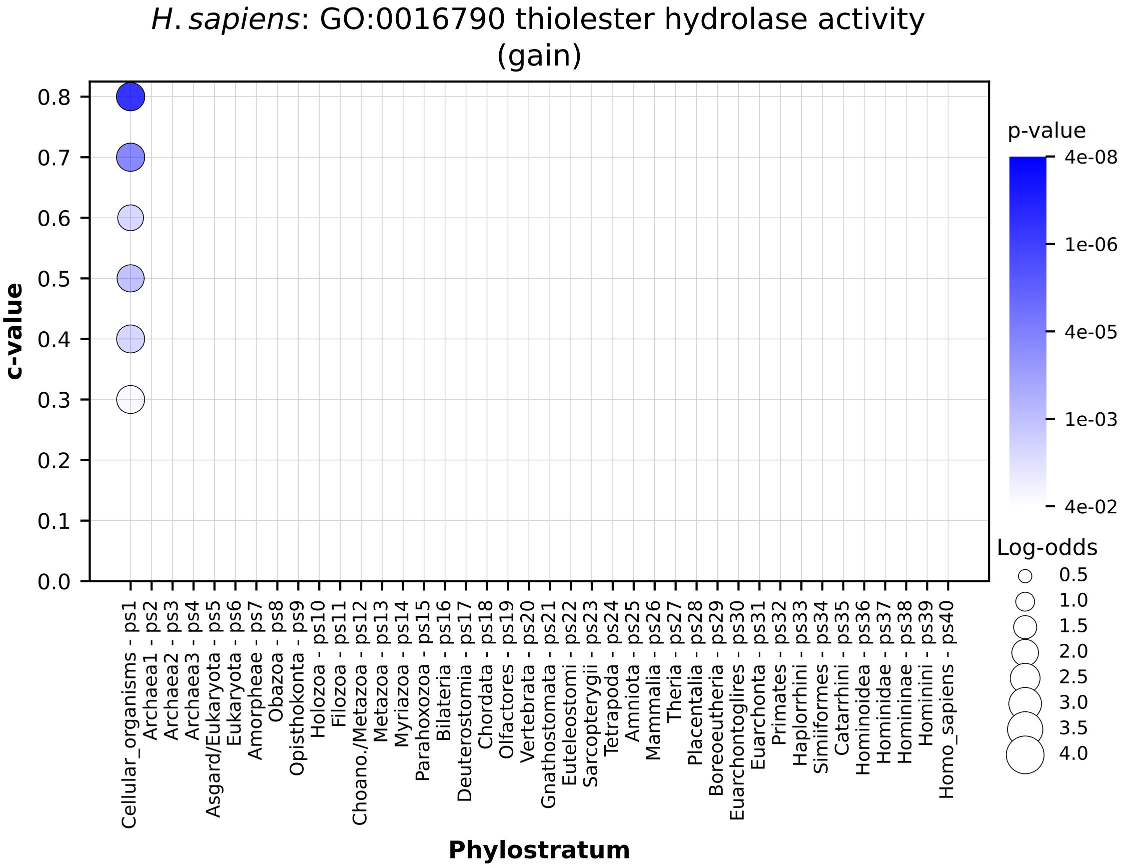

H. sapiens: GO:0016790 thiolester hydrolase activity (gain)
c-value
0.0
0.1
0.2
0.3
0.4
0.5
0.6
0.7
0.8
Phylostratum
Cellular_organisms - ps1
Archaea1 - ps2
Archaea2 - ps3
Archaea3 - ps4
Asgard/Eukaryota - ps5
Eukaryota - ps6
Amorpheae - ps7
Obazoa - ps8
Opisthokonta - ps9
Holozoa - ps10
Filozoa - ps11
Choano./Metazoa - ps12
Metazoa - ps13
Myriazoa - ps14
Parahoxozoa - ps15
Bilateria - ps16
Deuterostomia - ps17
Chordata - ps18
Olfactores - ps19
Vertebrata - ps20
Gnathostomata - ps21
Euteleostomi - ps22
Sarcopterygii - ps23
Tetrapoda - ps24
Amniota - ps25
Mammalia - ps26
Theria - ps27
Placentalia - ps28
Boreoeutheria - ps29
Euarchontoglires - ps30
Euarchonta - ps31
Primates - ps32
Haplorrhini - ps33
Simiiformes - ps34
Catarrhini - ps35
Hominoidea - ps36
Hominidae - ps37
Homininae - ps38
Hominini - ps39
Homo_sapiens - ps40
p-value
4e-08
1e-06
4e-05
1e-03
4e-02
Log-odds
0.5
1.0
1.5
2.0
2.5
3.0
3.5
4.0

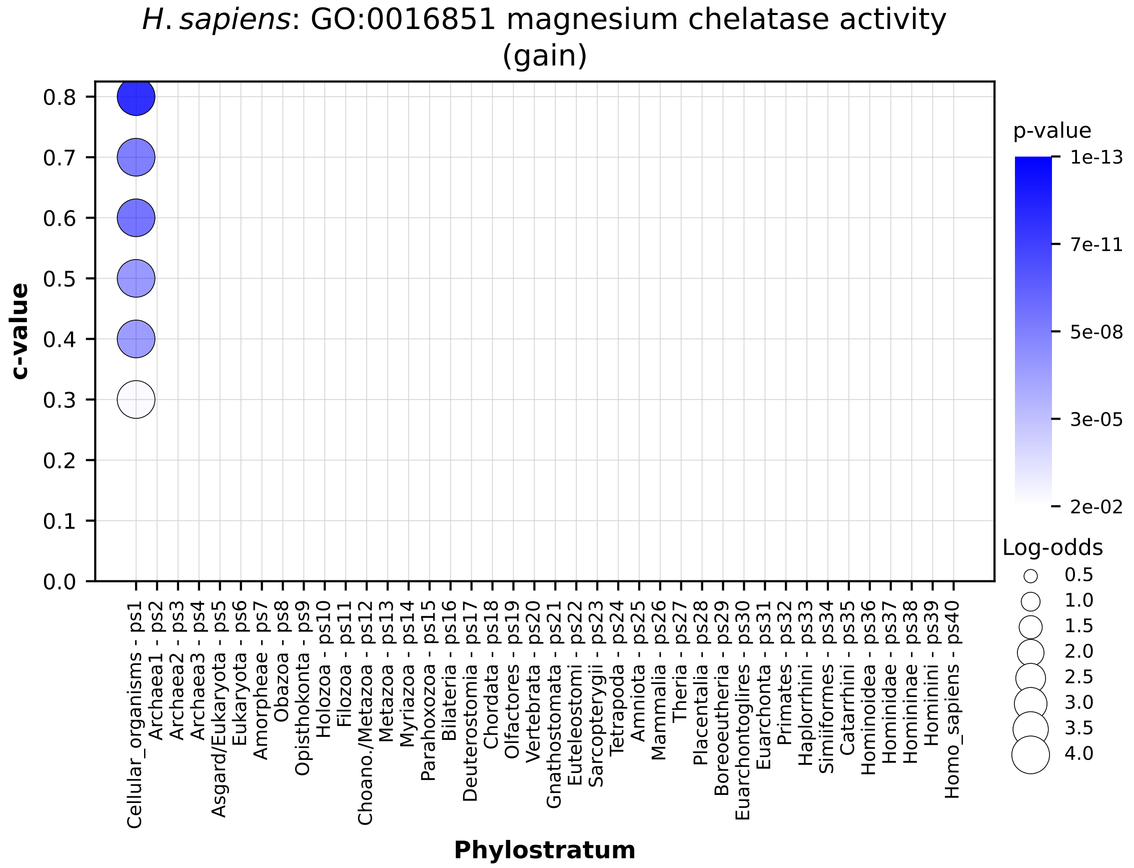

H. sapiens: GO:0016851 magnesium chelatase activity (gain)
c-value
0.8
0.7
0.6
0.5
0.4
0.3
0.2
0.1
0.0
Phylostratum
Cellular_organisms - ps1
Archaea1 - ps2
Archaea2 - ps3
Archaea3 - ps4
Asgard/Eukaryota - ps5
Eukaryota - ps6
Amorpheae - ps7
Obazoa - ps8
Opisthokonta - ps9
Holozoa - ps10
Filozoa - ps11
Choano./Metazoa - ps12
Metazoa - ps13
Myriazoa - ps14
Parahoxozoa - ps15
Bilateria - ps16
Deuterostomia - ps17
Chordata - ps18
Olfactores - ps19
Vertebrata - ps20
Gnathostomata - ps21
Euteleostomi - ps22
Sarcopterygii - ps23
Tetrapoda - ps24
Amniota - ps25
Mammalia - ps26
Theria - ps27
Placentalia - ps28
Boreoeutheria - ps29
Euarchontoglires - ps30
Euarchonta - ps31
Primates - ps32
Haplorrhini - ps33
Simiiformes - ps34
Catarrhini - ps35
Hominoidea - ps36
Hominidae - ps37
Homininae - ps38
Hominini - ps39
Homo_sapiens - ps40
p-value
1e-13
7e-11
5e-08
3e-05
2e-02
Log-odds
0.5
1.0
1.5
2.0
2.5
3.0
3.5
4.0

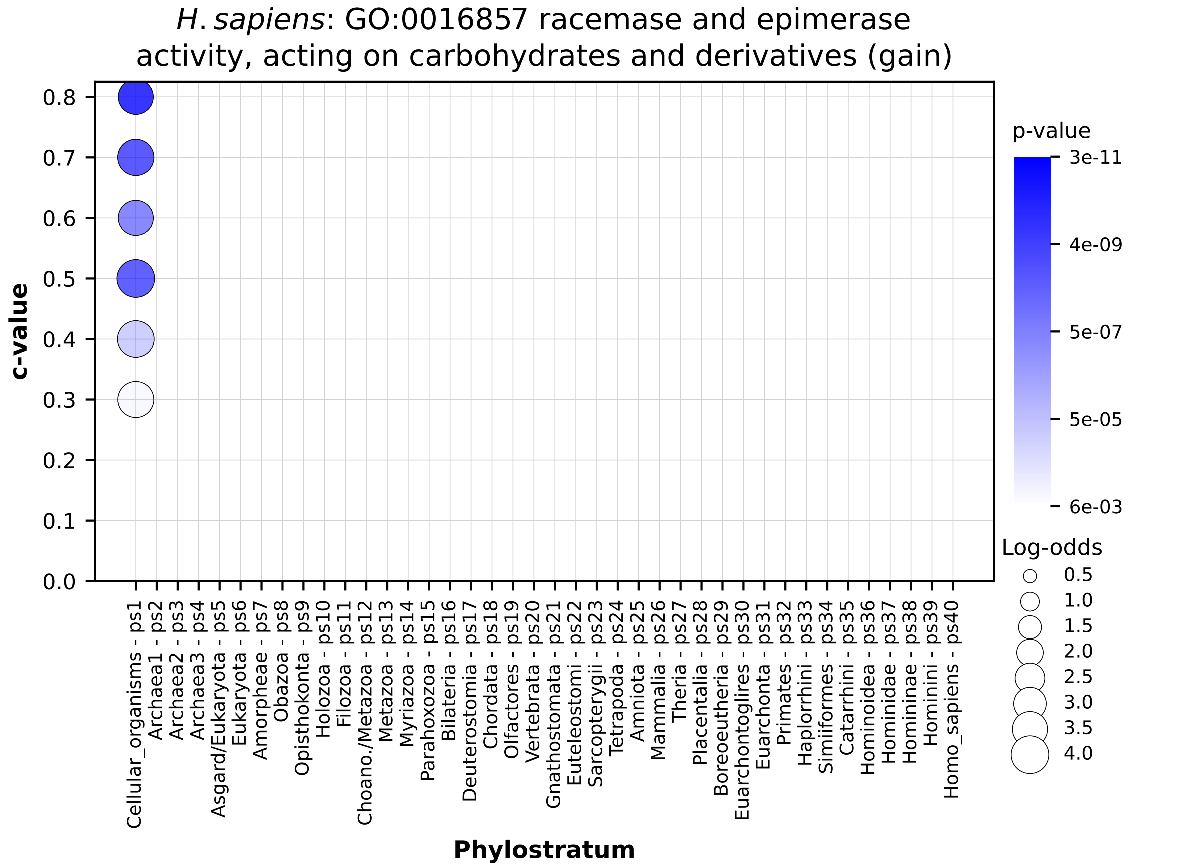

H. sapiens: GO:0016857 racemase and epimerase activity, acting on carbohydrates and derivatives (gain)
c-value
0.0
0.1
0.2
0.3
0.4
0.5
0.6
0.7
0.8
Phylostratum
Cellular_organisms - ps1
Archaea1 - ps2
Archaea2 - ps3
Archaea3 - ps4
Asgard/Eukaryota - ps5
Eukaryota - ps6
Amorpheae - ps7
Obazoa - ps8
Opisthokonta - ps9
Holozoa - ps10
Filozoa - ps11
Choano./Metazoa - ps12
Metazoa - ps13
Myriazoa - ps14
Parahoxozoa - ps15
Bilateria - ps16
Deuterostomia - ps17
Chordata - ps18
Olfactores - ps19
Vertebrata - ps20
Gnathostomata - ps21
Euteleostomi - ps22
Sarcopterygii - ps23
Tetrapoda - ps24
Amniota - ps25
Mammalia - ps26
Theria - ps27
Placentalia - ps28
Boreoeutheria - ps29
Euarchontoglires - ps30
Euarchonta - ps31
Primates - ps32
Haplorrhini - ps33
Simiiformes - ps34
Catarrhini - ps35
Hominoidea - ps36
Hominidae - ps37
Homininae - ps38
Hominini - ps39
Homo_sapiens - ps40
p-value
3e-11
4e-09
5e-07
5e-05
6e-03
Log-odds
0.5
1.0
1.5
2.0
2.5
3.0
3.5
4.0

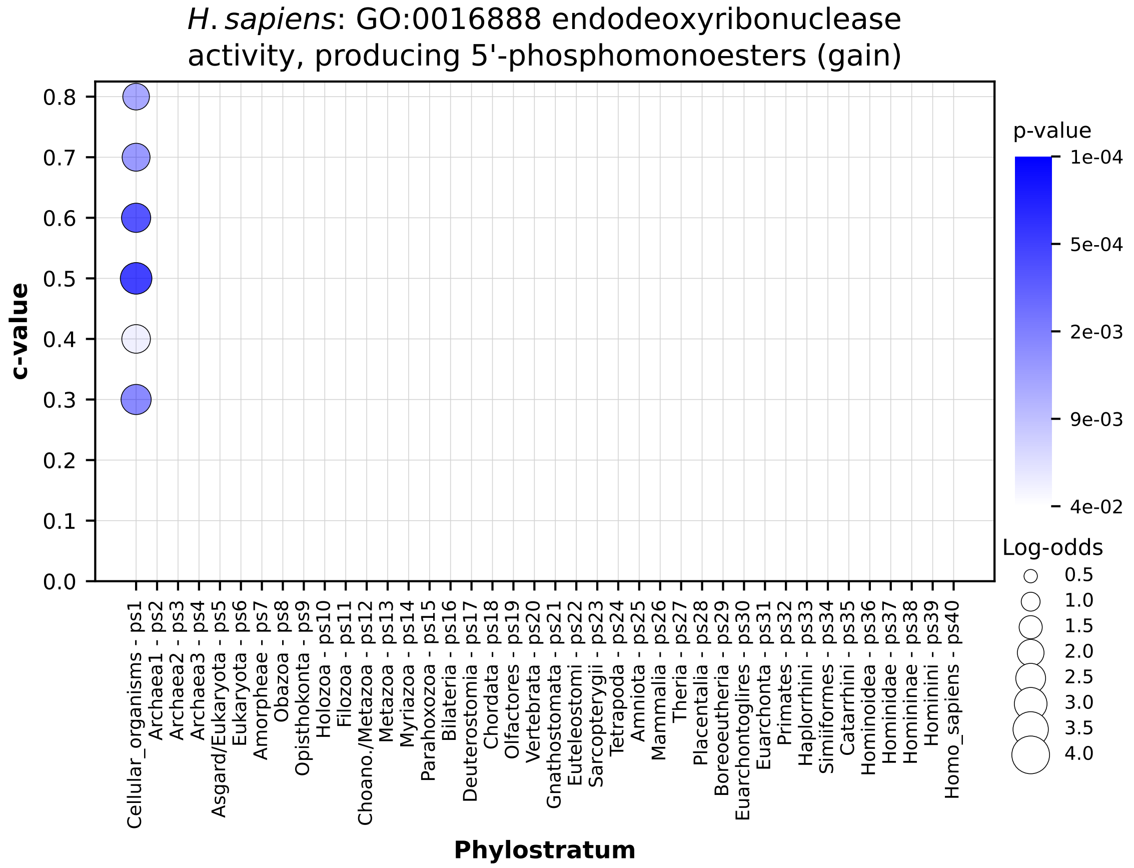

H. sapiens: GO:0016888 endodeoxyribonuclease activity, producing 5'-phosphomonoesters (gain)
c-value
0.8
0.7
0.6
0.5
0.4
0.3
0.2
0.1
0.0
Phylostratum
Cellular_organisms - ps1
Archaea1 - ps2
Archaea2 - ps3
Archaea3 - ps4
Asgard/Eukaryota - ps5
Eukaryota - ps6
Amorpheae - ps7
Obazoa - ps8
Opisthokonta - ps9
Holozoa - ps10
Filozoa - ps11
Choano./Metazoa - ps12
Metazoa - ps13
Myriazoa - ps14
Parahoxozoa - ps15
Bilateria - ps16
Deuterostomia - ps17
Chordata - ps18
Olfactores - ps19
Vertebrata - ps20
Gnathostomata - ps21
Euteleostomi - ps22
Sarcopterygii - ps23
Tetrapoda - ps24
Amniota - ps25
Mammalia - ps26
Theria - ps27
Placentalia - ps28
Boreoeutheria - ps29
Euarchontoglires - ps30
Euarchonta - ps31
Primates - ps32
Haplorrhini - ps33
Simiiformes - ps34
Catarrhini - ps35
Hominoidea - ps36
Hominidae - ps37
Homininae - ps38
Hominini - ps39
Homo_sapiens - ps40
p-value
1e-04
5e-04
2e-03
9e-03
4e-02
Log-odds
0.5
1.0
1.5
2.0
2.5
3.0
3.5
4.0

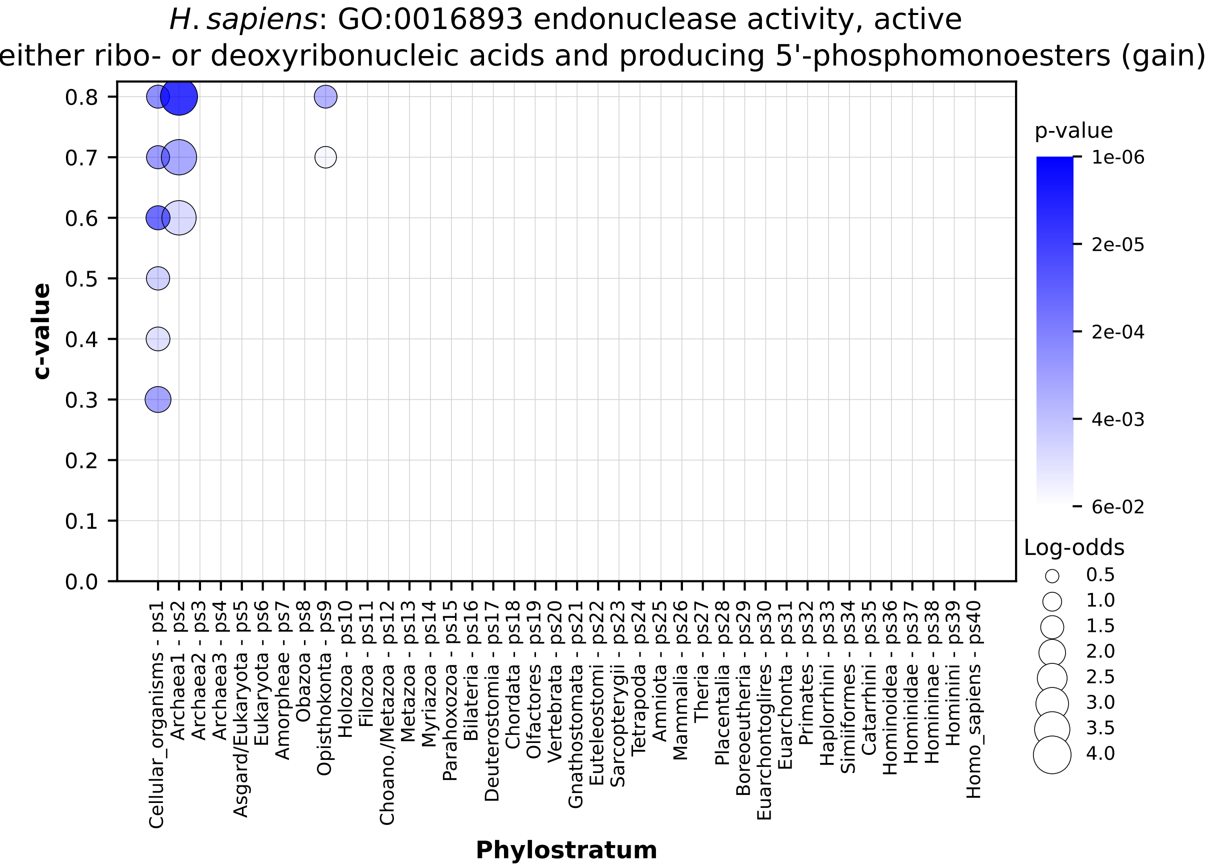

H. sapiens: GO:0016893 endonuclease activity, active
either ribo- or deoxyribonucleic acids and producing 5'-phosphomonoesters (gain)
c-value
Phylostratum
p-value
1e-06
2e-05
2e-04
4e-03
6e-02
Log-odds
0.5
1.0
1.5
2.0
2.5
3.0
3.5
4.0
Cellular_organisms - ps1
Archaea1 - ps2
Archaea2 - ps3
Archaea3 - ps4
Asgard/Eukaryota - ps5
Eukaryota - ps6
Amorpheae - ps7
Obazoa - ps8
Opisthokonta - ps9
Holozoa - ps10
Filozoa - ps11
Choano./Metazoa - ps12
Metazoa - ps13
Myriazoa - ps14
Parahoxozoa - ps15
Bilateria - ps16
Deuterostomia - ps17
Chordata - ps18
Olfactores - ps19
Vertebrata - ps20
Gnathostomata - ps21
Euteleostomi - ps22
Sarcopterygii - ps23
Tetrapoda - ps24
Amniota - ps25
Mammalia - ps26
Theria - ps27
Placentalia - ps28
Boreoeutheria - ps29
Euarchontoglires - ps30
Euarchonta - ps31
Primates - ps32
Haplorrhini - ps33
Simiiformes - ps34
Catarrhini - ps35
Hominoidea - ps36
Hominidae - ps37
Homininae - ps38
Hominini - ps39
Homo_sapiens - ps40

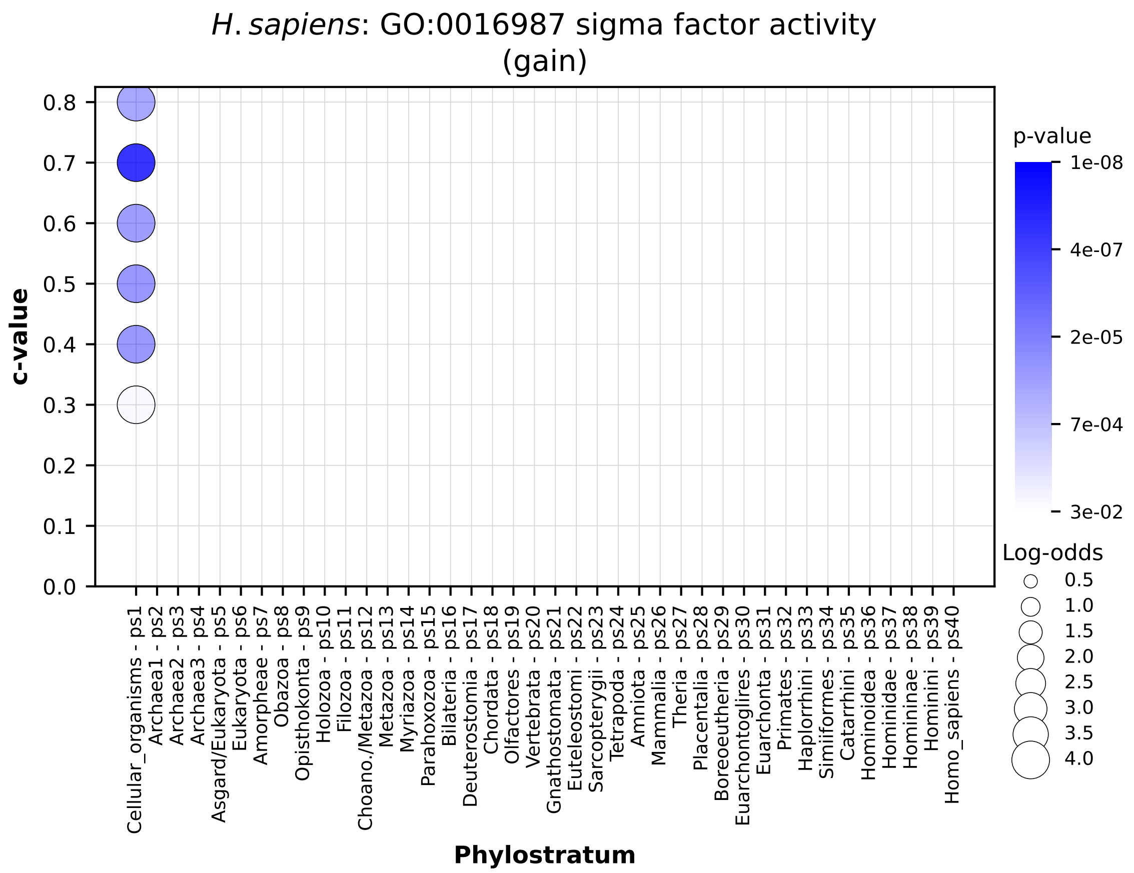

*H. sapiens*: GO:0016987 sigma factor activity
(gain)
c-value
0.8
0.7
0.6
0.5
0.4
0.3
0.2
0.1
0.0
Phylostratum
Cellular_organisms - ps1
Archaea1 - ps2
Archaea2 - ps3
Archaea3 - ps4
Asgard/Eukaryota - ps5
Eukaryota - ps6
Amorpheae - ps7
Obazoa - ps8
Opisthokonta - ps9
Holozoa - ps10
Filozoa - ps11
Choano./Metazoa - ps12
Metazoa - ps13
Myriazoa - ps14
Parahoxozoa - ps15
Bilateria - ps16
Deuterostomia - ps17
Chordata - ps18
Olfactores - ps19
Vertebrata - ps20
Gnathostomata - ps21
Euteleostomi - ps22
Sarcopterygii - ps23
Tetrapoda - ps24
Amniota - ps25
Mammalia - ps26
Theria - ps27
Placentalia - ps28
Boreoeutheria - ps29
Euarchontoglires - ps30
Euarchonta - ps31
Primates - ps32
Haplorrhini - ps33
Simiiformes - ps34
Catarrhini - ps35
Hominoidea - ps36
Hominidae - ps37
Homininae - ps38
Hominini - ps39
Homo_sapiens - ps40
p-value
1e-08
4e-07
2e-05
7e-04
3e-02
Log-odds
0.5
1.0
1.5
2.0
2.5
3.0
3.5
4.0

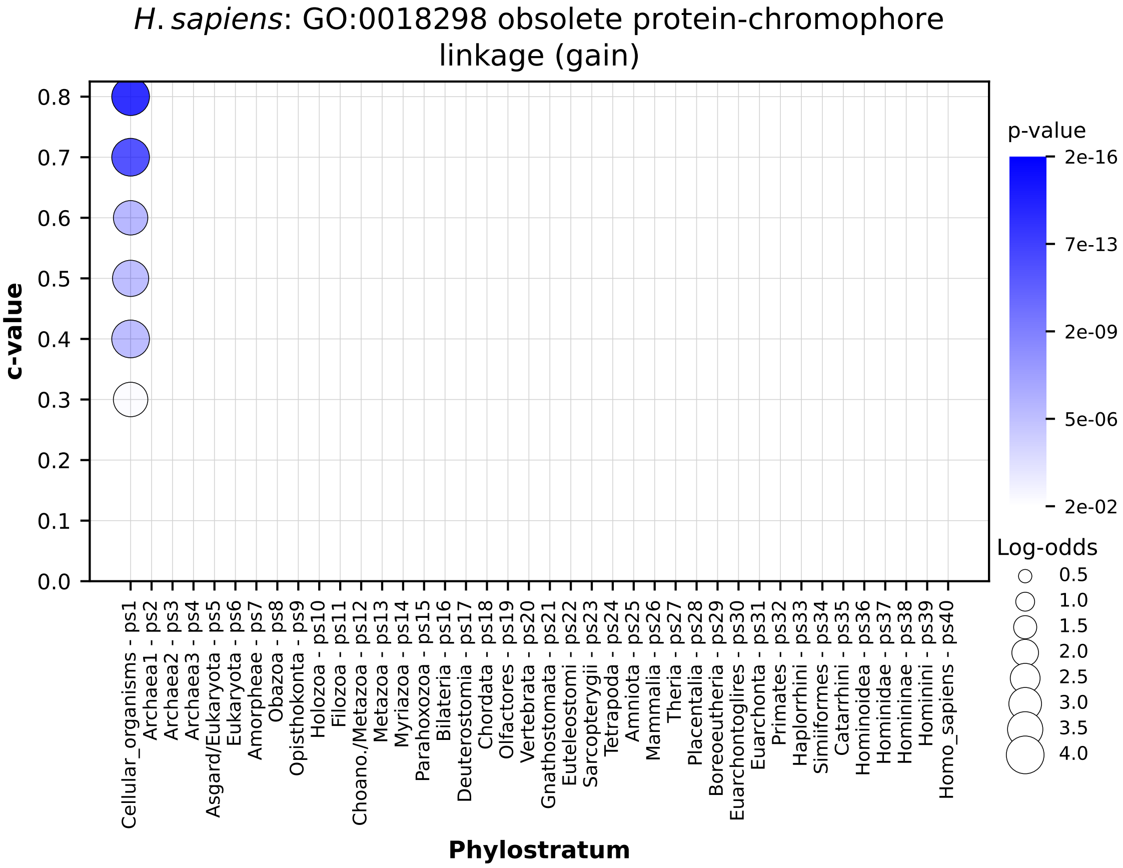
H. sapiens: GO:0018298 obsolete protein-chromophore linkage (gain)
c-value
0.0
0.1
0.2
0.3
0.4
0.5
0.6
0.7
0.8
Phylostratum
Cellular_organisms - ps1
Archaea1 - ps2
Archaea2 - ps3
Archaea3 - ps4
Asgard/Eukaryota - ps5
Eukaryota - ps6
Amorpheae - ps7
Obazoa - ps8
Opisthokonta - ps9
Holozoa - ps10
Filozoa - ps11
Choano./Metazoa - ps12
Metazoa - ps13
Myriazoa - ps14
Parahoxozoa - ps15
Bilateria - ps16
Deuterostomia - ps17
Chordata - ps18
Olfactores - ps19
Vertebrata - ps20
Gnathostomata - ps21
Euteleostomi - ps22
Sarcopterygii - ps23
Tetrapoda - ps24
Amniota - ps25
Mammalia - ps26
Theria - ps27
Placentalia - ps28
Boreoeutheria - ps29
Euarchontoglires - ps30
Euarchonta - ps31
Primates - ps32
Haplorrhini - ps33
Simiiformes - ps34
Catarrhini - ps35
Hominoidea - ps36
Hominidae - ps37
Homininae - ps38
Hominini - ps39
Homo_sapiens - ps40
p-value
2e-16
7e-13
2e-09
5e-06
2e-02
Log-odds
0.5
1.0
1.5
2.0
2.5
3.0
3.5
4.0

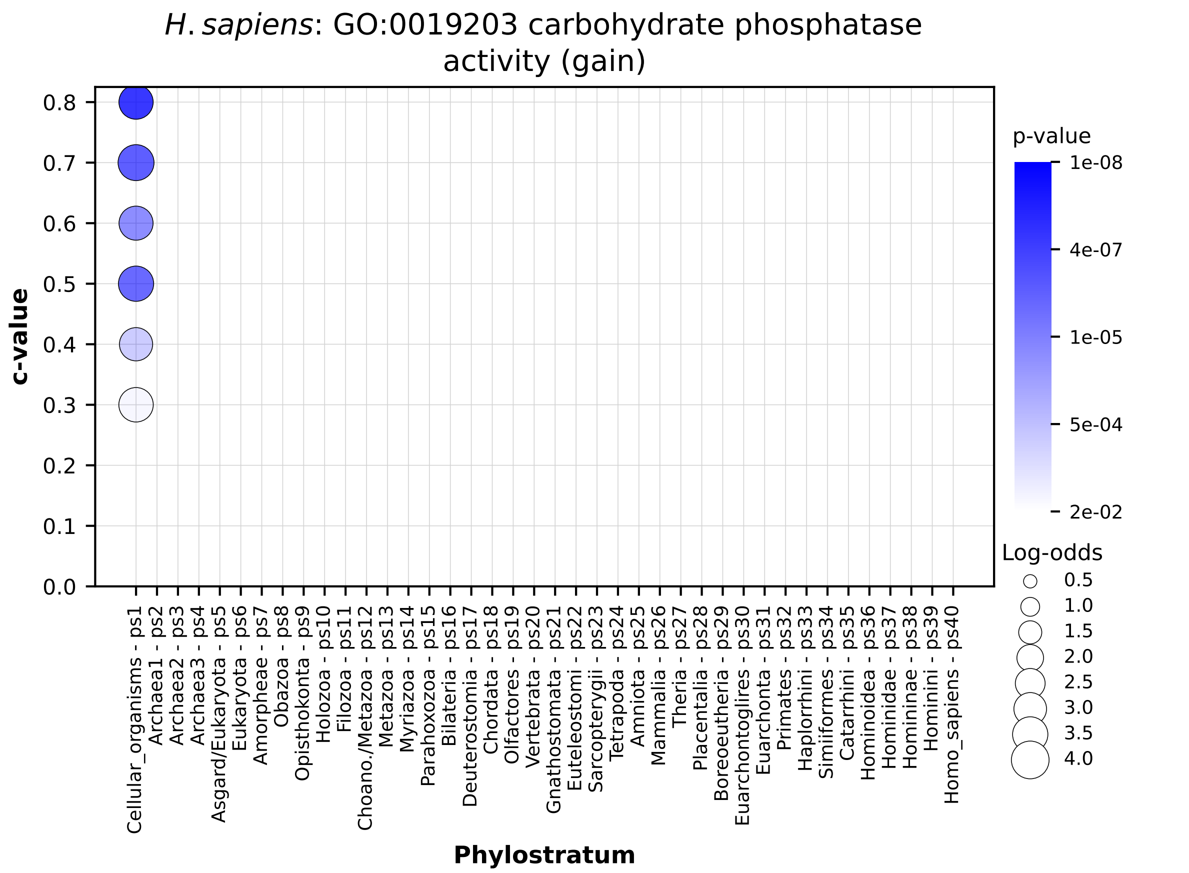
H. sapiens: GO:0019203 carbohydrate phosphatase activity (gain)
c-value
0.8
0.7
0.6
0.5
0.4
0.3
0.2
0.1
0.0
p-value
1e-08
4e-07
1e-05
5e-04
2e-02
Log-odds
0.5
1.0
1.5
2.0
2.5
3.0
3.5
4.0
Cellular_organisms - ps1
Archaea1 - ps2
Archaea2 - ps3
Archaea3 - ps4
Asgard/Eukaryota - ps5
Eukaryota - ps6
Amorpheae - ps7
Obazoa - ps8
Opisthokonta - ps9
Holozoa - ps10
Filozoa - ps11
Choano./Metazoa - ps12
Metazoa - ps13
Myriazoa - ps14
Parahoxozoa - ps15
Bilateria - ps16
Deuterostomia - ps17
Chordata - ps18
Olfactores - ps19
Vertebrata - ps20
Gnathostomata - ps21
Euteleostomi - ps22
Sarcopterygii - ps23
Tetrapoda - ps24
Amniota - ps25
Mammalia - ps26
Theria - ps27
Placentalia - ps28
Boreoeutheria - ps29
Euarchontoglires - ps30
Euarchonta - ps31
Primates - ps32
Haplorrhini - ps33
Simiiformes - ps34
Catarrhini - ps35
Hominoidea - ps36
Hominidae - ps37
Homininae - ps38
Hominini - ps39
Homo_sapiens - ps40
Phylostratum

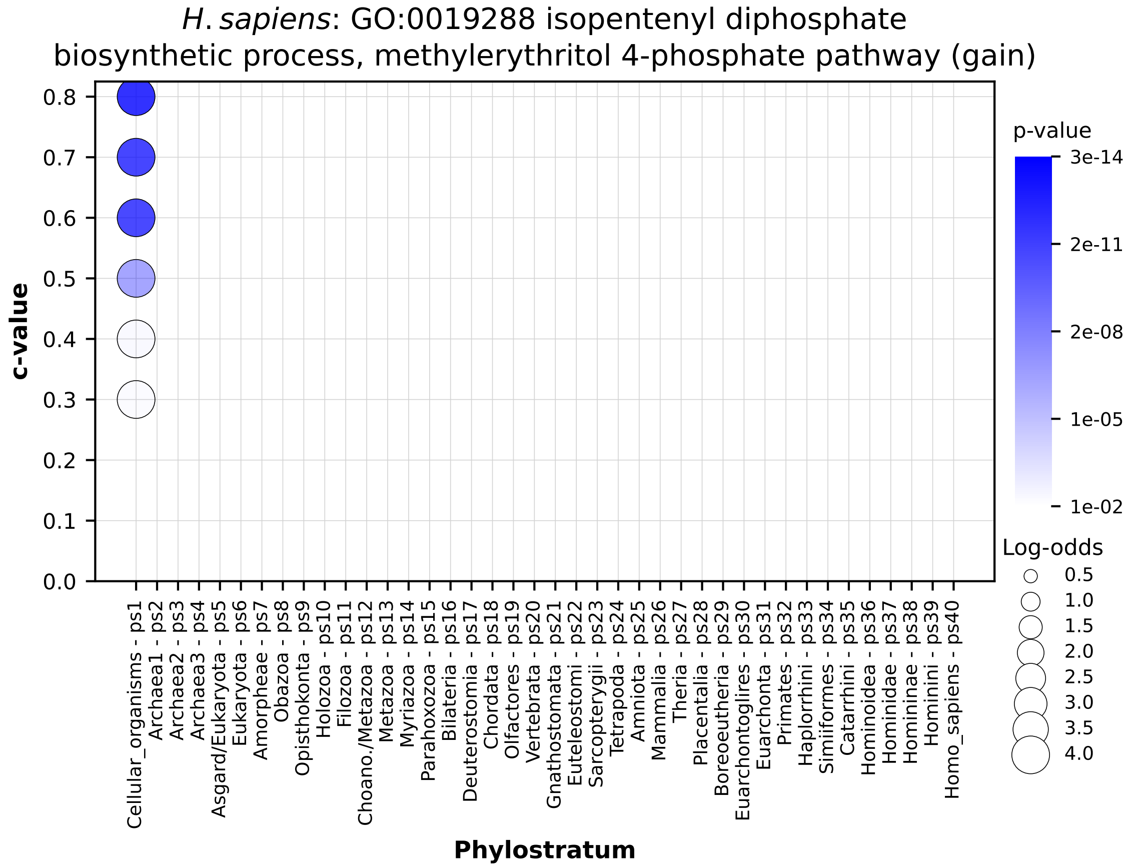

H. sapiens: GO:0019288 isopentenyl diphosphate biosynthetic process, methylerythritol 4-phosphate pathway (gain)
c-value
0.0
0.1
0.2
0.3
0.4
0.5
0.6
0.7
0.8
Phylostratum
Cellular_organisms - ps1
Archaea1 - ps2
Archaea2 - ps3
Archaea3 - ps4
Asgard/Eukaryota - ps5
Eukaryota - ps6
Amorpheae - ps7
Obazoa - ps8
Opisthokonta - ps9
Holozoa - ps10
Filozoa - ps11
Choano./Metazoa - ps12
Metazoa - ps13
Myriazoa - ps14
Parahoxozoa - ps15
Bilateria - ps16
Deuterostomia - ps17
Chordata - ps18
Olfactores - ps19
Vertebrata - ps20
Gnathostomata - ps21
Euteleostomi - ps22
Sarcopterygii - ps23
Tetrapoda - ps24
Amniota - ps25
Mammalia - ps26
Theria - ps27
Placentalia - ps28
Boreoeutheria - ps29
Euarchontoglires - ps30
Euarchonta - ps31
Primates - ps32
Haplorrhini - ps33
Simiiformes - ps34
Catarrhini - ps35
Hominoidea - ps36
Hominidae - ps37
Homininae - ps38
Hominini - ps39
Homo_sapiens - ps40
p-value
3e-14
2e-11
2e-08
1e-05
1e-02
Log-odds
0.5
1.0
1.5
2.0
2.5
3.0
3.5
4.0

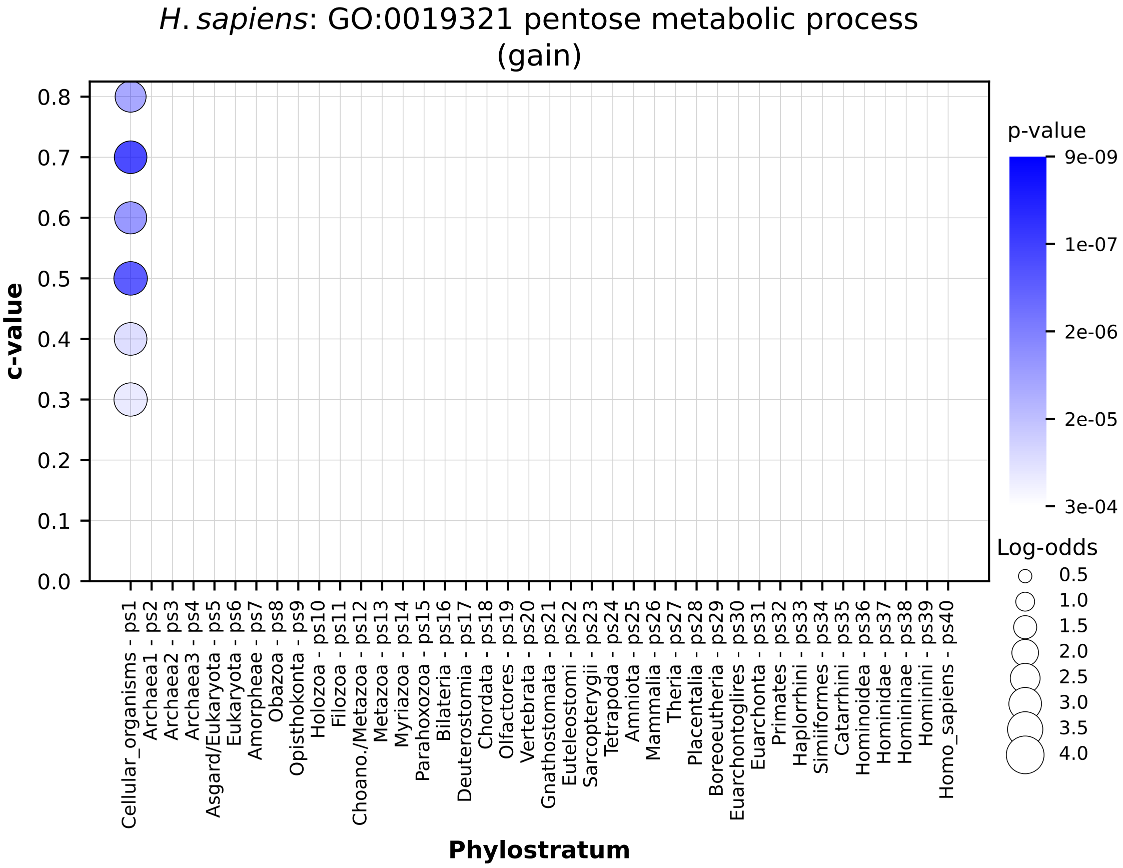

H. sapiens: GO:0019321 pentose metabolic process
(gain)
c-value
0.0
0.1
0.2
0.3
0.4
0.5
0.6
0.7
0.8
p-value
9e-09
1e-07
2e-06
2e-05
3e-04
Log-odds
0.5
1.0
1.5
2.0
2.5
3.0
3.5
4.0
Cellular_organisms - ps1
Archaea1 - ps2
Archaea2 - ps3
Archaea3 - ps4
Asgard/Eukaryota - ps5
Eukaryota - ps6
Amorpheae - ps7
Obazoa - ps8
Opisthokonta - ps9
Holozoa - ps10
Filozoa - ps11
Choano./Metazoa - ps12
Metazoa - ps13
Myriazoa - ps14
Parahoxozoa - ps15
Bilateria - ps16
Deuterostomia - ps17
Chordata - ps18
Olfactores - ps19
Vertebrata - ps20
Gnathostomata - ps21
Euteleostomi - ps22
Sarcopterygii - ps23
Tetrapoda - ps24
Amniota - ps25
Mammalia - ps26
Theria - ps27
Placentalia - ps28
Boreoeutheria - ps29
Euarchontoglires - ps30
Euarchonta - ps31
Primates - ps32
Haplorrhini - ps33
Simiiformes - ps34
Catarrhini - ps35
Hominoidea - ps36
Hominidae - ps37
Homininae - ps38
Hominini - ps39
Homo_sapiens - ps40
Phylostratum

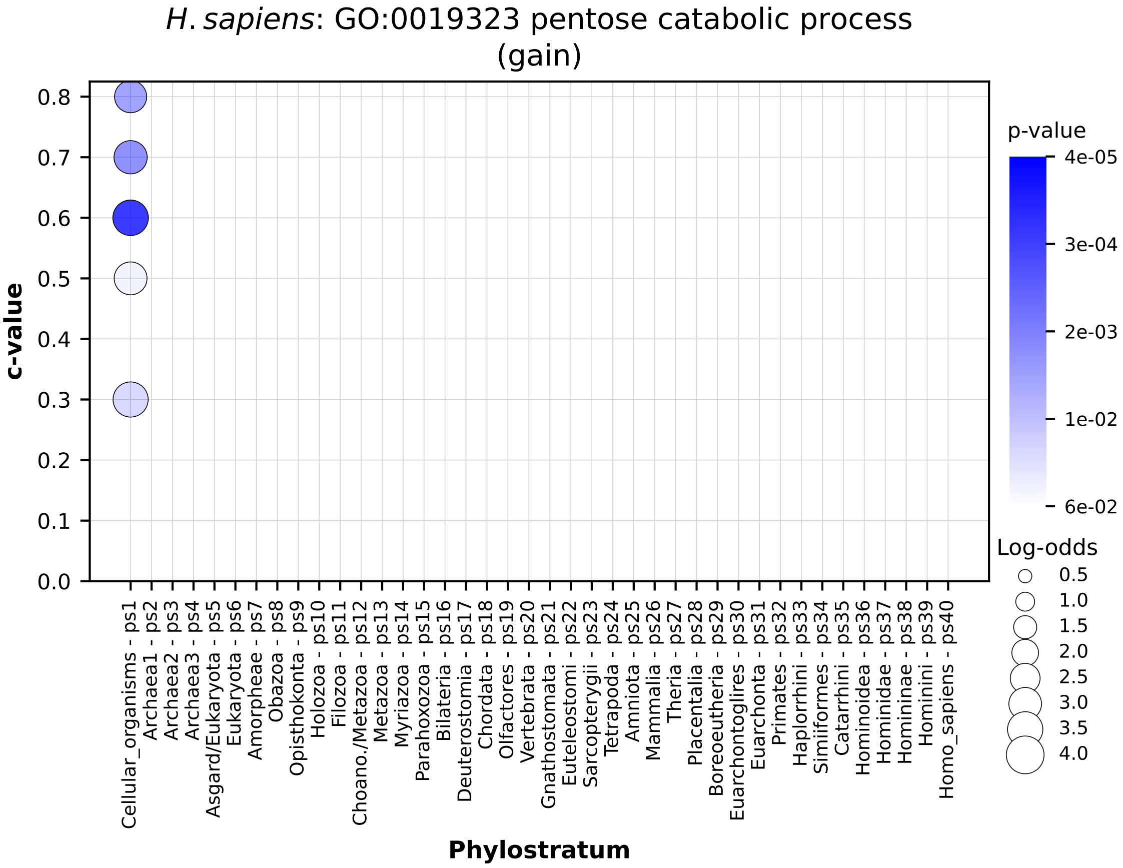

H. sapiens: GO:0019323 pentose catabolic process
(gain)
c-value
0.0
0.1
0.2
0.3
0.4
0.5
0.6
0.7
0.8
Phylostratum
Cellular_organisms - ps1
Archaea1 - ps2
Archaea2 - ps3
Archaea3 - ps4
Asgard/Eukaryota - ps5
Eukaryota - ps6
Amorpheae - ps7
Obazoa - ps8
Opisthokonta - ps9
Holozoa - ps10
Filozoa - ps11
Choano./Metazoa - ps12
Metazoa - ps13
Myriazoa - ps14
Parahoxozoa - ps15
Bilateria - ps16
Deuterostomia - ps17
Chordata - ps18
Olfactores - ps19
Vertebrata - ps20
Gnathostomata - ps21
Euteleostomi - ps22
Sarcopterygii - ps23
Tetrapoda - ps24
Amniota - ps25
Mammalia - ps26
Theria - ps27
Placentalia - ps28
Boreoeutheria - ps29
Euarchontoglires - ps30
Euarchonta - ps31
Primates - ps32
Haplorrhini - ps33
Simiiformes - ps34
Catarrhini - ps35
Hominoidea - ps36
Hominidae - ps37
Homininae - ps38
Hominini - ps39
Homo_sapiens - ps40
p-value
4e-05
3e-04
2e-03
1e-02
6e-02
Log-odds
0.5
1.0
1.5
2.0
2.5
3.0
3.5
4.0

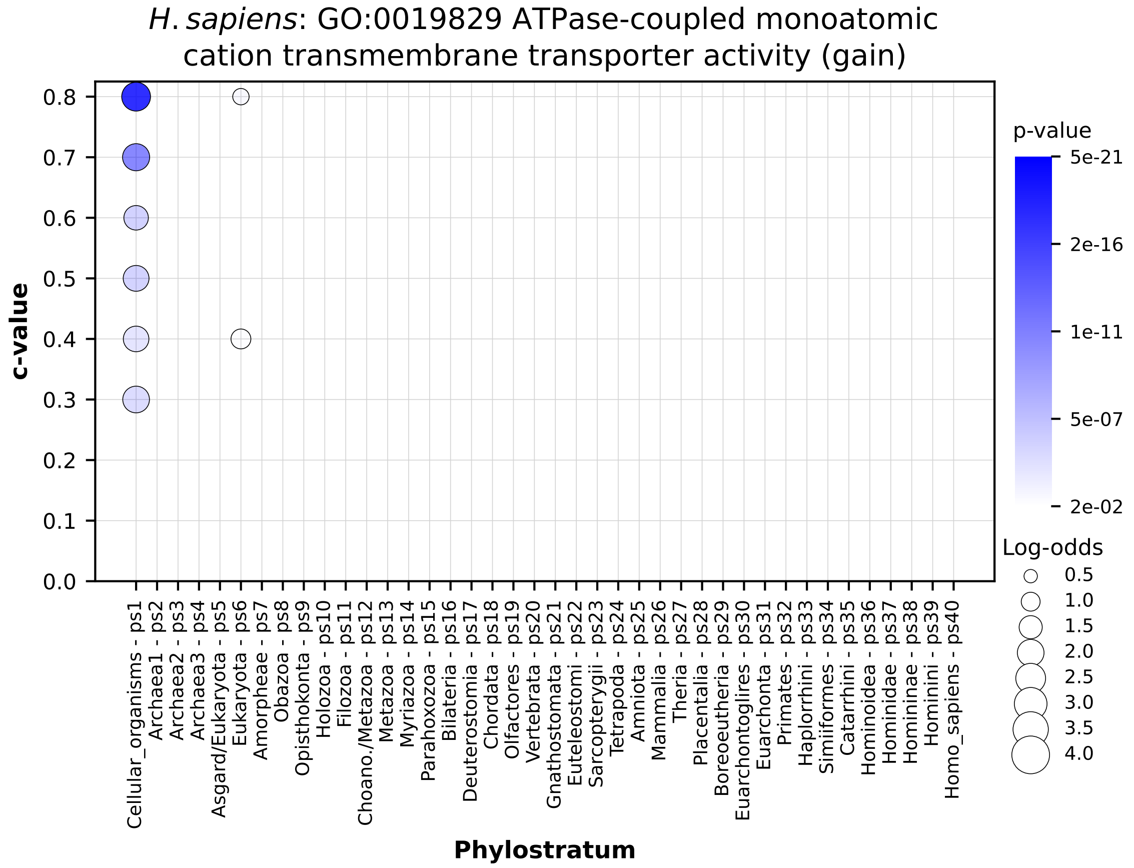

H. sapiens: GO:0019829 ATPase-coupled monoatomic cation transmembrane transporter activity (gain)
c-value
0.8
0.7
0.6
0.5
0.4
0.3
0.2
0.1
0.0
Phylostratum
Cellular_organisms - ps1
Archaea1 - ps2
Archaea2 - ps3
Archaea3 - ps4
Asgard/Eukaryota - ps5
Eukaryota - ps6
Amorpheae - ps7
Obazoa - ps8
Opisthokonta - ps9
Holozoa - ps10
Filozoa - ps11
Choano./Metazoa - ps12
Metazoa - ps13
Myriazoa - ps14
Parahoxozoa - ps15
Bilateria - ps16
Deuterostomia - ps17
Chordata - ps18
Olfactores - ps19
Vertebrata - ps20
Gnathostomata - ps21
Euteleostomi - ps22
Sarcopterygii - ps23
Tetrapoda - ps24
Amniota - ps25
Mammalia - ps26
Theria - ps27
Placentalia - ps28
Boreoeutheria - ps29
Euarchontoglires - ps30
Euarchonta - ps31
Primates - ps32
Haplorrhini - ps33
Simiiformes - ps34
Catarrhini - ps35
Hominoidea - ps36
Hominidae - ps37
Homininae - ps38
Hominini - ps39
Homo_sapiens - ps40
p-value
5e-21
2e-16
1e-11
5e-07
2e-02
Log-odds
0.5
1.0
1.5
2.0
2.5
3.0
3.5
4.0

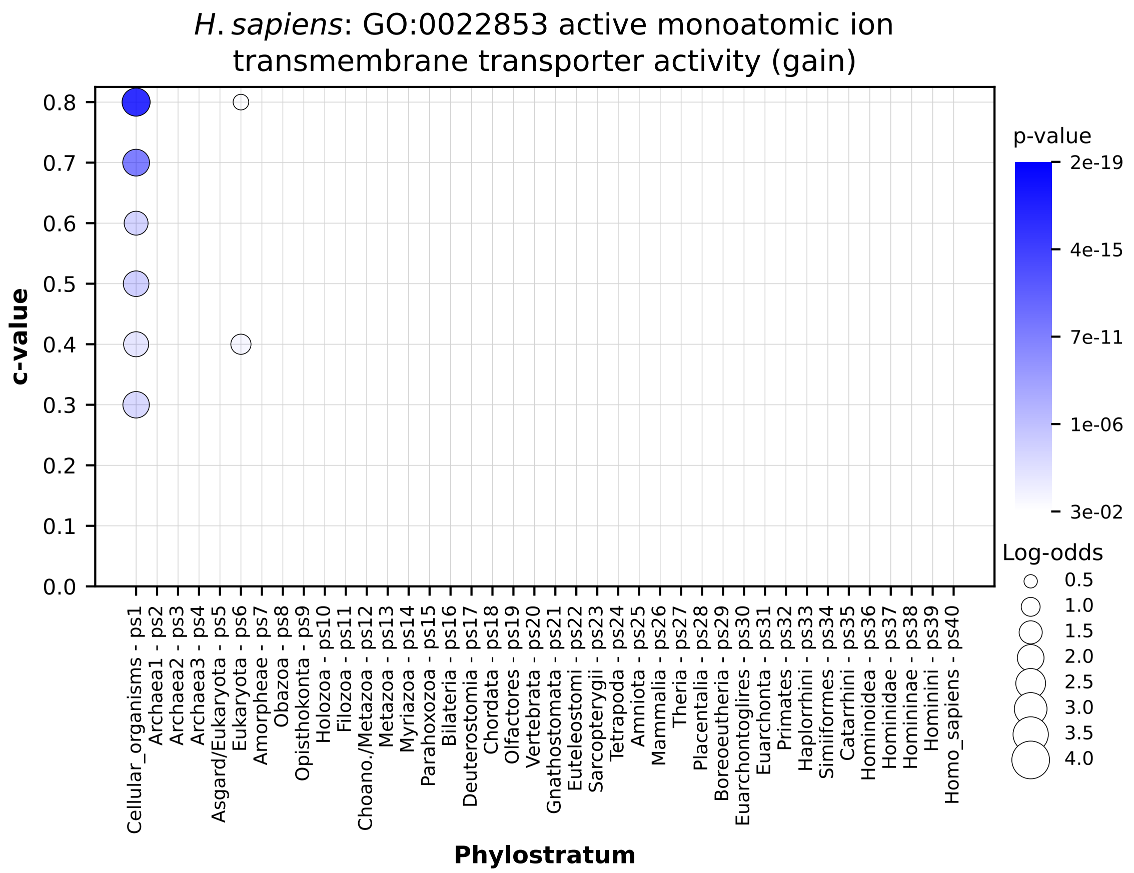

H. sapiens: GO:0022853 active monoatomic ion transmembrane transporter activity (gain)
c-value
0.0
0.1
0.2
0.3
0.4
0.5
0.6
0.7
0.8
Phylostratum
Cellular_organisms - ps1
Archaea1 - ps2
Archaea2 - ps3
Archaea3 - ps4
Asgard/Eukaryota - ps5
Eukaryota - ps6
Amorpheae - ps7
Obazoa - ps8
Opisthokonta - ps9
Holozoa - ps10
Filozoa - ps11
Choano./Metazoa - ps12
Metazoa - ps13
Myriazoa - ps14
Parahoxozoa - ps15
Bilateria - ps16
Deuterostomia - ps17
Chordata - ps18
Olfactores - ps19
Vertebrata - ps20
Gnathostomata - ps21
Euteleostomi - ps22
Sarcopterygii - ps23
Tetrapoda - ps24
Amniota - ps25
Mammalia - ps26
Theria - ps27
Placentalia - ps28
Boreoeutheria - ps29
Euarchontoglires - ps30
Euarchonta - ps31
Primates - ps32
Haplorrhini - ps33
Simiiformes - ps34
Catarrhini - ps35
Hominoidea - ps36
Hominidae - ps37
Homininae - ps38
Hominini - ps39
Homo_sapiens - ps40
p-value
2e-19
4e-15
7e-11
1e-06
3e-02
Log-odds
0.5
1.0
1.5
2.0
2.5
3.0
3.5
4.0

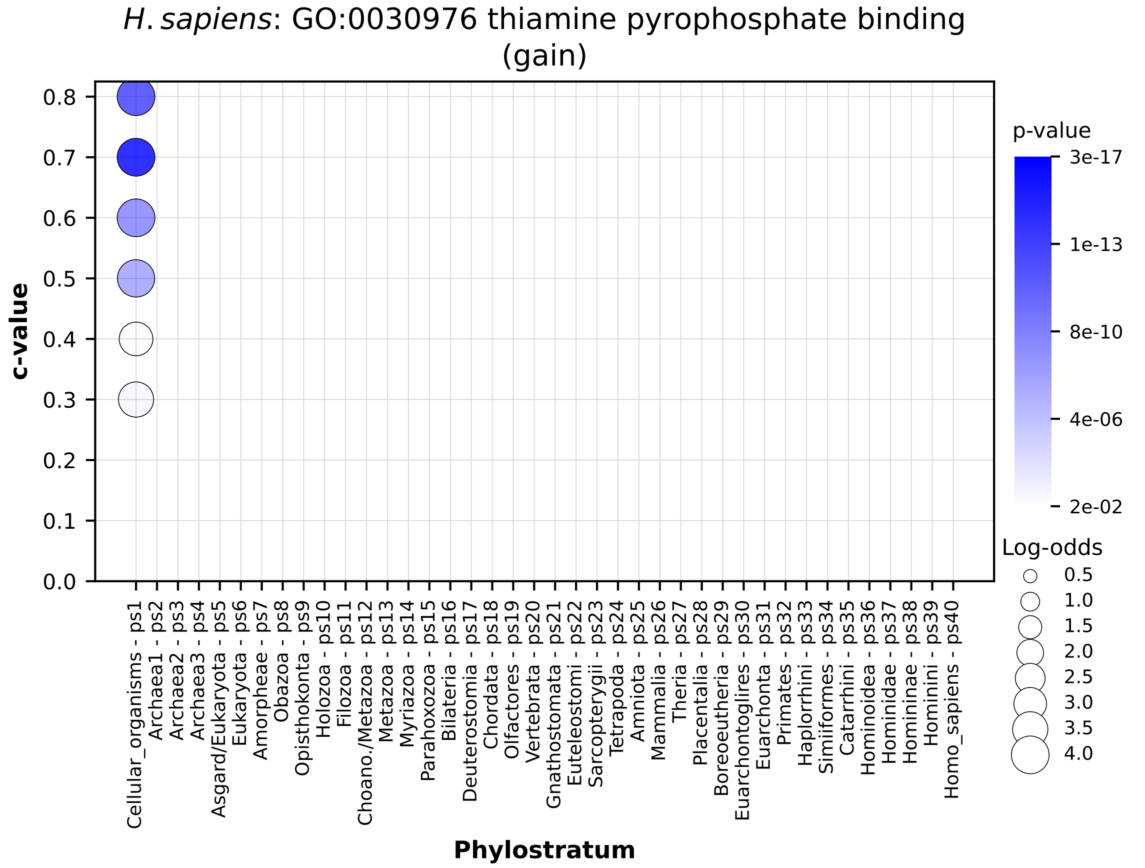

H. sapiens: GO:0030976 thiamine pyrophosphate binding
(gain)
c-value
0.0
0.1
0.2
0.3
0.4
0.5
0.6
0.7
0.8
Phylostratum
Cellular_organisms - ps1
Archaea1 - ps2
Archaea2 - ps3
Archaea3 - ps4
Asgard/Eukaryota - ps5
Eukaryota - ps6
Amorpheae - ps7
Obazoa - ps8
Opisthokonta - ps9
Holozoa - ps10
Filozoa - ps11
Choano./Metazoa - ps12
Metazoa - ps13
Myriazoa - ps14
Parahoxozoa - ps15
Bilateria - ps16
Deuterostomia - ps17
Chordata - ps18
Olfactores - ps19
Vertebrata - ps20
Gnathostomata - ps21
Euteleostomi - ps22
Sarcopterygii - ps23
Tetrapoda - ps24
Amniota - ps25
Mammalia - ps26
Theria - ps27
Placentalia - ps28
Boreoeutheria - ps29
Euarchontoglires - ps30
Euarchonta - ps31
Primates - ps32
Haplorrhini - ps33
Simiiformes - ps34
Catarrhini - ps35
Hominoidea - ps36
Hominidae - ps37
Homininae - ps38
Hominini - ps39
Homo_sapiens - ps40
p-value
3e-17
1e-13
8e-10
4e-06
2e-02
Log-odds
0.5
1.0
1.5
2.0
2.5
3.0
3.5
4.0

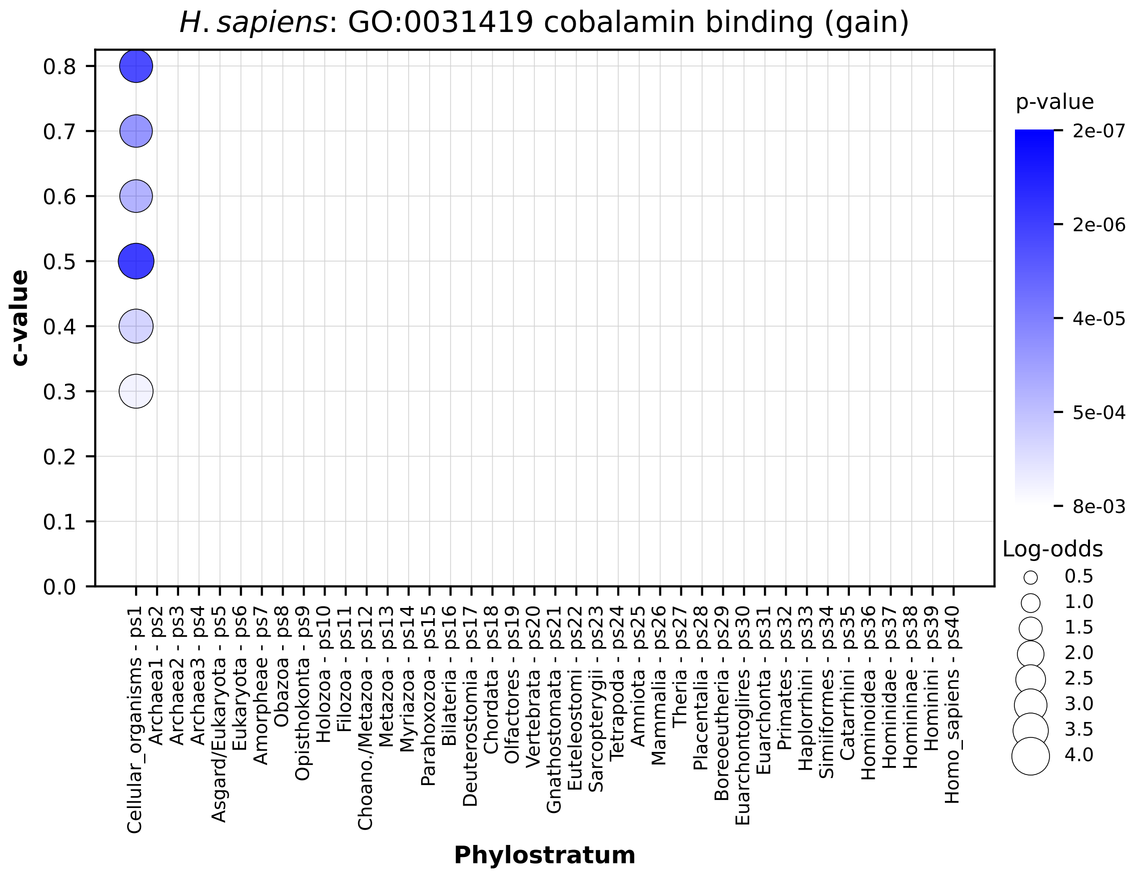

H. sapiens: GO:0031419 cobalamin binding (gain)
c-value
0.0
0.1
0.2
0.3
0.4
0.5
0.6
0.7
0.8
Phylostratum
Cellular_organisms - ps1
Archaea1 - ps2
Archaea2 - ps3
Archaea3 - ps4
Asgard/Eukaryota - ps5
Eukaryota - ps6
Amorpheae - ps7
Obazoa - ps8
Opisthokonta - ps9
Holozoa - ps10
Filozoa - ps11
Choano./Metazoa - ps12
Metazoa - ps13
Myriazoa - ps14
Parahoxozoa - ps15
Bilateria - ps16
Deuterostomia - ps17
Chordata - ps18
Olfactores - ps19
Vertebrata - ps20
Gnathostomata - ps21
Euteleostomi - ps22
Sarcopterygii - ps23
Tetrapoda - ps24
Amniota - ps25
Mammalia - ps26
Theria - ps27
Placentalia - ps28
Boreoeutheria - ps29
Euarchontoglires - ps30
Euarchonta - ps31
Primates - ps32
Haplorrhini - ps33
Simiiformes - ps34
Catarrhini - ps35
Hominoidea - ps36
Hominidae - ps37
Homininae - ps38
Hominini - ps39
Homo_sapiens - ps40
p-value
2e-07
2e-06
4e-05
5e-04
8e-03
Log-odds
0.5
1.0
1.5
2.0
2.5
3.0
3.5
4.0

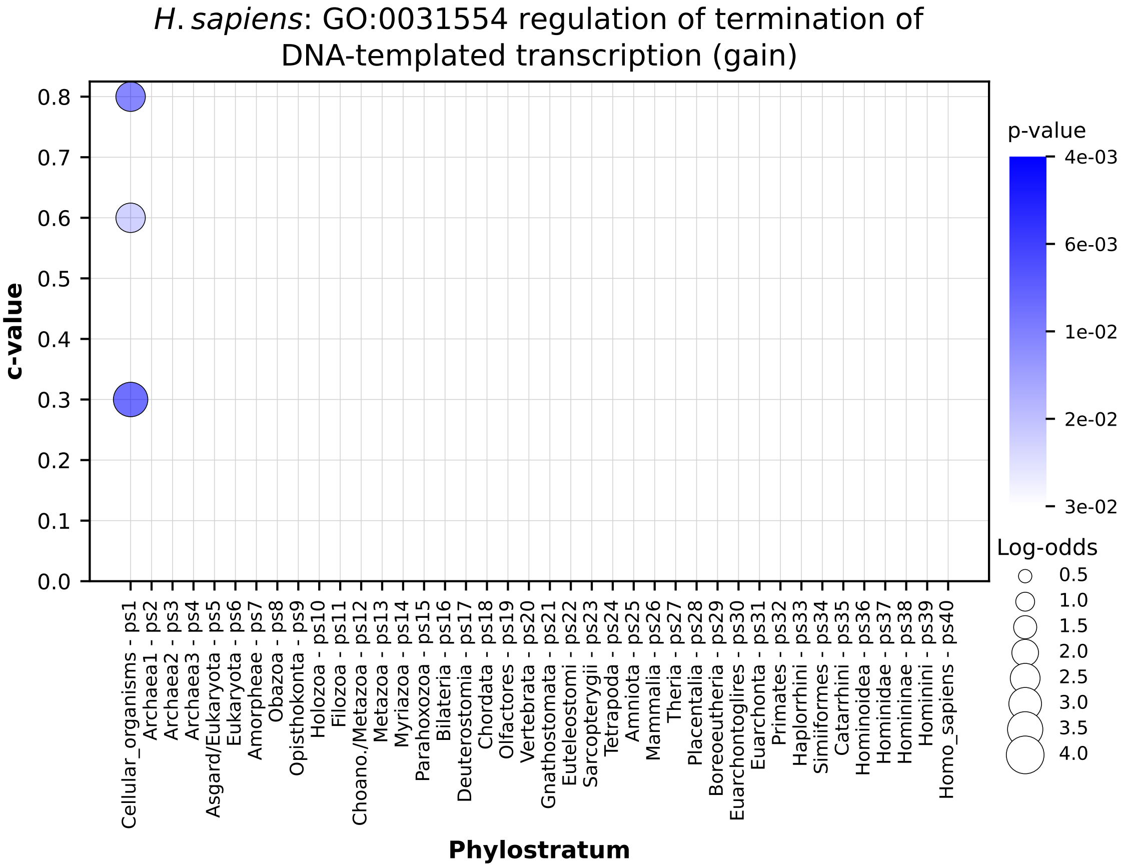

*H. sapiens*: GO:0031554 regulation of termination of DNA-templated transcription (gain)
c-value
0.0
0.1
0.2
0.3
0.4
0.5
0.6
0.7
0.8
Phylostratum
Cellular_organisms - ps1
Archaea1 - ps2
Archaea2 - ps3
Archaea3 - ps4
Asgard/Eukaryota - ps5
Eukaryota - ps6
Amorpheae - ps7
Obazoa - ps8
Opisthokonta - ps9
Holozoa - ps10
Filozoa - ps11
Choano./Metazoa - ps12
Metazoa - ps13
Myriazoa - ps14
Parahoxozoa - ps15
Bilateria - ps16
Deuterostomia - ps17
Chordata - ps18
Olfactores - ps19
Vertebrata - ps20
Gnathostomata - ps21
Euteleostomi - ps22
Sarcopterygii - ps23
Tetrapoda - ps24
Amniota - ps25
Mammalia - ps26
Theria - ps27
Placentalia - ps28
Boreoeutheria - ps29
Euarchontoglires - ps30
Euarchonta - ps31
Primates - ps32
Haplorrhini - ps33
Simiiformes - ps34
Catarrhini - ps35
Hominoidea - ps36
Hominidae - ps37
Homininae - ps38
Hominini - ps39
Homo_sapiens - ps40
p-value
4e-03
6e-03
1e-02
2e-02
3e-02
Log-odds
0.5
1.0
1.5
2.0
2.5
3.0
3.5
4.0

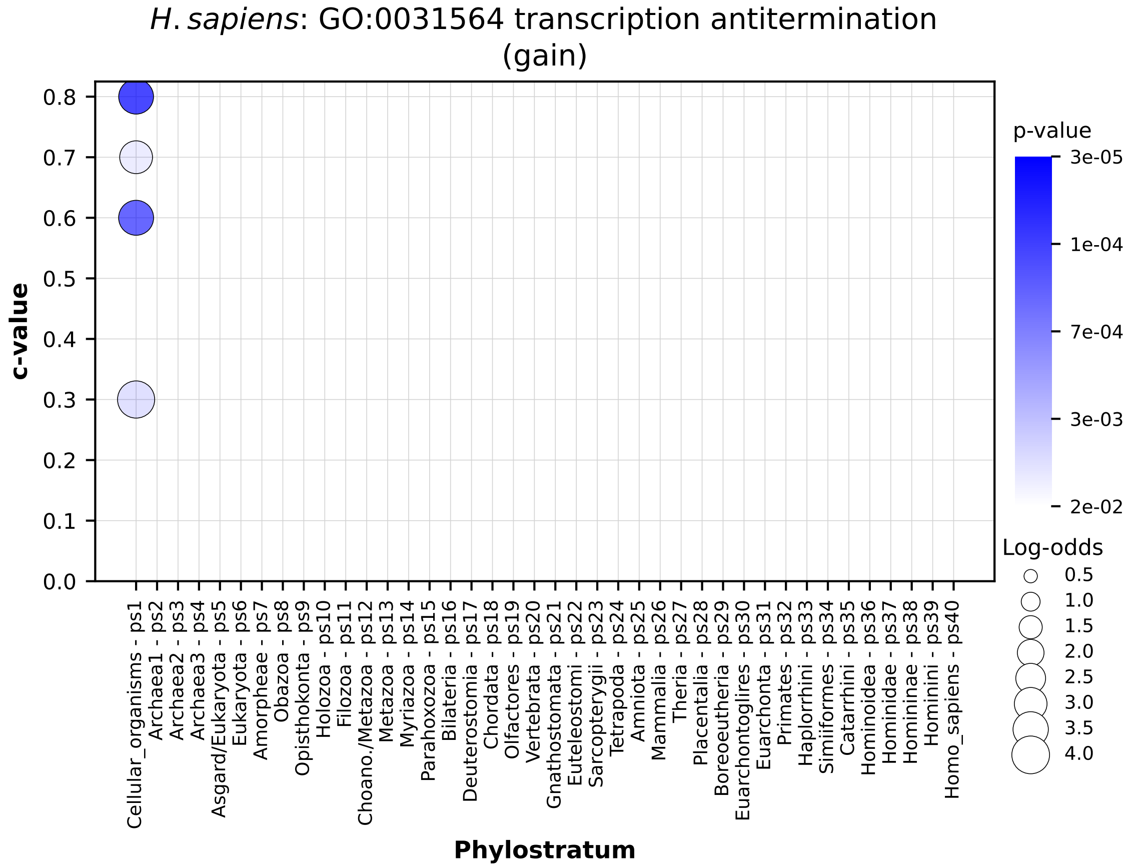
H. sapiens: GO:0031564 transcription antitermination
(gain)
c-value
0.0
0.1
0.2
0.3
0.4
0.5
0.6
0.7
0.8
p-value
3e-05
1e-04
7e-04
3e-03
2e-02
Log-odds
0.5
1.0
1.5
2.0
2.5
3.0
3.5
4.0
Cellular_organisms - ps1
Archaea1 - ps2
Archaea2 - ps3
Archaea3 - ps4
Asgard/Eukaryota - ps5
Eukaryota - ps6
Amorpheae - ps7
Obazoa - ps8
Opisthokonta - ps9
Holozoa - ps10
Filozoa - ps11
Choano./Metazoa - ps12
Metazoa - ps13
Myriazoa - ps14
Parahoxozoa - ps15
Bilateria - ps16
Deuterostomia - ps17
Chordata - ps18
Olfactores - ps19
Vertebrata - ps20
Gnathostomata - ps21
Euteleostomi - ps22
Sarcopterygii - ps23
Tetrapoda - ps24
Amniota - ps25
Mammalia - ps26
Theria - ps27
Placentalia - ps28
Boreoeutheria - ps29
Euarchontoglires - ps30
Euarchonta - ps31
Primates - ps32
Haplorrhini - ps33
Simiiformes - ps34
Catarrhini - ps35
Hominoidea - ps36
Hominidae - ps37
Homininae - ps38
Hominini - ps39
Homo_sapiens - ps40
Phylostratum

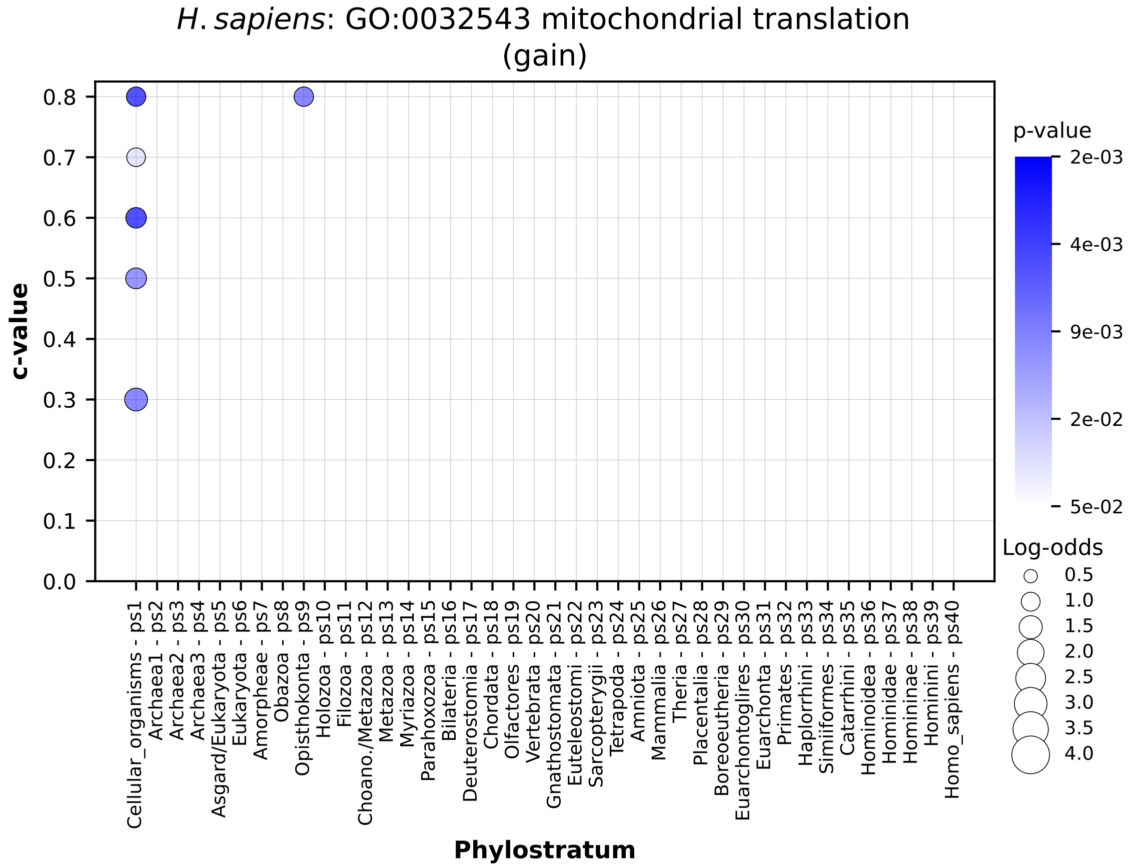

H. sapiens: GO:0032543 mitochondrial translation
(gain)
c-value
0.0
0.1
0.2
0.3
0.4
0.5
0.6
0.7
0.8
Phylostratum
Cellular_organisms - ps1
Archaea1 - ps2
Archaea2 - ps3
Archaea3 - ps4
Asgard/Eukaryota - ps5
Eukaryota - ps6
Amorpheae - ps7
Obazoa - ps8
Opisthokonta - ps9
Holozoa - ps10
Filozoa - ps11
Choano./Metazoa - ps12
Metazoa - ps13
Myriazoa - ps14
Parahoxozoa - ps15
Bilateria - ps16
Deuterostomia - ps17
Chordata - ps18
Olfactores - ps19
Vertebrata - ps20
Gnathostomata - ps21
Euteleostomi - ps22
Sarcopterygii - ps23
Tetrapoda - ps24
Amniota - ps25
Mammalia - ps26
Theria - ps27
Placentalia - ps28
Boreoeutheria - ps29
Euarchontoglires - ps30
Euarchonta - ps31
Primates - ps32
Haplorrhini - ps33
Simiiformes - ps34
Catarrhini - ps35
Hominoidea - ps36
Hominidae - ps37
Homininae - ps38
Hominini - ps39
Homo_sapiens - ps40
p-value
2e-03
4e-03
9e-03
2e-02
5e-02
Log-odds
0.5
1.0
1.5
2.0
2.5
3.0
3.5
4.0

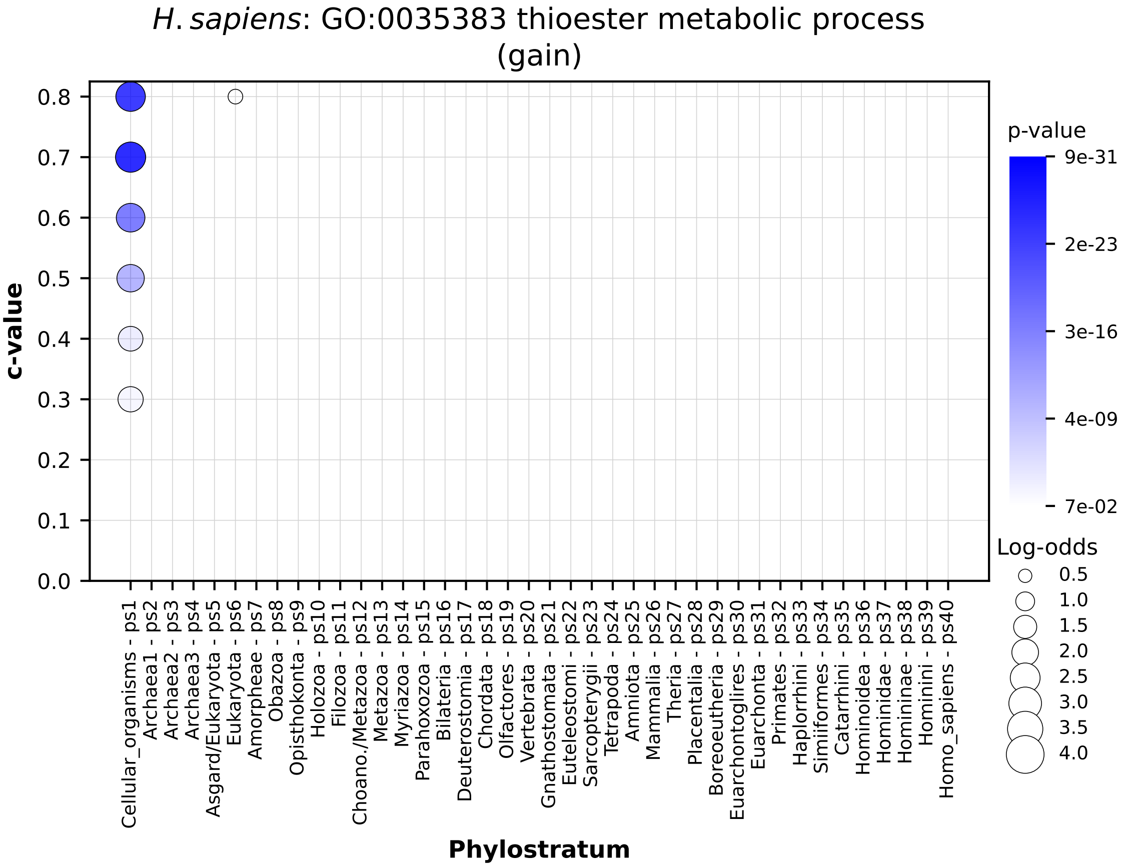

H. sapiens: GO:0035383 thioester metabolic process
(gain)
c-value
0.0
0.1
0.2
0.3
0.4
0.5
0.6
0.7
0.8
Phylostratum
Cellular_organisms - ps1
Archaea1 - ps2
Archaea2 - ps3
Archaea3 - ps4
Asgard/Eukaryota - ps5
Eukaryota - ps6
Amorpheae - ps7
Obazoa - ps8
Opisthokonta - ps9
Holozoa - ps10
Filozoa - ps11
Choano./Metazoa - ps12
Metazoa - ps13
Myriazoa - ps14
Parahoxozoa - ps15
Bilateria - ps16
Deuterostomia - ps17
Chordata - ps18
Olfactores - ps19
Vertebrata - ps20
Gnathostomata - ps21
Euteleostomi - ps22
Sarcopterygii - ps23
Tetrapoda - ps24
Amniota - ps25
Mammalia - ps26
Theria - ps27
Placentalia - ps28
Boreoeutheria - ps29
Euarchontoglires - ps30
Euarchonta - ps31
Primates - ps32
Haplorrhini - ps33
Simiiformes - ps34
Catarrhini - ps35
Hominoidea - ps36
Hominidae - ps37
Homininae - ps38
Hominini - ps39
Homo_sapiens - ps40
p-value
9e-31
2e-23
3e-16
4e-09
7e-02
Log-odds
0.5
1.0
1.5
2.0
2.5
3.0
3.5
4.0

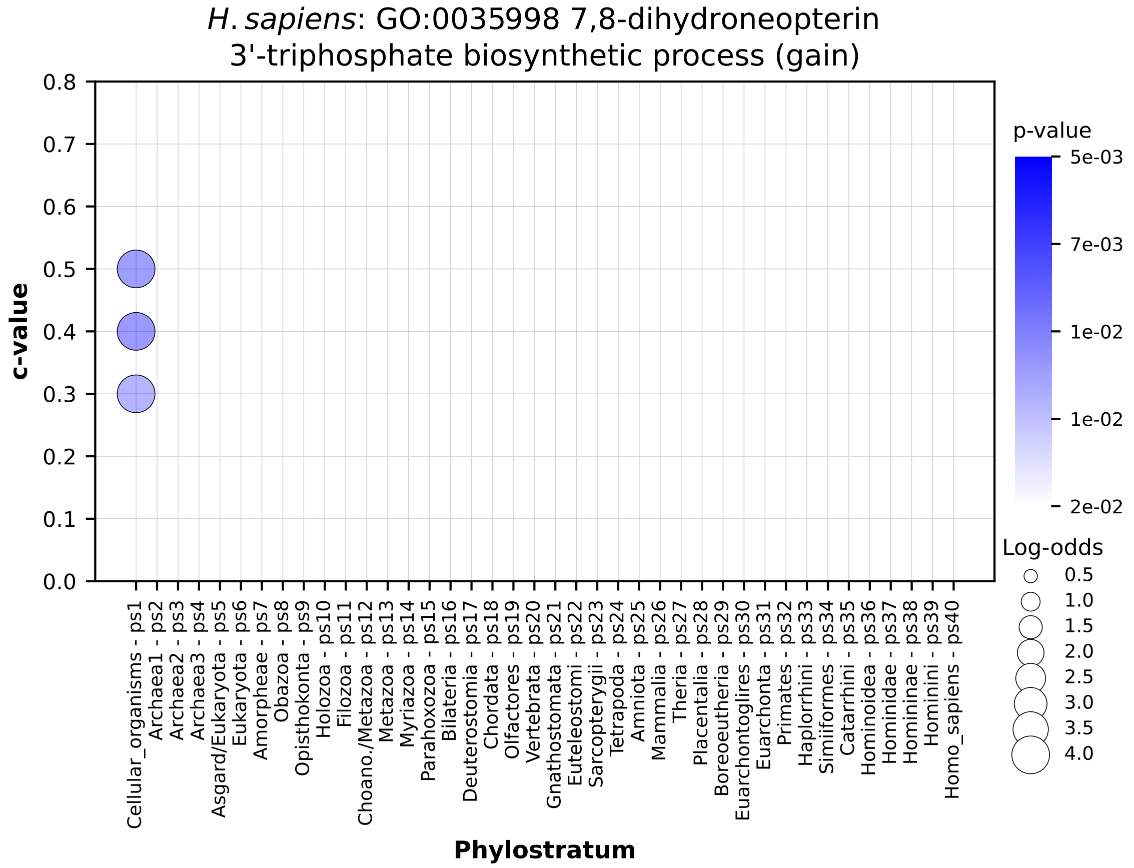

*H. sapiens*: GO:0035998 7,8-dihydroneopterin 3'-triphosphate biosynthetic process (gain)
c-value
0.8
0.7
0.6
0.5
0.4
0.3
0.2
0.1
0.0
Phylostratum
Cellular_organisms - ps1
Archaea1 - ps2
Archaea2 - ps3
Archaea3 - ps4
Asgard/Eukaryota - ps5
Eukaryota - ps6
Amorpheae - ps7
Obazoa - ps8
Opisthokonta - ps9
Holozoa - ps10
Filozoa - ps11
Choano./Metazoa - ps12
Metazoa - ps13
Myriazoa - ps14
Parahoxozoa - ps15
Bilateria - ps16
Deuterostomia - ps17
Chordata - ps18
Olfactores - ps19
Vertebrata - ps20
Gnathostomata - ps21
Euteleostomi - ps22
Sarcopterygii - ps23
Tetrapoda - ps24
Amniota - ps25
Mammalia - ps26
Theria - ps27
Placentalia - ps28
Boreoeutheria - ps29
Euarchontoglires - ps30
Euarchonta - ps31
Primates - ps32
Haplorrhini - ps33
Simiiformes - ps34
Catarrhini - ps35
Hominoidea - ps36
Hominidae - ps37
Homininae - ps38
Hominini - ps39
Homo_sapiens - ps40
p-value
5e-03
7e-03
1e-02
1e-02
2e-02
Log-odds
0.5
1.0
1.5
2.0
2.5
3.0
3.5
4.0

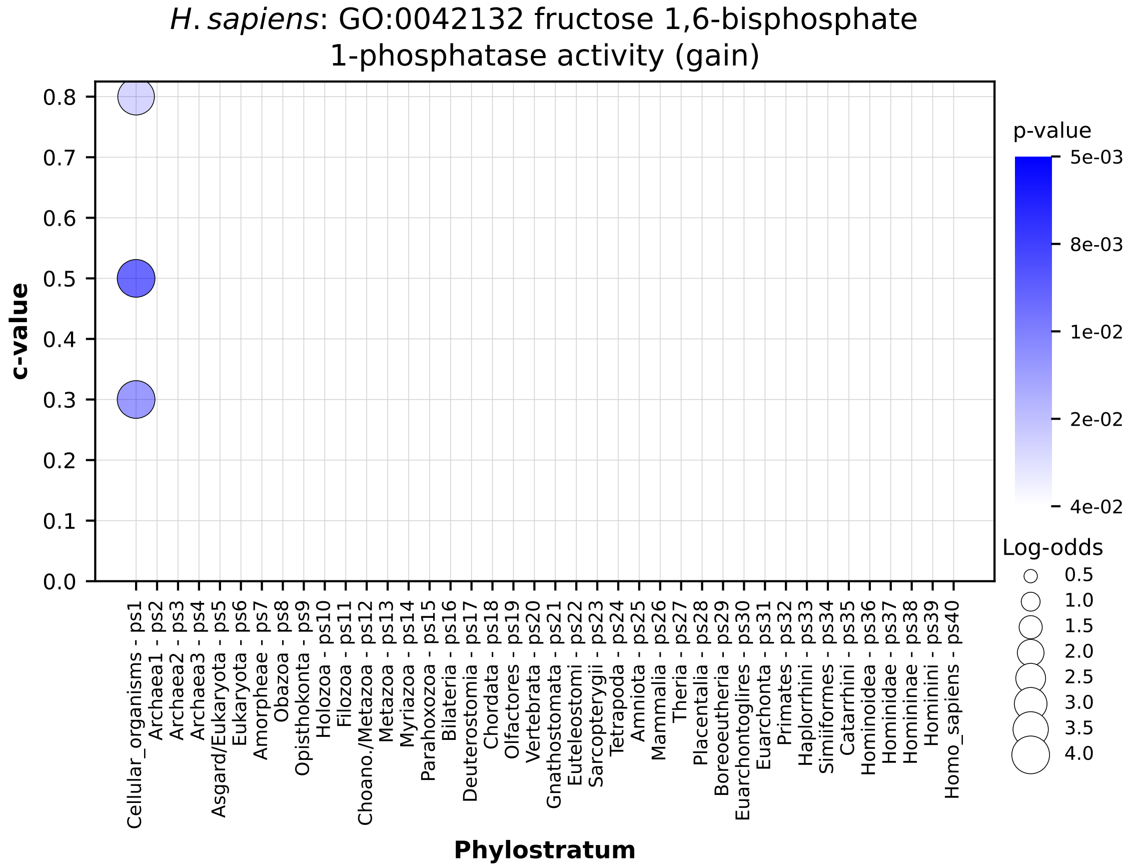

*H. sapiens*: GO:0042132 fructose 1,6-bisphosphate
1-phosphatase activity (gain)
c-value
0.0
0.1
0.2
0.3
0.4
0.5
0.6
0.7
0.8
Phylostratum
Cellular_organisms - ps1
Archaea1 - ps2
Archaea2 - ps3
Archaea3 - ps4
Asgard/Eukaryota - ps5
Eukaryota - ps6
Amorpheae - ps7
Obazoa - ps8
Opisthokonta - ps9
Holozoa - ps10
Filozoa - ps11
Choano./Metazoa - ps12
Metazoa - ps13
Myriazoa - ps14
Parahoxozoa - ps15
Bilateria - ps16
Deuterostomia - ps17
Chordata - ps18
Olfactores - ps19
Vertebrata - ps20
Gnathostomata - ps21
Euteleostomi - ps22
Sarcopterygii - ps23
Tetrapoda - ps24
Amniota - ps25
Mammalia - ps26
Theria - ps27
Placentalia - ps28
Boreoeutheria - ps29
Euarchontoglires - ps30
Euarchonta - ps31
Primates - ps32
Haplorrhini - ps33
Simiiformes - ps34
Catarrhini - ps35
Hominoidea - ps36
Hominidae - ps37
Homininae - ps38
Hominini - ps39
Homo_sapiens - ps40
p-value
5e-03
8e-03
1e-02
2e-02
4e-02
Log-odds
0.5
1.0
1.5
2.0
2.5
3.0
3.5
4.0

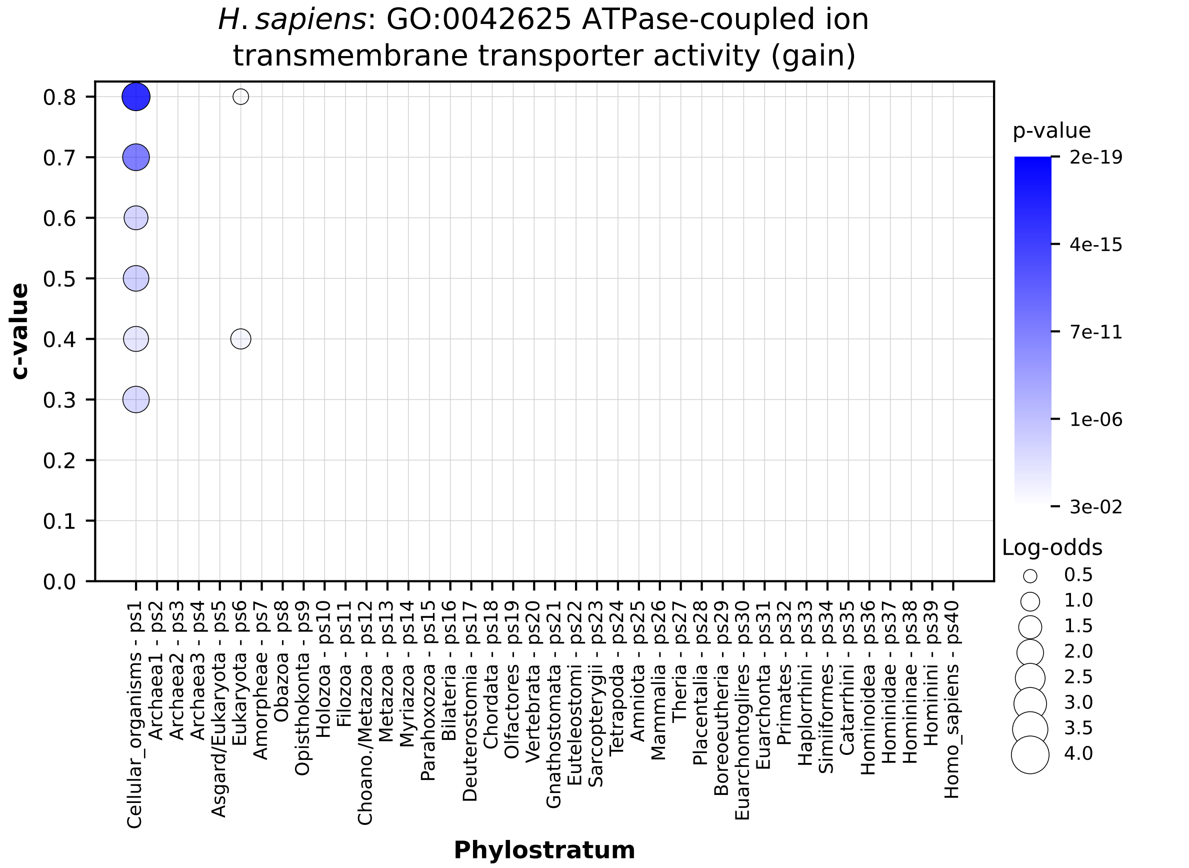

H. sapiens: GO:0042625 ATPase-coupled ion transmembrane transporter activity (gain)
c-value
0.8
0.7
0.6
0.5
0.4
0.3
0.2
0.1
0.0
p-value
2e-19
4e-15
7e-11
1e-06
3e-02
Log-odds
0.5
1.0
1.5
2.0
2.5
3.0
3.5
4.0
Cellular_organisms - ps1
Archaea1 - ps2
Archaea2 - ps3
Archaea3 - ps4
Asgard/Eukaryota - ps5
Eukaryota - ps6
Amorpheae - ps7
Obazoa - ps8
Opisthokonta - ps9
Holozoa - ps10
Filozoa - ps11
Choano./Metazoa - ps12
Metazoa - ps13
Myriazoa - ps14
Parahoxozoa - ps15
Bilateria - ps16
Deuterostomia - ps17
Chordata - ps18
Olfactores - ps19
Vertebrata - ps20
Gnathostomata - ps21
Euteleostomi - ps22
Sarcopterygii - ps23
Tetrapoda - ps24
Amniota - ps25
Mammalia - ps26
Theria - ps27
Placentalia - ps28
Boreoeutheria - ps29
Euarchontoglires - ps30
Euarchonta - ps31
Primates - ps32
Haplorrhini - ps33
Simiiformes - ps34
Catarrhini - ps35
Hominoidea - ps36
Hominidae - ps37
Homininae - ps38
Hominini - ps39
Homo_sapiens - ps40
Phylostratum

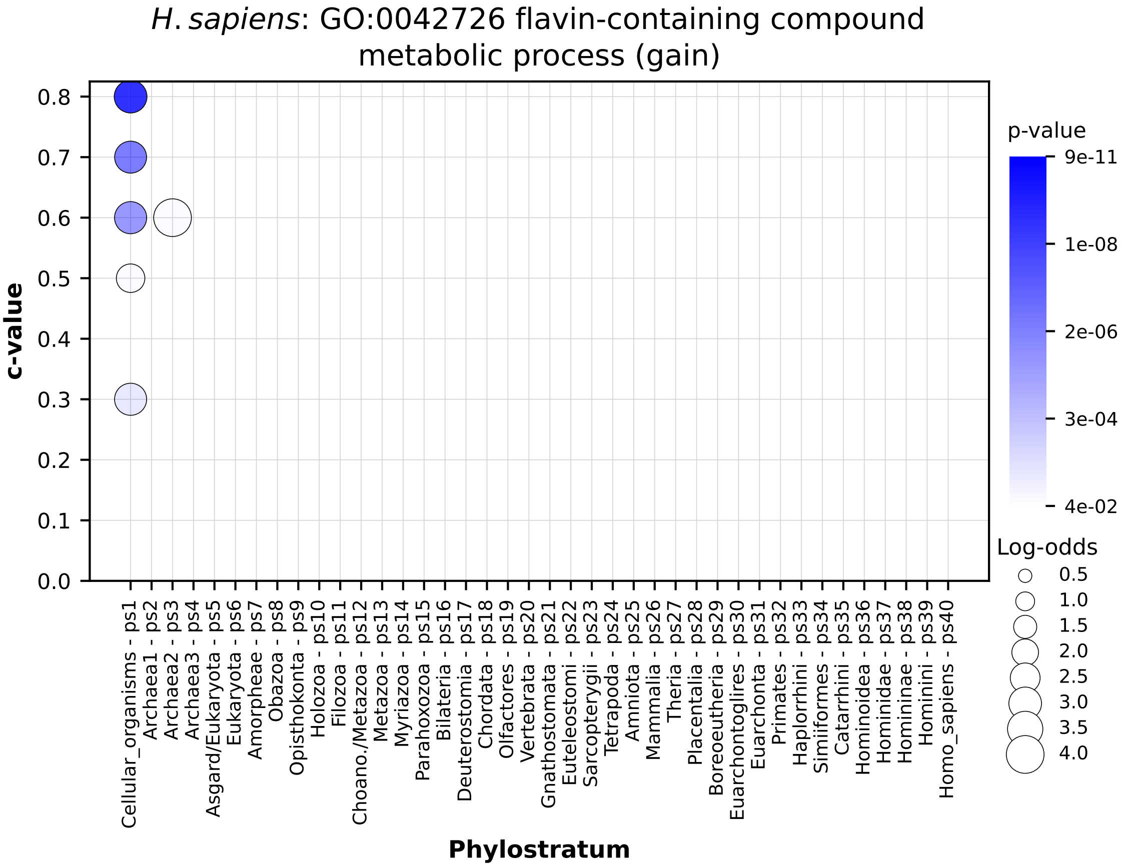

H. sapiens: GO:0042726 flavin-containing compound metabolic process (gain)
c-value
0.8
0.7
0.6
0.5
0.4
0.3
0.2
0.1
0.0
Phylostratum
Cellular_organisms - ps1
Archaea1 - ps2
Archaea2 - ps3
Archaea3 - ps4
Asgard/Eukaryota - ps5
Eukaryota - ps6
Amorpheae - ps7
Obazoa - ps8
Opisthokonta - ps9
Holozoa - ps10
Filozoa - ps11
Choano./Metazoa - ps12
Metazoa - ps13
Myriazoa - ps14
Parahoxozoa - ps15
Bilateria - ps16
Deuterostomia - ps17
Chordata - ps18
Olfactores - ps19
Vertebrata - ps20
Gnathostomata - ps21
Euteleostomi - ps22
Sarcopterygii - ps23
Tetrapoda - ps24
Amniota - ps25
Mammalia - ps26
Theria - ps27
Placentalia - ps28
Boreoeutheria - ps29
Euarchontoglires - ps30
Euarchonta - ps31
Primates - ps32
Haplorrhini - ps33
Simiiformes - ps34
Catarrhini - ps35
Hominoidea - ps36
Hominidae - ps37
Homininae - ps38
Hominini - ps39
Homo_sapiens - ps40
p-value
9e-11
1e-08
2e-06
3e-04
4e-02
Log-odds
0.5
1.0
1.5
2.0
2.5
3.0
3.5
4.0

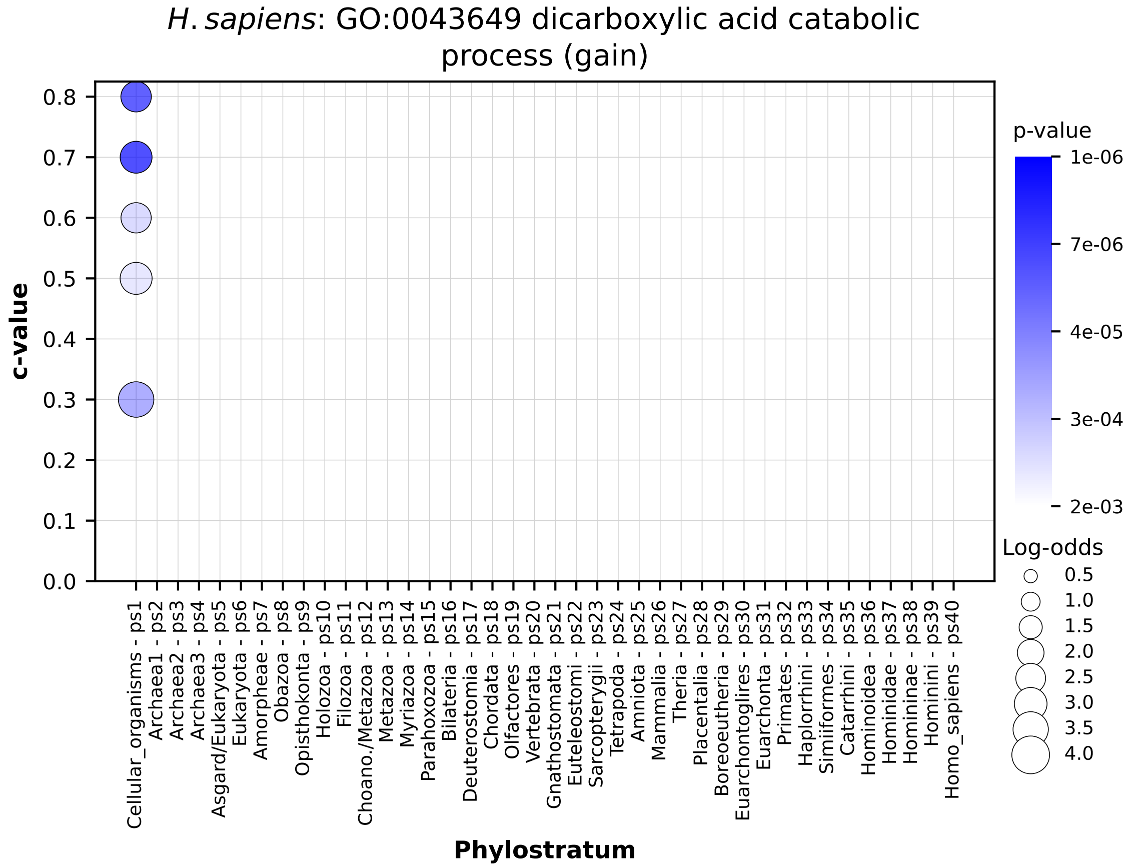

H. sapiens: GO:0043649 dicarboxylic acid catabolic process (gain)
c-value
0.0
0.1
0.2
0.3
0.4
0.5
0.6
0.7
0.8
Phylostratum
Cellular_organisms - ps1
Archaea1 - ps2
Archaea2 - ps3
Archaea3 - ps4
Asgard/Eukaryota - ps5
Eukaryota - ps6
Amorpheae - ps7
Obazoa - ps8
Opisthokonta - ps9
Holozoa - ps10
Filozoa - ps11
Choano./Metazoa - ps12
Metazoa - ps13
Myriazoa - ps14
Parahoxozoa - ps15
Bilateria - ps16
Deuterostomia - ps17
Chordata - ps18
Olfactores - ps19
Vertebrata - ps20
Gnathostomata - ps21
Euteleostomi - ps22
Sarcopterygii - ps23
Tetrapoda - ps24
Amniota - ps25
Mammalia - ps26
Theria - ps27
Placentalia - ps28
Boreoeutheria - ps29
Euarchontoglires - ps30
Euarchonta - ps31
Primates - ps32
Haplorrhini - ps33
Simiiformes - ps34
Catarrhini - ps35
Hominoidea - ps36
Hominidae - ps37
Homininae - ps38
Hominini - ps39
Homo_sapiens - ps40
p-value
1e-06
7e-06
4e-05
3e-04
2e-03
Log-odds
0.5
1.0
1.5
2.0
2.5
3.0
3.5
4.0

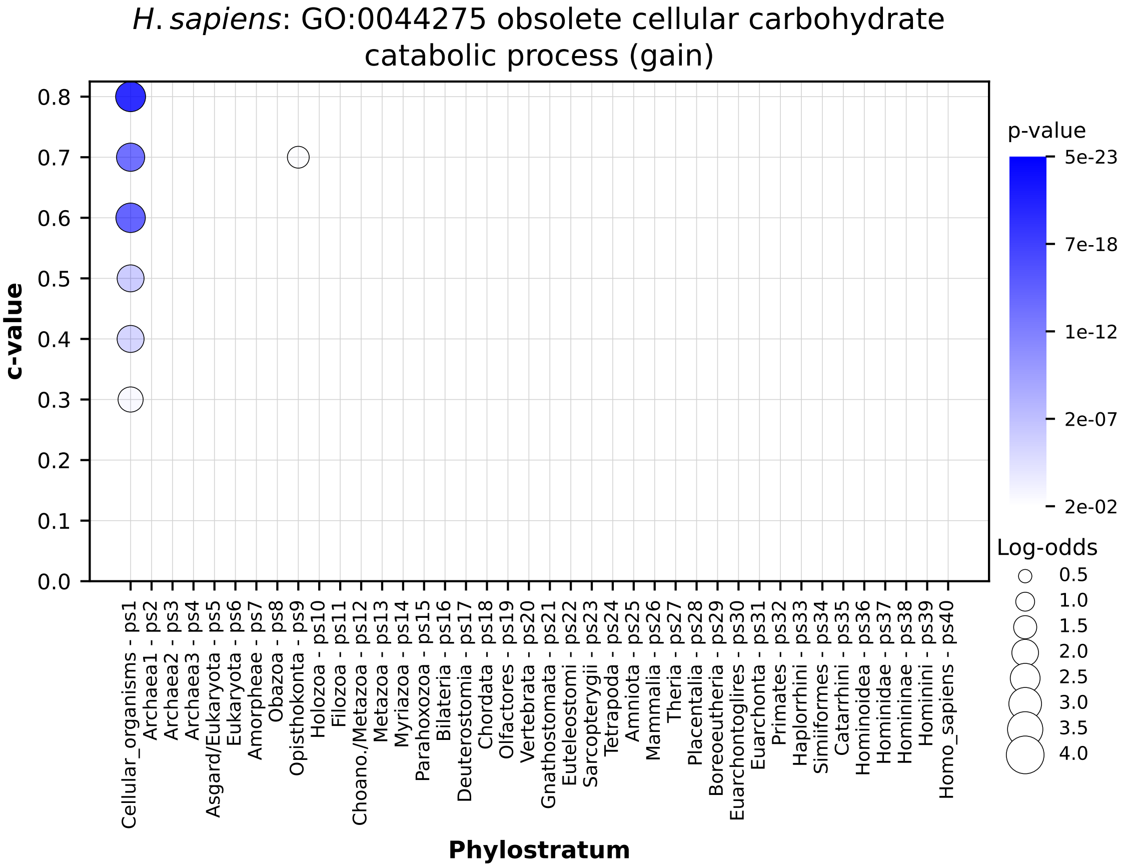

H. sapiens: GO:0044275 obsolete cellular carbohydrate catabolic process (gain)
c-value
0.0
0.1
0.2
0.3
0.4
0.5
0.6
0.7
0.8
Phylostratum
Cellular_organisms - ps1
Archaea1 - ps2
Archaea2 - ps3
Archaea3 - ps4
Asgard/Eukaryota - ps5
Eukaryota - ps6
Amorpheae - ps7
Obazoa - ps8
Opisthokonta - ps9
Holozoa - ps10
Filozoa - ps11
Choano./Metazoa - ps12
Metazoa - ps13
Myriazoa - ps14
Parahoxozoa - ps15
Bilateria - ps16
Deuterostomia - ps17
Chordata - ps18
Olfactores - ps19
Vertebrata - ps20
Gnathostomata - ps21
Euteleostomi - ps22
Sarcopterygii - ps23
Tetrapoda - ps24
Amniota - ps25
Mammalia - ps26
Theria - ps27
Placentalia - ps28
Boreoeutheria - ps29
Euarchontoglires - ps30
Euarchonta - ps31
Primates - ps32
Haplorrhini - ps33
Simiiformes - ps34
Catarrhini - ps35
Hominoidea - ps36
Hominidae - ps37
Homininae - ps38
Hominini - ps39
Homo_sapiens - ps40
p-value
5e-23
7e-18
1e-12
2e-07
2e-02
Log-odds
0.5
1.0
1.5
2.0
2.5
3.0
3.5
4.0

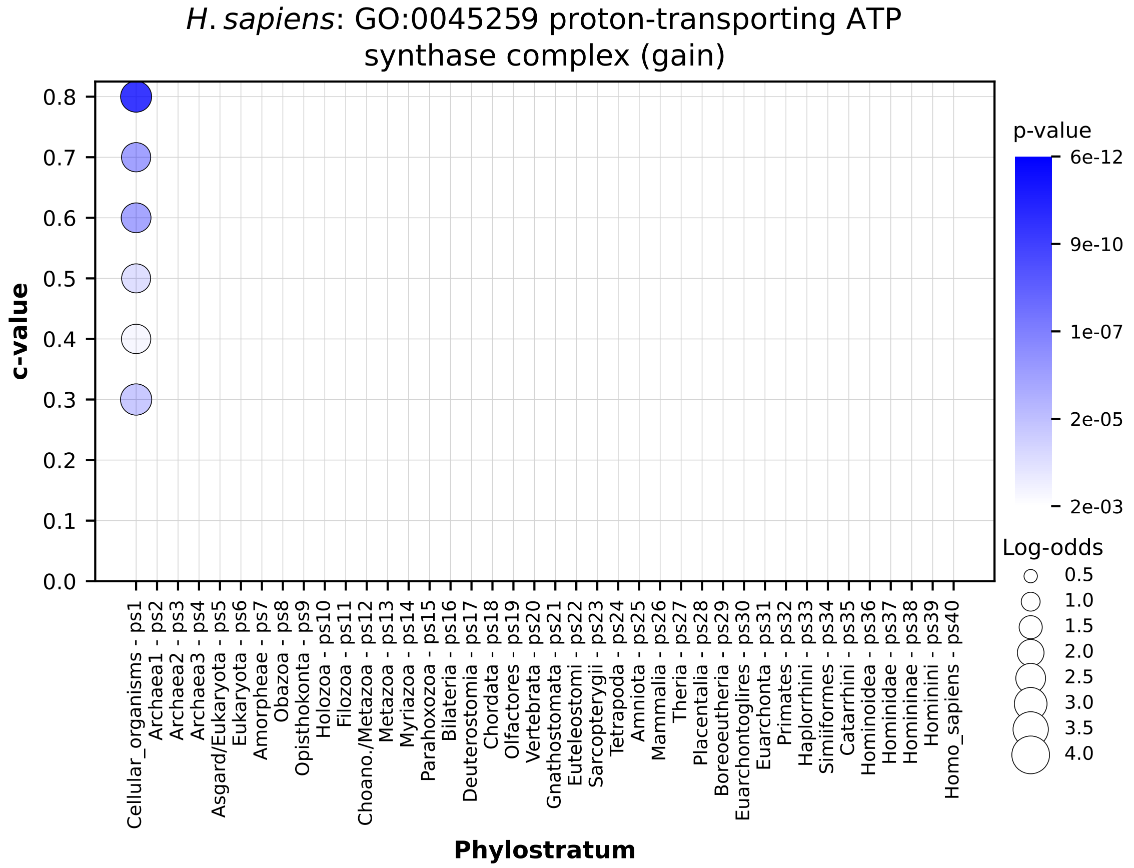
H. sapiens: GO:0045259 proton-transporting ATP synthase complex (gain)
c-value
0.0
0.1
0.2
0.3
0.4
0.5
0.6
0.7
0.8
Phylostratum
Cellular_organisms - ps1
Archaea1 - ps2
Archaea2 - ps3
Archaea3 - ps4
Asgard/Eukaryota - ps5
Eukaryota - ps6
Amorpheae - ps7
Obazoa - ps8
Opisthokonta - ps9
Holozoa - ps10
Filozoa - ps11
Choano./Metazoa - ps12
Metazoa - ps13
Myriazoa - ps14
Parahoxozoa - ps15
Bilateria - ps16
Deuterostomia - ps17
Chordata - ps18
Olfactores - ps19
Vertebrata - ps20
Gnathostomata - ps21
Euteleostomi - ps22
Sarcopterygii - ps23
Tetrapoda - ps24
Amniota - ps25
Mammalia - ps26
Theria - ps27
Placentalia - ps28
Boreoeutheria - ps29
Euarchontoglires - ps30
Euarchonta - ps31
Primates - ps32
Haplorrhini - ps33
Simiiformes - ps34
Catarrhini - ps35
Hominoidea - ps36
Hominidae - ps37
Homininae - ps38
Hominini - ps39
Homo_sapiens - ps40
p-value
6e-12
9e-10
1e-07
2e-05
2e-03
Log-odds
0.5
1.0
1.5
2.0
2.5
3.0
3.5
4.0

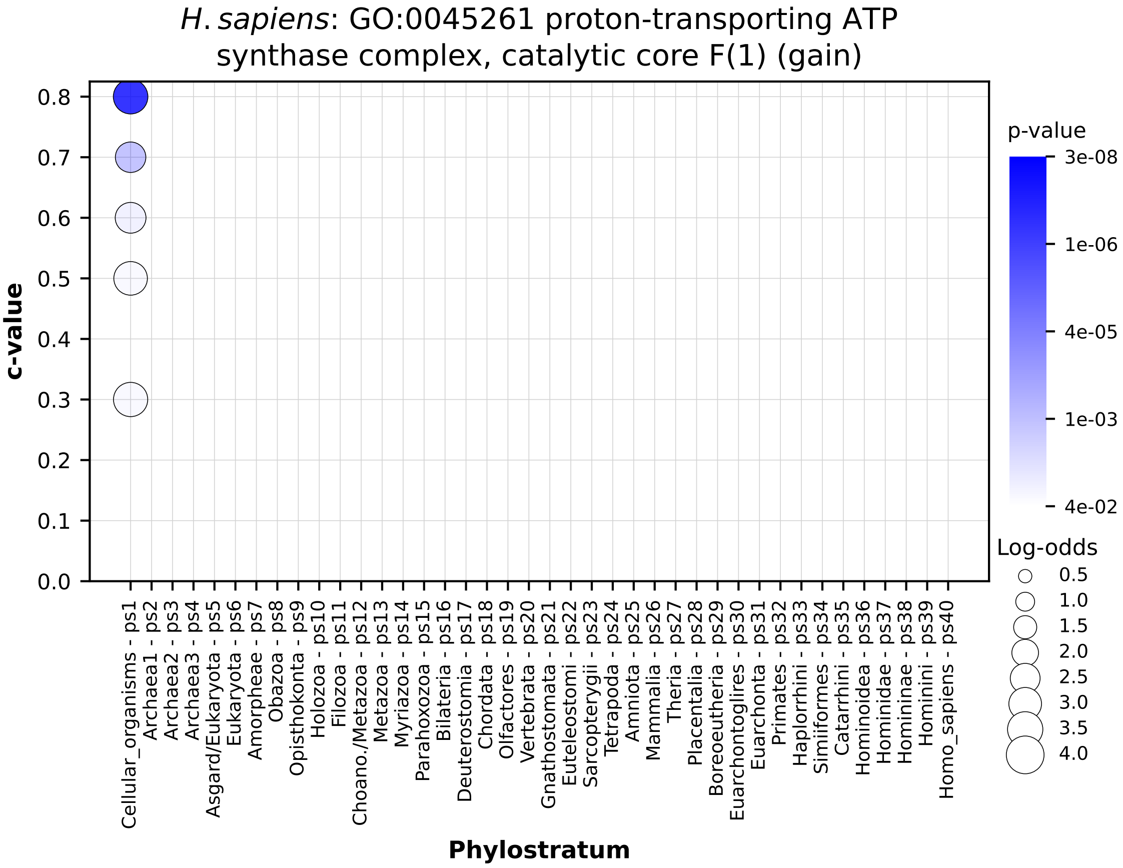

H. sapiens: GO:0045261 proton-transporting ATP synthase complex, catalytic core F(1) (gain)
c-value
0.0
0.1
0.2
0.3
0.4
0.5
0.6
0.7
0.8
Phylostratum
Cellular_organisms - ps1
Archaea1 - ps2
Archaea2 - ps3
Archaea3 - ps4
Asgard/Eukaryota - ps5
Eukaryota - ps6
Amorpheae - ps7
Obazoa - ps8
Opisthokonta - ps9
Holozoa - ps10
Filozoa - ps11
Choano./Metazoa - ps12
Metazoa - ps13
Myriazoa - ps14
Parahoxozoa - ps15
Bilateria - ps16
Deuterostomia - ps17
Chordata - ps18
Olfactores - ps19
Vertebrata - ps20
Gnathostomata - ps21
Euteleostomi - ps22
Sarcopterygii - ps23
Tetrapoda - ps24
Amniota - ps25
Mammalia - ps26
Theria - ps27
Placentalia - ps28
Boreoeutheria - ps29
Euarchontoglires - ps30
Euarchonta - ps31
Primates - ps32
Haplorrhini - ps33
Simiiformes - ps34
Catarrhini - ps35
Hominoidea - ps36
Hominidae - ps37
Homininae - ps38
Hominini - ps39
Homo_sapiens - ps40
p-value
3e-08
1e-06
4e-05
1e-03
4e-02
Log-odds
0.5
1.0
1.5
2.0
2.5
3.0
3.5
4.0

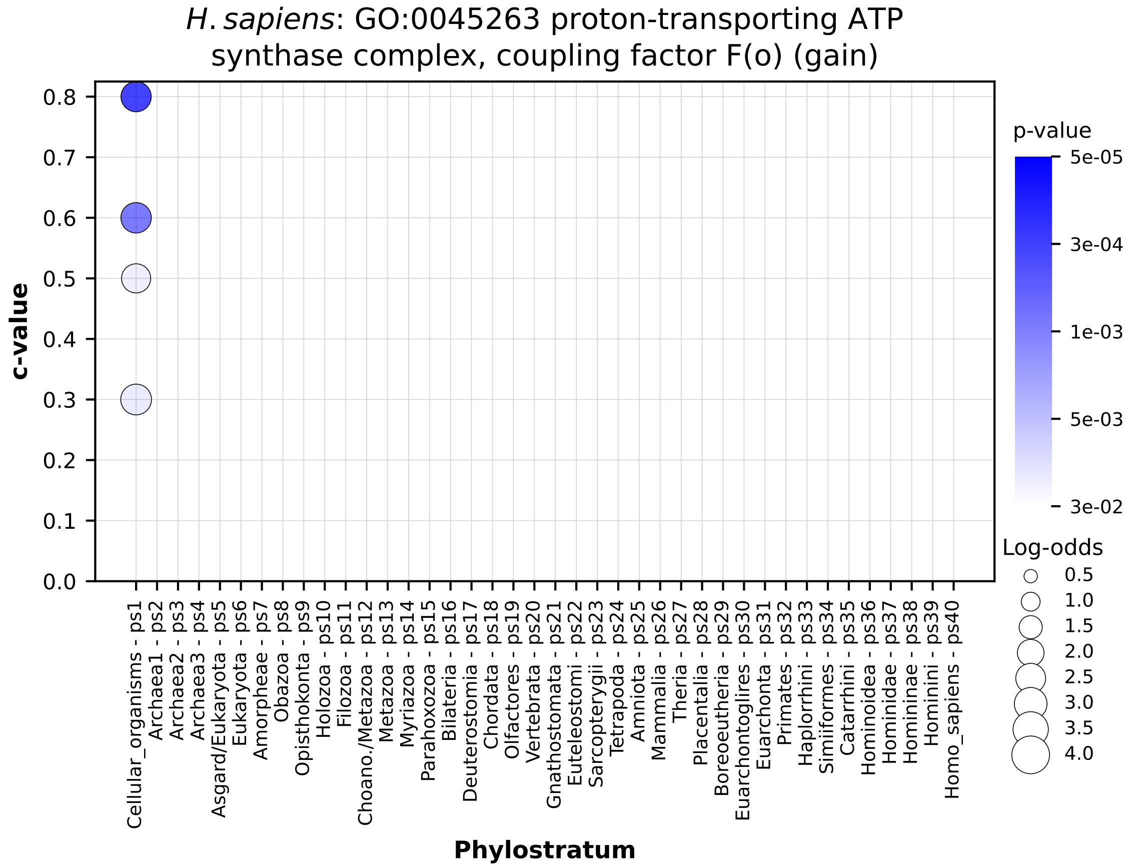

H. sapiens: GO:0045263 proton-transporting ATP synthase complex, coupling factor F(o) (gain)
c-value
0.0
0.1
0.2
0.3
0.4
0.5
0.6
0.7
0.8
Phylostratum
Cellular_organisms - ps1
Archaea1 - ps2
Archaea2 - ps3
Archaea3 - ps4
Asgard/Eukaryota - ps5
Eukaryota - ps6
Amorpheae - ps7
Obazoa - ps8
Opisthokonta - ps9
Holozoa - ps10
Filozoa - ps11
Choano./Metazoa - ps12
Metazoa - ps13
Myriazoa - ps14
Parahoxozoa - ps15
Bilateria - ps16
Deuterostomia - ps17
Chordata - ps18
Olfactores - ps19
Vertebrata - ps20
Gnathostomata - ps21
Euteleostomi - ps22
Sarcopterygii - ps23
Tetrapoda - ps24
Amniota - ps25
Mammalia - ps26
Theria - ps27
Placentalia - ps28
Boreoeutheria - ps29
Euarchontoglires - ps30
Euarchonta - ps31
Primates - ps32
Haplorrhini - ps33
Simiiformes - ps34
Catarrhini - ps35
Hominoidea - ps36
Hominidae - ps37
Homininae - ps38
Hominini - ps39
Homo_sapiens - ps40
p-value
5e-05
3e-04
1e-03
5e-03
3e-02
Log-odds
0.5
1.0
1.5
2.0
2.5
3.0
3.5
4.0

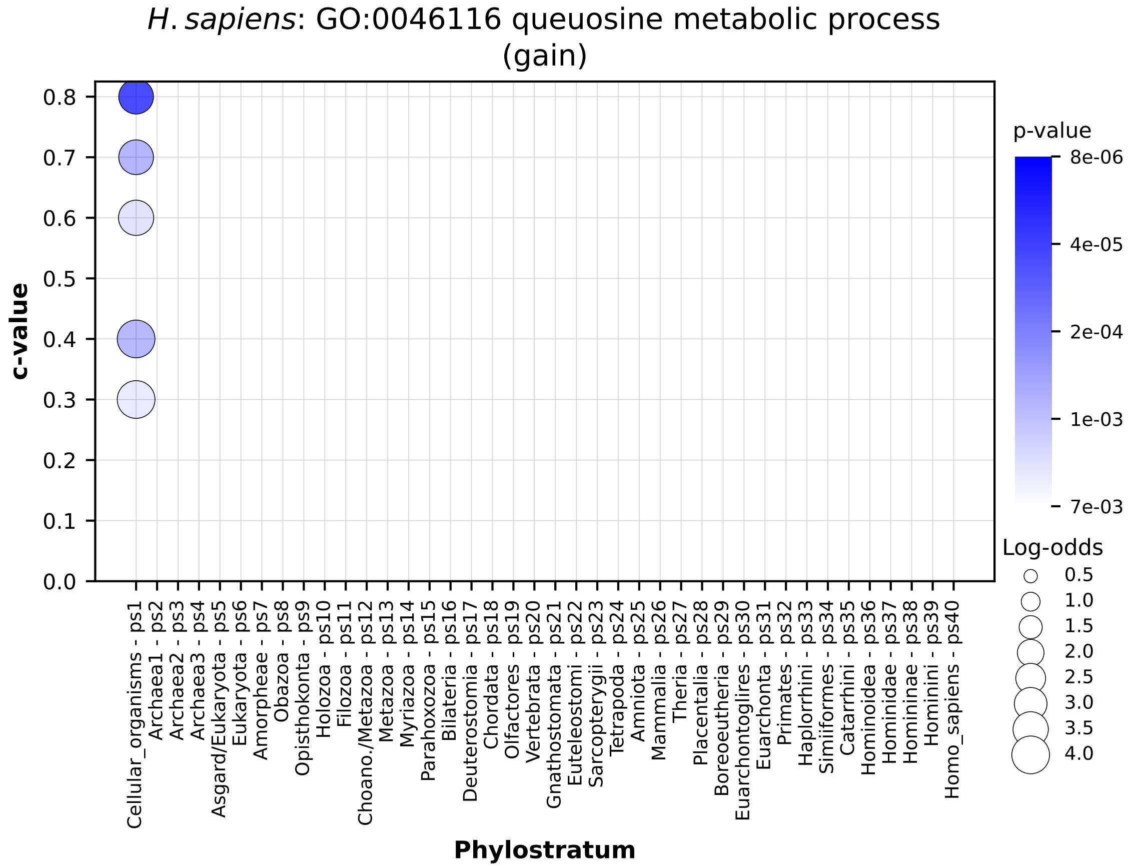

H. sapiens: GO:0046116 queuosine metabolic process
(gain)
c-value
0.0
0.1
0.2
0.3
0.4
0.5
0.6
0.7
0.8
Phylostratum
Cellular_organisms - ps1
Archaea1 - ps2
Archaea2 - ps3
Archaea3 - ps4
Asgard/Eukaryota - ps5
Eukaryota - ps6
Amorpheae - ps7
Obazoa - ps8
Opisthokonta - ps9
Holozoa - ps10
Filozoa - ps11
Choano./Metazoa - ps12
Metazoa - ps13
Myriazoa - ps14
Parahoxozoa - ps15
Bilateria - ps16
Deuterostomia - ps17
Chordata - ps18
Olfactores - ps19
Vertebrata - ps20
Gnathostomata - ps21
Euteleostomi - ps22
Sarcopterygii - ps23
Tetrapoda - ps24
Amniota - ps25
Mammalia - ps26
Theria - ps27
Placentalia - ps28
Boreoeutheria - ps29
Euarchontoglires - ps30
Euarchonta - ps31
Primates - ps32
Haplorrhini - ps33
Simiiformes - ps34
Catarrhini - ps35
Hominoidea - ps36
Hominidae - ps37
Homininae - ps38
Hominini - ps39
Homo_sapiens - ps40
p-value
8e-06
4e-05
2e-04
1e-03
7e-03
Log-odds
0.5
1.0
1.5
2.0
2.5
3.0
3.5
4.0

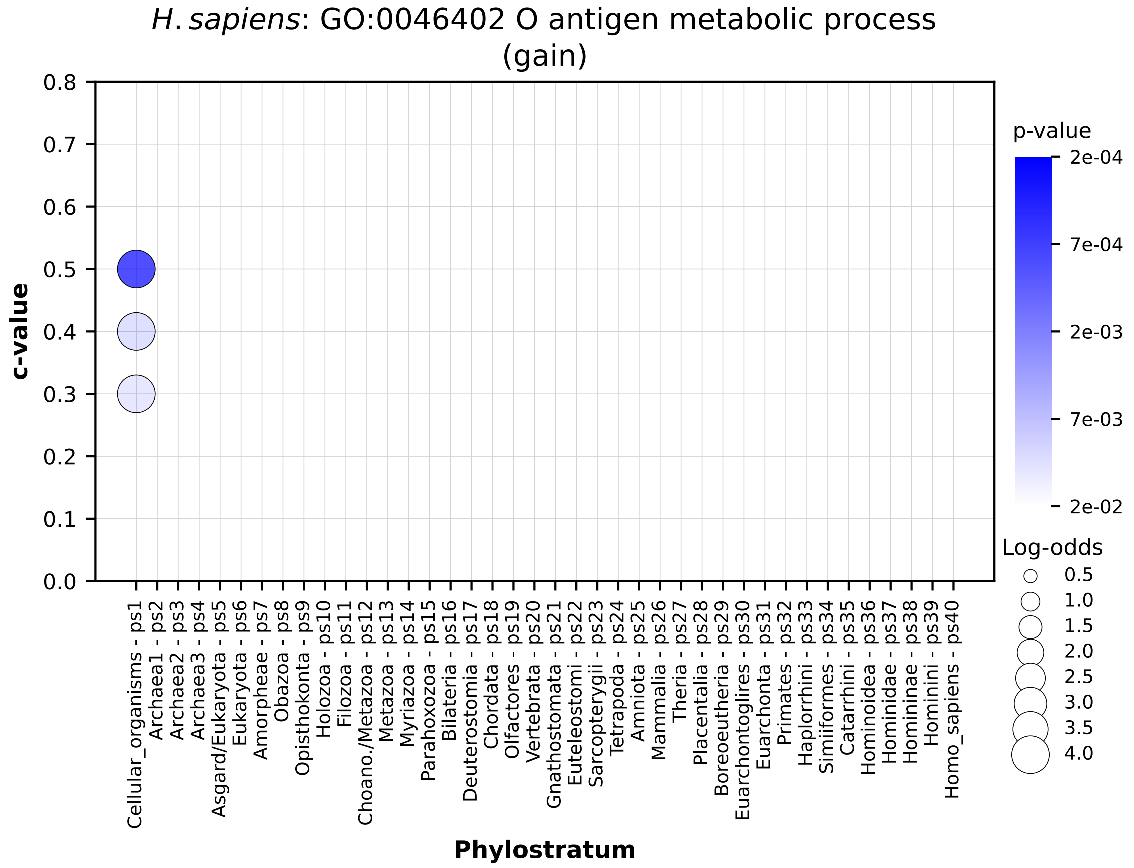
H. sapiens: GO:0046402 O antigen metabolic process
(gain)
c-value
0.0
0.1
0.2
0.3
0.4
0.5
0.6
0.7
0.8
Phylostratum
Cellular_organisms - ps1
Archaea1 - ps2
Archaea2 - ps3
Archaea3 - ps4
Asgard/Eukaryota - ps5
Eukaryota - ps6
Amorpheae - ps7
Obazoa - ps8
Opisthokonta - ps9
Holozoa - ps10
Filozoa - ps11
Choano./Metazoa - ps12
Metazoa - ps13
Myriazoa - ps14
Parahoxozoa - ps15
Bilateria - ps16
Deuterostomia - ps17
Chordata - ps18
Olfactores - ps19
Vertebrata - ps20
Gnathostomata - ps21
Euteleostomi - ps22
Sarcopterygii - ps23
Tetrapoda - ps24
Amniota - ps25
Mammalia - ps26
Theria - ps27
Placentalia - ps28
Boreoeutheria - ps29
Euarchontoglires - ps30
Euarchonta - ps31
Primates - ps32
Haplorrhini - ps33
Simiiformes - ps34
Catarrhini - ps35
Hominoidea - ps36
Hominidae - ps37
Homininae - ps38
Hominini - ps39
Homo_sapiens - ps40
p-value
2e-04
7e-04
2e-03
7e-03
2e-02
Log-odds
0.5
1.0
1.5
2.0
2.5
3.0
3.5
4.0

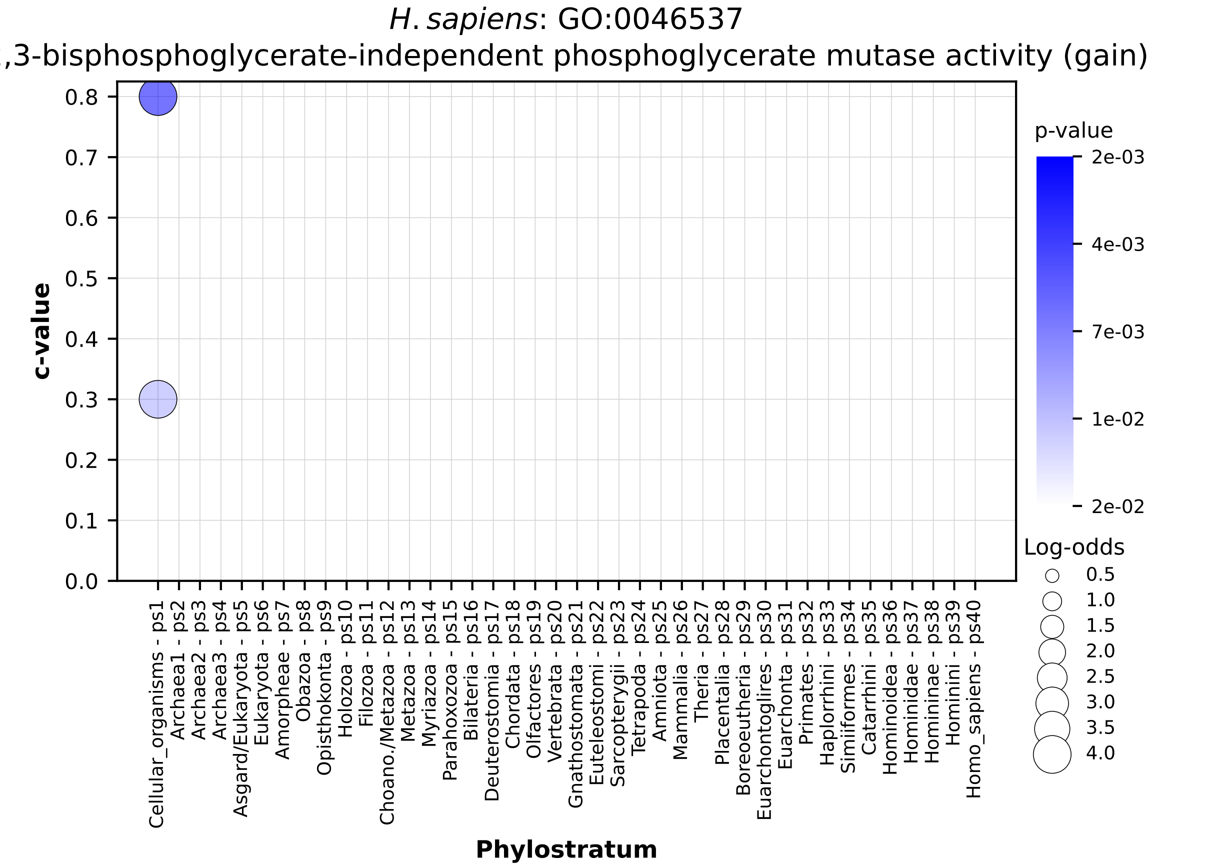

*H. sapiens*: GO:0046537
[illegible],3-bisphosphoglycerate-independent phosphoglycerate mutase activity (gain)
c-value
0.8
0.7
0.6
0.5
0.4
0.3
0.2
0.1
0.0
p-value
2e-03
4e-03
7e-03
1e-02
2e-02
Log-odds
0.5
1.0
1.5
2.0
2.5
3.0
3.5
4.0
Cellular_organisms - ps1
Archaea1 - ps2
Archaea2 - ps3
Archaea3 - ps4
Asgard/Eukaryota - ps5
Eukaryota - ps6
Amorpheae - ps7
Obazoa - ps8
Opisthokonta - ps9
Holozoa - ps10
Filozoa - ps11
Choano./Metazoa - ps12
Metazoa - ps13
Myriazoa - ps14
Parahoxozoa - ps15
Bilateria - ps16
Deuterostomia - ps17
Chordata - ps18
Olfactores - ps19
Vertebrata - ps20
Gnathostomata - ps21
Euteleostomi - ps22
Sarcopterygii - ps23
Tetrapoda - ps24
Amniota - ps25
Mammalia - ps26
Theria - ps27
Placentalia - ps28
Boreoeutheria - ps29
Euarchontoglires - ps30
Euarchonta - ps31
Primates - ps32
Haplorrhini - ps33
Simiiformes - ps34
Catarrhini - ps35
Hominoidea - ps36
Hominidae - ps37
Homininae - ps38
Hominini - ps39
Homo_sapiens - ps40
Phylostratum

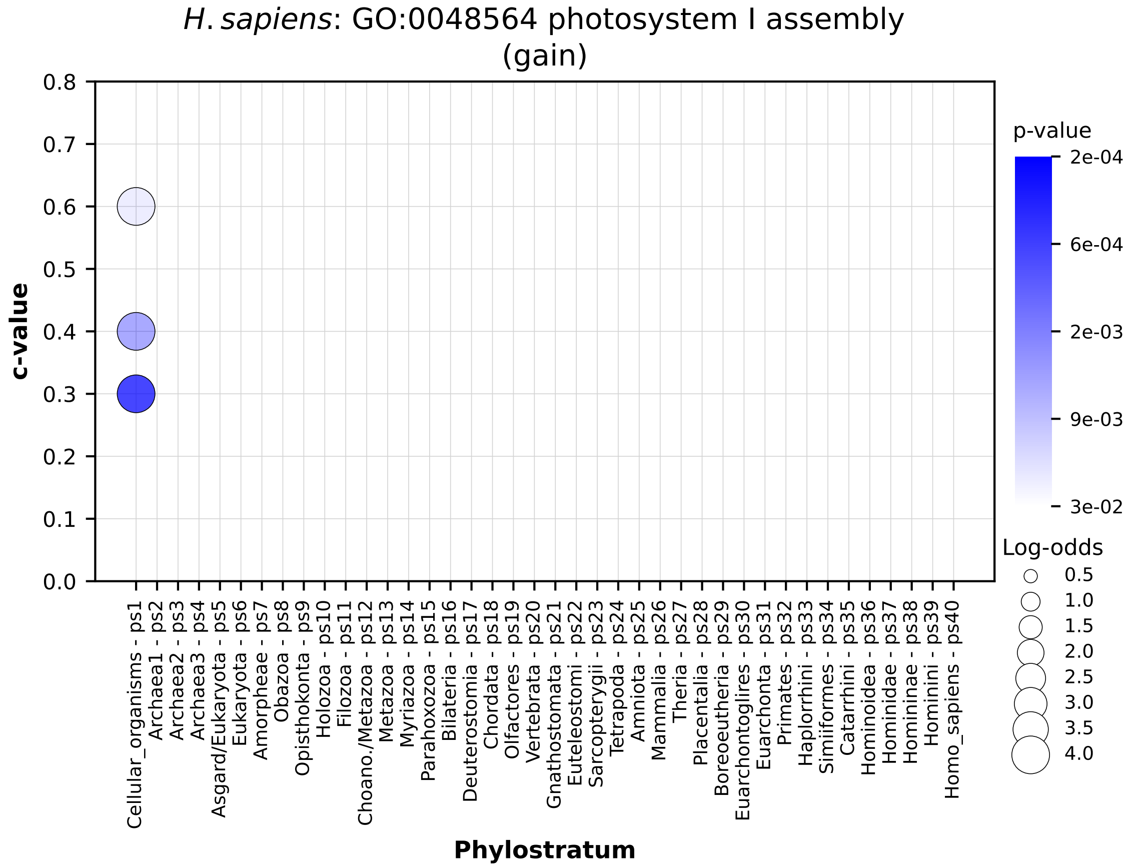

H. sapiens: GO:0048564 photosystem I assembly
(gain)
c-value
0.8
0.7
0.6
0.5
0.4
0.3
0.2
0.1
0.0
p-value
2e-04
6e-04
2e-03
9e-03
3e-02
Log-odds
0.5
1.0
1.5
2.0
2.5
3.0
3.5
4.0
Cellular_organisms - ps1
Archaea1 - ps2
Archaea2 - ps3
Archaea3 - ps4
Asgard/Eukaryota - ps5
Eukaryota - ps6
Amorpheae - ps7
Obazoa - ps8
Opisthokonta - ps9
Holozoa - ps10
Filozoa - ps11
Choano./Metazoa - ps12
Metazoa - ps13
Myriazoa - ps14
Parahoxozoa - ps15
Bilateria - ps16
Deuterostomia - ps17
Chordata - ps18
Olfactores - ps19
Vertebrata - ps20
Gnathostomata - ps21
Euteleostomi - ps22
Sarcopterygii - ps23
Tetrapoda - ps24
Amniota - ps25
Mammalia - ps26
Theria - ps27
Placentalia - ps28
Boreoeutheria - ps29
Euarchontoglires - ps30
Euarchonta - ps31
Primates - ps32
Haplorrhini - ps33
Simiiformes - ps34
Catarrhini - ps35
Hominoidea - ps36
Hominidae - ps37
Homininae - ps38
Hominini - ps39
Homo_sapiens - ps40
Phylostratum

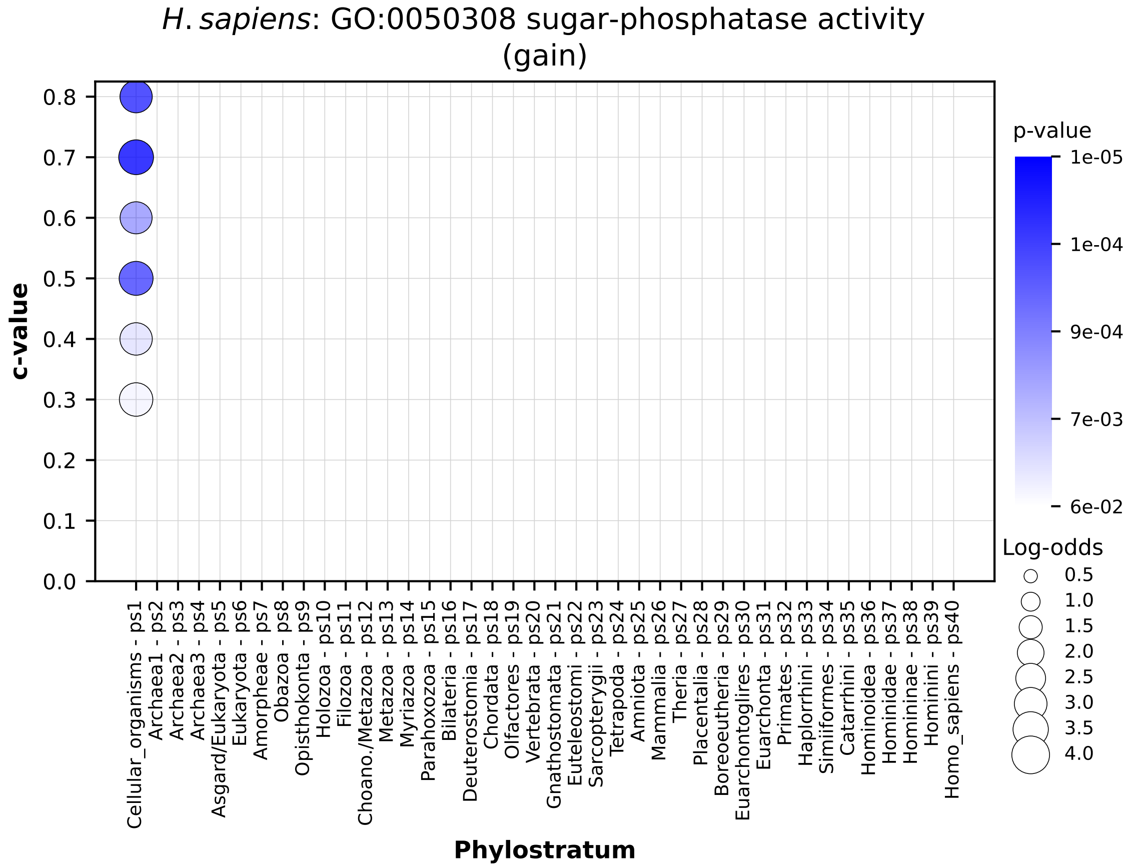

*H. sapiens*: GO:0050308 sugar-phosphatase activity
(gain)
c-value
0.8
0.7
0.6
0.5
0.4
0.3
0.2
0.1
0.0
p-value
1e-05
1e-04
9e-04
7e-03
6e-02
Log-odds
0.5
1.0
1.5
2.0
2.5
3.0
3.5
4.0
Cellular_organisms - ps1
Archaea1 - ps2
Archaea2 - ps3
Archaea3 - ps4
Asgard/Eukaryota - ps5
Eukaryota - ps6
Amorpheae - ps7
Obazoa - ps8
Opisthokonta - ps9
Holozoa - ps10
Filozoa - ps11
Choano./Metazoa - ps12
Metazoa - ps13
Myriazoa - ps14
Parahoxozoa - ps15
Bilateria - ps16
Deuterostomia - ps17
Chordata - ps18
Olfactores - ps19
Vertebrata - ps20
Gnathostomata - ps21
Euteleostomi - ps22
Sarcopterygii - ps23
Tetrapoda - ps24
Amniota - ps25
Mammalia - ps26
Theria - ps27
Placentalia - ps28
Boreoeutheria - ps29
Euarchontoglires - ps30
Euarchonta - ps31
Primates - ps32
Haplorrhini - ps33
Simiiformes - ps34
Catarrhini - ps35
Hominoidea - ps36
Hominidae - ps37
Homininae - ps38
Hominini - ps39
Homo_sapiens - ps40
Phylostratum

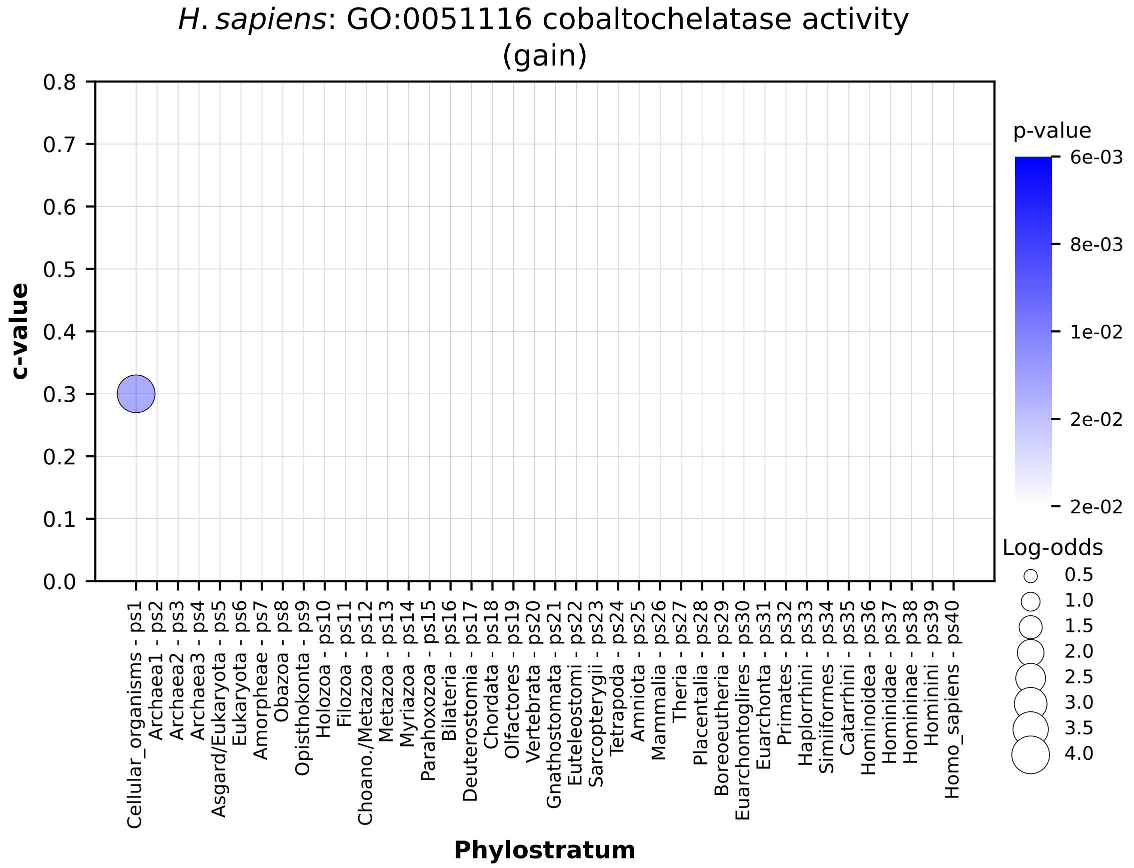

H. sapiens: GO:0051116 cobaltochelatase activity
(gain)
c-value
0.0
0.1
0.2
0.3
0.4
0.5
0.6
0.7
0.8
p-value
6e-03
8e-03
1e-02
2e-02
2e-02
Log-odds
0.5
1.0
1.5
2.0
2.5
3.0
3.5
4.0
Cellular_organisms - ps1
Archaea1 - ps2
Archaea2 - ps3
Archaea3 - ps4
Asgard/Eukaryota - ps5
Eukaryota - ps6
Amorpheae - ps7
Obazoa - ps8
Opisthokonta - ps9
Holozoa - ps10
Filozoa - ps11
Choano./Metazoa - ps12
Metazoa - ps13
Myriazoa - ps14
Parahoxozoa - ps15
Bilateria - ps16
Deuterostomia - ps17
Chordata - ps18
Olfactores - ps19
Vertebrata - ps20
Gnathostomata - ps21
Euteleostomi - ps22
Sarcopterygii - ps23
Tetrapoda - ps24
Amniota - ps25
Mammalia - ps26
Theria - ps27
Placentalia - ps28
Boreoeutheria - ps29
Euarchontoglires - ps30
Euarchonta - ps31
Primates - ps32
Haplorrhini - ps33
Simiiformes - ps34
Catarrhini - ps35
Hominoidea - ps36
Hominidae - ps37
Homininae - ps38
Hominini - ps39
Homo_sapiens - ps40
Phylostratum

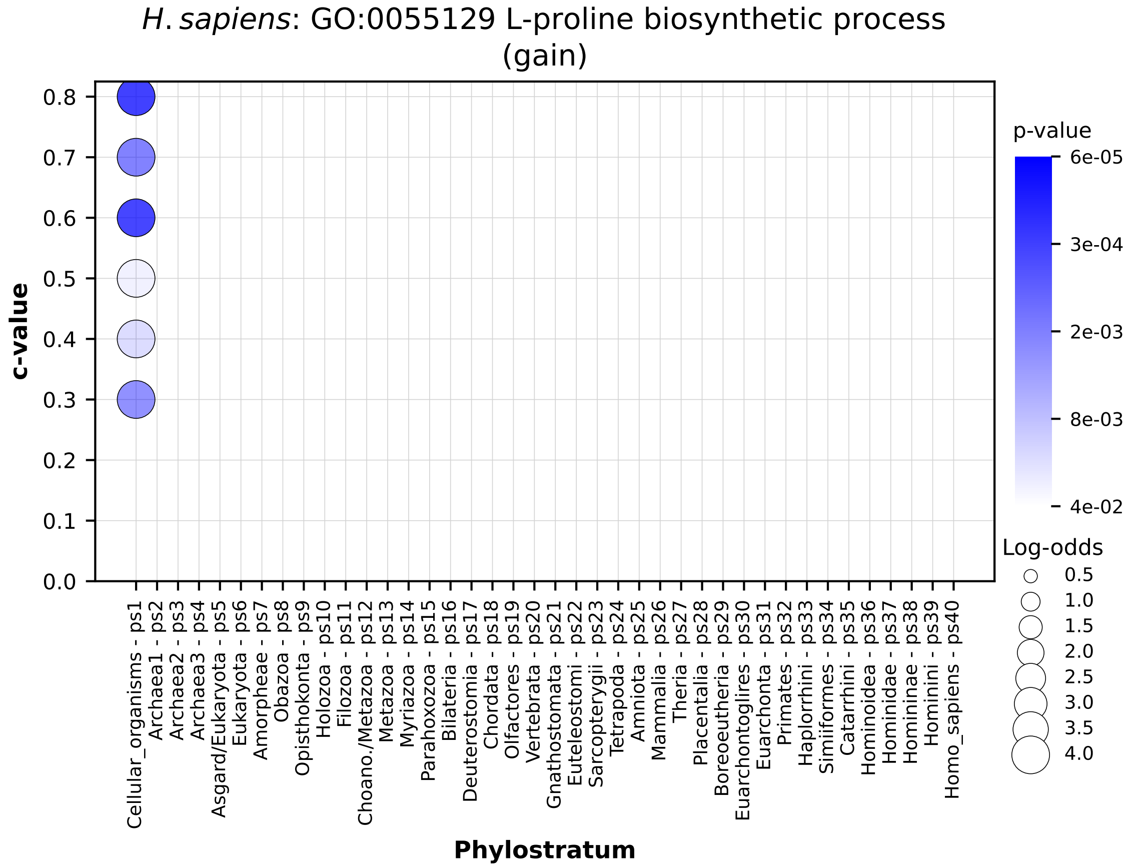

H. sapiens: GO:0055129 L-proline biosynthetic process
(gain)
c-value
0.0
0.1
0.2
0.3
0.4
0.5
0.6
0.7
0.8
Phylostratum
Cellular_organisms - ps1
Archaea1 - ps2
Archaea2 - ps3
Archaea3 - ps4
Asgard/Eukaryota - ps5
Eukaryota - ps6
Amorpheae - ps7
Obazoa - ps8
Opisthokonta - ps9
Holozoa - ps10
Filozoa - ps11
Choano./Metazoa - ps12
Metazoa - ps13
Myriazoa - ps14
Parahoxozoa - ps15
Bilateria - ps16
Deuterostomia - ps17
Chordata - ps18
Olfactores - ps19
Vertebrata - ps20
Gnathostomata - ps21
Euteleostomi - ps22
Sarcopterygii - ps23
Tetrapoda - ps24
Amniota - ps25
Mammalia - ps26
Theria - ps27
Placentalia - ps28
Boreoeutheria - ps29
Euarchontoglires - ps30
Euarchonta - ps31
Primates - ps32
Haplorrhini - ps33
Simiiformes - ps34
Catarrhini - ps35
Hominoidea - ps36
Hominidae - ps37
Homininae - ps38
Hominini - ps39
Homo_sapiens - ps40
p-value
6e-05
3e-04
2e-03
8e-03
4e-02
Log-odds
0.5
1.0
1.5
2.0
2.5
3.0
3.5
4.0

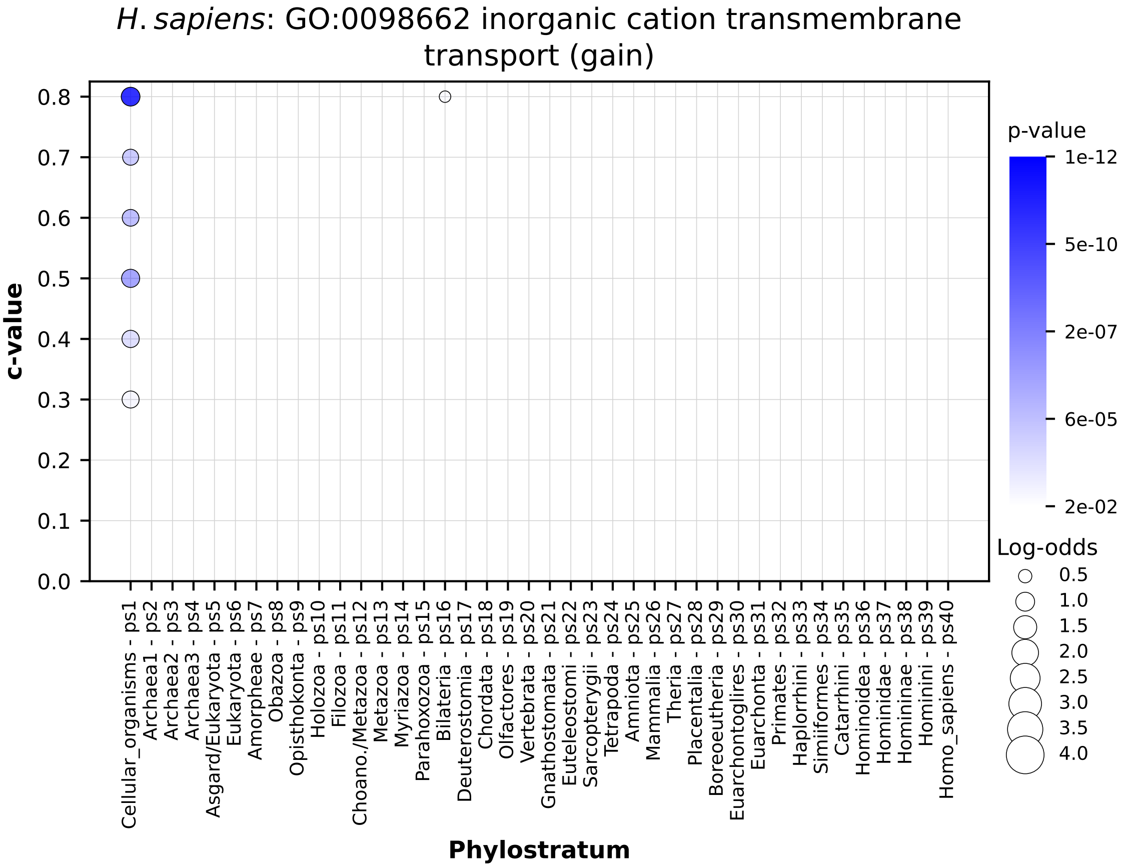
H. sapiens: GO:0098662 inorganic cation transmembrane transport (gain)
c-value
0.0
0.1
0.2
0.3
0.4
0.5
0.6
0.7
0.8
Phylostratum
Cellular_organisms - ps1
Archaea1 - ps2
Archaea2 - ps3
Archaea3 - ps4
Asgard/Eukaryota - ps5
Eukaryota - ps6
Amorpheae - ps7
Obazoa - ps8
Opisthokonta - ps9
Holozoa - ps10
Filozoa - ps11
Choano./Metazoa - ps12
Metazoa - ps13
Myriazoa - ps14
Parahoxozoa - ps15
Bilateria - ps16
Deuterostomia - ps17
Chordata - ps18
Olfactores - ps19
Vertebrata - ps20
Gnathostomata - ps21
Euteleostomi - ps22
Sarcopterygii - ps23
Tetrapoda - ps24
Amniota - ps25
Mammalia - ps26
Theria - ps27
Placentalia - ps28
Boreoeutheria - ps29
Euarchontoglires - ps30
Euarchonta - ps31
Primates - ps32
Haplorrhini - ps33
Simiiformes - ps34
Catarrhini - ps35
Hominoidea - ps36
Hominidae - ps37
Homininae - ps38
Hominini - ps39
Homo_sapiens - ps40
p-value
1e-12
5e-10
2e-07
6e-05
2e-02
Log-odds
0.5
1.0
1.5
2.0
2.5
3.0
3.5
4.0

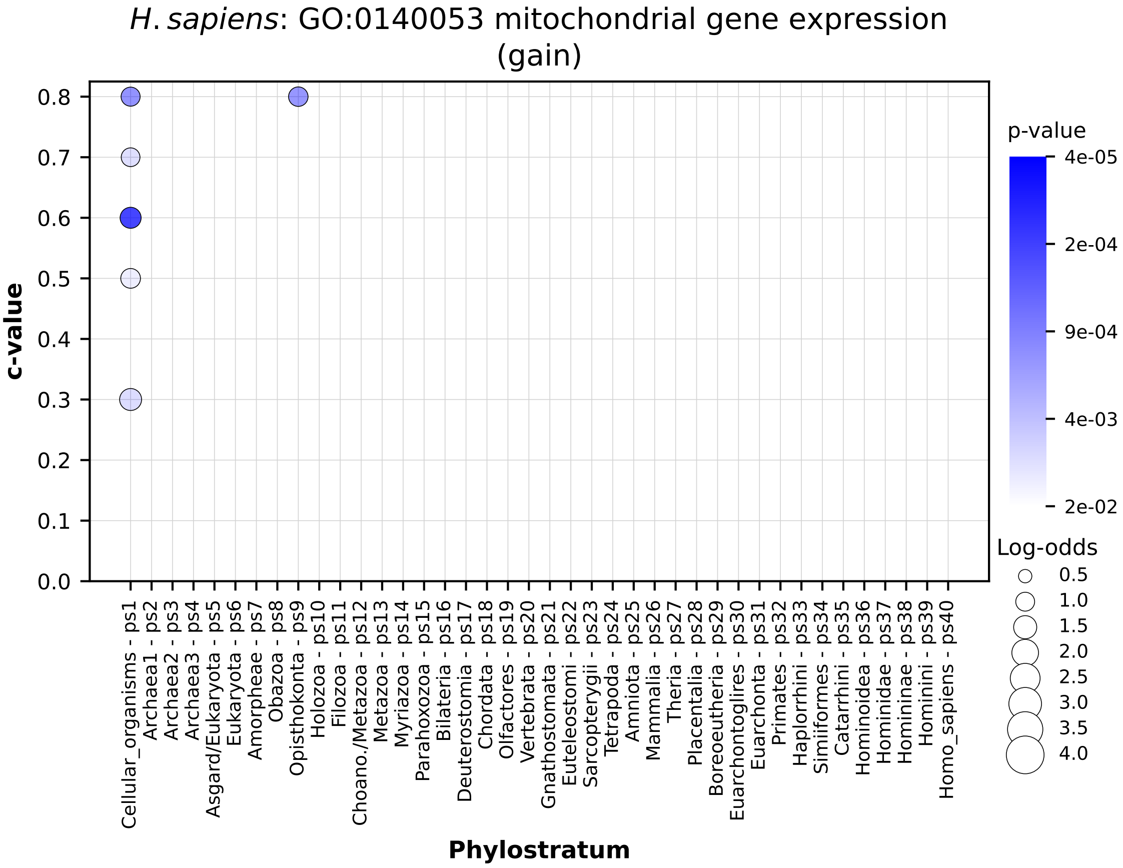

H. sapiens: GO:0140053 mitochondrial gene expression (gain)
c-value
0.0
0.1
0.2
0.3
0.4
0.5
0.6
0.7
0.8
p-value
4e-05
2e-04
9e-04
4e-03
2e-02
Log-odds
0.5
1.0
1.5
2.0
2.5
3.0
3.5
4.0
Cellular_organisms - ps1
Archaea1 - ps2
Archaea2 - ps3
Archaea3 - ps4
Asgard/Eukaryota - ps5
Eukaryota - ps6
Amorpheae - ps7
Obazoa - ps8
Opisthokonta - ps9
Holozoa - ps10
Filozoa - ps11
Choano./Metazoa - ps12
Metazoa - ps13
Myriazoa - ps14
Parahoxozoa - ps15
Bilateria - ps16
Deuterostomia - ps17
Chordata - ps18
Olfactores - ps19
Vertebrata - ps20
Gnathostomata - ps21
Euteleostomi - ps22
Sarcopterygii - ps23
Tetrapoda - ps24
Amniota - ps25
Mammalia - ps26
Theria - ps27
Placentalia - ps28
Boreoeutheria - ps29
Euarchontoglires - ps30
Euarchonta - ps31
Primates - ps32
Haplorrhini - ps33
Simiiformes - ps34
Catarrhini - ps35
Hominoidea - ps36
Hominidae - ps37
Homininae - ps38
Hominini - ps39
Homo_sapiens - ps40
Phylostratum

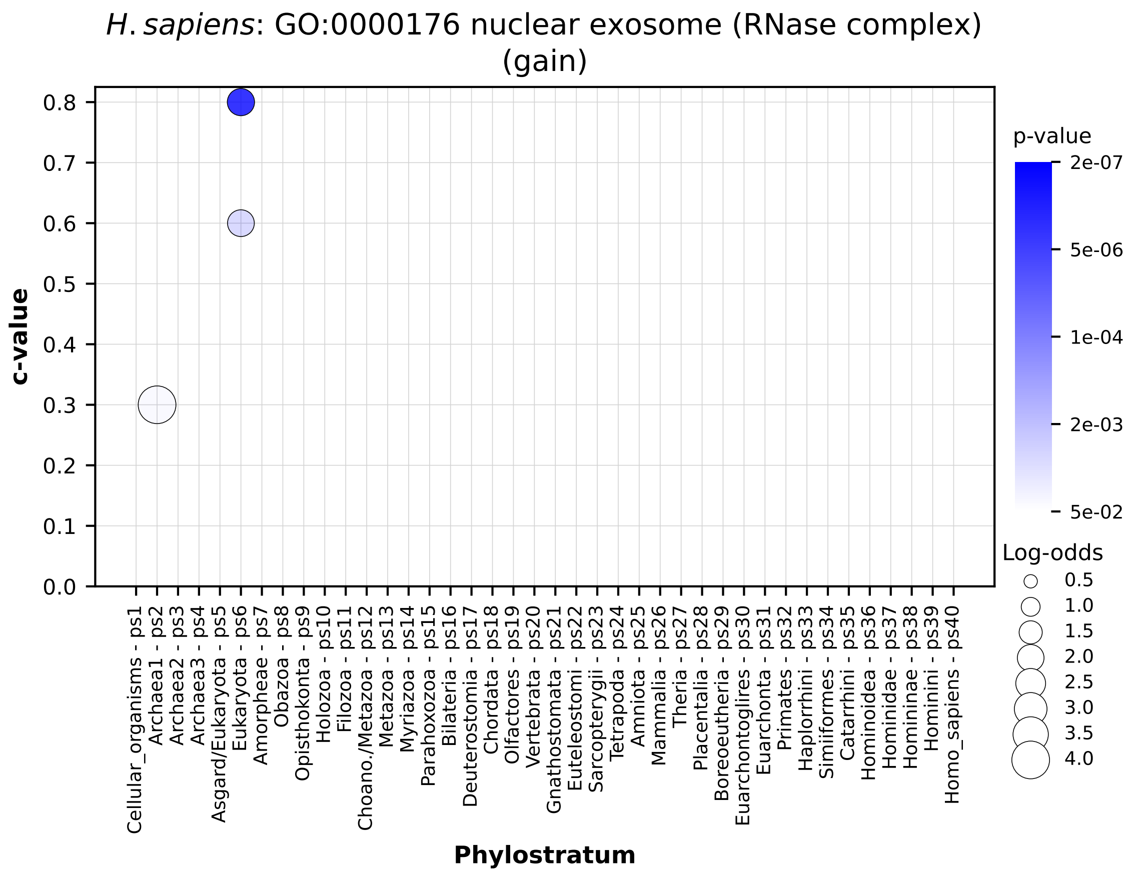

*H. sapiens*: GO:0000176 nuclear exosome (RNase complex) (gain)
c-value
0.0
0.1
0.2
0.3
0.4
0.5
0.6
0.7
0.8
Phylostratum
Cellular_organisms - ps1
Archaea1 - ps2
Archaea2 - ps3
Archaea3 - ps4
Asgard/Eukaryota - ps5
Eukaryota - ps6
Amorpheae - ps7
Obazoa - ps8
Opisthokonta - ps9
Holozoa - ps10
Filozoa - ps11
Choano./Metazoa - ps12
Metazoa - ps13
Myriazoa - ps14
Parahoxozoa - ps15
Bilateria - ps16
Deuterostomia - ps17
Chordata - ps18
Olfactores - ps19
Vertebrata - ps20
Gnathostomata - ps21
Euteleostomi - ps22
Sarcopterygii - ps23
Tetrapoda - ps24
Amniota - ps25
Mammalia - ps26
Theria - ps27
Placentalia - ps28
Boreoeutheria - ps29
Euarchontoglires - ps30
Euarchonta - ps31
Primates - ps32
Haplorrhini - ps33
Simiiformes - ps34
Catarrhini - ps35
Hominoidea - ps36
Hominidae - ps37
Homininae - ps38
Hominini - ps39
Homo_sapiens - ps40
p-value
2e-07
5e-06
1e-04
2e-03
5e-02
Log-odds
0.5
1.0
1.5
2.0
2.5
3.0
3.5
4.0

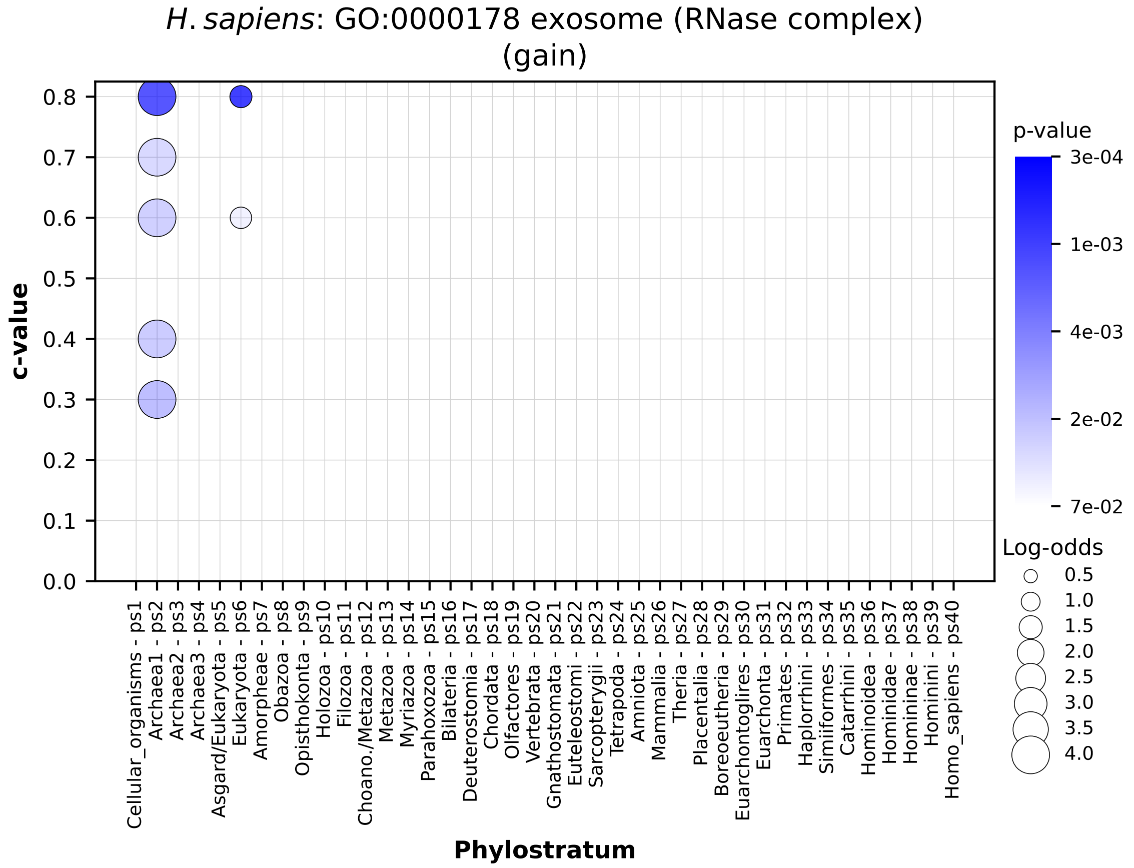

*H. sapiens*: GO:0000178 exosome (RNase complex) (gain)
c-value
0.0
0.1
0.2
0.3
0.4
0.5
0.6
0.7
0.8
Phylostratum
Cellular_organisms - ps1
Archaea1 - ps2
Archaea2 - ps3
Archaea3 - ps4
Asgard/Eukaryota - ps5
Eukaryota - ps6
Amorpheae - ps7
Obazoa - ps8
Opisthokonta - ps9
Holozoa - ps10
Filozoa - ps11
Choano./Metazoa - ps12
Metazoa - ps13
Myriazoa - ps14
Parahoxozoa - ps15
Bilateria - ps16
Deuterostomia - ps17
Chordata - ps18
Olfactores - ps19
Vertebrata - ps20
Gnathostomata - ps21
Euteleostomi - ps22
Sarcopterygii - ps23
Tetrapoda - ps24
Amniota - ps25
Mammalia - ps26
Theria - ps27
Placentalia - ps28
Boreoeutheria - ps29
Euarchontoglires - ps30
Euarchonta - ps31
Primates - ps32
Haplorrhini - ps33
Simiiformes - ps34
Catarrhini - ps35
Hominoidea - ps36
Hominidae - ps37
Homininae - ps38
Hominini - ps39
Homo_sapiens - ps40
p-value
3e-04
1e-03
4e-03
2e-02
7e-02
Log-odds
0.5
1.0
1.5
2.0
2.5
3.0
3.5
4.0

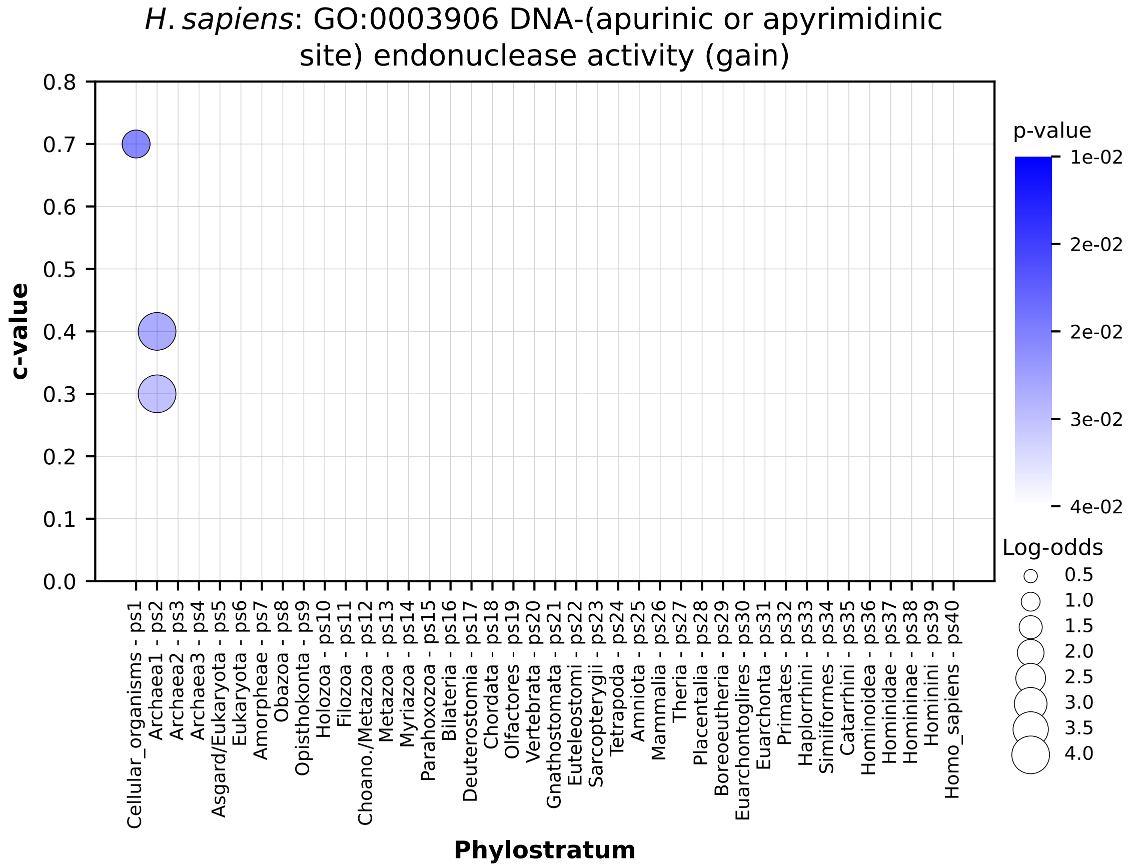
H. sapiens: GO:0003906 DNA-(apurinic or apyrimidinic site) endonuclease activity (gain)
c-value
0.0
0.1
0.2
0.3
0.4
0.5
0.6
0.7
0.8
Phylostratum
Cellular_organisms - ps1
Archaea1 - ps2
Archaea2 - ps3
Archaea3 - ps4
Asgard/Eukaryota - ps5
Eukaryota - ps6
Amorpheae - ps7
Obazoa - ps8
Opisthokonta - ps9
Holozoa - ps10
Filozoa - ps11
Choano./Metazoa - ps12
Metazoa - ps13
Myriazoa - ps14
Parahoxozoa - ps15
Bilateria - ps16
Deuterostomia - ps17
Chordata - ps18
Olfactores - ps19
Vertebrata - ps20
Gnathostomata - ps21
Euteleostomi - ps22
Sarcopterygii - ps23
Tetrapoda - ps24
Amniota - ps25
Mammalia - ps26
Theria - ps27
Placentalia - ps28
Boreoeutheria - ps29
Euarchontoglires - ps30
Euarchonta - ps31
Primates - ps32
Haplorrhini - ps33
Simiiformes - ps34
Catarrhini - ps35
Hominoidea - ps36
Hominidae - ps37
Homininae - ps38
Hominini - ps39
Homo_sapiens - ps40
p-value
1e-02
2e-02
2e-02
3e-02
4e-02
Log-odds
0.5
1.0
1.5
2.0
2.5
3.0
3.5
4.0

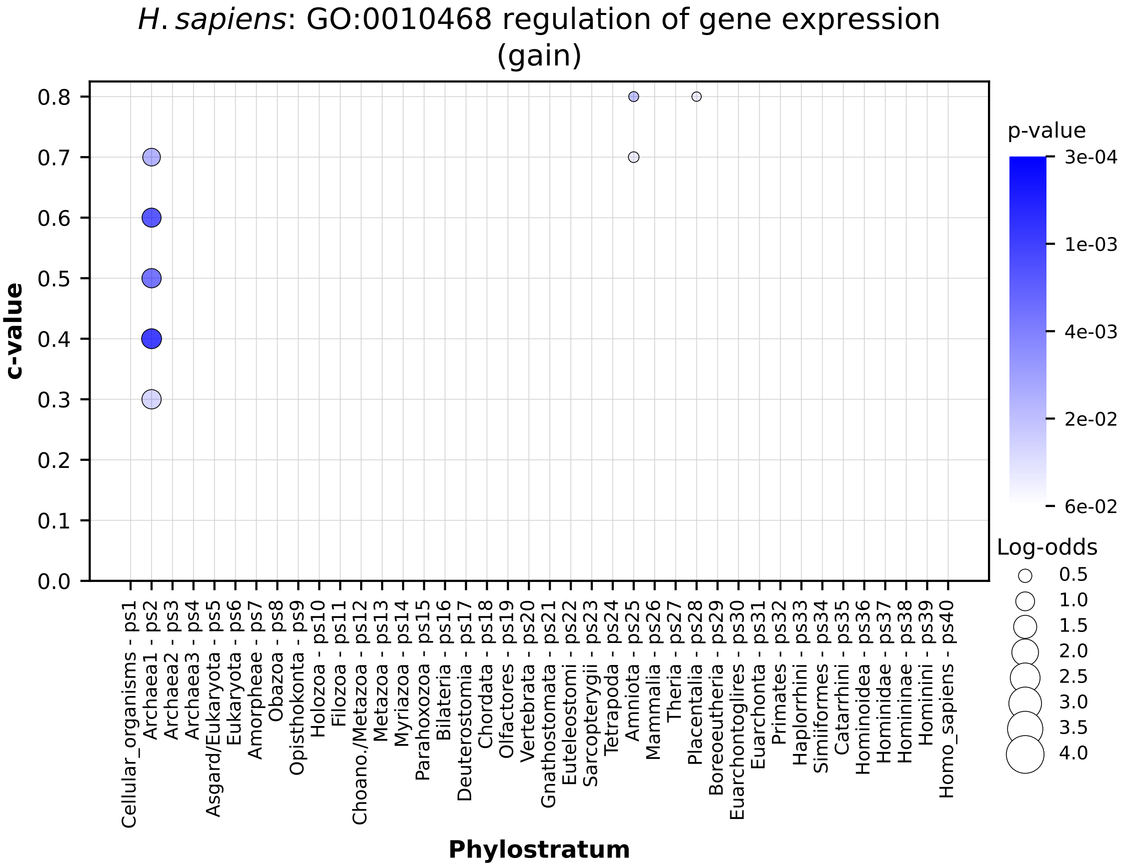

*H. sapiens*: GO:0010468 regulation of gene expression
(gain)
c-value
0.0
0.1
0.2
0.3
0.4
0.5
0.6
0.7
0.8
p-value
3e-04
1e-03
4e-03
2e-02
6e-02
Log-odds
0.5
1.0
1.5
2.0
2.5
3.0
3.5
4.0
Cellular_organisms - ps1
Archaea1 - ps2
Archaea2 - ps3
Archaea3 - ps4
Asgard/Eukaryota - ps5
Eukaryota - ps6
Amorpheae - ps7
Obazoa - ps8
Opisthokonta - ps9
Holozoa - ps10
Filozoa - ps11
Choano./Metazoa - ps12
Metazoa - ps13
Myriazoa - ps14
Parahoxozoa - ps15
Bilateria - ps16
Deuterostomia - ps17
Chordata - ps18
Olfactores - ps19
Vertebrata - ps20
Gnathostomata - ps21
Euteleostomi - ps22
Sarcopterygii - ps23
Tetrapoda - ps24
Amniota - ps25
Mammalia - ps26
Theria - ps27
Placentalia - ps28
Boreoeutheria - ps29
Euarchontoglires - ps30
Euarchonta - ps31
Primates - ps32
Haplorrhini - ps33
Simiiformes - ps34
Catarrhini - ps35
Hominoidea - ps36
Hominidae - ps37
Homininae - ps38
Hominini - ps39
Homo_sapiens - ps40
Phylostratum

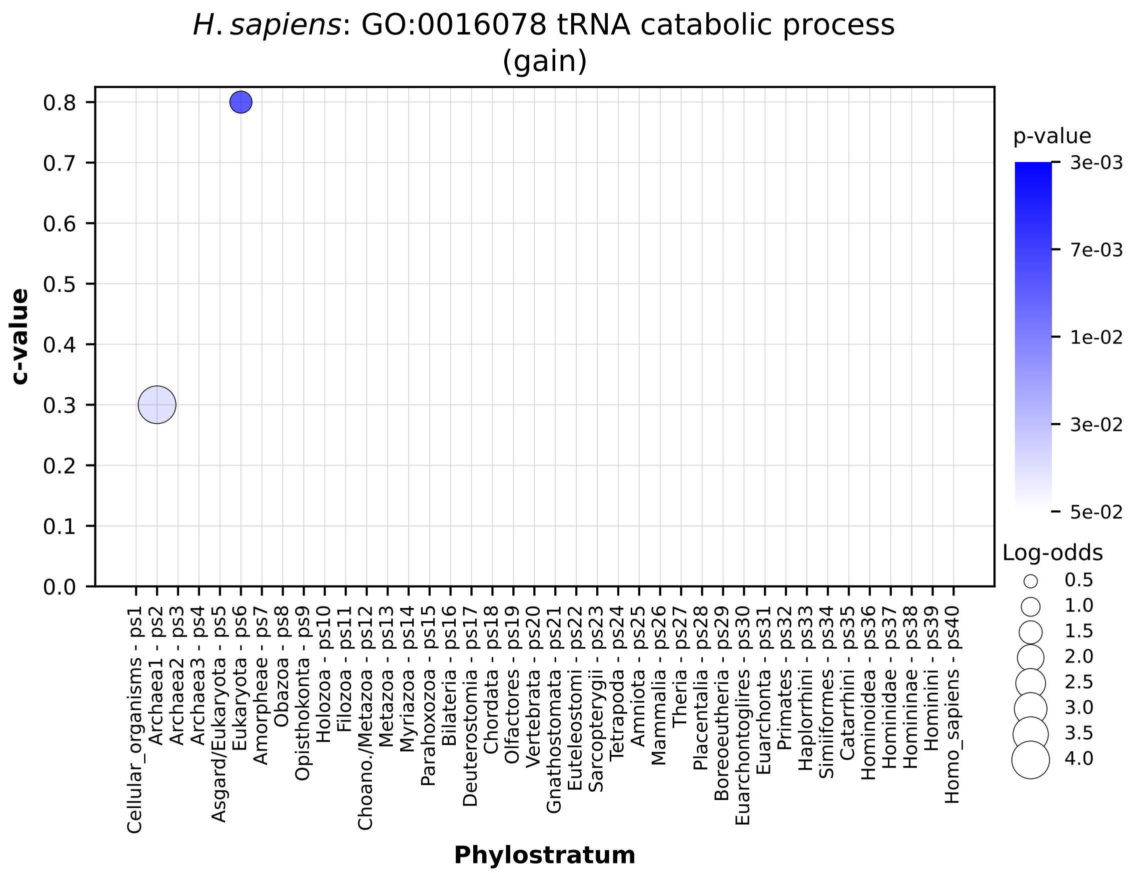

H. sapiens: GO:0016078 tRNA catabolic process
(gain)
c-value
0.0
0.1
0.2
0.3
0.4
0.5
0.6
0.7
0.8
Phylostratum
Cellular_organisms - ps1
Archaea1 - ps2
Archaea2 - ps3
Archaea3 - ps4
Asgard/Eukaryota - ps5
Eukaryota - ps6
Amorpheae - ps7
Obazoa - ps8
Opisthokonta - ps9
Holozoa - ps10
Filozoa - ps11
Choano./Metazoa - ps12
Metazoa - ps13
Myriazoa - ps14
Parahoxozoa - ps15
Bilateria - ps16
Deuterostomia - ps17
Chordata - ps18
Olfactores - ps19
Vertebrata - ps20
Gnathostomata - ps21
Euteleostomi - ps22
Sarcopterygii - ps23
Tetrapoda - ps24
Amniota - ps25
Mammalia - ps26
Theria - ps27
Placentalia - ps28
Boreoeutheria - ps29
Euarchontoglires - ps30
Euarchonta - ps31
Primates - ps32
Haplorrhini - ps33
Simiiformes - ps34
Catarrhini - ps35
Hominoidea - ps36
Hominidae - ps37
Homininae - ps38
Hominini - ps39
Homo_sapiens - ps40
p-value
3e-03
7e-03
1e-02
3e-02
5e-02
Log-odds
0.5
1.0
1.5
2.0
2.5
3.0
3.5
4.0

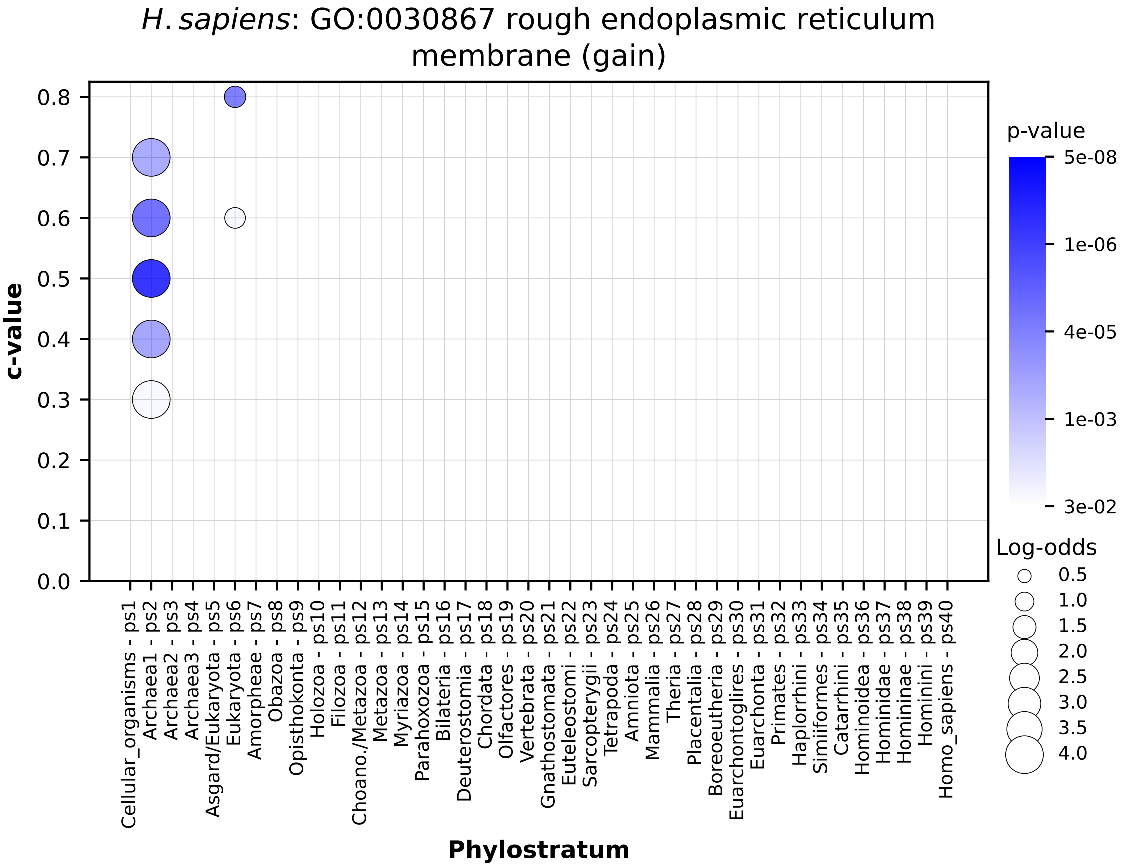

H. sapiens: GO:0030867 rough endoplasmic reticulum membrane (gain)
c-value
0.0
0.1
0.2
0.3
0.4
0.5
0.6
0.7
0.8
Phylostratum
Cellular_organisms - ps1
Archaea1 - ps2
Archaea2 - ps3
Archaea3 - ps4
Asgard/Eukaryota - ps5
Eukaryota - ps6
Amorpheae - ps7
Obazoa - ps8
Opisthokonta - ps9
Holozoa - ps10
Filozoa - ps11
Choano./Metazoa - ps12
Metazoa - ps13
Myriazoa - ps14
Parahoxozoa - ps15
Bilateria - ps16
Deuterostomia - ps17
Chordata - ps18
Olfactores - ps19
Vertebrata - ps20
Gnathostomata - ps21
Euteleostomi - ps22
Sarcopterygii - ps23
Tetrapoda - ps24
Amniota - ps25
Mammalia - ps26
Theria - ps27
Placentalia - ps28
Boreoeutheria - ps29
Euarchontoglires - ps30
Euarchonta - ps31
Primates - ps32
Haplorrhini - ps33
Simiiformes - ps34
Catarrhini - ps35
Hominoidea - ps36
Hominidae - ps37
Homininae - ps38
Hominini - ps39
Homo_sapiens - ps40
p-value
5e-08
1e-06
4e-05
1e-03
3e-02
Log-odds
0.5
1.0
1.5
2.0
2.5
3.0
3.5
4.0

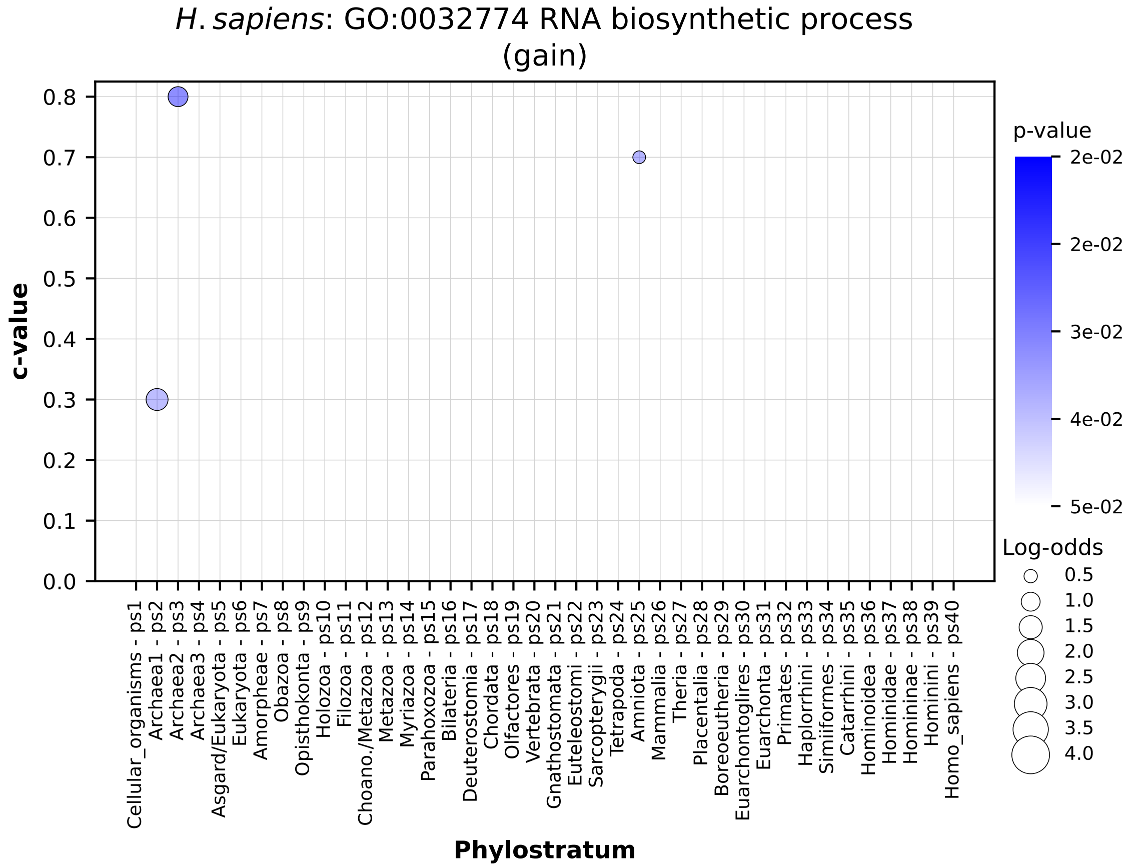

*H. sapiens*: GO:0032774 RNA biosynthetic process
(gain)
c-value
0.0
0.1
0.2
0.3
0.4
0.5
0.6
0.7
0.8
Phylostratum
Cellular_organisms - ps1
Archaea1 - ps2
Archaea2 - ps3
Archaea3 - ps4
Asgard/Eukaryota - ps5
Eukaryota - ps6
Amorpheae - ps7
Obazoa - ps8
Opisthokonta - ps9
Holozoa - ps10
Filozoa - ps11
Choano./Metazoa - ps12
Metazoa - ps13
Myriazoa - ps14
Parahoxozoa - ps15
Bilateria - ps16
Deuterostomia - ps17
Chordata - ps18
Olfactores - ps19
Vertebrata - ps20
Gnathostomata - ps21
Euteleostomi - ps22
Sarcopterygii - ps23
Tetrapoda - ps24
Amniota - ps25
Mammalia - ps26
Theria - ps27
Placentalia - ps28
Boreoeutheria - ps29
Euarchontoglires - ps30
Euarchonta - ps31
Primates - ps32
Haplorrhini - ps33
Simiiformes - ps34
Catarrhini - ps35
Hominoidea - ps36
Hominidae - ps37
Homininae - ps38
Hominini - ps39
Homo_sapiens - ps40
p-value
2e-02
2e-02
3e-02
4e-02
5e-02
Log-odds
0.5
1.0
1.5
2.0
2.5
3.0
3.5
4.0

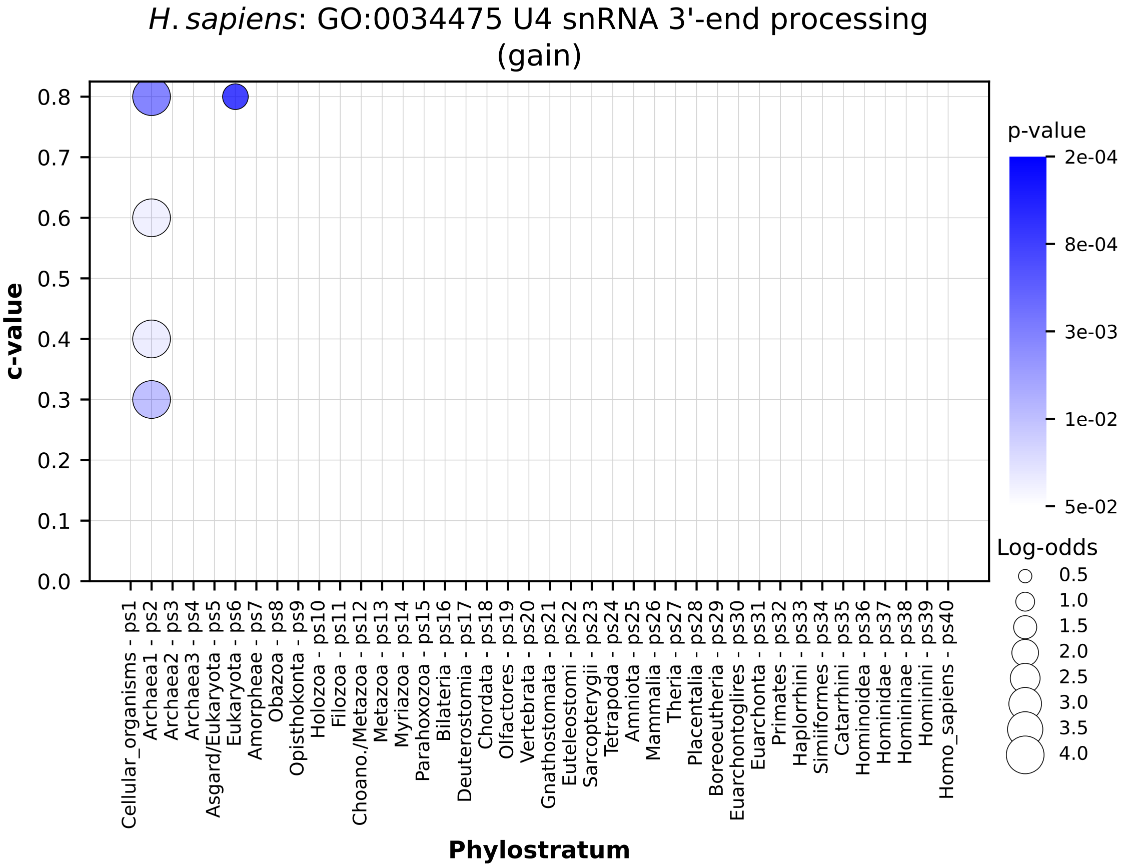

H. sapiens: GO:0034475 U4 snRNA 3'-end processing
(gain)
c-value
0.0
0.1
0.2
0.3
0.4
0.5
0.6
0.7
0.8
Phylostratum
Cellular_organisms - ps1
Archaea1 - ps2
Archaea2 - ps3
Archaea3 - ps4
Asgard/Eukaryota - ps5
Eukaryota - ps6
Amorpheae - ps7
Obazoa - ps8
Opisthokonta - ps9
Holozoa - ps10
Filozoa - ps11
Choano./Metazoa - ps12
Metazoa - ps13
Myriazoa - ps14
Parahoxozoa - ps15
Bilateria - ps16
Deuterostomia - ps17
Chordata - ps18
Olfactores - ps19
Vertebrata - ps20
Gnathostomata - ps21
Euteleostomi - ps22
Sarcopterygii - ps23
Tetrapoda - ps24
Amniota - ps25
Mammalia - ps26
Theria - ps27
Placentalia - ps28
Boreoeutheria - ps29
Euarchontoglires - ps30
Euarchonta - ps31
Primates - ps32
Haplorrhini - ps33
Simiiformes - ps34
Catarrhini - ps35
Hominoidea - ps36
Hominidae - ps37
Homininae - ps38
Hominini - ps39
Homo_sapiens - ps40
p-value
2e-04
8e-04
3e-03
1e-02
5e-02
Log-odds
0.5
1.0
1.5
2.0
2.5
3.0
3.5
4.0

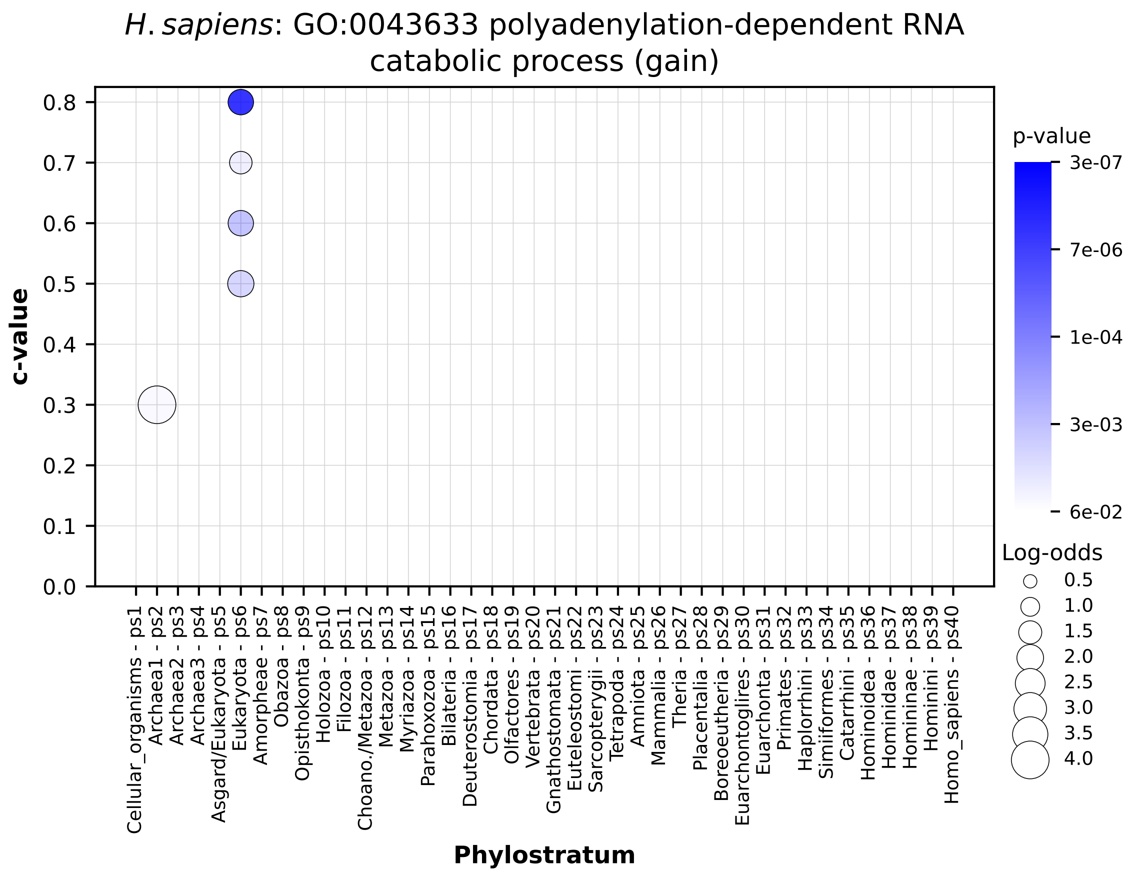

*H. sapiens*: GO:0043633 polyadenylation-dependent RNA catabolic process (gain)
c-value
0.0
0.1
0.2
0.3
0.4
0.5
0.6
0.7
0.8
Phylostratum
Cellular_organisms - ps1
Archaea1 - ps2
Archaea2 - ps3
Archaea3 - ps4
Asgard/Eukaryota - ps5
Eukaryota - ps6
Amorpheae - ps7
Obazoa - ps8
Opisthokonta - ps9
Holozoa - ps10
Filozoa - ps11
Choano./Metazoa - ps12
Metazoa - ps13
Myriazoa - ps14
Parahoxozoa - ps15
Bilateria - ps16
Deuterostomia - ps17
Chordata - ps18
Olfactores - ps19
Vertebrata - ps20
Gnathostomata - ps21
Euteleostomi - ps22
Sarcopterygii - ps23
Tetrapoda - ps24
Amniota - ps25
Mammalia - ps26
Theria - ps27
Placentalia - ps28
Boreoeutheria - ps29
Euarchontoglires - ps30
Euarchonta - ps31
Primates - ps32
Haplorrhini - ps33
Simiiformes - ps34
Catarrhini - ps35
Hominoidea - ps36
Hominidae - ps37
Homininae - ps38
Hominini - ps39
Homo_sapiens - ps40
p-value
3e-07
7e-06
1e-04
3e-03
6e-02
Log-odds
0.5
1.0
1.5
2.0
2.5
3.0
3.5
4.0

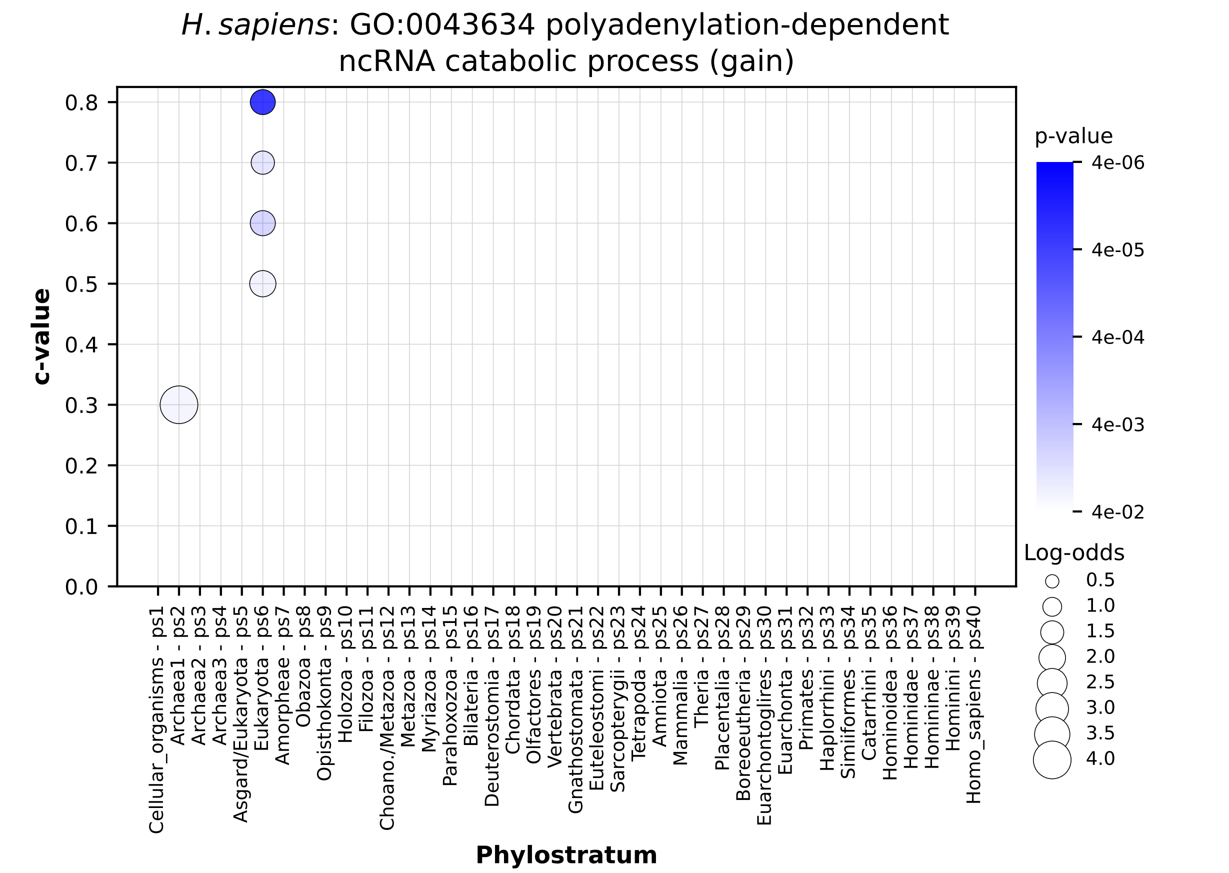

H. sapiens: GO:0043634 polyadenylation-dependent ncRNA catabolic process (gain)
c-value
0.0
0.1
0.2
0.3
0.4
0.5
0.6
0.7
0.8
Phylostratum
Cellular_organisms - ps1
Archaea1 - ps2
Archaea2 - ps3
Archaea3 - ps4
Asgard/Eukaryota - ps5
Eukaryota - ps6
Amorpheae - ps7
Obazoa - ps8
Opisthokonta - ps9
Holozoa - ps10
Filozoa - ps11
Choano./Metazoa - ps12
Metazoa - ps13
Myriazoa - ps14
Parahoxozoa - ps15
Bilateria - ps16
Deuterostomia - ps17
Chordata - ps18
Olfactores - ps19
Vertebrata - ps20
Gnathostomata - ps21
Euteleostomi - ps22
Sarcopterygii - ps23
Tetrapoda - ps24
Amniota - ps25
Mammalia - ps26
Theria - ps27
Placentalia - ps28
Boreoeutheria - ps29
Euarchontoglires - ps30
Euarchonta - ps31
Primates - ps32
Haplorrhini - ps33
Simiiformes - ps34
Catarrhini - ps35
Hominoidea - ps36
Hominidae - ps37
Homininae - ps38
Hominini - ps39
Homo_sapiens - ps40
p-value
4e-06
4e-05
4e-04
4e-03
4e-02
Log-odds
0.5
1.0
1.5
2.0
2.5
3.0
3.5
4.0

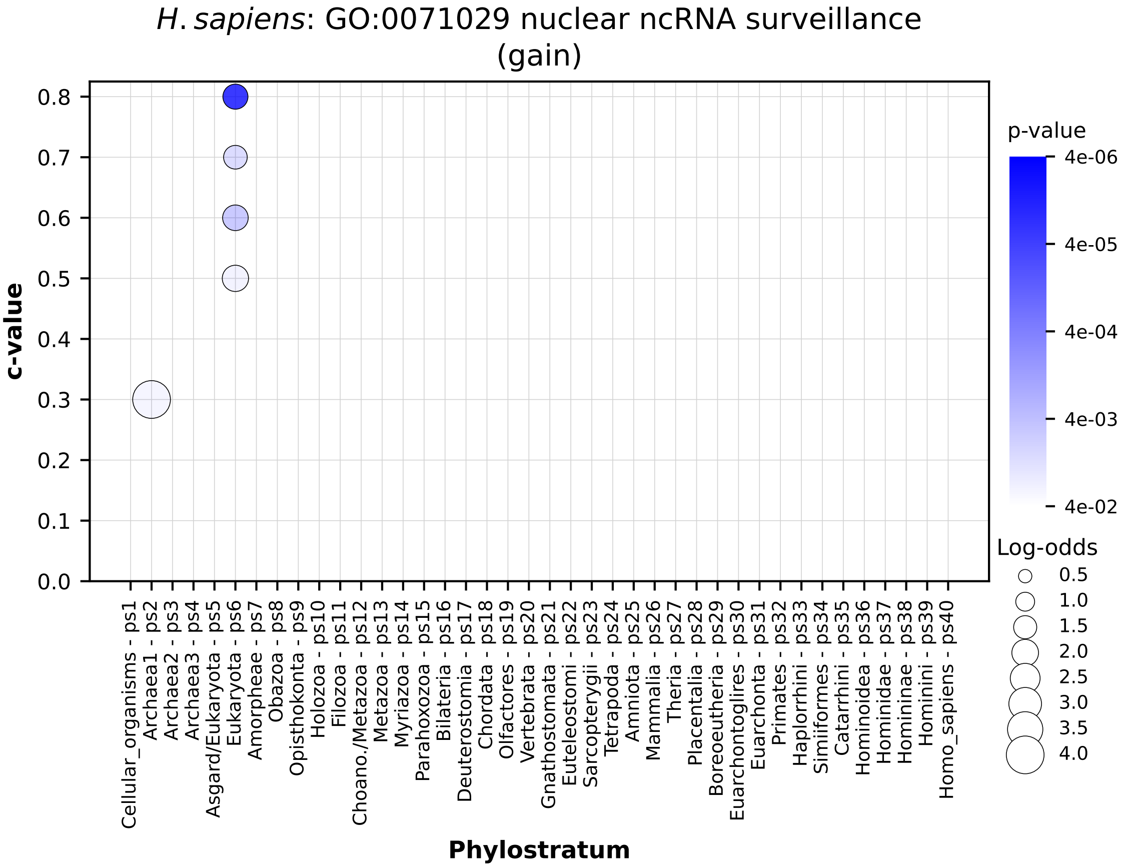

*H. sapiens*: GO:0071029 nuclear ncRNA surveillance
(gain)
c-value
0.8
0.7
0.6
0.5
0.4
0.3
0.2
0.1
0.0
Phylostratum
Cellular_organisms - ps1
Archaea1 - ps2
Archaea2 - ps3
Archaea3 - ps4
Asgard/Eukaryota - ps5
Eukaryota - ps6
Amorpheae - ps7
Obazoa - ps8
Opisthokonta - ps9
Holozoa - ps10
Filozoa - ps11
Choano./Metazoa - ps12
Metazoa - ps13
Myriazoa - ps14
Parahoxozoa - ps15
Bilateria - ps16
Deuterostomia - ps17
Chordata - ps18
Olfactores - ps19
Vertebrata - ps20
Gnathostomata - ps21
Euteleostomi - ps22
Sarcopterygii - ps23
Tetrapoda - ps24
Amniota - ps25
Mammalia - ps26
Theria - ps27
Placentalia - ps28
Boreoeutheria - ps29
Euarchontoglires - ps30
Euarchonta - ps31
Primates - ps32
Haplorrhini - ps33
Simiiformes - ps34
Catarrhini - ps35
Hominoidea - ps36
Hominidae - ps37
Homininae - ps38
Hominini - ps39
Homo_sapiens - ps40
p-value
4e-06
4e-05
4e-04
4e-03
4e-02
Log-odds
0.5
1.0
1.5
2.0
2.5
3.0
3.5
4.0

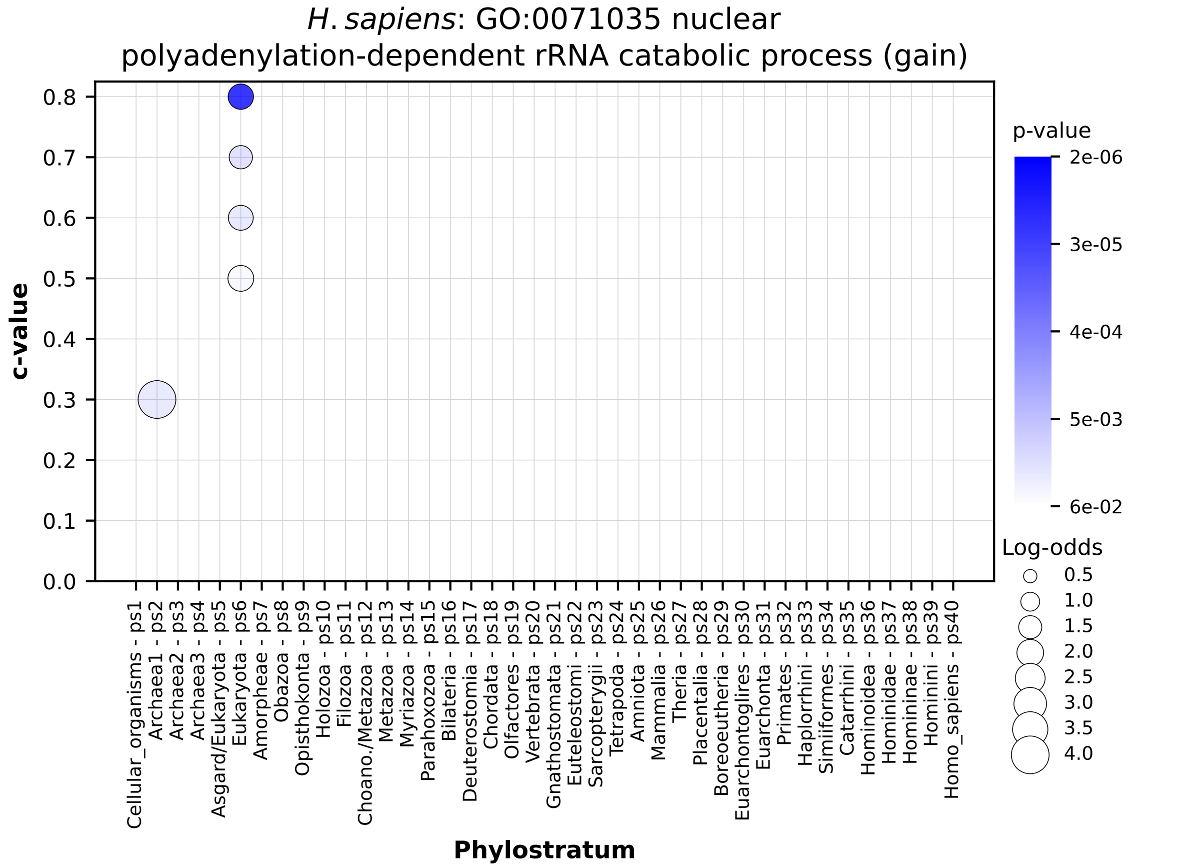

*H. sapiens*: GO:0071035 nuclear polyadenylation-dependent rRNA catabolic process (gain)
c-value
0.8
0.7
0.6
0.5
0.4
0.3
0.2
0.1
0.0
Phylostratum
Cellular_organisms - ps1
Archaea1 - ps2
Archaea2 - ps3
Archaea3 - ps4
Asgard/Eukaryota - ps5
Eukaryota - ps6
Amorpheae - ps7
Obazoa - ps8
Opisthokonta - ps9
Holozoa - ps10
Filozoa - ps11
Choano./Metazoa - ps12
Metazoa - ps13
Myriazoa - ps14
Parahoxozoa - ps15
Bilateria - ps16
Deuterostomia - ps17
Chordata - ps18
Olfactores - ps19
Vertebrata - ps20
Gnathostomata - ps21
Euteleostomi - ps22
Sarcopterygii - ps23
Tetrapoda - ps24
Amniota - ps25
Mammalia - ps26
Theria - ps27
Placentalia - ps28
Boreoeutheria - ps29
Euarchontoglires - ps30
Euarchonta - ps31
Primates - ps32
Haplorrhini - ps33
Simiiformes - ps34
Catarrhini - ps35
Hominoidea - ps36
Hominidae - ps37
Homininae - ps38
Hominini - ps39
Homo_sapiens - ps40
p-value
2e-06
3e-05
4e-04
5e-03
6e-02
Log-odds
0.5
1.0
1.5
2.0
2.5
3.0
3.5
4.0

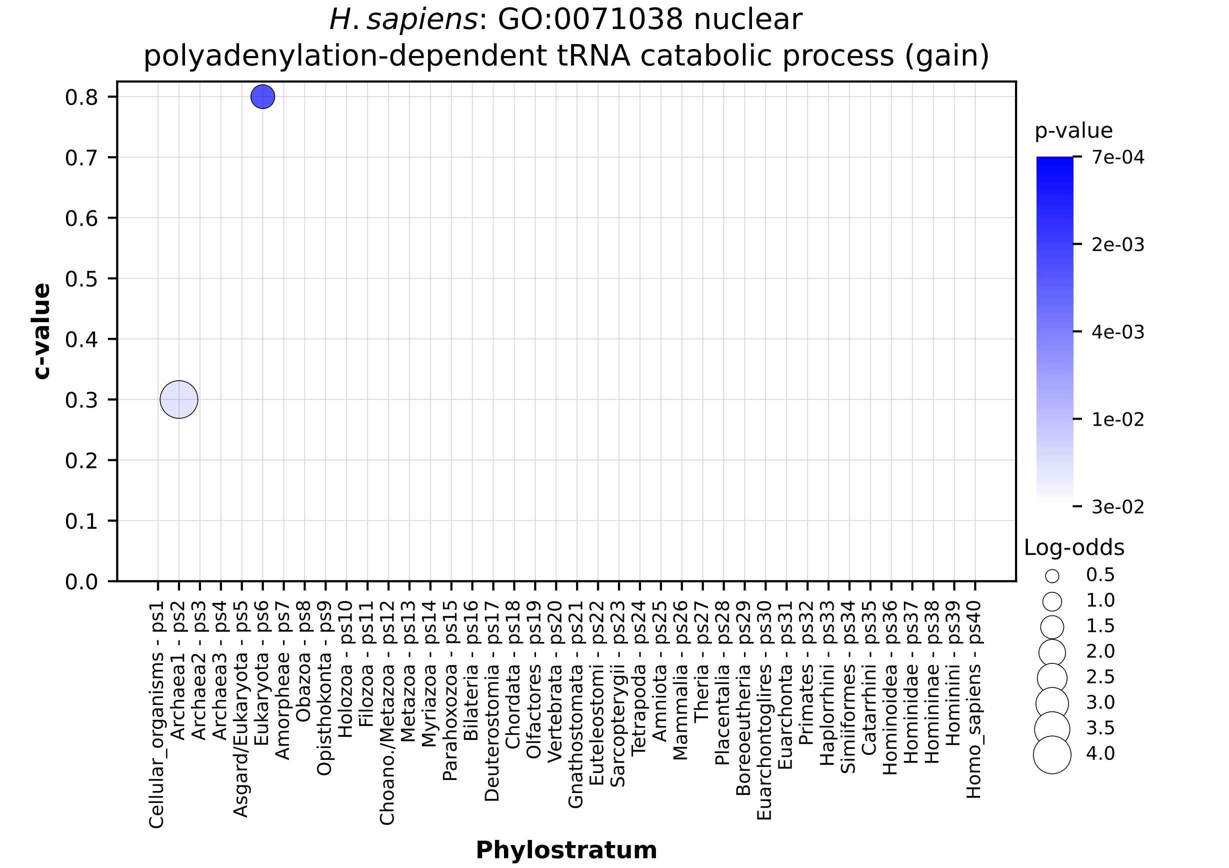

*H. sapiens*: GO:0071038 nuclear polyadenylation-dependent tRNA catabolic process (gain)
c-value
0.8
0.7
0.6
0.5
0.4
0.3
0.2
0.1
0.0
Cellular_organisms - ps1
Archaea1 - ps2
Archaea2 - ps3
Archaea3 - ps4
Asgard/Eukaryota - ps5
Eukaryota - ps6
Amorpheae - ps7
Obazoa - ps8
Opisthokonta - ps9
Holozoa - ps10
Filozoa - ps11
Choano./Metazoa - ps12
Metazoa - ps13
Myriazoa - ps14
Parahoxozoa - ps15
Bilateria - ps16
Deuterostomia - ps17
Chordata - ps18
Olfactores - ps19
Vertebrata - ps20
Gnathostomata - ps21
Euteleostomi - ps22
Sarcopterygii - ps23
Tetrapoda - ps24
Amniota - ps25
Mammalia - ps26
Theria - ps27
Placentalia - ps28
Boreoeutheria - ps29
Euarchontoglires - ps30
Euarchonta - ps31
Primates - ps32
Haplorrhini - ps33
Simiiformes - ps34
Catarrhini - ps35
Hominoidea - ps36
Hominidae - ps37
Homininae - ps38
Hominini - ps39
Homo_sapiens - ps40
Phylostratum
p-value
7e-04
2e-03
4e-03
1e-02
3e-02
Log-odds
0.5
1.0
1.5
2.0
2.5
3.0
3.5
4.0

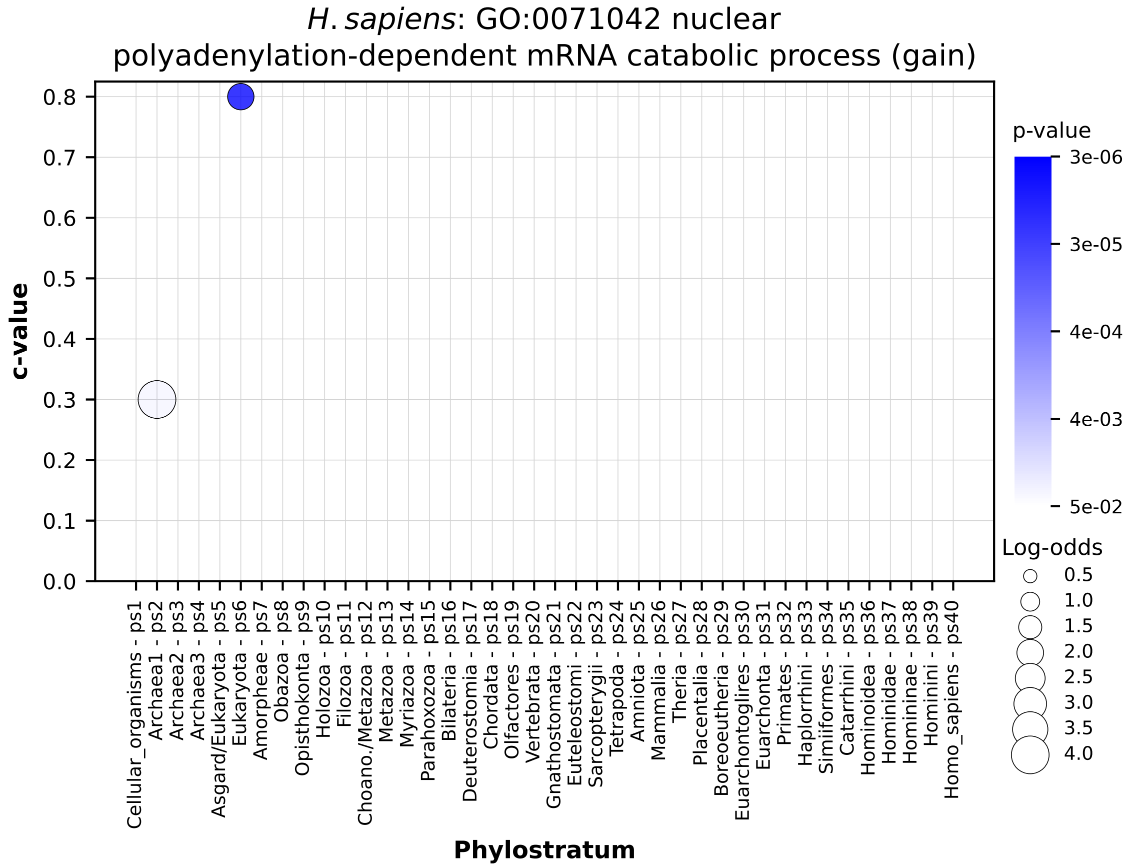

H. sapiens: GO:0071042 nuclear polyadenylation-dependent mRNA catabolic process (gain)
c-value
0.0
0.1
0.2
0.3
0.4
0.5
0.6
0.7
0.8
Phylostratum
Cellular_organisms - ps1
Archaea1 - ps2
Archaea2 - ps3
Archaea3 - ps4
Asgard/Eukaryota - ps5
Eukaryota - ps6
Amorpheae - ps7
Obazoa - ps8
Opisthokonta - ps9
Holozoa - ps10
Filozoa - ps11
Choano./Metazoa - ps12
Metazoa - ps13
Myriazoa - ps14
Parahoxozoa - ps15
Bilateria - ps16
Deuterostomia - ps17
Chordata - ps18
Olfactores - ps19
Vertebrata - ps20
Gnathostomata - ps21
Euteleostomi - ps22
Sarcopterygii - ps23
Tetrapoda - ps24
Amniota - ps25
Mammalia - ps26
Theria - ps27
Placentalia - ps28
Boreoeutheria - ps29
Euarchontoglires - ps30
Euarchonta - ps31
Primates - ps32
Haplorrhini - ps33
Simiiformes - ps34
Catarrhini - ps35
Hominoidea - ps36
Hominidae - ps37
Homininae - ps38
Hominini - ps39
Homo_sapiens - ps40
p-value
3e-06
3e-05
4e-04
4e-03
5e-02
Log-odds
0.5
1.0
1.5
2.0
2.5
3.0
3.5
4.0

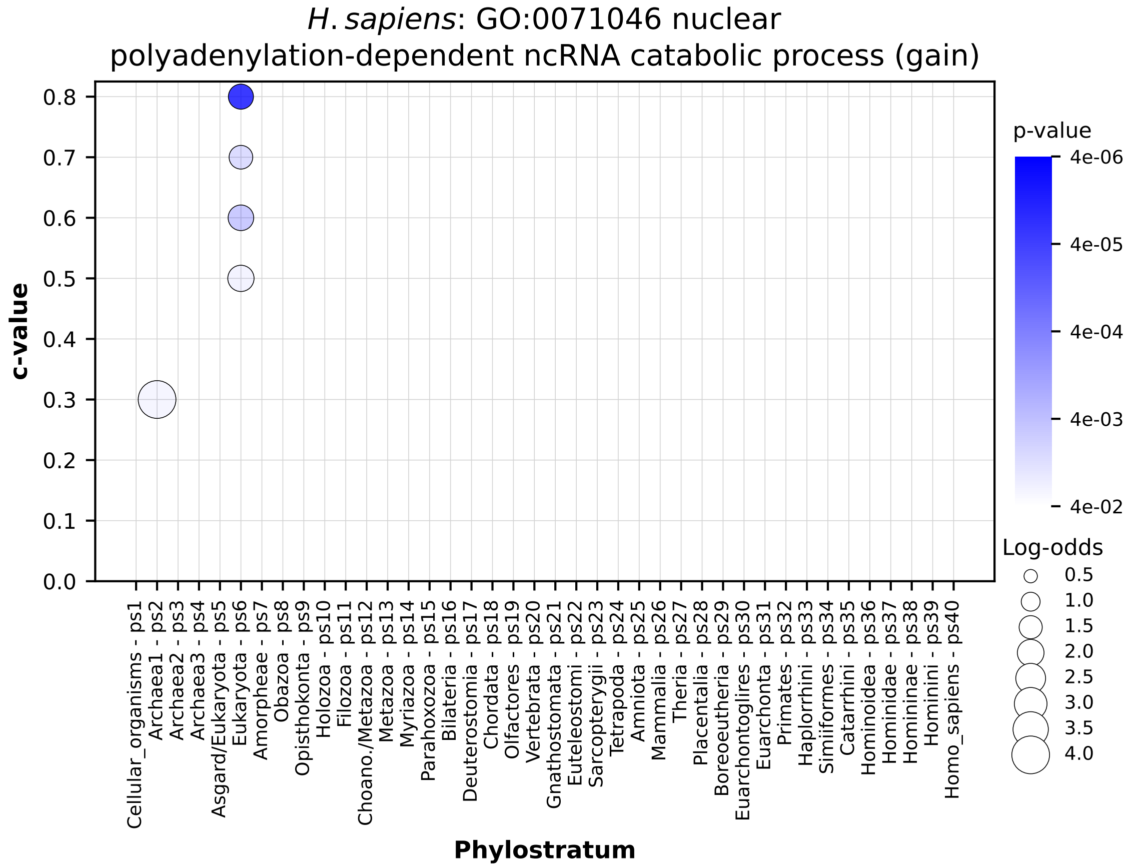

H. sapiens: GO:0071046 nuclear polyadenylation-dependent ncRNA catabolic process (gain)
c-value
0.0
0.1
0.2
0.3
0.4
0.5
0.6
0.7
0.8
Phylostratum
Cellular_organisms - ps1
Archaea1 - ps2
Archaea2 - ps3
Archaea3 - ps4
Asgard/Eukaryota - ps5
Eukaryota - ps6
Amorpheae - ps7
Obazoa - ps8
Opisthokonta - ps9
Holozoa - ps10
Filozoa - ps11
Choano./Metazoa - ps12
Metazoa - ps13
Myriazoa - ps14
Parahoxozoa - ps15
Bilateria - ps16
Deuterostomia - ps17
Chordata - ps18
Olfactores - ps19
Vertebrata - ps20
Gnathostomata - ps21
Euteleostomi - ps22
Sarcopterygii - ps23
Tetrapoda - ps24
Amniota - ps25
Mammalia - ps26
Theria - ps27
Placentalia - ps28
Boreoeutheria - ps29
Euarchontoglires - ps30
Euarchonta - ps31
Primates - ps32
Haplorrhini - ps33
Simiiformes - ps34
Catarrhini - ps35
Hominoidea - ps36
Hominidae - ps37
Homininae - ps38
Hominini - ps39
Homo_sapiens - ps40
p-value
4e-06
4e-05
4e-04
4e-03
4e-02
Log-odds
0.5
1.0
1.5
2.0
2.5
3.0
3.5
4.0

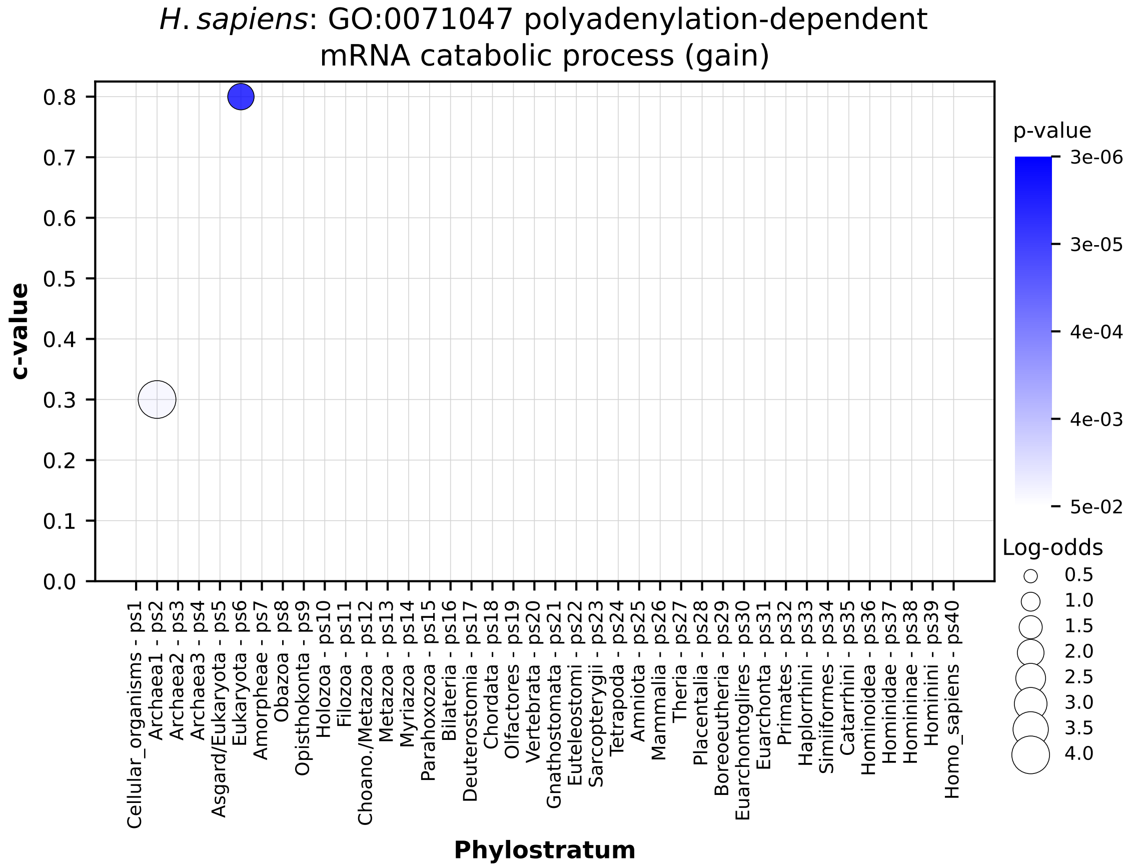

*H. sapiens*: GO:0071047 polyadenylation-dependent mRNA catabolic process (gain)
c-value
0.0
0.1
0.2
0.3
0.4
0.5
0.6
0.7
0.8
Cellular_organisms - ps1
Archaea1 - ps2
Archaea2 - ps3
Archaea3 - ps4
Asgard/Eukaryota - ps5
Eukaryota - ps6
Amorpheae - ps7
Obazoa - ps8
Opisthokonta - ps9
Holozoa - ps10
Filozoa - ps11
Choano./Metazoa - ps12
Metazoa - ps13
Myriazoa - ps14
Parahoxozoa - ps15
Bilateria - ps16
Deuterostomia - ps17
Chordata - ps18
Olfactores - ps19
Vertebrata - ps20
Gnathostomata - ps21
Euteleostomi - ps22
Sarcopterygii - ps23
Tetrapoda - ps24
Amniota - ps25
Mammalia - ps26
Theria - ps27
Placentalia - ps28
Boreoeutheria - ps29
Euarchontoglires - ps30
Euarchonta - ps31
Primates - ps32
Haplorrhini - ps33
Simiiformes - ps34
Catarrhini - ps35
Hominoidea - ps36
Hominidae - ps37
Homininae - ps38
Hominini - ps39
Homo_sapiens - ps40
Phylostratum
p-value
3e-06
3e-05
4e-04
4e-03
5e-02
Log-odds
0.5
1.0
1.5
2.0
2.5
3.0
3.5
4.0

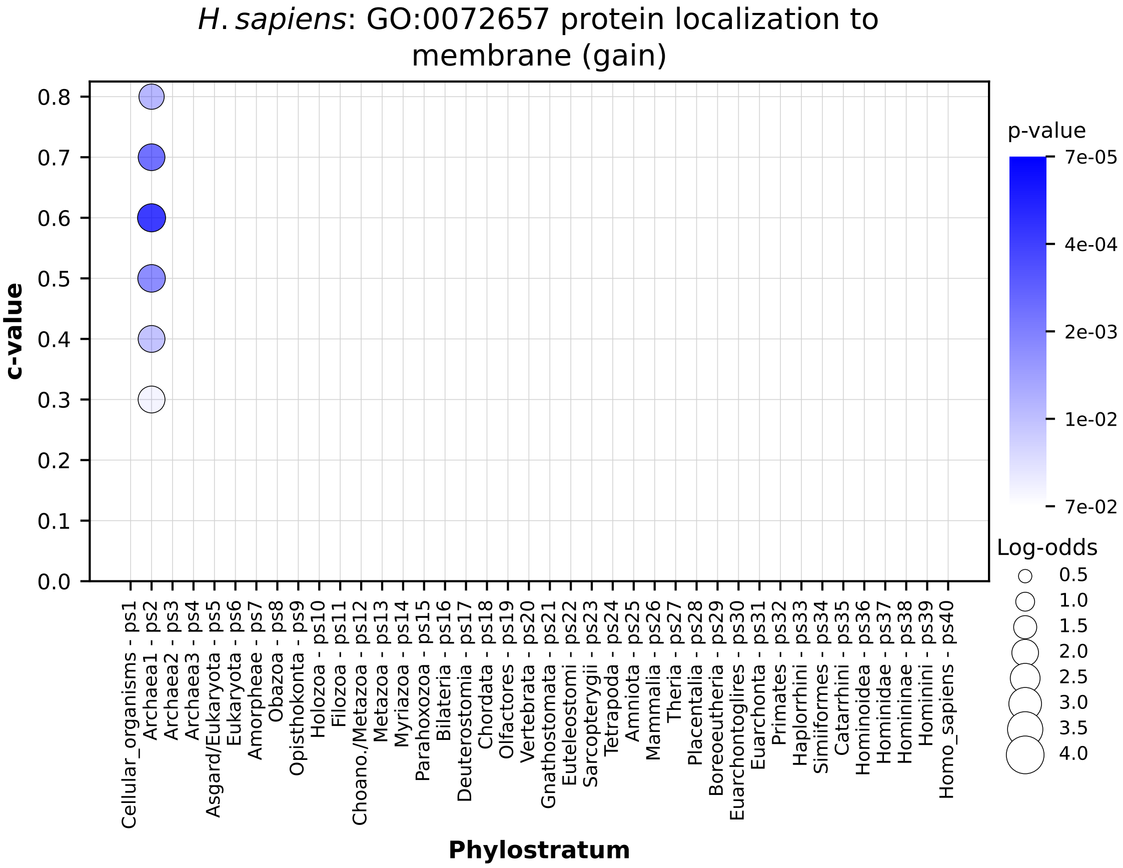

*H. sapiens*: GO:0072657 protein localization to membrane (gain)
c-value
0.8
0.7
0.6
0.5
0.4
0.3
0.2
0.1
0.0
Phylostratum
Cellular_organisms - ps1
Archaea1 - ps2
Archaea2 - ps3
Archaea3 - ps4
Asgard/Eukaryota - ps5
Eukaryota - ps6
Amorpheae - ps7
Obazoa - ps8
Opisthokonta - ps9
Holozoa - ps10
Filozoa - ps11
Choano./Metazoa - ps12
Metazoa - ps13
Myriazoa - ps14
Parahoxozoa - ps15
Bilateria - ps16
Deuterostomia - ps17
Chordata - ps18
Olfactores - ps19
Vertebrata - ps20
Gnathostomata - ps21
Euteleostomi - ps22
Sarcopterygii - ps23
Tetrapoda - ps24
Amniota - ps25
Mammalia - ps26
Theria - ps27
Placentalia - ps28
Boreoeutheria - ps29
Euarchontoglires - ps30
Euarchonta - ps31
Primates - ps32
Haplorrhini - ps33
Simiiformes - ps34
Catarrhini - ps35
Hominoidea - ps36
Hominidae - ps37
Homininae - ps38
Hominini - ps39
Homo_sapiens - ps40
p-value
7e-05
4e-04
2e-03
1e-02
7e-02
Log-odds
0.5
1.0
1.5
2.0
2.5
3.0
3.5
4.0

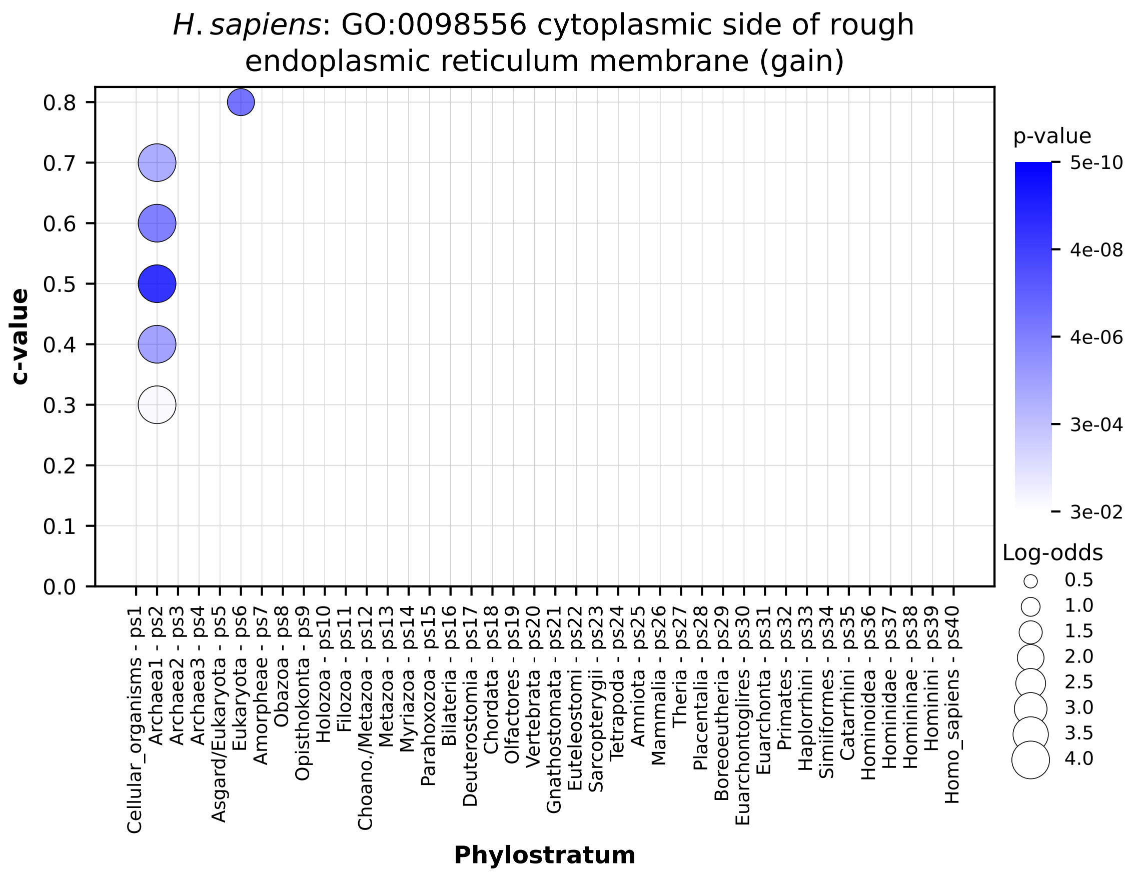

H. sapiens: GO:0098556 cytoplasmic side of rough endoplasmic reticulum membrane (gain)
c-value
0.0
0.1
0.2
0.3
0.4
0.5
0.6
0.7
0.8
Phylostratum
Cellular_organisms - ps1
Archaea1 - ps2
Archaea2 - ps3
Archaea3 - ps4
Asgard/Eukaryota - ps5
Eukaryota - ps6
Amorpheae - ps7
Obazoa - ps8
Opisthokonta - ps9
Holozoa - ps10
Filozoa - ps11
Choano./Metazoa - ps12
Metazoa - ps13
Myriazoa - ps14
Parahoxozoa - ps15
Bilateria - ps16
Deuterostomia - ps17
Chordata - ps18
Olfactores - ps19
Vertebrata - ps20
Gnathostomata - ps21
Euteleostomi - ps22
Sarcopterygii - ps23
Tetrapoda - ps24
Amniota - ps25
Mammalia - ps26
Theria - ps27
Placentalia - ps28
Boreoeutheria - ps29
Euarchontoglires - ps30
Euarchonta - ps31
Primates - ps32
Haplorrhini - ps33
Simiiformes - ps34
Catarrhini - ps35
Hominoidea - ps36
Hominidae - ps37
Homininae - ps38
Hominini - ps39
Homo_sapiens - ps40
p-value
5e-10
4e-08
4e-06
3e-04
3e-02
Log-odds
0.5
1.0
1.5
2.0
2.5
3.0
3.5
4.0

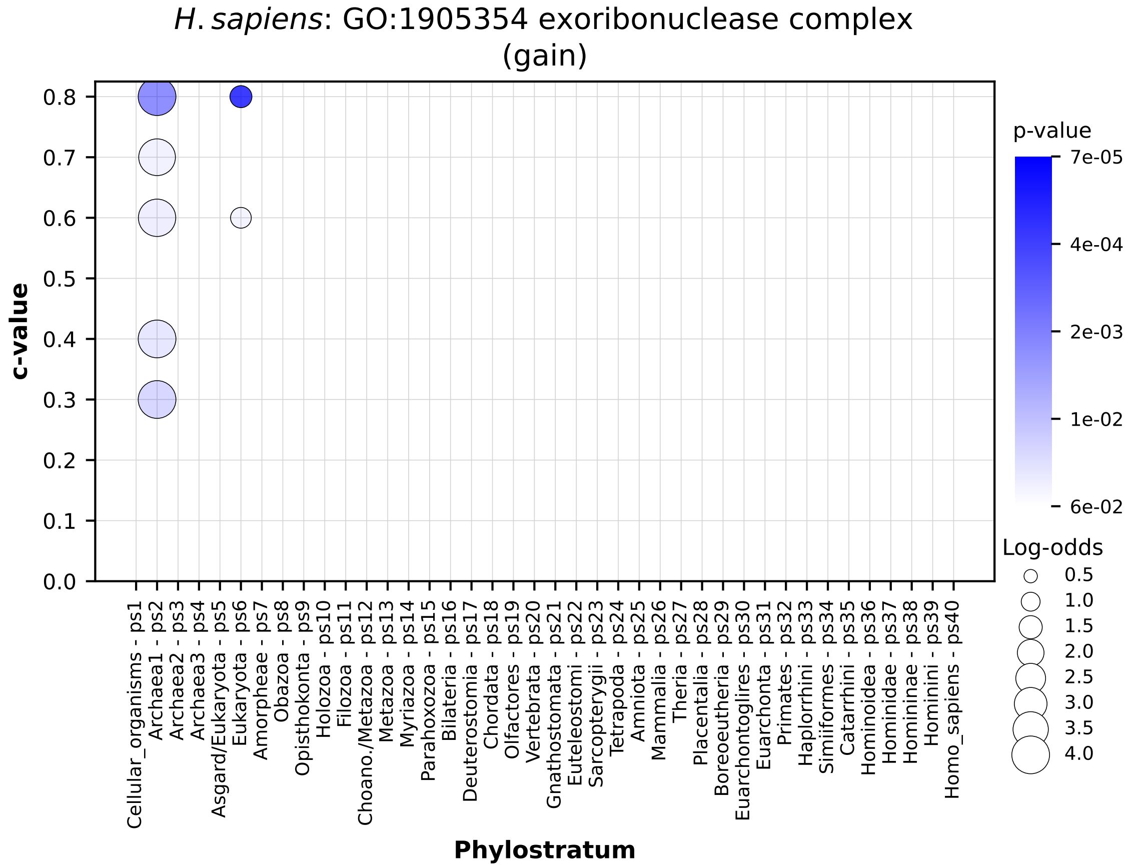
H. sapiens: GO:1905354 exoribonuclease complex
(gain)
c-value
0.8
0.7
0.6
0.5
0.4
0.3
0.2
0.1
0.0
Phylostratum
Cellular_organisms - ps1
Archaea1 - ps2
Archaea2 - ps3
Archaea3 - ps4
Asgard/Eukaryota - ps5
Eukaryota - ps6
Amorpheae - ps7
Obazoa - ps8
Opisthokonta - ps9
Holozoa - ps10
Filozoa - ps11
Choano./Metazoa - ps12
Metazoa - ps13
Myriazoa - ps14
Parahoxozoa - ps15
Bilateria - ps16
Deuterostomia - ps17
Chordata - ps18
Olfactores - ps19
Vertebrata - ps20
Gnathostomata - ps21
Euteleostomi - ps22
Sarcopterygii - ps23
Tetrapoda - ps24
Amniota - ps25
Mammalia - ps26
Theria - ps27
Placentalia - ps28
Boreoeutheria - ps29
Euarchontoglires - ps30
Euarchonta - ps31
Primates - ps32
Haplorrhini - ps33
Simiiformes - ps34
Catarrhini - ps35
Hominoidea - ps36
Hominidae - ps37
Homininae - ps38
Hominini - ps39
Homo_sapiens - ps40
p-value
7e-05
4e-04
2e-03
1e-02
6e-02
Log-odds
0.5
1.0
1.5
2.0
2.5
3.0
3.5
4.0

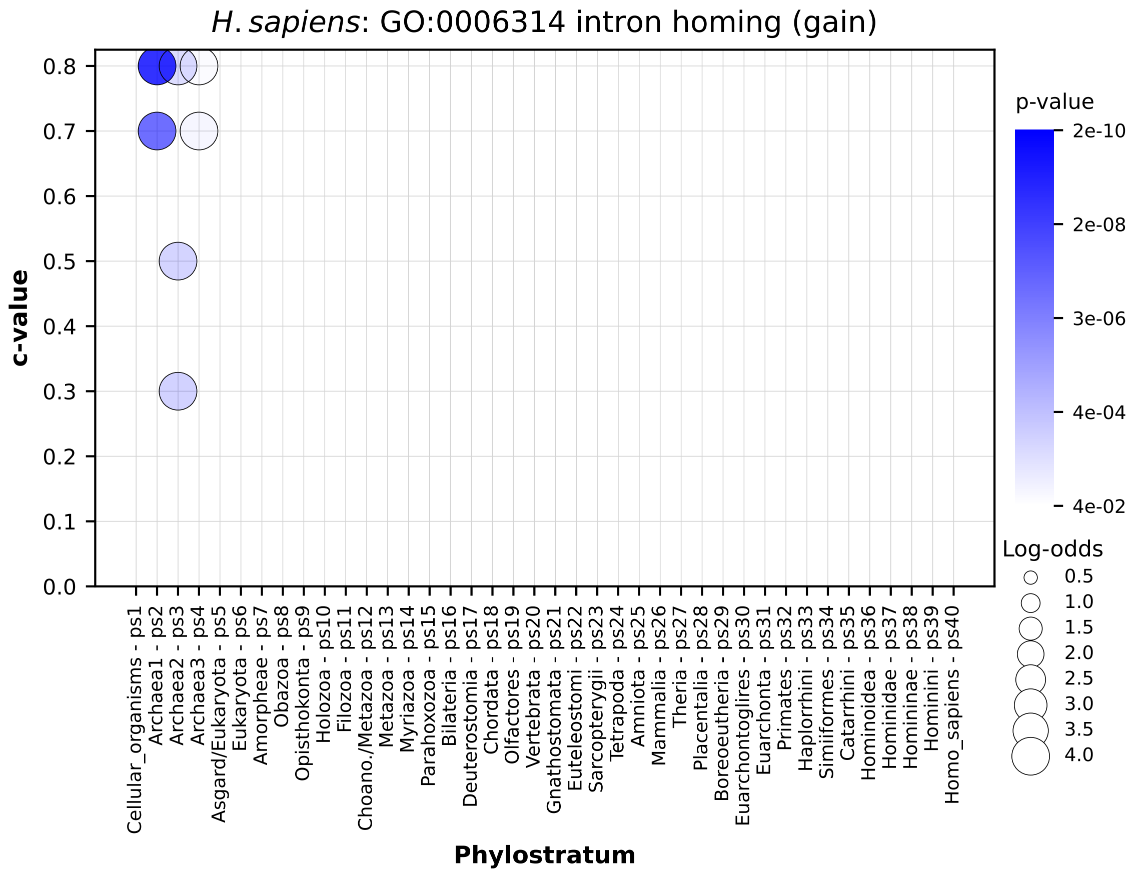

*H. sapiens*: GO:0006314 intron homing (gain)
c-value
0.0
0.1
0.2
0.3
0.4
0.5
0.6
0.7
0.8
Phylostratum
Cellular_organisms - ps1
Archaea1 - ps2
Archaea2 - ps3
Archaea3 - ps4
Asgard/Eukaryota - ps5
Eukaryota - ps6
Amorpheae - ps7
Obazoa - ps8
Opisthokonta - ps9
Holozoa - ps10
Filozoa - ps11
Choano./Metazoa - ps12
Metazoa - ps13
Myriazoa - ps14
Parahoxozoa - ps15
Bilateria - ps16
Deuterostomia - ps17
Chordata - ps18
Olfactores - ps19
Vertebrata - ps20
Gnathostomata - ps21
Euteleostomi - ps22
Sarcopterygii - ps23
Tetrapoda - ps24
Amniota - ps25
Mammalia - ps26
Theria - ps27
Placentalia - ps28
Boreoeutheria - ps29
Euarchontoglires - ps30
Euarchonta - ps31
Primates - ps32
Haplorrhini - ps33
Simiiformes - ps34
Catarrhini - ps35
Hominoidea - ps36
Hominidae - ps37
Homininae - ps38
Hominini - ps39
Homo_sapiens - ps40
p-value
2e-10
2e-08
3e-06
4e-04
4e-02
Log-odds
0.5
1.0
1.5
2.0
2.5
3.0
3.5
4.0

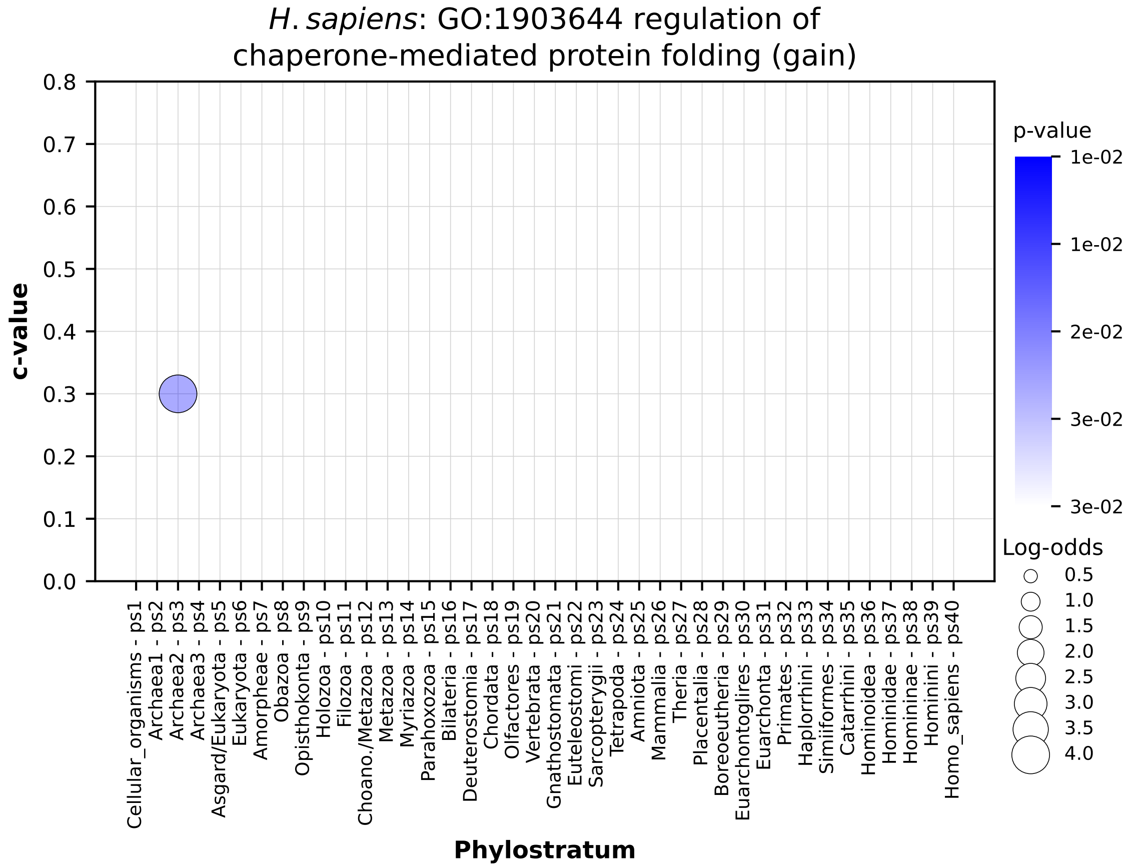

H. sapiens: GO:1903644 regulation of chaperone-mediated protein folding (gain)
c-value
0.0
0.1
0.2
0.3
0.4
0.5
0.6
0.7
0.8
Phylostratum
Cellular_organisms - ps1
Archaea1 - ps2
Archaea2 - ps3
Archaea3 - ps4
Asgard/Eukaryota - ps5
Eukaryota - ps6
Amorpheae - ps7
Obazoa - ps8
Opisthokonta - ps9
Holozoa - ps10
Filozoa - ps11
Choano./Metazoa - ps12
Metazoa - ps13
Myriazoa - ps14
Parahoxozoa - ps15
Bilateria - ps16
Deuterostomia - ps17
Chordata - ps18
Olfactores - ps19
Vertebrata - ps20
Gnathostomata - ps21
Euteleostomi - ps22
Sarcopterygii - ps23
Tetrapoda - ps24
Amniota - ps25
Mammalia - ps26
Theria - ps27
Placentalia - ps28
Boreoeutheria - ps29
Euarchontoglires - ps30
Euarchonta - ps31
Primates - ps32
Haplorrhini - ps33
Simiiformes - ps34
Catarrhini - ps35
Hominoidea - ps36
Hominidae - ps37
Homininae - ps38
Hominini - ps39
Homo_sapiens - ps40
p-value
1e-02
1e-02
2e-02
3e-02
3e-02
Log-odds
0.5
1.0
1.5
2.0
2.5
3.0
3.5
4.0

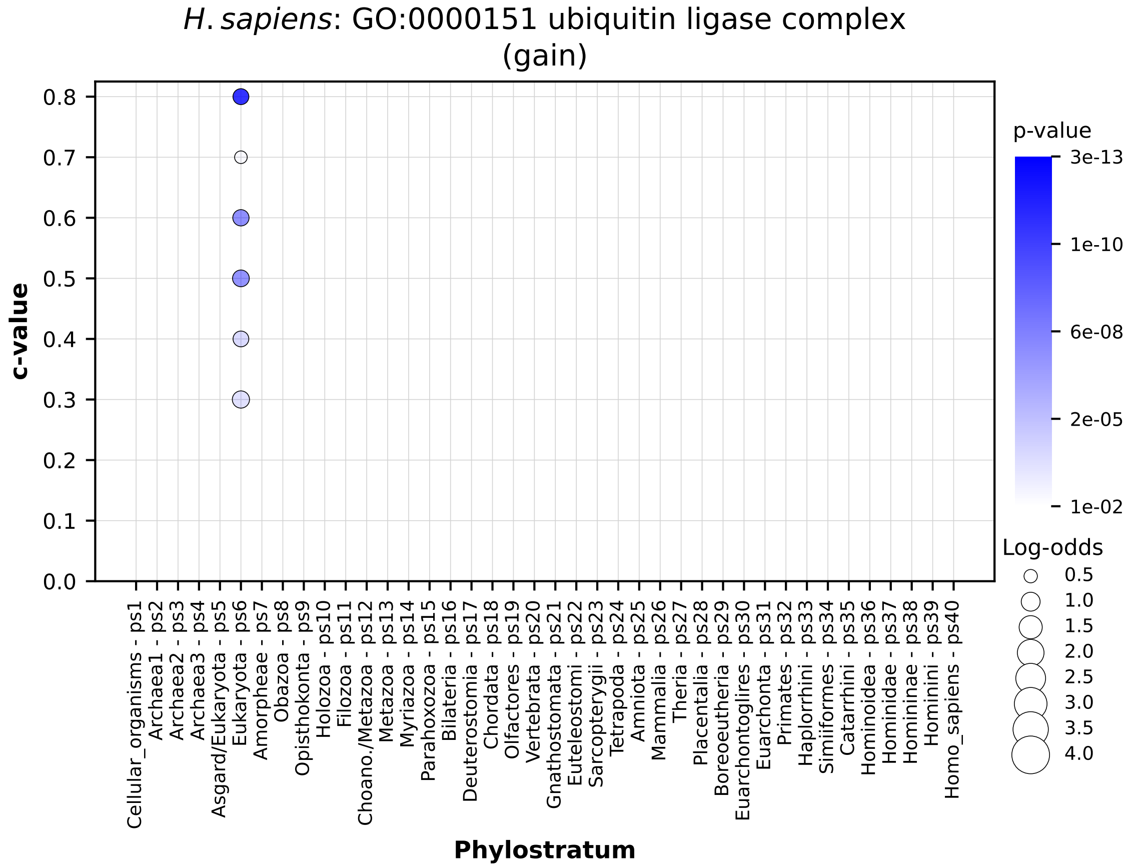

H. sapiens: GO:0000151 ubiquitin ligase complex
(gain)
c-value
0.0
0.1
0.2
0.3
0.4
0.5
0.6
0.7
0.8
Phylostratum
Cellular_organisms - ps1
Archaea1 - ps2
Archaea2 - ps3
Archaea3 - ps4
Asgard/Eukaryota - ps5
Eukaryota - ps6
Amorpheae - ps7
Obazoa - ps8
Opisthokonta - ps9
Holozoa - ps10
Filozoa - ps11
Choano./Metazoa - ps12
Metazoa - ps13
Myriazoa - ps14
Parahoxozoa - ps15
Bilateria - ps16
Deuterostomia - ps17
Chordata - ps18
Olfactores - ps19
Vertebrata - ps20
Gnathostomata - ps21
Euteleostomi - ps22
Sarcopterygii - ps23
Tetrapoda - ps24
Amniota - ps25
Mammalia - ps26
Theria - ps27
Placentalia - ps28
Boreoeutheria - ps29
Euarchontoglires - ps30
Euarchonta - ps31
Primates - ps32
Haplorrhini - ps33
Simiiformes - ps34
Catarrhini - ps35
Hominoidea - ps36
Hominidae - ps37
Homininae - ps38
Hominini - ps39
Homo_sapiens - ps40
p-value
3e-13
1e-10
6e-08
2e-05
1e-02
Log-odds
0.5
1.0
1.5
2.0
2.5
3.0
3.5
4.0

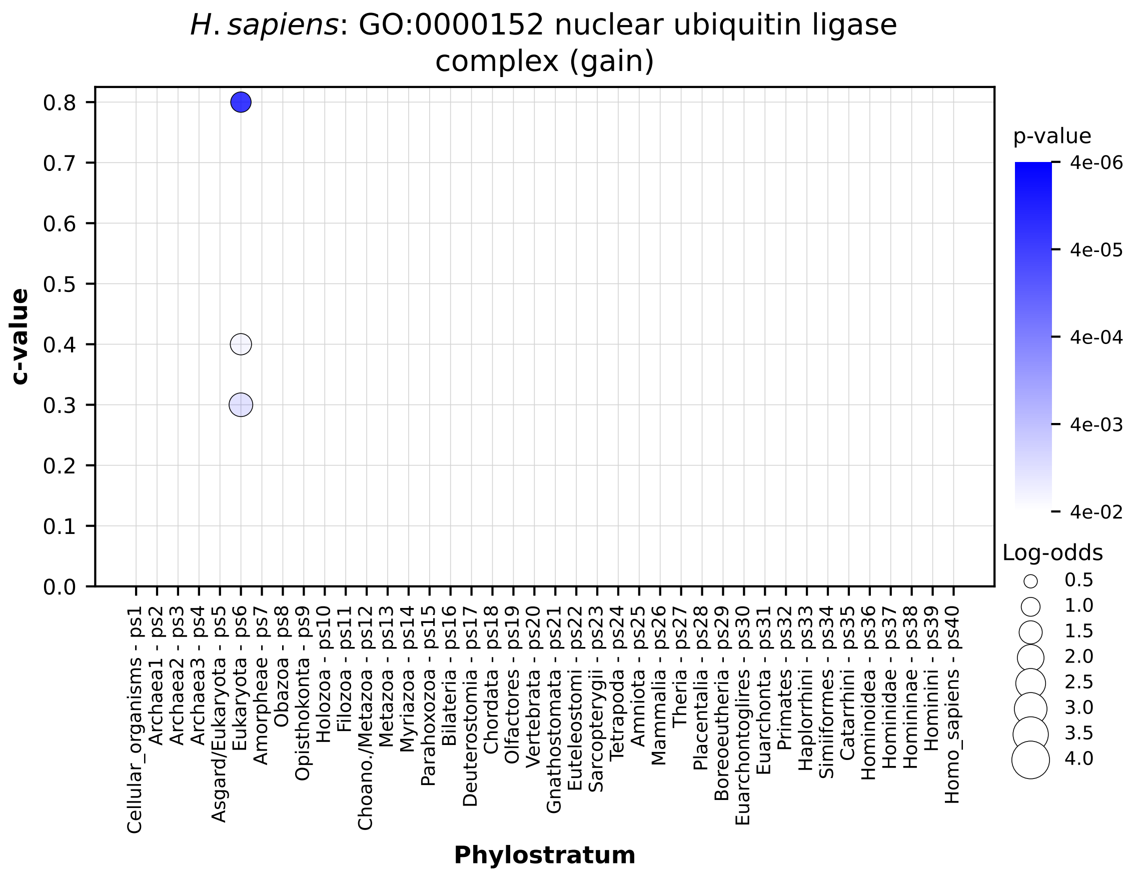

*H. sapiens*: GO:0000152 nuclear ubiquitin ligase complex (gain)
c-value
0.0
0.1
0.2
0.3
0.4
0.5
0.6
0.7
0.8
Phylostratum
Cellular_organisms - ps1
Archaea1 - ps2
Archaea2 - ps3
Archaea3 - ps4
Asgard/Eukaryota - ps5
Eukaryota - ps6
Amorpheae - ps7
Obazoa - ps8
Opisthokonta - ps9
Holozoa - ps10
Filozoa - ps11
Choano./Metazoa - ps12
Metazoa - ps13
Myriazoa - ps14
Parahoxozoa - ps15
Bilateria - ps16
Deuterostomia - ps17
Chordata - ps18
Olfactores - ps19
Vertebrata - ps20
Gnathostomata - ps21
Euteleostomi - ps22
Sarcopterygii - ps23
Tetrapoda - ps24
Amniota - ps25
Mammalia - ps26
Theria - ps27
Placentalia - ps28
Boreoeutheria - ps29
Euarchontoglires - ps30
Euarchonta - ps31
Primates - ps32
Haplorrhini - ps33
Simiiformes - ps34
Catarrhini - ps35
Hominoidea - ps36
Hominidae - ps37
Homininae - ps38
Hominini - ps39
Homo_sapiens - ps40
p-value
4e-06
4e-05
4e-04
4e-03
4e-02
Log-odds
0.5
1.0
1.5
2.0
2.5
3.0
3.5
4.0

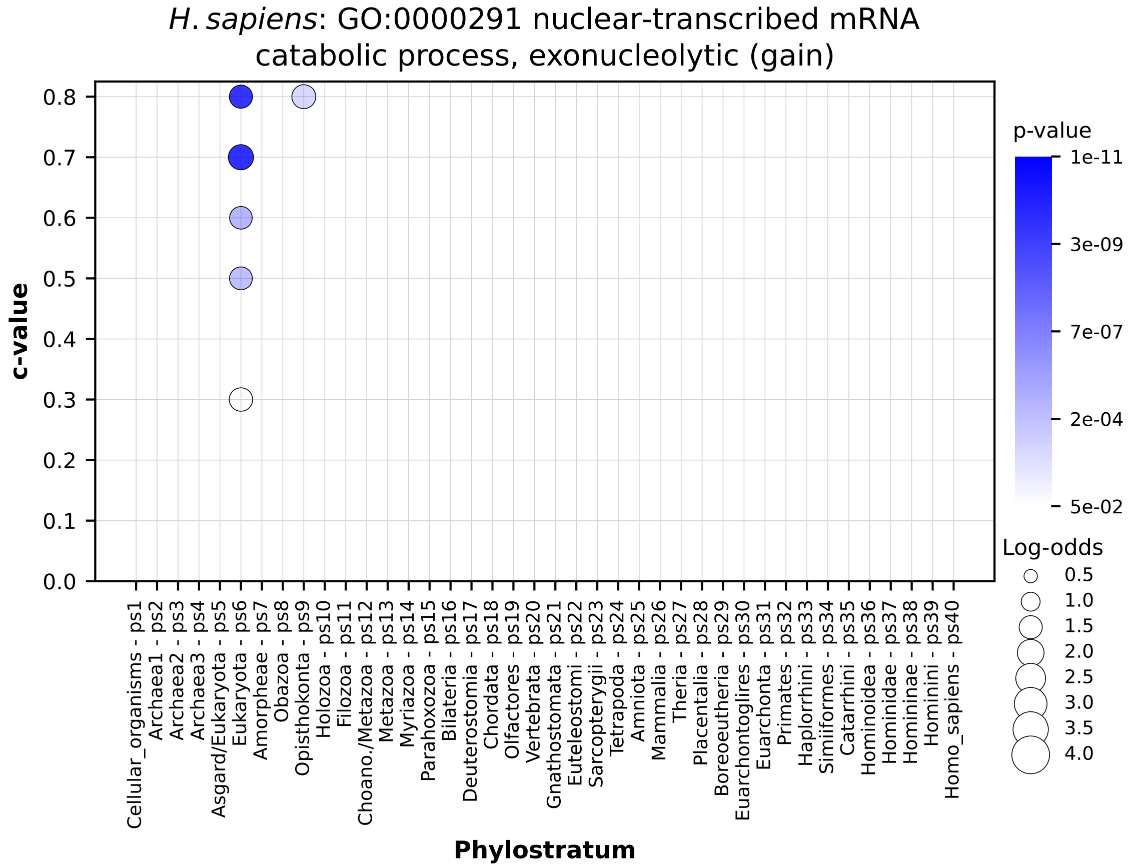

H. sapiens: GO:0000291 nuclear-transcribed mRNA catabolic process, exonucleolytic (gain)
c-value
0.0
0.1
0.2
0.3
0.4
0.5
0.6
0.7
0.8
Phylostratum
Cellular_organisms - ps1
Archaea1 - ps2
Archaea2 - ps3
Archaea3 - ps4
Asgard/Eukaryota - ps5
Eukaryota - ps6
Amorpheae - ps7
Obazoa - ps8
Opisthokonta - ps9
Holozoa - ps10
Filozoa - ps11
Choano./Metazoa - ps12
Metazoa - ps13
Myriazoa - ps14
Parahoxozoa - ps15
Bilateria - ps16
Deuterostomia - ps17
Chordata - ps18
Olfactores - ps19
Vertebrata - ps20
Gnathostomata - ps21
Euteleostomi - ps22
Sarcopterygii - ps23
Tetrapoda - ps24
Amniota - ps25
Mammalia - ps26
Theria - ps27
Placentalia - ps28
Boreoeutheria - ps29
Euarchontoglires - ps30
Euarchonta - ps31
Primates - ps32
[truncated: 502,489 more chars]
